# Supplementary material for: Astrin-SKAP complex reconstitution reveals its kinetochore interaction with microtubule-bound Ndc80
Source: eLife. 2017 Aug 25;6:e26866. doi: 10.7554/eLife.26866 (PMC5602300; doi:10.7554/eLife.26866)
Supplement: Source data 1. — Complete mass spectrometry searches using methods described in (Washburn et al., 2001) for affinity purification/mass spectrometry data sets described in this paper (data from this study; [Kern et al., 2016] [Gascoigne et al., 2011]). Individual Astrin cross-linking immunoprecipitations are listed based on the order in Figure 4—figure supplement 1. These samples have not been pruned for common or antibody-specific contaminants. [file elife-26866-data1.zip › Astrin_Crosslinking#1.html]

D NLDAstrin
DTASelect v2.0.21  
/nfs/cheeseman\_massspec/David/NLDAstrin  
/nfs/cheeseman\_massspec/Databases/NCBI-RefSeq\_human\_na\_04-13-2009\_con\_reversed.fasta  
SEQUEST 3.0 in SQT format.  
-p 1  
 Jump  to the summary table.  
  
sequest.params modifications:

|  |  |  |
| --- | --- | --- |
| \* | S | 80.0 |
| # | T | 80.0 |
| @ | K | 12.0 |
| Static | C | 57.0 |

|  |  |
| --- | --- |
| true | Use criteria |
| 0.0 | Minimum peptide confidence |
| 0.05 | Peptide false positive rate |
| 0.0 | Minimum protein confidence |
| 1.0 | Protein false positive rate |
| 1 | Minimum charge state |
| 16 | Maximum charge state |
| 0.0 | Minimum ion proportion |
| 1000 | Maximum Sp rank |
| -1.0 | Minimum Sp score |
| Include | Modified peptide inclusion |
| Any | Tryptic status requirement |
| false | Multiple, ambiguous IDs allowed |
| Ignore | Peptide validation handling |
| XCorr | Purge duplicate peptides by protein |
| false | Include only loci with unique peptide |
| true | Remove subset proteins |
| Ignore | Locus validation handling |
| 0 | Minimum modified peptides per locus |
| 1000 | Minimum redundancy for low coverage loci |
| 1 | Minimum peptides per locus |

#### Locus Key:

|  |  |  |  |  |  |  |  |  |
| --- | --- | --- | --- | --- | --- | --- | --- | --- |
| Validation Status | Locus | Sequence Count | Spectrum Count | Sequence Coverage | Length | MolWt | pI | Descriptive Name |

#### Similarity Key:

|  |  |  |
| --- | --- | --- |
| Locus | # of identical peptides | # of differing peptides |

---

|  |  |  |  |  |  |  |  |  |
| --- | --- | --- | --- | --- | --- | --- | --- | --- |
| U | *gi|154800483|ref|NP\_0* | 59 | 488 | 94.1% | 339 | 39541 | 9.1 | centromere protein N isoform 2 [Homo sapiens] |

| Filename XCorr DeltCN Conf% ObsM+H+ CalcM+H+ SpR ZScore Ion% # Sequence  | | | | | | | | | | | | |
| --- | --- | --- | --- | --- | --- | --- | --- | --- | --- | --- | --- | --- |
|  | Astrin\_NLD\_STLC\_tube2\_021014\_01.09740.09740.2 | 3.6546 | 0.4522 | 100.0% | 1182.9722 | 1183.3618 | 1 | 8.176 | 77.8% | 4 | -.MDETVAEFIK.R | 22 |
|  | Astrin\_NLD\_STLC\_031014\_02.06572.06572.3 | 2.4158 | 0.2871 | 97.8% | 1338.5643 | 1339.5493 | 477 | 5.488 | 32.5% | 1 | -.MDETVAEFIKR.T | 3 |
|  | Astrin\_NLD\_STLC\_tube2\_021014\_01.08414.08414.2 | 3.2945 | 0.3467 | 100.0% | 1338.9321 | 1339.5493 | 1 | 6.946 | 75.0% | 13 | -.MDETVAEFIKR.T | 22 |
|  | Astrin\_NLD\_STLC\_tube2\_021014\_01.12992.12992.2 | 3.4768 | 0.2917 | 100.0% | 1728.4922 | 1729.1719 | 2 | 5.034 | 60.7% | 7 | R.TILKIPMNELTTILK.A | 22 |
|  | Astrin\_NLD\_STLC\_031014\_01.11240.11240.3 | 4.4357 | 0.4426 | 100.0% | 1729.9143 | 1729.1719 | 14 | 6.628 | 46.4% | 8 | R.TILKIPMNELTTILK.A | 33 |
|  | Astrin\_NLD\_STLC\_tube2\_021014\_01.17877.17877.3 | 6.2108 | 0.4843 | 100.0% | 3677.9644 | 3679.3123 | 1 | 8.134 | 35.0% | 1 | R.TILKIPMNELTTILKAWDFLSENQLQTVNFR.Q | 33 |
|  | Astrin\_NLD\_STLC\_tube2\_021014\_01.11900.11900.2 | 3.6942 | 0.417 | 100.0% | 1273.2922 | 1273.5737 | 1 | 7.132 | 85.0% | 27 | K.IPMNELTTILK.A | 22 |
|  | Astrin\_NLD\_STLC\_tube2\_021014\_01.12981.12981.1 | 2.1951 | 0.5538 | 100.0% | 1967.88 | 1969.1637 | 3 | 8.043 | 40.0% | 1 | K.AWDFLSENQLQTVNFR.Q | 11 |
|  | Astrin\_NLD\_STLC\_031014\_02.09548.09548.3 | 4.7469 | 0.4349 | 100.0% | 1968.8344 | 1969.1637 | 1 | 8.505 | 46.7% | 10 | K.AWDFLSENQLQTVNFR.Q | 33 |
|  | Astrin\_NLD\_STLC\_031014\_02.09554.09554.2 | 5.0274 | 0.4062 | 100.0% | 1969.3922 | 1969.1637 | 1 | 8.645 | 76.7% | 31 | K.AWDFLSENQLQTVNFR.Q | 22 |
|  | Astrin\_NLD\_STLC\_031014\_01.09758.09758.3 | 3.732 | 0.2028 | 99.4% | 1849.5844 | 1850.0912 | 1 | 5.154 | 42.9% | 1 | R.KESVVQHLIHLCEEK.R | 33 |
|  | Astrin\_NLD\_STLC\_031014\_01.10187.10187.2 | 3.5328 | 0.3523 | 100.0% | 1720.7922 | 1721.9171 | 1 | 6.518 | 57.7% | 1 | K.ESVVQHLIHLCEEK.R | 22 |
|  | Astrin\_NLD\_STLC\_tube2\_021014\_01.12916.12916.3 | 6.8447 | 0.4011 | 100.0% | 2586.2344 | 2586.9714 | 1 | 8.212 | 38.1% | 2 | K.RASISDAALLDIIYMQFHQHQK.V | 33 |
|  | Astrin\_NLD\_STLC\_tube2\_021014\_01.13785.13785.2 | 4.7865 | 0.4513 | 100.0% | 2429.652 | 2430.784 | 1 | 8.408 | 50.0% | 1 | R.ASISDAALLDIIYMQFHQHQK.V | 22 |
|  | Astrin\_NLD\_STLC\_tube2\_021014\_01.13844.13844.3 | 3.1585 | 0.266 | 98.4% | 2429.9944 | 2430.784 | 1 | 5.157 | 35.0% | 3 | R.ASISDAALLDIIYMQFHQHQK.V | 33 |
|  | Astrin\_NLD\_STLC\_tube2\_021014\_01.15286.15286.3 | 4.5068 | 0.415 | 100.0% | 3551.3342 | 3552.103 | 1 | 6.204 | 26.7% | 1 | R.ASISDAALLDIIYMQFHQHQKVWDVFQMSK.G | 33 |
|  | Astrin\_NLD\_STLC\_tube2\_021014\_01.11547.11547.1 | 2.0847 | 0.3667 | 100.0% | 1139.49 | 1140.3423 | 1 | 6.837 | 75.0% | 2 | K.VWDVFQMSK.G | 11 |
|  | Astrin\_NLD\_STLC\_tube2\_021014\_01.11516.11516.2 | 3.399 | 0.3196 | 100.0% | 1140.0521 | 1140.3423 | 1 | 6.892 | 81.2% | 6 | K.VWDVFQMSK.G | 22 |
|  | Astrin\_NLD\_STLC\_tube2\_021014\_01.10488.10488.1 | 2.6404 | 0.387 | 100.0% | 1322.45 | 1323.4602 | 2 | 8.362 | 59.1% | 1 | K.GPGEDVDLFDMK.Q | 11 |
|  | Astrin\_NLD\_STLC\_tube2\_021014\_01.10502.10502.2 | 4.1308 | 0.5977 | 100.0% | 1323.0122 | 1323.4602 | 1 | 10.286 | 68.2% | 19 | K.GPGEDVDLFDMK.Q | 22 |
|  | Astrin\_NLD\_STLC\_tube2\_021014\_01.10685.10685.2 | 4.5421 | 0.4915 | 100.0% | 1726.1921 | 1726.9415 | 1 | 7.831 | 64.3% | 1 | K.GPGEDVDLFDMKQFK.N | 22 |
|  | Astrin\_NLD\_STLC\_tube2\_021014\_01.10688.10688.3 | 3.4703 | 0.5038 | 100.0% | 1726.8544 | 1726.9415 | 2 | 7.142 | 44.6% | 1 | K.GPGEDVDLFDMKQFK.N | 33 |
|  | Astrin\_NLD\_STLC\_tube2\_021014\_01.06052.06052.2 | 3.5049 | 0.3337 | 100.0% | 1135.4521 | 1135.3518 | 5 | 7.113 | 77.8% | 2 | R.ALKNVTVSFR.E | 22 |
|  | Astrin\_NLD\_STLC\_tube2\_021014\_01.07694.07694.1 | 1.8497 | 0.2567 | 98.3% | 1246.58 | 1247.3501 | 230 | 6.095 | 50.0% | 1 | R.ETEENAVWIR.I | 11 |
|  | Astrin\_NLD\_STLC\_tube2\_021014\_01.07724.07724.2 | 2.8236 | 0.3418 | 100.0% | 1247.0122 | 1247.3501 | 7 | 5.842 | 77.8% | 5 | R.ETEENAVWIR.I | 22 |
|  | Astrin\_NLD\_STLC\_tube2\_021014\_01.11631.11631.3 | 5.959 | 0.4887 | 100.0% | 4141.9443 | 4142.675 | 1 | 7.631 | 27.2% | 3 | R.IAWGTQYTKPNQYKPTYVVYYSQTPYAFTSSSMLR.R | 33 |
|  | Astrin\_NLD\_STLC\_tube2\_021014\_01.09380.09380.2 | 4.4996 | 0.4962 | 100.0% | 1583.2722 | 1583.8717 | 1 | 8.02 | 71.4% | 9 | R.RNTPLLGQALTIASK.H | 22 |
|  | Astrin\_NLD\_STLC\_tube2\_021014\_01.09268.09268.3 | 4.5168 | 0.4501 | 100.0% | 1584.4744 | 1583.8717 | 1 | 7.835 | 44.6% | 3 | R.RNTPLLGQALTIASK.H | 33 |
|  | Astrin\_NLD\_STLC\_031014\_01.10493.10493.1 | 2.5031 | 0.1558 | 98.4% | 1427.75 | 1427.6842 | 2 | 4.634 | 53.8% | 5 | R.NTPLLGQALTIASK.H | 11 |
|  | Astrin\_NLD\_STLC\_tube2\_021014\_01.11392.11392.2 | 4.5659 | 0.5253 | 100.0% | 1428.4922 | 1427.6842 | 1 | 8.647 | 80.8% | 46 | R.NTPLLGQALTIASK.H | 22 |
|  | Astrin\_NLD\_STLC\_tube2\_021014\_01.10924.10924.3 | 3.4434 | 0.2154 | 99.1% | 1429.0443 | 1427.6842 | 1 | 5.448 | 51.9% | 1 | R.NTPLLGQALTIASK.H | 33 |
|  | Astrin\_NLD\_STLC\_031014\_01.09180.09180.3 | 2.6584 | 0.3029 | 99.8% | 1278.2943 | 1277.5303 | 8 | 4.899 | 38.9% | 1 | K.HHQIVKMDLR.S | 33 |
|  | Astrin\_NLD\_STLC\_031014\_01.09153.09153.2 | 2.7419 | 0.1846 | 99.7% | 981.9922 | 982.12463 | 2 | 4.094 | 85.7% | 3 | R.SRYLDSLK.A | 22 |
|  | Astrin\_NLD\_STLC\_031014\_01.09605.09605.3 | 5.1117 | 0.4057 | 100.0% | 2753.2444 | 2754.0312 | 1 | 6.858 | 34.1% | 2 | K.AIVFKQYNQTFETHNSTTPLQER.S | 33 |
|  | Astrin\_NLD\_STLC\_tube2\_021014\_01.05517.05517.2 | 3.3667 | 0.5209 | 100.0% | 2194.392 | 2195.3098 | 1 | 7.519 | 50.0% | 4 | K.QYNQTFETHNSTTPLQER.S | 22 |
|  | Astrin\_NLD\_STLC\_tube2\_021014\_01.05397.05397.3 | 3.7725 | 0.399 | 100.0% | 2194.6743 | 2195.3098 | 2 | 6.537 | 35.3% | 4 | K.QYNQTFETHNSTTPLQER.S | 33 |
|  | Astrin\_NLD\_STLC\_tube2\_021014\_01.09226.09226.1 | 2.5736 | 0.5097 | 100.0% | 1220.53 | 1221.371 | 1 | 7.395 | 70.0% | 6 | R.SLGLDINMDSR.I | 11 |
|  | Astrin\_NLD\_STLC\_tube2\_021014\_01.09284.09284.2 | 4.1376 | 0.5111 | 100.0% | 1221.1721 | 1221.371 | 1 | 8.089 | 90.0% | 113 | R.SLGLDINMDSR.I | 22 |
|  | Astrin\_NLD\_STLC\_031014\_01.08974.08974.1 | 2.3599 | 0.2128 | 100.0% | 1094.56 | 1095.284 | 2 | 5.322 | 68.8% | 1 | R.IIHENIVEK.E | 11 |
|  | Astrin\_NLD\_STLC\_031014\_01.08972.08972.2 | 2.4044 | 0.2891 | 99.8% | 1095.2122 | 1095.284 | 1 | 5.446 | 87.5% | 2 | R.IIHENIVEK.E | 22 |
|  | Astrin\_NLD\_STLC\_tube2\_021014\_01.04112.04112.2 | 3.6068 | 0.4155 | 100.0% | 1379.7922 | 1380.587 | 1 | 7.489 | 85.0% | 4 | R.IIHENIVEKER.V | 22 |
|  | Astrin\_NLD\_STLC\_031014\_01.09148.09148.3 | 2.9882 | 0.3121 | 100.0% | 1380.0543 | 1380.587 | 1 | 5.512 | 45.0% | 4 | R.IIHENIVEKER.V | 33 |
|  | Astrin\_NLD\_STLC\_031014\_01.10464.10464.2 | 5.8669 | 0.5523 | 100.0% | 2403.912 | 2404.638 | 1 | 10.859 | 65.8% | 19 | R.ITQETFGDYPQPQLEFAQYK.L | 22 |
|  | Astrin\_NLD\_STLC\_031014\_01.10479.10479.3 | 5.0854 | 0.4505 | 100.0% | 2406.0842 | 2404.638 | 1 | 7.92 | 43.4% | 8 | R.ITQETFGDYPQPQLEFAQYK.L | 33 |
|  | Astrin\_NLD\_STLC\_031014\_01.10454.10454.2 | 4.886 | 0.5122 | 100.0% | 2875.5923 | 2876.192 | 1 | 9.971 | 43.5% | 3 | R.ITQETFGDYPQPQLEFAQYKLETK.F | 22 |
|  | Astrin\_NLD\_STLC\_031014\_01.10466.10466.3 | 6.1483 | 0.5449 | 100.0% | 2876.0344 | 2876.192 | 1 | 8.838 | 42.4% | 10 | R.ITQETFGDYPQPQLEFAQYKLETK.F | 33 |
|  | Astrin\_NLD\_STLC\_tube2\_021014\_02.05792.05792.2 | 3.464 | 0.3926 | 100.0% | 1391.8322 | 1392.598 | 1 | 6.903 | 79.2% | 4 | K.FKSGLNGSILAER.E | 22 |
|  | Astrin\_NLD\_STLC\_031014\_02.05832.05832.3 | 4.0889 | 0.3354 | 100.0% | 1392.0243 | 1392.598 | 1 | 6.271 | 52.1% | 2 | K.FKSGLNGSILAER.E | 33 |
|  | Astrin\_NLD\_STLC\_031014\_01.09611.09611.2 | 3.5811 | 0.235 | 100.0% | 2016.6522 | 2017.2926 | 80 | 5.4 | 35.3% | 1 | K.FKSGLNGSILAEREEPLR.C | 22 |
|  | Astrin\_NLD\_STLC\_tube2\_021014\_01.07227.07227.1 | 2.0669 | 0.2647 | 100.0% | 1116.69 | 1117.2474 | 157 | 4.789 | 45.0% | 2 | K.SGLNGSILAER.E | 11 |
|  | Astrin\_NLD\_STLC\_tube2\_021014\_01.07274.07274.2 | 3.4635 | 0.3447 | 100.0% | 1117.1322 | 1117.2474 | 1 | 7.452 | 85.0% | 22 | K.SGLNGSILAER.E | 22 |
|  | Astrin\_NLD\_STLC\_tube2\_021014\_01.07886.07886.2 | 3.1578 | 0.2282 | 99.7% | 1741.3522 | 1741.9419 | 69 | 4.684 | 40.0% | 7 | K.SGLNGSILAEREEPLR.C | 22 |
|  | Astrin\_NLD\_STLC\_031014\_01.09680.09680.3 | 3.267 | 0.2989 | 100.0% | 1742.0343 | 1741.9419 | 1 | 5.25 | 40.0% | 11 | K.SGLNGSILAEREEPLR.C | 33 |
|  | Astrin\_NLD\_STLC\_tube2\_021014\_01.08934.08934.1 | 2.2654 | 0.2482 | 100.0% | 1241.62 | 1242.4606 | 1 | 4.955 | 55.0% | 3 | K.FSSPHLLEALK.S | 11 |
|  | Astrin\_NLD\_STLC\_tube2\_021014\_01.09356.09356.2 | 3.2793 | 0.3937 | 100.0% | 1242.3522 | 1242.4606 | 1 | 6.693 | 85.0% | 31 | K.FSSPHLLEALK.S | 22 |
|  | Astrin\_NLD\_STLC\_tube2\_021014\_01.09339.09339.3 | 2.2147 | 0.2802 | 95.4% | 1242.4744 | 1242.4606 | 2 | 5.171 | 47.5% | 1 | K.FSSPHLLEALK.S | 33 |
| \* | Astrin\_NLD\_STLC\_031014\_01.10766.10766.3 | 2.5487 | 0.2867 | 95.5% | 2375.0044 | 2376.768 | 4 | 5.563 | 29.5% | 1 | K.SLAPAGIADAPLSPLLTCIPNKR.M | 3 |
| \* | Astrin\_NLD\_STLC\_031014\_01.10755.10755.2 | 4.4336 | 0.5067 | 100.0% | 2375.7122 | 2376.768 | 1 | 9.105 | 43.2% | 1 | K.SLAPAGIADAPLSPLLTCIPNKR.M | 2 |
| \* | Astrin\_NLD\_STLC\_tube2\_021014\_01.08979.08979.2 | 2.1014 | 0.2217 | 97.2% | 972.27216 | 972.1931 | 45 | 5.363 | 66.7% | 1 | R.MNYFKIR.D | 2 |

Similarities:
gi|154800485|ref|NP\_0(55:4)  

---

|  |  |  |  |  |  |  |  |  |
| --- | --- | --- | --- | --- | --- | --- | --- | --- |
| U | *gi|154800485|ref|NP\_0* | 57 | 490 | 81.9% | 353 | 41180 | 8.9 | centromere protein N isoform 1 [Homo sapiens] |

| Filename XCorr DeltCN Conf% ObsM+H+ CalcM+H+ SpR ZScore Ion% # Sequence  | | | | | | | | | | | | |
| --- | --- | --- | --- | --- | --- | --- | --- | --- | --- | --- | --- | --- |
|  | Astrin\_NLD\_STLC\_tube2\_021014\_01.09740.09740.2 | 3.6546 | 0.4522 | 100.0% | 1182.9722 | 1183.3618 | 1 | 8.176 | 77.8% | 4 | -.MDETVAEFIK.R | 22 |
|  | Astrin\_NLD\_STLC\_tube2\_021014\_01.08414.08414.2 | 3.2945 | 0.3467 | 100.0% | 1338.9321 | 1339.5493 | 1 | 6.946 | 75.0% | 13 | -.MDETVAEFIKR.T | 22 |
|  | Astrin\_NLD\_STLC\_tube2\_021014\_01.08673.08673.3 | 2.5121 | 0.3029 | 99.4% | 1340.2743 | 1339.5493 | 177 | 5.187 | 37.5% | 4 | -.MDETVAEFIKR.T | 3 |
|  | Astrin\_NLD\_STLC\_tube2\_021014\_01.12992.12992.2 | 3.4768 | 0.2917 | 100.0% | 1728.4922 | 1729.1719 | 2 | 5.034 | 60.7% | 7 | R.TILKIPMNELTTILK.A | 22 |
|  | Astrin\_NLD\_STLC\_031014\_01.11240.11240.3 | 4.4357 | 0.4426 | 100.0% | 1729.9143 | 1729.1719 | 14 | 6.628 | 46.4% | 8 | R.TILKIPMNELTTILK.A | 33 |
|  | Astrin\_NLD\_STLC\_tube2\_021014\_01.17877.17877.3 | 6.2108 | 0.4843 | 100.0% | 3677.9644 | 3679.3123 | 1 | 8.134 | 35.0% | 1 | R.TILKIPMNELTTILKAWDFLSENQLQTVNFR.Q | 33 |
|  | Astrin\_NLD\_STLC\_tube2\_021014\_01.11900.11900.2 | 3.6942 | 0.417 | 100.0% | 1273.2922 | 1273.5737 | 1 | 7.132 | 85.0% | 27 | K.IPMNELTTILK.A | 22 |
|  | Astrin\_NLD\_STLC\_tube2\_021014\_01.12981.12981.1 | 2.1951 | 0.5538 | 100.0% | 1967.88 | 1969.1637 | 3 | 8.043 | 40.0% | 1 | K.AWDFLSENQLQTVNFR.Q | 11 |
|  | Astrin\_NLD\_STLC\_031014\_02.09548.09548.3 | 4.7469 | 0.4349 | 100.0% | 1968.8344 | 1969.1637 | 1 | 8.505 | 46.7% | 10 | K.AWDFLSENQLQTVNFR.Q | 33 |
|  | Astrin\_NLD\_STLC\_031014\_02.09554.09554.2 | 5.0274 | 0.4062 | 100.0% | 1969.3922 | 1969.1637 | 1 | 8.645 | 76.7% | 31 | K.AWDFLSENQLQTVNFR.Q | 22 |
|  | Astrin\_NLD\_STLC\_031014\_01.09758.09758.3 | 3.732 | 0.2028 | 99.4% | 1849.5844 | 1850.0912 | 1 | 5.154 | 42.9% | 1 | R.KESVVQHLIHLCEEK.R | 33 |
|  | Astrin\_NLD\_STLC\_031014\_01.10187.10187.2 | 3.5328 | 0.3523 | 100.0% | 1720.7922 | 1721.9171 | 1 | 6.518 | 57.7% | 1 | K.ESVVQHLIHLCEEK.R | 22 |
|  | Astrin\_NLD\_STLC\_tube2\_021014\_01.12916.12916.3 | 6.8447 | 0.4011 | 100.0% | 2586.2344 | 2586.9714 | 1 | 8.212 | 38.1% | 2 | K.RASISDAALLDIIYMQFHQHQK.V | 33 |
|  | Astrin\_NLD\_STLC\_tube2\_021014\_01.13785.13785.2 | 4.7865 | 0.4513 | 100.0% | 2429.652 | 2430.784 | 1 | 8.408 | 50.0% | 1 | R.ASISDAALLDIIYMQFHQHQK.V | 22 |
|  | Astrin\_NLD\_STLC\_tube2\_021014\_01.13844.13844.3 | 3.1585 | 0.266 | 98.4% | 2429.9944 | 2430.784 | 1 | 5.157 | 35.0% | 3 | R.ASISDAALLDIIYMQFHQHQK.V | 33 |
|  | Astrin\_NLD\_STLC\_tube2\_021014\_01.15286.15286.3 | 4.5068 | 0.415 | 100.0% | 3551.3342 | 3552.103 | 1 | 6.204 | 26.7% | 1 | R.ASISDAALLDIIYMQFHQHQKVWDVFQMSK.G | 33 |
|  | Astrin\_NLD\_STLC\_tube2\_021014\_01.11547.11547.1 | 2.0847 | 0.3667 | 100.0% | 1139.49 | 1140.3423 | 1 | 6.837 | 75.0% | 2 | K.VWDVFQMSK.G | 11 |
|  | Astrin\_NLD\_STLC\_tube2\_021014\_01.11516.11516.2 | 3.399 | 0.3196 | 100.0% | 1140.0521 | 1140.3423 | 1 | 6.892 | 81.2% | 6 | K.VWDVFQMSK.G | 22 |
|  | Astrin\_NLD\_STLC\_tube2\_021014\_01.10488.10488.1 | 2.6404 | 0.387 | 100.0% | 1322.45 | 1323.4602 | 2 | 8.362 | 59.1% | 1 | K.GPGEDVDLFDMK.Q | 11 |
|  | Astrin\_NLD\_STLC\_tube2\_021014\_01.10502.10502.2 | 4.1308 | 0.5977 | 100.0% | 1323.0122 | 1323.4602 | 1 | 10.286 | 68.2% | 19 | K.GPGEDVDLFDMK.Q | 22 |
|  | Astrin\_NLD\_STLC\_tube2\_021014\_01.10685.10685.2 | 4.5421 | 0.4915 | 100.0% | 1726.1921 | 1726.9415 | 1 | 7.831 | 64.3% | 1 | K.GPGEDVDLFDMKQFK.N | 22 |
|  | Astrin\_NLD\_STLC\_tube2\_021014\_01.10688.10688.3 | 3.4703 | 0.5038 | 100.0% | 1726.8544 | 1726.9415 | 2 | 7.142 | 44.6% | 1 | K.GPGEDVDLFDMKQFK.N | 33 |
|  | Astrin\_NLD\_STLC\_tube2\_021014\_01.06052.06052.2 | 3.5049 | 0.3337 | 100.0% | 1135.4521 | 1135.3518 | 5 | 7.113 | 77.8% | 2 | R.ALKNVTVSFR.E | 22 |
|  | Astrin\_NLD\_STLC\_tube2\_021014\_01.07694.07694.1 | 1.8497 | 0.2567 | 98.3% | 1246.58 | 1247.3501 | 230 | 6.095 | 50.0% | 1 | R.ETEENAVWIR.I | 11 |
|  | Astrin\_NLD\_STLC\_tube2\_021014\_01.07724.07724.2 | 2.8236 | 0.3418 | 100.0% | 1247.0122 | 1247.3501 | 7 | 5.842 | 77.8% | 5 | R.ETEENAVWIR.I | 22 |
|  | Astrin\_NLD\_STLC\_tube2\_021014\_01.11631.11631.3 | 5.959 | 0.4887 | 100.0% | 4141.9443 | 4142.675 | 1 | 7.631 | 27.2% | 3 | R.IAWGTQYTKPNQYKPTYVVYYSQTPYAFTSSSMLR.R | 33 |
|  | Astrin\_NLD\_STLC\_tube2\_021014\_01.09380.09380.2 | 4.4996 | 0.4962 | 100.0% | 1583.2722 | 1583.8717 | 1 | 8.02 | 71.4% | 9 | R.RNTPLLGQALTIASK.H | 22 |
|  | Astrin\_NLD\_STLC\_tube2\_021014\_01.09268.09268.3 | 4.5168 | 0.4501 | 100.0% | 1584.4744 | 1583.8717 | 1 | 7.835 | 44.6% | 3 | R.RNTPLLGQALTIASK.H | 33 |
|  | Astrin\_NLD\_STLC\_031014\_01.10493.10493.1 | 2.5031 | 0.1558 | 98.4% | 1427.75 | 1427.6842 | 2 | 4.634 | 53.8% | 5 | R.NTPLLGQALTIASK.H | 11 |
|  | Astrin\_NLD\_STLC\_tube2\_021014\_01.11392.11392.2 | 4.5659 | 0.5253 | 100.0% | 1428.4922 | 1427.6842 | 1 | 8.647 | 80.8% | 46 | R.NTPLLGQALTIASK.H | 22 |
|  | Astrin\_NLD\_STLC\_tube2\_021014\_01.10924.10924.3 | 3.4434 | 0.2154 | 99.1% | 1429.0443 | 1427.6842 | 1 | 5.448 | 51.9% | 1 | R.NTPLLGQALTIASK.H | 33 |
|  | Astrin\_NLD\_STLC\_031014\_01.09180.09180.3 | 2.6584 | 0.3029 | 99.8% | 1278.2943 | 1277.5303 | 8 | 4.899 | 38.9% | 1 | K.HHQIVKMDLR.S | 33 |
|  | Astrin\_NLD\_STLC\_031014\_01.09153.09153.2 | 2.7419 | 0.1846 | 99.7% | 981.9922 | 982.12463 | 2 | 4.094 | 85.7% | 3 | R.SRYLDSLK.A | 22 |
|  | Astrin\_NLD\_STLC\_031014\_01.09605.09605.3 | 5.1117 | 0.4057 | 100.0% | 2753.2444 | 2754.0312 | 1 | 6.858 | 34.1% | 2 | K.AIVFKQYNQTFETHNSTTPLQER.S | 33 |
|  | Astrin\_NLD\_STLC\_tube2\_021014\_01.05517.05517.2 | 3.3667 | 0.5209 | 100.0% | 2194.392 | 2195.3098 | 1 | 7.519 | 50.0% | 4 | K.QYNQTFETHNSTTPLQER.S | 22 |
|  | Astrin\_NLD\_STLC\_tube2\_021014\_01.05397.05397.3 | 3.7725 | 0.399 | 100.0% | 2194.6743 | 2195.3098 | 2 | 6.537 | 35.3% | 4 | K.QYNQTFETHNSTTPLQER.S | 33 |
|  | Astrin\_NLD\_STLC\_tube2\_021014\_01.09226.09226.1 | 2.5736 | 0.5097 | 100.0% | 1220.53 | 1221.371 | 1 | 7.395 | 70.0% | 6 | R.SLGLDINMDSR.I | 11 |
|  | Astrin\_NLD\_STLC\_tube2\_021014\_01.09284.09284.2 | 4.1376 | 0.5111 | 100.0% | 1221.1721 | 1221.371 | 1 | 8.089 | 90.0% | 113 | R.SLGLDINMDSR.I | 22 |
|  | Astrin\_NLD\_STLC\_031014\_01.08974.08974.1 | 2.3599 | 0.2128 | 100.0% | 1094.56 | 1095.284 | 2 | 5.322 | 68.8% | 1 | R.IIHENIVEK.E | 11 |
|  | Astrin\_NLD\_STLC\_031014\_01.08972.08972.2 | 2.4044 | 0.2891 | 99.8% | 1095.2122 | 1095.284 | 1 | 5.446 | 87.5% | 2 | R.IIHENIVEK.E | 22 |
|  | Astrin\_NLD\_STLC\_tube2\_021014\_01.04112.04112.2 | 3.6068 | 0.4155 | 100.0% | 1379.7922 | 1380.587 | 1 | 7.489 | 85.0% | 4 | R.IIHENIVEKER.V | 22 |
|  | Astrin\_NLD\_STLC\_031014\_01.09148.09148.3 | 2.9882 | 0.3121 | 100.0% | 1380.0543 | 1380.587 | 1 | 5.512 | 45.0% | 4 | R.IIHENIVEKER.V | 33 |
|  | Astrin\_NLD\_STLC\_031014\_01.10464.10464.2 | 5.8669 | 0.5523 | 100.0% | 2403.912 | 2404.638 | 1 | 10.859 | 65.8% | 19 | R.ITQETFGDYPQPQLEFAQYK.L | 22 |
|  | Astrin\_NLD\_STLC\_031014\_01.10479.10479.3 | 5.0854 | 0.4505 | 100.0% | 2406.0842 | 2404.638 | 1 | 7.92 | 43.4% | 8 | R.ITQETFGDYPQPQLEFAQYK.L | 33 |
|  | Astrin\_NLD\_STLC\_031014\_01.10454.10454.2 | 4.886 | 0.5122 | 100.0% | 2875.5923 | 2876.192 | 1 | 9.971 | 43.5% | 3 | R.ITQETFGDYPQPQLEFAQYKLETK.F | 22 |
|  | Astrin\_NLD\_STLC\_031014\_01.10466.10466.3 | 6.1483 | 0.5449 | 100.0% | 2876.0344 | 2876.192 | 1 | 8.838 | 42.4% | 10 | R.ITQETFGDYPQPQLEFAQYKLETK.F | 33 |
|  | Astrin\_NLD\_STLC\_tube2\_021014\_02.05792.05792.2 | 3.464 | 0.3926 | 100.0% | 1391.8322 | 1392.598 | 1 | 6.903 | 79.2% | 4 | K.FKSGLNGSILAER.E | 22 |
|  | Astrin\_NLD\_STLC\_031014\_02.05832.05832.3 | 4.0889 | 0.3354 | 100.0% | 1392.0243 | 1392.598 | 1 | 6.271 | 52.1% | 2 | K.FKSGLNGSILAER.E | 33 |
|  | Astrin\_NLD\_STLC\_031014\_01.09611.09611.2 | 3.5811 | 0.235 | 100.0% | 2016.6522 | 2017.2926 | 80 | 5.4 | 35.3% | 1 | K.FKSGLNGSILAEREEPLR.C | 22 |
| \* | Astrin\_NLD\_STLC\_031014\_02.06338.06338.3 | 2.7321 | 0.263 | 96.5% | 2017.7943 | 2017.2926 | 181 | 4.364 | 27.9% | 2 | K.FKSGLNGSILAEREEPLR.C | 3 |
|  | Astrin\_NLD\_STLC\_tube2\_021014\_01.07227.07227.1 | 2.0669 | 0.2647 | 100.0% | 1116.69 | 1117.2474 | 157 | 4.789 | 45.0% | 2 | K.SGLNGSILAER.E | 11 |
|  | Astrin\_NLD\_STLC\_tube2\_021014\_01.07274.07274.2 | 3.4635 | 0.3447 | 100.0% | 1117.1322 | 1117.2474 | 1 | 7.452 | 85.0% | 22 | K.SGLNGSILAER.E | 22 |
|  | Astrin\_NLD\_STLC\_tube2\_021014\_01.07886.07886.2 | 3.1578 | 0.2282 | 99.7% | 1741.3522 | 1741.9419 | 69 | 4.684 | 40.0% | 7 | K.SGLNGSILAEREEPLR.C | 22 |
|  | Astrin\_NLD\_STLC\_031014\_01.09680.09680.3 | 3.267 | 0.2989 | 100.0% | 1742.0343 | 1741.9419 | 1 | 5.25 | 40.0% | 11 | K.SGLNGSILAEREEPLR.C | 33 |
|  | Astrin\_NLD\_STLC\_tube2\_021014\_01.08934.08934.1 | 2.2654 | 0.2482 | 100.0% | 1241.62 | 1242.4606 | 1 | 4.955 | 55.0% | 3 | K.FSSPHLLEALK.S | 11 |
|  | Astrin\_NLD\_STLC\_tube2\_021014\_01.09356.09356.2 | 3.2793 | 0.3937 | 100.0% | 1242.3522 | 1242.4606 | 1 | 6.693 | 85.0% | 31 | K.FSSPHLLEALK.S | 22 |
|  | Astrin\_NLD\_STLC\_tube2\_021014\_01.09339.09339.3 | 2.2147 | 0.2802 | 95.4% | 1242.4744 | 1242.4606 | 2 | 5.171 | 47.5% | 1 | K.FSSPHLLEALK.S | 33 |

Similarities:
gi|154800483|ref|NP\_0(55:2)  

---

|  |  |  |  |  |  |  |  |  |
| --- | --- | --- | --- | --- | --- | --- | --- | --- |
| U | *gi|4504919|ref|NP\_002* | 39 | 103 | 60.9% | 483 | 53704 | 5.6 | keratin 8 [Homo sapiens] |

| Filename XCorr DeltCN Conf% ObsM+H+ CalcM+H+ SpR ZScore Ion% # Sequence  | | | | | | | | | | | | |
| --- | --- | --- | --- | --- | --- | --- | --- | --- | --- | --- | --- | --- |
| \* | Astrin\_NLD\_STLC\_tube2\_021014\_01.15436.15436.3 | 4.9228 | 0.3462 | 100.0% | 3926.5444 | 3927.465 | 1 | 6.555 | 21.2% | 1 | R.GGLGGGYGGASGMGGITAVTVNQSLLSPLVLEVDPNIQAVR.T | 3 |
|  | Astrin\_NLD\_STLC\_tube2\_021014\_01.07623.07623.2 | 2.9741 | 0.1656 | 99.6% | 1082.8121 | 1083.2755 | 7 | 7.028 | 75.0% | 2 | K.FASFIDKVR.F | 22222 |
|  | Astrin\_NLD\_STLC\_tube2\_021014\_01.08486.08486.2 | 3.0583 | 0.1206 | 99.2% | 1031.2722 | 1031.1997 | 2 | 3.753 | 92.9% | 2 | K.WSLLQQQK.T | 2 |
|  | Astrin\_NLD\_STLC\_tube2\_021014\_01.13679.13679.2 | 3.7561 | 0.3979 | 100.0% | 1849.3322 | 1849.0431 | 1 | 6.537 | 50.0% | 1 | R.SNMDNMFESYINNLR.R | 2 |
|  | Astrin\_NLD\_STLC\_tube2\_021014\_01.13073.13073.3 | 4.1695 | 0.2551 | 100.0% | 2034.8644 | 2035.363 | 1 | 7.564 | 39.7% | 2 | K.LKLEAELGNMQGLVEDFK.N | 3 |
|  | Astrin\_NLD\_STLC\_tube2\_021014\_01.10467.10467.2 | 3.1934 | 0.3889 | 100.0% | 1353.6522 | 1353.5732 | 1 | 6.778 | 75.0% | 2 | R.TEMENEFVLIK.K | 2 |
|  | Astrin\_NLD\_STLC\_tube2\_021014\_02.06556.06556.2 | 3.7042 | 0.3079 | 100.0% | 1481.2522 | 1481.7473 | 1 | 5.659 | 72.7% | 6 | R.TEMENEFVLIKK.D | 2 |
|  | Astrin\_NLD\_STLC\_tube2\_021014\_01.06185.06185.3 | 3.4783 | 0.3467 | 100.0% | 1926.2943 | 1927.1365 | 1 | 5.771 | 35.0% | 3 | K.KDVDEAYMNKVELESR.L | 3 |
|  | Astrin\_NLD\_STLC\_tube2\_021014\_02.05920.05920.2 | 4.3587 | 0.5213 | 100.0% | 1798.2122 | 1798.9623 | 1 | 8.186 | 71.4% | 2 | K.DVDEAYMNKVELESR.L | 2 |
|  | Astrin\_NLD\_STLC\_031014\_02.05984.05984.3 | 4.4231 | 0.3742 | 100.0% | 1799.2444 | 1798.9623 | 1 | 6.008 | 51.8% | 8 | K.DVDEAYMNKVELESR.L | 3 |
|  | Astrin\_NLD\_STLC\_tube2\_021014\_01.12351.12351.2 | 4.101 | 0.4913 | 100.0% | 1420.2122 | 1420.6055 | 1 | 8.699 | 90.9% | 9 | R.LEGLTDEINFLR.Q | 2 |
|  | Astrin\_NLD\_STLC\_tube2\_021014\_01.09720.09720.2 | 5.3645 | 0.6279 | 100.0% | 2109.3123 | 2110.3008 | 1 | 10.724 | 63.9% | 1 | R.ELQSQISDTSVVLSMDNSR.S | 2 |
|  | Astrin\_NLD\_STLC\_tube2\_021014\_01.12083.12083.1 | 2.3513 | 0.2799 | 100.0% | 1320.54 | 1321.5286 | 6 | 5.772 | 54.5% | 1 | R.SLDMDSIIAEVK.A | 1 |
|  | Astrin\_NLD\_STLC\_tube2\_021014\_01.12059.12059.2 | 4.2423 | 0.4753 | 100.0% | 1321.3322 | 1321.5286 | 1 | 8.544 | 77.3% | 4 | R.SLDMDSIIAEVK.A | 2 |
|  | Astrin\_NLD\_STLC\_tube2\_021014\_01.15390.15390.2 | 2.8267 | 0.3141 | 99.9% | 2382.2522 | 2382.6477 | 12 | 5.22 | 32.5% | 1 | R.SLDMDSIIAEVKAQYEDIANR.S | 2 |
|  | Astrin\_NLD\_STLC\_tube2\_021014\_01.04317.04317.1 | 1.9579 | 0.2777 | 100.0% | 1079.45 | 1080.1423 | 4 | 5.773 | 56.2% | 2 | K.AQYEDIANR.S | 11 |
|  | Astrin\_NLD\_STLC\_031014\_01.09014.09014.2 | 3.2681 | 0.2878 | 100.0% | 1079.9722 | 1080.1423 | 2 | 6.731 | 75.0% | 3 | K.AQYEDIANR.S | 22 |
|  | Astrin\_NLD\_STLC\_tube2\_021014\_01.04886.04886.2 | 3.2267 | 0.2784 | 100.0% | 1414.0922 | 1413.5884 | 1 | 5.961 | 72.7% | 1 | R.SRAEAESMYQIK.Y | 2 |
|  | Astrin\_NLD\_STLC\_031014\_02.07017.07017.3 | 5.6314 | 0.3368 | 100.0% | 2533.2244 | 2532.828 | 1 | 5.959 | 34.5% | 3 | R.SRAEAESMYQIKYEELQSLAGK.H | 3 |
|  | Astrin\_NLD\_STLC\_tube2\_021014\_01.05788.05788.2 | 3.391 | 0.4615 | 100.0% | 1170.0521 | 1170.3228 | 1 | 7.159 | 77.8% | 2 | R.AEAESMYQIK.Y | 2 |
|  | Astrin\_NLD\_STLC\_031014\_02.07472.07472.3 | 5.1406 | 0.4228 | 100.0% | 2289.2344 | 2289.5623 | 1 | 7.887 | 32.9% | 3 | R.AEAESMYQIKYEELQSLAGK.H | 3 |
|  | Astrin\_NLD\_STLC\_tube2\_021014\_01.06752.06752.2 | 3.8055 | 0.0737 | 99.6% | 1138.1122 | 1138.2627 | 2 | 7.092 | 77.8% | 3 | K.YEELQSLAGK.H | 2 |
|  | Astrin\_NLD\_STLC\_031014\_01.09467.09467.2 | 2.8034 | 0.2732 | 100.0% | 1001.1722 | 1001.168 | 52 | 5.12 | 75.0% | 2 | R.LQAEIEGLK.G | 2 |
|  | Astrin\_NLD\_STLC\_031014\_01.09182.09182.2 | 3.542 | 0.2992 | 100.0% | 1342.1522 | 1342.5381 | 1 | 7.359 | 77.3% | 3 | R.LQAEIEGLKGQR.A | 2 |
|  | Astrin\_NLD\_STLC\_tube2\_021014\_01.09368.09368.2 | 3.7831 | 0.4347 | 100.0% | 1344.6921 | 1345.452 | 1 | 7.453 | 70.8% | 3 | R.ASLEAAIADAEQR.G | 2 |
|  | Astrin\_NLD\_STLC\_031014\_02.08646.08646.3 | 3.4241 | 0.3593 | 100.0% | 1956.8644 | 1957.1912 | 1 | 5.923 | 38.9% | 1 | R.ASLEAAIADAEQRGELAIK.D | 3 |
|  | Astrin\_NLD\_STLC\_tube2\_021014\_01.11879.11879.2 | 5.4067 | 0.423 | 100.0% | 1957.4521 | 1957.1912 | 1 | 7.653 | 55.6% | 3 | R.ASLEAAIADAEQRGELAIK.D | 2 |
|  | Astrin\_NLD\_STLC\_031014\_01.10870.10870.3 | 5.2916 | 0.4732 | 100.0% | 2455.4644 | 2456.7153 | 1 | 7.754 | 32.6% | 3 | R.ASLEAAIADAEQRGELAIKDANAK.L | 3 |
|  | Astrin\_NLD\_STLC\_031014\_01.09888.09888.1 | 2.1905 | 0.296 | 100.0% | 1129.57 | 1130.2865 | 1 | 5.118 | 55.6% | 2 | K.LSELEAALQR.A | 1 |
|  | Astrin\_NLD\_STLC\_tube2\_021014\_01.08426.08426.2 | 4.343 | 0.2502 | 100.0% | 1130.1921 | 1130.2865 | 32 | 6.039 | 72.2% | 5 | K.LSELEAALQR.A | 2 |
|  | Astrin\_NLD\_STLC\_tube2\_021014\_01.06941.06941.1 | 2.2327 | 0.2314 | 100.0% | 1153.42 | 1154.3234 | 400 | 5.93 | 50.0% | 1 | R.EYQELMNVK.L | 11 |
|  | Astrin\_NLD\_STLC\_tube2\_021014\_01.06962.06962.2 | 2.6071 | 0.2243 | 99.6% | 1154.0521 | 1154.3234 | 7 | 6.457 | 62.5% | 1 | R.EYQELMNVK.L | 22 |
|  | Astrin\_NLD\_STLC\_tube2\_021014\_01.09447.09447.2 | 2.9328 | 0.1879 | 98.7% | 1406.3722 | 1406.6653 | 4 | 4.937 | 68.2% | 1 | K.LALDIEIATYRK.L | 222 |
|  | Astrin\_NLD\_STLC\_tube2\_021014\_01.08200.08200.3 | 5.273 | 0.4907 | 100.0% | 2518.2844 | 2518.8628 | 1 | 7.947 | 38.1% | 2 | R.KLLEGEESRLESGMQNMSIHTK.T | 3 |
|  | Astrin\_NLD\_STLC\_tube2\_021014\_01.08823.08823.3 | 5.6545 | 0.4176 | 100.0% | 2391.0244 | 2390.6887 | 1 | 8.236 | 37.5% | 2 | K.LLEGEESRLESGMQNMSIHTK.T | 3 |
|  | Astrin\_NLD\_STLC\_031014\_01.09158.09158.2 | 4.0999 | 0.48 | 100.0% | 1476.2322 | 1476.7058 | 1 | 8.072 | 83.3% | 2 | R.LESGMQNMSIHTK.T | 2 |
|  | Astrin\_NLD\_STLC\_tube2\_021014\_01.05038.05038.3 | 3.4427 | 0.3061 | 100.0% | 1478.5443 | 1476.7058 | 4 | 4.801 | 41.7% | 1 | R.LESGMQNMSIHTK.T | 3 |
|  | Astrin\_NLD\_STLC\_tube2\_021014\_01.06256.06256.2 | 3.7761 | 0.3793 | 100.0% | 1474.1921 | 1474.6512 | 1 | 7.267 | 65.4% | 2 | R.DGKLVSESSDVLPK.- | 2 |
|  | Astrin\_NLD\_STLC\_tube2\_021014\_01.05948.05948.2 | 3.3865 | 0.4415 | 100.0% | 1173.7922 | 1174.3367 | 1 | 8.126 | 80.0% | 7 | K.LVSESSDVLPK.- | 2 |

Similarities:
gi|67782365|ref|NP\_00(2:37)  
gi|47132620|ref|NP\_00(1:38)  
gi|119703753|ref|NP\_0(3:36)  
gi|153791158|ref|NP\_0(3:36)  
contaminant\_KERATIN16(1:38)  

---

|  |  |  |  |  |  |  |  |  |
| --- | --- | --- | --- | --- | --- | --- | --- | --- |
| U | *gi|10800130|ref|NP\_06* | 8 | 13 | 60.0% | 130 | 14107 | 10.9 | histone cluster 1, H2ad [Homo sapiens] |
| U | *gi|4504249|ref|NP\_003* | 7 | 11 | 60.0% | 130 | 14091 | 10.9 | histone cluster 1, H2am [Homo sapiens] |
| U | *gi|4504243|ref|NP\_003* | 8 | 13 | 60.0% | 130 | 14091 | 10.9 | histone cluster 1, H2al [Homo sapiens] |
| U | *gi|4504241|ref|NP\_003* | 6 | 10 | 60.0% | 130 | 14091 | 10.9 | histone cluster 1, H2ak [Homo sapiens] |
| U | *gi|4504239|ref|NP\_003* | 8 | 13 | 60.0% | 130 | 14091 | 10.9 | histone cluster 1, H2ai [Homo sapiens] |
| U | *gi|29553970|ref|NP\_80* | 6 | 10 | 60.5% | 129 | 14019 | 10.9 | H2A histone family, member J [Homo sapiens] |
| U | *gi|18105045|ref|NP\_54* | 8 | 13 | 60.9% | 128 | 13906 | 10.9 | histone cluster 1, H2ah [Homo sapiens] |
| U | *gi|10800144|ref|NP\_06* | 8 | 13 | 60.9% | 128 | 13936 | 10.9 | histone cluster 1, H2aj [Homo sapiens] |
| U | *gi|10800132|ref|NP\_06* | 8 | 13 | 60.0% | 130 | 14091 | 10.9 | histone cluster 1, H2ag [Homo sapiens] |

| Filename XCorr DeltCN Conf% ObsM+H+ CalcM+H+ SpR ZScore Ion% # Sequence  | | | | | | | | | | | | |
| --- | --- | --- | --- | --- | --- | --- | --- | --- | --- | --- | --- | --- |
|  | Astrin\_NLD\_STLC\_tube2\_021014\_01.06609.06609.2 | 2.5698 | 0.1841 | 96.0% | 1275.3322 | 1275.4531 | 1 | 4.246 | 68.2% | 1 | R.SSRAGLQFPVGR.V | 22 |
|  | Astrin\_NLD\_STLC\_tube2\_021014\_01.08300.08300.2 | 3.0539 | 0.3056 | 100.0% | 945.0722 | 945.1093 | 3 | 5.864 | 81.2% | 2 | R.AGLQFPVGR.V | 22 |
|  | Astrin\_NLD\_STLC\_tube2\_021014\_01.20649.20649.2 | 5.5115 | 0.5301 | 100.0% | 2917.0322 | 2917.3752 | 1 | 10.916 | 42.9% | 2 | R.VGAGAPVYLAAVLEYLTAEILELAGNAAR.D | 2 |
|  | Astrin\_NLD\_STLC\_031014\_01.09255.09255.2 | 2.4025 | 0.1444 | 96.8% | 850.83215 | 851.0396 | 1 | 5.215 | 91.7% | 1 | R.HLQLAIR.N | 22 |
|  | Astrin\_NLD\_STLC\_tube2\_021014\_02.04988.04988.3 | 2.8283 | 0.3285 | 100.0% | 1693.9443 | 1693.9004 | 4 | 5.785 | 42.3% | 2 | R.HLQLAIRNDEELNK.L | 33 |
|  | Astrin\_NLD\_STLC\_tube2\_021014\_01.09371.09371.3 | 3.3578 | 0.3109 | 100.0% | 2104.0745 | 2105.4453 | 1 | 6.536 | 33.8% | 1 | R.HLQLAIRNDEELNKLLGK.V | 33 |
|  | Astrin\_NLD\_STLC\_tube2\_021014\_01.08174.08174.2 | 3.1651 | 0.2903 | 100.0% | 1273.3121 | 1273.4288 | 1 | 5.193 | 70.0% | 2 | R.NDEELNKLLGK.V | 22 |
|  | Astrin\_NLD\_STLC\_tube2\_021014\_01.13730.13730.2 | 4.9626 | 0.4911 | 100.0% | 1931.8522 | 1932.3573 | 1 | 8.543 | 66.7% | 2 | K.VTIAQGGVLPNIQAVLLPK.K | 22 |

Similarities:
gi|106775678|ref|NP\_0(7:1)  

---

|  |  |  |  |  |  |  |  |  |
| --- | --- | --- | --- | --- | --- | --- | --- | --- |
| U | *gi|106775678|ref|NP\_0* | 9 | 15 | 60.0% | 130 | 14095 | 10.9 | histone cluster 2, H2aa4 [Homo sapiens] |
| U | *gi|4504251|ref|NP\_003* | 9 | 15 | 60.0% | 130 | 14095 | 10.9 | histone cluster 2, H2aa3 [Homo sapiens] |
| U | *gi|24638446|ref|NP\_00* | 9 | 15 | 60.5% | 129 | 13988 | 10.9 | histone cluster 2, H2ac [Homo sapiens] |

| Filename XCorr DeltCN Conf% ObsM+H+ CalcM+H+ SpR ZScore Ion% # Sequence  | | | | | | | | | | | | |
| --- | --- | --- | --- | --- | --- | --- | --- | --- | --- | --- | --- | --- |
|  | Astrin\_NLD\_STLC\_tube2\_021014\_01.06609.06609.2 | 2.5698 | 0.1841 | 96.0% | 1275.3322 | 1275.4531 | 1 | 4.246 | 68.2% | 1 | R.SSRAGLQFPVGR.V | 22 |
|  | Astrin\_NLD\_STLC\_tube2\_021014\_01.08300.08300.2 | 3.0539 | 0.3056 | 100.0% | 945.0722 | 945.1093 | 3 | 5.864 | 81.2% | 2 | R.AGLQFPVGR.V | 22 |
|  | Astrin\_NLD\_STLC\_tube2\_021014\_01.20498.20498.3 | 3.8023 | 0.2427 | 99.4% | 2935.1643 | 2935.4082 | 1 | 4.418 | 24.1% | 2 | R.VGAGAPVYMAAVLEYLTAEILELAGNAAR.D | 3 |
|  | Astrin\_NLD\_STLC\_tube2\_021014\_01.20505.20505.2 | 5.5694 | 0.5404 | 100.0% | 2935.7722 | 2935.4082 | 1 | 9.66 | 44.6% | 2 | R.VGAGAPVYMAAVLEYLTAEILELAGNAAR.D | 2 |
|  | Astrin\_NLD\_STLC\_031014\_01.09255.09255.2 | 2.4025 | 0.1444 | 96.8% | 850.83215 | 851.0396 | 1 | 5.215 | 91.7% | 1 | R.HLQLAIR.N | 22 |
|  | Astrin\_NLD\_STLC\_tube2\_021014\_02.04988.04988.3 | 2.8283 | 0.3285 | 100.0% | 1693.9443 | 1693.9004 | 4 | 5.785 | 42.3% | 2 | R.HLQLAIRNDEELNK.L | 33 |
|  | Astrin\_NLD\_STLC\_tube2\_021014\_01.09371.09371.3 | 3.3578 | 0.3109 | 100.0% | 2104.0745 | 2105.4453 | 1 | 6.536 | 33.8% | 1 | R.HLQLAIRNDEELNKLLGK.V | 33 |
|  | Astrin\_NLD\_STLC\_tube2\_021014\_01.08174.08174.2 | 3.1651 | 0.2903 | 100.0% | 1273.3121 | 1273.4288 | 1 | 5.193 | 70.0% | 2 | R.NDEELNKLLGK.V | 22 |
|  | Astrin\_NLD\_STLC\_tube2\_021014\_01.13730.13730.2 | 4.9626 | 0.4911 | 100.0% | 1931.8522 | 1932.3573 | 1 | 8.543 | 66.7% | 2 | K.VTIAQGGVLPNIQAVLLPK.K | 22 |

Similarities:
gi|10800130|ref|NP\_06(7:2)  

---

|  |  |  |  |  |  |  |  |  |
| --- | --- | --- | --- | --- | --- | --- | --- | --- |
| U | *gi|40354195|ref|NP\_95* | 25 | 106 | 59.3% | 430 | 48058 | 5.5 | keratin 18 [Homo sapiens] |
| U | *gi|4557888|ref|NP\_000* | 25 | 106 | 59.3% | 430 | 48058 | 5.5 | keratin 18 [Homo sapiens] |

| Filename XCorr DeltCN Conf% ObsM+H+ CalcM+H+ SpR ZScore Ion% # Sequence  | | | | | | | | | | | | |
| --- | --- | --- | --- | --- | --- | --- | --- | --- | --- | --- | --- | --- |
|  | Astrin\_NLD\_STLC\_031014\_02.06081.06081.3 | 5.207 | 0.5785 | 100.0% | 2855.3943 | 2856.0813 | 1 | 9.903 | 29.2% | 15 | R.SLGSVQAPSYGARPVSSAASVYAGAGGSGSR.I | 3 |
|  | Astrin\_NLD\_STLC\_tube2\_021014\_01.07491.07491.2 | 4.5549 | 0.4658 | 100.0% | 2855.8123 | 2856.0813 | 2 | 7.148 | 30.0% | 1 | R.SLGSVQAPSYGARPVSSAASVYAGAGGSGSR.I | 2 |
|  | Astrin\_NLD\_STLC\_tube2\_021014\_01.11445.11445.3 | 6.0106 | 0.5619 | 100.0% | 3337.8245 | 3337.7224 | 1 | 8.455 | 27.2% | 2 | R.GGMGSGGLATGIAGGLAGMGGIQNEKETMQSLNDR.L | 3 |
|  | Astrin\_NLD\_STLC\_tube2\_021014\_01.05040.05040.2 | 1.9022 | 0.2598 | 96.6% | 837.2322 | 837.9511 | 20 | 4.708 | 83.3% | 3 | R.LASYLDR.V | 2 |
|  | Astrin\_NLD\_STLC\_tube2\_021014\_01.06450.06450.1 | 2.2261 | 0.4378 | 100.0% | 1319.41 | 1320.4478 | 15 | 6.781 | 45.5% | 1 | R.AQIFANTVDNAR.I | 1 |
|  | Astrin\_NLD\_STLC\_tube2\_021014\_01.06440.06440.2 | 3.9945 | 0.5258 | 100.0% | 1320.1721 | 1320.4478 | 1 | 9.011 | 72.7% | 9 | R.AQIFANTVDNAR.I | 2 |
|  | Astrin\_NLD\_STLC\_tube2\_021014\_01.07084.07084.1 | 2.2993 | 0.2542 | 100.0% | 1041.64 | 1042.2235 | 1 | 6.129 | 75.0% | 1 | R.IVLQIDNAR.L | 11 |
|  | Astrin\_NLD\_STLC\_tube2\_021014\_01.07100.07100.2 | 3.2063 | 0.1355 | 99.7% | 1042.1122 | 1042.2235 | 1 | 6.273 | 87.5% | 5 | R.IVLQIDNAR.L | 22 |
|  | Astrin\_NLD\_STLC\_tube2\_021014\_01.05060.05060.2 | 2.4574 | 0.2931 | 100.0% | 807.9122 | 807.8815 | 9 | 6.526 | 75.0% | 2 | R.LAADDFR.V | 22222 |
|  | Astrin\_NLD\_STLC\_031014\_01.09236.09236.2 | 2.9933 | 0.3866 | 100.0% | 1240.1721 | 1240.4601 | 5 | 6.933 | 77.8% | 4 | R.VKYETELAMR.Q | 2 |
|  | Astrin\_NLD\_STLC\_tube2\_021014\_02.04946.04946.3 | 3.329 | 0.1842 | 98.4% | 1240.3744 | 1240.4601 | 43 | 4.76 | 50.0% | 1 | R.VKYETELAMR.Q | 3 |
|  | Astrin\_NLD\_STLC\_031014\_01.08912.08912.2 | 3.0829 | 0.1829 | 99.8% | 1176.2522 | 1175.3274 | 6 | 5.49 | 72.2% | 1 | R.KVIDDTNITR.L | 2 |
|  | Astrin\_NLD\_STLC\_031014\_01.12798.12798.2 | 5.718 | 0.5062 | 100.0% | 2178.652 | 2178.589 | 1 | 8.771 | 58.8% | 3 | R.LQLETEIEALKEELLFMK.K | 2 |
|  | Astrin\_NLD\_STLC\_tube2\_021014\_01.15716.15716.3 | 2.8355 | 0.2727 | 97.7% | 2178.9844 | 2178.589 | 67 | 4.974 | 32.4% | 3 | R.LQLETEIEALKEELLFMK.K | 3 |
|  | Astrin\_NLD\_STLC\_tube2\_021014\_01.09276.09276.3 | 5.4614 | 0.4461 | 100.0% | 2750.2144 | 2751.0227 | 1 | 7.739 | 32.0% | 1 | K.NHEEEVKGLQAQIASSGLTVEVDAPK.S | 3 |
|  | Astrin\_NLD\_STLC\_tube2\_021014\_01.04583.04583.1 | 1.6007 | 0.2947 | 100.0% | 965.35 | 966.0385 | 85 | 4.406 | 50.0% | 1 | R.AQYDELAR.K | 1 |
|  | Astrin\_NLD\_STLC\_tube2\_021014\_01.11054.11054.2 | 4.0177 | 0.468 | 100.0% | 1508.2122 | 1507.699 | 1 | 8.516 | 75.0% | 7 | R.TVQSLEIDLDSMR.N | 2 |
|  | Astrin\_NLD\_STLC\_tube2\_021014\_01.04694.04694.2 | 2.5134 | 0.1975 | 98.8% | 889.8522 | 889.9841 | 15 | 5.361 | 85.7% | 1 | K.ASLENSLR.E | 2 |
|  | Astrin\_NLD\_STLC\_031014\_01.13641.13641.3 | 6.2592 | 0.4315 | 100.0% | 2671.6143 | 2672.0715 | 1 | 8.353 | 36.4% | 32 | R.YALQMEQLNGILLHLESELAQTR.A | 3 |
|  | Astrin\_NLD\_STLC\_tube2\_021014\_01.16709.16709.2 | 5.6556 | 0.4659 | 100.0% | 2672.3323 | 2672.0715 | 1 | 8.413 | 50.0% | 1 | R.YALQMEQLNGILLHLESELAQTR.A | 2 |
|  | Astrin\_NLD\_STLC\_tube2\_021014\_01.10197.10197.2 | 3.1691 | 0.3583 | 100.0% | 1420.1122 | 1420.6055 | 71 | 5.861 | 59.1% | 2 | R.QAQEYEALLNIK.V | 2 |
|  | Astrin\_NLD\_STLC\_031014\_02.05720.05720.2 | 2.8565 | 0.3377 | 100.0% | 1292.3722 | 1293.5059 | 1 | 6.221 | 80.0% | 4 | K.VKLEAEIATYR.R | 2 |
|  | Astrin\_NLD\_STLC\_tube2\_021014\_01.06179.06179.1 | 2.0548 | 0.2887 | 100.0% | 1065.53 | 1066.1992 | 3 | 5.231 | 62.5% | 1 | K.LEAEIATYR.R | 1 |
|  | Astrin\_NLD\_STLC\_tube2\_021014\_01.06206.06206.2 | 3.0487 | 0.4066 | 100.0% | 1066.1122 | 1066.1992 | 4 | 6.952 | 81.2% | 2 | K.LEAEIATYR.R | 2 |
|  | Astrin\_NLD\_STLC\_031014\_01.06272.06272.3 | 3.1775 | 0.3293 | 100.0% | 1661.7544 | 1661.8528 | 1 | 6.689 | 42.9% | 3 | R.RIVDGKVVSETNDTK.V | 3 |

Similarities:
gi|4557701|ref|NP\_000(1:24)  
contaminant\_KERATIN03(1:24)  
contaminant\_KERATIN05(1:24)  
gi|24234699|ref|NP\_00(3:22)  

---

|  |  |  |  |  |  |  |  |  |
| --- | --- | --- | --- | --- | --- | --- | --- | --- |
| U | *gi|58743363|ref|NP\_20* | 29 | 173 | 56.4% | 344 | 38998 | 6.5 | centromere protein L isoform 2 [Homo sapiens] |

| Filename XCorr DeltCN Conf% ObsM+H+ CalcM+H+ SpR ZScore Ion% # Sequence  | | | | | | | | | | | | |
| --- | --- | --- | --- | --- | --- | --- | --- | --- | --- | --- | --- | --- |
|  | Astrin\_NLD\_STLC\_tube2\_021014\_01.09915.09915.3 | 5.4419 | 0.4444 | 100.0% | 3133.1042 | 3134.398 | 1 | 7.751 | 33.9% | 14 | -.MDSYSAPESTPSASSRPEDYFIGATPLQK.R | 3 |
|  | Astrin\_NLD\_STLC\_tube2\_021014\_01.09735.09735.2 | 4.0764 | 0.5197 | 100.0% | 3134.372 | 3134.398 | 1 | 7.389 | 37.5% | 4 | -.MDSYSAPESTPSASSRPEDYFIGATPLQK.R | 2 |
|  | Astrin\_NLD\_STLC\_tube2\_021014\_01.09057.09057.3 | 5.0803 | 0.3926 | 100.0% | 3288.8342 | 3290.5854 | 1 | 6.645 | 25.9% | 3 | -.MDSYSAPESTPSASSRPEDYFIGATPLQKR.L | 3 |
|  | Astrin\_NLD\_STLC\_031014\_01.09603.09603.2 | 3.2777 | 0.3596 | 100.0% | 1274.3522 | 1274.5057 | 1 | 6.008 | 70.0% | 16 | R.KQSSFILTPPR.R | 2 |
|  | Astrin\_NLD\_STLC\_tube2\_021014\_01.05375.05375.2 | 2.3799 | 0.2319 | 97.1% | 1430.6122 | 1430.6932 | 5 | 4.865 | 59.1% | 1 | R.KQSSFILTPPRR.K | 2 |
|  | Astrin\_NLD\_STLC\_031014\_01.09220.09220.3 | 3.0635 | 0.2543 | 99.6% | 1431.1444 | 1430.6932 | 19 | 5.743 | 38.6% | 4 | R.KQSSFILTPPRR.K | 3 |
|  | Astrin\_NLD\_STLC\_031014\_01.09938.09938.2 | 3.0631 | 0.3633 | 100.0% | 1146.1322 | 1146.3317 | 1 | 6.183 | 83.3% | 13 | K.QSSFILTPPR.R | 2 |
|  | Astrin\_NLD\_STLC\_031014\_01.09381.09381.2 | 3.9284 | 0.2973 | 100.0% | 1785.6122 | 1785.9147 | 1 | 6.447 | 75.0% | 1 | K.IPQCSQLQEDVDPQK.V | 2 |
|  | Astrin\_NLD\_STLC\_tube2\_021014\_01.06339.06339.2 | 2.2896 | 0.1549 | 95.4% | 827.7522 | 828.04517 | 3 | 5.234 | 83.3% | 2 | K.VAFLLHK.Q | 2 |
|  | Astrin\_NLD\_STLC\_tube2\_021014\_01.12443.12443.3 | 3.7774 | 0.3228 | 100.0% | 2322.0842 | 2322.7983 | 1 | 5.579 | 31.9% | 1 | K.VAFLLHKQWTLYSLTPLYK.F | 3 |
|  | Astrin\_NLD\_STLC\_tube2\_021014\_01.13494.13494.1 | 2.1704 | 0.4412 | 100.0% | 1512.59 | 1513.7765 | 1 | 6.647 | 54.5% | 1 | K.QWTLYSLTPLYK.F | 1 |
|  | Astrin\_NLD\_STLC\_031014\_01.11434.11434.2 | 3.3237 | 0.3971 | 100.0% | 1513.3522 | 1513.7765 | 1 | 6.924 | 72.7% | 5 | K.QWTLYSLTPLYK.F | 2 |
|  | Astrin\_NLD\_STLC\_tube2\_021014\_01.06632.06632.2 | 3.5919 | 0.4094 | 100.0% | 1394.1921 | 1394.5266 | 1 | 7.537 | 75.0% | 4 | K.FSYSNLKEYSR.L | 2 |
|  | Astrin\_NLD\_STLC\_031014\_01.10449.10449.1 | 2.1006 | 0.2964 | 100.0% | 1117.6 | 1118.3617 | 3 | 5.399 | 66.7% | 4 | R.LLNAFIVAEK.Q | 1 |
|  | Astrin\_NLD\_STLC\_031014\_02.07809.07809.2 | 3.3264 | 0.3709 | 100.0% | 1118.3322 | 1118.3617 | 2 | 6.753 | 72.2% | 29 | R.LLNAFIVAEK.Q | 2 |
|  | Astrin\_NLD\_STLC\_tube2\_021014\_01.08601.08601.2 | 3.3731 | 0.316 | 100.0% | 1374.3121 | 1374.6665 | 1 | 6.565 | 72.7% | 1 | R.LLNAFIVAEKQK.G | 2 |
|  | Astrin\_NLD\_STLC\_031014\_02.06982.06982.3 | 3.5939 | 0.1954 | 98.1% | 1647.4143 | 1647.8687 | 206 | 4.864 | 37.5% | 1 | K.QKGLAVEVGEDFNIK.V | 3 |
|  | Astrin\_NLD\_STLC\_tube2\_021014\_01.10490.10490.2 | 4.6642 | 0.4804 | 100.0% | 1391.2322 | 1391.5638 | 1 | 7.761 | 79.2% | 16 | K.GLAVEVGEDFNIK.V | 2 |
|  | Astrin\_NLD\_STLC\_tube2\_021014\_01.12419.12419.1 | 1.7295 | 0.3539 | 100.0% | 1108.62 | 1109.4125 | 7 | 6.016 | 50.0% | 1 | K.VIFSTLLGMK.G | 1 |
|  | Astrin\_NLD\_STLC\_031014\_01.11093.11093.2 | 3.3838 | 0.4607 | 100.0% | 1109.5322 | 1109.4125 | 1 | 6.813 | 77.8% | 7 | K.VIFSTLLGMK.G | 2 |
| \* | Astrin\_NLD\_STLC\_031014\_01.10732.10732.2 | 4.9511 | 0.3349 | 100.0% | 1788.4521 | 1789.0415 | 1 | 7.185 | 66.7% | 5 | K.GTQRDPEAFLVQIVSK.S | 2 |
| \* | Astrin\_NLD\_STLC\_tube2\_021014\_01.11750.11750.3 | 4.9766 | 0.2946 | 100.0% | 1788.6244 | 1789.0415 | 1 | 6.901 | 50.0% | 6 | K.GTQRDPEAFLVQIVSK.S | 3 |
| \* | Astrin\_NLD\_STLC\_tube2\_021014\_01.13637.13637.1 | 2.7251 | 0.3344 | 100.0% | 1345.51 | 1346.5663 | 2 | 5.679 | 68.2% | 3 | R.DPEAFLVQIVSK.S | 1 |
| \* | Astrin\_NLD\_STLC\_tube2\_021014\_01.13706.13706.2 | 4.4481 | 0.403 | 100.0% | 1346.3722 | 1346.5663 | 1 | 8.3 | 68.2% | 18 | R.DPEAFLVQIVSK.S | 2 |
|  | Astrin\_NLD\_STLC\_tube2\_021014\_01.06418.06418.1 | 1.9546 | 0.1894 | 97.1% | 955.52 | 956.0892 | 5 | 4.338 | 71.4% | 1 | K.ALWDSVHK.T | 1 |
|  | Astrin\_NLD\_STLC\_tube2\_021014\_01.06338.06338.2 | 2.4255 | 0.2335 | 99.3% | 956.0522 | 956.0892 | 4 | 4.616 | 78.6% | 1 | K.ALWDSVHK.T | 2 |
|  | Astrin\_NLD\_STLC\_031014\_01.12364.12364.3 | 4.7712 | 0.3817 | 100.0% | 2813.2444 | 2812.0486 | 1 | 6.051 | 39.8% | 2 | K.TPGEVTQEEVDLFMDCLYSHFHR.H | 3 |
|  | Astrin\_NLD\_STLC\_tube2\_021014\_02.05048.05048.3 | 3.4761 | 0.4254 | 100.0% | 1210.8243 | 1210.4244 | 3 | 7.284 | 50.0% | 2 | R.HFKIHLSATR.L | 3 |
|  | Astrin\_NLD\_STLC\_031014\_01.06423.06423.2 | 3.5559 | 0.3853 | 100.0% | 1501.2722 | 1501.6799 | 1 | 6.597 | 53.6% | 7 | R.VSTSVASAHTDGKIK.I | 2 |

---

|  |  |  |  |  |  |  |  |  |
| --- | --- | --- | --- | --- | --- | --- | --- | --- |
| U | *gi|13129022|ref|NP\_07* | 8 | 34 | 53.3% | 180 | 19737 | 7.2 | centromere protein M isoform a [Homo sapiens] |

| Filename XCorr DeltCN Conf% ObsM+H+ CalcM+H+ SpR ZScore Ion% # Sequence  | | | | | | | | | | | | |
| --- | --- | --- | --- | --- | --- | --- | --- | --- | --- | --- | --- | --- |
|  | Astrin\_NLD\_STLC\_031014\_01.16166.16166.3 | 4.3415 | 0.3774 | 100.0% | 2939.4844 | 2940.4658 | 1 | 7.293 | 27.8% | 2 | K.LPGLNTATILLVGTEDALLQQLADSMLK.E | 3 |
|  | Astrin\_NLD\_STLC\_031014\_01.09248.09248.2 | 2.9914 | 0.3336 | 100.0% | 1323.2122 | 1323.5381 | 2 | 5.333 | 63.6% | 4 | K.SLPLPSSVNRPR.I | 2 |
|  | Astrin\_NLD\_STLC\_tube2\_021014\_01.13745.13745.2 | 3.9129 | 0.5109 | 100.0% | 1497.2322 | 1497.821 | 1 | 8.898 | 79.2% | 5 | R.IDLIVFVVNLHSK.Y | 2 |
|  | Astrin\_NLD\_STLC\_031014\_02.10204.10204.3 | 3.8269 | 0.2228 | 100.0% | 1497.9543 | 1497.821 | 18 | 5.576 | 37.5% | 3 | R.IDLIVFVVNLHSK.Y | 3 |
|  | Astrin\_NLD\_STLC\_031014\_01.09479.09479.2 | 3.2708 | 0.2929 | 100.0% | 1339.9922 | 1340.4325 | 1 | 8.37 | 75.0% | 4 | K.YSLQNTEESLR.H | 2 |
|  | Astrin\_NLD\_STLC\_tube2\_021014\_01.08948.08948.2 | 3.3721 | 0.5493 | 100.0% | 1121.1522 | 1121.281 | 1 | 9.061 | 72.2% | 13 | R.HVDASFFLGK.V | 2 |
|  | Astrin\_NLD\_STLC\_031014\_01.12580.12580.2 | 3.4366 | 0.4169 | 100.0% | 2330.4722 | 2331.7734 | 1 | 7.419 | 40.5% | 1 | R.VLQICAGHVPGVSALNLLSLLR.S | 2 |
|  | Astrin\_NLD\_STLC\_031014\_01.12596.12596.3 | 4.3254 | 0.3384 | 100.0% | 2331.5942 | 2331.7734 | 1 | 6.964 | 39.3% | 2 | R.VLQICAGHVPGVSALNLLSLLR.S | 3 |

---

|  |  |  |  |  |  |  |  |  |
| --- | --- | --- | --- | --- | --- | --- | --- | --- |
| U | *gi|11415030|ref|NP\_06* | 7 | 18 | 48.5% | 103 | 11367 | 11.4 | histone cluster 1, H4j [Homo sapiens] |
| U | *gi|77539758|ref|NP\_00* | 7 | 18 | 48.5% | 103 | 11367 | 11.4 | histone cluster 2, H4b [Homo sapiens] |
| U | *gi|4504323|ref|NP\_003* | 7 | 18 | 48.5% | 103 | 11367 | 11.4 | histone cluster 2, H4a [Homo sapiens] |
| U | *gi|4504321|ref|NP\_003* | 7 | 18 | 48.5% | 103 | 11367 | 11.4 | histone cluster 1, H4i [Homo sapiens] |
| U | *gi|4504317|ref|NP\_003* | 7 | 18 | 48.5% | 103 | 11367 | 11.4 | histone cluster 1, H4l [Homo sapiens] |
| U | *gi|4504315|ref|NP\_003* | 7 | 18 | 48.5% | 103 | 11367 | 11.4 | histone cluster 1, H4e [Homo sapiens] |
| U | *gi|4504313|ref|NP\_003* | 7 | 18 | 48.5% | 103 | 11367 | 11.4 | histone cluster 1, H4b [Homo sapiens] |
| U | *gi|4504311|ref|NP\_003* | 7 | 18 | 48.5% | 103 | 11367 | 11.4 | histone cluster 1, H4h [Homo sapiens] |
| U | *gi|4504309|ref|NP\_003* | 7 | 18 | 48.5% | 103 | 11367 | 11.4 | histone cluster 1, H4c [Homo sapiens] |
| U | *gi|4504307|ref|NP\_003* | 7 | 18 | 48.5% | 103 | 11367 | 11.4 | histone cluster 1, H4k [Homo sapiens] |
| U | *gi|4504305|ref|NP\_003* | 7 | 18 | 48.5% | 103 | 11367 | 11.4 | histone cluster 1, H4f [Homo sapiens] |
| U | *gi|4504303|ref|NP\_003* | 7 | 18 | 48.5% | 103 | 11367 | 11.4 | histone cluster 1, H4d [Homo sapiens] |
| U | *gi|4504301|ref|NP\_003* | 7 | 18 | 48.5% | 103 | 11367 | 11.4 | histone cluster 1, H4a [Homo sapiens] |
| U | *gi|28173560|ref|NP\_77* | 7 | 18 | 48.5% | 103 | 11367 | 11.4 | histone cluster 4, H4 [Homo sapiens] |

| Filename XCorr DeltCN Conf% ObsM+H+ CalcM+H+ SpR ZScore Ion% # Sequence  | | | | | | | | | | | | |
| --- | --- | --- | --- | --- | --- | --- | --- | --- | --- | --- | --- | --- |
|  | Astrin\_NLD\_STLC\_tube2\_021014\_01.05259.05259.2 | 3.5328 | 0.2393 | 100.0% | 1326.5322 | 1326.5387 | 1 | 6.522 | 81.8% | 3 | R.DNIQGITKPAIR.R | 2 |
|  | Astrin\_NLD\_STLC\_tube2\_021014\_01.07595.07595.1 | 2.3977 | 0.2288 | 100.0% | 1180.55 | 1181.3312 | 4 | 5.109 | 66.7% | 1 | R.ISGLIYEETR.G | 1 |
|  | Astrin\_NLD\_STLC\_tube2\_021014\_02.06017.06017.2 | 3.6741 | 0.383 | 100.0% | 1181.0922 | 1181.3312 | 1 | 7.368 | 88.9% | 7 | R.ISGLIYEETR.G | 2 |
|  | Astrin\_NLD\_STLC\_tube2\_021014\_01.09759.09759.2 | 2.8309 | 0.2056 | 99.8% | 990.1922 | 990.19055 | 1 | 5.227 | 85.7% | 3 | K.VFLENVIR.D | 2 |
|  | Astrin\_NLD\_STLC\_tube2\_021014\_01.12010.12010.2 | 3.3679 | 0.375 | 100.0% | 1311.8522 | 1311.5793 | 1 | 6.509 | 72.7% | 1 | K.TVTAMDVVYALK.R | 2 |
|  | Astrin\_NLD\_STLC\_tube2\_021014\_01.10792.10792.2 | 3.361 | 0.432 | 100.0% | 1468.6522 | 1467.7667 | 1 | 7.175 | 75.0% | 2 | K.TVTAMDVVYALKR.Q | 2 |
|  | Astrin\_NLD\_STLC\_tube2\_021014\_01.09518.09518.1 | 1.7469 | 0.4069 | 100.0% | 714.41 | 714.796 | 1 | 6.146 | 66.7% | 1 | R.TLYGFGG.- | 1 |

---

|  |  |  |  |  |  |  |  |  |
| --- | --- | --- | --- | --- | --- | --- | --- | --- |
| U | *contaminant\_gi|746301* | 17 | 194 | 42.8% | 269 | 27961 | 6.7 | lysyl endopeptidase (EC 3.4.21.50) - Lysobacter enzymogenes |

| Filename XCorr DeltCN Conf% ObsM+H+ CalcM+H+ SpR ZScore Ion% # Sequence  | | | | | | | | | | | | |
| --- | --- | --- | --- | --- | --- | --- | --- | --- | --- | --- | --- | --- |
| \* | Astrin\_NLD\_STLC\_031014\_01.03735.03735.1 | 1.9528 | 0.257 | 100.0% | 725.86 | 725.8198 | 1 | 5.169 | 66.7% | 1 | R.SVAAYSK.Q | 1 |
| \* | Astrin\_NLD\_STLC\_tube2\_021014\_01.04779.04779.2 | 6.1139 | 0.5989 | 100.0% | 2261.0923 | 2262.355 | 1 | 11.117 | 56.2% | 18 | R.APGSSSSGANGDGSLAQSQTGAVVR.A | 2 |
| \* | Astrin\_NLD\_STLC\_031014\_01.09083.09083.3 | 5.0972 | 0.4292 | 100.0% | 2262.1443 | 2262.355 | 1 | 7.495 | 37.5% | 24 | R.APGSSSSGANGDGSLAQSQTGAVVR.A | 3 |
| \* | Astrin\_NLD\_STLC\_031014\_02.12422.12422.3 | 6.4532 | 0.4802 | 100.0% | 3315.7144 | 3315.6257 | 1 | 7.903 | 27.6% | 3 | R.ATNAASDFTLLELNTAANPAYNLFWAGWDR.R | 3 |
| \* | Astrin\_NLD\_STLC\_tube2\_021014\_01.17010.17010.2 | 5.1465 | 0.4615 | 100.0% | 3315.872 | 3315.6257 | 1 | 10.222 | 39.7% | 4 | R.ATNAASDFTLLELNTAANPAYNLFWAGWDR.R | 2 |
| \* | Astrin\_NLD\_STLC\_tube2\_021014\_01.15998.15998.3 | 6.7063 | 0.4961 | 100.0% | 3470.8442 | 3471.813 | 1 | 9.888 | 29.2% | 49 | R.ATNAASDFTLLELNTAANPAYNLFWAGWDRR.D | 3 |
| \* | Astrin\_NLD\_STLC\_tube2\_021014\_01.04454.04454.3 | 4.7755 | 0.2696 | 100.0% | 2077.4944 | 2077.2668 | 1 | 6.546 | 50.0% | 3 | R.RDQNFAGATAIHHPNVAEK.R | 3 |
| \* | Astrin\_NLD\_STLC\_031014\_01.08907.08907.3 | 4.8561 | 0.3694 | 100.0% | 2233.4644 | 2233.4543 | 1 | 6.291 | 39.5% | 3 | R.RDQNFAGATAIHHPNVAEKR.I | 3 |
| \* | Astrin\_NLD\_STLC\_tube2\_021014\_01.04192.04192.2 | 5.9375 | 0.5266 | 100.0% | 2233.5522 | 2233.4543 | 1 | 9.318 | 60.5% | 2 | R.RDQNFAGATAIHHPNVAEKR.I | 2 |
| \* | Astrin\_NLD\_STLC\_tube2\_021014\_01.04936.04936.2 | 4.7174 | 0.5311 | 100.0% | 1919.5122 | 1921.0793 | 1 | 8.171 | 58.8% | 3 | R.DQNFAGATAIHHPNVAEK.R | 2 |
| \* | Astrin\_NLD\_STLC\_031014\_01.09092.09092.3 | 2.5159 | 0.3374 | 99.6% | 1920.7743 | 1921.0793 | 11 | 5.691 | 33.8% | 1 | R.DQNFAGATAIHHPNVAEK.R | 3 |
| \* | Astrin\_NLD\_STLC\_031014\_01.09000.09000.2 | 5.2272 | 0.4027 | 100.0% | 2076.6921 | 2077.2668 | 1 | 7.151 | 58.3% | 6 | R.DQNFAGATAIHHPNVAEKR.I | 2 |
| \* | Astrin\_NLD\_STLC\_tube2\_021014\_01.04520.04520.3 | 4.2425 | 0.4758 | 100.0% | 2076.8943 | 2077.2668 | 1 | 7.953 | 41.7% | 8 | R.DQNFAGATAIHHPNVAEKR.I | 3 |
| \* | Astrin\_NLD\_STLC\_031014\_01.09015.09015.2 | 4.8879 | 0.4086 | 100.0% | 1870.0322 | 1870.983 | 1 | 7.99 | 66.7% | 2 | R.VLGQLHGGPSSCSATGADR.S | 2 |
| \* | Astrin\_NLD\_STLC\_031014\_01.09009.09009.3 | 4.41 | 0.4726 | 100.0% | 1870.4043 | 1870.983 | 1 | 7.682 | 38.9% | 2 | R.VLGQLHGGPSSCSATGADR.S | 3 |
| \* | Astrin\_NLD\_STLC\_tube2\_021014\_01.07809.07809.2 | 5.1869 | 0.4699 | 100.0% | 1429.4321 | 1428.5443 | 1 | 8.767 | 73.1% | 62 | R.VFTSWTGGGTSATR.L | 2 |
| \* | Astrin\_NLD\_STLC\_tube2\_021014\_01.07194.07194.1 | 2.2431 | 0.2262 | 100.0% | 1429.52 | 1428.5443 | 14 | 4.198 | 42.3% | 3 | R.VFTSWTGGGTSATR.L | 1 |

---

|  |  |  |  |  |  |  |  |  |
| --- | --- | --- | --- | --- | --- | --- | --- | --- |
| U | *gi|5174457|ref|NP\_006* | 26 | 42 | 41.3% | 642 | 73913 | 5.6 | kinetochore associated 2 [Homo sapiens] |

| Filename XCorr DeltCN Conf% ObsM+H+ CalcM+H+ SpR ZScore Ion% # Sequence  | | | | | | | | | | | | |
| --- | --- | --- | --- | --- | --- | --- | --- | --- | --- | --- | --- | --- |
| \* | Astrin\_NLD\_STLC\_031014\_01.08957.08957.2 | 4.0437 | 0.4579 | 100.0% | 1707.4122 | 1707.8809 | 1 | 8.281 | 57.1% | 1 | R.SQDVNKQGLYTPQTK.E | 2 |
| \* | Astrin\_NLD\_STLC\_031014\_01.08648.08648.2 | 2.5354 | 0.2615 | 99.7% | 1145.4321 | 1145.3011 | 1 | 5.272 | 77.8% | 2 | K.LSINKPTSER.K | 2 |
| \* | Astrin\_NLD\_STLC\_tube2\_021014\_01.08037.08037.2 | 2.8764 | 0.1961 | 98.7% | 1299.1322 | 1297.4075 | 5 | 4.813 | 63.6% | 2 | R.NSQLGIFSSSEK.I | 2 |
| \* | Astrin\_NLD\_STLC\_tube2\_021014\_01.10764.10764.2 | 4.2097 | 0.4127 | 100.0% | 1499.5721 | 1499.793 | 1 | 6.872 | 79.2% | 1 | R.IFKDLGYPFALSK.S | 2 |
| \* | Astrin\_NLD\_STLC\_tube2\_021014\_01.10739.10739.2 | 2.6376 | 0.2237 | 99.8% | 1013.03217 | 1013.2218 | 1 | 5.192 | 85.7% | 1 | K.LFLDYTIK.C | 2 |
| \* | Astrin\_NLD\_STLC\_tube2\_021014\_01.11858.11858.3 | 4.5825 | 0.3212 | 100.0% | 2081.9944 | 2081.4167 | 1 | 6.88 | 44.1% | 1 | K.LKDLFNVDAFKLESLEAK.N | 3 |
| \* | Astrin\_NLD\_STLC\_tube2\_021014\_01.12731.12731.2 | 3.3875 | 0.3629 | 100.0% | 1840.1322 | 1840.0831 | 8 | 5.711 | 43.3% | 1 | K.DLFNVDAFKLESLEAK.N | 2 |
| \* | Astrin\_NLD\_STLC\_tube2\_021014\_01.04272.04272.3 | 3.1118 | 0.2613 | 99.4% | 2026.8544 | 2027.2449 | 63 | 5.201 | 30.0% | 2 | R.LEQEREKEPNRLESLR.K | 3 |
| \* | Astrin\_NLD\_STLC\_031014\_01.08961.08961.2 | 2.6053 | 0.204 | 98.0% | 1372.2522 | 1371.5363 | 35 | 4.513 | 60.0% | 1 | R.EKEPNRLESLR.K | 2 |
| \* | Astrin\_NLD\_STLC\_031014\_02.06584.06584.3 | 3.1659 | 0.4302 | 100.0% | 2098.3743 | 2099.3228 | 9 | 7.269 | 33.8% | 4 | K.YQAYMSNLESHSAILDQK.L | 3 |
| \* | Astrin\_NLD\_STLC\_tube2\_021014\_01.08622.08622.2 | 5.3796 | 0.5391 | 100.0% | 2098.5322 | 2099.3228 | 1 | 8.937 | 61.8% | 1 | K.YQAYMSNLESHSAILDQK.L | 2 |
| \* | Astrin\_NLD\_STLC\_tube2\_021014\_01.06080.06080.2 | 3.2631 | 0.3785 | 100.0% | 1128.5521 | 1129.2584 | 3 | 7.075 | 77.8% | 4 | K.LNGLNEEIAR.V | 2 |
| \* | Astrin\_NLD\_STLC\_tube2\_021014\_01.04790.04790.2 | 3.1566 | 0.1868 | 99.9% | 1086.2122 | 1086.2334 | 7 | 4.71 | 75.0% | 1 | R.LQNIIDNQK.Y | 2 |
| \* | Astrin\_NLD\_STLC\_tube2\_021014\_01.06309.06309.2 | 2.6049 | 0.3926 | 100.0% | 952.3522 | 953.03973 | 1 | 7.157 | 85.7% | 2 | K.YSVADIER.I | 2 |
| \* | Astrin\_NLD\_STLC\_031014\_01.08922.08922.2 | 4.3474 | 0.3365 | 100.0% | 1738.5122 | 1737.9132 | 1 | 6.252 | 73.1% | 1 | R.INHERNELQQTINK.L | 2 |
| \* | Astrin\_NLD\_STLC\_031014\_01.08920.08920.3 | 4.0299 | 0.2198 | 100.0% | 1739.0343 | 1737.9132 | 2 | 4.711 | 46.2% | 1 | R.INHERNELQQTINK.L | 3 |
| \* | Astrin\_NLD\_STLC\_tube2\_021014\_01.07492.07492.3 | 3.9761 | 0.3568 | 100.0% | 2078.9944 | 2080.3518 | 1 | 6.359 | 37.5% | 1 | R.INHERNELQQTINKLTK.D | 3 |
| \* | Astrin\_NLD\_STLC\_tube2\_021014\_01.09846.09846.3 | 2.9182 | 0.2661 | 98.4% | 1874.3644 | 1874.0575 | 104 | 4.54 | 32.1% | 1 | K.DLEAEQQKLWNEELK.Y | 3 |
| \* | Astrin\_NLD\_STLC\_tube2\_021014\_02.04805.04805.3 | 4.4636 | 0.4011 | 100.0% | 1618.6743 | 1617.7991 | 4 | 6.702 | 44.2% | 3 | R.GKEAIETQLAEYHK.L | 3 |
| \* | Astrin\_NLD\_STLC\_tube2\_021014\_01.13724.13724.2 | 5.2272 | 0.4431 | 100.0% | 2359.5322 | 2360.6665 | 1 | 7.884 | 57.9% | 1 | R.AQVYVPLKELLNETEEEINK.A | 2 |
| \* | Astrin\_NLD\_STLC\_031014\_01.11481.11481.3 | 4.7675 | 0.4288 | 100.0% | 2360.7844 | 2360.6665 | 1 | 6.717 | 36.8% | 2 | R.AQVYVPLKELLNETEEEINK.A | 3 |
| \* | Astrin\_NLD\_STLC\_tube2\_021014\_01.07271.07271.2 | 3.6965 | 0.3624 | 100.0% | 1461.5122 | 1461.5658 | 1 | 6.505 | 77.3% | 1 | K.ELLNETEEEINK.A | 2 |
| \* | Astrin\_NLD\_STLC\_031014\_01.12207.12207.3 | 4.0452 | 0.3342 | 100.0% | 2180.6943 | 2180.4963 | 1 | 7.137 | 38.9% | 3 | K.MGLEDTLEQLNAMITESKR.S | 3 |
| \* | Astrin\_NLD\_STLC\_tube2\_021014\_01.09605.09605.3 | 3.2178 | 0.2376 | 98.4% | 1979.4844 | 1979.2377 | 7 | 4.397 | 35.0% | 1 | R.TLKEEVQKLDDLYQQK.I | 3 |
| \* | Astrin\_NLD\_STLC\_tube2\_021014\_02.05789.05789.2 | 4.1977 | 0.4835 | 100.0% | 1595.9922 | 1596.7344 | 1 | 9.513 | 75.0% | 1 | R.EYQLVVQTTTEER.R | 2 |
| \* | Astrin\_NLD\_STLC\_tube2\_021014\_02.06129.06129.2 | 4.1338 | 0.3732 | 100.0% | 1513.6122 | 1513.7925 | 1 | 6.731 | 73.1% | 2 | R.LLEMVATHVGSVEK.H | 2 |

---

|  |  |  |  |  |  |  |  |  |
| --- | --- | --- | --- | --- | --- | --- | --- | --- |
| U | *gi|29788785|ref|NP\_82* | 18 | 52 | 39.0% | 444 | 49671 | 4.9 | tubulin, beta [Homo sapiens] |

| Filename XCorr DeltCN Conf% ObsM+H+ CalcM+H+ SpR ZScore Ion% # Sequence  | | | | | | | | | | | | |
| --- | --- | --- | --- | --- | --- | --- | --- | --- | --- | --- | --- | --- |
| \* | Astrin\_NLD\_STLC\_tube2\_021014\_01.05931.05931.2 | 3.6407 | 0.5487 | 100.0% | 1301.9122 | 1302.4265 | 1 | 9.21 | 86.4% | 4 | R.ISVYYNEATGGK.Y | 2 |
| \* | Astrin\_NLD\_STLC\_tube2\_021014\_02.05636.05636.3 | 2.6121 | 0.2599 | 95.4% | 1818.0543 | 1818.0392 | 7 | 5.01 | 38.3% | 1 | R.ISVYYNEATGGKYVPR.A | 3 |
|  | Astrin\_NLD\_STLC\_031014\_01.10481.10481.2 | 4.3846 | 0.483 | 100.0% | 1617.0922 | 1616.8701 | 1 | 8.766 | 67.9% | 5 | R.AILVDLEPGTMDSVR.S | 22 |
|  | Astrin\_NLD\_STLC\_031014\_01.11183.11183.3 | 5.6192 | 0.4239 | 100.0% | 2799.1143 | 2800.0647 | 1 | 7.423 | 32.0% | 6 | R.SGPFGQIFRPDNFVFGQSGAGNNWAK.G | 33 |
|  | Astrin\_NLD\_STLC\_031014\_01.11441.11441.3 | 3.3688 | 0.1916 | 95.4% | 1959.4143 | 1960.151 | 1 | 5.674 | 39.7% | 1 | K.GHYTEGAELVDSVLDVVR.K | 333 |
|  | Astrin\_NLD\_STLC\_tube2\_021014\_01.13540.13540.2 | 6.8342 | 0.5802 | 100.0% | 1959.5122 | 1960.151 | 1 | 10.762 | 79.4% | 1 | K.GHYTEGAELVDSVLDVVR.K | 222 |
|  | Astrin\_NLD\_STLC\_tube2\_021014\_01.12447.12447.3 | 4.5141 | 0.4937 | 100.0% | 2088.3542 | 2088.325 | 1 | 7.055 | 37.5% | 3 | K.GHYTEGAELVDSVLDVVRK.E | 333 |
|  | Astrin\_NLD\_STLC\_tube2\_021014\_01.08906.08906.2 | 3.931 | 0.4413 | 100.0% | 1320.0322 | 1320.5896 | 1 | 8.255 | 72.7% | 7 | R.IMNTFSVVPSPK.V | 222 |
|  | Astrin\_NLD\_STLC\_tube2\_021014\_01.07730.07730.2 | 2.8655 | 0.2256 | 99.8% | 1130.6322 | 1131.2767 | 5 | 4.422 | 77.8% | 4 | R.FPGQLNADLR.K | 222 |
|  | Astrin\_NLD\_STLC\_tube2\_021014\_01.08946.08946.2 | 3.5321 | 0.3443 | 100.0% | 1272.1522 | 1272.5945 | 1 | 6.817 | 70.0% | 1 | R.KLAVNMVPFPR.L | 222 |
|  | Astrin\_NLD\_STLC\_tube2\_021014\_01.10448.10448.2 | 3.6872 | 0.512 | 100.0% | 1144.1322 | 1144.4204 | 1 | 8.442 | 88.9% | 3 | K.LAVNMVPFPR.L | 222 |
|  | Astrin\_NLD\_STLC\_031014\_01.11120.11120.2 | 3.5059 | 0.3473 | 100.0% | 1622.0322 | 1621.9403 | 1 | 7.187 | 65.4% | 3 | R.LHFFMPGFAPLTSR.G | 22 |
|  | Astrin\_NLD\_STLC\_tube2\_021014\_01.12707.12707.3 | 3.9064 | 0.3323 | 100.0% | 1622.1843 | 1621.9403 | 2 | 6.479 | 50.0% | 2 | R.LHFFMPGFAPLTSR.G | 33 |
| \* | Astrin\_NLD\_STLC\_tube2\_021014\_01.12335.12335.2 | 3.0673 | 0.2774 | 99.8% | 1660.5721 | 1660.9078 | 2 | 6.434 | 53.6% | 1 | R.ALTVPELTQQVFDAK.N | 2 |
|  | Astrin\_NLD\_STLC\_tube2\_021014\_01.10960.10960.2 | 2.7155 | 0.3513 | 100.0% | 1041.3522 | 1040.2505 | 1 | 6.323 | 87.5% | 2 | R.YLTVAAVFR.G | 22 |
|  | Astrin\_NLD\_STLC\_031014\_02.06596.06596.3 | 4.0469 | 0.2858 | 100.0% | 1924.9744 | 1925.2405 | 6 | 5.946 | 40.0% | 1 | R.MSMKEVDEQMLNVQNK.N | 33 |
|  | Astrin\_NLD\_STLC\_tube2\_021014\_01.06237.06237.2 | 4.3052 | 0.2375 | 100.0% | 1448.2522 | 1447.6031 | 6 | 5.817 | 63.6% | 1 | K.EVDEQMLNVQNK.N | 22 |
|  | Astrin\_NLD\_STLC\_tube2\_021014\_01.10736.10736.2 | 3.4182 | 0.4473 | 100.0% | 1229.9321 | 1230.4241 | 1 | 7.21 | 94.4% | 6 | R.ISEQFTAMFR.R | 222 |

Similarities:
gi|5174735|ref|NP\_006(14:4)  
gi|50592996|ref|NP\_00(9:9)  

---

|  |  |  |  |  |  |  |  |  |
| --- | --- | --- | --- | --- | --- | --- | --- | --- |
| U | *gi|5174735|ref|NP\_006* | 17 | 45 | 38.0% | 445 | 49831 | 4.9 | tubulin, beta, 2 [Homo sapiens] |

| Filename XCorr DeltCN Conf% ObsM+H+ CalcM+H+ SpR ZScore Ion% # Sequence  | | | | | | | | | | | | |
| --- | --- | --- | --- | --- | --- | --- | --- | --- | --- | --- | --- | --- |
| \* | Astrin\_NLD\_STLC\_tube2\_021014\_01.05969.05969.2 | 3.2544 | 0.3911 | 100.0% | 1329.5122 | 1329.4521 | 1 | 7.905 | 81.8% | 1 | R.INVYYNEATGGK.Y | 2 |
|  | Astrin\_NLD\_STLC\_tube2\_021014\_01.10178.10178.2 | 4.3072 | 0.4394 | 100.0% | 1603.2722 | 1602.8431 | 1 | 8.26 | 75.0% | 2 | R.AVLVDLEPGTMDSVR.S | 2 |
|  | Astrin\_NLD\_STLC\_031014\_01.11183.11183.3 | 5.6192 | 0.4239 | 100.0% | 2799.1143 | 2800.0647 | 1 | 7.423 | 32.0% | 6 | R.SGPFGQIFRPDNFVFGQSGAGNNWAK.G | 33 |
|  | Astrin\_NLD\_STLC\_031014\_01.11441.11441.3 | 3.3688 | 0.1916 | 95.4% | 1959.4143 | 1960.151 | 1 | 5.674 | 39.7% | 1 | K.GHYTEGAELVDSVLDVVR.K | 333 |
|  | Astrin\_NLD\_STLC\_tube2\_021014\_01.13540.13540.2 | 6.8342 | 0.5802 | 100.0% | 1959.5122 | 1960.151 | 1 | 10.762 | 79.4% | 1 | K.GHYTEGAELVDSVLDVVR.K | 222 |
|  | Astrin\_NLD\_STLC\_tube2\_021014\_01.12447.12447.3 | 4.5141 | 0.4937 | 100.0% | 2088.3542 | 2088.325 | 1 | 7.055 | 37.5% | 3 | K.GHYTEGAELVDSVLDVVRK.E | 333 |
|  | Astrin\_NLD\_STLC\_tube2\_021014\_01.08906.08906.2 | 3.931 | 0.4413 | 100.0% | 1320.0322 | 1320.5896 | 1 | 8.255 | 72.7% | 7 | R.IMNTFSVVPSPK.V | 222 |
|  | Astrin\_NLD\_STLC\_tube2\_021014\_01.07730.07730.2 | 2.8655 | 0.2256 | 99.8% | 1130.6322 | 1131.2767 | 5 | 4.422 | 77.8% | 4 | R.FPGQLNADLR.K | 222 |
|  | Astrin\_NLD\_STLC\_tube2\_021014\_01.08946.08946.2 | 3.5321 | 0.3443 | 100.0% | 1272.1522 | 1272.5945 | 1 | 6.817 | 70.0% | 1 | R.KLAVNMVPFPR.L | 222 |
|  | Astrin\_NLD\_STLC\_tube2\_021014\_01.10448.10448.2 | 3.6872 | 0.512 | 100.0% | 1144.1322 | 1144.4204 | 1 | 8.442 | 88.9% | 3 | K.LAVNMVPFPR.L | 222 |
|  | Astrin\_NLD\_STLC\_031014\_01.11120.11120.2 | 3.5059 | 0.3473 | 100.0% | 1622.0322 | 1621.9403 | 1 | 7.187 | 65.4% | 3 | R.LHFFMPGFAPLTSR.G | 22 |
|  | Astrin\_NLD\_STLC\_tube2\_021014\_01.12707.12707.3 | 3.9064 | 0.3323 | 100.0% | 1622.1843 | 1621.9403 | 2 | 6.479 | 50.0% | 2 | R.LHFFMPGFAPLTSR.G | 33 |
|  | Astrin\_NLD\_STLC\_tube2\_021014\_01.12653.12653.2 | 3.2588 | 0.3888 | 100.0% | 1692.6322 | 1692.9678 | 1 | 6.595 | 71.4% | 1 | R.ALTVPELTQQMFDAK.N | 22 |
|  | Astrin\_NLD\_STLC\_tube2\_021014\_01.10960.10960.2 | 2.7155 | 0.3513 | 100.0% | 1041.3522 | 1040.2505 | 1 | 6.323 | 87.5% | 2 | R.YLTVAAVFR.G | 22 |
|  | Astrin\_NLD\_STLC\_031014\_02.06596.06596.3 | 4.0469 | 0.2858 | 100.0% | 1924.9744 | 1925.2405 | 6 | 5.946 | 40.0% | 1 | R.MSMKEVDEQMLNVQNK.N | 33 |
|  | Astrin\_NLD\_STLC\_tube2\_021014\_01.06237.06237.2 | 4.3052 | 0.2375 | 100.0% | 1448.2522 | 1447.6031 | 6 | 5.817 | 63.6% | 1 | K.EVDEQMLNVQNK.N | 22 |
|  | Astrin\_NLD\_STLC\_tube2\_021014\_01.10736.10736.2 | 3.4182 | 0.4473 | 100.0% | 1229.9321 | 1230.4241 | 1 | 7.21 | 94.4% | 6 | R.ISEQFTAMFR.R | 222 |

Similarities:
gi|29788785|ref|NP\_82(14:3)  
gi|50592996|ref|NP\_00(9:8)  

---

|  |  |  |  |  |  |  |  |  |
| --- | --- | --- | --- | --- | --- | --- | --- | --- |
| U | *gi|73623035|ref|NP\_00* | 49 | 146 | 36.7% | 1193 | 134422 | 5.0 | sperm associated antigen 5 [Homo sapiens] |

| Filename XCorr DeltCN Conf% ObsM+H+ CalcM+H+ SpR ZScore Ion% # Sequence  | | | | | | | | | | | | |
| --- | --- | --- | --- | --- | --- | --- | --- | --- | --- | --- | --- | --- |
| \* | Astrin\_NLD\_STLC\_tube2\_021014\_01.03771.03771.3 | 3.3991 | 0.2235 | 99.4% | 1618.5243 | 1618.7031 | 106 | 5.29 | 32.7% | 1 | K.RTDLSSEHFSHSSK.W | 3 |
| \* | Astrin\_NLD\_STLC\_tube2\_021014\_01.03894.03894.2 | 3.8054 | 0.3962 | 100.0% | 1463.0122 | 1462.5156 | 1 | 6.235 | 70.8% | 1 | R.TDLSSEHFSHSSK.W | 2 |
| \* | Astrin\_NLD\_STLC\_tube2\_021014\_01.15512.15512.3 | 6.1907 | 0.2218 | 100.0% | 3858.8044 | 3858.2102 | 1 | 6.438 | 28.7% | 1 | R.TEAVREDLVPSESNAFLPSSVLWLS\*PSTALAADFR.V | 3 |
| \* | Astrin\_NLD\_STLC\_031014\_02.05823.05823.3 | 4.3852 | 0.2972 | 100.0% | 2220.5044 | 2220.3752 | 1 | 5.945 | 38.9% | 6 | R.VNHVDPEEEIVEHGAMEER.E | 3 |
| \* | Astrin\_NLD\_STLC\_tube2\_021014\_01.16173.16173.2 | 4.0707 | 0.569 | 100.0% | 2062.672 | 2064.3606 | 1 | 10.067 | 55.9% | 2 | R.ILGSDTESWMSPLAWLEK.G | 2 |
| \* | Astrin\_NLD\_STLC\_tube2\_021014\_01.16763.16763.2 | 3.2168 | 0.3798 | 100.0% | 2143.0322 | 2144.3606 | 1 | 6.309 | 41.2% | 1 | R.ILGSDTESWMS\*PLAWLEK.G | 2 |
| \* | Astrin\_NLD\_STLC\_tube2\_021014\_01.03957.03957.2 | 3.6103 | 0.5046 | 100.0% | 1294.4122 | 1294.4044 | 1 | 8.093 | 70.8% | 1 | K.STNTSQTGLVGTK.H | 2 |
| \* | Astrin\_NLD\_STLC\_tube2\_021014\_01.17976.17976.2 | 7.4185 | 0.6208 | 100.0% | 2165.6921 | 2166.4795 | 1 | 11.427 | 69.4% | 6 | R.HDLEDNLLSSLVILEVLSR.Q | 2 |
| \* | Astrin\_NLD\_STLC\_031014\_01.08975.08975.2 | 3.9642 | 0.402 | 100.0% | 1485.1322 | 1485.6146 | 1 | 6.432 | 62.5% | 2 | K.ESHEMGQALQQAR.N | 2 |
| \* | Astrin\_NLD\_STLC\_tube2\_021014\_01.11537.11537.2 | 2.5644 | 0.375 | 100.0% | 1305.0721 | 1305.578 | 1 | 6.527 | 70.0% | 1 | R.NVMQSWVLISK.E | 2 |
| \* | Astrin\_NLD\_STLC\_tube2\_021014\_01.10424.10424.2 | 3.6261 | 0.147 | 99.8% | 1390.8121 | 1391.5823 | 2 | 6.466 | 68.2% | 2 | R.ISQLEQDLASMR.E | 2 |
| \* | Astrin\_NLD\_STLC\_tube2\_021014\_01.09386.09386.3 | 3.3978 | 0.2595 | 99.6% | 2126.4243 | 2125.4788 | 4 | 4.811 | 33.3% | 1 | R.EFRGLLKDAQTQLVGLHAK.Q | 3 |
| \* | Astrin\_NLD\_STLC\_tube2\_021014\_01.08294.08294.3 | 3.7696 | 0.3217 | 100.0% | 1692.9243 | 1692.9994 | 5 | 5.772 | 41.7% | 1 | R.GLLKDAQTQLVGLHAK.Q | 3 |
| \* | Astrin\_NLD\_STLC\_tube2\_021014\_01.05145.05145.2 | 2.511 | 0.2457 | 98.7% | 1280.8922 | 1281.4545 | 7 | 4.696 | 59.1% | 1 | K.DAQTQLVGLHAK.Q | 2 |
| \* | Astrin\_NLD\_STLC\_tube2\_021014\_02.04524.04524.3 | 4.0551 | 0.2551 | 100.0% | 2344.2244 | 2344.5437 | 2 | 4.89 | 34.2% | 1 | K.SQQALQERDVAIEEKQEVSR.V | 3 |
| \* | Astrin\_NLD\_STLC\_031014\_01.08921.08921.2 | 3.7945 | 0.4391 | 100.0% | 1403.3322 | 1403.5321 | 2 | 7.204 | 72.7% | 3 | R.DVAIEEKQEVSR.V | 2 |
| \* | Astrin\_NLD\_STLC\_tube2\_021014\_02.03930.03930.3 | 2.2781 | 0.3865 | 100.0% | 1404.8344 | 1403.5321 | 1 | 5.339 | 43.2% | 2 | R.DVAIEEKQEVSR.V | 3 |
| \* | Astrin\_NLD\_STLC\_tube2\_021014\_01.05084.05084.2 | 4.2425 | 0.4284 | 100.0% | 1404.0322 | 1404.4764 | 9 | 7.272 | 63.6% | 1 | K.GQTEQLELENSR.L | 2 |
| \* | Astrin\_NLD\_STLC\_031014\_02.08272.08272.2 | 4.8426 | 0.3436 | 100.0% | 1573.7922 | 1573.848 | 1 | 7.272 | 76.9% | 5 | R.AQLQILANMDSQLK.E | 2 |
| \* | Astrin\_NLD\_STLC\_031014\_01.09170.09170.2 | 5.4726 | 0.5402 | 100.0% | 1723.3922 | 1723.9879 | 1 | 9.799 | 82.1% | 5 | K.HMQAELQQQQAVLAK.E | 2 |
| \* | Astrin\_NLD\_STLC\_tube2\_021014\_02.04664.04664.3 | 5.128 | 0.3193 | 100.0% | 1724.4243 | 1723.9879 | 1 | 5.786 | 51.8% | 16 | K.HMQAELQQQQAVLAK.E | 3 |
| \* | Astrin\_NLD\_STLC\_031014\_01.09952.09952.3 | 3.4722 | 0.1983 | 97.0% | 1964.1244 | 1964.1462 | 1 | 4.228 | 40.6% | 1 | R.SLQCENLKDTVENLTAK.L | 3 |
| \* | Astrin\_NLD\_STLC\_tube2\_021014\_01.05678.05678.2 | 5.0084 | 0.4301 | 100.0% | 1675.3522 | 1675.7899 | 1 | 7.362 | 71.4% | 5 | K.LASTIADNQEQDLEK.T | 2 |
| \* | Astrin\_NLD\_STLC\_tube2\_021014\_01.05537.05537.2 | 3.9242 | 0.442 | 100.0% | 1932.4521 | 1933.0825 | 1 | 8.093 | 59.4% | 1 | K.LASTIADNQEQDLEKTR.Q | 2 |
| \* | Astrin\_NLD\_STLC\_031014\_02.12458.12458.3 | 4.9525 | 0.4354 | 100.0% | 2047.1943 | 2047.443 | 1 | 7.603 | 48.5% | 4 | K.LGLLTEQLQSLTLFLQTK.L | 3 |
| \* | Astrin\_NLD\_STLC\_tube2\_021014\_01.17015.17015.2 | 5.2614 | 0.4311 | 100.0% | 2047.3922 | 2047.443 | 1 | 8.266 | 67.6% | 15 | K.LGLLTEQLQSLTLFLQTK.L | 2 |
| \* | Astrin\_NLD\_STLC\_tube2\_021014\_01.16629.16629.2 | 5.4394 | 0.5089 | 100.0% | 2787.7922 | 2788.121 | 1 | 8.744 | 40.4% | 6 | R.TFLGSILTAVADEEPESTPVPLLGSDK.S | 2 |
| \* | Astrin\_NLD\_STLC\_tube2\_021014\_01.16511.16511.3 | 4.8387 | 0.4261 | 100.0% | 3429.0544 | 3430.7473 | 1 | 5.944 | 28.2% | 1 | R.TFLGSILTAVADEEPESTPVPLLGSDKSAFT#R.V | 3 |
| \* | Astrin\_NLD\_STLC\_tube2\_021014\_01.16577.16577.3 | 3.8911 | 0.334 | 100.0% | 3429.3542 | 3430.7473 | 1 | 6.239 | 25.0% | 2 | R.TFLGSILTAVADEEPESTPVPLLGS\*DKSAFTR.V | 3 |
| \* | Astrin\_NLD\_STLC\_tube2\_021014\_01.03630.03630.2 | 4.6621 | 0.3597 | 100.0% | 1595.2522 | 1595.7092 | 1 | 6.967 | 87.5% | 1 | R.LQAQEEQHQEVQK.A | 2 |
| \* | Astrin\_NLD\_STLC\_031014\_01.09208.09208.3 | 5.7684 | 0.3157 | 100.0% | 2148.4744 | 2149.3652 | 1 | 6.021 | 50.0% | 2 | R.YKNEKELQEVIQQQNEK.I | 3 |
| \* | Astrin\_NLD\_STLC\_tube2\_021014\_01.05634.05634.2 | 6.3816 | 0.3913 | 100.0% | 2148.7922 | 2149.3652 | 1 | 7.744 | 68.8% | 2 | R.YKNEKELQEVIQQQNEK.I | 2 |
| \* | Astrin\_NLD\_STLC\_tube2\_021014\_01.05085.05085.2 | 4.8324 | 0.2354 | 100.0% | 1486.3121 | 1486.622 | 1 | 6.368 | 86.4% | 4 | K.ELQEVIQQQNEK.I | 2 |
| \* | Astrin\_NLD\_STLC\_tube2\_021014\_01.11366.11366.3 | 5.0034 | 0.2126 | 100.0% | 2681.6643 | 2681.0618 | 1 | 5.53 | 35.2% | 1 | K.ILEQIDKSGELISLREEVTHLTR.S | 3 |
| \* | Astrin\_NLD\_STLC\_tube2\_021014\_01.07515.07515.2 | 2.5088 | 0.1879 | 98.6% | 874.5522 | 875.0128 | 8 | 5.172 | 85.7% | 1 | K.SGELISLR.E | 2 |
| \* | Astrin\_NLD\_STLC\_tube2\_021014\_01.09971.09971.2 | 4.0419 | 0.405 | 100.0% | 1840.3522 | 1841.0745 | 1 | 6.991 | 56.7% | 3 | K.SGELISLREEVTHLTR.S | 2 |
| \* | Astrin\_NLD\_STLC\_tube2\_021014\_01.10010.10010.3 | 4.9568 | 0.3465 | 100.0% | 1841.4543 | 1841.0745 | 1 | 6.874 | 50.0% | 10 | K.SGELISLREEVTHLTR.S | 3 |
| \* | Astrin\_NLD\_STLC\_tube2\_021014\_01.07421.07421.2 | 2.3287 | 0.2324 | 98.3% | 1103.6122 | 1104.248 | 389 | 5.284 | 62.5% | 1 | K.VWLSQEVDK.L | 2 |
| \* | Astrin\_NLD\_STLC\_tube2\_021014\_01.09285.09285.2 | 2.9907 | 0.2255 | 99.8% | 1373.2322 | 1373.595 | 1 | 5.819 | 85.0% | 3 | K.VWLSQEVDKLR.V | 2 |
| \* | Astrin\_NLD\_STLC\_tube2\_021014\_01.10070.10070.2 | 2.5247 | 0.3353 | 100.0% | 898.15216 | 898.16644 | 1 | 6.979 | 91.7% | 2 | R.VMFLEMK.N | 2 |
| \* | Astrin\_NLD\_STLC\_tube2\_021014\_01.07782.07782.2 | 2.6837 | 0.3599 | 100.0% | 1268.9722 | 1269.5598 | 1 | 6.393 | 72.2% | 2 | R.VMFLEMKNEK.E | 2 |
| \* | Astrin\_NLD\_STLC\_031014\_01.09642.09642.1 | 2.1504 | 0.1978 | 100.0% | 1000.44 | 1001.1277 | 5 | 4.2 | 71.4% | 2 | R.NILEENLR.R | 1 |
| \* | Astrin\_NLD\_STLC\_tube2\_021014\_01.10131.10131.3 | 5.554 | 0.4412 | 100.0% | 2230.6443 | 2230.526 | 1 | 7.735 | 41.2% | 3 | R.RSDKELEKLDDIVQHIYK.T | 3 |
| \* | Astrin\_NLD\_STLC\_tube2\_021014\_01.10905.10905.3 | 5.032 | 0.328 | 100.0% | 2074.8843 | 2074.3384 | 1 | 6.563 | 43.8% | 2 | R.SDKELEKLDDIVQHIYK.T | 3 |
| \* | Astrin\_NLD\_STLC\_tube2\_021014\_01.10367.10367.3 | 3.1497 | 0.26 | 99.6% | 1743.8344 | 1743.9977 | 1 | 5.596 | 44.2% | 1 | K.ELEKLDDIVQHIYK.T | 3 |
| \* | Astrin\_NLD\_STLC\_tube2\_021014\_01.07527.07527.2 | 3.4068 | 0.3543 | 100.0% | 1244.3322 | 1244.4331 | 1 | 7.033 | 72.2% | 2 | K.LDDIVQHIYK.T | 2 |
| \* | Astrin\_NLD\_STLC\_031014\_01.10494.10494.1 | 1.7012 | 0.2579 | 98.5% | 1126.67 | 1127.3696 | 263 | 5.096 | 38.9% | 1 | K.TLLSIPEVVR.G | 1 |
| \* | Astrin\_NLD\_STLC\_031014\_01.10563.10563.2 | 2.8309 | 0.3132 | 100.0% | 1127.3322 | 1127.3696 | 4 | 6.252 | 72.2% | 4 | K.TLLSIPEVVR.G | 2 |
| \* | Astrin\_NLD\_STLC\_031014\_01.13590.13590.1 | 1.9685 | 0.3639 | 100.0% | 1148.57 | 1149.3293 | 1 | 5.763 | 61.1% | 4 | K.ELQGLLEFLS.- | 1 |

---

|  |  |  |  |  |  |  |  |  |
| --- | --- | --- | --- | --- | --- | --- | --- | --- |
| U | *gi|4501885|ref|NP\_001* | 10 | 50 | 34.9% | 375 | 41737 | 5.5 | beta actin [Homo sapiens] |
| U | *gi|4501887|ref|NP\_001* | 10 | 50 | 34.9% | 375 | 41793 | 5.5 | actin, gamma 1 propeptide [Homo sapiens] |

| Filename XCorr DeltCN Conf% ObsM+H+ CalcM+H+ SpR ZScore Ion% # Sequence  | | | | | | | | | | | | |
| --- | --- | --- | --- | --- | --- | --- | --- | --- | --- | --- | --- | --- |
|  | Astrin\_NLD\_STLC\_031014\_01.06884.06884.2 | 3.2662 | 0.4547 | 100.0% | 976.33215 | 977.02136 | 2 | 7.839 | 72.2% | 30 | K.AGFAGDDAPR.A | 22 |
|  | Astrin\_NLD\_STLC\_031014\_01.09627.09627.2 | 2.5872 | 0.2875 | 99.8% | 1199.2722 | 1199.4415 | 5 | 5.809 | 65.0% | 2 | R.AVFPSIVGRPR.H | 22 |
|  | Astrin\_NLD\_STLC\_031014\_01.09447.09447.2 | 3.2367 | 0.43 | 100.0% | 1516.2122 | 1516.7019 | 1 | 6.454 | 80.0% | 2 | K.IWHHTFYNELR.V | 22 |
|  | Astrin\_NLD\_STLC\_tube2\_021014\_01.08314.08314.2 | 4.3895 | 0.3226 | 100.0% | 1955.1122 | 1955.2615 | 1 | 7.205 | 55.9% | 3 | R.VAPEEHPVLLTEAPLNPK.A | 2 |
|  | Astrin\_NLD\_STLC\_tube2\_021014\_01.11351.11351.3 | 6.4807 | 0.5198 | 100.0% | 3185.2144 | 3185.622 | 1 | 9.582 | 31.0% | 2 | R.TTGIVMDSGDGVTHTVPIYEGYALPHAILR.L | 3 |
|  | Astrin\_NLD\_STLC\_tube2\_021014\_01.11475.11475.2 | 2.9524 | 0.2059 | 98.7% | 1624.5322 | 1624.8927 | 2 | 5.245 | 57.7% | 1 | R.LDLAGRDLTDYLMK.I | 222 |
|  | Astrin\_NLD\_STLC\_tube2\_021014\_01.05628.05628.2 | 2.6241 | 0.3917 | 100.0% | 1133.1921 | 1133.2029 | 1 | 7.819 | 66.7% | 3 | R.GYSFTTTAER.E | 2 |
|  | Astrin\_NLD\_STLC\_tube2\_021014\_01.10676.10676.2 | 4.8426 | 0.3035 | 100.0% | 1791.2922 | 1791.9554 | 1 | 8.531 | 83.3% | 3 | K.SYELPDGQVITIGNER.F | 222 |
|  | Astrin\_NLD\_STLC\_tube2\_021014\_01.06791.06791.1 | 2.5336 | 0.4765 | 100.0% | 1161.6 | 1162.3868 | 1 | 7.532 | 55.0% | 1 | K.EITALAPSTMK.I | 11 |
|  | Astrin\_NLD\_STLC\_tube2\_021014\_01.06812.06812.2 | 2.6074 | 0.3641 | 100.0% | 1162.0521 | 1162.3868 | 1 | 6.597 | 80.0% | 3 | K.EITALAPSTMK.I | 22 |

Similarities:
gi|4501881|ref|NP\_001(7:3)  
gi|63055057|ref|NP\_00(2:8)  

---

|  |  |  |  |  |  |  |  |  |
| --- | --- | --- | --- | --- | --- | --- | --- | --- |
| U | *gi|20127519|ref|NP\_03* | 25 | 38 | 34.1% | 747 | 85653 | 9.2 | TPX2, microtubule-associated protein homolog [Homo sapiens] |

| Filename XCorr DeltCN Conf% ObsM+H+ CalcM+H+ SpR ZScore Ion% # Sequence  | | | | | | | | | | | | |
| --- | --- | --- | --- | --- | --- | --- | --- | --- | --- | --- | --- | --- |
| \* | Astrin\_NLD\_STLC\_tube2\_021014\_01.09297.09297.3 | 3.4271 | 0.3515 | 100.0% | 2277.0842 | 2277.6255 | 4 | 6.414 | 27.6% | 1 | K.ANLQQAIVTPLKPVDNTYYK.E | 3 |
| \* | Astrin\_NLD\_STLC\_tube2\_021014\_01.05120.05120.2 | 2.7365 | 0.4068 | 100.0% | 1149.4122 | 1150.3534 | 5 | 6.591 | 75.0% | 1 | K.MQQEVVEMR.K | 2 |
| \* | Astrin\_NLD\_STLC\_tube2\_021014\_01.08153.08153.2 | 2.6021 | 0.3467 | 100.0% | 1066.9922 | 1067.317 | 23 | 6.062 | 60.0% | 1 | K.LALAGIGQPVK.K | 2 |
| \* | Astrin\_NLD\_STLC\_tube2\_021014\_01.06420.06420.2 | 3.0275 | 0.2824 | 100.0% | 1195.1921 | 1195.4911 | 21 | 6.838 | 59.1% | 1 | K.LALAGIGQPVKK.S | 2 |
| \* | Astrin\_NLD\_STLC\_tube2\_021014\_02.06206.06206.3 | 4.3198 | 0.3965 | 100.0% | 1886.1244 | 1887.013 | 1 | 7.17 | 42.9% | 4 | K.NQEEYKEVNFTSELR.K | 3 |
| \* | Astrin\_NLD\_STLC\_tube2\_021014\_01.08026.08026.2 | 4.7078 | 0.3942 | 100.0% | 1886.5322 | 1887.013 | 1 | 7.212 | 78.6% | 1 | K.NQEEYKEVNFTSELR.K | 2 |
| \* | Astrin\_NLD\_STLC\_tube2\_021014\_01.06587.06587.2 | 3.3611 | 0.2463 | 100.0% | 1349.1522 | 1349.4344 | 1 | 5.328 | 77.3% | 1 | K.STAELEAEELEK.L | 2 |
| \* | Astrin\_NLD\_STLC\_tube2\_021014\_01.07988.07988.2 | 2.9845 | 0.3229 | 100.0% | 1037.1721 | 1037.2877 | 1 | 5.232 | 66.7% | 2 | R.ILEGGPILPK.K | 2 |
| \* | Astrin\_NLD\_STLC\_tube2\_021014\_01.09058.09058.3 | 4.1786 | 0.3902 | 100.0% | 2135.3044 | 2135.5083 | 1 | 6.469 | 37.5% | 1 | K.KPPVKPPTEPIGFDLEIEK.R | 3 |
| \* | Astrin\_NLD\_STLC\_031014\_01.08832.08832.3 | 3.4451 | 0.2687 | 99.8% | 2273.7544 | 2274.4639 | 3 | 4.872 | 35.3% | 1 | K.KKTEDEHFEFHSRPCPTK.I | 3 |
| \* | Astrin\_NLD\_STLC\_tube2\_021014\_01.08001.08001.2 | 2.7793 | 0.3182 | 100.0% | 1199.2322 | 1198.402 | 1 | 6.108 | 80.0% | 1 | K.ILEDVVGVPEK.K | 2 |
| \* | Astrin\_NLD\_STLC\_tube2\_021014\_01.06677.06677.2 | 3.3191 | 0.2694 | 100.0% | 1326.0922 | 1326.576 | 1 | 6.164 | 77.3% | 1 | K.ILEDVVGVPEKK.V | 2 |
| \* | Astrin\_NLD\_STLC\_tube2\_021014\_01.06698.06698.3 | 3.5301 | 0.3012 | 100.0% | 1327.0144 | 1326.576 | 116 | 5.112 | 40.9% | 1 | K.ILEDVVGVPEKK.V | 3 |
| \* | Astrin\_NLD\_STLC\_031014\_01.10452.10452.2 | 3.5213 | 0.4227 | 100.0% | 1661.6122 | 1661.9823 | 1 | 7.074 | 64.3% | 2 | K.VLPITVPKS\*PAFALK.N | 2 |
| \* | Astrin\_NLD\_STLC\_tube2\_021014\_01.06436.06436.3 | 4.2745 | 0.3597 | 100.0% | 2159.7544 | 2158.4285 | 2 | 6.308 | 36.8% | 1 | R.IRMPTKEDEEEDEPVVIK.A | 3 |
| \* | Astrin\_NLD\_STLC\_tube2\_021014\_01.05548.05548.3 | 4.3362 | 0.2562 | 100.0% | 1888.7043 | 1889.0815 | 2 | 5.479 | 43.3% | 3 | R.MPTKEDEEEDEPVVIK.A | 3 |
| \* | Astrin\_NLD\_STLC\_031014\_01.09678.09678.3 | 2.7445 | 0.2987 | 98.4% | 2132.2144 | 2132.473 | 25 | 5.17 | 30.6% | 2 | K.AQPVPHYGVPFKPQIPEAR.T | 3 |
| \* | Astrin\_NLD\_STLC\_031014\_01.08010.08010.2 | 2.7091 | 0.2531 | 100.0% | 1053.5521 | 1054.1478 | 2 | 5.117 | 78.6% | 5 | K.HQLEEELR.Q | 2 |
| \* | Astrin\_NLD\_STLC\_tube2\_021014\_01.07263.07263.3 | 2.9766 | 0.3111 | 100.0% | 1684.5243 | 1683.9481 | 1 | 5.306 | 41.1% | 1 | K.ARPNTVISQEPFVPK.K | 3 |
| \* | Astrin\_NLD\_STLC\_tube2\_021014\_01.05770.05770.3 | 4.3896 | 0.3402 | 100.0% | 1811.6044 | 1812.1222 | 1 | 7.038 | 45.0% | 1 | K.ARPNTVISQEPFVPKK.E | 3 |
| \* | Astrin\_NLD\_STLC\_tube2\_021014\_01.10245.10245.3 | 3.3739 | 0.231 | 97.1% | 2474.5745 | 2475.8044 | 6 | 5.438 | 29.5% | 1 | K.KSVAEGLSGSLVQEPFQLATEKR.A | 3 |
| \* | Astrin\_NLD\_STLC\_tube2\_021014\_01.10979.10979.3 | 4.0005 | 0.3753 | 100.0% | 2346.9844 | 2347.6304 | 7 | 5.417 | 31.0% | 1 | K.SVAEGLSGSLVQEPFQLATEKR.A | 3 |
| \* | Astrin\_NLD\_STLC\_tube2\_021014\_01.11500.11500.3 | 3.3841 | 0.2233 | 96.8% | 2427.7144 | 2427.6304 | 153 | 4.733 | 25.0% | 1 | K.SVAEGLSGS\*LVQEPFQLATEKR.A | 3 |
| \* | Astrin\_NLD\_STLC\_tube2\_021014\_01.05513.05513.3 | 3.7814 | 0.2238 | 99.6% | 1832.9043 | 1832.0386 | 9 | 5.648 | 36.7% | 1 | R.MAEVEAQKAQQLEEAR.L | 3 |
| \* | Astrin\_NLD\_STLC\_tube2\_021014\_01.06924.06924.2 | 3.2111 | 0.3548 | 100.0% | 1435.2722 | 1435.5309 | 1 | 6.199 | 75.0% | 2 | K.SSDQPLTVPVS\*PK.F | 2 |

---

|  |  |  |  |  |  |  |  |  |
| --- | --- | --- | --- | --- | --- | --- | --- | --- |
| U | *gi|10800138|ref|NP\_06* | 5 | 13 | 33.3% | 126 | 13936 | 10.3 | histone cluster 1, H2bd [Homo sapiens] |
| U | *gi|66912162|ref|NP\_00* | 5 | 13 | 33.3% | 126 | 13920 | 10.3 | histone cluster 2, H2bf [Homo sapiens] |
| U | *gi|4504277|ref|NP\_003* | 5 | 13 | 33.3% | 126 | 13920 | 10.3 | histone cluster 2, H2be [Homo sapiens] |
| U | *gi|4504271|ref|NP\_003* | 5 | 13 | 33.3% | 126 | 13906 | 10.3 | histone cluster 1, H2bi [Homo sapiens] |
| U | *gi|4504269|ref|NP\_003* | 5 | 13 | 33.3% | 126 | 13892 | 10.3 | histone cluster 1, H2bh [Homo sapiens] |
| U | *gi|4504265|ref|NP\_003* | 5 | 13 | 33.3% | 126 | 13906 | 10.3 | histone cluster 1, H2bf [Homo sapiens] |
| U | *gi|4504263|ref|NP\_003* | 5 | 13 | 33.3% | 126 | 13989 | 10.3 | histone cluster 1, H2bm [Homo sapiens] |
| U | *gi|4504261|ref|NP\_003* | 5 | 13 | 33.3% | 126 | 13922 | 10.3 | histone cluster 1, H2bn [Homo sapiens] |
| U | *gi|4504257|ref|NP\_003* | 5 | 13 | 33.3% | 126 | 13906 | 10.3 | histone cluster 1, H2bg [Homo sapiens] |
| U | *gi|21396484|ref|NP\_00* | 5 | 13 | 33.3% | 126 | 13906 | 10.3 | histone cluster 1, H2be [Homo sapiens] |
| U | *gi|21166389|ref|NP\_00* | 5 | 13 | 33.3% | 126 | 13906 | 10.3 | histone cluster 1, H2bc [Homo sapiens] |
| U | *gi|20336754|ref|NP\_06* | 5 | 13 | 33.3% | 126 | 13904 | 10.3 | histone cluster 1, H2bj [Homo sapiens] |
| U | *gi|20336752|ref|NP\_61* | 5 | 13 | 33.3% | 126 | 13936 | 10.3 | histone cluster 1, H2bd [Homo sapiens] |
| U | *gi|18105048|ref|NP\_54* | 5 | 13 | 33.3% | 126 | 13890 | 10.3 | histone cluster 1, H2bk [Homo sapiens] |
| U | *gi|16306566|ref|NP\_00* | 5 | 13 | 33.3% | 126 | 13906 | 10.3 | histone cluster 1, H2bo [Homo sapiens] |
| U | *gi|10800140|ref|NP\_06* | 5 | 13 | 33.3% | 126 | 13950 | 10.3 | histone cluster 1, H2bb [Homo sapiens] |

| Filename XCorr DeltCN Conf% ObsM+H+ CalcM+H+ SpR ZScore Ion% # Sequence  | | | | | | | | | | | | |
| --- | --- | --- | --- | --- | --- | --- | --- | --- | --- | --- | --- | --- |
|  | Astrin\_NLD\_STLC\_tube2\_021014\_01.15002.15002.2 | 5.0364 | 0.5092 | 100.0% | 1744.4122 | 1745.0211 | 1 | 9.608 | 78.6% | 9 | K.AMGIMNSFVNDIFER.I | 2 |
|  | Astrin\_NLD\_STLC\_tube2\_021014\_01.17006.17006.3 | 3.2816 | 0.2993 | 99.8% | 2430.8342 | 2429.7712 | 3 | 5.063 | 32.1% | 1 | K.AMGIMNSFVNDIFERIAGEASR.L | 3 |
|  | Astrin\_NLD\_STLC\_031014\_01.08852.08852.3 | 3.7836 | 0.348 | 100.0% | 1587.0243 | 1586.7941 | 1 | 6.046 | 46.2% | 1 | R.IAGEASRLAHYNKR.S | 3 |
|  | Astrin\_NLD\_STLC\_tube2\_021014\_01.05123.05123.3 | 2.6019 | 0.2651 | 97.1% | 1461.2644 | 1462.6462 | 16 | 4.48 | 37.5% | 1 | R.STITSREIQTAVR.L | 3 |
|  | Astrin\_NLD\_STLC\_tube2\_021014\_01.05126.05126.2 | 3.4493 | 0.3375 | 100.0% | 1462.0922 | 1462.6462 | 1 | 6.537 | 58.3% | 1 | R.STITSREIQTAVR.L | 2 |

---

|  |  |  |  |  |  |  |  |  |
| --- | --- | --- | --- | --- | --- | --- | --- | --- |
| U | *gi|4506901|ref|NP\_003* | 5 | 5 | 32.9% | 164 | 19330 | 11.6 | splicing factor, arginine/serine-rich 3 [Homo sapiens] |

| Filename XCorr DeltCN Conf% ObsM+H+ CalcM+H+ SpR ZScore Ion% # Sequence  | | | | | | | | | | | | |
| --- | --- | --- | --- | --- | --- | --- | --- | --- | --- | --- | --- | --- |
| \* | Astrin\_NLD\_STLC\_tube2\_021014\_01.05555.05555.2 | 4.1265 | 0.404 | 100.0% | 1877.2922 | 1878.0519 | 1 | 8.126 | 59.4% | 1 | K.VYVGNLGNNGNKTELER.A | 2 |
| \* | Astrin\_NLD\_STLC\_tube2\_021014\_01.08889.08889.1 | 1.8643 | 0.2613 | 100.0% | 1043.56 | 1044.198 | 93 | 5.102 | 43.8% | 1 | R.AFGYYGPLR.S | 1 |
| \* | Astrin\_NLD\_STLC\_tube2\_021014\_01.08866.08866.2 | 2.4285 | 0.2695 | 99.7% | 1044.3922 | 1044.198 | 1 | 5.184 | 81.2% | 1 | R.AFGYYGPLR.S | 2 |
|  | Astrin\_NLD\_STLC\_tube2\_021014\_01.12198.12198.2 | 2.6152 | 0.2101 | 97.0% | 1622.1522 | 1622.7771 | 1 | 4.635 | 61.5% | 1 | R.NPPGFAFVEFEDPR.D | 22 |
| \* | Astrin\_NLD\_STLC\_031014\_02.06932.06932.3 | 3.1176 | 0.1909 | 95.3% | 1835.7544 | 1832.9338 | 2 | 4.831 | 38.5% | 1 | R.ERSLS\*RERNHKPSR.S | 3 |

Similarities:
gi|72534660|ref|NP\_00(1:4)  

---

|  |  |  |  |  |  |  |  |  |
| --- | --- | --- | --- | --- | --- | --- | --- | --- |
| U | *gi|119395750|ref|NP\_0* | 12 | 15 | 32.6% | 644 | 66039 | 8.1 | keratin 1 [Homo sapiens] |

| Filename XCorr DeltCN Conf% ObsM+H+ CalcM+H+ SpR ZScore Ion% # Sequence  | | | | | | | | | | | | |
| --- | --- | --- | --- | --- | --- | --- | --- | --- | --- | --- | --- | --- |
|  | Astrin\_NLD\_STLC\_tube2\_021014\_01.10652.10652.2 | 3.4583 | 0.4378 | 100.0% | 1384.0922 | 1384.5315 | 2 | 7.096 | 59.1% | 1 | K.SLNNQFASFIDK.V | 2 |
|  | Astrin\_NLD\_STLC\_tube2\_021014\_01.06434.06434.2 | 4.4159 | 0.0626 | 99.8% | 1476.3722 | 1476.6726 | 1 | 6.932 | 90.9% | 1 | R.FLEQQNQVLQTK.W | 22 |
|  | Astrin\_NLD\_STLC\_tube2\_021014\_01.13704.13704.2 | 3.2918 | 0.3599 | 100.0% | 1994.2122 | 1995.2017 | 1 | 5.808 | 56.7% | 2 | R.THNLEPYFESFINNLR.R | 2 |
|  | Astrin\_NLD\_STLC\_tube2\_021014\_01.07334.07334.2 | 2.9649 | 0.275 | 100.0% | 1266.1921 | 1266.3934 | 1 | 5.904 | 75.0% | 1 | R.TNAENEFVTIK.K | 2 |
| \* | Astrin\_NLD\_STLC\_tube2\_021014\_01.13360.13360.2 | 4.0754 | 0.4517 | 100.0% | 1303.4922 | 1303.4955 | 1 | 8.777 | 86.4% | 1 | R.SLDLDSIIAEVK.A | 2 |
|  | Astrin\_NLD\_STLC\_tube2\_021014\_02.05543.05543.2 | 3.4163 | 0.3238 | 100.0% | 1180.2122 | 1180.303 | 1 | 7.435 | 83.3% | 2 | K.YEELQITAGR.H | 22 |
|  | Astrin\_NLD\_STLC\_tube2\_021014\_01.06564.06564.2 | 2.9459 | 0.0753 | 97.1% | 974.1322 | 974.102 | 175 | 4.257 | 64.3% | 1 | K.IEISELNR.V | 22 |
| \* | Astrin\_NLD\_STLC\_tube2\_021014\_01.12202.12202.3 | 4.8778 | 0.4232 | 100.0% | 2185.1643 | 2185.399 | 1 | 6.791 | 48.6% | 1 | K.NKLNDLEDALQQAKEDLAR.L | 3 |
|  | Astrin\_NLD\_STLC\_tube2\_021014\_01.08040.08040.3 | 2.8193 | 0.2094 | 95.3% | 1524.2344 | 1524.7754 | 66 | 4.582 | 34.1% | 1 | R.LLRDYQELMNTK.L | 3 |
|  | Astrin\_NLD\_STLC\_tube2\_021014\_01.04131.04131.2 | 6.1204 | 0.5987 | 100.0% | 2384.2122 | 2385.298 | 1 | 11.749 | 41.7% | 1 | R.GGGGGGYGSGGSSYGSGGGSYGSGGGGGGGR.G | 2 |
| \* | Astrin\_NLD\_STLC\_tube2\_021014\_02.04121.04121.3 | 3.7446 | 0.2968 | 100.0% | 3314.4243 | 3314.2085 | 1 | 5.738 | 21.1% | 1 | R.GSYGSGGSSYGSGGGSYGSGGGGGGHGSYGSGSSSGGYR.G | 3 |
| \* | Astrin\_NLD\_STLC\_031014\_01.10116.10116.3 | 4.0585 | 0.2109 | 98.4% | 2240.3943 | 2241.0396 | 1 | 4.434 | 36.1% | 2 | R.GGSGGGGGGS\*S\*GGRGSGGGSSGGSIGGR.G | 3 |

Similarities:
gi|47132620|ref|NP\_00(2:10)  
gi|119703753|ref|NP\_0(1:11)  

---

|  |  |  |  |  |  |  |  |  |
| --- | --- | --- | --- | --- | --- | --- | --- | --- |
| U | *gi|4557701|ref|NP\_000* | 15 | 27 | 31.9% | 432 | 48106 | 5.0 | keratin 17 [Homo sapiens] |

| Filename XCorr DeltCN Conf% ObsM+H+ CalcM+H+ SpR ZScore Ion% # Sequence  | | | | | | | | | | | | |
| --- | --- | --- | --- | --- | --- | --- | --- | --- | --- | --- | --- | --- |
|  | Astrin\_NLD\_STLC\_tube2\_021014\_01.05645.05645.2 | 2.9461 | 0.1436 | 98.9% | 1065.2122 | 1065.2578 | 50 | 6.07 | 62.5% | 2 | R.LASYLDKVR.A | 2222 |
|  | Astrin\_NLD\_STLC\_tube2\_021014\_02.05198.05198.2 | 4.0318 | 0.4298 | 100.0% | 1346.4122 | 1346.4772 | 1 | 7.983 | 72.7% | 7 | R.ALEEANTELEVK.I | 2 |
|  | Astrin\_NLD\_STLC\_tube2\_021014\_01.05060.05060.2 | 2.4574 | 0.2931 | 100.0% | 807.9122 | 807.8815 | 9 | 6.526 | 75.0% | 2 | R.LAADDFR.T | 22222 |
|  | Astrin\_NLD\_STLC\_tube2\_021014\_01.04334.04334.2 | 2.6976 | 0.3418 | 100.0% | 1223.0122 | 1223.3715 | 19 | 5.786 | 66.7% | 1 | R.TKFETEQALR.L | 22 |
|  | Astrin\_NLD\_STLC\_tube2\_021014\_02.06634.06634.2 | 3.2897 | 0.3272 | 100.0% | 1187.2922 | 1187.3384 | 1 | 7.29 | 80.0% | 2 | R.LSVEADINGLR.R | 2 |
|  | Astrin\_NLD\_STLC\_tube2\_021014\_01.07816.07816.2 | 2.407 | 0.225 | 98.0% | 1186.8522 | 1186.397 | 4 | 5.0 | 72.2% | 1 | R.RVLDELTLAR.A | 222 |
|  | Astrin\_NLD\_STLC\_tube2\_021014\_01.08553.08553.2 | 3.5532 | 0.3938 | 100.0% | 1030.2322 | 1030.2096 | 1 | 7.35 | 87.5% | 2 | R.VLDELTLAR.A | 222 |
|  | Astrin\_NLD\_STLC\_tube2\_021014\_01.05013.05013.2 | 3.1274 | 0.1615 | 99.1% | 1439.5922 | 1439.6263 | 1 | 4.739 | 75.0% | 2 | R.ILNEMRDQYEK.M | 22 |
|  | Astrin\_NLD\_STLC\_031014\_01.08982.08982.2 | 3.168 | 0.4267 | 100.0% | 1361.0122 | 1362.4796 | 1 | 7.174 | 66.7% | 1 | R.EVATNSELVQSGK.S | 22 |
|  | Astrin\_NLD\_STLC\_tube2\_021014\_01.05760.05760.2 | 4.0567 | 0.4237 | 100.0% | 1405.0922 | 1404.4764 | 1 | 7.95 | 70.8% | 1 | K.ASLEGNLAETENR.Y | 2 |
|  | Astrin\_NLD\_STLC\_tube2\_021014\_01.06844.06844.2 | 3.547 | 0.3505 | 100.0% | 1381.3922 | 1380.5437 | 1 | 6.085 | 70.0% | 1 | K.TRLEQEIATYR.R | 22 |
|  | Astrin\_NLD\_STLC\_tube2\_021014\_01.05813.05813.3 | 3.1264 | 0.2733 | 100.0% | 1536.1743 | 1536.7311 | 2 | 5.027 | 38.6% | 1 | K.TRLEQEIATYRR.L | 33 |
|  | Astrin\_NLD\_STLC\_tube2\_021014\_01.06066.06066.2 | 3.4411 | 0.3785 | 100.0% | 1516.1921 | 1517.6787 | 1 | 8.165 | 70.8% | 2 | R.LLEGEDAHLTQYK.K | 2 |
|  | Astrin\_NLD\_STLC\_tube2\_021014\_01.05164.05164.3 | 4.5016 | 0.3981 | 100.0% | 2329.1042 | 2329.6152 | 1 | 7.25 | 39.5% | 1 | R.LLEGEDAHLTQYKKEPVTTR.Q | 3 |
|  | Astrin\_NLD\_STLC\_tube2\_021014\_01.05175.05175.2 | 2.7251 | 0.2799 | 99.9% | 1118.2722 | 1118.2291 | 1 | 5.73 | 72.2% | 1 | R.TIVEEVQDGK.V | 2 |

Similarities:
gi|40354195|ref|NP\_95(1:14)  
contaminant\_KERATIN03(2:13)  
contaminant\_KERATIN05(8:7)  
gi|24234699|ref|NP\_00(5:10)  

---

|  |  |  |  |  |  |  |  |  |
| --- | --- | --- | --- | --- | --- | --- | --- | --- |
| U | *gi|14389309|ref|NP\_11* | 11 | 30 | 31.8% | 449 | 49895 | 5.1 | tubulin alpha 6 [Homo sapiens] |
| U | *gi|57013276|ref|NP\_00* | 11 | 30 | 31.7% | 451 | 50152 | 5.1 | tubulin, alpha, ubiquitous [Homo sapiens] |

| Filename XCorr DeltCN Conf% ObsM+H+ CalcM+H+ SpR ZScore Ion% # Sequence  | | | | | | | | | | | | |
| --- | --- | --- | --- | --- | --- | --- | --- | --- | --- | --- | --- | --- |
|  | Astrin\_NLD\_STLC\_031014\_02.08351.08351.2 | 5.288 | 0.6315 | 100.0% | 2008.2722 | 2009.093 | 1 | 11.527 | 57.9% | 3 | K.TIGGGDDSFNTFFSETGAGK.H | 2 |
|  | Astrin\_NLD\_STLC\_tube2\_021014\_01.12238.12238.2 | 4.479 | 0.4135 | 100.0% | 1702.3722 | 1702.9451 | 1 | 7.737 | 60.7% | 5 | R.AVFVDLEPTVIDEVR.T | 2 |
|  | Astrin\_NLD\_STLC\_tube2\_021014\_01.08564.08564.3 | 3.5392 | 0.2676 | 99.6% | 2416.1943 | 2416.6555 | 1 | 6.372 | 32.5% | 3 | R.QLFHPEQLITGKEDAANNYAR.G | 3 |
|  | Astrin\_NLD\_STLC\_tube2\_021014\_01.11716.11716.2 | 2.1351 | 0.2872 | 98.7% | 1086.0721 | 1086.2737 | 10 | 5.735 | 75.0% | 1 | K.EIIDLVLDR.I | 2 |
|  | Astrin\_NLD\_STLC\_031014\_02.11068.11068.2 | 4.1309 | 0.5548 | 100.0% | 1488.3522 | 1488.7678 | 1 | 8.98 | 69.2% | 5 | R.LISQIVSSITASLR.F | 2 |
|  | Astrin\_NLD\_STLC\_tube2\_021014\_01.11012.11012.3 | 2.9963 | 0.228 | 96.4% | 1757.8444 | 1758.0703 | 35 | 4.452 | 36.7% | 1 | R.IHFPLATYAPVISAEK.A | 3 |
|  | Astrin\_NLD\_STLC\_tube2\_021014\_01.10952.10952.2 | 3.8958 | 0.5172 | 100.0% | 1758.4722 | 1758.0703 | 1 | 8.476 | 66.7% | 5 | R.IHFPLATYAPVISAEK.A | 2 |
|  | Astrin\_NLD\_STLC\_031014\_01.09542.09542.1 | 1.7555 | 0.3013 | 100.0% | 1015.51 | 1016.1827 | 1 | 5.348 | 66.7% | 1 | K.DVNAAIATIK.T | 1 |
|  | Astrin\_NLD\_STLC\_tube2\_021014\_01.07138.07138.2 | 2.9637 | 0.3261 | 100.0% | 1016.3522 | 1016.1827 | 1 | 7.319 | 88.9% | 2 | K.DVNAAIATIK.T | 2 |
|  | Astrin\_NLD\_STLC\_tube2\_021014\_01.09214.09214.2 | 3.8868 | 0.4587 | 100.0% | 1826.4722 | 1826.1027 | 1 | 7.355 | 64.7% | 3 | K.VGINYQPPTVVPGGDLAK.V | 2 |
|  | Astrin\_NLD\_STLC\_tube2\_021014\_01.10917.10917.3 | 3.8725 | 0.3218 | 100.0% | 2331.8342 | 2331.5208 | 1 | 5.894 | 35.5% | 1 | R.AFVHWYVGEGMEEGEFSEAR.E | 3 |

---

|  |  |  |  |  |  |  |  |  |
| --- | --- | --- | --- | --- | --- | --- | --- | --- |
| U | *gi|4826998|ref|NP\_005* | 20 | 46 | 30.7% | 707 | 76150 | 9.4 | splicing factor proline/glutamine rich (polypyrimidine tract binding protein associated) [Homo sapiens] |

| Filename XCorr DeltCN Conf% ObsM+H+ CalcM+H+ SpR ZScore Ion% # Sequence  | | | | | | | | | | | | |
| --- | --- | --- | --- | --- | --- | --- | --- | --- | --- | --- | --- | --- |
| \* | Astrin\_NLD\_STLC\_031014\_01.08885.08885.3 | 4.5679 | 0.3873 | 100.0% | 2371.2844 | 2371.725 | 1 | 6.95 | 34.4% | 1 | K.MPGGPKPGGGPGLSTPGGHPKPPHR.G | 3 |
| \* | Astrin\_NLD\_STLC\_tube2\_021014\_02.06792.06792.3 | 3.6043 | 0.3208 | 100.0% | 2124.2944 | 2124.3555 | 1 | 5.681 | 38.9% | 1 | R.SEEKISDSEGFKANLSLLR.R | 3 |
| \* | Astrin\_NLD\_STLC\_tube2\_021014\_01.12428.12428.2 | 2.7875 | 0.349 | 100.0% | 1808.0122 | 1809.0258 | 14 | 6.048 | 43.3% | 2 | R.LFVGNLPADITEDEFK.R | 2 |
| \* | Astrin\_NLD\_STLC\_tube2\_021014\_01.07648.07648.2 | 3.5824 | 0.4683 | 100.0% | 1254.2122 | 1253.3971 | 5 | 7.353 | 60.0% | 3 | K.YGEPGEVFINK.G | 2 |
| \* | Astrin\_NLD\_STLC\_tube2\_021014\_01.09422.09422.3 | 3.4497 | 0.3101 | 100.0% | 1744.5844 | 1745.0007 | 2 | 5.824 | 43.3% | 1 | R.ALAEIAKAELDDTPMR.G | 3 |
| \* | Astrin\_NLD\_STLC\_tube2\_021014\_01.04878.04878.1 | 1.5919 | 0.2516 | 97.1% | 1047.52 | 1048.1559 | 46 | 3.923 | 43.8% | 1 | K.AELDDTPMR.G | 1 |
| \* | Astrin\_NLD\_STLC\_031014\_01.09147.09147.1 | 2.2164 | 0.4345 | 100.0% | 1143.61 | 1144.3188 | 1 | 6.516 | 60.0% | 2 | R.FATHAAALSVR.N | 1 |
| \* | Astrin\_NLD\_STLC\_tube2\_021014\_01.04868.04868.2 | 3.1891 | 0.421 | 100.0% | 1144.2322 | 1144.3188 | 1 | 7.927 | 90.0% | 2 | R.FATHAAALSVR.N | 2 |
| \* | Astrin\_NLD\_STLC\_tube2\_021014\_01.15780.15780.2 | 4.9141 | 0.5291 | 100.0% | 2640.652 | 2640.9092 | 1 | 9.133 | 47.7% | 3 | R.NLSPYVSNELLEEAFSQFGPIER.A | 2 |
|  | Astrin\_NLD\_STLC\_tube2\_021014\_01.04656.04656.1 | 2.1922 | 0.3804 | 100.0% | 886.44 | 887.0238 | 23 | 6.739 | 64.3% | 2 | R.AVVIVDDR.G | 11 |
|  | Astrin\_NLD\_STLC\_tube2\_021014\_01.04706.04706.2 | 2.3101 | 0.2583 | 99.3% | 886.47217 | 887.0238 | 1 | 6.189 | 78.6% | 1 | R.AVVIVDDR.G | 22 |
| \* | Astrin\_NLD\_STLC\_031014\_01.09130.09130.3 | 2.8129 | 0.3365 | 100.0% | 1619.1543 | 1619.8613 | 1 | 5.606 | 38.3% | 1 | R.STGKGIVEFASKPAAR.K | 3 |
| \* | Astrin\_NLD\_STLC\_031014\_01.09250.09250.2 | 2.8399 | 0.3557 | 100.0% | 1246.1322 | 1246.452 | 1 | 6.958 | 72.7% | 2 | K.GIVEFASKPAAR.K | 2 |
| \* | Astrin\_NLD\_STLC\_031014\_02.05315.05315.3 | 4.6466 | 0.3561 | 100.0% | 1762.1044 | 1763.8632 | 2 | 6.454 | 42.3% | 9 | R.FAQHGTFEYEYSQR.W | 3 |
| \* | Astrin\_NLD\_STLC\_tube2\_021014\_02.05219.05219.2 | 4.6092 | 0.4974 | 100.0% | 1762.5721 | 1763.8632 | 1 | 8.028 | 69.2% | 2 | R.FAQHGTFEYEYSQR.W | 2 |
| \* | Astrin\_NLD\_STLC\_031014\_01.09880.09880.3 | 5.9824 | 0.4465 | 100.0% | 2429.4844 | 2429.6233 | 1 | 8.472 | 42.1% | 3 | K.DKLESEMEDAYHEHQANLLR.Q | 3 |
| \* | Astrin\_NLD\_STLC\_031014\_01.08504.08504.2 | 3.9628 | 0.352 | 100.0% | 1417.2522 | 1417.5946 | 1 | 6.181 | 85.0% | 3 | R.MEELHNQEMQK.R | 2 |
| \* | Astrin\_NLD\_STLC\_031014\_01.08526.08526.3 | 2.598 | 0.2336 | 95.4% | 1418.1843 | 1417.5946 | 7 | 4.588 | 47.5% | 1 | R.MEELHNQEMQK.R | 3 |
| \* | Astrin\_NLD\_STLC\_tube2\_021014\_01.04425.04425.2 | 4.0713 | 0.5897 | 100.0% | 1342.0922 | 1342.4569 | 1 | 9.39 | 78.6% | 5 | R.FGQGGAGPVGGQGPR.G | 2 |
| \* | Astrin\_NLD\_STLC\_tube2\_021014\_01.04508.04508.2 | 2.9289 | 0.3445 | 100.0% | 1120.8322 | 1121.2561 | 10 | 7.923 | 63.6% | 1 | R.GMGPGTPAGYGR.G | 2 |

Similarities:
gi|224028244|ref|NP\_0(2:18)  

---

|  |  |  |  |  |  |  |  |  |
| --- | --- | --- | --- | --- | --- | --- | --- | --- |
| U | *gi|57242777|ref|NP\_03* | 2 | 2 | 30.1% | 103 | 11967 | 5.9 | c-myc binding protein [Homo sapiens] |

| Filename XCorr DeltCN Conf% ObsM+H+ CalcM+H+ SpR ZScore Ion% # Sequence  | | | | | | | | | | | | |
| --- | --- | --- | --- | --- | --- | --- | --- | --- | --- | --- | --- | --- |
| \* | Astrin\_NLD\_STLC\_tube2\_021014\_01.12086.12086.3 | 3.5664 | 0.2374 | 99.1% | 2276.0942 | 2276.6348 | 1 | 5.25 | 28.9% | 1 | K.VLVALYEEPEKPNSALDFLK.H | 3 |
| \* | Astrin\_NLD\_STLC\_tube2\_021014\_01.04456.04456.2 | 2.8822 | 0.2778 | 100.0% | 1332.2722 | 1332.4528 | 6 | 4.993 | 60.0% | 1 | K.LAQYEPPQEEK.R | 2 |

---

|  |  |  |  |  |  |  |  |  |
| --- | --- | --- | --- | --- | --- | --- | --- | --- |
| U | *gi|12667788|ref|NP\_00* | 48 | 90 | 29.1% | 1960 | 226530 | 5.6 | myosin, heavy polypeptide 9, non-muscle [Homo sapiens] |

| Filename XCorr DeltCN Conf% ObsM+H+ CalcM+H+ SpR ZScore Ion% # Sequence  | | | | | | | | | | | | |
| --- | --- | --- | --- | --- | --- | --- | --- | --- | --- | --- | --- | --- |
| \* | Astrin\_NLD\_STLC\_tube2\_021014\_01.11429.11429.2 | 3.5043 | 0.3919 | 100.0% | 1672.4321 | 1673.8687 | 1 | 7.691 | 67.9% | 1 | K.NFINNPLAQADWAAK.K | 2 |
| \* | Astrin\_NLD\_STLC\_tube2\_021014\_01.06706.06706.2 | 2.2909 | 0.2504 | 98.7% | 1072.1921 | 1072.2926 | 55 | 4.482 | 50.0% | 1 | K.KLVWVPSDK.S | 2 |
| \* | Astrin\_NLD\_STLC\_tube2\_021014\_01.07286.07286.3 | 3.5884 | 0.3387 | 100.0% | 1916.5743 | 1916.1614 | 7 | 5.592 | 35.0% | 5 | R.HEMPPHIYAITDTAYR.S | 3 |
| \* | Astrin\_NLD\_STLC\_tube2\_021014\_01.08230.08230.3 | 3.0737 | 0.3262 | 100.0% | 1609.5844 | 1607.8931 | 1 | 5.092 | 44.2% | 1 | K.KVIQYLAYVASSHK.S | 3 |
| \* | Astrin\_NLD\_STLC\_031014\_02.06789.06789.2 | 2.837 | 0.2962 | 99.9% | 1479.7322 | 1479.719 | 1 | 6.146 | 66.7% | 1 | K.VIQYLAYVASSHK.S | 2 |
| \* | Astrin\_NLD\_STLC\_tube2\_021014\_02.06765.06765.3 | 2.2277 | 0.3176 | 97.5% | 1480.1344 | 1479.719 | 2 | 4.998 | 37.5% | 1 | K.VIQYLAYVASSHK.S | 3 |
| \* | Astrin\_NLD\_STLC\_tube2\_021014\_01.13605.13605.3 | 4.5795 | 0.4242 | 100.0% | 1996.2544 | 1997.3037 | 1 | 7.41 | 50.0% | 1 | R.TFHIFYYLLSGAGEHLK.T | 3 |
| \* | Astrin\_NLD\_STLC\_tube2\_021014\_01.11272.11272.2 | 3.3069 | 0.2913 | 100.0% | 1617.2322 | 1616.9313 | 1 | 6.086 | 76.9% | 3 | R.IMGIPEEEQMGLLR.V | 2 |
| \* | Astrin\_NLD\_STLC\_tube2\_021014\_01.12222.12222.2 | 3.3439 | 0.2477 | 99.9% | 1616.4321 | 1616.0 | 1 | 4.688 | 64.3% | 1 | R.VISGVLQLGNIVFKK.E | 2 |
|  | Astrin\_NLD\_STLC\_031014\_01.05930.05930.2 | 5.1525 | 0.4536 | 100.0% | 1593.3322 | 1592.6776 | 1 | 7.792 | 75.0% | 18 | R.NTDQASMPDNTAAQK.V | 2 |
| \* | Astrin\_NLD\_STLC\_tube2\_021014\_01.10649.10649.3 | 3.4825 | 0.2979 | 100.0% | 1572.3844 | 1572.8044 | 1 | 5.311 | 53.8% | 1 | K.VSHLLGINVTDFTR.G | 3 |
| \* | Astrin\_NLD\_STLC\_tube2\_021014\_01.10668.10668.2 | 4.2189 | 0.4152 | 100.0% | 1573.2522 | 1572.8044 | 1 | 7.856 | 76.9% | 1 | K.VSHLLGINVTDFTR.G | 2 |
| \* | Astrin\_NLD\_STLC\_tube2\_021014\_01.13282.13282.2 | 4.4183 | 0.4114 | 100.0% | 2019.3722 | 2019.3636 | 1 | 8.374 | 50.0% | 1 | R.IIGLDQVAGMSETALPGAFK.T | 2 |
| \* | Astrin\_NLD\_STLC\_tube2\_021014\_01.08075.08075.2 | 2.1691 | 0.3019 | 99.8% | 925.1922 | 925.07513 | 1 | 5.593 | 75.0% | 2 | R.VVFQEFR.Q | 2 |
| \* | Astrin\_NLD\_STLC\_tube2\_021014\_01.08614.08614.2 | 3.142 | 0.4161 | 100.0% | 1194.1322 | 1194.33 | 1 | 7.445 | 72.2% | 1 | K.ALELDSNLYR.I | 2 |
|  | Astrin\_NLD\_STLC\_tube2\_021014\_01.06784.06784.2 | 2.8625 | 0.3603 | 100.0% | 1223.3322 | 1224.3591 | 1 | 6.86 | 70.0% | 1 | R.AGVLAHLEEER.D | 22 |
| \* | Astrin\_NLD\_STLC\_tube2\_021014\_02.06219.06219.2 | 4.7394 | 0.4748 | 100.0% | 1654.0322 | 1654.7681 | 1 | 7.995 | 76.9% | 2 | R.IAEFTTNLTEEEEK.S | 2 |
| \* | Astrin\_NLD\_STLC\_tube2\_021014\_01.06574.06574.2 | 2.2756 | 0.3594 | 99.8% | 1343.7922 | 1344.4822 | 2 | 6.258 | 65.0% | 1 | K.HEAMITDLEER.L | 2 |
|  | Astrin\_NLD\_STLC\_tube2\_021014\_01.05199.05199.2 | 2.9172 | 0.2546 | 99.8% | 1258.3522 | 1258.4172 | 2 | 4.643 | 80.0% | 1 | K.KEEELQAALAR.V | 2 |
| \* | Astrin\_NLD\_STLC\_031014\_01.09438.09438.3 | 3.3802 | 0.2189 | 97.8% | 2043.9844 | 2044.2439 | 2 | 5.252 | 35.9% | 1 | K.TLEEEAKTHEAQIQEMR.Q | 3 |
| \* | Astrin\_NLD\_STLC\_tube2\_021014\_01.08684.08684.3 | 4.7033 | 0.4805 | 100.0% | 1996.5844 | 1997.1722 | 1 | 7.545 | 37.5% | 2 | K.HSQAVEELAEQLEQTKR.V | 3 |
| \* | Astrin\_NLD\_STLC\_031014\_01.09119.09119.3 | 3.39 | 0.2143 | 97.7% | 1930.5243 | 1930.1252 | 1 | 4.324 | 42.2% | 1 | K.AKQTLENERGELANEVK.V | 3 |
| \* | Astrin\_NLD\_STLC\_tube2\_021014\_01.06154.06154.2 | 3.853 | 0.3698 | 100.0% | 1413.4321 | 1413.6573 | 1 | 6.741 | 77.3% | 1 | K.KVEAQLQELQVK.F | 2 |
| \* | Astrin\_NLD\_STLC\_031014\_02.08595.08595.2 | 3.4089 | 0.3875 | 100.0% | 1947.9122 | 1947.1498 | 2 | 5.821 | 47.1% | 1 | K.LQVELDNVTGLLSQSDSK.S | 2 |
| \* | Astrin\_NLD\_STLC\_031014\_01.08837.08837.2 | 3.0217 | 0.2137 | 99.7% | 1492.3722 | 1493.6598 | 1 | 4.968 | 72.7% | 1 | K.LKQVEDEKNSFR.E | 2 |
| \* | Astrin\_NLD\_STLC\_tube2\_021014\_01.12648.12648.3 | 3.4309 | 0.2902 | 100.0% | 1951.4944 | 1951.1436 | 2 | 5.415 | 43.3% | 1 | R.LQQELDDLLVDLDHQR.Q | 3 |
|  | Astrin\_NLD\_STLC\_tube2\_021014\_01.06402.06402.2 | 2.798 | 0.1367 | 97.1% | 1221.2922 | 1221.3959 | 6 | 4.448 | 66.7% | 1 | K.KFDQLLAEEK.T | 22 |
|  | Astrin\_NLD\_STLC\_tube2\_021014\_01.06868.06868.2 | 3.1864 | 0.2186 | 100.0% | 1093.1122 | 1093.2218 | 1 | 5.928 | 81.2% | 1 | K.FDQLLAEEK.T | 22 |
| \* | Astrin\_NLD\_STLC\_031014\_02.05462.05462.3 | 3.5643 | 0.365 | 100.0% | 1647.7444 | 1647.8407 | 1 | 6.363 | 55.8% | 3 | R.ALEEAMEQKAELER.L | 3 |
| \* | Astrin\_NLD\_STLC\_tube2\_021014\_02.05332.05332.2 | 3.6914 | 0.3268 | 100.0% | 1647.7522 | 1647.8407 | 1 | 6.509 | 61.5% | 3 | R.ALEEAMEQKAELER.L | 2 |
| \* | Astrin\_NLD\_STLC\_tube2\_021014\_01.07383.07383.2 | 2.3517 | 0.266 | 98.9% | 1170.7722 | 1171.3229 | 18 | 6.377 | 61.1% | 1 | R.TEMEDLMSSK.D | 2 |
| \* | Astrin\_NLD\_STLC\_tube2\_021014\_01.06195.06195.2 | 2.9748 | 0.4138 | 100.0% | 1204.2522 | 1205.3685 | 3 | 7.746 | 66.7% | 2 | R.ALEQQVEEMK.T | 2 |
| \* | Astrin\_NLD\_STLC\_tube2\_021014\_01.15297.15297.3 | 4.6822 | 0.407 | 100.0% | 3148.1343 | 3149.4048 | 2 | 7.685 | 26.0% | 1 | R.ALEQQVEEMKTQLEELEDELQATEDAK.L | 3 |
| \* | Astrin\_NLD\_STLC\_tube2\_021014\_02.06834.06834.2 | 2.4423 | 0.2864 | 99.7% | 1313.9321 | 1315.6171 | 3 | 5.836 | 60.0% | 1 | K.LRLEVNLQAMK.A | 2 |
| \* | Astrin\_NLD\_STLC\_tube2\_021014\_01.05244.05244.2 | 2.2652 | 0.2371 | 97.5% | 1252.0521 | 1251.3075 | 2 | 4.493 | 72.2% | 1 | R.EMEAELEDER.K | 2 |
| \* | Astrin\_NLD\_STLC\_tube2\_021014\_01.04332.04332.2 | 3.4196 | 0.4899 | 100.0% | 1212.3121 | 1213.2896 | 1 | 9.3 | 75.0% | 1 | K.DLEAHIDSANK.N | 2 |
| \* | Astrin\_NLD\_STLC\_tube2\_021014\_01.13919.13919.2 | 4.1472 | 0.4239 | 100.0% | 2049.0923 | 2050.3064 | 1 | 7.744 | 50.0% | 1 | K.SMEAEMIQLQEELAAAER.A | 2 |
| \* | Astrin\_NLD\_STLC\_tube2\_021014\_01.10475.10475.3 | 4.6469 | 0.3625 | 100.0% | 2473.4043 | 2473.6099 | 1 | 7.371 | 38.8% | 3 | R.IAQLEEELEEEQGNTELINDR.L | 3 |
| \* | Astrin\_NLD\_STLC\_tube2\_021014\_01.09255.09255.3 | 3.4457 | 0.2572 | 99.6% | 1999.1643 | 1999.2314 | 3 | 4.868 | 34.4% | 1 | K.KANLQIDQINTDLNLER.S | 3 |
| \* | Astrin\_NLD\_STLC\_tube2\_021014\_01.10498.10498.2 | 5.1494 | 0.5643 | 100.0% | 1870.2722 | 1871.0574 | 1 | 9.716 | 73.3% | 1 | K.ANLQIDQINTDLNLER.S | 2 |
| \* | Astrin\_NLD\_STLC\_tube2\_021014\_01.05201.05201.2 | 2.4213 | 0.3631 | 100.0% | 904.0722 | 904.05133 | 1 | 6.72 | 68.8% | 1 | K.ASITALEAK.I | 2 |
| \* | Astrin\_NLD\_STLC\_031014\_01.09454.09454.3 | 2.7295 | 0.3286 | 99.8% | 1816.6743 | 1816.9628 | 210 | 5.248 | 30.4% | 1 | K.IAQLEEQLDNETKER.Q | 3 |
| \* | Astrin\_NLD\_STLC\_031014\_01.05478.05478.2 | 3.0491 | 0.3724 | 100.0% | 1725.1721 | 1725.8125 | 7 | 7.401 | 46.4% | 1 | R.NAEQYKDQADKASTR.L | 2 |
| \* | Astrin\_NLD\_STLC\_031014\_01.05498.05498.3 | 3.9237 | 0.4273 | 100.0% | 1726.1044 | 1725.8125 | 8 | 7.033 | 39.3% | 8 | R.NAEQYKDQADKASTR.L | 3 |
| \* | Astrin\_NLD\_STLC\_031014\_01.08807.08807.2 | 4.3254 | 0.304 | 100.0% | 1488.5521 | 1488.5541 | 1 | 6.775 | 77.3% | 1 | K.RQLEEAEEEAQR.A | 2 |
| \* | Astrin\_NLD\_STLC\_tube2\_021014\_01.05723.05723.2 | 4.5793 | 0.5497 | 100.0% | 1566.1122 | 1566.6367 | 1 | 9.208 | 65.4% | 1 | R.ELEDATETADAMNR.E | 2 |
| \* | Astrin\_NLD\_STLC\_tube2\_021014\_01.08360.08360.2 | 3.6135 | 0.2217 | 100.0% | 1156.1122 | 1156.3732 | 1 | 5.673 | 88.9% | 3 | R.RGDLPFVVPR.R | 2 |
| \* | Astrin\_NLD\_STLC\_031014\_01.08696.08696.3 | 4.3239 | 0.3019 | 100.0% | 2384.1543 | 2384.3428 | 4 | 5.867 | 29.3% | 1 | R.KGAGDGS\*DEEVDGKADGAEAKPAE.- | 3 |

Similarities:
gi|41406064|ref|NP\_00(3:45)  

---

|  |  |  |  |  |  |  |  |  |
| --- | --- | --- | --- | --- | --- | --- | --- | --- |
| U | *gi|150456457|ref|NP\_9* | 9 | 27 | 28.8% | 347 | 39929 | 5.6 | HMT1 hnRNP methyltransferase-like 2 isoform 2 [Homo sapiens] |
| U | *gi|154759421|ref|NP\_0* | 9 | 27 | 27.0% | 371 | 42462 | 5.3 | HMT1 hnRNP methyltransferase-like 2 isoform 1 [Homo sapiens] |
| U | *gi|151301219|ref|NP\_9* | 9 | 27 | 28.3% | 353 | 40548 | 5.5 | HMT1 hnRNP methyltransferase-like 2 isoform 3 [Homo sapiens] |

| Filename XCorr DeltCN Conf% ObsM+H+ CalcM+H+ SpR ZScore Ion% # Sequence  | | | | | | | | | | | | |
| --- | --- | --- | --- | --- | --- | --- | --- | --- | --- | --- | --- | --- |
|  | Astrin\_NLD\_STLC\_tube2\_021014\_01.05938.05938.2 | 3.2647 | 0.2518 | 100.0% | 1351.3722 | 1351.6322 | 1 | 5.723 | 68.2% | 1 | K.ANKLDHVVTIIK.G | 2 |
|  | Astrin\_NLD\_STLC\_tube2\_021014\_01.05922.05922.3 | 3.7427 | 0.4317 | 100.0% | 1352.2743 | 1351.6322 | 1 | 7.064 | 54.5% | 1 | K.ANKLDHVVTIIK.G | 3 |
|  | Astrin\_NLD\_STLC\_031014\_01.10752.10752.2 | 3.6312 | 0.2703 | 100.0% | 1644.5721 | 1643.8827 | 1 | 5.987 | 65.4% | 3 | R.DKWLAPDGLIFPDR.A | 2 |
|  | Astrin\_NLD\_STLC\_tube2\_021014\_02.06548.06548.2 | 3.6804 | 0.4664 | 100.0% | 1253.2522 | 1252.4099 | 1 | 8.195 | 80.0% | 13 | R.ATLYVTAIEDR.Q | 2 |
|  | Astrin\_NLD\_STLC\_tube2\_021014\_01.09382.09382.2 | 3.9554 | 0.36 | 100.0% | 1637.4122 | 1637.914 | 1 | 7.031 | 71.4% | 1 | K.DVAIKEPLVDVVDPK.Q | 2 |
|  | Astrin\_NLD\_STLC\_031014\_02.11583.11583.2 | 2.4887 | 0.2419 | 97.5% | 2073.2322 | 2073.3152 | 4 | 4.751 | 37.5% | 1 | R.NDYVHALVAYFNIEFTR.C | 2 |
|  | Astrin\_NLD\_STLC\_tube2\_021014\_01.07419.07419.2 | 3.4134 | 0.4759 | 100.0% | 1725.2122 | 1725.8547 | 1 | 7.035 | 57.1% | 2 | R.TGFSTSPESPYTHWK.Q | 2 |
|  | Astrin\_NLD\_STLC\_tube2\_021014\_01.08804.08804.2 | 3.8306 | 0.4468 | 100.0% | 1721.2722 | 1721.969 | 1 | 7.239 | 60.0% | 1 | K.TGEEIFGTIGMRPNAK.N | 2 |
|  | Astrin\_NLD\_STLC\_031014\_02.06770.06770.3 | 3.2131 | 0.347 | 100.0% | 1721.9944 | 1721.969 | 7 | 5.65 | 33.3% | 4 | K.TGEEIFGTIGMRPNAK.N | 3 |

---

|  |  |  |  |  |  |  |  |  |
| --- | --- | --- | --- | --- | --- | --- | --- | --- |
| U | *gi|62414289|ref|NP\_00* | 10 | 10 | 25.8% | 466 | 53652 | 5.1 | vimentin [Homo sapiens] |

| Filename XCorr DeltCN Conf% ObsM+H+ CalcM+H+ SpR ZScore Ion% # Sequence  | | | | | | | | | | | | |
| --- | --- | --- | --- | --- | --- | --- | --- | --- | --- | --- | --- | --- |
| \* | Astrin\_NLD\_STLC\_tube2\_021014\_01.07073.07073.2 | 3.2047 | 0.3857 | 100.0% | 1508.9922 | 1509.5724 | 1 | 6.12 | 61.5% | 1 | R.SLYASS\*PGGVYATR.S | 2 |
| \* | Astrin\_NLD\_STLC\_tube2\_021014\_01.11856.11856.2 | 2.6046 | 0.3192 | 100.0% | 1170.1721 | 1170.4349 | 5 | 6.205 | 66.7% | 1 | K.ILLAELEQLK.G | 2 |
| \* | Astrin\_NLD\_STLC\_031014\_01.10211.10211.2 | 2.9287 | 0.303 | 100.0% | 1541.0721 | 1540.8436 | 26 | 5.308 | 42.3% | 1 | K.ILLAELEQLKGQGK.S | 2 |
| \* | Astrin\_NLD\_STLC\_tube2\_021014\_01.06840.06840.2 | 2.2394 | 0.2794 | 98.4% | 1323.7922 | 1324.3898 | 56 | 5.875 | 50.0% | 1 | R.EEAENTLQSFR.Q | 2 |
| \* | Astrin\_NLD\_STLC\_tube2\_021014\_01.10712.10712.2 | 3.0937 | 0.3139 | 100.0% | 1533.8522 | 1534.793 | 12 | 5.598 | 54.2% | 1 | R.KVESLQEEIAFLK.K | 2 |
|  | Astrin\_NLD\_STLC\_tube2\_021014\_01.08318.08318.2 | 2.9398 | 0.284 | 100.0% | 1310.2522 | 1310.4056 | 2 | 4.79 | 72.2% | 1 | K.NLQEAEEWYK.S | 2 |
| \* | Astrin\_NLD\_STLC\_tube2\_021014\_01.06321.06321.3 | 2.7323 | 0.2722 | 97.5% | 1777.5243 | 1777.8912 | 4 | 5.449 | 35.0% | 1 | K.FADLSEAANRNNDALR.Q | 3 |
| \* | Astrin\_NLD\_STLC\_tube2\_021014\_01.07614.07614.2 | 3.9209 | 0.4337 | 100.0% | 1735.0922 | 1735.9679 | 1 | 7.419 | 69.2% | 1 | R.LQDEIQNMKEEMAR.H | 2 |
|  | Astrin\_NLD\_STLC\_tube2\_021014\_01.07344.07344.3 | 3.3255 | 0.304 | 100.0% | 1528.9744 | 1528.7513 | 1 | 5.551 | 52.3% | 1 | R.HLREYQDLLNVK.M | 3 |
| \* | Astrin\_NLD\_STLC\_tube2\_021014\_01.04668.04668.2 | 3.5498 | 0.3457 | 100.0% | 1837.2722 | 1837.854 | 2 | 5.804 | 50.0% | 1 | R.DGQVINETSQHHDDLE.- | 2 |

---

|  |  |  |  |  |  |  |  |  |
| --- | --- | --- | --- | --- | --- | --- | --- | --- |
| U | *gi|224028244|ref|NP\_0* | 14 | 32 | 25.7% | 471 | 54232 | 8.9 | non-POU domain containing, octamer-binding isoform 1 [Homo sapiens] |
| U | *gi|34932414|ref|NP\_03* | 14 | 32 | 25.7% | 471 | 54232 | 8.9 | non-POU domain containing, octamer-binding isoform 1 [Homo sapiens] |
| U | *gi|224028246|ref|NP\_0* | 14 | 32 | 25.7% | 471 | 54232 | 8.9 | non-POU domain containing, octamer-binding isoform 1 [Homo sapiens] |

| Filename XCorr DeltCN Conf% ObsM+H+ CalcM+H+ SpR ZScore Ion% # Sequence  | | | | | | | | | | | | |
| --- | --- | --- | --- | --- | --- | --- | --- | --- | --- | --- | --- | --- |
|  | Astrin\_NLD\_STLC\_tube2\_021014\_01.11350.11350.2 | 3.081 | 0.4207 | 100.0% | 1860.4922 | 1861.12 | 12 | 6.387 | 53.3% | 2 | R.LFVGNLPPDITEEEMR.K | 2 |
|  | Astrin\_NLD\_STLC\_tube2\_021014\_01.12233.12233.2 | 4.5915 | 0.5215 | 100.0% | 1813.1921 | 1814.1504 | 1 | 8.9 | 66.7% | 1 | R.TLAEIAKVELDNMPLR.G | 2 |
|  | Astrin\_NLD\_STLC\_tube2\_021014\_01.12216.12216.3 | 3.5748 | 0.3564 | 100.0% | 1814.3944 | 1814.1504 | 1 | 6.096 | 46.7% | 1 | R.TLAEIAKVELDNMPLR.G | 3 |
|  | Astrin\_NLD\_STLC\_tube2\_021014\_01.08000.08000.2 | 2.8741 | 0.2766 | 100.0% | 1087.1122 | 1087.2793 | 6 | 5.233 | 81.2% | 1 | K.VELDNMPLR.G | 2 |
|  | Astrin\_NLD\_STLC\_031014\_01.15010.15010.2 | 5.5932 | 0.5177 | 100.0% | 2668.7922 | 2669.9507 | 1 | 10.852 | 54.5% | 3 | R.NLPQYVSNELLEEAFSVFGQVER.A | 2 |
|  | Astrin\_NLD\_STLC\_tube2\_021014\_01.18632.18632.3 | 4.6495 | 0.4788 | 100.0% | 2669.6943 | 2669.9507 | 1 | 7.847 | 38.6% | 2 | R.NLPQYVSNELLEEAFSVFGQVER.A | 3 |
|  | Astrin\_NLD\_STLC\_tube2\_021014\_01.04656.04656.1 | 2.1922 | 0.3804 | 100.0% | 886.44 | 887.0238 | 23 | 6.739 | 64.3% | 2 | R.AVVIVDDR.G | 11 |
|  | Astrin\_NLD\_STLC\_tube2\_021014\_01.04706.04706.2 | 2.3101 | 0.2583 | 99.3% | 886.47217 | 887.0238 | 1 | 6.189 | 78.6% | 1 | R.AVVIVDDR.G | 22 |
|  | Astrin\_NLD\_STLC\_tube2\_021014\_01.04250.04250.3 | 3.0065 | 0.2286 | 95.4% | 1816.6743 | 1815.0854 | 40 | 4.659 | 29.4% | 1 | R.GRPSGKGIVEFSGKPAAR.K | 3 |
|  | Astrin\_NLD\_STLC\_tube2\_021014\_01.05096.05096.2 | 2.9676 | 0.3669 | 100.0% | 1232.0922 | 1232.4252 | 1 | 6.923 | 77.3% | 2 | K.GIVEFSGKPAAR.K | 2 |
|  | Astrin\_NLD\_STLC\_tube2\_021014\_01.09597.09597.2 | 3.9549 | 0.5226 | 100.0% | 1696.6522 | 1696.8744 | 1 | 8.538 | 57.7% | 6 | R.FAQPGSFEYEYAMR.W | 2 |
|  | Astrin\_NLD\_STLC\_tube2\_021014\_01.05799.05799.2 | 2.929 | 0.4605 | 100.0% | 1336.8522 | 1337.5488 | 1 | 7.001 | 75.0% | 4 | R.EKLEMEMEAAR.H | 2 |
|  | Astrin\_NLD\_STLC\_031014\_02.05982.05982.2 | 4.3332 | 0.4879 | 100.0% | 1539.0721 | 1539.8441 | 1 | 9.541 | 67.9% | 5 | R.MGQMAMGGAMGINNR.G | 2 |
|  | Astrin\_NLD\_STLC\_tube2\_021014\_02.05948.05948.3 | 3.1536 | 0.3095 | 100.0% | 1539.5044 | 1539.8441 | 4 | 6.049 | 39.3% | 1 | R.MGQMAMGGAMGINNR.G | 3 |

Similarities:
gi|4826998|ref|NP\_005(2:12)  

---

|  |  |  |  |  |  |  |  |  |
| --- | --- | --- | --- | --- | --- | --- | --- | --- |
| U | *contaminant\_KERATIN03* | 14 | 19 | 24.8% | 593 | 59519 | 5.2 | no description |
| U | *gi|195972866|ref|NP\_0* | 14 | 19 | 25.2% | 584 | 58801 | 5.2 | keratin 10 [Homo sapiens] |

| Filename XCorr DeltCN Conf% ObsM+H+ CalcM+H+ SpR ZScore Ion% # Sequence  | | | | | | | | | | | | |
| --- | --- | --- | --- | --- | --- | --- | --- | --- | --- | --- | --- | --- |
|  | Astrin\_NLD\_STLC\_tube2\_021014\_02.06794.06794.2 | 5.1863 | 0.4837 | 100.0% | 1708.2722 | 1708.7844 | 1 | 8.498 | 63.9% | 1 | K.GSLGGGFSSGGFSGGSFSR.G | 2 |
|  | Astrin\_NLD\_STLC\_tube2\_021014\_01.04526.04526.2 | 2.8206 | 0.328 | 100.0% | 1090.9922 | 1091.2273 | 46 | 6.066 | 68.8% | 1 | K.VTMQNLNDR.L | 22 |
|  | Astrin\_NLD\_STLC\_tube2\_021014\_01.05645.05645.2 | 2.9461 | 0.1436 | 98.9% | 1065.2122 | 1065.2578 | 50 | 6.07 | 62.5% | 2 | R.LASYLDKVR.A | 2222 |
|  | Astrin\_NLD\_STLC\_tube2\_021014\_01.05768.05768.2 | 3.62 | 0.417 | 100.0% | 1382.0922 | 1382.4668 | 1 | 8.46 | 68.2% | 2 | R.ALEESNYELEGK.I | 2 |
|  | Astrin\_NLD\_STLC\_tube2\_021014\_01.15460.15460.3 | 3.9303 | 0.3028 | 100.0% | 3053.6343 | 3054.4277 | 1 | 5.56 | 27.9% | 1 | K.TIDDLKNQILNLTTDNANILLQIDNAR.L | 3 |
|  | Astrin\_NLD\_STLC\_tube2\_021014\_01.05060.05060.2 | 2.4574 | 0.2931 | 100.0% | 807.9122 | 807.8815 | 9 | 6.526 | 75.0% | 2 | R.LAADDFR.L | 22222 |
|  | Astrin\_NLD\_STLC\_tube2\_021014\_01.05398.05398.2 | 2.7893 | 0.4008 | 100.0% | 1234.6122 | 1235.4258 | 6 | 6.67 | 72.2% | 2 | R.LKYENEVALR.Q | 2 |
|  | Astrin\_NLD\_STLC\_tube2\_021014\_01.07449.07449.2 | 2.6343 | 0.2881 | 99.9% | 1188.2722 | 1188.4099 | 1 | 5.736 | 77.8% | 1 | R.RVLDELTLTK.A | 2 |
|  | Astrin\_NLD\_STLC\_tube2\_021014\_01.08194.08194.2 | 2.8458 | 0.4078 | 100.0% | 1031.6522 | 1032.2224 | 1 | 7.283 | 87.5% | 1 | R.VLDELTLTK.A | 2 |
|  | Astrin\_NLD\_STLC\_tube2\_021014\_01.04660.04660.2 | 3.9455 | 0.3756 | 100.0% | 1366.2122 | 1366.43 | 6 | 6.829 | 65.0% | 1 | R.SQYEQLAEQNR.K | 2 |
|  | Astrin\_NLD\_STLC\_tube2\_021014\_01.08760.08760.2 | 2.793 | 0.1416 | 98.0% | 1111.2322 | 1110.1681 | 2 | 5.579 | 75.0% | 1 | K.DAEAWFNEK.S | 2 |
|  | Astrin\_NLD\_STLC\_tube2\_021014\_02.06034.06034.2 | 3.2373 | 0.4325 | 100.0% | 1391.1721 | 1391.4778 | 1 | 7.261 | 75.0% | 2 | K.QSLEASLAETEGR.Y | 2 |
|  | Astrin\_NLD\_STLC\_tube2\_021014\_01.06789.06789.2 | 3.275 | 0.3521 | 100.0% | 1435.0521 | 1435.623 | 1 | 6.305 | 80.0% | 1 | K.IRLENEIQTYR.S | 2 |
|  | Astrin\_NLD\_STLC\_tube2\_021014\_01.05109.05109.2 | 3.3136 | 0.0973 | 99.0% | 1165.9521 | 1166.2761 | 1 | 5.98 | 87.5% | 1 | R.LENEIQTYR.S | 2 |

Similarities:
gi|40354195|ref|NP\_95(1:13)  
gi|4557701|ref|NP\_000(2:12)  
contaminant\_KERATIN05(3:11)  
gi|24234699|ref|NP\_00(2:12)  

---

|  |  |  |  |  |  |  |  |  |
| --- | --- | --- | --- | --- | --- | --- | --- | --- |
| U | *gi|14043070|ref|NP\_11* | 7 | 16 | 24.7% | 372 | 38747 | 9.1 | heterogeneous nuclear ribonucleoprotein A1 isoform b [Homo sapiens] |
| U | *gi|4504445|ref|NP\_002* | 7 | 16 | 28.8% | 320 | 34196 | 9.2 | heterogeneous nuclear ribonucleoprotein A1 isoform a [Homo sapiens] |

| Filename XCorr DeltCN Conf% ObsM+H+ CalcM+H+ SpR ZScore Ion% # Sequence  | | | | | | | | | | | | |
| --- | --- | --- | --- | --- | --- | --- | --- | --- | --- | --- | --- | --- |
|  | Astrin\_NLD\_STLC\_031014\_01.06768.06768.2 | 2.52 | 0.2405 | 98.7% | 1300.3121 | 1300.4111 | 3 | 4.823 | 65.0% | 4 | K.SESPKEPEQLR.K | 2 |
|  | Astrin\_NLD\_STLC\_031014\_02.08403.08403.2 | 2.2939 | 0.3284 | 99.1% | 1911.5721 | 1914.1656 | 28 | 5.577 | 40.6% | 1 | R.KLFIGGLSFETTDESLR.S | 2 |
|  | Astrin\_NLD\_STLC\_031014\_02.09387.09387.2 | 4.8253 | 0.472 | 100.0% | 1786.1721 | 1785.9916 | 1 | 8.321 | 63.3% | 1 | K.LFIGGLSFETTDESLR.S | 2 |
|  | Astrin\_NLD\_STLC\_tube2\_021014\_01.10892.10892.2 | 3.5806 | 0.4048 | 100.0% | 1219.3121 | 1219.4387 | 1 | 7.495 | 88.9% | 1 | K.IEVIEIMTDR.G | 2 |
|  | Astrin\_NLD\_STLC\_031014\_02.08145.08145.3 | 3.6816 | 0.231 | 99.3% | 2281.8542 | 2282.5579 | 1 | 5.976 | 34.2% | 1 | R.GFAFVTFDDHDSVDKIVIQK.Y | 3 |
|  | Astrin\_NLD\_STLC\_031014\_01.09200.09200.2 | 3.7252 | 0.4316 | 100.0% | 1629.2122 | 1629.7721 | 1 | 7.045 | 63.3% | 1 | R.SSGPYGGGGQYFAKPR.N | 2 |
|  | Astrin\_NLD\_STLC\_031014\_01.05216.05216.2 | 4.87 | 0.5279 | 100.0% | 1695.3322 | 1695.6561 | 1 | 10.254 | 64.7% | 7 | R.NQGGYGGSSSSSSYGSGR.R | 2 |

---

|  |  |  |  |  |  |  |  |  |
| --- | --- | --- | --- | --- | --- | --- | --- | --- |
| U | *gi|4506743|ref|NP\_001* | 5 | 6 | 24.0% | 208 | 24205 | 10.3 | ribosomal protein S8 [Homo sapiens] |

| Filename XCorr DeltCN Conf% ObsM+H+ CalcM+H+ SpR ZScore Ion% # Sequence  | | | | | | | | | | | | |
| --- | --- | --- | --- | --- | --- | --- | --- | --- | --- | --- | --- | --- |
| \* | Astrin\_NLD\_STLC\_031014\_01.08871.08871.2 | 2.6286 | 0.3809 | 100.0% | 1220.0122 | 1220.3707 | 1 | 6.0 | 75.0% | 1 | K.YELGRPAANTK.I | 2 |
| \* | Astrin\_NLD\_STLC\_031014\_02.07056.07056.2 | 3.2977 | 0.332 | 100.0% | 1719.4521 | 1719.9353 | 1 | 5.577 | 57.1% | 1 | R.IIDVVYNASNNELVR.T | 2 |
| \* | Astrin\_NLD\_STLC\_031014\_02.07062.07062.3 | 3.7983 | 0.3201 | 100.0% | 1720.2544 | 1719.9353 | 1 | 5.888 | 42.9% | 1 | R.IIDVVYNASNNELVR.T | 3 |
|  | Astrin\_NLD\_STLC\_tube2\_021014\_01.07287.07287.2 | 2.5702 | 0.3622 | 100.0% | 1315.4122 | 1315.4631 | 188 | 6.622 | 55.0% | 1 | K.LTPEEEEILNK.K | 2 |
| \* | Astrin\_NLD\_STLC\_tube2\_021014\_01.08871.08871.2 | 4.2275 | 0.4411 | 100.0% | 1507.6721 | 1507.6836 | 1 | 7.435 | 79.2% | 2 | K.ISSLLEEQFQQGK.L | 2 |

---

|  |  |  |  |  |  |  |  |  |
| --- | --- | --- | --- | --- | --- | --- | --- | --- |
| U | *gi|4504517|ref|NP\_001* | 3 | 3 | 23.9% | 205 | 22783 | 6.4 | heat shock protein beta-1 [Homo sapiens] |

| Filename XCorr DeltCN Conf% ObsM+H+ CalcM+H+ SpR ZScore Ion% # Sequence  | | | | | | | | | | | | |
| --- | --- | --- | --- | --- | --- | --- | --- | --- | --- | --- | --- | --- |
| \* | Astrin\_NLD\_STLC\_tube2\_021014\_01.11399.11399.3 | 2.8214 | 0.3026 | 99.6% | 1904.2743 | 1904.0537 | 1 | 5.196 | 44.6% | 1 | R.GPSWDPFRDWYPHSR.L | 3 |
| \* | Astrin\_NLD\_STLC\_tube2\_021014\_01.10420.10420.2 | 2.7287 | 0.2112 | 97.7% | 1907.7122 | 1907.1307 | 1 | 5.134 | 46.9% | 1 | K.LATQSNEITIPVTFESR.A | 2 |
| \* | Astrin\_NLD\_STLC\_031014\_01.07510.07510.2 | 3.3963 | 0.5073 | 100.0% | 1644.2122 | 1644.7789 | 1 | 9.311 | 62.5% | 1 | R.AQLGGPEAAKSDETAAK.- | 2 |

---

|  |  |  |  |  |  |  |  |  |
| --- | --- | --- | --- | --- | --- | --- | --- | --- |
| U | *gi|27436946|ref|NP\_73* | 14 | 19 | 23.8% | 664 | 74140 | 7.0 | lamin A/C isoform 1 precursor [Homo sapiens] |

| Filename XCorr DeltCN Conf% ObsM+H+ CalcM+H+ SpR ZScore Ion% # Sequence  | | | | | | | | | | | | |
| --- | --- | --- | --- | --- | --- | --- | --- | --- | --- | --- | --- | --- |
|  | Astrin\_NLD\_STLC\_031014\_01.08848.08848.2 | 3.6563 | 0.4903 | 100.0% | 1361.2922 | 1360.4667 | 1 | 8.105 | 57.7% | 1 | R.SGAQASSTPLSPTR.I | 2 |
|  | Astrin\_NLD\_STLC\_tube2\_021014\_02.04823.04823.3 | 3.74 | 0.2033 | 99.8% | 1418.3344 | 1418.5901 | 19 | 4.984 | 45.5% | 1 | R.LRITESEEVVSR.E | 3 |
|  | Astrin\_NLD\_STLC\_031014\_02.04925.04925.2 | 3.0639 | 0.3106 | 100.0% | 1418.5322 | 1418.5901 | 2 | 6.126 | 54.5% | 1 | R.LRITESEEVVSR.E | 2 |
|  | Astrin\_NLD\_STLC\_031014\_01.08956.08956.2 | 3.0164 | 0.4687 | 100.0% | 1149.2322 | 1149.2432 | 1 | 8.847 | 83.3% | 1 | R.ITESEEVVSR.E | 2 |
|  | Astrin\_NLD\_STLC\_tube2\_021014\_01.07485.07485.2 | 2.5636 | 0.3098 | 100.0% | 1182.9321 | 1183.3066 | 12 | 5.368 | 72.2% | 1 | R.TLEGELHDLR.G | 2 |
|  | Astrin\_NLD\_STLC\_tube2\_021014\_01.08177.08177.2 | 2.6256 | 0.213 | 99.2% | 1028.4122 | 1029.1814 | 1 | 5.408 | 87.5% | 1 | R.LADALQELR.A | 2 |
|  | Astrin\_NLD\_STLC\_tube2\_021014\_01.06210.06210.2 | 5.0424 | 0.5227 | 100.0% | 1753.1921 | 1753.8693 | 1 | 8.622 | 70.0% | 1 | R.NSNLVGAAHEELQQSR.I | 2 |
|  | Astrin\_NLD\_STLC\_tube2\_021014\_01.06072.06072.2 | 2.6119 | 0.1786 | 97.7% | 1189.0122 | 1188.3262 | 15 | 4.364 | 66.7% | 1 | K.LRDLEDSLAR.E | 2 |
|  | Astrin\_NLD\_STLC\_tube2\_021014\_01.11986.11986.2 | 2.7825 | 0.2646 | 99.5% | 1894.4321 | 1895.1346 | 1 | 4.845 | 57.1% | 1 | R.MQQQLDEYQELLDIK.L | 2 |
|  | Astrin\_NLD\_STLC\_031014\_01.09363.09363.2 | 3.8148 | 0.4265 | 100.0% | 1606.2922 | 1606.7728 | 1 | 7.303 | 57.7% | 2 | R.VAVEEVDEEGKFVR.L | 2 |
|  | Astrin\_NLD\_STLC\_031014\_02.05456.05456.3 | 2.3613 | 0.2792 | 95.4% | 1606.8243 | 1606.7728 | 5 | 5.328 | 38.5% | 3 | R.VAVEEVDEEGKFVR.L | 3 |
|  | Astrin\_NLD\_STLC\_tube2\_021014\_02.05775.05775.2 | 4.2382 | 0.5671 | 100.0% | 1491.9521 | 1492.6874 | 1 | 9.569 | 76.9% | 2 | R.TALINSTGEEVAMR.K | 2 |
|  | Astrin\_NLD\_STLC\_tube2\_021014\_02.04828.04828.3 | 4.6464 | 0.4856 | 100.0% | 2366.4243 | 2366.504 | 1 | 8.809 | 35.6% | 1 | K.ASASGSGAQVGGPISSGSSASSVTVTR.S | 3 |
|  | Astrin\_NLD\_STLC\_031014\_02.05752.05752.2 | 3.5127 | 0.4567 | 100.0% | 1567.1522 | 1567.6555 | 136 | 6.902 | 37.5% | 2 | R.SVGGSGGGSFGDNLVTR.S | 2 |

---

|  |  |  |  |  |  |  |  |  |
| --- | --- | --- | --- | --- | --- | --- | --- | --- |
| U | *gi|32189392|ref|NP\_00* | 3 | 3 | 23.7% | 198 | 21892 | 6.0 | peroxiredoxin 2 isoform a [Homo sapiens] |

| Filename XCorr DeltCN Conf% ObsM+H+ CalcM+H+ SpR ZScore Ion% # Sequence  | | | | | | | | | | | | |
| --- | --- | --- | --- | --- | --- | --- | --- | --- | --- | --- | --- | --- |
| \* | Astrin\_NLD\_STLC\_tube2\_021014\_01.13503.13503.2 | 2.9674 | 0.3434 | 100.0% | 1863.1122 | 1864.1954 | 3 | 5.844 | 38.2% | 1 | R.KEGGLGPLNIPLLADVTR.R | 2 |
| \* | Astrin\_NLD\_STLC\_tube2\_021014\_02.05937.05937.3 | 3.5663 | 0.3328 | 100.0% | 2086.2244 | 2086.309 | 1 | 5.677 | 33.8% | 1 | R.RLSEDYGVLKTDEGIAYR.G | 3 |
|  | Astrin\_NLD\_STLC\_tube2\_021014\_01.07878.07878.2 | 2.63 | 0.2523 | 99.6% | 1212.0721 | 1212.3915 | 37 | 5.263 | 70.0% | 1 | R.QITVNDLPVGR.S | 22 |

Similarities:
gi|32455264|ref|NP\_85(1:2)  

---

|  |  |  |  |  |  |  |  |  |
| --- | --- | --- | --- | --- | --- | --- | --- | --- |
| U | *gi|4501881|ref|NP\_001* | 9 | 44 | 23.6% | 377 | 42051 | 5.4 | actin, alpha 1, skeletal muscle [Homo sapiens] |
| U | *gi|4885049|ref|NP\_005* | 9 | 44 | 23.6% | 377 | 42019 | 5.4 | cardiac muscle alpha actin 1 proprotein [Homo sapiens] |

| Filename XCorr DeltCN Conf% ObsM+H+ CalcM+H+ SpR ZScore Ion% # Sequence  | | | | | | | | | | | | |
| --- | --- | --- | --- | --- | --- | --- | --- | --- | --- | --- | --- | --- |
|  | Astrin\_NLD\_STLC\_031014\_01.06884.06884.2 | 3.2662 | 0.4547 | 100.0% | 976.33215 | 977.02136 | 2 | 7.839 | 72.2% | 30 | K.AGFAGDDAPR.A | 22 |
|  | Astrin\_NLD\_STLC\_031014\_01.09627.09627.2 | 2.5872 | 0.2875 | 99.8% | 1199.2722 | 1199.4415 | 5 | 5.809 | 65.0% | 2 | R.AVFPSIVGRPR.H | 22 |
|  | Astrin\_NLD\_STLC\_tube2\_021014\_01.09419.09419.2 | 3.9624 | 0.389 | 100.0% | 1961.4521 | 1962.1841 | 1 | 7.013 | 63.3% | 1 | K.YPIEHGIITNWDDMEK.I | 2 |
|  | Astrin\_NLD\_STLC\_tube2\_021014\_01.09402.09402.3 | 4.3625 | 0.2297 | 100.0% | 1962.5944 | 1962.1841 | 52 | 4.981 | 40.0% | 1 | K.YPIEHGIITNWDDMEK.I | 3 |
|  | Astrin\_NLD\_STLC\_031014\_01.09447.09447.2 | 3.2367 | 0.43 | 100.0% | 1516.2122 | 1516.7019 | 1 | 6.454 | 80.0% | 2 | K.IWHHTFYNELR.V | 22 |
|  | Astrin\_NLD\_STLC\_tube2\_021014\_01.11475.11475.2 | 2.9524 | 0.2059 | 98.7% | 1624.5322 | 1624.8927 | 2 | 5.245 | 57.7% | 1 | R.LDLAGRDLTDYLMK.I | 222 |
|  | Astrin\_NLD\_STLC\_tube2\_021014\_01.10676.10676.2 | 4.8426 | 0.3035 | 100.0% | 1791.2922 | 1791.9554 | 1 | 8.531 | 83.3% | 3 | K.SYELPDGQVITIGNER.F | 222 |
|  | Astrin\_NLD\_STLC\_tube2\_021014\_01.06791.06791.1 | 2.5336 | 0.4765 | 100.0% | 1161.6 | 1162.3868 | 1 | 7.532 | 55.0% | 1 | K.EITALAPSTMK.I | 11 |
|  | Astrin\_NLD\_STLC\_tube2\_021014\_01.06812.06812.2 | 2.6074 | 0.3641 | 100.0% | 1162.0521 | 1162.3868 | 1 | 6.597 | 80.0% | 3 | K.EITALAPSTMK.I | 22 |

Similarities:
gi|4501885|ref|NP\_001(7:2)  
gi|63055057|ref|NP\_00(2:7)  

---

|  |  |  |  |  |  |  |  |  |
| --- | --- | --- | --- | --- | --- | --- | --- | --- |
| U | *contaminant\_INT-STD1* | 17 | 56 | 22.6% | 607 | 69271 | 6.1 | BSA |

| Filename XCorr DeltCN Conf% ObsM+H+ CalcM+H+ SpR ZScore Ion% # Sequence  | | | | | | | | | | | | |
| --- | --- | --- | --- | --- | --- | --- | --- | --- | --- | --- | --- | --- |
| \* | Astrin\_NLD\_STLC\_tube2\_021014\_01.09227.09227.2 | 3.2727 | 0.2478 | 100.0% | 1165.2122 | 1164.344 | 1 | 6.428 | 88.9% | 3 | K.LVNELTEFAK.T | 2 |
|  | Astrin\_NLD\_STLC\_tube2\_021014\_01.07281.07281.1 | 1.7678 | 0.2045 | 97.1% | 927.39 | 928.0758 | 2 | 4.563 | 75.0% | 1 | K.YLYEIAR.R | 1 |
|  | Astrin\_NLD\_STLC\_tube2\_021014\_01.07298.07298.2 | 2.2925 | 0.3128 | 100.0% | 928.1322 | 928.0758 | 3 | 6.317 | 83.3% | 1 | K.YLYEIAR.R | 2 |
| \* | Astrin\_NLD\_STLC\_031014\_01.10420.10420.3 | 3.6678 | 0.2717 | 100.0% | 2046.2644 | 2046.3354 | 1 | 5.71 | 48.3% | 3 | R.RHPYFYAPELLYYANK.Y | 3 |
| \* | Astrin\_NLD\_STLC\_tube2\_021014\_01.05134.05134.1 | 1.9382 | 0.2146 | 98.4% | 922.46 | 923.05383 | 10 | 4.51 | 57.1% | 1 | K.AEFVEVTK.L | 1 |
| \* | Astrin\_NLD\_STLC\_tube2\_021014\_01.05169.05169.2 | 2.3412 | 0.2107 | 98.1% | 922.5522 | 923.05383 | 1 | 5.35 | 85.7% | 1 | K.AEFVEVTK.L | 2 |
| \* | Astrin\_NLD\_STLC\_tube2\_021014\_01.13974.13974.2 | 4.328 | 0.4914 | 100.0% | 1568.4321 | 1568.7258 | 1 | 8.517 | 79.2% | 2 | K.DAFLGSFLYEYSR.R | 2 |
| \* | Astrin\_NLD\_STLC\_tube2\_021014\_01.07726.07726.2 | 3.0795 | 0.4171 | 100.0% | 1440.3322 | 1440.6884 | 4 | 7.205 | 59.1% | 3 | R.RHPEYAVSVLLR.L | 2 |
| \* | Astrin\_NLD\_STLC\_tube2\_021014\_02.06260.06260.3 | 4.4472 | 0.2993 | 100.0% | 1441.1643 | 1440.6884 | 1 | 5.386 | 59.1% | 10 | R.RHPEYAVSVLLR.L | 3 |
| \* | Astrin\_NLD\_STLC\_031014\_01.09420.09420.2 | 3.3641 | 0.4473 | 100.0% | 1306.2122 | 1306.5046 | 1 | 7.595 | 70.0% | 4 | K.HLVDEPQNLIK.Q | 2 |
| \* | Astrin\_NLD\_STLC\_031014\_02.08120.08120.2 | 4.1916 | 0.4401 | 100.0% | 1480.2322 | 1480.7068 | 1 | 8.494 | 79.2% | 11 | K.LGEYGFQNALIVR.Y | 2 |
|  | Astrin\_NLD\_STLC\_tube2\_021014\_01.07310.07310.3 | 3.8413 | 0.3284 | 100.0% | 1640.3944 | 1640.9205 | 2 | 7.452 | 48.2% | 4 | R.KVPQVSTPTLVEVSR.S | 3 |
|  | Astrin\_NLD\_STLC\_tube2\_021014\_01.07328.07328.2 | 4.0026 | 0.4425 | 100.0% | 1640.4922 | 1640.9205 | 1 | 8.54 | 60.7% | 3 | R.KVPQVSTPTLVEVSR.S | 2 |
|  | Astrin\_NLD\_STLC\_tube2\_021014\_01.08284.08284.2 | 2.9449 | 0.4363 | 100.0% | 1512.0721 | 1512.7465 | 1 | 8.143 | 61.5% | 1 | K.VPQVSTPTLVEVSR.S | 2 |
| \* | Astrin\_NLD\_STLC\_tube2\_021014\_01.08538.08538.2 | 2.3867 | 0.2071 | 96.9% | 1143.0922 | 1143.4124 | 28 | 4.54 | 66.7% | 1 | K.KQTALVELLK.H | 2 |
| \* | Astrin\_NLD\_STLC\_tube2\_021014\_01.13077.13077.2 | 3.865 | 0.5614 | 100.0% | 1400.1721 | 1400.6324 | 1 | 8.968 | 77.3% | 6 | K.TVMENFVAFVDK.C | 2 |
| \* | Astrin\_NLD\_STLC\_tube2\_021014\_02.06104.06104.2 | 2.4822 | 0.3604 | 100.0% | 1002.89215 | 1003.1839 | 6 | 6.42 | 66.7% | 1 | K.LVVSTQTALA.- | 2 |

---

|  |  |  |  |  |  |  |  |  |
| --- | --- | --- | --- | --- | --- | --- | --- | --- |
| U | *contaminant\_KERATIN05* | 11 | 15 | 22.3% | 471 | 51531 | 5.2 | no description |
| U | *gi|15431310|ref|NP\_00* | 11 | 15 | 22.2% | 472 | 51622 | 5.2 | keratin 14 [Homo sapiens] |

| Filename XCorr DeltCN Conf% ObsM+H+ CalcM+H+ SpR ZScore Ion% # Sequence  | | | | | | | | | | | | |
| --- | --- | --- | --- | --- | --- | --- | --- | --- | --- | --- | --- | --- |
|  | Astrin\_NLD\_STLC\_tube2\_021014\_01.04526.04526.2 | 2.8206 | 0.328 | 100.0% | 1090.9922 | 1091.2273 | 46 | 6.066 | 68.8% | 1 | K.VTMQNLNDR.L | 22 |
|  | Astrin\_NLD\_STLC\_tube2\_021014\_01.05645.05645.2 | 2.9461 | 0.1436 | 98.9% | 1065.2122 | 1065.2578 | 50 | 6.07 | 62.5% | 2 | R.LASYLDKVR.A | 2222 |
|  | Astrin\_NLD\_STLC\_tube2\_021014\_02.05150.05150.2 | 3.5297 | 0.3564 | 100.0% | 1301.4521 | 1302.4241 | 2 | 7.873 | 63.6% | 1 | R.ALEEANADLEVK.I | 2 |
|  | Astrin\_NLD\_STLC\_tube2\_021014\_01.05060.05060.2 | 2.4574 | 0.2931 | 100.0% | 807.9122 | 807.8815 | 9 | 6.526 | 75.0% | 2 | R.LAADDFR.T | 22222 |
|  | Astrin\_NLD\_STLC\_tube2\_021014\_01.07816.07816.2 | 2.407 | 0.225 | 98.0% | 1186.8522 | 1186.397 | 4 | 5.0 | 72.2% | 1 | R.RVLDELTLAR.A | 222 |
|  | Astrin\_NLD\_STLC\_tube2\_021014\_01.08553.08553.2 | 3.5532 | 0.3938 | 100.0% | 1030.2322 | 1030.2096 | 1 | 7.35 | 87.5% | 2 | R.VLDELTLAR.A | 222 |
|  | Astrin\_NLD\_STLC\_tube2\_021014\_01.05013.05013.2 | 3.1274 | 0.1615 | 99.1% | 1439.5922 | 1439.6263 | 1 | 4.739 | 75.0% | 2 | R.ILNEMRDQYEK.M | 22 |
|  | Astrin\_NLD\_STLC\_031014\_01.08982.08982.2 | 3.168 | 0.4267 | 100.0% | 1361.0122 | 1362.4796 | 1 | 7.174 | 66.7% | 1 | R.EVATNSELVQSGK.S | 22 |
|  | Astrin\_NLD\_STLC\_tube2\_021014\_01.06844.06844.2 | 3.547 | 0.3505 | 100.0% | 1381.3922 | 1380.5437 | 1 | 6.085 | 70.0% | 1 | K.TRLEQEIATYR.R | 22 |
|  | Astrin\_NLD\_STLC\_tube2\_021014\_01.05813.05813.3 | 3.1264 | 0.2733 | 100.0% | 1536.1743 | 1536.7311 | 2 | 5.027 | 38.6% | 1 | K.TRLEQEIATYRR.L | 33 |
|  | Astrin\_NLD\_STLC\_tube2\_021014\_02.05234.05234.3 | 5.64 | 0.5171 | 100.0% | 2310.4143 | 2310.396 | 1 | 7.992 | 39.3% | 1 | R.LLEGEDAHLSSSQFSSGSQSSR.D | 3 |

Similarities:
gi|40354195|ref|NP\_95(1:10)  
gi|4557701|ref|NP\_000(8:3)  
contaminant\_KERATIN03(3:8)  
gi|24234699|ref|NP\_00(4:7)  

---

|  |  |  |  |  |  |  |  |  |
| --- | --- | --- | --- | --- | --- | --- | --- | --- |
| U | *gi|218505827|ref|NP\_1* | 4 | 8 | 21.8% | 316 | 35438 | 6.3 | TRAF4 associated factor 1 isoform a [Homo sapiens] |
| U | *gi|218505831|ref|NP\_0* | 4 | 8 | 24.1% | 286 | 31880 | 7.1 | TRAF4 associated factor 1 isoform b [Homo sapiens] |

| Filename XCorr DeltCN Conf% ObsM+H+ CalcM+H+ SpR ZScore Ion% # Sequence  | | | | | | | | | | | | |
| --- | --- | --- | --- | --- | --- | --- | --- | --- | --- | --- | --- | --- |
|  | Astrin\_NLD\_STLC\_tube2\_021014\_01.08463.08463.2 | 5.0529 | 0.5001 | 100.0% | 2274.5522 | 2275.4802 | 1 | 8.211 | 59.5% | 1 | K.TVYSLQPPSALSGGQPADTQTR.A | 2 |
|  | Astrin\_NLD\_STLC\_tube2\_021014\_01.03730.03730.2 | 2.166 | 0.4906 | 100.0% | 1382.1122 | 1382.4728 | 4 | 7.844 | 58.3% | 1 | K.QLHSGGPENDVTK.I | 2 |
|  | Astrin\_NLD\_STLC\_tube2\_021014\_01.10948.10948.3 | 4.7239 | 0.3185 | 100.0% | 2315.6042 | 2316.6543 | 2 | 6.115 | 36.8% | 2 | K.LTETQGELKDLTQKVELLEK.F | 3 |
|  | Astrin\_NLD\_STLC\_tube2\_021014\_01.07864.07864.2 | 3.6335 | 0.5808 | 100.0% | 1386.5922 | 1387.5327 | 1 | 10.215 | 69.2% | 4 | K.GLDPALGSETLASR.Q | 2 |

---

|  |  |  |  |  |  |  |  |  |
| --- | --- | --- | --- | --- | --- | --- | --- | --- |
| U | *gi|4506699|ref|NP\_001* | 1 | 1 | 21.7% | 83 | 9111 | 8.5 | ribosomal protein S21 [Homo sapiens] |

| Filename XCorr DeltCN Conf% ObsM+H+ CalcM+H+ SpR ZScore Ion% # Sequence  | | | | | | | | | | | | |
| --- | --- | --- | --- | --- | --- | --- | --- | --- | --- | --- | --- | --- |
| \* | Astrin\_NLD\_STLC\_tube2\_021014\_02.06050.06050.3 | 2.8883 | 0.2486 | 96.4% | 1969.5543 | 1971.1956 | 7 | 4.562 | 33.8% | 1 | K.DHASIQMNVAEVDKVTGR.F | 3 |

---

|  |  |  |  |  |  |  |  |  |
| --- | --- | --- | --- | --- | --- | --- | --- | --- |
| U | *gi|72534660|ref|NP\_00* | 4 | 4 | 21.4% | 238 | 27367 | 11.8 | splicing factor, arginine/serine-rich 7 [Homo sapiens] |

| Filename XCorr DeltCN Conf% ObsM+H+ CalcM+H+ SpR ZScore Ion% # Sequence  | | | | | | | | | | | | |
| --- | --- | --- | --- | --- | --- | --- | --- | --- | --- | --- | --- | --- |
| \* | Astrin\_NLD\_STLC\_tube2\_021014\_02.05157.05157.3 | 2.2925 | 0.3248 | 97.5% | 1721.2144 | 1720.923 | 1 | 6.644 | 43.8% | 1 | K.VYVGNLGTGAGKGELER.A | 3 |
| \* | Astrin\_NLD\_STLC\_tube2\_021014\_01.08769.08769.2 | 2.1217 | 0.2828 | 98.6% | 1073.6721 | 1074.2242 | 3 | 6.239 | 75.0% | 1 | R.AFSYYGPLR.T | 2 |
|  | Astrin\_NLD\_STLC\_tube2\_021014\_01.12198.12198.2 | 2.6152 | 0.2101 | 97.0% | 1622.1522 | 1622.7771 | 1 | 4.635 | 61.5% | 1 | R.NPPGFAFVEFEDPR.D | 22 |
| \* | Astrin\_NLD\_STLC\_tube2\_021014\_01.05926.05926.2 | 2.7488 | 0.3513 | 100.0% | 1244.4722 | 1245.4827 | 4 | 5.876 | 60.0% | 1 | R.VRVELSTGMPR.R | 2 |

Similarities:
gi|4506901|ref|NP\_003(1:3)  

---

|  |  |  |  |  |  |  |  |  |
| --- | --- | --- | --- | --- | --- | --- | --- | --- |
| U | *gi|14165435|ref|NP\_11* | 6 | 7 | 21.2% | 463 | 50976 | 5.5 | heterogeneous nuclear ribonucleoprotein K isoform b [Homo sapiens] |
| U | *gi|14165439|ref|NP\_00* | 6 | 7 | 21.1% | 464 | 51028 | 5.3 | heterogeneous nuclear ribonucleoprotein K isoform a [Homo sapiens] |
| U | *gi|14165437|ref|NP\_11* | 6 | 7 | 21.1% | 464 | 51028 | 5.3 | heterogeneous nuclear ribonucleoprotein K isoform a [Homo sapiens] |

| Filename XCorr DeltCN Conf% ObsM+H+ CalcM+H+ SpR ZScore Ion% # Sequence  | | | | | | | | | | | | |
| --- | --- | --- | --- | --- | --- | --- | --- | --- | --- | --- | --- | --- |
|  | Astrin\_NLD\_STLC\_tube2\_021014\_01.05662.05662.2 | 3.3469 | 0.5186 | 100.0% | 1781.3522 | 1781.8302 | 10 | 8.323 | 50.0% | 1 | R.TDYNASVSVPDSSGPER.I | 2 |
|  | Astrin\_NLD\_STLC\_tube2\_021014\_01.16504.16504.2 | 3.97 | 0.4381 | 100.0% | 1716.3922 | 1716.0251 | 1 | 8.443 | 63.3% | 1 | R.ILSISADIETIGEILK.K | 2 |
|  | Astrin\_NLD\_STLC\_tube2\_021014\_01.09408.09408.2 | 3.7603 | 0.4449 | 100.0% | 1519.3922 | 1519.8711 | 1 | 8.025 | 71.4% | 1 | R.LLIHQSLAGGIIGVK.G | 2 |
|  | Astrin\_NLD\_STLC\_tube2\_021014\_01.12600.12600.2 | 3.3803 | 0.3074 | 100.0% | 1342.3322 | 1341.6311 | 1 | 5.832 | 68.2% | 1 | K.IILDLISESPIK.G | 2 |
|  | Astrin\_NLD\_STLC\_tube2\_021014\_01.10666.10666.2 | 5.3361 | 0.552 | 100.0% | 1917.5922 | 1918.1974 | 1 | 9.927 | 61.1% | 2 | R.GSYGDLGGPIITTQVTIPK.D | 2 |
|  | Astrin\_NLD\_STLC\_tube2\_021014\_01.05956.05956.3 | 2.5452 | 0.2846 | 96.2% | 2069.1543 | 2070.1772 | 52 | 4.711 | 29.2% | 1 | R.HESGASIKIDEPLEGSEDR.I | 3 |

---

|  |  |  |  |  |  |  |  |  |
| --- | --- | --- | --- | --- | --- | --- | --- | --- |
| U | *gi|67782365|ref|NP\_00* | 7 | 11 | 20.9% | 469 | 51386 | 5.5 | keratin 7 [Homo sapiens] |

| Filename XCorr DeltCN Conf% ObsM+H+ CalcM+H+ SpR ZScore Ion% # Sequence  | | | | | | | | | | | | |
| --- | --- | --- | --- | --- | --- | --- | --- | --- | --- | --- | --- | --- |
|  | Astrin\_NLD\_STLC\_tube2\_021014\_01.07005.07005.3 | 3.9413 | 0.3693 | 100.0% | 2245.6443 | 2247.519 | 2 | 6.417 | 30.7% | 2 | R.LSSARPGGLGSSSLYGLGASRPR.V | 3 |
|  | Astrin\_NLD\_STLC\_tube2\_021014\_01.05405.05405.2 | 3.2323 | 0.3647 | 100.0% | 1104.3922 | 1105.2388 | 1 | 6.593 | 72.7% | 2 | R.SAYGGPVGAGIR.E | 2 |
|  | Astrin\_NLD\_STLC\_tube2\_021014\_01.07623.07623.2 | 2.9741 | 0.1656 | 99.6% | 1082.8121 | 1083.2755 | 7 | 7.028 | 75.0% | 2 | K.FASFIDKVR.F | 22222 |
| \* | Astrin\_NLD\_STLC\_031014\_02.07883.07883.3 | 3.3806 | 0.3604 | 100.0% | 1954.5243 | 1955.1783 | 1 | 6.216 | 38.2% | 2 | R.GQLEALQVDGGRLEAELR.S | 3 |
|  | Astrin\_NLD\_STLC\_tube2\_021014\_01.11499.11499.2 | 3.6439 | 0.1333 | 99.7% | 1419.4321 | 1419.5773 | 1 | 8.288 | 77.3% | 1 | K.VDALNDEINFLR.T | 2 |
|  | Astrin\_NLD\_STLC\_tube2\_021014\_01.05670.05670.2 | 3.755 | 0.3599 | 100.0% | 1385.5721 | 1386.548 | 1 | 6.985 | 72.7% | 1 | R.AKQEELEAALQR.G | 2 |
|  | Astrin\_NLD\_STLC\_tube2\_021014\_01.09447.09447.2 | 2.9328 | 0.1879 | 98.7% | 1406.3722 | 1406.6653 | 4 | 4.937 | 68.2% | 1 | K.LALDIEIATYRK.L | 222 |

Similarities:
gi|4504919|ref|NP\_002(2:5)  
gi|47132620|ref|NP\_00(1:6)  
gi|119703753|ref|NP\_0(1:6)  
gi|153791158|ref|NP\_0(1:6)  
contaminant\_KERATIN16(1:6)  

---

|  |  |  |  |  |  |  |  |  |
| --- | --- | --- | --- | --- | --- | --- | --- | --- |
| U | *gi|56699409|ref|NP\_00* | 6 | 15 | 20.5% | 391 | 42332 | 10.1 | RNA binding motif protein, X-linked [Homo sapiens] |

| Filename XCorr DeltCN Conf% ObsM+H+ CalcM+H+ SpR ZScore Ion% # Sequence  | | | | | | | | | | | | |
| --- | --- | --- | --- | --- | --- | --- | --- | --- | --- | --- | --- | --- |
|  | Astrin\_NLD\_STLC\_tube2\_021014\_01.08810.08810.2 | 2.7877 | 0.3076 | 99.9% | 1435.2522 | 1436.6049 | 2 | 5.709 | 70.8% | 2 | K.LFIGGLNTETNEK.A | 2 |
|  | Astrin\_NLD\_STLC\_tube2\_021014\_01.09981.09981.2 | 2.1949 | 0.3024 | 99.7% | 944.9122 | 945.2488 | 63 | 5.261 | 64.3% | 1 | R.IVEVLLMK.D | 2 |
|  | Astrin\_NLD\_STLC\_tube2\_021014\_01.11590.11590.2 | 3.9588 | 0.416 | 100.0% | 1487.1322 | 1487.6519 | 1 | 9.383 | 69.2% | 1 | R.GFAFVTFESPADAK.D | 2 |
| \* | Astrin\_NLD\_STLC\_031014\_01.09039.09039.3 | 3.7804 | 0.2563 | 100.0% | 1749.6244 | 1748.9768 | 1 | 6.693 | 41.7% | 2 | K.AIKVEQATKPSFESGR.R | 3 |
|  | Astrin\_NLD\_STLC\_tube2\_021014\_02.06068.06068.3 | 4.2516 | 0.3581 | 100.0% | 2050.9443 | 2051.1873 | 2 | 7.131 | 36.1% | 2 | R.GGHMDDGGYSMNFNMSSSR.G | 3 |
|  | Astrin\_NLD\_STLC\_031014\_01.06534.06534.2 | 2.8932 | 0.3918 | 100.0% | 1178.1522 | 1178.1571 | 1 | 5.897 | 72.2% | 7 | R.DSYESYGNSR.S | 2 |

---

|  |  |  |  |  |  |  |  |  |
| --- | --- | --- | --- | --- | --- | --- | --- | --- |
| U | *gi|50592996|ref|NP\_00* | 10 | 32 | 20.4% | 450 | 50433 | 4.9 | tubulin, beta, 4 [Homo sapiens] |

| Filename XCorr DeltCN Conf% ObsM+H+ CalcM+H+ SpR ZScore Ion% # Sequence  | | | | | | | | | | | | |
| --- | --- | --- | --- | --- | --- | --- | --- | --- | --- | --- | --- | --- |
|  | Astrin\_NLD\_STLC\_031014\_01.10481.10481.2 | 4.3846 | 0.483 | 100.0% | 1617.0922 | 1616.8701 | 1 | 8.766 | 67.9% | 5 | R.AILVDLEPGTMDSVR.S | 22 |
|  | Astrin\_NLD\_STLC\_031014\_01.11441.11441.3 | 3.3688 | 0.1916 | 95.4% | 1959.4143 | 1960.151 | 1 | 5.674 | 39.7% | 1 | K.GHYTEGAELVDSVLDVVR.K | 333 |
|  | Astrin\_NLD\_STLC\_tube2\_021014\_01.13540.13540.2 | 6.8342 | 0.5802 | 100.0% | 1959.5122 | 1960.151 | 1 | 10.762 | 79.4% | 1 | K.GHYTEGAELVDSVLDVVR.K | 222 |
|  | Astrin\_NLD\_STLC\_tube2\_021014\_01.12447.12447.3 | 4.5141 | 0.4937 | 100.0% | 2088.3542 | 2088.325 | 1 | 7.055 | 37.5% | 3 | K.GHYTEGAELVDSVLDVVRK.E | 333 |
|  | Astrin\_NLD\_STLC\_tube2\_021014\_01.08906.08906.2 | 3.931 | 0.4413 | 100.0% | 1320.0322 | 1320.5896 | 1 | 8.255 | 72.7% | 7 | R.IMNTFSVVPSPK.V | 222 |
|  | Astrin\_NLD\_STLC\_tube2\_021014\_01.07730.07730.2 | 2.8655 | 0.2256 | 99.8% | 1130.6322 | 1131.2767 | 5 | 4.422 | 77.8% | 4 | R.FPGQLNADLR.K | 222 |
|  | Astrin\_NLD\_STLC\_tube2\_021014\_01.08946.08946.2 | 3.5321 | 0.3443 | 100.0% | 1272.1522 | 1272.5945 | 1 | 6.817 | 70.0% | 1 | R.KLAVNMVPFPR.L | 222 |
|  | Astrin\_NLD\_STLC\_tube2\_021014\_01.10448.10448.2 | 3.6872 | 0.512 | 100.0% | 1144.1322 | 1144.4204 | 1 | 8.442 | 88.9% | 3 | K.LAVNMVPFPR.L | 222 |
|  | Astrin\_NLD\_STLC\_tube2\_021014\_01.12653.12653.2 | 3.2588 | 0.3888 | 100.0% | 1692.6322 | 1692.9678 | 1 | 6.595 | 71.4% | 1 | R.ALTVPELTQQMFDAK.N | 22 |
|  | Astrin\_NLD\_STLC\_tube2\_021014\_01.10736.10736.2 | 3.4182 | 0.4473 | 100.0% | 1229.9321 | 1230.4241 | 1 | 7.21 | 94.4% | 6 | R.ISEQFTAMFR.R | 222 |

Similarities:
gi|29788785|ref|NP\_82(9:1)  
gi|5174735|ref|NP\_006(9:1)  

---

|  |  |  |  |  |  |  |  |  |
| --- | --- | --- | --- | --- | --- | --- | --- | --- |
| U | *gi|5031753|ref|NP\_005* | 6 | 7 | 20.0% | 449 | 49229 | 6.3 | heterogeneous nuclear ribonucleoprotein H1 [Homo sapiens] |

| Filename XCorr DeltCN Conf% ObsM+H+ CalcM+H+ SpR ZScore Ion% # Sequence  | | | | | | | | | | | | |
| --- | --- | --- | --- | --- | --- | --- | --- | --- | --- | --- | --- | --- |
| \* | Astrin\_NLD\_STLC\_tube2\_021014\_02.06448.06448.3 | 3.6979 | 0.1852 | 96.7% | 2109.2043 | 2108.2231 | 2 | 4.056 | 37.5% | 1 | R.EGRPSGEAFVELESEDEVK.L | 3 |
| \* | Astrin\_NLD\_STLC\_031014\_02.07924.07924.2 | 3.0379 | 0.3308 | 100.0% | 1335.3522 | 1335.5176 | 1 | 5.608 | 80.0% | 1 | K.SNNVEMDWVLK.H | 2 |
|  | Astrin\_NLD\_STLC\_tube2\_021014\_01.04445.04445.2 | 3.4422 | 0.5192 | 100.0% | 1686.3722 | 1685.7501 | 1 | 8.106 | 76.7% | 1 | K.HTGPNSPDTANDGFVR.L | 2 |
|  | Astrin\_NLD\_STLC\_tube2\_021014\_01.11806.11806.2 | 5.1994 | 0.4738 | 100.0% | 1842.3121 | 1843.0001 | 1 | 9.375 | 68.8% | 1 | R.STGEAFVQFASQEIAEK.A | 2 |
|  | Astrin\_NLD\_STLC\_tube2\_021014\_01.13943.13943.2 | 3.4978 | 0.3535 | 100.0% | 1997.4521 | 1998.2023 | 2 | 7.159 | 43.8% | 1 | R.ATENDIYNFFSPLNPVR.V | 22 |
|  | Astrin\_NLD\_STLC\_tube2\_021014\_01.05140.05140.2 | 2.7007 | 0.3228 | 100.0% | 1092.4922 | 1093.2278 | 1 | 7.099 | 83.3% | 2 | R.VHIEIGPDGR.V | 22 |

Similarities:
gi|148470397|ref|NP\_0(2:4)  

---

|  |  |  |  |  |  |  |  |  |
| --- | --- | --- | --- | --- | --- | --- | --- | --- |
| U | *gi|169212778|ref|XP\_0* | 4 | 4 | 19.5% | 266 | 30042 | 10.6 | PREDICTED: similar to ribosomal protein L7a [Homo sapiens] |
| U | *gi|4506661|ref|NP\_000* | 4 | 4 | 19.5% | 266 | 29996 | 10.6 | ribosomal protein L7a [Homo sapiens] |
| U | *gi|169213130|ref|XP\_0* | 4 | 4 | 19.5% | 266 | 30042 | 10.6 | PREDICTED: similar to ribosomal protein L7a [Homo sapiens] |
| U | *gi|169212940|ref|XP\_0* | 4 | 4 | 19.5% | 266 | 30028 | 10.6 | PREDICTED: similar to ribosomal protein L7a [Homo sapiens] |

| Filename XCorr DeltCN Conf% ObsM+H+ CalcM+H+ SpR ZScore Ion% # Sequence  | | | | | | | | | | | | |
| --- | --- | --- | --- | --- | --- | --- | --- | --- | --- | --- | --- | --- |
|  | Astrin\_NLD\_STLC\_tube2\_021014\_01.07509.07509.2 | 2.4764 | 0.2037 | 97.1% | 1217.6721 | 1217.3672 | 2 | 5.624 | 65.0% | 1 | K.NFGIGQDIQPK.R | 2 |
|  | Astrin\_NLD\_STLC\_tube2\_021014\_01.11608.11608.3 | 3.5782 | 0.3816 | 100.0% | 1812.3243 | 1812.1222 | 2 | 6.629 | 45.0% | 1 | R.LKVPPAINQFTQALDR.Q | 3 |
|  | Astrin\_NLD\_STLC\_tube2\_021014\_01.07718.07718.2 | 2.6595 | 0.3026 | 99.8% | 1346.1721 | 1346.5236 | 77 | 5.588 | 50.0% | 1 | R.AGVNTVTTLVENK.K | 2 |
|  | Astrin\_NLD\_STLC\_tube2\_021014\_01.04221.04221.3 | 3.4287 | 0.159 | 96.2% | 1615.7043 | 1615.7025 | 15 | 5.1 | 45.5% | 1 | R.TNYNDRYDEIRR.H | 3 |

---

|  |  |  |  |  |  |  |  |  |
| --- | --- | --- | --- | --- | --- | --- | --- | --- |
| U | *gi|14249348|ref|NP\_11* | 2 | 3 | 19.5% | 123 | 13941 | 5.5 | thioredoxin-like 5 [Homo sapiens] |

| Filename XCorr DeltCN Conf% ObsM+H+ CalcM+H+ SpR ZScore Ion% # Sequence  | | | | | | | | | | | | |
| --- | --- | --- | --- | --- | --- | --- | --- | --- | --- | --- | --- | --- |
| \* | Astrin\_NLD\_STLC\_tube2\_021014\_02.06776.06776.3 | 2.767 | 0.2423 | 96.4% | 1715.9644 | 1715.8162 | 4 | 5.282 | 40.4% | 2 | R.YEEVSVSGFEEFHR.A | 3 |
| \* | Astrin\_NLD\_STLC\_tube2\_021014\_01.11482.11482.2 | 1.9471 | 0.3741 | 99.7% | 1135.1721 | 1135.3049 | 30 | 5.877 | 50.0% | 1 | K.TIFAYFTGSK.D | 2 |

---

|  |  |  |  |  |  |  |  |  |
| --- | --- | --- | --- | --- | --- | --- | --- | --- |
| U | *gi|7669492|ref|NP\_002* | 4 | 11 | 19.4% | 335 | 36053 | 8.5 | glyceraldehyde-3-phosphate dehydrogenase [Homo sapiens] |

| Filename XCorr DeltCN Conf% ObsM+H+ CalcM+H+ SpR ZScore Ion% # Sequence  | | | | | | | | | | | | |
| --- | --- | --- | --- | --- | --- | --- | --- | --- | --- | --- | --- | --- |
| \* | Astrin\_NLD\_STLC\_tube2\_021014\_01.14940.14940.3 | 5.8452 | 0.4834 | 100.0% | 2598.4443 | 2597.0044 | 1 | 8.746 | 37.0% | 3 | K.VIHDNFGIVEGLMTTVHAITATQK.T | 3 |
| \* | Astrin\_NLD\_STLC\_tube2\_021014\_01.08303.08303.2 | 3.5042 | 0.3336 | 100.0% | 1412.3722 | 1412.6292 | 2 | 5.618 | 64.3% | 3 | R.GALQNIIPASTGAAK.A | 2 |
|  | Astrin\_NLD\_STLC\_tube2\_021014\_01.11308.11308.2 | 3.6073 | 0.55 | 100.0% | 1764.1322 | 1764.8914 | 1 | 9.339 | 61.5% | 2 | K.LISWYDNEFGYSNR.V | 2 |
| \* | Astrin\_NLD\_STLC\_tube2\_021014\_01.07354.07354.2 | 3.3941 | 0.4492 | 100.0% | 1331.2922 | 1331.5879 | 1 | 8.068 | 72.7% | 3 | R.VVDLMAHMASKE.- | 2 |

---

|  |  |  |  |  |  |  |  |  |
| --- | --- | --- | --- | --- | --- | --- | --- | --- |
| U | *gi|4506613|ref|NP\_000* | 2 | 2 | 18.8% | 128 | 14787 | 9.2 | ribosomal protein L22 proprotein [Homo sapiens] |

| Filename XCorr DeltCN Conf% ObsM+H+ CalcM+H+ SpR ZScore Ion% # Sequence  | | | | | | | | | | | | |
| --- | --- | --- | --- | --- | --- | --- | --- | --- | --- | --- | --- | --- |
| \* | Astrin\_NLD\_STLC\_tube2\_021014\_01.06768.06768.2 | 3.3457 | 0.3599 | 100.0% | 1244.2922 | 1243.4056 | 1 | 5.63 | 66.7% | 1 | K.AGNLGGGVVTIER.S | 2 |
| \* | Astrin\_NLD\_STLC\_tube2\_021014\_01.08057.08057.2 | 2.1827 | 0.3468 | 99.7% | 1207.5122 | 1208.3971 | 18 | 5.625 | 60.0% | 1 | K.ITVTSEVPFSK.R | 2 |

---

|  |  |  |  |  |  |  |  |  |
| --- | --- | --- | --- | --- | --- | --- | --- | --- |
| U | *gi|47132620|ref|NP\_00* | 11 | 15 | 18.0% | 639 | 65433 | 8.0 | keratin 2 [Homo sapiens] |

| Filename XCorr DeltCN Conf% ObsM+H+ CalcM+H+ SpR ZScore Ion% # Sequence  | | | | | | | | | | | | |
| --- | --- | --- | --- | --- | --- | --- | --- | --- | --- | --- | --- | --- |
|  | Astrin\_NLD\_STLC\_tube2\_021014\_01.04533.04533.2 | 3.3624 | 0.406 | 100.0% | 1256.4122 | 1255.3298 | 1 | 7.47 | 69.2% | 1 | R.GFSSGSAVVSGGSR.R | 2 |
|  | Astrin\_NLD\_STLC\_tube2\_021014\_01.05289.05289.1 | 1.5882 | 0.2502 | 97.2% | 831.34 | 831.9878 | 434 | 5.16 | 37.5% | 1 | R.SLVGLGGTK.S | 1 |
|  | Astrin\_NLD\_STLC\_tube2\_021014\_01.07623.07623.2 | 2.9741 | 0.1656 | 99.6% | 1082.8121 | 1083.2755 | 7 | 7.028 | 75.0% | 2 | K.FASFIDKVR.F | 22222 |
|  | Astrin\_NLD\_STLC\_tube2\_021014\_01.06434.06434.2 | 4.4159 | 0.0626 | 99.8% | 1476.3722 | 1476.6726 | 1 | 6.932 | 90.9% | 1 | R.FLEQQNQVLQTK.W | 22 |
|  | Astrin\_NLD\_STLC\_tube2\_021014\_01.06866.06866.2 | 2.9544 | 0.3384 | 100.0% | 1038.1322 | 1038.1454 | 1 | 6.526 | 87.5% | 2 | R.YLDGLTAER.T | 2 |
|  | Astrin\_NLD\_STLC\_tube2\_021014\_01.07499.07499.2 | 2.4179 | 0.2734 | 99.1% | 1209.3121 | 1209.3416 | 4 | 5.422 | 70.0% | 1 | R.TAAENDFVTLK.K | 2 |
|  | Astrin\_NLD\_STLC\_tube2\_021014\_01.12972.12972.2 | 3.7648 | 0.3089 | 100.0% | 1461.3522 | 1461.6982 | 2 | 7.072 | 72.7% | 1 | K.VDLLNQEIEFLK.V | 2 |
|  | Astrin\_NLD\_STLC\_tube2\_021014\_01.13338.13338.2 | 4.1957 | 0.3905 | 100.0% | 1331.0922 | 1330.5211 | 1 | 7.868 | 86.4% | 1 | R.NLDLDSIIAEVK.A | 222 |
|  | Astrin\_NLD\_STLC\_tube2\_021014\_01.04328.04328.2 | 3.2426 | 0.0852 | 98.5% | 1108.1122 | 1108.196 | 2 | 6.567 | 75.0% | 1 | K.AQYEEIAQR.S | 222 |
|  | Astrin\_NLD\_STLC\_tube2\_021014\_02.05640.05640.2 | 2.8479 | 0.4634 | 100.0% | 1193.2922 | 1194.33 | 1 | 8.388 | 77.8% | 3 | K.YEELQVTVGR.H | 2 |
|  | Astrin\_NLD\_STLC\_tube2\_021014\_01.06564.06564.2 | 2.9459 | 0.0753 | 97.1% | 974.1322 | 974.102 | 175 | 4.257 | 64.3% | 1 | K.IEISELNR.V | 22 |

Similarities:
gi|4504919|ref|NP\_002(1:10)  
gi|119395750|ref|NP\_0(2:9)  
gi|67782365|ref|NP\_00(1:10)  
gi|119703753|ref|NP\_0(3:8)  
gi|153791158|ref|NP\_0(2:9)  
contaminant\_KERATIN16(1:10)  

---

|  |  |  |  |  |  |  |  |  |
| --- | --- | --- | --- | --- | --- | --- | --- | --- |
| U | *gi|15718687|ref|NP\_00* | 3 | 6 | 17.7% | 243 | 26688 | 9.7 | ribosomal protein S3 [Homo sapiens] |

| Filename XCorr DeltCN Conf% ObsM+H+ CalcM+H+ SpR ZScore Ion% # Sequence  | | | | | | | | | | | | |
| --- | --- | --- | --- | --- | --- | --- | --- | --- | --- | --- | --- | --- |
| \* | Astrin\_NLD\_STLC\_031014\_02.05410.05410.2 | 3.2423 | 0.3618 | 100.0% | 1424.2122 | 1424.5071 | 1 | 7.103 | 70.8% | 4 | R.ELAEDGYSGVEVR.V | 2 |
| \* | Astrin\_NLD\_STLC\_tube2\_021014\_01.08898.08898.3 | 3.0347 | 0.2674 | 99.6% | 1585.3744 | 1584.8998 | 1 | 5.697 | 51.9% | 1 | R.VTPTRTEIIILATR.T | 3 |
| \* | Astrin\_NLD\_STLC\_tube2\_021014\_01.06700.06700.2 | 3.4169 | 0.262 | 100.0% | 1574.5322 | 1574.8352 | 4 | 5.478 | 53.3% | 1 | K.GGKPEPPAMPQPVPTA.- | 2 |

---

|  |  |  |  |  |  |  |  |  |
| --- | --- | --- | --- | --- | --- | --- | --- | --- |
| U | *gi|5902102|ref|NP\_008* | 1 | 1 | 16.8% | 119 | 13282 | 11.6 | small nuclear ribonucleoprotein D1 polypeptide 16kDa [Homo sapiens] |

| Filename XCorr DeltCN Conf% ObsM+H+ CalcM+H+ SpR ZScore Ion% # Sequence  | | | | | | | | | | | | |
| --- | --- | --- | --- | --- | --- | --- | --- | --- | --- | --- | --- | --- |
| \* | Astrin\_NLD\_STLC\_031014\_01.14194.14194.2 | 3.3207 | 0.3316 | 100.0% | 2287.7922 | 2288.6863 | 1 | 6.074 | 42.1% | 1 | R.YFILPDSLPLDTLLVDVEPK.V | 23 |

---

|  |  |  |  |  |  |  |  |  |
| --- | --- | --- | --- | --- | --- | --- | --- | --- |
| U | *gi|169201338|ref|XP\_0* | 3 | 9 | 16.2% | 160 | 18565 | 10.5 | PREDICTED: hypothetical protein [Homo sapiens] |
| U | *gi|89040203|ref|XP\_93* | 3 | 9 | 16.2% | 160 | 18593 | 10.5 | PREDICTED: hypothetical protein [Homo sapiens] |
| U | *gi|18104948|ref|NP\_00* | 3 | 9 | 16.2% | 160 | 18565 | 10.5 | ribosomal protein L21 [Homo sapiens] |
| U | *gi|169213854|ref|XP\_0* | 3 | 9 | 16.2% | 160 | 18790 | 10.3 | PREDICTED: hypothetical protein [Homo sapiens] |
| U | *gi|169210381|ref|XP\_0* | 3 | 9 | 16.2% | 160 | 18535 | 10.6 | PREDICTED: hypothetical protein isoform 2 [Homo sapiens] |
| U | *gi|169210379|ref|XP\_0* | 3 | 9 | 16.2% | 160 | 18535 | 10.6 | PREDICTED: hypothetical protein isoform 3 [Homo sapiens] |
| U | *gi|169210377|ref|XP\_0* | 3 | 9 | 16.2% | 160 | 18535 | 10.6 | PREDICTED: hypothetical protein isoform 1 [Homo sapiens] |
| U | *gi|169202779|ref|XP\_0* | 3 | 9 | 16.2% | 160 | 18521 | 10.5 | PREDICTED: similar to ribosomal protein L21 isoform 1 [Homo sapiens] |
| U | *gi|169202777|ref|XP\_0* | 3 | 9 | 16.2% | 160 | 18521 | 10.5 | PREDICTED: similar to ribosomal protein L21 isoform 2 [Homo sapiens] |
| U | *gi|169201750|ref|XP\_0* | 3 | 9 | 16.2% | 160 | 18550 | 10.5 | PREDICTED: hypothetical protein [Homo sapiens] |

| Filename XCorr DeltCN Conf% ObsM+H+ CalcM+H+ SpR ZScore Ion% # Sequence  | | | | | | | | | | | | |
| --- | --- | --- | --- | --- | --- | --- | --- | --- | --- | --- | --- | --- |
|  | Astrin\_NLD\_STLC\_tube2\_021014\_01.07793.07793.2 | 2.9445 | 0.4733 | 100.0% | 1244.2922 | 1244.4973 | 1 | 6.705 | 80.0% | 2 | K.HGVVPLATYMR.I | 2 |
|  | Astrin\_NLD\_STLC\_tube2\_021014\_02.05730.05730.2 | 4.8658 | 0.4124 | 100.0% | 1642.4122 | 1641.9108 | 1 | 8.103 | 82.1% | 2 | R.VYNVTQHAVGIVVNK.Q | 2 |
|  | Astrin\_NLD\_STLC\_031014\_02.05780.05780.3 | 4.4485 | 0.4375 | 100.0% | 1642.6444 | 1641.9108 | 1 | 8.53 | 50.0% | 5 | R.VYNVTQHAVGIVVNK.Q | 3 |

---

|  |  |  |  |  |  |  |  |  |
| --- | --- | --- | --- | --- | --- | --- | --- | --- |
| U | *gi|14141152|ref|NP\_00* | 9 | 18 | 16.0% | 730 | 77516 | 8.7 | heterogeneous nuclear ribonucleoprotein M isoform a [Homo sapiens] |
| U | *gi|157412270|ref|NP\_1* | 9 | 18 | 16.9% | 691 | 73621 | 8.8 | heterogeneous nuclear ribonucleoprotein M isoform b [Homo sapiens] |

| Filename XCorr DeltCN Conf% ObsM+H+ CalcM+H+ SpR ZScore Ion% # Sequence  | | | | | | | | | | | | |
| --- | --- | --- | --- | --- | --- | --- | --- | --- | --- | --- | --- | --- |
|  | Astrin\_NLD\_STLC\_tube2\_021014\_01.09840.09840.2 | 2.5213 | 0.3309 | 99.8% | 1717.6721 | 1715.9724 | 2 | 5.037 | 40.6% | 1 | K.MGGMEGPFGGGMENMGR.F | 2 |
|  | Astrin\_NLD\_STLC\_tube2\_021014\_01.04996.04996.2 | 1.8326 | 0.3134 | 96.9% | 956.4922 | 957.11017 | 29 | 6.704 | 62.5% | 1 | R.FGSGMNMGR.I | 2 |
|  | Astrin\_NLD\_STLC\_tube2\_021014\_01.09250.09250.2 | 4.5873 | 0.4929 | 100.0% | 1614.2722 | 1614.875 | 1 | 8.942 | 75.0% | 1 | R.MGPLGLDHMASSIER.M | 2 |
|  | Astrin\_NLD\_STLC\_tube2\_021014\_01.09274.09274.3 | 3.9711 | 0.4338 | 100.0% | 1614.5643 | 1614.875 | 1 | 7.76 | 42.9% | 1 | R.MGPLGLDHMASSIER.M | 3 |
|  | Astrin\_NLD\_STLC\_tube2\_021014\_02.06720.06720.2 | 3.4898 | 0.4738 | 100.0% | 1126.5721 | 1126.3337 | 1 | 8.888 | 80.0% | 3 | R.MGAGMGFGLER.M | 2 |
|  | Astrin\_NLD\_STLC\_tube2\_021014\_01.06839.06839.2 | 3.0605 | 0.4796 | 100.0% | 1189.3322 | 1189.4333 | 1 | 7.712 | 77.3% | 2 | R.MVPAGMGAGLER.M | 2 |
|  | Astrin\_NLD\_STLC\_tube2\_021014\_01.08504.08504.2 | 2.9128 | 0.1803 | 97.5% | 1428.0721 | 1428.7076 | 1 | 6.191 | 67.9% | 1 | R.MGPAMGPALGAGIER.M | 2 |
|  | Astrin\_NLD\_STLC\_tube2\_021014\_02.06245.06245.2 | 3.4597 | 0.4824 | 100.0% | 1384.0922 | 1384.5677 | 1 | 7.388 | 67.9% | 5 | R.MGLAMGGGGGASFDR.A | 2 |
|  | Astrin\_NLD\_STLC\_tube2\_021014\_02.06404.06404.3 | 3.9794 | 0.4499 | 100.0% | 2035.4043 | 2036.1735 | 1 | 7.122 | 34.1% | 3 | R.GNFGGSFAGSFGGAGGHAPGVAR.K | 3 |

---

|  |  |  |  |  |  |  |  |  |
| --- | --- | --- | --- | --- | --- | --- | --- | --- |
| U | *gi|34098946|ref|NP\_00* | 2 | 2 | 16.0% | 324 | 35924 | 9.9 | nuclease sensitive element binding protein 1 [Homo sapiens] |

| Filename XCorr DeltCN Conf% ObsM+H+ CalcM+H+ SpR ZScore Ion% # Sequence  | | | | | | | | | | | | |
| --- | --- | --- | --- | --- | --- | --- | --- | --- | --- | --- | --- | --- |
| \* | Astrin\_NLD\_STLC\_tube2\_021014\_01.03993.03993.3 | 5.7879 | 0.5193 | 100.0% | 3258.4744 | 3259.2566 | 1 | 6.938 | 32.1% | 1 | R.NYQQNYQNSESGEKNEGSESAPEGQAQQR.R | 3 |
| \* | Astrin\_NLD\_STLC\_031014\_01.05030.05030.3 | 4.1415 | 0.3514 | 100.0% | 2628.9243 | 2629.5835 | 1 | 6.334 | 33.0% | 1 | R.EDGNEEDKENQGDETQGQQPPQR.R | 3 |

---

|  |  |  |  |  |  |  |  |  |
| --- | --- | --- | --- | --- | --- | --- | --- | --- |
| U | *gi|209862831|ref|NP\_0* | 4 | 4 | 15.9% | 339 | 38604 | 7.8 | annexin A2 isoform 2 [Homo sapiens] |
| U | *gi|50845388|ref|NP\_00* | 4 | 4 | 15.1% | 357 | 40411 | 8.4 | annexin A2 isoform 1 [Homo sapiens] |
| U | *gi|50845386|ref|NP\_00* | 4 | 4 | 15.9% | 339 | 38604 | 7.8 | annexin A2 isoform 2 [Homo sapiens] |
| U | *gi|4757756|ref|NP\_004* | 4 | 4 | 15.9% | 339 | 38604 | 7.8 | annexin A2 isoform 2 [Homo sapiens] |

| Filename XCorr DeltCN Conf% ObsM+H+ CalcM+H+ SpR ZScore Ion% # Sequence  | | | | | | | | | | | | |
| --- | --- | --- | --- | --- | --- | --- | --- | --- | --- | --- | --- | --- |
|  | Astrin\_NLD\_STLC\_tube2\_021014\_01.14878.14878.2 | 4.2155 | 0.4348 | 100.0% | 1651.6921 | 1651.9872 | 1 | 9.067 | 56.7% | 1 | K.SALSGHLETVILGLLK.T | 2 |
|  | Astrin\_NLD\_STLC\_tube2\_021014\_01.04839.04839.2 | 3.069 | 0.3801 | 100.0% | 1223.0922 | 1223.3251 | 1 | 7.117 | 70.0% | 1 | K.TPAQYDASELK.A | 2 |
|  | Astrin\_NLD\_STLC\_tube2\_021014\_01.04596.04596.2 | 2.5966 | 0.1998 | 98.3% | 1244.6322 | 1245.3347 | 1 | 5.523 | 77.8% | 1 | R.TNQELQEINR.V | 2 |
|  | Astrin\_NLD\_STLC\_tube2\_021014\_01.08886.08886.3 | 4.1326 | 0.4796 | 100.0% | 1941.2344 | 1941.102 | 2 | 7.583 | 43.8% | 1 | K.TDLEKDIISDTSGDFRK.L | 3 |

---

|  |  |  |  |  |  |  |  |  |
| --- | --- | --- | --- | --- | --- | --- | --- | --- |
| U | *gi|5032051|ref|NP\_005* | 2 | 3 | 15.9% | 151 | 16273 | 10.1 | ribosomal protein S14 [Homo sapiens] |
| U | *gi|68160922|ref|NP\_00* | 2 | 3 | 15.9% | 151 | 16273 | 10.1 | ribosomal protein S14 [Homo sapiens] |
| U | *gi|68160915|ref|NP\_00* | 2 | 3 | 15.9% | 151 | 16273 | 10.1 | ribosomal protein S14 [Homo sapiens] |

| Filename XCorr DeltCN Conf% ObsM+H+ CalcM+H+ SpR ZScore Ion% # Sequence  | | | | | | | | | | | | |
| --- | --- | --- | --- | --- | --- | --- | --- | --- | --- | --- | --- | --- |
|  | Astrin\_NLD\_STLC\_031014\_01.08787.08787.2 | 3.1492 | 0.3511 | 100.0% | 1055.3722 | 1055.179 | 2 | 7.726 | 65.0% | 2 | K.TPGPGAQSALR.A | 2 |
|  | Astrin\_NLD\_STLC\_tube2\_021014\_01.06040.06040.2 | 3.3583 | 0.4274 | 100.0% | 1430.5521 | 1430.5547 | 1 | 6.809 | 75.0% | 1 | R.IEDVTPIPSDSTR.R | 2 |

---

|  |  |  |  |  |  |  |  |  |
| --- | --- | --- | --- | --- | --- | --- | --- | --- |
| U | *gi|169160598|ref|XP\_0* | 1 | 1 | 15.5% | 84 | 9461 | 9.5 | PREDICTED: similar to hCG1783679 [Homo sapiens] |
| U | *gi|7705706|ref|NP\_057* | 1 | 1 | 15.5% | 84 | 9477 | 9.5 | ribosomal protein S27-like [Homo sapiens] |
| U | *gi|4506711|ref|NP\_001* | 1 | 1 | 15.5% | 84 | 9461 | 9.5 | ribosomal protein S27 [Homo sapiens] |
| U | *gi|169214231|ref|XP\_0* | 1 | 1 | 15.7% | 83 | 9378 | 9.6 | PREDICTED: similar to hCG2027326 [Homo sapiens] |
| U | *gi|169213802|ref|XP\_0* | 1 | 1 | 15.7% | 83 | 9378 | 9.6 | PREDICTED: similar to hCG2027326 [Homo sapiens] |
| U | *gi|169213575|ref|XP\_0* | 1 | 1 | 15.7% | 83 | 9378 | 9.6 | PREDICTED: similar to hCG2027326 [Homo sapiens] |
| U | *gi|169166679|ref|XP\_0* | 1 | 1 | 15.5% | 84 | 9450 | 9.4 | PREDICTED: similar to metallopanstimulin [Homo sapiens] |
| U | *gi|169166621|ref|XP\_0* | 1 | 1 | 15.5% | 84 | 9465 | 9.5 | PREDICTED: similar to metallopanstimulin [Homo sapiens] |
| U | *gi|169166508|ref|XP\_0* | 1 | 1 | 15.5% | 84 | 9450 | 9.4 | PREDICTED: similar to metallopanstimulin [Homo sapiens] |
| U | *gi|169161552|ref|XP\_0* | 1 | 1 | 15.5% | 84 | 9461 | 9.5 | PREDICTED: similar to hCG1783679 [Homo sapiens] |
| U | *gi|169161255|ref|XP\_0* | 1 | 1 | 15.5% | 84 | 9461 | 9.5 | PREDICTED: hypothetical protein [Homo sapiens] |

| Filename XCorr DeltCN Conf% ObsM+H+ CalcM+H+ SpR ZScore Ion% # Sequence  | | | | | | | | | | | | |
| --- | --- | --- | --- | --- | --- | --- | --- | --- | --- | --- | --- | --- |
|  | Astrin\_NLD\_STLC\_tube2\_021014\_01.09429.09429.2 | 3.7553 | 0.3924 | 100.0% | 1528.2722 | 1528.7632 | 1 | 7.446 | 79.2% | 1 | R.LVQSPNSYFMDVK.C | 2 |

---

|  |  |  |  |  |  |  |  |  |
| --- | --- | --- | --- | --- | --- | --- | --- | --- |
| U | *gi|4503471|ref|NP\_001* | 5 | 20 | 15.2% | 462 | 50141 | 9.0 | eukaryotic translation elongation factor 1 alpha 1 [Homo sapiens] |

| Filename XCorr DeltCN Conf% ObsM+H+ CalcM+H+ SpR ZScore Ion% # Sequence  | | | | | | | | | | | | |
| --- | --- | --- | --- | --- | --- | --- | --- | --- | --- | --- | --- | --- |
|  | Astrin\_NLD\_STLC\_tube2\_021014\_02.05702.05702.3 | 4.5243 | 0.4618 | 100.0% | 1590.1144 | 1589.835 | 1 | 7.642 | 46.4% | 12 | K.THINIVVIGHVDSGK.S | 3 |
|  | Astrin\_NLD\_STLC\_031014\_02.06989.06989.2 | 2.2712 | 0.2518 | 97.1% | 1316.5322 | 1315.5553 | 4 | 4.66 | 54.5% | 1 | R.EHALLAYTLGVK.Q | 2 |
|  | Astrin\_NLD\_STLC\_tube2\_021014\_01.07437.07437.2 | 2.2984 | 0.3002 | 99.8% | 976.6322 | 976.1607 | 14 | 5.672 | 71.4% | 1 | R.LPLQDVYK.I | 2 |
|  | Astrin\_NLD\_STLC\_tube2\_021014\_01.06572.06572.2 | 3.4306 | 0.3868 | 100.0% | 1026.0922 | 1026.2241 | 2 | 7.325 | 75.0% | 5 | K.IGGIGTVPVGR.V | 2 |
| \* | Astrin\_NLD\_STLC\_tube2\_021014\_01.11294.11294.3 | 4.5126 | 0.3726 | 100.0% | 2516.6943 | 2516.999 | 1 | 6.307 | 34.8% | 1 | R.VETGVLKPGMVVTFAPVNVTTEVK.S | 3 |

---

|  |  |  |  |  |  |  |  |  |
| --- | --- | --- | --- | --- | --- | --- | --- | --- |
| U | *gi|16905517|ref|NP\_47* | 3 | 3 | 14.9% | 262 | 31301 | 11.3 | FUS interacting protein (serine-arginine rich) 1 isoform 2 [Homo sapiens] |
| U | *gi|5730079|ref|NP\_006* | 3 | 3 | 21.3% | 183 | 22222 | 10.3 | FUS interacting protein (serine-arginine rich) 1 isoform 1 [Homo sapiens] |
| U | *gi|169161980|ref|XP\_0* | 3 | 3 | 21.5% | 181 | 22022 | 10.3 | PREDICTED: hypothetical protein, partial [Homo sapiens] |
| U | *gi|169161109|ref|XP\_0* | 3 | 3 | 21.3% | 183 | 22222 | 10.3 | PREDICTED: hypothetical protein LOC642558 [Homo sapiens] |
| U | *gi|169161107|ref|XP\_0* | 3 | 3 | 14.9% | 262 | 31301 | 11.3 | PREDICTED: hypothetical protein LOC642558 [Homo sapiens] |

| Filename XCorr DeltCN Conf% ObsM+H+ CalcM+H+ SpR ZScore Ion% # Sequence  | | | | | | | | | | | | |
| --- | --- | --- | --- | --- | --- | --- | --- | --- | --- | --- | --- | --- |
|  | Astrin\_NLD\_STLC\_tube2\_021014\_01.08529.08529.3 | 3.3721 | 0.2727 | 100.0% | 1463.4543 | 1463.7227 | 1 | 5.894 | 59.1% | 1 | R.YLRPPNTSLFVR.N | 3 |
|  | Astrin\_NLD\_STLC\_tube2\_021014\_01.14176.14176.2 | 2.7508 | 0.3473 | 100.0% | 1917.1122 | 1918.1992 | 1 | 5.751 | 50.0% | 1 | R.YGPIVDVYVPLDFYTR.R | 2 |
|  | Astrin\_NLD\_STLC\_tube2\_021014\_01.10325.10325.2 | 2.4305 | 0.269 | 99.1% | 1331.9722 | 1331.4705 | 21 | 4.746 | 55.0% | 1 | R.GFAYVQFEDVR.D | 2 |

---

|  |  |  |  |  |  |  |  |  |
| --- | --- | --- | --- | --- | --- | --- | --- | --- |
| U | *gi|5454064|ref|NP\_006* | 7 | 10 | 14.8% | 669 | 69492 | 9.7 | RNA binding motif protein 14 [Homo sapiens] |

| Filename XCorr DeltCN Conf% ObsM+H+ CalcM+H+ SpR ZScore Ion% # Sequence  | | | | | | | | | | | | |
| --- | --- | --- | --- | --- | --- | --- | --- | --- | --- | --- | --- | --- |
| \* | Astrin\_NLD\_STLC\_tube2\_021014\_01.04263.04263.3 | 3.2954 | 0.3267 | 100.0% | 1556.4844 | 1556.7677 | 3 | 5.193 | 38.5% | 1 | R.AIEALHGHELRPGR.A | 3 |
| \* | Astrin\_NLD\_STLC\_tube2\_021014\_01.07517.07517.2 | 4.0231 | 0.4514 | 100.0% | 1610.4122 | 1609.8223 | 1 | 7.418 | 71.4% | 1 | R.ASYVAPLTAQPATYR.A | 2 |
| \* | Astrin\_NLD\_STLC\_tube2\_021014\_01.06336.06336.2 | 2.5147 | 0.2267 | 97.9% | 1221.2122 | 1220.3707 | 6 | 4.944 | 59.1% | 1 | R.AQPSVSLGAAYR.A | 2 |
| \* | Astrin\_NLD\_STLC\_tube2\_021014\_01.04527.04527.2 | 2.9166 | 0.4039 | 100.0% | 1324.9521 | 1325.482 | 1 | 6.819 | 72.7% | 1 | R.TQPMTAQAASYR.A | 2 |
| \* | Astrin\_NLD\_STLC\_tube2\_021014\_01.06569.06569.2 | 2.5226 | 0.4133 | 100.0% | 1246.0521 | 1246.4087 | 1 | 6.661 | 63.6% | 1 | R.AQPSVSLGAPYR.G | 2 |
| \* | Astrin\_NLD\_STLC\_tube2\_021014\_02.05126.05126.3 | 4.2381 | 0.4907 | 100.0% | 2465.5444 | 2466.6292 | 1 | 8.529 | 33.7% | 4 | R.TQSSASLAASYAAQQHPQAAASYR.G | 3 |
| \* | Astrin\_NLD\_STLC\_tube2\_021014\_01.06886.06886.2 | 2.6625 | 0.3341 | 100.0% | 1238.0122 | 1238.2988 | 1 | 8.148 | 66.7% | 1 | R.YSGSYNDYLR.A | 2 |

---

|  |  |  |  |  |  |  |  |  |
| --- | --- | --- | --- | --- | --- | --- | --- | --- |
| U | *gi|5729877|ref|NP\_006* | 7 | 9 | 14.7% | 646 | 70898 | 5.5 | heat shock 70kDa protein 8 isoform 1 [Homo sapiens] |

| Filename XCorr DeltCN Conf% ObsM+H+ CalcM+H+ SpR ZScore Ion% # Sequence  | | | | | | | | | | | | |
| --- | --- | --- | --- | --- | --- | --- | --- | --- | --- | --- | --- | --- |
|  | Astrin\_NLD\_STLC\_tube2\_021014\_01.07839.07839.2 | 3.2184 | 0.2936 | 100.0% | 1488.1721 | 1488.5939 | 1 | 5.063 | 62.5% | 2 | R.TTPSYVAFTDTER.L | 22 |
|  | Astrin\_NLD\_STLC\_tube2\_021014\_01.03838.03838.2 | 2.3256 | 0.2652 | 98.8% | 1181.0721 | 1181.3312 | 71 | 4.615 | 61.1% | 1 | K.VQVEYKGETK.S | 2 |
|  | Astrin\_NLD\_STLC\_tube2\_021014\_01.10414.10414.2 | 4.7786 | 0.4625 | 100.0% | 1661.2522 | 1660.9078 | 1 | 8.509 | 73.3% | 2 | R.IINEPTAAAIAYGLDK.K | 2 |
|  | Astrin\_NLD\_STLC\_tube2\_021014\_01.09900.09900.2 | 2.5271 | 0.1922 | 96.1% | 1480.4122 | 1481.6511 | 7 | 4.834 | 54.5% | 1 | R.ARFEELNADLFR.G | 2 |
|  | Astrin\_NLD\_STLC\_tube2\_021014\_02.05919.05919.3 | 3.6612 | 0.3088 | 100.0% | 1838.3944 | 1839.1019 | 55 | 5.447 | 31.2% | 1 | K.LDKSQIHDIVLVGGSTR.I | 3 |
| \* | Astrin\_NLD\_STLC\_031014\_01.06324.06324.3 | 3.4228 | 0.2187 | 98.4% | 1982.8444 | 1983.2036 | 6 | 5.781 | 36.7% | 1 | R.MVQEAEKYKAEDEKQR.D | 3 |
| \* | Astrin\_NLD\_STLC\_tube2\_021014\_01.09411.09411.2 | 3.4611 | 0.4435 | 100.0% | 1304.2122 | 1304.4602 | 2 | 7.909 | 80.0% | 1 | K.NSLESYAFNMK.A | 2 |

Similarities:
gi|167466173|ref|NP\_0(1:6)  

---

|  |  |  |  |  |  |  |  |  |
| --- | --- | --- | --- | --- | --- | --- | --- | --- |
| U | *gi|4506623|ref|NP\_000* | 2 | 2 | 14.7% | 136 | 15798 | 10.6 | ribosomal protein L27 [Homo sapiens] |

| Filename XCorr DeltCN Conf% ObsM+H+ CalcM+H+ SpR ZScore Ion% # Sequence  | | | | | | | | | | | | |
| --- | --- | --- | --- | --- | --- | --- | --- | --- | --- | --- | --- | --- |
|  | Astrin\_NLD\_STLC\_tube2\_021014\_01.06604.06604.2 | 3.1232 | 0.1733 | 99.5% | 1408.3722 | 1408.6177 | 2 | 5.577 | 75.0% | 1 | K.VYNYNHLMPTR.Y | 2 |
| \* | Astrin\_NLD\_STLC\_tube2\_021014\_01.08306.08306.2 | 2.2155 | 0.3333 | 99.8% | 1049.7522 | 1050.1968 | 42 | 6.058 | 50.0% | 1 | R.YSVDIPLDK.T | 2 |

---

|  |  |  |  |  |  |  |  |  |
| --- | --- | --- | --- | --- | --- | --- | --- | --- |
| U | *gi|10440560|ref|NP\_06* | 2 | 3 | 14.7% | 136 | 15404 | 11.1 | histone cluster 1, H3f [Homo sapiens] |
| U | *gi|88976633|ref|XP\_93* | 2 | 3 | 14.7% | 136 | 15226 | 11.0 | PREDICTED: hypothetical protein [Homo sapiens] |
| U | *gi|53793688|ref|NP\_00* | 2 | 3 | 14.7% | 136 | 15388 | 11.3 | histone cluster 2, H3a [Homo sapiens] |
| U | *gi|4885385|ref|NP\_005* | 2 | 3 | 14.7% | 136 | 15328 | 11.3 | H3 histone, family 3B [Homo sapiens] |
| U | *gi|4504299|ref|NP\_003* | 2 | 3 | 14.7% | 136 | 15508 | 11.1 | histone cluster 3, H3 [Homo sapiens] |
| U | *gi|4504297|ref|NP\_003* | 2 | 3 | 14.7% | 136 | 15404 | 11.1 | histone cluster 1, H3b [Homo sapiens] |
| U | *gi|4504295|ref|NP\_003* | 2 | 3 | 14.7% | 136 | 15404 | 11.1 | histone cluster 1, H3h [Homo sapiens] |
| U | *gi|4504293|ref|NP\_003* | 2 | 3 | 14.7% | 136 | 15404 | 11.1 | histone cluster 1, H3j [Homo sapiens] |
| U | *gi|4504291|ref|NP\_003* | 2 | 3 | 14.7% | 136 | 15404 | 11.1 | H3 histone family, member H [Homo sapiens] |
| U | *gi|4504289|ref|NP\_003* | 2 | 3 | 14.7% | 136 | 15404 | 11.1 | histone cluster 1, H3i [Homo sapiens] |
| U | *gi|4504287|ref|NP\_003* | 2 | 3 | 14.7% | 136 | 15404 | 11.1 | histone cluster 1, H3e [Homo sapiens] |
| U | *gi|4504285|ref|NP\_003* | 2 | 3 | 14.7% | 136 | 15404 | 11.1 | histone cluster 1, H3c [Homo sapiens] |
| U | *gi|4504281|ref|NP\_003* | 2 | 3 | 14.7% | 136 | 15404 | 11.1 | histone cluster 1, H3a [Homo sapiens] |
| U | *gi|4504279|ref|NP\_002* | 2 | 3 | 14.7% | 136 | 15328 | 11.3 | H3 histone, family 3A [Homo sapiens] |
| U | *gi|31742503|ref|NP\_06* | 2 | 3 | 14.7% | 136 | 15388 | 11.3 | histone cluster 2, H3c [Homo sapiens] |
| U | *gi|183076548|ref|NP\_0* | 2 | 3 | 14.7% | 136 | 15388 | 11.3 | histone cluster 2, H3d [Homo sapiens] |
| U | *gi|169167131|ref|XP\_0* | 2 | 3 | 14.7% | 136 | 15226 | 11.0 | PREDICTED: hypothetical protein [Homo sapiens] |
| U | *gi|113416274|ref|XP\_0* | 2 | 3 | 14.7% | 136 | 15226 | 11.0 | PREDICTED: hypothetical protein [Homo sapiens] |

| Filename XCorr DeltCN Conf% ObsM+H+ CalcM+H+ SpR ZScore Ion% # Sequence  | | | | | | | | | | | | |
| --- | --- | --- | --- | --- | --- | --- | --- | --- | --- | --- | --- | --- |
|  | Astrin\_NLD\_STLC\_031014\_01.09021.09021.2 | 2.2531 | 0.2629 | 98.7% | 1032.7522 | 1033.2186 | 296 | 4.699 | 56.2% | 1 | R.YRPGTVALR.E | 2 |
|  | Astrin\_NLD\_STLC\_tube2\_021014\_01.06610.06610.2 | 2.8657 | 0.3672 | 100.0% | 1336.1522 | 1336.4875 | 2 | 6.169 | 70.0% | 2 | R.EIAQDFKTDLR.F | 2 |

---

|  |  |  |  |  |  |  |  |  |
| --- | --- | --- | --- | --- | --- | --- | --- | --- |
| U | *gi|20149594|ref|NP\_03* | 8 | 11 | 14.6% | 724 | 83264 | 5.0 | heat shock 90kDa protein 1, beta [Homo sapiens] |

| Filename XCorr DeltCN Conf% ObsM+H+ CalcM+H+ SpR ZScore Ion% # Sequence  | | | | | | | | | | | | |
| --- | --- | --- | --- | --- | --- | --- | --- | --- | --- | --- | --- | --- |
|  | Astrin\_NLD\_STLC\_031014\_02.05927.05927.3 | 3.7325 | 0.4142 | 100.0% | 2015.7244 | 2016.2584 | 1 | 6.73 | 40.0% | 1 | K.VILHLKEDQTEYLEER.R | 3 |
|  | Astrin\_NLD\_STLC\_tube2\_021014\_01.04584.04584.2 | 2.4536 | 0.1736 | 96.8% | 1152.0521 | 1152.2462 | 2 | 4.902 | 81.2% | 1 | K.YIDQEELNK.T | 2 |
| \* | Astrin\_NLD\_STLC\_tube2\_021014\_02.06621.06621.2 | 3.6354 | 0.3033 | 100.0% | 1848.2522 | 1848.9171 | 1 | 5.839 | 67.9% | 1 | R.NPDDITQEEYGEFYK.S | 2 |
|  | Astrin\_NLD\_STLC\_tube2\_021014\_02.06472.06472.2 | 3.0916 | 0.4043 | 100.0% | 1349.2722 | 1349.4886 | 1 | 6.76 | 65.0% | 2 | K.HFSVEGQLEFR.A | 2 |
| \* | Astrin\_NLD\_STLC\_tube2\_021014\_02.06506.06506.3 | 4.448 | 0.3684 | 100.0% | 2179.1943 | 2178.2915 | 1 | 6.389 | 36.1% | 3 | R.YHTSQSGDEMTSLSEYVSR.M | 3 |
| \* | Astrin\_NLD\_STLC\_tube2\_021014\_01.06894.06894.2 | 2.1488 | 0.3197 | 99.3% | 1161.9922 | 1161.297 | 29 | 5.324 | 55.6% | 1 | K.SIYYITGESK.E | 2 |
| \* | Astrin\_NLD\_STLC\_tube2\_021014\_01.05094.05094.2 | 2.3297 | 0.3616 | 99.9% | 1250.0922 | 1250.3538 | 165 | 6.082 | 50.0% | 1 | K.EQVANSAFVER.V | 2 |
| \* | Astrin\_NLD\_STLC\_tube2\_021014\_01.07688.07688.3 | 3.6665 | 0.4604 | 100.0% | 1784.2444 | 1784.025 | 1 | 7.121 | 48.2% | 1 | K.HLEINPDHPIVETLR.Q | 3 |

---

|  |  |  |  |  |  |  |  |  |
| --- | --- | --- | --- | --- | --- | --- | --- | --- |
| U | *gi|14043072|ref|NP\_11* | 3 | 4 | 14.4% | 353 | 37430 | 8.9 | heterogeneous nuclear ribonucleoprotein A2/B1 isoform B1 [Homo sapiens] |
| U | *gi|4504447|ref|NP\_002* | 3 | 4 | 15.0% | 341 | 36006 | 8.6 | heterogeneous nuclear ribonucleoprotein A2/B1 isoform A2 [Homo sapiens] |

| Filename XCorr DeltCN Conf% ObsM+H+ CalcM+H+ SpR ZScore Ion% # Sequence  | | | | | | | | | | | | |
| --- | --- | --- | --- | --- | --- | --- | --- | --- | --- | --- | --- | --- |
|  | Astrin\_NLD\_STLC\_tube2\_021014\_01.12964.12964.2 | 4.1868 | 0.3029 | 100.0% | 1800.7122 | 1800.0184 | 1 | 6.032 | 63.3% | 1 | K.LFIGGLSFETTEESLR.N | 2 |
|  | Astrin\_NLD\_STLC\_tube2\_021014\_01.11607.11607.3 | 3.2112 | 0.3871 | 100.0% | 2278.7644 | 2278.5693 | 1 | 6.285 | 34.2% | 2 | R.GFGFVTFDDHDPVDKIVLQK.Y | 3 |
|  | Astrin\_NLD\_STLC\_tube2\_021014\_01.06022.06022.2 | 3.1089 | 0.468 | 100.0% | 1377.9722 | 1378.4465 | 1 | 7.207 | 57.1% | 1 | R.GGGGNFGPGPGSNFR.G | 2 |

---

|  |  |  |  |  |  |  |  |  |
| --- | --- | --- | --- | --- | --- | --- | --- | --- |
| U | *gi|10835063|ref|NP\_00* | 2 | 2 | 14.3% | 294 | 32575 | 4.8 | nucleophosmin 1 isoform 1 [Homo sapiens] |
| U | *gi|40353734|ref|NP\_95* | 2 | 2 | 15.8% | 265 | 29465 | 4.6 | nucleophosmin 1 isoform 2 [Homo sapiens] |

| Filename XCorr DeltCN Conf% ObsM+H+ CalcM+H+ SpR ZScore Ion% # Sequence  | | | | | | | | | | | | |
| --- | --- | --- | --- | --- | --- | --- | --- | --- | --- | --- | --- | --- |
|  | Astrin\_NLD\_STLC\_tube2\_021014\_01.11933.11933.3 | 5.4874 | 0.4468 | 100.0% | 2931.1443 | 2931.2874 | 1 | 7.843 | 32.4% | 1 | R.TVSLGAGAKDELHIVEAEAMNYEGSPIK.V | 3 |
|  | Astrin\_NLD\_STLC\_tube2\_021014\_01.13773.13773.2 | 3.311 | 0.2644 | 100.0% | 1820.1721 | 1821.0172 | 1 | 5.87 | 61.5% | 1 | R.MTDQEAIQDLWQWR.K | 2 |

---

|  |  |  |  |  |  |  |  |  |
| --- | --- | --- | --- | --- | --- | --- | --- | --- |
| U | *gi|16753227|ref|NP\_00* | 3 | 3 | 14.2% | 288 | 32728 | 10.6 | ribosomal protein L6 [Homo sapiens] |
| U | *gi|67189747|ref|NP\_00* | 3 | 3 | 14.2% | 288 | 32728 | 10.6 | ribosomal protein L6 [Homo sapiens] |

| Filename XCorr DeltCN Conf% ObsM+H+ CalcM+H+ SpR ZScore Ion% # Sequence  | | | | | | | | | | | | |
| --- | --- | --- | --- | --- | --- | --- | --- | --- | --- | --- | --- | --- |
|  | Astrin\_NLD\_STLC\_031014\_01.08940.08940.2 | 2.5387 | 0.3982 | 100.0% | 1284.3722 | 1285.5266 | 9 | 6.593 | 54.2% | 1 | K.VLATVTKPVGGDK.N | 2 |
|  | Astrin\_NLD\_STLC\_tube2\_021014\_01.05270.05270.2 | 2.5377 | 0.3519 | 100.0% | 995.2522 | 995.1228 | 4 | 6.689 | 71.4% | 1 | K.HLTDAYFK.K | 2 |
|  | Astrin\_NLD\_STLC\_tube2\_021014\_02.05346.05346.3 | 3.9997 | 0.415 | 100.0% | 2510.0645 | 2510.6763 | 1 | 5.951 | 38.2% | 1 | R.HQEGEIFDTEKEKYEITEQR.K | 3 |

---

|  |  |  |  |  |  |  |  |  |
| --- | --- | --- | --- | --- | --- | --- | --- | --- |
| U | *gi|21396489|ref|NP\_00* | 9 | 12 | 14.0% | 959 | 106489 | 6.4 | mitochondrial lon peptidase 1 [Homo sapiens] |

| Filename XCorr DeltCN Conf% ObsM+H+ CalcM+H+ SpR ZScore Ion% # Sequence  | | | | | | | | | | | | |
| --- | --- | --- | --- | --- | --- | --- | --- | --- | --- | --- | --- | --- |
| \* | Astrin\_NLD\_STLC\_tube2\_021014\_01.10926.10926.2 | 2.5752 | 0.2318 | 98.7% | 1235.0322 | 1235.512 | 2 | 6.045 | 70.0% | 1 | R.LAQPYVGVFLK.R | 2 |
| \* | Astrin\_NLD\_STLC\_tube2\_021014\_01.11896.11896.2 | 2.5123 | 0.2 | 95.7% | 1558.1721 | 1558.8644 | 2 | 4.057 | 58.3% | 1 | K.TIRDIIALNPLYR.E | 2 |
| \* | Astrin\_NLD\_STLC\_tube2\_021014\_01.10115.10115.2 | 2.7686 | 0.2105 | 98.7% | 1378.1322 | 1378.6044 | 3 | 5.263 | 63.6% | 1 | R.ESVLQMMQAGQR.V | 2 |
| \* | Astrin\_NLD\_STLC\_tube2\_021014\_01.08164.08164.2 | 3.8813 | 0.5257 | 100.0% | 1401.2122 | 1401.5745 | 1 | 9.352 | 77.3% | 1 | K.HVMDVVDEELSK.L | 2 |
| \* | Astrin\_NLD\_STLC\_tube2\_021014\_02.06656.06656.3 | 4.7187 | 0.4163 | 100.0% | 1703.2444 | 1702.8644 | 1 | 7.051 | 42.9% | 3 | K.LGLLDNHSSEFNVTR.N | 3 |
| \* | Astrin\_NLD\_STLC\_tube2\_021014\_01.15963.15963.2 | 2.9557 | 0.3142 | 100.0% | 1593.2122 | 1593.8223 | 1 | 5.718 | 62.5% | 2 | R.NYLDWLTSIPWGK.Y | 2 |
| \* | Astrin\_NLD\_STLC\_tube2\_021014\_01.05740.05740.2 | 3.1533 | 0.3038 | 100.0% | 1195.1522 | 1195.2743 | 1 | 6.695 | 77.8% | 1 | K.YSNENLDLAR.A | 2 |
| \* | Astrin\_NLD\_STLC\_tube2\_021014\_01.09702.09702.2 | 2.4819 | 0.2624 | 98.7% | 1354.2722 | 1354.561 | 5 | 4.904 | 58.3% | 1 | R.FSVGGMTDVAEIK.G | 2 |
| \* | Astrin\_NLD\_STLC\_tube2\_021014\_01.17115.17115.3 | 5.5539 | 0.566 | 100.0% | 3874.5842 | 3875.2373 | 1 | 9.682 | 26.5% | 1 | R.GYQGDPSSALLELLDPEQNANFLDHYLDVPVDLSK.V | 3 |

---

|  |  |  |  |  |  |  |  |  |
| --- | --- | --- | --- | --- | --- | --- | --- | --- |
| U | *gi|4506607|ref|NP\_000* | 2 | 5 | 13.8% | 188 | 21634 | 11.7 | ribosomal protein L18 [Homo sapiens] |

| Filename XCorr DeltCN Conf% ObsM+H+ CalcM+H+ SpR ZScore Ion% # Sequence  | | | | | | | | | | | | |
| --- | --- | --- | --- | --- | --- | --- | --- | --- | --- | --- | --- | --- |
| \* | Astrin\_NLD\_STLC\_tube2\_021014\_02.06194.06194.2 | 3.5023 | 0.4594 | 100.0% | 1346.4122 | 1346.5236 | 1 | 8.244 | 75.0% | 4 | K.TAVVVGTITDDVR.V | 2 |
| \* | Astrin\_NLD\_STLC\_tube2\_021014\_01.11723.11723.2 | 3.2085 | 0.4719 | 100.0% | 1460.6322 | 1461.6982 | 1 | 8.082 | 62.5% | 1 | K.ILTFDQLALDSPK.G | 2 |

---

|  |  |  |  |  |  |  |  |  |
| --- | --- | --- | --- | --- | --- | --- | --- | --- |
| U | *gi|4504523|ref|NP\_002* | 1 | 1 | 13.7% | 102 | 10932 | 8.9 | heat shock 10kDa protein 1 [Homo sapiens] |

| Filename XCorr DeltCN Conf% ObsM+H+ CalcM+H+ SpR ZScore Ion% # Sequence  | | | | | | | | | | | | |
| --- | --- | --- | --- | --- | --- | --- | --- | --- | --- | --- | --- | --- |
| \* | Astrin\_NLD\_STLC\_tube2\_021014\_02.05181.05181.2 | 2.4657 | 0.4517 | 100.0% | 1315.5922 | 1316.5406 | 1 | 6.816 | 57.7% | 1 | K.VLQATVVAVGSGSK.G | 2 |

---

|  |  |  |  |  |  |  |  |  |
| --- | --- | --- | --- | --- | --- | --- | --- | --- |
| U | *gi|63055057|ref|NP\_00* | 3 | 5 | 13.6% | 376 | 42003 | 5.6 | actin, beta-like 2 [Homo sapiens] |

| Filename XCorr DeltCN Conf% ObsM+H+ CalcM+H+ SpR ZScore Ion% # Sequence  | | | | | | | | | | | | |
| --- | --- | --- | --- | --- | --- | --- | --- | --- | --- | --- | --- | --- |
| \* | Astrin\_NLD\_STLC\_tube2\_021014\_01.08750.08750.3 | 3.3675 | 0.2062 | 95.4% | 2339.0942 | 2338.7122 | 2 | 4.763 | 33.8% | 1 | R.VAPDEHPILLTEAPLNPKINR.E | 3 |
|  | Astrin\_NLD\_STLC\_tube2\_021014\_01.11475.11475.2 | 2.9524 | 0.2059 | 98.7% | 1624.5322 | 1624.8927 | 2 | 5.245 | 57.7% | 1 | R.LDLAGRDLTDYLMK.I | 222 |
|  | Astrin\_NLD\_STLC\_tube2\_021014\_01.10676.10676.2 | 4.8426 | 0.3035 | 100.0% | 1791.2922 | 1791.9554 | 1 | 8.531 | 83.3% | 3 | R.SYELPDGQVITIGNER.F | 222 |

Similarities:
gi|4501885|ref|NP\_001(2:1)  
gi|4501881|ref|NP\_001(2:1)  

---

|  |  |  |  |  |  |  |  |  |
| --- | --- | --- | --- | --- | --- | --- | --- | --- |
| U | *gi|169214003|ref|XP\_0* | 1 | 1 | 13.4% | 97 | 10967 | 11.1 | PREDICTED: similar to ribosomal protein L37 [Homo sapiens] |
| U | *gi|89057609|ref|XP\_94* | 1 | 1 | 13.4% | 97 | 10967 | 11.1 | PREDICTED: similar to ribosomal protein L37 [Homo sapiens] |
| U | *gi|51474779|ref|XP\_49* | 1 | 1 | 13.4% | 97 | 10967 | 11.1 | PREDICTED: similar to ribosomal protein L37 [Homo sapiens] |

| Filename XCorr DeltCN Conf% ObsM+H+ CalcM+H+ SpR ZScore Ion% # Sequence  | | | | | | | | | | | | |
| --- | --- | --- | --- | --- | --- | --- | --- | --- | --- | --- | --- | --- |
|  | Astrin\_NLD\_STLC\_031014\_01.06598.06598.2 | 2.5324 | 0.2154 | 97.1% | 1402.2922 | 1401.5834 | 5 | 4.518 | 54.2% | 1 | R.QNTTGTGGMRHLK.I | 2 |

---

|  |  |  |  |  |  |  |  |  |
| --- | --- | --- | --- | --- | --- | --- | --- | --- |
| U | *gi|113426225|ref|XP\_0* | 1 | 1 | 13.3% | 166 | 17477 | 8.5 | PREDICTED: FLJ44904 protein [Homo sapiens] |
| U | *gi|169209551|ref|XP\_0* | 1 | 1 | 13.3% | 166 | 17477 | 8.5 | PREDICTED: FLJ44904 protein [Homo sapiens] |

| Filename XCorr DeltCN Conf% ObsM+H+ CalcM+H+ SpR ZScore Ion% # Sequence  | | | | | | | | | | | | |
| --- | --- | --- | --- | --- | --- | --- | --- | --- | --- | --- | --- | --- |
|  | Astrin\_NLD\_STLC\_tube2\_021014\_01.23591.23591.3 | 2.7581 | 0.3045 | 98.4% | 2381.4844 | 2380.4106 | 190 | 4.617 | 27.4% | 1 | R.S\*S\*SSALGLRLCALAGSGLGS\*FK.F | 3 |

---

|  |  |  |  |  |  |  |  |  |
| --- | --- | --- | --- | --- | --- | --- | --- | --- |
| U | *gi|15431306|ref|NP\_15* | 3 | 3 | 13.2% | 257 | 28025 | 11.0 | ribosomal protein L8 [Homo sapiens] |
| U | *gi|4506663|ref|NP\_000* | 3 | 3 | 13.2% | 257 | 28025 | 11.0 | ribosomal protein L8 [Homo sapiens] |

| Filename XCorr DeltCN Conf% ObsM+H+ CalcM+H+ SpR ZScore Ion% # Sequence  | | | | | | | | | | | | |
| --- | --- | --- | --- | --- | --- | --- | --- | --- | --- | --- | --- | --- |
|  | Astrin\_NLD\_STLC\_tube2\_021014\_01.04925.04925.2 | 4.1925 | 0.5014 | 100.0% | 1689.3922 | 1689.8223 | 1 | 8.004 | 60.0% | 1 | R.ASGNYATVISHNPETK.K | 2 |
|  | Astrin\_NLD\_STLC\_031014\_01.09056.09056.1 | 2.6091 | 0.4522 | 100.0% | 941.62 | 942.1062 | 15 | 7.54 | 50.0% | 1 | R.AVVGVVAGGGR.I | 1 |
|  | Astrin\_NLD\_STLC\_031014\_02.05590.05590.3 | 2.726 | 0.307 | 99.1% | 1750.4644 | 1750.138 | 1 | 4.731 | 42.6% | 1 | R.AVVGVVAGGGRIDKPILK.A | 3 |

---

|  |  |  |  |  |  |  |  |  |
| --- | --- | --- | --- | --- | --- | --- | --- | --- |
| U | *gi|4503529|ref|NP\_001* | 4 | 4 | 13.1% | 406 | 46154 | 5.5 | eukaryotic translation initiation factor 4A isoform 1 [Homo sapiens] |

| Filename XCorr DeltCN Conf% ObsM+H+ CalcM+H+ SpR ZScore Ion% # Sequence  | | | | | | | | | | | | |
| --- | --- | --- | --- | --- | --- | --- | --- | --- | --- | --- | --- | --- |
|  | Astrin\_NLD\_STLC\_tube2\_021014\_01.07902.07902.2 | 4.6949 | 0.516 | 100.0% | 1828.5721 | 1829.0654 | 1 | 8.512 | 73.3% | 1 | R.GIYAYGFEKPSAIQQR.A | 2 |
| \* | Astrin\_NLD\_STLC\_tube2\_021014\_01.08319.08319.3 | 3.2215 | 0.2228 | 97.8% | 1619.2743 | 1619.9225 | 65 | 4.88 | 33.9% | 1 | K.LQMEAPHIIVGTPGR.V | 3 |
| \* | Astrin\_NLD\_STLC\_tube2\_021014\_01.11021.11021.2 | 2.8263 | 0.1325 | 95.1% | 1503.8322 | 1502.71 | 2 | 4.362 | 59.1% | 1 | R.GFKDQIYDIFQK.L | 2 |
|  | Astrin\_NLD\_STLC\_tube2\_021014\_01.10412.10412.2 | 2.2991 | 0.3559 | 99.9% | 1115.1122 | 1115.3585 | 2 | 5.803 | 72.2% | 1 | R.VLITTDLLAR.G | 2 |

---

|  |  |  |  |  |  |  |  |  |
| --- | --- | --- | --- | --- | --- | --- | --- | --- |
| U | *gi|4885375|ref|NP\_005* | 3 | 6 | 13.1% | 213 | 21365 | 10.9 | histone cluster 1, H1c [Homo sapiens] |
| U | *gi|4885379|ref|NP\_005* | 3 | 6 | 12.8% | 219 | 21865 | 11.0 | histone cluster 1, H1e [Homo sapiens] |
| U | *gi|4885377|ref|NP\_005* | 3 | 6 | 12.7% | 221 | 22350 | 11.0 | histone cluster 1, H1d [Homo sapiens] |

| Filename XCorr DeltCN Conf% ObsM+H+ CalcM+H+ SpR ZScore Ion% # Sequence  | | | | | | | | | | | | |
| --- | --- | --- | --- | --- | --- | --- | --- | --- | --- | --- | --- | --- |
|  | Astrin\_NLD\_STLC\_031014\_01.09394.09394.2 | 3.5805 | 0.4076 | 100.0% | 1327.2922 | 1327.5638 | 1 | 6.76 | 75.0% | 3 | R.KASGPPVSELITK.A | 2 |
|  | Astrin\_NLD\_STLC\_tube2\_021014\_01.05177.05177.2 | 2.852 | 0.266 | 99.9% | 1109.0721 | 1108.2365 | 5 | 5.964 | 65.0% | 1 | K.ALAAAGYDVEK.N | 2 |
|  | Astrin\_NLD\_STLC\_tube2\_021014\_01.04505.04505.2 | 4.0855 | 0.4439 | 100.0% | 1579.3922 | 1579.7098 | 1 | 8.835 | 75.0% | 2 | K.ALAAAGYDVEKNNSR.I | 2 |

---

|  |  |  |  |  |  |  |  |  |
| --- | --- | --- | --- | --- | --- | --- | --- | --- |
| U | *gi|117189975|ref|NP\_1* | 3 | 4 | 12.7% | 306 | 33670 | 5.1 | heterogeneous nuclear ribonucleoprotein C isoform a [Homo sapiens] |
| U | *gi|117190254|ref|NP\_0* | 3 | 4 | 13.3% | 293 | 32338 | 5.1 | heterogeneous nuclear ribonucleoprotein C isoform b [Homo sapiens] |
| U | *gi|117190192|ref|NP\_0* | 3 | 4 | 12.7% | 306 | 33670 | 5.1 | heterogeneous nuclear ribonucleoprotein C isoform a [Homo sapiens] |
| U | *gi|117190174|ref|NP\_0* | 3 | 4 | 13.3% | 293 | 32338 | 5.1 | heterogeneous nuclear ribonucleoprotein C isoform b [Homo sapiens] |

| Filename XCorr DeltCN Conf% ObsM+H+ CalcM+H+ SpR ZScore Ion% # Sequence  | | | | | | | | | | | | |
| --- | --- | --- | --- | --- | --- | --- | --- | --- | --- | --- | --- | --- |
|  | Astrin\_NLD\_STLC\_031014\_02.08225.08225.2 | 3.3705 | 0.2153 | 99.9% | 1317.2522 | 1317.6145 | 1 | 6.393 | 77.3% | 2 | R.VFIGNLNTLVVK.K | 2 |
|  | Astrin\_NLD\_STLC\_tube2\_021014\_01.10630.10630.2 | 3.8638 | 0.4346 | 100.0% | 1331.3922 | 1330.4857 | 1 | 7.018 | 80.0% | 1 | K.GFAFVQYVNER.N | 2 |
|  | Astrin\_NLD\_STLC\_031014\_02.08248.08248.2 | 4.0925 | 0.3777 | 100.0% | 1683.3722 | 1684.0038 | 1 | 7.693 | 76.7% | 1 | R.MIAGQVLDINLAAEPK.V | 2 |

---

|  |  |  |  |  |  |  |  |  |
| --- | --- | --- | --- | --- | --- | --- | --- | --- |
| U | *gi|16418397|ref|NP\_44* | 1 | 1 | 12.5% | 176 | 19125 | 6.2 | MAL2 proteolipid protein [Homo sapiens] |

| Filename XCorr DeltCN Conf% ObsM+H+ CalcM+H+ SpR ZScore Ion% # Sequence  | | | | | | | | | | | | |
| --- | --- | --- | --- | --- | --- | --- | --- | --- | --- | --- | --- | --- |
| \* | Astrin\_NLD\_STLC\_031014\_02.10689.10689.2 | 2.5191 | 0.2672 | 98.6% | 2213.9722 | 2211.457 | 10 | 4.2 | 28.6% | 1 | -.MSAGGASVPPPPNPAVS\*FPPPR.V | 2 |

---

|  |  |  |  |  |  |  |  |  |
| --- | --- | --- | --- | --- | --- | --- | --- | --- |
| U | *gi|221307584|ref|NP\_0* | 3 | 13 | 12.4% | 299 | 33296 | 9.8 | prohibitin 2 isoform 1 [Homo sapiens] |
| U | *gi|6005854|ref|NP\_009* | 3 | 13 | 12.4% | 299 | 33296 | 9.8 | prohibitin 2 isoform 2 [Homo sapiens] |

| Filename XCorr DeltCN Conf% ObsM+H+ CalcM+H+ SpR ZScore Ion% # Sequence  | | | | | | | | | | | | |
| --- | --- | --- | --- | --- | --- | --- | --- | --- | --- | --- | --- | --- |
|  | Astrin\_NLD\_STLC\_tube2\_021014\_01.10796.10796.3 | 3.7386 | 0.3893 | 100.0% | 1855.1344 | 1855.1038 | 1 | 6.584 | 43.8% | 1 | R.IGGVQQDTILAEGLHFR.I | 3 |
|  | Astrin\_NLD\_STLC\_tube2\_021014\_01.06501.06501.2 | 2.2541 | 0.3451 | 100.0% | 995.3122 | 995.077 | 1 | 6.764 | 85.7% | 1 | R.LGLDYEER.V | 2 |
|  | Astrin\_NLD\_STLC\_031014\_01.06956.06956.2 | 3.2321 | 0.416 | 100.0% | 1215.4321 | 1216.3336 | 1 | 8.011 | 68.2% | 11 | K.IVQAEGEAEAAK.M | 2 |

---

|  |  |  |  |  |  |  |  |  |
| --- | --- | --- | --- | --- | --- | --- | --- | --- |
| U | *gi|15431295|ref|NP\_15* | 3 | 3 | 12.3% | 211 | 24261 | 11.7 | ribosomal protein L13 [Homo sapiens] |
| U | *gi|15431297|ref|NP\_00* | 3 | 3 | 12.3% | 211 | 24261 | 11.7 | ribosomal protein L13 [Homo sapiens] |

| Filename XCorr DeltCN Conf% ObsM+H+ CalcM+H+ SpR ZScore Ion% # Sequence  | | | | | | | | | | | | |
| --- | --- | --- | --- | --- | --- | --- | --- | --- | --- | --- | --- | --- |
|  | Astrin\_NLD\_STLC\_031014\_01.08555.08555.2 | 2.3169 | 0.3225 | 99.6% | 1475.0322 | 1475.6017 | 14 | 5.987 | 50.0% | 1 | R.NKSTESLQANVQR.L | 2 |
|  | Astrin\_NLD\_STLC\_031014\_01.08943.08943.2 | 3.0026 | 0.3673 | 100.0% | 1232.5521 | 1233.3237 | 1 | 6.274 | 85.0% | 1 | K.STESLQANVQR.L | 2 |
|  | Astrin\_NLD\_STLC\_tube2\_021014\_01.08506.08506.2 | 2.8892 | 0.2525 | 99.8% | 1384.1921 | 1383.6923 | 3 | 4.889 | 58.3% | 1 | K.LATQLTGPVMPVR.N | 2 |

---

|  |  |  |  |  |  |  |  |  |
| --- | --- | --- | --- | --- | --- | --- | --- | --- |
| U | *gi|34740329|ref|NP\_91* | 3 | 3 | 12.2% | 378 | 39595 | 9.0 | heterogeneous nuclear ribonucleoprotein A3 [Homo sapiens] |

| Filename XCorr DeltCN Conf% ObsM+H+ CalcM+H+ SpR ZScore Ion% # Sequence  | | | | | | | | | | | | |
| --- | --- | --- | --- | --- | --- | --- | --- | --- | --- | --- | --- | --- |
| \* | Astrin\_NLD\_STLC\_tube2\_021014\_02.06459.06459.3 | 4.0223 | 0.3032 | 100.0% | 1884.7743 | 1884.096 | 1 | 6.375 | 41.7% | 1 | K.IFVGGIKEDTEEYNLR.D | 3 |
| \* | Astrin\_NLD\_STLC\_tube2\_021014\_01.07974.07974.2 | 2.937 | 0.362 | 100.0% | 1234.9722 | 1235.3948 | 1 | 6.683 | 77.8% | 1 | K.IETIEVMEDR.Q | 2 |
|  | Astrin\_NLD\_STLC\_031014\_02.07984.07984.3 | 2.8367 | 0.2952 | 98.4% | 2281.0444 | 2282.5579 | 1 | 4.401 | 31.6% | 1 | R.GFAFVTFDDHDTVDKIVVQK.Y | 3 |

---

|  |  |  |  |  |  |  |  |  |
| --- | --- | --- | --- | --- | --- | --- | --- | --- |
| U | *gi|56847610|ref|NP\_00* | 1 | 1 | 12.1% | 182 | 20071 | 8.7 | transmembrane and coiled-coil domains 2 [Homo sapiens] |

| Filename XCorr DeltCN Conf% ObsM+H+ CalcM+H+ SpR ZScore Ion% # Sequence  | | | | | | | | | | | | |
| --- | --- | --- | --- | --- | --- | --- | --- | --- | --- | --- | --- | --- |
| \* | Astrin\_NLD\_STLC\_tube2\_021014\_01.20457.20457.3 | 2.5946 | 0.3377 | 99.4% | 2867.4243 | 2869.3123 | 99 | 2.944 | 23.8% | 1 | K.RSIQS\*IQKT#LLFVITLYK@LYKK@.G | 3 |

---

|  |  |  |  |  |  |  |  |  |
| --- | --- | --- | --- | --- | --- | --- | --- | --- |
| U | *gi|4758792|ref|NP\_004* | 1 | 2 | 12.1% | 124 | 13712 | 8.3 | NADH dehydrogenase (ubiquinone) Fe-S protein 6, 13kDa (NADH-coenzyme Q reductase) [Homo sapiens] |

| Filename XCorr DeltCN Conf% ObsM+H+ CalcM+H+ SpR ZScore Ion% # Sequence  | | | | | | | | | | | | |
| --- | --- | --- | --- | --- | --- | --- | --- | --- | --- | --- | --- | --- |
| \* | Astrin\_NLD\_STLC\_031014\_01.05938.05938.3 | 2.8398 | 0.2631 | 98.0% | 1853.1543 | 1853.989 | 160 | 5.231 | 32.1% | 2 | K.VTHTGQVYDDKDYRR.I | 3 |

---

|  |  |  |  |  |  |  |  |  |
| --- | --- | --- | --- | --- | --- | --- | --- | --- |
| U | *gi|15055539|ref|NP\_00* | 3 | 3 | 11.6% | 293 | 31324 | 10.2 | ribosomal protein S2 [Homo sapiens] |
| U | *gi|169205508|ref|XP\_0* | 3 | 3 | 14.6% | 233 | 25545 | 9.9 | PREDICTED: hypothetical protein isoform 2 [Homo sapiens] |
| U | *gi|169205506|ref|XP\_0* | 3 | 3 | 11.6% | 293 | 31364 | 10.2 | PREDICTED: hypothetical protein isoform 1 [Homo sapiens] |
| U | *gi|169204986|ref|XP\_0* | 3 | 3 | 14.6% | 233 | 25545 | 9.9 | PREDICTED: hypothetical protein isoform 2 [Homo sapiens] |
| U | *gi|169204984|ref|XP\_0* | 3 | 3 | 11.6% | 293 | 31364 | 10.2 | PREDICTED: hypothetical protein isoform 1 [Homo sapiens] |
| U | *gi|169204456|ref|XP\_0* | 3 | 3 | 14.6% | 233 | 25619 | 9.8 | PREDICTED: hypothetical protein isoform 2 [Homo sapiens] |
| U | *gi|169204454|ref|XP\_0* | 3 | 3 | 11.6% | 293 | 31438 | 10.2 | PREDICTED: hypothetical protein isoform 1 [Homo sapiens] |

| Filename XCorr DeltCN Conf% ObsM+H+ CalcM+H+ SpR ZScore Ion% # Sequence  | | | | | | | | | | | | |
| --- | --- | --- | --- | --- | --- | --- | --- | --- | --- | --- | --- | --- |
|  | Astrin\_NLD\_STLC\_tube2\_021014\_01.05202.05202.2 | 2.4001 | 0.269 | 98.9% | 1026.0521 | 1026.2212 | 38 | 5.565 | 60.0% | 1 | R.GTGIVSAPVPK.K | 2 |
|  | Astrin\_NLD\_STLC\_tube2\_021014\_01.11571.11571.2 | 2.3464 | 0.4452 | 100.0% | 1386.8922 | 1387.575 | 4 | 6.6 | 55.0% | 1 | K.TYSYLTPDLWK.E | 2 |
|  | Astrin\_NLD\_STLC\_tube2\_021014\_01.08903.08903.3 | 2.6913 | 0.2756 | 98.9% | 1464.9543 | 1464.6177 | 1 | 5.526 | 47.7% | 1 | K.SPYQEFTDHLVK.T | 3 |

---

|  |  |  |  |  |  |  |  |  |
| --- | --- | --- | --- | --- | --- | --- | --- | --- |
| U | *gi|15809016|ref|NP\_29* | 1 | 1 | 11.6% | 172 | 19779 | 4.8 | myosin regulatory light chain MRCL2 isoform A [Homo sapiens] |
| U | *gi|5453740|ref|NP\_006* | 1 | 1 | 11.7% | 171 | 19794 | 4.8 | myosin, light chain 12A, regulatory, non-sarcomeric [Homo sapiens] |
| U | *gi|222144328|ref|NP\_0* | 1 | 1 | 13.0% | 154 | 17757 | 4.4 | myosin regulatory light chain MRCL2 isoform B [Homo sapiens] |
| U | *gi|222144326|ref|NP\_0* | 1 | 1 | 11.6% | 172 | 19779 | 4.8 | myosin regulatory light chain MRCL2 isoform A [Homo sapiens] |
| U | *gi|222144324|ref|NP\_0* | 1 | 1 | 11.6% | 172 | 19779 | 4.8 | myosin regulatory light chain MRCL2 isoform A [Homo sapiens] |

| Filename XCorr DeltCN Conf% ObsM+H+ CalcM+H+ SpR ZScore Ion% # Sequence  | | | | | | | | | | | | |
| --- | --- | --- | --- | --- | --- | --- | --- | --- | --- | --- | --- | --- |
|  | Astrin\_NLD\_STLC\_tube2\_021014\_01.11734.11734.3 | 3.9553 | 0.3759 | 100.0% | 2433.1743 | 2433.649 | 1 | 6.801 | 36.8% | 1 | R.ELLTTMGDRFTDEEVDELYR.E | 3 |

---

|  |  |  |  |  |  |  |  |  |
| --- | --- | --- | --- | --- | --- | --- | --- | --- |
| U | *gi|31542947|ref|NP\_00* | 4 | 6 | 11.2% | 573 | 61055 | 5.9 | chaperonin [Homo sapiens] |
| U | *gi|41399285|ref|NP\_95* | 4 | 6 | 11.2% | 573 | 61055 | 5.9 | chaperonin [Homo sapiens] |

| Filename XCorr DeltCN Conf% ObsM+H+ CalcM+H+ SpR ZScore Ion% # Sequence  | | | | | | | | | | | | |
| --- | --- | --- | --- | --- | --- | --- | --- | --- | --- | --- | --- | --- |
|  | Astrin\_NLD\_STLC\_tube2\_021014\_02.05594.05594.3 | 3.8113 | 0.511 | 100.0% | 2561.5444 | 2561.7222 | 1 | 7.848 | 31.2% | 1 | K.LVQDVANNTNEEAGDGTTTATVLAR.S | 3 |
|  | Astrin\_NLD\_STLC\_tube2\_021014\_02.06635.06635.3 | 3.032 | 0.2976 | 99.8% | 1631.0044 | 1631.9684 | 3 | 5.895 | 39.3% | 1 | K.VGEVIVTKDDAMLLK.G | 3 |
|  | Astrin\_NLD\_STLC\_031014\_01.05726.05726.2 | 2.6436 | 0.4257 | 100.0% | 1234.3522 | 1234.3055 | 12 | 6.724 | 59.1% | 3 | K.VGGTSDVEVNEK.K | 2 |
|  | Astrin\_NLD\_STLC\_tube2\_021014\_02.05440.05440.2 | 2.7129 | 0.3033 | 99.9% | 1215.4922 | 1216.377 | 1 | 5.749 | 72.7% | 1 | K.NAGVEGSLIVEK.I | 2 |

---

|  |  |  |  |  |  |  |  |  |
| --- | --- | --- | --- | --- | --- | --- | --- | --- |
| U | *gi|24234699|ref|NP\_00* | 7 | 14 | 11.2% | 400 | 44106 | 5.1 | keratin 19 [Homo sapiens] |

| Filename XCorr DeltCN Conf% ObsM+H+ CalcM+H+ SpR ZScore Ion% # Sequence  | | | | | | | | | | | | |
| --- | --- | --- | --- | --- | --- | --- | --- | --- | --- | --- | --- | --- |
|  | Astrin\_NLD\_STLC\_tube2\_021014\_01.05645.05645.2 | 2.9461 | 0.1436 | 98.9% | 1065.2122 | 1065.2578 | 50 | 6.07 | 62.5% | 2 | R.LASYLDKVR.A | 2222 |
|  | Astrin\_NLD\_STLC\_tube2\_021014\_01.07084.07084.1 | 2.2993 | 0.2542 | 100.0% | 1041.64 | 1042.2235 | 1 | 6.129 | 75.0% | 1 | R.IVLQIDNAR.L | 11 |
|  | Astrin\_NLD\_STLC\_tube2\_021014\_01.07100.07100.2 | 3.2063 | 0.1355 | 99.7% | 1042.1122 | 1042.2235 | 1 | 6.273 | 87.5% | 5 | R.IVLQIDNAR.L | 22 |
|  | Astrin\_NLD\_STLC\_tube2\_021014\_01.05060.05060.2 | 2.4574 | 0.2931 | 100.0% | 807.9122 | 807.8815 | 9 | 6.526 | 75.0% | 2 | R.LAADDFR.T | 22222 |
|  | Astrin\_NLD\_STLC\_tube2\_021014\_01.04334.04334.2 | 2.6976 | 0.3418 | 100.0% | 1223.0122 | 1223.3715 | 19 | 5.786 | 66.7% | 1 | R.TKFETEQALR.M | 22 |
|  | Astrin\_NLD\_STLC\_tube2\_021014\_01.07816.07816.2 | 2.407 | 0.225 | 98.0% | 1186.8522 | 1186.397 | 4 | 5.0 | 72.2% | 1 | R.RVLDELTLAR.T | 222 |
|  | Astrin\_NLD\_STLC\_tube2\_021014\_01.08553.08553.2 | 3.5532 | 0.3938 | 100.0% | 1030.2322 | 1030.2096 | 1 | 7.35 | 87.5% | 2 | R.VLDELTLAR.T | 222 |

Similarities:
gi|40354195|ref|NP\_95(3:4)  
gi|4557701|ref|NP\_000(5:2)  
contaminant\_KERATIN03(2:5)  
contaminant\_KERATIN05(4:3)  

---

|  |  |  |  |  |  |  |  |  |
| --- | --- | --- | --- | --- | --- | --- | --- | --- |
| U | *gi|32455264|ref|NP\_85* | 2 | 2 | 11.1% | 199 | 22110 | 8.1 | peroxiredoxin 1 [Homo sapiens] |
| U | *gi|4505591|ref|NP\_002* | 2 | 2 | 11.1% | 199 | 22110 | 8.1 | peroxiredoxin 1 [Homo sapiens] |
| U | *gi|32455266|ref|NP\_85* | 2 | 2 | 11.1% | 199 | 22110 | 8.1 | peroxiredoxin 1 [Homo sapiens] |

| Filename XCorr DeltCN Conf% ObsM+H+ CalcM+H+ SpR ZScore Ion% # Sequence  | | | | | | | | | | | | |
| --- | --- | --- | --- | --- | --- | --- | --- | --- | --- | --- | --- | --- |
|  | Astrin\_NLD\_STLC\_tube2\_021014\_01.06035.06035.2 | 2.4305 | 0.2986 | 99.7% | 1164.8722 | 1165.3496 | 9 | 5.883 | 60.0% | 1 | K.ATAVMPDGQFK.D | 2 |
|  | Astrin\_NLD\_STLC\_tube2\_021014\_01.07878.07878.2 | 2.63 | 0.2523 | 99.6% | 1212.0721 | 1212.3915 | 37 | 5.263 | 70.0% | 1 | R.QITVNDLPVGR.S | 22 |

Similarities:
gi|32189392|ref|NP\_00(1:1)  

---

|  |  |  |  |  |  |  |  |  |
| --- | --- | --- | --- | --- | --- | --- | --- | --- |
| U | *gi|169164494|ref|XP\_0* | 1 | 1 | 11.0% | 100 | 11493 | 10.1 | PREDICTED: similar to ribosomal protein L10 [Homo sapiens] |
| U | *gi|41151097|ref|XP\_20* | 1 | 1 | 5.1% | 214 | 24627 | 10.1 | PREDICTED: similar to QM protein isoform 1 [Homo sapiens] |
| U | *gi|223890243|ref|NP\_0* | 1 | 1 | 5.1% | 214 | 24604 | 10.1 | ribosomal protein L10 [Homo sapiens] |
| U | *gi|18152783|ref|NP\_54* | 1 | 1 | 5.1% | 214 | 24519 | 10.0 | ribosomal protein L10-like protein [Homo sapiens] |
| U | *gi|169213734|ref|XP\_0* | 1 | 1 | 6.6% | 167 | 19409 | 9.9 | PREDICTED: similar to Q1Z 7F5 isoform 2 [Homo sapiens] |
| U | *gi|169213732|ref|XP\_0* | 1 | 1 | 5.1% | 214 | 24600 | 10.1 | PREDICTED: similar to Q1Z 7F5 isoform 1 [Homo sapiens] |
| U | *gi|169213538|ref|XP\_0* | 1 | 1 | 6.6% | 167 | 19436 | 9.9 | PREDICTED: similar to QM protein isoform 2 [Homo sapiens] |
| U | *gi|169213536|ref|XP\_0* | 1 | 1 | 5.1% | 214 | 24627 | 10.1 | PREDICTED: similar to QM protein isoform 1 [Homo sapiens] |

| Filename XCorr DeltCN Conf% ObsM+H+ CalcM+H+ SpR ZScore Ion% # Sequence  | | | | | | | | | | | | |
| --- | --- | --- | --- | --- | --- | --- | --- | --- | --- | --- | --- | --- |
|  | Astrin\_NLD\_STLC\_tube2\_021014\_01.09238.09238.2 | 2.6544 | 0.4687 | 100.0% | 1253.3322 | 1253.5486 | 1 | 7.914 | 70.0% | 1 | R.VHIGQVIMSIR.T | 2 |

---

|  |  |  |  |  |  |  |  |  |
| --- | --- | --- | --- | --- | --- | --- | --- | --- |
| U | *gi|14165469|ref|NP\_00* | 1 | 1 | 10.8% | 130 | 14839 | 10.1 | ribosomal protein S15a [Homo sapiens] |
| U | *gi|71772415|ref|NP\_00* | 1 | 1 | 10.8% | 130 | 14839 | 10.1 | ribosomal protein S15a [Homo sapiens] |

| Filename XCorr DeltCN Conf% ObsM+H+ CalcM+H+ SpR ZScore Ion% # Sequence  | | | | | | | | | | | | |
| --- | --- | --- | --- | --- | --- | --- | --- | --- | --- | --- | --- | --- |
|  | Astrin\_NLD\_STLC\_tube2\_021014\_02.06724.06724.3 | 3.601 | 0.1927 | 98.4% | 1702.1943 | 1701.8357 | 48 | 4.568 | 36.5% | 1 | K.HGYIGEFEIIDDHR.A | 3 |

---

|  |  |  |  |  |  |  |  |  |
| --- | --- | --- | --- | --- | --- | --- | --- | --- |
| U | *gi|4506605|ref|NP\_000* | 1 | 5 | 10.7% | 140 | 14865 | 10.5 | ribosomal protein L23 [Homo sapiens] |

| Filename XCorr DeltCN Conf% ObsM+H+ CalcM+H+ SpR ZScore Ion% # Sequence  | | | | | | | | | | | | |
| --- | --- | --- | --- | --- | --- | --- | --- | --- | --- | --- | --- | --- |
| \* | Astrin\_NLD\_STLC\_tube2\_021014\_01.10746.10746.2 | 3.6426 | 0.4953 | 100.0% | 1460.3522 | 1460.7902 | 1 | 8.943 | 75.0% | 5 | R.LPAAGVGDMVMATVK.K | 2 |

---

|  |  |  |  |  |  |  |  |  |
| --- | --- | --- | --- | --- | --- | --- | --- | --- |
| U | *gi|148470397|ref|NP\_0* | 3 | 5 | 10.6% | 415 | 45672 | 5.6 | heterogeneous nuclear ribonucleoprotein F [Homo sapiens] |
| U | *gi|4826760|ref|NP\_004* | 3 | 5 | 10.6% | 415 | 45672 | 5.6 | heterogeneous nuclear ribonucleoprotein F [Homo sapiens] |
| U | *gi|148470406|ref|NP\_0* | 3 | 5 | 10.6% | 415 | 45672 | 5.6 | heterogeneous nuclear ribonucleoprotein F [Homo sapiens] |
| U | *gi|148470404|ref|NP\_0* | 3 | 5 | 10.6% | 415 | 45672 | 5.6 | heterogeneous nuclear ribonucleoprotein F [Homo sapiens] |
| U | *gi|148470402|ref|NP\_0* | 3 | 5 | 10.6% | 415 | 45672 | 5.6 | heterogeneous nuclear ribonucleoprotein F [Homo sapiens] |
| U | *gi|148470400|ref|NP\_0* | 3 | 5 | 10.6% | 415 | 45672 | 5.6 | heterogeneous nuclear ribonucleoprotein F [Homo sapiens] |

| Filename XCorr DeltCN Conf% ObsM+H+ CalcM+H+ SpR ZScore Ion% # Sequence  | | | | | | | | | | | | |
| --- | --- | --- | --- | --- | --- | --- | --- | --- | --- | --- | --- | --- |
|  | Astrin\_NLD\_STLC\_tube2\_021014\_01.13403.13403.2 | 2.6509 | 0.2106 | 96.8% | 1867.3922 | 1869.0813 | 19 | 4.346 | 37.5% | 2 | K.ITGEAFVQFASQELAEK.A | 2 |
|  | Astrin\_NLD\_STLC\_tube2\_021014\_01.13943.13943.2 | 3.4978 | 0.3535 | 100.0% | 1997.4521 | 1998.2023 | 2 | 7.159 | 43.8% | 1 | K.ATENDIYNFFSPLNPVR.V | 22 |
|  | Astrin\_NLD\_STLC\_tube2\_021014\_01.05140.05140.2 | 2.7007 | 0.3228 | 100.0% | 1092.4922 | 1093.2278 | 1 | 7.099 | 83.3% | 2 | R.VHIEIGPDGR.V | 22 |

Similarities:
gi|5031753|ref|NP\_005(2:1)  

---

|  |  |  |  |  |  |  |  |  |
| --- | --- | --- | --- | --- | --- | --- | --- | --- |
| U | *Reverse\_gi|18254468|r* | 1 | 1 | 10.6% | 235 | 25701 | 8.5 | diffuse panbronchiolitis critical region 1 protein [Homo sapiens] |

| Filename XCorr DeltCN Conf% ObsM+H+ CalcM+H+ SpR ZScore Ion% # Sequence  | | | | | | | | | | | | |
| --- | --- | --- | --- | --- | --- | --- | --- | --- | --- | --- | --- | --- |
| \* | Astrin\_NLD\_STLC\_tube2\_021014\_01.15141.15141.3 | 2.8477 | 0.2684 | 95.8% | 2826.1443 | 2826.9988 | 170 | 4.386 | 20.8% | 1 | K.DGTVKVSPK@ITK@TVAATK@EPNET#S\*K.V | 3 |

---

|  |  |  |  |  |  |  |  |  |
| --- | --- | --- | --- | --- | --- | --- | --- | --- |
| U | *Reverse\_gi|17986258|r* | 1 | 7 | 10.6% | 151 | 16930 | 4.7 | myosin, light chain 6, alkali, smooth muscle and non-muscle isoform 1 [Homo sapiens] |
| U | *Reverse\_gi|88999583|r* | 1 | 7 | 10.6% | 151 | 16961 | 4.6 | myosin, light chain 6, alkali, smooth muscle and non-muscle isoform 2 [Homo sapiens] |

| Filename XCorr DeltCN Conf% ObsM+H+ CalcM+H+ SpR ZScore Ion% # Sequence  | | | | | | | | | | | | |
| --- | --- | --- | --- | --- | --- | --- | --- | --- | --- | --- | --- | --- |
|  | Astrin\_NLD\_STLC\_tube2\_021014\_01.10516.10516.2 | 4.2123 | 0.1647 | 99.9% | 1801.3722 | 1800.9905 | 166 | 5.203 | 40.0% | 7 | R.MVDGCQSYLIKGDGTR.D | 2 |

---

|  |  |  |  |  |  |  |  |  |
| --- | --- | --- | --- | --- | --- | --- | --- | --- |
| U | *gi|118582269|ref|NP\_0* | 2 | 2 | 10.4% | 201 | 22460 | 8.0 | splicing factor, arginine/serine-rich 1 isoform 2 [Homo sapiens] |
| U | *gi|5902076|ref|NP\_008* | 2 | 2 | 8.5% | 248 | 27745 | 10.4 | splicing factor, arginine/serine-rich 1 isoform 1 [Homo sapiens] |

| Filename XCorr DeltCN Conf% ObsM+H+ CalcM+H+ SpR ZScore Ion% # Sequence  | | | | | | | | | | | | |
| --- | --- | --- | --- | --- | --- | --- | --- | --- | --- | --- | --- | --- |
|  | Astrin\_NLD\_STLC\_tube2\_021014\_01.08668.08668.2 | 3.1051 | 0.3263 | 100.0% | 1257.3722 | 1257.4752 | 1 | 6.048 | 80.0% | 1 | R.IYVGNLPPDIR.T | 2 |
|  | Astrin\_NLD\_STLC\_tube2\_021014\_01.07749.07749.2 | 2.6889 | 0.2009 | 98.8% | 1257.4122 | 1258.4137 | 1 | 5.185 | 77.8% | 1 | R.TKDIEDVFYK.Y | 2 |

---

|  |  |  |  |  |  |  |  |  |
| --- | --- | --- | --- | --- | --- | --- | --- | --- |
| U | *gi|25777713|ref|NP\_73* | 1 | 1 | 10.4% | 163 | 18658 | 4.5 | S-phase kinase-associated protein 1 isoform b [Homo sapiens] |

| Filename XCorr DeltCN Conf% ObsM+H+ CalcM+H+ SpR ZScore Ion% # Sequence  | | | | | | | | | | | | |
| --- | --- | --- | --- | --- | --- | --- | --- | --- | --- | --- | --- | --- |
| \* | Astrin\_NLD\_STLC\_tube2\_021014\_02.06581.06581.3 | 3.7332 | 0.2681 | 100.0% | 2071.8245 | 2071.2078 | 1 | 4.836 | 39.1% | 1 | K.TFNIKNDFTEEEEAQVR.K | 3 |

---

|  |  |  |  |  |  |  |  |  |
| --- | --- | --- | --- | --- | --- | --- | --- | --- |
| U | *gi|4757714|ref|NP\_004* | 1 | 1 | 10.1% | 158 | 18042 | 6.8 | acid phosphatase 1, soluble isoform c [Homo sapiens] |
| U | *gi|6005988|ref|NP\_009* | 1 | 1 | 10.1% | 158 | 17977 | 6.9 | acid phosphatase 1, soluble isoform b [Homo sapiens] |

| Filename XCorr DeltCN Conf% ObsM+H+ CalcM+H+ SpR ZScore Ion% # Sequence  | | | | | | | | | | | | |
| --- | --- | --- | --- | --- | --- | --- | --- | --- | --- | --- | --- | --- |
|  | Astrin\_NLD\_STLC\_tube2\_021014\_01.10988.10988.3 | 3.095 | 0.3034 | 100.0% | 1851.6843 | 1852.104 | 117 | 4.878 | 31.7% | 1 | K.TCKAKIELLGSYDPQK.Q | 3 |

---

|  |  |  |  |  |  |  |  |  |
| --- | --- | --- | --- | --- | --- | --- | --- | --- |
| U | *gi|4506629|ref|NP\_000* | 2 | 2 | 10.1% | 159 | 17752 | 11.7 | ribosomal protein L29 [Homo sapiens] |

| Filename XCorr DeltCN Conf% ObsM+H+ CalcM+H+ SpR ZScore Ion% # Sequence  | | | | | | | | | | | | |
| --- | --- | --- | --- | --- | --- | --- | --- | --- | --- | --- | --- | --- |
|  | Astrin\_NLD\_STLC\_tube2\_021014\_01.04178.04178.2 | 4.4043 | 0.2862 | 100.0% | 1378.3322 | 1378.5712 | 1 | 8.212 | 71.4% | 1 | K.AQAAAPASVPAQAPK.R | 2 |
| \* | Astrin\_NLD\_STLC\_031014\_01.08783.08783.2 | 3.2299 | 0.2602 | 99.9% | 1535.9521 | 1534.7587 | 1 | 4.774 | 66.7% | 1 | K.AQAAAPASVPAQAPKR.T | 2 |

---

|  |  |  |  |  |  |  |  |  |
| --- | --- | --- | --- | --- | --- | --- | --- | --- |
| U | *gi|4506725|ref|NP\_000* | 2 | 2 | 9.9% | 263 | 29598 | 10.2 | ribosomal protein S4, X-linked X isoform [Homo sapiens] |

| Filename XCorr DeltCN Conf% ObsM+H+ CalcM+H+ SpR ZScore Ion% # Sequence  | | | | | | | | | | | | |
| --- | --- | --- | --- | --- | --- | --- | --- | --- | --- | --- | --- | --- |
| \* | Astrin\_NLD\_STLC\_031014\_02.06599.06599.2 | 3.4631 | 0.3554 | 100.0% | 1446.9722 | 1446.5975 | 1 | 6.868 | 66.7% | 1 | K.VNDTIQIDLETGK.I | 2 |
|  | Astrin\_NLD\_STLC\_tube2\_021014\_01.04523.04523.3 | 3.1793 | 0.2882 | 100.0% | 1508.1543 | 1507.692 | 5 | 5.982 | 43.8% | 1 | R.ERHPGSFDVVHVK.D | 3 |

---

|  |  |  |  |  |  |  |  |  |
| --- | --- | --- | --- | --- | --- | --- | --- | --- |
| U | *gi|87196351|ref|NP\_00* | 5 | 7 | 9.8% | 662 | 73244 | 7.2 | DEAD/H (Asp-Glu-Ala-Asp/His) box polypeptide 3 [Homo sapiens] |

| Filename XCorr DeltCN Conf% ObsM+H+ CalcM+H+ SpR ZScore Ion% # Sequence  | | | | | | | | | | | | |
| --- | --- | --- | --- | --- | --- | --- | --- | --- | --- | --- | --- | --- |
|  | Astrin\_NLD\_STLC\_tube2\_021014\_02.06797.06797.2 | 3.171 | 0.3351 | 100.0% | 1322.1322 | 1321.4729 | 2 | 6.05 | 80.0% | 1 | R.ELAVQIYEEAR.K | 2 |
|  | Astrin\_NLD\_STLC\_tube2\_021014\_01.10677.10677.2 | 3.2162 | 0.3627 | 100.0% | 1337.2922 | 1337.5946 | 1 | 6.715 | 85.0% | 3 | R.MLDMGFEPQIR.R | 22 |
|  | Astrin\_NLD\_STLC\_tube2\_021014\_02.06401.06401.2 | 3.0232 | 0.3028 | 100.0% | 1170.6322 | 1169.4099 | 1 | 5.431 | 72.7% | 1 | K.SPILVATAVAAR.G | 2 |
|  | Astrin\_NLD\_STLC\_tube2\_021014\_01.11931.11931.3 | 4.8804 | 0.3492 | 100.0% | 2084.9644 | 2084.2957 | 1 | 7.562 | 48.4% | 1 | K.HVINFDLPSDIEEYVHR.I | 3 |
| \* | Astrin\_NLD\_STLC\_tube2\_021014\_01.12105.12105.2 | 3.1832 | 0.3611 | 100.0% | 1526.2722 | 1525.7043 | 1 | 6.495 | 69.2% | 1 | R.VGNLGLATSFFNER.N | 2 |

Similarities:
gi|4758138|ref|NP\_004(1:4)  

---

|  |  |  |  |  |  |  |  |  |
| --- | --- | --- | --- | --- | --- | --- | --- | --- |
| U | *gi|4758138|ref|NP\_004* | 6 | 13 | 9.6% | 614 | 69148 | 8.9 | DEAD (Asp-Glu-Ala-Asp) box polypeptide 5 [Homo sapiens] |

| Filename XCorr DeltCN Conf% ObsM+H+ CalcM+H+ SpR ZScore Ion% # Sequence  | | | | | | | | | | | | |
| --- | --- | --- | --- | --- | --- | --- | --- | --- | --- | --- | --- | --- |
| \* | Astrin\_NLD\_STLC\_tube2\_021014\_01.08696.08696.2 | 3.5811 | 0.5219 | 100.0% | 1296.0721 | 1296.4198 | 1 | 9.265 | 75.0% | 2 | R.TTYLVLDEADR.M | 2 |
|  | Astrin\_NLD\_STLC\_tube2\_021014\_01.10677.10677.2 | 3.2162 | 0.3627 | 100.0% | 1337.2922 | 1337.5946 | 1 | 6.715 | 85.0% | 3 | R.MLDMGFEPQIR.K | 22 |
| \* | Astrin\_NLD\_STLC\_tube2\_021014\_01.05285.05285.2 | 2.1695 | 0.2641 | 98.3% | 1093.1921 | 1093.3115 | 382 | 4.668 | 56.2% | 1 | K.TIVFVETKR.R | 2 |
|  | Astrin\_NLD\_STLC\_tube2\_021014\_01.08331.08331.2 | 3.6106 | 0.2817 | 100.0% | 1227.2522 | 1227.4465 | 2 | 7.2 | 77.3% | 4 | K.APILIATDVASR.G | 2 |
|  | Astrin\_NLD\_STLC\_tube2\_021014\_01.05238.05238.1 | 1.9714 | 0.2367 | 100.0% | 874.51 | 874.96643 | 3 | 5.852 | 71.4% | 1 | R.GLDVEDVK.F | 1 |
| \* | Astrin\_NLD\_STLC\_tube2\_021014\_01.07962.07962.2 | 2.7053 | 0.2163 | 99.8% | 986.2522 | 986.1564 | 1 | 5.774 | 85.7% | 2 | K.LLQLVEDR.G | 2 |

Similarities:
gi|87196351|ref|NP\_00(1:5)  

---

|  |  |  |  |  |  |  |  |  |
| --- | --- | --- | --- | --- | --- | --- | --- | --- |
| U | *gi|4506903|ref|NP\_003* | 2 | 2 | 9.5% | 221 | 25542 | 8.6 | splicing factor, arginine/serine-rich 9 [Homo sapiens] |

| Filename XCorr DeltCN Conf% ObsM+H+ CalcM+H+ SpR ZScore Ion% # Sequence  | | | | | | | | | | | | |
| --- | --- | --- | --- | --- | --- | --- | --- | --- | --- | --- | --- | --- |
| \* | Astrin\_NLD\_STLC\_tube2\_021014\_01.08199.08199.2 | 3.2839 | 0.2534 | 100.0% | 1247.2922 | 1247.4368 | 20 | 5.623 | 65.0% | 1 | R.IYVGNLPTDVR.E | 2 |
| \* | Astrin\_NLD\_STLC\_tube2\_021014\_01.10548.10548.2 | 2.1989 | 0.3135 | 99.5% | 1142.9321 | 1143.3768 | 48 | 6.544 | 55.6% | 1 | R.HGLVPFAFVR.F | 2 |

---

|  |  |  |  |  |  |  |  |  |
| --- | --- | --- | --- | --- | --- | --- | --- | --- |
| U | *Reverse\_gi|29824427|r* | 1 | 1 | 9.5% | 201 | 22433 | 7.4 | dynein 2 light intermediate chain isoform 2 [Homo sapiens] |
| U | *Reverse\_gi|61102732|r* | 1 | 1 | 5.7% | 334 | 37500 | 7.5 | dynein 2 light intermediate chain isoform 3 [Homo sapiens] |
| U | *Reverse\_gi|40548413|r* | 1 | 1 | 5.4% | 351 | 39625 | 7.5 | dynein 2 light intermediate chain isoform 1 [Homo sapiens] |

| Filename XCorr DeltCN Conf% ObsM+H+ CalcM+H+ SpR ZScore Ion% # Sequence  | | | | | | | | | | | | |
| --- | --- | --- | --- | --- | --- | --- | --- | --- | --- | --- | --- | --- |
|  | Astrin\_NLD\_STLC\_tube2\_021014\_01.11680.11680.3 | 3.2447 | 0.256 | 98.7% | 2328.6243 | 2329.619 | 25 | 4.357 | 31.9% | 1 | K.QRMES\*VAK@ANT#K@GLK@MIVK.D | 3 |

---

|  |  |  |  |  |  |  |  |  |
| --- | --- | --- | --- | --- | --- | --- | --- | --- |
| U | *gi|222352151|ref|NP\_0* | 2 | 2 | 9.3% | 356 | 37498 | 7.1 | poly(rC) binding protein 1 [Homo sapiens] |

| Filename XCorr DeltCN Conf% ObsM+H+ CalcM+H+ SpR ZScore Ion% # Sequence  | | | | | | | | | | | | |
| --- | --- | --- | --- | --- | --- | --- | --- | --- | --- | --- | --- | --- |
| \* | Astrin\_NLD\_STLC\_tube2\_021014\_01.11108.11108.2 | 2.716 | 0.3656 | 100.0% | 1389.3121 | 1389.6781 | 1 | 7.546 | 58.3% | 1 | R.IITLTGPTNAIFK.A | 2 |
|  | Astrin\_NLD\_STLC\_tube2\_021014\_02.05969.05969.3 | 3.6803 | 0.2737 | 100.0% | 2091.0842 | 2091.2573 | 2 | 5.557 | 42.1% | 1 | R.ESTGAQVQVAGDMLPNSTER.A | 3 |

---

|  |  |  |  |  |  |  |  |  |
| --- | --- | --- | --- | --- | --- | --- | --- | --- |
| U | *gi|215490089|ref|NP\_0* | 1 | 1 | 9.3% | 172 | 19526 | 9.8 | Sin3A-associated protein, 18kDa [Homo sapiens] |

| Filename XCorr DeltCN Conf% ObsM+H+ CalcM+H+ SpR ZScore Ion% # Sequence  | | | | | | | | | | | | |
| --- | --- | --- | --- | --- | --- | --- | --- | --- | --- | --- | --- | --- |
| \* | Astrin\_NLD\_STLC\_031014\_01.08742.08742.3 | 3.2516 | 0.236 | 98.4% | 1938.9844 | 1940.2041 | 438 | 4.292 | 28.3% | 1 | R.VTQEEIKKEPEKPIDR.E | 3 |

---

|  |  |  |  |  |  |  |  |  |
| --- | --- | --- | --- | --- | --- | --- | --- | --- |
| U | *Reverse\_gi|10864013|r* | 1 | 1 | 9.1% | 243 | 27186 | 7.4 | pleckstrin homology domain containing, family B (evectins) member 1 isoform a [Homo sapiens] |
| U | *Reverse\_gi|194097469|* | 1 | 1 | 9.8% | 224 | 25233 | 8.4 | pleckstrin homology domain containing, family B (evectins) member 1 isoform b [Homo sapiens] |

| Filename XCorr DeltCN Conf% ObsM+H+ CalcM+H+ SpR ZScore Ion% # Sequence  | | | | | | | | | | | | |
| --- | --- | --- | --- | --- | --- | --- | --- | --- | --- | --- | --- | --- |
|  | Astrin\_NLD\_STLC\_031014\_01.16103.16103.2 | 1.9574 | 0.322 | 96.6% | 2229.112 | 2230.44 | 229 | 4.308 | 23.8% | 1 | R.SRPPVTAGAPAPTSNAELLAT#K.W | 2 |

---

|  |  |  |  |  |  |  |  |  |
| --- | --- | --- | --- | --- | --- | --- | --- | --- |
| U | *gi|119703753|ref|NP\_0* | 6 | 8 | 8.7% | 564 | 60067 | 8.0 | keratin 6B [Homo sapiens] |

| Filename XCorr DeltCN Conf% ObsM+H+ CalcM+H+ SpR ZScore Ion% # Sequence  | | | | | | | | | | | | |
| --- | --- | --- | --- | --- | --- | --- | --- | --- | --- | --- | --- | --- |
|  | Astrin\_NLD\_STLC\_tube2\_021014\_01.07623.07623.2 | 2.9741 | 0.1656 | 99.6% | 1082.8121 | 1083.2755 | 7 | 7.028 | 75.0% | 2 | K.FASFIDKVR.F | 22222 |
|  | Astrin\_NLD\_STLC\_tube2\_021014\_01.13338.13338.2 | 4.1957 | 0.3905 | 100.0% | 1331.0922 | 1330.5211 | 1 | 7.868 | 86.4% | 1 | R.NLDLDSIIAEVK.A | 222 |
|  | Astrin\_NLD\_STLC\_tube2\_021014\_01.04328.04328.2 | 3.2426 | 0.0852 | 98.5% | 1108.1122 | 1108.196 | 2 | 6.567 | 75.0% | 1 | K.AQYEEIAQR.S | 222 |
|  | Astrin\_NLD\_STLC\_tube2\_021014\_02.05543.05543.2 | 3.4163 | 0.3238 | 100.0% | 1180.2122 | 1180.303 | 1 | 7.435 | 83.3% | 2 | K.YEELQITAGR.H | 22 |
|  | Astrin\_NLD\_STLC\_tube2\_021014\_01.06941.06941.1 | 2.2327 | 0.2314 | 100.0% | 1153.42 | 1154.3234 | 400 | 5.93 | 50.0% | 1 | K.EYQELMNVK.L | 11 |
|  | Astrin\_NLD\_STLC\_tube2\_021014\_01.06962.06962.2 | 2.6071 | 0.2243 | 99.6% | 1154.0521 | 1154.3234 | 7 | 6.457 | 62.5% | 1 | K.EYQELMNVK.L | 22 |

Similarities:
gi|4504919|ref|NP\_002(3:3)  
gi|119395750|ref|NP\_0(1:5)  
gi|67782365|ref|NP\_00(1:5)  
gi|47132620|ref|NP\_00(3:3)  
gi|153791158|ref|NP\_0(2:4)  
contaminant\_KERATIN16(1:5)  

---

|  |  |  |  |  |  |  |  |  |
| --- | --- | --- | --- | --- | --- | --- | --- | --- |
| U | *Reverse\_gi|20149598|r* | 2 | 2 | 8.7% | 287 | 31282 | 9.5 | solute carrier family 25 (mitochondrial carrier; dicarboxylate transporter), member 10 [Homo sapiens] |

| Filename XCorr DeltCN Conf% ObsM+H+ CalcM+H+ SpR ZScore Ion% # Sequence  | | | | | | | | | | | | |
| --- | --- | --- | --- | --- | --- | --- | --- | --- | --- | --- | --- | --- |
| \* | Astrin\_NLD\_STLC\_tube2\_021014\_01.20288.20288.3 | 2.7517 | 0.285 | 96.6% | 2858.0044 | 2859.147 | 151 | 5.198 | 22.9% | 1 | R.TLSYTMQRCLS\*ASLGSYLALIGDTR.V | 3 |
| \* | Astrin\_NLD\_STLC\_tube2\_021014\_01.10724.10724.2 | 3.2095 | 0.2023 | 99.2% | 1797.1921 | 1798.0123 | 9 | 4.125 | 43.8% | 1 | R.CLSASLGSYLALIGDTR.V | 2 |

---

|  |  |  |  |  |  |  |  |  |
| --- | --- | --- | --- | --- | --- | --- | --- | --- |
| U | *gi|94538362|ref|NP\_00* | 3 | 3 | 8.6% | 428 | 47064 | 5.3 | flotillin 2 [Homo sapiens] |

| Filename XCorr DeltCN Conf% ObsM+H+ CalcM+H+ SpR ZScore Ion% # Sequence  | | | | | | | | | | | | |
| --- | --- | --- | --- | --- | --- | --- | --- | --- | --- | --- | --- | --- |
| \* | Astrin\_NLD\_STLC\_tube2\_021014\_02.05694.05694.2 | 2.182 | 0.2596 | 97.7% | 1124.0721 | 1124.2358 | 1 | 5.327 | 66.7% | 1 | K.SAFSEEVNIK.T | 2 |
| \* | Astrin\_NLD\_STLC\_tube2\_021014\_02.06755.06755.2 | 4.1988 | 0.3596 | 100.0% | 1522.4521 | 1521.6702 | 1 | 7.418 | 80.8% | 1 | K.TAEAQLAYELQGAR.E | 2 |
| \* | Astrin\_NLD\_STLC\_tube2\_021014\_01.07911.07911.2 | 2.3548 | 0.2396 | 96.9% | 1375.1721 | 1375.5187 | 1 | 5.409 | 62.5% | 1 | K.VDEIVVLSGDNSK.V | 2 |

---

|  |  |  |  |  |  |  |  |  |
| --- | --- | --- | --- | --- | --- | --- | --- | --- |
| U | *gi|17986258|ref|NP\_06* | 1 | 1 | 8.6% | 151 | 16930 | 4.7 | myosin, light chain 6, alkali, smooth muscle and non-muscle isoform 1 [Homo sapiens] |
| U | *gi|88999583|ref|NP\_52* | 1 | 1 | 8.6% | 151 | 16961 | 4.6 | myosin, light chain 6, alkali, smooth muscle and non-muscle isoform 2 [Homo sapiens] |
| U | *gi|4505303|ref|NP\_002* | 1 | 1 | 6.2% | 208 | 22764 | 5.7 | smooth muscle and non-muscle myosin alkali light chain 6B [Homo sapiens] |

| Filename XCorr DeltCN Conf% ObsM+H+ CalcM+H+ SpR ZScore Ion% # Sequence  | | | | | | | | | | | | |
| --- | --- | --- | --- | --- | --- | --- | --- | --- | --- | --- | --- | --- |
|  | Astrin\_NLD\_STLC\_tube2\_021014\_01.05705.05705.2 | 3.6548 | 0.3657 | 100.0% | 1355.4122 | 1355.5339 | 1 | 6.243 | 66.7% | 1 | R.ALGQNPTNAEVLK.V | 2 |

---

|  |  |  |  |  |  |  |  |  |
| --- | --- | --- | --- | --- | --- | --- | --- | --- |
| U | *gi|16117794|ref|NP\_05* | 1 | 1 | 8.6% | 105 | 12254 | 11.6 | ribosomal protein L36 [Homo sapiens] |
| U | *gi|16117796|ref|NP\_37* | 1 | 1 | 8.6% | 105 | 12254 | 11.6 | ribosomal protein L36 [Homo sapiens] |

| Filename XCorr DeltCN Conf% ObsM+H+ CalcM+H+ SpR ZScore Ion% # Sequence  | | | | | | | | | | | | |
| --- | --- | --- | --- | --- | --- | --- | --- | --- | --- | --- | --- | --- |
|  | Astrin\_NLD\_STLC\_tube2\_021014\_01.06646.06646.2 | 2.0736 | 0.2779 | 97.9% | 993.2322 | 993.20905 | 54 | 5.874 | 56.2% | 1 | R.YPMAVGLNK.G | 2 |

---

|  |  |  |  |  |  |  |  |  |
| --- | --- | --- | --- | --- | --- | --- | --- | --- |
| U | *gi|4506619|ref|NP\_000* | 1 | 1 | 8.3% | 157 | 17779 | 11.3 | ribosomal protein L24 [Homo sapiens] |

| Filename XCorr DeltCN Conf% ObsM+H+ CalcM+H+ SpR ZScore Ion% # Sequence  | | | | | | | | | | | | |
| --- | --- | --- | --- | --- | --- | --- | --- | --- | --- | --- | --- | --- |
| \* | Astrin\_NLD\_STLC\_tube2\_021014\_01.10372.10372.2 | 4.0736 | 0.456 | 100.0% | 1262.2322 | 1262.5072 | 1 | 9.486 | 79.2% | 1 | R.AITGASLADIMAK.R | 2 |

---

|  |  |  |  |  |  |  |  |  |
| --- | --- | --- | --- | --- | --- | --- | --- | --- |
| U | *gi|17105394|ref|NP\_00* | 1 | 1 | 8.3% | 156 | 17695 | 10.4 | ribosomal protein L23a [Homo sapiens] |

| Filename XCorr DeltCN Conf% ObsM+H+ CalcM+H+ SpR ZScore Ion% # Sequence  | | | | | | | | | | | | |
| --- | --- | --- | --- | --- | --- | --- | --- | --- | --- | --- | --- | --- |
| \* | Astrin\_NLD\_STLC\_tube2\_021014\_01.08114.08114.2 | 3.1128 | 0.4082 | 100.0% | 1405.2722 | 1405.5474 | 6 | 6.672 | 58.3% | 1 | R.LAPDYDALDVANK.I | 2 |

---

|  |  |  |  |  |  |  |  |  |
| --- | --- | --- | --- | --- | --- | --- | --- | --- |
| U | *gi|19920317|ref|NP\_00* | 3 | 5 | 8.1% | 602 | 66023 | 5.9 | cytoskeleton-associated protein 4 [Homo sapiens] |

| Filename XCorr DeltCN Conf% ObsM+H+ CalcM+H+ SpR ZScore Ion% # Sequence  | | | | | | | | | | | | |
| --- | --- | --- | --- | --- | --- | --- | --- | --- | --- | --- | --- | --- |
| \* | Astrin\_NLD\_STLC\_tube2\_021014\_02.06096.06096.3 | 3.4304 | 0.2335 | 98.9% | 1839.9243 | 1841.0923 | 1 | 5.746 | 40.6% | 2 | R.LQHVEDGVLSMQVASAR.Q | 3 |
| \* | Astrin\_NLD\_STLC\_tube2\_021014\_01.08074.08074.2 | 2.8881 | 0.2568 | 99.7% | 1474.3121 | 1474.6512 | 8 | 4.578 | 57.7% | 1 | R.SVGELPSTVESLQK.V | 2 |
| \* | Astrin\_NLD\_STLC\_tube2\_021014\_02.05081.05081.3 | 5.3313 | 0.4634 | 100.0% | 2022.2043 | 2023.2131 | 1 | 8.503 | 38.2% | 2 | K.VQEQVHTLLSQDQAQAAR.L | 3 |

---

|  |  |  |  |  |  |  |  |  |
| --- | --- | --- | --- | --- | --- | --- | --- | --- |
| U | *gi|11415026|ref|NP\_00* | 1 | 1 | 8.0% | 176 | 20762 | 10.7 | ribosomal protein L18a [Homo sapiens] |
| U | *gi|88954757|ref|XP\_94* | 1 | 1 | 8.0% | 176 | 20767 | 10.7 | PREDICTED: similar to ribosomal protein L18a isoform 4 [Homo sapiens] |
| U | *gi|27480190|ref|XP\_20* | 1 | 1 | 8.0% | 176 | 20767 | 10.7 | PREDICTED: similar to ribosomal protein L18a isoform 1 [Homo sapiens] |
| U | *gi|169163931|ref|XP\_0* | 1 | 1 | 8.0% | 176 | 20767 | 10.7 | PREDICTED: similar to ribosomal protein L18a isoform 1 [Homo sapiens] |

| Filename XCorr DeltCN Conf% ObsM+H+ CalcM+H+ SpR ZScore Ion% # Sequence  | | | | | | | | | | | | |
| --- | --- | --- | --- | --- | --- | --- | --- | --- | --- | --- | --- | --- |
|  | Astrin\_NLD\_STLC\_031014\_02.06309.06309.2 | 2.9971 | 0.3573 | 100.0% | 1602.8322 | 1603.7351 | 10 | 5.698 | 50.0% | 1 | K.SSGEIVYCGQVFEK.S | 2 |

---

|  |  |  |  |  |  |  |  |  |
| --- | --- | --- | --- | --- | --- | --- | --- | --- |
| U | *Reverse\_gi|208431824|* | 1 | 1 | 7.9% | 420 | 43326 | 8.3 | forkhead box I3 [Homo sapiens] |

| Filename XCorr DeltCN Conf% ObsM+H+ CalcM+H+ SpR ZScore Ion% # Sequence  | | | | | | | | | | | | |
| --- | --- | --- | --- | --- | --- | --- | --- | --- | --- | --- | --- | --- |
| \* | Astrin\_NLD\_STLC\_tube2\_021014\_01.14784.14784.3 | 3.4956 | 0.2623 | 99.3% | 3346.7644 | 3346.268 | 304 | 4.333 | 18.0% | 1 | K.GGVGS\*GLGSSLGEES\*K@ST#GAAVTSGNSAESRRK@.R | 3 |

---

|  |  |  |  |  |  |  |  |  |
| --- | --- | --- | --- | --- | --- | --- | --- | --- |
| U | *gi|15431290|ref|NP\_00* | 1 | 1 | 7.9% | 178 | 20252 | 9.6 | ribosomal protein L11 [Homo sapiens] |

| Filename XCorr DeltCN Conf% ObsM+H+ CalcM+H+ SpR ZScore Ion% # Sequence  | | | | | | | | | | | | |
| --- | --- | --- | --- | --- | --- | --- | --- | --- | --- | --- | --- | --- |
| \* | Astrin\_NLD\_STLC\_tube2\_021014\_01.09006.09006.2 | 3.7241 | 0.5302 | 100.0% | 1547.3522 | 1547.7917 | 1 | 9.394 | 65.4% | 1 | K.VLEQLTGQTPVFSK.A | 2 |

---

|  |  |  |  |  |  |  |  |  |
| --- | --- | --- | --- | --- | --- | --- | --- | --- |
| U | *gi|4759160|ref|NP\_004* | 1 | 1 | 7.9% | 126 | 13916 | 10.3 | small nuclear ribonucleoprotein polypeptide D3 [Homo sapiens] |

| Filename XCorr DeltCN Conf% ObsM+H+ CalcM+H+ SpR ZScore Ion% # Sequence  | | | | | | | | | | | | |
| --- | --- | --- | --- | --- | --- | --- | --- | --- | --- | --- | --- | --- |
| \* | Astrin\_NLD\_STLC\_tube2\_021014\_01.07947.07947.2 | 3.0144 | 0.2897 | 100.0% | 1219.4122 | 1219.4264 | 1 | 5.914 | 77.8% | 1 | R.VAQLEQVYIR.G | 2 |

---

|  |  |  |  |  |  |  |  |  |
| --- | --- | --- | --- | --- | --- | --- | --- | --- |
| U | *gi|4758086|ref|NP\_004* | 1 | 1 | 7.8% | 193 | 20567 | 8.6 | cysteine and glycine-rich protein 1 isoform 1 [Homo sapiens] |

| Filename XCorr DeltCN Conf% ObsM+H+ CalcM+H+ SpR ZScore Ion% # Sequence  | | | | | | | | | | | | |
| --- | --- | --- | --- | --- | --- | --- | --- | --- | --- | --- | --- | --- |
| \* | Astrin\_NLD\_STLC\_031014\_02.07318.07318.2 | 2.4634 | 0.2641 | 98.1% | 1434.3121 | 1434.551 | 240 | 4.978 | 42.9% | 1 | K.GFGFGQGAGALVHSE.- | 2 |

---

|  |  |  |  |  |  |  |  |  |
| --- | --- | --- | --- | --- | --- | --- | --- | --- |
| U | *gi|41393577|ref|NP\_07* | 1 | 2 | 7.8% | 167 | 18824 | 5.1 | gemin 6 [Homo sapiens] |

| Filename XCorr DeltCN Conf% ObsM+H+ CalcM+H+ SpR ZScore Ion% # Sequence  | | | | | | | | | | | | |
| --- | --- | --- | --- | --- | --- | --- | --- | --- | --- | --- | --- | --- |
| \* | Astrin\_NLD\_STLC\_tube2\_021014\_02.05951.05951.2 | 3.5862 | 0.3147 | 100.0% | 1412.4521 | 1411.5547 | 1 | 6.839 | 75.0% | 2 | R.VQDLIEGHLTASQ.- | 2 |

---

|  |  |  |  |  |  |  |  |  |
| --- | --- | --- | --- | --- | --- | --- | --- | --- |
| U | *Reverse\_gi|113425967|* | 1 | 1 | 7.8% | 167 | 17483 | 9.4 | PREDICTED: similar to TP53TG3b isoform 1 [Homo sapiens] |
| U | *Reverse\_gi|169209862|* | 1 | 1 | 7.8% | 167 | 17455 | 9.4 | PREDICTED: similar to TP53TG3b isoform 3 [Homo sapiens] |

| Filename XCorr DeltCN Conf% ObsM+H+ CalcM+H+ SpR ZScore Ion% # Sequence  | | | | | | | | | | | | |
| --- | --- | --- | --- | --- | --- | --- | --- | --- | --- | --- | --- | --- |
|  | Astrin\_NLD\_STLC\_tube2\_021014\_01.19772.19772.2 | 2.0356 | 0.2779 | 95.1% | 1587.3722 | 1587.5919 | 1 | 4.585 | 54.2% | 1 | R.K@IVS\*TPFDGHS\*AR.G | 2 |

---

|  |  |  |  |  |  |  |  |  |
| --- | --- | --- | --- | --- | --- | --- | --- | --- |
| U | *gi|153791632|ref|NP\_0* | 1 | 1 | 7.8% | 154 | 16773 | 5.0 | eukaryotic translation initiation factor 5A-like 1 [Homo sapiens] |
| U | *gi|9966867|ref|NP\_065* | 1 | 1 | 7.8% | 153 | 16793 | 5.6 | eIF-5A2 protein [Homo sapiens] |
| U | *gi|4503545|ref|NP\_001* | 1 | 1 | 7.8% | 154 | 16832 | 5.2 | eukaryotic translation initiation factor 5A isoform B [Homo sapiens] |
| U | *gi|219555712|ref|NP\_0* | 1 | 1 | 7.8% | 154 | 16832 | 5.2 | eukaryotic translation initiation factor 5A isoform B [Homo sapiens] |
| U | *gi|219555710|ref|NP\_0* | 1 | 1 | 7.8% | 154 | 16832 | 5.2 | eukaryotic translation initiation factor 5A isoform B [Homo sapiens] |
| U | *gi|219555707|ref|NP\_0* | 1 | 1 | 6.5% | 184 | 20170 | 7.0 | eukaryotic translation initiation factor 5A isoform A [Homo sapiens] |

| Filename XCorr DeltCN Conf% ObsM+H+ CalcM+H+ SpR ZScore Ion% # Sequence  | | | | | | | | | | | | |
| --- | --- | --- | --- | --- | --- | --- | --- | --- | --- | --- | --- | --- |
|  | Astrin\_NLD\_STLC\_tube2\_021014\_01.11067.11067.2 | 3.1286 | 0.4504 | 100.0% | 1299.1122 | 1299.5559 | 1 | 8.659 | 72.7% | 1 | K.VHLVGIDIFTGK.K | 2 |

---

|  |  |  |  |  |  |  |  |  |
| --- | --- | --- | --- | --- | --- | --- | --- | --- |
| U | *gi|36287110|ref|NP\_91* | 2 | 2 | 7.7% | 379 | 40907 | 4.6 | FGFR1 oncogene partner isoform b [Homo sapiens] |
| U | *gi|5901954|ref|NP\_008* | 2 | 2 | 7.3% | 399 | 43065 | 4.8 | FGFR1 oncogene partner isoform a [Homo sapiens] |

| Filename XCorr DeltCN Conf% ObsM+H+ CalcM+H+ SpR ZScore Ion% # Sequence  | | | | | | | | | | | | |
| --- | --- | --- | --- | --- | --- | --- | --- | --- | --- | --- | --- | --- |
|  | Astrin\_NLD\_STLC\_031014\_01.13182.13182.2 | 2.8062 | 0.3208 | 99.9% | 2165.8123 | 2165.5352 | 220 | 5.801 | 25.0% | 1 | R.DLGIIEAEGTVGGPLLLEVIR.R | 2 |
|  | Astrin\_NLD\_STLC\_tube2\_021014\_01.06860.06860.2 | 2.2177 | 0.3726 | 100.0% | 894.1722 | 894.0183 | 33 | 6.733 | 64.3% | 1 | K.IGSFLSNR.T | 2 |

---

|  |  |  |  |  |  |  |  |  |
| --- | --- | --- | --- | --- | --- | --- | --- | --- |
| U | *gi|93277122|ref|NP\_00* | 1 | 1 | 7.7% | 364 | 40314 | 7.1 | RNA binding motif protein 4 [Homo sapiens] |

| Filename XCorr DeltCN Conf% ObsM+H+ CalcM+H+ SpR ZScore Ion% # Sequence  | | | | | | | | | | | | |
| --- | --- | --- | --- | --- | --- | --- | --- | --- | --- | --- | --- | --- |
| \* | Astrin\_NLD\_STLC\_tube2\_021014\_02.05878.05878.3 | 3.001 | 0.3621 | 100.0% | 2777.6943 | 2777.964 | 1 | 5.968 | 27.8% | 1 | R.ATAPVPTVGEGYGYGHESELSQASAAAR.N | 3 |

---

|  |  |  |  |  |  |  |  |  |
| --- | --- | --- | --- | --- | --- | --- | --- | --- |
| U | *gi|47271443|ref|NP\_00* | 1 | 1 | 7.7% | 221 | 25476 | 11.9 | splicing factor, arginine/serine-rich 2 [Homo sapiens] |

| Filename XCorr DeltCN Conf% ObsM+H+ CalcM+H+ SpR ZScore Ion% # Sequence  | | | | | | | | | | | | |
| --- | --- | --- | --- | --- | --- | --- | --- | --- | --- | --- | --- | --- |
| \* | Astrin\_NLD\_STLC\_tube2\_021014\_01.10869.10869.2 | 3.5155 | 0.4236 | 100.0% | 1752.1322 | 1752.8654 | 1 | 7.68 | 59.4% | 1 | R.DAEDAMDAMDGAVLDGR.E | 2 |

---

|  |  |  |  |  |  |  |  |  |
| --- | --- | --- | --- | --- | --- | --- | --- | --- |
| U | *gi|4826734|ref|NP\_004* | 2 | 4 | 7.6% | 526 | 53426 | 9.4 | fusion (involved in t(12;16) in malignant liposarcoma) [Homo sapiens] |

| Filename XCorr DeltCN Conf% ObsM+H+ CalcM+H+ SpR ZScore Ion% # Sequence  | | | | | | | | | | | | |
| --- | --- | --- | --- | --- | --- | --- | --- | --- | --- | --- | --- | --- |
| \* | Astrin\_NLD\_STLC\_tube2\_021014\_02.04912.04912.3 | 4.0479 | 0.3091 | 100.0% | 1662.5643 | 1662.837 | 1 | 5.519 | 50.0% | 3 | K.LKGEATVSFDDPPSAK.A | 3 |
| \* | Astrin\_NLD\_STLC\_tube2\_021014\_01.04007.04007.3 | 3.3951 | 0.3897 | 100.0% | 2253.9844 | 2254.355 | 1 | 6.36 | 37.0% | 1 | K.APKPDGPGGGPGGSHMGGNYGDDR.R | 3 |

---

|  |  |  |  |  |  |  |  |  |
| --- | --- | --- | --- | --- | --- | --- | --- | --- |
| U | *Reverse\_gi|215820622|* | 1 | 1 | 7.6% | 263 | 30279 | 9.1 | mixed lineage kinase domain-like isoform 2 [Homo sapiens] |

| Filename XCorr DeltCN Conf% ObsM+H+ CalcM+H+ SpR ZScore Ion% # Sequence  | | | | | | | | | | | | |
| --- | --- | --- | --- | --- | --- | --- | --- | --- | --- | --- | --- | --- |
| \* | Astrin\_NLD\_STLC\_031014\_02.14534.14534.3 | 2.7395 | 0.2678 | 96.2% | 2474.5144 | 2476.5332 | 97 | 4.069 | 28.9% | 1 | K.VKFRSIELPS\*KGS\*S\*SELSQR.L | 3 |

---

|  |  |  |  |  |  |  |  |  |
| --- | --- | --- | --- | --- | --- | --- | --- | --- |
| U | *gi|38201714|ref|NP\_00* | 2 | 2 | 7.4% | 326 | 36092 | 9.2 | ELAV-like 1 [Homo sapiens] |

| Filename XCorr DeltCN Conf% ObsM+H+ CalcM+H+ SpR ZScore Ion% # Sequence  | | | | | | | | | | | | |
| --- | --- | --- | --- | --- | --- | --- | --- | --- | --- | --- | --- | --- |
| \* | Astrin\_NLD\_STLC\_tube2\_021014\_01.10607.10607.2 | 3.1163 | 0.3502 | 100.0% | 1354.6322 | 1354.4998 | 7 | 6.241 | 54.2% | 1 | R.SLFSSIGEVESAK.L | 2 |
| \* | Astrin\_NLD\_STLC\_tube2\_021014\_01.05542.05542.2 | 2.1989 | 0.281 | 98.3% | 1189.2522 | 1189.3542 | 2 | 5.121 | 60.0% | 1 | R.VLVDQTTGLSR.G | 2 |

---

|  |  |  |  |  |  |  |  |  |
| --- | --- | --- | --- | --- | --- | --- | --- | --- |
| U | *gi|4506625|ref|NP\_000* | 1 | 1 | 7.4% | 148 | 16561 | 11.0 | ribosomal protein L27a [Homo sapiens] |

| Filename XCorr DeltCN Conf% ObsM+H+ CalcM+H+ SpR ZScore Ion% # Sequence  | | | | | | | | | | | | |
| --- | --- | --- | --- | --- | --- | --- | --- | --- | --- | --- | --- | --- |
| \* | Astrin\_NLD\_STLC\_tube2\_021014\_01.08469.08469.2 | 2.5231 | 0.3266 | 99.9% | 1111.3922 | 1112.3146 | 1 | 6.767 | 95.0% | 1 | K.TGAAPIIDVVR.S | 2 |

---

|  |  |  |  |  |  |  |  |  |
| --- | --- | --- | --- | --- | --- | --- | --- | --- |
| U | *gi|10863927|ref|NP\_06* | 2 | 2 | 7.3% | 165 | 18012 | 7.8 | peptidylprolyl isomerase A [Homo sapiens] |
| U | *gi|169215435|ref|XP\_0* | 2 | 2 | 5.4% | 223 | 24376 | 6.9 | PREDICTED: similar to peptidylprolyl isomerase A-like [Homo sapiens] |

| Filename XCorr DeltCN Conf% ObsM+H+ CalcM+H+ SpR ZScore Ion% # Sequence  | | | | | | | | | | | | |
| --- | --- | --- | --- | --- | --- | --- | --- | --- | --- | --- | --- | --- |
|  | Astrin\_NLD\_STLC\_tube2\_021014\_01.10332.10332.3 | 2.7666 | 0.3171 | 100.0% | 1380.0844 | 1380.6268 | 53 | 5.737 | 38.6% | 1 | R.VSFELFADKVPK.T | 3 |
|  | Astrin\_NLD\_STLC\_tube2\_021014\_01.10359.10359.2 | 3.1667 | 0.3416 | 100.0% | 1380.1921 | 1380.6268 | 1 | 6.141 | 72.7% | 1 | R.VSFELFADKVPK.T | 2 |

---

|  |  |  |  |  |  |  |  |  |
| --- | --- | --- | --- | --- | --- | --- | --- | --- |
| U | *gi|16117791|ref|NP\_00* | 1 | 1 | 7.3% | 110 | 12538 | 11.1 | ribosomal protein L35a [Homo sapiens] |

| Filename XCorr DeltCN Conf% ObsM+H+ CalcM+H+ SpR ZScore Ion% # Sequence  | | | | | | | | | | | | |
| --- | --- | --- | --- | --- | --- | --- | --- | --- | --- | --- | --- | --- |
| \* | Astrin\_NLD\_STLC\_tube2\_021014\_01.08822.08822.2 | 2.491 | 0.2412 | 99.7% | 979.2522 | 979.2255 | 4 | 6.042 | 78.6% | 1 | R.VMLYPSRI.- | 2 |

---

|  |  |  |  |  |  |  |  |  |
| --- | --- | --- | --- | --- | --- | --- | --- | --- |
| U | *gi|11968182|ref|NP\_07* | 1 | 1 | 7.2% | 152 | 17719 | 11.0 | ribosomal protein S18 [Homo sapiens] |
| U | *gi|169168597|ref|XP\_0* | 1 | 1 | 7.2% | 152 | 17719 | 11.0 | PREDICTED: hypothetical protein [Homo sapiens] |

| Filename XCorr DeltCN Conf% ObsM+H+ CalcM+H+ SpR ZScore Ion% # Sequence  | | | | | | | | | | | | |
| --- | --- | --- | --- | --- | --- | --- | --- | --- | --- | --- | --- | --- |
|  | Astrin\_NLD\_STLC\_tube2\_021014\_01.04608.04608.2 | 2.4117 | 0.2517 | 98.7% | 1248.5322 | 1248.2891 | 6 | 5.074 | 60.0% | 1 | R.AGELTEDEVER.V | 2 |

---

|  |  |  |  |  |  |  |  |  |
| --- | --- | --- | --- | --- | --- | --- | --- | --- |
| U | *gi|113430469|ref|XP\_9* | 1 | 1 | 7.2% | 153 | 16681 | 10.0 | PREDICTED: similar to ribosomal protein S23 [Homo sapiens] |
| U | *gi|4506701|ref|NP\_001* | 1 | 1 | 7.7% | 143 | 15808 | 10.5 | ribosomal protein S23 [Homo sapiens] |
| U | *gi|169217106|ref|XP\_0* | 1 | 1 | 8.9% | 124 | 13528 | 9.9 | PREDICTED: similar to 40S ribosomal protein S23 [Homo sapiens] |
| U | *gi|169216671|ref|XP\_0* | 1 | 1 | 8.5% | 129 | 14049 | 10.0 | PREDICTED: similar to hCG1990955 [Homo sapiens] |

| Filename XCorr DeltCN Conf% ObsM+H+ CalcM+H+ SpR ZScore Ion% # Sequence  | | | | | | | | | | | | |
| --- | --- | --- | --- | --- | --- | --- | --- | --- | --- | --- | --- | --- |
|  | Astrin\_NLD\_STLC\_tube2\_021014\_01.10925.10925.2 | 3.6357 | 0.5209 | 100.0% | 1191.4521 | 1191.4563 | 1 | 9.816 | 80.0% | 1 | K.VANVSLLALYK.G | 2 |

---

|  |  |  |  |  |  |  |  |  |
| --- | --- | --- | --- | --- | --- | --- | --- | --- |
| U | *Reverse\_gi|10834992|r* | 1 | 1 | 7.1% | 380 | 44177 | 5.0 | interleukin 13 receptor, alpha 2 precursor [Homo sapiens] |

| Filename XCorr DeltCN Conf% ObsM+H+ CalcM+H+ SpR ZScore Ion% # Sequence  | | | | | | | | | | | | |
| --- | --- | --- | --- | --- | --- | --- | --- | --- | --- | --- | --- | --- |
| \* | Astrin\_NLD\_STLC\_tube2\_021014\_01.14675.14675.3 | 3.4074 | 0.231 | 96.8% | 3358.4644 | 3360.6885 | 22 | 4.026 | 22.1% | 1 | K.NLDFGDK@YHLNKTIITK@WT#ESGINRYK.L | 3 |

---

|  |  |  |  |  |  |  |  |  |
| --- | --- | --- | --- | --- | --- | --- | --- | --- |
| U | *gi|163965362|ref|NP\_0* | 1 | 1 | 7.0% | 215 | 23384 | 4.6 | nascent polypeptide-associated complex alpha subunit isoform b [Homo sapiens] |
| U | *gi|5031931|ref|NP\_005* | 1 | 1 | 7.0% | 215 | 23384 | 4.6 | nascent polypeptide-associated complex alpha subunit isoform b [Homo sapiens] |
| U | *gi|163965366|ref|NP\_0* | 1 | 1 | 0.7% | 2078 | 205419 | 9.6 | nascent polypeptide-associated complex alpha subunit isoform a [Homo sapiens] |
| U | *gi|163965364|ref|NP\_0* | 1 | 1 | 7.0% | 215 | 23384 | 4.6 | nascent polypeptide-associated complex alpha subunit isoform b [Homo sapiens] |

| Filename XCorr DeltCN Conf% ObsM+H+ CalcM+H+ SpR ZScore Ion% # Sequence  | | | | | | | | | | | | |
| --- | --- | --- | --- | --- | --- | --- | --- | --- | --- | --- | --- | --- |
|  | Astrin\_NLD\_STLC\_031014\_02.05626.05626.2 | 3.3862 | 0.2489 | 99.9% | 1614.5322 | 1615.7808 | 8 | 6.312 | 50.0% | 1 | K.IEDLSQQAQLAAAEK.F | 2 |

---

|  |  |  |  |  |  |  |  |  |
| --- | --- | --- | --- | --- | --- | --- | --- | --- |
| U | *gi|4506681|ref|NP\_001* | 1 | 1 | 7.0% | 158 | 18431 | 10.3 | ribosomal protein S11 [Homo sapiens] |

| Filename XCorr DeltCN Conf% ObsM+H+ CalcM+H+ SpR ZScore Ion% # Sequence  | | | | | | | | | | | | |
| --- | --- | --- | --- | --- | --- | --- | --- | --- | --- | --- | --- | --- |
| \* | Astrin\_NLD\_STLC\_tube2\_021014\_01.04538.04538.2 | 2.364 | 0.2615 | 98.7% | 1267.5122 | 1267.4216 | 88 | 4.319 | 65.0% | 1 | K.EAIEGTYIDKK.C | 2 |

---

|  |  |  |  |  |  |  |  |  |
| --- | --- | --- | --- | --- | --- | --- | --- | --- |
| U | *gi|148746199|ref|NP\_0* | 1 | 1 | 7.0% | 128 | 14632 | 10.8 | ribosomal protein L31 isoform 2 [Homo sapiens] |
| U | *gi|4506633|ref|NP\_000* | 1 | 1 | 7.2% | 125 | 14463 | 10.5 | ribosomal protein L31 isoform 1 [Homo sapiens] |
| U | *gi|153252132|ref|NP\_0* | 1 | 1 | 7.4% | 121 | 13995 | 10.8 | ribosomal protein L31 isoform 3 [Homo sapiens] |

| Filename XCorr DeltCN Conf% ObsM+H+ CalcM+H+ SpR ZScore Ion% # Sequence  | | | | | | | | | | | | |
| --- | --- | --- | --- | --- | --- | --- | --- | --- | --- | --- | --- | --- |
|  | Astrin\_NLD\_STLC\_tube2\_021014\_01.04911.04911.2 | 2.5066 | 0.2743 | 99.8% | 988.5122 | 989.11664 | 1 | 5.824 | 81.2% | 1 | R.SAINEVVTR.E | 2 |

---

|  |  |  |  |  |  |  |  |  |
| --- | --- | --- | --- | --- | --- | --- | --- | --- |
| U | *gi|109240550|ref|NP\_0* | 3 | 3 | 6.9% | 523 | 58744 | 6.7 | paraspeckle protein 1 [Homo sapiens] |

| Filename XCorr DeltCN Conf% ObsM+H+ CalcM+H+ SpR ZScore Ion% # Sequence  | | | | | | | | | | | | |
| --- | --- | --- | --- | --- | --- | --- | --- | --- | --- | --- | --- | --- |
| \* | Astrin\_NLD\_STLC\_tube2\_021014\_01.07848.07848.2 | 2.7438 | 0.4759 | 100.0% | 1310.8922 | 1311.4368 | 1 | 7.381 | 65.0% | 1 | R.YGEPSEVFINR.D | 2 |
| \* | Astrin\_NLD\_STLC\_tube2\_021014\_01.04558.04558.2 | 2.0483 | 0.4169 | 99.9% | 1115.5721 | 1116.3054 | 73 | 6.03 | 55.0% | 1 | R.FATHGAALTVK.N | 2 |
| \* | Astrin\_NLD\_STLC\_tube2\_021014\_01.09920.09920.2 | 2.7047 | 0.3853 | 100.0% | 1650.1721 | 1650.7875 | 1 | 5.286 | 61.5% | 1 | R.FAQPGTFEFEYASR.W | 2 |

---

|  |  |  |  |  |  |  |  |  |
| --- | --- | --- | --- | --- | --- | --- | --- | --- |
| U | *gi|22749351|ref|NP\_68* | 1 | 1 | 6.7% | 179 | 20730 | 9.0 | Tctex1 domain containing 1 [Homo sapiens] |

| Filename XCorr DeltCN Conf% ObsM+H+ CalcM+H+ SpR ZScore Ion% # Sequence  | | | | | | | | | | | | |
| --- | --- | --- | --- | --- | --- | --- | --- | --- | --- | --- | --- | --- |
| \* | Astrin\_NLD\_STLC\_031014\_02.06344.06344.2 | 2.3976 | 0.2419 | 97.8% | 1373.2522 | 1375.7006 | 98 | 4.882 | 45.5% | 1 | K.LIVIVHIGQLNR.Q | 2 |

---

|  |  |  |  |  |  |  |  |  |
| --- | --- | --- | --- | --- | --- | --- | --- | --- |
| U | *gi|5453555|ref|NP\_006* | 1 | 1 | 6.5% | 216 | 24423 | 7.5 | ras-related nuclear protein [Homo sapiens] |

| Filename XCorr DeltCN Conf% ObsM+H+ CalcM+H+ SpR ZScore Ion% # Sequence  | | | | | | | | | | | | |
| --- | --- | --- | --- | --- | --- | --- | --- | --- | --- | --- | --- | --- |
| \* | Astrin\_NLD\_STLC\_tube2\_021014\_01.12618.12618.3 | 3.6714 | 0.4579 | 100.0% | 1786.2843 | 1786.0427 | 1 | 7.458 | 46.2% | 1 | K.SNYNFEKPFLWLAR.K | 3 |

---

|  |  |  |  |  |  |  |  |  |
| --- | --- | --- | --- | --- | --- | --- | --- | --- |
| U | *Reverse\_gi|4501853|re* | 1 | 1 | 6.4% | 424 | 44292 | 8.4 | acetyl-Coenzyme A acyltransferase 1 isoform a [Homo sapiens] |

| Filename XCorr DeltCN Conf% ObsM+H+ CalcM+H+ SpR ZScore Ion% # Sequence  | | | | | | | | | | | | |
| --- | --- | --- | --- | --- | --- | --- | --- | --- | --- | --- | --- | --- |
| \* | Astrin\_NLD\_STLC\_031014\_02.07755.07755.3 | 2.6229 | 0.3149 | 97.7% | 2740.6743 | 2742.92 | 1 | 5.295 | 26.9% | 1 | K.S\*RRALLIAAAGDSVQSSNGATTSGDKK.F | 3 |

---

|  |  |  |  |  |  |  |  |  |
| --- | --- | --- | --- | --- | --- | --- | --- | --- |
| U | *gi|23957680|ref|NP\_70* | 1 | 3 | 6.3% | 473 | 52346 | 7.9 | ral guanine nucleotide dissociation stimulator-like 4 [Homo sapiens] |

| Filename XCorr DeltCN Conf% ObsM+H+ CalcM+H+ SpR ZScore Ion% # Sequence  | | | | | | | | | | | | |
| --- | --- | --- | --- | --- | --- | --- | --- | --- | --- | --- | --- | --- |
| \* | Astrin\_NLD\_STLC\_031014\_01.11098.11098.3 | 4.0306 | 0.2077 | 98.3% | 3340.5544 | 3341.6128 | 241 | 4.256 | 19.0% | 3 | K.GVVPFLGDFLTELQRLDS\*AIPDDLDGNTNK.R | 3 |

---

|  |  |  |  |  |  |  |  |  |
| --- | --- | --- | --- | --- | --- | --- | --- | --- |
| U | *gi|7706333|ref|NP\_057* | 1 | 1 | 6.1% | 212 | 23935 | 9.0 | mitochondrial ribosomal protein L48 [Homo sapiens] |

| Filename XCorr DeltCN Conf% ObsM+H+ CalcM+H+ SpR ZScore Ion% # Sequence  | | | | | | | | | | | | |
| --- | --- | --- | --- | --- | --- | --- | --- | --- | --- | --- | --- | --- |
| \* | Astrin\_NLD\_STLC\_tube2\_021014\_01.19197.19197.1 | 2.1884 | 0.212 | 98.3% | 1633.3 | 1631.6396 | 1 | 5.124 | 41.7% | 1 | K.T#IEVLQLQDQGS\*K@.M | 1 |

---

|  |  |  |  |  |  |  |  |  |
| --- | --- | --- | --- | --- | --- | --- | --- | --- |
| U | *gi|33469968|ref|NP\_00* | 3 | 3 | 6.0% | 719 | 81308 | 6.5 | minichromosome maintenance complex component 7 isoform 1 [Homo sapiens] |

| Filename XCorr DeltCN Conf% ObsM+H+ CalcM+H+ SpR ZScore Ion% # Sequence  | | | | | | | | | | | | |
| --- | --- | --- | --- | --- | --- | --- | --- | --- | --- | --- | --- | --- |
| \* | Astrin\_NLD\_STLC\_tube2\_021014\_02.06659.06659.3 | 3.3081 | 0.3534 | 100.0% | 1829.3043 | 1829.063 | 1 | 6.019 | 42.9% | 1 | R.EVVNKDVLDVYIEHR.L | 3 |
|  | Astrin\_NLD\_STLC\_tube2\_021014\_02.06484.06484.2 | 2.9401 | 0.2593 | 99.8% | 1188.0521 | 1188.3695 | 1 | 4.757 | 62.5% | 1 | R.GSSGVGLTAAVLR.D | 2 |
|  | Astrin\_NLD\_STLC\_tube2\_021014\_02.05513.05513.3 | 4.3088 | 0.3195 | 100.0% | 1746.7444 | 1746.9733 | 1 | 6.064 | 48.2% | 1 | R.MVDVVEKEDVNEAIR.L | 3 |

---

|  |  |  |  |  |  |  |  |  |
| --- | --- | --- | --- | --- | --- | --- | --- | --- |
| U | *gi|7705716|ref|NP\_057* | 1 | 1 | 6.0% | 301 | 33172 | 8.8 | nitric oxide synthase interacting protein [Homo sapiens] |

| Filename XCorr DeltCN Conf% ObsM+H+ CalcM+H+ SpR ZScore Ion% # Sequence  | | | | | | | | | | | | |
| --- | --- | --- | --- | --- | --- | --- | --- | --- | --- | --- | --- | --- |
| \* | Astrin\_NLD\_STLC\_tube2\_021014\_01.19776.19776.2 | 2.3347 | 0.2858 | 98.1% | 1866.5521 | 1868.0458 | 36 | 5.879 | 35.3% | 1 | R.DIIVLQRGGT#GFAGSGVK@.L | 2 |

---

|  |  |  |  |  |  |  |  |  |
| --- | --- | --- | --- | --- | --- | --- | --- | --- |
| U | *gi|4507357|ref|NP\_003* | 1 | 1 | 6.0% | 199 | 22391 | 8.2 | transgelin 2 [Homo sapiens] |

| Filename XCorr DeltCN Conf% ObsM+H+ CalcM+H+ SpR ZScore Ion% # Sequence  | | | | | | | | | | | | |
| --- | --- | --- | --- | --- | --- | --- | --- | --- | --- | --- | --- | --- |
| \* | Astrin\_NLD\_STLC\_tube2\_021014\_01.10605.10605.2 | 3.1005 | 0.3311 | 100.0% | 1216.7322 | 1216.4845 | 1 | 6.984 | 72.7% | 1 | R.TLMNLGGLAVAR.D | 2 |

---

|  |  |  |  |  |  |  |  |  |
| --- | --- | --- | --- | --- | --- | --- | --- | --- |
| U | *Reverse\_gi|169166806|* | 1 | 1 | 5.9% | 492 | 55854 | 8.7 | PREDICTED: similar to hCG2036814 [Homo sapiens] |
| U | *Reverse\_gi|169167068|* | 1 | 1 | 5.9% | 492 | 55854 | 8.7 | PREDICTED: similar to hCG2036814 [Homo sapiens] |

| Filename XCorr DeltCN Conf% ObsM+H+ CalcM+H+ SpR ZScore Ion% # Sequence  | | | | | | | | | | | | |
| --- | --- | --- | --- | --- | --- | --- | --- | --- | --- | --- | --- | --- |
|  | Astrin\_NLD\_STLC\_031014\_01.17252.17252.3 | 3.0783 | 0.2693 | 97.5% | 3641.7844 | 3642.6746 | 25 | 4.877 | 19.6% | 1 | K.HQISNLLT#K@LT#DS\*AEHLYKDET#LGAEEIK@.E | 3 |

---

|  |  |  |  |  |  |  |  |  |
| --- | --- | --- | --- | --- | --- | --- | --- | --- |
| U | *gi|12056465|ref|NP\_00* | 1 | 1 | 5.9% | 321 | 33784 | 10.2 | fibrillarin [Homo sapiens] |

| Filename XCorr DeltCN Conf% ObsM+H+ CalcM+H+ SpR ZScore Ion% # Sequence  | | | | | | | | | | | | |
| --- | --- | --- | --- | --- | --- | --- | --- | --- | --- | --- | --- | --- |
| \* | Astrin\_NLD\_STLC\_tube2\_021014\_02.06749.06749.3 | 3.029 | 0.2623 | 97.9% | 1872.4744 | 1873.2491 | 64 | 4.494 | 30.6% | 1 | K.LAAAILGGVDQIHIKPGAK.V | 3 |

---

|  |  |  |  |  |  |  |  |  |
| --- | --- | --- | --- | --- | --- | --- | --- | --- |
| U | *Reverse\_gi|169168324|* | 1 | 1 | 5.9% | 152 | 15862 | 4.3 | PREDICTED: hypothetical protein [Homo sapiens] |

| Filename XCorr DeltCN Conf% ObsM+H+ CalcM+H+ SpR ZScore Ion% # Sequence  | | | | | | | | | | | | |
| --- | --- | --- | --- | --- | --- | --- | --- | --- | --- | --- | --- | --- |
| \* | Astrin\_NLD\_STLC\_031014\_01.18999.18999.1 | 1.3331 | 0.3456 | 98.2% | 856.45 | 859.0716 | 207 | 4.474 | 43.8% | 1 | K.VAALVSAPM.- | 1 |

---

|  |  |  |  |  |  |  |  |  |
| --- | --- | --- | --- | --- | --- | --- | --- | --- |
| U | *gi|33286418|ref|NP\_00* | 2 | 2 | 5.8% | 531 | 57937 | 7.8 | pyruvate kinase, muscle isoform M2 [Homo sapiens] |
| U | *gi|33286422|ref|NP\_87* | 2 | 2 | 5.8% | 531 | 58062 | 7.7 | pyruvate kinase, muscle isoform M1 [Homo sapiens] |
| U | *gi|33286420|ref|NP\_87* | 2 | 2 | 5.8% | 531 | 58062 | 7.7 | pyruvate kinase, muscle isoform M1 [Homo sapiens] |

| Filename XCorr DeltCN Conf% ObsM+H+ CalcM+H+ SpR ZScore Ion% # Sequence  | | | | | | | | | | | | |
| --- | --- | --- | --- | --- | --- | --- | --- | --- | --- | --- | --- | --- |
|  | Astrin\_NLD\_STLC\_tube2\_021014\_01.11956.11956.3 | 2.87 | 0.2649 | 96.5% | 2466.6843 | 2466.7937 | 14 | 4.755 | 23.9% | 1 | R.TATESFASDPILYRPVAVALDTK.G | 3 |
|  | Astrin\_NLD\_STLC\_tube2\_021014\_01.05291.05291.1 | 1.734 | 0.3165 | 100.0% | 840.5 | 841.0415 | 27 | 5.158 | 57.1% | 1 | R.APIIAVTR.N | 1 |

---

|  |  |  |  |  |  |  |  |  |
| --- | --- | --- | --- | --- | --- | --- | --- | --- |
| U | *Reverse\_gi|9910346|re* | 1 | 1 | 5.8% | 242 | 26471 | 9.2 | chromosome 15 open reading frame 24 [Homo sapiens] |

| Filename XCorr DeltCN Conf% ObsM+H+ CalcM+H+ SpR ZScore Ion% # Sequence  | | | | | | | | | | | | |
| --- | --- | --- | --- | --- | --- | --- | --- | --- | --- | --- | --- | --- |
| \* | Astrin\_NLD\_STLC\_031014\_02.03756.03756.3 | 2.2224 | 0.3048 | 96.1% | 1377.0844 | 1377.3538 | 123 | 4.982 | 32.7% | 1 | K.GTKSSGSSSKGSS\*K@.S | 3 |

---

|  |  |  |  |  |  |  |  |  |
| --- | --- | --- | --- | --- | --- | --- | --- | --- |
| U | *gi|78000181|ref|NP\_00* | 1 | 1 | 5.6% | 215 | 23432 | 10.9 | ribosomal protein L14 [Homo sapiens] |
| U | *gi|78000183|ref|NP\_00* | 1 | 1 | 5.6% | 215 | 23432 | 10.9 | ribosomal protein L14 [Homo sapiens] |

| Filename XCorr DeltCN Conf% ObsM+H+ CalcM+H+ SpR ZScore Ion% # Sequence  | | | | | | | | | | | | |
| --- | --- | --- | --- | --- | --- | --- | --- | --- | --- | --- | --- | --- |
|  | Astrin\_NLD\_STLC\_031014\_02.08201.08201.2 | 2.9799 | 0.3373 | 100.0% | 1355.5521 | 1355.5773 | 1 | 6.663 | 63.6% | 1 | K.LVAIVDVIDQNR.A | 2 |

---

|  |  |  |  |  |  |  |  |  |
| --- | --- | --- | --- | --- | --- | --- | --- | --- |
| U | *gi|7706254|ref|NP\_057* | 2 | 2 | 5.5% | 529 | 59578 | 8.9 | NOP58 ribonucleoprotein homolog [Homo sapiens] |

| Filename XCorr DeltCN Conf% ObsM+H+ CalcM+H+ SpR ZScore Ion% # Sequence  | | | | | | | | | | | | |
| --- | --- | --- | --- | --- | --- | --- | --- | --- | --- | --- | --- | --- |
| \* | Astrin\_NLD\_STLC\_tube2\_021014\_01.09346.09346.2 | 2.5274 | 0.2916 | 99.7% | 1399.5322 | 1399.6049 | 397 | 5.534 | 45.8% | 1 | R.SQMDGLIPGVEPR.E | 2 |
| \* | Astrin\_NLD\_STLC\_tube2\_021014\_02.06258.06258.3 | 4.1102 | 0.4467 | 100.0% | 1735.0144 | 1734.9927 | 1 | 7.311 | 40.0% | 1 | K.YGLIYHASLVGQTSPK.H | 3 |

---

|  |  |  |  |  |  |  |  |  |
| --- | --- | --- | --- | --- | --- | --- | --- | --- |
| U | *gi|169216167|ref|XP\_9* | 1 | 1 | 5.5% | 201 | 23351 | 5.2 | PREDICTED: similar to RanBP1 [Homo sapiens] |
| U | *gi|4506407|ref|NP\_002* | 1 | 1 | 5.5% | 201 | 23310 | 5.3 | RAN binding protein 1 [Homo sapiens] |
| U | *gi|169216989|ref|XP\_0* | 1 | 1 | 6.1% | 180 | 20655 | 5.1 | PREDICTED: hypothetical protein [Homo sapiens] |
| U | *gi|169216776|ref|XP\_3* | 1 | 1 | 5.5% | 201 | 23351 | 5.2 | PREDICTED: similar to RanBP1 [Homo sapiens] |

| Filename XCorr DeltCN Conf% ObsM+H+ CalcM+H+ SpR ZScore Ion% # Sequence  | | | | | | | | | | | | |
| --- | --- | --- | --- | --- | --- | --- | --- | --- | --- | --- | --- | --- |
|  | Astrin\_NLD\_STLC\_tube2\_021014\_01.10427.10427.2 | 2.4121 | 0.2524 | 98.7% | 1383.0521 | 1382.4639 | 62 | 4.213 | 55.0% | 1 | K.TLEEDEEELFK.M | 2 |

---

|  |  |  |  |  |  |  |  |  |
| --- | --- | --- | --- | --- | --- | --- | --- | --- |
| U | *gi|153791158|ref|NP\_0* | 4 | 8 | 5.4% | 551 | 59560 | 7.7 | keratin 75 [Homo sapiens] |

| Filename XCorr DeltCN Conf% ObsM+H+ CalcM+H+ SpR ZScore Ion% # Sequence  | | | | | | | | | | | | |
| --- | --- | --- | --- | --- | --- | --- | --- | --- | --- | --- | --- | --- |
|  | Astrin\_NLD\_STLC\_tube2\_021014\_01.07623.07623.2 | 2.9741 | 0.1656 | 99.6% | 1082.8121 | 1083.2755 | 7 | 7.028 | 75.0% | 2 | K.FASFIDKVR.F | 22222 |
|  | Astrin\_NLD\_STLC\_tube2\_021014\_01.13338.13338.2 | 4.1957 | 0.3905 | 100.0% | 1331.0922 | 1330.5211 | 1 | 7.868 | 86.4% | 1 | R.NLDLDSIIAEVK.A | 222 |
|  | Astrin\_NLD\_STLC\_tube2\_021014\_01.04317.04317.1 | 1.9579 | 0.2777 | 100.0% | 1079.45 | 1080.1423 | 4 | 5.773 | 56.2% | 2 | K.AQYEDIANR.S | 11 |
|  | Astrin\_NLD\_STLC\_031014\_01.09014.09014.2 | 3.2681 | 0.2878 | 100.0% | 1079.9722 | 1080.1423 | 2 | 6.731 | 75.0% | 3 | K.AQYEDIANR.S | 22 |

Similarities:
gi|4504919|ref|NP\_002(3:1)  
gi|67782365|ref|NP\_00(1:3)  
gi|47132620|ref|NP\_00(2:2)  
gi|119703753|ref|NP\_0(2:2)  

---

|  |  |  |  |  |  |  |  |  |
| --- | --- | --- | --- | --- | --- | --- | --- | --- |
| U | *gi|6912634|ref|NP\_036* | 1 | 2 | 5.4% | 203 | 23577 | 10.9 | ribosomal protein L13a [Homo sapiens] |

| Filename XCorr DeltCN Conf% ObsM+H+ CalcM+H+ SpR ZScore Ion% # Sequence  | | | | | | | | | | | | |
| --- | --- | --- | --- | --- | --- | --- | --- | --- | --- | --- | --- | --- |
| \* | Astrin\_NLD\_STLC\_tube2\_021014\_01.05943.05943.2 | 2.6436 | 0.4204 | 100.0% | 1253.1322 | 1253.3947 | 1 | 6.158 | 65.0% | 2 | K.YQAVTATLEEK.R | 2 |

---

|  |  |  |  |  |  |  |  |  |
| --- | --- | --- | --- | --- | --- | --- | --- | --- |
| U | *gi|4503483|ref|NP\_001* | 3 | 4 | 5.2% | 858 | 95338 | 6.8 | eukaryotic translation elongation factor 2 [Homo sapiens] |

| Filename XCorr DeltCN Conf% ObsM+H+ CalcM+H+ SpR ZScore Ion% # Sequence  | | | | | | | | | | | | |
| --- | --- | --- | --- | --- | --- | --- | --- | --- | --- | --- | --- | --- |
| \* | Astrin\_NLD\_STLC\_tube2\_021014\_01.08842.08842.2 | 2.3159 | 0.2829 | 98.9% | 1107.3722 | 1108.3231 | 2 | 5.551 | 65.0% | 1 | R.VFSGLVSTGLK.V | 2 |
| \* | Astrin\_NLD\_STLC\_031014\_02.06594.06594.3 | 3.6314 | 0.3273 | 100.0% | 2143.2844 | 2144.3489 | 15 | 5.147 | 30.3% | 2 | K.ARPFPDGLAEDIDKGEVSAR.Q | 3 |
| \* | Astrin\_NLD\_STLC\_tube2\_021014\_02.06512.06512.3 | 2.7367 | 0.3039 | 99.6% | 1743.3844 | 1743.9133 | 3 | 5.449 | 44.2% | 1 | R.YLAEKYEWDVAEAR.K | 3 |

---

|  |  |  |  |  |  |  |  |  |
| --- | --- | --- | --- | --- | --- | --- | --- | --- |
| U | *gi|4557303|ref|NP\_000* | 2 | 2 | 5.2% | 485 | 54848 | 7.9 | aldehyde dehydrogenase 3A2 isoform 2 [Homo sapiens] |
| U | *gi|73466520|ref|NP\_00* | 2 | 2 | 4.9% | 508 | 57669 | 8.9 | aldehyde dehydrogenase 3A2 isoform 1 [Homo sapiens] |

| Filename XCorr DeltCN Conf% ObsM+H+ CalcM+H+ SpR ZScore Ion% # Sequence  | | | | | | | | | | | | |
| --- | --- | --- | --- | --- | --- | --- | --- | --- | --- | --- | --- | --- |
|  | Astrin\_NLD\_STLC\_tube2\_021014\_01.09354.09354.2 | 2.965 | 0.204 | 99.0% | 1432.1522 | 1432.6567 | 1 | 5.121 | 70.8% | 1 | R.YIAPTVLTDVDPK.T | 2 |
|  | Astrin\_NLD\_STLC\_tube2\_021014\_01.09917.09917.2 | 3.0097 | 0.3167 | 100.0% | 1433.9722 | 1434.5486 | 1 | 5.965 | 77.3% | 1 | K.NVDEAINFINER.E | 2 |

---

|  |  |  |  |  |  |  |  |  |
| --- | --- | --- | --- | --- | --- | --- | --- | --- |
| U | *gi|16579885|ref|NP\_00* | 1 | 1 | 5.2% | 427 | 47697 | 11.1 | ribosomal protein L4 [Homo sapiens] |

| Filename XCorr DeltCN Conf% ObsM+H+ CalcM+H+ SpR ZScore Ion% # Sequence  | | | | | | | | | | | | |
| --- | --- | --- | --- | --- | --- | --- | --- | --- | --- | --- | --- | --- |
| \* | Astrin\_NLD\_STLC\_tube2\_021014\_02.06222.06222.3 | 2.684 | 0.2673 | 95.2% | 2333.9043 | 2333.479 | 1 | 4.451 | 27.4% | 1 | R.QPYAVSELAGHQTSAESWGTGR.A | 3 |

---

|  |  |  |  |  |  |  |  |  |
| --- | --- | --- | --- | --- | --- | --- | --- | --- |
| U | *gi|20127499|ref|NP\_00* | 2 | 3 | 5.2% | 344 | 39587 | 11.4 | arginine/serine-rich splicing factor 6 [Homo sapiens] |

| Filename XCorr DeltCN Conf% ObsM+H+ CalcM+H+ SpR ZScore Ion% # Sequence  | | | | | | | | | | | | |
| --- | --- | --- | --- | --- | --- | --- | --- | --- | --- | --- | --- | --- |
|  | Astrin\_NLD\_STLC\_tube2\_021014\_01.06682.06682.2 | 3.1225 | 0.3037 | 100.0% | 1030.7722 | 1031.1973 | 1 | 6.397 | 93.8% | 2 | R.LIVENLSSR.C | 2 |
| \* | Astrin\_NLD\_STLC\_tube2\_021014\_01.07265.07265.2 | 2.8887 | 0.3641 | 100.0% | 1065.2122 | 1065.171 | 1 | 7.13 | 93.8% | 1 | R.TNEGVIEFR.S | 2 |

---

|  |  |  |  |  |  |  |  |  |
| --- | --- | --- | --- | --- | --- | --- | --- | --- |
| U | *gi|5803036|ref|NP\_006* | 1 | 2 | 5.2% | 305 | 30841 | 9.3 | heterogeneous nuclear ribonucleoprotein A0 [Homo sapiens] |

| Filename XCorr DeltCN Conf% ObsM+H+ CalcM+H+ SpR ZScore Ion% # Sequence  | | | | | | | | | | | | |
| --- | --- | --- | --- | --- | --- | --- | --- | --- | --- | --- | --- | --- |
| \* | Astrin\_NLD\_STLC\_031014\_02.08128.08128.2 | 4.2895 | 0.4154 | 100.0% | 1691.2722 | 1691.9248 | 1 | 7.372 | 66.7% | 2 | K.LFIGGLNVQTSESGLR.G | 2 |

---

|  |  |  |  |  |  |  |  |  |
| --- | --- | --- | --- | --- | --- | --- | --- | --- |
| U | *gi|4507163|ref|NP\_003* | 1 | 1 | 5.1% | 474 | 47263 | 7.3 | SRY (sex determining region Y)-box 4 [Homo sapiens] |

| Filename XCorr DeltCN Conf% ObsM+H+ CalcM+H+ SpR ZScore Ion% # Sequence  | | | | | | | | | | | | |
| --- | --- | --- | --- | --- | --- | --- | --- | --- | --- | --- | --- | --- |
| \* | Astrin\_NLD\_STLC\_tube2\_021014\_01.10310.10310.3 | 3.157 | 0.2529 | 97.1% | 2531.9644 | 2529.399 | 277 | 4.29 | 23.9% | 1 | K.VK@SGNANSSSS\*AAAS\*S\*KPGEKGDK@.V | 3 |

---

|  |  |  |  |  |  |  |  |  |
| --- | --- | --- | --- | --- | --- | --- | --- | --- |
| U | *Reverse\_gi|20149695|r* | 1 | 1 | 5.1% | 257 | 29395 | 9.7 | receptor accessory protein 4 [Homo sapiens] |

| Filename XCorr DeltCN Conf% ObsM+H+ CalcM+H+ SpR ZScore Ion% # Sequence  | | | | | | | | | | | | |
| --- | --- | --- | --- | --- | --- | --- | --- | --- | --- | --- | --- | --- |
| \* | Astrin\_NLD\_STLC\_tube2\_021014\_01.10349.10349.2 | 2.2613 | 0.2377 | 95.3% | 1616.2522 | 1616.8936 | 4 | 4.619 | 50.0% | 1 | R.K@VVRLSQS\*RILPK.E | 2 |

---

|  |  |  |  |  |  |  |  |  |
| --- | --- | --- | --- | --- | --- | --- | --- | --- |
| U | *gi|155030232|ref|NP\_0* | 3 | 3 | 5.0% | 1130 | 119700 | 4.3 | proline, glutamic acid and leucine rich protein 1 [Homo sapiens] |

| Filename XCorr DeltCN Conf% ObsM+H+ CalcM+H+ SpR ZScore Ion% # Sequence  | | | | | | | | | | | | |
| --- | --- | --- | --- | --- | --- | --- | --- | --- | --- | --- | --- | --- |
| \* | Astrin\_NLD\_STLC\_031014\_01.08817.08817.2 | 2.3745 | 0.443 | 100.0% | 1303.5521 | 1303.4636 | 1 | 6.566 | 62.5% | 1 | R.TGSAVAPVHPPNR.S | 2 |
| \* | Astrin\_NLD\_STLC\_031014\_01.08674.08674.2 | 4.2226 | 0.4469 | 100.0% | 1527.1122 | 1527.6744 | 1 | 7.679 | 66.7% | 1 | R.GSPDGSLQTGKPSAPK.K | 2 |
| \* | Astrin\_NLD\_STLC\_tube2\_021014\_01.13352.13352.3 | 2.7922 | 0.2822 | 96.7% | 2883.5942 | 2884.3203 | 1 | 4.542 | 26.0% | 1 | R.LHDLVLPLVMGVQQGEVLGSSPYTSSR.C | 3 |

---

|  |  |  |  |  |  |  |  |  |
| --- | --- | --- | --- | --- | --- | --- | --- | --- |
| U | *gi|32698748|ref|NP\_06* | 1 | 1 | 5.0% | 579 | 67087 | 8.2 | zinc finger protein 248 [Homo sapiens] |

| Filename XCorr DeltCN Conf% ObsM+H+ CalcM+H+ SpR ZScore Ion% # Sequence  | | | | | | | | | | | | |
| --- | --- | --- | --- | --- | --- | --- | --- | --- | --- | --- | --- | --- |
| \* | Astrin\_NLD\_STLC\_031014\_02.11477.11477.3 | 3.2756 | 0.2261 | 95.4% | 3387.4443 | 3388.5344 | 4 | 3.933 | 21.4% | 1 | K.TVS\*VENGDRGSKT#FNLGTDPVSLRNYPYK@.I | 3 |

---

|  |  |  |  |  |  |  |  |  |
| --- | --- | --- | --- | --- | --- | --- | --- | --- |
| U | *gi|108936958|ref|NP\_0* | 1 | 1 | 5.0% | 342 | 38926 | 5.5 | WD-repeat protein [Homo sapiens] |

| Filename XCorr DeltCN Conf% ObsM+H+ CalcM+H+ SpR ZScore Ion% # Sequence  | | | | | | | | | | | | |
| --- | --- | --- | --- | --- | --- | --- | --- | --- | --- | --- | --- | --- |
| \* | Astrin\_NLD\_STLC\_tube2\_021014\_02.06630.06630.3 | 3.9474 | 0.3687 | 100.0% | 2006.4844 | 2007.2535 | 1 | 6.56 | 43.8% | 1 | K.TQLIAHDKEVYDIAFSR.A | 3 |

---

|  |  |  |  |  |  |  |  |  |
| --- | --- | --- | --- | --- | --- | --- | --- | --- |
| U | *gi|218749820|ref|NP\_0* | 1 | 1 | 5.0% | 340 | 37613 | 7.4 | hypothetical protein LOC84221 isoform 1 [Homo sapiens] |
| U | *gi|23346427|ref|NP\_11* | 1 | 1 | 9.1% | 186 | 21049 | 9.2 | hypothetical protein LOC84221 isoform 2 [Homo sapiens] |

| Filename XCorr DeltCN Conf% ObsM+H+ CalcM+H+ SpR ZScore Ion% # Sequence  | | | | | | | | | | | | |
| --- | --- | --- | --- | --- | --- | --- | --- | --- | --- | --- | --- | --- |
|  | Astrin\_NLD\_STLC\_031014\_02.06110.06110.3 | 2.5106 | 0.2746 | 95.4% | 2141.6943 | 2141.4355 | 225 | 4.322 | 28.1% | 1 | K.LRELT#QRYLALSARLEK.L | 3 |

---

|  |  |  |  |  |  |  |  |  |
| --- | --- | --- | --- | --- | --- | --- | --- | --- |
| U | *gi|9558733|ref|NP\_037* | 1 | 1 | 5.0% | 282 | 32689 | 11.3 | transformer-2 alpha [Homo sapiens] |

| Filename XCorr DeltCN Conf% ObsM+H+ CalcM+H+ SpR ZScore Ion% # Sequence  | | | | | | | | | | | | |
| --- | --- | --- | --- | --- | --- | --- | --- | --- | --- | --- | --- | --- |
| \* | Astrin\_NLD\_STLC\_tube2\_021014\_01.09370.09370.2 | 2.8866 | 0.3146 | 100.0% | 1567.5122 | 1567.7416 | 1 | 5.613 | 65.4% | 1 | R.YGPLSGVNVVYDQR.T | 2 |

---

|  |  |  |  |  |  |  |  |  |
| --- | --- | --- | --- | --- | --- | --- | --- | --- |
| U | *Reverse\_gi|169208198|* | 1 | 1 | 5.0% | 258 | 30145 | 8.6 | PREDICTED: hypothetical protein [Homo sapiens] |
| U | *Reverse\_gi|169208965|* | 1 | 1 | 5.5% | 235 | 26929 | 8.5 | PREDICTED: hypothetical protein [Homo sapiens] |
| U | *Reverse\_gi|169208711|* | 1 | 1 | 5.0% | 261 | 29911 | 7.5 | PREDICTED: hypothetical protein [Homo sapiens] |

| Filename XCorr DeltCN Conf% ObsM+H+ CalcM+H+ SpR ZScore Ion% # Sequence  | | | | | | | | | | | | |
| --- | --- | --- | --- | --- | --- | --- | --- | --- | --- | --- | --- | --- |
|  | Astrin\_NLD\_STLC\_031014\_01.13353.13353.2 | 2.4105 | 0.2253 | 96.6% | 1772.7122 | 1772.8723 | 3 | 3.774 | 50.0% | 1 | R.RITELTT#HKFHT#K.S | 2 |

---

|  |  |  |  |  |  |  |  |  |
| --- | --- | --- | --- | --- | --- | --- | --- | --- |
| U | *gi|52632383|ref|NP\_00* | 2 | 2 | 4.9% | 589 | 64133 | 8.2 | heterogeneous nuclear ribonucleoprotein L isoform a [Homo sapiens] |
| U | *gi|52632385|ref|NP\_00* | 2 | 2 | 6.4% | 456 | 50561 | 7.5 | heterogeneous nuclear ribonucleoprotein L isoform b [Homo sapiens] |

| Filename XCorr DeltCN Conf% ObsM+H+ CalcM+H+ SpR ZScore Ion% # Sequence  | | | | | | | | | | | | |
| --- | --- | --- | --- | --- | --- | --- | --- | --- | --- | --- | --- | --- |
|  | Astrin\_NLD\_STLC\_031014\_02.06466.06466.3 | 3.0715 | 0.3736 | 100.0% | 1868.7244 | 1869.1176 | 11 | 5.545 | 32.4% | 1 | K.SKPGAAMVEMADGYAVDR.A | 3 |
|  | Astrin\_NLD\_STLC\_tube2\_021014\_01.06732.06732.2 | 2.1907 | 0.2999 | 98.7% | 1264.2322 | 1264.4233 | 94 | 4.721 | 45.0% | 1 | K.NPNGPYPYTLK.L | 2 |

---

|  |  |  |  |  |  |  |  |  |
| --- | --- | --- | --- | --- | --- | --- | --- | --- |
| U | *Reverse\_gi|4505651|re* | 1 | 1 | 4.9% | 389 | 43835 | 6.9 | phosphate cytidylyltransferase 2, ethanolamine [Homo sapiens] |

| Filename XCorr DeltCN Conf% ObsM+H+ CalcM+H+ SpR ZScore Ion% # Sequence  | | | | | | | | | | | | |
| --- | --- | --- | --- | --- | --- | --- | --- | --- | --- | --- | --- | --- |
| \* | Astrin\_NLD\_STLC\_031014\_01.16661.16661.2 | 2.1996 | 0.2745 | 96.5% | 2158.2122 | 2156.4624 | 1 | 5.024 | 38.9% | 1 | K.T#VLLMRGVLDTTSVGQTRK.C | 2 |

---

|  |  |  |  |  |  |  |  |  |
| --- | --- | --- | --- | --- | --- | --- | --- | --- |
| U | *gi|55741475|ref|NP\_06* | 1 | 1 | 4.8% | 336 | 36418 | 5.1 | Nedd4 family interacting protein 2 [Homo sapiens] |

| Filename XCorr DeltCN Conf% ObsM+H+ CalcM+H+ SpR ZScore Ion% # Sequence  | | | | | | | | | | | | |
| --- | --- | --- | --- | --- | --- | --- | --- | --- | --- | --- | --- | --- |
| \* | Astrin\_NLD\_STLC\_031014\_01.14984.14984.2 | 2.8224 | 0.2177 | 98.3% | 1802.2922 | 1801.8951 | 1 | 4.683 | 50.0% | 1 | R.SQRVCAS\*GPSMLNSAR.G | 2 |

---

|  |  |  |  |  |  |  |  |  |
| --- | --- | --- | --- | --- | --- | --- | --- | --- |
| U | *gi|21626466|ref|NP\_06* | 2 | 2 | 4.7% | 847 | 94623 | 6.3 | matrin 3 [Homo sapiens] |
| U | *gi|62750354|ref|NP\_95* | 2 | 2 | 4.7% | 847 | 94623 | 6.3 | matrin 3 [Homo sapiens] |

| Filename XCorr DeltCN Conf% ObsM+H+ CalcM+H+ SpR ZScore Ion% # Sequence  | | | | | | | | | | | | |
| --- | --- | --- | --- | --- | --- | --- | --- | --- | --- | --- | --- | --- |
|  | Astrin\_NLD\_STLC\_tube2\_021014\_01.15438.15438.3 | 3.5997 | 0.2592 | 99.6% | 2438.6643 | 2439.9036 | 63 | 5.911 | 27.5% | 1 | R.YQLLQLVEPFGVISNHLILNK.I | 3 |
|  | Astrin\_NLD\_STLC\_tube2\_021014\_01.07550.07550.3 | 3.5815 | 0.2825 | 100.0% | 2038.3744 | 2038.3109 | 1 | 5.417 | 38.9% | 1 | R.VIHLSNLPHSGYSDSAVLK.L | 3 |

---

|  |  |  |  |  |  |  |  |  |
| --- | --- | --- | --- | --- | --- | --- | --- | --- |
| U | *Reverse\_gi|29540562|r* | 1 | 1 | 4.7% | 632 | 65683 | 8.5 | ALEX2 protein [Homo sapiens] |
| U | *Reverse\_gi|7662162|re* | 1 | 1 | 4.7% | 632 | 65683 | 8.5 | ALEX2 protein [Homo sapiens] |

| Filename XCorr DeltCN Conf% ObsM+H+ CalcM+H+ SpR ZScore Ion% # Sequence  | | | | | | | | | | | | |
| --- | --- | --- | --- | --- | --- | --- | --- | --- | --- | --- | --- | --- |
|  | Astrin\_NLD\_STLC\_tube2\_021014\_01.15396.15396.3 | 3.2498 | 0.2877 | 99.4% | 2967.7444 | 2966.075 | 1 | 4.577 | 25.0% | 1 | R.T#VGKGGGK@PVAGTASTAK@PIAGT#HAGPT#AK@.K | 3 |

---

|  |  |  |  |  |  |  |  |  |
| --- | --- | --- | --- | --- | --- | --- | --- | --- |
| U | *Reverse\_gi|34365783|r* | 1 | 1 | 4.7% | 361 | 39411 | 7.6 | ceh-10 homeo domain containing homolog [Homo sapiens] |

| Filename XCorr DeltCN Conf% ObsM+H+ CalcM+H+ SpR ZScore Ion% # Sequence  | | | | | | | | | | | | |
| --- | --- | --- | --- | --- | --- | --- | --- | --- | --- | --- | --- | --- |
| \* | Astrin\_NLD\_STLC\_tube2\_021014\_02.05658.05658.3 | 2.6515 | 0.2937 | 97.9% | 1893.2344 | 1892.8119 | 84 | 4.725 | 32.8% | 1 | R.APAGGST#SK@AVT#ES\*K@PK@.S | 3 |

---

|  |  |  |  |  |  |  |  |  |
| --- | --- | --- | --- | --- | --- | --- | --- | --- |
| U | *gi|14602427|ref|NP\_12* | 1 | 2 | 4.7% | 277 | 31293 | 5.2 | ZW10 interactor isoform a [Homo sapiens] |
| U | *gi|14602429|ref|NP\_00* | 1 | 2 | 4.7% | 277 | 31293 | 5.2 | ZW10 interactor isoform a [Homo sapiens] |

| Filename XCorr DeltCN Conf% ObsM+H+ CalcM+H+ SpR ZScore Ion% # Sequence  | | | | | | | | | | | | |
| --- | --- | --- | --- | --- | --- | --- | --- | --- | --- | --- | --- | --- |
|  | Astrin\_NLD\_STLC\_031014\_02.04664.04664.3 | 4.0746 | 0.3415 | 100.0% | 1490.2444 | 1489.6743 | 1 | 6.954 | 47.9% | 2 | K.HLQHLAEVSAEVR.E | 3 |

---

|  |  |  |  |  |  |  |  |  |
| --- | --- | --- | --- | --- | --- | --- | --- | --- |
| U | *gi|4557553|ref|NP\_000* | 1 | 1 | 4.7% | 254 | 28994 | 5.5 | emerin [Homo sapiens] |

| Filename XCorr DeltCN Conf% ObsM+H+ CalcM+H+ SpR ZScore Ion% # Sequence  | | | | | | | | | | | | |
| --- | --- | --- | --- | --- | --- | --- | --- | --- | --- | --- | --- | --- |
| \* | Astrin\_NLD\_STLC\_031014\_01.07319.07319.2 | 2.0302 | 0.3356 | 98.7% | 1251.2722 | 1251.295 | 4 | 6.137 | 54.5% | 1 | R.TYGEPESAGPSR.A | 2 |

---

|  |  |  |  |  |  |  |  |  |
| --- | --- | --- | --- | --- | --- | --- | --- | --- |
| U | *gi|114155140|ref|NP\_6* | 1 | 2 | 4.6% | 285 | 32950 | 4.7 | tropomyosin 3 isoform 1 [Homo sapiens] |
| U | *gi|63252906|ref|NP\_00* | 1 | 2 | 4.6% | 284 | 32817 | 4.8 | tropomyosin 1 alpha chain isoform 7 [Homo sapiens] |
| U | *gi|63252904|ref|NP\_00* | 1 | 2 | 5.3% | 245 | 28385 | 4.8 | tropomyosin 1 alpha chain isoform 6 [Homo sapiens] |
| U | *gi|63252902|ref|NP\_00* | 1 | 2 | 4.6% | 284 | 32678 | 4.7 | tropomyosin 1 alpha chain isoform 2 [Homo sapiens] |
| U | *gi|63252900|ref|NP\_00* | 1 | 2 | 4.6% | 284 | 32876 | 4.8 | tropomyosin 1 alpha chain isoform 4 [Homo sapiens] |
| U | *gi|63252898|ref|NP\_00* | 1 | 2 | 4.6% | 284 | 32709 | 4.7 | tropomyosin 1 alpha chain isoform 1 [Homo sapiens] |
| U | *gi|63252896|ref|NP\_00* | 1 | 2 | 4.6% | 284 | 32737 | 4.8 | tropomyosin 1 alpha chain isoform 3 [Homo sapiens] |
| U | *gi|47519616|ref|NP\_99* | 1 | 2 | 4.6% | 284 | 32990 | 4.7 | tropomyosin 2 (beta) isoform 2 [Homo sapiens] |
| U | *gi|4507651|ref|NP\_003* | 1 | 2 | 5.2% | 248 | 28522 | 4.7 | tropomyosin 4 isoform 2 [Homo sapiens] |
| U | *gi|42476296|ref|NP\_00* | 1 | 2 | 4.6% | 284 | 32851 | 4.7 | tropomyosin 2 (beta) isoform 1 [Homo sapiens] |
| U | *gi|27597085|ref|NP\_00* | 1 | 2 | 4.6% | 284 | 32866 | 4.8 | tropomyosin 1 alpha chain isoform 5 [Homo sapiens] |
| U | *gi|24119203|ref|NP\_70* | 1 | 2 | 5.2% | 248 | 29033 | 4.8 | tropomyosin 3 isoform 2 [Homo sapiens] |
| U | *gi|223555975|ref|NP\_0* | 1 | 2 | 4.6% | 284 | 32723 | 4.7 | tropomyosin 4 isoform 1 [Homo sapiens] |
| U | *gi|114155148|ref|NP\_0* | 1 | 2 | 5.3% | 247 | 28793 | 4.8 | tropomyosin 3 isoform 5 [Homo sapiens] |
| U | *gi|114155146|ref|NP\_0* | 1 | 2 | 5.3% | 247 | 28955 | 4.8 | tropomyosin 3 isoform 3 [Homo sapiens] |
| U | *gi|114155144|ref|NP\_0* | 1 | 2 | 5.2% | 248 | 28870 | 4.8 | tropomyosin 3 isoform 4 [Homo sapiens] |

| Filename XCorr DeltCN Conf% ObsM+H+ CalcM+H+ SpR ZScore Ion% # Sequence  | | | | | | | | | | | | |
| --- | --- | --- | --- | --- | --- | --- | --- | --- | --- | --- | --- | --- |
|  | Astrin\_NLD\_STLC\_031014\_01.06035.06035.2 | 3.6527 | 0.4189 | 100.0% | 1477.6721 | 1477.5248 | 2 | 6.354 | 62.5% | 2 | K.LEEAEKAADESER.G | 2 |

---

|  |  |  |  |  |  |  |  |  |
| --- | --- | --- | --- | --- | --- | --- | --- | --- |
| U | *gi|14110414|ref|NP\_00* | 1 | 1 | 4.6% | 306 | 32835 | 8.2 | heterogeneous nuclear ribonucleoprotein D isoform c [Homo sapiens] |
| U | *gi|51477708|ref|NP\_00* | 1 | 1 | 4.9% | 287 | 30672 | 8.4 | heterogeneous nuclear ribonucleoprotein D isoform d [Homo sapiens] |
| U | *gi|14110420|ref|NP\_11* | 1 | 1 | 3.9% | 355 | 38434 | 7.8 | heterogeneous nuclear ribonucleoprotein D isoform a [Homo sapiens] |
| U | *gi|14110417|ref|NP\_11* | 1 | 1 | 4.2% | 336 | 36272 | 8.1 | heterogeneous nuclear ribonucleoprotein D isoform b [Homo sapiens] |

| Filename XCorr DeltCN Conf% ObsM+H+ CalcM+H+ SpR ZScore Ion% # Sequence  | | | | | | | | | | | | |
| --- | --- | --- | --- | --- | --- | --- | --- | --- | --- | --- | --- | --- |
|  | Astrin\_NLD\_STLC\_tube2\_021014\_01.08237.08237.2 | 4.0469 | 0.385 | 100.0% | 1490.0721 | 1489.6653 | 1 | 6.294 | 69.2% | 1 | K.IFVGGLSPDTPEEK.I | 2 |

---

|  |  |  |  |  |  |  |  |  |
| --- | --- | --- | --- | --- | --- | --- | --- | --- |
| U | *Reverse\_gi|194097356|* | 1 | 5 | 4.6% | 262 | 29747 | 5.7 | phosphomannomutase 1 [Homo sapiens] |

| Filename XCorr DeltCN Conf% ObsM+H+ CalcM+H+ SpR ZScore Ion% # Sequence  | | | | | | | | | | | | |
| --- | --- | --- | --- | --- | --- | --- | --- | --- | --- | --- | --- | --- |
| \* | Astrin\_NLD\_STLC\_031014\_01.11403.11403.2 | 2.9779 | 0.21 | 99.6% | 1340.6522 | 1340.562 | 13 | 4.386 | 59.1% | 5 | K.GAFETKLAEVFK.E | 2 |

---

|  |  |  |  |  |  |  |  |  |
| --- | --- | --- | --- | --- | --- | --- | --- | --- |
| U | *gi|32483377|ref|NP\_05* | 1 | 1 | 4.6% | 238 | 25839 | 7.5 | peroxiredoxin 3 isoform b [Homo sapiens] |
| U | *gi|5802974|ref|NP\_006* | 1 | 1 | 4.3% | 256 | 27693 | 7.8 | peroxiredoxin 3 isoform a precursor [Homo sapiens] |

| Filename XCorr DeltCN Conf% ObsM+H+ CalcM+H+ SpR ZScore Ion% # Sequence  | | | | | | | | | | | | |
| --- | --- | --- | --- | --- | --- | --- | --- | --- | --- | --- | --- | --- |
|  | Astrin\_NLD\_STLC\_tube2\_021014\_01.06598.06598.2 | 2.6131 | 0.3094 | 99.9% | 1206.6122 | 1207.375 | 10 | 6.345 | 55.0% | 1 | K.HLSVNDLPVGR.S | 2 |

---

|  |  |  |  |  |  |  |  |  |
| --- | --- | --- | --- | --- | --- | --- | --- | --- |
| U | *gi|4506609|ref|NP\_000* | 1 | 1 | 4.6% | 196 | 23466 | 11.5 | ribosomal protein L19 [Homo sapiens] |

| Filename XCorr DeltCN Conf% ObsM+H+ CalcM+H+ SpR ZScore Ion% # Sequence  | | | | | | | | | | | | |
| --- | --- | --- | --- | --- | --- | --- | --- | --- | --- | --- | --- | --- |
| \* | Astrin\_NLD\_STLC\_031014\_01.06904.06904.2 | 2.4952 | 0.2162 | 98.7% | 987.15216 | 987.10077 | 27 | 5.13 | 68.8% | 1 | K.LLADQAEAR.R | 2 |

---

|  |  |  |  |  |  |  |  |  |
| --- | --- | --- | --- | --- | --- | --- | --- | --- |
| U | *gi|167466173|ref|NP\_0* | 2 | 3 | 4.5% | 641 | 70052 | 5.6 | heat shock 70kDa protein 1B [Homo sapiens] |
| U | *gi|34419635|ref|NP\_00* | 2 | 3 | 4.5% | 643 | 71028 | 6.1 | heat shock 70kDa protein 6 (HSP70B') [Homo sapiens] |
| U | *gi|194248072|ref|NP\_0* | 2 | 3 | 4.5% | 641 | 70052 | 5.6 | heat shock 70kDa protein 1A [Homo sapiens] |

| Filename XCorr DeltCN Conf% ObsM+H+ CalcM+H+ SpR ZScore Ion% # Sequence  | | | | | | | | | | | | |
| --- | --- | --- | --- | --- | --- | --- | --- | --- | --- | --- | --- | --- |
|  | Astrin\_NLD\_STLC\_tube2\_021014\_01.07839.07839.2 | 3.2184 | 0.2936 | 100.0% | 1488.1721 | 1488.5939 | 1 | 5.063 | 62.5% | 2 | R.TTPSYVAFTDTER.L | 22 |
|  | Astrin\_NLD\_STLC\_tube2\_021014\_01.10733.10733.2 | 4.4966 | 0.4585 | 100.0% | 1688.6122 | 1688.9213 | 1 | 9.287 | 70.0% | 1 | R.IINEPTAAAIAYGLDR.T | 2 |

Similarities:
gi|5729877|ref|NP\_006(1:1)  

---

|  |  |  |  |  |  |  |  |  |
| --- | --- | --- | --- | --- | --- | --- | --- | --- |
| U | *gi|117968353|ref|NP\_1* | 2 | 2 | 4.5% | 464 | 54304 | 8.3 | NUF2, NDC80 kinetochore complex component [Homo sapiens] |
| U | *gi|117968420|ref|NP\_6* | 2 | 2 | 4.5% | 464 | 54304 | 8.3 | NUF2, NDC80 kinetochore complex component [Homo sapiens] |

| Filename XCorr DeltCN Conf% ObsM+H+ CalcM+H+ SpR ZScore Ion% # Sequence  | | | | | | | | | | | | |
| --- | --- | --- | --- | --- | --- | --- | --- | --- | --- | --- | --- | --- |
|  | Astrin\_NLD\_STLC\_tube2\_021014\_01.10782.10782.2 | 3.2356 | 0.4984 | 100.0% | 1327.0721 | 1327.5693 | 1 | 8.307 | 80.0% | 1 | R.YNVAEIVIHIR.N | 2 |
|  | Astrin\_NLD\_STLC\_tube2\_021014\_01.04610.04610.2 | 2.2848 | 0.3681 | 100.0% | 1173.6721 | 1174.3396 | 187 | 6.32 | 61.1% | 1 | R.VTTINQEIQK.I | 2 |

---

|  |  |  |  |  |  |  |  |  |
| --- | --- | --- | --- | --- | --- | --- | --- | --- |
| U | *gi|114796640|ref|NP\_0* | 1 | 2 | 4.5% | 421 | 44969 | 7.5 | regulator of chromosome condensation 1 [Homo sapiens] |
| U | *gi|4502801|ref|NP\_001* | 1 | 2 | 4.5% | 421 | 44969 | 7.5 | regulator of chromosome condensation 1 isoform c [Homo sapiens] |
| U | *gi|114796648|ref|NP\_0* | 1 | 2 | 4.5% | 421 | 44969 | 7.5 | regulator of chromosome condensation 1 [Homo sapiens] |
| U | *gi|114796646|ref|NP\_0* | 1 | 2 | 4.3% | 438 | 46753 | 8.1 | regulator of chromosome condensation 1 isoform b [Homo sapiens] |
| U | *gi|114796644|ref|NP\_0* | 1 | 2 | 4.2% | 452 | 48146 | 8.2 | regulator of chromosome condensation 1 isoform a [Homo sapiens] |
| U | *gi|114796642|ref|NP\_0* | 1 | 2 | 4.5% | 421 | 44969 | 7.5 | regulator of chromosome condensation 1 [Homo sapiens] |

| Filename XCorr DeltCN Conf% ObsM+H+ CalcM+H+ SpR ZScore Ion% # Sequence  | | | | | | | | | | | | |
| --- | --- | --- | --- | --- | --- | --- | --- | --- | --- | --- | --- | --- |
|  | Astrin\_NLD\_STLC\_tube2\_021014\_02.04431.04431.3 | 4.4547 | 0.4604 | 100.0% | 1899.6244 | 1900.0122 | 1 | 7.377 | 41.7% | 2 | K.VVQVSAGDSHTAALTDDGR.V | 3 |

---

|  |  |  |  |  |  |  |  |  |
| --- | --- | --- | --- | --- | --- | --- | --- | --- |
| U | *Reverse\_gi|21553335|r* | 1 | 1 | 4.5% | 309 | 35434 | 5.5 | DnaJ (Hsp40) homolog, subfamily B, member 7 [Homo sapiens] |

| Filename XCorr DeltCN Conf% ObsM+H+ CalcM+H+ SpR ZScore Ion% # Sequence  | | | | | | | | | | | | |
| --- | --- | --- | --- | --- | --- | --- | --- | --- | --- | --- | --- | --- |
| \* | Astrin\_NLD\_STLC\_tube2\_021014\_01.11025.11025.2 | 2.4255 | 0.2569 | 97.8% | 1676.6921 | 1676.688 | 1 | 4.361 | 57.7% | 1 | K.EDNS\*LVEYAEAVEK.F | 2 |

---

|  |  |  |  |  |  |  |  |  |
| --- | --- | --- | --- | --- | --- | --- | --- | --- |
| U | *gi|154355000|ref|NP\_0* | 1 | 1 | 4.4% | 711 | 73115 | 7.3 | KH-type splicing regulatory protein (FUSE binding protein 2) [Homo sapiens] |

| Filename XCorr DeltCN Conf% ObsM+H+ CalcM+H+ SpR ZScore Ion% # Sequence  | | | | | | | | | | | | |
| --- | --- | --- | --- | --- | --- | --- | --- | --- | --- | --- | --- | --- |
| \* | Astrin\_NLD\_STLC\_tube2\_021014\_01.04058.04058.3 | 3.6407 | 0.3276 | 100.0% | 2399.1543 | 2399.5447 | 6 | 5.054 | 29.2% | 1 | R.GGGGPGGGGPGGGSAGGPSQPPGGGGPGIRK.D | 3 |

---

|  |  |  |  |  |  |  |  |  |
| --- | --- | --- | --- | --- | --- | --- | --- | --- |
| U | *gi|30089954|ref|NP\_05* | 1 | 1 | 4.4% | 597 | 66852 | 5.4 | H326 [Homo sapiens] |

| Filename XCorr DeltCN Conf% ObsM+H+ CalcM+H+ SpR ZScore Ion% # Sequence  | | | | | | | | | | | | |
| --- | --- | --- | --- | --- | --- | --- | --- | --- | --- | --- | --- | --- |
| \* | Astrin\_NLD\_STLC\_031014\_02.09192.09192.3 | 2.9704 | 0.3297 | 99.8% | 2927.0942 | 2927.7014 | 2 | 3.308 | 27.0% | 1 | R.ET#S\*SGIEVEASDLSLS\*LT#GDDGGPNR.T | 3 |

---

|  |  |  |  |  |  |  |  |  |
| --- | --- | --- | --- | --- | --- | --- | --- | --- |
| U | *gi|25282469|ref|NP\_74* | 1 | 1 | 4.4% | 341 | 38087 | 7.4 | retinol dehydrogenase 10 [Homo sapiens] |

| Filename XCorr DeltCN Conf% ObsM+H+ CalcM+H+ SpR ZScore Ion% # Sequence  | | | | | | | | | | | | |
| --- | --- | --- | --- | --- | --- | --- | --- | --- | --- | --- | --- | --- |
| \* | Astrin\_NLD\_STLC\_tube2\_021014\_02.06473.06473.3 | 2.4402 | 0.3052 | 97.8% | 1685.9343 | 1686.9078 | 14 | 5.03 | 32.1% | 1 | K.FGVVGFHESLSHELK.A | 3 |

---

|  |  |  |  |  |  |  |  |  |
| --- | --- | --- | --- | --- | --- | --- | --- | --- |
| U | *gi|15431301|ref|NP\_00* | 1 | 1 | 4.4% | 248 | 29226 | 10.7 | ribosomal protein L7 [Homo sapiens] |
| U | *gi|88988289|ref|XP\_94* | 1 | 1 | 4.2% | 259 | 30508 | 10.8 | PREDICTED: hypothetical protein LOC648000 isoform 3 [Homo sapiens] |
| U | *gi|169171881|ref|XP\_0* | 1 | 1 | 4.5% | 247 | 29037 | 10.7 | PREDICTED: hypothetical protein [Homo sapiens] |
| U | *gi|169171450|ref|XP\_0* | 1 | 1 | 4.5% | 247 | 28971 | 10.6 | PREDICTED: hypothetical protein [Homo sapiens] |
| U | *gi|169171114|ref|XP\_0* | 1 | 1 | 4.5% | 247 | 29037 | 10.7 | PREDICTED: hypothetical protein [Homo sapiens] |
| U | *gi|169170622|ref|XP\_0* | 1 | 1 | 4.5% | 247 | 29009 | 10.7 | PREDICTED: hypothetical protein [Homo sapiens] |
| U | *gi|169168181|ref|XP\_0* | 1 | 1 | 4.2% | 259 | 30508 | 10.8 | PREDICTED: hypothetical protein LOC648000 [Homo sapiens] |
| U | *gi|169167651|ref|XP\_0* | 1 | 1 | 4.2% | 259 | 30508 | 10.8 | PREDICTED: hypothetical protein LOC648000 [Homo sapiens] |

| Filename XCorr DeltCN Conf% ObsM+H+ CalcM+H+ SpR ZScore Ion% # Sequence  | | | | | | | | | | | | |
| --- | --- | --- | --- | --- | --- | --- | --- | --- | --- | --- | --- | --- |
|  | Astrin\_NLD\_STLC\_tube2\_021014\_02.06657.06657.2 | 3.2775 | 0.2096 | 99.9% | 1172.4122 | 1171.3823 | 1 | 6.453 | 75.0% | 1 | R.IALTDNALIAR.S | 2 |

---

|  |  |  |  |  |  |  |  |  |
| --- | --- | --- | --- | --- | --- | --- | --- | --- |
| U | *gi|94721342|ref|NP\_00* | 1 | 2 | 4.3% | 441 | 50141 | 7.2 | zinc finger and BTB domain containing 8A [Homo sapiens] |

| Filename XCorr DeltCN Conf% ObsM+H+ CalcM+H+ SpR ZScore Ion% # Sequence  | | | | | | | | | | | | |
| --- | --- | --- | --- | --- | --- | --- | --- | --- | --- | --- | --- | --- |
| \* | Astrin\_NLD\_STLC\_tube2\_021014\_01.10690.10690.3 | 3.7041 | 0.2228 | 99.1% | 2399.9343 | 2401.4814 | 17 | 4.023 | 30.6% | 2 | K.CKRHVT#DLTGQVVQEGT#RR.Y | 3 |

---

|  |  |  |  |  |  |  |  |  |
| --- | --- | --- | --- | --- | --- | --- | --- | --- |
| U | *gi|170296790|ref|NP\_0* | 1 | 3 | 4.3% | 304 | 32499 | 7.5 | mesotrypsin isoform 1 preproprotein [Homo sapiens] |
| U | *gi|21536452|ref|NP\_00* | 1 | 3 | 5.3% | 247 | 26697 | 6.1 | mesotrypsin isoform 2 preproprotein [Homo sapiens] |

| Filename XCorr DeltCN Conf% ObsM+H+ CalcM+H+ SpR ZScore Ion% # Sequence  | | | | | | | | | | | | |
| --- | --- | --- | --- | --- | --- | --- | --- | --- | --- | --- | --- | --- |
|  | Astrin\_NLD\_STLC\_031014\_01.09494.09494.2 | 4.1477 | 0.4428 | 100.0% | 1432.9521 | 1433.6041 | 1 | 10.01 | 70.8% | 3 | K.VLEGNEQFINAAK.I | 2 |

---

|  |  |  |  |  |  |  |  |  |
| --- | --- | --- | --- | --- | --- | --- | --- | --- |
| U | *gi|21361122|ref|NP\_00* | 1 | 1 | 4.3% | 280 | 31895 | 8.3 | four and a half LIM domains 1 [Homo sapiens] |

| Filename XCorr DeltCN Conf% ObsM+H+ CalcM+H+ SpR ZScore Ion% # Sequence  | | | | | | | | | | | | |
| --- | --- | --- | --- | --- | --- | --- | --- | --- | --- | --- | --- | --- |
| \* | Astrin\_NLD\_STLC\_tube2\_021014\_01.04912.04912.2 | 2.7928 | 0.2964 | 99.9% | 1308.0521 | 1307.4459 | 1 | 6.161 | 68.2% | 1 | K.AIVAGDQNVEYK.G | 2 |

---

|  |  |  |  |  |  |  |  |  |
| --- | --- | --- | --- | --- | --- | --- | --- | --- |
| U | *gi|55770864|ref|NP\_00* | 1 | 1 | 4.3% | 257 | 26888 | 11.2 | THO complex 4 [Homo sapiens] |

| Filename XCorr DeltCN Conf% ObsM+H+ CalcM+H+ SpR ZScore Ion% # Sequence  | | | | | | | | | | | | |
| --- | --- | --- | --- | --- | --- | --- | --- | --- | --- | --- | --- | --- |
| \* | Astrin\_NLD\_STLC\_tube2\_021014\_01.05856.05856.2 | 2.7911 | 0.2451 | 99.8% | 1232.4722 | 1232.3384 | 26 | 6.598 | 60.0% | 1 | R.SLGTADVHFER.K | 2 |

---

|  |  |  |  |  |  |  |  |  |
| --- | --- | --- | --- | --- | --- | --- | --- | --- |
| U | *gi|37620194|ref|NP\_93* | 1 | 1 | 4.3% | 184 | 20749 | 8.0 | glucosamine-phosphate N-acetyltransferase 1 [Homo sapiens] |

| Filename XCorr DeltCN Conf% ObsM+H+ CalcM+H+ SpR ZScore Ion% # Sequence  | | | | | | | | | | | | |
| --- | --- | --- | --- | --- | --- | --- | --- | --- | --- | --- | --- | --- |
| \* | Astrin\_NLD\_STLC\_tube2\_021014\_01.07300.07300.2 | 2.1288 | 0.2444 | 97.8% | 1111.1721 | 1111.1577 | 8 | 4.665 | 64.3% | 1 | K.FIHS\*CAK@R.G | 2 |

---

|  |  |  |  |  |  |  |  |  |
| --- | --- | --- | --- | --- | --- | --- | --- | --- |
| U | *Reverse\_gi|23510410|r* | 1 | 1 | 4.2% | 506 | 56094 | 7.1 | solute carrier family 22 member 1 isoform b [Homo sapiens] |
| U | *Reverse\_gi|4506999|re* | 1 | 1 | 3.8% | 554 | 61154 | 6.8 | solute carrier family 22 member 1 isoform a [Homo sapiens] |

| Filename XCorr DeltCN Conf% ObsM+H+ CalcM+H+ SpR ZScore Ion% # Sequence  | | | | | | | | | | | | |
| --- | --- | --- | --- | --- | --- | --- | --- | --- | --- | --- | --- | --- |
|  | Astrin\_NLD\_STLC\_031014\_02.08447.08447.3 | 2.8287 | 0.2862 | 97.8% | 2494.6443 | 2492.746 | 48 | 4.127 | 26.2% | 1 | R.FLDAFS\*PSLKETVDEELSLMK@.L | 3 |

---

|  |  |  |  |  |  |  |  |  |
| --- | --- | --- | --- | --- | --- | --- | --- | --- |
| U | *gi|21071014|ref|NP\_07* | 1 | 1 | 4.1% | 563 | 63502 | 7.7 | epidermal growth factor receptor pathway substrate 8-like protein 3 isoform c [Homo sapiens] |
| U | *gi|21071018|ref|NP\_62* | 1 | 1 | 3.9% | 594 | 66948 | 8.0 | epidermal growth factor receptor pathway substrate 8-like protein 3 isoform a [Homo sapiens] |
| U | *gi|21071016|ref|NP\_57* | 1 | 1 | 3.9% | 593 | 66861 | 8.0 | epidermal growth factor receptor pathway substrate 8-like protein 3 isoform b [Homo sapiens] |

| Filename XCorr DeltCN Conf% ObsM+H+ CalcM+H+ SpR ZScore Ion% # Sequence  | | | | | | | | | | | | |
| --- | --- | --- | --- | --- | --- | --- | --- | --- | --- | --- | --- | --- |
|  | Astrin\_NLD\_STLC\_031014\_01.14098.14098.3 | 3.0174 | 0.2354 | 95.0% | 2720.7544 | 2717.8657 | 1 | 4.342 | 27.3% | 1 | R.LSS\*RPEEVTDWLQAENFSTATVR.T | 3 |

---

|  |  |  |  |  |  |  |  |  |
| --- | --- | --- | --- | --- | --- | --- | --- | --- |
| U | *gi|169172218|ref|XP\_0* | 1 | 2 | 4.1% | 365 | 43591 | 8.1 | PREDICTED: hypothetical protein [Homo sapiens] |

| Filename XCorr DeltCN Conf% ObsM+H+ CalcM+H+ SpR ZScore Ion% # Sequence  | | | | | | | | | | | | |
| --- | --- | --- | --- | --- | --- | --- | --- | --- | --- | --- | --- | --- |
| \* | Astrin\_NLD\_STLC\_031014\_02.08550.08550.2 | 2.7121 | 0.2138 | 97.7% | 1798.2522 | 1798.1375 | 26 | 3.946 | 39.3% | 2 | K.KLGYVESLFWAGWIK.S | 2 |

---

|  |  |  |  |  |  |  |  |  |
| --- | --- | --- | --- | --- | --- | --- | --- | --- |
| U | *gi|134152672|ref|NP\_5* | 1 | 1 | 4.1% | 292 | 31594 | 9.1 | syndecan binding protein 2 isoform a [Homo sapiens] |
| U | *gi|38016916|ref|NP\_05* | 1 | 1 | 5.8% | 207 | 22664 | 9.8 | syndecan binding protein 2 isoform b [Homo sapiens] |

| Filename XCorr DeltCN Conf% ObsM+H+ CalcM+H+ SpR ZScore Ion% # Sequence  | | | | | | | | | | | | |
| --- | --- | --- | --- | --- | --- | --- | --- | --- | --- | --- | --- | --- |
|  | Astrin\_NLD\_STLC\_031014\_01.10880.10880.2 | 2.1938 | 0.309 | 98.7% | 1361.6921 | 1360.4587 | 1 | 5.357 | 50.0% | 1 | K.AHQVVKK@AS\*GDK.I | 2 |

---

|  |  |  |  |  |  |  |  |  |
| --- | --- | --- | --- | --- | --- | --- | --- | --- |
| U | *Reverse\_gi|221136939|* | 1 | 1 | 4.0% | 499 | 55456 | 5.8 | pre-mRNA processing factor 31 homolog [Homo sapiens] |

| Filename XCorr DeltCN Conf% ObsM+H+ CalcM+H+ SpR ZScore Ion% # Sequence  | | | | | | | | | | | | |
| --- | --- | --- | --- | --- | --- | --- | --- | --- | --- | --- | --- | --- |
| \* | Astrin\_NLD\_STLC\_tube2\_021014\_01.23567.23567.3 | 2.6467 | 0.2747 | 95.9% | 2408.8145 | 2408.6199 | 125 | 4.527 | 26.3% | 1 | R.IT#SKGGYVVSQK@QLT#RQLTK.S | 3 |

---

|  |  |  |  |  |  |  |  |  |
| --- | --- | --- | --- | --- | --- | --- | --- | --- |
| U | *gi|14591909|ref|NP\_00* | 1 | 1 | 4.0% | 297 | 34363 | 9.7 | ribosomal protein L5 [Homo sapiens] |

| Filename XCorr DeltCN Conf% ObsM+H+ CalcM+H+ SpR ZScore Ion% # Sequence  | | | | | | | | | | | | |
| --- | --- | --- | --- | --- | --- | --- | --- | --- | --- | --- | --- | --- |
| \* | Astrin\_NLD\_STLC\_tube2\_021014\_01.07175.07175.2 | 3.1097 | 0.4705 | 100.0% | 1436.0322 | 1435.6587 | 1 | 7.819 | 77.3% | 1 | K.HIMGQNVADYMR.Y | 2 |

---

|  |  |  |  |  |  |  |  |  |
| --- | --- | --- | --- | --- | --- | --- | --- | --- |
| U | *contaminant\_KERATIN16* | 2 | 2 | 3.9% | 534 | 57265 | 6.6 | no description |
| U | *gi|109255249|ref|NP\_0* | 2 | 2 | 3.5% | 594 | 63911 | 8.3 | keratin 4 [Homo sapiens] |

| Filename XCorr DeltCN Conf% ObsM+H+ CalcM+H+ SpR ZScore Ion% # Sequence  | | | | | | | | | | | | |
| --- | --- | --- | --- | --- | --- | --- | --- | --- | --- | --- | --- | --- |
|  | Astrin\_NLD\_STLC\_tube2\_021014\_01.04328.04328.2 | 3.2426 | 0.0852 | 98.5% | 1108.1122 | 1108.196 | 2 | 6.567 | 75.0% | 1 | R.AQYEEIAQR.S | 222 |
|  | Astrin\_NLD\_STLC\_tube2\_021014\_01.09447.09447.2 | 2.9328 | 0.1879 | 98.7% | 1406.3722 | 1406.6653 | 4 | 4.937 | 68.2% | 1 | K.LALDIEIATYRK.L | 222 |

Similarities:
gi|4504919|ref|NP\_002(1:1)  
gi|67782365|ref|NP\_00(1:1)  
gi|47132620|ref|NP\_00(1:1)  
gi|119703753|ref|NP\_0(1:1)  

---

|  |  |  |  |  |  |  |  |  |
| --- | --- | --- | --- | --- | --- | --- | --- | --- |
| U | *gi|24234688|ref|NP\_00* | 2 | 2 | 3.8% | 679 | 73681 | 6.2 | heat shock 70kDa protein 9 precursor [Homo sapiens] |

| Filename XCorr DeltCN Conf% ObsM+H+ CalcM+H+ SpR ZScore Ion% # Sequence  | | | | | | | | | | | | |
| --- | --- | --- | --- | --- | --- | --- | --- | --- | --- | --- | --- | --- |
| \* | Astrin\_NLD\_STLC\_tube2\_021014\_01.07943.07943.2 | 3.0009 | 0.2527 | 99.8% | 1451.3722 | 1451.576 | 1 | 5.295 | 57.7% | 1 | R.TTPSVVAFTADGER.L | 2 |
| \* | Astrin\_NLD\_STLC\_031014\_01.11146.11146.2 | 2.2157 | 0.3839 | 99.9% | 1362.0922 | 1362.5687 | 84 | 6.303 | 50.0% | 1 | R.AQFEGIVTDLIR.R | 2 |

---

|  |  |  |  |  |  |  |  |  |
| --- | --- | --- | --- | --- | --- | --- | --- | --- |
| U | *Reverse\_gi|8922631|re* | 1 | 1 | 3.8% | 496 | 56346 | 8.3 | exonuclease 3'-5' domain containing 2 [Homo sapiens] |

| Filename XCorr DeltCN Conf% ObsM+H+ CalcM+H+ SpR ZScore Ion% # Sequence  | | | | | | | | | | | | |
| --- | --- | --- | --- | --- | --- | --- | --- | --- | --- | --- | --- | --- |
| \* | Astrin\_NLD\_STLC\_tube2\_021014\_01.19080.19080.2 | 2.5276 | 0.2187 | 96.3% | 2214.112 | 2214.4602 | 126 | 3.502 | 27.8% | 1 | R.GQHNGPVMGDMK@S\*KKNRPK@.Q | 2 |

---

|  |  |  |  |  |  |  |  |  |
| --- | --- | --- | --- | --- | --- | --- | --- | --- |
| U | *gi|29826294|ref|NP\_05* | 1 | 1 | 3.8% | 452 | 47145 | 4.8 | golgi reassembly stacking protein 2 [Homo sapiens] |

| Filename XCorr DeltCN Conf% ObsM+H+ CalcM+H+ SpR ZScore Ion% # Sequence  | | | | | | | | | | | | |
| --- | --- | --- | --- | --- | --- | --- | --- | --- | --- | --- | --- | --- |
| \* | Astrin\_NLD\_STLC\_031014\_01.10433.10433.3 | 2.7714 | 0.2588 | 96.7% | 2018.9644 | 2017.1534 | 20 | 4.505 | 28.1% | 1 | K.K@IS\*LPGQMAGT#PIT#PLK@.D | 3 |

---

|  |  |  |  |  |  |  |  |  |
| --- | --- | --- | --- | --- | --- | --- | --- | --- |
| U | *gi|116812577|ref|NP\_0* | 1 | 1 | 3.8% | 392 | 46514 | 10.0 | LUC7-like 2 [Homo sapiens] |

| Filename XCorr DeltCN Conf% ObsM+H+ CalcM+H+ SpR ZScore Ion% # Sequence  | | | | | | | | | | | | |
| --- | --- | --- | --- | --- | --- | --- | --- | --- | --- | --- | --- | --- |
| \* | Astrin\_NLD\_STLC\_tube2\_021014\_02.05394.05394.2 | 3.8123 | 0.4843 | 100.0% | 1588.7522 | 1589.7399 | 1 | 8.708 | 67.9% | 1 | R.LAETQEEISAEVAAK.A | 2 |

---

|  |  |  |  |  |  |  |  |  |
| --- | --- | --- | --- | --- | --- | --- | --- | --- |
| U | *gi|193794814|ref|NP\_0* | 1 | 1 | 3.8% | 364 | 39420 | 8.1 | fructose-bisphosphate aldolase A [Homo sapiens] |
| U | *gi|4557305|ref|NP\_000* | 1 | 1 | 3.8% | 364 | 39420 | 8.1 | fructose-bisphosphate aldolase A [Homo sapiens] |
| U | *gi|34577112|ref|NP\_90* | 1 | 1 | 3.8% | 364 | 39420 | 8.1 | fructose-bisphosphate aldolase A [Homo sapiens] |
| U | *gi|34577110|ref|NP\_90* | 1 | 1 | 3.8% | 364 | 39420 | 8.1 | fructose-bisphosphate aldolase A [Homo sapiens] |

| Filename XCorr DeltCN Conf% ObsM+H+ CalcM+H+ SpR ZScore Ion% # Sequence  | | | | | | | | | | | | |
| --- | --- | --- | --- | --- | --- | --- | --- | --- | --- | --- | --- | --- |
|  | Astrin\_NLD\_STLC\_tube2\_021014\_02.05291.05291.2 | 2.6059 | 0.3646 | 100.0% | 1333.4321 | 1333.4814 | 10 | 6.117 | 53.8% | 1 | K.GILAADESTGSIAK.R | 2 |

---

|  |  |  |  |  |  |  |  |  |
| --- | --- | --- | --- | --- | --- | --- | --- | --- |
| U | *gi|156523262|ref|NP\_4* | 1 | 1 | 3.8% | 213 | 23922 | 4.8 | hypothetical protein LOC112752 isoform 1 [Homo sapiens] |
| U | *gi|156523264|ref|NP\_0* | 1 | 1 | 3.8% | 208 | 23529 | 4.7 | hypothetical protein LOC112752 isoform 2 [Homo sapiens] |

| Filename XCorr DeltCN Conf% ObsM+H+ CalcM+H+ SpR ZScore Ion% # Sequence  | | | | | | | | | | | | |
| --- | --- | --- | --- | --- | --- | --- | --- | --- | --- | --- | --- | --- |
|  | Astrin\_NLD\_STLC\_tube2\_021014\_01.13824.13824.1 | 2.4457 | 0.1494 | 98.4% | 963.95 | 964.07837 | 100 | 4.499 | 57.1% | 1 | R.DLDNDLMK.Y | 1 |

---

|  |  |  |  |  |  |  |  |  |
| --- | --- | --- | --- | --- | --- | --- | --- | --- |
| U | *gi|4885225|ref|NP\_005* | 1 | 1 | 3.7% | 656 | 68478 | 9.3 | Ewing sarcoma breakpoint region 1 isoform EWS [Homo sapiens] |

| Filename XCorr DeltCN Conf% ObsM+H+ CalcM+H+ SpR ZScore Ion% # Sequence  | | | | | | | | | | | | |
| --- | --- | --- | --- | --- | --- | --- | --- | --- | --- | --- | --- | --- |
| \* | Astrin\_NLD\_STLC\_tube2\_021014\_02.05205.05205.3 | 3.8792 | 0.3944 | 100.0% | 2481.1743 | 2481.572 | 5 | 7.21 | 26.1% | 1 | R.QDHPSSMGVYGQESGGFSGPGENR.S | 3 |

---

|  |  |  |  |  |  |  |  |  |
| --- | --- | --- | --- | --- | --- | --- | --- | --- |
| U | *Reverse\_gi|47716512|r* | 1 | 1 | 3.7% | 569 | 58832 | 6.9 | mex-3 homolog B [Homo sapiens] |

| Filename XCorr DeltCN Conf% ObsM+H+ CalcM+H+ SpR ZScore Ion% # Sequence  | | | | | | | | | | | | |
| --- | --- | --- | --- | --- | --- | --- | --- | --- | --- | --- | --- | --- |
| \* | Astrin\_NLD\_STLC\_tube2\_021014\_01.12234.12234.2 | 2.4239 | 0.2477 | 96.9% | 1982.4122 | 1982.7562 | 2 | 4.83 | 35.0% | 1 | R.QDDLTEGGGS\*SGGGGGGS\*GNR.E | 2 |

---

|  |  |  |  |  |  |  |  |  |
| --- | --- | --- | --- | --- | --- | --- | --- | --- |
| U | *gi|189083844|ref|NP\_0* | 1 | 1 | 3.7% | 463 | 51842 | 7.0 | cathepsin C isoform a preproprotein [Homo sapiens] |

| Filename XCorr DeltCN Conf% ObsM+H+ CalcM+H+ SpR ZScore Ion% # Sequence  | | | | | | | | | | | | |
| --- | --- | --- | --- | --- | --- | --- | --- | --- | --- | --- | --- | --- |
| \* | Astrin\_NLD\_STLC\_031014\_02.05026.05026.3 | 2.5507 | 0.2701 | 95.4% | 2002.3143 | 2004.0665 | 11 | 4.291 | 34.4% | 1 | K.KVGT#AS\*ENVYVNTAHLK@.N | 3 |

---

|  |  |  |  |  |  |  |  |  |
| --- | --- | --- | --- | --- | --- | --- | --- | --- |
| U | *gi|4505773|ref|NP\_002* | 1 | 1 | 3.7% | 272 | 29804 | 5.8 | prohibitin [Homo sapiens] |

| Filename XCorr DeltCN Conf% ObsM+H+ CalcM+H+ SpR ZScore Ion% # Sequence  | | | | | | | | | | | | |
| --- | --- | --- | --- | --- | --- | --- | --- | --- | --- | --- | --- | --- |
| \* | Astrin\_NLD\_STLC\_tube2\_021014\_01.08678.08678.2 | 2.6173 | 0.2193 | 98.9% | 1150.3522 | 1150.2767 | 273 | 4.713 | 55.6% | 1 | R.FDAGELITQR.E | 2 |

---

|  |  |  |  |  |  |  |  |  |
| --- | --- | --- | --- | --- | --- | --- | --- | --- |
| U | *gi|221316723|ref|NP\_0* | 3 | 3 | 3.5% | 1025 | 115704 | 8.3 | N-acetyltransferase 10 isoform a [Homo sapiens] |
| U | *gi|221316741|ref|NP\_0* | 3 | 3 | 3.8% | 953 | 107271 | 7.0 | N-acetyltransferase 10 isoform b [Homo sapiens] |

| Filename XCorr DeltCN Conf% ObsM+H+ CalcM+H+ SpR ZScore Ion% # Sequence  | | | | | | | | | | | | |
| --- | --- | --- | --- | --- | --- | --- | --- | --- | --- | --- | --- | --- |
|  | Astrin\_NLD\_STLC\_tube2\_021014\_02.05368.05368.2 | 2.3701 | 0.3261 | 99.8% | 1411.9922 | 1412.5858 | 1 | 5.4 | 68.2% | 1 | R.TLHEVSLQESIR.Y | 2 |
|  | Astrin\_NLD\_STLC\_tube2\_021014\_01.10860.10860.2 | 3.1773 | 0.359 | 100.0% | 1454.1921 | 1454.6659 | 1 | 7.671 | 70.8% | 1 | R.LDYLGVSYGLTPR.L | 2 |
|  | Astrin\_NLD\_STLC\_tube2\_021014\_01.07689.07689.2 | 2.3688 | 0.2659 | 98.7% | 1071.3121 | 1071.2621 | 2 | 4.788 | 75.0% | 1 | K.AGPNASIISLK.S | 2 |

---

|  |  |  |  |  |  |  |  |  |
| --- | --- | --- | --- | --- | --- | --- | --- | --- |
| U | *gi|205277463|ref|NP\_0* | 1 | 1 | 3.5% | 623 | 67878 | 7.7 | transketolase isoform 1 [Homo sapiens] |
| U | *gi|4507521|ref|NP\_001* | 1 | 1 | 3.5% | 623 | 67878 | 7.7 | transketolase isoform 1 [Homo sapiens] |
| U | *gi|205277465|ref|NP\_0* | 1 | 1 | 4.1% | 540 | 58982 | 7.7 | transketolase isoform 2 [Homo sapiens] |

| Filename XCorr DeltCN Conf% ObsM+H+ CalcM+H+ SpR ZScore Ion% # Sequence  | | | | | | | | | | | | |
| --- | --- | --- | --- | --- | --- | --- | --- | --- | --- | --- | --- | --- |
|  | Astrin\_NLD\_STLC\_tube2\_021014\_02.05843.05843.3 | 5.4193 | 0.371 | 100.0% | 2510.2144 | 2509.6946 | 1 | 6.423 | 39.3% | 1 | R.TSRPENAIIYNNNEDFQVGQAK.V | 3 |

---

|  |  |  |  |  |  |  |  |  |
| --- | --- | --- | --- | --- | --- | --- | --- | --- |
| U | *Reverse\_gi|45580709|r* | 1 | 1 | 3.5% | 597 | 66631 | 7.0 | unc-93 homolog B1 [Homo sapiens] |

| Filename XCorr DeltCN Conf% ObsM+H+ CalcM+H+ SpR ZScore Ion% # Sequence  | | | | | | | | | | | | |
| --- | --- | --- | --- | --- | --- | --- | --- | --- | --- | --- | --- | --- |
| \* | Astrin\_NLD\_STLC\_tube2\_021014\_01.13442.13442.3 | 3.5404 | 0.229 | 98.3% | 2474.0645 | 2475.8093 | 1 | 4.5 | 33.8% | 1 | K.FPLQFINGWGVSRLDIEETPR.Y | 3 |

---

|  |  |  |  |  |  |  |  |  |
| --- | --- | --- | --- | --- | --- | --- | --- | --- |
| U | *gi|49472835|ref|NP\_05* | 1 | 1 | 3.5% | 565 | 61874 | 5.1 | bridging integrator 2 [Homo sapiens] |

| Filename XCorr DeltCN Conf% ObsM+H+ CalcM+H+ SpR ZScore Ion% # Sequence  | | | | | | | | | | | | |
| --- | --- | --- | --- | --- | --- | --- | --- | --- | --- | --- | --- | --- |
| \* | Astrin\_NLD\_STLC\_tube2\_021014\_01.13104.13104.3 | 2.5413 | 0.2766 | 95.3% | 2389.7043 | 2388.2317 | 27 | 3.066 | 27.6% | 1 | R.TAT#VSSPLTSPT#S\*PS\*T#LSLK@.S | 3 |

---

|  |  |  |  |  |  |  |  |  |
| --- | --- | --- | --- | --- | --- | --- | --- | --- |
| U | *gi|22547212|ref|NP\_68* | 1 | 1 | 3.5% | 520 | 58823 | 8.8 | peptidylprolyl isomerase-like 2 isoform a [Homo sapiens] |
| U | *gi|7657473|ref|NP\_055* | 1 | 1 | 3.5% | 520 | 58823 | 8.8 | peptidylprolyl isomerase-like 2 isoform a [Homo sapiens] |
| U | *gi|22547215|ref|NP\_68* | 1 | 1 | 3.4% | 527 | 59458 | 8.7 | peptidylprolyl isomerase-like 2 isoform b [Homo sapiens] |

| Filename XCorr DeltCN Conf% ObsM+H+ CalcM+H+ SpR ZScore Ion% # Sequence  | | | | | | | | | | | | |
| --- | --- | --- | --- | --- | --- | --- | --- | --- | --- | --- | --- | --- |
|  | Astrin\_NLD\_STLC\_031014\_01.15538.15538.2 | 3.42 | 0.2035 | 99.8% | 2213.912 | 2214.159 | 4 | 3.806 | 41.2% | 1 | K.YGT#NPS\*NGEK@LDGRS\*LIK@.L | 2 |

---

|  |  |  |  |  |  |  |  |  |
| --- | --- | --- | --- | --- | --- | --- | --- | --- |
| U | *gi|34147630|ref|NP\_00* | 1 | 1 | 3.5% | 455 | 49875 | 7.6 | Tu translation elongation factor, mitochondrial precursor [Homo sapiens] |

| Filename XCorr DeltCN Conf% ObsM+H+ CalcM+H+ SpR ZScore Ion% # Sequence  | | | | | | | | | | | | |
| --- | --- | --- | --- | --- | --- | --- | --- | --- | --- | --- | --- | --- |
| \* | Astrin\_NLD\_STLC\_tube2\_021014\_02.05463.05463.3 | 2.7658 | 0.3177 | 99.6% | 1674.8944 | 1674.854 | 36 | 5.114 | 30.0% | 1 | R.GITINAAHVEYSTAAR.H | 3 |

---

|  |  |  |  |  |  |  |  |  |
| --- | --- | --- | --- | --- | --- | --- | --- | --- |
| U | *gi|38327562|ref|NP\_00* | 1 | 1 | 3.5% | 403 | 45823 | 9.4 | serine/threonine protein kinase 6 [Homo sapiens] |
| U | *gi|38327572|ref|NP\_94* | 1 | 1 | 3.5% | 403 | 45823 | 9.4 | serine/threonine protein kinase 6 [Homo sapiens] |
| U | *gi|38327570|ref|NP\_94* | 1 | 1 | 3.5% | 403 | 45823 | 9.4 | serine/threonine protein kinase 6 [Homo sapiens] |
| U | *gi|38327568|ref|NP\_94* | 1 | 1 | 3.5% | 403 | 45823 | 9.4 | serine/threonine protein kinase 6 [Homo sapiens] |
| U | *gi|38327566|ref|NP\_94* | 1 | 1 | 3.5% | 403 | 45823 | 9.4 | serine/threonine protein kinase 6 [Homo sapiens] |
| U | *gi|38327564|ref|NP\_94* | 1 | 1 | 3.5% | 403 | 45823 | 9.4 | serine/threonine protein kinase 6 [Homo sapiens] |

| Filename XCorr DeltCN Conf% ObsM+H+ CalcM+H+ SpR ZScore Ion% # Sequence  | | | | | | | | | | | | |
| --- | --- | --- | --- | --- | --- | --- | --- | --- | --- | --- | --- | --- |
|  | Astrin\_NLD\_STLC\_tube2\_021014\_01.13168.13168.2 | 2.5043 | 0.3339 | 99.8% | 1614.6122 | 1615.7826 | 285 | 5.962 | 34.6% | 1 | R.VEFTFPDFVTEGAR.D | 2 |

---

|  |  |  |  |  |  |  |  |  |
| --- | --- | --- | --- | --- | --- | --- | --- | --- |
| U | *gi|14141157|ref|NP\_03* | 1 | 2 | 3.5% | 346 | 36926 | 6.9 | heterogeneous nuclear ribonucleoprotein H3 isoform a [Homo sapiens] |
| U | *gi|14141159|ref|NP\_06* | 1 | 2 | 3.6% | 331 | 35239 | 6.9 | heterogeneous nuclear ribonucleoprotein H3 isoform b [Homo sapiens] |

| Filename XCorr DeltCN Conf% ObsM+H+ CalcM+H+ SpR ZScore Ion% # Sequence  | | | | | | | | | | | | |
| --- | --- | --- | --- | --- | --- | --- | --- | --- | --- | --- | --- | --- |
|  | Astrin\_NLD\_STLC\_031014\_02.06524.06524.2 | 2.9314 | 0.3316 | 100.0% | 1272.1921 | 1272.4001 | 1 | 7.128 | 59.1% | 2 | R.STGEAFVQFASK.E | 2 |

---

|  |  |  |  |  |  |  |  |  |
| --- | --- | --- | --- | --- | --- | --- | --- | --- |
| U | *gi|4759098|ref|NP\_004* | 1 | 1 | 3.5% | 288 | 33666 | 11.2 | splicing factor, arginine/serine-rich 10 [Homo sapiens] |

| Filename XCorr DeltCN Conf% ObsM+H+ CalcM+H+ SpR ZScore Ion% # Sequence  | | | | | | | | | | | | |
| --- | --- | --- | --- | --- | --- | --- | --- | --- | --- | --- | --- | --- |
| \* | Astrin\_NLD\_STLC\_tube2\_021014\_01.04246.04246.3 | 2.3694 | 0.4568 | 100.0% | 1310.1543 | 1310.322 | 1 | 7.018 | 41.7% | 1 | R.GYDDRDYYSR.S | 3 |

---

|  |  |  |  |  |  |  |  |  |
| --- | --- | --- | --- | --- | --- | --- | --- | --- |
| U | *Reverse\_gi|112420958|* | 1 | 1 | 3.4% | 900 | 100307 | 8.3 | polymerase (DNA directed) nu [Homo sapiens] |

| Filename XCorr DeltCN Conf% ObsM+H+ CalcM+H+ SpR ZScore Ion% # Sequence  | | | | | | | | | | | | |
| --- | --- | --- | --- | --- | --- | --- | --- | --- | --- | --- | --- | --- |
| \* | Astrin\_NLD\_STLC\_tube2\_021014\_01.16792.16792.3 | 3.2103 | 0.2677 | 98.1% | 3562.0745 | 3559.5347 | 308 | 3.975 | 17.5% | 1 | -.LCFS\*PSFHLPPPQTSAPS\*GPAALSNS\*PSET#R.C | 3 |

---

|  |  |  |  |  |  |  |  |  |
| --- | --- | --- | --- | --- | --- | --- | --- | --- |
| U | *Reverse\_gi|19923167|r* | 1 | 1 | 3.4% | 561 | 62091 | 9.5 | tubby isoform a [Homo sapiens] |
| U | *Reverse\_gi|29826279|r* | 1 | 1 | 3.8% | 506 | 55651 | 8.8 | tubby isoform b [Homo sapiens] |

| Filename XCorr DeltCN Conf% ObsM+H+ CalcM+H+ SpR ZScore Ion% # Sequence  | | | | | | | | | | | | |
| --- | --- | --- | --- | --- | --- | --- | --- | --- | --- | --- | --- | --- |
|  | Astrin\_NLD\_STLC\_031014\_01.14717.14717.2 | 1.9627 | 0.3247 | 96.8% | 2002.8322 | 2002.0573 | 1 | 5.649 | 38.9% | 1 | K.EKRAAGGQGGAT#AAAK@T#RK.A | 2 |

---

|  |  |  |  |  |  |  |  |  |
| --- | --- | --- | --- | --- | --- | --- | --- | --- |
| U | *Reverse\_gi|116284398|* | 1 | 1 | 3.4% | 503 | 51259 | 11.5 | WAS/WASL interacting protein family, member 1 [Homo sapiens] |
| U | *Reverse\_gi|38373695|r* | 1 | 1 | 3.4% | 503 | 51259 | 11.5 | WAS/WASL interacting protein family, member 1 [Homo sapiens] |

| Filename XCorr DeltCN Conf% ObsM+H+ CalcM+H+ SpR ZScore Ion% # Sequence  | | | | | | | | | | | | |
| --- | --- | --- | --- | --- | --- | --- | --- | --- | --- | --- | --- | --- |
|  | Astrin\_NLD\_STLC\_tube2\_021014\_02.05769.05769.3 | 2.9842 | 0.2187 | 95.1% | 2073.2344 | 2073.956 | 17 | 4.273 | 31.2% | 1 | R.S\*PLQPT#APLARS\*TS\*GNR.S | 3 |

---

|  |  |  |  |  |  |  |  |  |
| --- | --- | --- | --- | --- | --- | --- | --- | --- |
| U | *gi|154800487|ref|NP\_0* | 1 | 1 | 3.4% | 348 | 39171 | 7.9 | ER lipid raft associated 1 [Homo sapiens] |
| U | *gi|6005721|ref|NP\_009* | 1 | 1 | 3.5% | 339 | 37840 | 5.6 | ER lipid raft associated 2 isoform 1 [Homo sapiens] |
| U | *gi|154800489|ref|NP\_0* | 1 | 1 | 3.4% | 348 | 39171 | 7.9 | ER lipid raft associated 1 [Homo sapiens] |

| Filename XCorr DeltCN Conf% ObsM+H+ CalcM+H+ SpR ZScore Ion% # Sequence  | | | | | | | | | | | | |
| --- | --- | --- | --- | --- | --- | --- | --- | --- | --- | --- | --- | --- |
|  | Astrin\_NLD\_STLC\_tube2\_021014\_01.09867.09867.2 | 2.771 | 0.1668 | 96.9% | 1335.7922 | 1335.4998 | 1 | 4.593 | 72.7% | 1 | R.ISEIEDAAFLAR.E | 2 |

---

|  |  |  |  |  |  |  |  |  |
| --- | --- | --- | --- | --- | --- | --- | --- | --- |
| U | *gi|156071459|ref|NP\_0* | 1 | 1 | 3.4% | 298 | 32852 | 9.7 | solute carrier family 25, member 5 [Homo sapiens] |
| U | *gi|55749577|ref|NP\_00* | 1 | 1 | 3.4% | 298 | 33064 | 9.8 | solute carrier family 25 (mitochondrial carrier; adenine nucleotide translocator), member 4 [Homo sapiens] |
| U | *gi|156071462|ref|NP\_0* | 1 | 1 | 3.4% | 298 | 32866 | 9.7 | solute carrier family 25, member A6 [Homo sapiens] |

| Filename XCorr DeltCN Conf% ObsM+H+ CalcM+H+ SpR ZScore Ion% # Sequence  | | | | | | | | | | | | |
| --- | --- | --- | --- | --- | --- | --- | --- | --- | --- | --- | --- | --- |
|  | Astrin\_NLD\_STLC\_tube2\_021014\_01.05021.05021.2 | 2.6374 | 0.3106 | 100.0% | 1136.8722 | 1137.3677 | 6 | 5.171 | 72.2% | 1 | K.LLLQVQHASK.Q | 2 |

---

|  |  |  |  |  |  |  |  |  |
| --- | --- | --- | --- | --- | --- | --- | --- | --- |
| U | *Reverse\_gi|38372911|r* | 1 | 1 | 3.3% | 665 | 73102 | 7.5 | dual specificity phosphatase 16 [Homo sapiens] |

| Filename XCorr DeltCN Conf% ObsM+H+ CalcM+H+ SpR ZScore Ion% # Sequence  | | | | | | | | | | | | |
| --- | --- | --- | --- | --- | --- | --- | --- | --- | --- | --- | --- | --- |
| \* | Astrin\_NLD\_STLC\_tube2\_021014\_01.12296.12296.3 | 3.0479 | 0.2863 | 98.9% | 2499.9543 | 2499.9167 | 1 | 4.344 | 33.3% | 1 | K.ELHLLKLKSKPGS\*AGTQNKIKK.E | 3 |

---

|  |  |  |  |  |  |  |  |  |
| --- | --- | --- | --- | --- | --- | --- | --- | --- |
| U | *gi|167466272|ref|NP\_6* | 2 | 2 | 3.2% | 745 | 83587 | 9.8 | cytoskeleton associated protein 2-like [Homo sapiens] |

| Filename XCorr DeltCN Conf% ObsM+H+ CalcM+H+ SpR ZScore Ion% # Sequence  | | | | | | | | | | | | |
| --- | --- | --- | --- | --- | --- | --- | --- | --- | --- | --- | --- | --- |
| \* | Astrin\_NLD\_STLC\_tube2\_021014\_01.06935.06935.2 | 2.9619 | 0.1788 | 98.7% | 1327.4122 | 1326.5791 | 73 | 4.013 | 59.1% | 1 | R.KPVGSLNIEQLK.T | 2 |
| \* | Astrin\_NLD\_STLC\_tube2\_021014\_01.07883.07883.2 | 2.3635 | 0.3072 | 99.6% | 1398.4521 | 1399.6334 | 11 | 5.134 | 59.1% | 1 | R.KVVLNILQDSNR.T | 2 |

---

|  |  |  |  |  |  |  |  |  |
| --- | --- | --- | --- | --- | --- | --- | --- | --- |
| U | *gi|24475816|ref|NP\_61* | 1 | 1 | 3.2% | 308 | 36034 | 9.4 | glycoprotein, synaptic 2 [Homo sapiens] |

| Filename XCorr DeltCN Conf% ObsM+H+ CalcM+H+ SpR ZScore Ion% # Sequence  | | | | | | | | | | | | |
| --- | --- | --- | --- | --- | --- | --- | --- | --- | --- | --- | --- | --- |
| \* | Astrin\_NLD\_STLC\_tube2\_021014\_01.07113.07113.2 | 2.1711 | 0.3577 | 99.8% | 1216.3722 | 1217.3641 | 7 | 5.997 | 66.7% | 1 | K.HYEVEILDAK.T | 2 |

---

|  |  |  |  |  |  |  |  |  |
| --- | --- | --- | --- | --- | --- | --- | --- | --- |
| U | *gi|117935038|ref|NP\_0* | 1 | 1 | 3.1% | 651 | 74242 | 9.7 | cylicin, basic protein of sperm head cytoskeleton 1 [Homo sapiens] |

| Filename XCorr DeltCN Conf% ObsM+H+ CalcM+H+ SpR ZScore Ion% # Sequence  | | | | | | | | | | | | |
| --- | --- | --- | --- | --- | --- | --- | --- | --- | --- | --- | --- | --- |
| \* | Astrin\_NLD\_STLC\_tube2\_021014\_01.13500.13500.2 | 2.021 | 0.2931 | 95.1% | 2375.0522 | 2375.2085 | 32 | 3.732 | 31.6% | 1 | K.YPES\*T#DTESGDAKDARNDSR.N | 2 |

---

|  |  |  |  |  |  |  |  |  |
| --- | --- | --- | --- | --- | --- | --- | --- | --- |
| U | *Reverse\_gi|116812628|* | 1 | 1 | 3.1% | 604 | 70978 | 6.2 | coiled-coil domain containing 67 [Homo sapiens] |

| Filename XCorr DeltCN Conf% ObsM+H+ CalcM+H+ SpR ZScore Ion% # Sequence  | | | | | | | | | | | | |
| --- | --- | --- | --- | --- | --- | --- | --- | --- | --- | --- | --- | --- |
| \* | Astrin\_NLD\_STLC\_031014\_01.10695.10695.2 | 3.3609 | 0.1725 | 98.9% | 2256.2722 | 2256.518 | 2 | 3.897 | 47.2% | 1 | R.S\*LAIENVASKLKEIIFEDR.E | 2 |

---

|  |  |  |  |  |  |  |  |  |
| --- | --- | --- | --- | --- | --- | --- | --- | --- |
| U | *gi|10864047|ref|NP\_06* | 1 | 1 | 3.0% | 864 | 94255 | 5.1 | epidermal growth factor receptor pathway substrate 15-like 1 [Homo sapiens] |

| Filename XCorr DeltCN Conf% ObsM+H+ CalcM+H+ SpR ZScore Ion% # Sequence  | | | | | | | | | | | | |
| --- | --- | --- | --- | --- | --- | --- | --- | --- | --- | --- | --- | --- |
| \* | Astrin\_NLD\_STLC\_tube2\_021014\_01.09324.09324.3 | 3.564 | 0.2123 | 96.5% | 2797.0444 | 2799.7917 | 2 | 4.735 | 26.0% | 1 | K.DPFVPSSAAKPSKAS\*AS\*GFADFTSVS\*.- | 3 |

---

|  |  |  |  |  |  |  |  |  |
| --- | --- | --- | --- | --- | --- | --- | --- | --- |
| U | *Reverse\_gi|7657265|re* | 1 | 1 | 3.0% | 627 | 70264 | 6.6 | fem-1 homolog b [Homo sapiens] |

| Filename XCorr DeltCN Conf% ObsM+H+ CalcM+H+ SpR ZScore Ion% # Sequence  | | | | | | | | | | | | |
| --- | --- | --- | --- | --- | --- | --- | --- | --- | --- | --- | --- | --- |
| \* | Astrin\_NLD\_STLC\_tube2\_021014\_01.11583.11583.2 | 3.4357 | 0.0938 | 95.7% | 2239.5322 | 2240.4783 | 15 | 3.837 | 47.2% | 1 | K.NAIS\*INANNEVLYKVIDLR.G | 2 |

---

|  |  |  |  |  |  |  |  |  |
| --- | --- | --- | --- | --- | --- | --- | --- | --- |
| U | *gi|169204626|ref|XP\_0* | 1 | 4 | 3.0% | 501 | 56923 | 5.8 | PREDICTED: hypothetical protein [Homo sapiens] |

| Filename XCorr DeltCN Conf% ObsM+H+ CalcM+H+ SpR ZScore Ion% # Sequence  | | | | | | | | | | | | |
| --- | --- | --- | --- | --- | --- | --- | --- | --- | --- | --- | --- | --- |
| \* | Astrin\_NLD\_STLC\_tube2\_021014\_01.04731.04731.2 | 3.175 | 0.2634 | 99.8% | 1814.4122 | 1814.9653 | 1 | 5.245 | 53.6% | 4 | K.S\*MIVAQSKQLETENR.A | 2 |

---

|  |  |  |  |  |  |  |  |  |
| --- | --- | --- | --- | --- | --- | --- | --- | --- |
| U | *gi|4503481|ref|NP\_001* | 1 | 3 | 3.0% | 437 | 50119 | 6.7 | eukaryotic translation elongation factor 1 gamma [Homo sapiens] |

| Filename XCorr DeltCN Conf% ObsM+H+ CalcM+H+ SpR ZScore Ion% # Sequence  | | | | | | | | | | | | |
| --- | --- | --- | --- | --- | --- | --- | --- | --- | --- | --- | --- | --- |
| \* | Astrin\_NLD\_STLC\_tube2\_021014\_02.05466.05466.2 | 3.74 | 0.4137 | 100.0% | 1347.8922 | 1348.5448 | 13 | 7.055 | 58.3% | 3 | K.ALIAAQYSGAQVR.V | 2 |

---

|  |  |  |  |  |  |  |  |  |
| --- | --- | --- | --- | --- | --- | --- | --- | --- |
| U | *Reverse\_gi|4885101|re* | 1 | 1 | 3.0% | 264 | 29658 | 7.4 | carbonic anhydrase VII isoform 1 [Homo sapiens] |

| Filename XCorr DeltCN Conf% ObsM+H+ CalcM+H+ SpR ZScore Ion% # Sequence  | | | | | | | | | | | | |
| --- | --- | --- | --- | --- | --- | --- | --- | --- | --- | --- | --- | --- |
| \* | Astrin\_NLD\_STLC\_tube2\_021014\_01.07952.07952.2 | 2.4553 | 0.1881 | 98.1% | 1031.1721 | 1031.2206 | 67 | 4.278 | 57.1% | 1 | R.FNNVMHIR.E | 2 |

---

|  |  |  |  |  |  |  |  |  |
| --- | --- | --- | --- | --- | --- | --- | --- | --- |
| U | *gi|190684710|ref|NP\_0* | 1 | 1 | 2.9% | 817 | 92487 | 6.7 | EFR3 homolog B [Homo sapiens] |

| Filename XCorr DeltCN Conf% ObsM+H+ CalcM+H+ SpR ZScore Ion% # Sequence  | | | | | | | | | | | | |
| --- | --- | --- | --- | --- | --- | --- | --- | --- | --- | --- | --- | --- |
| \* | Astrin\_NLD\_STLC\_031014\_01.16203.16203.3 | 3.0432 | 0.2504 | 96.2% | 2905.0745 | 2908.0093 | 101 | 4.584 | 22.8% | 1 | R.S\*PSPLQAPEK@EK@ES\*PAELAERCLR.E | 3 |

---

|  |  |  |  |  |  |  |  |  |
| --- | --- | --- | --- | --- | --- | --- | --- | --- |
| U | *Reverse\_gi|223633919|* | 2 | 5 | 2.9% | 657 | 76034 | 8.1 | hypothetical protein LOC285550 [Homo sapiens] |

| Filename XCorr DeltCN Conf% ObsM+H+ CalcM+H+ SpR ZScore Ion% # Sequence  | | | | | | | | | | | | |
| --- | --- | --- | --- | --- | --- | --- | --- | --- | --- | --- | --- | --- |
| \* | Astrin\_NLD\_STLC\_tube2\_021014\_01.07655.07655.2 | 2.8456 | 0.1729 | 96.4% | 2177.2922 | 2179.1812 | 2 | 3.847 | 41.7% | 1 | R.S\*HKGTMTAT#GDSTIGK@CNK@.W | 2 |
| \* | Astrin\_NLD\_STLC\_tube2\_021014\_01.07124.07124.2 | 3.1614 | 0.2146 | 99.5% | 2177.5122 | 2179.1812 | 1 | 4.517 | 41.7% | 4 | R.SHKGTMT#AT#GDSTIGK@CNK@.W | 2 |

---

|  |  |  |  |  |  |  |  |  |
| --- | --- | --- | --- | --- | --- | --- | --- | --- |
| U | *Reverse\_gi|28827807|r* | 1 | 6 | 2.9% | 655 | 75032 | 7.2 | NLR family, pyrin domain containing 10 [Homo sapiens] |

| Filename XCorr DeltCN Conf% ObsM+H+ CalcM+H+ SpR ZScore Ion% # Sequence  | | | | | | | | | | | | |
| --- | --- | --- | --- | --- | --- | --- | --- | --- | --- | --- | --- | --- |
| \* | Astrin\_NLD\_STLC\_tube2\_021014\_01.09578.09578.2 | 4.3494 | 0.0525 | 98.6% | 2215.2122 | 2215.2212 | 11 | 4.144 | 44.4% | 6 | K.QT#GAINDKGEKQGHVS\*PCK.Q | 2 |

---

|  |  |  |  |  |  |  |  |  |
| --- | --- | --- | --- | --- | --- | --- | --- | --- |
| U | *gi|40789249|ref|NP\_06* | 1 | 1 | 2.9% | 645 | 73563 | 8.0 | aspartyl-tRNA synthetase 2, mitochondrial [Homo sapiens] |

| Filename XCorr DeltCN Conf% ObsM+H+ CalcM+H+ SpR ZScore Ion% # Sequence  | | | | | | | | | | | | |
| --- | --- | --- | --- | --- | --- | --- | --- | --- | --- | --- | --- | --- |
| \* | Astrin\_NLD\_STLC\_tube2\_021014\_02.06644.06644.3 | 3.4836 | 0.2735 | 99.8% | 2029.9443 | 2030.2046 | 1 | 5.42 | 36.1% | 1 | R.SQHYDLVLNGNEIGGGSIR.I | 3 |

---

|  |  |  |  |  |  |  |  |  |
| --- | --- | --- | --- | --- | --- | --- | --- | --- |
| U | *gi|13129078|ref|NP\_07* | 1 | 1 | 2.9% | 553 | 60183 | 6.6 | alveolar soft part sarcoma chromosome region, candidate 1 [Homo sapiens] |

| Filename XCorr DeltCN Conf% ObsM+H+ CalcM+H+ SpR ZScore Ion% # Sequence  | | | | | | | | | | | | |
| --- | --- | --- | --- | --- | --- | --- | --- | --- | --- | --- | --- | --- |
| \* | Astrin\_NLD\_STLC\_031014\_02.08705.08705.2 | 2.454 | 0.2916 | 98.9% | 1611.6122 | 1610.7263 | 1 | 5.497 | 46.7% | 1 | R.APAAAPFVPFS\*GGGQR.L | 2 |

---

|  |  |  |  |  |  |  |  |  |
| --- | --- | --- | --- | --- | --- | --- | --- | --- |
| U | *gi|89001096|ref|NP\_00* | 1 | 1 | 2.9% | 381 | 43841 | 8.7 | zinc finger protein 550 [Homo sapiens] |

| Filename XCorr DeltCN Conf% ObsM+H+ CalcM+H+ SpR ZScore Ion% # Sequence  | | | | | | | | | | | | |
| --- | --- | --- | --- | --- | --- | --- | --- | --- | --- | --- | --- | --- |
| \* | Astrin\_NLD\_STLC\_tube2\_021014\_01.04180.04180.2 | 2.9272 | 0.2696 | 100.0% | 1445.4122 | 1444.5901 | 57 | 4.867 | 60.0% | 1 | R.EEWRQLDLAQR.T | 2 |

---

|  |  |  |  |  |  |  |  |  |
| --- | --- | --- | --- | --- | --- | --- | --- | --- |
| U | *gi|169203523|ref|XP\_0* | 1 | 1 | 2.9% | 346 | 40111 | 6.2 | PREDICTED: similar to oocyte-secreted protein [Homo sapiens] |

| Filename XCorr DeltCN Conf% ObsM+H+ CalcM+H+ SpR ZScore Ion% # Sequence  | | | | | | | | | | | | |
| --- | --- | --- | --- | --- | --- | --- | --- | --- | --- | --- | --- | --- |
| \* | Astrin\_NLD\_STLC\_tube2\_021014\_01.04720.04720.2 | 2.3829 | 0.2153 | 97.6% | 1201.4922 | 1202.5468 | 3 | 5.002 | 72.2% | 1 | R.FPIILVMRGR.E | 2 |

---

|  |  |  |  |  |  |  |  |  |
| --- | --- | --- | --- | --- | --- | --- | --- | --- |
| U | *gi|126273519|ref|NP\_7* | 1 | 1 | 2.8% | 675 | 73855 | 9.1 | exonuclease GOR [Homo sapiens] |

| Filename XCorr DeltCN Conf% ObsM+H+ CalcM+H+ SpR ZScore Ion% # Sequence  | | | | | | | | | | | | |
| --- | --- | --- | --- | --- | --- | --- | --- | --- | --- | --- | --- | --- |
| \* | Astrin\_NLD\_STLC\_tube2\_021014\_01.12783.12783.2 | 3.66 | 0.0677 | 95.7% | 2322.6921 | 2321.7292 | 2 | 3.498 | 38.9% | 1 | R.IVRRASLPS\*LS\*LKKPIILR.S | 2 |

---

|  |  |  |  |  |  |  |  |  |
| --- | --- | --- | --- | --- | --- | --- | --- | --- |
| U | *gi|4506675|ref|NP\_002* | 1 | 1 | 2.8% | 607 | 68569 | 6.4 | ribophorin I precursor [Homo sapiens] |

| Filename XCorr DeltCN Conf% ObsM+H+ CalcM+H+ SpR ZScore Ion% # Sequence  | | | | | | | | | | | | |
| --- | --- | --- | --- | --- | --- | --- | --- | --- | --- | --- | --- | --- |
| \* | Astrin\_NLD\_STLC\_tube2\_021014\_02.06886.06886.3 | 3.1158 | 0.3825 | 100.0% | 1654.8243 | 1654.8638 | 2 | 6.328 | 35.9% | 1 | K.VTAEVVLAHLGGGSTSR.A | 3 |

---

|  |  |  |  |  |  |  |  |  |
| --- | --- | --- | --- | --- | --- | --- | --- | --- |
| U | *gi|115511026|ref|NP\_4* | 1 | 1 | 2.8% | 577 | 65273 | 8.3 | acyl-CoA synthetase medium-chain family member 1 [Homo sapiens] |

| Filename XCorr DeltCN Conf% ObsM+H+ CalcM+H+ SpR ZScore Ion% # Sequence  | | | | | | | | | | | | |
| --- | --- | --- | --- | --- | --- | --- | --- | --- | --- | --- | --- | --- |
| \* | Astrin\_NLD\_STLC\_031014\_01.19937.19937.2 | 3.0605 | 0.2149 | 99.1% | 1798.5322 | 1799.9437 | 5 | 4.273 | 40.0% | 1 | K.HS\*HGLALQPSFPGSRK.L | 2 |

---

|  |  |  |  |  |  |  |  |  |
| --- | --- | --- | --- | --- | --- | --- | --- | --- |
| U | *gi|35493811|ref|NP\_90* | 1 | 1 | 2.8% | 530 | 59380 | 10.1 | RNA binding motif protein 39 isoform a [Homo sapiens] |
| U | *gi|4757926|ref|NP\_004* | 1 | 1 | 2.9% | 524 | 58657 | 10.1 | RNA binding motif protein 39 isoform b [Homo sapiens] |

| Filename XCorr DeltCN Conf% ObsM+H+ CalcM+H+ SpR ZScore Ion% # Sequence  | | | | | | | | | | | | |
| --- | --- | --- | --- | --- | --- | --- | --- | --- | --- | --- | --- | --- |
|  | Astrin\_NLD\_STLC\_tube2\_021014\_01.10710.10710.2 | 2.1955 | 0.2632 | 95.3% | 1552.7322 | 1552.8546 | 1 | 4.768 | 53.6% | 1 | R.VLGVPIIVQASQAEK.N | 2 |

---

|  |  |  |  |  |  |  |  |  |
| --- | --- | --- | --- | --- | --- | --- | --- | --- |
| U | *gi|4503571|ref|NP\_001* | 1 | 1 | 2.8% | 434 | 47169 | 7.4 | enolase 1 [Homo sapiens] |

| Filename XCorr DeltCN Conf% ObsM+H+ CalcM+H+ SpR ZScore Ion% # Sequence  | | | | | | | | | | | | |
| --- | --- | --- | --- | --- | --- | --- | --- | --- | --- | --- | --- | --- |
| \* | Astrin\_NLD\_STLC\_tube2\_021014\_01.10798.10798.2 | 3.3825 | 0.1967 | 99.8% | 1427.2722 | 1426.6091 | 2 | 6.088 | 68.2% | 1 | R.YISPDQLADLYK.S | 2 |

---

|  |  |  |  |  |  |  |  |  |
| --- | --- | --- | --- | --- | --- | --- | --- | --- |
| U | *gi|5174447|ref|NP\_006* | 1 | 1 | 2.8% | 317 | 35077 | 7.7 | guanine nucleotide binding protein (G protein), beta polypeptide 2-like 1 [Homo sapiens] |

| Filename XCorr DeltCN Conf% ObsM+H+ CalcM+H+ SpR ZScore Ion% # Sequence  | | | | | | | | | | | | |
| --- | --- | --- | --- | --- | --- | --- | --- | --- | --- | --- | --- | --- |
| \* | Astrin\_NLD\_STLC\_tube2\_021014\_01.08387.08387.2 | 2.9109 | 0.3538 | 100.0% | 1060.2122 | 1060.2412 | 1 | 6.965 | 87.5% | 1 | R.VWQVTIGTR.- | 2 |

---

|  |  |  |  |  |  |  |  |  |
| --- | --- | --- | --- | --- | --- | --- | --- | --- |
| U | *gi|78000163|ref|NP\_00* | 1 | 1 | 2.7% | 1292 | 142497 | 6.8 | sorbin and SH3 domain containing 1 isoform 3 [Homo sapiens] |
| U | *gi|78000169|ref|NP\_07* | 1 | 1 | 4.5% | 781 | 87183 | 8.1 | sorbin and SH3 domain containing 1 isoform 6 [Homo sapiens] |
| U | *gi|78000167|ref|NP\_00* | 1 | 1 | 3.9% | 905 | 101066 | 7.4 | sorbin and SH3 domain containing 1 isoform 5 [Homo sapiens] |
| U | *gi|78000165|ref|NP\_00* | 1 | 1 | 3.0% | 1151 | 127260 | 6.6 | sorbin and SH3 domain containing 1 isoform 4 [Homo sapiens] |

| Filename XCorr DeltCN Conf% ObsM+H+ CalcM+H+ SpR ZScore Ion% # Sequence  | | | | | | | | | | | | |
| --- | --- | --- | --- | --- | --- | --- | --- | --- | --- | --- | --- | --- |
|  | Astrin\_NLD\_STLC\_031014\_02.12150.12150.3 | 3.1393 | 0.2552 | 97.0% | 3960.1143 | 3961.208 | 256 | 4.03 | 16.2% | 1 | K.TVK@NASGLVLPTDMDPT#KICTGK@GAVT#LRAS\*SSYR.E | 3 |

---

|  |  |  |  |  |  |  |  |  |
| --- | --- | --- | --- | --- | --- | --- | --- | --- |
| U | *gi|195976782|ref|NP\_0* | 1 | 3 | 2.7% | 710 | 80314 | 8.2 | Paf1/RNA polymerase II complex component [Homo sapiens] |

| Filename XCorr DeltCN Conf% ObsM+H+ CalcM+H+ SpR ZScore Ion% # Sequence  | | | | | | | | | | | | |
| --- | --- | --- | --- | --- | --- | --- | --- | --- | --- | --- | --- | --- |
| \* | Astrin\_NLD\_STLC\_tube2\_021014\_01.09573.09573.2 | 3.3259 | 0.1136 | 96.6% | 2265.652 | 2266.4631 | 16 | 3.57 | 38.9% | 3 | K.KT#QLLKEK@AMAEDLGDQDK@.A | 2 |

---

|  |  |  |  |  |  |  |  |  |
| --- | --- | --- | --- | --- | --- | --- | --- | --- |
| U | *gi|163792208|ref|NP\_0* | 1 | 2 | 2.7% | 709 | 80105 | 7.4 | Rho guanine exchange factor 16 [Homo sapiens] |

| Filename XCorr DeltCN Conf% ObsM+H+ CalcM+H+ SpR ZScore Ion% # Sequence  | | | | | | | | | | | | |
| --- | --- | --- | --- | --- | --- | --- | --- | --- | --- | --- | --- | --- |
| \* | Astrin\_NLD\_STLC\_031014\_01.10533.10533.2 | 3.2706 | 0.1138 | 95.9% | 2287.6921 | 2288.3796 | 31 | 3.824 | 33.3% | 2 | R.DPK@LLPAPS\*FS\*LDDMDVDK@.D | 2 |

---

|  |  |  |  |  |  |  |  |  |
| --- | --- | --- | --- | --- | --- | --- | --- | --- |
| U | *Reverse\_gi|113419070|* | 1 | 1 | 2.7% | 519 | 52392 | 12.3 | PREDICTED: hypothetical protein [Homo sapiens] |

| Filename XCorr DeltCN Conf% ObsM+H+ CalcM+H+ SpR ZScore Ion% # Sequence  | | | | | | | | | | | | |
| --- | --- | --- | --- | --- | --- | --- | --- | --- | --- | --- | --- | --- |
| \* | Astrin\_NLD\_STLC\_tube2\_021014\_02.06440.06440.3 | 2.266 | 0.2908 | 95.3% | 1530.5044 | 1532.5833 | 1 | 4.866 | 36.5% | 1 | K.K@DGGGS\*RK@AAK@QGR.W | 3 |

---

|  |  |  |  |  |  |  |  |  |
| --- | --- | --- | --- | --- | --- | --- | --- | --- |
| U | *gi|5802970|ref|NP\_006* | 1 | 1 | 2.6% | 797 | 88484 | 8.7 | AFG3 ATPase family gene 3-like 2 [Homo sapiens] |

| Filename XCorr DeltCN Conf% ObsM+H+ CalcM+H+ SpR ZScore Ion% # Sequence  | | | | | | | | | | | | |
| --- | --- | --- | --- | --- | --- | --- | --- | --- | --- | --- | --- | --- |
| \* | Astrin\_NLD\_STLC\_tube2\_021014\_01.17921.17921.2 | 2.2942 | 0.2483 | 95.4% | 2044.5521 | 2043.9828 | 255 | 4.286 | 27.5% | 1 | K.K@ES\*K@PAATTRS\*SGGGGGGGGK@.R | 2 |

---

|  |  |  |  |  |  |  |  |  |
| --- | --- | --- | --- | --- | --- | --- | --- | --- |
| U | *gi|40255119|ref|NP\_68* | 1 | 1 | 2.6% | 740 | 82879 | 5.1 | RAS and EF-hand domain containing [Homo sapiens] |

| Filename XCorr DeltCN Conf% ObsM+H+ CalcM+H+ SpR ZScore Ion% # Sequence  | | | | | | | | | | | | |
| --- | --- | --- | --- | --- | --- | --- | --- | --- | --- | --- | --- | --- |
| \* | Astrin\_NLD\_STLC\_031014\_01.12699.12699.2 | 2.148 | 0.2913 | 96.9% | 2253.152 | 2254.2322 | 253 | 5.082 | 25.0% | 1 | R.T#DK@DDSRSIT#NLTGTNSKK.S | 2 |

---

|  |  |  |  |  |  |  |  |  |
| --- | --- | --- | --- | --- | --- | --- | --- | --- |
| U | *Reverse\_gi|76881819|r* | 1 | 1 | 2.6% | 681 | 72678 | 7.7 | sterile alpha motif domain containing 11 [Homo sapiens] |

| Filename XCorr DeltCN Conf% ObsM+H+ CalcM+H+ SpR ZScore Ion% # Sequence  | | | | | | | | | | | | |
| --- | --- | --- | --- | --- | --- | --- | --- | --- | --- | --- | --- | --- |
| \* | Astrin\_NLD\_STLC\_tube2\_021014\_01.12831.12831.3 | 2.9741 | 0.3178 | 99.8% | 2028.9844 | 2030.9688 | 11 | 4.926 | 32.4% | 1 | K.S\*ES\*PRAS\*APGPGGK@RPAR.R | 3 |

---

|  |  |  |  |  |  |  |  |  |
| --- | --- | --- | --- | --- | --- | --- | --- | --- |
| U | *gi|16753207|ref|NP\_03* | 1 | 2 | 2.6% | 624 | 65696 | 5.2 | ubiquilin 2 [Homo sapiens] |

| Filename XCorr DeltCN Conf% ObsM+H+ CalcM+H+ SpR ZScore Ion% # Sequence  | | | | | | | | | | | | |
| --- | --- | --- | --- | --- | --- | --- | --- | --- | --- | --- | --- | --- |
| \* | Astrin\_NLD\_STLC\_031014\_01.05909.05909.2 | 3.8777 | 0.4833 | 100.0% | 1394.5122 | 1394.5272 | 1 | 8.169 | 60.0% | 2 | R.GPAAAQGSAAAPAEPK.I | 2 |

---

|  |  |  |  |  |  |  |  |  |
| --- | --- | --- | --- | --- | --- | --- | --- | --- |
| U | *Reverse\_gi|22325392|r* | 1 | 1 | 2.6% | 549 | 63236 | 8.7 | tigger transposable element derived 7 [Homo sapiens] |

| Filename XCorr DeltCN Conf% ObsM+H+ CalcM+H+ SpR ZScore Ion% # Sequence  | | | | | | | | | | | | |
| --- | --- | --- | --- | --- | --- | --- | --- | --- | --- | --- | --- | --- |
| \* | Astrin\_NLD\_STLC\_tube2\_021014\_01.05663.05663.2 | 3.5728 | 0.0707 | 96.0% | 1678.3922 | 1677.9408 | 1 | 3.976 | 69.2% | 1 | K.YLLNEWANAITIQK.V | 2 |

---

|  |  |  |  |  |  |  |  |  |
| --- | --- | --- | --- | --- | --- | --- | --- | --- |
| U | *gi|7669490|ref|NP\_053* | 1 | 1 | 2.6% | 583 | 61287 | 9.1 | Ewing sarcoma breakpoint region 1 isoform EWS-b [Homo sapiens] |

| Filename XCorr DeltCN Conf% ObsM+H+ CalcM+H+ SpR ZScore Ion% # Sequence  | | | | | | | | | | | | |
| --- | --- | --- | --- | --- | --- | --- | --- | --- | --- | --- | --- | --- |
| \* | Astrin\_NLD\_STLC\_031014\_01.08939.08939.2 | 2.4723 | 0.2679 | 98.3% | 1208.3722 | 1208.3373 | 124 | 4.98 | 46.4% | 1 | R.GGPGGPGGPGGPMGR.M | 2 |

---

|  |  |  |  |  |  |  |  |  |
| --- | --- | --- | --- | --- | --- | --- | --- | --- |
| U | *gi|5031699|ref|NP\_005* | 1 | 1 | 2.6% | 427 | 47355 | 7.5 | flotillin 1 [Homo sapiens] |

| Filename XCorr DeltCN Conf% ObsM+H+ CalcM+H+ SpR ZScore Ion% # Sequence  | | | | | | | | | | | | |
| --- | --- | --- | --- | --- | --- | --- | --- | --- | --- | --- | --- | --- |
| \* | Astrin\_NLD\_STLC\_tube2\_021014\_01.10356.10356.2 | 2.3426 | 0.2447 | 97.9% | 1216.1721 | 1216.4636 | 27 | 5.364 | 60.0% | 1 | R.ISLNTLTLNVK.S | 2 |

---

|  |  |  |  |  |  |  |  |  |
| --- | --- | --- | --- | --- | --- | --- | --- | --- |
| U | *gi|156630995|ref|NP\_0* | 1 | 1 | 2.6% | 270 | 30800 | 9.4 | neurotrophin 3 isoform 1 preproprotein [Homo sapiens] |
| U | *gi|4505469|ref|NP\_002* | 1 | 1 | 2.7% | 257 | 29355 | 9.4 | neurotrophin 3 isoform 2 preproprotein [Homo sapiens] |

| Filename XCorr DeltCN Conf% ObsM+H+ CalcM+H+ SpR ZScore Ion% # Sequence  | | | | | | | | | | | | |
| --- | --- | --- | --- | --- | --- | --- | --- | --- | --- | --- | --- | --- |
|  | Astrin\_NLD\_STLC\_031014\_02.17315.17315.1 | 1.1801 | 0.3239 | 95.9% | 794.53 | 794.7856 | 1 | 5.018 | 66.7% | 1 | K.T#GNSPVK@.Q | 1 |

---

|  |  |  |  |  |  |  |  |  |
| --- | --- | --- | --- | --- | --- | --- | --- | --- |
| U | *Reverse\_gi|148612838|* | 2 | 3 | 2.5% | 2103 | 228085 | 9.0 | hypothetical protein LOC158358 [Homo sapiens] |

| Filename XCorr DeltCN Conf% ObsM+H+ CalcM+H+ SpR ZScore Ion% # Sequence  | | | | | | | | | | | | |
| --- | --- | --- | --- | --- | --- | --- | --- | --- | --- | --- | --- | --- |
| \* | Astrin\_NLD\_STLC\_tube2\_021014\_01.09771.09771.2 | 3.6916 | 0.1302 | 98.9% | 2226.612 | 2227.4338 | 8 | 3.841 | 42.1% | 2 | K.S\*APTNITNLIAGEPS\*VLLKK.V | 2 |
| \* | Astrin\_NLD\_STLC\_tube2\_021014\_01.14578.14578.3 | 3.8161 | 0.2385 | 99.1% | 3513.5344 | 3510.8613 | 166 | 4.335 | 16.9% | 1 | K.IPSAEPPS\*LVPK@EVALANRGTSPPLQNTLT#VK@.N | 3 |

---

|  |  |  |  |  |  |  |  |  |
| --- | --- | --- | --- | --- | --- | --- | --- | --- |
| U | *Reverse\_gi|50980301|r* | 1 | 1 | 2.5% | 1025 | 116533 | 6.2 | myosin phosphatase-Rho interacting protein isoform 2 [Homo sapiens] |
| U | *Reverse\_gi|50980307|r* | 1 | 1 | 2.5% | 1038 | 118103 | 6.4 | myosin phosphatase-Rho interacting protein isoform 1 [Homo sapiens] |

| Filename XCorr DeltCN Conf% ObsM+H+ CalcM+H+ SpR ZScore Ion% # Sequence  | | | | | | | | | | | | |
| --- | --- | --- | --- | --- | --- | --- | --- | --- | --- | --- | --- | --- |
|  | Astrin\_NLD\_STLC\_031014\_02.09920.09920.3 | 2.349 | 0.3366 | 97.2% | 2737.0144 | 2739.6885 | 175 | 2.708 | 24.0% | 1 | K.TT#PVKEASPISSSSSSS\*SSSS\*TVAVK.A | 3 |

---

|  |  |  |  |  |  |  |  |  |
| --- | --- | --- | --- | --- | --- | --- | --- | --- |
| U | *gi|223555955|ref|NP\_0* | 1 | 1 | 2.5% | 393 | 44825 | 5.2 | defective in sister chromatid cohesion 1 [Homo sapiens] |

| Filename XCorr DeltCN Conf% ObsM+H+ CalcM+H+ SpR ZScore Ion% # Sequence  | | | | | | | | | | | | |
| --- | --- | --- | --- | --- | --- | --- | --- | --- | --- | --- | --- | --- |
| \* | Astrin\_NLD\_STLC\_tube2\_021014\_01.09213.09213.2 | 2.0882 | 0.2723 | 97.5% | 1398.8522 | 1401.4325 | 32 | 4.195 | 50.0% | 1 | R.FNS\*LFS\*LREK.W | 2 |

---

|  |  |  |  |  |  |  |  |  |
| --- | --- | --- | --- | --- | --- | --- | --- | --- |
| U | *gi|19482158|ref|NP\_06* | 1 | 2 | 2.5% | 396 | 43577 | 9.3 | desert hedgehog preproprotein [Homo sapiens] |

| Filename XCorr DeltCN Conf% ObsM+H+ CalcM+H+ SpR ZScore Ion% # Sequence  | | | | | | | | | | | | |
| --- | --- | --- | --- | --- | --- | --- | --- | --- | --- | --- | --- | --- |
| \* | Astrin\_NLD\_STLC\_031014\_01.08679.08679.2 | 2.6831 | 0.1669 | 97.7% | 1189.1921 | 1188.381 | 3 | 4.152 | 66.7% | 2 | R.GPVGRRRYAR.K | 2 |

---

|  |  |  |  |  |  |  |  |  |
| --- | --- | --- | --- | --- | --- | --- | --- | --- |
| U | *gi|31563507|ref|NP\_85* | 2 | 2 | 2.4% | 1684 | 195909 | 5.1 | GRIP and coiled-coil domain-containing 2 isoform a [Homo sapiens] |
| U | *gi|7662062|ref|NP\_055* | 2 | 2 | 2.5% | 1583 | 184657 | 5.1 | GRIP and coiled-coil domain-containing 2 isoform b [Homo sapiens] |

| Filename XCorr DeltCN Conf% ObsM+H+ CalcM+H+ SpR ZScore Ion% # Sequence  | | | | | | | | | | | | |
| --- | --- | --- | --- | --- | --- | --- | --- | --- | --- | --- | --- | --- |
|  | Astrin\_NLD\_STLC\_031014\_01.14194.14194.3 | 3.4678 | 0.2504 | 98.4% | 3431.1843 | 3433.3289 | 30 | 4.359 | 21.0% | 1 | R.EVQS\*LK@EQHQK@EISELNET#FLS\*DS\*EK@.E | 23 |
|  | Astrin\_NLD\_STLC\_tube2\_021014\_02.05164.05164.3 | 2.5225 | 0.2589 | 95.5% | 1546.6144 | 1546.7582 | 1 | 4.738 | 46.2% | 1 | K.VLSEDKEVLSAEVK.S | 3 |

---

|  |  |  |  |  |  |  |  |  |
| --- | --- | --- | --- | --- | --- | --- | --- | --- |
| U | *gi|42716280|ref|NP\_97* | 2 | 2 | 2.4% | 1268 | 141439 | 6.9 | high density lipoprotein binding protein [Homo sapiens] |
| U | *gi|4885409|ref|NP\_005* | 2 | 2 | 2.4% | 1268 | 141439 | 6.9 | high density lipoprotein binding protein [Homo sapiens] |

| Filename XCorr DeltCN Conf% ObsM+H+ CalcM+H+ SpR ZScore Ion% # Sequence  | | | | | | | | | | | | |
| --- | --- | --- | --- | --- | --- | --- | --- | --- | --- | --- | --- | --- |
|  | Astrin\_NLD\_STLC\_031014\_02.05154.05154.2 | 2.6286 | 0.205 | 97.5% | 1327.8522 | 1328.5516 | 37 | 4.528 | 54.2% | 1 | R.LQTQASATVAIPK.E | 2 |
|  | Astrin\_NLD\_STLC\_tube2\_021014\_02.06474.06474.3 | 3.7428 | 0.3419 | 100.0% | 1991.3644 | 1991.2517 | 1 | 7.081 | 36.8% | 1 | R.TEIVFTGEKEQLAQAVAR.I | 3 |

---

|  |  |  |  |  |  |  |  |  |
| --- | --- | --- | --- | --- | --- | --- | --- | --- |
| U | *gi|148762963|ref|NP\_1* | 1 | 1 | 2.4% | 867 | 96866 | 8.2 | armadillo repeat containing 2 [Homo sapiens] |

| Filename XCorr DeltCN Conf% ObsM+H+ CalcM+H+ SpR ZScore Ion% # Sequence  | | | | | | | | | | | | |
| --- | --- | --- | --- | --- | --- | --- | --- | --- | --- | --- | --- | --- |
| \* | Astrin\_NLD\_STLC\_tube2\_021014\_01.20189.20189.2 | 2.2968 | 0.2702 | 97.1% | 2298.8323 | 2301.7075 | 80 | 4.787 | 25.0% | 1 | K.LVDVGS\*DSLSLK@LAKIILALK@.V | 2 |

---

|  |  |  |  |  |  |  |  |  |
| --- | --- | --- | --- | --- | --- | --- | --- | --- |
| U | *gi|18641360|ref|NP\_56* | 1 | 1 | 2.4% | 742 | 81525 | 5.7 | collectin sub-family member 12 [Homo sapiens] |

| Filename XCorr DeltCN Conf% ObsM+H+ CalcM+H+ SpR ZScore Ion% # Sequence  | | | | | | | | | | | | |
| --- | --- | --- | --- | --- | --- | --- | --- | --- | --- | --- | --- | --- |
| \* | Astrin\_NLD\_STLC\_tube2\_021014\_01.14093.14093.2 | 2.7655 | 0.2636 | 99.4% | 2228.0522 | 2226.3926 | 1 | 4.467 | 41.2% | 1 | R.S\*RLDTEVANLS\*VIMEEMK.L | 2 |

---

|  |  |  |  |  |  |  |  |  |
| --- | --- | --- | --- | --- | --- | --- | --- | --- |
| U | *gi|28372563|ref|NP\_77* | 1 | 1 | 2.4% | 694 | 75214 | 9.6 | growth arrest-specific 2 like 3 [Homo sapiens] |

| Filename XCorr DeltCN Conf% ObsM+H+ CalcM+H+ SpR ZScore Ion% # Sequence  | | | | | | | | | | | | |
| --- | --- | --- | --- | --- | --- | --- | --- | --- | --- | --- | --- | --- |
| \* | Astrin\_NLD\_STLC\_031014\_01.13569.13569.2 | 2.1046 | 0.2936 | 96.6% | 1914.1322 | 1915.2003 | 22 | 4.847 | 37.5% | 1 | K.SKDKNIVSATKKQPQNK.S | 2 |

---

|  |  |  |  |  |  |  |  |  |
| --- | --- | --- | --- | --- | --- | --- | --- | --- |
| U | *gi|39930517|ref|NP\_61* | 1 | 1 | 2.4% | 538 | 56052 | 7.6 | sterile alpha motif domain containing 1 [Homo sapiens] |

| Filename XCorr DeltCN Conf% ObsM+H+ CalcM+H+ SpR ZScore Ion% # Sequence  | | | | | | | | | | | | |
| --- | --- | --- | --- | --- | --- | --- | --- | --- | --- | --- | --- | --- |
| \* | Astrin\_NLD\_STLC\_031014\_01.06030.06030.3 | 2.9388 | 0.3243 | 100.0% | 1424.8143 | 1424.5625 | 1 | 5.725 | 41.7% | 1 | R.RGAT#PPAPPRAPR.G | 3 |

---

|  |  |  |  |  |  |  |  |  |
| --- | --- | --- | --- | --- | --- | --- | --- | --- |
| U | *Reverse\_gi|195546915|* | 1 | 1 | 2.4% | 419 | 46420 | 6.8 | hypothetical protein LOC125704 [Homo sapiens] |

| Filename XCorr DeltCN Conf% ObsM+H+ CalcM+H+ SpR ZScore Ion% # Sequence  | | | | | | | | | | | | |
| --- | --- | --- | --- | --- | --- | --- | --- | --- | --- | --- | --- | --- |
| \* | Astrin\_NLD\_STLC\_tube2\_021014\_02.05669.05669.2 | 2.1569 | 0.2397 | 96.2% | 1165.3121 | 1165.3805 | 235 | 4.673 | 50.0% | 1 | R.LKWFVSSAAR.R | 2 |

---

|  |  |  |  |  |  |  |  |  |
| --- | --- | --- | --- | --- | --- | --- | --- | --- |
| U | *Reverse\_gi|34147456|r* | 1 | 1 | 2.3% | 830 | 93726 | 6.4 | TBC domain-containing protein kinase-like [Homo sapiens] |

| Filename XCorr DeltCN Conf% ObsM+H+ CalcM+H+ SpR ZScore Ion% # Sequence  | | | | | | | | | | | | |
| --- | --- | --- | --- | --- | --- | --- | --- | --- | --- | --- | --- | --- |
| \* | Astrin\_NLD\_STLC\_tube2\_021014\_01.21088.21088.2 | 3.1249 | 0.15 | 97.2% | 2213.5322 | 2214.578 | 5 | 3.613 | 36.1% | 1 | R.IDVVLLKPKSSK@TKK@SPT#K@.F | 2 |

---

|  |  |  |  |  |  |  |  |  |
| --- | --- | --- | --- | --- | --- | --- | --- | --- |
| U | *Reverse\_gi|80861486|r* | 1 | 1 | 2.3% | 834 | 93548 | 6.9 | granule cell antiserum positive 14 [Homo sapiens] |

| Filename XCorr DeltCN Conf% ObsM+H+ CalcM+H+ SpR ZScore Ion% # Sequence  | | | | | | | | | | | | |
| --- | --- | --- | --- | --- | --- | --- | --- | --- | --- | --- | --- | --- |
| \* | Astrin\_NLD\_STLC\_tube2\_021014\_01.11192.11192.2 | 2.6081 | 0.1954 | 95.2% | 2283.612 | 2284.2498 | 37 | 4.356 | 36.1% | 1 | R.GLLT#GKT#FK@GLNS\*PGSFS\*K@.Q | 2 |

---

|  |  |  |  |  |  |  |  |  |
| --- | --- | --- | --- | --- | --- | --- | --- | --- |
| U | *gi|13376259|ref|NP\_07* | 1 | 1 | 2.3% | 656 | 75019 | 5.6 | nucleoporin 85 [Homo sapiens] |

| Filename XCorr DeltCN Conf% ObsM+H+ CalcM+H+ SpR ZScore Ion% # Sequence  | | | | | | | | | | | | |
| --- | --- | --- | --- | --- | --- | --- | --- | --- | --- | --- | --- | --- |
| \* | Astrin\_NLD\_STLC\_tube2\_021014\_01.09378.09378.3 | 3.4674 | 0.3348 | 100.0% | 1759.3744 | 1760.0488 | 2 | 5.895 | 41.1% | 1 | R.KLFNESHGIFLGLQR.I | 3 |

---

|  |  |  |  |  |  |  |  |  |
| --- | --- | --- | --- | --- | --- | --- | --- | --- |
| U | *gi|27436951|ref|NP\_11* | 1 | 1 | 2.3% | 600 | 67689 | 5.3 | lamin B2 [Homo sapiens] |

| Filename XCorr DeltCN Conf% ObsM+H+ CalcM+H+ SpR ZScore Ion% # Sequence  | | | | | | | | | | | | |
| --- | --- | --- | --- | --- | --- | --- | --- | --- | --- | --- | --- | --- |
| \* | Astrin\_NLD\_STLC\_tube2\_021014\_02.05734.05734.2 | 2.1842 | 0.2705 | 96.4% | 1504.3322 | 1504.6984 | 2 | 5.173 | 50.0% | 1 | R.TVLVNADGEEVAMR.T | 2 |

---

|  |  |  |  |  |  |  |  |  |
| --- | --- | --- | --- | --- | --- | --- | --- | --- |
| U | *gi|156151392|ref|NP\_0* | 1 | 1 | 2.3% | 532 | 59682 | 9.2 | heterogeneous nuclear ribonucleoprotein R isoform 4 [Homo sapiens] |
| U | *gi|5031755|ref|NP\_005* | 1 | 1 | 1.9% | 633 | 70943 | 8.1 | heterogeneous nuclear ribonucleoprotein R isoform 2 [Homo sapiens] |
| U | *gi|156151396|ref|NP\_0* | 1 | 1 | 2.2% | 535 | 59953 | 9.2 | heterogeneous nuclear ribonucleoprotein R isoform 3 [Homo sapiens] |
| U | *gi|156151394|ref|NP\_0* | 1 | 1 | 1.9% | 636 | 71214 | 8.1 | heterogeneous nuclear ribonucleoprotein R isoform 1 [Homo sapiens] |

| Filename XCorr DeltCN Conf% ObsM+H+ CalcM+H+ SpR ZScore Ion% # Sequence  | | | | | | | | | | | | |
| --- | --- | --- | --- | --- | --- | --- | --- | --- | --- | --- | --- | --- |
|  | Astrin\_NLD\_STLC\_tube2\_021014\_02.06242.06242.3 | 3.0158 | 0.1973 | 95.6% | 1540.8844 | 1540.7184 | 1 | 5.402 | 50.0% | 1 | K.LKDYAFVHFEDR.G | 3 |

---

|  |  |  |  |  |  |  |  |  |
| --- | --- | --- | --- | --- | --- | --- | --- | --- |
| U | *gi|209862881|ref|NP\_0* | 1 | 1 | 2.3% | 471 | 52050 | 8.0 | pre-mRNA cleavage factor I, 59 kDa subunit isoform 2 [Homo sapiens] |
| U | *gi|217035107|ref|NP\_0* | 1 | 1 | 2.4% | 462 | 51096 | 7.8 | pre-mRNA cleavage factor I, 59 kDa subunit isoform 3 [Homo sapiens] |
| U | *gi|217035102|ref|NP\_0* | 1 | 1 | 2.1% | 514 | 56375 | 8.9 | pre-mRNA cleavage factor I, 59 kDa subunit isoform 1 [Homo sapiens] |

| Filename XCorr DeltCN Conf% ObsM+H+ CalcM+H+ SpR ZScore Ion% # Sequence  | | | | | | | | | | | | |
| --- | --- | --- | --- | --- | --- | --- | --- | --- | --- | --- | --- | --- |
|  | Astrin\_NLD\_STLC\_tube2\_021014\_01.06101.06101.2 | 2.7272 | 0.3108 | 100.0% | 1292.3322 | 1292.3939 | 182 | 4.868 | 55.0% | 1 | R.QNLSQFEAQAR.K | 2 |

---

|  |  |  |  |  |  |  |  |  |
| --- | --- | --- | --- | --- | --- | --- | --- | --- |
| U | *Reverse\_gi|19923233|r* | 1 | 1 | 2.2% | 547 | 58994 | 6.9 | sterol carrier protein 2 isoform 1 proprotein [Homo sapiens] |
| U | *Reverse\_gi|55956779|r* | 1 | 1 | 8.6% | 140 | 15079 | 8.9 | sterol carrier protein 2 isoform 2 precursor [Homo sapiens] |
| U | *Reverse\_gi|55956777|r* | 1 | 1 | 8.4% | 143 | 15401 | 8.9 | sterol carrier protein 2 isoform 1 precursor [Homo sapiens] |

| Filename XCorr DeltCN Conf% ObsM+H+ CalcM+H+ SpR ZScore Ion% # Sequence  | | | | | | | | | | | | |
| --- | --- | --- | --- | --- | --- | --- | --- | --- | --- | --- | --- | --- |
|  | Astrin\_NLD\_STLC\_031014\_02.09785.09785.2 | 2.2019 | 0.2544 | 96.7% | 1377.3922 | 1376.3365 | 9 | 4.661 | 45.5% | 1 | K.DS\*NPLVS\*GKGNK.V | 2 |

---

|  |  |  |  |  |  |  |  |  |
| --- | --- | --- | --- | --- | --- | --- | --- | --- |
| U | *Reverse\_gi|11386193|r* | 1 | 1 | 2.1% | 1029 | 118847 | 8.6 | zinc finger protein 197 isoform 1 [Homo sapiens] |

| Filename XCorr DeltCN Conf% ObsM+H+ CalcM+H+ SpR ZScore Ion% # Sequence  | | | | | | | | | | | | |
| --- | --- | --- | --- | --- | --- | --- | --- | --- | --- | --- | --- | --- |
| \* | Astrin\_NLD\_STLC\_tube2\_021014\_01.18144.18144.3 | 3.0865 | 0.2374 | 95.3% | 2840.6643 | 2839.096 | 256 | 4.276 | 23.8% | 1 | K.ET#HVRQHLLLSK@S\*RIFVKGCEK.C | 3 |

---

|  |  |  |  |  |  |  |  |  |
| --- | --- | --- | --- | --- | --- | --- | --- | --- |
| U | *gi|50053795|ref|NP\_00* | 1 | 1 | 2.1% | 611 | 69151 | 5.7 | eukaryotic translation initiation factor 4B [Homo sapiens] |

| Filename XCorr DeltCN Conf% ObsM+H+ CalcM+H+ SpR ZScore Ion% # Sequence  | | | | | | | | | | | | |
| --- | --- | --- | --- | --- | --- | --- | --- | --- | --- | --- | --- | --- |
| \* | Astrin\_NLD\_STLC\_031014\_01.05830.05830.2 | 2.4944 | 0.4225 | 100.0% | 1294.8121 | 1295.3947 | 3 | 6.373 | 58.3% | 1 | K.VAPAQPSEEGPGR.K | 2 |

---

|  |  |  |  |  |  |  |  |  |
| --- | --- | --- | --- | --- | --- | --- | --- | --- |
| U | *gi|27436988|ref|NP\_00* | 1 | 1 | 2.1% | 513 | 57913 | 6.3 | potassium voltage-gated channel, subfamily G, member 1 [Homo sapiens] |

| Filename XCorr DeltCN Conf% ObsM+H+ CalcM+H+ SpR ZScore Ion% # Sequence  | | | | | | | | | | | | |
| --- | --- | --- | --- | --- | --- | --- | --- | --- | --- | --- | --- | --- |
| \* | Astrin\_NLD\_STLC\_031014\_01.09134.09134.2 | 2.5017 | 0.201 | 97.2% | 1423.3322 | 1423.5657 | 3 | 4.483 | 60.0% | 1 | R.SYLELKQEQER.V | 2 |

---

|  |  |  |  |  |  |  |  |  |
| --- | --- | --- | --- | --- | --- | --- | --- | --- |
| U | *Reverse\_gi|190014620|* | 1 | 1 | 2.0% | 744 | 85098 | 8.6 | zinc finger protein 483 isoform a [Homo sapiens] |

| Filename XCorr DeltCN Conf% ObsM+H+ CalcM+H+ SpR ZScore Ion% # Sequence  | | | | | | | | | | | | |
| --- | --- | --- | --- | --- | --- | --- | --- | --- | --- | --- | --- | --- |
| \* | Astrin\_NLD\_STLC\_031014\_02.11607.11607.2 | 2.5874 | 0.2105 | 96.4% | 1799.0122 | 1798.7295 | 45 | 4.953 | 35.7% | 1 | K.SCKHT#K@EGSENGEDK.N | 2 |

---

|  |  |  |  |  |  |  |  |  |
| --- | --- | --- | --- | --- | --- | --- | --- | --- |
| U | *gi|55956788|ref|NP\_00* | 1 | 1 | 2.0% | 710 | 76615 | 4.7 | nucleolin [Homo sapiens] |

| Filename XCorr DeltCN Conf% ObsM+H+ CalcM+H+ SpR ZScore Ion% # Sequence  | | | | | | | | | | | | |
| --- | --- | --- | --- | --- | --- | --- | --- | --- | --- | --- | --- | --- |
| \* | Astrin\_NLD\_STLC\_031014\_02.08075.08075.2 | 2.9198 | 0.3952 | 100.0% | 1561.3922 | 1562.6323 | 1 | 7.376 | 53.8% | 1 | K.GFGFVDFNSEEDAK.A | 2 |

---

|  |  |  |  |  |  |  |  |  |
| --- | --- | --- | --- | --- | --- | --- | --- | --- |
| U | *gi|61744475|ref|NP\_00* | 1 | 1 | 2.0% | 661 | 71123 | 5.0 | solute carrier family 3 (activators of dibasic and neutral amino acid transport), member 2 isoform a [Homo sapiens] |
| U | *gi|65506891|ref|NP\_00* | 1 | 1 | 2.1% | 630 | 67994 | 5.0 | solute carrier family 3 (activators of dibasic and neutral amino acid transport), member 2 isoform c [Homo sapiens] |
| U | *gi|61744483|ref|NP\_00* | 1 | 1 | 2.5% | 529 | 57945 | 5.3 | solute carrier family 3 (activators of dibasic and neutral amino acid transport), member 2 isoform f [Homo sapiens] |
| U | *gi|61744481|ref|NP\_00* | 1 | 1 | 2.3% | 568 | 61816 | 5.2 | solute carrier family 3 (activators of dibasic and neutral amino acid transport), member 2 isoform e [Homo sapiens] |
| U | *gi|61744479|ref|NP\_00* | 1 | 1 | 2.2% | 599 | 64873 | 5.1 | solute carrier family 3 (activators of dibasic and neutral amino acid transport), member 2 isoform d [Homo sapiens] |
| U | *gi|61744477|ref|NP\_00* | 1 | 1 | 2.1% | 631 | 68101 | 5.0 | solute carrier family 3 (activators of dibasic and neutral amino acid transport), member 2 isoform b [Homo sapiens] |

| Filename XCorr DeltCN Conf% ObsM+H+ CalcM+H+ SpR ZScore Ion% # Sequence  | | | | | | | | | | | | |
| --- | --- | --- | --- | --- | --- | --- | --- | --- | --- | --- | --- | --- |
|  | Astrin\_NLD\_STLC\_tube2\_021014\_01.04133.04133.2 | 2.2807 | 0.4409 | 100.0% | 1245.1522 | 1246.3165 | 5 | 6.495 | 58.3% | 1 | K.VAEDEAEAAAAAK.F | 2 |

---

|  |  |  |  |  |  |  |  |  |
| --- | --- | --- | --- | --- | --- | --- | --- | --- |
| U | *gi|17402900|ref|NP\_00* | 1 | 1 | 2.0% | 644 | 67560 | 7.6 | far upstream element-binding protein [Homo sapiens] |

| Filename XCorr DeltCN Conf% ObsM+H+ CalcM+H+ SpR ZScore Ion% # Sequence  | | | | | | | | | | | | |
| --- | --- | --- | --- | --- | --- | --- | --- | --- | --- | --- | --- | --- |
| \* | Astrin\_NLD\_STLC\_tube2\_021014\_01.08748.08748.2 | 2.8289 | 0.4195 | 100.0% | 1337.4521 | 1337.5187 | 1 | 6.801 | 62.5% | 1 | R.IGGNEGIDVPIPR.F | 2 |

---

|  |  |  |  |  |  |  |  |  |
| --- | --- | --- | --- | --- | --- | --- | --- | --- |
| U | *Reverse\_gi|109255249|* | 1 | 1 | 2.0% | 594 | 63911 | 8.3 | keratin 4 [Homo sapiens] |

| Filename XCorr DeltCN Conf% ObsM+H+ CalcM+H+ SpR ZScore Ion% # Sequence  | | | | | | | | | | | | |
| --- | --- | --- | --- | --- | --- | --- | --- | --- | --- | --- | --- | --- |
| \* | Astrin\_NLD\_STLC\_031014\_01.12324.12324.2 | 1.9191 | 0.3048 | 95.7% | 1523.3121 | 1523.7219 | 43 | 4.452 | 45.5% | 1 | -.RRK@NLT#TTSIIK.S | 2 |

---

|  |  |  |  |  |  |  |  |  |
| --- | --- | --- | --- | --- | --- | --- | --- | --- |
| U | *gi|41406064|ref|NP\_00* | 4 | 4 | 1.9% | 1976 | 228997 | 5.5 | myosin, heavy polypeptide 10, non-muscle [Homo sapiens] |

| Filename XCorr DeltCN Conf% ObsM+H+ CalcM+H+ SpR ZScore Ion% # Sequence  | | | | | | | | | | | | |
| --- | --- | --- | --- | --- | --- | --- | --- | --- | --- | --- | --- | --- |
|  | Astrin\_NLD\_STLC\_tube2\_021014\_01.06784.06784.2 | 2.8625 | 0.3603 | 100.0% | 1223.3322 | 1224.3591 | 1 | 6.86 | 70.0% | 1 | R.AGVLAHLEEER.D | 22 |
| \* | Astrin\_NLD\_STLC\_tube2\_021014\_01.10242.10242.3 | 2.8325 | 0.2319 | 95.0% | 1953.6843 | 1954.1472 | 1 | 4.987 | 32.8% | 1 | R.HATALEELSEQLEQAKR.F | 3 |
|  | Astrin\_NLD\_STLC\_tube2\_021014\_01.06402.06402.2 | 2.798 | 0.1367 | 97.1% | 1221.2922 | 1221.3959 | 6 | 4.448 | 66.7% | 1 | K.KFDQLLAEEK.S | 22 |
|  | Astrin\_NLD\_STLC\_tube2\_021014\_01.06868.06868.2 | 3.1864 | 0.2186 | 100.0% | 1093.1122 | 1093.2218 | 1 | 5.928 | 81.2% | 1 | K.FDQLLAEEK.S | 22 |

Similarities:
gi|12667788|ref|NP\_00(3:1)  

---

|  |  |  |  |  |  |  |  |  |
| --- | --- | --- | --- | --- | --- | --- | --- | --- |
| U | *gi|7657269|ref|NP\_055* | 1 | 1 | 1.9% | 1447 | 164666 | 8.5 | PDS5, regulator of cohesion maintenance, homolog B [Homo sapiens] |

| Filename XCorr DeltCN Conf% ObsM+H+ CalcM+H+ SpR ZScore Ion% # Sequence  | | | | | | | | | | | | |
| --- | --- | --- | --- | --- | --- | --- | --- | --- | --- | --- | --- | --- |
| \* | Astrin\_NLD\_STLC\_tube2\_021014\_01.17678.17678.3 | 3.3403 | 0.2254 | 95.6% | 2937.5942 | 2940.1716 | 19 | 5.134 | 23.1% | 1 | K.QSQTKSSRMETVSNASSSSNPSSPGRIK.G | 3 |

---

|  |  |  |  |  |  |  |  |  |
| --- | --- | --- | --- | --- | --- | --- | --- | --- |
| U | *gi|106049292|ref|NP\_0* | 1 | 1 | 1.9% | 1178 | 129634 | 6.8 | pyruvate carboxylase precursor [Homo sapiens] |
| U | *gi|106049528|ref|NP\_0* | 1 | 1 | 1.9% | 1178 | 129634 | 6.8 | pyruvate carboxylase precursor [Homo sapiens] |
| U | *gi|106049295|ref|NP\_0* | 1 | 1 | 1.9% | 1178 | 129634 | 6.8 | pyruvate carboxylase precursor [Homo sapiens] |

| Filename XCorr DeltCN Conf% ObsM+H+ CalcM+H+ SpR ZScore Ion% # Sequence  | | | | | | | | | | | | |
| --- | --- | --- | --- | --- | --- | --- | --- | --- | --- | --- | --- | --- |
|  | Astrin\_NLD\_STLC\_tube2\_021014\_01.10751.10751.3 | 3.081 | 0.2349 | 95.4% | 2346.5044 | 2346.645 | 32 | 4.905 | 28.6% | 1 | R.LDNASAFQGAVISPHYDSLLVK.V | 3 |

---

|  |  |  |  |  |  |  |  |  |
| --- | --- | --- | --- | --- | --- | --- | --- | --- |
| U | *Reverse\_gi|163310743|* | 1 | 1 | 1.9% | 1057 | 119078 | 6.3 | LIM and calponin homology domains 1 isoform b [Homo sapiens] |
| U | *Reverse\_gi|163310749|* | 1 | 1 | 2.2% | 890 | 100734 | 6.3 | LIM and calponin homology domains 1 isoform e [Homo sapiens] |
| U | *Reverse\_gi|163310747|* | 1 | 1 | 2.2% | 902 | 102022 | 6.2 | LIM and calponin homology domains 1 isoform d [Homo sapiens] |
| U | *Reverse\_gi|163310745|* | 1 | 1 | 1.9% | 1056 | 118950 | 6.3 | LIM and calponin homology domains 1 isoform c [Homo sapiens] |

| Filename XCorr DeltCN Conf% ObsM+H+ CalcM+H+ SpR ZScore Ion% # Sequence  | | | | | | | | | | | | |
| --- | --- | --- | --- | --- | --- | --- | --- | --- | --- | --- | --- | --- |
|  | Astrin\_NLD\_STLC\_031014\_02.09462.09462.3 | 3.2005 | 0.2208 | 95.5% | 2388.1143 | 2386.565 | 211 | 4.401 | 27.6% | 1 | K.K@GS\*ISKRRESPSEIQHNISK@.D | 3 |

---

|  |  |  |  |  |  |  |  |  |
| --- | --- | --- | --- | --- | --- | --- | --- | --- |
| U | *gi|127138957|ref|NP\_0* | 1 | 1 | 1.9% | 1081 | 118566 | 7.2 | zinc finger protein 537 [Homo sapiens] |

| Filename XCorr DeltCN Conf% ObsM+H+ CalcM+H+ SpR ZScore Ion% # Sequence  | | | | | | | | | | | | |
| --- | --- | --- | --- | --- | --- | --- | --- | --- | --- | --- | --- | --- |
| \* | Astrin\_NLD\_STLC\_tube2\_021014\_01.11634.11634.3 | 2.9364 | 0.2683 | 97.7% | 2107.5244 | 2107.2249 | 1 | 4.779 | 35.5% | 1 | K.KASLELELPSSPDS\*TGGTPK@.A | 3 |

---

|  |  |  |  |  |  |  |  |  |
| --- | --- | --- | --- | --- | --- | --- | --- | --- |
| U | *Reverse\_gi|163937854|* | 1 | 1 | 1.9% | 861 | 91514 | 9.8 | ataxin 7-like 1 isoform 1 [Homo sapiens] |
| U | *Reverse\_gi|211971080|* | 1 | 1 | 2.2% | 738 | 77874 | 10.0 | ataxin 7-like 1 isoform 3 [Homo sapiens] |

| Filename XCorr DeltCN Conf% ObsM+H+ CalcM+H+ SpR ZScore Ion% # Sequence  | | | | | | | | | | | | |
| --- | --- | --- | --- | --- | --- | --- | --- | --- | --- | --- | --- | --- |
|  | Astrin\_NLD\_STLC\_tube2\_021014\_01.07432.07432.3 | 2.7283 | 0.242 | 95.2% | 1961.8444 | 1963.956 | 172 | 4.626 | 28.3% | 1 | K.KNS\*PS\*EDSRTSLDK@VK.S | 3 |

---

|  |  |  |  |  |  |  |  |  |
| --- | --- | --- | --- | --- | --- | --- | --- | --- |
| U | *gi|14141161|ref|NP\_00* | 1 | 2 | 1.9% | 806 | 88980 | 5.8 | heterogeneous nuclear ribonucleoprotein U isoform b [Homo sapiens] |
| U | *gi|74136883|ref|NP\_11* | 1 | 2 | 1.8% | 825 | 90585 | 6.0 | heterogeneous nuclear ribonucleoprotein U isoform a [Homo sapiens] |

| Filename XCorr DeltCN Conf% ObsM+H+ CalcM+H+ SpR ZScore Ion% # Sequence  | | | | | | | | | | | | |
| --- | --- | --- | --- | --- | --- | --- | --- | --- | --- | --- | --- | --- |
|  | Astrin\_NLD\_STLC\_031014\_02.06639.06639.2 | 4.7413 | 0.4196 | 100.0% | 1648.2322 | 1648.816 | 1 | 7.938 | 82.1% | 2 | R.NFILDQTNVSAAAQR.R | 2 |

---

|  |  |  |  |  |  |  |  |  |
| --- | --- | --- | --- | --- | --- | --- | --- | --- |
| U | *gi|5032179|ref|NP\_005* | 1 | 1 | 1.9% | 835 | 88550 | 5.8 | tripartite motif-containing 28 protein [Homo sapiens] |

| Filename XCorr DeltCN Conf% ObsM+H+ CalcM+H+ SpR ZScore Ion% # Sequence  | | | | | | | | | | | | |
| --- | --- | --- | --- | --- | --- | --- | --- | --- | --- | --- | --- | --- |
| \* | Astrin\_NLD\_STLC\_tube2\_021014\_02.06538.06538.3 | 2.9549 | 0.215 | 95.0% | 1788.3544 | 1787.0232 | 1 | 4.548 | 38.3% | 1 | K.LTEDKADVQSIIGLQR.F | 3 |

---

|  |  |  |  |  |  |  |  |  |
| --- | --- | --- | --- | --- | --- | --- | --- | --- |
| U | *gi|148664201|ref|NP\_0* | 1 | 6 | 1.9% | 683 | 76987 | 9.4 | cytoskeleton associated protein 2 isoform 2 [Homo sapiens] |
| U | *gi|148664244|ref|NP\_0* | 1 | 6 | 1.9% | 682 | 76900 | 9.4 | cytoskeleton associated protein 2 isoform 1 [Homo sapiens] |

| Filename XCorr DeltCN Conf% ObsM+H+ CalcM+H+ SpR ZScore Ion% # Sequence  | | | | | | | | | | | | |
| --- | --- | --- | --- | --- | --- | --- | --- | --- | --- | --- | --- | --- |
|  | Astrin\_NLD\_STLC\_031014\_01.06275.06275.2 | 3.988 | 0.5156 | 100.0% | 1420.2522 | 1420.5162 | 1 | 8.836 | 75.0% | 6 | R.VVTSEDQVQEGTK.V | 2 |

---

|  |  |  |  |  |  |  |  |  |
| --- | --- | --- | --- | --- | --- | --- | --- | --- |
| U | *gi|194239723|ref|NP\_0* | 1 | 1 | 1.9% | 647 | 71408 | 6.4 | eukaryotic translation elongation factor 1 delta isoform 1 [Homo sapiens] |
| U | *gi|25453474|ref|NP\_11* | 1 | 1 | 1.9% | 647 | 71408 | 6.4 | eukaryotic translation elongation factor 1 delta isoform 1 [Homo sapiens] |
| U | *gi|25453472|ref|NP\_00* | 1 | 1 | 4.3% | 281 | 31122 | 5.0 | eukaryotic translation elongation factor 1 delta isoform 2 [Homo sapiens] |
| U | *gi|194239731|ref|NP\_0* | 1 | 1 | 4.3% | 281 | 31122 | 5.0 | eukaryotic translation elongation factor 1 delta isoform 2 [Homo sapiens] |
| U | *gi|194239729|ref|NP\_0* | 1 | 1 | 4.7% | 257 | 28558 | 4.9 | eukaryotic translation elongation factor 1 delta isoform 4 [Homo sapiens] |
| U | *gi|194239727|ref|NP\_0* | 1 | 1 | 4.3% | 281 | 31122 | 5.0 | eukaryotic translation elongation factor 1 delta isoform 2 [Homo sapiens] |
| U | *gi|194239725|ref|NP\_0* | 1 | 1 | 1.9% | 646 | 71266 | 6.4 | eukaryotic translation elongation factor 1 delta isoform 3 [Homo sapiens] |

| Filename XCorr DeltCN Conf% ObsM+H+ CalcM+H+ SpR ZScore Ion% # Sequence  | | | | | | | | | | | | |
| --- | --- | --- | --- | --- | --- | --- | --- | --- | --- | --- | --- | --- |
|  | Astrin\_NLD\_STLC\_tube2\_021014\_01.07061.07061.2 | 3.0118 | 0.2511 | 99.8% | 1359.5922 | 1359.5223 | 2 | 5.19 | 63.6% | 1 | R.IASLEVENQSLR.G | 2 |

---

|  |  |  |  |  |  |  |  |  |
| --- | --- | --- | --- | --- | --- | --- | --- | --- |
| U | *gi|20143967|ref|NP\_61* | 1 | 1 | 1.8% | 960 | 110059 | 8.5 | kinesin family member 23 isoform 1 [Homo sapiens] |
| U | *gi|6754472|ref|NP\_004* | 1 | 1 | 2.0% | 856 | 98105 | 8.5 | kinesin family member 23 isoform 2 [Homo sapiens] |

| Filename XCorr DeltCN Conf% ObsM+H+ CalcM+H+ SpR ZScore Ion% # Sequence  | | | | | | | | | | | | |
| --- | --- | --- | --- | --- | --- | --- | --- | --- | --- | --- | --- | --- |
|  | Astrin\_NLD\_STLC\_tube2\_021014\_02.05530.05530.3 | 4.2625 | 0.3202 | 100.0% | 1966.4944 | 1966.17 | 1 | 6.014 | 39.1% | 1 | K.YMLTHQELASDGEIETK.L | 3 |

---

|  |  |  |  |  |  |  |  |  |
| --- | --- | --- | --- | --- | --- | --- | --- | --- |
| U | *Reverse\_gi|153791535|* | 1 | 1 | 1.8% | 837 | 93926 | 9.0 | GCN5 general control of amino-acid synthesis 5-like 2 [Homo sapiens] |
| U | *Reverse\_gi|40805843|r* | 1 | 1 | 1.8% | 832 | 93013 | 9.0 | K(lysine) acetyltransferase 2B [Homo sapiens] |

| Filename XCorr DeltCN Conf% ObsM+H+ CalcM+H+ SpR ZScore Ion% # Sequence  | | | | | | | | | | | | |
| --- | --- | --- | --- | --- | --- | --- | --- | --- | --- | --- | --- | --- |
|  | Astrin\_NLD\_STLC\_031014\_01.06086.06086.3 | 2.6678 | 0.2709 | 97.4% | 1838.4844 | 1839.2754 | 19 | 4.633 | 37.5% | 1 | R.IQAQKREILKKIIEK.Q | 3 |

---

|  |  |  |  |  |  |  |  |  |
| --- | --- | --- | --- | --- | --- | --- | --- | --- |
| U | *gi|19923268|ref|NP\_00* | 1 | 1 | 1.8% | 761 | 87704 | 5.9 | protein-kinase, interferon-inducible double stranded RNA dependent inhibitor, repressor of (P58 repressor) [Homo sapiens] |

| Filename XCorr DeltCN Conf% ObsM+H+ CalcM+H+ SpR ZScore Ion% # Sequence  | | | | | | | | | | | | |
| --- | --- | --- | --- | --- | --- | --- | --- | --- | --- | --- | --- | --- |
| \* | Astrin\_NLD\_STLC\_031014\_01.09808.09808.3 | 2.8942 | 0.2538 | 98.1% | 1780.5844 | 1780.9376 | 417 | 4.448 | 32.7% | 1 | R.KSTQS\*DLAFFRFPR.D | 3 |

---

|  |  |  |  |  |  |  |  |  |
| --- | --- | --- | --- | --- | --- | --- | --- | --- |
| U | *Reverse\_gi|166706917|* | 1 | 1 | 1.8% | 728 | 82070 | 8.4 | hypothetical protein LOC199920 [Homo sapiens] |

| Filename XCorr DeltCN Conf% ObsM+H+ CalcM+H+ SpR ZScore Ion% # Sequence  | | | | | | | | | | | | |
| --- | --- | --- | --- | --- | --- | --- | --- | --- | --- | --- | --- | --- |
| \* | Astrin\_NLD\_STLC\_031014\_01.11144.11144.2 | 2.2511 | 0.2472 | 96.0% | 1591.0922 | 1593.8319 | 62 | 4.371 | 41.7% | 1 | K.TKPLHHHRVDPQK.E | 2 |

---

|  |  |  |  |  |  |  |  |  |
| --- | --- | --- | --- | --- | --- | --- | --- | --- |
| U | *gi|148277065|ref|NP\_0* | 1 | 1 | 1.8% | 551 | 60269 | 6.7 | thioredoxin reductase 1 isoform 1 [Homo sapiens] |
| U | *gi|33519430|ref|NP\_87* | 1 | 1 | 2.0% | 499 | 54604 | 6.5 | thioredoxin reductase 1 isoform 2 [Homo sapiens] |
| U | *gi|33519428|ref|NP\_87* | 1 | 1 | 2.0% | 499 | 54604 | 6.5 | thioredoxin reductase 1 isoform 2 [Homo sapiens] |
| U | *gi|33519426|ref|NP\_87* | 1 | 1 | 2.0% | 499 | 54604 | 6.5 | thioredoxin reductase 1 isoform 2 [Homo sapiens] |
| U | *gi|148277071|ref|NP\_0* | 1 | 1 | 1.5% | 649 | 70756 | 7.4 | thioredoxin reductase 1 isoform 3 [Homo sapiens] |

| Filename XCorr DeltCN Conf% ObsM+H+ CalcM+H+ SpR ZScore Ion% # Sequence  | | | | | | | | | | | | |
| --- | --- | --- | --- | --- | --- | --- | --- | --- | --- | --- | --- | --- |
|  | Astrin\_NLD\_STLC\_tube2\_021014\_01.05729.05729.2 | 2.5172 | 0.2128 | 98.3% | 1160.5122 | 1160.3616 | 4 | 4.526 | 72.2% | 1 | R.FLIATGERPR.Y | 2 |

---

|  |  |  |  |  |  |  |  |  |
| --- | --- | --- | --- | --- | --- | --- | --- | --- |
| U | *gi|40255180|ref|NP\_77* | 1 | 1 | 1.8% | 444 | 52409 | 5.7 | coiled-coil domain containing 83 [Homo sapiens] |

| Filename XCorr DeltCN Conf% ObsM+H+ CalcM+H+ SpR ZScore Ion% # Sequence  | | | | | | | | | | | | |
| --- | --- | --- | --- | --- | --- | --- | --- | --- | --- | --- | --- | --- |
| \* | Astrin\_NLD\_STLC\_031014\_02.06281.06281.2 | 2.042 | 0.2614 | 97.7% | 1055.0521 | 1056.1179 | 14 | 4.098 | 64.3% | 1 | K.IT#LEDTRK.K | 2 |

---

|  |  |  |  |  |  |  |  |  |
| --- | --- | --- | --- | --- | --- | --- | --- | --- |
| U | *Reverse\_gi|55741845|r* | 1 | 1 | 1.7% | 1063 | 118462 | 7.0 | valyl-tRNA synthetase 2, mitochondrial [Homo sapiens] |

| Filename XCorr DeltCN Conf% ObsM+H+ CalcM+H+ SpR ZScore Ion% # Sequence  | | | | | | | | | | | | |
| --- | --- | --- | --- | --- | --- | --- | --- | --- | --- | --- | --- | --- |
| \* | Astrin\_NLD\_STLC\_031014\_01.12172.12172.2 | 2.1647 | 0.3146 | 98.0% | 1917.5322 | 1917.0751 | 1 | 5.484 | 38.2% | 1 | K.K@QAAAVIALEAPDLNGS\*R.L | 2 |

---

|  |  |  |  |  |  |  |  |  |
| --- | --- | --- | --- | --- | --- | --- | --- | --- |
| U | *gi|9966881|ref|NP\_065* | 1 | 1 | 1.7% | 925 | 106374 | 5.4 | nucleoporin 107kDa [Homo sapiens] |

| Filename XCorr DeltCN Conf% ObsM+H+ CalcM+H+ SpR ZScore Ion% # Sequence  | | | | | | | | | | | | |
| --- | --- | --- | --- | --- | --- | --- | --- | --- | --- | --- | --- | --- |
| \* | Astrin\_NLD\_STLC\_tube2\_021014\_01.16954.16954.2 | 2.8023 | 0.3797 | 100.0% | 1813.5521 | 1814.1035 | 2 | 6.54 | 43.3% | 1 | K.FLILGDIDGLMDEFSK.W | 2 |

---

|  |  |  |  |  |  |  |  |  |
| --- | --- | --- | --- | --- | --- | --- | --- | --- |
| U | *gi|50659082|ref|NP\_06* | 1 | 1 | 1.7% | 885 | 99188 | 8.8 | suppressor of variegation 4-20 homolog 1 isoform 1 [Homo sapiens] |

| Filename XCorr DeltCN Conf% ObsM+H+ CalcM+H+ SpR ZScore Ion% # Sequence  | | | | | | | | | | | | |
| --- | --- | --- | --- | --- | --- | --- | --- | --- | --- | --- | --- | --- |
| \* | Astrin\_NLD\_STLC\_tube2\_021014\_02.06608.06608.3 | 2.6291 | 0.2655 | 96.7% | 1866.2043 | 1866.78 | 110 | 4.165 | 32.1% | 1 | K.NNAT#S\*NRK@SS\*VGVK@K@.N | 3 |

---

|  |  |  |  |  |  |  |  |  |
| --- | --- | --- | --- | --- | --- | --- | --- | --- |
| U | *gi|110225358|ref|NP\_0* | 1 | 1 | 1.7% | 858 | 96185 | 6.4 | tetratricopeptide repeat domain 7A [Homo sapiens] |

| Filename XCorr DeltCN Conf% ObsM+H+ CalcM+H+ SpR ZScore Ion% # Sequence  | | | | | | | | | | | | |
| --- | --- | --- | --- | --- | --- | --- | --- | --- | --- | --- | --- | --- |
| \* | Astrin\_NLD\_STLC\_031014\_02.05314.05314.3 | 2.8805 | 0.2745 | 98.9% | 1676.0643 | 1675.8387 | 6 | 4.651 | 35.7% | 1 | K.GAHGSYLKVESELER.C | 3 |

---

|  |  |  |  |  |  |  |  |  |
| --- | --- | --- | --- | --- | --- | --- | --- | --- |
| U | *gi|76150623|ref|NP\_00* | 1 | 1 | 1.7% | 808 | 88973 | 9.2 | nucleolar protein 1, 120kDa [Homo sapiens] |
| U | *gi|76150625|ref|NP\_00* | 1 | 1 | 1.7% | 808 | 88973 | 9.2 | nucleolar protein 1, 120kDa [Homo sapiens] |

| Filename XCorr DeltCN Conf% ObsM+H+ CalcM+H+ SpR ZScore Ion% # Sequence  | | | | | | | | | | | | |
| --- | --- | --- | --- | --- | --- | --- | --- | --- | --- | --- | --- | --- |
|  | Astrin\_NLD\_STLC\_tube2\_021014\_02.06220.06220.3 | 3.0461 | 0.2451 | 98.4% | 1547.1543 | 1546.7233 | 4 | 5.404 | 36.5% | 1 | R.LGVTNTIISHYDGR.Q | 3 |

---

|  |  |  |  |  |  |  |  |  |
| --- | --- | --- | --- | --- | --- | --- | --- | --- |
| U | *Reverse\_gi|156546890|* | 1 | 1 | 1.6% | 1192 | 133891 | 5.5 | WW and C2 domain containing 2 [Homo sapiens] |

| Filename XCorr DeltCN Conf% ObsM+H+ CalcM+H+ SpR ZScore Ion% # Sequence  | | | | | | | | | | | | |
| --- | --- | --- | --- | --- | --- | --- | --- | --- | --- | --- | --- | --- |
| \* | Astrin\_NLD\_STLC\_tube2\_021014\_01.13472.13472.2 | 2.6718 | 0.216 | 97.6% | 2218.0122 | 2217.3896 | 39 | 4.422 | 30.6% | 1 | R.VFLSK@KALTS\*SDSDSRNLR.C | 2 |

---

|  |  |  |  |  |  |  |  |  |
| --- | --- | --- | --- | --- | --- | --- | --- | --- |
| U | *gi|21237725|ref|NP\_00* | 1 | 4 | 1.6% | 1102 | 126454 | 7.5 | phosphoinositide-3-kinase, catalytic, gamma polypeptide [Homo sapiens] |

| Filename XCorr DeltCN Conf% ObsM+H+ CalcM+H+ SpR ZScore Ion% # Sequence  | | | | | | | | | | | | |
| --- | --- | --- | --- | --- | --- | --- | --- | --- | --- | --- | --- | --- |
| \* | Astrin\_NLD\_STLC\_031014\_01.10113.10113.2 | 4.2865 | 0.0638 | 98.7% | 2215.4521 | 2215.3857 | 1 | 4.258 | 55.9% | 4 | K.K@S\*LMDIPESQSEQDFVLR.V | 2 |

---

|  |  |  |  |  |  |  |  |  |
| --- | --- | --- | --- | --- | --- | --- | --- | --- |
| U | *gi|5032013|ref|NP\_005* | 1 | 1 | 1.6% | 890 | 100278 | 6.9 | kinesin family member 20A [Homo sapiens] |

| Filename XCorr DeltCN Conf% ObsM+H+ CalcM+H+ SpR ZScore Ion% # Sequence  | | | | | | | | | | | | |
| --- | --- | --- | --- | --- | --- | --- | --- | --- | --- | --- | --- | --- |
| \* | Astrin\_NLD\_STLC\_tube2\_021014\_01.05166.05166.2 | 2.8862 | 0.1583 | 96.4% | 1475.4321 | 1474.6543 | 6 | 4.536 | 53.8% | 1 | R.LAASASTQQLQEVK.A | 2 |

---

|  |  |  |  |  |  |  |  |  |
| --- | --- | --- | --- | --- | --- | --- | --- | --- |
| U | *gi|68509926|ref|NP\_00* | 1 | 1 | 1.6% | 795 | 90933 | 7.5 | DEAH (Asp-Glu-Ala-His) box polypeptide 15 [Homo sapiens] |

| Filename XCorr DeltCN Conf% ObsM+H+ CalcM+H+ SpR ZScore Ion% # Sequence  | | | | | | | | | | | | |
| --- | --- | --- | --- | --- | --- | --- | --- | --- | --- | --- | --- | --- |
| \* | Astrin\_NLD\_STLC\_031014\_02.08027.08027.2 | 2.7832 | 0.2487 | 99.6% | 1430.0122 | 1429.7434 | 1 | 4.495 | 62.5% | 1 | R.IRVESLLVTAISK.A | 2 |

---

|  |  |  |  |  |  |  |  |  |
| --- | --- | --- | --- | --- | --- | --- | --- | --- |
| U | *gi|23510448|ref|NP\_00* | 1 | 1 | 1.6% | 734 | 82286 | 8.4 | minichromosome maintenance complex component 5 [Homo sapiens] |

| Filename XCorr DeltCN Conf% ObsM+H+ CalcM+H+ SpR ZScore Ion% # Sequence  | | | | | | | | | | | | |
| --- | --- | --- | --- | --- | --- | --- | --- | --- | --- | --- | --- | --- |
| \* | Astrin\_NLD\_STLC\_tube2\_021014\_01.10439.10439.2 | 2.251 | 0.3793 | 99.9% | 1180.5122 | 1181.4642 | 1 | 6.498 | 77.3% | 1 | K.IPGIIIAASAVR.A | 2 |

---

|  |  |  |  |  |  |  |  |  |
| --- | --- | --- | --- | --- | --- | --- | --- | --- |
| U | *Reverse\_gi|33636756|r* | 1 | 1 | 1.6% | 688 | 75321 | 9.8 | MAP/microtubule affinity-regulating kinase 4 [Homo sapiens] |

| Filename XCorr DeltCN Conf% ObsM+H+ CalcM+H+ SpR ZScore Ion% # Sequence  | | | | | | | | | | | | |
| --- | --- | --- | --- | --- | --- | --- | --- | --- | --- | --- | --- | --- |
| \* | Astrin\_NLD\_STLC\_tube2\_021014\_01.10547.10547.2 | 2.2384 | 0.2598 | 97.7% | 1227.7922 | 1228.2151 | 4 | 4.627 | 70.0% | 1 | K.LEAEGTS\*TPSR.K | 2 |

---

|  |  |  |  |  |  |  |  |  |
| --- | --- | --- | --- | --- | --- | --- | --- | --- |
| U | *gi|116089325|ref|NP\_0* | 1 | 1 | 1.6% | 624 | 71650 | 10.2 | splicing factor, arginine/serine-rich 12 isoform a [Homo sapiens] |
| U | *gi|21040255|ref|NP\_63* | 1 | 1 | 2.0% | 508 | 59380 | 10.4 | splicing factor, arginine/serine-rich 12 isoform b [Homo sapiens] |

| Filename XCorr DeltCN Conf% ObsM+H+ CalcM+H+ SpR ZScore Ion% # Sequence  | | | | | | | | | | | | |
| --- | --- | --- | --- | --- | --- | --- | --- | --- | --- | --- | --- | --- |
|  | Astrin\_NLD\_STLC\_031014\_01.10145.10145.2 | 2.2624 | 0.2177 | 96.1% | 1317.1921 | 1317.4257 | 45 | 3.96 | 55.6% | 1 | K.KDK@K@S\*RTPPR.S | 2 |

---

|  |  |  |  |  |  |  |  |  |
| --- | --- | --- | --- | --- | --- | --- | --- | --- |
| U | *contaminant\_NRL\_1MCOH* | 1 | 1 | 1.6% | 428 | 46852 | 8.9 | owl|| Immunoglobulin g1 (igg1) (mcg) with a hinge deletion, chain H... |

| Filename XCorr DeltCN Conf% ObsM+H+ CalcM+H+ SpR ZScore Ion% # Sequence  | | | | | | | | | | | | |
| --- | --- | --- | --- | --- | --- | --- | --- | --- | --- | --- | --- | --- |
| \* | Astrin\_NLD\_STLC\_tube2\_021014\_01.05345.05345.1 | 1.9092 | 0.2197 | 98.2% | 835.58 | 835.994 | 280 | 5.029 | 41.7% | 1 | K.DTLMISR.T | 1 |

---

|  |  |  |  |  |  |  |  |  |
| --- | --- | --- | --- | --- | --- | --- | --- | --- |
| U | *gi|224589071|ref|NP\_0* | 1 | 1 | 1.5% | 1282 | 145807 | 6.4 | BMS1-like, ribosome assembly protein [Homo sapiens] |

| Filename XCorr DeltCN Conf% ObsM+H+ CalcM+H+ SpR ZScore Ion% # Sequence  | | | | | | | | | | | | |
| --- | --- | --- | --- | --- | --- | --- | --- | --- | --- | --- | --- | --- |
| \* | Astrin\_NLD\_STLC\_tube2\_021014\_01.16223.16223.2 | 2.3604 | 0.2614 | 97.4% | 2219.7722 | 2217.6213 | 268 | 5.086 | 27.8% | 1 | R.K@ILALLDALSTVHS\*QKMKK.A | 2 |

---

|  |  |  |  |  |  |  |  |  |
| --- | --- | --- | --- | --- | --- | --- | --- | --- |
| U | *gi|82830424|ref|NP\_00* | 1 | 1 | 1.4% | 2240 | 248547 | 5.0 | gon-4-like isoform a [Homo sapiens] |
| U | *gi|82830428|ref|NP\_11* | 1 | 1 | 2.0% | 1529 | 169106 | 5.0 | gon-4-like isoform b [Homo sapiens] |

| Filename XCorr DeltCN Conf% ObsM+H+ CalcM+H+ SpR ZScore Ion% # Sequence  | | | | | | | | | | | | |
| --- | --- | --- | --- | --- | --- | --- | --- | --- | --- | --- | --- | --- |
|  | Astrin\_NLD\_STLC\_tube2\_021014\_01.16995.16995.3 | 3.3482 | 0.2494 | 97.7% | 3316.6443 | 3316.5203 | 230 | 4.44 | 16.7% | 1 | R.DICDDIKVEHAVELDTGAPSEELSSAGEVTK.Q | 3 |

---

|  |  |  |  |  |  |  |  |  |
| --- | --- | --- | --- | --- | --- | --- | --- | --- |
| U | *Reverse\_gi|209862849|* | 1 | 1 | 1.4% | 1474 | 160795 | 7.1 | Nance-Horan syndrome protein isoform 2 [Homo sapiens] |
| U | *Reverse\_gi|38093637|r* | 1 | 1 | 1.3% | 1630 | 176699 | 6.8 | Nance-Horan syndrome protein isoform 1 [Homo sapiens] |

| Filename XCorr DeltCN Conf% ObsM+H+ CalcM+H+ SpR ZScore Ion% # Sequence  | | | | | | | | | | | | |
| --- | --- | --- | --- | --- | --- | --- | --- | --- | --- | --- | --- | --- |
|  | Astrin\_NLD\_STLC\_tube2\_021014\_01.08664.08664.3 | 3.2663 | 0.2194 | 95.6% | 2275.5544 | 2273.2317 | 25 | 3.901 | 27.5% | 1 | K.LSAEDVNGQTEEAKSNDSAS\*K@.A | 3 |

---

|  |  |  |  |  |  |  |  |  |
| --- | --- | --- | --- | --- | --- | --- | --- | --- |
| U | *Reverse\_gi|116256336|* | 1 | 1 | 1.4% | 1106 | 121651 | 6.9 | SEC31 homolog A isoform 4 [Homo sapiens] |
| U | *Reverse\_gi|41349441|r* | 1 | 1 | 1.3% | 1181 | 129036 | 7.4 | SEC31 homolog A isoform 2 [Homo sapiens] |
| U | *Reverse\_gi|41349439|r* | 1 | 1 | 1.2% | 1220 | 133014 | 6.9 | SEC31 homolog A isoform 1 [Homo sapiens] |
| U | *Reverse\_gi|116256340|* | 1 | 1 | 1.2% | 1205 | 131534 | 6.9 | SEC31 homolog A isoform 3 [Homo sapiens] |
| U | *Reverse\_gi|116256338|* | 1 | 1 | 1.2% | 1220 | 133014 | 6.9 | SEC31 homolog A isoform 1 [Homo sapiens] |

| Filename XCorr DeltCN Conf% ObsM+H+ CalcM+H+ SpR ZScore Ion% # Sequence  | | | | | | | | | | | | |
| --- | --- | --- | --- | --- | --- | --- | --- | --- | --- | --- | --- | --- |
|  | Astrin\_NLD\_STLC\_tube2\_021014\_01.09968.09968.2 | 2.4556 | 0.229 | 96.1% | 1636.5122 | 1636.7742 | 1 | 4.302 | 53.6% | 1 | R.LGDTSGGMISYVS\*IR.G | 2 |

---

|  |  |  |  |  |  |  |  |  |
| --- | --- | --- | --- | --- | --- | --- | --- | --- |
| U | *Reverse\_gi|197276600|* | 1 | 1 | 1.4% | 1135 | 119958 | 6.2 | microtubule-associated protein 4 isoform 4 [Homo sapiens] |
| U | *Reverse\_gi|47519675|r* | 1 | 1 | 1.8% | 887 | 92745 | 9.4 | microtubule-associated protein 4 isoform 2 [Homo sapiens] |
| U | *Reverse\_gi|47519639|r* | 1 | 1 | 1.4% | 1152 | 121005 | 5.4 | microtubule-associated protein 4 isoform 1 [Homo sapiens] |
| U | *Reverse\_gi|197276602|* | 1 | 1 | 2.0% | 817 | 85534 | 9.3 | microtubule-associated protein 4 isoform 5 [Homo sapiens] |

| Filename XCorr DeltCN Conf% ObsM+H+ CalcM+H+ SpR ZScore Ion% # Sequence  | | | | | | | | | | | | |
| --- | --- | --- | --- | --- | --- | --- | --- | --- | --- | --- | --- | --- |
|  | Astrin\_NLD\_STLC\_031014\_02.06232.06232.3 | 2.9641 | 0.2671 | 98.4% | 1949.1244 | 1950.9492 | 25 | 4.647 | 33.3% | 1 | R.SLRPT#TSSPKAS\*T#PK@K@.D | 3 |

---

|  |  |  |  |  |  |  |  |  |
| --- | --- | --- | --- | --- | --- | --- | --- | --- |
| U | *gi|7661936|ref|NP\_055* | 1 | 1 | 1.4% | 953 | 107474 | 6.2 | scaffold attachment factor B2 [Homo sapiens] |

| Filename XCorr DeltCN Conf% ObsM+H+ CalcM+H+ SpR ZScore Ion% # Sequence  | | | | | | | | | | | | |
| --- | --- | --- | --- | --- | --- | --- | --- | --- | --- | --- | --- | --- |
| \* | Astrin\_NLD\_STLC\_tube2\_021014\_01.05591.05591.2 | 2.2777 | 0.2597 | 97.2% | 1292.2322 | 1292.4119 | 19 | 5.211 | 62.5% | 1 | R.AWQGAMDAGAASR.E | 2 |

---

|  |  |  |  |  |  |  |  |  |
| --- | --- | --- | --- | --- | --- | --- | --- | --- |
| U | *gi|116256464|ref|NP\_9* | 1 | 1 | 1.4% | 962 | 102157 | 8.0 | hypothetical protein LOC343990 [Homo sapiens] |

| Filename XCorr DeltCN Conf% ObsM+H+ CalcM+H+ SpR ZScore Ion% # Sequence  | | | | | | | | | | | | |
| --- | --- | --- | --- | --- | --- | --- | --- | --- | --- | --- | --- | --- |
| \* | Astrin\_NLD\_STLC\_tube2\_021014\_01.06110.06110.2 | 2.1861 | 0.3002 | 98.1% | 1343.8522 | 1344.2885 | 17 | 4.461 | 45.8% | 1 | R.EAAEGLGEDST#GK.K | 2 |

---

|  |  |  |  |  |  |  |  |  |
| --- | --- | --- | --- | --- | --- | --- | --- | --- |
| U | *gi|50659095|ref|NP\_00* | 1 | 1 | 1.4% | 783 | 87344 | 9.3 | DEAD (Asp-Glu-Ala-Asp) box polypeptide 21 [Homo sapiens] |

| Filename XCorr DeltCN Conf% ObsM+H+ CalcM+H+ SpR ZScore Ion% # Sequence  | | | | | | | | | | | | |
| --- | --- | --- | --- | --- | --- | --- | --- | --- | --- | --- | --- | --- |
| \* | Astrin\_NLD\_STLC\_tube2\_021014\_01.08240.08240.2 | 2.2919 | 0.3578 | 99.8% | 1165.2522 | 1165.4215 | 2 | 5.647 | 65.0% | 1 | R.APQVLVLAPTR.E | 2 |

---

|  |  |  |  |  |  |  |  |  |
| --- | --- | --- | --- | --- | --- | --- | --- | --- |
| U | *Reverse\_gi|194239662|* | 1 | 1 | 1.4% | 583 | 62822 | 6.4 | SHC (Src homology 2 domain containing) transforming protein 1 isoform 1 [Homo sapiens] |
| U | *Reverse\_gi|32261324|r* | 1 | 1 | 1.7% | 474 | 51682 | 7.2 | SHC (Src homology 2 domain containing) transforming protein 1 isoform 2 [Homo sapiens] |
| U | *Reverse\_gi|194239668|* | 1 | 1 | 1.7% | 473 | 51611 | 7.2 | SHC (Src homology 2 domain containing) transforming protein 1 isoform 4 [Homo sapiens] |
| U | *Reverse\_gi|194239664|* | 1 | 1 | 1.4% | 584 | 62893 | 6.4 | SHC (Src homology 2 domain containing) transforming protein 1 isoform 3 [Homo sapiens] |

| Filename XCorr DeltCN Conf% ObsM+H+ CalcM+H+ SpR ZScore Ion% # Sequence  | | | | | | | | | | | | |
| --- | --- | --- | --- | --- | --- | --- | --- | --- | --- | --- | --- | --- |
|  | Astrin\_NLD\_STLC\_tube2\_021014\_01.05924.05924.2 | 1.9777 | 0.279 | 97.7% | 842.9922 | 843.01404 | 11 | 5.254 | 78.6% | 1 | R.GLISSLPR.S | 2 |

---

|  |  |  |  |  |  |  |  |  |
| --- | --- | --- | --- | --- | --- | --- | --- | --- |
| U | *gi|41322908|ref|NP\_95* | 4 | 4 | 1.3% | 4525 | 513712 | 5.8 | plectin 1 isoform 3 [Homo sapiens] |
| U | *gi|47607492|ref|NP\_00* | 4 | 4 | 1.3% | 4574 | 518478 | 5.7 | plectin 1 isoform 1 [Homo sapiens] |
| U | *gi|41322923|ref|NP\_95* | 4 | 4 | 1.3% | 4547 | 516204 | 5.8 | plectin 1 isoform 11 [Homo sapiens] |
| U | *gi|41322919|ref|NP\_95* | 4 | 4 | 1.3% | 4547 | 516282 | 5.8 | plectin 1 isoform 8 [Homo sapiens] |
| U | *gi|41322916|ref|NP\_95* | 4 | 4 | 1.3% | 4684 | 531796 | 6.0 | plectin 1 isoform 6 [Homo sapiens] |
| U | *gi|41322914|ref|NP\_95* | 4 | 4 | 1.3% | 4551 | 516484 | 5.8 | plectin 1 isoform 10 [Homo sapiens] |
| U | *gi|41322912|ref|NP\_95* | 4 | 4 | 1.3% | 4533 | 514780 | 5.7 | plectin 1 isoform 2 [Homo sapiens] |
| U | *gi|41322910|ref|NP\_95* | 4 | 4 | 1.3% | 4515 | 512609 | 5.8 | plectin 1 isoform 7 [Homo sapiens] |

| Filename XCorr DeltCN Conf% ObsM+H+ CalcM+H+ SpR ZScore Ion% # Sequence  | | | | | | | | | | | | |
| --- | --- | --- | --- | --- | --- | --- | --- | --- | --- | --- | --- | --- |
|  | Astrin\_NLD\_STLC\_tube2\_021014\_01.06676.06676.3 | 3.2986 | 0.1948 | 97.4% | 1691.5144 | 1692.9156 | 42 | 3.638 | 41.7% | 1 | R.LREQLQLLEEQHR.A | 3 |
|  | Astrin\_NLD\_STLC\_031014\_02.04655.04655.3 | 2.7961 | 0.3074 | 99.6% | 1783.9443 | 1784.964 | 29 | 5.607 | 30.9% | 1 | R.AALAHSEEVTASQVAATK.T | 3 |
|  | Astrin\_NLD\_STLC\_tube2\_021014\_02.06261.06261.3 | 3.6221 | 0.1891 | 96.4% | 2015.2144 | 2014.292 | 1 | 5.13 | 36.1% | 1 | R.LLEAQIATGGVIDPVHSHR.V | 3 |
|  | Astrin\_NLD\_STLC\_tube2\_021014\_01.07235.07235.2 | 1.8893 | 0.3735 | 99.6% | 1161.9521 | 1161.2311 | 1 | 6.388 | 75.0% | 1 | R.GYFDEEMNR.V | 2 |

---

|  |  |  |  |  |  |  |  |  |
| --- | --- | --- | --- | --- | --- | --- | --- | --- |
| U | *Reverse\_gi|7656967|re* | 1 | 1 | 1.3% | 3014 | 329486 | 5.9 | cadherin EGF LAG seven-pass G-type receptor 1 [Homo sapiens] |

| Filename XCorr DeltCN Conf% ObsM+H+ CalcM+H+ SpR ZScore Ion% # Sequence  | | | | | | | | | | | | |
| --- | --- | --- | --- | --- | --- | --- | --- | --- | --- | --- | --- | --- |
| \* | Astrin\_NLD\_STLC\_tube2\_021014\_01.12551.12551.3 | 3.6864 | 0.209 | 96.7% | 4763.9346 | 4765.9536 | 129 | 4.515 | 15.1% | 1 | R.ERYES\*QEFVPS\*HDNTDK@VLVTIYTTAS\*RPPTSYDVAK@VR.L | 3 |

---

|  |  |  |  |  |  |  |  |  |
| --- | --- | --- | --- | --- | --- | --- | --- | --- |
| U | *gi|148491080|ref|NP\_0* | 1 | 1 | 1.3% | 1723 | 182814 | 7.0 | trinucleotide repeat containing 6B isoform 1 [Homo sapiens] |
| U | *gi|67782330|ref|NP\_00* | 1 | 1 | 2.2% | 1029 | 109345 | 7.3 | trinucleotide repeat containing 6B isoform 2 [Homo sapiens] |

| Filename XCorr DeltCN Conf% ObsM+H+ CalcM+H+ SpR ZScore Ion% # Sequence  | | | | | | | | | | | | |
| --- | --- | --- | --- | --- | --- | --- | --- | --- | --- | --- | --- | --- |
|  | Astrin\_NLD\_STLC\_031014\_01.12186.12186.3 | 3.2645 | 0.2231 | 95.4% | 2801.3643 | 2799.8865 | 4 | 4.589 | 28.4% | 1 | K.FPDYKS\*TWSPDPIGHNPTHLS\*NK.M | 3 |

---

|  |  |  |  |  |  |  |  |  |
| --- | --- | --- | --- | --- | --- | --- | --- | --- |
| U | *gi|217272892|ref|NP\_0* | 1 | 1 | 1.3% | 972 | 109436 | 5.0 | elongation factor Tu GTP binding domain containing 2 isoform a [Homo sapiens] |
| U | *gi|217272894|ref|NP\_0* | 1 | 1 | 1.4% | 937 | 105384 | 5.2 | elongation factor Tu GTP binding domain containing 2 isoform b [Homo sapiens] |

| Filename XCorr DeltCN Conf% ObsM+H+ CalcM+H+ SpR ZScore Ion% # Sequence  | | | | | | | | | | | | |
| --- | --- | --- | --- | --- | --- | --- | --- | --- | --- | --- | --- | --- |
|  | Astrin\_NLD\_STLC\_tube2\_021014\_01.04319.04319.2 | 2.2886 | 0.2979 | 98.7% | 1307.3121 | 1307.5356 | 14 | 6.405 | 54.2% | 1 | R.VLSGTIHAGQPVK.V | 2 |

---

|  |  |  |  |  |  |  |  |  |
| --- | --- | --- | --- | --- | --- | --- | --- | --- |
| U | *gi|10835220|ref|NP\_00* | 1 | 1 | 1.3% | 994 | 109283 | 5.2 | ATPase, Ca++ transporting, fast twitch 1 isoform b [Homo sapiens] |
| U | *gi|4502285|ref|NP\_001* | 1 | 1 | 1.3% | 997 | 109691 | 5.4 | ATPase, Ca++ transporting, slow twitch 2 isoform 2 [Homo sapiens] |
| U | *gi|27886529|ref|NP\_77* | 1 | 1 | 1.3% | 1001 | 110252 | 5.2 | ATPase, Ca++ transporting, fast twitch 1 isoform a [Homo sapiens] |
| U | *gi|24638454|ref|NP\_73* | 1 | 1 | 1.2% | 1042 | 114757 | 5.3 | ATPase, Ca++ transporting, slow twitch 2 isoform 1 [Homo sapiens] |
| U | *gi|209413709|ref|NP\_0* | 1 | 1 | 1.3% | 1015 | 111847 | 5.3 | ATPase, Ca++ transporting, slow twitch 2 isoform 3 [Homo sapiens] |

| Filename XCorr DeltCN Conf% ObsM+H+ CalcM+H+ SpR ZScore Ion% # Sequence  | | | | | | | | | | | | |
| --- | --- | --- | --- | --- | --- | --- | --- | --- | --- | --- | --- | --- |
|  | Astrin\_NLD\_STLC\_tube2\_021014\_02.06828.06828.2 | 2.5069 | 0.225 | 97.6% | 1325.5922 | 1324.5377 | 1 | 3.975 | 62.5% | 1 | K.NMLFSGTNIAAGK.A | 2 |

---

|  |  |  |  |  |  |  |  |  |
| --- | --- | --- | --- | --- | --- | --- | --- | --- |
| U | *gi|21264343|ref|NP\_00* | 1 | 2 | 1.3% | 915 | 102642 | 5.5 | scaffold attachment factor B [Homo sapiens] |

| Filename XCorr DeltCN Conf% ObsM+H+ CalcM+H+ SpR ZScore Ion% # Sequence  | | | | | | | | | | | | |
| --- | --- | --- | --- | --- | --- | --- | --- | --- | --- | --- | --- | --- |
| \* | Astrin\_NLD\_STLC\_tube2\_021014\_01.10218.10218.2 | 2.8621 | 0.3878 | 100.0% | 1355.5322 | 1355.4929 | 1 | 6.495 | 63.6% | 2 | R.NFWVSGLSSTTR.A | 2 |

---

|  |  |  |  |  |  |  |  |  |
| --- | --- | --- | --- | --- | --- | --- | --- | --- |
| U | *gi|28373065|ref|NP\_06* | 1 | 1 | 1.3% | 932 | 102179 | 9.5 | potassium voltage-gated channel, KQT-like subfamily, member 5 [Homo sapiens] |

| Filename XCorr DeltCN Conf% ObsM+H+ CalcM+H+ SpR ZScore Ion% # Sequence  | | | | | | | | | | | | |
| --- | --- | --- | --- | --- | --- | --- | --- | --- | --- | --- | --- | --- |
| \* | Astrin\_NLD\_STLC\_031014\_01.07124.07124.2 | 2.0717 | 0.2715 | 95.7% | 994.09216 | 996.96936 | 50 | 4.926 | 54.5% | 1 | K.S\*GAAAAAAGGGR.L | 2 |

---

|  |  |  |  |  |  |  |  |  |
| --- | --- | --- | --- | --- | --- | --- | --- | --- |
| U | *gi|117320527|ref|NP\_0* | 1 | 1 | 1.3% | 899 | 96678 | 6.2 | nuclear factor of kappa light polypeptide gene enhancer in B-cells 2 isoform b [Homo sapiens] |
| U | *gi|117320540|ref|NP\_0* | 1 | 1 | 1.3% | 899 | 96678 | 6.2 | nuclear factor of kappa light polypeptide gene enhancer in B-cells 2 isoform b [Homo sapiens] |
| U | *gi|117320531|ref|NP\_0* | 1 | 1 | 1.3% | 900 | 96749 | 6.2 | nuclear factor of kappa light polypeptide gene enhancer in B-cells 2 isoform a [Homo sapiens] |

| Filename XCorr DeltCN Conf% ObsM+H+ CalcM+H+ SpR ZScore Ion% # Sequence  | | | | | | | | | | | | |
| --- | --- | --- | --- | --- | --- | --- | --- | --- | --- | --- | --- | --- |
|  | Astrin\_NLD\_STLC\_tube2\_021014\_02.06412.06412.2 | 2.6486 | 0.2237 | 98.7% | 1265.0322 | 1265.4087 | 18 | 5.059 | 59.1% | 1 | R.ALLDYGVTADAR.A | 2 |

---

|  |  |  |  |  |  |  |  |  |
| --- | --- | --- | --- | --- | --- | --- | --- | --- |
| U | *gi|21264592|ref|NP\_05* | 1 | 1 | 1.2% | 1704 | 193451 | 7.4 | ArfGAP with RhoGAP domain, ankyrin repeat and PH domain 2 [Homo sapiens] |

| Filename XCorr DeltCN Conf% ObsM+H+ CalcM+H+ SpR ZScore Ion% # Sequence  | | | | | | | | | | | | |
| --- | --- | --- | --- | --- | --- | --- | --- | --- | --- | --- | --- | --- |
| \* | Astrin\_NLD\_STLC\_tube2\_021014\_01.14519.14519.3 | 3.1681 | 0.2232 | 95.3% | 2654.8442 | 2655.5059 | 84 | 3.644 | 28.9% | 1 | R.K@T#EDRNS\*K@AT#LDS\*DHK@LPSR.V | 3 |

---

|  |  |  |  |  |  |  |  |  |
| --- | --- | --- | --- | --- | --- | --- | --- | --- |
| U | *gi|222537754|ref|NP\_0* | 1 | 1 | 1.2% | 1692 | 190926 | 7.9 | hypothetical protein LOC375337 [Homo sapiens] |

| Filename XCorr DeltCN Conf% ObsM+H+ CalcM+H+ SpR ZScore Ion% # Sequence  | | | | | | | | | | | | |
| --- | --- | --- | --- | --- | --- | --- | --- | --- | --- | --- | --- | --- |
| \* | Astrin\_NLD\_STLC\_tube2\_021014\_01.19629.19629.2 | 2.3604 | 0.3075 | 98.9% | 2374.6921 | 2372.468 | 33 | 4.808 | 28.9% | 1 | K.RKVT#EAS\*SDDPQPGLDLVRK.E | 2 |

---

|  |  |  |  |  |  |  |  |  |
| --- | --- | --- | --- | --- | --- | --- | --- | --- |
| U | *Reverse\_gi|153945755|* | 1 | 1 | 1.2% | 1512 | 162208 | 8.8 | zinc finger protein 608 [Homo sapiens] |

| Filename XCorr DeltCN Conf% ObsM+H+ CalcM+H+ SpR ZScore Ion% # Sequence  | | | | | | | | | | | | |
| --- | --- | --- | --- | --- | --- | --- | --- | --- | --- | --- | --- | --- |
| \* | Astrin\_NLD\_STLC\_tube2\_021014\_01.11751.11751.2 | 3.6228 | 0.1351 | 98.7% | 1791.4122 | 1792.0392 | 16 | 4.401 | 47.1% | 1 | K.GTSSIEPIGYLAASPLSK.N | 2 |

---

|  |  |  |  |  |  |  |  |  |
| --- | --- | --- | --- | --- | --- | --- | --- | --- |
| U | *gi|4885399|ref|NP\_005* | 1 | 1 | 1.2% | 1217 | 141541 | 7.2 | structural maintenance of chromosomes 3 [Homo sapiens] |

| Filename XCorr DeltCN Conf% ObsM+H+ CalcM+H+ SpR ZScore Ion% # Sequence  | | | | | | | | | | | | |
| --- | --- | --- | --- | --- | --- | --- | --- | --- | --- | --- | --- | --- |
| \* | Astrin\_NLD\_STLC\_tube2\_021014\_02.06221.06221.3 | 3.2022 | 0.432 | 100.0% | 1653.6843 | 1653.8291 | 7 | 6.205 | 34.6% | 1 | R.LFYHIVDSDEVSTK.I | 3 |

---

|  |  |  |  |  |  |  |  |  |
| --- | --- | --- | --- | --- | --- | --- | --- | --- |
| U | *Reverse\_gi|59814247|r* | 1 | 1 | 1.2% | 1050 | 119517 | 5.3 | budding uninhibited by benzimidazoles 1 beta [Homo sapiens] |

| Filename XCorr DeltCN Conf% ObsM+H+ CalcM+H+ SpR ZScore Ion% # Sequence  | | | | | | | | | | | | |
| --- | --- | --- | --- | --- | --- | --- | --- | --- | --- | --- | --- | --- |
| \* | Astrin\_NLD\_STLC\_031014\_02.05200.05200.3 | 2.4249 | 0.3137 | 98.6% | 1503.0543 | 1502.7354 | 7 | 4.118 | 39.6% | 1 | R.IIPARATK@KGK@S\*K.L | 3 |

---

|  |  |  |  |  |  |  |  |  |
| --- | --- | --- | --- | --- | --- | --- | --- | --- |
| U | *Reverse\_gi|4507281|re* | 1 | 1 | 1.2% | 1030 | 115538 | 9.5 | cyclin-dependent kinase-like 5 [Homo sapiens] |
| U | *Reverse\_gi|83367069|r* | 1 | 1 | 1.2% | 1030 | 115538 | 9.5 | cyclin-dependent kinase-like 5 [Homo sapiens] |

| Filename XCorr DeltCN Conf% ObsM+H+ CalcM+H+ SpR ZScore Ion% # Sequence  | | | | | | | | | | | | |
| --- | --- | --- | --- | --- | --- | --- | --- | --- | --- | --- | --- | --- |
|  | Astrin\_NLD\_STLC\_tube2\_021014\_01.11984.11984.2 | 2.5472 | 0.2055 | 97.3% | 1617.3922 | 1618.829 | 376 | 4.457 | 50.0% | 1 | K.KK@MS\*RFFGQK@EK.E | 2 |

---

|  |  |  |  |  |  |  |  |  |
| --- | --- | --- | --- | --- | --- | --- | --- | --- |
| U | *gi|4504141|ref|NP\_000* | 1 | 1 | 1.2% | 912 | 101868 | 8.8 | glutamate receptor, metabotropic 4 [Homo sapiens] |

| Filename XCorr DeltCN Conf% ObsM+H+ CalcM+H+ SpR ZScore Ion% # Sequence  | | | | | | | | | | | | |
| --- | --- | --- | --- | --- | --- | --- | --- | --- | --- | --- | --- | --- |
| \* | Astrin\_NLD\_STLC\_tube2\_021014\_01.06386.06386.3 | 1.7889 | 0.3775 | 97.5% | 1264.0144 | 1265.2836 | 77 | 5.41 | 30.0% | 1 | K.AVVT#AATMS\*NK@.F | 3 |

---

|  |  |  |  |  |  |  |  |  |
| --- | --- | --- | --- | --- | --- | --- | --- | --- |
| U | *gi|215598688|ref|NP\_0* | 1 | 1 | 1.2% | 863 | 97327 | 5.5 | ubiquitin specific protease 13 [Homo sapiens] |

| Filename XCorr DeltCN Conf% ObsM+H+ CalcM+H+ SpR ZScore Ion% # Sequence  | | | | | | | | | | | | |
| --- | --- | --- | --- | --- | --- | --- | --- | --- | --- | --- | --- | --- |
| \* | Astrin\_NLD\_STLC\_tube2\_021014\_01.04347.04347.1 | 1.8408 | 0.2368 | 98.5% | 1178.37 | 1179.4513 | 38 | 4.418 | 50.0% | 1 | R.RPLPELVRAK.I | 1 |

---

|  |  |  |  |  |  |  |  |  |
| --- | --- | --- | --- | --- | --- | --- | --- | --- |
| U | *gi|57863301|ref|NP\_05* | 1 | 1 | 1.1% | 1506 | 164886 | 8.2 | CLIP-associating protein 2 [Homo sapiens] |

| Filename XCorr DeltCN Conf% ObsM+H+ CalcM+H+ SpR ZScore Ion% # Sequence  | | | | | | | | | | | | |
| --- | --- | --- | --- | --- | --- | --- | --- | --- | --- | --- | --- | --- |
| \* | Astrin\_NLD\_STLC\_031014\_01.05374.05374.2 | 3.6735 | 0.3765 | 100.0% | 1551.8322 | 1551.5638 | 1 | 7.17 | 56.7% | 1 | R.AGGDATDSSQTALDNK.A | 2 |

---

|  |  |  |  |  |  |  |  |  |
| --- | --- | --- | --- | --- | --- | --- | --- | --- |
| U | *gi|116686122|ref|NP\_0* | 1 | 2 | 1.1% | 1232 | 139881 | 6.3 | kinesin family member 4 [Homo sapiens] |
| U | *gi|150010604|ref|NP\_0* | 1 | 2 | 1.1% | 1234 | 140035 | 6.2 | kinesin family member 4B [Homo sapiens] |

| Filename XCorr DeltCN Conf% ObsM+H+ CalcM+H+ SpR ZScore Ion% # Sequence  | | | | | | | | | | | | |
| --- | --- | --- | --- | --- | --- | --- | --- | --- | --- | --- | --- | --- |
|  | Astrin\_NLD\_STLC\_031014\_01.05622.05622.2 | 2.489 | 0.3327 | 99.8% | 1341.1721 | 1340.4509 | 1 | 5.816 | 58.3% | 2 | R.TVASTAMNSQSSR.S | 2 |

---

|  |  |  |  |  |  |  |  |  |
| --- | --- | --- | --- | --- | --- | --- | --- | --- |
| U | *gi|197276600|ref|NP\_0* | 1 | 1 | 1.1% | 1135 | 119958 | 6.2 | microtubule-associated protein 4 isoform 4 [Homo sapiens] |
| U | *gi|47519639|ref|NP\_00* | 1 | 1 | 1.1% | 1152 | 121005 | 5.4 | microtubule-associated protein 4 isoform 1 [Homo sapiens] |

| Filename XCorr DeltCN Conf% ObsM+H+ CalcM+H+ SpR ZScore Ion% # Sequence  | | | | | | | | | | | | |
| --- | --- | --- | --- | --- | --- | --- | --- | --- | --- | --- | --- | --- |
|  | Astrin\_NLD\_STLC\_tube2\_021014\_02.05956.05956.2 | 2.7745 | 0.3156 | 100.0% | 1347.1921 | 1346.5236 | 2 | 5.375 | 66.7% | 1 | K.VALSSETEVALAR.D | 2 |

---

|  |  |  |  |  |  |  |  |  |
| --- | --- | --- | --- | --- | --- | --- | --- | --- |
| U | *Reverse\_gi|24476013|r* | 1 | 1 | 1.1% | 1052 | 119233 | 6.6 | PTK2 protein tyrosine kinase 2 isoform a [Homo sapiens] |

| Filename XCorr DeltCN Conf% ObsM+H+ CalcM+H+ SpR ZScore Ion% # Sequence  | | | | | | | | | | | | |
| --- | --- | --- | --- | --- | --- | --- | --- | --- | --- | --- | --- | --- |
| \* | Astrin\_NLD\_STLC\_tube2\_021014\_01.11916.11916.2 | 2.399 | 0.2072 | 95.5% | 1342.0322 | 1343.5259 | 43 | 4.148 | 54.5% | 1 | R.DISGRSLRVDPK.L | 2 |

---

|  |  |  |  |  |  |  |  |  |
| --- | --- | --- | --- | --- | --- | --- | --- | --- |
| U | *gi|4507877|ref|NP\_003* | 1 | 1 | 1.1% | 1066 | 116722 | 6.1 | vinculin isoform VCL [Homo sapiens] |
| U | *gi|7669550|ref|NP\_054* | 1 | 1 | 1.1% | 1134 | 123799 | 5.7 | vinculin isoform meta-VCL [Homo sapiens] |

| Filename XCorr DeltCN Conf% ObsM+H+ CalcM+H+ SpR ZScore Ion% # Sequence  | | | | | | | | | | | | |
| --- | --- | --- | --- | --- | --- | --- | --- | --- | --- | --- | --- | --- |
|  | Astrin\_NLD\_STLC\_tube2\_021014\_02.06279.06279.2 | 2.371 | 0.2847 | 98.9% | 1293.3121 | 1293.4749 | 1 | 5.44 | 68.2% | 1 | K.MTGLVDEAIDTK.S | 2 |

---

|  |  |  |  |  |  |  |  |  |
| --- | --- | --- | --- | --- | --- | --- | --- | --- |
| U | *gi|22538461|ref|NP\_00* | 1 | 1 | 1.0% | 2440 | 270207 | 7.1 | nuclear receptor co-repressor 1 [Homo sapiens] |

| Filename XCorr DeltCN Conf% ObsM+H+ CalcM+H+ SpR ZScore Ion% # Sequence  | | | | | | | | | | | | |
| --- | --- | --- | --- | --- | --- | --- | --- | --- | --- | --- | --- | --- |
| \* | Astrin\_NLD\_STLC\_tube2\_021014\_01.15989.15989.3 | 2.6648 | 0.3351 | 99.4% | 2736.0244 | 2738.9578 | 1 | 5.15 | 26.1% | 1 | R.K@T#PEVVQS\*TRPIIEGSISQGTPIK.F | 3 |

---

|  |  |  |  |  |  |  |  |  |
| --- | --- | --- | --- | --- | --- | --- | --- | --- |
| U | *gi|169163292|ref|XP\_0* | 1 | 1 | 1.0% | 1778 | 199747 | 7.8 | PREDICTED: protein immuno-reactive with anti-PTH polyclonal antibodies [Homo sapiens] |
| U | *gi|169163816|ref|XP\_0* | 1 | 1 | 0.9% | 1938 | 215129 | 8.5 | PREDICTED: protein immuno-reactive with anti-PTH polyclonal antibodies [Homo sapiens] |

| Filename XCorr DeltCN Conf% ObsM+H+ CalcM+H+ SpR ZScore Ion% # Sequence  | | | | | | | | | | | | |
| --- | --- | --- | --- | --- | --- | --- | --- | --- | --- | --- | --- | --- |
|  | Astrin\_NLD\_STLC\_tube2\_021014\_02.06351.06351.3 | 2.9254 | 0.2361 | 96.1% | 2111.0942 | 2110.1367 | 19 | 4.14 | 31.2% | 1 | R.K@K@HEELSINS\*NPVSS\*QK.Q | 3 |

---

|  |  |  |  |  |  |  |  |  |
| --- | --- | --- | --- | --- | --- | --- | --- | --- |
| U | *Reverse\_gi|33620716|r* | 1 | 13 | 1.0% | 1758 | 197286 | 9.6 | retinoblastoma-binding protein 6 isoform 2 [Homo sapiens] |
| U | *Reverse\_gi|33620769|r* | 1 | 13 | 0.9% | 1792 | 201563 | 9.6 | retinoblastoma-binding protein 6 isoform 1 [Homo sapiens] |

| Filename XCorr DeltCN Conf% ObsM+H+ CalcM+H+ SpR ZScore Ion% # Sequence  | | | | | | | | | | | | |
| --- | --- | --- | --- | --- | --- | --- | --- | --- | --- | --- | --- | --- |
|  | Astrin\_NLD\_STLC\_tube2\_021014\_01.11691.11691.2 | 3.6883 | 0.0817 | 96.8% | 2213.612 | 2213.2517 | 21 | 4.209 | 40.6% | 13 | K.EEKT#T#LKESKVDK@SYDK@.K | 2 |

---

|  |  |  |  |  |  |  |  |  |
| --- | --- | --- | --- | --- | --- | --- | --- | --- |
| U | *Reverse\_gi|33620745|r* | 2 | 2 | 1.0% | 1555 | 173049 | 8.5 | pre-mRNA cleavage complex II protein Pcf11 [Homo sapiens] |

| Filename XCorr DeltCN Conf% ObsM+H+ CalcM+H+ SpR ZScore Ion% # Sequence  | | | | | | | | | | | | |
| --- | --- | --- | --- | --- | --- | --- | --- | --- | --- | --- | --- | --- |
| \* | Astrin\_NLD\_STLC\_031014\_01.08286.08286.1 | 1.5233 | 0.2568 | 97.0% | 813.48 | 812.8149 | 12 | 3.99 | 66.7% | 1 | R.T#SSRGPK.T | 1 |
| \* | Astrin\_NLD\_STLC\_tube2\_021014\_01.07088.07088.2 | 2.2517 | 0.1917 | 96.2% | 971.2322 | 971.0556 | 20 | 4.295 | 71.4% | 1 | K.KTIKEGS\*K.S | 2 |

---

|  |  |  |  |  |  |  |  |  |
| --- | --- | --- | --- | --- | --- | --- | --- | --- |
| U | *Reverse\_gi|41872673|r* | 1 | 1 | 1.0% | 1258 | 142828 | 5.9 | NIMA-related kinase 1 [Homo sapiens] |

| Filename XCorr DeltCN Conf% ObsM+H+ CalcM+H+ SpR ZScore Ion% # Sequence  | | | | | | | | | | | | |
| --- | --- | --- | --- | --- | --- | --- | --- | --- | --- | --- | --- | --- |
| \* | Astrin\_NLD\_STLC\_031014\_01.07335.07335.3 | 2.5765 | 0.2606 | 96.4% | 1643.9343 | 1645.8618 | 6 | 4.361 | 37.5% | 1 | K.AKIQQRENFNQLR.I | 3 |

---

|  |  |  |  |  |  |  |  |  |
| --- | --- | --- | --- | --- | --- | --- | --- | --- |
| U | *gi|70166852|ref|NP\_00* | 1 | 2 | 1.0% | 1226 | 135967 | 8.6 | adenosine deaminase, RNA-specific isoform a [Homo sapiens] |
| U | *gi|70167113|ref|NP\_00* | 1 | 2 | 1.3% | 931 | 103642 | 8.7 | adenosine deaminase, RNA-specific isoform d [Homo sapiens] |
| U | *gi|70167032|ref|NP\_05* | 1 | 2 | 1.0% | 1181 | 131070 | 8.6 | adenosine deaminase, RNA-specific isoform c [Homo sapiens] |
| U | *gi|70166944|ref|NP\_05* | 1 | 2 | 1.0% | 1200 | 133175 | 8.5 | adenosine deaminase, RNA-specific isoform b [Homo sapiens] |

| Filename XCorr DeltCN Conf% ObsM+H+ CalcM+H+ SpR ZScore Ion% # Sequence  | | | | | | | | | | | | |
| --- | --- | --- | --- | --- | --- | --- | --- | --- | --- | --- | --- | --- |
|  | Astrin\_NLD\_STLC\_031014\_01.06107.06107.2 | 2.4475 | 0.2546 | 98.6% | 1304.1721 | 1304.4453 | 3 | 5.112 | 54.5% | 2 | K.LVDQSGPPHEPK.F | 2 |

---

|  |  |  |  |  |  |  |  |  |
| --- | --- | --- | --- | --- | --- | --- | --- | --- |
| U | *gi|116805322|ref|NP\_0* | 1 | 1 | 0.9% | 2725 | 291020 | 6.0 | gamma filamin isoform a [Homo sapiens] |
| U | *gi|89027550|ref|XP\_49* | 1 | 1 | 8.2% | 306 | 32808 | 8.1 | PREDICTED: hypothetical protein [Homo sapiens] |
| U | *gi|188595687|ref|NP\_0* | 1 | 1 | 0.9% | 2692 | 287279 | 6.0 | gamma filamin isoform b [Homo sapiens] |

| Filename XCorr DeltCN Conf% ObsM+H+ CalcM+H+ SpR ZScore Ion% # Sequence  | | | | | | | | | | | | |
| --- | --- | --- | --- | --- | --- | --- | --- | --- | --- | --- | --- | --- |
|  | Astrin\_NLD\_STLC\_031014\_02.10430.10430.3 | 2.2276 | 0.3476 | 97.1% | 2766.5942 | 2768.8247 | 155 | 4.785 | 20.8% | 1 | K.SSSSRGSS\*YSS\*IPKFSSDASKVVTR.G | 3 |

---

|  |  |  |  |  |  |  |  |  |
| --- | --- | --- | --- | --- | --- | --- | --- | --- |
| U | *gi|41872631|ref|NP\_00* | 1 | 1 | 0.9% | 2511 | 273424 | 6.4 | fatty acid synthase [Homo sapiens] |

| Filename XCorr DeltCN Conf% ObsM+H+ CalcM+H+ SpR ZScore Ion% # Sequence  | | | | | | | | | | | | |
| --- | --- | --- | --- | --- | --- | --- | --- | --- | --- | --- | --- | --- |
| \* | Astrin\_NLD\_STLC\_tube2\_021014\_01.16948.16948.3 | 2.6457 | 0.2752 | 95.4% | 2423.9343 | 2423.769 | 3 | 4.338 | 28.4% | 1 | R.TLLEGSGLESIISIIHSSLAEPR.V | 3 |

---

|  |  |  |  |  |  |  |  |  |
| --- | --- | --- | --- | --- | --- | --- | --- | --- |
| U | *gi|218083800|ref|NP\_0* | 1 | 1 | 0.9% | 2087 | 230527 | 6.7 | Rho GTPase-activating protein isoform 1 [Homo sapiens] |
| U | *gi|29469071|ref|NP\_05* | 1 | 1 | 1.1% | 1738 | 190969 | 6.9 | Rho GTPase-activating protein isoform 2 [Homo sapiens] |

| Filename XCorr DeltCN Conf% ObsM+H+ CalcM+H+ SpR ZScore Ion% # Sequence  | | | | | | | | | | | | |
| --- | --- | --- | --- | --- | --- | --- | --- | --- | --- | --- | --- | --- |
|  | Astrin\_NLD\_STLC\_tube2\_021014\_01.16181.16181.2 | 2.7582 | 0.19 | 96.8% | 2213.672 | 2214.0776 | 284 | 4.542 | 27.8% | 1 | R.S\*AKS\*EESLTSLHAVDGDS\*K@.L | 2 |

---

|  |  |  |  |  |  |  |  |  |
| --- | --- | --- | --- | --- | --- | --- | --- | --- |
| U | *gi|94538366|ref|NP\_00* | 1 | 2 | 0.9% | 1603 | 182178 | 6.8 | transient receptor potential cation channel, subfamily M, member 1 [Homo sapiens] |

| Filename XCorr DeltCN Conf% ObsM+H+ CalcM+H+ SpR ZScore Ion% # Sequence  | | | | | | | | | | | | |
| --- | --- | --- | --- | --- | --- | --- | --- | --- | --- | --- | --- | --- |
| \* | Astrin\_NLD\_STLC\_031014\_02.09693.09693.3 | 3.97 | 0.1404 | 96.4% | 2024.5144 | 2024.1295 | 4 | 4.385 | 42.9% | 2 | R.EQLLVT#IQK@T#FNYNK@.A | 3 |

---

|  |  |  |  |  |  |  |  |  |
| --- | --- | --- | --- | --- | --- | --- | --- | --- |
| U | *Reverse\_gi|194440660|* | 1 | 1 | 0.9% | 1522 | 170678 | 7.0 | topoisomerase (DNA) II binding protein 1 [Homo sapiens] |

| Filename XCorr DeltCN Conf% ObsM+H+ CalcM+H+ SpR ZScore Ion% # Sequence  | | | | | | | | | | | | |
| --- | --- | --- | --- | --- | --- | --- | --- | --- | --- | --- | --- | --- |
| \* | Astrin\_NLD\_STLC\_tube2\_021014\_01.05027.05027.3 | 2.5348 | 0.2766 | 97.0% | 1589.3344 | 1587.3905 | 12 | 4.458 | 38.5% | 1 | R.TSDPTSS\*ASNCGS\*R.S | 3 |

---

|  |  |  |  |  |  |  |  |  |
| --- | --- | --- | --- | --- | --- | --- | --- | --- |
| U | *Reverse\_gi|151301137|* | 1 | 1 | 0.9% | 1439 | 155139 | 6.3 | AT-hook transcription factor [Homo sapiens] |

| Filename XCorr DeltCN Conf% ObsM+H+ CalcM+H+ SpR ZScore Ion% # Sequence  | | | | | | | | | | | | |
| --- | --- | --- | --- | --- | --- | --- | --- | --- | --- | --- | --- | --- |
| \* | Astrin\_NLD\_STLC\_031014\_01.09504.09504.3 | 2.5725 | 0.2699 | 97.1% | 1457.2743 | 1456.5125 | 45 | 4.856 | 37.5% | 1 | R.TT#SRVSEAAQVAR.S | 3 |

---

|  |  |  |  |  |  |  |  |  |
| --- | --- | --- | --- | --- | --- | --- | --- | --- |
| U | *Reverse\_gi|110347418|* | 1 | 6 | 0.9% | 1197 | 135656 | 8.4 | structural maintenance of chromosomes 2 [Homo sapiens] |
| U | *Reverse\_gi|110347425|* | 1 | 6 | 0.9% | 1197 | 135656 | 8.4 | structural maintenance of chromosomes 2 [Homo sapiens] |
| U | *Reverse\_gi|110347420|* | 1 | 6 | 0.9% | 1197 | 135656 | 8.4 | structural maintenance of chromosomes 2 [Homo sapiens] |

| Filename XCorr DeltCN Conf% ObsM+H+ CalcM+H+ SpR ZScore Ion% # Sequence  | | | | | | | | | | | | |
| --- | --- | --- | --- | --- | --- | --- | --- | --- | --- | --- | --- | --- |
|  | Astrin\_NLD\_STLC\_tube2\_021014\_01.09950.09950.2 | 3.1986 | 0.2151 | 99.8% | 1288.9122 | 1289.4686 | 4 | 4.248 | 70.0% | 6 | K.QDLDEITTLIK.S | 2 |

---

|  |  |  |  |  |  |  |  |  |
| --- | --- | --- | --- | --- | --- | --- | --- | --- |
| U | *Reverse\_gi|21359945|r* | 1 | 1 | 0.9% | 1015 | 114334 | 5.6 | chromosome condensation protein G [Homo sapiens] |

| Filename XCorr DeltCN Conf% ObsM+H+ CalcM+H+ SpR ZScore Ion% # Sequence  | | | | | | | | | | | | |
| --- | --- | --- | --- | --- | --- | --- | --- | --- | --- | --- | --- | --- |
| \* | Astrin\_NLD\_STLC\_tube2\_021014\_02.05698.05698.2 | 2.031 | 0.2615 | 96.7% | 924.1122 | 925.93097 | 20 | 5.476 | 50.0% | 1 | K.ALGEAAGT#R.L | 2 |

---

|  |  |  |  |  |  |  |  |  |
| --- | --- | --- | --- | --- | --- | --- | --- | --- |
| U | *gi|116063573|ref|NP\_0* | 1 | 1 | 0.8% | 2639 | 280016 | 6.0 | filamin A, alpha isoform 1 [Homo sapiens] |
| U | *gi|160420317|ref|NP\_0* | 1 | 1 | 0.8% | 2647 | 280737 | 6.1 | filamin A, alpha isoform 2 [Homo sapiens] |

| Filename XCorr DeltCN Conf% ObsM+H+ CalcM+H+ SpR ZScore Ion% # Sequence  | | | | | | | | | | | | |
| --- | --- | --- | --- | --- | --- | --- | --- | --- | --- | --- | --- | --- |
|  | Astrin\_NLD\_STLC\_031014\_02.06574.06574.3 | 3.2674 | 0.2489 | 98.4% | 2202.5044 | 2201.4412 | 1 | 4.706 | 32.9% | 1 | R.LVSNHSLHETSSVFVDSLTK.A | 3 |

---

|  |  |  |  |  |  |  |  |  |
| --- | --- | --- | --- | --- | --- | --- | --- | --- |
| U | *gi|122937514|ref|NP\_0* | 1 | 1 | 0.8% | 2214 | 250909 | 5.9 | unc-13 homolog C [Homo sapiens] |

| Filename XCorr DeltCN Conf% ObsM+H+ CalcM+H+ SpR ZScore Ion% # Sequence  | | | | | | | | | | | | |
| --- | --- | --- | --- | --- | --- | --- | --- | --- | --- | --- | --- | --- |
| \* | Astrin\_NLD\_STLC\_031014\_02.11222.11222.2 | 2.1368 | 0.2964 | 96.9% | 1926.8522 | 1924.1179 | 129 | 4.852 | 26.5% | 1 | K.DKTGSSDPYVTVQVGKNK.R | 2 |

---

|  |  |  |  |  |  |  |  |  |
| --- | --- | --- | --- | --- | --- | --- | --- | --- |
| U | *gi|38604073|ref|NP\_07* | 1 | 1 | 0.8% | 2095 | 236515 | 6.6 | hypothetical protein LOC65250 [Homo sapiens] |

| Filename XCorr DeltCN Conf% ObsM+H+ CalcM+H+ SpR ZScore Ion% # Sequence  | | | | | | | | | | | | |
| --- | --- | --- | --- | --- | --- | --- | --- | --- | --- | --- | --- | --- |
| \* | Astrin\_NLD\_STLC\_031014\_01.14812.14812.2 | 2.3961 | 0.2569 | 97.5% | 1769.6721 | 1770.038 | 2 | 4.961 | 40.0% | 1 | K.TFSPGDGFPLLQFKSK.Q | 2 |

---

|  |  |  |  |  |  |  |  |  |
| --- | --- | --- | --- | --- | --- | --- | --- | --- |
| U | *Reverse\_gi|14211536|r* | 1 | 1 | 0.8% | 1712 | 184981 | 5.9 | neurexin 2 isoform alpha-1 precursor [Homo sapiens] |
| U | *Reverse\_gi|21166380|r* | 1 | 1 | 0.9% | 1642 | 177292 | 5.8 | neurexin 2 isoform alpha-2 precursor [Homo sapiens] |

| Filename XCorr DeltCN Conf% ObsM+H+ CalcM+H+ SpR ZScore Ion% # Sequence  | | | | | | | | | | | | |
| --- | --- | --- | --- | --- | --- | --- | --- | --- | --- | --- | --- | --- |
|  | Astrin\_NLD\_STLC\_tube2\_021014\_01.14718.14718.2 | 2.1913 | 0.2823 | 97.1% | 1533.9321 | 1533.7245 | 1 | 5.528 | 50.0% | 1 | R.GQSFLLLGNPETTR.F | 2 |

---

|  |  |  |  |  |  |  |  |  |
| --- | --- | --- | --- | --- | --- | --- | --- | --- |
| U | *Reverse\_gi|22094135|r* | 1 | 1 | 0.8% | 1537 | 164855 | 9.2 | DOT1-like, histone H3 methyltransferase [Homo sapiens] |

| Filename XCorr DeltCN Conf% ObsM+H+ CalcM+H+ SpR ZScore Ion% # Sequence  | | | | | | | | | | | | |
| --- | --- | --- | --- | --- | --- | --- | --- | --- | --- | --- | --- | --- |
| \* | Astrin\_NLD\_STLC\_tube2\_021014\_01.05025.05025.2 | 2.336 | 0.2431 | 97.4% | 1276.3522 | 1277.4526 | 7 | 4.474 | 54.5% | 1 | R.AKAAK@GSLLPT#K.D | 2 |

---

|  |  |  |  |  |  |  |  |  |
| --- | --- | --- | --- | --- | --- | --- | --- | --- |
| U | *Reverse\_gi|40254439|r* | 1 | 1 | 0.8% | 870 | 96459 | 6.3 | endothelial PAS domain protein 1 [Homo sapiens] |

| Filename XCorr DeltCN Conf% ObsM+H+ CalcM+H+ SpR ZScore Ion% # Sequence  | | | | | | | | | | | | |
| --- | --- | --- | --- | --- | --- | --- | --- | --- | --- | --- | --- | --- |
| \* | Astrin\_NLD\_STLC\_031014\_01.08973.08973.2 | 2.3485 | 0.1632 | 96.9% | 868.9122 | 868.9652 | 1 | 4.197 | 100.0% | 1 | R.HEPETKK.S | 2 |

---

|  |  |  |  |  |  |  |  |  |
| --- | --- | --- | --- | --- | --- | --- | --- | --- |
| U | *Reverse\_gi|87578396|r* | 1 | 1 | 0.7% | 1827 | 199525 | 4.9 | microtubule-associated protein 2 isoform 1 [Homo sapiens] |

| Filename XCorr DeltCN Conf% ObsM+H+ CalcM+H+ SpR ZScore Ion% # Sequence  | | | | | | | | | | | | |
| --- | --- | --- | --- | --- | --- | --- | --- | --- | --- | --- | --- | --- |
| \* | Astrin\_NLD\_STLC\_031014\_01.11415.11415.2 | 2.6022 | 0.2401 | 98.8% | 1368.9122 | 1368.5356 | 32 | 4.772 | 54.5% | 1 | K.RSPSHAQVETKK.A | 2 |

---

|  |  |  |  |  |  |  |  |  |
| --- | --- | --- | --- | --- | --- | --- | --- | --- |
| U | *gi|7662238|ref|NP\_055* | 1 | 1 | 0.7% | 1341 | 151887 | 6.4 | apoptotic chromatin condensation inducer 1 [Homo sapiens] |

| Filename XCorr DeltCN Conf% ObsM+H+ CalcM+H+ SpR ZScore Ion% # Sequence  | | | | | | | | | | | | |
| --- | --- | --- | --- | --- | --- | --- | --- | --- | --- | --- | --- | --- |
| \* | Astrin\_NLD\_STLC\_031014\_02.06939.06939.2 | 2.9833 | 0.1433 | 98.6% | 1276.2922 | 1276.0464 | 42 | 4.309 | 61.1% | 1 | R.SRS\*PDS\*S\*GSR.S | 2 |

---

|  |  |  |  |  |  |  |  |  |
| --- | --- | --- | --- | --- | --- | --- | --- | --- |
| U | *Reverse\_gi|91718902|r* | 1 | 1 | 0.6% | 4911 | 541375 | 6.5 | myeloid/lymphoid or mixed-lineage leukemia 3 [Homo sapiens] |

| Filename XCorr DeltCN Conf% ObsM+H+ CalcM+H+ SpR ZScore Ion% # Sequence  | | | | | | | | | | | | |
| --- | --- | --- | --- | --- | --- | --- | --- | --- | --- | --- | --- | --- |
| \* | Astrin\_NLD\_STLC\_031014\_01.12844.12844.3 | 3.9912 | 0.2508 | 99.6% | 3419.1543 | 3420.2864 | 9 | 4.304 | 21.3% | 1 | K.IETEVIT#ET#ETTELGDMS\*DEDEVAT#KGR.S | 3 |

---

|  |  |  |  |  |  |  |  |  |
| --- | --- | --- | --- | --- | --- | --- | --- | --- |
| U | *Reverse\_gi|31652242|r* | 1 | 1 | 0.6% | 1938 | 209652 | 9.1 | transcription factor 20 isoform 2 [Homo sapiens] |
| U | *Reverse\_gi|31652244|r* | 1 | 1 | 0.6% | 1960 | 211769 | 9.0 | transcription factor 20 isoform 1 [Homo sapiens] |

| Filename XCorr DeltCN Conf% ObsM+H+ CalcM+H+ SpR ZScore Ion% # Sequence  | | | | | | | | | | | | |
| --- | --- | --- | --- | --- | --- | --- | --- | --- | --- | --- | --- | --- |
|  | Astrin\_NLD\_STLC\_tube2\_021014\_02.05489.05489.2 | 2.2185 | 0.2544 | 97.3% | 1252.3322 | 1251.2847 | 17 | 4.446 | 60.0% | 1 | R.LSGPSK@S\*PETR.S | 2 |

---

|  |  |  |  |  |  |  |  |  |
| --- | --- | --- | --- | --- | --- | --- | --- | --- |
| U | *Reverse\_gi|154275767|* | 1 | 1 | 0.6% | 1762 | 193408 | 7.8 | ADAMTS-like 1 isoform 4 precursor [Homo sapiens] |

| Filename XCorr DeltCN Conf% ObsM+H+ CalcM+H+ SpR ZScore Ion% # Sequence  | | | | | | | | | | | | |
| --- | --- | --- | --- | --- | --- | --- | --- | --- | --- | --- | --- | --- |
| \* | Astrin\_NLD\_STLC\_031014\_01.10080.10080.2 | 2.1979 | 0.2251 | 95.6% | 1095.6122 | 1095.2437 | 1 | 4.659 | 72.2% | 1 | K.HPSLTVPSTR.R | 2 |

---

|  |  |  |  |  |  |  |  |  |
| --- | --- | --- | --- | --- | --- | --- | --- | --- |
| U | *gi|19882241|ref|NP\_06* | 1 | 1 | 0.6% | 1250 | 134210 | 6.2 | immunoglobulin superfamily, DCC subclass, member 4 [Homo sapiens] |

| Filename XCorr DeltCN Conf% ObsM+H+ CalcM+H+ SpR ZScore Ion% # Sequence  | | | | | | | | | | | | |
| --- | --- | --- | --- | --- | --- | --- | --- | --- | --- | --- | --- | --- |
| \* | Astrin\_NLD\_STLC\_031014\_01.06209.06209.2 | 1.9257 | 0.2674 | 97.4% | 941.7322 | 943.0067 | 27 | 4.919 | 83.3% | 1 | R.FS\*PWGLR.N | 2 |

---

|  |  |  |  |  |  |  |  |  |
| --- | --- | --- | --- | --- | --- | --- | --- | --- |
| U | *Reverse\_gi|150170670|* | 1 | 1 | 0.5% | 4935 | 537896 | 6.3 | piccolo isoform 2 [Homo sapiens] |
| U | *Reverse\_gi|150378539|* | 1 | 1 | 0.5% | 5142 | 560710 | 6.5 | piccolo isoform 1 [Homo sapiens] |

| Filename XCorr DeltCN Conf% ObsM+H+ CalcM+H+ SpR ZScore Ion% # Sequence  | | | | | | | | | | | | |
| --- | --- | --- | --- | --- | --- | --- | --- | --- | --- | --- | --- | --- |
|  | Astrin\_NLD\_STLC\_tube2\_021014\_01.18082.18082.3 | 3.2536 | 0.2332 | 96.3% | 2881.4343 | 2879.067 | 11 | 4.093 | 25.0% | 1 | K.VDMT#SASLDICK@DTTK@VEVKPVT#R.L | 3 |

---

|  |  |  |  |  |  |  |  |  |
| --- | --- | --- | --- | --- | --- | --- | --- | --- |
| U | *gi|126012573|ref|NP\_0* | 1 | 1 | 0.5% | 4655 | 521961 | 5.1 | low density lipoprotein-related protein 2 [Homo sapiens] |

| Filename XCorr DeltCN Conf% ObsM+H+ CalcM+H+ SpR ZScore Ion% # Sequence  | | | | | | | | | | | | |
| --- | --- | --- | --- | --- | --- | --- | --- | --- | --- | --- | --- | --- |
| \* | Astrin\_NLD\_STLC\_tube2\_021014\_01.11415.11415.3 | 3.9789 | 0.1785 | 96.4% | 3080.3643 | 3082.248 | 10 | 4.301 | 26.1% | 1 | K.NLYWTDSHYKS\*IS\*VMRLADKT#RR.T | 3 |

---

|  |  |  |  |  |  |  |  |  |
| --- | --- | --- | --- | --- | --- | --- | --- | --- |
| U | *Reverse\_gi|171184451|* | 1 | 1 | 0.5% | 3117 | 350931 | 6.3 | centrosome-associated protein 350 [Homo sapiens] |

| Filename XCorr DeltCN Conf% ObsM+H+ CalcM+H+ SpR ZScore Ion% # Sequence  | | | | | | | | | | | | |
| --- | --- | --- | --- | --- | --- | --- | --- | --- | --- | --- | --- | --- |
| \* | Astrin\_NLD\_STLC\_tube2\_021014\_01.07653.07653.2 | 3.3233 | 0.1033 | 95.6% | 1728.2322 | 1729.928 | 1 | 4.774 | 57.1% | 1 | K.KQDDGLLEQNSLQIK.E | 2 |

---

|  |  |  |  |  |  |  |  |  |
| --- | --- | --- | --- | --- | --- | --- | --- | --- |
| U | *Reverse\_gi|169218268|* | 1 | 2 | 0.5% | 2934 | 336126 | 6.5 | PREDICTED: similar to mutated in ataxia telangiectasia, partial [Homo sapiens] |

| Filename XCorr DeltCN Conf% ObsM+H+ CalcM+H+ SpR ZScore Ion% # Sequence  | | | | | | | | | | | | |
| --- | --- | --- | --- | --- | --- | --- | --- | --- | --- | --- | --- | --- |
| \* | Astrin\_NLD\_STLC\_031014\_02.07637.07637.2 | 3.4327 | 0.1999 | 99.7% | 1771.2922 | 1771.0046 | 34 | 4.81 | 43.3% | 2 | K.FLTISEGACQMLSNAK.Q | 2 |

---

|  |  |  |  |  |  |  |  |  |
| --- | --- | --- | --- | --- | --- | --- | --- | --- |
| U | *gi|118572613|ref|NP\_0* | 1 | 1 | 0.5% | 2752 | 299616 | 12.1 | splicing coactivator subunit SRm300 [Homo sapiens] |

| Filename XCorr DeltCN Conf% ObsM+H+ CalcM+H+ SpR ZScore Ion% # Sequence  | | | | | | | | | | | | |
| --- | --- | --- | --- | --- | --- | --- | --- | --- | --- | --- | --- | --- |
| \* | Astrin\_NLD\_STLC\_tube2\_021014\_01.06137.06137.2 | 2.1717 | 0.3972 | 99.8% | 1239.6122 | 1239.4172 | 5 | 6.196 | 58.3% | 1 | R.TPTAPAVNLAGAR.T | 2 |

---

|  |  |  |  |  |  |  |  |  |
| --- | --- | --- | --- | --- | --- | --- | --- | --- |
| U | *gi|19923586|ref|NP\_07* | 1 | 1 | 0.5% | 2696 | 296651 | 8.0 | nuclear receptor binding SET domain protein 1 isoform b [Homo sapiens] |

| Filename XCorr DeltCN Conf% ObsM+H+ CalcM+H+ SpR ZScore Ion% # Sequence  | | | | | | | | | | | | |
| --- | --- | --- | --- | --- | --- | --- | --- | --- | --- | --- | --- | --- |
| \* | Astrin\_NLD\_STLC\_031014\_01.15657.15657.2 | 1.9501 | 0.3308 | 97.3% | 1633.3922 | 1630.7179 | 17 | 5.226 | 38.5% | 1 | K.QEPSCNNSPELQVK.V | 2 |

---

|  |  |  |  |  |  |  |  |  |
| --- | --- | --- | --- | --- | --- | --- | --- | --- |
| U | *Reverse\_gi|190194412|* | 1 | 1 | 0.5% | 1979 | 227584 | 5.3 | thyroid hormone receptor interactor 11 [Homo sapiens] |

| Filename XCorr DeltCN Conf% ObsM+H+ CalcM+H+ SpR ZScore Ion% # Sequence  | | | | | | | | | | | | |
| --- | --- | --- | --- | --- | --- | --- | --- | --- | --- | --- | --- | --- |
| \* | Astrin\_NLD\_STLC\_tube2\_021014\_01.04632.04632.2 | 2.3614 | 0.1967 | 95.9% | 1205.0122 | 1205.3103 | 2 | 4.85 | 66.7% | 1 | K.DLEEQLKSSR.E | 2 |

---

|  |  |  |  |  |  |  |  |  |
| --- | --- | --- | --- | --- | --- | --- | --- | --- |
| U | *gi|31657092|ref|NP\_68* | 1 | 1 | 0.4% | 5058 | 576166 | 6.4 | ATP binding cassette, sub-family A (ABC1), member 13 [Homo sapiens] |

| Filename XCorr DeltCN Conf% ObsM+H+ CalcM+H+ SpR ZScore Ion% # Sequence  | | | | | | | | | | | | |
| --- | --- | --- | --- | --- | --- | --- | --- | --- | --- | --- | --- | --- |
| \* | Astrin\_NLD\_STLC\_tube2\_021014\_01.17079.17079.3 | 3.1494 | 0.3356 | 100.0% | 2383.8542 | 2384.6619 | 111 | 5.674 | 30.6% | 1 | K.DMACVT#S\*LIKIYIPQAFLK@.D | 3 |

---

|  |  |  |  |  |  |  |  |  |
| --- | --- | --- | --- | --- | --- | --- | --- | --- |
| U | *gi|188536004|ref|NP\_0* | 1 | 1 | 0.4% | 3925 | 410207 | 7.7 | zinc finger protein 469 [Homo sapiens] |

| Filename XCorr DeltCN Conf% ObsM+H+ CalcM+H+ SpR ZScore Ion% # Sequence  | | | | | | | | | | | | |
| --- | --- | --- | --- | --- | --- | --- | --- | --- | --- | --- | --- | --- |
| \* | Astrin\_NLD\_STLC\_tube2\_021014\_01.19410.19410.2 | 2.158 | 0.2787 | 96.0% | 1659.5521 | 1660.8247 | 11 | 4.804 | 34.4% | 1 | R.AT#SPPLAGAVSPSVAVR.A | 2 |

---

|  |  |  |  |  |  |  |  |  |
| --- | --- | --- | --- | --- | --- | --- | --- | --- |
| U | *gi|171184451|ref|NP\_0* | 1 | 1 | 0.4% | 3117 | 350931 | 6.3 | centrosome-associated protein 350 [Homo sapiens] |

| Filename XCorr DeltCN Conf% ObsM+H+ CalcM+H+ SpR ZScore Ion% # Sequence  | | | | | | | | | | | | |
| --- | --- | --- | --- | --- | --- | --- | --- | --- | --- | --- | --- | --- |
| \* | Astrin\_NLD\_STLC\_tube2\_021014\_01.09497.09497.2 | 2.4894 | 0.3349 | 99.9% | 1278.9321 | 1279.5686 | 2 | 6.191 | 63.6% | 1 | R.VLIGNVQPGILR.F | 2 |

---

|  |  |  |  |  |  |  |  |  |
| --- | --- | --- | --- | --- | --- | --- | --- | --- |
| U | *gi|56549696|ref|NP\_05* | 1 | 1 | 0.4% | 3122 | 339693 | 9.2 | E1A binding protein p400 [Homo sapiens] |

| Filename XCorr DeltCN Conf% ObsM+H+ CalcM+H+ SpR ZScore Ion% # Sequence  | | | | | | | | | | | | |
| --- | --- | --- | --- | --- | --- | --- | --- | --- | --- | --- | --- | --- |
| \* | Astrin\_NLD\_STLC\_031014\_02.07635.07635.2 | 2.1737 | 0.2649 | 97.5% | 1362.6721 | 1363.546 | 1 | 4.871 | 65.0% | 1 | K.LLK@NGT#KDLIR.E | 2 |

---

|  |  |  |  |  |  |  |  |  |
| --- | --- | --- | --- | --- | --- | --- | --- | --- |
| U | *gi|58530840|ref|NP\_00* | 1 | 1 | 0.4% | 2871 | 331774 | 6.8 | desmoplakin isoform I [Homo sapiens] |

| Filename XCorr DeltCN Conf% ObsM+H+ CalcM+H+ SpR ZScore Ion% # Sequence  | | | | | | | | | | | | |
| --- | --- | --- | --- | --- | --- | --- | --- | --- | --- | --- | --- | --- |
| \* | Astrin\_NLD\_STLC\_tube2\_021014\_01.07734.07734.2 | 2.2386 | 0.2461 | 96.6% | 1388.2522 | 1388.5205 | 1 | 4.682 | 68.2% | 1 | R.LNDSILQATEQR.R | 2 |

---

|  |  |  |  |  |  |  |  |  |
| --- | --- | --- | --- | --- | --- | --- | --- | --- |
| U | *gi|148762969|ref|NP\_0* | 1 | 1 | 0.2% | 5537 | 593399 | 5.6 | myeloid/lymphoid or mixed-lineage leukemia 2 [Homo sapiens] |

| Filename XCorr DeltCN Conf% ObsM+H+ CalcM+H+ SpR ZScore Ion% # Sequence  | | | | | | | | | | | | |
| --- | --- | --- | --- | --- | --- | --- | --- | --- | --- | --- | --- | --- |
| \* | Astrin\_NLD\_STLC\_031014\_01.12624.12624.2 | 2.0683 | 0.2891 | 96.8% | 1658.9122 | 1659.7194 | 381 | 4.621 | 37.5% | 1 | R.RS\*SPARS\*RIKQGR.S | 2 |

---

|  |  |  |  |  |  |  |  |  |
| --- | --- | --- | --- | --- | --- | --- | --- | --- |
| U | *gi|110349713|ref|NP\_5* | 2 | 4 | 0.1% | 27051 | 3006786 | 6.8 | titin isoform novex-1 [Homo sapiens] |
| U | *gi|110349719|ref|NP\_5* | 2 | 4 | 0.1% | 33423 | 3713573 | 6.5 | titin isoform N2-A [Homo sapiens] |
| U | *gi|110349717|ref|NP\_5* | 2 | 4 | 0.1% | 27118 | 3013987 | 6.7 | titin isoform novex-2 [Homo sapiens] |
| U | *gi|110349715|ref|NP\_0* | 2 | 4 | 0.1% | 26926 | 2992971 | 6.7 | titin isoform N2-B [Homo sapiens] |

| Filename XCorr DeltCN Conf% ObsM+H+ CalcM+H+ SpR ZScore Ion% # Sequence  | | | | | | | | | | | | |
| --- | --- | --- | --- | --- | --- | --- | --- | --- | --- | --- | --- | --- |
|  | Astrin\_NLD\_STLC\_tube2\_021014\_01.07558.07558.2 | 2.5804 | 0.212 | 97.8% | 1355.3522 | 1355.5458 | 1 | 5.127 | 63.6% | 2 | K.FDEVTAEAMTLK.W | 2 |
|  | Astrin\_NLD\_STLC\_tube2\_021014\_01.09675.09675.2 | 2.3531 | 0.2145 | 96.6% | 1364.8522 | 1364.3777 | 19 | 4.566 | 50.0% | 2 | K.SYSTAT#TKCHK.C | 2 |

---

|  |  |  |  |  |  |  |  |  |
| --- | --- | --- | --- | --- | --- | --- | --- | --- |
| U | *contaminant\_UBIQUITIN* | 4 | 4 | 0.0% | 1118 | 127523 | 8.5 | no description |

| Filename XCorr DeltCN Conf% ObsM+H+ CalcM+H+ SpR ZScore Ion% # Sequence  | | | | | | | | | | | | |
| --- | --- | --- | --- | --- | --- | --- | --- | --- | --- | --- | --- | --- |
|  | Astrin\_NLD\_STLC\_031014\_01.07086.07086.2 | 2.5408 | 0.2803 | 99.4% | 1524.2122 | 1524.6738 | 1 | 5.271 | 62.5% | 1 | K.IQDKEGIPPDQQR.L | 2 |
|  | Astrin\_NLD\_STLC\_tube2\_021014\_01.09353.09353.2 | 4.3008 | 0.4693 | 100.0% | 1789.1522 | 1788.9897 | 1 | 8.187 | 70.0% | 1 | K.TITLEVEPSDTIENVK.A | 2 |
|  | Astrin\_NLD\_STLC\_tube2\_021014\_02.06887.06887.3 | 3.3188 | 0.3622 | 100.0% | 2131.8843 | 2131.4368 | 4 | 5.179 | 33.8% | 1 | R.TLSDYNIQKESTLHLVLR.L | 3 |
|  | Astrin\_NLD\_STLC\_tube2\_021014\_01.15232.15232.2 | 2.8229 | 0.3645 | 100.0% | 2601.5522 | 2601.7454 | 1 | 6.021 | 34.1% | 1 | R.IYDDDFFQNLDGVANALDNVDAR.M | 2 |

|  |  |  |  |
| --- | --- | --- | --- |
|  | Proteins | Peptide IDs | Spectra |
| Unfiltered | 42561 | 82121 | 139793 |
| Filtered | 387 | 1056 | 2590 |
| Forward matches | 307 | 972 | 2469 |
| Decoy matches | 80 | 84 | 121 |
| Forward FP rate | 26.06% | 8.64% | 4.9% |

  
/nfs/cheeseman\_massspec/David/NLDAstrin
