# Supplementary material for: Astrin-SKAP complex reconstitution reveals its kinetochore interaction with microtubule-bound Ndc80
Source: eLife. 2017 Aug 25;6:e26866. doi: 10.7554/eLife.26866 (PMC5602300; doi:10.7554/eLife.26866)
Supplement: Source data 1. — Complete mass spectrometry searches using methods described in (Washburn et al., 2001) for affinity purification/mass spectrometry data sets described in this paper (data from this study; [Kern et al., 2016] [Gascoigne et al., 2011]). Individual Astrin cross-linking immunoprecipitations are listed based on the order in Figure 4—figure supplement 1. These samples have not been pruned for common or antibody-specific contaminants. [file elife-26866-data1.zip › Astrin_Crosslinking#2.html]

D Astrin\_STLCLD20
DTASelect v2.0.21  
/nfs/cheeseman\_massspec/David/Astrin\_STLCLD20  
/nfs/cheeseman\_massspec/Databases/NCBI-RefSeq\_human\_na\_04-13-2009\_con\_reversed.fasta  
SEQUEST 3.0 in SQT format.  
  
 Jump  to the summary table.  
  
sequest.params modifications:

|  |  |  |
| --- | --- | --- |
| \* | S | 80.0 |
| # | T | 80.0 |
| @ | K | 12.0 |
| Static | C | 57.0 |

|  |  |
| --- | --- |
| true | Use criteria |
| 0.0 | Minimum peptide confidence |
| 0.05 | Peptide false positive rate |
| 0.0 | Minimum protein confidence |
| 1.0 | Protein false positive rate |
| 1 | Minimum charge state |
| 16 | Maximum charge state |
| 0.0 | Minimum ion proportion |
| 1000 | Maximum Sp rank |
| -1.0 | Minimum Sp score |
| Include | Modified peptide inclusion |
| Any | Tryptic status requirement |
| false | Multiple, ambiguous IDs allowed |
| Ignore | Peptide validation handling |
| XCorr | Purge duplicate peptides by protein |
| false | Include only loci with unique peptide |
| true | Remove subset proteins |
| Ignore | Locus validation handling |
| 0 | Minimum modified peptides per locus |
| 1000 | Minimum redundancy for low coverage loci |
| 2 | Minimum peptides per locus |

#### Locus Key:

|  |  |  |  |  |  |  |  |  |
| --- | --- | --- | --- | --- | --- | --- | --- | --- |
| Validation Status | Locus | Sequence Count | Spectrum Count | Sequence Coverage | Length | MolWt | pI | Descriptive Name |

#### Similarity Key:

|  |  |  |
| --- | --- | --- |
| Locus | # of identical peptides | # of differing peptides |

---

|  |  |  |  |  |  |  |  |  |
| --- | --- | --- | --- | --- | --- | --- | --- | --- |
| U | *gi|40354195|ref|NP\_95* | 39 | 172 | 65.3% | 430 | 48058 | 5.5 | keratin 18 [Homo sapiens] |
| U | *gi|4557888|ref|NP\_000* | 39 | 172 | 65.3% | 430 | 48058 | 5.5 | keratin 18 [Homo sapiens] |

| Filename XCorr DeltCN Conf% ObsM+H+ CalcM+H+ SpR ZScore Ion% # Sequence  | | | | | | | | | | | | |
| --- | --- | --- | --- | --- | --- | --- | --- | --- | --- | --- | --- | --- |
|  | Astrin\_STLCLD20\_112214\_tube2\_01.09368.09368.2 | 5.1021 | 0.501 | 100.0% | 2855.8523 | 2856.0813 | 1 | 8.057 | 30.0% | 3 | R.SLGSVQAPSYGARPVSSAASVYAGAGGSGSR.I | 2 |
|  | Astrin\_STLCLD20\_112214\_01.08540.08540.3 | 4.9552 | 0.5431 | 100.0% | 2856.0842 | 2856.0813 | 1 | 8.854 | 30.0% | 15 | R.SLGSVQAPSYGARPVSSAASVYAGAGGSGSR.I | 3 |
|  | Astrin\_STLCLD20\_112214\_02.09957.09957.3 | 3.8771 | 0.3909 | 99.8% | 2935.5244 | 2936.0813 | 2 | 5.917 | 26.7% | 1 | R.SLGSVQAPSYGARPVSSAASVYAGAGGS\*GSR.I | 3 |
|  | Astrin\_STLCLD20\_112214\_tube2\_02.08818.08818.2 | 5.5902 | 0.5825 | 100.0% | 2261.5322 | 2262.561 | 1 | 10.735 | 48.0% | 3 | R.GGMGSGGLATGIAGGLAGMGGIQNEK.E | 2 |
|  | Astrin\_STLCLD20\_112214\_tube2\_02.08814.08814.3 | 4.6793 | 0.31 | 99.8% | 2265.1443 | 2262.561 | 1 | 6.114 | 38.0% | 3 | R.GGMGSGGLATGIAGGLAGMGGIQNEK.E | 3 |
|  | Astrin\_STLCLD20\_112214\_tube2\_01.14294.14294.3 | 6.0631 | 0.5355 | 100.0% | 3337.4944 | 3337.7224 | 1 | 8.68 | 27.2% | 8 | R.GGMGSGGLATGIAGGLAGMGGIQNEKETMQSLNDR.L | 3 |
|  | Astrin\_STLCLD20\_112214\_01.10024.10024.1 | 1.7507 | 0.3024 | 100.0% | 982.5 | 983.0709 | 5 | 5.045 | 58.3% | 2 | R.DWSHYFK.I | 1 |
|  | Astrin\_STLCLD20\_112214\_02.12161.12161.3 | 3.8851 | 0.3999 | 99.7% | 2059.4944 | 2060.3176 | 4 | 6.977 | 36.8% | 1 | K.IIEDLRAQIFANTVDNAR.I | 3 |
|  | Astrin\_STLCLD20\_112214\_01.07252.07252.1 | 2.1656 | 0.3464 | 100.0% | 1319.59 | 1320.4478 | 135 | 6.06 | 40.9% | 2 | R.AQIFANTVDNAR.I | 1 |
|  | Astrin\_STLCLD20\_112214\_01.07189.07189.2 | 4.1827 | 0.4578 | 100.0% | 1320.3121 | 1320.4478 | 1 | 8.414 | 77.3% | 11 | R.AQIFANTVDNAR.I | 2 |
|  | Astrin\_STLCLD20\_112214\_tube2\_01.09495.09495.1 | 2.6834 | 0.3062 | 100.0% | 1041.62 | 1042.2235 | 1 | 7.063 | 68.8% | 3 | R.IVLQIDNAR.L | 11 |
|  | Astrin\_STLCLD20\_112214\_tube2\_01.09470.09470.2 | 3.1917 | 0.1503 | 99.4% | 1042.1921 | 1042.2235 | 3 | 6.148 | 87.5% | 8 | R.IVLQIDNAR.L | 22 |
|  | Astrin\_STLCLD20\_112214\_tube2\_01.07546.07546.2 | 2.2441 | 0.2268 | 98.6% | 807.7322 | 807.8815 | 35 | 5.587 | 66.7% | 3 | R.LAADDFR.V | 22222 |
|  | Astrin\_STLCLD20\_112214\_01.05707.05707.2 | 3.073 | 0.4746 | 100.0% | 1240.3121 | 1240.4601 | 16 | 7.561 | 72.2% | 7 | R.VKYETELAMR.Q | 2 |
|  | Astrin\_STLCLD20\_112214\_tube2\_01.06293.06293.2 | 3.0568 | 0.2057 | 99.4% | 1176.2122 | 1175.3274 | 4 | 5.727 | 72.2% | 2 | R.KVIDDTNITR.L | 2 |
|  | Astrin\_STLCLD20\_112214\_tube2\_01.06578.06578.2 | 2.3695 | 0.3345 | 99.4% | 1046.4722 | 1047.1533 | 6 | 7.068 | 68.8% | 1 | K.VIDDTNITR.L | 2 |
|  | Astrin\_STLCLD20\_112214\_tube2\_01.06584.06584.1 | 1.8182 | 0.3081 | 100.0% | 1046.56 | 1047.1533 | 2 | 5.245 | 62.5% | 1 | K.VIDDTNITR.L | 1 |
|  | Astrin\_STLCLD20\_112214\_01.15206.15206.2 | 6.4218 | 0.5637 | 100.0% | 2177.7322 | 2178.589 | 1 | 10.167 | 67.6% | 5 | R.LQLETEIEALKEELLFMK.K | 2 |
|  | Astrin\_STLCLD20\_112214\_tube2\_02.11539.11539.3 | 3.2119 | 0.4364 | 99.7% | 2178.1143 | 2178.589 | 67 | 6.355 | 33.8% | 3 | R.LQLETEIEALKEELLFMK.K | 3 |
|  | Astrin\_STLCLD20\_112214\_01.10426.10426.3 | 5.3076 | 0.3458 | 100.0% | 2750.7544 | 2751.0227 | 1 | 7.623 | 35.0% | 6 | K.NHEEEVKGLQAQIASSGLTVEVDAPK.S | 3 |
|  | Astrin\_STLCLD20\_112214\_tube2\_01.11565.11565.2 | 6.4403 | 0.5816 | 100.0% | 2750.872 | 2751.0227 | 1 | 11.094 | 56.0% | 1 | K.NHEEEVKGLQAQIASSGLTVEVDAPK.S | 2 |
|  | Astrin\_STLCLD20\_112214\_tube2\_01.12294.12294.2 | 4.8914 | 0.519 | 100.0% | 1884.5922 | 1885.1246 | 1 | 9.854 | 66.7% | 4 | K.GLQAQIASSGLTVEVDAPK.S | 2 |
|  | Astrin\_STLCLD20\_112214\_tube2\_01.06986.06986.1 | 1.9887 | 0.2102 | 98.0% | 965.61 | 966.0385 | 63 | 5.014 | 50.0% | 3 | R.AQYDELAR.K | 1 |
|  | Astrin\_STLCLD20\_112214\_tube2\_01.12106.12106.2 | 3.5869 | 0.4313 | 100.0% | 1664.3722 | 1663.8865 | 1 | 7.071 | 73.1% | 2 | R.RTVQSLEIDLDSMR.N | 2 |
|  | Astrin\_STLCLD20\_112214\_tube2\_01.14206.14206.1 | 2.2803 | 0.4271 | 100.0% | 1506.63 | 1507.699 | 1 | 6.577 | 66.7% | 4 | R.TVQSLEIDLDSMR.N | 1 |
|  | Astrin\_STLCLD20\_112214\_01.11641.11641.2 | 4.4726 | 0.494 | 100.0% | 1508.5322 | 1507.699 | 1 | 9.071 | 75.0% | 12 | R.TVQSLEIDLDSMR.N | 2 |
|  | Astrin\_STLCLD20\_112214\_01.07821.07821.2 | 2.2496 | 0.1995 | 95.6% | 1244.1721 | 1245.4215 | 100 | 4.086 | 55.0% | 1 | R.NLKASLENSLR.E | 2 |
|  | Astrin\_STLCLD20\_112214\_tube2\_01.07078.07078.2 | 2.4396 | 0.3187 | 99.7% | 890.33215 | 889.9841 | 46 | 6.057 | 78.6% | 1 | K.ASLENSLR.E | 2 |
|  | Astrin\_STLCLD20\_112214\_01.08857.08857.2 | 2.7572 | 0.1465 | 96.9% | 1473.8722 | 1474.6139 | 1 | 4.465 | 58.3% | 1 | K.ASLENSLREVEAR.Y | 2 |
|  | Astrin\_STLCLD20\_112214\_tube2\_01.09820.09820.3 | 2.6679 | 0.2342 | 95.6% | 1475.4543 | 1474.6139 | 27 | 5.197 | 37.5% | 1 | K.ASLENSLREVEAR.Y | 3 |
|  | Astrin\_STLCLD20\_112214\_tube2\_02.12146.12146.2 | 6.175 | 0.4456 | 100.0% | 2671.7122 | 2672.0715 | 1 | 9.736 | 54.5% | 7 | R.YALQMEQLNGILLHLESELAQTR.A | 2 |
|  | Astrin\_STLCLD20\_112214\_02.17163.17163.3 | 6.6694 | 0.512 | 100.0% | 2672.5444 | 2672.0715 | 1 | 9.274 | 46.6% | 19 | R.YALQMEQLNGILLHLESELAQTR.A | 3 |
|  | Astrin\_STLCLD20\_112214\_tube2\_02.08295.08295.2 | 3.5934 | 0.4014 | 100.0% | 1421.5322 | 1420.6055 | 1 | 5.934 | 77.3% | 7 | R.QAQEYEALLNIK.V | 2 |
|  | Astrin\_STLCLD20\_112214\_tube2\_02.06601.06601.2 | 3.5685 | 0.3479 | 100.0% | 1293.4521 | 1293.5059 | 1 | 7.01 | 75.0% | 7 | K.VKLEAEIATYR.R | 2 |
|  | Astrin\_STLCLD20\_112214\_tube2\_01.08554.08554.1 | 2.3173 | 0.3228 | 100.0% | 1065.55 | 1066.1992 | 72 | 5.913 | 56.2% | 1 | K.LEAEIATYR.R | 1 |
|  | Astrin\_STLCLD20\_112214\_01.06829.06829.2 | 3.5529 | 0.3712 | 100.0% | 1066.2722 | 1066.1992 | 1 | 6.82 | 87.5% | 5 | K.LEAEIATYR.R | 2 |
|  | Astrin\_STLCLD20\_112214\_02.12562.12562.3 | 5.335 | 0.3869 | 100.0% | 2897.6643 | 2898.128 | 1 | 6.627 | 29.0% | 4 | R.RLLEDGEDFNLGDALDSSNSMQTIQK.T | 3 |
|  | Astrin\_STLCLD20\_112214\_tube2\_01.14951.14951.2 | 6.0933 | 0.6171 | 100.0% | 2740.652 | 2741.9404 | 1 | 12.384 | 52.1% | 3 | R.LLEDGEDFNLGDALDSSNSMQTIQK.T | 2 |
|  | Astrin\_STLCLD20\_112214\_tube2\_02.09108.09108.3 | 4.2513 | 0.3372 | 99.8% | 2741.9043 | 2741.9404 | 2 | 5.888 | 27.1% | 1 | R.LLEDGEDFNLGDALDSSNSMQTIQK.T | 3 |

Similarities:
gi|4557701|ref|NP\_000(1:38)  
contaminant\_KERATIN05(1:38)  
contaminant\_KERATIN03(1:38)  
gi|24234699|ref|NP\_00(3:36)  

---

|  |  |  |  |  |  |  |  |  |
| --- | --- | --- | --- | --- | --- | --- | --- | --- |
| U | *gi|106775678|ref|NP\_0* | 11 | 29 | 62.3% | 130 | 14095 | 10.9 | histone cluster 2, H2aa4 [Homo sapiens] |
| U | *gi|4504251|ref|NP\_003* | 11 | 29 | 62.3% | 130 | 14095 | 10.9 | histone cluster 2, H2aa3 [Homo sapiens] |
| U | *gi|24638446|ref|NP\_00* | 11 | 29 | 62.8% | 129 | 13988 | 10.9 | histone cluster 2, H2ac [Homo sapiens] |

| Filename XCorr DeltCN Conf% ObsM+H+ CalcM+H+ SpR ZScore Ion% # Sequence  | | | | | | | | | | | | |
| --- | --- | --- | --- | --- | --- | --- | --- | --- | --- | --- | --- | --- |
|  | Astrin\_STLCLD20\_112214\_tube2\_01.08619.08619.2 | 2.8086 | 0.2147 | 98.8% | 1275.5521 | 1275.4531 | 1 | 5.206 | 77.3% | 1 | R.SSRAGLQFPVGR.V | 222 |
|  | Astrin\_STLCLD20\_112214\_01.09511.09511.2 | 2.9305 | 0.285 | 99.9% | 945.4122 | 945.1093 | 2 | 5.81 | 81.2% | 6 | R.AGLQFPVGR.V | 2222 |
|  | Astrin\_STLCLD20\_112214\_01.19304.19304.2 | 5.3728 | 0.5704 | 100.0% | 2934.5923 | 2935.4082 | 1 | 10.366 | 42.9% | 4 | R.VGAGAPVYMAAVLEYLTAEILELAGNAAR.D | 2 |
|  | Astrin\_STLCLD20\_112214\_tube2\_02.00464.00464.3 | 4.7352 | 0.3446 | 99.7% | 2935.4644 | 2935.4082 | 1 | 7.289 | 30.4% | 2 | R.VGAGAPVYMAAVLEYLTAEILELAGNAAR.D | 3 |
|  | Astrin\_STLCLD20\_112214\_tube2\_02.00326.00326.3 | 3.9845 | 0.3733 | 99.8% | 3293.1243 | 3292.7747 | 1 | 6.832 | 25.8% | 1 | R.VGAGAPVYMAAVLEYLTAEILELAGNAARDNK.K | 3 |
|  | Astrin\_STLCLD20\_112214\_tube2\_01.07832.07832.2 | 2.6508 | 0.2524 | 99.7% | 851.2922 | 851.0396 | 4 | 6.019 | 83.3% | 3 | R.HLQLAIR.N | 2222 |
|  | Astrin\_STLCLD20\_112214\_tube2\_02.05964.05964.3 | 3.0828 | 0.3511 | 99.8% | 1695.0543 | 1693.9004 | 1 | 5.873 | 46.2% | 2 | R.HLQLAIRNDEELNK.L | 333 |
|  | Astrin\_STLCLD20\_112214\_tube2\_01.11649.11649.3 | 4.8389 | 0.5071 | 100.0% | 2106.0244 | 2105.4453 | 1 | 7.497 | 39.7% | 3 | R.HLQLAIRNDEELNKLLGK.V | 33 |
|  | Astrin\_STLCLD20\_112214\_01.09259.09259.2 | 3.407 | 0.3327 | 100.0% | 1272.7522 | 1273.4288 | 9 | 6.016 | 65.0% | 4 | R.NDEELNKLLGK.V | 22 |
|  | Astrin\_STLCLD20\_112214\_tube2\_01.17512.17512.1 | 2.388 | 0.2221 | 97.8% | 1931.12 | 1932.3573 | 40 | 5.511 | 33.3% | 1 | K.VTIAQGGVLPNIQAVLLPK.K | 111 |
|  | Astrin\_STLCLD20\_112214\_01.13480.13480.2 | 4.493 | 0.4772 | 100.0% | 1932.6522 | 1932.3573 | 1 | 7.3 | 66.7% | 2 | K.VTIAQGGVLPNIQAVLLPK.K | 222 |

Similarities:
gi|10645195|ref|NP\_06(6:5)  
gi|10800130|ref|NP\_06(8:3)  
gi|20357599|ref|NP\_61(2:9)  

---

|  |  |  |  |  |  |  |  |  |
| --- | --- | --- | --- | --- | --- | --- | --- | --- |
| U | *gi|10645195|ref|NP\_06* | 10 | 22 | 60.0% | 130 | 14135 | 11.1 | histone cluster 1, H2ae [Homo sapiens] |
| U | *gi|4504245|ref|NP\_003* | 9 | 16 | 60.0% | 130 | 14105 | 11.1 | histone cluster 1, H2ac [Homo sapiens] |
| U | *gi|19557656|ref|NP\_00* | 10 | 22 | 60.0% | 130 | 14135 | 11.1 | histone cluster 1, H2ab [Homo sapiens] |
| U | *gi|15617199|ref|NP\_25* | 10 | 22 | 60.0% | 130 | 14121 | 11.1 | histone cluster 3, H2a [Homo sapiens] |

| Filename XCorr DeltCN Conf% ObsM+H+ CalcM+H+ SpR ZScore Ion% # Sequence  | | | | | | | | | | | | |
| --- | --- | --- | --- | --- | --- | --- | --- | --- | --- | --- | --- | --- |
|  | Astrin\_STLCLD20\_112214\_tube2\_01.08619.08619.2 | 2.8086 | 0.2147 | 98.8% | 1275.5521 | 1275.4531 | 1 | 5.206 | 77.3% | 1 | R.SSRAGLQFPVGR.V | 222 |
|  | Astrin\_STLCLD20\_112214\_01.09511.09511.2 | 2.9305 | 0.285 | 99.9% | 945.4122 | 945.1093 | 2 | 5.81 | 81.2% | 6 | R.AGLQFPVGR.V | 2222 |
|  | Astrin\_STLCLD20\_112214\_01.19383.19383.2 | 5.6359 | 0.5114 | 100.0% | 2917.8523 | 2917.3752 | 1 | 9.662 | 42.9% | 2 | R.VGAGAPVYLAAVLEYLTAEILELAGNAAR.D | 22 |
|  | Astrin\_STLCLD20\_112214\_tube2\_02.00560.00560.3 | 6.2608 | 0.5052 | 100.0% | 2917.9143 | 2917.3752 | 1 | 8.493 | 33.0% | 2 | R.VGAGAPVYLAAVLEYLTAEILELAGNAAR.D | 33 |
|  | Astrin\_STLCLD20\_112214\_tube2\_01.07832.07832.2 | 2.6508 | 0.2524 | 99.7% | 851.2922 | 851.0396 | 4 | 6.019 | 83.3% | 3 | R.HLQLAIR.N | 2222 |
|  | Astrin\_STLCLD20\_112214\_tube2\_02.05964.05964.3 | 3.0828 | 0.3511 | 99.8% | 1695.0543 | 1693.9004 | 1 | 5.873 | 46.2% | 2 | R.HLQLAIRNDEELNK.L | 333 |
|  | Astrin\_STLCLD20\_112214\_01.10678.10678.3 | 4.1655 | 0.4581 | 100.0% | 2132.6643 | 2133.4587 | 1 | 7.186 | 39.7% | 2 | R.HLQLAIRNDEELNKLLGR.V | 3 |
|  | Astrin\_STLCLD20\_112214\_01.09729.09729.2 | 2.5942 | 0.2495 | 98.9% | 1300.6721 | 1301.4423 | 12 | 5.215 | 65.0% | 1 | R.NDEELNKLLGR.V | 2 |
|  | Astrin\_STLCLD20\_112214\_tube2\_01.17512.17512.1 | 2.388 | 0.2221 | 97.8% | 1931.12 | 1932.3573 | 40 | 5.511 | 33.3% | 1 | R.VTIAQGGVLPNIQAVLLPK.K | 111 |
|  | Astrin\_STLCLD20\_112214\_01.13480.13480.2 | 4.493 | 0.4772 | 100.0% | 1932.6522 | 1932.3573 | 1 | 7.3 | 66.7% | 2 | R.VTIAQGGVLPNIQAVLLPK.K | 222 |

Similarities:
gi|106775678|ref|NP\_0(6:4)  
gi|10800130|ref|NP\_06(8:2)  
gi|20357599|ref|NP\_61(2:8)  

---

|  |  |  |  |  |  |  |  |  |
| --- | --- | --- | --- | --- | --- | --- | --- | --- |
| U | *gi|10800130|ref|NP\_06* | 10 | 26 | 60.0% | 130 | 14107 | 10.9 | histone cluster 1, H2ad [Homo sapiens] |
| U | *gi|4504249|ref|NP\_003* | 9 | 20 | 60.0% | 130 | 14091 | 10.9 | histone cluster 1, H2am [Homo sapiens] |
| U | *gi|4504243|ref|NP\_003* | 10 | 26 | 60.0% | 130 | 14091 | 10.9 | histone cluster 1, H2al [Homo sapiens] |
| U | *gi|4504241|ref|NP\_003* | 8 | 17 | 60.0% | 130 | 14091 | 10.9 | histone cluster 1, H2ak [Homo sapiens] |
| U | *gi|4504239|ref|NP\_003* | 10 | 26 | 60.0% | 130 | 14091 | 10.9 | histone cluster 1, H2ai [Homo sapiens] |
| U | *gi|29553970|ref|NP\_80* | 8 | 17 | 60.5% | 129 | 14019 | 10.9 | H2A histone family, member J [Homo sapiens] |
| U | *gi|18105045|ref|NP\_54* | 10 | 26 | 60.9% | 128 | 13906 | 10.9 | histone cluster 1, H2ah [Homo sapiens] |
| U | *gi|10800144|ref|NP\_06* | 10 | 26 | 60.9% | 128 | 13936 | 10.9 | histone cluster 1, H2aj [Homo sapiens] |
| U | *gi|10800132|ref|NP\_06* | 10 | 26 | 60.0% | 130 | 14091 | 10.9 | histone cluster 1, H2ag [Homo sapiens] |

| Filename XCorr DeltCN Conf% ObsM+H+ CalcM+H+ SpR ZScore Ion% # Sequence  | | | | | | | | | | | | |
| --- | --- | --- | --- | --- | --- | --- | --- | --- | --- | --- | --- | --- |
|  | Astrin\_STLCLD20\_112214\_tube2\_01.08619.08619.2 | 2.8086 | 0.2147 | 98.8% | 1275.5521 | 1275.4531 | 1 | 5.206 | 77.3% | 1 | R.SSRAGLQFPVGR.V | 222 |
|  | Astrin\_STLCLD20\_112214\_01.09511.09511.2 | 2.9305 | 0.285 | 99.9% | 945.4122 | 945.1093 | 2 | 5.81 | 81.2% | 6 | R.AGLQFPVGR.V | 2222 |
|  | Astrin\_STLCLD20\_112214\_01.19383.19383.2 | 5.6359 | 0.5114 | 100.0% | 2917.8523 | 2917.3752 | 1 | 9.662 | 42.9% | 2 | R.VGAGAPVYLAAVLEYLTAEILELAGNAAR.D | 22 |
|  | Astrin\_STLCLD20\_112214\_tube2\_02.00560.00560.3 | 6.2608 | 0.5052 | 100.0% | 2917.9143 | 2917.3752 | 1 | 8.493 | 33.0% | 2 | R.VGAGAPVYLAAVLEYLTAEILELAGNAAR.D | 33 |
|  | Astrin\_STLCLD20\_112214\_tube2\_01.07832.07832.2 | 2.6508 | 0.2524 | 99.7% | 851.2922 | 851.0396 | 4 | 6.019 | 83.3% | 3 | R.HLQLAIR.N | 2222 |
|  | Astrin\_STLCLD20\_112214\_tube2\_02.05964.05964.3 | 3.0828 | 0.3511 | 99.8% | 1695.0543 | 1693.9004 | 1 | 5.873 | 46.2% | 2 | R.HLQLAIRNDEELNK.L | 333 |
|  | Astrin\_STLCLD20\_112214\_tube2\_01.11649.11649.3 | 4.8389 | 0.5071 | 100.0% | 2106.0244 | 2105.4453 | 1 | 7.497 | 39.7% | 3 | R.HLQLAIRNDEELNKLLGK.V | 33 |
|  | Astrin\_STLCLD20\_112214\_01.09259.09259.2 | 3.407 | 0.3327 | 100.0% | 1272.7522 | 1273.4288 | 9 | 6.016 | 65.0% | 4 | R.NDEELNKLLGK.V | 22 |
|  | Astrin\_STLCLD20\_112214\_tube2\_01.17512.17512.1 | 2.388 | 0.2221 | 97.8% | 1931.12 | 1932.3573 | 40 | 5.511 | 33.3% | 1 | K.VTIAQGGVLPNIQAVLLPK.K | 111 |
|  | Astrin\_STLCLD20\_112214\_01.13480.13480.2 | 4.493 | 0.4772 | 100.0% | 1932.6522 | 1932.3573 | 1 | 7.3 | 66.7% | 2 | K.VTIAQGGVLPNIQAVLLPK.K | 222 |

Similarities:
gi|106775678|ref|NP\_0(8:2)  
gi|10645195|ref|NP\_06(8:2)  
gi|20357599|ref|NP\_61(2:8)  

---

|  |  |  |  |  |  |  |  |  |
| --- | --- | --- | --- | --- | --- | --- | --- | --- |
| U | *gi|4504919|ref|NP\_002* | 46 | 162 | 58.4% | 483 | 53704 | 5.6 | keratin 8 [Homo sapiens] |

| Filename XCorr DeltCN Conf% ObsM+H+ CalcM+H+ SpR ZScore Ion% # Sequence  | | | | | | | | | | | | |
| --- | --- | --- | --- | --- | --- | --- | --- | --- | --- | --- | --- | --- |
| \* | Astrin\_STLCLD20\_112214\_01.14989.14989.3 | 4.388 | 0.3719 | 99.7% | 3927.1143 | 3927.465 | 11 | 5.688 | 17.5% | 1 | R.GGLGGGYGGASGMGGITAVTVNQSLLSPLVLEVDPNIQAVR.T | 3 |
|  | Astrin\_STLCLD20\_112214\_01.08756.08756.2 | 2.5869 | 0.1788 | 98.4% | 1082.6522 | 1083.2755 | 3 | 6.37 | 75.0% | 2 | K.FASFIDKVR.F | 2222 |
|  | Astrin\_STLCLD20\_112214\_01.09691.09691.2 | 3.0026 | 0.0918 | 98.8% | 1031.3322 | 1031.1997 | 2 | 3.719 | 92.9% | 3 | K.WSLLQQQK.T | 2 |
|  | Astrin\_STLCLD20\_112214\_tube2\_01.16650.16650.2 | 5.033 | 0.3575 | 100.0% | 2034.4922 | 2035.363 | 1 | 9.481 | 61.8% | 1 | K.LKLEAELGNMQGLVEDFK.N | 2 |
|  | Astrin\_STLCLD20\_112214\_tube2\_02.09914.09914.3 | 4.9087 | 0.1983 | 99.7% | 2035.1943 | 2035.363 | 1 | 8.559 | 42.6% | 3 | K.LKLEAELGNMQGLVEDFK.N | 3 |
|  | Astrin\_STLCLD20\_112214\_tube2\_01.16520.16520.2 | 3.9658 | 0.2823 | 100.0% | 1793.9922 | 1794.0295 | 2 | 8.639 | 50.0% | 5 | K.LEAELGNMQGLVEDFK.N | 2 |
|  | Astrin\_STLCLD20\_112214\_tube2\_01.13413.13413.2 | 3.5674 | 0.4238 | 100.0% | 1353.4122 | 1353.5732 | 1 | 7.877 | 85.0% | 8 | R.TEMENEFVLIK.K | 2 |
|  | Astrin\_STLCLD20\_112214\_tube2\_01.10959.10959.2 | 3.5142 | 0.2554 | 100.0% | 1481.5122 | 1481.7473 | 1 | 6.171 | 77.3% | 6 | R.TEMENEFVLIKK.D | 2 |
|  | Astrin\_STLCLD20\_112214\_tube2\_02.06158.06158.3 | 3.1641 | 0.3605 | 99.8% | 1926.1444 | 1927.1365 | 1 | 6.001 | 36.7% | 1 | K.KDVDEAYMNKVELESR.L | 3 |
|  | Astrin\_STLCLD20\_112214\_tube2\_01.09696.09696.2 | 4.2333 | 0.4871 | 100.0% | 1798.2922 | 1798.9623 | 1 | 8.535 | 71.4% | 7 | K.DVDEAYMNKVELESR.L | 2 |
|  | Astrin\_STLCLD20\_112214\_tube2\_02.06816.06816.3 | 4.5861 | 0.3449 | 100.0% | 1799.6643 | 1798.9623 | 1 | 6.319 | 55.4% | 2 | K.DVDEAYMNKVELESR.L | 3 |
|  | Astrin\_STLCLD20\_112214\_02.15702.15702.3 | 3.9037 | 0.3448 | 99.8% | 3200.0344 | 3200.5444 | 1 | 6.266 | 26.9% | 1 | K.DVDEAYMNKVELESRLEGLTDEINFLR.Q | 3 |
|  | Astrin\_STLCLD20\_112214\_01.12742.12742.1 | 2.9453 | 0.2941 | 100.0% | 1419.6 | 1420.6055 | 1 | 5.852 | 63.6% | 3 | R.LEGLTDEINFLR.Q | 1 |
|  | Astrin\_STLCLD20\_112214\_tube2\_01.16046.16046.2 | 4.1456 | 0.5011 | 100.0% | 1420.5122 | 1420.6055 | 1 | 8.872 | 86.4% | 9 | R.LEGLTDEINFLR.Q | 2 |
|  | Astrin\_STLCLD20\_112214\_tube2\_02.07909.07909.2 | 5.3916 | 0.559 | 100.0% | 2109.2922 | 2110.3008 | 1 | 9.18 | 61.1% | 2 | R.ELQSQISDTSVVLSMDNSR.S | 2 |
|  | Astrin\_STLCLD20\_112214\_tube2\_01.15461.15461.1 | 2.5272 | 0.3514 | 100.0% | 1320.64 | 1321.5286 | 4 | 6.314 | 59.1% | 3 | R.SLDMDSIIAEVK.A | 1 |
|  | Astrin\_STLCLD20\_112214\_tube2\_01.15494.15494.2 | 4.3102 | 0.4883 | 100.0% | 1321.0721 | 1321.5286 | 1 | 8.294 | 77.3% | 7 | R.SLDMDSIIAEVK.A | 2 |
|  | Astrin\_STLCLD20\_112214\_01.14918.14918.2 | 2.7552 | 0.3191 | 99.4% | 2381.632 | 2382.6477 | 49 | 5.275 | 30.0% | 1 | R.SLDMDSIIAEVKAQYEDIANR.S | 2 |
|  | Astrin\_STLCLD20\_112214\_tube2\_01.06596.06596.1 | 1.9314 | 0.3393 | 100.0% | 1079.4 | 1080.1423 | 6 | 6.565 | 56.2% | 1 | K.AQYEDIANR.S | 1 |
|  | Astrin\_STLCLD20\_112214\_tube2\_02.05388.05388.2 | 3.2333 | 0.2191 | 99.9% | 1080.1322 | 1080.1423 | 1 | 6.554 | 75.0% | 4 | K.AQYEDIANR.S | 2 |
|  | Astrin\_STLCLD20\_112214\_tube2\_01.07305.07305.2 | 3.4487 | 0.2 | 99.7% | 1414.2522 | 1413.5884 | 1 | 5.608 | 77.3% | 3 | R.SRAEAESMYQIK.Y | 2 |
|  | Astrin\_STLCLD20\_112214\_02.11017.11017.3 | 6.0844 | 0.4341 | 100.0% | 2532.3245 | 2532.828 | 1 | 7.119 | 40.5% | 5 | R.SRAEAESMYQIKYEELQSLAGK.H | 3 |
|  | Astrin\_STLCLD20\_112214\_01.05845.05845.2 | 3.2804 | 0.3032 | 100.0% | 1170.2922 | 1170.3228 | 6 | 6.548 | 83.3% | 3 | R.AEAESMYQIK.Y | 2 |
|  | Astrin\_STLCLD20\_112214\_02.11686.11686.3 | 5.1538 | 0.4307 | 100.0% | 2289.2344 | 2289.5623 | 1 | 8.344 | 34.2% | 4 | R.AEAESMYQIKYEELQSLAGK.H | 3 |
|  | Astrin\_STLCLD20\_112214\_tube2\_01.08973.08973.2 | 3.4809 | 0.0738 | 98.9% | 1137.8322 | 1138.2627 | 2 | 7.332 | 77.8% | 7 | K.YEELQSLAGK.H | 2 |
|  | Astrin\_STLCLD20\_112214\_tube2\_01.08957.08957.1 | 2.0941 | 0.2302 | 97.9% | 1000.5 | 1001.168 | 10 | 5.469 | 62.5% | 2 | R.LQAEIEGLK.G | 1 |
|  | Astrin\_STLCLD20\_112214\_tube2\_01.08900.08900.2 | 3.2154 | 0.2175 | 99.9% | 1001.3122 | 1001.168 | 3 | 5.211 | 87.5% | 3 | R.LQAEIEGLK.G | 2 |
|  | Astrin\_STLCLD20\_112214\_01.05091.05091.2 | 3.6977 | 0.2385 | 100.0% | 1342.2922 | 1342.5381 | 1 | 6.616 | 77.3% | 5 | R.LQAEIEGLKGQR.A | 2 |
|  | Astrin\_STLCLD20\_112214\_tube2\_01.12674.12674.3 | 4.1554 | 0.4053 | 99.7% | 2669.7544 | 2668.967 | 1 | 7.6 | 31.2% | 1 | R.LQAEIEGLKGQRASLEAAIADAEQR.G | 3 |
|  | Astrin\_STLCLD20\_112214\_01.10636.10636.1 | 2.8357 | 0.5447 | 100.0% | 1344.6 | 1345.452 | 1 | 8.051 | 62.5% | 1 | R.ASLEAAIADAEQR.G | 1 |
|  | Astrin\_STLCLD20\_112214\_01.10616.10616.2 | 4.3054 | 0.4007 | 100.0% | 1346.3522 | 1345.452 | 1 | 7.313 | 70.8% | 6 | R.ASLEAAIADAEQR.G | 2 |
|  | Astrin\_STLCLD20\_112214\_tube2\_01.14822.14822.2 | 5.1535 | 0.4515 | 100.0% | 1956.7122 | 1957.1912 | 1 | 8.427 | 61.1% | 5 | R.ASLEAAIADAEQRGELAIK.D | 2 |
|  | Astrin\_STLCLD20\_112214\_02.13493.13493.3 | 3.6053 | 0.3203 | 99.6% | 1957.4343 | 1957.1912 | 24 | 6.57 | 34.7% | 3 | R.ASLEAAIADAEQRGELAIK.D | 3 |
|  | Astrin\_STLCLD20\_112214\_tube2\_01.15478.15478.3 | 5.8266 | 0.4404 | 100.0% | 2456.6343 | 2456.7153 | 1 | 7.699 | 33.7% | 6 | R.ASLEAAIADAEQRGELAIKDANAK.L | 3 |
|  | Astrin\_STLCLD20\_112214\_01.09673.09673.1 | 2.1044 | 0.2633 | 98.8% | 1129.54 | 1130.2865 | 6 | 5.28 | 55.6% | 2 | K.LSELEAALQR.A | 1 |
|  | Astrin\_STLCLD20\_112214\_tube2\_01.11018.11018.2 | 4.2332 | 0.2608 | 100.0% | 1130.3522 | 1130.2865 | 1 | 6.149 | 83.3% | 7 | K.LSELEAALQR.A | 2 |
|  | Astrin\_STLCLD20\_112214\_tube2\_01.10042.10042.2 | 3.7422 | 0.2042 | 99.9% | 1551.3121 | 1551.801 | 1 | 5.206 | 72.7% | 2 | R.QLREYQELMNVK.L | 2 |
|  | Astrin\_STLCLD20\_112214\_tube2\_01.09255.09255.1 | 2.466 | 0.2121 | 98.9% | 1153.62 | 1154.3234 | 15 | 5.518 | 68.8% | 2 | R.EYQELMNVK.L | 11 |
|  | Astrin\_STLCLD20\_112214\_tube2\_01.09290.09290.2 | 2.4474 | 0.2352 | 98.7% | 1154.3322 | 1154.3234 | 15 | 6.215 | 62.5% | 3 | R.EYQELMNVK.L | 22 |
|  | Astrin\_STLCLD20\_112214\_tube2\_01.12144.12144.2 | 2.7748 | 0.2075 | 98.6% | 1406.3922 | 1406.6653 | 2 | 5.0 | 68.2% | 1 | K.LALDIEIATYRK.L | 22 |
|  | Astrin\_STLCLD20\_112214\_tube2\_01.10205.10205.3 | 4.7417 | 0.4134 | 100.0% | 2518.0144 | 2518.8628 | 1 | 6.553 | 34.5% | 2 | R.KLLEGEESRLESGMQNMSIHTK.T | 3 |
|  | Astrin\_STLCLD20\_112214\_tube2\_01.10989.10989.3 | 5.2374 | 0.3028 | 99.7% | 2391.0842 | 2390.6887 | 1 | 6.798 | 36.2% | 2 | K.LLEGEESRLESGMQNMSIHTK.T | 3 |
|  | Astrin\_STLCLD20\_112214\_tube2\_01.07485.07485.2 | 3.9735 | 0.4377 | 100.0% | 1476.3522 | 1476.7058 | 1 | 7.747 | 83.3% | 2 | R.LESGMQNMSIHTK.T | 2 |
|  | Astrin\_STLCLD20\_112214\_tube2\_01.07472.07472.3 | 2.9709 | 0.3348 | 99.5% | 1476.8344 | 1476.7058 | 13 | 6.144 | 37.5% | 1 | R.LESGMQNMSIHTK.T | 3 |
|  | Astrin\_STLCLD20\_112214\_01.07169.07169.2 | 3.7622 | 0.3314 | 100.0% | 1474.3322 | 1474.6512 | 1 | 7.321 | 73.1% | 3 | R.DGKLVSESSDVLPK.- | 2 |
|  | Astrin\_STLCLD20\_112214\_01.06139.06139.2 | 3.5584 | 0.4345 | 100.0% | 1174.1522 | 1174.3367 | 1 | 7.499 | 80.0% | 11 | K.LVSESSDVLPK.- | 2 |

Similarities:
gi|67782365|ref|NP\_00(2:44)  
gi|47132620|ref|NP\_00(1:45)  
gi|119703753|ref|NP\_0(3:43)  

---

|  |  |  |  |  |  |  |  |  |
| --- | --- | --- | --- | --- | --- | --- | --- | --- |
| U | *gi|73623035|ref|NP\_00* | 86 | 350 | 56.1% | 1193 | 134422 | 5.0 | sperm associated antigen 5 [Homo sapiens] |

| Filename XCorr DeltCN Conf% ObsM+H+ CalcM+H+ SpR ZScore Ion% # Sequence  | | | | | | | | | | | | |
| --- | --- | --- | --- | --- | --- | --- | --- | --- | --- | --- | --- | --- |
| \* | Astrin\_STLCLD20\_112214\_tube2\_01.05944.05944.2 | 3.5391 | 0.3781 | 100.0% | 1462.1921 | 1462.5156 | 1 | 6.291 | 66.7% | 1 | R.TDLSSEHFSHSSK.W | 2 |
| \* | Astrin\_STLCLD20\_112214\_01.10850.10850.2 | 3.9524 | 0.365 | 100.0% | 1653.5122 | 1653.8445 | 1 | 8.171 | 67.9% | 3 | K.TSEEAVDPLGNYMVK.T | 2 |
| \* | Astrin\_STLCLD20\_112214\_tube2\_01.17284.17284.2 | 3.3186 | 0.5459 | 100.0% | 2323.5923 | 2323.6262 | 1 | 8.408 | 42.1% | 2 | K.TIVLVPS\*PLGQQQDMIFEAR.L | 2 |
| \* | Astrin\_STLCLD20\_112214\_02.11457.11457.2 | 5.1743 | 0.4952 | 100.0% | 1832.2922 | 1833.0668 | 1 | 8.616 | 65.6% | 7 | R.LDTMAETNSISLNGPLR.T | 2 |
| \* | Astrin\_STLCLD20\_112214\_tube2\_02.08271.08271.3 | 4.1338 | 0.4293 | 100.0% | 2532.3843 | 2532.8286 | 1 | 7.263 | 34.1% | 4 | R.LDTMAETNSISLNGPLRTDDLVR.E | 3 |
| \* | Astrin\_STLCLD20\_112214\_tube2\_01.19553.19553.3 | 6.1013 | 0.4712 | 100.0% | 3778.6443 | 3778.2102 | 1 | 7.846 | 23.5% | 1 | R.TEAVREDLVPSESNAFLPSSVLWLSPSTALAADFR.V | 3 |
| \* | Astrin\_STLCLD20\_112214\_tube2\_02.11436.11436.3 | 4.6436 | 0.2379 | 99.5% | 3857.0344 | 3858.2102 | 2 | 5.468 | 22.8% | 1 | R.TEAVREDLVPSESNAFLPSSVLWLSPST#ALAADFR.V | 3 |
| \* | Astrin\_STLCLD20\_112214\_01.15097.15097.3 | 5.6025 | 0.2446 | 99.8% | 3858.0842 | 3858.2102 | 2 | 6.411 | 27.9% | 1 | R.TEAVREDLVPSESNAFLPSSVLWLSPS\*TALAADFR.V | 3 |
| \* | Astrin\_STLCLD20\_112214\_01.15035.15035.3 | 6.0953 | 0.1971 | 99.8% | 3859.1042 | 3858.2102 | 1 | 6.058 | 29.4% | 3 | R.TEAVREDLVPSESNAFLPSSVLWLS\*PSTALAADFR.V | 3 |
| \* | Astrin\_STLCLD20\_112214\_tube2\_01.09070.09070.3 | 5.1026 | 0.4147 | 100.0% | 2219.3643 | 2220.3752 | 1 | 7.114 | 45.8% | 10 | R.VNHVDPEEEIVEHGAMEER.E | 3 |
| \* | Astrin\_STLCLD20\_112214\_tube2\_01.09050.09050.2 | 5.8438 | 0.5651 | 100.0% | 2219.6921 | 2220.3752 | 1 | 9.622 | 72.2% | 2 | R.VNHVDPEEEIVEHGAMEER.E | 2 |
| \* | Astrin\_STLCLD20\_112214\_tube2\_01.20054.20054.2 | 5.1826 | 0.5688 | 100.0% | 2064.132 | 2064.3606 | 1 | 9.338 | 58.8% | 3 | R.ILGSDTESWMSPLAWLEK.G | 2 |
| \* | Astrin\_STLCLD20\_112214\_tube2\_01.20486.20486.2 | 4.7872 | 0.5115 | 100.0% | 2143.392 | 2144.3606 | 1 | 8.46 | 61.8% | 4 | R.ILGSDTESWMS\*PLAWLEK.G | 2 |
| \* | Astrin\_STLCLD20\_112214\_tube2\_01.12158.12158.2 | 3.3134 | 0.4624 | 100.0% | 1332.7922 | 1333.5457 | 1 | 7.786 | 81.8% | 3 | K.GVNTSVMLENLR.Q | 2 |
| \* | Astrin\_STLCLD20\_112214\_tube2\_01.13191.13191.2 | 2.2102 | 0.2081 | 96.4% | 1132.6522 | 1132.3635 | 56 | 4.914 | 61.1% | 1 | R.QSLSLPSMLR.D | 2 |
| \* | Astrin\_STLCLD20\_112214\_tube2\_01.00602.00602.2 | 7.5875 | 0.6387 | 100.0% | 2165.7722 | 2166.4795 | 1 | 11.53 | 72.2% | 19 | R.HDLEDNLLSSLVILEVLSR.Q | 2 |
| \* | Astrin\_STLCLD20\_112214\_tube2\_01.00584.00584.3 | 3.4163 | 0.3521 | 99.8% | 2167.0444 | 2166.4795 | 4 | 6.465 | 36.1% | 4 | R.HDLEDNLLSSLVILEVLSR.Q | 3 |
| \* | Astrin\_STLCLD20\_112214\_01.05011.05011.3 | 5.2468 | 0.4639 | 100.0% | 2868.3843 | 2868.0 | 1 | 7.471 | 33.7% | 4 | K.SQLAVPHPETQDSSTQTDTSHSGITNK.L | 3 |
| \* | Astrin\_STLCLD20\_112214\_tube2\_01.06624.06624.3 | 4.0024 | 0.2379 | 99.5% | 2104.7344 | 2105.3794 | 3 | 5.318 | 42.6% | 1 | K.LQHLKESHEMGQALQQAR.N | 3 |
| \* | Astrin\_STLCLD20\_112214\_tube2\_01.06368.06368.2 | 3.8012 | 0.3447 | 100.0% | 1485.6122 | 1485.6146 | 1 | 6.998 | 70.8% | 2 | K.ESHEMGQALQQAR.N | 2 |
| \* | Astrin\_STLCLD20\_112214\_tube2\_01.14876.14876.2 | 3.5588 | 0.4082 | 100.0% | 1305.6721 | 1305.578 | 1 | 6.935 | 75.0% | 8 | R.NVMQSWVLISK.E | 2 |
| \* | Astrin\_STLCLD20\_112214\_tube2\_01.16263.16263.3 | 6.2339 | 0.4205 | 100.0% | 2891.5144 | 2892.2793 | 1 | 6.667 | 34.4% | 3 | K.ELISLLHLSLLHLEEDKTTVSQESR.R | 3 |
| \* | Astrin\_STLCLD20\_112214\_tube2\_01.15131.15131.3 | 3.7659 | 0.4106 | 99.7% | 2074.5244 | 2074.267 | 1 | 6.615 | 31.9% | 3 | R.GKDAAEIVLEAFCAHASQR.I | 3 |
| \* | Astrin\_STLCLD20\_112214\_tube2\_01.13140.13140.1 | 2.4811 | 0.4938 | 100.0% | 1390.58 | 1391.5823 | 4 | 7.486 | 54.5% | 2 | R.ISQLEQDLASMR.E | 1 |
| \* | Astrin\_STLCLD20\_112214\_tube2\_01.13196.13196.2 | 3.8244 | 0.3755 | 100.0% | 1391.2522 | 1391.5823 | 1 | 8.253 | 68.2% | 7 | R.ISQLEQDLASMR.E | 2 |
| \* | Astrin\_STLCLD20\_112214\_tube2\_01.14393.14393.3 | 2.7911 | 0.2251 | 95.3% | 1823.6044 | 1824.0618 | 6 | 4.662 | 37.5% | 1 | R.ISQLEQDLASMREFR.G | 3 |
| \* | Astrin\_STLCLD20\_112214\_tube2\_01.11734.11734.3 | 4.8788 | 0.5072 | 100.0% | 2124.8643 | 2125.4788 | 1 | 8.156 | 40.3% | 3 | R.EFRGLLKDAQTQLVGLHAK.Q | 3 |
| \* | Astrin\_STLCLD20\_112214\_01.09409.09409.2 | 3.886 | 0.3525 | 100.0% | 1692.7522 | 1692.9994 | 1 | 6.81 | 60.0% | 1 | R.GLLKDAQTQLVGLHAK.Q | 2 |
| \* | Astrin\_STLCLD20\_112214\_01.09433.09433.3 | 3.9096 | 0.2966 | 99.8% | 1693.2843 | 1692.9994 | 8 | 5.405 | 40.0% | 1 | R.GLLKDAQTQLVGLHAK.Q | 3 |
| \* | Astrin\_STLCLD20\_112214\_tube2\_01.07730.07730.2 | 3.6744 | 0.4439 | 100.0% | 1280.6921 | 1281.4545 | 1 | 7.255 | 68.2% | 2 | K.DAQTQLVGLHAK.Q | 2 |
| \* | Astrin\_STLCLD20\_112214\_tube2\_01.17201.17201.2 | 5.0867 | 0.5326 | 100.0% | 2390.6921 | 2390.612 | 1 | 8.921 | 50.0% | 4 | K.QEELVQQTVSLTSTLQQDWR.S | 2 |
| \* | Astrin\_STLCLD20\_112214\_02.15251.15251.3 | 4.0911 | 0.4276 | 100.0% | 2391.3245 | 2390.612 | 1 | 6.242 | 35.5% | 2 | K.QEELVQQTVSLTSTLQQDWR.S | 3 |
| \* | Astrin\_STLCLD20\_112214\_tube2\_01.18215.18215.1 | 2.2791 | 0.1827 | 98.1% | 1786.86 | 1787.0405 | 1 | 4.769 | 46.4% | 1 | R.SMQLDYTTWTALLSR.S | 1 |
| \* | Astrin\_STLCLD20\_112214\_tube2\_01.18255.18255.2 | 5.4153 | 0.4536 | 100.0% | 1787.4922 | 1787.0405 | 1 | 8.786 | 78.6% | 6 | R.SMQLDYTTWTALLSR.S | 2 |
| \* | Astrin\_STLCLD20\_112214\_tube2\_01.07226.07226.3 | 3.6587 | 0.1959 | 97.8% | 2344.4644 | 2344.5437 | 1 | 5.04 | 36.8% | 1 | K.SQQALQERDVAIEEKQEVSR.V | 3 |
| \* | Astrin\_STLCLD20\_112214\_tube2\_02.05371.05371.2 | 3.7281 | 0.471 | 100.0% | 1403.9722 | 1403.5321 | 3 | 8.008 | 68.2% | 10 | R.DVAIEEKQEVSR.V | 2 |
| \* | Astrin\_STLCLD20\_112214\_tube2\_02.06710.06710.2 | 3.8452 | 0.474 | 100.0% | 1533.1522 | 1533.6849 | 1 | 8.359 | 70.8% | 2 | R.VLEQVSAQLEECK.G | 2 |
| \* | Astrin\_STLCLD20\_112214\_tube2\_02.07473.07473.3 | 5.0764 | 0.4245 | 100.0% | 2919.1143 | 2919.1382 | 1 | 8.597 | 35.4% | 3 | R.VLEQVSAQLEECKGQTEQLELENSR.L | 3 |
| \* | Astrin\_STLCLD20\_112214\_tube2\_01.07600.07600.2 | 4.0245 | 0.4226 | 100.0% | 1404.1122 | 1404.4764 | 1 | 7.53 | 72.7% | 4 | K.GQTEQLELENSR.L | 2 |
| \* | Astrin\_STLCLD20\_112214\_tube2\_02.08883.08883.3 | 3.8198 | 0.4167 | 100.0% | 1574.3344 | 1573.848 | 2 | 7.232 | 46.2% | 1 | R.AQLQILANMDSQLK.E | 3 |
| \* | Astrin\_STLCLD20\_112214\_tube2\_01.14447.14447.2 | 5.6941 | 0.3751 | 100.0% | 1574.5521 | 1573.848 | 1 | 7.272 | 76.9% | 15 | R.AQLQILANMDSQLK.E | 2 |
| \* | Astrin\_STLCLD20\_112214\_02.08673.08673.3 | 4.7835 | 0.275 | 99.7% | 1724.2144 | 1723.9879 | 1 | 5.383 | 51.8% | 5 | K.HMQAELQQQQAVLAK.E | 3 |
| \* | Astrin\_STLCLD20\_112214\_01.05265.05265.2 | 5.4932 | 0.5308 | 100.0% | 1724.4521 | 1723.9879 | 1 | 9.378 | 82.1% | 11 | K.HMQAELQQQQAVLAK.E | 2 |
| \* | Astrin\_STLCLD20\_112214\_tube2\_02.06391.06391.3 | 3.7142 | 0.4353 | 100.0% | 2108.1843 | 2108.4233 | 1 | 6.886 | 39.7% | 1 | K.HMQAELQQQQAVLAKEVR.D | 3 |
| \* | Astrin\_STLCLD20\_112214\_tube2\_02.10047.10047.3 | 5.7987 | 0.477 | 100.0% | 3287.3643 | 3286.5862 | 1 | 8.361 | 32.4% | 3 | R.DLKETLEFADQENQVAHLELGQVECQLK.T | 3 |
| \* | Astrin\_STLCLD20\_112214\_tube2\_01.08762.08762.1 | 1.6191 | 0.2292 | 95.5% | 831.57 | 831.9878 | 184 | 4.297 | 50.0% | 1 | K.TTLEVLR.E | 1 |
| \* | Astrin\_STLCLD20\_112214\_01.06937.06937.2 | 2.1675 | 0.1666 | 96.5% | 832.21216 | 831.9878 | 8 | 4.595 | 83.3% | 2 | K.TTLEVLR.E | 2 |
| \* | Astrin\_STLCLD20\_112214\_tube2\_01.11487.11487.2 | 4.8751 | 0.4855 | 100.0% | 1963.7522 | 1964.1462 | 1 | 8.859 | 68.8% | 1 | R.SLQCENLKDTVENLTAK.L | 2 |
| \* | Astrin\_STLCLD20\_112214\_tube2\_01.11504.11504.3 | 4.3754 | 0.418 | 100.0% | 1964.7244 | 1964.1462 | 1 | 7.109 | 46.9% | 2 | R.SLQCENLKDTVENLTAK.L | 3 |
| \* | Astrin\_STLCLD20\_112214\_tube2\_01.07992.07992.1 | 2.6097 | 0.3571 | 100.0% | 1674.85 | 1675.7899 | 1 | 6.876 | 53.6% | 1 | K.LASTIADNQEQDLEK.T | 1 |
| \* | Astrin\_STLCLD20\_112214\_01.05893.05893.2 | 4.9181 | 0.4366 | 100.0% | 1675.4122 | 1675.7899 | 1 | 7.793 | 71.4% | 11 | K.LASTIADNQEQDLEK.T | 2 |
| \* | Astrin\_STLCLD20\_112214\_tube2\_01.07742.07742.2 | 4.3765 | 0.5366 | 100.0% | 1932.5922 | 1933.0825 | 1 | 8.354 | 62.5% | 2 | K.LASTIADNQEQDLEKTR.Q | 2 |
| \* | Astrin\_STLCLD20\_112214\_tube2\_01.20468.20468.2 | 5.9673 | 0.4892 | 100.0% | 2046.8922 | 2047.443 | 1 | 9.578 | 61.8% | 26 | K.LGLLTEQLQSLTLFLQTK.L | 2 |
| \* | Astrin\_STLCLD20\_112214\_tube2\_02.12384.12384.3 | 5.4456 | 0.3899 | 100.0% | 2048.6343 | 2047.443 | 1 | 8.349 | 52.9% | 5 | K.LGLLTEQLQSLTLFLQTK.L | 3 |
| \* | Astrin\_STLCLD20\_112214\_02.17230.17230.2 | 5.3039 | 0.4946 | 100.0% | 2787.3123 | 2788.121 | 1 | 8.386 | 38.5% | 12 | R.TFLGSILTAVADEEPESTPVPLLGSDK.S | 2 |
| \* | Astrin\_STLCLD20\_112214\_tube2\_01.20228.20228.3 | 4.9896 | 0.5187 | 100.0% | 2788.4043 | 2788.121 | 1 | 7.984 | 32.7% | 1 | R.TFLGSILTAVADEEPESTPVPLLGSDK.S | 3 |
| \* | Astrin\_STLCLD20\_112214\_01.16711.16711.2 | 4.6126 | 0.5463 | 100.0% | 2868.0723 | 2868.121 | 1 | 8.66 | 46.2% | 3 | R.TFLGSILTAVADEEPESTPVPLLGS\*DK.S | 2 |
| \* | Astrin\_STLCLD20\_112214\_01.15506.15506.3 | 3.7448 | 0.3607 | 99.8% | 3350.2144 | 3350.7473 | 1 | 5.654 | 22.6% | 1 | R.TFLGSILTAVADEEPESTPVPLLGSDKSAFTR.V | 3 |
| \* | Astrin\_STLCLD20\_112214\_tube2\_01.20102.20102.3 | 4.9757 | 0.4221 | 100.0% | 3429.3542 | 3430.7473 | 2 | 7.39 | 28.2% | 4 | R.TFLGSILTAVADEEPESTPVPLLGSDKS\*AFTR.V | 3 |
| \* | Astrin\_STLCLD20\_112214\_01.15961.15961.3 | 4.1767 | 0.3315 | 99.8% | 3430.4944 | 3430.7473 | 2 | 6.642 | 25.8% | 1 | R.TFLGSILTAVADEEPESTPVPLLGS\*DKSAFTR.V | 3 |
| \* | Astrin\_STLCLD20\_112214\_02.17089.17089.3 | 4.3827 | 0.3058 | 99.8% | 3431.0344 | 3430.7473 | 1 | 5.465 | 23.4% | 1 | R.TFLGSILTAVADEEPESTPVPLLGSDKSAFT#R.V | 3 |
| \* | Astrin\_STLCLD20\_112214\_tube2\_01.05642.05642.2 | 4.6908 | 0.413 | 100.0% | 1595.3922 | 1595.7092 | 1 | 7.726 | 83.3% | 3 | R.LQAQEEQHQEVQK.A | 2 |
| \* | Astrin\_STLCLD20\_112214\_01.05780.05780.2 | 6.0944 | 0.3922 | 100.0% | 2148.5522 | 2149.3652 | 1 | 8.064 | 68.8% | 2 | R.YKNEKELQEVIQQQNEK.I | 2 |
| \* | Astrin\_STLCLD20\_112214\_01.05821.05821.3 | 6.054 | 0.3 | 100.0% | 2149.2244 | 2149.3652 | 1 | 6.537 | 53.1% | 5 | R.YKNEKELQEVIQQQNEK.I | 3 |
| \* | Astrin\_STLCLD20\_112214\_01.05057.05057.1 | 3.3999 | 0.2453 | 100.0% | 1485.72 | 1486.622 | 1 | 6.034 | 72.7% | 1 | K.ELQEVIQQQNEK.I | 1 |
| \* | Astrin\_STLCLD20\_112214\_01.05023.05023.2 | 4.696 | 0.2242 | 100.0% | 1486.6322 | 1486.622 | 1 | 6.56 | 86.4% | 9 | K.ELQEVIQQQNEK.I | 2 |
| \* | Astrin\_STLCLD20\_112214\_tube2\_01.12134.12134.2 | 3.8845 | 0.3436 | 100.0% | 1714.6122 | 1715.0 | 1 | 6.314 | 60.7% | 1 | K.ILEQIDKSGELISLR.E | 2 |
| \* | Astrin\_STLCLD20\_112214\_tube2\_01.13996.13996.3 | 5.0916 | 0.2947 | 99.8% | 2680.3743 | 2681.0618 | 1 | 6.413 | 40.9% | 4 | K.ILEQIDKSGELISLREEVTHLTR.S | 3 |
| \* | Astrin\_STLCLD20\_112214\_tube2\_01.12387.12387.2 | 4.1255 | 0.4074 | 100.0% | 1840.6721 | 1841.0745 | 1 | 6.812 | 56.7% | 4 | K.SGELISLREEVTHLTR.S | 2 |
| \* | Astrin\_STLCLD20\_112214\_tube2\_01.12356.12356.3 | 5.048 | 0.3687 | 100.0% | 1841.6344 | 1841.0745 | 1 | 6.602 | 53.3% | 13 | K.SGELISLREEVTHLTR.S | 3 |
| \* | Astrin\_STLCLD20\_112214\_tube2\_01.09807.09807.2 | 2.8019 | 0.2287 | 99.4% | 1104.1721 | 1104.248 | 16 | 5.894 | 75.0% | 2 | K.VWLSQEVDK.L | 2 |
| \* | Astrin\_STLCLD20\_112214\_tube2\_01.11774.11774.2 | 3.4549 | 0.312 | 100.0% | 1373.2922 | 1373.595 | 1 | 6.164 | 80.0% | 8 | K.VWLSQEVDKLR.V | 2 |
| \* | Astrin\_STLCLD20\_112214\_01.11097.11097.2 | 2.572 | 0.3484 | 100.0% | 898.1122 | 898.16644 | 1 | 7.17 | 91.7% | 3 | R.VMFLEMK.N | 2 |
| \* | Astrin\_STLCLD20\_112214\_01.08917.08917.2 | 2.8217 | 0.3663 | 100.0% | 1269.3922 | 1269.5598 | 1 | 6.237 | 83.3% | 4 | R.VMFLEMKNEK.E | 2 |
| \* | Astrin\_STLCLD20\_112214\_tube2\_01.09748.09748.1 | 2.4559 | 0.2094 | 98.7% | 1000.48 | 1001.1277 | 7 | 4.668 | 71.4% | 6 | R.NILEENLR.R | 1 |
| \* | Astrin\_STLCLD20\_112214\_01.08473.08473.2 | 2.7832 | 0.041 | 96.7% | 1001.1122 | 1001.1277 | 2 | 4.345 | 85.7% | 1 | R.NILEENLR.R | 2 |
| \* | Astrin\_STLCLD20\_112214\_01.10858.10858.2 | 5.4045 | 0.4677 | 100.0% | 2229.9922 | 2230.526 | 1 | 8.234 | 70.6% | 2 | R.RSDKELEKLDDIVQHIYK.T | 2 |
| \* | Astrin\_STLCLD20\_112214\_tube2\_01.12357.12357.3 | 5.5516 | 0.4508 | 100.0% | 2230.8245 | 2230.526 | 1 | 8.023 | 39.7% | 5 | R.RSDKELEKLDDIVQHIYK.T | 3 |
| \* | Astrin\_STLCLD20\_112214\_tube2\_01.13635.13635.3 | 5.3612 | 0.3134 | 100.0% | 2074.1643 | 2074.3384 | 1 | 6.803 | 48.4% | 5 | R.SDKELEKLDDIVQHIYK.T | 3 |
| \* | Astrin\_STLCLD20\_112214\_01.11344.11344.2 | 5.2585 | 0.535 | 100.0% | 2074.632 | 2074.3384 | 1 | 9.406 | 68.8% | 2 | R.SDKELEKLDDIVQHIYK.T | 2 |
| \* | Astrin\_STLCLD20\_112214\_tube2\_01.12887.12887.3 | 2.821 | 0.3264 | 99.6% | 1743.5643 | 1743.9977 | 1 | 5.427 | 44.2% | 1 | K.ELEKLDDIVQHIYK.T | 3 |
| \* | Astrin\_STLCLD20\_112214\_01.11006.11006.2 | 2.5153 | 0.3116 | 99.0% | 1743.5922 | 1743.9977 | 1 | 5.642 | 57.7% | 1 | K.ELEKLDDIVQHIYK.T | 2 |
| \* | Astrin\_STLCLD20\_112214\_01.08659.08659.2 | 3.4257 | 0.3789 | 100.0% | 1244.5122 | 1244.4331 | 44 | 6.931 | 61.1% | 4 | K.LDDIVQHIYK.T | 2 |
| \* | Astrin\_STLCLD20\_112214\_tube2\_01.13808.13808.2 | 2.6383 | 0.3226 | 99.7% | 1127.3922 | 1127.3696 | 3 | 5.416 | 83.3% | 8 | K.TLLSIPEVVR.G | 2 |
| \* | Astrin\_STLCLD20\_112214\_tube2\_01.19313.19313.2 | 2.5142 | 0.3093 | 99.1% | 1493.9122 | 1494.6941 | 1 | 5.882 | 54.2% | 1 | R.GCKELQGLLEFLS.- | 2 |
| \* | Astrin\_STLCLD20\_112214\_01.16099.16099.1 | 1.9036 | 0.305 | 100.0% | 1148.54 | 1149.3293 | 1 | 5.871 | 61.1% | 5 | K.ELQGLLEFLS.- | 1 |

---

|  |  |  |  |  |  |  |  |  |
| --- | --- | --- | --- | --- | --- | --- | --- | --- |
| U | *gi|29788785|ref|NP\_82* | 31 | 175 | 53.2% | 444 | 49671 | 4.9 | tubulin, beta [Homo sapiens] |

| Filename XCorr DeltCN Conf% ObsM+H+ CalcM+H+ SpR ZScore Ion% # Sequence  | | | | | | | | | | | | |
| --- | --- | --- | --- | --- | --- | --- | --- | --- | --- | --- | --- | --- |
| \* | Astrin\_STLCLD20\_112214\_02.12189.12189.3 | 5.5702 | 0.3792 | 100.0% | 3104.2744 | 3104.2725 | 1 | 7.468 | 32.7% | 6 | K.FWEVISDEHGIDPTGTYHGDSDLQLDR.I | 3 |
| \* | Astrin\_STLCLD20\_112214\_01.06379.06379.2 | 3.8259 | 0.5649 | 100.0% | 1301.8922 | 1302.4265 | 1 | 9.861 | 86.4% | 14 | R.ISVYYNEATGGK.Y | 2 |
|  | Astrin\_STLCLD20\_112214\_01.11522.11522.2 | 4.511 | 0.4938 | 100.0% | 1617.3121 | 1616.8701 | 1 | 8.254 | 64.3% | 14 | R.AILVDLEPGTMDSVR.S | 222 |
|  | Astrin\_STLCLD20\_112214\_tube2\_01.16521.16521.2 | 5.3152 | 0.5218 | 100.0% | 2799.0322 | 2800.0647 | 1 | 8.506 | 44.0% | 6 | R.SGPFGQIFRPDNFVFGQSGAGNNWAK.G | 222 |
|  | Astrin\_STLCLD20\_112214\_tube2\_01.16562.16562.3 | 6.8919 | 0.438 | 100.0% | 2799.8342 | 2800.0647 | 1 | 8.685 | 39.0% | 8 | R.SGPFGQIFRPDNFVFGQSGAGNNWAK.G | 333 |
|  | Astrin\_STLCLD20\_112214\_tube2\_01.17294.17294.2 | 6.9168 | 0.5867 | 100.0% | 1959.5922 | 1960.151 | 1 | 11.206 | 79.4% | 2 | K.GHYTEGAELVDSVLDVVR.K | 2222 |
|  | Astrin\_STLCLD20\_112214\_01.13379.13379.3 | 3.6233 | 0.2439 | 99.6% | 1960.6144 | 1960.151 | 1 | 5.03 | 38.2% | 3 | K.GHYTEGAELVDSVLDVVR.K | 3333 |
|  | Astrin\_STLCLD20\_112214\_01.12767.12767.2 | 5.5584 | 0.5301 | 100.0% | 2087.7522 | 2088.325 | 1 | 9.725 | 72.2% | 3 | K.GHYTEGAELVDSVLDVVRK.E | 2222 |
|  | Astrin\_STLCLD20\_112214\_tube2\_01.15854.15854.3 | 4.7062 | 0.4415 | 100.0% | 2088.8943 | 2088.325 | 1 | 7.599 | 41.7% | 5 | K.GHYTEGAELVDSVLDVVRK.E | 3333 |
|  | Astrin\_STLCLD20\_112214\_tube2\_01.05867.05867.2 | 2.3753 | 0.2512 | 98.9% | 1078.1522 | 1078.1698 | 1 | 5.048 | 85.7% | 1 | K.IREEYPDR.I | 222 |
|  | Astrin\_STLCLD20\_112214\_01.10173.10173.1 | 2.1231 | 0.2434 | 97.9% | 1319.72 | 1320.5896 | 53 | 5.055 | 45.5% | 2 | R.IMNTFSVVPSPK.V | 111 |
|  | Astrin\_STLCLD20\_112214\_tube2\_01.11552.11552.2 | 4.4232 | 0.4158 | 100.0% | 1320.1721 | 1320.5896 | 1 | 8.424 | 77.3% | 12 | R.IMNTFSVVPSPK.V | 222 |
|  | Astrin\_STLCLD20\_112214\_tube2\_01.09993.09993.2 | 3.1291 | 0.339 | 100.0% | 1131.4922 | 1131.2767 | 11 | 5.764 | 77.8% | 19 | R.FPGQLNADLR.K | 22222 |
|  | Astrin\_STLCLD20\_112214\_tube2\_01.08446.08446.2 | 2.7132 | 0.2665 | 99.3% | 1259.5122 | 1259.4508 | 43 | 5.338 | 60.0% | 3 | R.FPGQLNADLRK.L | 22222 |
|  | Astrin\_STLCLD20\_112214\_tube2\_01.11456.11456.2 | 3.6404 | 0.3874 | 100.0% | 1272.3522 | 1272.5945 | 1 | 6.64 | 70.0% | 4 | R.KLAVNMVPFPR.L | 22222 |
|  | Astrin\_STLCLD20\_112214\_01.11192.11192.1 | 2.2399 | 0.2147 | 98.0% | 1143.63 | 1144.4204 | 6 | 6.378 | 61.1% | 2 | K.LAVNMVPFPR.L | 11111 |
|  | Astrin\_STLCLD20\_112214\_01.11233.11233.2 | 3.8296 | 0.4984 | 100.0% | 1144.4722 | 1144.4204 | 1 | 8.878 | 94.4% | 6 | K.LAVNMVPFPR.L | 22222 |
|  | Astrin\_STLCLD20\_112214\_tube2\_01.16406.16406.2 | 3.7237 | 0.4102 | 100.0% | 1621.0322 | 1621.9403 | 1 | 8.877 | 73.1% | 7 | R.LHFFMPGFAPLTSR.G | 2222 |
|  | Astrin\_STLCLD20\_112214\_tube2\_01.16287.16287.3 | 4.1587 | 0.348 | 99.7% | 1622.1543 | 1621.9403 | 2 | 6.383 | 50.0% | 4 | R.LHFFMPGFAPLTSR.G | 3333 |
| \* | Astrin\_STLCLD20\_112214\_tube2\_01.15897.15897.2 | 4.0211 | 0.4382 | 100.0% | 1661.4521 | 1660.9078 | 1 | 7.621 | 67.9% | 8 | R.ALTVPELTQQVFDAK.N | 2 |
|  | Astrin\_STLCLD20\_112214\_tube2\_01.14066.14066.1 | 1.7627 | 0.4097 | 100.0% | 1039.58 | 1040.2505 | 13 | 6.463 | 56.2% | 6 | R.YLTVAAVFR.G | 11 |
|  | Astrin\_STLCLD20\_112214\_01.11702.11702.2 | 2.7866 | 0.3903 | 100.0% | 1041.7322 | 1040.2505 | 1 | 5.881 | 87.5% | 6 | R.YLTVAAVFR.G | 22 |
|  | Astrin\_STLCLD20\_112214\_tube2\_02.07332.07332.3 | 4.839 | 0.2248 | 99.8% | 1926.3243 | 1925.2405 | 1 | 5.81 | 50.0% | 2 | R.MSMKEVDEQMLNVQNK.N | 333 |
|  | Astrin\_STLCLD20\_112214\_tube2\_01.08570.08570.2 | 4.1176 | 0.2811 | 100.0% | 1447.3322 | 1447.6031 | 1 | 6.064 | 81.8% | 4 | K.EVDEQMLNVQNK.N | 222 |
|  | Astrin\_STLCLD20\_112214\_tube2\_01.15262.15262.2 | 3.3078 | 0.3064 | 99.9% | 1698.6122 | 1697.8877 | 1 | 5.236 | 61.5% | 3 | K.NSSYFVEWIPNNVK.T | 22222 |
| \* | Astrin\_STLCLD20\_112214\_01.14077.14077.2 | 4.2661 | 0.4475 | 100.0% | 1870.8922 | 1871.2018 | 1 | 8.012 | 62.5% | 3 | K.MAVTFIGNSTAIQELFK.R | 2 |
| \* | Astrin\_STLCLD20\_112214\_tube2\_01.16989.16989.2 | 5.2038 | 0.5156 | 100.0% | 2026.4521 | 2027.3893 | 1 | 8.599 | 70.6% | 1 | K.MAVTFIGNSTAIQELFKR.I | 2 |
| \* | Astrin\_STLCLD20\_112214\_02.15201.15201.3 | 3.6006 | 0.3355 | 99.8% | 2027.3043 | 2027.3893 | 1 | 6.97 | 41.2% | 5 | K.MAVTFIGNSTAIQELFKR.I | 3 |
|  | Astrin\_STLCLD20\_112214\_tube2\_01.12078.12078.2 | 3.0386 | 0.2531 | 99.7% | 1385.6921 | 1386.6116 | 84 | 5.63 | 55.0% | 5 | K.RISEQFTAMFR.R | 2222 |
|  | Astrin\_STLCLD20\_112214\_tube2\_01.13623.13623.1 | 1.9755 | 0.2993 | 100.0% | 1229.5 | 1230.4241 | 1 | 5.342 | 61.1% | 1 | R.ISEQFTAMFR.R | 1111 |
|  | Astrin\_STLCLD20\_112214\_02.12843.12843.2 | 3.8745 | 0.4739 | 100.0% | 1230.1921 | 1230.4241 | 1 | 8.01 | 94.4% | 10 | R.ISEQFTAMFR.R | 2222 |

Similarities:
gi|5174735|ref|NP\_006(24:7)  
gi|29788768|ref|NP\_82(21:10)  
gi|50592996|ref|NP\_00(16:15)  
gi|14210536|ref|NP\_11(8:23)  

---

|  |  |  |  |  |  |  |  |  |
| --- | --- | --- | --- | --- | --- | --- | --- | --- |
| U | *gi|5174735|ref|NP\_006* | 29 | 140 | 53.0% | 445 | 49831 | 4.9 | tubulin, beta, 2 [Homo sapiens] |

| Filename XCorr DeltCN Conf% ObsM+H+ CalcM+H+ SpR ZScore Ion% # Sequence  | | | | | | | | | | | | |
| --- | --- | --- | --- | --- | --- | --- | --- | --- | --- | --- | --- | --- |
|  | Astrin\_STLCLD20\_112214\_02.12119.12119.3 | 6.0814 | 0.3723 | 100.0% | 3118.4043 | 3118.2996 | 1 | 7.723 | 33.7% | 1 | K.FWEVISDEHGIDPTGTYHGDSDLQLER.I | 3 |
| \* | Astrin\_STLCLD20\_112214\_01.06439.06439.2 | 3.4957 | 0.376 | 100.0% | 1328.7322 | 1329.4521 | 1 | 7.329 | 86.4% | 4 | R.INVYYNEATGGK.Y | 2 |
|  | Astrin\_STLCLD20\_112214\_tube2\_01.12946.12946.2 | 4.312 | 0.3215 | 100.0% | 1604.5322 | 1602.8431 | 1 | 6.404 | 67.9% | 6 | R.AVLVDLEPGTMDSVR.S | 2 |
|  | Astrin\_STLCLD20\_112214\_tube2\_01.16521.16521.2 | 5.3152 | 0.5218 | 100.0% | 2799.0322 | 2800.0647 | 1 | 8.506 | 44.0% | 6 | R.SGPFGQIFRPDNFVFGQSGAGNNWAK.G | 222 |
|  | Astrin\_STLCLD20\_112214\_tube2\_01.16562.16562.3 | 6.8919 | 0.438 | 100.0% | 2799.8342 | 2800.0647 | 1 | 8.685 | 39.0% | 8 | R.SGPFGQIFRPDNFVFGQSGAGNNWAK.G | 333 |
|  | Astrin\_STLCLD20\_112214\_tube2\_01.17294.17294.2 | 6.9168 | 0.5867 | 100.0% | 1959.5922 | 1960.151 | 1 | 11.206 | 79.4% | 2 | K.GHYTEGAELVDSVLDVVR.K | 2222 |
|  | Astrin\_STLCLD20\_112214\_01.13379.13379.3 | 3.6233 | 0.2439 | 99.6% | 1960.6144 | 1960.151 | 1 | 5.03 | 38.2% | 3 | K.GHYTEGAELVDSVLDVVR.K | 3333 |
|  | Astrin\_STLCLD20\_112214\_01.12767.12767.2 | 5.5584 | 0.5301 | 100.0% | 2087.7522 | 2088.325 | 1 | 9.725 | 72.2% | 3 | K.GHYTEGAELVDSVLDVVRK.E | 2222 |
|  | Astrin\_STLCLD20\_112214\_tube2\_01.15854.15854.3 | 4.7062 | 0.4415 | 100.0% | 2088.8943 | 2088.325 | 1 | 7.599 | 41.7% | 5 | K.GHYTEGAELVDSVLDVVRK.E | 3333 |
|  | Astrin\_STLCLD20\_112214\_tube2\_01.05867.05867.2 | 2.3753 | 0.2512 | 98.9% | 1078.1522 | 1078.1698 | 1 | 5.048 | 85.7% | 1 | K.IREEYPDR.I | 222 |
|  | Astrin\_STLCLD20\_112214\_01.10173.10173.1 | 2.1231 | 0.2434 | 97.9% | 1319.72 | 1320.5896 | 53 | 5.055 | 45.5% | 2 | R.IMNTFSVVPSPK.V | 111 |
|  | Astrin\_STLCLD20\_112214\_tube2\_01.11552.11552.2 | 4.4232 | 0.4158 | 100.0% | 1320.1721 | 1320.5896 | 1 | 8.424 | 77.3% | 12 | R.IMNTFSVVPSPK.V | 222 |
|  | Astrin\_STLCLD20\_112214\_tube2\_01.09993.09993.2 | 3.1291 | 0.339 | 100.0% | 1131.4922 | 1131.2767 | 11 | 5.764 | 77.8% | 19 | R.FPGQLNADLR.K | 22222 |
|  | Astrin\_STLCLD20\_112214\_tube2\_01.08446.08446.2 | 2.7132 | 0.2665 | 99.3% | 1259.5122 | 1259.4508 | 43 | 5.338 | 60.0% | 3 | R.FPGQLNADLRK.L | 22222 |
|  | Astrin\_STLCLD20\_112214\_tube2\_01.11456.11456.2 | 3.6404 | 0.3874 | 100.0% | 1272.3522 | 1272.5945 | 1 | 6.64 | 70.0% | 4 | R.KLAVNMVPFPR.L | 22222 |
|  | Astrin\_STLCLD20\_112214\_01.11192.11192.1 | 2.2399 | 0.2147 | 98.0% | 1143.63 | 1144.4204 | 6 | 6.378 | 61.1% | 2 | K.LAVNMVPFPR.L | 11111 |
|  | Astrin\_STLCLD20\_112214\_01.11233.11233.2 | 3.8296 | 0.4984 | 100.0% | 1144.4722 | 1144.4204 | 1 | 8.878 | 94.4% | 6 | K.LAVNMVPFPR.L | 22222 |
|  | Astrin\_STLCLD20\_112214\_tube2\_01.16406.16406.2 | 3.7237 | 0.4102 | 100.0% | 1621.0322 | 1621.9403 | 1 | 8.877 | 73.1% | 7 | R.LHFFMPGFAPLTSR.G | 2222 |
|  | Astrin\_STLCLD20\_112214\_tube2\_01.16287.16287.3 | 4.1587 | 0.348 | 99.7% | 1622.1543 | 1621.9403 | 2 | 6.383 | 50.0% | 4 | R.LHFFMPGFAPLTSR.G | 3333 |
|  | Astrin\_STLCLD20\_112214\_tube2\_01.16373.16373.2 | 4.1702 | 0.4839 | 100.0% | 1692.2322 | 1692.9678 | 1 | 8.782 | 78.6% | 4 | R.ALTVPELTQQMFDAK.N | 22 |
|  | Astrin\_STLCLD20\_112214\_tube2\_01.14066.14066.1 | 1.7627 | 0.4097 | 100.0% | 1039.58 | 1040.2505 | 13 | 6.463 | 56.2% | 6 | R.YLTVAAVFR.G | 11 |
|  | Astrin\_STLCLD20\_112214\_01.11702.11702.2 | 2.7866 | 0.3903 | 100.0% | 1041.7322 | 1040.2505 | 1 | 5.881 | 87.5% | 6 | R.YLTVAAVFR.G | 22 |
|  | Astrin\_STLCLD20\_112214\_tube2\_02.07332.07332.3 | 4.839 | 0.2248 | 99.8% | 1926.3243 | 1925.2405 | 1 | 5.81 | 50.0% | 2 | R.MSMKEVDEQMLNVQNK.N | 333 |
|  | Astrin\_STLCLD20\_112214\_tube2\_01.08570.08570.2 | 4.1176 | 0.2811 | 100.0% | 1447.3322 | 1447.6031 | 1 | 6.064 | 81.8% | 4 | K.EVDEQMLNVQNK.N | 222 |
|  | Astrin\_STLCLD20\_112214\_tube2\_01.15262.15262.2 | 3.3078 | 0.3064 | 99.9% | 1698.6122 | 1697.8877 | 1 | 5.236 | 61.5% | 3 | K.NSSYFVEWIPNNVK.T | 22222 |
|  | Astrin\_STLCLD20\_112214\_02.14525.14525.3 | 3.4924 | 0.1623 | 95.1% | 2016.4443 | 2015.335 | 1 | 5.966 | 32.4% | 1 | K.MSATFIGNSTAIQELFKR.I | 33 |
|  | Astrin\_STLCLD20\_112214\_tube2\_01.12078.12078.2 | 3.0386 | 0.2531 | 99.7% | 1385.6921 | 1386.6116 | 84 | 5.63 | 55.0% | 5 | K.RISEQFTAMFR.R | 2222 |
|  | Astrin\_STLCLD20\_112214\_tube2\_01.13623.13623.1 | 1.9755 | 0.2993 | 100.0% | 1229.5 | 1230.4241 | 1 | 5.342 | 61.1% | 1 | R.ISEQFTAMFR.R | 1111 |
|  | Astrin\_STLCLD20\_112214\_02.12843.12843.2 | 3.8745 | 0.4739 | 100.0% | 1230.1921 | 1230.4241 | 1 | 8.01 | 94.4% | 10 | R.ISEQFTAMFR.R | 2222 |

Similarities:
gi|29788785|ref|NP\_82(24:5)  
gi|29788768|ref|NP\_82(21:8)  
gi|50592996|ref|NP\_00(16:13)  
gi|14210536|ref|NP\_11(8:21)  

---

|  |  |  |  |  |  |  |  |  |
| --- | --- | --- | --- | --- | --- | --- | --- | --- |
| U | *gi|62414289|ref|NP\_00* | 27 | 45 | 51.7% | 466 | 53652 | 5.1 | vimentin [Homo sapiens] |

| Filename XCorr DeltCN Conf% ObsM+H+ CalcM+H+ SpR ZScore Ion% # Sequence  | | | | | | | | | | | | |
| --- | --- | --- | --- | --- | --- | --- | --- | --- | --- | --- | --- | --- |
| \* | Astrin\_STLCLD20\_112214\_tube2\_01.06398.06398.2 | 2.6618 | 0.2317 | 98.0% | 1496.2522 | 1495.6531 | 199 | 4.474 | 39.3% | 1 | R.MFGGPGTASRPSSSR.S | 2 |
| \* | Astrin\_STLCLD20\_112214\_tube2\_01.08566.08566.3 | 2.6309 | 0.3632 | 99.5% | 1496.6344 | 1496.6633 | 4 | 5.97 | 36.5% | 1 | R.TYSLGSALRPSTSR.S | 3 |
| \* | Astrin\_STLCLD20\_112214\_01.07441.07441.2 | 2.2012 | 0.3149 | 98.0% | 1497.3322 | 1496.6633 | 99 | 5.096 | 42.3% | 1 | R.TYSLGSALRPSTSR.S | 2 |
| \* | Astrin\_STLCLD20\_112214\_tube2\_01.08585.08585.2 | 3.4496 | 0.2935 | 100.0% | 1429.2522 | 1429.5724 | 1 | 5.546 | 69.2% | 2 | R.SLYASSPGGVYATR.S | 2 |
| \* | Astrin\_STLCLD20\_112214\_01.08125.08125.2 | 3.8456 | 0.3379 | 100.0% | 1510.3722 | 1509.5724 | 1 | 5.431 | 69.2% | 4 | R.SLYASS\*PGGVYATR.S | 2 |
|  | Astrin\_STLCLD20\_112214\_01.05755.05755.2 | 4.0579 | 0.4725 | 100.0% | 1588.5721 | 1588.7147 | 1 | 7.735 | 79.2% | 1 | R.TNEKVELQELNDR.F | 2 |
|  | Astrin\_STLCLD20\_112214\_01.05740.05740.3 | 3.2733 | 0.2085 | 98.5% | 1589.2444 | 1588.7147 | 2 | 5.213 | 50.0% | 1 | R.TNEKVELQELNDR.F | 3 |
| \* | Astrin\_STLCLD20\_112214\_01.06446.06446.2 | 2.1568 | 0.4069 | 99.7% | 1126.3522 | 1126.3005 | 111 | 6.366 | 62.5% | 1 | R.FANYIDKVR.F | 2 |
| \* | Astrin\_STLCLD20\_112214\_01.10841.10841.2 | 3.3857 | 0.4495 | 100.0% | 1539.9321 | 1540.8436 | 1 | 8.963 | 65.4% | 1 | K.ILLAELEQLKGQGK.S | 2 |
| \* | Astrin\_STLCLD20\_112214\_tube2\_01.10439.10439.2 | 3.0329 | 0.365 | 100.0% | 1498.3322 | 1498.6508 | 1 | 6.624 | 77.3% | 1 | K.SRLGDLYEEEMR.E | 2 |
| \* | Astrin\_STLCLD20\_112214\_tube2\_01.10162.10162.2 | 3.6185 | 0.4899 | 100.0% | 1255.3922 | 1255.385 | 1 | 7.589 | 77.8% | 5 | R.LGDLYEEEMR.E | 2 |
| \* | Astrin\_STLCLD20\_112214\_tube2\_01.11066.11066.3 | 2.902 | 0.2367 | 97.5% | 1689.6244 | 1689.881 | 4 | 4.699 | 40.4% | 1 | R.VEVERDNLAEDIMR.L | 3 |
| \* | Astrin\_STLCLD20\_112214\_tube2\_01.11614.11614.3 | 3.3778 | 0.1885 | 95.8% | 2352.0244 | 2352.581 | 25 | 5.003 | 31.9% | 1 | K.LQEEMLQREEAENTLQSFR.Q | 3 |
| \* | Astrin\_STLCLD20\_112214\_tube2\_01.09155.09155.2 | 2.29 | 0.306 | 98.7% | 1324.1721 | 1324.3898 | 74 | 5.481 | 50.0% | 2 | R.EEAENTLQSFR.Q | 2 |
| \* | Astrin\_STLCLD20\_112214\_tube2\_01.12725.12725.3 | 3.0053 | 0.2617 | 97.4% | 2394.2644 | 2394.5168 | 4 | 5.627 | 30.0% | 1 | R.EEAENTLQSFRQDVDNASLAR.L | 3 |
| \* | Astrin\_STLCLD20\_112214\_tube2\_01.13305.13305.2 | 4.6213 | 0.4259 | 100.0% | 1535.5922 | 1534.793 | 1 | 8.079 | 70.8% | 5 | R.KVESLQEEIAFLK.K | 2 |
| \* | Astrin\_STLCLD20\_112214\_01.10371.10371.3 | 3.5289 | 0.278 | 99.5% | 1663.2843 | 1662.967 | 4 | 5.53 | 40.4% | 1 | R.KVESLQEEIAFLKK.L | 3 |
|  | Astrin\_STLCLD20\_112214\_01.09596.09596.2 | 2.7851 | 0.2045 | 99.0% | 1311.0122 | 1310.4056 | 1 | 4.339 | 77.8% | 3 | K.NLQEAEEWYK.S | 2 |
| \* | Astrin\_STLCLD20\_112214\_01.05585.05585.2 | 3.1341 | 0.2545 | 99.9% | 1094.4321 | 1094.1692 | 2 | 6.774 | 72.2% | 3 | K.FADLSEAANR.N | 2 |
| \* | Astrin\_STLCLD20\_112214\_02.11982.11982.2 | 4.7386 | 0.4725 | 100.0% | 2187.4922 | 2188.33 | 1 | 8.958 | 58.3% | 1 | R.EMEENFAVEAANYQDTIGR.L | 2 |
| \* | Astrin\_STLCLD20\_112214\_02.12046.12046.3 | 2.8698 | 0.3179 | 99.5% | 2187.9844 | 2188.33 | 87 | 5.303 | 29.2% | 1 | R.EMEENFAVEAANYQDTIGR.L | 3 |
| \* | Astrin\_STLCLD20\_112214\_tube2\_01.09653.09653.2 | 4.5021 | 0.4789 | 100.0% | 1734.4722 | 1735.9679 | 1 | 8.788 | 76.9% | 2 | R.LQDEIQNMKEEMAR.H | 2 |
|  | Astrin\_STLCLD20\_112214\_01.08361.08361.2 | 3.0768 | 0.2236 | 99.4% | 1528.5922 | 1528.7513 | 1 | 4.891 | 68.2% | 1 | R.HLREYQDLLNVK.M | 2 |
|  | Astrin\_STLCLD20\_112214\_01.08344.08344.3 | 3.5739 | 0.3364 | 99.8% | 1529.4844 | 1528.7513 | 2 | 5.877 | 54.5% | 1 | R.HLREYQDLLNVK.M | 3 |
|  | Astrin\_STLCLD20\_112214\_tube2\_02.08709.08709.2 | 3.5361 | 0.3788 | 100.0% | 1296.6721 | 1296.5243 | 1 | 8.989 | 85.0% | 1 | K.MALDIEIATYR.K | 2 |
|  | Astrin\_STLCLD20\_112214\_tube2\_02.07794.07794.3 | 2.8535 | 0.2242 | 97.4% | 1424.8143 | 1424.6984 | 1 | 5.103 | 45.5% | 1 | K.MALDIEIATYRK.L | 3 |
| \* | Astrin\_STLCLD20\_112214\_tube2\_01.06893.06893.2 | 3.6633 | 0.4247 | 100.0% | 1837.3922 | 1837.854 | 1 | 6.206 | 50.0% | 1 | R.DGQVINETSQHHDDLE.- | 2 |

---

|  |  |  |  |  |  |  |  |  |
| --- | --- | --- | --- | --- | --- | --- | --- | --- |
| U | *gi|57013276|ref|NP\_00* | 30 | 137 | 49.7% | 451 | 50152 | 5.1 | tubulin, alpha, ubiquitous [Homo sapiens] |

| Filename XCorr DeltCN Conf% ObsM+H+ CalcM+H+ SpR ZScore Ion% # Sequence  | | | | | | | | | | | | |
| --- | --- | --- | --- | --- | --- | --- | --- | --- | --- | --- | --- | --- |
|  | Astrin\_STLCLD20\_112214\_tube2\_01.14672.14672.2 | 5.6344 | 0.6328 | 100.0% | 2008.3322 | 2009.093 | 1 | 11.791 | 57.9% | 9 | K.TIGGGDDSFNTFFSETGAGK.H | 2 |
|  | Astrin\_STLCLD20\_112214\_01.12753.12753.2 | 5.108 | 0.4717 | 100.0% | 1703.4722 | 1702.9451 | 1 | 8.242 | 78.6% | 15 | R.AVFVDLEPTVIDEVR.T | 2 |
|  | Astrin\_STLCLD20\_112214\_tube2\_01.15864.15864.1 | 3.0432 | 0.325 | 100.0% | 1703.81 | 1702.9451 | 1 | 5.3 | 50.0% | 4 | R.AVFVDLEPTVIDEVR.T | 1 |
|  | Astrin\_STLCLD20\_112214\_02.14376.14376.3 | 4.4306 | 0.4215 | 100.0% | 1704.2644 | 1702.9451 | 1 | 6.97 | 50.0% | 2 | R.AVFVDLEPTVIDEVR.T | 3 |
|  | Astrin\_STLCLD20\_112214\_01.10293.10293.2 | 1.9386 | 0.3075 | 96.4% | 1411.5521 | 1411.6439 | 367 | 5.745 | 50.0% | 2 | R.QLFHPEQLITGK.E | 22 |
|  | Astrin\_STLCLD20\_112214\_tube2\_01.10802.10802.3 | 3.9221 | 0.4709 | 100.0% | 2416.4644 | 2416.6555 | 2 | 7.043 | 30.0% | 4 | R.QLFHPEQLITGKEDAANNYAR.G | 33 |
|  | Astrin\_STLCLD20\_112214\_tube2\_01.10767.10767.2 | 2.6947 | 0.3834 | 99.7% | 2416.5723 | 2416.6555 | 6 | 5.895 | 37.5% | 2 | R.QLFHPEQLITGKEDAANNYAR.G | 22 |
|  | Astrin\_STLCLD20\_112214\_01.13269.13269.3 | 4.8135 | 0.4526 | 100.0% | 1843.2544 | 1843.1332 | 2 | 7.617 | 43.3% | 3 | R.GHYTIGKEIIDLVLDR.I | 3 |
|  | Astrin\_STLCLD20\_112214\_01.13243.13243.2 | 3.1114 | 0.3337 | 99.9% | 1843.6522 | 1843.1332 | 1 | 5.332 | 53.3% | 1 | R.GHYTIGKEIIDLVLDR.I | 2 |
|  | Astrin\_STLCLD20\_112214\_tube2\_01.15296.15296.1 | 2.1784 | 0.3293 | 100.0% | 1085.77 | 1086.2737 | 2 | 6.033 | 62.5% | 3 | K.EIIDLVLDR.I | 1 |
|  | Astrin\_STLCLD20\_112214\_tube2\_01.15250.15250.2 | 2.4174 | 0.3619 | 99.8% | 1085.9521 | 1086.2737 | 1 | 5.361 | 81.2% | 3 | K.EIIDLVLDR.I | 2 |
|  | Astrin\_STLCLD20\_112214\_tube2\_01.08110.08110.3 | 3.4495 | 0.2115 | 98.6% | 1876.0144 | 1876.0824 | 3 | 4.994 | 41.1% | 2 | R.RNLDIERPTYTNLNR.L | 33 |
|  | Astrin\_STLCLD20\_112214\_01.08119.08119.2 | 2.8999 | 0.1481 | 97.3% | 1719.4521 | 1719.8949 | 1 | 4.855 | 57.7% | 4 | R.NLDIERPTYTNLNR.L | 22 |
|  | Astrin\_STLCLD20\_112214\_01.08131.08131.3 | 2.6874 | 0.2989 | 98.8% | 1720.3744 | 1719.8949 | 31 | 5.026 | 40.4% | 4 | R.NLDIERPTYTNLNR.L | 33 |
|  | Astrin\_STLCLD20\_112214\_01.14491.14491.2 | 4.841 | 0.4865 | 100.0% | 1488.6921 | 1488.7678 | 1 | 9.307 | 76.9% | 14 | R.LISQIVSSITASLR.F | 22 |
|  | Astrin\_STLCLD20\_112214\_tube2\_01.19137.19137.1 | 3.2824 | 0.3949 | 100.0% | 1488.85 | 1488.7678 | 2 | 6.981 | 50.0% | 2 | R.LISQIVSSITASLR.F | 11 |
|  | Astrin\_STLCLD20\_112214\_02.16335.16335.3 | 4.1559 | 0.3309 | 99.7% | 1488.9844 | 1488.7678 | 2 | 6.853 | 51.9% | 2 | R.LISQIVSSITASLR.F | 33 |
|  | Astrin\_STLCLD20\_112214\_tube2\_02.10440.10440.2 | 5.451 | 0.5225 | 100.0% | 2410.0122 | 2410.6885 | 1 | 9.459 | 50.0% | 13 | R.FDGALNVDLTEFQTNLVPYPR.I | 22 |
|  | Astrin\_STLCLD20\_112214\_tube2\_02.10416.10416.3 | 3.7941 | 0.4445 | 100.0% | 2410.8245 | 2410.6885 | 1 | 7.732 | 36.2% | 1 | R.FDGALNVDLTEFQTNLVPYPR.I | 33 |
|  | Astrin\_STLCLD20\_112214\_tube2\_01.13778.13778.2 | 4.406 | 0.5301 | 100.0% | 1758.4321 | 1758.0703 | 1 | 9.532 | 76.7% | 12 | R.IHFPLATYAPVISAEK.A | 22 |
|  | Astrin\_STLCLD20\_112214\_tube2\_01.13851.13851.3 | 3.9489 | 0.3932 | 100.0% | 1758.7444 | 1758.0703 | 7 | 6.803 | 45.0% | 3 | R.IHFPLATYAPVISAEK.A | 33 |
|  | Astrin\_STLCLD20\_112214\_tube2\_01.11561.11561.2 | 2.2349 | 0.358 | 99.4% | 1249.8121 | 1250.4304 | 45 | 5.945 | 56.2% | 2 | K.YMACCLLYR.G | 22 |
|  | Astrin\_STLCLD20\_112214\_tube2\_01.09555.09555.1 | 1.7892 | 0.4094 | 100.0% | 1015.62 | 1016.1827 | 2 | 6.574 | 66.7% | 4 | K.DVNAAIATIK.T | 1 |
|  | Astrin\_STLCLD20\_112214\_tube2\_01.09616.09616.2 | 3.1029 | 0.3365 | 100.0% | 1016.1922 | 1016.1827 | 1 | 7.24 | 88.9% | 3 | K.DVNAAIATIK.T | 2 |
|  | Astrin\_STLCLD20\_112214\_tube2\_01.11630.11630.2 | 4.3829 | 0.4725 | 100.0% | 1825.4722 | 1826.1027 | 1 | 8.801 | 67.6% | 10 | K.VGINYQPPTVVPGGDLAK.V | 22 |
|  | Astrin\_STLCLD20\_112214\_02.14556.14556.2 | 2.552 | 0.236 | 97.7% | 1867.2922 | 1866.1084 | 4 | 5.551 | 37.5% | 1 | R.AVCMLSNTTAIAEAWAR.L | 22 |
|  | Astrin\_STLCLD20\_112214\_01.08110.08110.2 | 3.5435 | 0.367 | 100.0% | 1382.0521 | 1381.6324 | 1 | 6.667 | 65.0% | 2 | R.LDHKFDLMYAK.R | 22 |
|  | Astrin\_STLCLD20\_112214\_tube2\_01.09242.09242.3 | 4.085 | 0.3888 | 100.0% | 1382.3344 | 1381.6324 | 14 | 6.31 | 55.0% | 4 | R.LDHKFDLMYAK.R | 33 |
|  | Astrin\_STLCLD20\_112214\_tube2\_02.08007.08007.3 | 4.4979 | 0.3173 | 99.8% | 2488.4343 | 2487.7083 | 1 | 6.132 | 35.0% | 2 | K.RAFVHWYVGEGMEEGEFSEAR.E | 33 |
|  | Astrin\_STLCLD20\_112214\_02.12735.12735.3 | 4.7846 | 0.4381 | 100.0% | 2331.8044 | 2331.5208 | 1 | 7.169 | 40.8% | 4 | R.AFVHWYVGEGMEEGEFSEAR.E | 33 |

Similarities:
gi|17921989|ref|NP\_00(20:10)  

---

|  |  |  |  |  |  |  |  |  |
| --- | --- | --- | --- | --- | --- | --- | --- | --- |
| U | *gi|11415030|ref|NP\_06* | 8 | 36 | 49.5% | 103 | 11367 | 11.4 | histone cluster 1, H4j [Homo sapiens] |
| U | *gi|77539758|ref|NP\_00* | 8 | 36 | 49.5% | 103 | 11367 | 11.4 | histone cluster 2, H4b [Homo sapiens] |
| U | *gi|4504323|ref|NP\_003* | 7 | 34 | 49.5% | 103 | 11367 | 11.4 | histone cluster 2, H4a [Homo sapiens] |
| U | *gi|4504321|ref|NP\_003* | 8 | 36 | 49.5% | 103 | 11367 | 11.4 | histone cluster 1, H4i [Homo sapiens] |
| U | *gi|4504317|ref|NP\_003* | 8 | 36 | 49.5% | 103 | 11367 | 11.4 | histone cluster 1, H4l [Homo sapiens] |
| U | *gi|4504315|ref|NP\_003* | 8 | 36 | 49.5% | 103 | 11367 | 11.4 | histone cluster 1, H4e [Homo sapiens] |
| U | *gi|4504313|ref|NP\_003* | 8 | 36 | 49.5% | 103 | 11367 | 11.4 | histone cluster 1, H4b [Homo sapiens] |
| U | *gi|4504311|ref|NP\_003* | 8 | 36 | 49.5% | 103 | 11367 | 11.4 | histone cluster 1, H4h [Homo sapiens] |
| U | *gi|4504309|ref|NP\_003* | 8 | 36 | 49.5% | 103 | 11367 | 11.4 | histone cluster 1, H4c [Homo sapiens] |
| U | *gi|4504307|ref|NP\_003* | 8 | 36 | 49.5% | 103 | 11367 | 11.4 | histone cluster 1, H4k [Homo sapiens] |
| U | *gi|4504305|ref|NP\_003* | 8 | 36 | 49.5% | 103 | 11367 | 11.4 | histone cluster 1, H4f [Homo sapiens] |
| U | *gi|4504303|ref|NP\_003* | 8 | 36 | 49.5% | 103 | 11367 | 11.4 | histone cluster 1, H4d [Homo sapiens] |
| U | *gi|4504301|ref|NP\_003* | 8 | 36 | 49.5% | 103 | 11367 | 11.4 | histone cluster 1, H4a [Homo sapiens] |
| U | *gi|28173560|ref|NP\_77* | 8 | 36 | 49.5% | 103 | 11367 | 11.4 | histone cluster 4, H4 [Homo sapiens] |

| Filename XCorr DeltCN Conf% ObsM+H+ CalcM+H+ SpR ZScore Ion% # Sequence  | | | | | | | | | | | | |
| --- | --- | --- | --- | --- | --- | --- | --- | --- | --- | --- | --- | --- |
|  | Astrin\_STLCLD20\_112214\_01.05432.05432.2 | 3.2883 | 0.1947 | 99.4% | 1326.3722 | 1326.5387 | 1 | 6.49 | 81.8% | 7 | R.DNIQGITKPAIR.R | 2 |
|  | Astrin\_STLCLD20\_112214\_01.08653.08653.1 | 2.6057 | 0.2463 | 100.0% | 1180.57 | 1181.3312 | 3 | 5.159 | 66.7% | 3 | R.ISGLIYEETR.G | 1 |
|  | Astrin\_STLCLD20\_112214\_tube2\_01.10004.10004.2 | 3.9194 | 0.3998 | 100.0% | 1181.0521 | 1181.3312 | 1 | 7.38 | 88.9% | 12 | R.ISGLIYEETR.G | 2 |
|  | Astrin\_STLCLD20\_112214\_tube2\_01.12584.12584.2 | 3.0222 | 0.2779 | 100.0% | 989.6122 | 990.19055 | 2 | 5.968 | 85.7% | 3 | K.VFLENVIR.D | 2 |
|  | Astrin\_STLCLD20\_112214\_01.10903.10903.1 | 2.0827 | 0.3308 | 100.0% | 989.64 | 990.19055 | 8 | 6.091 | 64.3% | 2 | K.VFLENVIR.D | 1 |
|  | Astrin\_STLCLD20\_112214\_01.10647.10647.3 | 3.3963 | 0.4033 | 99.7% | 1595.8444 | 1595.9409 | 6 | 6.331 | 42.3% | 1 | R.KTVTAMDVVYALKR.Q | 3 |
|  | Astrin\_STLCLD20\_112214\_tube2\_01.13589.13589.2 | 3.4715 | 0.4623 | 100.0% | 1467.9922 | 1467.7667 | 1 | 7.492 | 70.8% | 4 | K.TVTAMDVVYALKR.Q | 2 |
|  | Astrin\_STLCLD20\_112214\_tube2\_01.12728.12728.1 | 1.775 | 0.5072 | 100.0% | 714.29 | 714.796 | 1 | 8.167 | 75.0% | 4 | R.TLYGFGG.- | 1 |

---

|  |  |  |  |  |  |  |  |  |
| --- | --- | --- | --- | --- | --- | --- | --- | --- |
| U | *gi|10800140|ref|NP\_06* | 6 | 19 | 48.4% | 126 | 13950 | 10.3 | histone cluster 1, H2bb [Homo sapiens] |
| U | *gi|4504277|ref|NP\_003* | 6 | 19 | 48.4% | 126 | 13920 | 10.3 | histone cluster 2, H2be [Homo sapiens] |
| U | *gi|20336754|ref|NP\_06* | 6 | 19 | 48.4% | 126 | 13904 | 10.3 | histone cluster 1, H2bj [Homo sapiens] |
| U | *gi|16306566|ref|NP\_00* | 6 | 19 | 48.4% | 126 | 13906 | 10.3 | histone cluster 1, H2bo [Homo sapiens] |

| Filename XCorr DeltCN Conf% ObsM+H+ CalcM+H+ SpR ZScore Ion% # Sequence  | | | | | | | | | | | | |
| --- | --- | --- | --- | --- | --- | --- | --- | --- | --- | --- | --- | --- |
|  | Astrin\_STLCLD20\_112214\_tube2\_01.08565.08565.2 | 2.3869 | 0.2779 | 98.8% | 1280.0521 | 1280.4631 | 18 | 5.608 | 61.1% | 1 | R.KESYSIYVYK.V | 2 |
|  | Astrin\_STLCLD20\_112214\_tube2\_01.19358.19358.2 | 5.4029 | 0.5019 | 100.0% | 1744.1721 | 1745.0211 | 1 | 9.93 | 82.1% | 10 | K.AMGIMNSFVNDIFER.I | 22 |
|  | Astrin\_STLCLD20\_112214\_tube2\_01.05998.05998.3 | 3.4354 | 0.3307 | 99.8% | 1586.7843 | 1586.7941 | 1 | 5.915 | 46.2% | 1 | R.IAGEASRLAHYNKR.S | 33 |
|  | Astrin\_STLCLD20\_112214\_tube2\_01.07574.07574.2 | 3.8004 | 0.4522 | 100.0% | 1462.4321 | 1462.6462 | 1 | 7.074 | 66.7% | 5 | R.STITSREIQTAVR.L | 22 |
|  | Astrin\_STLCLD20\_112214\_tube2\_01.07562.07562.3 | 3.0124 | 0.2375 | 98.3% | 1463.1244 | 1462.6462 | 5 | 4.944 | 41.7% | 1 | R.STITSREIQTAVR.L | 33 |
|  | Astrin\_STLCLD20\_112214\_tube2\_01.12044.12044.2 | 2.5857 | 0.152 | 97.7% | 954.27216 | 954.19794 | 17 | 4.077 | 68.8% | 1 | R.LLLPGELAK.H | 22 |

Similarities:
gi|10800138|ref|NP\_06(5:1)  

---

|  |  |  |  |  |  |  |  |  |
| --- | --- | --- | --- | --- | --- | --- | --- | --- |
| U | *gi|10800138|ref|NP\_06* | 6 | 20 | 48.4% | 126 | 13936 | 10.3 | histone cluster 1, H2bd [Homo sapiens] |
| U | *gi|66912162|ref|NP\_00* | 6 | 20 | 48.4% | 126 | 13920 | 10.3 | histone cluster 2, H2bf [Homo sapiens] |
| U | *gi|4504271|ref|NP\_003* | 6 | 20 | 48.4% | 126 | 13906 | 10.3 | histone cluster 1, H2bi [Homo sapiens] |
| U | *gi|4504269|ref|NP\_003* | 6 | 20 | 48.4% | 126 | 13892 | 10.3 | histone cluster 1, H2bh [Homo sapiens] |
| U | *gi|4504265|ref|NP\_003* | 6 | 20 | 48.4% | 126 | 13906 | 10.3 | histone cluster 1, H2bf [Homo sapiens] |
| U | *gi|4504263|ref|NP\_003* | 6 | 20 | 48.4% | 126 | 13989 | 10.3 | histone cluster 1, H2bm [Homo sapiens] |
| U | *gi|4504261|ref|NP\_003* | 6 | 20 | 48.4% | 126 | 13922 | 10.3 | histone cluster 1, H2bn [Homo sapiens] |
| U | *gi|4504257|ref|NP\_003* | 6 | 20 | 48.4% | 126 | 13906 | 10.3 | histone cluster 1, H2bg [Homo sapiens] |
| U | *gi|21396484|ref|NP\_00* | 6 | 20 | 48.4% | 126 | 13906 | 10.3 | histone cluster 1, H2be [Homo sapiens] |
| U | *gi|21166389|ref|NP\_00* | 6 | 20 | 48.4% | 126 | 13906 | 10.3 | histone cluster 1, H2bc [Homo sapiens] |
| U | *gi|20336752|ref|NP\_61* | 6 | 20 | 48.4% | 126 | 13936 | 10.3 | histone cluster 1, H2bd [Homo sapiens] |
| U | *gi|18105048|ref|NP\_54* | 6 | 20 | 48.4% | 126 | 13890 | 10.3 | histone cluster 1, H2bk [Homo sapiens] |

| Filename XCorr DeltCN Conf% ObsM+H+ CalcM+H+ SpR ZScore Ion% # Sequence  | | | | | | | | | | | | |
| --- | --- | --- | --- | --- | --- | --- | --- | --- | --- | --- | --- | --- |
|  | Astrin\_STLCLD20\_112214\_01.05702.05702.2 | 2.5795 | 0.2669 | 99.3% | 1266.6122 | 1266.4363 | 3 | 5.057 | 72.2% | 2 | R.KESYSVYVYK.V | 2 |
|  | Astrin\_STLCLD20\_112214\_tube2\_01.19358.19358.2 | 5.4029 | 0.5019 | 100.0% | 1744.1721 | 1745.0211 | 1 | 9.93 | 82.1% | 10 | K.AMGIMNSFVNDIFER.I | 22 |
|  | Astrin\_STLCLD20\_112214\_tube2\_01.05998.05998.3 | 3.4354 | 0.3307 | 99.8% | 1586.7843 | 1586.7941 | 1 | 5.915 | 46.2% | 1 | R.IAGEASRLAHYNKR.S | 33 |
|  | Astrin\_STLCLD20\_112214\_tube2\_01.07574.07574.2 | 3.8004 | 0.4522 | 100.0% | 1462.4321 | 1462.6462 | 1 | 7.074 | 66.7% | 5 | R.STITSREIQTAVR.L | 22 |
|  | Astrin\_STLCLD20\_112214\_tube2\_01.07562.07562.3 | 3.0124 | 0.2375 | 98.3% | 1463.1244 | 1462.6462 | 5 | 4.944 | 41.7% | 1 | R.STITSREIQTAVR.L | 33 |
|  | Astrin\_STLCLD20\_112214\_tube2\_01.12044.12044.2 | 2.5857 | 0.152 | 97.7% | 954.27216 | 954.19794 | 17 | 4.077 | 68.8% | 1 | R.LLLPGELAK.H | 22 |

Similarities:
gi|10800140|ref|NP\_06(5:1)  

---

|  |  |  |  |  |  |  |  |  |
| --- | --- | --- | --- | --- | --- | --- | --- | --- |
| U | *gi|57242777|ref|NP\_03* | 4 | 5 | 46.6% | 103 | 11967 | 5.9 | c-myc binding protein [Homo sapiens] |

| Filename XCorr DeltCN Conf% ObsM+H+ CalcM+H+ SpR ZScore Ion% # Sequence  | | | | | | | | | | | | |
| --- | --- | --- | --- | --- | --- | --- | --- | --- | --- | --- | --- | --- |
| \* | Astrin\_STLCLD20\_112214\_01.12430.12430.2 | 4.5601 | 0.4139 | 100.0% | 2275.6921 | 2276.6348 | 1 | 7.695 | 44.7% | 2 | K.VLVALYEEPEKPNSALDFLK.H | 2 |
| \* | Astrin\_STLCLD20\_112214\_tube2\_01.09737.09737.2 | 4.217 | 0.4143 | 100.0% | 1897.2522 | 1898.1289 | 1 | 7.352 | 71.9% | 1 | K.HHLGAATPENPEIELLR.L | 2 |
| \* | Astrin\_STLCLD20\_112214\_01.08882.08882.3 | 3.4942 | 0.2266 | 99.0% | 1897.7943 | 1898.1289 | 211 | 4.705 | 34.4% | 1 | K.HHLGAATPENPEIELLR.L | 3 |
| \* | Astrin\_STLCLD20\_112214\_tube2\_01.06716.06716.2 | 2.9957 | 0.3209 | 100.0% | 1332.1721 | 1332.4528 | 4 | 5.32 | 65.0% | 1 | K.LAQYEPPQEEK.R | 2 |

---

|  |  |  |  |  |  |  |  |  |
| --- | --- | --- | --- | --- | --- | --- | --- | --- |
| U | *gi|150456457|ref|NP\_9* | 18 | 67 | 46.4% | 347 | 39929 | 5.6 | HMT1 hnRNP methyltransferase-like 2 isoform 2 [Homo sapiens] |
| U | *gi|154759421|ref|NP\_0* | 18 | 67 | 43.4% | 371 | 42462 | 5.3 | HMT1 hnRNP methyltransferase-like 2 isoform 1 [Homo sapiens] |
| U | *gi|151301219|ref|NP\_9* | 18 | 67 | 45.6% | 353 | 40548 | 5.5 | HMT1 hnRNP methyltransferase-like 2 isoform 3 [Homo sapiens] |

| Filename XCorr DeltCN Conf% ObsM+H+ CalcM+H+ SpR ZScore Ion% # Sequence  | | | | | | | | | | | | |
| --- | --- | --- | --- | --- | --- | --- | --- | --- | --- | --- | --- | --- |
|  | Astrin\_STLCLD20\_112214\_tube2\_02.08493.08493.3 | 3.3663 | 0.3392 | 99.5% | 2765.5444 | 2766.0132 | 2 | 5.581 | 26.2% | 1 | K.DYYFDSYAHFGIHEEMLKDEVR.T | 3 |
|  | Astrin\_STLCLD20\_112214\_01.06351.06351.2 | 3.3349 | 0.189 | 99.4% | 1352.0721 | 1351.6322 | 5 | 5.227 | 68.2% | 5 | K.ANKLDHVVTIIK.G | 2 |
|  | Astrin\_STLCLD20\_112214\_01.06397.06397.3 | 4.0569 | 0.4132 | 100.0% | 1352.5144 | 1351.6322 | 1 | 7.17 | 63.6% | 3 | K.ANKLDHVVTIIK.G | 3 |
|  | Astrin\_STLCLD20\_112214\_01.06626.06626.2 | 3.3001 | 0.4164 | 100.0% | 1356.3922 | 1356.559 | 1 | 6.728 | 72.7% | 1 | K.GKVEEVELPVEK.V | 2 |
|  | Astrin\_STLCLD20\_112214\_tube2\_01.14894.14894.2 | 3.9991 | 0.4029 | 100.0% | 1643.5521 | 1643.8827 | 1 | 7.156 | 73.1% | 7 | R.DKWLAPDGLIFPDR.A | 2 |
|  | Astrin\_STLCLD20\_112214\_tube2\_01.16576.16576.2 | 3.3191 | 0.463 | 100.0% | 1400.4122 | 1400.6201 | 1 | 7.057 | 77.3% | 3 | K.WLAPDGLIFPDR.A | 2 |
|  | Astrin\_STLCLD20\_112214\_tube2\_01.11110.11110.1 | 2.0704 | 0.3927 | 100.0% | 1251.55 | 1252.4099 | 2 | 6.621 | 60.0% | 2 | R.ATLYVTAIEDR.Q | 1 |
|  | Astrin\_STLCLD20\_112214\_02.10911.10911.2 | 3.8036 | 0.4711 | 100.0% | 1253.2722 | 1252.4099 | 1 | 8.283 | 75.0% | 16 | R.ATLYVTAIEDR.Q | 2 |
|  | Astrin\_STLCLD20\_112214\_01.10580.10580.2 | 3.3359 | 0.2888 | 99.8% | 1637.9521 | 1637.914 | 1 | 6.141 | 64.3% | 4 | K.DVAIKEPLVDVVDPK.Q | 2 |
|  | Astrin\_STLCLD20\_112214\_tube2\_01.18464.18464.2 | 3.1424 | 0.3674 | 100.0% | 2229.6921 | 2229.5027 | 4 | 5.42 | 41.2% | 2 | K.RNDYVHALVAYFNIEFTR.C | 2 |
|  | Astrin\_STLCLD20\_112214\_02.15965.15965.3 | 5.1918 | 0.3604 | 100.0% | 2230.1643 | 2229.5027 | 1 | 8.991 | 50.0% | 2 | K.RNDYVHALVAYFNIEFTR.C | 3 |
|  | Astrin\_STLCLD20\_112214\_tube2\_01.19751.19751.2 | 5.6507 | 0.5334 | 100.0% | 2072.9321 | 2073.3152 | 1 | 9.27 | 65.6% | 3 | R.NDYVHALVAYFNIEFTR.C | 2 |
|  | Astrin\_STLCLD20\_112214\_tube2\_01.09482.09482.2 | 3.598 | 0.495 | 100.0% | 1725.1721 | 1725.8547 | 1 | 8.107 | 60.7% | 4 | R.TGFSTSPESPYTHWK.Q | 2 |
|  | Astrin\_STLCLD20\_112214\_01.08541.08541.3 | 2.4168 | 0.2912 | 96.4% | 1726.5844 | 1725.8547 | 1 | 5.438 | 33.9% | 1 | R.TGFSTSPESPYTHWK.Q | 3 |
|  | Astrin\_STLCLD20\_112214\_02.14441.14441.2 | 4.2178 | 0.4677 | 100.0% | 1638.5521 | 1637.8878 | 1 | 8.309 | 75.0% | 4 | K.QTVFYMEDYLTVK.T | 2 |
|  | Astrin\_STLCLD20\_112214\_tube2\_01.11270.11270.2 | 3.9443 | 0.4578 | 100.0% | 1721.5721 | 1721.969 | 1 | 7.811 | 60.0% | 5 | K.TGEEIFGTIGMRPNAK.N | 2 |
|  | Astrin\_STLCLD20\_112214\_01.09994.09994.3 | 3.0608 | 0.2413 | 98.0% | 1722.7743 | 1721.969 | 8 | 5.548 | 36.7% | 3 | K.TGEEIFGTIGMRPNAK.N | 3 |
|  | Astrin\_STLCLD20\_112214\_tube2\_01.08688.08688.2 | 3.5922 | 0.3292 | 100.0% | 1590.1921 | 1589.6313 | 1 | 6.455 | 62.5% | 1 | K.GQLCELSCSTDYR.M | 2 |

---

|  |  |  |  |  |  |  |  |  |
| --- | --- | --- | --- | --- | --- | --- | --- | --- |
| U | *gi|7669492|ref|NP\_002* | 10 | 38 | 43.9% | 335 | 36053 | 8.5 | glyceraldehyde-3-phosphate dehydrogenase [Homo sapiens] |

| Filename XCorr DeltCN Conf% ObsM+H+ CalcM+H+ SpR ZScore Ion% # Sequence  | | | | | | | | | | | | |
| --- | --- | --- | --- | --- | --- | --- | --- | --- | --- | --- | --- | --- |
| \* | Astrin\_STLCLD20\_112214\_tube2\_01.21141.21141.3 | 4.2436 | 0.3449 | 99.8% | 3310.9744 | 3310.7634 | 7 | 5.574 | 24.1% | 3 | K.VDIVAINDPFIDLNYMVYMFQYDSTHGK.F | 3 |
| \* | Astrin\_STLCLD20\_112214\_tube2\_01.15888.15888.2 | 3.632 | 0.4113 | 100.0% | 1614.8322 | 1614.8851 | 1 | 7.927 | 61.5% | 4 | K.LVINGNPITIFQER.D | 2 |
| \* | Astrin\_STLCLD20\_112214\_01.11543.11543.2 | 2.9456 | 0.3341 | 99.7% | 2042.4521 | 2042.3427 | 86 | 5.955 | 29.4% | 1 | K.LVINGNPITIFQERDPSK.I | 2 |
| \* | Astrin\_STLCLD20\_112214\_01.10694.10694.3 | 4.9672 | 0.3163 | 99.7% | 2371.5544 | 2370.79 | 1 | 6.111 | 40.5% | 2 | K.RVIISAPSADAPMFVMGVNHEK.Y | 3 |
| \* | Astrin\_STLCLD20\_112214\_01.14481.14481.3 | 6.2219 | 0.6409 | 100.0% | 2596.9143 | 2597.0044 | 1 | 10.704 | 39.1% | 5 | K.VIHDNFGIVEGLMTTVHAITATQK.T | 3 |
| \* | Astrin\_STLCLD20\_112214\_tube2\_01.19043.19043.2 | 5.9089 | 0.509 | 100.0% | 2597.3123 | 2597.0044 | 1 | 9.752 | 47.8% | 2 | K.VIHDNFGIVEGLMTTVHAITATQK.T | 2 |
| \* | Astrin\_STLCLD20\_112214\_tube2\_01.10850.10850.2 | 4.0214 | 0.4181 | 100.0% | 1412.5122 | 1412.6292 | 1 | 6.909 | 67.9% | 7 | R.GALQNIIPASTGAAK.A | 2 |
| \* | Astrin\_STLCLD20\_112214\_01.09829.09829.2 | 3.2203 | 0.368 | 100.0% | 1531.7122 | 1531.7155 | 13 | 6.808 | 50.0% | 2 | R.VPTANVSVVDLTCR.L | 2 |
|  | Astrin\_STLCLD20\_112214\_tube2\_01.14618.14618.2 | 4.4208 | 0.4852 | 100.0% | 1764.3922 | 1764.8914 | 1 | 9.011 | 65.4% | 5 | K.LISWYDNEFGYSNR.V | 2 |
| \* | Astrin\_STLCLD20\_112214\_01.08446.08446.2 | 3.7795 | 0.3968 | 100.0% | 1332.4722 | 1331.5879 | 1 | 7.057 | 68.2% | 7 | R.VVDLMAHMASKE.- | 2 |

---

|  |  |  |  |  |  |  |  |  |
| --- | --- | --- | --- | --- | --- | --- | --- | --- |
| U | *contaminant\_gi|746301* | 15 | 104 | 42.8% | 269 | 27961 | 6.7 | lysyl endopeptidase (EC 3.4.21.50) - Lysobacter enzymogenes |

| Filename XCorr DeltCN Conf% ObsM+H+ CalcM+H+ SpR ZScore Ion% # Sequence  | | | | | | | | | | | | |
| --- | --- | --- | --- | --- | --- | --- | --- | --- | --- | --- | --- | --- |
| \* | Astrin\_STLCLD20\_112214\_tube2\_01.04187.04187.1 | 1.6812 | 0.3322 | 100.0% | 726.03 | 725.8198 | 1 | 5.679 | 66.7% | 1 | R.SVAAYSK.Q | 1 |
| \* | Astrin\_STLCLD20\_112214\_tube2\_01.07130.07130.2 | 6.2535 | 0.6409 | 100.0% | 2261.5322 | 2262.355 | 1 | 11.042 | 54.2% | 15 | R.APGSSSSGANGDGSLAQSQTGAVVR.A | 2 |
| \* | Astrin\_STLCLD20\_112214\_01.04903.04903.3 | 4.6982 | 0.4246 | 100.0% | 2262.6843 | 2262.355 | 1 | 7.408 | 42.7% | 9 | R.APGSSSSGANGDGSLAQSQTGAVVR.A | 3 |
| \* | Astrin\_STLCLD20\_112214\_tube2\_01.00010.00010.2 | 5.3858 | 0.4731 | 100.0% | 3315.912 | 3315.6257 | 1 | 10.569 | 39.7% | 7 | R.ATNAASDFTLLELNTAANPAYNLFWAGWDR.R | 2 |
| \* | Astrin\_STLCLD20\_112214\_tube2\_02.12348.12348.3 | 5.5274 | 0.3295 | 99.7% | 3316.7944 | 3315.6257 | 1 | 5.164 | 25.0% | 6 | R.ATNAASDFTLLELNTAANPAYNLFWAGWDR.R | 3 |
| \* | Astrin\_STLCLD20\_112214\_01.15409.15409.3 | 6.4682 | 0.5014 | 100.0% | 3471.8044 | 3471.813 | 1 | 8.853 | 28.3% | 15 | R.ATNAASDFTLLELNTAANPAYNLFWAGWDRR.D | 3 |
| \* | Astrin\_STLCLD20\_112214\_tube2\_01.06520.06520.3 | 4.148 | 0.2933 | 99.8% | 2076.7144 | 2077.2668 | 1 | 5.926 | 41.7% | 2 | R.RDQNFAGATAIHHPNVAEK.R | 3 |
| \* | Astrin\_STLCLD20\_112214\_tube2\_01.06176.06176.3 | 4.07 | 0.1498 | 97.0% | 2236.3743 | 2233.4543 | 1 | 4.468 | 36.8% | 2 | R.RDQNFAGATAIHHPNVAEKR.I | 3 |
| \* | Astrin\_STLCLD20\_112214\_tube2\_01.07262.07262.2 | 4.7404 | 0.5683 | 100.0% | 1920.6122 | 1921.0793 | 1 | 8.828 | 61.8% | 5 | R.DQNFAGATAIHHPNVAEK.R | 2 |
| \* | Astrin\_STLCLD20\_112214\_tube2\_01.07310.07310.3 | 2.5866 | 0.3294 | 98.6% | 1920.9243 | 1921.0793 | 3 | 5.46 | 33.8% | 1 | R.DQNFAGATAIHHPNVAEK.R | 3 |
| \* | Astrin\_STLCLD20\_112214\_tube2\_01.06617.06617.2 | 5.4608 | 0.4663 | 100.0% | 2076.652 | 2077.2668 | 1 | 7.914 | 52.8% | 3 | R.DQNFAGATAIHHPNVAEKR.I | 2 |
| \* | Astrin\_STLCLD20\_112214\_tube2\_01.06668.06668.3 | 4.093 | 0.4775 | 100.0% | 2077.6443 | 2077.2668 | 1 | 7.53 | 40.3% | 8 | R.DQNFAGATAIHHPNVAEKR.I | 3 |
| \* | Astrin\_STLCLD20\_112214\_tube2\_01.06635.06635.3 | 3.1792 | 0.227 | 97.1% | 1870.4043 | 1870.983 | 1 | 5.618 | 36.1% | 1 | R.VLGQLHGGPSSCSATGADR.S | 3 |
| \* | Astrin\_STLCLD20\_112214\_01.08305.08305.2 | 4.7548 | 0.5286 | 100.0% | 1428.2922 | 1428.5443 | 1 | 8.762 | 69.2% | 25 | R.VFTSWTGGGTSATR.L | 2 |
| \* | Astrin\_STLCLD20\_112214\_tube2\_01.09540.09540.1 | 2.6307 | 0.1733 | 98.0% | 1429.62 | 1428.5443 | 23 | 4.616 | 38.5% | 4 | R.VFTSWTGGGTSATR.L | 1 |

---

|  |  |  |  |  |  |  |  |  |
| --- | --- | --- | --- | --- | --- | --- | --- | --- |
| U | *gi|4501885|ref|NP\_001* | 18 | 60 | 41.1% | 375 | 41737 | 5.5 | beta actin [Homo sapiens] |
| U | *gi|4501887|ref|NP\_001* | 18 | 60 | 41.1% | 375 | 41793 | 5.5 | actin, gamma 1 propeptide [Homo sapiens] |

| Filename XCorr DeltCN Conf% ObsM+H+ CalcM+H+ SpR ZScore Ion% # Sequence  | | | | | | | | | | | | |
| --- | --- | --- | --- | --- | --- | --- | --- | --- | --- | --- | --- | --- |
|  | Astrin\_STLCLD20\_112214\_01.08519.08519.2 | 2.8688 | 0.378 | 100.0% | 1199.3522 | 1199.4415 | 19 | 6.226 | 60.0% | 5 | R.AVFPSIVGRPR.H | 22 |
|  | Astrin\_STLCLD20\_112214\_tube2\_01.06642.06642.1 | 2.0003 | 0.2433 | 98.0% | 1171.74 | 1172.4058 | 1 | 5.709 | 65.0% | 1 | R.HQGVMVGMGQK.D | 11 |
|  | Astrin\_STLCLD20\_112214\_tube2\_01.06474.06474.2 | 2.7079 | 0.0917 | 95.2% | 1173.2722 | 1172.4058 | 1 | 4.414 | 75.0% | 1 | R.HQGVMVGMGQK.D | 22 |
|  | Astrin\_STLCLD20\_112214\_tube2\_01.08564.08564.2 | 3.1715 | 0.4269 | 100.0% | 1516.3322 | 1516.7019 | 1 | 6.715 | 75.0% | 3 | K.IWHHTFYNELR.V | 22 |
|  | Astrin\_STLCLD20\_112214\_01.07657.07657.3 | 3.1919 | 0.2456 | 99.6% | 1517.2743 | 1516.7019 | 1 | 5.871 | 55.0% | 4 | K.IWHHTFYNELR.V | 33 |
|  | Astrin\_STLCLD20\_112214\_tube2\_01.10730.10730.3 | 4.2988 | 0.0975 | 95.6% | 1954.4944 | 1955.2615 | 2 | 7.035 | 39.7% | 1 | R.VAPEEHPVLLTEAPLNPK.A | 3 |
|  | Astrin\_STLCLD20\_112214\_01.09541.09541.2 | 4.46 | 0.3636 | 100.0% | 1954.6522 | 1955.2615 | 1 | 8.501 | 52.9% | 4 | R.VAPEEHPVLLTEAPLNPK.A | 2 |
|  | Astrin\_STLCLD20\_112214\_tube2\_01.14018.14018.3 | 6.925 | 0.4464 | 100.0% | 3186.7744 | 3185.622 | 1 | 7.981 | 33.6% | 8 | R.TTGIVMDSGDGVTHTVPIYEGYALPHAILR.L | 3 |
|  | Astrin\_STLCLD20\_112214\_01.11773.11773.2 | 2.8328 | 0.1328 | 96.4% | 1625.0721 | 1624.8927 | 1 | 4.697 | 65.4% | 2 | R.LDLAGRDLTDYLMK.I | 22 |
|  | Astrin\_STLCLD20\_112214\_tube2\_01.13721.13721.1 | 2.0707 | 0.2892 | 100.0% | 998.5 | 999.167 | 2 | 5.437 | 71.4% | 2 | R.DLTDYLMK.I | 11 |
|  | Astrin\_STLCLD20\_112214\_tube2\_01.13726.13726.2 | 2.0606 | 0.342 | 99.0% | 999.21216 | 999.167 | 16 | 5.639 | 71.4% | 1 | R.DLTDYLMK.I | 22 |
|  | Astrin\_STLCLD20\_112214\_01.05720.05720.2 | 2.9636 | 0.4383 | 100.0% | 1133.1921 | 1133.2029 | 1 | 8.271 | 77.8% | 8 | R.GYSFTTTAER.E | 2 |
|  | Astrin\_STLCLD20\_112214\_tube2\_01.13478.13478.2 | 4.7344 | 0.3284 | 100.0% | 1792.1322 | 1791.9554 | 1 | 8.421 | 83.3% | 6 | K.SYELPDGQVITIGNER.F | 22 |
|  | Astrin\_STLCLD20\_112214\_tube2\_02.07957.07957.3 | 5.353 | 0.4954 | 100.0% | 2344.5544 | 2344.6448 | 1 | 7.659 | 38.1% | 4 | R.KDLYANTVLSGGTTMYPGIADR.M | 3 |
|  | Astrin\_STLCLD20\_112214\_tube2\_01.14519.14519.2 | 4.4383 | 0.4928 | 100.0% | 2216.3523 | 2216.4705 | 1 | 8.792 | 45.0% | 3 | K.DLYANTVLSGGTTMYPGIADR.M | 2 |
|  | Astrin\_STLCLD20\_112214\_02.13135.13135.3 | 3.9604 | 0.4186 | 99.7% | 2216.3943 | 2216.4705 | 1 | 6.825 | 41.2% | 1 | K.DLYANTVLSGGTTMYPGIADR.M | 3 |
|  | Astrin\_STLCLD20\_112214\_tube2\_01.09000.09000.1 | 2.2724 | 0.3842 | 100.0% | 1161.6 | 1162.3868 | 1 | 6.273 | 60.0% | 1 | K.EITALAPSTMK.I | 11 |
|  | Astrin\_STLCLD20\_112214\_tube2\_01.09039.09039.2 | 2.8112 | 0.3091 | 99.7% | 1162.4122 | 1162.3868 | 13 | 5.941 | 55.0% | 5 | K.EITALAPSTMK.I | 22 |

Similarities:
gi|4501881|ref|NP\_001(11:7)  

---

|  |  |  |  |  |  |  |  |  |
| --- | --- | --- | --- | --- | --- | --- | --- | --- |
| U | *gi|20127519|ref|NP\_03* | 40 | 102 | 41.0% | 747 | 85653 | 9.2 | TPX2, microtubule-associated protein homolog [Homo sapiens] |

| Filename XCorr DeltCN Conf% ObsM+H+ CalcM+H+ SpR ZScore Ion% # Sequence  | | | | | | | | | | | | |
| --- | --- | --- | --- | --- | --- | --- | --- | --- | --- | --- | --- | --- |
| \* | Astrin\_STLCLD20\_112214\_01.09244.09244.3 | 4.2341 | 0.4146 | 100.0% | 2405.8743 | 2405.7996 | 1 | 6.742 | 38.8% | 2 | R.KANLQQAIVTPLKPVDNTYYK.E | 3 |
| \* | Astrin\_STLCLD20\_112214\_tube2\_01.11662.11662.3 | 3.6563 | 0.4146 | 99.7% | 2276.7844 | 2277.6255 | 1 | 6.598 | 31.6% | 3 | K.ANLQQAIVTPLKPVDNTYYK.E | 3 |
| \* | Astrin\_STLCLD20\_112214\_tube2\_01.07684.07684.2 | 3.3911 | 0.2839 | 100.0% | 1151.1122 | 1150.3534 | 3 | 5.765 | 75.0% | 1 | K.MQQEVVEMR.K | 2 |
| \* | Astrin\_STLCLD20\_112214\_01.09303.09303.2 | 2.7259 | 0.3711 | 100.0% | 1067.3922 | 1067.317 | 4 | 7.208 | 65.0% | 2 | K.LALAGIGQPVK.K | 2 |
| \* | Astrin\_STLCLD20\_112214\_tube2\_01.08577.08577.2 | 3.0903 | 0.3117 | 99.9% | 1195.5721 | 1195.4911 | 19 | 6.33 | 59.1% | 4 | K.LALAGIGQPVKK.S | 2 |
| \* | Astrin\_STLCLD20\_112214\_01.07591.07591.2 | 2.1992 | 0.3647 | 99.7% | 908.0722 | 908.0043 | 3 | 6.183 | 83.3% | 1 | K.SVDFHFR.T | 2 |
| \* | Astrin\_STLCLD20\_112214\_01.09141.09141.2 | 4.951 | 0.5085 | 100.0% | 1886.3922 | 1887.013 | 1 | 9.19 | 78.6% | 3 | K.NQEEYKEVNFTSELR.K | 2 |
| \* | Astrin\_STLCLD20\_112214\_01.09181.09181.3 | 4.3796 | 0.4169 | 100.0% | 1886.8143 | 1887.013 | 1 | 6.8 | 46.4% | 7 | K.NQEEYKEVNFTSELR.K | 3 |
| \* | Astrin\_STLCLD20\_112214\_01.07835.07835.3 | 3.4626 | 0.2577 | 99.6% | 2015.0944 | 2015.187 | 17 | 5.854 | 31.7% | 1 | K.NQEEYKEVNFTSELRK.H | 3 |
| \* | Astrin\_STLCLD20\_112214\_01.09193.09193.2 | 2.1509 | 0.2919 | 98.5% | 1096.3322 | 1095.1974 | 9 | 5.223 | 62.5% | 1 | K.EVNFTSELR.K | 2 |
| \* | Astrin\_STLCLD20\_112214\_tube2\_01.07426.07426.2 | 2.9998 | 0.1647 | 98.6% | 1358.4321 | 1358.5779 | 2 | 5.294 | 72.7% | 1 | R.SKKDDINLLPSK.S | 2 |
| \* | Astrin\_STLCLD20\_112214\_01.07561.07561.2 | 3.8276 | 0.2713 | 100.0% | 1349.5922 | 1349.4344 | 1 | 5.811 | 81.8% | 4 | K.STAELEAEELEK.L | 2 |
| \* | Astrin\_STLCLD20\_112214\_tube2\_01.12225.12225.3 | 3.0024 | 0.2708 | 98.6% | 2010.4143 | 2010.2053 | 11 | 4.979 | 34.4% | 2 | K.STAELEAEELEKLQQYK.F | 3 |
| \* | Astrin\_STLCLD20\_112214\_01.10762.10762.2 | 5.4142 | 0.2964 | 100.0% | 2010.4321 | 2010.2053 | 1 | 7.229 | 62.5% | 1 | K.STAELEAEELEKLQQYK.F | 2 |
| \* | Astrin\_STLCLD20\_112214\_01.09115.09115.2 | 3.2402 | 0.2471 | 99.9% | 1037.3522 | 1037.2877 | 1 | 5.468 | 72.2% | 5 | R.ILEGGPILPK.K | 2 |
| \* | Astrin\_STLCLD20\_112214\_tube2\_01.11330.11330.3 | 3.6454 | 0.366 | 99.8% | 2135.4844 | 2135.5083 | 1 | 6.204 | 38.9% | 3 | K.KPPVKPPTEPIGFDLEIEK.R | 3 |
| \* | Astrin\_STLCLD20\_112214\_tube2\_01.10149.10149.3 | 4.5667 | 0.4854 | 100.0% | 2291.6042 | 2291.6958 | 1 | 7.469 | 40.8% | 3 | K.KPPVKPPTEPIGFDLEIEKR.I | 3 |
| \* | Astrin\_STLCLD20\_112214\_tube2\_01.10466.10466.2 | 3.5848 | 0.3396 | 100.0% | 1198.4122 | 1198.402 | 1 | 7.719 | 80.0% | 4 | K.ILEDVVGVPEK.K | 2 |
| \* | Astrin\_STLCLD20\_112214\_tube2\_01.08721.08721.2 | 3.7069 | 0.245 | 100.0% | 1327.5922 | 1326.576 | 10 | 4.96 | 68.2% | 4 | K.ILEDVVGVPEKK.V | 2 |
| \* | Astrin\_STLCLD20\_112214\_tube2\_01.13604.13604.2 | 3.3669 | 0.4512 | 100.0% | 1661.5322 | 1661.9823 | 1 | 7.207 | 64.3% | 4 | K.VLPITVPKS\*PAFALK.N | 2 |
| \* | Astrin\_STLCLD20\_112214\_01.07436.07436.3 | 4.5356 | 0.2957 | 99.8% | 2157.9844 | 2158.4285 | 1 | 6.681 | 39.7% | 3 | R.IRMPTKEDEEEDEPVVIK.A | 3 |
| \* | Astrin\_STLCLD20\_112214\_tube2\_01.08044.08044.2 | 5.2133 | 0.3839 | 100.0% | 1889.1122 | 1889.0815 | 1 | 7.553 | 83.3% | 3 | R.MPTKEDEEEDEPVVIK.A | 2 |
| \* | Astrin\_STLCLD20\_112214\_01.05767.05767.3 | 5.5648 | 0.2988 | 100.0% | 1889.5743 | 1889.0815 | 1 | 6.346 | 56.7% | 7 | R.MPTKEDEEEDEPVVIK.A | 3 |
| \* | Astrin\_STLCLD20\_112214\_01.09100.09100.2 | 3.7769 | 0.4376 | 100.0% | 2131.8323 | 2132.473 | 1 | 8.115 | 61.1% | 2 | K.AQPVPHYGVPFKPQIPEAR.T | 2 |
| \* | Astrin\_STLCLD20\_112214\_01.09170.09170.3 | 3.046 | 0.355 | 99.5% | 2132.8145 | 2132.473 | 1 | 5.925 | 31.9% | 3 | K.AQPVPHYGVPFKPQIPEAR.T | 3 |
| \* | Astrin\_STLCLD20\_112214\_tube2\_01.13964.13964.2 | 2.9604 | 0.3961 | 100.0% | 1458.2922 | 1458.5769 | 1 | 6.818 | 77.3% | 2 | R.TVEICPFSFDSR.D | 2 |
| \* | Astrin\_STLCLD20\_112214\_01.11372.11372.2 | 3.0068 | 0.3583 | 99.9% | 1705.4122 | 1705.9945 | 1 | 5.627 | 50.0% | 2 | K.ALPLPHFDTINLPEK.K | 2 |
| \* | Astrin\_STLCLD20\_112214\_tube2\_02.05370.05370.2 | 2.4917 | 0.1647 | 98.2% | 1054.2722 | 1054.1478 | 7 | 4.673 | 64.3% | 2 | K.HQLEEELR.Q | 2 |
| \* | Astrin\_STLCLD20\_112214\_tube2\_01.09310.09310.2 | 3.0209 | 0.2925 | 99.5% | 1682.8722 | 1683.9481 | 20 | 5.85 | 50.0% | 3 | K.ARPNTVISQEPFVPK.K | 2 |
| \* | Astrin\_STLCLD20\_112214\_01.08307.08307.3 | 2.8663 | 0.214 | 95.1% | 1684.4944 | 1683.9481 | 1 | 4.752 | 37.5% | 1 | K.ARPNTVISQEPFVPK.K | 3 |
| \* | Astrin\_STLCLD20\_112214\_01.06009.06009.3 | 4.247 | 0.3361 | 99.7% | 1811.8444 | 1812.1222 | 1 | 7.16 | 41.7% | 3 | K.ARPNTVISQEPFVPKK.E | 3 |
| \* | Astrin\_STLCLD20\_112214\_tube2\_01.07376.07376.3 | 3.4547 | 0.2565 | 99.6% | 2069.3044 | 2069.4116 | 154 | 5.326 | 30.9% | 1 | K.ARPNTVISQEPFVPKKEK.K | 3 |
| \* | Astrin\_STLCLD20\_112214\_tube2\_01.12658.12658.3 | 3.4756 | 0.3325 | 99.5% | 2474.2744 | 2475.8044 | 6 | 5.399 | 28.4% | 1 | K.KSVAEGLSGSLVQEPFQLATEKR.A | 3 |
| \* | Astrin\_STLCLD20\_112214\_tube2\_01.15162.15162.2 | 4.59 | 0.5379 | 100.0% | 2190.3123 | 2191.4429 | 1 | 9.331 | 47.5% | 1 | K.SVAEGLSGSLVQEPFQLATEK.R | 2 |
| \* | Astrin\_STLCLD20\_112214\_tube2\_01.13860.13860.2 | 4.701 | 0.5485 | 100.0% | 2346.7522 | 2347.6304 | 1 | 9.595 | 40.5% | 1 | K.SVAEGLSGSLVQEPFQLATEKR.A | 2 |
| \* | Astrin\_STLCLD20\_112214\_tube2\_01.13845.13845.3 | 3.5583 | 0.4727 | 100.0% | 2347.4644 | 2347.6304 | 8 | 7.712 | 26.2% | 2 | K.SVAEGLSGSLVQEPFQLATEKR.A | 3 |
| \* | Astrin\_STLCLD20\_112214\_01.05632.05632.3 | 3.4863 | 0.2677 | 99.5% | 1832.3043 | 1832.0386 | 1 | 6.222 | 41.7% | 3 | R.MAEVEAQKAQQLEEAR.L | 3 |
| \* | Astrin\_STLCLD20\_112214\_tube2\_01.05726.05726.3 | 3.207 | 0.2671 | 99.6% | 1629.5944 | 1630.7954 | 1 | 5.945 | 54.2% | 2 | R.LQEEEQKKEELAR.L | 3 |
| \* | Astrin\_STLCLD20\_112214\_tube2\_01.05727.05727.2 | 5.1049 | 0.3282 | 100.0% | 1630.2722 | 1630.7954 | 1 | 8.296 | 79.2% | 1 | R.LQEEEQKKEELAR.L | 2 |
| \* | Astrin\_STLCLD20\_112214\_01.07796.07796.2 | 3.4199 | 0.2947 | 100.0% | 1437.4521 | 1435.5309 | 1 | 5.415 | 75.0% | 3 | K.SSDQPLTVPVS\*PK.F | 2 |

---

|  |  |  |  |  |  |  |  |  |
| --- | --- | --- | --- | --- | --- | --- | --- | --- |
| U | *gi|67782365|ref|NP\_00* | 15 | 25 | 39.7% | 469 | 51386 | 5.5 | keratin 7 [Homo sapiens] |

| Filename XCorr DeltCN Conf% ObsM+H+ CalcM+H+ SpR ZScore Ion% # Sequence  | | | | | | | | | | | | |
| --- | --- | --- | --- | --- | --- | --- | --- | --- | --- | --- | --- | --- |
|  | Astrin\_STLCLD20\_112214\_01.08062.08062.3 | 4.4152 | 0.4135 | 100.0% | 2249.2144 | 2247.519 | 4 | 7.024 | 30.7% | 3 | R.LSSARPGGLGSSSLYGLGASRPR.V | 3 |
|  | Astrin\_STLCLD20\_112214\_01.05175.05175.2 | 3.3806 | 0.4733 | 100.0% | 1104.8922 | 1105.2388 | 1 | 8.066 | 77.3% | 2 | R.SAYGGPVGAGIR.E | 2 |
|  | Astrin\_STLCLD20\_112214\_tube2\_01.15480.15480.3 | 3.0959 | 0.2473 | 97.0% | 2451.3843 | 2450.7979 | 3 | 4.596 | 28.6% | 1 | R.EVTINQSLLAPLRLDADPSLQR.V | 3 |
|  | Astrin\_STLCLD20\_112214\_01.08756.08756.2 | 2.5869 | 0.1788 | 98.4% | 1082.6522 | 1083.2755 | 3 | 6.37 | 75.0% | 2 | K.FASFIDKVR.F | 2222 |
|  | Astrin\_STLCLD20\_112214\_tube2\_01.16786.16786.2 | 4.0728 | 0.4839 | 100.0% | 1443.6921 | 1443.686 | 3 | 8.556 | 62.5% | 4 | R.LPDIFEAQIAGLR.G | 2 |
|  | Astrin\_STLCLD20\_112214\_tube2\_01.08830.08830.2 | 2.5418 | 0.346 | 99.5% | 1243.3722 | 1243.3622 | 11 | 6.002 | 54.5% | 1 | R.GQLEALQVDGGR.L | 2 |
| \* | Astrin\_STLCLD20\_112214\_tube2\_01.13338.13338.2 | 3.2423 | 0.1977 | 99.0% | 1954.5721 | 1955.1783 | 2 | 5.197 | 41.2% | 1 | R.GQLEALQVDGGRLEAELR.S | 2 |
| \* | Astrin\_STLCLD20\_112214\_tube2\_02.08499.08499.3 | 4.0432 | 0.4357 | 100.0% | 1955.2144 | 1955.1783 | 1 | 7.188 | 41.2% | 2 | R.GQLEALQVDGGRLEAELR.S | 3 |
|  | Astrin\_STLCLD20\_112214\_tube2\_01.14806.14806.2 | 3.4353 | 0.1702 | 99.4% | 1419.2922 | 1419.5773 | 1 | 7.194 | 72.7% | 3 | K.VDALNDEINFLR.T | 2 |
|  | Astrin\_STLCLD20\_112214\_tube2\_01.16433.16433.2 | 3.3848 | 0.4027 | 100.0% | 1273.4722 | 1273.4692 | 1 | 8.166 | 72.7% | 1 | R.SLDLDGIIAEVK.A | 2 |
|  | Astrin\_STLCLD20\_112214\_01.06345.06345.2 | 2.3708 | 0.1992 | 97.2% | 1196.4122 | 1197.2897 | 6 | 5.586 | 66.7% | 1 | R.AEAEAWYQTK.F | 2 |
| \* | Astrin\_STLCLD20\_112214\_02.12118.12118.3 | 3.773 | 0.3258 | 99.8% | 2013.9844 | 2013.2987 | 1 | 5.24 | 37.5% | 1 | R.AKLEAAIAEAEERGELALK.D | 3 |
|  | Astrin\_STLCLD20\_112214\_01.05824.05824.2 | 4.1189 | 0.3595 | 100.0% | 1386.2522 | 1386.548 | 1 | 7.488 | 72.7% | 1 | R.AKQEELEAALQR.G | 2 |
|  | Astrin\_STLCLD20\_112214\_01.09243.09243.2 | 2.7639 | 0.2529 | 99.1% | 1524.2522 | 1524.7754 | 24 | 5.185 | 59.1% | 1 | R.QLREYQELMSVK.L | 2 |
|  | Astrin\_STLCLD20\_112214\_tube2\_01.12144.12144.2 | 2.7748 | 0.2075 | 98.6% | 1406.3922 | 1406.6653 | 2 | 5.0 | 68.2% | 1 | K.LALDIEIATYRK.L | 22 |

Similarities:
gi|4504919|ref|NP\_002(2:13)  
gi|47132620|ref|NP\_00(1:14)  
gi|119703753|ref|NP\_0(1:14)  

---

|  |  |  |  |  |  |  |  |  |
| --- | --- | --- | --- | --- | --- | --- | --- | --- |
| U | *gi|17921989|ref|NP\_00* | 21 | 91 | 39.7% | 448 | 49924 | 5.1 | tubulin, alpha 4a [Homo sapiens] |

| Filename XCorr DeltCN Conf% ObsM+H+ CalcM+H+ SpR ZScore Ion% # Sequence  | | | | | | | | | | | | |
| --- | --- | --- | --- | --- | --- | --- | --- | --- | --- | --- | --- | --- |
| \* | Astrin\_STLCLD20\_112214\_tube2\_01.16911.16911.2 | 2.8673 | 0.2831 | 99.3% | 1716.7522 | 1716.9719 | 1 | 5.276 | 64.3% | 1 | R.AVFVDLEPTVIDEIR.N | 2 |
|  | Astrin\_STLCLD20\_112214\_01.10293.10293.2 | 1.9386 | 0.3075 | 96.4% | 1411.5521 | 1411.6439 | 367 | 5.745 | 50.0% | 2 | R.QLFHPEQLITGK.E | 22 |
|  | Astrin\_STLCLD20\_112214\_tube2\_01.10802.10802.3 | 3.9221 | 0.4709 | 100.0% | 2416.4644 | 2416.6555 | 2 | 7.043 | 30.0% | 4 | R.QLFHPEQLITGKEDAANNYAR.G | 33 |
|  | Astrin\_STLCLD20\_112214\_tube2\_01.10767.10767.2 | 2.6947 | 0.3834 | 99.7% | 2416.5723 | 2416.6555 | 6 | 5.895 | 37.5% | 2 | R.QLFHPEQLITGKEDAANNYAR.G | 22 |
|  | Astrin\_STLCLD20\_112214\_tube2\_01.08110.08110.3 | 3.4495 | 0.2115 | 98.6% | 1876.0144 | 1876.0824 | 3 | 4.994 | 41.1% | 2 | R.RNLDIERPTYTNLNR.L | 33 |
|  | Astrin\_STLCLD20\_112214\_01.08119.08119.2 | 2.8999 | 0.1481 | 97.3% | 1719.4521 | 1719.8949 | 1 | 4.855 | 57.7% | 4 | R.NLDIERPTYTNLNR.L | 22 |
|  | Astrin\_STLCLD20\_112214\_01.08131.08131.3 | 2.6874 | 0.2989 | 98.8% | 1720.3744 | 1719.8949 | 31 | 5.026 | 40.4% | 4 | R.NLDIERPTYTNLNR.L | 33 |
|  | Astrin\_STLCLD20\_112214\_01.14491.14491.2 | 4.841 | 0.4865 | 100.0% | 1488.6921 | 1488.7678 | 1 | 9.307 | 76.9% | 14 | R.LISQIVSSITASLR.F | 22 |
|  | Astrin\_STLCLD20\_112214\_tube2\_01.19137.19137.1 | 3.2824 | 0.3949 | 100.0% | 1488.85 | 1488.7678 | 2 | 6.981 | 50.0% | 2 | R.LISQIVSSITASLR.F | 11 |
|  | Astrin\_STLCLD20\_112214\_02.16335.16335.3 | 4.1559 | 0.3309 | 99.7% | 1488.9844 | 1488.7678 | 2 | 6.853 | 51.9% | 2 | R.LISQIVSSITASLR.F | 33 |
|  | Astrin\_STLCLD20\_112214\_tube2\_02.10440.10440.2 | 5.451 | 0.5225 | 100.0% | 2410.0122 | 2410.6885 | 1 | 9.459 | 50.0% | 13 | R.FDGALNVDLTEFQTNLVPYPR.I | 22 |
|  | Astrin\_STLCLD20\_112214\_tube2\_02.10416.10416.3 | 3.7941 | 0.4445 | 100.0% | 2410.8245 | 2410.6885 | 1 | 7.732 | 36.2% | 1 | R.FDGALNVDLTEFQTNLVPYPR.I | 33 |
|  | Astrin\_STLCLD20\_112214\_tube2\_01.13778.13778.2 | 4.406 | 0.5301 | 100.0% | 1758.4321 | 1758.0703 | 1 | 9.532 | 76.7% | 12 | R.IHFPLATYAPVISAEK.A | 22 |
|  | Astrin\_STLCLD20\_112214\_tube2\_01.13851.13851.3 | 3.9489 | 0.3932 | 100.0% | 1758.7444 | 1758.0703 | 7 | 6.803 | 45.0% | 3 | R.IHFPLATYAPVISAEK.A | 33 |
|  | Astrin\_STLCLD20\_112214\_tube2\_01.11561.11561.2 | 2.2349 | 0.358 | 99.4% | 1249.8121 | 1250.4304 | 45 | 5.945 | 56.2% | 2 | K.YMACCLLYR.G | 22 |
|  | Astrin\_STLCLD20\_112214\_tube2\_01.11630.11630.2 | 4.3829 | 0.4725 | 100.0% | 1825.4722 | 1826.1027 | 1 | 8.801 | 67.6% | 10 | K.VGINYQPPTVVPGGDLAK.V | 22 |
|  | Astrin\_STLCLD20\_112214\_02.14556.14556.2 | 2.552 | 0.236 | 97.7% | 1867.2922 | 1866.1084 | 4 | 5.551 | 37.5% | 1 | R.AVCMLSNTTAIAEAWAR.L | 22 |
|  | Astrin\_STLCLD20\_112214\_01.08110.08110.2 | 3.5435 | 0.367 | 100.0% | 1382.0521 | 1381.6324 | 1 | 6.667 | 65.0% | 2 | R.LDHKFDLMYAK.R | 22 |
|  | Astrin\_STLCLD20\_112214\_tube2\_01.09242.09242.3 | 4.085 | 0.3888 | 100.0% | 1382.3344 | 1381.6324 | 14 | 6.31 | 55.0% | 4 | R.LDHKFDLMYAK.R | 33 |
|  | Astrin\_STLCLD20\_112214\_tube2\_02.08007.08007.3 | 4.4979 | 0.3173 | 99.8% | 2488.4343 | 2487.7083 | 1 | 6.132 | 35.0% | 2 | K.RAFVHWYVGEGMEEGEFSEAR.E | 33 |
|  | Astrin\_STLCLD20\_112214\_02.12735.12735.3 | 4.7846 | 0.4381 | 100.0% | 2331.8044 | 2331.5208 | 1 | 7.169 | 40.8% | 4 | R.AFVHWYVGEGMEEGEFSEAR.E | 33 |

Similarities:
gi|57013276|ref|NP\_00(20:1)  

---

|  |  |  |  |  |  |  |  |  |
| --- | --- | --- | --- | --- | --- | --- | --- | --- |
| U | *gi|20357599|ref|NP\_61* | 3 | 10 | 39.5% | 114 | 12146 | 10.5 | H2A histone family, member V isoform 2 [Homo sapiens] |
| U | *gi|6912616|ref|NP\_036* | 3 | 10 | 35.2% | 128 | 13509 | 10.6 | H2A histone family, member V isoform 1 [Homo sapiens] |
| U | *gi|4504255|ref|NP\_002* | 3 | 10 | 35.2% | 128 | 13553 | 10.6 | H2A histone family, member Z [Homo sapiens] |

| Filename XCorr DeltCN Conf% ObsM+H+ CalcM+H+ SpR ZScore Ion% # Sequence  | | | | | | | | | | | | |
| --- | --- | --- | --- | --- | --- | --- | --- | --- | --- | --- | --- | --- |
|  | Astrin\_STLCLD20\_112214\_01.09511.09511.2 | 2.9305 | 0.285 | 99.9% | 945.4122 | 945.1093 | 2 | 5.81 | 81.2% | 6 | R.AGLQFPVGR.I | 2222 |
|  | Astrin\_STLCLD20\_112214\_01.19147.19147.2 | 2.8134 | 0.2206 | 98.4% | 2896.0723 | 2897.2952 | 72 | 4.788 | 23.2% | 1 | R.VGATAAVYSAAILEYLTAEVLELAGNASK.D | 2 |
|  | Astrin\_STLCLD20\_112214\_tube2\_01.07832.07832.2 | 2.6508 | 0.2524 | 99.7% | 851.2922 | 851.0396 | 4 | 6.019 | 83.3% | 3 | R.HLQLAIR.G | 2222 |

Similarities:
gi|106775678|ref|NP\_0(2:1)  
gi|10645195|ref|NP\_06(2:1)  
gi|10800130|ref|NP\_06(2:1)  

---

|  |  |  |  |  |  |  |  |  |
| --- | --- | --- | --- | --- | --- | --- | --- | --- |
| U | *GST* | 11 | 28 | 39.3% | 244 | 28430 | 6.2 | no description |

| Filename XCorr DeltCN Conf% ObsM+H+ CalcM+H+ SpR ZScore Ion% # Sequence  | | | | | | | | | | | | |
| --- | --- | --- | --- | --- | --- | --- | --- | --- | --- | --- | --- | --- |
| \* | Astrin\_STLCLD20\_112214\_tube2\_01.16154.16154.3 | 4.356 | 0.2701 | 99.8% | 2270.4844 | 2270.5437 | 1 | 7.169 | 39.1% | 3 | R.LLLEYLEEKYEEHLYER.D | 3 |
| \* | Astrin\_STLCLD20\_112214\_01.12117.12117.3 | 4.5262 | 0.4061 | 100.0% | 3156.9243 | 3157.4631 | 2 | 6.517 | 28.3% | 2 | R.LLLEYLEEKYEEHLYERDEGDKWR.N | 3 |
| \* | Astrin\_STLCLD20\_112214\_01.12859.12859.3 | 4.5631 | 0.3678 | 99.7% | 2601.1743 | 2600.9739 | 1 | 6.711 | 41.7% | 1 | R.NKKFELGLEFPNLPYYIDGDVK.L | 3 |
| \* | Astrin\_STLCLD20\_112214\_01.14750.14750.2 | 4.5319 | 0.3891 | 100.0% | 2230.632 | 2230.522 | 1 | 7.242 | 50.0% | 3 | K.FELGLEFPNLPYYIDGDVK.L | 2 |
| \* | Astrin\_STLCLD20\_112214\_tube2\_01.09198.09198.1 | 1.8273 | 0.2961 | 98.8% | 1032.51 | 1033.2744 | 21 | 5.316 | 56.2% | 1 | K.LTQSMAIIR.Y | 1 |
| \* | Astrin\_STLCLD20\_112214\_tube2\_01.09266.09266.2 | 3.1021 | 0.228 | 99.9% | 1033.1921 | 1033.2744 | 2 | 5.747 | 81.2% | 4 | K.LTQSMAIIR.Y | 2 |
| \* | Astrin\_STLCLD20\_112214\_tube2\_01.17315.17315.2 | 4.8964 | 0.3311 | 100.0% | 1518.5322 | 1517.7809 | 1 | 6.924 | 73.1% | 5 | R.AEISMLEGAVLDIR.Y | 2 |
| \* | Astrin\_STLCLD20\_112214\_tube2\_01.13656.13656.3 | 3.6225 | 0.3785 | 99.7% | 2005.7043 | 2005.3184 | 1 | 5.965 | 34.4% | 2 | R.IAYSKDFETLKVDFLSK.L | 3 |
| \* | Astrin\_STLCLD20\_112214\_01.11925.11925.2 | 3.6655 | 0.3331 | 100.0% | 1443.6122 | 1442.6519 | 1 | 6.182 | 72.7% | 2 | K.DFETLKVDFLSK.L | 2 |
| \* | Astrin\_STLCLD20\_112214\_01.06206.06206.2 | 2.475 | 0.223 | 98.4% | 1182.6522 | 1183.3934 | 1 | 4.599 | 83.3% | 3 | K.RIEAIPQIDK.Y | 2 |
| \* | Astrin\_STLCLD20\_112214\_01.07862.07862.2 | 2.425 | 0.143 | 96.4% | 1027.3922 | 1027.2059 | 50 | 4.547 | 56.2% | 2 | R.IEAIPQIDK.Y | 2 |

---

|  |  |  |  |  |  |  |  |  |
| --- | --- | --- | --- | --- | --- | --- | --- | --- |
| U | *gi|29788768|ref|NP\_82* | 23 | 114 | 38.9% | 445 | 49953 | 4.9 | tubulin, beta 2B [Homo sapiens] |
| U | *gi|4507729|ref|NP\_001* | 23 | 115 | 38.9% | 445 | 49907 | 4.9 | tubulin, beta 2 [Homo sapiens] |

| Filename XCorr DeltCN Conf% ObsM+H+ CalcM+H+ SpR ZScore Ion% # Sequence  | | | | | | | | | | | | |
| --- | --- | --- | --- | --- | --- | --- | --- | --- | --- | --- | --- | --- |
|  | Astrin\_STLCLD20\_112214\_01.11522.11522.2 | 4.511 | 0.4938 | 100.0% | 1617.3121 | 1616.8701 | 1 | 8.254 | 64.3% | 14 | R.AILVDLEPGTMDSVR.S | 222 |
|  | Astrin\_STLCLD20\_112214\_tube2\_01.16521.16521.2 | 5.3152 | 0.5218 | 100.0% | 2799.0322 | 2800.0647 | 1 | 8.506 | 44.0% | 6 | R.SGPFGQIFRPDNFVFGQSGAGNNWAK.G | 222 |
|  | Astrin\_STLCLD20\_112214\_tube2\_01.16562.16562.3 | 6.8919 | 0.438 | 100.0% | 2799.8342 | 2800.0647 | 1 | 8.685 | 39.0% | 8 | R.SGPFGQIFRPDNFVFGQSGAGNNWAK.G | 333 |
|  | Astrin\_STLCLD20\_112214\_tube2\_01.17294.17294.2 | 6.9168 | 0.5867 | 100.0% | 1959.5922 | 1960.151 | 1 | 11.206 | 79.4% | 2 | K.GHYTEGAELVDSVLDVVR.K | 2222 |
|  | Astrin\_STLCLD20\_112214\_01.13379.13379.3 | 3.6233 | 0.2439 | 99.6% | 1960.6144 | 1960.151 | 1 | 5.03 | 38.2% | 3 | K.GHYTEGAELVDSVLDVVR.K | 3333 |
|  | Astrin\_STLCLD20\_112214\_01.12767.12767.2 | 5.5584 | 0.5301 | 100.0% | 2087.7522 | 2088.325 | 1 | 9.725 | 72.2% | 3 | K.GHYTEGAELVDSVLDVVRK.E | 2222 |
|  | Astrin\_STLCLD20\_112214\_tube2\_01.15854.15854.3 | 4.7062 | 0.4415 | 100.0% | 2088.8943 | 2088.325 | 1 | 7.599 | 41.7% | 5 | K.GHYTEGAELVDSVLDVVRK.E | 3333 |
|  | Astrin\_STLCLD20\_112214\_tube2\_01.05867.05867.2 | 2.3753 | 0.2512 | 98.9% | 1078.1522 | 1078.1698 | 1 | 5.048 | 85.7% | 1 | K.IREEYPDR.I | 222 |
|  | Astrin\_STLCLD20\_112214\_01.10486.10486.2 | 3.3502 | 0.4172 | 100.0% | 1352.5322 | 1352.6497 | 1 | 6.771 | 63.6% | 1 | R.IMNTFSVMPSPK.V | 22 |
|  | Astrin\_STLCLD20\_112214\_tube2\_01.09993.09993.2 | 3.1291 | 0.339 | 100.0% | 1131.4922 | 1131.2767 | 11 | 5.764 | 77.8% | 19 | R.FPGQLNADLR.K | 22222 |
|  | Astrin\_STLCLD20\_112214\_tube2\_01.08446.08446.2 | 2.7132 | 0.2665 | 99.3% | 1259.5122 | 1259.4508 | 43 | 5.338 | 60.0% | 3 | R.FPGQLNADLRK.L | 22222 |
|  | Astrin\_STLCLD20\_112214\_tube2\_01.11456.11456.2 | 3.6404 | 0.3874 | 100.0% | 1272.3522 | 1272.5945 | 1 | 6.64 | 70.0% | 4 | R.KLAVNMVPFPR.L | 22222 |
|  | Astrin\_STLCLD20\_112214\_01.11192.11192.1 | 2.2399 | 0.2147 | 98.0% | 1143.63 | 1144.4204 | 6 | 6.378 | 61.1% | 2 | K.LAVNMVPFPR.L | 11111 |
|  | Astrin\_STLCLD20\_112214\_01.11233.11233.2 | 3.8296 | 0.4984 | 100.0% | 1144.4722 | 1144.4204 | 1 | 8.878 | 94.4% | 6 | K.LAVNMVPFPR.L | 22222 |
|  | Astrin\_STLCLD20\_112214\_tube2\_01.16406.16406.2 | 3.7237 | 0.4102 | 100.0% | 1621.0322 | 1621.9403 | 1 | 8.877 | 73.1% | 7 | R.LHFFMPGFAPLTSR.G | 2222 |
|  | Astrin\_STLCLD20\_112214\_tube2\_01.16287.16287.3 | 4.1587 | 0.348 | 99.7% | 1622.1543 | 1621.9403 | 2 | 6.383 | 50.0% | 4 | R.LHFFMPGFAPLTSR.G | 3333 |
|  | Astrin\_STLCLD20\_112214\_tube2\_02.07332.07332.3 | 4.839 | 0.2248 | 99.8% | 1926.3243 | 1925.2405 | 1 | 5.81 | 50.0% | 2 | R.MSMKEVDEQMLNVQNK.N | 333 |
|  | Astrin\_STLCLD20\_112214\_tube2\_01.08570.08570.2 | 4.1176 | 0.2811 | 100.0% | 1447.3322 | 1447.6031 | 1 | 6.064 | 81.8% | 4 | K.EVDEQMLNVQNK.N | 222 |
|  | Astrin\_STLCLD20\_112214\_tube2\_01.15262.15262.2 | 3.3078 | 0.3064 | 99.9% | 1698.6122 | 1697.8877 | 1 | 5.236 | 61.5% | 3 | K.NSSYFVEWIPNNVK.T | 22222 |
|  | Astrin\_STLCLD20\_112214\_02.14525.14525.3 | 3.4924 | 0.1623 | 95.1% | 2016.4443 | 2015.335 | 1 | 5.966 | 32.4% | 1 | K.MSATFIGNSTAIQELFKR.I | 33 |
|  | Astrin\_STLCLD20\_112214\_tube2\_01.12078.12078.2 | 3.0386 | 0.2531 | 99.7% | 1385.6921 | 1386.6116 | 84 | 5.63 | 55.0% | 5 | K.RISEQFTAMFR.R | 2222 |
|  | Astrin\_STLCLD20\_112214\_tube2\_01.13623.13623.1 | 1.9755 | 0.2993 | 100.0% | 1229.5 | 1230.4241 | 1 | 5.342 | 61.1% | 1 | R.ISEQFTAMFR.R | 1111 |
|  | Astrin\_STLCLD20\_112214\_02.12843.12843.2 | 3.8745 | 0.4739 | 100.0% | 1230.1921 | 1230.4241 | 1 | 8.01 | 94.4% | 10 | R.ISEQFTAMFR.R | 2222 |

Similarities:
gi|29788785|ref|NP\_82(21:2)  
gi|5174735|ref|NP\_006(21:2)  
gi|50592996|ref|NP\_00(14:9)  
gi|14210536|ref|NP\_11(9:14)  

---

|  |  |  |  |  |  |  |  |  |
| --- | --- | --- | --- | --- | --- | --- | --- | --- |
| U | *gi|4503529|ref|NP\_001* | 12 | 17 | 38.2% | 406 | 46154 | 5.5 | eukaryotic translation initiation factor 4A isoform 1 [Homo sapiens] |

| Filename XCorr DeltCN Conf% ObsM+H+ CalcM+H+ SpR ZScore Ion% # Sequence  | | | | | | | | | | | | |
| --- | --- | --- | --- | --- | --- | --- | --- | --- | --- | --- | --- | --- |
| \* | Astrin\_STLCLD20\_112214\_tube2\_01.20542.20542.3 | 4.9976 | 0.4117 | 100.0% | 4169.994 | 4169.451 | 1 | 6.889 | 22.2% | 3 | R.SRDNGPDGMEPEGVIESNWNEIVDSFDDMNLSESLLR.G | 3 |
|  | Astrin\_STLCLD20\_112214\_01.09010.09010.2 | 4.6068 | 0.5611 | 100.0% | 1828.3922 | 1829.0654 | 1 | 9.405 | 70.0% | 2 | R.GIYAYGFEKPSAIQQR.A | 22 |
|  | Astrin\_STLCLD20\_112214\_01.09019.09019.3 | 2.9649 | 0.219 | 95.9% | 1830.8043 | 1829.0654 | 4 | 4.311 | 38.3% | 1 | R.GIYAYGFEKPSAIQQR.A | 33 |
|  | Astrin\_STLCLD20\_112214\_01.06633.06633.2 | 2.3861 | 0.2832 | 98.2% | 1394.3322 | 1395.512 | 40 | 4.942 | 50.0% | 1 | K.GYDVIAQAQSGTGK.T | 2 |
| \* | Astrin\_STLCLD20\_112214\_01.09521.09521.2 | 3.7186 | 0.3161 | 100.0% | 1619.5521 | 1619.9225 | 2 | 7.215 | 64.3% | 1 | K.LQMEAPHIIVGTPGR.V | 2 |
| \* | Astrin\_STLCLD20\_112214\_01.09484.09484.3 | 3.4431 | 0.39 | 99.7% | 1620.4744 | 1619.9225 | 1 | 6.088 | 48.2% | 1 | K.LQMEAPHIIVGTPGR.V | 3 |
|  | Astrin\_STLCLD20\_112214\_tube2\_01.17031.17031.2 | 5.1714 | 0.5104 | 100.0% | 1557.3922 | 1556.789 | 1 | 9.616 | 79.2% | 2 | K.MFVLDEADEMLSR.G | 2 |
| \* | Astrin\_STLCLD20\_112214\_tube2\_01.14045.14045.2 | 3.6601 | 0.2674 | 100.0% | 1503.5122 | 1502.71 | 5 | 6.016 | 63.6% | 2 | R.GFKDQIYDIFQK.L | 2 |
|  | Astrin\_STLCLD20\_112214\_tube2\_01.13348.13348.1 | 1.6928 | 0.2508 | 97.2% | 1114.59 | 1115.3585 | 7 | 5.286 | 55.6% | 1 | R.VLITTDLLAR.G | 1 |
|  | Astrin\_STLCLD20\_112214\_tube2\_01.13318.13318.2 | 2.6763 | 0.3086 | 99.7% | 1115.1322 | 1115.3585 | 1 | 5.594 | 77.8% | 1 | R.VLITTDLLAR.G | 2 |
| \* | Astrin\_STLCLD20\_112214\_tube2\_01.07329.07329.3 | 3.7809 | 0.3615 | 99.7% | 1591.5243 | 1590.8352 | 1 | 6.213 | 44.2% | 1 | R.KGVAINMVTEEDKR.T | 3 |
| \* | Astrin\_STLCLD20\_112214\_01.16750.16750.2 | 2.561 | 0.3149 | 99.0% | 2797.7722 | 2799.1653 | 1 | 6.251 | 34.8% | 1 | R.TLRDIETFYNTSIEEMPLNVADLI.- | 2 |

Similarities:
gi|7661920|ref|NP\_055(2:10)  

---

|  |  |  |  |  |  |  |  |  |
| --- | --- | --- | --- | --- | --- | --- | --- | --- |
| U | *gi|12667788|ref|NP\_00* | 68 | 156 | 37.9% | 1960 | 226530 | 5.6 | myosin, heavy polypeptide 9, non-muscle [Homo sapiens] |

| Filename XCorr DeltCN Conf% ObsM+H+ CalcM+H+ SpR ZScore Ion% # Sequence  | | | | | | | | | | | | |
| --- | --- | --- | --- | --- | --- | --- | --- | --- | --- | --- | --- | --- |
| \* | Astrin\_STLCLD20\_112214\_tube2\_01.14602.14602.2 | 3.9986 | 0.2871 | 100.0% | 1673.5122 | 1673.8687 | 1 | 6.584 | 71.4% | 4 | K.NFINNPLAQADWAAK.K | 2 |
| \* | Astrin\_STLCLD20\_112214\_01.07504.07504.2 | 2.1575 | 0.2439 | 97.4% | 1072.3922 | 1072.2926 | 14 | 5.294 | 62.5% | 2 | K.KLVWVPSDK.S | 2 |
| \* | Astrin\_STLCLD20\_112214\_tube2\_01.15735.15735.2 | 2.4809 | 0.2311 | 97.3% | 1728.6921 | 1728.9978 | 11 | 5.623 | 42.3% | 1 | K.NLPIYSEEIVEMYK.G | 2 |
| \* | Astrin\_STLCLD20\_112214\_tube2\_01.08433.08433.3 | 4.3809 | 0.3901 | 100.0% | 2072.3342 | 2072.3489 | 1 | 7.218 | 45.3% | 2 | K.RHEMPPHIYAITDTAYR.S | 3 |
| \* | Astrin\_STLCLD20\_112214\_01.08240.08240.3 | 4.3084 | 0.4868 | 100.0% | 1916.1543 | 1916.1614 | 1 | 8.118 | 53.3% | 10 | R.HEMPPHIYAITDTAYR.S | 3 |
| \* | Astrin\_STLCLD20\_112214\_tube2\_02.07593.07593.3 | 2.1084 | 0.3763 | 98.6% | 1479.2043 | 1479.719 | 1 | 5.875 | 37.5% | 1 | K.VIQYLAYVASSHK.S | 3 |
| \* | Astrin\_STLCLD20\_112214\_tube2\_01.11262.11262.2 | 3.8502 | 0.4132 | 100.0% | 1479.5721 | 1479.719 | 1 | 8.266 | 75.0% | 2 | K.VIQYLAYVASSHK.S | 2 |
|  | Astrin\_STLCLD20\_112214\_01.13430.13430.2 | 4.9502 | 0.4604 | 100.0% | 1727.9321 | 1728.0012 | 1 | 9.572 | 63.3% | 2 | R.QLLQANPILEAFGNAK.T | 2 |
| \* | Astrin\_STLCLD20\_112214\_tube2\_02.10189.10189.3 | 4.9487 | 0.4022 | 100.0% | 1997.6344 | 1997.3037 | 1 | 7.988 | 46.9% | 4 | R.TFHIFYYLLSGAGEHLK.T | 3 |
| \* | Astrin\_STLCLD20\_112214\_tube2\_01.17004.17004.2 | 4.3074 | 0.3687 | 100.0% | 1997.8522 | 1997.3037 | 1 | 7.166 | 59.4% | 1 | R.TFHIFYYLLSGAGEHLK.T | 2 |
| \* | Astrin\_STLCLD20\_112214\_01.11324.11324.3 | 4.6822 | 0.1701 | 99.1% | 3011.8743 | 3012.4 | 1 | 5.046 | 32.0% | 1 | R.FLSNGHVTIPGQQDKDMFQETMEAMR.I | 3 |
| \* | Astrin\_STLCLD20\_112214\_01.11827.11827.2 | 4.0814 | 0.4199 | 100.0% | 1616.5122 | 1616.9313 | 1 | 8.078 | 80.8% | 6 | R.IMGIPEEEQMGLLR.V | 2 |
| \* | Astrin\_STLCLD20\_112214\_tube2\_01.15566.15566.2 | 3.8874 | 0.2548 | 100.0% | 1616.1522 | 1616.0 | 1 | 5.467 | 67.9% | 2 | R.VISGVLQLGNIVFKK.E | 2 |
| \* | Astrin\_STLCLD20\_112214\_tube2\_01.13328.13328.2 | 4.1825 | 0.406 | 100.0% | 1572.5122 | 1572.8044 | 1 | 8.19 | 76.9% | 5 | K.VSHLLGINVTDFTR.G | 2 |
| \* | Astrin\_STLCLD20\_112214\_tube2\_01.13337.13337.3 | 3.3337 | 0.3367 | 99.8% | 1573.2244 | 1572.8044 | 5 | 5.612 | 46.2% | 1 | K.VSHLLGINVTDFTR.G | 3 |
| \* | Astrin\_STLCLD20\_112214\_tube2\_01.17058.17058.2 | 4.6739 | 0.6498 | 100.0% | 2018.3722 | 2019.3636 | 1 | 11.134 | 57.9% | 3 | R.IIGLDQVAGMSETALPGAFK.T | 2 |
| \* | Astrin\_STLCLD20\_112214\_tube2\_01.07731.07731.2 | 2.9455 | 0.2813 | 99.7% | 1378.1721 | 1378.6115 | 8 | 5.025 | 54.5% | 2 | R.TVGQLYKEQLAK.L | 2 |
|  | Astrin\_STLCLD20\_112214\_tube2\_01.12982.12982.2 | 2.3095 | 0.3423 | 99.3% | 1319.8722 | 1319.5468 | 23 | 5.288 | 60.0% | 2 | K.LDPHLVLDQLR.C | 22 |
| \* | Astrin\_STLCLD20\_112214\_tube2\_01.11349.11349.2 | 2.7434 | 0.2948 | 99.5% | 1275.4521 | 1275.4875 | 1 | 6.763 | 65.0% | 3 | R.YEILTPNSIPK.G | 2 |
| \* | Astrin\_STLCLD20\_112214\_01.09799.09799.2 | 2.8667 | 0.3983 | 100.0% | 1194.3522 | 1194.33 | 4 | 7.152 | 72.2% | 4 | K.ALELDSNLYR.I | 2 |
|  | Astrin\_STLCLD20\_112214\_tube2\_01.08715.08715.2 | 3.1383 | 0.3993 | 100.0% | 1224.5122 | 1224.3591 | 1 | 7.007 | 70.0% | 2 | R.AGVLAHLEEER.D | 22 |
| \* | Astrin\_STLCLD20\_112214\_tube2\_01.13828.13828.2 | 5.0055 | 0.4568 | 100.0% | 1752.3922 | 1753.0358 | 1 | 7.721 | 64.3% | 5 | R.LTEMETLQSQLMAEK.L | 2 |
| \* | Astrin\_STLCLD20\_112214\_tube2\_01.13979.13979.2 | 6.079 | 0.4628 | 100.0% | 2333.5522 | 2334.4736 | 1 | 9.889 | 66.7% | 1 | K.MQQNIQELEEQLEEEESAR.Q | 2 |
| \* | Astrin\_STLCLD20\_112214\_02.10467.10467.2 | 4.7294 | 0.477 | 100.0% | 1655.2722 | 1654.7681 | 1 | 7.77 | 80.8% | 5 | R.IAEFTTNLTEEEEK.S | 2 |
| \* | Astrin\_STLCLD20\_112214\_tube2\_01.09180.09180.2 | 3.9685 | 0.3047 | 100.0% | 1870.5122 | 1870.0203 | 1 | 5.918 | 53.3% | 1 | R.IAEFTTNLTEEEEKSK.S | 2 |
| \* | Astrin\_STLCLD20\_112214\_tube2\_01.07839.07839.2 | 3.1375 | 0.1804 | 98.9% | 1587.5721 | 1586.76 | 2 | 6.838 | 62.5% | 1 | K.NKHEAMITDLEER.L | 2 |
| \* | Astrin\_STLCLD20\_112214\_01.07474.07474.2 | 2.6176 | 0.4724 | 100.0% | 1343.9521 | 1344.4822 | 1 | 7.711 | 75.0% | 1 | K.HEAMITDLEER.L | 2 |
|  | Astrin\_STLCLD20\_112214\_01.05162.05162.2 | 3.2255 | 0.3083 | 100.0% | 1258.1921 | 1258.4172 | 5 | 5.34 | 75.0% | 2 | K.KEEELQAALAR.V | 2 |
| \* | Astrin\_STLCLD20\_112214\_01.11944.11944.3 | 5.011 | 0.3452 | 100.0% | 2305.1343 | 2304.473 | 1 | 6.165 | 38.9% | 1 | K.IRELESQISELQEDLESER.A | 3 |
| \* | Astrin\_STLCLD20\_112214\_tube2\_01.15195.15195.2 | 3.9789 | 0.4771 | 100.0% | 2034.2122 | 2035.126 | 1 | 8.323 | 59.4% | 1 | R.ELESQISELQEDLESER.A | 2 |
| \* | Astrin\_STLCLD20\_112214\_01.16693.16693.3 | 4.1211 | 0.3383 | 99.8% | 3018.2944 | 3019.2434 | 1 | 6.406 | 35.6% | 2 | R.DLGEELEALKTELEDTLDSTAAQQELR.S | 3 |
| \* | Astrin\_STLCLD20\_112214\_01.16679.16679.2 | 5.1368 | 0.5418 | 100.0% | 3019.152 | 3019.2434 | 1 | 10.356 | 44.2% | 2 | R.DLGEELEALKTELEDTLDSTAAQQELR.S | 2 |
| \* | Astrin\_STLCLD20\_112214\_01.08039.08039.3 | 3.6435 | 0.4576 | 100.0% | 2043.0844 | 2044.2439 | 1 | 6.603 | 40.6% | 1 | K.TLEEEAKTHEAQIQEMR.Q | 3 |
| \* | Astrin\_STLCLD20\_112214\_tube2\_01.10922.10922.3 | 5.3902 | 0.3925 | 100.0% | 1997.0944 | 1997.1722 | 1 | 7.878 | 43.8% | 4 | K.HSQAVEELAEQLEQTKR.V | 3 |
| \* | Astrin\_STLCLD20\_112214\_01.04990.04990.3 | 3.7062 | 0.2806 | 99.5% | 1930.3444 | 1930.1252 | 1 | 4.772 | 42.2% | 1 | K.AKQTLENERGELANEVK.V | 3 |
| \* | Astrin\_STLCLD20\_112214\_tube2\_01.07534.07534.3 | 2.9398 | 0.201 | 95.8% | 1544.2144 | 1541.8314 | 354 | 4.462 | 37.5% | 1 | R.KKVEAQLQELQVK.F | 3 |
| \* | Astrin\_STLCLD20\_112214\_01.06953.06953.2 | 3.9367 | 0.3241 | 100.0% | 1413.8922 | 1413.6573 | 1 | 6.179 | 72.7% | 1 | K.KVEAQLQELQVK.F | 2 |
| \* | Astrin\_STLCLD20\_112214\_01.08309.08309.2 | 3.5521 | 0.3183 | 100.0% | 1286.0322 | 1285.4833 | 1 | 6.283 | 85.0% | 1 | K.VEAQLQELQVK.F | 2 |
| \* | Astrin\_STLCLD20\_112214\_tube2\_02.09156.09156.2 | 5.6644 | 0.5434 | 100.0% | 1946.8121 | 1947.1498 | 1 | 10.073 | 73.5% | 2 | K.LQVELDNVTGLLSQSDSK.S | 2 |
| \* | Astrin\_STLCLD20\_112214\_tube2\_01.06059.06059.3 | 2.442 | 0.2606 | 95.7% | 1493.9043 | 1493.6598 | 2 | 5.22 | 45.5% | 1 | K.LKQVEDEKNSFR.E | 3 |
| \* | Astrin\_STLCLD20\_112214\_01.12899.12899.2 | 5.0288 | 0.4855 | 100.0% | 1950.3322 | 1951.1436 | 1 | 9.727 | 76.7% | 2 | R.LQQELDDLLVDLDHQR.Q | 2 |
| \* | Astrin\_STLCLD20\_112214\_tube2\_01.16215.16215.3 | 3.564 | 0.2624 | 99.5% | 1951.5844 | 1951.1436 | 1 | 4.912 | 48.3% | 3 | R.LQQELDDLLVDLDHQR.Q | 3 |
|  | Astrin\_STLCLD20\_112214\_tube2\_01.08607.08607.2 | 2.7025 | 0.1787 | 98.5% | 1222.3922 | 1221.3959 | 5 | 4.236 | 66.7% | 1 | K.KFDQLLAEEK.T | 22 |
|  | Astrin\_STLCLD20\_112214\_tube2\_01.09251.09251.2 | 3.0319 | 0.2398 | 99.8% | 1094.3922 | 1093.2218 | 1 | 6.362 | 81.2% | 2 | K.FDQLLAEEK.T | 22 |
| \* | Astrin\_STLCLD20\_112214\_02.09303.09303.3 | 4.0355 | 0.4133 | 100.0% | 1647.6843 | 1647.8407 | 1 | 6.99 | 51.9% | 2 | R.ALEEAMEQKAELER.L | 3 |
| \* | Astrin\_STLCLD20\_112214\_01.07549.07549.2 | 3.6395 | 0.4003 | 100.0% | 1647.9122 | 1647.8407 | 1 | 7.35 | 69.2% | 3 | R.ALEEAMEQKAELER.L | 2 |
| \* | Astrin\_STLCLD20\_112214\_01.08177.08177.2 | 3.6382 | 0.5277 | 100.0% | 1685.1322 | 1685.8586 | 1 | 9.306 | 71.4% | 2 | R.TEMEDLMSSKDDVGK.S | 2 |
| \* | Astrin\_STLCLD20\_112214\_01.06967.06967.2 | 3.1455 | 0.3717 | 100.0% | 1205.3522 | 1205.3685 | 3 | 7.05 | 77.8% | 4 | R.ALEQQVEEMK.T | 2 |
| \* | Astrin\_STLCLD20\_112214\_tube2\_01.19353.19353.3 | 4.0863 | 0.1903 | 98.2% | 3150.3843 | 3149.4048 | 7 | 5.46 | 24.0% | 1 | R.ALEQQVEEMKTQLEELEDELQATEDAK.L | 3 |
| \* | Astrin\_STLCLD20\_112214\_tube2\_01.11390.11390.2 | 2.8321 | 0.2545 | 99.4% | 1315.5122 | 1315.6171 | 2 | 5.666 | 65.0% | 3 | K.LRLEVNLQAMK.A | 2 |
| \* | Astrin\_STLCLD20\_112214\_tube2\_01.10553.10553.2 | 2.0419 | 0.3191 | 98.5% | 1046.4321 | 1046.2701 | 3 | 5.385 | 75.0% | 1 | R.LEVNLQAMK.A | 2 |
| \* | Astrin\_STLCLD20\_112214\_tube2\_01.07773.07773.2 | 2.61 | 0.3203 | 99.7% | 1251.1721 | 1251.3075 | 1 | 6.21 | 72.2% | 2 | R.EMEAELEDER.K | 2 |
| \* | Astrin\_STLCLD20\_112214\_tube2\_01.06603.06603.2 | 2.334 | 0.1879 | 96.2% | 1380.3722 | 1379.4816 | 18 | 4.015 | 55.0% | 1 | R.EMEAELEDERK.Q | 2 |
| \* | Astrin\_STLCLD20\_112214\_tube2\_01.06416.06416.2 | 2.9415 | 0.1937 | 99.0% | 1216.1921 | 1216.3799 | 2 | 4.959 | 70.0% | 1 | R.ASREEILAQAK.E | 2 |
| \* | Astrin\_STLCLD20\_112214\_tube2\_01.06051.06051.3 | 3.573 | 0.2372 | 99.6% | 1844.4844 | 1845.0629 | 12 | 4.739 | 33.3% | 1 | R.ASREEILAQAKENEKK.L | 3 |
| \* | Astrin\_STLCLD20\_112214\_01.13648.13648.2 | 4.046 | 0.5077 | 100.0% | 2049.6921 | 2050.3064 | 1 | 8.081 | 55.9% | 1 | K.SMEAEMIQLQEELAAAER.A | 2 |
| \* | Astrin\_STLCLD20\_112214\_01.07594.07594.3 | 3.8507 | 0.4253 | 100.0% | 2090.5444 | 2090.168 | 1 | 6.569 | 36.1% | 2 | R.QAQQERDELADEIANSSGK.G | 3 |
| \* | Astrin\_STLCLD20\_112214\_tube2\_02.08328.08328.3 | 4.5556 | 0.3965 | 100.0% | 2473.1343 | 2473.6099 | 1 | 7.537 | 37.5% | 3 | R.IAQLEEELEEEQGNTELINDR.L | 3 |
| \* | Astrin\_STLCLD20\_112214\_tube2\_01.13109.13109.2 | 5.9838 | 0.4485 | 100.0% | 2473.6921 | 2473.6099 | 1 | 8.928 | 62.5% | 2 | R.IAQLEEELEEEQGNTELINDR.L | 2 |
| \* | Astrin\_STLCLD20\_112214\_01.11114.11114.2 | 5.1949 | 0.5244 | 100.0% | 1870.4122 | 1871.0574 | 1 | 10.375 | 66.7% | 6 | K.ANLQIDQINTDLNLER.S | 2 |
| \* | Astrin\_STLCLD20\_112214\_01.08248.08248.2 | 4.4012 | 0.4517 | 100.0% | 1531.2322 | 1531.6598 | 1 | 7.969 | 79.2% | 3 | K.IAQLEEQLDNETK.E | 2 |
| \* | Astrin\_STLCLD20\_112214\_01.07952.07952.2 | 4.9376 | 0.505 | 100.0% | 1816.4922 | 1816.9628 | 1 | 8.37 | 78.6% | 2 | K.IAQLEEQLDNETKER.Q | 2 |
| \* | Astrin\_STLCLD20\_112214\_tube2\_01.05704.05704.3 | 3.3629 | 0.3316 | 99.8% | 1726.4043 | 1725.8125 | 6 | 6.031 | 35.7% | 1 | R.NAEQYKDQADKASTR.L | 3 |
| \* | Astrin\_STLCLD20\_112214\_tube2\_01.05997.05997.2 | 4.1877 | 0.3543 | 100.0% | 1487.3722 | 1488.5541 | 1 | 7.095 | 81.8% | 1 | K.RQLEEAEEEAQR.A | 2 |
| \* | Astrin\_STLCLD20\_112214\_tube2\_01.06171.06171.2 | 3.5123 | 0.342 | 100.0% | 1333.3322 | 1332.3666 | 1 | 6.31 | 80.0% | 1 | R.QLEEAEEEAQR.A | 2 |
| \* | Astrin\_STLCLD20\_112214\_01.05926.05926.2 | 3.7362 | 0.5951 | 100.0% | 1565.7722 | 1566.6367 | 1 | 10.219 | 73.1% | 5 | R.ELEDATETADAMNR.E | 2 |
| \* | Astrin\_STLCLD20\_112214\_tube2\_01.10814.10814.2 | 3.4401 | 0.1042 | 99.3% | 1156.3322 | 1156.3732 | 1 | 5.822 | 88.9% | 5 | R.RGDLPFVVPR.R | 2 |
| \* | Astrin\_STLCLD20\_112214\_tube2\_01.05817.05817.3 | 4.7448 | 0.362 | 99.7% | 2384.5745 | 2384.3428 | 1 | 7.269 | 33.7% | 1 | R.KGAGDGS\*DEEVDGKADGAEAKPAE.- | 3 |

Similarities:
gi|41406064|ref|NP\_00(4:64)  

---

|  |  |  |  |  |  |  |  |  |
| --- | --- | --- | --- | --- | --- | --- | --- | --- |
| U | *gi|4557701|ref|NP\_000* | 19 | 38 | 37.5% | 432 | 48106 | 5.0 | keratin 17 [Homo sapiens] |

| Filename XCorr DeltCN Conf% ObsM+H+ CalcM+H+ SpR ZScore Ion% # Sequence  | | | | | | | | | | | | |
| --- | --- | --- | --- | --- | --- | --- | --- | --- | --- | --- | --- | --- |
|  | Astrin\_STLCLD20\_112214\_01.05697.05697.2 | 2.9151 | 0.1301 | 98.6% | 1065.6322 | 1065.2578 | 20 | 6.487 | 68.8% | 1 | R.LASYLDKVR.A | 2222 |
|  | Astrin\_STLCLD20\_112214\_01.06919.06919.2 | 3.7296 | 0.4247 | 100.0% | 1346.1322 | 1346.4772 | 4 | 7.636 | 63.6% | 7 | R.ALEEANTELEVK.I | 2 |
|  | Astrin\_STLCLD20\_112214\_02.14227.14227.3 | 4.6244 | 0.3541 | 99.7% | 2070.4443 | 2069.366 | 1 | 6.58 | 44.4% | 1 | K.ILTATVDNANILLQIDNAR.L | 3 |
|  | Astrin\_STLCLD20\_112214\_tube2\_01.07546.07546.2 | 2.2441 | 0.2268 | 98.6% | 807.7322 | 807.8815 | 35 | 5.587 | 66.7% | 3 | R.LAADDFR.T | 22222 |
|  | Astrin\_STLCLD20\_112214\_tube2\_01.11273.11273.2 | 2.5013 | 0.2335 | 98.4% | 1186.5322 | 1187.3384 | 1 | 5.184 | 80.0% | 1 | R.LSVEADINGLR.R | 2 |
|  | Astrin\_STLCLD20\_112214\_01.08975.08975.2 | 2.9226 | 0.3172 | 100.0% | 1186.2522 | 1186.397 | 1 | 5.85 | 83.3% | 2 | R.RVLDELTLAR.A | 222 |
|  | Astrin\_STLCLD20\_112214\_01.09782.09782.1 | 2.1585 | 0.3224 | 100.0% | 1029.49 | 1030.2096 | 10 | 5.815 | 56.2% | 1 | R.VLDELTLAR.A | 111 |
|  | Astrin\_STLCLD20\_112214\_01.09757.09757.2 | 3.0933 | 0.3041 | 100.0% | 1030.3522 | 1030.2096 | 2 | 6.471 | 81.2% | 3 | R.VLDELTLAR.A | 222 |
|  | Astrin\_STLCLD20\_112214\_01.05006.05006.2 | 3.4896 | 0.2654 | 100.0% | 1439.7322 | 1439.6263 | 1 | 5.072 | 80.0% | 2 | R.ILNEMRDQYEK.M | 22 |
|  | Astrin\_STLCLD20\_112214\_tube2\_01.14853.14853.2 | 2.9049 | 0.314 | 99.5% | 1887.4922 | 1888.0001 | 1 | 6.515 | 50.0% | 2 | K.DAEDWFFSKTEELNR.E | 2 |
|  | Astrin\_STLCLD20\_112214\_01.06820.06820.3 | 3.5479 | 0.1718 | 95.8% | 2105.6042 | 2105.2664 | 54 | 4.942 | 30.6% | 2 | K.TEELNREVATNSELVQSGK.S | 33 |
|  | Astrin\_STLCLD20\_112214\_tube2\_01.06412.06412.2 | 3.7182 | 0.4945 | 100.0% | 1361.8522 | 1362.4796 | 4 | 8.36 | 58.3% | 1 | R.EVATNSELVQSGK.S | 22 |
|  | Astrin\_STLCLD20\_112214\_01.06017.06017.2 | 3.8976 | 0.3908 | 100.0% | 1405.4321 | 1404.4764 | 1 | 7.368 | 75.0% | 3 | K.ASLEGNLAETENR.Y | 2 |
|  | Astrin\_STLCLD20\_112214\_tube2\_01.08813.08813.2 | 2.9233 | 0.2777 | 99.7% | 1380.0122 | 1380.5437 | 1 | 5.774 | 70.0% | 3 | K.TRLEQEIATYR.R | 22 |
|  | Astrin\_STLCLD20\_112214\_01.06103.06103.3 | 3.1185 | 0.2697 | 99.6% | 1536.5643 | 1536.7311 | 3 | 4.784 | 36.4% | 1 | K.TRLEQEIATYRR.L | 33 |
|  | Astrin\_STLCLD20\_112214\_01.05125.05125.2 | 3.1188 | 0.3235 | 100.0% | 1123.3922 | 1123.2511 | 2 | 6.193 | 81.2% | 1 | R.LEQEIATYR.R | 222 |
|  | Astrin\_STLCLD20\_112214\_01.06881.06881.2 | 4.3342 | 0.3729 | 100.0% | 1518.7922 | 1517.6787 | 1 | 7.328 | 79.2% | 2 | R.LLEGEDAHLTQYK.K | 2 |
|  | Astrin\_STLCLD20\_112214\_01.05290.05290.3 | 4.1664 | 0.4284 | 100.0% | 2328.8044 | 2329.6152 | 2 | 6.773 | 36.8% | 1 | R.LLEGEDAHLTQYKKEPVTTR.Q | 3 |
|  | Astrin\_STLCLD20\_112214\_tube2\_01.07751.07751.2 | 2.662 | 0.1506 | 97.8% | 1118.1322 | 1118.2291 | 9 | 5.011 | 61.1% | 1 | R.TIVEEVQDGK.V | 2 |

Similarities:
gi|40354195|ref|NP\_95(1:18)  
contaminant\_KERATIN05(11:8)  
contaminant\_KERATIN03(2:17)  
gi|24234699|ref|NP\_00(6:13)  

---

|  |  |  |  |  |  |  |  |  |
| --- | --- | --- | --- | --- | --- | --- | --- | --- |
| U | *gi|154800483|ref|NP\_0* | 16 | 44 | 37.5% | 339 | 39541 | 9.1 | centromere protein N isoform 2 [Homo sapiens] |
| U | *gi|154800485|ref|NP\_0* | 16 | 44 | 36.0% | 353 | 41180 | 8.9 | centromere protein N isoform 1 [Homo sapiens] |

| Filename XCorr DeltCN Conf% ObsM+H+ CalcM+H+ SpR ZScore Ion% # Sequence  | | | | | | | | | | | | |
| --- | --- | --- | --- | --- | --- | --- | --- | --- | --- | --- | --- | --- |
|  | Astrin\_STLCLD20\_112214\_tube2\_01.14878.14878.2 | 3.4721 | 0.3599 | 100.0% | 1273.3522 | 1273.5737 | 1 | 6.931 | 80.0% | 2 | K.IPMNELTTILK.A | 2 |
|  | Astrin\_STLCLD20\_112214\_01.13149.13149.2 | 4.8254 | 0.4912 | 100.0% | 1968.4521 | 1969.1637 | 1 | 9.024 | 70.0% | 5 | K.AWDFLSENQLQTVNFR.Q | 2 |
|  | Astrin\_STLCLD20\_112214\_01.11305.11305.2 | 3.2772 | 0.4539 | 100.0% | 1323.0322 | 1323.4602 | 1 | 8.495 | 59.1% | 3 | K.GPGEDVDLFDMK.Q | 2 |
|  | Astrin\_STLCLD20\_112214\_tube2\_01.11776.11776.2 | 3.398 | 0.3901 | 100.0% | 1584.4722 | 1583.8717 | 3 | 7.031 | 53.6% | 2 | R.RNTPLLGQALTIASK.H | 2 |
|  | Astrin\_STLCLD20\_112214\_tube2\_01.13928.13928.2 | 4.177 | 0.5693 | 100.0% | 1427.2922 | 1427.6842 | 1 | 9.501 | 88.5% | 5 | R.NTPLLGQALTIASK.H | 2 |
|  | Astrin\_STLCLD20\_112214\_tube2\_01.11943.11943.1 | 2.2123 | 0.4038 | 100.0% | 1220.54 | 1221.371 | 1 | 6.422 | 55.0% | 2 | R.SLGLDINMDSR.I | 1 |
|  | Astrin\_STLCLD20\_112214\_02.11391.11391.2 | 3.922 | 0.4943 | 100.0% | 1221.9722 | 1221.371 | 1 | 8.438 | 90.0% | 12 | R.SLGLDINMDSR.I | 2 |
|  | Astrin\_STLCLD20\_112214\_tube2\_01.06399.06399.2 | 2.369 | 0.3481 | 99.7% | 1095.0521 | 1095.284 | 1 | 5.04 | 75.0% | 1 | R.IIHENIVEK.E | 2 |
|  | Astrin\_STLCLD20\_112214\_tube2\_01.06152.06152.2 | 3.6573 | 0.3712 | 100.0% | 1380.3722 | 1380.587 | 1 | 7.537 | 85.0% | 1 | R.IIHENIVEKER.V | 2 |
|  | Astrin\_STLCLD20\_112214\_tube2\_01.06146.06146.3 | 2.6434 | 0.2504 | 97.6% | 1380.8043 | 1380.587 | 1 | 4.982 | 50.0% | 1 | R.IIHENIVEKER.V | 3 |
|  | Astrin\_STLCLD20\_112214\_tube2\_01.13931.13931.2 | 5.8502 | 0.5656 | 100.0% | 2404.2922 | 2404.638 | 1 | 10.172 | 65.8% | 2 | R.ITQETFGDYPQPQLEFAQYK.L | 2 |
|  | Astrin\_STLCLD20\_112214\_tube2\_01.13708.13708.3 | 3.6703 | 0.3698 | 99.8% | 2875.3145 | 2876.192 | 1 | 5.755 | 30.4% | 1 | R.ITQETFGDYPQPQLEFAQYKLETK.F | 3 |
|  | Astrin\_STLCLD20\_112214\_01.08296.08296.2 | 2.6986 | 0.2316 | 98.9% | 1117.4122 | 1117.2474 | 2 | 6.369 | 70.0% | 1 | K.SGLNGSILAER.E | 2 |
|  | Astrin\_STLCLD20\_112214\_tube2\_01.09879.09879.2 | 3.1044 | 0.127 | 97.2% | 1741.0721 | 1741.9419 | 74 | 4.3 | 40.0% | 2 | K.SGLNGSILAEREEPLR.C | 2 |
|  | Astrin\_STLCLD20\_112214\_01.08942.08942.3 | 2.917 | 0.2193 | 95.4% | 1742.4543 | 1741.9419 | 30 | 4.5 | 36.7% | 1 | K.SGLNGSILAEREEPLR.C | 3 |
|  | Astrin\_STLCLD20\_112214\_01.10219.10219.2 | 3.0407 | 0.2314 | 99.4% | 1243.6921 | 1242.4606 | 1 | 4.731 | 70.0% | 3 | K.FSSPHLLEALK.S | 2 |

---

|  |  |  |  |  |  |  |  |  |
| --- | --- | --- | --- | --- | --- | --- | --- | --- |
| U | *gi|224028244|ref|NP\_0* | 19 | 73 | 36.7% | 471 | 54232 | 8.9 | non-POU domain containing, octamer-binding isoform 1 [Homo sapiens] |
| U | *gi|34932414|ref|NP\_03* | 19 | 73 | 36.7% | 471 | 54232 | 8.9 | non-POU domain containing, octamer-binding isoform 1 [Homo sapiens] |
| U | *gi|224028246|ref|NP\_0* | 19 | 73 | 36.7% | 471 | 54232 | 8.9 | non-POU domain containing, octamer-binding isoform 1 [Homo sapiens] |

| Filename XCorr DeltCN Conf% ObsM+H+ CalcM+H+ SpR ZScore Ion% # Sequence  | | | | | | | | | | | | |
| --- | --- | --- | --- | --- | --- | --- | --- | --- | --- | --- | --- | --- |
|  | Astrin\_STLCLD20\_112214\_01.11551.11551.2 | 3.8157 | 0.5133 | 100.0% | 1860.3922 | 1861.12 | 1 | 8.105 | 70.0% | 9 | R.LFVGNLPPDITEEEMR.K | 2 |
|  | Astrin\_STLCLD20\_112214\_tube2\_01.15689.15689.2 | 5.1098 | 0.5731 | 100.0% | 1813.5322 | 1814.1504 | 1 | 10.145 | 73.3% | 3 | R.TLAEIAKVELDNMPLR.G | 2 |
|  | Astrin\_STLCLD20\_112214\_01.12586.12586.3 | 3.7575 | 0.3587 | 99.7% | 1814.3344 | 1814.1504 | 4 | 6.432 | 40.0% | 1 | R.TLAEIAKVELDNMPLR.G | 3 |
|  | Astrin\_STLCLD20\_112214\_01.11429.11429.3 | 3.421 | 0.3744 | 99.8% | 1998.6843 | 1999.3765 | 1 | 6.36 | 39.7% | 1 | R.TLAEIAKVELDNMPLRGK.Q | 3 |
|  | Astrin\_STLCLD20\_112214\_01.09075.09075.2 | 2.7206 | 0.3069 | 99.9% | 1087.4722 | 1087.2793 | 18 | 5.692 | 75.0% | 4 | K.VELDNMPLR.G | 2 |
|  | Astrin\_STLCLD20\_112214\_01.07006.07006.2 | 2.4881 | 0.2 | 97.6% | 1271.7722 | 1272.5052 | 1 | 5.466 | 80.0% | 1 | K.VELDNMPLRGK.Q | 2 |
|  | Astrin\_STLCLD20\_112214\_tube2\_01.21479.21479.2 | 5.3993 | 0.545 | 100.0% | 2669.0122 | 2669.9507 | 1 | 11.315 | 54.5% | 10 | R.NLPQYVSNELLEEAFSVFGQVER.A | 2 |
|  | Astrin\_STLCLD20\_112214\_01.17785.17785.3 | 5.0303 | 0.3864 | 100.0% | 2670.5344 | 2669.9507 | 1 | 8.075 | 39.8% | 4 | R.NLPQYVSNELLEEAFSVFGQVER.A | 3 |
|  | Astrin\_STLCLD20\_112214\_tube2\_01.07018.07018.1 | 1.9202 | 0.2757 | 98.7% | 886.57 | 887.0238 | 69 | 5.975 | 57.1% | 2 | R.AVVIVDDR.G | 11 |
|  | Astrin\_STLCLD20\_112214\_tube2\_01.07612.07612.2 | 3.0678 | 0.3722 | 100.0% | 1232.1921 | 1232.4252 | 1 | 7.863 | 72.7% | 2 | K.GIVEFSGKPAAR.K | 2 |
|  | Astrin\_STLCLD20\_112214\_tube2\_01.12293.12293.2 | 4.6923 | 0.5562 | 100.0% | 1696.5122 | 1696.8744 | 1 | 10.467 | 76.9% | 8 | R.FAQPGSFEYEYAMR.W | 2 |
|  | Astrin\_STLCLD20\_112214\_tube2\_01.08214.08214.2 | 2.9856 | 0.3427 | 100.0% | 1337.2322 | 1337.5488 | 1 | 6.604 | 75.0% | 5 | R.EKLEMEMEAAR.H | 2 |
|  | Astrin\_STLCLD20\_112214\_tube2\_01.06486.06486.2 | 2.2656 | 0.2388 | 98.0% | 1180.6522 | 1181.4161 | 1 | 5.523 | 68.8% | 1 | R.HEHQVMLMR.Q | 2 |
|  | Astrin\_STLCLD20\_112214\_tube2\_01.05771.05771.2 | 3.9157 | 0.3321 | 100.0% | 1541.2122 | 1541.7222 | 1 | 6.254 | 72.7% | 1 | R.RMEELHNQEVQK.R | 2 |
|  | Astrin\_STLCLD20\_112214\_tube2\_01.05681.05681.2 | 4.6847 | 0.2587 | 100.0% | 1697.3522 | 1697.9097 | 1 | 5.697 | 70.8% | 1 | R.RMEELHNQEVQKR.K | 2 |
|  | Astrin\_STLCLD20\_112214\_02.10185.10185.2 | 4.5346 | 0.5636 | 100.0% | 1538.7322 | 1539.8441 | 1 | 10.03 | 71.4% | 12 | R.MGQMAMGGAMGINNR.G | 2 |
|  | Astrin\_STLCLD20\_112214\_tube2\_02.08637.08637.2 | 4.6748 | 0.5394 | 100.0% | 2243.6921 | 2244.4436 | 1 | 9.39 | 52.4% | 2 | R.FGQAATMEGIGAIGGT#PPAFNR.A | 2 |
|  | Astrin\_STLCLD20\_112214\_tube2\_02.08617.08617.3 | 4.2158 | 0.4155 | 100.0% | 2243.9343 | 2244.4436 | 1 | 6.861 | 44.0% | 4 | R.FGQAATMEGIGAIGGT#PPAFNR.A | 3 |
|  | Astrin\_STLCLD20\_112214\_tube2\_01.06364.06364.2 | 2.2854 | 0.2556 | 97.5% | 1229.2322 | 1229.3811 | 1 | 4.6 | 72.7% | 2 | R.AAPGAEFAPNKR.R | 2 |

Similarities:
gi|4826998|ref|NP\_005(1:18)  

---

|  |  |  |  |  |  |  |  |  |
| --- | --- | --- | --- | --- | --- | --- | --- | --- |
| U | *gi|218505827|ref|NP\_1* | 9 | 23 | 35.4% | 316 | 35438 | 6.3 | TRAF4 associated factor 1 isoform a [Homo sapiens] |
| U | *gi|218505831|ref|NP\_0* | 10 | 24 | 39.2% | 286 | 31880 | 7.1 | TRAF4 associated factor 1 isoform b [Homo sapiens] |

| Filename XCorr DeltCN Conf% ObsM+H+ CalcM+H+ SpR ZScore Ion% # Sequence  | | | | | | | | | | | | |
| --- | --- | --- | --- | --- | --- | --- | --- | --- | --- | --- | --- | --- |
|  | Astrin\_STLCLD20\_112214\_tube2\_01.10773.10773.2 | 6.1531 | 0.5839 | 100.0% | 2274.7522 | 2275.4802 | 1 | 9.807 | 61.9% | 4 | K.TVYSLQPPSALSGGQPADTQTR.A | 2 |
|  | Astrin\_STLCLD20\_112214\_tube2\_01.10820.10820.3 | 3.9688 | 0.4057 | 99.7% | 2274.8342 | 2275.4802 | 1 | 5.85 | 36.9% | 1 | K.TVYSLQPPSALSGGQPADTQTR.A | 3 |
|  | Astrin\_STLCLD20\_112214\_01.09561.09561.3 | 3.4029 | 0.2939 | 99.6% | 3411.6243 | 3408.7502 | 26 | 4.306 | 21.0% | 1 | K.TVYSLQPPSALSGGQPADTQTRATSKS\*LLPVR.S | 3 |
|  | Astrin\_STLCLD20\_112214\_01.07513.07513.3 | 4.9092 | 0.4299 | 100.0% | 1989.3243 | 1988.2023 | 1 | 7.025 | 42.2% | 2 | K.SEEELKDKNQLLEAVNK.Q | 3 |
|  | Astrin\_STLCLD20\_112214\_01.06850.06850.2 | 3.7699 | 0.3354 | 100.0% | 1604.7122 | 1604.7979 | 1 | 6.092 | 69.2% | 1 | K.LTETQGELKDLTQK.V | 2 |
|  | Astrin\_STLCLD20\_112214\_01.11345.11345.3 | 5.0263 | 0.3527 | 100.0% | 2316.4143 | 2316.6543 | 1 | 6.722 | 38.2% | 2 | K.LTETQGELKDLTQKVELLEK.F | 3 |
|  | Astrin\_STLCLD20\_112214\_tube2\_01.13703.13703.2 | 5.4086 | 0.4003 | 100.0% | 2316.912 | 2316.6543 | 1 | 7.418 | 60.5% | 1 | K.LTETQGELKDLTQKVELLEK.F | 2 |
|  | Astrin\_STLCLD20\_112214\_01.08995.08995.2 | 4.3186 | 0.5406 | 100.0% | 1387.2722 | 1387.5327 | 1 | 9.581 | 65.4% | 8 | K.GLDPALGSETLASR.Q | 2 |
|  | Astrin\_STLCLD20\_112214\_01.15753.15753.3 | 5.0676 | 0.3904 | 100.0% | 3395.5444 | 3396.8062 | 1 | 6.961 | 27.7% | 3 | R.QESTTDHMDSMLLLETLQEELKLFNETAK.K | 3 |

---

|  |  |  |  |  |  |  |  |  |
| --- | --- | --- | --- | --- | --- | --- | --- | --- |
| U | *gi|14043072|ref|NP\_11* | 12 | 36 | 33.4% | 353 | 37430 | 8.9 | heterogeneous nuclear ribonucleoprotein A2/B1 isoform B1 [Homo sapiens] |
| U | *gi|4504447|ref|NP\_002* | 12 | 35 | 34.6% | 341 | 36006 | 8.6 | heterogeneous nuclear ribonucleoprotein A2/B1 isoform A2 [Homo sapiens] |

| Filename XCorr DeltCN Conf% ObsM+H+ CalcM+H+ SpR ZScore Ion% # Sequence  | | | | | | | | | | | | |
| --- | --- | --- | --- | --- | --- | --- | --- | --- | --- | --- | --- | --- |
|  | Astrin\_STLCLD20\_112214\_tube2\_01.14544.14544.2 | 3.7088 | 0.2688 | 100.0% | 1928.9321 | 1928.1925 | 2 | 5.517 | 56.2% | 3 | R.KLFIGGLSFETTEESLR.N | 2 |
|  | Astrin\_STLCLD20\_112214\_tube2\_01.16983.16983.2 | 3.4321 | 0.3705 | 100.0% | 1799.2722 | 1800.0184 | 1 | 5.719 | 66.7% | 5 | K.LFIGGLSFETTEESLR.N | 2 |
|  | Astrin\_STLCLD20\_112214\_01.07525.07525.2 | 2.1615 | 0.295 | 98.8% | 1088.1522 | 1088.1644 | 9 | 5.304 | 71.4% | 1 | R.NYYEQWGK.L | 2 |
|  | Astrin\_STLCLD20\_112214\_tube2\_01.12449.12449.2 | 3.3941 | 0.4952 | 100.0% | 1189.3322 | 1189.3513 | 4 | 7.966 | 77.8% | 1 | K.IDTIEIITDR.Q | 2 |
|  | Astrin\_STLCLD20\_112214\_01.11173.11173.2 | 3.6407 | 0.4217 | 100.0% | 1696.4321 | 1696.8132 | 1 | 8.234 | 64.3% | 1 | R.GFGFVTFDDHDPVDK.I | 2 |
|  | Astrin\_STLCLD20\_112214\_tube2\_01.14595.14595.3 | 3.7297 | 0.4395 | 100.0% | 2279.1243 | 2278.5693 | 6 | 7.502 | 32.9% | 4 | R.GFGFVTFDDHDPVDKIVLQK.Y | 3 |
|  | Astrin\_STLCLD20\_112214\_tube2\_01.05752.05752.2 | 3.4351 | 0.3521 | 100.0% | 1411.4922 | 1411.5198 | 1 | 7.011 | 81.8% | 1 | K.YHTINGHNAEVR.K | 2 |
|  | Astrin\_STLCLD20\_112214\_tube2\_01.05622.05622.2 | 2.7917 | 0.1615 | 97.4% | 1540.4122 | 1539.6938 | 1 | 4.982 | 66.7% | 1 | K.YHTINGHNAEVRK.A | 2 |
|  | Astrin\_STLCLD20\_112214\_tube2\_01.08646.08646.2 | 2.4727 | 0.1657 | 96.9% | 1014.1922 | 1014.0421 | 2 | 4.934 | 72.2% | 2 | R.GGNFGFGDSR.G | 2 |
|  | Astrin\_STLCLD20\_112214\_tube2\_01.08471.08471.2 | 3.1269 | 0.4721 | 100.0% | 1378.0922 | 1378.4465 | 1 | 6.934 | 53.6% | 9 | R.GGGGNFGPGPGSNFR.G | 2 |
|  | Astrin\_STLCLD20\_112214\_01.06461.06461.2 | 5.9547 | 0.6315 | 100.0% | 2190.3323 | 2191.2554 | 1 | 11.333 | 54.2% | 5 | R.NMGGPYGGGNYGPGGSGGSGGYGGR.S | 2 |
|  | Astrin\_STLCLD20\_112214\_01.06530.06530.3 | 4.9634 | 0.5095 | 100.0% | 2190.7144 | 2191.2554 | 1 | 8.872 | 36.5% | 3 | R.NMGGPYGGGNYGPGGSGGSGGYGGR.S | 3 |

---

|  |  |  |  |  |  |  |  |  |
| --- | --- | --- | --- | --- | --- | --- | --- | --- |
| U | *gi|5174457|ref|NP\_006* | 18 | 41 | 33.0% | 642 | 73913 | 5.6 | kinetochore associated 2 [Homo sapiens] |

| Filename XCorr DeltCN Conf% ObsM+H+ CalcM+H+ SpR ZScore Ion% # Sequence  | | | | | | | | | | | | |
| --- | --- | --- | --- | --- | --- | --- | --- | --- | --- | --- | --- | --- |
| \* | Astrin\_STLCLD20\_112214\_01.09195.09195.2 | 2.6012 | 0.2764 | 99.1% | 1297.7522 | 1297.4075 | 1 | 5.231 | 68.2% | 1 | R.NSQLGIFSSSEK.I | 2 |
| \* | Astrin\_STLCLD20\_112214\_tube2\_01.13484.13484.2 | 3.8052 | 0.2953 | 100.0% | 1500.6921 | 1499.793 | 1 | 5.952 | 66.7% | 1 | R.IFKDLGYPFALSK.S | 2 |
| \* | Astrin\_STLCLD20\_112214\_tube2\_01.14793.14793.3 | 4.2777 | 0.4896 | 100.0% | 2081.3342 | 2081.4167 | 1 | 7.66 | 39.7% | 3 | K.LKDLFNVDAFKLESLEAK.N | 3 |
| \* | Astrin\_STLCLD20\_112214\_tube2\_02.07344.07344.3 | 3.33 | 0.369 | 99.8% | 2098.5842 | 2099.3228 | 1 | 6.871 | 35.3% | 2 | K.YQAYMSNLESHSAILDQK.L | 3 |
| \* | Astrin\_STLCLD20\_112214\_tube2\_01.08525.08525.2 | 3.1229 | 0.2325 | 99.7% | 1130.1322 | 1129.2584 | 1 | 5.019 | 83.3% | 3 | K.LNGLNEEIAR.V | 2 |
| \* | Astrin\_STLCLD20\_112214\_tube2\_01.08615.08615.2 | 2.1415 | 0.3869 | 99.7% | 953.03217 | 953.03973 | 1 | 6.546 | 85.7% | 2 | K.YSVADIER.I | 2 |
| \* | Astrin\_STLCLD20\_112214\_tube2\_01.06172.06172.2 | 3.9374 | 0.3499 | 100.0% | 1737.7322 | 1737.9132 | 1 | 6.028 | 73.1% | 1 | R.INHERNELQQTINK.L | 2 |
| \* | Astrin\_STLCLD20\_112214\_tube2\_01.07726.07726.2 | 3.1604 | 0.4599 | 100.0% | 1616.4122 | 1617.7991 | 1 | 7.731 | 57.7% | 1 | R.GKEAIETQLAEYHK.L | 2 |
| \* | Astrin\_STLCLD20\_112214\_tube2\_01.17378.17378.2 | 4.7337 | 0.5055 | 100.0% | 2359.7122 | 2360.6665 | 1 | 8.297 | 57.9% | 1 | R.AQVYVPLKELLNETEEEINK.A | 2 |
| \* | Astrin\_STLCLD20\_112214\_tube2\_01.19950.19950.2 | 2.4291 | 0.2739 | 97.9% | 2024.0721 | 2024.3088 | 2 | 5.699 | 41.2% | 1 | K.MGLEDTLEQLNAMITESK.R | 2 |
| \* | Astrin\_STLCLD20\_112214\_tube2\_01.19080.19080.2 | 5.0434 | 0.5388 | 100.0% | 2179.7322 | 2180.4963 | 1 | 9.359 | 58.3% | 1 | K.MGLEDTLEQLNAMITESKR.S | 2 |
| \* | Astrin\_STLCLD20\_112214\_tube2\_01.19070.19070.3 | 4.84 | 0.4471 | 100.0% | 2180.0044 | 2180.4963 | 1 | 7.939 | 43.1% | 2 | K.MGLEDTLEQLNAMITESKR.S | 3 |
| \* | Astrin\_STLCLD20\_112214\_tube2\_01.12110.12110.3 | 4.7894 | 0.3146 | 99.7% | 1979.1843 | 1979.2377 | 1 | 7.301 | 45.0% | 4 | R.TLKEEVQKLDDLYQQK.I | 3 |
| \* | Astrin\_STLCLD20\_112214\_tube2\_01.16977.16977.2 | 5.7535 | 0.5461 | 100.0% | 2554.9321 | 2555.8223 | 1 | 10.084 | 59.1% | 1 | K.HLLESTVNQGLSEAMNELDAVQR.E | 2 |
| \* | Astrin\_STLCLD20\_112214\_tube2\_01.16974.16974.3 | 3.2034 | 0.3282 | 99.6% | 2555.3943 | 2555.8223 | 4 | 5.668 | 27.3% | 1 | K.HLLESTVNQGLSEAMNELDAVQR.E | 3 |
| \* | Astrin\_STLCLD20\_112214\_tube2\_02.06757.06757.2 | 4.3994 | 0.4295 | 100.0% | 1596.8922 | 1596.7344 | 1 | 8.439 | 75.0% | 6 | R.EYQLVVQTTTEER.R | 2 |
| \* | Astrin\_STLCLD20\_112214\_tube2\_01.09980.09980.2 | 4.1984 | 0.3831 | 100.0% | 1513.4922 | 1513.7925 | 1 | 7.56 | 76.9% | 8 | R.LLEMVATHVGSVEK.H | 2 |
| \* | Astrin\_STLCLD20\_112214\_tube2\_01.09966.09966.3 | 3.0067 | 0.458 | 99.7% | 1513.7644 | 1513.7925 | 2 | 7.097 | 42.3% | 2 | R.LLEMVATHVGSVEK.H | 3 |

---

|  |  |  |  |  |  |  |  |  |
| --- | --- | --- | --- | --- | --- | --- | --- | --- |
| U | *gi|18087855|ref|NP\_54* | 2 | 2 | 32.6% | 89 | 10350 | 7.4 | dynein, light chain, LC8-type 2 [Homo sapiens] |
| U | *gi|83267868|ref|NP\_00* | 2 | 2 | 32.6% | 89 | 10366 | 7.4 | dynein light chain 1 [Homo sapiens] |
| U | *gi|83267866|ref|NP\_00* | 2 | 2 | 32.6% | 89 | 10366 | 7.4 | dynein light chain 1 [Homo sapiens] |
| U | *gi|4505813|ref|NP\_003* | 2 | 2 | 32.6% | 89 | 10366 | 7.4 | dynein light chain 1 [Homo sapiens] |

| Filename XCorr DeltCN Conf% ObsM+H+ CalcM+H+ SpR ZScore Ion% # Sequence  | | | | | | | | | | | | |
| --- | --- | --- | --- | --- | --- | --- | --- | --- | --- | --- | --- | --- |
|  | Astrin\_STLCLD20\_112214\_tube2\_01.07452.07452.2 | 2.6585 | 0.3579 | 99.9% | 1284.2322 | 1283.383 | 3 | 5.737 | 60.0% | 1 | R.NFGSYVTHETK.H | 2 |
|  | Astrin\_STLCLD20\_112214\_tube2\_01.20860.20860.3 | 4.2952 | 0.1552 | 97.1% | 3381.0544 | 3381.9011 | 1 | 5.102 | 25.9% | 1 | R.NFGSYVTHETKHFIYFYLGQVAILLFKSG.- | 3 |

---

|  |  |  |  |  |  |  |  |  |
| --- | --- | --- | --- | --- | --- | --- | --- | --- |
| U | *gi|14165435|ref|NP\_11* | 13 | 27 | 32.2% | 463 | 50976 | 5.5 | heterogeneous nuclear ribonucleoprotein K isoform b [Homo sapiens] |
| U | *gi|14165439|ref|NP\_00* | 13 | 27 | 32.1% | 464 | 51028 | 5.3 | heterogeneous nuclear ribonucleoprotein K isoform a [Homo sapiens] |
| U | *gi|14165437|ref|NP\_11* | 13 | 27 | 32.1% | 464 | 51028 | 5.3 | heterogeneous nuclear ribonucleoprotein K isoform a [Homo sapiens] |

| Filename XCorr DeltCN Conf% ObsM+H+ CalcM+H+ SpR ZScore Ion% # Sequence  | | | | | | | | | | | | |
| --- | --- | --- | --- | --- | --- | --- | --- | --- | --- | --- | --- | --- |
|  | Astrin\_STLCLD20\_112214\_tube2\_01.06615.06615.3 | 4.0547 | 0.3536 | 99.7% | 1736.7244 | 1736.8969 | 1 | 5.851 | 46.2% | 2 | K.RPAEDMEEEQAFKR.S | 3 |
|  | Astrin\_STLCLD20\_112214\_tube2\_01.07581.07581.2 | 3.3204 | 0.3255 | 100.0% | 1350.2522 | 1350.4894 | 1 | 5.87 | 80.0% | 1 | R.SRNTDEMVELR.I | 2 |
|  | Astrin\_STLCLD20\_112214\_01.06733.06733.2 | 2.8448 | 0.3794 | 100.0% | 1107.3922 | 1107.2238 | 1 | 6.833 | 81.2% | 4 | R.NTDEMVELR.I | 2 |
|  | Astrin\_STLCLD20\_112214\_01.05876.05876.2 | 4.1582 | 0.5045 | 100.0% | 1781.2322 | 1781.8302 | 3 | 8.669 | 56.2% | 3 | R.TDYNASVSVPDSSGPER.I | 2 |
|  | Astrin\_STLCLD20\_112214\_02.17237.17237.2 | 4.3464 | 0.5109 | 100.0% | 1716.0922 | 1716.0251 | 1 | 9.141 | 60.0% | 1 | R.ILSISADIETIGEILK.K | 2 |
|  | Astrin\_STLCLD20\_112214\_tube2\_01.19446.19446.2 | 3.2268 | 0.3788 | 100.0% | 1843.3722 | 1844.1992 | 6 | 6.48 | 37.5% | 1 | R.ILSISADIETIGEILKK.I | 2 |
|  | Astrin\_STLCLD20\_112214\_tube2\_01.11945.11945.2 | 3.8957 | 0.4866 | 100.0% | 1519.6322 | 1519.8711 | 1 | 8.302 | 67.9% | 4 | R.LLIHQSLAGGIIGVK.G | 2 |
|  | Astrin\_STLCLD20\_112214\_tube2\_02.07874.07874.3 | 4.6156 | 0.4768 | 100.0% | 1520.1543 | 1519.8711 | 1 | 7.829 | 50.0% | 3 | R.LLIHQSLAGGIIGVK.G | 3 |
|  | Astrin\_STLCLD20\_112214\_tube2\_01.16395.16395.2 | 3.4211 | 0.4516 | 100.0% | 1341.3722 | 1341.6311 | 1 | 7.871 | 72.7% | 2 | K.IILDLISESPIK.G | 2 |
|  | Astrin\_STLCLD20\_112214\_tube2\_01.13829.13829.2 | 3.0609 | 0.2125 | 98.9% | 1554.4521 | 1554.8705 | 2 | 5.784 | 61.5% | 1 | K.IILDLISESPIKGR.A | 2 |
|  | Astrin\_STLCLD20\_112214\_tube2\_01.13436.13436.2 | 5.1402 | 0.4799 | 100.0% | 1918.5322 | 1918.1974 | 1 | 9.567 | 58.3% | 3 | R.GSYGDLGGPIITTQVTIPK.D | 2 |
|  | Astrin\_STLCLD20\_112214\_02.09090.09090.3 | 2.8797 | 0.2408 | 95.8% | 2069.7544 | 2070.1772 | 1 | 4.365 | 37.5% | 1 | R.HESGASIKIDEPLEGSEDR.I | 3 |
|  | Astrin\_STLCLD20\_112214\_tube2\_01.16102.16102.2 | 5.6302 | 0.537 | 100.0% | 2590.9922 | 2590.9365 | 1 | 9.445 | 52.3% | 1 | R.IITITGTQDQIQNAQYLLQNSVK.Q | 2 |

---

|  |  |  |  |  |  |  |  |  |
| --- | --- | --- | --- | --- | --- | --- | --- | --- |
| U | *gi|4503571|ref|NP\_001* | 8 | 17 | 31.8% | 434 | 47169 | 7.4 | enolase 1 [Homo sapiens] |

| Filename XCorr DeltCN Conf% ObsM+H+ CalcM+H+ SpR ZScore Ion% # Sequence  | | | | | | | | | | | | |
| --- | --- | --- | --- | --- | --- | --- | --- | --- | --- | --- | --- | --- |
|  | Astrin\_STLCLD20\_112214\_tube2\_01.14748.14748.2 | 3.9668 | 0.4336 | 100.0% | 1806.0521 | 1806.0258 | 1 | 8.08 | 61.8% | 3 | R.AAVPSGASTGIYEALELR.D | 2 |
| \* | Astrin\_STLCLD20\_112214\_01.11993.11993.3 | 4.982 | 0.3726 | 100.0% | 3012.1743 | 3013.383 | 4 | 6.02 | 25.9% | 2 | R.HIADLAGNSEVILPVPAFNVINGGSHAGNK.L | 3 |
| \* | Astrin\_STLCLD20\_112214\_01.14282.14282.2 | 4.5006 | 0.4715 | 100.0% | 1909.5721 | 1909.3148 | 1 | 7.834 | 62.5% | 1 | K.LAMQEFMILPVGAANFR.E | 2 |
| \* | Astrin\_STLCLD20\_112214\_tube2\_01.07101.07101.2 | 2.8002 | 0.2438 | 99.4% | 1145.3121 | 1144.3158 | 23 | 4.66 | 61.1% | 1 | R.IGAEVYHNLK.N | 2 |
| \* | Astrin\_STLCLD20\_112214\_01.06779.06779.3 | 3.4238 | 0.3141 | 99.7% | 1829.4243 | 1827.9451 | 1 | 5.181 | 40.0% | 2 | R.SGKYDLDFKSPDDPSR.Y | 3 |
| \* | Astrin\_STLCLD20\_112214\_tube2\_01.13724.13724.2 | 3.4674 | 0.4559 | 100.0% | 1426.3121 | 1426.6091 | 2 | 6.992 | 72.7% | 4 | R.YISPDQLADLYK.S | 2 |
| \* | Astrin\_STLCLD20\_112214\_tube2\_02.07890.07890.3 | 4.326 | 0.3663 | 99.7% | 2191.7043 | 2190.4612 | 2 | 6.524 | 32.5% | 3 | K.FTASAGIQVVGDDLTVTNPKR.I | 3 |
| \* | Astrin\_STLCLD20\_112214\_01.08806.08806.2 | 2.9646 | 0.2833 | 99.4% | 1526.2522 | 1526.7563 | 1 | 4.914 | 65.4% | 1 | K.LAQANGWGVMVSHR.S | 2 |

---

|  |  |  |  |  |  |  |  |  |
| --- | --- | --- | --- | --- | --- | --- | --- | --- |
| U | *gi|4826898|ref|NP\_005* | 3 | 5 | 31.4% | 140 | 15054 | 8.3 | profilin 1 [Homo sapiens] |

| Filename XCorr DeltCN Conf% ObsM+H+ CalcM+H+ SpR ZScore Ion% # Sequence  | | | | | | | | | | | | |
| --- | --- | --- | --- | --- | --- | --- | --- | --- | --- | --- | --- | --- |
| \* | Astrin\_STLCLD20\_112214\_tube2\_01.16176.16176.2 | 3.8876 | 0.3527 | 100.0% | 1644.4521 | 1644.9518 | 1 | 7.431 | 66.7% | 2 | K.TFVNITPAEVGVLVGK.D | 2 |
| \* | Astrin\_STLCLD20\_112214\_tube2\_01.12378.12378.2 | 3.9425 | 0.5238 | 100.0% | 1471.5322 | 1471.6531 | 1 | 8.483 | 73.1% | 2 | R.SSFYVNGLTLGGQK.C | 2 |
| \* | Astrin\_STLCLD20\_112214\_02.14119.14119.2 | 3.3518 | 0.1492 | 98.8% | 1628.1921 | 1626.7784 | 1 | 5.672 | 73.1% | 1 | R.DSLLQDGEFSMDLR.T | 2 |

---

|  |  |  |  |  |  |  |  |  |
| --- | --- | --- | --- | --- | --- | --- | --- | --- |
| U | *gi|209862831|ref|NP\_0* | 8 | 16 | 31.3% | 339 | 38604 | 7.8 | annexin A2 isoform 2 [Homo sapiens] |
| U | *gi|50845388|ref|NP\_00* | 8 | 16 | 29.7% | 357 | 40411 | 8.4 | annexin A2 isoform 1 [Homo sapiens] |
| U | *gi|50845386|ref|NP\_00* | 8 | 16 | 31.3% | 339 | 38604 | 7.8 | annexin A2 isoform 2 [Homo sapiens] |
| U | *gi|4757756|ref|NP\_004* | 8 | 15 | 31.3% | 339 | 38604 | 7.8 | annexin A2 isoform 2 [Homo sapiens] |

| Filename XCorr DeltCN Conf% ObsM+H+ CalcM+H+ SpR ZScore Ion% # Sequence  | | | | | | | | | | | | |
| --- | --- | --- | --- | --- | --- | --- | --- | --- | --- | --- | --- | --- |
|  | Astrin\_STLCLD20\_112214\_01.13384.13384.2 | 3.7393 | 0.4068 | 100.0% | 1544.0122 | 1543.7605 | 1 | 7.363 | 57.7% | 2 | K.GVDEVTIVNILTNR.S | 2 |
|  | Astrin\_STLCLD20\_112214\_01.08049.08049.2 | 1.9779 | 0.2613 | 96.4% | 1112.3922 | 1112.2303 | 1 | 4.521 | 75.0% | 1 | R.QDIAFAYQR.R | 2 |
|  | Astrin\_STLCLD20\_112214\_tube2\_01.19079.19079.2 | 4.9535 | 0.4297 | 100.0% | 1651.6721 | 1651.9872 | 1 | 8.778 | 70.0% | 2 | K.SALSGHLETVILGLLK.T | 2 |
|  | Astrin\_STLCLD20\_112214\_tube2\_01.07251.07251.2 | 2.9899 | 0.4457 | 100.0% | 1222.6921 | 1223.3251 | 1 | 7.772 | 70.0% | 1 | K.TPAQYDASELK.A | 2 |
|  | Astrin\_STLCLD20\_112214\_tube2\_01.06930.06930.2 | 3.0456 | 0.1604 | 99.1% | 1245.5122 | 1245.3347 | 4 | 5.522 | 77.8% | 2 | R.TNQELQEINR.V | 2 |
|  | Astrin\_STLCLD20\_112214\_01.10029.10029.3 | 4.0162 | 0.4429 | 100.0% | 1940.9043 | 1941.102 | 1 | 7.446 | 40.6% | 3 | K.TDLEKDIISDTSGDFRK.L | 3 |
|  | Astrin\_STLCLD20\_112214\_01.10295.10295.3 | 4.504 | 0.3014 | 99.8% | 2066.6943 | 2066.1887 | 1 | 5.951 | 52.9% | 4 | R.RAEDGSVIDYELIDQDAR.D | 3 |
|  | Astrin\_STLCLD20\_112214\_tube2\_01.11715.11715.2 | 2.5552 | 0.277 | 99.1% | 1422.9321 | 1422.5774 | 1 | 5.066 | 75.0% | 1 | K.SLYYYIQQDTK.G | 2 |

---

|  |  |  |  |  |  |  |  |  |
| --- | --- | --- | --- | --- | --- | --- | --- | --- |
| U | *gi|27436946|ref|NP\_73* | 18 | 43 | 30.6% | 664 | 74140 | 7.0 | lamin A/C isoform 1 precursor [Homo sapiens] |

| Filename XCorr DeltCN Conf% ObsM+H+ CalcM+H+ SpR ZScore Ion% # Sequence  | | | | | | | | | | | | |
| --- | --- | --- | --- | --- | --- | --- | --- | --- | --- | --- | --- | --- |
|  | Astrin\_STLCLD20\_112214\_tube2\_01.07804.07804.3 | 3.6249 | 0.301 | 99.6% | 2001.7144 | 2001.204 | 1 | 5.341 | 38.3% | 1 | R.ITRLQEKEDLQELNDR.L | 3 |
|  | Astrin\_STLCLD20\_112214\_01.05247.05247.2 | 4.6903 | 0.3466 | 100.0% | 1630.4722 | 1630.7521 | 1 | 7.695 | 79.2% | 2 | R.LQEKEDLQELNDR.L | 2 |
|  | Astrin\_STLCLD20\_112214\_tube2\_01.07499.07499.3 | 3.1133 | 0.2622 | 99.6% | 1631.4543 | 1630.7521 | 10 | 4.711 | 45.8% | 1 | R.LQEKEDLQELNDR.L | 3 |
|  | Astrin\_STLCLD20\_112214\_tube2\_01.06881.06881.2 | 2.5224 | 0.2097 | 98.4% | 1089.5521 | 1090.1783 | 12 | 5.101 | 66.7% | 1 | R.SLETENAGLR.L | 2 |
|  | Astrin\_STLCLD20\_112214\_tube2\_01.06297.06297.2 | 2.7956 | 0.4126 | 100.0% | 1148.4922 | 1149.2432 | 1 | 8.369 | 72.2% | 1 | R.ITESEEVVSR.E | 2 |
|  | Astrin\_STLCLD20\_112214\_01.05174.05174.2 | 2.72 | 0.3718 | 100.0% | 1166.2922 | 1166.2328 | 4 | 5.835 | 70.0% | 1 | K.AAYEAELGDAR.K | 2 |
|  | Astrin\_STLCLD20\_112214\_tube2\_01.12057.12057.2 | 3.4708 | 0.3768 | 100.0% | 1244.4122 | 1244.474 | 1 | 6.959 | 80.0% | 1 | R.LKDLEALLNSK.E | 2 |
|  | Astrin\_STLCLD20\_112214\_01.08570.08570.2 | 3.0528 | 0.2967 | 100.0% | 1183.5922 | 1183.3066 | 6 | 6.442 | 77.8% | 2 | R.TLEGELHDLR.G | 2 |
|  | Astrin\_STLCLD20\_112214\_01.09344.09344.2 | 3.2705 | 0.3365 | 100.0% | 1028.6721 | 1029.1814 | 1 | 6.408 | 93.8% | 3 | R.LADALQELR.A | 2 |
|  | Astrin\_STLCLD20\_112214\_tube2\_01.08445.08445.2 | 4.8872 | 0.4973 | 100.0% | 1752.6522 | 1753.8693 | 1 | 8.81 | 66.7% | 3 | R.NSNLVGAAHEELQQSR.I | 2 |
|  | Astrin\_STLCLD20\_112214\_01.10593.10593.2 | 2.6175 | 0.1904 | 97.2% | 1431.1322 | 1431.6293 | 4 | 4.084 | 62.5% | 1 | R.IDSLSAQLSQLQK.Q | 2 |
|  | Astrin\_STLCLD20\_112214\_tube2\_01.08483.08483.2 | 2.8691 | 0.2971 | 99.9% | 1188.2722 | 1188.3262 | 5 | 5.203 | 72.2% | 4 | K.LRDLEDSLAR.E | 2 |
|  | Astrin\_STLCLD20\_112214\_tube2\_01.15236.15236.2 | 3.2282 | 0.3851 | 100.0% | 1894.4321 | 1895.1346 | 1 | 6.593 | 64.3% | 2 | R.MQQQLDEYQELLDIK.L | 2 |
|  | Astrin\_STLCLD20\_112214\_tube2\_01.08522.08522.2 | 4.0918 | 0.5225 | 100.0% | 1606.4321 | 1606.7728 | 1 | 8.937 | 69.2% | 5 | R.VAVEEVDEEGKFVR.L | 2 |
|  | Astrin\_STLCLD20\_112214\_02.09285.09285.3 | 2.6683 | 0.3403 | 99.6% | 1607.6643 | 1606.7728 | 63 | 5.17 | 34.6% | 1 | R.VAVEEVDEEGKFVR.L | 3 |
|  | Astrin\_STLCLD20\_112214\_tube2\_02.06672.06672.2 | 4.3609 | 0.5222 | 100.0% | 1492.4122 | 1492.6874 | 1 | 9.129 | 76.9% | 8 | R.TALINSTGEEVAMR.K | 2 |
|  | Astrin\_STLCLD20\_112214\_02.08823.08823.3 | 3.4948 | 0.3446 | 99.7% | 2367.9844 | 2366.504 | 1 | 5.681 | 28.8% | 1 | K.ASASGSGAQVGGPISSGSSASSVTVTR.S | 3 |
|  | Astrin\_STLCLD20\_112214\_tube2\_01.09384.09384.2 | 4.2525 | 0.5209 | 100.0% | 1567.5322 | 1567.6555 | 1 | 9.039 | 50.0% | 5 | R.SVGGSGGGSFGDNLVTR.S | 2 |

---

|  |  |  |  |  |  |  |  |  |
| --- | --- | --- | --- | --- | --- | --- | --- | --- |
| U | *gi|34098946|ref|NP\_00* | 4 | 5 | 30.2% | 324 | 35924 | 9.9 | nuclease sensitive element binding protein 1 [Homo sapiens] |

| Filename XCorr DeltCN Conf% ObsM+H+ CalcM+H+ SpR ZScore Ion% # Sequence  | | | | | | | | | | | | |
| --- | --- | --- | --- | --- | --- | --- | --- | --- | --- | --- | --- | --- |
|  | Astrin\_STLCLD20\_112214\_tube2\_01.07565.07565.3 | 2.9988 | 0.1978 | 95.1% | 1746.3243 | 1745.9298 | 1 | 4.527 | 42.9% | 1 | R.NDTKEDVFVHQTAIK.K | 3 |
| \* | Astrin\_STLCLD20\_112214\_tube2\_01.05934.05934.3 | 5.6458 | 0.4834 | 100.0% | 3258.0244 | 3259.2566 | 1 | 9.481 | 33.9% | 1 | R.NYQQNYQNSESGEKNEGSESAPEGQAQQR.R | 3 |
| \* | Astrin\_STLCLD20\_112214\_tube2\_01.07647.07647.3 | 5.3307 | 0.3882 | 100.0% | 3224.4844 | 3225.4795 | 1 | 6.441 | 27.6% | 2 | R.RPQYSNPPVQGEVMEGADNQGAGEQGRPVR.Q | 3 |
| \* | Astrin\_STLCLD20\_112214\_tube2\_01.05595.05595.3 | 3.0194 | 0.3757 | 99.5% | 2785.0444 | 2785.771 | 1 | 5.514 | 26.1% | 1 | R.EDGNEEDKENQGDETQGQQPPQRR.Y | 3 |

---

|  |  |  |  |  |  |  |  |  |
| --- | --- | --- | --- | --- | --- | --- | --- | --- |
| U | *gi|4826998|ref|NP\_005* | 20 | 59 | 30.1% | 707 | 76150 | 9.4 | splicing factor proline/glutamine rich (polypyrimidine tract binding protein associated) [Homo sapiens] |

| Filename XCorr DeltCN Conf% ObsM+H+ CalcM+H+ SpR ZScore Ion% # Sequence  | | | | | | | | | | | | |
| --- | --- | --- | --- | --- | --- | --- | --- | --- | --- | --- | --- | --- |
| \* | Astrin\_STLCLD20\_112214\_tube2\_01.06933.06933.2 | 2.2186 | 0.3038 | 98.3% | 1268.1921 | 1268.4332 | 3 | 6.656 | 59.1% | 2 | R.SPPPGMGLNQNR.G | 2 |
| \* | Astrin\_STLCLD20\_112214\_tube2\_01.05523.05523.3 | 3.3624 | 0.4263 | 99.8% | 2404.1343 | 2404.5747 | 1 | 6.416 | 34.5% | 2 | R.QHHPPYHQQHHQGPPPGGPGGR.S | 3 |
| \* | Astrin\_STLCLD20\_112214\_02.11436.11436.3 | 3.3725 | 0.3602 | 99.8% | 1650.7144 | 1650.8723 | 6 | 5.593 | 37.5% | 4 | K.ISDSEGFKANLSLLR.R | 3 |
| \* | Astrin\_STLCLD20\_112214\_tube2\_01.16137.16137.2 | 3.5934 | 0.3997 | 100.0% | 1808.7122 | 1809.0258 | 1 | 6.208 | 53.3% | 2 | R.LFVGNLPADITEDEFK.R | 2 |
| \* | Astrin\_STLCLD20\_112214\_tube2\_01.14229.14229.2 | 4.4517 | 0.5094 | 100.0% | 1964.5922 | 1965.2133 | 1 | 9.002 | 65.6% | 3 | R.LFVGNLPADITEDEFKR.L | 2 |
| \* | Astrin\_STLCLD20\_112214\_01.11665.11665.3 | 2.5277 | 0.2931 | 96.9% | 1965.7444 | 1965.2133 | 12 | 4.249 | 34.4% | 3 | R.LFVGNLPADITEDEFKR.L | 3 |
| \* | Astrin\_STLCLD20\_112214\_01.08697.08697.2 | 3.5552 | 0.4313 | 100.0% | 1254.3121 | 1253.3971 | 1 | 6.626 | 60.0% | 6 | K.YGEPGEVFINK.G | 2 |
| \* | Astrin\_STLCLD20\_112214\_tube2\_02.05492.05492.2 | 2.2597 | 0.2831 | 98.7% | 1048.4722 | 1048.1559 | 1 | 5.807 | 75.0% | 1 | K.AELDDTPMR.G | 2 |
| \* | Astrin\_STLCLD20\_112214\_tube2\_01.07288.07288.1 | 2.1404 | 0.3911 | 100.0% | 1143.54 | 1144.3188 | 1 | 6.291 | 70.0% | 2 | R.FATHAAALSVR.N | 1 |
| \* | Astrin\_STLCLD20\_112214\_tube2\_01.07232.07232.2 | 3.1083 | 0.4229 | 100.0% | 1145.3722 | 1144.3188 | 1 | 7.406 | 90.0% | 2 | R.FATHAAALSVR.N | 2 |
| \* | Astrin\_STLCLD20\_112214\_tube2\_01.18612.18612.3 | 5.5508 | 0.3735 | 100.0% | 3767.8145 | 3766.2046 | 1 | 6.603 | 25.8% | 1 | R.FATHAAALSVRNLSPYVSNELLEEAFSQFGPIER.A | 3 |
| \* | Astrin\_STLCLD20\_112214\_01.15302.15302.2 | 5.2212 | 0.5868 | 100.0% | 2640.5522 | 2640.9092 | 1 | 9.563 | 47.7% | 3 | R.NLSPYVSNELLEEAFSQFGPIER.A | 2 |
|  | Astrin\_STLCLD20\_112214\_tube2\_01.07018.07018.1 | 1.9202 | 0.2757 | 98.7% | 886.57 | 887.0238 | 69 | 5.975 | 57.1% | 2 | R.AVVIVDDR.G | 11 |
| \* | Astrin\_STLCLD20\_112214\_tube2\_01.08014.08014.2 | 2.4368 | 0.3556 | 99.4% | 1246.2522 | 1246.452 | 2 | 6.534 | 59.1% | 3 | K.GIVEFASKPAAR.K | 2 |
| \* | Astrin\_STLCLD20\_112214\_02.09279.09279.3 | 4.474 | 0.3378 | 100.0% | 1763.2144 | 1763.8632 | 1 | 6.189 | 44.2% | 10 | R.FAQHGTFEYEYSQR.W | 3 |
| \* | Astrin\_STLCLD20\_112214\_02.09252.09252.2 | 4.4791 | 0.4657 | 100.0% | 1764.3722 | 1763.8632 | 1 | 8.924 | 69.2% | 3 | R.FAQHGTFEYEYSQR.W | 2 |
| \* | Astrin\_STLCLD20\_112214\_tube2\_01.11069.11069.3 | 4.4886 | 0.3487 | 99.7% | 2432.1543 | 2429.6233 | 1 | 5.333 | 43.4% | 3 | K.DKLESEMEDAYHEHQANLLR.Q | 3 |
| \* | Astrin\_STLCLD20\_112214\_tube2\_01.05859.05859.2 | 3.5096 | 0.1883 | 99.7% | 1573.4922 | 1573.7821 | 1 | 5.32 | 72.7% | 1 | R.RMEELHNQEMQK.R | 2 |
| \* | Astrin\_STLCLD20\_112214\_tube2\_01.06620.06620.2 | 3.8993 | 0.532 | 100.0% | 1342.1721 | 1342.4569 | 1 | 9.349 | 82.1% | 5 | R.FGQGGAGPVGGQGPR.G | 2 |
| \* | Astrin\_STLCLD20\_112214\_tube2\_01.06797.06797.2 | 2.1952 | 0.3238 | 98.5% | 1122.3121 | 1121.2561 | 17 | 5.738 | 54.5% | 1 | R.GMGPGTPAGYGR.G | 2 |

Similarities:
gi|224028244|ref|NP\_0(1:19)  

---

|  |  |  |  |  |  |  |  |  |
| --- | --- | --- | --- | --- | --- | --- | --- | --- |
| U | *gi|5031699|ref|NP\_005* | 9 | 18 | 30.0% | 427 | 47355 | 7.5 | flotillin 1 [Homo sapiens] |

| Filename XCorr DeltCN Conf% ObsM+H+ CalcM+H+ SpR ZScore Ion% # Sequence  | | | | | | | | | | | | |
| --- | --- | --- | --- | --- | --- | --- | --- | --- | --- | --- | --- | --- |
| \* | Astrin\_STLCLD20\_112214\_tube2\_01.14283.14283.3 | 4.2895 | 0.4239 | 100.0% | 2020.7043 | 2019.2217 | 1 | 7.346 | 42.6% | 1 | K.TEAEIAHIALETLEGHQR.A | 3 |
| \* | Astrin\_STLCLD20\_112214\_02.10325.10325.3 | 3.1934 | 0.3426 | 99.8% | 1808.0643 | 1808.1206 | 1 | 5.613 | 41.1% | 1 | R.AIMAHMTVEEIYKDR.Q | 3 |
| \* | Astrin\_STLCLD20\_112214\_tube2\_02.07720.07720.2 | 3.5734 | 0.429 | 100.0% | 1469.8722 | 1469.693 | 1 | 7.624 | 62.5% | 1 | K.VSAQYLSEIEMAK.A | 2 |
| \* | Astrin\_STLCLD20\_112214\_tube2\_02.07069.07069.2 | 4.2647 | 0.4972 | 100.0% | 1420.0922 | 1419.6206 | 1 | 9.137 | 79.2% | 2 | R.AQADLAYQLQVAK.T | 2 |
| \* | Astrin\_STLCLD20\_112214\_02.11917.11917.2 | 4.4846 | 0.5031 | 100.0% | 1604.5521 | 1604.8187 | 1 | 8.917 | 67.9% | 3 | K.SQLIMQAEAEAASVR.M | 2 |
| \* | Astrin\_STLCLD20\_112214\_tube2\_01.08621.08621.2 | 2.9807 | 0.1371 | 97.8% | 1381.2922 | 1379.5768 | 1 | 4.969 | 79.2% | 4 | R.MRGEAEAFAIGAR.A | 2 |
| \* | Astrin\_STLCLD20\_112214\_tube2\_02.06217.06217.2 | 4.6119 | 0.4677 | 100.0% | 1380.4122 | 1380.5994 | 1 | 8.728 | 78.6% | 2 | K.ITLVSSGSGTMGAAK.V | 2 |
| \* | Astrin\_STLCLD20\_112214\_01.11732.11732.2 | 2.8353 | 0.3533 | 100.0% | 1216.9122 | 1216.4203 | 1 | 6.667 | 85.0% | 3 | K.VTGEVLDILTR.L | 2 |
| \* | Astrin\_STLCLD20\_112214\_tube2\_01.07527.07527.2 | 3.1161 | 0.2975 | 99.7% | 1649.9722 | 1649.934 | 1 | 5.777 | 53.6% | 1 | R.LTGVSISQVNHKPLR.T | 2 |

---

|  |  |  |  |  |  |  |  |  |
| --- | --- | --- | --- | --- | --- | --- | --- | --- |
| U | *gi|21396489|ref|NP\_00* | 17 | 59 | 28.9% | 959 | 106489 | 6.4 | mitochondrial lon peptidase 1 [Homo sapiens] |

| Filename XCorr DeltCN Conf% ObsM+H+ CalcM+H+ SpR ZScore Ion% # Sequence  | | | | | | | | | | | | |
| --- | --- | --- | --- | --- | --- | --- | --- | --- | --- | --- | --- | --- |
| \* | Astrin\_STLCLD20\_112214\_tube2\_01.13929.13929.2 | 3.1352 | 0.4476 | 100.0% | 1235.7122 | 1235.512 | 1 | 8.055 | 75.0% | 1 | R.LAQPYVGVFLK.R | 2 |
| \* | Astrin\_STLCLD20\_112214\_01.11131.11131.2 | 2.9442 | 0.2162 | 98.6% | 1796.4321 | 1796.8809 | 1 | 4.548 | 57.1% | 9 | K.S\*K@RGKKEAEDELSAR.H | 2 |
| \* | Astrin\_STLCLD20\_112214\_01.12195.12195.2 | 3.4342 | 0.2748 | 99.9% | 1558.9122 | 1558.8644 | 2 | 5.71 | 58.3% | 2 | K.TIRDIIALNPLYR.E | 2 |
| \* | Astrin\_STLCLD20\_112214\_01.14803.14803.3 | 5.0179 | 0.467 | 100.0% | 3670.6443 | 3671.0674 | 1 | 8.375 | 28.0% | 2 | R.VVDNPIYLSDMGAALTGAESHELQDVLEETNIPK.R | 3 |
| \* | Astrin\_STLCLD20\_112214\_tube2\_01.10458.10458.2 | 3.9022 | 0.4998 | 100.0% | 1402.5322 | 1401.5745 | 1 | 9.115 | 72.7% | 4 | K.HVMDVVDEELSK.L | 2 |
| \* | Astrin\_STLCLD20\_112214\_tube2\_01.11147.11147.2 | 3.634 | 0.3713 | 100.0% | 1702.1522 | 1702.8644 | 6 | 7.706 | 46.4% | 2 | K.LGLLDNHSSEFNVTR.N | 2 |
| \* | Astrin\_STLCLD20\_112214\_tube2\_01.11174.11174.3 | 4.2273 | 0.3065 | 99.8% | 1703.5743 | 1702.8644 | 2 | 6.305 | 39.3% | 4 | K.LGLLDNHSSEFNVTR.N | 3 |
| \* | Astrin\_STLCLD20\_112214\_tube2\_01.20000.20000.2 | 3.4817 | 0.35 | 100.0% | 1593.3522 | 1593.8223 | 1 | 6.623 | 75.0% | 3 | R.NYLDWLTSIPWGK.Y | 2 |
| \* | Astrin\_STLCLD20\_112214\_01.05905.05905.2 | 3.289 | 0.3324 | 100.0% | 1195.2922 | 1195.2743 | 1 | 7.207 | 77.8% | 5 | K.YSNENLDLAR.A | 2 |
| \* | Astrin\_STLCLD20\_112214\_02.14686.14686.2 | 3.6864 | 0.2845 | 100.0% | 1289.3322 | 1289.5608 | 1 | 5.791 | 80.0% | 6 | R.ILEFIAVSQLR.G | 2 |
| \* | Astrin\_STLCLD20\_112214\_tube2\_01.12424.12424.2 | 3.3629 | 0.4183 | 100.0% | 1354.2522 | 1354.561 | 1 | 6.471 | 62.5% | 4 | R.FSVGGMTDVAEIK.G | 2 |
| \* | Astrin\_STLCLD20\_112214\_01.13279.13279.2 | 4.3277 | 0.4636 | 100.0% | 1825.1921 | 1826.1002 | 1 | 8.541 | 66.7% | 3 | K.TENPLILIDEVDKIGR.G | 2 |
| \* | Astrin\_STLCLD20\_112214\_tube2\_01.20307.20307.3 | 5.3431 | 0.4974 | 100.0% | 3875.4243 | 3875.2373 | 1 | 7.193 | 29.4% | 3 | R.GYQGDPSSALLELLDPEQNANFLDHYLDVPVDLSK.V | 3 |
| \* | Astrin\_STLCLD20\_112214\_02.11481.11481.2 | 5.0713 | 0.4466 | 100.0% | 1600.3722 | 1599.8574 | 1 | 7.976 | 76.9% | 4 | R.MEMINVSGYVAQEK.L | 2 |
| \* | Astrin\_STLCLD20\_112214\_tube2\_01.15598.15598.3 | 4.3217 | 0.4133 | 100.0% | 3176.2444 | 3177.5352 | 2 | 6.321 | 25.9% | 3 | K.IVSGEAESVEVTPENLQDFVGKPVFTVER.M | 3 |
| \* | Astrin\_STLCLD20\_112214\_tube2\_02.07028.07028.2 | 3.9176 | 0.4712 | 100.0% | 1450.2922 | 1449.6624 | 3 | 8.12 | 57.7% | 3 | R.QNLAMTGEVSLTGK.I | 2 |
| \* | Astrin\_STLCLD20\_112214\_tube2\_01.00634.00634.3 | 3.3213 | 0.2739 | 99.1% | 2671.7644 | 2672.956 | 12 | 4.922 | 27.4% | 1 | K.DFYDLAAFITEGLEVHFVEHYR.E | 3 |

---

|  |  |  |  |  |  |  |  |  |
| --- | --- | --- | --- | --- | --- | --- | --- | --- |
| U | *gi|4506901|ref|NP\_003* | 5 | 10 | 28.7% | 164 | 19330 | 11.6 | splicing factor, arginine/serine-rich 3 [Homo sapiens] |

| Filename XCorr DeltCN Conf% ObsM+H+ CalcM+H+ SpR ZScore Ion% # Sequence  | | | | | | | | | | | | |
| --- | --- | --- | --- | --- | --- | --- | --- | --- | --- | --- | --- | --- |
| \* | Astrin\_STLCLD20\_112214\_01.05734.05734.2 | 4.4632 | 0.3896 | 100.0% | 1877.5521 | 1878.0519 | 1 | 7.386 | 65.6% | 2 | K.VYVGNLGNNGNKTELER.A | 2 |
| \* | Astrin\_STLCLD20\_112214\_tube2\_01.11609.11609.2 | 2.7267 | 0.4244 | 100.0% | 1044.3722 | 1044.198 | 1 | 7.324 | 81.2% | 3 | R.AFGYYGPLR.S | 2 |
|  | Astrin\_STLCLD20\_112214\_tube2\_01.15758.15758.2 | 3.3718 | 0.37 | 100.0% | 1622.3522 | 1622.7771 | 1 | 6.459 | 65.4% | 2 | R.NPPGFAFVEFEDPR.D | 22 |
| \* | Astrin\_STLCLD20\_112214\_tube2\_01.14967.14967.2 | 3.4948 | 0.4018 | 100.0% | 2320.652 | 2321.5107 | 11 | 6.421 | 40.0% | 1 | R.NPPGFAFVEFEDPRDAADAVR.E | 2 |
| \* | Astrin\_STLCLD20\_112214\_01.12193.12193.3 | 2.8981 | 0.3195 | 99.5% | 2322.1443 | 2321.5107 | 1 | 5.096 | 33.8% | 2 | R.NPPGFAFVEFEDPRDAADAVR.E | 3 |

Similarities:
gi|72534660|ref|NP\_00(1:4)  

---

|  |  |  |  |  |  |  |  |  |
| --- | --- | --- | --- | --- | --- | --- | --- | --- |
| U | *gi|4501881|ref|NP\_001* | 15 | 38 | 28.6% | 377 | 42051 | 5.4 | actin, alpha 1, skeletal muscle [Homo sapiens] |
| U | *gi|4885049|ref|NP\_005* | 15 | 38 | 28.6% | 377 | 42019 | 5.4 | cardiac muscle alpha actin 1 proprotein [Homo sapiens] |

| Filename XCorr DeltCN Conf% ObsM+H+ CalcM+H+ SpR ZScore Ion% # Sequence  | | | | | | | | | | | | |
| --- | --- | --- | --- | --- | --- | --- | --- | --- | --- | --- | --- | --- |
|  | Astrin\_STLCLD20\_112214\_01.08519.08519.2 | 2.8688 | 0.378 | 100.0% | 1199.3522 | 1199.4415 | 19 | 6.226 | 60.0% | 5 | R.AVFPSIVGRPR.H | 22 |
|  | Astrin\_STLCLD20\_112214\_tube2\_01.06642.06642.1 | 2.0003 | 0.2433 | 98.0% | 1171.74 | 1172.4058 | 1 | 5.709 | 65.0% | 1 | R.HQGVMVGMGQK.D | 11 |
|  | Astrin\_STLCLD20\_112214\_tube2\_01.06474.06474.2 | 2.7079 | 0.0917 | 95.2% | 1173.2722 | 1172.4058 | 1 | 4.414 | 75.0% | 1 | R.HQGVMVGMGQK.D | 22 |
|  | Astrin\_STLCLD20\_112214\_tube2\_01.12063.12063.2 | 4.5689 | 0.4291 | 100.0% | 1961.2522 | 1962.1841 | 1 | 7.923 | 63.3% | 3 | K.YPIEHGIITNWDDMEK.I | 2 |
|  | Astrin\_STLCLD20\_112214\_01.10669.10669.3 | 3.9198 | 0.1624 | 98.3% | 1962.0543 | 1962.1841 | 1 | 4.829 | 43.3% | 1 | K.YPIEHGIITNWDDMEK.I | 3 |
|  | Astrin\_STLCLD20\_112214\_01.13180.13180.3 | 5.0812 | 0.3412 | 99.7% | 3459.3843 | 3459.8628 | 1 | 5.511 | 32.7% | 2 | K.YPIEHGIITNWDDMEKIWHHTFYNELR.V | 3 |
|  | Astrin\_STLCLD20\_112214\_tube2\_01.08564.08564.2 | 3.1715 | 0.4269 | 100.0% | 1516.3322 | 1516.7019 | 1 | 6.715 | 75.0% | 3 | K.IWHHTFYNELR.V | 22 |
|  | Astrin\_STLCLD20\_112214\_01.07657.07657.3 | 3.1919 | 0.2456 | 99.6% | 1517.2743 | 1516.7019 | 1 | 5.871 | 55.0% | 4 | K.IWHHTFYNELR.V | 33 |
|  | Astrin\_STLCLD20\_112214\_01.09520.09520.2 | 4.9868 | 0.4013 | 100.0% | 1957.7122 | 1957.234 | 1 | 7.383 | 64.7% | 1 | R.VAPEEHPTLLTEAPLNPK.A | 2 |
|  | Astrin\_STLCLD20\_112214\_01.11773.11773.2 | 2.8328 | 0.1328 | 96.4% | 1625.0721 | 1624.8927 | 1 | 4.697 | 65.4% | 2 | R.LDLAGRDLTDYLMK.I | 22 |
|  | Astrin\_STLCLD20\_112214\_tube2\_01.13721.13721.1 | 2.0707 | 0.2892 | 100.0% | 998.5 | 999.167 | 2 | 5.437 | 71.4% | 2 | R.DLTDYLMK.I | 11 |
|  | Astrin\_STLCLD20\_112214\_tube2\_01.13726.13726.2 | 2.0606 | 0.342 | 99.0% | 999.21216 | 999.167 | 16 | 5.639 | 71.4% | 1 | R.DLTDYLMK.I | 22 |
|  | Astrin\_STLCLD20\_112214\_tube2\_01.13478.13478.2 | 4.7344 | 0.3284 | 100.0% | 1792.1322 | 1791.9554 | 1 | 8.421 | 83.3% | 6 | K.SYELPDGQVITIGNER.F | 22 |
|  | Astrin\_STLCLD20\_112214\_tube2\_01.09000.09000.1 | 2.2724 | 0.3842 | 100.0% | 1161.6 | 1162.3868 | 1 | 6.273 | 60.0% | 1 | K.EITALAPSTMK.I | 11 |
|  | Astrin\_STLCLD20\_112214\_tube2\_01.09039.09039.2 | 2.8112 | 0.3091 | 99.7% | 1162.4122 | 1162.3868 | 13 | 5.941 | 55.0% | 5 | K.EITALAPSTMK.I | 22 |

Similarities:
gi|4501885|ref|NP\_001(11:4)  

---

|  |  |  |  |  |  |  |  |  |
| --- | --- | --- | --- | --- | --- | --- | --- | --- |
| U | *gi|4504517|ref|NP\_001* | 5 | 11 | 28.3% | 205 | 22783 | 6.4 | heat shock protein beta-1 [Homo sapiens] |

| Filename XCorr DeltCN Conf% ObsM+H+ CalcM+H+ SpR ZScore Ion% # Sequence  | | | | | | | | | | | | |
| --- | --- | --- | --- | --- | --- | --- | --- | --- | --- | --- | --- | --- |
| \* | Astrin\_STLCLD20\_112214\_01.11786.11786.3 | 3.004 | 0.3622 | 99.6% | 1904.3344 | 1904.0537 | 4 | 6.217 | 37.5% | 3 | R.GPSWDPFRDWYPHSR.L | 3 |
| \* | Astrin\_STLCLD20\_112214\_tube2\_01.14056.14056.2 | 3.2529 | 0.36 | 100.0% | 1165.3922 | 1164.3494 | 1 | 6.62 | 88.9% | 4 | R.LFDQAFGLPR.L | 2 |
| \* | Astrin\_STLCLD20\_112214\_tube2\_01.12213.12213.2 | 4.8386 | 0.4427 | 100.0% | 1784.5922 | 1785.0068 | 1 | 8.434 | 60.0% | 2 | R.VSLDVNHFAPDELTVK.T | 2 |
| \* | Astrin\_STLCLD20\_112214\_tube2\_01.12236.12236.3 | 2.9535 | 0.369 | 99.6% | 1784.8143 | 1785.0068 | 4 | 6.029 | 35.0% | 1 | R.VSLDVNHFAPDELTVK.T | 3 |
| \* | Astrin\_STLCLD20\_112214\_01.11115.11115.2 | 3.7385 | 0.527 | 100.0% | 1906.5922 | 1907.1307 | 1 | 8.434 | 50.0% | 1 | K.LATQSNEITIPVTFESR.A | 2 |

---

|  |  |  |  |  |  |  |  |  |
| --- | --- | --- | --- | --- | --- | --- | --- | --- |
| U | *gi|4506687|ref|NP\_001* | 2 | 3 | 28.3% | 145 | 17040 | 10.4 | ribosomal protein S15 [Homo sapiens] |

| Filename XCorr DeltCN Conf% ObsM+H+ CalcM+H+ SpR ZScore Ion% # Sequence  | | | | | | | | | | | | |
| --- | --- | --- | --- | --- | --- | --- | --- | --- | --- | --- | --- | --- |
|  | Astrin\_STLCLD20\_112214\_01.17566.17566.2 | 3.8631 | 0.5116 | 100.0% | 2588.5522 | 2589.938 | 1 | 8.707 | 45.2% | 2 | R.GVDLDQLLDMSYEQLMQLYSAR.Q | 2 |
| \* | Astrin\_STLCLD20\_112214\_01.15038.15038.2 | 2.9751 | 0.4218 | 100.0% | 2054.1921 | 2054.4856 | 1 | 6.719 | 47.2% | 1 | R.DMIILPEMVGSMVGVYNGK.T | 2 |

---

|  |  |  |  |  |  |  |  |  |
| --- | --- | --- | --- | --- | --- | --- | --- | --- |
| U | *gi|4758086|ref|NP\_004* | 3 | 4 | 28.0% | 193 | 20567 | 8.6 | cysteine and glycine-rich protein 1 isoform 1 [Homo sapiens] |

| Filename XCorr DeltCN Conf% ObsM+H+ CalcM+H+ SpR ZScore Ion% # Sequence  | | | | | | | | | | | | |
| --- | --- | --- | --- | --- | --- | --- | --- | --- | --- | --- | --- | --- |
|  | Astrin\_STLCLD20\_112214\_tube2\_02.06603.06603.3 | 2.5741 | 0.2817 | 95.1% | 2159.5444 | 2160.3452 | 2 | 4.599 | 28.6% | 1 | K.GYGYGQGAGTLSTDKGESLGIK.H | 3 |
|  | Astrin\_STLCLD20\_112214\_tube2\_01.05512.05512.2 | 3.3109 | 0.4094 | 100.0% | 1842.8322 | 1843.9535 | 1 | 6.957 | 59.4% | 1 | K.HEEAPGHRPTTNPNASK.F | 2 |
| \* | Astrin\_STLCLD20\_112214\_tube2\_02.07960.07960.2 | 3.3288 | 0.422 | 100.0% | 1434.3722 | 1434.551 | 2 | 7.966 | 60.7% | 2 | K.GFGFGQGAGALVHSE.- | 2 |

---

|  |  |  |  |  |  |  |  |  |
| --- | --- | --- | --- | --- | --- | --- | --- | --- |
| U | *gi|5902102|ref|NP\_008* | 2 | 5 | 27.7% | 119 | 13282 | 11.6 | small nuclear ribonucleoprotein D1 polypeptide 16kDa [Homo sapiens] |

| Filename XCorr DeltCN Conf% ObsM+H+ CalcM+H+ SpR ZScore Ion% # Sequence  | | | | | | | | | | | | |
| --- | --- | --- | --- | --- | --- | --- | --- | --- | --- | --- | --- | --- |
|  | Astrin\_STLCLD20\_112214\_01.09279.09279.2 | 3.6488 | 0.3364 | 100.0% | 1556.3922 | 1555.7745 | 10 | 6.072 | 58.3% | 1 | K.NREPVQLETLSIR.G | 2 |
| \* | Astrin\_STLCLD20\_112214\_tube2\_01.20769.20769.2 | 4.7631 | 0.5653 | 100.0% | 2287.652 | 2288.6863 | 1 | 8.697 | 63.2% | 4 | R.YFILPDSLPLDTLLVDVEPK.V | 2 |

---

|  |  |  |  |  |  |  |  |  |
| --- | --- | --- | --- | --- | --- | --- | --- | --- |
| U | *gi|118582269|ref|NP\_0* | 4 | 9 | 25.4% | 201 | 22460 | 8.0 | splicing factor, arginine/serine-rich 1 isoform 2 [Homo sapiens] |
| U | *gi|5902076|ref|NP\_008* | 4 | 9 | 20.6% | 248 | 27745 | 10.4 | splicing factor, arginine/serine-rich 1 isoform 1 [Homo sapiens] |

| Filename XCorr DeltCN Conf% ObsM+H+ CalcM+H+ SpR ZScore Ion% # Sequence  | | | | | | | | | | | | |
| --- | --- | --- | --- | --- | --- | --- | --- | --- | --- | --- | --- | --- |
|  | Astrin\_STLCLD20\_112214\_tube2\_01.11264.11264.2 | 2.961 | 0.2312 | 99.4% | 1257.4321 | 1257.4752 | 1 | 6.224 | 75.0% | 4 | R.IYVGNLPPDIR.T | 2 |
|  | Astrin\_STLCLD20\_112214\_01.08852.08852.2 | 3.4968 | 0.3227 | 100.0% | 1259.3121 | 1258.4137 | 1 | 5.93 | 83.3% | 3 | R.TKDIEDVFYK.Y | 2 |
|  | Astrin\_STLCLD20\_112214\_tube2\_01.16248.16248.2 | 3.1318 | 0.1409 | 97.8% | 2541.7522 | 2542.7234 | 1 | 4.28 | 38.6% | 1 | R.GGPPFAFVEFEDPRDAEDAVYGR.D | 2 |
|  | Astrin\_STLCLD20\_112214\_01.07489.07489.2 | 2.3638 | 0.1102 | 96.4% | 917.47217 | 917.0989 | 2 | 4.174 | 83.3% | 1 | R.LRVEFPR.S | 22 |

Similarities:
gi|4506903|ref|NP\_003(1:3)  

---

|  |  |  |  |  |  |  |  |  |
| --- | --- | --- | --- | --- | --- | --- | --- | --- |
| U | *gi|222352151|ref|NP\_0* | 5 | 9 | 25.0% | 356 | 37498 | 7.1 | poly(rC) binding protein 1 [Homo sapiens] |

| Filename XCorr DeltCN Conf% ObsM+H+ CalcM+H+ SpR ZScore Ion% # Sequence  | | | | | | | | | | | | |
| --- | --- | --- | --- | --- | --- | --- | --- | --- | --- | --- | --- | --- |
| \* | Astrin\_STLCLD20\_112214\_tube2\_01.14316.14316.2 | 3.5479 | 0.3875 | 100.0% | 1390.4722 | 1389.6781 | 2 | 6.884 | 66.7% | 1 | R.IITLTGPTNAIFK.A | 2 |
| \* | Astrin\_STLCLD20\_112214\_01.14134.14134.3 | 4.4566 | 0.3426 | 99.8% | 3380.0645 | 3380.8562 | 1 | 5.911 | 29.2% | 3 | K.AFAMIIDKLEEDINSSMTNSTAASRPPVTLR.L | 3 |
|  | Astrin\_STLCLD20\_112214\_02.10069.10069.2 | 4.9019 | 0.5556 | 100.0% | 2091.4922 | 2091.2573 | 1 | 9.838 | 57.9% | 3 | R.ESTGAQVQVAGDMLPNSTER.A | 22 |
|  | Astrin\_STLCLD20\_112214\_tube2\_02.06836.06836.3 | 4.1418 | 0.4486 | 100.0% | 2091.8342 | 2091.2573 | 1 | 7.273 | 39.5% | 1 | R.ESTGAQVQVAGDMLPNSTER.A | 33 |
| \* | Astrin\_STLCLD20\_112214\_tube2\_02.07244.07244.3 | 2.9638 | 0.2691 | 96.9% | 2688.1743 | 2687.875 | 2 | 4.541 | 29.2% | 1 | R.QQSHFAMMHGGTGFAGIDSS\*SPEVK.G | 3 |

Similarities:
gi|14141166|ref|NP\_11(2:3)  

---

|  |  |  |  |  |  |  |  |  |
| --- | --- | --- | --- | --- | --- | --- | --- | --- |
| U | *gi|94538362|ref|NP\_00* | 8 | 16 | 24.8% | 428 | 47064 | 5.3 | flotillin 2 [Homo sapiens] |

| Filename XCorr DeltCN Conf% ObsM+H+ CalcM+H+ SpR ZScore Ion% # Sequence  | | | | | | | | | | | | |
| --- | --- | --- | --- | --- | --- | --- | --- | --- | --- | --- | --- | --- |
| \* | Astrin\_STLCLD20\_112214\_tube2\_01.12927.12927.2 | 3.1387 | 0.4533 | 100.0% | 1378.8922 | 1379.6023 | 1 | 7.786 | 77.3% | 1 | K.NVVLQTLEGHLR.S | 2 |
| \* | Astrin\_STLCLD20\_112214\_tube2\_02.06655.06655.2 | 2.079 | 0.249 | 96.5% | 1123.9722 | 1124.2358 | 239 | 4.607 | 50.0% | 1 | K.SAFSEEVNIK.T | 2 |
| \* | Astrin\_STLCLD20\_112214\_tube2\_01.11495.11495.2 | 3.7743 | 0.3312 | 100.0% | 1522.3121 | 1521.6702 | 1 | 5.977 | 73.1% | 6 | K.TAEAQLAYELQGAR.E | 2 |
| \* | Astrin\_STLCLD20\_112214\_02.09964.09964.3 | 3.4694 | 0.1661 | 97.5% | 1642.0443 | 1641.865 | 1 | 5.258 | 50.0% | 1 | K.IRQEEIEIEVVQR.K | 3 |
| \* | Astrin\_STLCLD20\_112214\_02.11136.11136.2 | 2.105 | 0.2651 | 96.9% | 1270.3922 | 1270.4716 | 273 | 4.573 | 55.0% | 1 | K.QIAVEAQEILR.T | 2 |
| \* | Astrin\_STLCLD20\_112214\_01.11337.11337.2 | 1.973 | 0.2977 | 95.6% | 1389.0122 | 1389.6068 | 52 | 5.031 | 42.3% | 1 | K.IGEAEAAVIEAMGK.A | 2 |
| \* | Astrin\_STLCLD20\_112214\_01.09003.09003.2 | 3.5627 | 0.4266 | 100.0% | 1376.3522 | 1375.5187 | 1 | 7.56 | 75.0% | 2 | K.VDEIVVLSGDNSK.V | 2 |
| \* | Astrin\_STLCLD20\_112214\_01.11996.11996.2 | 4.1248 | 0.3682 | 100.0% | 1935.7522 | 1935.2712 | 1 | 7.175 | 58.3% | 3 | R.LLAELPASVHALTGVDLSK.I | 2 |

---

|  |  |  |  |  |  |  |  |  |
| --- | --- | --- | --- | --- | --- | --- | --- | --- |
| U | *gi|15431295|ref|NP\_15* | 5 | 7 | 24.6% | 211 | 24261 | 11.7 | ribosomal protein L13 [Homo sapiens] |
| U | *gi|15431297|ref|NP\_00* | 5 | 7 | 24.6% | 211 | 24261 | 11.7 | ribosomal protein L13 [Homo sapiens] |

| Filename XCorr DeltCN Conf% ObsM+H+ CalcM+H+ SpR ZScore Ion% # Sequence  | | | | | | | | | | | | |
| --- | --- | --- | --- | --- | --- | --- | --- | --- | --- | --- | --- | --- |
|  | Astrin\_STLCLD20\_112214\_tube2\_01.11000.11000.2 | 2.9143 | 0.3397 | 100.0% | 1190.3922 | 1190.3469 | 10 | 6.018 | 72.2% | 1 | R.VATWFNQPAR.K | 2 |
|  | Astrin\_STLCLD20\_112214\_tube2\_01.12533.12533.2 | 2.2876 | 0.3844 | 99.8% | 951.3522 | 951.0672 | 2 | 6.824 | 78.6% | 1 | R.GFSLEELR.V | 2 |
|  | Astrin\_STLCLD20\_112214\_tube2\_01.06262.06262.2 | 2.5543 | 0.2512 | 98.8% | 1233.0721 | 1233.3237 | 11 | 4.694 | 60.0% | 1 | K.STESLQANVQR.L | 2 |
|  | Astrin\_STLCLD20\_112214\_tube2\_01.11006.11006.2 | 2.9831 | 0.4398 | 100.0% | 1383.4521 | 1383.6923 | 1 | 7.052 | 70.8% | 3 | K.LATQLTGPVMPVR.N | 2 |
|  | Astrin\_STLCLD20\_112214\_tube2\_01.06371.06371.2 | 2.4302 | 0.2733 | 98.9% | 1237.1721 | 1237.3953 | 50 | 5.003 | 61.1% | 1 | R.VITEEEKNFK.A | 2 |

---

|  |  |  |  |  |  |  |  |  |
| --- | --- | --- | --- | --- | --- | --- | --- | --- |
| U | *gi|10863927|ref|NP\_06* | 4 | 8 | 24.2% | 165 | 18012 | 7.8 | peptidylprolyl isomerase A [Homo sapiens] |
| U | *gi|169215435|ref|XP\_0* | 4 | 8 | 17.9% | 223 | 24376 | 6.9 | PREDICTED: similar to peptidylprolyl isomerase A-like [Homo sapiens] |

| Filename XCorr DeltCN Conf% ObsM+H+ CalcM+H+ SpR ZScore Ion% # Sequence  | | | | | | | | | | | | |
| --- | --- | --- | --- | --- | --- | --- | --- | --- | --- | --- | --- | --- |
|  | Astrin\_STLCLD20\_112214\_tube2\_01.13060.13060.2 | 3.168 | 0.308 | 100.0% | 1380.4922 | 1380.6268 | 1 | 6.062 | 72.7% | 2 | R.VSFELFADKVPK.T | 2 |
|  | Astrin\_STLCLD20\_112214\_01.10942.10942.2 | 4.4902 | 0.3905 | 100.0% | 1832.7322 | 1833.0477 | 1 | 6.635 | 60.7% | 2 | K.SIYGEKFEDENFILK.H | 2 |
|  | Astrin\_STLCLD20\_112214\_01.09839.09839.2 | 3.8121 | 0.0891 | 99.0% | 1506.2522 | 1506.7755 | 2 | 8.373 | 75.0% | 2 | K.VKEGMNIVEAMER.F | 2 |
|  | Astrin\_STLCLD20\_112214\_tube2\_01.13011.13011.2 | 2.63 | 0.2519 | 98.9% | 1279.0922 | 1279.4689 | 1 | 6.616 | 70.0% | 2 | K.EGMNIVEAMER.F | 2 |

---

|  |  |  |  |  |  |  |  |  |
| --- | --- | --- | --- | --- | --- | --- | --- | --- |
| U | *gi|169164494|ref|XP\_0* | 2 | 3 | 24.0% | 100 | 11493 | 10.1 | PREDICTED: similar to ribosomal protein L10 [Homo sapiens] |
| U | *gi|41151097|ref|XP\_20* | 2 | 3 | 11.2% | 214 | 24627 | 10.1 | PREDICTED: similar to QM protein isoform 1 [Homo sapiens] |
| U | *gi|223890243|ref|NP\_0* | 2 | 3 | 11.2% | 214 | 24604 | 10.1 | ribosomal protein L10 [Homo sapiens] |
| U | *gi|169213734|ref|XP\_0* | 2 | 3 | 14.4% | 167 | 19409 | 9.9 | PREDICTED: similar to Q1Z 7F5 isoform 2 [Homo sapiens] |
| U | *gi|169213732|ref|XP\_0* | 2 | 3 | 11.2% | 214 | 24600 | 10.1 | PREDICTED: similar to Q1Z 7F5 isoform 1 [Homo sapiens] |
| U | *gi|169213538|ref|XP\_0* | 2 | 3 | 14.4% | 167 | 19436 | 9.9 | PREDICTED: similar to QM protein isoform 2 [Homo sapiens] |
| U | *gi|169213536|ref|XP\_0* | 2 | 3 | 11.2% | 214 | 24627 | 10.1 | PREDICTED: similar to QM protein isoform 1 [Homo sapiens] |

| Filename XCorr DeltCN Conf% ObsM+H+ CalcM+H+ SpR ZScore Ion% # Sequence  | | | | | | | | | | | | |
| --- | --- | --- | --- | --- | --- | --- | --- | --- | --- | --- | --- | --- |
|  | Astrin\_STLCLD20\_112214\_01.10352.10352.2 | 3.2788 | 0.5335 | 100.0% | 1253.4722 | 1253.5486 | 2 | 9.089 | 65.0% | 2 | R.VHIGQVIMSIR.T | 2 |
|  | Astrin\_STLCLD20\_112214\_tube2\_01.13890.13890.2 | 2.8264 | 0.2093 | 98.6% | 1545.5521 | 1545.6606 | 1 | 6.025 | 66.7% | 1 | K.FNADEFEDMVAEK.W | 2 |

---

|  |  |  |  |  |  |  |  |  |
| --- | --- | --- | --- | --- | --- | --- | --- | --- |
| U | *gi|50592996|ref|NP\_00* | 17 | 97 | 23.8% | 450 | 50433 | 4.9 | tubulin, beta, 4 [Homo sapiens] |

| Filename XCorr DeltCN Conf% ObsM+H+ CalcM+H+ SpR ZScore Ion% # Sequence  | | | | | | | | | | | | |
| --- | --- | --- | --- | --- | --- | --- | --- | --- | --- | --- | --- | --- |
|  | Astrin\_STLCLD20\_112214\_01.11522.11522.2 | 4.511 | 0.4938 | 100.0% | 1617.3121 | 1616.8701 | 1 | 8.254 | 64.3% | 14 | R.AILVDLEPGTMDSVR.S | 222 |
|  | Astrin\_STLCLD20\_112214\_tube2\_01.17294.17294.2 | 6.9168 | 0.5867 | 100.0% | 1959.5922 | 1960.151 | 1 | 11.206 | 79.4% | 2 | K.GHYTEGAELVDSVLDVVR.K | 2222 |
|  | Astrin\_STLCLD20\_112214\_01.13379.13379.3 | 3.6233 | 0.2439 | 99.6% | 1960.6144 | 1960.151 | 1 | 5.03 | 38.2% | 3 | K.GHYTEGAELVDSVLDVVR.K | 3333 |
|  | Astrin\_STLCLD20\_112214\_01.12767.12767.2 | 5.5584 | 0.5301 | 100.0% | 2087.7522 | 2088.325 | 1 | 9.725 | 72.2% | 3 | K.GHYTEGAELVDSVLDVVRK.E | 2222 |
|  | Astrin\_STLCLD20\_112214\_tube2\_01.15854.15854.3 | 4.7062 | 0.4415 | 100.0% | 2088.8943 | 2088.325 | 1 | 7.599 | 41.7% | 5 | K.GHYTEGAELVDSVLDVVRK.E | 3333 |
|  | Astrin\_STLCLD20\_112214\_01.10173.10173.1 | 2.1231 | 0.2434 | 97.9% | 1319.72 | 1320.5896 | 53 | 5.055 | 45.5% | 2 | R.IMNTFSVVPSPK.V | 111 |
|  | Astrin\_STLCLD20\_112214\_tube2\_01.11552.11552.2 | 4.4232 | 0.4158 | 100.0% | 1320.1721 | 1320.5896 | 1 | 8.424 | 77.3% | 12 | R.IMNTFSVVPSPK.V | 222 |
|  | Astrin\_STLCLD20\_112214\_tube2\_01.09993.09993.2 | 3.1291 | 0.339 | 100.0% | 1131.4922 | 1131.2767 | 11 | 5.764 | 77.8% | 19 | R.FPGQLNADLR.K | 22222 |
|  | Astrin\_STLCLD20\_112214\_tube2\_01.08446.08446.2 | 2.7132 | 0.2665 | 99.3% | 1259.5122 | 1259.4508 | 43 | 5.338 | 60.0% | 3 | R.FPGQLNADLRK.L | 22222 |
|  | Astrin\_STLCLD20\_112214\_tube2\_01.11456.11456.2 | 3.6404 | 0.3874 | 100.0% | 1272.3522 | 1272.5945 | 1 | 6.64 | 70.0% | 4 | R.KLAVNMVPFPR.L | 22222 |
|  | Astrin\_STLCLD20\_112214\_01.11192.11192.1 | 2.2399 | 0.2147 | 98.0% | 1143.63 | 1144.4204 | 6 | 6.378 | 61.1% | 2 | K.LAVNMVPFPR.L | 11111 |
|  | Astrin\_STLCLD20\_112214\_01.11233.11233.2 | 3.8296 | 0.4984 | 100.0% | 1144.4722 | 1144.4204 | 1 | 8.878 | 94.4% | 6 | K.LAVNMVPFPR.L | 22222 |
|  | Astrin\_STLCLD20\_112214\_tube2\_01.16373.16373.2 | 4.1702 | 0.4839 | 100.0% | 1692.2322 | 1692.9678 | 1 | 8.782 | 78.6% | 4 | R.ALTVPELTQQMFDAK.N | 22 |
|  | Astrin\_STLCLD20\_112214\_tube2\_01.15262.15262.2 | 3.3078 | 0.3064 | 99.9% | 1698.6122 | 1697.8877 | 1 | 5.236 | 61.5% | 3 | K.NSSYFVEWIPNNVK.V | 22222 |
|  | Astrin\_STLCLD20\_112214\_tube2\_01.12078.12078.2 | 3.0386 | 0.2531 | 99.7% | 1385.6921 | 1386.6116 | 84 | 5.63 | 55.0% | 4 | K.RISEQFTAMFR.R | 2222 |
|  | Astrin\_STLCLD20\_112214\_tube2\_01.13623.13623.1 | 1.9755 | 0.2993 | 100.0% | 1229.5 | 1230.4241 | 1 | 5.342 | 61.1% | 1 | R.ISEQFTAMFR.R | 1111 |
|  | Astrin\_STLCLD20\_112214\_02.12843.12843.2 | 3.8745 | 0.4739 | 100.0% | 1230.1921 | 1230.4241 | 1 | 8.01 | 94.4% | 10 | R.ISEQFTAMFR.R | 2222 |

Similarities:
gi|29788785|ref|NP\_82(16:1)  
gi|5174735|ref|NP\_006(16:1)  
gi|29788768|ref|NP\_82(14:3)  
gi|14210536|ref|NP\_11(6:11)  

---

|  |  |  |  |  |  |  |  |  |
| --- | --- | --- | --- | --- | --- | --- | --- | --- |
| U | *gi|10835063|ref|NP\_00* | 3 | 4 | 23.8% | 294 | 32575 | 4.8 | nucleophosmin 1 isoform 1 [Homo sapiens] |
| U | *gi|83641870|ref|NP\_00* | 3 | 4 | 27.0% | 259 | 28400 | 4.7 | nucleophosmin 1 isoform 3 [Homo sapiens] |
| U | *gi|40353734|ref|NP\_95* | 3 | 4 | 26.4% | 265 | 29465 | 4.6 | nucleophosmin 1 isoform 2 [Homo sapiens] |

| Filename XCorr DeltCN Conf% ObsM+H+ CalcM+H+ SpR ZScore Ion% # Sequence  | | | | | | | | | | | | |
| --- | --- | --- | --- | --- | --- | --- | --- | --- | --- | --- | --- | --- |
|  | Astrin\_STLCLD20\_112214\_01.05853.05853.3 | 3.9254 | 0.224 | 99.6% | 2573.9343 | 2574.7258 | 21 | 5.307 | 28.8% | 1 | K.ADKDYHFKVDNDENEHQLSLR.T | 3 |
|  | Astrin\_STLCLD20\_112214\_tube2\_01.14770.14770.3 | 5.0365 | 0.4581 | 100.0% | 2929.8542 | 2931.2874 | 1 | 8.172 | 26.9% | 2 | R.TVSLGAGAKDELHIVEAEAMNYEGSPIK.V | 3 |
|  | Astrin\_STLCLD20\_112214\_01.13580.13580.2 | 3.6036 | 0.441 | 100.0% | 2228.5522 | 2228.655 | 1 | 6.547 | 40.0% | 1 | K.MSVQPTVSLGGFEITPPVVLR.L | 2 |

---

|  |  |  |  |  |  |  |  |  |
| --- | --- | --- | --- | --- | --- | --- | --- | --- |
| U | *gi|16905517|ref|NP\_47* | 7 | 10 | 23.7% | 262 | 31301 | 11.3 | FUS interacting protein (serine-arginine rich) 1 isoform 2 [Homo sapiens] |
| U | *gi|5730079|ref|NP\_006* | 7 | 10 | 33.9% | 183 | 22222 | 10.3 | FUS interacting protein (serine-arginine rich) 1 isoform 1 [Homo sapiens] |
| U | *gi|169161980|ref|XP\_0* | 7 | 10 | 34.3% | 181 | 22022 | 10.3 | PREDICTED: hypothetical protein, partial [Homo sapiens] |
| U | *gi|169161109|ref|XP\_0* | 7 | 10 | 33.9% | 183 | 22222 | 10.3 | PREDICTED: hypothetical protein LOC642558 [Homo sapiens] |
| U | *gi|169161107|ref|XP\_0* | 7 | 10 | 23.7% | 262 | 31301 | 11.3 | PREDICTED: hypothetical protein LOC642558 [Homo sapiens] |

| Filename XCorr DeltCN Conf% ObsM+H+ CalcM+H+ SpR ZScore Ion% # Sequence  | | | | | | | | | | | | |
| --- | --- | --- | --- | --- | --- | --- | --- | --- | --- | --- | --- | --- |
|  | Astrin\_STLCLD20\_112214\_tube2\_01.10824.10824.3 | 3.4885 | 0.2611 | 99.5% | 1463.8143 | 1463.7227 | 1 | 5.723 | 54.5% | 2 | R.YLRPPNTSLFVR.N | 3 |
|  | Astrin\_STLCLD20\_112214\_tube2\_01.18545.18545.2 | 4.3356 | 0.5495 | 100.0% | 1918.0922 | 1918.1992 | 1 | 9.21 | 60.0% | 1 | R.YGPIVDVYVPLDFYTR.R | 2 |
|  | Astrin\_STLCLD20\_112214\_tube2\_01.13052.13052.2 | 2.5592 | 0.278 | 99.1% | 1331.7322 | 1331.4705 | 9 | 5.204 | 60.0% | 1 | R.GFAYVQFEDVR.D | 2 |
|  | Astrin\_STLCLD20\_112214\_tube2\_01.15809.15809.3 | 4.1756 | 0.4364 | 100.0% | 2580.5942 | 2581.7605 | 1 | 7.225 | 34.5% | 1 | R.GFAYVQFEDVRDAEDALHNLDR.K | 3 |
|  | Astrin\_STLCLD20\_112214\_tube2\_01.14609.14609.3 | 4.1533 | 0.3468 | 99.8% | 2709.5942 | 2709.9346 | 1 | 6.223 | 37.5% | 2 | R.GFAYVQFEDVRDAEDALHNLDRK.W | 3 |
|  | Astrin\_STLCLD20\_112214\_01.07516.07516.2 | 2.1582 | 0.3058 | 98.3% | 1269.2922 | 1269.3134 | 1 | 4.903 | 75.0% | 1 | R.DAEDALHNLDR.K | 2 |
|  | Astrin\_STLCLD20\_112214\_tube2\_01.10443.10443.2 | 2.5955 | 0.3366 | 99.7% | 1305.4521 | 1305.4331 | 363 | 5.49 | 50.0% | 2 | R.QIEIQFAQGDR.K | 2 |

---

|  |  |  |  |  |  |  |  |  |
| --- | --- | --- | --- | --- | --- | --- | --- | --- |
| U | *gi|16306492|ref|NP\_20* | 4 | 4 | 23.3% | 240 | 27503 | 7.1 | cell division cycle 2 isoform 2 [Homo sapiens] |
| U | *gi|4502709|ref|NP\_001* | 4 | 4 | 18.9% | 297 | 34095 | 8.4 | cell division cycle 2 isoform 1 [Homo sapiens] |
| U | *gi|195927041|ref|NP\_0* | 4 | 4 | 18.9% | 297 | 34081 | 8.4 | cell division cycle 2 isoform 3 [Homo sapiens] |

| Filename XCorr DeltCN Conf% ObsM+H+ CalcM+H+ SpR ZScore Ion% # Sequence  | | | | | | | | | | | | |
| --- | --- | --- | --- | --- | --- | --- | --- | --- | --- | --- | --- | --- |
|  | Astrin\_STLCLD20\_112214\_tube2\_01.08728.08728.2 | 2.915 | 0.4024 | 100.0% | 1186.8722 | 1186.3501 | 1 | 7.374 | 70.0% | 1 | K.IGEGTYGVVYK.G | 2 |
|  | Astrin\_STLCLD20\_112214\_01.07199.07199.2 | 2.2808 | 0.2193 | 97.1% | 1029.2922 | 1029.1814 | 1 | 5.295 | 83.3% | 1 | R.SPEVLLGSAR.Y | 2 |
|  | Astrin\_STLCLD20\_112214\_tube2\_02.13686.13686.2 | 3.4901 | 0.4905 | 100.0% | 2214.0322 | 2213.5352 | 1 | 7.699 | 39.5% | 1 | R.YSTPVDIWSIGTIFAELATK.K | 2 |
|  | Astrin\_STLCLD20\_112214\_tube2\_01.12462.12462.3 | 3.753 | 0.3114 | 99.8% | 1802.5743 | 1803.0275 | 1 | 5.594 | 50.0% | 1 | K.KPLFHGDSEIDQLFR.I | 3 |

---

|  |  |  |  |  |  |  |  |  |
| --- | --- | --- | --- | --- | --- | --- | --- | --- |
| U | *gi|32189392|ref|NP\_00* | 3 | 8 | 23.2% | 198 | 21892 | 6.0 | peroxiredoxin 2 isoform a [Homo sapiens] |

| Filename XCorr DeltCN Conf% ObsM+H+ CalcM+H+ SpR ZScore Ion% # Sequence  | | | | | | | | | | | | |
| --- | --- | --- | --- | --- | --- | --- | --- | --- | --- | --- | --- | --- |
| \* | Astrin\_STLCLD20\_112214\_tube2\_01.17223.17223.2 | 4.3767 | 0.5141 | 100.0% | 1863.9922 | 1864.1954 | 1 | 8.409 | 58.8% | 2 | R.KEGGLGPLNIPLLADVTR.R | 2 |
| \* | Astrin\_STLCLD20\_112214\_02.10372.10372.3 | 2.8448 | 0.3437 | 99.6% | 1930.2244 | 1930.1217 | 1 | 5.688 | 37.5% | 1 | R.LSEDYGVLKTDEGIAYR.G | 3 |
|  | Astrin\_STLCLD20\_112214\_tube2\_01.10262.10262.2 | 2.9791 | 0.2073 | 99.3% | 1212.5122 | 1212.3915 | 3 | 6.522 | 75.0% | 5 | R.QITVNDLPVGR.S | 22 |

Similarities:
gi|32455264|ref|NP\_85(1:2)  

---

|  |  |  |  |  |  |  |  |  |
| --- | --- | --- | --- | --- | --- | --- | --- | --- |
| U | *gi|4503471|ref|NP\_001* | 11 | 42 | 22.7% | 462 | 50141 | 9.0 | eukaryotic translation elongation factor 1 alpha 1 [Homo sapiens] |

| Filename XCorr DeltCN Conf% ObsM+H+ CalcM+H+ SpR ZScore Ion% # Sequence  | | | | | | | | | | | | |
| --- | --- | --- | --- | --- | --- | --- | --- | --- | --- | --- | --- | --- |
|  | Astrin\_STLCLD20\_112214\_tube2\_01.08751.08751.2 | 2.8665 | 0.2046 | 98.3% | 1589.6721 | 1589.835 | 1 | 5.092 | 57.1% | 2 | K.THINIVVIGHVDSGK.S | 2 |
|  | Astrin\_STLCLD20\_112214\_02.09717.09717.3 | 4.8998 | 0.4611 | 100.0% | 1590.3544 | 1589.835 | 1 | 8.331 | 51.8% | 13 | K.THINIVVIGHVDSGK.S | 3 |
|  | Astrin\_STLCLD20\_112214\_01.07457.07457.3 | 2.8286 | 0.3334 | 99.5% | 1613.3043 | 1611.8506 | 14 | 5.88 | 34.6% | 1 | R.TIEKFEKEAAEMGK.G | 3 |
| \* | Astrin\_STLCLD20\_112214\_01.08984.08984.2 | 3.0738 | 0.3348 | 100.0% | 1405.1322 | 1405.5962 | 1 | 5.929 | 72.7% | 3 | K.YYVTIIDAPGHR.D | 2 |
|  | Astrin\_STLCLD20\_112214\_tube2\_01.11613.11613.2 | 3.4317 | 0.4258 | 100.0% | 1315.7122 | 1315.5553 | 1 | 8.224 | 72.7% | 5 | R.EHALLAYTLGVK.Q | 2 |
|  | Astrin\_STLCLD20\_112214\_01.08528.08528.2 | 2.2809 | 0.2456 | 98.6% | 976.59216 | 976.1607 | 13 | 5.678 | 78.6% | 1 | R.LPLQDVYK.I | 2 |
|  | Astrin\_STLCLD20\_112214\_tube2\_01.08742.08742.1 | 1.7253 | 0.3275 | 100.0% | 1025.64 | 1026.2241 | 3 | 5.554 | 55.0% | 1 | K.IGGIGTVPVGR.V | 1 |
|  | Astrin\_STLCLD20\_112214\_01.07327.07327.2 | 3.7298 | 0.3353 | 100.0% | 1027.1921 | 1026.2241 | 1 | 6.092 | 80.0% | 7 | K.IGGIGTVPVGR.V | 2 |
| \* | Astrin\_STLCLD20\_112214\_tube2\_01.14037.14037.2 | 5.0601 | 0.4766 | 100.0% | 2515.892 | 2516.999 | 1 | 9.878 | 52.2% | 3 | R.VETGVLKPGMVVTFAPVNVTTEVK.S | 2 |
| \* | Astrin\_STLCLD20\_112214\_tube2\_01.14084.14084.3 | 4.7835 | 0.4081 | 100.0% | 2517.2644 | 2516.999 | 1 | 6.311 | 35.9% | 5 | R.VETGVLKPGMVVTFAPVNVTTEVK.S | 3 |
|  | Astrin\_STLCLD20\_112214\_tube2\_01.07851.07851.1 | 1.9061 | 0.3959 | 100.0% | 914.56 | 915.1209 | 60 | 5.532 | 56.2% | 1 | R.QTVAVGVIK.A | 1 |

---

|  |  |  |  |  |  |  |  |  |
| --- | --- | --- | --- | --- | --- | --- | --- | --- |
| U | *gi|5032051|ref|NP\_005* | 2 | 5 | 22.5% | 151 | 16273 | 10.1 | ribosomal protein S14 [Homo sapiens] |
| U | *gi|68160922|ref|NP\_00* | 2 | 5 | 22.5% | 151 | 16273 | 10.1 | ribosomal protein S14 [Homo sapiens] |
| U | *gi|68160915|ref|NP\_00* | 2 | 5 | 22.5% | 151 | 16273 | 10.1 | ribosomal protein S14 [Homo sapiens] |

| Filename XCorr DeltCN Conf% ObsM+H+ CalcM+H+ SpR ZScore Ion% # Sequence  | | | | | | | | | | | | |
| --- | --- | --- | --- | --- | --- | --- | --- | --- | --- | --- | --- | --- |
|  | Astrin\_STLCLD20\_112214\_tube2\_01.11889.11889.3 | 3.6945 | 0.3807 | 99.8% | 2266.6443 | 2266.4473 | 1 | 5.984 | 41.2% | 1 | K.ADRDESSPYAAMLAAQDVAQR.C | 3 |
|  | Astrin\_STLCLD20\_112214\_01.06518.06518.2 | 3.8788 | 0.4443 | 100.0% | 1430.4122 | 1430.5547 | 1 | 8.188 | 75.0% | 4 | R.IEDVTPIPSDSTR.R | 2 |

---

|  |  |  |  |  |  |  |  |  |
| --- | --- | --- | --- | --- | --- | --- | --- | --- |
| U | *gi|10440560|ref|NP\_06* | 3 | 4 | 22.1% | 136 | 15404 | 11.1 | histone cluster 1, H3f [Homo sapiens] |
| U | *gi|53793688|ref|NP\_00* | 3 | 4 | 22.1% | 136 | 15388 | 11.3 | histone cluster 2, H3a [Homo sapiens] |
| U | *gi|4885385|ref|NP\_005* | 3 | 4 | 22.1% | 136 | 15328 | 11.3 | H3 histone, family 3B [Homo sapiens] |
| U | *gi|4504299|ref|NP\_003* | 3 | 4 | 22.1% | 136 | 15508 | 11.1 | histone cluster 3, H3 [Homo sapiens] |
| U | *gi|4504297|ref|NP\_003* | 3 | 4 | 22.1% | 136 | 15404 | 11.1 | histone cluster 1, H3b [Homo sapiens] |
| U | *gi|4504295|ref|NP\_003* | 3 | 4 | 22.1% | 136 | 15404 | 11.1 | histone cluster 1, H3h [Homo sapiens] |
| U | *gi|4504293|ref|NP\_003* | 3 | 4 | 22.1% | 136 | 15404 | 11.1 | histone cluster 1, H3j [Homo sapiens] |
| U | *gi|4504291|ref|NP\_003* | 3 | 4 | 22.1% | 136 | 15404 | 11.1 | H3 histone family, member H [Homo sapiens] |
| U | *gi|4504289|ref|NP\_003* | 3 | 4 | 22.1% | 136 | 15404 | 11.1 | histone cluster 1, H3i [Homo sapiens] |
| U | *gi|4504287|ref|NP\_003* | 3 | 4 | 22.1% | 136 | 15404 | 11.1 | histone cluster 1, H3e [Homo sapiens] |
| U | *gi|4504285|ref|NP\_003* | 3 | 4 | 22.1% | 136 | 15404 | 11.1 | histone cluster 1, H3c [Homo sapiens] |
| U | *gi|4504281|ref|NP\_003* | 3 | 4 | 22.1% | 136 | 15404 | 11.1 | histone cluster 1, H3a [Homo sapiens] |
| U | *gi|4504279|ref|NP\_002* | 3 | 4 | 22.1% | 136 | 15328 | 11.3 | H3 histone, family 3A [Homo sapiens] |
| U | *gi|31742503|ref|NP\_06* | 3 | 4 | 22.1% | 136 | 15388 | 11.3 | histone cluster 2, H3c [Homo sapiens] |
| U | *gi|21071021|ref|NP\_00* | 3 | 4 | 22.1% | 136 | 15404 | 11.1 | histone cluster 1, H3d [Homo sapiens] |
| U | *gi|183076548|ref|NP\_0* | 3 | 4 | 22.1% | 136 | 15388 | 11.3 | histone cluster 2, H3d [Homo sapiens] |

| Filename XCorr DeltCN Conf% ObsM+H+ CalcM+H+ SpR ZScore Ion% # Sequence  | | | | | | | | | | | | |
| --- | --- | --- | --- | --- | --- | --- | --- | --- | --- | --- | --- | --- |
|  | Astrin\_STLCLD20\_112214\_tube2\_01.06626.06626.2 | 2.3346 | 0.2004 | 97.5% | 1032.9321 | 1033.2186 | 18 | 4.911 | 68.8% | 1 | R.YRPGTVALR.E | 2 |
|  | Astrin\_STLCLD20\_112214\_01.05207.05207.2 | 2.2973 | 0.1987 | 96.8% | 1252.0521 | 1251.4685 | 1 | 5.567 | 88.9% | 1 | R.YQKSTELLIR.K | 2 |
|  | Astrin\_STLCLD20\_112214\_tube2\_01.08714.08714.2 | 3.0084 | 0.3456 | 100.0% | 1336.4922 | 1336.4875 | 1 | 5.672 | 80.0% | 2 | R.EIAQDFKTDLR.F | 2 |

---

|  |  |  |  |  |  |  |  |  |
| --- | --- | --- | --- | --- | --- | --- | --- | --- |
| U | *gi|5729877|ref|NP\_006* | 11 | 27 | 21.4% | 646 | 70898 | 5.5 | heat shock 70kDa protein 8 isoform 1 [Homo sapiens] |

| Filename XCorr DeltCN Conf% ObsM+H+ CalcM+H+ SpR ZScore Ion% # Sequence  | | | | | | | | | | | | |
| --- | --- | --- | --- | --- | --- | --- | --- | --- | --- | --- | --- | --- |
|  | Astrin\_STLCLD20\_112214\_01.09043.09043.2 | 3.6782 | 0.4657 | 100.0% | 1488.2922 | 1488.5939 | 1 | 9.028 | 75.0% | 7 | R.TTPSYVAFTDTER.L | 22 |
|  | Astrin\_STLCLD20\_112214\_tube2\_01.10332.10332.2 | 4.0807 | 0.4782 | 100.0% | 1650.4321 | 1650.8468 | 1 | 8.41 | 71.4% | 4 | K.NQVAMNPTNTVFDAK.R | 2 |
|  | Astrin\_STLCLD20\_112214\_tube2\_01.08080.08080.2 | 2.6932 | 0.3318 | 99.7% | 1411.4722 | 1411.5725 | 1 | 6.362 | 72.7% | 1 | R.RFDDAVVQSDMK.H | 2 |
|  | Astrin\_STLCLD20\_112214\_tube2\_01.13115.13115.2 | 4.1946 | 0.2878 | 100.0% | 1662.5322 | 1660.9078 | 1 | 7.371 | 83.3% | 2 | R.IINEPTAAAIAYGLDK.K | 22 |
|  | Astrin\_STLCLD20\_112214\_tube2\_01.07186.07186.2 | 4.0096 | 0.4701 | 100.0% | 1692.0122 | 1692.6958 | 1 | 8.338 | 60.0% | 2 | K.STAGDTHLGGEDFDNR.M | 2 |
|  | Astrin\_STLCLD20\_112214\_01.09548.09548.2 | 2.7215 | 0.4138 | 100.0% | 1235.6921 | 1236.4741 | 1 | 6.694 | 83.3% | 1 | R.MVNHFIAEFK.R | 2 |
|  | Astrin\_STLCLD20\_112214\_01.10897.10897.2 | 3.5925 | 0.3267 | 100.0% | 1482.4321 | 1481.6511 | 1 | 6.485 | 77.3% | 3 | R.ARFEELNADLFR.G | 2 |
|  | Astrin\_STLCLD20\_112214\_02.09943.09943.3 | 4.5177 | 0.2797 | 99.8% | 1839.0844 | 1839.1019 | 1 | 6.452 | 37.5% | 2 | K.LDKSQIHDIVLVGGSTR.I | 3 |
|  | Astrin\_STLCLD20\_112214\_01.07954.07954.2 | 3.3794 | 0.4885 | 100.0% | 1481.7122 | 1482.6798 | 1 | 8.132 | 76.9% | 2 | K.SQIHDIVLVGGSTR.I | 2 |
| \* | Astrin\_STLCLD20\_112214\_tube2\_01.05751.05751.3 | 4.0546 | 0.3064 | 99.8% | 1983.3544 | 1983.2036 | 3 | 6.357 | 40.0% | 1 | R.MVQEAEKYKAEDEKQR.D | 3 |
| \* | Astrin\_STLCLD20\_112214\_tube2\_01.12108.12108.2 | 3.8702 | 0.4388 | 100.0% | 1305.2922 | 1304.4602 | 1 | 6.787 | 85.0% | 2 | K.NSLESYAFNMK.A | 2 |

Similarities:
gi|167466173|ref|NP\_0(1:10)  
contaminant\_GR78\_HUMA(1:10)  

---

|  |  |  |  |  |  |  |  |  |
| --- | --- | --- | --- | --- | --- | --- | --- | --- |
| U | *gi|72534660|ref|NP\_00* | 5 | 11 | 21.4% | 238 | 27367 | 11.8 | splicing factor, arginine/serine-rich 7 [Homo sapiens] |

| Filename XCorr DeltCN Conf% ObsM+H+ CalcM+H+ SpR ZScore Ion% # Sequence  | | | | | | | | | | | | |
| --- | --- | --- | --- | --- | --- | --- | --- | --- | --- | --- | --- | --- |
| \* | Astrin\_STLCLD20\_112214\_01.06891.06891.2 | 3.8301 | 0.4885 | 100.0% | 1720.4922 | 1720.923 | 1 | 8.375 | 68.8% | 2 | K.VYVGNLGTGAGKGELER.A | 2 |
| \* | Astrin\_STLCLD20\_112214\_tube2\_02.06138.06138.3 | 2.279 | 0.3058 | 95.3% | 1721.1543 | 1720.923 | 33 | 4.789 | 31.2% | 2 | K.VYVGNLGTGAGKGELER.A | 3 |
| \* | Astrin\_STLCLD20\_112214\_tube2\_01.11373.11373.2 | 2.5498 | 0.3219 | 99.7% | 1074.2522 | 1074.2242 | 19 | 6.915 | 68.8% | 1 | R.AFSYYGPLR.T | 2 |
|  | Astrin\_STLCLD20\_112214\_tube2\_01.15758.15758.2 | 3.3718 | 0.37 | 100.0% | 1622.3522 | 1622.7771 | 1 | 6.459 | 65.4% | 2 | R.NPPGFAFVEFEDPR.D | 22 |
| \* | Astrin\_STLCLD20\_112214\_tube2\_01.08337.08337.2 | 3.1958 | 0.342 | 100.0% | 1245.5521 | 1245.4827 | 5 | 6.127 | 65.0% | 4 | R.VRVELSTGMPR.R | 2 |

Similarities:
gi|4506901|ref|NP\_003(1:4)  

---

|  |  |  |  |  |  |  |  |  |
| --- | --- | --- | --- | --- | --- | --- | --- | --- |
| U | *gi|4506607|ref|NP\_000* | 3 | 9 | 21.3% | 188 | 21634 | 11.7 | ribosomal protein L18 [Homo sapiens] |

| Filename XCorr DeltCN Conf% ObsM+H+ CalcM+H+ SpR ZScore Ion% # Sequence  | | | | | | | | | | | | |
| --- | --- | --- | --- | --- | --- | --- | --- | --- | --- | --- | --- | --- |
| \* | Astrin\_STLCLD20\_112214\_tube2\_02.07076.07076.2 | 4.1965 | 0.4117 | 100.0% | 1347.6322 | 1346.5236 | 1 | 7.494 | 83.3% | 6 | K.TAVVVGTITDDVR.V | 2 |
| \* | Astrin\_STLCLD20\_112214\_01.12163.12163.2 | 3.6788 | 0.5086 | 100.0% | 1461.4922 | 1461.6982 | 1 | 9.455 | 75.0% | 2 | K.ILTFDQLALDSPK.G | 2 |
| \* | Astrin\_STLCLD20\_112214\_tube2\_01.05580.05580.3 | 3.0457 | 0.3925 | 99.8% | 1549.9143 | 1548.7446 | 1 | 6.13 | 46.2% | 1 | K.APGTPHSHTKPYVR.S | 3 |

---

|  |  |  |  |  |  |  |  |  |
| --- | --- | --- | --- | --- | --- | --- | --- | --- |
| U | *gi|20149594|ref|NP\_03* | 12 | 32 | 21.1% | 724 | 83264 | 5.0 | heat shock 90kDa protein 1, beta [Homo sapiens] |

| Filename XCorr DeltCN Conf% ObsM+H+ CalcM+H+ SpR ZScore Ion% # Sequence  | | | | | | | | | | | | |
| --- | --- | --- | --- | --- | --- | --- | --- | --- | --- | --- | --- | --- |
|  | Astrin\_STLCLD20\_112214\_01.07856.07856.2 | 2.5924 | 0.3021 | 99.3% | 1276.4922 | 1276.3861 | 14 | 5.747 | 54.5% | 1 | R.ELISNASDALDK.I | 2 |
|  | Astrin\_STLCLD20\_112214\_tube2\_01.11159.11159.2 | 3.7146 | 0.4163 | 100.0% | 1545.5721 | 1545.733 | 1 | 6.528 | 73.1% | 2 | R.ELISNASDALDKIR.Y | 2 |
| \* | Astrin\_STLCLD20\_112214\_01.10736.10736.2 | 2.144 | 0.3129 | 97.9% | 1350.3322 | 1350.6135 | 181 | 5.701 | 41.7% | 1 | R.TLTLVDTGIGMTK.A | 2 |
|  | Astrin\_STLCLD20\_112214\_tube2\_01.12254.12254.2 | 3.4834 | 0.4327 | 100.0% | 1243.4521 | 1243.4459 | 1 | 7.724 | 81.8% | 3 | K.ADLINNLGTIAK.S | 22 |
|  | Astrin\_STLCLD20\_112214\_02.09754.09754.3 | 3.9778 | 0.4595 | 100.0% | 2015.8444 | 2016.2584 | 1 | 7.162 | 43.3% | 6 | K.VILHLKEDQTEYLEER.R | 33 |
| \* | Astrin\_STLCLD20\_112214\_tube2\_01.15354.15354.2 | 4.5798 | 0.4949 | 100.0% | 1810.2322 | 1810.1027 | 1 | 8.36 | 71.4% | 1 | K.HSQFIGYPITLYLEK.E | 2 |
| \* | Astrin\_STLCLD20\_112214\_01.10025.10025.2 | 4.7676 | 0.5328 | 100.0% | 1848.4521 | 1848.9171 | 1 | 9.763 | 82.1% | 2 | R.NPDDITQEEYGEFYK.S | 2 |
|  | Astrin\_STLCLD20\_112214\_01.09962.09962.2 | 4.3602 | 0.4022 | 100.0% | 1528.3922 | 1528.6616 | 1 | 7.855 | 70.8% | 2 | K.SLTNDWEDHLAVK.H | 22 |
|  | Astrin\_STLCLD20\_112214\_02.10618.10618.2 | 2.964 | 0.4331 | 100.0% | 1349.4521 | 1349.4886 | 1 | 6.95 | 70.0% | 5 | K.HFSVEGQLEFR.A | 22 |
| \* | Astrin\_STLCLD20\_112214\_tube2\_01.12466.12466.2 | 2.7135 | 0.2089 | 98.9% | 1237.3922 | 1237.4008 | 3 | 4.694 | 72.2% | 1 | R.RAPFDLFENK.K | 2 |
| \* | Astrin\_STLCLD20\_112214\_02.10624.10624.3 | 4.4877 | 0.4473 | 100.0% | 2178.8643 | 2178.2915 | 1 | 7.707 | 37.5% | 4 | R.YHTSQSGDEMTSLSEYVSR.M | 3 |
| \* | Astrin\_STLCLD20\_112214\_tube2\_01.09629.09629.3 | 3.894 | 0.4858 | 100.0% | 1784.3344 | 1784.025 | 1 | 7.423 | 50.0% | 4 | K.HLEINPDHPIVETLR.Q | 3 |

Similarities:
gi|153792590|ref|NP\_0(4:8)  

---

|  |  |  |  |  |  |  |  |  |
| --- | --- | --- | --- | --- | --- | --- | --- | --- |
| U | *gi|14141152|ref|NP\_00* | 11 | 37 | 21.1% | 730 | 77516 | 8.7 | heterogeneous nuclear ribonucleoprotein M isoform a [Homo sapiens] |
| U | *gi|157412270|ref|NP\_1* | 11 | 37 | 22.3% | 691 | 73621 | 8.8 | heterogeneous nuclear ribonucleoprotein M isoform b [Homo sapiens] |

| Filename XCorr DeltCN Conf% ObsM+H+ CalcM+H+ SpR ZScore Ion% # Sequence  | | | | | | | | | | | | |
| --- | --- | --- | --- | --- | --- | --- | --- | --- | --- | --- | --- | --- |
|  | Astrin\_STLCLD20\_112214\_02.12070.12070.2 | 4.079 | 0.4854 | 100.0% | 1427.5721 | 1427.6403 | 1 | 8.279 | 58.3% | 2 | R.LGSTVFVANLDYK.V | 2 |
|  | Astrin\_STLCLD20\_112214\_02.14731.14731.2 | 2.7006 | 0.3273 | 99.4% | 2179.9922 | 2179.5752 | 1 | 5.832 | 31.8% | 1 | K.GIGMGNIGPAGMGMEGIGFGINK.M | 2 |
|  | Astrin\_STLCLD20\_112214\_tube2\_01.12773.12773.2 | 3.208 | 0.4006 | 100.0% | 1715.3121 | 1715.9724 | 1 | 6.737 | 53.1% | 1 | K.MGGMEGPFGGGMENMGR.F | 2 |
|  | Astrin\_STLCLD20\_112214\_tube2\_01.11279.11279.2 | 3.0001 | 0.248 | 99.7% | 1114.3922 | 1115.3152 | 35 | 4.975 | 61.1% | 2 | R.INEILSNALK.R | 2 |
|  | Astrin\_STLCLD20\_112214\_01.10383.10383.2 | 4.732 | 0.4895 | 100.0% | 1614.3722 | 1614.875 | 1 | 9.627 | 75.0% | 3 | R.MGPLGLDHMASSIER.M | 2 |
|  | Astrin\_STLCLD20\_112214\_tube2\_01.11624.11624.3 | 3.6006 | 0.4619 | 100.0% | 1615.7043 | 1614.875 | 1 | 7.853 | 50.0% | 3 | R.MGPLGLDHMASSIER.M | 3 |
|  | Astrin\_STLCLD20\_112214\_tube2\_02.07596.07596.2 | 3.2856 | 0.5906 | 100.0% | 1126.0322 | 1126.3337 | 1 | 9.586 | 75.0% | 6 | R.MGAGMGFGLER.M | 2 |
|  | Astrin\_STLCLD20\_112214\_tube2\_01.08920.08920.2 | 2.3122 | 0.433 | 99.8% | 1189.3922 | 1189.4333 | 7 | 6.217 | 68.2% | 3 | R.MVPAGMGAGLER.M | 2 |
|  | Astrin\_STLCLD20\_112214\_tube2\_01.10958.10958.2 | 3.2367 | 0.3215 | 99.9% | 1428.3121 | 1428.7076 | 1 | 5.413 | 64.3% | 3 | R.MGPAMGPALGAGIER.M | 2 |
|  | Astrin\_STLCLD20\_112214\_02.10587.10587.2 | 4.111 | 0.5118 | 100.0% | 1384.3121 | 1384.5677 | 1 | 9.846 | 75.0% | 10 | R.MGLAMGGGGGASFDR.A | 2 |
|  | Astrin\_STLCLD20\_112214\_02.10491.10491.3 | 3.6694 | 0.4714 | 100.0% | 2035.8544 | 2036.1735 | 1 | 6.57 | 34.1% | 3 | R.GNFGGSFAGSFGGAGGHAPGVAR.K | 3 |

---

|  |  |  |  |  |  |  |  |  |
| --- | --- | --- | --- | --- | --- | --- | --- | --- |
| U | *gi|14043070|ref|NP\_11* | 8 | 28 | 20.7% | 372 | 38747 | 9.1 | heterogeneous nuclear ribonucleoprotein A1 isoform b [Homo sapiens] |
| U | *gi|88958985|ref|XP\_94* | 8 | 28 | 25.6% | 301 | 32399 | 9.0 | PREDICTED: similar to heterogeneous nuclear ribonucleoprotein A1 [Homo sapiens] |
| U | *gi|88953883|ref|XP\_93* | 8 | 28 | 25.6% | 301 | 32380 | 8.9 | PREDICTED: similar to heterogeneous nuclear ribonucleoprotein A1 [Homo sapiens] |
| U | *gi|4504445|ref|NP\_002* | 8 | 28 | 24.1% | 320 | 34196 | 9.2 | heterogeneous nuclear ribonucleoprotein A1 isoform a [Homo sapiens] |
| U | *gi|169164476|ref|XP\_0* | 8 | 28 | 25.6% | 301 | 32399 | 9.0 | PREDICTED: similar to heterogeneous nuclear ribonucleoprotein A1 [Homo sapiens] |

| Filename XCorr DeltCN Conf% ObsM+H+ CalcM+H+ SpR ZScore Ion% # Sequence  | | | | | | | | | | | | |
| --- | --- | --- | --- | --- | --- | --- | --- | --- | --- | --- | --- | --- |
|  | Astrin\_STLCLD20\_112214\_tube2\_01.14348.14348.2 | 4.7721 | 0.4437 | 100.0% | 1913.6522 | 1914.1656 | 1 | 7.581 | 59.4% | 2 | R.KLFIGGLSFETTDESLR.S | 2 |
|  | Astrin\_STLCLD20\_112214\_02.13241.13241.3 | 3.918 | 0.4172 | 100.0% | 1913.8744 | 1914.1656 | 1 | 6.302 | 42.2% | 1 | R.KLFIGGLSFETTDESLR.S | 3 |
|  | Astrin\_STLCLD20\_112214\_02.15045.15045.2 | 5.2919 | 0.5374 | 100.0% | 1786.4922 | 1785.9916 | 1 | 8.909 | 70.0% | 10 | K.LFIGGLSFETTDESLR.S | 2 |
|  | Astrin\_STLCLD20\_112214\_tube2\_01.05668.05668.2 | 2.7633 | 0.2421 | 98.7% | 1566.5521 | 1566.7574 | 9 | 4.408 | 53.8% | 1 | R.EDSQRPGAHLTVKK.I | 2 |
|  | Astrin\_STLCLD20\_112214\_tube2\_01.13930.13930.2 | 3.3393 | 0.4234 | 100.0% | 1219.1721 | 1219.4387 | 1 | 7.693 | 88.9% | 6 | K.IEVIEIMTDR.G | 2 |
|  | Astrin\_STLCLD20\_112214\_tube2\_01.13103.13103.2 | 3.5452 | 0.2203 | 99.6% | 1701.5322 | 1700.8016 | 1 | 5.102 | 57.1% | 1 | R.GFAFVTFDDHDSVDK.I | 2 |
|  | Astrin\_STLCLD20\_112214\_02.12657.12657.3 | 3.9932 | 0.2856 | 99.7% | 2282.0645 | 2282.5579 | 1 | 6.697 | 36.8% | 2 | R.GFAFVTFDDHDSVDKIVIQK.Y | 3 |
|  | Astrin\_STLCLD20\_112214\_tube2\_01.07743.07743.2 | 3.8363 | 0.3779 | 100.0% | 1630.1322 | 1629.7721 | 1 | 7.415 | 60.0% | 5 | R.SSGPYGGGGQYFAKPR.N | 2 |

---

|  |  |  |  |  |  |  |  |  |
| --- | --- | --- | --- | --- | --- | --- | --- | --- |
| U | *gi|4506623|ref|NP\_000* | 3 | 4 | 20.6% | 136 | 15798 | 10.6 | ribosomal protein L27 [Homo sapiens] |

| Filename XCorr DeltCN Conf% ObsM+H+ CalcM+H+ SpR ZScore Ion% # Sequence  | | | | | | | | | | | | |
| --- | --- | --- | --- | --- | --- | --- | --- | --- | --- | --- | --- | --- |
| \* | Astrin\_STLCLD20\_112214\_01.08674.08674.1 | 1.8258 | 0.2726 | 97.8% | 826.56 | 827.058 | 1 | 5.575 | 71.4% | 1 | K.VVLVLAGR.Y | 1 |
|  | Astrin\_STLCLD20\_112214\_tube2\_01.08604.08604.2 | 3.2147 | 0.3582 | 100.0% | 1408.1522 | 1408.6177 | 1 | 7.302 | 75.0% | 2 | K.VYNYNHLMPTR.Y | 2 |
| \* | Astrin\_STLCLD20\_112214\_tube2\_01.10821.10821.2 | 2.008 | 0.2927 | 97.7% | 1050.3322 | 1050.1968 | 56 | 6.512 | 50.0% | 1 | R.YSVDIPLDK.T | 2 |

---

|  |  |  |  |  |  |  |  |  |
| --- | --- | --- | --- | --- | --- | --- | --- | --- |
| U | *gi|11024714|ref|NP\_06* | 4 | 10 | 20.5% | 229 | 25762 | 7.4 | ubiquitin B precursor [Homo sapiens] |
| U | *gi|77539055|ref|NP\_00* | 4 | 10 | 36.7% | 128 | 14728 | 9.8 | ubiquitin and ribosomal protein L40 precursor [Homo sapiens] |
| U | *gi|67191208|ref|NP\_06* | 4 | 10 | 6.9% | 685 | 77029 | 7.7 | ubiquitin C [Homo sapiens] |
| U | *gi|4507761|ref|NP\_003* | 4 | 10 | 36.7% | 128 | 14728 | 9.8 | ubiquitin and ribosomal protein L40 precursor [Homo sapiens] |
| U | *gi|4506713|ref|NP\_002* | 4 | 10 | 30.1% | 156 | 17965 | 9.6 | ubiquitin and ribosomal protein S27a precursor [Homo sapiens] |
| U | *gi|208022622|ref|NP\_0* | 4 | 10 | 30.1% | 156 | 17965 | 9.6 | ubiquitin and ribosomal protein S27a precursor [Homo sapiens] |

| Filename XCorr DeltCN Conf% ObsM+H+ CalcM+H+ SpR ZScore Ion% # Sequence  | | | | | | | | | | | | |
| --- | --- | --- | --- | --- | --- | --- | --- | --- | --- | --- | --- | --- |
|  | Astrin\_STLCLD20\_112214\_tube2\_01.11918.11918.2 | 3.9571 | 0.4315 | 100.0% | 1788.5122 | 1788.9897 | 1 | 7.304 | 56.7% | 6 | K.TITLEVEPSDTIENVK.A | 2 |
|  | Astrin\_STLCLD20\_112214\_tube2\_01.05832.05832.2 | 3.587 | 0.3876 | 100.0% | 1524.4122 | 1524.6738 | 1 | 7.514 | 79.2% | 1 | K.IQDKEGIPPDQQR.L | 2 |
|  | Astrin\_STLCLD20\_112214\_tube2\_01.07493.07493.2 | 1.923 | 0.2687 | 96.2% | 1082.3322 | 1082.1986 | 57 | 4.875 | 56.2% | 1 | R.TLSDYNIQK.E | 2 |
|  | Astrin\_STLCLD20\_112214\_tube2\_01.09656.09656.2 | 2.9144 | 0.3629 | 100.0% | 1068.2522 | 1068.2615 | 2 | 5.969 | 87.5% | 2 | K.ESTLHLVLR.L | 2 |

---

|  |  |  |  |  |  |  |  |  |
| --- | --- | --- | --- | --- | --- | --- | --- | --- |
| U | *gi|4758792|ref|NP\_004* | 4 | 4 | 20.2% | 124 | 13712 | 8.3 | NADH dehydrogenase (ubiquinone) Fe-S protein 6, 13kDa (NADH-coenzyme Q reductase) [Homo sapiens] |

| Filename XCorr DeltCN Conf% ObsM+H+ CalcM+H+ SpR ZScore Ion% # Sequence  | | | | | | | | | | | | |
| --- | --- | --- | --- | --- | --- | --- | --- | --- | --- | --- | --- | --- |
| \* | Astrin\_STLCLD20\_112214\_tube2\_01.05955.05955.3 | 3.2878 | 0.3325 | 99.8% | 1697.9944 | 1697.8015 | 13 | 5.01 | 36.5% | 1 | K.VTHTGQVYDDKDYR.R | 3 |
| \* | Astrin\_STLCLD20\_112214\_tube2\_01.05781.05781.2 | 3.2194 | 0.3586 | 100.0% | 1853.2522 | 1853.989 | 1 | 6.006 | 60.7% | 1 | K.VTHTGQVYDDKDYRR.I | 2 |
| \* | Astrin\_STLCLD20\_112214\_tube2\_01.05777.05777.3 | 2.8601 | 0.4235 | 99.8% | 1853.6044 | 1853.989 | 1 | 6.026 | 41.1% | 1 | K.VTHTGQVYDDKDYRR.I | 3 |
| \* | Astrin\_STLCLD20\_112214\_01.05161.05161.2 | 3.1012 | 0.2489 | 99.8% | 1223.3722 | 1223.4117 | 1 | 5.907 | 72.2% | 1 | K.VYINLDKETK.T | 2 |

---

|  |  |  |  |  |  |  |  |  |
| --- | --- | --- | --- | --- | --- | --- | --- | --- |
| U | *gi|56549636|ref|NP\_00* | 4 | 4 | 19.7% | 361 | 41487 | 6.6 | septin 2 [Homo sapiens] |
| U | *gi|56549640|ref|NP\_00* | 4 | 4 | 19.7% | 361 | 41487 | 6.6 | septin 2 [Homo sapiens] |
| U | *gi|56549638|ref|NP\_00* | 4 | 4 | 19.7% | 361 | 41487 | 6.6 | septin 2 [Homo sapiens] |

| Filename XCorr DeltCN Conf% ObsM+H+ CalcM+H+ SpR ZScore Ion% # Sequence  | | | | | | | | | | | | |
| --- | --- | --- | --- | --- | --- | --- | --- | --- | --- | --- | --- | --- |
|  | Astrin\_STLCLD20\_112214\_tube2\_02.06873.06873.2 | 3.8589 | 0.4 | 100.0% | 1604.0922 | 1604.7545 | 1 | 7.987 | 73.1% | 1 | R.TVQIEASTVEIEER.G | 2 |
|  | Astrin\_STLCLD20\_112214\_01.07247.07247.2 | 2.2217 | 0.1979 | 95.0% | 1352.2522 | 1353.515 | 62 | 4.716 | 60.0% | 1 | R.ILDEIEEHNIK.I | 2 |
|  | Astrin\_STLCLD20\_112214\_tube2\_01.15051.15051.3 | 3.7424 | 0.2454 | 99.5% | 2865.2344 | 2864.2202 | 23 | 5.412 | 29.5% | 1 | R.TMLITHMQDLQEVTQDLHYENFR.S | 3 |
|  | Astrin\_STLCLD20\_112214\_tube2\_02.06512.06512.3 | 4.5773 | 0.3482 | 99.7% | 2384.3342 | 2385.6675 | 1 | 7.289 | 36.4% | 1 | R.MQAQMQMQMQGGDGDGGALGHHV.- | 3 |

---

|  |  |  |  |  |  |  |  |  |
| --- | --- | --- | --- | --- | --- | --- | --- | --- |
| U | *gi|117189975|ref|NP\_1* | 4 | 14 | 19.6% | 306 | 33670 | 5.1 | heterogeneous nuclear ribonucleoprotein C isoform a [Homo sapiens] |
| U | *gi|117190254|ref|NP\_0* | 4 | 14 | 20.5% | 293 | 32338 | 5.1 | heterogeneous nuclear ribonucleoprotein C isoform b [Homo sapiens] |
| U | *gi|117190192|ref|NP\_0* | 4 | 14 | 19.6% | 306 | 33670 | 5.1 | heterogeneous nuclear ribonucleoprotein C isoform a [Homo sapiens] |
| U | *gi|117190174|ref|NP\_0* | 4 | 14 | 20.5% | 293 | 32338 | 5.1 | heterogeneous nuclear ribonucleoprotein C isoform b [Homo sapiens] |

| Filename XCorr DeltCN Conf% ObsM+H+ CalcM+H+ SpR ZScore Ion% # Sequence  | | | | | | | | | | | | |
| --- | --- | --- | --- | --- | --- | --- | --- | --- | --- | --- | --- | --- |
|  | Astrin\_STLCLD20\_112214\_tube2\_01.14291.14291.2 | 3.6881 | 0.2662 | 100.0% | 1318.3522 | 1317.6145 | 1 | 6.797 | 77.3% | 3 | R.VFIGNLNTLVVK.K | 2 |
|  | Astrin\_STLCLD20\_112214\_tube2\_02.08534.08534.2 | 3.6412 | 0.5197 | 100.0% | 1330.2922 | 1330.4857 | 1 | 8.244 | 80.0% | 4 | K.GFAFVQYVNER.N | 2 |
|  | Astrin\_STLCLD20\_112214\_02.13110.13110.2 | 4.6539 | 0.4564 | 100.0% | 1684.2922 | 1684.0038 | 1 | 8.233 | 80.0% | 6 | R.MIAGQVLDINLAAEPK.V | 2 |
|  | Astrin\_STLCLD20\_112214\_tube2\_01.05634.05634.3 | 4.0924 | 0.343 | 99.8% | 2369.3943 | 2369.4583 | 1 | 5.555 | 45.0% | 1 | K.NDKSEEEQSSSSVKKDETNVK.M | 3 |

---

|  |  |  |  |  |  |  |  |  |
| --- | --- | --- | --- | --- | --- | --- | --- | --- |
| U | *gi|32455264|ref|NP\_85* | 4 | 14 | 19.6% | 199 | 22110 | 8.1 | peroxiredoxin 1 [Homo sapiens] |
| U | *gi|4505591|ref|NP\_002* | 4 | 14 | 19.6% | 199 | 22110 | 8.1 | peroxiredoxin 1 [Homo sapiens] |
| U | *gi|32455266|ref|NP\_85* | 4 | 14 | 19.6% | 199 | 22110 | 8.1 | peroxiredoxin 1 [Homo sapiens] |

| Filename XCorr DeltCN Conf% ObsM+H+ CalcM+H+ SpR ZScore Ion% # Sequence  | | | | | | | | | | | | |
| --- | --- | --- | --- | --- | --- | --- | --- | --- | --- | --- | --- | --- |
|  | Astrin\_STLCLD20\_112214\_tube2\_01.09722.09722.2 | 3.0434 | 0.2851 | 99.9% | 1108.1921 | 1108.2798 | 1 | 6.364 | 83.3% | 3 | R.TIAQDYGVLK.A | 2 |
|  | Astrin\_STLCLD20\_112214\_02.11777.11777.3 | 3.6397 | 0.4218 | 99.7% | 1984.6444 | 1984.2163 | 1 | 6.198 | 42.6% | 3 | R.TIAQDYGVLKADEGISFR.G | 3 |
|  | Astrin\_STLCLD20\_112214\_tube2\_01.10262.10262.2 | 2.9791 | 0.2073 | 99.3% | 1212.5122 | 1212.3915 | 3 | 6.522 | 75.0% | 5 | R.QITVNDLPVGR.S | 22 |
|  | Astrin\_STLCLD20\_112214\_tube2\_01.11576.11576.2 | 3.3148 | 0.4837 | 100.0% | 1197.6122 | 1197.3763 | 1 | 7.744 | 88.9% | 3 | R.LVQAFQFTDK.H | 2 |

Similarities:
gi|32189392|ref|NP\_00(1:3)  

---

|  |  |  |  |  |  |  |  |  |
| --- | --- | --- | --- | --- | --- | --- | --- | --- |
| U | *gi|17986258|ref|NP\_06* | 2 | 4 | 19.2% | 151 | 16930 | 4.7 | myosin, light chain 6, alkali, smooth muscle and non-muscle isoform 1 [Homo sapiens] |
| U | *gi|88999583|ref|NP\_52* | 2 | 4 | 19.2% | 151 | 16961 | 4.6 | myosin, light chain 6, alkali, smooth muscle and non-muscle isoform 2 [Homo sapiens] |

| Filename XCorr DeltCN Conf% ObsM+H+ CalcM+H+ SpR ZScore Ion% # Sequence  | | | | | | | | | | | | |
| --- | --- | --- | --- | --- | --- | --- | --- | --- | --- | --- | --- | --- |
|  | Astrin\_STLCLD20\_112214\_01.05887.05887.2 | 3.3552 | 0.3479 | 100.0% | 1355.1721 | 1355.5339 | 1 | 5.895 | 66.7% | 3 | R.ALGQNPTNAEVLK.V | 2 |
|  | Astrin\_STLCLD20\_112214\_01.14357.14357.2 | 3.2261 | 0.3214 | 99.9% | 1889.8121 | 1889.2628 | 1 | 6.422 | 50.0% | 1 | K.VLDFEHFLPMLQTVAK.N | 2 |

---

|  |  |  |  |  |  |  |  |  |
| --- | --- | --- | --- | --- | --- | --- | --- | --- |
| U | *gi|87196351|ref|NP\_00* | 10 | 21 | 18.9% | 662 | 73244 | 7.2 | DEAD/H (Asp-Glu-Ala-Asp/His) box polypeptide 3 [Homo sapiens] |

| Filename XCorr DeltCN Conf% ObsM+H+ CalcM+H+ SpR ZScore Ion% # Sequence  | | | | | | | | | | | | |
| --- | --- | --- | --- | --- | --- | --- | --- | --- | --- | --- | --- | --- |
| \* | Astrin\_STLCLD20\_112214\_tube2\_01.20427.20427.2 | 4.5757 | 0.5178 | 100.0% | 2332.8523 | 2333.6897 | 1 | 9.311 | 47.6% | 2 | K.TAAFLLPILSQIYSDGPGEALR.A | 2 |
| \* | Astrin\_STLCLD20\_112214\_tube2\_01.20415.20415.3 | 3.8626 | 0.2726 | 99.5% | 2333.6643 | 2333.6897 | 5 | 5.303 | 29.8% | 1 | K.TAAFLLPILSQIYSDGPGEALR.A | 3 |
|  | Astrin\_STLCLD20\_112214\_tube2\_02.07622.07622.2 | 3.2353 | 0.4493 | 100.0% | 1321.3722 | 1321.4729 | 1 | 7.761 | 80.0% | 2 | R.ELAVQIYEEAR.K | 2 |
|  | Astrin\_STLCLD20\_112214\_01.08672.08672.2 | 2.2595 | 0.1903 | 96.7% | 1094.3322 | 1094.2096 | 1 | 4.808 | 81.2% | 1 | K.YLVLDEADR.M | 2 |
|  | Astrin\_STLCLD20\_112214\_01.11336.11336.2 | 4.1408 | 0.4316 | 100.0% | 1338.4122 | 1337.5946 | 1 | 7.877 | 85.0% | 4 | R.MLDMGFEPQIR.R | 22 |
|  | Astrin\_STLCLD20\_112214\_tube2\_02.12001.12001.2 | 2.6797 | 0.3731 | 99.8% | 1558.4321 | 1558.774 | 9 | 7.036 | 50.0% | 1 | R.DFLDEYIFLAVGR.V | 2 |
|  | Astrin\_STLCLD20\_112214\_tube2\_02.07282.07282.2 | 3.145 | 0.2981 | 99.9% | 1168.5521 | 1169.4099 | 1 | 7.12 | 68.2% | 3 | K.SPILVATAVAAR.G | 2 |
|  | Astrin\_STLCLD20\_112214\_01.12056.12056.3 | 4.6302 | 0.475 | 100.0% | 2084.3943 | 2084.2957 | 1 | 7.668 | 42.2% | 3 | K.HVINFDLPSDIEEYVHR.I | 3 |
| \* | Astrin\_STLCLD20\_112214\_tube2\_01.15556.15556.2 | 4.1252 | 0.4429 | 100.0% | 1526.2522 | 1525.7043 | 1 | 7.419 | 73.1% | 3 | R.VGNLGLATSFFNER.N | 2 |
| \* | Astrin\_STLCLD20\_112214\_tube2\_01.19626.19626.2 | 2.9849 | 0.4816 | 100.0% | 1812.8322 | 1813.1448 | 6 | 8.139 | 46.7% | 1 | R.NINITKDLLDLLVEAK.Q | 2 |

Similarities:
gi|4758138|ref|NP\_004(1:9)  

---

|  |  |  |  |  |  |  |  |  |
| --- | --- | --- | --- | --- | --- | --- | --- | --- |
| U | *gi|19920317|ref|NP\_00* | 7 | 12 | 18.8% | 602 | 66023 | 5.9 | cytoskeleton-associated protein 4 [Homo sapiens] |

| Filename XCorr DeltCN Conf% ObsM+H+ CalcM+H+ SpR ZScore Ion% # Sequence  | | | | | | | | | | | | |
| --- | --- | --- | --- | --- | --- | --- | --- | --- | --- | --- | --- | --- |
| \* | Astrin\_STLCLD20\_112214\_tube2\_01.19055.19055.2 | 3.7145 | 0.4323 | 100.0% | 1797.7922 | 1798.049 | 1 | 7.181 | 53.3% | 2 | K.VQSLQATFGTFESILR.S | 2 |
| \* | Astrin\_STLCLD20\_112214\_tube2\_01.07522.07522.2 | 2.9519 | 0.3464 | 100.0% | 1288.0521 | 1288.4026 | 31 | 5.914 | 65.0% | 1 | R.HSEAFEALQQK.S | 2 |
| \* | Astrin\_STLCLD20\_112214\_02.10188.10188.3 | 3.3559 | 0.3786 | 99.8% | 1842.1144 | 1841.0923 | 1 | 6.135 | 42.2% | 1 | R.LQHVEDGVLSMQVASAR.Q | 3 |
| \* | Astrin\_STLCLD20\_112214\_02.10215.10215.2 | 2.9398 | 0.3595 | 99.9% | 1906.1721 | 1906.0135 | 1 | 6.86 | 41.7% | 1 | R.LEGLGSSEADQDGLASTVR.S | 2 |
| \* | Astrin\_STLCLD20\_112214\_tube2\_02.08778.08778.3 | 2.9238 | 0.2936 | 99.0% | 2051.4844 | 2050.3164 | 10 | 4.949 | 30.9% | 1 | R.SLGETQLVLYGDVEELKR.S | 3 |
| \* | Astrin\_STLCLD20\_112214\_01.09213.09213.2 | 3.2408 | 0.3424 | 100.0% | 1474.3722 | 1474.6512 | 10 | 5.592 | 53.8% | 2 | R.SVGELPSTVESLQK.V | 2 |
| \* | Astrin\_STLCLD20\_112214\_tube2\_02.06056.06056.3 | 3.8499 | 0.2715 | 99.5% | 2023.2544 | 2023.2131 | 8 | 4.799 | 32.4% | 4 | K.VQEQVHTLLSQDQAQAAR.L | 3 |

---

|  |  |  |  |  |  |  |  |  |
| --- | --- | --- | --- | --- | --- | --- | --- | --- |
| U | *gi|4505773|ref|NP\_002* | 3 | 3 | 18.8% | 272 | 29804 | 5.8 | prohibitin [Homo sapiens] |

| Filename XCorr DeltCN Conf% ObsM+H+ CalcM+H+ SpR ZScore Ion% # Sequence  | | | | | | | | | | | | |
| --- | --- | --- | --- | --- | --- | --- | --- | --- | --- | --- | --- | --- |
| \* | Astrin\_STLCLD20\_112214\_01.09515.09515.2 | 2.8121 | 0.2586 | 99.3% | 1398.0521 | 1397.7067 | 4 | 5.089 | 63.6% | 1 | R.ILFRPVASQLPR.I | 2 |
| \* | Astrin\_STLCLD20\_112214\_01.09895.09895.2 | 2.6741 | 0.2631 | 99.3% | 1150.6522 | 1150.2767 | 7 | 6.162 | 66.7% | 1 | R.FDAGELITQR.E | 2 |
| \* | Astrin\_STLCLD20\_112214\_tube2\_02.11209.11209.3 | 3.8672 | 0.2809 | 99.5% | 3126.6843 | 3125.5457 | 2 | 4.698 | 25.0% | 1 | R.AATFGLILDDVSLTHLTFGKEFTEAVEAK.Q | 3 |

---

|  |  |  |  |  |  |  |  |  |
| --- | --- | --- | --- | --- | --- | --- | --- | --- |
| U | *gi|4506743|ref|NP\_001* | 3 | 9 | 18.8% | 208 | 24205 | 10.3 | ribosomal protein S8 [Homo sapiens] |

| Filename XCorr DeltCN Conf% ObsM+H+ CalcM+H+ SpR ZScore Ion% # Sequence  | | | | | | | | | | | | |
| --- | --- | --- | --- | --- | --- | --- | --- | --- | --- | --- | --- | --- |
| \* | Astrin\_STLCLD20\_112214\_tube2\_02.07838.07838.2 | 2.3495 | 0.2804 | 97.7% | 1720.3322 | 1719.9353 | 1 | 4.572 | 50.0% | 1 | R.IIDVVYNASNNELVR.T | 2 |
|  | Astrin\_STLCLD20\_112214\_01.08348.08348.2 | 2.5987 | 0.3235 | 99.5% | 1315.2522 | 1315.4631 | 2 | 5.185 | 65.0% | 1 | K.LTPEEEEILNK.K | 2 |
| \* | Astrin\_STLCLD20\_112214\_tube2\_02.07575.07575.2 | 3.9291 | 0.4041 | 100.0% | 1508.5322 | 1507.6836 | 1 | 6.99 | 75.0% | 7 | K.ISSLLEEQFQQGK.L | 2 |

---

|  |  |  |  |  |  |  |  |  |
| --- | --- | --- | --- | --- | --- | --- | --- | --- |
| U | *gi|33286418|ref|NP\_00* | 6 | 10 | 18.6% | 531 | 57937 | 7.8 | pyruvate kinase, muscle isoform M2 [Homo sapiens] |
| U | *gi|33286422|ref|NP\_87* | 6 | 10 | 18.6% | 531 | 58062 | 7.7 | pyruvate kinase, muscle isoform M1 [Homo sapiens] |
| U | *gi|33286420|ref|NP\_87* | 6 | 10 | 18.6% | 531 | 58062 | 7.7 | pyruvate kinase, muscle isoform M1 [Homo sapiens] |

| Filename XCorr DeltCN Conf% ObsM+H+ CalcM+H+ SpR ZScore Ion% # Sequence  | | | | | | | | | | | | |
| --- | --- | --- | --- | --- | --- | --- | --- | --- | --- | --- | --- | --- |
|  | Astrin\_STLCLD20\_112214\_tube2\_01.09890.09890.2 | 3.3459 | 0.3238 | 100.0% | 1198.4722 | 1198.3617 | 2 | 6.845 | 80.0% | 3 | R.LDIDSPPITAR.N | 2 |
|  | Astrin\_STLCLD20\_112214\_tube2\_01.06616.06616.3 | 3.5863 | 0.3871 | 99.7% | 1884.3544 | 1885.0458 | 1 | 6.34 | 41.7% | 1 | R.LNFSHGTHEYHAETIK.N | 3 |
|  | Astrin\_STLCLD20\_112214\_tube2\_01.13895.13895.2 | 2.8935 | 0.274 | 99.3% | 1780.5322 | 1780.9292 | 3 | 4.885 | 44.1% | 1 | K.GADFLVTEVENGGSLGSK.K | 2 |
|  | Astrin\_STLCLD20\_112214\_tube2\_01.20558.20558.2 | 3.6399 | 0.5348 | 100.0% | 1860.7122 | 1861.1224 | 1 | 8.662 | 66.7% | 3 | K.FGVEQDVDMVFASFIR.K | 2 |
|  | Astrin\_STLCLD20\_112214\_01.11403.11403.2 | 2.8914 | 0.1979 | 98.3% | 1823.9521 | 1823.0741 | 1 | 4.637 | 53.3% | 1 | R.RFDEILEASDGIMVAR.G | 2 |
|  | Astrin\_STLCLD20\_112214\_tube2\_01.13672.13672.3 | 3.7788 | 0.1958 | 97.8% | 2392.7043 | 2392.7815 | 1 | 4.149 | 36.9% | 1 | K.KGDVVIVLTGWRPGSGFTNTMR.V | 3 |

---

|  |  |  |  |  |  |  |  |  |
| --- | --- | --- | --- | --- | --- | --- | --- | --- |
| U | *gi|4506741|ref|NP\_001* | 2 | 2 | 18.6% | 194 | 22127 | 10.1 | ribosomal protein S7 [Homo sapiens] |

| Filename XCorr DeltCN Conf% ObsM+H+ CalcM+H+ SpR ZScore Ion% # Sequence  | | | | | | | | | | | | |
| --- | --- | --- | --- | --- | --- | --- | --- | --- | --- | --- | --- | --- |
| \* | Astrin\_STLCLD20\_112214\_tube2\_01.14750.14750.2 | 2.8793 | 0.1971 | 98.6% | 1466.1522 | 1466.8937 | 2 | 5.165 | 58.3% | 1 | R.KAIIIFVPVPQLK.S | 2 |
| \* | Astrin\_STLCLD20\_112214\_tube2\_01.17279.17279.3 | 4.9758 | 0.4322 | 100.0% | 2523.8342 | 2524.92 | 1 | 7.689 | 37.5% | 1 | R.TLTAVHDAILEDLVFPSEIVGKR.I | 3 |

---

|  |  |  |  |  |  |  |  |  |
| --- | --- | --- | --- | --- | --- | --- | --- | --- |
| U | *gi|148470397|ref|NP\_0* | 4 | 10 | 18.3% | 415 | 45672 | 5.6 | heterogeneous nuclear ribonucleoprotein F [Homo sapiens] |
| U | *gi|4826760|ref|NP\_004* | 4 | 10 | 18.3% | 415 | 45672 | 5.6 | heterogeneous nuclear ribonucleoprotein F [Homo sapiens] |
| U | *gi|148470406|ref|NP\_0* | 4 | 10 | 18.3% | 415 | 45672 | 5.6 | heterogeneous nuclear ribonucleoprotein F [Homo sapiens] |
| U | *gi|148470404|ref|NP\_0* | 4 | 10 | 18.3% | 415 | 45672 | 5.6 | heterogeneous nuclear ribonucleoprotein F [Homo sapiens] |
| U | *gi|148470402|ref|NP\_0* | 4 | 10 | 18.3% | 415 | 45672 | 5.6 | heterogeneous nuclear ribonucleoprotein F [Homo sapiens] |
| U | *gi|148470400|ref|NP\_0* | 4 | 10 | 18.3% | 415 | 45672 | 5.6 | heterogeneous nuclear ribonucleoprotein F [Homo sapiens] |

| Filename XCorr DeltCN Conf% ObsM+H+ CalcM+H+ SpR ZScore Ion% # Sequence  | | | | | | | | | | | | |
| --- | --- | --- | --- | --- | --- | --- | --- | --- | --- | --- | --- | --- |
|  | Astrin\_STLCLD20\_112214\_tube2\_01.17202.17202.2 | 5.5366 | 0.3923 | 100.0% | 1869.4922 | 1869.0813 | 1 | 8.299 | 62.5% | 3 | K.ITGEAFVQFASQELAEK.A | 2 |
|  | Astrin\_STLCLD20\_112214\_tube2\_02.09829.09829.3 | 6.785 | 0.5165 | 100.0% | 3476.4243 | 3476.7114 | 1 | 8.588 | 30.6% | 3 | R.MRPGAYSTGYGGYEEYSGLSDGYGFTTDLFGR.D | 3 |
|  | Astrin\_STLCLD20\_112214\_tube2\_01.18082.18082.2 | 3.6227 | 0.441 | 100.0% | 1998.5721 | 1998.2023 | 1 | 7.571 | 53.1% | 2 | K.ATENDIYNFFSPLNPVR.V | 22 |
|  | Astrin\_STLCLD20\_112214\_tube2\_01.07737.07737.2 | 3.2067 | 0.508 | 100.0% | 1093.4122 | 1093.2278 | 1 | 8.327 | 83.3% | 2 | R.VHIEIGPDGR.V | 22 |

Similarities:
gi|5031753|ref|NP\_005(2:2)  

---

|  |  |  |  |  |  |  |  |  |
| --- | --- | --- | --- | --- | --- | --- | --- | --- |
| U | *gi|21464101|ref|NP\_03* | 2 | 3 | 18.2% | 247 | 28303 | 4.9 | tyrosine 3-monooxygenase/tryptophan 5-monooxygenase activation protein, gamma polypeptide [Homo sapiens] |

| Filename XCorr DeltCN Conf% ObsM+H+ CalcM+H+ SpR ZScore Ion% # Sequence  | | | | | | | | | | | | |
| --- | --- | --- | --- | --- | --- | --- | --- | --- | --- | --- | --- | --- |
| \* | Astrin\_STLCLD20\_112214\_tube2\_02.08598.08598.2 | 3.0354 | 0.1316 | 97.1% | 1797.4722 | 1798.8473 | 67 | 3.4 | 43.3% | 1 | R.VISS\*IEQK@TSADGNEK.K | 2 |
| \* | Astrin\_STLCLD20\_112214\_01.17873.17873.3 | 4.3857 | 0.3018 | 99.8% | 3304.6143 | 3303.6626 | 1 | 6.084 | 27.7% | 2 | K.TAFDDAIAELDTLNEDSYKDSTLIMQLLR.D | 3 |

---

|  |  |  |  |  |  |  |  |  |
| --- | --- | --- | --- | --- | --- | --- | --- | --- |
| U | *gi|5031753|ref|NP\_005* | 5 | 11 | 18.0% | 449 | 49229 | 6.3 | heterogeneous nuclear ribonucleoprotein H1 [Homo sapiens] |

| Filename XCorr DeltCN Conf% ObsM+H+ CalcM+H+ SpR ZScore Ion% # Sequence  | | | | | | | | | | | | |
| --- | --- | --- | --- | --- | --- | --- | --- | --- | --- | --- | --- | --- |
|  | Astrin\_STLCLD20\_112214\_tube2\_01.06706.06706.2 | 4.0054 | 0.5992 | 100.0% | 1685.1322 | 1685.7501 | 1 | 9.839 | 76.7% | 1 | K.HTGPNSPDTANDGFVR.L | 2 |
|  | Astrin\_STLCLD20\_112214\_tube2\_01.14956.14956.2 | 4.7741 | 0.5255 | 100.0% | 1842.2322 | 1843.0001 | 1 | 9.469 | 65.6% | 5 | R.STGEAFVQFASQEIAEK.A | 2 |
|  | Astrin\_STLCLD20\_112214\_tube2\_01.18082.18082.2 | 3.6227 | 0.441 | 100.0% | 1998.5721 | 1998.2023 | 1 | 7.571 | 53.1% | 2 | R.ATENDIYNFFSPLNPVR.V | 22 |
|  | Astrin\_STLCLD20\_112214\_tube2\_01.07737.07737.2 | 3.2067 | 0.508 | 100.0% | 1093.4122 | 1093.2278 | 1 | 8.327 | 83.3% | 2 | R.VHIEIGPDGR.V | 22 |
| \* | Astrin\_STLCLD20\_112214\_02.10489.10489.3 | 2.6858 | 0.3843 | 99.5% | 2179.8245 | 2179.363 | 1 | 5.319 | 36.2% | 1 | R.VTGEADVEFATHEDAVAAMSK.D | 3 |

Similarities:
gi|148470397|ref|NP\_0(2:3)  

---

|  |  |  |  |  |  |  |  |  |
| --- | --- | --- | --- | --- | --- | --- | --- | --- |
| U | *gi|15809016|ref|NP\_29* | 3 | 6 | 18.0% | 172 | 19779 | 4.8 | myosin regulatory light chain MRCL2 isoform A [Homo sapiens] |
| U | *gi|5453740|ref|NP\_006* | 3 | 6 | 18.1% | 171 | 19794 | 4.8 | myosin, light chain 12A, regulatory, non-sarcomeric [Homo sapiens] |
| U | *gi|222144328|ref|NP\_0* | 3 | 6 | 20.1% | 154 | 17757 | 4.4 | myosin regulatory light chain MRCL2 isoform B [Homo sapiens] |
| U | *gi|222144326|ref|NP\_0* | 3 | 6 | 18.0% | 172 | 19779 | 4.8 | myosin regulatory light chain MRCL2 isoform A [Homo sapiens] |
| U | *gi|222144324|ref|NP\_0* | 3 | 6 | 18.0% | 172 | 19779 | 4.8 | myosin regulatory light chain MRCL2 isoform A [Homo sapiens] |

| Filename XCorr DeltCN Conf% ObsM+H+ CalcM+H+ SpR ZScore Ion% # Sequence  | | | | | | | | | | | | |
| --- | --- | --- | --- | --- | --- | --- | --- | --- | --- | --- | --- | --- |
|  | Astrin\_STLCLD20\_112214\_tube2\_01.08741.08741.2 | 2.8659 | 0.2328 | 99.3% | 1229.5922 | 1229.3324 | 6 | 5.195 | 65.0% | 1 | K.LNGTDPEDVIR.N | 2 |
|  | Astrin\_STLCLD20\_112214\_tube2\_01.14668.14668.3 | 4.4127 | 0.3918 | 100.0% | 2433.4744 | 2433.649 | 1 | 6.504 | 38.2% | 3 | R.ELLTTMGDRFTDEEVDELYR.E | 3 |
|  | Astrin\_STLCLD20\_112214\_01.09869.09869.2 | 3.3094 | 0.3842 | 100.0% | 1417.2122 | 1416.4839 | 1 | 6.479 | 65.0% | 2 | R.FTDEEVDELYR.E | 2 |

---

|  |  |  |  |  |  |  |  |  |
| --- | --- | --- | --- | --- | --- | --- | --- | --- |
| U | *gi|4503483|ref|NP\_001* | 12 | 21 | 17.9% | 858 | 95338 | 6.8 | eukaryotic translation elongation factor 2 [Homo sapiens] |

| Filename XCorr DeltCN Conf% ObsM+H+ CalcM+H+ SpR ZScore Ion% # Sequence  | | | | | | | | | | | | |
| --- | --- | --- | --- | --- | --- | --- | --- | --- | --- | --- | --- | --- |
| \* | Astrin\_STLCLD20\_112214\_tube2\_01.06542.06542.2 | 2.6912 | 0.309 | 99.4% | 1307.3922 | 1308.4979 | 133 | 6.142 | 54.5% | 1 | R.NMSVIAHVDHGK.S | 2 |
| \* | Astrin\_STLCLD20\_112214\_01.14132.14132.2 | 3.3478 | 0.2499 | 99.7% | 2222.652 | 2221.5151 | 5 | 4.938 | 41.2% | 1 | R.ALLELQLEPEELYQTFQR.I | 2 |
| \* | Astrin\_STLCLD20\_112214\_tube2\_01.19284.19284.3 | 5.1017 | 0.3406 | 99.7% | 2757.9243 | 2758.2976 | 1 | 6.244 | 36.5% | 1 | R.RWLPAGDALLQMITIHLPSPVTAQK.Y | 3 |
| \* | Astrin\_STLCLD20\_112214\_tube2\_01.12167.12167.2 | 1.7851 | 0.3789 | 98.0% | 1040.6122 | 1040.3241 | 66 | 6.005 | 56.2% | 1 | K.GPLMMYISK.M | 2 |
| \* | Astrin\_STLCLD20\_112214\_01.10083.10083.2 | 3.0172 | 0.3714 | 100.0% | 1108.1522 | 1108.3231 | 1 | 7.223 | 75.0% | 3 | R.VFSGLVSTGLK.V | 2 |
| \* | Astrin\_STLCLD20\_112214\_tube2\_01.07274.07274.2 | 4.2458 | 0.5054 | 100.0% | 1616.2122 | 1616.7917 | 1 | 8.957 | 57.7% | 1 | K.TGTITTFEHAHNMR.V | 2 |
| \* | Astrin\_STLCLD20\_112214\_01.09825.09825.3 | 5.5735 | 0.4055 | 100.0% | 2143.7344 | 2144.3489 | 1 | 8.269 | 43.4% | 7 | K.ARPFPDGLAEDIDKGEVSAR.Q | 3 |
| \* | Astrin\_STLCLD20\_112214\_02.10126.10126.3 | 3.2024 | 0.2954 | 99.6% | 1969.4343 | 1971.1796 | 57 | 4.538 | 33.3% | 1 | R.ARYLAEKYEWDVAEAR.K | 3 |
| \* | Astrin\_STLCLD20\_112214\_tube2\_02.07335.07335.3 | 2.8702 | 0.3433 | 99.5% | 1743.7144 | 1743.9133 | 1 | 5.731 | 51.9% | 1 | R.YLAEKYEWDVAEAR.K | 3 |
| \* | Astrin\_STLCLD20\_112214\_tube2\_01.10478.10478.2 | 1.9764 | 0.3184 | 98.0% | 1139.0122 | 1139.2096 | 7 | 5.832 | 75.0% | 1 | K.YEWDVAEAR.K | 2 |
| \* | Astrin\_STLCLD20\_112214\_02.14511.14511.2 | 3.3201 | 0.4104 | 100.0% | 1801.4122 | 1801.0087 | 1 | 7.185 | 66.7% | 1 | K.AYLPVNESFGFTADLR.S | 2 |
| \* | Astrin\_STLCLD20\_112214\_tube2\_01.19799.19799.2 | 3.3217 | 0.4781 | 100.0% | 1446.6522 | 1445.6555 | 1 | 7.089 | 62.5% | 2 | K.EGIPALDNFLDKL.- | 2 |

---

|  |  |  |  |  |  |  |  |  |
| --- | --- | --- | --- | --- | --- | --- | --- | --- |
| U | *gi|15718687|ref|NP\_00* | 3 | 8 | 17.7% | 243 | 26688 | 9.7 | ribosomal protein S3 [Homo sapiens] |

| Filename XCorr DeltCN Conf% ObsM+H+ CalcM+H+ SpR ZScore Ion% # Sequence  | | | | | | | | | | | | |
| --- | --- | --- | --- | --- | --- | --- | --- | --- | --- | --- | --- | --- |
| \* | Astrin\_STLCLD20\_112214\_tube2\_01.08662.08662.2 | 3.8919 | 0.3792 | 100.0% | 1424.2122 | 1424.5071 | 1 | 8.321 | 83.3% | 5 | R.ELAEDGYSGVEVR.V | 2 |
| \* | Astrin\_STLCLD20\_112214\_tube2\_01.13858.13858.2 | 2.5851 | 0.2747 | 98.7% | 1573.5721 | 1573.7423 | 1 | 6.282 | 69.2% | 1 | R.FGFPEGSVELYAEK.V | 2 |
| \* | Astrin\_STLCLD20\_112214\_01.07665.07665.2 | 3.165 | 0.3893 | 100.0% | 1574.4521 | 1574.8352 | 1 | 5.915 | 56.7% | 2 | K.GGKPEPPAMPQPVPTA.- | 2 |

---

|  |  |  |  |  |  |  |  |  |
| --- | --- | --- | --- | --- | --- | --- | --- | --- |
| U | *gi|4758138|ref|NP\_004* | 10 | 23 | 17.4% | 614 | 69148 | 8.9 | DEAD (Asp-Glu-Ala-Asp) box polypeptide 5 [Homo sapiens] |

| Filename XCorr DeltCN Conf% ObsM+H+ CalcM+H+ SpR ZScore Ion% # Sequence  | | | | | | | | | | | | |
| --- | --- | --- | --- | --- | --- | --- | --- | --- | --- | --- | --- | --- |
| \* | Astrin\_STLCLD20\_112214\_01.05816.05816.2 | 3.1001 | 0.3796 | 100.0% | 1389.5122 | 1390.4978 | 1 | 6.866 | 65.0% | 1 | K.NFYQEHPDLAR.R | 2 |
| \* | Astrin\_STLCLD20\_112214\_tube2\_01.05714.05714.3 | 2.7174 | 0.2256 | 96.7% | 1410.2644 | 1409.5448 | 4 | 5.271 | 37.5% | 1 | R.RTAQEVETYRR.S | 3 |
| \* | Astrin\_STLCLD20\_112214\_tube2\_01.11259.11259.2 | 3.535 | 0.4847 | 100.0% | 1296.1921 | 1296.4198 | 1 | 8.492 | 75.0% | 4 | R.TTYLVLDEADR.M | 2 |
|  | Astrin\_STLCLD20\_112214\_01.11336.11336.2 | 4.1408 | 0.4316 | 100.0% | 1338.4122 | 1337.5946 | 1 | 7.877 | 85.0% | 4 | R.MLDMGFEPQIR.K | 22 |
|  | Astrin\_STLCLD20\_112214\_tube2\_01.11244.11244.2 | 2.6476 | 0.2499 | 98.8% | 1466.4521 | 1465.7688 | 406 | 4.569 | 45.5% | 1 | R.MLDMGFEPQIRK.I | 2 |
|  | Astrin\_STLCLD20\_112214\_01.11839.11839.2 | 2.3141 | 0.2075 | 96.7% | 1350.0122 | 1349.5902 | 128 | 4.927 | 50.0% | 1 | R.QTLMWSATWPK.E | 2 |
|  | Astrin\_STLCLD20\_112214\_tube2\_01.10916.10916.2 | 4.1393 | 0.3247 | 100.0% | 1228.5922 | 1227.4465 | 1 | 6.899 | 77.3% | 7 | K.APILIATDVASR.G | 2 |
| \* | Astrin\_STLCLD20\_112214\_01.10442.10442.3 | 3.3089 | 0.1841 | 95.9% | 2133.0544 | 2133.281 | 194 | 4.553 | 29.7% | 1 | K.FVINYDYPNSSEDYIHR.I | 3 |
| \* | Astrin\_STLCLD20\_112214\_01.11093.11093.2 | 2.9017 | 0.3042 | 99.5% | 1576.1721 | 1575.7612 | 1 | 6.012 | 65.4% | 2 | K.TGTAYTFFTPNNIK.Q | 2 |
| \* | Astrin\_STLCLD20\_112214\_01.09097.09097.2 | 2.4326 | 0.1691 | 97.9% | 986.1322 | 986.1564 | 1 | 5.318 | 85.7% | 1 | K.LLQLVEDR.G | 2 |

Similarities:
gi|87196351|ref|NP\_00(1:9)  

---

|  |  |  |  |  |  |  |  |  |
| --- | --- | --- | --- | --- | --- | --- | --- | --- |
| U | *gi|4506903|ref|NP\_003* | 4 | 6 | 17.2% | 221 | 25542 | 8.6 | splicing factor, arginine/serine-rich 9 [Homo sapiens] |

| Filename XCorr DeltCN Conf% ObsM+H+ CalcM+H+ SpR ZScore Ion% # Sequence  | | | | | | | | | | | | |
| --- | --- | --- | --- | --- | --- | --- | --- | --- | --- | --- | --- | --- |
| \* | Astrin\_STLCLD20\_112214\_tube2\_01.10526.10526.2 | 3.1087 | 0.3355 | 100.0% | 1247.4722 | 1247.4368 | 1 | 6.661 | 75.0% | 3 | R.IYVGNLPTDVR.E | 2 |
| \* | Astrin\_STLCLD20\_112214\_tube2\_01.12360.12360.2 | 2.6465 | 0.2784 | 99.4% | 1300.1322 | 1300.4509 | 82 | 5.778 | 55.6% | 1 | R.EKDLEDLFYK.Y | 2 |
| \* | Astrin\_STLCLD20\_112214\_01.11308.11308.2 | 2.3433 | 0.4045 | 99.8% | 1143.8922 | 1143.3768 | 3 | 6.396 | 66.7% | 1 | R.HGLVPFAFVR.F | 2 |
|  | Astrin\_STLCLD20\_112214\_01.07489.07489.2 | 2.3638 | 0.1102 | 96.4% | 917.47217 | 917.0989 | 2 | 4.174 | 83.3% | 1 | R.LRVEFPR.T | 22 |

Similarities:
gi|118582269|ref|NP\_0(1:3)  

---

|  |  |  |  |  |  |  |  |  |
| --- | --- | --- | --- | --- | --- | --- | --- | --- |
| U | *contaminant\_KERATIN05* | 12 | 21 | 17.0% | 471 | 51531 | 5.2 | no description |
| U | *gi|15431310|ref|NP\_00* | 12 | 21 | 16.9% | 472 | 51622 | 5.2 | keratin 14 [Homo sapiens] |

| Filename XCorr DeltCN Conf% ObsM+H+ CalcM+H+ SpR ZScore Ion% # Sequence  | | | | | | | | | | | | |
| --- | --- | --- | --- | --- | --- | --- | --- | --- | --- | --- | --- | --- |
|  | Astrin\_STLCLD20\_112214\_01.05697.05697.2 | 2.9151 | 0.1301 | 98.6% | 1065.6322 | 1065.2578 | 20 | 6.487 | 68.8% | 1 | R.LASYLDKVR.A | 2222 |
|  | Astrin\_STLCLD20\_112214\_02.09281.09281.2 | 2.612 | 0.2844 | 99.2% | 1301.8722 | 1302.4241 | 4 | 6.246 | 59.1% | 1 | R.ALEEANADLEVK.I | 2 |
|  | Astrin\_STLCLD20\_112214\_tube2\_01.07546.07546.2 | 2.2441 | 0.2268 | 98.6% | 807.7322 | 807.8815 | 35 | 5.587 | 66.7% | 3 | R.LAADDFR.T | 22222 |
|  | Astrin\_STLCLD20\_112214\_01.08975.08975.2 | 2.9226 | 0.3172 | 100.0% | 1186.2522 | 1186.397 | 1 | 5.85 | 83.3% | 2 | R.RVLDELTLAR.A | 222 |
|  | Astrin\_STLCLD20\_112214\_01.09782.09782.1 | 2.1585 | 0.3224 | 100.0% | 1029.49 | 1030.2096 | 10 | 5.815 | 56.2% | 1 | R.VLDELTLAR.A | 111 |
|  | Astrin\_STLCLD20\_112214\_01.09757.09757.2 | 3.0933 | 0.3041 | 100.0% | 1030.3522 | 1030.2096 | 2 | 6.471 | 81.2% | 3 | R.VLDELTLAR.A | 222 |
|  | Astrin\_STLCLD20\_112214\_01.05006.05006.2 | 3.4896 | 0.2654 | 100.0% | 1439.7322 | 1439.6263 | 1 | 5.072 | 80.0% | 2 | R.ILNEMRDQYEK.M | 22 |
|  | Astrin\_STLCLD20\_112214\_01.06820.06820.3 | 3.5479 | 0.1718 | 95.8% | 2105.6042 | 2105.2664 | 54 | 4.942 | 30.6% | 2 | K.TEELNREVATNSELVQSGK.S | 33 |
|  | Astrin\_STLCLD20\_112214\_tube2\_01.06412.06412.2 | 3.7182 | 0.4945 | 100.0% | 1361.8522 | 1362.4796 | 4 | 8.36 | 58.3% | 1 | R.EVATNSELVQSGK.S | 22 |
|  | Astrin\_STLCLD20\_112214\_tube2\_01.08813.08813.2 | 2.9233 | 0.2777 | 99.7% | 1380.0122 | 1380.5437 | 1 | 5.774 | 70.0% | 3 | K.TRLEQEIATYR.R | 22 |
|  | Astrin\_STLCLD20\_112214\_01.06103.06103.3 | 3.1185 | 0.2697 | 99.6% | 1536.5643 | 1536.7311 | 3 | 4.784 | 36.4% | 1 | K.TRLEQEIATYRR.L | 33 |
|  | Astrin\_STLCLD20\_112214\_01.05125.05125.2 | 3.1188 | 0.3235 | 100.0% | 1123.3922 | 1123.2511 | 2 | 6.193 | 81.2% | 1 | R.LEQEIATYR.R | 222 |

Similarities:
gi|40354195|ref|NP\_95(1:11)  
gi|4557701|ref|NP\_000(11:1)  
contaminant\_KERATIN03(2:10)  
gi|24234699|ref|NP\_00(6:6)  

---

|  |  |  |  |  |  |  |  |  |
| --- | --- | --- | --- | --- | --- | --- | --- | --- |
| U | *gi|14210536|ref|NP\_11* | 10 | 52 | 17.0% | 446 | 49857 | 4.9 | tubulin, beta 6 [Homo sapiens] |

| Filename XCorr DeltCN Conf% ObsM+H+ CalcM+H+ SpR ZScore Ion% # Sequence  | | | | | | | | | | | | |
| --- | --- | --- | --- | --- | --- | --- | --- | --- | --- | --- | --- | --- |
| \* | Astrin\_STLCLD20\_112214\_tube2\_01.12347.12347.2 | 3.2606 | 0.3769 | 100.0% | 1574.3322 | 1574.7894 | 1 | 7.123 | 57.1% | 3 | R.AALVDLEPGTMDSVR.S | 2 |
|  | Astrin\_STLCLD20\_112214\_01.10486.10486.2 | 3.3502 | 0.4172 | 100.0% | 1352.5322 | 1352.6497 | 1 | 6.771 | 63.6% | 1 | R.IMNTFSVMPSPK.V | 22 |
|  | Astrin\_STLCLD20\_112214\_tube2\_01.09993.09993.2 | 3.1291 | 0.339 | 100.0% | 1131.4922 | 1131.2767 | 11 | 5.764 | 77.8% | 19 | R.FPGQLNADLR.K | 22222 |
|  | Astrin\_STLCLD20\_112214\_tube2\_01.08446.08446.2 | 2.7132 | 0.2665 | 99.3% | 1259.5122 | 1259.4508 | 43 | 5.338 | 60.0% | 3 | R.FPGQLNADLRK.L | 22222 |
|  | Astrin\_STLCLD20\_112214\_tube2\_01.11456.11456.2 | 3.6404 | 0.3874 | 100.0% | 1272.3522 | 1272.5945 | 1 | 6.64 | 70.0% | 4 | R.KLAVNMVPFPR.L | 22222 |
|  | Astrin\_STLCLD20\_112214\_01.11192.11192.1 | 2.2399 | 0.2147 | 98.0% | 1143.63 | 1144.4204 | 6 | 6.378 | 61.1% | 2 | K.LAVNMVPFPR.L | 11111 |
|  | Astrin\_STLCLD20\_112214\_01.11233.11233.2 | 3.8296 | 0.4984 | 100.0% | 1144.4722 | 1144.4204 | 1 | 8.878 | 94.4% | 6 | K.LAVNMVPFPR.L | 22222 |
|  | Astrin\_STLCLD20\_112214\_tube2\_01.16406.16406.2 | 3.7237 | 0.4102 | 100.0% | 1621.0322 | 1621.9403 | 1 | 8.877 | 73.1% | 7 | R.LHFFMPGFAPLTSR.G | 2222 |
|  | Astrin\_STLCLD20\_112214\_tube2\_01.16287.16287.3 | 4.1587 | 0.348 | 99.7% | 1622.1543 | 1621.9403 | 2 | 6.383 | 50.0% | 4 | R.LHFFMPGFAPLTSR.G | 3333 |
|  | Astrin\_STLCLD20\_112214\_tube2\_01.15262.15262.2 | 3.3078 | 0.3064 | 99.9% | 1698.6122 | 1697.8877 | 1 | 5.236 | 61.5% | 3 | K.NSSYFVEWIPNNVK.V | 22222 |

Similarities:
gi|29788785|ref|NP\_82(8:2)  
gi|5174735|ref|NP\_006(8:2)  
gi|29788768|ref|NP\_82(9:1)  
gi|50592996|ref|NP\_00(6:4)  

---

|  |  |  |  |  |  |  |  |  |
| --- | --- | --- | --- | --- | --- | --- | --- | --- |
| U | *gi|119395750|ref|NP\_0* | 9 | 21 | 16.8% | 644 | 66039 | 8.1 | keratin 1 [Homo sapiens] |

| Filename XCorr DeltCN Conf% ObsM+H+ CalcM+H+ SpR ZScore Ion% # Sequence  | | | | | | | | | | | | |
| --- | --- | --- | --- | --- | --- | --- | --- | --- | --- | --- | --- | --- |
| \* | Astrin\_STLCLD20\_112214\_02.10081.10081.2 | 3.8494 | 0.3805 | 100.0% | 1658.1322 | 1658.7678 | 1 | 6.303 | 62.5% | 2 | R.SGGGFSSGSAGIINYQR.R | 2 |
|  | Astrin\_STLCLD20\_112214\_01.11307.11307.2 | 2.2891 | 0.3333 | 98.9% | 1383.6322 | 1384.5315 | 250 | 5.224 | 40.9% | 2 | K.SLNNQFASFIDK.V | 2 |
|  | Astrin\_STLCLD20\_112214\_01.11787.11787.2 | 2.6999 | 0.3446 | 99.6% | 1640.3322 | 1639.8516 | 8 | 4.891 | 42.3% | 1 | K.SLNNQFASFIDKVR.F | 2 |
|  | Astrin\_STLCLD20\_112214\_01.07333.07333.2 | 4.5527 | 0.0972 | 99.9% | 1476.4922 | 1476.6726 | 1 | 7.289 | 90.9% | 3 | R.FLEQQNQVLQTK.W | 22 |
|  | Astrin\_STLCLD20\_112214\_02.12445.12445.2 | 4.0184 | 0.4955 | 100.0% | 1476.4922 | 1476.6293 | 1 | 8.225 | 86.4% | 4 | K.WELLQQVDTSTR.T | 2 |
|  | Astrin\_STLCLD20\_112214\_tube2\_01.07780.07780.2 | 2.8999 | 0.1001 | 96.7% | 1394.3322 | 1394.5675 | 7 | 4.44 | 59.1% | 1 | R.TNAENEFVTIKK.D | 2 |
| \* | Astrin\_STLCLD20\_112214\_01.13391.13391.2 | 4.0524 | 0.4915 | 100.0% | 1303.4722 | 1303.4955 | 1 | 9.19 | 77.3% | 2 | R.SLDLDSIIAEVK.A | 2 |
|  | Astrin\_STLCLD20\_112214\_01.07803.07803.2 | 3.2051 | 0.3213 | 100.0% | 1180.5122 | 1180.303 | 1 | 7.983 | 83.3% | 5 | K.YEELQITAGR.H | 22 |
| \* | Astrin\_STLCLD20\_112214\_01.12530.12530.3 | 4.8373 | 0.3806 | 100.0% | 2186.0044 | 2185.399 | 1 | 6.205 | 38.9% | 1 | K.NKLNDLEDALQQAKEDLAR.L | 3 |

Similarities:
gi|47132620|ref|NP\_00(1:8)  
gi|119703753|ref|NP\_0(1:8)  

---

|  |  |  |  |  |  |  |  |  |
| --- | --- | --- | --- | --- | --- | --- | --- | --- |
| U | *gi|5803225|ref|NP\_006* | 2 | 2 | 16.5% | 255 | 29174 | 4.7 | tyrosine 3/tryptophan 5 -monooxygenase activation protein, epsilon polypeptide [Homo sapiens] |

| Filename XCorr DeltCN Conf% ObsM+H+ CalcM+H+ SpR ZScore Ion% # Sequence  | | | | | | | | | | | | |
| --- | --- | --- | --- | --- | --- | --- | --- | --- | --- | --- | --- | --- |
| \* | Astrin\_STLCLD20\_112214\_tube2\_02.07515.07515.2 | 3.4006 | 0.2066 | 99.5% | 1449.6721 | 1448.6312 | 1 | 6.107 | 75.0% | 1 | K.VAGMDVELTVEER.N | 2 |
| \* | Astrin\_STLCLD20\_112214\_tube2\_02.00161.00161.3 | 3.8137 | 0.3154 | 99.7% | 3259.0444 | 3260.6375 | 1 | 4.817 | 25.9% | 1 | K.AAFDDAIAELDTLSEESYKDSTLIMQLLR.D | 3 |

---

|  |  |  |  |  |  |  |  |  |
| --- | --- | --- | --- | --- | --- | --- | --- | --- |
| U | *gi|169201338|ref|XP\_0* | 3 | 12 | 16.2% | 160 | 18565 | 10.5 | PREDICTED: hypothetical protein [Homo sapiens] |
| U | *gi|89040203|ref|XP\_93* | 3 | 12 | 16.2% | 160 | 18593 | 10.5 | PREDICTED: hypothetical protein [Homo sapiens] |
| U | *gi|18104948|ref|NP\_00* | 3 | 12 | 16.2% | 160 | 18565 | 10.5 | ribosomal protein L21 [Homo sapiens] |
| U | *gi|169213854|ref|XP\_0* | 3 | 12 | 16.2% | 160 | 18790 | 10.3 | PREDICTED: hypothetical protein [Homo sapiens] |
| U | *gi|169210381|ref|XP\_0* | 3 | 12 | 16.2% | 160 | 18535 | 10.6 | PREDICTED: hypothetical protein isoform 2 [Homo sapiens] |
| U | *gi|169210379|ref|XP\_0* | 3 | 12 | 16.2% | 160 | 18535 | 10.6 | PREDICTED: hypothetical protein isoform 3 [Homo sapiens] |
| U | *gi|169210377|ref|XP\_0* | 3 | 12 | 16.2% | 160 | 18535 | 10.6 | PREDICTED: hypothetical protein isoform 1 [Homo sapiens] |
| U | *gi|169202779|ref|XP\_0* | 3 | 12 | 16.2% | 160 | 18521 | 10.5 | PREDICTED: similar to ribosomal protein L21 isoform 1 [Homo sapiens] |
| U | *gi|169202777|ref|XP\_0* | 3 | 12 | 16.2% | 160 | 18521 | 10.5 | PREDICTED: similar to ribosomal protein L21 isoform 2 [Homo sapiens] |
| U | *gi|169201750|ref|XP\_0* | 3 | 12 | 16.2% | 160 | 18550 | 10.5 | PREDICTED: hypothetical protein [Homo sapiens] |

| Filename XCorr DeltCN Conf% ObsM+H+ CalcM+H+ SpR ZScore Ion% # Sequence  | | | | | | | | | | | | |
| --- | --- | --- | --- | --- | --- | --- | --- | --- | --- | --- | --- | --- |
|  | Astrin\_STLCLD20\_112214\_01.08926.08926.2 | 2.6824 | 0.4261 | 100.0% | 1243.8922 | 1244.4973 | 1 | 7.798 | 75.0% | 4 | K.HGVVPLATYMR.I | 2 |
|  | Astrin\_STLCLD20\_112214\_01.08151.08151.2 | 4.457 | 0.4035 | 100.0% | 1641.3522 | 1641.9108 | 1 | 8.701 | 85.7% | 3 | R.VYNVTQHAVGIVVNK.Q | 2 |
|  | Astrin\_STLCLD20\_112214\_tube2\_02.06589.06589.3 | 3.7793 | 0.3874 | 99.7% | 1642.2244 | 1641.9108 | 1 | 8.272 | 50.0% | 5 | R.VYNVTQHAVGIVVNK.Q | 3 |

---

|  |  |  |  |  |  |  |  |  |
| --- | --- | --- | --- | --- | --- | --- | --- | --- |
| U | *gi|4506625|ref|NP\_000* | 2 | 3 | 16.2% | 148 | 16561 | 11.0 | ribosomal protein L27a [Homo sapiens] |

| Filename XCorr DeltCN Conf% ObsM+H+ CalcM+H+ SpR ZScore Ion% # Sequence  | | | | | | | | | | | | |
| --- | --- | --- | --- | --- | --- | --- | --- | --- | --- | --- | --- | --- |
| \* | Astrin\_STLCLD20\_112214\_01.08506.08506.2 | 2.2541 | 0.2232 | 95.8% | 1586.2322 | 1586.7899 | 2 | 4.469 | 50.0% | 1 | R.INFDKYHPGYFGK.V | 2 |
| \* | Astrin\_STLCLD20\_112214\_tube2\_01.11060.11060.2 | 2.607 | 0.314 | 99.4% | 1111.7922 | 1112.3146 | 1 | 6.51 | 85.0% | 2 | K.TGAAPIIDVVR.S | 2 |

---

|  |  |  |  |  |  |  |  |  |
| --- | --- | --- | --- | --- | --- | --- | --- | --- |
| U | *gi|4502891|ref|NP\_001* | 2 | 4 | 16.0% | 237 | 26215 | 4.1 | chloride channel, nucleotide-sensitive, 1A [Homo sapiens] |

| Filename XCorr DeltCN Conf% ObsM+H+ CalcM+H+ SpR ZScore Ion% # Sequence  | | | | | | | | | | | | |
| --- | --- | --- | --- | --- | --- | --- | --- | --- | --- | --- | --- | --- |
| \* | Astrin\_STLCLD20\_112214\_02.10829.10829.2 | 2.8312 | 0.4876 | 100.0% | 1337.8121 | 1338.5034 | 2 | 7.949 | 66.7% | 3 | K.GLGTGTLYIAESR.L | 2 |
| \* | Astrin\_STLCLD20\_112214\_tube2\_02.11212.11212.3 | 3.2681 | 0.2394 | 97.0% | 2720.8145 | 2721.085 | 21 | 4.828 | 24.0% | 1 | R.LSWLDGSGLGFSLEYPTISLHALSR.D | 3 |

---

|  |  |  |  |  |  |  |  |  |
| --- | --- | --- | --- | --- | --- | --- | --- | --- |
| U | *gi|38201714|ref|NP\_00* | 3 | 3 | 15.6% | 326 | 36092 | 9.2 | ELAV-like 1 [Homo sapiens] |

| Filename XCorr DeltCN Conf% ObsM+H+ CalcM+H+ SpR ZScore Ion% # Sequence  | | | | | | | | | | | | |
| --- | --- | --- | --- | --- | --- | --- | --- | --- | --- | --- | --- | --- |
| \* | Astrin\_STLCLD20\_112214\_01.12364.12364.2 | 3.2114 | 0.4459 | 100.0% | 2162.9521 | 2163.4534 | 105 | 6.497 | 32.4% | 1 | R.TNLIVNYLPQNMTQDELR.S | 2 |
| \* | Astrin\_STLCLD20\_112214\_02.11121.11121.3 | 3.3174 | 0.2756 | 99.6% | 2027.8444 | 2027.2871 | 1 | 5.14 | 37.5% | 1 | R.DKVAGHSLGYGFVNYVTAK.D | 3 |
| \* | Astrin\_STLCLD20\_112214\_tube2\_01.12957.12957.2 | 2.6 | 0.1932 | 97.0% | 1649.8121 | 1649.8038 | 1 | 4.904 | 57.7% | 1 | K.NVALLSQLYHS\*PAR.R | 2 |

---

|  |  |  |  |  |  |  |  |  |
| --- | --- | --- | --- | --- | --- | --- | --- | --- |
| U | *gi|4507357|ref|NP\_003* | 2 | 2 | 15.1% | 199 | 22391 | 8.2 | transgelin 2 [Homo sapiens] |

| Filename XCorr DeltCN Conf% ObsM+H+ CalcM+H+ SpR ZScore Ion% # Sequence  | | | | | | | | | | | | |
| --- | --- | --- | --- | --- | --- | --- | --- | --- | --- | --- | --- | --- |
| \* | Astrin\_STLCLD20\_112214\_tube2\_01.19211.19211.2 | 3.6544 | 0.4141 | 100.0% | 2100.132 | 2101.3203 | 29 | 7.175 | 35.3% | 1 | R.YGINTTDIFQTVDLWEGK.N | 2 |
| \* | Astrin\_STLCLD20\_112214\_02.12729.12729.2 | 2.2999 | 0.2988 | 98.5% | 1215.8522 | 1216.4845 | 1 | 5.698 | 63.6% | 1 | R.TLMNLGGLAVAR.D | 2 |

---

|  |  |  |  |  |  |  |  |  |
| --- | --- | --- | --- | --- | --- | --- | --- | --- |
| U | *gi|5031635|ref|NP\_005* | 2 | 3 | 15.1% | 166 | 18502 | 8.1 | cofilin 1 (non-muscle) [Homo sapiens] |

| Filename XCorr DeltCN Conf% ObsM+H+ CalcM+H+ SpR ZScore Ion% # Sequence  | | | | | | | | | | | | |
| --- | --- | --- | --- | --- | --- | --- | --- | --- | --- | --- | --- | --- |
|  | Astrin\_STLCLD20\_112214\_tube2\_01.09730.09730.2 | 3.2835 | 0.4616 | 100.0% | 1338.1322 | 1338.4564 | 1 | 7.473 | 65.0% | 2 | R.YALYDATYETK.E | 2 |
| \* | Astrin\_STLCLD20\_112214\_02.12051.12051.2 | 2.3314 | 0.2763 | 97.7% | 1341.7522 | 1341.5907 | 1 | 5.109 | 61.5% | 1 | K.LGGSAVISLEGKPL.- | 2 |

---

|  |  |  |  |  |  |  |  |  |
| --- | --- | --- | --- | --- | --- | --- | --- | --- |
| U | *gi|5454064|ref|NP\_006* | 8 | 17 | 14.8% | 669 | 69492 | 9.7 | RNA binding motif protein 14 [Homo sapiens] |

| Filename XCorr DeltCN Conf% ObsM+H+ CalcM+H+ SpR ZScore Ion% # Sequence  | | | | | | | | | | | | |
| --- | --- | --- | --- | --- | --- | --- | --- | --- | --- | --- | --- | --- |
| \* | Astrin\_STLCLD20\_112214\_tube2\_01.06377.06377.2 | 2.6501 | 0.1418 | 95.2% | 1556.2922 | 1556.7677 | 1 | 4.747 | 57.7% | 1 | R.AIEALHGHELRPGR.A | 2 |
| \* | Astrin\_STLCLD20\_112214\_tube2\_01.06362.06362.3 | 3.5703 | 0.4672 | 100.0% | 1557.5643 | 1556.7677 | 1 | 7.219 | 44.2% | 1 | R.AIEALHGHELRPGR.A | 3 |
| \* | Astrin\_STLCLD20\_112214\_tube2\_01.10102.10102.3 | 3.0415 | 0.2464 | 98.2% | 1898.6044 | 1898.2767 | 1 | 4.934 | 38.3% | 1 | R.ALVVEMSRPRPLNTWK.I | 3 |
| \* | Astrin\_STLCLD20\_112214\_tube2\_01.09675.09675.2 | 3.4567 | 0.4166 | 100.0% | 1609.5521 | 1609.8223 | 1 | 7.321 | 75.0% | 3 | R.ASYVAPLTAQPATYR.A | 2 |
| \* | Astrin\_STLCLD20\_112214\_tube2\_01.08381.08381.3 | 4.3253 | 0.4229 | 100.0% | 2466.0244 | 2466.6292 | 1 | 7.322 | 35.9% | 7 | R.TQSSASLAASYAAQQHPQAAASYR.G | 3 |
| \* | Astrin\_STLCLD20\_112214\_01.08293.08293.2 | 2.0733 | 0.327 | 98.6% | 1067.4122 | 1067.1869 | 1 | 6.587 | 75.0% | 1 | R.LSESQLSFR.R | 2 |
| \* | Astrin\_STLCLD20\_112214\_tube2\_01.05927.05927.2 | 2.3917 | 0.2693 | 98.6% | 1300.5922 | 1301.4043 | 22 | 5.101 | 55.0% | 1 | R.RLPDAHSDYAR.Y | 2 |
| \* | Astrin\_STLCLD20\_112214\_tube2\_01.09147.09147.2 | 2.7254 | 0.3651 | 100.0% | 1238.2722 | 1238.2988 | 1 | 8.449 | 66.7% | 2 | R.YSGSYNDYLR.A | 2 |

---

|  |  |  |  |  |  |  |  |  |
| --- | --- | --- | --- | --- | --- | --- | --- | --- |
| U | *gi|15431293|ref|NP\_00* | 2 | 2 | 14.7% | 204 | 24146 | 11.6 | ribosomal protein L15 [Homo sapiens] |
| U | *gi|88998868|ref|XP\_94* | 2 | 2 | 14.7% | 204 | 24174 | 11.6 | PREDICTED: hypothetical protein isoform 4 [Homo sapiens] |
| U | *gi|88992455|ref|XP\_93* | 2 | 2 | 14.7% | 204 | 24174 | 11.6 | PREDICTED: hypothetical protein isoform 1 [Homo sapiens] |
| U | *gi|169169711|ref|XP\_0* | 2 | 2 | 14.7% | 204 | 24174 | 11.6 | PREDICTED: hypothetical protein [Homo sapiens] |

| Filename XCorr DeltCN Conf% ObsM+H+ CalcM+H+ SpR ZScore Ion% # Sequence  | | | | | | | | | | | | |
| --- | --- | --- | --- | --- | --- | --- | --- | --- | --- | --- | --- | --- |
|  | Astrin\_STLCLD20\_112214\_tube2\_01.05703.05703.3 | 2.9679 | 0.3352 | 99.5% | 1706.5743 | 1706.945 | 6 | 5.652 | 35.0% | 1 | K.GATYGKPVHHGVNQLK.F | 3 |
|  | Astrin\_STLCLD20\_112214\_01.10327.10327.2 | 3.3473 | 0.4581 | 100.0% | 1661.1921 | 1661.8083 | 1 | 7.943 | 65.4% | 1 | R.VLNSYWVGEDSTYK.F | 2 |

---

|  |  |  |  |  |  |  |  |  |
| --- | --- | --- | --- | --- | --- | --- | --- | --- |
| U | *gi|31542947|ref|NP\_00* | 5 | 5 | 14.3% | 573 | 61055 | 5.9 | chaperonin [Homo sapiens] |
| U | *gi|41399285|ref|NP\_95* | 5 | 5 | 14.3% | 573 | 61055 | 5.9 | chaperonin [Homo sapiens] |

| Filename XCorr DeltCN Conf% ObsM+H+ CalcM+H+ SpR ZScore Ion% # Sequence  | | | | | | | | | | | | |
| --- | --- | --- | --- | --- | --- | --- | --- | --- | --- | --- | --- | --- |
|  | Astrin\_STLCLD20\_112214\_01.17625.17625.2 | 2.8206 | 0.2404 | 98.7% | 2116.0122 | 2114.5667 | 12 | 4.907 | 32.5% | 1 | R.ALMLQGVDLLADAVAVTMGPK.G | 2 |
|  | Astrin\_STLCLD20\_112214\_tube2\_01.18668.18668.2 | 2.2203 | 0.2822 | 97.0% | 1557.6522 | 1557.9324 | 2 | 5.215 | 46.4% | 1 | R.GVMLAVDAVIAELKK.Q | 2 |
|  | Astrin\_STLCLD20\_112214\_tube2\_02.07460.07460.3 | 3.2314 | 0.3878 | 99.8% | 1631.9644 | 1631.9684 | 2 | 6.293 | 41.1% | 1 | K.VGEVIVTKDDAMLLK.G | 3 |
|  | Astrin\_STLCLD20\_112214\_tube2\_02.08629.08629.3 | 3.5924 | 0.2646 | 99.6% | 2297.6042 | 2296.5334 | 445 | 4.324 | 33.3% | 1 | R.IQEIIEQLDVTTSEYEKEK.L | 3 |
|  | Astrin\_STLCLD20\_112214\_02.09691.09691.2 | 2.9257 | 0.3545 | 100.0% | 1216.2722 | 1216.377 | 1 | 6.806 | 77.3% | 1 | K.NAGVEGSLIVEK.I | 2 |

---

|  |  |  |  |  |  |  |  |  |
| --- | --- | --- | --- | --- | --- | --- | --- | --- |
| U | *gi|56699409|ref|NP\_00* | 4 | 10 | 14.3% | 391 | 42332 | 10.1 | RNA binding motif protein, X-linked [Homo sapiens] |

| Filename XCorr DeltCN Conf% ObsM+H+ CalcM+H+ SpR ZScore Ion% # Sequence  | | | | | | | | | | | | |
| --- | --- | --- | --- | --- | --- | --- | --- | --- | --- | --- | --- | --- |
|  | Astrin\_STLCLD20\_112214\_01.10077.10077.2 | 3.445 | 0.2489 | 99.9% | 1437.3922 | 1436.6049 | 1 | 5.357 | 75.0% | 4 | K.LFIGGLNTETNEK.A | 2 |
|  | Astrin\_STLCLD20\_112214\_01.09551.09551.2 | 2.5882 | 0.1692 | 97.7% | 1216.4521 | 1216.5249 | 6 | 4.476 | 66.7% | 1 | R.IVEVLLMKDR.E | 2 |
|  | Astrin\_STLCLD20\_112214\_01.12130.12130.2 | 2.8061 | 0.4141 | 100.0% | 1486.4722 | 1487.6519 | 1 | 6.159 | 69.2% | 2 | R.GFAFVTFESPADAK.D | 2 |
|  | Astrin\_STLCLD20\_112214\_tube2\_02.06967.06967.3 | 3.8492 | 0.4265 | 100.0% | 2049.9543 | 2051.1873 | 1 | 7.24 | 36.1% | 3 | R.GGHMDDGGYSMNFNMSSSR.G | 3 |

---

|  |  |  |  |  |  |  |  |  |
| --- | --- | --- | --- | --- | --- | --- | --- | --- |
| U | *gi|16753227|ref|NP\_00* | 3 | 4 | 14.2% | 288 | 32728 | 10.6 | ribosomal protein L6 [Homo sapiens] |
| U | *gi|67189747|ref|NP\_00* | 3 | 4 | 14.2% | 288 | 32728 | 10.6 | ribosomal protein L6 [Homo sapiens] |

| Filename XCorr DeltCN Conf% ObsM+H+ CalcM+H+ SpR ZScore Ion% # Sequence  | | | | | | | | | | | | |
| --- | --- | --- | --- | --- | --- | --- | --- | --- | --- | --- | --- | --- |
|  | Astrin\_STLCLD20\_112214\_01.05191.05191.2 | 2.5442 | 0.3402 | 99.9% | 995.15216 | 995.1228 | 1 | 6.838 | 71.4% | 1 | K.HLTDAYFK.K | 2 |
|  | Astrin\_STLCLD20\_112214\_02.09220.09220.3 | 3.1676 | 0.2652 | 98.6% | 2509.8843 | 2510.6763 | 2 | 4.27 | 35.5% | 1 | R.HQEGEIFDTEKEKYEITEQR.K | 3 |
|  | Astrin\_STLCLD20\_112214\_tube2\_01.12094.12094.2 | 3.0575 | 0.3533 | 100.0% | 1448.3522 | 1447.6769 | 6 | 6.699 | 50.0% | 2 | R.SVFALTNGIYPHK.L | 2 |

---

|  |  |  |  |  |  |  |  |  |
| --- | --- | --- | --- | --- | --- | --- | --- | --- |
| U | *gi|52632383|ref|NP\_00* | 5 | 8 | 13.8% | 589 | 64133 | 8.2 | heterogeneous nuclear ribonucleoprotein L isoform a [Homo sapiens] |

| Filename XCorr DeltCN Conf% ObsM+H+ CalcM+H+ SpR ZScore Ion% # Sequence  | | | | | | | | | | | | |
| --- | --- | --- | --- | --- | --- | --- | --- | --- | --- | --- | --- | --- |
| \* | Astrin\_STLCLD20\_112214\_tube2\_02.00288.00288.3 | 6.3031 | 0.5261 | 100.0% | 3088.9744 | 3089.6143 | 1 | 9.213 | 36.6% | 2 | R.GLIDGVVEADLVEALQEFGPISYVVVMPK.K | 3 |
|  | Astrin\_STLCLD20\_112214\_01.09713.09713.2 | 3.7755 | 0.4222 | 100.0% | 1635.4722 | 1635.881 | 1 | 7.246 | 61.5% | 1 | R.AITHLNNNFMFGQK.L | 2 |
|  | Astrin\_STLCLD20\_112214\_tube2\_01.11663.11663.2 | 3.0216 | 0.4133 | 100.0% | 1223.4321 | 1223.3251 | 1 | 7.221 | 75.0% | 3 | R.SSSGLLEWESK.S | 2 |
|  | Astrin\_STLCLD20\_112214\_01.12143.12143.2 | 2.4827 | 0.2126 | 96.7% | 1868.3722 | 1868.1144 | 1 | 4.589 | 43.3% | 1 | K.SDALETLGFLNHYQMK.N | 2 |
|  | Astrin\_STLCLD20\_112214\_01.07678.07678.2 | 2.4747 | 0.2996 | 99.2% | 1264.2122 | 1264.4233 | 1 | 6.041 | 55.0% | 1 | K.NPNGPYPYTLK.L | 2 |

---

|  |  |  |  |  |  |  |  |  |
| --- | --- | --- | --- | --- | --- | --- | --- | --- |
| U | *gi|36287110|ref|NP\_91* | 4 | 7 | 13.5% | 379 | 40907 | 4.6 | FGFR1 oncogene partner isoform b [Homo sapiens] |
| U | *gi|5901954|ref|NP\_008* | 4 | 7 | 12.8% | 399 | 43065 | 4.8 | FGFR1 oncogene partner isoform a [Homo sapiens] |

| Filename XCorr DeltCN Conf% ObsM+H+ CalcM+H+ SpR ZScore Ion% # Sequence  | | | | | | | | | | | | |
| --- | --- | --- | --- | --- | --- | --- | --- | --- | --- | --- | --- | --- |
|  | Astrin\_STLCLD20\_112214\_tube2\_01.19959.19959.2 | 4.3682 | 0.4461 | 100.0% | 2164.7922 | 2165.5352 | 1 | 7.94 | 50.0% | 1 | R.DLGIIEAEGTVGGPLLLEVIR.R | 2 |
|  | Astrin\_STLCLD20\_112214\_tube2\_02.10959.10959.3 | 3.4274 | 0.23 | 97.7% | 2322.7444 | 2321.7227 | 5 | 4.994 | 31.0% | 3 | R.DLGIIEAEGTVGGPLLLEVIRR.C | 3 |
|  | Astrin\_STLCLD20\_112214\_01.08091.08091.2 | 3.2469 | 0.2453 | 99.4% | 1584.1921 | 1583.8253 | 1 | 5.232 | 56.7% | 2 | R.KQAGSLASLSDAPPLK.S | 2 |
|  | Astrin\_STLCLD20\_112214\_01.09295.09295.2 | 2.1497 | 0.2424 | 95.5% | 1188.0721 | 1188.3666 | 13 | 4.91 | 58.3% | 1 | K.SGLSSLAGAPSLK.D | 2 |

---

|  |  |  |  |  |  |  |  |  |
| --- | --- | --- | --- | --- | --- | --- | --- | --- |
| U | *gi|214830438|ref|NP\_0* | 2 | 3 | 13.5% | 356 | 38629 | 5.5 | sequestosome 1 isoform 2 [Homo sapiens] |
| U | *gi|4505571|ref|NP\_003* | 2 | 3 | 10.9% | 440 | 47687 | 5.2 | sequestosome 1 isoform 1 [Homo sapiens] |
| U | *gi|214830451|ref|NP\_0* | 2 | 3 | 13.5% | 356 | 38629 | 5.5 | sequestosome 1 isoform 2 [Homo sapiens] |

| Filename XCorr DeltCN Conf% ObsM+H+ CalcM+H+ SpR ZScore Ion% # Sequence  | | | | | | | | | | | | |
| --- | --- | --- | --- | --- | --- | --- | --- | --- | --- | --- | --- | --- |
|  | Astrin\_STLCLD20\_112214\_01.08915.08915.3 | 4.2133 | 0.3474 | 99.8% | 2571.4744 | 2572.7478 | 1 | 5.908 | 30.0% | 2 | R.AGEARPGPTAESASGPSEDPSVNFLK.N | 3 |
|  | Astrin\_STLCLD20\_112214\_tube2\_02.12048.12048.2 | 2.7959 | 0.3386 | 99.7% | 2458.1921 | 2458.8066 | 1 | 5.108 | 38.1% | 1 | R.LIESLSQMLSMGFSDEGGWLTR.L | 2 |

---

|  |  |  |  |  |  |  |  |  |
| --- | --- | --- | --- | --- | --- | --- | --- | --- |
| U | *gi|4759098|ref|NP\_004* | 3 | 7 | 13.5% | 288 | 33666 | 11.2 | splicing factor, arginine/serine-rich 10 [Homo sapiens] |

| Filename XCorr DeltCN Conf% ObsM+H+ CalcM+H+ SpR ZScore Ion% # Sequence  | | | | | | | | | | | | |
| --- | --- | --- | --- | --- | --- | --- | --- | --- | --- | --- | --- | --- |
| \* | Astrin\_STLCLD20\_112214\_02.11875.11875.2 | 4.6096 | 0.553 | 100.0% | 1811.3121 | 1811.989 | 1 | 8.883 | 63.3% | 4 | K.YGPIADVSIVYDQQSR.R | 2 |
| \* | Astrin\_STLCLD20\_112214\_02.15029.15029.2 | 3.5992 | 0.4348 | 100.0% | 1622.0322 | 1622.774 | 1 | 7.069 | 65.4% | 1 | R.GFAFVYFENVDDAK.E | 2 |
| \* | Astrin\_STLCLD20\_112214\_tube2\_01.09894.09894.2 | 2.7358 | 0.2695 | 99.7% | 1078.5322 | 1079.2847 | 1 | 5.2 | 68.8% | 2 | R.IRVDFSITK.R | 2 |

---

|  |  |  |  |  |  |  |  |  |
| --- | --- | --- | --- | --- | --- | --- | --- | --- |
| U | *gi|5453555|ref|NP\_006* | 3 | 5 | 13.4% | 216 | 24423 | 7.5 | ras-related nuclear protein [Homo sapiens] |

| Filename XCorr DeltCN Conf% ObsM+H+ CalcM+H+ SpR ZScore Ion% # Sequence  | | | | | | | | | | | | |
| --- | --- | --- | --- | --- | --- | --- | --- | --- | --- | --- | --- | --- |
| \* | Astrin\_STLCLD20\_112214\_01.10414.10414.2 | 3.3742 | 0.3505 | 100.0% | 1690.1522 | 1690.8962 | 1 | 5.903 | 60.7% | 1 | R.GPIKFNVWDTAGQEK.F | 2 |
| \* | Astrin\_STLCLD20\_112214\_01.09711.09711.2 | 2.8096 | 0.3328 | 99.9% | 1295.9321 | 1295.394 | 1 | 5.766 | 75.0% | 1 | K.FNVWDTAGQEK.F | 2 |
| \* | Astrin\_STLCLD20\_112214\_tube2\_01.16278.16278.2 | 3.3987 | 0.2865 | 99.9% | 1786.3322 | 1786.0427 | 1 | 6.636 | 65.4% | 3 | K.SNYNFEKPFLWLAR.K | 2 |

---

|  |  |  |  |  |  |  |  |  |
| --- | --- | --- | --- | --- | --- | --- | --- | --- |
| U | *gi|108936958|ref|NP\_0* | 3 | 6 | 13.2% | 342 | 38926 | 5.5 | WD-repeat protein [Homo sapiens] |

| Filename XCorr DeltCN Conf% ObsM+H+ CalcM+H+ SpR ZScore Ion% # Sequence  | | | | | | | | | | | | |
| --- | --- | --- | --- | --- | --- | --- | --- | --- | --- | --- | --- | --- |
| \* | Astrin\_STLCLD20\_112214\_tube2\_01.13415.13415.2 | 3.7572 | 0.4429 | 100.0% | 1484.4122 | 1484.6488 | 1 | 7.213 | 58.3% | 3 | R.LALGSFVEEYNNK.V | 2 |
| \* | Astrin\_STLCLD20\_112214\_tube2\_01.15244.15244.2 | 2.5171 | 0.3335 | 99.1% | 1640.3322 | 1640.8333 | 1 | 5.648 | 46.4% | 1 | K.GVYPDLLATSGDYLR.V | 2 |
| \* | Astrin\_STLCLD20\_112214\_02.10636.10636.3 | 3.8965 | 0.4143 | 100.0% | 2007.5643 | 2007.2535 | 1 | 7.04 | 40.6% | 2 | K.TQLIAHDKEVYDIAFSR.A | 3 |

---

|  |  |  |  |  |  |  |  |  |
| --- | --- | --- | --- | --- | --- | --- | --- | --- |
| U | *gi|11968182|ref|NP\_07* | 2 | 2 | 13.2% | 152 | 17719 | 11.0 | ribosomal protein S18 [Homo sapiens] |
| U | *gi|169168597|ref|XP\_0* | 2 | 2 | 13.2% | 152 | 17719 | 11.0 | PREDICTED: hypothetical protein [Homo sapiens] |

| Filename XCorr DeltCN Conf% ObsM+H+ CalcM+H+ SpR ZScore Ion% # Sequence  | | | | | | | | | | | | |
| --- | --- | --- | --- | --- | --- | --- | --- | --- | --- | --- | --- | --- |
|  | Astrin\_STLCLD20\_112214\_tube2\_01.06882.06882.2 | 2.5243 | 0.1977 | 97.8% | 1248.5322 | 1248.2891 | 2 | 4.597 | 70.0% | 1 | R.AGELTEDEVER.V | 2 |
|  | Astrin\_STLCLD20\_112214\_01.07300.07300.2 | 2.0896 | 0.3581 | 99.1% | 1072.2122 | 1072.311 | 150 | 5.338 | 62.5% | 1 | R.VITIMQNPR.Q | 2 |

---

|  |  |  |  |  |  |  |  |  |
| --- | --- | --- | --- | --- | --- | --- | --- | --- |
| U | *gi|4885375|ref|NP\_005* | 4 | 9 | 13.1% | 213 | 21365 | 10.9 | histone cluster 1, H1c [Homo sapiens] |
| U | *gi|4885379|ref|NP\_005* | 4 | 9 | 12.8% | 219 | 21865 | 11.0 | histone cluster 1, H1e [Homo sapiens] |
| U | *gi|4885377|ref|NP\_005* | 4 | 9 | 12.7% | 221 | 22350 | 11.0 | histone cluster 1, H1d [Homo sapiens] |

| Filename XCorr DeltCN Conf% ObsM+H+ CalcM+H+ SpR ZScore Ion% # Sequence  | | | | | | | | | | | | |
| --- | --- | --- | --- | --- | --- | --- | --- | --- | --- | --- | --- | --- |
|  | Astrin\_STLCLD20\_112214\_tube2\_01.08475.08475.2 | 3.6798 | 0.5046 | 100.0% | 1327.4922 | 1327.5638 | 2 | 7.608 | 58.3% | 4 | R.KASGPPVSELITK.A | 2 |
|  | Astrin\_STLCLD20\_112214\_tube2\_01.10022.10022.2 | 2.7392 | 0.399 | 100.0% | 1199.5521 | 1199.3898 | 1 | 6.503 | 68.2% | 2 | K.ASGPPVSELITK.A | 2 |
|  | Astrin\_STLCLD20\_112214\_tube2\_01.07986.07986.1 | 2.6116 | 0.4079 | 100.0% | 1108.09 | 1108.2365 | 217 | 6.902 | 45.0% | 2 | K.ALAAAGYDVEK.N | 1 |
|  | Astrin\_STLCLD20\_112214\_tube2\_01.06738.06738.2 | 4.0702 | 0.4623 | 100.0% | 1579.5922 | 1579.7098 | 1 | 8.389 | 75.0% | 1 | K.ALAAAGYDVEKNNSR.I | 2 |

---

|  |  |  |  |  |  |  |  |  |
| --- | --- | --- | --- | --- | --- | --- | --- | --- |
| U | *gi|23397427|ref|NP\_00* | 5 | 7 | 13.0% | 623 | 69633 | 8.6 | synaptotagmin binding, cytoplasmic RNA interacting protein [Homo sapiens] |

| Filename XCorr DeltCN Conf% ObsM+H+ CalcM+H+ SpR ZScore Ion% # Sequence  | | | | | | | | | | | | |
| --- | --- | --- | --- | --- | --- | --- | --- | --- | --- | --- | --- | --- |
| \* | Astrin\_STLCLD20\_112214\_02.12574.12574.3 | 4.2116 | 0.3293 | 99.8% | 2443.6443 | 2443.716 | 1 | 5.938 | 34.5% | 2 | K.VAEKLDEIYVAGLVAHSDLDER.A | 3 |
| \* | Astrin\_STLCLD20\_112214\_tube2\_01.17495.17495.2 | 3.1153 | 0.4152 | 100.0% | 2335.2522 | 2335.6624 | 2 | 6.123 | 35.0% | 1 | R.AIEALKEFNEDGALAVLQQFK.D | 2 |
|  | Astrin\_STLCLD20\_112214\_01.07394.07394.2 | 2.8159 | 0.3712 | 100.0% | 1312.3922 | 1312.4221 | 1 | 6.943 | 68.2% | 2 | R.TGYTLDVTTGQR.K | 22 |
| \* | Astrin\_STLCLD20\_112214\_tube2\_01.19472.19472.2 | 2.7108 | 0.3604 | 99.7% | 1594.2522 | 1594.8016 | 3 | 6.213 | 50.0% | 1 | R.DLFEDELVPLFEK.A | 2 |
| \* | Astrin\_STLCLD20\_112214\_tube2\_01.14886.14886.2 | 3.9333 | 0.4145 | 100.0% | 1474.6921 | 1474.6512 | 1 | 8.528 | 75.0% | 1 | R.NLANTVTEEILEK.A | 2 |

Similarities:
gi|156151392|ref|NP\_0(1:4)  

---

|  |  |  |  |  |  |  |  |  |
| --- | --- | --- | --- | --- | --- | --- | --- | --- |
| U | *contaminant\_KERATIN03* | 6 | 10 | 13.0% | 593 | 59519 | 5.2 | no description |
| U | *gi|195972866|ref|NP\_0* | 6 | 10 | 13.2% | 584 | 58801 | 5.2 | keratin 10 [Homo sapiens] |

| Filename XCorr DeltCN Conf% ObsM+H+ CalcM+H+ SpR ZScore Ion% # Sequence  | | | | | | | | | | | | |
| --- | --- | --- | --- | --- | --- | --- | --- | --- | --- | --- | --- | --- |
|  | Astrin\_STLCLD20\_112214\_01.05697.05697.2 | 2.9151 | 0.1301 | 98.6% | 1065.6322 | 1065.2578 | 20 | 6.487 | 68.8% | 1 | R.LASYLDKVR.A | 2222 |
|  | Astrin\_STLCLD20\_112214\_01.06026.06026.2 | 3.6248 | 0.46 | 100.0% | 1382.3522 | 1382.4668 | 1 | 7.97 | 63.6% | 2 | R.ALEESNYELEGK.I | 2 |
|  | Astrin\_STLCLD20\_112214\_tube2\_01.19536.19536.3 | 4.4437 | 0.2611 | 99.5% | 3053.9944 | 3054.4277 | 15 | 6.229 | 22.1% | 1 | K.TIDDLKNQILNLTTDNANILLQIDNAR.L | 3 |
|  | Astrin\_STLCLD20\_112214\_tube2\_01.07546.07546.2 | 2.2441 | 0.2268 | 98.6% | 807.7322 | 807.8815 | 35 | 5.587 | 66.7% | 3 | R.LAADDFR.L | 22222 |
|  | Astrin\_STLCLD20\_112214\_01.05560.05560.2 | 2.8917 | 0.3716 | 100.0% | 1235.7922 | 1235.4258 | 6 | 6.199 | 72.2% | 2 | R.LKYENEVALR.Q | 2 |
|  | Astrin\_STLCLD20\_112214\_tube2\_01.06234.06234.2 | 3.0523 | 0.2143 | 99.3% | 1495.4922 | 1494.6041 | 1 | 5.032 | 68.2% | 1 | R.SQYEQLAEQNRK.D | 2 |

Similarities:
gi|40354195|ref|NP\_95(1:5)  
gi|4557701|ref|NP\_000(2:4)  
contaminant\_KERATIN05(2:4)  
gi|24234699|ref|NP\_00(2:4)  

---

|  |  |  |  |  |  |  |  |  |
| --- | --- | --- | --- | --- | --- | --- | --- | --- |
| U | *gi|169165117|ref|XP\_0* | 2 | 3 | 13.0% | 184 | 21343 | 10.1 | PREDICTED: hypothetical protein [Homo sapiens] |
| U | *gi|78000186|ref|NP\_00* | 2 | 3 | 13.0% | 184 | 21397 | 10.2 | ribosomal protein L17 [Homo sapiens] |
| U | *gi|4506617|ref|NP\_000* | 2 | 3 | 13.0% | 184 | 21397 | 10.2 | ribosomal protein L17 [Homo sapiens] |
| U | *gi|169212979|ref|XP\_0* | 2 | 3 | 13.0% | 184 | 21397 | 10.2 | PREDICTED: hypothetical protein [Homo sapiens] |
| U | *gi|169166152|ref|XP\_0* | 2 | 3 | 13.0% | 184 | 21343 | 10.1 | PREDICTED: hypothetical protein [Homo sapiens] |
| U | *gi|169165785|ref|XP\_0* | 2 | 3 | 13.0% | 184 | 21343 | 10.1 | PREDICTED: hypothetical protein [Homo sapiens] |

| Filename XCorr DeltCN Conf% ObsM+H+ CalcM+H+ SpR ZScore Ion% # Sequence  | | | | | | | | | | | | |
| --- | --- | --- | --- | --- | --- | --- | --- | --- | --- | --- | --- | --- |
|  | Astrin\_STLCLD20\_112214\_01.06526.06526.2 | 2.1912 | 0.2103 | 96.2% | 1164.2122 | 1164.2572 | 2 | 6.247 | 55.6% | 2 | R.YSLDPENPTK.S | 2 |
|  | Astrin\_STLCLD20\_112214\_tube2\_01.07060.07060.2 | 3.2596 | 0.2644 | 99.7% | 1624.0922 | 1624.8314 | 1 | 5.722 | 57.7% | 1 | K.EQIVPKPEEEVAQK.K | 2 |

---

|  |  |  |  |  |  |  |  |  |
| --- | --- | --- | --- | --- | --- | --- | --- | --- |
| U | *gi|14141161|ref|NP\_00* | 7 | 18 | 12.7% | 806 | 88980 | 5.8 | heterogeneous nuclear ribonucleoprotein U isoform b [Homo sapiens] |
| U | *gi|74136883|ref|NP\_11* | 7 | 18 | 12.4% | 825 | 90585 | 6.0 | heterogeneous nuclear ribonucleoprotein U isoform a [Homo sapiens] |

| Filename XCorr DeltCN Conf% ObsM+H+ CalcM+H+ SpR ZScore Ion% # Sequence  | | | | | | | | | | | | |
| --- | --- | --- | --- | --- | --- | --- | --- | --- | --- | --- | --- | --- |
|  | Astrin\_STLCLD20\_112214\_tube2\_01.10493.10493.3 | 4.1452 | 0.3969 | 99.7% | 3127.3442 | 3128.311 | 1 | 5.681 | 28.2% | 1 | R.LQAALDDEEAGGRPAMEPGNGSLDLGGDSAGR.S | 3 |
|  | Astrin\_STLCLD20\_112214\_01.09897.09897.2 | 3.5193 | 0.4855 | 100.0% | 1698.2922 | 1698.8291 | 1 | 7.708 | 75.0% | 1 | R.GYFEYIEENKYSR.A | 2 |
|  | Astrin\_STLCLD20\_112214\_01.12995.12995.3 | 3.711 | 0.2509 | 99.6% | 2726.0645 | 2726.0576 | 2 | 4.806 | 26.2% | 2 | K.EKPYFPIPEEYTFIQNVPLEDR.V | 3 |
|  | Astrin\_STLCLD20\_112214\_01.08888.08888.3 | 4.2038 | 0.3512 | 99.7% | 2188.8245 | 2188.4631 | 1 | 6.858 | 36.8% | 2 | K.HAAENPGKYNILGTNTIMDK.M | 3 |
|  | Astrin\_STLCLD20\_112214\_01.10827.10827.2 | 2.663 | 0.1804 | 97.7% | 1383.8522 | 1383.6025 | 1 | 5.168 | 63.6% | 2 | K.YNILGTNTIMDK.M | 2 |
|  | Astrin\_STLCLD20\_112214\_tube2\_02.07436.07436.3 | 4.085 | 0.2053 | 99.5% | 1648.7943 | 1648.816 | 1 | 5.543 | 46.4% | 2 | R.NFILDQTNVSAAAQR.R | 3 |
|  | Astrin\_STLCLD20\_112214\_tube2\_02.07423.07423.2 | 5.041 | 0.4088 | 100.0% | 1649.3522 | 1648.816 | 1 | 7.932 | 75.0% | 8 | R.NFILDQTNVSAAAQR.R | 2 |

---

|  |  |  |  |  |  |  |  |  |
| --- | --- | --- | --- | --- | --- | --- | --- | --- |
| U | *gi|11136628|ref|NP\_06* | 2 | 4 | 12.4% | 225 | 24764 | 4.7 | eukaryotic translation elongation factor 1 beta 2 [Homo sapiens] |
| U | *gi|83376130|ref|NP\_00* | 2 | 4 | 12.4% | 225 | 24764 | 4.7 | eukaryotic translation elongation factor 1 beta 2 [Homo sapiens] |
| U | *gi|4503477|ref|NP\_001* | 2 | 4 | 12.4% | 225 | 24764 | 4.7 | eukaryotic translation elongation factor 1 beta 2 [Homo sapiens] |

| Filename XCorr DeltCN Conf% ObsM+H+ CalcM+H+ SpR ZScore Ion% # Sequence  | | | | | | | | | | | | |
| --- | --- | --- | --- | --- | --- | --- | --- | --- | --- | --- | --- | --- |
|  | Astrin\_STLCLD20\_112214\_tube2\_01.15359.15359.2 | 2.8737 | 0.2656 | 99.1% | 1605.7322 | 1604.8003 | 188 | 5.732 | 35.7% | 1 | K.SPAGLQVLNDYLADK.S | 2 |
|  | Astrin\_STLCLD20\_112214\_tube2\_02.07606.07606.2 | 3.0664 | 0.4165 | 100.0% | 1348.6322 | 1348.4985 | 1 | 8.244 | 70.8% | 3 | R.SIQADGLVWGSSK.L | 2 |

---

|  |  |  |  |  |  |  |  |  |
| --- | --- | --- | --- | --- | --- | --- | --- | --- |
| U | *gi|153792590|ref|NP\_0* | 8 | 20 | 12.3% | 854 | 98161 | 5.2 | heat shock 90kDa protein 1, alpha isoform 1 [Homo sapiens] |
| U | *gi|154146191|ref|NP\_0* | 8 | 20 | 14.3% | 732 | 84660 | 5.0 | heat shock 90kDa protein 1, alpha isoform 2 [Homo sapiens] |

| Filename XCorr DeltCN Conf% ObsM+H+ CalcM+H+ SpR ZScore Ion% # Sequence  | | | | | | | | | | | | |
| --- | --- | --- | --- | --- | --- | --- | --- | --- | --- | --- | --- | --- |
|  | Astrin\_STLCLD20\_112214\_tube2\_01.12254.12254.2 | 3.4834 | 0.4327 | 100.0% | 1243.4521 | 1243.4459 | 1 | 7.724 | 81.8% | 3 | K.ADLINNLGTIAK.S | 22 |
|  | Astrin\_STLCLD20\_112214\_02.09754.09754.3 | 3.9778 | 0.4595 | 100.0% | 2015.8444 | 2016.2584 | 1 | 7.162 | 43.3% | 6 | K.VILHLKEDQTEYLEER.R | 33 |
|  | Astrin\_STLCLD20\_112214\_01.11969.11969.3 | 4.4434 | 0.3415 | 99.7% | 2066.8442 | 2065.3794 | 1 | 6.369 | 37.5% | 1 | K.HSQFIGYPITLFVEKER.D | 3 |
|  | Astrin\_STLCLD20\_112214\_01.09962.09962.2 | 4.3602 | 0.4022 | 100.0% | 1528.3922 | 1528.6616 | 1 | 7.855 | 70.8% | 2 | K.SLTNDWEDHLAVK.H | 22 |
|  | Astrin\_STLCLD20\_112214\_02.10618.10618.2 | 2.964 | 0.4331 | 100.0% | 1349.4521 | 1349.4886 | 1 | 6.95 | 70.0% | 5 | K.HFSVEGQLEFR.A | 22 |
|  | Astrin\_STLCLD20\_112214\_tube2\_01.12694.12694.2 | 2.1497 | 0.3204 | 98.6% | 1265.7322 | 1265.4142 | 5 | 5.612 | 61.1% | 1 | R.RAPFDLFENR.K | 2 |
|  | Astrin\_STLCLD20\_112214\_01.05137.05137.2 | 2.5728 | 0.2789 | 99.2% | 1236.1122 | 1236.3268 | 1 | 5.31 | 75.0% | 1 | K.DQVANSAFVER.L | 2 |
|  | Astrin\_STLCLD20\_112214\_tube2\_01.10924.10924.3 | 2.9647 | 0.4648 | 99.7% | 1789.2244 | 1788.0134 | 1 | 6.708 | 42.9% | 1 | K.HLEINPDHSIIETLR.Q | 3 |

Similarities:
gi|20149594|ref|NP\_03(4:4)  

---

|  |  |  |  |  |  |  |  |  |
| --- | --- | --- | --- | --- | --- | --- | --- | --- |
| U | *contaminant\_INT-STD1* | 7 | 28 | 12.2% | 607 | 69271 | 6.1 | BSA |

| Filename XCorr DeltCN Conf% ObsM+H+ CalcM+H+ SpR ZScore Ion% # Sequence  | | | | | | | | | | | | |
| --- | --- | --- | --- | --- | --- | --- | --- | --- | --- | --- | --- | --- |
| \* | Astrin\_STLCLD20\_112214\_tube2\_01.11936.11936.2 | 3.1112 | 0.2971 | 100.0% | 1164.1322 | 1164.344 | 6 | 6.307 | 72.2% | 3 | K.LVNELTEFAK.T | 2 |
| \* | Astrin\_STLCLD20\_112214\_02.15899.15899.2 | 3.7186 | 0.4393 | 100.0% | 1568.3121 | 1568.7258 | 1 | 8.079 | 75.0% | 2 | K.DAFLGSFLYEYSR.R | 2 |
| \* | Astrin\_STLCLD20\_112214\_tube2\_01.09741.09741.2 | 3.0358 | 0.3424 | 100.0% | 1440.3322 | 1440.6884 | 4 | 6.41 | 59.1% | 2 | R.RHPEYAVSVLLR.L | 2 |
| \* | Astrin\_STLCLD20\_112214\_tube2\_02.07041.07041.3 | 4.3348 | 0.204 | 99.8% | 1440.6843 | 1440.6884 | 1 | 5.172 | 59.1% | 7 | R.RHPEYAVSVLLR.L | 3 |
| \* | Astrin\_STLCLD20\_112214\_01.07592.07592.2 | 3.0126 | 0.423 | 100.0% | 1305.9321 | 1306.5046 | 2 | 7.564 | 60.0% | 2 | K.HLVDEPQNLIK.Q | 2 |
| \* | Astrin\_STLCLD20\_112214\_tube2\_02.08720.08720.2 | 4.2558 | 0.4291 | 100.0% | 1481.4922 | 1480.7068 | 1 | 8.038 | 75.0% | 8 | K.LGEYGFQNALIVR.Y | 2 |
|  | Astrin\_STLCLD20\_112214\_01.08360.08360.2 | 3.9455 | 0.5541 | 100.0% | 1641.5922 | 1640.9205 | 1 | 8.932 | 67.9% | 4 | R.KVPQVSTPTLVEVSR.S | 2 |

---

|  |  |  |  |  |  |  |  |  |
| --- | --- | --- | --- | --- | --- | --- | --- | --- |
| U | *gi|33469968|ref|NP\_00* | 6 | 8 | 12.1% | 719 | 81308 | 6.5 | minichromosome maintenance complex component 7 isoform 1 [Homo sapiens] |

| Filename XCorr DeltCN Conf% ObsM+H+ CalcM+H+ SpR ZScore Ion% # Sequence  | | | | | | | | | | | | |
| --- | --- | --- | --- | --- | --- | --- | --- | --- | --- | --- | --- | --- |
| \* | Astrin\_STLCLD20\_112214\_02.10756.10756.3 | 3.824 | 0.272 | 99.6% | 1829.8143 | 1829.063 | 2 | 6.633 | 41.1% | 2 | R.EVVNKDVLDVYIEHR.L | 3 |
| \* | Astrin\_STLCLD20\_112214\_01.08954.08954.3 | 2.8231 | 0.2548 | 97.6% | 1827.6244 | 1827.0525 | 47 | 4.613 | 32.1% | 1 | R.RFELYFQGPSSNKPR.V | 3 |
|  | Astrin\_STLCLD20\_112214\_tube2\_02.06751.06751.3 | 3.3061 | 0.217 | 98.2% | 1799.9944 | 1800.0802 | 15 | 4.156 | 33.3% | 1 | R.TAIHEVMEQQTISIAK.A | 3 |
|  | Astrin\_STLCLD20\_112214\_tube2\_01.05764.05764.3 | 2.7562 | 0.2449 | 97.4% | 1591.1344 | 1590.7849 | 1 | 4.512 | 47.9% | 1 | R.LAQHITYVHQHSR.Q | 3 |
|  | Astrin\_STLCLD20\_112214\_tube2\_02.06428.06428.3 | 3.6267 | 0.3081 | 99.8% | 1746.7743 | 1746.9733 | 3 | 5.044 | 42.9% | 2 | R.MVDVVEKEDVNEAIR.L | 3 |
|  | Astrin\_STLCLD20\_112214\_01.10018.10018.2 | 2.3187 | 0.2115 | 95.9% | 1475.7322 | 1474.703 | 39 | 4.459 | 50.0% | 1 | R.TQRPADVIFATVR.E | 2 |

---

|  |  |  |  |  |  |  |  |  |
| --- | --- | --- | --- | --- | --- | --- | --- | --- |
| U | *gi|24234688|ref|NP\_00* | 6 | 11 | 12.1% | 679 | 73681 | 6.2 | heat shock 70kDa protein 9 precursor [Homo sapiens] |

| Filename XCorr DeltCN Conf% ObsM+H+ CalcM+H+ SpR ZScore Ion% # Sequence  | | | | | | | | | | | | |
| --- | --- | --- | --- | --- | --- | --- | --- | --- | --- | --- | --- | --- |
| \* | Astrin\_STLCLD20\_112214\_01.09099.09099.2 | 3.2314 | 0.3856 | 100.0% | 1451.4722 | 1451.576 | 1 | 6.397 | 65.4% | 2 | R.TTPSVVAFTADGER.L | 2 |
| \* | Astrin\_STLCLD20\_112214\_tube2\_01.17133.17133.2 | 2.6871 | 0.2419 | 98.5% | 1554.1522 | 1554.8878 | 1 | 5.253 | 65.4% | 1 | K.LYSPSQIGAFVLMK.M | 2 |
| \* | Astrin\_STLCLD20\_112214\_01.10406.10406.2 | 3.1683 | 0.2979 | 99.9% | 1243.6122 | 1243.4056 | 1 | 6.176 | 68.2% | 1 | K.DAGQISGLNVLR.V | 2 |
| \* | Astrin\_STLCLD20\_112214\_tube2\_01.15821.15821.2 | 4.3515 | 0.4823 | 100.0% | 2057.0522 | 2057.181 | 1 | 7.872 | 47.2% | 1 | K.STNGDTFLGGEDFDQALLR.H | 2 |
| \* | Astrin\_STLCLD20\_112214\_tube2\_01.16337.16337.2 | 4.0023 | 0.4104 | 100.0% | 1362.5322 | 1362.5687 | 1 | 8.152 | 63.6% | 4 | R.AQFEGIVTDLIR.R | 2 |
| \* | Astrin\_STLCLD20\_112214\_01.10292.10292.2 | 3.1056 | 0.3543 | 100.0% | 1291.4922 | 1291.4496 | 1 | 7.93 | 75.0% | 2 | K.VQQTVQDLFGR.A | 2 |

---

|  |  |  |  |  |  |  |  |  |
| --- | --- | --- | --- | --- | --- | --- | --- | --- |
| U | *gi|113412878|ref|XP\_0* | 2 | 2 | 11.9% | 293 | 31479 | 7.6 | PREDICTED: similar to voltage-dependent anion channel [Homo sapiens] |
| U | *gi|42476281|ref|NP\_00* | 2 | 2 | 11.9% | 294 | 31566 | 7.6 | voltage-dependent anion channel 2 [Homo sapiens] |
| U | *gi|169164151|ref|XP\_0* | 2 | 2 | 11.9% | 293 | 31445 | 7.6 | PREDICTED: similar to voltage-dependent anion channel [Homo sapiens] |

| Filename XCorr DeltCN Conf% ObsM+H+ CalcM+H+ SpR ZScore Ion% # Sequence  | | | | | | | | | | | | |
| --- | --- | --- | --- | --- | --- | --- | --- | --- | --- | --- | --- | --- |
|  | Astrin\_STLCLD20\_112214\_tube2\_02.07422.07422.3 | 4.6319 | 0.378 | 99.7% | 2529.6243 | 2529.682 | 1 | 7.178 | 39.8% | 1 | R.TGDFQLHTNVNDGTEFGGSIYQK.V | 3 |
|  | Astrin\_STLCLD20\_112214\_tube2\_01.09372.09372.2 | 2.1427 | 0.3035 | 97.9% | 1294.6322 | 1294.4473 | 17 | 5.282 | 50.0% | 1 | K.YQLDPTASISAK.V | 2 |

---

|  |  |  |  |  |  |  |  |  |
| --- | --- | --- | --- | --- | --- | --- | --- | --- |
| U | *gi|14249348|ref|NP\_11* | 2 | 4 | 11.4% | 123 | 13941 | 5.5 | thioredoxin-like 5 [Homo sapiens] |

| Filename XCorr DeltCN Conf% ObsM+H+ CalcM+H+ SpR ZScore Ion% # Sequence  | | | | | | | | | | | | |
| --- | --- | --- | --- | --- | --- | --- | --- | --- | --- | --- | --- | --- |
| \* | Astrin\_STLCLD20\_112214\_02.10919.10919.2 | 3.1524 | 0.4605 | 100.0% | 1715.4722 | 1715.8162 | 15 | 8.099 | 46.2% | 1 | R.YEEVSVSGFEEFHR.A | 2 |
| \* | Astrin\_STLCLD20\_112214\_tube2\_01.11408.11408.3 | 3.1867 | 0.4485 | 99.7% | 1716.2344 | 1715.8162 | 1 | 7.349 | 46.2% | 3 | R.YEEVSVSGFEEFHR.A | 3 |

---

|  |  |  |  |  |  |  |  |  |
| --- | --- | --- | --- | --- | --- | --- | --- | --- |
| U | *gi|47271443|ref|NP\_00* | 2 | 3 | 11.3% | 221 | 25476 | 11.9 | splicing factor, arginine/serine-rich 2 [Homo sapiens] |

| Filename XCorr DeltCN Conf% ObsM+H+ CalcM+H+ SpR ZScore Ion% # Sequence  | | | | | | | | | | | | |
| --- | --- | --- | --- | --- | --- | --- | --- | --- | --- | --- | --- | --- |
|  | Astrin\_STLCLD20\_112214\_tube2\_01.08657.08657.2 | 2.6262 | 0.1034 | 97.3% | 919.4322 | 919.0684 | 3 | 4.065 | 85.7% | 1 | R.VGDVYIPR.D | 2 |
| \* | Astrin\_STLCLD20\_112214\_tube2\_01.13959.13959.2 | 4.0783 | 0.3912 | 100.0% | 1752.1721 | 1752.8654 | 1 | 6.767 | 59.4% | 2 | R.DAEDAMDAMDGAVLDGR.E | 2 |

---

|  |  |  |  |  |  |  |  |  |
| --- | --- | --- | --- | --- | --- | --- | --- | --- |
| U | *gi|78000181|ref|NP\_00* | 2 | 3 | 11.2% | 215 | 23432 | 10.9 | ribosomal protein L14 [Homo sapiens] |
| U | *gi|78000183|ref|NP\_00* | 2 | 3 | 11.2% | 215 | 23432 | 10.9 | ribosomal protein L14 [Homo sapiens] |

| Filename XCorr DeltCN Conf% ObsM+H+ CalcM+H+ SpR ZScore Ion% # Sequence  | | | | | | | | | | | | |
| --- | --- | --- | --- | --- | --- | --- | --- | --- | --- | --- | --- | --- |
|  | Astrin\_STLCLD20\_112214\_tube2\_01.08453.08453.2 | 2.6742 | 0.4948 | 100.0% | 1233.5322 | 1233.4124 | 1 | 7.732 | 63.6% | 2 | R.VAYVSFGPHAGK.L | 2 |
|  | Astrin\_STLCLD20\_112214\_tube2\_02.08806.08806.2 | 3.1662 | 0.2777 | 99.9% | 1356.8322 | 1355.5773 | 1 | 6.124 | 72.7% | 1 | K.LVAIVDVIDQNR.A | 2 |

---

|  |  |  |  |  |  |  |  |  |
| --- | --- | --- | --- | --- | --- | --- | --- | --- |
| U | *gi|24234699|ref|NP\_00* | 8 | 22 | 11.0% | 400 | 44106 | 5.1 | keratin 19 [Homo sapiens] |

| Filename XCorr DeltCN Conf% ObsM+H+ CalcM+H+ SpR ZScore Ion% # Sequence  | | | | | | | | | | | | |
| --- | --- | --- | --- | --- | --- | --- | --- | --- | --- | --- | --- | --- |
|  | Astrin\_STLCLD20\_112214\_01.05697.05697.2 | 2.9151 | 0.1301 | 98.6% | 1065.6322 | 1065.2578 | 20 | 6.487 | 68.8% | 1 | R.LASYLDKVR.A | 2222 |
|  | Astrin\_STLCLD20\_112214\_tube2\_01.09495.09495.1 | 2.6834 | 0.3062 | 100.0% | 1041.62 | 1042.2235 | 1 | 7.063 | 68.8% | 3 | R.IVLQIDNAR.L | 11 |
|  | Astrin\_STLCLD20\_112214\_tube2\_01.09470.09470.2 | 3.1917 | 0.1503 | 99.4% | 1042.1921 | 1042.2235 | 3 | 6.148 | 87.5% | 8 | R.IVLQIDNAR.L | 22 |
|  | Astrin\_STLCLD20\_112214\_tube2\_01.07546.07546.2 | 2.2441 | 0.2268 | 98.6% | 807.7322 | 807.8815 | 35 | 5.587 | 66.7% | 3 | R.LAADDFR.T | 22222 |
|  | Astrin\_STLCLD20\_112214\_01.08975.08975.2 | 2.9226 | 0.3172 | 100.0% | 1186.2522 | 1186.397 | 1 | 5.85 | 83.3% | 2 | R.RVLDELTLAR.T | 222 |
|  | Astrin\_STLCLD20\_112214\_01.09782.09782.1 | 2.1585 | 0.3224 | 100.0% | 1029.49 | 1030.2096 | 10 | 5.815 | 56.2% | 1 | R.VLDELTLAR.T | 111 |
|  | Astrin\_STLCLD20\_112214\_01.09757.09757.2 | 3.0933 | 0.3041 | 100.0% | 1030.3522 | 1030.2096 | 2 | 6.471 | 81.2% | 3 | R.VLDELTLAR.T | 222 |
|  | Astrin\_STLCLD20\_112214\_01.05125.05125.2 | 3.1188 | 0.3235 | 100.0% | 1123.3922 | 1123.2511 | 2 | 6.193 | 81.2% | 1 | R.LEQEIATYR.S | 222 |

Similarities:
gi|40354195|ref|NP\_95(3:5)  
gi|4557701|ref|NP\_000(6:2)  
contaminant\_KERATIN05(6:2)  
contaminant\_KERATIN03(2:6)  

---

|  |  |  |  |  |  |  |  |  |
| --- | --- | --- | --- | --- | --- | --- | --- | --- |
| U | *gi|14141157|ref|NP\_03* | 2 | 4 | 11.0% | 346 | 36926 | 6.9 | heterogeneous nuclear ribonucleoprotein H3 isoform a [Homo sapiens] |
| U | *gi|14141159|ref|NP\_06* | 2 | 4 | 11.5% | 331 | 35239 | 6.9 | heterogeneous nuclear ribonucleoprotein H3 isoform b [Homo sapiens] |

| Filename XCorr DeltCN Conf% ObsM+H+ CalcM+H+ SpR ZScore Ion% # Sequence  | | | | | | | | | | | | |
| --- | --- | --- | --- | --- | --- | --- | --- | --- | --- | --- | --- | --- |
|  | Astrin\_STLCLD20\_112214\_02.10767.10767.2 | 3.3936 | 0.429 | 100.0% | 1272.5721 | 1272.4001 | 1 | 7.799 | 81.8% | 3 | R.STGEAFVQFASK.E | 2 |
|  | Astrin\_STLCLD20\_112214\_01.05368.05368.3 | 5.0538 | 0.5125 | 100.0% | 2544.5645 | 2544.7437 | 1 | 8.377 | 38.0% | 1 | R.GMGGHGYGGAGDASSGFHGGHFVHMR.G | 3 |

---

|  |  |  |  |  |  |  |  |  |
| --- | --- | --- | --- | --- | --- | --- | --- | --- |
| U | *gi|38016911|ref|NP\_00* | 2 | 2 | 10.8% | 288 | 31731 | 7.9 | stomatin isoform a [Homo sapiens] |

| Filename XCorr DeltCN Conf% ObsM+H+ CalcM+H+ SpR ZScore Ion% # Sequence  | | | | | | | | | | | | |
| --- | --- | --- | --- | --- | --- | --- | --- | --- | --- | --- | --- | --- |
| \* | Astrin\_STLCLD20\_112214\_02.10439.10439.2 | 5.2381 | 0.3371 | 100.0% | 1932.1921 | 1931.113 | 1 | 9.317 | 55.6% | 1 | R.VQNATLAVANITNADSATR.L | 2 |
|  | Astrin\_STLCLD20\_112214\_02.10869.10869.2 | 2.4659 | 0.2881 | 98.8% | 1352.5521 | 1352.5707 | 5 | 5.679 | 54.5% | 1 | R.YLQTLTTIAAEK.N | 2 |

---

|  |  |  |  |  |  |  |  |  |
| --- | --- | --- | --- | --- | --- | --- | --- | --- |
| U | *gi|32483377|ref|NP\_05* | 2 | 5 | 10.5% | 238 | 25839 | 7.5 | peroxiredoxin 3 isoform b [Homo sapiens] |
| U | *gi|5802974|ref|NP\_006* | 2 | 5 | 9.8% | 256 | 27693 | 7.8 | peroxiredoxin 3 isoform a precursor [Homo sapiens] |

| Filename XCorr DeltCN Conf% ObsM+H+ CalcM+H+ SpR ZScore Ion% # Sequence  | | | | | | | | | | | | |
| --- | --- | --- | --- | --- | --- | --- | --- | --- | --- | --- | --- | --- |
|  | Astrin\_STLCLD20\_112214\_02.14232.14232.2 | 4.646 | 0.5015 | 100.0% | 1463.4922 | 1463.6738 | 1 | 9.13 | 80.8% | 3 | R.DYGVLLEGSGLALR.G | 2 |
|  | Astrin\_STLCLD20\_112214\_tube2\_01.08630.08630.2 | 3.0466 | 0.3499 | 100.0% | 1207.5122 | 1207.375 | 2 | 7.156 | 65.0% | 2 | K.HLSVNDLPVGR.S | 2 |

---

|  |  |  |  |  |  |  |  |  |
| --- | --- | --- | --- | --- | --- | --- | --- | --- |
| U | *gi|117968353|ref|NP\_1* | 4 | 6 | 10.3% | 464 | 54304 | 8.3 | NUF2, NDC80 kinetochore complex component [Homo sapiens] |
| U | *gi|117968420|ref|NP\_6* | 4 | 6 | 10.3% | 464 | 54304 | 8.3 | NUF2, NDC80 kinetochore complex component [Homo sapiens] |

| Filename XCorr DeltCN Conf% ObsM+H+ CalcM+H+ SpR ZScore Ion% # Sequence  | | | | | | | | | | | | |
| --- | --- | --- | --- | --- | --- | --- | --- | --- | --- | --- | --- | --- |
|  | Astrin\_STLCLD20\_112214\_tube2\_02.08691.08691.2 | 2.7574 | 0.3804 | 100.0% | 1327.6522 | 1327.5693 | 1 | 6.828 | 75.0% | 2 | R.YNVAEIVIHIR.N | 2 |
|  | Astrin\_STLCLD20\_112214\_01.05494.05494.2 | 3.6178 | 0.3934 | 100.0% | 1612.8322 | 1613.8903 | 1 | 6.775 | 73.1% | 1 | K.MQQLNAAHQEALMK.L | 2 |
|  | Astrin\_STLCLD20\_112214\_01.08368.08368.2 | 3.0497 | 0.3734 | 100.0% | 1549.4321 | 1549.6746 | 1 | 5.867 | 62.5% | 2 | R.LDSVPVEEQEEFK.Q | 2 |
|  | Astrin\_STLCLD20\_112214\_tube2\_01.06951.06951.2 | 2.4662 | 0.3376 | 99.5% | 1173.7122 | 1174.3396 | 304 | 5.355 | 61.1% | 1 | R.VTTINQEIQK.I | 2 |

---

|  |  |  |  |  |  |  |  |  |
| --- | --- | --- | --- | --- | --- | --- | --- | --- |
| U | *gi|4826734|ref|NP\_004* | 4 | 5 | 10.1% | 526 | 53426 | 9.4 | fusion (involved in t(12;16) in malignant liposarcoma) [Homo sapiens] |

| Filename XCorr DeltCN Conf% ObsM+H+ CalcM+H+ SpR ZScore Ion% # Sequence  | | | | | | | | | | | | |
| --- | --- | --- | --- | --- | --- | --- | --- | --- | --- | --- | --- | --- |
| \* | Astrin\_STLCLD20\_112214\_01.09795.09795.2 | 2.4682 | 0.1846 | 96.7% | 1410.3922 | 1409.6 | 1 | 4.261 | 63.6% | 1 | K.TGQPMINLYTDR.E | 2 |
| \* | Astrin\_STLCLD20\_112214\_tube2\_01.14507.14507.2 | 3.9055 | 0.3647 | 100.0% | 1896.5521 | 1896.1094 | 1 | 6.531 | 59.4% | 2 | K.AAIDWFDGKEFSGNPIK.V | 2 |
| \* | Astrin\_STLCLD20\_112214\_tube2\_01.05992.05992.2 | 5.1609 | 0.6038 | 100.0% | 2253.2522 | 2254.355 | 1 | 10.519 | 52.2% | 1 | K.APKPDGPGGGPGGSHMGGNYGDDR.R | 2 |
| \* | Astrin\_STLCLD20\_112214\_tube2\_01.05981.05981.3 | 3.3506 | 0.3694 | 99.8% | 2253.6543 | 2254.355 | 1 | 6.284 | 35.9% | 1 | K.APKPDGPGGGPGGSHMGGNYGDDR.R | 3 |

---

|  |  |  |  |  |  |  |  |  |
| --- | --- | --- | --- | --- | --- | --- | --- | --- |
| U | *gi|15431301|ref|NP\_00* | 2 | 5 | 10.1% | 248 | 29226 | 10.7 | ribosomal protein L7 [Homo sapiens] |
| U | *gi|88988289|ref|XP\_94* | 2 | 5 | 9.7% | 259 | 30508 | 10.8 | PREDICTED: hypothetical protein LOC648000 isoform 3 [Homo sapiens] |
| U | *gi|169171881|ref|XP\_0* | 2 | 5 | 10.1% | 247 | 29037 | 10.7 | PREDICTED: hypothetical protein [Homo sapiens] |
| U | *gi|169171450|ref|XP\_0* | 2 | 5 | 10.1% | 247 | 28971 | 10.6 | PREDICTED: hypothetical protein [Homo sapiens] |
| U | *gi|169171114|ref|XP\_0* | 2 | 5 | 10.1% | 247 | 29037 | 10.7 | PREDICTED: hypothetical protein [Homo sapiens] |
| U | *gi|169170622|ref|XP\_0* | 2 | 5 | 10.1% | 247 | 29009 | 10.7 | PREDICTED: hypothetical protein [Homo sapiens] |
| U | *gi|169168181|ref|XP\_0* | 2 | 5 | 9.7% | 259 | 30508 | 10.8 | PREDICTED: hypothetical protein LOC648000 [Homo sapiens] |
| U | *gi|169167651|ref|XP\_0* | 2 | 5 | 9.7% | 259 | 30508 | 10.8 | PREDICTED: hypothetical protein LOC648000 [Homo sapiens] |

| Filename XCorr DeltCN Conf% ObsM+H+ CalcM+H+ SpR ZScore Ion% # Sequence  | | | | | | | | | | | | |
| --- | --- | --- | --- | --- | --- | --- | --- | --- | --- | --- | --- | --- |
|  | Astrin\_STLCLD20\_112214\_01.12712.12712.2 | 2.7244 | 0.3085 | 99.3% | 1663.6921 | 1663.9567 | 11 | 5.521 | 50.0% | 2 | R.IVEPYIAWGYPNLK.S | 2 |
|  | Astrin\_STLCLD20\_112214\_tube2\_01.11343.11343.2 | 3.4449 | 0.3762 | 100.0% | 1171.3722 | 1171.3823 | 1 | 7.901 | 90.0% | 3 | R.IALTDNALIAR.S | 2 |

---

|  |  |  |  |  |  |  |  |  |
| --- | --- | --- | --- | --- | --- | --- | --- | --- |
| U | *gi|221307584|ref|NP\_0* | 3 | 9 | 10.0% | 299 | 33296 | 9.8 | prohibitin 2 isoform 1 [Homo sapiens] |
| U | *gi|6005854|ref|NP\_009* | 3 | 9 | 10.0% | 299 | 33296 | 9.8 | prohibitin 2 isoform 2 [Homo sapiens] |

| Filename XCorr DeltCN Conf% ObsM+H+ CalcM+H+ SpR ZScore Ion% # Sequence  | | | | | | | | | | | | |
| --- | --- | --- | --- | --- | --- | --- | --- | --- | --- | --- | --- | --- |
|  | Astrin\_STLCLD20\_112214\_01.11302.11302.2 | 3.0964 | 0.2653 | 99.4% | 1854.8722 | 1855.1038 | 12 | 5.06 | 40.6% | 2 | R.IGGVQQDTILAEGLHFR.I | 2 |
|  | Astrin\_STLCLD20\_112214\_tube2\_02.08632.08632.3 | 4.2214 | 0.4757 | 100.0% | 1855.0443 | 1855.1038 | 1 | 7.223 | 45.3% | 5 | R.IGGVQQDTILAEGLHFR.I | 3 |
|  | Astrin\_STLCLD20\_112214\_tube2\_01.19502.19502.2 | 4.1275 | 0.3611 | 100.0% | 1724.6522 | 1725.0428 | 1 | 7.228 | 66.7% | 2 | R.IPWFQYPIIYDIR.A | 2 |

---

|  |  |  |  |  |  |  |  |  |
| --- | --- | --- | --- | --- | --- | --- | --- | --- |
| U | *gi|38327564|ref|NP\_94* | 3 | 4 | 9.9% | 403 | 45823 | 9.4 | serine/threonine protein kinase 6 [Homo sapiens] |
| U | *gi|38327572|ref|NP\_94* | 3 | 4 | 9.9% | 403 | 45823 | 9.4 | serine/threonine protein kinase 6 [Homo sapiens] |
| U | *gi|38327570|ref|NP\_94* | 3 | 4 | 9.9% | 403 | 45823 | 9.4 | serine/threonine protein kinase 6 [Homo sapiens] |
| U | *gi|38327568|ref|NP\_94* | 3 | 4 | 9.9% | 403 | 45823 | 9.4 | serine/threonine protein kinase 6 [Homo sapiens] |
| U | *gi|38327566|ref|NP\_94* | 3 | 4 | 9.9% | 403 | 45823 | 9.4 | serine/threonine protein kinase 6 [Homo sapiens] |

| Filename XCorr DeltCN Conf% ObsM+H+ CalcM+H+ SpR ZScore Ion% # Sequence  | | | | | | | | | | | | |
| --- | --- | --- | --- | --- | --- | --- | --- | --- | --- | --- | --- | --- |
|  | Astrin\_STLCLD20\_112214\_01.07147.07147.2 | 2.1943 | 0.2001 | 95.7% | 1244.0521 | 1243.3641 | 1 | 5.062 | 77.8% | 1 | R.LYGYFHDATR.V | 2 |
|  | Astrin\_STLCLD20\_112214\_tube2\_01.13649.13649.2 | 2.463 | 0.207 | 96.3% | 1697.5521 | 1697.9695 | 58 | 4.222 | 43.3% | 1 | R.DIKPENLLLGSAGELK.I | 2 |
|  | Astrin\_STLCLD20\_112214\_01.13255.13255.2 | 2.9086 | 0.3161 | 99.7% | 1615.2122 | 1615.7826 | 11 | 6.617 | 50.0% | 2 | R.VEFTFPDFVTEGAR.D | 2 |

---

|  |  |  |  |  |  |  |  |  |
| --- | --- | --- | --- | --- | --- | --- | --- | --- |
| U | *gi|14602427|ref|NP\_12* | 2 | 4 | 9.7% | 277 | 31293 | 5.2 | ZW10 interactor isoform a [Homo sapiens] |
| U | *gi|14602429|ref|NP\_00* | 2 | 4 | 9.7% | 277 | 31293 | 5.2 | ZW10 interactor isoform a [Homo sapiens] |

| Filename XCorr DeltCN Conf% ObsM+H+ CalcM+H+ SpR ZScore Ion% # Sequence  | | | | | | | | | | | | |
| --- | --- | --- | --- | --- | --- | --- | --- | --- | --- | --- | --- | --- |
|  | Astrin\_STLCLD20\_112214\_tube2\_02.05577.05577.3 | 3.8198 | 0.4008 | 100.0% | 1489.4343 | 1489.6743 | 1 | 7.001 | 50.0% | 2 | K.HLQHLAEVSAEVR.E | 3 |
|  | Astrin\_STLCLD20\_112214\_tube2\_01.20096.20096.2 | 2.2366 | 0.3437 | 98.6% | 1716.1122 | 1717.0178 | 10 | 5.548 | 38.5% | 2 | R.YQTFLQLLYTLQGK.L | 2 |

---

|  |  |  |  |  |  |  |  |  |
| --- | --- | --- | --- | --- | --- | --- | --- | --- |
| U | *gi|20357552|ref|NP\_00* | 3 | 4 | 9.6% | 550 | 61586 | 5.4 | cortactin isoform a [Homo sapiens] |
| U | *gi|20357556|ref|NP\_61* | 3 | 4 | 10.3% | 513 | 57467 | 5.3 | cortactin isoform b [Homo sapiens] |

| Filename XCorr DeltCN Conf% ObsM+H+ CalcM+H+ SpR ZScore Ion% # Sequence  | | | | | | | | | | | | |
| --- | --- | --- | --- | --- | --- | --- | --- | --- | --- | --- | --- | --- |
|  | Astrin\_STLCLD20\_112214\_02.10110.10110.3 | 3.7089 | 0.3556 | 99.8% | 2250.1743 | 2250.442 | 4 | 6.358 | 34.2% | 2 | R.MDKNASTFEDVTQVSSAYQK.T | 3 |
|  | Astrin\_STLCLD20\_112214\_tube2\_01.11252.11252.2 | 2.4743 | 0.25 | 97.6% | 2155.5723 | 2156.3284 | 5 | 5.003 | 42.1% | 1 | R.GPVSGTEPEPVYSMEAADYR.E | 2 |
|  | Astrin\_STLCLD20\_112214\_01.12159.12159.2 | 2.8298 | 0.3843 | 100.0% | 1570.4922 | 1570.7881 | 1 | 6.67 | 50.0% | 1 | R.YGLFPANYVELRQ.- | 2 |

---

|  |  |  |  |  |  |  |  |  |
| --- | --- | --- | --- | --- | --- | --- | --- | --- |
| U | *gi|169212778|ref|XP\_0* | 4 | 7 | 9.4% | 266 | 30042 | 10.6 | PREDICTED: similar to ribosomal protein L7a [Homo sapiens] |
| U | *gi|4506661|ref|NP\_000* | 4 | 7 | 9.4% | 266 | 29996 | 10.6 | ribosomal protein L7a [Homo sapiens] |
| U | *gi|169213130|ref|XP\_0* | 4 | 7 | 9.4% | 266 | 30042 | 10.6 | PREDICTED: similar to ribosomal protein L7a [Homo sapiens] |
| U | *gi|169212940|ref|XP\_0* | 4 | 7 | 9.4% | 266 | 30028 | 10.6 | PREDICTED: similar to ribosomal protein L7a [Homo sapiens] |

| Filename XCorr DeltCN Conf% ObsM+H+ CalcM+H+ SpR ZScore Ion% # Sequence  | | | | | | | | | | | | |
| --- | --- | --- | --- | --- | --- | --- | --- | --- | --- | --- | --- | --- |
|  | Astrin\_STLCLD20\_112214\_tube2\_01.09764.09764.2 | 2.6581 | 0.1589 | 97.4% | 1217.7922 | 1217.3672 | 26 | 5.43 | 60.0% | 2 | K.NFGIGQDIQPK.R | 2 |
|  | Astrin\_STLCLD20\_112214\_01.08908.08908.2 | 3.5536 | 0.2533 | 99.9% | 1347.4122 | 1346.5236 | 2 | 5.292 | 62.5% | 3 | R.AGVNTVTTLVENK.K | 2 |
|  | Astrin\_STLCLD20\_112214\_01.07407.07407.2 | 2.8089 | 0.2659 | 99.1% | 1474.5322 | 1474.6976 | 24 | 4.827 | 53.8% | 1 | R.AGVNTVTTLVENKK.A | 2 |
|  | Astrin\_STLCLD20\_112214\_tube2\_01.08536.08536.3 | 2.8712 | 0.3074 | 99.6% | 1475.3644 | 1474.6976 | 15 | 5.931 | 32.7% | 1 | R.AGVNTVTTLVENKK.A | 3 |

---

|  |  |  |  |  |  |  |  |  |
| --- | --- | --- | --- | --- | --- | --- | --- | --- |
| U | *gi|148277065|ref|NP\_0* | 3 | 6 | 9.1% | 551 | 60269 | 6.7 | thioredoxin reductase 1 isoform 1 [Homo sapiens] |
| U | *gi|33519430|ref|NP\_87* | 3 | 6 | 10.0% | 499 | 54604 | 6.5 | thioredoxin reductase 1 isoform 2 [Homo sapiens] |
| U | *gi|33519428|ref|NP\_87* | 3 | 6 | 10.0% | 499 | 54604 | 6.5 | thioredoxin reductase 1 isoform 2 [Homo sapiens] |
| U | *gi|33519426|ref|NP\_87* | 3 | 6 | 10.0% | 499 | 54604 | 6.5 | thioredoxin reductase 1 isoform 2 [Homo sapiens] |
| U | *gi|148277071|ref|NP\_0* | 3 | 6 | 7.7% | 649 | 70756 | 7.4 | thioredoxin reductase 1 isoform 3 [Homo sapiens] |

| Filename XCorr DeltCN Conf% ObsM+H+ CalcM+H+ SpR ZScore Ion% # Sequence  | | | | | | | | | | | | |
| --- | --- | --- | --- | --- | --- | --- | --- | --- | --- | --- | --- | --- |
|  | Astrin\_STLCLD20\_112214\_tube2\_02.08104.08104.3 | 2.6974 | 0.2935 | 98.2% | 1989.2943 | 1989.2592 | 8 | 5.15 | 34.4% | 1 | R.MIEAVQNHIGSLNWGYR.V | 3 |
|  | Astrin\_STLCLD20\_112214\_01.05842.05842.2 | 2.7685 | 0.2645 | 99.4% | 1160.8522 | 1160.3616 | 9 | 5.674 | 66.7% | 4 | R.FLIATGERPR.Y | 2 |
|  | Astrin\_STLCLD20\_112214\_tube2\_01.19575.19575.3 | 4.1406 | 0.3415 | 99.8% | 2901.3245 | 2901.2065 | 7 | 5.353 | 28.4% | 1 | K.FGEENIEVYHSYFWPLEWTIPSR.D | 3 |

---

|  |  |  |  |  |  |  |  |  |
| --- | --- | --- | --- | --- | --- | --- | --- | --- |
| U | *gi|14141166|ref|NP\_11* | 3 | 8 | 9.1% | 362 | 38222 | 6.8 | poly(rC) binding protein 2 isoform b [Homo sapiens] |
| U | *gi|193083114|ref|NP\_0* | 3 | 8 | 10.4% | 318 | 33497 | 8.2 | poly(rC) binding protein 2 isoform g [Homo sapiens] |
| U | *gi|193083112|ref|NP\_0* | 3 | 8 | 9.9% | 335 | 35347 | 8.0 | poly(rC) binding protein 2 isoform f [Homo sapiens] |
| U | *gi|193083110|ref|NP\_0* | 3 | 8 | 9.1% | 361 | 38151 | 6.8 | poly(rC) binding protein 2 isoform e [Homo sapiens] |
| U | *gi|193083108|ref|NP\_0* | 3 | 8 | 9.0% | 365 | 38580 | 6.8 | poly(rC) binding protein 2 isoform d [Homo sapiens] |
| U | *gi|148833484|ref|NP\_0* | 3 | 8 | 10.0% | 331 | 34917 | 8.0 | poly(rC) binding protein 2 isoform c [Homo sapiens] |
| U | *gi|14141168|ref|NP\_00* | 3 | 8 | 9.0% | 366 | 38651 | 6.8 | poly(rC) binding protein 2 isoform a [Homo sapiens] |

| Filename XCorr DeltCN Conf% ObsM+H+ CalcM+H+ SpR ZScore Ion% # Sequence  | | | | | | | | | | | | |
| --- | --- | --- | --- | --- | --- | --- | --- | --- | --- | --- | --- | --- |
|  | Astrin\_STLCLD20\_112214\_01.11809.11809.2 | 3.4369 | 0.3953 | 100.0% | 1360.4321 | 1359.6519 | 1 | 6.818 | 58.3% | 4 | R.IITLAGPTNAIFK.A | 2 |
|  | Astrin\_STLCLD20\_112214\_02.10069.10069.2 | 4.9019 | 0.5556 | 100.0% | 2091.4922 | 2091.2573 | 1 | 9.838 | 57.9% | 3 | R.ESTGAQVQVAGDMLPNSTER.A | 22 |
|  | Astrin\_STLCLD20\_112214\_tube2\_02.06836.06836.3 | 4.1418 | 0.4486 | 100.0% | 2091.8342 | 2091.2573 | 1 | 7.273 | 39.5% | 1 | R.ESTGAQVQVAGDMLPNSTER.A | 33 |

Similarities:
gi|222352151|ref|NP\_0(2:1)  

---

|  |  |  |  |  |  |  |  |  |
| --- | --- | --- | --- | --- | --- | --- | --- | --- |
| U | *gi|32454741|ref|NP\_00* | 2 | 2 | 8.9% | 418 | 46441 | 8.7 | serine (or cysteine) proteinase inhibitor, clade H, member 1 precursor [Homo sapiens] |

| Filename XCorr DeltCN Conf% ObsM+H+ CalcM+H+ SpR ZScore Ion% # Sequence  | | | | | | | | | | | | |
| --- | --- | --- | --- | --- | --- | --- | --- | --- | --- | --- | --- | --- |
| \* | Astrin\_STLCLD20\_112214\_tube2\_01.13704.13704.3 | 3.6481 | 0.293 | 99.5% | 2406.0244 | 2406.7012 | 2 | 5.34 | 29.8% | 1 | K.AVLSAEQLRDEEVHAGLGELLR.S | 3 |
| \* | Astrin\_STLCLD20\_112214\_tube2\_01.14733.14733.2 | 3.2007 | 0.3602 | 100.0% | 1660.4722 | 1660.8235 | 4 | 6.169 | 46.4% | 1 | R.LYGPSSVSFADDFVR.S | 2 |

---

|  |  |  |  |  |  |  |  |  |
| --- | --- | --- | --- | --- | --- | --- | --- | --- |
| U | *gi|4503481|ref|NP\_001* | 3 | 6 | 8.5% | 437 | 50119 | 6.7 | eukaryotic translation elongation factor 1 gamma [Homo sapiens] |

| Filename XCorr DeltCN Conf% ObsM+H+ CalcM+H+ SpR ZScore Ion% # Sequence  | | | | | | | | | | | | |
| --- | --- | --- | --- | --- | --- | --- | --- | --- | --- | --- | --- | --- |
| \* | Astrin\_STLCLD20\_112214\_tube2\_02.06445.06445.2 | 3.8983 | 0.4351 | 100.0% | 1348.1522 | 1348.5448 | 1 | 8.226 | 70.8% | 3 | K.ALIAAQYSGAQVR.V | 2 |
| \* | Astrin\_STLCLD20\_112214\_tube2\_01.11338.11338.2 | 2.1893 | 0.3706 | 99.3% | 1242.3922 | 1242.4172 | 3 | 5.831 | 61.1% | 2 | K.STFVLDEFKR.K | 2 |
| \* | Astrin\_STLCLD20\_112214\_tube2\_01.07642.07642.2 | 3.3251 | 0.2391 | 99.6% | 1574.2122 | 1573.7434 | 1 | 5.325 | 65.4% | 1 | R.KLDPGSEETQTLVR.E | 2 |

---

|  |  |  |  |  |  |  |  |  |
| --- | --- | --- | --- | --- | --- | --- | --- | --- |
| U | *gi|167466173|ref|NP\_0* | 4 | 12 | 8.4% | 641 | 70052 | 5.6 | heat shock 70kDa protein 1B [Homo sapiens] |
| U | *gi|194248072|ref|NP\_0* | 4 | 12 | 8.4% | 641 | 70052 | 5.6 | heat shock 70kDa protein 1A [Homo sapiens] |

| Filename XCorr DeltCN Conf% ObsM+H+ CalcM+H+ SpR ZScore Ion% # Sequence  | | | | | | | | | | | | |
| --- | --- | --- | --- | --- | --- | --- | --- | --- | --- | --- | --- | --- |
|  | Astrin\_STLCLD20\_112214\_01.09043.09043.2 | 3.6782 | 0.4657 | 100.0% | 1488.2922 | 1488.5939 | 1 | 9.028 | 75.0% | 7 | R.TTPSYVAFTDTER.L | 22 |
|  | Astrin\_STLCLD20\_112214\_01.09389.09389.2 | 3.9187 | 0.2615 | 100.0% | 1660.3121 | 1659.8394 | 1 | 6.93 | 60.7% | 1 | K.NQVALNPQNTVFDAK.R | 2 |
|  | Astrin\_STLCLD20\_112214\_tube2\_01.13556.13556.2 | 4.9624 | 0.4574 | 100.0% | 1688.5122 | 1688.9213 | 1 | 9.611 | 76.7% | 3 | R.IINEPTAAAIAYGLDR.T | 2 |
|  | Astrin\_STLCLD20\_112214\_tube2\_01.09964.09964.2 | 2.2563 | 0.2563 | 98.0% | 1262.5721 | 1262.4508 | 1 | 5.332 | 72.2% | 1 | R.LVNHFVEEFK.R | 2 |

Similarities:
gi|5729877|ref|NP\_006(1:3)  

---

|  |  |  |  |  |  |  |  |  |
| --- | --- | --- | --- | --- | --- | --- | --- | --- |
| U | *gi|24430151|ref|NP\_00* | 2 | 3 | 8.4% | 440 | 49185 | 6.2 | proteasome 26S ATPase subunit 1 [Homo sapiens] |

| Filename XCorr DeltCN Conf% ObsM+H+ CalcM+H+ SpR ZScore Ion% # Sequence  | | | | | | | | | | | | |
| --- | --- | --- | --- | --- | --- | --- | --- | --- | --- | --- | --- | --- |
| \* | Astrin\_STLCLD20\_112214\_tube2\_01.15112.15112.3 | 4.1804 | 0.3197 | 99.8% | 2410.6743 | 2411.7173 | 2 | 6.329 | 29.8% | 2 | R.VAEEHAPSIVFIDEIDAIGTKR.Y | 3 |
| \* | Astrin\_STLCLD20\_112214\_tube2\_01.19455.19455.2 | 3.778 | 0.4412 | 100.0% | 1752.6322 | 1752.98 | 1 | 8.1 | 53.6% | 1 | R.TMLELLNQLDGFDSR.G | 2 |

---

|  |  |  |  |  |  |  |  |  |
| --- | --- | --- | --- | --- | --- | --- | --- | --- |
| U | *gi|171906575|ref|NP\_0* | 3 | 4 | 8.4% | 439 | 49042 | 5.0 | drebrin-like isoform c [Homo sapiens] |
| U | *gi|62198235|ref|NP\_00* | 3 | 4 | 8.6% | 430 | 48207 | 5.0 | drebrin-like isoform b [Homo sapiens] |
| U | *gi|21361670|ref|NP\_05* | 3 | 4 | 8.6% | 431 | 48294 | 5.0 | drebrin-like isoform a [Homo sapiens] |

| Filename XCorr DeltCN Conf% ObsM+H+ CalcM+H+ SpR ZScore Ion% # Sequence  | | | | | | | | | | | | |
| --- | --- | --- | --- | --- | --- | --- | --- | --- | --- | --- | --- | --- |
|  | Astrin\_STLCLD20\_112214\_01.06443.06443.2 | 2.871 | 0.3506 | 100.0% | 1218.4922 | 1218.3549 | 11 | 5.652 | 70.0% | 2 | R.NGPALQEAYVR.V | 2 |
|  | Astrin\_STLCLD20\_112214\_02.14710.14710.2 | 2.7048 | 0.4199 | 99.9% | 1719.6322 | 1719.9377 | 3 | 6.976 | 46.4% | 1 | K.FVLINWTGEGVNDVR.K | 2 |
|  | Astrin\_STLCLD20\_112214\_01.05061.05061.2 | 3.3316 | 0.3294 | 100.0% | 1390.1322 | 1390.4954 | 2 | 6.067 | 80.0% | 1 | R.TWEQQQEVVSR.N | 2 |

---

|  |  |  |  |  |  |  |  |  |
| --- | --- | --- | --- | --- | --- | --- | --- | --- |
| U | *gi|156071459|ref|NP\_0* | 2 | 4 | 8.4% | 298 | 32852 | 9.7 | solute carrier family 25, member 5 [Homo sapiens] |

| Filename XCorr DeltCN Conf% ObsM+H+ CalcM+H+ SpR ZScore Ion% # Sequence  | | | | | | | | | | | | |
| --- | --- | --- | --- | --- | --- | --- | --- | --- | --- | --- | --- | --- |
| \* | Astrin\_STLCLD20\_112214\_02.12583.12583.2 | 3.5412 | 0.4394 | 100.0% | 1220.4722 | 1220.4111 | 1 | 8.242 | 79.2% | 3 | K.DFLAGGVAAAISK.T | 2 |
|  | Astrin\_STLCLD20\_112214\_tube2\_01.16740.16740.2 | 2.6214 | 0.402 | 99.9% | 1447.4122 | 1447.6763 | 1 | 6.425 | 68.2% | 1 | R.YFPTQALNFAFK.D | 2 |

---

|  |  |  |  |  |  |  |  |  |
| --- | --- | --- | --- | --- | --- | --- | --- | --- |
| U | *gi|21626466|ref|NP\_06* | 4 | 6 | 8.3% | 847 | 94623 | 6.3 | matrin 3 [Homo sapiens] |
| U | *gi|62750354|ref|NP\_95* | 4 | 6 | 8.3% | 847 | 94623 | 6.3 | matrin 3 [Homo sapiens] |

| Filename XCorr DeltCN Conf% ObsM+H+ CalcM+H+ SpR ZScore Ion% # Sequence  | | | | | | | | | | | | |
| --- | --- | --- | --- | --- | --- | --- | --- | --- | --- | --- | --- | --- |
|  | Astrin\_STLCLD20\_112214\_01.09935.09935.3 | 3.4683 | 0.1816 | 95.3% | 2363.4243 | 2363.462 | 5 | 4.088 | 32.5% | 1 | R.DSFDDRGPSLNPVLDYDHGSR.S | 3 |
|  | Astrin\_STLCLD20\_112214\_01.08516.08516.2 | 2.242 | 0.1708 | 95.6% | 1145.2922 | 1145.3647 | 1 | 5.3 | 75.0% | 1 | R.VVHIMDFQR.G | 2 |
|  | Astrin\_STLCLD20\_112214\_01.14905.14905.3 | 4.6495 | 0.4325 | 100.0% | 2439.6843 | 2439.9036 | 4 | 7.029 | 31.2% | 2 | R.YQLLQLVEPFGVISNHLILNK.I | 3 |
|  | Astrin\_STLCLD20\_112214\_01.08630.08630.3 | 3.4259 | 0.4076 | 99.8% | 2037.2344 | 2038.3109 | 2 | 6.722 | 31.9% | 2 | R.VIHLSNLPHSGYSDSAVLK.L | 3 |

---

|  |  |  |  |  |  |  |  |  |
| --- | --- | --- | --- | --- | --- | --- | --- | --- |
| U | *gi|9558733|ref|NP\_037* | 2 | 4 | 8.2% | 282 | 32689 | 11.3 | transformer-2 alpha [Homo sapiens] |

| Filename XCorr DeltCN Conf% ObsM+H+ CalcM+H+ SpR ZScore Ion% # Sequence  | | | | | | | | | | | | |
| --- | --- | --- | --- | --- | --- | --- | --- | --- | --- | --- | --- | --- |
| \* | Astrin\_STLCLD20\_112214\_tube2\_01.11961.11961.2 | 3.6585 | 0.3649 | 100.0% | 1568.4122 | 1567.7416 | 1 | 6.35 | 69.2% | 2 | R.YGPLSGVNVVYDQR.T | 2 |
| \* | Astrin\_STLCLD20\_112214\_tube2\_01.15959.15959.2 | 2.0704 | 0.2651 | 97.3% | 1136.5122 | 1136.295 | 56 | 5.735 | 62.5% | 2 | R.GFAFVYFER.I | 2 |

---

|  |  |  |  |  |  |  |  |  |
| --- | --- | --- | --- | --- | --- | --- | --- | --- |
| U | *gi|14591909|ref|NP\_00* | 2 | 4 | 8.1% | 297 | 34363 | 9.7 | ribosomal protein L5 [Homo sapiens] |

| Filename XCorr DeltCN Conf% ObsM+H+ CalcM+H+ SpR ZScore Ion% # Sequence  | | | | | | | | | | | | |
| --- | --- | --- | --- | --- | --- | --- | --- | --- | --- | --- | --- | --- |
| \* | Astrin\_STLCLD20\_112214\_01.08218.08218.2 | 2.6508 | 0.4115 | 100.0% | 1435.1921 | 1435.6587 | 1 | 7.084 | 77.3% | 3 | K.HIMGQNVADYMR.Y | 2 |
| \* | Astrin\_STLCLD20\_112214\_01.05635.05635.2 | 2.4868 | 0.3802 | 99.7% | 1533.2122 | 1534.6777 | 9 | 6.338 | 54.5% | 1 | R.YLMEEDEDAYKK.Q | 2 |

---

|  |  |  |  |  |  |  |  |  |
| --- | --- | --- | --- | --- | --- | --- | --- | --- |
| U | *gi|17158044|ref|NP\_00* | 2 | 2 | 8.0% | 249 | 28681 | 10.8 | ribosomal protein S6 [Homo sapiens] |

| Filename XCorr DeltCN Conf% ObsM+H+ CalcM+H+ SpR ZScore Ion% # Sequence  | | | | | | | | | | | | |
| --- | --- | --- | --- | --- | --- | --- | --- | --- | --- | --- | --- | --- |
| \* | Astrin\_STLCLD20\_112214\_tube2\_01.12880.12880.2 | 2.3296 | 0.2659 | 97.2% | 1621.3722 | 1621.8022 | 1 | 4.676 | 53.6% | 1 | R.MATEVAADALGEEWK.G | 2 |
| \* | Astrin\_STLCLD20\_112214\_02.12948.12948.3 | 3.3941 | 0.3335 | 99.7% | 2196.3843 | 2196.4827 | 1 | 5.692 | 34.2% | 1 | R.MATEVAADALGEEWKGYVVR.I | 3 |

---

|  |  |  |  |  |  |  |  |  |
| --- | --- | --- | --- | --- | --- | --- | --- | --- |
| U | *gi|5174447|ref|NP\_006* | 2 | 3 | 7.9% | 317 | 35077 | 7.7 | guanine nucleotide binding protein (G protein), beta polypeptide 2-like 1 [Homo sapiens] |

| Filename XCorr DeltCN Conf% ObsM+H+ CalcM+H+ SpR ZScore Ion% # Sequence  | | | | | | | | | | | | |
| --- | --- | --- | --- | --- | --- | --- | --- | --- | --- | --- | --- | --- |
| \* | Astrin\_STLCLD20\_112214\_tube2\_01.11706.11706.2 | 3.6857 | 0.232 | 99.8% | 1789.5521 | 1790.0642 | 1 | 6.024 | 60.0% | 2 | K.IIVDELKQEVISTSSK.A | 2 |
| \* | Astrin\_STLCLD20\_112214\_tube2\_01.10973.10973.2 | 2.0756 | 0.2792 | 97.7% | 1060.3322 | 1060.2412 | 8 | 5.143 | 68.8% | 1 | R.VWQVTIGTR.- | 2 |

---

|  |  |  |  |  |  |  |  |  |
| --- | --- | --- | --- | --- | --- | --- | --- | --- |
| U | *gi|47132620|ref|NP\_00* | 4 | 7 | 7.8% | 639 | 65433 | 8.0 | keratin 2 [Homo sapiens] |

| Filename XCorr DeltCN Conf% ObsM+H+ CalcM+H+ SpR ZScore Ion% # Sequence  | | | | | | | | | | | | |
| --- | --- | --- | --- | --- | --- | --- | --- | --- | --- | --- | --- | --- |
|  | Astrin\_STLCLD20\_112214\_01.08756.08756.2 | 2.5869 | 0.1788 | 98.4% | 1082.6522 | 1083.2755 | 3 | 6.37 | 75.0% | 2 | K.FASFIDKVR.F | 2222 |
|  | Astrin\_STLCLD20\_112214\_01.07333.07333.2 | 4.5527 | 0.0972 | 99.9% | 1476.4922 | 1476.6726 | 1 | 7.289 | 90.9% | 3 | R.FLEQQNQVLQTK.W | 22 |
|  | Astrin\_STLCLD20\_112214\_tube2\_02.06602.06602.2 | 2.8848 | 0.4186 | 100.0% | 1194.8322 | 1194.33 | 1 | 6.654 | 83.3% | 1 | K.YEELQVTVGR.H | 2 |
|  | Astrin\_STLCLD20\_112214\_tube2\_01.15761.15761.3 | 4.4833 | 0.3719 | 100.0% | 2199.5344 | 2199.4258 | 2 | 6.021 | 36.1% | 1 | R.NKLNDLEEALQQAKEDLAR.L | 3 |

Similarities:
gi|4504919|ref|NP\_002(1:3)  
gi|67782365|ref|NP\_00(1:3)  
gi|119395750|ref|NP\_0(1:3)  
gi|119703753|ref|NP\_0(1:3)  

---

|  |  |  |  |  |  |  |  |  |
| --- | --- | --- | --- | --- | --- | --- | --- | --- |
| U | *gi|34740329|ref|NP\_91* | 3 | 4 | 7.7% | 378 | 39595 | 9.0 | heterogeneous nuclear ribonucleoprotein A3 [Homo sapiens] |

| Filename XCorr DeltCN Conf% ObsM+H+ CalcM+H+ SpR ZScore Ion% # Sequence  | | | | | | | | | | | | |
| --- | --- | --- | --- | --- | --- | --- | --- | --- | --- | --- | --- | --- |
| \* | Astrin\_STLCLD20\_112214\_02.15077.15077.2 | 2.5633 | 0.3066 | 98.9% | 1772.1921 | 1771.9646 | 326 | 5.41 | 33.3% | 1 | K.LFIGGLSFETTDDSLR.E | 2 |
| \* | Astrin\_STLCLD20\_112214\_01.08883.08883.2 | 3.0923 | 0.3813 | 100.0% | 1583.4321 | 1583.7968 | 1 | 5.927 | 70.8% | 1 | K.YGKIETIEVMEDR.Q | 2 |
| \* | Astrin\_STLCLD20\_112214\_tube2\_01.10398.10398.2 | 2.8625 | 0.4276 | 100.0% | 1235.1921 | 1235.3948 | 22 | 7.113 | 72.2% | 2 | K.IETIEVMEDR.Q | 2 |

---

|  |  |  |  |  |  |  |  |  |
| --- | --- | --- | --- | --- | --- | --- | --- | --- |
| U | *gi|4557303|ref|NP\_000* | 3 | 4 | 7.6% | 485 | 54848 | 7.9 | aldehyde dehydrogenase 3A2 isoform 2 [Homo sapiens] |
| U | *gi|73466520|ref|NP\_00* | 3 | 4 | 7.3% | 508 | 57669 | 8.9 | aldehyde dehydrogenase 3A2 isoform 1 [Homo sapiens] |

| Filename XCorr DeltCN Conf% ObsM+H+ CalcM+H+ SpR ZScore Ion% # Sequence  | | | | | | | | | | | | |
| --- | --- | --- | --- | --- | --- | --- | --- | --- | --- | --- | --- | --- |
|  | Astrin\_STLCLD20\_112214\_01.05636.05636.2 | 2.675 | 0.2324 | 98.7% | 1268.3522 | 1267.3379 | 2 | 4.968 | 68.2% | 1 | K.IAFGGETDEATR.Y | 2 |
|  | Astrin\_STLCLD20\_112214\_01.10526.10526.2 | 3.5562 | 0.4102 | 100.0% | 1433.5721 | 1432.6567 | 1 | 6.784 | 79.2% | 2 | R.YIAPTVLTDVDPK.T | 2 |
|  | Astrin\_STLCLD20\_112214\_tube2\_01.12746.12746.2 | 2.9341 | 0.4098 | 100.0% | 1434.4122 | 1434.5486 | 1 | 7.353 | 77.3% | 1 | K.NVDEAINFINER.E | 2 |

---

|  |  |  |  |  |  |  |  |  |
| --- | --- | --- | --- | --- | --- | --- | --- | --- |
| U | *gi|14327896|ref|NP\_11* | 2 | 2 | 7.6% | 433 | 48337 | 7.5 | cyclin B1 [Homo sapiens] |

| Filename XCorr DeltCN Conf% ObsM+H+ CalcM+H+ SpR ZScore Ion% # Sequence  | | | | | | | | | | | | |
| --- | --- | --- | --- | --- | --- | --- | --- | --- | --- | --- | --- | --- |
| \* | Astrin\_STLCLD20\_112214\_tube2\_01.06078.06078.2 | 3.1621 | 0.3031 | 99.7% | 1619.2322 | 1619.9077 | 1 | 5.513 | 63.3% | 1 | R.VPTAPAATSKPGLRPR.T | 2 |
| \* | Astrin\_STLCLD20\_112214\_tube2\_01.16209.16209.2 | 3.5661 | 0.5407 | 100.0% | 1811.9722 | 1812.1167 | 1 | 8.105 | 56.2% | 1 | K.ISTLPQLNSALVQDLAK.A | 2 |

---

|  |  |  |  |  |  |  |  |  |
| --- | --- | --- | --- | --- | --- | --- | --- | --- |
| U | *gi|4502101|ref|NP\_000* | 2 | 2 | 7.5% | 346 | 38714 | 7.0 | annexin I [Homo sapiens] |

| Filename XCorr DeltCN Conf% ObsM+H+ CalcM+H+ SpR ZScore Ion% # Sequence  | | | | | | | | | | | | |
| --- | --- | --- | --- | --- | --- | --- | --- | --- | --- | --- | --- | --- |
| \* | Astrin\_STLCLD20\_112214\_tube2\_01.19451.19451.2 | 3.6905 | 0.2646 | 99.9% | 1607.4722 | 1606.9463 | 1 | 6.223 | 64.3% | 1 | K.ALTGHLEEVVLALLK.T | 2 |
| \* | Astrin\_STLCLD20\_112214\_tube2\_01.09302.09302.2 | 2.996 | 0.2525 | 99.7% | 1263.2322 | 1263.3495 | 1 | 4.536 | 80.0% | 1 | K.TPAQFDADELR.A | 2 |

---

|  |  |  |  |  |  |  |  |  |
| --- | --- | --- | --- | --- | --- | --- | --- | --- |
| U | *gi|221316723|ref|NP\_0* | 4 | 4 | 7.4% | 1025 | 115704 | 8.3 | N-acetyltransferase 10 isoform a [Homo sapiens] |

| Filename XCorr DeltCN Conf% ObsM+H+ CalcM+H+ SpR ZScore Ion% # Sequence  | | | | | | | | | | | | |
| --- | --- | --- | --- | --- | --- | --- | --- | --- | --- | --- | --- | --- |
| \* | Astrin\_STLCLD20\_112214\_01.06169.06169.2 | 2.297 | 0.2478 | 98.0% | 1114.2922 | 1114.2871 | 430 | 4.855 | 55.6% | 1 | R.ILIENGVAER.Q | 2 |
|  | Astrin\_STLCLD20\_112214\_tube2\_01.13797.13797.2 | 2.4531 | 0.3265 | 99.1% | 1454.7922 | 1454.6659 | 5 | 5.34 | 50.0% | 1 | R.LDYLGVSYGLTPR.L | 2 |
|  | Astrin\_STLCLD20\_112214\_tube2\_01.16331.16331.3 | 2.9818 | 0.2781 | 97.4% | 2831.8442 | 2832.2874 | 1 | 5.15 | 28.1% | 1 | R.NMGKPAQPALSREELEALFLPYDLK.R | 3 |
|  | Astrin\_STLCLD20\_112214\_01.19087.19087.3 | 4.8884 | 0.5032 | 100.0% | 2969.4844 | 2969.4973 | 1 | 7.808 | 32.4% | 1 | R.IYFLNQLGDLALSAAQSALLLGIGLQHK.S | 3 |

---

|  |  |  |  |  |  |  |  |  |
| --- | --- | --- | --- | --- | --- | --- | --- | --- |
| U | *gi|7661920|ref|NP\_055* | 3 | 4 | 7.1% | 411 | 46871 | 6.7 | eukaryotic translation initiation factor 4A, isoform 3 [Homo sapiens] |

| Filename XCorr DeltCN Conf% ObsM+H+ CalcM+H+ SpR ZScore Ion% # Sequence  | | | | | | | | | | | | |
| --- | --- | --- | --- | --- | --- | --- | --- | --- | --- | --- | --- | --- |
|  | Astrin\_STLCLD20\_112214\_01.09010.09010.2 | 4.6068 | 0.5611 | 100.0% | 1828.3922 | 1829.0654 | 1 | 9.405 | 70.0% | 2 | R.GIYAYGFEKPSAIQQR.A | 22 |
|  | Astrin\_STLCLD20\_112214\_01.09019.09019.3 | 2.9649 | 0.219 | 95.9% | 1830.8043 | 1829.0654 | 4 | 4.311 | 38.3% | 1 | R.GIYAYGFEKPSAIQQR.A | 33 |
| \* | Astrin\_STLCLD20\_112214\_01.12404.12404.2 | 3.2337 | 0.3007 | 99.9% | 1521.8121 | 1521.784 | 31 | 5.773 | 54.2% | 1 | K.MLVLDEADEMLNK.G | 2 |

Similarities:
gi|4503529|ref|NP\_001(2:1)  

---

|  |  |  |  |  |  |  |  |  |
| --- | --- | --- | --- | --- | --- | --- | --- | --- |
| U | *gi|4504511|ref|NP\_001* | 2 | 2 | 7.1% | 397 | 44868 | 7.1 | DnaJ (Hsp40) homolog, subfamily A, member 1 [Homo sapiens] |

| Filename XCorr DeltCN Conf% ObsM+H+ CalcM+H+ SpR ZScore Ion% # Sequence  | | | | | | | | | | | | |
| --- | --- | --- | --- | --- | --- | --- | --- | --- | --- | --- | --- | --- |
| \* | Astrin\_STLCLD20\_112214\_01.06093.06093.2 | 2.4504 | 0.3525 | 99.3% | 1392.5521 | 1393.6696 | 17 | 6.22 | 54.2% | 1 | R.TIVITSHPGQIVK.H | 2 |
| \* | Astrin\_STLCLD20\_112214\_tube2\_01.05685.05685.3 | 3.7478 | 0.4026 | 99.7% | 1869.9243 | 1869.8607 | 1 | 7.017 | 44.6% | 1 | R.HYNGEAYEDDEHHPR.G | 3 |

---

|  |  |  |  |  |  |  |  |  |
| --- | --- | --- | --- | --- | --- | --- | --- | --- |
| U | *gi|23308577|ref|NP\_00* | 2 | 4 | 6.9% | 533 | 56651 | 6.7 | phosphoglycerate dehydrogenase [Homo sapiens] |

| Filename XCorr DeltCN Conf% ObsM+H+ CalcM+H+ SpR ZScore Ion% # Sequence  | | | | | | | | | | | | |
| --- | --- | --- | --- | --- | --- | --- | --- | --- | --- | --- | --- | --- |
| \* | Astrin\_STLCLD20\_112214\_01.07613.07613.2 | 3.6817 | 0.494 | 100.0% | 1489.2322 | 1489.5822 | 1 | 8.907 | 64.3% | 2 | R.AGTGVDNVDLEAATR.K | 2 |
| \* | Astrin\_STLCLD20\_112214\_01.15904.15904.2 | 3.8684 | 0.4564 | 100.0% | 2273.0322 | 2273.668 | 1 | 7.686 | 47.6% | 2 | R.TQTSDPAMLPTMIGLLAEAGVR.L | 2 |

---

|  |  |  |  |  |  |  |  |  |
| --- | --- | --- | --- | --- | --- | --- | --- | --- |
| U | *gi|21327708|ref|NP\_63* | 2 | 3 | 6.9% | 391 | 45374 | 4.5 | nucleosome assembly protein 1-like 1 [Homo sapiens] |
| U | *gi|4758756|ref|NP\_004* | 2 | 3 | 6.9% | 391 | 45374 | 4.5 | nucleosome assembly protein 1-like 1 [Homo sapiens] |

| Filename XCorr DeltCN Conf% ObsM+H+ CalcM+H+ SpR ZScore Ion% # Sequence  | | | | | | | | | | | | |
| --- | --- | --- | --- | --- | --- | --- | --- | --- | --- | --- | --- | --- |
|  | Astrin\_STLCLD20\_112214\_tube2\_01.14864.14864.2 | 3.1212 | 0.1467 | 97.9% | 1860.4122 | 1861.102 | 3 | 4.446 | 43.8% | 1 | R.LDGLVETPTGYIESLPR.V | 2 |
|  | Astrin\_STLCLD20\_112214\_01.07187.07187.2 | 2.652 | 0.3699 | 100.0% | 1337.5922 | 1337.4314 | 1 | 6.195 | 77.8% | 2 | K.FYEEVHDLER.K | 2 |

---

|  |  |  |  |  |  |  |  |  |
| --- | --- | --- | --- | --- | --- | --- | --- | --- |
| U | *gi|16753203|ref|NP\_03* | 3 | 4 | 6.8% | 589 | 62519 | 5.1 | ubiquilin 1 isoform 1 [Homo sapiens] |
| U | *gi|16753205|ref|NP\_44* | 3 | 4 | 7.1% | 561 | 59220 | 5.1 | ubiquilin 1 isoform 2 [Homo sapiens] |

| Filename XCorr DeltCN Conf% ObsM+H+ CalcM+H+ SpR ZScore Ion% # Sequence  | | | | | | | | | | | | |
| --- | --- | --- | --- | --- | --- | --- | --- | --- | --- | --- | --- | --- |
|  | Astrin\_STLCLD20\_112214\_tube2\_01.13380.13380.2 | 3.0007 | 0.2463 | 99.1% | 1812.4521 | 1813.1865 | 1 | 4.723 | 57.1% | 2 | R.QLIMANPQMQQLIQR.N | 2 |
|  | Astrin\_STLCLD20\_112214\_01.09989.09989.2 | 3.822 | 0.3697 | 100.0% | 1781.4321 | 1782.0424 | 1 | 7.827 | 67.9% | 1 | R.NPEISHMLNNPDIMR.Q | 2 |
|  | Astrin\_STLCLD20\_112214\_01.09338.09338.2 | 1.9374 | 0.3496 | 98.2% | 1238.4722 | 1239.5265 | 141 | 5.723 | 61.1% | 1 | R.NPAMMQEMMR.N | 2 |

---

|  |  |  |  |  |  |  |  |  |
| --- | --- | --- | --- | --- | --- | --- | --- | --- |
| U | *gi|156151392|ref|NP\_0* | 3 | 6 | 6.8% | 532 | 59682 | 9.2 | heterogeneous nuclear ribonucleoprotein R isoform 4 [Homo sapiens] |
| U | *gi|5031755|ref|NP\_005* | 3 | 6 | 5.7% | 633 | 70943 | 8.1 | heterogeneous nuclear ribonucleoprotein R isoform 2 [Homo sapiens] |
| U | *gi|156151396|ref|NP\_0* | 3 | 6 | 6.7% | 535 | 59953 | 9.2 | heterogeneous nuclear ribonucleoprotein R isoform 3 [Homo sapiens] |
| U | *gi|156151394|ref|NP\_0* | 3 | 6 | 5.7% | 636 | 71214 | 8.1 | heterogeneous nuclear ribonucleoprotein R isoform 1 [Homo sapiens] |

| Filename XCorr DeltCN Conf% ObsM+H+ CalcM+H+ SpR ZScore Ion% # Sequence  | | | | | | | | | | | | |
| --- | --- | --- | --- | --- | --- | --- | --- | --- | --- | --- | --- | --- |
|  | Astrin\_STLCLD20\_112214\_01.07394.07394.2 | 2.8159 | 0.3712 | 100.0% | 1312.3922 | 1312.4221 | 1 | 6.943 | 68.2% | 2 | R.TGYTLDVTTGQR.K | 22 |
|  | Astrin\_STLCLD20\_112214\_tube2\_01.11138.11138.2 | 2.7863 | 0.3491 | 99.9% | 1262.0322 | 1262.4846 | 1 | 6.3 | 70.0% | 1 | R.LMMDPLSGQNR.G | 2 |
|  | Astrin\_STLCLD20\_112214\_01.13162.13162.2 | 3.3795 | 0.4021 | 100.0% | 1461.6721 | 1461.6525 | 2 | 7.224 | 58.3% | 3 | R.NLATTVTEEILEK.S | 2 |

Similarities:
gi|23397427|ref|NP\_00(1:2)  

---

|  |  |  |  |  |  |  |  |  |
| --- | --- | --- | --- | --- | --- | --- | --- | --- |
| U | *gi|4757810|ref|NP\_004* | 2 | 5 | 6.7% | 553 | 59751 | 9.1 | ATP synthase, H+ transporting, mitochondrial F1 complex, alpha subunit precursor [Homo sapiens] |
| U | *gi|50345984|ref|NP\_00* | 2 | 6 | 6.7% | 553 | 59751 | 9.1 | ATP synthase, H+ transporting, mitochondrial F1 complex, alpha subunit precursor [Homo sapiens] |

| Filename XCorr DeltCN Conf% ObsM+H+ CalcM+H+ SpR ZScore Ion% # Sequence  | | | | | | | | | | | | |
| --- | --- | --- | --- | --- | --- | --- | --- | --- | --- | --- | --- | --- |
|  | Astrin\_STLCLD20\_112214\_tube2\_02.07020.07020.2 | 3.8692 | 0.5095 | 100.0% | 1577.0922 | 1576.7007 | 1 | 8.917 | 67.9% | 4 | R.ILGADTSVDLEETGR.V | 2 |
|  | Astrin\_STLCLD20\_112214\_tube2\_01.19860.19860.2 | 3.4227 | 0.3322 | 100.0% | 2340.2922 | 2339.567 | 1 | 5.938 | 33.3% | 1 | R.EVAAFAQFGSDLDAATQQLLSR.G | 2 |

---

|  |  |  |  |  |  |  |  |  |
| --- | --- | --- | --- | --- | --- | --- | --- | --- |
| U | *gi|4505763|ref|NP\_000* | 2 | 2 | 6.7% | 417 | 44615 | 8.1 | phosphoglycerate kinase 1 [Homo sapiens] |

| Filename XCorr DeltCN Conf% ObsM+H+ CalcM+H+ SpR ZScore Ion% # Sequence  | | | | | | | | | | | | |
| --- | --- | --- | --- | --- | --- | --- | --- | --- | --- | --- | --- | --- |
|  | Astrin\_STLCLD20\_112214\_01.09335.09335.2 | 3.105 | 0.3453 | 99.9% | 1637.4321 | 1635.7764 | 1 | 6.488 | 67.9% | 1 | K.LGDVYVNDAFGTAHR.A | 2 |
| \* | Astrin\_STLCLD20\_112214\_tube2\_01.07696.07696.2 | 2.7449 | 0.4232 | 100.0% | 1369.9922 | 1368.5939 | 1 | 6.416 | 62.5% | 1 | R.AHSSMVGVNLPQK.A | 2 |

---

|  |  |  |  |  |  |  |  |  |
| --- | --- | --- | --- | --- | --- | --- | --- | --- |
| U | *gi|66933016|ref|NP\_00* | 2 | 3 | 6.6% | 514 | 55805 | 6.9 | inosine monophosphate dehydrogenase 2 [Homo sapiens] |

| Filename XCorr DeltCN Conf% ObsM+H+ CalcM+H+ SpR ZScore Ion% # Sequence  | | | | | | | | | | | | |
| --- | --- | --- | --- | --- | --- | --- | --- | --- | --- | --- | --- | --- |
| \* | Astrin\_STLCLD20\_112214\_tube2\_01.11100.11100.2 | 2.8087 | 0.1356 | 96.4% | 1482.5322 | 1482.7635 | 12 | 4.034 | 50.0% | 1 | K.REDLVVAPAGITLK.E | 2 |
| \* | Astrin\_STLCLD20\_112214\_01.10197.10197.3 | 4.478 | 0.4022 | 100.0% | 2050.5842 | 2049.3835 | 1 | 6.433 | 42.1% | 2 | R.RFGVPVIADGGIQNVGHIAK.A | 3 |

---

|  |  |  |  |  |  |  |  |  |
| --- | --- | --- | --- | --- | --- | --- | --- | --- |
| U | *Reverse\_gi|20336305|r* | 2 | 2 | 6.5% | 835 | 91197 | 6.6 | B-cell CLL/lymphoma 11A isoform 1 [Homo sapiens] |
| U | *Reverse\_gi|20336307|r* | 2 | 2 | 7.0% | 773 | 83860 | 6.3 | B-cell CLL/lymphoma 11A isoform 2 [Homo sapiens] |

| Filename XCorr DeltCN Conf% ObsM+H+ CalcM+H+ SpR ZScore Ion% # Sequence  | | | | | | | | | | | | |
| --- | --- | --- | --- | --- | --- | --- | --- | --- | --- | --- | --- | --- |
|  | Astrin\_STLCLD20\_112214\_02.16345.16345.3 | 3.279 | 0.2128 | 95.1% | 3354.5344 | 3354.2468 | 180 | 4.453 | 17.7% | 1 | K.AVVSKLAS\*S\*ASGVLDST#GPEPS\*SATSLGDDSK@.V | 3 |
|  | Astrin\_STLCLD20\_112214\_01.18591.18591.2 | 2.4111 | 0.2234 | 96.4% | 2780.8123 | 2781.724 | 20 | 3.298 | 28.6% | 1 | K.SAQT#CAHDCLNCKYPKEGT#HSR.R | 2 |

---

|  |  |  |  |  |  |  |  |  |
| --- | --- | --- | --- | --- | --- | --- | --- | --- |
| U | *gi|7661936|ref|NP\_055* | 4 | 4 | 6.3% | 953 | 107474 | 6.2 | scaffold attachment factor B2 [Homo sapiens] |

| Filename XCorr DeltCN Conf% ObsM+H+ CalcM+H+ SpR ZScore Ion% # Sequence  | | | | | | | | | | | | |
| --- | --- | --- | --- | --- | --- | --- | --- | --- | --- | --- | --- | --- |
|  | Astrin\_STLCLD20\_112214\_tube2\_01.09958.09958.3 | 4.5243 | 0.3263 | 99.8% | 2849.2144 | 2849.9788 | 1 | 6.612 | 35.0% | 1 | K.SEPVKEESSELEQPFAQDTSSVGPDR.K | 33 |
|  | Astrin\_STLCLD20\_112214\_01.09904.09904.1 | 1.3685 | 0.2878 | 95.5% | 915.56 | 916.1057 | 11 | 4.659 | 50.0% | 1 | K.ADSLLAVVK.R | 11 |
| \* | Astrin\_STLCLD20\_112214\_tube2\_01.12314.12314.2 | 2.3202 | 0.1969 | 95.7% | 1321.5721 | 1321.4758 | 1 | 5.252 | 63.6% | 1 | R.NLWVSGLSSTTR.A | 2 |
| \* | Astrin\_STLCLD20\_112214\_01.05669.05669.2 | 2.3025 | 0.3063 | 98.5% | 1293.2922 | 1292.4119 | 6 | 5.269 | 58.3% | 1 | R.AWQGAMDAGAASR.E | 2 |

Similarities:
gi|21264343|ref|NP\_00(2:2)  

---

|  |  |  |  |  |  |  |  |  |
| --- | --- | --- | --- | --- | --- | --- | --- | --- |
| U | *gi|46367787|ref|NP\_00* | 2 | 3 | 6.3% | 636 | 70671 | 9.5 | poly(A) binding protein, cytoplasmic 1 [Homo sapiens] |

| Filename XCorr DeltCN Conf% ObsM+H+ CalcM+H+ SpR ZScore Ion% # Sequence  | | | | | | | | | | | | |
| --- | --- | --- | --- | --- | --- | --- | --- | --- | --- | --- | --- | --- |
|  | Astrin\_STLCLD20\_112214\_tube2\_01.19562.19562.3 | 3.5631 | 0.2484 | 98.6% | 2743.2244 | 2742.175 | 1 | 4.654 | 31.5% | 2 | K.ITGMLLEIDNSELLHMLESPESLR.S | 3 |
|  | Astrin\_STLCLD20\_112214\_01.07855.07855.3 | 4.1786 | 0.3623 | 99.7% | 1695.0844 | 1694.9285 | 1 | 6.49 | 53.3% | 1 | R.SKVDEAVAVLQAHQAK.E | 3 |

---

|  |  |  |  |  |  |  |  |  |
| --- | --- | --- | --- | --- | --- | --- | --- | --- |
| U | *gi|34147630|ref|NP\_00* | 2 | 2 | 6.2% | 455 | 49875 | 7.6 | Tu translation elongation factor, mitochondrial precursor [Homo sapiens] |

| Filename XCorr DeltCN Conf% ObsM+H+ CalcM+H+ SpR ZScore Ion% # Sequence  | | | | | | | | | | | | |
| --- | --- | --- | --- | --- | --- | --- | --- | --- | --- | --- | --- | --- |
| \* | Astrin\_STLCLD20\_112214\_02.09459.09459.3 | 3.102 | 0.3572 | 99.6% | 1675.2843 | 1674.854 | 2 | 5.645 | 33.3% | 1 | R.GITINAAHVEYSTAAR.H | 3 |
| \* | Astrin\_STLCLD20\_112214\_tube2\_01.10008.10008.2 | 3.1709 | 0.064 | 97.0% | 1186.3322 | 1186.3103 | 1 | 4.822 | 77.3% | 1 | R.AEAGDNLGALVR.G | 2 |

---

|  |  |  |  |  |  |  |  |  |
| --- | --- | --- | --- | --- | --- | --- | --- | --- |
| U | *gi|4507877|ref|NP\_003* | 5 | 6 | 5.9% | 1066 | 116722 | 6.1 | vinculin isoform VCL [Homo sapiens] |
| U | *gi|7669550|ref|NP\_054* | 5 | 6 | 5.6% | 1134 | 123799 | 5.7 | vinculin isoform meta-VCL [Homo sapiens] |

| Filename XCorr DeltCN Conf% ObsM+H+ CalcM+H+ SpR ZScore Ion% # Sequence  | | | | | | | | | | | | |
| --- | --- | --- | --- | --- | --- | --- | --- | --- | --- | --- | --- | --- |
|  | Astrin\_STLCLD20\_112214\_01.10216.10216.2 | 3.1648 | 0.2388 | 99.4% | 1478.4722 | 1478.7217 | 95 | 5.964 | 54.2% | 2 | K.MLGQMTDQVADLR.A | 2 |
|  | Astrin\_STLCLD20\_112214\_01.09292.09292.2 | 2.2159 | 0.3348 | 98.5% | 1458.5122 | 1458.6549 | 1 | 5.215 | 53.8% | 1 | K.AQQVSQGLDVLTAK.V | 2 |
|  | Astrin\_STLCLD20\_112214\_01.07373.07373.2 | 2.4175 | 0.2698 | 98.8% | 1231.5122 | 1231.3073 | 1 | 4.678 | 77.8% | 1 | R.WIDNPTVDDR.G | 2 |
|  | Astrin\_STLCLD20\_112214\_tube2\_02.07155.07155.2 | 2.3701 | 0.1997 | 96.4% | 1295.2922 | 1293.4749 | 170 | 4.484 | 54.5% | 1 | K.MTGLVDEAIDTK.S | 2 |
|  | Astrin\_STLCLD20\_112214\_tube2\_01.10644.10644.2 | 3.1322 | 0.3095 | 99.8% | 1519.2722 | 1519.8354 | 12 | 5.309 | 53.8% | 1 | K.AGEVINQPMMMAAR.Q | 2 |

---

|  |  |  |  |  |  |  |  |  |
| --- | --- | --- | --- | --- | --- | --- | --- | --- |
| U | *contaminant\_KERATIN02* | 3 | 4 | 5.9% | 622 | 61987 | 5.2 | no description |
| U | *gi|55956899|ref|NP\_00* | 3 | 4 | 5.9% | 623 | 62064 | 5.2 | keratin 9 [Homo sapiens] |

| Filename XCorr DeltCN Conf% ObsM+H+ CalcM+H+ SpR ZScore Ion% # Sequence  | | | | | | | | | | | | |
| --- | --- | --- | --- | --- | --- | --- | --- | --- | --- | --- | --- | --- |
|  | Astrin\_STLCLD20\_112214\_01.08911.08911.2 | 2.315 | 0.3241 | 99.3% | 1060.6322 | 1061.1802 | 1 | 5.487 | 87.5% | 1 | K.TLLDIDNTR.M | 2 |
|  | Astrin\_STLCLD20\_112214\_01.07406.07406.2 | 3.0642 | 0.3213 | 100.0% | 1158.4922 | 1158.2566 | 8 | 5.376 | 65.0% | 1 | R.QGVDADINGLR.Q | 2 |
|  | Astrin\_STLCLD20\_112214\_02.11724.11724.3 | 5.6819 | 0.4051 | 100.0% | 1967.4243 | 1967.2297 | 1 | 7.486 | 48.4% | 2 | R.HGVQELEIELQSQLSKK.A | 3 |

---

|  |  |  |  |  |  |  |  |  |
| --- | --- | --- | --- | --- | --- | --- | --- | --- |
| U | *gi|13376259|ref|NP\_07* | 3 | 3 | 5.8% | 656 | 75019 | 5.6 | nucleoporin 85 [Homo sapiens] |

| Filename XCorr DeltCN Conf% ObsM+H+ CalcM+H+ SpR ZScore Ion% # Sequence  | | | | | | | | | | | | |
| --- | --- | --- | --- | --- | --- | --- | --- | --- | --- | --- | --- | --- |
| \* | Astrin\_STLCLD20\_112214\_01.11024.11024.2 | 2.5242 | 0.1697 | 97.3% | 1091.0521 | 1090.2676 | 1 | 6.309 | 72.2% | 1 | R.LGSALSWSIR.A | 2 |
| \* | Astrin\_STLCLD20\_112214\_tube2\_01.09641.09641.2 | 2.4061 | 0.3565 | 99.3% | 1364.7322 | 1365.529 | 2 | 5.623 | 58.3% | 1 | R.AKDAAFATLVSDR.F | 2 |
| \* | Astrin\_STLCLD20\_112214\_tube2\_02.12549.12549.2 | 3.0036 | 0.3169 | 99.7% | 1596.5122 | 1596.8822 | 1 | 6.046 | 50.0% | 1 | R.FADAASLLLSLMTSR.I | 2 |

---

|  |  |  |  |  |  |  |  |  |
| --- | --- | --- | --- | --- | --- | --- | --- | --- |
| U | *gi|4506209|ref|NP\_002* | 3 | 3 | 5.8% | 433 | 48634 | 5.9 | proteasome 26S ATPase subunit 2 [Homo sapiens] |

| Filename XCorr DeltCN Conf% ObsM+H+ CalcM+H+ SpR ZScore Ion% # Sequence  | | | | | | | | | | | | |
| --- | --- | --- | --- | --- | --- | --- | --- | --- | --- | --- | --- | --- |
| \* | Astrin\_STLCLD20\_112214\_tube2\_01.12390.12390.2 | 2.4356 | 0.1949 | 97.1% | 1158.2122 | 1158.3373 | 32 | 5.402 | 60.0% | 1 | R.ALDEGDIALLK.T | 2 |
| \* | Astrin\_STLCLD20\_112214\_01.07967.07967.3 | 3.7565 | 0.3041 | 99.8% | 1689.6244 | 1688.9675 | 132 | 6.511 | 36.5% | 1 | K.LREVVETPLLHPER.F | 3 |
| \* | Astrin\_STLCLD20\_112214\_tube2\_01.09114.09114.2 | 2.3832 | 0.245 | 97.7% | 1419.5122 | 1419.6206 | 14 | 5.26 | 50.0% | 1 | R.EVVETPLLHPER.F | 2 |

---

|  |  |  |  |  |  |  |  |  |
| --- | --- | --- | --- | --- | --- | --- | --- | --- |
| U | *gi|32698730|ref|NP\_06* | 3 | 3 | 5.6% | 695 | 76121 | 8.7 | nuclear fragile X mental retardation protein interacting protein 2 [Homo sapiens] |

| Filename XCorr DeltCN Conf% ObsM+H+ CalcM+H+ SpR ZScore Ion% # Sequence  | | | | | | | | | | | | |
| --- | --- | --- | --- | --- | --- | --- | --- | --- | --- | --- | --- | --- |
| \* | Astrin\_STLCLD20\_112214\_01.05014.05014.2 | 2.9763 | 0.3443 | 99.9% | 1405.2922 | 1404.4764 | 2 | 5.772 | 62.5% | 1 | K.NLSSDEATNPISR.V | 2 |
| \* | Astrin\_STLCLD20\_112214\_01.06417.06417.2 | 3.0846 | 0.3416 | 99.9% | 1515.8322 | 1515.7068 | 1 | 6.843 | 65.4% | 1 | R.VLNGNQQVVDTSLK.Q | 2 |
| \* | Astrin\_STLCLD20\_112214\_01.07306.07306.2 | 2.5184 | 0.2361 | 98.3% | 1376.3522 | 1375.6233 | 170 | 4.766 | 50.0% | 1 | K.IMQQETSVPTLK.Q | 2 |

---

|  |  |  |  |  |  |  |  |  |
| --- | --- | --- | --- | --- | --- | --- | --- | --- |
| U | *gi|41322908|ref|NP\_95* | 15 | 30 | 5.5% | 4525 | 513712 | 5.8 | plectin 1 isoform 3 [Homo sapiens] |
| U | *gi|47607492|ref|NP\_00* | 15 | 30 | 5.5% | 4574 | 518478 | 5.7 | plectin 1 isoform 1 [Homo sapiens] |
| U | *gi|41322923|ref|NP\_95* | 15 | 30 | 5.5% | 4547 | 516204 | 5.8 | plectin 1 isoform 11 [Homo sapiens] |
| U | *gi|41322919|ref|NP\_95* | 15 | 30 | 5.5% | 4547 | 516282 | 5.8 | plectin 1 isoform 8 [Homo sapiens] |
| U | *gi|41322916|ref|NP\_95* | 15 | 30 | 5.3% | 4684 | 531796 | 6.0 | plectin 1 isoform 6 [Homo sapiens] |
| U | *gi|41322914|ref|NP\_95* | 15 | 30 | 5.5% | 4551 | 516484 | 5.8 | plectin 1 isoform 10 [Homo sapiens] |
| U | *gi|41322912|ref|NP\_95* | 15 | 30 | 5.5% | 4533 | 514780 | 5.7 | plectin 1 isoform 2 [Homo sapiens] |
| U | *gi|41322910|ref|NP\_95* | 15 | 30 | 5.5% | 4515 | 512609 | 5.8 | plectin 1 isoform 7 [Homo sapiens] |

| Filename XCorr DeltCN Conf% ObsM+H+ CalcM+H+ SpR ZScore Ion% # Sequence  | | | | | | | | | | | | |
| --- | --- | --- | --- | --- | --- | --- | --- | --- | --- | --- | --- | --- |
|  | Astrin\_STLCLD20\_112214\_tube2\_01.17500.17500.2 | 3.5822 | 0.5092 | 100.0% | 1914.0521 | 1915.1155 | 1 | 7.808 | 57.1% | 1 | R.YLQDLLAWVEENQHR.V | 2 |
|  | Astrin\_STLCLD20\_112214\_tube2\_01.14472.14472.3 | 2.9948 | 0.2688 | 97.8% | 2224.0144 | 2223.4067 | 75 | 4.851 | 27.5% | 1 | R.VDGAEWGVDLPSVEAQLGSHR.G | 3 |
|  | Astrin\_STLCLD20\_112214\_01.10564.10564.2 | 2.4273 | 0.3022 | 98.5% | 1532.2922 | 1532.8235 | 86 | 5.665 | 42.9% | 1 | K.VLALPEPSPAAPTLR.S | 2 |
|  | Astrin\_STLCLD20\_112214\_tube2\_01.08595.08595.3 | 3.7837 | 0.2308 | 99.5% | 1692.4143 | 1692.9156 | 115 | 4.964 | 41.7% | 2 | R.LREQLQLLEEQHR.A | 3 |
|  | Astrin\_STLCLD20\_112214\_02.08457.08457.3 | 3.5923 | 0.411 | 99.7% | 1785.0844 | 1784.964 | 1 | 7.153 | 38.2% | 1 | R.AALAHSEEVTASQVAATK.T | 3 |
|  | Astrin\_STLCLD20\_112214\_tube2\_02.07588.07588.2 | 4.8025 | 0.3447 | 100.0% | 1558.4922 | 1557.744 | 1 | 7.423 | 76.9% | 4 | R.LQEAGILSAEELQR.L | 2 |
|  | Astrin\_STLCLD20\_112214\_tube2\_01.06867.06867.2 | 3.3575 | 0.466 | 100.0% | 1411.4521 | 1411.5577 | 1 | 7.532 | 75.0% | 1 | R.LAQGHTTVDELAR.R | 2 |
|  | Astrin\_STLCLD20\_112214\_tube2\_02.07104.07104.3 | 3.7517 | 0.308 | 99.6% | 2013.5343 | 2014.292 | 1 | 6.201 | 38.9% | 1 | R.LLEAQIATGGVIDPVHSHR.V | 33 |
|  | Astrin\_STLCLD20\_112214\_02.10979.10979.2 | 3.6859 | 0.4143 | 100.0% | 1614.1721 | 1614.8363 | 1 | 7.44 | 66.7% | 1 | R.LLDAQLSTGGIVDPSK.S | 2 |
|  | Astrin\_STLCLD20\_112214\_tube2\_01.14259.14259.2 | 4.1113 | 0.5078 | 100.0% | 1566.9122 | 1567.8259 | 1 | 8.705 | 73.3% | 4 | R.APVPASELLASGVLSR.A | 2 |
|  | Astrin\_STLCLD20\_112214\_tube2\_02.07936.07936.2 | 3.6283 | 0.4121 | 100.0% | 1539.2522 | 1539.7721 | 1 | 7.606 | 71.4% | 2 | R.LLDAQLATGGIVDPR.L | 2 |
|  | Astrin\_STLCLD20\_112214\_tube2\_01.14390.14390.2 | 2.78 | 0.1895 | 98.1% | 1544.3722 | 1543.8088 | 1 | 4.863 | 62.5% | 2 | R.LGFHLPLEVAYQR.G | 2 |
|  | Astrin\_STLCLD20\_112214\_tube2\_01.11325.11325.2 | 2.9263 | 0.351 | 99.9% | 1461.8922 | 1462.6611 | 28 | 5.996 | 62.5% | 4 | R.SQVMDEATALQLR.E | 2 |
|  | Astrin\_STLCLD20\_112214\_01.14902.14902.2 | 4.5478 | 0.4689 | 100.0% | 2116.9321 | 2116.3533 | 1 | 7.206 | 47.5% | 3 | R.AGTLSITEFADMLSGNAGGFR.S | 2 |
|  | Astrin\_STLCLD20\_112214\_tube2\_02.10249.10249.3 | 4.1584 | 0.3354 | 99.8% | 3128.1843 | 3128.5522 | 1 | 5.571 | 32.4% | 2 | R.FLEVQYLTGGLIEPDTPGRVPLDEALQR.G | 3 |

Similarities:
gi|207452735|ref|NP\_1(1:14)  

---

|  |  |  |  |  |  |  |  |  |
| --- | --- | --- | --- | --- | --- | --- | --- | --- |
| U | *gi|14141193|ref|NP\_00* | 2 | 3 | 5.2% | 194 | 22591 | 10.7 | ribosomal protein S9 [Homo sapiens] |

| Filename XCorr DeltCN Conf% ObsM+H+ CalcM+H+ SpR ZScore Ion% # Sequence  | | | | | | | | | | | | |
| --- | --- | --- | --- | --- | --- | --- | --- | --- | --- | --- | --- | --- |
| \* | Astrin\_STLCLD20\_112214\_tube2\_01.10376.10376.2 | 3.3931 | 0.2893 | 100.0% | 1189.5721 | 1189.4031 | 1 | 5.071 | 77.8% | 2 | R.RLFEGNALLR.R | 2 |
| \* | Astrin\_STLCLD20\_112214\_tube2\_01.11834.11834.2 | 2.6586 | 0.1945 | 98.8% | 1033.0322 | 1033.2156 | 48 | 5.188 | 68.8% | 1 | R.LFEGNALLR.R | 2 |

---

|  |  |  |  |  |  |  |  |  |
| --- | --- | --- | --- | --- | --- | --- | --- | --- |
| U | *gi|21264343|ref|NP\_00* | 3 | 4 | 5.1% | 915 | 102642 | 5.5 | scaffold attachment factor B [Homo sapiens] |

| Filename XCorr DeltCN Conf% ObsM+H+ CalcM+H+ SpR ZScore Ion% # Sequence  | | | | | | | | | | | | |
| --- | --- | --- | --- | --- | --- | --- | --- | --- | --- | --- | --- | --- |
|  | Astrin\_STLCLD20\_112214\_tube2\_01.09958.09958.3 | 4.5243 | 0.3263 | 99.8% | 2849.2144 | 2849.9788 | 1 | 6.612 | 35.0% | 1 | K.SEPVKEESSELEQPFAQDTSSVGPDR.K | 33 |
|  | Astrin\_STLCLD20\_112214\_01.09904.09904.1 | 1.3685 | 0.2878 | 95.5% | 915.56 | 916.1057 | 11 | 4.659 | 50.0% | 1 | K.ADSLLAVVK.R | 11 |
| \* | Astrin\_STLCLD20\_112214\_tube2\_01.13094.13094.2 | 3.1239 | 0.4111 | 100.0% | 1355.1522 | 1355.4929 | 1 | 6.459 | 81.8% | 2 | R.NFWVSGLSSTTR.A | 2 |

Similarities:
gi|7661936|ref|NP\_055(2:1)  

---

|  |  |  |  |  |  |  |  |  |
| --- | --- | --- | --- | --- | --- | --- | --- | --- |
| U | *gi|41406064|ref|NP\_00* | 9 | 13 | 5.0% | 1976 | 228997 | 5.5 | myosin, heavy polypeptide 10, non-muscle [Homo sapiens] |

| Filename XCorr DeltCN Conf% ObsM+H+ CalcM+H+ SpR ZScore Ion% # Sequence  | | | | | | | | | | | | |
| --- | --- | --- | --- | --- | --- | --- | --- | --- | --- | --- | --- | --- |
|  | Astrin\_STLCLD20\_112214\_tube2\_01.12982.12982.2 | 2.3095 | 0.3423 | 99.3% | 1319.8722 | 1319.5468 | 23 | 5.288 | 60.0% | 2 | K.LDPHLVLDQLR.C | 22 |
|  | Astrin\_STLCLD20\_112214\_tube2\_01.08715.08715.2 | 3.1383 | 0.3993 | 100.0% | 1224.5122 | 1224.3591 | 1 | 7.007 | 70.0% | 2 | R.AGVLAHLEEER.D | 22 |
|  | Astrin\_STLCLD20\_112214\_tube2\_01.08607.08607.2 | 2.7025 | 0.1787 | 98.5% | 1222.3922 | 1221.3959 | 5 | 4.236 | 66.7% | 1 | K.KFDQLLAEEK.S | 22 |
|  | Astrin\_STLCLD20\_112214\_tube2\_01.09251.09251.2 | 3.0319 | 0.2398 | 99.8% | 1094.3922 | 1093.2218 | 1 | 6.362 | 81.2% | 2 | K.FDQLLAEEK.S | 22 |
| \* | Astrin\_STLCLD20\_112214\_02.10245.10245.3 | 2.8493 | 0.2915 | 99.5% | 1665.3844 | 1664.8094 | 1 | 5.311 | 48.1% | 1 | R.ALEEALEAKEEFER.Q | 3 |
| \* | Astrin\_STLCLD20\_112214\_01.07574.07574.2 | 2.906 | 0.2639 | 99.7% | 1233.5122 | 1233.382 | 1 | 6.123 | 77.8% | 2 | R.ALEQQVEEMR.T | 2 |
| \* | Astrin\_STLCLD20\_112214\_02.11461.11461.2 | 2.6798 | 0.1657 | 96.4% | 1745.7722 | 1745.9274 | 1 | 5.576 | 46.7% | 1 | K.TTLQVDTLNAELAAER.S | 2 |
| \* | Astrin\_STLCLD20\_112214\_tube2\_01.10618.10618.2 | 3.3063 | 0.3353 | 100.0% | 1515.4722 | 1515.6604 | 1 | 6.723 | 70.8% | 1 | K.IGQLEEQLEQEAK.E | 2 |
| \* | Astrin\_STLCLD20\_112214\_01.04988.04988.2 | 4.2721 | 0.4197 | 100.0% | 1520.3322 | 1520.5498 | 1 | 7.994 | 73.1% | 1 | R.ELDDATEANEGLSR.E | 2 |

Similarities:
gi|12667788|ref|NP\_00(4:5)  

---

|  |  |  |  |  |  |  |  |  |
| --- | --- | --- | --- | --- | --- | --- | --- | --- |
| U | *gi|119703753|ref|NP\_0* | 4 | 12 | 5.0% | 564 | 60067 | 8.0 | keratin 6B [Homo sapiens] |

| Filename XCorr DeltCN Conf% ObsM+H+ CalcM+H+ SpR ZScore Ion% # Sequence  | | | | | | | | | | | | |
| --- | --- | --- | --- | --- | --- | --- | --- | --- | --- | --- | --- | --- |
|  | Astrin\_STLCLD20\_112214\_01.08756.08756.2 | 2.5869 | 0.1788 | 98.4% | 1082.6522 | 1083.2755 | 3 | 6.37 | 75.0% | 2 | K.FASFIDKVR.F | 2222 |
|  | Astrin\_STLCLD20\_112214\_01.07803.07803.2 | 3.2051 | 0.3213 | 100.0% | 1180.5122 | 1180.303 | 1 | 7.983 | 83.3% | 5 | K.YEELQITAGR.H | 22 |
|  | Astrin\_STLCLD20\_112214\_tube2\_01.09255.09255.1 | 2.466 | 0.2121 | 98.9% | 1153.62 | 1154.3234 | 15 | 5.518 | 68.8% | 2 | K.EYQELMNVK.L | 11 |
|  | Astrin\_STLCLD20\_112214\_tube2\_01.09290.09290.2 | 2.4474 | 0.2352 | 98.7% | 1154.3322 | 1154.3234 | 15 | 6.215 | 62.5% | 3 | K.EYQELMNVK.L | 22 |

Similarities:
gi|4504919|ref|NP\_002(3:1)  
gi|67782365|ref|NP\_00(1:3)  
gi|119395750|ref|NP\_0(1:3)  
gi|47132620|ref|NP\_00(1:3)  

---

|  |  |  |  |  |  |  |  |  |
| --- | --- | --- | --- | --- | --- | --- | --- | --- |
| U | *gi|154355000|ref|NP\_0* | 3 | 5 | 4.9% | 711 | 73115 | 7.3 | KH-type splicing regulatory protein (FUSE binding protein 2) [Homo sapiens] |

| Filename XCorr DeltCN Conf% ObsM+H+ CalcM+H+ SpR ZScore Ion% # Sequence  | | | | | | | | | | | | |
| --- | --- | --- | --- | --- | --- | --- | --- | --- | --- | --- | --- | --- |
| \* | Astrin\_STLCLD20\_112214\_tube2\_01.10217.10217.2 | 3.1719 | 0.4686 | 100.0% | 1080.3522 | 1080.2725 | 1 | 8.497 | 95.0% | 2 | R.IGGGIDVPVPR.H | 2 |
| \* | Astrin\_STLCLD20\_112214\_01.11885.11885.2 | 2.9032 | 0.3677 | 100.0% | 1185.2922 | 1185.4093 | 1 | 6.371 | 72.2% | 1 | R.IINDLLQSLR.S | 2 |
| \* | Astrin\_STLCLD20\_112214\_02.10297.10297.2 | 3.9685 | 0.414 | 100.0% | 1534.4521 | 1534.7123 | 1 | 7.898 | 69.2% | 2 | K.AINQQTGAFVEISR.Q | 2 |

---

|  |  |  |  |  |  |  |  |  |
| --- | --- | --- | --- | --- | --- | --- | --- | --- |
| U | *gi|50053795|ref|NP\_00* | 2 | 2 | 4.9% | 611 | 69151 | 5.7 | eukaryotic translation initiation factor 4B [Homo sapiens] |

| Filename XCorr DeltCN Conf% ObsM+H+ CalcM+H+ SpR ZScore Ion% # Sequence  | | | | | | | | | | | | |
| --- | --- | --- | --- | --- | --- | --- | --- | --- | --- | --- | --- | --- |
| \* | Astrin\_STLCLD20\_112214\_tube2\_01.19874.19874.3 | 3.8762 | 0.4032 | 99.7% | 3344.7244 | 3345.6904 | 1 | 5.816 | 26.7% | 1 | R.LKGFGYAEFEDLDSLLSALSLNEESLGNRR.I | 3 |
| \* | Astrin\_STLCLD20\_112214\_tube2\_01.20295.20295.3 | 3.0161 | 0.3375 | 99.6% | 3103.8843 | 3104.357 | 18 | 5.007 | 20.4% | 1 | K.GFGYAEFEDLDSLLSALSLNEESLGNRR.I | 3 |

---

|  |  |  |  |  |  |  |  |  |
| --- | --- | --- | --- | --- | --- | --- | --- | --- |
| U | *gi|4505257|ref|NP\_002* | 2 | 2 | 4.7% | 577 | 67820 | 6.4 | moesin [Homo sapiens] |

| Filename XCorr DeltCN Conf% ObsM+H+ CalcM+H+ SpR ZScore Ion% # Sequence  | | | | | | | | | | | | |
| --- | --- | --- | --- | --- | --- | --- | --- | --- | --- | --- | --- | --- |
| \* | Astrin\_STLCLD20\_112214\_tube2\_01.19246.19246.2 | 2.7545 | 0.3207 | 99.4% | 2082.412 | 2083.259 | 8 | 5.818 | 34.4% | 1 | K.FYPEDVSEELIQDITQR.L | 2 |
|  | Astrin\_STLCLD20\_112214\_tube2\_01.13506.13506.2 | 3.3469 | 0.427 | 100.0% | 1183.3121 | 1183.3519 | 1 | 7.487 | 77.8% | 1 | K.APDFVFYAPR.L | 2 |

---

|  |  |  |  |  |  |  |  |  |
| --- | --- | --- | --- | --- | --- | --- | --- | --- |
| U | *gi|38569421|ref|NP\_00* | 3 | 4 | 4.6% | 1101 | 120839 | 7.3 | ATP citrate lyase isoform 1 [Homo sapiens] |
| U | *gi|38569423|ref|NP\_94* | 3 | 4 | 4.7% | 1091 | 119772 | 7.3 | ATP citrate lyase isoform 2 [Homo sapiens] |

| Filename XCorr DeltCN Conf% ObsM+H+ CalcM+H+ SpR ZScore Ion% # Sequence  | | | | | | | | | | | | |
| --- | --- | --- | --- | --- | --- | --- | --- | --- | --- | --- | --- | --- |
|  | Astrin\_STLCLD20\_112214\_02.10317.10317.3 | 3.8761 | 0.4276 | 99.7% | 2259.2644 | 2259.3936 | 13 | 7.093 | 27.5% | 1 | R.EGDYVLFHHEGGVDVGDVDAK.A | 3 |
|  | Astrin\_STLCLD20\_112214\_tube2\_01.11079.11079.3 | 2.946 | 0.2221 | 95.9% | 1931.4844 | 1932.1863 | 1 | 4.631 | 33.3% | 1 | R.DYQGPLKEHEVTIFVR.R | 3 |
|  | Astrin\_STLCLD20\_112214\_tube2\_01.12111.12111.2 | 3.5526 | 0.3752 | 100.0% | 1492.2122 | 1492.647 | 3 | 6.055 | 57.7% | 2 | R.SGGMSNELNNIISR.T | 2 |

---

|  |  |  |  |  |  |  |  |  |
| --- | --- | --- | --- | --- | --- | --- | --- | --- |
| U | *gi|23510448|ref|NP\_00* | 2 | 3 | 4.6% | 734 | 82286 | 8.4 | minichromosome maintenance complex component 5 [Homo sapiens] |

| Filename XCorr DeltCN Conf% ObsM+H+ CalcM+H+ SpR ZScore Ion% # Sequence  | | | | | | | | | | | | |
| --- | --- | --- | --- | --- | --- | --- | --- | --- | --- | --- | --- | --- |
| \* | Astrin\_STLCLD20\_112214\_tube2\_01.12908.12908.3 | 3.026 | 0.2301 | 96.2% | 2089.6743 | 2088.4326 | 1 | 4.33 | 38.9% | 1 | R.NTLTNIAMRPGLEGYALPR.K | 3 |
| \* | Astrin\_STLCLD20\_112214\_tube2\_01.14561.14561.2 | 3.2775 | 0.3377 | 100.0% | 1690.0322 | 1689.8627 | 1 | 6.369 | 57.1% | 2 | K.LQPFATEADVEEALR.L | 2 |

---

|  |  |  |  |  |  |  |  |  |
| --- | --- | --- | --- | --- | --- | --- | --- | --- |
| U | *contaminant\_GR78\_HUMA* | 2 | 4 | 4.6% | 653 | 72116 | 5.1 | owl|P11021| 78 KD GLUCOSE REGULATED PROTEIN PRECURSOR (GRP 78) (IMMUNOGLOBULIN... |
| U | *gi|16507237|ref|NP\_00* | 2 | 4 | 4.6% | 654 | 72333 | 5.2 | heat shock 70kDa protein 5 [Homo sapiens] |
| U | *contaminant\_GR78\_RAT* | 2 | 4 | 4.6% | 654 | 72347 | 5.2 | owl|P06761| 78 KD GLUCOSE REGULATED PROTEIN PRECURSOR (GRP 78) (IMMUNOGLOBULIN... |
| U | *contaminant\_GR78\_MOUS* | 2 | 4 | 4.6% | 655 | 72421 | 5.2 | owl|P20029| 78 KD GLUCOSE REGULATED PROTEIN PRECURSOR (GRP 78) (IMMUNOGLOBULIN... |
| U | *contaminant\_GR78\_MESA* | 2 | 4 | 4.6% | 654 | 72379 | 5.2 | owl|P07823| 78 KD GLUCOSE REGULATED PROTEIN PRECURSOR (GRP 78) (IMMUNOGLOBULIN... |

| Filename XCorr DeltCN Conf% ObsM+H+ CalcM+H+ SpR ZScore Ion% # Sequence  | | | | | | | | | | | | |
| --- | --- | --- | --- | --- | --- | --- | --- | --- | --- | --- | --- | --- |
|  | Astrin\_STLCLD20\_112214\_01.09968.09968.2 | 3.0644 | 0.3705 | 100.0% | 1567.4122 | 1567.7386 | 1 | 7.015 | 57.7% | 2 | R.ITPSYVAFTPEGER.L | 2 |
|  | Astrin\_STLCLD20\_112214\_tube2\_01.13115.13115.2 | 4.1946 | 0.2878 | 100.0% | 1662.5322 | 1660.9078 | 1 | 7.371 | 83.3% | 2 | R.IINEPTAAAIAYGLDK.R | 22 |

Similarities:
gi|5729877|ref|NP\_006(1:1)  

---

|  |  |  |  |  |  |  |  |  |
| --- | --- | --- | --- | --- | --- | --- | --- | --- |
| U | *gi|50659095|ref|NP\_00* | 2 | 2 | 4.5% | 783 | 87344 | 9.3 | DEAD (Asp-Glu-Ala-Asp) box polypeptide 21 [Homo sapiens] |

| Filename XCorr DeltCN Conf% ObsM+H+ CalcM+H+ SpR ZScore Ion% # Sequence  | | | | | | | | | | | | |
| --- | --- | --- | --- | --- | --- | --- | --- | --- | --- | --- | --- | --- |
| \* | Astrin\_STLCLD20\_112214\_01.07955.07955.2 | 2.5564 | 0.2485 | 98.4% | 1377.7122 | 1378.6609 | 326 | 4.819 | 45.8% | 1 | R.GRAPQVLVLAPTR.E | 2 |
| \* | Astrin\_STLCLD20\_112214\_tube2\_01.18696.18696.2 | 3.7242 | 0.5049 | 100.0% | 2106.9722 | 2109.347 | 3 | 9.598 | 33.3% | 1 | K.GAVEALAAALAHISGATSVDQR.S | 2 |

---

|  |  |  |  |  |  |  |  |  |
| --- | --- | --- | --- | --- | --- | --- | --- | --- |
| U | *gi|40353736|ref|NP\_95* | 2 | 2 | 4.4% | 653 | 72400 | 6.3 | c-Mpl binding protein isoform c [Homo sapiens] |
| U | *gi|40353740|ref|NP\_95* | 2 | 2 | 4.0% | 730 | 81244 | 6.6 | c-Mpl binding protein isoform b [Homo sapiens] |
| U | *gi|40353738|ref|NP\_44* | 2 | 2 | 4.0% | 724 | 80624 | 6.6 | c-Mpl binding protein isoform a [Homo sapiens] |

| Filename XCorr DeltCN Conf% ObsM+H+ CalcM+H+ SpR ZScore Ion% # Sequence  | | | | | | | | | | | | |
| --- | --- | --- | --- | --- | --- | --- | --- | --- | --- | --- | --- | --- |
|  | Astrin\_STLCLD20\_112214\_tube2\_01.18681.18681.2 | 2.4491 | 0.2471 | 97.9% | 1498.3322 | 1498.76 | 4 | 5.229 | 45.8% | 1 | K.LTTDPDLILEVLR.S | 2 |
|  | Astrin\_STLCLD20\_112214\_tube2\_01.06129.06129.3 | 2.5023 | 0.316 | 98.2% | 1881.0844 | 1882.0427 | 413 | 5.572 | 25.0% | 1 | R.HNPTVTGHQEQTYLQK.E | 3 |

---

|  |  |  |  |  |  |  |  |  |
| --- | --- | --- | --- | --- | --- | --- | --- | --- |
| U | *gi|9966881|ref|NP\_065* | 5 | 8 | 4.3% | 925 | 106374 | 5.4 | nucleoporin 107kDa [Homo sapiens] |

| Filename XCorr DeltCN Conf% ObsM+H+ CalcM+H+ SpR ZScore Ion% # Sequence  | | | | | | | | | | | | |
| --- | --- | --- | --- | --- | --- | --- | --- | --- | --- | --- | --- | --- |
| \* | Astrin\_STLCLD20\_112214\_tube2\_01.12268.12268.2 | 2.7403 | 0.3509 | 99.8% | 1329.5122 | 1329.4093 | 1 | 6.234 | 63.6% | 2 | R.SGFGEISS\*PVIR.E | 2 |
| \* | Astrin\_STLCLD20\_112214\_tube2\_01.09648.09648.2 | 4.9121 | 0.4936 | 100.0% | 1890.4922 | 1891.0476 | 1 | 9.854 | 68.8% | 3 | R.VLLQASQDENFGNTTPR.N | 2 |
| \* | Astrin\_STLCLD20\_112214\_tube2\_02.06870.06870.3 | 3.6004 | 0.319 | 99.8% | 1971.2344 | 1971.0476 | 1 | 5.472 | 37.5% | 1 | R.VLLQASQDENFGNTT#PR.N | 3 |
| \* | Astrin\_STLCLD20\_112214\_02.10230.10230.2 | 4.674 | 0.4057 | 100.0% | 1971.4122 | 1971.0476 | 1 | 7.124 | 62.5% | 1 | R.VLLQASQDENFGNTT#PR.N | 2 |
| \* | Astrin\_STLCLD20\_112214\_tube2\_01.10822.10822.2 | 2.5823 | 0.2968 | 99.3% | 1122.1522 | 1121.3219 | 1 | 5.996 | 80.0% | 1 | R.AIYAALSGNLK.Q | 2 |

---

|  |  |  |  |  |  |  |  |  |
| --- | --- | --- | --- | --- | --- | --- | --- | --- |
| U | *gi|32483374|ref|NP\_00* | 2 | 2 | 4.2% | 594 | 66050 | 9.2 | nucleolar protein 5A [Homo sapiens] |

| Filename XCorr DeltCN Conf% ObsM+H+ CalcM+H+ SpR ZScore Ion% # Sequence  | | | | | | | | | | | | |
| --- | --- | --- | --- | --- | --- | --- | --- | --- | --- | --- | --- | --- |
| \* | Astrin\_STLCLD20\_112214\_01.06863.06863.2 | 2.6508 | 0.2513 | 98.7% | 1309.5322 | 1309.5516 | 18 | 5.496 | 54.2% | 1 | R.LIAHAGSLTNLAK.Y | 2 |
| \* | Astrin\_STLCLD20\_112214\_tube2\_01.13152.13152.2 | 2.2595 | 0.2673 | 97.7% | 1411.6721 | 1411.6462 | 1 | 4.989 | 59.1% | 1 | K.YGLIFHSTFIGR.A | 2 |

---

|  |  |  |  |  |  |  |  |  |
| --- | --- | --- | --- | --- | --- | --- | --- | --- |
| U | *gi|48762932|ref|NP\_00* | 2 | 2 | 4.2% | 548 | 59621 | 5.6 | chaperonin containing TCP1, subunit 8 (theta) [Homo sapiens] |

| Filename XCorr DeltCN Conf% ObsM+H+ CalcM+H+ SpR ZScore Ion% # Sequence  | | | | | | | | | | | | |
| --- | --- | --- | --- | --- | --- | --- | --- | --- | --- | --- | --- | --- |
| \* | Astrin\_STLCLD20\_112214\_01.08115.08115.2 | 2.1197 | 0.3317 | 98.5% | 1308.2322 | 1308.4362 | 47 | 5.774 | 60.0% | 1 | K.HFSGLEEAVYR.N | 2 |
| \* | Astrin\_STLCLD20\_112214\_tube2\_01.13155.13155.2 | 2.42 | 0.2358 | 97.7% | 1334.6522 | 1334.5583 | 1 | 5.249 | 63.6% | 1 | K.LFVTNDAATILR.E | 2 |

---

|  |  |  |  |  |  |  |  |  |
| --- | --- | --- | --- | --- | --- | --- | --- | --- |
| U | *gi|7657307|ref|NP\_055* | 3 | 4 | 4.1% | 676 | 72190 | 6.7 | LIM domains containing 1 [Homo sapiens] |

| Filename XCorr DeltCN Conf% ObsM+H+ CalcM+H+ SpR ZScore Ion% # Sequence  | | | | | | | | | | | | |
| --- | --- | --- | --- | --- | --- | --- | --- | --- | --- | --- | --- | --- |
| \* | Astrin\_STLCLD20\_112214\_tube2\_01.06303.06303.2 | 3.2029 | 0.4009 | 100.0% | 1462.4521 | 1463.5039 | 1 | 7.212 | 63.3% | 2 | R.SSEGSLGGQNSGIGGR.S | 2 |
| \* | Astrin\_STLCLD20\_112214\_tube2\_01.05787.05787.2 | 2.5125 | 0.285 | 98.9% | 1418.2922 | 1418.5576 | 53 | 5.121 | 54.5% | 1 | K.RPSSTALHQHHF.- | 2 |
| \* | Astrin\_STLCLD20\_112214\_tube2\_01.05774.05774.3 | 2.8951 | 0.2167 | 97.1% | 1419.2943 | 1418.5576 | 19 | 4.666 | 40.9% | 1 | K.RPSSTALHQHHF.- | 3 |

---

|  |  |  |  |  |  |  |  |  |
| --- | --- | --- | --- | --- | --- | --- | --- | --- |
| U | *gi|17402900|ref|NP\_00* | 2 | 5 | 4.0% | 644 | 67560 | 7.6 | far upstream element-binding protein [Homo sapiens] |

| Filename XCorr DeltCN Conf% ObsM+H+ CalcM+H+ SpR ZScore Ion% # Sequence  | | | | | | | | | | | | |
| --- | --- | --- | --- | --- | --- | --- | --- | --- | --- | --- | --- | --- |
| \* | Astrin\_STLCLD20\_112214\_01.07832.07832.2 | 2.6655 | 0.1946 | 97.8% | 1354.2922 | 1353.5181 | 2 | 5.155 | 62.5% | 1 | K.IQIAPDSGGLPER.S | 2 |
| \* | Astrin\_STLCLD20\_112214\_tube2\_01.11276.11276.2 | 3.2123 | 0.4672 | 100.0% | 1336.4521 | 1337.5187 | 1 | 7.798 | 79.2% | 4 | R.IGGNEGIDVPIPR.F | 2 |

---

|  |  |  |  |  |  |  |  |  |
| --- | --- | --- | --- | --- | --- | --- | --- | --- |
| U | *gi|150418007|ref|NP\_0* | 8 | 11 | 3.9% | 3224 | 358201 | 6.2 | RAN binding protein 2 [Homo sapiens] |

| Filename XCorr DeltCN Conf% ObsM+H+ CalcM+H+ SpR ZScore Ion% # Sequence  | | | | | | | | | | | | |
| --- | --- | --- | --- | --- | --- | --- | --- | --- | --- | --- | --- | --- |
|  | Astrin\_STLCLD20\_112214\_tube2\_02.14061.14061.3 | 3.2224 | 0.3469 | 99.5% | 2310.5645 | 2309.7266 | 1 | 5.825 | 36.2% | 1 | R.ATNTDLLLAYANLMLLTLSTR.D | 33 |
|  | Astrin\_STLCLD20\_112214\_01.12728.12728.2 | 2.6044 | 0.2601 | 98.6% | 1565.6122 | 1565.7637 | 246 | 5.876 | 38.5% | 1 | R.ELLQSFDSALQSVK.S | 2 |
|  | Astrin\_STLCLD20\_112214\_tube2\_02.07051.07051.3 | 3.082 | 0.2845 | 99.6% | 1457.0343 | 1456.7031 | 14 | 4.886 | 35.4% | 1 | R.LSQSGHMLLNLSR.G | 33 |
|  | Astrin\_STLCLD20\_112214\_01.07970.07970.2 | 3.1769 | 0.3327 | 100.0% | 1337.4321 | 1336.5773 | 1 | 5.928 | 80.0% | 3 | R.LLVQHEINTLR.A | 22 |
| \* | Astrin\_STLCLD20\_112214\_tube2\_02.06999.06999.2 | 3.8554 | 0.531 | 100.0% | 1815.3121 | 1814.9647 | 1 | 9.059 | 62.5% | 1 | K.NVSGISFTENMGSSQQK.N | 2 |
| \* | Astrin\_STLCLD20\_112214\_tube2\_01.15641.15641.3 | 4.85 | 0.4587 | 100.0% | 2599.4944 | 2599.9487 | 1 | 6.824 | 35.7% | 2 | R.SFVWHALDYADELPKPEQLAIR.F | 3 |
| \* | Astrin\_STLCLD20\_112214\_tube2\_01.06906.06906.2 | 3.6099 | 0.4243 | 100.0% | 1487.6122 | 1487.6738 | 1 | 6.977 | 64.3% | 1 | K.APGTNVAMASNQAVR.I | 2 |
| \* | Astrin\_STLCLD20\_112214\_tube2\_02.08037.08037.2 | 2.9057 | 0.1517 | 97.4% | 1540.4122 | 1540.6726 | 1 | 4.959 | 69.2% | 1 | K.SDAGNLNFEFQVAK.K | 2 |

Similarities:
gi|211059431|ref|NP\_8(3:5)  

---

|  |  |  |  |  |  |  |  |  |
| --- | --- | --- | --- | --- | --- | --- | --- | --- |
| U | *gi|55956788|ref|NP\_00* | 2 | 4 | 3.9% | 710 | 76615 | 4.7 | nucleolin [Homo sapiens] |

| Filename XCorr DeltCN Conf% ObsM+H+ CalcM+H+ SpR ZScore Ion% # Sequence  | | | | | | | | | | | | |
| --- | --- | --- | --- | --- | --- | --- | --- | --- | --- | --- | --- | --- |
| \* | Astrin\_STLCLD20\_112214\_02.12958.12958.2 | 2.8815 | 0.2453 | 99.0% | 1649.3922 | 1649.751 | 1 | 5.224 | 53.8% | 2 | K.FGYVDFESAEDLEK.A | 2 |
| \* | Astrin\_STLCLD20\_112214\_02.13039.13039.2 | 3.2472 | 0.3908 | 100.0% | 1563.0122 | 1562.6323 | 1 | 6.994 | 61.5% | 2 | K.GFGFVDFNSEEDAK.A | 2 |

---

|  |  |  |  |  |  |  |  |  |
| --- | --- | --- | --- | --- | --- | --- | --- | --- |
| U | *gi|19923142|ref|NP\_00* | 2 | 3 | 3.7% | 876 | 97170 | 4.8 | karyopherin beta 1 [Homo sapiens] |

| Filename XCorr DeltCN Conf% ObsM+H+ CalcM+H+ SpR ZScore Ion% # Sequence  | | | | | | | | | | | | |
| --- | --- | --- | --- | --- | --- | --- | --- | --- | --- | --- | --- | --- |
| \* | Astrin\_STLCLD20\_112214\_tube2\_01.16216.16216.2 | 2.5239 | 0.2789 | 98.5% | 1606.1522 | 1606.8595 | 20 | 5.539 | 46.4% | 1 | K.LAATNALLNSLEFTK.A | 2 |
| \* | Astrin\_STLCLD20\_112214\_02.09933.09933.3 | 3.3868 | 0.3038 | 99.5% | 2001.6543 | 2001.2218 | 1 | 6.255 | 42.2% | 2 | R.LQQVLQMESHIQSTSDR.I | 3 |

---

|  |  |  |  |  |  |  |  |  |
| --- | --- | --- | --- | --- | --- | --- | --- | --- |
| U | *gi|211059431|ref|NP\_8* | 4 | 6 | 3.6% | 1758 | 197287 | 6.3 | RANBP2-like and GRIP domain containing 4 [Homo sapiens] |
| U | *gi|221307607|ref|NP\_0* | 4 | 6 | 3.6% | 1758 | 197486 | 6.3 | RANBP2-like and GRIP domain containing 3 [Homo sapiens] |

| Filename XCorr DeltCN Conf% ObsM+H+ CalcM+H+ SpR ZScore Ion% # Sequence  | | | | | | | | | | | | |
| --- | --- | --- | --- | --- | --- | --- | --- | --- | --- | --- | --- | --- |
|  | Astrin\_STLCLD20\_112214\_tube2\_02.14061.14061.3 | 3.2224 | 0.3469 | 99.5% | 2310.5645 | 2309.7266 | 1 | 5.825 | 36.2% | 1 | R.ATNTDLLLAYANLMLLTLSTR.D | 33 |
|  | Astrin\_STLCLD20\_112214\_tube2\_02.07051.07051.3 | 3.082 | 0.2845 | 99.6% | 1457.0343 | 1456.7031 | 14 | 4.886 | 35.4% | 1 | R.LSQSGHMLLNLSR.G | 33 |
|  | Astrin\_STLCLD20\_112214\_01.07970.07970.2 | 3.1769 | 0.3327 | 100.0% | 1337.4321 | 1336.5773 | 1 | 5.928 | 80.0% | 3 | R.LLVQHEINTLR.A | 22 |
|  | Astrin\_STLCLD20\_112214\_tube2\_01.09866.09866.3 | 3.6538 | 0.1491 | 95.4% | 2192.6643 | 2192.325 | 3 | 3.676 | 41.2% | 1 | K.EEMQELKLNSSKSAS\*HHR.W | 3 |

Similarities:
gi|150418007|ref|NP\_0(3:1)  

---

|  |  |  |  |  |  |  |  |  |
| --- | --- | --- | --- | --- | --- | --- | --- | --- |
| U | *gi|21361368|ref|NP\_00* | 2 | 3 | 3.6% | 795 | 87302 | 7.1 | pyrroline-5-carboxylate synthetase isoform 1 [Homo sapiens] |
| U | *gi|62912457|ref|NP\_00* | 2 | 3 | 3.7% | 793 | 87089 | 7.1 | pyrroline-5-carboxylate synthetase isoform 2 [Homo sapiens] |

| Filename XCorr DeltCN Conf% ObsM+H+ CalcM+H+ SpR ZScore Ion% # Sequence  | | | | | | | | | | | | |
| --- | --- | --- | --- | --- | --- | --- | --- | --- | --- | --- | --- | --- |
|  | Astrin\_STLCLD20\_112214\_tube2\_02.07990.07990.3 | 4.1012 | 0.2476 | 99.7% | 1772.6943 | 1773.1289 | 1 | 5.945 | 45.0% | 2 | R.ILHLLTQEALSIHGVK.E | 3 |
|  | Astrin\_STLCLD20\_112214\_02.11065.11065.2 | 2.8249 | 0.4181 | 100.0% | 1294.2122 | 1294.4502 | 2 | 7.694 | 54.2% | 1 | R.FGLGAEVGISTSR.I | 2 |

---

|  |  |  |  |  |  |  |  |  |
| --- | --- | --- | --- | --- | --- | --- | --- | --- |
| U | *gi|155030232|ref|NP\_0* | 2 | 2 | 3.5% | 1130 | 119700 | 4.3 | proline, glutamic acid and leucine rich protein 1 [Homo sapiens] |

| Filename XCorr DeltCN Conf% ObsM+H+ CalcM+H+ SpR ZScore Ion% # Sequence  | | | | | | | | | | | | |
| --- | --- | --- | --- | --- | --- | --- | --- | --- | --- | --- | --- | --- |
| \* | Astrin\_STLCLD20\_112214\_tube2\_01.12528.12528.2 | 2.0609 | 0.2886 | 96.6% | 1275.4521 | 1275.4906 | 19 | 4.787 | 50.0% | 1 | R.LPSLGAGFSQGLK.H | 2 |
| \* | Astrin\_STLCLD20\_112214\_tube2\_01.17072.17072.3 | 4.5034 | 0.4784 | 100.0% | 2885.2444 | 2884.3203 | 1 | 7.377 | 28.8% | 1 | R.LHDLVLPLVMGVQQGEVLGSSPYTSSR.C | 3 |

---

|  |  |  |  |  |  |  |  |  |
| --- | --- | --- | --- | --- | --- | --- | --- | --- |
| U | *gi|116063573|ref|NP\_0* | 6 | 10 | 3.4% | 2639 | 280016 | 6.0 | filamin A, alpha isoform 1 [Homo sapiens] |
| U | *gi|160420317|ref|NP\_0* | 6 | 10 | 3.4% | 2647 | 280737 | 6.1 | filamin A, alpha isoform 2 [Homo sapiens] |

| Filename XCorr DeltCN Conf% ObsM+H+ CalcM+H+ SpR ZScore Ion% # Sequence  | | | | | | | | | | | | |
| --- | --- | --- | --- | --- | --- | --- | --- | --- | --- | --- | --- | --- |
|  | Astrin\_STLCLD20\_112214\_tube2\_01.12015.12015.2 | 3.1116 | 0.427 | 100.0% | 1416.3322 | 1416.5742 | 1 | 7.507 | 79.2% | 1 | R.IANLQTDLSDGLR.L | 2 |
|  | Astrin\_STLCLD20\_112214\_tube2\_01.13751.13751.2 | 2.0808 | 0.2712 | 96.9% | 1286.8322 | 1286.5167 | 43 | 5.339 | 55.0% | 1 | K.LPQLPITNFSR.D | 2 |
|  | Astrin\_STLCLD20\_112214\_02.10969.10969.2 | 2.7136 | 0.3081 | 99.3% | 1501.8722 | 1501.6335 | 2 | 6.104 | 53.3% | 2 | K.DAGEGGLSLAIEGPSK.A | 2 |
|  | Astrin\_STLCLD20\_112214\_tube2\_01.15221.15221.2 | 2.3183 | 0.2801 | 97.7% | 1534.1122 | 1534.7117 | 4 | 4.469 | 50.0% | 1 | R.AEAGVPAEFSIWTR.E | 2 |
|  | Astrin\_STLCLD20\_112214\_tube2\_02.06862.06862.2 | 3.701 | 0.3916 | 100.0% | 1427.5122 | 1427.5974 | 1 | 6.796 | 60.0% | 2 | R.EAGAGGLAIAVEGPSK.A | 2 |
|  | Astrin\_STLCLD20\_112214\_02.10533.10533.3 | 4.427 | 0.4887 | 100.0% | 2201.6343 | 2201.4412 | 1 | 7.343 | 36.8% | 3 | R.LVSNHSLHETSSVFVDSLTK.A | 3 |

---

|  |  |  |  |  |  |  |  |  |
| --- | --- | --- | --- | --- | --- | --- | --- | --- |
| U | *gi|153792294|ref|NP\_1* | 2 | 2 | 3.3% | 1320 | 145257 | 6.8 | myopalladin [Homo sapiens] |

| Filename XCorr DeltCN Conf% ObsM+H+ CalcM+H+ SpR ZScore Ion% # Sequence  | | | | | | | | | | | | |
| --- | --- | --- | --- | --- | --- | --- | --- | --- | --- | --- | --- | --- |
| \* | Astrin\_STLCLD20\_112214\_02.09504.09504.3 | 3.8121 | 0.3644 | 99.8% | 2548.3743 | 2546.71 | 1 | 5.68 | 27.2% | 1 | R.VHFNLPEDDKGSEASSEAGVVTTR.Q | 3 |
| \* | Astrin\_STLCLD20\_112214\_01.09739.09739.3 | 3.8144 | 0.3948 | 99.7% | 2213.3643 | 2213.5242 | 1 | 6.274 | 45.8% | 1 | R.FFRPHFLQAPGDMVAHEGR.L | 3 |

---

|  |  |  |  |  |  |  |  |  |
| --- | --- | --- | --- | --- | --- | --- | --- | --- |
| U | *gi|167466272|ref|NP\_6* | 2 | 4 | 3.2% | 745 | 83587 | 9.8 | cytoskeleton associated protein 2-like [Homo sapiens] |

| Filename XCorr DeltCN Conf% ObsM+H+ CalcM+H+ SpR ZScore Ion% # Sequence  | | | | | | | | | | | | |
| --- | --- | --- | --- | --- | --- | --- | --- | --- | --- | --- | --- | --- |
| \* | Astrin\_STLCLD20\_112214\_01.07919.07919.2 | 3.6076 | 0.3066 | 100.0% | 1326.3922 | 1326.5791 | 3 | 5.662 | 63.6% | 2 | R.KPVGSLNIEQLK.T | 2 |
| \* | Astrin\_STLCLD20\_112214\_01.09029.09029.2 | 3.6 | 0.4371 | 100.0% | 1399.3722 | 1399.6334 | 1 | 7.286 | 63.6% | 2 | R.KVVLNILQDSNR.T | 2 |

---

|  |  |  |  |  |  |  |  |  |
| --- | --- | --- | --- | --- | --- | --- | --- | --- |
| U | *gi|41872631|ref|NP\_00* | 6 | 9 | 3.1% | 2511 | 273424 | 6.4 | fatty acid synthase [Homo sapiens] |

| Filename XCorr DeltCN Conf% ObsM+H+ CalcM+H+ SpR ZScore Ion% # Sequence  | | | | | | | | | | | | |
| --- | --- | --- | --- | --- | --- | --- | --- | --- | --- | --- | --- | --- |
| \* | Astrin\_STLCLD20\_112214\_01.10781.10781.2 | 2.4979 | 0.3121 | 99.3% | 1253.4521 | 1252.4148 | 1 | 5.872 | 60.0% | 2 | R.FDASFFGVHPK.Q | 2 |
| \* | Astrin\_STLCLD20\_112214\_tube2\_02.07023.07023.2 | 2.9236 | 0.2999 | 99.4% | 1550.8922 | 1550.7086 | 27 | 5.732 | 42.9% | 1 | R.AFEVSENGNLVVSGK.V | 2 |
| \* | Astrin\_STLCLD20\_112214\_tube2\_01.06653.06653.2 | 2.413 | 0.2247 | 97.4% | 1331.3722 | 1331.5173 | 1 | 4.888 | 63.6% | 1 | R.VTAIHIDPATHR.Q | 2 |
| \* | Astrin\_STLCLD20\_112214\_01.10150.10150.2 | 3.6475 | 0.412 | 100.0% | 1470.2122 | 1470.5815 | 1 | 6.837 | 79.2% | 2 | R.FPQLDSTSFANSR.D | 2 |
| \* | Astrin\_STLCLD20\_112214\_01.17063.17063.2 | 2.458 | 0.238 | 97.8% | 1406.2922 | 1406.6682 | 2 | 5.507 | 54.2% | 1 | R.DLVEAVAHILGIR.D | 2 |
| \* | Astrin\_STLCLD20\_112214\_01.10917.10917.2 | 2.5057 | 0.202 | 97.0% | 1428.2722 | 1427.702 | 1 | 4.398 | 62.5% | 2 | R.SLLVNPEGPTLMR.L | 2 |

---

|  |  |  |  |  |  |  |  |  |
| --- | --- | --- | --- | --- | --- | --- | --- | --- |
| U | *gi|20143967|ref|NP\_61* | 2 | 3 | 3.0% | 960 | 110059 | 8.5 | kinesin family member 23 isoform 1 [Homo sapiens] |
| U | *gi|6754472|ref|NP\_004* | 2 | 3 | 3.4% | 856 | 98105 | 8.5 | kinesin family member 23 isoform 2 [Homo sapiens] |

| Filename XCorr DeltCN Conf% ObsM+H+ CalcM+H+ SpR ZScore Ion% # Sequence  | | | | | | | | | | | | |
| --- | --- | --- | --- | --- | --- | --- | --- | --- | --- | --- | --- | --- |
|  | Astrin\_STLCLD20\_112214\_tube2\_01.10127.10127.2 | 2.6027 | 0.4029 | 99.9% | 1498.1522 | 1497.6196 | 1 | 6.233 | 59.1% | 1 | K.AEDYEENLQVMR.F | 2 |
|  | Astrin\_STLCLD20\_112214\_02.09487.09487.3 | 3.9719 | 0.3556 | 99.7% | 1967.0944 | 1966.17 | 103 | 6.164 | 29.7% | 2 | K.YMLTHQELASDGEIETK.L | 3 |

---

|  |  |  |  |  |  |  |  |  |
| --- | --- | --- | --- | --- | --- | --- | --- | --- |
| U | *gi|126722969|ref|NP\_0* | 2 | 4 | 2.9% | 561 | 60423 | 6.6 | centromere protein T [Homo sapiens] |

| Filename XCorr DeltCN Conf% ObsM+H+ CalcM+H+ SpR ZScore Ion% # Sequence  | | | | | | | | | | | | |
| --- | --- | --- | --- | --- | --- | --- | --- | --- | --- | --- | --- | --- |
| \* | Astrin\_STLCLD20\_112214\_tube2\_01.06020.06020.2 | 5.9175 | 0.5425 | 100.0% | 1761.2722 | 1761.8918 | 1 | 9.816 | 66.7% | 1 | R.SAHIQASGHLEEQTPR.T | 2 |
| \* | Astrin\_STLCLD20\_112214\_02.07605.07605.3 | 3.6704 | 0.3347 | 99.8% | 1762.0443 | 1761.8918 | 1 | 6.561 | 46.7% | 3 | R.SAHIQASGHLEEQTPR.T | 3 |

---

|  |  |  |  |  |  |  |  |  |
| --- | --- | --- | --- | --- | --- | --- | --- | --- |
| U | *gi|156523968|ref|NP\_0* | 2 | 4 | 2.8% | 1014 | 113084 | 8.9 | poly (ADP-ribose) polymerase family, member 1 [Homo sapiens] |

| Filename XCorr DeltCN Conf% ObsM+H+ CalcM+H+ SpR ZScore Ion% # Sequence  | | | | | | | | | | | | |
| --- | --- | --- | --- | --- | --- | --- | --- | --- | --- | --- | --- | --- |
| \* | Astrin\_STLCLD20\_112214\_02.12120.12120.2 | 4.2298 | 0.527 | 100.0% | 1625.1522 | 1625.7728 | 1 | 10.009 | 67.9% | 2 | R.VVSEDFLQDVSASTK.S | 2 |
| \* | Astrin\_STLCLD20\_112214\_01.12116.12116.2 | 2.9855 | 0.3681 | 100.0% | 1378.6122 | 1378.5712 | 1 | 6.934 | 58.3% | 2 | R.TTNFAGILSQGLR.I | 2 |

---

|  |  |  |  |  |  |  |  |  |
| --- | --- | --- | --- | --- | --- | --- | --- | --- |
| U | *gi|167234419|ref|NP\_0* | 2 | 2 | 2.8% | 955 | 108666 | 10.2 | thyroid hormone receptor associated protein 3 [Homo sapiens] |

| Filename XCorr DeltCN Conf% ObsM+H+ CalcM+H+ SpR ZScore Ion% # Sequence  | | | | | | | | | | | | |
| --- | --- | --- | --- | --- | --- | --- | --- | --- | --- | --- | --- | --- |
| \* | Astrin\_STLCLD20\_112214\_01.06819.06819.2 | 3.277 | 0.3757 | 100.0% | 1530.3722 | 1530.6835 | 1 | 5.982 | 66.7% | 1 | R.SIFQHIQSAQSQR.S | 2 |
| \* | Astrin\_STLCLD20\_112214\_tube2\_01.06197.06197.2 | 2.5893 | 0.3481 | 99.4% | 1626.0922 | 1625.7588 | 46 | 5.663 | 50.0% | 1 | K.EHHFGSSGMTLHER.F | 2 |

---

|  |  |  |  |  |  |  |  |  |
| --- | --- | --- | --- | --- | --- | --- | --- | --- |
| U | *gi|106049292|ref|NP\_0* | 2 | 3 | 2.6% | 1178 | 129634 | 6.8 | pyruvate carboxylase precursor [Homo sapiens] |
| U | *gi|106049528|ref|NP\_0* | 2 | 3 | 2.6% | 1178 | 129634 | 6.8 | pyruvate carboxylase precursor [Homo sapiens] |
| U | *gi|106049295|ref|NP\_0* | 2 | 3 | 2.6% | 1178 | 129634 | 6.8 | pyruvate carboxylase precursor [Homo sapiens] |

| Filename XCorr DeltCN Conf% ObsM+H+ CalcM+H+ SpR ZScore Ion% # Sequence  | | | | | | | | | | | | |
| --- | --- | --- | --- | --- | --- | --- | --- | --- | --- | --- | --- | --- |
|  | Astrin\_STLCLD20\_112214\_tube2\_02.09343.09343.3 | 3.3478 | 0.2431 | 98.3% | 2411.0044 | 2411.7227 | 23 | 5.224 | 30.3% | 2 | R.HIEVQILGDQYGNILHLYER.D | 3 |
|  | Astrin\_STLCLD20\_112214\_tube2\_02.07941.07941.2 | 2.9735 | 0.2268 | 99.4% | 1364.9122 | 1364.4949 | 4 | 5.386 | 70.0% | 1 | K.IAEEFEVELER.G | 2 |

---

|  |  |  |  |  |  |  |  |  |
| --- | --- | --- | --- | --- | --- | --- | --- | --- |
| U | *gi|12025678|ref|NP\_00* | 2 | 2 | 2.6% | 911 | 104854 | 5.4 | actinin, alpha 4 [Homo sapiens] |
| U | *gi|4501891|ref|NP\_001* | 2 | 2 | 2.7% | 892 | 103058 | 5.4 | actinin, alpha 1 isoform b [Homo sapiens] |
| U | *gi|194097352|ref|NP\_0* | 2 | 2 | 2.7% | 887 | 102709 | 5.5 | actinin, alpha 1 isoform c [Homo sapiens] |
| U | *gi|194097350|ref|NP\_0* | 2 | 2 | 2.6% | 914 | 105568 | 5.4 | actinin, alpha 1 isoform a [Homo sapiens] |

| Filename XCorr DeltCN Conf% ObsM+H+ CalcM+H+ SpR ZScore Ion% # Sequence  | | | | | | | | | | | | |
| --- | --- | --- | --- | --- | --- | --- | --- | --- | --- | --- | --- | --- |
|  | Astrin\_STLCLD20\_112214\_tube2\_01.18845.18845.2 | 3.8066 | 0.3899 | 100.0% | 1387.2122 | 1387.6218 | 1 | 7.208 | 63.6% | 1 | R.VGWEQLLTTIAR.T | 2 |
|  | Astrin\_STLCLD20\_112214\_tube2\_01.11157.11157.2 | 2.8636 | 0.2508 | 99.3% | 1430.4922 | 1430.6011 | 13 | 5.518 | 59.1% | 1 | R.TINEVENQILTR.D | 2 |

---

|  |  |  |  |  |  |  |  |  |
| --- | --- | --- | --- | --- | --- | --- | --- | --- |
| U | *gi|74048514|ref|NP\_73* | 3 | 3 | 2.5% | 2342 | 265290 | 5.4 | cancer susceptibility candidate 5 isoform 1 [Homo sapiens] |
| U | *gi|74048554|ref|NP\_65* | 3 | 3 | 2.5% | 2316 | 262530 | 5.4 | cancer susceptibility candidate 5 isoform 2 [Homo sapiens] |

| Filename XCorr DeltCN Conf% ObsM+H+ CalcM+H+ SpR ZScore Ion% # Sequence  | | | | | | | | | | | | |
| --- | --- | --- | --- | --- | --- | --- | --- | --- | --- | --- | --- | --- |
|  | Astrin\_STLCLD20\_112214\_tube2\_01.13548.13548.2 | 3.5296 | 0.5062 | 100.0% | 1772.2722 | 1772.9963 | 1 | 7.916 | 56.7% | 1 | R.IQQSLSNPLSISLTDR.K | 2 |
|  | Astrin\_STLCLD20\_112214\_01.14708.14708.3 | 4.4466 | 0.4403 | 100.0% | 2793.6543 | 2793.2354 | 1 | 7.027 | 33.3% | 1 | K.TGEFLAFQTVHLPPLPEQLLELGNK.A | 3 |
|  | Astrin\_STLCLD20\_112214\_tube2\_01.10445.10445.3 | 3.9522 | 0.3733 | 99.7% | 2174.5144 | 2174.3696 | 3 | 6.326 | 32.4% | 1 | R.AAEKELEQLKTEEEELQR.N | 3 |

---

|  |  |  |  |  |  |  |  |  |
| --- | --- | --- | --- | --- | --- | --- | --- | --- |
| U | *gi|30581135|ref|NP\_00* | 2 | 2 | 2.4% | 1233 | 143233 | 7.6 | structural maintenance of chromosomes 1A [Homo sapiens] |

| Filename XCorr DeltCN Conf% ObsM+H+ CalcM+H+ SpR ZScore Ion% # Sequence  | | | | | | | | | | | | |
| --- | --- | --- | --- | --- | --- | --- | --- | --- | --- | --- | --- | --- |
| \* | Astrin\_STLCLD20\_112214\_tube2\_02.07600.07600.2 | 2.4681 | 0.392 | 99.5% | 1689.2922 | 1690.8219 | 9 | 6.286 | 53.6% | 1 | R.AFVSMVYSEEGAEDR.T | 2 |
| \* | Astrin\_STLCLD20\_112214\_tube2\_01.08548.08548.2 | 2.3788 | 0.2999 | 98.5% | 1674.7522 | 1673.7332 | 152 | 4.494 | 46.2% | 1 | R.DKFQETSDEFEAAR.K | 2 |

---

|  |  |  |  |  |  |  |  |  |
| --- | --- | --- | --- | --- | --- | --- | --- | --- |
| U | *gi|100913206|ref|NP\_0* | 2 | 2 | 2.3% | 1270 | 140958 | 6.8 | DEAH (Asp-Glu-Ala-His) box polypeptide 9 [Homo sapiens] |

| Filename XCorr DeltCN Conf% ObsM+H+ CalcM+H+ SpR ZScore Ion% # Sequence  | | | | | | | | | | | | |
| --- | --- | --- | --- | --- | --- | --- | --- | --- | --- | --- | --- | --- |
| \* | Astrin\_STLCLD20\_112214\_tube2\_01.08632.08632.3 | 3.327 | 0.313 | 99.8% | 1505.1543 | 1504.686 | 1 | 6.169 | 47.9% | 1 | R.GISHVIVDEIHER.D | 3 |
| \* | Astrin\_STLCLD20\_112214\_tube2\_01.13144.13144.2 | 3.0898 | 0.2268 | 99.1% | 1743.3522 | 1742.8804 | 1 | 5.017 | 60.0% | 1 | R.ELDALDANDELTPLGR.I | 2 |

---

|  |  |  |  |  |  |  |  |  |
| --- | --- | --- | --- | --- | --- | --- | --- | --- |
| U | *gi|118572613|ref|NP\_0* | 4 | 5 | 2.1% | 2752 | 299616 | 12.1 | splicing coactivator subunit SRm300 [Homo sapiens] |

| Filename XCorr DeltCN Conf% ObsM+H+ CalcM+H+ SpR ZScore Ion% # Sequence  | | | | | | | | | | | | |
| --- | --- | --- | --- | --- | --- | --- | --- | --- | --- | --- | --- | --- |
| \* | Astrin\_STLCLD20\_112214\_tube2\_01.10450.10450.2 | 2.5714 | 0.2498 | 98.1% | 1721.6721 | 1720.92 | 2 | 4.451 | 46.9% | 1 | R.SSTGPEPPAPTPLLAER.H | 2 |
| \* | Astrin\_STLCLD20\_112214\_01.09275.09275.2 | 3.8102 | 0.5609 | 100.0% | 1414.9122 | 1415.6506 | 1 | 9.02 | 75.0% | 2 | R.IPAASAAAMNLASAR.T | 2 |
| \* | Astrin\_STLCLD20\_112214\_01.09544.09544.2 | 2.7365 | 0.3542 | 99.7% | 1426.5322 | 1426.6127 | 72 | 6.116 | 53.8% | 1 | R.TPAIPTAVNLADSR.T | 2 |
| \* | Astrin\_STLCLD20\_112214\_01.06875.06875.2 | 2.1351 | 0.339 | 98.4% | 1240.0922 | 1239.4172 | 18 | 5.42 | 54.2% | 1 | R.TPTAPAVNLAGAR.T | 2 |

---

|  |  |  |  |  |  |  |  |  |
| --- | --- | --- | --- | --- | --- | --- | --- | --- |
| U | *gi|21626468|ref|NP\_05* | 2 | 2 | 2.0% | 1978 | 220623 | 6.4 | zinc finger protein 638 [Homo sapiens] |
| U | *gi|62526045|ref|NP\_00* | 2 | 2 | 2.0% | 1978 | 220623 | 6.4 | zinc finger protein 638 [Homo sapiens] |

| Filename XCorr DeltCN Conf% ObsM+H+ CalcM+H+ SpR ZScore Ion% # Sequence  | | | | | | | | | | | | |
| --- | --- | --- | --- | --- | --- | --- | --- | --- | --- | --- | --- | --- |
|  | Astrin\_STLCLD20\_112214\_02.09237.09237.3 | 3.8777 | 0.3351 | 99.8% | 2308.1343 | 2307.479 | 1 | 6.222 | 31.0% | 1 | R.NKETLGSEAVSSNVIDYGHASK.Y | 3 |
|  | Astrin\_STLCLD20\_112214\_tube2\_01.07508.07508.3 | 2.7126 | 0.2845 | 97.8% | 1842.5044 | 1843.0525 | 252 | 4.361 | 32.8% | 1 | R.IPHEPVINSSNVHVGSR.G | 3 |

---

|  |  |  |  |  |  |  |  |  |
| --- | --- | --- | --- | --- | --- | --- | --- | --- |
| U | *gi|42716280|ref|NP\_97* | 2 | 3 | 2.0% | 1268 | 141439 | 6.9 | high density lipoprotein binding protein [Homo sapiens] |
| U | *gi|4885409|ref|NP\_005* | 2 | 3 | 2.0% | 1268 | 141439 | 6.9 | high density lipoprotein binding protein [Homo sapiens] |

| Filename XCorr DeltCN Conf% ObsM+H+ CalcM+H+ SpR ZScore Ion% # Sequence  | | | | | | | | | | | | |
| --- | --- | --- | --- | --- | --- | --- | --- | --- | --- | --- | --- | --- |
|  | Astrin\_STLCLD20\_112214\_02.09137.09137.2 | 2.7713 | 0.2748 | 99.3% | 1328.0521 | 1328.5516 | 24 | 4.722 | 54.2% | 1 | R.LQTQASATVAIPK.E | 2 |
|  | Astrin\_STLCLD20\_112214\_01.08311.08311.2 | 3.1157 | 0.478 | 100.0% | 1334.4521 | 1334.5304 | 1 | 9.707 | 72.7% | 2 | R.LVGEIMQETGTR.I | 2 |

---

|  |  |  |  |  |  |  |  |  |
| --- | --- | --- | --- | --- | --- | --- | --- | --- |
| U | *gi|207452735|ref|NP\_1* | 5 | 5 | 1.5% | 5090 | 555629 | 5.6 | epiplakin 1 [Homo sapiens] |

| Filename XCorr DeltCN Conf% ObsM+H+ CalcM+H+ SpR ZScore Ion% # Sequence  | | | | | | | | | | | | |
| --- | --- | --- | --- | --- | --- | --- | --- | --- | --- | --- | --- | --- |
|  | Astrin\_STLCLD20\_112214\_tube2\_02.07104.07104.3 | 3.7517 | 0.308 | 99.6% | 2013.5343 | 2014.292 | 1 | 6.201 | 38.9% | 1 | R.LLEAQIATGGVIDPVHSHR.V | 33 |
| \* | Astrin\_STLCLD20\_112214\_tube2\_01.08406.08406.2 | 2.7493 | 0.3452 | 99.8% | 1326.3722 | 1326.452 | 2 | 5.848 | 72.7% | 1 | R.GSAVHQLSEELR.C | 2 |
| \* | Astrin\_STLCLD20\_112214\_01.15476.15476.2 | 2.811 | 0.2161 | 98.4% | 2396.2522 | 2395.7202 | 1 | 4.817 | 38.1% | 1 | R.VTPGSGALQGQSVSVWELLFYR.E | 2 |
| \* | Astrin\_STLCLD20\_112214\_01.17089.17089.3 | 4.124 | 0.2 | 98.6% | 2514.5942 | 2514.8386 | 1 | 6.191 | 37.5% | 1 | R.AGTLTVEELGATLTSLLAQAQAQAR.A | 3 |
| \* | Astrin\_STLCLD20\_112214\_tube2\_01.20880.20880.2 | 4.0097 | 0.4525 | 100.0% | 2514.9521 | 2514.8386 | 2 | 7.352 | 31.2% | 1 | R.AGTLTVEELGATLTSLLAQAQAQAR.A | 2 |

Similarities:
gi|41322908|ref|NP\_95(1:4)  

---

|  |  |  |  |  |  |  |  |  |
| --- | --- | --- | --- | --- | --- | --- | --- | --- |
| U | *gi|71361682|ref|NP\_00* | 2 | 3 | 1.4% | 2115 | 238257 | 5.8 | nuclear mitotic apparatus protein 1 [Homo sapiens] |

| Filename XCorr DeltCN Conf% ObsM+H+ CalcM+H+ SpR ZScore Ion% # Sequence  | | | | | | | | | | | | |
| --- | --- | --- | --- | --- | --- | --- | --- | --- | --- | --- | --- | --- |
| \* | Astrin\_STLCLD20\_112214\_01.10684.10684.2 | 3.2884 | 0.4326 | 100.0% | 1712.9922 | 1712.9437 | 1 | 7.675 | 64.3% | 1 | R.LQAQLNELQAQLSQK.E | 2 |
| \* | Astrin\_STLCLD20\_112214\_01.08038.08038.2 | 4.1913 | 0.4351 | 100.0% | 1566.5721 | 1566.7141 | 1 | 6.563 | 61.5% | 2 | R.SLEAQVAHADQQLR.D | 2 |

---

|  |  |  |  |  |  |  |  |  |
| --- | --- | --- | --- | --- | --- | --- | --- | --- |
| U | *gi|163965366|ref|NP\_0* | 2 | 2 | 1.4% | 2078 | 205419 | 9.6 | nascent polypeptide-associated complex alpha subunit isoform a [Homo sapiens] |

| Filename XCorr DeltCN Conf% ObsM+H+ CalcM+H+ SpR ZScore Ion% # Sequence  | | | | | | | | | | | | |
| --- | --- | --- | --- | --- | --- | --- | --- | --- | --- | --- | --- | --- |
| \* | Astrin\_STLCLD20\_112214\_tube2\_01.13181.13181.2 | 1.9626 | 0.3235 | 96.7% | 1317.5721 | 1317.5278 | 7 | 5.277 | 50.0% | 1 | K.GAPTPPAVTPPSPK.G | 2 |
|  | Astrin\_STLCLD20\_112214\_02.09607.09607.2 | 2.8469 | 0.1702 | 97.3% | 1617.5521 | 1615.7808 | 1 | 4.63 | 64.3% | 1 | K.IEDLSQQAQLAAAEK.F | 2 |

---

|  |  |  |  |  |  |  |  |  |
| --- | --- | --- | --- | --- | --- | --- | --- | --- |
| U | *gi|58530840|ref|NP\_00* | 3 | 4 | 1.3% | 2871 | 331774 | 6.8 | desmoplakin isoform I [Homo sapiens] |

| Filename XCorr DeltCN Conf% ObsM+H+ CalcM+H+ SpR ZScore Ion% # Sequence  | | | | | | | | | | | | |
| --- | --- | --- | --- | --- | --- | --- | --- | --- | --- | --- | --- | --- |
|  | Astrin\_STLCLD20\_112214\_tube2\_01.19390.19390.2 | 2.9161 | 0.2431 | 99.1% | 1588.6721 | 1587.9153 | 2 | 4.84 | 53.8% | 1 | R.ALLQAILQTEDMLK.V | 2 |
| \* | Astrin\_STLCLD20\_112214\_01.08865.08865.2 | 3.1794 | 0.2355 | 99.7% | 1388.4521 | 1388.5205 | 1 | 6.124 | 72.7% | 2 | R.LNDSILQATEQR.R | 2 |
| \* | Astrin\_STLCLD20\_112214\_tube2\_02.07017.07017.2 | 2.686 | 0.2716 | 99.3% | 1337.5122 | 1337.4723 | 1 | 5.179 | 65.0% | 1 | K.NQFETEINITK.T | 2 |

---

|  |  |  |  |  |  |  |  |  |
| --- | --- | --- | --- | --- | --- | --- | --- | --- |
| U | *gi|57164942|ref|NP\_00* | 2 | 2 | 1.3% | 2032 | 225493 | 7.8 | colonic and hepatic tumor over-expressed protein isoform a [Homo sapiens] |
| U | *gi|57222563|ref|NP\_05* | 2 | 2 | 1.3% | 1972 | 218524 | 8.1 | colonic and hepatic tumor over-expressed protein isoform b [Homo sapiens] |

| Filename XCorr DeltCN Conf% ObsM+H+ CalcM+H+ SpR ZScore Ion% # Sequence  | | | | | | | | | | | | |
| --- | --- | --- | --- | --- | --- | --- | --- | --- | --- | --- | --- | --- |
|  | Astrin\_STLCLD20\_112214\_02.10590.10590.2 | 2.4035 | 0.2115 | 96.4% | 1550.0922 | 1550.7466 | 1 | 4.962 | 65.4% | 1 | R.TEISDKITSELVSK.I | 2 |
|  | Astrin\_STLCLD20\_112214\_01.05169.05169.2 | 2.8918 | 0.2143 | 99.0% | 1319.6921 | 1319.4784 | 1 | 5.876 | 68.2% | 1 | R.AQNISSNANMLR.K | 2 |

---

|  |  |  |  |  |  |  |  |  |
| --- | --- | --- | --- | --- | --- | --- | --- | --- |
| U | *gi|21264365|ref|NP\_05* | 2 | 2 | 1.3% | 1800 | 195816 | 6.4 | nucleoporin 98kD isoform 1 [Homo sapiens] |

| Filename XCorr DeltCN Conf% ObsM+H+ CalcM+H+ SpR ZScore Ion% # Sequence  | | | | | | | | | | | | |
| --- | --- | --- | --- | --- | --- | --- | --- | --- | --- | --- | --- | --- |
| \* | Astrin\_STLCLD20\_112214\_01.10634.10634.2 | 2.6441 | 0.3306 | 99.7% | 1398.5122 | 1398.5614 | 13 | 5.317 | 65.0% | 1 | R.HYDLNQLLEPR.S | 2 |
|  | Astrin\_STLCLD20\_112214\_tube2\_01.13835.13835.2 | 2.5694 | 0.3245 | 99.3% | 1580.3722 | 1580.811 | 1 | 5.638 | 62.5% | 1 | R.LPMPEDYAMDELR.S | 2 |

---

|  |  |  |  |  |  |  |  |  |
| --- | --- | --- | --- | --- | --- | --- | --- | --- |
| U | *gi|67551265|ref|NP\_05* | 2 | 2 | 1.1% | 2266 | 252496 | 6.6 | transcription factor ELYS [Homo sapiens] |

| Filename XCorr DeltCN Conf% ObsM+H+ CalcM+H+ SpR ZScore Ion% # Sequence  | | | | | | | | | | | | |
| --- | --- | --- | --- | --- | --- | --- | --- | --- | --- | --- | --- | --- |
| \* | Astrin\_STLCLD20\_112214\_tube2\_01.13114.13114.2 | 2.8575 | 0.2428 | 98.9% | 1548.6522 | 1549.851 | 2 | 5.343 | 53.8% | 1 | R.ASELHLLETPLVVK.K | 2 |
| \* | Astrin\_STLCLD20\_112214\_01.07114.07114.2 | 2.9439 | 0.3073 | 99.9% | 1300.5122 | 1300.454 | 9 | 5.986 | 65.0% | 1 | R.ISFVEEDVHPK.W | 2 |

---

|  |  |  |  |  |  |  |  |  |
| --- | --- | --- | --- | --- | --- | --- | --- | --- |
| U | *gi|171184451|ref|NP\_0* | 2 | 3 | 0.8% | 3117 | 350931 | 6.3 | centrosome-associated protein 350 [Homo sapiens] |

| Filename XCorr DeltCN Conf% ObsM+H+ CalcM+H+ SpR ZScore Ion% # Sequence  | | | | | | | | | | | | |
| --- | --- | --- | --- | --- | --- | --- | --- | --- | --- | --- | --- | --- |
| \* | Astrin\_STLCLD20\_112214\_02.10422.10422.2 | 3.3264 | 0.3003 | 99.9% | 1519.3121 | 1518.7045 | 1 | 5.565 | 57.7% | 2 | K.LVLEQGDSSEILSK.K | 2 |
| \* | Astrin\_STLCLD20\_112214\_tube2\_01.12250.12250.2 | 2.8643 | 0.3119 | 99.7% | 1279.2322 | 1279.5686 | 1 | 7.452 | 59.1% | 1 | R.VLIGNVQPGILR.F | 2 |

|  |  |  |  |
| --- | --- | --- | --- |
|  | Proteins | Peptide IDs | Spectra |
| Unfiltered | 46071 | 98844 | 171654 |
| Filtered | 222 | 1306 | 3363 |
| Forward matches | 221 | 1304 | 3361 |
| Decoy matches | 1 | 2 | 2 |
| Forward FP rate | 0.45% | 0.15% | 0.06% |

  
/nfs/cheeseman\_massspec/David/Astrin\_STLCLD20
