# Supplementary material for: Astrin-SKAP complex reconstitution reveals its kinetochore interaction with microtubule-bound Ndc80
Source: eLife. 2017 Aug 25;6:e26866. doi: 10.7554/eLife.26866 (PMC5602300; doi:10.7554/eLife.26866)
Supplement: Source data 1. — Complete mass spectrometry searches using methods described in (Washburn et al., 2001) for affinity purification/mass spectrometry data sets described in this paper (data from this study; [Kern et al., 2016] [Gascoigne et al., 2011]). Individual Astrin cross-linking immunoprecipitations are listed based on the order in Figure 4—figure supplement 1. These samples have not been pruned for common or antibody-specific contaminants. [file elife-26866-data1.zip › Astrin_Crosslinking#3.html]

D Astrin\_STLCHLD
DTASelect v2.0.21  
/nfs/cheeseman\_massspec/David/Astrin\_STLCHLD  
/nfs/cheeseman\_massspec/Databases/NCBI-RefSeq\_human\_na\_04-13-2009\_con\_reversed.fasta  
SEQUEST 3.0 in SQT format.  
  
 Jump  to the summary table.  
  
sequest.params modifications:

|  |  |  |
| --- | --- | --- |
| \* | S | 80.0 |
| # | T | 80.0 |
| @ | K | 12.0 |
| Static | C | 57.0 |

|  |  |
| --- | --- |
| true | Use criteria |
| 0.0 | Minimum peptide confidence |
| 0.05 | Peptide false positive rate |
| 0.0 | Minimum protein confidence |
| 1.0 | Protein false positive rate |
| 1 | Minimum charge state |
| 16 | Maximum charge state |
| 0.0 | Minimum ion proportion |
| 1000 | Maximum Sp rank |
| -1.0 | Minimum Sp score |
| Include | Modified peptide inclusion |
| Any | Tryptic status requirement |
| false | Multiple, ambiguous IDs allowed |
| Ignore | Peptide validation handling |
| XCorr | Purge duplicate peptides by protein |
| false | Include only loci with unique peptide |
| true | Remove subset proteins |
| Ignore | Locus validation handling |
| 0 | Minimum modified peptides per locus |
| 1000 | Minimum redundancy for low coverage loci |
| 2 | Minimum peptides per locus |

#### Locus Key:

|  |  |  |  |  |  |  |  |  |
| --- | --- | --- | --- | --- | --- | --- | --- | --- |
| Validation Status | Locus | Sequence Count | Spectrum Count | Sequence Coverage | Length | MolWt | pI | Descriptive Name |

#### Similarity Key:

|  |  |  |
| --- | --- | --- |
| Locus | # of identical peptides | # of differing peptides |

---

|  |  |  |  |  |  |  |  |  |
| --- | --- | --- | --- | --- | --- | --- | --- | --- |
| U | *gi|57013276|ref|NP\_00* | 35 | 155 | 65.2% | 451 | 50152 | 5.1 | tubulin, alpha, ubiquitous [Homo sapiens] |

| Filename XCorr DeltCN Conf% ObsM+H+ CalcM+H+ SpR ZScore Ion% # Sequence  | | | | | | | | | | | | |
| --- | --- | --- | --- | --- | --- | --- | --- | --- | --- | --- | --- | --- |
|  | Astrin\_STLCHLD\_tube2\_050114\_01.10939.10939.2 | 5.614 | 0.6543 | 100.0% | 2008.4321 | 2009.093 | 1 | 11.783 | 57.9% | 8 | K.TIGGGDDSFNTFFSETGAGK.H | 22 |
|  | Astrin\_STLCHLD\_050114\_02.08865.08865.3 | 3.8391 | 0.2974 | 100.0% | 2009.6044 | 2009.093 | 12 | 5.573 | 31.6% | 1 | K.TIGGGDDSFNTFFSETGAGK.H | 33 |
|  | Astrin\_STLCHLD\_050114\_01.12436.12436.1 | 2.6238 | 0.525 | 100.0% | 1701.85 | 1702.9451 | 1 | 7.571 | 50.0% | 2 | R.AVFVDLEPTVIDEVR.T | 11 |
|  | Astrin\_STLCHLD\_tube2\_050114\_01.11695.11695.2 | 4.7671 | 0.4744 | 100.0% | 1702.5521 | 1702.9451 | 1 | 7.991 | 78.6% | 7 | R.AVFVDLEPTVIDEVR.T | 22 |
|  | Astrin\_STLCHLD\_tube2\_050114\_01.11676.11676.3 | 4.6957 | 0.4534 | 100.0% | 1704.2943 | 1702.9451 | 1 | 7.889 | 51.8% | 3 | R.AVFVDLEPTVIDEVR.T | 33 |
|  | Astrin\_STLCHLD\_tube2\_050114\_01.08551.08551.2 | 1.7735 | 0.3588 | 96.6% | 1411.4922 | 1411.6439 | 213 | 5.569 | 59.1% | 1 | R.QLFHPEQLITGK.E | 222 |
|  | Astrin\_STLCHLD\_tube2\_050114\_01.07937.07937.3 | 3.8211 | 0.4011 | 100.0% | 2416.9443 | 2416.6555 | 1 | 6.467 | 31.2% | 3 | R.QLFHPEQLITGKEDAANNYAR.G | 333 |
|  | Astrin\_STLCHLD\_tube2\_050114\_01.12647.12647.2 | 5.0104 | 0.5165 | 100.0% | 1843.7322 | 1843.1332 | 1 | 8.617 | 70.0% | 4 | R.GHYTIGKEIIDLVLDR.I | 22 |
|  | Astrin\_STLCHLD\_tube2\_050114\_01.12601.12601.3 | 4.4043 | 0.3166 | 100.0% | 1844.5443 | 1843.1332 | 4 | 6.558 | 43.3% | 1 | R.GHYTIGKEIIDLVLDR.I | 33 |
|  | Astrin\_STLCHLD\_tube2\_050114\_01.11209.11209.1 | 1.9634 | 0.3261 | 100.0% | 1085.65 | 1086.2737 | 2 | 5.985 | 68.8% | 2 | K.EIIDLVLDR.I | 11 |
|  | Astrin\_STLCHLD\_tube2\_050114\_01.11230.11230.2 | 2.9303 | 0.379 | 100.0% | 1086.6322 | 1086.2737 | 8 | 5.697 | 81.2% | 2 | K.EIIDLVLDR.I | 22 |
|  | Astrin\_STLCHLD\_tube2\_050114\_02.11004.11004.3 | 3.9515 | 0.2893 | 100.0% | 3392.8145 | 3392.7769 | 1 | 5.149 | 23.4% | 1 | K.LADQCTGLQGFLVFHSFGGGTGSGFTSLLMER.L | 33 |
|  | Astrin\_STLCHLD\_050114\_01.09799.09799.3 | 3.3823 | 0.333 | 100.0% | 1876.1943 | 1876.0824 | 2 | 5.44 | 41.1% | 2 | R.RNLDIERPTYTNLNR.L | 333 |
|  | Astrin\_STLCHLD\_050114\_01.11147.11147.2 | 3.4174 | 0.0864 | 98.0% | 1719.6322 | 1719.8949 | 1 | 5.134 | 61.5% | 2 | R.NLDIERPTYTNLNR.L | 222 |
|  | Astrin\_STLCHLD\_tube2\_050114\_01.06465.06465.3 | 2.9056 | 0.311 | 100.0% | 1721.3644 | 1719.8949 | 2 | 5.652 | 46.2% | 3 | R.NLDIERPTYTNLNR.L | 333 |
|  | Astrin\_STLCHLD\_050114\_01.13856.13856.1 | 3.7074 | 0.3194 | 100.0% | 1488.91 | 1488.7678 | 3 | 6.528 | 50.0% | 5 | R.LISQIVSSITASLR.F | 111 |
|  | Astrin\_STLCHLD\_tube2\_050114\_02.10976.10976.2 | 4.8703 | 0.4751 | 100.0% | 1490.2522 | 1488.7678 | 1 | 9.358 | 73.1% | 26 | R.LISQIVSSITASLR.F | 222 |
|  | Astrin\_STLCHLD\_tube2\_050114\_01.13991.13991.3 | 4.7196 | 0.3289 | 100.0% | 1490.3344 | 1488.7678 | 2 | 6.09 | 50.0% | 6 | R.LISQIVSSITASLR.F | 333 |
|  | Astrin\_STLCHLD\_tube2\_050114\_02.10115.10115.2 | 5.7616 | 0.571 | 100.0% | 2410.1921 | 2410.6885 | 1 | 10.015 | 52.5% | 20 | R.FDGALNVDLTEFQTNLVPYPR.I | 222 |
|  | Astrin\_STLCHLD\_tube2\_050114\_02.10073.10073.3 | 3.3301 | 0.2622 | 99.8% | 2413.4343 | 2410.6885 | 1 | 5.258 | 36.2% | 3 | R.FDGALNVDLTEFQTNLVPYPR.I | 333 |
|  | Astrin\_STLCHLD\_tube2\_050114\_01.10237.10237.2 | 4.4468 | 0.5586 | 100.0% | 1757.4722 | 1758.0703 | 1 | 9.549 | 76.7% | 8 | R.IHFPLATYAPVISAEK.A | 222 |
|  | Astrin\_STLCHLD\_050114\_01.12271.12271.3 | 4.3329 | 0.4459 | 100.0% | 1757.9644 | 1758.0703 | 1 | 7.63 | 51.7% | 9 | R.IHFPLATYAPVISAEK.A | 333 |
|  | Astrin\_STLCHLD\_050114\_02.08102.08102.3 | 4.2224 | 0.3505 | 100.0% | 2751.6543 | 2752.0369 | 5 | 6.013 | 27.2% | 1 | K.AYHEQLSVAEITNACFEPANQMVK.C | 33 |
|  | Astrin\_STLCHLD\_050114\_01.12021.12021.2 | 2.5571 | 0.4159 | 99.9% | 1250.3722 | 1250.4304 | 1 | 6.932 | 75.0% | 1 | K.YMACCLLYR.G | 222 |
|  | Astrin\_STLCHLD\_tube2\_050114\_01.08558.08558.2 | 2.9016 | 0.2756 | 99.2% | 1611.1322 | 1611.879 | 15 | 5.949 | 46.7% | 1 | R.GDVVPKDVNAAIATIK.T | 22 |
|  | Astrin\_STLCHLD\_050114\_01.11279.11279.2 | 3.242 | 0.4486 | 100.0% | 1016.15216 | 1016.1827 | 1 | 7.729 | 94.4% | 6 | K.DVNAAIATIK.T | 22 |
|  | Astrin\_STLCHLD\_tube2\_050114\_01.06721.06721.1 | 1.9783 | 0.1958 | 98.7% | 1017.55 | 1016.1827 | 47 | 4.032 | 50.0% | 5 | K.DVNAAIATIK.T | 11 |
|  | Astrin\_STLCHLD\_050114\_01.11948.11948.2 | 4.3001 | 0.3901 | 100.0% | 1825.6122 | 1826.1027 | 1 | 7.994 | 61.8% | 4 | K.VGINYQPPTVVPGGDLAK.V | 222 |
|  | Astrin\_STLCHLD\_050114\_02.09635.09635.2 | 4.2429 | 0.3899 | 100.0% | 1865.7122 | 1866.1084 | 1 | 8.618 | 56.2% | 1 | R.AVCMLSNTTAIAEAWAR.L | 22 |
|  | Astrin\_STLCHLD\_050114\_01.11149.11149.3 | 4.1633 | 0.3999 | 100.0% | 1381.6743 | 1381.6324 | 1 | 6.474 | 62.5% | 4 | R.LDHKFDLMYAK.R | 333 |
|  | Astrin\_STLCHLD\_050114\_01.09772.09772.3 | 2.6871 | 0.2432 | 98.1% | 1538.2444 | 1537.82 | 69 | 4.407 | 40.9% | 1 | R.LDHKFDLMYAKR.A | 333 |
|  | Astrin\_STLCHLD\_050114\_02.07700.07700.3 | 6.6327 | 0.4886 | 100.0% | 2487.6843 | 2487.7083 | 1 | 9.145 | 48.8% | 3 | K.RAFVHWYVGEGMEEGEFSEAR.E | 333 |
|  | Astrin\_STLCHLD\_050114\_02.08531.08531.2 | 5.3032 | 0.4677 | 100.0% | 2331.412 | 2331.5208 | 1 | 8.46 | 65.8% | 1 | R.AFVHWYVGEGMEEGEFSEAR.E | 222 |
|  | Astrin\_STLCHLD\_050114\_02.08576.08576.3 | 4.9127 | 0.279 | 100.0% | 2334.1743 | 2331.5208 | 1 | 4.983 | 43.4% | 6 | R.AFVHWYVGEGMEEGEFSEAR.E | 333 |
|  | Astrin\_STLCHLD\_tube2\_050114\_01.11503.11503.3 | 4.258 | 0.3976 | 100.0% | 3219.3843 | 3219.524 | 1 | 7.266 | 31.5% | 2 | R.AFVHWYVGEGMEEGEFSEAREDMAALEK.D | 333 |

Similarities:
gi|14389309|ref|NP\_11(33:2)  
gi|17921989|ref|NP\_00(22:13)  

---

|  |  |  |  |  |  |  |  |  |
| --- | --- | --- | --- | --- | --- | --- | --- | --- |
| U | *gi|40354195|ref|NP\_95* | 32 | 125 | 64.9% | 430 | 48058 | 5.5 | keratin 18 [Homo sapiens] |
| U | *gi|4557888|ref|NP\_000* | 32 | 125 | 64.9% | 430 | 48058 | 5.5 | keratin 18 [Homo sapiens] |

| Filename XCorr DeltCN Conf% ObsM+H+ CalcM+H+ SpR ZScore Ion% # Sequence  | | | | | | | | | | | | |
| --- | --- | --- | --- | --- | --- | --- | --- | --- | --- | --- | --- | --- |
|  | Astrin\_STLCHLD\_050114\_01.05293.05293.2 | 1.8227 | 0.3647 | 98.6% | 975.9922 | 976.0336 | 1 | 6.297 | 78.6% | 3 | R.STFSTNYR.S | 2 |
|  | Astrin\_STLCHLD\_tube2\_050114\_01.06946.06946.2 | 4.4746 | 0.419 | 100.0% | 2854.912 | 2856.0813 | 3 | 7.352 | 26.7% | 2 | R.SLGSVQAPSYGARPVSSAASVYAGAGGSGSR.I | 2 |
|  | Astrin\_STLCHLD\_tube2\_050114\_02.06020.06020.3 | 4.9956 | 0.5787 | 100.0% | 2856.2644 | 2856.0813 | 1 | 10.205 | 32.5% | 7 | R.SLGSVQAPSYGARPVSSAASVYAGAGGSGSR.I | 3 |
|  | Astrin\_STLCHLD\_tube2\_050114\_02.08289.08289.2 | 5.9754 | 0.5229 | 100.0% | 2262.7322 | 2262.561 | 1 | 8.534 | 52.0% | 1 | R.GGMGSGGLATGIAGGLAGMGGIQNEK.E | 2 |
|  | Astrin\_STLCHLD\_050114\_02.08696.08696.3 | 3.2764 | 0.2657 | 99.3% | 2263.6743 | 2262.561 | 1 | 4.571 | 32.0% | 1 | R.GGMGSGGLATGIAGGLAGMGGIQNEK.E | 3 |
|  | Astrin\_STLCHLD\_tube2\_050114\_02.08372.08372.3 | 6.0422 | 0.4766 | 100.0% | 3336.7144 | 3337.7224 | 1 | 8.172 | 27.2% | 4 | R.GGMGSGGLATGIAGGLAGMGGIQNEKETMQSLNDR.L | 3 |
|  | Astrin\_STLCHLD\_050114\_01.06770.06770.1 | 1.7938 | 0.1806 | 96.2% | 837.48 | 837.9511 | 52 | 5.093 | 58.3% | 1 | R.LASYLDR.V | 1 |
|  | Astrin\_STLCHLD\_050114\_01.06577.06577.2 | 1.5228 | 0.3968 | 96.7% | 837.6322 | 837.9511 | 1 | 5.793 | 91.7% | 2 | R.LASYLDR.V | 2 |
|  | Astrin\_STLCHLD\_050114\_01.10477.10477.2 | 3.9092 | 0.4554 | 100.0% | 1320.3121 | 1320.4478 | 1 | 9.006 | 72.7% | 12 | R.AQIFANTVDNAR.I | 22 |
|  | Astrin\_STLCHLD\_050114\_01.11190.11190.1 | 2.5285 | 0.3185 | 100.0% | 1041.64 | 1042.2235 | 1 | 6.721 | 75.0% | 3 | R.IVLQIDNAR.L | 11 |
|  | Astrin\_STLCHLD\_tube2\_050114\_01.06537.06537.2 | 3.0568 | 0.1895 | 99.6% | 1042.2922 | 1042.2235 | 2 | 6.339 | 87.5% | 4 | R.IVLQIDNAR.L | 22 |
|  | Astrin\_STLCHLD\_050114\_01.06505.06505.2 | 2.4865 | 0.2457 | 99.3% | 808.09216 | 807.8815 | 46 | 6.454 | 66.7% | 10 | R.LAADDFR.V | 22222 |
|  | Astrin\_STLCHLD\_050114\_01.08846.08846.2 | 3.0644 | 0.4762 | 100.0% | 1240.2922 | 1240.4601 | 5 | 8.122 | 72.2% | 10 | R.VKYETELAMR.Q | 2 |
|  | Astrin\_STLCHLD\_tube2\_050114\_01.05102.05102.3 | 2.9924 | 0.2433 | 99.9% | 1240.6144 | 1240.4601 | 13 | 4.612 | 52.8% | 1 | R.VKYETELAMR.Q | 3 |
|  | Astrin\_STLCHLD\_050114\_01.08619.08619.2 | 1.8966 | 0.5107 | 99.9% | 1013.6722 | 1013.1535 | 40 | 6.936 | 71.4% | 1 | K.YETELAMR.Q | 2 |
|  | Astrin\_STLCHLD\_050114\_01.04682.04682.2 | 2.795 | 0.315 | 99.9% | 1175.1122 | 1175.3274 | 2 | 6.138 | 77.8% | 1 | R.KVIDDTNITR.L | 2 |
|  | Astrin\_STLCHLD\_tube2\_050114\_01.14893.14893.2 | 5.8637 | 0.5097 | 100.0% | 2178.0522 | 2178.589 | 1 | 8.999 | 64.7% | 6 | R.LQLETEIEALKEELLFMK.K | 22 |
|  | Astrin\_STLCHLD\_050114\_01.14664.14664.3 | 2.9045 | 0.2699 | 99.2% | 2179.4944 | 2178.589 | 8 | 4.647 | 41.2% | 2 | R.LQLETEIEALKEELLFMK.K | 33 |
|  | Astrin\_STLCHLD\_tube2\_050114\_01.08716.08716.3 | 5.8449 | 0.5194 | 100.0% | 2750.7544 | 2751.0227 | 1 | 9.699 | 35.0% | 4 | K.NHEEEVKGLQAQIASSGLTVEVDAPK.S | 3 |
|  | Astrin\_STLCHLD\_tube2\_050114\_02.07353.07353.2 | 3.6305 | 0.4433 | 100.0% | 1884.4521 | 1885.1246 | 1 | 8.705 | 58.3% | 1 | K.GLQAQIASSGLTVEVDAPK.S | 2 |
|  | Astrin\_STLCHLD\_050114\_01.05548.05548.1 | 1.7698 | 0.2711 | 100.0% | 965.44 | 966.0385 | 87 | 5.442 | 50.0% | 1 | R.AQYDELAR.K | 1 |
|  | Astrin\_STLCHLD\_tube2\_050114\_01.10480.10480.1 | 2.3465 | 0.4692 | 100.0% | 1506.68 | 1507.699 | 1 | 7.556 | 62.5% | 1 | R.TVQSLEIDLDSMR.N | 1 |
|  | Astrin\_STLCHLD\_tube2\_050114\_01.10509.10509.2 | 4.0774 | 0.4549 | 100.0% | 1507.4321 | 1507.699 | 1 | 8.306 | 75.0% | 8 | R.TVQSLEIDLDSMR.N | 2 |
|  | Astrin\_STLCHLD\_050114\_01.05607.05607.2 | 2.6608 | 0.2448 | 99.5% | 891.1122 | 889.9841 | 107 | 5.434 | 71.4% | 4 | K.ASLENSLR.E | 2 |
|  | Astrin\_STLCHLD\_tube2\_050114\_01.07107.07107.3 | 2.6518 | 0.2924 | 99.7% | 1475.1244 | 1474.6139 | 1 | 5.991 | 41.7% | 1 | K.ASLENSLREVEAR.Y | 3 |
|  | Astrin\_STLCHLD\_050114\_01.15424.15424.2 | 5.834 | 0.5031 | 100.0% | 2670.9321 | 2672.0715 | 1 | 10.072 | 54.5% | 6 | R.YALQMEQLNGILLHLESELAQTR.A | 2 |
|  | Astrin\_STLCHLD\_050114\_01.15459.15459.3 | 6.447 | 0.5109 | 100.0% | 2671.5544 | 2672.0715 | 1 | 8.789 | 48.9% | 14 | R.YALQMEQLNGILLHLESELAQTR.A | 3 |
|  | Astrin\_STLCHLD\_tube2\_050114\_01.09622.09622.2 | 3.3533 | 0.3373 | 99.9% | 1420.4521 | 1420.6055 | 3 | 6.143 | 68.2% | 3 | R.QAQEYEALLNIK.V | 2 |
|  | Astrin\_STLCHLD\_050114\_02.06194.06194.2 | 3.7113 | 0.3085 | 100.0% | 1293.7522 | 1293.5059 | 1 | 6.541 | 75.0% | 4 | K.VKLEAEIATYR.R | 2 |
|  | Astrin\_STLCHLD\_tube2\_050114\_01.05672.05672.2 | 2.7687 | 0.3844 | 99.9% | 1065.4722 | 1066.1992 | 1 | 6.474 | 81.2% | 5 | K.LEAEIATYR.R | 2 |
|  | Astrin\_STLCHLD\_tube2\_050114\_02.08131.08131.3 | 3.6626 | 0.2937 | 100.0% | 2897.4543 | 2898.128 | 32 | 5.401 | 23.0% | 1 | R.RLLEDGEDFNLGDALDSSNSMQTIQK.T | 3 |
|  | Astrin\_STLCHLD\_tube2\_050114\_02.08597.08597.2 | 6.0254 | 0.5824 | 100.0% | 2741.5522 | 2741.9404 | 1 | 11.731 | 54.2% | 1 | R.LLEDGEDFNLGDALDSSNSMQTIQK.T | 2 |

Similarities:
gi|4557701|ref|NP\_000(1:31)  
gi|15431310|ref|NP\_00(1:31)  
gi|24234699|ref|NP\_00(3:29)  
contaminant\_KERATIN03(1:31)  
gi|169164037|ref|XP\_0(3:29)  

---

|  |  |  |  |  |  |  |  |  |
| --- | --- | --- | --- | --- | --- | --- | --- | --- |
| U | *gi|29788785|ref|NP\_82* | 43 | 191 | 64.6% | 444 | 49671 | 4.9 | tubulin, beta [Homo sapiens] |

| Filename XCorr DeltCN Conf% ObsM+H+ CalcM+H+ SpR ZScore Ion% # Sequence  | | | | | | | | | | | | |
| --- | --- | --- | --- | --- | --- | --- | --- | --- | --- | --- | --- | --- |
| \* | Astrin\_STLCHLD\_050114\_02.08224.08224.3 | 6.5782 | 0.409 | 100.0% | 3103.5842 | 3104.2725 | 1 | 9.131 | 36.5% | 7 | K.FWEVISDEHGIDPTGTYHGDSDLQLDR.I | 3 |
| \* | Astrin\_STLCHLD\_050114\_01.09485.09485.1 | 2.2901 | 0.4397 | 100.0% | 1301.61 | 1302.4265 | 3 | 6.483 | 54.5% | 3 | R.ISVYYNEATGGK.Y | 1 |
| \* | Astrin\_STLCHLD\_tube2\_050114\_01.05498.05498.2 | 3.9324 | 0.5962 | 100.0% | 1302.2122 | 1302.4265 | 1 | 9.872 | 86.4% | 17 | R.ISVYYNEATGGK.Y | 2 |
| \* | Astrin\_STLCHLD\_tube2\_050114\_02.05798.05798.3 | 2.3338 | 0.2962 | 96.3% | 1817.1543 | 1818.0392 | 5 | 5.224 | 38.3% | 1 | R.ISVYYNEATGGKYVPR.A | 3 |
| \* | Astrin\_STLCHLD\_tube2\_050114\_02.05813.05813.2 | 3.94 | 0.4671 | 100.0% | 1817.7522 | 1818.0392 | 1 | 7.821 | 56.7% | 1 | R.ISVYYNEATGGKYVPR.A | 2 |
|  | Astrin\_STLCHLD\_tube2\_050114\_01.10300.10300.1 | 1.9796 | 0.4452 | 100.0% | 1615.8 | 1616.8701 | 25 | 6.88 | 42.9% | 1 | R.AILVDLEPGTMDSVR.S | 11 |
|  | Astrin\_STLCHLD\_tube2\_050114\_01.10255.10255.2 | 4.3551 | 0.5096 | 100.0% | 1616.3322 | 1616.8701 | 1 | 8.913 | 75.0% | 6 | R.AILVDLEPGTMDSVR.S | 22 |
|  | Astrin\_STLCHLD\_tube2\_050114\_01.12338.12338.2 | 5.6846 | 0.5176 | 100.0% | 2798.8523 | 2800.0647 | 1 | 8.445 | 40.0% | 6 | R.SGPFGQIFRPDNFVFGQSGAGNNWAK.G | 222 |
|  | Astrin\_STLCHLD\_tube2\_050114\_01.12242.12242.3 | 6.768 | 0.5498 | 100.0% | 2799.6543 | 2800.0647 | 1 | 8.573 | 38.0% | 7 | R.SGPFGQIFRPDNFVFGQSGAGNNWAK.G | 333 |
|  | Astrin\_STLCHLD\_tube2\_050114\_01.12857.12857.2 | 7.2534 | 0.4819 | 100.0% | 1960.4722 | 1960.151 | 1 | 9.656 | 79.4% | 5 | K.GHYTEGAELVDSVLDVVR.K | 222 |
|  | Astrin\_STLCHLD\_tube2\_050114\_01.12839.12839.3 | 4.3447 | 0.3812 | 100.0% | 1960.7644 | 1960.151 | 1 | 7.142 | 45.6% | 3 | K.GHYTEGAELVDSVLDVVR.K | 333 |
|  | Astrin\_STLCHLD\_tube2\_050114\_01.11581.11581.3 | 4.7423 | 0.5057 | 100.0% | 2088.3245 | 2088.325 | 1 | 7.853 | 40.3% | 2 | K.GHYTEGAELVDSVLDVVRK.E | 333 |
|  | Astrin\_STLCHLD\_050114\_01.12405.12405.2 | 6.3217 | 0.4514 | 100.0% | 2088.9321 | 2088.325 | 1 | 8.891 | 66.7% | 2 | K.GHYTEGAELVDSVLDVVRK.E | 222 |
|  | Astrin\_STLCHLD\_050114\_01.04328.04328.2 | 2.6855 | 0.1744 | 99.0% | 1078.0122 | 1078.1698 | 9 | 4.186 | 78.6% | 1 | K.IREEYPDR.I | 22 |
|  | Astrin\_STLCHLD\_050114\_01.11995.11995.1 | 2.1993 | 0.2639 | 100.0% | 1319.69 | 1320.5896 | 245 | 5.053 | 40.9% | 3 | R.IMNTFSVVPSPK.V | 1111 |
|  | Astrin\_STLCHLD\_tube2\_050114\_01.08305.08305.2 | 4.34 | 0.4185 | 100.0% | 1320.4321 | 1320.5896 | 1 | 7.953 | 72.7% | 8 | R.IMNTFSVVPSPK.V | 2222 |
|  | Astrin\_STLCHLD\_050114\_01.12590.12590.3 | 4.1941 | 0.3268 | 100.0% | 2710.8843 | 2710.0405 | 1 | 6.714 | 30.2% | 1 | K.LTTPTYGDLNHLVSATMSGVTTCLR.F | 333 |
|  | Astrin\_STLCHLD\_tube2\_050114\_01.07179.07179.2 | 3.0568 | 0.2949 | 99.9% | 1131.2522 | 1131.2767 | 7 | 5.17 | 77.8% | 13 | R.FPGQLNADLR.K | 22222 |
|  | Astrin\_STLCHLD\_tube2\_050114\_01.05601.05601.2 | 2.7143 | 0.2756 | 99.3% | 1258.5521 | 1259.4508 | 11 | 5.358 | 65.0% | 13 | R.FPGQLNADLRK.L | 22222 |
|  | Astrin\_STLCHLD\_tube2\_050114\_01.05583.05583.3 | 3.152 | 0.3056 | 100.0% | 1259.8744 | 1259.4508 | 8 | 5.284 | 45.0% | 12 | R.FPGQLNADLRK.L | 33333 |
|  | Astrin\_STLCHLD\_tube2\_050114\_01.08365.08365.2 | 3.793 | 0.3681 | 100.0% | 1271.8722 | 1272.5945 | 1 | 7.682 | 80.0% | 5 | R.KLAVNMVPFPR.L | 22222 |
|  | Astrin\_STLCHLD\_tube2\_050114\_01.09803.09803.1 | 2.0853 | 0.2613 | 100.0% | 1143.58 | 1144.4204 | 13 | 6.348 | 61.1% | 1 | K.LAVNMVPFPR.L | 11111 |
|  | Astrin\_STLCHLD\_tube2\_050114\_01.09839.09839.2 | 3.9889 | 0.4725 | 100.0% | 1144.4122 | 1144.4204 | 1 | 8.55 | 94.4% | 3 | K.LAVNMVPFPR.L | 22222 |
|  | Astrin\_STLCHLD\_050114\_01.12456.12456.2 | 3.7154 | 0.3929 | 100.0% | 1621.7122 | 1621.9403 | 1 | 8.432 | 76.9% | 3 | R.LHFFMPGFAPLTSR.G | 2222 |
|  | Astrin\_STLCHLD\_050114\_01.12457.12457.3 | 4.2506 | 0.3686 | 100.0% | 1622.0643 | 1621.9403 | 1 | 6.108 | 53.8% | 4 | R.LHFFMPGFAPLTSR.G | 3333 |
| \* | Astrin\_STLCHLD\_tube2\_050114\_01.11701.11701.2 | 4.2031 | 0.5417 | 100.0% | 1660.4321 | 1660.9078 | 1 | 9.309 | 67.9% | 4 | R.ALTVPELTQQVFDAK.N | 2 |
| \* | Astrin\_STLCHLD\_tube2\_050114\_01.11774.11774.3 | 3.158 | 0.3724 | 100.0% | 1660.8243 | 1660.9078 | 1 | 6.247 | 39.3% | 1 | R.ALTVPELTQQVFDAK.N | 3 |
|  | Astrin\_STLCHLD\_050114\_01.04952.04952.2 | 2.8672 | 0.3941 | 100.0% | 1066.2322 | 1066.2013 | 2 | 7.174 | 68.8% | 2 | K.NMMAACDPR.H | 22222 |
|  | Astrin\_STLCHLD\_050114\_02.06747.06747.3 | 4.3076 | 0.2581 | 100.0% | 1391.1244 | 1390.631 | 1 | 7.084 | 47.7% | 2 | R.HGRYLTVAAVFR.G | 333 |
|  | Astrin\_STLCHLD\_tube2\_050114\_01.10402.10402.1 | 1.7815 | 0.4511 | 100.0% | 1039.71 | 1040.2505 | 1 | 6.99 | 81.2% | 4 | R.YLTVAAVFR.G | 111 |
|  | Astrin\_STLCHLD\_tube2\_050114\_01.10269.10269.2 | 3.417 | 0.3601 | 100.0% | 1041.3322 | 1040.2505 | 1 | 6.884 | 87.5% | 9 | R.YLTVAAVFR.G | 222 |
|  | Astrin\_STLCHLD\_tube2\_050114\_02.06679.06679.2 | 5.6297 | 0.5004 | 100.0% | 1924.5322 | 1925.2405 | 1 | 8.712 | 56.7% | 2 | R.MSMKEVDEQMLNVQNK.N | 22 |
|  | Astrin\_STLCHLD\_050114\_02.06995.06995.3 | 4.822 | 0.2537 | 100.0% | 1926.3844 | 1925.2405 | 1 | 5.685 | 46.7% | 3 | R.MSMKEVDEQMLNVQNK.N | 33 |
|  | Astrin\_STLCHLD\_tube2\_050114\_01.05776.05776.1 | 2.9126 | 0.2315 | 100.0% | 1446.63 | 1447.6031 | 1 | 5.343 | 68.2% | 1 | K.EVDEQMLNVQNK.N | 11 |
|  | Astrin\_STLCHLD\_050114\_01.10279.10279.2 | 4.2441 | 0.2595 | 100.0% | 1447.4122 | 1447.6031 | 4 | 6.023 | 68.2% | 8 | K.EVDEQMLNVQNK.N | 22 |
|  | Astrin\_STLCHLD\_tube2\_050114\_01.11339.11339.2 | 3.5338 | 0.3838 | 100.0% | 1696.7722 | 1697.8877 | 1 | 7.601 | 57.7% | 2 | K.NSSYFVEWIPNNVK.T | 22222 |
|  | Astrin\_STLCHLD\_050114\_01.05029.05029.2 | 2.2588 | 0.224 | 97.9% | 1029.1921 | 1029.1473 | 9 | 4.44 | 62.5% | 1 | K.TAVCDIPPR.G | 222 |
| \* | Astrin\_STLCHLD\_050114\_01.13027.13027.2 | 5.1805 | 0.4144 | 100.0% | 1871.3121 | 1871.2018 | 1 | 7.839 | 78.1% | 4 | K.MAVTFIGNSTAIQELFK.R | 2 |
| \* | Astrin\_STLCHLD\_tube2\_050114\_01.12602.12602.2 | 4.8788 | 0.5206 | 100.0% | 2027.5721 | 2027.3893 | 1 | 8.908 | 50.0% | 1 | K.MAVTFIGNSTAIQELFKR.I | 2 |
| \* | Astrin\_STLCHLD\_tube2\_050114\_01.12570.12570.3 | 3.3429 | 0.289 | 100.0% | 2027.8444 | 2027.3893 | 1 | 7.125 | 38.2% | 4 | K.MAVTFIGNSTAIQELFKR.I | 3 |
|  | Astrin\_STLCHLD\_050114\_02.07650.07650.2 | 3.8098 | 0.3921 | 100.0% | 1386.7522 | 1386.6116 | 1 | 7.005 | 75.0% | 4 | K.RISEQFTAMFR.R | 2222 |
|  | Astrin\_STLCHLD\_tube2\_050114\_02.06787.06787.3 | 3.1948 | 0.3082 | 100.0% | 1543.3143 | 1542.7991 | 1 | 5.726 | 52.3% | 1 | K.RISEQFTAMFRR.K | 3333 |
|  | Astrin\_STLCHLD\_tube2\_050114\_02.08042.08042.2 | 4.0657 | 0.4262 | 100.0% | 1231.3121 | 1230.4241 | 1 | 6.725 | 94.4% | 10 | R.ISEQFTAMFR.R | 2222 |

Similarities:
gi|5174735|ref|NP\_006(31:12)  
gi|21361322|ref|NP\_00(22:21)  
gi|50592996|ref|NP\_00(19:24)  
gi|14210536|ref|NP\_11(10:33)  

---

|  |  |  |  |  |  |  |  |  |
| --- | --- | --- | --- | --- | --- | --- | --- | --- |
| U | *gi|14389309|ref|NP\_11* | 34 | 154 | 61.7% | 449 | 49895 | 5.1 | tubulin alpha 6 [Homo sapiens] |

| Filename XCorr DeltCN Conf% ObsM+H+ CalcM+H+ SpR ZScore Ion% # Sequence  | | | | | | | | | | | | |
| --- | --- | --- | --- | --- | --- | --- | --- | --- | --- | --- | --- | --- |
|  | Astrin\_STLCHLD\_tube2\_050114\_01.10939.10939.2 | 5.614 | 0.6543 | 100.0% | 2008.4321 | 2009.093 | 1 | 11.783 | 57.9% | 8 | K.TIGGGDDSFNTFFSETGAGK.H | 22 |
|  | Astrin\_STLCHLD\_050114\_02.08865.08865.3 | 3.8391 | 0.2974 | 100.0% | 2009.6044 | 2009.093 | 12 | 5.573 | 31.6% | 1 | K.TIGGGDDSFNTFFSETGAGK.H | 33 |
|  | Astrin\_STLCHLD\_050114\_01.12436.12436.1 | 2.6238 | 0.525 | 100.0% | 1701.85 | 1702.9451 | 1 | 7.571 | 50.0% | 2 | R.AVFVDLEPTVIDEVR.T | 11 |
|  | Astrin\_STLCHLD\_tube2\_050114\_01.11695.11695.2 | 4.7671 | 0.4744 | 100.0% | 1702.5521 | 1702.9451 | 1 | 7.991 | 78.6% | 7 | R.AVFVDLEPTVIDEVR.T | 22 |
|  | Astrin\_STLCHLD\_tube2\_050114\_01.11676.11676.3 | 4.6957 | 0.4534 | 100.0% | 1704.2943 | 1702.9451 | 1 | 7.889 | 51.8% | 3 | R.AVFVDLEPTVIDEVR.T | 33 |
|  | Astrin\_STLCHLD\_tube2\_050114\_01.08551.08551.2 | 1.7735 | 0.3588 | 96.6% | 1411.4922 | 1411.6439 | 213 | 5.569 | 59.1% | 1 | R.QLFHPEQLITGK.E | 222 |
|  | Astrin\_STLCHLD\_tube2\_050114\_01.07937.07937.3 | 3.8211 | 0.4011 | 100.0% | 2416.9443 | 2416.6555 | 1 | 6.467 | 31.2% | 3 | R.QLFHPEQLITGKEDAANNYAR.G | 333 |
|  | Astrin\_STLCHLD\_tube2\_050114\_01.12647.12647.2 | 5.0104 | 0.5165 | 100.0% | 1843.7322 | 1843.1332 | 1 | 8.617 | 70.0% | 4 | R.GHYTIGKEIIDLVLDR.I | 22 |
|  | Astrin\_STLCHLD\_tube2\_050114\_01.12601.12601.3 | 4.4043 | 0.3166 | 100.0% | 1844.5443 | 1843.1332 | 4 | 6.558 | 43.3% | 1 | R.GHYTIGKEIIDLVLDR.I | 33 |
|  | Astrin\_STLCHLD\_tube2\_050114\_01.11209.11209.1 | 1.9634 | 0.3261 | 100.0% | 1085.65 | 1086.2737 | 2 | 5.985 | 68.8% | 2 | K.EIIDLVLDR.I | 11 |
|  | Astrin\_STLCHLD\_tube2\_050114\_01.11230.11230.2 | 2.9303 | 0.379 | 100.0% | 1086.6322 | 1086.2737 | 8 | 5.697 | 81.2% | 2 | K.EIIDLVLDR.I | 22 |
|  | Astrin\_STLCHLD\_tube2\_050114\_02.11004.11004.3 | 3.9515 | 0.2893 | 100.0% | 3392.8145 | 3392.7769 | 1 | 5.149 | 23.4% | 1 | K.LADQCTGLQGFLVFHSFGGGTGSGFTSLLMER.L | 33 |
|  | Astrin\_STLCHLD\_050114\_01.09799.09799.3 | 3.3823 | 0.333 | 100.0% | 1876.1943 | 1876.0824 | 2 | 5.44 | 41.1% | 2 | R.RNLDIERPTYTNLNR.L | 333 |
|  | Astrin\_STLCHLD\_050114\_01.11147.11147.2 | 3.4174 | 0.0864 | 98.0% | 1719.6322 | 1719.8949 | 1 | 5.134 | 61.5% | 2 | R.NLDIERPTYTNLNR.L | 222 |
|  | Astrin\_STLCHLD\_tube2\_050114\_01.06465.06465.3 | 2.9056 | 0.311 | 100.0% | 1721.3644 | 1719.8949 | 2 | 5.652 | 46.2% | 3 | R.NLDIERPTYTNLNR.L | 333 |
|  | Astrin\_STLCHLD\_050114\_01.13856.13856.1 | 3.7074 | 0.3194 | 100.0% | 1488.91 | 1488.7678 | 3 | 6.528 | 50.0% | 5 | R.LISQIVSSITASLR.F | 111 |
|  | Astrin\_STLCHLD\_tube2\_050114\_02.10976.10976.2 | 4.8703 | 0.4751 | 100.0% | 1490.2522 | 1488.7678 | 1 | 9.358 | 73.1% | 26 | R.LISQIVSSITASLR.F | 222 |
|  | Astrin\_STLCHLD\_tube2\_050114\_01.13991.13991.3 | 4.7196 | 0.3289 | 100.0% | 1490.3344 | 1488.7678 | 2 | 6.09 | 50.0% | 6 | R.LISQIVSSITASLR.F | 333 |
|  | Astrin\_STLCHLD\_tube2\_050114\_02.10115.10115.2 | 5.7616 | 0.571 | 100.0% | 2410.1921 | 2410.6885 | 1 | 10.015 | 52.5% | 20 | R.FDGALNVDLTEFQTNLVPYPR.I | 222 |
|  | Astrin\_STLCHLD\_tube2\_050114\_02.10073.10073.3 | 3.3301 | 0.2622 | 99.8% | 2413.4343 | 2410.6885 | 1 | 5.258 | 36.2% | 3 | R.FDGALNVDLTEFQTNLVPYPR.I | 333 |
|  | Astrin\_STLCHLD\_tube2\_050114\_01.10237.10237.2 | 4.4468 | 0.5586 | 100.0% | 1757.4722 | 1758.0703 | 1 | 9.549 | 76.7% | 8 | R.IHFPLATYAPVISAEK.A | 222 |
|  | Astrin\_STLCHLD\_050114\_01.12271.12271.3 | 4.3329 | 0.4459 | 100.0% | 1757.9644 | 1758.0703 | 1 | 7.63 | 51.7% | 9 | R.IHFPLATYAPVISAEK.A | 333 |
| \* | Astrin\_STLCHLD\_050114\_02.08192.08192.3 | 3.8453 | 0.2905 | 100.0% | 2765.9944 | 2766.064 | 343 | 5.097 | 23.9% | 1 | K.AYHEQLTVAEITNACFEPANQMVK.C | 3 |
|  | Astrin\_STLCHLD\_050114\_01.12021.12021.2 | 2.5571 | 0.4159 | 99.9% | 1250.3722 | 1250.4304 | 1 | 6.932 | 75.0% | 1 | K.YMACCLLYR.G | 222 |
|  | Astrin\_STLCHLD\_tube2\_050114\_01.08558.08558.2 | 2.9016 | 0.2756 | 99.2% | 1611.1322 | 1611.879 | 15 | 5.949 | 46.7% | 1 | R.GDVVPKDVNAAIATIK.T | 22 |
|  | Astrin\_STLCHLD\_050114\_01.11279.11279.2 | 3.242 | 0.4486 | 100.0% | 1016.15216 | 1016.1827 | 1 | 7.729 | 94.4% | 6 | K.DVNAAIATIK.T | 22 |
|  | Astrin\_STLCHLD\_tube2\_050114\_01.06721.06721.1 | 1.9783 | 0.1958 | 98.7% | 1017.55 | 1016.1827 | 47 | 4.032 | 50.0% | 5 | K.DVNAAIATIK.T | 11 |
|  | Astrin\_STLCHLD\_050114\_01.11948.11948.2 | 4.3001 | 0.3901 | 100.0% | 1825.6122 | 1826.1027 | 1 | 7.994 | 61.8% | 4 | K.VGINYQPPTVVPGGDLAK.V | 222 |
|  | Astrin\_STLCHLD\_050114\_01.11149.11149.3 | 4.1633 | 0.3999 | 100.0% | 1381.6743 | 1381.6324 | 1 | 6.474 | 62.5% | 4 | R.LDHKFDLMYAK.R | 333 |
|  | Astrin\_STLCHLD\_050114\_01.09772.09772.3 | 2.6871 | 0.2432 | 98.1% | 1538.2444 | 1537.82 | 69 | 4.407 | 40.9% | 1 | R.LDHKFDLMYAKR.A | 333 |
|  | Astrin\_STLCHLD\_050114\_02.07700.07700.3 | 6.6327 | 0.4886 | 100.0% | 2487.6843 | 2487.7083 | 1 | 9.145 | 48.8% | 3 | K.RAFVHWYVGEGMEEGEFSEAR.E | 333 |
|  | Astrin\_STLCHLD\_050114\_02.08531.08531.2 | 5.3032 | 0.4677 | 100.0% | 2331.412 | 2331.5208 | 1 | 8.46 | 65.8% | 1 | R.AFVHWYVGEGMEEGEFSEAR.E | 222 |
|  | Astrin\_STLCHLD\_050114\_02.08576.08576.3 | 4.9127 | 0.279 | 100.0% | 2334.1743 | 2331.5208 | 1 | 4.983 | 43.4% | 6 | R.AFVHWYVGEGMEEGEFSEAR.E | 333 |
|  | Astrin\_STLCHLD\_tube2\_050114\_01.11503.11503.3 | 4.258 | 0.3976 | 100.0% | 3219.3843 | 3219.524 | 1 | 7.266 | 31.5% | 2 | R.AFVHWYVGEGMEEGEFSEAREDMAALEK.D | 333 |

Similarities:
gi|57013276|ref|NP\_00(33:1)  
gi|17921989|ref|NP\_00(20:14)  

---

|  |  |  |  |  |  |  |  |  |
| --- | --- | --- | --- | --- | --- | --- | --- | --- |
| U | *gi|11415030|ref|NP\_06* | 10 | 31 | 60.2% | 103 | 11367 | 11.4 | histone cluster 1, H4j [Homo sapiens] |
| U | *gi|77539758|ref|NP\_00* | 10 | 31 | 60.2% | 103 | 11367 | 11.4 | histone cluster 2, H4b [Homo sapiens] |
| U | *gi|4504323|ref|NP\_003* | 9 | 30 | 60.2% | 103 | 11367 | 11.4 | histone cluster 2, H4a [Homo sapiens] |
| U | *gi|4504321|ref|NP\_003* | 10 | 31 | 60.2% | 103 | 11367 | 11.4 | histone cluster 1, H4i [Homo sapiens] |
| U | *gi|4504317|ref|NP\_003* | 10 | 31 | 60.2% | 103 | 11367 | 11.4 | histone cluster 1, H4l [Homo sapiens] |
| U | *gi|4504315|ref|NP\_003* | 10 | 31 | 60.2% | 103 | 11367 | 11.4 | histone cluster 1, H4e [Homo sapiens] |
| U | *gi|4504313|ref|NP\_003* | 10 | 31 | 60.2% | 103 | 11367 | 11.4 | histone cluster 1, H4b [Homo sapiens] |
| U | *gi|4504311|ref|NP\_003* | 10 | 31 | 60.2% | 103 | 11367 | 11.4 | histone cluster 1, H4h [Homo sapiens] |
| U | *gi|4504309|ref|NP\_003* | 10 | 31 | 60.2% | 103 | 11367 | 11.4 | histone cluster 1, H4c [Homo sapiens] |
| U | *gi|4504307|ref|NP\_003* | 10 | 31 | 60.2% | 103 | 11367 | 11.4 | histone cluster 1, H4k [Homo sapiens] |
| U | *gi|4504305|ref|NP\_003* | 10 | 31 | 60.2% | 103 | 11367 | 11.4 | histone cluster 1, H4f [Homo sapiens] |
| U | *gi|4504303|ref|NP\_003* | 10 | 31 | 60.2% | 103 | 11367 | 11.4 | histone cluster 1, H4d [Homo sapiens] |
| U | *gi|4504301|ref|NP\_003* | 10 | 31 | 60.2% | 103 | 11367 | 11.4 | histone cluster 1, H4a [Homo sapiens] |
| U | *gi|28173560|ref|NP\_77* | 10 | 31 | 60.2% | 103 | 11367 | 11.4 | histone cluster 4, H4 [Homo sapiens] |

| Filename XCorr DeltCN Conf% ObsM+H+ CalcM+H+ SpR ZScore Ion% # Sequence  | | | | | | | | | | | | |
| --- | --- | --- | --- | --- | --- | --- | --- | --- | --- | --- | --- | --- |
|  | Astrin\_STLCHLD\_050114\_01.08137.08137.2 | 3.6336 | 0.2083 | 99.9% | 1326.2122 | 1326.5387 | 1 | 6.833 | 81.8% | 10 | R.DNIQGITKPAIR.R | 2 |
|  | Astrin\_STLCHLD\_tube2\_050114\_01.05855.05855.2 | 2.9559 | 0.2856 | 99.8% | 1337.2922 | 1337.5187 | 13 | 5.64 | 60.0% | 1 | K.RISGLIYEETR.G | 2 |
|  | Astrin\_STLCHLD\_tube2\_050114\_01.07045.07045.1 | 2.4606 | 0.2204 | 100.0% | 1180.71 | 1181.3312 | 2 | 5.176 | 66.7% | 1 | R.ISGLIYEETR.G | 1 |
|  | Astrin\_STLCHLD\_tube2\_050114\_01.07065.07065.2 | 3.7543 | 0.3747 | 100.0% | 1181.2322 | 1181.3312 | 1 | 7.131 | 88.9% | 7 | R.ISGLIYEETR.G | 2 |
|  | Astrin\_STLCHLD\_tube2\_050114\_01.09231.09231.1 | 2.0088 | 0.2421 | 100.0% | 989.67 | 990.19055 | 130 | 5.468 | 57.1% | 1 | K.VFLENVIR.D | 1 |
|  | Astrin\_STLCHLD\_050114\_01.12198.12198.2 | 2.9098 | 0.2438 | 99.9% | 990.3122 | 990.19055 | 1 | 5.159 | 85.7% | 4 | K.VFLENVIR.D | 2 |
|  | Astrin\_STLCHLD\_tube2\_050114\_01.13015.13015.3 | 4.7061 | 0.4251 | 100.0% | 2106.6543 | 2106.386 | 1 | 7.019 | 44.1% | 1 | K.VFLENVIRDAVTYTEHAK.R | 3 |
|  | Astrin\_STLCHLD\_tube2\_050114\_01.08799.08799.3 | 3.8216 | 0.4229 | 100.0% | 1595.3944 | 1595.9409 | 5 | 5.954 | 38.5% | 1 | R.KTVTAMDVVYALKR.Q | 3 |
|  | Astrin\_STLCHLD\_050114\_01.12252.12252.2 | 3.6021 | 0.4683 | 100.0% | 1467.4922 | 1467.7667 | 1 | 7.764 | 62.5% | 4 | K.TVTAMDVVYALKR.Q | 2 |
|  | Astrin\_STLCHLD\_tube2\_050114\_01.09111.09111.1 | 1.7631 | 0.3841 | 100.0% | 714.4 | 714.796 | 1 | 6.414 | 75.0% | 1 | R.TLYGFGG.- | 1 |

---

|  |  |  |  |  |  |  |  |  |
| --- | --- | --- | --- | --- | --- | --- | --- | --- |
| U | *gi|10645195|ref|NP\_06* | 9 | 18 | 60.0% | 130 | 14135 | 11.1 | histone cluster 1, H2ae [Homo sapiens] |
| U | *gi|4504245|ref|NP\_003* | 7 | 11 | 60.0% | 130 | 14105 | 11.1 | histone cluster 1, H2ac [Homo sapiens] |
| U | *gi|19557656|ref|NP\_00* | 9 | 18 | 60.0% | 130 | 14135 | 11.1 | histone cluster 1, H2ab [Homo sapiens] |
| U | *gi|15617199|ref|NP\_25* | 9 | 18 | 60.0% | 130 | 14121 | 11.1 | histone cluster 3, H2a [Homo sapiens] |

| Filename XCorr DeltCN Conf% ObsM+H+ CalcM+H+ SpR ZScore Ion% # Sequence  | | | | | | | | | | | | |
| --- | --- | --- | --- | --- | --- | --- | --- | --- | --- | --- | --- | --- |
|  | Astrin\_STLCHLD\_050114\_01.10552.10552.2 | 2.9962 | 0.2759 | 99.7% | 1276.0322 | 1275.4531 | 1 | 5.752 | 77.3% | 3 | R.SSRAGLQFPVGR.V | 222 |
|  | Astrin\_STLCHLD\_tube2\_050114\_01.07675.07675.1 | 1.8957 | 0.2671 | 100.0% | 944.47 | 945.1093 | 9 | 4.882 | 56.2% | 2 | R.AGLQFPVGR.V | 111 |
|  | Astrin\_STLCHLD\_050114\_01.11880.11880.2 | 3.1855 | 0.3006 | 99.9% | 946.1722 | 945.1093 | 1 | 6.103 | 81.2% | 5 | R.AGLQFPVGR.V | 222 |
|  | Astrin\_STLCHLD\_050114\_01.18621.18621.2 | 5.597 | 0.5112 | 100.0% | 2918.5923 | 2917.3752 | 1 | 9.738 | 44.6% | 2 | R.VGAGAPVYLAAVLEYLTAEILELAGNAAR.D | 22 |
|  | Astrin\_STLCHLD\_050114\_01.18655.18655.3 | 3.6932 | 0.2377 | 99.8% | 2919.5942 | 2917.3752 | 1 | 4.66 | 26.8% | 1 | R.VGAGAPVYLAAVLEYLTAEILELAGNAAR.D | 33 |
|  | Astrin\_STLCHLD\_050114\_01.07129.07129.2 | 2.4319 | 0.0855 | 96.4% | 853.1722 | 851.0396 | 4 | 4.226 | 83.3% | 1 | R.HLQLAIR.N | 222 |
|  | Astrin\_STLCHLD\_tube2\_050114\_02.05234.05234.3 | 3.0808 | 0.31 | 100.0% | 1695.3544 | 1693.9004 | 1 | 5.449 | 48.1% | 1 | R.HLQLAIRNDEELNK.L | 333 |
|  | Astrin\_STLCHLD\_tube2\_050114\_01.07926.07926.2 | 3.3565 | 0.3178 | 99.9% | 1301.4321 | 1301.4423 | 2 | 6.011 | 70.0% | 1 | R.NDEELNKLLGR.V | 2 |
|  | Astrin\_STLCHLD\_050114\_01.12591.12591.2 | 4.4953 | 0.4374 | 100.0% | 1932.1921 | 1932.3573 | 1 | 7.882 | 63.9% | 2 | R.VTIAQGGVLPNIQAVLLPK.K | 222 |

Similarities:
gi|10800130|ref|NP\_06(8:1)  
gi|106775678|ref|NP\_0(6:3)  

---

|  |  |  |  |  |  |  |  |  |
| --- | --- | --- | --- | --- | --- | --- | --- | --- |
| U | *gi|10800130|ref|NP\_06* | 10 | 21 | 60.0% | 130 | 14107 | 10.9 | histone cluster 1, H2ad [Homo sapiens] |
| U | *gi|4504249|ref|NP\_003* | 8 | 14 | 60.0% | 130 | 14091 | 10.9 | histone cluster 1, H2am [Homo sapiens] |
| U | *gi|4504243|ref|NP\_003* | 10 | 21 | 60.0% | 130 | 14091 | 10.9 | histone cluster 1, H2al [Homo sapiens] |
| U | *gi|4504241|ref|NP\_003* | 7 | 13 | 60.0% | 130 | 14091 | 10.9 | histone cluster 1, H2ak [Homo sapiens] |
| U | *gi|4504239|ref|NP\_003* | 10 | 21 | 60.0% | 130 | 14091 | 10.9 | histone cluster 1, H2ai [Homo sapiens] |
| U | *gi|29553970|ref|NP\_80* | 7 | 13 | 60.5% | 129 | 14019 | 10.9 | H2A histone family, member J [Homo sapiens] |
| U | *gi|18105045|ref|NP\_54* | 10 | 21 | 60.9% | 128 | 13906 | 10.9 | histone cluster 1, H2ah [Homo sapiens] |
| U | *gi|10800144|ref|NP\_06* | 10 | 21 | 60.9% | 128 | 13936 | 10.9 | histone cluster 1, H2aj [Homo sapiens] |
| U | *gi|10800132|ref|NP\_06* | 10 | 21 | 60.0% | 130 | 14091 | 10.9 | histone cluster 1, H2ag [Homo sapiens] |

| Filename XCorr DeltCN Conf% ObsM+H+ CalcM+H+ SpR ZScore Ion% # Sequence  | | | | | | | | | | | | |
| --- | --- | --- | --- | --- | --- | --- | --- | --- | --- | --- | --- | --- |
|  | Astrin\_STLCHLD\_050114\_01.10552.10552.2 | 2.9962 | 0.2759 | 99.7% | 1276.0322 | 1275.4531 | 1 | 5.752 | 77.3% | 3 | R.SSRAGLQFPVGR.V | 222 |
|  | Astrin\_STLCHLD\_tube2\_050114\_01.07675.07675.1 | 1.8957 | 0.2671 | 100.0% | 944.47 | 945.1093 | 9 | 4.882 | 56.2% | 2 | R.AGLQFPVGR.V | 111 |
|  | Astrin\_STLCHLD\_050114\_01.11880.11880.2 | 3.1855 | 0.3006 | 99.9% | 946.1722 | 945.1093 | 1 | 6.103 | 81.2% | 5 | R.AGLQFPVGR.V | 222 |
|  | Astrin\_STLCHLD\_050114\_01.18621.18621.2 | 5.597 | 0.5112 | 100.0% | 2918.5923 | 2917.3752 | 1 | 9.738 | 44.6% | 2 | R.VGAGAPVYLAAVLEYLTAEILELAGNAAR.D | 22 |
|  | Astrin\_STLCHLD\_050114\_01.18655.18655.3 | 3.6932 | 0.2377 | 99.8% | 2919.5942 | 2917.3752 | 1 | 4.66 | 26.8% | 1 | R.VGAGAPVYLAAVLEYLTAEILELAGNAAR.D | 33 |
|  | Astrin\_STLCHLD\_050114\_01.07129.07129.2 | 2.4319 | 0.0855 | 96.4% | 853.1722 | 851.0396 | 4 | 4.226 | 83.3% | 1 | R.HLQLAIR.N | 222 |
|  | Astrin\_STLCHLD\_tube2\_050114\_02.05234.05234.3 | 3.0808 | 0.31 | 100.0% | 1695.3544 | 1693.9004 | 1 | 5.449 | 48.1% | 1 | R.HLQLAIRNDEELNK.L | 333 |
|  | Astrin\_STLCHLD\_tube2\_050114\_01.08724.08724.3 | 4.4291 | 0.4313 | 100.0% | 2106.1143 | 2105.4453 | 1 | 6.638 | 38.2% | 2 | R.HLQLAIRNDEELNKLLGK.V | 33 |
|  | Astrin\_STLCHLD\_050114\_01.11766.11766.2 | 3.4579 | 0.3649 | 100.0% | 1273.4321 | 1273.4288 | 1 | 5.883 | 70.0% | 2 | R.NDEELNKLLGK.V | 22 |
|  | Astrin\_STLCHLD\_050114\_01.12591.12591.2 | 4.4953 | 0.4374 | 100.0% | 1932.1921 | 1932.3573 | 1 | 7.882 | 63.9% | 2 | K.VTIAQGGVLPNIQAVLLPK.K | 222 |

Similarities:
gi|10645195|ref|NP\_06(8:2)  
gi|106775678|ref|NP\_0(8:2)  

---

|  |  |  |  |  |  |  |  |  |
| --- | --- | --- | --- | --- | --- | --- | --- | --- |
| U | *gi|106775678|ref|NP\_0* | 9 | 20 | 60.0% | 130 | 14095 | 10.9 | histone cluster 2, H2aa4 [Homo sapiens] |
| U | *gi|4504251|ref|NP\_003* | 9 | 20 | 60.0% | 130 | 14095 | 10.9 | histone cluster 2, H2aa3 [Homo sapiens] |
| U | *gi|24638446|ref|NP\_00* | 9 | 20 | 60.5% | 129 | 13988 | 10.9 | histone cluster 2, H2ac [Homo sapiens] |

| Filename XCorr DeltCN Conf% ObsM+H+ CalcM+H+ SpR ZScore Ion% # Sequence  | | | | | | | | | | | | |
| --- | --- | --- | --- | --- | --- | --- | --- | --- | --- | --- | --- | --- |
|  | Astrin\_STLCHLD\_050114\_01.10552.10552.2 | 2.9962 | 0.2759 | 99.7% | 1276.0322 | 1275.4531 | 1 | 5.752 | 77.3% | 3 | R.SSRAGLQFPVGR.V | 222 |
|  | Astrin\_STLCHLD\_tube2\_050114\_01.07675.07675.1 | 1.8957 | 0.2671 | 100.0% | 944.47 | 945.1093 | 9 | 4.882 | 56.2% | 2 | R.AGLQFPVGR.V | 111 |
|  | Astrin\_STLCHLD\_050114\_01.11880.11880.2 | 3.1855 | 0.3006 | 99.9% | 946.1722 | 945.1093 | 1 | 6.103 | 81.2% | 5 | R.AGLQFPVGR.V | 222 |
|  | Astrin\_STLCHLD\_050114\_01.18547.18547.2 | 4.8497 | 0.5735 | 100.0% | 2934.3523 | 2935.4082 | 1 | 10.123 | 37.5% | 2 | R.VGAGAPVYMAAVLEYLTAEILELAGNAAR.D | 2 |
|  | Astrin\_STLCHLD\_050114\_01.07129.07129.2 | 2.4319 | 0.0855 | 96.4% | 853.1722 | 851.0396 | 4 | 4.226 | 83.3% | 1 | R.HLQLAIR.N | 222 |
|  | Astrin\_STLCHLD\_tube2\_050114\_02.05234.05234.3 | 3.0808 | 0.31 | 100.0% | 1695.3544 | 1693.9004 | 1 | 5.449 | 48.1% | 1 | R.HLQLAIRNDEELNK.L | 333 |
|  | Astrin\_STLCHLD\_tube2\_050114\_01.08724.08724.3 | 4.4291 | 0.4313 | 100.0% | 2106.1143 | 2105.4453 | 1 | 6.638 | 38.2% | 2 | R.HLQLAIRNDEELNKLLGK.V | 33 |
|  | Astrin\_STLCHLD\_050114\_01.11766.11766.2 | 3.4579 | 0.3649 | 100.0% | 1273.4321 | 1273.4288 | 1 | 5.883 | 70.0% | 2 | R.NDEELNKLLGK.V | 22 |
|  | Astrin\_STLCHLD\_050114\_01.12591.12591.2 | 4.4953 | 0.4374 | 100.0% | 1932.1921 | 1932.3573 | 1 | 7.882 | 63.9% | 2 | K.VTIAQGGVLPNIQAVLLPK.K | 222 |

Similarities:
gi|10645195|ref|NP\_06(6:3)  
gi|10800130|ref|NP\_06(8:1)  

---

|  |  |  |  |  |  |  |  |  |
| --- | --- | --- | --- | --- | --- | --- | --- | --- |
| U | *gi|5174735|ref|NP\_006* | 36 | 156 | 59.8% | 445 | 49831 | 4.9 | tubulin, beta, 2 [Homo sapiens] |

| Filename XCorr DeltCN Conf% ObsM+H+ CalcM+H+ SpR ZScore Ion% # Sequence  | | | | | | | | | | | | |
| --- | --- | --- | --- | --- | --- | --- | --- | --- | --- | --- | --- | --- |
|  | Astrin\_STLCHLD\_050114\_02.08218.08218.3 | 6.3129 | 0.4919 | 100.0% | 3117.2344 | 3118.2996 | 1 | 8.572 | 35.6% | 3 | K.FWEVISDEHGIDPTGTYHGDSDLQLER.I | 33 |
| \* | Astrin\_STLCHLD\_050114\_01.09653.09653.1 | 2.0865 | 0.2983 | 100.0% | 1328.61 | 1329.4521 | 1 | 6.346 | 54.5% | 1 | R.INVYYNEATGGK.Y | 1 |
| \* | Astrin\_STLCHLD\_050114\_01.09643.09643.2 | 3.9256 | 0.4196 | 100.0% | 1328.7522 | 1329.4521 | 1 | 7.909 | 81.8% | 5 | R.INVYYNEATGGK.Y | 2 |
|  | Astrin\_STLCHLD\_050114\_02.08076.08076.2 | 3.8623 | 0.4318 | 100.0% | 1603.4321 | 1602.8431 | 1 | 7.878 | 64.3% | 3 | R.AVLVDLEPGTMDSVR.S | 22 |
|  | Astrin\_STLCHLD\_tube2\_050114\_01.12338.12338.2 | 5.6846 | 0.5176 | 100.0% | 2798.8523 | 2800.0647 | 1 | 8.445 | 40.0% | 6 | R.SGPFGQIFRPDNFVFGQSGAGNNWAK.G | 222 |
|  | Astrin\_STLCHLD\_tube2\_050114\_01.12242.12242.3 | 6.768 | 0.5498 | 100.0% | 2799.6543 | 2800.0647 | 1 | 8.573 | 38.0% | 7 | R.SGPFGQIFRPDNFVFGQSGAGNNWAK.G | 333 |
|  | Astrin\_STLCHLD\_tube2\_050114\_01.12857.12857.2 | 7.2534 | 0.4819 | 100.0% | 1960.4722 | 1960.151 | 1 | 9.656 | 79.4% | 5 | K.GHYTEGAELVDSVLDVVR.K | 222 |
|  | Astrin\_STLCHLD\_tube2\_050114\_01.12839.12839.3 | 4.3447 | 0.3812 | 100.0% | 1960.7644 | 1960.151 | 1 | 7.142 | 45.6% | 3 | K.GHYTEGAELVDSVLDVVR.K | 333 |
|  | Astrin\_STLCHLD\_tube2\_050114\_01.11581.11581.3 | 4.7423 | 0.5057 | 100.0% | 2088.3245 | 2088.325 | 1 | 7.853 | 40.3% | 2 | K.GHYTEGAELVDSVLDVVRK.E | 333 |
|  | Astrin\_STLCHLD\_050114\_01.12405.12405.2 | 6.3217 | 0.4514 | 100.0% | 2088.9321 | 2088.325 | 1 | 8.891 | 66.7% | 2 | K.GHYTEGAELVDSVLDVVRK.E | 222 |
|  | Astrin\_STLCHLD\_050114\_01.04328.04328.2 | 2.6855 | 0.1744 | 99.0% | 1078.0122 | 1078.1698 | 9 | 4.186 | 78.6% | 1 | K.IREEYPDR.I | 22 |
|  | Astrin\_STLCHLD\_050114\_01.11995.11995.1 | 2.1993 | 0.2639 | 100.0% | 1319.69 | 1320.5896 | 245 | 5.053 | 40.9% | 3 | R.IMNTFSVVPSPK.V | 1111 |
|  | Astrin\_STLCHLD\_tube2\_050114\_01.08305.08305.2 | 4.34 | 0.4185 | 100.0% | 1320.4321 | 1320.5896 | 1 | 7.953 | 72.7% | 8 | R.IMNTFSVVPSPK.V | 2222 |
|  | Astrin\_STLCHLD\_050114\_01.12590.12590.3 | 4.1941 | 0.3268 | 100.0% | 2710.8843 | 2710.0405 | 1 | 6.714 | 30.2% | 1 | K.LTTPTYGDLNHLVSATMSGVTTCLR.F | 333 |
|  | Astrin\_STLCHLD\_tube2\_050114\_01.07179.07179.2 | 3.0568 | 0.2949 | 99.9% | 1131.2522 | 1131.2767 | 7 | 5.17 | 77.8% | 13 | R.FPGQLNADLR.K | 22222 |
|  | Astrin\_STLCHLD\_tube2\_050114\_01.05601.05601.2 | 2.7143 | 0.2756 | 99.3% | 1258.5521 | 1259.4508 | 11 | 5.358 | 65.0% | 13 | R.FPGQLNADLRK.L | 22222 |
|  | Astrin\_STLCHLD\_tube2\_050114\_01.05583.05583.3 | 3.152 | 0.3056 | 100.0% | 1259.8744 | 1259.4508 | 8 | 5.284 | 45.0% | 12 | R.FPGQLNADLRK.L | 33333 |
|  | Astrin\_STLCHLD\_tube2\_050114\_01.08365.08365.2 | 3.793 | 0.3681 | 100.0% | 1271.8722 | 1272.5945 | 1 | 7.682 | 80.0% | 5 | R.KLAVNMVPFPR.L | 22222 |
|  | Astrin\_STLCHLD\_tube2\_050114\_01.09803.09803.1 | 2.0853 | 0.2613 | 100.0% | 1143.58 | 1144.4204 | 13 | 6.348 | 61.1% | 1 | K.LAVNMVPFPR.L | 11111 |
|  | Astrin\_STLCHLD\_tube2\_050114\_01.09839.09839.2 | 3.9889 | 0.4725 | 100.0% | 1144.4122 | 1144.4204 | 1 | 8.55 | 94.4% | 3 | K.LAVNMVPFPR.L | 22222 |
|  | Astrin\_STLCHLD\_050114\_01.12456.12456.2 | 3.7154 | 0.3929 | 100.0% | 1621.7122 | 1621.9403 | 1 | 8.432 | 76.9% | 3 | R.LHFFMPGFAPLTSR.G | 2222 |
|  | Astrin\_STLCHLD\_050114\_01.12457.12457.3 | 4.2506 | 0.3686 | 100.0% | 1622.0643 | 1621.9403 | 1 | 6.108 | 53.8% | 4 | R.LHFFMPGFAPLTSR.G | 3333 |
|  | Astrin\_STLCHLD\_tube2\_050114\_01.12007.12007.2 | 4.2206 | 0.404 | 100.0% | 1693.3722 | 1692.9678 | 1 | 7.262 | 78.6% | 3 | R.ALTVPELTQQMFDAK.N | 222 |
|  | Astrin\_STLCHLD\_050114\_01.04952.04952.2 | 2.8672 | 0.3941 | 100.0% | 1066.2322 | 1066.2013 | 2 | 7.174 | 68.8% | 2 | K.NMMAACDPR.H | 22222 |
|  | Astrin\_STLCHLD\_050114\_02.06747.06747.3 | 4.3076 | 0.2581 | 100.0% | 1391.1244 | 1390.631 | 1 | 7.084 | 47.7% | 2 | R.HGRYLTVAAVFR.G | 333 |
|  | Astrin\_STLCHLD\_tube2\_050114\_01.10402.10402.1 | 1.7815 | 0.4511 | 100.0% | 1039.71 | 1040.2505 | 1 | 6.99 | 81.2% | 4 | R.YLTVAAVFR.G | 111 |
|  | Astrin\_STLCHLD\_tube2\_050114\_01.10269.10269.2 | 3.417 | 0.3601 | 100.0% | 1041.3322 | 1040.2505 | 1 | 6.884 | 87.5% | 9 | R.YLTVAAVFR.G | 222 |
|  | Astrin\_STLCHLD\_tube2\_050114\_02.06679.06679.2 | 5.6297 | 0.5004 | 100.0% | 1924.5322 | 1925.2405 | 1 | 8.712 | 56.7% | 2 | R.MSMKEVDEQMLNVQNK.N | 22 |
|  | Astrin\_STLCHLD\_050114\_02.06995.06995.3 | 4.822 | 0.2537 | 100.0% | 1926.3844 | 1925.2405 | 1 | 5.685 | 46.7% | 3 | R.MSMKEVDEQMLNVQNK.N | 33 |
|  | Astrin\_STLCHLD\_tube2\_050114\_01.05776.05776.1 | 2.9126 | 0.2315 | 100.0% | 1446.63 | 1447.6031 | 1 | 5.343 | 68.2% | 1 | K.EVDEQMLNVQNK.N | 11 |
|  | Astrin\_STLCHLD\_050114\_01.10279.10279.2 | 4.2441 | 0.2595 | 100.0% | 1447.4122 | 1447.6031 | 4 | 6.023 | 68.2% | 8 | K.EVDEQMLNVQNK.N | 22 |
|  | Astrin\_STLCHLD\_tube2\_050114\_01.11339.11339.2 | 3.5338 | 0.3838 | 100.0% | 1696.7722 | 1697.8877 | 1 | 7.601 | 57.7% | 2 | K.NSSYFVEWIPNNVK.T | 22222 |
|  | Astrin\_STLCHLD\_050114\_01.05029.05029.2 | 2.2588 | 0.224 | 97.9% | 1029.1921 | 1029.1473 | 9 | 4.44 | 62.5% | 1 | K.TAVCDIPPR.G | 222 |
|  | Astrin\_STLCHLD\_050114\_02.07650.07650.2 | 3.8098 | 0.3921 | 100.0% | 1386.7522 | 1386.6116 | 1 | 7.005 | 75.0% | 4 | K.RISEQFTAMFR.R | 2222 |
|  | Astrin\_STLCHLD\_tube2\_050114\_02.06787.06787.3 | 3.1948 | 0.3082 | 100.0% | 1543.3143 | 1542.7991 | 1 | 5.726 | 52.3% | 1 | K.RISEQFTAMFRR.K | 3333 |
|  | Astrin\_STLCHLD\_tube2\_050114\_02.08042.08042.2 | 4.0657 | 0.4262 | 100.0% | 1231.3121 | 1230.4241 | 1 | 6.725 | 94.4% | 10 | R.ISEQFTAMFR.R | 2222 |

Similarities:
gi|29788785|ref|NP\_82(31:5)  
gi|21361322|ref|NP\_00(25:11)  
gi|50592996|ref|NP\_00(18:18)  
gi|14210536|ref|NP\_11(10:26)  

---

|  |  |  |  |  |  |  |  |  |
| --- | --- | --- | --- | --- | --- | --- | --- | --- |
| U | *gi|8923110|ref|NP\_060* | 16 | 118 | 56.4% | 335 | 37721 | 5.2 | nuclear distribution gene E homolog 1 [Homo sapiens] |

| Filename XCorr DeltCN Conf% ObsM+H+ CalcM+H+ SpR ZScore Ion% # Sequence  | | | | | | | | | | | | |
| --- | --- | --- | --- | --- | --- | --- | --- | --- | --- | --- | --- | --- |
|  | Astrin\_STLCHLD\_050114\_02.05486.05486.3 | 2.9283 | 0.2483 | 99.1% | 1923.3844 | 1923.9908 | 1 | 5.254 | 40.0% | 1 | R.AENTQEELREFQEGSR.E | 33 |
| \* | Astrin\_STLCHLD\_050114\_01.05239.05239.3 | 3.1744 | 0.2708 | 100.0% | 1501.8544 | 1500.6578 | 225 | 4.789 | 38.6% | 1 | R.NRDLLSENNRLR.M | 3 |
|  | Astrin\_STLCHLD\_050114\_02.04517.04517.2 | 3.1405 | 0.3403 | 99.9% | 1251.6721 | 1252.3286 | 1 | 5.421 | 72.2% | 1 | K.FEVQHSEGYR.Q | 22 |
|  | Astrin\_STLCHLD\_050114\_01.11316.11316.2 | 3.6075 | 0.1861 | 99.6% | 1434.3922 | 1432.5707 | 1 | 6.027 | 66.7% | 3 | R.QISALEDDLAQTK.A | 22 |
|  | Astrin\_STLCHLD\_050114\_02.05610.05610.3 | 3.5797 | 0.2107 | 99.9% | 1763.5443 | 1764.8894 | 5 | 5.143 | 42.3% | 1 | K.YIRELEQANDDLER.A | 33 |
|  | Astrin\_STLCHLD\_050114\_02.04664.04664.2 | 3.3547 | 0.381 | 100.0% | 1332.0922 | 1332.3666 | 1 | 6.314 | 75.0% | 6 | R.ELEQANDDLER.A | 22 |
|  | Astrin\_STLCHLD\_050114\_02.08097.08097.3 | 4.5724 | 0.3229 | 100.0% | 2364.7444 | 2364.5713 | 1 | 7.224 | 38.2% | 2 | R.NAFLESELDEKENLLESVQR.L | 33 |
|  | Astrin\_STLCHLD\_tube2\_050114\_02.04838.04838.2 | 2.8859 | 0.0714 | 96.3% | 1328.4922 | 1328.5112 | 24 | 4.327 | 55.0% | 4 | R.DLRQELAVQQK.Q | 22 |
|  | Astrin\_STLCHLD\_050114\_02.05216.05216.3 | 3.9148 | 0.1358 | 99.8% | 1329.2943 | 1328.5112 | 7 | 5.119 | 52.5% | 1 | R.DLRQELAVQQK.Q | 33 |
|  | Astrin\_STLCHLD\_050114\_01.10937.10937.3 | 4.4531 | 0.3325 | 100.0% | 3095.0344 | 3095.4114 | 1 | 6.548 | 30.2% | 1 | R.TPMPSSVEAERTDTAVQATGSVPSTPIAHR.G | 33 |
|  | Astrin\_STLCHLD\_tube2\_050114\_02.04502.04502.3 | 3.213 | 0.3653 | 100.0% | 1911.0844 | 1910.0941 | 1 | 6.378 | 38.9% | 2 | R.TDTAVQATGSVPSTPIAHR.G | 33 |
|  | Astrin\_STLCHLD\_tube2\_050114\_02.04648.04648.2 | 3.3929 | 0.4412 | 100.0% | 1307.3322 | 1307.4056 | 1 | 8.037 | 58.3% | 4 | R.GPSSSLNTPGSFR.R | 22 |
|  | Astrin\_STLCHLD\_050114\_01.07725.07725.2 | 4.603 | 0.504 | 100.0% | 1529.8121 | 1529.647 | 1 | 9.106 | 63.3% | 6 | R.GLDDSTGGTPLTPAAR.I | 22 |
|  | Astrin\_STLCHLD\_050114\_01.13333.13333.2 | 4.1749 | 0.42 | 100.0% | 1284.0521 | 1284.5419 | 1 | 7.891 | 90.9% | 2 | R.ISALNIVGDLLR.K | 22 |
|  | Astrin\_STLCHLD\_tube2\_050114\_02.18050.18050.2 | 2.9916 | 0.3024 | 99.9% | 1206.1721 | 1206.3005 | 13 | 5.784 | 66.7% | 81 | R.NLVYDQSPNR.T | 22 |
|  | Astrin\_STLCHLD\_050114\_02.04400.04400.2 | 2.64 | 0.3593 | 99.8% | 1244.2122 | 1244.3904 | 1 | 6.649 | 68.2% | 2 | R.RPSSTSVPLGDK.G | 22 |

Similarities:
gi|221316642|ref|NP\_0(15:1)  

---

|  |  |  |  |  |  |  |  |  |
| --- | --- | --- | --- | --- | --- | --- | --- | --- |
| U | *gi|4504919|ref|NP\_002* | 42 | 116 | 56.1% | 483 | 53704 | 5.6 | keratin 8 [Homo sapiens] |

| Filename XCorr DeltCN Conf% ObsM+H+ CalcM+H+ SpR ZScore Ion% # Sequence  | | | | | | | | | | | | |
| --- | --- | --- | --- | --- | --- | --- | --- | --- | --- | --- | --- | --- |
|  | Astrin\_STLCHLD\_050114\_01.10456.10456.2 | 2.4721 | 0.2629 | 98.1% | 1618.0521 | 1618.7465 | 73 | 4.409 | 39.3% | 1 | R.ISSSSFSRVGSSNFR.G | 2 |
|  | Astrin\_STLCHLD\_tube2\_050114\_01.06813.06813.2 | 2.2879 | 0.2249 | 98.9% | 828.2322 | 827.95544 | 7 | 5.23 | 91.7% | 2 | K.FASFIDK.V | 2222 |
|  | Astrin\_STLCHLD\_tube2\_050114\_01.06993.06993.2 | 2.7851 | 0.1943 | 99.2% | 1083.4122 | 1083.2755 | 4 | 6.85 | 81.2% | 2 | K.FASFIDKVR.F | 2222 |
|  | Astrin\_STLCHLD\_tube2\_050114\_01.07872.07872.2 | 3.1307 | 0.1777 | 99.8% | 1030.6522 | 1031.1997 | 3 | 4.008 | 92.9% | 1 | K.WSLLQQQK.T | 2 |
|  | Astrin\_STLCHLD\_tube2\_050114\_01.13035.13035.2 | 3.5024 | 0.3216 | 99.9% | 1849.5922 | 1849.0431 | 1 | 5.835 | 50.0% | 2 | R.SNMDNMFESYINNLR.R | 2 |
|  | Astrin\_STLCHLD\_050114\_02.09917.09917.3 | 4.9914 | 0.2895 | 100.0% | 2036.6044 | 2035.363 | 1 | 7.594 | 45.6% | 1 | K.LKLEAELGNMQGLVEDFK.N | 3 |
|  | Astrin\_STLCHLD\_tube2\_050114\_01.09872.09872.2 | 3.382 | 0.445 | 100.0% | 1353.6721 | 1353.5732 | 1 | 8.014 | 80.0% | 5 | R.TEMENEFVLIK.K | 2 |
|  | Astrin\_STLCHLD\_050114\_02.07112.07112.2 | 3.3374 | 0.168 | 99.3% | 1482.5721 | 1481.7473 | 6 | 4.764 | 63.6% | 3 | R.TEMENEFVLIKK.D | 2 |
|  | Astrin\_STLCHLD\_tube2\_050114\_01.07946.07946.3 | 2.3312 | 0.3006 | 98.3% | 1483.3744 | 1481.7473 | 16 | 4.946 | 40.9% | 1 | R.TEMENEFVLIKK.D | 3 |
|  | Astrin\_STLCHLD\_050114\_02.05722.05722.3 | 3.982 | 0.4171 | 100.0% | 1926.6244 | 1927.1365 | 1 | 6.668 | 36.7% | 1 | K.KDVDEAYMNKVELESR.L | 3 |
|  | Astrin\_STLCHLD\_050114\_01.05252.05252.2 | 2.2896 | 0.3194 | 99.2% | 1084.7322 | 1085.1737 | 1 | 5.518 | 81.2% | 1 | K.DVDEAYMNK.V | 2 |
|  | Astrin\_STLCHLD\_tube2\_050114\_01.07050.07050.2 | 4.4449 | 0.5029 | 100.0% | 1798.2922 | 1798.9623 | 1 | 8.594 | 75.0% | 5 | K.DVDEAYMNKVELESR.L | 2 |
|  | Astrin\_STLCHLD\_050114\_02.06418.06418.3 | 4.3402 | 0.3703 | 100.0% | 1799.6643 | 1798.9623 | 2 | 6.154 | 48.2% | 5 | K.DVDEAYMNKVELESR.L | 3 |
|  | Astrin\_STLCHLD\_tube2\_050114\_01.11719.11719.2 | 4.101 | 0.4997 | 100.0% | 1420.3722 | 1420.6055 | 1 | 8.885 | 86.4% | 4 | R.LEGLTDEINFLR.Q | 2 |
|  | Astrin\_STLCHLD\_050114\_02.07643.07643.2 | 5.8514 | 0.5637 | 100.0% | 2109.6921 | 2110.3008 | 1 | 10.001 | 72.2% | 2 | R.ELQSQISDTSVVLSMDNSR.S | 2 |
|  | Astrin\_STLCHLD\_tube2\_050114\_01.11498.11498.1 | 2.3515 | 0.2658 | 100.0% | 1320.74 | 1321.5286 | 10 | 5.827 | 54.5% | 1 | R.SLDMDSIIAEVK.A | 1 |
|  | Astrin\_STLCHLD\_tube2\_050114\_01.11509.11509.2 | 4.4156 | 0.4933 | 100.0% | 1321.5922 | 1321.5286 | 1 | 8.546 | 77.3% | 3 | R.SLDMDSIIAEVK.A | 2 |
|  | Astrin\_STLCHLD\_tube2\_050114\_01.14632.14632.2 | 3.4678 | 0.4072 | 100.0% | 2381.9321 | 2382.6477 | 1 | 6.067 | 40.0% | 1 | R.SLDMDSIIAEVKAQYEDIANR.S | 2 |
|  | Astrin\_STLCHLD\_050114\_01.05089.05089.1 | 1.8908 | 0.3223 | 100.0% | 1079.51 | 1080.1423 | 4 | 5.333 | 62.5% | 1 | K.AQYEDIANR.S | 1 |
|  | Astrin\_STLCHLD\_050114\_01.05041.05041.2 | 3.2047 | 0.2176 | 99.9% | 1080.0721 | 1080.1423 | 2 | 6.441 | 75.0% | 6 | K.AQYEDIANR.S | 2 |
|  | Astrin\_STLCHLD\_050114\_01.06423.06423.2 | 3.5727 | 0.2997 | 99.9% | 1412.6721 | 1413.5884 | 1 | 6.14 | 81.8% | 4 | R.SRAEAESMYQIK.Y | 2 |
|  | Astrin\_STLCHLD\_050114\_02.07454.07454.3 | 6.3252 | 0.4019 | 100.0% | 2533.1343 | 2532.828 | 1 | 7.97 | 38.1% | 3 | R.SRAEAESMYQIKYEELQSLAGK.H | 3 |
|  | Astrin\_STLCHLD\_050114\_01.08797.08797.2 | 2.7835 | 0.3696 | 99.9% | 1170.3121 | 1170.3228 | 5 | 6.308 | 72.2% | 3 | R.AEAESMYQIK.Y | 2 |
|  | Astrin\_STLCHLD\_050114\_02.07904.07904.3 | 5.1439 | 0.4636 | 100.0% | 2289.3245 | 2289.5623 | 1 | 8.599 | 36.8% | 2 | R.AEAESMYQIKYEELQSLAGK.H | 3 |
|  | Astrin\_STLCHLD\_tube2\_050114\_01.06176.06176.2 | 3.309 | 0.0659 | 98.8% | 1138.3121 | 1138.2627 | 2 | 6.95 | 77.8% | 6 | K.YEELQSLAGK.H | 2 |
|  | Astrin\_STLCHLD\_tube2\_050114\_01.06082.06082.1 | 2.1935 | 0.1761 | 100.0% | 1000.61 | 1001.168 | 6 | 5.784 | 68.8% | 2 | R.LQAEIEGLK.G | 1 |
|  | Astrin\_STLCHLD\_tube2\_050114\_01.06128.06128.2 | 2.9827 | 0.3015 | 99.9% | 1001.47217 | 1001.168 | 30 | 5.31 | 81.2% | 5 | R.LQAEIEGLK.G | 2 |
|  | Astrin\_STLCHLD\_tube2\_050114\_01.04833.04833.2 | 3.3774 | 0.307 | 99.9% | 1342.5322 | 1342.5381 | 1 | 6.363 | 77.3% | 4 | R.LQAEIEGLKGQR.A | 2 |
|  | Astrin\_STLCHLD\_tube2\_050114\_01.08847.08847.2 | 3.9824 | 0.4435 | 100.0% | 1345.3121 | 1345.452 | 1 | 7.255 | 66.7% | 4 | R.ASLEAAIADAEQR.G | 2 |
|  | Astrin\_STLCHLD\_050114\_01.12327.12327.3 | 3.6899 | 0.4202 | 100.0% | 1956.8944 | 1957.1912 | 1 | 6.481 | 41.7% | 2 | R.ASLEAAIADAEQRGELAIK.D | 3 |
|  | Astrin\_STLCHLD\_tube2\_050114\_01.11157.11157.2 | 5.0942 | 0.3967 | 100.0% | 1957.0322 | 1957.1912 | 1 | 7.753 | 58.3% | 3 | R.ASLEAAIADAEQRGELAIK.D | 2 |
|  | Astrin\_STLCHLD\_050114\_01.12358.12358.3 | 5.4126 | 0.5114 | 100.0% | 2456.1843 | 2456.7153 | 1 | 8.35 | 34.8% | 2 | R.ASLEAAIADAEQRGELAIKDANAK.L | 3 |
|  | Astrin\_STLCHLD\_050114\_01.11905.11905.1 | 2.1243 | 0.2671 | 100.0% | 1129.61 | 1130.2865 | 33 | 5.579 | 50.0% | 2 | K.LSELEAALQR.A | 1 |
|  | Astrin\_STLCHLD\_tube2\_050114\_01.07938.07938.2 | 4.3186 | 0.2539 | 100.0% | 1130.1921 | 1130.2865 | 25 | 5.846 | 72.2% | 3 | K.LSELEAALQR.A | 2 |
|  | Astrin\_STLCHLD\_tube2\_050114\_01.07295.07295.2 | 3.4109 | 0.219 | 99.8% | 1551.5322 | 1551.801 | 1 | 4.882 | 72.7% | 1 | R.QLREYQELMNVK.L | 2 |
|  | Astrin\_STLCHLD\_050114\_01.11141.11141.2 | 2.8705 | 0.2502 | 99.7% | 1154.1322 | 1154.3234 | 15 | 6.116 | 62.5% | 1 | R.EYQELMNVK.L | 22 |
|  | Astrin\_STLCHLD\_tube2\_050114\_01.08928.08928.2 | 3.0067 | 0.3256 | 99.9% | 1406.2922 | 1406.6653 | 6 | 6.062 | 63.6% | 3 | K.LALDIEIATYRK.L | 22 |
|  | Astrin\_STLCHLD\_tube2\_050114\_01.08199.08199.3 | 5.3838 | 0.3787 | 100.0% | 2390.5144 | 2390.6887 | 1 | 8.239 | 42.5% | 1 | K.LLEGEESRLESGMQNMSIHTK.T | 3 |
|  | Astrin\_STLCHLD\_050114\_01.07004.07004.3 | 2.8386 | 0.3992 | 100.0% | 1474.7943 | 1476.7058 | 10 | 5.8 | 41.7% | 5 | R.LESGMQNMSIHTK.T | 3 |
|  | Astrin\_STLCHLD\_tube2\_050114\_01.04716.04716.2 | 4.0742 | 0.471 | 100.0% | 1476.4722 | 1476.7058 | 1 | 7.456 | 83.3% | 3 | R.LESGMQNMSIHTK.T | 2 |
|  | Astrin\_STLCHLD\_tube2\_050114\_01.05736.05736.2 | 2.6314 | 0.3423 | 99.4% | 1473.3322 | 1474.6512 | 10 | 6.53 | 50.0% | 1 | R.DGKLVSESSDVLPK.- | 2 |
|  | Astrin\_STLCHLD\_tube2\_050114\_01.05480.05480.2 | 3.5787 | 0.4495 | 100.0% | 1175.1721 | 1174.3367 | 1 | 7.381 | 75.0% | 8 | K.LVSESSDVLPK.- | 2 |

Similarities:
gi|67782365|ref|NP\_00(3:39)  
gi|119703753|ref|NP\_0(3:39)  
gi|47132620|ref|NP\_00(2:40)  

---

|  |  |  |  |  |  |  |  |  |
| --- | --- | --- | --- | --- | --- | --- | --- | --- |
| U | *gi|221316642|ref|NP\_0* | 16 | 118 | 55.5% | 335 | 37721 | 5.2 | nuclear distribution gene E homolog 1 [Homo sapiens] |

| Filename XCorr DeltCN Conf% ObsM+H+ CalcM+H+ SpR ZScore Ion% # Sequence  | | | | | | | | | | | | |
| --- | --- | --- | --- | --- | --- | --- | --- | --- | --- | --- | --- | --- |
|  | Astrin\_STLCHLD\_050114\_02.05486.05486.3 | 2.9283 | 0.2483 | 99.1% | 1923.3844 | 1923.9908 | 1 | 5.254 | 40.0% | 1 | R.AENTQEELREFQEGSR.E | 33 |
| \* | Astrin\_STLCHLD\_050114\_01.05381.05381.2 | 2.215 | 0.1714 | 95.2% | 1120.7722 | 1121.3344 | 337 | 4.445 | 56.2% | 1 | R.MELETIKEK.F | 2 |
|  | Astrin\_STLCHLD\_050114\_02.04517.04517.2 | 3.1405 | 0.3403 | 99.9% | 1251.6721 | 1252.3286 | 1 | 5.421 | 72.2% | 1 | K.FEVQHSEGYR.Q | 22 |
|  | Astrin\_STLCHLD\_050114\_01.11316.11316.2 | 3.6075 | 0.1861 | 99.6% | 1434.3922 | 1432.5707 | 1 | 6.027 | 66.7% | 3 | R.QISALEDDLAQTK.A | 22 |
|  | Astrin\_STLCHLD\_050114\_02.05610.05610.3 | 3.5797 | 0.2107 | 99.9% | 1763.5443 | 1764.8894 | 5 | 5.143 | 42.3% | 1 | K.YIRELEQANDDLER.A | 33 |
|  | Astrin\_STLCHLD\_050114\_02.04664.04664.2 | 3.3547 | 0.381 | 100.0% | 1332.0922 | 1332.3666 | 1 | 6.314 | 75.0% | 6 | R.ELEQANDDLER.A | 22 |
|  | Astrin\_STLCHLD\_050114\_02.08097.08097.3 | 4.5724 | 0.3229 | 100.0% | 2364.7444 | 2364.5713 | 1 | 7.224 | 38.2% | 2 | R.NAFLESELDEKENLLESVQR.L | 33 |
|  | Astrin\_STLCHLD\_tube2\_050114\_02.04838.04838.2 | 2.8859 | 0.0714 | 96.3% | 1328.4922 | 1328.5112 | 24 | 4.327 | 55.0% | 4 | R.DLRQELAVQQK.Q | 22 |
|  | Astrin\_STLCHLD\_050114\_02.05216.05216.3 | 3.9148 | 0.1358 | 99.8% | 1329.2943 | 1328.5112 | 7 | 5.119 | 52.5% | 1 | R.DLRQELAVQQK.Q | 33 |
|  | Astrin\_STLCHLD\_050114\_01.10937.10937.3 | 4.4531 | 0.3325 | 100.0% | 3095.0344 | 3095.4114 | 1 | 6.548 | 30.2% | 1 | R.TPMPSSVEAERTDTAVQATGSVPSTPIAHR.G | 33 |
|  | Astrin\_STLCHLD\_tube2\_050114\_02.04502.04502.3 | 3.213 | 0.3653 | 100.0% | 1911.0844 | 1910.0941 | 1 | 6.378 | 38.9% | 2 | R.TDTAVQATGSVPSTPIAHR.G | 33 |
|  | Astrin\_STLCHLD\_tube2\_050114\_02.04648.04648.2 | 3.3929 | 0.4412 | 100.0% | 1307.3322 | 1307.4056 | 1 | 8.037 | 58.3% | 4 | R.GPSSSLNTPGSFR.R | 22 |
|  | Astrin\_STLCHLD\_050114\_01.07725.07725.2 | 4.603 | 0.504 | 100.0% | 1529.8121 | 1529.647 | 1 | 9.106 | 63.3% | 6 | R.GLDDSTGGTPLTPAAR.I | 22 |
|  | Astrin\_STLCHLD\_050114\_01.13333.13333.2 | 4.1749 | 0.42 | 100.0% | 1284.0521 | 1284.5419 | 1 | 7.891 | 90.9% | 2 | R.ISALNIVGDLLR.K | 22 |
|  | Astrin\_STLCHLD\_tube2\_050114\_02.18050.18050.2 | 2.9916 | 0.3024 | 99.9% | 1206.1721 | 1206.3005 | 13 | 5.784 | 66.7% | 81 | R.NLVYDQSPNR.T | 22 |
|  | Astrin\_STLCHLD\_050114\_02.04400.04400.2 | 2.64 | 0.3593 | 99.8% | 1244.2122 | 1244.3904 | 1 | 6.649 | 68.2% | 2 | R.RPSSTSVPLGDK.G | 22 |

Similarities:
gi|8923110|ref|NP\_060(15:1)  

---

|  |  |  |  |  |  |  |  |  |
| --- | --- | --- | --- | --- | --- | --- | --- | --- |
| U | *gi|223890147|ref|NP\_0* | 24 | 92 | 54.2% | 356 | 40067 | 7.0 | DSN1, MIND kinetochore complex component, homolog isoform 1 [Homo sapiens] |
| U | *gi|223972618|ref|NP\_0* | 24 | 92 | 54.2% | 356 | 40067 | 7.0 | DSN1, MIND kinetochore complex component, homolog isoform 1 [Homo sapiens] |
| U | *gi|223890149|ref|NP\_0* | 24 | 92 | 54.2% | 356 | 40067 | 7.0 | DSN1, MIND kinetochore complex component, homolog isoform 1 [Homo sapiens] |

| Filename XCorr DeltCN Conf% ObsM+H+ CalcM+H+ SpR ZScore Ion% # Sequence  | | | | | | | | | | | | |
| --- | --- | --- | --- | --- | --- | --- | --- | --- | --- | --- | --- | --- |
|  | Astrin\_STLCHLD\_tube2\_050114\_01.04899.04899.2 | 4.2495 | 0.3785 | 100.0% | 1433.4521 | 1433.6599 | 1 | 7.196 | 75.0% | 8 | R.SEIIDEKGPVMSK.T | 2 |
|  | Astrin\_STLCHLD\_tube2\_050114\_01.08214.08214.2 | 6.5535 | 0.6047 | 100.0% | 2024.6522 | 2025.2255 | 1 | 11.316 | 79.4% | 3 | K.THDHQLESSLSPVEVFAK.T | 2 |
|  | Astrin\_STLCHLD\_tube2\_050114\_01.08202.08202.3 | 5.4903 | 0.3636 | 100.0% | 2025.8344 | 2025.2255 | 1 | 7.09 | 47.1% | 4 | K.THDHQLESSLSPVEVFAK.T | 3 |
|  | Astrin\_STLCHLD\_050114\_02.05258.05258.2 | 4.7966 | 0.4593 | 100.0% | 1638.2722 | 1638.7467 | 1 | 8.859 | 57.1% | 10 | K.TSASLEMNQGVSEER.I | 2 |
|  | Astrin\_STLCHLD\_tube2\_050114\_01.04714.04714.2 | 5.4626 | 0.5507 | 100.0% | 1846.5922 | 1846.9512 | 1 | 9.901 | 66.7% | 2 | K.SLHLSPQEQSASYQDR.R | 2 |
|  | Astrin\_STLCHLD\_050114\_01.07135.07135.3 | 4.6132 | 0.3599 | 100.0% | 1847.3944 | 1846.9512 | 3 | 6.422 | 41.7% | 5 | K.SLHLSPQEQSASYQDR.R | 3 |
|  | Astrin\_STLCHLD\_tube2\_050114\_01.04385.04385.2 | 3.9021 | 0.3923 | 100.0% | 2002.4122 | 2003.1387 | 1 | 6.383 | 56.2% | 1 | K.SLHLSPQEQSASYQDRR.Q | 2 |
|  | Astrin\_STLCHLD\_050114\_01.05332.05332.3 | 3.7003 | 0.4868 | 100.0% | 1717.2843 | 1716.981 | 1 | 7.415 | 46.4% | 1 | R.KSLHPIHQGITELSR.S | 3 |
|  | Astrin\_STLCHLD\_tube2\_050114\_01.04896.04896.2 | 3.8435 | 0.4583 | 100.0% | 1588.6322 | 1588.8069 | 1 | 7.934 | 84.6% | 4 | K.SLHPIHQGITELSR.S | 2 |
|  | Astrin\_STLCHLD\_tube2\_050114\_01.04892.04892.3 | 4.1868 | 0.5459 | 100.0% | 1589.5443 | 1588.8069 | 1 | 8.734 | 51.9% | 13 | K.SLHPIHQGITELSR.S | 3 |
|  | Astrin\_STLCHLD\_050114\_01.09977.09977.2 | 2.4377 | 0.3293 | 99.4% | 1049.6522 | 1049.1663 | 1 | 6.253 | 77.8% | 3 | R.SISVDLAESK.R | 2 |
|  | Astrin\_STLCHLD\_050114\_01.07346.07346.2 | 3.1436 | 0.3608 | 100.0% | 1204.9122 | 1205.3538 | 1 | 6.463 | 80.0% | 9 | R.SISVDLAESKR.L | 2 |
|  | Astrin\_STLCHLD\_tube2\_050114\_01.09538.09538.1 | 1.7298 | 0.3129 | 100.0% | 942.45 | 943.04706 | 99 | 5.318 | 50.0% | 2 | K.GFSLESFR.A | 1 |
|  | Astrin\_STLCHLD\_tube2\_050114\_01.09502.09502.2 | 2.5362 | 0.4913 | 100.0% | 943.09216 | 943.04706 | 2 | 7.305 | 71.4% | 2 | K.GFSLESFR.A | 2 |
|  | Astrin\_STLCHLD\_050114\_01.06038.06038.2 | 2.7127 | 0.3453 | 99.9% | 964.1922 | 964.06055 | 304 | 6.882 | 56.2% | 2 | K.ASSLSEELK.H | 2 |
|  | Astrin\_STLCHLD\_tube2\_050114\_01.11721.11721.3 | 6.1593 | 0.4367 | 100.0% | 2477.7244 | 2477.6873 | 1 | 8.469 | 39.8% | 2 | K.ASSLSEELKHFADGLETDGTLQK.C | 3 |
|  | Astrin\_STLCHLD\_050114\_01.09850.09850.2 | 4.0074 | 0.5199 | 100.0% | 1532.3121 | 1532.65 | 1 | 8.582 | 80.8% | 4 | K.HFADGLETDGTLQK.C | 2 |
|  | Astrin\_STLCHLD\_tube2\_050114\_02.07744.07744.2 | 4.6703 | 0.4649 | 100.0% | 1486.6921 | 1485.6489 | 1 | 8.56 | 73.1% | 3 | K.ASDFSLEASVAEMK.E | 2 |
|  | Astrin\_STLCHLD\_tube2\_050114\_01.12679.12679.2 | 4.8989 | 0.4961 | 100.0% | 2120.5122 | 2120.379 | 1 | 9.053 | 52.8% | 1 | K.ASDFSLEASVAEMKEYITK.F | 2 |
|  | Astrin\_STLCHLD\_tube2\_050114\_02.09718.09718.3 | 3.2296 | 0.272 | 99.8% | 2120.9644 | 2120.379 | 6 | 5.504 | 33.3% | 1 | K.ASDFSLEASVAEMKEYITK.F | 3 |
|  | Astrin\_STLCHLD\_tube2\_050114\_01.10517.10517.2 | 2.444 | 0.2969 | 98.7% | 1901.7722 | 1902.1167 | 210 | 4.394 | 39.3% | 1 | R.QTWDQLLLHYQQEAK.E | 2 |
|  | Astrin\_STLCHLD\_tube2\_050114\_01.08664.08664.3 | 6.7309 | 0.4988 | 100.0% | 3212.9043 | 3213.627 | 1 | 8.034 | 36.1% | 1 | K.ITEVKVEPMTYLGSSQNEVLNTKPDYQK.I | 3 |
|  | Astrin\_STLCHLD\_tube2\_050114\_01.08302.08302.3 | 2.1374 | 0.3443 | 95.5% | 2641.7944 | 2642.9404 | 1 | 4.781 | 30.7% | 1 | K.VEPMTYLGSSQNEVLNTKPDYQK.I | 3 |
|  | Astrin\_STLCHLD\_050114\_01.05955.05955.2 | 4.1475 | 0.3478 | 100.0% | 1230.0922 | 1230.3813 | 1 | 6.874 | 85.0% | 9 | R.SMQQLDPSPAR.K | 2 |

---

|  |  |  |  |  |  |  |  |  |
| --- | --- | --- | --- | --- | --- | --- | --- | --- |
| U | *gi|73623035|ref|NP\_00* | 81 | 300 | 53.6% | 1193 | 134422 | 5.0 | sperm associated antigen 5 [Homo sapiens] |

| Filename XCorr DeltCN Conf% ObsM+H+ CalcM+H+ SpR ZScore Ion% # Sequence  | | | | | | | | | | | | |
| --- | --- | --- | --- | --- | --- | --- | --- | --- | --- | --- | --- | --- |
| \* | Astrin\_STLCHLD\_tube2\_050114\_01.09172.09172.2 | 3.7973 | 0.3591 | 100.0% | 1653.5922 | 1653.8445 | 1 | 8.288 | 64.3% | 1 | K.TSEEAVDPLGNYMVK.T | 2 |
| \* | Astrin\_STLCHLD\_tube2\_050114\_01.12828.12828.2 | 3.4052 | 0.502 | 100.0% | 2323.2922 | 2323.6262 | 1 | 8.634 | 52.6% | 2 | K.TIVLVPS\*PLGQQQDMIFEAR.L | 2 |
| \* | Astrin\_STLCHLD\_tube2\_050114\_02.07279.07279.2 | 4.7132 | 0.4024 | 100.0% | 1832.4521 | 1833.0668 | 1 | 8.194 | 68.8% | 2 | R.LDTMAETNSISLNGPLR.T | 2 |
| \* | Astrin\_STLCHLD\_tube2\_050114\_01.09753.09753.3 | 3.4773 | 0.2215 | 99.1% | 2533.6743 | 2532.8286 | 10 | 4.043 | 27.3% | 2 | R.LDTMAETNSISLNGPLRTDDLVR.E | 3 |
| \* | Astrin\_STLCHLD\_050114\_01.14591.14591.3 | 6.0521 | 0.2454 | 100.0% | 3857.5745 | 3858.2102 | 1 | 6.562 | 30.9% | 6 | R.TEAVREDLVPSESNAFLPSSVLWLS\*PSTALAADFR.V | 3 |
| \* | Astrin\_STLCHLD\_tube2\_050114\_01.06545.06545.2 | 5.7813 | 0.549 | 100.0% | 2220.2922 | 2220.3752 | 1 | 8.973 | 72.2% | 1 | R.VNHVDPEEEIVEHGAMEER.E | 2 |
| \* | Astrin\_STLCHLD\_050114\_02.06255.06255.3 | 5.1837 | 0.3493 | 100.0% | 2220.6543 | 2220.3752 | 1 | 6.247 | 43.1% | 8 | R.VNHVDPEEEIVEHGAMEER.E | 3 |
| \* | Astrin\_STLCHLD\_050114\_01.15103.15103.2 | 5.2318 | 0.4745 | 100.0% | 2064.672 | 2064.3606 | 1 | 7.651 | 52.9% | 3 | R.ILGSDTESWMSPLAWLEK.G | 2 |
| \* | Astrin\_STLCHLD\_tube2\_050114\_01.15962.15962.2 | 4.7517 | 0.4985 | 100.0% | 2143.4922 | 2144.3606 | 1 | 7.97 | 64.7% | 1 | R.ILGSDTESWMS\*PLAWLEK.G | 2 |
| \* | Astrin\_STLCHLD\_050114\_01.16471.16471.2 | 7.565 | 0.6026 | 100.0% | 2166.4722 | 2166.4795 | 1 | 10.614 | 72.2% | 9 | R.HDLEDNLLSSLVILEVLSR.Q | 2 |
| \* | Astrin\_STLCHLD\_050114\_01.16469.16469.3 | 3.2929 | 0.3223 | 100.0% | 2167.1343 | 2166.4795 | 10 | 5.743 | 33.3% | 2 | R.HDLEDNLLSSLVILEVLSR.Q | 3 |
| \* | Astrin\_STLCHLD\_050114\_01.07125.07125.3 | 4.7886 | 0.4569 | 100.0% | 2867.5444 | 2868.0 | 1 | 7.946 | 31.7% | 5 | K.SQLAVPHPETQDSSTQTDTSHSGITNK.L | 3 |
| \* | Astrin\_STLCHLD\_tube2\_050114\_01.04396.04396.2 | 5.4276 | 0.4906 | 100.0% | 2105.5522 | 2105.3794 | 1 | 8.697 | 70.6% | 1 | K.LQHLKESHEMGQALQQAR.N | 2 |
| \* | Astrin\_STLCHLD\_tube2\_050114\_01.04389.04389.3 | 5.4181 | 0.3977 | 100.0% | 2106.5942 | 2105.3794 | 1 | 6.693 | 47.1% | 1 | K.LQHLKESHEMGQALQQAR.N | 3 |
| \* | Astrin\_STLCHLD\_050114\_01.04909.04909.2 | 3.8418 | 0.3859 | 100.0% | 1485.3722 | 1485.6146 | 1 | 6.637 | 62.5% | 2 | K.ESHEMGQALQQAR.N | 2 |
| \* | Astrin\_STLCHLD\_050114\_01.04885.04885.3 | 4.0989 | 0.0781 | 97.7% | 1486.5243 | 1485.6146 | 13 | 4.774 | 47.9% | 1 | K.ESHEMGQALQQAR.N | 3 |
| \* | Astrin\_STLCHLD\_tube2\_050114\_01.10987.10987.2 | 3.9091 | 0.3893 | 100.0% | 1306.4722 | 1305.578 | 1 | 6.815 | 80.0% | 3 | R.NVMQSWVLISK.E | 2 |
| \* | Astrin\_STLCHLD\_050114\_01.12426.12426.3 | 6.364 | 0.5078 | 100.0% | 2891.5144 | 2892.2793 | 1 | 7.949 | 35.4% | 5 | K.ELISLLHLSLLHLEEDKTTVSQESR.R | 3 |
| \* | Astrin\_STLCHLD\_050114\_02.09958.09958.2 | 4.2047 | 0.3751 | 100.0% | 1889.4922 | 1889.041 | 1 | 6.93 | 62.5% | 1 | K.DAAEIVLEAFCAHASQR.I | 2 |
| \* | Astrin\_STLCHLD\_tube2\_050114\_01.09771.09771.1 | 2.1711 | 0.4302 | 100.0% | 1390.7 | 1391.5823 | 16 | 6.278 | 50.0% | 1 | R.ISQLEQDLASMR.E | 1 |
| \* | Astrin\_STLCHLD\_050114\_01.12218.12218.2 | 3.3298 | 0.3682 | 100.0% | 1391.0122 | 1391.5823 | 1 | 7.405 | 68.2% | 3 | R.ISQLEQDLASMR.E | 2 |
| \* | Astrin\_STLCHLD\_tube2\_050114\_01.08741.08741.3 | 4.7868 | 0.3988 | 100.0% | 2125.8843 | 2125.4788 | 5 | 7.4 | 33.3% | 2 | R.EFRGLLKDAQTQLVGLHAK.Q | 3 |
| \* | Astrin\_STLCHLD\_tube2\_050114\_01.07601.07601.3 | 3.9998 | 0.2924 | 100.0% | 1691.8444 | 1692.9994 | 3 | 5.602 | 41.7% | 1 | R.GLLKDAQTQLVGLHAK.Q | 3 |
| \* | Astrin\_STLCHLD\_tube2\_050114\_01.04834.04834.2 | 3.5567 | 0.4024 | 100.0% | 1281.5721 | 1281.4545 | 1 | 6.362 | 81.8% | 4 | K.DAQTQLVGLHAK.Q | 2 |
| \* | Astrin\_STLCHLD\_tube2\_050114\_02.09698.09698.2 | 3.0895 | 0.4481 | 100.0% | 2389.632 | 2390.612 | 5 | 6.592 | 28.9% | 1 | K.QEELVQQTVSLTSTLQQDWR.S | 2 |
| \* | Astrin\_STLCHLD\_050114\_01.12858.12858.2 | 5.4031 | 0.4639 | 100.0% | 1786.3322 | 1787.0405 | 1 | 8.989 | 67.9% | 7 | R.SMQLDYTTWTALLSR.S | 2 |
| \* | Astrin\_STLCHLD\_tube2\_050114\_01.02086.02086.2 | 2.3831 | 0.2282 | 98.9% | 960.4122 | 960.0348 | 137 | 4.74 | 64.3% | 1 | K.SQQALQER.D | 2 |
| \* | Astrin\_STLCHLD\_tube2\_050114\_02.04668.04668.3 | 4.8302 | 0.2887 | 100.0% | 2344.3442 | 2344.5437 | 1 | 6.05 | 36.8% | 1 | K.SQQALQERDVAIEEKQEVSR.V | 3 |
| \* | Astrin\_STLCHLD\_050114\_01.04801.04801.2 | 3.9368 | 0.3945 | 100.0% | 1403.1322 | 1403.5321 | 1 | 6.994 | 72.7% | 8 | R.DVAIEEKQEVSR.V | 2 |
| \* | Astrin\_STLCHLD\_050114\_02.04570.04570.3 | 2.6614 | 0.3367 | 100.0% | 1405.3744 | 1403.5321 | 1 | 5.732 | 43.2% | 2 | R.DVAIEEKQEVSR.V | 3 |
| \* | Astrin\_STLCHLD\_050114\_02.06351.06351.2 | 4.1465 | 0.4471 | 100.0% | 1533.2722 | 1533.6849 | 1 | 8.43 | 70.8% | 4 | R.VLEQVSAQLEECK.G | 2 |
| \* | Astrin\_STLCHLD\_tube2\_050114\_02.06831.06831.3 | 4.6733 | 0.4097 | 100.0% | 2918.8145 | 2919.1382 | 1 | 7.068 | 32.3% | 2 | R.VLEQVSAQLEECKGQTEQLELENSR.L | 3 |
| \* | Astrin\_STLCHLD\_050114\_01.06979.06979.2 | 4.3543 | 0.4622 | 100.0% | 1404.3121 | 1404.4764 | 1 | 8.036 | 68.2% | 8 | K.GQTEQLELENSR.L | 2 |
| \* | Astrin\_STLCHLD\_050114\_02.08750.08750.3 | 5.3272 | 0.3177 | 100.0% | 1574.6044 | 1573.848 | 1 | 6.912 | 51.9% | 2 | R.AQLQILANMDSQLK.E | 3 |
| \* | Astrin\_STLCHLD\_050114\_01.12318.12318.2 | 5.5602 | 0.3606 | 100.0% | 1574.8121 | 1573.848 | 1 | 6.697 | 76.9% | 13 | R.AQLQILANMDSQLK.E | 2 |
| \* | Astrin\_STLCHLD\_tube2\_050114\_01.04895.04895.2 | 5.6943 | 0.5635 | 100.0% | 1722.7122 | 1723.9879 | 1 | 10.394 | 82.1% | 13 | K.HMQAELQQQQAVLAK.E | 2 |
| \* | Astrin\_STLCHLD\_050114\_02.05204.05204.3 | 5.1866 | 0.3005 | 100.0% | 1724.5443 | 1723.9879 | 1 | 5.834 | 50.0% | 13 | K.HMQAELQQQQAVLAK.E | 3 |
| \* | Astrin\_STLCHLD\_050114\_02.05957.05957.3 | 4.9848 | 0.4934 | 100.0% | 2107.4644 | 2108.4233 | 1 | 8.772 | 47.1% | 3 | K.HMQAELQQQQAVLAKEVR.D | 3 |
| \* | Astrin\_STLCHLD\_050114\_02.08849.08849.3 | 3.2023 | 0.2962 | 99.8% | 2931.1443 | 2930.1638 | 16 | 4.866 | 25.0% | 1 | K.ETLEFADQENQVAHLELGQVECQLK.T | 3 |
| \* | Astrin\_STLCHLD\_050114\_01.09823.09823.2 | 2.2633 | 0.2319 | 98.9% | 832.47217 | 831.9878 | 21 | 5.008 | 83.3% | 2 | K.TTLEVLR.E | 2 |
| \* | Astrin\_STLCHLD\_050114\_01.11925.11925.3 | 4.0232 | 0.2312 | 100.0% | 1965.2344 | 1964.1462 | 1 | 5.603 | 46.9% | 1 | R.SLQCENLKDTVENLTAK.L | 3 |
| \* | Astrin\_STLCHLD\_tube2\_050114\_01.05264.05264.2 | 4.629 | 0.4078 | 100.0% | 1675.4722 | 1675.7899 | 1 | 8.053 | 67.9% | 14 | K.LASTIADNQEQDLEK.T | 2 |
| \* | Astrin\_STLCHLD\_tube2\_050114\_01.05045.05045.2 | 4.0899 | 0.4808 | 100.0% | 1932.4521 | 1933.0825 | 1 | 8.315 | 62.5% | 1 | K.LASTIADNQEQDLEKTR.Q | 2 |
| \* | Astrin\_STLCHLD\_tube2\_050114\_01.05090.05090.3 | 2.3525 | 0.2975 | 96.3% | 1933.4944 | 1933.0825 | 140 | 4.854 | 32.8% | 1 | K.LASTIADNQEQDLEKTR.Q | 3 |
| \* | Astrin\_STLCHLD\_050114\_02.15328.15328.3 | 3.2405 | 0.2337 | 97.8% | 2762.6943 | 2762.1326 | 20 | 4.65 | 26.1% | 1 | R.QYS\*QKLGLLTEQLQSLTLFLQTK.L | 3 |
| \* | Astrin\_STLCHLD\_050114\_02.12775.12775.2 | 6.2077 | 0.513 | 100.0% | 2046.7922 | 2047.443 | 1 | 9.979 | 67.6% | 25 | K.LGLLTEQLQSLTLFLQTK.L | 2 |
| \* | Astrin\_STLCHLD\_tube2\_050114\_02.12526.12526.3 | 5.3639 | 0.3865 | 100.0% | 2048.3943 | 2047.443 | 1 | 7.961 | 50.0% | 8 | K.LGLLTEQLQSLTLFLQTK.L | 3 |
| \* | Astrin\_STLCHLD\_tube2\_050114\_01.15850.15850.2 | 5.4955 | 0.4967 | 100.0% | 2787.6921 | 2788.121 | 1 | 9.007 | 42.3% | 9 | R.TFLGSILTAVADEEPESTPVPLLGSDK.S | 2 |
| \* | Astrin\_STLCHLD\_tube2\_050114\_01.15772.15772.3 | 5.4049 | 0.5346 | 100.0% | 2788.3145 | 2788.121 | 1 | 8.687 | 29.8% | 1 | R.TFLGSILTAVADEEPESTPVPLLGSDK.S | 3 |
| \* | Astrin\_STLCHLD\_050114\_01.16015.16015.2 | 4.7113 | 0.4389 | 100.0% | 2868.672 | 2868.121 | 1 | 7.327 | 46.2% | 4 | R.TFLGSILTAVADEEPESTPVPLLGS\*DK.S | 2 |
| \* | Astrin\_STLCHLD\_tube2\_050114\_01.15226.15226.3 | 4.5468 | 0.3527 | 100.0% | 3350.5745 | 3350.7473 | 1 | 5.944 | 23.4% | 2 | R.TFLGSILTAVADEEPESTPVPLLGSDKSAFTR.V | 3 |
| \* | Astrin\_STLCHLD\_050114\_01.15369.15369.3 | 4.6331 | 0.2578 | 100.0% | 3429.0842 | 3430.7473 | 1 | 7.303 | 28.2% | 3 | R.TFLGSILTAVADEEPESTPVPLLGS\*DKSAFTR.V | 3 |
| \* | Astrin\_STLCHLD\_tube2\_050114\_01.15688.15688.3 | 4.5669 | 0.4432 | 100.0% | 3430.1343 | 3430.7473 | 2 | 6.846 | 29.8% | 1 | R.TFLGSILTAVADEEPESTPVPLLGSDKS\*AFTR.V | 3 |
| \* | Astrin\_STLCHLD\_050114\_01.04143.04143.2 | 3.8815 | 0.3368 | 100.0% | 1595.5721 | 1595.7092 | 1 | 5.48 | 75.0% | 1 | R.LQAQEEQHQEVQK.A | 2 |
| \* | Astrin\_STLCHLD\_050114\_01.09140.09140.2 | 2.4527 | 0.2997 | 99.6% | 988.27216 | 988.1412 | 1 | 5.219 | 92.9% | 2 | K.LNQALCLR.Y | 2 |
| \* | Astrin\_STLCHLD\_tube2\_050114\_01.05100.05100.2 | 6.2636 | 0.3639 | 100.0% | 2148.652 | 2149.3652 | 1 | 7.476 | 65.6% | 1 | R.YKNEKELQEVIQQQNEK.I | 2 |
| \* | Astrin\_STLCHLD\_tube2\_050114\_01.05098.05098.3 | 6.2748 | 0.2843 | 100.0% | 2149.7344 | 2149.3652 | 1 | 5.996 | 51.6% | 3 | R.YKNEKELQEVIQQQNEK.I | 3 |
| \* | Astrin\_STLCHLD\_050114\_01.07406.07406.2 | 4.7687 | 0.1475 | 99.9% | 1487.2722 | 1486.622 | 1 | 5.91 | 86.4% | 14 | K.ELQEVIQQQNEK.I | 2 |
| \* | Astrin\_STLCHLD\_050114\_01.05869.05869.2 | 2.7162 | 0.0506 | 97.6% | 859.03217 | 859.01044 | 15 | 3.939 | 83.3% | 2 | K.ILEQIDK.S | 2 |
| \* | Astrin\_STLCHLD\_050114\_01.12037.12037.2 | 3.0219 | 0.3024 | 99.6% | 1714.2322 | 1715.0 | 1 | 6.32 | 57.1% | 1 | K.ILEQIDKSGELISLR.E | 2 |
| \* | Astrin\_STLCHLD\_tube2\_050114\_01.09000.09000.3 | 3.7176 | 0.3409 | 100.0% | 1714.9143 | 1715.0 | 4 | 6.429 | 42.9% | 2 | K.ILEQIDKSGELISLR.E | 3 |
| \* | Astrin\_STLCHLD\_tube2\_050114\_01.10703.10703.3 | 5.0916 | 0.3148 | 100.0% | 2681.9343 | 2681.0618 | 1 | 6.59 | 37.5% | 2 | K.ILEQIDKSGELISLREEVTHLTR.S | 3 |
| \* | Astrin\_STLCHLD\_tube2\_050114\_01.06852.06852.1 | 1.8653 | 0.1818 | 96.1% | 874.51 | 875.0128 | 4 | 4.375 | 71.4% | 1 | K.SGELISLR.E | 1 |
| \* | Astrin\_STLCHLD\_050114\_01.11435.11435.2 | 2.9641 | 0.1782 | 99.5% | 875.15216 | 875.0128 | 6 | 5.622 | 85.7% | 2 | K.SGELISLR.E | 2 |
| \* | Astrin\_STLCHLD\_tube2\_050114\_01.09280.09280.2 | 4.0663 | 0.4195 | 100.0% | 1840.6122 | 1841.0745 | 1 | 6.507 | 60.0% | 4 | K.SGELISLREEVTHLTR.S | 2 |
| \* | Astrin\_STLCHLD\_050114\_02.07917.07917.3 | 4.9271 | 0.4344 | 100.0% | 1840.7344 | 1841.0745 | 1 | 7.199 | 50.0% | 7 | K.SGELISLREEVTHLTR.S | 3 |
| \* | Astrin\_STLCHLD\_tube2\_050114\_01.08666.08666.2 | 3.2604 | 0.3524 | 100.0% | 1372.7922 | 1373.595 | 1 | 6.998 | 80.0% | 3 | K.VWLSQEVDKLR.V | 2 |
| \* | Astrin\_STLCHLD\_050114\_01.12266.12266.1 | 2.3236 | 0.186 | 100.0% | 897.65 | 898.16644 | 1 | 4.715 | 75.0% | 1 | R.VMFLEMK.N | 1 |
| \* | Astrin\_STLCHLD\_050114\_01.12262.12262.2 | 2.8071 | 0.2616 | 99.9% | 898.09216 | 898.16644 | 1 | 6.901 | 91.7% | 4 | R.VMFLEMK.N | 2 |
| \* | Astrin\_STLCHLD\_tube2\_050114\_01.07158.07158.2 | 2.6617 | 0.4016 | 99.9% | 1268.6522 | 1269.5598 | 1 | 6.24 | 72.2% | 2 | R.VMFLEMKNEK.E | 2 |
| \* | Astrin\_STLCHLD\_tube2\_050114\_01.06875.06875.1 | 2.5079 | 0.2754 | 100.0% | 1000.5 | 1001.1277 | 4 | 4.839 | 71.4% | 5 | R.NILEENLR.R | 1 |
| \* | Astrin\_STLCHLD\_tube2\_050114\_01.09328.09328.3 | 5.8334 | 0.4605 | 100.0% | 2231.3342 | 2230.526 | 1 | 8.389 | 51.5% | 3 | R.RSDKELEKLDDIVQHIYK.T | 3 |
| \* | Astrin\_STLCHLD\_tube2\_050114\_01.10311.10311.3 | 5.3002 | 0.3937 | 100.0% | 2073.9543 | 2074.3384 | 1 | 7.603 | 45.3% | 2 | R.SDKELEKLDDIVQHIYK.T | 3 |
| \* | Astrin\_STLCHLD\_tube2\_050114\_01.09691.09691.2 | 2.7641 | 0.1917 | 98.0% | 1744.0122 | 1743.9977 | 2 | 4.947 | 50.0% | 1 | K.ELEKLDDIVQHIYK.T | 2 |
| \* | Astrin\_STLCHLD\_050114\_01.12158.12158.3 | 3.0229 | 0.3747 | 100.0% | 1744.2544 | 1743.9977 | 2 | 6.048 | 44.2% | 1 | K.ELEKLDDIVQHIYK.T | 3 |
| \* | Astrin\_STLCHLD\_050114\_01.11473.11473.2 | 3.4178 | 0.3809 | 100.0% | 1244.3522 | 1244.4331 | 15 | 7.362 | 66.7% | 4 | K.LDDIVQHIYK.T | 2 |
| \* | Astrin\_STLCHLD\_050114\_01.11483.11483.3 | 2.5959 | 0.2643 | 99.3% | 1244.6943 | 1244.4331 | 60 | 4.98 | 44.4% | 1 | K.LDDIVQHIYK.T | 3 |
| \* | Astrin\_STLCHLD\_050114\_01.12307.12307.1 | 1.8279 | 0.2628 | 100.0% | 1126.71 | 1127.3696 | 7 | 5.51 | 55.6% | 1 | K.TLLSIPEVVR.G | 1 |
| \* | Astrin\_STLCHLD\_tube2\_050114\_01.10224.10224.2 | 2.6689 | 0.306 | 99.7% | 1127.0322 | 1127.3696 | 2 | 5.489 | 72.2% | 3 | K.TLLSIPEVVR.G | 2 |
| \* | Astrin\_STLCHLD\_tube2\_050114\_01.15567.15567.1 | 1.8318 | 0.286 | 100.0% | 1148.63 | 1149.3293 | 10 | 5.352 | 50.0% | 6 | K.ELQGLLEFLS.- | 1 |
| \* | Astrin\_STLCHLD\_tube2\_050114\_01.15586.15586.2 | 1.9953 | 0.2828 | 96.8% | 1148.8922 | 1149.3293 | 1 | 5.429 | 66.7% | 2 | K.ELQGLLEFLS.- | 2 |

---

|  |  |  |  |  |  |  |  |  |
| --- | --- | --- | --- | --- | --- | --- | --- | --- |
| U | *gi|21361322|ref|NP\_00* | 26 | 124 | 51.1% | 444 | 49586 | 4.9 | tubulin, beta 4 [Homo sapiens] |

| Filename XCorr DeltCN Conf% ObsM+H+ CalcM+H+ SpR ZScore Ion% # Sequence  | | | | | | | | | | | | |
| --- | --- | --- | --- | --- | --- | --- | --- | --- | --- | --- | --- | --- |
|  | Astrin\_STLCHLD\_050114\_02.08218.08218.3 | 6.3129 | 0.4919 | 100.0% | 3117.2344 | 3118.2996 | 1 | 8.572 | 35.6% | 3 | K.FWEVISDEHGIDPTGTYHGDSDLQLER.I | 33 |
| \* | Astrin\_STLCHLD\_050114\_02.06892.06892.3 | 2.6021 | 0.361 | 100.0% | 1909.8544 | 1910.9945 | 2 | 5.146 | 33.3% | 1 | R.INVYYNEAT#GGNYVPR.A | 3 |
|  | Astrin\_STLCHLD\_050114\_02.08076.08076.2 | 3.8623 | 0.4318 | 100.0% | 1603.4321 | 1602.8431 | 1 | 7.878 | 64.3% | 3 | R.AVLVDLEPGTMDSVR.S | 22 |
|  | Astrin\_STLCHLD\_tube2\_050114\_01.12338.12338.2 | 5.6846 | 0.5176 | 100.0% | 2798.8523 | 2800.0647 | 1 | 8.445 | 40.0% | 6 | R.SGPFGQIFRPDNFVFGQSGAGNNWAK.G | 222 |
|  | Astrin\_STLCHLD\_tube2\_050114\_01.12242.12242.3 | 6.768 | 0.5498 | 100.0% | 2799.6543 | 2800.0647 | 1 | 8.573 | 38.0% | 7 | R.SGPFGQIFRPDNFVFGQSGAGNNWAK.G | 333 |
|  | Astrin\_STLCHLD\_050114\_01.11995.11995.1 | 2.1993 | 0.2639 | 100.0% | 1319.69 | 1320.5896 | 245 | 5.053 | 40.9% | 3 | R.IMNTFSVVPSPK.V | 1111 |
|  | Astrin\_STLCHLD\_tube2\_050114\_01.08305.08305.2 | 4.34 | 0.4185 | 100.0% | 1320.4321 | 1320.5896 | 1 | 7.953 | 72.7% | 8 | R.IMNTFSVVPSPK.V | 2222 |
|  | Astrin\_STLCHLD\_050114\_01.12590.12590.3 | 4.1941 | 0.3268 | 100.0% | 2710.8843 | 2710.0405 | 1 | 6.714 | 30.2% | 1 | K.LTTPTYGDLNHLVSATMSGVTTCLR.F | 333 |
|  | Astrin\_STLCHLD\_tube2\_050114\_01.07179.07179.2 | 3.0568 | 0.2949 | 99.9% | 1131.2522 | 1131.2767 | 7 | 5.17 | 77.8% | 13 | R.FPGQLNADLR.K | 22222 |
|  | Astrin\_STLCHLD\_tube2\_050114\_01.05601.05601.2 | 2.7143 | 0.2756 | 99.3% | 1258.5521 | 1259.4508 | 11 | 5.358 | 65.0% | 13 | R.FPGQLNADLRK.L | 22222 |
|  | Astrin\_STLCHLD\_tube2\_050114\_01.05583.05583.3 | 3.152 | 0.3056 | 100.0% | 1259.8744 | 1259.4508 | 8 | 5.284 | 45.0% | 12 | R.FPGQLNADLRK.L | 33333 |
|  | Astrin\_STLCHLD\_tube2\_050114\_01.08365.08365.2 | 3.793 | 0.3681 | 100.0% | 1271.8722 | 1272.5945 | 1 | 7.682 | 80.0% | 5 | R.KLAVNMVPFPR.L | 22222 |
|  | Astrin\_STLCHLD\_tube2\_050114\_01.09803.09803.1 | 2.0853 | 0.2613 | 100.0% | 1143.58 | 1144.4204 | 13 | 6.348 | 61.1% | 1 | K.LAVNMVPFPR.L | 11111 |
|  | Astrin\_STLCHLD\_tube2\_050114\_01.09839.09839.2 | 3.9889 | 0.4725 | 100.0% | 1144.4122 | 1144.4204 | 1 | 8.55 | 94.4% | 3 | K.LAVNMVPFPR.L | 22222 |
|  | Astrin\_STLCHLD\_050114\_01.12456.12456.2 | 3.7154 | 0.3929 | 100.0% | 1621.7122 | 1621.9403 | 1 | 8.432 | 76.9% | 3 | R.LHFFMPGFAPLTSR.G | 2222 |
|  | Astrin\_STLCHLD\_050114\_01.12457.12457.3 | 4.2506 | 0.3686 | 100.0% | 1622.0643 | 1621.9403 | 1 | 6.108 | 53.8% | 4 | R.LHFFMPGFAPLTSR.G | 3333 |
|  | Astrin\_STLCHLD\_tube2\_050114\_01.12007.12007.2 | 4.2206 | 0.404 | 100.0% | 1693.3722 | 1692.9678 | 1 | 7.262 | 78.6% | 3 | R.ALTVPELTQQMFDAK.N | 222 |
|  | Astrin\_STLCHLD\_050114\_01.04952.04952.2 | 2.8672 | 0.3941 | 100.0% | 1066.2322 | 1066.2013 | 2 | 7.174 | 68.8% | 2 | K.NMMAACDPR.H | 22222 |
|  | Astrin\_STLCHLD\_050114\_02.06747.06747.3 | 4.3076 | 0.2581 | 100.0% | 1391.1244 | 1390.631 | 1 | 7.084 | 47.7% | 2 | R.HGRYLTVAAVFR.G | 333 |
|  | Astrin\_STLCHLD\_tube2\_050114\_01.10402.10402.1 | 1.7815 | 0.4511 | 100.0% | 1039.71 | 1040.2505 | 1 | 6.99 | 81.2% | 4 | R.YLTVAAVFR.G | 111 |
|  | Astrin\_STLCHLD\_tube2\_050114\_01.10269.10269.2 | 3.417 | 0.3601 | 100.0% | 1041.3322 | 1040.2505 | 1 | 6.884 | 87.5% | 9 | R.YLTVAAVFR.G | 222 |
|  | Astrin\_STLCHLD\_tube2\_050114\_01.11339.11339.2 | 3.5338 | 0.3838 | 100.0% | 1696.7722 | 1697.8877 | 1 | 7.601 | 57.7% | 2 | K.NSSYFVEWIPNNVK.T | 22222 |
|  | Astrin\_STLCHLD\_050114\_01.05029.05029.2 | 2.2588 | 0.224 | 97.9% | 1029.1921 | 1029.1473 | 9 | 4.44 | 62.5% | 1 | K.TAVCDIPPR.G | 222 |
|  | Astrin\_STLCHLD\_050114\_02.07650.07650.2 | 3.8098 | 0.3921 | 100.0% | 1386.7522 | 1386.6116 | 1 | 7.005 | 75.0% | 4 | K.RISEQFTAMFR.R | 2222 |
|  | Astrin\_STLCHLD\_tube2\_050114\_02.06787.06787.3 | 3.1948 | 0.3082 | 100.0% | 1543.3143 | 1542.7991 | 1 | 5.726 | 52.3% | 1 | K.RISEQFTAMFRR.K | 3333 |
|  | Astrin\_STLCHLD\_tube2\_050114\_02.08042.08042.2 | 4.0657 | 0.4262 | 100.0% | 1231.3121 | 1230.4241 | 1 | 6.725 | 94.4% | 10 | R.ISEQFTAMFR.R | 2222 |

Similarities:
gi|29788785|ref|NP\_82(22:4)  
gi|5174735|ref|NP\_006(25:1)  
gi|50592996|ref|NP\_00(14:12)  
gi|14210536|ref|NP\_11(10:16)  

---

|  |  |  |  |  |  |  |  |  |
| --- | --- | --- | --- | --- | --- | --- | --- | --- |
| U | *GFP* | 16 | 227 | 50.0% | 238 | 26813 | 5.8 | no description |

| Filename XCorr DeltCN Conf% ObsM+H+ CalcM+H+ SpR ZScore Ion% # Sequence  | | | | | | | | | | | | |
| --- | --- | --- | --- | --- | --- | --- | --- | --- | --- | --- | --- | --- |
| \* | Astrin\_STLCHLD\_tube2\_050114\_01.00077.00077.3 | 4.5694 | 0.4756 | 100.0% | 3922.7644 | 3924.2666 | 1 | 8.431 | 21.6% | 1 | K.GEELFTGVVPILVELDGDVNGHKFSVSGEGEGDATYGK.L | 3 |
| \* | Astrin\_STLCHLD\_tube2\_050114\_02.04556.04556.2 | 4.3666 | 0.5894 | 100.0% | 1504.3922 | 1504.5499 | 1 | 9.676 | 71.4% | 45 | K.FSVSGEGEGDATYGK.L | 2 |
| \* | Astrin\_STLCHLD\_050114\_02.04848.04848.3 | 2.775 | 0.2722 | 99.8% | 1593.2943 | 1593.7998 | 1 | 4.311 | 52.3% | 1 | R.YPDHMKQHDFFK.S | 3 |
| \* | Astrin\_STLCHLD\_tube2\_050114\_02.04526.04526.2 | 3.1874 | 0.4521 | 100.0% | 1267.1122 | 1267.399 | 1 | 7.704 | 80.0% | 58 | K.SAMPEGYVQER.T | 2 |
| \* | Astrin\_STLCHLD\_050114\_02.05606.05606.2 | 3.4397 | 0.4891 | 100.0% | 1348.3522 | 1348.4979 | 1 | 7.845 | 85.0% | 22 | R.TIFFKDDGNYK.T | 2 |
| \* | Astrin\_STLCHLD\_tube2\_050114\_02.04785.04785.3 | 2.6865 | 0.2446 | 97.6% | 1606.6444 | 1605.7905 | 1 | 4.649 | 41.7% | 3 | R.TIFFKDDGNYKTR.A | 3 |
| \* | Astrin\_STLCHLD\_050114\_02.05099.05099.3 | 3.0692 | 0.2348 | 99.2% | 1736.4543 | 1735.9376 | 106 | 4.237 | 32.1% | 1 | K.TRAEVKFEGDTLVNR.I | 3 |
| \* | Astrin\_STLCHLD\_050114\_02.05481.05481.2 | 4.2464 | 0.4888 | 100.0% | 1478.3322 | 1478.6451 | 1 | 8.587 | 75.0% | 8 | R.AEVKFEGDTLVNR.I | 2 |
| \* | Astrin\_STLCHLD\_050114\_02.05518.05518.3 | 3.0655 | 0.3424 | 100.0% | 1479.9844 | 1478.6451 | 2 | 5.668 | 41.7% | 8 | R.AEVKFEGDTLVNR.I | 3 |
| \* | Astrin\_STLCHLD\_050114\_01.06460.06460.1 | 1.7312 | 0.4474 | 100.0% | 1050.52 | 1051.1442 | 1 | 6.189 | 56.2% | 1 | K.FEGDTLVNR.I | 1 |
| \* | Astrin\_STLCHLD\_050114\_02.05006.05006.2 | 3.4221 | 0.4217 | 100.0% | 1051.2322 | 1051.1442 | 1 | 7.609 | 87.5% | 52 | K.FEGDTLVNR.I | 2 |
| \* | Astrin\_STLCHLD\_050114\_01.10953.10953.2 | 4.0302 | 0.3871 | 100.0% | 1543.4521 | 1543.7196 | 2 | 6.937 | 65.4% | 3 | K.GIDFKEDGNILGHK.L | 2 |
| \* | Astrin\_STLCHLD\_tube2\_050114\_02.05504.05504.3 | 4.3541 | 0.3373 | 100.0% | 1544.3644 | 1543.7196 | 12 | 5.868 | 42.3% | 13 | K.GIDFKEDGNILGHK.L | 3 |
| \* | Astrin\_STLCHLD\_tube2\_050114\_02.05732.05732.2 | 4.9532 | 0.502 | 100.0% | 1974.5721 | 1975.1829 | 1 | 9.76 | 70.0% | 2 | K.LEYNYNSHNVYIMADK.Q | 2 |
| \* | Astrin\_STLCHLD\_050114\_02.06298.06298.3 | 3.0052 | 0.2915 | 99.9% | 1974.8944 | 1975.1829 | 32 | 5.373 | 30.0% | 7 | K.LEYNYNSHNVYIMADK.Q | 3 |
| \* | Astrin\_STLCHLD\_050114\_02.05604.05604.3 | 4.4255 | 0.4436 | 100.0% | 2233.3442 | 2231.4875 | 19 | 6.853 | 32.4% | 2 | K.LEYNYNSHNVYIMADKQK.N | 3 |

---

|  |  |  |  |  |  |  |  |  |
| --- | --- | --- | --- | --- | --- | --- | --- | --- |
| U | *gi|17921989|ref|NP\_00* | 23 | 113 | 46.9% | 448 | 49924 | 5.1 | tubulin, alpha 4a [Homo sapiens] |

| Filename XCorr DeltCN Conf% ObsM+H+ CalcM+H+ SpR ZScore Ion% # Sequence  | | | | | | | | | | | | |
| --- | --- | --- | --- | --- | --- | --- | --- | --- | --- | --- | --- | --- |
| \* | Astrin\_STLCHLD\_tube2\_050114\_01.12483.12483.2 | 3.1905 | 0.287 | 99.7% | 1716.6921 | 1716.9719 | 1 | 6.486 | 75.0% | 1 | R.AVFVDLEPTVIDEIR.N | 2 |
|  | Astrin\_STLCHLD\_tube2\_050114\_01.08551.08551.2 | 1.7735 | 0.3588 | 96.6% | 1411.4922 | 1411.6439 | 213 | 5.569 | 59.1% | 1 | R.QLFHPEQLITGK.E | 222 |
|  | Astrin\_STLCHLD\_tube2\_050114\_01.07937.07937.3 | 3.8211 | 0.4011 | 100.0% | 2416.9443 | 2416.6555 | 1 | 6.467 | 31.2% | 3 | R.QLFHPEQLITGKEDAANNYAR.G | 333 |
|  | Astrin\_STLCHLD\_050114\_01.09799.09799.3 | 3.3823 | 0.333 | 100.0% | 1876.1943 | 1876.0824 | 2 | 5.44 | 41.1% | 2 | R.RNLDIERPTYTNLNR.L | 333 |
|  | Astrin\_STLCHLD\_050114\_01.11147.11147.2 | 3.4174 | 0.0864 | 98.0% | 1719.6322 | 1719.8949 | 1 | 5.134 | 61.5% | 2 | R.NLDIERPTYTNLNR.L | 222 |
|  | Astrin\_STLCHLD\_tube2\_050114\_01.06465.06465.3 | 2.9056 | 0.311 | 100.0% | 1721.3644 | 1719.8949 | 2 | 5.652 | 46.2% | 3 | R.NLDIERPTYTNLNR.L | 333 |
|  | Astrin\_STLCHLD\_050114\_01.13856.13856.1 | 3.7074 | 0.3194 | 100.0% | 1488.91 | 1488.7678 | 3 | 6.528 | 50.0% | 5 | R.LISQIVSSITASLR.F | 111 |
|  | Astrin\_STLCHLD\_tube2\_050114\_02.10976.10976.2 | 4.8703 | 0.4751 | 100.0% | 1490.2522 | 1488.7678 | 1 | 9.358 | 73.1% | 26 | R.LISQIVSSITASLR.F | 222 |
|  | Astrin\_STLCHLD\_tube2\_050114\_01.13991.13991.3 | 4.7196 | 0.3289 | 100.0% | 1490.3344 | 1488.7678 | 2 | 6.09 | 50.0% | 6 | R.LISQIVSSITASLR.F | 333 |
|  | Astrin\_STLCHLD\_tube2\_050114\_02.10115.10115.2 | 5.7616 | 0.571 | 100.0% | 2410.1921 | 2410.6885 | 1 | 10.015 | 52.5% | 20 | R.FDGALNVDLTEFQTNLVPYPR.I | 222 |
|  | Astrin\_STLCHLD\_tube2\_050114\_02.10073.10073.3 | 3.3301 | 0.2622 | 99.8% | 2413.4343 | 2410.6885 | 1 | 5.258 | 36.2% | 3 | R.FDGALNVDLTEFQTNLVPYPR.I | 333 |
|  | Astrin\_STLCHLD\_tube2\_050114\_01.10237.10237.2 | 4.4468 | 0.5586 | 100.0% | 1757.4722 | 1758.0703 | 1 | 9.549 | 76.7% | 8 | R.IHFPLATYAPVISAEK.A | 222 |
|  | Astrin\_STLCHLD\_050114\_01.12271.12271.3 | 4.3329 | 0.4459 | 100.0% | 1757.9644 | 1758.0703 | 1 | 7.63 | 51.7% | 9 | R.IHFPLATYAPVISAEK.A | 333 |
|  | Astrin\_STLCHLD\_050114\_02.08102.08102.3 | 4.2224 | 0.3505 | 100.0% | 2751.6543 | 2752.0369 | 5 | 6.013 | 27.2% | 1 | K.AYHEQLSVAEITNACFEPANQMVK.C | 33 |
|  | Astrin\_STLCHLD\_050114\_01.12021.12021.2 | 2.5571 | 0.4159 | 99.9% | 1250.3722 | 1250.4304 | 1 | 6.932 | 75.0% | 1 | K.YMACCLLYR.G | 222 |
|  | Astrin\_STLCHLD\_050114\_01.11948.11948.2 | 4.3001 | 0.3901 | 100.0% | 1825.6122 | 1826.1027 | 1 | 7.994 | 61.8% | 4 | K.VGINYQPPTVVPGGDLAK.V | 222 |
|  | Astrin\_STLCHLD\_050114\_02.09635.09635.2 | 4.2429 | 0.3899 | 100.0% | 1865.7122 | 1866.1084 | 1 | 8.618 | 56.2% | 1 | R.AVCMLSNTTAIAEAWAR.L | 22 |
|  | Astrin\_STLCHLD\_050114\_01.11149.11149.3 | 4.1633 | 0.3999 | 100.0% | 1381.6743 | 1381.6324 | 1 | 6.474 | 62.5% | 4 | R.LDHKFDLMYAK.R | 333 |
|  | Astrin\_STLCHLD\_050114\_01.09772.09772.3 | 2.6871 | 0.2432 | 98.1% | 1538.2444 | 1537.82 | 69 | 4.407 | 40.9% | 1 | R.LDHKFDLMYAKR.A | 333 |
|  | Astrin\_STLCHLD\_050114\_02.07700.07700.3 | 6.6327 | 0.4886 | 100.0% | 2487.6843 | 2487.7083 | 1 | 9.145 | 48.8% | 3 | K.RAFVHWYVGEGMEEGEFSEAR.E | 333 |
|  | Astrin\_STLCHLD\_050114\_02.08531.08531.2 | 5.3032 | 0.4677 | 100.0% | 2331.412 | 2331.5208 | 1 | 8.46 | 65.8% | 1 | R.AFVHWYVGEGMEEGEFSEAR.E | 222 |
|  | Astrin\_STLCHLD\_050114\_02.08576.08576.3 | 4.9127 | 0.279 | 100.0% | 2334.1743 | 2331.5208 | 1 | 4.983 | 43.4% | 6 | R.AFVHWYVGEGMEEGEFSEAR.E | 333 |
|  | Astrin\_STLCHLD\_tube2\_050114\_01.11503.11503.3 | 4.258 | 0.3976 | 100.0% | 3219.3843 | 3219.524 | 1 | 7.266 | 31.5% | 2 | R.AFVHWYVGEGMEEGEFSEAREDMAALEK.D | 333 |

Similarities:
gi|57013276|ref|NP\_00(22:1)  
gi|14389309|ref|NP\_11(20:3)  

---

|  |  |  |  |  |  |  |  |  |
| --- | --- | --- | --- | --- | --- | --- | --- | --- |
| U | *gi|57242777|ref|NP\_03* | 4 | 6 | 46.6% | 103 | 11967 | 5.9 | c-myc binding protein [Homo sapiens] |

| Filename XCorr DeltCN Conf% ObsM+H+ CalcM+H+ SpR ZScore Ion% # Sequence  | | | | | | | | | | | | |
| --- | --- | --- | --- | --- | --- | --- | --- | --- | --- | --- | --- | --- |
| \* | Astrin\_STLCHLD\_tube2\_050114\_01.11423.11423.3 | 4.7952 | 0.4795 | 100.0% | 2276.6343 | 2276.6348 | 1 | 7.923 | 39.5% | 1 | K.VLVALYEEPEKPNSALDFLK.H | 3 |
| \* | Astrin\_STLCHLD\_tube2\_050114\_01.07083.07083.2 | 4.1212 | 0.3245 | 100.0% | 1898.0122 | 1898.1289 | 1 | 6.14 | 62.5% | 1 | K.HHLGAATPENPEIELLR.L | 2 |
| \* | Astrin\_STLCHLD\_tube2\_050114\_01.07047.07047.3 | 3.9444 | 0.2725 | 100.0% | 1898.3043 | 1898.1289 | 15 | 4.577 | 39.1% | 1 | K.HHLGAATPENPEIELLR.L | 3 |
| \* | Astrin\_STLCHLD\_050114\_01.05491.05491.2 | 3.1578 | 0.2815 | 99.9% | 1333.0521 | 1332.4528 | 76 | 5.237 | 55.0% | 3 | K.LAQYEPPQEEK.R | 2 |

---

|  |  |  |  |  |  |  |  |  |
| --- | --- | --- | --- | --- | --- | --- | --- | --- |
| U | *gi|4505813|ref|NP\_003* | 5 | 9 | 46.1% | 89 | 10366 | 7.4 | dynein light chain 1 [Homo sapiens] |
| U | *gi|83267868|ref|NP\_00* | 5 | 9 | 46.1% | 89 | 10366 | 7.4 | dynein light chain 1 [Homo sapiens] |
| U | *gi|83267866|ref|NP\_00* | 5 | 9 | 46.1% | 89 | 10366 | 7.4 | dynein light chain 1 [Homo sapiens] |

| Filename XCorr DeltCN Conf% ObsM+H+ CalcM+H+ SpR ZScore Ion% # Sequence  | | | | | | | | | | | | |
| --- | --- | --- | --- | --- | --- | --- | --- | --- | --- | --- | --- | --- |
|  | Astrin\_STLCHLD\_tube2\_050114\_01.07313.07313.3 | 2.7371 | 0.2719 | 99.7% | 1415.8444 | 1415.6322 | 3 | 4.743 | 47.7% | 1 | K.YNIEKDIAAHIK.K | 3 |
|  | Astrin\_STLCHLD\_tube2\_050114\_01.07279.07279.2 | 3.1321 | 0.3495 | 99.9% | 1415.8922 | 1415.6322 | 1 | 6.407 | 68.2% | 1 | K.YNIEKDIAAHIK.K | 2 |
|  | Astrin\_STLCHLD\_050114\_01.06503.06503.2 | 3.232 | 0.3623 | 100.0% | 1283.2922 | 1283.383 | 8 | 6.356 | 60.0% | 3 | R.NFGSYVTHETK.H | 2 |
|  | Astrin\_STLCHLD\_050114\_01.16517.16517.3 | 3.9645 | 0.2972 | 100.0% | 3237.4443 | 3237.771 | 1 | 5.752 | 28.8% | 2 | R.NFGSYVTHETKHFIYFYLGQVAILLFK.S | 3 |
|  | Astrin\_STLCHLD\_050114\_02.13524.13524.3 | 3.8172 | 0.2234 | 99.7% | 3381.5942 | 3381.9011 | 1 | 4.851 | 25.9% | 2 | R.NFGSYVTHETKHFIYFYLGQVAILLFKSG.- | 3 |

---

|  |  |  |  |  |  |  |  |  |
| --- | --- | --- | --- | --- | --- | --- | --- | --- |
| U | *gi|4501885|ref|NP\_001* | 12 | 38 | 45.6% | 375 | 41737 | 5.5 | beta actin [Homo sapiens] |
| U | *gi|4501887|ref|NP\_001* | 12 | 38 | 45.6% | 375 | 41793 | 5.5 | actin, gamma 1 propeptide [Homo sapiens] |

| Filename XCorr DeltCN Conf% ObsM+H+ CalcM+H+ SpR ZScore Ion% # Sequence  | | | | | | | | | | | | |
| --- | --- | --- | --- | --- | --- | --- | --- | --- | --- | --- | --- | --- |
|  | Astrin\_STLCHLD\_tube2\_050114\_01.06957.06957.2 | 2.7548 | 0.3244 | 99.8% | 1199.3322 | 1199.4415 | 6 | 5.746 | 65.0% | 3 | R.AVFPSIVGRPR.H | 22 |
|  | Astrin\_STLCHLD\_tube2\_050114\_01.05846.05846.3 | 3.4498 | 0.3159 | 100.0% | 1515.5643 | 1516.7019 | 1 | 6.429 | 55.0% | 2 | K.IWHHTFYNELR.V | 33 |
|  | Astrin\_STLCHLD\_tube2\_050114\_01.05795.05795.2 | 3.3616 | 0.4634 | 100.0% | 1516.2722 | 1516.7019 | 1 | 7.494 | 80.0% | 2 | K.IWHHTFYNELR.V | 22 |
|  | Astrin\_STLCHLD\_tube2\_050114\_01.07740.07740.2 | 4.5586 | 0.3648 | 100.0% | 1954.7122 | 1955.2615 | 1 | 7.903 | 61.8% | 3 | R.VAPEEHPVLLTEAPLNPK.A | 2 |
|  | Astrin\_STLCHLD\_050114\_02.15382.15382.3 | 3.6962 | 0.1804 | 96.6% | 3257.0044 | 3255.8325 | 1 | 4.968 | 22.3% | 1 | K.MTQIMFETFNTPAMYVAIQAVLSLYASGR.T | 3 |
|  | Astrin\_STLCHLD\_tube2\_050114\_01.10740.10740.3 | 6.0122 | 0.462 | 100.0% | 3185.0942 | 3185.622 | 1 | 8.764 | 36.2% | 3 | R.TTGIVMDSGDGVTHTVPIYEGYALPHAILR.L | 3 |
|  | Astrin\_STLCHLD\_050114\_01.08528.08528.2 | 3.1329 | 0.5222 | 100.0% | 1132.9722 | 1133.2029 | 1 | 8.979 | 77.8% | 11 | R.GYSFTTTAER.E | 2 |
|  | Astrin\_STLCHLD\_tube2\_050114\_01.10092.10092.2 | 3.6111 | 0.3284 | 99.9% | 1790.9722 | 1791.9554 | 1 | 6.687 | 66.7% | 5 | K.SYELPDGQVITIGNER.F | 222 |
|  | Astrin\_STLCHLD\_tube2\_050114\_02.07358.07358.3 | 3.9522 | 0.3455 | 100.0% | 2343.7444 | 2344.6448 | 5 | 5.682 | 29.8% | 1 | R.KDLYANTVLSGGTTMYPGIADR.M | 3 |
|  | Astrin\_STLCHLD\_tube2\_050114\_02.08325.08325.2 | 3.3491 | 0.5511 | 100.0% | 2215.5322 | 2216.4705 | 1 | 8.465 | 47.5% | 1 | K.DLYANTVLSGGTTMYPGIADR.M | 2 |
|  | Astrin\_STLCHLD\_tube2\_050114\_01.06200.06200.2 | 2.6991 | 0.3752 | 99.9% | 1162.3722 | 1162.3868 | 1 | 6.418 | 75.0% | 5 | K.EITALAPSTMK.I | 22 |
|  | Astrin\_STLCHLD\_tube2\_050114\_01.04500.04500.3 | 2.417 | 0.3928 | 100.0% | 1517.7244 | 1517.595 | 1 | 6.263 | 43.8% | 1 | K.QEYDESGPSIVHR.K | 3 |

Similarities:
gi|4501881|ref|NP\_001(5:7)  
gi|63055057|ref|NP\_00(1:11)  

---

|  |  |  |  |  |  |  |  |  |
| --- | --- | --- | --- | --- | --- | --- | --- | --- |
| U | *gi|118582269|ref|NP\_0* | 7 | 15 | 45.3% | 201 | 22460 | 8.0 | splicing factor, arginine/serine-rich 1 isoform 2 [Homo sapiens] |
| U | *gi|5902076|ref|NP\_008* | 7 | 15 | 36.7% | 248 | 27745 | 10.4 | splicing factor, arginine/serine-rich 1 isoform 1 [Homo sapiens] |

| Filename XCorr DeltCN Conf% ObsM+H+ CalcM+H+ SpR ZScore Ion% # Sequence  | | | | | | | | | | | | |
| --- | --- | --- | --- | --- | --- | --- | --- | --- | --- | --- | --- | --- |
|  | Astrin\_STLCHLD\_050114\_01.11913.11913.2 | 3.0678 | 0.3651 | 99.9% | 1257.6322 | 1257.4752 | 1 | 7.451 | 85.0% | 6 | R.IYVGNLPPDIR.T | 2 |
|  | Astrin\_STLCHLD\_tube2\_050114\_01.07060.07060.2 | 2.9546 | 0.3798 | 100.0% | 1258.3922 | 1258.4137 | 2 | 6.165 | 72.2% | 2 | R.TKDIEDVFYK.Y | 2 |
|  | Astrin\_STLCHLD\_tube2\_050114\_01.12047.12047.2 | 2.8008 | 0.2283 | 98.7% | 2541.9922 | 2542.7234 | 1 | 4.647 | 38.6% | 1 | R.GGPPFAFVEFEDPRDAEDAVYGR.D | 2 |
|  | Astrin\_STLCHLD\_050114\_01.08651.08651.2 | 2.5253 | 0.3205 | 99.7% | 1123.9521 | 1124.1083 | 1 | 5.309 | 75.0% | 2 | R.DGYDYDGYR.L | 2 |
|  | Astrin\_STLCHLD\_050114\_01.10723.10723.2 | 2.3397 | 0.1688 | 98.1% | 917.2322 | 917.0989 | 3 | 4.406 | 83.3% | 1 | R.LRVEFPR.S | 22 |
|  | Astrin\_STLCHLD\_tube2\_050114\_01.06284.06284.2 | 2.5582 | 0.2258 | 98.5% | 1418.7922 | 1418.4688 | 1 | 4.303 | 68.2% | 2 | R.EAGDVCYADVYR.D | 2 |
|  | Astrin\_STLCHLD\_tube2\_050114\_02.07461.07461.3 | 3.5217 | 0.3421 | 100.0% | 2173.1943 | 2173.4485 | 1 | 5.329 | 36.1% | 1 | R.DGTGVVEFVRKEDMTYAVR.K | 3 |

Similarities:
gi|4506903|ref|NP\_003(1:6)  

---

|  |  |  |  |  |  |  |  |  |
| --- | --- | --- | --- | --- | --- | --- | --- | --- |
| U | *TEV-Speptide* | 5 | 124 | 45.1% | 51 | 5423 | 9.4 | no description |

| Filename XCorr DeltCN Conf% ObsM+H+ CalcM+H+ SpR ZScore Ion% # Sequence  | | | | | | | | | | | | |
| --- | --- | --- | --- | --- | --- | --- | --- | --- | --- | --- | --- | --- |
| \* | Astrin\_STLCHLD\_tube2\_050114\_02.00065.00065.2 | 4.3888 | 0.3797 | 100.0% | 1384.8121 | 1384.5345 | 1 | 7.387 | 68.2% | 59 | R.SRENLYFQGAAK.F | 2 |
| \* | Astrin\_STLCHLD\_050114\_01.05803.05803.3 | 3.9215 | 0.3863 | 100.0% | 1385.1244 | 1384.5345 | 1 | 6.95 | 52.3% | 53 | R.SRENLYFQGAAK.F | 3 |
| \* | Astrin\_STLCHLD\_050114\_02.05378.05378.2 | 3.0698 | 0.261 | 99.9% | 1140.6522 | 1141.2688 | 3 | 5.398 | 72.2% | 10 | R.ENLYFQGAAK.F | 2 |
| \* | Astrin\_STLCHLD\_050114\_02.04496.04496.2 | 2.85 | 0.3403 | 99.9% | 1299.1721 | 1298.4844 | 1 | 6.368 | 70.0% | 1 | K.FKETAAAKFER.Q | 2 |
| \* | Astrin\_STLCHLD\_050114\_01.04651.04651.3 | 3.5284 | 0.2032 | 100.0% | 1299.6244 | 1298.4844 | 1 | 5.632 | 55.0% | 1 | K.FKETAAAKFER.Q | 3 |

---

|  |  |  |  |  |  |  |  |  |
| --- | --- | --- | --- | --- | --- | --- | --- | --- |
| U | *gi|20127519|ref|NP\_03* | 44 | 122 | 44.7% | 747 | 85653 | 9.2 | TPX2, microtubule-associated protein homolog [Homo sapiens] |

| Filename XCorr DeltCN Conf% ObsM+H+ CalcM+H+ SpR ZScore Ion% # Sequence  | | | | | | | | | | | | |
| --- | --- | --- | --- | --- | --- | --- | --- | --- | --- | --- | --- | --- |
| \* | Astrin\_STLCHLD\_tube2\_050114\_01.07479.07479.3 | 4.2752 | 0.2365 | 100.0% | 2407.3743 | 2405.7996 | 1 | 5.939 | 41.2% | 1 | R.KANLQQAIVTPLKPVDNTYYK.E | 3 |
| \* | Astrin\_STLCHLD\_tube2\_050114\_01.08675.08675.3 | 3.7185 | 0.316 | 100.0% | 2278.1343 | 2277.6255 | 1 | 5.545 | 36.8% | 1 | K.ANLQQAIVTPLKPVDNTYYK.E | 3 |
| \* | Astrin\_STLCHLD\_050114\_01.07153.07153.2 | 3.5532 | 0.4055 | 100.0% | 1150.1721 | 1150.3534 | 1 | 7.323 | 81.2% | 9 | K.MQQEVVEMR.K | 2 |
| \* | Astrin\_STLCHLD\_tube2\_050114\_01.07642.07642.2 | 3.3083 | 0.4009 | 100.0% | 1067.2522 | 1067.317 | 1 | 8.242 | 65.0% | 3 | K.LALAGIGQPVK.K | 2 |
| \* | Astrin\_STLCHLD\_050114\_01.10574.10574.2 | 3.3019 | 0.3182 | 99.9% | 1195.4321 | 1195.4911 | 18 | 6.89 | 59.1% | 7 | K.LALAGIGQPVKK.S | 2 |
| \* | Astrin\_STLCHLD\_tube2\_050114\_01.05876.05876.2 | 1.853 | 0.2458 | 95.5% | 907.6922 | 908.0043 | 123 | 5.065 | 66.7% | 1 | K.SVDFHFR.T | 2 |
| \* | Astrin\_STLCHLD\_tube2\_050114\_01.07379.07379.2 | 5.1042 | 0.4608 | 100.0% | 1887.4521 | 1887.013 | 1 | 7.895 | 67.9% | 3 | K.NQEEYKEVNFTSELR.K | 2 |
| \* | Astrin\_STLCHLD\_tube2\_050114\_01.07389.07389.3 | 4.4329 | 0.3811 | 100.0% | 1887.6543 | 1887.013 | 7 | 6.215 | 39.3% | 6 | K.NQEEYKEVNFTSELR.K | 3 |
| \* | Astrin\_STLCHLD\_050114\_01.11039.11039.3 | 4.1704 | 0.4179 | 100.0% | 2015.5144 | 2015.187 | 1 | 6.159 | 38.3% | 1 | K.NQEEYKEVNFTSELRK.H | 3 |
| \* | Astrin\_STLCHLD\_tube2\_050114\_01.10518.10518.3 | 3.5177 | 0.3841 | 100.0% | 2611.2844 | 2611.8706 | 1 | 5.681 | 31.0% | 1 | R.TFDETVSTYVPLAQQVEDFHKR.T | 3 |
| \* | Astrin\_STLCHLD\_050114\_01.06237.06237.3 | 3.6722 | 0.1461 | 99.3% | 1359.8644 | 1358.5779 | 159 | 4.557 | 43.2% | 3 | R.SKKDDINLLPSK.S | 3 |
| \* | Astrin\_STLCHLD\_050114\_01.07163.07163.3 | 3.2821 | 0.1726 | 95.9% | 1941.0243 | 1941.146 | 11 | 3.994 | 37.5% | 1 | R.SKKDDINLLPSKSS\*VTK.I | 3 |
| \* | Astrin\_STLCHLD\_050114\_01.05060.05060.2 | 2.3717 | 0.188 | 96.9% | 1207.2122 | 1207.2828 | 1 | 5.109 | 77.8% | 1 | R.DPQT#PVLQTK.H | 2 |
| \* | Astrin\_STLCHLD\_050114\_01.10827.10827.2 | 3.7832 | 0.1322 | 99.5% | 1350.3522 | 1349.4344 | 1 | 4.493 | 81.8% | 5 | K.STAELEAEELEK.L | 2 |
| \* | Astrin\_STLCHLD\_tube2\_050114\_01.09154.09154.3 | 2.8802 | 0.3092 | 99.9% | 2010.2344 | 2010.2053 | 30 | 5.949 | 34.4% | 1 | K.STAELEAEELEKLQQYK.F | 3 |
| \* | Astrin\_STLCHLD\_tube2\_050114\_01.09145.09145.2 | 5.1968 | 0.3033 | 100.0% | 2010.3522 | 2010.2053 | 1 | 7.24 | 65.6% | 1 | K.STAELEAEELEKLQQYK.F | 2 |
| \* | Astrin\_STLCHLD\_tube2\_050114\_01.07461.07461.2 | 3.4002 | 0.3369 | 100.0% | 1037.3722 | 1037.2877 | 1 | 5.997 | 72.2% | 4 | R.ILEGGPILPK.K | 2 |
| \* | Astrin\_STLCHLD\_tube2\_050114\_01.08348.08348.3 | 3.5208 | 0.3298 | 100.0% | 2135.8145 | 2135.5083 | 1 | 6.405 | 33.3% | 4 | K.KPPVKPPTEPIGFDLEIEK.R | 3 |
| \* | Astrin\_STLCHLD\_050114\_01.11684.11684.3 | 5.2407 | 0.5365 | 100.0% | 2291.7544 | 2291.6958 | 1 | 8.875 | 39.5% | 2 | K.KPPVKPPTEPIGFDLEIEKR.I | 3 |
| \* | Astrin\_STLCHLD\_tube2\_050114\_01.07539.07539.2 | 3.1106 | 0.4139 | 100.0% | 1197.6921 | 1198.402 | 1 | 7.06 | 80.0% | 3 | K.ILEDVVGVPEK.K | 2 |
| \* | Astrin\_STLCHLD\_tube2\_050114\_01.06140.06140.2 | 3.8866 | 0.3407 | 100.0% | 1326.3322 | 1326.576 | 2 | 6.104 | 72.7% | 6 | K.ILEDVVGVPEKK.V | 2 |
| \* | Astrin\_STLCHLD\_050114\_01.10903.10903.3 | 3.2753 | 0.3767 | 100.0% | 1327.1943 | 1326.576 | 37 | 5.74 | 47.7% | 3 | K.ILEDVVGVPEKK.V | 3 |
| \* | Astrin\_STLCHLD\_tube2\_050114\_01.08911.08911.3 | 3.1289 | 0.2999 | 100.0% | 1790.6643 | 1790.1564 | 4 | 5.418 | 38.3% | 1 | K.KVLPITVPKS\*PAFALK.N | 3 |
| \* | Astrin\_STLCHLD\_tube2\_050114\_01.10252.10252.2 | 3.872 | 0.4272 | 100.0% | 1661.6122 | 1661.9823 | 1 | 7.698 | 67.9% | 3 | K.VLPITVPKS\*PAFALK.N | 2 |
| \* | Astrin\_STLCHLD\_tube2\_050114\_01.05788.05788.3 | 4.6899 | 0.2624 | 100.0% | 2158.4644 | 2158.4285 | 1 | 6.367 | 35.3% | 3 | R.IRMPTKEDEEEDEPVVIK.A | 3 |
| \* | Astrin\_STLCHLD\_050114\_01.09074.09074.3 | 5.4374 | 0.334 | 100.0% | 1888.7644 | 1889.0815 | 1 | 6.585 | 50.0% | 8 | R.MPTKEDEEEDEPVVIK.A | 3 |
| \* | Astrin\_STLCHLD\_050114\_01.08996.08996.2 | 5.0796 | 0.3675 | 100.0% | 1889.4722 | 1889.0815 | 1 | 7.088 | 73.3% | 7 | R.MPTKEDEEEDEPVVIK.A | 2 |
| \* | Astrin\_STLCHLD\_tube2\_050114\_01.07431.07431.3 | 2.8631 | 0.3989 | 100.0% | 2132.0044 | 2132.473 | 3 | 5.617 | 31.9% | 3 | K.AQPVPHYGVPFKPQIPEAR.T | 3 |
| \* | Astrin\_STLCHLD\_050114\_01.12322.12322.2 | 2.3235 | 0.2903 | 98.7% | 1458.6921 | 1458.5769 | 101 | 5.292 | 50.0% | 1 | R.TVEICPFSFDSR.D | 2 |
| \* | Astrin\_STLCHLD\_tube2\_050114\_01.10361.10361.2 | 3.4232 | 0.3986 | 100.0% | 1705.1721 | 1705.9945 | 1 | 7.85 | 67.9% | 1 | K.ALPLPHFDTINLPEK.K | 2 |
| \* | Astrin\_STLCHLD\_tube2\_050114\_01.09246.09246.2 | 3.2188 | 0.4602 | 100.0% | 1833.5922 | 1834.1686 | 3 | 7.08 | 46.7% | 1 | K.ALPLPHFDTINLPEKK.V | 2 |
| \* | Astrin\_STLCHLD\_050114\_01.11370.11370.2 | 3.249 | 0.2573 | 99.6% | 1683.3722 | 1683.9481 | 2 | 5.918 | 53.6% | 3 | K.ARPNTVISQEPFVPK.K | 2 |
| \* | Astrin\_STLCHLD\_050114\_01.11333.11333.3 | 3.8155 | 0.1787 | 99.8% | 1684.9143 | 1683.9481 | 1 | 5.598 | 44.6% | 4 | K.ARPNTVISQEPFVPK.K | 3 |
| \* | Astrin\_STLCHLD\_050114\_01.09548.09548.3 | 4.5148 | 0.3862 | 100.0% | 1812.2943 | 1812.1222 | 1 | 6.8 | 45.0% | 5 | K.ARPNTVISQEPFVPKK.E | 3 |
| \* | Astrin\_STLCHLD\_050114\_01.12111.12111.3 | 4.7169 | 0.3978 | 100.0% | 2475.4143 | 2475.8044 | 1 | 6.377 | 37.5% | 2 | K.KSVAEGLSGSLVQEPFQLATEKR.A | 3 |
| \* | Astrin\_STLCHLD\_tube2\_050114\_01.10427.10427.2 | 4.9567 | 0.4993 | 100.0% | 2347.0322 | 2347.6304 | 1 | 9.444 | 45.2% | 1 | K.SVAEGLSGSLVQEPFQLATEKR.A | 2 |
| \* | Astrin\_STLCHLD\_tube2\_050114\_01.10409.10409.3 | 3.6816 | 0.3816 | 100.0% | 2347.4644 | 2347.6304 | 1 | 6.81 | 29.8% | 1 | K.SVAEGLSGSLVQEPFQLATEKR.A | 3 |
| \* | Astrin\_STLCHLD\_tube2\_050114\_01.10978.10978.3 | 3.9127 | 0.5041 | 100.0% | 2427.3542 | 2427.6304 | 1 | 7.625 | 39.3% | 1 | K.SVAEGLSGS\*LVQEPFQLATEKR.A | 3 |
| \* | Astrin\_STLCHLD\_tube2\_050114\_01.05018.05018.2 | 5.2937 | 0.5472 | 100.0% | 1831.5322 | 1832.0386 | 1 | 9.62 | 73.3% | 1 | R.MAEVEAQKAQQLEEAR.L | 2 |
| \* | Astrin\_STLCHLD\_tube2\_050114\_01.05015.05015.3 | 5.1008 | 0.4613 | 100.0% | 1832.2144 | 1832.0386 | 1 | 8.668 | 48.3% | 1 | R.MAEVEAQKAQQLEEAR.L | 3 |
| \* | Astrin\_STLCHLD\_050114\_01.03613.03613.2 | 2.6639 | 0.1714 | 99.0% | 944.7322 | 945.02014 | 4 | 4.723 | 78.6% | 3 | K.AQQLEEAR.L | 2 |
| \* | Astrin\_STLCHLD\_050114\_01.04229.04229.2 | 4.844 | 0.2711 | 100.0% | 1630.6322 | 1630.7954 | 1 | 7.581 | 75.0% | 1 | R.LQEEEQKKEELAR.L | 2 |
| \* | Astrin\_STLCHLD\_050114\_01.04216.04216.3 | 3.2147 | 0.2639 | 100.0% | 1630.9143 | 1630.7954 | 1 | 5.53 | 47.9% | 1 | R.LQEEEQKKEELAR.L | 3 |
| \* | Astrin\_STLCHLD\_tube2\_050114\_01.06617.06617.2 | 3.6328 | 0.4166 | 100.0% | 1436.4122 | 1435.5309 | 1 | 6.327 | 79.2% | 3 | K.SSDQPLTVPVS\*PK.F | 2 |

---

|  |  |  |  |  |  |  |  |  |
| --- | --- | --- | --- | --- | --- | --- | --- | --- |
| U | *gi|183076548|ref|NP\_0* | 4 | 6 | 43.4% | 136 | 15388 | 11.3 | histone cluster 2, H3d [Homo sapiens] |
| U | *gi|53793688|ref|NP\_00* | 4 | 6 | 43.4% | 136 | 15388 | 11.3 | histone cluster 2, H3a [Homo sapiens] |
| U | *gi|31742503|ref|NP\_06* | 4 | 6 | 43.4% | 136 | 15388 | 11.3 | histone cluster 2, H3c [Homo sapiens] |

| Filename XCorr DeltCN Conf% ObsM+H+ CalcM+H+ SpR ZScore Ion% # Sequence  | | | | | | | | | | | | |
| --- | --- | --- | --- | --- | --- | --- | --- | --- | --- | --- | --- | --- |
|  | Astrin\_STLCHLD\_050114\_01.04999.04999.2 | 2.293 | 0.2425 | 98.6% | 1033.4521 | 1033.2186 | 36 | 4.599 | 62.5% | 2 | R.YRPGTVALR.E | 2 |
|  | Astrin\_STLCHLD\_050114\_01.09625.09625.2 | 2.0376 | 0.195 | 96.3% | 831.9922 | 831.9878 | 3 | 4.613 | 83.3% | 1 | K.STELLIR.K | 2 |
|  | Astrin\_STLCHLD\_tube2\_050114\_01.06003.06003.2 | 2.9554 | 0.3691 | 99.9% | 1336.3522 | 1336.4875 | 1 | 6.553 | 75.0% | 2 | R.EIAQDFKTDLR.F | 2 |
|  | Astrin\_STLCHLD\_tube2\_050114\_01.16857.16857.3 | 3.4445 | 0.3219 | 100.0% | 3516.1143 | 3515.9258 | 2 | 4.604 | 21.0% | 1 | R.FQSSAVMALQEASEAYLVGLFEDTNLCAIHAK.R | 3 |

---

|  |  |  |  |  |  |  |  |  |
| --- | --- | --- | --- | --- | --- | --- | --- | --- |
| U | *gi|62414289|ref|NP\_00* | 17 | 25 | 42.7% | 466 | 53652 | 5.1 | vimentin [Homo sapiens] |

| Filename XCorr DeltCN Conf% ObsM+H+ CalcM+H+ SpR ZScore Ion% # Sequence  | | | | | | | | | | | | |
| --- | --- | --- | --- | --- | --- | --- | --- | --- | --- | --- | --- | --- |
| \* | Astrin\_STLCHLD\_tube2\_050114\_01.06697.06697.2 | 3.9865 | 0.3548 | 100.0% | 1509.4321 | 1509.5724 | 1 | 6.371 | 61.5% | 1 | R.SLYASS\*PGGVYATR.S | 2 |
|  | Astrin\_STLCHLD\_tube2\_050114\_01.05117.05117.2 | 4.1023 | 0.4856 | 100.0% | 1588.2722 | 1588.7147 | 1 | 7.801 | 79.2% | 1 | R.TNEKVELQELNDR.F | 2 |
|  | Astrin\_STLCHLD\_tube2\_050114\_01.05114.05114.3 | 3.677 | 0.3006 | 100.0% | 1590.0543 | 1588.7147 | 1 | 5.749 | 47.9% | 1 | R.TNEKVELQELNDR.F | 3 |
| \* | Astrin\_STLCHLD\_050114\_01.07387.07387.2 | 2.1513 | 0.285 | 99.0% | 871.2922 | 870.9805 | 3 | 4.865 | 91.7% | 1 | R.FANYIDK.V | 2 |
| \* | Astrin\_STLCHLD\_tube2\_050114\_01.05457.05457.2 | 2.9656 | 0.3507 | 99.9% | 1125.3121 | 1126.3005 | 2 | 6.675 | 75.0% | 1 | R.FANYIDKVR.F | 2 |
| \* | Astrin\_STLCHLD\_tube2\_050114\_01.09109.09109.2 | 2.4959 | 0.3315 | 99.2% | 1539.4922 | 1540.8436 | 32 | 5.362 | 38.5% | 1 | K.ILLAELEQLKGQGK.S | 2 |
| \* | Astrin\_STLCHLD\_tube2\_050114\_01.07191.07191.2 | 3.1255 | 0.4614 | 100.0% | 1254.5122 | 1255.385 | 1 | 8.452 | 77.8% | 2 | R.LGDLYEEEMR.E | 2 |
| \* | Astrin\_STLCHLD\_tube2\_050114\_01.08030.08030.2 | 3.2495 | 0.2607 | 99.7% | 1690.4321 | 1689.881 | 1 | 5.238 | 53.8% | 1 | R.VEVERDNLAEDIMR.L | 2 |
| \* | Astrin\_STLCHLD\_tube2\_050114\_02.07094.07094.3 | 3.7256 | 0.2547 | 100.0% | 2353.4644 | 2352.581 | 2 | 5.007 | 34.7% | 1 | K.LQEEMLQREEAENTLQSFR.Q | 3 |
|  | Astrin\_STLCHLD\_tube2\_050114\_01.07782.07782.2 | 3.1061 | 0.2515 | 99.9% | 1311.0322 | 1310.4056 | 2 | 4.977 | 77.8% | 1 | K.NLQEAEEWYK.S | 2 |
| \* | Astrin\_STLCHLD\_tube2\_050114\_01.05064.05064.2 | 3.4768 | 0.4107 | 100.0% | 1095.2922 | 1094.1692 | 1 | 7.784 | 77.8% | 4 | K.FADLSEAANR.N | 2 |
| \* | Astrin\_STLCHLD\_050114\_02.08058.08058.2 | 6.0084 | 0.5746 | 100.0% | 2187.8323 | 2188.33 | 1 | 11.216 | 66.7% | 2 | R.EMEENFAVEAANYQDTIGR.L | 2 |
| \* | Astrin\_STLCHLD\_tube2\_050114\_01.06983.06983.2 | 4.6638 | 0.5108 | 100.0% | 1734.6122 | 1735.9679 | 1 | 8.873 | 76.9% | 2 | R.LQDEIQNMKEEMAR.H | 2 |
|  | Astrin\_STLCHLD\_tube2\_050114\_01.06643.06643.3 | 3.3387 | 0.198 | 99.8% | 1529.2144 | 1528.7513 | 1 | 4.263 | 54.5% | 1 | R.HLREYQDLLNVK.M | 3 |
|  | Astrin\_STLCHLD\_050114\_02.08578.08578.2 | 3.9851 | 0.4267 | 100.0% | 1296.1921 | 1296.5243 | 1 | 9.369 | 85.0% | 2 | K.MALDIEIATYR.K | 2 |
| \* | Astrin\_STLCHLD\_tube2\_050114\_01.13125.13125.2 | 2.2562 | 0.3078 | 98.3% | 1571.7722 | 1571.8601 | 3 | 5.488 | 57.7% | 1 | R.ISLPLPNFSSLNLR.E | 2 |
| \* | Astrin\_STLCHLD\_050114\_01.06021.06021.2 | 3.8523 | 0.4076 | 100.0% | 1837.2922 | 1837.854 | 1 | 7.163 | 56.7% | 2 | R.DGQVINETSQHHDDLE.- | 2 |

---

|  |  |  |  |  |  |  |  |  |
| --- | --- | --- | --- | --- | --- | --- | --- | --- |
| U | *gi|224028244|ref|NP\_0* | 21 | 52 | 42.3% | 471 | 54232 | 8.9 | non-POU domain containing, octamer-binding isoform 1 [Homo sapiens] |
| U | *gi|34932414|ref|NP\_03* | 21 | 52 | 42.3% | 471 | 54232 | 8.9 | non-POU domain containing, octamer-binding isoform 1 [Homo sapiens] |
| U | *gi|224028246|ref|NP\_0* | 21 | 52 | 42.3% | 471 | 54232 | 8.9 | non-POU domain containing, octamer-binding isoform 1 [Homo sapiens] |

| Filename XCorr DeltCN Conf% ObsM+H+ CalcM+H+ SpR ZScore Ion% # Sequence  | | | | | | | | | | | | |
| --- | --- | --- | --- | --- | --- | --- | --- | --- | --- | --- | --- | --- |
|  | Astrin\_STLCHLD\_tube2\_050114\_01.10753.10753.2 | 3.4296 | 0.26 | 99.8% | 1861.4722 | 1861.12 | 3 | 5.557 | 53.3% | 3 | R.LFVGNLPPDITEEEMR.K | 2 |
|  | Astrin\_STLCHLD\_tube2\_050114\_01.11539.11539.2 | 5.1585 | 0.5425 | 100.0% | 1813.0122 | 1814.1504 | 1 | 9.765 | 73.3% | 1 | R.TLAEIAKVELDNMPLR.G | 2 |
|  | Astrin\_STLCHLD\_tube2\_050114\_01.11529.11529.3 | 4.2254 | 0.4053 | 100.0% | 1814.2743 | 1814.1504 | 1 | 6.705 | 48.3% | 1 | R.TLAEIAKVELDNMPLR.G | 3 |
|  | Astrin\_STLCHLD\_tube2\_050114\_01.10397.10397.3 | 3.4973 | 0.3719 | 100.0% | 1999.8544 | 1999.3765 | 1 | 5.755 | 35.3% | 1 | R.TLAEIAKVELDNMPLRGK.Q | 3 |
|  | Astrin\_STLCHLD\_tube2\_050114\_01.07449.07449.2 | 3.0057 | 0.2635 | 99.9% | 1087.2722 | 1087.2793 | 73 | 5.381 | 68.8% | 2 | K.VELDNMPLR.G | 2 |
|  | Astrin\_STLCHLD\_tube2\_050114\_01.05652.05652.2 | 2.7312 | 0.1537 | 98.0% | 1271.8922 | 1272.5052 | 1 | 4.174 | 80.0% | 1 | K.VELDNMPLRGK.Q | 2 |
|  | Astrin\_STLCHLD\_050114\_01.05543.05543.3 | 2.9639 | 0.2589 | 99.9% | 1250.0044 | 1249.3782 | 13 | 5.138 | 42.5% | 1 | R.FACHSASLTVR.N | 3 |
|  | Astrin\_STLCHLD\_050114\_01.16989.16989.3 | 4.1799 | 0.3893 | 100.0% | 2668.7944 | 2669.9507 | 1 | 6.852 | 35.2% | 2 | R.NLPQYVSNELLEEAFSVFGQVER.A | 3 |
|  | Astrin\_STLCHLD\_tube2\_050114\_02.13654.13654.2 | 5.2511 | 0.5746 | 100.0% | 2669.8123 | 2669.9507 | 1 | 11.883 | 54.5% | 9 | R.NLPQYVSNELLEEAFSVFGQVER.A | 2 |
|  | Astrin\_STLCHLD\_050114\_01.05576.05576.1 | 2.1052 | 0.2457 | 100.0% | 886.5 | 887.0238 | 27 | 6.277 | 64.3% | 2 | R.AVVIVDDR.G | 11 |
|  | Astrin\_STLCHLD\_050114\_01.05475.05475.2 | 2.2217 | 0.2079 | 97.7% | 887.09216 | 887.0238 | 1 | 7.125 | 92.9% | 3 | R.AVVIVDDR.G | 22 |
|  | Astrin\_STLCHLD\_050114\_01.07069.07069.2 | 2.634 | 0.3075 | 99.3% | 1232.1122 | 1232.4252 | 2 | 6.71 | 59.1% | 3 | K.GIVEFSGKPAAR.K | 2 |
|  | Astrin\_STLCHLD\_tube2\_050114\_01.08952.08952.2 | 3.9668 | 0.592 | 100.0% | 1696.4521 | 1696.8744 | 1 | 9.255 | 76.9% | 5 | R.FAQPGSFEYEYAMR.W | 2 |
|  | Astrin\_STLCHLD\_tube2\_050114\_01.05164.05164.3 | 2.7696 | 0.3303 | 100.0% | 1832.2144 | 1832.0386 | 1 | 4.799 | 35.7% | 1 | K.ALIEMEKQQQDQVDR.N | 3 |
|  | Astrin\_STLCHLD\_tube2\_050114\_01.05237.05237.2 | 3.1846 | 0.4268 | 100.0% | 1337.3322 | 1337.5488 | 1 | 7.137 | 80.0% | 3 | R.EKLEMEMEAAR.H | 2 |
|  | Astrin\_STLCHLD\_tube2\_050114\_02.06202.06202.3 | 4.2419 | 0.2807 | 100.0% | 2499.3245 | 2499.942 | 1 | 5.713 | 32.9% | 1 | R.EKLEMEMEAARHEHQVMLMR.Q | 3 |
|  | Astrin\_STLCHLD\_050114\_01.04185.04185.3 | 3.651 | 0.1176 | 96.9% | 1697.8143 | 1697.9097 | 1 | 4.722 | 54.2% | 1 | R.RMEELHNQEVQKR.K | 3 |
|  | Astrin\_STLCHLD\_050114\_02.06501.06501.3 | 3.353 | 0.3897 | 100.0% | 1539.5343 | 1539.8441 | 2 | 5.802 | 42.9% | 1 | R.MGQMAMGGAMGINNR.G | 3 |
|  | Astrin\_STLCHLD\_050114\_01.11546.11546.2 | 4.4353 | 0.4863 | 100.0% | 1539.9521 | 1539.8441 | 1 | 7.829 | 71.4% | 8 | R.MGQMAMGGAMGINNR.G | 2 |
|  | Astrin\_STLCHLD\_050114\_02.08475.08475.3 | 3.4062 | 0.2912 | 100.0% | 2244.7444 | 2244.4436 | 423 | 5.201 | 27.4% | 2 | R.FGQAATMEGIGAIGGT#PPAFNR.A | 3 |
|  | Astrin\_STLCHLD\_050114\_01.04708.04708.2 | 2.2463 | 0.3887 | 99.3% | 1230.2922 | 1229.3811 | 1 | 6.04 | 68.2% | 1 | R.AAPGAEFAPNKR.R | 2 |

Similarities:
gi|4826998|ref|NP\_005(2:19)  

---

|  |  |  |  |  |  |  |  |  |
| --- | --- | --- | --- | --- | --- | --- | --- | --- |
| U | *gi|5174457|ref|NP\_006* | 33 | 79 | 42.2% | 642 | 73913 | 5.6 | kinetochore associated 2 [Homo sapiens] |

| Filename XCorr DeltCN Conf% ObsM+H+ CalcM+H+ SpR ZScore Ion% # Sequence  | | | | | | | | | | | | |
| --- | --- | --- | --- | --- | --- | --- | --- | --- | --- | --- | --- | --- |
| \* | Astrin\_STLCHLD\_tube2\_050114\_02.05643.05643.3 | 3.333 | 0.2712 | 100.0% | 1803.2043 | 1802.914 | 1 | 5.096 | 39.1% | 1 | R.SSVSSGGAGRLS\*MQELR.S | 3 |
| \* | Astrin\_STLCHLD\_050114\_01.04528.04528.2 | 2.4 | 0.2319 | 98.5% | 1145.5122 | 1145.3011 | 13 | 4.803 | 61.1% | 1 | K.LSINKPTSER.K | 2 |
| \* | Astrin\_STLCHLD\_tube2\_050114\_01.07438.07438.2 | 2.7906 | 0.2227 | 99.0% | 1297.1122 | 1297.4075 | 2 | 5.97 | 68.2% | 1 | R.NSQLGIFSSSEK.I | 2 |
| \* | Astrin\_STLCHLD\_050114\_01.05834.05834.2 | 2.2876 | 0.3155 | 99.6% | 906.4122 | 905.9829 | 1 | 4.853 | 83.3% | 2 | K.FEEEVPR.I | 2 |
| \* | Astrin\_STLCHLD\_tube2\_050114\_01.10071.10071.2 | 3.8044 | 0.3476 | 100.0% | 1499.7522 | 1499.793 | 2 | 6.477 | 66.7% | 1 | R.IFKDLGYPFALSK.S | 2 |
| \* | Astrin\_STLCHLD\_tube2\_050114\_01.10152.10152.2 | 2.0787 | 0.2332 | 97.1% | 1013.3522 | 1013.2218 | 10 | 4.968 | 78.6% | 1 | K.LFLDYTIK.C | 2 |
| \* | Astrin\_STLCHLD\_tube2\_050114\_01.11104.11104.3 | 5.319 | 0.5269 | 100.0% | 2081.1543 | 2081.4167 | 1 | 8.552 | 51.5% | 1 | K.LKDLFNVDAFKLESLEAK.N | 3 |
| \* | Astrin\_STLCHLD\_tube2\_050114\_01.12083.12083.2 | 3.1105 | 0.2985 | 99.7% | 1840.5721 | 1840.0831 | 1 | 6.124 | 50.0% | 1 | K.DLFNVDAFKLESLEAK.N | 2 |
| \* | Astrin\_STLCHLD\_tube2\_050114\_01.07991.07991.2 | 5.5815 | 0.506 | 100.0% | 2098.5122 | 2099.3228 | 1 | 9.397 | 64.7% | 2 | K.YQAYMSNLESHSAILDQK.L | 2 |
| \* | Astrin\_STLCHLD\_050114\_02.07010.07010.3 | 3.4858 | 0.4813 | 100.0% | 2099.5444 | 2099.3228 | 3 | 7.011 | 33.8% | 4 | K.YQAYMSNLESHSAILDQK.L | 3 |
| \* | Astrin\_STLCHLD\_050114\_01.09668.09668.2 | 3.214 | 0.3057 | 99.9% | 1129.1921 | 1129.2584 | 1 | 6.488 | 77.8% | 4 | K.LNGLNEEIAR.V | 2 |
| \* | Astrin\_STLCHLD\_050114\_01.05942.05942.2 | 3.0723 | 0.1572 | 99.3% | 1086.1522 | 1086.2334 | 41 | 4.611 | 68.8% | 4 | R.LQNIIDNQK.Y | 2 |
| \* | Astrin\_STLCHLD\_tube2\_050114\_01.07915.07915.3 | 3.5864 | 0.3533 | 100.0% | 2021.2144 | 2020.2499 | 1 | 5.623 | 39.1% | 1 | R.LQNIIDNQKYSVADIER.I | 3 |
| \* | Astrin\_STLCHLD\_050114\_01.10153.10153.2 | 2.7647 | 0.4499 | 100.0% | 952.4922 | 953.03973 | 1 | 7.426 | 85.7% | 6 | K.YSVADIER.I | 2 |
| \* | Astrin\_STLCHLD\_050114\_01.04592.04592.3 | 4.565 | 0.3836 | 100.0% | 1738.1943 | 1737.9132 | 1 | 5.965 | 51.9% | 1 | R.INHERNELQQTINK.L | 3 |
| \* | Astrin\_STLCHLD\_050114\_01.05066.05066.2 | 2.9083 | 0.1383 | 98.9% | 1088.2322 | 1088.2058 | 1 | 5.085 | 81.2% | 1 | R.NELQQTINK.L | 2 |
| \* | Astrin\_STLCHLD\_tube2\_050114\_01.04944.04944.2 | 4.1119 | 0.4631 | 100.0% | 1617.5122 | 1617.7991 | 2 | 8.165 | 61.5% | 1 | R.GKEAIETQLAEYHK.L | 2 |
| \* | Astrin\_STLCHLD\_050114\_01.08678.08678.3 | 4.3225 | 0.2879 | 100.0% | 1617.9844 | 1617.7991 | 4 | 6.95 | 42.3% | 6 | R.GKEAIETQLAEYHK.L | 3 |
| \* | Astrin\_STLCHLD\_050114\_01.11235.11235.2 | 2.1734 | 0.26 | 96.8% | 1320.2722 | 1320.454 | 1 | 4.664 | 77.3% | 1 | K.FNPEAGANCLVK.Y | 2 |
| \* | Astrin\_STLCHLD\_tube2\_050114\_01.13032.13032.3 | 4.3494 | 0.4562 | 100.0% | 2360.3342 | 2360.6665 | 1 | 7.283 | 39.5% | 2 | R.AQVYVPLKELLNETEEEINK.A | 3 |
| \* | Astrin\_STLCHLD\_050114\_01.12524.12524.2 | 5.0362 | 0.4266 | 100.0% | 2360.4922 | 2360.6665 | 1 | 7.881 | 57.9% | 2 | R.AQVYVPLKELLNETEEEINK.A | 2 |
| \* | Astrin\_STLCHLD\_tube2\_050114\_01.06798.06798.2 | 3.581 | 0.3485 | 100.0% | 1461.3522 | 1461.5658 | 1 | 5.914 | 81.8% | 2 | K.ELLNETEEEINK.A | 2 |
| \* | Astrin\_STLCHLD\_050114\_01.15012.15012.2 | 4.8879 | 0.4708 | 100.0% | 2024.8121 | 2024.3088 | 1 | 8.436 | 64.7% | 2 | K.MGLEDTLEQLNAMITESK.R | 2 |
| \* | Astrin\_STLCHLD\_tube2\_050114\_01.14125.14125.2 | 5.0525 | 0.5754 | 100.0% | 2179.7922 | 2180.4963 | 1 | 9.591 | 63.9% | 1 | K.MGLEDTLEQLNAMITESKR.S | 2 |
| \* | Astrin\_STLCHLD\_050114\_01.13696.13696.3 | 4.498 | 0.4601 | 100.0% | 2180.9944 | 2180.4963 | 1 | 8.324 | 41.7% | 3 | K.MGLEDTLEQLNAMITESKR.S | 3 |
| \* | Astrin\_STLCHLD\_tube2\_050114\_01.08922.08922.2 | 4.6593 | 0.4127 | 100.0% | 1979.6721 | 1979.2377 | 1 | 8.128 | 63.3% | 1 | R.TLKEEVQKLDDLYQQK.I | 2 |
| \* | Astrin\_STLCHLD\_tube2\_050114\_01.08913.08913.3 | 4.2522 | 0.3087 | 100.0% | 1980.0543 | 1979.2377 | 1 | 6.525 | 43.3% | 1 | R.TLKEEVQKLDDLYQQK.I | 3 |
| \* | Astrin\_STLCHLD\_050114\_01.05660.05660.2 | 2.7565 | 0.33 | 99.9% | 1023.4322 | 1023.1308 | 1 | 5.677 | 85.7% | 1 | K.LDDLYQQK.I | 2 |
| \* | Astrin\_STLCHLD\_tube2\_050114\_02.06003.06003.2 | 4.6226 | 0.4061 | 100.0% | 1597.4922 | 1596.7344 | 1 | 8.874 | 79.2% | 12 | R.EYQLVVQTTTEER.R | 2 |
| \* | Astrin\_STLCHLD\_050114\_02.05789.05789.2 | 2.9395 | 0.22 | 99.0% | 1753.0521 | 1752.9219 | 7 | 5.281 | 46.2% | 2 | R.EYQLVVQTTTEERR.K | 2 |
| \* | Astrin\_STLCHLD\_tube2\_050114\_01.07229.07229.2 | 4.3027 | 0.4147 | 100.0% | 1513.3322 | 1513.7925 | 1 | 7.513 | 76.9% | 5 | R.LLEMVATHVGSVEK.H | 2 |
| \* | Astrin\_STLCHLD\_tube2\_050114\_02.06402.06402.3 | 3.6309 | 0.38 | 100.0% | 1514.6044 | 1513.7925 | 1 | 6.536 | 44.2% | 4 | R.LLEMVATHVGSVEK.H | 3 |
| \* | Astrin\_STLCHLD\_050114\_02.06885.06885.3 | 3.3791 | 0.3574 | 100.0% | 2245.9744 | 2247.3584 | 1 | 5.933 | 45.6% | 1 | K.VDREYEECMSEDLSENIK.E | 3 |

---

|  |  |  |  |  |  |  |  |  |
| --- | --- | --- | --- | --- | --- | --- | --- | --- |
| U | *gi|10800138|ref|NP\_06* | 5 | 16 | 41.3% | 126 | 13936 | 10.3 | histone cluster 1, H2bd [Homo sapiens] |
| U | *gi|66912162|ref|NP\_00* | 5 | 16 | 41.3% | 126 | 13920 | 10.3 | histone cluster 2, H2bf [Homo sapiens] |
| U | *gi|4504271|ref|NP\_003* | 5 | 16 | 41.3% | 126 | 13906 | 10.3 | histone cluster 1, H2bi [Homo sapiens] |
| U | *gi|4504269|ref|NP\_003* | 5 | 16 | 41.3% | 126 | 13892 | 10.3 | histone cluster 1, H2bh [Homo sapiens] |
| U | *gi|4504265|ref|NP\_003* | 5 | 16 | 41.3% | 126 | 13906 | 10.3 | histone cluster 1, H2bf [Homo sapiens] |
| U | *gi|4504263|ref|NP\_003* | 5 | 16 | 41.3% | 126 | 13989 | 10.3 | histone cluster 1, H2bm [Homo sapiens] |
| U | *gi|4504261|ref|NP\_003* | 5 | 16 | 41.3% | 126 | 13922 | 10.3 | histone cluster 1, H2bn [Homo sapiens] |
| U | *gi|4504257|ref|NP\_003* | 5 | 16 | 41.3% | 126 | 13906 | 10.3 | histone cluster 1, H2bg [Homo sapiens] |
| U | *gi|21396484|ref|NP\_00* | 5 | 16 | 41.3% | 126 | 13906 | 10.3 | histone cluster 1, H2be [Homo sapiens] |
| U | *gi|21166389|ref|NP\_00* | 5 | 16 | 41.3% | 126 | 13906 | 10.3 | histone cluster 1, H2bc [Homo sapiens] |
| U | *gi|20336752|ref|NP\_61* | 5 | 16 | 41.3% | 126 | 13936 | 10.3 | histone cluster 1, H2bd [Homo sapiens] |
| U | *gi|18105048|ref|NP\_54* | 5 | 16 | 41.3% | 126 | 13890 | 10.3 | histone cluster 1, H2bk [Homo sapiens] |

| Filename XCorr DeltCN Conf% ObsM+H+ CalcM+H+ SpR ZScore Ion% # Sequence  | | | | | | | | | | | | |
| --- | --- | --- | --- | --- | --- | --- | --- | --- | --- | --- | --- | --- |
|  | Astrin\_STLCHLD\_tube2\_050114\_01.05016.05016.2 | 3.2571 | 0.3992 | 100.0% | 1266.4321 | 1266.4363 | 1 | 6.337 | 77.8% | 3 | R.KESYSVYVYK.V | 2 |
|  | Astrin\_STLCHLD\_tube2\_050114\_01.14217.14217.2 | 4.9634 | 0.4506 | 100.0% | 1744.6522 | 1745.0211 | 1 | 9.82 | 75.0% | 5 | K.AMGIMNSFVNDIFER.I | 2 |
|  | Astrin\_STLCHLD\_050114\_01.04453.04453.3 | 3.7699 | 0.3764 | 100.0% | 1586.4243 | 1586.7941 | 1 | 6.726 | 51.9% | 1 | R.IAGEASRLAHYNKR.S | 3 |
|  | Astrin\_STLCHLD\_tube2\_050114\_01.04814.04814.2 | 3.3866 | 0.3912 | 100.0% | 1462.2322 | 1462.6462 | 1 | 6.9 | 62.5% | 3 | R.STITSREIQTAVR.L | 2 |
|  | Astrin\_STLCHLD\_tube2\_050114\_01.04809.04809.3 | 3.6418 | 0.3964 | 100.0% | 1463.3644 | 1462.6462 | 86 | 6.688 | 37.5% | 4 | R.STITSREIQTAVR.L | 3 |

---

|  |  |  |  |  |  |  |  |  |
| --- | --- | --- | --- | --- | --- | --- | --- | --- |
| U | *gi|5031699|ref|NP\_005* | 15 | 22 | 41.0% | 427 | 47355 | 7.5 | flotillin 1 [Homo sapiens] |

| Filename XCorr DeltCN Conf% ObsM+H+ CalcM+H+ SpR ZScore Ion% # Sequence  | | | | | | | | | | | | |
| --- | --- | --- | --- | --- | --- | --- | --- | --- | --- | --- | --- | --- |
| \* | Astrin\_STLCHLD\_tube2\_050114\_01.09747.09747.2 | 3.1121 | 0.3557 | 99.9% | 1216.5521 | 1216.4636 | 2 | 7.288 | 70.0% | 1 | R.ISLNTLTLNVK.S | 2 |
| \* | Astrin\_STLCHLD\_tube2\_050114\_01.06985.06985.2 | 2.8035 | 0.4339 | 99.9% | 1406.2722 | 1406.6682 | 1 | 6.761 | 61.5% | 1 | R.HGVPISVTGIAQVK.I | 2 |
| \* | Astrin\_STLCHLD\_tube2\_050114\_01.10692.10692.3 | 4.3487 | 0.4111 | 100.0% | 2018.2444 | 2019.2217 | 1 | 8.424 | 45.6% | 1 | K.TEAEIAHIALETLEGHQR.A | 3 |
| \* | Astrin\_STLCHLD\_050114\_02.06765.06765.3 | 3.1076 | 0.3117 | 100.0% | 1807.7344 | 1808.1206 | 1 | 6.134 | 50.0% | 1 | R.AIMAHMTVEEIYKDR.Q | 3 |
| \* | Astrin\_STLCHLD\_050114\_02.07443.07443.2 | 4.0736 | 0.4621 | 100.0% | 1470.0721 | 1469.693 | 2 | 8.311 | 62.5% | 2 | K.VSAQYLSEIEMAK.A | 2 |
| \* | Astrin\_STLCHLD\_tube2\_050114\_02.05820.05820.3 | 3.0215 | 0.309 | 100.0% | 1576.4944 | 1575.8081 | 1 | 5.381 | 44.2% | 1 | R.RAQADLAYQLQVAK.T | 3 |
| \* | Astrin\_STLCHLD\_050114\_02.06744.06744.2 | 3.5921 | 0.5229 | 100.0% | 1418.3922 | 1419.6206 | 1 | 9.562 | 79.2% | 1 | R.AQADLAYQLQVAK.T | 2 |
| \* | Astrin\_STLCHLD\_050114\_01.05061.05061.2 | 2.2668 | 0.1521 | 96.3% | 956.4122 | 957.11786 | 4 | 5.157 | 78.6% | 1 | R.VQVQVVER.A | 2 |
| \* | Astrin\_STLCHLD\_050114\_01.06520.06520.2 | 4.7613 | 0.4519 | 100.0% | 1470.3121 | 1470.6255 | 1 | 7.908 | 70.8% | 2 | R.AQQVAVQEQEIAR.R | 2 |
| \* | Astrin\_STLCHLD\_tube2\_050114\_02.07635.07635.2 | 5.3854 | 0.5966 | 100.0% | 1604.4722 | 1604.8187 | 1 | 10.241 | 71.4% | 2 | K.SQLIMQAEAEAASVR.M | 2 |
| \* | Astrin\_STLCHLD\_tube2\_050114\_01.05790.05790.2 | 3.8045 | 0.3974 | 100.0% | 1379.5521 | 1379.5768 | 1 | 6.886 | 79.2% | 3 | R.MRGEAEAFAIGAR.A | 2 |
| \* | Astrin\_STLCHLD\_050114\_02.05997.05997.3 | 2.9975 | 0.2396 | 99.5% | 1379.8444 | 1379.5768 | 2 | 5.171 | 47.9% | 1 | R.MRGEAEAFAIGAR.A | 3 |
| \* | Astrin\_STLCHLD\_tube2\_050114\_02.05433.05433.2 | 4.7378 | 0.5073 | 100.0% | 1380.4722 | 1380.5994 | 1 | 8.571 | 82.1% | 3 | K.ITLVSSGSGTMGAAK.V | 2 |
| \* | Astrin\_STLCHLD\_tube2\_050114\_01.10553.10553.2 | 3.8674 | 0.3758 | 100.0% | 1216.2522 | 1216.4203 | 1 | 6.928 | 80.0% | 1 | K.VTGEVLDILTR.L | 2 |
| \* | Astrin\_STLCHLD\_tube2\_050114\_01.04842.04842.2 | 2.8694 | 0.3597 | 99.9% | 1649.6122 | 1649.934 | 67 | 5.732 | 39.3% | 1 | R.LTGVSISQVNHKPLR.T | 2 |

---

|  |  |  |  |  |  |  |  |  |
| --- | --- | --- | --- | --- | --- | --- | --- | --- |
| U | *contaminant\_gi|746301* | 15 | 109 | 40.1% | 269 | 27961 | 6.7 | lysyl endopeptidase (EC 3.4.21.50) - Lysobacter enzymogenes |

| Filename XCorr DeltCN Conf% ObsM+H+ CalcM+H+ SpR ZScore Ion% # Sequence  | | | | | | | | | | | | |
| --- | --- | --- | --- | --- | --- | --- | --- | --- | --- | --- | --- | --- |
| \* | Astrin\_STLCHLD\_050114\_01.06523.06523.2 | 6.3901 | 0.6156 | 100.0% | 2261.4922 | 2262.355 | 1 | 11.178 | 56.2% | 34 | R.APGSSSSGANGDGSLAQSQTGAVVR.A | 2 |
| \* | Astrin\_STLCHLD\_050114\_01.06551.06551.3 | 5.0368 | 0.4181 | 100.0% | 2263.7344 | 2262.355 | 1 | 6.544 | 35.4% | 10 | R.APGSSSSGANGDGSLAQSQTGAVVR.A | 3 |
| \* | Astrin\_STLCHLD\_tube2\_050114\_02.12345.12345.3 | 4.8215 | 0.2751 | 100.0% | 3316.0444 | 3315.6257 | 1 | 5.091 | 25.9% | 3 | R.ATNAASDFTLLELNTAANPAYNLFWAGWDR.R | 3 |
| \* | Astrin\_STLCHLD\_050114\_02.12788.12788.2 | 5.4228 | 0.4616 | 100.0% | 3316.2722 | 3315.6257 | 1 | 8.99 | 39.7% | 6 | R.ATNAASDFTLLELNTAANPAYNLFWAGWDR.R | 2 |
| \* | Astrin\_STLCHLD\_050114\_01.14857.14857.3 | 6.3248 | 0.5243 | 100.0% | 3471.3245 | 3471.813 | 1 | 9.117 | 27.5% | 6 | R.ATNAASDFTLLELNTAANPAYNLFWAGWDRR.D | 3 |
| \* | Astrin\_STLCHLD\_tube2\_050114\_01.04346.04346.3 | 4.7789 | 0.313 | 100.0% | 2077.1042 | 2077.2668 | 1 | 6.482 | 47.2% | 3 | R.RDQNFAGATAIHHPNVAEK.R | 3 |
| \* | Astrin\_STLCHLD\_050114\_01.04706.04706.3 | 4.3052 | 0.3274 | 100.0% | 2234.3943 | 2233.4543 | 6 | 5.571 | 34.2% | 1 | R.RDQNFAGATAIHHPNVAEKR.I | 3 |
| \* | Astrin\_STLCHLD\_tube2\_050114\_01.04670.04670.2 | 4.9285 | 0.5221 | 100.0% | 1920.7522 | 1921.0793 | 1 | 8.428 | 58.8% | 6 | R.DQNFAGATAIHHPNVAEK.R | 2 |
| \* | Astrin\_STLCHLD\_050114\_01.07117.07117.3 | 3.0045 | 0.3658 | 100.0% | 1921.3744 | 1921.0793 | 1 | 5.83 | 38.2% | 3 | R.DQNFAGATAIHHPNVAEK.R | 3 |
| \* | Astrin\_STLCHLD\_tube2\_050114\_01.04448.04448.2 | 5.5433 | 0.4268 | 100.0% | 2076.5322 | 2077.2668 | 1 | 7.603 | 55.6% | 4 | R.DQNFAGATAIHHPNVAEKR.I | 2 |
| \* | Astrin\_STLCHLD\_tube2\_050114\_01.04412.04412.3 | 4.5726 | 0.4477 | 100.0% | 2078.3044 | 2077.2668 | 1 | 8.101 | 43.1% | 10 | R.DQNFAGATAIHHPNVAEKR.I | 3 |
| \* | Astrin\_STLCHLD\_050114\_01.05450.05450.2 | 4.3348 | 0.429 | 100.0% | 1870.5122 | 1870.983 | 1 | 6.911 | 58.3% | 1 | R.VLGQLHGGPSSCSATGADR.S | 2 |
| \* | Astrin\_STLCHLD\_050114\_01.05455.05455.3 | 4.2431 | 0.4642 | 100.0% | 1870.6743 | 1870.983 | 1 | 7.824 | 41.7% | 2 | R.VLGQLHGGPSSCSATGADR.S | 3 |
| \* | Astrin\_STLCHLD\_050114\_01.11195.11195.2 | 4.9574 | 0.5058 | 100.0% | 1428.5322 | 1428.5443 | 1 | 9.309 | 69.2% | 17 | R.VFTSWTGGGTSATR.L | 2 |
| \* | Astrin\_STLCHLD\_tube2\_050114\_01.06605.06605.1 | 2.2914 | 0.1681 | 100.0% | 1429.65 | 1428.5443 | 209 | 4.034 | 34.6% | 3 | R.VFTSWTGGGTSATR.L | 1 |

---

|  |  |  |  |  |  |  |  |  |
| --- | --- | --- | --- | --- | --- | --- | --- | --- |
| U | *gi|21396489|ref|NP\_00* | 29 | 64 | 39.6% | 959 | 106489 | 6.4 | mitochondrial lon peptidase 1 [Homo sapiens] |

| Filename XCorr DeltCN Conf% ObsM+H+ CalcM+H+ SpR ZScore Ion% # Sequence  | | | | | | | | | | | | |
| --- | --- | --- | --- | --- | --- | --- | --- | --- | --- | --- | --- | --- |
| \* | Astrin\_STLCHLD\_tube2\_050114\_01.10342.10342.2 | 2.5299 | 0.3088 | 99.3% | 1235.2522 | 1235.512 | 2 | 6.577 | 65.0% | 1 | R.LAQPYVGVFLK.R | 2 |
| \* | Astrin\_STLCHLD\_tube2\_050114\_01.08787.08787.2 | 2.4845 | 0.1608 | 95.9% | 1391.2322 | 1391.6995 | 2 | 4.921 | 63.6% | 1 | R.LAQPYVGVFLKR.D | 2 |
| \* | Astrin\_STLCHLD\_050114\_01.13543.13543.2 | 2.8474 | 0.2793 | 99.2% | 1796.6522 | 1796.8809 | 11 | 5.453 | 42.9% | 1 | K.S\*K@RGKKEAEDELSAR.H | 2 |
| \* | Astrin\_STLCHLD\_tube2\_050114\_01.11192.11192.2 | 3.4215 | 0.3101 | 99.9% | 1558.6721 | 1558.8644 | 2 | 5.716 | 58.3% | 1 | K.TIRDIIALNPLYR.E | 2 |
| \* | Astrin\_STLCHLD\_tube2\_050114\_01.10954.10954.1 | 1.9918 | 0.2879 | 100.0% | 1187.74 | 1188.4124 | 90 | 5.389 | 50.0% | 1 | R.DIIALNPLYR.E | 1 |
| \* | Astrin\_STLCHLD\_tube2\_050114\_01.10910.10910.2 | 2.7246 | 0.3869 | 99.9% | 1188.3322 | 1188.4124 | 1 | 6.156 | 77.8% | 1 | R.DIIALNPLYR.E | 2 |
| \* | Astrin\_STLCHLD\_050114\_01.14265.14265.3 | 4.9125 | 0.2603 | 100.0% | 3671.6042 | 3671.0674 | 1 | 6.724 | 29.5% | 4 | R.VVDNPIYLSDMGAALTGAESHELQDVLEETNIPK.R | 3 |
| \* | Astrin\_STLCHLD\_050114\_01.06242.06242.2 | 2.5661 | 0.2257 | 99.2% | 926.0122 | 926.18756 | 3 | 4.45 | 85.7% | 1 | R.LKELVVPK.H | 2 |
| \* | Astrin\_STLCHLD\_050114\_01.11719.11719.2 | 3.7812 | 0.5417 | 100.0% | 1401.5521 | 1401.5745 | 1 | 9.897 | 72.7% | 1 | K.HVMDVVDEELSK.L | 2 |
| \* | Astrin\_STLCHLD\_tube2\_050114\_01.13058.13058.3 | 6.0468 | 0.4902 | 100.0% | 3085.5842 | 3085.4155 | 1 | 8.446 | 38.5% | 1 | K.HVMDVVDEELSKLGLLDNHSSEFNVTR.N | 3 |
| \* | Astrin\_STLCHLD\_050114\_02.07166.07166.2 | 4.1233 | 0.3618 | 100.0% | 1702.2922 | 1702.8644 | 6 | 7.708 | 50.0% | 3 | K.LGLLDNHSSEFNVTR.N | 2 |
| \* | Astrin\_STLCHLD\_050114\_02.07133.07133.3 | 4.2904 | 0.4152 | 100.0% | 1703.0944 | 1702.8644 | 1 | 7.056 | 42.9% | 5 | K.LGLLDNHSSEFNVTR.N | 3 |
| \* | Astrin\_STLCHLD\_tube2\_050114\_01.15217.15217.2 | 4.7028 | 0.407 | 100.0% | 1594.6721 | 1593.8223 | 1 | 7.016 | 70.8% | 4 | R.NYLDWLTSIPWGK.Y | 2 |
| \* | Astrin\_STLCHLD\_tube2\_050114\_01.05217.05217.2 | 3.3792 | 0.3859 | 100.0% | 1195.3322 | 1195.2743 | 1 | 7.069 | 77.8% | 7 | K.YSNENLDLAR.A | 2 |
| \* | Astrin\_STLCHLD\_050114\_01.11054.11054.2 | 5.1178 | 0.5263 | 100.0% | 1834.3722 | 1834.9954 | 1 | 10.475 | 70.0% | 3 | R.AQAVLEEDHYGMEDVK.K | 2 |
| \* | Astrin\_STLCHLD\_tube2\_050114\_01.11606.11606.2 | 3.6998 | 0.2623 | 99.9% | 1289.3922 | 1289.5608 | 1 | 5.776 | 75.0% | 3 | R.ILEFIAVSQLR.G | 2 |
| \* | Astrin\_STLCHLD\_tube2\_050114\_02.07411.07411.2 | 3.5125 | 0.528 | 100.0% | 1354.1721 | 1354.561 | 1 | 8.08 | 70.8% | 4 | R.FSVGGMTDVAEIK.G | 2 |
| \* | Astrin\_STLCHLD\_050114\_01.05191.05191.2 | 2.2434 | 0.4689 | 99.9% | 923.5522 | 924.1028 | 19 | 7.648 | 68.8% | 1 | R.TYVGAMPGK.I | 2 |
| \* | Astrin\_STLCHLD\_tube2\_050114\_01.11238.11238.3 | 4.4153 | 0.3296 | 100.0% | 2055.1743 | 2055.3794 | 1 | 6.054 | 42.6% | 1 | K.TKTENPLILIDEVDKIGR.G | 3 |
| \* | Astrin\_STLCHLD\_tube2\_050114\_01.12654.12654.2 | 4.0317 | 0.4931 | 100.0% | 1824.9521 | 1826.1002 | 1 | 7.631 | 63.3% | 3 | K.TENPLILIDEVDKIGR.G | 2 |
| \* | Astrin\_STLCHLD\_050114\_01.15733.15733.3 | 6.0052 | 0.4575 | 100.0% | 3875.6643 | 3875.2373 | 1 | 8.951 | 30.1% | 4 | R.GYQGDPSSALLELLDPEQNANFLDHYLDVPVDLSK.V | 3 |
| \* | Astrin\_STLCHLD\_050114\_02.07682.07682.2 | 4.9899 | 0.4981 | 100.0% | 1599.3922 | 1599.8574 | 1 | 8.952 | 76.9% | 3 | R.MEMINVSGYVAQEK.L | 2 |
| \* | Astrin\_STLCHLD\_tube2\_050114\_01.11674.11674.3 | 4.729 | 0.5291 | 100.0% | 3177.1743 | 3177.5352 | 1 | 8.586 | 25.9% | 2 | K.IVSGEAESVEVTPENLQDFVGKPVFTVER.M | 3 |
| \* | Astrin\_STLCHLD\_050114\_01.18383.18383.2 | 3.634 | 0.585 | 100.0% | 3187.3523 | 3188.7422 | 1 | 9.846 | 36.2% | 1 | R.MYDVTPPGVVMGLAWTAMGGSTLFVETSLR.R | 2 |
| \* | Astrin\_STLCHLD\_tube2\_050114\_02.06728.06728.3 | 3.5822 | 0.2829 | 100.0% | 1863.9243 | 1864.0778 | 1 | 5.552 | 42.6% | 2 | K.GDKDGSLEVTGQLGEVMK.E | 3 |
| \* | Astrin\_STLCHLD\_050114\_02.07766.07766.2 | 2.6133 | 0.2707 | 98.8% | 1564.5322 | 1563.7632 | 26 | 5.122 | 46.4% | 1 | K.DGSLEVTGQLGEVMK.E | 2 |
| \* | Astrin\_STLCHLD\_tube2\_050114\_02.06352.06352.2 | 3.7567 | 0.4657 | 100.0% | 1449.2322 | 1449.6624 | 1 | 8.323 | 73.1% | 2 | R.QNLAMTGEVSLTGK.I | 2 |
| \* | Astrin\_STLCHLD\_050114\_02.13692.13692.3 | 3.1613 | 0.3808 | 100.0% | 2672.3342 | 2672.956 | 96 | 6.027 | 23.8% | 1 | K.DFYDLAAFITEGLEVHFVEHYR.E | 3 |
| \* | Astrin\_STLCHLD\_tube2\_050114\_01.12894.12894.2 | 3.6061 | 0.2195 | 99.7% | 2165.9521 | 2164.3765 | 8 | 4.471 | 38.9% | 1 | R.EIFDIAFPDEQAEALAVER.- | 2 |

---

|  |  |  |  |  |  |  |  |  |
| --- | --- | --- | --- | --- | --- | --- | --- | --- |
| U | *gi|27436946|ref|NP\_73* | 27 | 68 | 39.0% | 664 | 74140 | 7.0 | lamin A/C isoform 1 precursor [Homo sapiens] |

| Filename XCorr DeltCN Conf% ObsM+H+ CalcM+H+ SpR ZScore Ion% # Sequence  | | | | | | | | | | | | |
| --- | --- | --- | --- | --- | --- | --- | --- | --- | --- | --- | --- | --- |
|  | Astrin\_STLCHLD\_tube2\_050114\_01.04823.04823.3 | 4.0271 | 0.236 | 100.0% | 1630.5543 | 1630.7521 | 28 | 4.825 | 47.9% | 3 | R.LQEKEDLQELNDR.L | 3 |
|  | Astrin\_STLCHLD\_tube2\_050114\_01.04829.04829.2 | 4.7687 | 0.3346 | 100.0% | 1630.5721 | 1630.7521 | 1 | 7.693 | 75.0% | 1 | R.LQEKEDLQELNDR.L | 2 |
|  | Astrin\_STLCHLD\_050114\_01.05409.05409.2 | 2.9416 | 0.2321 | 99.5% | 1091.3121 | 1090.1783 | 1 | 4.863 | 77.8% | 3 | R.SLETENAGLR.L | 2 |
|  | Astrin\_STLCHLD\_050114\_02.05327.05327.2 | 3.3283 | 0.4352 | 100.0% | 1417.5322 | 1418.5901 | 1 | 6.767 | 63.6% | 1 | R.LRITESEEVVSR.E | 2 |
|  | Astrin\_STLCHLD\_tube2\_050114\_02.05049.05049.3 | 3.6076 | 0.2477 | 100.0% | 1418.6943 | 1418.5901 | 10 | 5.141 | 47.7% | 2 | R.LRITESEEVVSR.E | 3 |
|  | Astrin\_STLCHLD\_050114\_01.04789.04789.2 | 3.471 | 0.4691 | 100.0% | 1149.0721 | 1149.2432 | 2 | 8.35 | 77.8% | 2 | R.ITESEEVVSR.E | 2 |
|  | Astrin\_STLCHLD\_050114\_01.07574.07574.2 | 3.406 | 0.4553 | 100.0% | 1165.9722 | 1166.2328 | 1 | 7.373 | 85.0% | 6 | K.AAYEAELGDAR.K | 2 |
|  | Astrin\_STLCHLD\_050114\_01.05674.05674.2 | 2.9068 | 0.3053 | 99.9% | 1044.2322 | 1044.1527 | 16 | 5.992 | 66.7% | 2 | K.EGDLIAAQAR.L | 2 |
|  | Astrin\_STLCHLD\_tube2\_050114\_01.08818.08818.2 | 3.2528 | 0.3081 | 99.9% | 1244.4722 | 1244.474 | 1 | 6.157 | 70.0% | 2 | R.LKDLEALLNSK.E | 2 |
|  | Astrin\_STLCHLD\_tube2\_050114\_01.06803.06803.2 | 2.9453 | 0.2613 | 99.8% | 1183.4722 | 1183.3066 | 5 | 5.699 | 77.8% | 3 | R.TLEGELHDLR.G | 2 |
|  | Astrin\_STLCHLD\_tube2\_050114\_01.06528.06528.2 | 3.8853 | 0.3583 | 100.0% | 1510.2722 | 1510.7455 | 3 | 6.884 | 63.6% | 1 | R.LQTMKEELDFQK.N | 2 |
|  | Astrin\_STLCHLD\_tube2\_050114\_01.04920.04920.2 | 2.6856 | 0.2747 | 99.3% | 1381.5521 | 1382.5132 | 63 | 5.129 | 55.0% | 1 | K.NIYSEELRETK.R | 2 |
|  | Astrin\_STLCHLD\_tube2\_050114\_01.07648.07648.2 | 3.6507 | 0.3318 | 100.0% | 1028.9722 | 1029.1814 | 1 | 6.38 | 87.5% | 3 | R.LADALQELR.A | 2 |
|  | Astrin\_STLCHLD\_050114\_01.10645.10645.2 | 4.8137 | 0.4969 | 100.0% | 1753.6522 | 1753.8693 | 1 | 8.858 | 70.0% | 4 | R.NSNLVGAAHEELQQSR.I | 2 |
|  | Astrin\_STLCHLD\_tube2\_050114\_02.05426.05426.3 | 2.6082 | 0.2666 | 96.9% | 1753.7943 | 1753.8693 | 13 | 5.844 | 35.0% | 1 | R.NSNLVGAAHEELQQSR.I | 3 |
|  | Astrin\_STLCHLD\_050114\_01.12115.12115.2 | 4.6168 | 0.4308 | 100.0% | 1700.6522 | 1700.9762 | 1 | 7.861 | 71.4% | 2 | R.IRIDSLSAQLSQLQK.Q | 2 |
|  | Astrin\_STLCHLD\_050114\_01.09764.09764.2 | 2.9308 | 0.245 | 99.6% | 1188.3922 | 1188.3262 | 2 | 5.383 | 77.8% | 4 | K.LRDLEDSLAR.E | 2 |
|  | Astrin\_STLCHLD\_tube2\_050114\_01.11328.11328.2 | 4.1349 | 0.4293 | 100.0% | 1893.8522 | 1895.1346 | 1 | 8.808 | 67.9% | 1 | R.MQQQLDEYQELLDIK.L | 2 |
|  | Astrin\_STLCHLD\_050114\_02.07234.07234.2 | 2.6902 | 0.4308 | 99.9% | 1332.2922 | 1332.5603 | 1 | 7.215 | 55.0% | 1 | K.LALDMEIHAYR.K | 2 |
|  | Astrin\_STLCHLD\_050114\_01.06712.06712.2 | 2.4118 | 0.2949 | 99.0% | 1402.1921 | 1402.4208 | 45 | 4.984 | 60.0% | 2 | R.LRLS\*PSPT#SQR.S | 2 |
|  | Astrin\_STLCHLD\_tube2\_050114\_01.04672.04672.2 | 2.8028 | 0.3221 | 99.9% | 1402.2322 | 1402.4208 | 12 | 5.193 | 65.0% | 2 | R.LRLS\*PS\*PTSQR.S | 2 |
|  | Astrin\_STLCHLD\_050114\_01.04970.04970.2 | 2.8172 | 0.4066 | 99.9% | 1204.1921 | 1204.2762 | 1 | 7.56 | 75.0% | 1 | R.VAVEEVDEEGK.F | 2 |
|  | Astrin\_STLCHLD\_tube2\_050114\_01.05812.05812.2 | 4.3557 | 0.5296 | 100.0% | 1606.3522 | 1606.7728 | 1 | 9.048 | 73.1% | 4 | R.VAVEEVDEEGKFVR.L | 2 |
|  | Astrin\_STLCHLD\_tube2\_050114\_02.05534.05534.3 | 2.7507 | 0.3238 | 100.0% | 1606.7943 | 1606.7728 | 24 | 6.534 | 38.5% | 3 | R.VAVEEVDEEGKFVR.L | 3 |
|  | Astrin\_STLCHLD\_tube2\_050114\_01.06783.06783.2 | 4.1568 | 0.5531 | 100.0% | 1492.3121 | 1492.6874 | 1 | 9.2 | 73.1% | 6 | R.TALINSTGEEVAMR.K | 2 |
|  | Astrin\_STLCHLD\_tube2\_050114\_02.05010.05010.3 | 4.2543 | 0.4839 | 100.0% | 2366.7544 | 2366.504 | 1 | 7.658 | 32.7% | 2 | K.ASASGSGAQVGGPISSGSSASSVTVTR.S | 3 |
|  | Astrin\_STLCHLD\_tube2\_050114\_01.06601.06601.2 | 4.5228 | 0.5756 | 100.0% | 1567.2322 | 1567.6555 | 1 | 9.503 | 53.1% | 5 | R.SVGGSGGGSFGDNLVTR.S | 2 |

---

|  |  |  |  |  |  |  |  |  |
| --- | --- | --- | --- | --- | --- | --- | --- | --- |
| U | *gi|150456457|ref|NP\_9* | 18 | 50 | 38.9% | 347 | 39929 | 5.6 | HMT1 hnRNP methyltransferase-like 2 isoform 2 [Homo sapiens] |
| U | *gi|154759421|ref|NP\_0* | 18 | 50 | 36.4% | 371 | 42462 | 5.3 | HMT1 hnRNP methyltransferase-like 2 isoform 1 [Homo sapiens] |
| U | *gi|151301219|ref|NP\_9* | 18 | 50 | 38.2% | 353 | 40548 | 5.5 | HMT1 hnRNP methyltransferase-like 2 isoform 3 [Homo sapiens] |

| Filename XCorr DeltCN Conf% ObsM+H+ CalcM+H+ SpR ZScore Ion% # Sequence  | | | | | | | | | | | | |
| --- | --- | --- | --- | --- | --- | --- | --- | --- | --- | --- | --- | --- |
|  | Astrin\_STLCHLD\_050114\_02.08259.08259.3 | 4.9772 | 0.4767 | 100.0% | 2764.9443 | 2766.0132 | 1 | 8.839 | 33.3% | 1 | K.DYYFDSYAHFGIHEEMLKDEVR.T | 3 |
|  | Astrin\_STLCHLD\_tube2\_050114\_01.05380.05380.2 | 3.4675 | 0.2305 | 99.9% | 1351.5521 | 1351.6322 | 4 | 5.821 | 68.2% | 3 | K.ANKLDHVVTIIK.G | 2 |
|  | Astrin\_STLCHLD\_050114\_01.09817.09817.3 | 4.2349 | 0.4163 | 100.0% | 1351.8844 | 1351.6322 | 2 | 7.84 | 52.3% | 4 | K.ANKLDHVVTIIK.G | 3 |
|  | Astrin\_STLCHLD\_050114\_01.09831.09831.2 | 3.3185 | 0.3983 | 100.0% | 1356.1322 | 1356.559 | 1 | 6.365 | 72.7% | 1 | K.GKVEEVELPVEK.V | 2 |
|  | Astrin\_STLCHLD\_tube2\_050114\_01.06750.06750.2 | 2.4186 | 0.2674 | 99.0% | 1171.3522 | 1171.333 | 100 | 5.696 | 55.6% | 1 | K.VEEVELPVEK.V | 2 |
|  | Astrin\_STLCHLD\_tube2\_050114\_01.11124.11124.2 | 3.9561 | 0.3995 | 100.0% | 1643.4321 | 1643.8827 | 1 | 6.919 | 65.4% | 3 | R.DKWLAPDGLIFPDR.A | 2 |
|  | Astrin\_STLCHLD\_tube2\_050114\_01.11119.11119.3 | 3.9731 | 0.4103 | 100.0% | 1644.1444 | 1643.8827 | 2 | 6.113 | 46.2% | 1 | R.DKWLAPDGLIFPDR.A | 3 |
|  | Astrin\_STLCHLD\_tube2\_050114\_01.12034.12034.2 | 3.2233 | 0.345 | 99.9% | 1401.3522 | 1400.6201 | 1 | 6.563 | 77.3% | 1 | K.WLAPDGLIFPDR.A | 2 |
|  | Astrin\_STLCHLD\_050114\_01.11929.11929.1 | 2.2431 | 0.3953 | 100.0% | 1251.66 | 1252.4099 | 1 | 7.365 | 60.0% | 2 | R.ATLYVTAIEDR.Q | 1 |
|  | Astrin\_STLCHLD\_050114\_02.07118.07118.2 | 3.9077 | 0.4948 | 100.0% | 1252.0521 | 1252.4099 | 1 | 8.151 | 75.0% | 9 | R.ATLYVTAIEDR.Q | 2 |
|  | Astrin\_STLCHLD\_050114\_01.12033.12033.2 | 3.9196 | 0.3403 | 100.0% | 1637.6322 | 1637.914 | 1 | 6.511 | 67.9% | 2 | K.DVAIKEPLVDVVDPK.Q | 2 |
|  | Astrin\_STLCHLD\_tube2\_050114\_02.10609.10609.3 | 4.0344 | 0.3245 | 100.0% | 2229.0842 | 2229.5027 | 1 | 8.028 | 39.7% | 3 | K.RNDYVHALVAYFNIEFTR.C | 3 |
|  | Astrin\_STLCHLD\_050114\_02.11816.11816.3 | 4.5928 | 0.3813 | 100.0% | 2073.4744 | 2073.3152 | 1 | 6.78 | 40.6% | 2 | R.NDYVHALVAYFNIEFTR.C | 3 |
|  | Astrin\_STLCHLD\_tube2\_050114\_01.14961.14961.2 | 4.3643 | 0.2986 | 100.0% | 2074.4722 | 2073.3152 | 1 | 6.158 | 59.4% | 3 | R.NDYVHALVAYFNIEFTR.C | 2 |
|  | Astrin\_STLCHLD\_tube2\_050114\_01.06838.06838.2 | 3.5514 | 0.3849 | 100.0% | 1726.3121 | 1725.8547 | 2 | 7.153 | 53.6% | 4 | R.TGFSTSPESPYTHWK.Q | 2 |
|  | Astrin\_STLCHLD\_050114\_01.11447.11447.3 | 2.8391 | 0.2619 | 99.2% | 1726.4043 | 1725.8547 | 2 | 5.781 | 35.7% | 1 | R.TGFSTSPESPYTHWK.Q | 3 |
|  | Astrin\_STLCHLD\_tube2\_050114\_01.08245.08245.2 | 3.8348 | 0.4511 | 100.0% | 1721.5322 | 1721.969 | 1 | 7.166 | 56.7% | 6 | K.TGEEIFGTIGMRPNAK.N | 2 |
|  | Astrin\_STLCHLD\_tube2\_050114\_02.06912.06912.3 | 3.3111 | 0.4109 | 100.0% | 1721.9343 | 1721.969 | 5 | 6.459 | 35.0% | 3 | K.TGEEIFGTIGMRPNAK.N | 3 |

---

|  |  |  |  |  |  |  |  |  |
| --- | --- | --- | --- | --- | --- | --- | --- | --- |
| U | *gi|4503529|ref|NP\_001* | 13 | 18 | 38.2% | 406 | 46154 | 5.5 | eukaryotic translation initiation factor 4A isoform 1 [Homo sapiens] |

| Filename XCorr DeltCN Conf% ObsM+H+ CalcM+H+ SpR ZScore Ion% # Sequence  | | | | | | | | | | | | |
| --- | --- | --- | --- | --- | --- | --- | --- | --- | --- | --- | --- | --- |
| \* | Astrin\_STLCHLD\_tube2\_050114\_01.16426.16426.3 | 4.5917 | 0.2351 | 100.0% | 4170.9243 | 4169.451 | 1 | 4.969 | 24.3% | 1 | R.SRDNGPDGMEPEGVIESNWNEIVDSFDDMNLSESLLR.G | 3 |
|  | Astrin\_STLCHLD\_tube2\_050114\_01.07276.07276.3 | 2.9102 | 0.3762 | 100.0% | 1829.1244 | 1829.0654 | 1 | 5.769 | 40.0% | 2 | R.GIYAYGFEKPSAIQQR.A | 33 |
|  | Astrin\_STLCHLD\_tube2\_050114\_01.07223.07223.2 | 4.427 | 0.4297 | 100.0% | 1829.7122 | 1829.0654 | 1 | 7.72 | 70.0% | 3 | R.GIYAYGFEKPSAIQQR.A | 22 |
|  | Astrin\_STLCHLD\_tube2\_050114\_01.05626.05626.2 | 4.2106 | 0.5396 | 100.0% | 1395.3121 | 1395.512 | 1 | 9.128 | 76.9% | 1 | K.GYDVIAQAQSGTGK.T | 2 |
| \* | Astrin\_STLCHLD\_tube2\_050114\_01.07802.07802.2 | 3.2648 | 0.3859 | 99.9% | 1618.5322 | 1619.9225 | 1 | 6.408 | 60.7% | 1 | K.LQMEAPHIIVGTPGR.V | 2 |
| \* | Astrin\_STLCHLD\_tube2\_050114\_01.07757.07757.3 | 2.5852 | 0.2558 | 96.3% | 1618.7344 | 1619.9225 | 3 | 4.425 | 39.3% | 1 | K.LQMEAPHIIVGTPGR.V | 3 |
|  | Astrin\_STLCHLD\_tube2\_050114\_01.12540.12540.2 | 4.4307 | 0.5259 | 100.0% | 1556.3922 | 1556.789 | 1 | 9.956 | 83.3% | 1 | K.MFVLDEADEMLSR.G | 2 |
| \* | Astrin\_STLCHLD\_tube2\_050114\_01.10464.10464.2 | 3.7722 | 0.2826 | 100.0% | 1501.8322 | 1502.71 | 1 | 6.134 | 68.2% | 1 | R.GFKDQIYDIFQK.L | 2 |
| \* | Astrin\_STLCHLD\_050114\_01.11173.11173.2 | 2.5097 | 0.247 | 99.0% | 1188.3522 | 1188.3666 | 1 | 4.66 | 83.3% | 1 | K.KEELTLEGIR.Q | 2 |
|  | Astrin\_STLCHLD\_tube2\_050114\_01.06438.06438.2 | 3.0458 | 0.2519 | 99.3% | 1581.9922 | 1582.7432 | 1 | 5.715 | 65.4% | 1 | R.DFTVSAMHGDMDQK.E | 2 |
|  | Astrin\_STLCHLD\_tube2\_050114\_01.09870.09870.2 | 2.8784 | 0.407 | 100.0% | 1115.7722 | 1115.3585 | 1 | 7.634 | 83.3% | 1 | R.VLITTDLLAR.G | 2 |
| \* | Astrin\_STLCHLD\_tube2\_050114\_01.04677.04677.3 | 3.5242 | 0.3237 | 100.0% | 1591.5543 | 1590.8352 | 41 | 5.14 | 40.4% | 3 | R.KGVAINMVTEEDKR.T | 3 |
|  | Astrin\_STLCHLD\_tube2\_050114\_01.05526.05526.2 | 2.517 | 0.1776 | 96.4% | 1463.7322 | 1462.6611 | 12 | 4.242 | 62.5% | 1 | K.GVAINMVTEEDKR.T | 2 |

Similarities:
gi|7661920|ref|NP\_055(2:11)  

---

|  |  |  |  |  |  |  |  |  |
| --- | --- | --- | --- | --- | --- | --- | --- | --- |
| U | *gi|218505827|ref|NP\_1* | 10 | 18 | 35.4% | 316 | 35438 | 6.3 | TRAF4 associated factor 1 isoform a [Homo sapiens] |

| Filename XCorr DeltCN Conf% ObsM+H+ CalcM+H+ SpR ZScore Ion% # Sequence  | | | | | | | | | | | | |
| --- | --- | --- | --- | --- | --- | --- | --- | --- | --- | --- | --- | --- |
|  | Astrin\_STLCHLD\_050114\_01.11810.11810.2 | 5.3994 | 0.5257 | 100.0% | 2274.7722 | 2275.4802 | 1 | 8.278 | 59.5% | 3 | K.TVYSLQPPSALSGGQPADTQTR.A | 2 |
|  | Astrin\_STLCHLD\_tube2\_050114\_01.07949.07949.3 | 3.9766 | 0.3637 | 100.0% | 2276.3044 | 2275.4802 | 1 | 6.55 | 36.9% | 1 | K.TVYSLQPPSALSGGQPADTQTR.A | 3 |
|  | Astrin\_STLCHLD\_tube2\_050114\_01.05848.05848.3 | 4.9753 | 0.4362 | 100.0% | 1989.5044 | 1988.2023 | 1 | 6.989 | 45.3% | 2 | K.SEEELKDKNQLLEAVNK.Q | 3 |
|  | Astrin\_STLCHLD\_tube2\_050114\_01.05554.05554.2 | 3.7359 | 0.3786 | 100.0% | 1604.8322 | 1604.7979 | 1 | 6.375 | 69.2% | 1 | K.LTETQGELKDLTQK.V | 2 |
|  | Astrin\_STLCHLD\_tube2\_050114\_01.10334.10334.3 | 4.6128 | 0.3185 | 100.0% | 2316.3843 | 2316.6543 | 1 | 5.784 | 35.5% | 1 | K.LTETQGELKDLTQKVELLEK.F | 3 |
|  | Astrin\_STLCHLD\_050114\_02.06850.06850.2 | 2.3446 | 0.174 | 95.5% | 1316.0322 | 1316.5376 | 3 | 4.278 | 65.0% | 1 | K.DLTQKVELLEK.F | 2 |
|  | Astrin\_STLCHLD\_tube2\_050114\_01.07377.07377.2 | 3.9514 | 0.6088 | 100.0% | 1386.7322 | 1387.5327 | 1 | 11.365 | 69.2% | 4 | K.GLDPALGSETLASR.Q | 2 |
|  | Astrin\_STLCHLD\_050114\_01.13533.13533.2 | 3.2031 | 0.1119 | 97.5% | 2593.7922 | 2592.8928 | 2 | 3.906 | 33.3% | 1 | R.QESTTDHMDSMLLLETLQEELK.L | 2 |
|  | Astrin\_STLCHLD\_tube2\_050114\_01.15451.15451.3 | 5.4297 | 0.3737 | 100.0% | 3397.2544 | 3396.8062 | 2 | 6.702 | 26.8% | 3 | R.QESTTDHMDSMLLLETLQEELKLFNETAK.K | 3 |
| \* | Astrin\_STLCHLD\_050114\_01.07264.07264.2 | 3.0147 | 0.2102 | 99.4% | 1217.7522 | 1218.454 | 35 | 4.894 | 66.7% | 1 | K.KQMEELQALK.V | 2 |

---

|  |  |  |  |  |  |  |  |  |
| --- | --- | --- | --- | --- | --- | --- | --- | --- |
| U | *gi|5902102|ref|NP\_008* | 2 | 4 | 34.5% | 119 | 13282 | 11.6 | small nuclear ribonucleoprotein D1 polypeptide 16kDa [Homo sapiens] |

| Filename XCorr DeltCN Conf% ObsM+H+ CalcM+H+ SpR ZScore Ion% # Sequence  | | | | | | | | | | | | |
| --- | --- | --- | --- | --- | --- | --- | --- | --- | --- | --- | --- | --- |
| \* | Astrin\_STLCHLD\_050114\_02.05975.05975.3 | 4.6454 | 0.3889 | 100.0% | 2211.0244 | 2210.47 | 1 | 7.581 | 38.8% | 1 | K.NGTQVHGTITGVDVSMNTHLK.A | 3 |
| \* | Astrin\_STLCHLD\_tube2\_050114\_01.16557.16557.2 | 4.4467 | 0.5212 | 100.0% | 2287.7922 | 2288.6863 | 1 | 8.988 | 60.5% | 3 | R.YFILPDSLPLDTLLVDVEPK.V | 2 |

---

|  |  |  |  |  |  |  |  |  |
| --- | --- | --- | --- | --- | --- | --- | --- | --- |
| U | *gi|7669492|ref|NP\_002* | 7 | 20 | 34.3% | 335 | 36053 | 8.5 | glyceraldehyde-3-phosphate dehydrogenase [Homo sapiens] |

| Filename XCorr DeltCN Conf% ObsM+H+ CalcM+H+ SpR ZScore Ion% # Sequence  | | | | | | | | | | | | |
| --- | --- | --- | --- | --- | --- | --- | --- | --- | --- | --- | --- | --- |
| \* | Astrin\_STLCHLD\_050114\_01.06145.06145.2 | 2.424 | 0.2581 | 99.2% | 806.27216 | 805.912 | 15 | 5.995 | 71.4% | 3 | K.VGVNGFGR.I | 2 |
| \* | Astrin\_STLCHLD\_050114\_02.13705.13705.3 | 4.2906 | 0.4006 | 100.0% | 3310.0444 | 3310.7634 | 7 | 6.463 | 24.1% | 1 | K.VDIVAINDPFIDLNYMVYMFQYDSTHGK.F | 3 |
| \* | Astrin\_STLCHLD\_050114\_01.13819.13819.3 | 6.0548 | 0.5642 | 100.0% | 2597.8145 | 2597.0044 | 1 | 9.774 | 39.1% | 5 | K.VIHDNFGIVEGLMTTVHAITATQK.T | 3 |
| \* | Astrin\_STLCHLD\_tube2\_050114\_01.07787.07787.2 | 3.8885 | 0.4065 | 100.0% | 1412.5122 | 1412.6292 | 1 | 6.557 | 67.9% | 3 | R.GALQNIIPASTGAAK.A | 2 |
| \* | Astrin\_STLCHLD\_050114\_01.11901.11901.2 | 2.4804 | 0.2678 | 98.6% | 1531.8121 | 1531.7155 | 8 | 5.443 | 53.8% | 1 | R.VPTANVSVVDLTCR.L | 2 |
|  | Astrin\_STLCHLD\_tube2\_050114\_02.08416.08416.2 | 4.3608 | 0.4812 | 100.0% | 1765.2722 | 1764.8914 | 1 | 9.239 | 69.2% | 2 | K.LISWYDNEFGYSNR.V | 2 |
| \* | Astrin\_STLCHLD\_tube2\_050114\_01.06777.06777.2 | 4.0811 | 0.4896 | 100.0% | 1331.4321 | 1331.5879 | 1 | 8.771 | 77.3% | 5 | R.VVDLMAHMASKE.- | 2 |

---

|  |  |  |  |  |  |  |  |  |
| --- | --- | --- | --- | --- | --- | --- | --- | --- |
| U | *gi|4503571|ref|NP\_001* | 9 | 14 | 33.4% | 434 | 47169 | 7.4 | enolase 1 [Homo sapiens] |

| Filename XCorr DeltCN Conf% ObsM+H+ CalcM+H+ SpR ZScore Ion% # Sequence  | | | | | | | | | | | | |
| --- | --- | --- | --- | --- | --- | --- | --- | --- | --- | --- | --- | --- |
| \* | Astrin\_STLCHLD\_tube2\_050114\_02.07513.07513.2 | 2.9936 | 0.3516 | 99.9% | 1407.5122 | 1407.5634 | 1 | 8.024 | 62.5% | 2 | R.GNPTVEVDLFTSK.G | 2 |
|  | Astrin\_STLCHLD\_tube2\_050114\_02.08527.08527.2 | 4.2542 | 0.4175 | 100.0% | 1805.9521 | 1806.0258 | 11 | 7.927 | 41.2% | 4 | R.AAVPSGASTGIYEALELR.D | 2 |
| \* | Astrin\_STLCHLD\_tube2\_050114\_01.06970.06970.2 | 2.302 | 0.2358 | 97.5% | 1281.7122 | 1281.4817 | 5 | 5.403 | 60.0% | 1 | K.LMIEMDGTENK.S | 2 |
| \* | Astrin\_STLCHLD\_tube2\_050114\_01.11073.11073.3 | 3.7041 | 0.291 | 100.0% | 3012.2644 | 3013.383 | 6 | 5.003 | 21.6% | 1 | R.HIADLAGNSEVILPVPAFNVINGGSHAGNK.L | 3 |
| \* | Astrin\_STLCHLD\_050114\_01.06059.06059.2 | 2.888 | 0.2163 | 99.3% | 1145.3922 | 1144.3158 | 4 | 4.703 | 72.2% | 1 | R.IGAEVYHNLK.N | 2 |
| \* | Astrin\_STLCHLD\_tube2\_050114\_01.05556.05556.3 | 3.3017 | 0.2938 | 100.0% | 1828.1643 | 1827.9451 | 1 | 6.714 | 45.0% | 1 | R.SGKYDLDFKSPDDPSR.Y | 3 |
| \* | Astrin\_STLCHLD\_tube2\_050114\_01.10250.10250.2 | 3.7409 | 0.4546 | 100.0% | 1426.4722 | 1426.6091 | 2 | 7.537 | 77.3% | 2 | R.YISPDQLADLYK.S | 2 |
| \* | Astrin\_STLCHLD\_050114\_02.07569.07569.3 | 3.1413 | 0.343 | 100.0% | 2190.6843 | 2190.4612 | 1 | 5.596 | 36.2% | 1 | K.FTASAGIQVVGDDLTVTNPKR.I | 3 |
| \* | Astrin\_STLCHLD\_050114\_02.06598.06598.3 | 3.1429 | 0.3106 | 100.0% | 1526.9343 | 1526.7563 | 1 | 5.693 | 50.0% | 1 | K.LAQANGWGVMVSHR.S | 3 |

---

|  |  |  |  |  |  |  |  |  |
| --- | --- | --- | --- | --- | --- | --- | --- | --- |
| U | *gi|12667788|ref|NP\_00* | 50 | 74 | 32.7% | 1960 | 226530 | 5.6 | myosin, heavy polypeptide 9, non-muscle [Homo sapiens] |

| Filename XCorr DeltCN Conf% ObsM+H+ CalcM+H+ SpR ZScore Ion% # Sequence  | | | | | | | | | | | | |
| --- | --- | --- | --- | --- | --- | --- | --- | --- | --- | --- | --- | --- |
| \* | Astrin\_STLCHLD\_tube2\_050114\_01.10850.10850.2 | 3.7742 | 0.2805 | 99.9% | 1673.5721 | 1673.8687 | 1 | 7.206 | 67.9% | 1 | K.NFINNPLAQADWAAK.K | 2 |
| \* | Astrin\_STLCHLD\_050114\_01.11256.11256.3 | 3.9183 | 0.4777 | 100.0% | 1916.8744 | 1916.1614 | 1 | 8.102 | 50.0% | 7 | R.HEMPPHIYAITDTAYR.S | 3 |
| \* | Astrin\_STLCHLD\_050114\_02.07278.07278.3 | 2.226 | 0.3761 | 99.8% | 1479.2943 | 1479.719 | 84 | 5.961 | 31.2% | 1 | K.VIQYLAYVASSHK.S | 3 |
|  | Astrin\_STLCHLD\_tube2\_050114\_01.12825.12825.2 | 2.4754 | 0.3095 | 98.9% | 1727.5721 | 1728.0012 | 1 | 5.616 | 46.7% | 1 | R.QLLQANPILEAFGNAK.T | 2 |
| \* | Astrin\_STLCHLD\_tube2\_050114\_02.11081.11081.2 | 4.1881 | 0.4969 | 100.0% | 2385.9321 | 2386.7068 | 1 | 9.329 | 42.5% | 1 | R.INFDVNGYIVGANIETYLLEK.S | 2 |
| \* | Astrin\_STLCHLD\_050114\_02.10203.10203.3 | 5.0084 | 0.4593 | 100.0% | 1997.3944 | 1997.3037 | 1 | 8.252 | 45.3% | 1 | R.TFHIFYYLLSGAGEHLK.T | 3 |
| \* | Astrin\_STLCHLD\_tube2\_050114\_01.10830.10830.2 | 3.6471 | 0.4923 | 100.0% | 1616.5322 | 1616.9313 | 1 | 8.168 | 76.9% | 2 | R.IMGIPEEEQMGLLR.V | 2 |
| \* | Astrin\_STLCHLD\_050114\_01.12674.12674.2 | 4.0689 | 0.5232 | 100.0% | 1487.7522 | 1487.8259 | 1 | 9.075 | 69.2% | 2 | R.VISGVLQLGNIVFK.K | 2 |
| \* | Astrin\_STLCHLD\_tube2\_050114\_01.09924.09924.2 | 4.0737 | 0.4551 | 100.0% | 1572.6522 | 1572.8044 | 1 | 7.985 | 76.9% | 1 | K.VSHLLGINVTDFTR.G | 2 |
| \* | Astrin\_STLCHLD\_tube2\_050114\_01.12703.12703.2 | 4.6844 | 0.4506 | 100.0% | 2018.6921 | 2019.3636 | 1 | 9.411 | 57.9% | 1 | R.IIGLDQVAGMSETALPGAFK.T | 2 |
| \* | Astrin\_STLCHLD\_050114\_01.11817.11817.2 | 2.3691 | 0.3028 | 99.6% | 925.5722 | 925.07513 | 1 | 6.503 | 75.0% | 1 | R.VVFQEFR.Q | 2 |
| \* | Astrin\_STLCHLD\_tube2\_050114\_01.08106.08106.2 | 2.2038 | 0.2962 | 98.7% | 1193.5922 | 1194.33 | 3 | 5.637 | 72.2% | 1 | K.ALELDSNLYR.I | 2 |
|  | Astrin\_STLCHLD\_tube2\_050114\_01.05992.05992.2 | 3.3196 | 0.4629 | 100.0% | 1224.0521 | 1224.3591 | 1 | 7.862 | 80.0% | 2 | R.AGVLAHLEEER.D | 22 |
| \* | Astrin\_STLCHLD\_tube2\_050114\_02.08132.08132.2 | 5.0968 | 0.4607 | 100.0% | 1752.4722 | 1753.0358 | 1 | 8.123 | 67.9% | 2 | R.LTEMETLQSQLMAEK.L | 2 |
| \* | Astrin\_STLCHLD\_tube2\_050114\_01.10626.10626.2 | 6.3834 | 0.4357 | 100.0% | 2333.7522 | 2334.4736 | 1 | 9.573 | 66.7% | 1 | K.MQQNIQELEEQLEEEESAR.Q | 2 |
| \* | Astrin\_STLCHLD\_050114\_02.06750.06750.2 | 4.8512 | 0.4747 | 100.0% | 1654.3722 | 1654.7681 | 1 | 8.353 | 73.1% | 3 | R.IAEFTTNLTEEEEK.S | 2 |
| \* | Astrin\_STLCHLD\_tube2\_050114\_01.04702.04702.3 | 3.2573 | 0.352 | 100.0% | 1828.0743 | 1828.0936 | 11 | 5.453 | 37.5% | 1 | K.LKNKHEAMITDLEER.L | 3 |
| \* | Astrin\_STLCHLD\_050114\_01.10661.10661.2 | 2.0939 | 0.2332 | 95.2% | 1345.9521 | 1344.4822 | 81 | 4.94 | 50.0% | 1 | K.HEAMITDLEER.L | 2 |
| \* | Astrin\_STLCHLD\_tube2\_050114\_01.11067.11067.3 | 4.3293 | 0.3831 | 100.0% | 2303.5144 | 2304.473 | 1 | 6.556 | 36.1% | 1 | K.IRELESQISELQEDLESER.A | 3 |
| \* | Astrin\_STLCHLD\_050114\_01.16040.16040.2 | 4.9401 | 0.5271 | 100.0% | 3020.2322 | 3019.2434 | 1 | 8.581 | 44.2% | 1 | R.DLGEELEALKTELEDTLDSTAAQQELR.S | 2 |
| \* | Astrin\_STLCHLD\_tube2\_050114\_01.06329.06329.3 | 3.4566 | 0.3255 | 100.0% | 2044.0443 | 2044.2439 | 1 | 5.641 | 35.9% | 1 | K.TLEEEAKTHEAQIQEMR.Q | 3 |
| \* | Astrin\_STLCHLD\_tube2\_050114\_01.08039.08039.3 | 5.1801 | 0.5069 | 100.0% | 1997.0343 | 1997.1722 | 1 | 8.014 | 43.8% | 3 | K.HSQAVEELAEQLEQTKR.V | 3 |
| \* | Astrin\_STLCHLD\_tube2\_050114\_01.04668.04668.3 | 3.2522 | 0.2905 | 100.0% | 1930.4644 | 1930.1252 | 2 | 5.007 | 37.5% | 1 | K.AKQTLENERGELANEVK.V | 3 |
| \* | Astrin\_STLCHLD\_050114\_01.07318.07318.3 | 3.1659 | 0.2247 | 99.8% | 1542.1444 | 1541.8314 | 197 | 4.784 | 35.4% | 1 | R.KKVEAQLQELQVK.F | 3 |
| \* | Astrin\_STLCHLD\_tube2\_050114\_02.08692.08692.2 | 5.5379 | 0.5875 | 100.0% | 1946.2922 | 1947.1498 | 1 | 10.413 | 64.7% | 1 | K.LQVELDNVTGLLSQSDSK.S | 2 |
| \* | Astrin\_STLCHLD\_050114\_01.04487.04487.2 | 2.9436 | 0.1561 | 98.7% | 1492.6522 | 1493.6598 | 1 | 5.14 | 63.6% | 1 | K.LKQVEDEKNSFR.E | 2 |
| \* | Astrin\_STLCHLD\_050114\_01.04478.04478.3 | 3.0958 | 0.3149 | 100.0% | 1493.6943 | 1493.6598 | 14 | 5.673 | 40.9% | 1 | K.LKQVEDEKNSFR.E | 3 |
| \* | Astrin\_STLCHLD\_050114\_01.12440.12440.3 | 3.2748 | 0.3057 | 100.0% | 1951.5243 | 1951.1436 | 1 | 5.356 | 51.7% | 1 | R.LQQELDDLLVDLDHQR.Q | 3 |
| \* | Astrin\_STLCHLD\_tube2\_050114\_01.11996.11996.2 | 4.4825 | 0.2915 | 100.0% | 1951.8322 | 1951.1436 | 1 | 7.227 | 73.3% | 1 | R.LQQELDDLLVDLDHQR.Q | 2 |
|  | Astrin\_STLCHLD\_tube2\_050114\_01.05727.05727.2 | 2.6149 | 0.2047 | 98.8% | 1221.6122 | 1221.3959 | 12 | 4.433 | 66.7% | 2 | K.KFDQLLAEEK.T | 22 |
| \* | Astrin\_STLCHLD\_tube2\_050114\_02.05512.05512.2 | 4.4595 | 0.4129 | 100.0% | 1647.0322 | 1647.8407 | 1 | 7.937 | 76.9% | 1 | R.ALEEAMEQKAELER.L | 2 |
| \* | Astrin\_STLCHLD\_050114\_02.05837.05837.3 | 2.9833 | 0.2298 | 99.1% | 1647.5044 | 1647.8407 | 3 | 4.885 | 44.2% | 1 | R.ALEEAMEQKAELER.L | 3 |
| \* | Astrin\_STLCHLD\_tube2\_050114\_01.06575.06575.2 | 3.8653 | 0.5138 | 100.0% | 1685.0521 | 1685.8586 | 1 | 8.499 | 67.9% | 1 | R.TEMEDLMSSKDDVGK.S | 2 |
| \* | Astrin\_STLCHLD\_tube2\_050114\_01.05654.05654.2 | 3.4135 | 0.4385 | 100.0% | 1204.9722 | 1205.3685 | 4 | 7.91 | 66.7% | 3 | R.ALEQQVEEMK.T | 2 |
| \* | Astrin\_STLCHLD\_050114\_01.14224.14224.3 | 4.1861 | 0.2592 | 100.0% | 3149.6343 | 3149.4048 | 1 | 5.78 | 26.0% | 1 | R.ALEQQVEEMKTQLEELEDELQATEDAK.L | 3 |
| \* | Astrin\_STLCHLD\_050114\_02.07322.07322.2 | 2.694 | 0.2753 | 99.3% | 1315.7122 | 1315.6171 | 1 | 5.485 | 60.0% | 1 | K.LRLEVNLQAMK.A | 2 |
| \* | Astrin\_STLCHLD\_tube2\_050114\_01.07576.07576.2 | 1.9546 | 0.2475 | 95.4% | 1045.8522 | 1046.2701 | 1 | 5.122 | 68.8% | 1 | R.LEVNLQAMK.A | 2 |
| \* | Astrin\_STLCHLD\_050114\_01.07624.07624.2 | 2.5983 | 0.2161 | 98.9% | 1251.0721 | 1251.3075 | 48 | 5.274 | 50.0% | 3 | R.EMEAELEDER.K | 2 |
| \* | Astrin\_STLCHLD\_050114\_01.05099.05099.2 | 3.0115 | 0.4783 | 100.0% | 1212.6721 | 1213.2896 | 1 | 7.795 | 80.0% | 1 | K.DLEAHIDSANK.N | 2 |
| \* | Astrin\_STLCHLD\_050114\_01.04815.04815.3 | 2.9432 | 0.2167 | 99.2% | 1217.2444 | 1216.3799 | 180 | 4.533 | 35.0% | 1 | R.ASREEILAQAK.E | 3 |
| \* | Astrin\_STLCHLD\_050114\_02.08115.08115.3 | 4.642 | 0.3696 | 100.0% | 2474.3643 | 2473.6099 | 1 | 6.897 | 38.8% | 2 | R.IAQLEEELEEEQGNTELINDR.L | 3 |
| \* | Astrin\_STLCHLD\_tube2\_050114\_01.09926.09926.2 | 3.9953 | 0.4113 | 100.0% | 1870.3322 | 1871.0574 | 1 | 6.547 | 63.3% | 1 | K.ANLQIDQINTDLNLER.S | 2 |
| \* | Astrin\_STLCHLD\_050114\_01.11329.11329.2 | 4.6611 | 0.4513 | 100.0% | 1531.4122 | 1531.6598 | 1 | 8.23 | 79.2% | 2 | K.IAQLEEQLDNETK.E | 2 |
| \* | Astrin\_STLCHLD\_tube2\_050114\_01.06314.06314.3 | 2.9142 | 0.2397 | 98.5% | 1817.4543 | 1816.9628 | 132 | 5.35 | 32.1% | 1 | K.IAQLEEQLDNETKER.Q | 3 |
| \* | Astrin\_STLCHLD\_tube2\_050114\_01.05897.05897.3 | 2.99 | 0.2733 | 99.9% | 1601.0944 | 1599.8278 | 1 | 5.898 | 50.0% | 1 | K.LKDVLLQVDDERR.N | 3 |
| \* | Astrin\_STLCHLD\_050114\_01.04462.04462.2 | 4.4929 | 0.3654 | 100.0% | 1488.2722 | 1488.5541 | 1 | 6.954 | 81.8% | 1 | K.RQLEEAEEEAQR.A | 2 |
| \* | Astrin\_STLCHLD\_050114\_01.04615.04615.2 | 3.3172 | 0.3751 | 100.0% | 1332.0521 | 1332.3666 | 1 | 5.874 | 80.0% | 1 | R.QLEEAEEEAQR.A | 2 |
| \* | Astrin\_STLCHLD\_050114\_01.09098.09098.2 | 4.0938 | 0.5494 | 100.0% | 1566.2522 | 1566.6367 | 1 | 9.873 | 65.4% | 2 | R.ELEDATETADAMNR.E | 2 |
| \* | Astrin\_STLCHLD\_050114\_01.11858.11858.2 | 3.6954 | 0.2591 | 100.0% | 1156.6921 | 1156.3732 | 1 | 6.507 | 88.9% | 3 | R.RGDLPFVVPR.R | 2 |
| \* | Astrin\_STLCHLD\_050114\_01.04310.04310.3 | 4.2115 | 0.3008 | 100.0% | 2385.8943 | 2384.3428 | 1 | 5.912 | 33.7% | 1 | R.KGAGDGS\*DEEVDGKADGAEAKPAE.- | 3 |

Similarities:
gi|41406064|ref|NP\_00(2:48)  

---

|  |  |  |  |  |  |  |  |  |
| --- | --- | --- | --- | --- | --- | --- | --- | --- |
| U | *gi|94538362|ref|NP\_00* | 11 | 16 | 32.5% | 428 | 47064 | 5.3 | flotillin 2 [Homo sapiens] |

| Filename XCorr DeltCN Conf% ObsM+H+ CalcM+H+ SpR ZScore Ion% # Sequence  | | | | | | | | | | | | |
| --- | --- | --- | --- | --- | --- | --- | --- | --- | --- | --- | --- | --- |
| \* | Astrin\_STLCHLD\_tube2\_050114\_01.09592.09592.2 | 3.0921 | 0.0704 | 96.8% | 1379.7522 | 1379.6023 | 1 | 4.913 | 81.8% | 1 | K.NVVLQTLEGHLR.S | 2 |
| \* | Astrin\_STLCHLD\_tube2\_050114\_01.15887.15887.3 | 5.476 | 0.5188 | 100.0% | 2835.2344 | 2836.313 | 3 | 8.944 | 29.2% | 1 | R.MGIEILSFTIKDVYDKVDYLSSLGK.T | 3 |
| \* | Astrin\_STLCHLD\_050114\_01.06652.06652.2 | 2.9759 | 0.3144 | 99.9% | 1145.7122 | 1146.1992 | 1 | 5.146 | 80.0% | 2 | R.DADIGVAEAER.D | 2 |
| \* | Astrin\_STLCHLD\_050114\_02.06252.06252.2 | 2.4332 | 0.2805 | 99.1% | 1124.6322 | 1124.2358 | 1 | 5.793 | 72.2% | 1 | K.SAFSEEVNIK.T | 2 |
| \* | Astrin\_STLCHLD\_tube2\_050114\_02.06907.06907.2 | 4.6022 | 0.4009 | 100.0% | 1521.5322 | 1521.6702 | 1 | 7.677 | 76.9% | 3 | K.TAEAQLAYELQGAR.E | 2 |
| \* | Astrin\_STLCHLD\_050114\_02.07300.07300.3 | 3.7467 | 0.2962 | 100.0% | 1523.0343 | 1521.6702 | 1 | 5.213 | 59.6% | 2 | K.TAEAQLAYELQGAR.E | 3 |
| \* | Astrin\_STLCHLD\_tube2\_050114\_02.06074.06074.3 | 3.8638 | 0.1519 | 99.8% | 1642.8544 | 1641.865 | 18 | 4.499 | 45.8% | 2 | K.IRQEEIEIEVVQR.K | 3 |
| \* | Astrin\_STLCHLD\_tube2\_050114\_01.06977.06977.2 | 2.4072 | 0.1788 | 96.0% | 1398.2922 | 1398.6456 | 9 | 4.096 | 59.1% | 1 | K.KQIAVEAQEILR.T | 2 |
| \* | Astrin\_STLCHLD\_050114\_01.05918.05918.2 | 2.3794 | 0.2066 | 97.8% | 1146.1721 | 1146.3292 | 13 | 4.303 | 66.7% | 1 | R.TDKELIATVR.R | 2 |
| \* | Astrin\_STLCHLD\_tube2\_050114\_01.07366.07366.2 | 2.9967 | 0.4502 | 100.0% | 1375.3922 | 1375.5187 | 1 | 6.971 | 66.7% | 1 | K.VDEIVVLSGDNSK.V | 2 |
| \* | Astrin\_STLCHLD\_tube2\_050114\_01.11110.11110.2 | 3.1402 | 0.342 | 99.9% | 1934.0322 | 1935.2712 | 1 | 5.224 | 41.7% | 1 | R.LLAELPASVHALTGVDLSK.I | 2 |

---

|  |  |  |  |  |  |  |  |  |
| --- | --- | --- | --- | --- | --- | --- | --- | --- |
| U | *gi|24234686|ref|NP\_69* | 15 | 24 | 31.2% | 493 | 53518 | 5.9 | heat shock 70kDa protein 8 isoform 2 [Homo sapiens] |
| U | *gi|5729877|ref|NP\_006* | 15 | 24 | 23.8% | 646 | 70898 | 5.5 | heat shock 70kDa protein 8 isoform 1 [Homo sapiens] |

| Filename XCorr DeltCN Conf% ObsM+H+ CalcM+H+ SpR ZScore Ion% # Sequence  | | | | | | | | | | | | |
| --- | --- | --- | --- | --- | --- | --- | --- | --- | --- | --- | --- | --- |
|  | Astrin\_STLCHLD\_tube2\_050114\_01.07314.07314.2 | 3.523 | 0.5545 | 100.0% | 1488.4922 | 1488.5939 | 1 | 9.144 | 75.0% | 3 | R.TTPSYVAFTDTER.L | 222 |
|  | Astrin\_STLCHLD\_050114\_01.11713.11713.2 | 3.0385 | 0.3215 | 99.8% | 1649.8922 | 1650.8468 | 1 | 6.341 | 57.1% | 1 | K.NQVAMNPTNTVFDAK.R | 2 |
|  | Astrin\_STLCHLD\_050114\_01.08992.08992.2 | 2.9837 | 0.3636 | 99.9% | 1410.8121 | 1411.5725 | 1 | 6.38 | 72.7% | 2 | R.RFDDAVVQSDMK.H | 2 |
|  | Astrin\_STLCHLD\_tube2\_050114\_01.08079.08079.3 | 2.6784 | 0.2552 | 97.7% | 1655.5743 | 1654.9298 | 200 | 4.781 | 30.8% | 1 | K.HWPFMVVNDAGRPK.V | 3 |
|  | Astrin\_STLCHLD\_050114\_02.06054.06054.3 | 2.954 | 0.2753 | 100.0% | 1253.6643 | 1253.4993 | 30 | 4.959 | 42.5% | 1 | K.MKEIAEAYLGK.T | 3 |
|  | Astrin\_STLCHLD\_tube2\_050114\_01.05915.05915.2 | 3.0165 | 0.1928 | 99.2% | 1254.2922 | 1253.4993 | 1 | 4.57 | 80.0% | 1 | K.MKEIAEAYLGK.T | 2 |
|  | Astrin\_STLCHLD\_tube2\_050114\_01.09337.09337.2 | 3.0032 | 0.3503 | 99.9% | 1983.3522 | 1983.1882 | 3 | 5.942 | 44.1% | 1 | K.TVTNAVVTVPAYFNDSQR.Q | 2 |
|  | Astrin\_STLCHLD\_tube2\_050114\_01.09822.09822.2 | 3.9832 | 0.4639 | 100.0% | 1660.4922 | 1660.9078 | 1 | 8.122 | 63.3% | 1 | R.IINEPTAAAIAYGLDK.K | 222 |
|  | Astrin\_STLCHLD\_tube2\_050114\_01.04599.04599.2 | 4.3472 | 0.4848 | 100.0% | 1692.2522 | 1692.6958 | 1 | 9.085 | 63.3% | 2 | K.STAGDTHLGGEDFDNR.M | 2 |
|  | Astrin\_STLCHLD\_050114\_01.06475.06475.3 | 2.9132 | 0.4213 | 100.0% | 1692.3544 | 1692.6958 | 2 | 6.602 | 36.7% | 3 | K.STAGDTHLGGEDFDNR.M | 3 |
|  | Astrin\_STLCHLD\_tube2\_050114\_01.07664.07664.2 | 2.7309 | 0.4118 | 99.9% | 1235.4922 | 1236.4741 | 1 | 7.784 | 83.3% | 1 | R.MVNHFIAEFK.R | 2 |
|  | Astrin\_STLCHLD\_tube2\_050114\_01.09239.09239.2 | 3.6423 | 0.337 | 100.0% | 1482.3922 | 1481.6511 | 1 | 6.341 | 77.3% | 3 | R.ARFEELNADLFR.G | 2 |
|  | Astrin\_STLCHLD\_tube2\_050114\_01.10366.10366.2 | 2.5598 | 0.3508 | 99.9% | 1254.4321 | 1254.3849 | 1 | 6.058 | 83.3% | 1 | R.FEELNADLFR.G | 2 |
|  | Astrin\_STLCHLD\_050114\_02.06412.06412.3 | 4.3266 | 0.3878 | 100.0% | 1838.2743 | 1839.1019 | 1 | 7.519 | 42.2% | 1 | K.LDKSQIHDIVLVGGSTR.I | 3 |
|  | Astrin\_STLCHLD\_050114\_01.11161.11161.2 | 2.9582 | 0.3967 | 99.9% | 1483.7722 | 1482.6798 | 1 | 6.544 | 57.7% | 2 | K.SQIHDIVLVGGSTR.I | 2 |

Similarities:
gi|167466173|ref|NP\_0(1:14)  
gi|124256496|ref|NP\_0(2:13)  
contaminant\_GR78\_HUMA(1:14)  

---

|  |  |  |  |  |  |  |  |  |
| --- | --- | --- | --- | --- | --- | --- | --- | --- |
| U | *gi|4826998|ref|NP\_005* | 23 | 57 | 31.0% | 707 | 76150 | 9.4 | splicing factor proline/glutamine rich (polypyrimidine tract binding protein associated) [Homo sapiens] |

| Filename XCorr DeltCN Conf% ObsM+H+ CalcM+H+ SpR ZScore Ion% # Sequence  | | | | | | | | | | | | |
| --- | --- | --- | --- | --- | --- | --- | --- | --- | --- | --- | --- | --- |
| \* | Astrin\_STLCHLD\_tube2\_050114\_01.04173.04173.3 | 3.1936 | 0.2827 | 99.7% | 2451.6843 | 2451.725 | 7 | 4.652 | 28.1% | 1 | K.MPGGPKPGGGPGLS\*TPGGHPKPPHR.G | 3 |
| \* | Astrin\_STLCHLD\_050114\_01.04865.04865.3 | 3.0292 | 0.3215 | 100.0% | 1358.0343 | 1356.429 | 2 | 5.852 | 38.6% | 1 | R.SEEKISDSEGFK.A | 3 |
| \* | Astrin\_STLCHLD\_tube2\_050114\_01.08873.08873.2 | 3.4427 | 0.282 | 99.9% | 1649.7922 | 1650.8723 | 2 | 5.022 | 57.1% | 1 | K.ISDSEGFKANLSLLR.R | 2 |
| \* | Astrin\_STLCHLD\_tube2\_050114\_02.07346.07346.3 | 4.1233 | 0.444 | 100.0% | 1650.9543 | 1650.8723 | 1 | 6.804 | 42.9% | 3 | K.ISDSEGFKANLSLLR.R | 3 |
| \* | Astrin\_STLCHLD\_tube2\_050114\_01.11855.11855.2 | 2.7615 | 0.3981 | 99.9% | 1808.8322 | 1809.0258 | 1 | 6.299 | 53.3% | 1 | R.LFVGNLPADITEDEFK.R | 2 |
| \* | Astrin\_STLCHLD\_tube2\_050114\_01.10662.10662.2 | 4.1348 | 0.4112 | 100.0% | 1964.6522 | 1965.2133 | 1 | 7.669 | 62.5% | 1 | R.LFVGNLPADITEDEFKR.L | 2 |
| \* | Astrin\_STLCHLD\_tube2\_050114\_01.10659.10659.3 | 2.3625 | 0.3099 | 97.1% | 1964.9944 | 1965.2133 | 11 | 4.276 | 34.4% | 1 | R.LFVGNLPADITEDEFKR.L | 3 |
| \* | Astrin\_STLCHLD\_050114\_01.11589.11589.2 | 2.9317 | 0.4774 | 100.0% | 1252.8922 | 1253.3971 | 1 | 7.974 | 65.0% | 3 | K.YGEPGEVFINK.G | 2 |
| \* | Astrin\_STLCHLD\_tube2\_050114\_01.08887.08887.2 | 4.3463 | 0.4497 | 100.0% | 1745.5122 | 1745.0007 | 1 | 7.132 | 63.3% | 1 | R.ALAEIAKAELDDTPMR.G | 2 |
| \* | Astrin\_STLCHLD\_050114\_01.06235.06235.2 | 2.5667 | 0.2665 | 99.3% | 1048.1122 | 1048.1559 | 1 | 5.81 | 81.2% | 2 | K.AELDDTPMR.G | 2 |
| \* | Astrin\_STLCHLD\_050114\_01.06320.06320.2 | 3.4433 | 0.3813 | 100.0% | 1144.4922 | 1144.3188 | 1 | 7.598 | 90.0% | 5 | R.FATHAAALSVR.N | 2 |
| \* | Astrin\_STLCHLD\_tube2\_050114\_01.14975.14975.3 | 3.6456 | 0.4038 | 100.0% | 2640.3245 | 2640.9092 | 1 | 6.545 | 30.7% | 1 | R.NLSPYVSNELLEEAFSQFGPIER.A | 3 |
| \* | Astrin\_STLCHLD\_tube2\_050114\_01.14982.14982.2 | 4.8856 | 0.5794 | 100.0% | 2640.4321 | 2640.9092 | 1 | 10.107 | 47.7% | 4 | R.NLSPYVSNELLEEAFSQFGPIER.A | 2 |
|  | Astrin\_STLCHLD\_050114\_01.05576.05576.1 | 2.1052 | 0.2457 | 100.0% | 886.5 | 887.0238 | 27 | 6.277 | 64.3% | 2 | R.AVVIVDDR.G | 11 |
|  | Astrin\_STLCHLD\_050114\_01.05475.05475.2 | 2.2217 | 0.2079 | 97.7% | 887.09216 | 887.0238 | 1 | 7.125 | 92.9% | 3 | R.AVVIVDDR.G | 22 |
| \* | Astrin\_STLCHLD\_050114\_01.08540.08540.2 | 2.8269 | 0.2883 | 99.5% | 1247.1921 | 1246.452 | 3 | 6.296 | 63.6% | 5 | K.GIVEFASKPAAR.K | 2 |
| \* | Astrin\_STLCHLD\_050114\_02.05730.05730.2 | 4.2222 | 0.4722 | 100.0% | 1762.9521 | 1763.8632 | 1 | 8.977 | 69.2% | 3 | R.FAQHGTFEYEYSQR.W | 2 |
| \* | Astrin\_STLCHLD\_050114\_02.05822.05822.3 | 4.793 | 0.3652 | 100.0% | 1764.1743 | 1763.8632 | 2 | 6.493 | 44.2% | 9 | R.FAQHGTFEYEYSQR.W | 3 |
| \* | Astrin\_STLCHLD\_tube2\_050114\_01.08754.08754.3 | 3.758 | 0.2126 | 99.2% | 2743.3743 | 2743.9648 | 122 | 5.074 | 25.0% | 1 | K.DAKDKLESEMEDAYHEHQANLLR.Q | 3 |
| \* | Astrin\_STLCHLD\_050114\_01.11822.11822.3 | 5.7197 | 0.4776 | 100.0% | 2430.7444 | 2429.6233 | 1 | 7.708 | 40.8% | 2 | K.DKLESEMEDAYHEHQANLLR.Q | 3 |
| \* | Astrin\_STLCHLD\_050114\_01.04232.04232.3 | 3.8661 | 0.1659 | 99.8% | 1730.0044 | 1729.9696 | 2 | 4.784 | 41.7% | 1 | R.RMEELHNQEMQKR.K | 3 |
| \* | Astrin\_STLCHLD\_050114\_01.05311.05311.2 | 4.2654 | 0.571 | 100.0% | 1342.3522 | 1342.4569 | 1 | 9.217 | 78.6% | 5 | R.FGQGGAGPVGGQGPR.G | 2 |
| \* | Astrin\_STLCHLD\_050114\_01.05577.05577.2 | 2.4155 | 0.347 | 99.3% | 1120.0922 | 1121.2561 | 65 | 5.668 | 59.1% | 1 | R.GMGPGTPAGYGR.G | 2 |

Similarities:
gi|224028244|ref|NP\_0(2:21)  

---

|  |  |  |  |  |  |  |  |  |
| --- | --- | --- | --- | --- | --- | --- | --- | --- |
| U | *gi|117968353|ref|NP\_1* | 13 | 21 | 30.8% | 464 | 54304 | 8.3 | NUF2, NDC80 kinetochore complex component [Homo sapiens] |
| U | *gi|117968420|ref|NP\_6* | 13 | 21 | 30.8% | 464 | 54304 | 8.3 | NUF2, NDC80 kinetochore complex component [Homo sapiens] |

| Filename XCorr DeltCN Conf% ObsM+H+ CalcM+H+ SpR ZScore Ion% # Sequence  | | | | | | | | | | | | |
| --- | --- | --- | --- | --- | --- | --- | --- | --- | --- | --- | --- | --- |
|  | Astrin\_STLCHLD\_050114\_02.08458.08458.2 | 3.7181 | 0.4326 | 100.0% | 1327.1522 | 1327.5693 | 1 | 9.25 | 85.0% | 4 | R.YNVAEIVIHIR.N | 2 |
|  | Astrin\_STLCHLD\_050114\_02.08451.08451.3 | 3.3316 | 0.4684 | 100.0% | 1327.8544 | 1327.5693 | 4 | 6.781 | 50.0% | 2 | R.YNVAEIVIHIR.N | 3 |
|  | Astrin\_STLCHLD\_050114\_01.12172.12172.3 | 3.0581 | 0.3165 | 100.0% | 2231.3643 | 2231.6355 | 3 | 5.377 | 32.4% | 1 | K.NDLYPNPKPEVLHMIYMR.A | 3 |
|  | Astrin\_STLCHLD\_tube2\_050114\_01.08644.08644.2 | 2.4365 | 0.289 | 99.3% | 1033.5122 | 1033.259 | 3 | 5.793 | 75.0% | 1 | R.ALQIVYGIR.L | 2 |
|  | Astrin\_STLCHLD\_050114\_01.12633.12633.2 | 4.2653 | 0.4713 | 100.0% | 1465.3522 | 1464.7532 | 1 | 9.118 | 68.2% | 2 | R.FLSGIINFIHFR.E | 2 |
|  | Astrin\_STLCHLD\_tube2\_050114\_01.04935.04935.2 | 4.475 | 0.4713 | 100.0% | 1612.7122 | 1613.8903 | 1 | 8.087 | 76.9% | 1 | K.MQQLNAAHQEALMK.L | 2 |
|  | Astrin\_STLCHLD\_tube2\_050114\_01.06804.06804.2 | 3.0124 | 0.4104 | 99.9% | 1549.1322 | 1549.6746 | 2 | 6.821 | 62.5% | 1 | R.LDSVPVEEQEEFK.Q | 2 |
|  | Astrin\_STLCHLD\_050114\_01.14745.14745.3 | 3.5958 | 0.2083 | 98.0% | 3888.2944 | 3888.193 | 1 | 4.321 | 24.2% | 1 | R.LDSVPVEEQEEFKQLSDGIQELQQSLNQDFHQK.T | 3 |
|  | Astrin\_STLCHLD\_050114\_01.04897.04897.2 | 1.9462 | 0.2557 | 95.9% | 1108.5122 | 1109.2218 | 3 | 5.309 | 75.0% | 1 | K.IVDS\*PEKLK.N | 2 |
|  | Astrin\_STLCHLD\_050114\_02.08524.08524.2 | 3.0454 | 0.3328 | 99.9% | 1979.2722 | 1979.0588 | 16 | 6.027 | 37.5% | 1 | K.ESLNLEDQIESDESELK.K | 2 |
|  | Astrin\_STLCHLD\_tube2\_050114\_01.09516.09516.2 | 3.4745 | 0.3725 | 100.0% | 2106.3523 | 2107.233 | 1 | 6.249 | 41.2% | 1 | K.ESLNLEDQIESDESELKK.L | 2 |
|  | Astrin\_STLCHLD\_050114\_01.05665.05665.2 | 3.4211 | 0.4159 | 100.0% | 1174.1921 | 1174.3396 | 1 | 6.642 | 77.8% | 4 | R.VTTINQEIQK.I | 2 |
|  | Astrin\_STLCHLD\_tube2\_050114\_01.09183.09183.2 | 2.5067 | 0.237 | 99.0% | 1092.1921 | 1092.2804 | 1 | 6.68 | 81.2% | 1 | K.SQEIFLNLK.T | 2 |

---

|  |  |  |  |  |  |  |  |  |
| --- | --- | --- | --- | --- | --- | --- | --- | --- |
| U | *gi|209862831|ref|NP\_0* | 11 | 20 | 30.7% | 339 | 38604 | 7.8 | annexin A2 isoform 2 [Homo sapiens] |
| U | *gi|50845388|ref|NP\_00* | 11 | 20 | 29.1% | 357 | 40411 | 8.4 | annexin A2 isoform 1 [Homo sapiens] |
| U | *gi|50845386|ref|NP\_00* | 11 | 20 | 30.7% | 339 | 38604 | 7.8 | annexin A2 isoform 2 [Homo sapiens] |
| U | *gi|4757756|ref|NP\_004* | 11 | 20 | 30.7% | 339 | 38604 | 7.8 | annexin A2 isoform 2 [Homo sapiens] |

| Filename XCorr DeltCN Conf% ObsM+H+ CalcM+H+ SpR ZScore Ion% # Sequence  | | | | | | | | | | | | |
| --- | --- | --- | --- | --- | --- | --- | --- | --- | --- | --- | --- | --- |
|  | Astrin\_STLCHLD\_tube2\_050114\_01.12738.12738.2 | 2.599 | 0.2365 | 98.3% | 1543.5521 | 1543.7605 | 1 | 5.097 | 53.8% | 1 | K.GVDEVTIVNILTNR.S | 2 |
|  | Astrin\_STLCHLD\_050114\_01.11177.11177.2 | 2.8515 | 0.2602 | 99.8% | 1112.1322 | 1112.2303 | 1 | 4.998 | 68.8% | 1 | R.QDIAFAYQR.R | 2 |
|  | Astrin\_STLCHLD\_050114\_01.13726.13726.2 | 3.2516 | 0.4487 | 100.0% | 1650.3522 | 1651.9872 | 1 | 8.742 | 53.3% | 2 | K.SALSGHLETVILGLLK.T | 2 |
|  | Astrin\_STLCHLD\_050114\_01.13731.13731.3 | 4.2852 | 0.309 | 100.0% | 1651.9744 | 1651.9872 | 23 | 5.874 | 41.7% | 2 | K.SALSGHLETVILGLLK.T | 3 |
|  | Astrin\_STLCHLD\_050114\_01.06446.06446.2 | 3.6633 | 0.4393 | 100.0% | 1223.4521 | 1223.3251 | 1 | 7.66 | 80.0% | 4 | K.TPAQYDASELK.A | 2 |
|  | Astrin\_STLCHLD\_050114\_01.05768.05768.2 | 3.1872 | 0.2786 | 99.9% | 1245.0122 | 1245.3347 | 1 | 6.28 | 83.3% | 4 | R.TNQELQEINR.V | 2 |
|  | Astrin\_STLCHLD\_tube2\_050114\_01.09366.09366.2 | 3.5243 | 0.4317 | 100.0% | 1812.5521 | 1812.928 | 1 | 7.877 | 56.7% | 1 | K.TDLEKDIISDTSGDFR.K | 2 |
|  | Astrin\_STLCHLD\_tube2\_050114\_01.08256.08256.3 | 4.4259 | 0.4288 | 100.0% | 1941.2644 | 1941.102 | 1 | 7.34 | 43.8% | 2 | K.TDLEKDIISDTSGDFRK.L | 3 |
|  | Astrin\_STLCHLD\_050114\_02.07349.07349.3 | 3.7947 | 0.2121 | 99.8% | 2065.6443 | 2066.1887 | 1 | 5.161 | 45.6% | 1 | R.RAEDGSVIDYELIDQDAR.D | 3 |
|  | Astrin\_STLCHLD\_tube2\_050114\_01.09590.09590.2 | 3.0368 | 0.4258 | 99.9% | 1909.6522 | 1910.0013 | 2 | 6.959 | 46.9% | 1 | R.AEDGSVIDYELIDQDAR.D | 2 |
|  | Astrin\_STLCHLD\_050114\_01.05119.05119.2 | 1.9942 | 0.2843 | 97.5% | 1037.0721 | 1037.1606 | 156 | 4.812 | 62.5% | 1 | R.DLYDAGVKR.K | 2 |

---

|  |  |  |  |  |  |  |  |  |
| --- | --- | --- | --- | --- | --- | --- | --- | --- |
| U | *gi|67782365|ref|NP\_00* | 12 | 26 | 30.5% | 469 | 51386 | 5.5 | keratin 7 [Homo sapiens] |

| Filename XCorr DeltCN Conf% ObsM+H+ CalcM+H+ SpR ZScore Ion% # Sequence  | | | | | | | | | | | | |
| --- | --- | --- | --- | --- | --- | --- | --- | --- | --- | --- | --- | --- |
|  | Astrin\_STLCHLD\_050114\_01.11142.11142.3 | 4.0671 | 0.2638 | 100.0% | 2247.1743 | 2247.519 | 27 | 5.643 | 28.4% | 1 | R.LSSARPGGLGSSSLYGLGASRPR.V | 3 |
|  | Astrin\_STLCHLD\_tube2\_050114\_01.04937.04937.2 | 3.4126 | 0.4558 | 100.0% | 1106.3121 | 1105.2388 | 1 | 6.855 | 86.4% | 6 | R.SAYGGPVGAGIR.E | 2 |
|  | Astrin\_STLCHLD\_tube2\_050114\_01.06813.06813.2 | 2.2879 | 0.2249 | 98.9% | 828.2322 | 827.95544 | 7 | 5.23 | 91.7% | 2 | K.FASFIDK.V | 2222 |
|  | Astrin\_STLCHLD\_tube2\_050114\_01.06993.06993.2 | 2.7851 | 0.1943 | 99.2% | 1083.4122 | 1083.2755 | 4 | 6.85 | 81.2% | 2 | K.FASFIDKVR.F | 2222 |
|  | Astrin\_STLCHLD\_tube2\_050114\_01.12328.12328.2 | 4.4723 | 0.4584 | 100.0% | 1443.2522 | 1443.686 | 2 | 8.868 | 62.5% | 1 | R.LPDIFEAQIAGLR.G | 2 |
| \* | Astrin\_STLCHLD\_tube2\_050114\_02.07958.07958.3 | 4.0729 | 0.4898 | 100.0% | 1955.6344 | 1955.1783 | 1 | 7.39 | 45.6% | 2 | R.GQLEALQVDGGRLEAELR.S | 3 |
|  | Astrin\_STLCHLD\_tube2\_050114\_01.10958.10958.2 | 3.3331 | 0.1645 | 99.2% | 1419.3722 | 1419.5773 | 1 | 6.15 | 72.7% | 1 | K.VDALNDEINFLR.T | 2 |
|  | Astrin\_STLCHLD\_tube2\_050114\_02.07310.07310.3 | 3.6715 | 0.1748 | 97.7% | 2272.3145 | 2271.4912 | 11 | 4.506 | 31.6% | 1 | R.AEAEAWYQTKFETLQAQAGK.H | 3 |
|  | Astrin\_STLCHLD\_050114\_01.05710.05710.2 | 2.015 | 0.2999 | 97.7% | 1092.9122 | 1093.2249 | 1 | 5.15 | 72.2% | 1 | K.FETLQAQAGK.H | 2 |
|  | Astrin\_STLCHLD\_tube2\_050114\_01.04607.04607.3 | 2.3556 | 0.2927 | 97.8% | 1442.6943 | 1442.6151 | 4 | 5.166 | 40.9% | 1 | R.LQAEIDNIKNQR.A | 3 |
|  | Astrin\_STLCHLD\_050114\_01.08986.08986.2 | 3.8426 | 0.3914 | 100.0% | 1386.2122 | 1386.548 | 1 | 7.238 | 77.3% | 5 | R.AKQEELEAALQR.G | 2 |
|  | Astrin\_STLCHLD\_tube2\_050114\_01.08928.08928.2 | 3.0067 | 0.3256 | 99.9% | 1406.2922 | 1406.6653 | 6 | 6.062 | 63.6% | 3 | K.LALDIEIATYRK.L | 22 |

Similarities:
gi|4504919|ref|NP\_002(3:9)  
gi|119703753|ref|NP\_0(2:10)  
gi|47132620|ref|NP\_00(2:10)  

---

|  |  |  |  |  |  |  |  |  |
| --- | --- | --- | --- | --- | --- | --- | --- | --- |
| U | *gi|4506901|ref|NP\_003* | 4 | 5 | 28.7% | 164 | 19330 | 11.6 | splicing factor, arginine/serine-rich 3 [Homo sapiens] |

| Filename XCorr DeltCN Conf% ObsM+H+ CalcM+H+ SpR ZScore Ion% # Sequence  | | | | | | | | | | | | |
| --- | --- | --- | --- | --- | --- | --- | --- | --- | --- | --- | --- | --- |
| \* | Astrin\_STLCHLD\_tube2\_050114\_01.05087.05087.2 | 4.5127 | 0.3686 | 100.0% | 1877.4521 | 1878.0519 | 1 | 7.18 | 68.8% | 1 | K.VYVGNLGNNGNKTELER.A | 2 |
| \* | Astrin\_STLCHLD\_tube2\_050114\_01.08359.08359.2 | 2.932 | 0.3909 | 100.0% | 1044.2722 | 1044.198 | 1 | 7.82 | 87.5% | 2 | R.AFGYYGPLR.S | 2 |
|  | Astrin\_STLCHLD\_tube2\_050114\_01.11524.11524.2 | 2.5541 | 0.1729 | 96.2% | 1622.5322 | 1622.7771 | 15 | 4.375 | 50.0% | 1 | R.NPPGFAFVEFEDPR.D | 22 |
| \* | Astrin\_STLCHLD\_tube2\_050114\_01.11231.11231.3 | 2.5792 | 0.3092 | 98.3% | 2322.1443 | 2321.5107 | 17 | 4.955 | 28.8% | 1 | R.NPPGFAFVEFEDPRDAADAVR.E | 3 |

Similarities:
gi|72534660|ref|NP\_00(1:3)  

---

|  |  |  |  |  |  |  |  |  |
| --- | --- | --- | --- | --- | --- | --- | --- | --- |
| U | *gi|4557701|ref|NP\_000* | 14 | 36 | 28.2% | 432 | 48106 | 5.0 | keratin 17 [Homo sapiens] |

| Filename XCorr DeltCN Conf% ObsM+H+ CalcM+H+ SpR ZScore Ion% # Sequence  | | | | | | | | | | | | |
| --- | --- | --- | --- | --- | --- | --- | --- | --- | --- | --- | --- | --- |
|  | Astrin\_STLCHLD\_tube2\_050114\_01.05063.05063.2 | 3.0476 | 0.142 | 99.2% | 1064.9722 | 1065.2578 | 35 | 6.057 | 62.5% | 1 | R.LASYLDKVR.A | 2222 |
|  | Astrin\_STLCHLD\_tube2\_050114\_01.05684.05684.2 | 3.9799 | 0.4353 | 100.0% | 1346.3322 | 1346.4772 | 1 | 7.185 | 68.2% | 4 | R.ALEEANTELEVK.I | 2 |
|  | Astrin\_STLCHLD\_050114\_01.06505.06505.2 | 2.4865 | 0.2457 | 99.3% | 808.09216 | 807.8815 | 46 | 6.454 | 66.7% | 10 | R.LAADDFR.T | 22222 |
|  | Astrin\_STLCHLD\_050114\_01.05083.05083.2 | 2.6635 | 0.3252 | 99.8% | 1223.2722 | 1223.3715 | 47 | 5.486 | 66.7% | 1 | R.TKFETEQALR.L | 22 |
|  | Astrin\_STLCHLD\_tube2\_050114\_01.07270.07270.2 | 2.7001 | 0.1527 | 98.1% | 1185.9722 | 1186.397 | 1 | 4.645 | 83.3% | 1 | R.RVLDELTLAR.A | 222 |
|  | Astrin\_STLCHLD\_tube2\_050114\_01.08094.08094.2 | 3.8958 | 0.4282 | 100.0% | 1030.5721 | 1030.2096 | 1 | 7.786 | 93.8% | 3 | R.VLDELTLAR.A | 222 |
|  | Astrin\_STLCHLD\_tube2\_050114\_01.04733.04733.2 | 2.999 | 0.2859 | 99.9% | 1439.0122 | 1439.6263 | 1 | 5.252 | 75.0% | 1 | R.ILNEMRDQYEK.M | 22 |
|  | Astrin\_STLCHLD\_tube2\_050114\_01.11020.11020.2 | 3.7708 | 0.4345 | 100.0% | 1887.4722 | 1888.0001 | 1 | 6.784 | 53.6% | 1 | K.DAEDWFFSKTEELNR.E | 2 |
|  | Astrin\_STLCHLD\_050114\_01.04933.04933.2 | 4.0231 | 0.5067 | 100.0% | 1362.1122 | 1362.4796 | 1 | 9.592 | 75.0% | 2 | R.EVATNSELVQSGK.S | 22 |
|  | Astrin\_STLCHLD\_050114\_01.09059.09059.2 | 3.684 | 0.3745 | 100.0% | 1404.3722 | 1404.4764 | 8 | 6.775 | 58.3% | 3 | K.ASLEGNLAETENR.Y | 2 |
|  | Astrin\_STLCHLD\_tube2\_050114\_01.06167.06167.2 | 3.6142 | 0.3306 | 100.0% | 1381.3722 | 1380.5437 | 4 | 5.939 | 65.0% | 2 | K.TRLEQEIATYR.R | 22 |
|  | Astrin\_STLCHLD\_tube2\_050114\_01.05246.05246.3 | 3.0259 | 0.1746 | 96.5% | 1537.2544 | 1536.7311 | 3 | 4.38 | 40.9% | 1 | K.TRLEQEIATYRR.L | 33 |
|  | Astrin\_STLCHLD\_050114\_01.07538.07538.2 | 3.1047 | 0.317 | 99.9% | 1123.3322 | 1123.2511 | 3 | 5.706 | 81.2% | 3 | R.LEQEIATYR.R | 222 |
|  | Astrin\_STLCHLD\_050114\_01.07274.07274.2 | 2.4641 | 0.4093 | 99.9% | 1118.1921 | 1118.2291 | 1 | 6.406 | 77.8% | 3 | R.TIVEEVQDGK.V | 2 |

Similarities:
gi|40354195|ref|NP\_95(1:13)  
gi|15431310|ref|NP\_00(9:5)  
gi|24234699|ref|NP\_00(6:8)  
contaminant\_KERATIN03(2:12)  

---

|  |  |  |  |  |  |  |  |  |
| --- | --- | --- | --- | --- | --- | --- | --- | --- |
| U | *gi|34098946|ref|NP\_00* | 4 | 8 | 28.1% | 324 | 35924 | 9.9 | nuclease sensitive element binding protein 1 [Homo sapiens] |

| Filename XCorr DeltCN Conf% ObsM+H+ CalcM+H+ SpR ZScore Ion% # Sequence  | | | | | | | | | | | | |
| --- | --- | --- | --- | --- | --- | --- | --- | --- | --- | --- | --- | --- |
|  | Astrin\_STLCHLD\_tube2\_050114\_01.04841.04841.3 | 3.4665 | 0.2201 | 99.8% | 1745.1843 | 1745.9298 | 1 | 5.08 | 44.6% | 2 | R.NDTKEDVFVHQTAIK.K | 3 |
|  | Astrin\_STLCHLD\_tube2\_050114\_02.06963.06963.2 | 2.9444 | 0.2222 | 98.9% | 1796.7122 | 1796.8822 | 2 | 5.297 | 46.9% | 3 | R.SVGDGETVEFDVVEGEK.G | 2 |
| \* | Astrin\_STLCHLD\_050114\_01.04435.04435.3 | 6.1553 | 0.5007 | 100.0% | 3258.5344 | 3259.2566 | 1 | 9.553 | 33.9% | 1 | R.NYQQNYQNSESGEKNEGSESAPEGQAQQR.R | 3 |
| \* | Astrin\_STLCHLD\_tube2\_050114\_01.04967.04967.3 | 4.42 | 0.2704 | 100.0% | 3225.4744 | 3225.4795 | 3 | 5.417 | 24.1% | 2 | R.RPQYSNPPVQGEVMEGADNQGAGEQGRPVR.Q | 3 |

---

|  |  |  |  |  |  |  |  |  |
| --- | --- | --- | --- | --- | --- | --- | --- | --- |
| U | *gi|14043070|ref|NP\_11* | 9 | 23 | 26.9% | 372 | 38747 | 9.1 | heterogeneous nuclear ribonucleoprotein A1 isoform b [Homo sapiens] |
| U | *gi|4504445|ref|NP\_002* | 9 | 23 | 31.2% | 320 | 34196 | 9.2 | heterogeneous nuclear ribonucleoprotein A1 isoform a [Homo sapiens] |

| Filename XCorr DeltCN Conf% ObsM+H+ CalcM+H+ SpR ZScore Ion% # Sequence  | | | | | | | | | | | | |
| --- | --- | --- | --- | --- | --- | --- | --- | --- | --- | --- | --- | --- |
|  | Astrin\_STLCHLD\_050114\_02.08789.08789.2 | 3.8091 | 0.4012 | 100.0% | 1913.9722 | 1914.1656 | 1 | 7.068 | 53.1% | 2 | R.KLFIGGLSFETTDESLR.S | 2 |
|  | Astrin\_STLCHLD\_050114\_02.08804.08804.3 | 3.8333 | 0.2834 | 100.0% | 1914.4143 | 1914.1656 | 1 | 5.612 | 46.9% | 2 | R.KLFIGGLSFETTDESLR.S | 3 |
|  | Astrin\_STLCHLD\_tube2\_050114\_02.09412.09412.2 | 4.9522 | 0.5207 | 100.0% | 1785.9722 | 1785.9916 | 1 | 9.232 | 70.0% | 2 | K.LFIGGLSFETTDESLR.S | 2 |
|  | Astrin\_STLCHLD\_tube2\_050114\_02.09551.09551.3 | 2.863 | 0.2574 | 96.5% | 2511.9243 | 2511.8145 | 7 | 5.066 | 28.4% | 1 | R.GFGFVTYATVEEVDAAMNARPHK.V | 3 |
|  | Astrin\_STLCHLD\_050114\_01.04321.04321.2 | 2.8506 | 0.0927 | 95.2% | 1438.4922 | 1438.5834 | 1 | 4.766 | 66.7% | 1 | R.EDSQRPGAHLTVK.K | 2 |
|  | Astrin\_STLCHLD\_tube2\_050114\_01.10352.10352.2 | 3.5073 | 0.3986 | 100.0% | 1219.6122 | 1219.4387 | 1 | 7.181 | 88.9% | 4 | K.IEVIEIMTDR.G | 2 |
|  | Astrin\_STLCHLD\_tube2\_050114\_02.07174.07174.3 | 4.4243 | 0.4236 | 100.0% | 1857.5643 | 1856.989 | 1 | 7.404 | 43.3% | 2 | K.RGFAFVTFDDHDSVDK.I | 3 |
|  | Astrin\_STLCHLD\_050114\_02.08540.08540.3 | 4.2942 | 0.261 | 100.0% | 2283.3542 | 2282.5579 | 1 | 6.841 | 35.5% | 3 | R.GFAFVTFDDHDSVDKIVIQK.Y | 3 |
|  | Astrin\_STLCHLD\_050114\_01.08305.08305.2 | 4.1816 | 0.5067 | 100.0% | 1628.7922 | 1629.7721 | 1 | 8.198 | 66.7% | 6 | R.SSGPYGGGGQYFAKPR.N | 2 |

---

|  |  |  |  |  |  |  |  |  |
| --- | --- | --- | --- | --- | --- | --- | --- | --- |
| U | *gi|14043072|ref|NP\_11* | 7 | 16 | 26.9% | 353 | 37430 | 8.9 | heterogeneous nuclear ribonucleoprotein A2/B1 isoform B1 [Homo sapiens] |
| U | *gi|4504447|ref|NP\_002* | 7 | 16 | 27.9% | 341 | 36006 | 8.6 | heterogeneous nuclear ribonucleoprotein A2/B1 isoform A2 [Homo sapiens] |

| Filename XCorr DeltCN Conf% ObsM+H+ CalcM+H+ SpR ZScore Ion% # Sequence  | | | | | | | | | | | | |
| --- | --- | --- | --- | --- | --- | --- | --- | --- | --- | --- | --- | --- |
|  | Astrin\_STLCHLD\_tube2\_050114\_01.10899.10899.2 | 3.9211 | 0.2938 | 99.9% | 1928.0322 | 1928.1925 | 1 | 5.951 | 59.4% | 3 | R.KLFIGGLSFETTEESLR.N | 2 |
|  | Astrin\_STLCHLD\_050114\_02.09946.09946.2 | 4.6563 | 0.4658 | 100.0% | 1799.7122 | 1800.0184 | 1 | 8.038 | 76.7% | 2 | K.LFIGGLSFETTEESLR.N | 2 |
|  | Astrin\_STLCHLD\_050114\_01.10760.10760.2 | 2.4711 | 0.3508 | 99.9% | 1088.0721 | 1088.1644 | 1 | 5.901 | 85.7% | 1 | R.NYYEQWGK.L | 2 |
|  | Astrin\_STLCHLD\_tube2\_050114\_01.09102.09102.2 | 3.2421 | 0.3674 | 100.0% | 1189.5521 | 1189.3513 | 3 | 6.992 | 72.2% | 1 | K.IDTIEIITDR.Q | 2 |
|  | Astrin\_STLCHLD\_tube2\_050114\_01.10969.10969.3 | 4.3152 | 0.4963 | 100.0% | 2278.1343 | 2278.5693 | 1 | 7.586 | 39.5% | 1 | R.GFGFVTFDDHDPVDKIVLQK.Y | 3 |
|  | Astrin\_STLCHLD\_050114\_01.09699.09699.2 | 3.0404 | 0.4619 | 100.0% | 1378.3722 | 1378.4465 | 3 | 6.723 | 57.1% | 5 | R.GGGGNFGPGPGSNFR.G | 2 |
|  | Astrin\_STLCHLD\_tube2\_050114\_01.05513.05513.2 | 6.0916 | 0.682 | 100.0% | 2190.4521 | 2191.2554 | 1 | 11.531 | 54.2% | 3 | R.NMGGPYGGGNYGPGGSGGSGGYGGR.S | 2 |

---

|  |  |  |  |  |  |  |  |  |
| --- | --- | --- | --- | --- | --- | --- | --- | --- |
| U | *gi|4502709|ref|NP\_001* | 7 | 13 | 26.9% | 297 | 34095 | 8.4 | cell division cycle 2 isoform 1 [Homo sapiens] |

| Filename XCorr DeltCN Conf% ObsM+H+ CalcM+H+ SpR ZScore Ion% # Sequence  | | | | | | | | | | | | |
| --- | --- | --- | --- | --- | --- | --- | --- | --- | --- | --- | --- | --- |
|  | Astrin\_STLCHLD\_050114\_01.10755.10755.2 | 3.1102 | 0.4672 | 100.0% | 1186.4321 | 1186.3501 | 1 | 8.533 | 70.0% | 2 | K.IGEGTYGVVYK.G | 2 |
| \* | Astrin\_STLCHLD\_050114\_02.07818.07818.3 | 3.9341 | 0.2219 | 100.0% | 1567.1344 | 1566.7997 | 3 | 5.963 | 50.0% | 1 | R.VYTHEVVTLWYR.S | 3 |
|  | Astrin\_STLCHLD\_050114\_01.10261.10261.2 | 2.9548 | 0.3379 | 99.9% | 1029.1721 | 1029.1814 | 1 | 6.28 | 83.3% | 6 | R.SPEVLLGSAR.Y | 2 |
|  | Astrin\_STLCHLD\_050114\_01.17256.17256.2 | 3.5076 | 0.4603 | 100.0% | 2213.672 | 2213.5352 | 1 | 7.503 | 39.5% | 1 | R.YSTPVDIWSIGTIFAELATK.K | 2 |
|  | Astrin\_STLCHLD\_050114\_01.12117.12117.2 | 3.1535 | 0.2461 | 99.3% | 1802.6322 | 1803.0275 | 1 | 5.522 | 57.1% | 1 | K.KPLFHGDSEIDQLFR.I | 2 |
|  | Astrin\_STLCHLD\_tube2\_050114\_01.09313.09313.3 | 4.0191 | 0.2843 | 100.0% | 1803.3544 | 1803.0275 | 1 | 5.901 | 46.4% | 1 | K.KPLFHGDSEIDQLFR.I | 3 |
|  | Astrin\_STLCHLD\_tube2\_050114\_01.09396.09396.2 | 2.9369 | 0.3927 | 99.9% | 1331.2122 | 1331.4656 | 58 | 6.796 | 45.5% | 1 | K.NLDENGLDLLSK.M | 2 |

---

|  |  |  |  |  |  |  |  |  |
| --- | --- | --- | --- | --- | --- | --- | --- | --- |
| U | *gi|50592996|ref|NP\_00* | 20 | 99 | 26.0% | 450 | 50433 | 4.9 | tubulin, beta, 4 [Homo sapiens] |

| Filename XCorr DeltCN Conf% ObsM+H+ CalcM+H+ SpR ZScore Ion% # Sequence  | | | | | | | | | | | | |
| --- | --- | --- | --- | --- | --- | --- | --- | --- | --- | --- | --- | --- |
|  | Astrin\_STLCHLD\_tube2\_050114\_01.10300.10300.1 | 1.9796 | 0.4452 | 100.0% | 1615.8 | 1616.8701 | 25 | 6.88 | 42.9% | 1 | R.AILVDLEPGTMDSVR.S | 11 |
|  | Astrin\_STLCHLD\_tube2\_050114\_01.10255.10255.2 | 4.3551 | 0.5096 | 100.0% | 1616.3322 | 1616.8701 | 1 | 8.913 | 75.0% | 6 | R.AILVDLEPGTMDSVR.S | 22 |
|  | Astrin\_STLCHLD\_tube2\_050114\_01.12857.12857.2 | 7.2534 | 0.4819 | 100.0% | 1960.4722 | 1960.151 | 1 | 9.656 | 79.4% | 5 | K.GHYTEGAELVDSVLDVVR.K | 222 |
|  | Astrin\_STLCHLD\_tube2\_050114\_01.12839.12839.3 | 4.3447 | 0.3812 | 100.0% | 1960.7644 | 1960.151 | 1 | 7.142 | 45.6% | 3 | K.GHYTEGAELVDSVLDVVR.K | 333 |
|  | Astrin\_STLCHLD\_tube2\_050114\_01.11581.11581.3 | 4.7423 | 0.5057 | 100.0% | 2088.3245 | 2088.325 | 1 | 7.853 | 40.3% | 2 | K.GHYTEGAELVDSVLDVVRK.E | 333 |
|  | Astrin\_STLCHLD\_050114\_01.12405.12405.2 | 6.3217 | 0.4514 | 100.0% | 2088.9321 | 2088.325 | 1 | 8.891 | 66.7% | 2 | K.GHYTEGAELVDSVLDVVRK.E | 222 |
|  | Astrin\_STLCHLD\_050114\_01.11995.11995.1 | 2.1993 | 0.2639 | 100.0% | 1319.69 | 1320.5896 | 245 | 5.053 | 40.9% | 3 | R.IMNTFSVVPSPK.V | 1111 |
|  | Astrin\_STLCHLD\_tube2\_050114\_01.08305.08305.2 | 4.34 | 0.4185 | 100.0% | 1320.4321 | 1320.5896 | 1 | 7.953 | 72.7% | 8 | R.IMNTFSVVPSPK.V | 2222 |
|  | Astrin\_STLCHLD\_tube2\_050114\_01.07179.07179.2 | 3.0568 | 0.2949 | 99.9% | 1131.2522 | 1131.2767 | 7 | 5.17 | 77.8% | 13 | R.FPGQLNADLR.K | 22222 |
|  | Astrin\_STLCHLD\_tube2\_050114\_01.05601.05601.2 | 2.7143 | 0.2756 | 99.3% | 1258.5521 | 1259.4508 | 11 | 5.358 | 65.0% | 13 | R.FPGQLNADLRK.L | 22222 |
|  | Astrin\_STLCHLD\_tube2\_050114\_01.05583.05583.3 | 3.152 | 0.3056 | 100.0% | 1259.8744 | 1259.4508 | 8 | 5.284 | 45.0% | 12 | R.FPGQLNADLRK.L | 33333 |
|  | Astrin\_STLCHLD\_tube2\_050114\_01.08365.08365.2 | 3.793 | 0.3681 | 100.0% | 1271.8722 | 1272.5945 | 1 | 7.682 | 80.0% | 5 | R.KLAVNMVPFPR.L | 22222 |
|  | Astrin\_STLCHLD\_tube2\_050114\_01.09803.09803.1 | 2.0853 | 0.2613 | 100.0% | 1143.58 | 1144.4204 | 13 | 6.348 | 61.1% | 1 | K.LAVNMVPFPR.L | 11111 |
|  | Astrin\_STLCHLD\_tube2\_050114\_01.09839.09839.2 | 3.9889 | 0.4725 | 100.0% | 1144.4122 | 1144.4204 | 1 | 8.55 | 94.4% | 3 | K.LAVNMVPFPR.L | 22222 |
|  | Astrin\_STLCHLD\_tube2\_050114\_01.12007.12007.2 | 4.2206 | 0.404 | 100.0% | 1693.3722 | 1692.9678 | 1 | 7.262 | 78.6% | 3 | R.ALTVPELTQQMFDAK.N | 222 |
|  | Astrin\_STLCHLD\_050114\_01.04952.04952.2 | 2.8672 | 0.3941 | 100.0% | 1066.2322 | 1066.2013 | 2 | 7.174 | 68.8% | 2 | K.NMMAACDPR.H | 22222 |
|  | Astrin\_STLCHLD\_tube2\_050114\_01.11339.11339.2 | 3.5338 | 0.3838 | 100.0% | 1696.7722 | 1697.8877 | 1 | 7.601 | 57.7% | 2 | K.NSSYFVEWIPNNVK.V | 22222 |
|  | Astrin\_STLCHLD\_050114\_02.07650.07650.2 | 3.8098 | 0.3921 | 100.0% | 1386.7522 | 1386.6116 | 1 | 7.005 | 75.0% | 4 | K.RISEQFTAMFR.R | 2222 |
|  | Astrin\_STLCHLD\_tube2\_050114\_02.06787.06787.3 | 3.1948 | 0.3082 | 100.0% | 1543.3143 | 1542.7991 | 1 | 5.726 | 52.3% | 1 | K.RISEQFTAMFRR.K | 3333 |
|  | Astrin\_STLCHLD\_tube2\_050114\_02.08042.08042.2 | 4.0657 | 0.4262 | 100.0% | 1231.3121 | 1230.4241 | 1 | 6.725 | 94.4% | 10 | R.ISEQFTAMFR.R | 2222 |

Similarities:
gi|29788785|ref|NP\_82(19:1)  
gi|5174735|ref|NP\_006(18:2)  
gi|21361322|ref|NP\_00(14:6)  
gi|14210536|ref|NP\_11(8:12)  

---

|  |  |  |  |  |  |  |  |  |
| --- | --- | --- | --- | --- | --- | --- | --- | --- |
| U | *gi|148470397|ref|NP\_0* | 6 | 14 | 26.0% | 415 | 45672 | 5.6 | heterogeneous nuclear ribonucleoprotein F [Homo sapiens] |
| U | *gi|4826760|ref|NP\_004* | 6 | 14 | 26.0% | 415 | 45672 | 5.6 | heterogeneous nuclear ribonucleoprotein F [Homo sapiens] |
| U | *gi|148470406|ref|NP\_0* | 6 | 14 | 26.0% | 415 | 45672 | 5.6 | heterogeneous nuclear ribonucleoprotein F [Homo sapiens] |
| U | *gi|148470404|ref|NP\_0* | 6 | 14 | 26.0% | 415 | 45672 | 5.6 | heterogeneous nuclear ribonucleoprotein F [Homo sapiens] |
| U | *gi|148470402|ref|NP\_0* | 6 | 14 | 26.0% | 415 | 45672 | 5.6 | heterogeneous nuclear ribonucleoprotein F [Homo sapiens] |
| U | *gi|148470400|ref|NP\_0* | 6 | 14 | 26.0% | 415 | 45672 | 5.6 | heterogeneous nuclear ribonucleoprotein F [Homo sapiens] |

| Filename XCorr DeltCN Conf% ObsM+H+ CalcM+H+ SpR ZScore Ion% # Sequence  | | | | | | | | | | | | |
| --- | --- | --- | --- | --- | --- | --- | --- | --- | --- | --- | --- | --- |
|  | Astrin\_STLCHLD\_050114\_02.07005.07005.2 | 3.4015 | 0.3848 | 100.0% | 1710.2722 | 1710.7919 | 1 | 6.499 | 53.3% | 1 | R.QSGEAFVELGSEDDVK.M | 2 |
|  | Astrin\_STLCHLD\_050114\_01.04711.04711.3 | 3.0961 | 0.2811 | 99.9% | 1631.4543 | 1631.6584 | 332 | 4.94 | 30.0% | 1 | K.HSGPNSADSANDGFVR.L | 3 |
|  | Astrin\_STLCHLD\_tube2\_050114\_02.09724.09724.2 | 4.8902 | 0.4455 | 100.0% | 1868.6322 | 1869.0813 | 1 | 9.405 | 68.8% | 2 | K.ITGEAFVQFASQELAEK.A | 2 |
|  | Astrin\_STLCHLD\_tube2\_050114\_02.09436.09436.3 | 7.2757 | 0.48 | 100.0% | 3477.2344 | 3476.7114 | 1 | 9.04 | 33.1% | 2 | R.MRPGAYSTGYGGYEEYSGLSDGYGFTTDLFGR.D | 3 |
|  | Astrin\_STLCHLD\_050114\_01.12769.12769.2 | 4.1076 | 0.4915 | 100.0% | 1997.4521 | 1998.2023 | 1 | 7.45 | 59.4% | 3 | K.ATENDIYNFFSPLNPVR.V | 22 |
|  | Astrin\_STLCHLD\_050114\_01.07357.07357.2 | 3.1623 | 0.5656 | 100.0% | 1092.7322 | 1093.2278 | 1 | 8.032 | 77.8% | 5 | R.VHIEIGPDGR.V | 22 |

Similarities:
gi|5031753|ref|NP\_005(2:4)  

---

|  |  |  |  |  |  |  |  |  |
| --- | --- | --- | --- | --- | --- | --- | --- | --- |
| U | *gi|32455264|ref|NP\_85* | 5 | 10 | 25.6% | 199 | 22110 | 8.1 | peroxiredoxin 1 [Homo sapiens] |
| U | *gi|4505591|ref|NP\_002* | 5 | 10 | 25.6% | 199 | 22110 | 8.1 | peroxiredoxin 1 [Homo sapiens] |
| U | *gi|32455266|ref|NP\_85* | 5 | 10 | 25.6% | 199 | 22110 | 8.1 | peroxiredoxin 1 [Homo sapiens] |

| Filename XCorr DeltCN Conf% ObsM+H+ CalcM+H+ SpR ZScore Ion% # Sequence  | | | | | | | | | | | | |
| --- | --- | --- | --- | --- | --- | --- | --- | --- | --- | --- | --- | --- |
|  | Astrin\_STLCHLD\_tube2\_050114\_01.06934.06934.2 | 2.6826 | 0.2777 | 99.4% | 1107.6921 | 1108.2798 | 1 | 6.169 | 77.8% | 1 | R.TIAQDYGVLK.A | 2 |
|  | Astrin\_STLCHLD\_tube2\_050114\_02.07585.07585.3 | 3.2187 | 0.5019 | 100.0% | 1983.3544 | 1984.2163 | 1 | 7.019 | 44.1% | 1 | R.TIAQDYGVLKADEGISFR.G | 3 |
|  | Astrin\_STLCHLD\_tube2\_050114\_01.10778.10778.2 | 3.2519 | 0.2212 | 99.5% | 1361.9122 | 1360.6395 | 1 | 5.696 | 68.2% | 2 | R.GLFIIDDKGILR.Q | 2 |
|  | Astrin\_STLCHLD\_tube2\_050114\_01.07383.07383.2 | 2.9696 | 0.3359 | 99.9% | 1212.5521 | 1212.3915 | 4 | 6.228 | 75.0% | 3 | R.QITVNDLPVGR.S | 22 |
|  | Astrin\_STLCHLD\_tube2\_050114\_01.08337.08337.2 | 2.9667 | 0.4428 | 100.0% | 1197.5721 | 1197.3763 | 1 | 7.311 | 88.9% | 3 | R.LVQAFQFTDK.H | 2 |

Similarities:
gi|32189392|ref|NP\_00(1:4)  

---

|  |  |  |  |  |  |  |  |  |
| --- | --- | --- | --- | --- | --- | --- | --- | --- |
| U | *gi|222352151|ref|NP\_0* | 6 | 8 | 25.0% | 356 | 37498 | 7.1 | poly(rC) binding protein 1 [Homo sapiens] |

| Filename XCorr DeltCN Conf% ObsM+H+ CalcM+H+ SpR ZScore Ion% # Sequence  | | | | | | | | | | | | |
| --- | --- | --- | --- | --- | --- | --- | --- | --- | --- | --- | --- | --- |
| \* | Astrin\_STLCHLD\_tube2\_050114\_01.10606.10606.2 | 3.453 | 0.3872 | 100.0% | 1389.6522 | 1389.6781 | 1 | 7.68 | 70.8% | 1 | R.IITLTGPTNAIFK.A | 2 |
| \* | Astrin\_STLCHLD\_tube2\_050114\_01.13743.13743.3 | 4.7225 | 0.3663 | 100.0% | 3380.4543 | 3380.8562 | 1 | 6.483 | 25.8% | 1 | K.AFAMIIDKLEEDINSSMTNSTAASRPPVTLR.L | 3 |
|  | Astrin\_STLCHLD\_050114\_02.06494.06494.2 | 4.6947 | 0.5727 | 100.0% | 2090.2722 | 2091.2573 | 1 | 10.461 | 50.0% | 3 | R.ESTGAQVQVAGDMLPNSTER.A | 22 |
|  | Astrin\_STLCHLD\_tube2\_050114\_02.06140.06140.3 | 3.4459 | 0.2808 | 100.0% | 2091.1743 | 2091.2573 | 3 | 4.976 | 36.8% | 1 | R.ESTGAQVQVAGDMLPNSTER.A | 33 |
| \* | Astrin\_STLCHLD\_tube2\_050114\_02.06340.06340.3 | 3.612 | 0.3227 | 100.0% | 2606.8145 | 2607.875 | 1 | 5.849 | 29.2% | 1 | R.QQSHFAMMHGGTGFAGIDSSSPEVK.G | 3 |
| \* | Astrin\_STLCHLD\_050114\_02.06918.06918.3 | 3.6103 | 0.239 | 99.5% | 2687.6042 | 2687.875 | 1 | 6.076 | 30.2% | 1 | R.QQSHFAMMHGGTGFAGIDSSS\*PEVK.G | 3 |

Similarities:
gi|14141166|ref|NP\_11(2:4)  

---

|  |  |  |  |  |  |  |  |  |
| --- | --- | --- | --- | --- | --- | --- | --- | --- |
| U | *gi|5031753|ref|NP\_005* | 8 | 19 | 24.9% | 449 | 49229 | 6.3 | heterogeneous nuclear ribonucleoprotein H1 [Homo sapiens] |

| Filename XCorr DeltCN Conf% ObsM+H+ CalcM+H+ SpR ZScore Ion% # Sequence  | | | | | | | | | | | | |
| --- | --- | --- | --- | --- | --- | --- | --- | --- | --- | --- | --- | --- |
| \* | Astrin\_STLCHLD\_050114\_02.08410.08410.2 | 2.7344 | 0.3435 | 99.9% | 1335.3322 | 1335.5176 | 1 | 6.75 | 65.0% | 3 | K.SNNVEMDWVLK.H | 2 |
|  | Astrin\_STLCHLD\_050114\_01.05415.05415.2 | 4.1137 | 0.511 | 100.0% | 1685.3722 | 1685.7501 | 1 | 8.232 | 70.0% | 1 | K.HTGPNSPDTANDGFVR.L | 2 |
|  | Astrin\_STLCHLD\_050114\_01.05353.05353.3 | 3.9638 | 0.2195 | 100.0% | 1686.9543 | 1685.7501 | 2 | 4.836 | 48.3% | 2 | K.HTGPNSPDTANDGFVR.L | 3 |
|  | Astrin\_STLCHLD\_tube2\_050114\_01.11245.11245.2 | 4.2646 | 0.4244 | 100.0% | 1843.3922 | 1843.0001 | 1 | 7.517 | 56.2% | 3 | R.STGEAFVQFASQEIAEK.A | 2 |
|  | Astrin\_STLCHLD\_050114\_01.12769.12769.2 | 4.1076 | 0.4915 | 100.0% | 1997.4521 | 1998.2023 | 1 | 7.45 | 59.4% | 3 | R.ATENDIYNFFSPLNPVR.V | 22 |
|  | Astrin\_STLCHLD\_050114\_01.07357.07357.2 | 3.1623 | 0.5656 | 100.0% | 1092.7322 | 1093.2278 | 1 | 8.032 | 77.8% | 5 | R.VHIEIGPDGR.V | 22 |
| \* | Astrin\_STLCHLD\_tube2\_050114\_02.06613.06613.3 | 2.9466 | 0.3218 | 99.9% | 2179.8245 | 2179.363 | 4 | 5.308 | 28.8% | 1 | R.VTGEADVEFATHEDAVAAMSK.D | 3 |
| \* | Astrin\_STLCHLD\_050114\_02.07676.07676.3 | 3.2425 | 0.3422 | 100.0% | 2144.0645 | 2143.32 | 1 | 6.494 | 36.8% | 1 | R.YVELFLNSTAGASGGAYEHR.Y | 3 |

Similarities:
gi|148470397|ref|NP\_0(2:6)  

---

|  |  |  |  |  |  |  |  |  |
| --- | --- | --- | --- | --- | --- | --- | --- | --- |
| U | *gi|14165435|ref|NP\_11* | 11 | 20 | 24.8% | 463 | 50976 | 5.5 | heterogeneous nuclear ribonucleoprotein K isoform b [Homo sapiens] |
| U | *gi|14165439|ref|NP\_00* | 11 | 20 | 24.8% | 464 | 51028 | 5.3 | heterogeneous nuclear ribonucleoprotein K isoform a [Homo sapiens] |
| U | *gi|14165437|ref|NP\_11* | 11 | 20 | 24.8% | 464 | 51028 | 5.3 | heterogeneous nuclear ribonucleoprotein K isoform a [Homo sapiens] |

| Filename XCorr DeltCN Conf% ObsM+H+ CalcM+H+ SpR ZScore Ion% # Sequence  | | | | | | | | | | | | |
| --- | --- | --- | --- | --- | --- | --- | --- | --- | --- | --- | --- | --- |
|  | Astrin\_STLCHLD\_050114\_01.05408.05408.3 | 4.015 | 0.275 | 100.0% | 1737.1743 | 1736.8969 | 1 | 5.466 | 50.0% | 2 | K.RPAEDMEEEQAFKR.S | 3 |
|  | Astrin\_STLCHLD\_tube2\_050114\_01.05338.05338.2 | 3.7175 | 0.4082 | 100.0% | 1782.2922 | 1781.8302 | 1 | 6.873 | 56.2% | 3 | R.TDYNASVSVPDSSGPER.I | 2 |
|  | Astrin\_STLCHLD\_tube2\_050114\_01.15664.15664.2 | 2.5361 | 0.3171 | 99.0% | 1716.3522 | 1716.0251 | 9 | 5.711 | 46.7% | 2 | R.ILSISADIETIGEILK.K | 2 |
|  | Astrin\_STLCHLD\_050114\_01.14188.14188.2 | 2.2281 | 0.295 | 97.6% | 1844.1522 | 1844.1992 | 161 | 4.831 | 28.1% | 1 | R.ILSISADIETIGEILKK.I | 2 |
|  | Astrin\_STLCHLD\_050114\_02.07559.07559.3 | 4.3015 | 0.5211 | 100.0% | 1519.7043 | 1519.8711 | 1 | 8.552 | 55.4% | 3 | R.LLIHQSLAGGIIGVK.G | 3 |
|  | Astrin\_STLCHLD\_tube2\_050114\_02.07330.07330.2 | 3.9984 | 0.4974 | 100.0% | 1519.9122 | 1519.8711 | 1 | 8.471 | 71.4% | 2 | R.LLIHQSLAGGIIGVK.G | 2 |
|  | Astrin\_STLCHLD\_tube2\_050114\_01.11973.11973.2 | 2.9911 | 0.3721 | 99.9% | 1341.7522 | 1341.6311 | 1 | 6.696 | 72.7% | 1 | K.IILDLISESPIK.G | 2 |
|  | Astrin\_STLCHLD\_tube2\_050114\_01.10348.10348.2 | 2.8534 | 0.2559 | 99.1% | 1554.4922 | 1554.8705 | 3 | 5.639 | 50.0% | 1 | K.IILDLISESPIKGR.A | 2 |
|  | Astrin\_STLCHLD\_tube2\_050114\_01.10110.10110.2 | 4.5125 | 0.379 | 100.0% | 1918.6921 | 1918.1974 | 1 | 7.83 | 50.0% | 1 | R.GSYGDLGGPIITTQVTIPK.D | 2 |
|  | Astrin\_STLCHLD\_050114\_02.05618.05618.3 | 3.84 | 0.3195 | 100.0% | 2070.2344 | 2070.1772 | 1 | 5.731 | 38.9% | 1 | R.HESGASIKIDEPLEGSEDR.I | 3 |
|  | Astrin\_STLCHLD\_050114\_01.06543.06543.2 | 3.3909 | 0.4183 | 100.0% | 1260.1122 | 1260.3 | 1 | 6.946 | 85.0% | 3 | K.IDEPLEGSEDR.I | 2 |

---

|  |  |  |  |  |  |  |  |  |
| --- | --- | --- | --- | --- | --- | --- | --- | --- |
| U | *gi|10863927|ref|NP\_06* | 3 | 5 | 23.0% | 165 | 18012 | 7.8 | peptidylprolyl isomerase A [Homo sapiens] |
| U | *gi|169215435|ref|XP\_0* | 3 | 5 | 17.0% | 223 | 24376 | 6.9 | PREDICTED: similar to peptidylprolyl isomerase A-like [Homo sapiens] |

| Filename XCorr DeltCN Conf% ObsM+H+ CalcM+H+ SpR ZScore Ion% # Sequence  | | | | | | | | | | | | |
| --- | --- | --- | --- | --- | --- | --- | --- | --- | --- | --- | --- | --- |
|  | Astrin\_STLCHLD\_050114\_01.12201.12201.2 | 3.4543 | 0.3089 | 99.9% | 1380.6721 | 1380.6268 | 3 | 6.616 | 59.1% | 3 | R.VSFELFADKVPK.T | 2 |
|  | Astrin\_STLCHLD\_tube2\_050114\_01.09421.09421.2 | 3.4138 | 0.4091 | 100.0% | 1832.4321 | 1833.0477 | 1 | 7.042 | 60.7% | 1 | K.SIYGEKFEDENFILK.H | 2 |
|  | Astrin\_STLCHLD\_tube2\_050114\_01.09598.09598.2 | 2.7521 | 0.1799 | 98.7% | 1279.2722 | 1279.4689 | 2 | 7.026 | 70.0% | 1 | K.EGMNIVEAMER.F | 2 |

---

|  |  |  |  |  |  |  |  |  |
| --- | --- | --- | --- | --- | --- | --- | --- | --- |
| U | *gi|14210536|ref|NP\_11* | 13 | 63 | 22.4% | 446 | 49857 | 4.9 | tubulin, beta 6 [Homo sapiens] |

| Filename XCorr DeltCN Conf% ObsM+H+ CalcM+H+ SpR ZScore Ion% # Sequence  | | | | | | | | | | | | |
| --- | --- | --- | --- | --- | --- | --- | --- | --- | --- | --- | --- | --- |
| \* | Astrin\_STLCHLD\_tube2\_050114\_01.09148.09148.2 | 3.6548 | 0.403 | 100.0% | 1575.3121 | 1574.7894 | 1 | 6.653 | 60.7% | 2 | R.AALVDLEPGTMDSVR.S | 2 |
|  | Astrin\_STLCHLD\_tube2\_050114\_01.07179.07179.2 | 3.0568 | 0.2949 | 99.9% | 1131.2522 | 1131.2767 | 7 | 5.17 | 77.8% | 13 | R.FPGQLNADLR.K | 22222 |
|  | Astrin\_STLCHLD\_tube2\_050114\_01.05601.05601.2 | 2.7143 | 0.2756 | 99.3% | 1258.5521 | 1259.4508 | 11 | 5.358 | 65.0% | 13 | R.FPGQLNADLRK.L | 22222 |
|  | Astrin\_STLCHLD\_tube2\_050114\_01.05583.05583.3 | 3.152 | 0.3056 | 100.0% | 1259.8744 | 1259.4508 | 8 | 5.284 | 45.0% | 12 | R.FPGQLNADLRK.L | 33333 |
|  | Astrin\_STLCHLD\_tube2\_050114\_01.08365.08365.2 | 3.793 | 0.3681 | 100.0% | 1271.8722 | 1272.5945 | 1 | 7.682 | 80.0% | 5 | R.KLAVNMVPFPR.L | 22222 |
|  | Astrin\_STLCHLD\_tube2\_050114\_01.09803.09803.1 | 2.0853 | 0.2613 | 100.0% | 1143.58 | 1144.4204 | 13 | 6.348 | 61.1% | 1 | K.LAVNMVPFPR.L | 11111 |
|  | Astrin\_STLCHLD\_tube2\_050114\_01.09839.09839.2 | 3.9889 | 0.4725 | 100.0% | 1144.4122 | 1144.4204 | 1 | 8.55 | 94.4% | 3 | K.LAVNMVPFPR.L | 22222 |
|  | Astrin\_STLCHLD\_050114\_01.12456.12456.2 | 3.7154 | 0.3929 | 100.0% | 1621.7122 | 1621.9403 | 1 | 8.432 | 76.9% | 3 | R.LHFFMPGFAPLTSR.G | 2222 |
|  | Astrin\_STLCHLD\_050114\_01.12457.12457.3 | 4.2506 | 0.3686 | 100.0% | 1622.0643 | 1621.9403 | 1 | 6.108 | 53.8% | 4 | R.LHFFMPGFAPLTSR.G | 3333 |
|  | Astrin\_STLCHLD\_050114\_01.04952.04952.2 | 2.8672 | 0.3941 | 100.0% | 1066.2322 | 1066.2013 | 2 | 7.174 | 68.8% | 2 | R.NMMAACDPR.H | 22222 |
|  | Astrin\_STLCHLD\_tube2\_050114\_01.11339.11339.2 | 3.5338 | 0.3838 | 100.0% | 1696.7722 | 1697.8877 | 1 | 7.601 | 57.7% | 2 | K.NSSYFVEWIPNNVK.V | 22222 |
| \* | Astrin\_STLCHLD\_050114\_01.12639.12639.2 | 3.5667 | 0.0027 | 95.2% | 1859.0721 | 1859.1475 | 1 | 6.278 | 59.4% | 1 | K.MASTFIGNSTAIQELFK.R | 2 |
| \* | Astrin\_STLCHLD\_tube2\_050114\_01.09654.09654.2 | 2.4815 | 0.2046 | 98.2% | 1215.9922 | 1216.3972 | 7 | 4.948 | 66.7% | 2 | R.ISEQFSAMFR.R | 2 |

Similarities:
gi|29788785|ref|NP\_82(10:3)  
gi|5174735|ref|NP\_006(10:3)  
gi|21361322|ref|NP\_00(10:3)  
gi|50592996|ref|NP\_00(8:5)  

---

|  |  |  |  |  |  |  |  |  |
| --- | --- | --- | --- | --- | --- | --- | --- | --- |
| U | *gi|10835063|ref|NP\_00* | 3 | 4 | 21.4% | 294 | 32575 | 4.8 | nucleophosmin 1 isoform 1 [Homo sapiens] |
| U | *gi|40353734|ref|NP\_95* | 3 | 4 | 23.8% | 265 | 29465 | 4.6 | nucleophosmin 1 isoform 2 [Homo sapiens] |

| Filename XCorr DeltCN Conf% ObsM+H+ CalcM+H+ SpR ZScore Ion% # Sequence  | | | | | | | | | | | | |
| --- | --- | --- | --- | --- | --- | --- | --- | --- | --- | --- | --- | --- |
|  | Astrin\_STLCHLD\_050114\_02.09039.09039.3 | 3.817 | 0.2301 | 99.8% | 2932.8245 | 2931.2874 | 1 | 4.49 | 25.9% | 1 | R.TVSLGAGAKDELHIVEAEAMNYEGSPIK.V | 3 |
|  | Astrin\_STLCHLD\_tube2\_050114\_01.13190.13190.2 | 4.6233 | 0.5332 | 100.0% | 2229.112 | 2228.655 | 1 | 8.229 | 52.5% | 2 | K.MSVQPTVSLGGFEITPPVVLR.L | 2 |
|  | Astrin\_STLCHLD\_050114\_01.12661.12661.2 | 3.6322 | 0.2054 | 99.7% | 1820.6322 | 1821.0172 | 1 | 5.438 | 53.8% | 1 | R.MTDQEAIQDLWQWR.K | 2 |

---

|  |  |  |  |  |  |  |  |  |
| --- | --- | --- | --- | --- | --- | --- | --- | --- |
| U | *contaminant\_INT-STD1* | 13 | 46 | 21.3% | 607 | 69271 | 6.1 | BSA |

| Filename XCorr DeltCN Conf% ObsM+H+ CalcM+H+ SpR ZScore Ion% # Sequence  | | | | | | | | | | | | |
| --- | --- | --- | --- | --- | --- | --- | --- | --- | --- | --- | --- | --- |
| \* | Astrin\_STLCHLD\_tube2\_050114\_01.08720.08720.2 | 3.5324 | 0.3784 | 100.0% | 1164.1721 | 1164.344 | 3 | 6.967 | 77.8% | 3 | K.LVNELTEFAK.T | 2 |
|  | Astrin\_STLCHLD\_tube2\_050114\_01.06729.06729.2 | 2.4104 | 0.2791 | 99.6% | 927.5722 | 928.0758 | 1 | 5.881 | 83.3% | 1 | K.YLYEIAR.R | 2 |
| \* | Astrin\_STLCHLD\_tube2\_050114\_01.09871.09871.3 | 3.5824 | 0.3922 | 100.0% | 2046.2344 | 2046.3354 | 1 | 6.054 | 48.3% | 1 | R.RHPYFYAPELLYYANK.Y | 3 |
| \* | Astrin\_STLCHLD\_tube2\_050114\_02.10227.10227.2 | 3.7452 | 0.4815 | 100.0% | 1567.9122 | 1568.7258 | 1 | 7.991 | 70.8% | 4 | K.DAFLGSFLYEYSR.R | 2 |
| \* | Astrin\_STLCHLD\_tube2\_050114\_01.07049.07049.2 | 3.2494 | 0.3408 | 99.9% | 1441.3722 | 1440.6884 | 1 | 6.315 | 68.2% | 4 | R.RHPEYAVSVLLR.L | 2 |
| \* | Astrin\_STLCHLD\_050114\_02.06710.06710.3 | 4.621 | 0.2762 | 100.0% | 1442.1843 | 1440.6884 | 1 | 5.33 | 59.1% | 8 | R.RHPEYAVSVLLR.L | 3 |
| \* | Astrin\_STLCHLD\_tube2\_050114\_01.05978.05978.2 | 3.3761 | 0.413 | 100.0% | 1306.3522 | 1306.5046 | 1 | 7.769 | 70.0% | 5 | K.HLVDEPQNLIK.Q | 2 |
| \* | Astrin\_STLCHLD\_tube2\_050114\_02.08219.08219.2 | 4.7237 | 0.3588 | 100.0% | 1482.0322 | 1480.7068 | 1 | 7.228 | 75.0% | 8 | K.LGEYGFQNALIVR.Y | 2 |
|  | Astrin\_STLCHLD\_tube2\_050114\_01.06747.06747.3 | 4.3564 | 0.4316 | 100.0% | 1640.9644 | 1640.9205 | 2 | 7.441 | 44.6% | 2 | R.KVPQVSTPTLVEVSR.S | 3 |
|  | Astrin\_STLCHLD\_tube2\_050114\_01.06773.06773.2 | 4.0709 | 0.3843 | 100.0% | 1641.4922 | 1640.9205 | 1 | 8.109 | 60.7% | 4 | R.KVPQVSTPTLVEVSR.S | 2 |
| \* | Astrin\_STLCHLD\_tube2\_050114\_01.07832.07832.2 | 2.7361 | 0.3021 | 99.8% | 1143.4521 | 1143.4124 | 1 | 6.076 | 77.8% | 1 | K.KQTALVELLK.H | 2 |
| \* | Astrin\_STLCHLD\_tube2\_050114\_01.12457.12457.2 | 4.0676 | 0.5221 | 100.0% | 1401.2722 | 1400.6324 | 1 | 8.136 | 77.3% | 3 | K.TVMENFVAFVDK.C | 2 |
| \* | Astrin\_STLCHLD\_050114\_02.06674.06674.2 | 2.3477 | 0.5105 | 100.0% | 1003.1122 | 1003.1839 | 6 | 6.859 | 66.7% | 2 | K.LVVSTQTALA.- | 2 |

---

|  |  |  |  |  |  |  |  |  |
| --- | --- | --- | --- | --- | --- | --- | --- | --- |
| U | *gi|15431295|ref|NP\_15* | 5 | 8 | 21.3% | 211 | 24261 | 11.7 | ribosomal protein L13 [Homo sapiens] |
| U | *gi|15431297|ref|NP\_00* | 5 | 8 | 21.3% | 211 | 24261 | 11.7 | ribosomal protein L13 [Homo sapiens] |

| Filename XCorr DeltCN Conf% ObsM+H+ CalcM+H+ SpR ZScore Ion% # Sequence  | | | | | | | | | | | | |
| --- | --- | --- | --- | --- | --- | --- | --- | --- | --- | --- | --- | --- |
|  | Astrin\_STLCHLD\_050114\_01.11000.11000.2 | 2.5357 | 0.1586 | 96.7% | 1347.3121 | 1346.5344 | 2 | 4.1 | 70.0% | 1 | R.RVATWFNQPAR.K | 2 |
|  | Astrin\_STLCHLD\_050114\_01.11911.11911.2 | 3.1878 | 0.2922 | 99.9% | 1190.2722 | 1190.3469 | 45 | 6.134 | 66.7% | 3 | R.VATWFNQPAR.K | 2 |
|  | Astrin\_STLCHLD\_050114\_01.04715.04715.2 | 3.8243 | 0.3532 | 100.0% | 1234.4521 | 1233.3237 | 1 | 5.771 | 80.0% | 1 | K.STESLQANVQR.L | 2 |
|  | Astrin\_STLCHLD\_050114\_01.11890.11890.2 | 2.4436 | 0.2809 | 98.7% | 1383.3722 | 1383.6923 | 78 | 5.145 | 45.8% | 2 | K.LATQLTGPVMPVR.N | 2 |
|  | Astrin\_STLCHLD\_050114\_01.04858.04858.2 | 2.5146 | 0.258 | 99.0% | 1238.0721 | 1237.3953 | 19 | 4.469 | 61.1% | 1 | R.VITEEEKNFK.A | 2 |

---

|  |  |  |  |  |  |  |  |  |
| --- | --- | --- | --- | --- | --- | --- | --- | --- |
| U | *gi|15431293|ref|NP\_00* | 3 | 3 | 20.6% | 204 | 24146 | 11.6 | ribosomal protein L15 [Homo sapiens] |
| U | *gi|88998868|ref|XP\_94* | 3 | 3 | 20.6% | 204 | 24174 | 11.6 | PREDICTED: hypothetical protein isoform 4 [Homo sapiens] |
| U | *gi|88992455|ref|XP\_93* | 3 | 3 | 20.6% | 204 | 24174 | 11.6 | PREDICTED: hypothetical protein isoform 1 [Homo sapiens] |
| U | *gi|169169711|ref|XP\_0* | 3 | 3 | 20.6% | 204 | 24174 | 11.6 | PREDICTED: hypothetical protein [Homo sapiens] |

| Filename XCorr DeltCN Conf% ObsM+H+ CalcM+H+ SpR ZScore Ion% # Sequence  | | | | | | | | | | | | |
| --- | --- | --- | --- | --- | --- | --- | --- | --- | --- | --- | --- | --- |
|  | Astrin\_STLCHLD\_050114\_01.04201.04201.3 | 3.2348 | 0.3619 | 100.0% | 1706.7544 | 1706.945 | 3 | 6.462 | 40.0% | 1 | K.GATYGKPVHHGVNQLK.F | 3 |
|  | Astrin\_STLCHLD\_tube2\_050114\_01.08525.08525.2 | 3.195 | 0.4379 | 100.0% | 1661.6721 | 1661.8083 | 1 | 7.056 | 73.1% | 1 | R.VLNSYWVGEDSTYK.F | 2 |
|  | Astrin\_STLCHLD\_050114\_01.12909.12909.2 | 2.7043 | 0.2142 | 98.7% | 1506.8322 | 1505.7997 | 14 | 4.569 | 50.0% | 1 | K.FFEVILIDPFHK.A | 2 |

---

|  |  |  |  |  |  |  |  |  |
| --- | --- | --- | --- | --- | --- | --- | --- | --- |
| U | *gi|11024714|ref|NP\_06* | 5 | 11 | 20.5% | 229 | 25762 | 7.4 | ubiquitin B precursor [Homo sapiens] |
| U | *gi|77539055|ref|NP\_00* | 5 | 11 | 36.7% | 128 | 14728 | 9.8 | ubiquitin and ribosomal protein L40 precursor [Homo sapiens] |
| U | *gi|67191208|ref|NP\_06* | 5 | 11 | 6.9% | 685 | 77029 | 7.7 | ubiquitin C [Homo sapiens] |
| U | *gi|4507761|ref|NP\_003* | 5 | 11 | 36.7% | 128 | 14728 | 9.8 | ubiquitin and ribosomal protein L40 precursor [Homo sapiens] |
| U | *gi|4506713|ref|NP\_002* | 5 | 11 | 30.1% | 156 | 17965 | 9.6 | ubiquitin and ribosomal protein S27a precursor [Homo sapiens] |
| U | *gi|208022622|ref|NP\_0* | 5 | 11 | 30.1% | 156 | 17965 | 9.6 | ubiquitin and ribosomal protein S27a precursor [Homo sapiens] |

| Filename XCorr DeltCN Conf% ObsM+H+ CalcM+H+ SpR ZScore Ion% # Sequence  | | | | | | | | | | | | |
| --- | --- | --- | --- | --- | --- | --- | --- | --- | --- | --- | --- | --- |
|  | Astrin\_STLCHLD\_050114\_02.07464.07464.2 | 3.789 | 0.305 | 99.9% | 1788.7322 | 1788.9897 | 6 | 6.275 | 46.7% | 3 | K.TITLEVEPSDTIENVK.A | 2 |
|  | Astrin\_STLCHLD\_050114\_01.04343.04343.2 | 2.5334 | 0.2956 | 99.0% | 1523.5922 | 1524.6738 | 2 | 5.279 | 70.8% | 1 | K.IQDKEGIPPDQQR.L | 2 |
|  | Astrin\_STLCHLD\_050114\_01.06895.06895.2 | 2.5347 | 0.2384 | 99.1% | 1082.3722 | 1082.1986 | 2 | 5.538 | 75.0% | 4 | R.TLSDYNIQK.E | 2 |
|  | Astrin\_STLCHLD\_tube2\_050114\_02.07117.07117.3 | 3.373 | 0.449 | 100.0% | 2130.3542 | 2131.4368 | 1 | 6.513 | 38.2% | 2 | R.TLSDYNIQKESTLHLVLR.L | 3 |
|  | Astrin\_STLCHLD\_tube2\_050114\_01.06771.06771.2 | 2.854 | 0.2743 | 99.9% | 1068.3322 | 1068.2615 | 2 | 5.151 | 87.5% | 1 | K.ESTLHLVLR.L | 2 |

---

|  |  |  |  |  |  |  |  |  |
| --- | --- | --- | --- | --- | --- | --- | --- | --- |
| U | *gi|33286418|ref|NP\_00* | 7 | 11 | 20.2% | 531 | 57937 | 7.8 | pyruvate kinase, muscle isoform M2 [Homo sapiens] |

| Filename XCorr DeltCN Conf% ObsM+H+ CalcM+H+ SpR ZScore Ion% # Sequence  | | | | | | | | | | | | |
| --- | --- | --- | --- | --- | --- | --- | --- | --- | --- | --- | --- | --- |
|  | Astrin\_STLCHLD\_tube2\_050114\_01.07161.07161.2 | 2.8667 | 0.3211 | 99.9% | 1198.7922 | 1198.3617 | 10 | 5.562 | 65.0% | 3 | R.LDIDSPPITAR.N | 2 |
|  | Astrin\_STLCHLD\_tube2\_050114\_01.04382.04382.3 | 4.0463 | 0.357 | 100.0% | 1885.6144 | 1885.0458 | 1 | 7.06 | 45.0% | 2 | R.LNFSHGTHEYHAETIK.N | 3 |
|  | Astrin\_STLCHLD\_tube2\_050114\_01.10809.10809.2 | 2.7415 | 0.2655 | 99.1% | 1463.3922 | 1463.7142 | 35 | 5.347 | 50.0% | 1 | K.IYVDDGLISLQVK.Q | 2 |
|  | Astrin\_STLCHLD\_tube2\_050114\_02.08128.08128.2 | 3.6162 | 0.4909 | 100.0% | 1780.4122 | 1780.9292 | 1 | 9.054 | 55.9% | 1 | K.GADFLVTEVENGGSLGSK.K | 2 |
|  | Astrin\_STLCHLD\_050114\_01.15876.15876.2 | 4.0523 | 0.5159 | 100.0% | 1860.8722 | 1861.1224 | 1 | 8.684 | 66.7% | 2 | K.FGVEQDVDMVFASFIR.K | 2 |
|  | Astrin\_STLCHLD\_050114\_02.08494.08494.3 | 3.1215 | 0.2548 | 99.8% | 1823.3644 | 1823.0741 | 1 | 5.303 | 41.7% | 1 | R.RFDEILEASDGIMVAR.G | 3 |
| \* | Astrin\_STLCHLD\_tube2\_050114\_02.08443.08443.3 | 2.5772 | 0.3543 | 99.9% | 2090.1543 | 2089.3586 | 3 | 5.262 | 35.9% | 1 | R.EAEAAIYHLQLFEELRR.L | 3 |

---

|  |  |  |  |  |  |  |  |  |
| --- | --- | --- | --- | --- | --- | --- | --- | --- |
| U | *gi|21464101|ref|NP\_03* | 4 | 5 | 20.2% | 247 | 28303 | 4.9 | tyrosine 3-monooxygenase/tryptophan 5-monooxygenase activation protein, gamma polypeptide [Homo sapiens] |

| Filename XCorr DeltCN Conf% ObsM+H+ CalcM+H+ SpR ZScore Ion% # Sequence  | | | | | | | | | | | | |
| --- | --- | --- | --- | --- | --- | --- | --- | --- | --- | --- | --- | --- |
|  | Astrin\_STLCHLD\_050114\_01.06965.06965.2 | 2.0972 | 0.249 | 97.2% | 1016.2522 | 1016.172 | 1 | 4.78 | 68.8% | 2 | R.YDDMAAAMK.N | 2 |
| \* | Astrin\_STLCHLD\_050114\_01.10814.10814.2 | 2.9059 | 0.2571 | 99.2% | 1644.6721 | 1644.7356 | 4 | 5.482 | 46.2% | 1 | K.NVTELNEPLSNEER.N | 2 |
| \* | Astrin\_STLCHLD\_050114\_02.09672.09672.2 | 2.9714 | 0.2058 | 98.8% | 1798.8522 | 1798.8473 | 79 | 3.36 | 40.0% | 1 | R.VISS\*IEQK@TSADGNEK.K | 2 |
| \* | Astrin\_STLCHLD\_050114\_01.04990.04990.2 | 2.4955 | 0.148 | 96.2% | 1237.2722 | 1237.3983 | 3 | 4.716 | 65.0% | 1 | R.YLAEVATGEKR.A | 2 |

---

|  |  |  |  |  |  |  |  |  |
| --- | --- | --- | --- | --- | --- | --- | --- | --- |
| U | *gi|4758792|ref|NP\_004* | 2 | 3 | 20.2% | 124 | 13712 | 8.3 | NADH dehydrogenase (ubiquinone) Fe-S protein 6, 13kDa (NADH-coenzyme Q reductase) [Homo sapiens] |

| Filename XCorr DeltCN Conf% ObsM+H+ CalcM+H+ SpR ZScore Ion% # Sequence  | | | | | | | | | | | | |
| --- | --- | --- | --- | --- | --- | --- | --- | --- | --- | --- | --- | --- |
| \* | Astrin\_STLCHLD\_050114\_01.04263.04263.3 | 2.8238 | 0.2423 | 97.8% | 1853.5443 | 1853.989 | 2 | 5.279 | 37.5% | 1 | K.VTHTGQVYDDKDYRR.I | 3 |
| \* | Astrin\_STLCHLD\_050114\_01.07499.07499.2 | 3.1894 | 0.2778 | 99.9% | 1222.9521 | 1223.4117 | 1 | 5.99 | 77.8% | 2 | K.VYINLDKETK.T | 2 |

---

|  |  |  |  |  |  |  |  |  |
| --- | --- | --- | --- | --- | --- | --- | --- | --- |
| U | *gi|4504517|ref|NP\_001* | 3 | 4 | 20.0% | 205 | 22783 | 6.4 | heat shock protein beta-1 [Homo sapiens] |

| Filename XCorr DeltCN Conf% ObsM+H+ CalcM+H+ SpR ZScore Ion% # Sequence  | | | | | | | | | | | | |
| --- | --- | --- | --- | --- | --- | --- | --- | --- | --- | --- | --- | --- |
| \* | Astrin\_STLCHLD\_tube2\_050114\_01.10764.10764.3 | 3.3775 | 0.2942 | 100.0% | 1904.8744 | 1904.0537 | 1 | 5.649 | 41.1% | 1 | R.GPSWDPFRDWYPHSR.L | 3 |
| \* | Astrin\_STLCHLD\_tube2\_050114\_01.10418.10418.2 | 4.0182 | 0.4491 | 100.0% | 1164.1322 | 1164.3494 | 1 | 8.048 | 83.3% | 2 | R.LFDQAFGLPR.L | 2 |
| \* | Astrin\_STLCHLD\_tube2\_050114\_01.09162.09162.2 | 4.6078 | 0.5358 | 100.0% | 1784.3722 | 1785.0068 | 1 | 9.389 | 66.7% | 1 | R.VSLDVNHFAPDELTVK.T | 2 |

---

|  |  |  |  |  |  |  |  |  |
| --- | --- | --- | --- | --- | --- | --- | --- | --- |
| U | *gi|24234688|ref|NP\_00* | 10 | 16 | 19.6% | 679 | 73681 | 6.2 | heat shock 70kDa protein 9 precursor [Homo sapiens] |

| Filename XCorr DeltCN Conf% ObsM+H+ CalcM+H+ SpR ZScore Ion% # Sequence  | | | | | | | | | | | | |
| --- | --- | --- | --- | --- | --- | --- | --- | --- | --- | --- | --- | --- |
| \* | Astrin\_STLCHLD\_tube2\_050114\_01.07373.07373.2 | 3.2381 | 0.4267 | 100.0% | 1451.1522 | 1451.576 | 1 | 6.766 | 61.5% | 2 | R.TTPSVVAFTADGER.L | 2 |
| \* | Astrin\_STLCHLD\_tube2\_050114\_01.12606.12606.2 | 2.307 | 0.2569 | 97.1% | 1555.7322 | 1554.8878 | 1 | 4.459 | 57.7% | 1 | K.LYSPSQIGAFVLMK.M | 2 |
| \* | Astrin\_STLCHLD\_tube2\_050114\_01.09437.09437.2 | 2.9344 | 0.3783 | 99.9% | 1696.4722 | 1695.8723 | 1 | 5.875 | 60.7% | 1 | K.NAVITVPAYFNDSQR.Q | 2 |
| \* | Astrin\_STLCHLD\_tube2\_050114\_01.08744.08744.2 | 3.4737 | 0.3321 | 100.0% | 1243.5922 | 1243.4056 | 1 | 6.368 | 77.3% | 2 | K.DAGQISGLNVLR.V | 2 |
| \* | Astrin\_STLCHLD\_050114\_01.11035.11035.2 | 2.336 | 0.2327 | 96.4% | 1692.5721 | 1691.8969 | 2 | 4.51 | 57.1% | 1 | R.ETGVDLTKDNMALQR.V | 2 |
| \* | Astrin\_STLCHLD\_tube2\_050114\_01.11938.11938.2 | 3.5052 | 0.3996 | 100.0% | 1362.2122 | 1362.5687 | 1 | 8.523 | 63.6% | 3 | R.AQFEGIVTDLIR.R | 2 |
| \* | Astrin\_STLCHLD\_tube2\_050114\_01.08521.08521.2 | 2.9079 | 0.3166 | 99.9% | 1291.7922 | 1291.4496 | 1 | 5.952 | 70.0% | 2 | K.VQQTVQDLFGR.A | 2 |
| \* | Astrin\_STLCHLD\_050114\_02.05636.05636.3 | 4.162 | 0.2243 | 100.0% | 1939.2544 | 1938.1448 | 7 | 5.177 | 36.8% | 1 | K.KSQVFSTAADGQTQVEIK.V | 3 |
| \* | Astrin\_STLCHLD\_tube2\_050114\_02.05938.05938.2 | 3.2332 | 0.2911 | 99.8% | 1810.7922 | 1809.9707 | 2 | 5.204 | 46.9% | 1 | K.SQVFSTAADGQTQVEIK.V | 2 |
| \* | Astrin\_STLCHLD\_tube2\_050114\_02.07076.07076.3 | 3.1888 | 0.3113 | 100.0% | 2421.0842 | 2419.7095 | 4 | 5.909 | 29.8% | 2 | R.EQQIVIQSSGGLSKDDIENMVK.N | 3 |

---

|  |  |  |  |  |  |  |  |  |
| --- | --- | --- | --- | --- | --- | --- | --- | --- |
| U | *gi|16753227|ref|NP\_00* | 4 | 6 | 19.4% | 288 | 32728 | 10.6 | ribosomal protein L6 [Homo sapiens] |
| U | *gi|67189747|ref|NP\_00* | 4 | 6 | 19.4% | 288 | 32728 | 10.6 | ribosomal protein L6 [Homo sapiens] |

| Filename XCorr DeltCN Conf% ObsM+H+ CalcM+H+ SpR ZScore Ion% # Sequence  | | | | | | | | | | | | |
| --- | --- | --- | --- | --- | --- | --- | --- | --- | --- | --- | --- | --- |
|  | Astrin\_STLCHLD\_050114\_01.15124.15124.2 | 3.4033 | 0.5136 | 100.0% | 1526.4922 | 1526.8601 | 1 | 9.07 | 75.0% | 2 | R.ASITPGTILIILTGR.H | 2 |
|  | Astrin\_STLCHLD\_050114\_01.07599.07599.2 | 2.11 | 0.2998 | 98.9% | 994.4522 | 995.1228 | 2 | 5.269 | 71.4% | 1 | K.HLTDAYFK.K | 2 |
|  | Astrin\_STLCHLD\_tube2\_050114\_02.05508.05508.3 | 4.4273 | 0.4352 | 100.0% | 2510.2744 | 2510.6763 | 1 | 7.25 | 42.1% | 2 | R.HQEGEIFDTEKEKYEITEQR.K | 3 |
|  | Astrin\_STLCHLD\_tube2\_050114\_01.08895.08895.2 | 3.1901 | 0.3874 | 100.0% | 1447.0922 | 1447.6769 | 4 | 7.303 | 54.2% | 1 | R.SVFALTNGIYPHK.L | 2 |

---

|  |  |  |  |  |  |  |  |  |
| --- | --- | --- | --- | --- | --- | --- | --- | --- |
| U | *gi|4758086|ref|NP\_004* | 2 | 8 | 19.2% | 193 | 20567 | 8.6 | cysteine and glycine-rich protein 1 isoform 1 [Homo sapiens] |

| Filename XCorr DeltCN Conf% ObsM+H+ CalcM+H+ SpR ZScore Ion% # Sequence  | | | | | | | | | | | | |
| --- | --- | --- | --- | --- | --- | --- | --- | --- | --- | --- | --- | --- |
|  | Astrin\_STLCHLD\_050114\_02.06182.06182.3 | 3.0079 | 0.3548 | 100.0% | 2159.4844 | 2160.3452 | 3 | 5.301 | 31.0% | 1 | K.GYGYGQGAGTLSTDKGESLGIK.H | 3 |
| \* | Astrin\_STLCHLD\_050114\_01.12136.12136.2 | 3.6879 | 0.373 | 100.0% | 1434.3522 | 1434.551 | 2 | 7.625 | 64.3% | 7 | K.GFGFGQGAGALVHSE.- | 2 |

---

|  |  |  |  |  |  |  |  |  |
| --- | --- | --- | --- | --- | --- | --- | --- | --- |
| U | *gi|17986258|ref|NP\_06* | 2 | 3 | 19.2% | 151 | 16930 | 4.7 | myosin, light chain 6, alkali, smooth muscle and non-muscle isoform 1 [Homo sapiens] |
| U | *gi|88999583|ref|NP\_52* | 2 | 3 | 19.2% | 151 | 16961 | 4.6 | myosin, light chain 6, alkali, smooth muscle and non-muscle isoform 2 [Homo sapiens] |

| Filename XCorr DeltCN Conf% ObsM+H+ CalcM+H+ SpR ZScore Ion% # Sequence  | | | | | | | | | | | | |
| --- | --- | --- | --- | --- | --- | --- | --- | --- | --- | --- | --- | --- |
|  | Astrin\_STLCHLD\_tube2\_050114\_01.05236.05236.2 | 3.1836 | 0.2625 | 99.7% | 1355.3722 | 1355.5339 | 1 | 5.972 | 62.5% | 2 | R.ALGQNPTNAEVLK.V | 2 |
|  | Astrin\_STLCHLD\_050114\_01.13667.13667.2 | 2.9647 | 0.2297 | 99.0% | 1889.7122 | 1889.2628 | 2 | 4.559 | 36.7% | 1 | K.VLDFEHFLPMLQTVAK.N | 2 |

---

|  |  |  |  |  |  |  |  |  |
| --- | --- | --- | --- | --- | --- | --- | --- | --- |
| U | *gi|221307584|ref|NP\_0* | 4 | 7 | 19.1% | 299 | 33296 | 9.8 | prohibitin 2 isoform 1 [Homo sapiens] |
| U | *gi|6005854|ref|NP\_009* | 4 | 7 | 19.1% | 299 | 33296 | 9.8 | prohibitin 2 isoform 2 [Homo sapiens] |

| Filename XCorr DeltCN Conf% ObsM+H+ CalcM+H+ SpR ZScore Ion% # Sequence  | | | | | | | | | | | | |
| --- | --- | --- | --- | --- | --- | --- | --- | --- | --- | --- | --- | --- |
|  | Astrin\_STLCHLD\_050114\_02.08445.08445.3 | 4.5089 | 0.5137 | 100.0% | 1855.5543 | 1855.1038 | 1 | 8.476 | 50.0% | 3 | R.IGGVQQDTILAEGLHFR.I | 3 |
|  | Astrin\_STLCHLD\_050114\_01.14243.14243.2 | 4.167 | 0.4088 | 100.0% | 1724.5122 | 1725.0428 | 1 | 7.427 | 66.7% | 2 | R.IPWFQYPIIYDIR.A | 2 |
|  | Astrin\_STLCHLD\_tube2\_050114\_01.05828.05828.2 | 1.6431 | 0.3457 | 96.0% | 995.0522 | 995.077 | 1 | 5.913 | 78.6% | 1 | R.LGLDYEER.V | 2 |
|  | Astrin\_STLCHLD\_tube2\_050114\_02.09942.09942.2 | 2.554 | 0.2094 | 96.9% | 2226.3123 | 2226.4912 | 6 | 4.795 | 38.9% | 1 | R.IYLTADNLVLNLQDESFTR.G | 2 |

---

|  |  |  |  |  |  |  |  |  |
| --- | --- | --- | --- | --- | --- | --- | --- | --- |
| U | *gi|219555707|ref|NP\_0* | 2 | 2 | 19.0% | 184 | 20170 | 7.0 | eukaryotic translation initiation factor 5A isoform A [Homo sapiens] |
| U | *gi|4503545|ref|NP\_001* | 2 | 2 | 22.7% | 154 | 16832 | 5.2 | eukaryotic translation initiation factor 5A isoform B [Homo sapiens] |
| U | *gi|219555712|ref|NP\_0* | 2 | 2 | 22.7% | 154 | 16832 | 5.2 | eukaryotic translation initiation factor 5A isoform B [Homo sapiens] |
| U | *gi|219555710|ref|NP\_0* | 2 | 2 | 22.7% | 154 | 16832 | 5.2 | eukaryotic translation initiation factor 5A isoform B [Homo sapiens] |

| Filename XCorr DeltCN Conf% ObsM+H+ CalcM+H+ SpR ZScore Ion% # Sequence  | | | | | | | | | | | | |
| --- | --- | --- | --- | --- | --- | --- | --- | --- | --- | --- | --- | --- |
|  | Astrin\_STLCHLD\_tube2\_050114\_01.10463.10463.2 | 2.9051 | 0.3509 | 99.9% | 1300.5922 | 1299.5559 | 1 | 6.368 | 72.7% | 1 | K.VHLVGIDIFTGK.K | 2 |
|  | Astrin\_STLCHLD\_050114\_02.11271.11271.2 | 5.0697 | 0.4686 | 100.0% | 2581.8323 | 2581.8418 | 1 | 9.659 | 47.7% | 1 | R.NDFQLIGIQDGYLSLLQDSGEVR.E | 2 |

---

|  |  |  |  |  |  |  |  |  |
| --- | --- | --- | --- | --- | --- | --- | --- | --- |
| U | *gi|56699409|ref|NP\_00* | 5 | 10 | 18.9% | 391 | 42332 | 10.1 | RNA binding motif protein, X-linked [Homo sapiens] |

| Filename XCorr DeltCN Conf% ObsM+H+ CalcM+H+ SpR ZScore Ion% # Sequence  | | | | | | | | | | | | |
| --- | --- | --- | --- | --- | --- | --- | --- | --- | --- | --- | --- | --- |
|  | Astrin\_STLCHLD\_tube2\_050114\_01.08312.08312.2 | 3.1613 | 0.2573 | 99.6% | 1435.6721 | 1436.6049 | 1 | 6.375 | 70.8% | 3 | K.LFIGGLNTETNEK.A | 2 |
|  | Astrin\_STLCHLD\_tube2\_050114\_01.11028.11028.2 | 4.438 | 0.4571 | 100.0% | 1487.3121 | 1487.6519 | 1 | 8.812 | 73.1% | 1 | R.GFAFVTFESPADAK.D | 2 |
| \* | Astrin\_STLCHLD\_050114\_01.05601.05601.3 | 3.1606 | 0.2747 | 99.9% | 1750.5844 | 1748.9768 | 4 | 4.953 | 36.7% | 1 | K.AIKVEQATKPSFESGR.R | 3 |
|  | Astrin\_STLCHLD\_tube2\_050114\_02.06211.06211.3 | 4.0011 | 0.315 | 100.0% | 2050.7944 | 2051.1873 | 1 | 6.158 | 40.3% | 4 | R.GGHMDDGGYSMNFNMSSSR.G | 3 |
|  | Astrin\_STLCHLD\_tube2\_050114\_01.04769.04769.2 | 2.9954 | 0.4334 | 100.0% | 1514.3322 | 1514.6372 | 35 | 6.897 | 54.5% | 1 | R.DYAPPPRDYTYR.D | 2 |

---

|  |  |  |  |  |  |  |  |  |
| --- | --- | --- | --- | --- | --- | --- | --- | --- |
| U | *gi|4757880|ref|NP\_004* | 4 | 6 | 18.9% | 328 | 37155 | 6.8 | budding uninhibited by benzimidazoles 3 isoform a [Homo sapiens] |
| U | *gi|56550081|ref|NP\_00* | 4 | 6 | 19.0% | 326 | 36955 | 6.8 | budding uninhibited by benzimidazoles 3 isoform b [Homo sapiens] |

| Filename XCorr DeltCN Conf% ObsM+H+ CalcM+H+ SpR ZScore Ion% # Sequence  | | | | | | | | | | | | |
| --- | --- | --- | --- | --- | --- | --- | --- | --- | --- | --- | --- | --- |
|  | Astrin\_STLCHLD\_050114\_01.07263.07263.2 | 3.0556 | 0.2433 | 99.3% | 1384.6522 | 1384.529 | 27 | 5.344 | 54.2% | 2 | K.LNQPPEDGISSVK.F | 2 |
|  | Astrin\_STLCHLD\_tube2\_050114\_02.09785.09785.2 | 3.5043 | 0.4444 | 100.0% | 2172.9321 | 2172.402 | 1 | 7.047 | 44.4% | 1 | K.FSPNTSQFLLVSSWDTSVR.L | 2 |
|  | Astrin\_STLCHLD\_tube2\_050114\_01.05711.05711.2 | 2.4974 | 0.2568 | 99.0% | 1166.1721 | 1166.3373 | 17 | 4.866 | 66.7% | 1 | R.LYDVPANSMR.L | 2 |
|  | Astrin\_STLCHLD\_050114\_01.10786.10786.3 | 5.11 | 0.4458 | 100.0% | 2276.2144 | 2277.4736 | 1 | 8.026 | 42.1% | 2 | K.MHDLNTDQENLVGTHDAPIR.C | 3 |

---

|  |  |  |  |  |  |  |  |  |
| --- | --- | --- | --- | --- | --- | --- | --- | --- |
| U | *gi|4506613|ref|NP\_000* | 2 | 4 | 18.8% | 128 | 14787 | 9.2 | ribosomal protein L22 proprotein [Homo sapiens] |

| Filename XCorr DeltCN Conf% ObsM+H+ CalcM+H+ SpR ZScore Ion% # Sequence  | | | | | | | | | | | | |
| --- | --- | --- | --- | --- | --- | --- | --- | --- | --- | --- | --- | --- |
| \* | Astrin\_STLCHLD\_tube2\_050114\_01.06258.06258.2 | 3.2445 | 0.4286 | 100.0% | 1243.0521 | 1243.4056 | 2 | 7.055 | 66.7% | 3 | K.AGNLGGGVVTIER.S | 2 |
| \* | Astrin\_STLCHLD\_tube2\_050114\_01.07525.07525.2 | 2.4014 | 0.3155 | 99.2% | 1208.3922 | 1208.3971 | 14 | 5.563 | 65.0% | 1 | K.ITVTSEVPFSK.R | 2 |

---

|  |  |  |  |  |  |  |  |  |
| --- | --- | --- | --- | --- | --- | --- | --- | --- |
| U | *gi|87196351|ref|NP\_00* | 9 | 17 | 18.4% | 662 | 73244 | 7.2 | DEAD/H (Asp-Glu-Ala-Asp/His) box polypeptide 3 [Homo sapiens] |

| Filename XCorr DeltCN Conf% ObsM+H+ CalcM+H+ SpR ZScore Ion% # Sequence  | | | | | | | | | | | | |
| --- | --- | --- | --- | --- | --- | --- | --- | --- | --- | --- | --- | --- |
| \* | Astrin\_STLCHLD\_050114\_01.15770.15770.2 | 4.5694 | 0.5526 | 100.0% | 2333.3523 | 2333.6897 | 1 | 10.026 | 54.8% | 3 | K.TAAFLLPILSQIYSDGPGEALR.A | 2 |
|  | Astrin\_STLCHLD\_tube2\_050114\_02.06996.06996.2 | 3.2324 | 0.4907 | 100.0% | 1321.1721 | 1321.4729 | 2 | 8.05 | 75.0% | 3 | R.ELAVQIYEEAR.K | 2 |
|  | Astrin\_STLCHLD\_tube2\_050114\_01.09962.09962.2 | 3.4719 | 0.4712 | 100.0% | 1336.7922 | 1337.5946 | 1 | 7.799 | 85.0% | 2 | R.MLDMGFEPQIR.R | 222 |
|  | Astrin\_STLCHLD\_tube2\_050114\_02.11896.11896.2 | 2.7157 | 0.3014 | 99.3% | 1558.2122 | 1558.774 | 10 | 6.473 | 54.2% | 1 | R.DFLDEYIFLAVGR.V | 2 |
| \* | Astrin\_STLCHLD\_050114\_01.13718.13718.2 | 3.6688 | 0.522 | 100.0% | 1292.3322 | 1292.5181 | 1 | 10.171 | 81.8% | 1 | R.SFLLDLLNATGK.D | 2 |
|  | Astrin\_STLCHLD\_050114\_01.05005.05005.3 | 2.9733 | 0.164 | 96.7% | 1302.2043 | 1301.4043 | 1 | 4.155 | 47.2% | 1 | R.DREEALHQFR.S | 3 |
|  | Astrin\_STLCHLD\_050114\_02.06951.06951.2 | 3.8789 | 0.5069 | 100.0% | 1169.4321 | 1169.4099 | 1 | 7.497 | 81.8% | 4 | K.SPILVATAVAAR.G | 2 |
|  | Astrin\_STLCHLD\_tube2\_050114\_01.11176.11176.3 | 4.3503 | 0.3258 | 100.0% | 2084.1843 | 2084.2957 | 1 | 7.249 | 46.9% | 1 | K.HVINFDLPSDIEEYVHR.I | 3 |
| \* | Astrin\_STLCHLD\_tube2\_050114\_01.11424.11424.2 | 4.0909 | 0.4095 | 100.0% | 1526.4521 | 1525.7043 | 1 | 7.106 | 73.1% | 1 | R.VGNLGLATSFFNER.N | 2 |

Similarities:
gi|4758138|ref|NP\_004(1:8)  
gi|148613856|ref|NP\_0(1:8)  

---

|  |  |  |  |  |  |  |  |  |
| --- | --- | --- | --- | --- | --- | --- | --- | --- |
| U | *gi|38327562|ref|NP\_00* | 6 | 7 | 18.4% | 403 | 45823 | 9.4 | serine/threonine protein kinase 6 [Homo sapiens] |
| U | *gi|38327572|ref|NP\_94* | 6 | 7 | 18.4% | 403 | 45823 | 9.4 | serine/threonine protein kinase 6 [Homo sapiens] |
| U | *gi|38327570|ref|NP\_94* | 6 | 7 | 18.4% | 403 | 45823 | 9.4 | serine/threonine protein kinase 6 [Homo sapiens] |
| U | *gi|38327568|ref|NP\_94* | 6 | 7 | 18.4% | 403 | 45823 | 9.4 | serine/threonine protein kinase 6 [Homo sapiens] |
| U | *gi|38327566|ref|NP\_94* | 6 | 7 | 18.4% | 403 | 45823 | 9.4 | serine/threonine protein kinase 6 [Homo sapiens] |
| U | *gi|38327564|ref|NP\_94* | 6 | 7 | 18.4% | 403 | 45823 | 9.4 | serine/threonine protein kinase 6 [Homo sapiens] |

| Filename XCorr DeltCN Conf% ObsM+H+ CalcM+H+ SpR ZScore Ion% # Sequence  | | | | | | | | | | | | |
| --- | --- | --- | --- | --- | --- | --- | --- | --- | --- | --- | --- | --- |
|  | Astrin\_STLCHLD\_050114\_01.10582.10582.3 | 3.9156 | 0.3095 | 100.0% | 2167.4343 | 2166.3496 | 3 | 5.137 | 32.9% | 1 | K.SKQPLPSAPENNPEEELASK.Q | 3 |
|  | Astrin\_STLCHLD\_050114\_01.10382.10382.2 | 2.768 | 0.362 | 99.9% | 1243.3322 | 1243.3641 | 1 | 6.331 | 83.3% | 2 | R.LYGYFHDATR.V | 2 |
|  | Astrin\_STLCHLD\_tube2\_050114\_01.10298.10298.2 | 2.6322 | 0.2373 | 98.2% | 1696.8522 | 1697.9695 | 1 | 6.419 | 50.0% | 1 | R.DIKPENLLLGSAGELK.I | 2 |
|  | Astrin\_STLCHLD\_tube2\_050114\_01.08101.08101.3 | 2.047 | 0.4221 | 99.9% | 1530.4744 | 1530.683 | 2 | 5.998 | 38.5% | 1 | K.IADFGWSVHAPSSR.R | 3 |
|  | Astrin\_STLCHLD\_tube2\_050114\_01.08085.08085.2 | 3.1272 | 0.3426 | 99.9% | 1531.8722 | 1530.683 | 3 | 5.443 | 53.8% | 1 | K.IADFGWSVHAPSSR.R | 2 |
|  | Astrin\_STLCHLD\_tube2\_050114\_01.12575.12575.2 | 3.0768 | 0.3971 | 99.9% | 1614.9722 | 1615.7826 | 2 | 7.077 | 57.7% | 1 | R.VEFTFPDFVTEGAR.D | 2 |

---

|  |  |  |  |  |  |  |  |  |
| --- | --- | --- | --- | --- | --- | --- | --- | --- |
| U | *gi|5454064|ref|NP\_006* | 10 | 25 | 18.1% | 669 | 69492 | 9.7 | RNA binding motif protein 14 [Homo sapiens] |

| Filename XCorr DeltCN Conf% ObsM+H+ CalcM+H+ SpR ZScore Ion% # Sequence  | | | | | | | | | | | | |
| --- | --- | --- | --- | --- | --- | --- | --- | --- | --- | --- | --- | --- |
| \* | Astrin\_STLCHLD\_050114\_01.04961.04961.3 | 3.0787 | 0.3099 | 100.0% | 1556.8444 | 1556.7677 | 1 | 5.062 | 44.2% | 2 | R.AIEALHGHELRPGR.A | 3 |
| \* | Astrin\_STLCHLD\_tube2\_050114\_01.07035.07035.2 | 3.5964 | 0.4396 | 100.0% | 1609.8922 | 1609.8223 | 1 | 8.286 | 67.9% | 2 | R.ASYVAPLTAQPATYR.A | 2 |
| \* | Astrin\_STLCHLD\_tube2\_050114\_01.05794.05794.2 | 2.377 | 0.3014 | 98.9% | 1220.3322 | 1220.3707 | 17 | 5.069 | 54.5% | 1 | R.AQPSVSLGAAYR.A | 2 |
| \* | Astrin\_STLCHLD\_tube2\_050114\_01.05150.05150.2 | 2.5876 | 0.3317 | 99.5% | 1206.2322 | 1206.3439 | 1 | 6.208 | 68.2% | 1 | R.AQPSASLGVGYR.T | 2 |
| \* | Astrin\_STLCHLD\_050114\_01.05588.05588.2 | 2.7262 | 0.2643 | 99.2% | 1325.1322 | 1325.482 | 2 | 6.158 | 68.2% | 3 | R.TQPMTAQAASYR.A | 2 |
| \* | Astrin\_STLCHLD\_tube2\_050114\_01.05991.05991.2 | 2.624 | 0.3236 | 99.5% | 1246.1322 | 1246.4087 | 1 | 5.455 | 63.6% | 3 | R.AQPSVSLGAPYR.G | 2 |
| \* | Astrin\_STLCHLD\_tube2\_050114\_01.05584.05584.3 | 4.3935 | 0.5019 | 100.0% | 2466.1743 | 2466.6292 | 1 | 7.96 | 37.0% | 7 | R.TQSSASLAASYAAQQHPQAAASYR.G | 3 |
| \* | Astrin\_STLCHLD\_tube2\_050114\_01.05340.05340.2 | 2.4842 | 0.1197 | 95.1% | 1223.2522 | 1223.3744 | 10 | 4.79 | 72.2% | 1 | R.RLSESQLSFR.R | 2 |
| \* | Astrin\_STLCHLD\_tube2\_050114\_01.06705.06705.2 | 2.9759 | 0.3875 | 100.0% | 1068.2922 | 1067.1869 | 1 | 6.177 | 81.2% | 2 | R.LSESQLSFR.R | 2 |
| \* | Astrin\_STLCHLD\_050114\_01.11120.11120.2 | 3.1048 | 0.3939 | 100.0% | 1237.8322 | 1238.2988 | 1 | 7.871 | 83.3% | 3 | R.YSGSYNDYLR.A | 2 |

---

|  |  |  |  |  |  |  |  |  |
| --- | --- | --- | --- | --- | --- | --- | --- | --- |
| U | *gi|20149594|ref|NP\_03* | 11 | 22 | 18.0% | 724 | 83264 | 5.0 | heat shock 90kDa protein 1, beta [Homo sapiens] |

| Filename XCorr DeltCN Conf% ObsM+H+ CalcM+H+ SpR ZScore Ion% # Sequence  | | | | | | | | | | | | |
| --- | --- | --- | --- | --- | --- | --- | --- | --- | --- | --- | --- | --- |
|  | Astrin\_STLCHLD\_tube2\_050114\_01.08227.08227.2 | 2.3194 | 0.3076 | 98.7% | 1545.1522 | 1545.733 | 22 | 4.62 | 46.2% | 1 | R.ELISNASDALDKIR.Y | 2 |
|  | Astrin\_STLCHLD\_tube2\_050114\_01.08950.08950.2 | 2.5774 | 0.3972 | 99.9% | 1242.6322 | 1243.4459 | 1 | 6.893 | 68.2% | 1 | K.ADLINNLGTIAK.S | 22 |
|  | Astrin\_STLCHLD\_050114\_02.06302.06302.3 | 4.6555 | 0.5081 | 100.0% | 2016.2344 | 2016.2584 | 1 | 8.269 | 48.3% | 2 | K.VILHLKEDQTEYLEER.R | 33 |
|  | Astrin\_STLCHLD\_050114\_01.05627.05627.2 | 2.9558 | 0.3011 | 99.9% | 1152.4722 | 1152.2462 | 1 | 5.822 | 81.2% | 3 | K.YIDQEELNK.T | 22 |
|  | Astrin\_STLCHLD\_tube2\_050114\_01.08112.08112.2 | 3.8262 | 0.4294 | 100.0% | 1527.9722 | 1528.6616 | 1 | 7.894 | 75.0% | 2 | K.SLTNDWEDHLAVK.H | 22 |
|  | Astrin\_STLCHLD\_050114\_02.06964.06964.3 | 2.8425 | 0.2263 | 99.2% | 1349.2444 | 1349.4886 | 308 | 5.284 | 40.0% | 2 | K.HFSVEGQLEFR.A | 33 |
|  | Astrin\_STLCHLD\_tube2\_050114\_02.06687.06687.2 | 2.9963 | 0.4425 | 100.0% | 1349.4321 | 1349.4886 | 1 | 6.469 | 70.0% | 4 | K.HFSVEGQLEFR.A | 22 |
| \* | Astrin\_STLCHLD\_tube2\_050114\_02.06678.06678.3 | 4.4679 | 0.3728 | 100.0% | 2178.6243 | 2178.2915 | 5 | 6.914 | 34.7% | 2 | R.YHTSQSGDEMTSLSEYVSR.M | 3 |
| \* | Astrin\_STLCHLD\_tube2\_050114\_01.06338.06338.2 | 1.9797 | 0.2544 | 95.4% | 1162.1522 | 1161.297 | 9 | 4.689 | 61.1% | 1 | K.SIYYITGESK.E | 2 |
| \* | Astrin\_STLCHLD\_050114\_01.07091.07091.2 | 2.6319 | 0.3153 | 99.6% | 1250.3922 | 1250.3538 | 245 | 7.101 | 50.0% | 2 | K.EQVANSAFVER.V | 2 |
| \* | Astrin\_STLCHLD\_tube2\_050114\_01.07024.07024.3 | 4.1236 | 0.5214 | 100.0% | 1783.4944 | 1784.025 | 1 | 8.673 | 46.4% | 2 | K.HLEINPDHPIVETLR.Q | 3 |

Similarities:
gi|153792590|ref|NP\_0(6:5)  

---

|  |  |  |  |  |  |  |  |  |
| --- | --- | --- | --- | --- | --- | --- | --- | --- |
| U | *gi|15809016|ref|NP\_29* | 2 | 2 | 17.4% | 172 | 19779 | 4.8 | myosin regulatory light chain MRCL2 isoform A [Homo sapiens] |
| U | *gi|5453740|ref|NP\_006* | 2 | 2 | 17.5% | 171 | 19794 | 4.8 | myosin, light chain 12A, regulatory, non-sarcomeric [Homo sapiens] |
| U | *gi|222144328|ref|NP\_0* | 2 | 2 | 19.5% | 154 | 17757 | 4.4 | myosin regulatory light chain MRCL2 isoform B [Homo sapiens] |
| U | *gi|222144326|ref|NP\_0* | 2 | 2 | 17.4% | 172 | 19779 | 4.8 | myosin regulatory light chain MRCL2 isoform A [Homo sapiens] |
| U | *gi|222144324|ref|NP\_0* | 2 | 2 | 17.4% | 172 | 19779 | 4.8 | myosin regulatory light chain MRCL2 isoform A [Homo sapiens] |

| Filename XCorr DeltCN Conf% ObsM+H+ CalcM+H+ SpR ZScore Ion% # Sequence  | | | | | | | | | | | | |
| --- | --- | --- | --- | --- | --- | --- | --- | --- | --- | --- | --- | --- |
|  | Astrin\_STLCHLD\_tube2\_050114\_02.08584.08584.3 | 3.2574 | 0.2125 | 97.7% | 2434.9443 | 2433.649 | 6 | 5.274 | 27.6% | 1 | R.ELLTTMGDRFTDEEVDELYR.E | 3 |
|  | Astrin\_STLCHLD\_tube2\_050114\_01.09245.09245.2 | 3.075 | 0.2808 | 99.9% | 1262.2322 | 1261.3794 | 1 | 6.864 | 66.7% | 1 | K.GNFNYIEFTR.I | 2 |

---

|  |  |  |  |  |  |  |  |  |
| --- | --- | --- | --- | --- | --- | --- | --- | --- |
| U | *gi|4501881|ref|NP\_001* | 7 | 20 | 17.2% | 377 | 42051 | 5.4 | actin, alpha 1, skeletal muscle [Homo sapiens] |
| U | *gi|4885049|ref|NP\_005* | 7 | 20 | 17.2% | 377 | 42019 | 5.4 | cardiac muscle alpha actin 1 proprotein [Homo sapiens] |

| Filename XCorr DeltCN Conf% ObsM+H+ CalcM+H+ SpR ZScore Ion% # Sequence  | | | | | | | | | | | | |
| --- | --- | --- | --- | --- | --- | --- | --- | --- | --- | --- | --- | --- |
|  | Astrin\_STLCHLD\_tube2\_050114\_01.06957.06957.2 | 2.7548 | 0.3244 | 99.8% | 1199.3322 | 1199.4415 | 6 | 5.746 | 65.0% | 3 | R.AVFPSIVGRPR.H | 22 |
|  | Astrin\_STLCHLD\_tube2\_050114\_01.08855.08855.2 | 4.0249 | 0.2415 | 99.9% | 1961.4722 | 1962.1841 | 1 | 6.456 | 60.0% | 1 | K.YPIEHGIITNWDDMEK.I | 2 |
|  | Astrin\_STLCHLD\_050114\_02.07078.07078.3 | 3.8086 | 0.1092 | 95.6% | 1962.0543 | 1962.1841 | 1 | 4.566 | 41.7% | 2 | K.YPIEHGIITNWDDMEK.I | 3 |
|  | Astrin\_STLCHLD\_tube2\_050114\_01.05846.05846.3 | 3.4498 | 0.3159 | 100.0% | 1515.5643 | 1516.7019 | 1 | 6.429 | 55.0% | 2 | K.IWHHTFYNELR.V | 33 |
|  | Astrin\_STLCHLD\_tube2\_050114\_01.05795.05795.2 | 3.3616 | 0.4634 | 100.0% | 1516.2722 | 1516.7019 | 1 | 7.494 | 80.0% | 2 | K.IWHHTFYNELR.V | 22 |
|  | Astrin\_STLCHLD\_tube2\_050114\_01.10092.10092.2 | 3.6111 | 0.3284 | 99.9% | 1790.9722 | 1791.9554 | 1 | 6.687 | 66.7% | 5 | K.SYELPDGQVITIGNER.F | 222 |
|  | Astrin\_STLCHLD\_tube2\_050114\_01.06200.06200.2 | 2.6991 | 0.3752 | 99.9% | 1162.3722 | 1162.3868 | 1 | 6.418 | 75.0% | 5 | K.EITALAPSTMK.I | 22 |

Similarities:
gi|4501885|ref|NP\_001(5:2)  
gi|63055057|ref|NP\_00(1:6)  

---

|  |  |  |  |  |  |  |  |  |
| --- | --- | --- | --- | --- | --- | --- | --- | --- |
| U | *gi|14141152|ref|NP\_00* | 10 | 26 | 17.0% | 730 | 77516 | 8.7 | heterogeneous nuclear ribonucleoprotein M isoform a [Homo sapiens] |
| U | *gi|157412270|ref|NP\_1* | 10 | 26 | 17.9% | 691 | 73621 | 8.8 | heterogeneous nuclear ribonucleoprotein M isoform b [Homo sapiens] |

| Filename XCorr DeltCN Conf% ObsM+H+ CalcM+H+ SpR ZScore Ion% # Sequence  | | | | | | | | | | | | |
| --- | --- | --- | --- | --- | --- | --- | --- | --- | --- | --- | --- | --- |
|  | Astrin\_STLCHLD\_050114\_01.06481.06481.2 | 2.0128 | 0.3171 | 98.5% | 957.21216 | 957.11017 | 5 | 6.066 | 75.0% | 1 | R.FGSGMNMGR.I | 2 |
|  | Astrin\_STLCHLD\_tube2\_050114\_01.08139.08139.2 | 2.3532 | 0.2431 | 98.5% | 1115.2722 | 1115.3152 | 195 | 5.137 | 61.1% | 1 | R.INEILSNALK.R | 2 |
|  | Astrin\_STLCHLD\_050114\_01.09317.09317.2 | 2.3259 | 0.3439 | 99.9% | 822.1722 | 822.0283 | 2 | 6.503 | 83.3% | 1 | R.MGLVMDR.M | 2 |
|  | Astrin\_STLCHLD\_tube2\_050114\_01.08679.08679.3 | 3.5101 | 0.5001 | 100.0% | 1615.1643 | 1614.875 | 1 | 7.925 | 44.6% | 3 | R.MGPLGLDHMASSIER.M | 3 |
|  | Astrin\_STLCHLD\_050114\_02.07302.07302.2 | 3.6724 | 0.4778 | 100.0% | 1126.3322 | 1126.3337 | 1 | 9.623 | 75.0% | 5 | R.MGAGMGFGLER.M | 2 |
|  | Astrin\_STLCHLD\_050114\_01.07514.07514.2 | 2.2174 | 0.2001 | 98.0% | 825.2322 | 824.00085 | 1 | 6.176 | 66.7% | 2 | R.MGLSMER.M | 2 |
|  | Astrin\_STLCHLD\_tube2\_050114\_01.06279.06279.2 | 3.0318 | 0.4497 | 100.0% | 1189.1322 | 1189.4333 | 1 | 7.879 | 77.3% | 2 | R.MVPAGMGAGLER.M | 2 |
|  | Astrin\_STLCHLD\_tube2\_050114\_01.07985.07985.2 | 3.119 | 0.2115 | 99.0% | 1429.7322 | 1428.7076 | 1 | 4.673 | 64.3% | 3 | R.MGPAMGPALGAGIER.M | 2 |
|  | Astrin\_STLCHLD\_tube2\_050114\_02.06504.06504.2 | 3.2311 | 0.2917 | 99.8% | 1384.3522 | 1384.5677 | 1 | 5.494 | 67.9% | 3 | R.MGLAMGGGGGASFDR.A | 2 |
|  | Astrin\_STLCHLD\_050114\_02.06902.06902.3 | 3.616 | 0.4032 | 100.0% | 2037.0243 | 2036.1735 | 1 | 6.789 | 34.1% | 5 | R.GNFGGSFAGSFGGAGGHAPGVAR.K | 3 |

---

|  |  |  |  |  |  |  |  |  |
| --- | --- | --- | --- | --- | --- | --- | --- | --- |
| U | *gi|15718687|ref|NP\_00* | 3 | 6 | 16.9% | 243 | 26688 | 9.7 | ribosomal protein S3 [Homo sapiens] |

| Filename XCorr DeltCN Conf% ObsM+H+ CalcM+H+ SpR ZScore Ion% # Sequence  | | | | | | | | | | | | |
| --- | --- | --- | --- | --- | --- | --- | --- | --- | --- | --- | --- | --- |
| \* | Astrin\_STLCHLD\_050114\_02.05867.05867.2 | 3.7259 | 0.3946 | 100.0% | 1425.4922 | 1424.5071 | 1 | 7.922 | 83.3% | 3 | R.ELAEDGYSGVEVR.V | 2 |
| \* | Astrin\_STLCHLD\_tube2\_050114\_02.07100.07100.3 | 2.6666 | 0.2968 | 99.7% | 1587.5044 | 1584.8998 | 6 | 5.217 | 40.4% | 1 | R.VTPTRTEIIILATR.T | 3 |
| \* | Astrin\_STLCHLD\_tube2\_050114\_02.08135.08135.2 | 2.7249 | 0.2792 | 99.1% | 1574.7922 | 1573.7423 | 1 | 5.115 | 50.0% | 2 | R.FGFPEGSVELYAEK.V | 2 |

---

|  |  |  |  |  |  |  |  |  |
| --- | --- | --- | --- | --- | --- | --- | --- | --- |
| U | *gi|208973238|ref|NP\_0* | 2 | 2 | 16.7% | 245 | 27745 | 4.8 | tyrosine 3/tryptophan 5 -monooxygenase activation protein, zeta polypeptide [Homo sapiens] |
| U | *gi|4507953|ref|NP\_003* | 2 | 2 | 16.7% | 245 | 27745 | 4.8 | tyrosine 3/tryptophan 5 -monooxygenase activation protein, zeta polypeptide [Homo sapiens] |
| U | *gi|21735625|ref|NP\_66* | 2 | 2 | 16.7% | 245 | 27745 | 4.8 | tyrosine 3/tryptophan 5 -monooxygenase activation protein, zeta polypeptide [Homo sapiens] |
| U | *gi|208973244|ref|NP\_0* | 2 | 2 | 16.7% | 245 | 27745 | 4.8 | tyrosine 3/tryptophan 5 -monooxygenase activation protein, zeta polypeptide [Homo sapiens] |
| U | *gi|208973242|ref|NP\_0* | 2 | 2 | 16.7% | 245 | 27745 | 4.8 | tyrosine 3/tryptophan 5 -monooxygenase activation protein, zeta polypeptide [Homo sapiens] |
| U | *gi|208973240|ref|NP\_0* | 2 | 2 | 16.7% | 245 | 27745 | 4.8 | tyrosine 3/tryptophan 5 -monooxygenase activation protein, zeta polypeptide [Homo sapiens] |

| Filename XCorr DeltCN Conf% ObsM+H+ CalcM+H+ SpR ZScore Ion% # Sequence  | | | | | | | | | | | | |
| --- | --- | --- | --- | --- | --- | --- | --- | --- | --- | --- | --- | --- |
|  | Astrin\_STLCHLD\_050114\_01.04787.04787.2 | 2.3869 | 0.2659 | 98.6% | 1280.2922 | 1280.4203 | 1 | 5.456 | 68.2% | 1 | R.YLAEVAAGDDKK.G | 2 |
|  | Astrin\_STLCHLD\_050114\_01.18307.18307.2 | 3.6207 | 0.4753 | 100.0% | 3305.0723 | 3304.6907 | 1 | 8.951 | 28.6% | 1 | K.TAFDEAIAELDTLSEESYKDSTLIMQLLR.D | 2 |

---

|  |  |  |  |  |  |  |  |  |
| --- | --- | --- | --- | --- | --- | --- | --- | --- |
| U | *gi|4758158|ref|NP\_004* | 3 | 5 | 16.6% | 361 | 41487 | 6.6 | septin 2 [Homo sapiens] |
| U | *gi|56549640|ref|NP\_00* | 3 | 5 | 16.6% | 361 | 41487 | 6.6 | septin 2 [Homo sapiens] |
| U | *gi|56549638|ref|NP\_00* | 3 | 5 | 16.6% | 361 | 41487 | 6.6 | septin 2 [Homo sapiens] |
| U | *gi|56549636|ref|NP\_00* | 3 | 5 | 16.6% | 361 | 41487 | 6.6 | septin 2 [Homo sapiens] |

| Filename XCorr DeltCN Conf% ObsM+H+ CalcM+H+ SpR ZScore Ion% # Sequence  | | | | | | | | | | | | |
| --- | --- | --- | --- | --- | --- | --- | --- | --- | --- | --- | --- | --- |
|  | Astrin\_STLCHLD\_tube2\_050114\_02.06173.06173.2 | 4.1252 | 0.3983 | 100.0% | 1604.3922 | 1604.7545 | 1 | 7.633 | 80.8% | 2 | R.TVQIEASTVEIEER.G | 2 |
|  | Astrin\_STLCHLD\_tube2\_050114\_01.11357.11357.3 | 3.9932 | 0.3301 | 100.0% | 2863.9443 | 2864.2202 | 2 | 6.149 | 29.5% | 1 | R.TMLITHMQDLQEVTQDLHYENFR.S | 3 |
|  | Astrin\_STLCHLD\_050114\_02.06094.06094.3 | 5.0925 | 0.4597 | 100.0% | 2384.2744 | 2385.6675 | 1 | 7.576 | 37.5% | 2 | R.MQAQMQMQMQGGDGDGGALGHHV.- | 3 |

---

|  |  |  |  |  |  |  |  |  |
| --- | --- | --- | --- | --- | --- | --- | --- | --- |
| U | *gi|15431310|ref|NP\_00* | 10 | 25 | 16.3% | 472 | 51622 | 5.2 | keratin 14 [Homo sapiens] |

| Filename XCorr DeltCN Conf% ObsM+H+ CalcM+H+ SpR ZScore Ion% # Sequence  | | | | | | | | | | | | |
| --- | --- | --- | --- | --- | --- | --- | --- | --- | --- | --- | --- | --- |
| \* | Astrin\_STLCHLD\_tube2\_050114\_01.04863.04863.2 | 2.1145 | 0.3273 | 97.9% | 1426.0521 | 1426.526 | 28 | 5.388 | 39.3% | 1 | R.APSTYGGGLSVSSSR.F | 2 |
|  | Astrin\_STLCHLD\_tube2\_050114\_01.05063.05063.2 | 3.0476 | 0.142 | 99.2% | 1064.9722 | 1065.2578 | 35 | 6.057 | 62.5% | 1 | R.LASYLDKVR.A | 2222 |
|  | Astrin\_STLCHLD\_050114\_01.06505.06505.2 | 2.4865 | 0.2457 | 99.3% | 808.09216 | 807.8815 | 46 | 6.454 | 66.7% | 10 | R.LAADDFR.T | 22222 |
|  | Astrin\_STLCHLD\_tube2\_050114\_01.07270.07270.2 | 2.7001 | 0.1527 | 98.1% | 1185.9722 | 1186.397 | 1 | 4.645 | 83.3% | 1 | R.RVLDELTLAR.A | 222 |
|  | Astrin\_STLCHLD\_tube2\_050114\_01.08094.08094.2 | 3.8958 | 0.4282 | 100.0% | 1030.5721 | 1030.2096 | 1 | 7.786 | 93.8% | 3 | R.VLDELTLAR.A | 222 |
|  | Astrin\_STLCHLD\_tube2\_050114\_01.04733.04733.2 | 2.999 | 0.2859 | 99.9% | 1439.0122 | 1439.6263 | 1 | 5.252 | 75.0% | 1 | R.ILNEMRDQYEK.M | 22 |
|  | Astrin\_STLCHLD\_050114\_01.04933.04933.2 | 4.0231 | 0.5067 | 100.0% | 1362.1122 | 1362.4796 | 1 | 9.592 | 75.0% | 2 | R.EVATNSELVQSGK.S | 22 |
|  | Astrin\_STLCHLD\_tube2\_050114\_01.06167.06167.2 | 3.6142 | 0.3306 | 100.0% | 1381.3722 | 1380.5437 | 4 | 5.939 | 65.0% | 2 | K.TRLEQEIATYR.R | 22 |
|  | Astrin\_STLCHLD\_tube2\_050114\_01.05246.05246.3 | 3.0259 | 0.1746 | 96.5% | 1537.2544 | 1536.7311 | 3 | 4.38 | 40.9% | 1 | K.TRLEQEIATYRR.L | 33 |
|  | Astrin\_STLCHLD\_050114\_01.07538.07538.2 | 3.1047 | 0.317 | 99.9% | 1123.3322 | 1123.2511 | 3 | 5.706 | 81.2% | 3 | R.LEQEIATYR.R | 222 |

Similarities:
gi|40354195|ref|NP\_95(1:9)  
gi|4557701|ref|NP\_000(9:1)  
gi|24234699|ref|NP\_00(5:5)  
contaminant\_KERATIN03(2:8)  

---

|  |  |  |  |  |  |  |  |  |
| --- | --- | --- | --- | --- | --- | --- | --- | --- |
| U | *gi|32698866|ref|NP\_87* | 2 | 5 | 16.2% | 197 | 22443 | 4.7 | spindle pole body component 24 homolog [Homo sapiens] |

| Filename XCorr DeltCN Conf% ObsM+H+ CalcM+H+ SpR ZScore Ion% # Sequence  | | | | | | | | | | | | |
| --- | --- | --- | --- | --- | --- | --- | --- | --- | --- | --- | --- | --- |
| \* | Astrin\_STLCHLD\_tube2\_050114\_01.15665.15665.2 | 3.1429 | 0.3664 | 99.9% | 1814.7522 | 1815.0336 | 4 | 6.692 | 53.1% | 2 | R.DIEEVSQGLLSLLGANR.A | 2 |
| \* | Astrin\_STLCHLD\_050114\_02.13037.13037.2 | 3.3941 | 0.4218 | 100.0% | 1903.3322 | 1903.1412 | 1 | 6.95 | 50.0% | 3 | R.KFISDYLWSLVDTEW.- | 2 |

---

|  |  |  |  |  |  |  |  |  |
| --- | --- | --- | --- | --- | --- | --- | --- | --- |
| U | *gi|169201338|ref|XP\_0* | 3 | 10 | 16.2% | 160 | 18565 | 10.5 | PREDICTED: hypothetical protein [Homo sapiens] |
| U | *gi|89040203|ref|XP\_93* | 3 | 10 | 16.2% | 160 | 18593 | 10.5 | PREDICTED: hypothetical protein [Homo sapiens] |
| U | *gi|18104948|ref|NP\_00* | 3 | 10 | 16.2% | 160 | 18565 | 10.5 | ribosomal protein L21 [Homo sapiens] |
| U | *gi|169213854|ref|XP\_0* | 3 | 10 | 16.2% | 160 | 18790 | 10.3 | PREDICTED: hypothetical protein [Homo sapiens] |
| U | *gi|169210381|ref|XP\_0* | 3 | 10 | 16.2% | 160 | 18535 | 10.6 | PREDICTED: hypothetical protein isoform 2 [Homo sapiens] |
| U | *gi|169210379|ref|XP\_0* | 3 | 10 | 16.2% | 160 | 18535 | 10.6 | PREDICTED: hypothetical protein isoform 3 [Homo sapiens] |
| U | *gi|169210377|ref|XP\_0* | 3 | 10 | 16.2% | 160 | 18535 | 10.6 | PREDICTED: hypothetical protein isoform 1 [Homo sapiens] |
| U | *gi|169202779|ref|XP\_0* | 3 | 10 | 16.2% | 160 | 18521 | 10.5 | PREDICTED: similar to ribosomal protein L21 isoform 1 [Homo sapiens] |
| U | *gi|169202777|ref|XP\_0* | 3 | 10 | 16.2% | 160 | 18521 | 10.5 | PREDICTED: similar to ribosomal protein L21 isoform 2 [Homo sapiens] |
| U | *gi|169201750|ref|XP\_0* | 3 | 10 | 16.2% | 160 | 18550 | 10.5 | PREDICTED: hypothetical protein [Homo sapiens] |

| Filename XCorr DeltCN Conf% ObsM+H+ CalcM+H+ SpR ZScore Ion% # Sequence  | | | | | | | | | | | | |
| --- | --- | --- | --- | --- | --- | --- | --- | --- | --- | --- | --- | --- |
|  | Astrin\_STLCHLD\_tube2\_050114\_01.07140.07140.2 | 2.9194 | 0.4855 | 100.0% | 1244.2922 | 1244.4973 | 1 | 7.761 | 85.0% | 3 | K.HGVVPLATYMR.I | 2 |
|  | Astrin\_STLCHLD\_050114\_02.06204.06204.2 | 4.8275 | 0.4228 | 100.0% | 1641.3522 | 1641.9108 | 1 | 8.659 | 85.7% | 2 | R.VYNVTQHAVGIVVNK.Q | 2 |
|  | Astrin\_STLCHLD\_tube2\_050114\_02.05908.05908.3 | 4.6601 | 0.445 | 100.0% | 1642.1044 | 1641.9108 | 1 | 9.183 | 57.1% | 5 | R.VYNVTQHAVGIVVNK.Q | 3 |

---

|  |  |  |  |  |  |  |  |  |
| --- | --- | --- | --- | --- | --- | --- | --- | --- |
| U | *gi|4506625|ref|NP\_000* | 2 | 3 | 16.2% | 148 | 16561 | 11.0 | ribosomal protein L27a [Homo sapiens] |

| Filename XCorr DeltCN Conf% ObsM+H+ CalcM+H+ SpR ZScore Ion% # Sequence  | | | | | | | | | | | | |
| --- | --- | --- | --- | --- | --- | --- | --- | --- | --- | --- | --- | --- |
| \* | Astrin\_STLCHLD\_tube2\_050114\_01.06768.06768.3 | 2.8238 | 0.2707 | 99.8% | 1587.8644 | 1586.7899 | 7 | 5.439 | 45.8% | 1 | R.INFDKYHPGYFGK.V | 3 |
| \* | Astrin\_STLCHLD\_tube2\_050114\_01.07983.07983.2 | 2.9649 | 0.2554 | 99.5% | 1112.5521 | 1112.3146 | 1 | 6.156 | 90.0% | 2 | K.TGAAPIIDVVR.S | 2 |

---

|  |  |  |  |  |  |  |  |  |
| --- | --- | --- | --- | --- | --- | --- | --- | --- |
| U | *gi|4503471|ref|NP\_001* | 10 | 39 | 16.0% | 462 | 50141 | 9.0 | eukaryotic translation elongation factor 1 alpha 1 [Homo sapiens] |

| Filename XCorr DeltCN Conf% ObsM+H+ CalcM+H+ SpR ZScore Ion% # Sequence  | | | | | | | | | | | | |
| --- | --- | --- | --- | --- | --- | --- | --- | --- | --- | --- | --- | --- |
|  | Astrin\_STLCHLD\_050114\_02.06164.06164.3 | 4.9619 | 0.4802 | 100.0% | 1590.0543 | 1589.835 | 1 | 7.934 | 48.2% | 14 | K.THINIVVIGHVDSGK.S | 3 |
|  | Astrin\_STLCHLD\_tube2\_050114\_02.05919.05919.2 | 4.232 | 0.5034 | 100.0% | 1590.5521 | 1589.835 | 1 | 8.679 | 71.4% | 2 | K.THINIVVIGHVDSGK.S | 2 |
| \* | Astrin\_STLCHLD\_tube2\_050114\_01.07228.07228.3 | 2.8697 | 0.3243 | 100.0% | 1405.4644 | 1405.5962 | 25 | 5.77 | 40.9% | 2 | K.YYVTIIDAPGHR.D | 3 |
| \* | Astrin\_STLCHLD\_tube2\_050114\_01.07277.07277.2 | 3.2302 | 0.4753 | 100.0% | 1405.5322 | 1405.5962 | 1 | 8.302 | 72.7% | 3 | K.YYVTIIDAPGHR.D | 2 |
|  | Astrin\_STLCHLD\_tube2\_050114\_01.08464.08464.2 | 3.2414 | 0.4472 | 100.0% | 1315.3121 | 1315.5553 | 1 | 8.019 | 77.3% | 4 | R.EHALLAYTLGVK.Q | 2 |
|  | Astrin\_STLCHLD\_050114\_02.07432.07432.3 | 3.5808 | 0.3459 | 100.0% | 1316.2444 | 1315.5553 | 4 | 5.481 | 45.5% | 2 | R.EHALLAYTLGVK.Q | 3 |
|  | Astrin\_STLCHLD\_tube2\_050114\_01.05968.05968.1 | 1.6438 | 0.3374 | 100.0% | 1025.58 | 1026.2241 | 5 | 5.937 | 50.0% | 2 | K.IGGIGTVPVGR.V | 1 |
|  | Astrin\_STLCHLD\_tube2\_050114\_01.05972.05972.2 | 3.4852 | 0.4217 | 100.0% | 1026.1921 | 1026.2241 | 1 | 7.925 | 80.0% | 7 | K.IGGIGTVPVGR.V | 2 |
| \* | Astrin\_STLCHLD\_tube2\_050114\_01.10709.10709.3 | 4.7135 | 0.3654 | 100.0% | 2517.2644 | 2516.999 | 1 | 6.632 | 37.0% | 2 | R.VETGVLKPGMVVTFAPVNVTTEVK.S | 3 |
| \* | Astrin\_STLCHLD\_tube2\_050114\_01.10679.10679.2 | 5.2973 | 0.4452 | 100.0% | 2517.4521 | 2516.999 | 1 | 7.528 | 47.8% | 1 | R.VETGVLKPGMVVTFAPVNVTTEVK.S | 2 |

---

|  |  |  |  |  |  |  |  |  |
| --- | --- | --- | --- | --- | --- | --- | --- | --- |
| U | *gi|4885375|ref|NP\_005* | 5 | 8 | 16.0% | 213 | 21365 | 10.9 | histone cluster 1, H1c [Homo sapiens] |
| U | *gi|4885379|ref|NP\_005* | 5 | 8 | 15.5% | 219 | 21865 | 11.0 | histone cluster 1, H1e [Homo sapiens] |
| U | *gi|4885377|ref|NP\_005* | 5 | 8 | 15.4% | 221 | 22350 | 11.0 | histone cluster 1, H1d [Homo sapiens] |

| Filename XCorr DeltCN Conf% ObsM+H+ CalcM+H+ SpR ZScore Ion% # Sequence  | | | | | | | | | | | | |
| --- | --- | --- | --- | --- | --- | --- | --- | --- | --- | --- | --- | --- |
|  | Astrin\_STLCHLD\_050114\_01.09803.09803.2 | 2.968 | 0.3149 | 99.8% | 1327.0322 | 1327.5638 | 1 | 5.76 | 75.0% | 1 | R.KASGPPVSELITK.A | 2 |
|  | Astrin\_STLCHLD\_tube2\_050114\_01.05486.05486.3 | 2.8438 | 0.2643 | 99.7% | 1328.1843 | 1327.5638 | 1 | 4.67 | 47.9% | 1 | R.KASGPPVSELITK.A | 3 |
|  | Astrin\_STLCHLD\_050114\_01.11658.11658.2 | 2.7397 | 0.3717 | 99.9% | 1199.2122 | 1199.3898 | 1 | 6.5 | 68.2% | 1 | K.ASGPPVSELITK.A | 2 |
|  | Astrin\_STLCHLD\_050114\_01.07187.07187.2 | 2.9073 | 0.1523 | 98.9% | 974.4322 | 974.1887 | 6 | 4.872 | 77.8% | 1 | R.SGVSLAALKK.A | 2 |
|  | Astrin\_STLCHLD\_tube2\_050114\_01.04839.04839.2 | 3.213 | 0.3866 | 100.0% | 1109.2722 | 1108.2365 | 3 | 6.976 | 70.0% | 4 | K.ALAAAGYDVEK.N | 2 |

---

|  |  |  |  |  |  |  |  |  |
| --- | --- | --- | --- | --- | --- | --- | --- | --- |
| U | *gi|119395750|ref|NP\_0* | 7 | 10 | 15.8% | 644 | 66039 | 8.1 | keratin 1 [Homo sapiens] |

| Filename XCorr DeltCN Conf% ObsM+H+ CalcM+H+ SpR ZScore Ion% # Sequence  | | | | | | | | | | | | |
| --- | --- | --- | --- | --- | --- | --- | --- | --- | --- | --- | --- | --- |
| \* | Astrin\_STLCHLD\_tube2\_050114\_02.06138.06138.2 | 3.6824 | 0.3206 | 99.9% | 1658.6122 | 1658.7678 | 1 | 6.389 | 56.2% | 1 | R.SGGGFSSGSAGIINYQR.R | 2 |
|  | Astrin\_STLCHLD\_tube2\_050114\_01.10675.10675.2 | 2.673 | 0.2371 | 98.7% | 1639.4321 | 1639.8516 | 7 | 5.001 | 46.2% | 1 | K.SLNNQFASFIDKVR.F | 2 |
|  | Astrin\_STLCHLD\_tube2\_050114\_01.05886.05886.2 | 4.2621 | 0.08 | 99.6% | 1476.3522 | 1476.6726 | 1 | 6.871 | 86.4% | 3 | R.FLEQQNQVLQTK.W | 22 |
|  | Astrin\_STLCHLD\_tube2\_050114\_01.04962.04962.2 | 2.9535 | 0.2244 | 99.2% | 1394.8522 | 1394.5675 | 4 | 5.232 | 63.6% | 1 | R.TNAENEFVTIKK.D | 2 |
|  | Astrin\_STLCHLD\_tube2\_050114\_01.06291.06291.2 | 3.0202 | 0.2969 | 99.9% | 1180.3522 | 1180.303 | 1 | 7.562 | 77.8% | 2 | K.YEELQITAGR.H | 22 |
|  | Astrin\_STLCHLD\_050114\_01.05411.05411.2 | 2.2176 | 0.202 | 96.7% | 1034.3522 | 1034.1112 | 67 | 5.011 | 62.5% | 1 | R.TLLEGEESR.M | 2 |
| \* | Astrin\_STLCHLD\_050114\_02.07685.07685.3 | 3.6894 | 0.2391 | 99.8% | 2239.3145 | 2241.0396 | 1 | 4.233 | 30.6% | 1 | R.GGSGGGGGGS\*S\*GGRGSGGGSSGGSIGGR.G | 3 |

Similarities:
gi|119703753|ref|NP\_0(1:6)  
gi|47132620|ref|NP\_00(1:6)  

---

|  |  |  |  |  |  |  |  |  |
| --- | --- | --- | --- | --- | --- | --- | --- | --- |
| U | *gi|117189975|ref|NP\_1* | 4 | 8 | 15.7% | 306 | 33670 | 5.1 | heterogeneous nuclear ribonucleoprotein C isoform a [Homo sapiens] |
| U | *gi|117190254|ref|NP\_0* | 4 | 8 | 16.4% | 293 | 32338 | 5.1 | heterogeneous nuclear ribonucleoprotein C isoform b [Homo sapiens] |
| U | *gi|117190192|ref|NP\_0* | 4 | 8 | 15.7% | 306 | 33670 | 5.1 | heterogeneous nuclear ribonucleoprotein C isoform a [Homo sapiens] |
| U | *gi|117190174|ref|NP\_0* | 4 | 8 | 16.4% | 293 | 32338 | 5.1 | heterogeneous nuclear ribonucleoprotein C isoform b [Homo sapiens] |

| Filename XCorr DeltCN Conf% ObsM+H+ CalcM+H+ SpR ZScore Ion% # Sequence  | | | | | | | | | | | | |
| --- | --- | --- | --- | --- | --- | --- | --- | --- | --- | --- | --- | --- |
|  | Astrin\_STLCHLD\_050114\_02.08697.08697.2 | 3.6803 | 0.2867 | 99.9% | 1317.3922 | 1317.6145 | 1 | 7.452 | 77.3% | 3 | R.VFIGNLNTLVVK.K | 2 |
|  | Astrin\_STLCHLD\_tube2\_050114\_02.07996.07996.2 | 3.4073 | 0.4907 | 100.0% | 1330.7722 | 1330.4857 | 1 | 8.867 | 75.0% | 2 | K.GFAFVQYVNER.N | 2 |
|  | Astrin\_STLCHLD\_050114\_02.08688.08688.2 | 4.2415 | 0.4244 | 100.0% | 1683.3121 | 1684.0038 | 1 | 8.157 | 80.0% | 2 | R.MIAGQVLDINLAAEPK.V | 2 |
|  | Astrin\_STLCHLD\_050114\_01.06009.06009.2 | 2.1321 | 0.3654 | 99.3% | 943.7922 | 944.1649 | 3 | 7.008 | 68.8% | 1 | R.VPPPPPIAR.A | 2 |

---

|  |  |  |  |  |  |  |  |  |
| --- | --- | --- | --- | --- | --- | --- | --- | --- |
| U | *gi|4503483|ref|NP\_001* | 10 | 18 | 15.6% | 858 | 95338 | 6.8 | eukaryotic translation elongation factor 2 [Homo sapiens] |

| Filename XCorr DeltCN Conf% ObsM+H+ CalcM+H+ SpR ZScore Ion% # Sequence  | | | | | | | | | | | | |
| --- | --- | --- | --- | --- | --- | --- | --- | --- | --- | --- | --- | --- |
| \* | Astrin\_STLCHLD\_050114\_01.13058.13058.2 | 4.0366 | 0.3752 | 100.0% | 2221.612 | 2221.5151 | 1 | 5.773 | 50.0% | 3 | R.ALLELQLEPEELYQTFQR.I | 2 |
| \* | Astrin\_STLCHLD\_050114\_01.15437.15437.2 | 4.8207 | 0.4994 | 100.0% | 2602.2522 | 2602.11 | 1 | 8.118 | 47.8% | 1 | R.WLPAGDALLQMITIHLPSPVTAQK.Y | 2 |
| \* | Astrin\_STLCHLD\_tube2\_050114\_01.08861.08861.2 | 1.9221 | 0.3881 | 99.0% | 1038.9122 | 1040.3241 | 3 | 6.554 | 68.8% | 1 | K.GPLMMYISK.M | 2 |
| \* | Astrin\_STLCHLD\_tube2\_050114\_01.08324.08324.2 | 2.8668 | 0.3811 | 99.9% | 1108.1522 | 1108.3231 | 2 | 6.763 | 70.0% | 2 | R.VFSGLVSTGLK.V | 2 |
| \* | Astrin\_STLCHLD\_tube2\_050114\_01.08063.08063.3 | 5.6602 | 0.4501 | 100.0% | 2144.2444 | 2144.3489 | 1 | 8.117 | 52.6% | 4 | K.ARPFPDGLAEDIDKGEVSAR.Q | 3 |
| \* | Astrin\_STLCHLD\_tube2\_050114\_02.06727.06727.2 | 4.8937 | 0.5816 | 100.0% | 1743.2122 | 1743.9133 | 1 | 9.72 | 80.8% | 1 | R.YLAEKYEWDVAEAR.K | 2 |
| \* | Astrin\_STLCHLD\_050114\_02.07028.07028.3 | 3.7729 | 0.4121 | 100.0% | 1744.7043 | 1743.9133 | 1 | 7.107 | 51.9% | 2 | R.YLAEKYEWDVAEAR.K | 3 |
| \* | Astrin\_STLCHLD\_tube2\_050114\_02.06065.06065.3 | 3.0634 | 0.2002 | 96.9% | 1872.7444 | 1872.0874 | 1 | 4.844 | 42.9% | 1 | R.YLAEKYEWDVAEARK.I | 3 |
| \* | Astrin\_STLCHLD\_tube2\_050114\_01.13914.13914.2 | 4.7915 | 0.5382 | 100.0% | 2353.912 | 2354.6677 | 1 | 9.87 | 52.5% | 1 | K.GVQYLNEIKDSVVAGFQWATK.E | 2 |
| \* | Astrin\_STLCHLD\_tube2\_050114\_01.11728.11728.2 | 4.1779 | 0.3978 | 100.0% | 1800.6921 | 1801.0087 | 2 | 7.46 | 56.7% | 2 | K.AYLPVNESFGFTADLR.S | 2 |

---

|  |  |  |  |  |  |  |  |  |
| --- | --- | --- | --- | --- | --- | --- | --- | --- |
| U | *gi|41393577|ref|NP\_07* | 2 | 3 | 15.6% | 167 | 18824 | 5.1 | gemin 6 [Homo sapiens] |

| Filename XCorr DeltCN Conf% ObsM+H+ CalcM+H+ SpR ZScore Ion% # Sequence  | | | | | | | | | | | | |
| --- | --- | --- | --- | --- | --- | --- | --- | --- | --- | --- | --- | --- |
| \* | Astrin\_STLCHLD\_050114\_01.04911.04911.3 | 3.342 | 0.2756 | 100.0% | 1449.5643 | 1448.5785 | 16 | 5.012 | 41.7% | 1 | K.NHIPITEQGDAPR.T | 3 |
| \* | Astrin\_STLCHLD\_tube2\_050114\_02.06120.06120.2 | 3.5971 | 0.3524 | 100.0% | 1412.3722 | 1411.5547 | 1 | 6.883 | 70.8% | 2 | R.VQDLIEGHLTASQ.- | 2 |

---

|  |  |  |  |  |  |  |  |  |
| --- | --- | --- | --- | --- | --- | --- | --- | --- |
| U | *gi|32483377|ref|NP\_05* | 3 | 8 | 15.5% | 238 | 25839 | 7.5 | peroxiredoxin 3 isoform b [Homo sapiens] |
| U | *gi|5802974|ref|NP\_006* | 3 | 8 | 14.5% | 256 | 27693 | 7.8 | peroxiredoxin 3 isoform a precursor [Homo sapiens] |

| Filename XCorr DeltCN Conf% ObsM+H+ CalcM+H+ SpR ZScore Ion% # Sequence  | | | | | | | | | | | | |
| --- | --- | --- | --- | --- | --- | --- | --- | --- | --- | --- | --- | --- |
|  | Astrin\_STLCHLD\_tube2\_050114\_01.11584.11584.2 | 4.1178 | 0.5168 | 100.0% | 1463.4722 | 1463.6738 | 1 | 8.761 | 76.9% | 2 | R.DYGVLLEGSGLALR.G | 2 |
|  | Astrin\_STLCHLD\_tube2\_050114\_01.11792.11792.2 | 2.9692 | 0.2839 | 99.7% | 1286.5322 | 1286.5571 | 27 | 5.944 | 54.5% | 1 | R.GLFIIDPNGVIK.H | 2 |
|  | Astrin\_STLCHLD\_tube2\_050114\_01.05948.05948.2 | 3.0336 | 0.4125 | 100.0% | 1206.8922 | 1207.375 | 1 | 7.203 | 70.0% | 5 | K.HLSVNDLPVGR.S | 2 |

---

|  |  |  |  |  |  |  |  |  |
| --- | --- | --- | --- | --- | --- | --- | --- | --- |
| U | *gi|4757834|ref|NP\_004* | 2 | 3 | 15.2% | 211 | 23772 | 6.7 | BCL2-associated athanogene 2 [Homo sapiens] |

| Filename XCorr DeltCN Conf% ObsM+H+ CalcM+H+ SpR ZScore Ion% # Sequence  | | | | | | | | | | | | |
| --- | --- | --- | --- | --- | --- | --- | --- | --- | --- | --- | --- | --- |
| \* | Astrin\_STLCHLD\_tube2\_050114\_01.10755.10755.2 | 2.2783 | 0.2808 | 98.6% | 1329.5721 | 1329.5364 | 1 | 5.217 | 70.0% | 1 | R.LLESLDQLELR.V | 2 |
| \* | Astrin\_STLCHLD\_tube2\_050114\_02.06715.06715.3 | 4.2846 | 0.3538 | 100.0% | 2400.9243 | 2400.6917 | 6 | 6.11 | 35.0% | 2 | R.TLTVEVSVETIRNPQQQESLK.H | 3 |

---

|  |  |  |  |  |  |  |  |  |
| --- | --- | --- | --- | --- | --- | --- | --- | --- |
| U | *gi|88900509|ref|NP\_00* | 2 | 2 | 15.1% | 205 | 23339 | 5.5 | polyamine-modulated factor 1 [Homo sapiens] |

| Filename XCorr DeltCN Conf% ObsM+H+ CalcM+H+ SpR ZScore Ion% # Sequence  | | | | | | | | | | | | |
| --- | --- | --- | --- | --- | --- | --- | --- | --- | --- | --- | --- | --- |
| \* | Astrin\_STLCHLD\_050114\_02.06927.06927.2 | 2.8168 | 0.3107 | 99.9% | 1176.9521 | 1177.3892 | 1 | 5.916 | 88.9% | 1 | K.FIAQLQTSIR.E | 2 |
| \* | Astrin\_STLCHLD\_tube2\_050114\_02.08001.08001.3 | 3.4704 | 0.2924 | 100.0% | 2532.4744 | 2532.8215 | 3 | 5.13 | 31.2% | 1 | R.QVEELQLQVQAQQQAWQALHR.E | 3 |

---

|  |  |  |  |  |  |  |  |  |
| --- | --- | --- | --- | --- | --- | --- | --- | --- |
| U | *gi|5031635|ref|NP\_005* | 2 | 2 | 15.1% | 166 | 18502 | 8.1 | cofilin 1 (non-muscle) [Homo sapiens] |

| Filename XCorr DeltCN Conf% ObsM+H+ CalcM+H+ SpR ZScore Ion% # Sequence  | | | | | | | | | | | | |
| --- | --- | --- | --- | --- | --- | --- | --- | --- | --- | --- | --- | --- |
|  | Astrin\_STLCHLD\_tube2\_050114\_01.06929.06929.2 | 2.4452 | 0.3947 | 99.9% | 1337.4321 | 1338.4564 | 1 | 6.459 | 65.0% | 1 | R.YALYDATYETK.E | 2 |
| \* | Astrin\_STLCHLD\_tube2\_050114\_02.07639.07639.2 | 2.4873 | 0.2497 | 98.0% | 1342.6122 | 1341.5907 | 2 | 4.456 | 53.8% | 1 | K.LGGSAVISLEGKPL.- | 2 |

---

|  |  |  |  |  |  |  |  |  |
| --- | --- | --- | --- | --- | --- | --- | --- | --- |
| U | *gi|169212778|ref|XP\_0* | 5 | 7 | 15.0% | 266 | 30042 | 10.6 | PREDICTED: similar to ribosomal protein L7a [Homo sapiens] |
| U | *gi|4506661|ref|NP\_000* | 5 | 7 | 15.0% | 266 | 29996 | 10.6 | ribosomal protein L7a [Homo sapiens] |
| U | *gi|169213130|ref|XP\_0* | 5 | 7 | 15.0% | 266 | 30042 | 10.6 | PREDICTED: similar to ribosomal protein L7a [Homo sapiens] |
| U | *gi|169212940|ref|XP\_0* | 5 | 7 | 15.0% | 266 | 30028 | 10.6 | PREDICTED: similar to ribosomal protein L7a [Homo sapiens] |

| Filename XCorr DeltCN Conf% ObsM+H+ CalcM+H+ SpR ZScore Ion% # Sequence  | | | | | | | | | | | | |
| --- | --- | --- | --- | --- | --- | --- | --- | --- | --- | --- | --- | --- |
|  | Astrin\_STLCHLD\_tube2\_050114\_01.06941.06941.2 | 2.3729 | 0.2877 | 98.9% | 1217.3922 | 1217.3672 | 43 | 5.002 | 55.0% | 1 | K.NFGIGQDIQPK.R | 2 |
|  | Astrin\_STLCHLD\_tube2\_050114\_01.10915.10915.2 | 2.5138 | 0.3246 | 99.1% | 1812.1522 | 1812.1222 | 12 | 4.965 | 46.7% | 1 | R.LKVPPAINQFTQALDR.Q | 2 |
|  | Astrin\_STLCHLD\_tube2\_050114\_01.10929.10929.3 | 5.2131 | 0.3967 | 100.0% | 1812.3243 | 1812.1222 | 1 | 7.107 | 46.7% | 1 | R.LKVPPAINQFTQALDR.Q | 3 |
|  | Astrin\_STLCHLD\_tube2\_050114\_01.10555.10555.2 | 2.2917 | 0.2614 | 97.2% | 1571.6522 | 1570.7886 | 34 | 5.075 | 42.3% | 1 | K.VPPAINQFTQALDR.Q | 2 |
|  | Astrin\_STLCHLD\_050114\_01.11635.11635.2 | 3.5757 | 0.3672 | 100.0% | 1346.2522 | 1346.5236 | 1 | 6.062 | 66.7% | 3 | R.AGVNTVTTLVENK.K | 2 |

---

|  |  |  |  |  |  |  |  |  |
| --- | --- | --- | --- | --- | --- | --- | --- | --- |
| U | *gi|16905517|ref|NP\_47* | 3 | 7 | 14.9% | 262 | 31301 | 11.3 | FUS interacting protein (serine-arginine rich) 1 isoform 2 [Homo sapiens] |
| U | *gi|5730079|ref|NP\_006* | 3 | 7 | 21.3% | 183 | 22222 | 10.3 | FUS interacting protein (serine-arginine rich) 1 isoform 1 [Homo sapiens] |
| U | *gi|169161980|ref|XP\_0* | 3 | 7 | 21.5% | 181 | 22022 | 10.3 | PREDICTED: hypothetical protein, partial [Homo sapiens] |
| U | *gi|169161109|ref|XP\_0* | 3 | 7 | 21.3% | 183 | 22222 | 10.3 | PREDICTED: hypothetical protein LOC642558 [Homo sapiens] |
| U | *gi|169161107|ref|XP\_0* | 3 | 7 | 14.9% | 262 | 31301 | 11.3 | PREDICTED: hypothetical protein LOC642558 [Homo sapiens] |

| Filename XCorr DeltCN Conf% ObsM+H+ CalcM+H+ SpR ZScore Ion% # Sequence  | | | | | | | | | | | | |
| --- | --- | --- | --- | --- | --- | --- | --- | --- | --- | --- | --- | --- |
|  | Astrin\_STLCHLD\_tube2\_050114\_01.07925.07925.3 | 3.6532 | 0.2325 | 100.0% | 1463.7244 | 1463.7227 | 1 | 5.848 | 52.3% | 2 | R.YLRPPNTSLFVR.N | 3 |
|  | Astrin\_STLCHLD\_050114\_01.13086.13086.2 | 3.4865 | 0.3337 | 99.9% | 1917.8922 | 1918.1992 | 1 | 6.349 | 50.0% | 3 | R.YGPIVDVYVPLDFYTR.R | 2 |
|  | Astrin\_STLCHLD\_tube2\_050114\_01.09681.09681.2 | 3.0659 | 0.3946 | 100.0% | 1331.4321 | 1331.4705 | 1 | 6.279 | 65.0% | 2 | R.GFAYVQFEDVR.D | 2 |

---

|  |  |  |  |  |  |  |  |  |
| --- | --- | --- | --- | --- | --- | --- | --- | --- |
| U | *gi|32189392|ref|NP\_00* | 3 | 5 | 14.6% | 198 | 21892 | 6.0 | peroxiredoxin 2 isoform a [Homo sapiens] |

| Filename XCorr DeltCN Conf% ObsM+H+ CalcM+H+ SpR ZScore Ion% # Sequence  | | | | | | | | | | | | |
| --- | --- | --- | --- | --- | --- | --- | --- | --- | --- | --- | --- | --- |
| \* | Astrin\_STLCHLD\_tube2\_050114\_02.06111.06111.3 | 4.2187 | 0.3145 | 100.0% | 2086.1943 | 2086.309 | 1 | 5.88 | 36.8% | 1 | R.RLSEDYGVLKTDEGIAYR.G | 3 |
| \* | Astrin\_STLCHLD\_tube2\_050114\_02.06492.06492.3 | 3.9077 | 0.4421 | 100.0% | 1930.4043 | 1930.1217 | 1 | 8.124 | 37.5% | 1 | R.LSEDYGVLKTDEGIAYR.G | 3 |
|  | Astrin\_STLCHLD\_tube2\_050114\_01.07383.07383.2 | 2.9696 | 0.3359 | 99.9% | 1212.5521 | 1212.3915 | 4 | 6.228 | 75.0% | 3 | R.QITVNDLPVGR.S | 22 |

Similarities:
gi|32455264|ref|NP\_85(1:2)  

---

|  |  |  |  |  |  |  |  |  |
| --- | --- | --- | --- | --- | --- | --- | --- | --- |
| U | *gi|72534660|ref|NP\_00* | 4 | 4 | 14.3% | 238 | 27367 | 11.8 | splicing factor, arginine/serine-rich 7 [Homo sapiens] |

| Filename XCorr DeltCN Conf% ObsM+H+ CalcM+H+ SpR ZScore Ion% # Sequence  | | | | | | | | | | | | |
| --- | --- | --- | --- | --- | --- | --- | --- | --- | --- | --- | --- | --- |
| \* | Astrin\_STLCHLD\_tube2\_050114\_01.08251.08251.2 | 2.1305 | 0.268 | 98.0% | 1074.1522 | 1074.2242 | 1 | 6.081 | 81.2% | 1 | R.AFSYYGPLR.T | 2 |
|  | Astrin\_STLCHLD\_tube2\_050114\_01.11524.11524.2 | 2.5541 | 0.1729 | 96.2% | 1622.5322 | 1622.7771 | 15 | 4.375 | 50.0% | 1 | R.NPPGFAFVEFEDPR.D | 22 |
| \* | Astrin\_STLCHLD\_tube2\_050114\_01.05487.05487.2 | 2.7929 | 0.2782 | 99.5% | 1245.4122 | 1245.4827 | 1 | 5.904 | 70.0% | 1 | R.VRVELSTGMPR.R | 2 |
| \* | Astrin\_STLCHLD\_050114\_01.08723.08723.2 | 2.1797 | 0.2127 | 96.7% | 991.0522 | 990.1626 | 88 | 3.732 | 68.8% | 1 | R.VELSTGMPR.R | 2 |

Similarities:
gi|4506901|ref|NP\_003(1:3)  

---

|  |  |  |  |  |  |  |  |  |
| --- | --- | --- | --- | --- | --- | --- | --- | --- |
| U | *gi|36287110|ref|NP\_91* | 4 | 5 | 14.2% | 379 | 40907 | 4.6 | FGFR1 oncogene partner isoform b [Homo sapiens] |
| U | *gi|5901954|ref|NP\_008* | 4 | 5 | 13.5% | 399 | 43065 | 4.8 | FGFR1 oncogene partner isoform a [Homo sapiens] |

| Filename XCorr DeltCN Conf% ObsM+H+ CalcM+H+ SpR ZScore Ion% # Sequence  | | | | | | | | | | | | |
| --- | --- | --- | --- | --- | --- | --- | --- | --- | --- | --- | --- | --- |
|  | Astrin\_STLCHLD\_050114\_02.12009.12009.2 | 3.5433 | 0.4664 | 100.0% | 2164.612 | 2165.5352 | 1 | 7.763 | 45.0% | 1 | R.DLGIIEAEGTVGGPLLLEVIR.R | 2 |
|  | Astrin\_STLCHLD\_tube2\_050114\_02.10732.10732.3 | 3.3978 | 0.2765 | 99.9% | 2321.9343 | 2321.7227 | 1 | 6.187 | 33.3% | 2 | R.DLGIIEAEGTVGGPLLLEVIRR.C | 3 |
|  | Astrin\_STLCHLD\_050114\_01.10586.10586.2 | 2.1605 | 0.1664 | 95.2% | 893.4122 | 894.0183 | 13 | 5.417 | 71.4% | 1 | K.IGSFLSNR.T | 2 |
|  | Astrin\_STLCHLD\_tube2\_050114\_01.09113.09113.3 | 4.1855 | 0.3134 | 100.0% | 2571.3542 | 2571.63 | 10 | 5.463 | 26.1% | 1 | K.IGSLGLGTGEDDDYVDDFNSTSHR.S | 3 |

---

|  |  |  |  |  |  |  |  |  |
| --- | --- | --- | --- | --- | --- | --- | --- | --- |
| U | *gi|11415026|ref|NP\_00* | 2 | 2 | 14.2% | 176 | 20762 | 10.7 | ribosomal protein L18a [Homo sapiens] |
| U | *gi|88954757|ref|XP\_94* | 2 | 2 | 14.2% | 176 | 20767 | 10.7 | PREDICTED: similar to ribosomal protein L18a isoform 4 [Homo sapiens] |
| U | *gi|27480190|ref|XP\_20* | 2 | 2 | 14.2% | 176 | 20767 | 10.7 | PREDICTED: similar to ribosomal protein L18a isoform 1 [Homo sapiens] |
| U | *gi|169163931|ref|XP\_0* | 2 | 2 | 14.2% | 176 | 20767 | 10.7 | PREDICTED: similar to ribosomal protein L18a isoform 1 [Homo sapiens] |

| Filename XCorr DeltCN Conf% ObsM+H+ CalcM+H+ SpR ZScore Ion% # Sequence  | | | | | | | | | | | | |
| --- | --- | --- | --- | --- | --- | --- | --- | --- | --- | --- | --- | --- |
|  | Astrin\_STLCHLD\_tube2\_050114\_01.11186.11186.2 | 2.8206 | 0.1528 | 98.3% | 1462.6921 | 1461.7062 | 1 | 3.746 | 70.0% | 1 | K.SRFWYFVSQLK.K | 2 |
|  | Astrin\_STLCHLD\_050114\_02.06773.06773.2 | 2.6494 | 0.4036 | 99.9% | 1602.5521 | 1603.7351 | 44 | 6.918 | 46.2% | 1 | K.SSGEIVYCGQVFEK.S | 2 |

---

|  |  |  |  |  |  |  |  |  |
| --- | --- | --- | --- | --- | --- | --- | --- | --- |
| U | *gi|19920317|ref|NP\_00* | 6 | 7 | 13.8% | 602 | 66023 | 5.9 | cytoskeleton-associated protein 4 [Homo sapiens] |

| Filename XCorr DeltCN Conf% ObsM+H+ CalcM+H+ SpR ZScore Ion% # Sequence  | | | | | | | | | | | | |
| --- | --- | --- | --- | --- | --- | --- | --- | --- | --- | --- | --- | --- |
| \* | Astrin\_STLCHLD\_tube2\_050114\_01.07062.07062.2 | 2.6292 | 0.188 | 98.7% | 1293.1522 | 1293.44 | 3 | 4.677 | 66.7% | 1 | K.SREWDMEALR.S | 2 |
| \* | Astrin\_STLCHLD\_050114\_02.06638.06638.3 | 3.8558 | 0.4332 | 100.0% | 1841.3644 | 1841.0923 | 1 | 7.302 | 46.9% | 2 | R.LQHVEDGVLSMQVASAR.Q | 3 |
| \* | Astrin\_STLCHLD\_tube2\_050114\_01.07589.07589.2 | 2.6978 | 0.1441 | 96.1% | 1474.3322 | 1474.6512 | 68 | 4.596 | 46.2% | 1 | R.SVGELPSTVESLQK.V | 2 |
| \* | Astrin\_STLCHLD\_tube2\_050114\_01.05444.05444.3 | 5.1372 | 0.3316 | 100.0% | 2023.1643 | 2023.2131 | 1 | 6.9 | 41.2% | 1 | K.VQEQVHTLLSQDQAQAAR.L | 3 |
| \* | Astrin\_STLCHLD\_tube2\_050114\_02.06931.06931.2 | 2.1003 | 0.3091 | 97.9% | 1253.5122 | 1253.4381 | 5 | 5.727 | 59.1% | 1 | R.TAVDSLVAYSVK.I | 2 |
| \* | Astrin\_STLCHLD\_050114\_01.04658.04658.2 | 3.2078 | 0.4433 | 100.0% | 1361.8121 | 1362.4363 | 1 | 7.454 | 72.7% | 1 | K.IETNENNLESAK.G | 2 |

---

|  |  |  |  |  |  |  |  |  |
| --- | --- | --- | --- | --- | --- | --- | --- | --- |
| U | *gi|4506607|ref|NP\_000* | 2 | 7 | 13.8% | 188 | 21634 | 11.7 | ribosomal protein L18 [Homo sapiens] |

| Filename XCorr DeltCN Conf% ObsM+H+ CalcM+H+ SpR ZScore Ion% # Sequence  | | | | | | | | | | | | |
| --- | --- | --- | --- | --- | --- | --- | --- | --- | --- | --- | --- | --- |
| \* | Astrin\_STLCHLD\_tube2\_050114\_02.06373.06373.2 | 3.5074 | 0.3654 | 100.0% | 1345.8722 | 1346.5236 | 1 | 9.491 | 79.2% | 6 | K.TAVVVGTITDDVR.V | 2 |
| \* | Astrin\_STLCHLD\_tube2\_050114\_01.11109.11109.2 | 3.4714 | 0.4722 | 100.0% | 1461.8121 | 1461.6982 | 1 | 8.543 | 70.8% | 1 | K.ILTFDQLALDSPK.G | 2 |

---

|  |  |  |  |  |  |  |  |  |
| --- | --- | --- | --- | --- | --- | --- | --- | --- |
| U | *gi|4505763|ref|NP\_000* | 5 | 7 | 13.7% | 417 | 44615 | 8.1 | phosphoglycerate kinase 1 [Homo sapiens] |

| Filename XCorr DeltCN Conf% ObsM+H+ CalcM+H+ SpR ZScore Ion% # Sequence  | | | | | | | | | | | | |
| --- | --- | --- | --- | --- | --- | --- | --- | --- | --- | --- | --- | --- |
|  | Astrin\_STLCHLD\_tube2\_050114\_01.07584.07584.2 | 3.7833 | 0.4641 | 100.0% | 1635.2922 | 1635.7764 | 1 | 9.598 | 75.0% | 1 | K.LGDVYVNDAFGTAHR.A | 2 |
|  | Astrin\_STLCHLD\_050114\_02.06788.06788.3 | 2.9443 | 0.2571 | 99.2% | 1636.4043 | 1635.7764 | 1 | 5.783 | 42.9% | 2 | K.LGDVYVNDAFGTAHR.A | 3 |
| \* | Astrin\_STLCHLD\_tube2\_050114\_01.14689.14689.2 | 2.3809 | 0.2514 | 97.2% | 2105.8123 | 2106.3909 | 344 | 4.991 | 26.5% | 1 | K.QIVWNGPVGVFEWEAFAR.G | 2 |
|  | Astrin\_STLCHLD\_050114\_02.07043.07043.3 | 4.7217 | 0.4617 | 100.0% | 2516.1243 | 2515.7393 | 1 | 6.915 | 39.1% | 2 | K.WNTEDKVSHVSTGGGASLELLEGK.V | 3 |
|  | Astrin\_STLCHLD\_050114\_02.06795.06795.3 | 3.1642 | 0.3733 | 100.0% | 1741.7943 | 1741.939 | 1 | 6.234 | 35.3% | 1 | K.VSHVSTGGGASLELLEGK.V | 3 |

---

|  |  |  |  |  |  |  |  |  |
| --- | --- | --- | --- | --- | --- | --- | --- | --- |
| U | *gi|24234699|ref|NP\_00* | 8 | 26 | 13.5% | 400 | 44106 | 5.1 | keratin 19 [Homo sapiens] |

| Filename XCorr DeltCN Conf% ObsM+H+ CalcM+H+ SpR ZScore Ion% # Sequence  | | | | | | | | | | | | |
| --- | --- | --- | --- | --- | --- | --- | --- | --- | --- | --- | --- | --- |
|  | Astrin\_STLCHLD\_tube2\_050114\_01.05063.05063.2 | 3.0476 | 0.142 | 99.2% | 1064.9722 | 1065.2578 | 35 | 6.057 | 62.5% | 1 | R.LASYLDKVR.A | 2222 |
|  | Astrin\_STLCHLD\_050114\_01.11190.11190.1 | 2.5285 | 0.3185 | 100.0% | 1041.64 | 1042.2235 | 1 | 6.721 | 75.0% | 3 | R.IVLQIDNAR.L | 11 |
|  | Astrin\_STLCHLD\_tube2\_050114\_01.06537.06537.2 | 3.0568 | 0.1895 | 99.6% | 1042.2922 | 1042.2235 | 2 | 6.339 | 87.5% | 4 | R.IVLQIDNAR.L | 22 |
|  | Astrin\_STLCHLD\_050114\_01.06505.06505.2 | 2.4865 | 0.2457 | 99.3% | 808.09216 | 807.8815 | 46 | 6.454 | 66.7% | 10 | R.LAADDFR.T | 22222 |
|  | Astrin\_STLCHLD\_050114\_01.05083.05083.2 | 2.6635 | 0.3252 | 99.8% | 1223.2722 | 1223.3715 | 47 | 5.486 | 66.7% | 1 | R.TKFETEQALR.M | 22 |
|  | Astrin\_STLCHLD\_tube2\_050114\_01.07270.07270.2 | 2.7001 | 0.1527 | 98.1% | 1185.9722 | 1186.397 | 1 | 4.645 | 83.3% | 1 | R.RVLDELTLAR.T | 222 |
|  | Astrin\_STLCHLD\_tube2\_050114\_01.08094.08094.2 | 3.8958 | 0.4282 | 100.0% | 1030.5721 | 1030.2096 | 1 | 7.786 | 93.8% | 3 | R.VLDELTLAR.T | 222 |
|  | Astrin\_STLCHLD\_050114\_01.07538.07538.2 | 3.1047 | 0.317 | 99.9% | 1123.3322 | 1123.2511 | 3 | 5.706 | 81.2% | 3 | R.LEQEIATYR.S | 222 |

Similarities:
gi|40354195|ref|NP\_95(3:5)  
gi|4557701|ref|NP\_000(6:2)  
gi|15431310|ref|NP\_00(5:3)  
contaminant\_KERATIN03(2:6)  

---

|  |  |  |  |  |  |  |  |  |
| --- | --- | --- | --- | --- | --- | --- | --- | --- |
| U | *gi|4506743|ref|NP\_001* | 3 | 5 | 13.5% | 208 | 24205 | 10.3 | ribosomal protein S8 [Homo sapiens] |

| Filename XCorr DeltCN Conf% ObsM+H+ CalcM+H+ SpR ZScore Ion% # Sequence  | | | | | | | | | | | | |
| --- | --- | --- | --- | --- | --- | --- | --- | --- | --- | --- | --- | --- |
| \* | Astrin\_STLCHLD\_tube2\_050114\_01.08893.08893.2 | 3.9335 | 0.3459 | 100.0% | 1719.6522 | 1719.9353 | 1 | 7.16 | 64.3% | 2 | R.IIDVVYNASNNELVR.T | 2 |
| \* | Astrin\_STLCHLD\_050114\_02.07556.07556.3 | 4.0032 | 0.1435 | 99.4% | 1720.7943 | 1719.9353 | 4 | 5.28 | 44.6% | 1 | R.IIDVVYNASNNELVR.T | 3 |
| \* | Astrin\_STLCHLD\_tube2\_050114\_01.08362.08362.2 | 4.1213 | 0.4659 | 100.0% | 1507.5922 | 1507.6836 | 1 | 8.098 | 75.0% | 2 | K.ISSLLEEQFQQGK.L | 2 |

---

|  |  |  |  |  |  |  |  |  |
| --- | --- | --- | --- | --- | --- | --- | --- | --- |
| U | *gi|14602427|ref|NP\_12* | 3 | 4 | 13.4% | 277 | 31293 | 5.2 | ZW10 interactor isoform a [Homo sapiens] |
| U | *gi|14602429|ref|NP\_00* | 3 | 4 | 13.4% | 277 | 31293 | 5.2 | ZW10 interactor isoform a [Homo sapiens] |

| Filename XCorr DeltCN Conf% ObsM+H+ CalcM+H+ SpR ZScore Ion% # Sequence  | | | | | | | | | | | | |
| --- | --- | --- | --- | --- | --- | --- | --- | --- | --- | --- | --- | --- |
|  | Astrin\_STLCHLD\_050114\_01.05097.05097.2 | 2.6717 | 0.2842 | 99.5% | 1177.4722 | 1177.3177 | 2 | 5.649 | 77.8% | 1 | K.ALTQMEEAQR.K | 2 |
|  | Astrin\_STLCHLD\_050114\_02.04976.04976.3 | 4.236 | 0.3708 | 100.0% | 1490.0044 | 1489.6743 | 1 | 6.617 | 56.2% | 2 | K.HLQHLAEVSAEVR.E | 3 |
|  | Astrin\_STLCHLD\_050114\_01.15267.15267.2 | 3.1844 | 0.2973 | 99.9% | 1718.6921 | 1717.0178 | 1 | 6.049 | 50.0% | 1 | R.YQTFLQLLYTLQGK.L | 2 |

---

|  |  |  |  |  |  |  |  |  |
| --- | --- | --- | --- | --- | --- | --- | --- | --- |
| U | *gi|4506619|ref|NP\_000* | 2 | 2 | 13.4% | 157 | 17779 | 11.3 | ribosomal protein L24 [Homo sapiens] |

| Filename XCorr DeltCN Conf% ObsM+H+ CalcM+H+ SpR ZScore Ion% # Sequence  | | | | | | | | | | | | |
| --- | --- | --- | --- | --- | --- | --- | --- | --- | --- | --- | --- | --- |
| \* | Astrin\_STLCHLD\_tube2\_050114\_01.08927.08927.2 | 2.373 | 0.1982 | 98.5% | 966.9922 | 967.15576 | 1 | 4.794 | 92.9% | 1 | K.VFQFLNAK.C | 2 |
| \* | Astrin\_STLCHLD\_tube2\_050114\_01.09799.09799.2 | 3.4047 | 0.4639 | 100.0% | 1262.5122 | 1262.5072 | 1 | 8.886 | 66.7% | 1 | R.AITGASLADIMAK.R | 2 |

---

|  |  |  |  |  |  |  |  |  |
| --- | --- | --- | --- | --- | --- | --- | --- | --- |
| U | *gi|11968182|ref|NP\_07* | 2 | 2 | 13.2% | 152 | 17719 | 11.0 | ribosomal protein S18 [Homo sapiens] |
| U | *gi|169168597|ref|XP\_0* | 2 | 2 | 13.2% | 152 | 17719 | 11.0 | PREDICTED: hypothetical protein [Homo sapiens] |

| Filename XCorr DeltCN Conf% ObsM+H+ CalcM+H+ SpR ZScore Ion% # Sequence  | | | | | | | | | | | | |
| --- | --- | --- | --- | --- | --- | --- | --- | --- | --- | --- | --- | --- |
|  | Astrin\_STLCHLD\_050114\_01.05764.05764.2 | 2.9265 | 0.3359 | 99.9% | 1248.2922 | 1248.2891 | 1 | 6.0 | 70.0% | 1 | R.AGELTEDEVER.V | 2 |
|  | Astrin\_STLCHLD\_050114\_01.10451.10451.2 | 2.1926 | 0.3112 | 99.0% | 1072.1921 | 1072.311 | 23 | 5.63 | 68.8% | 1 | R.VITIMQNPR.Q | 2 |

---

|  |  |  |  |  |  |  |  |  |
| --- | --- | --- | --- | --- | --- | --- | --- | --- |
| U | *gi|59859885|ref|NP\_00* | 3 | 3 | 12.9% | 295 | 32854 | 4.9 | ribosomal protein SA [Homo sapiens] |
| U | *gi|9845502|ref|NP\_002* | 3 | 3 | 12.9% | 295 | 32854 | 4.9 | ribosomal protein SA [Homo sapiens] |

| Filename XCorr DeltCN Conf% ObsM+H+ CalcM+H+ SpR ZScore Ion% # Sequence  | | | | | | | | | | | | |
| --- | --- | --- | --- | --- | --- | --- | --- | --- | --- | --- | --- | --- |
|  | Astrin\_STLCHLD\_tube2\_050114\_01.09409.09409.2 | 2.382 | 0.3245 | 98.9% | 1741.8522 | 1741.9823 | 1 | 5.707 | 46.9% | 1 | R.AIVAIENPADVSVISSR.N | 2 |
|  | Astrin\_STLCHLD\_tube2\_050114\_01.04869.04869.2 | 2.4553 | 0.2181 | 97.2% | 1205.4521 | 1204.3713 | 1 | 4.646 | 70.8% | 1 | K.FAAATGATPIAGR.F | 2 |
|  | Astrin\_STLCHLD\_050114\_01.10051.10051.2 | 1.9876 | 0.2446 | 96.6% | 914.3122 | 913.10504 | 9 | 4.811 | 78.6% | 1 | R.LLVVTDPR.A | 2 |

---

|  |  |  |  |  |  |  |  |  |
| --- | --- | --- | --- | --- | --- | --- | --- | --- |
| U | *gi|55956919|ref|NP\_11* | 3 | 3 | 12.7% | 332 | 35968 | 6.9 | heterogeneous nuclear ribonucleoprotein A/B isoform a [Homo sapiens] |
| U | *gi|55956921|ref|NP\_00* | 3 | 3 | 14.7% | 285 | 30588 | 7.9 | heterogeneous nuclear ribonucleoprotein A/B isoform b [Homo sapiens] |

| Filename XCorr DeltCN Conf% ObsM+H+ CalcM+H+ SpR ZScore Ion% # Sequence  | | | | | | | | | | | | |
| --- | --- | --- | --- | --- | --- | --- | --- | --- | --- | --- | --- | --- |
|  | Astrin\_STLCHLD\_tube2\_050114\_01.08771.08771.2 | 2.1732 | 0.2879 | 97.5% | 1456.3121 | 1456.6996 | 7 | 4.971 | 50.0% | 1 | K.MFVGGLSWDTSKK.D | 2 |
|  | Astrin\_STLCHLD\_tube2\_050114\_01.07884.07884.2 | 3.7774 | 0.4371 | 100.0% | 1503.9922 | 1504.6799 | 1 | 7.653 | 65.4% | 1 | K.IFVGGLNPEATEEK.I | 2 |
|  | Astrin\_STLCHLD\_050114\_02.07764.07764.3 | 3.4815 | 0.3919 | 100.0% | 1827.6843 | 1827.1332 | 1 | 5.978 | 37.5% | 1 | R.RGFVFITFKEEEPVK.K | 3 |

---

|  |  |  |  |  |  |  |  |  |
| --- | --- | --- | --- | --- | --- | --- | --- | --- |
| U | *gi|4506903|ref|NP\_003* | 3 | 3 | 12.7% | 221 | 25542 | 8.6 | splicing factor, arginine/serine-rich 9 [Homo sapiens] |

| Filename XCorr DeltCN Conf% ObsM+H+ CalcM+H+ SpR ZScore Ion% # Sequence  | | | | | | | | | | | | |
| --- | --- | --- | --- | --- | --- | --- | --- | --- | --- | --- | --- | --- |
| \* | Astrin\_STLCHLD\_tube2\_050114\_01.07672.07672.2 | 2.6509 | 0.253 | 99.1% | 1247.2122 | 1247.4368 | 80 | 4.997 | 60.0% | 1 | R.IYVGNLPTDVR.E | 2 |
| \* | Astrin\_STLCHLD\_tube2\_050114\_01.09905.09905.2 | 2.7046 | 0.4609 | 100.0% | 1143.9722 | 1143.3768 | 1 | 7.781 | 72.2% | 1 | R.HGLVPFAFVR.F | 2 |
|  | Astrin\_STLCHLD\_050114\_01.10723.10723.2 | 2.3397 | 0.1688 | 98.1% | 917.2322 | 917.0989 | 3 | 4.406 | 83.3% | 1 | R.LRVEFPR.T | 22 |

Similarities:
gi|118582269|ref|NP\_0(1:2)  

---

|  |  |  |  |  |  |  |  |  |
| --- | --- | --- | --- | --- | --- | --- | --- | --- |
| U | *gi|108936958|ref|NP\_0* | 3 | 4 | 12.6% | 342 | 38926 | 5.5 | WD-repeat protein [Homo sapiens] |

| Filename XCorr DeltCN Conf% ObsM+H+ CalcM+H+ SpR ZScore Ion% # Sequence  | | | | | | | | | | | | |
| --- | --- | --- | --- | --- | --- | --- | --- | --- | --- | --- | --- | --- |
| \* | Astrin\_STLCHLD\_tube2\_050114\_01.10013.10013.2 | 2.7591 | 0.3153 | 99.5% | 1484.9722 | 1484.6488 | 95 | 5.279 | 41.7% | 1 | R.LALGSFVEEYNNK.V | 2 |
| \* | Astrin\_STLCHLD\_tube2\_050114\_02.06816.06816.3 | 4.8716 | 0.4469 | 100.0% | 2007.5044 | 2007.2535 | 1 | 7.881 | 48.4% | 2 | K.TQLIAHDKEVYDIAFSR.A | 3 |
| \* | Astrin\_STLCHLD\_050114\_02.06965.06965.2 | 2.8875 | 0.4537 | 100.0% | 1312.3121 | 1312.44 | 1 | 7.853 | 70.8% | 1 | R.DMFASVGADGSVR.M | 2 |

---

|  |  |  |  |  |  |  |  |  |
| --- | --- | --- | --- | --- | --- | --- | --- | --- |
| U | *gi|13376259|ref|NP\_07* | 6 | 8 | 12.5% | 656 | 75019 | 5.6 | nucleoporin 85 [Homo sapiens] |

| Filename XCorr DeltCN Conf% ObsM+H+ CalcM+H+ SpR ZScore Ion% # Sequence  | | | | | | | | | | | | |
| --- | --- | --- | --- | --- | --- | --- | --- | --- | --- | --- | --- | --- |
| \* | Astrin\_STLCHLD\_tube2\_050114\_01.09989.09989.3 | 2.678 | 0.3029 | 99.3% | 2052.7444 | 2051.3037 | 4 | 5.063 | 35.3% | 1 | R.YLQDSTFATSPHLESLLK.I | 3 |
| \* | Astrin\_STLCHLD\_tube2\_050114\_01.06947.06947.2 | 2.6865 | 0.1288 | 98.0% | 1096.3522 | 1096.2719 | 4 | 4.892 | 68.8% | 1 | R.VSLELHIER.I | 2 |
| \* | Astrin\_STLCHLD\_050114\_02.08025.08025.2 | 2.8532 | 0.0571 | 96.2% | 1090.2722 | 1090.2676 | 9 | 5.93 | 72.2% | 1 | R.LGSALSWSIR.A | 2 |
| \* | Astrin\_STLCHLD\_tube2\_050114\_01.06840.06840.2 | 3.964 | 0.5177 | 100.0% | 1365.8121 | 1365.529 | 1 | 9.609 | 79.2% | 1 | R.AKDAAFATLVSDR.F | 2 |
| \* | Astrin\_STLCHLD\_050114\_01.16111.16111.2 | 4.0308 | 0.4449 | 100.0% | 1597.2722 | 1596.8822 | 1 | 7.946 | 60.7% | 3 | R.FADAASLLLSLMTSR.I | 2 |
| \* | Astrin\_STLCHLD\_050114\_01.17848.17848.2 | 3.112 | 0.4069 | 99.9% | 2006.9722 | 2007.3955 | 1 | 7.373 | 50.0% | 1 | R.SFWMTLLTDALPLLEQK.Q | 2 |

---

|  |  |  |  |  |  |  |  |  |
| --- | --- | --- | --- | --- | --- | --- | --- | --- |
| U | *gi|34740329|ref|NP\_91* | 3 | 4 | 12.2% | 378 | 39595 | 9.0 | heterogeneous nuclear ribonucleoprotein A3 [Homo sapiens] |

| Filename XCorr DeltCN Conf% ObsM+H+ CalcM+H+ SpR ZScore Ion% # Sequence  | | | | | | | | | | | | |
| --- | --- | --- | --- | --- | --- | --- | --- | --- | --- | --- | --- | --- |
| \* | Astrin\_STLCHLD\_050114\_02.06944.06944.3 | 3.9623 | 0.4637 | 100.0% | 1884.3844 | 1884.096 | 1 | 6.7 | 46.7% | 1 | K.IFVGGIKEDTEEYNLR.D | 3 |
| \* | Astrin\_STLCHLD\_tube2\_050114\_01.07404.07404.2 | 2.4916 | 0.2955 | 99.3% | 1235.0721 | 1235.3948 | 1 | 6.03 | 77.8% | 1 | K.IETIEVMEDR.Q | 2 |
|  | Astrin\_STLCHLD\_050114\_02.08408.08408.3 | 3.9927 | 0.3425 | 100.0% | 2283.9243 | 2282.5579 | 2 | 5.869 | 31.6% | 2 | R.GFAFVTFDDHDTVDKIVVQK.Y | 3 |

---

|  |  |  |  |  |  |  |  |  |
| --- | --- | --- | --- | --- | --- | --- | --- | --- |
| U | *gi|14141166|ref|NP\_11* | 4 | 7 | 12.2% | 362 | 38222 | 6.8 | poly(rC) binding protein 2 isoform b [Homo sapiens] |
| U | *gi|193083114|ref|NP\_0* | 4 | 7 | 13.8% | 318 | 33497 | 8.2 | poly(rC) binding protein 2 isoform g [Homo sapiens] |
| U | *gi|193083112|ref|NP\_0* | 4 | 7 | 13.1% | 335 | 35347 | 8.0 | poly(rC) binding protein 2 isoform f [Homo sapiens] |
| U | *gi|193083110|ref|NP\_0* | 4 | 7 | 12.2% | 361 | 38151 | 6.8 | poly(rC) binding protein 2 isoform e [Homo sapiens] |
| U | *gi|193083108|ref|NP\_0* | 4 | 7 | 12.1% | 365 | 38580 | 6.8 | poly(rC) binding protein 2 isoform d [Homo sapiens] |
| U | *gi|148833484|ref|NP\_0* | 4 | 7 | 13.3% | 331 | 34917 | 8.0 | poly(rC) binding protein 2 isoform c [Homo sapiens] |
| U | *gi|14141168|ref|NP\_00* | 4 | 7 | 12.0% | 366 | 38651 | 6.8 | poly(rC) binding protein 2 isoform a [Homo sapiens] |

| Filename XCorr DeltCN Conf% ObsM+H+ CalcM+H+ SpR ZScore Ion% # Sequence  | | | | | | | | | | | | |
| --- | --- | --- | --- | --- | --- | --- | --- | --- | --- | --- | --- | --- |
|  | Astrin\_STLCHLD\_tube2\_050114\_01.10704.10704.2 | 3.0541 | 0.2535 | 99.4% | 1360.3922 | 1359.6519 | 5 | 6.131 | 58.3% | 1 | R.IITLAGPTNAIFK.A | 2 |
|  | Astrin\_STLCHLD\_050114\_02.06494.06494.2 | 4.6947 | 0.5727 | 100.0% | 2090.2722 | 2091.2573 | 1 | 10.461 | 50.0% | 3 | R.ESTGAQVQVAGDMLPNSTER.A | 22 |
|  | Astrin\_STLCHLD\_tube2\_050114\_02.06140.06140.3 | 3.4459 | 0.2808 | 100.0% | 2091.1743 | 2091.2573 | 3 | 4.976 | 36.8% | 1 | R.ESTGAQVQVAGDMLPNSTER.A | 33 |
|  | Astrin\_STLCHLD\_050114\_01.05035.05035.2 | 2.6046 | 0.3966 | 99.9% | 1159.0122 | 1159.2413 | 1 | 6.689 | 75.0% | 2 | K.IANPVEGSTDR.Q | 2 |

Similarities:
gi|222352151|ref|NP\_0(2:2)  

---

|  |  |  |  |  |  |  |  |  |
| --- | --- | --- | --- | --- | --- | --- | --- | --- |
| U | *gi|4759098|ref|NP\_004* | 3 | 7 | 12.2% | 288 | 33666 | 11.2 | splicing factor, arginine/serine-rich 10 [Homo sapiens] |

| Filename XCorr DeltCN Conf% ObsM+H+ CalcM+H+ SpR ZScore Ion% # Sequence  | | | | | | | | | | | | |
| --- | --- | --- | --- | --- | --- | --- | --- | --- | --- | --- | --- | --- |
| \* | Astrin\_STLCHLD\_050114\_02.07954.07954.2 | 4.4806 | 0.5224 | 100.0% | 1812.5322 | 1811.989 | 1 | 8.48 | 60.0% | 4 | K.YGPIADVSIVYDQQSR.R | 2 |
| \* | Astrin\_STLCHLD\_tube2\_050114\_01.07189.07189.2 | 2.2218 | 0.2632 | 98.6% | 1079.3522 | 1079.2847 | 7 | 4.765 | 62.5% | 2 | R.IRVDFSITK.R | 2 |
| \* | Astrin\_STLCHLD\_050114\_01.04940.04940.3 | 2.2028 | 0.2852 | 96.5% | 1308.7444 | 1310.322 | 41 | 5.567 | 36.1% | 1 | R.GYDDRDYYSR.S | 3 |

---

|  |  |  |  |  |  |  |  |  |
| --- | --- | --- | --- | --- | --- | --- | --- | --- |
| U | *gi|126722969|ref|NP\_0* | 8 | 13 | 11.9% | 561 | 60423 | 6.6 | centromere protein T [Homo sapiens] |

| Filename XCorr DeltCN Conf% ObsM+H+ CalcM+H+ SpR ZScore Ion% # Sequence  | | | | | | | | | | | | |
| --- | --- | --- | --- | --- | --- | --- | --- | --- | --- | --- | --- | --- |
| \* | Astrin\_STLCHLD\_050114\_01.05013.05013.2 | 3.3265 | 0.4268 | 100.0% | 1113.9122 | 1114.29 | 1 | 7.116 | 94.4% | 1 | R.RALLETASPR.K | 2 |
| \* | Astrin\_STLCHLD\_050114\_01.06278.06278.2 | 2.1419 | 0.2412 | 97.4% | 958.7522 | 958.1026 | 44 | 5.329 | 68.8% | 2 | R.ALLETASPR.K | 2 |
| \* | Astrin\_STLCHLD\_tube2\_050114\_01.04109.04109.2 | 5.3277 | 0.465 | 100.0% | 1762.7122 | 1761.8918 | 1 | 8.15 | 66.7% | 2 | R.SAHIQASGHLEEQTPR.T | 2 |
| \* | Astrin\_STLCHLD\_tube2\_050114\_02.04094.04094.3 | 3.4574 | 0.3575 | 100.0% | 1762.9443 | 1761.8918 | 1 | 6.064 | 50.0% | 1 | R.SAHIQASGHLEEQTPR.T | 3 |
| \* | Astrin\_STLCHLD\_tube2\_050114\_01.10588.10588.3 | 7.2787 | 0.4074 | 100.0% | 3302.9944 | 3303.6133 | 1 | 7.458 | 38.3% | 1 | R.LRLSVFQQGVDQGLSLSQEPQGNADASSLTR.S | 3 |
| \* | Astrin\_STLCHLD\_tube2\_050114\_01.10695.10695.3 | 4.7975 | 0.205 | 100.0% | 3036.0544 | 3034.2664 | 1 | 4.437 | 28.6% | 2 | R.LSVFQQGVDQGLSLSQEPQGNADASSLTR.S | 3 |
| \* | Astrin\_STLCHLD\_tube2\_050114\_01.07874.07874.2 | 3.2117 | 0.3602 | 100.0% | 1103.8522 | 1104.2974 | 1 | 7.07 | 83.3% | 3 | R.RAVDVGAFLR.D | 2 |
| \* | Astrin\_STLCHLD\_tube2\_050114\_01.09656.09656.2 | 2.7129 | 0.4105 | 100.0% | 948.3122 | 948.1099 | 1 | 7.716 | 81.2% | 1 | R.AVDVGAFLR.D | 2 |

---

|  |  |  |  |  |  |  |  |  |
| --- | --- | --- | --- | --- | --- | --- | --- | --- |
| U | *gi|16579885|ref|NP\_00* | 4 | 7 | 11.9% | 427 | 47697 | 11.1 | ribosomal protein L4 [Homo sapiens] |

| Filename XCorr DeltCN Conf% ObsM+H+ CalcM+H+ SpR ZScore Ion% # Sequence  | | | | | | | | | | | | |
| --- | --- | --- | --- | --- | --- | --- | --- | --- | --- | --- | --- | --- |
| \* | Astrin\_STLCHLD\_tube2\_050114\_01.09018.09018.3 | 4.9375 | 0.4156 | 100.0% | 1863.5944 | 1863.1727 | 1 | 7.623 | 41.7% | 3 | K.APIRPDIVNFVHTNLR.K | 3 |
| \* | Astrin\_STLCHLD\_050114\_02.06240.06240.3 | 5.0234 | 0.3842 | 100.0% | 2717.0942 | 2717.874 | 1 | 5.95 | 30.2% | 2 | K.NNRQPYAVSELAGHQTSAESWGTGR.A | 3 |
| \* | Astrin\_STLCHLD\_tube2\_050114\_02.06375.06375.3 | 3.082 | 0.3013 | 99.8% | 2333.7244 | 2333.479 | 1 | 5.828 | 32.1% | 1 | R.QPYAVSELAGHQTSAESWGTGR.A | 3 |
| \* | Astrin\_STLCHLD\_tube2\_050114\_01.07591.07591.2 | 2.8917 | 0.1839 | 99.1% | 1282.5521 | 1281.4539 | 112 | 4.5 | 61.1% | 1 | R.KLDELYGTWR.K | 2 |

---

|  |  |  |  |  |  |  |  |  |
| --- | --- | --- | --- | --- | --- | --- | --- | --- |
| U | *gi|4506649|ref|NP\_000* | 3 | 5 | 11.7% | 403 | 46109 | 10.2 | ribosomal protein L3 isoform a [Homo sapiens] |
| U | *gi|76496472|ref|NP\_00* | 3 | 5 | 13.3% | 354 | 40152 | 10.2 | ribosomal protein L3 isoform b [Homo sapiens] |

| Filename XCorr DeltCN Conf% ObsM+H+ CalcM+H+ SpR ZScore Ion% # Sequence  | | | | | | | | | | | | |
| --- | --- | --- | --- | --- | --- | --- | --- | --- | --- | --- | --- | --- |
|  | Astrin\_STLCHLD\_tube2\_050114\_01.06225.06225.2 | 2.7036 | 0.4898 | 100.0% | 984.21216 | 984.14594 | 2 | 8.521 | 68.8% | 3 | R.HGSLGFLPR.K | 2 |
|  | Astrin\_STLCHLD\_050114\_02.06138.06138.2 | 3.1165 | 0.337 | 99.9% | 1698.6522 | 1697.947 | 9 | 5.788 | 43.3% | 1 | K.AHLMEIQVNGGTVAEK.L | 2 |
|  | Astrin\_STLCHLD\_050114\_02.10293.10293.3 | 4.0021 | 0.4025 | 100.0% | 2439.1143 | 2438.8035 | 1 | 6.378 | 34.5% | 1 | K.SINPLGGFVHYGEVTNDFVMLK.G | 3 |

---

|  |  |  |  |  |  |  |  |  |
| --- | --- | --- | --- | --- | --- | --- | --- | --- |
| U | *gi|167466173|ref|NP\_0* | 5 | 11 | 11.5% | 641 | 70052 | 5.6 | heat shock 70kDa protein 1B [Homo sapiens] |
| U | *gi|194248072|ref|NP\_0* | 5 | 11 | 11.5% | 641 | 70052 | 5.6 | heat shock 70kDa protein 1A [Homo sapiens] |

| Filename XCorr DeltCN Conf% ObsM+H+ CalcM+H+ SpR ZScore Ion% # Sequence  | | | | | | | | | | | | |
| --- | --- | --- | --- | --- | --- | --- | --- | --- | --- | --- | --- | --- |
|  | Astrin\_STLCHLD\_tube2\_050114\_01.07314.07314.2 | 3.523 | 0.5545 | 100.0% | 1488.4922 | 1488.5939 | 1 | 9.144 | 75.0% | 3 | R.TTPSYVAFTDTER.L | 222 |
|  | Astrin\_STLCHLD\_050114\_02.08631.08631.2 | 3.3212 | 0.3399 | 99.9% | 1198.6721 | 1198.408 | 3 | 6.667 | 72.7% | 4 | K.DAGVIAGLNVLR.I | 22 |
|  | Astrin\_STLCHLD\_tube2\_050114\_01.10082.10082.2 | 4.1972 | 0.3829 | 100.0% | 1690.1522 | 1688.9213 | 1 | 8.009 | 63.3% | 2 | R.IINEPTAAAIAYGLDR.T | 2 |
|  | Astrin\_STLCHLD\_tube2\_050114\_01.04613.04613.3 | 2.8229 | 0.3291 | 100.0% | 1678.3744 | 1676.6964 | 20 | 5.364 | 35.0% | 1 | K.ATAGDTHLGGEDFDNR.L | 33 |
|  | Astrin\_STLCHLD\_tube2\_050114\_02.06418.06418.3 | 3.4057 | 0.3029 | 100.0% | 1823.6344 | 1823.1025 | 3 | 6.304 | 37.5% | 1 | K.LDKAQIHDLVLVGGSTR.I | 3 |

Similarities:
gi|24234686|ref|NP\_69(1:4)  
gi|124256496|ref|NP\_0(3:2)  

---

|  |  |  |  |  |  |  |  |  |
| --- | --- | --- | --- | --- | --- | --- | --- | --- |
| U | *gi|214830438|ref|NP\_0* | 2 | 3 | 11.5% | 356 | 38629 | 5.5 | sequestosome 1 isoform 2 [Homo sapiens] |
| U | *gi|4505571|ref|NP\_003* | 2 | 3 | 9.3% | 440 | 47687 | 5.2 | sequestosome 1 isoform 1 [Homo sapiens] |
| U | *gi|214830451|ref|NP\_0* | 2 | 3 | 11.5% | 356 | 38629 | 5.5 | sequestosome 1 isoform 2 [Homo sapiens] |

| Filename XCorr DeltCN Conf% ObsM+H+ CalcM+H+ SpR ZScore Ion% # Sequence  | | | | | | | | | | | | |
| --- | --- | --- | --- | --- | --- | --- | --- | --- | --- | --- | --- | --- |
|  | Astrin\_STLCHLD\_tube2\_050114\_01.07156.07156.3 | 4.7292 | 0.3878 | 100.0% | 2572.5544 | 2572.7478 | 1 | 5.7 | 32.0% | 1 | R.AGEARPGPTAESASGPSEDPSVNFLK.N | 3 |
|  | Astrin\_STLCHLD\_tube2\_050114\_02.08111.08111.2 | 4.0816 | 0.5202 | 100.0% | 1672.5521 | 1672.8322 | 1 | 8.035 | 64.3% | 2 | K.NYDIGAALDTIQYSK.H | 2 |

---

|  |  |  |  |  |  |  |  |  |
| --- | --- | --- | --- | --- | --- | --- | --- | --- |
| U | *gi|4503609|ref|NP\_001* | 2 | 2 | 11.4% | 255 | 27844 | 8.1 | electron-transfer-flavoprotein, beta polypeptide isoform 1 [Homo sapiens] |
| U | *gi|62420877|ref|NP\_00* | 2 | 2 | 8.4% | 346 | 37434 | 7.2 | electron-transfer-flavoprotein, beta polypeptide isoform 2 [Homo sapiens] |

| Filename XCorr DeltCN Conf% ObsM+H+ CalcM+H+ SpR ZScore Ion% # Sequence  | | | | | | | | | | | | |
| --- | --- | --- | --- | --- | --- | --- | --- | --- | --- | --- | --- | --- |
|  | Astrin\_STLCHLD\_tube2\_050114\_02.06472.06472.3 | 2.9202 | 0.2755 | 99.8% | 1555.7344 | 1555.8986 | 53 | 4.826 | 38.5% | 1 | K.LAEKEKVDLVLLGK.Q | 3 |
|  | Astrin\_STLCHLD\_050114\_02.05369.05369.3 | 2.5765 | 0.2729 | 97.7% | 1561.4944 | 1561.7728 | 68 | 5.214 | 33.9% | 1 | R.TAGVKVETTEDLVAK.L | 3 |

---

|  |  |  |  |  |  |  |  |  |
| --- | --- | --- | --- | --- | --- | --- | --- | --- |
| U | *gi|14249348|ref|NP\_11* | 2 | 5 | 11.4% | 123 | 13941 | 5.5 | thioredoxin-like 5 [Homo sapiens] |

| Filename XCorr DeltCN Conf% ObsM+H+ CalcM+H+ SpR ZScore Ion% # Sequence  | | | | | | | | | | | | |
| --- | --- | --- | --- | --- | --- | --- | --- | --- | --- | --- | --- | --- |
| \* | Astrin\_STLCHLD\_tube2\_050114\_02.06924.06924.3 | 3.1114 | 0.4605 | 100.0% | 1714.7644 | 1715.8162 | 3 | 7.554 | 38.5% | 4 | R.YEEVSVSGFEEFHR.A | 3 |
| \* | Astrin\_STLCHLD\_tube2\_050114\_02.06930.06930.2 | 3.7022 | 0.4959 | 100.0% | 1715.3722 | 1715.8162 | 8 | 8.031 | 46.2% | 1 | R.YEEVSVSGFEEFHR.A | 2 |

---

|  |  |  |  |  |  |  |  |  |
| --- | --- | --- | --- | --- | --- | --- | --- | --- |
| U | *gi|9966881|ref|NP\_065* | 9 | 13 | 11.0% | 925 | 106374 | 5.4 | nucleoporin 107kDa [Homo sapiens] |

| Filename XCorr DeltCN Conf% ObsM+H+ CalcM+H+ SpR ZScore Ion% # Sequence  | | | | | | | | | | | | |
| --- | --- | --- | --- | --- | --- | --- | --- | --- | --- | --- | --- | --- |
| \* | Astrin\_STLCHLD\_tube2\_050114\_01.08967.08967.2 | 2.6744 | 0.2933 | 99.3% | 1329.4321 | 1329.4093 | 1 | 6.63 | 68.2% | 1 | R.SGFGEISS\*PVIR.E | 2 |
| \* | Astrin\_STLCHLD\_050114\_02.06380.06380.2 | 5.4062 | 0.5284 | 100.0% | 1890.3722 | 1891.0476 | 1 | 10.531 | 68.8% | 3 | R.VLLQASQDENFGNTTPR.N | 2 |
| \* | Astrin\_STLCHLD\_tube2\_050114\_02.06166.06166.2 | 4.6012 | 0.5 | 100.0% | 1971.3522 | 1971.0476 | 1 | 7.696 | 62.5% | 1 | R.VLLQASQDENFGNTT#PR.N | 2 |
| \* | Astrin\_STLCHLD\_tube2\_050114\_01.05448.05448.3 | 3.0298 | 0.4213 | 100.0% | 1611.4744 | 1609.7819 | 7 | 7.096 | 38.5% | 1 | R.TPSSFRQPFTPTSR.S | 3 |
| \* | Astrin\_STLCHLD\_tube2\_050114\_01.09373.09373.2 | 2.307 | 0.3188 | 99.3% | 1063.1522 | 1063.242 | 2 | 5.813 | 68.8% | 1 | K.TVVEALFQR.D | 2 |
| \* | Astrin\_STLCHLD\_tube2\_050114\_01.08294.08294.2 | 3.4707 | 0.4093 | 100.0% | 1491.3922 | 1491.6866 | 1 | 6.697 | 63.6% | 1 | K.SVYWENTLHTLK.Q | 2 |
| \* | Astrin\_STLCHLD\_050114\_02.06957.06957.2 | 3.4525 | 0.3563 | 100.0% | 1122.5721 | 1121.3219 | 1 | 6.986 | 75.0% | 3 | R.AIYAALSGNLK.Q | 2 |
| \* | Astrin\_STLCHLD\_050114\_02.07763.07763.2 | 2.546 | 0.222 | 98.6% | 1325.2322 | 1324.476 | 29 | 5.292 | 55.0% | 1 | R.EYLGANWTLEK.V | 2 |
| \* | Astrin\_STLCHLD\_tube2\_050114\_01.16037.16037.2 | 4.7782 | 0.4857 | 100.0% | 1814.6322 | 1814.1035 | 1 | 8.768 | 70.0% | 1 | K.FLILGDIDGLMDEFSK.W | 2 |

---

|  |  |  |  |  |  |  |  |  |
| --- | --- | --- | --- | --- | --- | --- | --- | --- |
| U | *contaminant\_KERATIN03* | 7 | 15 | 11.0% | 593 | 59519 | 5.2 | no description |
| U | *gi|195972866|ref|NP\_0* | 7 | 17 | 11.1% | 584 | 58801 | 5.2 | keratin 10 [Homo sapiens] |

| Filename XCorr DeltCN Conf% ObsM+H+ CalcM+H+ SpR ZScore Ion% # Sequence  | | | | | | | | | | | | |
| --- | --- | --- | --- | --- | --- | --- | --- | --- | --- | --- | --- | --- |
|  | Astrin\_STLCHLD\_tube2\_050114\_02.06980.06980.2 | 4.2346 | 0.4733 | 100.0% | 1709.3322 | 1708.7844 | 1 | 7.896 | 47.2% | 1 | K.GSLGGGFSSGGFSGGSFSR.G | 2 |
|  | Astrin\_STLCHLD\_tube2\_050114\_01.05063.05063.2 | 3.0476 | 0.142 | 99.2% | 1064.9722 | 1065.2578 | 35 | 6.057 | 62.5% | 1 | R.LASYLDKVR.A | 2222 |
|  | Astrin\_STLCHLD\_050114\_01.06505.06505.2 | 2.4865 | 0.2457 | 99.3% | 808.09216 | 807.8815 | 46 | 6.454 | 66.7% | 8 | R.LAADDFR.L | 22222 |
|  | Astrin\_STLCHLD\_tube2\_050114\_01.04985.04985.2 | 2.0515 | 0.263 | 96.7% | 1236.3121 | 1235.4258 | 26 | 4.939 | 61.1% | 1 | R.LKYENEVALR.Q | 2 |
|  | Astrin\_STLCHLD\_tube2\_050114\_01.07632.07632.2 | 2.0303 | 0.2375 | 96.3% | 1032.5721 | 1032.2224 | 7 | 4.585 | 68.8% | 1 | R.VLDELTLTK.A | 2 |
|  | Astrin\_STLCHLD\_050114\_01.11085.11085.2 | 2.9694 | 0.2873 | 99.9% | 1435.6322 | 1435.623 | 1 | 5.276 | 70.0% | 1 | K.IRLENEIQTYR.S | 2 |
|  | Astrin\_STLCHLD\_tube2\_050114\_01.04778.04778.2 | 3.44 | 0.0158 | 98.7% | 1167.3722 | 1166.2761 | 1 | 4.539 | 87.5% | 2 | R.LENEIQTYR.S | 2 |

Similarities:
gi|40354195|ref|NP\_95(1:6)  
gi|4557701|ref|NP\_000(2:5)  
gi|15431310|ref|NP\_00(2:5)  
gi|24234699|ref|NP\_00(2:5)  

---

|  |  |  |  |  |  |  |  |  |
| --- | --- | --- | --- | --- | --- | --- | --- | --- |
| U | *gi|4758138|ref|NP\_004* | 6 | 9 | 10.9% | 614 | 69148 | 8.9 | DEAD (Asp-Glu-Ala-Asp) box polypeptide 5 [Homo sapiens] |

| Filename XCorr DeltCN Conf% ObsM+H+ CalcM+H+ SpR ZScore Ion% # Sequence  | | | | | | | | | | | | |
| --- | --- | --- | --- | --- | --- | --- | --- | --- | --- | --- | --- | --- |
| \* | Astrin\_STLCHLD\_050114\_01.08955.08955.2 | 3.4659 | 0.412 | 100.0% | 1390.3121 | 1390.4978 | 1 | 7.145 | 65.0% | 1 | K.NFYQEHPDLAR.R | 2 |
| \* | Astrin\_STLCHLD\_tube2\_050114\_01.08105.08105.2 | 3.0907 | 0.4261 | 100.0% | 1297.5521 | 1296.4198 | 1 | 7.68 | 75.0% | 2 | R.TTYLVLDEADR.M | 2 |
|  | Astrin\_STLCHLD\_tube2\_050114\_01.09962.09962.2 | 3.4719 | 0.4712 | 100.0% | 1336.7922 | 1337.5946 | 1 | 7.799 | 85.0% | 2 | R.MLDMGFEPQIR.K | 222 |
|  | Astrin\_STLCHLD\_tube2\_050114\_01.07865.07865.2 | 2.5978 | 0.2518 | 98.9% | 1227.3722 | 1227.4465 | 4 | 5.353 | 59.1% | 1 | K.APILIATDVASR.G | 22 |
| \* | Astrin\_STLCHLD\_tube2\_050114\_01.09655.09655.2 | 3.4412 | 0.3084 | 99.9% | 1575.6122 | 1575.7612 | 1 | 6.327 | 69.2% | 1 | K.TGTAYTFFTPNNIK.Q | 2 |
| \* | Astrin\_STLCHLD\_tube2\_050114\_01.07456.07456.2 | 2.487 | 0.21 | 99.0% | 986.47217 | 986.1564 | 1 | 5.759 | 78.6% | 2 | K.LLQLVEDR.G | 2 |

Similarities:
gi|87196351|ref|NP\_00(1:5)  
gi|148613856|ref|NP\_0(2:4)  

---

|  |  |  |  |  |  |  |  |  |
| --- | --- | --- | --- | --- | --- | --- | --- | --- |
| U | *gi|153792590|ref|NP\_0* | 8 | 16 | 10.7% | 854 | 98161 | 5.2 | heat shock 90kDa protein 1, alpha isoform 1 [Homo sapiens] |
| U | *gi|154146191|ref|NP\_0* | 8 | 16 | 12.4% | 732 | 84660 | 5.0 | heat shock 90kDa protein 1, alpha isoform 2 [Homo sapiens] |

| Filename XCorr DeltCN Conf% ObsM+H+ CalcM+H+ SpR ZScore Ion% # Sequence  | | | | | | | | | | | | |
| --- | --- | --- | --- | --- | --- | --- | --- | --- | --- | --- | --- | --- |
|  | Astrin\_STLCHLD\_tube2\_050114\_01.08950.08950.2 | 2.5774 | 0.3972 | 99.9% | 1242.6322 | 1243.4459 | 1 | 6.893 | 68.2% | 1 | K.ADLINNLGTIAK.S | 22 |
|  | Astrin\_STLCHLD\_050114\_02.06302.06302.3 | 4.6555 | 0.5081 | 100.0% | 2016.2344 | 2016.2584 | 1 | 8.269 | 48.3% | 2 | K.VILHLKEDQTEYLEER.R | 33 |
|  | Astrin\_STLCHLD\_050114\_01.05627.05627.2 | 2.9558 | 0.3011 | 99.9% | 1152.4722 | 1152.2462 | 1 | 5.822 | 81.2% | 3 | K.YIDQEELNK.T | 22 |
|  | Astrin\_STLCHLD\_050114\_02.07073.07073.2 | 2.7322 | 0.3456 | 99.6% | 1833.3322 | 1834.8903 | 12 | 6.582 | 42.9% | 1 | R.NPDDITNEEYGEFYK.S | 2 |
|  | Astrin\_STLCHLD\_tube2\_050114\_01.08112.08112.2 | 3.8262 | 0.4294 | 100.0% | 1527.9722 | 1528.6616 | 1 | 7.894 | 75.0% | 2 | K.SLTNDWEDHLAVK.H | 22 |
|  | Astrin\_STLCHLD\_050114\_02.06964.06964.3 | 2.8425 | 0.2263 | 99.2% | 1349.2444 | 1349.4886 | 308 | 5.284 | 40.0% | 2 | K.HFSVEGQLEFR.A | 33 |
|  | Astrin\_STLCHLD\_tube2\_050114\_02.06687.06687.2 | 2.9963 | 0.4425 | 100.0% | 1349.4321 | 1349.4886 | 1 | 6.469 | 70.0% | 4 | K.HFSVEGQLEFR.A | 22 |
|  | Astrin\_STLCHLD\_tube2\_050114\_01.08041.08041.3 | 3.0232 | 0.4207 | 100.0% | 1788.1444 | 1788.0134 | 1 | 6.404 | 41.1% | 1 | K.HLEINPDHSIIETLR.Q | 3 |

Similarities:
gi|20149594|ref|NP\_03(6:2)  

---

|  |  |  |  |  |  |  |  |  |
| --- | --- | --- | --- | --- | --- | --- | --- | --- |
| U | *gi|13128994|ref|NP\_07* | 2 | 2 | 10.7% | 205 | 24140 | 5.7 | MIS12 homolog [Homo sapiens] |

| Filename XCorr DeltCN Conf% ObsM+H+ CalcM+H+ SpR ZScore Ion% # Sequence  | | | | | | | | | | | | |
| --- | --- | --- | --- | --- | --- | --- | --- | --- | --- | --- | --- | --- |
| \* | Astrin\_STLCHLD\_tube2\_050114\_01.08456.08456.2 | 3.0413 | 0.3858 | 100.0% | 1239.6122 | 1239.4546 | 1 | 6.985 | 80.0% | 1 | R.IPSNILLPEDK.C | 2 |
| \* | Astrin\_STLCHLD\_tube2\_050114\_01.08890.08890.2 | 2.2545 | 0.1903 | 95.2% | 1234.2722 | 1232.3794 | 6 | 3.999 | 55.0% | 1 | R.ESLVSLVQNSR.K | 2 |

---

|  |  |  |  |  |  |  |  |  |
| --- | --- | --- | --- | --- | --- | --- | --- | --- |
| U | *gi|23397427|ref|NP\_00* | 5 | 6 | 10.6% | 623 | 69633 | 8.6 | synaptotagmin binding, cytoplasmic RNA interacting protein [Homo sapiens] |

| Filename XCorr DeltCN Conf% ObsM+H+ CalcM+H+ SpR ZScore Ion% # Sequence  | | | | | | | | | | | | |
| --- | --- | --- | --- | --- | --- | --- | --- | --- | --- | --- | --- | --- |
| \* | Astrin\_STLCHLD\_tube2\_050114\_02.08199.08199.3 | 4.7003 | 0.3688 | 100.0% | 2443.2244 | 2443.716 | 1 | 6.12 | 40.5% | 2 | K.VAEKLDEIYVAGLVAHSDLDER.A | 3 |
|  | Astrin\_STLCHLD\_tube2\_050114\_01.05973.05973.2 | 3.1513 | 0.4152 | 100.0% | 1312.5122 | 1312.4221 | 1 | 8.018 | 72.7% | 1 | R.TGYTLDVTTGQR.K | 22 |
|  | Astrin\_STLCHLD\_tube2\_050114\_01.09307.09307.2 | 2.2068 | 0.2436 | 98.5% | 928.1122 | 928.0788 | 1 | 5.984 | 71.4% | 1 | K.AGPIWDLR.L | 22 |
| \* | Astrin\_STLCHLD\_tube2\_050114\_01.10412.10412.2 | 2.2285 | 0.2836 | 98.3% | 1261.8522 | 1261.5403 | 166 | 5.817 | 50.0% | 1 | R.LMMDPLTGLNR.G | 2 |
| \* | Astrin\_STLCHLD\_tube2\_050114\_01.11023.11023.2 | 2.7901 | 0.2784 | 99.3% | 1475.4122 | 1474.6512 | 25 | 6.27 | 50.0% | 1 | R.NLANTVTEEILEK.A | 2 |

Similarities:
gi|156151392|ref|NP\_0(2:3)  

---

|  |  |  |  |  |  |  |  |  |
| --- | --- | --- | --- | --- | --- | --- | --- | --- |
| U | *gi|63055057|ref|NP\_00* | 2 | 7 | 9.8% | 376 | 42003 | 5.6 | actin, beta-like 2 [Homo sapiens] |

| Filename XCorr DeltCN Conf% ObsM+H+ CalcM+H+ SpR ZScore Ion% # Sequence  | | | | | | | | | | | | |
| --- | --- | --- | --- | --- | --- | --- | --- | --- | --- | --- | --- | --- |
| \* | Astrin\_STLCHLD\_tube2\_050114\_01.08154.08154.3 | 3.1767 | 0.2161 | 96.7% | 2338.9443 | 2338.7122 | 2 | 4.742 | 35.0% | 2 | R.VAPDEHPILLTEAPLNPKINR.E | 3 |
|  | Astrin\_STLCHLD\_tube2\_050114\_01.10092.10092.2 | 3.6111 | 0.3284 | 99.9% | 1790.9722 | 1791.9554 | 1 | 6.687 | 66.7% | 5 | R.SYELPDGQVITIGNER.F | 222 |

Similarities:
gi|4501885|ref|NP\_001(1:1)  
gi|4501881|ref|NP\_001(1:1)  

---

|  |  |  |  |  |  |  |  |  |
| --- | --- | --- | --- | --- | --- | --- | --- | --- |
| U | *gi|7661920|ref|NP\_055* | 4 | 7 | 9.5% | 411 | 46871 | 6.7 | eukaryotic translation initiation factor 4A, isoform 3 [Homo sapiens] |

| Filename XCorr DeltCN Conf% ObsM+H+ CalcM+H+ SpR ZScore Ion% # Sequence  | | | | | | | | | | | | |
| --- | --- | --- | --- | --- | --- | --- | --- | --- | --- | --- | --- | --- |
|  | Astrin\_STLCHLD\_tube2\_050114\_01.07276.07276.3 | 2.9102 | 0.3762 | 100.0% | 1829.1244 | 1829.0654 | 1 | 5.769 | 40.0% | 2 | R.GIYAYGFEKPSAIQQR.A | 33 |
|  | Astrin\_STLCHLD\_tube2\_050114\_01.07223.07223.2 | 4.427 | 0.4297 | 100.0% | 1829.7122 | 1829.0654 | 1 | 7.72 | 70.0% | 3 | R.GIYAYGFEKPSAIQQR.A | 22 |
| \* | Astrin\_STLCHLD\_tube2\_050114\_01.11393.11393.2 | 2.7215 | 0.2755 | 99.2% | 1521.2722 | 1521.784 | 1 | 5.381 | 62.5% | 1 | K.MLVLDEADEMLNK.G | 2 |
| \* | Astrin\_STLCHLD\_tube2\_050114\_01.09316.09316.2 | 2.6027 | 0.3795 | 99.9% | 1160.7722 | 1160.3585 | 4 | 6.786 | 61.1% | 1 | R.VLISTDVWAR.G | 2 |

Similarities:
gi|4503529|ref|NP\_001(2:2)  

---

|  |  |  |  |  |  |  |  |  |
| --- | --- | --- | --- | --- | --- | --- | --- | --- |
| U | *gi|14141193|ref|NP\_00* | 2 | 3 | 9.3% | 194 | 22591 | 10.7 | ribosomal protein S9 [Homo sapiens] |

| Filename XCorr DeltCN Conf% ObsM+H+ CalcM+H+ SpR ZScore Ion% # Sequence  | | | | | | | | | | | | |
| --- | --- | --- | --- | --- | --- | --- | --- | --- | --- | --- | --- | --- |
| \* | Astrin\_STLCHLD\_tube2\_050114\_01.06802.06802.2 | 2.337 | 0.2514 | 99.0% | 921.3522 | 921.0843 | 13 | 5.457 | 85.7% | 1 | K.LIGEYGLR.N | 2 |
| \* | Astrin\_STLCHLD\_050114\_01.11756.11756.2 | 3.7557 | 0.3401 | 100.0% | 1190.1122 | 1189.4031 | 1 | 6.198 | 88.9% | 2 | R.RLFEGNALLR.R | 2 |

---

|  |  |  |  |  |  |  |  |  |
| --- | --- | --- | --- | --- | --- | --- | --- | --- |
| U | *gi|148277065|ref|NP\_0* | 3 | 3 | 9.1% | 551 | 60269 | 6.7 | thioredoxin reductase 1 isoform 1 [Homo sapiens] |
| U | *gi|33519430|ref|NP\_87* | 3 | 3 | 10.0% | 499 | 54604 | 6.5 | thioredoxin reductase 1 isoform 2 [Homo sapiens] |
| U | *gi|33519428|ref|NP\_87* | 3 | 3 | 10.0% | 499 | 54604 | 6.5 | thioredoxin reductase 1 isoform 2 [Homo sapiens] |
| U | *gi|33519426|ref|NP\_87* | 3 | 3 | 10.0% | 499 | 54604 | 6.5 | thioredoxin reductase 1 isoform 2 [Homo sapiens] |
| U | *gi|148277071|ref|NP\_0* | 3 | 3 | 7.7% | 649 | 70756 | 7.4 | thioredoxin reductase 1 isoform 3 [Homo sapiens] |

| Filename XCorr DeltCN Conf% ObsM+H+ CalcM+H+ SpR ZScore Ion% # Sequence  | | | | | | | | | | | | |
| --- | --- | --- | --- | --- | --- | --- | --- | --- | --- | --- | --- | --- |
|  | Astrin\_STLCHLD\_050114\_02.07828.07828.3 | 3.4138 | 0.4069 | 100.0% | 1989.3844 | 1989.2592 | 2 | 6.415 | 39.1% | 1 | R.MIEAVQNHIGSLNWGYR.V | 3 |
|  | Astrin\_STLCHLD\_tube2\_050114\_01.05180.05180.2 | 2.3592 | 0.2675 | 98.9% | 1160.6522 | 1160.3616 | 3 | 4.973 | 72.2% | 1 | R.FLIATGERPR.Y | 2 |
|  | Astrin\_STLCHLD\_050114\_01.14467.14467.3 | 4.7258 | 0.3761 | 100.0% | 2901.2644 | 2901.2065 | 2 | 6.247 | 30.7% | 1 | K.FGEENIEVYHSYFWPLEWTIPSR.D | 3 |

---

|  |  |  |  |  |  |  |  |  |
| --- | --- | --- | --- | --- | --- | --- | --- | --- |
| U | *gi|4506725|ref|NP\_000* | 2 | 2 | 9.1% | 263 | 29598 | 10.2 | ribosomal protein S4, X-linked X isoform [Homo sapiens] |

| Filename XCorr DeltCN Conf% ObsM+H+ CalcM+H+ SpR ZScore Ion% # Sequence  | | | | | | | | | | | | |
| --- | --- | --- | --- | --- | --- | --- | --- | --- | --- | --- | --- | --- |
| \* | Astrin\_STLCHLD\_050114\_02.07050.07050.2 | 4.3736 | 0.504 | 100.0% | 1446.1322 | 1446.5975 | 1 | 8.58 | 70.8% | 1 | K.VNDTIQIDLETGK.I | 2 |
|  | Astrin\_STLCHLD\_050114\_01.06213.06213.3 | 2.8712 | 0.3275 | 100.0% | 1222.9443 | 1222.3892 | 2 | 5.577 | 47.5% | 1 | R.HPGSFDVVHVK.D | 3 |

---

|  |  |  |  |  |  |  |  |  |
| --- | --- | --- | --- | --- | --- | --- | --- | --- |
| U | *gi|154355000|ref|NP\_0* | 4 | 5 | 9.0% | 711 | 73115 | 7.3 | KH-type splicing regulatory protein (FUSE binding protein 2) [Homo sapiens] |

| Filename XCorr DeltCN Conf% ObsM+H+ CalcM+H+ SpR ZScore Ion% # Sequence  | | | | | | | | | | | | |
| --- | --- | --- | --- | --- | --- | --- | --- | --- | --- | --- | --- | --- |
| \* | Astrin\_STLCHLD\_tube2\_050114\_01.04391.04391.2 | 4.409 | 0.6195 | 100.0% | 2269.112 | 2271.3706 | 1 | 11.488 | 39.7% | 1 | R.GGGGPGGGGPGGGSAGGPSQPPGGGGPGIR.K | 2 |
| \* | Astrin\_STLCHLD\_tube2\_050114\_01.09035.09035.2 | 2.5515 | 0.2339 | 99.0% | 1080.8121 | 1080.3026 | 56 | 4.945 | 62.5% | 1 | K.MMLDDIVSR.G | 2 |
| \* | Astrin\_STLCHLD\_tube2\_050114\_01.07414.07414.2 | 3.6155 | 0.4633 | 100.0% | 1081.7122 | 1080.2725 | 1 | 7.336 | 75.0% | 1 | R.IGGGIDVPVPR.H | 2 |
| \* | Astrin\_STLCHLD\_050114\_02.06623.06623.2 | 3.9677 | 0.2951 | 100.0% | 1535.2722 | 1534.7123 | 1 | 6.207 | 69.2% | 2 | K.AINQQTGAFVEISR.Q | 2 |

---

|  |  |  |  |  |  |  |  |  |
| --- | --- | --- | --- | --- | --- | --- | --- | --- |
| U | *gi|18765752|ref|NP\_56* | 4 | 4 | 8.9% | 754 | 84557 | 8.8 | dual-specificity tyrosine-(Y)-phosphorylation regulated kinase 1A isoform 2 [Homo sapiens] |

| Filename XCorr DeltCN Conf% ObsM+H+ CalcM+H+ SpR ZScore Ion% # Sequence  | | | | | | | | | | | | |
| --- | --- | --- | --- | --- | --- | --- | --- | --- | --- | --- | --- | --- |
| \* | Astrin\_STLCHLD\_tube2\_050114\_02.06554.06554.3 | 4.1215 | 0.3554 | 100.0% | 3115.6443 | 3116.3745 | 1 | 6.233 | 26.9% | 1 | R.RQPNISDQQVSALSYSDQIQQPLTNQR.R | 3 |
|  | Astrin\_STLCHLD\_050114\_02.06996.06996.2 | 2.5478 | 0.2875 | 99.2% | 1288.5322 | 1289.477 | 80 | 6.893 | 55.0% | 1 | K.AFLNQAQIEVR.L | 2 |
|  | Astrin\_STLCHLD\_tube2\_050114\_01.05219.05219.3 | 2.9504 | 0.2857 | 99.8% | 1607.0944 | 1605.8374 | 1 | 5.013 | 45.0% | 1 | R.KLHNILGVETGGPGGR.R | 3 |
|  | Astrin\_STLCHLD\_050114\_01.10001.10001.2 | 2.4479 | 0.1711 | 95.2% | 1348.3121 | 1348.4552 | 3 | 4.737 | 62.5% | 1 | R.AGESGHTVADYLK.F | 2 |

---

|  |  |  |  |  |  |  |  |  |
| --- | --- | --- | --- | --- | --- | --- | --- | --- |
| U | *gi|124256496|ref|NP\_0* | 4 | 9 | 8.9% | 641 | 70375 | 6.0 | heat shock 70kDa protein 1-like [Homo sapiens] |

| Filename XCorr DeltCN Conf% ObsM+H+ CalcM+H+ SpR ZScore Ion% # Sequence  | | | | | | | | | | | | |
| --- | --- | --- | --- | --- | --- | --- | --- | --- | --- | --- | --- | --- |
|  | Astrin\_STLCHLD\_tube2\_050114\_01.07314.07314.2 | 3.523 | 0.5545 | 100.0% | 1488.4922 | 1488.5939 | 1 | 9.144 | 75.0% | 3 | R.TTPSYVAFTDTER.L | 222 |
|  | Astrin\_STLCHLD\_050114\_02.08631.08631.2 | 3.3212 | 0.3399 | 99.9% | 1198.6721 | 1198.408 | 3 | 6.667 | 72.7% | 4 | K.DAGVIAGLNVLR.I | 22 |
|  | Astrin\_STLCHLD\_tube2\_050114\_01.09822.09822.2 | 3.9832 | 0.4639 | 100.0% | 1660.4922 | 1660.9078 | 1 | 8.122 | 63.3% | 1 | R.IINEPTAAAIAYGLDK.G | 222 |
|  | Astrin\_STLCHLD\_tube2\_050114\_01.04613.04613.3 | 2.8229 | 0.3291 | 100.0% | 1678.3744 | 1676.6964 | 20 | 5.364 | 35.0% | 1 | K.ATAGDTHLGGEDFDNR.L | 33 |

Similarities:
gi|24234686|ref|NP\_69(2:2)  
gi|167466173|ref|NP\_0(3:1)  
contaminant\_GR78\_HUMA(1:3)  

---

|  |  |  |  |  |  |  |  |  |
| --- | --- | --- | --- | --- | --- | --- | --- | --- |
| U | *gi|106049292|ref|NP\_0* | 8 | 11 | 8.8% | 1178 | 129634 | 6.8 | pyruvate carboxylase precursor [Homo sapiens] |
| U | *gi|106049528|ref|NP\_0* | 8 | 11 | 8.8% | 1178 | 129634 | 6.8 | pyruvate carboxylase precursor [Homo sapiens] |
| U | *gi|106049295|ref|NP\_0* | 8 | 11 | 8.8% | 1178 | 129634 | 6.8 | pyruvate carboxylase precursor [Homo sapiens] |

| Filename XCorr DeltCN Conf% ObsM+H+ CalcM+H+ SpR ZScore Ion% # Sequence  | | | | | | | | | | | | |
| --- | --- | --- | --- | --- | --- | --- | --- | --- | --- | --- | --- | --- |
|  | Astrin\_STLCHLD\_tube2\_050114\_01.06356.06356.3 | 3.4072 | 0.2463 | 100.0% | 1768.7644 | 1768.8773 | 43 | 4.629 | 42.3% | 1 | R.VVHSYEELEENYTR.A | 3 |
|  | Astrin\_STLCHLD\_tube2\_050114\_02.08937.08937.3 | 4.0834 | 0.4975 | 100.0% | 2409.5344 | 2411.7227 | 1 | 6.717 | 36.8% | 2 | R.HIEVQILGDQYGNILHLYER.D | 3 |
|  | Astrin\_STLCHLD\_050114\_01.09821.09821.2 | 2.2461 | 0.2133 | 97.4% | 1165.1122 | 1165.2938 | 5 | 5.3 | 68.8% | 1 | K.HYFIEVNSR.L | 2 |
|  | Astrin\_STLCHLD\_tube2\_050114\_01.09811.09811.2 | 3.3039 | 0.5687 | 100.0% | 1515.1721 | 1515.7704 | 1 | 8.798 | 62.5% | 1 | R.NHPGLLLMDTTFR.D | 2 |
|  | Astrin\_STLCHLD\_050114\_01.14422.14422.2 | 2.6191 | 0.3046 | 99.2% | 1584.7122 | 1584.9604 | 15 | 5.61 | 58.3% | 1 | R.ELIPNIPFQMLLR.G | 2 |
|  | Astrin\_STLCHLD\_tube2\_050114\_01.10158.10158.2 | 2.4877 | 0.2816 | 99.0% | 1522.0322 | 1522.6134 | 1 | 4.442 | 68.2% | 1 | R.VFDYSEYWEGAR.G | 2 |
|  | Astrin\_STLCHLD\_tube2\_050114\_01.07561.07561.2 | 2.9913 | 0.3921 | 99.9% | 1548.1522 | 1548.6494 | 1 | 7.591 | 69.2% | 3 | R.AEAEAQAEELSFPR.S | 2 |
|  | Astrin\_STLCHLD\_050114\_01.08315.08315.2 | 2.0547 | 0.3395 | 98.9% | 959.47217 | 959.0904 | 234 | 5.557 | 62.5% | 1 | K.ALAVSDLNR.A | 2 |

---

|  |  |  |  |  |  |  |  |  |
| --- | --- | --- | --- | --- | --- | --- | --- | --- |
| U | *gi|113429348|ref|XP\_3* | 2 | 3 | 8.8% | 171 | 19863 | 10.1 | PREDICTED: similar to ribosomal protein S10 [Homo sapiens] |
| U | *gi|4506679|ref|NP\_001* | 2 | 3 | 9.1% | 165 | 18898 | 10.2 | ribosomal protein S10 [Homo sapiens] |
| U | *gi|113429532|ref|XP\_9* | 2 | 3 | 8.8% | 171 | 19863 | 10.1 | PREDICTED: similar to ribosomal protein S10 [Homo sapiens] |

| Filename XCorr DeltCN Conf% ObsM+H+ CalcM+H+ SpR ZScore Ion% # Sequence  | | | | | | | | | | | | |
| --- | --- | --- | --- | --- | --- | --- | --- | --- | --- | --- | --- | --- |
|  | Astrin\_STLCHLD\_tube2\_050114\_02.05621.05621.3 | 3.0958 | 0.191 | 96.6% | 1570.8544 | 1570.7019 | 8 | 4.007 | 41.1% | 1 | K.KAEAGAGSATEFQFR.G | 3 |
|  | Astrin\_STLCHLD\_050114\_02.06612.06612.2 | 3.1556 | 0.4771 | 100.0% | 1442.1522 | 1442.5278 | 1 | 7.19 | 57.7% | 2 | K.AEAGAGSATEFQFR.G | 2 |

---

|  |  |  |  |  |  |  |  |  |
| --- | --- | --- | --- | --- | --- | --- | --- | --- |
| U | *gi|17402900|ref|NP\_00* | 4 | 7 | 8.7% | 644 | 67560 | 7.6 | far upstream element-binding protein [Homo sapiens] |

| Filename XCorr DeltCN Conf% ObsM+H+ CalcM+H+ SpR ZScore Ion% # Sequence  | | | | | | | | | | | | |
| --- | --- | --- | --- | --- | --- | --- | --- | --- | --- | --- | --- | --- |
| \* | Astrin\_STLCHLD\_tube2\_050114\_01.12060.12060.2 | 2.6204 | 0.3282 | 99.3% | 2229.912 | 2229.6143 | 1 | 5.437 | 39.5% | 1 | R.SVMTEEYKVPDGMVGFIIGR.G | 2 |
| \* | Astrin\_STLCHLD\_tube2\_050114\_01.06359.06359.2 | 2.5258 | 0.2556 | 98.7% | 1354.4722 | 1353.5181 | 90 | 4.809 | 50.0% | 1 | K.IQIAPDSGGLPER.S | 2 |
| \* | Astrin\_STLCHLD\_tube2\_050114\_01.08217.08217.2 | 3.3523 | 0.4033 | 100.0% | 1337.7122 | 1337.5187 | 1 | 6.597 | 58.3% | 3 | R.IGGNEGIDVPIPR.F | 2 |
| \* | Astrin\_STLCHLD\_050114\_01.06045.06045.2 | 2.4718 | 0.2948 | 99.2% | 1068.0922 | 1068.218 | 1 | 5.911 | 72.2% | 2 | R.IAQITGPPDR.C | 2 |

---

|  |  |  |  |  |  |  |  |  |
| --- | --- | --- | --- | --- | --- | --- | --- | --- |
| U | *gi|50345984|ref|NP\_00* | 3 | 4 | 8.7% | 553 | 59751 | 9.1 | ATP synthase, H+ transporting, mitochondrial F1 complex, alpha subunit precursor [Homo sapiens] |

| Filename XCorr DeltCN Conf% ObsM+H+ CalcM+H+ SpR ZScore Ion% # Sequence  | | | | | | | | | | | | |
| --- | --- | --- | --- | --- | --- | --- | --- | --- | --- | --- | --- | --- |
|  | Astrin\_STLCHLD\_tube2\_050114\_02.06354.06354.2 | 4.9206 | 0.5757 | 100.0% | 1576.3322 | 1576.7007 | 1 | 9.949 | 78.6% | 2 | R.ILGADTSVDLEETGR.V | 2 |
|  | Astrin\_STLCHLD\_tube2\_050114\_01.07670.07670.2 | 2.6411 | 0.3498 | 99.9% | 1288.4722 | 1288.4863 | 5 | 6.951 | 60.0% | 1 | K.HALIIYDDLSK.Q | 2 |
| \* | Astrin\_STLCHLD\_tube2\_050114\_01.15097.15097.2 | 2.7208 | 0.3038 | 99.2% | 2340.7722 | 2339.567 | 8 | 5.571 | 31.0% | 1 | R.EVAAFAQFGSDLDAATQQLLSR.G | 2 |

---

|  |  |  |  |  |  |  |  |  |
| --- | --- | --- | --- | --- | --- | --- | --- | --- |
| U | *gi|4557303|ref|NP\_000* | 3 | 3 | 8.7% | 485 | 54848 | 7.9 | aldehyde dehydrogenase 3A2 isoform 2 [Homo sapiens] |
| U | *gi|73466520|ref|NP\_00* | 3 | 3 | 8.3% | 508 | 57669 | 8.9 | aldehyde dehydrogenase 3A2 isoform 1 [Homo sapiens] |

| Filename XCorr DeltCN Conf% ObsM+H+ CalcM+H+ SpR ZScore Ion% # Sequence  | | | | | | | | | | | | |
| --- | --- | --- | --- | --- | --- | --- | --- | --- | --- | --- | --- | --- |
|  | Astrin\_STLCHLD\_tube2\_050114\_01.05145.05145.2 | 3.5203 | 0.4649 | 100.0% | 1267.0122 | 1267.3379 | 1 | 8.39 | 86.4% | 1 | K.IAFGGETDEATR.Y | 2 |
|  | Astrin\_STLCHLD\_tube2\_050114\_01.08829.08829.2 | 3.1607 | 0.2116 | 99.3% | 1432.5322 | 1432.6567 | 2 | 5.19 | 66.7% | 1 | R.YIAPTVLTDVDPK.T | 2 |
|  | Astrin\_STLCHLD\_050114\_01.13087.13087.2 | 2.9815 | 0.2407 | 99.1% | 1910.9321 | 1910.3655 | 1 | 5.383 | 50.0% | 1 | K.VMQEEIFGPILPIVPVK.N | 2 |

---

|  |  |  |  |  |  |  |  |  |
| --- | --- | --- | --- | --- | --- | --- | --- | --- |
| U | *gi|46367787|ref|NP\_00* | 3 | 4 | 8.6% | 636 | 70671 | 9.5 | poly(A) binding protein, cytoplasmic 1 [Homo sapiens] |

| Filename XCorr DeltCN Conf% ObsM+H+ CalcM+H+ SpR ZScore Ion% # Sequence  | | | | | | | | | | | | |
| --- | --- | --- | --- | --- | --- | --- | --- | --- | --- | --- | --- | --- |
| \* | Astrin\_STLCHLD\_050114\_02.06863.06863.3 | 2.9941 | 0.4051 | 100.0% | 1742.3644 | 1741.857 | 37 | 6.233 | 33.9% | 1 | K.GYGFVHFETQEAAER.A | 3 |
|  | Astrin\_STLCHLD\_tube2\_050114\_01.14726.14726.3 | 4.8699 | 0.3738 | 100.0% | 2741.8743 | 2742.175 | 1 | 6.659 | 29.3% | 2 | K.ITGMLLEIDNSELLHMLESPESLR.S | 3 |
|  | Astrin\_STLCHLD\_050114\_01.11027.11027.3 | 4.4461 | 0.2829 | 100.0% | 1694.7544 | 1694.9285 | 1 | 5.99 | 53.3% | 1 | R.SKVDEAVAVLQAHQAK.E | 3 |

---

|  |  |  |  |  |  |  |  |  |
| --- | --- | --- | --- | --- | --- | --- | --- | --- |
| U | *gi|4506411|ref|NP\_002* | 4 | 4 | 8.5% | 587 | 63542 | 4.7 | Ran GTPase activating protein 1 [Homo sapiens] |

| Filename XCorr DeltCN Conf% ObsM+H+ CalcM+H+ SpR ZScore Ion% # Sequence  | | | | | | | | | | | | |
| --- | --- | --- | --- | --- | --- | --- | --- | --- | --- | --- | --- | --- |
| \* | Astrin\_STLCHLD\_050114\_01.05295.05295.2 | 2.2738 | 0.2884 | 98.5% | 1216.0322 | 1216.3365 | 9 | 5.523 | 54.5% | 1 | R.LEGNTVGVEAAR.V | 2 |
| \* | Astrin\_STLCHLD\_tube2\_050114\_01.09196.09196.2 | 3.9305 | 0.3996 | 100.0% | 1748.3322 | 1748.8931 | 3 | 6.917 | 53.3% | 1 | R.NRLENDGATALAEAFR.V | 2 |
| \* | Astrin\_STLCHLD\_tube2\_050114\_01.07416.07416.2 | 3.0549 | 0.3175 | 99.9% | 1408.4122 | 1408.551 | 1 | 6.076 | 72.7% | 1 | R.VINLNDNTFTEK.G | 2 |
| \* | Astrin\_STLCHLD\_tube2\_050114\_01.09028.09028.2 | 2.2979 | 0.341 | 99.3% | 1202.1721 | 1202.4392 | 9 | 5.992 | 66.7% | 1 | R.HSLLQTLYKV.- | 2 |

---

|  |  |  |  |  |  |  |  |  |
| --- | --- | --- | --- | --- | --- | --- | --- | --- |
| U | *gi|20357552|ref|NP\_00* | 3 | 4 | 8.4% | 550 | 61586 | 5.4 | cortactin isoform a [Homo sapiens] |
| U | *gi|20357556|ref|NP\_61* | 3 | 4 | 9.0% | 513 | 57467 | 5.3 | cortactin isoform b [Homo sapiens] |

| Filename XCorr DeltCN Conf% ObsM+H+ CalcM+H+ SpR ZScore Ion% # Sequence  | | | | | | | | | | | | |
| --- | --- | --- | --- | --- | --- | --- | --- | --- | --- | --- | --- | --- |
|  | Astrin\_STLCHLD\_050114\_01.07021.07021.3 | 3.4728 | 0.314 | 100.0% | 1644.2644 | 1642.8552 | 22 | 5.741 | 41.7% | 1 | K.LRENVFQEHQTLK.E | 3 |
|  | Astrin\_STLCHLD\_tube2\_050114\_02.06224.06224.3 | 4.0303 | 0.3606 | 100.0% | 2250.2344 | 2250.442 | 1 | 7.018 | 38.2% | 2 | R.MDKNASTFEDVTQVSSAYQK.T | 3 |
|  | Astrin\_STLCHLD\_tube2\_050114\_01.11084.11084.2 | 2.7801 | 0.3583 | 99.9% | 1570.8922 | 1570.7881 | 1 | 5.534 | 66.7% | 1 | R.YGLFPANYVELRQ.- | 2 |

---

|  |  |  |  |  |  |  |  |  |
| --- | --- | --- | --- | --- | --- | --- | --- | --- |
| U | *gi|15082258|ref|NP\_00* | 2 | 2 | 7.7% | 183 | 20811 | 5.3 | chromobox homolog 3 [Homo sapiens] |
| U | *gi|89038889|ref|XP\_94* | 2 | 2 | 7.7% | 183 | 20811 | 5.3 | PREDICTED: similar to Chromobox homolog 3 (HP1 gamma homolog, Drosophila) [Homo sapiens] |
| U | *gi|20544151|ref|NP\_05* | 2 | 2 | 7.7% | 183 | 20811 | 5.3 | chromobox homolog 3 [Homo sapiens] |

| Filename XCorr DeltCN Conf% ObsM+H+ CalcM+H+ SpR ZScore Ion% # Sequence  | | | | | | | | | | | | |
| --- | --- | --- | --- | --- | --- | --- | --- | --- | --- | --- | --- | --- |
|  | Astrin\_STLCHLD\_tube2\_050114\_01.05974.05974.2 | 2.9204 | 0.2356 | 99.0% | 1662.3722 | 1662.834 | 5 | 5.205 | 65.4% | 1 | K.KVEEAEPEEFVVEK.V | 2 |
|  | Astrin\_STLCHLD\_tube2\_050114\_01.07264.07264.2 | 2.6981 | 0.1955 | 98.0% | 1533.5521 | 1534.6599 | 1 | 5.104 | 58.3% | 1 | K.VEEAEPEEFVVEK.V | 2 |

---

|  |  |  |  |  |  |  |  |  |
| --- | --- | --- | --- | --- | --- | --- | --- | --- |
| U | *gi|74048514|ref|NP\_73* | 14 | 25 | 7.6% | 2342 | 265290 | 5.4 | cancer susceptibility candidate 5 isoform 1 [Homo sapiens] |
| U | *gi|74048554|ref|NP\_65* | 14 | 25 | 7.7% | 2316 | 262530 | 5.4 | cancer susceptibility candidate 5 isoform 2 [Homo sapiens] |

| Filename XCorr DeltCN Conf% ObsM+H+ CalcM+H+ SpR ZScore Ion% # Sequence  | | | | | | | | | | | | |
| --- | --- | --- | --- | --- | --- | --- | --- | --- | --- | --- | --- | --- |
|  | Astrin\_STLCHLD\_tube2\_050114\_01.10812.10812.2 | 3.0271 | 0.4508 | 100.0% | 1223.4521 | 1223.4117 | 1 | 7.147 | 80.0% | 1 | K.IDTTSFLANLK.L | 2 |
|  | Astrin\_STLCHLD\_tube2\_050114\_01.04914.04914.3 | 3.559 | 0.2252 | 99.9% | 1717.6144 | 1717.8944 | 3 | 4.84 | 41.1% | 2 | K.TNLEHTTGQLTTMNR.Q | 3 |
|  | Astrin\_STLCHLD\_050114\_01.07487.07487.2 | 3.1615 | 0.3356 | 99.9% | 1363.3121 | 1363.4728 | 1 | 5.848 | 75.0% | 1 | R.TDRPNFELSQR.K | 2 |
|  | Astrin\_STLCHLD\_050114\_01.14097.14097.3 | 5.9444 | 0.4637 | 100.0% | 2793.8342 | 2793.2354 | 1 | 7.631 | 40.6% | 2 | K.TGEFLAFQTVHLPPLPEQLLELGNK.A | 3 |
|  | Astrin\_STLCHLD\_050114\_01.14127.14127.2 | 2.8325 | 0.1662 | 97.3% | 2794.412 | 2793.2354 | 15 | 4.686 | 27.1% | 1 | K.TGEFLAFQTVHLPPLPEQLLELGNK.A | 2 |
|  | Astrin\_STLCHLD\_050114\_02.06749.06749.3 | 5.7205 | 0.5161 | 100.0% | 2672.0645 | 2671.991 | 1 | 9.594 | 37.0% | 1 | K.AHNDMHIVQATEIHNINIISSNAK.D | 3 |
|  | Astrin\_STLCHLD\_tube2\_050114\_01.06593.06593.2 | 2.3151 | 0.1808 | 96.4% | 1213.5521 | 1213.3508 | 1 | 4.001 | 72.2% | 1 | R.SSQMESQFLR.D | 2 |
|  | Astrin\_STLCHLD\_tube2\_050114\_01.07734.07734.3 | 4.7353 | 0.4267 | 100.0% | 2174.3643 | 2174.3696 | 1 | 8.608 | 36.8% | 1 | R.AAEKELEQLKTEEEELQR.N | 3 |
|  | Astrin\_STLCHLD\_tube2\_050114\_01.12184.12184.3 | 3.4362 | 0.254 | 99.7% | 2666.6042 | 2666.0032 | 1 | 5.077 | 27.2% | 1 | K.IVDVNFQSLLDEDQAPPSSLLVHK.L | 3 |
|  | Astrin\_STLCHLD\_050114\_01.12182.12182.2 | 3.1356 | 0.3654 | 100.0% | 1169.3922 | 1169.3629 | 1 | 6.35 | 81.2% | 2 | K.LIFQYVEEK.E | 2 |
|  | Astrin\_STLCHLD\_tube2\_050114\_01.09605.09605.2 | 3.0019 | 0.2059 | 99.3% | 1208.5721 | 1207.4094 | 1 | 6.244 | 66.7% | 1 | R.LLGEEIEYLK.R | 2 |
|  | Astrin\_STLCHLD\_050114\_01.11898.11898.2 | 3.9381 | 0.3312 | 100.0% | 1363.5122 | 1363.5968 | 3 | 7.509 | 70.0% | 3 | R.LLGEEIEYLKR.W | 2 |
|  | Astrin\_STLCHLD\_050114\_02.07895.07895.2 | 2.9699 | 0.3383 | 99.9% | 1142.1921 | 1142.3403 | 1 | 7.33 | 85.0% | 2 | R.LLFSSSAAFAK.F | 2 |
|  | Astrin\_STLCHLD\_050114\_01.11117.11117.2 | 2.8884 | 0.3744 | 100.0% | 1090.3322 | 1090.2645 | 3 | 6.408 | 75.0% | 6 | K.VPLENNYLK.N | 2 |

---

|  |  |  |  |  |  |  |  |  |
| --- | --- | --- | --- | --- | --- | --- | --- | --- |
| U | *gi|4826734|ref|NP\_004* | 2 | 4 | 7.6% | 526 | 53426 | 9.4 | fusion (involved in t(12;16) in malignant liposarcoma) [Homo sapiens] |

| Filename XCorr DeltCN Conf% ObsM+H+ CalcM+H+ SpR ZScore Ion% # Sequence  | | | | | | | | | | | | |
| --- | --- | --- | --- | --- | --- | --- | --- | --- | --- | --- | --- | --- |
| \* | Astrin\_STLCHLD\_050114\_02.05420.05420.3 | 3.5854 | 0.2402 | 100.0% | 1662.8344 | 1662.837 | 1 | 5.29 | 43.3% | 3 | K.LKGEATVSFDDPPSAK.A | 3 |
| \* | Astrin\_STLCHLD\_050114\_01.04459.04459.3 | 3.9767 | 0.4337 | 100.0% | 2254.0745 | 2254.355 | 1 | 7.007 | 40.2% | 1 | K.APKPDGPGGGPGGSHMGGNYGDDR.R | 3 |

---

|  |  |  |  |  |  |  |  |  |
| --- | --- | --- | --- | --- | --- | --- | --- | --- |
| U | *gi|14141157|ref|NP\_03* | 2 | 4 | 7.5% | 346 | 36926 | 6.9 | heterogeneous nuclear ribonucleoprotein H3 isoform a [Homo sapiens] |
| U | *gi|14141159|ref|NP\_06* | 2 | 4 | 7.9% | 331 | 35239 | 6.9 | heterogeneous nuclear ribonucleoprotein H3 isoform b [Homo sapiens] |

| Filename XCorr DeltCN Conf% ObsM+H+ CalcM+H+ SpR ZScore Ion% # Sequence  | | | | | | | | | | | | |
| --- | --- | --- | --- | --- | --- | --- | --- | --- | --- | --- | --- | --- |
|  | Astrin\_STLCHLD\_050114\_02.07025.07025.2 | 2.9929 | 0.4519 | 100.0% | 1271.5122 | 1272.4001 | 1 | 8.491 | 68.2% | 2 | R.STGEAFVQFASK.E | 2 |
|  | Astrin\_STLCHLD\_050114\_01.05698.05698.2 | 2.9896 | 0.3715 | 99.9% | 1412.3121 | 1413.4613 | 1 | 6.639 | 76.9% | 2 | R.DGMDNQGGYGSVGR.M | 2 |

---

|  |  |  |  |  |  |  |  |  |
| --- | --- | --- | --- | --- | --- | --- | --- | --- |
| U | *gi|4826686|ref|NP\_004* | 3 | 3 | 7.2% | 740 | 82432 | 7.2 | DEAD (Asp-Glu-Ala-Asp) box polypeptide 1 [Homo sapiens] |

| Filename XCorr DeltCN Conf% ObsM+H+ CalcM+H+ SpR ZScore Ion% # Sequence  | | | | | | | | | | | | |
| --- | --- | --- | --- | --- | --- | --- | --- | --- | --- | --- | --- | --- |
| \* | Astrin\_STLCHLD\_050114\_01.15074.15074.2 | 2.5998 | 0.3548 | 99.3% | 2374.8523 | 2373.6245 | 57 | 5.829 | 30.0% | 1 | R.FLVLDEADGLLSQGYSDFINR.M | 2 |
| \* | Astrin\_STLCHLD\_050114\_01.04631.04631.3 | 3.6157 | 0.2715 | 100.0% | 1612.6144 | 1611.8162 | 5 | 5.347 | 38.5% | 1 | R.MHNQIPQVTSDGKR.L | 3 |
| \* | Astrin\_STLCHLD\_tube2\_050114\_01.11411.11411.2 | 2.4165 | 0.2402 | 97.0% | 1904.6522 | 1905.2017 | 21 | 4.425 | 38.2% | 1 | K.GHVDILAPTVQELAALEK.E | 2 |

---

|  |  |  |  |  |  |  |  |  |
| --- | --- | --- | --- | --- | --- | --- | --- | --- |
| U | *gi|148613856|ref|NP\_0* | 4 | 5 | 7.1% | 731 | 80458 | 8.4 | DEAD box polypeptide 17 isoform 3 [Homo sapiens] |
| U | *gi|38201710|ref|NP\_00* | 4 | 5 | 7.1% | 729 | 80273 | 8.3 | DEAD box polypeptide 17 isoform 1 [Homo sapiens] |

| Filename XCorr DeltCN Conf% ObsM+H+ CalcM+H+ SpR ZScore Ion% # Sequence  | | | | | | | | | | | | |
| --- | --- | --- | --- | --- | --- | --- | --- | --- | --- | --- | --- | --- |
|  | Astrin\_STLCHLD\_tube2\_050114\_01.08500.08500.2 | 4.2729 | 0.4859 | 100.0% | 1692.4122 | 1692.8229 | 1 | 8.611 | 67.9% | 1 | R.ELAQQVQQVADDYGK.C | 2 |
|  | Astrin\_STLCHLD\_tube2\_050114\_01.09962.09962.2 | 3.4719 | 0.4712 | 100.0% | 1336.7922 | 1337.5946 | 1 | 7.799 | 85.0% | 2 | R.MLDMGFEPQIR.K | 222 |
|  | Astrin\_STLCHLD\_tube2\_050114\_01.07865.07865.2 | 2.5978 | 0.2518 | 98.9% | 1227.3722 | 1227.4465 | 4 | 5.353 | 59.1% | 1 | K.APILIATDVASR.G | 22 |
|  | Astrin\_STLCHLD\_050114\_01.06155.06155.2 | 4.0598 | 0.4875 | 100.0% | 1456.3322 | 1456.5118 | 1 | 8.173 | 76.9% | 1 | R.SSQSSSQQFSGIGR.S | 2 |

Similarities:
gi|87196351|ref|NP\_00(1:3)  
gi|4758138|ref|NP\_004(2:2)  

---

|  |  |  |  |  |  |  |  |  |
| --- | --- | --- | --- | --- | --- | --- | --- | --- |
| U | *gi|23308577|ref|NP\_00* | 2 | 2 | 6.9% | 533 | 56651 | 6.7 | phosphoglycerate dehydrogenase [Homo sapiens] |

| Filename XCorr DeltCN Conf% ObsM+H+ CalcM+H+ SpR ZScore Ion% # Sequence  | | | | | | | | | | | | |
| --- | --- | --- | --- | --- | --- | --- | --- | --- | --- | --- | --- | --- |
| \* | Astrin\_STLCHLD\_tube2\_050114\_01.06183.06183.2 | 4.2508 | 0.5469 | 100.0% | 1489.0122 | 1489.5822 | 1 | 9.327 | 64.3% | 1 | R.AGTGVDNVDLEAATR.K | 2 |
| \* | Astrin\_STLCHLD\_050114\_01.15314.15314.2 | 3.2298 | 0.4336 | 100.0% | 2272.5723 | 2273.668 | 2 | 6.985 | 35.7% | 1 | R.TQTSDPAMLPTMIGLLAEAGVR.L | 2 |

---

|  |  |  |  |  |  |  |  |  |
| --- | --- | --- | --- | --- | --- | --- | --- | --- |
| U | *gi|21327708|ref|NP\_63* | 2 | 2 | 6.9% | 391 | 45374 | 4.5 | nucleosome assembly protein 1-like 1 [Homo sapiens] |
| U | *gi|4758756|ref|NP\_004* | 2 | 2 | 6.9% | 391 | 45374 | 4.5 | nucleosome assembly protein 1-like 1 [Homo sapiens] |

| Filename XCorr DeltCN Conf% ObsM+H+ CalcM+H+ SpR ZScore Ion% # Sequence  | | | | | | | | | | | | |
| --- | --- | --- | --- | --- | --- | --- | --- | --- | --- | --- | --- | --- |
|  | Astrin\_STLCHLD\_tube2\_050114\_01.11152.11152.2 | 3.348 | 0.0488 | 96.3% | 1861.6921 | 1861.102 | 3 | 4.061 | 43.8% | 1 | R.LDGLVETPTGYIESLPR.V | 2 |
|  | Astrin\_STLCHLD\_050114\_01.10492.10492.2 | 2.6848 | 0.3558 | 99.9% | 1337.5322 | 1337.4314 | 1 | 6.13 | 77.8% | 1 | K.FYEEVHDLER.K | 2 |

---

|  |  |  |  |  |  |  |  |  |
| --- | --- | --- | --- | --- | --- | --- | --- | --- |
| U | *gi|221316723|ref|NP\_0* | 5 | 6 | 6.6% | 1025 | 115704 | 8.3 | N-acetyltransferase 10 isoform a [Homo sapiens] |
| U | *gi|221316741|ref|NP\_0* | 5 | 6 | 7.1% | 953 | 107271 | 7.0 | N-acetyltransferase 10 isoform b [Homo sapiens] |

| Filename XCorr DeltCN Conf% ObsM+H+ CalcM+H+ SpR ZScore Ion% # Sequence  | | | | | | | | | | | | |
| --- | --- | --- | --- | --- | --- | --- | --- | --- | --- | --- | --- | --- |
|  | Astrin\_STLCHLD\_050114\_01.10652.10652.2 | 2.1756 | 0.2565 | 96.8% | 1412.3522 | 1412.5858 | 1 | 5.662 | 68.2% | 1 | R.TLHEVSLQESIR.Y | 2 |
|  | Astrin\_STLCHLD\_tube2\_050114\_02.05582.05582.3 | 3.5923 | 0.1645 | 99.7% | 1412.9343 | 1412.5858 | 31 | 4.148 | 43.2% | 2 | R.TLHEVSLQESIR.Y | 3 |
|  | Astrin\_STLCHLD\_tube2\_050114\_01.13915.13915.2 | 4.2404 | 0.4651 | 100.0% | 2020.1721 | 2020.4304 | 1 | 8.673 | 46.9% | 1 | R.NMVDYHLIMDMIPAISR.I | 2 |
|  | Astrin\_STLCHLD\_050114\_01.18340.18340.3 | 3.3629 | 0.3558 | 100.0% | 2968.5544 | 2969.4973 | 1 | 5.64 | 27.8% | 1 | R.IYFLNQLGDLALSAAQSALLLGIGLQHK.S | 3 |
|  | Astrin\_STLCHLD\_tube2\_050114\_01.07168.07168.2 | 2.2525 | 0.2826 | 98.5% | 1071.5122 | 1071.2621 | 1 | 5.335 | 75.0% | 1 | K.AGPNASIISLK.S | 2 |

---

|  |  |  |  |  |  |  |  |  |
| --- | --- | --- | --- | --- | --- | --- | --- | --- |
| U | *gi|5453555|ref|NP\_006* | 2 | 2 | 6.5% | 216 | 24423 | 7.5 | ras-related nuclear protein [Homo sapiens] |

| Filename XCorr DeltCN Conf% ObsM+H+ CalcM+H+ SpR ZScore Ion% # Sequence  | | | | | | | | | | | | |
| --- | --- | --- | --- | --- | --- | --- | --- | --- | --- | --- | --- | --- |
| \* | Astrin\_STLCHLD\_tube2\_050114\_01.11842.11842.2 | 3.3532 | 0.3111 | 99.9% | 1785.6122 | 1786.0427 | 1 | 6.056 | 65.4% | 1 | K.SNYNFEKPFLWLAR.K | 2 |
| \* | Astrin\_STLCHLD\_tube2\_050114\_01.11818.11818.3 | 2.3277 | 0.3163 | 98.4% | 1786.3444 | 1786.0427 | 3 | 4.993 | 38.5% | 1 | K.SNYNFEKPFLWLAR.K | 3 |

---

|  |  |  |  |  |  |  |  |  |
| --- | --- | --- | --- | --- | --- | --- | --- | --- |
| U | *gi|33469968|ref|NP\_00* | 3 | 5 | 6.4% | 719 | 81308 | 6.5 | minichromosome maintenance complex component 7 isoform 1 [Homo sapiens] |

| Filename XCorr DeltCN Conf% ObsM+H+ CalcM+H+ SpR ZScore Ion% # Sequence  | | | | | | | | | | | | |
| --- | --- | --- | --- | --- | --- | --- | --- | --- | --- | --- | --- | --- |
| \* | Astrin\_STLCHLD\_tube2\_050114\_02.06871.06871.3 | 4.1025 | 0.4403 | 100.0% | 1829.3944 | 1829.063 | 1 | 7.373 | 42.9% | 2 | R.EVVNKDVLDVYIEHR.L | 3 |
|  | Astrin\_STLCHLD\_050114\_02.06352.06352.3 | 3.4904 | 0.2713 | 100.0% | 1799.8744 | 1800.0802 | 20 | 5.293 | 33.3% | 1 | R.TAIHEVMEQQTISIAK.A | 3 |
|  | Astrin\_STLCHLD\_050114\_02.06009.06009.3 | 4.337 | 0.3394 | 100.0% | 1747.1943 | 1746.9733 | 1 | 6.071 | 48.2% | 2 | R.MVDVVEKEDVNEAIR.L | 3 |

---

|  |  |  |  |  |  |  |  |  |
| --- | --- | --- | --- | --- | --- | --- | --- | --- |
| U | *gi|116812577|ref|NP\_0* | 2 | 2 | 6.4% | 392 | 46514 | 10.0 | LUC7-like 2 [Homo sapiens] |

| Filename XCorr DeltCN Conf% ObsM+H+ CalcM+H+ SpR ZScore Ion% # Sequence  | | | | | | | | | | | | |
| --- | --- | --- | --- | --- | --- | --- | --- | --- | --- | --- | --- | --- |
| \* | Astrin\_STLCHLD\_tube2\_050114\_02.05565.05565.2 | 3.8229 | 0.4408 | 100.0% | 1588.9521 | 1589.7399 | 1 | 8.277 | 60.7% | 1 | R.LAETQEEISAEVAAK.A | 2 |
|  | Astrin\_STLCHLD\_050114\_01.04835.04835.2 | 2.6198 | 0.4289 | 99.9% | 1168.3121 | 1168.292 | 2 | 6.624 | 72.2% | 1 | R.VHELNEEIGK.L | 2 |

---

|  |  |  |  |  |  |  |  |  |
| --- | --- | --- | --- | --- | --- | --- | --- | --- |
| U | *gi|4507943|ref|NP\_003* | 4 | 4 | 6.3% | 1071 | 123386 | 6.1 | exportin 1 [Homo sapiens] |

| Filename XCorr DeltCN Conf% ObsM+H+ CalcM+H+ SpR ZScore Ion% # Sequence  | | | | | | | | | | | | |
| --- | --- | --- | --- | --- | --- | --- | --- | --- | --- | --- | --- | --- |
| \* | Astrin\_STLCHLD\_tube2\_050114\_01.06937.06937.3 | 3.4239 | 0.3271 | 100.0% | 2163.8342 | 2163.4587 | 1 | 5.632 | 33.8% | 1 | R.MAQEVLTHLKEHPDAWTR.V | 3 |
| \* | Astrin\_STLCHLD\_050114\_02.06622.06622.3 | 3.9928 | 0.3558 | 100.0% | 2143.0144 | 2142.4321 | 1 | 5.967 | 38.9% | 1 | R.MAKPEEVLVVENDQGEVVR.E | 3 |
| \* | Astrin\_STLCHLD\_tube2\_050114\_01.15449.15449.2 | 4.0928 | 0.3424 | 100.0% | 2149.8123 | 2149.5127 | 1 | 7.051 | 58.8% | 1 | K.YMLLPNQVWDSIIQQATK.N | 2 |
| \* | Astrin\_STLCHLD\_tube2\_050114\_01.08125.08125.2 | 2.2365 | 0.2123 | 95.5% | 1294.0521 | 1294.5424 | 1 | 4.44 | 63.6% | 1 | K.AVGHPFVIQLGR.I | 2 |

---

|  |  |  |  |  |  |  |  |  |
| --- | --- | --- | --- | --- | --- | --- | --- | --- |
| U | *gi|31542947|ref|NP\_00* | 2 | 2 | 6.3% | 573 | 61055 | 5.9 | chaperonin [Homo sapiens] |
| U | *gi|41399285|ref|NP\_95* | 2 | 2 | 6.3% | 573 | 61055 | 5.9 | chaperonin [Homo sapiens] |

| Filename XCorr DeltCN Conf% ObsM+H+ CalcM+H+ SpR ZScore Ion% # Sequence  | | | | | | | | | | | | |
| --- | --- | --- | --- | --- | --- | --- | --- | --- | --- | --- | --- | --- |
|  | Astrin\_STLCHLD\_050114\_01.16836.16836.2 | 2.8042 | 0.4533 | 99.9% | 2113.8323 | 2114.5667 | 2 | 7.709 | 37.5% | 1 | R.ALMLQGVDLLADAVAVTMGPK.G | 2 |
|  | Astrin\_STLCHLD\_tube2\_050114\_02.06856.06856.3 | 3.0481 | 0.341 | 100.0% | 1632.1144 | 1631.9684 | 6 | 7.373 | 35.7% | 1 | K.VGEVIVTKDDAMLLK.G | 3 |

---

|  |  |  |  |  |  |  |  |  |
| --- | --- | --- | --- | --- | --- | --- | --- | --- |
| U | *gi|14141161|ref|NP\_00* | 5 | 9 | 6.2% | 806 | 88980 | 5.8 | heterogeneous nuclear ribonucleoprotein U isoform b [Homo sapiens] |
| U | *gi|74136883|ref|NP\_11* | 5 | 9 | 6.1% | 825 | 90585 | 6.0 | heterogeneous nuclear ribonucleoprotein U isoform a [Homo sapiens] |

| Filename XCorr DeltCN Conf% ObsM+H+ CalcM+H+ SpR ZScore Ion% # Sequence  | | | | | | | | | | | | |
| --- | --- | --- | --- | --- | --- | --- | --- | --- | --- | --- | --- | --- |
|  | Astrin\_STLCHLD\_tube2\_050114\_01.08048.08048.2 | 3.1375 | 0.4531 | 100.0% | 1697.8922 | 1698.8291 | 1 | 6.737 | 54.2% | 1 | R.GYFEYIEENKYSR.A | 2 |
|  | Astrin\_STLCHLD\_tube2\_050114\_02.06824.06824.3 | 2.2817 | 0.3841 | 100.0% | 1698.5944 | 1698.8291 | 1 | 5.673 | 37.5% | 1 | R.GYFEYIEENKYSR.A | 3 |
|  | Astrin\_STLCHLD\_tube2\_050114\_01.12270.12270.3 | 3.3334 | 0.2336 | 99.1% | 2726.6643 | 2726.0576 | 1 | 4.472 | 32.1% | 1 | K.EKPYFPIPEEYTFIQNVPLEDR.V | 3 |
|  | Astrin\_STLCHLD\_tube2\_050114\_02.06781.06781.2 | 4.796 | 0.435 | 100.0% | 1648.9122 | 1648.816 | 1 | 8.229 | 75.0% | 5 | R.NFILDQTNVSAAAQR.R | 2 |
|  | Astrin\_STLCHLD\_050114\_02.07156.07156.3 | 4.1283 | 0.2981 | 100.0% | 1649.9043 | 1648.816 | 1 | 5.994 | 48.2% | 1 | R.NFILDQTNVSAAAQR.R | 3 |

---

|  |  |  |  |  |  |  |  |  |
| --- | --- | --- | --- | --- | --- | --- | --- | --- |
| U | *gi|5032013|ref|NP\_005* | 4 | 6 | 6.1% | 890 | 100278 | 6.9 | kinesin family member 20A [Homo sapiens] |

| Filename XCorr DeltCN Conf% ObsM+H+ CalcM+H+ SpR ZScore Ion% # Sequence  | | | | | | | | | | | | |
| --- | --- | --- | --- | --- | --- | --- | --- | --- | --- | --- | --- | --- |
| \* | Astrin\_STLCHLD\_tube2\_050114\_01.06991.06991.2 | 2.1169 | 0.2256 | 95.1% | 1309.0521 | 1309.5516 | 2 | 4.721 | 55.0% | 1 | R.VRPLLPSELER.Q | 2 |
| \* | Astrin\_STLCHLD\_050114\_02.06431.06431.3 | 3.566 | 0.372 | 100.0% | 1420.1344 | 1419.6641 | 1 | 6.488 | 45.8% | 2 | R.ILHLQGEGDIVPK.I | 3 |
| \* | Astrin\_STLCHLD\_tube2\_050114\_02.05177.05177.3 | 4.2459 | 0.4073 | 100.0% | 1724.8744 | 1724.9578 | 1 | 7.044 | 46.7% | 2 | R.LKEAGNINTSLHTLGR.C | 3 |
| \* | Astrin\_STLCHLD\_tube2\_050114\_01.04883.04883.2 | 3.8577 | 0.426 | 100.0% | 1474.4321 | 1474.6543 | 1 | 7.107 | 69.2% | 1 | R.LAASASTQQLQEVK.A | 2 |

---

|  |  |  |  |  |  |  |  |  |
| --- | --- | --- | --- | --- | --- | --- | --- | --- |
| U | *gi|5730023|ref|NP\_006* | 2 | 2 | 6.0% | 463 | 51157 | 5.6 | RuvB-like 2 [Homo sapiens] |

| Filename XCorr DeltCN Conf% ObsM+H+ CalcM+H+ SpR ZScore Ion% # Sequence  | | | | | | | | | | | | |
| --- | --- | --- | --- | --- | --- | --- | --- | --- | --- | --- | --- | --- |
| \* | Astrin\_STLCHLD\_tube2\_050114\_01.07892.07892.2 | 2.5561 | 0.2451 | 98.9% | 1214.7722 | 1215.4998 | 1 | 4.81 | 70.0% | 1 | R.RAAGVVLEMIR.E | 2 |
| \* | Astrin\_STLCHLD\_050114\_02.06778.06778.3 | 3.2498 | 0.4311 | 100.0% | 1948.0743 | 1948.1863 | 2 | 6.91 | 35.9% | 1 | K.EVVHTVSLHEIDVINSR.T | 3 |

---

|  |  |  |  |  |  |  |  |  |
| --- | --- | --- | --- | --- | --- | --- | --- | --- |
| U | *gi|34147630|ref|NP\_00* | 3 | 3 | 5.9% | 455 | 49875 | 7.6 | Tu translation elongation factor, mitochondrial precursor [Homo sapiens] |

| Filename XCorr DeltCN Conf% ObsM+H+ CalcM+H+ SpR ZScore Ion% # Sequence  | | | | | | | | | | | | |
| --- | --- | --- | --- | --- | --- | --- | --- | --- | --- | --- | --- | --- |
| \* | Astrin\_STLCHLD\_050114\_01.11017.11017.2 | 3.2872 | 0.3865 | 99.9% | 1674.9321 | 1674.854 | 5 | 5.826 | 50.0% | 1 | R.GITINAAHVEYSTAAR.H | 2 |
| \* | Astrin\_STLCHLD\_tube2\_050114\_02.05661.05661.3 | 3.7822 | 0.3659 | 100.0% | 1676.1244 | 1674.854 | 1 | 6.71 | 40.0% | 1 | R.GITINAAHVEYSTAAR.H | 3 |
| \* | Astrin\_STLCHLD\_tube2\_050114\_01.07633.07633.2 | 2.4866 | 0.3023 | 99.2% | 1262.7922 | 1262.5096 | 29 | 5.339 | 55.0% | 1 | R.TVVTGIEMFHK.S | 2 |

---

|  |  |  |  |  |  |  |  |  |
| --- | --- | --- | --- | --- | --- | --- | --- | --- |
| U | *gi|205277463|ref|NP\_0* | 2 | 3 | 5.8% | 623 | 67878 | 7.7 | transketolase isoform 1 [Homo sapiens] |
| U | *gi|4507521|ref|NP\_001* | 2 | 3 | 5.8% | 623 | 67878 | 7.7 | transketolase isoform 1 [Homo sapiens] |
| U | *gi|205277465|ref|NP\_0* | 2 | 3 | 6.7% | 540 | 58982 | 7.7 | transketolase isoform 2 [Homo sapiens] |

| Filename XCorr DeltCN Conf% ObsM+H+ CalcM+H+ SpR ZScore Ion% # Sequence  | | | | | | | | | | | | |
| --- | --- | --- | --- | --- | --- | --- | --- | --- | --- | --- | --- | --- |
|  | Astrin\_STLCHLD\_tube2\_050114\_02.06004.06004.3 | 4.92 | 0.4164 | 100.0% | 2508.8643 | 2509.6946 | 1 | 6.277 | 41.7% | 2 | R.TSRPENAIIYNNNEDFQVGQAK.V | 3 |
|  | Astrin\_STLCHLD\_tube2\_050114\_01.09157.09157.2 | 2.6823 | 0.2652 | 99.0% | 1563.8922 | 1563.815 | 1 | 5.059 | 57.7% | 1 | K.MFGIDRDAIAQAVR.G | 2 |

---

|  |  |  |  |  |  |  |  |  |
| --- | --- | --- | --- | --- | --- | --- | --- | --- |
| U | *gi|5031877|ref|NP\_005* | 2 | 2 | 5.8% | 586 | 66408 | 5.2 | lamin B1 [Homo sapiens] |

| Filename XCorr DeltCN Conf% ObsM+H+ CalcM+H+ SpR ZScore Ion% # Sequence  | | | | | | | | | | | | |
| --- | --- | --- | --- | --- | --- | --- | --- | --- | --- | --- | --- | --- |
|  | Astrin\_STLCHLD\_tube2\_050114\_01.04526.04526.2 | 3.3794 | 0.3149 | 99.9% | 1672.9321 | 1672.8357 | 1 | 5.334 | 66.7% | 1 | R.LQEKEELRELNDR.L | 2 |
| \* | Astrin\_STLCHLD\_tube2\_050114\_02.06740.06740.3 | 3.5273 | 0.31 | 100.0% | 2373.5645 | 2372.5842 | 6 | 5.284 | 33.8% | 1 | R.LSSEMNTSTVNSAREELMESR.M | 3 |

---

|  |  |  |  |  |  |  |  |  |
| --- | --- | --- | --- | --- | --- | --- | --- | --- |
| U | *gi|156151392|ref|NP\_0* | 3 | 3 | 5.8% | 532 | 59682 | 9.2 | heterogeneous nuclear ribonucleoprotein R isoform 4 [Homo sapiens] |
| U | *gi|5031755|ref|NP\_005* | 3 | 3 | 4.9% | 633 | 70943 | 8.1 | heterogeneous nuclear ribonucleoprotein R isoform 2 [Homo sapiens] |
| U | *gi|156151396|ref|NP\_0* | 3 | 3 | 5.8% | 535 | 59953 | 9.2 | heterogeneous nuclear ribonucleoprotein R isoform 3 [Homo sapiens] |
| U | *gi|156151394|ref|NP\_0* | 3 | 3 | 4.9% | 636 | 71214 | 8.1 | heterogeneous nuclear ribonucleoprotein R isoform 1 [Homo sapiens] |

| Filename XCorr DeltCN Conf% ObsM+H+ CalcM+H+ SpR ZScore Ion% # Sequence  | | | | | | | | | | | | |
| --- | --- | --- | --- | --- | --- | --- | --- | --- | --- | --- | --- | --- |
|  | Astrin\_STLCHLD\_tube2\_050114\_01.05973.05973.2 | 3.1513 | 0.4152 | 100.0% | 1312.5122 | 1312.4221 | 1 | 8.018 | 72.7% | 1 | R.TGYTLDVTTGQR.K | 22 |
|  | Astrin\_STLCHLD\_tube2\_050114\_01.09307.09307.2 | 2.2068 | 0.2436 | 98.5% | 928.1122 | 928.0788 | 1 | 5.984 | 71.4% | 1 | K.AGPIWDLR.L | 22 |
|  | Astrin\_STLCHLD\_tube2\_050114\_01.07974.07974.2 | 2.4248 | 0.297 | 99.0% | 1263.1721 | 1262.4846 | 8 | 4.8 | 60.0% | 1 | R.LMMDPLSGQNR.G | 2 |

Similarities:
gi|23397427|ref|NP\_00(2:1)  

---

|  |  |  |  |  |  |  |  |  |
| --- | --- | --- | --- | --- | --- | --- | --- | --- |
| U | *gi|5031857|ref|NP\_005* | 2 | 4 | 5.7% | 332 | 36689 | 8.3 | L-lactate dehydrogenase A isoform 1 [Homo sapiens] |

| Filename XCorr DeltCN Conf% ObsM+H+ CalcM+H+ SpR ZScore Ion% # Sequence  | | | | | | | | | | | | |
| --- | --- | --- | --- | --- | --- | --- | --- | --- | --- | --- | --- | --- |
| \* | Astrin\_STLCHLD\_tube2\_050114\_01.06018.06018.2 | 2.2139 | 0.2803 | 98.8% | 913.53217 | 914.13617 | 1 | 5.831 | 75.0% | 1 | K.LVIITAGAR.Q | 2 |
|  | Astrin\_STLCHLD\_050114\_01.05067.05067.2 | 3.2533 | 0.4333 | 100.0% | 1135.1522 | 1135.2163 | 9 | 8.004 | 66.7% | 3 | K.VTLTSEEEAR.L | 2 |

---

|  |  |  |  |  |  |  |  |  |
| --- | --- | --- | --- | --- | --- | --- | --- | --- |
| U | *gi|156523260|ref|NP\_6* | 2 | 2 | 5.6% | 412 | 46373 | 5.1 | hypothetical protein LOC221150 [Homo sapiens] |

| Filename XCorr DeltCN Conf% ObsM+H+ CalcM+H+ SpR ZScore Ion% # Sequence  | | | | | | | | | | | | |
| --- | --- | --- | --- | --- | --- | --- | --- | --- | --- | --- | --- | --- |
| \* | Astrin\_STLCHLD\_tube2\_050114\_01.08203.08203.2 | 2.992 | 0.3588 | 99.9% | 1306.4521 | 1306.4583 | 1 | 5.836 | 75.0% | 1 | R.LENQEGIDFIK.A | 2 |
| \* | Astrin\_STLCHLD\_tube2\_050114\_01.07468.07468.2 | 2.6748 | 0.131 | 96.4% | 1386.5322 | 1385.5168 | 3 | 4.016 | 68.2% | 1 | K.YNSNLAT#PIAIK.A | 2 |

---

|  |  |  |  |  |  |  |  |  |
| --- | --- | --- | --- | --- | --- | --- | --- | --- |
| U | *gi|4503481|ref|NP\_001* | 2 | 3 | 5.3% | 437 | 50119 | 6.7 | eukaryotic translation elongation factor 1 gamma [Homo sapiens] |

| Filename XCorr DeltCN Conf% ObsM+H+ CalcM+H+ SpR ZScore Ion% # Sequence  | | | | | | | | | | | | |
| --- | --- | --- | --- | --- | --- | --- | --- | --- | --- | --- | --- | --- |
| \* | Astrin\_STLCHLD\_tube2\_050114\_01.06191.06191.2 | 3.2567 | 0.3998 | 100.0% | 1348.2922 | 1348.5448 | 4 | 8.057 | 62.5% | 2 | K.ALIAAQYSGAQVR.V | 2 |
| \* | Astrin\_STLCHLD\_tube2\_050114\_01.08270.08270.2 | 2.6714 | 0.3498 | 99.9% | 1242.4722 | 1242.4172 | 1 | 7.565 | 72.2% | 1 | K.STFVLDEFKR.K | 2 |

---

|  |  |  |  |  |  |  |  |  |
| --- | --- | --- | --- | --- | --- | --- | --- | --- |
| U | *gi|20127499|ref|NP\_00* | 2 | 4 | 5.2% | 344 | 39587 | 11.4 | arginine/serine-rich splicing factor 6 [Homo sapiens] |

| Filename XCorr DeltCN Conf% ObsM+H+ CalcM+H+ SpR ZScore Ion% # Sequence  | | | | | | | | | | | | |
| --- | --- | --- | --- | --- | --- | --- | --- | --- | --- | --- | --- | --- |
|  | Astrin\_STLCHLD\_tube2\_050114\_01.06008.06008.2 | 2.6765 | 0.3312 | 99.9% | 1030.7122 | 1031.1973 | 1 | 6.699 | 93.8% | 1 | R.LIVENLSSR.C | 22 |
| \* | Astrin\_STLCHLD\_tube2\_050114\_01.06637.06637.2 | 2.8871 | 0.333 | 99.9% | 1065.4922 | 1065.171 | 1 | 7.031 | 93.8% | 3 | R.TNEGVIEFR.S | 2 |

Similarities:
gi|21361282|ref|NP\_00(1:1)  

---

|  |  |  |  |  |  |  |  |  |
| --- | --- | --- | --- | --- | --- | --- | --- | --- |
| U | *gi|150418007|ref|NP\_0* | 14 | 18 | 5.1% | 3224 | 358201 | 6.2 | RAN binding protein 2 [Homo sapiens] |

| Filename XCorr DeltCN Conf% ObsM+H+ CalcM+H+ SpR ZScore Ion% # Sequence  | | | | | | | | | | | | |
| --- | --- | --- | --- | --- | --- | --- | --- | --- | --- | --- | --- | --- |
|  | Astrin\_STLCHLD\_050114\_01.17655.17655.2 | 3.3438 | 0.319 | 99.9% | 2310.4722 | 2309.7266 | 1 | 6.286 | 40.0% | 1 | R.ATNTDLLLAYANLMLLTLSTR.D | 2 |
|  | Astrin\_STLCHLD\_050114\_01.11667.11667.2 | 2.6511 | 0.2281 | 98.7% | 1456.3922 | 1456.7031 | 2 | 4.472 | 58.3% | 1 | R.LSQSGHMLLNLSR.G | 2 |
|  | Astrin\_STLCHLD\_tube2\_050114\_02.06416.06416.3 | 3.7307 | 0.4678 | 100.0% | 1456.7344 | 1456.7031 | 9 | 7.274 | 37.5% | 2 | R.LSQSGHMLLNLSR.G | 3 |
|  | Astrin\_STLCHLD\_tube2\_050114\_01.10902.10902.2 | 3.2858 | 0.3447 | 99.9% | 1787.3322 | 1786.9359 | 1 | 5.413 | 43.8% | 1 | K.SGQSALYDALFSSQSPK.D | 2 |
|  | Astrin\_STLCHLD\_050114\_01.11124.11124.3 | 2.5569 | 0.3128 | 99.9% | 1336.7943 | 1336.5773 | 58 | 6.029 | 37.5% | 2 | R.LLVQHEINTLR.A | 3 |
|  | Astrin\_STLCHLD\_tube2\_050114\_01.06267.06267.2 | 3.1758 | 0.4175 | 100.0% | 1336.9521 | 1336.5773 | 1 | 6.36 | 80.0% | 2 | R.LLVQHEINTLR.A | 2 |
|  | Astrin\_STLCHLD\_tube2\_050114\_01.06661.06661.2 | 2.978 | 0.2773 | 99.7% | 1345.3922 | 1345.4111 | 1 | 5.517 | 63.6% | 2 | K.TGSGLNSFYDQR.E | 2 |
| \* | Astrin\_STLCHLD\_tube2\_050114\_01.06106.06106.3 | 3.2075 | 0.183 | 98.3% | 1494.8043 | 1495.6526 | 55 | 4.226 | 37.5% | 1 | R.RSDDMFTFHGPGK.S | 3 |
| \* | Astrin\_STLCHLD\_050114\_01.12382.12382.3 | 4.522 | 0.3582 | 100.0% | 2599.5842 | 2599.9487 | 1 | 6.513 | 33.3% | 1 | R.SFVWHALDYADELPKPEQLAIR.F | 3 |
| \* | Astrin\_STLCHLD\_050114\_01.05951.05951.2 | 4.287 | 0.4882 | 100.0% | 1487.4922 | 1487.6738 | 1 | 8.266 | 64.3% | 1 | K.APGTNVAMASNQAVR.I | 2 |
| \* | Astrin\_STLCHLD\_050114\_02.07776.07776.2 | 2.7071 | 0.2949 | 99.2% | 1539.3922 | 1540.6726 | 1 | 5.57 | 57.7% | 1 | K.SDAGNLNFEFQVAK.K | 2 |
| \* | Astrin\_STLCHLD\_tube2\_050114\_01.07679.07679.3 | 3.1239 | 0.3381 | 100.0% | 1691.1843 | 1691.8839 | 5 | 6.055 | 37.5% | 1 | K.FGQGDLPKPINSDFR.S | 3 |
| \* | Astrin\_STLCHLD\_tube2\_050114\_01.08628.08628.2 | 2.2892 | 0.4258 | 99.9% | 1003.59216 | 1004.14557 | 2 | 6.354 | 68.8% | 1 | K.SGFEGMFTK.K | 2 |
| \* | Astrin\_STLCHLD\_tube2\_050114\_01.06829.06829.2 | 2.2623 | 0.2441 | 98.0% | 1132.6322 | 1132.3197 | 1 | 4.996 | 72.2% | 1 | K.SGFEGMFTKK.E | 2 |

---

|  |  |  |  |  |  |  |  |  |
| --- | --- | --- | --- | --- | --- | --- | --- | --- |
| U | *gi|194328685|ref|NP\_0* | 3 | 3 | 5.1% | 1136 | 131985 | 9.4 | myosin IB isoform 1 [Homo sapiens] |
| U | *gi|44889481|ref|NP\_03* | 3 | 3 | 5.4% | 1078 | 124951 | 9.2 | myosin IB isoform 2 [Homo sapiens] |

| Filename XCorr DeltCN Conf% ObsM+H+ CalcM+H+ SpR ZScore Ion% # Sequence  | | | | | | | | | | | | |
| --- | --- | --- | --- | --- | --- | --- | --- | --- | --- | --- | --- | --- |
|  | Astrin\_STLCHLD\_050114\_01.18505.18505.2 | 3.2006 | 0.1089 | 97.5% | 2643.5122 | 2644.1194 | 17 | 5.184 | 25.0% | 1 | R.NAMQIVGFMDHEAESVLAVVAAVLK.L | 2 |
|  | Astrin\_STLCHLD\_tube2\_050114\_02.06591.06591.2 | 2.7221 | 0.3528 | 99.7% | 1557.5322 | 1557.7465 | 3 | 6.461 | 53.8% | 1 | K.VSTTLNVAQAYYAR.D | 2 |
|  | Astrin\_STLCHLD\_050114\_02.11426.11426.2 | 2.6002 | 0.2287 | 98.0% | 2425.3123 | 2423.7686 | 1 | 4.945 | 33.3% | 1 | K.LQQIFIELTLKEEQEEYIR.E | 2 |

---

|  |  |  |  |  |  |  |  |  |
| --- | --- | --- | --- | --- | --- | --- | --- | --- |
| U | *gi|5032179|ref|NP\_005* | 3 | 3 | 5.1% | 835 | 88550 | 5.8 | tripartite motif-containing 28 protein [Homo sapiens] |

| Filename XCorr DeltCN Conf% ObsM+H+ CalcM+H+ SpR ZScore Ion% # Sequence  | | | | | | | | | | | | |
| --- | --- | --- | --- | --- | --- | --- | --- | --- | --- | --- | --- | --- |
| \* | Astrin\_STLCHLD\_tube2\_050114\_01.04468.04468.3 | 3.0489 | 0.2083 | 96.9% | 1815.2344 | 1814.9933 | 1 | 5.869 | 43.3% | 1 | R.VLVNDAQKVTEGQQER.L | 3 |
| \* | Astrin\_STLCHLD\_tube2\_050114\_01.05638.05638.2 | 2.3362 | 0.225 | 97.5% | 1122.2122 | 1122.2384 | 29 | 5.372 | 60.0% | 1 | R.SGEGEVSGLMR.K | 2 |
| \* | Astrin\_STLCHLD\_050114\_02.07060.07060.3 | 3.5917 | 0.2179 | 99.8% | 1787.1843 | 1787.0232 | 1 | 5.208 | 41.7% | 1 | K.LTEDKADVQSIIGLQR.F | 3 |

---

|  |  |  |  |  |  |  |  |  |
| --- | --- | --- | --- | --- | --- | --- | --- | --- |
| U | *Reverse\_gi|116812628|* | 2 | 2 | 5.1% | 604 | 70978 | 6.2 | coiled-coil domain containing 67 [Homo sapiens] |

| Filename XCorr DeltCN Conf% ObsM+H+ CalcM+H+ SpR ZScore Ion% # Sequence  | | | | | | | | | | | | |
| --- | --- | --- | --- | --- | --- | --- | --- | --- | --- | --- | --- | --- |
| \* | Astrin\_STLCHLD\_050114\_01.11432.11432.2 | 3.0841 | 0.0688 | 96.8% | 1554.6322 | 1554.6108 | 156 | 3.309 | 54.5% | 1 | K.IS\*PLYSS\*ELRTK.N | 2 |
| \* | Astrin\_STLCHLD\_tube2\_050114\_01.10945.10945.2 | 3.2882 | 0.0944 | 97.4% | 2255.7322 | 2256.518 | 11 | 3.132 | 44.4% | 1 | R.S\*LAIENVASKLKEIIFEDR.E | 2 |

---

|  |  |  |  |  |  |  |  |  |
| --- | --- | --- | --- | --- | --- | --- | --- | --- |
| U | *gi|21361282|ref|NP\_00* | 2 | 2 | 5.1% | 494 | 56678 | 11.5 | splicing factor, arginine/serine-rich 4 [Homo sapiens] |

| Filename XCorr DeltCN Conf% ObsM+H+ CalcM+H+ SpR ZScore Ion% # Sequence  | | | | | | | | | | | | |
| --- | --- | --- | --- | --- | --- | --- | --- | --- | --- | --- | --- | --- |
|  | Astrin\_STLCHLD\_tube2\_050114\_01.06008.06008.2 | 2.6765 | 0.3312 | 99.9% | 1030.7122 | 1031.1973 | 1 | 6.699 | 93.8% | 1 | R.LIVENLSSR.C | 22 |
| \* | Astrin\_STLCHLD\_050114\_02.07797.07797.3 | 2.9036 | 0.2037 | 95.1% | 1893.8043 | 1893.8306 | 1 | 4.043 | 38.3% | 1 | R.SRSGSSKSSHSK@S\*RS\*R.S | 3 |

Similarities:
gi|20127499|ref|NP\_00(1:1)  

---

|  |  |  |  |  |  |  |  |  |
| --- | --- | --- | --- | --- | --- | --- | --- | --- |
| U | *gi|21264343|ref|NP\_00* | 3 | 4 | 5.0% | 915 | 102642 | 5.5 | scaffold attachment factor B [Homo sapiens] |

| Filename XCorr DeltCN Conf% ObsM+H+ CalcM+H+ SpR ZScore Ion% # Sequence  | | | | | | | | | | | | |
| --- | --- | --- | --- | --- | --- | --- | --- | --- | --- | --- | --- | --- |
| \* | Astrin\_STLCHLD\_050114\_01.04258.04258.3 | 2.5181 | 0.3141 | 97.7% | 2648.2744 | 2648.5098 | 2 | 4.983 | 29.5% | 1 | K.ESSTSEGADQKMS\*S\*PEDDSDTKR.L | 3 |
| \* | Astrin\_STLCHLD\_tube2\_050114\_01.09634.09634.2 | 3.0243 | 0.4591 | 100.0% | 1354.8722 | 1355.4929 | 1 | 7.74 | 77.3% | 2 | R.NFWVSGLSSTTR.A | 2 |
|  | Astrin\_STLCHLD\_050114\_01.05603.05603.2 | 2.8304 | 0.289 | 99.7% | 1198.2722 | 1198.237 | 7 | 5.073 | 60.0% | 1 | R.DGWGGYGSDKR.M | 22 |

Similarities:
gi|7661936|ref|NP\_055(1:2)  

---

|  |  |  |  |  |  |  |  |  |
| --- | --- | --- | --- | --- | --- | --- | --- | --- |
| U | *gi|119703753|ref|NP\_0* | 4 | 7 | 5.0% | 564 | 60067 | 8.0 | keratin 6B [Homo sapiens] |

| Filename XCorr DeltCN Conf% ObsM+H+ CalcM+H+ SpR ZScore Ion% # Sequence  | | | | | | | | | | | | |
| --- | --- | --- | --- | --- | --- | --- | --- | --- | --- | --- | --- | --- |
|  | Astrin\_STLCHLD\_tube2\_050114\_01.06813.06813.2 | 2.2879 | 0.2249 | 98.9% | 828.2322 | 827.95544 | 7 | 5.23 | 91.7% | 2 | K.FASFIDK.V | 2222 |
|  | Astrin\_STLCHLD\_tube2\_050114\_01.06993.06993.2 | 2.7851 | 0.1943 | 99.2% | 1083.4122 | 1083.2755 | 4 | 6.85 | 81.2% | 2 | K.FASFIDKVR.F | 2222 |
|  | Astrin\_STLCHLD\_tube2\_050114\_01.06291.06291.2 | 3.0202 | 0.2969 | 99.9% | 1180.3522 | 1180.303 | 1 | 7.562 | 77.8% | 2 | K.YEELQITAGR.H | 22 |
|  | Astrin\_STLCHLD\_050114\_01.11141.11141.2 | 2.8705 | 0.2502 | 99.7% | 1154.1322 | 1154.3234 | 15 | 6.116 | 62.5% | 1 | K.EYQELMNVK.L | 22 |

Similarities:
gi|4504919|ref|NP\_002(3:1)  
gi|67782365|ref|NP\_00(2:2)  
gi|119395750|ref|NP\_0(1:3)  
gi|47132620|ref|NP\_00(2:2)  

---

|  |  |  |  |  |  |  |  |  |
| --- | --- | --- | --- | --- | --- | --- | --- | --- |
| U | *gi|21361368|ref|NP\_00* | 3 | 3 | 4.9% | 795 | 87302 | 7.1 | pyrroline-5-carboxylate synthetase isoform 1 [Homo sapiens] |
| U | *gi|62912457|ref|NP\_00* | 3 | 3 | 4.9% | 793 | 87089 | 7.1 | pyrroline-5-carboxylate synthetase isoform 2 [Homo sapiens] |

| Filename XCorr DeltCN Conf% ObsM+H+ CalcM+H+ SpR ZScore Ion% # Sequence  | | | | | | | | | | | | |
| --- | --- | --- | --- | --- | --- | --- | --- | --- | --- | --- | --- | --- |
|  | Astrin\_STLCHLD\_tube2\_050114\_01.05866.05866.2 | 2.7463 | 0.2584 | 99.3% | 1188.5322 | 1188.3844 | 6 | 5.87 | 72.2% | 1 | R.MLATLEPEQR.A | 2 |
|  | Astrin\_STLCHLD\_050114\_02.07661.07661.3 | 3.9232 | 0.4532 | 100.0% | 1773.6244 | 1773.1289 | 1 | 7.126 | 46.7% | 1 | R.ILHLLTQEALSIHGVK.E | 3 |
|  | Astrin\_STLCHLD\_tube2\_050114\_02.06951.06951.2 | 2.5689 | 0.5295 | 100.0% | 1293.8522 | 1294.4502 | 2 | 7.31 | 54.2% | 1 | R.FGLGAEVGISTSR.I | 2 |

---

|  |  |  |  |  |  |  |  |  |
| --- | --- | --- | --- | --- | --- | --- | --- | --- |
| U | *gi|154800453|ref|NP\_0* | 2 | 3 | 4.9% | 778 | 83857 | 7.4 | tastin isoform 1 [Homo sapiens] |

| Filename XCorr DeltCN Conf% ObsM+H+ CalcM+H+ SpR ZScore Ion% # Sequence  | | | | | | | | | | | | |
| --- | --- | --- | --- | --- | --- | --- | --- | --- | --- | --- | --- | --- |
| \* | Astrin\_STLCHLD\_tube2\_050114\_02.05780.05780.3 | 2.873 | 0.3265 | 99.9% | 1925.4543 | 1926.0935 | 12 | 4.853 | 31.9% | 1 | R.EVVTHSDEGGVASLGLAQR.V | 3 |
| \* | Astrin\_STLCHLD\_050114\_02.05853.05853.3 | 3.601 | 0.2073 | 99.3% | 1991.0643 | 1991.2113 | 18 | 4.859 | 31.9% | 2 | R.TLNATEHNSGTSHLPGLLK.H | 3 |

---

|  |  |  |  |  |  |  |  |  |
| --- | --- | --- | --- | --- | --- | --- | --- | --- |
| U | *gi|50053795|ref|NP\_00* | 2 | 2 | 4.9% | 611 | 69151 | 5.7 | eukaryotic translation initiation factor 4B [Homo sapiens] |

| Filename XCorr DeltCN Conf% ObsM+H+ CalcM+H+ SpR ZScore Ion% # Sequence  | | | | | | | | | | | | |
| --- | --- | --- | --- | --- | --- | --- | --- | --- | --- | --- | --- | --- |
| \* | Astrin\_STLCHLD\_tube2\_050114\_01.15285.15285.3 | 4.3443 | 0.3358 | 100.0% | 3344.8442 | 3345.6904 | 1 | 6.149 | 26.7% | 1 | R.LKGFGYAEFEDLDSLLSALSLNEESLGNRR.I | 3 |
| \* | Astrin\_STLCHLD\_tube2\_050114\_01.16026.16026.3 | 4.1854 | 0.2664 | 100.0% | 3105.2944 | 3104.357 | 17 | 5.675 | 22.2% | 1 | K.GFGYAEFEDLDSLLSALSLNEESLGNRR.I | 3 |

---

|  |  |  |  |  |  |  |  |  |
| --- | --- | --- | --- | --- | --- | --- | --- | --- |
| U | *gi|52632383|ref|NP\_00* | 2 | 2 | 4.9% | 589 | 64133 | 8.2 | heterogeneous nuclear ribonucleoprotein L isoform a [Homo sapiens] |
| U | *gi|52632385|ref|NP\_00* | 2 | 2 | 6.4% | 456 | 50561 | 7.5 | heterogeneous nuclear ribonucleoprotein L isoform b [Homo sapiens] |

| Filename XCorr DeltCN Conf% ObsM+H+ CalcM+H+ SpR ZScore Ion% # Sequence  | | | | | | | | | | | | |
| --- | --- | --- | --- | --- | --- | --- | --- | --- | --- | --- | --- | --- |
|  | Astrin\_STLCHLD\_tube2\_050114\_02.06669.06669.3 | 3.0785 | 0.2553 | 99.3% | 1868.7244 | 1869.1176 | 254 | 5.313 | 27.9% | 1 | K.SKPGAAMVEMADGYAVDR.A | 3 |
|  | Astrin\_STLCHLD\_tube2\_050114\_01.08410.08410.2 | 2.2357 | 0.3674 | 99.3% | 1223.3522 | 1223.3251 | 28 | 6.367 | 55.0% | 1 | R.SSSGLLEWESK.S | 2 |

---

|  |  |  |  |  |  |  |  |  |
| --- | --- | --- | --- | --- | --- | --- | --- | --- |
| U | *gi|217272802|ref|NP\_0* | 2 | 3 | 4.8% | 725 | 84229 | 5.9 | hyaluronan-mediated motility receptor isoform a [Homo sapiens] |
| U | *gi|217416398|ref|NP\_0* | 2 | 3 | 4.9% | 709 | 82301 | 5.9 | hyaluronan-mediated motility receptor isoform c [Homo sapiens] |
| U | *gi|217416394|ref|NP\_0* | 2 | 3 | 4.8% | 724 | 84100 | 5.8 | hyaluronan-mediated motility receptor isoform b [Homo sapiens] |
| U | *gi|217272804|ref|NP\_0* | 2 | 3 | 5.5% | 638 | 74495 | 5.4 | hyaluronan-mediated motility receptor isoform d [Homo sapiens] |

| Filename XCorr DeltCN Conf% ObsM+H+ CalcM+H+ SpR ZScore Ion% # Sequence  | | | | | | | | | | | | |
| --- | --- | --- | --- | --- | --- | --- | --- | --- | --- | --- | --- | --- |
|  | Astrin\_STLCHLD\_tube2\_050114\_02.05544.05544.3 | 4.3668 | 0.2484 | 100.0% | 1598.8143 | 1598.7966 | 2 | 6.369 | 44.6% | 2 | K.SSAAHTQATLLLQEK.Y | 3 |
|  | Astrin\_STLCHLD\_tube2\_050114\_02.06745.06745.3 | 4.1136 | 0.3552 | 100.0% | 2218.9443 | 2219.451 | 6 | 5.596 | 31.6% | 1 | K.ALTASEIEDLKLENSSLQEK.A | 3 |

---

|  |  |  |  |  |  |  |  |  |
| --- | --- | --- | --- | --- | --- | --- | --- | --- |
| U | *gi|109240550|ref|NP\_0* | 2 | 2 | 4.8% | 523 | 58744 | 6.7 | paraspeckle protein 1 [Homo sapiens] |

| Filename XCorr DeltCN Conf% ObsM+H+ CalcM+H+ SpR ZScore Ion% # Sequence  | | | | | | | | | | | | |
| --- | --- | --- | --- | --- | --- | --- | --- | --- | --- | --- | --- | --- |
| \* | Astrin\_STLCHLD\_tube2\_050114\_01.07348.07348.2 | 3.2017 | 0.4876 | 100.0% | 1311.4722 | 1311.4368 | 1 | 8.55 | 70.0% | 1 | R.YGEPSEVFINR.D | 2 |
| \* | Astrin\_STLCHLD\_tube2\_050114\_01.09432.09432.2 | 3.5782 | 0.4474 | 100.0% | 1650.3522 | 1650.7875 | 1 | 7.297 | 69.2% | 1 | R.FAQPGTFEFEYASR.W | 2 |

---

|  |  |  |  |  |  |  |  |  |
| --- | --- | --- | --- | --- | --- | --- | --- | --- |
| U | *gi|20143967|ref|NP\_61* | 3 | 3 | 4.7% | 960 | 110059 | 8.5 | kinesin family member 23 isoform 1 [Homo sapiens] |
| U | *gi|6754472|ref|NP\_004* | 3 | 3 | 5.3% | 856 | 98105 | 8.5 | kinesin family member 23 isoform 2 [Homo sapiens] |

| Filename XCorr DeltCN Conf% ObsM+H+ CalcM+H+ SpR ZScore Ion% # Sequence  | | | | | | | | | | | | |
| --- | --- | --- | --- | --- | --- | --- | --- | --- | --- | --- | --- | --- |
|  | Astrin\_STLCHLD\_tube2\_050114\_01.07253.07253.2 | 2.6815 | 0.3267 | 99.5% | 1497.3922 | 1497.6196 | 1 | 6.427 | 68.2% | 1 | K.AEDYEENLQVMR.F | 2 |
|  | Astrin\_STLCHLD\_050114\_02.05973.05973.3 | 2.4579 | 0.2921 | 97.5% | 1818.1743 | 1818.0367 | 2 | 5.321 | 36.7% | 1 | R.FAEVTQEVEVARPVDK.A | 3 |
|  | Astrin\_STLCHLD\_050114\_02.06020.06020.3 | 4.4321 | 0.285 | 100.0% | 1966.9143 | 1966.17 | 2 | 6.271 | 37.5% | 1 | K.YMLTHQELASDGEIETK.L | 3 |

---

|  |  |  |  |  |  |  |  |  |
| --- | --- | --- | --- | --- | --- | --- | --- | --- |
| U | *gi|47132620|ref|NP\_00* | 4 | 8 | 4.7% | 639 | 65433 | 8.0 | keratin 2 [Homo sapiens] |

| Filename XCorr DeltCN Conf% ObsM+H+ CalcM+H+ SpR ZScore Ion% # Sequence  | | | | | | | | | | | | |
| --- | --- | --- | --- | --- | --- | --- | --- | --- | --- | --- | --- | --- |
|  | Astrin\_STLCHLD\_tube2\_050114\_01.06813.06813.2 | 2.2879 | 0.2249 | 98.9% | 828.2322 | 827.95544 | 7 | 5.23 | 91.7% | 2 | K.FASFIDK.V | 2222 |
|  | Astrin\_STLCHLD\_tube2\_050114\_01.06993.06993.2 | 2.7851 | 0.1943 | 99.2% | 1083.4122 | 1083.2755 | 4 | 6.85 | 81.2% | 2 | K.FASFIDKVR.F | 2222 |
|  | Astrin\_STLCHLD\_tube2\_050114\_01.05886.05886.2 | 4.2621 | 0.08 | 99.6% | 1476.3522 | 1476.6726 | 1 | 6.871 | 86.4% | 3 | R.FLEQQNQVLQTK.W | 22 |
|  | Astrin\_STLCHLD\_tube2\_050114\_01.06305.06305.2 | 2.3482 | 0.1857 | 97.3% | 1037.6921 | 1038.1454 | 1 | 4.742 | 81.2% | 1 | R.YLDGLTAER.T | 2 |

Similarities:
gi|4504919|ref|NP\_002(2:2)  
gi|67782365|ref|NP\_00(2:2)  
gi|119395750|ref|NP\_0(1:3)  
gi|119703753|ref|NP\_0(2:2)  

---

|  |  |  |  |  |  |  |  |  |
| --- | --- | --- | --- | --- | --- | --- | --- | --- |
| U | *gi|41322908|ref|NP\_95* | 16 | 24 | 4.6% | 4525 | 513712 | 5.8 | plectin 1 isoform 3 [Homo sapiens] |
| U | *gi|47607492|ref|NP\_00* | 16 | 24 | 4.5% | 4574 | 518478 | 5.7 | plectin 1 isoform 1 [Homo sapiens] |
| U | *gi|41322923|ref|NP\_95* | 16 | 24 | 4.6% | 4547 | 516204 | 5.8 | plectin 1 isoform 11 [Homo sapiens] |
| U | *gi|41322919|ref|NP\_95* | 16 | 24 | 4.6% | 4547 | 516282 | 5.8 | plectin 1 isoform 8 [Homo sapiens] |
| U | *gi|41322916|ref|NP\_95* | 16 | 24 | 4.4% | 4684 | 531796 | 6.0 | plectin 1 isoform 6 [Homo sapiens] |
| U | *gi|41322914|ref|NP\_95* | 16 | 24 | 4.6% | 4551 | 516484 | 5.8 | plectin 1 isoform 10 [Homo sapiens] |
| U | *gi|41322912|ref|NP\_95* | 16 | 24 | 4.6% | 4533 | 514780 | 5.7 | plectin 1 isoform 2 [Homo sapiens] |
| U | *gi|41322910|ref|NP\_95* | 16 | 24 | 4.6% | 4515 | 512609 | 5.8 | plectin 1 isoform 7 [Homo sapiens] |

| Filename XCorr DeltCN Conf% ObsM+H+ CalcM+H+ SpR ZScore Ion% # Sequence  | | | | | | | | | | | | |
| --- | --- | --- | --- | --- | --- | --- | --- | --- | --- | --- | --- | --- |
|  | Astrin\_STLCHLD\_050114\_01.05459.05459.2 | 2.5797 | 0.195 | 98.6% | 1216.6921 | 1216.3365 | 19 | 4.687 | 61.1% | 1 | R.RPELEDSTLR.Y | 2 |
|  | Astrin\_STLCHLD\_tube2\_050114\_01.13063.13063.2 | 2.7067 | 0.4189 | 99.9% | 1914.2522 | 1915.1155 | 1 | 6.638 | 46.4% | 1 | R.YLQDLLAWVEENQHR.V | 2 |
|  | Astrin\_STLCHLD\_050114\_01.10646.10646.2 | 2.7097 | 0.1591 | 98.8% | 1043.4321 | 1043.2083 | 1 | 5.09 | 93.8% | 1 | R.LAAEQELIR.L | 2 |
|  | Astrin\_STLCHLD\_tube2\_050114\_01.05878.05878.3 | 3.9707 | 0.2147 | 100.0% | 1692.5343 | 1692.9156 | 17 | 5.269 | 45.8% | 3 | R.LREQLQLLEEQHR.A | 3 |
|  | Astrin\_STLCHLD\_050114\_02.05048.05048.3 | 3.2395 | 0.2304 | 99.2% | 1784.0343 | 1784.964 | 1 | 6.22 | 35.3% | 1 | R.AALAHSEEVTASQVAATK.T | 3 |
|  | Astrin\_STLCHLD\_050114\_02.05034.05034.2 | 2.1898 | 0.343 | 98.6% | 1784.4521 | 1784.964 | 24 | 6.058 | 35.3% | 1 | R.AALAHSEEVTASQVAATK.T | 2 |
|  | Astrin\_STLCHLD\_050114\_02.07295.07295.2 | 4.5579 | 0.4558 | 100.0% | 1557.6122 | 1557.744 | 1 | 8.436 | 80.8% | 3 | R.LQEAGILSAEELQR.L | 2 |
|  | Astrin\_STLCHLD\_tube2\_050114\_02.06462.06462.3 | 4.5575 | 0.3987 | 100.0% | 2013.9543 | 2014.292 | 1 | 6.59 | 41.7% | 2 | R.LLEAQIATGGVIDPVHSHR.V | 33 |
|  | Astrin\_STLCHLD\_050114\_01.07276.07276.2 | 2.3554 | 0.3397 | 99.7% | 919.59216 | 919.0684 | 26 | 6.34 | 64.3% | 2 | R.VPVDVAYR.R | 22 |
|  | Astrin\_STLCHLD\_tube2\_050114\_01.06755.06755.2 | 1.9277 | 0.3093 | 97.6% | 1162.4521 | 1161.2311 | 1 | 6.107 | 75.0% | 1 | R.GYFDEEMNR.V | 22 |
|  | Astrin\_STLCHLD\_tube2\_050114\_02.06908.06908.2 | 3.7663 | 0.4555 | 100.0% | 1614.7522 | 1614.8363 | 1 | 8.052 | 70.0% | 2 | R.LLDAQLSTGGIVDPSK.S | 2 |
|  | Astrin\_STLCHLD\_050114\_02.07160.07160.3 | 3.9362 | 0.2784 | 100.0% | 2028.5343 | 2028.3188 | 3 | 6.001 | 34.7% | 1 | R.LLEAQIATGGIIDPVHSHR.V | 33 |
|  | Astrin\_STLCHLD\_050114\_01.06925.06925.2 | 2.1763 | 0.2561 | 98.0% | 1047.2122 | 1047.1991 | 3 | 5.441 | 75.0% | 1 | R.VPVDVAYQR.G | 2 |
|  | Astrin\_STLCHLD\_050114\_02.07692.07692.2 | 3.149 | 0.3653 | 99.9% | 1539.8322 | 1539.7721 | 1 | 6.859 | 57.1% | 1 | R.LLDAQLATGGIVDPR.L | 2 |
|  | Astrin\_STLCHLD\_050114\_02.07222.07222.2 | 3.1066 | 0.2315 | 99.3% | 1462.9722 | 1462.6611 | 1 | 6.15 | 66.7% | 2 | R.SQVMDEATALQLR.E | 2 |
|  | Astrin\_STLCHLD\_tube2\_050114\_01.14523.14523.2 | 4.6556 | 0.5148 | 100.0% | 2116.4722 | 2116.3533 | 1 | 8.848 | 50.0% | 1 | R.AGTLSITEFADMLSGNAGGFR.S | 2 |

Similarities:
gi|207452735|ref|NP\_1(4:12)  

---

|  |  |  |  |  |  |  |  |  |
| --- | --- | --- | --- | --- | --- | --- | --- | --- |
| U | *gi|38569421|ref|NP\_00* | 3 | 3 | 4.6% | 1101 | 120839 | 7.3 | ATP citrate lyase isoform 1 [Homo sapiens] |
| U | *gi|38569423|ref|NP\_94* | 3 | 3 | 4.7% | 1091 | 119772 | 7.3 | ATP citrate lyase isoform 2 [Homo sapiens] |

| Filename XCorr DeltCN Conf% ObsM+H+ CalcM+H+ SpR ZScore Ion% # Sequence  | | | | | | | | | | | | |
| --- | --- | --- | --- | --- | --- | --- | --- | --- | --- | --- | --- | --- |
|  | Astrin\_STLCHLD\_tube2\_050114\_02.06496.06496.3 | 3.353 | 0.2853 | 100.0% | 2259.1443 | 2259.3936 | 86 | 5.061 | 27.5% | 1 | R.EGDYVLFHHEGGVDVGDVDAK.A | 3 |
|  | Astrin\_STLCHLD\_tube2\_050114\_02.06933.06933.2 | 3.9713 | 0.4359 | 100.0% | 1881.3722 | 1882.07 | 1 | 8.054 | 56.7% | 1 | R.SAYDSTMETMNYAQIR.T | 2 |
|  | Astrin\_STLCHLD\_tube2\_050114\_01.08896.08896.2 | 3.4455 | 0.3539 | 99.9% | 1492.3322 | 1492.647 | 1 | 6.812 | 65.4% | 1 | R.SGGMSNELNNIISR.T | 2 |

---

|  |  |  |  |  |  |  |  |  |
| --- | --- | --- | --- | --- | --- | --- | --- | --- |
| U | *gi|4506003|ref|NP\_002* | 2 | 3 | 4.5% | 330 | 37512 | 6.3 | protein phosphatase 1, catalytic subunit, alpha isoform 1 [Homo sapiens] |
| U | *gi|56790945|ref|NP\_00* | 2 | 3 | 4.4% | 341 | 38631 | 6.6 | protein phosphatase 1, catalytic subunit, alpha isoform 3 [Homo sapiens] |
| U | *gi|46249376|ref|NP\_99* | 2 | 3 | 4.6% | 327 | 37187 | 6.2 | protein phosphatase 1, catalytic subunit, beta isoform 1 [Homo sapiens] |
| U | *gi|45827798|ref|NP\_99* | 2 | 3 | 5.2% | 286 | 32595 | 6.1 | protein phosphatase 1, catalytic subunit, alpha isoform 2 [Homo sapiens] |
| U | *gi|4506007|ref|NP\_002* | 2 | 3 | 4.6% | 323 | 36984 | 6.5 | protein phosphatase 1, catalytic subunit, gamma isoform [Homo sapiens] |
| U | *gi|4506005|ref|NP\_002* | 2 | 3 | 4.6% | 327 | 37187 | 6.2 | protein phosphatase 1, catalytic subunit, beta isoform 1 [Homo sapiens] |

| Filename XCorr DeltCN Conf% ObsM+H+ CalcM+H+ SpR ZScore Ion% # Sequence  | | | | | | | | | | | | |
| --- | --- | --- | --- | --- | --- | --- | --- | --- | --- | --- | --- | --- |
|  | Astrin\_STLCHLD\_tube2\_050114\_02.06991.06991.3 | 3.8242 | 0.1583 | 99.7% | 1641.6244 | 1640.7924 | 2 | 6.291 | 46.2% | 2 | R.AHQVVEDGYEFFAK.R | 3 |
|  | Astrin\_STLCHLD\_tube2\_050114\_01.07610.07610.3 | 2.5642 | 0.2725 | 97.6% | 1795.9443 | 1796.9799 | 238 | 5.696 | 32.1% | 1 | R.AHQVVEDGYEFFAKR.Q | 3 |

---

|  |  |  |  |  |  |  |  |  |
| --- | --- | --- | --- | --- | --- | --- | --- | --- |
| U | *gi|148664201|ref|NP\_0* | 2 | 4 | 4.4% | 683 | 76987 | 9.4 | cytoskeleton associated protein 2 isoform 2 [Homo sapiens] |
| U | *gi|148664244|ref|NP\_0* | 2 | 4 | 4.4% | 682 | 76900 | 9.4 | cytoskeleton associated protein 2 isoform 1 [Homo sapiens] |

| Filename XCorr DeltCN Conf% ObsM+H+ CalcM+H+ SpR ZScore Ion% # Sequence  | | | | | | | | | | | | |
| --- | --- | --- | --- | --- | --- | --- | --- | --- | --- | --- | --- | --- |
|  | Astrin\_STLCHLD\_tube2\_050114\_01.05970.05970.3 | 2.7527 | 0.3591 | 100.0% | 1946.3944 | 1945.229 | 147 | 5.219 | 28.1% | 2 | K.FVSTTSQNTQLVRPPIR.S | 3 |
|  | Astrin\_STLCHLD\_050114\_01.11886.11886.2 | 3.0023 | 0.2131 | 99.1% | 1399.3722 | 1399.6483 | 1 | 5.787 | 70.8% | 2 | K.AILAGAQPIEEMR.H | 2 |

---

|  |  |  |  |  |  |  |  |  |
| --- | --- | --- | --- | --- | --- | --- | --- | --- |
| U | *gi|222831647|ref|NP\_0* | 2 | 2 | 4.3% | 900 | 99510 | 7.8 | similar to KIAA1680 protein isoform 1 [Homo sapiens] |
| U | *gi|46575934|ref|NP\_99* | 2 | 2 | 5.8% | 677 | 74288 | 6.9 | similar to KIAA1680 protein isoform 2 [Homo sapiens] |

| Filename XCorr DeltCN Conf% ObsM+H+ CalcM+H+ SpR ZScore Ion% # Sequence  | | | | | | | | | | | | |
| --- | --- | --- | --- | --- | --- | --- | --- | --- | --- | --- | --- | --- |
|  | Astrin\_STLCHLD\_tube2\_050114\_01.17187.17187.3 | 3.4692 | 0.1913 | 96.1% | 4172.8145 | 4171.098 | 100 | 3.734 | 17.1% | 1 | R.S\*INRRHDSLPSSPS\*SSNTVGVHSSSPS\*STNSSSGSTGKR.R | 3 |
|  | Astrin\_STLCHLD\_tube2\_050114\_01.14058.14058.3 | 3.6901 | 0.1778 | 96.4% | 4172.9644 | 4171.098 | 321 | 4.259 | 17.1% | 1 | R.S\*INRRHDSLPSSPSSSNT#VGVHSSSPS\*STNSSSGSTGKR.R | 3 |

---

|  |  |  |  |  |  |  |  |  |
| --- | --- | --- | --- | --- | --- | --- | --- | --- |
| U | *contaminant\_GR78\_HUMA* | 2 | 2 | 4.3% | 653 | 72116 | 5.1 | owl|P11021| 78 KD GLUCOSE REGULATED PROTEIN PRECURSOR (GRP 78) (IMMUNOGLOBULIN... |
| U | *gi|16507237|ref|NP\_00* | 2 | 2 | 4.3% | 654 | 72333 | 5.2 | heat shock 70kDa protein 5 [Homo sapiens] |
| U | *contaminant\_GR78\_RAT* | 2 | 2 | 4.3% | 654 | 72347 | 5.2 | owl|P06761| 78 KD GLUCOSE REGULATED PROTEIN PRECURSOR (GRP 78) (IMMUNOGLOBULIN... |
| U | *contaminant\_GR78\_MOUS* | 2 | 2 | 4.3% | 655 | 72421 | 5.2 | owl|P20029| 78 KD GLUCOSE REGULATED PROTEIN PRECURSOR (GRP 78) (IMMUNOGLOBULIN... |
| U | *contaminant\_GR78\_MESA* | 2 | 2 | 4.3% | 654 | 72379 | 5.2 | owl|P07823| 78 KD GLUCOSE REGULATED PROTEIN PRECURSOR (GRP 78) (IMMUNOGLOBULIN... |

| Filename XCorr DeltCN Conf% ObsM+H+ CalcM+H+ SpR ZScore Ion% # Sequence  | | | | | | | | | | | | |
| --- | --- | --- | --- | --- | --- | --- | --- | --- | --- | --- | --- | --- |
|  | Astrin\_STLCHLD\_050114\_02.04540.04540.3 | 3.896 | 0.2844 | 100.0% | 1370.1543 | 1369.619 | 1 | 5.875 | 50.0% | 1 | K.MKETAEAYLGKK.V | 3 |
|  | Astrin\_STLCHLD\_tube2\_050114\_01.09822.09822.2 | 3.9832 | 0.4639 | 100.0% | 1660.4922 | 1660.9078 | 1 | 8.122 | 63.3% | 1 | R.IINEPTAAAIAYGLDK.R | 222 |

Similarities:
gi|24234686|ref|NP\_69(1:1)  
gi|124256496|ref|NP\_0(1:1)  

---

|  |  |  |  |  |  |  |  |  |
| --- | --- | --- | --- | --- | --- | --- | --- | --- |
| U | *gi|167466272|ref|NP\_6* | 3 | 4 | 4.2% | 745 | 83587 | 9.8 | cytoskeleton associated protein 2-like [Homo sapiens] |

| Filename XCorr DeltCN Conf% ObsM+H+ CalcM+H+ SpR ZScore Ion% # Sequence  | | | | | | | | | | | | |
| --- | --- | --- | --- | --- | --- | --- | --- | --- | --- | --- | --- | --- |
| \* | Astrin\_STLCHLD\_tube2\_050114\_01.06239.06239.2 | 3.5576 | 0.3902 | 100.0% | 1326.3322 | 1326.5791 | 1 | 6.426 | 72.7% | 2 | R.KPVGSLNIEQLK.T | 2 |
| \* | Astrin\_STLCHLD\_tube2\_050114\_01.08742.08742.2 | 2.6172 | 0.2431 | 99.0% | 1271.5521 | 1271.4594 | 39 | 5.369 | 50.0% | 1 | K.VVLNILQDSNR.T | 2 |
| \* | Astrin\_STLCHLD\_tube2\_050114\_01.07222.07222.2 | 2.2055 | 0.1866 | 96.8% | 894.6322 | 894.10504 | 88 | 4.133 | 64.3% | 1 | K.LQIGPIPR.I | 2 |

---

|  |  |  |  |  |  |  |  |  |
| --- | --- | --- | --- | --- | --- | --- | --- | --- |
| U | *gi|27477136|ref|NP\_06* | 2 | 4 | 4.1% | 902 | 101431 | 8.4 | zinc finger antiviral protein isoform 1 [Homo sapiens] |
| U | *gi|27477138|ref|NP\_07* | 2 | 4 | 5.3% | 699 | 77903 | 8.4 | zinc finger antiviral protein isoform 2 [Homo sapiens] |

| Filename XCorr DeltCN Conf% ObsM+H+ CalcM+H+ SpR ZScore Ion% # Sequence  | | | | | | | | | | | | |
| --- | --- | --- | --- | --- | --- | --- | --- | --- | --- | --- | --- | --- |
|  | Astrin\_STLCHLD\_050114\_02.07144.07144.2 | 3.2545 | 0.4014 | 100.0% | 1449.0721 | 1449.647 | 1 | 7.468 | 61.5% | 2 | R.FVVLETGGEAGITR.S | 2 |
|  | Astrin\_STLCHLD\_tube2\_050114\_01.13682.13682.2 | 3.2554 | 0.258 | 99.6% | 2784.3123 | 2785.9714 | 21 | 4.749 | 27.3% | 2 | R.EHGLNPDVVQNIQDICNS\*KHMQK.N | 2 |

---

|  |  |  |  |  |  |  |  |  |
| --- | --- | --- | --- | --- | --- | --- | --- | --- |
| U | *gi|21626466|ref|NP\_06* | 2 | 2 | 4.0% | 847 | 94623 | 6.3 | matrin 3 [Homo sapiens] |
| U | *gi|62750354|ref|NP\_95* | 2 | 2 | 4.0% | 847 | 94623 | 6.3 | matrin 3 [Homo sapiens] |

| Filename XCorr DeltCN Conf% ObsM+H+ CalcM+H+ SpR ZScore Ion% # Sequence  | | | | | | | | | | | | |
| --- | --- | --- | --- | --- | --- | --- | --- | --- | --- | --- | --- | --- |
|  | Astrin\_STLCHLD\_050114\_01.04707.04707.3 | 2.9896 | 0.2984 | 100.0% | 1522.5844 | 1522.6609 | 3 | 5.48 | 43.8% | 1 | R.RRTEEGPTLSYGR.D | 3 |
|  | Astrin\_STLCHLD\_050114\_01.14345.14345.3 | 3.9779 | 0.314 | 100.0% | 2440.4644 | 2439.9036 | 1 | 6.196 | 35.0% | 1 | R.YQLLQLVEPFGVISNHLILNK.I | 3 |

---

|  |  |  |  |  |  |  |  |  |
| --- | --- | --- | --- | --- | --- | --- | --- | --- |
| U | *gi|148727341|ref|NP\_0* | 2 | 2 | 4.0% | 350 | 38438 | 5.1 | serine/threonine kinase receptor associated protein [Homo sapiens] |

| Filename XCorr DeltCN Conf% ObsM+H+ CalcM+H+ SpR ZScore Ion% # Sequence  | | | | | | | | | | | | |
| --- | --- | --- | --- | --- | --- | --- | --- | --- | --- | --- | --- | --- |
| \* | Astrin\_STLCHLD\_tube2\_050114\_01.08459.08459.2 | 2.2033 | 0.2754 | 96.9% | 1486.1322 | 1485.7233 | 7 | 5.126 | 50.0% | 1 | R.SIAFHSAVSLDPIK.S | 2 |
| \* | Astrin\_STLCHLD\_050114\_02.07361.07361.3 | 2.6441 | 0.2562 | 97.7% | 1486.8243 | 1485.7233 | 190 | 4.561 | 26.9% | 1 | R.SIAFHSAVSLDPIK.S | 3 |

---

|  |  |  |  |  |  |  |  |  |
| --- | --- | --- | --- | --- | --- | --- | --- | --- |
| U | *gi|55956788|ref|NP\_00* | 2 | 5 | 3.9% | 710 | 76615 | 4.7 | nucleolin [Homo sapiens] |

| Filename XCorr DeltCN Conf% ObsM+H+ CalcM+H+ SpR ZScore Ion% # Sequence  | | | | | | | | | | | | |
| --- | --- | --- | --- | --- | --- | --- | --- | --- | --- | --- | --- | --- |
| \* | Astrin\_STLCHLD\_tube2\_050114\_02.08138.08138.2 | 3.5416 | 0.4177 | 100.0% | 1649.8922 | 1649.751 | 1 | 7.947 | 69.2% | 3 | K.FGYVDFESAEDLEK.A | 2 |
| \* | Astrin\_STLCHLD\_tube2\_050114\_02.08171.08171.2 | 3.8217 | 0.4949 | 100.0% | 1562.2922 | 1562.6323 | 1 | 7.704 | 69.2% | 2 | K.GFGFVDFNSEEDAK.A | 2 |

---

|  |  |  |  |  |  |  |  |  |
| --- | --- | --- | --- | --- | --- | --- | --- | --- |
| U | *gi|194239723|ref|NP\_0* | 2 | 4 | 3.9% | 647 | 71408 | 6.4 | eukaryotic translation elongation factor 1 delta isoform 1 [Homo sapiens] |
| U | *gi|25453474|ref|NP\_11* | 2 | 4 | 3.9% | 647 | 71408 | 6.4 | eukaryotic translation elongation factor 1 delta isoform 1 [Homo sapiens] |
| U | *gi|25453472|ref|NP\_00* | 2 | 4 | 8.9% | 281 | 31122 | 5.0 | eukaryotic translation elongation factor 1 delta isoform 2 [Homo sapiens] |
| U | *gi|194239731|ref|NP\_0* | 2 | 4 | 8.9% | 281 | 31122 | 5.0 | eukaryotic translation elongation factor 1 delta isoform 2 [Homo sapiens] |
| U | *gi|194239729|ref|NP\_0* | 2 | 4 | 9.7% | 257 | 28558 | 4.9 | eukaryotic translation elongation factor 1 delta isoform 4 [Homo sapiens] |
| U | *gi|194239727|ref|NP\_0* | 2 | 4 | 8.9% | 281 | 31122 | 5.0 | eukaryotic translation elongation factor 1 delta isoform 2 [Homo sapiens] |
| U | *gi|194239725|ref|NP\_0* | 2 | 4 | 3.9% | 646 | 71266 | 6.4 | eukaryotic translation elongation factor 1 delta isoform 3 [Homo sapiens] |

| Filename XCorr DeltCN Conf% ObsM+H+ CalcM+H+ SpR ZScore Ion% # Sequence  | | | | | | | | | | | | |
| --- | --- | --- | --- | --- | --- | --- | --- | --- | --- | --- | --- | --- |
|  | Astrin\_STLCHLD\_tube2\_050114\_01.06573.06573.2 | 3.3123 | 0.3247 | 99.9% | 1360.4722 | 1359.5223 | 2 | 6.522 | 72.7% | 3 | R.IASLEVENQSLR.G | 2 |
|  | Astrin\_STLCHLD\_050114\_02.09052.09052.2 | 2.472 | 0.3787 | 99.5% | 1374.4521 | 1374.5797 | 6 | 5.764 | 50.0% | 1 | R.SIQLDGLVWGASK.L | 2 |

---

|  |  |  |  |  |  |  |  |  |
| --- | --- | --- | --- | --- | --- | --- | --- | --- |
| U | *gi|71361682|ref|NP\_00* | 7 | 11 | 3.7% | 2115 | 238257 | 5.8 | nuclear mitotic apparatus protein 1 [Homo sapiens] |

| Filename XCorr DeltCN Conf% ObsM+H+ CalcM+H+ SpR ZScore Ion% # Sequence  | | | | | | | | | | | | |
| --- | --- | --- | --- | --- | --- | --- | --- | --- | --- | --- | --- | --- |
| \* | Astrin\_STLCHLD\_tube2\_050114\_02.05211.05211.3 | 3.2818 | 0.3902 | 100.0% | 1526.6643 | 1526.6897 | 8 | 6.396 | 38.5% | 2 | K.VAGIESHSELQISR.Q | 3 |
| \* | Astrin\_STLCHLD\_050114\_01.06064.06064.2 | 2.6603 | 0.2846 | 99.3% | 1306.1921 | 1306.3899 | 37 | 5.093 | 50.0% | 2 | R.EAEQMGNELER.L | 2 |
| \* | Astrin\_STLCHLD\_050114\_01.04718.04718.2 | 3.1354 | 0.1759 | 98.7% | 1654.3322 | 1654.78 | 1 | 5.296 | 63.3% | 1 | K.AQELGHSQSALASAQR.E | 2 |
| \* | Astrin\_STLCHLD\_050114\_01.04738.04738.3 | 3.1806 | 0.3551 | 100.0% | 1655.2743 | 1654.78 | 3 | 6.188 | 36.7% | 2 | K.AQELGHSQSALASAQR.E | 3 |
| \* | Astrin\_STLCHLD\_050114\_01.04825.04825.3 | 3.5902 | 0.2029 | 100.0% | 1542.0844 | 1542.692 | 18 | 4.011 | 45.5% | 1 | K.KQQNQELQEQLR.S | 3 |
| \* | Astrin\_STLCHLD\_050114\_01.05565.05565.2 | 2.3801 | 0.2686 | 98.8% | 1194.7322 | 1194.3762 | 1 | 6.003 | 70.0% | 1 | R.LGHELQQAGLK.T | 2 |
| \* | Astrin\_STLCHLD\_tube2\_050114\_01.06363.06363.2 | 4.5223 | 0.441 | 100.0% | 1567.3922 | 1566.7141 | 1 | 7.693 | 73.1% | 2 | R.SLEAQVAHADQQLR.D | 2 |

---

|  |  |  |  |  |  |  |  |  |
| --- | --- | --- | --- | --- | --- | --- | --- | --- |
| U | *gi|19923142|ref|NP\_00* | 2 | 2 | 3.7% | 876 | 97170 | 4.8 | karyopherin beta 1 [Homo sapiens] |

| Filename XCorr DeltCN Conf% ObsM+H+ CalcM+H+ SpR ZScore Ion% # Sequence  | | | | | | | | | | | | |
| --- | --- | --- | --- | --- | --- | --- | --- | --- | --- | --- | --- | --- |
| \* | Astrin\_STLCHLD\_050114\_01.13708.13708.2 | 4.1756 | 0.4063 | 100.0% | 1660.3522 | 1659.9231 | 1 | 6.986 | 64.3% | 1 | R.AAVENLPTFLVELSR.V | 2 |
| \* | Astrin\_STLCHLD\_050114\_02.06384.06384.3 | 3.2954 | 0.309 | 100.0% | 2002.0743 | 2001.2218 | 1 | 5.043 | 40.6% | 1 | R.LQQVLQMESHIQSTSDR.I | 3 |

---

|  |  |  |  |  |  |  |  |  |
| --- | --- | --- | --- | --- | --- | --- | --- | --- |
| U | *gi|32698730|ref|NP\_06* | 2 | 2 | 3.6% | 695 | 76121 | 8.7 | nuclear fragile X mental retardation protein interacting protein 2 [Homo sapiens] |

| Filename XCorr DeltCN Conf% ObsM+H+ CalcM+H+ SpR ZScore Ion% # Sequence  | | | | | | | | | | | | |
| --- | --- | --- | --- | --- | --- | --- | --- | --- | --- | --- | --- | --- |
| \* | Astrin\_STLCHLD\_050114\_01.05399.05399.2 | 2.8472 | 0.3495 | 99.9% | 1337.9122 | 1338.3763 | 25 | 5.667 | 54.2% | 1 | K.TGYGELNGNAGER.E | 2 |
| \* | Astrin\_STLCHLD\_tube2\_050114\_01.05856.05856.2 | 2.6701 | 0.323 | 99.6% | 1376.3322 | 1375.6233 | 1 | 5.346 | 68.2% | 1 | K.IMQQETSVPTLK.Q | 2 |

---

|  |  |  |  |  |  |  |  |  |
| --- | --- | --- | --- | --- | --- | --- | --- | --- |
| U | *gi|42716280|ref|NP\_97* | 3 | 4 | 3.4% | 1268 | 141439 | 6.9 | high density lipoprotein binding protein [Homo sapiens] |
| U | *gi|4885409|ref|NP\_005* | 3 | 4 | 3.4% | 1268 | 141439 | 6.9 | high density lipoprotein binding protein [Homo sapiens] |

| Filename XCorr DeltCN Conf% ObsM+H+ CalcM+H+ SpR ZScore Ion% # Sequence  | | | | | | | | | | | | |
| --- | --- | --- | --- | --- | --- | --- | --- | --- | --- | --- | --- | --- |
|  | Astrin\_STLCHLD\_tube2\_050114\_02.05260.05260.2 | 2.4162 | 0.1995 | 96.4% | 1329.1921 | 1328.5516 | 1 | 3.99 | 70.8% | 1 | R.LQTQASATVAIPK.E | 2 |
|  | Astrin\_STLCHLD\_tube2\_050114\_01.06766.06766.2 | 2.4609 | 0.1667 | 96.0% | 1335.7322 | 1334.5304 | 2 | 4.772 | 63.6% | 1 | R.LVGEIMQETGTR.I | 2 |
|  | Astrin\_STLCHLD\_050114\_02.06941.06941.3 | 3.4869 | 0.27 | 100.0% | 1991.2743 | 1991.2517 | 3 | 5.021 | 36.8% | 2 | R.TEIVFTGEKEQLAQAVAR.I | 3 |

---

|  |  |  |  |  |  |  |  |  |
| --- | --- | --- | --- | --- | --- | --- | --- | --- |
| U | *gi|4757878|ref|NP\_004* | 3 | 3 | 3.4% | 1085 | 122375 | 6.5 | budding uninhibited by benzimidazoles 1 [Homo sapiens] |

| Filename XCorr DeltCN Conf% ObsM+H+ CalcM+H+ SpR ZScore Ion% # Sequence  | | | | | | | | | | | | |
| --- | --- | --- | --- | --- | --- | --- | --- | --- | --- | --- | --- | --- |
| \* | Astrin\_STLCHLD\_050114\_01.04679.04679.3 | 2.3085 | 0.2752 | 96.5% | 1238.1843 | 1237.4012 | 15 | 4.888 | 40.0% | 1 | R.LTETHLPAQAR.T | 3 |
| \* | Astrin\_STLCHLD\_050114\_01.06778.06778.2 | 2.8848 | 0.2531 | 99.3% | 1289.7922 | 1289.4491 | 8 | 5.579 | 59.1% | 1 | R.MGPSVGSQQELR.A | 2 |
| \* | Astrin\_STLCHLD\_050114\_02.07199.07199.2 | 2.6254 | 0.4407 | 99.9% | 1457.1122 | 1456.6538 | 1 | 7.168 | 65.4% | 1 | K.AQTVTDSMFAVASK.D | 2 |

---

|  |  |  |  |  |  |  |  |  |
| --- | --- | --- | --- | --- | --- | --- | --- | --- |
| U | *gi|4507877|ref|NP\_003* | 3 | 3 | 3.4% | 1066 | 116722 | 6.1 | vinculin isoform VCL [Homo sapiens] |
| U | *gi|7669550|ref|NP\_054* | 3 | 3 | 3.2% | 1134 | 123799 | 5.7 | vinculin isoform meta-VCL [Homo sapiens] |

| Filename XCorr DeltCN Conf% ObsM+H+ CalcM+H+ SpR ZScore Ion% # Sequence  | | | | | | | | | | | | |
| --- | --- | --- | --- | --- | --- | --- | --- | --- | --- | --- | --- | --- |
|  | Astrin\_STLCHLD\_tube2\_050114\_02.06877.06877.2 | 3.3008 | 0.1296 | 99.2% | 1176.4922 | 1176.3734 | 1 | 6.071 | 83.3% | 1 | K.MSAEINEIIR.V | 2 |
|  | Astrin\_STLCHLD\_050114\_01.11793.11793.2 | 2.4925 | 0.2323 | 98.2% | 1294.3121 | 1293.4749 | 5 | 4.61 | 68.2% | 1 | K.MTGLVDEAIDTK.S | 2 |
|  | Astrin\_STLCHLD\_tube2\_050114\_01.07669.07669.2 | 2.6565 | 0.2287 | 98.5% | 1518.6122 | 1519.8354 | 10 | 5.175 | 53.8% | 1 | K.AGEVINQPMMMAAR.Q | 2 |

---

|  |  |  |  |  |  |  |  |  |
| --- | --- | --- | --- | --- | --- | --- | --- | --- |
| U | *gi|41872631|ref|NP\_00* | 6 | 8 | 3.3% | 2511 | 273424 | 6.4 | fatty acid synthase [Homo sapiens] |

| Filename XCorr DeltCN Conf% ObsM+H+ CalcM+H+ SpR ZScore Ion% # Sequence  | | | | | | | | | | | | |
| --- | --- | --- | --- | --- | --- | --- | --- | --- | --- | --- | --- | --- |
| \* | Astrin\_STLCHLD\_050114\_01.07571.07571.2 | 2.4369 | 0.2258 | 98.8% | 922.4922 | 922.0287 | 25 | 6.536 | 68.8% | 1 | R.GYAVLGGER.G | 2 |
| \* | Astrin\_STLCHLD\_050114\_02.05607.05607.3 | 3.4514 | 0.3598 | 100.0% | 1891.0443 | 1889.1215 | 1 | 6.003 | 34.7% | 1 | R.VTVAGGVHISGLHTESAPR.R | 3 |
| \* | Astrin\_STLCHLD\_tube2\_050114\_01.08417.08417.2 | 3.1598 | 0.2331 | 99.3% | 1471.4321 | 1470.5815 | 1 | 5.547 | 62.5% | 1 | R.FPQLDSTSFANSR.D | 2 |
| \* | Astrin\_STLCHLD\_tube2\_050114\_02.05950.05950.3 | 3.2685 | 0.2337 | 99.2% | 2118.2944 | 2117.3267 | 30 | 4.959 | 31.6% | 1 | R.RQGVQVQVSTSNISSLEGAR.G | 3 |
| \* | Astrin\_STLCHLD\_050114\_01.15677.15677.2 | 4.6309 | 0.5137 | 100.0% | 2423.152 | 2423.769 | 1 | 8.476 | 40.9% | 2 | R.TLLEGSGLESIISIIHSSLAEPR.V | 2 |
| \* | Astrin\_STLCHLD\_tube2\_050114\_01.15988.15988.3 | 3.5484 | 0.2667 | 99.9% | 2424.3245 | 2423.769 | 1 | 5.802 | 33.0% | 2 | R.TLLEGSGLESIISIIHSSLAEPR.V | 3 |

---

|  |  |  |  |  |  |  |  |  |
| --- | --- | --- | --- | --- | --- | --- | --- | --- |
| U | *gi|153792294|ref|NP\_1* | 2 | 2 | 3.3% | 1320 | 145257 | 6.8 | myopalladin [Homo sapiens] |

| Filename XCorr DeltCN Conf% ObsM+H+ CalcM+H+ SpR ZScore Ion% # Sequence  | | | | | | | | | | | | |
| --- | --- | --- | --- | --- | --- | --- | --- | --- | --- | --- | --- | --- |
| \* | Astrin\_STLCHLD\_050114\_02.06069.06069.3 | 3.1506 | 0.3609 | 100.0% | 2545.5244 | 2546.71 | 1 | 5.98 | 29.3% | 1 | R.VHFNLPEDDKGSEASSEAGVVTTR.Q | 3 |
| \* | Astrin\_STLCHLD\_tube2\_050114\_01.07928.07928.3 | 4.7045 | 0.3899 | 100.0% | 2213.3643 | 2213.5242 | 1 | 6.243 | 45.8% | 1 | R.FFRPHFLQAPGDMVAHEGR.L | 3 |

---

|  |  |  |  |  |  |  |  |  |
| --- | --- | --- | --- | --- | --- | --- | --- | --- |
| U | *gi|169164037|ref|XP\_0* | 4 | 21 | 3.2% | 929 | 104892 | 6.3 | PREDICTED: similar to hCG21219 [Homo sapiens] |

| Filename XCorr DeltCN Conf% ObsM+H+ CalcM+H+ SpR ZScore Ion% # Sequence  | | | | | | | | | | | | |
| --- | --- | --- | --- | --- | --- | --- | --- | --- | --- | --- | --- | --- |
| \* | Astrin\_STLCHLD\_tube2\_050114\_01.05820.05820.1 | 2.3571 | 0.4345 | 100.0% | 1319.69 | 1320.4478 | 41 | 6.651 | 40.9% | 1 | R.AQIFANTVDNAR.I | 1 |
|  | Astrin\_STLCHLD\_050114\_01.10477.10477.2 | 3.9092 | 0.4554 | 100.0% | 1320.3121 | 1320.4478 | 1 | 9.006 | 72.7% | 12 | R.AQIFANTVDNAR.I | 22 |
|  | Astrin\_STLCHLD\_tube2\_050114\_01.14893.14893.2 | 5.8637 | 0.5097 | 100.0% | 2178.0522 | 2178.589 | 1 | 8.999 | 64.7% | 6 | R.LQLETEIEALKEELLFMK.K | 22 |
|  | Astrin\_STLCHLD\_050114\_01.14664.14664.3 | 2.9045 | 0.2699 | 99.2% | 2179.4944 | 2178.589 | 8 | 4.647 | 41.2% | 2 | R.LQLETEIEALKEELLFMK.K | 33 |

Similarities:
gi|40354195|ref|NP\_95(3:1)  

---

|  |  |  |  |  |  |  |  |  |
| --- | --- | --- | --- | --- | --- | --- | --- | --- |
| U | *gi|156523968|ref|NP\_0* | 2 | 4 | 2.8% | 1014 | 113084 | 8.9 | poly (ADP-ribose) polymerase family, member 1 [Homo sapiens] |

| Filename XCorr DeltCN Conf% ObsM+H+ CalcM+H+ SpR ZScore Ion% # Sequence  | | | | | | | | | | | | |
| --- | --- | --- | --- | --- | --- | --- | --- | --- | --- | --- | --- | --- |
| \* | Astrin\_STLCHLD\_tube2\_050114\_02.07688.07688.2 | 4.7079 | 0.564 | 100.0% | 1625.6522 | 1625.7728 | 1 | 10.542 | 67.9% | 3 | R.VVSEDFLQDVSASTK.S | 2 |
| \* | Astrin\_STLCHLD\_tube2\_050114\_01.11029.11029.2 | 2.909 | 0.4668 | 100.0% | 1378.2722 | 1378.5712 | 1 | 7.437 | 66.7% | 1 | R.TTNFAGILSQGLR.I | 2 |

---

|  |  |  |  |  |  |  |  |  |
| --- | --- | --- | --- | --- | --- | --- | --- | --- |
| U | *gi|32189394|ref|NP\_00* | 2 | 3 | 2.8% | 529 | 56560 | 5.4 | mitochondrial ATP synthase beta subunit precursor [Homo sapiens] |

| Filename XCorr DeltCN Conf% ObsM+H+ CalcM+H+ SpR ZScore Ion% # Sequence  | | | | | | | | | | | | |
| --- | --- | --- | --- | --- | --- | --- | --- | --- | --- | --- | --- | --- |
| \* | Astrin\_STLCHLD\_050114\_02.07382.07382.2 | 3.2142 | 0.454 | 100.0% | 1651.1122 | 1651.9034 | 1 | 7.405 | 60.7% | 2 | R.LVLEVAQHLGESTVR.T | 2 |
| \* | Astrin\_STLCHLD\_050114\_02.07391.07391.3 | 3.0947 | 0.4815 | 100.0% | 1652.0343 | 1651.9034 | 1 | 6.738 | 42.9% | 1 | R.LVLEVAQHLGESTVR.T | 3 |

---

|  |  |  |  |  |  |  |  |  |
| --- | --- | --- | --- | --- | --- | --- | --- | --- |
| U | *gi|7661936|ref|NP\_055* | 2 | 2 | 2.5% | 953 | 107474 | 6.2 | scaffold attachment factor B2 [Homo sapiens] |

| Filename XCorr DeltCN Conf% ObsM+H+ CalcM+H+ SpR ZScore Ion% # Sequence  | | | | | | | | | | | | |
| --- | --- | --- | --- | --- | --- | --- | --- | --- | --- | --- | --- | --- |
|  | Astrin\_STLCHLD\_050114\_01.05603.05603.2 | 2.8304 | 0.289 | 99.7% | 1198.2722 | 1198.237 | 7 | 5.073 | 60.0% | 1 | R.DGWGGYGSDKR.L | 22 |
| \* | Astrin\_STLCHLD\_050114\_02.05499.05499.2 | 2.4086 | 0.3054 | 98.9% | 1292.2722 | 1292.4119 | 1 | 5.557 | 66.7% | 1 | R.AWQGAMDAGAASR.E | 2 |

Similarities:
gi|21264343|ref|NP\_00(1:1)  

---

|  |  |  |  |  |  |  |  |  |
| --- | --- | --- | --- | --- | --- | --- | --- | --- |
| U | *gi|222136639|ref|NP\_0* | 2 | 2 | 2.5% | 935 | 101531 | 7.3 | methylenetetrahydrofolate dehydrogenase 1 [Homo sapiens] |

| Filename XCorr DeltCN Conf% ObsM+H+ CalcM+H+ SpR ZScore Ion% # Sequence  | | | | | | | | | | | | |
| --- | --- | --- | --- | --- | --- | --- | --- | --- | --- | --- | --- | --- |
| \* | Astrin\_STLCHLD\_tube2\_050114\_01.06281.06281.2 | 2.0964 | 0.3504 | 98.9% | 1200.3121 | 1200.3342 | 52 | 5.658 | 55.0% | 1 | K.TPVPSDIDISR.S | 2 |
| \* | Astrin\_STLCHLD\_050114\_02.07314.07314.2 | 2.9307 | 0.1474 | 98.3% | 1448.9722 | 1449.6439 | 8 | 5.321 | 63.6% | 1 | K.AYIQENLELVEK.G | 2 |

---

|  |  |  |  |  |  |  |  |  |
| --- | --- | --- | --- | --- | --- | --- | --- | --- |
| U | *gi|207452735|ref|NP\_1* | 7 | 9 | 2.4% | 5090 | 555629 | 5.6 | epiplakin 1 [Homo sapiens] |

| Filename XCorr DeltCN Conf% ObsM+H+ CalcM+H+ SpR ZScore Ion% # Sequence  | | | | | | | | | | | | |
| --- | --- | --- | --- | --- | --- | --- | --- | --- | --- | --- | --- | --- |
|  | Astrin\_STLCHLD\_tube2\_050114\_02.06462.06462.3 | 4.5575 | 0.3987 | 100.0% | 2013.9543 | 2014.292 | 1 | 6.59 | 41.7% | 2 | R.LLEAQIATGGVIDPVHSHR.V | 33 |
|  | Astrin\_STLCHLD\_050114\_01.07276.07276.2 | 2.3554 | 0.3397 | 99.7% | 919.59216 | 919.0684 | 26 | 6.34 | 64.3% | 2 | R.VPVDVAYR.R | 22 |
|  | Astrin\_STLCHLD\_050114\_02.07160.07160.3 | 3.9362 | 0.2784 | 100.0% | 2028.5343 | 2028.3188 | 3 | 6.001 | 34.7% | 1 | R.LLEAQIATGGIIDPVHSHR.V | 33 |
|  | Astrin\_STLCHLD\_tube2\_050114\_01.06755.06755.2 | 1.9277 | 0.3093 | 97.6% | 1162.4521 | 1161.2311 | 1 | 6.107 | 75.0% | 1 | C.GYFDEEMNR.I | 22 |
| \* | Astrin\_STLCHLD\_tube2\_050114\_01.15021.15021.2 | 3.0045 | 0.2396 | 99.1% | 2395.632 | 2395.7202 | 3 | 5.071 | 33.3% | 1 | R.VTPGSGALQGQSVSVWELLFYR.E | 2 |
| \* | Astrin\_STLCHLD\_050114\_01.16357.16357.3 | 3.9422 | 0.3118 | 100.0% | 2516.0344 | 2514.8386 | 1 | 5.616 | 33.3% | 1 | R.AGTLTVEELGATLTSLLAQAQAQAR.A | 3 |
| \* | Astrin\_STLCHLD\_tube2\_050114\_02.08315.08315.2 | 2.6996 | 0.1894 | 97.2% | 1886.4722 | 1886.1094 | 1 | 4.921 | 38.9% | 1 | R.LSVEEAVAAGVVGGEIQEK.L | 2 |

Similarities:
gi|41322908|ref|NP\_95(4:3)  

---

|  |  |  |  |  |  |  |  |  |
| --- | --- | --- | --- | --- | --- | --- | --- | --- |
| U | *gi|26051235|ref|NP\_06* | 3 | 4 | 2.4% | 1156 | 128979 | 5.1 | nucleoporin 133kDa [Homo sapiens] |

| Filename XCorr DeltCN Conf% ObsM+H+ CalcM+H+ SpR ZScore Ion% # Sequence  | | | | | | | | | | | | |
| --- | --- | --- | --- | --- | --- | --- | --- | --- | --- | --- | --- | --- |
| \* | Astrin\_STLCHLD\_050114\_01.14351.14351.2 | 4.9913 | 0.583 | 100.0% | 1975.4122 | 1976.3062 | 1 | 11.333 | 62.5% | 1 | K.AHSFLMDFIHQVGLFGR.L | 2 |
| \* | Astrin\_STLCHLD\_tube2\_050114\_01.14489.14489.3 | 4.1678 | 0.4587 | 100.0% | 1975.9443 | 1976.3062 | 1 | 7.219 | 40.6% | 2 | K.AHSFLMDFIHQVGLFGR.L | 3 |
| \* | Astrin\_STLCHLD\_tube2\_050114\_01.08416.08416.2 | 3.0632 | 0.2947 | 99.9% | 1446.2722 | 1446.6147 | 12 | 5.744 | 60.0% | 1 | R.YDNLEMEYLQK.R | 2 |

---

|  |  |  |  |  |  |  |  |  |
| --- | --- | --- | --- | --- | --- | --- | --- | --- |
| U | *gi|57863301|ref|NP\_05* | 2 | 2 | 2.3% | 1506 | 164886 | 8.2 | CLIP-associating protein 2 [Homo sapiens] |

| Filename XCorr DeltCN Conf% ObsM+H+ CalcM+H+ SpR ZScore Ion% # Sequence  | | | | | | | | | | | | |
| --- | --- | --- | --- | --- | --- | --- | --- | --- | --- | --- | --- | --- |
| \* | Astrin\_STLCHLD\_tube2\_050114\_01.16739.16739.2 | 3.2297 | 0.3744 | 99.9% | 2391.9521 | 2392.6733 | 1 | 6.537 | 36.1% | 1 | R.SFEFLDLLLQEWQTHSLER.H | 2 |
| \* | Astrin\_STLCHLD\_tube2\_050114\_01.07547.07547.2 | 2.3787 | 0.2866 | 98.2% | 1621.0922 | 1620.8436 | 1 | 4.898 | 50.0% | 1 | R.VLTTTALSTVSSGVQR.V | 2 |

---

|  |  |  |  |  |  |  |  |  |
| --- | --- | --- | --- | --- | --- | --- | --- | --- |
| U | *gi|171184451|ref|NP\_0* | 4 | 4 | 2.1% | 3117 | 350931 | 6.3 | centrosome-associated protein 350 [Homo sapiens] |

| Filename XCorr DeltCN Conf% ObsM+H+ CalcM+H+ SpR ZScore Ion% # Sequence  | | | | | | | | | | | | |
| --- | --- | --- | --- | --- | --- | --- | --- | --- | --- | --- | --- | --- |
| \* | Astrin\_STLCHLD\_tube2\_050114\_02.05996.05996.3 | 3.5652 | 0.1732 | 96.9% | 2147.4543 | 2146.4692 | 24 | 4.405 | 31.6% | 1 | K.LGHIDHPVMVVNVDNSVTAK.V | 3 |
| \* | Astrin\_STLCHLD\_050114\_02.08055.08055.3 | 3.8806 | 0.3837 | 100.0% | 2188.3442 | 2188.316 | 10 | 6.312 | 30.0% | 1 | R.SRGSLESIAEHVDASLSGSER.S | 3 |
| \* | Astrin\_STLCHLD\_050114\_02.06755.06755.2 | 3.5219 | 0.4175 | 100.0% | 1518.6921 | 1518.7045 | 1 | 6.265 | 65.4% | 1 | K.LVLEQGDSSEILSK.K | 2 |
| \* | Astrin\_STLCHLD\_tube2\_050114\_01.10849.10849.2 | 2.2606 | 0.2554 | 97.5% | 1322.4922 | 1322.5474 | 1 | 5.769 | 68.2% | 1 | K.DLVGLAIENLHK.S | 2 |

---

|  |  |  |  |  |  |  |  |  |
| --- | --- | --- | --- | --- | --- | --- | --- | --- |
| U | *gi|54859722|ref|NP\_05* | 2 | 2 | 1.9% | 1436 | 162121 | 5.5 | nucleoporin 160kDa [Homo sapiens] |

| Filename XCorr DeltCN Conf% ObsM+H+ CalcM+H+ SpR ZScore Ion% # Sequence  | | | | | | | | | | | | |
| --- | --- | --- | --- | --- | --- | --- | --- | --- | --- | --- | --- | --- |
| \* | Astrin\_STLCHLD\_050114\_01.11184.11184.2 | 2.4241 | 0.337 | 99.5% | 1095.1921 | 1095.1974 | 1 | 6.262 | 72.2% | 1 | R.SFVELSGAER.E | 2 |
| \* | Astrin\_STLCHLD\_050114\_01.17138.17138.2 | 4.5611 | 0.5506 | 100.0% | 2097.8523 | 2098.4062 | 1 | 9.152 | 58.8% | 1 | R.FVSSPQTIVELFFQEVAR.K | 2 |

---

|  |  |  |  |  |  |  |  |  |
| --- | --- | --- | --- | --- | --- | --- | --- | --- |
| U | *gi|41406064|ref|NP\_00* | 3 | 5 | 1.7% | 1976 | 228997 | 5.5 | myosin, heavy polypeptide 10, non-muscle [Homo sapiens] |

| Filename XCorr DeltCN Conf% ObsM+H+ CalcM+H+ SpR ZScore Ion% # Sequence  | | | | | | | | | | | | |
| --- | --- | --- | --- | --- | --- | --- | --- | --- | --- | --- | --- | --- |
| \* | Astrin\_STLCHLD\_050114\_01.04691.04691.3 | 2.5546 | 0.2544 | 97.7% | 1375.8844 | 1374.496 | 31 | 4.677 | 40.9% | 1 | R.HGFEAASIKEER.G | 3 |
|  | Astrin\_STLCHLD\_tube2\_050114\_01.05992.05992.2 | 3.3196 | 0.4629 | 100.0% | 1224.0521 | 1224.3591 | 1 | 7.862 | 80.0% | 2 | R.AGVLAHLEEER.D | 22 |
|  | Astrin\_STLCHLD\_tube2\_050114\_01.05727.05727.2 | 2.6149 | 0.2047 | 98.8% | 1221.6122 | 1221.3959 | 12 | 4.433 | 66.7% | 2 | K.KFDQLLAEEK.S | 22 |

Similarities:
gi|12667788|ref|NP\_00(2:1)  

---

|  |  |  |  |  |  |  |  |  |
| --- | --- | --- | --- | --- | --- | --- | --- | --- |
| U | *gi|100913206|ref|NP\_0* | 2 | 2 | 1.7% | 1270 | 140958 | 6.8 | DEAH (Asp-Glu-Ala-His) box polypeptide 9 [Homo sapiens] |

| Filename XCorr DeltCN Conf% ObsM+H+ CalcM+H+ SpR ZScore Ion% # Sequence  | | | | | | | | | | | | |
| --- | --- | --- | --- | --- | --- | --- | --- | --- | --- | --- | --- | --- |
| \* | Astrin\_STLCHLD\_050114\_01.05948.05948.2 | 2.1913 | 0.2258 | 97.2% | 1076.2922 | 1076.1973 | 1 | 5.331 | 68.8% | 1 | K.LAQFEPSQR.Q | 2 |
| \* | Astrin\_STLCHLD\_tube2\_050114\_01.05998.05998.3 | 2.8249 | 0.2133 | 96.7% | 1505.0044 | 1504.686 | 6 | 4.073 | 43.8% | 1 | R.GISHVIVDEIHER.D | 3 |

---

|  |  |  |  |  |  |  |  |  |
| --- | --- | --- | --- | --- | --- | --- | --- | --- |
| U | *gi|57164942|ref|NP\_00* | 2 | 2 | 1.6% | 2032 | 225493 | 7.8 | colonic and hepatic tumor over-expressed protein isoform a [Homo sapiens] |
| U | *gi|57222563|ref|NP\_05* | 2 | 2 | 1.6% | 1972 | 218524 | 8.1 | colonic and hepatic tumor over-expressed protein isoform b [Homo sapiens] |

| Filename XCorr DeltCN Conf% ObsM+H+ CalcM+H+ SpR ZScore Ion% # Sequence  | | | | | | | | | | | | |
| --- | --- | --- | --- | --- | --- | --- | --- | --- | --- | --- | --- | --- |
|  | Astrin\_STLCHLD\_tube2\_050114\_01.10064.10064.2 | 2.3941 | 0.2622 | 98.5% | 1207.5122 | 1207.369 | 305 | 5.407 | 40.9% | 1 | R.DAAFEALGTALK.V | 2 |
|  | Astrin\_STLCHLD\_tube2\_050114\_02.08297.08297.3 | 3.0742 | 0.3895 | 100.0% | 2204.8442 | 2204.4905 | 407 | 6.678 | 22.4% | 1 | R.NAALNTIVTVYNVHGDQVFK.L | 3 |

---

|  |  |  |  |  |  |  |  |  |
| --- | --- | --- | --- | --- | --- | --- | --- | --- |
| U | *Reverse\_gi|119226260|* | 3 | 4 | 1.6% | 916 | 103702 | 9.0 | calcium homeostasis endoplasmic reticulum protein [Homo sapiens] |

| Filename XCorr DeltCN Conf% ObsM+H+ CalcM+H+ SpR ZScore Ion% # Sequence  | | | | | | | | | | | | |
| --- | --- | --- | --- | --- | --- | --- | --- | --- | --- | --- | --- | --- |
| \* | Astrin\_STLCHLD\_tube2\_050114\_02.12286.12286.2 | 3.0437 | 0.1334 | 97.4% | 1799.3522 | 1799.7286 | 39 | 4.032 | 35.7% | 1 | K.SSRS\*NS\*RSSSRGRSK.S | 2 |
| \* | Astrin\_STLCHLD\_050114\_02.15568.15568.2 | 3.0643 | 0.1285 | 97.3% | 1799.4321 | 1799.7286 | 1 | 4.104 | 46.4% | 2 | K.S\*S\*RSNSRSSSRGRSK.S | 2 |
| \* | Astrin\_STLCHLD\_tube2\_050114\_02.08381.08381.2 | 3.0214 | 0.0918 | 95.6% | 1800.2122 | 1799.7286 | 2 | 3.599 | 50.0% | 1 | K.SS\*RS\*NSRSSSRGRSK.S | 2 |

---

|  |  |  |  |  |  |  |  |  |
| --- | --- | --- | --- | --- | --- | --- | --- | --- |
| U | *gi|58530840|ref|NP\_00* | 3 | 3 | 1.5% | 2871 | 331774 | 6.8 | desmoplakin isoform I [Homo sapiens] |
| U | *gi|58530842|ref|NP\_00* | 3 | 3 | 1.9% | 2272 | 260116 | 7.0 | desmoplakin isoform II [Homo sapiens] |

| Filename XCorr DeltCN Conf% ObsM+H+ CalcM+H+ SpR ZScore Ion% # Sequence  | | | | | | | | | | | | |
| --- | --- | --- | --- | --- | --- | --- | --- | --- | --- | --- | --- | --- |
|  | Astrin\_STLCHLD\_050114\_01.11605.11605.2 | 2.2735 | 0.2806 | 98.9% | 1159.0521 | 1159.3892 | 17 | 5.33 | 68.8% | 1 | R.LLQLQEQMR.A | 2 |
|  | Astrin\_STLCHLD\_050114\_01.14092.14092.2 | 3.6715 | 0.3475 | 100.0% | 1588.4521 | 1587.9153 | 1 | 6.768 | 53.8% | 1 | R.ALLQAILQTEDMLK.V | 2 |
|  | Astrin\_STLCHLD\_050114\_02.08039.08039.3 | 2.6334 | 0.2859 | 97.6% | 2125.2844 | 2124.4197 | 105 | 4.741 | 26.3% | 1 | R.TMIQSPSGVILQEAADVHAR.Y | 3 |

---

|  |  |  |  |  |  |  |  |  |
| --- | --- | --- | --- | --- | --- | --- | --- | --- |
| U | *gi|55770834|ref|NP\_05* | 2 | 2 | 1.1% | 3114 | 357527 | 5.1 | centromere protein F [Homo sapiens] |

| Filename XCorr DeltCN Conf% ObsM+H+ CalcM+H+ SpR ZScore Ion% # Sequence  | | | | | | | | | | | | |
| --- | --- | --- | --- | --- | --- | --- | --- | --- | --- | --- | --- | --- |
| \* | Astrin\_STLCHLD\_tube2\_050114\_02.06550.06550.3 | 2.6809 | 0.2463 | 97.0% | 1706.1843 | 1705.9238 | 1 | 5.087 | 38.5% | 1 | R.TLEMDRENLSVEIR.N | 3 |
| \* | Astrin\_STLCHLD\_tube2\_050114\_01.04587.04587.3 | 3.9942 | 0.2494 | 100.0% | 2300.1543 | 2299.4563 | 1 | 5.364 | 36.2% | 1 | R.TNQEHAALEAENSKGEVETLK.A | 3 |

---

|  |  |  |  |  |  |  |  |  |
| --- | --- | --- | --- | --- | --- | --- | --- | --- |
| U | *Reverse\_gi|157885806|* | 2 | 2 | 0.4% | 3996 | 439346 | 6.2 | chromosome 12 open reading frame 51 [Homo sapiens] |

| Filename XCorr DeltCN Conf% ObsM+H+ CalcM+H+ SpR ZScore Ion% # Sequence  | | | | | | | | | | | | |
| --- | --- | --- | --- | --- | --- | --- | --- | --- | --- | --- | --- | --- |
| \* | Astrin\_STLCHLD\_tube2\_050114\_02.09122.09122.2 | 2.8356 | 0.1637 | 97.2% | 2043.2922 | 2043.1222 | 21 | 3.97 | 37.5% | 1 | K.K@ESLAT#RSVS\*NLVAK@DK@.A | 2 |
| \* | Astrin\_STLCHLD\_050114\_02.09552.09552.2 | 3.113 | 0.1321 | 97.8% | 2044.0322 | 2043.1222 | 402 | 3.934 | 31.2% | 1 | K.K@ES\*LATRSVS\*NLVAK@DK@.A | 2 |

|  |  |  |  |
| --- | --- | --- | --- |
|  | Proteins | Peptide IDs | Spectra |
| Unfiltered | 43646 | 87762 | 149148 |
| Filtered | 229 | 1418 | 3582 |
| Forward matches | 226 | 1411 | 3574 |
| Decoy matches | 3 | 7 | 8 |
| Forward FP rate | 1.33% | 0.5% | 0.22% |

  
/nfs/cheeseman\_massspec/David/Astrin\_STLCHLD
