# Supplementary material for: Astrin-SKAP complex reconstitution reveals its kinetochore interaction with microtubule-bound Ndc80
Source: eLife. 2017 Aug 25;6:e26866. doi: 10.7554/eLife.26866 (PMC5602300; doi:10.7554/eLife.26866)
Supplement: Source data 1. — Complete mass spectrometry searches using methods described in (Washburn et al., 2001) for affinity purification/mass spectrometry data sets described in this paper (data from this study; [Kern et al., 2016] [Gascoigne et al., 2011]). Individual Astrin cross-linking immunoprecipitations are listed based on the order in Figure 4—figure supplement 1. These samples have not been pruned for common or antibody-specific contaminants. [file elife-26866-data1.zip › Astrin_Crosslinking#4.html]

D 2NLDAstrin
DTASelect v2.0.21  
/nfs/cheeseman\_massspec/David/2NLDAstrin  
/nfs/cheeseman\_massspec/Databases/NCBI-RefSeq\_human\_na\_04-13-2009\_con\_reversed.fasta  
SEQUEST 3.0 in SQT format.  
  
 Jump  to the summary table.  
  
sequest.params modifications:

|  |  |  |
| --- | --- | --- |
| \* | S | 80.0 |
| # | T | 80.0 |
| @ | K | 12.0 |
| Static | C | 57.0 |

|  |  |
| --- | --- |
| true | Use criteria |
| 0.0 | Minimum peptide confidence |
| 0.05 | Peptide false positive rate |
| 0.0 | Minimum protein confidence |
| 1.0 | Protein false positive rate |
| 1 | Minimum charge state |
| 16 | Maximum charge state |
| 0.0 | Minimum ion proportion |
| 1000 | Maximum Sp rank |
| -1.0 | Minimum Sp score |
| Include | Modified peptide inclusion |
| Any | Tryptic status requirement |
| false | Multiple, ambiguous IDs allowed |
| Ignore | Peptide validation handling |
| XCorr | Purge duplicate peptides by protein |
| false | Include only loci with unique peptide |
| true | Remove subset proteins |
| Ignore | Locus validation handling |
| 0 | Minimum modified peptides per locus |
| 1000 | Minimum redundancy for low coverage loci |
| 2 | Minimum peptides per locus |

#### Locus Key:

|  |  |  |  |  |  |  |  |  |
| --- | --- | --- | --- | --- | --- | --- | --- | --- |
| Validation Status | Locus | Sequence Count | Spectrum Count | Sequence Coverage | Length | MolWt | pI | Descriptive Name |

#### Similarity Key:

|  |  |  |
| --- | --- | --- |
| Locus | # of identical peptides | # of differing peptides |

---

|  |  |  |  |  |  |  |  |  |
| --- | --- | --- | --- | --- | --- | --- | --- | --- |
| U | *gi|4504919|ref|NP\_002* | 47 | 149 | 68.1% | 483 | 53704 | 5.6 | keratin 8 [Homo sapiens] |

| Filename XCorr DeltCN Conf% ObsM+H+ CalcM+H+ SpR ZScore Ion% # Sequence  | | | | | | | | | | | | |
| --- | --- | --- | --- | --- | --- | --- | --- | --- | --- | --- | --- | --- |
| \* | IPAstrin\_STLCD\_032114\_01.14733.14733.3 | 5.9211 | 0.4884 | 100.0% | 3928.2844 | 3927.465 | 1 | 8.875 | 22.5% | 3 | R.GGLGGGYGGASGMGGITAVTVNQSLLSPLVLEVDPNIQAVR.T | 3 |
|  | IPAstrin\_STLCD\_tube2\_032114\_01.06068.06068.2 | 2.3494 | 0.2074 | 99.3% | 828.0122 | 827.95544 | 6 | 5.13 | 91.7% | 1 | K.FASFIDK.V | 222222 |
|  | IPAstrin\_STLCD\_032114\_01.07575.07575.2 | 2.7065 | 0.1841 | 99.4% | 1082.3121 | 1083.2755 | 6 | 6.916 | 75.0% | 2 | K.FASFIDKVR.F | 222222 |
|  | IPAstrin\_STLCD\_tube2\_032114\_01.07226.07226.2 | 3.2153 | 0.1215 | 99.8% | 1031.1921 | 1031.1997 | 2 | 3.989 | 92.9% | 6 | K.WSLLQQQK.T | 2 |
|  | IPAstrin\_STLCD\_tube2\_032114\_01.12420.12420.2 | 4.0136 | 0.5196 | 100.0% | 1847.6721 | 1849.0431 | 1 | 7.78 | 67.9% | 2 | R.SNMDNMFESYINNLR.R | 2 |
|  | IPAstrin\_STLCD\_tube2\_032114\_01.11889.11889.2 | 6.2704 | 0.324 | 100.0% | 2034.4521 | 2035.363 | 1 | 10.855 | 73.5% | 2 | K.LKLEAELGNMQGLVEDFK.N | 2 |
|  | IPAstrin\_STLCD\_tube2\_032114\_02.08416.08416.3 | 5.2965 | 0.2784 | 100.0% | 2035.1643 | 2035.363 | 1 | 8.818 | 47.1% | 5 | K.LKLEAELGNMQGLVEDFK.N | 3 |
|  | IPAstrin\_STLCD\_032114\_01.12802.12802.2 | 3.5237 | 0.2378 | 99.9% | 1792.9521 | 1794.0295 | 1 | 7.96 | 56.7% | 2 | K.LEAELGNMQGLVEDFK.N | 2 |
|  | IPAstrin\_STLCD\_032114\_01.03831.03831.2 | 2.6693 | 0.1152 | 96.7% | 1310.2122 | 1309.4215 | 1 | 4.407 | 77.8% | 1 | K.NKYEDEINKR.T | 222 |
|  | IPAstrin\_STLCD\_tube2\_032114\_01.09242.09242.2 | 3.426 | 0.4642 | 100.0% | 1352.7922 | 1353.5732 | 1 | 7.394 | 75.0% | 6 | R.TEMENEFVLIK.K | 2 |
|  | IPAstrin\_STLCD\_tube2\_032114\_01.07212.07212.2 | 3.5215 | 0.2373 | 99.9% | 1482.0122 | 1481.7473 | 4 | 5.598 | 63.6% | 5 | R.TEMENEFVLIKK.D | 2 |
|  | IPAstrin\_STLCD\_032114\_02.06206.06206.3 | 2.2582 | 0.3816 | 99.4% | 1482.2043 | 1481.7473 | 50 | 6.734 | 34.1% | 1 | R.TEMENEFVLIKK.D | 3 |
|  | IPAstrin\_STLCD\_tube2\_032114\_01.05117.05117.3 | 3.0577 | 0.2603 | 98.1% | 1927.6444 | 1927.1365 | 1 | 4.267 | 36.7% | 1 | K.KDVDEAYMNKVELESR.L | 3 |
|  | IPAstrin\_STLCD\_tube2\_032114\_01.06347.06347.2 | 4.3686 | 0.5208 | 100.0% | 1797.6921 | 1798.9623 | 1 | 8.481 | 71.4% | 7 | K.DVDEAYMNKVELESR.L | 2 |
|  | IPAstrin\_STLCD\_032114\_02.05580.05580.3 | 3.9306 | 0.3829 | 100.0% | 1798.7043 | 1798.9623 | 1 | 6.42 | 44.6% | 3 | K.DVDEAYMNKVELESR.L | 3 |
|  | IPAstrin\_STLCD\_tube2\_032114\_01.11045.11045.1 | 2.4102 | 0.3355 | 100.0% | 1419.6 | 1420.6055 | 1 | 5.751 | 72.7% | 1 | R.LEGLTDEINFLR.Q | 1 |
|  | IPAstrin\_STLCD\_tube2\_032114\_01.11019.11019.2 | 4.2098 | 0.4673 | 100.0% | 1421.4122 | 1420.6055 | 1 | 8.411 | 86.4% | 6 | R.LEGLTDEINFLR.Q | 2 |
|  | IPAstrin\_STLCD\_tube2\_032114\_01.04570.04570.2 | 1.9758 | 0.3205 | 99.3% | 1079.6721 | 1080.1827 | 20 | 4.739 | 64.3% | 1 | R.QLYEEEIR.E | 2 |
|  | IPAstrin\_STLCD\_032114\_02.06666.06666.3 | 3.4895 | 0.3044 | 99.4% | 2109.5344 | 2110.3008 | 1 | 6.241 | 36.1% | 2 | R.ELQSQISDTSVVLSMDNSR.S | 3 |
|  | IPAstrin\_STLCD\_032114\_02.06675.06675.2 | 5.8547 | 0.5392 | 100.0% | 2110.7122 | 2110.3008 | 1 | 9.179 | 77.8% | 6 | R.ELQSQISDTSVVLSMDNSR.S | 2 |
|  | IPAstrin\_STLCD\_032114\_01.12368.12368.1 | 2.4739 | 0.3155 | 100.0% | 1320.52 | 1321.5286 | 4 | 6.1 | 59.1% | 2 | R.SLDMDSIIAEVK.A | 1 |
|  | IPAstrin\_STLCD\_032114\_01.12402.12402.2 | 4.407 | 0.4384 | 100.0% | 1321.2322 | 1321.5286 | 1 | 8.384 | 77.3% | 4 | R.SLDMDSIIAEVK.A | 2 |
|  | IPAstrin\_STLCD\_032114\_01.04178.04178.2 | 2.3538 | 0.2742 | 99.6% | 1080.2122 | 1080.1423 | 66 | 5.963 | 56.2% | 1 | K.AQYEDIANR.S | 22 |
|  | IPAstrin\_STLCD\_032114\_01.04448.04448.2 | 3.3318 | 0.1598 | 99.6% | 1413.0122 | 1413.5884 | 1 | 5.109 | 86.4% | 3 | R.SRAEAESMYQIK.Y | 2 |
|  | IPAstrin\_STLCD\_tube2\_032114\_02.06275.06275.3 | 5.489 | 0.3991 | 100.0% | 2532.3542 | 2532.828 | 1 | 7.084 | 38.1% | 3 | R.SRAEAESMYQIKYEELQSLAGK.H | 3 |
|  | IPAstrin\_STLCD\_tube2\_032114\_01.04430.04430.2 | 3.3085 | 0.4563 | 100.0% | 1169.9321 | 1170.3228 | 1 | 6.971 | 83.3% | 5 | R.AEAESMYQIK.Y | 2 |
|  | IPAstrin\_STLCD\_032114\_02.07010.07010.3 | 4.8881 | 0.3998 | 100.0% | 2289.2644 | 2289.5623 | 1 | 7.138 | 34.2% | 4 | R.AEAESMYQIKYEELQSLAGK.H | 3 |
|  | IPAstrin\_STLCD\_tube2\_032114\_01.05498.05498.2 | 3.6987 | 0.0555 | 99.4% | 1138.0721 | 1138.2627 | 1 | 7.092 | 77.8% | 8 | K.YEELQSLAGK.H | 2 |
|  | IPAstrin\_STLCD\_032114\_01.03817.03817.2 | 2.7306 | 0.2124 | 99.5% | 1209.2522 | 1209.36 | 1 | 5.404 | 72.2% | 2 | R.TKTEISEMNR.N | 2 |
|  | IPAstrin\_STLCD\_tube2\_032114\_01.05624.05624.2 | 2.6856 | 0.2262 | 99.8% | 1000.5722 | 1001.168 | 139 | 5.523 | 75.0% | 1 | R.LQAEIEGLK.G | 2 |
|  | IPAstrin\_STLCD\_tube2\_032114\_01.04089.04089.2 | 3.2006 | 0.2812 | 99.9% | 1341.7522 | 1342.5381 | 1 | 7.317 | 68.2% | 2 | R.LQAEIEGLKGQR.A | 2 |
|  | IPAstrin\_STLCD\_tube2\_032114\_01.08086.08086.1 | 2.5796 | 0.4397 | 100.0% | 1344.54 | 1345.452 | 1 | 7.331 | 54.2% | 2 | R.ASLEAAIADAEQR.G | 1 |
|  | IPAstrin\_STLCD\_tube2\_032114\_01.08068.08068.2 | 4.3219 | 0.4468 | 100.0% | 1345.2722 | 1345.452 | 1 | 8.305 | 70.8% | 4 | R.ASLEAAIADAEQR.G | 2 |
|  | IPAstrin\_STLCD\_tube2\_032114\_01.10576.10576.2 | 4.7382 | 0.3321 | 100.0% | 1957.5521 | 1957.1912 | 1 | 6.558 | 52.8% | 3 | R.ASLEAAIADAEQRGELAIK.D | 2 |
|  | IPAstrin\_STLCD\_032114\_01.12217.12217.3 | 4.0163 | 0.2857 | 99.6% | 1957.8243 | 1957.1912 | 1 | 6.012 | 38.9% | 2 | R.ASLEAAIADAEQRGELAIK.D | 3 |
|  | IPAstrin\_STLCD\_032114\_01.12462.12462.3 | 5.2018 | 0.3765 | 100.0% | 2456.9343 | 2456.7153 | 1 | 7.202 | 30.4% | 2 | R.ASLEAAIADAEQRGELAIKDANAK.L | 3 |
|  | IPAstrin\_STLCD\_032114\_01.08701.08701.1 | 2.381 | 0.2873 | 100.0% | 1129.62 | 1130.2865 | 13 | 5.859 | 50.0% | 4 | K.LSELEAALQR.A | 1 |
|  | IPAstrin\_STLCD\_tube2\_032114\_01.07184.07184.2 | 4.219 | 0.2142 | 100.0% | 1130.2922 | 1130.2865 | 1 | 6.109 | 88.9% | 5 | K.LSELEAALQR.A | 2 |
|  | IPAstrin\_STLCD\_032114\_01.08211.08211.2 | 3.2494 | 0.2142 | 99.9% | 1551.1921 | 1551.801 | 1 | 4.546 | 77.3% | 1 | R.QLREYQELMNVK.L | 2 |
|  | IPAstrin\_STLCD\_tube2\_032114\_01.05698.05698.1 | 2.4645 | 0.2588 | 100.0% | 1153.4 | 1154.3234 | 148 | 6.038 | 56.2% | 2 | R.EYQELMNVK.L | 11 |
|  | IPAstrin\_STLCD\_032114\_01.06540.06540.2 | 2.6868 | 0.2337 | 99.8% | 1154.0521 | 1154.3234 | 8 | 6.442 | 68.8% | 4 | R.EYQELMNVK.L | 22 |
|  | IPAstrin\_STLCD\_tube2\_032114\_01.08175.08175.2 | 3.1566 | 0.2642 | 99.9% | 1406.3121 | 1406.6653 | 2 | 5.702 | 72.7% | 2 | K.LALDIEIATYRK.L | 22 |
|  | IPAstrin\_STLCD\_tube2\_032114\_01.06942.06942.3 | 4.0974 | 0.4181 | 100.0% | 2518.0444 | 2518.8628 | 1 | 6.157 | 33.3% | 1 | R.KLLEGEESRLESGMQNMSIHTK.T | 3 |
|  | IPAstrin\_STLCD\_032114\_01.04656.04656.3 | 3.0948 | 0.3108 | 99.4% | 1476.0243 | 1476.7058 | 146 | 5.726 | 35.4% | 1 | R.LESGMQNMSIHTK.T | 3 |
|  | IPAstrin\_STLCD\_tube2\_032114\_01.04242.04242.2 | 4.0434 | 0.4642 | 100.0% | 1476.3322 | 1476.7058 | 1 | 7.785 | 83.3% | 4 | R.LESGMQNMSIHTK.T | 2 |
|  | IPAstrin\_STLCD\_tube2\_032114\_01.04950.04950.3 | 2.8044 | 0.2916 | 98.1% | 1973.9043 | 1974.2188 | 1 | 5.369 | 33.8% | 1 | K.IETRDGKLVSESSDVLPK.- | 3 |
|  | IPAstrin\_STLCD\_032114\_01.05449.05449.2 | 3.3132 | 0.4871 | 100.0% | 1174.1921 | 1174.3367 | 2 | 7.712 | 70.0% | 13 | K.LVSESSDVLPK.- | 2 |

Similarities:
gi|67782365|ref|NP\_00(3:44)  
gi|119395750|ref|NP\_0(1:46)  
gi|47132620|ref|NP\_00(2:45)  
gi|119703753|ref|NP\_0(5:42)  
gi|32567786|ref|NP\_78(2:45)  
gi|153791158|ref|NP\_0(3:44)  

---

|  |  |  |  |  |  |  |  |  |
| --- | --- | --- | --- | --- | --- | --- | --- | --- |
| U | *gi|11415030|ref|NP\_06* | 8 | 29 | 59.2% | 103 | 11367 | 11.4 | histone cluster 1, H4j [Homo sapiens] |
| U | *gi|77539758|ref|NP\_00* | 8 | 29 | 59.2% | 103 | 11367 | 11.4 | histone cluster 2, H4b [Homo sapiens] |
| U | *gi|4504323|ref|NP\_003* | 8 | 29 | 59.2% | 103 | 11367 | 11.4 | histone cluster 2, H4a [Homo sapiens] |
| U | *gi|4504321|ref|NP\_003* | 8 | 29 | 59.2% | 103 | 11367 | 11.4 | histone cluster 1, H4i [Homo sapiens] |
| U | *gi|4504317|ref|NP\_003* | 8 | 29 | 59.2% | 103 | 11367 | 11.4 | histone cluster 1, H4l [Homo sapiens] |
| U | *gi|4504315|ref|NP\_003* | 8 | 29 | 59.2% | 103 | 11367 | 11.4 | histone cluster 1, H4e [Homo sapiens] |
| U | *gi|4504313|ref|NP\_003* | 8 | 29 | 59.2% | 103 | 11367 | 11.4 | histone cluster 1, H4b [Homo sapiens] |
| U | *gi|4504311|ref|NP\_003* | 8 | 29 | 59.2% | 103 | 11367 | 11.4 | histone cluster 1, H4h [Homo sapiens] |
| U | *gi|4504309|ref|NP\_003* | 8 | 29 | 59.2% | 103 | 11367 | 11.4 | histone cluster 1, H4c [Homo sapiens] |
| U | *gi|4504307|ref|NP\_003* | 8 | 29 | 59.2% | 103 | 11367 | 11.4 | histone cluster 1, H4k [Homo sapiens] |
| U | *gi|4504305|ref|NP\_003* | 8 | 29 | 59.2% | 103 | 11367 | 11.4 | histone cluster 1, H4f [Homo sapiens] |
| U | *gi|4504303|ref|NP\_003* | 8 | 29 | 59.2% | 103 | 11367 | 11.4 | histone cluster 1, H4d [Homo sapiens] |
| U | *gi|4504301|ref|NP\_003* | 8 | 29 | 59.2% | 103 | 11367 | 11.4 | histone cluster 1, H4a [Homo sapiens] |
| U | *gi|28173560|ref|NP\_77* | 8 | 29 | 59.2% | 103 | 11367 | 11.4 | histone cluster 4, H4 [Homo sapiens] |

| Filename XCorr DeltCN Conf% ObsM+H+ CalcM+H+ SpR ZScore Ion% # Sequence  | | | | | | | | | | | | |
| --- | --- | --- | --- | --- | --- | --- | --- | --- | --- | --- | --- | --- |
|  | IPAstrin\_STLCD\_tube2\_032114\_01.04310.04310.2 | 3.3311 | 0.2007 | 99.9% | 1326.3121 | 1326.5387 | 1 | 6.016 | 77.3% | 5 | R.DNIQGITKPAIR.R | 2 |
|  | IPAstrin\_STLCD\_tube2\_032114\_01.06358.06358.1 | 2.5908 | 0.2447 | 100.0% | 1180.54 | 1181.3312 | 5 | 5.391 | 66.7% | 1 | R.ISGLIYEETR.G | 1 |
|  | IPAstrin\_STLCD\_tube2\_032114\_01.06296.06296.2 | 3.8514 | 0.4012 | 100.0% | 1181.1522 | 1181.3312 | 1 | 7.375 | 88.9% | 9 | R.ISGLIYEETR.G | 2 |
|  | IPAstrin\_STLCD\_tube2\_032114\_01.08367.08367.2 | 2.9072 | 0.2256 | 99.9% | 990.1322 | 990.19055 | 1 | 5.733 | 92.9% | 5 | K.VFLENVIR.D | 2 |
|  | IPAstrin\_STLCD\_032114\_01.03694.03694.2 | 2.6085 | 0.4205 | 100.0% | 1291.1721 | 1291.4062 | 7 | 6.259 | 60.0% | 1 | R.DAVTYTEHAKR.K | 2 |
|  | IPAstrin\_STLCD\_tube2\_032114\_01.10714.10714.2 | 3.134 | 0.4911 | 100.0% | 1311.1322 | 1311.5793 | 1 | 7.723 | 81.8% | 1 | K.TVTAMDVVYALK.R | 2 |
|  | IPAstrin\_STLCD\_tube2\_032114\_01.09492.09492.2 | 3.5305 | 0.4591 | 100.0% | 1468.4922 | 1467.7667 | 1 | 7.306 | 70.8% | 3 | K.TVTAMDVVYALKR.Q | 2 |
|  | IPAstrin\_STLCD\_032114\_01.09732.09732.1 | 1.8039 | 0.4124 | 100.0% | 714.4 | 714.796 | 1 | 6.397 | 75.0% | 4 | R.TLYGFGG.- | 1 |

---

|  |  |  |  |  |  |  |  |  |
| --- | --- | --- | --- | --- | --- | --- | --- | --- |
| U | *contaminant\_KERATIN09* | 26 | 123 | 58.3% | 429 | 47927 | 5.5 | no description |
| U | *gi|4557888|ref|NP\_000* | 26 | 123 | 58.1% | 430 | 48058 | 5.5 | keratin 18 [Homo sapiens] |
| U | *gi|40354195|ref|NP\_95* | 26 | 123 | 58.1% | 430 | 48058 | 5.5 | keratin 18 [Homo sapiens] |

| Filename XCorr DeltCN Conf% ObsM+H+ CalcM+H+ SpR ZScore Ion% # Sequence  | | | | | | | | | | | | |
| --- | --- | --- | --- | --- | --- | --- | --- | --- | --- | --- | --- | --- |
|  | IPAstrin\_STLCD\_032114\_01.07828.07828.2 | 4.7823 | 0.4589 | 100.0% | 2854.7522 | 2856.0813 | 1 | 7.217 | 31.7% | 2 | R.SLGSVQAPSYGARPVSSAASVYAGAGGSGSR.I | 2 |
|  | IPAstrin\_STLCD\_tube2\_032114\_02.05211.05211.3 | 5.222 | 0.5528 | 100.0% | 2854.9744 | 2856.0813 | 1 | 9.206 | 35.0% | 9 | R.SLGSVQAPSYGARPVSSAASVYAGAGGSGSR.I | 3 |
|  | IPAstrin\_STLCD\_tube2\_032114\_01.10046.10046.2 | 5.5773 | 0.5714 | 100.0% | 2261.632 | 2262.561 | 1 | 10.859 | 52.0% | 2 | R.GGMGSGGLATGIAGGLAGMGGIQNEK.E | 2 |
|  | IPAstrin\_STLCD\_tube2\_032114\_02.07276.07276.3 | 5.4834 | 0.3921 | 100.0% | 2263.8843 | 2262.561 | 1 | 7.181 | 41.0% | 1 | R.GGMGSGGLATGIAGGLAGMGGIQNEK.E | 3 |
|  | IPAstrin\_STLCD\_tube2\_032114\_02.07380.07380.3 | 5.3986 | 0.449 | 100.0% | 3338.0645 | 3337.7224 | 1 | 8.402 | 27.9% | 4 | R.GGMGSGGLATGIAGGLAGMGGIQNEKETMQSLNDR.L | 3 |
|  | IPAstrin\_STLCD\_032114\_01.09121.09121.2 | 2.0098 | 0.2158 | 97.2% | 982.89215 | 983.0709 | 2 | 4.446 | 83.3% | 1 | R.DWSHYFK.I | 2 |
|  | IPAstrin\_STLCD\_tube2\_032114\_02.06916.06916.3 | 3.2639 | 0.3284 | 99.6% | 2059.9744 | 2060.3176 | 309 | 5.864 | 27.9% | 1 | K.IIEDLRAQIFANTVDNAR.I | 3 |
|  | IPAstrin\_STLCD\_tube2\_032114\_01.05228.05228.2 | 3.888 | 0.4647 | 100.0% | 1320.0922 | 1320.4478 | 1 | 8.368 | 72.7% | 10 | R.AQIFANTVDNAR.I | 2 |
|  | IPAstrin\_STLCD\_tube2\_032114\_01.05762.05762.1 | 2.4421 | 0.3161 | 100.0% | 1041.45 | 1042.2235 | 11 | 6.413 | 62.5% | 3 | R.IVLQIDNAR.L | 11 |
|  | IPAstrin\_STLCD\_032114\_01.06636.06636.2 | 3.2213 | 0.1416 | 99.8% | 1042.1721 | 1042.2235 | 3 | 6.071 | 87.5% | 7 | R.IVLQIDNAR.L | 22 |
|  | IPAstrin\_STLCD\_032114\_01.05089.05089.2 | 2.9384 | 0.471 | 100.0% | 1240.2522 | 1240.4601 | 9 | 7.307 | 72.2% | 6 | R.VKYETELAMR.Q | 2 |
|  | IPAstrin\_STLCD\_tube2\_032114\_01.04413.04413.2 | 2.6718 | 0.3311 | 100.0% | 1013.03217 | 1013.1535 | 98 | 6.216 | 64.3% | 1 | K.YETELAMR.Q | 2 |
|  | IPAstrin\_STLCD\_tube2\_032114\_01.04071.04071.2 | 1.8494 | 0.2818 | 95.4% | 1268.3522 | 1268.372 | 5 | 5.139 | 70.0% | 1 | R.QSVENDIHGLR.K | 2 |
|  | IPAstrin\_STLCD\_032114\_01.14988.14988.2 | 6.1312 | 0.48 | 100.0% | 2178.7922 | 2178.589 | 1 | 8.869 | 58.8% | 5 | R.LQLETEIEALKEELLFMK.K | 2 |
|  | IPAstrin\_STLCD\_032114\_01.15042.15042.3 | 2.7429 | 0.2795 | 97.3% | 2179.3442 | 2178.589 | 8 | 4.606 | 41.2% | 3 | R.LQLETEIEALKEELLFMK.K | 3 |
|  | IPAstrin\_STLCD\_032114\_02.06534.06534.3 | 4.8039 | 0.4682 | 100.0% | 2750.6042 | 2751.0227 | 1 | 8.305 | 33.0% | 4 | K.NHEEEVKGLQAQIASSGLTVEVDAPK.S | 3 |
|  | IPAstrin\_STLCD\_032114\_02.06726.06726.2 | 6.2265 | 0.563 | 100.0% | 1884.3322 | 1885.1246 | 1 | 10.342 | 66.7% | 3 | K.GLQAQIASSGLTVEVDAPK.S | 2 |
|  | IPAstrin\_STLCD\_032114\_01.10022.10022.2 | 3.9311 | 0.4968 | 100.0% | 1663.4122 | 1663.8865 | 1 | 8.215 | 73.1% | 2 | R.RTVQSLEIDLDSMR.N | 2 |
|  | IPAstrin\_STLCD\_tube2\_032114\_01.09788.09788.1 | 2.1291 | 0.47 | 100.0% | 1506.57 | 1507.699 | 1 | 6.475 | 62.5% | 2 | R.TVQSLEIDLDSMR.N | 1 |
|  | IPAstrin\_STLCD\_032114\_01.11634.11634.2 | 3.9538 | 0.5287 | 100.0% | 1507.1522 | 1507.699 | 1 | 9.627 | 75.0% | 10 | R.TVQSLEIDLDSMR.N | 2 |
|  | IPAstrin\_STLCD\_032114\_01.16016.16016.2 | 5.8249 | 0.4976 | 100.0% | 2670.7122 | 2672.0715 | 1 | 10.033 | 52.3% | 6 | R.YALQMEQLNGILLHLESELAQTR.A | 2 |
|  | IPAstrin\_STLCD\_032114\_02.11543.11543.3 | 6.2667 | 0.5321 | 100.0% | 2672.3943 | 2672.0715 | 1 | 8.379 | 44.3% | 21 | R.YALQMEQLNGILLHLESELAQTR.A | 3 |
|  | IPAstrin\_STLCD\_tube2\_032114\_01.08924.08924.2 | 3.5322 | 0.4446 | 100.0% | 1420.2322 | 1420.6055 | 3 | 6.519 | 68.2% | 5 | R.QAQEYEALLNIK.V | 2 |
|  | IPAstrin\_STLCD\_032114\_01.06727.06727.2 | 3.6388 | 0.3521 | 100.0% | 1294.3522 | 1293.5059 | 1 | 6.411 | 75.0% | 5 | K.VKLEAEIATYR.R | 2 |
|  | IPAstrin\_STLCD\_tube2\_032114\_01.04974.04974.2 | 3.1487 | 0.3705 | 100.0% | 1065.9922 | 1066.1992 | 4 | 5.992 | 81.2% | 7 | K.LEAEIATYR.R | 2 |
|  | IPAstrin\_STLCD\_tube2\_032114\_01.10564.10564.2 | 6.0287 | 0.5628 | 100.0% | 2740.7122 | 2741.9404 | 1 | 11.523 | 54.2% | 2 | R.LLEDGEDFNLGDALDSSNSMQTIQK.T | 2 |

Similarities:
contaminant\_KERATIN10(2:24)  

---

|  |  |  |  |  |  |  |  |  |
| --- | --- | --- | --- | --- | --- | --- | --- | --- |
| U | *gi|10645195|ref|NP\_06* | 9 | 19 | 57.7% | 130 | 14135 | 11.1 | histone cluster 1, H2ae [Homo sapiens] |
| U | *gi|19557656|ref|NP\_00* | 9 | 19 | 57.7% | 130 | 14135 | 11.1 | histone cluster 1, H2ab [Homo sapiens] |
| U | *gi|15617199|ref|NP\_25* | 9 | 19 | 57.7% | 130 | 14121 | 11.1 | histone cluster 3, H2a [Homo sapiens] |

| Filename XCorr DeltCN Conf% ObsM+H+ CalcM+H+ SpR ZScore Ion% # Sequence  | | | | | | | | | | | | |
| --- | --- | --- | --- | --- | --- | --- | --- | --- | --- | --- | --- | --- |
|  | IPAstrin\_STLCD\_032114\_01.08424.08424.2 | 3.3184 | 0.3544 | 100.0% | 944.65216 | 945.1093 | 1 | 5.961 | 81.2% | 5 | R.AGLQFPVGR.V | 222 |
|  | IPAstrin\_STLCD\_032114\_01.19659.19659.2 | 5.4117 | 0.57 | 100.0% | 2916.872 | 2917.3752 | 1 | 11.413 | 44.6% | 3 | R.VGAGAPVYLAAVLEYLTAEILELAGNAAR.D | 22 |
|  | IPAstrin\_STLCD\_tube2\_032114\_01.18866.18866.3 | 6.2954 | 0.5503 | 100.0% | 2917.4043 | 2917.3752 | 1 | 9.412 | 34.8% | 2 | R.VGAGAPVYLAAVLEYLTAEILELAGNAAR.D | 33 |
|  | IPAstrin\_STLCD\_032114\_01.05084.05084.2 | 2.1568 | 0.1899 | 97.6% | 851.3522 | 851.0396 | 11 | 5.259 | 75.0% | 1 | R.HLQLAIR.N | 222 |
|  | IPAstrin\_STLCD\_032114\_01.05242.05242.2 | 2.6629 | 0.2876 | 99.7% | 1692.1322 | 1693.9004 | 1 | 5.879 | 53.8% | 1 | R.HLQLAIRNDEELNK.L | 222 |
|  | IPAstrin\_STLCD\_tube2\_032114\_02.04420.04420.3 | 3.3392 | 0.2896 | 99.4% | 1694.1244 | 1693.9004 | 7 | 5.584 | 38.5% | 2 | R.HLQLAIRNDEELNK.L | 333 |
|  | IPAstrin\_STLCD\_032114\_01.08878.08878.2 | 2.4969 | 0.3487 | 99.9% | 1301.4722 | 1301.4423 | 3 | 5.824 | 60.0% | 1 | R.NDEELNKLLGR.V | 2 |
|  | IPAstrin\_STLCD\_tube2\_032114\_01.12512.12512.3 | 5.2627 | 0.3994 | 100.0% | 1932.5944 | 1932.3573 | 1 | 7.678 | 48.6% | 1 | R.VTIAQGGVLPNIQAVLLPK.K | 333 |
|  | IPAstrin\_STLCD\_tube2\_032114\_01.12464.12464.2 | 4.9421 | 0.4351 | 100.0% | 1932.7122 | 1932.3573 | 1 | 7.266 | 61.1% | 3 | R.VTIAQGGVLPNIQAVLLPK.K | 222 |

Similarities:
gi|10800130|ref|NP\_06(8:1)  
gi|106775678|ref|NP\_0(6:3)  

---

|  |  |  |  |  |  |  |  |  |
| --- | --- | --- | --- | --- | --- | --- | --- | --- |
| U | *gi|10800130|ref|NP\_06* | 9 | 23 | 57.7% | 130 | 14107 | 10.9 | histone cluster 1, H2ad [Homo sapiens] |
| U | *gi|4504243|ref|NP\_003* | 9 | 23 | 57.7% | 130 | 14091 | 10.9 | histone cluster 1, H2al [Homo sapiens] |
| U | *gi|4504239|ref|NP\_003* | 9 | 23 | 57.7% | 130 | 14091 | 10.9 | histone cluster 1, H2ai [Homo sapiens] |
| U | *gi|18105045|ref|NP\_54* | 9 | 23 | 58.6% | 128 | 13906 | 10.9 | histone cluster 1, H2ah [Homo sapiens] |
| U | *gi|10800144|ref|NP\_06* | 9 | 23 | 58.6% | 128 | 13936 | 10.9 | histone cluster 1, H2aj [Homo sapiens] |
| U | *gi|10800132|ref|NP\_06* | 9 | 23 | 57.7% | 130 | 14091 | 10.9 | histone cluster 1, H2ag [Homo sapiens] |

| Filename XCorr DeltCN Conf% ObsM+H+ CalcM+H+ SpR ZScore Ion% # Sequence  | | | | | | | | | | | | |
| --- | --- | --- | --- | --- | --- | --- | --- | --- | --- | --- | --- | --- |
|  | IPAstrin\_STLCD\_032114\_01.08424.08424.2 | 3.3184 | 0.3544 | 100.0% | 944.65216 | 945.1093 | 1 | 5.961 | 81.2% | 5 | R.AGLQFPVGR.V | 222 |
|  | IPAstrin\_STLCD\_032114\_01.19659.19659.2 | 5.4117 | 0.57 | 100.0% | 2916.872 | 2917.3752 | 1 | 11.413 | 44.6% | 3 | R.VGAGAPVYLAAVLEYLTAEILELAGNAAR.D | 22 |
|  | IPAstrin\_STLCD\_tube2\_032114\_01.18866.18866.3 | 6.2954 | 0.5503 | 100.0% | 2917.4043 | 2917.3752 | 1 | 9.412 | 34.8% | 2 | R.VGAGAPVYLAAVLEYLTAEILELAGNAAR.D | 33 |
|  | IPAstrin\_STLCD\_032114\_01.05084.05084.2 | 2.1568 | 0.1899 | 97.6% | 851.3522 | 851.0396 | 11 | 5.259 | 75.0% | 1 | R.HLQLAIR.N | 222 |
|  | IPAstrin\_STLCD\_032114\_01.05242.05242.2 | 2.6629 | 0.2876 | 99.7% | 1692.1322 | 1693.9004 | 1 | 5.879 | 53.8% | 1 | R.HLQLAIRNDEELNK.L | 222 |
|  | IPAstrin\_STLCD\_tube2\_032114\_02.04420.04420.3 | 3.3392 | 0.2896 | 99.4% | 1694.1244 | 1693.9004 | 7 | 5.584 | 38.5% | 2 | R.HLQLAIRNDEELNK.L | 333 |
|  | IPAstrin\_STLCD\_032114\_01.08366.08366.2 | 3.854 | 0.3884 | 100.0% | 1273.5122 | 1273.4288 | 1 | 6.531 | 75.0% | 5 | R.NDEELNKLLGK.V | 22 |
|  | IPAstrin\_STLCD\_tube2\_032114\_01.12512.12512.3 | 5.2627 | 0.3994 | 100.0% | 1932.5944 | 1932.3573 | 1 | 7.678 | 48.6% | 1 | K.VTIAQGGVLPNIQAVLLPK.K | 333 |
|  | IPAstrin\_STLCD\_tube2\_032114\_01.12464.12464.2 | 4.9421 | 0.4351 | 100.0% | 1932.7122 | 1932.3573 | 1 | 7.266 | 61.1% | 3 | K.VTIAQGGVLPNIQAVLLPK.K | 222 |

Similarities:
gi|10645195|ref|NP\_06(8:1)  
gi|106775678|ref|NP\_0(7:2)  

---

|  |  |  |  |  |  |  |  |  |
| --- | --- | --- | --- | --- | --- | --- | --- | --- |
| U | *gi|106775678|ref|NP\_0* | 9 | 21 | 57.7% | 130 | 14095 | 10.9 | histone cluster 2, H2aa4 [Homo sapiens] |
| U | *gi|4504251|ref|NP\_003* | 9 | 21 | 57.7% | 130 | 14095 | 10.9 | histone cluster 2, H2aa3 [Homo sapiens] |
| U | *gi|24638446|ref|NP\_00* | 9 | 21 | 58.1% | 129 | 13988 | 10.9 | histone cluster 2, H2ac [Homo sapiens] |

| Filename XCorr DeltCN Conf% ObsM+H+ CalcM+H+ SpR ZScore Ion% # Sequence  | | | | | | | | | | | | |
| --- | --- | --- | --- | --- | --- | --- | --- | --- | --- | --- | --- | --- |
|  | IPAstrin\_STLCD\_032114\_01.08424.08424.2 | 3.3184 | 0.3544 | 100.0% | 944.65216 | 945.1093 | 1 | 5.961 | 81.2% | 5 | R.AGLQFPVGR.V | 222 |
|  | IPAstrin\_STLCD\_tube2\_032114\_01.18716.18716.2 | 5.6033 | 0.5879 | 100.0% | 2934.6921 | 2935.4082 | 1 | 9.686 | 41.1% | 2 | R.VGAGAPVYMAAVLEYLTAEILELAGNAAR.D | 2 |
|  | IPAstrin\_STLCD\_tube2\_032114\_01.18711.18711.3 | 4.5125 | 0.4035 | 100.0% | 2935.1943 | 2935.4082 | 1 | 7.055 | 25.0% | 1 | R.VGAGAPVYMAAVLEYLTAEILELAGNAAR.D | 3 |
|  | IPAstrin\_STLCD\_032114\_01.05084.05084.2 | 2.1568 | 0.1899 | 97.6% | 851.3522 | 851.0396 | 11 | 5.259 | 75.0% | 1 | R.HLQLAIR.N | 222 |
|  | IPAstrin\_STLCD\_032114\_01.05242.05242.2 | 2.6629 | 0.2876 | 99.7% | 1692.1322 | 1693.9004 | 1 | 5.879 | 53.8% | 1 | R.HLQLAIRNDEELNK.L | 222 |
|  | IPAstrin\_STLCD\_tube2\_032114\_02.04420.04420.3 | 3.3392 | 0.2896 | 99.4% | 1694.1244 | 1693.9004 | 7 | 5.584 | 38.5% | 2 | R.HLQLAIRNDEELNK.L | 333 |
|  | IPAstrin\_STLCD\_032114\_01.08366.08366.2 | 3.854 | 0.3884 | 100.0% | 1273.5122 | 1273.4288 | 1 | 6.531 | 75.0% | 5 | R.NDEELNKLLGK.V | 22 |
|  | IPAstrin\_STLCD\_tube2\_032114\_01.12512.12512.3 | 5.2627 | 0.3994 | 100.0% | 1932.5944 | 1932.3573 | 1 | 7.678 | 48.6% | 1 | K.VTIAQGGVLPNIQAVLLPK.K | 333 |
|  | IPAstrin\_STLCD\_tube2\_032114\_01.12464.12464.2 | 4.9421 | 0.4351 | 100.0% | 1932.7122 | 1932.3573 | 1 | 7.266 | 61.1% | 3 | K.VTIAQGGVLPNIQAVLLPK.K | 222 |

Similarities:
gi|10645195|ref|NP\_06(6:3)  
gi|10800130|ref|NP\_06(7:2)  

---

|  |  |  |  |  |  |  |  |  |
| --- | --- | --- | --- | --- | --- | --- | --- | --- |
| U | *gi|73623035|ref|NP\_00* | 77 | 310 | 53.3% | 1193 | 134422 | 5.0 | sperm associated antigen 5 [Homo sapiens] |

| Filename XCorr DeltCN Conf% ObsM+H+ CalcM+H+ SpR ZScore Ion% # Sequence  | | | | | | | | | | | | |
| --- | --- | --- | --- | --- | --- | --- | --- | --- | --- | --- | --- | --- |
| \* | IPAstrin\_STLCD\_032114\_01.03792.03792.2 | 3.8668 | 0.5182 | 100.0% | 1462.1322 | 1462.5156 | 1 | 8.062 | 75.0% | 1 | R.TDLSSEHFSHSSK.W | 2 |
| \* | IPAstrin\_STLCD\_032114\_01.10128.10128.2 | 4.1984 | 0.4251 | 100.0% | 1653.2722 | 1653.8445 | 1 | 9.08 | 67.9% | 4 | K.TSEEAVDPLGNYMVK.T | 2 |
| \* | IPAstrin\_STLCD\_tube2\_032114\_01.12046.12046.2 | 3.5099 | 0.4626 | 100.0% | 2322.5923 | 2323.6262 | 1 | 8.048 | 50.0% | 2 | K.TIVLVPS\*PLGQQQDMIFEAR.L | 2 |
| \* | IPAstrin\_STLCD\_tube2\_032114\_01.08156.08156.2 | 5.5406 | 0.5011 | 100.0% | 1833.4521 | 1833.0668 | 1 | 7.806 | 75.0% | 5 | R.LDTMAETNSISLNGPLR.T | 2 |
| \* | IPAstrin\_STLCD\_tube2\_032114\_02.06340.06340.3 | 3.8327 | 0.2375 | 98.9% | 1834.0443 | 1833.0668 | 1 | 5.866 | 40.6% | 1 | R.LDTMAETNSISLNGPLR.T | 3 |
| \* | IPAstrin\_STLCD\_tube2\_032114\_01.09028.09028.3 | 3.346 | 0.3035 | 98.9% | 2532.1143 | 2532.8286 | 5 | 5.186 | 29.5% | 1 | R.LDTMAETNSISLNGPLRTDDLVR.E | 3 |
| \* | IPAstrin\_STLCD\_tube2\_032114\_01.14229.14229.3 | 5.4024 | 0.3438 | 100.0% | 3777.5942 | 3778.2102 | 1 | 6.929 | 24.3% | 1 | R.TEAVREDLVPSESNAFLPSSVLWLSPSTALAADFR.V | 3 |
| \* | IPAstrin\_STLCD\_tube2\_032114\_01.14294.14294.3 | 6.0315 | 0.172 | 99.4% | 3858.5044 | 3858.2102 | 1 | 6.468 | 33.8% | 4 | R.TEAVREDLVPSESNAFLPSSVLWLS\*PSTALAADFR.V | 3 |
| \* | IPAstrin\_STLCD\_tube2\_032114\_01.05963.05963.2 | 6.0044 | 0.5387 | 100.0% | 2219.5122 | 2220.3752 | 1 | 9.435 | 72.2% | 3 | R.VNHVDPEEEIVEHGAMEER.E | 2 |
| \* | IPAstrin\_STLCD\_tube2\_032114\_01.05912.05912.3 | 5.3518 | 0.4365 | 100.0% | 2220.4744 | 2220.3752 | 1 | 6.634 | 43.1% | 10 | R.VNHVDPEEEIVEHGAMEER.E | 3 |
| \* | IPAstrin\_STLCD\_tube2\_032114\_01.14782.14782.2 | 5.5383 | 0.4982 | 100.0% | 2063.7122 | 2064.3606 | 1 | 8.36 | 58.8% | 4 | R.ILGSDTESWMSPLAWLEK.G | 2 |
| \* | IPAstrin\_STLCD\_tube2\_032114\_01.15332.15332.2 | 4.2921 | 0.4138 | 100.0% | 2144.4922 | 2144.3606 | 1 | 6.754 | 61.8% | 5 | R.ILGSDTESWMS\*PLAWLEK.G | 2 |
| \* | IPAstrin\_STLCD\_tube2\_032114\_01.08124.08124.2 | 3.2172 | 0.2863 | 100.0% | 1333.4321 | 1333.5457 | 1 | 6.566 | 77.3% | 2 | K.GVNTSVMLENLR.Q | 2 |
| \* | IPAstrin\_STLCD\_032114\_01.03903.03903.2 | 3.1048 | 0.3938 | 100.0% | 1293.9521 | 1294.4044 | 1 | 6.795 | 66.7% | 1 | K.STNTSQTGLVGTK.H | 2 |
| \* | IPAstrin\_STLCD\_032114\_01.09838.09838.3 | 3.1478 | 0.2678 | 97.7% | 2469.8643 | 2469.68 | 20 | 4.447 | 27.4% | 1 | K.HSTSETEQLLCGRPPDLTALSR.H | 3 |
| \* | IPAstrin\_STLCD\_tube2\_032114\_01.16419.16419.3 | 3.5883 | 0.4447 | 100.0% | 2165.3943 | 2166.4795 | 2 | 6.387 | 36.1% | 6 | R.HDLEDNLLSSLVILEVLSR.Q | 3 |
| \* | IPAstrin\_STLCD\_032114\_01.17334.17334.2 | 7.6923 | 0.6182 | 100.0% | 2166.5723 | 2166.4795 | 1 | 10.702 | 72.2% | 18 | R.HDLEDNLLSSLVILEVLSR.Q | 2 |
| \* | IPAstrin\_STLCD\_tube2\_032114\_01.03928.03928.3 | 5.382 | 0.4792 | 100.0% | 2866.7944 | 2868.0 | 1 | 7.78 | 35.6% | 4 | K.SQLAVPHPETQDSSTQTDTSHSGITNK.L | 3 |
| \* | IPAstrin\_STLCD\_tube2\_032114\_01.03990.03990.2 | 4.1665 | 0.4935 | 100.0% | 2867.5923 | 2868.0 | 1 | 8.172 | 44.2% | 1 | K.SQLAVPHPETQDSSTQTDTSHSGITNK.L | 2 |
| \* | IPAstrin\_STLCD\_032114\_01.04057.04057.2 | 3.5412 | 0.339 | 100.0% | 1485.2922 | 1485.6146 | 4 | 6.486 | 58.3% | 2 | K.ESHEMGQALQQAR.N | 2 |
| \* | IPAstrin\_STLCD\_tube2\_032114\_01.10248.10248.2 | 4.0496 | 0.3929 | 100.0% | 1306.3722 | 1305.578 | 1 | 6.408 | 80.0% | 6 | R.NVMQSWVLISK.E | 2 |
| \* | IPAstrin\_STLCD\_tube2\_032114\_01.11661.11661.3 | 5.6203 | 0.3702 | 100.0% | 2891.9043 | 2892.2793 | 1 | 6.967 | 27.1% | 4 | K.ELISLLHLSLLHLEEDKTTVSQESR.R | 3 |
| \* | IPAstrin\_STLCD\_032114\_01.03715.03715.2 | 2.1291 | 0.2315 | 98.2% | 1042.3121 | 1042.201 | 7 | 5.03 | 71.4% | 1 | R.HREEMALR.G | 2 |
| \* | IPAstrin\_STLCD\_032114\_01.10899.10899.1 | 2.1613 | 0.4208 | 100.0% | 1390.61 | 1391.5823 | 15 | 6.212 | 50.0% | 2 | R.ISQLEQDLASMR.E | 1 |
| \* | IPAstrin\_STLCD\_032114\_01.10902.10902.2 | 3.9345 | 0.4035 | 100.0% | 1391.2322 | 1391.5823 | 1 | 8.179 | 68.2% | 6 | R.ISQLEQDLASMR.E | 2 |
| \* | IPAstrin\_STLCD\_032114\_01.09981.09981.3 | 5.2496 | 0.4532 | 100.0% | 2125.4944 | 2125.4788 | 1 | 7.888 | 36.1% | 1 | R.EFRGLLKDAQTQLVGLHAK.Q | 3 |
| \* | IPAstrin\_STLCD\_032114\_01.08866.08866.3 | 3.7231 | 0.1984 | 97.8% | 1692.7144 | 1692.9994 | 138 | 4.582 | 35.0% | 2 | R.GLLKDAQTQLVGLHAK.Q | 3 |
| \* | IPAstrin\_STLCD\_032114\_01.04887.04887.2 | 3.3452 | 0.4307 | 100.0% | 1281.0521 | 1281.4545 | 1 | 6.868 | 68.2% | 2 | K.DAQTQLVGLHAK.Q | 2 |
| \* | IPAstrin\_STLCD\_tube2\_032114\_01.12172.12172.2 | 5.4441 | 0.5937 | 100.0% | 2389.8523 | 2390.612 | 1 | 10.745 | 63.2% | 4 | K.QEELVQQTVSLTSTLQQDWR.S | 2 |
| \* | IPAstrin\_STLCD\_tube2\_032114\_01.12171.12171.3 | 4.7237 | 0.4346 | 100.0% | 2390.7544 | 2390.612 | 8 | 7.265 | 31.6% | 3 | K.QEELVQQTVSLTSTLQQDWR.S | 3 |
| \* | IPAstrin\_STLCD\_032114\_02.09805.09805.2 | 4.4708 | 0.3162 | 100.0% | 1786.7922 | 1787.0405 | 1 | 7.978 | 71.4% | 4 | R.SMQLDYTTWTALLSR.S | 2 |
| \* | IPAstrin\_STLCD\_tube2\_032114\_01.12832.12832.1 | 2.5003 | 0.3771 | 100.0% | 1786.88 | 1787.0405 | 5 | 5.706 | 35.7% | 1 | R.SMQLDYTTWTALLSR.S | 1 |
| \* | IPAstrin\_STLCD\_tube2\_032114\_01.03567.03567.2 | 3.1933 | 0.4184 | 100.0% | 1402.4321 | 1403.5321 | 1 | 7.665 | 68.2% | 3 | R.DVAIEEKQEVSR.V | 2 |
| \* | IPAstrin\_STLCD\_032114\_01.07442.07442.2 | 3.5897 | 0.3886 | 100.0% | 1533.5322 | 1533.6849 | 6 | 7.734 | 62.5% | 2 | R.VLEQVSAQLEECK.G | 2 |
| \* | IPAstrin\_STLCD\_032114\_02.06296.06296.3 | 4.9803 | 0.4131 | 100.0% | 2919.4443 | 2919.1382 | 1 | 7.152 | 34.4% | 2 | R.VLEQVSAQLEECKGQTEQLELENSR.L | 3 |
| \* | IPAstrin\_STLCD\_032114\_01.04545.04545.2 | 4.1347 | 0.4208 | 100.0% | 1404.0922 | 1404.4764 | 13 | 7.287 | 59.1% | 2 | K.GQTEQLELENSR.L | 2 |
| \* | IPAstrin\_STLCD\_tube2\_032114\_01.09950.09950.2 | 5.6145 | 0.4394 | 100.0% | 1573.3121 | 1573.848 | 1 | 7.754 | 76.9% | 14 | R.AQLQILANMDSQLK.E | 2 |
| \* | IPAstrin\_STLCD\_032114\_02.04367.04367.3 | 5.0696 | 0.342 | 100.0% | 1722.9844 | 1723.9879 | 1 | 6.549 | 51.8% | 6 | K.HMQAELQQQQAVLAK.E | 3 |
| \* | IPAstrin\_STLCD\_tube2\_032114\_01.04306.04306.2 | 5.3915 | 0.5287 | 100.0% | 1723.1122 | 1723.9879 | 1 | 9.947 | 82.1% | 11 | K.HMQAELQQQQAVLAK.E | 2 |
| \* | IPAstrin\_STLCD\_032114\_01.06192.06192.2 | 2.4753 | 0.2255 | 99.8% | 832.2322 | 831.9878 | 2 | 4.977 | 91.7% | 2 | K.TTLEVLR.E | 2 |
| \* | IPAstrin\_STLCD\_032114\_01.09750.09750.3 | 3.6999 | 0.2851 | 99.4% | 1964.4543 | 1964.1462 | 2 | 5.733 | 34.4% | 1 | R.SLQCENLKDTVENLTAK.L | 3 |
| \* | IPAstrin\_STLCD\_tube2\_032114\_01.04576.04576.3 | 3.7901 | 0.2227 | 98.8% | 1676.0343 | 1675.7899 | 5 | 5.088 | 41.1% | 1 | K.LASTIADNQEQDLEK.T | 3 |
| \* | IPAstrin\_STLCD\_tube2\_032114\_01.04526.04526.2 | 4.8854 | 0.3908 | 100.0% | 1676.3722 | 1675.7899 | 1 | 7.284 | 64.3% | 8 | K.LASTIADNQEQDLEK.T | 2 |
| \* | IPAstrin\_STLCD\_tube2\_032114\_01.04478.04478.3 | 2.8972 | 0.3654 | 99.6% | 1932.2344 | 1933.0825 | 148 | 6.479 | 31.2% | 1 | K.LASTIADNQEQDLEKTR.Q | 3 |
| \* | IPAstrin\_STLCD\_tube2\_032114\_01.15572.15572.2 | 5.878 | 0.5096 | 100.0% | 2046.5322 | 2047.443 | 1 | 9.403 | 67.6% | 37 | K.LGLLTEQLQSLTLFLQTK.L | 2 |
| \* | IPAstrin\_STLCD\_032114\_02.11831.11831.3 | 5.1671 | 0.418 | 100.0% | 2047.3744 | 2047.443 | 1 | 8.599 | 51.5% | 10 | K.LGLLTEQLQSLTLFLQTK.L | 3 |
| \* | IPAstrin\_STLCD\_tube2\_032114\_01.15248.15248.3 | 3.6669 | 0.3804 | 100.0% | 2786.9944 | 2788.121 | 1 | 6.909 | 28.8% | 2 | R.TFLGSILTAVADEEPESTPVPLLGSDK.S | 3 |
| \* | IPAstrin\_STLCD\_032114\_01.15847.15847.2 | 5.7129 | 0.5202 | 100.0% | 2787.8523 | 2788.121 | 1 | 8.617 | 40.4% | 8 | R.TFLGSILTAVADEEPESTPVPLLGSDK.S | 2 |
| \* | IPAstrin\_STLCD\_tube2\_032114\_01.15668.15668.2 | 4.5381 | 0.3932 | 100.0% | 2868.9321 | 2868.121 | 1 | 7.515 | 42.3% | 1 | R.TFLGSILTAVADEEPESTPVPLLGS\*DK.S | 2 |
| \* | IPAstrin\_STLCD\_tube2\_032114\_01.15164.15164.3 | 5.0644 | 0.3489 | 100.0% | 3429.2344 | 3430.7473 | 1 | 8.257 | 29.8% | 3 | R.TFLGSILTAVADEEPESTPVPLLGSDKS\*AFTR.V | 3 |
| \* | IPAstrin\_STLCD\_032114\_01.15738.15738.3 | 4.3949 | 0.3708 | 100.0% | 3430.3145 | 3430.7473 | 1 | 6.944 | 23.4% | 1 | R.TFLGSILTAVADEEPESTPVPLLGS\*DKSAFTR.V | 3 |
| \* | IPAstrin\_STLCD\_tube2\_032114\_01.15098.15098.3 | 4.0944 | 0.3171 | 99.8% | 3430.9744 | 3430.7473 | 1 | 5.491 | 21.0% | 1 | R.TFLGSILTAVADEEPESTPVPLLGSDKSAFT#R.V | 3 |
| \* | IPAstrin\_STLCD\_tube2\_032114\_01.03252.03252.2 | 4.6297 | 0.3509 | 100.0% | 1595.1122 | 1595.7092 | 1 | 6.732 | 83.3% | 5 | R.LQAQEEQHQEVQK.A | 2 |
| \* | IPAstrin\_STLCD\_tube2\_032114\_01.04538.04538.2 | 5.7078 | 0.3641 | 100.0% | 2148.4321 | 2149.3652 | 1 | 8.138 | 71.9% | 1 | R.YKNEKELQEVIQQQNEK.I | 2 |
| \* | IPAstrin\_STLCD\_tube2\_032114\_01.04521.04521.3 | 5.6446 | 0.2383 | 100.0% | 2149.4944 | 2149.3652 | 4 | 6.159 | 48.4% | 3 | R.YKNEKELQEVIQQQNEK.I | 3 |
| \* | IPAstrin\_STLCD\_tube2\_032114\_01.03945.03945.2 | 4.3536 | 0.2803 | 100.0% | 1486.0721 | 1486.622 | 1 | 6.677 | 86.4% | 4 | K.ELQEVIQQQNEK.I | 2 |
| \* | IPAstrin\_STLCD\_032114\_01.10048.10048.2 | 3.5065 | 0.3827 | 100.0% | 1713.7122 | 1715.0 | 1 | 7.716 | 60.7% | 2 | K.ILEQIDKSGELISLR.E | 2 |
| \* | IPAstrin\_STLCD\_tube2\_032114\_01.10124.10124.3 | 5.1868 | 0.3164 | 100.0% | 2679.9844 | 2681.0618 | 1 | 6.15 | 37.5% | 2 | K.ILEQIDKSGELISLREEVTHLTR.S | 3 |
| \* | IPAstrin\_STLCD\_tube2\_032114\_01.06010.06010.2 | 2.9513 | 0.1807 | 99.9% | 875.2322 | 875.0128 | 10 | 5.328 | 85.7% | 3 | K.SGELISLR.E | 2 |
| \* | IPAstrin\_STLCD\_tube2\_032114\_01.08714.08714.2 | 3.9337 | 0.3211 | 100.0% | 1840.2922 | 1841.0745 | 1 | 5.986 | 53.3% | 2 | K.SGELISLREEVTHLTR.S | 2 |
| \* | IPAstrin\_STLCD\_032114\_02.07079.07079.3 | 4.9432 | 0.4157 | 100.0% | 1840.4043 | 1841.0745 | 1 | 6.981 | 50.0% | 8 | K.SGELISLREEVTHLTR.S | 3 |
| \* | IPAstrin\_STLCD\_tube2\_032114\_01.06080.06080.2 | 2.5933 | 0.1398 | 98.0% | 1104.1921 | 1104.248 | 24 | 5.091 | 68.8% | 5 | K.VWLSQEVDK.L | 2 |
| \* | IPAstrin\_STLCD\_tube2\_032114\_01.07994.07994.2 | 3.4552 | 0.3471 | 100.0% | 1373.1522 | 1373.595 | 1 | 6.274 | 90.0% | 4 | K.VWLSQEVDKLR.V | 2 |
| \* | IPAstrin\_STLCD\_032114\_01.10419.10419.2 | 2.732 | 0.2622 | 100.0% | 897.9122 | 898.16644 | 1 | 6.823 | 91.7% | 4 | R.VMFLEMK.N | 2 |
| \* | IPAstrin\_STLCD\_tube2\_032114\_01.06474.06474.2 | 2.3395 | 0.1574 | 95.4% | 1271.1322 | 1269.5598 | 3 | 3.857 | 66.7% | 1 | R.VMFLEMKNEK.E | 2 |
| \* | IPAstrin\_STLCD\_tube2\_032114\_01.06135.06135.1 | 2.388 | 0.2618 | 100.0% | 1000.5 | 1001.1277 | 9 | 4.721 | 71.4% | 8 | R.NILEENLR.R | 1 |
| \* | IPAstrin\_STLCD\_tube2\_032114\_01.06176.06176.2 | 2.6869 | 0.054 | 95.5% | 1000.71216 | 1001.1277 | 5 | 4.209 | 85.7% | 1 | R.NILEENLR.R | 2 |
| \* | IPAstrin\_STLCD\_tube2\_032114\_01.04965.04965.2 | 2.398 | 0.143 | 95.8% | 1156.7722 | 1157.3152 | 10 | 3.786 | 81.2% | 1 | R.NILEENLRR.S | 2 |
| \* | IPAstrin\_STLCD\_tube2\_032114\_01.08828.08828.3 | 5.8775 | 0.4608 | 100.0% | 2230.4944 | 2230.526 | 1 | 8.147 | 47.1% | 5 | R.RSDKELEKLDDIVQHIYK.T | 3 |
| \* | IPAstrin\_STLCD\_tube2\_032114\_01.09717.09717.2 | 5.7808 | 0.5367 | 100.0% | 2073.612 | 2074.3384 | 1 | 10.409 | 68.8% | 2 | R.SDKELEKLDDIVQHIYK.T | 2 |
| \* | IPAstrin\_STLCD\_032114\_01.11533.11533.3 | 5.228 | 0.2856 | 100.0% | 2074.2844 | 2074.3384 | 1 | 7.164 | 46.9% | 4 | R.SDKELEKLDDIVQHIYK.T | 3 |
| \* | IPAstrin\_STLCD\_tube2\_032114\_01.09096.09096.2 | 3.1982 | 0.2784 | 99.9% | 1743.2522 | 1743.9977 | 1 | 5.516 | 73.1% | 2 | K.ELEKLDDIVQHIYK.T | 2 |
| \* | IPAstrin\_STLCD\_tube2\_032114\_01.09039.09039.3 | 3.5041 | 0.3635 | 100.0% | 1743.8043 | 1743.9977 | 1 | 6.168 | 46.2% | 2 | K.ELEKLDDIVQHIYK.T | 3 |
| \* | IPAstrin\_STLCD\_tube2\_032114\_01.06308.06308.2 | 3.7445 | 0.37 | 100.0% | 1244.5322 | 1244.4331 | 4 | 6.806 | 72.2% | 5 | K.LDDIVQHIYK.T | 2 |
| \* | IPAstrin\_STLCD\_032114\_01.11388.11388.1 | 1.9045 | 0.3021 | 100.0% | 1126.46 | 1127.3696 | 90 | 5.72 | 44.4% | 1 | K.TLLSIPEVVR.G | 1 |
| \* | IPAstrin\_STLCD\_032114\_01.11376.11376.2 | 2.8998 | 0.4175 | 100.0% | 1127.2122 | 1127.3696 | 2 | 6.588 | 77.8% | 5 | K.TLLSIPEVVR.G | 2 |
| \* | IPAstrin\_STLCD\_032114\_01.15522.15522.1 | 1.9361 | 0.3324 | 100.0% | 1148.45 | 1149.3293 | 23 | 5.792 | 44.4% | 2 | K.ELQGLLEFLS.- | 1 |

---

|  |  |  |  |  |  |  |  |  |
| --- | --- | --- | --- | --- | --- | --- | --- | --- |
| U | *gi|29788785|ref|NP\_82* | 26 | 116 | 51.4% | 444 | 49671 | 4.9 | tubulin, beta [Homo sapiens] |

| Filename XCorr DeltCN Conf% ObsM+H+ CalcM+H+ SpR ZScore Ion% # Sequence  | | | | | | | | | | | | |
| --- | --- | --- | --- | --- | --- | --- | --- | --- | --- | --- | --- | --- |
| \* | IPAstrin\_STLCD\_tube2\_032114\_01.09394.09394.3 | 6.3866 | 0.3968 | 100.0% | 3103.6143 | 3104.2725 | 1 | 8.55 | 32.7% | 4 | K.FWEVISDEHGIDPTGTYHGDSDLQLDR.I | 3 |
| \* | IPAstrin\_STLCD\_032114\_01.05568.05568.2 | 3.711 | 0.5204 | 100.0% | 1302.2522 | 1302.4265 | 1 | 9.102 | 81.8% | 10 | R.ISVYYNEATGGK.Y | 2 |
|  | IPAstrin\_STLCD\_tube2\_032114\_01.09521.09521.2 | 4.4557 | 0.4927 | 100.0% | 1616.3922 | 1616.8701 | 2 | 8.148 | 64.3% | 6 | R.AILVDLEPGTMDSVR.S | 22 |
|  | IPAstrin\_STLCD\_tube2\_032114\_01.11814.11814.2 | 4.6413 | 0.5074 | 100.0% | 2798.5122 | 2800.0647 | 1 | 8.552 | 40.0% | 2 | R.SGPFGQIFRPDNFVFGQSGAGNNWAK.G | 22 |
|  | IPAstrin\_STLCD\_tube2\_032114\_01.11876.11876.3 | 7.2984 | 0.5263 | 100.0% | 2800.0745 | 2800.0647 | 1 | 9.699 | 37.0% | 7 | R.SGPFGQIFRPDNFVFGQSGAGNNWAK.G | 33 |
|  | IPAstrin\_STLCD\_tube2\_032114\_01.12326.12326.3 | 3.5451 | 0.3347 | 99.8% | 1960.1044 | 1960.151 | 1 | 6.352 | 39.7% | 3 | K.GHYTEGAELVDSVLDVVR.K | 333 |
|  | IPAstrin\_STLCD\_tube2\_032114\_01.12274.12274.2 | 7.3835 | 0.5359 | 100.0% | 1960.3121 | 1960.151 | 1 | 10.083 | 79.4% | 3 | K.GHYTEGAELVDSVLDVVR.K | 222 |
|  | IPAstrin\_STLCD\_tube2\_032114\_01.11199.11199.3 | 4.8341 | 0.4769 | 100.0% | 2088.1143 | 2088.325 | 1 | 8.071 | 40.3% | 4 | K.GHYTEGAELVDSVLDVVRK.E | 333 |
|  | IPAstrin\_STLCD\_tube2\_032114\_01.07706.07706.2 | 4.1678 | 0.4142 | 100.0% | 1320.0322 | 1320.5896 | 2 | 7.752 | 72.7% | 10 | R.IMNTFSVVPSPK.V | 222 |
|  | IPAstrin\_STLCD\_tube2\_032114\_01.06530.06530.2 | 2.998 | 0.3386 | 100.0% | 1131.3322 | 1131.2767 | 133 | 5.952 | 61.1% | 14 | R.FPGQLNADLR.K | 2222 |
|  | IPAstrin\_STLCD\_tube2\_032114\_01.05058.05058.2 | 2.4459 | 0.1587 | 95.7% | 1259.4521 | 1259.4508 | 80 | 4.442 | 55.0% | 1 | R.FPGQLNADLRK.L | 2222 |
|  | IPAstrin\_STLCD\_tube2\_032114\_01.07702.07702.2 | 3.6879 | 0.347 | 100.0% | 1272.4321 | 1272.5945 | 3 | 7.18 | 65.0% | 4 | R.KLAVNMVPFPR.L | 2222 |
|  | IPAstrin\_STLCD\_tube2\_032114\_01.09071.09071.1 | 2.021 | 0.2752 | 100.0% | 1143.62 | 1144.4204 | 9 | 5.967 | 61.1% | 1 | K.LAVNMVPFPR.L | 1111 |
|  | IPAstrin\_STLCD\_tube2\_032114\_01.09111.09111.2 | 3.5075 | 0.4273 | 100.0% | 1143.6522 | 1144.4204 | 1 | 8.353 | 94.4% | 4 | K.LAVNMVPFPR.L | 2222 |
|  | IPAstrin\_STLCD\_032114\_01.12828.12828.2 | 3.4639 | 0.3347 | 100.0% | 1621.3322 | 1621.9403 | 1 | 7.353 | 80.8% | 6 | R.LHFFMPGFAPLTSR.G | 222 |
|  | IPAstrin\_STLCD\_tube2\_032114\_01.11356.11356.3 | 4.2941 | 0.3603 | 100.0% | 1621.6743 | 1621.9403 | 1 | 6.6 | 50.0% | 2 | R.LHFFMPGFAPLTSR.G | 333 |
| \* | IPAstrin\_STLCD\_032114\_01.12584.12584.2 | 3.4199 | 0.4796 | 100.0% | 1660.1921 | 1660.9078 | 1 | 7.977 | 57.1% | 3 | R.ALTVPELTQQVFDAK.N | 2 |
|  | IPAstrin\_STLCD\_032114\_01.11383.11383.1 | 1.6593 | 0.4206 | 100.0% | 1039.62 | 1040.2505 | 2 | 6.348 | 68.8% | 3 | R.YLTVAAVFR.G | 11 |
|  | IPAstrin\_STLCD\_tube2\_032114\_01.09626.09626.2 | 3.1367 | 0.4645 | 100.0% | 1040.1721 | 1040.2505 | 1 | 7.776 | 93.8% | 6 | R.YLTVAAVFR.G | 22 |
|  | IPAstrin\_STLCD\_tube2\_032114\_02.05848.05848.3 | 3.8439 | 0.3065 | 99.8% | 1923.8944 | 1925.2405 | 48 | 5.881 | 35.0% | 1 | R.MSMKEVDEQMLNVQNK.N | 33 |
|  | IPAstrin\_STLCD\_tube2\_032114\_01.05205.05205.2 | 3.5221 | 0.2626 | 100.0% | 1447.1921 | 1447.6031 | 2 | 5.781 | 68.2% | 5 | K.EVDEQMLNVQNK.N | 22 |
|  | IPAstrin\_STLCD\_tube2\_032114\_01.10725.10725.2 | 2.6091 | 0.3156 | 99.8% | 1696.4922 | 1697.8877 | 1 | 5.145 | 61.5% | 2 | K.NSSYFVEWIPNNVK.T | 2222 |
| \* | IPAstrin\_STLCD\_tube2\_032114\_01.13040.13040.2 | 4.1246 | 0.3008 | 100.0% | 1871.6322 | 1871.2018 | 1 | 6.897 | 62.5% | 5 | K.MAVTFIGNSTAIQELFK.R | 2 |
| \* | IPAstrin\_STLCD\_032114\_02.09230.09230.3 | 3.3996 | 0.3426 | 99.8% | 2027.6943 | 2027.3893 | 1 | 7.095 | 41.2% | 2 | K.MAVTFIGNSTAIQELFKR.I | 3 |
|  | IPAstrin\_STLCD\_tube2\_032114\_01.08130.08130.2 | 3.1632 | 0.3675 | 100.0% | 1386.3522 | 1386.6116 | 1 | 6.603 | 70.0% | 2 | K.RISEQFTAMFR.R | 222 |
|  | IPAstrin\_STLCD\_tube2\_032114\_01.09399.09399.2 | 3.8735 | 0.4603 | 100.0% | 1230.9922 | 1230.4241 | 1 | 7.297 | 94.4% | 6 | R.ISEQFTAMFR.R | 222 |

Similarities:
gi|5174735|ref|NP\_006(20:6)  
gi|50592996|ref|NP\_00(13:13)  
gi|14210536|ref|NP\_11(8:18)  

---

|  |  |  |  |  |  |  |  |  |
| --- | --- | --- | --- | --- | --- | --- | --- | --- |
| U | *gi|57013276|ref|NP\_00* | 23 | 80 | 50.3% | 451 | 50152 | 5.1 | tubulin, alpha, ubiquitous [Homo sapiens] |

| Filename XCorr DeltCN Conf% ObsM+H+ CalcM+H+ SpR ZScore Ion% # Sequence  | | | | | | | | | | | | |
| --- | --- | --- | --- | --- | --- | --- | --- | --- | --- | --- | --- | --- |
|  | IPAstrin\_STLCD\_032114\_01.11899.11899.2 | 5.7183 | 0.6449 | 100.0% | 2008.1721 | 2009.093 | 1 | 11.702 | 63.2% | 5 | K.TIGGGDDSFNTFFSETGAGK.H | 2 |
|  | IPAstrin\_STLCD\_032114\_01.12558.12558.2 | 4.4735 | 0.4452 | 100.0% | 1703.3322 | 1702.9451 | 1 | 7.409 | 82.1% | 5 | R.AVFVDLEPTVIDEVR.T | 2 |
|  | IPAstrin\_STLCD\_032114\_01.09666.09666.2 | 2.3859 | 0.456 | 100.0% | 1411.3722 | 1411.6439 | 434 | 6.257 | 54.5% | 2 | R.QLFHPEQLITGK.E | 22 |
|  | IPAstrin\_STLCD\_tube2\_032114\_01.07352.07352.3 | 3.7535 | 0.3903 | 100.0% | 2415.8943 | 2416.6555 | 1 | 6.125 | 33.8% | 5 | R.QLFHPEQLITGKEDAANNYAR.G | 33 |
|  | IPAstrin\_STLCD\_032114\_01.13207.13207.2 | 3.9921 | 0.4354 | 100.0% | 1843.5721 | 1843.1332 | 3 | 7.122 | 53.3% | 1 | R.GHYTIGKEIIDLVLDR.I | 2 |
|  | IPAstrin\_STLCD\_032114\_01.12092.12092.2 | 3.0486 | 0.3662 | 100.0% | 1086.2122 | 1086.2737 | 7 | 5.666 | 75.0% | 3 | K.EIIDLVLDR.I | 2 |
|  | IPAstrin\_STLCD\_tube2\_032114\_01.04664.04664.3 | 3.4981 | 0.3002 | 99.6% | 1875.3844 | 1876.0824 | 1 | 5.227 | 42.9% | 1 | R.RNLDIERPTYTNLNR.L | 33 |
|  | IPAstrin\_STLCD\_032114\_01.06942.06942.2 | 3.2791 | 0.1294 | 98.7% | 1719.2722 | 1719.8949 | 3 | 5.129 | 53.8% | 2 | R.NLDIERPTYTNLNR.L | 22 |
|  | IPAstrin\_STLCD\_032114\_01.06936.06936.3 | 2.9305 | 0.4019 | 100.0% | 1719.8644 | 1719.8949 | 18 | 5.943 | 40.4% | 2 | R.NLDIERPTYTNLNR.L | 33 |
|  | IPAstrin\_STLCD\_032114\_01.14217.14217.3 | 3.7027 | 0.3777 | 100.0% | 1489.0443 | 1488.7678 | 1 | 6.866 | 50.0% | 2 | R.LISQIVSSITASLR.F | 33 |
|  | IPAstrin\_STLCD\_032114\_01.14244.14244.2 | 4.6874 | 0.4701 | 100.0% | 1489.5521 | 1488.7678 | 1 | 9.068 | 73.1% | 8 | R.LISQIVSSITASLR.F | 22 |
|  | IPAstrin\_STLCD\_032114\_02.09542.09542.2 | 5.1919 | 0.4807 | 100.0% | 2410.6921 | 2410.6885 | 1 | 8.564 | 47.5% | 5 | R.FDGALNVDLTEFQTNLVPYPR.I | 22 |
|  | IPAstrin\_STLCD\_032114\_01.11640.11640.2 | 4.0663 | 0.4653 | 100.0% | 1757.3522 | 1758.0703 | 1 | 8.398 | 66.7% | 9 | R.IHFPLATYAPVISAEK.A | 22 |
|  | IPAstrin\_STLCD\_032114\_01.11562.11562.3 | 3.7045 | 0.3746 | 100.0% | 1757.9944 | 1758.0703 | 4 | 5.616 | 41.7% | 2 | R.IHFPLATYAPVISAEK.A | 33 |
|  | IPAstrin\_STLCD\_tube2\_032114\_01.05884.05884.2 | 2.6398 | 0.3118 | 99.9% | 1015.5522 | 1016.1827 | 1 | 7.056 | 88.9% | 3 | K.DVNAAIATIK.T | 2 |
|  | IPAstrin\_STLCD\_032114\_01.06788.06788.1 | 1.8278 | 0.3649 | 100.0% | 1015.59 | 1016.1827 | 3 | 6.058 | 61.1% | 3 | K.DVNAAIATIK.T | 1 |
|  | IPAstrin\_STLCD\_tube2\_032114\_01.07898.07898.2 | 4.1434 | 0.4055 | 100.0% | 1826.4722 | 1826.1027 | 1 | 7.0 | 70.6% | 5 | K.VGINYQPPTVVPGGDLAK.V | 22 |
|  | IPAstrin\_STLCD\_tube2\_032114\_01.05871.05871.2 | 3.3035 | 0.4303 | 100.0% | 1381.4122 | 1381.6324 | 1 | 6.829 | 65.0% | 4 | R.LDHKFDLMYAK.R | 22 |
|  | IPAstrin\_STLCD\_032114\_01.06853.06853.3 | 3.8515 | 0.3833 | 100.0% | 1381.7943 | 1381.6324 | 1 | 6.871 | 55.0% | 3 | R.LDHKFDLMYAK.R | 33 |
|  | IPAstrin\_STLCD\_tube2\_032114\_02.06575.06575.3 | 3.2218 | 0.2377 | 97.0% | 2486.9343 | 2487.7083 | 2 | 4.323 | 31.2% | 1 | K.RAFVHWYVGEGMEEGEFSEAR.E | 33 |
|  | IPAstrin\_STLCD\_032114\_02.07649.07649.3 | 4.3179 | 0.4047 | 100.0% | 2330.2744 | 2331.5208 | 1 | 7.38 | 40.8% | 4 | R.AFVHWYVGEGMEEGEFSEAR.E | 33 |
|  | IPAstrin\_STLCD\_tube2\_032114\_02.08005.08005.3 | 4.0064 | 0.3519 | 100.0% | 3219.0244 | 3219.524 | 1 | 5.546 | 26.9% | 1 | R.AFVHWYVGEGMEEGEFSEAREDMAALEK.D | 33 |
|  | IPAstrin\_STLCD\_tube2\_032114\_02.05436.05436.2 | 3.2003 | 0.4756 | 100.0% | 2349.2522 | 2350.2751 | 1 | 7.961 | 42.5% | 4 | K.DYEEVGVDSVEGEGEEEGEEY.- | 2 |

Similarities:
gi|17921989|ref|NP\_00(16:7)  

---

|  |  |  |  |  |  |  |  |  |
| --- | --- | --- | --- | --- | --- | --- | --- | --- |
| U | *contaminant\_gi|746301* | 18 | 128 | 49.8% | 269 | 27961 | 6.7 | lysyl endopeptidase (EC 3.4.21.50) - Lysobacter enzymogenes |

| Filename XCorr DeltCN Conf% ObsM+H+ CalcM+H+ SpR ZScore Ion% # Sequence  | | | | | | | | | | | | |
| --- | --- | --- | --- | --- | --- | --- | --- | --- | --- | --- | --- | --- |
| \* | IPAstrin\_STLCD\_tube2\_032114\_01.03860.03860.2 | 6.1099 | 0.6125 | 100.0% | 2261.5522 | 2262.355 | 1 | 11.042 | 54.2% | 16 | R.APGSSSSGANGDGSLAQSQTGAVVR.A | 2 |
| \* | IPAstrin\_STLCD\_tube2\_032114\_01.03866.03866.3 | 4.814 | 0.4169 | 100.0% | 2262.5645 | 2262.355 | 1 | 7.19 | 40.6% | 12 | R.APGSSSSGANGDGSLAQSQTGAVVR.A | 3 |
| \* | IPAstrin\_STLCD\_tube2\_032114\_02.10815.10815.3 | 6.9029 | 0.4974 | 100.0% | 3315.4744 | 3315.6257 | 1 | 9.809 | 30.2% | 11 | R.ATNAASDFTLLELNTAANPAYNLFWAGWDR.R | 3 |
| \* | IPAstrin\_STLCD\_032114\_02.11707.11707.2 | 5.2755 | 0.437 | 100.0% | 3315.912 | 3315.6257 | 1 | 8.75 | 39.7% | 12 | R.ATNAASDFTLLELNTAANPAYNLFWAGWDR.R | 2 |
| \* | IPAstrin\_STLCD\_032114\_01.15300.15300.3 | 6.4816 | 0.5094 | 100.0% | 3472.0144 | 3471.813 | 1 | 10.485 | 29.2% | 12 | R.ATNAASDFTLLELNTAANPAYNLFWAGWDRR.D | 3 |
| \* | IPAstrin\_STLCD\_032114\_01.04134.04134.2 | 4.01 | 0.4071 | 100.0% | 2076.5122 | 2077.2668 | 1 | 6.595 | 55.6% | 2 | R.RDQNFAGATAIHHPNVAEK.R | 2 |
| \* | IPAstrin\_STLCD\_tube2\_032114\_01.03680.03680.3 | 4.7189 | 0.2903 | 100.0% | 2077.6743 | 2077.2668 | 1 | 5.797 | 43.1% | 3 | R.RDQNFAGATAIHHPNVAEK.R | 3 |
| \* | IPAstrin\_STLCD\_tube2\_032114\_01.03596.03596.3 | 4.9427 | 0.3748 | 100.0% | 2232.3843 | 2233.4543 | 1 | 7.39 | 46.1% | 4 | R.RDQNFAGATAIHHPNVAEKR.I | 3 |
| \* | IPAstrin\_STLCD\_032114\_01.03955.03955.2 | 5.2886 | 0.5232 | 100.0% | 2232.4521 | 2233.4543 | 1 | 9.115 | 44.7% | 1 | R.RDQNFAGATAIHHPNVAEKR.I | 2 |
| \* | IPAstrin\_STLCD\_032114\_01.04602.04602.2 | 4.8316 | 0.5173 | 100.0% | 1920.3121 | 1921.0793 | 1 | 9.664 | 64.7% | 6 | R.DQNFAGATAIHHPNVAEK.R | 2 |
| \* | IPAstrin\_STLCD\_032114\_01.04596.04596.3 | 3.0355 | 0.46 | 100.0% | 1920.4744 | 1921.0793 | 1 | 6.734 | 39.7% | 1 | R.DQNFAGATAIHHPNVAEK.R | 3 |
| \* | IPAstrin\_STLCD\_tube2\_032114\_01.03717.03717.2 | 5.2609 | 0.4065 | 100.0% | 2076.5522 | 2077.2668 | 1 | 7.572 | 61.1% | 4 | R.DQNFAGATAIHHPNVAEKR.I | 2 |
| \* | IPAstrin\_STLCD\_032114\_01.04248.04248.3 | 4.0893 | 0.4865 | 100.0% | 2077.4343 | 2077.2668 | 1 | 8.218 | 43.1% | 8 | R.DQNFAGATAIHHPNVAEKR.I | 3 |
| \* | IPAstrin\_STLCD\_032114\_01.04173.04173.2 | 4.6047 | 0.4022 | 100.0% | 1870.3322 | 1870.983 | 1 | 7.316 | 61.1% | 1 | R.VLGQLHGGPSSCSATGADR.S | 2 |
| \* | IPAstrin\_STLCD\_tube2\_032114\_01.03758.03758.3 | 2.7945 | 0.2743 | 97.1% | 1870.4343 | 1870.983 | 4 | 4.815 | 31.9% | 1 | R.VLGQLHGGPSSCSATGADR.S | 3 |
| \* | IPAstrin\_STLCD\_tube2\_032114\_01.06075.06075.2 | 5.1389 | 0.5317 | 100.0% | 1428.0922 | 1428.5443 | 1 | 9.143 | 69.2% | 29 | R.VFTSWTGGGTSATR.L | 2 |
| \* | IPAstrin\_STLCD\_tube2\_032114\_01.05970.05970.1 | 2.2933 | 0.2381 | 100.0% | 1429.54 | 1428.5443 | 23 | 4.062 | 38.5% | 4 | R.VFTSWTGGGTSATR.L | 1 |
| \* | IPAstrin\_STLCD\_032114\_01.14659.14659.2 | 2.4622 | 0.2772 | 99.2% | 2605.0723 | 2605.8174 | 54 | 4.711 | 24.0% | 1 | R.LSDWLDAAGTGAQFIDGLDSTGTPPV.- | 2 |

---

|  |  |  |  |  |  |  |  |  |
| --- | --- | --- | --- | --- | --- | --- | --- | --- |
| U | *gi|5174735|ref|NP\_006* | 24 | 100 | 47.4% | 445 | 49831 | 4.9 | tubulin, beta, 2 [Homo sapiens] |

| Filename XCorr DeltCN Conf% ObsM+H+ CalcM+H+ SpR ZScore Ion% # Sequence  | | | | | | | | | | | | |
| --- | --- | --- | --- | --- | --- | --- | --- | --- | --- | --- | --- | --- |
|  | IPAstrin\_STLCD\_032114\_01.11218.11218.3 | 5.399 | 0.4076 | 100.0% | 3117.8943 | 3118.2996 | 1 | 6.621 | 31.7% | 1 | K.FWEVISDEHGIDPTGTYHGDSDLQLER.I | 3 |
| \* | IPAstrin\_STLCD\_tube2\_032114\_01.04856.04856.2 | 3.4453 | 0.3819 | 100.0% | 1329.0922 | 1329.4521 | 1 | 7.247 | 77.3% | 6 | R.INVYYNEATGGK.Y | 2 |
|  | IPAstrin\_STLCD\_032114\_01.10645.10645.2 | 4.2996 | 0.4243 | 100.0% | 1602.3922 | 1602.8431 | 1 | 8.782 | 71.4% | 4 | R.AVLVDLEPGTMDSVR.S | 2 |
|  | IPAstrin\_STLCD\_tube2\_032114\_01.11814.11814.2 | 4.6413 | 0.5074 | 100.0% | 2798.5122 | 2800.0647 | 1 | 8.552 | 40.0% | 2 | R.SGPFGQIFRPDNFVFGQSGAGNNWAK.G | 22 |
|  | IPAstrin\_STLCD\_tube2\_032114\_01.11876.11876.3 | 7.2984 | 0.5263 | 100.0% | 2800.0745 | 2800.0647 | 1 | 9.699 | 37.0% | 7 | R.SGPFGQIFRPDNFVFGQSGAGNNWAK.G | 33 |
|  | IPAstrin\_STLCD\_tube2\_032114\_01.12326.12326.3 | 3.5451 | 0.3347 | 99.8% | 1960.1044 | 1960.151 | 1 | 6.352 | 39.7% | 3 | K.GHYTEGAELVDSVLDVVR.K | 333 |
|  | IPAstrin\_STLCD\_tube2\_032114\_01.12274.12274.2 | 7.3835 | 0.5359 | 100.0% | 1960.3121 | 1960.151 | 1 | 10.083 | 79.4% | 3 | K.GHYTEGAELVDSVLDVVR.K | 222 |
|  | IPAstrin\_STLCD\_tube2\_032114\_01.11199.11199.3 | 4.8341 | 0.4769 | 100.0% | 2088.1143 | 2088.325 | 1 | 8.071 | 40.3% | 4 | K.GHYTEGAELVDSVLDVVRK.E | 333 |
|  | IPAstrin\_STLCD\_tube2\_032114\_01.07706.07706.2 | 4.1678 | 0.4142 | 100.0% | 1320.0322 | 1320.5896 | 2 | 7.752 | 72.7% | 10 | R.IMNTFSVVPSPK.V | 222 |
|  | IPAstrin\_STLCD\_tube2\_032114\_01.06530.06530.2 | 2.998 | 0.3386 | 100.0% | 1131.3322 | 1131.2767 | 133 | 5.952 | 61.1% | 14 | R.FPGQLNADLR.K | 2222 |
|  | IPAstrin\_STLCD\_tube2\_032114\_01.05058.05058.2 | 2.4459 | 0.1587 | 95.7% | 1259.4521 | 1259.4508 | 80 | 4.442 | 55.0% | 1 | R.FPGQLNADLRK.L | 2222 |
|  | IPAstrin\_STLCD\_tube2\_032114\_01.07702.07702.2 | 3.6879 | 0.347 | 100.0% | 1272.4321 | 1272.5945 | 3 | 7.18 | 65.0% | 4 | R.KLAVNMVPFPR.L | 2222 |
|  | IPAstrin\_STLCD\_tube2\_032114\_01.09071.09071.1 | 2.021 | 0.2752 | 100.0% | 1143.62 | 1144.4204 | 9 | 5.967 | 61.1% | 1 | K.LAVNMVPFPR.L | 1111 |
|  | IPAstrin\_STLCD\_tube2\_032114\_01.09111.09111.2 | 3.5075 | 0.4273 | 100.0% | 1143.6522 | 1144.4204 | 1 | 8.353 | 94.4% | 4 | K.LAVNMVPFPR.L | 2222 |
|  | IPAstrin\_STLCD\_032114\_01.12828.12828.2 | 3.4639 | 0.3347 | 100.0% | 1621.3322 | 1621.9403 | 1 | 7.353 | 80.8% | 6 | R.LHFFMPGFAPLTSR.G | 222 |
|  | IPAstrin\_STLCD\_tube2\_032114\_01.11356.11356.3 | 4.2941 | 0.3603 | 100.0% | 1621.6743 | 1621.9403 | 1 | 6.6 | 50.0% | 2 | R.LHFFMPGFAPLTSR.G | 333 |
|  | IPAstrin\_STLCD\_tube2\_032114\_01.11334.11334.2 | 3.3711 | 0.4597 | 100.0% | 1692.4922 | 1692.9678 | 1 | 8.146 | 67.9% | 3 | R.ALTVPELTQQMFDAK.N | 22 |
|  | IPAstrin\_STLCD\_032114\_01.11383.11383.1 | 1.6593 | 0.4206 | 100.0% | 1039.62 | 1040.2505 | 2 | 6.348 | 68.8% | 3 | R.YLTVAAVFR.G | 11 |
|  | IPAstrin\_STLCD\_tube2\_032114\_01.09626.09626.2 | 3.1367 | 0.4645 | 100.0% | 1040.1721 | 1040.2505 | 1 | 7.776 | 93.8% | 6 | R.YLTVAAVFR.G | 22 |
|  | IPAstrin\_STLCD\_tube2\_032114\_02.05848.05848.3 | 3.8439 | 0.3065 | 99.8% | 1923.8944 | 1925.2405 | 48 | 5.881 | 35.0% | 1 | R.MSMKEVDEQMLNVQNK.N | 33 |
|  | IPAstrin\_STLCD\_tube2\_032114\_01.05205.05205.2 | 3.5221 | 0.2626 | 100.0% | 1447.1921 | 1447.6031 | 2 | 5.781 | 68.2% | 5 | K.EVDEQMLNVQNK.N | 22 |
|  | IPAstrin\_STLCD\_tube2\_032114\_01.10725.10725.2 | 2.6091 | 0.3156 | 99.8% | 1696.4922 | 1697.8877 | 1 | 5.145 | 61.5% | 2 | K.NSSYFVEWIPNNVK.T | 2222 |
|  | IPAstrin\_STLCD\_tube2\_032114\_01.08130.08130.2 | 3.1632 | 0.3675 | 100.0% | 1386.3522 | 1386.6116 | 1 | 6.603 | 70.0% | 2 | K.RISEQFTAMFR.R | 222 |
|  | IPAstrin\_STLCD\_tube2\_032114\_01.09399.09399.2 | 3.8735 | 0.4603 | 100.0% | 1230.9922 | 1230.4241 | 1 | 7.297 | 94.4% | 6 | R.ISEQFTAMFR.R | 222 |

Similarities:
gi|29788785|ref|NP\_82(20:4)  
gi|50592996|ref|NP\_00(13:11)  
gi|14210536|ref|NP\_11(8:16)  

---

|  |  |  |  |  |  |  |  |  |
| --- | --- | --- | --- | --- | --- | --- | --- | --- |
| U | *gi|150456457|ref|NP\_9* | 20 | 70 | 42.7% | 347 | 39929 | 5.6 | HMT1 hnRNP methyltransferase-like 2 isoform 2 [Homo sapiens] |
| U | *gi|154759421|ref|NP\_0* | 20 | 71 | 39.9% | 371 | 42462 | 5.3 | HMT1 hnRNP methyltransferase-like 2 isoform 1 [Homo sapiens] |
| U | *gi|151301219|ref|NP\_9* | 20 | 71 | 41.9% | 353 | 40548 | 5.5 | HMT1 hnRNP methyltransferase-like 2 isoform 3 [Homo sapiens] |

| Filename XCorr DeltCN Conf% ObsM+H+ CalcM+H+ SpR ZScore Ion% # Sequence  | | | | | | | | | | | | |
| --- | --- | --- | --- | --- | --- | --- | --- | --- | --- | --- | --- | --- |
|  | IPAstrin\_STLCD\_tube2\_032114\_01.09431.09431.3 | 4.4006 | 0.2519 | 99.4% | 2764.5544 | 2766.0132 | 1 | 9.574 | 34.5% | 2 | K.DYYFDSYAHFGIHEEMLKDEVR.T | 3 |
|  | IPAstrin\_STLCD\_032114\_01.05617.05617.2 | 3.2948 | 0.2983 | 100.0% | 1351.3722 | 1351.6322 | 6 | 5.861 | 63.6% | 3 | K.ANKLDHVVTIIK.G | 2 |
|  | IPAstrin\_STLCD\_tube2\_032114\_01.04875.04875.3 | 4.2498 | 0.4424 | 100.0% | 1351.6144 | 1351.6322 | 2 | 7.833 | 54.5% | 4 | K.ANKLDHVVTIIK.G | 3 |
|  | IPAstrin\_STLCD\_tube2\_032114\_01.04883.04883.2 | 3.4928 | 0.3837 | 100.0% | 1356.5322 | 1356.559 | 1 | 7.128 | 72.7% | 2 | K.GKVEEVELPVEK.V | 2 |
|  | IPAstrin\_STLCD\_tube2\_032114\_01.05945.05945.2 | 2.9302 | 0.256 | 99.9% | 1171.1522 | 1171.333 | 67 | 6.027 | 66.7% | 2 | K.VEEVELPVEK.V | 2 |
|  | IPAstrin\_STLCD\_032114\_01.12090.12090.2 | 3.9897 | 0.3952 | 100.0% | 1644.3722 | 1643.8827 | 1 | 6.291 | 76.9% | 3 | R.DKWLAPDGLIFPDR.A | 2 |
|  | IPAstrin\_STLCD\_tube2\_032114\_01.11325.11325.2 | 3.6807 | 0.4203 | 100.0% | 1400.1522 | 1400.6201 | 1 | 7.516 | 72.7% | 2 | K.WLAPDGLIFPDR.A | 2 |
|  | IPAstrin\_STLCD\_tube2\_032114\_01.07368.07368.1 | 2.7362 | 0.3227 | 100.0% | 1252.56 | 1252.4099 | 7 | 6.003 | 55.0% | 4 | R.ATLYVTAIEDR.Q | 1 |
|  | IPAstrin\_STLCD\_tube2\_032114\_01.07307.07307.2 | 3.9093 | 0.3354 | 100.0% | 1253.9521 | 1252.4099 | 1 | 6.732 | 75.0% | 9 | R.ATLYVTAIEDR.Q | 2 |
|  | IPAstrin\_STLCD\_tube2\_032114\_01.08081.08081.3 | 3.3624 | 0.3005 | 99.4% | 1637.7843 | 1637.914 | 12 | 6.164 | 37.5% | 1 | K.DVAIKEPLVDVVDPK.Q | 3 |
|  | IPAstrin\_STLCD\_032114\_01.09936.09936.2 | 3.9911 | 0.4052 | 100.0% | 1638.5322 | 1637.914 | 1 | 6.876 | 60.7% | 2 | K.DVAIKEPLVDVVDPK.Q | 2 |
|  | IPAstrin\_STLCD\_tube2\_032114\_01.13178.13178.2 | 3.6198 | 0.343 | 100.0% | 2228.672 | 2229.5027 | 2 | 6.818 | 47.1% | 2 | K.RNDYVHALVAYFNIEFTR.C | 2 |
|  | IPAstrin\_STLCD\_tube2\_032114\_02.09389.09389.3 | 4.8908 | 0.3637 | 100.0% | 2229.0842 | 2229.5027 | 1 | 8.226 | 47.1% | 2 | K.RNDYVHALVAYFNIEFTR.C | 3 |
|  | IPAstrin\_STLCD\_tube2\_032114\_01.14487.14487.2 | 5.7627 | 0.5557 | 100.0% | 2072.612 | 2073.3152 | 1 | 11.027 | 78.1% | 5 | R.NDYVHALVAYFNIEFTR.C | 2 |
|  | IPAstrin\_STLCD\_tube2\_032114\_01.14486.14486.3 | 4.8082 | 0.4008 | 100.0% | 2073.5942 | 2073.3152 | 1 | 6.532 | 43.8% | 3 | R.NDYVHALVAYFNIEFTR.C | 3 |
|  | IPAstrin\_STLCD\_032114\_01.07470.07470.2 | 3.6681 | 0.4153 | 100.0% | 1725.3322 | 1725.8547 | 1 | 7.654 | 53.6% | 5 | R.TGFSTSPESPYTHWK.Q | 2 |
|  | IPAstrin\_STLCD\_032114\_01.07500.07500.3 | 2.7093 | 0.3218 | 98.8% | 1725.4443 | 1725.8547 | 1 | 5.515 | 39.3% | 1 | R.TGFSTSPESPYTHWK.Q | 3 |
|  | IPAstrin\_STLCD\_032114\_02.08531.08531.2 | 3.8246 | 0.4444 | 100.0% | 1637.2322 | 1637.8878 | 1 | 7.233 | 62.5% | 7 | K.QTVFYMEDYLTVK.T | 2 |
|  | IPAstrin\_STLCD\_032114\_01.09300.09300.2 | 4.741 | 0.4421 | 100.0% | 1721.8322 | 1721.969 | 1 | 7.458 | 63.3% | 6 | K.TGEEIFGTIGMRPNAK.N | 2 |
|  | IPAstrin\_STLCD\_tube2\_032114\_01.07550.07550.3 | 3.2095 | 0.3977 | 100.0% | 1722.1144 | 1721.969 | 9 | 6.184 | 33.3% | 5 | K.TGEEIFGTIGMRPNAK.N | 3 |

---

|  |  |  |  |  |  |  |  |  |
| --- | --- | --- | --- | --- | --- | --- | --- | --- |
| U | *gi|12667788|ref|NP\_00* | 75 | 167 | 41.4% | 1960 | 226530 | 5.6 | myosin, heavy polypeptide 9, non-muscle [Homo sapiens] |

| Filename XCorr DeltCN Conf% ObsM+H+ CalcM+H+ SpR ZScore Ion% # Sequence  | | | | | | | | | | | | |
| --- | --- | --- | --- | --- | --- | --- | --- | --- | --- | --- | --- | --- |
| \* | IPAstrin\_STLCD\_tube2\_032114\_01.10144.10144.2 | 4.4609 | 0.4216 | 100.0% | 1673.3922 | 1673.8687 | 1 | 7.908 | 78.6% | 3 | K.NFINNPLAQADWAAK.K | 2 |
| \* | IPAstrin\_STLCD\_tube2\_032114\_01.05398.05398.2 | 2.2498 | 0.3302 | 99.8% | 1072.1122 | 1072.2926 | 4 | 5.429 | 62.5% | 1 | K.KLVWVPSDK.S | 2 |
| \* | IPAstrin\_STLCD\_tube2\_032114\_01.10774.10774.3 | 5.9838 | 0.4647 | 100.0% | 2790.3245 | 2790.0967 | 1 | 8.105 | 36.0% | 1 | K.SGFEPASLKEEVGEEAIVELVENGKK.V | 3 |
| \* | IPAstrin\_STLCD\_tube2\_032114\_01.10937.10937.2 | 3.1257 | 0.4601 | 100.0% | 1728.3722 | 1728.9978 | 1 | 8.47 | 73.1% | 1 | K.NLPIYSEEIVEMYK.G | 2 |
| \* | IPAstrin\_STLCD\_032114\_01.07060.07060.3 | 3.8948 | 0.461 | 100.0% | 1916.6344 | 1916.1614 | 1 | 7.571 | 46.7% | 11 | R.HEMPPHIYAITDTAYR.S | 3 |
| \* | IPAstrin\_STLCD\_tube2\_032114\_01.07534.07534.2 | 4.0575 | 0.416 | 100.0% | 1479.2722 | 1479.719 | 1 | 7.497 | 79.2% | 2 | K.VIQYLAYVASSHK.S | 2 |
|  | IPAstrin\_STLCD\_tube2\_032114\_01.12201.12201.2 | 4.1223 | 0.353 | 100.0% | 1728.5521 | 1728.0012 | 1 | 6.381 | 66.7% | 2 | R.QLLQANPILEAFGNAK.T | 2 |
| \* | IPAstrin\_STLCD\_tube2\_032114\_02.09784.09784.2 | 5.0059 | 0.4517 | 100.0% | 2385.9922 | 2386.7068 | 1 | 8.42 | 47.5% | 1 | R.INFDVNGYIVGANIETYLLEK.S | 2 |
| \* | IPAstrin\_STLCD\_tube2\_032114\_01.12237.12237.3 | 4.643 | 0.3143 | 100.0% | 1996.2544 | 1997.3037 | 6 | 6.339 | 42.2% | 3 | R.TFHIFYYLLSGAGEHLK.T | 3 |
| \* | IPAstrin\_STLCD\_tube2\_032114\_01.07602.07602.2 | 2.9713 | 0.4182 | 100.0% | 1206.1921 | 1206.3812 | 1 | 6.933 | 77.8% | 2 | K.TDLLLEPYNK.Y | 2 |
| \* | IPAstrin\_STLCD\_032114\_01.11581.11581.3 | 4.1223 | 0.2922 | 99.6% | 3009.5942 | 3012.4 | 4 | 5.049 | 23.0% | 2 | R.FLSNGHVTIPGQQDKDMFQETMEAMR.I | 3 |
| \* | IPAstrin\_STLCD\_tube2\_032114\_01.10041.10041.2 | 3.8475 | 0.4364 | 100.0% | 1616.3121 | 1616.9313 | 1 | 8.057 | 84.6% | 5 | R.IMGIPEEEQMGLLR.V | 2 |
| \* | IPAstrin\_STLCD\_tube2\_032114\_01.12594.12594.2 | 4.7868 | 0.4715 | 100.0% | 1488.5521 | 1487.8259 | 1 | 7.268 | 80.8% | 2 | R.VISGVLQLGNIVFK.K | 2 |
|  | IPAstrin\_STLCD\_032114\_01.03758.03758.2 | 5.1367 | 0.4783 | 100.0% | 1592.4321 | 1592.6776 | 1 | 7.34 | 71.4% | 1 | R.NTDQASMPDNTAAQK.V | 2 |
| \* | IPAstrin\_STLCD\_032114\_01.11172.11172.2 | 4.1068 | 0.4371 | 100.0% | 1572.3121 | 1572.8044 | 1 | 8.774 | 76.9% | 4 | K.VSHLLGINVTDFTR.G | 2 |
| \* | IPAstrin\_STLCD\_032114\_01.11180.11180.3 | 3.8947 | 0.3368 | 100.0% | 1573.4043 | 1572.8044 | 7 | 5.297 | 46.2% | 2 | K.VSHLLGINVTDFTR.G | 3 |
|  | IPAstrin\_STLCD\_032114\_01.08546.08546.2 | 2.0209 | 0.2799 | 97.5% | 1295.8322 | 1295.4467 | 58 | 4.588 | 55.0% | 1 | K.ADFCIIHYAGK.V | 22 |
|  | IPAstrin\_STLCD\_032114\_01.09322.09322.2 | 2.6793 | 0.2294 | 99.4% | 1398.1122 | 1398.6166 | 1 | 4.657 | 80.0% | 2 | K.VDYKADEWLMK.N | 22 |
| \* | IPAstrin\_STLCD\_032114\_01.13068.13068.2 | 4.4652 | 0.3529 | 100.0% | 2019.6522 | 2019.3636 | 1 | 7.899 | 50.0% | 2 | R.IIGLDQVAGMSETALPGAFK.T | 2 |
|  | IPAstrin\_STLCD\_032114\_01.10813.10813.2 | 3.0077 | 0.3601 | 100.0% | 1319.5122 | 1319.5468 | 33 | 5.895 | 65.0% | 2 | K.LDPHLVLDQLR.C | 22 |
| \* | IPAstrin\_STLCD\_tube2\_032114\_01.06681.06681.2 | 1.9017 | 0.3772 | 99.8% | 925.1922 | 925.07513 | 1 | 6.775 | 75.0% | 1 | R.VVFQEFR.Q | 2 |
| \* | IPAstrin\_STLCD\_032114\_01.07851.07851.2 | 2.6044 | 0.1832 | 97.5% | 1559.1122 | 1559.8058 | 22 | 4.05 | 58.3% | 1 | R.QRYEILTPNSIPK.G | 2 |
| \* | IPAstrin\_STLCD\_tube2\_032114\_01.07512.07512.2 | 3.0319 | 0.2528 | 99.9% | 1276.3522 | 1275.4875 | 6 | 5.402 | 60.0% | 2 | R.YEILTPNSIPK.G | 2 |
| \* | IPAstrin\_STLCD\_032114\_01.08948.08948.2 | 3.2905 | 0.3995 | 100.0% | 1194.1322 | 1194.33 | 14 | 7.508 | 72.2% | 4 | K.ALELDSNLYR.I | 2 |
|  | IPAstrin\_STLCD\_032114\_01.06412.06412.2 | 3.7363 | 0.4361 | 100.0% | 1224.1122 | 1224.3591 | 1 | 7.776 | 75.0% | 4 | R.AGVLAHLEEER.D | 22 |
| \* | IPAstrin\_STLCD\_tube2\_032114\_01.09692.09692.2 | 4.6119 | 0.4312 | 100.0% | 1752.3722 | 1753.0358 | 1 | 8.378 | 71.4% | 4 | R.LTEMETLQSQLMAEK.L | 2 |
| \* | IPAstrin\_STLCD\_tube2\_032114\_01.09893.09893.2 | 5.797 | 0.4326 | 100.0% | 2333.2922 | 2334.4736 | 1 | 10.273 | 66.7% | 1 | K.MQQNIQELEEQLEEEESAR.Q | 2 |
| \* | IPAstrin\_STLCD\_tube2\_032114\_01.06824.06824.2 | 4.7599 | 0.4678 | 100.0% | 1654.2122 | 1654.7681 | 1 | 8.051 | 73.1% | 6 | R.IAEFTTNLTEEEEK.S | 2 |
| \* | IPAstrin\_STLCD\_tube2\_032114\_01.05838.05838.2 | 3.7551 | 0.3983 | 100.0% | 1869.9922 | 1870.0203 | 1 | 7.28 | 53.3% | 1 | R.IAEFTTNLTEEEEKSK.S | 2 |
| \* | IPAstrin\_STLCD\_032114\_01.05085.05085.2 | 3.503 | 0.4777 | 100.0% | 1586.2322 | 1586.76 | 1 | 8.8 | 70.8% | 1 | K.NKHEAMITDLEER.L | 2 |
| \* | IPAstrin\_STLCD\_tube2\_032114\_01.04593.04593.3 | 3.1764 | 0.336 | 99.8% | 1587.0844 | 1586.76 | 1 | 5.912 | 47.9% | 1 | K.NKHEAMITDLEER.L | 3 |
| \* | IPAstrin\_STLCD\_032114\_01.06116.06116.2 | 2.8622 | 0.4507 | 100.0% | 1344.0721 | 1344.4822 | 1 | 7.555 | 80.0% | 3 | K.HEAMITDLEER.L | 2 |
| \* | IPAstrin\_STLCD\_tube2\_032114\_01.10708.10708.2 | 4.6298 | 0.5133 | 100.0% | 2034.3522 | 2035.126 | 1 | 9.875 | 68.8% | 1 | R.ELESQISELQEDLESER.A | 2 |
| \* | IPAstrin\_STLCD\_tube2\_032114\_01.15860.15860.2 | 4.8689 | 0.5774 | 100.0% | 3018.0122 | 3019.2434 | 1 | 10.566 | 48.1% | 2 | R.DLGEELEALKTELEDTLDSTAAQQELR.S | 2 |
| \* | IPAstrin\_STLCD\_032114\_01.06961.06961.3 | 2.855 | 0.2976 | 98.5% | 2043.1743 | 2044.2439 | 1 | 5.085 | 34.4% | 1 | K.TLEEEAKTHEAQIQEMR.Q | 3 |
| \* | IPAstrin\_STLCD\_tube2\_032114\_01.05771.05771.2 | 2.7579 | 0.1388 | 95.5% | 2043.5922 | 2044.2439 | 372 | 3.888 | 34.4% | 1 | K.TLEEEAKTHEAQIQEMR.Q | 2 |
| \* | IPAstrin\_STLCD\_032114\_01.10035.10035.2 | 2.6057 | 0.2341 | 98.5% | 1841.9521 | 1840.9847 | 1 | 5.201 | 56.7% | 1 | K.HSQAVEELAEQLEQTK.R | 2 |
| \* | IPAstrin\_STLCD\_tube2\_032114\_01.07424.07424.3 | 5.439 | 0.5173 | 100.0% | 1996.1344 | 1997.1722 | 1 | 8.786 | 45.3% | 3 | K.HSQAVEELAEQLEQTKR.V | 3 |
| \* | IPAstrin\_STLCD\_tube2\_032114\_01.04605.04605.2 | 2.5252 | 0.2728 | 99.3% | 1730.1522 | 1730.8723 | 3 | 4.573 | 50.0% | 1 | K.QTLENERGELANEVK.V | 2 |
| \* | IPAstrin\_STLCD\_tube2\_032114\_01.05093.05093.2 | 3.4499 | 0.2863 | 100.0% | 1414.0721 | 1413.6573 | 2 | 5.607 | 72.7% | 2 | K.KVEAQLQELQVK.F | 2 |
| \* | IPAstrin\_STLCD\_032114\_01.07034.07034.2 | 3.1099 | 0.2914 | 100.0% | 1285.3922 | 1285.4833 | 1 | 5.986 | 90.0% | 2 | K.VEAQLQELQVK.F | 2 |
| \* | IPAstrin\_STLCD\_032114\_01.12260.12260.2 | 5.7578 | 0.5323 | 100.0% | 1946.4122 | 1947.1498 | 1 | 9.399 | 67.6% | 3 | K.LQVELDNVTGLLSQSDSK.S | 2 |
| \* | IPAstrin\_STLCD\_tube2\_032114\_01.12551.12551.3 | 3.7816 | 0.3121 | 99.8% | 2493.9844 | 2494.631 | 8 | 6.225 | 28.8% | 1 | K.DFSALESQLQDTQELLQEENR.Q | 3 |
| \* | IPAstrin\_STLCD\_tube2\_032114\_01.12567.12567.2 | 5.3801 | 0.4729 | 100.0% | 2494.172 | 2494.631 | 1 | 8.981 | 62.5% | 1 | K.DFSALESQLQDTQELLQEENR.Q | 2 |
| \* | IPAstrin\_STLCD\_032114\_01.03858.03858.2 | 3.0093 | 0.1372 | 98.5% | 1492.8121 | 1493.6598 | 2 | 4.491 | 68.2% | 1 | K.LKQVEDEKNSFR.E | 2 |
| \* | IPAstrin\_STLCD\_032114\_01.04171.04171.2 | 4.0382 | 0.3407 | 100.0% | 1855.2722 | 1855.953 | 1 | 6.24 | 60.7% | 2 | R.EQLEEEEEAKHNLEK.Q | 2 |
| \* | IPAstrin\_STLCD\_tube2\_032114\_01.04698.04698.2 | 2.7191 | 0.3714 | 100.0% | 1425.4122 | 1426.674 | 30 | 6.355 | 50.0% | 1 | K.QIATLHAQVADMK.K | 2 |
| \* | IPAstrin\_STLCD\_tube2\_032114\_01.12612.12612.3 | 5.8078 | 0.2671 | 100.0% | 2208.7744 | 2208.436 | 1 | 6.99 | 45.6% | 1 | K.TRLQQELDDLLVDLDHQR.Q | 3 |
| \* | IPAstrin\_STLCD\_032114\_01.12810.12810.3 | 3.5598 | 0.2619 | 99.3% | 1951.1044 | 1951.1436 | 1 | 5.028 | 48.3% | 3 | R.LQQELDDLLVDLDHQR.Q | 3 |
| \* | IPAstrin\_STLCD\_tube2\_032114\_01.11459.11459.2 | 5.1851 | 0.3283 | 100.0% | 1951.3322 | 1951.1436 | 1 | 8.527 | 76.7% | 2 | R.LQQELDDLLVDLDHQR.Q | 2 |
|  | IPAstrin\_STLCD\_032114\_01.05947.05947.2 | 2.6189 | 0.1364 | 97.3% | 1220.7722 | 1221.3959 | 34 | 4.604 | 55.6% | 2 | K.KFDQLLAEEK.T | 22 |
|  | IPAstrin\_STLCD\_tube2\_032114\_01.05541.05541.2 | 2.6717 | 0.0723 | 95.3% | 1093.1322 | 1093.2218 | 1 | 4.929 | 87.5% | 4 | K.FDQLLAEEK.T | 22 |
| \* | IPAstrin\_STLCD\_032114\_02.05019.05019.3 | 2.8431 | 0.324 | 99.4% | 1647.6843 | 1647.8407 | 1 | 5.308 | 44.2% | 1 | R.ALEEAMEQKAELER.L | 3 |
| \* | IPAstrin\_STLCD\_tube2\_032114\_01.06102.06102.2 | 2.6156 | 0.4034 | 100.0% | 1171.2322 | 1171.3229 | 1 | 6.236 | 72.2% | 1 | R.TEMEDLMSSK.D | 2 |
| \* | IPAstrin\_STLCD\_032114\_01.06878.06878.2 | 4.2679 | 0.4572 | 100.0% | 1685.7522 | 1685.8586 | 1 | 7.961 | 75.0% | 2 | R.TEMEDLMSSKDDVGK.S | 2 |
| \* | IPAstrin\_STLCD\_tube2\_032114\_01.05054.05054.2 | 3.192 | 0.4026 | 100.0% | 1205.1721 | 1205.3685 | 4 | 6.86 | 66.7% | 3 | R.ALEQQVEEMK.T | 2 |
| \* | IPAstrin\_STLCD\_tube2\_032114\_01.14079.14079.3 | 4.3547 | 0.37 | 100.0% | 3149.0044 | 3149.4048 | 1 | 7.037 | 26.9% | 2 | R.ALEQQVEEMKTQLEELEDELQATEDAK.L | 3 |
|  | IPAstrin\_STLCD\_tube2\_032114\_01.10646.10646.2 | 6.0427 | 0.5791 | 100.0% | 1962.1721 | 1963.0594 | 1 | 10.358 | 71.9% | 1 | K.TQLEELEDELQATEDAK.L | 22 |
| \* | IPAstrin\_STLCD\_tube2\_032114\_01.07736.07736.2 | 2.7422 | 0.304 | 99.9% | 1314.8722 | 1315.6171 | 142 | 5.998 | 60.0% | 2 | K.LRLEVNLQAMK.A | 2 |
| \* | IPAstrin\_STLCD\_tube2\_032114\_01.06915.06915.2 | 2.8376 | 0.3012 | 100.0% | 1046.0721 | 1046.2701 | 4 | 6.467 | 68.8% | 4 | R.LEVNLQAMK.A | 2 |
| \* | IPAstrin\_STLCD\_tube2\_032114\_01.04107.04107.2 | 2.8466 | 0.304 | 99.9% | 1251.0122 | 1251.3075 | 1 | 6.546 | 88.9% | 1 | R.EMEAELEDER.K | 2 |
| \* | IPAstrin\_STLCD\_032114\_01.13455.13455.2 | 5.6666 | 0.5047 | 100.0% | 2049.3523 | 2050.3064 | 1 | 9.365 | 64.7% | 2 | K.SMEAEMIQLQEELAAAER.A | 2 |
| \* | IPAstrin\_STLCD\_tube2\_032114\_01.12729.12729.3 | 3.7962 | 0.3963 | 100.0% | 2050.5842 | 2050.3064 | 1 | 6.548 | 44.1% | 1 | K.SMEAEMIQLQEELAAAER.A | 3 |
| \* | IPAstrin\_STLCD\_tube2\_032114\_01.05429.05429.2 | 3.7043 | 0.3781 | 100.0% | 2089.3523 | 2090.168 | 1 | 6.913 | 55.6% | 1 | R.QAQQERDELADEIANSSGK.G | 2 |
| \* | IPAstrin\_STLCD\_032114\_01.06252.06252.3 | 4.5219 | 0.4448 | 100.0% | 2090.6042 | 2090.168 | 1 | 7.266 | 41.7% | 4 | R.QAQQERDELADEIANSSGK.G | 3 |
| \* | IPAstrin\_STLCD\_tube2\_032114\_01.05769.05769.2 | 3.238 | 0.4667 | 100.0% | 1348.3922 | 1349.394 | 1 | 7.681 | 70.8% | 1 | R.DELADEIANSSGK.G | 2 |
| \* | IPAstrin\_STLCD\_tube2\_032114\_01.09208.09208.2 | 5.6915 | 0.4986 | 100.0% | 2472.652 | 2473.6099 | 1 | 9.254 | 62.5% | 2 | R.IAQLEEELEEEQGNTELINDR.L | 2 |
| \* | IPAstrin\_STLCD\_032114\_01.11035.11035.3 | 5.0125 | 0.4159 | 100.0% | 2474.2744 | 2473.6099 | 1 | 7.487 | 40.0% | 4 | R.IAQLEEELEEEQGNTELINDR.L | 3 |
| \* | IPAstrin\_STLCD\_032114\_01.11091.11091.2 | 5.4438 | 0.4887 | 100.0% | 1871.4122 | 1871.0574 | 1 | 8.338 | 70.0% | 4 | K.ANLQIDQINTDLNLER.S | 2 |
| \* | IPAstrin\_STLCD\_tube2\_032114\_01.05985.05985.2 | 4.8934 | 0.4615 | 100.0% | 1531.0521 | 1531.6598 | 1 | 8.404 | 79.2% | 4 | K.IAQLEEQLDNETK.E | 2 |
| \* | IPAstrin\_STLCD\_tube2\_032114\_01.05679.05679.2 | 4.7384 | 0.415 | 100.0% | 1816.3722 | 1816.9628 | 1 | 7.693 | 75.0% | 1 | K.IAQLEEQLDNETKER.Q | 2 |
| \* | IPAstrin\_STLCD\_tube2\_032114\_01.05722.05722.3 | 2.6455 | 0.2593 | 95.8% | 1817.3344 | 1816.9628 | 243 | 4.563 | 30.4% | 1 | K.IAQLEEQLDNETKER.Q | 3 |
| \* | IPAstrin\_STLCD\_032114\_01.06289.06289.3 | 2.9112 | 0.2544 | 98.0% | 1600.1943 | 1599.8278 | 3 | 5.738 | 43.8% | 1 | K.LKDVLLQVDDERR.N | 3 |
| \* | IPAstrin\_STLCD\_tube2\_032114\_01.04616.04616.2 | 4.5064 | 0.5259 | 100.0% | 1566.1322 | 1566.6367 | 1 | 9.488 | 73.1% | 5 | R.ELEDATETADAMNR.E | 2 |
| \* | IPAstrin\_STLCD\_tube2\_032114\_01.07125.07125.2 | 3.785 | 0.2874 | 100.0% | 1156.2522 | 1156.3732 | 1 | 6.278 | 88.9% | 5 | R.RGDLPFVVPR.R | 2 |

Similarities:
gi|41406064|ref|NP\_00(7:68)  

---

|  |  |  |  |  |  |  |  |  |
| --- | --- | --- | --- | --- | --- | --- | --- | --- |
| U | *gi|4501885|ref|NP\_001* | 15 | 63 | 39.5% | 375 | 41737 | 5.5 | beta actin [Homo sapiens] |
| U | *gi|4501887|ref|NP\_001* | 15 | 62 | 39.5% | 375 | 41793 | 5.5 | actin, gamma 1 propeptide [Homo sapiens] |

| Filename XCorr DeltCN Conf% ObsM+H+ CalcM+H+ SpR ZScore Ion% # Sequence  | | | | | | | | | | | | |
| --- | --- | --- | --- | --- | --- | --- | --- | --- | --- | --- | --- | --- |
|  | IPAstrin\_STLCD\_tube2\_032114\_01.06184.06184.2 | 2.9176 | 0.3032 | 99.9% | 1200.3121 | 1199.4415 | 1 | 5.206 | 70.0% | 5 | R.AVFPSIVGRPR.H | 22 |
|  | IPAstrin\_STLCD\_032114\_01.06444.06444.2 | 3.1096 | 0.3417 | 100.0% | 1516.1122 | 1516.7019 | 1 | 5.977 | 65.0% | 7 | K.IWHHTFYNELR.V | 22 |
|  | IPAstrin\_STLCD\_tube2\_032114\_01.05558.05558.3 | 3.3425 | 0.2701 | 99.4% | 1516.9143 | 1516.7019 | 1 | 6.452 | 57.5% | 3 | K.IWHHTFYNELR.V | 33 |
|  | IPAstrin\_STLCD\_032114\_01.08844.08844.2 | 4.5654 | 0.3904 | 100.0% | 1954.5122 | 1955.2615 | 1 | 8.532 | 61.8% | 6 | R.VAPEEHPVLLTEAPLNPK.A | 2 |
|  | IPAstrin\_STLCD\_tube2\_032114\_01.10120.10120.3 | 6.6034 | 0.5099 | 100.0% | 3185.2444 | 3185.622 | 1 | 8.504 | 33.6% | 4 | R.TTGIVMDSGDGVTHTVPIYEGYALPHAILR.L | 3 |
|  | IPAstrin\_STLCD\_tube2\_032114\_01.10210.10210.2 | 2.9411 | 0.1875 | 99.1% | 1624.4521 | 1624.8927 | 2 | 5.353 | 53.8% | 2 | R.LDLAGRDLTDYLMK.I | 22 |
|  | IPAstrin\_STLCD\_032114\_01.10946.10946.1 | 2.0232 | 0.2376 | 100.0% | 998.51 | 999.167 | 1 | 5.803 | 71.4% | 2 | R.DLTDYLMK.I | 11 |
|  | IPAstrin\_STLCD\_032114\_01.10965.10965.2 | 2.0349 | 0.2647 | 98.4% | 999.0722 | 999.167 | 11 | 5.496 | 64.3% | 2 | R.DLTDYLMK.I | 22 |
|  | IPAstrin\_STLCD\_032114\_01.15333.15333.2 | 2.7053 | 0.3393 | 99.9% | 1611.5322 | 1611.8939 | 1 | 6.323 | 58.3% | 1 | R.DLTDYLMKILTER.G | 22 |
|  | IPAstrin\_STLCD\_tube2\_032114\_01.04346.04346.2 | 3.1013 | 0.4735 | 100.0% | 1133.1122 | 1133.2029 | 1 | 8.625 | 83.3% | 8 | R.GYSFTTTAER.E | 2 |
|  | IPAstrin\_STLCD\_tube2\_032114\_01.09428.09428.2 | 4.9451 | 0.3138 | 100.0% | 1792.4521 | 1791.9554 | 1 | 8.164 | 80.0% | 6 | K.SYELPDGQVITIGNER.F | 22 |
|  | IPAstrin\_STLCD\_tube2\_032114\_01.08510.08510.3 | 5.3106 | 0.4656 | 100.0% | 2345.0344 | 2344.6448 | 1 | 6.84 | 36.9% | 4 | R.KDLYANTVLSGGTTMYPGIADR.M | 3 |
|  | IPAstrin\_STLCD\_tube2\_032114\_02.07278.07278.2 | 4.6427 | 0.5671 | 100.0% | 2215.5723 | 2216.4705 | 1 | 9.336 | 52.5% | 4 | K.DLYANTVLSGGTTMYPGIADR.M | 2 |
|  | IPAstrin\_STLCD\_032114\_01.06277.06277.1 | 2.747 | 0.455 | 100.0% | 1161.58 | 1162.3868 | 1 | 8.392 | 55.0% | 3 | K.EITALAPSTMK.I | 11 |
|  | IPAstrin\_STLCD\_032114\_01.06288.06288.2 | 2.6163 | 0.3984 | 100.0% | 1161.9321 | 1162.3868 | 1 | 6.234 | 70.0% | 6 | K.EITALAPSTMK.I | 22 |

Similarities:
gi|4501881|ref|NP\_001(10:5)  

---

|  |  |  |  |  |  |  |  |  |
| --- | --- | --- | --- | --- | --- | --- | --- | --- |
| U | *gi|14043072|ref|NP\_11* | 12 | 30 | 37.7% | 353 | 37430 | 8.9 | heterogeneous nuclear ribonucleoprotein A2/B1 isoform B1 [Homo sapiens] |
| U | *gi|4504447|ref|NP\_002* | 12 | 30 | 39.0% | 341 | 36006 | 8.6 | heterogeneous nuclear ribonucleoprotein A2/B1 isoform A2 [Homo sapiens] |

| Filename XCorr DeltCN Conf% ObsM+H+ CalcM+H+ SpR ZScore Ion% # Sequence  | | | | | | | | | | | | |
| --- | --- | --- | --- | --- | --- | --- | --- | --- | --- | --- | --- | --- |
|  | IPAstrin\_STLCD\_032114\_01.11965.11965.2 | 3.1288 | 0.3018 | 99.9% | 1928.1721 | 1928.1925 | 21 | 4.931 | 34.4% | 2 | R.KLFIGGLSFETTEESLR.N | 2 |
|  | IPAstrin\_STLCD\_tube2\_032114\_01.11819.11819.2 | 4.6651 | 0.375 | 100.0% | 1800.1522 | 1800.0184 | 1 | 7.715 | 70.0% | 6 | K.LFIGGLSFETTEESLR.N | 2 |
|  | IPAstrin\_STLCD\_tube2\_032114\_01.05405.05405.2 | 2.2469 | 0.1766 | 97.2% | 1089.3121 | 1088.1644 | 368 | 4.933 | 64.3% | 1 | R.NYYEQWGK.L | 2 |
|  | IPAstrin\_STLCD\_tube2\_032114\_01.04398.04398.3 | 2.9257 | 0.3534 | 99.6% | 1881.9243 | 1881.0984 | 113 | 6.146 | 30.0% | 1 | K.LFVGGIKEDTEEHHLR.D | 3 |
|  | IPAstrin\_STLCD\_tube2\_032114\_01.08290.08290.2 | 3.2474 | 0.4952 | 100.0% | 1189.2522 | 1189.3513 | 1 | 8.088 | 77.8% | 2 | K.IDTIEIITDR.Q | 2 |
|  | IPAstrin\_STLCD\_032114\_01.10941.10941.2 | 3.9881 | 0.4384 | 100.0% | 1696.2722 | 1696.8132 | 1 | 8.306 | 60.7% | 2 | R.GFGFVTFDDHDPVDK.I | 2 |
|  | IPAstrin\_STLCD\_tube2\_032114\_01.10400.10400.3 | 3.6215 | 0.3865 | 100.0% | 2278.9744 | 2278.5693 | 11 | 6.446 | 28.9% | 2 | R.GFGFVTFDDHDPVDKIVLQK.Y | 3 |
|  | IPAstrin\_STLCD\_032114\_01.03693.03693.2 | 3.2457 | 0.2952 | 100.0% | 1411.2522 | 1411.5198 | 1 | 5.986 | 72.7% | 2 | K.YHTINGHNAEVR.K | 2 |
|  | IPAstrin\_STLCD\_tube2\_032114\_01.04479.04479.2 | 2.8007 | 0.2537 | 99.9% | 1014.3122 | 1014.0421 | 2 | 4.961 | 77.8% | 1 | R.GGNFGFGDSR.G | 2 |
|  | IPAstrin\_STLCD\_tube2\_032114\_01.04839.04839.2 | 2.9828 | 0.4135 | 100.0% | 1378.1721 | 1378.4465 | 1 | 6.539 | 53.6% | 7 | R.GGGGNFGPGPGSNFR.G | 2 |
|  | IPAstrin\_STLCD\_032114\_01.05509.05509.3 | 3.1916 | 0.2528 | 97.0% | 2190.1743 | 2191.2554 | 304 | 4.527 | 22.9% | 1 | R.NMGGPYGGGNYGPGGSGGSGGYGGR.S | 3 |
|  | IPAstrin\_STLCD\_tube2\_032114\_01.04822.04822.2 | 5.6193 | 0.649 | 100.0% | 2190.412 | 2191.2554 | 1 | 11.332 | 43.8% | 3 | R.NMGGPYGGGNYGPGGSGGSGGYGGR.S | 2 |

---

|  |  |  |  |  |  |  |  |  |
| --- | --- | --- | --- | --- | --- | --- | --- | --- |
| U | *gi|10800140|ref|NP\_06* | 4 | 10 | 37.3% | 126 | 13950 | 10.3 | histone cluster 1, H2bb [Homo sapiens] |
| U | *gi|4504277|ref|NP\_003* | 4 | 10 | 37.3% | 126 | 13920 | 10.3 | histone cluster 2, H2be [Homo sapiens] |
| U | *gi|20336754|ref|NP\_06* | 4 | 10 | 37.3% | 126 | 13904 | 10.3 | histone cluster 1, H2bj [Homo sapiens] |
| U | *gi|16306566|ref|NP\_00* | 4 | 10 | 37.3% | 126 | 13906 | 10.3 | histone cluster 1, H2bo [Homo sapiens] |

| Filename XCorr DeltCN Conf% ObsM+H+ CalcM+H+ SpR ZScore Ion% # Sequence  | | | | | | | | | | | | |
| --- | --- | --- | --- | --- | --- | --- | --- | --- | --- | --- | --- | --- |
|  | IPAstrin\_STLCD\_032114\_01.05980.05980.2 | 2.1213 | 0.2579 | 98.1% | 1280.2922 | 1280.4631 | 210 | 4.551 | 55.6% | 1 | R.KESYSIYVYK.V | 2 |
|  | IPAstrin\_STLCD\_tube2\_032114\_01.13724.13724.2 | 5.3014 | 0.4776 | 100.0% | 1744.5122 | 1745.0211 | 1 | 9.586 | 78.6% | 4 | K.AMGIMNSFVNDIFER.I | 2 |
|  | IPAstrin\_STLCD\_tube2\_032114\_01.04348.04348.2 | 3.2061 | 0.3553 | 100.0% | 1462.5322 | 1462.6462 | 7 | 6.151 | 54.2% | 4 | R.STITSREIQTAVR.L | 2 |
|  | IPAstrin\_STLCD\_tube2\_032114\_01.07934.07934.2 | 2.6828 | 0.2098 | 99.6% | 954.2322 | 954.19794 | 4 | 4.722 | 81.2% | 1 | R.LLLPGELAK.H | 2 |

---

|  |  |  |  |  |  |  |  |  |
| --- | --- | --- | --- | --- | --- | --- | --- | --- |
| U | *gi|62414289|ref|NP\_00* | 16 | 30 | 36.7% | 466 | 53652 | 5.1 | vimentin [Homo sapiens] |

| Filename XCorr DeltCN Conf% ObsM+H+ CalcM+H+ SpR ZScore Ion% # Sequence  | | | | | | | | | | | | |
| --- | --- | --- | --- | --- | --- | --- | --- | --- | --- | --- | --- | --- |
| \* | IPAstrin\_STLCD\_tube2\_032114\_01.03642.03642.2 | 2.3647 | 0.2811 | 98.7% | 1494.9722 | 1495.6531 | 50 | 4.878 | 42.9% | 1 | R.MFGGPGTASRPSSSR.S | 2 |
| \* | IPAstrin\_STLCD\_tube2\_032114\_01.05219.05219.2 | 3.2664 | 0.3948 | 100.0% | 1429.1921 | 1429.5724 | 1 | 5.849 | 73.1% | 2 | R.SLYASSPGGVYATR.S | 2 |
| \* | IPAstrin\_STLCD\_032114\_01.06721.06721.2 | 3.8147 | 0.4086 | 100.0% | 1509.4922 | 1509.5724 | 1 | 6.276 | 69.2% | 4 | R.SLYASS\*PGGVYATR.S | 2 |
|  | IPAstrin\_STLCD\_tube2\_032114\_01.04524.04524.2 | 3.6593 | 0.4526 | 100.0% | 1587.3922 | 1588.7147 | 1 | 7.566 | 75.0% | 1 | R.TNEKVELQELNDR.F | 2 |
| \* | IPAstrin\_STLCD\_tube2\_032114\_01.06971.06971.2 | 2.7437 | 0.306 | 99.9% | 1498.4722 | 1498.6508 | 1 | 5.967 | 68.2% | 1 | K.SRLGDLYEEEMR.E | 2 |
| \* | IPAstrin\_STLCD\_tube2\_032114\_01.06532.06532.2 | 3.342 | 0.4562 | 100.0% | 1255.1522 | 1255.385 | 1 | 7.651 | 77.8% | 3 | R.LGDLYEEEMR.E | 2 |
| \* | IPAstrin\_STLCD\_tube2\_032114\_01.07386.07386.2 | 2.9237 | 0.1907 | 99.1% | 1689.3522 | 1689.881 | 2 | 4.299 | 57.7% | 1 | R.VEVERDNLAEDIMR.L | 2 |
| \* | IPAstrin\_STLCD\_tube2\_032114\_01.05604.05604.2 | 2.7771 | 0.3802 | 100.0% | 1324.0521 | 1324.3898 | 1 | 6.798 | 65.0% | 1 | R.EEAENTLQSFR.Q | 2 |
| \* | IPAstrin\_STLCD\_tube2\_032114\_01.07943.07943.3 | 4.2721 | 0.3517 | 100.0% | 1662.8043 | 1662.967 | 2 | 6.167 | 42.3% | 2 | R.KVESLQEEIAFLKK.L | 3 |
|  | IPAstrin\_STLCD\_tube2\_032114\_01.07095.07095.2 | 3.0394 | 0.273 | 99.9% | 1310.2722 | 1310.4056 | 1 | 5.112 | 77.8% | 2 | K.NLQEAEEWYK.S | 2 |
| \* | IPAstrin\_STLCD\_tube2\_032114\_01.04300.04300.2 | 3.4035 | 0.4509 | 100.0% | 1094.0122 | 1094.1692 | 1 | 7.775 | 77.8% | 2 | K.FADLSEAANR.N | 2 |
| \* | IPAstrin\_STLCD\_tube2\_032114\_02.06726.06726.2 | 5.8514 | 0.5766 | 100.0% | 2187.3323 | 2188.33 | 1 | 11.496 | 69.4% | 1 | R.EMEENFAVEAANYQDTIGR.L | 2 |
| \* | IPAstrin\_STLCD\_tube2\_032114\_02.06712.06712.3 | 4.3579 | 0.4346 | 100.0% | 2187.6843 | 2188.33 | 1 | 7.053 | 41.7% | 1 | R.EMEENFAVEAANYQDTIGR.L | 3 |
| \* | IPAstrin\_STLCD\_032114\_01.07717.07717.2 | 4.0442 | 0.3769 | 100.0% | 1736.1322 | 1735.9679 | 1 | 7.222 | 73.1% | 2 | R.LQDEIQNMKEEMAR.H | 2 |
|  | IPAstrin\_STLCD\_032114\_02.07565.07565.2 | 3.7247 | 0.408 | 100.0% | 1296.2122 | 1296.5243 | 1 | 9.119 | 85.0% | 4 | K.MALDIEIATYR.K | 2 |
| \* | IPAstrin\_STLCD\_tube2\_032114\_01.12552.12552.2 | 3.0578 | 0.3176 | 99.9% | 1572.5721 | 1571.8601 | 1 | 6.074 | 69.2% | 2 | R.ISLPLPNFSSLNLR.E | 2 |

---

|  |  |  |  |  |  |  |  |  |
| --- | --- | --- | --- | --- | --- | --- | --- | --- |
| U | *gi|57242777|ref|NP\_03* | 2 | 3 | 35.9% | 103 | 11967 | 5.9 | c-myc binding protein [Homo sapiens] |

| Filename XCorr DeltCN Conf% ObsM+H+ CalcM+H+ SpR ZScore Ion% # Sequence  | | | | | | | | | | | | |
| --- | --- | --- | --- | --- | --- | --- | --- | --- | --- | --- | --- | --- |
| \* | IPAstrin\_STLCD\_032114\_01.12386.12386.3 | 4.2166 | 0.4357 | 100.0% | 2276.3943 | 2276.6348 | 1 | 6.897 | 36.8% | 2 | K.VLVALYEEPEKPNSALDFLK.H | 3 |
| \* | IPAstrin\_STLCD\_tube2\_032114\_01.06531.06531.3 | 4.6493 | 0.2708 | 100.0% | 1899.0844 | 1898.1289 | 1 | 4.989 | 51.6% | 1 | K.HHLGAATPENPEIELLR.L | 3 |

---

|  |  |  |  |  |  |  |  |  |
| --- | --- | --- | --- | --- | --- | --- | --- | --- |
| U | *gi|17921989|ref|NP\_00* | 17 | 57 | 35.7% | 448 | 49924 | 5.1 | tubulin, alpha 4a [Homo sapiens] |

| Filename XCorr DeltCN Conf% ObsM+H+ CalcM+H+ SpR ZScore Ion% # Sequence  | | | | | | | | | | | | |
| --- | --- | --- | --- | --- | --- | --- | --- | --- | --- | --- | --- | --- |
| \* | IPAstrin\_STLCD\_tube2\_032114\_01.11861.11861.2 | 3.7211 | 0.2651 | 100.0% | 1716.4722 | 1716.9719 | 1 | 5.498 | 75.0% | 1 | R.AVFVDLEPTVIDEIR.N | 2 |
|  | IPAstrin\_STLCD\_032114\_01.09666.09666.2 | 2.3859 | 0.456 | 100.0% | 1411.3722 | 1411.6439 | 434 | 6.257 | 54.5% | 2 | R.QLFHPEQLITGK.E | 22 |
|  | IPAstrin\_STLCD\_tube2\_032114\_01.07352.07352.3 | 3.7535 | 0.3903 | 100.0% | 2415.8943 | 2416.6555 | 1 | 6.125 | 33.8% | 5 | R.QLFHPEQLITGKEDAANNYAR.G | 33 |
|  | IPAstrin\_STLCD\_tube2\_032114\_01.04664.04664.3 | 3.4981 | 0.3002 | 99.6% | 1875.3844 | 1876.0824 | 1 | 5.227 | 42.9% | 1 | R.RNLDIERPTYTNLNR.L | 33 |
|  | IPAstrin\_STLCD\_032114\_01.06942.06942.2 | 3.2791 | 0.1294 | 98.7% | 1719.2722 | 1719.8949 | 3 | 5.129 | 53.8% | 2 | R.NLDIERPTYTNLNR.L | 22 |
|  | IPAstrin\_STLCD\_032114\_01.06936.06936.3 | 2.9305 | 0.4019 | 100.0% | 1719.8644 | 1719.8949 | 18 | 5.943 | 40.4% | 2 | R.NLDIERPTYTNLNR.L | 33 |
|  | IPAstrin\_STLCD\_032114\_01.14217.14217.3 | 3.7027 | 0.3777 | 100.0% | 1489.0443 | 1488.7678 | 1 | 6.866 | 50.0% | 2 | R.LISQIVSSITASLR.F | 33 |
|  | IPAstrin\_STLCD\_032114\_01.14244.14244.2 | 4.6874 | 0.4701 | 100.0% | 1489.5521 | 1488.7678 | 1 | 9.068 | 73.1% | 8 | R.LISQIVSSITASLR.F | 22 |
|  | IPAstrin\_STLCD\_032114\_02.09542.09542.2 | 5.1919 | 0.4807 | 100.0% | 2410.6921 | 2410.6885 | 1 | 8.564 | 47.5% | 5 | R.FDGALNVDLTEFQTNLVPYPR.I | 22 |
|  | IPAstrin\_STLCD\_032114\_01.11640.11640.2 | 4.0663 | 0.4653 | 100.0% | 1757.3522 | 1758.0703 | 1 | 8.398 | 66.7% | 9 | R.IHFPLATYAPVISAEK.A | 22 |
|  | IPAstrin\_STLCD\_032114\_01.11562.11562.3 | 3.7045 | 0.3746 | 100.0% | 1757.9944 | 1758.0703 | 4 | 5.616 | 41.7% | 2 | R.IHFPLATYAPVISAEK.A | 33 |
|  | IPAstrin\_STLCD\_tube2\_032114\_01.07898.07898.2 | 4.1434 | 0.4055 | 100.0% | 1826.4722 | 1826.1027 | 1 | 7.0 | 70.6% | 5 | K.VGINYQPPTVVPGGDLAK.V | 22 |
|  | IPAstrin\_STLCD\_tube2\_032114\_01.05871.05871.2 | 3.3035 | 0.4303 | 100.0% | 1381.4122 | 1381.6324 | 1 | 6.829 | 65.0% | 4 | R.LDHKFDLMYAK.R | 22 |
|  | IPAstrin\_STLCD\_032114\_01.06853.06853.3 | 3.8515 | 0.3833 | 100.0% | 1381.7943 | 1381.6324 | 1 | 6.871 | 55.0% | 3 | R.LDHKFDLMYAK.R | 33 |
|  | IPAstrin\_STLCD\_tube2\_032114\_02.06575.06575.3 | 3.2218 | 0.2377 | 97.0% | 2486.9343 | 2487.7083 | 2 | 4.323 | 31.2% | 1 | K.RAFVHWYVGEGMEEGEFSEAR.E | 33 |
|  | IPAstrin\_STLCD\_032114\_02.07649.07649.3 | 4.3179 | 0.4047 | 100.0% | 2330.2744 | 2331.5208 | 1 | 7.38 | 40.8% | 4 | R.AFVHWYVGEGMEEGEFSEAR.E | 33 |
|  | IPAstrin\_STLCD\_tube2\_032114\_02.08005.08005.3 | 4.0064 | 0.3519 | 100.0% | 3219.0244 | 3219.524 | 1 | 5.546 | 26.9% | 1 | R.AFVHWYVGEGMEEGEFSEAREDMAALEK.D | 33 |

Similarities:
gi|57013276|ref|NP\_00(16:1)  

---

|  |  |  |  |  |  |  |  |  |
| --- | --- | --- | --- | --- | --- | --- | --- | --- |
| U | *gi|224028244|ref|NP\_0* | 18 | 79 | 34.6% | 471 | 54232 | 8.9 | non-POU domain containing, octamer-binding isoform 1 [Homo sapiens] |
| U | *gi|34932414|ref|NP\_03* | 18 | 79 | 34.6% | 471 | 54232 | 8.9 | non-POU domain containing, octamer-binding isoform 1 [Homo sapiens] |
| U | *gi|224028246|ref|NP\_0* | 18 | 79 | 34.6% | 471 | 54232 | 8.9 | non-POU domain containing, octamer-binding isoform 1 [Homo sapiens] |

| Filename XCorr DeltCN Conf% ObsM+H+ CalcM+H+ SpR ZScore Ion% # Sequence  | | | | | | | | | | | | |
| --- | --- | --- | --- | --- | --- | --- | --- | --- | --- | --- | --- | --- |
|  | IPAstrin\_STLCD\_tube2\_032114\_01.09807.09807.2 | 3.8997 | 0.4305 | 100.0% | 1860.2922 | 1861.12 | 1 | 6.63 | 70.0% | 9 | R.LFVGNLPPDITEEEMR.K | 2 |
|  | IPAstrin\_STLCD\_tube2\_032114\_01.10986.10986.2 | 4.9 | 0.5844 | 100.0% | 1813.3722 | 1814.1504 | 1 | 9.873 | 73.3% | 2 | R.TLAEIAKVELDNMPLR.G | 2 |
|  | IPAstrin\_STLCD\_tube2\_032114\_01.10980.10980.3 | 5.1601 | 0.4959 | 100.0% | 1814.1843 | 1814.1504 | 1 | 8.343 | 48.3% | 2 | R.TLAEIAKVELDNMPLR.G | 3 |
|  | IPAstrin\_STLCD\_tube2\_032114\_01.06706.06706.2 | 3.0285 | 0.2931 | 100.0% | 1086.7122 | 1087.2793 | 3 | 5.979 | 81.2% | 5 | K.VELDNMPLR.G | 2 |
|  | IPAstrin\_STLCD\_tube2\_032114\_01.16910.16910.2 | 5.7356 | 0.4787 | 100.0% | 2669.652 | 2669.9507 | 1 | 11.789 | 54.5% | 12 | R.NLPQYVSNELLEEAFSVFGQVER.A | 2 |
|  | IPAstrin\_STLCD\_tube2\_032114\_02.11985.11985.3 | 4.9338 | 0.4788 | 100.0% | 2669.9644 | 2669.9507 | 1 | 7.83 | 36.4% | 8 | R.NLPQYVSNELLEEAFSVFGQVER.A | 3 |
|  | IPAstrin\_STLCD\_032114\_01.04669.04669.2 | 2.5561 | 0.2794 | 99.6% | 1232.3121 | 1232.4252 | 1 | 6.128 | 72.7% | 3 | K.GIVEFSGKPAAR.K | 2 |
|  | IPAstrin\_STLCD\_032114\_01.10029.10029.2 | 4.1295 | 0.5472 | 100.0% | 1697.4521 | 1696.8744 | 1 | 9.458 | 61.5% | 8 | R.FAQPGSFEYEYAMR.W | 2 |
|  | IPAstrin\_STLCD\_tube2\_032114\_01.04791.04791.2 | 3.2378 | 0.4029 | 100.0% | 1336.5721 | 1337.5488 | 1 | 7.208 | 75.0% | 8 | R.EKLEMEMEAAR.H | 2 |
|  | IPAstrin\_STLCD\_tube2\_032114\_01.03696.03696.2 | 2.2418 | 0.1891 | 96.8% | 1181.0122 | 1181.4161 | 6 | 4.544 | 75.0% | 2 | R.HEHQVMLMR.Q | 2 |
|  | IPAstrin\_STLCD\_032114\_01.03692.03692.2 | 3.8147 | 0.2724 | 100.0% | 1541.3922 | 1541.7222 | 1 | 5.306 | 81.8% | 1 | R.RMEELHNQEVQK.R | 2 |
|  | IPAstrin\_STLCD\_032114\_01.03639.03639.2 | 4.3194 | 0.2345 | 100.0% | 1697.1721 | 1697.9097 | 1 | 5.121 | 66.7% | 1 | R.RMEELHNQEVQKR.K | 2 |
|  | IPAstrin\_STLCD\_032114\_01.03781.03781.2 | 3.566 | 0.2854 | 100.0% | 1385.2922 | 1385.5347 | 1 | 5.464 | 80.0% | 1 | R.MEELHNQEVQK.R | 2 |
|  | IPAstrin\_STLCD\_032114\_02.05633.05633.2 | 4.5426 | 0.4849 | 100.0% | 1538.9922 | 1539.8441 | 1 | 8.372 | 64.3% | 12 | R.MGQMAMGGAMGINNR.G | 2 |
|  | IPAstrin\_STLCD\_tube2\_032114\_02.06947.06947.3 | 3.0611 | 0.2925 | 98.5% | 2164.7644 | 2164.4436 | 1 | 6.455 | 34.5% | 1 | R.FGQAATMEGIGAIGGTPPAFNR.A | 3 |
|  | IPAstrin\_STLCD\_032114\_01.11522.11522.2 | 5.0181 | 0.5236 | 100.0% | 2243.652 | 2244.4436 | 1 | 10.293 | 59.5% | 1 | R.FGQAATMEGIGAIGGT#PPAFNR.A | 2 |
|  | IPAstrin\_STLCD\_tube2\_032114\_02.07015.07015.3 | 4.2268 | 0.2349 | 98.9% | 2246.1543 | 2244.4436 | 1 | 5.212 | 41.7% | 2 | R.FGQAATMEGIGAIGGT#PPAFNR.A | 3 |
|  | IPAstrin\_STLCD\_tube2\_032114\_01.03603.03603.2 | 2.406 | 0.3552 | 99.9% | 1229.2322 | 1229.3811 | 1 | 5.804 | 68.2% | 1 | R.AAPGAEFAPNKR.R | 2 |

---

|  |  |  |  |  |  |  |  |  |
| --- | --- | --- | --- | --- | --- | --- | --- | --- |
| U | *gi|67782365|ref|NP\_00* | 15 | 29 | 34.3% | 469 | 51386 | 5.5 | keratin 7 [Homo sapiens] |

| Filename XCorr DeltCN Conf% ObsM+H+ CalcM+H+ SpR ZScore Ion% # Sequence  | | | | | | | | | | | | |
| --- | --- | --- | --- | --- | --- | --- | --- | --- | --- | --- | --- | --- |
|  | IPAstrin\_STLCD\_032114\_01.06867.06867.3 | 3.912 | 0.3369 | 99.7% | 2246.6643 | 2247.519 | 10 | 5.934 | 30.7% | 1 | R.LSSARPGGLGSSSLYGLGASRPR.V | 3 |
|  | IPAstrin\_STLCD\_tube2\_032114\_01.04199.04199.2 | 3.3601 | 0.459 | 100.0% | 1104.9521 | 1105.2388 | 1 | 7.476 | 77.3% | 4 | R.SAYGGPVGAGIR.E | 2 |
|  | IPAstrin\_STLCD\_tube2\_032114\_01.09713.09713.2 | 2.9779 | 0.3499 | 100.0% | 1454.1721 | 1454.7098 | 221 | 6.085 | 50.0% | 2 | R.EVTINQSLLAPLR.L | 2 |
|  | IPAstrin\_STLCD\_tube2\_032114\_01.06068.06068.2 | 2.3494 | 0.2074 | 99.3% | 828.0122 | 827.95544 | 6 | 5.13 | 91.7% | 1 | K.FASFIDK.V | 222222 |
|  | IPAstrin\_STLCD\_032114\_01.07575.07575.2 | 2.7065 | 0.1841 | 99.4% | 1082.3121 | 1083.2755 | 6 | 6.916 | 75.0% | 2 | K.FASFIDKVR.F | 222222 |
|  | IPAstrin\_STLCD\_tube2\_032114\_01.07284.07284.2 | 2.8703 | 0.2014 | 99.9% | 1046.2722 | 1046.2114 | 2 | 5.387 | 78.6% | 2 | K.WTLLQEQK.S | 2 |
|  | IPAstrin\_STLCD\_tube2\_032114\_01.11655.11655.2 | 4.1429 | 0.4667 | 100.0% | 1443.7722 | 1443.686 | 1 | 8.79 | 70.8% | 4 | R.LPDIFEAQIAGLR.G | 2 |
|  | IPAstrin\_STLCD\_032114\_01.06178.06178.2 | 3.3117 | 0.4095 | 100.0% | 1243.1322 | 1243.3622 | 1 | 7.249 | 72.7% | 1 | R.GQLEALQVDGGR.L | 2 |
| \* | IPAstrin\_STLCD\_032114\_01.11304.11304.3 | 3.3552 | 0.3916 | 100.0% | 1955.1843 | 1955.1783 | 1 | 6.26 | 39.7% | 2 | R.GQLEALQVDGGRLEAELR.S | 3 |
|  | IPAstrin\_STLCD\_032114\_01.11884.11884.2 | 3.8149 | 0.1062 | 99.8% | 1419.1322 | 1419.5773 | 1 | 7.825 | 77.3% | 3 | K.VDALNDEINFLR.T | 2 |
|  | IPAstrin\_STLCD\_tube2\_032114\_01.04722.04722.2 | 3.0223 | 0.2726 | 99.9% | 1197.2122 | 1197.2897 | 1 | 6.082 | 77.8% | 1 | R.AEAEAWYQTK.F | 22 |
| \* | IPAstrin\_STLCD\_tube2\_032114\_01.05668.05668.2 | 2.1195 | 0.2551 | 96.4% | 1402.2322 | 1401.5596 | 3 | 4.737 | 58.3% | 1 | R.AKLEAAIAEAEER.G | 2 |
| \* | IPAstrin\_STLCD\_tube2\_032114\_02.07002.07002.3 | 3.6948 | 0.2578 | 98.9% | 2013.0543 | 2013.2987 | 1 | 5.323 | 43.1% | 1 | R.AKLEAAIAEAEERGELALK.D | 3 |
|  | IPAstrin\_STLCD\_tube2\_032114\_01.04539.04539.2 | 3.4155 | 0.3588 | 100.0% | 1385.9521 | 1386.548 | 1 | 6.352 | 81.8% | 2 | R.AKQEELEAALQR.G | 2 |
|  | IPAstrin\_STLCD\_tube2\_032114\_01.08175.08175.2 | 3.1566 | 0.2642 | 99.9% | 1406.3121 | 1406.6653 | 2 | 5.702 | 72.7% | 2 | K.LALDIEIATYRK.L | 22 |

Similarities:
gi|4504919|ref|NP\_002(3:12)  
gi|47132620|ref|NP\_00(2:13)  
gi|119703753|ref|NP\_0(2:13)  
gi|32567786|ref|NP\_78(3:12)  
gi|153791158|ref|NP\_0(2:13)  

---

|  |  |  |  |  |  |  |  |  |
| --- | --- | --- | --- | --- | --- | --- | --- | --- |
| U | *gi|17986258|ref|NP\_06* | 4 | 6 | 33.8% | 151 | 16930 | 4.7 | myosin, light chain 6, alkali, smooth muscle and non-muscle isoform 1 [Homo sapiens] |
| U | *gi|88999583|ref|NP\_52* | 4 | 6 | 33.8% | 151 | 16961 | 4.6 | myosin, light chain 6, alkali, smooth muscle and non-muscle isoform 2 [Homo sapiens] |

| Filename XCorr DeltCN Conf% ObsM+H+ CalcM+H+ SpR ZScore Ion% # Sequence  | | | | | | | | | | | | |
| --- | --- | --- | --- | --- | --- | --- | --- | --- | --- | --- | --- | --- |
|  | IPAstrin\_STLCD\_tube2\_032114\_01.04562.04562.2 | 3.4134 | 0.2912 | 100.0% | 1355.2922 | 1355.5339 | 1 | 5.119 | 66.7% | 3 | R.ALGQNPTNAEVLK.V | 2 |
|  | IPAstrin\_STLCD\_tube2\_032114\_01.13469.13469.2 | 3.0672 | 0.3082 | 99.9% | 1888.6122 | 1889.2628 | 1 | 5.733 | 46.7% | 1 | K.VLDFEHFLPMLQTVAK.N | 2 |
|  | IPAstrin\_STLCD\_032114\_01.09884.09884.2 | 2.8555 | 0.2251 | 99.4% | 1545.5521 | 1545.6024 | 4 | 4.917 | 62.5% | 1 | K.DQGTYEDYVEGLR.V | 2 |
|  | IPAstrin\_STLCD\_032114\_01.04730.04730.2 | 2.1725 | 0.2402 | 98.2% | 996.0522 | 996.1949 | 171 | 5.327 | 56.2% | 1 | R.HVLVTLGEK.M | 2 |

---

|  |  |  |  |  |  |  |  |  |
| --- | --- | --- | --- | --- | --- | --- | --- | --- |
| U | *gi|20127519|ref|NP\_03* | 31 | 73 | 33.3% | 747 | 85653 | 9.2 | TPX2, microtubule-associated protein homolog [Homo sapiens] |

| Filename XCorr DeltCN Conf% ObsM+H+ CalcM+H+ SpR ZScore Ion% # Sequence  | | | | | | | | | | | | |
| --- | --- | --- | --- | --- | --- | --- | --- | --- | --- | --- | --- | --- |
| \* | IPAstrin\_STLCD\_tube2\_032114\_01.06888.06888.3 | 4.385 | 0.3997 | 100.0% | 2405.4543 | 2405.7996 | 1 | 6.253 | 33.8% | 1 | R.KANLQQAIVTPLKPVDNTYYK.E | 3 |
| \* | IPAstrin\_STLCD\_tube2\_032114\_01.08016.08016.2 | 4.4849 | 0.5195 | 100.0% | 2276.612 | 2277.6255 | 1 | 9.164 | 47.4% | 1 | K.ANLQQAIVTPLKPVDNTYYK.E | 2 |
| \* | IPAstrin\_STLCD\_tube2\_032114\_01.07984.07984.3 | 3.9339 | 0.3319 | 100.0% | 2278.1343 | 2277.6255 | 1 | 5.885 | 34.2% | 1 | K.ANLQQAIVTPLKPVDNTYYK.E | 3 |
| \* | IPAstrin\_STLCD\_032114\_01.04614.04614.2 | 2.9387 | 0.3745 | 100.0% | 1150.1322 | 1150.3534 | 3 | 6.871 | 75.0% | 4 | K.MQQEVVEMR.K | 2 |
| \* | IPAstrin\_STLCD\_tube2\_032114\_01.05373.05373.2 | 2.8165 | 0.3185 | 99.9% | 1195.2322 | 1195.4911 | 7 | 6.395 | 59.1% | 3 | K.LALAGIGQPVKK.S | 2 |
| \* | IPAstrin\_STLCD\_tube2\_032114\_01.06738.06738.2 | 5.0389 | 0.5142 | 100.0% | 1886.1522 | 1887.013 | 1 | 8.446 | 78.6% | 3 | K.NQEEYKEVNFTSELR.K | 2 |
| \* | IPAstrin\_STLCD\_032114\_01.08352.08352.3 | 4.4373 | 0.3901 | 100.0% | 1886.8744 | 1887.013 | 23 | 6.543 | 37.5% | 3 | K.NQEEYKEVNFTSELR.K | 3 |
| \* | IPAstrin\_STLCD\_tube2\_032114\_01.05445.05445.2 | 3.7726 | 0.2228 | 100.0% | 1349.2122 | 1349.4344 | 1 | 5.573 | 72.7% | 4 | K.STAELEAEELEK.L | 2 |
| \* | IPAstrin\_STLCD\_tube2\_032114\_01.08542.08542.2 | 4.6573 | 0.4003 | 100.0% | 2009.5322 | 2010.2053 | 1 | 8.734 | 53.1% | 1 | K.STAELEAEELEKLQQYK.F | 2 |
| \* | IPAstrin\_STLCD\_tube2\_032114\_01.06680.06680.2 | 3.1075 | 0.2665 | 100.0% | 1037.1522 | 1037.2877 | 1 | 5.202 | 66.7% | 5 | R.ILEGGPILPK.K | 2 |
| \* | IPAstrin\_STLCD\_tube2\_032114\_01.07820.07820.3 | 3.7403 | 0.4444 | 100.0% | 2135.2744 | 2135.5083 | 1 | 6.796 | 40.3% | 1 | K.KPPVKPPTEPIGFDLEIEK.R | 3 |
| \* | IPAstrin\_STLCD\_032114\_01.08648.08648.3 | 4.5566 | 0.4967 | 100.0% | 2290.8245 | 2291.6958 | 1 | 7.61 | 38.2% | 2 | K.KPPVKPPTEPIGFDLEIEKR.I | 3 |
| \* | IPAstrin\_STLCD\_tube2\_032114\_01.06719.06719.2 | 3.3064 | 0.3935 | 100.0% | 1198.3922 | 1198.402 | 2 | 6.588 | 75.0% | 4 | K.ILEDVVGVPEK.K | 2 |
| \* | IPAstrin\_STLCD\_tube2\_032114\_01.05470.05470.2 | 3.7869 | 0.249 | 100.0% | 1327.2722 | 1326.576 | 9 | 5.535 | 68.2% | 4 | K.ILEDVVGVPEKK.V | 2 |
| \* | IPAstrin\_STLCD\_tube2\_032114\_01.09616.09616.2 | 4.2401 | 0.5086 | 100.0% | 1661.2522 | 1661.9823 | 1 | 7.906 | 64.3% | 4 | K.VLPITVPKS\*PAFALK.N | 2 |
| \* | IPAstrin\_STLCD\_tube2\_032114\_01.05288.05288.3 | 4.7831 | 0.3723 | 100.0% | 2158.9744 | 2158.4285 | 1 | 7.172 | 38.2% | 2 | R.IRMPTKEDEEEDEPVVIK.A | 3 |
| \* | IPAstrin\_STLCD\_tube2\_032114\_01.04533.04533.2 | 5.3806 | 0.4009 | 100.0% | 1888.2522 | 1889.0815 | 1 | 7.984 | 76.7% | 2 | R.MPTKEDEEEDEPVVIK.A | 2 |
| \* | IPAstrin\_STLCD\_tube2\_032114\_01.04556.04556.3 | 5.4544 | 0.3271 | 100.0% | 1888.8544 | 1889.0815 | 1 | 6.426 | 51.7% | 5 | R.MPTKEDEEEDEPVVIK.A | 3 |
| \* | IPAstrin\_STLCD\_032114\_01.08509.08509.2 | 3.3785 | 0.4912 | 100.0% | 2131.392 | 2132.473 | 1 | 7.757 | 58.3% | 1 | K.AQPVPHYGVPFKPQIPEAR.T | 2 |
| \* | IPAstrin\_STLCD\_tube2\_032114\_01.06725.06725.3 | 2.6556 | 0.2983 | 97.6% | 2131.8245 | 2132.473 | 2 | 5.955 | 31.9% | 3 | K.AQPVPHYGVPFKPQIPEAR.T | 3 |
| \* | IPAstrin\_STLCD\_tube2\_032114\_01.09749.09749.2 | 2.9261 | 0.1262 | 96.0% | 1705.3722 | 1705.9945 | 2 | 5.916 | 53.6% | 1 | K.ALPLPHFDTINLPEK.K | 2 |
| \* | IPAstrin\_STLCD\_tube2\_032114\_01.08550.08550.2 | 2.4625 | 0.2562 | 98.5% | 1833.5721 | 1834.1686 | 65 | 5.46 | 36.7% | 1 | K.ALPLPHFDTINLPEKK.V | 2 |
| \* | IPAstrin\_STLCD\_tube2\_032114\_01.06012.06012.3 | 2.4439 | 0.3201 | 98.1% | 1683.2943 | 1683.9481 | 8 | 5.245 | 33.9% | 1 | K.ARPNTVISQEPFVPK.K | 3 |
| \* | IPAstrin\_STLCD\_032114\_01.07088.07088.2 | 3.2911 | 0.3356 | 100.0% | 1683.3121 | 1683.9481 | 3 | 5.853 | 53.6% | 3 | K.ARPNTVISQEPFVPK.K | 2 |
| \* | IPAstrin\_STLCD\_032114\_01.05343.05343.3 | 3.9505 | 0.2728 | 99.6% | 1811.8444 | 1812.1222 | 9 | 6.17 | 40.0% | 2 | K.ARPNTVISQEPFVPKK.E | 3 |
| \* | IPAstrin\_STLCD\_tube2\_032114\_01.09696.09696.3 | 4.2393 | 0.172 | 96.9% | 2320.4043 | 2319.617 | 1 | 4.813 | 35.7% | 1 | K.KSVAEGLSGSLVQEPFQLATEK.R | 3 |
| \* | IPAstrin\_STLCD\_tube2\_032114\_01.08944.08944.3 | 3.8877 | 0.3022 | 99.6% | 2475.1743 | 2475.8044 | 73 | 6.552 | 25.0% | 2 | K.KSVAEGLSGSLVQEPFQLATEKR.A | 3 |
| \* | IPAstrin\_STLCD\_tube2\_032114\_01.09760.09760.3 | 4.3082 | 0.373 | 100.0% | 2347.0144 | 2347.6304 | 1 | 7.37 | 31.0% | 2 | K.SVAEGLSGSLVQEPFQLATEKR.A | 3 |
| \* | IPAstrin\_STLCD\_tube2\_032114\_01.03273.03273.2 | 3.903 | 0.3234 | 100.0% | 1630.3121 | 1630.7954 | 1 | 6.858 | 75.0% | 1 | R.LQEEEQKKEELAR.L | 2 |
| \* | IPAstrin\_STLCD\_032114\_01.05854.05854.2 | 2.9272 | 0.0999 | 95.7% | 1355.8722 | 1355.5309 | 1 | 4.139 | 66.7% | 1 | K.SSDQPLTVPVSPK.F | 2 |
| \* | IPAstrin\_STLCD\_032114\_01.06516.06516.2 | 3.3629 | 0.4458 | 100.0% | 1435.3121 | 1435.5309 | 1 | 6.919 | 79.2% | 4 | K.SSDQPLTVPVS\*PK.F | 2 |

---

|  |  |  |  |  |  |  |  |  |
| --- | --- | --- | --- | --- | --- | --- | --- | --- |
| U | *gi|27436946|ref|NP\_73* | 19 | 51 | 32.5% | 664 | 74140 | 7.0 | lamin A/C isoform 1 precursor [Homo sapiens] |

| Filename XCorr DeltCN Conf% ObsM+H+ CalcM+H+ SpR ZScore Ion% # Sequence  | | | | | | | | | | | | |
| --- | --- | --- | --- | --- | --- | --- | --- | --- | --- | --- | --- | --- |
|  | IPAstrin\_STLCD\_tube2\_032114\_01.04164.04164.2 | 4.6829 | 0.3932 | 100.0% | 1630.2322 | 1630.7521 | 1 | 7.731 | 79.2% | 2 | R.LQEKEDLQELNDR.L | 2 |
|  | IPAstrin\_STLCD\_tube2\_032114\_01.04178.04178.3 | 3.3873 | 0.1956 | 97.3% | 1631.2144 | 1630.7521 | 2 | 4.4 | 50.0% | 1 | R.LQEKEDLQELNDR.L | 3 |
|  | IPAstrin\_STLCD\_032114\_02.04490.04490.2 | 2.5279 | 0.2036 | 98.2% | 1417.7522 | 1418.5901 | 6 | 5.18 | 54.5% | 1 | R.LRITESEEVVSR.E | 2 |
|  | IPAstrin\_STLCD\_032114\_01.04046.04046.2 | 2.4409 | 0.3596 | 99.9% | 1149.4521 | 1149.2432 | 71 | 6.319 | 66.7% | 1 | R.ITESEEVVSR.E | 2 |
|  | IPAstrin\_STLCD\_tube2\_032114\_01.04064.04064.2 | 3.1868 | 0.3678 | 100.0% | 1166.9122 | 1166.2328 | 1 | 6.076 | 85.0% | 2 | K.AAYEAELGDAR.K | 2 |
|  | IPAstrin\_STLCD\_tube2\_032114\_01.08118.08118.2 | 3.4257 | 0.2779 | 100.0% | 1243.3522 | 1244.474 | 1 | 6.433 | 75.0% | 2 | R.LKDLEALLNSK.E | 2 |
|  | IPAstrin\_STLCD\_tube2\_032114\_01.06168.06168.2 | 2.8244 | 0.3158 | 100.0% | 1183.2922 | 1183.3066 | 7 | 5.693 | 77.8% | 2 | R.TLEGELHDLR.G | 2 |
|  | IPAstrin\_STLCD\_tube2\_032114\_01.06944.06944.2 | 3.7608 | 0.293 | 100.0% | 1029.1122 | 1029.1814 | 3 | 6.01 | 87.5% | 5 | R.LADALQELR.A | 2 |
|  | IPAstrin\_STLCD\_032114\_01.05816.05816.2 | 4.7704 | 0.3898 | 100.0% | 1752.5721 | 1753.8693 | 1 | 7.45 | 63.3% | 4 | R.NSNLVGAAHEELQQSR.I | 2 |
|  | IPAstrin\_STLCD\_tube2\_032114\_01.05168.05168.3 | 2.8032 | 0.3392 | 99.4% | 1752.9844 | 1753.8693 | 27 | 5.7 | 33.3% | 1 | R.NSNLVGAAHEELQQSR.I | 3 |
|  | IPAstrin\_STLCD\_tube2\_032114\_01.08768.08768.2 | 4.2729 | 0.4071 | 100.0% | 1699.9722 | 1700.9762 | 1 | 6.743 | 67.9% | 4 | R.IRIDSLSAQLSQLQK.Q | 2 |
|  | IPAstrin\_STLCD\_tube2\_032114\_01.04889.04889.2 | 2.7091 | 0.2888 | 99.9% | 1188.4122 | 1188.3262 | 14 | 4.93 | 72.2% | 3 | K.LRDLEDSLAR.E | 2 |
|  | IPAstrin\_STLCD\_tube2\_032114\_01.10654.10654.2 | 4.7646 | 0.4907 | 100.0% | 1894.4321 | 1895.1346 | 1 | 9.073 | 75.0% | 2 | R.MQQQLDEYQELLDIK.L | 2 |
|  | IPAstrin\_STLCD\_tube2\_032114\_02.06031.06031.2 | 2.8212 | 0.4351 | 100.0% | 1332.0122 | 1332.5603 | 11 | 6.911 | 50.0% | 1 | K.LALDMEIHAYR.K | 2 |
|  | IPAstrin\_STLCD\_032114\_01.06001.06001.2 | 4.112 | 0.5355 | 100.0% | 1606.1721 | 1606.7728 | 1 | 9.323 | 73.1% | 3 | R.VAVEEVDEEGKFVR.L | 2 |
|  | IPAstrin\_STLCD\_tube2\_032114\_01.06633.06633.2 | 2.3736 | 0.2226 | 98.2% | 1292.4122 | 1292.391 | 38 | 5.134 | 55.0% | 3 | R.QNGDDPLLTYR.F | 2 |
|  | IPAstrin\_STLCD\_tube2\_032114\_02.05107.05107.2 | 4.127 | 0.52 | 100.0% | 1491.8722 | 1492.6874 | 1 | 8.551 | 69.2% | 7 | R.TALINSTGEEVAMR.K | 2 |
|  | IPAstrin\_STLCD\_tube2\_032114\_02.04204.04204.3 | 3.3336 | 0.3609 | 99.8% | 2367.4443 | 2366.504 | 1 | 5.808 | 30.8% | 1 | K.ASASGSGAQVGGPISSGSSASSVTVTR.S | 3 |
|  | IPAstrin\_STLCD\_tube2\_032114\_01.05867.05867.2 | 4.3164 | 0.5083 | 100.0% | 1568.1322 | 1567.6555 | 1 | 9.547 | 50.0% | 6 | R.SVGGSGGGSFGDNLVTR.S | 2 |

---

|  |  |  |  |  |  |  |  |  |
| --- | --- | --- | --- | --- | --- | --- | --- | --- |
| U | *gi|4758792|ref|NP\_004* | 2 | 4 | 31.5% | 124 | 13712 | 8.3 | NADH dehydrogenase (ubiquinone) Fe-S protein 6, 13kDa (NADH-coenzyme Q reductase) [Homo sapiens] |

| Filename XCorr DeltCN Conf% ObsM+H+ CalcM+H+ SpR ZScore Ion% # Sequence  | | | | | | | | | | | | |
| --- | --- | --- | --- | --- | --- | --- | --- | --- | --- | --- | --- | --- |
| \* | IPAstrin\_STLCD\_tube2\_032114\_01.03304.03304.3 | 2.7471 | 0.2749 | 97.6% | 1854.1444 | 1853.989 | 130 | 4.843 | 32.1% | 1 | K.VTHTGQVYDDKDYRR.I | 3 |
| \* | IPAstrin\_STLCD\_tube2\_032114\_02.07518.07518.3 | 4.6095 | 0.361 | 100.0% | 2758.7644 | 2760.03 | 1 | 6.141 | 32.6% | 3 | R.QKEVNENFAIDLIAEQPVSEVETR.V | 3 |

---

|  |  |  |  |  |  |  |  |  |
| --- | --- | --- | --- | --- | --- | --- | --- | --- |
| U | *gi|118582269|ref|NP\_0* | 5 | 14 | 30.3% | 201 | 22460 | 8.0 | splicing factor, arginine/serine-rich 1 isoform 2 [Homo sapiens] |
| U | *gi|5902076|ref|NP\_008* | 5 | 14 | 24.6% | 248 | 27745 | 10.4 | splicing factor, arginine/serine-rich 1 isoform 1 [Homo sapiens] |

| Filename XCorr DeltCN Conf% ObsM+H+ CalcM+H+ SpR ZScore Ion% # Sequence  | | | | | | | | | | | | |
| --- | --- | --- | --- | --- | --- | --- | --- | --- | --- | --- | --- | --- |
|  | IPAstrin\_STLCD\_tube2\_032114\_01.07371.07371.2 | 3.1306 | 0.2986 | 100.0% | 1257.6721 | 1257.4752 | 1 | 6.766 | 85.0% | 5 | R.IYVGNLPPDIR.T | 2 |
|  | IPAstrin\_STLCD\_032114\_01.07758.07758.2 | 3.3823 | 0.3711 | 100.0% | 1258.3922 | 1258.4137 | 1 | 5.992 | 83.3% | 3 | R.TKDIEDVFYK.Y | 2 |
|  | IPAstrin\_STLCD\_tube2\_032114\_01.11448.11448.3 | 2.9699 | 0.2983 | 98.4% | 2542.6443 | 2542.7234 | 1 | 4.855 | 27.3% | 1 | R.GGPPFAFVEFEDPRDAEDAVYGR.D | 3 |
|  | IPAstrin\_STLCD\_032114\_01.06174.06174.2 | 2.4635 | 0.0956 | 96.7% | 917.4122 | 917.0989 | 4 | 4.102 | 75.0% | 2 | R.LRVEFPR.S | 22 |
|  | IPAstrin\_STLCD\_032114\_01.08276.08276.2 | 2.7229 | 0.1795 | 99.3% | 1079.1522 | 1079.198 | 1 | 4.54 | 88.9% | 3 | R.DGTGVVEFVR.K | 2 |

Similarities:
gi|4506903|ref|NP\_003(1:4)  

---

|  |  |  |  |  |  |  |  |  |
| --- | --- | --- | --- | --- | --- | --- | --- | --- |
| U | *gi|7669492|ref|NP\_002* | 7 | 21 | 30.1% | 335 | 36053 | 8.5 | glyceraldehyde-3-phosphate dehydrogenase [Homo sapiens] |

| Filename XCorr DeltCN Conf% ObsM+H+ CalcM+H+ SpR ZScore Ion% # Sequence  | | | | | | | | | | | | |
| --- | --- | --- | --- | --- | --- | --- | --- | --- | --- | --- | --- | --- |
| \* | IPAstrin\_STLCD\_032114\_01.12512.12512.2 | 3.032 | 0.3101 | 99.9% | 1614.2322 | 1614.8851 | 1 | 6.5 | 65.4% | 1 | K.LVINGNPITIFQER.D | 2 |
| \* | IPAstrin\_STLCD\_tube2\_032114\_01.08169.08169.3 | 5.3159 | 0.3965 | 100.0% | 2369.9043 | 2370.79 | 1 | 7.094 | 35.7% | 1 | K.RVIISAPSADAPMFVMGVNHEK.Y | 3 |
| \* | IPAstrin\_STLCD\_032114\_01.14305.14305.3 | 7.5148 | 0.6033 | 100.0% | 2596.2544 | 2597.0044 | 1 | 10.858 | 43.5% | 4 | K.VIHDNFGIVEGLMTTVHAITATQK.T | 3 |
| \* | IPAstrin\_STLCD\_tube2\_032114\_01.07082.07082.2 | 4.0584 | 0.392 | 100.0% | 1412.3922 | 1412.6292 | 1 | 6.236 | 67.9% | 6 | R.GALQNIIPASTGAAK.A | 2 |
|  | IPAstrin\_STLCD\_032114\_01.11757.11757.2 | 4.1557 | 0.5454 | 100.0% | 1764.9521 | 1764.8914 | 1 | 9.205 | 69.2% | 5 | K.LISWYDNEFGYSNR.V | 2 |
| \* | IPAstrin\_STLCD\_032114\_01.06738.06738.2 | 2.4165 | 0.2326 | 98.6% | 1202.3522 | 1202.4724 | 83 | 4.527 | 65.0% | 1 | R.VVDLMAHMASK.E | 2 |
| \* | IPAstrin\_STLCD\_032114\_01.07184.07184.2 | 3.4133 | 0.4904 | 100.0% | 1331.0721 | 1331.5879 | 1 | 8.032 | 68.2% | 3 | R.VVDLMAHMASKE.- | 2 |

---

|  |  |  |  |  |  |  |  |  |
| --- | --- | --- | --- | --- | --- | --- | --- | --- |
| U | *gi|4826998|ref|NP\_005* | 18 | 63 | 28.4% | 707 | 76150 | 9.4 | splicing factor proline/glutamine rich (polypyrimidine tract binding protein associated) [Homo sapiens] |

| Filename XCorr DeltCN Conf% ObsM+H+ CalcM+H+ SpR ZScore Ion% # Sequence  | | | | | | | | | | | | |
| --- | --- | --- | --- | --- | --- | --- | --- | --- | --- | --- | --- | --- |
| \* | IPAstrin\_STLCD\_032114\_01.03900.03900.3 | 5.1424 | 0.4987 | 100.0% | 2372.4543 | 2371.725 | 1 | 8.209 | 35.4% | 1 | K.MPGGPKPGGGPGLSTPGGHPKPPHR.G | 3 |
| \* | IPAstrin\_STLCD\_tube2\_032114\_01.08154.08154.3 | 3.8349 | 0.3846 | 100.0% | 1651.5243 | 1650.8723 | 1 | 6.147 | 42.9% | 3 | K.ISDSEGFKANLSLLR.R | 3 |
| \* | IPAstrin\_STLCD\_032114\_01.13707.13707.3 | 3.1521 | 0.241 | 95.7% | 3010.7644 | 3011.244 | 2 | 4.419 | 25.0% | 1 | K.T#YTQRCRLFVGNLPADITEDEFKR.L | 3 |
| \* | IPAstrin\_STLCD\_tube2\_032114\_01.11157.11157.2 | 2.6406 | 0.2339 | 98.7% | 1808.1721 | 1809.0258 | 8 | 4.774 | 46.7% | 1 | R.LFVGNLPADITEDEFK.R | 2 |
| \* | IPAstrin\_STLCD\_032114\_01.11685.11685.2 | 4.7931 | 0.491 | 100.0% | 1964.3522 | 1965.2133 | 1 | 8.703 | 65.6% | 4 | R.LFVGNLPADITEDEFKR.L | 2 |
| \* | IPAstrin\_STLCD\_032114\_01.07484.07484.2 | 3.6805 | 0.4284 | 100.0% | 1253.2322 | 1253.3971 | 1 | 7.393 | 75.0% | 5 | K.YGEPGEVFINK.G | 2 |
| \* | IPAstrin\_STLCD\_032114\_01.04440.04440.1 | 2.3405 | 0.3694 | 100.0% | 1143.56 | 1144.3188 | 1 | 6.998 | 75.0% | 2 | R.FATHAAALSVR.N | 1 |
| \* | IPAstrin\_STLCD\_032114\_01.04569.04569.2 | 3.4305 | 0.4793 | 100.0% | 1143.6921 | 1144.3188 | 1 | 7.978 | 95.0% | 7 | R.FATHAAALSVR.N | 2 |
| \* | IPAstrin\_STLCD\_tube2\_032114\_01.14403.14403.2 | 5.1518 | 0.5974 | 100.0% | 2639.7922 | 2640.9092 | 1 | 9.974 | 47.7% | 5 | R.NLSPYVSNELLEEAFSQFGPIER.A | 2 |
| \* | IPAstrin\_STLCD\_032114\_01.05124.05124.2 | 2.2514 | 0.3682 | 99.8% | 1246.0521 | 1246.452 | 6 | 6.38 | 54.5% | 3 | K.GIVEFASKPAAR.K | 2 |
| \* | IPAstrin\_STLCD\_tube2\_032114\_01.05104.05104.2 | 4.7783 | 0.4529 | 100.0% | 1764.3121 | 1763.8632 | 1 | 8.615 | 76.9% | 6 | R.FAQHGTFEYEYSQR.W | 2 |
| \* | IPAstrin\_STLCD\_tube2\_032114\_02.04641.04641.3 | 4.9362 | 0.2452 | 100.0% | 1764.4143 | 1763.8632 | 8 | 6.618 | 40.4% | 9 | R.FAQHGTFEYEYSQR.W | 3 |
| \* | IPAstrin\_STLCD\_032114\_01.09655.09655.3 | 5.6459 | 0.4067 | 100.0% | 2431.0444 | 2429.6233 | 1 | 7.613 | 40.8% | 4 | K.DKLESEMEDAYHEHQANLLR.Q | 3 |
| \* | IPAstrin\_STLCD\_032114\_01.03740.03740.2 | 3.7596 | 0.23 | 100.0% | 1573.3522 | 1573.7821 | 1 | 5.591 | 77.3% | 2 | R.RMEELHNQEMQK.R | 2 |
| \* | IPAstrin\_STLCD\_tube2\_032114\_01.03333.03333.3 | 3.3126 | 0.2886 | 99.6% | 1573.4043 | 1573.7821 | 1 | 4.853 | 45.5% | 2 | R.RMEELHNQEMQK.R | 3 |
| \* | IPAstrin\_STLCD\_032114\_01.03861.03861.2 | 3.6675 | 0.349 | 100.0% | 1417.0122 | 1417.5946 | 1 | 6.04 | 75.0% | 1 | R.MEELHNQEMQK.R | 2 |
| \* | IPAstrin\_STLCD\_tube2\_032114\_01.05064.05064.2 | 4.2263 | 0.4831 | 100.0% | 1773.1721 | 1772.9631 | 1 | 7.53 | 55.6% | 1 | R.MGGGGAMNMGDPYGSGGQK.F | 2 |
| \* | IPAstrin\_STLCD\_tube2\_032114\_01.03806.03806.2 | 3.7892 | 0.5487 | 100.0% | 1341.8922 | 1342.4569 | 1 | 8.65 | 71.4% | 6 | R.FGQGGAGPVGGQGPR.G | 2 |

---

|  |  |  |  |  |  |  |  |  |
| --- | --- | --- | --- | --- | --- | --- | --- | --- |
| U | *gi|5902102|ref|NP\_008* | 2 | 4 | 27.7% | 119 | 13282 | 11.6 | small nuclear ribonucleoprotein D1 polypeptide 16kDa [Homo sapiens] |

| Filename XCorr DeltCN Conf% ObsM+H+ CalcM+H+ SpR ZScore Ion% # Sequence  | | | | | | | | | | | | |
| --- | --- | --- | --- | --- | --- | --- | --- | --- | --- | --- | --- | --- |
|  | IPAstrin\_STLCD\_tube2\_032114\_01.06879.06879.2 | 3.5717 | 0.3769 | 100.0% | 1555.3322 | 1555.7745 | 8 | 6.347 | 58.3% | 1 | K.NREPVQLETLSIR.G | 2 |
| \* | IPAstrin\_STLCD\_tube2\_032114\_01.15981.15981.2 | 4.1189 | 0.4611 | 100.0% | 2288.372 | 2288.6863 | 1 | 7.373 | 55.3% | 3 | R.YFILPDSLPLDTLLVDVEPK.V | 2 |

---

|  |  |  |  |  |  |  |  |  |
| --- | --- | --- | --- | --- | --- | --- | --- | --- |
| U | *gi|148470397|ref|NP\_0* | 7 | 16 | 27.2% | 415 | 45672 | 5.6 | heterogeneous nuclear ribonucleoprotein F [Homo sapiens] |
| U | *gi|4826760|ref|NP\_004* | 7 | 16 | 27.2% | 415 | 45672 | 5.6 | heterogeneous nuclear ribonucleoprotein F [Homo sapiens] |
| U | *gi|148470406|ref|NP\_0* | 7 | 16 | 27.2% | 415 | 45672 | 5.6 | heterogeneous nuclear ribonucleoprotein F [Homo sapiens] |
| U | *gi|148470404|ref|NP\_0* | 7 | 16 | 27.2% | 415 | 45672 | 5.6 | heterogeneous nuclear ribonucleoprotein F [Homo sapiens] |
| U | *gi|148470402|ref|NP\_0* | 7 | 16 | 27.2% | 415 | 45672 | 5.6 | heterogeneous nuclear ribonucleoprotein F [Homo sapiens] |
| U | *gi|148470400|ref|NP\_0* | 7 | 16 | 27.2% | 415 | 45672 | 5.6 | heterogeneous nuclear ribonucleoprotein F [Homo sapiens] |

| Filename XCorr DeltCN Conf% ObsM+H+ CalcM+H+ SpR ZScore Ion% # Sequence  | | | | | | | | | | | | |
| --- | --- | --- | --- | --- | --- | --- | --- | --- | --- | --- | --- | --- |
|  | IPAstrin\_STLCD\_tube2\_032114\_02.05743.05743.2 | 3.3114 | 0.1846 | 99.5% | 1711.3922 | 1710.7919 | 1 | 6.389 | 60.0% | 3 | R.QSGEAFVELGSEDDVK.M | 2 |
|  | IPAstrin\_STLCD\_tube2\_032114\_01.12137.12137.2 | 5.5612 | 0.4252 | 100.0% | 1868.4922 | 1869.0813 | 1 | 9.225 | 71.9% | 3 | K.ITGEAFVQFASQELAEK.A | 2 |
|  | IPAstrin\_STLCD\_tube2\_032114\_02.08328.08328.3 | 6.6304 | 0.4818 | 100.0% | 3476.0344 | 3476.7114 | 1 | 9.208 | 30.6% | 1 | R.MRPGAYSTGYGGYEEYSGLSDGYGFTTDLFGR.D | 3 |
|  | IPAstrin\_STLCD\_032114\_01.13498.13498.2 | 3.7193 | 0.4677 | 100.0% | 1998.0521 | 1998.2023 | 1 | 7.585 | 50.0% | 2 | K.ATENDIYNFFSPLNPVR.V | 22 |
|  | IPAstrin\_STLCD\_tube2\_032114\_01.12737.12737.3 | 3.779 | 0.1862 | 97.3% | 1998.5044 | 1998.2023 | 17 | 4.461 | 34.4% | 1 | K.ATENDIYNFFSPLNPVR.V | 33 |
|  | IPAstrin\_STLCD\_tube2\_032114\_01.04458.04458.2 | 2.8245 | 0.4221 | 100.0% | 1093.3922 | 1093.2278 | 1 | 8.46 | 83.3% | 5 | R.VHIEIGPDGR.V | 222 |
|  | IPAstrin\_STLCD\_tube2\_032114\_02.05991.05991.3 | 3.1174 | 0.2685 | 97.8% | 2192.3943 | 2193.39 | 3 | 5.261 | 27.5% | 1 | R.VTGEADVEFATHEEAVAAMSK.D | 3 |

Similarities:
gi|5031753|ref|NP\_005(3:4)  
gi|74099697|ref|NP\_00(1:6)  

---

|  |  |  |  |  |  |  |  |  |
| --- | --- | --- | --- | --- | --- | --- | --- | --- |
| U | *gi|218505827|ref|NP\_1* | 6 | 15 | 27.2% | 316 | 35438 | 6.3 | TRAF4 associated factor 1 isoform a [Homo sapiens] |
| U | *gi|218505831|ref|NP\_0* | 6 | 15 | 30.1% | 286 | 31880 | 7.1 | TRAF4 associated factor 1 isoform b [Homo sapiens] |

| Filename XCorr DeltCN Conf% ObsM+H+ CalcM+H+ SpR ZScore Ion% # Sequence  | | | | | | | | | | | | |
| --- | --- | --- | --- | --- | --- | --- | --- | --- | --- | --- | --- | --- |
|  | IPAstrin\_STLCD\_tube2\_032114\_01.07232.07232.2 | 5.7309 | 0.5443 | 100.0% | 2275.4321 | 2275.4802 | 1 | 9.239 | 59.5% | 4 | K.TVYSLQPPSALSGGQPADTQTR.A | 2 |
|  | IPAstrin\_STLCD\_032114\_01.03679.03679.2 | 2.2199 | 0.4447 | 99.9% | 1382.5122 | 1382.4728 | 3 | 7.9 | 62.5% | 1 | K.QLHSGGPENDVTK.I | 2 |
|  | IPAstrin\_STLCD\_032114\_01.06166.06166.3 | 3.0508 | 0.3546 | 99.8% | 1988.9043 | 1988.2023 | 1 | 5.589 | 37.5% | 1 | K.SEEELKDKNQLLEAVNK.Q | 3 |
|  | IPAstrin\_STLCD\_tube2\_032114\_01.04990.04990.2 | 4.4638 | 0.392 | 100.0% | 1605.2722 | 1604.7979 | 4 | 6.745 | 61.5% | 1 | K.LTETQGELKDLTQK.V | 2 |
|  | IPAstrin\_STLCD\_tube2\_032114\_01.09758.09758.3 | 4.6813 | 0.3232 | 100.0% | 2317.7344 | 2316.6543 | 1 | 6.0 | 36.8% | 2 | K.LTETQGELKDLTQKVELLEK.F | 3 |
|  | IPAstrin\_STLCD\_tube2\_032114\_01.06626.06626.2 | 4.4208 | 0.4643 | 100.0% | 1388.2122 | 1387.5327 | 1 | 9.262 | 65.4% | 6 | K.GLDPALGSETLASR.Q | 2 |

---

|  |  |  |  |  |  |  |  |  |
| --- | --- | --- | --- | --- | --- | --- | --- | --- |
| U | *gi|4501881|ref|NP\_001* | 12 | 41 | 27.1% | 377 | 42051 | 5.4 | actin, alpha 1, skeletal muscle [Homo sapiens] |
| U | *gi|4885049|ref|NP\_005* | 12 | 41 | 27.1% | 377 | 42019 | 5.4 | cardiac muscle alpha actin 1 proprotein [Homo sapiens] |

| Filename XCorr DeltCN Conf% ObsM+H+ CalcM+H+ SpR ZScore Ion% # Sequence  | | | | | | | | | | | | |
| --- | --- | --- | --- | --- | --- | --- | --- | --- | --- | --- | --- | --- |
|  | IPAstrin\_STLCD\_tube2\_032114\_01.06184.06184.2 | 2.9176 | 0.3032 | 99.9% | 1200.3121 | 1199.4415 | 1 | 5.206 | 70.0% | 5 | R.AVFPSIVGRPR.H | 22 |
|  | IPAstrin\_STLCD\_tube2\_032114\_01.08128.08128.2 | 3.9703 | 0.26 | 100.0% | 1962.2722 | 1962.1841 | 1 | 6.066 | 56.7% | 2 | K.YPIEHGIITNWDDMEK.I | 2 |
|  | IPAstrin\_STLCD\_032114\_01.06444.06444.2 | 3.1096 | 0.3417 | 100.0% | 1516.1122 | 1516.7019 | 1 | 5.977 | 65.0% | 7 | K.IWHHTFYNELR.V | 22 |
|  | IPAstrin\_STLCD\_tube2\_032114\_01.05558.05558.3 | 3.3425 | 0.2701 | 99.4% | 1516.9143 | 1516.7019 | 1 | 6.452 | 57.5% | 3 | K.IWHHTFYNELR.V | 33 |
|  | IPAstrin\_STLCD\_tube2\_032114\_01.07097.07097.2 | 4.8637 | 0.3989 | 100.0% | 1957.5721 | 1957.234 | 1 | 7.609 | 55.9% | 2 | R.VAPEEHPTLLTEAPLNPK.A | 2 |
|  | IPAstrin\_STLCD\_tube2\_032114\_01.10210.10210.2 | 2.9411 | 0.1875 | 99.1% | 1624.4521 | 1624.8927 | 2 | 5.353 | 53.8% | 2 | R.LDLAGRDLTDYLMK.I | 22 |
|  | IPAstrin\_STLCD\_032114\_01.10946.10946.1 | 2.0232 | 0.2376 | 100.0% | 998.51 | 999.167 | 1 | 5.803 | 71.4% | 2 | R.DLTDYLMK.I | 11 |
|  | IPAstrin\_STLCD\_032114\_01.10965.10965.2 | 2.0349 | 0.2647 | 98.4% | 999.0722 | 999.167 | 11 | 5.496 | 64.3% | 2 | R.DLTDYLMK.I | 22 |
|  | IPAstrin\_STLCD\_032114\_01.15333.15333.2 | 2.7053 | 0.3393 | 99.9% | 1611.5322 | 1611.8939 | 1 | 6.323 | 58.3% | 1 | R.DLTDYLMKILTER.G | 22 |
|  | IPAstrin\_STLCD\_tube2\_032114\_01.09428.09428.2 | 4.9451 | 0.3138 | 100.0% | 1792.4521 | 1791.9554 | 1 | 8.164 | 80.0% | 6 | K.SYELPDGQVITIGNER.F | 22 |
|  | IPAstrin\_STLCD\_032114\_01.06277.06277.1 | 2.747 | 0.455 | 100.0% | 1161.58 | 1162.3868 | 1 | 8.392 | 55.0% | 3 | K.EITALAPSTMK.I | 11 |
|  | IPAstrin\_STLCD\_032114\_01.06288.06288.2 | 2.6163 | 0.3984 | 100.0% | 1161.9321 | 1162.3868 | 1 | 6.234 | 70.0% | 6 | K.EITALAPSTMK.I | 22 |

Similarities:
gi|4501885|ref|NP\_001(10:2)  

---

|  |  |  |  |  |  |  |  |  |
| --- | --- | --- | --- | --- | --- | --- | --- | --- |
| U | *gi|209862831|ref|NP\_0* | 8 | 12 | 26.8% | 339 | 38604 | 7.8 | annexin A2 isoform 2 [Homo sapiens] |
| U | *gi|50845388|ref|NP\_00* | 8 | 12 | 25.5% | 357 | 40411 | 8.4 | annexin A2 isoform 1 [Homo sapiens] |
| U | *gi|50845386|ref|NP\_00* | 8 | 12 | 26.8% | 339 | 38604 | 7.8 | annexin A2 isoform 2 [Homo sapiens] |
| U | *gi|4757756|ref|NP\_004* | 8 | 12 | 26.8% | 339 | 38604 | 7.8 | annexin A2 isoform 2 [Homo sapiens] |

| Filename XCorr DeltCN Conf% ObsM+H+ CalcM+H+ SpR ZScore Ion% # Sequence  | | | | | | | | | | | | |
| --- | --- | --- | --- | --- | --- | --- | --- | --- | --- | --- | --- | --- |
|  | IPAstrin\_STLCD\_tube2\_032114\_01.07404.07404.2 | 2.6018 | 0.2183 | 99.4% | 1088.4122 | 1088.2462 | 4 | 5.245 | 66.7% | 1 | R.DALNIETAIK.T | 2 |
|  | IPAstrin\_STLCD\_032114\_01.06601.06601.2 | 2.6804 | 0.1921 | 99.4% | 1112.0122 | 1112.2303 | 1 | 4.257 | 75.0% | 3 | R.QDIAFAYQR.R | 2 |
|  | IPAstrin\_STLCD\_032114\_01.14288.14288.2 | 4.8022 | 0.4657 | 100.0% | 1651.6721 | 1651.9872 | 1 | 8.287 | 66.7% | 2 | K.SALSGHLETVILGLLK.T | 2 |
|  | IPAstrin\_STLCD\_032114\_01.04383.04383.2 | 2.8835 | 0.1479 | 99.2% | 1245.3722 | 1245.3347 | 1 | 4.552 | 72.2% | 1 | R.TNQELQEINR.V | 2 |
|  | IPAstrin\_STLCD\_tube2\_032114\_01.07637.07637.3 | 3.5049 | 0.2242 | 97.9% | 1940.7843 | 1941.102 | 44 | 5.329 | 34.4% | 2 | K.TDLEKDIISDTSGDFRK.L | 3 |
|  | IPAstrin\_STLCD\_tube2\_032114\_01.07851.07851.2 | 3.9233 | 0.4307 | 100.0% | 2065.2722 | 2066.1887 | 1 | 7.079 | 47.1% | 1 | R.RAEDGSVIDYELIDQDAR.D | 2 |
|  | IPAstrin\_STLCD\_tube2\_032114\_02.06132.06132.3 | 3.5938 | 0.1934 | 96.9% | 2066.0942 | 2066.1887 | 1 | 4.876 | 44.1% | 1 | R.RAEDGSVIDYELIDQDAR.D | 3 |
|  | IPAstrin\_STLCD\_tube2\_032114\_01.07895.07895.2 | 2.8662 | 0.3238 | 100.0% | 1422.1721 | 1422.5774 | 1 | 5.902 | 75.0% | 1 | K.SLYYYIQQDTK.G | 2 |

---

|  |  |  |  |  |  |  |  |  |
| --- | --- | --- | --- | --- | --- | --- | --- | --- |
| U | *gi|15718687|ref|NP\_00* | 5 | 10 | 26.7% | 243 | 26688 | 9.7 | ribosomal protein S3 [Homo sapiens] |

| Filename XCorr DeltCN Conf% ObsM+H+ CalcM+H+ SpR ZScore Ion% # Sequence  | | | | | | | | | | | | |
| --- | --- | --- | --- | --- | --- | --- | --- | --- | --- | --- | --- | --- |
| \* | IPAstrin\_STLCD\_tube2\_032114\_01.07792.07792.2 | 2.6173 | 0.2648 | 99.9% | 1093.3322 | 1093.2249 | 1 | 6.48 | 81.2% | 1 | K.AELNEFLTR.E | 2 |
| \* | IPAstrin\_STLCD\_tube2\_032114\_01.05320.05320.2 | 3.5876 | 0.3999 | 100.0% | 1424.1122 | 1424.5071 | 1 | 7.581 | 83.3% | 4 | R.ELAEDGYSGVEVR.V | 2 |
| \* | IPAstrin\_STLCD\_032114\_01.11431.11431.2 | 3.1528 | 0.3431 | 100.0% | 1573.7522 | 1573.7423 | 1 | 6.018 | 61.5% | 3 | R.FGFPEGSVELYAEK.V | 2 |
| \* | IPAstrin\_STLCD\_tube2\_032114\_01.06692.06692.2 | 2.6859 | 0.2159 | 99.0% | 1471.6122 | 1471.6476 | 15 | 4.484 | 66.7% | 1 | K.DEILPTTPISEQK.G | 2 |
| \* | IPAstrin\_STLCD\_tube2\_032114\_01.05471.05471.2 | 2.858 | 0.3074 | 99.9% | 1574.5122 | 1574.8352 | 40 | 5.101 | 43.3% | 1 | K.GGKPEPPAMPQPVPTA.- | 2 |

---

|  |  |  |  |  |  |  |  |  |
| --- | --- | --- | --- | --- | --- | --- | --- | --- |
| U | *gi|5729877|ref|NP\_006* | 14 | 27 | 26.3% | 646 | 70898 | 5.5 | heat shock 70kDa protein 8 isoform 1 [Homo sapiens] |

| Filename XCorr DeltCN Conf% ObsM+H+ CalcM+H+ SpR ZScore Ion% # Sequence  | | | | | | | | | | | | |
| --- | --- | --- | --- | --- | --- | --- | --- | --- | --- | --- | --- | --- |
|  | IPAstrin\_STLCD\_tube2\_032114\_01.06587.06587.2 | 3.1471 | 0.4339 | 100.0% | 1489.0521 | 1488.5939 | 1 | 8.802 | 70.8% | 7 | R.TTPSYVAFTDTER.L | 222 |
|  | IPAstrin\_STLCD\_032114\_01.08110.08110.2 | 4.3487 | 0.4831 | 100.0% | 1650.3121 | 1650.8468 | 1 | 9.533 | 78.6% | 2 | K.NQVAMNPTNTVFDAK.R | 2 |
|  | IPAstrin\_STLCD\_tube2\_032114\_01.05266.05266.2 | 2.4871 | 0.2952 | 99.8% | 1255.2322 | 1255.385 | 1 | 5.544 | 75.0% | 1 | R.FDDAVVQSDMK.H | 2 |
|  | IPAstrin\_STLCD\_tube2\_032114\_01.07492.07492.3 | 3.3417 | 0.3069 | 99.6% | 1654.7943 | 1654.9298 | 12 | 5.276 | 34.6% | 1 | K.HWPFMVVNDAGRPK.V | 3 |
|  | IPAstrin\_STLCD\_032114\_01.03762.03762.2 | 2.3853 | 0.2313 | 99.0% | 1180.9321 | 1181.3312 | 79 | 4.697 | 55.6% | 1 | K.VQVEYKGETK.S | 22 |
|  | IPAstrin\_STLCD\_tube2\_032114\_01.10179.10179.2 | 2.6962 | 0.1984 | 98.2% | 1617.1522 | 1617.8542 | 3 | 5.045 | 57.7% | 1 | K.SFYPEEVSSMVLTK.M | 2 |
|  | IPAstrin\_STLCD\_032114\_01.06208.06208.2 | 2.6151 | 0.2683 | 99.8% | 1253.6721 | 1253.4993 | 6 | 4.636 | 70.0% | 1 | K.MKEIAEAYLGK.T | 2 |
|  | IPAstrin\_STLCD\_tube2\_032114\_01.08578.08578.2 | 3.3693 | 0.3296 | 100.0% | 1983.5322 | 1983.1882 | 2 | 5.524 | 44.1% | 1 | K.TVTNAVVTVPAYFNDSQR.Q | 2 |
|  | IPAstrin\_STLCD\_032114\_01.10974.10974.2 | 4.4218 | 0.485 | 100.0% | 1660.4922 | 1660.9078 | 1 | 8.781 | 70.0% | 3 | R.IINEPTAAAIAYGLDK.K | 222 |
|  | IPAstrin\_STLCD\_tube2\_032114\_01.08004.08004.2 | 3.8742 | 0.2692 | 100.0% | 1788.4722 | 1789.0819 | 1 | 6.977 | 62.5% | 1 | R.IINEPTAAAIAYGLDKK.V | 22 |
|  | IPAstrin\_STLCD\_tube2\_032114\_01.07068.07068.2 | 2.9222 | 0.3858 | 100.0% | 1237.1522 | 1236.4741 | 1 | 6.549 | 77.8% | 2 | R.MVNHFIAEFK.R | 2 |
|  | IPAstrin\_STLCD\_032114\_01.10381.10381.2 | 3.304 | 0.3653 | 100.0% | 1481.2922 | 1481.6511 | 1 | 6.572 | 72.7% | 2 | R.ARFEELNADLFR.G | 22 |
|  | IPAstrin\_STLCD\_032114\_01.06591.06591.2 | 4.5011 | 0.5289 | 100.0% | 1482.0922 | 1482.6798 | 1 | 9.546 | 80.8% | 3 | K.SQIHDIVLVGGSTR.I | 2 |
| \* | IPAstrin\_STLCD\_tube2\_032114\_01.08135.08135.2 | 3.0702 | 0.41 | 100.0% | 1304.0721 | 1304.4602 | 2 | 7.121 | 75.0% | 1 | K.NSLESYAFNMK.A | 2 |

Similarities:
gi|13676857|ref|NP\_06(5:9)  
contaminant\_GR78\_HUMA(1:13)  
gi|167466173|ref|NP\_0(1:13)  

---

|  |  |  |  |  |  |  |  |  |
| --- | --- | --- | --- | --- | --- | --- | --- | --- |
| U | *gi|20149594|ref|NP\_03* | 13 | 29 | 25.8% | 724 | 83264 | 5.0 | heat shock 90kDa protein 1, beta [Homo sapiens] |

| Filename XCorr DeltCN Conf% ObsM+H+ CalcM+H+ SpR ZScore Ion% # Sequence  | | | | | | | | | | | | |
| --- | --- | --- | --- | --- | --- | --- | --- | --- | --- | --- | --- | --- |
|  | IPAstrin\_STLCD\_tube2\_032114\_01.05650.05650.2 | 3.1292 | 0.3573 | 100.0% | 1276.0922 | 1276.3861 | 3 | 5.739 | 59.1% | 1 | R.ELISNASDALDK.I | 2 |
|  | IPAstrin\_STLCD\_032114\_01.09438.09438.2 | 3.2877 | 0.3466 | 100.0% | 1546.3522 | 1545.733 | 1 | 5.95 | 73.1% | 1 | R.ELISNASDALDKIR.Y | 2 |
|  | IPAstrin\_STLCD\_tube2\_032114\_01.08184.08184.2 | 3.5154 | 0.4169 | 100.0% | 1242.6921 | 1243.4459 | 1 | 7.317 | 77.3% | 2 | K.ADLINNLGTIAK.S | 2 |
|  | IPAstrin\_STLCD\_tube2\_032114\_02.05899.05899.3 | 3.9424 | 0.424 | 100.0% | 2257.1943 | 2257.294 | 1 | 6.621 | 35.5% | 1 | K.HNDDEQYAWESSAGGSFTVR.A | 3 |
|  | IPAstrin\_STLCD\_tube2\_032114\_01.06111.06111.3 | 3.9792 | 0.4318 | 100.0% | 2015.6943 | 2016.2584 | 1 | 7.263 | 45.0% | 3 | K.VILHLKEDQTEYLEER.R | 3 |
| \* | IPAstrin\_STLCD\_tube2\_032114\_01.10823.10823.2 | 3.8762 | 0.4203 | 100.0% | 1809.5122 | 1810.1027 | 1 | 8.288 | 53.6% | 2 | K.HSQFIGYPITLYLEK.E | 2 |
| \* | IPAstrin\_STLCD\_tube2\_032114\_01.07558.07558.2 | 4.7677 | 0.4671 | 100.0% | 1848.1721 | 1848.9171 | 1 | 8.505 | 78.6% | 3 | R.NPDDITQEEYGEFYK.S | 2 |
|  | IPAstrin\_STLCD\_tube2\_032114\_01.07444.07444.2 | 3.7787 | 0.4278 | 100.0% | 1527.6522 | 1528.6616 | 1 | 7.73 | 62.5% | 2 | K.SLTNDWEDHLAVK.H | 2 |
|  | IPAstrin\_STLCD\_tube2\_032114\_02.05831.05831.2 | 2.9491 | 0.2759 | 99.9% | 1350.3322 | 1349.4886 | 5 | 5.568 | 55.0% | 4 | K.HFSVEGQLEFR.A | 2 |
| \* | IPAstrin\_STLCD\_tube2\_032114\_01.08374.08374.2 | 2.6602 | 0.1724 | 98.7% | 1236.9922 | 1237.4008 | 1 | 4.918 | 77.8% | 1 | R.RAPFDLFENK.K | 2 |
| \* | IPAstrin\_STLCD\_032114\_01.09069.09069.3 | 3.8677 | 0.3554 | 100.0% | 2178.2344 | 2178.2915 | 1 | 6.127 | 36.1% | 3 | R.YHTSQSGDEMTSLSEYVSR.M | 3 |
| \* | IPAstrin\_STLCD\_032114\_01.07946.07946.3 | 4.2594 | 0.4702 | 100.0% | 1784.1543 | 1784.025 | 1 | 8.217 | 48.2% | 5 | K.HLEINPDHPIVETLR.Q | 3 |
| \* | IPAstrin\_STLCD\_tube2\_032114\_01.17798.17798.3 | 3.6968 | 0.3836 | 100.0% | 2989.0444 | 2990.3398 | 1 | 6.124 | 26.0% | 1 | K.DLVVLLFETALLSSGFSLEDPQTHSNR.I | 3 |

---

|  |  |  |  |  |  |  |  |  |
| --- | --- | --- | --- | --- | --- | --- | --- | --- |
| U | *gi|21396489|ref|NP\_00* | 16 | 31 | 25.4% | 959 | 106489 | 6.4 | mitochondrial lon peptidase 1 [Homo sapiens] |

| Filename XCorr DeltCN Conf% ObsM+H+ CalcM+H+ SpR ZScore Ion% # Sequence  | | | | | | | | | | | | |
| --- | --- | --- | --- | --- | --- | --- | --- | --- | --- | --- | --- | --- |
| \* | IPAstrin\_STLCD\_tube2\_032114\_01.08830.08830.2 | 2.7919 | 0.0956 | 95.3% | 1378.5322 | 1378.6044 | 2 | 5.425 | 63.6% | 2 | R.ESVLQMMQAGQR.V | 2 |
| \* | IPAstrin\_STLCD\_tube2\_032114\_01.14093.14093.3 | 3.7792 | 0.2575 | 98.6% | 3672.1443 | 3671.0674 | 16 | 4.731 | 19.7% | 2 | R.VVDNPIYLSDMGAALTGAESHELQDVLEETNIPK.R | 3 |
| \* | IPAstrin\_STLCD\_tube2\_032114\_01.06887.06887.2 | 3.7144 | 0.5202 | 100.0% | 1401.3922 | 1401.5745 | 1 | 9.182 | 72.7% | 2 | K.HVMDVVDEELSK.L | 2 |
| \* | IPAstrin\_STLCD\_tube2\_032114\_02.05949.05949.2 | 2.447 | 0.2011 | 95.8% | 1701.9321 | 1702.8644 | 410 | 5.341 | 35.7% | 1 | K.LGLLDNHSSEFNVTR.N | 2 |
| \* | IPAstrin\_STLCD\_tube2\_032114\_02.05964.05964.3 | 4.4438 | 0.3901 | 100.0% | 1702.6444 | 1702.8644 | 2 | 6.574 | 41.1% | 2 | K.LGLLDNHSSEFNVTR.N | 3 |
| \* | IPAstrin\_STLCD\_tube2\_032114\_01.14651.14651.2 | 3.7879 | 0.4637 | 100.0% | 1593.3722 | 1593.8223 | 1 | 7.383 | 70.8% | 2 | R.NYLDWLTSIPWGK.Y | 2 |
| \* | IPAstrin\_STLCD\_tube2\_032114\_01.04486.04486.2 | 2.4963 | 0.3336 | 99.9% | 1194.1322 | 1195.2743 | 1 | 6.009 | 72.2% | 2 | K.YSNENLDLAR.A | 2 |
| \* | IPAstrin\_STLCD\_tube2\_032114\_01.11008.11008.2 | 2.8938 | 0.2731 | 99.9% | 1289.1122 | 1289.5608 | 1 | 5.942 | 70.0% | 1 | R.ILEFIAVSQLR.G | 2 |
| \* | IPAstrin\_STLCD\_032114\_01.10179.10179.2 | 3.7259 | 0.3612 | 100.0% | 1354.1122 | 1354.561 | 1 | 7.645 | 66.7% | 2 | R.FSVGGMTDVAEIK.G | 2 |
| \* | IPAstrin\_STLCD\_032114\_01.13130.13130.2 | 3.7625 | 0.377 | 100.0% | 1825.3722 | 1826.1002 | 1 | 7.154 | 66.7% | 2 | K.TENPLILIDEVDKIGR.G | 2 |
| \* | IPAstrin\_STLCD\_tube2\_032114\_01.15574.15574.3 | 5.2661 | 0.489 | 100.0% | 3874.4944 | 3875.2373 | 1 | 8.785 | 26.5% | 3 | R.GYQGDPSSALLELLDPEQNANFLDHYLDVPVDLSK.V | 3 |
| \* | IPAstrin\_STLCD\_032114\_02.06741.06741.2 | 3.8076 | 0.3406 | 100.0% | 1598.8322 | 1599.8574 | 1 | 6.849 | 65.4% | 3 | R.MEMINVSGYVAQEK.L | 2 |
| \* | IPAstrin\_STLCD\_tube2\_032114\_01.12150.12150.2 | 3.2058 | 0.3029 | 100.0% | 1202.3322 | 1202.4772 | 1 | 6.816 | 80.0% | 1 | K.LSSDVLTLLIK.Q | 2 |
| \* | IPAstrin\_STLCD\_tube2\_032114\_02.06438.06438.2 | 2.2484 | 0.2461 | 96.5% | 1563.6522 | 1563.7632 | 1 | 4.64 | 53.6% | 1 | K.DGSLEVTGQLGEVMK.E | 2 |
| \* | IPAstrin\_STLCD\_tube2\_032114\_02.05473.05473.2 | 3.6521 | 0.5161 | 100.0% | 1448.7122 | 1449.6624 | 1 | 7.886 | 65.4% | 4 | R.QNLAMTGEVSLTGK.I | 2 |
| \* | IPAstrin\_STLCD\_tube2\_032114\_01.12312.12312.2 | 3.3988 | 0.3916 | 100.0% | 2164.7722 | 2164.3765 | 15 | 5.857 | 33.3% | 1 | R.EIFDIAFPDEQAEALAVER.- | 2 |

---

|  |  |  |  |  |  |  |  |  |
| --- | --- | --- | --- | --- | --- | --- | --- | --- |
| U | *gi|4503529|ref|NP\_001* | 6 | 12 | 25.4% | 406 | 46154 | 5.5 | eukaryotic translation initiation factor 4A isoform 1 [Homo sapiens] |

| Filename XCorr DeltCN Conf% ObsM+H+ CalcM+H+ SpR ZScore Ion% # Sequence  | | | | | | | | | | | | |
| --- | --- | --- | --- | --- | --- | --- | --- | --- | --- | --- | --- | --- |
| \* | IPAstrin\_STLCD\_tube2\_032114\_01.15869.15869.3 | 5.1292 | 0.3615 | 100.0% | 4170.0244 | 4169.451 | 1 | 5.869 | 21.5% | 2 | R.SRDNGPDGMEPEGVIESNWNEIVDSFDDMNLSESLLR.G | 3 |
|  | IPAstrin\_STLCD\_032114\_01.08190.08190.2 | 4.8086 | 0.5811 | 100.0% | 1828.5122 | 1829.0654 | 1 | 9.069 | 76.7% | 3 | R.GIYAYGFEKPSAIQQR.A | 22 |
| \* | IPAstrin\_STLCD\_tube2\_032114\_01.07119.07119.3 | 4.237 | 0.511 | 100.0% | 1619.7244 | 1619.9225 | 1 | 8.342 | 50.0% | 2 | K.LQMEAPHIIVGTPGR.V | 3 |
|  | IPAstrin\_STLCD\_tube2\_032114\_01.11907.11907.2 | 4.7766 | 0.531 | 100.0% | 1556.2722 | 1556.789 | 1 | 9.983 | 79.2% | 1 | K.MFVLDEADEMLSR.G | 2 |
| \* | IPAstrin\_STLCD\_tube2\_032114\_01.09797.09797.2 | 3.8037 | 0.2909 | 100.0% | 1501.7922 | 1502.71 | 2 | 6.017 | 68.2% | 1 | R.GFKDQIYDIFQK.L | 2 |
|  | IPAstrin\_STLCD\_tube2\_032114\_01.09131.09131.2 | 2.4343 | 0.2714 | 99.6% | 1115.0721 | 1115.3585 | 15 | 5.003 | 66.7% | 3 | R.VLITTDLLAR.G | 2 |

Similarities:
gi|7661920|ref|NP\_055(1:5)  

---

|  |  |  |  |  |  |  |  |  |
| --- | --- | --- | --- | --- | --- | --- | --- | --- |
| U | *gi|14043070|ref|NP\_11* | 8 | 28 | 25.3% | 372 | 38747 | 9.1 | heterogeneous nuclear ribonucleoprotein A1 isoform b [Homo sapiens] |
| U | *gi|4504445|ref|NP\_002* | 8 | 28 | 29.4% | 320 | 34196 | 9.2 | heterogeneous nuclear ribonucleoprotein A1 isoform a [Homo sapiens] |

| Filename XCorr DeltCN Conf% ObsM+H+ CalcM+H+ SpR ZScore Ion% # Sequence  | | | | | | | | | | | | |
| --- | --- | --- | --- | --- | --- | --- | --- | --- | --- | --- | --- | --- |
|  | IPAstrin\_STLCD\_tube2\_032114\_01.09994.09994.2 | 4.5633 | 0.4164 | 100.0% | 1913.1122 | 1914.1656 | 1 | 7.327 | 56.2% | 2 | R.KLFIGGLSFETTDESLR.S | 2 |
|  | IPAstrin\_STLCD\_tube2\_032114\_01.11502.11502.2 | 5.2296 | 0.4703 | 100.0% | 1785.7122 | 1785.9916 | 1 | 8.981 | 66.7% | 9 | K.LFIGGLSFETTDESLR.S | 2 |
|  | IPAstrin\_STLCD\_032114\_01.03751.03751.2 | 2.834 | 0.1785 | 98.6% | 1437.5922 | 1438.5834 | 1 | 5.196 | 66.7% | 1 | R.EDSQRPGAHLTVK.K | 2 |
|  | IPAstrin\_STLCD\_tube2\_032114\_01.09650.09650.2 | 3.7216 | 0.3926 | 100.0% | 1219.3121 | 1219.4387 | 1 | 7.213 | 94.4% | 6 | K.IEVIEIMTDR.G | 2 |
|  | IPAstrin\_STLCD\_032114\_01.10746.10746.2 | 3.6048 | 0.4086 | 100.0% | 1699.7122 | 1700.8016 | 2 | 7.092 | 50.0% | 3 | R.GFAFVTFDDHDSVDK.I | 2 |
|  | IPAstrin\_STLCD\_032114\_02.07603.07603.3 | 3.4599 | 0.384 | 100.0% | 2282.6042 | 2282.5579 | 1 | 6.407 | 34.2% | 1 | R.GFAFVTFDDHDSVDKIVIQK.Y | 3 |
|  | IPAstrin\_STLCD\_032114\_01.04987.04987.2 | 3.8458 | 0.483 | 100.0% | 1628.9521 | 1629.7721 | 1 | 7.663 | 63.3% | 5 | R.SSGPYGGGGQYFAKPR.N | 2 |
|  | IPAstrin\_STLCD\_032114\_01.03733.03733.2 | 4.9749 | 0.4855 | 100.0% | 1695.2122 | 1695.6561 | 1 | 9.656 | 64.7% | 1 | R.NQGGYGGSSSSSSYGSGR.R | 2 |

---

|  |  |  |  |  |  |  |  |  |
| --- | --- | --- | --- | --- | --- | --- | --- | --- |
| U | *gi|4506743|ref|NP\_001* | 5 | 9 | 25.0% | 208 | 24205 | 10.3 | ribosomal protein S8 [Homo sapiens] |

| Filename XCorr DeltCN Conf% ObsM+H+ CalcM+H+ SpR ZScore Ion% # Sequence  | | | | | | | | | | | | |
| --- | --- | --- | --- | --- | --- | --- | --- | --- | --- | --- | --- | --- |
| \* | IPAstrin\_STLCD\_tube2\_032114\_02.06294.06294.3 | 4.111 | 0.3751 | 100.0% | 1719.7743 | 1719.9353 | 1 | 6.565 | 50.0% | 1 | R.IIDVVYNASNNELVR.T | 3 |
| \* | IPAstrin\_STLCD\_tube2\_032114\_02.06286.06286.2 | 3.9291 | 0.442 | 100.0% | 1720.2122 | 1719.9353 | 1 | 7.771 | 71.4% | 3 | R.IIDVVYNASNNELVR.T | 2 |
| \* | IPAstrin\_STLCD\_032114\_01.09931.09931.2 | 2.5519 | 0.3029 | 99.8% | 1620.1921 | 1620.8076 | 18 | 5.814 | 58.3% | 1 | R.QWYESHYALPLGR.K | 2 |
|  | IPAstrin\_STLCD\_032114\_01.07093.07093.2 | 2.7702 | 0.211 | 99.4% | 1315.2122 | 1315.4631 | 31 | 4.825 | 60.0% | 2 | K.LTPEEEEILNK.K | 2 |
| \* | IPAstrin\_STLCD\_032114\_01.09372.09372.2 | 3.9869 | 0.4356 | 100.0% | 1507.4122 | 1507.6836 | 1 | 7.658 | 75.0% | 2 | K.ISSLLEEQFQQGK.L | 2 |

---

|  |  |  |  |  |  |  |  |  |
| --- | --- | --- | --- | --- | --- | --- | --- | --- |
| U | *gi|14165435|ref|NP\_11* | 9 | 22 | 24.2% | 463 | 50976 | 5.5 | heterogeneous nuclear ribonucleoprotein K isoform b [Homo sapiens] |
| U | *gi|14165439|ref|NP\_00* | 9 | 21 | 24.1% | 464 | 51028 | 5.3 | heterogeneous nuclear ribonucleoprotein K isoform a [Homo sapiens] |
| U | *gi|14165437|ref|NP\_11* | 9 | 22 | 24.1% | 464 | 51028 | 5.3 | heterogeneous nuclear ribonucleoprotein K isoform a [Homo sapiens] |

| Filename XCorr DeltCN Conf% ObsM+H+ CalcM+H+ SpR ZScore Ion% # Sequence  | | | | | | | | | | | | |
| --- | --- | --- | --- | --- | --- | --- | --- | --- | --- | --- | --- | --- |
|  | IPAstrin\_STLCD\_tube2\_032114\_01.04880.04880.2 | 2.6041 | 0.3224 | 99.9% | 1106.9922 | 1107.2238 | 1 | 6.573 | 81.2% | 2 | R.NTDEMVELR.I | 2 |
|  | IPAstrin\_STLCD\_032114\_01.05240.05240.2 | 4.2362 | 0.4493 | 100.0% | 1782.2122 | 1781.8302 | 1 | 8.029 | 59.4% | 2 | R.TDYNASVSVPDSSGPER.I | 2 |
|  | IPAstrin\_STLCD\_tube2\_032114\_01.15152.15152.2 | 3.3505 | 0.4347 | 100.0% | 1716.1522 | 1716.0251 | 1 | 6.756 | 56.7% | 4 | R.ILSISADIETIGEILK.K | 2 |
|  | IPAstrin\_STLCD\_tube2\_032114\_01.13948.13948.2 | 3.7357 | 0.4371 | 100.0% | 1843.9922 | 1844.1992 | 1 | 8.005 | 43.8% | 1 | R.ILSISADIETIGEILKK.I | 2 |
|  | IPAstrin\_STLCD\_tube2\_032114\_01.08100.08100.2 | 4.0076 | 0.4657 | 100.0% | 1519.7722 | 1519.8711 | 1 | 7.884 | 78.6% | 5 | R.LLIHQSLAGGIIGVK.G | 2 |
|  | IPAstrin\_STLCD\_032114\_02.06738.06738.3 | 4.7124 | 0.4218 | 100.0% | 1520.0643 | 1519.8711 | 1 | 6.759 | 55.4% | 1 | R.LLIHQSLAGGIIGVK.G | 3 |
|  | IPAstrin\_STLCD\_tube2\_032114\_01.11350.11350.2 | 4.0238 | 0.494 | 100.0% | 1341.4122 | 1341.6311 | 1 | 7.924 | 86.4% | 3 | K.IILDLISESPIK.G | 2 |
|  | IPAstrin\_STLCD\_032114\_01.11179.11179.2 | 5.0837 | 0.3915 | 100.0% | 1918.6921 | 1918.1974 | 1 | 8.86 | 55.6% | 3 | R.GSYGDLGGPIITTQVTIPK.D | 2 |
|  | IPAstrin\_STLCD\_tube2\_032114\_01.11396.11396.3 | 3.4088 | 0.2926 | 98.9% | 2590.4644 | 2590.9365 | 1 | 5.417 | 28.4% | 1 | R.IITITGTQDQIQNAQYLLQNSVK.Q | 3 |

---

|  |  |  |  |  |  |  |  |  |
| --- | --- | --- | --- | --- | --- | --- | --- | --- |
| U | *gi|10863927|ref|NP\_06* | 4 | 8 | 24.2% | 165 | 18012 | 7.8 | peptidylprolyl isomerase A [Homo sapiens] |
| U | *gi|169215435|ref|XP\_0* | 4 | 8 | 17.9% | 223 | 24376 | 6.9 | PREDICTED: similar to peptidylprolyl isomerase A-like [Homo sapiens] |

| Filename XCorr DeltCN Conf% ObsM+H+ CalcM+H+ SpR ZScore Ion% # Sequence  | | | | | | | | | | | | |
| --- | --- | --- | --- | --- | --- | --- | --- | --- | --- | --- | --- | --- |
|  | IPAstrin\_STLCD\_tube2\_032114\_01.09020.09020.2 | 3.4592 | 0.3543 | 100.0% | 1379.9722 | 1380.6268 | 1 | 6.864 | 68.2% | 2 | R.VSFELFADKVPK.T | 2 |
|  | IPAstrin\_STLCD\_tube2\_032114\_01.08835.08835.2 | 5.0031 | 0.4542 | 100.0% | 1832.2722 | 1833.0477 | 1 | 8.39 | 64.3% | 3 | K.SIYGEKFEDENFILK.H | 2 |
|  | IPAstrin\_STLCD\_tube2\_032114\_01.07347.07347.2 | 3.9107 | 0.0413 | 99.0% | 1506.0521 | 1506.7755 | 1 | 8.446 | 75.0% | 2 | K.VKEGMNIVEAMER.F | 2 |
|  | IPAstrin\_STLCD\_tube2\_032114\_01.08793.08793.2 | 2.7262 | 0.1228 | 96.8% | 1279.1921 | 1279.4689 | 9 | 5.716 | 55.0% | 1 | K.EGMNIVEAMER.F | 2 |

---

|  |  |  |  |  |  |  |  |  |
| --- | --- | --- | --- | --- | --- | --- | --- | --- |
| U | *gi|4506619|ref|NP\_000* | 3 | 4 | 24.2% | 157 | 17779 | 11.3 | ribosomal protein L24 [Homo sapiens] |

| Filename XCorr DeltCN Conf% ObsM+H+ CalcM+H+ SpR ZScore Ion% # Sequence  | | | | | | | | | | | | |
| --- | --- | --- | --- | --- | --- | --- | --- | --- | --- | --- | --- | --- |
| \* | IPAstrin\_STLCD\_tube2\_032114\_01.09844.09844.2 | 2.0274 | 0.2624 | 97.9% | 1192.4722 | 1193.391 | 14 | 4.758 | 75.0% | 1 | R.QINWTVLYR.R | 2 |
| \* | IPAstrin\_STLCD\_032114\_01.10833.10833.2 | 3.9012 | 0.4325 | 100.0% | 1263.2922 | 1262.5072 | 1 | 7.705 | 75.0% | 2 | R.AITGASLADIMAK.R | 2 |
| \* | IPAstrin\_STLCD\_032114\_01.13058.13058.2 | 2.1966 | 0.2705 | 97.3% | 1661.7522 | 1660.9072 | 10 | 4.998 | 40.0% | 1 | K.K@TAMAAAK@APT#KAAPK.Q | 2 |

---

|  |  |  |  |  |  |  |  |  |
| --- | --- | --- | --- | --- | --- | --- | --- | --- |
| U | *gi|50592996|ref|NP\_00* | 14 | 63 | 23.8% | 450 | 50433 | 4.9 | tubulin, beta, 4 [Homo sapiens] |

| Filename XCorr DeltCN Conf% ObsM+H+ CalcM+H+ SpR ZScore Ion% # Sequence  | | | | | | | | | | | | |
| --- | --- | --- | --- | --- | --- | --- | --- | --- | --- | --- | --- | --- |
|  | IPAstrin\_STLCD\_tube2\_032114\_01.09521.09521.2 | 4.4557 | 0.4927 | 100.0% | 1616.3922 | 1616.8701 | 2 | 8.148 | 64.3% | 6 | R.AILVDLEPGTMDSVR.S | 22 |
|  | IPAstrin\_STLCD\_tube2\_032114\_01.12326.12326.3 | 3.5451 | 0.3347 | 99.8% | 1960.1044 | 1960.151 | 1 | 6.352 | 39.7% | 3 | K.GHYTEGAELVDSVLDVVR.K | 333 |
|  | IPAstrin\_STLCD\_tube2\_032114\_01.12274.12274.2 | 7.3835 | 0.5359 | 100.0% | 1960.3121 | 1960.151 | 1 | 10.083 | 79.4% | 3 | K.GHYTEGAELVDSVLDVVR.K | 222 |
|  | IPAstrin\_STLCD\_tube2\_032114\_01.11199.11199.3 | 4.8341 | 0.4769 | 100.0% | 2088.1143 | 2088.325 | 1 | 8.071 | 40.3% | 4 | K.GHYTEGAELVDSVLDVVRK.E | 333 |
|  | IPAstrin\_STLCD\_tube2\_032114\_01.07706.07706.2 | 4.1678 | 0.4142 | 100.0% | 1320.0322 | 1320.5896 | 2 | 7.752 | 72.7% | 10 | R.IMNTFSVVPSPK.V | 222 |
|  | IPAstrin\_STLCD\_tube2\_032114\_01.06530.06530.2 | 2.998 | 0.3386 | 100.0% | 1131.3322 | 1131.2767 | 133 | 5.952 | 61.1% | 14 | R.FPGQLNADLR.K | 2222 |
|  | IPAstrin\_STLCD\_tube2\_032114\_01.05058.05058.2 | 2.4459 | 0.1587 | 95.7% | 1259.4521 | 1259.4508 | 80 | 4.442 | 55.0% | 1 | R.FPGQLNADLRK.L | 2222 |
|  | IPAstrin\_STLCD\_tube2\_032114\_01.07702.07702.2 | 3.6879 | 0.347 | 100.0% | 1272.4321 | 1272.5945 | 3 | 7.18 | 65.0% | 4 | R.KLAVNMVPFPR.L | 2222 |
|  | IPAstrin\_STLCD\_tube2\_032114\_01.09071.09071.1 | 2.021 | 0.2752 | 100.0% | 1143.62 | 1144.4204 | 9 | 5.967 | 61.1% | 1 | K.LAVNMVPFPR.L | 1111 |
|  | IPAstrin\_STLCD\_tube2\_032114\_01.09111.09111.2 | 3.5075 | 0.4273 | 100.0% | 1143.6522 | 1144.4204 | 1 | 8.353 | 94.4% | 4 | K.LAVNMVPFPR.L | 2222 |
|  | IPAstrin\_STLCD\_tube2\_032114\_01.11334.11334.2 | 3.3711 | 0.4597 | 100.0% | 1692.4922 | 1692.9678 | 1 | 8.146 | 67.9% | 3 | R.ALTVPELTQQMFDAK.N | 22 |
|  | IPAstrin\_STLCD\_tube2\_032114\_01.10725.10725.2 | 2.6091 | 0.3156 | 99.8% | 1696.4922 | 1697.8877 | 1 | 5.145 | 61.5% | 2 | K.NSSYFVEWIPNNVK.V | 2222 |
|  | IPAstrin\_STLCD\_tube2\_032114\_01.08130.08130.2 | 3.1632 | 0.3675 | 100.0% | 1386.3522 | 1386.6116 | 1 | 6.603 | 70.0% | 2 | K.RISEQFTAMFR.R | 222 |
|  | IPAstrin\_STLCD\_tube2\_032114\_01.09399.09399.2 | 3.8735 | 0.4603 | 100.0% | 1230.9922 | 1230.4241 | 1 | 7.297 | 94.4% | 6 | R.ISEQFTAMFR.R | 222 |

Similarities:
gi|29788785|ref|NP\_82(13:1)  
gi|5174735|ref|NP\_006(13:1)  
gi|14210536|ref|NP\_11(6:8)  

---

|  |  |  |  |  |  |  |  |  |
| --- | --- | --- | --- | --- | --- | --- | --- | --- |
| U | *gi|32455264|ref|NP\_85* | 4 | 12 | 23.6% | 199 | 22110 | 8.1 | peroxiredoxin 1 [Homo sapiens] |
| U | *gi|4505591|ref|NP\_002* | 4 | 12 | 23.6% | 199 | 22110 | 8.1 | peroxiredoxin 1 [Homo sapiens] |
| U | *gi|32455266|ref|NP\_85* | 4 | 12 | 23.6% | 199 | 22110 | 8.1 | peroxiredoxin 1 [Homo sapiens] |

| Filename XCorr DeltCN Conf% ObsM+H+ CalcM+H+ SpR ZScore Ion% # Sequence  | | | | | | | | | | | | |
| --- | --- | --- | --- | --- | --- | --- | --- | --- | --- | --- | --- | --- |
|  | IPAstrin\_STLCD\_032114\_01.11521.11521.2 | 2.9288 | 0.3441 | 99.9% | 1623.7122 | 1623.908 | 1 | 5.618 | 53.3% | 1 | K.QGGLGPMNIPLVSDPK.R | 2 |
|  | IPAstrin\_STLCD\_032114\_01.07317.07317.2 | 2.4533 | 0.3164 | 99.9% | 1108.3322 | 1108.2798 | 2 | 5.604 | 77.8% | 3 | R.TIAQDYGVLK.A | 2 |
|  | IPAstrin\_STLCD\_tube2\_032114\_01.06621.06621.2 | 2.8438 | 0.3153 | 99.9% | 1212.1322 | 1212.3915 | 15 | 6.103 | 70.0% | 4 | R.QITVNDLPVGR.S | 22 |
|  | IPAstrin\_STLCD\_032114\_01.09284.09284.2 | 3.3962 | 0.4374 | 100.0% | 1197.2322 | 1197.3763 | 1 | 8.161 | 83.3% | 4 | R.LVQAFQFTDK.H | 2 |

Similarities:
gi|32189392|ref|NP\_00(1:3)  

---

|  |  |  |  |  |  |  |  |  |
| --- | --- | --- | --- | --- | --- | --- | --- | --- |
| U | *gi|14141152|ref|NP\_00* | 14 | 50 | 23.2% | 730 | 77516 | 8.7 | heterogeneous nuclear ribonucleoprotein M isoform a [Homo sapiens] |
| U | *gi|157412270|ref|NP\_1* | 14 | 50 | 24.5% | 691 | 73621 | 8.8 | heterogeneous nuclear ribonucleoprotein M isoform b [Homo sapiens] |

| Filename XCorr DeltCN Conf% ObsM+H+ CalcM+H+ SpR ZScore Ion% # Sequence  | | | | | | | | | | | | |
| --- | --- | --- | --- | --- | --- | --- | --- | --- | --- | --- | --- | --- |
|  | IPAstrin\_STLCD\_032114\_01.12123.12123.2 | 3.2431 | 0.4196 | 100.0% | 1265.7122 | 1265.4949 | 1 | 6.376 | 80.0% | 2 | R.AFITNIPFDVK.W | 2 |
|  | IPAstrin\_STLCD\_tube2\_032114\_01.12426.12426.2 | 4.3303 | 0.5061 | 100.0% | 1753.2722 | 1754.0051 | 1 | 8.957 | 63.3% | 2 | K.VGEVTYVELLMDAEGK.S | 2 |
|  | IPAstrin\_STLCD\_032114\_02.06984.06984.2 | 3.5705 | 0.5024 | 100.0% | 1427.2522 | 1427.6403 | 10 | 7.747 | 45.8% | 5 | R.LGSTVFVANLDYK.V | 2 |
|  | IPAstrin\_STLCD\_tube2\_032114\_01.08609.08609.2 | 3.3296 | 0.1631 | 99.3% | 1716.3121 | 1715.9724 | 1 | 5.399 | 46.9% | 3 | K.MGGMEGPFGGGMENMGR.F | 2 |
|  | IPAstrin\_STLCD\_032114\_01.09048.09048.2 | 2.8917 | 0.2942 | 99.9% | 1115.3121 | 1115.3152 | 21 | 5.578 | 66.7% | 3 | R.INEILSNALK.R | 2 |
|  | IPAstrin\_STLCD\_032114\_01.04062.04062.2 | 2.7635 | 0.2761 | 99.9% | 1103.1522 | 1102.2714 | 1 | 5.074 | 65.0% | 1 | R.MGAGLGHGMDR.V | 2 |
|  | IPAstrin\_STLCD\_032114\_01.09846.09846.3 | 3.6682 | 0.5295 | 100.0% | 1614.1743 | 1614.875 | 1 | 7.862 | 48.2% | 4 | R.MGPLGLDHMASSIER.M | 3 |
|  | IPAstrin\_STLCD\_032114\_01.09790.09790.2 | 4.3642 | 0.446 | 100.0% | 1614.2522 | 1614.875 | 1 | 8.549 | 75.0% | 3 | R.MGPLGLDHMASSIER.M | 2 |
|  | IPAstrin\_STLCD\_tube2\_032114\_02.06033.06033.2 | 3.6866 | 0.5202 | 100.0% | 1126.0322 | 1126.3337 | 2 | 9.22 | 70.0% | 5 | R.MGAGMGFGLER.M | 2 |
|  | IPAstrin\_STLCD\_tube2\_032114\_01.05608.05608.2 | 2.7147 | 0.516 | 100.0% | 1189.1721 | 1189.4333 | 1 | 7.489 | 77.3% | 2 | R.MVPAGMGAGLER.M | 2 |
|  | IPAstrin\_STLCD\_tube2\_032114\_01.07172.07172.2 | 3.3401 | 0.3054 | 100.0% | 1429.2122 | 1428.7076 | 1 | 6.221 | 67.9% | 4 | R.MGPAMGPALGAGIER.M | 2 |
|  | IPAstrin\_STLCD\_tube2\_032114\_02.05613.05613.2 | 4.0326 | 0.4477 | 100.0% | 1384.1921 | 1384.5677 | 1 | 8.984 | 78.6% | 10 | R.MGLAMGGGGGASFDR.A | 2 |
|  | IPAstrin\_STLCD\_032114\_01.08744.08744.2 | 4.7383 | 0.4992 | 100.0% | 2035.8522 | 2036.1735 | 2 | 9.359 | 38.6% | 2 | R.GNFGGSFAGSFGGAGGHAPGVAR.K | 2 |
|  | IPAstrin\_STLCD\_032114\_02.06059.06059.3 | 3.5467 | 0.4023 | 100.0% | 2036.4543 | 2036.1735 | 1 | 6.054 | 28.4% | 4 | R.GNFGGSFAGSFGGAGGHAPGVAR.K | 3 |

---

|  |  |  |  |  |  |  |  |  |
| --- | --- | --- | --- | --- | --- | --- | --- | --- |
| U | *gi|119395750|ref|NP\_0* | 11 | 18 | 23.1% | 644 | 66039 | 8.1 | keratin 1 [Homo sapiens] |

| Filename XCorr DeltCN Conf% ObsM+H+ CalcM+H+ SpR ZScore Ion% # Sequence  | | | | | | | | | | | | |
| --- | --- | --- | --- | --- | --- | --- | --- | --- | --- | --- | --- | --- |
| \* | IPAstrin\_STLCD\_032114\_02.05557.05557.2 | 4.0805 | 0.4176 | 100.0% | 1658.3121 | 1658.7678 | 1 | 6.447 | 59.4% | 1 | R.SGGGFSSGSAGIINYQR.R | 2 |
|  | IPAstrin\_STLCD\_tube2\_032114\_01.09329.09329.2 | 3.2957 | 0.4182 | 100.0% | 1384.2922 | 1384.5315 | 1 | 7.063 | 63.6% | 2 | K.SLNNQFASFIDK.V | 2 |
|  | IPAstrin\_STLCD\_tube2\_032114\_01.05296.05296.2 | 4.2794 | 0.067 | 99.8% | 1476.2722 | 1476.6726 | 1 | 6.963 | 90.9% | 3 | R.FLEQQNQVLQTK.W | 22 |
|  | IPAstrin\_STLCD\_032114\_01.10864.10864.2 | 3.0853 | 0.3844 | 100.0% | 1475.3722 | 1476.6293 | 1 | 6.563 | 72.7% | 2 | K.WELLQQVDTSTR.T | 2 |
|  | IPAstrin\_STLCD\_tube2\_032114\_01.12489.12489.2 | 3.369 | 0.46 | 100.0% | 1993.8922 | 1995.2017 | 1 | 6.746 | 60.0% | 1 | R.THNLEPYFESFINNLR.R | 2 |
|  | IPAstrin\_STLCD\_032114\_01.03831.03831.2 | 2.6693 | 0.1152 | 96.7% | 1310.2122 | 1309.4215 | 1 | 4.407 | 77.8% | 1 | R.NKYEDEINKR.T | 222 |
|  | IPAstrin\_STLCD\_tube2\_032114\_01.04347.04347.2 | 2.9772 | 0.2389 | 99.8% | 1393.9922 | 1394.5675 | 26 | 4.688 | 54.5% | 1 | R.TNAENEFVTIKK.D | 2 |
| \* | IPAstrin\_STLCD\_tube2\_032114\_01.12110.12110.2 | 3.7734 | 0.4586 | 100.0% | 1302.6522 | 1303.4955 | 1 | 8.613 | 81.8% | 1 | R.SLDLDSIIAEVK.A | 2 |
|  | IPAstrin\_STLCD\_tube2\_032114\_01.05621.05621.2 | 3.2069 | 0.3302 | 100.0% | 1180.3121 | 1180.303 | 1 | 7.894 | 83.3% | 3 | K.YEELQITAGR.H | 22 |
|  | IPAstrin\_STLCD\_tube2\_032114\_01.05304.05304.2 | 2.5054 | 0.1212 | 97.3% | 974.2922 | 974.102 | 123 | 4.93 | 71.4% | 1 | K.IEISELNR.V | 22 |
| \* | IPAstrin\_STLCD\_tube2\_032114\_01.08529.08529.3 | 4.0718 | 0.2666 | 99.4% | 2239.6443 | 2241.0396 | 1 | 4.326 | 37.0% | 2 | R.GGSGGGGGGS\*S\*GGRGSGGGSSGGSIGGR.G | 3 |

Similarities:
gi|4504919|ref|NP\_002(1:10)  
gi|47132620|ref|NP\_00(2:9)  
gi|119703753|ref|NP\_0(2:9)  

---

|  |  |  |  |  |  |  |  |  |
| --- | --- | --- | --- | --- | --- | --- | --- | --- |
| U | *gi|5031753|ref|NP\_005* | 7 | 16 | 22.5% | 449 | 49229 | 6.3 | heterogeneous nuclear ribonucleoprotein H1 [Homo sapiens] |

| Filename XCorr DeltCN Conf% ObsM+H+ CalcM+H+ SpR ZScore Ion% # Sequence  | | | | | | | | | | | | |
| --- | --- | --- | --- | --- | --- | --- | --- | --- | --- | --- | --- | --- |
|  | IPAstrin\_STLCD\_032114\_01.04140.04140.2 | 3.9174 | 0.3891 | 100.0% | 1686.2922 | 1685.7501 | 1 | 6.574 | 63.3% | 1 | K.HTGPNSPDTANDGFVR.L | 22 |
|  | IPAstrin\_STLCD\_tube2\_032114\_02.07582.07582.2 | 5.0394 | 0.5534 | 100.0% | 1842.2122 | 1843.0001 | 1 | 10.261 | 65.6% | 4 | R.STGEAFVQFASQEIAEK.A | 22 |
|  | IPAstrin\_STLCD\_032114\_01.13498.13498.2 | 3.7193 | 0.4677 | 100.0% | 1998.0521 | 1998.2023 | 1 | 7.585 | 50.0% | 2 | R.ATENDIYNFFSPLNPVR.V | 22 |
|  | IPAstrin\_STLCD\_tube2\_032114\_01.12737.12737.3 | 3.779 | 0.1862 | 97.3% | 1998.5044 | 1998.2023 | 17 | 4.461 | 34.4% | 1 | R.ATENDIYNFFSPLNPVR.V | 33 |
|  | IPAstrin\_STLCD\_tube2\_032114\_01.04458.04458.2 | 2.8245 | 0.4221 | 100.0% | 1093.3922 | 1093.2278 | 1 | 8.46 | 83.3% | 5 | R.VHIEIGPDGR.V | 222 |
| \* | IPAstrin\_STLCD\_032114\_02.06053.06053.3 | 3.1945 | 0.3949 | 100.0% | 2180.4243 | 2179.363 | 2 | 6.365 | 30.0% | 2 | R.VTGEADVEFATHEDAVAAMSK.D | 3 |
| \* | IPAstrin\_STLCD\_032114\_02.06785.06785.3 | 2.6505 | 0.2883 | 97.0% | 2143.4043 | 2143.32 | 1 | 4.937 | 31.6% | 1 | R.YVELFLNSTAGASGGAYEHR.Y | 3 |

Similarities:
gi|148470397|ref|NP\_0(3:4)  
gi|74099697|ref|NP\_00(3:4)  

---

|  |  |  |  |  |  |  |  |  |
| --- | --- | --- | --- | --- | --- | --- | --- | --- |
| U | *contaminant\_KERATIN12* | 9 | 24 | 22.3% | 431 | 47974 | 5.0 | no description |
| U | *gi|4557701|ref|NP\_000* | 9 | 24 | 22.2% | 432 | 48106 | 5.0 | keratin 17 [Homo sapiens] |

| Filename XCorr DeltCN Conf% ObsM+H+ CalcM+H+ SpR ZScore Ion% # Sequence  | | | | | | | | | | | | |
| --- | --- | --- | --- | --- | --- | --- | --- | --- | --- | --- | --- | --- |
|  | IPAstrin\_STLCD\_032114\_01.05754.05754.2 | 3.7632 | 0.4018 | 100.0% | 1346.2722 | 1346.4772 | 1 | 7.529 | 77.3% | 7 | R.ALEEANTELEVK.I | 2 |
|  | IPAstrin\_STLCD\_032114\_02.08423.08423.3 | 4.202 | 0.3132 | 100.0% | 2069.7844 | 2069.366 | 1 | 6.49 | 40.3% | 1 | K.ILTATVDNANILLQIDNAR.L | 3 |
|  | IPAstrin\_STLCD\_032114\_01.08774.08774.2 | 3.4852 | 0.4075 | 100.0% | 1030.1522 | 1030.2096 | 1 | 6.822 | 81.2% | 3 | R.VLDELTLAR.A | 222 |
|  | IPAstrin\_STLCD\_tube2\_032114\_01.11645.11645.2 | 2.6103 | 0.2746 | 99.4% | 2278.8123 | 2279.6538 | 9 | 4.815 | 33.3% | 1 | R.ADLEMQIENLKEELAYLKK.N | 2 |
|  | IPAstrin\_STLCD\_tube2\_032114\_01.11590.11590.3 | 3.7948 | 0.2812 | 99.4% | 2280.2644 | 2279.6538 | 1 | 5.487 | 36.1% | 2 | R.ADLEMQIENLKEELAYLKK.N | 3 |
|  | IPAstrin\_STLCD\_tube2\_032114\_01.04594.04594.2 | 3.8756 | 0.4569 | 100.0% | 1404.3121 | 1404.4764 | 1 | 7.781 | 70.8% | 3 | K.ASLEGNLAETENR.Y | 2 |
|  | IPAstrin\_STLCD\_tube2\_032114\_01.05660.05660.2 | 3.1546 | 0.0813 | 98.1% | 1382.6122 | 1380.5437 | 3 | 4.115 | 70.0% | 4 | K.TRLEQEIATYR.R | 22 |
|  | IPAstrin\_STLCD\_tube2\_032114\_01.04053.04053.2 | 3.0883 | 0.297 | 100.0% | 1122.9521 | 1123.2511 | 1 | 6.048 | 81.2% | 1 | R.LEQEIATYR.R | 222 |
|  | IPAstrin\_STLCD\_tube2\_032114\_01.04979.04979.2 | 3.6158 | 0.2859 | 100.0% | 1516.6122 | 1517.6787 | 1 | 7.533 | 58.3% | 2 | R.LLEGEDAHLTQYK.K | 2 |

Similarities:
contaminant\_KERATIN05(3:6)  
contaminant\_KERATIN10(2:7)  

---

|  |  |  |  |  |  |  |  |  |
| --- | --- | --- | --- | --- | --- | --- | --- | --- |
| U | *gi|33286418|ref|NP\_00* | 8 | 14 | 22.2% | 531 | 57937 | 7.8 | pyruvate kinase, muscle isoform M2 [Homo sapiens] |

| Filename XCorr DeltCN Conf% ObsM+H+ CalcM+H+ SpR ZScore Ion% # Sequence  | | | | | | | | | | | | |
| --- | --- | --- | --- | --- | --- | --- | --- | --- | --- | --- | --- | --- |
|  | IPAstrin\_STLCD\_032114\_01.07549.07549.2 | 2.9619 | 0.2336 | 99.9% | 1199.1122 | 1198.3617 | 12 | 5.23 | 65.0% | 3 | R.LDIDSPPITAR.N | 2 |
|  | IPAstrin\_STLCD\_032114\_01.07027.07027.2 | 2.2529 | 0.2446 | 97.3% | 1360.0521 | 1360.5193 | 85 | 4.747 | 50.0% | 1 | R.NTGIICTIGPASR.S | 2 |
|  | IPAstrin\_STLCD\_032114\_01.04197.04197.3 | 3.1137 | 0.3473 | 99.8% | 1883.8143 | 1885.0458 | 1 | 6.032 | 41.7% | 1 | R.LNFSHGTHEYHAETIK.N | 3 |
|  | IPAstrin\_STLCD\_tube2\_032114\_01.06116.06116.2 | 2.6597 | 0.3417 | 99.9% | 1198.2122 | 1198.3765 | 2 | 6.05 | 72.2% | 1 | K.ITLDNAYMEK.C | 2 |
|  | IPAstrin\_STLCD\_tube2\_032114\_01.10070.10070.2 | 3.0382 | 0.2688 | 99.9% | 1464.5122 | 1463.7142 | 2 | 5.364 | 62.5% | 2 | K.IYVDDGLISLQVK.Q | 2 |
|  | IPAstrin\_STLCD\_tube2\_032114\_01.15671.15671.2 | 4.3446 | 0.546 | 100.0% | 1860.8522 | 1861.1224 | 1 | 9.754 | 63.3% | 4 | K.FGVEQDVDMVFASFIR.K | 2 |
| \* | IPAstrin\_STLCD\_tube2\_032114\_02.07417.07417.3 | 2.4102 | 0.3281 | 98.0% | 2088.1443 | 2089.3586 | 1 | 5.431 | 31.2% | 1 | R.EAEAAIYHLQLFEELRR.L | 3 |
|  | IPAstrin\_STLCD\_tube2\_032114\_01.09768.09768.3 | 3.797 | 0.2545 | 98.8% | 2392.4043 | 2392.7815 | 1 | 4.652 | 38.1% | 1 | K.KGDVVIVLTGWRPGSGFTNTMR.V | 3 |

---

|  |  |  |  |  |  |  |  |  |
| --- | --- | --- | --- | --- | --- | --- | --- | --- |
| U | *gi|222352151|ref|NP\_0* | 4 | 5 | 22.2% | 356 | 37498 | 7.1 | poly(rC) binding protein 1 [Homo sapiens] |

| Filename XCorr DeltCN Conf% ObsM+H+ CalcM+H+ SpR ZScore Ion% # Sequence  | | | | | | | | | | | | |
| --- | --- | --- | --- | --- | --- | --- | --- | --- | --- | --- | --- | --- |
| \* | IPAstrin\_STLCD\_tube2\_032114\_01.09849.09849.2 | 3.654 | 0.3008 | 100.0% | 1390.3722 | 1389.6781 | 1 | 6.234 | 70.8% | 1 | R.IITLTGPTNAIFK.A | 2 |
|  | IPAstrin\_STLCD\_tube2\_032114\_02.05302.05302.2 | 4.2487 | 0.5519 | 100.0% | 2090.612 | 2091.2573 | 1 | 8.894 | 50.0% | 1 | R.ESTGAQVQVAGDMLPNSTER.A | 22 |
| \* | IPAstrin\_STLCD\_032114\_02.05792.05792.3 | 3.3848 | 0.2729 | 98.5% | 2606.0044 | 2607.875 | 1 | 5.19 | 26.0% | 1 | R.QQSHFAMMHGGTGFAGIDSSSPEVK.G | 3 |
| \* | IPAstrin\_STLCD\_tube2\_032114\_02.08785.08785.3 | 4.1392 | 0.3659 | 100.0% | 2178.3843 | 2178.4937 | 1 | 6.819 | 32.5% | 2 | R.QVTITGSAASISLAQYLINAR.L | 3 |

Similarities:
gi|14141166|ref|NP\_11(1:3)  

---

|  |  |  |  |  |  |  |  |  |
| --- | --- | --- | --- | --- | --- | --- | --- | --- |
| U | *gi|25777713|ref|NP\_73* | 3 | 7 | 22.1% | 163 | 18658 | 4.5 | S-phase kinase-associated protein 1 isoform b [Homo sapiens] |

| Filename XCorr DeltCN Conf% ObsM+H+ CalcM+H+ SpR ZScore Ion% # Sequence  | | | | | | | | | | | | |
| --- | --- | --- | --- | --- | --- | --- | --- | --- | --- | --- | --- | --- |
|  | IPAstrin\_STLCD\_tube2\_032114\_02.11038.11038.2 | 2.9032 | 0.3337 | 99.9% | 2136.412 | 2137.481 | 218 | 6.171 | 27.8% | 4 | K.VDQGTLFELILAANYLDIK.G | 2 |
| \* | IPAstrin\_STLCD\_032114\_02.06201.06201.3 | 4.5992 | 0.404 | 100.0% | 2071.4644 | 2071.2078 | 1 | 6.807 | 45.3% | 2 | K.TFNIKNDFTEEEEAQVR.K | 3 |
| \* | IPAstrin\_STLCD\_tube2\_032114\_01.04917.04917.2 | 2.1721 | 0.2432 | 96.9% | 1467.2122 | 1467.4888 | 22 | 5.133 | 63.6% | 1 | K.NDFTEEEEAQVR.K | 2 |

---

|  |  |  |  |  |  |  |  |  |
| --- | --- | --- | --- | --- | --- | --- | --- | --- |
| U | *gi|4506623|ref|NP\_000* | 2 | 3 | 22.1% | 136 | 15798 | 10.6 | ribosomal protein L27 [Homo sapiens] |

| Filename XCorr DeltCN Conf% ObsM+H+ CalcM+H+ SpR ZScore Ion% # Sequence  | | | | | | | | | | | | |
| --- | --- | --- | --- | --- | --- | --- | --- | --- | --- | --- | --- | --- |
| \* | IPAstrin\_STLCD\_032114\_01.06991.06991.3 | 3.2915 | 0.3006 | 98.9% | 2273.2444 | 2273.4238 | 1 | 5.597 | 32.5% | 1 | K.NIDDGTSDRPYSHALVAGIDR.Y | 3 |
| \* | IPAstrin\_STLCD\_tube2\_032114\_01.07043.07043.2 | 2.3248 | 0.3616 | 99.9% | 1050.3322 | 1050.1968 | 343 | 6.048 | 43.8% | 2 | R.YSVDIPLDK.T | 2 |

---

|  |  |  |  |  |  |  |  |  |
| --- | --- | --- | --- | --- | --- | --- | --- | --- |
| U | *gi|5031699|ref|NP\_005* | 7 | 11 | 22.0% | 427 | 47355 | 7.5 | flotillin 1 [Homo sapiens] |

| Filename XCorr DeltCN Conf% ObsM+H+ CalcM+H+ SpR ZScore Ion% # Sequence  | | | | | | | | | | | | |
| --- | --- | --- | --- | --- | --- | --- | --- | --- | --- | --- | --- | --- |
| \* | IPAstrin\_STLCD\_032114\_01.07606.07606.2 | 2.3921 | 0.3855 | 99.9% | 1405.5721 | 1406.6682 | 4 | 6.663 | 57.7% | 1 | R.HGVPISVTGIAQVK.I | 2 |
| \* | IPAstrin\_STLCD\_tube2\_032114\_02.05694.05694.3 | 2.8145 | 0.2889 | 98.5% | 1808.1244 | 1808.1206 | 4 | 5.722 | 41.1% | 1 | R.AIMAHMTVEEIYKDR.Q | 3 |
| \* | IPAstrin\_STLCD\_tube2\_032114\_01.08002.08002.2 | 2.4757 | 0.3246 | 99.8% | 1469.3922 | 1469.693 | 157 | 5.791 | 41.7% | 2 | K.VSAQYLSEIEMAK.A | 2 |
| \* | IPAstrin\_STLCD\_tube2\_032114\_02.05524.05524.2 | 4.3826 | 0.5151 | 100.0% | 1420.1921 | 1419.6206 | 1 | 9.63 | 79.2% | 2 | R.AQADLAYQLQVAK.T | 2 |
| \* | IPAstrin\_STLCD\_tube2\_032114\_02.06707.06707.2 | 4.1173 | 0.4962 | 100.0% | 1605.3922 | 1604.8187 | 1 | 8.795 | 67.9% | 2 | K.SQLIMQAEAEAASVR.M | 2 |
| \* | IPAstrin\_STLCD\_tube2\_032114\_01.05408.05408.2 | 2.8746 | 0.186 | 99.1% | 1380.4922 | 1379.5768 | 2 | 5.068 | 66.7% | 2 | R.MRGEAEAFAIGAR.A | 2 |
| \* | IPAstrin\_STLCD\_032114\_01.11613.11613.2 | 3.3766 | 0.2691 | 100.0% | 1216.4722 | 1216.4203 | 1 | 6.447 | 75.0% | 1 | K.VTGEVLDILTR.L | 2 |

---

|  |  |  |  |  |  |  |  |  |
| --- | --- | --- | --- | --- | --- | --- | --- | --- |
| U | *contaminant\_INT-STD1* | 19 | 82 | 21.6% | 607 | 69271 | 6.1 | BSA |

| Filename XCorr DeltCN Conf% ObsM+H+ CalcM+H+ SpR ZScore Ion% # Sequence  | | | | | | | | | | | | |
| --- | --- | --- | --- | --- | --- | --- | --- | --- | --- | --- | --- | --- |
| \* | IPAstrin\_STLCD\_032114\_01.09716.09716.1 | 2.2324 | 0.3845 | 100.0% | 1163.6 | 1164.344 | 1 | 6.861 | 72.2% | 2 | K.LVNELTEFAK.T | 1 |
| \* | IPAstrin\_STLCD\_tube2\_032114\_01.07958.07958.2 | 3.4503 | 0.3751 | 100.0% | 1163.6322 | 1164.344 | 3 | 6.674 | 77.8% | 3 | K.LVNELTEFAK.T | 2 |
| \* | IPAstrin\_STLCD\_032114\_01.09770.09770.2 | 2.5124 | 0.3215 | 99.9% | 1420.8322 | 1420.5713 | 3 | 5.571 | 54.5% | 1 | K.SLHTLFGDELCK.V | 2 |
|  | IPAstrin\_STLCD\_032114\_01.06889.06889.1 | 1.9998 | 0.2251 | 100.0% | 927.55 | 928.0758 | 2 | 4.408 | 75.0% | 3 | K.YLYEIAR.R | 1 |
|  | IPAstrin\_STLCD\_tube2\_032114\_01.05979.05979.2 | 2.6763 | 0.3803 | 100.0% | 928.1722 | 928.0758 | 1 | 5.81 | 83.3% | 4 | K.YLYEIAR.R | 2 |
| \* | IPAstrin\_STLCD\_032114\_01.11288.11288.2 | 5.1308 | 0.527 | 100.0% | 2045.3322 | 2046.3354 | 1 | 9.339 | 73.3% | 2 | R.RHPYFYAPELLYYANK.Y | 2 |
| \* | IPAstrin\_STLCD\_tube2\_032114\_01.09437.09437.3 | 4.5721 | 0.4354 | 100.0% | 2046.7144 | 2046.3354 | 1 | 7.11 | 50.0% | 3 | R.RHPYFYAPELLYYANK.Y | 3 |
| \* | IPAstrin\_STLCD\_tube2\_032114\_01.10552.10552.2 | 2.0348 | 0.3766 | 99.3% | 1889.3522 | 1890.148 | 1 | 5.233 | 53.6% | 1 | R.HPYFYAPELLYYANK.Y | 2 |
| \* | IPAstrin\_STLCD\_032114\_02.09672.09672.2 | 4.7309 | 0.4705 | 100.0% | 1569.3922 | 1568.7258 | 1 | 8.421 | 75.0% | 5 | K.DAFLGSFLYEYSR.R | 2 |
| \* | IPAstrin\_STLCD\_tube2\_032114\_01.06459.06459.2 | 3.202 | 0.4957 | 100.0% | 1439.5322 | 1440.6884 | 4 | 8.073 | 63.6% | 8 | R.RHPEYAVSVLLR.L | 2 |
| \* | IPAstrin\_STLCD\_tube2\_032114\_01.06394.06394.3 | 4.6449 | 0.283 | 100.0% | 1441.9443 | 1440.6884 | 1 | 5.676 | 56.8% | 12 | R.RHPEYAVSVLLR.L | 3 |
| \* | IPAstrin\_STLCD\_032114\_01.06228.06228.2 | 3.4243 | 0.5225 | 100.0% | 1305.9722 | 1306.5046 | 1 | 7.971 | 70.0% | 7 | K.HLVDEPQNLIK.Q | 2 |
| \* | IPAstrin\_STLCD\_tube2\_032114\_01.09674.09674.2 | 4.3909 | 0.42 | 100.0% | 1479.6322 | 1480.7068 | 1 | 8.276 | 70.8% | 10 | K.LGEYGFQNALIVR.Y | 2 |
|  | IPAstrin\_STLCD\_032114\_01.07224.07224.2 | 3.8674 | 0.4435 | 100.0% | 1640.4122 | 1640.9205 | 1 | 9.101 | 60.7% | 7 | R.KVPQVSTPTLVEVSR.S | 2 |
|  | IPAstrin\_STLCD\_tube2\_032114\_01.06074.06074.3 | 4.658 | 0.3531 | 100.0% | 1640.6943 | 1640.9205 | 1 | 7.792 | 55.4% | 5 | R.KVPQVSTPTLVEVSR.S | 3 |
|  | IPAstrin\_STLCD\_tube2\_032114\_01.07019.07019.2 | 2.9161 | 0.3376 | 99.9% | 1512.3121 | 1512.7465 | 2 | 5.755 | 57.7% | 3 | K.VPQVSTPTLVEVSR.S | 2 |
| \* | IPAstrin\_STLCD\_032114\_01.08773.08773.2 | 2.5637 | 0.1726 | 98.2% | 1143.3722 | 1143.4124 | 10 | 4.84 | 66.7% | 1 | K.KQTALVELLK.H | 2 |
| \* | IPAstrin\_STLCD\_tube2\_032114\_01.09010.09010.2 | 1.5973 | 0.38 | 97.6% | 1015.2322 | 1015.2383 | 7 | 6.445 | 75.0% | 1 | K.QTALVELLK.H | 2 |
| \* | IPAstrin\_STLCD\_032114\_01.13002.13002.2 | 3.7893 | 0.5575 | 100.0% | 1400.2522 | 1400.6324 | 1 | 9.767 | 72.7% | 4 | K.TVMENFVAFVDK.C | 2 |

---

|  |  |  |  |  |  |  |  |  |
| --- | --- | --- | --- | --- | --- | --- | --- | --- |
| U | *gi|6005854|ref|NP\_009* | 5 | 8 | 21.4% | 299 | 33296 | 9.8 | prohibitin 2 isoform 2 [Homo sapiens] |

| Filename XCorr DeltCN Conf% ObsM+H+ CalcM+H+ SpR ZScore Ion% # Sequence  | | | | | | | | | | | | |
| --- | --- | --- | --- | --- | --- | --- | --- | --- | --- | --- | --- | --- |
|  | IPAstrin\_STLCD\_tube2\_032114\_01.09501.09501.3 | 4.6636 | 0.4976 | 100.0% | 1854.8644 | 1855.1038 | 1 | 8.448 | 45.3% | 3 | R.IGGVQQDTILAEGLHFR.I | 3 |
|  | IPAstrin\_STLCD\_032114\_01.14534.14534.2 | 4.0542 | 0.3759 | 100.0% | 1724.0521 | 1725.0428 | 1 | 7.865 | 66.7% | 2 | R.IPWFQYPIIYDIR.A | 2 |
| \* | IPAstrin\_STLCD\_tube2\_032114\_01.05696.05696.3 | 3.4252 | 0.2342 | 98.4% | 1889.4844 | 1890.1675 | 174 | 4.502 | 36.7% | 1 | R.VLSRPNAQELPSMYQR.L | 3 |
|  | IPAstrin\_STLCD\_tube2\_032114\_01.05216.05216.2 | 2.1591 | 0.2754 | 99.3% | 993.8722 | 995.077 | 20 | 5.229 | 78.6% | 1 | R.LGLDYEER.V | 2 |
|  | IPAstrin\_STLCD\_032114\_01.08641.08641.2 | 2.7428 | 0.3583 | 100.0% | 1178.2522 | 1178.3335 | 1 | 6.055 | 77.8% | 1 | K.FNASQLITQR.A | 2 |

---

|  |  |  |  |  |  |  |  |  |
| --- | --- | --- | --- | --- | --- | --- | --- | --- |
| U | *gi|4826898|ref|NP\_005* | 2 | 3 | 21.4% | 140 | 15054 | 8.3 | profilin 1 [Homo sapiens] |

| Filename XCorr DeltCN Conf% ObsM+H+ CalcM+H+ SpR ZScore Ion% # Sequence  | | | | | | | | | | | | |
| --- | --- | --- | --- | --- | --- | --- | --- | --- | --- | --- | --- | --- |
| \* | IPAstrin\_STLCD\_032114\_01.12676.12676.2 | 3.0724 | 0.2634 | 99.9% | 1644.0521 | 1644.9518 | 2 | 5.028 | 53.3% | 2 | K.TFVNITPAEVGVLVGK.D | 2 |
| \* | IPAstrin\_STLCD\_tube2\_032114\_01.05733.05733.2 | 3.3347 | 0.3919 | 100.0% | 1380.0322 | 1380.5406 | 1 | 6.552 | 57.7% | 1 | K.STGGAPTFNVTVTK.T | 2 |

---

|  |  |  |  |  |  |  |  |  |
| --- | --- | --- | --- | --- | --- | --- | --- | --- |
| U | *gi|4504517|ref|NP\_001* | 4 | 6 | 21.0% | 205 | 22783 | 6.4 | heat shock protein beta-1 [Homo sapiens] |

| Filename XCorr DeltCN Conf% ObsM+H+ CalcM+H+ SpR ZScore Ion% # Sequence  | | | | | | | | | | | | |
| --- | --- | --- | --- | --- | --- | --- | --- | --- | --- | --- | --- | --- |
| \* | IPAstrin\_STLCD\_tube2\_032114\_01.09676.09676.2 | 3.6808 | 0.3789 | 100.0% | 1164.2922 | 1164.3494 | 1 | 7.729 | 83.3% | 2 | R.LFDQAFGLPR.L | 2 |
| \* | IPAstrin\_STLCD\_032114\_01.10192.10192.2 | 4.3858 | 0.469 | 100.0% | 1784.4722 | 1785.0068 | 1 | 7.902 | 53.3% | 2 | R.VSLDVNHFAPDELTVK.T | 2 |
| \* | IPAstrin\_STLCD\_tube2\_032114\_01.08450.08450.3 | 2.635 | 0.3364 | 98.8% | 1785.3544 | 1785.0068 | 240 | 5.154 | 26.7% | 1 | R.VSLDVNHFAPDELTVK.T | 3 |
| \* | IPAstrin\_STLCD\_tube2\_032114\_01.09149.09149.2 | 2.6255 | 0.3137 | 99.8% | 1906.4321 | 1907.1307 | 1 | 6.113 | 46.9% | 1 | K.LATQSNEITIPVTFESR.A | 2 |

---

|  |  |  |  |  |  |  |  |  |
| --- | --- | --- | --- | --- | --- | --- | --- | --- |
| U | *gi|36287110|ref|NP\_91* | 5 | 5 | 20.8% | 379 | 40907 | 4.6 | FGFR1 oncogene partner isoform b [Homo sapiens] |
| U | *gi|5901954|ref|NP\_008* | 4 | 4 | 19.8% | 399 | 43065 | 4.8 | FGFR1 oncogene partner isoform a [Homo sapiens] |

| Filename XCorr DeltCN Conf% ObsM+H+ CalcM+H+ SpR ZScore Ion% # Sequence  | | | | | | | | | | | | |
| --- | --- | --- | --- | --- | --- | --- | --- | --- | --- | --- | --- | --- |
|  | IPAstrin\_STLCD\_tube2\_032114\_01.14615.14615.2 | 3.8124 | 0.2359 | 99.9% | 2167.112 | 2165.5352 | 2 | 4.088 | 42.5% | 1 | R.DLGIIEAEGTVGGPLLLEVIR.R | 2 |
|  | IPAstrin\_STLCD\_tube2\_032114\_01.03834.03834.2 | 4.7564 | 0.5075 | 100.0% | 1892.4722 | 1892.928 | 1 | 9.262 | 58.8% | 1 | K.ANDEANQSDTSVSLSEPK.S | 2 |
|  | IPAstrin\_STLCD\_tube2\_032114\_01.05724.05724.2 | 3.1659 | 0.1457 | 98.4% | 1582.6122 | 1583.8253 | 6 | 5.147 | 53.3% | 1 | R.KQAGSLASLSDAPPLK.S | 2 |
| \* | IPAstrin\_STLCD\_tube2\_032114\_01.07060.07060.2 | 2.0325 | 0.2829 | 95.9% | 1455.6921 | 1455.6512 | 194 | 4.9 | 42.9% | 1 | K.QAGSLASLSDAPPLK.S | 2 |
|  | IPAstrin\_STLCD\_032114\_01.10270.10270.3 | 3.4857 | 0.347 | 99.8% | 2571.0842 | 2571.63 | 1 | 5.629 | 28.3% | 1 | K.IGSLGLGTGEDDDYVDDFNSTSHR.S | 3 |

---

|  |  |  |  |  |  |  |  |  |
| --- | --- | --- | --- | --- | --- | --- | --- | --- |
| U | *gi|87196351|ref|NP\_00* | 9 | 18 | 20.5% | 662 | 73244 | 7.2 | DEAD/H (Asp-Glu-Ala-Asp/His) box polypeptide 3 [Homo sapiens] |

| Filename XCorr DeltCN Conf% ObsM+H+ CalcM+H+ SpR ZScore Ion% # Sequence  | | | | | | | | | | | | |
| --- | --- | --- | --- | --- | --- | --- | --- | --- | --- | --- | --- | --- |
|  | IPAstrin\_STLCD\_tube2\_032114\_02.06709.06709.2 | 2.9474 | 0.1613 | 98.1% | 1884.4122 | 1884.0526 | 2 | 3.925 | 43.8% | 1 | R.LEQELFSGGNTGINFEK.Y | 2 |
| \* | IPAstrin\_STLCD\_tube2\_032114\_01.15527.15527.2 | 4.9951 | 0.461 | 100.0% | 2334.372 | 2333.6897 | 1 | 8.264 | 50.0% | 1 | K.TAAFLLPILSQIYSDGPGEALR.A | 2 |
|  | IPAstrin\_STLCD\_032114\_01.09529.09529.2 | 3.1951 | 0.306 | 100.0% | 1321.3522 | 1321.4729 | 1 | 5.512 | 85.0% | 3 | R.ELAVQIYEEAR.K | 2 |
|  | IPAstrin\_STLCD\_032114\_01.07425.07425.2 | 2.3232 | 0.1746 | 97.1% | 1094.3121 | 1094.2096 | 1 | 4.126 | 87.5% | 1 | K.YLVLDEADR.M | 2 |
|  | IPAstrin\_STLCD\_032114\_01.11119.11119.2 | 3.5919 | 0.4336 | 100.0% | 1337.1721 | 1337.5946 | 1 | 7.79 | 85.0% | 4 | R.MLDMGFEPQIR.R | 222 |
| \* | IPAstrin\_STLCD\_032114\_02.12276.12276.3 | 3.5923 | 0.2952 | 99.4% | 2525.1543 | 2525.945 | 119 | 5.789 | 23.9% | 1 | R.SFLLDLLNATGKDSLTLVFVETK.K | 3 |
|  | IPAstrin\_STLCD\_tube2\_032114\_01.07047.07047.2 | 4.0404 | 0.4449 | 100.0% | 1170.2922 | 1169.4099 | 1 | 7.397 | 77.3% | 3 | K.SPILVATAVAAR.G | 2 |
|  | IPAstrin\_STLCD\_tube2\_032114\_01.10661.10661.3 | 4.8202 | 0.377 | 100.0% | 2083.4343 | 2084.2957 | 1 | 7.733 | 45.3% | 2 | K.HVINFDLPSDIEEYVHR.I | 3 |
| \* | IPAstrin\_STLCD\_tube2\_032114\_01.10786.10786.2 | 4.3433 | 0.4405 | 100.0% | 1526.3922 | 1525.7043 | 1 | 7.577 | 73.1% | 2 | R.VGNLGLATSFFNER.N | 2 |

Similarities:
gi|4758138|ref|NP\_004(1:8)  
gi|148613856|ref|NP\_0(1:8)  

---

|  |  |  |  |  |  |  |  |  |
| --- | --- | --- | --- | --- | --- | --- | --- | --- |
| U | *gi|5174457|ref|NP\_006* | 11 | 20 | 20.2% | 642 | 73913 | 5.6 | kinetochore associated 2 [Homo sapiens] |

| Filename XCorr DeltCN Conf% ObsM+H+ CalcM+H+ SpR ZScore Ion% # Sequence  | | | | | | | | | | | | |
| --- | --- | --- | --- | --- | --- | --- | --- | --- | --- | --- | --- | --- |
| \* | IPAstrin\_STLCD\_032114\_01.07928.07928.2 | 2.4696 | 0.2994 | 99.6% | 1297.2922 | 1297.4075 | 2 | 5.01 | 68.2% | 2 | R.NSQLGIFSSSEK.I | 2 |
| \* | IPAstrin\_STLCD\_tube2\_032114\_02.05842.05842.3 | 2.6384 | 0.3022 | 97.7% | 2098.6143 | 2099.3228 | 4 | 4.979 | 36.8% | 1 | K.YQAYMSNLESHSAILDQK.L | 3 |
| \* | IPAstrin\_STLCD\_tube2\_032114\_01.04836.04836.2 | 2.5544 | 0.33 | 99.9% | 1128.4122 | 1129.2584 | 4 | 5.399 | 72.2% | 2 | K.LNGLNEEIAR.V | 2 |
| \* | IPAstrin\_STLCD\_tube2\_032114\_01.05085.05085.2 | 2.158 | 0.3486 | 99.9% | 952.2322 | 953.03973 | 1 | 6.465 | 85.7% | 2 | K.YSVADIER.I | 2 |
| \* | IPAstrin\_STLCD\_tube2\_032114\_01.12485.12485.3 | 3.8544 | 0.3011 | 99.6% | 2359.9744 | 2360.6665 | 1 | 5.443 | 38.2% | 1 | R.AQVYVPLKELLNETEEEINK.A | 3 |
| \* | IPAstrin\_STLCD\_tube2\_032114\_01.13578.13578.3 | 4.5312 | 0.4292 | 100.0% | 2179.7944 | 2180.4963 | 1 | 7.335 | 38.9% | 1 | K.MGLEDTLEQLNAMITESKR.S | 3 |
| \* | IPAstrin\_STLCD\_tube2\_032114\_01.08264.08264.2 | 2.3874 | 0.2708 | 98.5% | 1979.1522 | 1979.2377 | 115 | 4.806 | 33.3% | 1 | R.TLKEEVQKLDDLYQQK.I | 2 |
| \* | IPAstrin\_STLCD\_tube2\_032114\_01.08238.08238.3 | 2.6626 | 0.2572 | 95.3% | 1979.3644 | 1979.2377 | 16 | 4.612 | 31.7% | 1 | R.TLKEEVQKLDDLYQQK.I | 3 |
| \* | IPAstrin\_STLCD\_032114\_02.05375.05375.2 | 4.0738 | 0.4277 | 100.0% | 1596.2522 | 1596.7344 | 1 | 8.31 | 75.0% | 4 | R.EYQLVVQTTTEER.R | 2 |
| \* | IPAstrin\_STLCD\_tube2\_032114\_01.06585.06585.2 | 4.3493 | 0.3716 | 100.0% | 1513.3322 | 1513.7925 | 1 | 7.999 | 76.9% | 4 | R.LLEMVATHVGSVEK.H | 2 |
| \* | IPAstrin\_STLCD\_032114\_02.05786.05786.3 | 2.642 | 0.3384 | 99.3% | 1513.5844 | 1513.7925 | 1 | 5.603 | 38.5% | 1 | R.LLEMVATHVGSVEK.H | 3 |

---

|  |  |  |  |  |  |  |  |  |
| --- | --- | --- | --- | --- | --- | --- | --- | --- |
| U | *gi|4503571|ref|NP\_001* | 6 | 9 | 19.4% | 434 | 47169 | 7.4 | enolase 1 [Homo sapiens] |

| Filename XCorr DeltCN Conf% ObsM+H+ CalcM+H+ SpR ZScore Ion% # Sequence  | | | | | | | | | | | | |
| --- | --- | --- | --- | --- | --- | --- | --- | --- | --- | --- | --- | --- |
| \* | IPAstrin\_STLCD\_tube2\_032114\_01.08483.08483.2 | 2.9398 | 0.2339 | 99.8% | 1407.2122 | 1407.5634 | 145 | 6.037 | 45.8% | 1 | R.GNPTVEVDLFTSK.G | 2 |
|  | IPAstrin\_STLCD\_tube2\_032114\_01.10288.10288.2 | 3.6654 | 0.3938 | 100.0% | 1806.7322 | 1806.0258 | 1 | 7.135 | 52.9% | 2 | R.AAVPSGASTGIYEALELR.D | 2 |
| \* | IPAstrin\_STLCD\_032114\_01.14006.14006.2 | 3.8614 | 0.3679 | 100.0% | 1909.5521 | 1909.3148 | 1 | 6.636 | 50.0% | 1 | K.LAMQEFMILPVGAANFR.E | 2 |
| \* | IPAstrin\_STLCD\_032114\_01.04360.04360.2 | 2.859 | 0.2626 | 99.9% | 1144.1322 | 1144.3158 | 4 | 6.33 | 72.2% | 1 | R.IGAEVYHNLK.N | 2 |
| \* | IPAstrin\_STLCD\_tube2\_032114\_01.12118.12118.2 | 3.0829 | 0.29 | 99.9% | 1542.3922 | 1541.8053 | 2 | 6.313 | 53.8% | 1 | K.VVIGMDVAASEFFR.S | 2 |
| \* | IPAstrin\_STLCD\_tube2\_032114\_01.09561.09561.2 | 3.4593 | 0.4652 | 100.0% | 1426.3322 | 1426.6091 | 1 | 7.188 | 77.3% | 3 | R.YISPDQLADLYK.S | 2 |

---

|  |  |  |  |  |  |  |  |  |
| --- | --- | --- | --- | --- | --- | --- | --- | --- |
| U | *gi|16905517|ref|NP\_47* | 5 | 8 | 19.1% | 262 | 31301 | 11.3 | FUS interacting protein (serine-arginine rich) 1 isoform 2 [Homo sapiens] |
| U | *gi|5730079|ref|NP\_006* | 5 | 8 | 27.3% | 183 | 22222 | 10.3 | FUS interacting protein (serine-arginine rich) 1 isoform 1 [Homo sapiens] |
| U | *gi|169161980|ref|XP\_0* | 5 | 8 | 27.6% | 181 | 22022 | 10.3 | PREDICTED: hypothetical protein, partial [Homo sapiens] |
| U | *gi|169161109|ref|XP\_0* | 5 | 8 | 27.3% | 183 | 22222 | 10.3 | PREDICTED: hypothetical protein LOC642558 [Homo sapiens] |
| U | *gi|169161107|ref|XP\_0* | 5 | 8 | 19.1% | 262 | 31301 | 11.3 | PREDICTED: hypothetical protein LOC642558 [Homo sapiens] |

| Filename XCorr DeltCN Conf% ObsM+H+ CalcM+H+ SpR ZScore Ion% # Sequence  | | | | | | | | | | | | |
| --- | --- | --- | --- | --- | --- | --- | --- | --- | --- | --- | --- | --- |
|  | IPAstrin\_STLCD\_tube2\_032114\_01.07240.07240.3 | 3.6414 | 0.2734 | 99.8% | 1463.8744 | 1463.7227 | 1 | 5.844 | 61.4% | 1 | R.YLRPPNTSLFVR.N | 3 |
|  | IPAstrin\_STLCD\_032114\_01.08976.08976.2 | 2.3747 | 0.2851 | 99.3% | 1464.4922 | 1463.7227 | 6 | 4.772 | 54.5% | 1 | R.YLRPPNTSLFVR.N | 2 |
|  | IPAstrin\_STLCD\_032114\_01.13784.13784.2 | 3.6892 | 0.4459 | 100.0% | 1917.3922 | 1918.1992 | 1 | 7.193 | 50.0% | 2 | R.YGPIVDVYVPLDFYTR.R | 2 |
|  | IPAstrin\_STLCD\_032114\_01.10748.10748.2 | 2.7808 | 0.3076 | 99.9% | 1331.0721 | 1331.4705 | 10 | 5.433 | 60.0% | 2 | R.GFAYVQFEDVR.D | 2 |
|  | IPAstrin\_STLCD\_032114\_01.08086.08086.2 | 3.2189 | 0.3182 | 100.0% | 1305.3322 | 1305.4331 | 3 | 5.701 | 70.0% | 2 | R.QIEIQFAQGDR.K | 2 |

---

|  |  |  |  |  |  |  |  |  |
| --- | --- | --- | --- | --- | --- | --- | --- | --- |
| U | *gi|4506613|ref|NP\_000* | 2 | 3 | 18.8% | 128 | 14787 | 9.2 | ribosomal protein L22 proprotein [Homo sapiens] |

| Filename XCorr DeltCN Conf% ObsM+H+ CalcM+H+ SpR ZScore Ion% # Sequence  | | | | | | | | | | | | |
| --- | --- | --- | --- | --- | --- | --- | --- | --- | --- | --- | --- | --- |
| \* | IPAstrin\_STLCD\_032114\_01.06310.06310.2 | 3.3338 | 0.4316 | 100.0% | 1243.1721 | 1243.4056 | 1 | 6.739 | 70.8% | 2 | K.AGNLGGGVVTIER.S | 2 |
| \* | IPAstrin\_STLCD\_tube2\_032114\_01.06774.06774.2 | 2.5296 | 0.2881 | 99.8% | 1208.7322 | 1208.3971 | 21 | 5.958 | 70.0% | 1 | K.ITVTSEVPFSK.R | 2 |

---

|  |  |  |  |  |  |  |  |  |
| --- | --- | --- | --- | --- | --- | --- | --- | --- |
| U | *gi|4506901|ref|NP\_003* | 4 | 7 | 18.3% | 164 | 19330 | 11.6 | splicing factor, arginine/serine-rich 3 [Homo sapiens] |

| Filename XCorr DeltCN Conf% ObsM+H+ CalcM+H+ SpR ZScore Ion% # Sequence  | | | | | | | | | | | | |
| --- | --- | --- | --- | --- | --- | --- | --- | --- | --- | --- | --- | --- |
| \* | IPAstrin\_STLCD\_032114\_01.09229.09229.2 | 2.7614 | 0.3717 | 100.0% | 1044.0721 | 1044.198 | 1 | 6.817 | 87.5% | 4 | R.AFGYYGPLR.S | 2 |
|  | IPAstrin\_STLCD\_tube2\_032114\_01.10900.10900.2 | 2.5677 | 0.3378 | 99.9% | 1621.8322 | 1622.7771 | 1 | 4.778 | 57.7% | 1 | R.NPPGFAFVEFEDPR.D | 22 |
| \* | IPAstrin\_STLCD\_032114\_01.12236.12236.2 | 3.3053 | 0.3675 | 100.0% | 2320.4321 | 2321.5107 | 23 | 5.917 | 37.5% | 1 | R.NPPGFAFVEFEDPRDAADAVR.E | 2 |
| \* | IPAstrin\_STLCD\_032114\_01.12261.12261.3 | 2.3749 | 0.3134 | 96.9% | 2321.6042 | 2321.5107 | 2 | 4.914 | 31.2% | 1 | R.NPPGFAFVEFEDPRDAADAVR.E | 3 |

Similarities:
gi|72534660|ref|NP\_00(1:3)  

---

|  |  |  |  |  |  |  |  |  |
| --- | --- | --- | --- | --- | --- | --- | --- | --- |
| U | *gi|21464101|ref|NP\_03* | 2 | 2 | 18.2% | 247 | 28303 | 4.9 | tyrosine 3-monooxygenase/tryptophan 5-monooxygenase activation protein, gamma polypeptide [Homo sapiens] |

| Filename XCorr DeltCN Conf% ObsM+H+ CalcM+H+ SpR ZScore Ion% # Sequence  | | | | | | | | | | | | |
| --- | --- | --- | --- | --- | --- | --- | --- | --- | --- | --- | --- | --- |
| \* | IPAstrin\_STLCD\_032114\_02.07247.07247.2 | 3.169 | 0.0774 | 95.5% | 1799.1921 | 1798.8473 | 35 | 3.119 | 46.7% | 1 | R.VISS\*IEQK@TSADGNEK.K | 2 |
| \* | IPAstrin\_STLCD\_tube2\_032114\_01.17110.17110.3 | 4.3652 | 0.3366 | 100.0% | 3303.2043 | 3303.6626 | 5 | 6.816 | 23.2% | 1 | K.TAFDDAIAELDTLNEDSYKDSTLIMQLLR.D | 3 |

---

|  |  |  |  |  |  |  |  |  |
| --- | --- | --- | --- | --- | --- | --- | --- | --- |
| U | *gi|15809016|ref|NP\_29* | 3 | 6 | 18.0% | 172 | 19779 | 4.8 | myosin regulatory light chain MRCL2 isoform A [Homo sapiens] |
| U | *gi|5453740|ref|NP\_006* | 3 | 6 | 18.1% | 171 | 19794 | 4.8 | myosin, light chain 12A, regulatory, non-sarcomeric [Homo sapiens] |
| U | *gi|222144328|ref|NP\_0* | 3 | 6 | 20.1% | 154 | 17757 | 4.4 | myosin regulatory light chain MRCL2 isoform B [Homo sapiens] |
| U | *gi|222144326|ref|NP\_0* | 3 | 6 | 18.0% | 172 | 19779 | 4.8 | myosin regulatory light chain MRCL2 isoform A [Homo sapiens] |
| U | *gi|222144324|ref|NP\_0* | 3 | 6 | 18.0% | 172 | 19779 | 4.8 | myosin regulatory light chain MRCL2 isoform A [Homo sapiens] |

| Filename XCorr DeltCN Conf% ObsM+H+ CalcM+H+ SpR ZScore Ion% # Sequence  | | | | | | | | | | | | |
| --- | --- | --- | --- | --- | --- | --- | --- | --- | --- | --- | --- | --- |
|  | IPAstrin\_STLCD\_tube2\_032114\_01.05284.05284.2 | 2.715 | 0.1791 | 98.7% | 1228.9521 | 1229.3324 | 1 | 5.287 | 75.0% | 1 | K.LNGTDPEDVIR.N | 2 |
|  | IPAstrin\_STLCD\_032114\_01.12145.12145.3 | 4.0824 | 0.3606 | 100.0% | 2433.4744 | 2433.649 | 1 | 6.607 | 34.2% | 1 | R.ELLTTMGDRFTDEEVDELYR.E | 3 |
|  | IPAstrin\_STLCD\_032114\_01.09118.09118.2 | 3.4822 | 0.3491 | 100.0% | 1416.1921 | 1416.4839 | 1 | 7.623 | 70.0% | 4 | R.FTDEEVDELYR.E | 2 |

---

|  |  |  |  |  |  |  |  |  |
| --- | --- | --- | --- | --- | --- | --- | --- | --- |
| U | *gi|19920317|ref|NP\_00* | 7 | 11 | 17.9% | 602 | 66023 | 5.9 | cytoskeleton-associated protein 4 [Homo sapiens] |

| Filename XCorr DeltCN Conf% ObsM+H+ CalcM+H+ SpR ZScore Ion% # Sequence  | | | | | | | | | | | | |
| --- | --- | --- | --- | --- | --- | --- | --- | --- | --- | --- | --- | --- |
| \* | IPAstrin\_STLCD\_032114\_01.07719.07719.2 | 2.4371 | 0.1311 | 95.2% | 1292.3121 | 1293.44 | 98 | 4.271 | 55.6% | 1 | K.SREWDMEALR.S | 2 |
| \* | IPAstrin\_STLCD\_032114\_02.05750.05750.3 | 3.8646 | 0.341 | 100.0% | 1841.1543 | 1841.0923 | 1 | 6.022 | 42.2% | 2 | R.LQHVEDGVLSMQVASAR.Q | 3 |
| \* | IPAstrin\_STLCD\_032114\_01.08100.08100.2 | 5.1081 | 0.5609 | 100.0% | 1905.2722 | 1906.0135 | 1 | 10.086 | 44.4% | 2 | R.LEGLGSSEADQDGLASTVR.S | 2 |
| \* | IPAstrin\_STLCD\_tube2\_032114\_02.07235.07235.3 | 2.3988 | 0.2962 | 95.7% | 2050.1943 | 2050.3164 | 3 | 5.092 | 33.8% | 1 | R.SLGETQLVLYGDVEELKR.S | 3 |
| \* | IPAstrin\_STLCD\_032114\_01.08361.08361.2 | 2.9256 | 0.401 | 100.0% | 1473.3922 | 1474.6512 | 2 | 6.297 | 57.7% | 1 | R.SVGELPSTVESLQK.V | 2 |
| \* | IPAstrin\_STLCD\_032114\_01.05539.05539.3 | 3.4926 | 0.3229 | 99.8% | 2021.9944 | 2023.2131 | 1 | 5.686 | 33.8% | 1 | K.VQEQVHTLLSQDQAQAAR.L | 3 |
| \* | IPAstrin\_STLCD\_tube2\_032114\_02.05994.05994.2 | 3.1338 | 0.4866 | 100.0% | 1253.4722 | 1253.4381 | 1 | 7.797 | 63.6% | 3 | R.TAVDSLVAYSVK.I | 2 |

---

|  |  |  |  |  |  |  |  |  |
| --- | --- | --- | --- | --- | --- | --- | --- | --- |
| U | *gi|16306492|ref|NP\_20* | 3 | 3 | 17.9% | 240 | 27503 | 7.1 | cell division cycle 2 isoform 2 [Homo sapiens] |
| U | *gi|4502709|ref|NP\_001* | 3 | 3 | 14.5% | 297 | 34095 | 8.4 | cell division cycle 2 isoform 1 [Homo sapiens] |
| U | *gi|195927041|ref|NP\_0* | 3 | 3 | 14.5% | 297 | 34081 | 8.4 | cell division cycle 2 isoform 3 [Homo sapiens] |

| Filename XCorr DeltCN Conf% ObsM+H+ CalcM+H+ SpR ZScore Ion% # Sequence  | | | | | | | | | | | | |
| --- | --- | --- | --- | --- | --- | --- | --- | --- | --- | --- | --- | --- |
|  | IPAstrin\_STLCD\_032114\_01.06092.06092.2 | 2.858 | 0.2888 | 99.9% | 1187.2122 | 1186.3501 | 1 | 5.302 | 70.0% | 1 | K.IGEGTYGVVYK.G | 2 |
|  | IPAstrin\_STLCD\_tube2\_032114\_02.12175.12175.2 | 2.9072 | 0.3977 | 100.0% | 2212.5522 | 2213.5352 | 1 | 7.216 | 36.8% | 1 | R.YSTPVDIWSIGTIFAELATK.K | 2 |
|  | IPAstrin\_STLCD\_032114\_01.10263.10263.2 | 2.3629 | 0.3152 | 99.6% | 1330.3922 | 1331.4656 | 2 | 5.353 | 54.5% | 1 | K.NLDENGLDLLSK.M | 2 |

---

|  |  |  |  |  |  |  |  |  |
| --- | --- | --- | --- | --- | --- | --- | --- | --- |
| U | *gi|4503471|ref|NP\_001* | 9 | 48 | 17.7% | 462 | 50141 | 9.0 | eukaryotic translation elongation factor 1 alpha 1 [Homo sapiens] |

| Filename XCorr DeltCN Conf% ObsM+H+ CalcM+H+ SpR ZScore Ion% # Sequence  | | | | | | | | | | | | |
| --- | --- | --- | --- | --- | --- | --- | --- | --- | --- | --- | --- | --- |
|  | IPAstrin\_STLCD\_tube2\_032114\_02.05037.05037.3 | 4.8502 | 0.4564 | 100.0% | 1590.0543 | 1589.835 | 1 | 7.941 | 53.6% | 17 | K.THINIVVIGHVDSGK.S | 3 |
|  | IPAstrin\_STLCD\_032114\_01.06670.06670.2 | 3.3782 | 0.4924 | 100.0% | 1590.1721 | 1589.835 | 2 | 7.546 | 64.3% | 3 | K.THINIVVIGHVDSGK.S | 2 |
| \* | IPAstrin\_STLCD\_032114\_01.07873.07873.2 | 3.1671 | 0.3851 | 100.0% | 1405.5721 | 1405.5962 | 1 | 7.004 | 72.7% | 4 | K.YYVTIIDAPGHR.D | 2 |
| \* | IPAstrin\_STLCD\_032114\_01.07885.07885.3 | 3.1242 | 0.2466 | 98.7% | 1405.7644 | 1405.5962 | 27 | 5.004 | 45.5% | 2 | K.YYVTIIDAPGHR.D | 3 |
|  | IPAstrin\_STLCD\_032114\_02.06575.06575.2 | 3.1658 | 0.4393 | 100.0% | 1315.4321 | 1315.5553 | 1 | 7.698 | 77.3% | 4 | R.EHALLAYTLGVK.Q | 2 |
|  | IPAstrin\_STLCD\_tube2\_032114\_01.06170.06170.2 | 2.7768 | 0.1939 | 99.8% | 976.0122 | 976.1607 | 1 | 6.591 | 92.9% | 4 | R.LPLQDVYK.I | 2 |
|  | IPAstrin\_STLCD\_tube2\_032114\_01.05378.05378.2 | 3.611 | 0.405 | 100.0% | 1026.0922 | 1026.2241 | 1 | 7.55 | 80.0% | 6 | K.IGGIGTVPVGR.V | 2 |
| \* | IPAstrin\_STLCD\_032114\_01.11790.11790.3 | 4.648 | 0.3705 | 100.0% | 2516.5745 | 2516.999 | 1 | 6.683 | 32.6% | 7 | R.VETGVLKPGMVVTFAPVNVTTEVK.S | 3 |
| \* | IPAstrin\_STLCD\_tube2\_032114\_01.10010.10010.2 | 5.0288 | 0.4818 | 100.0% | 2517.2122 | 2516.999 | 1 | 8.253 | 50.0% | 1 | R.VETGVLKPGMVVTFAPVNVTTEVK.S | 2 |

---

|  |  |  |  |  |  |  |  |  |
| --- | --- | --- | --- | --- | --- | --- | --- | --- |
| U | *gi|72534660|ref|NP\_00* | 4 | 9 | 17.2% | 238 | 27367 | 11.8 | splicing factor, arginine/serine-rich 7 [Homo sapiens] |

| Filename XCorr DeltCN Conf% ObsM+H+ CalcM+H+ SpR ZScore Ion% # Sequence  | | | | | | | | | | | | |
| --- | --- | --- | --- | --- | --- | --- | --- | --- | --- | --- | --- | --- |
| \* | IPAstrin\_STLCD\_032114\_01.09073.09073.2 | 2.152 | 0.3443 | 99.8% | 1074.0721 | 1074.2242 | 2 | 6.372 | 75.0% | 3 | R.AFSYYGPLR.T | 2 |
|  | IPAstrin\_STLCD\_tube2\_032114\_01.10900.10900.2 | 2.5677 | 0.3378 | 99.9% | 1621.8322 | 1622.7771 | 1 | 4.778 | 57.7% | 1 | R.NPPGFAFVEFEDPR.D | 22 |
| \* | IPAstrin\_STLCD\_tube2\_032114\_01.10556.10556.3 | 2.6623 | 0.3617 | 99.3% | 2378.7244 | 2379.5474 | 1 | 5.626 | 37.5% | 1 | R.NPPGFAFVEFEDPRDAEDAVR.G | 3 |
| \* | IPAstrin\_STLCD\_tube2\_032114\_01.04906.04906.2 | 3.1523 | 0.332 | 100.0% | 1245.3522 | 1245.4827 | 85 | 5.488 | 50.0% | 4 | R.VRVELSTGMPR.R | 2 |

Similarities:
gi|4506901|ref|NP\_003(1:3)  

---

|  |  |  |  |  |  |  |  |  |
| --- | --- | --- | --- | --- | --- | --- | --- | --- |
| U | *gi|15431303|ref|NP\_00* | 2 | 2 | 17.2% | 192 | 21863 | 10.0 | ribosomal protein L9 [Homo sapiens] |
| U | *gi|67944630|ref|NP\_00* | 2 | 2 | 17.2% | 192 | 21863 | 10.0 | ribosomal protein L9 [Homo sapiens] |

| Filename XCorr DeltCN Conf% ObsM+H+ CalcM+H+ SpR ZScore Ion% # Sequence  | | | | | | | | | | | | |
| --- | --- | --- | --- | --- | --- | --- | --- | --- | --- | --- | --- | --- |
|  | IPAstrin\_STLCD\_tube2\_032114\_01.10050.10050.2 | 2.547 | 0.3291 | 99.8% | 2113.4521 | 2114.401 | 1 | 4.99 | 36.1% | 1 | K.TILSNQTVDIPENVDITLK.G | 2 |
|  | IPAstrin\_STLCD\_tube2\_032114\_01.10524.10524.2 | 2.7897 | 0.2351 | 99.4% | 1598.8522 | 1599.8271 | 1 | 5.114 | 53.8% | 1 | R.DFNHINVELSLLGK.K | 2 |

---

|  |  |  |  |  |  |  |  |  |
| --- | --- | --- | --- | --- | --- | --- | --- | --- |
| U | *contaminant\_KERATIN03* | 7 | 13 | 17.0% | 593 | 59519 | 5.2 | no description |
| U | *gi|195972866|ref|NP\_0* | 7 | 13 | 17.3% | 584 | 58801 | 5.2 | keratin 10 [Homo sapiens] |

| Filename XCorr DeltCN Conf% ObsM+H+ CalcM+H+ SpR ZScore Ion% # Sequence  | | | | | | | | | | | | |
| --- | --- | --- | --- | --- | --- | --- | --- | --- | --- | --- | --- | --- |
|  | IPAstrin\_STLCD\_tube2\_032114\_01.07842.07842.2 | 4.9105 | 0.4847 | 100.0% | 1708.0721 | 1708.7844 | 1 | 8.411 | 58.3% | 3 | K.GSLGGGFSSGGFSGGSFSR.G | 2 |
|  | IPAstrin\_STLCD\_tube2\_032114\_01.04628.04628.2 | 3.8034 | 0.4132 | 100.0% | 1382.0521 | 1382.4668 | 1 | 7.463 | 68.2% | 1 | R.ALEESNYELEGK.I | 2 |
|  | IPAstrin\_STLCD\_tube2\_032114\_01.14226.14226.3 | 5.0502 | 0.4709 | 100.0% | 3053.7544 | 3054.4277 | 1 | 7.517 | 29.8% | 1 | K.TIDDLKNQILNLTTDNANILLQIDNAR.L | 3 |
|  | IPAstrin\_STLCD\_tube2\_032114\_01.04402.04402.2 | 2.436 | 0.2986 | 99.8% | 1235.3322 | 1235.4258 | 52 | 5.31 | 66.7% | 2 | R.LKYENEVALR.Q | 2 |
|  | IPAstrin\_STLCD\_tube2\_032114\_01.06926.06926.2 | 2.923 | 0.407 | 100.0% | 1032.1721 | 1032.2224 | 2 | 6.744 | 75.0% | 3 | R.VLDELTLTK.A | 2 |
|  | IPAstrin\_STLCD\_tube2\_032114\_01.06453.06453.2 | 3.9301 | 0.422 | 100.0% | 1391.5322 | 1391.4778 | 1 | 7.624 | 66.7% | 2 | K.QSLEASLAETEGR.Y | 2 |
|  | IPAstrin\_STLCD\_tube2\_032114\_01.05578.05578.2 | 2.7505 | 0.3441 | 100.0% | 1434.2122 | 1435.623 | 2 | 5.609 | 75.0% | 1 | K.IRLENEIQTYR.S | 2 |

---

|  |  |  |  |  |  |  |  |  |
| --- | --- | --- | --- | --- | --- | --- | --- | --- |
| U | *gi|14210536|ref|NP\_11* | 10 | 36 | 17.0% | 446 | 49857 | 4.9 | tubulin, beta 6 [Homo sapiens] |

| Filename XCorr DeltCN Conf% ObsM+H+ CalcM+H+ SpR ZScore Ion% # Sequence  | | | | | | | | | | | | |
| --- | --- | --- | --- | --- | --- | --- | --- | --- | --- | --- | --- | --- |
|  | IPAstrin\_STLCD\_tube2\_032114\_01.06530.06530.2 | 2.998 | 0.3386 | 100.0% | 1131.3322 | 1131.2767 | 133 | 5.952 | 61.1% | 14 | R.FPGQLNADLR.K | 2222 |
|  | IPAstrin\_STLCD\_tube2\_032114\_01.05058.05058.2 | 2.4459 | 0.1587 | 95.7% | 1259.4521 | 1259.4508 | 80 | 4.442 | 55.0% | 1 | R.FPGQLNADLRK.L | 2222 |
|  | IPAstrin\_STLCD\_tube2\_032114\_01.07702.07702.2 | 3.6879 | 0.347 | 100.0% | 1272.4321 | 1272.5945 | 3 | 7.18 | 65.0% | 4 | R.KLAVNMVPFPR.L | 2222 |
|  | IPAstrin\_STLCD\_tube2\_032114\_01.09071.09071.1 | 2.021 | 0.2752 | 100.0% | 1143.62 | 1144.4204 | 9 | 5.967 | 61.1% | 1 | K.LAVNMVPFPR.L | 1111 |
|  | IPAstrin\_STLCD\_tube2\_032114\_01.09111.09111.2 | 3.5075 | 0.4273 | 100.0% | 1143.6522 | 1144.4204 | 1 | 8.353 | 94.4% | 4 | K.LAVNMVPFPR.L | 2222 |
|  | IPAstrin\_STLCD\_032114\_01.12828.12828.2 | 3.4639 | 0.3347 | 100.0% | 1621.3322 | 1621.9403 | 1 | 7.353 | 80.8% | 6 | R.LHFFMPGFAPLTSR.G | 222 |
|  | IPAstrin\_STLCD\_tube2\_032114\_01.11356.11356.3 | 4.2941 | 0.3603 | 100.0% | 1621.6743 | 1621.9403 | 1 | 6.6 | 50.0% | 2 | R.LHFFMPGFAPLTSR.G | 333 |
|  | IPAstrin\_STLCD\_tube2\_032114\_01.10725.10725.2 | 2.6091 | 0.3156 | 99.8% | 1696.4922 | 1697.8877 | 1 | 5.145 | 61.5% | 2 | K.NSSYFVEWIPNNVK.V | 2222 |
| \* | IPAstrin\_STLCD\_tube2\_032114\_01.12520.12520.2 | 3.7003 | 0.0026 | 95.3% | 1858.3121 | 1859.1475 | 1 | 7.59 | 59.4% | 1 | K.MASTFIGNSTAIQELFK.R | 2 |
| \* | IPAstrin\_STLCD\_032114\_01.10657.10657.2 | 2.4055 | 0.2762 | 99.5% | 1216.4722 | 1216.3972 | 60 | 4.808 | 61.1% | 1 | R.ISEQFSAMFR.R | 2 |

Similarities:
gi|29788785|ref|NP\_82(8:2)  
gi|5174735|ref|NP\_006(8:2)  
gi|50592996|ref|NP\_00(6:4)  

---

|  |  |  |  |  |  |  |  |  |
| --- | --- | --- | --- | --- | --- | --- | --- | --- |
| U | *gi|4503483|ref|NP\_001* | 10 | 19 | 16.7% | 858 | 95338 | 6.8 | eukaryotic translation elongation factor 2 [Homo sapiens] |

| Filename XCorr DeltCN Conf% ObsM+H+ CalcM+H+ SpR ZScore Ion% # Sequence  | | | | | | | | | | | | |
| --- | --- | --- | --- | --- | --- | --- | --- | --- | --- | --- | --- | --- |
| \* | IPAstrin\_STLCD\_tube2\_032114\_01.03699.03699.2 | 3.134 | 0.395 | 100.0% | 1307.3922 | 1308.4979 | 19 | 7.812 | 59.1% | 1 | R.NMSVIAHVDHGK.S | 2 |
| \* | IPAstrin\_STLCD\_tube2\_032114\_01.15016.15016.2 | 2.642 | 0.3043 | 99.8% | 2205.3123 | 2205.4692 | 23 | 5.571 | 30.6% | 1 | K.STAISLFYELSENDLNFIK.Q | 2 |
| \* | IPAstrin\_STLCD\_tube2\_032114\_01.15252.15252.2 | 3.3927 | 0.2213 | 99.9% | 2603.152 | 2602.11 | 1 | 4.467 | 37.0% | 1 | R.WLPAGDALLQMITIHLPSPVTAQK.Y | 2 |
| \* | IPAstrin\_STLCD\_tube2\_032114\_01.07532.07532.2 | 3.1472 | 0.2841 | 100.0% | 1109.2122 | 1108.3231 | 1 | 6.334 | 80.0% | 1 | R.VFSGLVSTGLK.V | 2 |
| \* | IPAstrin\_STLCD\_032114\_01.04544.04544.2 | 3.9837 | 0.4184 | 100.0% | 1616.1721 | 1616.7917 | 1 | 7.856 | 57.7% | 1 | K.TGTITTFEHAHNMR.V | 2 |
| \* | IPAstrin\_STLCD\_tube2\_032114\_01.07374.07374.3 | 5.6114 | 0.4255 | 100.0% | 2143.7944 | 2144.3489 | 1 | 7.73 | 44.7% | 8 | K.ARPFPDGLAEDIDKGEVSAR.Q | 3 |
| \* | IPAstrin\_STLCD\_tube2\_032114\_02.05837.05837.3 | 3.9145 | 0.3179 | 100.0% | 1743.8644 | 1743.9133 | 1 | 6.268 | 50.0% | 1 | R.YLAEKYEWDVAEAR.K | 3 |
| \* | IPAstrin\_STLCD\_tube2\_032114\_01.06742.06742.2 | 2.5169 | 0.4214 | 100.0% | 1139.3722 | 1139.2096 | 1 | 7.327 | 81.2% | 2 | K.YEWDVAEAR.K | 2 |
| \* | IPAstrin\_STLCD\_032114\_01.12591.12591.2 | 2.0974 | 0.3127 | 98.2% | 1801.0322 | 1801.0087 | 6 | 5.517 | 43.3% | 1 | K.AYLPVNESFGFTADLR.S | 2 |
| \* | IPAstrin\_STLCD\_032114\_01.15022.15022.2 | 2.8889 | 0.4005 | 100.0% | 1445.4122 | 1445.6555 | 5 | 6.968 | 54.2% | 2 | K.EGIPALDNFLDKL.- | 2 |

---

|  |  |  |  |  |  |  |  |  |
| --- | --- | --- | --- | --- | --- | --- | --- | --- |
| U | *gi|117189975|ref|NP\_1* | 4 | 14 | 16.7% | 306 | 33670 | 5.1 | heterogeneous nuclear ribonucleoprotein C isoform a [Homo sapiens] |
| U | *gi|117190254|ref|NP\_0* | 4 | 14 | 17.4% | 293 | 32338 | 5.1 | heterogeneous nuclear ribonucleoprotein C isoform b [Homo sapiens] |
| U | *gi|117190192|ref|NP\_0* | 4 | 14 | 16.7% | 306 | 33670 | 5.1 | heterogeneous nuclear ribonucleoprotein C isoform a [Homo sapiens] |
| U | *gi|117190174|ref|NP\_0* | 4 | 14 | 17.4% | 293 | 32338 | 5.1 | heterogeneous nuclear ribonucleoprotein C isoform b [Homo sapiens] |

| Filename XCorr DeltCN Conf% ObsM+H+ CalcM+H+ SpR ZScore Ion% # Sequence  | | | | | | | | | | | | |
| --- | --- | --- | --- | --- | --- | --- | --- | --- | --- | --- | --- | --- |
|  | IPAstrin\_STLCD\_tube2\_032114\_01.09831.09831.2 | 3.714 | 0.2874 | 100.0% | 1317.5521 | 1317.6145 | 1 | 7.723 | 81.8% | 3 | R.VFIGNLNTLVVK.K | 2 |
|  | IPAstrin\_STLCD\_tube2\_032114\_01.09255.09255.2 | 3.1492 | 0.3435 | 100.0% | 1331.2322 | 1330.4857 | 1 | 5.726 | 80.0% | 3 | K.GFAFVQYVNER.N | 2 |
|  | IPAstrin\_STLCD\_tube2\_032114\_01.09884.09884.2 | 4.7485 | 0.3932 | 100.0% | 1682.9922 | 1684.0038 | 1 | 7.699 | 76.7% | 7 | R.MIAGQVLDINLAAEPK.V | 2 |
|  | IPAstrin\_STLCD\_032114\_01.09543.09543.2 | 2.6665 | 0.179 | 98.2% | 1417.3922 | 1416.6146 | 213 | 3.893 | 54.5% | 1 | K.QKVDSLLENLEK.I | 2 |

---

|  |  |  |  |  |  |  |  |  |
| --- | --- | --- | --- | --- | --- | --- | --- | --- |
| U | *gi|15431295|ref|NP\_15* | 4 | 7 | 16.6% | 211 | 24261 | 11.7 | ribosomal protein L13 [Homo sapiens] |
| U | *gi|15431297|ref|NP\_00* | 4 | 7 | 16.6% | 211 | 24261 | 11.7 | ribosomal protein L13 [Homo sapiens] |

| Filename XCorr DeltCN Conf% ObsM+H+ CalcM+H+ SpR ZScore Ion% # Sequence  | | | | | | | | | | | | |
| --- | --- | --- | --- | --- | --- | --- | --- | --- | --- | --- | --- | --- |
|  | IPAstrin\_STLCD\_tube2\_032114\_01.05649.05649.2 | 2.5543 | 0.1895 | 98.2% | 1346.0721 | 1346.5344 | 2 | 4.201 | 70.0% | 2 | R.RVATWFNQPAR.K | 2 |
|  | IPAstrin\_STLCD\_tube2\_032114\_01.07022.07022.2 | 2.4781 | 0.1609 | 97.2% | 1189.9922 | 1190.3469 | 146 | 3.816 | 61.1% | 1 | R.VATWFNQPAR.K | 2 |
|  | IPAstrin\_STLCD\_032114\_01.04034.04034.2 | 2.5493 | 0.2292 | 99.3% | 1233.3522 | 1233.3237 | 1 | 4.273 | 75.0% | 1 | K.STESLQANVQR.L | 2 |
|  | IPAstrin\_STLCD\_tube2\_032114\_01.07222.07222.2 | 3.0755 | 0.3569 | 100.0% | 1383.2522 | 1383.6923 | 2 | 5.99 | 62.5% | 3 | K.LATQLTGPVMPVR.N | 2 |

---

|  |  |  |  |  |  |  |  |  |
| --- | --- | --- | --- | --- | --- | --- | --- | --- |
| U | *gi|5803225|ref|NP\_006* | 2 | 4 | 16.5% | 255 | 29174 | 4.7 | tyrosine 3/tryptophan 5 -monooxygenase activation protein, epsilon polypeptide [Homo sapiens] |

| Filename XCorr DeltCN Conf% ObsM+H+ CalcM+H+ SpR ZScore Ion% # Sequence  | | | | | | | | | | | | |
| --- | --- | --- | --- | --- | --- | --- | --- | --- | --- | --- | --- | --- |
| \* | IPAstrin\_STLCD\_032114\_02.06236.06236.2 | 3.4953 | 0.2895 | 100.0% | 1448.9122 | 1448.6312 | 2 | 7.501 | 66.7% | 2 | K.VAGMDVELTVEER.N | 2 |
| \* | IPAstrin\_STLCD\_tube2\_032114\_01.18057.18057.3 | 5.1986 | 0.45 | 100.0% | 3258.7744 | 3260.6375 | 1 | 8.992 | 30.4% | 2 | K.AAFDDAIAELDTLSEESYKDSTLIMQLLR.D | 3 |

---

|  |  |  |  |  |  |  |  |  |
| --- | --- | --- | --- | --- | --- | --- | --- | --- |
| U | *gi|169201338|ref|XP\_0* | 3 | 11 | 16.2% | 160 | 18565 | 10.5 | PREDICTED: hypothetical protein [Homo sapiens] |
| U | *gi|89040203|ref|XP\_93* | 3 | 11 | 16.2% | 160 | 18593 | 10.5 | PREDICTED: hypothetical protein [Homo sapiens] |
| U | *gi|18104948|ref|NP\_00* | 3 | 11 | 16.2% | 160 | 18565 | 10.5 | ribosomal protein L21 [Homo sapiens] |
| U | *gi|169213854|ref|XP\_0* | 3 | 11 | 16.2% | 160 | 18790 | 10.3 | PREDICTED: hypothetical protein [Homo sapiens] |
| U | *gi|169210381|ref|XP\_0* | 3 | 11 | 16.2% | 160 | 18535 | 10.6 | PREDICTED: hypothetical protein isoform 2 [Homo sapiens] |
| U | *gi|169210379|ref|XP\_0* | 3 | 11 | 16.2% | 160 | 18535 | 10.6 | PREDICTED: hypothetical protein isoform 3 [Homo sapiens] |
| U | *gi|169210377|ref|XP\_0* | 3 | 11 | 16.2% | 160 | 18535 | 10.6 | PREDICTED: hypothetical protein isoform 1 [Homo sapiens] |
| U | *gi|169202779|ref|XP\_0* | 3 | 11 | 16.2% | 160 | 18521 | 10.5 | PREDICTED: similar to ribosomal protein L21 isoform 1 [Homo sapiens] |
| U | *gi|169202777|ref|XP\_0* | 3 | 11 | 16.2% | 160 | 18521 | 10.5 | PREDICTED: similar to ribosomal protein L21 isoform 2 [Homo sapiens] |
| U | *gi|169201750|ref|XP\_0* | 3 | 11 | 16.2% | 160 | 18550 | 10.5 | PREDICTED: hypothetical protein [Homo sapiens] |

| Filename XCorr DeltCN Conf% ObsM+H+ CalcM+H+ SpR ZScore Ion% # Sequence  | | | | | | | | | | | | |
| --- | --- | --- | --- | --- | --- | --- | --- | --- | --- | --- | --- | --- |
|  | IPAstrin\_STLCD\_tube2\_032114\_01.06578.06578.2 | 2.7812 | 0.3724 | 100.0% | 1244.0521 | 1244.4973 | 1 | 6.827 | 75.0% | 5 | K.HGVVPLATYMR.I | 2 |
|  | IPAstrin\_STLCD\_tube2\_032114\_01.05808.05808.2 | 4.4964 | 0.4078 | 100.0% | 1641.7322 | 1641.9108 | 1 | 7.461 | 85.7% | 3 | R.VYNVTQHAVGIVVNK.Q | 2 |
|  | IPAstrin\_STLCD\_032114\_02.05359.05359.3 | 3.8811 | 0.3241 | 100.0% | 1642.4944 | 1641.9108 | 1 | 7.398 | 44.6% | 3 | R.VYNVTQHAVGIVVNK.Q | 3 |

---

|  |  |  |  |  |  |  |  |  |
| --- | --- | --- | --- | --- | --- | --- | --- | --- |
| U | *gi|94538362|ref|NP\_00* | 6 | 10 | 16.1% | 428 | 47064 | 5.3 | flotillin 2 [Homo sapiens] |

| Filename XCorr DeltCN Conf% ObsM+H+ CalcM+H+ SpR ZScore Ion% # Sequence  | | | | | | | | | | | | |
| --- | --- | --- | --- | --- | --- | --- | --- | --- | --- | --- | --- | --- |
| \* | IPAstrin\_STLCD\_tube2\_032114\_01.08956.08956.2 | 3.3651 | 0.3926 | 100.0% | 1379.6721 | 1379.6023 | 1 | 7.297 | 77.3% | 2 | K.NVVLQTLEGHLR.S | 2 |
| \* | IPAstrin\_STLCD\_tube2\_032114\_01.07695.07695.2 | 4.0412 | 0.4098 | 100.0% | 1522.2522 | 1521.6702 | 1 | 7.087 | 76.9% | 4 | K.TAEAQLAYELQGAR.E | 2 |
| \* | IPAstrin\_STLCD\_tube2\_032114\_02.06064.06064.2 | 2.2522 | 0.1942 | 95.5% | 1270.2322 | 1270.4716 | 300 | 4.805 | 50.0% | 1 | K.QIAVEAQEILR.T | 2 |
| \* | IPAstrin\_STLCD\_tube2\_032114\_01.06688.06688.2 | 3.0842 | 0.3763 | 100.0% | 1375.5322 | 1375.5187 | 1 | 6.762 | 66.7% | 1 | K.VDEIVVLSGDNSK.V | 2 |
| \* | IPAstrin\_STLCD\_tube2\_032114\_01.10472.10472.2 | 3.2007 | 0.4144 | 100.0% | 1934.5721 | 1935.2712 | 1 | 6.762 | 41.7% | 1 | R.LLAELPASVHALTGVDLSK.I | 2 |
| \* | IPAstrin\_STLCD\_032114\_01.12150.12150.3 | 3.0451 | 0.2574 | 97.3% | 1935.2943 | 1935.2712 | 33 | 4.732 | 30.6% | 1 | R.LLAELPASVHALTGVDLSK.I | 3 |

---

|  |  |  |  |  |  |  |  |  |
| --- | --- | --- | --- | --- | --- | --- | --- | --- |
| U | *gi|219555707|ref|NP\_0* | 2 | 4 | 15.8% | 184 | 20170 | 7.0 | eukaryotic translation initiation factor 5A isoform A [Homo sapiens] |
| U | *gi|4503545|ref|NP\_001* | 2 | 4 | 18.8% | 154 | 16832 | 5.2 | eukaryotic translation initiation factor 5A isoform B [Homo sapiens] |
| U | *gi|219555712|ref|NP\_0* | 2 | 4 | 18.8% | 154 | 16832 | 5.2 | eukaryotic translation initiation factor 5A isoform B [Homo sapiens] |
| U | *gi|219555710|ref|NP\_0* | 2 | 4 | 18.8% | 154 | 16832 | 5.2 | eukaryotic translation initiation factor 5A isoform B [Homo sapiens] |

| Filename XCorr DeltCN Conf% ObsM+H+ CalcM+H+ SpR ZScore Ion% # Sequence  | | | | | | | | | | | | |
| --- | --- | --- | --- | --- | --- | --- | --- | --- | --- | --- | --- | --- |
|  | IPAstrin\_STLCD\_tube2\_032114\_01.09826.09826.2 | 3.3536 | 0.4651 | 100.0% | 1298.9521 | 1299.5559 | 1 | 8.896 | 72.7% | 2 | K.VHLVGIDIFTGK.K | 2 |
|  | IPAstrin\_STLCD\_tube2\_032114\_01.06881.06881.3 | 3.6352 | 0.1762 | 95.5% | 1969.8243 | 1970.187 | 3 | 5.201 | 34.4% | 2 | R.EDLRLPEGDLGKEIEQK.Y | 3 |

---

|  |  |  |  |  |  |  |  |  |
| --- | --- | --- | --- | --- | --- | --- | --- | --- |
| U | *gi|14141193|ref|NP\_00* | 3 | 3 | 14.9% | 194 | 22591 | 10.7 | ribosomal protein S9 [Homo sapiens] |

| Filename XCorr DeltCN Conf% ObsM+H+ CalcM+H+ SpR ZScore Ion% # Sequence  | | | | | | | | | | | | |
| --- | --- | --- | --- | --- | --- | --- | --- | --- | --- | --- | --- | --- |
| \* | IPAstrin\_STLCD\_032114\_01.08277.08277.2 | 3.2061 | 0.2743 | 100.0% | 1189.2522 | 1189.4031 | 1 | 5.088 | 77.8% | 1 | R.RLFEGNALLR.R | 2 |
| \* | IPAstrin\_STLCD\_tube2\_032114\_01.08392.08392.2 | 2.5081 | 0.155 | 95.5% | 1400.0922 | 1400.7074 | 40 | 4.069 | 54.5% | 1 | R.KQVVNIPSFIVR.L | 2 |
| \* | IPAstrin\_STLCD\_032114\_01.06579.06579.2 | 2.1014 | 0.1841 | 96.7% | 888.0722 | 888.01404 | 5 | 4.829 | 75.0% | 1 | K.HIDFSLR.S | 2 |

---

|  |  |  |  |  |  |  |  |  |
| --- | --- | --- | --- | --- | --- | --- | --- | --- |
| U | *gi|13376259|ref|NP\_07* | 8 | 10 | 14.8% | 656 | 75019 | 5.6 | nucleoporin 85 [Homo sapiens] |

| Filename XCorr DeltCN Conf% ObsM+H+ CalcM+H+ SpR ZScore Ion% # Sequence  | | | | | | | | | | | | |
| --- | --- | --- | --- | --- | --- | --- | --- | --- | --- | --- | --- | --- |
| \* | IPAstrin\_STLCD\_tube2\_032114\_01.08079.08079.3 | 4.0046 | 0.2559 | 99.6% | 1760.2743 | 1760.0488 | 2 | 5.284 | 44.6% | 1 | R.KLFNESHGIFLGLQR.I | 3 |
| \* | IPAstrin\_STLCD\_032114\_01.11109.11109.2 | 3.6559 | 0.4264 | 100.0% | 1631.1522 | 1631.8748 | 1 | 8.24 | 69.2% | 1 | K.LFNESHGIFLGLQR.I | 2 |
| \* | IPAstrin\_STLCD\_tube2\_032114\_01.09330.09330.3 | 4.1077 | 0.4727 | 100.0% | 2051.3643 | 2051.3037 | 1 | 8.232 | 38.2% | 2 | R.YLQDSTFATSPHLESLLK.I | 3 |
| \* | IPAstrin\_STLCD\_032114\_01.07466.07466.2 | 2.5712 | 0.1269 | 97.2% | 1097.4521 | 1096.2719 | 11 | 5.256 | 68.8% | 1 | R.VSLELHIER.I | 2 |
| \* | IPAstrin\_STLCD\_032114\_01.10353.10353.2 | 2.8339 | 0.3155 | 100.0% | 1090.7122 | 1090.2676 | 5 | 6.727 | 66.7% | 1 | R.LGSALSWSIR.A | 2 |
| \* | IPAstrin\_STLCD\_tube2\_032114\_01.06172.06172.2 | 3.0426 | 0.3658 | 100.0% | 1366.6122 | 1365.529 | 1 | 6.79 | 79.2% | 1 | R.AKDAAFATLVSDR.F | 2 |
| \* | IPAstrin\_STLCD\_032114\_01.16587.16587.2 | 3.9338 | 0.3966 | 100.0% | 1597.2722 | 1596.8822 | 1 | 7.507 | 67.9% | 2 | R.FADAASLLLSLMTSR.I | 2 |
| \* | IPAstrin\_STLCD\_tube2\_032114\_01.17912.17912.2 | 3.2869 | 0.2215 | 99.8% | 2007.7722 | 2007.3955 | 2 | 5.979 | 43.8% | 1 | R.SFWMTLLTDALPLLEQK.Q | 2 |

---

|  |  |  |  |  |  |  |  |  |
| --- | --- | --- | --- | --- | --- | --- | --- | --- |
| U | *gi|169212778|ref|XP\_0* | 4 | 9 | 14.7% | 266 | 30042 | 10.6 | PREDICTED: similar to ribosomal protein L7a [Homo sapiens] |
| U | *gi|4506661|ref|NP\_000* | 4 | 9 | 14.7% | 266 | 29996 | 10.6 | ribosomal protein L7a [Homo sapiens] |
| U | *gi|169213130|ref|XP\_0* | 4 | 9 | 14.7% | 266 | 30042 | 10.6 | PREDICTED: similar to ribosomal protein L7a [Homo sapiens] |
| U | *gi|169212940|ref|XP\_0* | 4 | 9 | 14.7% | 266 | 30028 | 10.6 | PREDICTED: similar to ribosomal protein L7a [Homo sapiens] |

| Filename XCorr DeltCN Conf% ObsM+H+ CalcM+H+ SpR ZScore Ion% # Sequence  | | | | | | | | | | | | |
| --- | --- | --- | --- | --- | --- | --- | --- | --- | --- | --- | --- | --- |
|  | IPAstrin\_STLCD\_tube2\_032114\_01.06192.06192.2 | 2.9207 | 0.3273 | 100.0% | 1217.7522 | 1217.3672 | 13 | 5.548 | 60.0% | 3 | K.NFGIGQDIQPK.R | 2 |
|  | IPAstrin\_STLCD\_tube2\_032114\_01.09880.09880.2 | 2.4719 | 0.4063 | 99.9% | 1570.6721 | 1570.7886 | 8 | 6.776 | 50.0% | 2 | K.VPPAINQFTQALDR.Q | 2 |
|  | IPAstrin\_STLCD\_tube2\_032114\_01.06489.06489.2 | 3.5868 | 0.337 | 100.0% | 1346.1921 | 1346.5236 | 4 | 5.791 | 62.5% | 3 | R.AGVNTVTTLVENK.K | 2 |
|  | IPAstrin\_STLCD\_032114\_01.06056.06056.2 | 2.6301 | 0.3097 | 99.8% | 1474.1322 | 1474.6976 | 131 | 5.193 | 42.3% | 1 | R.AGVNTVTTLVENKK.A | 2 |

---

|  |  |  |  |  |  |  |  |  |
| --- | --- | --- | --- | --- | --- | --- | --- | --- |
| U | *gi|15431293|ref|NP\_00* | 2 | 4 | 14.7% | 204 | 24146 | 11.6 | ribosomal protein L15 [Homo sapiens] |
| U | *gi|88998868|ref|XP\_94* | 2 | 4 | 14.7% | 204 | 24174 | 11.6 | PREDICTED: hypothetical protein isoform 4 [Homo sapiens] |
| U | *gi|88992455|ref|XP\_93* | 2 | 4 | 14.7% | 204 | 24174 | 11.6 | PREDICTED: hypothetical protein isoform 1 [Homo sapiens] |
| U | *gi|169169711|ref|XP\_0* | 2 | 4 | 14.7% | 204 | 24174 | 11.6 | PREDICTED: hypothetical protein [Homo sapiens] |

| Filename XCorr DeltCN Conf% ObsM+H+ CalcM+H+ SpR ZScore Ion% # Sequence  | | | | | | | | | | | | |
| --- | --- | --- | --- | --- | --- | --- | --- | --- | --- | --- | --- | --- |
|  | IPAstrin\_STLCD\_tube2\_032114\_01.03264.03264.2 | 2.3799 | 0.3057 | 99.3% | 1704.8722 | 1706.945 | 99 | 5.545 | 36.7% | 1 | K.GATYGKPVHHGVNQLK.F | 2 |
|  | IPAstrin\_STLCD\_tube2\_032114\_01.07811.07811.2 | 4.1384 | 0.5195 | 100.0% | 1661.2722 | 1661.8083 | 1 | 8.965 | 76.9% | 3 | R.VLNSYWVGEDSTYK.F | 2 |

---

|  |  |  |  |  |  |  |  |  |
| --- | --- | --- | --- | --- | --- | --- | --- | --- |
| U | *gi|4759098|ref|NP\_004* | 4 | 9 | 14.6% | 288 | 33666 | 11.2 | splicing factor, arginine/serine-rich 10 [Homo sapiens] |

| Filename XCorr DeltCN Conf% ObsM+H+ CalcM+H+ SpR ZScore Ion% # Sequence  | | | | | | | | | | | | |
| --- | --- | --- | --- | --- | --- | --- | --- | --- | --- | --- | --- | --- |
| \* | IPAstrin\_STLCD\_tube2\_032114\_01.08824.08824.2 | 4.6981 | 0.4545 | 100.0% | 1812.2322 | 1811.989 | 1 | 7.282 | 56.7% | 6 | K.YGPIADVSIVYDQQSR.R | 2 |
| \* | IPAstrin\_STLCD\_tube2\_032114\_02.08274.08274.2 | 2.295 | 0.2687 | 98.2% | 1621.6122 | 1622.774 | 38 | 5.126 | 42.3% | 1 | R.GFAFVYFENVDDAK.E | 2 |
| \* | IPAstrin\_STLCD\_032114\_01.09966.09966.2 | 2.7417 | 0.1568 | 96.3% | 1950.3522 | 1951.1425 | 14 | 3.762 | 40.6% | 1 | R.GFAFVYFENVDDAKEAK.E | 2 |
| \* | IPAstrin\_STLCD\_tube2\_032114\_01.06503.06503.2 | 2.0612 | 0.3648 | 99.8% | 1078.7322 | 1079.2847 | 1 | 5.441 | 68.8% | 1 | R.IRVDFSITK.R | 2 |

---

|  |  |  |  |  |  |  |  |  |
| --- | --- | --- | --- | --- | --- | --- | --- | --- |
| U | *gi|32189392|ref|NP\_00* | 2 | 5 | 14.6% | 198 | 21892 | 6.0 | peroxiredoxin 2 isoform a [Homo sapiens] |

| Filename XCorr DeltCN Conf% ObsM+H+ CalcM+H+ SpR ZScore Ion% # Sequence  | | | | | | | | | | | | |
| --- | --- | --- | --- | --- | --- | --- | --- | --- | --- | --- | --- | --- |
| \* | IPAstrin\_STLCD\_tube2\_032114\_01.12228.12228.2 | 3.1251 | 0.4138 | 100.0% | 1862.4321 | 1864.1954 | 5 | 7.284 | 38.2% | 1 | R.KEGGLGPLNIPLLADVTR.R | 2 |
|  | IPAstrin\_STLCD\_tube2\_032114\_01.06621.06621.2 | 2.8438 | 0.3153 | 99.9% | 1212.1322 | 1212.3915 | 15 | 6.103 | 70.0% | 4 | R.QITVNDLPVGR.S | 22 |

Similarities:
gi|32455264|ref|NP\_85(1:1)  

---

|  |  |  |  |  |  |  |  |  |
| --- | --- | --- | --- | --- | --- | --- | --- | --- |
| U | *gi|34098946|ref|NP\_00* | 2 | 3 | 14.5% | 324 | 35924 | 9.9 | nuclease sensitive element binding protein 1 [Homo sapiens] |

| Filename XCorr DeltCN Conf% ObsM+H+ CalcM+H+ SpR ZScore Ion% # Sequence  | | | | | | | | | | | | |
| --- | --- | --- | --- | --- | --- | --- | --- | --- | --- | --- | --- | --- |
|  | IPAstrin\_STLCD\_tube2\_032114\_02.06011.06011.2 | 4.0469 | 0.4897 | 100.0% | 1796.4122 | 1796.8822 | 1 | 9.037 | 46.9% | 2 | R.SVGDGETVEFDVVEGEK.G | 2 |
| \* | IPAstrin\_STLCD\_tube2\_032114\_01.04408.04408.3 | 4.5714 | 0.326 | 100.0% | 3224.2144 | 3225.4795 | 1 | 5.318 | 27.6% | 1 | R.RPQYSNPPVQGEVMEGADNQGAGEQGRPVR.Q | 3 |

---

|  |  |  |  |  |  |  |  |  |
| --- | --- | --- | --- | --- | --- | --- | --- | --- |
| U | *gi|74099697|ref|NP\_00* | 4 | 11 | 14.3% | 449 | 49264 | 6.3 | heterogeneous nuclear ribonucleoprotein H2 [Homo sapiens] |
| U | *gi|9624998|ref|NP\_062* | 4 | 11 | 14.3% | 449 | 49264 | 6.3 | heterogeneous nuclear ribonucleoprotein H2 [Homo sapiens] |

| Filename XCorr DeltCN Conf% ObsM+H+ CalcM+H+ SpR ZScore Ion% # Sequence  | | | | | | | | | | | | |
| --- | --- | --- | --- | --- | --- | --- | --- | --- | --- | --- | --- | --- |
|  | IPAstrin\_STLCD\_032114\_01.04140.04140.2 | 3.9174 | 0.3891 | 100.0% | 1686.2922 | 1685.7501 | 1 | 6.574 | 63.3% | 1 | K.HTGPNSPDTANDGFVR.L | 22 |
|  | IPAstrin\_STLCD\_tube2\_032114\_02.07582.07582.2 | 5.0394 | 0.5534 | 100.0% | 1842.2122 | 1843.0001 | 1 | 10.261 | 65.6% | 4 | R.STGEAFVQFASQEIAEK.A | 22 |
|  | IPAstrin\_STLCD\_tube2\_032114\_01.04458.04458.2 | 2.8245 | 0.4221 | 100.0% | 1093.3922 | 1093.2278 | 1 | 8.46 | 83.3% | 5 | R.VHIEIGPDGR.V | 222 |
|  | IPAstrin\_STLCD\_032114\_02.06212.06212.3 | 2.9241 | 0.2485 | 95.1% | 2161.3145 | 2163.3638 | 20 | 4.354 | 28.8% | 1 | R.VTGEADVEFATHEDAVAAMAK.D | 3 |

Similarities:
gi|148470397|ref|NP\_0(1:3)  
gi|5031753|ref|NP\_005(3:1)  

---

|  |  |  |  |  |  |  |  |  |
| --- | --- | --- | --- | --- | --- | --- | --- | --- |
| U | *gi|4506625|ref|NP\_000* | 2 | 5 | 14.2% | 148 | 16561 | 11.0 | ribosomal protein L27a [Homo sapiens] |

| Filename XCorr DeltCN Conf% ObsM+H+ CalcM+H+ SpR ZScore Ion% # Sequence  | | | | | | | | | | | | |
| --- | --- | --- | --- | --- | --- | --- | --- | --- | --- | --- | --- | --- |
| \* | IPAstrin\_STLCD\_tube2\_032114\_01.08219.08219.2 | 2.0154 | 0.2845 | 98.1% | 1234.5521 | 1233.4099 | 19 | 4.807 | 61.1% | 1 | K.LWTLVSEQTR.V | 2 |
| \* | IPAstrin\_STLCD\_tube2\_032114\_01.07146.07146.2 | 2.625 | 0.178 | 98.2% | 1113.2922 | 1112.3146 | 1 | 5.743 | 80.0% | 4 | K.TGAAPIIDVVR.S | 2 |

---

|  |  |  |  |  |  |  |  |  |
| --- | --- | --- | --- | --- | --- | --- | --- | --- |
| U | *gi|52632383|ref|NP\_00* | 7 | 10 | 14.1% | 589 | 64133 | 8.2 | heterogeneous nuclear ribonucleoprotein L isoform a [Homo sapiens] |

| Filename XCorr DeltCN Conf% ObsM+H+ CalcM+H+ SpR ZScore Ion% # Sequence  | | | | | | | | | | | | |
| --- | --- | --- | --- | --- | --- | --- | --- | --- | --- | --- | --- | --- |
| \* | IPAstrin\_STLCD\_032114\_01.18972.18972.3 | 6.1199 | 0.4649 | 100.0% | 3090.1143 | 3089.6143 | 1 | 8.102 | 37.5% | 2 | R.GLIDGVVEADLVEALQEFGPISYVVVMPK.K | 3 |
|  | IPAstrin\_STLCD\_032114\_01.08870.08870.2 | 3.1947 | 0.3189 | 99.9% | 1868.4521 | 1869.1176 | 8 | 5.37 | 44.1% | 2 | K.SKPGAAMVEMADGYAVDR.A | 2 |
|  | IPAstrin\_STLCD\_tube2\_032114\_02.05777.05777.3 | 2.8846 | 0.3185 | 98.8% | 1868.9644 | 1869.1176 | 16 | 5.193 | 29.4% | 1 | K.SKPGAAMVEMADGYAVDR.A | 3 |
|  | IPAstrin\_STLCD\_tube2\_032114\_01.07205.07205.2 | 4.1058 | 0.4518 | 100.0% | 1635.3722 | 1635.881 | 1 | 7.309 | 65.4% | 2 | R.AITHLNNNFMFGQK.L | 2 |
|  | IPAstrin\_STLCD\_tube2\_032114\_01.07199.07199.3 | 3.2408 | 0.2887 | 99.4% | 1636.9143 | 1635.881 | 1 | 4.723 | 40.4% | 1 | R.AITHLNNNFMFGQK.L | 3 |
|  | IPAstrin\_STLCD\_tube2\_032114\_01.07659.07659.2 | 2.4397 | 0.1903 | 97.5% | 1222.5922 | 1223.3251 | 7 | 5.248 | 55.0% | 1 | R.SSSGLLEWESK.S | 2 |
|  | IPAstrin\_STLCD\_tube2\_032114\_01.05482.05482.2 | 2.0832 | 0.268 | 97.5% | 1264.3121 | 1264.4233 | 4 | 4.639 | 55.0% | 1 | K.NPNGPYPYTLK.L | 2 |

---

|  |  |  |  |  |  |  |  |  |
| --- | --- | --- | --- | --- | --- | --- | --- | --- |
| U | *gi|16579885|ref|NP\_00* | 5 | 7 | 14.1% | 427 | 47697 | 11.1 | ribosomal protein L4 [Homo sapiens] |

| Filename XCorr DeltCN Conf% ObsM+H+ CalcM+H+ SpR ZScore Ion% # Sequence  | | | | | | | | | | | | |
| --- | --- | --- | --- | --- | --- | --- | --- | --- | --- | --- | --- | --- |
| \* | IPAstrin\_STLCD\_032114\_01.10174.10174.3 | 4.369 | 0.4108 | 100.0% | 1863.2644 | 1863.1727 | 5 | 7.083 | 40.0% | 2 | K.APIRPDIVNFVHTNLR.K | 3 |
| \* | IPAstrin\_STLCD\_032114\_01.09420.09420.3 | 3.7734 | 0.2515 | 99.3% | 1991.3944 | 1991.3468 | 1 | 5.337 | 42.2% | 1 | K.APIRPDIVNFVHTNLRK.N | 3 |
| \* | IPAstrin\_STLCD\_032114\_01.08533.08533.3 | 3.2417 | 0.3084 | 98.9% | 2333.8743 | 2333.479 | 1 | 5.233 | 31.0% | 1 | R.QPYAVSELAGHQTSAESWGTGR.A | 3 |
| \* | IPAstrin\_STLCD\_tube2\_032114\_01.06981.06981.2 | 2.2775 | 0.1903 | 96.4% | 1281.5922 | 1281.4539 | 39 | 4.154 | 61.1% | 2 | R.KLDELYGTWR.K | 2 |
| \* | IPAstrin\_STLCD\_032114\_01.03975.03975.1 | 2.1504 | 0.3776 | 100.0% | 956.35 | 957.11786 | 1 | 6.66 | 70.0% | 1 | K.AAAAAAALQAK.S | 1 |

---

|  |  |  |  |  |  |  |  |  |
| --- | --- | --- | --- | --- | --- | --- | --- | --- |
| U | *gi|56699409|ref|NP\_00* | 4 | 8 | 13.8% | 391 | 42332 | 10.1 | RNA binding motif protein, X-linked [Homo sapiens] |

| Filename XCorr DeltCN Conf% ObsM+H+ CalcM+H+ SpR ZScore Ion% # Sequence  | | | | | | | | | | | | |
| --- | --- | --- | --- | --- | --- | --- | --- | --- | --- | --- | --- | --- |
|  | IPAstrin\_STLCD\_032114\_01.09122.09122.2 | 3.8502 | 0.1915 | 99.9% | 1437.5122 | 1436.6049 | 2 | 5.588 | 70.8% | 4 | K.LFIGGLNTETNEK.A | 2 |
|  | IPAstrin\_STLCD\_032114\_01.12028.12028.2 | 4.0737 | 0.3945 | 100.0% | 1488.3121 | 1487.6519 | 1 | 8.363 | 76.9% | 2 | R.GFAFVTFESPADAK.D | 2 |
| \* | IPAstrin\_STLCD\_032114\_01.03574.03574.2 | 2.3604 | 0.2585 | 99.6% | 873.4522 | 874.0335 | 4 | 5.543 | 78.6% | 1 | R.RGPPPPPR.S | 2 |
|  | IPAstrin\_STLCD\_032114\_02.05687.05687.3 | 4.0004 | 0.301 | 99.8% | 2050.4644 | 2051.1873 | 3 | 6.478 | 34.7% | 1 | R.GGHMDDGGYSMNFNMSSSR.G | 3 |

---

|  |  |  |  |  |  |  |  |  |
| --- | --- | --- | --- | --- | --- | --- | --- | --- |
| U | *gi|4506607|ref|NP\_000* | 2 | 8 | 13.8% | 188 | 21634 | 11.7 | ribosomal protein L18 [Homo sapiens] |

| Filename XCorr DeltCN Conf% ObsM+H+ CalcM+H+ SpR ZScore Ion% # Sequence  | | | | | | | | | | | | |
| --- | --- | --- | --- | --- | --- | --- | --- | --- | --- | --- | --- | --- |
| \* | IPAstrin\_STLCD\_032114\_02.05774.05774.2 | 3.7389 | 0.4436 | 100.0% | 1346.0922 | 1346.5236 | 1 | 8.729 | 75.0% | 6 | K.TAVVVGTITDDVR.V | 2 |
| \* | IPAstrin\_STLCD\_tube2\_032114\_01.10408.10408.2 | 4.2727 | 0.4739 | 100.0% | 1461.5322 | 1461.6982 | 1 | 8.712 | 79.2% | 2 | K.ILTFDQLALDSPK.G | 2 |

---

|  |  |  |  |  |  |  |  |  |
| --- | --- | --- | --- | --- | --- | --- | --- | --- |
| U | *gi|47132620|ref|NP\_00* | 8 | 12 | 13.5% | 639 | 65433 | 8.0 | keratin 2 [Homo sapiens] |

| Filename XCorr DeltCN Conf% ObsM+H+ CalcM+H+ SpR ZScore Ion% # Sequence  | | | | | | | | | | | | |
| --- | --- | --- | --- | --- | --- | --- | --- | --- | --- | --- | --- | --- |
|  | IPAstrin\_STLCD\_tube2\_032114\_02.06599.06599.2 | 3.6599 | 0.3573 | 100.0% | 1839.9922 | 1840.0055 | 1 | 6.666 | 45.2% | 1 | K.SISISVAGGGGGFGAAGGFGGR.G | 2 |
|  | IPAstrin\_STLCD\_tube2\_032114\_01.06068.06068.2 | 2.3494 | 0.2074 | 99.3% | 828.0122 | 827.95544 | 6 | 5.13 | 91.7% | 1 | K.FASFIDK.V | 222222 |
|  | IPAstrin\_STLCD\_032114\_01.07575.07575.2 | 2.7065 | 0.1841 | 99.4% | 1082.3121 | 1083.2755 | 6 | 6.916 | 75.0% | 2 | K.FASFIDKVR.F | 222222 |
|  | IPAstrin\_STLCD\_tube2\_032114\_01.05296.05296.2 | 4.2794 | 0.067 | 99.8% | 1476.2722 | 1476.6726 | 1 | 6.963 | 90.9% | 3 | R.FLEQQNQVLQTK.W | 22 |
|  | IPAstrin\_STLCD\_tube2\_032114\_01.11807.11807.2 | 3.2089 | 0.3007 | 100.0% | 1461.2322 | 1461.6982 | 3 | 5.62 | 68.2% | 1 | K.VDLLNQEIEFLK.V | 2 |
|  | IPAstrin\_STLCD\_tube2\_032114\_01.12105.12105.2 | 3.6519 | 0.4065 | 100.0% | 1330.4321 | 1330.5211 | 1 | 7.885 | 86.4% | 1 | R.NLDLDSIIAEVK.A | 2222 |
|  | IPAstrin\_STLCD\_tube2\_032114\_01.05304.05304.2 | 2.5054 | 0.1212 | 97.3% | 974.2922 | 974.102 | 123 | 4.93 | 71.4% | 1 | K.IEISELNR.V | 22 |
|  | IPAstrin\_STLCD\_tube2\_032114\_01.09230.09230.2 | 2.7294 | 0.22 | 99.4% | 1264.4521 | 1264.4644 | 2 | 6.703 | 70.0% | 2 | K.LALDVEIATYR.K | 2222 |

Similarities:
gi|4504919|ref|NP\_002(2:6)  
gi|67782365|ref|NP\_00(2:6)  
gi|119395750|ref|NP\_0(2:6)  
gi|119703753|ref|NP\_0(4:4)  
gi|32567786|ref|NP\_78(4:4)  
gi|153791158|ref|NP\_0(4:4)  

---

|  |  |  |  |  |  |  |  |  |
| --- | --- | --- | --- | --- | --- | --- | --- | --- |
| U | *gi|13676857|ref|NP\_06* | 6 | 15 | 13.0% | 639 | 70021 | 5.7 | heat shock 70kDa protein 2 [Homo sapiens] |

| Filename XCorr DeltCN Conf% ObsM+H+ CalcM+H+ SpR ZScore Ion% # Sequence  | | | | | | | | | | | | |
| --- | --- | --- | --- | --- | --- | --- | --- | --- | --- | --- | --- | --- |
|  | IPAstrin\_STLCD\_tube2\_032114\_01.06587.06587.2 | 3.1471 | 0.4339 | 100.0% | 1489.0521 | 1488.5939 | 1 | 8.802 | 70.8% | 7 | R.TTPSYVAFTDTER.L | 222 |
|  | IPAstrin\_STLCD\_032114\_01.03762.03762.2 | 2.3853 | 0.2313 | 99.0% | 1180.9321 | 1181.3312 | 79 | 4.697 | 55.6% | 1 | K.VQVEYKGETK.T | 22 |
|  | IPAstrin\_STLCD\_032114\_01.10974.10974.2 | 4.4218 | 0.485 | 100.0% | 1660.4922 | 1660.9078 | 1 | 8.781 | 70.0% | 3 | R.IINEPTAAAIAYGLDK.K | 222 |
|  | IPAstrin\_STLCD\_tube2\_032114\_01.08004.08004.2 | 3.8742 | 0.2692 | 100.0% | 1788.4722 | 1789.0819 | 1 | 6.977 | 62.5% | 1 | R.IINEPTAAAIAYGLDKK.G | 22 |
|  | IPAstrin\_STLCD\_032114\_01.10381.10381.2 | 3.304 | 0.3653 | 100.0% | 1481.2922 | 1481.6511 | 1 | 6.572 | 72.7% | 2 | R.ARFEELNADLFR.G | 22 |
| \* | IPAstrin\_STLCD\_tube2\_032114\_01.18273.18273.3 | 3.5245 | 0.2604 | 98.4% | 3398.3943 | 3398.8047 | 29 | 4.161 | 20.8% | 1 | K.SENVQDLLLLDVTPLSLGIET#AGGVMT#PLIK.R | 3 |

Similarities:
gi|5729877|ref|NP\_006(5:1)  
contaminant\_GR78\_HUMA(1:5)  
gi|167466173|ref|NP\_0(1:5)  

---

|  |  |  |  |  |  |  |  |  |
| --- | --- | --- | --- | --- | --- | --- | --- | --- |
| U | *gi|5454064|ref|NP\_006* | 6 | 17 | 12.6% | 669 | 69492 | 9.7 | RNA binding motif protein 14 [Homo sapiens] |

| Filename XCorr DeltCN Conf% ObsM+H+ CalcM+H+ SpR ZScore Ion% # Sequence  | | | | | | | | | | | | |
| --- | --- | --- | --- | --- | --- | --- | --- | --- | --- | --- | --- | --- |
| \* | IPAstrin\_STLCD\_tube2\_032114\_01.03602.03602.3 | 3.262 | 0.3782 | 100.0% | 1556.0944 | 1556.7677 | 1 | 6.142 | 38.5% | 1 | R.AIEALHGHELRPGR.A | 3 |
| \* | IPAstrin\_STLCD\_tube2\_032114\_01.06251.06251.2 | 4.0464 | 0.4632 | 100.0% | 1610.2522 | 1609.8223 | 1 | 7.603 | 67.9% | 3 | R.ASYVAPLTAQPATYR.A | 2 |
| \* | IPAstrin\_STLCD\_tube2\_032114\_01.05290.05290.2 | 2.1716 | 0.2409 | 96.8% | 1220.2522 | 1220.3707 | 17 | 5.319 | 54.5% | 1 | R.AQPSVSLGAAYR.A | 2 |
| \* | IPAstrin\_STLCD\_tube2\_032114\_01.05072.05072.3 | 4.3437 | 0.5249 | 100.0% | 2466.2043 | 2466.6292 | 1 | 8.312 | 32.6% | 7 | R.TQSSASLAASYAAQQHPQAAASYR.G | 3 |
| \* | IPAstrin\_STLCD\_032114\_01.07003.07003.2 | 2.9127 | 0.3677 | 100.0% | 1066.7722 | 1067.1869 | 10 | 5.834 | 68.8% | 2 | R.LSESQLSFR.R | 2 |
| \* | IPAstrin\_STLCD\_tube2\_032114\_01.05657.05657.2 | 2.8636 | 0.2962 | 99.9% | 1239.1322 | 1238.2988 | 1 | 6.374 | 77.8% | 3 | R.YSGSYNDYLR.A | 2 |

---

|  |  |  |  |  |  |  |  |  |
| --- | --- | --- | --- | --- | --- | --- | --- | --- |
| U | *gi|4758138|ref|NP\_004* | 7 | 18 | 12.5% | 614 | 69148 | 8.9 | DEAD (Asp-Glu-Ala-Asp) box polypeptide 5 [Homo sapiens] |

| Filename XCorr DeltCN Conf% ObsM+H+ CalcM+H+ SpR ZScore Ion% # Sequence  | | | | | | | | | | | | |
| --- | --- | --- | --- | --- | --- | --- | --- | --- | --- | --- | --- | --- |
| \* | IPAstrin\_STLCD\_tube2\_032114\_01.04565.04565.2 | 3.6815 | 0.4164 | 100.0% | 1390.1721 | 1390.4978 | 2 | 7.406 | 70.0% | 1 | K.NFYQEHPDLAR.R | 2 |
| \* | IPAstrin\_STLCD\_tube2\_032114\_01.07355.07355.2 | 3.1346 | 0.4692 | 100.0% | 1295.9122 | 1296.4198 | 1 | 7.98 | 80.0% | 4 | R.TTYLVLDEADR.M | 2 |
|  | IPAstrin\_STLCD\_032114\_01.11119.11119.2 | 3.5919 | 0.4336 | 100.0% | 1337.1721 | 1337.5946 | 1 | 7.79 | 85.0% | 4 | R.MLDMGFEPQIR.K | 222 |
|  | IPAstrin\_STLCD\_tube2\_032114\_01.07053.07053.2 | 3.6861 | 0.2947 | 100.0% | 1228.0922 | 1227.4465 | 2 | 6.977 | 81.8% | 4 | K.APILIATDVASR.G | 22 |
| \* | IPAstrin\_STLCD\_032114\_01.10681.10681.2 | 3.453 | 0.4359 | 100.0% | 1575.1522 | 1575.7612 | 1 | 6.675 | 69.2% | 1 | K.TGTAYTFFTPNNIK.Q | 2 |
| \* | IPAstrin\_STLCD\_tube2\_032114\_01.09764.09764.2 | 2.0486 | 0.2992 | 98.6% | 1129.3522 | 1130.3298 | 2 | 4.96 | 66.7% | 1 | K.QVSDLISVLR.E | 2 |
| \* | IPAstrin\_STLCD\_032114\_01.07868.07868.2 | 2.6443 | 0.2139 | 99.8% | 986.03217 | 986.1564 | 1 | 5.178 | 85.7% | 3 | K.LLQLVEDR.G | 2 |

Similarities:
gi|87196351|ref|NP\_00(1:6)  
gi|148613856|ref|NP\_0(2:5)  

---

|  |  |  |  |  |  |  |  |  |
| --- | --- | --- | --- | --- | --- | --- | --- | --- |
| U | *gi|4757810|ref|NP\_004* | 5 | 8 | 12.3% | 553 | 59751 | 9.1 | ATP synthase, H+ transporting, mitochondrial F1 complex, alpha subunit precursor [Homo sapiens] |
| U | *gi|50345984|ref|NP\_00* | 5 | 8 | 12.3% | 553 | 59751 | 9.1 | ATP synthase, H+ transporting, mitochondrial F1 complex, alpha subunit precursor [Homo sapiens] |

| Filename XCorr DeltCN Conf% ObsM+H+ CalcM+H+ SpR ZScore Ion% # Sequence  | | | | | | | | | | | | |
| --- | --- | --- | --- | --- | --- | --- | --- | --- | --- | --- | --- | --- |
|  | IPAstrin\_STLCD\_tube2\_032114\_01.07449.07449.2 | 4.0755 | 0.4981 | 100.0% | 1424.1322 | 1424.5659 | 3 | 7.6 | 62.5% | 1 | K.TGTAEMSSILEER.I | 2 |
|  | IPAstrin\_STLCD\_tube2\_032114\_02.05518.05518.2 | 3.9327 | 0.5314 | 100.0% | 1576.1522 | 1576.7007 | 1 | 9.113 | 67.9% | 4 | R.ILGADTSVDLEETGR.V | 2 |
|  | IPAstrin\_STLCD\_tube2\_032114\_01.10275.10275.2 | 3.5611 | 0.2937 | 100.0% | 1625.3322 | 1625.8625 | 1 | 6.51 | 63.3% | 1 | R.TGAIVDVPVGEELLGR.V | 2 |
|  | IPAstrin\_STLCD\_032114\_01.08593.08593.2 | 2.6739 | 0.4221 | 100.0% | 1288.3722 | 1288.4863 | 1 | 6.712 | 75.0% | 1 | K.HALIIYDDLSK.Q | 2 |
|  | IPAstrin\_STLCD\_032114\_01.09494.09494.2 | 2.329 | 0.2205 | 96.9% | 1554.1721 | 1554.7019 | 3 | 5.382 | 54.2% | 1 | R.EAYPGDVFYLHSR.L | 2 |

---

|  |  |  |  |  |  |  |  |  |
| --- | --- | --- | --- | --- | --- | --- | --- | --- |
| U | *gi|16753227|ref|NP\_00* | 2 | 2 | 12.2% | 288 | 32728 | 10.6 | ribosomal protein L6 [Homo sapiens] |
| U | *gi|67189747|ref|NP\_00* | 2 | 2 | 12.2% | 288 | 32728 | 10.6 | ribosomal protein L6 [Homo sapiens] |

| Filename XCorr DeltCN Conf% ObsM+H+ CalcM+H+ SpR ZScore Ion% # Sequence  | | | | | | | | | | | | |
| --- | --- | --- | --- | --- | --- | --- | --- | --- | --- | --- | --- | --- |
|  | IPAstrin\_STLCD\_tube2\_032114\_01.14847.14847.2 | 3.0465 | 0.4053 | 100.0% | 1526.4722 | 1526.8601 | 2 | 7.006 | 53.6% | 1 | R.ASITPGTILIILTGR.H | 2 |
|  | IPAstrin\_STLCD\_tube2\_032114\_01.05310.05310.3 | 3.517 | 0.3747 | 100.0% | 2510.5745 | 2510.6763 | 1 | 5.704 | 38.2% | 1 | R.HQEGEIFDTEKEKYEITEQR.K | 3 |

---

|  |  |  |  |  |  |  |  |  |
| --- | --- | --- | --- | --- | --- | --- | --- | --- |
| U | *gi|4507879|ref|NP\_003* | 2 | 2 | 12.0% | 283 | 30773 | 8.5 | voltage-dependent anion channel 1 [Homo sapiens] |

| Filename XCorr DeltCN Conf% ObsM+H+ CalcM+H+ SpR ZScore Ion% # Sequence  | | | | | | | | | | | | |
| --- | --- | --- | --- | --- | --- | --- | --- | --- | --- | --- | --- | --- |
| \* | IPAstrin\_STLCD\_032114\_02.04717.04717.2 | 2.2242 | 0.3161 | 99.4% | 1214.0122 | 1214.3635 | 384 | 6.111 | 50.0% | 1 | R.VTQSNFAVGYK.T | 2 |
| \* | IPAstrin\_STLCD\_032114\_02.06405.06405.3 | 3.9371 | 0.2323 | 98.5% | 2601.0544 | 2601.7454 | 1 | 4.956 | 30.7% | 1 | K.TDEFQLHTNVNDGTEFGGSIYQK.V | 3 |

---

|  |  |  |  |  |  |  |  |  |
| --- | --- | --- | --- | --- | --- | --- | --- | --- |
| U | *gi|117968353|ref|NP\_1* | 4 | 5 | 11.9% | 464 | 54304 | 8.3 | NUF2, NDC80 kinetochore complex component [Homo sapiens] |
| U | *gi|117968420|ref|NP\_6* | 4 | 5 | 11.9% | 464 | 54304 | 8.3 | NUF2, NDC80 kinetochore complex component [Homo sapiens] |

| Filename XCorr DeltCN Conf% ObsM+H+ CalcM+H+ SpR ZScore Ion% # Sequence  | | | | | | | | | | | | |
| --- | --- | --- | --- | --- | --- | --- | --- | --- | --- | --- | --- | --- |
|  | IPAstrin\_STLCD\_tube2\_032114\_01.09455.09455.2 | 3.2744 | 0.5067 | 100.0% | 1327.0521 | 1327.5693 | 1 | 8.642 | 80.0% | 1 | R.YNVAEIVIHIR.N | 2 |
|  | IPAstrin\_STLCD\_tube2\_032114\_01.04361.04361.2 | 3.6815 | 0.3466 | 100.0% | 1613.0322 | 1613.8903 | 3 | 6.007 | 61.5% | 2 | K.MQQLNAAHQEALMK.L | 2 |
|  | IPAstrin\_STLCD\_tube2\_032114\_01.06072.06072.2 | 3.0236 | 0.3539 | 100.0% | 1550.2322 | 1549.6746 | 1 | 6.198 | 70.8% | 1 | R.LDSVPVEEQEEFK.Q | 2 |
|  | IPAstrin\_STLCD\_032114\_01.11550.11550.2 | 2.6295 | 0.1809 | 96.8% | 1978.7322 | 1979.0588 | 387 | 4.963 | 31.2% | 1 | K.ESLNLEDQIESDESELK.K | 2 |

---

|  |  |  |  |  |  |  |  |  |
| --- | --- | --- | --- | --- | --- | --- | --- | --- |
| U | *gi|4757880|ref|NP\_004* | 2 | 3 | 11.9% | 328 | 37155 | 6.8 | budding uninhibited by benzimidazoles 3 isoform a [Homo sapiens] |
| U | *gi|56550081|ref|NP\_00* | 2 | 3 | 12.0% | 326 | 36955 | 6.8 | budding uninhibited by benzimidazoles 3 isoform b [Homo sapiens] |

| Filename XCorr DeltCN Conf% ObsM+H+ CalcM+H+ SpR ZScore Ion% # Sequence  | | | | | | | | | | | | |
| --- | --- | --- | --- | --- | --- | --- | --- | --- | --- | --- | --- | --- |
|  | IPAstrin\_STLCD\_tube2\_032114\_01.12173.12173.2 | 2.5406 | 0.2265 | 98.1% | 2171.372 | 2172.402 | 31 | 4.885 | 30.6% | 1 | K.FSPNTSQFLLVSSWDTSVR.L | 2 |
|  | IPAstrin\_STLCD\_tube2\_032114\_01.05183.05183.3 | 4.8141 | 0.4409 | 100.0% | 2276.9343 | 2277.4736 | 1 | 7.526 | 46.1% | 2 | K.MHDLNTDQENLVGTHDAPIR.C | 3 |

---

|  |  |  |  |  |  |  |  |  |
| --- | --- | --- | --- | --- | --- | --- | --- | --- |
| U | *gi|10835063|ref|NP\_00* | 2 | 3 | 11.9% | 294 | 32575 | 4.8 | nucleophosmin 1 isoform 1 [Homo sapiens] |
| U | *gi|40353734|ref|NP\_95* | 2 | 3 | 13.2% | 265 | 29465 | 4.6 | nucleophosmin 1 isoform 2 [Homo sapiens] |

| Filename XCorr DeltCN Conf% ObsM+H+ CalcM+H+ SpR ZScore Ion% # Sequence  | | | | | | | | | | | | |
| --- | --- | --- | --- | --- | --- | --- | --- | --- | --- | --- | --- | --- |
|  | IPAstrin\_STLCD\_032114\_01.13432.13432.2 | 4.4001 | 0.5675 | 100.0% | 2228.3323 | 2228.655 | 1 | 10.045 | 42.5% | 2 | K.MSVQPTVSLGGFEITPPVVLR.L | 2 |
|  | IPAstrin\_STLCD\_tube2\_032114\_01.12557.12557.2 | 2.734 | 0.177 | 97.8% | 1820.2722 | 1821.0172 | 1 | 4.851 | 57.7% | 1 | R.MTDQEAIQDLWQWR.K | 2 |

---

|  |  |  |  |  |  |  |  |  |
| --- | --- | --- | --- | --- | --- | --- | --- | --- |
| U | *gi|113412878|ref|XP\_0* | 2 | 3 | 11.9% | 293 | 31479 | 7.6 | PREDICTED: similar to voltage-dependent anion channel [Homo sapiens] |
| U | *gi|42476281|ref|NP\_00* | 2 | 3 | 11.9% | 294 | 31566 | 7.6 | voltage-dependent anion channel 2 [Homo sapiens] |
| U | *gi|169164151|ref|XP\_0* | 2 | 3 | 11.9% | 293 | 31445 | 7.6 | PREDICTED: similar to voltage-dependent anion channel [Homo sapiens] |

| Filename XCorr DeltCN Conf% ObsM+H+ CalcM+H+ SpR ZScore Ion% # Sequence  | | | | | | | | | | | | |
| --- | --- | --- | --- | --- | --- | --- | --- | --- | --- | --- | --- | --- |
|  | IPAstrin\_STLCD\_tube2\_032114\_02.05901.05901.3 | 5.0953 | 0.4756 | 100.0% | 2528.6643 | 2529.682 | 1 | 7.416 | 36.4% | 2 | R.TGDFQLHTNVNDGTEFGGSIYQK.V | 3 |
|  | IPAstrin\_STLCD\_tube2\_032114\_01.05883.05883.2 | 2.7689 | 0.4058 | 100.0% | 1294.4122 | 1294.4473 | 1 | 6.222 | 68.2% | 1 | K.YQLDPTASISAK.V | 2 |

---

|  |  |  |  |  |  |  |  |  |
| --- | --- | --- | --- | --- | --- | --- | --- | --- |
| U | *gi|4506649|ref|NP\_000* | 4 | 5 | 11.7% | 403 | 46109 | 10.2 | ribosomal protein L3 isoform a [Homo sapiens] |
| U | *gi|76496472|ref|NP\_00* | 4 | 5 | 13.3% | 354 | 40152 | 10.2 | ribosomal protein L3 isoform b [Homo sapiens] |

| Filename XCorr DeltCN Conf% ObsM+H+ CalcM+H+ SpR ZScore Ion% # Sequence  | | | | | | | | | | | | |
| --- | --- | --- | --- | --- | --- | --- | --- | --- | --- | --- | --- | --- |
|  | IPAstrin\_STLCD\_032114\_01.06633.06633.2 | 2.7323 | 0.4241 | 100.0% | 984.1122 | 984.14594 | 1 | 8.087 | 75.0% | 1 | R.HGSLGFLPR.K | 2 |
|  | IPAstrin\_STLCD\_tube2\_032114\_02.05040.05040.2 | 3.4672 | 0.4662 | 100.0% | 1697.1721 | 1697.947 | 1 | 9.52 | 50.0% | 1 | K.AHLMEIQVNGGTVAEK.L | 2 |
|  | IPAstrin\_STLCD\_tube2\_032114\_02.05021.05021.3 | 3.8897 | 0.1732 | 97.3% | 1697.7843 | 1697.947 | 3 | 5.292 | 38.3% | 2 | K.AHLMEIQVNGGTVAEK.L | 3 |
|  | IPAstrin\_STLCD\_tube2\_032114\_01.12372.12372.3 | 2.9141 | 0.2592 | 96.3% | 2438.1843 | 2438.8035 | 16 | 4.344 | 28.6% | 1 | K.SINPLGGFVHYGEVTNDFVMLK.G | 3 |

---

|  |  |  |  |  |  |  |  |  |
| --- | --- | --- | --- | --- | --- | --- | --- | --- |
| U | *gi|14249348|ref|NP\_11* | 2 | 9 | 11.4% | 123 | 13941 | 5.5 | thioredoxin-like 5 [Homo sapiens] |

| Filename XCorr DeltCN Conf% ObsM+H+ CalcM+H+ SpR ZScore Ion% # Sequence  | | | | | | | | | | | | |
| --- | --- | --- | --- | --- | --- | --- | --- | --- | --- | --- | --- | --- |
| \* | IPAstrin\_STLCD\_032114\_01.09583.09583.3 | 3.0783 | 0.3487 | 99.8% | 1715.5443 | 1715.8162 | 1 | 6.101 | 40.4% | 6 | R.YEEVSVSGFEEFHR.A | 3 |
| \* | IPAstrin\_STLCD\_032114\_01.09609.09609.2 | 4.1275 | 0.4057 | 100.0% | 1716.3322 | 1715.8162 | 1 | 8.172 | 57.7% | 3 | R.YEEVSVSGFEEFHR.A | 2 |

---

|  |  |  |  |  |  |  |  |  |
| --- | --- | --- | --- | --- | --- | --- | --- | --- |
| U | *gi|4885375|ref|NP\_005* | 3 | 5 | 11.3% | 213 | 21365 | 10.9 | histone cluster 1, H1c [Homo sapiens] |
| U | *gi|4885379|ref|NP\_005* | 3 | 5 | 11.0% | 219 | 21865 | 11.0 | histone cluster 1, H1e [Homo sapiens] |
| U | *gi|4885377|ref|NP\_005* | 3 | 5 | 10.9% | 221 | 22350 | 11.0 | histone cluster 1, H1d [Homo sapiens] |

| Filename XCorr DeltCN Conf% ObsM+H+ CalcM+H+ SpR ZScore Ion% # Sequence  | | | | | | | | | | | | |
| --- | --- | --- | --- | --- | --- | --- | --- | --- | --- | --- | --- | --- |
|  | IPAstrin\_STLCD\_tube2\_032114\_01.05157.05157.2 | 2.5656 | 0.1698 | 96.4% | 1326.7322 | 1327.5638 | 22 | 5.155 | 54.2% | 3 | R.KASGPPVSELITK.A | 2 |
|  | IPAstrin\_STLCD\_tube2\_032114\_01.06446.06446.2 | 2.5526 | 0.2276 | 99.0% | 1199.3522 | 1199.3898 | 13 | 4.874 | 54.5% | 1 | K.ASGPPVSELITK.A | 2 |
|  | IPAstrin\_STLCD\_tube2\_032114\_01.04020.04020.2 | 2.8992 | 0.3245 | 100.0% | 1108.9922 | 1108.2365 | 131 | 6.08 | 50.0% | 1 | K.ALAAAGYDVEK.N | 2 |

---

|  |  |  |  |  |  |  |  |  |
| --- | --- | --- | --- | --- | --- | --- | --- | --- |
| U | *gi|78000181|ref|NP\_00* | 2 | 4 | 11.2% | 215 | 23432 | 10.9 | ribosomal protein L14 [Homo sapiens] |
| U | *gi|78000183|ref|NP\_00* | 2 | 4 | 11.2% | 215 | 23432 | 10.9 | ribosomal protein L14 [Homo sapiens] |

| Filename XCorr DeltCN Conf% ObsM+H+ CalcM+H+ SpR ZScore Ion% # Sequence  | | | | | | | | | | | | |
| --- | --- | --- | --- | --- | --- | --- | --- | --- | --- | --- | --- | --- |
|  | IPAstrin\_STLCD\_032114\_01.05613.05613.2 | 2.3401 | 0.3899 | 99.9% | 1233.3522 | 1233.4124 | 55 | 6.322 | 54.5% | 1 | R.VAYVSFGPHAGK.L | 2 |
|  | IPAstrin\_STLCD\_tube2\_032114\_01.09795.09795.2 | 4.0373 | 0.5082 | 100.0% | 1355.2922 | 1355.5773 | 1 | 8.491 | 81.8% | 3 | K.LVAIVDVIDQNR.A | 2 |

---

|  |  |  |  |  |  |  |  |  |
| --- | --- | --- | --- | --- | --- | --- | --- | --- |
| U | *gi|11024714|ref|NP\_06* | 2 | 6 | 10.9% | 229 | 25762 | 7.4 | ubiquitin B precursor [Homo sapiens] |
| U | *gi|77539055|ref|NP\_00* | 2 | 6 | 19.5% | 128 | 14728 | 9.8 | ubiquitin and ribosomal protein L40 precursor [Homo sapiens] |
| U | *gi|67191208|ref|NP\_06* | 2 | 6 | 3.6% | 685 | 77029 | 7.7 | ubiquitin C [Homo sapiens] |
| U | *gi|4507761|ref|NP\_003* | 2 | 6 | 19.5% | 128 | 14728 | 9.8 | ubiquitin and ribosomal protein L40 precursor [Homo sapiens] |
| U | *gi|4506713|ref|NP\_002* | 2 | 6 | 16.0% | 156 | 17965 | 9.6 | ubiquitin and ribosomal protein S27a precursor [Homo sapiens] |
| U | *gi|208022622|ref|NP\_0* | 2 | 6 | 16.0% | 156 | 17965 | 9.6 | ubiquitin and ribosomal protein S27a precursor [Homo sapiens] |

| Filename XCorr DeltCN Conf% ObsM+H+ CalcM+H+ SpR ZScore Ion% # Sequence  | | | | | | | | | | | | |
| --- | --- | --- | --- | --- | --- | --- | --- | --- | --- | --- | --- | --- |
|  | IPAstrin\_STLCD\_tube2\_032114\_01.08066.08066.2 | 4.3547 | 0.3789 | 100.0% | 1788.4521 | 1788.9897 | 1 | 8.03 | 70.0% | 3 | K.TITLEVEPSDTIENVK.A | 22 |
|  | IPAstrin\_STLCD\_tube2\_032114\_01.06098.06098.2 | 2.4778 | 0.1455 | 97.2% | 1068.1122 | 1068.2615 | 18 | 4.228 | 75.0% | 3 | K.ESTLHLVLR.L | 2 |

Similarities:
contaminant\_UBIQUITIN(1:1)  

---

|  |  |  |  |  |  |  |  |  |
| --- | --- | --- | --- | --- | --- | --- | --- | --- |
| U | *gi|24234688|ref|NP\_00* | 5 | 7 | 10.8% | 679 | 73681 | 6.2 | heat shock 70kDa protein 9 precursor [Homo sapiens] |

| Filename XCorr DeltCN Conf% ObsM+H+ CalcM+H+ SpR ZScore Ion% # Sequence  | | | | | | | | | | | | |
| --- | --- | --- | --- | --- | --- | --- | --- | --- | --- | --- | --- | --- |
| \* | IPAstrin\_STLCD\_032114\_01.08013.08013.2 | 2.7996 | 0.2582 | 99.6% | 1452.2722 | 1451.576 | 1 | 5.202 | 65.4% | 1 | R.TTPSVVAFTADGER.L | 2 |
| \* | IPAstrin\_STLCD\_tube2\_032114\_01.11999.11999.2 | 2.5026 | 0.1927 | 96.7% | 1555.7322 | 1554.8878 | 1 | 4.409 | 53.8% | 1 | K.LYSPSQIGAFVLMK.M | 2 |
| \* | IPAstrin\_STLCD\_tube2\_032114\_01.07920.07920.2 | 2.991 | 0.307 | 99.9% | 1243.4922 | 1243.4056 | 1 | 5.947 | 77.3% | 1 | K.DAGQISGLNVLR.V | 2 |
| \* | IPAstrin\_STLCD\_032114\_01.09382.09382.2 | 2.6095 | 0.2538 | 99.6% | 1291.4922 | 1291.4496 | 1 | 5.354 | 65.0% | 2 | K.VQQTVQDLFGR.A | 2 |
| \* | IPAstrin\_STLCD\_tube2\_032114\_02.06203.06203.3 | 3.0457 | 0.2666 | 97.3% | 2419.4644 | 2419.7095 | 5 | 5.543 | 31.0% | 2 | R.EQQIVIQSSGGLSKDDIENMVK.N | 3 |

---

|  |  |  |  |  |  |  |  |  |
| --- | --- | --- | --- | --- | --- | --- | --- | --- |
| U | *gi|119703753|ref|NP\_0* | 8 | 16 | 10.8% | 564 | 60067 | 8.0 | keratin 6B [Homo sapiens] |

| Filename XCorr DeltCN Conf% ObsM+H+ CalcM+H+ SpR ZScore Ion% # Sequence  | | | | | | | | | | | | |
| --- | --- | --- | --- | --- | --- | --- | --- | --- | --- | --- | --- | --- |
|  | IPAstrin\_STLCD\_tube2\_032114\_01.06068.06068.2 | 2.3494 | 0.2074 | 99.3% | 828.0122 | 827.95544 | 6 | 5.13 | 91.7% | 1 | K.FASFIDK.V | 222222 |
|  | IPAstrin\_STLCD\_032114\_01.07575.07575.2 | 2.7065 | 0.1841 | 99.4% | 1082.3121 | 1083.2755 | 6 | 6.916 | 75.0% | 2 | K.FASFIDKVR.F | 222222 |
|  | IPAstrin\_STLCD\_032114\_01.03831.03831.2 | 2.6693 | 0.1152 | 96.7% | 1310.2122 | 1309.4215 | 1 | 4.407 | 77.8% | 1 | K.NKYEDEINKR.T | 222 |
|  | IPAstrin\_STLCD\_tube2\_032114\_01.12105.12105.2 | 3.6519 | 0.4065 | 100.0% | 1330.4321 | 1330.5211 | 1 | 7.885 | 86.4% | 1 | R.NLDLDSIIAEVK.A | 2222 |
|  | IPAstrin\_STLCD\_tube2\_032114\_01.05621.05621.2 | 3.2069 | 0.3302 | 100.0% | 1180.3121 | 1180.303 | 1 | 7.894 | 83.3% | 3 | K.YEELQITAGR.H | 22 |
|  | IPAstrin\_STLCD\_tube2\_032114\_01.05698.05698.1 | 2.4645 | 0.2588 | 100.0% | 1153.4 | 1154.3234 | 148 | 6.038 | 56.2% | 2 | K.EYQELMNVK.L | 11 |
|  | IPAstrin\_STLCD\_032114\_01.06540.06540.2 | 2.6868 | 0.2337 | 99.8% | 1154.0521 | 1154.3234 | 8 | 6.442 | 68.8% | 4 | K.EYQELMNVK.L | 22 |
|  | IPAstrin\_STLCD\_tube2\_032114\_01.09230.09230.2 | 2.7294 | 0.22 | 99.4% | 1264.4521 | 1264.4644 | 2 | 6.703 | 70.0% | 2 | K.LALDVEIATYR.K | 2222 |

Similarities:
gi|4504919|ref|NP\_002(5:3)  
gi|67782365|ref|NP\_00(2:6)  
gi|119395750|ref|NP\_0(2:6)  
gi|47132620|ref|NP\_00(4:4)  
gi|32567786|ref|NP\_78(4:4)  
gi|153791158|ref|NP\_0(4:4)  

---

|  |  |  |  |  |  |  |  |  |
| --- | --- | --- | --- | --- | --- | --- | --- | --- |
| U | *gi|156151392|ref|NP\_0* | 5 | 8 | 10.7% | 532 | 59682 | 9.2 | heterogeneous nuclear ribonucleoprotein R isoform 4 [Homo sapiens] |
| U | *gi|5031755|ref|NP\_005* | 5 | 8 | 9.0% | 633 | 70943 | 8.1 | heterogeneous nuclear ribonucleoprotein R isoform 2 [Homo sapiens] |
| U | *gi|156151396|ref|NP\_0* | 5 | 8 | 10.7% | 535 | 59953 | 9.2 | heterogeneous nuclear ribonucleoprotein R isoform 3 [Homo sapiens] |
| U | *gi|156151394|ref|NP\_0* | 5 | 8 | 9.0% | 636 | 71214 | 8.1 | heterogeneous nuclear ribonucleoprotein R isoform 1 [Homo sapiens] |

| Filename XCorr DeltCN Conf% ObsM+H+ CalcM+H+ SpR ZScore Ion% # Sequence  | | | | | | | | | | | | |
| --- | --- | --- | --- | --- | --- | --- | --- | --- | --- | --- | --- | --- |
|  | IPAstrin\_STLCD\_tube2\_032114\_01.05297.05297.2 | 2.6218 | 0.3644 | 99.9% | 1311.7322 | 1312.4221 | 2 | 5.977 | 68.2% | 3 | R.TGYTLDVTTGQR.K | 22 |
|  | IPAstrin\_STLCD\_tube2\_032114\_01.07283.07283.2 | 2.5603 | 0.4041 | 100.0% | 1261.8922 | 1262.4846 | 6 | 6.359 | 60.0% | 2 | R.LMMDPLSGQNR.G | 2 |
|  | IPAstrin\_STLCD\_032114\_01.09105.09105.2 | 2.1623 | 0.2711 | 98.2% | 1338.1322 | 1338.5004 | 26 | 5.573 | 50.0% | 1 | K.TKENILEEFSK.V | 2 |
|  | IPAstrin\_STLCD\_tube2\_032114\_01.11718.11718.2 | 2.93 | 0.2665 | 99.9% | 1462.7322 | 1461.6525 | 1 | 5.102 | 66.7% | 1 | R.NLATTVTEEILEK.S | 2 |
|  | IPAstrin\_STLCD\_032114\_01.09614.09614.2 | 2.5889 | 0.2409 | 99.6% | 1299.4122 | 1299.3849 | 3 | 5.159 | 72.2% | 1 | K.DYAFVHFEDR.G | 2 |

Similarities:
gi|23397427|ref|NP\_00(1:4)  

---

|  |  |  |  |  |  |  |  |  |
| --- | --- | --- | --- | --- | --- | --- | --- | --- |
| U | *gi|9506437|ref|NP\_061* | 3 | 5 | 10.7% | 233 | 25863 | 5.2 | chromosome 21 open reading frame 45 [Homo sapiens] |

| Filename XCorr DeltCN Conf% ObsM+H+ CalcM+H+ SpR ZScore Ion% # Sequence  | | | | | | | | | | | | |
| --- | --- | --- | --- | --- | --- | --- | --- | --- | --- | --- | --- | --- |
| \* | IPAstrin\_STLCD\_032114\_01.09572.09572.2 | 3.4438 | 0.293 | 100.0% | 1806.9122 | 1807.9982 | 1 | 5.337 | 57.1% | 2 | K.QIVSEDKELFNLESR.V | 2 |
| \* | IPAstrin\_STLCD\_tube2\_032114\_01.07752.07752.3 | 3.3305 | 0.2392 | 98.5% | 1808.7843 | 1807.9982 | 2 | 5.054 | 42.9% | 2 | K.QIVSEDKELFNLESR.V | 3 |
| \* | IPAstrin\_STLCD\_tube2\_032114\_01.08106.08106.2 | 2.636 | 0.3671 | 100.0% | 1163.3922 | 1164.3594 | 1 | 6.664 | 66.7% | 1 | K.SLTQMEDVLK.A | 2 |

---

|  |  |  |  |  |  |  |  |  |
| --- | --- | --- | --- | --- | --- | --- | --- | --- |
| U | *gi|15431306|ref|NP\_15* | 2 | 2 | 10.5% | 257 | 28025 | 11.0 | ribosomal protein L8 [Homo sapiens] |
| U | *gi|4506663|ref|NP\_000* | 2 | 2 | 10.5% | 257 | 28025 | 11.0 | ribosomal protein L8 [Homo sapiens] |

| Filename XCorr DeltCN Conf% ObsM+H+ CalcM+H+ SpR ZScore Ion% # Sequence  | | | | | | | | | | | | |
| --- | --- | --- | --- | --- | --- | --- | --- | --- | --- | --- | --- | --- |
|  | IPAstrin\_STLCD\_tube2\_032114\_01.03922.03922.2 | 3.9154 | 0.4784 | 100.0% | 1689.3522 | 1689.8223 | 1 | 8.167 | 53.3% | 1 | R.ASGNYATVISHNPETK.K | 2 |
|  | IPAstrin\_STLCD\_032114\_01.04310.04310.1 | 1.886 | 0.3082 | 100.0% | 941.56 | 942.1062 | 304 | 5.733 | 40.0% | 1 | R.AVVGVVAGGGR.I | 1 |

---

|  |  |  |  |  |  |  |  |  |
| --- | --- | --- | --- | --- | --- | --- | --- | --- |
| U | *gi|32483377|ref|NP\_05* | 2 | 5 | 10.5% | 238 | 25839 | 7.5 | peroxiredoxin 3 isoform b [Homo sapiens] |
| U | *gi|5802974|ref|NP\_006* | 2 | 5 | 9.8% | 256 | 27693 | 7.8 | peroxiredoxin 3 isoform a precursor [Homo sapiens] |

| Filename XCorr DeltCN Conf% ObsM+H+ CalcM+H+ SpR ZScore Ion% # Sequence  | | | | | | | | | | | | |
| --- | --- | --- | --- | --- | --- | --- | --- | --- | --- | --- | --- | --- |
|  | IPAstrin\_STLCD\_032114\_01.12409.12409.2 | 2.957 | 0.2724 | 99.9% | 1464.2522 | 1463.6738 | 1 | 4.661 | 65.4% | 2 | R.DYGVLLEGSGLALR.G | 2 |
|  | IPAstrin\_STLCD\_tube2\_032114\_01.05397.05397.2 | 2.5769 | 0.2887 | 99.8% | 1206.3121 | 1207.375 | 2 | 6.813 | 65.0% | 3 | K.HLSVNDLPVGR.S | 2 |

---

|  |  |  |  |  |  |  |  |  |
| --- | --- | --- | --- | --- | --- | --- | --- | --- |
| U | *gi|34740329|ref|NP\_91* | 3 | 7 | 10.3% | 378 | 39595 | 9.0 | heterogeneous nuclear ribonucleoprotein A3 [Homo sapiens] |

| Filename XCorr DeltCN Conf% ObsM+H+ CalcM+H+ SpR ZScore Ion% # Sequence  | | | | | | | | | | | | |
| --- | --- | --- | --- | --- | --- | --- | --- | --- | --- | --- | --- | --- |
| \* | IPAstrin\_STLCD\_tube2\_032114\_01.03448.03448.2 | 2.6822 | 0.2868 | 99.8% | 1382.0922 | 1381.5718 | 16 | 4.822 | 54.2% | 1 | R.EDSVKPGAHLTVK.K | 2 |
| \* | IPAstrin\_STLCD\_tube2\_032114\_02.05775.05775.3 | 3.5186 | 0.3373 | 99.7% | 1884.0543 | 1884.096 | 1 | 6.28 | 43.3% | 2 | K.IFVGGIKEDTEEYNLR.D | 3 |
| \* | IPAstrin\_STLCD\_032114\_01.08029.08029.2 | 3.327 | 0.3132 | 100.0% | 1235.1122 | 1235.3948 | 3 | 6.973 | 83.3% | 4 | K.IETIEVMEDR.Q | 2 |

---

|  |  |  |  |  |  |  |  |  |
| --- | --- | --- | --- | --- | --- | --- | --- | --- |
| U | *gi|4758158|ref|NP\_004* | 2 | 2 | 10.2% | 361 | 41487 | 6.6 | septin 2 [Homo sapiens] |
| U | *gi|56549640|ref|NP\_00* | 2 | 2 | 10.2% | 361 | 41487 | 6.6 | septin 2 [Homo sapiens] |
| U | *gi|56549638|ref|NP\_00* | 2 | 2 | 10.2% | 361 | 41487 | 6.6 | septin 2 [Homo sapiens] |
| U | *gi|56549636|ref|NP\_00* | 2 | 2 | 10.2% | 361 | 41487 | 6.6 | septin 2 [Homo sapiens] |

| Filename XCorr DeltCN Conf% ObsM+H+ CalcM+H+ SpR ZScore Ion% # Sequence  | | | | | | | | | | | | |
| --- | --- | --- | --- | --- | --- | --- | --- | --- | --- | --- | --- | --- |
|  | IPAstrin\_STLCD\_tube2\_032114\_02.05327.05327.2 | 3.9632 | 0.3462 | 100.0% | 1605.4722 | 1604.7545 | 1 | 6.66 | 65.4% | 1 | R.TVQIEASTVEIEER.G | 2 |
|  | IPAstrin\_STLCD\_032114\_02.05259.05259.3 | 3.6295 | 0.3006 | 99.4% | 2386.2544 | 2385.6675 | 1 | 5.574 | 31.8% | 1 | R.MQAQMQMQMQGGDGDGGALGHHV.- | 3 |

---

|  |  |  |  |  |  |  |  |  |
| --- | --- | --- | --- | --- | --- | --- | --- | --- |
| U | *gi|31542947|ref|NP\_00* | 3 | 3 | 10.1% | 573 | 61055 | 5.9 | chaperonin [Homo sapiens] |
| U | *gi|41399285|ref|NP\_95* | 3 | 3 | 10.1% | 573 | 61055 | 5.9 | chaperonin [Homo sapiens] |

| Filename XCorr DeltCN Conf% ObsM+H+ CalcM+H+ SpR ZScore Ion% # Sequence  | | | | | | | | | | | | |
| --- | --- | --- | --- | --- | --- | --- | --- | --- | --- | --- | --- | --- |
|  | IPAstrin\_STLCD\_tube2\_032114\_01.16727.16727.2 | 2.8462 | 0.3493 | 99.9% | 2113.912 | 2114.5667 | 5 | 5.957 | 32.5% | 1 | R.ALMLQGVDLLADAVAVTMGPK.G | 2 |
|  | IPAstrin\_STLCD\_tube2\_032114\_02.04953.04953.3 | 3.3465 | 0.4346 | 100.0% | 2561.6042 | 2561.7222 | 1 | 6.68 | 29.2% | 1 | K.LVQDVANNTNEEAGDGTTTATVLAR.S | 3 |
|  | IPAstrin\_STLCD\_tube2\_032114\_02.04828.04828.2 | 2.8089 | 0.279 | 99.9% | 1216.1721 | 1216.377 | 1 | 6.277 | 68.2% | 1 | K.NAGVEGSLIVEK.I | 2 |

---

|  |  |  |  |  |  |  |  |  |
| --- | --- | --- | --- | --- | --- | --- | --- | --- |
| U | *gi|4502205|ref|NP\_001* | 2 | 2 | 10.0% | 180 | 20511 | 7.2 | ADP-ribosylation factor 4 [Homo sapiens] |

| Filename XCorr DeltCN Conf% ObsM+H+ CalcM+H+ SpR ZScore Ion% # Sequence  | | | | | | | | | | | | |
| --- | --- | --- | --- | --- | --- | --- | --- | --- | --- | --- | --- | --- |
| \* | IPAstrin\_STLCD\_tube2\_032114\_01.18263.18263.2 | 4.484 | 0.4235 | 100.0% | 2073.0522 | 2074.5298 | 1 | 8.732 | 58.8% | 1 | K.MLLVDELRDAVLLLFANK.Q | 2 |
| \* | IPAstrin\_STLCD\_tube2\_032114\_01.18264.18264.3 | 3.2697 | 0.3227 | 99.4% | 2075.2444 | 2074.5298 | 127 | 5.516 | 30.9% | 1 | K.MLLVDELRDAVLLLFANK.Q | 3 |

---

|  |  |  |  |  |  |  |  |  |
| --- | --- | --- | --- | --- | --- | --- | --- | --- |
| U | *gi|4826734|ref|NP\_004* | 3 | 4 | 9.9% | 526 | 53426 | 9.4 | fusion (involved in t(12;16) in malignant liposarcoma) [Homo sapiens] |

| Filename XCorr DeltCN Conf% ObsM+H+ CalcM+H+ SpR ZScore Ion% # Sequence  | | | | | | | | | | | | |
| --- | --- | --- | --- | --- | --- | --- | --- | --- | --- | --- | --- | --- |
| \* | IPAstrin\_STLCD\_tube2\_032114\_01.07310.07310.2 | 2.9031 | 0.3217 | 99.9% | 1409.5322 | 1409.6 | 1 | 5.294 | 68.2% | 2 | K.TGQPMINLYTDR.E | 2 |
| \* | IPAstrin\_STLCD\_tube2\_032114\_02.04331.04331.3 | 3.1925 | 0.2387 | 97.5% | 1662.6543 | 1662.837 | 1 | 5.272 | 43.3% | 1 | K.LKGEATVSFDDPPSAK.A | 3 |
| \* | IPAstrin\_STLCD\_tube2\_032114\_01.03401.03401.3 | 4.0766 | 0.3968 | 100.0% | 2254.4644 | 2254.355 | 1 | 6.486 | 40.2% | 1 | K.APKPDGPGGGPGGSHMGGNYGDDR.R | 3 |

---

|  |  |  |  |  |  |  |  |  |
| --- | --- | --- | --- | --- | --- | --- | --- | --- |
| U | *gi|5174447|ref|NP\_006* | 3 | 5 | 9.5% | 317 | 35077 | 7.7 | guanine nucleotide binding protein (G protein), beta polypeptide 2-like 1 [Homo sapiens] |

| Filename XCorr DeltCN Conf% ObsM+H+ CalcM+H+ SpR ZScore Ion% # Sequence  | | | | | | | | | | | | |
| --- | --- | --- | --- | --- | --- | --- | --- | --- | --- | --- | --- | --- |
| \* | IPAstrin\_STLCD\_tube2\_032114\_01.03996.03996.2 | 2.2397 | 0.1878 | 95.7% | 1193.4321 | 1193.2584 | 315 | 4.239 | 61.1% | 1 | R.DETNYGIPQR.A | 2 |
| \* | IPAstrin\_STLCD\_tube2\_032114\_01.07907.07907.2 | 2.3049 | 0.3013 | 99.4% | 1265.1322 | 1265.4087 | 1 | 5.611 | 65.0% | 2 | R.LWDLTTGTTTR.R | 2 |
| \* | IPAstrin\_STLCD\_tube2\_032114\_01.07120.07120.2 | 2.8578 | 0.3582 | 100.0% | 1060.4722 | 1060.2412 | 1 | 6.689 | 87.5% | 2 | R.VWQVTIGTR.- | 2 |

---

|  |  |  |  |  |  |  |  |  |
| --- | --- | --- | --- | --- | --- | --- | --- | --- |
| U | *gi|14141166|ref|NP\_11* | 2 | 3 | 9.1% | 362 | 38222 | 6.8 | poly(rC) binding protein 2 isoform b [Homo sapiens] |
| U | *gi|193083114|ref|NP\_0* | 2 | 3 | 10.4% | 318 | 33497 | 8.2 | poly(rC) binding protein 2 isoform g [Homo sapiens] |
| U | *gi|193083112|ref|NP\_0* | 2 | 3 | 9.9% | 335 | 35347 | 8.0 | poly(rC) binding protein 2 isoform f [Homo sapiens] |
| U | *gi|193083110|ref|NP\_0* | 2 | 3 | 9.1% | 361 | 38151 | 6.8 | poly(rC) binding protein 2 isoform e [Homo sapiens] |
| U | *gi|193083108|ref|NP\_0* | 2 | 3 | 9.0% | 365 | 38580 | 6.8 | poly(rC) binding protein 2 isoform d [Homo sapiens] |
| U | *gi|148833484|ref|NP\_0* | 2 | 3 | 10.0% | 331 | 34917 | 8.0 | poly(rC) binding protein 2 isoform c [Homo sapiens] |
| U | *gi|14141168|ref|NP\_00* | 2 | 3 | 9.0% | 366 | 38651 | 6.8 | poly(rC) binding protein 2 isoform a [Homo sapiens] |

| Filename XCorr DeltCN Conf% ObsM+H+ CalcM+H+ SpR ZScore Ion% # Sequence  | | | | | | | | | | | | |
| --- | --- | --- | --- | --- | --- | --- | --- | --- | --- | --- | --- | --- |
|  | IPAstrin\_STLCD\_tube2\_032114\_01.09952.09952.2 | 3.2237 | 0.3983 | 100.0% | 1359.5521 | 1359.6519 | 2 | 7.089 | 58.3% | 2 | R.IITLAGPTNAIFK.A | 2 |
|  | IPAstrin\_STLCD\_tube2\_032114\_02.05302.05302.2 | 4.2487 | 0.5519 | 100.0% | 2090.612 | 2091.2573 | 1 | 8.894 | 50.0% | 1 | R.ESTGAQVQVAGDMLPNSTER.A | 22 |

Similarities:
gi|222352151|ref|NP\_0(1:1)  

---

|  |  |  |  |  |  |  |  |  |
| --- | --- | --- | --- | --- | --- | --- | --- | --- |
| U | *gi|106049292|ref|NP\_0* | 7 | 13 | 8.9% | 1178 | 129634 | 6.8 | pyruvate carboxylase precursor [Homo sapiens] |
| U | *gi|106049528|ref|NP\_0* | 7 | 13 | 8.9% | 1178 | 129634 | 6.8 | pyruvate carboxylase precursor [Homo sapiens] |
| U | *gi|106049295|ref|NP\_0* | 7 | 13 | 8.9% | 1178 | 129634 | 6.8 | pyruvate carboxylase precursor [Homo sapiens] |

| Filename XCorr DeltCN Conf% ObsM+H+ CalcM+H+ SpR ZScore Ion% # Sequence  | | | | | | | | | | | | |
| --- | --- | --- | --- | --- | --- | --- | --- | --- | --- | --- | --- | --- |
|  | IPAstrin\_STLCD\_032114\_01.11484.11484.2 | 2.6037 | 0.1724 | 95.8% | 1797.2122 | 1797.9622 | 47 | 3.851 | 40.0% | 1 | K.QVGYENAGTVEFLVDR.H | 2 |
|  | IPAstrin\_STLCD\_tube2\_032114\_01.09477.09477.3 | 4.465 | 0.3823 | 100.0% | 2346.8643 | 2346.645 | 1 | 7.016 | 39.3% | 1 | R.LDNASAFQGAVISPHYDSLLVK.V | 3 |
|  | IPAstrin\_STLCD\_032114\_01.11053.11053.2 | 2.7615 | 0.4036 | 100.0% | 1515.2922 | 1515.7704 | 1 | 6.717 | 50.0% | 3 | R.NHPGLLLMDTTFR.D | 2 |
|  | IPAstrin\_STLCD\_032114\_01.07210.07210.2 | 2.5651 | 0.2034 | 98.3% | 1345.4922 | 1345.5103 | 112 | 4.071 | 54.5% | 2 | R.GTPLDTEVPMER.V | 2 |
|  | IPAstrin\_STLCD\_032114\_01.11191.11191.2 | 3.3767 | 0.3657 | 100.0% | 1523.1921 | 1522.6134 | 1 | 7.163 | 81.8% | 1 | R.VFDYSEYWEGAR.G | 2 |
|  | IPAstrin\_STLCD\_tube2\_032114\_01.11508.11508.2 | 4.3165 | 0.4797 | 100.0% | 1748.3322 | 1749.038 | 1 | 8.043 | 60.0% | 2 | K.IVGDLAQFMVQNGLSR.A | 2 |
|  | IPAstrin\_STLCD\_032114\_01.08319.08319.2 | 3.5945 | 0.3465 | 100.0% | 1548.0521 | 1548.6494 | 1 | 7.165 | 73.1% | 3 | R.AEAEAQAEELSFPR.S | 2 |

---

|  |  |  |  |  |  |  |  |  |
| --- | --- | --- | --- | --- | --- | --- | --- | --- |
| U | *gi|108936958|ref|NP\_0* | 2 | 2 | 8.8% | 342 | 38926 | 5.5 | WD-repeat protein [Homo sapiens] |

| Filename XCorr DeltCN Conf% ObsM+H+ CalcM+H+ SpR ZScore Ion% # Sequence  | | | | | | | | | | | | |
| --- | --- | --- | --- | --- | --- | --- | --- | --- | --- | --- | --- | --- |
| \* | IPAstrin\_STLCD\_tube2\_032114\_02.05939.05939.3 | 4.7222 | 0.437 | 100.0% | 2008.2244 | 2007.2535 | 1 | 7.493 | 45.3% | 1 | K.TQLIAHDKEVYDIAFSR.A | 3 |
| \* | IPAstrin\_STLCD\_tube2\_032114\_02.05656.05656.2 | 2.7865 | 0.4688 | 100.0% | 1312.1721 | 1312.44 | 1 | 7.235 | 58.3% | 1 | R.DMFASVGADGSVR.M | 2 |

---

|  |  |  |  |  |  |  |  |  |
| --- | --- | --- | --- | --- | --- | --- | --- | --- |
| U | *gi|14110414|ref|NP\_00* | 2 | 5 | 8.8% | 306 | 32835 | 8.2 | heterogeneous nuclear ribonucleoprotein D isoform c [Homo sapiens] |
| U | *gi|51477708|ref|NP\_00* | 2 | 5 | 9.4% | 287 | 30672 | 8.4 | heterogeneous nuclear ribonucleoprotein D isoform d [Homo sapiens] |
| U | *gi|14110420|ref|NP\_11* | 2 | 5 | 7.6% | 355 | 38434 | 7.8 | heterogeneous nuclear ribonucleoprotein D isoform a [Homo sapiens] |
| U | *gi|14110417|ref|NP\_11* | 2 | 5 | 8.0% | 336 | 36272 | 8.1 | heterogeneous nuclear ribonucleoprotein D isoform b [Homo sapiens] |

| Filename XCorr DeltCN Conf% ObsM+H+ CalcM+H+ SpR ZScore Ion% # Sequence  | | | | | | | | | | | | |
| --- | --- | --- | --- | --- | --- | --- | --- | --- | --- | --- | --- | --- |
|  | IPAstrin\_STLCD\_tube2\_032114\_01.08922.08922.2 | 2.9111 | 0.3394 | 99.9% | 1485.6322 | 1484.7534 | 30 | 5.883 | 54.2% | 1 | K.MFIGGLSWDTTKK.D | 2 |
|  | IPAstrin\_STLCD\_tube2\_032114\_01.06999.06999.2 | 4.2254 | 0.3708 | 100.0% | 1490.2522 | 1489.6653 | 1 | 6.399 | 73.1% | 4 | K.IFVGGLSPDTPEEK.I | 2 |

---

|  |  |  |  |  |  |  |  |  |
| --- | --- | --- | --- | --- | --- | --- | --- | --- |
| U | *gi|4505773|ref|NP\_002* | 2 | 2 | 8.8% | 272 | 29804 | 5.8 | prohibitin [Homo sapiens] |

| Filename XCorr DeltCN Conf% ObsM+H+ CalcM+H+ SpR ZScore Ion% # Sequence  | | | | | | | | | | | | |
| --- | --- | --- | --- | --- | --- | --- | --- | --- | --- | --- | --- | --- |
| \* | IPAstrin\_STLCD\_tube2\_032114\_01.07415.07415.2 | 2.5984 | 0.3713 | 100.0% | 1150.1122 | 1150.2767 | 5 | 6.151 | 72.2% | 1 | R.FDAGELITQR.E | 2 |
| \* | IPAstrin\_STLCD\_tube2\_032114\_01.08144.08144.2 | 2.5343 | 0.211 | 97.9% | 1608.0322 | 1607.804 | 42 | 4.76 | 50.0% | 1 | R.KLEAAEDIAYQLSR.S | 2 |

---

|  |  |  |  |  |  |  |  |  |
| --- | --- | --- | --- | --- | --- | --- | --- | --- |
| U | *gi|32189394|ref|NP\_00* | 3 | 4 | 8.5% | 529 | 56560 | 5.4 | mitochondrial ATP synthase beta subunit precursor [Homo sapiens] |

| Filename XCorr DeltCN Conf% ObsM+H+ CalcM+H+ SpR ZScore Ion% # Sequence  | | | | | | | | | | | | |
| --- | --- | --- | --- | --- | --- | --- | --- | --- | --- | --- | --- | --- |
| \* | IPAstrin\_STLCD\_tube2\_032114\_01.07890.07890.2 | 3.4463 | 0.4813 | 100.0% | 1651.4521 | 1651.9034 | 1 | 7.076 | 64.3% | 1 | R.LVLEVAQHLGESTVR.T | 2 |
| \* | IPAstrin\_STLCD\_032114\_01.17498.17498.2 | 2.9528 | 0.3614 | 100.0% | 1922.4122 | 1923.1326 | 11 | 6.083 | 40.0% | 2 | R.DQEGQDVLLFIDNIFR.F | 2 |
| \* | IPAstrin\_STLCD\_032114\_01.10726.10726.2 | 2.8827 | 0.2937 | 99.9% | 1437.3722 | 1436.6078 | 1 | 5.618 | 65.4% | 1 | R.FTQAGSEVSALLGR.I | 2 |

---

|  |  |  |  |  |  |  |  |  |
| --- | --- | --- | --- | --- | --- | --- | --- | --- |
| U | *gi|41406064|ref|NP\_00* | 13 | 22 | 8.4% | 1976 | 228997 | 5.5 | myosin, heavy polypeptide 10, non-muscle [Homo sapiens] |

| Filename XCorr DeltCN Conf% ObsM+H+ CalcM+H+ SpR ZScore Ion% # Sequence  | | | | | | | | | | | | |
| --- | --- | --- | --- | --- | --- | --- | --- | --- | --- | --- | --- | --- |
|  | IPAstrin\_STLCD\_032114\_01.08546.08546.2 | 2.0209 | 0.2799 | 97.5% | 1295.8322 | 1295.4467 | 58 | 4.588 | 55.0% | 1 | K.ADFCIIHYAGK.V | 22 |
|  | IPAstrin\_STLCD\_032114\_01.09322.09322.2 | 2.6793 | 0.2294 | 99.4% | 1398.1122 | 1398.6166 | 1 | 4.657 | 80.0% | 2 | K.VDYKADEWLMK.N | 22 |
|  | IPAstrin\_STLCD\_032114\_01.10813.10813.2 | 3.0077 | 0.3601 | 100.0% | 1319.5122 | 1319.5468 | 33 | 5.895 | 65.0% | 2 | K.LDPHLVLDQLR.C | 22 |
|  | IPAstrin\_STLCD\_032114\_01.06412.06412.2 | 3.7363 | 0.4361 | 100.0% | 1224.1122 | 1224.3591 | 1 | 7.776 | 75.0% | 4 | R.AGVLAHLEEER.D | 22 |
| \* | IPAstrin\_STLCD\_032114\_01.11349.11349.2 | 2.8853 | 0.1876 | 99.2% | 1620.9521 | 1620.8496 | 15 | 3.863 | 50.0% | 1 | K.KQQQLS\*ALKVLQR.N | 2 |
| \* | IPAstrin\_STLCD\_032114\_01.07009.07009.2 | 3.3541 | 0.3672 | 100.0% | 1591.2722 | 1591.7766 | 1 | 6.208 | 62.5% | 1 | R.NKQEVMISDLEER.L | 2 |
| \* | IPAstrin\_STLCD\_tube2\_032114\_01.11949.11949.2 | 2.7636 | 0.2389 | 99.3% | 2404.6921 | 2405.5347 | 54 | 4.571 | 30.0% | 1 | K.DAASLESQLQDTQELLQEETR.Q | 2 |
|  | IPAstrin\_STLCD\_032114\_01.05947.05947.2 | 2.6189 | 0.1364 | 97.3% | 1220.7722 | 1221.3959 | 34 | 4.604 | 55.6% | 2 | K.KFDQLLAEEK.S | 22 |
|  | IPAstrin\_STLCD\_tube2\_032114\_01.05541.05541.2 | 2.6717 | 0.0723 | 95.3% | 1093.1322 | 1093.2218 | 1 | 4.929 | 87.5% | 4 | K.FDQLLAEEK.S | 22 |
|  | IPAstrin\_STLCD\_tube2\_032114\_01.10646.10646.2 | 6.0427 | 0.5791 | 100.0% | 1962.1721 | 1963.0594 | 1 | 10.358 | 71.9% | 1 | R.TQLEELEDELQATEDAK.L | 22 |
| \* | IPAstrin\_STLCD\_tube2\_032114\_01.05376.05376.3 | 4.4972 | 0.3528 | 100.0% | 2200.7344 | 2201.2683 | 1 | 5.861 | 38.2% | 1 | R.HAEQERDELADEITNSASGK.S | 3 |
| \* | IPAstrin\_STLCD\_tube2\_032114\_01.07025.07025.2 | 3.6291 | 0.2687 | 100.0% | 1516.6122 | 1515.6604 | 1 | 5.9 | 75.0% | 1 | K.IGQLEEQLEQEAK.E | 2 |
| \* | IPAstrin\_STLCD\_tube2\_032114\_01.03964.03964.2 | 3.6882 | 0.408 | 100.0% | 1520.8522 | 1520.5498 | 1 | 7.343 | 69.2% | 1 | R.ELDDATEANEGLSR.E | 2 |

Similarities:
gi|12667788|ref|NP\_00(7:6)  

---

|  |  |  |  |  |  |  |  |  |
| --- | --- | --- | --- | --- | --- | --- | --- | --- |
| U | *contaminant\_KERATIN02* | 3 | 4 | 8.4% | 622 | 61987 | 5.2 | no description |
| U | *gi|55956899|ref|NP\_00* | 3 | 4 | 8.3% | 623 | 62064 | 5.2 | keratin 9 [Homo sapiens] |

| Filename XCorr DeltCN Conf% ObsM+H+ CalcM+H+ SpR ZScore Ion% # Sequence  | | | | | | | | | | | | |
| --- | --- | --- | --- | --- | --- | --- | --- | --- | --- | --- | --- | --- |
|  | IPAstrin\_STLCD\_tube2\_032114\_02.06731.06731.3 | 2.7396 | 0.2791 | 95.7% | 2706.5044 | 2706.7605 | 13 | 4.457 | 20.2% | 1 | R.GGGGSFGYSYGGGSGGGFSASSLGGGFGGGSR.G | 3 |
|  | IPAstrin\_STLCD\_tube2\_032114\_01.06455.06455.2 | 2.2176 | 0.2107 | 97.5% | 1061.3121 | 1061.1802 | 1 | 5.092 | 87.5% | 1 | K.TLLDIDNTR.M | 2 |
|  | IPAstrin\_STLCD\_tube2\_032114\_01.05289.05289.2 | 2.4994 | 0.2471 | 99.3% | 1158.0122 | 1158.2566 | 1 | 4.679 | 65.0% | 2 | R.QGVDADINGLR.Q | 2 |

---

|  |  |  |  |  |  |  |  |  |
| --- | --- | --- | --- | --- | --- | --- | --- | --- |
| U | *gi|34147630|ref|NP\_00* | 4 | 7 | 8.1% | 455 | 49875 | 7.6 | Tu translation elongation factor, mitochondrial precursor [Homo sapiens] |

| Filename XCorr DeltCN Conf% ObsM+H+ CalcM+H+ SpR ZScore Ion% # Sequence  | | | | | | | | | | | | |
| --- | --- | --- | --- | --- | --- | --- | --- | --- | --- | --- | --- | --- |
| \* | IPAstrin\_STLCD\_tube2\_032114\_02.04852.04852.2 | 2.1106 | 0.3072 | 98.1% | 1674.0122 | 1674.854 | 2 | 5.226 | 40.0% | 1 | R.GITINAAHVEYSTAAR.H | 2 |
| \* | IPAstrin\_STLCD\_032114\_02.05124.05124.3 | 3.0874 | 0.4233 | 100.0% | 1675.6144 | 1674.854 | 1 | 6.343 | 38.3% | 2 | R.GITINAAHVEYSTAAR.H | 3 |
| \* | IPAstrin\_STLCD\_tube2\_032114\_01.07029.07029.2 | 2.6354 | 0.2972 | 99.9% | 1262.3322 | 1262.5096 | 2 | 7.275 | 65.0% | 2 | R.TVVTGIEMFHK.S | 2 |
| \* | IPAstrin\_STLCD\_tube2\_032114\_01.06674.06674.2 | 2.6398 | 0.276 | 99.9% | 1151.0521 | 1150.3605 | 145 | 5.602 | 55.6% | 2 | K.VEAQVYILSK.E | 2 |

---

|  |  |  |  |  |  |  |  |  |
| --- | --- | --- | --- | --- | --- | --- | --- | --- |
| U | *gi|55956919|ref|NP\_11* | 2 | 3 | 8.1% | 332 | 35968 | 6.9 | heterogeneous nuclear ribonucleoprotein A/B isoform a [Homo sapiens] |
| U | *gi|55956921|ref|NP\_00* | 2 | 3 | 9.5% | 285 | 30588 | 7.9 | heterogeneous nuclear ribonucleoprotein A/B isoform b [Homo sapiens] |

| Filename XCorr DeltCN Conf% ObsM+H+ CalcM+H+ SpR ZScore Ion% # Sequence  | | | | | | | | | | | | |
| --- | --- | --- | --- | --- | --- | --- | --- | --- | --- | --- | --- | --- |
|  | IPAstrin\_STLCD\_032114\_01.09883.09883.2 | 2.5671 | 0.3567 | 99.9% | 1456.1122 | 1456.6996 | 1 | 5.634 | 58.3% | 1 | K.MFVGGLSWDTSKK.D | 2 |
|  | IPAstrin\_STLCD\_032114\_01.08671.08671.2 | 3.8656 | 0.3815 | 100.0% | 1504.3121 | 1504.6799 | 1 | 7.225 | 69.2% | 2 | K.IFVGGLNPEATEEK.I | 2 |

---

|  |  |  |  |  |  |  |  |  |
| --- | --- | --- | --- | --- | --- | --- | --- | --- |
| U | *gi|4506903|ref|NP\_003* | 2 | 5 | 8.1% | 221 | 25542 | 8.6 | splicing factor, arginine/serine-rich 9 [Homo sapiens] |

| Filename XCorr DeltCN Conf% ObsM+H+ CalcM+H+ SpR ZScore Ion% # Sequence  | | | | | | | | | | | | |
| --- | --- | --- | --- | --- | --- | --- | --- | --- | --- | --- | --- | --- |
| \* | IPAstrin\_STLCD\_tube2\_032114\_01.06903.06903.2 | 3.2162 | 0.26 | 99.9% | 1248.0521 | 1247.4368 | 7 | 5.698 | 70.0% | 3 | R.IYVGNLPTDVR.E | 2 |
|  | IPAstrin\_STLCD\_032114\_01.06174.06174.2 | 2.4635 | 0.0956 | 96.7% | 917.4122 | 917.0989 | 4 | 4.102 | 75.0% | 2 | R.LRVEFPR.T | 22 |

Similarities:
gi|118582269|ref|NP\_0(1:1)  

---

|  |  |  |  |  |  |  |  |  |
| --- | --- | --- | --- | --- | --- | --- | --- | --- |
| U | *gi|110225358|ref|NP\_0* | 4 | 7 | 8.0% | 858 | 96185 | 6.4 | tetratricopeptide repeat domain 7A [Homo sapiens] |

| Filename XCorr DeltCN Conf% ObsM+H+ CalcM+H+ SpR ZScore Ion% # Sequence  | | | | | | | | | | | | |
| --- | --- | --- | --- | --- | --- | --- | --- | --- | --- | --- | --- | --- |
| \* | IPAstrin\_STLCD\_tube2\_032114\_01.14727.14727.2 | 2.5718 | 0.3344 | 99.9% | 1662.0322 | 1662.9261 | 2 | 5.925 | 50.0% | 1 | R.ASWIAQVFLQELEK.T | 2 |
| \* | IPAstrin\_STLCD\_032114\_01.11604.11604.2 | 3.3754 | 0.3329 | 100.0% | 1765.3922 | 1766.075 | 1 | 6.425 | 46.7% | 2 | R.LEEAMSELTMPSSVLK.Q | 2 |
| \* | IPAstrin\_STLCD\_032114\_01.19044.19044.3 | 4.3616 | 0.4423 | 100.0% | 3398.3342 | 3399.9502 | 1 | 8.193 | 28.7% | 2 | K.QGPMQLWTTLEQIWLQAAELFMEQQHLK.E | 3 |
| \* | IPAstrin\_STLCD\_032114\_01.09536.09536.2 | 2.9953 | 0.3275 | 100.0% | 1259.3722 | 1258.583 | 1 | 6.181 | 75.0% | 2 | R.IMHSLGLMLSR.L | 2 |

---

|  |  |  |  |  |  |  |  |  |
| --- | --- | --- | --- | --- | --- | --- | --- | --- |
| U | *gi|32567786|ref|NP\_78* | 5 | 7 | 7.9% | 535 | 57836 | 7.2 | keratin 6L [Homo sapiens] |

| Filename XCorr DeltCN Conf% ObsM+H+ CalcM+H+ SpR ZScore Ion% # Sequence  | | | | | | | | | | | | |
| --- | --- | --- | --- | --- | --- | --- | --- | --- | --- | --- | --- | --- |
|  | IPAstrin\_STLCD\_tube2\_032114\_01.06068.06068.2 | 2.3494 | 0.2074 | 99.3% | 828.0122 | 827.95544 | 6 | 5.13 | 91.7% | 1 | K.FASFIDK.V | 222222 |
|  | IPAstrin\_STLCD\_032114\_01.07575.07575.2 | 2.7065 | 0.1841 | 99.4% | 1082.3121 | 1083.2755 | 6 | 6.916 | 75.0% | 2 | K.FASFIDKVR.F | 222222 |
|  | IPAstrin\_STLCD\_tube2\_032114\_01.12105.12105.2 | 3.6519 | 0.4065 | 100.0% | 1330.4321 | 1330.5211 | 1 | 7.885 | 86.4% | 1 | R.NLDLDSIIAEVK.A | 2222 |
|  | IPAstrin\_STLCD\_tube2\_032114\_01.04722.04722.2 | 3.0223 | 0.2726 | 99.9% | 1197.2122 | 1197.2897 | 1 | 6.082 | 77.8% | 1 | R.AEAEAWYQTK.Y | 22 |
|  | IPAstrin\_STLCD\_tube2\_032114\_01.09230.09230.2 | 2.7294 | 0.22 | 99.4% | 1264.4521 | 1264.4644 | 2 | 6.703 | 70.0% | 2 | K.LALDVEIATYR.K | 2222 |

Similarities:
gi|4504919|ref|NP\_002(2:3)  
gi|67782365|ref|NP\_00(3:2)  
gi|47132620|ref|NP\_00(4:1)  
gi|119703753|ref|NP\_0(4:1)  
gi|153791158|ref|NP\_0(4:1)  

---

|  |  |  |  |  |  |  |  |  |
| --- | --- | --- | --- | --- | --- | --- | --- | --- |
| U | *gi|5031873|ref|NP\_005* | 2 | 2 | 7.8% | 510 | 57549 | 6.8 | lectin, mannose-binding, 1 precursor [Homo sapiens] |

| Filename XCorr DeltCN Conf% ObsM+H+ CalcM+H+ SpR ZScore Ion% # Sequence  | | | | | | | | | | | | |
| --- | --- | --- | --- | --- | --- | --- | --- | --- | --- | --- | --- | --- |
| \* | IPAstrin\_STLCD\_032114\_01.10671.10671.3 | 3.6908 | 0.371 | 100.0% | 2958.0544 | 2959.2505 | 10 | 5.602 | 23.1% | 1 | K.GPHLVQSDGTVPFWAHAGNAIPSSDQIR.V | 3 |
| \* | IPAstrin\_STLCD\_032114\_01.06074.06074.2 | 2.3583 | 0.2398 | 98.2% | 1412.3522 | 1412.5828 | 95 | 5.537 | 50.0% | 1 | R.YVSSLTEEISKR.G | 2 |

---

|  |  |  |  |  |  |  |  |  |
| --- | --- | --- | --- | --- | --- | --- | --- | --- |
| U | *gi|14141161|ref|NP\_00* | 4 | 11 | 7.7% | 806 | 88980 | 5.8 | heterogeneous nuclear ribonucleoprotein U isoform b [Homo sapiens] |
| U | *gi|74136883|ref|NP\_11* | 4 | 11 | 7.5% | 825 | 90585 | 6.0 | heterogeneous nuclear ribonucleoprotein U isoform a [Homo sapiens] |

| Filename XCorr DeltCN Conf% ObsM+H+ CalcM+H+ SpR ZScore Ion% # Sequence  | | | | | | | | | | | | |
| --- | --- | --- | --- | --- | --- | --- | --- | --- | --- | --- | --- | --- |
|  | IPAstrin\_STLCD\_tube2\_032114\_01.07413.07413.2 | 3.7718 | 0.4507 | 100.0% | 1698.0322 | 1698.8291 | 1 | 7.831 | 70.8% | 2 | R.GYFEYIEENKYSR.A | 2 |
|  | IPAstrin\_STLCD\_tube2\_032114\_01.11759.11759.3 | 4.7961 | 0.3904 | 100.0% | 2726.1243 | 2726.0576 | 1 | 5.802 | 31.0% | 1 | K.EKPYFPIPEEYTFIQNVPLEDR.V | 3 |
|  | IPAstrin\_STLCD\_032114\_01.10075.10075.2 | 3.0403 | 0.2774 | 99.9% | 1383.4122 | 1383.6025 | 1 | 5.789 | 68.2% | 1 | K.YNILGTNTIMDK.M | 2 |
|  | IPAstrin\_STLCD\_tube2\_032114\_01.07420.07420.2 | 4.6147 | 0.4461 | 100.0% | 1648.3121 | 1648.816 | 1 | 7.814 | 82.1% | 7 | R.NFILDQTNVSAAAQR.R | 2 |

---

|  |  |  |  |  |  |  |  |  |
| --- | --- | --- | --- | --- | --- | --- | --- | --- |
| U | *gi|4557303|ref|NP\_000* | 3 | 7 | 7.6% | 485 | 54848 | 7.9 | aldehyde dehydrogenase 3A2 isoform 2 [Homo sapiens] |
| U | *gi|73466520|ref|NP\_00* | 3 | 7 | 7.3% | 508 | 57669 | 8.9 | aldehyde dehydrogenase 3A2 isoform 1 [Homo sapiens] |

| Filename XCorr DeltCN Conf% ObsM+H+ CalcM+H+ SpR ZScore Ion% # Sequence  | | | | | | | | | | | | |
| --- | --- | --- | --- | --- | --- | --- | --- | --- | --- | --- | --- | --- |
|  | IPAstrin\_STLCD\_tube2\_032114\_01.04360.04360.2 | 3.5862 | 0.4254 | 100.0% | 1267.2722 | 1267.3379 | 1 | 8.399 | 77.3% | 3 | K.IAFGGETDEATR.Y | 2 |
|  | IPAstrin\_STLCD\_tube2\_032114\_01.08049.08049.2 | 3.4687 | 0.3437 | 100.0% | 1432.1522 | 1432.6567 | 1 | 7.281 | 75.0% | 2 | R.YIAPTVLTDVDPK.T | 2 |
|  | IPAstrin\_STLCD\_032114\_01.10414.10414.2 | 2.7135 | 0.3539 | 99.9% | 1434.0122 | 1434.5486 | 5 | 6.011 | 63.6% | 2 | K.NVDEAINFINER.E | 2 |

---

|  |  |  |  |  |  |  |  |  |
| --- | --- | --- | --- | --- | --- | --- | --- | --- |
| U | *gi|23397427|ref|NP\_00* | 3 | 7 | 7.5% | 623 | 69633 | 8.6 | synaptotagmin binding, cytoplasmic RNA interacting protein [Homo sapiens] |

| Filename XCorr DeltCN Conf% ObsM+H+ CalcM+H+ SpR ZScore Ion% # Sequence  | | | | | | | | | | | | |
| --- | --- | --- | --- | --- | --- | --- | --- | --- | --- | --- | --- | --- |
| \* | IPAstrin\_STLCD\_032114\_02.07617.07617.3 | 4.2017 | 0.2303 | 98.8% | 2445.4744 | 2443.716 | 8 | 4.906 | 27.4% | 3 | K.VAEKLDEIYVAGLVAHSDLDER.A | 3 |
|  | IPAstrin\_STLCD\_tube2\_032114\_01.05297.05297.2 | 2.6218 | 0.3644 | 99.9% | 1311.7322 | 1312.4221 | 2 | 5.977 | 68.2% | 3 | R.TGYTLDVTTGQR.K | 22 |
| \* | IPAstrin\_STLCD\_tube2\_032114\_01.10432.10432.2 | 3.0911 | 0.2514 | 99.9% | 1474.4922 | 1474.6512 | 1 | 5.849 | 70.8% | 1 | R.NLANTVTEEILEK.A | 2 |

Similarities:
gi|156151392|ref|NP\_0(1:2)  

---

|  |  |  |  |  |  |  |  |  |
| --- | --- | --- | --- | --- | --- | --- | --- | --- |
| U | *gi|7661920|ref|NP\_055* | 3 | 5 | 7.5% | 411 | 46871 | 6.7 | eukaryotic translation initiation factor 4A, isoform 3 [Homo sapiens] |

| Filename XCorr DeltCN Conf% ObsM+H+ CalcM+H+ SpR ZScore Ion% # Sequence  | | | | | | | | | | | | |
| --- | --- | --- | --- | --- | --- | --- | --- | --- | --- | --- | --- | --- |
|  | IPAstrin\_STLCD\_032114\_01.08190.08190.2 | 4.8086 | 0.5811 | 100.0% | 1828.5122 | 1829.0654 | 1 | 9.069 | 76.7% | 3 | R.GIYAYGFEKPSAIQQR.A | 22 |
| \* | IPAstrin\_STLCD\_032114\_01.04144.04144.3 | 2.9521 | 0.3745 | 99.8% | 1598.9944 | 1598.802 | 3 | 5.684 | 35.7% | 1 | R.KLDYGQHVVAGTPGR.V | 3 |
| \* | IPAstrin\_STLCD\_tube2\_032114\_01.03918.03918.2 | 2.8963 | 0.2369 | 99.5% | 1471.4722 | 1470.6279 | 1 | 4.944 | 61.5% | 1 | K.LDYGQHVVAGTPGR.V | 2 |

Similarities:
gi|4503529|ref|NP\_001(1:2)  

---

|  |  |  |  |  |  |  |  |  |
| --- | --- | --- | --- | --- | --- | --- | --- | --- |
| U | *gi|153791158|ref|NP\_0* | 5 | 7 | 7.4% | 551 | 59560 | 7.7 | keratin 75 [Homo sapiens] |

| Filename XCorr DeltCN Conf% ObsM+H+ CalcM+H+ SpR ZScore Ion% # Sequence  | | | | | | | | | | | | |
| --- | --- | --- | --- | --- | --- | --- | --- | --- | --- | --- | --- | --- |
|  | IPAstrin\_STLCD\_tube2\_032114\_01.06068.06068.2 | 2.3494 | 0.2074 | 99.3% | 828.0122 | 827.95544 | 6 | 5.13 | 91.7% | 1 | K.FASFIDK.V | 222222 |
|  | IPAstrin\_STLCD\_032114\_01.07575.07575.2 | 2.7065 | 0.1841 | 99.4% | 1082.3121 | 1083.2755 | 6 | 6.916 | 75.0% | 2 | K.FASFIDKVR.F | 222222 |
|  | IPAstrin\_STLCD\_tube2\_032114\_01.12105.12105.2 | 3.6519 | 0.4065 | 100.0% | 1330.4321 | 1330.5211 | 1 | 7.885 | 86.4% | 1 | R.NLDLDSIIAEVK.A | 2222 |
|  | IPAstrin\_STLCD\_032114\_01.04178.04178.2 | 2.3538 | 0.2742 | 99.6% | 1080.2122 | 1080.1423 | 66 | 5.963 | 56.2% | 1 | K.AQYEDIANR.S | 22 |
|  | IPAstrin\_STLCD\_tube2\_032114\_01.09230.09230.2 | 2.7294 | 0.22 | 99.4% | 1264.4521 | 1264.4644 | 2 | 6.703 | 70.0% | 2 | K.LALDVEIATYR.K | 2222 |

Similarities:
gi|4504919|ref|NP\_002(3:2)  
gi|67782365|ref|NP\_00(2:3)  
gi|47132620|ref|NP\_00(4:1)  
gi|119703753|ref|NP\_0(4:1)  
gi|32567786|ref|NP\_78(4:1)  

---

|  |  |  |  |  |  |  |  |  |
| --- | --- | --- | --- | --- | --- | --- | --- | --- |
| U | *gi|38201714|ref|NP\_00* | 2 | 2 | 7.4% | 326 | 36092 | 9.2 | ELAV-like 1 [Homo sapiens] |

| Filename XCorr DeltCN Conf% ObsM+H+ CalcM+H+ SpR ZScore Ion% # Sequence  | | | | | | | | | | | | |
| --- | --- | --- | --- | --- | --- | --- | --- | --- | --- | --- | --- | --- |
| \* | IPAstrin\_STLCD\_tube2\_032114\_01.09315.09315.2 | 2.8806 | 0.2849 | 99.9% | 1354.5521 | 1354.4998 | 1 | 5.493 | 62.5% | 1 | R.SLFSSIGEVESAK.L | 2 |
| \* | IPAstrin\_STLCD\_tube2\_032114\_01.04324.04324.2 | 2.6763 | 0.3475 | 99.9% | 1188.5122 | 1189.3542 | 1 | 8.312 | 80.0% | 1 | R.VLVDQTTGLSR.G | 2 |

---

|  |  |  |  |  |  |  |  |  |
| --- | --- | --- | --- | --- | --- | --- | --- | --- |
| U | *gi|32698730|ref|NP\_06* | 4 | 8 | 7.3% | 695 | 76121 | 8.7 | nuclear fragile X mental retardation protein interacting protein 2 [Homo sapiens] |

| Filename XCorr DeltCN Conf% ObsM+H+ CalcM+H+ SpR ZScore Ion% # Sequence  | | | | | | | | | | | | |
| --- | --- | --- | --- | --- | --- | --- | --- | --- | --- | --- | --- | --- |
| \* | IPAstrin\_STLCD\_tube2\_032114\_01.04008.04008.2 | 3.813 | 0.3439 | 100.0% | 1404.8322 | 1404.4764 | 1 | 7.322 | 66.7% | 2 | K.NLSSDEATNPISR.V | 2 |
| \* | IPAstrin\_STLCD\_tube2\_032114\_01.04908.04908.2 | 2.9234 | 0.3004 | 99.9% | 1516.4321 | 1515.7068 | 4 | 5.992 | 53.8% | 3 | R.VLNGNQQVVDTSLK.Q | 2 |
| \* | IPAstrin\_STLCD\_tube2\_032114\_01.05273.05273.2 | 2.7351 | 0.2502 | 99.6% | 1376.0322 | 1375.6233 | 30 | 4.986 | 54.5% | 2 | K.IMQQETSVPTLK.Q | 2 |
| \* | IPAstrin\_STLCD\_032114\_01.09505.09505.2 | 2.5093 | 0.2149 | 98.3% | 1379.0322 | 1379.5565 | 1 | 4.302 | 72.7% | 1 | K.RTS\*PQVLGSILK.S | 2 |

---

|  |  |  |  |  |  |  |  |  |
| --- | --- | --- | --- | --- | --- | --- | --- | --- |
| U | *gi|162329583|ref|NP\_0* | 3 | 4 | 7.1% | 551 | 59210 | 7.2 | cleavage and polyadenylation specific factor 6, 68 kD subunit [Homo sapiens] |

| Filename XCorr DeltCN Conf% ObsM+H+ CalcM+H+ SpR ZScore Ion% # Sequence  | | | | | | | | | | | | |
| --- | --- | --- | --- | --- | --- | --- | --- | --- | --- | --- | --- | --- |
| \* | IPAstrin\_STLCD\_tube2\_032114\_01.10149.10149.2 | 4.5989 | 0.4652 | 100.0% | 1666.3522 | 1666.8433 | 1 | 9.905 | 80.8% | 1 | R.TPLSEAEFEEIMNR.N | 2 |
| \* | IPAstrin\_STLCD\_tube2\_032114\_01.17054.17054.2 | 5.081 | 0.5897 | 100.0% | 2452.5723 | 2453.749 | 1 | 10.527 | 43.8% | 2 | R.AVSDASAGDYGSAIETLVTAISLIK.Q | 2 |
| \* | IPAstrin\_STLCD\_tube2\_032114\_01.17055.17055.3 | 3.4592 | 0.2927 | 98.8% | 2453.3342 | 2453.749 | 1 | 5.55 | 30.2% | 1 | R.AVSDASAGDYGSAIETLVTAISLIK.Q | 3 |

---

|  |  |  |  |  |  |  |  |  |
| --- | --- | --- | --- | --- | --- | --- | --- | --- |
| U | *gi|66933016|ref|NP\_00* | 2 | 3 | 7.0% | 514 | 55805 | 6.9 | inosine monophosphate dehydrogenase 2 [Homo sapiens] |

| Filename XCorr DeltCN Conf% ObsM+H+ CalcM+H+ SpR ZScore Ion% # Sequence  | | | | | | | | | | | | |
| --- | --- | --- | --- | --- | --- | --- | --- | --- | --- | --- | --- | --- |
| \* | IPAstrin\_STLCD\_tube2\_032114\_01.12430.12430.2 | 2.4513 | 0.2735 | 99.0% | 1780.9922 | 1781.0593 | 326 | 5.679 | 30.0% | 1 | K.LPIVNEDDELVAIIAR.T | 2 |
| \* | IPAstrin\_STLCD\_032114\_01.09751.09751.3 | 4.539 | 0.3739 | 100.0% | 2049.2644 | 2049.3835 | 1 | 6.72 | 42.1% | 2 | R.RFGVPVIADGGIQNVGHIAK.A | 3 |

---

|  |  |  |  |  |  |  |  |  |
| --- | --- | --- | --- | --- | --- | --- | --- | --- |
| U | *gi|21327708|ref|NP\_63* | 2 | 2 | 6.9% | 391 | 45374 | 4.5 | nucleosome assembly protein 1-like 1 [Homo sapiens] |
| U | *gi|4758756|ref|NP\_004* | 2 | 2 | 6.9% | 391 | 45374 | 4.5 | nucleosome assembly protein 1-like 1 [Homo sapiens] |

| Filename XCorr DeltCN Conf% ObsM+H+ CalcM+H+ SpR ZScore Ion% # Sequence  | | | | | | | | | | | | |
| --- | --- | --- | --- | --- | --- | --- | --- | --- | --- | --- | --- | --- |
|  | IPAstrin\_STLCD\_tube2\_032114\_01.10498.10498.2 | 3.6316 | 0.267 | 100.0% | 1860.3722 | 1861.102 | 5 | 5.613 | 43.8% | 1 | R.LDGLVETPTGYIESLPR.V | 2 |
|  | IPAstrin\_STLCD\_tube2\_032114\_01.05169.05169.2 | 2.3402 | 0.2554 | 99.3% | 1337.1322 | 1337.4314 | 1 | 5.834 | 77.8% | 1 | K.FYEEVHDLER.K | 2 |

---

|  |  |  |  |  |  |  |  |  |
| --- | --- | --- | --- | --- | --- | --- | --- | --- |
| U | *gi|21626466|ref|NP\_06* | 3 | 7 | 6.8% | 847 | 94623 | 6.3 | matrin 3 [Homo sapiens] |
| U | *gi|62750354|ref|NP\_95* | 3 | 7 | 6.8% | 847 | 94623 | 6.3 | matrin 3 [Homo sapiens] |

| Filename XCorr DeltCN Conf% ObsM+H+ CalcM+H+ SpR ZScore Ion% # Sequence  | | | | | | | | | | | | |
| --- | --- | --- | --- | --- | --- | --- | --- | --- | --- | --- | --- | --- |
|  | IPAstrin\_STLCD\_tube2\_032114\_01.12174.12174.2 | 4.6636 | 0.4176 | 100.0% | 1794.5322 | 1793.931 | 1 | 7.671 | 64.7% | 2 | R.GDADQASNILASFGLSAR.D | 2 |
|  | IPAstrin\_STLCD\_tube2\_032114\_01.14162.14162.3 | 4.6021 | 0.3575 | 100.0% | 2440.3145 | 2439.9036 | 9 | 6.188 | 30.0% | 3 | R.YQLLQLVEPFGVISNHLILNK.I | 3 |
|  | IPAstrin\_STLCD\_tube2\_032114\_01.06306.06306.3 | 3.3215 | 0.3995 | 100.0% | 2037.0543 | 2038.3109 | 1 | 6.554 | 36.1% | 2 | R.VIHLSNLPHSGYSDSAVLK.L | 3 |

---

|  |  |  |  |  |  |  |  |  |
| --- | --- | --- | --- | --- | --- | --- | --- | --- |
| U | *gi|55956788|ref|NP\_00* | 3 | 3 | 6.8% | 710 | 76615 | 4.7 | nucleolin [Homo sapiens] |

| Filename XCorr DeltCN Conf% ObsM+H+ CalcM+H+ SpR ZScore Ion% # Sequence  | | | | | | | | | | | | |
| --- | --- | --- | --- | --- | --- | --- | --- | --- | --- | --- | --- | --- |
| \* | IPAstrin\_STLCD\_tube2\_032114\_01.09711.09711.2 | 2.9056 | 0.2758 | 99.8% | 1650.2322 | 1649.751 | 1 | 5.167 | 61.5% | 1 | K.FGYVDFESAEDLEK.A | 2 |
| \* | IPAstrin\_STLCD\_tube2\_032114\_02.06177.06177.3 | 3.0357 | 0.4088 | 100.0% | 2200.3442 | 2201.3057 | 1 | 6.32 | 32.9% | 1 | K.GLSEDTTEETLKESFDGSVR.A | 3 |
| \* | IPAstrin\_STLCD\_032114\_01.11389.11389.2 | 2.9396 | 0.2352 | 99.6% | 1563.6522 | 1562.6323 | 1 | 5.041 | 61.5% | 1 | K.GFGFVDFNSEEDAK.A | 2 |

---

|  |  |  |  |  |  |  |  |  |
| --- | --- | --- | --- | --- | --- | --- | --- | --- |
| U | *contaminant\_KERATIN05* | 4 | 9 | 6.8% | 471 | 51531 | 5.2 | no description |
| U | *gi|24430192|ref|NP\_00* | 4 | 9 | 6.8% | 473 | 51268 | 5.0 | keratin 16 [Homo sapiens] |
| U | *gi|24430190|ref|NP\_00* | 4 | 9 | 7.0% | 456 | 49198 | 4.8 | keratin 15 [Homo sapiens] |
| U | *gi|15431310|ref|NP\_00* | 4 | 9 | 6.8% | 472 | 51622 | 5.2 | keratin 14 [Homo sapiens] |
| U | *contaminant\_KERATIN08* | 4 | 9 | 6.8% | 469 | 50499 | 5.0 | no description |
| U | *contaminant\_KERATIN07* | 4 | 9 | 6.8% | 473 | 50915 | 5.5 | no description |
| U | *contaminant\_KERATIN06* | 4 | 9 | 7.0% | 456 | 49168 | 4.8 | no description |

| Filename XCorr DeltCN Conf% ObsM+H+ CalcM+H+ SpR ZScore Ion% # Sequence  | | | | | | | | | | | | |
| --- | --- | --- | --- | --- | --- | --- | --- | --- | --- | --- | --- | --- |
|  | IPAstrin\_STLCD\_tube2\_032114\_02.04549.04549.2 | 2.3551 | 0.198 | 96.4% | 1302.5521 | 1302.4241 | 4 | 5.201 | 54.5% | 1 | R.ALEEANADLEVK.I | 2 |
|  | IPAstrin\_STLCD\_032114\_01.08774.08774.2 | 3.4852 | 0.4075 | 100.0% | 1030.1522 | 1030.2096 | 1 | 6.822 | 81.2% | 3 | R.VLDELTLAR.A | 222 |
|  | IPAstrin\_STLCD\_tube2\_032114\_01.05660.05660.2 | 3.1546 | 0.0813 | 98.1% | 1382.6122 | 1380.5437 | 3 | 4.115 | 70.0% | 4 | K.TRLEQEIATYR.R | 22 |
|  | IPAstrin\_STLCD\_tube2\_032114\_01.04053.04053.2 | 3.0883 | 0.297 | 100.0% | 1122.9521 | 1123.2511 | 1 | 6.048 | 81.2% | 1 | R.LEQEIATYR.R | 222 |

Similarities:
contaminant\_KERATIN12(3:1)  
contaminant\_KERATIN10(2:2)  

---

|  |  |  |  |  |  |  |  |  |
| --- | --- | --- | --- | --- | --- | --- | --- | --- |
| U | *contaminant\_KERATIN10* | 4 | 14 | 6.8% | 400 | 44106 | 5.1 | no description |
| U | *gi|24234699|ref|NP\_00* | 4 | 14 | 6.8% | 400 | 44106 | 5.1 | keratin 19 [Homo sapiens] |

| Filename XCorr DeltCN Conf% ObsM+H+ CalcM+H+ SpR ZScore Ion% # Sequence  | | | | | | | | | | | | |
| --- | --- | --- | --- | --- | --- | --- | --- | --- | --- | --- | --- | --- |
|  | IPAstrin\_STLCD\_tube2\_032114\_01.05762.05762.1 | 2.4421 | 0.3161 | 100.0% | 1041.45 | 1042.2235 | 11 | 6.413 | 62.5% | 3 | R.IVLQIDNAR.L | 11 |
|  | IPAstrin\_STLCD\_032114\_01.06636.06636.2 | 3.2213 | 0.1416 | 99.8% | 1042.1721 | 1042.2235 | 3 | 6.071 | 87.5% | 7 | R.IVLQIDNAR.L | 22 |
|  | IPAstrin\_STLCD\_032114\_01.08774.08774.2 | 3.4852 | 0.4075 | 100.0% | 1030.1522 | 1030.2096 | 1 | 6.822 | 81.2% | 3 | R.VLDELTLAR.T | 222 |
|  | IPAstrin\_STLCD\_tube2\_032114\_01.04053.04053.2 | 3.0883 | 0.297 | 100.0% | 1122.9521 | 1123.2511 | 1 | 6.048 | 81.2% | 1 | R.LEQEIATYR.S | 222 |

Similarities:
contaminant\_KERATIN09(2:2)  
contaminant\_KERATIN12(2:2)  
contaminant\_KERATIN05(2:2)  

---

|  |  |  |  |  |  |  |  |  |
| --- | --- | --- | --- | --- | --- | --- | --- | --- |
| U | *gi|9966881|ref|NP\_065* | 6 | 6 | 6.7% | 925 | 106374 | 5.4 | nucleoporin 107kDa [Homo sapiens] |

| Filename XCorr DeltCN Conf% ObsM+H+ CalcM+H+ SpR ZScore Ion% # Sequence  | | | | | | | | | | | | |
| --- | --- | --- | --- | --- | --- | --- | --- | --- | --- | --- | --- | --- |
| \* | IPAstrin\_STLCD\_032114\_01.09794.09794.2 | 2.4871 | 0.2368 | 99.0% | 1328.8722 | 1329.4093 | 1 | 5.321 | 59.1% | 1 | R.SGFGEISS\*PVIR.E | 2 |
| \* | IPAstrin\_STLCD\_tube2\_032114\_02.05201.05201.2 | 4.3156 | 0.553 | 100.0% | 1890.2322 | 1891.0476 | 1 | 9.51 | 62.5% | 1 | R.VLLQASQDENFGNTTPR.N | 2 |
| \* | IPAstrin\_STLCD\_tube2\_032114\_02.05317.05317.2 | 3.9159 | 0.4452 | 100.0% | 1970.2122 | 1971.0476 | 1 | 6.969 | 59.4% | 1 | R.VLLQASQDENFGNTT#PR.N | 2 |
| \* | IPAstrin\_STLCD\_032114\_01.07563.07563.2 | 2.0737 | 0.2769 | 98.5% | 1125.4321 | 1125.2415 | 9 | 4.763 | 68.8% | 1 | R.MAEDELFNR.Y | 2 |
| \* | IPAstrin\_STLCD\_tube2\_032114\_01.05020.05020.2 | 2.6377 | 0.4057 | 100.0% | 1180.0322 | 1180.3 | 1 | 6.383 | 66.7% | 1 | K.VFEELQATDK.K | 2 |
| \* | IPAstrin\_STLCD\_032114\_01.15004.15004.2 | 2.6842 | 0.1476 | 95.7% | 1688.0322 | 1687.9359 | 3 | 5.264 | 46.2% | 1 | K.IDVIDWLVFDPAQR.A | 2 |

---

|  |  |  |  |  |  |  |  |  |
| --- | --- | --- | --- | --- | --- | --- | --- | --- |
| U | *gi|116812577|ref|NP\_0* | 2 | 3 | 6.6% | 392 | 46514 | 10.0 | LUC7-like 2 [Homo sapiens] |

| Filename XCorr DeltCN Conf% ObsM+H+ CalcM+H+ SpR ZScore Ion% # Sequence  | | | | | | | | | | | | |
| --- | --- | --- | --- | --- | --- | --- | --- | --- | --- | --- | --- | --- |
| \* | IPAstrin\_STLCD\_tube2\_032114\_01.08552.08552.2 | 1.9575 | 0.3195 | 98.2% | 1222.7722 | 1223.448 | 2 | 6.609 | 65.0% | 1 | R.AMLDQLMGTSR.D | 2 |
| \* | IPAstrin\_STLCD\_032114\_02.05004.05004.2 | 3.7511 | 0.402 | 100.0% | 1589.2922 | 1589.7399 | 1 | 7.698 | 53.6% | 2 | R.LAETQEEISAEVAAK.A | 2 |

---

|  |  |  |  |  |  |  |  |  |
| --- | --- | --- | --- | --- | --- | --- | --- | --- |
| U | *gi|5031877|ref|NP\_005* | 2 | 2 | 6.5% | 586 | 66408 | 5.2 | lamin B1 [Homo sapiens] |

| Filename XCorr DeltCN Conf% ObsM+H+ CalcM+H+ SpR ZScore Ion% # Sequence  | | | | | | | | | | | | |
| --- | --- | --- | --- | --- | --- | --- | --- | --- | --- | --- | --- | --- |
| \* | IPAstrin\_STLCD\_tube2\_032114\_01.15778.15778.3 | 3.6195 | 0.2666 | 98.6% | 2529.4143 | 2529.804 | 18 | 5.269 | 25.0% | 1 | K.SLEGDLEDLKDQIAQLEASLAAAK.K | 3 |
| \* | IPAstrin\_STLCD\_032114\_01.04090.04090.2 | 3.6294 | 0.3974 | 100.0% | 1498.1522 | 1497.6202 | 1 | 6.988 | 69.2% | 1 | R.LSSEMNTSTVNSAR.E | 2 |

---

|  |  |  |  |  |  |  |  |  |
| --- | --- | --- | --- | --- | --- | --- | --- | --- |
| U | *gi|33469968|ref|NP\_00* | 3 | 3 | 6.3% | 719 | 81308 | 6.5 | minichromosome maintenance complex component 7 isoform 1 [Homo sapiens] |

| Filename XCorr DeltCN Conf% ObsM+H+ CalcM+H+ SpR ZScore Ion% # Sequence  | | | | | | | | | | | | |
| --- | --- | --- | --- | --- | --- | --- | --- | --- | --- | --- | --- | --- |
| \* | IPAstrin\_STLCD\_tube2\_032114\_02.05982.05982.3 | 3.0492 | 0.3077 | 99.4% | 1829.1543 | 1829.063 | 59 | 5.566 | 35.7% | 1 | R.EVVNKDVLDVYIEHR.L | 3 |
|  | IPAstrin\_STLCD\_tube2\_032114\_01.10842.10842.2 | 3.4703 | 0.3262 | 100.0% | 1653.9922 | 1653.9193 | 1 | 6.596 | 53.6% | 1 | R.SLEQNIQLPAALLSR.F | 2 |
|  | IPAstrin\_STLCD\_032114\_02.05157.05157.3 | 3.7719 | 0.3576 | 100.0% | 1746.3844 | 1746.9733 | 2 | 6.111 | 42.9% | 1 | R.MVDVVEKEDVNEAIR.L | 3 |

---

|  |  |  |  |  |  |  |  |  |
| --- | --- | --- | --- | --- | --- | --- | --- | --- |
| U | *contaminant\_GR78\_HUMA* | 3 | 5 | 6.3% | 653 | 72116 | 5.1 | owl|P11021| 78 KD GLUCOSE REGULATED PROTEIN PRECURSOR (GRP 78) (IMMUNOGLOBULIN... |
| U | *gi|16507237|ref|NP\_00* | 3 | 5 | 6.3% | 654 | 72333 | 5.2 | heat shock 70kDa protein 5 [Homo sapiens] |
| U | *contaminant\_GR78\_RAT* | 3 | 5 | 6.3% | 654 | 72347 | 5.2 | owl|P06761| 78 KD GLUCOSE REGULATED PROTEIN PRECURSOR (GRP 78) (IMMUNOGLOBULIN... |
| U | *contaminant\_GR78\_MOUS* | 3 | 5 | 6.3% | 655 | 72421 | 5.2 | owl|P20029| 78 KD GLUCOSE REGULATED PROTEIN PRECURSOR (GRP 78) (IMMUNOGLOBULIN... |
| U | *contaminant\_GR78\_MESA* | 3 | 5 | 6.3% | 654 | 72379 | 5.2 | owl|P07823| 78 KD GLUCOSE REGULATED PROTEIN PRECURSOR (GRP 78) (IMMUNOGLOBULIN... |

| Filename XCorr DeltCN Conf% ObsM+H+ CalcM+H+ SpR ZScore Ion% # Sequence  | | | | | | | | | | | | |
| --- | --- | --- | --- | --- | --- | --- | --- | --- | --- | --- | --- | --- |
|  | IPAstrin\_STLCD\_tube2\_032114\_01.07475.07475.2 | 2.9676 | 0.4007 | 100.0% | 1567.3722 | 1567.7386 | 1 | 7.487 | 57.7% | 1 | R.ITPSYVAFTPEGER.L | 2 |
|  | IPAstrin\_STLCD\_032114\_01.10974.10974.2 | 4.4218 | 0.485 | 100.0% | 1660.4922 | 1660.9078 | 1 | 8.781 | 70.0% | 3 | R.IINEPTAAAIAYGLDK.R | 222 |
|  | IPAstrin\_STLCD\_032114\_01.09714.09714.2 | 2.5221 | 0.2011 | 98.4% | 1317.5721 | 1317.4381 | 2 | 4.236 | 75.0% | 1 | R.NELESYAYSLK.N | 2 |

Similarities:
gi|5729877|ref|NP\_006(1:2)  
gi|13676857|ref|NP\_06(1:2)  

---

|  |  |  |  |  |  |  |  |  |
| --- | --- | --- | --- | --- | --- | --- | --- | --- |
| U | *gi|77404397|ref|NP\_05* | 4 | 4 | 6.2% | 910 | 101997 | 7.2 | staphylococcal nuclease domain containing 1 [Homo sapiens] |

| Filename XCorr DeltCN Conf% ObsM+H+ CalcM+H+ SpR ZScore Ion% # Sequence  | | | | | | | | | | | | |
| --- | --- | --- | --- | --- | --- | --- | --- | --- | --- | --- | --- | --- |
| \* | IPAstrin\_STLCD\_tube2\_032114\_02.06511.06511.2 | 3.103 | 0.2691 | 99.9% | 1400.5521 | 1400.7197 | 2 | 5.996 | 62.5% | 1 | K.VMQVLNADAIVVK.L | 2 |
| \* | IPAstrin\_STLCD\_032114\_01.13048.13048.2 | 3.2857 | 0.3459 | 100.0% | 2306.372 | 2307.5623 | 5 | 6.285 | 35.0% | 1 | R.NLPGLVQEGEPFSEEATLFTK.E | 2 |
| \* | IPAstrin\_STLCD\_032114\_01.09849.09849.2 | 2.1848 | 0.21 | 95.6% | 1398.2722 | 1397.576 | 11 | 5.628 | 50.0% | 1 | K.IHVFYIDYGNR.E | 2 |
| \* | IPAstrin\_STLCD\_tube2\_032114\_01.04112.04112.2 | 2.4723 | 0.3374 | 99.9% | 1246.6322 | 1246.2322 | 1 | 5.441 | 75.0% | 1 | R.ADDADEFGYSR.- | 2 |

---

|  |  |  |  |  |  |  |  |  |
| --- | --- | --- | --- | --- | --- | --- | --- | --- |
| U | *gi|23308577|ref|NP\_00* | 2 | 2 | 6.2% | 533 | 56651 | 6.7 | phosphoglycerate dehydrogenase [Homo sapiens] |

| Filename XCorr DeltCN Conf% ObsM+H+ CalcM+H+ SpR ZScore Ion% # Sequence  | | | | | | | | | | | | |
| --- | --- | --- | --- | --- | --- | --- | --- | --- | --- | --- | --- | --- |
| \* | IPAstrin\_STLCD\_tube2\_032114\_01.07434.07434.2 | 2.8414 | 0.2347 | 99.8% | 1100.1522 | 1100.2603 | 116 | 5.382 | 60.0% | 1 | R.GGIVDEGALLR.A | 2 |
| \* | IPAstrin\_STLCD\_tube2\_032114\_01.15006.15006.2 | 3.5858 | 0.4329 | 100.0% | 2272.5723 | 2273.668 | 1 | 7.816 | 38.1% | 1 | R.TQTSDPAMLPTMIGLLAEAGVR.L | 2 |

---

|  |  |  |  |  |  |  |  |  |
| --- | --- | --- | --- | --- | --- | --- | --- | --- |
| U | *gi|167466173|ref|NP\_0* | 3 | 9 | 6.1% | 641 | 70052 | 5.6 | heat shock 70kDa protein 1B [Homo sapiens] |
| U | *gi|194248072|ref|NP\_0* | 3 | 9 | 6.1% | 641 | 70052 | 5.6 | heat shock 70kDa protein 1A [Homo sapiens] |

| Filename XCorr DeltCN Conf% ObsM+H+ CalcM+H+ SpR ZScore Ion% # Sequence  | | | | | | | | | | | | |
| --- | --- | --- | --- | --- | --- | --- | --- | --- | --- | --- | --- | --- |
|  | IPAstrin\_STLCD\_tube2\_032114\_01.06587.06587.2 | 3.1471 | 0.4339 | 100.0% | 1489.0521 | 1488.5939 | 1 | 8.802 | 70.8% | 7 | R.TTPSYVAFTDTER.L | 222 |
|  | IPAstrin\_STLCD\_tube2\_032114\_01.09418.09418.2 | 4.6093 | 0.4887 | 100.0% | 1688.3322 | 1688.9213 | 1 | 8.916 | 73.3% | 1 | R.IINEPTAAAIAYGLDR.T | 2 |
|  | IPAstrin\_STLCD\_tube2\_032114\_01.06569.06569.2 | 2.6615 | 0.3813 | 100.0% | 1262.0721 | 1262.4508 | 1 | 7.167 | 72.2% | 1 | R.LVNHFVEEFK.R | 2 |

Similarities:
gi|5729877|ref|NP\_006(1:2)  
gi|13676857|ref|NP\_06(1:2)  

---

|  |  |  |  |  |  |  |  |  |
| --- | --- | --- | --- | --- | --- | --- | --- | --- |
| U | *gi|118498359|ref|NP\_0* | 2 | 2 | 5.9% | 490 | 54973 | 10.1 | ribosomal L1 domain containing 1 [Homo sapiens] |

| Filename XCorr DeltCN Conf% ObsM+H+ CalcM+H+ SpR ZScore Ion% # Sequence  | | | | | | | | | | | | |
| --- | --- | --- | --- | --- | --- | --- | --- | --- | --- | --- | --- | --- |
| \* | IPAstrin\_STLCD\_tube2\_032114\_01.07092.07092.2 | 2.3612 | 0.1841 | 97.9% | 1022.9122 | 1025.2827 | 33 | 4.67 | 68.8% | 1 | R.RLLPSLIGR.H | 2 |
| \* | IPAstrin\_STLCD\_032114\_01.15169.15169.3 | 4.1407 | 0.3279 | 100.0% | 2202.3542 | 2202.62 | 3 | 5.68 | 31.6% | 1 | R.IGHVGMQIEHIIENIVAVTK.G | 3 |

---

|  |  |  |  |  |  |  |  |  |
| --- | --- | --- | --- | --- | --- | --- | --- | --- |
| U | *gi|27436951|ref|NP\_11* | 2 | 3 | 5.8% | 600 | 67689 | 5.3 | lamin B2 [Homo sapiens] |

| Filename XCorr DeltCN Conf% ObsM+H+ CalcM+H+ SpR ZScore Ion% # Sequence  | | | | | | | | | | | | |
| --- | --- | --- | --- | --- | --- | --- | --- | --- | --- | --- | --- | --- |
| \* | IPAstrin\_STLCD\_tube2\_032114\_01.10776.10776.2 | 3.4597 | 0.118 | 98.7% | 2369.7122 | 2370.452 | 4 | 4.044 | 40.0% | 1 | K.LSS\*DQNDKAASAAREELKEAR.M | 2 |
| \* | IPAstrin\_STLCD\_032114\_01.07052.07052.2 | 2.6777 | 0.2855 | 99.7% | 1505.5521 | 1504.6984 | 1 | 5.119 | 57.7% | 2 | R.TVLVNADGEEVAMR.T | 2 |

---

|  |  |  |  |  |  |  |  |  |
| --- | --- | --- | --- | --- | --- | --- | --- | --- |
| U | *gi|148613856|ref|NP\_0* | 4 | 11 | 5.6% | 731 | 80458 | 8.4 | DEAD box polypeptide 17 isoform 3 [Homo sapiens] |
| U | *gi|38201710|ref|NP\_00* | 4 | 11 | 5.6% | 729 | 80273 | 8.3 | DEAD box polypeptide 17 isoform 1 [Homo sapiens] |

| Filename XCorr DeltCN Conf% ObsM+H+ CalcM+H+ SpR ZScore Ion% # Sequence  | | | | | | | | | | | | |
| --- | --- | --- | --- | --- | --- | --- | --- | --- | --- | --- | --- | --- |
|  | IPAstrin\_STLCD\_tube2\_032114\_01.07804.07804.2 | 4.4927 | 0.4494 | 100.0% | 1691.9722 | 1692.8229 | 1 | 7.845 | 60.7% | 2 | R.ELAQQVQQVADDYGK.C | 2 |
|  | IPAstrin\_STLCD\_032114\_01.11119.11119.2 | 3.5919 | 0.4336 | 100.0% | 1337.1721 | 1337.5946 | 1 | 7.79 | 85.0% | 4 | R.MLDMGFEPQIR.K | 222 |
|  | IPAstrin\_STLCD\_032114\_01.07366.07366.2 | 2.9898 | 0.256 | 99.8% | 1500.1122 | 1499.7507 | 1 | 5.871 | 60.7% | 1 | R.SGKAPILIATDVASR.G | 2 |
|  | IPAstrin\_STLCD\_tube2\_032114\_01.07053.07053.2 | 3.6861 | 0.2947 | 100.0% | 1228.0922 | 1227.4465 | 2 | 6.977 | 81.8% | 4 | K.APILIATDVASR.G | 22 |

Similarities:
gi|87196351|ref|NP\_00(1:3)  
gi|4758138|ref|NP\_004(2:2)  

---

|  |  |  |  |  |  |  |  |  |
| --- | --- | --- | --- | --- | --- | --- | --- | --- |
| U | *gi|4504505|ref|NP\_000* | 2 | 2 | 5.6% | 736 | 79686 | 8.8 | hydroxysteroid (17-beta) dehydrogenase 4 [Homo sapiens] |

| Filename XCorr DeltCN Conf% ObsM+H+ CalcM+H+ SpR ZScore Ion% # Sequence  | | | | | | | | | | | | |
| --- | --- | --- | --- | --- | --- | --- | --- | --- | --- | --- | --- | --- |
| \* | IPAstrin\_STLCD\_032114\_02.05481.05481.2 | 2.6331 | 0.2797 | 99.6% | 1169.5922 | 1170.3976 | 1 | 6.217 | 66.7% | 1 | R.VVLVTGAGAGLGR.A | 2 |
| \* | IPAstrin\_STLCD\_tube2\_032114\_01.17727.17727.3 | 3.7991 | 0.4312 | 100.0% | 3046.2244 | 3046.5483 | 5 | 6.408 | 23.1% | 1 | K.LPPFSYAYTELEAIMYALGVGASIKDPK.D | 3 |

---

|  |  |  |  |  |  |  |  |  |
| --- | --- | --- | --- | --- | --- | --- | --- | --- |
| U | *gi|16753203|ref|NP\_03* | 2 | 2 | 5.3% | 589 | 62519 | 5.1 | ubiquilin 1 isoform 1 [Homo sapiens] |
| U | *gi|16753205|ref|NP\_44* | 2 | 2 | 5.5% | 561 | 59220 | 5.1 | ubiquilin 1 isoform 2 [Homo sapiens] |

| Filename XCorr DeltCN Conf% ObsM+H+ CalcM+H+ SpR ZScore Ion% # Sequence  | | | | | | | | | | | | |
| --- | --- | --- | --- | --- | --- | --- | --- | --- | --- | --- | --- | --- |
|  | IPAstrin\_STLCD\_032114\_01.11146.11146.2 | 3.2987 | 0.3781 | 100.0% | 1812.4122 | 1813.1865 | 1 | 6.043 | 57.1% | 1 | R.QLIMANPQMQQLIQR.N | 2 |
|  | IPAstrin\_STLCD\_tube2\_032114\_01.11147.11147.2 | 2.7202 | 0.3334 | 99.9% | 1910.4922 | 1911.1858 | 51 | 4.711 | 40.0% | 1 | R.FQQQLEQLSAMGFLNR.E | 2 |

---

|  |  |  |  |  |  |  |  |  |
| --- | --- | --- | --- | --- | --- | --- | --- | --- |
| U | *gi|4503481|ref|NP\_001* | 2 | 4 | 5.3% | 437 | 50119 | 6.7 | eukaryotic translation elongation factor 1 gamma [Homo sapiens] |

| Filename XCorr DeltCN Conf% ObsM+H+ CalcM+H+ SpR ZScore Ion% # Sequence  | | | | | | | | | | | | |
| --- | --- | --- | --- | --- | --- | --- | --- | --- | --- | --- | --- | --- |
| \* | IPAstrin\_STLCD\_032114\_02.05102.05102.2 | 3.7781 | 0.4487 | 100.0% | 1348.2922 | 1348.5448 | 1 | 8.793 | 66.7% | 2 | K.ALIAAQYSGAQVR.V | 2 |
| \* | IPAstrin\_STLCD\_tube2\_032114\_01.07577.07577.2 | 2.6715 | 0.375 | 100.0% | 1242.3522 | 1242.4172 | 1 | 7.042 | 72.2% | 2 | K.STFVLDEFKR.K | 2 |

---

|  |  |  |  |  |  |  |  |  |
| --- | --- | --- | --- | --- | --- | --- | --- | --- |
| U | *gi|221316723|ref|NP\_0* | 3 | 5 | 5.2% | 1025 | 115704 | 8.3 | N-acetyltransferase 10 isoform a [Homo sapiens] |
| U | *gi|221316741|ref|NP\_0* | 3 | 5 | 5.6% | 953 | 107271 | 7.0 | N-acetyltransferase 10 isoform b [Homo sapiens] |

| Filename XCorr DeltCN Conf% ObsM+H+ CalcM+H+ SpR ZScore Ion% # Sequence  | | | | | | | | | | | | |
| --- | --- | --- | --- | --- | --- | --- | --- | --- | --- | --- | --- | --- |
|  | IPAstrin\_STLCD\_tube2\_032114\_01.05265.05265.2 | 3.1321 | 0.3757 | 100.0% | 1412.2922 | 1412.5858 | 1 | 7.955 | 72.7% | 2 | R.TLHEVSLQESIR.Y | 2 |
|  | IPAstrin\_STLCD\_032114\_01.11366.11366.2 | 3.3997 | 0.3714 | 100.0% | 1455.0922 | 1454.6659 | 1 | 7.448 | 66.7% | 2 | R.LDYLGVSYGLTPR.L | 2 |
|  | IPAstrin\_STLCD\_032114\_01.19035.19035.3 | 4.7051 | 0.462 | 100.0% | 2967.9243 | 2969.4973 | 1 | 7.496 | 34.3% | 1 | R.IYFLNQLGDLALSAAQSALLLGIGLQHK.S | 3 |

---

|  |  |  |  |  |  |  |  |  |
| --- | --- | --- | --- | --- | --- | --- | --- | --- |
| U | *gi|20127499|ref|NP\_00* | 2 | 7 | 5.2% | 344 | 39587 | 11.4 | arginine/serine-rich splicing factor 6 [Homo sapiens] |

| Filename XCorr DeltCN Conf% ObsM+H+ CalcM+H+ SpR ZScore Ion% # Sequence  | | | | | | | | | | | | |
| --- | --- | --- | --- | --- | --- | --- | --- | --- | --- | --- | --- | --- |
|  | IPAstrin\_STLCD\_032114\_01.06096.06096.2 | 2.7483 | 0.2231 | 99.8% | 1031.1721 | 1031.1973 | 4 | 5.219 | 75.0% | 5 | R.LIVENLSSR.C | 2 |
| \* | IPAstrin\_STLCD\_032114\_01.06938.06938.2 | 2.4044 | 0.3068 | 99.9% | 1066.2722 | 1065.171 | 1 | 5.889 | 81.2% | 2 | R.TNEGVIEFR.S | 2 |

---

|  |  |  |  |  |  |  |  |  |
| --- | --- | --- | --- | --- | --- | --- | --- | --- |
| U | *gi|21264343|ref|NP\_00* | 2 | 3 | 4.8% | 915 | 102642 | 5.5 | scaffold attachment factor B [Homo sapiens] |

| Filename XCorr DeltCN Conf% ObsM+H+ CalcM+H+ SpR ZScore Ion% # Sequence  | | | | | | | | | | | | |
| --- | --- | --- | --- | --- | --- | --- | --- | --- | --- | --- | --- | --- |
|  | IPAstrin\_STLCD\_032114\_02.06519.06519.3 | 4.1253 | 0.3991 | 100.0% | 3431.0942 | 3430.5305 | 1 | 6.515 | 25.0% | 1 | K.LAEEEDLFDSAHPEEGDLDLASESTAHAQSSK.A | 3 |
| \* | IPAstrin\_STLCD\_032114\_01.10605.10605.2 | 2.7992 | 0.4321 | 100.0% | 1354.6921 | 1355.4929 | 1 | 6.711 | 77.3% | 2 | R.NFWVSGLSSTTR.A | 2 |

---

|  |  |  |  |  |  |  |  |  |
| --- | --- | --- | --- | --- | --- | --- | --- | --- |
| U | *Reverse\_gi|21536306|r* | 2 | 2 | 4.8% | 668 | 75325 | 9.3 | sciellin isoform a [Homo sapiens] |
| U | *Reverse\_gi|21536308|r* | 2 | 2 | 4.7% | 688 | 77551 | 9.3 | sciellin isoform b [Homo sapiens] |

| Filename XCorr DeltCN Conf% ObsM+H+ CalcM+H+ SpR ZScore Ion% # Sequence  | | | | | | | | | | | | |
| --- | --- | --- | --- | --- | --- | --- | --- | --- | --- | --- | --- | --- |
|  | IPAstrin\_STLCD\_tube2\_032114\_01.10125.10125.3 | 3.0987 | 0.2316 | 95.3% | 2367.1743 | 2367.5698 | 140 | 4.083 | 30.3% | 1 | K.IFNNLDKSGQNSRK@VEPT#VK.I | 3 |
|  | IPAstrin\_STLCD\_032114\_01.11682.11682.2 | 2.335 | 0.1735 | 95.1% | 1444.1522 | 1444.707 | 62 | 4.382 | 50.0% | 1 | K.TVELSRFMSMSR.N | 2 |

---

|  |  |  |  |  |  |  |  |  |
| --- | --- | --- | --- | --- | --- | --- | --- | --- |
| U | *Reverse\_gi|45446749|r* | 2 | 2 | 4.7% | 1032 | 117378 | 5.9 | kinesin family member 5A [Homo sapiens] |

| Filename XCorr DeltCN Conf% ObsM+H+ CalcM+H+ SpR ZScore Ion% # Sequence  | | | | | | | | | | | | |
| --- | --- | --- | --- | --- | --- | --- | --- | --- | --- | --- | --- | --- |
| \* | IPAstrin\_STLCD\_tube2\_032114\_01.12048.12048.3 | 3.692 | 0.2019 | 95.4% | 3385.4043 | 3387.8247 | 280 | 4.178 | 19.6% | 1 | K.K@WQEATLELNVSATNKITKARQGFMLT#SK.T | 3 |
| \* | IPAstrin\_STLCD\_032114\_01.18584.18584.2 | 2.5436 | 0.1968 | 96.8% | 2211.652 | 2210.2322 | 1 | 4.428 | 39.5% | 1 | K.GS\*STQGYAFIT#GNYGALVDK.V | 2 |

---

|  |  |  |  |  |  |  |  |  |
| --- | --- | --- | --- | --- | --- | --- | --- | --- |
| U | *gi|38016914|ref|NP\_05* | 2 | 3 | 4.5% | 626 | 72201 | 7.1 | SAM domain- and HD domain-containing protein 1 [Homo sapiens] |

| Filename XCorr DeltCN Conf% ObsM+H+ CalcM+H+ SpR ZScore Ion% # Sequence  | | | | | | | | | | | | |
| --- | --- | --- | --- | --- | --- | --- | --- | --- | --- | --- | --- | --- |
| \* | IPAstrin\_STLCD\_032114\_01.15885.15885.2 | 3.7058 | 0.4598 | 100.0% | 1651.4521 | 1651.9586 | 1 | 7.932 | 57.1% | 2 | K.VGNIIDTMITDAFLK.A | 2 |
| \* | IPAstrin\_STLCD\_tube2\_032114\_01.07887.07887.2 | 3.1076 | 0.2829 | 99.9% | 1458.4722 | 1458.6233 | 1 | 5.041 | 62.5% | 1 | R.ISTAIDDMEAYTK.L | 2 |

---

|  |  |  |  |  |  |  |  |  |
| --- | --- | --- | --- | --- | --- | --- | --- | --- |
| U | *gi|4507877|ref|NP\_003* | 4 | 4 | 4.3% | 1066 | 116722 | 6.1 | vinculin isoform VCL [Homo sapiens] |
| U | *gi|7669550|ref|NP\_054* | 4 | 4 | 4.1% | 1134 | 123799 | 5.7 | vinculin isoform meta-VCL [Homo sapiens] |

| Filename XCorr DeltCN Conf% ObsM+H+ CalcM+H+ SpR ZScore Ion% # Sequence  | | | | | | | | | | | | |
| --- | --- | --- | --- | --- | --- | --- | --- | --- | --- | --- | --- | --- |
|  | IPAstrin\_STLCD\_tube2\_032114\_01.06191.06191.2 | 2.279 | 0.3045 | 99.4% | 1236.3522 | 1236.5305 | 151 | 5.378 | 55.0% | 1 | R.VMLVNSMNTVK.E | 2 |
|  | IPAstrin\_STLCD\_tube2\_032114\_01.07482.07482.2 | 2.5456 | 0.2238 | 99.3% | 1176.5322 | 1176.3734 | 3 | 5.699 | 77.8% | 1 | K.MSAEINEIIR.V | 2 |
|  | IPAstrin\_STLCD\_tube2\_032114\_01.07762.07762.2 | 2.8404 | 0.1135 | 95.7% | 1479.8522 | 1478.7217 | 35 | 4.113 | 54.2% | 1 | K.MLGQMTDQVADLR.A | 2 |
|  | IPAstrin\_STLCD\_032114\_01.08313.08313.2 | 2.5334 | 0.4114 | 100.0% | 1292.3922 | 1293.4749 | 2 | 6.259 | 63.6% | 1 | K.MTGLVDEAIDTK.S | 2 |

---

|  |  |  |  |  |  |  |  |  |
| --- | --- | --- | --- | --- | --- | --- | --- | --- |
| U | *gi|46367787|ref|NP\_00* | 2 | 3 | 4.2% | 636 | 70671 | 9.5 | poly(A) binding protein, cytoplasmic 1 [Homo sapiens] |

| Filename XCorr DeltCN Conf% ObsM+H+ CalcM+H+ SpR ZScore Ion% # Sequence  | | | | | | | | | | | | |
| --- | --- | --- | --- | --- | --- | --- | --- | --- | --- | --- | --- | --- |
|  | IPAstrin\_STLCD\_tube2\_032114\_01.09359.09359.2 | 2.3148 | 0.1937 | 96.3% | 1267.0122 | 1267.4828 | 1 | 5.19 | 70.0% | 1 | R.ALDTMNFDVIK.G | 2 |
|  | IPAstrin\_STLCD\_tube2\_032114\_01.05619.05619.3 | 3.2269 | 0.3587 | 99.7% | 1694.0044 | 1694.9285 | 1 | 6.078 | 48.3% | 2 | R.SKVDEAVAVLQAHQAK.E | 3 |

---

|  |  |  |  |  |  |  |  |  |
| --- | --- | --- | --- | --- | --- | --- | --- | --- |
| U | *gi|27477136|ref|NP\_06* | 2 | 2 | 4.1% | 902 | 101431 | 8.4 | zinc finger antiviral protein isoform 1 [Homo sapiens] |
| U | *gi|27477138|ref|NP\_07* | 2 | 2 | 5.3% | 699 | 77903 | 8.4 | zinc finger antiviral protein isoform 2 [Homo sapiens] |

| Filename XCorr DeltCN Conf% ObsM+H+ CalcM+H+ SpR ZScore Ion% # Sequence  | | | | | | | | | | | | |
| --- | --- | --- | --- | --- | --- | --- | --- | --- | --- | --- | --- | --- |
|  | IPAstrin\_STLCD\_tube2\_032114\_02.05884.05884.2 | 2.3148 | 0.3282 | 99.4% | 1447.8722 | 1449.647 | 1 | 5.626 | 57.7% | 1 | R.FVVLETGGEAGITR.S | 2 |
|  | IPAstrin\_STLCD\_tube2\_032114\_01.12981.12981.2 | 2.65 | 0.2087 | 98.1% | 2784.2922 | 2785.9714 | 7 | 4.449 | 31.8% | 1 | R.EHGLNPDVVQNIQDICNS\*KHMQK.N | 2 |

---

|  |  |  |  |  |  |  |  |  |
| --- | --- | --- | --- | --- | --- | --- | --- | --- |
| U | *gi|17402900|ref|NP\_00* | 2 | 5 | 4.0% | 644 | 67560 | 7.6 | far upstream element-binding protein [Homo sapiens] |

| Filename XCorr DeltCN Conf% ObsM+H+ CalcM+H+ SpR ZScore Ion% # Sequence  | | | | | | | | | | | | |
| --- | --- | --- | --- | --- | --- | --- | --- | --- | --- | --- | --- | --- |
| \* | IPAstrin\_STLCD\_tube2\_032114\_01.05704.05704.2 | 2.3141 | 0.25 | 98.1% | 1354.1522 | 1353.5181 | 26 | 4.435 | 54.2% | 2 | K.IQIAPDSGGLPER.S | 2 |
| \* | IPAstrin\_STLCD\_032114\_01.09234.09234.2 | 3.387 | 0.4372 | 100.0% | 1337.4122 | 1337.5187 | 1 | 7.677 | 75.0% | 3 | R.IGGNEGIDVPIPR.F | 2 |

---

|  |  |  |  |  |  |  |  |  |
| --- | --- | --- | --- | --- | --- | --- | --- | --- |
| U | *gi|155030232|ref|NP\_0* | 3 | 3 | 3.6% | 1130 | 119700 | 4.3 | proline, glutamic acid and leucine rich protein 1 [Homo sapiens] |

| Filename XCorr DeltCN Conf% ObsM+H+ CalcM+H+ SpR ZScore Ion% # Sequence  | | | | | | | | | | | | |
| --- | --- | --- | --- | --- | --- | --- | --- | --- | --- | --- | --- | --- |
| \* | IPAstrin\_STLCD\_tube2\_032114\_01.03450.03450.2 | 2.6508 | 0.3552 | 99.9% | 1303.1921 | 1303.4636 | 1 | 5.762 | 62.5% | 1 | R.TGSAVAPVHPPNR.S | 2 |
| \* | IPAstrin\_STLCD\_tube2\_032114\_01.08518.08518.2 | 3.2916 | 0.4926 | 100.0% | 1275.6921 | 1275.4906 | 1 | 7.567 | 66.7% | 1 | R.LPSLGAGFSQGLK.H | 2 |
| \* | IPAstrin\_STLCD\_tube2\_032114\_01.05661.05661.3 | 3.2138 | 0.2732 | 98.8% | 1622.8143 | 1621.895 | 6 | 5.197 | 37.5% | 1 | K.LKLDVGEAMAPPSHR.K | 3 |

---

|  |  |  |  |  |  |  |  |  |
| --- | --- | --- | --- | --- | --- | --- | --- | --- |
| U | *gi|41872631|ref|NP\_00* | 6 | 7 | 3.5% | 2511 | 273424 | 6.4 | fatty acid synthase [Homo sapiens] |

| Filename XCorr DeltCN Conf% ObsM+H+ CalcM+H+ SpR ZScore Ion% # Sequence  | | | | | | | | | | | | |
| --- | --- | --- | --- | --- | --- | --- | --- | --- | --- | --- | --- | --- |
| \* | IPAstrin\_STLCD\_tube2\_032114\_01.15923.15923.3 | 4.6454 | 0.4093 | 100.0% | 3164.4543 | 3165.415 | 1 | 6.904 | 28.8% | 1 | K.LPESENLQEFWDNLIGGVDMVTDDDRR.W | 3 |
| \* | IPAstrin\_STLCD\_tube2\_032114\_01.04782.04782.2 | 2.7044 | 0.3857 | 100.0% | 1298.5521 | 1299.4264 | 2 | 5.84 | 68.2% | 1 | K.VGDPQELNGITR.A | 2 |
| \* | IPAstrin\_STLCD\_032114\_01.08106.08106.2 | 2.3778 | 0.3016 | 99.6% | 1263.6122 | 1264.5106 | 16 | 5.338 | 65.0% | 2 | R.LQVVDQPLPVR.G | 2 |
| \* | IPAstrin\_STLCD\_tube2\_032114\_02.06509.06509.2 | 2.3255 | 0.2159 | 97.2% | 1407.3121 | 1407.6709 | 3 | 4.785 | 59.1% | 1 | K.VLQGDLVMNVYR.D | 2 |
| \* | IPAstrin\_STLCD\_tube2\_032114\_01.07677.07677.2 | 3.7187 | 0.398 | 100.0% | 1470.1721 | 1470.5815 | 1 | 6.443 | 70.8% | 1 | R.FPQLDSTSFANSR.D | 2 |
| \* | IPAstrin\_STLCD\_032114\_01.10252.10252.2 | 2.48 | 0.2124 | 97.9% | 1427.7922 | 1427.702 | 407 | 4.829 | 41.7% | 1 | R.SLLVNPEGPTLMR.L | 2 |

---

|  |  |  |  |  |  |  |  |  |
| --- | --- | --- | --- | --- | --- | --- | --- | --- |
| U | *gi|4507555|ref|NP\_003* | 2 | 2 | 3.5% | 694 | 75492 | 7.7 | thymopoietin isoform alpha [Homo sapiens] |

| Filename XCorr DeltCN Conf% ObsM+H+ CalcM+H+ SpR ZScore Ion% # Sequence  | | | | | | | | | | | | |
| --- | --- | --- | --- | --- | --- | --- | --- | --- | --- | --- | --- | --- |
|  | IPAstrin\_STLCD\_032114\_01.06283.06283.2 | 2.5531 | 0.3251 | 99.9% | 1331.8722 | 1331.5143 | 1 | 4.88 | 62.5% | 1 | K.YGVNPGPIVGTTR.K | 2 |
| \* | IPAstrin\_STLCD\_032114\_01.07430.07430.2 | 2.4907 | 0.1617 | 96.4% | 1432.0721 | 1431.5449 | 8 | 4.432 | 70.0% | 1 | K.VIEEEWQQVDR.Q | 2 |

---

|  |  |  |  |  |  |  |  |  |
| --- | --- | --- | --- | --- | --- | --- | --- | --- |
| U | *gi|154355000|ref|NP\_0* | 2 | 6 | 3.5% | 711 | 73115 | 7.3 | KH-type splicing regulatory protein (FUSE binding protein 2) [Homo sapiens] |

| Filename XCorr DeltCN Conf% ObsM+H+ CalcM+H+ SpR ZScore Ion% # Sequence  | | | | | | | | | | | | |
| --- | --- | --- | --- | --- | --- | --- | --- | --- | --- | --- | --- | --- |
| \* | IPAstrin\_STLCD\_032114\_01.07978.07978.2 | 2.415 | 0.3156 | 99.8% | 1079.3522 | 1080.2725 | 56 | 5.563 | 65.0% | 3 | R.IGGGIDVPVPR.H | 2 |
| \* | IPAstrin\_STLCD\_tube2\_032114\_02.05393.05393.2 | 4.2147 | 0.3364 | 100.0% | 1534.1921 | 1534.7123 | 1 | 7.409 | 61.5% | 3 | K.AINQQTGAFVEISR.Q | 2 |

---

|  |  |  |  |  |  |  |  |  |
| --- | --- | --- | --- | --- | --- | --- | --- | --- |
| U | *gi|193083178|ref|NP\_0* | 2 | 2 | 3.4% | 524 | 60146 | 6.7 | cytochrome P450 family 4 subfamily F polypeptide 11 [Homo sapiens] |
| U | *gi|193083180|ref|NP\_0* | 2 | 2 | 3.4% | 524 | 60146 | 6.7 | cytochrome P450 family 4 subfamily F polypeptide 11 [Homo sapiens] |

| Filename XCorr DeltCN Conf% ObsM+H+ CalcM+H+ SpR ZScore Ion% # Sequence  | | | | | | | | | | | | |
| --- | --- | --- | --- | --- | --- | --- | --- | --- | --- | --- | --- | --- |
|  | IPAstrin\_STLCD\_tube2\_032114\_01.08507.08507.2 | 3.9454 | 0.0222 | 97.5% | 2229.3523 | 2230.4473 | 22 | 2.668 | 44.1% | 1 | K.NK@AKS\*K@TLDFIDVLLLS\*K@.D | 2 |
|  | IPAstrin\_STLCD\_tube2\_032114\_01.08438.08438.2 | 4.0517 | 0.0556 | 99.0% | 2229.4922 | 2230.4473 | 3 | 3.03 | 50.0% | 1 | K.NK@AK@SKT#LDFIDVLLLS\*K@.D | 2 |

---

|  |  |  |  |  |  |  |  |  |
| --- | --- | --- | --- | --- | --- | --- | --- | --- |
| U | *gi|100913206|ref|NP\_0* | 3 | 4 | 3.1% | 1270 | 140958 | 6.8 | DEAH (Asp-Glu-Ala-His) box polypeptide 9 [Homo sapiens] |

| Filename XCorr DeltCN Conf% ObsM+H+ CalcM+H+ SpR ZScore Ion% # Sequence  | | | | | | | | | | | | |
| --- | --- | --- | --- | --- | --- | --- | --- | --- | --- | --- | --- | --- |
| \* | IPAstrin\_STLCD\_032114\_01.06487.06487.3 | 3.1497 | 0.381 | 100.0% | 1504.4944 | 1504.686 | 1 | 5.811 | 43.8% | 2 | R.GISHVIVDEIHER.D | 3 |
| \* | IPAstrin\_STLCD\_tube2\_032114\_01.08634.08634.2 | 2.7044 | 0.2966 | 99.9% | 1004.15216 | 1004.21747 | 1 | 6.198 | 88.9% | 1 | R.LGGIGQFLAK.A | 2 |
| \* | IPAstrin\_STLCD\_tube2\_032114\_01.16415.16415.2 | 2.7159 | 0.2699 | 99.5% | 1733.9922 | 1733.1627 | 1 | 5.189 | 46.7% | 1 | K.GMTLVTPLQLLLFASK.K | 2 |

---

|  |  |  |  |  |  |  |  |  |
| --- | --- | --- | --- | --- | --- | --- | --- | --- |
| U | *gi|21361368|ref|NP\_00* | 2 | 2 | 3.0% | 795 | 87302 | 7.1 | pyrroline-5-carboxylate synthetase isoform 1 [Homo sapiens] |
| U | *gi|62912457|ref|NP\_00* | 2 | 2 | 3.0% | 793 | 87089 | 7.1 | pyrroline-5-carboxylate synthetase isoform 2 [Homo sapiens] |

| Filename XCorr DeltCN Conf% ObsM+H+ CalcM+H+ SpR ZScore Ion% # Sequence  | | | | | | | | | | | | |
| --- | --- | --- | --- | --- | --- | --- | --- | --- | --- | --- | --- | --- |
|  | IPAstrin\_STLCD\_tube2\_032114\_01.08590.08590.2 | 2.9994 | 0.2103 | 99.8% | 1280.6322 | 1280.4697 | 1 | 6.15 | 65.0% | 1 | R.NLNGTLHELLR.M | 2 |
|  | IPAstrin\_STLCD\_032114\_02.06386.06386.2 | 2.8249 | 0.3542 | 100.0% | 1293.3722 | 1294.4502 | 3 | 6.837 | 58.3% | 1 | R.FGLGAEVGISTSR.I | 2 |

---

|  |  |  |  |  |  |  |  |  |
| --- | --- | --- | --- | --- | --- | --- | --- | --- |
| U | *gi|41322908|ref|NP\_95* | 9 | 15 | 2.9% | 4525 | 513712 | 5.8 | plectin 1 isoform 3 [Homo sapiens] |
| U | *gi|47607492|ref|NP\_00* | 9 | 15 | 2.9% | 4574 | 518478 | 5.7 | plectin 1 isoform 1 [Homo sapiens] |
| U | *gi|41322923|ref|NP\_95* | 9 | 15 | 2.9% | 4547 | 516204 | 5.8 | plectin 1 isoform 11 [Homo sapiens] |
| U | *gi|41322916|ref|NP\_95* | 9 | 15 | 2.8% | 4684 | 531796 | 6.0 | plectin 1 isoform 6 [Homo sapiens] |
| U | *gi|41322914|ref|NP\_95* | 9 | 15 | 2.9% | 4551 | 516484 | 5.8 | plectin 1 isoform 10 [Homo sapiens] |
| U | *gi|41322912|ref|NP\_95* | 9 | 15 | 2.9% | 4533 | 514780 | 5.7 | plectin 1 isoform 2 [Homo sapiens] |
| U | *gi|41322910|ref|NP\_95* | 9 | 15 | 2.9% | 4515 | 512609 | 5.8 | plectin 1 isoform 7 [Homo sapiens] |

| Filename XCorr DeltCN Conf% ObsM+H+ CalcM+H+ SpR ZScore Ion% # Sequence  | | | | | | | | | | | | |
| --- | --- | --- | --- | --- | --- | --- | --- | --- | --- | --- | --- | --- |
|  | IPAstrin\_STLCD\_tube2\_032114\_01.07259.07259.2 | 2.5598 | 0.3192 | 99.8% | 1709.3722 | 1709.8474 | 4 | 5.973 | 46.4% | 1 | R.LLDPEDVDVPQPDEK.S | 2 |
|  | IPAstrin\_STLCD\_032114\_01.09930.09930.2 | 2.6443 | 0.2268 | 98.5% | 1532.1921 | 1532.8235 | 247 | 5.068 | 39.3% | 1 | K.VLALPEPSPAAPTLR.S | 2 |
|  | IPAstrin\_STLCD\_tube2\_032114\_01.07918.07918.2 | 3.2682 | 0.3625 | 100.0% | 1520.0122 | 1519.738 | 1 | 6.799 | 66.7% | 1 | K.AKLEQLFQDEVAK.A | 2 |
|  | IPAstrin\_STLCD\_tube2\_032114\_02.06053.06053.2 | 4.2414 | 0.2965 | 100.0% | 1558.2922 | 1557.744 | 1 | 6.766 | 61.5% | 2 | R.LQEAGILSAEELQR.L | 2 |
|  | IPAstrin\_STLCD\_tube2\_032114\_02.05619.05619.3 | 3.5822 | 0.3058 | 99.6% | 2013.2043 | 2014.292 | 3 | 5.644 | 36.1% | 4 | R.LLEAQIATGGVIDPVHSHR.V | 33 |
|  | IPAstrin\_STLCD\_032114\_01.07011.07011.2 | 2.0842 | 0.2891 | 99.1% | 1160.0122 | 1161.2311 | 29 | 4.989 | 68.8% | 2 | R.GYFDEEMNR.V | 22 |
|  | IPAstrin\_STLCD\_tube2\_032114\_02.06005.06005.2 | 4.6416 | 0.5223 | 100.0% | 1614.3121 | 1614.8363 | 1 | 9.058 | 76.7% | 2 | R.LLDAQLSTGGIVDPSK.S | 2 |
|  | IPAstrin\_STLCD\_tube2\_032114\_02.05998.05998.3 | 3.1588 | 0.349 | 99.6% | 2028.2344 | 2028.3188 | 50 | 6.004 | 29.2% | 1 | R.LLEAQIATGGIIDPVHSHR.V | 33 |
|  | IPAstrin\_STLCD\_032114\_01.09326.09326.2 | 2.8409 | 0.2574 | 99.8% | 1462.8322 | 1462.6611 | 1 | 5.032 | 62.5% | 1 | R.SQVMDEATALQLR.E | 2 |

Similarities:
gi|207452735|ref|NP\_1(3:6)  

---

|  |  |  |  |  |  |  |  |  |
| --- | --- | --- | --- | --- | --- | --- | --- | --- |
| U | *gi|222136639|ref|NP\_0* | 2 | 3 | 2.9% | 935 | 101531 | 7.3 | methylenetetrahydrofolate dehydrogenase 1 [Homo sapiens] |

| Filename XCorr DeltCN Conf% ObsM+H+ CalcM+H+ SpR ZScore Ion% # Sequence  | | | | | | | | | | | | |
| --- | --- | --- | --- | --- | --- | --- | --- | --- | --- | --- | --- | --- |
| \* | IPAstrin\_STLCD\_tube2\_032114\_01.09486.09486.2 | 3.2243 | 0.2553 | 99.9% | 1488.1322 | 1487.6525 | 4 | 5.88 | 63.6% | 2 | R.LDIDPETITWQR.V | 2 |
| \* | IPAstrin\_STLCD\_tube2\_032114\_01.11064.11064.2 | 2.6408 | 0.2199 | 98.3% | 1653.5122 | 1652.8877 | 37 | 5.076 | 42.9% | 1 | R.AAQAPSSFQLLYDLK.L | 2 |

---

|  |  |  |  |  |  |  |  |  |
| --- | --- | --- | --- | --- | --- | --- | --- | --- |
| U | *gi|26051235|ref|NP\_06* | 2 | 2 | 2.8% | 1156 | 128979 | 5.1 | nucleoporin 133kDa [Homo sapiens] |

| Filename XCorr DeltCN Conf% ObsM+H+ CalcM+H+ SpR ZScore Ion% # Sequence  | | | | | | | | | | | | |
| --- | --- | --- | --- | --- | --- | --- | --- | --- | --- | --- | --- | --- |
| \* | IPAstrin\_STLCD\_tube2\_032114\_01.06538.06538.3 | 3.6446 | 0.2875 | 99.4% | 1944.0844 | 1943.2773 | 3 | 5.777 | 35.3% | 1 | K.IHQHILPQGQGMLSGIGR.K | 3 |
| \* | IPAstrin\_STLCD\_032114\_01.09814.09814.2 | 2.1279 | 0.3372 | 99.3% | 1499.2722 | 1498.7405 | 2 | 5.722 | 53.8% | 1 | K.AHATLLGLANMETR.Y | 2 |

---

|  |  |  |  |  |  |  |  |  |
| --- | --- | --- | --- | --- | --- | --- | --- | --- |
| U | *gi|156523968|ref|NP\_0* | 2 | 4 | 2.8% | 1014 | 113084 | 8.9 | poly (ADP-ribose) polymerase family, member 1 [Homo sapiens] |

| Filename XCorr DeltCN Conf% ObsM+H+ CalcM+H+ SpR ZScore Ion% # Sequence  | | | | | | | | | | | | |
| --- | --- | --- | --- | --- | --- | --- | --- | --- | --- | --- | --- | --- |
| \* | IPAstrin\_STLCD\_tube2\_032114\_01.08950.08950.2 | 3.9834 | 0.4956 | 100.0% | 1625.4122 | 1625.7728 | 1 | 9.313 | 71.4% | 3 | R.VVSEDFLQDVSASTK.S | 2 |
| \* | IPAstrin\_STLCD\_tube2\_032114\_01.10388.10388.2 | 3.0048 | 0.4162 | 100.0% | 1378.1921 | 1378.5712 | 2 | 6.749 | 54.2% | 1 | R.TTNFAGILSQGLR.I | 2 |

---

|  |  |  |  |  |  |  |  |  |
| --- | --- | --- | --- | --- | --- | --- | --- | --- |
| U | *gi|24638454|ref|NP\_73* | 2 | 2 | 2.5% | 1042 | 114757 | 5.3 | ATPase, Ca++ transporting, slow twitch 2 isoform 1 [Homo sapiens] |
| U | *gi|4502285|ref|NP\_001* | 2 | 2 | 2.6% | 997 | 109691 | 5.4 | ATPase, Ca++ transporting, slow twitch 2 isoform 2 [Homo sapiens] |

| Filename XCorr DeltCN Conf% ObsM+H+ CalcM+H+ SpR ZScore Ion% # Sequence  | | | | | | | | | | | | |
| --- | --- | --- | --- | --- | --- | --- | --- | --- | --- | --- | --- | --- |
|  | IPAstrin\_STLCD\_tube2\_032114\_02.06155.06155.2 | 2.6195 | 0.2404 | 98.9% | 1574.5721 | 1575.7997 | 1 | 5.662 | 50.0% | 1 | R.VDQSILTGESVSVIK.H | 2 |
|  | IPAstrin\_STLCD\_tube2\_032114\_01.05268.05268.2 | 2.2781 | 0.237 | 98.1% | 1305.8922 | 1306.3745 | 13 | 3.777 | 55.0% | 1 | R.EFDELNPSAQR.D | 2 |

---

|  |  |  |  |  |  |  |  |  |
| --- | --- | --- | --- | --- | --- | --- | --- | --- |
| U | *gi|207452735|ref|NP\_1* | 6 | 10 | 2.2% | 5090 | 555629 | 5.6 | epiplakin 1 [Homo sapiens] |

| Filename XCorr DeltCN Conf% ObsM+H+ CalcM+H+ SpR ZScore Ion% # Sequence  | | | | | | | | | | | | |
| --- | --- | --- | --- | --- | --- | --- | --- | --- | --- | --- | --- | --- |
|  | IPAstrin\_STLCD\_tube2\_032114\_02.05619.05619.3 | 3.5822 | 0.3058 | 99.6% | 2013.2043 | 2014.292 | 3 | 5.644 | 36.1% | 4 | R.LLEAQIATGGVIDPVHSHR.V | 33 |
| \* | IPAstrin\_STLCD\_032114\_02.07046.07046.2 | 1.994 | 0.2982 | 96.5% | 2254.4321 | 2257.4111 | 23 | 4.415 | 32.4% | 1 | R.T#T#VPQLLASVQRWVQETK@.L | 2 |
|  | IPAstrin\_STLCD\_tube2\_032114\_02.05998.05998.3 | 3.1588 | 0.349 | 99.6% | 2028.2344 | 2028.3188 | 50 | 6.004 | 29.2% | 1 | R.LLEAQIATGGIIDPVHSHR.V | 33 |
|  | IPAstrin\_STLCD\_032114\_01.07011.07011.2 | 2.0842 | 0.2891 | 99.1% | 1160.0122 | 1161.2311 | 29 | 4.989 | 68.8% | 2 | C.GYFDEEMNR.I | 22 |
| \* | IPAstrin\_STLCD\_032114\_01.15123.15123.2 | 2.9644 | 0.3163 | 99.9% | 2394.8523 | 2395.7202 | 3 | 4.95 | 38.1% | 1 | R.VTPGSGALQGQSVSVWELLFYR.E | 2 |
| \* | IPAstrin\_STLCD\_032114\_01.17076.17076.3 | 4.4724 | 0.345 | 100.0% | 2515.2844 | 2514.8386 | 1 | 6.712 | 38.5% | 1 | R.AGTLTVEELGATLTSLLAQAQAQAR.A | 3 |

Similarities:
gi|41322908|ref|NP\_95(3:3)  

---

|  |  |  |  |  |  |  |  |  |
| --- | --- | --- | --- | --- | --- | --- | --- | --- |
| U | *gi|118572613|ref|NP\_0* | 4 | 7 | 2.1% | 2752 | 299616 | 12.1 | splicing coactivator subunit SRm300 [Homo sapiens] |

| Filename XCorr DeltCN Conf% ObsM+H+ CalcM+H+ SpR ZScore Ion% # Sequence  | | | | | | | | | | | | |
| --- | --- | --- | --- | --- | --- | --- | --- | --- | --- | --- | --- | --- |
| \* | IPAstrin\_STLCD\_tube2\_032114\_01.06784.06784.2 | 3.1807 | 0.3643 | 100.0% | 1720.0322 | 1720.92 | 2 | 5.723 | 46.9% | 2 | R.SSTGPEPPAPTPLLAER.H | 2 |
| \* | IPAstrin\_STLCD\_032114\_01.07360.07360.2 | 2.8291 | 0.3062 | 99.9% | 1231.8322 | 1231.4094 | 1 | 6.064 | 77.3% | 2 | R.SPGMLEPLGSSR.T | 2 |
| \* | IPAstrin\_STLCD\_032114\_01.08414.08414.2 | 3.8889 | 0.3281 | 100.0% | 1416.1522 | 1415.6506 | 1 | 7.471 | 67.9% | 2 | R.IPAASAAAMNLASAR.T | 2 |
| \* | IPAstrin\_STLCD\_tube2\_032114\_01.04414.04414.2 | 2.4484 | 0.3661 | 99.9% | 1379.3121 | 1379.559 | 1 | 6.582 | 57.7% | 1 | R.TPQAPASANLVGPR.S | 2 |

---

|  |  |  |  |  |  |  |  |  |
| --- | --- | --- | --- | --- | --- | --- | --- | --- |
| U | *gi|24430149|ref|NP\_70* | 2 | 2 | 2.1% | 1391 | 155199 | 6.2 | nucleoporin 155kDa isoform 1 [Homo sapiens] |
| U | *gi|4758844|ref|NP\_004* | 2 | 2 | 2.2% | 1332 | 149016 | 6.3 | nucleoporin 155kDa isoform 2 [Homo sapiens] |

| Filename XCorr DeltCN Conf% ObsM+H+ CalcM+H+ SpR ZScore Ion% # Sequence  | | | | | | | | | | | | |
| --- | --- | --- | --- | --- | --- | --- | --- | --- | --- | --- | --- | --- |
|  | IPAstrin\_STLCD\_tube2\_032114\_02.06125.06125.2 | 2.9202 | 0.2548 | 99.7% | 1729.6122 | 1728.9462 | 17 | 5.312 | 35.3% | 1 | R.VASVSQNAIVSAAGNIAR.T | 2 |
|  | IPAstrin\_STLCD\_tube2\_032114\_02.05908.05908.2 | 3.2507 | 0.0452 | 97.2% | 1370.1322 | 1370.5919 | 2 | 5.055 | 70.0% | 1 | R.IQLQIQETLQR.Q | 2 |

---

|  |  |  |  |  |  |  |  |  |
| --- | --- | --- | --- | --- | --- | --- | --- | --- |
| U | *gi|116063573|ref|NP\_0* | 3 | 4 | 1.8% | 2639 | 280016 | 6.0 | filamin A, alpha isoform 1 [Homo sapiens] |
| U | *gi|160420317|ref|NP\_0* | 3 | 4 | 1.8% | 2647 | 280737 | 6.1 | filamin A, alpha isoform 2 [Homo sapiens] |

| Filename XCorr DeltCN Conf% ObsM+H+ CalcM+H+ SpR ZScore Ion% # Sequence  | | | | | | | | | | | | |
| --- | --- | --- | --- | --- | --- | --- | --- | --- | --- | --- | --- | --- |
|  | IPAstrin\_STLCD\_tube2\_032114\_01.08062.08062.2 | 2.5653 | 0.4148 | 100.0% | 1416.0721 | 1416.5742 | 1 | 6.856 | 58.3% | 1 | R.IANLQTDLSDGLR.L | 2 |
|  | IPAstrin\_STLCD\_032114\_01.11131.11131.2 | 2.3845 | 0.2 | 95.5% | 1535.1921 | 1534.7496 | 1 | 4.746 | 50.0% | 1 | K.SPFSVAVSPSLDLSK.I | 2 |
|  | IPAstrin\_STLCD\_tube2\_032114\_02.05862.05862.3 | 3.7995 | 0.3273 | 99.8% | 2199.2944 | 2201.4412 | 1 | 6.354 | 32.9% | 2 | R.LVSNHSLHETSSVFVDSLTK.A | 3 |

---

|  |  |  |  |  |  |  |  |  |
| --- | --- | --- | --- | --- | --- | --- | --- | --- |
| U | *gi|58530840|ref|NP\_00* | 3 | 4 | 1.4% | 2871 | 331774 | 6.8 | desmoplakin isoform I [Homo sapiens] |

| Filename XCorr DeltCN Conf% ObsM+H+ CalcM+H+ SpR ZScore Ion% # Sequence  | | | | | | | | | | | | |
| --- | --- | --- | --- | --- | --- | --- | --- | --- | --- | --- | --- | --- |
|  | IPAstrin\_STLCD\_tube2\_032114\_01.06285.06285.2 | 1.9791 | 0.2209 | 95.2% | 1158.6721 | 1159.3892 | 32 | 4.422 | 68.8% | 1 | R.LLQLQEQMR.A | 2 |
| \* | IPAstrin\_STLCD\_tube2\_032114\_01.06498.06498.2 | 3.4353 | 0.4134 | 100.0% | 1388.4521 | 1388.5205 | 1 | 6.259 | 81.8% | 2 | R.LNDSILQATEQR.R | 2 |
|  | IPAstrin\_STLCD\_tube2\_032114\_01.12005.12005.3 | 3.2701 | 0.2408 | 97.4% | 2178.5344 | 2178.4521 | 54 | 5.253 | 30.6% | 1 | R.FLEFQYLTGGLVDPEVHGR.I | 3 |

---

|  |  |  |  |  |  |  |  |  |
| --- | --- | --- | --- | --- | --- | --- | --- | --- |
| U | *gi|21264365|ref|NP\_05* | 2 | 2 | 1.3% | 1800 | 195816 | 6.4 | nucleoporin 98kD isoform 1 [Homo sapiens] |

| Filename XCorr DeltCN Conf% ObsM+H+ CalcM+H+ SpR ZScore Ion% # Sequence  | | | | | | | | | | | | |
| --- | --- | --- | --- | --- | --- | --- | --- | --- | --- | --- | --- | --- |
| \* | IPAstrin\_STLCD\_032114\_01.09942.09942.2 | 2.76 | 0.3317 | 99.9% | 1398.7522 | 1398.5614 | 6 | 5.865 | 65.0% | 1 | R.HYDLNQLLEPR.S | 2 |
|  | IPAstrin\_STLCD\_tube2\_032114\_01.09660.09660.2 | 2.4315 | 0.2003 | 96.9% | 1580.2722 | 1580.811 | 1 | 5.175 | 62.5% | 1 | R.LPMPEDYAMDELR.S | 2 |

---

|  |  |  |  |  |  |  |  |  |
| --- | --- | --- | --- | --- | --- | --- | --- | --- |
| U | *Reverse\_gi|148612838|* | 2 | 3 | 1.0% | 2103 | 228085 | 9.0 | hypothetical protein LOC158358 [Homo sapiens] |

| Filename XCorr DeltCN Conf% ObsM+H+ CalcM+H+ SpR ZScore Ion% # Sequence  | | | | | | | | | | | | |
| --- | --- | --- | --- | --- | --- | --- | --- | --- | --- | --- | --- | --- |
| \* | IPAstrin\_STLCD\_032114\_01.10418.10418.2 | 3.3136 | 0.2095 | 99.8% | 2225.652 | 2227.4338 | 6 | 4.337 | 42.1% | 2 | K.S\*APTNITNLIAGEPS\*VLLKK.V | 2 |
| \* | IPAstrin\_STLCD\_032114\_01.10350.10350.2 | 3.1755 | 0.1667 | 99.2% | 2225.7722 | 2227.4338 | 5 | 3.809 | 42.1% | 1 | K.SAPT#NITNLIAGEPS\*VLLKK.V | 2 |

---

|  |  |  |  |  |  |  |  |  |
| --- | --- | --- | --- | --- | --- | --- | --- | --- |
| U | *gi|171184451|ref|NP\_0* | 2 | 3 | 0.8% | 3117 | 350931 | 6.3 | centrosome-associated protein 350 [Homo sapiens] |

| Filename XCorr DeltCN Conf% ObsM+H+ CalcM+H+ SpR ZScore Ion% # Sequence  | | | | | | | | | | | | |
| --- | --- | --- | --- | --- | --- | --- | --- | --- | --- | --- | --- | --- |
| \* | IPAstrin\_STLCD\_032114\_02.05394.05394.2 | 3.0249 | 0.1238 | 98.2% | 1432.5521 | 1432.385 | 9 | 4.117 | 54.5% | 2 | K.SCTSVSK@QES\*SK@.G | 2 |
| \* | IPAstrin\_STLCD\_tube2\_032114\_01.08198.08198.2 | 2.456 | 0.2888 | 99.5% | 1279.4321 | 1279.5686 | 2 | 5.966 | 63.6% | 1 | R.VLIGNVQPGILR.F | 2 |

---

|  |  |  |  |  |  |  |  |  |
| --- | --- | --- | --- | --- | --- | --- | --- | --- |
| U | *gi|150418007|ref|NP\_0* | 2 | 3 | 0.7% | 3224 | 358201 | 6.2 | RAN binding protein 2 [Homo sapiens] |
| U | *gi|169218183|ref|XP\_0* | 2 | 3 | 2.3% | 1022 | 115853 | 7.0 | PREDICTED: similar to nucleoporin [Homo sapiens] |

| Filename XCorr DeltCN Conf% ObsM+H+ CalcM+H+ SpR ZScore Ion% # Sequence  | | | | | | | | | | | | |
| --- | --- | --- | --- | --- | --- | --- | --- | --- | --- | --- | --- | --- |
|  | IPAstrin\_STLCD\_tube2\_032114\_01.05715.05715.2 | 3.0741 | 0.3478 | 100.0% | 1336.3922 | 1336.5773 | 1 | 5.645 | 80.0% | 2 | R.LLVQHEINTLR.A | 2 |
|  | IPAstrin\_STLCD\_032114\_01.06895.06895.2 | 2.2882 | 0.1904 | 95.4% | 1345.6122 | 1345.4111 | 1 | 3.91 | 63.6% | 1 | K.TGSGLNSFYDQR.E | 2 |

---

|  |  |  |  |  |  |  |  |  |
| --- | --- | --- | --- | --- | --- | --- | --- | --- |
| U | *gi|154277116|ref|NP\_8* | 2 | 2 | 0.3% | 8797 | 1011050 | 5.5 | spectrin repeat containing, nuclear envelope 1 isoform 1 [Homo sapiens] |
| U | *gi|23097308|ref|NP\_14* | 2 | 2 | 0.3% | 8749 | 1005204 | 5.5 | spectrin repeat containing, nuclear envelope 1 isoform 2 [Homo sapiens] |

| Filename XCorr DeltCN Conf% ObsM+H+ CalcM+H+ SpR ZScore Ion% # Sequence  | | | | | | | | | | | | |
| --- | --- | --- | --- | --- | --- | --- | --- | --- | --- | --- | --- | --- |
|  | IPAstrin\_STLCD\_032114\_02.07967.07967.2 | 2.1142 | 0.2536 | 95.5% | 1966.7322 | 1964.0651 | 32 | 5.654 | 40.0% | 1 | K.TDMES\*TVDKWLDVSEK.L | 2 |
|  | IPAstrin\_STLCD\_tube2\_032114\_01.07181.07181.2 | 3.2932 | 0.0486 | 98.8% | 1133.5322 | 1133.2157 | 63 | 3.334 | 68.8% | 1 | R.QSNNLCLQR.E | 2 |

---

|  |  |  |  |  |  |  |  |  |
| --- | --- | --- | --- | --- | --- | --- | --- | --- |
| U | *gi|110349713|ref|NP\_5* | 3 | 3 | 0.2% | 27051 | 3006786 | 6.8 | titin isoform novex-1 [Homo sapiens] |
| U | *gi|110349719|ref|NP\_5* | 3 | 3 | 0.2% | 33423 | 3713573 | 6.5 | titin isoform N2-A [Homo sapiens] |
| U | *gi|110349717|ref|NP\_5* | 3 | 3 | 0.2% | 27118 | 3013987 | 6.7 | titin isoform novex-2 [Homo sapiens] |
| U | *gi|110349715|ref|NP\_0* | 3 | 3 | 0.2% | 26926 | 2992971 | 6.7 | titin isoform N2-B [Homo sapiens] |

| Filename XCorr DeltCN Conf% ObsM+H+ CalcM+H+ SpR ZScore Ion% # Sequence  | | | | | | | | | | | | |
| --- | --- | --- | --- | --- | --- | --- | --- | --- | --- | --- | --- | --- |
|  | IPAstrin\_STLCD\_032114\_02.10217.10217.3 | 3.0648 | 0.2902 | 98.1% | 2836.4944 | 2838.1338 | 49 | 4.678 | 24.0% | 1 | K.ELSATSSAQKITKS\*VKAPTVK@PSETR.V | 3 |
|  | IPAstrin\_STLCD\_032114\_01.13251.13251.2 | 2.801 | 0.1232 | 95.2% | 2341.5923 | 2342.6238 | 70 | 3.974 | 31.6% | 1 | K.GRPTPK@SS\*WEFDGKAKKAMK.D | 2 |
|  | IPAstrin\_STLCD\_032114\_01.10318.10318.2 | 2.0842 | 0.2351 | 95.7% | 1362.9122 | 1364.3777 | 23 | 4.978 | 50.0% | 1 | K.SYSTAT#TKCHK.C | 2 |

---

|  |  |  |  |  |  |  |  |  |
| --- | --- | --- | --- | --- | --- | --- | --- | --- |
| U | *contaminant\_UBIQUITIN* | 2 | 4 | 0.0% | 1118 | 127523 | 8.5 | no description |

| Filename XCorr DeltCN Conf% ObsM+H+ CalcM+H+ SpR ZScore Ion% # Sequence  | | | | | | | | | | | | |
| --- | --- | --- | --- | --- | --- | --- | --- | --- | --- | --- | --- | --- |
|  | IPAstrin\_STLCD\_032114\_01.09380.09380.2 | 2.6874 | 0.2774 | 99.5% | 1656.5721 | 1656.7893 | 1 | 5.015 | 64.3% | 1 | R.YDGQVAVFGSDLQEK.L | 2 |
|  | IPAstrin\_STLCD\_tube2\_032114\_01.08066.08066.2 | 4.3547 | 0.3789 | 100.0% | 1788.4521 | 1788.9897 | 1 | 8.03 | 70.0% | 3 | K.TITLEVEPSDTIENVK.A | 22 |

Similarities:
gi|11024714|ref|NP\_06(1:1)  


|  |  |  |  |
| --- | --- | --- | --- |
|  | Proteins | Peptide IDs | Spectra |
| Unfiltered | 46281 | 94903 | 169555 |
| Filtered | 198 | 1137 | 2899 |
| Forward matches | 195 | 1131 | 2892 |
| Decoy matches | 3 | 6 | 7 |
| Forward FP rate | 1.54% | 0.53% | 0.24% |

  
/nfs/cheeseman\_massspec/David/2NLDAstrin
