# Supplementary material for: Astrin-SKAP complex reconstitution reveals its kinetochore interaction with microtubule-bound Ndc80
Source: eLife. 2017 Aug 25;6:e26866. doi: 10.7554/eLife.26866 (PMC5602300; doi:10.7554/eLife.26866)
Supplement: Source data 1. — Complete mass spectrometry searches using methods described in (Washburn et al., 2001) for affinity purification/mass spectrometry data sets described in this paper (data from this study; [Kern et al., 2016] [Gascoigne et al., 2011]). Individual Astrin cross-linking immunoprecipitations are listed based on the order in Figure 4—figure supplement 1. These samples have not been pruned for common or antibody-specific contaminants. [file elife-26866-data1.zip › Astrin_Crosslinking#5.html]

D Astrin\_STLCHLD2
DTASelect v2.0.21  
/nfs/cheeseman\_massspec/David/Astrin\_STLCHLD2  
/nfs/cheeseman\_massspec/Databases/NCBI-RefSeq\_human\_na\_04-13-2009\_con\_reversed.fasta  
SEQUEST 3.0 in SQT format.  
  
 Jump  to the summary table.  
  
sequest.params modifications:

|  |  |  |
| --- | --- | --- |
| \* | S | 80.0 |
| # | T | 80.0 |
| @ | K | 12.0 |
| Static | C | 57.0 |

|  |  |
| --- | --- |
| true | Use criteria |
| 0.0 | Minimum peptide confidence |
| 0.05 | Peptide false positive rate |
| 0.0 | Minimum protein confidence |
| 1.0 | Protein false positive rate |
| 1 | Minimum charge state |
| 16 | Maximum charge state |
| 0.0 | Minimum ion proportion |
| 1000 | Maximum Sp rank |
| -1.0 | Minimum Sp score |
| Include | Modified peptide inclusion |
| Any | Tryptic status requirement |
| false | Multiple, ambiguous IDs allowed |
| Ignore | Peptide validation handling |
| XCorr | Purge duplicate peptides by protein |
| false | Include only loci with unique peptide |
| true | Remove subset proteins |
| Ignore | Locus validation handling |
| 0 | Minimum modified peptides per locus |
| 1000 | Minimum redundancy for low coverage loci |
| 2 | Minimum peptides per locus |

#### Locus Key:

|  |  |  |  |  |  |  |  |  |
| --- | --- | --- | --- | --- | --- | --- | --- | --- |
| Validation Status | Locus | Sequence Count | Spectrum Count | Sequence Coverage | Length | MolWt | pI | Descriptive Name |

#### Similarity Key:

|  |  |  |
| --- | --- | --- |
| Locus | # of identical peptides | # of differing peptides |

---

|  |  |  |  |  |  |  |  |  |
| --- | --- | --- | --- | --- | --- | --- | --- | --- |
| U | *gi|10800130|ref|NP\_06* | 7 | 23 | 57.7% | 130 | 14107 | 10.9 | histone cluster 1, H2ad [Homo sapiens] |
| U | *gi|4504243|ref|NP\_003* | 7 | 23 | 57.7% | 130 | 14091 | 10.9 | histone cluster 1, H2al [Homo sapiens] |
| U | *gi|4504239|ref|NP\_003* | 7 | 23 | 57.7% | 130 | 14091 | 10.9 | histone cluster 1, H2ai [Homo sapiens] |
| U | *gi|18105045|ref|NP\_54* | 7 | 23 | 58.6% | 128 | 13906 | 10.9 | histone cluster 1, H2ah [Homo sapiens] |
| U | *gi|10800144|ref|NP\_06* | 7 | 23 | 58.6% | 128 | 13936 | 10.9 | histone cluster 1, H2aj [Homo sapiens] |
| U | *gi|10800132|ref|NP\_06* | 7 | 23 | 57.7% | 130 | 14091 | 10.9 | histone cluster 1, H2ag [Homo sapiens] |

| Filename XCorr DeltCN Conf% ObsM+H+ CalcM+H+ SpR ZScore Ion% # Sequence  | | | | | | | | | | | | |
| --- | --- | --- | --- | --- | --- | --- | --- | --- | --- | --- | --- | --- |
|  | Astrin\_STLCHLD\_tube2\_061314\_01.07517.07517.2 | 3.2091 | 0.3209 | 100.0% | 945.39215 | 945.1093 | 1 | 5.94 | 81.2% | 4 | R.AGLQFPVGR.V | 222 |
|  | Astrin\_STLCHLD\_tube2\_061314\_01.18624.18624.2 | 5.7651 | 0.5322 | 100.0% | 2917.172 | 2917.3752 | 1 | 11.331 | 44.6% | 3 | R.VGAGAPVYLAAVLEYLTAEILELAGNAAR.D | 2 |
|  | Astrin\_STLCHLD\_061214\_01.17076.17076.3 | 3.9577 | 0.2985 | 99.7% | 2917.7344 | 2917.3752 | 1 | 5.748 | 24.1% | 3 | R.VGAGAPVYLAAVLEYLTAEILELAGNAAR.D | 3 |
|  | Astrin\_STLCHLD\_tube2\_061314\_02.05192.05192.3 | 3.2474 | 0.3885 | 100.0% | 1694.3344 | 1693.9004 | 1 | 6.694 | 44.2% | 8 | R.HLQLAIRNDEELNK.L | 33 |
|  | Astrin\_STLCHLD\_tube2\_061314\_01.09187.09187.3 | 3.0933 | 0.377 | 100.0% | 2105.2444 | 2105.4453 | 1 | 5.437 | 36.8% | 1 | R.HLQLAIRNDEELNKLLGK.V | 33 |
|  | Astrin\_STLCHLD\_tube2\_061314\_01.07421.07421.2 | 3.6 | 0.3554 | 100.0% | 1273.4122 | 1273.4288 | 1 | 6.312 | 75.0% | 3 | R.NDEELNKLLGK.V | 22 |
|  | Astrin\_STLCHLD\_tube2\_061314\_01.12834.12834.2 | 4.6283 | 0.4647 | 100.0% | 1932.8722 | 1932.3573 | 1 | 7.283 | 66.7% | 1 | K.VTIAQGGVLPNIQAVLLPK.K | 22 |

Similarities:
gi|106775678|ref|NP\_0(5:2)  
gi|20357599|ref|NP\_61(1:6)  

---

|  |  |  |  |  |  |  |  |  |
| --- | --- | --- | --- | --- | --- | --- | --- | --- |
| U | *gi|106775678|ref|NP\_0* | 7 | 20 | 57.7% | 130 | 14095 | 10.9 | histone cluster 2, H2aa4 [Homo sapiens] |
| U | *gi|4504251|ref|NP\_003* | 7 | 20 | 57.7% | 130 | 14095 | 10.9 | histone cluster 2, H2aa3 [Homo sapiens] |
| U | *gi|24638446|ref|NP\_00* | 7 | 20 | 58.1% | 129 | 13988 | 10.9 | histone cluster 2, H2ac [Homo sapiens] |

| Filename XCorr DeltCN Conf% ObsM+H+ CalcM+H+ SpR ZScore Ion% # Sequence  | | | | | | | | | | | | |
| --- | --- | --- | --- | --- | --- | --- | --- | --- | --- | --- | --- | --- |
|  | Astrin\_STLCHLD\_tube2\_061314\_01.07517.07517.2 | 3.2091 | 0.3209 | 100.0% | 945.39215 | 945.1093 | 1 | 5.94 | 81.2% | 4 | R.AGLQFPVGR.V | 222 |
|  | Astrin\_STLCHLD\_tube2\_061314\_01.18505.18505.2 | 5.2781 | 0.5622 | 100.0% | 2934.9321 | 2935.4082 | 1 | 10.063 | 41.1% | 2 | R.VGAGAPVYMAAVLEYLTAEILELAGNAAR.D | 2 |
|  | Astrin\_STLCHLD\_tube2\_061314\_01.18563.18563.3 | 3.1594 | 0.2474 | 96.8% | 2935.1643 | 2935.4082 | 64 | 4.549 | 19.6% | 1 | R.VGAGAPVYMAAVLEYLTAEILELAGNAAR.D | 3 |
|  | Astrin\_STLCHLD\_tube2\_061314\_02.05192.05192.3 | 3.2474 | 0.3885 | 100.0% | 1694.3344 | 1693.9004 | 1 | 6.694 | 44.2% | 8 | R.HLQLAIRNDEELNK.L | 33 |
|  | Astrin\_STLCHLD\_tube2\_061314\_01.09187.09187.3 | 3.0933 | 0.377 | 100.0% | 2105.2444 | 2105.4453 | 1 | 5.437 | 36.8% | 1 | R.HLQLAIRNDEELNKLLGK.V | 33 |
|  | Astrin\_STLCHLD\_tube2\_061314\_01.07421.07421.2 | 3.6 | 0.3554 | 100.0% | 1273.4122 | 1273.4288 | 1 | 6.312 | 75.0% | 3 | R.NDEELNKLLGK.V | 22 |
|  | Astrin\_STLCHLD\_tube2\_061314\_01.12834.12834.2 | 4.6283 | 0.4647 | 100.0% | 1932.8722 | 1932.3573 | 1 | 7.283 | 66.7% | 1 | K.VTIAQGGVLPNIQAVLLPK.K | 22 |

Similarities:
gi|10800130|ref|NP\_06(5:2)  
gi|20357599|ref|NP\_61(1:6)  

---

|  |  |  |  |  |  |  |  |  |
| --- | --- | --- | --- | --- | --- | --- | --- | --- |
| U | *gi|4504919|ref|NP\_002* | 28 | 70 | 56.5% | 483 | 53704 | 5.6 | keratin 8 [Homo sapiens] |

| Filename XCorr DeltCN Conf% ObsM+H+ CalcM+H+ SpR ZScore Ion% # Sequence  | | | | | | | | | | | | |
| --- | --- | --- | --- | --- | --- | --- | --- | --- | --- | --- | --- | --- |
| \* | Astrin\_STLCHLD\_061214\_01.12787.12787.3 | 4.6616 | 0.2901 | 100.0% | 3929.0942 | 3927.465 | 1 | 5.915 | 19.4% | 1 | R.GGLGGGYGGASGMGGITAVTVNQSLLSPLVLEVDPNIQAVR.T | 3 |
|  | Astrin\_STLCHLD\_tube2\_061314\_01.06264.06264.2 | 2.4085 | 0.2136 | 97.8% | 828.2922 | 827.95544 | 5 | 5.059 | 91.7% | 1 | K.FASFIDK.V | 2222 |
|  | Astrin\_STLCHLD\_tube2\_061314\_01.07757.07757.2 | 2.9421 | 0.1283 | 98.0% | 1030.9122 | 1031.1997 | 2 | 3.74 | 92.9% | 3 | K.WSLLQQQK.T | 2 |
|  | Astrin\_STLCHLD\_tube2\_061314\_01.12083.12083.3 | 2.3488 | 0.3489 | 98.6% | 2005.2244 | 2005.2306 | 6 | 5.196 | 30.0% | 1 | R.SNMDNMFESYINNLRR.Q | 3 |
|  | Astrin\_STLCHLD\_tube2\_061314\_02.09177.09177.3 | 3.6053 | 0.2839 | 99.7% | 2034.3844 | 2035.363 | 1 | 6.879 | 41.2% | 1 | K.LKLEAELGNMQGLVEDFK.N | 3 |
|  | Astrin\_STLCHLD\_tube2\_061314\_01.10279.10279.2 | 3.219 | 0.4343 | 100.0% | 1353.5122 | 1353.5732 | 1 | 7.654 | 70.0% | 5 | R.TEMENEFVLIK.K | 2 |
|  | Astrin\_STLCHLD\_061214\_01.06962.06962.2 | 3.5 | 0.3044 | 100.0% | 1481.3922 | 1481.7473 | 1 | 6.002 | 68.2% | 3 | R.TEMENEFVLIKK.D | 2 |
|  | Astrin\_STLCHLD\_061214\_01.06991.06991.3 | 2.2614 | 0.3795 | 99.3% | 1482.1444 | 1481.7473 | 132 | 5.702 | 36.4% | 1 | R.TEMENEFVLIKK.D | 3 |
|  | Astrin\_STLCHLD\_061214\_01.05364.05364.3 | 3.3763 | 0.3513 | 100.0% | 1926.5343 | 1927.1365 | 1 | 5.957 | 38.3% | 1 | K.KDVDEAYMNKVELESR.L | 3 |
|  | Astrin\_STLCHLD\_061214\_01.06228.06228.2 | 4.1872 | 0.5042 | 100.0% | 1798.3922 | 1798.9623 | 1 | 8.264 | 67.9% | 5 | K.DVDEAYMNKVELESR.L | 2 |
|  | Astrin\_STLCHLD\_tube2\_061314\_02.06116.06116.3 | 4.3605 | 0.4118 | 100.0% | 1798.6144 | 1798.9623 | 1 | 6.97 | 55.4% | 6 | K.DVDEAYMNKVELESR.L | 3 |
|  | Astrin\_STLCHLD\_tube2\_061314\_01.12012.12012.2 | 3.9894 | 0.4787 | 100.0% | 1420.3922 | 1420.6055 | 1 | 8.427 | 86.4% | 2 | R.LEGLTDEINFLR.Q | 2 |
|  | Astrin\_STLCHLD\_tube2\_061314\_02.07244.07244.2 | 5.6101 | 0.6018 | 100.0% | 2109.5923 | 2110.3008 | 1 | 11.524 | 69.4% | 2 | R.ELQSQISDTSVVLSMDNSR.S | 2 |
|  | Astrin\_STLCHLD\_tube2\_061314\_01.11803.11803.2 | 4.4966 | 0.4215 | 100.0% | 1321.4922 | 1321.5286 | 1 | 8.047 | 72.7% | 1 | R.SLDMDSIIAEVK.A | 2 |
|  | Astrin\_STLCHLD\_061214\_02.07024.07024.3 | 5.1115 | 0.4039 | 100.0% | 2532.9543 | 2532.828 | 3 | 7.529 | 29.8% | 3 | R.SRAEAESMYQIKYEELQSLAGK.H | 3 |
|  | Astrin\_STLCHLD\_061214\_01.04747.04747.2 | 2.966 | 0.3824 | 100.0% | 1170.0521 | 1170.3228 | 4 | 6.777 | 77.8% | 1 | R.AEAESMYQIK.Y | 2 |
|  | Astrin\_STLCHLD\_061214\_02.07403.07403.3 | 4.8181 | 0.3865 | 100.0% | 2289.8643 | 2289.5623 | 1 | 6.961 | 43.4% | 4 | R.AEAESMYQIKYEELQSLAGK.H | 3 |
|  | Astrin\_STLCHLD\_061214\_01.05077.05077.2 | 3.7342 | 0.0616 | 98.3% | 1138.5721 | 1138.2627 | 2 | 7.572 | 77.8% | 6 | K.YEELQSLAGK.H | 2 |
|  | Astrin\_STLCHLD\_tube2\_061314\_01.05423.05423.2 | 2.9229 | 0.2248 | 98.8% | 1000.71216 | 1001.168 | 25 | 5.29 | 75.0% | 4 | R.LQAEIEGLK.G | 2 |
|  | Astrin\_STLCHLD\_061214\_01.04594.04594.2 | 3.5089 | 0.3188 | 100.0% | 1341.7922 | 1342.5381 | 1 | 7.344 | 72.7% | 1 | R.LQAEIEGLKGQR.A | 2 |
|  | Astrin\_STLCHLD\_tube2\_061314\_01.09221.09221.2 | 4.093 | 0.4119 | 100.0% | 1345.6522 | 1345.452 | 1 | 7.056 | 70.8% | 2 | R.ASLEAAIADAEQR.G | 2 |
|  | Astrin\_STLCHLD\_tube2\_061314\_01.11851.11851.3 | 5.266 | 0.4574 | 100.0% | 2457.5044 | 2456.7153 | 1 | 7.229 | 31.5% | 1 | R.ASLEAAIADAEQRGELAIKDANAK.L | 3 |
|  | Astrin\_STLCHLD\_tube2\_061314\_01.07979.07979.2 | 4.1758 | 0.2438 | 100.0% | 1130.2122 | 1130.2865 | 2 | 6.113 | 83.3% | 4 | K.LSELEAALQR.A | 2 |
|  | Astrin\_STLCHLD\_tube2\_061314\_01.05844.05844.2 | 2.4271 | 0.225 | 97.0% | 1154.3322 | 1154.3234 | 78 | 6.209 | 56.2% | 1 | R.EYQELMNVK.L | 22 |
|  | Astrin\_STLCHLD\_061214\_01.07862.07862.2 | 3.174 | 0.25 | 99.0% | 1407.6322 | 1406.6653 | 1 | 4.86 | 77.3% | 3 | K.LALDIEIATYRK.L | 22 |
|  | Astrin\_STLCHLD\_061214\_01.04520.04520.2 | 3.8354 | 0.3795 | 100.0% | 1476.3121 | 1476.7058 | 1 | 7.597 | 70.8% | 1 | R.LESGMQNMSIHTK.T | 2 |
|  | Astrin\_STLCHLD\_061214\_01.04516.04516.3 | 2.5462 | 0.3429 | 99.3% | 1476.5343 | 1476.7058 | 8 | 5.381 | 37.5% | 1 | R.LESGMQNMSIHTK.T | 3 |
|  | Astrin\_STLCHLD\_061214\_01.04812.04812.2 | 3.2834 | 0.4949 | 100.0% | 1174.3322 | 1174.3367 | 1 | 8.163 | 75.0% | 5 | K.LVSESSDVLPK.- | 2 |

Similarities:
gi|67782365|ref|NP\_00(2:26)  
gi|47132620|ref|NP\_00(1:27)  
gi|119703753|ref|NP\_0(2:26)  

---

|  |  |  |  |  |  |  |  |  |
| --- | --- | --- | --- | --- | --- | --- | --- | --- |
| U | *gi|29788785|ref|NP\_82* | 35 | 167 | 54.3% | 444 | 49671 | 4.9 | tubulin, beta [Homo sapiens] |

| Filename XCorr DeltCN Conf% ObsM+H+ CalcM+H+ SpR ZScore Ion% # Sequence  | | | | | | | | | | | | |
| --- | --- | --- | --- | --- | --- | --- | --- | --- | --- | --- | --- | --- |
| \* | Astrin\_STLCHLD\_061214\_02.07681.07681.3 | 5.8964 | 0.4394 | 100.0% | 3104.1543 | 3104.2725 | 1 | 7.518 | 34.6% | 8 | K.FWEVISDEHGIDPTGTYHGDSDLQLDR.I | 3 |
| \* | Astrin\_STLCHLD\_tube2\_061314\_01.04685.04685.2 | 3.8772 | 0.5572 | 100.0% | 1302.3322 | 1302.4265 | 1 | 10.206 | 86.4% | 7 | R.ISVYYNEATGGK.Y | 2 |
| \* | Astrin\_STLCHLD\_tube2\_061314\_01.04659.04659.1 | 2.4724 | 0.2415 | 100.0% | 1303.66 | 1302.4265 | 6 | 5.053 | 50.0% | 3 | R.ISVYYNEATGGK.Y | 1 |
| \* | Astrin\_STLCHLD\_061214\_01.05823.05823.2 | 4.198 | 0.518 | 100.0% | 1817.2722 | 1818.0392 | 1 | 9.098 | 70.0% | 1 | R.ISVYYNEATGGKYVPR.A | 2 |
|  | Astrin\_STLCHLD\_tube2\_061314\_01.10607.10607.2 | 4.4949 | 0.5136 | 100.0% | 1616.5521 | 1616.8701 | 1 | 8.677 | 71.4% | 9 | R.AILVDLEPGTMDSVR.S | 222 |
|  | Astrin\_STLCHLD\_tube2\_061314\_01.12390.12390.2 | 5.5984 | 0.5487 | 100.0% | 2798.9321 | 2800.0647 | 1 | 8.841 | 42.0% | 2 | R.SGPFGQIFRPDNFVFGQSGAGNNWAK.G | 222 |
|  | Astrin\_STLCHLD\_061214\_01.10677.10677.3 | 6.1862 | 0.4832 | 100.0% | 2800.4043 | 2800.0647 | 1 | 7.629 | 40.0% | 7 | R.SGPFGQIFRPDNFVFGQSGAGNNWAK.G | 333 |
|  | Astrin\_STLCHLD\_061214\_01.11227.11227.2 | 6.4727 | 0.5531 | 100.0% | 1959.4521 | 1960.151 | 1 | 10.56 | 73.5% | 4 | K.GHYTEGAELVDSVLDVVR.K | 2222 |
|  | Astrin\_STLCHLD\_061214\_01.11221.11221.3 | 4.0137 | 0.3325 | 100.0% | 1961.2144 | 1960.151 | 1 | 6.21 | 44.1% | 5 | K.GHYTEGAELVDSVLDVVR.K | 3333 |
|  | Astrin\_STLCHLD\_tube2\_061314\_01.12007.12007.2 | 5.7392 | 0.5731 | 100.0% | 2087.912 | 2088.325 | 1 | 10.188 | 69.4% | 1 | K.GHYTEGAELVDSVLDVVRK.E | 2222 |
|  | Astrin\_STLCHLD\_tube2\_061314\_01.11982.11982.3 | 4.7827 | 0.4838 | 100.0% | 2088.1143 | 2088.325 | 1 | 8.148 | 44.4% | 7 | K.GHYTEGAELVDSVLDVVRK.E | 3333 |
|  | Astrin\_STLCHLD\_tube2\_061314\_01.08593.08593.1 | 1.9485 | 0.2768 | 100.0% | 1319.74 | 1320.5896 | 79 | 4.843 | 45.5% | 2 | R.IMNTFSVVPSPK.V | 111 |
|  | Astrin\_STLCHLD\_tube2\_061314\_01.08597.08597.2 | 4.5471 | 0.3836 | 100.0% | 1320.4122 | 1320.5896 | 1 | 7.86 | 77.3% | 9 | R.IMNTFSVVPSPK.V | 222 |
|  | Astrin\_STLCHLD\_tube2\_061314\_01.07361.07361.2 | 3.2733 | 0.2391 | 99.5% | 1132.2322 | 1131.2767 | 1 | 5.073 | 83.3% | 14 | R.FPGQLNADLR.K | 22222 |
|  | Astrin\_STLCHLD\_tube2\_061314\_01.04904.04904.2 | 2.5627 | 0.1707 | 95.5% | 1259.2922 | 1259.4508 | 1 | 4.474 | 70.0% | 2 | R.FPGQLNADLRK.L | 22222 |
|  | Astrin\_STLCHLD\_tube2\_061314\_01.04842.04842.3 | 2.8966 | 0.3686 | 100.0% | 1260.2043 | 1259.4508 | 14 | 5.742 | 42.5% | 5 | R.FPGQLNADLRK.L | 33333 |
|  | Astrin\_STLCHLD\_tube2\_061314\_01.08730.08730.2 | 3.8464 | 0.3753 | 100.0% | 1272.4122 | 1272.5945 | 1 | 7.898 | 75.0% | 4 | R.KLAVNMVPFPR.L | 22222 |
|  | Astrin\_STLCHLD\_tube2\_061314\_01.10148.10148.1 | 2.1143 | 0.2239 | 96.6% | 1143.61 | 1144.4204 | 18 | 6.431 | 61.1% | 1 | K.LAVNMVPFPR.L | 11111 |
|  | Astrin\_STLCHLD\_tube2\_061314\_01.10247.10247.2 | 3.6473 | 0.5079 | 100.0% | 1145.2722 | 1144.4204 | 1 | 8.426 | 94.4% | 4 | K.LAVNMVPFPR.L | 22222 |
|  | Astrin\_STLCHLD\_tube2\_061314\_01.12143.12143.2 | 3.7666 | 0.4528 | 100.0% | 1621.8322 | 1621.9403 | 1 | 8.756 | 76.9% | 2 | R.LHFFMPGFAPLTSR.G | 2222 |
|  | Astrin\_STLCHLD\_tube2\_061314\_01.12204.12204.3 | 4.3867 | 0.3891 | 100.0% | 1622.3944 | 1621.9403 | 1 | 6.411 | 53.8% | 2 | R.LHFFMPGFAPLTSR.G | 3333 |
| \* | Astrin\_STLCHLD\_tube2\_061314\_01.11378.11378.3 | 2.9228 | 0.2689 | 97.3% | 2381.3044 | 2380.6628 | 1 | 4.399 | 30.0% | 1 | R.GSQQYRALTVPELTQQVFDAK.N | 3 |
| \* | Astrin\_STLCHLD\_tube2\_061314\_01.11900.11900.2 | 3.6723 | 0.4117 | 100.0% | 1660.7522 | 1660.9078 | 1 | 7.915 | 67.9% | 3 | R.ALTVPELTQQVFDAK.N | 2 |
|  | Astrin\_STLCHLD\_tube2\_061314\_02.06444.06444.3 | 3.2171 | 0.2673 | 99.6% | 1391.4243 | 1390.631 | 6 | 4.633 | 45.5% | 2 | R.HGRYLTVAAVFR.G | 33 |
|  | Astrin\_STLCHLD\_061214\_01.09126.09126.1 | 1.7694 | 0.4298 | 100.0% | 1039.6 | 1040.2505 | 3 | 6.392 | 56.2% | 6 | R.YLTVAAVFR.G | 11 |
|  | Astrin\_STLCHLD\_061214\_01.09102.09102.2 | 3.0215 | 0.4374 | 100.0% | 1040.2922 | 1040.2505 | 1 | 7.082 | 87.5% | 11 | R.YLTVAAVFR.G | 22 |
|  | Astrin\_STLCHLD\_061214\_01.06900.06900.2 | 5.2617 | 0.4991 | 100.0% | 1924.5122 | 1925.2405 | 1 | 9.197 | 60.0% | 2 | R.MSMKEVDEQMLNVQNK.N | 222 |
|  | Astrin\_STLCHLD\_tube2\_061314\_02.06675.06675.3 | 4.9405 | 0.1867 | 99.7% | 1926.6843 | 1925.2405 | 1 | 5.512 | 51.7% | 7 | R.MSMKEVDEQMLNVQNK.N | 333 |
|  | Astrin\_STLCHLD\_tube2\_061314\_01.05125.05125.2 | 4.2318 | 0.1226 | 99.5% | 1449.2122 | 1447.6031 | 1 | 4.856 | 72.7% | 3 | K.EVDEQMLNVQNK.N | 222 |
|  | Astrin\_STLCHLD\_tube2\_061314\_01.11781.11781.2 | 4.295 | 0.2978 | 100.0% | 1698.5322 | 1697.8877 | 1 | 7.708 | 73.1% | 2 | K.NSSYFVEWIPNNVK.T | 22222 |
| \* | Astrin\_STLCHLD\_tube2\_061314\_02.09998.09998.2 | 5.7383 | 0.5106 | 100.0% | 1870.6122 | 1871.2018 | 1 | 9.377 | 71.9% | 3 | K.MAVTFIGNSTAIQELFK.R | 2 |
| \* | Astrin\_STLCHLD\_061214\_01.10978.10978.2 | 4.8566 | 0.5314 | 100.0% | 2026.5922 | 2027.3893 | 1 | 8.775 | 61.8% | 1 | K.MAVTFIGNSTAIQELFKR.I | 2 |
| \* | Astrin\_STLCHLD\_061214\_02.09223.09223.3 | 3.6251 | 0.3561 | 100.0% | 2028.0543 | 2027.3893 | 1 | 6.713 | 41.2% | 5 | K.MAVTFIGNSTAIQELFKR.I | 3 |
|  | Astrin\_STLCHLD\_061214\_01.07812.07812.2 | 3.8437 | 0.3944 | 100.0% | 1386.3522 | 1386.6116 | 1 | 7.545 | 80.0% | 6 | K.RISEQFTAMFR.R | 2222 |
|  | Astrin\_STLCHLD\_tube2\_061314\_02.07946.07946.2 | 3.9325 | 0.4891 | 100.0% | 1229.9321 | 1230.4241 | 1 | 8.487 | 94.4% | 16 | R.ISEQFTAMFR.R | 2222 |

Similarities:
gi|5174735|ref|NP\_006(25:10)  
gi|29788768|ref|NP\_82(21:14)  
gi|50592996|ref|NP\_00(16:19)  
gi|14210536|ref|NP\_11(9:26)  

---

|  |  |  |  |  |  |  |  |  |
| --- | --- | --- | --- | --- | --- | --- | --- | --- |
| U | *gi|5174735|ref|NP\_006* | 30 | 148 | 51.9% | 445 | 49831 | 4.9 | tubulin, beta, 2 [Homo sapiens] |

| Filename XCorr DeltCN Conf% ObsM+H+ CalcM+H+ SpR ZScore Ion% # Sequence  | | | | | | | | | | | | |
| --- | --- | --- | --- | --- | --- | --- | --- | --- | --- | --- | --- | --- |
|  | Astrin\_STLCHLD\_061214\_01.08613.08613.3 | 6.218 | 0.3181 | 100.0% | 3119.0645 | 3118.2996 | 1 | 7.013 | 32.7% | 5 | K.FWEVISDEHGIDPTGTYHGDSDLQLER.I | 3 |
| \* | Astrin\_STLCHLD\_tube2\_061314\_01.04739.04739.2 | 4.0385 | 0.3962 | 100.0% | 1328.9722 | 1329.4521 | 1 | 8.671 | 81.8% | 5 | R.INVYYNEATGGK.Y | 2 |
|  | Astrin\_STLCHLD\_tube2\_061314\_02.07608.07608.2 | 4.1166 | 0.3404 | 100.0% | 1603.7322 | 1602.8431 | 1 | 7.556 | 67.9% | 9 | R.AVLVDLEPGTMDSVR.S | 2 |
|  | Astrin\_STLCHLD\_tube2\_061314\_01.12390.12390.2 | 5.5984 | 0.5487 | 100.0% | 2798.9321 | 2800.0647 | 1 | 8.841 | 42.0% | 2 | R.SGPFGQIFRPDNFVFGQSGAGNNWAK.G | 222 |
|  | Astrin\_STLCHLD\_061214\_01.10677.10677.3 | 6.1862 | 0.4832 | 100.0% | 2800.4043 | 2800.0647 | 1 | 7.629 | 40.0% | 7 | R.SGPFGQIFRPDNFVFGQSGAGNNWAK.G | 333 |
|  | Astrin\_STLCHLD\_061214\_01.11227.11227.2 | 6.4727 | 0.5531 | 100.0% | 1959.4521 | 1960.151 | 1 | 10.56 | 73.5% | 4 | K.GHYTEGAELVDSVLDVVR.K | 2222 |
|  | Astrin\_STLCHLD\_061214\_01.11221.11221.3 | 4.0137 | 0.3325 | 100.0% | 1961.2144 | 1960.151 | 1 | 6.21 | 44.1% | 5 | K.GHYTEGAELVDSVLDVVR.K | 3333 |
|  | Astrin\_STLCHLD\_tube2\_061314\_01.12007.12007.2 | 5.7392 | 0.5731 | 100.0% | 2087.912 | 2088.325 | 1 | 10.188 | 69.4% | 1 | K.GHYTEGAELVDSVLDVVRK.E | 2222 |
|  | Astrin\_STLCHLD\_tube2\_061314\_01.11982.11982.3 | 4.7827 | 0.4838 | 100.0% | 2088.1143 | 2088.325 | 1 | 8.148 | 44.4% | 7 | K.GHYTEGAELVDSVLDVVRK.E | 3333 |
|  | Astrin\_STLCHLD\_tube2\_061314\_01.08593.08593.1 | 1.9485 | 0.2768 | 100.0% | 1319.74 | 1320.5896 | 79 | 4.843 | 45.5% | 2 | R.IMNTFSVVPSPK.V | 111 |
|  | Astrin\_STLCHLD\_tube2\_061314\_01.08597.08597.2 | 4.5471 | 0.3836 | 100.0% | 1320.4122 | 1320.5896 | 1 | 7.86 | 77.3% | 9 | R.IMNTFSVVPSPK.V | 222 |
|  | Astrin\_STLCHLD\_tube2\_061314\_01.07361.07361.2 | 3.2733 | 0.2391 | 99.5% | 1132.2322 | 1131.2767 | 1 | 5.073 | 83.3% | 14 | R.FPGQLNADLR.K | 22222 |
|  | Astrin\_STLCHLD\_tube2\_061314\_01.04904.04904.2 | 2.5627 | 0.1707 | 95.5% | 1259.2922 | 1259.4508 | 1 | 4.474 | 70.0% | 2 | R.FPGQLNADLRK.L | 22222 |
|  | Astrin\_STLCHLD\_tube2\_061314\_01.04842.04842.3 | 2.8966 | 0.3686 | 100.0% | 1260.2043 | 1259.4508 | 14 | 5.742 | 42.5% | 5 | R.FPGQLNADLRK.L | 33333 |
|  | Astrin\_STLCHLD\_tube2\_061314\_01.08730.08730.2 | 3.8464 | 0.3753 | 100.0% | 1272.4122 | 1272.5945 | 1 | 7.898 | 75.0% | 4 | R.KLAVNMVPFPR.L | 22222 |
|  | Astrin\_STLCHLD\_tube2\_061314\_01.10148.10148.1 | 2.1143 | 0.2239 | 96.6% | 1143.61 | 1144.4204 | 18 | 6.431 | 61.1% | 1 | K.LAVNMVPFPR.L | 11111 |
|  | Astrin\_STLCHLD\_tube2\_061314\_01.10247.10247.2 | 3.6473 | 0.5079 | 100.0% | 1145.2722 | 1144.4204 | 1 | 8.426 | 94.4% | 4 | K.LAVNMVPFPR.L | 22222 |
|  | Astrin\_STLCHLD\_tube2\_061314\_01.12143.12143.2 | 3.7666 | 0.4528 | 100.0% | 1621.8322 | 1621.9403 | 1 | 8.756 | 76.9% | 2 | R.LHFFMPGFAPLTSR.G | 2222 |
|  | Astrin\_STLCHLD\_tube2\_061314\_01.12204.12204.3 | 4.3867 | 0.3891 | 100.0% | 1622.3944 | 1621.9403 | 1 | 6.411 | 53.8% | 2 | R.LHFFMPGFAPLTSR.G | 3333 |
|  | Astrin\_STLCHLD\_tube2\_061314\_01.12186.12186.2 | 4.648 | 0.4524 | 100.0% | 1692.6122 | 1692.9678 | 1 | 7.8 | 78.6% | 2 | R.ALTVPELTQQMFDAK.N | 22 |
|  | Astrin\_STLCHLD\_tube2\_061314\_02.06444.06444.3 | 3.2171 | 0.2673 | 99.6% | 1391.4243 | 1390.631 | 6 | 4.633 | 45.5% | 2 | R.HGRYLTVAAVFR.G | 33 |
|  | Astrin\_STLCHLD\_061214\_01.09126.09126.1 | 1.7694 | 0.4298 | 100.0% | 1039.6 | 1040.2505 | 3 | 6.392 | 56.2% | 6 | R.YLTVAAVFR.G | 11 |
|  | Astrin\_STLCHLD\_061214\_01.09102.09102.2 | 3.0215 | 0.4374 | 100.0% | 1040.2922 | 1040.2505 | 1 | 7.082 | 87.5% | 11 | R.YLTVAAVFR.G | 22 |
|  | Astrin\_STLCHLD\_061214\_01.06900.06900.2 | 5.2617 | 0.4991 | 100.0% | 1924.5122 | 1925.2405 | 1 | 9.197 | 60.0% | 2 | R.MSMKEVDEQMLNVQNK.N | 222 |
|  | Astrin\_STLCHLD\_tube2\_061314\_02.06675.06675.3 | 4.9405 | 0.1867 | 99.7% | 1926.6843 | 1925.2405 | 1 | 5.512 | 51.7% | 7 | R.MSMKEVDEQMLNVQNK.N | 333 |
|  | Astrin\_STLCHLD\_tube2\_061314\_01.05125.05125.2 | 4.2318 | 0.1226 | 99.5% | 1449.2122 | 1447.6031 | 1 | 4.856 | 72.7% | 3 | K.EVDEQMLNVQNK.N | 222 |
|  | Astrin\_STLCHLD\_tube2\_061314\_01.11781.11781.2 | 4.295 | 0.2978 | 100.0% | 1698.5322 | 1697.8877 | 1 | 7.708 | 73.1% | 2 | K.NSSYFVEWIPNNVK.T | 22222 |
|  | Astrin\_STLCHLD\_tube2\_061314\_02.09608.09608.2 | 3.6979 | 0.0138 | 95.3% | 1859.8522 | 1859.1475 | 1 | 5.87 | 50.0% | 1 | K.MSATFIGNSTAIQELFK.R | 22 |
|  | Astrin\_STLCHLD\_061214\_01.07812.07812.2 | 3.8437 | 0.3944 | 100.0% | 1386.3522 | 1386.6116 | 1 | 7.545 | 80.0% | 6 | K.RISEQFTAMFR.R | 2222 |
|  | Astrin\_STLCHLD\_tube2\_061314\_02.07946.07946.2 | 3.9325 | 0.4891 | 100.0% | 1229.9321 | 1230.4241 | 1 | 8.487 | 94.4% | 16 | R.ISEQFTAMFR.R | 2222 |

Similarities:
gi|29788785|ref|NP\_82(25:5)  
gi|29788768|ref|NP\_82(21:9)  
gi|50592996|ref|NP\_00(16:14)  
gi|14210536|ref|NP\_11(9:21)  

---

|  |  |  |  |  |  |  |  |  |
| --- | --- | --- | --- | --- | --- | --- | --- | --- |
| U | *gi|57013276|ref|NP\_00* | 28 | 131 | 50.3% | 451 | 50152 | 5.1 | tubulin, alpha, ubiquitous [Homo sapiens] |

| Filename XCorr DeltCN Conf% ObsM+H+ CalcM+H+ SpR ZScore Ion% # Sequence  | | | | | | | | | | | | |
| --- | --- | --- | --- | --- | --- | --- | --- | --- | --- | --- | --- | --- |
|  | Astrin\_STLCHLD\_061214\_01.09384.09384.2 | 6.1825 | 0.6572 | 100.0% | 2008.3922 | 2009.093 | 1 | 11.538 | 63.2% | 13 | K.TIGGGDDSFNTFFSETGAGK.H | 2 |
|  | Astrin\_STLCHLD\_tube2\_061314\_01.11935.11935.1 | 3.3623 | 0.5322 | 100.0% | 1702.95 | 1702.9451 | 1 | 8.203 | 57.1% | 1 | R.AVFVDLEPTVIDEVR.T | 1 |
|  | Astrin\_STLCHLD\_061214\_02.08717.08717.2 | 5.1706 | 0.51 | 100.0% | 1703.3522 | 1702.9451 | 1 | 8.59 | 78.6% | 15 | R.AVFVDLEPTVIDEVR.T | 2 |
|  | Astrin\_STLCHLD\_061214\_01.10140.10140.3 | 3.3117 | 0.3073 | 99.7% | 1704.1144 | 1702.9451 | 4 | 5.806 | 42.9% | 1 | R.AVFVDLEPTVIDEVR.T | 3 |
|  | Astrin\_STLCHLD\_tube2\_061314\_01.08855.08855.2 | 1.7582 | 0.466 | 97.3% | 1411.6122 | 1411.6439 | 23 | 6.372 | 59.1% | 1 | R.QLFHPEQLITGK.E | 22 |
|  | Astrin\_STLCHLD\_tube2\_061314\_01.08195.08195.3 | 3.8919 | 0.4186 | 100.0% | 2417.5144 | 2416.6555 | 1 | 6.286 | 36.2% | 2 | R.QLFHPEQLITGKEDAANNYAR.G | 33 |
|  | Astrin\_STLCHLD\_tube2\_061314\_01.12619.12619.3 | 4.286 | 0.4775 | 100.0% | 1843.4944 | 1843.1332 | 1 | 8.64 | 41.7% | 1 | R.GHYTIGKEIIDLVLDR.I | 3 |
|  | Astrin\_STLCHLD\_tube2\_061314\_01.11543.11543.2 | 3.0514 | 0.3519 | 100.0% | 1086.4722 | 1086.2737 | 9 | 6.025 | 81.2% | 2 | K.EIIDLVLDR.I | 2 |
|  | Astrin\_STLCHLD\_tube2\_061314\_01.04847.04847.3 | 3.6369 | 0.3589 | 100.0% | 1876.6144 | 1876.0824 | 1 | 5.792 | 48.2% | 2 | R.RNLDIERPTYTNLNR.L | 33 |
|  | Astrin\_STLCHLD\_tube2\_061314\_01.06192.06192.3 | 2.5081 | 0.4178 | 99.8% | 1719.2644 | 1719.8949 | 13 | 5.415 | 46.2% | 2 | R.NLDIERPTYTNLNR.L | 33 |
|  | Astrin\_STLCHLD\_tube2\_061314\_01.06234.06234.2 | 3.2301 | 0.1671 | 97.4% | 1719.3722 | 1719.8949 | 2 | 5.169 | 57.7% | 2 | R.NLDIERPTYTNLNR.L | 22 |
|  | Astrin\_STLCHLD\_061214\_01.12381.12381.1 | 2.936 | 0.2665 | 100.0% | 1487.87 | 1488.7678 | 8 | 7.187 | 46.2% | 2 | R.LISQIVSSITASLR.F | 11 |
|  | Astrin\_STLCHLD\_061214\_02.10179.10179.2 | 4.4551 | 0.492 | 100.0% | 1489.4122 | 1488.7678 | 1 | 8.895 | 76.9% | 20 | R.LISQIVSSITASLR.F | 22 |
|  | Astrin\_STLCHLD\_tube2\_061314\_01.13637.13637.3 | 4.5167 | 0.4146 | 100.0% | 1489.6144 | 1488.7678 | 1 | 7.584 | 57.7% | 4 | R.LISQIVSSITASLR.F | 33 |
|  | Astrin\_STLCHLD\_tube2\_061314\_02.09638.09638.2 | 5.8424 | 0.5235 | 100.0% | 2410.7122 | 2410.6885 | 1 | 10.298 | 50.0% | 13 | R.FDGALNVDLTEFQTNLVPYPR.I | 22 |
|  | Astrin\_STLCHLD\_061214\_01.11526.11526.3 | 3.5553 | 0.4671 | 100.0% | 2410.7344 | 2410.6885 | 1 | 8.003 | 37.5% | 2 | R.FDGALNVDLTEFQTNLVPYPR.I | 33 |
|  | Astrin\_STLCHLD\_tube2\_061314\_01.10737.10737.2 | 4.0361 | 0.5047 | 100.0% | 1758.6522 | 1758.0703 | 1 | 8.332 | 73.3% | 6 | R.IHFPLATYAPVISAEK.A | 22 |
|  | Astrin\_STLCHLD\_tube2\_061314\_01.10739.10739.3 | 3.8365 | 0.3294 | 100.0% | 1759.0743 | 1758.0703 | 1 | 6.275 | 46.7% | 5 | R.IHFPLATYAPVISAEK.A | 33 |
|  | Astrin\_STLCHLD\_tube2\_061314\_01.06093.06093.1 | 1.9394 | 0.2937 | 100.0% | 1015.66 | 1016.1827 | 1 | 5.809 | 77.8% | 2 | K.DVNAAIATIK.T | 1 |
|  | Astrin\_STLCHLD\_tube2\_061314\_01.06251.06251.2 | 3.4569 | 0.4018 | 100.0% | 1016.1922 | 1016.1827 | 1 | 7.757 | 88.9% | 4 | K.DVNAAIATIK.T | 2 |
|  | Astrin\_STLCHLD\_tube2\_061314\_01.08843.08843.2 | 4.4981 | 0.4675 | 100.0% | 1825.6721 | 1826.1027 | 1 | 7.437 | 67.6% | 5 | K.VGINYQPPTVVPGGDLAK.V | 22 |
|  | Astrin\_STLCHLD\_tube2\_061314\_01.06060.06060.3 | 4.3726 | 0.4192 | 100.0% | 1382.2144 | 1381.6324 | 1 | 6.674 | 60.0% | 5 | R.LDHKFDLMYAK.R | 33 |
|  | Astrin\_STLCHLD\_tube2\_061314\_01.08135.08135.2 | 2.4286 | 0.2419 | 98.2% | 887.8722 | 888.0692 | 1 | 4.937 | 91.7% | 1 | K.FDLMYAK.R | 22 |
|  | Astrin\_STLCHLD\_061214\_01.07863.07863.3 | 6.4401 | 0.5198 | 100.0% | 2488.7644 | 2487.7083 | 1 | 9.454 | 50.0% | 4 | K.RAFVHWYVGEGMEEGEFSEAR.E | 33 |
|  | Astrin\_STLCHLD\_tube2\_061314\_01.18852.18852.2 | 2.5758 | 0.2444 | 96.5% | 2331.2922 | 2331.5208 | 1 | 4.668 | 36.8% | 1 | R.AFVHWYVGEGMEEGEFSEAR.E | 22 |
|  | Astrin\_STLCHLD\_061214\_02.07921.07921.3 | 5.6027 | 0.4369 | 100.0% | 2332.3743 | 2331.5208 | 1 | 7.994 | 44.7% | 11 | R.AFVHWYVGEGMEEGEFSEAR.E | 33 |
|  | Astrin\_STLCHLD\_061214\_01.09956.09956.3 | 3.9499 | 0.2187 | 98.6% | 3220.7043 | 3219.524 | 1 | 4.394 | 28.7% | 1 | R.AFVHWYVGEGMEEGEFSEAREDMAALEK.D | 33 |
|  | Astrin\_STLCHLD\_tube2\_061314\_02.06330.06330.2 | 3.6552 | 0.5707 | 100.0% | 2349.612 | 2350.2751 | 1 | 10.384 | 52.5% | 3 | K.DYEEVGVDSVEGEGEEEGEEY.- | 2 |

Similarities:
gi|17921989|ref|NP\_00(19:9)  

---

|  |  |  |  |  |  |  |  |  |
| --- | --- | --- | --- | --- | --- | --- | --- | --- |
| U | *contaminant\_KERATIN09* | 19 | 70 | 46.9% | 429 | 47927 | 5.5 | no description |
| U | *gi|4557888|ref|NP\_000* | 19 | 70 | 46.7% | 430 | 48058 | 5.5 | keratin 18 [Homo sapiens] |
| U | *gi|40354195|ref|NP\_95* | 19 | 70 | 46.7% | 430 | 48058 | 5.5 | keratin 18 [Homo sapiens] |

| Filename XCorr DeltCN Conf% ObsM+H+ CalcM+H+ SpR ZScore Ion% # Sequence  | | | | | | | | | | | | |
| --- | --- | --- | --- | --- | --- | --- | --- | --- | --- | --- | --- | --- |
|  | Astrin\_STLCHLD\_061214\_02.05941.05941.3 | 4.9108 | 0.5613 | 100.0% | 2855.5144 | 2856.0813 | 1 | 9.755 | 32.5% | 10 | R.SLGSVQAPSYGARPVSSAASVYAGAGGSGSR.I | 3 |
|  | Astrin\_STLCHLD\_061214\_02.08073.08073.2 | 5.196 | 0.5609 | 100.0% | 2262.6921 | 2262.561 | 1 | 9.232 | 46.0% | 2 | R.GGMGSGGLATGIAGGLAGMGGIQNEK.E | 2 |
|  | Astrin\_STLCHLD\_tube2\_061314\_02.08139.08139.3 | 4.0467 | 0.2389 | 98.9% | 2265.4443 | 2262.561 | 1 | 5.191 | 35.0% | 1 | R.GGMGSGGLATGIAGGLAGMGGIQNEK.E | 3 |
|  | Astrin\_STLCHLD\_tube2\_061314\_02.08204.08204.3 | 5.0167 | 0.4905 | 100.0% | 3338.0942 | 3337.7224 | 1 | 7.332 | 24.3% | 5 | R.GGMGSGGLATGIAGGLAGMGGIQNEKETMQSLNDR.L | 3 |
|  | Astrin\_STLCHLD\_tube2\_061314\_01.05123.05123.2 | 4.1015 | 0.4736 | 100.0% | 1320.0721 | 1320.4478 | 1 | 8.79 | 77.3% | 7 | R.AQIFANTVDNAR.I | 2 |
|  | Astrin\_STLCHLD\_tube2\_061314\_01.05939.05939.2 | 3.1982 | 0.1994 | 99.3% | 1042.1921 | 1042.2235 | 2 | 6.692 | 87.5% | 3 | R.IVLQIDNAR.L | 22 |
|  | Astrin\_STLCHLD\_061214\_01.05097.05097.2 | 2.3723 | 0.415 | 99.3% | 1240.2522 | 1240.4601 | 25 | 6.191 | 72.2% | 1 | R.VKYETELAMR.Q | 2 |
|  | Astrin\_STLCHLD\_061214\_01.05124.05124.3 | 2.9589 | 0.1668 | 95.7% | 1241.2743 | 1240.4601 | 33 | 4.15 | 50.0% | 2 | R.VKYETELAMR.Q | 3 |
|  | Astrin\_STLCHLD\_tube2\_061314\_01.14199.14199.2 | 6.1165 | 0.5229 | 100.0% | 2178.172 | 2178.589 | 1 | 10.349 | 64.7% | 2 | R.LQLETEIEALKEELLFMK.K | 2 |
|  | Astrin\_STLCHLD\_tube2\_061314\_01.14250.14250.3 | 2.538 | 0.3277 | 98.3% | 2178.6543 | 2178.589 | 30 | 4.901 | 38.2% | 1 | R.LQLETEIEALKEELLFMK.K | 3 |
|  | Astrin\_STLCHLD\_tube2\_061314\_01.09105.09105.3 | 4.0718 | 0.3884 | 100.0% | 2751.3245 | 2751.0227 | 1 | 5.918 | 32.0% | 2 | K.NHEEEVKGLQAQIASSGLTVEVDAPK.S | 3 |
|  | Astrin\_STLCHLD\_tube2\_061314\_02.07277.07277.2 | 4.832 | 0.5654 | 100.0% | 1884.3722 | 1885.1246 | 1 | 10.947 | 63.9% | 3 | K.GLQAQIASSGLTVEVDAPK.S | 2 |
|  | Astrin\_STLCHLD\_061214\_01.07822.07822.2 | 3.0998 | 0.3408 | 99.6% | 1663.1921 | 1663.8865 | 1 | 5.85 | 50.0% | 1 | R.RTVQSLEIDLDSMR.N | 2 |
|  | Astrin\_STLCHLD\_tube2\_061314\_01.10914.10914.2 | 4.4331 | 0.524 | 100.0% | 1507.4321 | 1507.699 | 1 | 9.028 | 75.0% | 9 | R.TVQSLEIDLDSMR.N | 2 |
|  | Astrin\_STLCHLD\_tube2\_061314\_01.15008.15008.2 | 5.714 | 0.5657 | 100.0% | 2671.132 | 2672.0715 | 1 | 9.564 | 52.3% | 1 | R.YALQMEQLNGILLHLESELAQTR.A | 2 |
|  | Astrin\_STLCHLD\_tube2\_061314\_02.11354.11354.3 | 6.2202 | 0.5164 | 100.0% | 2672.8442 | 2672.0715 | 1 | 9.3 | 44.3% | 8 | R.YALQMEQLNGILLHLESELAQTR.A | 3 |
|  | Astrin\_STLCHLD\_tube2\_061314\_01.10035.10035.2 | 3.4588 | 0.3363 | 100.0% | 1421.7322 | 1420.6055 | 1 | 5.578 | 72.7% | 3 | R.QAQEYEALLNIK.V | 2 |
|  | Astrin\_STLCHLD\_061214\_01.05875.05875.2 | 3.0686 | 0.3395 | 100.0% | 1293.4722 | 1293.5059 | 1 | 6.079 | 75.0% | 6 | K.VKLEAEIATYR.R | 2 |
|  | Astrin\_STLCHLD\_tube2\_061314\_01.04795.04795.2 | 2.3813 | 0.2325 | 96.9% | 1066.5521 | 1066.1992 | 1 | 5.525 | 81.2% | 3 | K.LEAEIATYR.R | 2 |

Similarities:
contaminant\_KERATIN10(1:18)  

---

|  |  |  |  |  |  |  |  |  |
| --- | --- | --- | --- | --- | --- | --- | --- | --- |
| U | *gi|57242777|ref|NP\_03* | 4 | 5 | 44.7% | 103 | 11967 | 5.9 | c-myc binding protein [Homo sapiens] |

| Filename XCorr DeltCN Conf% ObsM+H+ CalcM+H+ SpR ZScore Ion% # Sequence  | | | | | | | | | | | | |
| --- | --- | --- | --- | --- | --- | --- | --- | --- | --- | --- | --- | --- |
| \* | Astrin\_STLCHLD\_tube2\_061314\_01.06689.06689.2 | 2.5818 | 0.2999 | 98.8% | 933.7522 | 934.07764 | 3 | 5.869 | 75.0% | 1 | K.SGVLDTLTK.V | 2 |
| \* | Astrin\_STLCHLD\_tube2\_061314\_01.16490.16490.3 | 4.8728 | 0.3685 | 100.0% | 3191.3342 | 3191.6892 | 1 | 5.676 | 23.2% | 1 | K.SGVLDTLTKVLVALYEEPEKPNSALDFLK.H | 3 |
| \* | Astrin\_STLCHLD\_tube2\_061314\_01.11723.11723.3 | 4.7286 | 0.4188 | 100.0% | 2277.4143 | 2276.6348 | 1 | 7.954 | 38.2% | 1 | K.VLVALYEEPEKPNSALDFLK.H | 3 |
| \* | Astrin\_STLCHLD\_tube2\_061314\_01.07005.07005.3 | 4.0617 | 0.3109 | 100.0% | 1896.8043 | 1898.1289 | 67 | 5.527 | 37.5% | 2 | K.HHLGAATPENPEIELLR.L | 3 |

---

|  |  |  |  |  |  |  |  |  |
| --- | --- | --- | --- | --- | --- | --- | --- | --- |
| U | *gi|73623035|ref|NP\_00* | 60 | 265 | 44.5% | 1193 | 134422 | 5.0 | sperm associated antigen 5 [Homo sapiens] |

| Filename XCorr DeltCN Conf% ObsM+H+ CalcM+H+ SpR ZScore Ion% # Sequence  | | | | | | | | | | | | |
| --- | --- | --- | --- | --- | --- | --- | --- | --- | --- | --- | --- | --- |
| \* | Astrin\_STLCHLD\_tube2\_061314\_01.09504.09504.2 | 4.3024 | 0.3726 | 100.0% | 1653.4321 | 1653.8445 | 1 | 9.01 | 71.4% | 3 | K.TSEEAVDPLGNYMVK.T | 2 |
| \* | Astrin\_STLCHLD\_tube2\_061314\_01.12601.12601.2 | 3.2119 | 0.4855 | 100.0% | 2322.672 | 2323.6262 | 1 | 8.442 | 55.3% | 1 | K.TIVLVPS\*PLGQQQDMIFEAR.L | 2 |
| \* | Astrin\_STLCHLD\_061214\_01.07765.07765.2 | 4.7973 | 0.482 | 100.0% | 1833.5721 | 1833.0668 | 1 | 8.628 | 68.8% | 8 | R.LDTMAETNSISLNGPLR.T | 2 |
| \* | Astrin\_STLCHLD\_tube2\_061314\_01.10111.10111.3 | 4.093 | 0.3906 | 100.0% | 2533.7043 | 2532.8286 | 1 | 7.267 | 33.0% | 5 | R.LDTMAETNSISLNGPLRTDDLVR.E | 3 |
| \* | Astrin\_STLCHLD\_tube2\_061314\_01.14202.14202.3 | 5.7038 | 0.222 | 100.0% | 3858.5645 | 3858.2102 | 1 | 6.763 | 32.4% | 7 | R.TEAVREDLVPSESNAFLPSSVLWLS\*PSTALAADFR.V | 3 |
| \* | Astrin\_STLCHLD\_061214\_01.05923.05923.3 | 5.1903 | 0.3857 | 100.0% | 2220.5645 | 2220.3752 | 1 | 6.272 | 40.3% | 12 | R.VNHVDPEEEIVEHGAMEER.E | 3 |
| \* | Astrin\_STLCHLD\_061214\_01.05943.05943.2 | 5.7916 | 0.5344 | 100.0% | 2220.8323 | 2220.3752 | 1 | 8.765 | 75.0% | 1 | R.VNHVDPEEEIVEHGAMEER.E | 2 |
| \* | Astrin\_STLCHLD\_tube2\_061314\_01.14687.14687.2 | 5.7115 | 0.5692 | 100.0% | 2063.9521 | 2064.3606 | 1 | 9.506 | 67.6% | 1 | R.ILGSDTESWMSPLAWLEK.G | 2 |
| \* | Astrin\_STLCHLD\_tube2\_061314\_01.15257.15257.2 | 4.7102 | 0.458 | 100.0% | 2143.7722 | 2144.3606 | 1 | 7.499 | 61.8% | 2 | R.ILGSDTESWMS\*PLAWLEK.G | 2 |
| \* | Astrin\_STLCHLD\_tube2\_061314\_01.09263.09263.2 | 3.4886 | 0.3311 | 100.0% | 1334.6721 | 1333.5457 | 1 | 6.338 | 81.8% | 3 | K.GVNTSVMLENLR.Q | 2 |
| \* | Astrin\_STLCHLD\_tube2\_061314\_01.16194.16194.2 | 7.3179 | 0.6078 | 100.0% | 2166.0322 | 2166.4795 | 1 | 11.538 | 72.2% | 17 | R.HDLEDNLLSSLVILEVLSR.Q | 2 |
| \* | Astrin\_STLCHLD\_061214\_01.14881.14881.3 | 3.6229 | 0.3024 | 99.7% | 2166.3843 | 2166.4795 | 7 | 5.81 | 33.3% | 5 | R.HDLEDNLLSSLVILEVLSR.Q | 3 |
| \* | Astrin\_STLCHLD\_061214\_01.04639.04639.3 | 4.4923 | 0.421 | 100.0% | 2867.5745 | 2868.0 | 4 | 6.548 | 30.8% | 1 | K.SQLAVPHPETQDSSTQTDTSHSGITNK.L | 3 |
| \* | Astrin\_STLCHLD\_tube2\_061314\_01.11369.11369.2 | 4.1386 | 0.4382 | 100.0% | 1305.5922 | 1305.578 | 1 | 7.437 | 80.0% | 4 | R.NVMQSWVLISK.E | 2 |
| \* | Astrin\_STLCHLD\_061214\_01.10670.10670.3 | 5.5017 | 0.3661 | 100.0% | 2892.2644 | 2892.2793 | 3 | 6.989 | 26.0% | 5 | K.ELISLLHLSLLHLEEDKTTVSQESR.R | 3 |
| \* | Astrin\_STLCHLD\_tube2\_061314\_01.10199.10199.2 | 3.7551 | 0.3442 | 100.0% | 1391.4321 | 1391.5823 | 1 | 7.872 | 68.2% | 4 | R.ISQLEQDLASMR.E | 2 |
| \* | Astrin\_STLCHLD\_tube2\_061314\_01.07865.07865.3 | 3.0616 | 0.2454 | 97.8% | 1692.2644 | 1692.9994 | 7 | 4.879 | 33.3% | 1 | R.GLLKDAQTQLVGLHAK.Q | 3 |
| \* | Astrin\_STLCHLD\_061214\_01.11054.11054.2 | 4.6365 | 0.5634 | 100.0% | 2390.0923 | 2390.612 | 1 | 9.702 | 50.0% | 4 | K.QEELVQQTVSLTSTLQQDWR.S | 2 |
| \* | Astrin\_STLCHLD\_061214\_02.09274.09274.3 | 3.9313 | 0.4671 | 100.0% | 2390.5745 | 2390.612 | 1 | 8.286 | 34.2% | 2 | K.QEELVQQTVSLTSTLQQDWR.S | 3 |
| \* | Astrin\_STLCHLD\_061214\_02.09740.09740.2 | 5.4525 | 0.4219 | 100.0% | 1786.7922 | 1787.0405 | 1 | 8.603 | 75.0% | 3 | R.SMQLDYTTWTALLSR.S | 2 |
| \* | Astrin\_STLCHLD\_tube2\_061314\_01.01591.01591.2 | 2.7269 | 0.1876 | 98.1% | 959.77216 | 960.0348 | 30 | 5.025 | 71.4% | 2 | K.SQQALQER.D | 2 |
| \* | Astrin\_STLCHLD\_061214\_01.04694.04694.3 | 5.0527 | 0.348 | 100.0% | 2344.4944 | 2344.5437 | 1 | 7.467 | 39.5% | 3 | K.SQQALQERDVAIEEKQEVSR.V | 3 |
| \* | Astrin\_STLCHLD\_tube2\_061314\_01.04021.04021.2 | 3.989 | 0.4907 | 100.0% | 1403.2522 | 1403.5321 | 1 | 8.1 | 68.2% | 6 | R.DVAIEEKQEVSR.V | 2 |
| \* | Astrin\_STLCHLD\_061214\_01.04236.04236.3 | 3.1118 | 0.4172 | 100.0% | 1404.1144 | 1403.5321 | 2 | 6.534 | 43.2% | 3 | R.DVAIEEKQEVSR.V | 3 |
| \* | Astrin\_STLCHLD\_061214\_01.09264.09264.2 | 5.4365 | 0.406 | 100.0% | 1573.6122 | 1573.848 | 1 | 7.65 | 76.9% | 21 | R.AQLQILANMDSQLK.E | 2 |
| \* | Astrin\_STLCHLD\_061214\_01.09289.09289.3 | 4.654 | 0.3824 | 100.0% | 1574.0944 | 1573.848 | 1 | 7.61 | 50.0% | 2 | R.AQLQILANMDSQLK.E | 3 |
| \* | Astrin\_STLCHLD\_061214\_01.04854.04854.2 | 5.4427 | 0.5601 | 100.0% | 1723.3922 | 1723.9879 | 1 | 9.809 | 82.1% | 11 | K.HMQAELQQQQAVLAK.E | 2 |
| \* | Astrin\_STLCHLD\_tube2\_061314\_02.04880.04880.3 | 4.9398 | 0.2297 | 100.0% | 1724.3944 | 1723.9879 | 1 | 5.673 | 55.4% | 12 | K.HMQAELQQQQAVLAK.E | 3 |
| \* | Astrin\_STLCHLD\_tube2\_061314\_02.05648.05648.3 | 4.7353 | 0.3687 | 100.0% | 2107.8843 | 2108.4233 | 1 | 7.221 | 41.2% | 2 | K.HMQAELQQQQAVLAKEVR.D | 3 |
| \* | Astrin\_STLCHLD\_tube2\_061314\_01.04601.04601.2 | 4.9208 | 0.4286 | 100.0% | 1676.4122 | 1675.7899 | 1 | 7.593 | 67.9% | 7 | K.LASTIADNQEQDLEK.T | 2 |
| \* | Astrin\_STLCHLD\_tube2\_061314\_01.04629.04629.2 | 4.0404 | 0.4612 | 100.0% | 1932.7922 | 1933.0825 | 1 | 7.998 | 56.2% | 2 | K.LASTIADNQEQDLEKTR.Q | 2 |
| \* | Astrin\_STLCHLD\_tube2\_061314\_02.13965.13965.3 | 4.0815 | 0.2186 | 98.7% | 2762.2744 | 2762.1326 | 1 | 5.579 | 29.5% | 1 | R.QYS\*QKLGLLTEQLQSLTLFLQTK.L | 3 |
| \* | Astrin\_STLCHLD\_tube2\_061314\_01.15383.15383.2 | 5.7496 | 0.4816 | 100.0% | 2046.9521 | 2047.443 | 1 | 9.693 | 64.7% | 26 | K.LGLLTEQLQSLTLFLQTK.L | 2 |
| \* | Astrin\_STLCHLD\_tube2\_061314\_01.15384.15384.3 | 5.5133 | 0.4316 | 100.0% | 2048.3044 | 2047.443 | 1 | 7.514 | 51.5% | 9 | K.LGLLTEQLQSLTLFLQTK.L | 3 |
| \* | Astrin\_STLCHLD\_tube2\_061314\_01.15006.15006.2 | 5.4069 | 0.4954 | 100.0% | 2788.152 | 2788.121 | 1 | 9.101 | 42.3% | 7 | R.TFLGSILTAVADEEPESTPVPLLGSDK.S | 2 |
| \* | Astrin\_STLCHLD\_tube2\_061314\_01.15035.15035.3 | 3.8684 | 0.2767 | 99.6% | 2788.6443 | 2788.121 | 1 | 5.031 | 28.8% | 2 | R.TFLGSILTAVADEEPESTPVPLLGSDK.S | 3 |
| \* | Astrin\_STLCHLD\_tube2\_061314\_01.15537.15537.2 | 4.841 | 0.5327 | 100.0% | 2868.4521 | 2868.121 | 1 | 8.704 | 44.2% | 2 | R.TFLGSILTAVADEEPESTPVPLLGS\*DK.S | 2 |
| \* | Astrin\_STLCHLD\_tube2\_061314\_01.14916.14916.3 | 5.2701 | 0.325 | 100.0% | 3430.3743 | 3430.7473 | 3 | 6.639 | 26.6% | 2 | R.TFLGSILTAVADEEPESTPVPLLGS\*DKSAFTR.V | 3 |
| \* | Astrin\_STLCHLD\_061214\_01.13669.13669.3 | 4.9469 | 0.4379 | 100.0% | 3430.8245 | 3430.7473 | 2 | 7.375 | 30.6% | 2 | R.TFLGSILTAVADEEPESTPVPLLGSDKS\*AFTR.V | 3 |
| \* | Astrin\_STLCHLD\_061214\_01.03957.03957.2 | 3.4428 | 0.2612 | 99.5% | 1595.7322 | 1595.7092 | 1 | 5.772 | 66.7% | 1 | R.LQAQEEQHQEVQK.A | 2 |
| \* | Astrin\_STLCHLD\_tube2\_061314\_01.04623.04623.2 | 5.2543 | 0.3509 | 100.0% | 2149.5723 | 2149.3652 | 1 | 6.436 | 62.5% | 1 | R.YKNEKELQEVIQQQNEK.I | 2 |
| \* | Astrin\_STLCHLD\_tube2\_061314\_01.04673.04673.3 | 6.0293 | 0.2501 | 100.0% | 2149.5842 | 2149.3652 | 2 | 6.08 | 46.9% | 2 | R.YKNEKELQEVIQQQNEK.I | 3 |
| \* | Astrin\_STLCHLD\_tube2\_061314\_01.09344.09344.2 | 4.2731 | 0.4963 | 100.0% | 1714.9321 | 1715.0 | 1 | 8.609 | 67.9% | 1 | K.ILEQIDKSGELISLR.E | 2 |
| \* | Astrin\_STLCHLD\_tube2\_061314\_02.07263.07263.3 | 4.1239 | 0.361 | 100.0% | 1715.2444 | 1715.0 | 14 | 7.42 | 39.3% | 2 | K.ILEQIDKSGELISLR.E | 3 |
| \* | Astrin\_STLCHLD\_061214\_01.09170.09170.3 | 4.5161 | 0.2736 | 99.8% | 2680.8542 | 2681.0618 | 1 | 6.197 | 38.6% | 3 | K.ILEQIDKSGELISLREEVTHLTR.S | 3 |
| \* | Astrin\_STLCHLD\_tube2\_061314\_01.06269.06269.2 | 3.1318 | 0.1818 | 99.3% | 875.0522 | 875.0128 | 36 | 4.94 | 78.6% | 2 | K.SGELISLR.E | 2 |
| \* | Astrin\_STLCHLD\_tube2\_061314\_01.09731.09731.2 | 3.745 | 0.3836 | 100.0% | 1840.8322 | 1841.0745 | 1 | 6.031 | 60.0% | 4 | K.SGELISLREEVTHLTR.S | 2 |
| \* | Astrin\_STLCHLD\_tube2\_061314\_02.07526.07526.3 | 5.2215 | 0.3986 | 100.0% | 1841.5743 | 1841.0745 | 1 | 7.207 | 53.3% | 13 | K.SGELISLREEVTHLTR.S | 3 |
| \* | Astrin\_STLCHLD\_tube2\_061314\_01.06395.06395.2 | 2.715 | 0.1992 | 98.0% | 1104.0521 | 1104.248 | 7 | 5.083 | 75.0% | 3 | K.VWLSQEVDK.L | 2 |
| \* | Astrin\_STLCHLD\_tube2\_061314\_01.09041.09041.2 | 3.6669 | 0.3763 | 100.0% | 1373.5322 | 1373.595 | 1 | 6.866 | 85.0% | 2 | K.VWLSQEVDKLR.V | 2 |
| \* | Astrin\_STLCHLD\_tube2\_061314\_01.09753.09753.1 | 1.9447 | 0.1317 | 95.3% | 897.67 | 898.16644 | 2 | 4.771 | 75.0% | 1 | R.VMFLEMK.N | 1 |
| \* | Astrin\_STLCHLD\_tube2\_061314\_01.09859.09859.2 | 2.695 | 0.3196 | 100.0% | 898.33215 | 898.16644 | 1 | 7.3 | 91.7% | 2 | R.VMFLEMK.N | 2 |
| \* | Astrin\_STLCHLD\_tube2\_061314\_01.06813.06813.2 | 2.9476 | 0.376 | 100.0% | 1270.5322 | 1269.5598 | 1 | 6.797 | 83.3% | 2 | R.VMFLEMKNEK.E | 2 |
| \* | Astrin\_STLCHLD\_tube2\_061314\_01.06421.06421.1 | 2.182 | 0.2349 | 100.0% | 1000.65 | 1001.1277 | 4 | 4.226 | 71.4% | 3 | R.NILEENLR.R | 1 |
| \* | Astrin\_STLCHLD\_tube2\_061314\_01.09882.09882.3 | 5.2858 | 0.4833 | 100.0% | 2230.8542 | 2230.526 | 1 | 8.777 | 41.2% | 2 | R.RSDKELEKLDDIVQHIYK.T | 3 |
| \* | Astrin\_STLCHLD\_tube2\_061314\_01.10770.10770.3 | 4.8457 | 0.3125 | 100.0% | 2074.2544 | 2074.3384 | 1 | 7.446 | 43.8% | 2 | R.SDKELEKLDDIVQHIYK.T | 3 |
| \* | Astrin\_STLCHLD\_tube2\_061314\_01.06674.06674.2 | 3.0322 | 0.3727 | 100.0% | 1243.8322 | 1244.4331 | 4 | 6.625 | 66.7% | 1 | K.LDDIVQHIYK.T | 2 |
| \* | Astrin\_STLCHLD\_tube2\_061314\_01.10566.10566.1 | 1.4618 | 0.2249 | 95.2% | 1126.78 | 1127.3696 | 405 | 4.382 | 44.4% | 1 | K.TLLSIPEVVR.G | 1 |
| \* | Astrin\_STLCHLD\_tube2\_061314\_01.10589.10589.2 | 2.8975 | 0.4307 | 100.0% | 1127.5521 | 1127.3696 | 3 | 6.64 | 83.3% | 2 | K.TLLSIPEVVR.G | 2 |
| \* | Astrin\_STLCHLD\_tube2\_061314\_01.15056.15056.1 | 1.8849 | 0.365 | 100.0% | 1148.89 | 1149.3293 | 6 | 6.226 | 50.0% | 4 | K.ELQGLLEFLS.- | 1 |

---

|  |  |  |  |  |  |  |  |  |
| --- | --- | --- | --- | --- | --- | --- | --- | --- |
| U | *gi|4505813|ref|NP\_003* | 2 | 4 | 43.8% | 89 | 10366 | 7.4 | dynein light chain 1 [Homo sapiens] |
| U | *gi|83267868|ref|NP\_00* | 2 | 4 | 43.8% | 89 | 10366 | 7.4 | dynein light chain 1 [Homo sapiens] |
| U | *gi|83267866|ref|NP\_00* | 2 | 4 | 43.8% | 89 | 10366 | 7.4 | dynein light chain 1 [Homo sapiens] |

| Filename XCorr DeltCN Conf% ObsM+H+ CalcM+H+ SpR ZScore Ion% # Sequence  | | | | | | | | | | | | |
| --- | --- | --- | --- | --- | --- | --- | --- | --- | --- | --- | --- | --- |
|  | Astrin\_STLCHLD\_tube2\_061314\_01.07322.07322.3 | 3.1616 | 0.3401 | 100.0% | 1417.3143 | 1415.6322 | 10 | 5.803 | 45.5% | 2 | K.YNIEKDIAAHIK.K | 3 |
|  | Astrin\_STLCHLD\_061214\_02.12477.12477.3 | 3.4984 | 0.203 | 96.2% | 3239.1243 | 3237.771 | 1 | 4.322 | 26.0% | 2 | R.NFGSYVTHETKHFIYFYLGQVAILLFK.S | 3 |

---

|  |  |  |  |  |  |  |  |  |
| --- | --- | --- | --- | --- | --- | --- | --- | --- |
| U | *gi|29788768|ref|NP\_82* | 23 | 107 | 41.6% | 445 | 49953 | 4.9 | tubulin, beta 2B [Homo sapiens] |
| U | *gi|4507729|ref|NP\_001* | 23 | 107 | 41.6% | 445 | 49907 | 4.9 | tubulin, beta 2 [Homo sapiens] |

| Filename XCorr DeltCN Conf% ObsM+H+ CalcM+H+ SpR ZScore Ion% # Sequence  | | | | | | | | | | | | |
| --- | --- | --- | --- | --- | --- | --- | --- | --- | --- | --- | --- | --- |
|  | Astrin\_STLCHLD\_tube2\_061314\_01.10607.10607.2 | 4.4949 | 0.5136 | 100.0% | 1616.5521 | 1616.8701 | 1 | 8.677 | 71.4% | 9 | R.AILVDLEPGTMDSVR.S | 222 |
|  | Astrin\_STLCHLD\_tube2\_061314\_01.12390.12390.2 | 5.5984 | 0.5487 | 100.0% | 2798.9321 | 2800.0647 | 1 | 8.841 | 42.0% | 2 | R.SGPFGQIFRPDNFVFGQSGAGNNWAK.G | 222 |
|  | Astrin\_STLCHLD\_061214\_01.10677.10677.3 | 6.1862 | 0.4832 | 100.0% | 2800.4043 | 2800.0647 | 1 | 7.629 | 40.0% | 7 | R.SGPFGQIFRPDNFVFGQSGAGNNWAK.G | 333 |
|  | Astrin\_STLCHLD\_061214\_01.11227.11227.2 | 6.4727 | 0.5531 | 100.0% | 1959.4521 | 1960.151 | 1 | 10.56 | 73.5% | 4 | K.GHYTEGAELVDSVLDVVR.K | 2222 |
|  | Astrin\_STLCHLD\_061214\_01.11221.11221.3 | 4.0137 | 0.3325 | 100.0% | 1961.2144 | 1960.151 | 1 | 6.21 | 44.1% | 5 | K.GHYTEGAELVDSVLDVVR.K | 3333 |
|  | Astrin\_STLCHLD\_tube2\_061314\_01.12007.12007.2 | 5.7392 | 0.5731 | 100.0% | 2087.912 | 2088.325 | 1 | 10.188 | 69.4% | 1 | K.GHYTEGAELVDSVLDVVRK.E | 2222 |
|  | Astrin\_STLCHLD\_tube2\_061314\_01.11982.11982.3 | 4.7827 | 0.4838 | 100.0% | 2088.1143 | 2088.325 | 1 | 8.148 | 44.4% | 7 | K.GHYTEGAELVDSVLDVVRK.E | 3333 |
|  | Astrin\_STLCHLD\_061214\_01.09505.09505.3 | 5.6867 | 0.3777 | 100.0% | 3327.7744 | 3329.5925 | 1 | 7.024 | 29.8% | 1 | K.ESESCDCLQGFQLTHSLGGGTGSGMGTLLISK.I | 3 |
|  | Astrin\_STLCHLD\_tube2\_061314\_01.07361.07361.2 | 3.2733 | 0.2391 | 99.5% | 1132.2322 | 1131.2767 | 1 | 5.073 | 83.3% | 14 | R.FPGQLNADLR.K | 22222 |
|  | Astrin\_STLCHLD\_tube2\_061314\_01.04904.04904.2 | 2.5627 | 0.1707 | 95.5% | 1259.2922 | 1259.4508 | 1 | 4.474 | 70.0% | 2 | R.FPGQLNADLRK.L | 22222 |
|  | Astrin\_STLCHLD\_tube2\_061314\_01.04842.04842.3 | 2.8966 | 0.3686 | 100.0% | 1260.2043 | 1259.4508 | 14 | 5.742 | 42.5% | 5 | R.FPGQLNADLRK.L | 33333 |
|  | Astrin\_STLCHLD\_tube2\_061314\_01.08730.08730.2 | 3.8464 | 0.3753 | 100.0% | 1272.4122 | 1272.5945 | 1 | 7.898 | 75.0% | 4 | R.KLAVNMVPFPR.L | 22222 |
|  | Astrin\_STLCHLD\_tube2\_061314\_01.10148.10148.1 | 2.1143 | 0.2239 | 96.6% | 1143.61 | 1144.4204 | 18 | 6.431 | 61.1% | 1 | K.LAVNMVPFPR.L | 11111 |
|  | Astrin\_STLCHLD\_tube2\_061314\_01.10247.10247.2 | 3.6473 | 0.5079 | 100.0% | 1145.2722 | 1144.4204 | 1 | 8.426 | 94.4% | 4 | K.LAVNMVPFPR.L | 22222 |
|  | Astrin\_STLCHLD\_tube2\_061314\_01.12143.12143.2 | 3.7666 | 0.4528 | 100.0% | 1621.8322 | 1621.9403 | 1 | 8.756 | 76.9% | 2 | R.LHFFMPGFAPLTSR.G | 2222 |
|  | Astrin\_STLCHLD\_tube2\_061314\_01.12204.12204.3 | 4.3867 | 0.3891 | 100.0% | 1622.3944 | 1621.9403 | 1 | 6.411 | 53.8% | 2 | R.LHFFMPGFAPLTSR.G | 3333 |
|  | Astrin\_STLCHLD\_061214\_01.06900.06900.2 | 5.2617 | 0.4991 | 100.0% | 1924.5122 | 1925.2405 | 1 | 9.197 | 60.0% | 2 | R.MSMKEVDEQMLNVQNK.N | 222 |
|  | Astrin\_STLCHLD\_tube2\_061314\_02.06675.06675.3 | 4.9405 | 0.1867 | 99.7% | 1926.6843 | 1925.2405 | 1 | 5.512 | 51.7% | 7 | R.MSMKEVDEQMLNVQNK.N | 333 |
|  | Astrin\_STLCHLD\_tube2\_061314\_01.05125.05125.2 | 4.2318 | 0.1226 | 99.5% | 1449.2122 | 1447.6031 | 1 | 4.856 | 72.7% | 3 | K.EVDEQMLNVQNK.N | 222 |
|  | Astrin\_STLCHLD\_tube2\_061314\_01.11781.11781.2 | 4.295 | 0.2978 | 100.0% | 1698.5322 | 1697.8877 | 1 | 7.708 | 73.1% | 2 | K.NSSYFVEWIPNNVK.T | 22222 |
|  | Astrin\_STLCHLD\_tube2\_061314\_02.09608.09608.2 | 3.6979 | 0.0138 | 95.3% | 1859.8522 | 1859.1475 | 1 | 5.87 | 50.0% | 1 | K.MSATFIGNSTAIQELFK.R | 22 |
|  | Astrin\_STLCHLD\_061214\_01.07812.07812.2 | 3.8437 | 0.3944 | 100.0% | 1386.3522 | 1386.6116 | 1 | 7.545 | 80.0% | 6 | K.RISEQFTAMFR.R | 2222 |
|  | Astrin\_STLCHLD\_tube2\_061314\_02.07946.07946.2 | 3.9325 | 0.4891 | 100.0% | 1229.9321 | 1230.4241 | 1 | 8.487 | 94.4% | 16 | R.ISEQFTAMFR.R | 2222 |

Similarities:
gi|29788785|ref|NP\_82(21:2)  
gi|5174735|ref|NP\_006(21:2)  
gi|50592996|ref|NP\_00(14:9)  
gi|14210536|ref|NP\_11(9:14)  

---

|  |  |  |  |  |  |  |  |  |
| --- | --- | --- | --- | --- | --- | --- | --- | --- |
| U | *gi|4826898|ref|NP\_005* | 4 | 7 | 41.4% | 140 | 15054 | 8.3 | profilin 1 [Homo sapiens] |

| Filename XCorr DeltCN Conf% ObsM+H+ CalcM+H+ SpR ZScore Ion% # Sequence  | | | | | | | | | | | | |
| --- | --- | --- | --- | --- | --- | --- | --- | --- | --- | --- | --- | --- |
| \* | Astrin\_STLCHLD\_tube2\_061314\_01.12103.12103.2 | 2.9266 | 0.3364 | 99.0% | 1644.7522 | 1644.9518 | 48 | 6.554 | 40.0% | 1 | K.TFVNITPAEVGVLVGK.D | 2 |
| \* | Astrin\_STLCHLD\_061214\_01.07917.07917.2 | 3.5931 | 0.4581 | 100.0% | 1471.5322 | 1471.6531 | 1 | 7.543 | 61.5% | 3 | R.SSFYVNGLTLGGQK.C | 2 |
| \* | Astrin\_STLCHLD\_tube2\_061314\_02.08597.08597.2 | 4.3052 | 0.4348 | 100.0% | 1628.0521 | 1626.7784 | 1 | 6.972 | 69.2% | 2 | R.DSLLQDGEFSMDLR.T | 2 |
| \* | Astrin\_STLCHLD\_tube2\_061314\_01.05958.05958.2 | 3.452 | 0.4581 | 100.0% | 1379.9922 | 1380.5406 | 1 | 7.537 | 69.2% | 1 | K.STGGAPTFNVTVTK.T | 2 |

---

|  |  |  |  |  |  |  |  |  |
| --- | --- | --- | --- | --- | --- | --- | --- | --- |
| U | *gi|4503529|ref|NP\_001* | 10 | 12 | 39.7% | 406 | 46154 | 5.5 | eukaryotic translation initiation factor 4A isoform 1 [Homo sapiens] |

| Filename XCorr DeltCN Conf% ObsM+H+ CalcM+H+ SpR ZScore Ion% # Sequence  | | | | | | | | | | | | |
| --- | --- | --- | --- | --- | --- | --- | --- | --- | --- | --- | --- | --- |
| \* | Astrin\_STLCHLD\_tube2\_061314\_01.15570.15570.3 | 4.6101 | 0.2142 | 99.4% | 4169.994 | 4169.451 | 1 | 5.649 | 22.9% | 2 | R.SRDNGPDGMEPEGVIESNWNEIVDSFDDMNLSESLLR.G | 3 |
|  | Astrin\_STLCHLD\_tube2\_061314\_01.07218.07218.2 | 4.8055 | 0.5196 | 100.0% | 1828.6721 | 1829.0654 | 1 | 8.339 | 70.0% | 1 | R.GIYAYGFEKPSAIQQR.A | 22 |
|  | Astrin\_STLCHLD\_tube2\_061314\_01.07230.07230.3 | 3.6443 | 0.3709 | 100.0% | 1829.9944 | 1829.0654 | 1 | 6.418 | 45.0% | 2 | R.GIYAYGFEKPSAIQQR.A | 33 |
|  | Astrin\_STLCHLD\_tube2\_061314\_01.04893.04893.2 | 4.1828 | 0.4804 | 100.0% | 1395.4122 | 1395.512 | 1 | 9.677 | 76.9% | 1 | K.GYDVIAQAQSGTGK.T | 2 |
| \* | Astrin\_STLCHLD\_tube2\_061314\_01.07853.07853.3 | 3.59 | 0.3972 | 100.0% | 1620.5044 | 1619.9225 | 1 | 6.559 | 48.2% | 1 | K.LQMEAPHIIVGTPGR.V | 3 |
|  | Astrin\_STLCHLD\_tube2\_061314\_01.12486.12486.2 | 4.2582 | 0.5279 | 100.0% | 1556.3121 | 1556.789 | 1 | 9.885 | 79.2% | 1 | K.MFVLDEADEMLSR.G | 2 |
| \* | Astrin\_STLCHLD\_tube2\_061314\_01.10863.10863.2 | 3.1382 | 0.1712 | 98.0% | 1503.4722 | 1502.71 | 6 | 4.56 | 59.1% | 1 | R.GFKDQIYDIFQK.L | 2 |
|  | Astrin\_STLCHLD\_tube2\_061314\_01.05057.05057.3 | 2.4889 | 0.284 | 96.8% | 1867.4343 | 1868.0461 | 1 | 4.89 | 40.0% | 1 | R.DFTVSAMHGDMDQKER.D | 3 |
| \* | Astrin\_STLCHLD\_061214\_01.04776.04776.3 | 3.1474 | 0.244 | 98.6% | 1591.6144 | 1590.8352 | 34 | 5.292 | 38.5% | 1 | R.KGVAINMVTEEDKR.T | 3 |
| \* | Astrin\_STLCHLD\_tube2\_061314\_01.15633.15633.2 | 3.9342 | 0.477 | 100.0% | 2799.132 | 2799.1653 | 1 | 8.452 | 47.8% | 1 | R.TLRDIETFYNTSIEEMPLNVADLI.- | 2 |

Similarities:
gi|7661920|ref|NP\_055(2:8)  

---

|  |  |  |  |  |  |  |  |  |
| --- | --- | --- | --- | --- | --- | --- | --- | --- |
| U | *gi|11415030|ref|NP\_06* | 5 | 22 | 37.9% | 103 | 11367 | 11.4 | histone cluster 1, H4j [Homo sapiens] |
| U | *gi|77539758|ref|NP\_00* | 5 | 22 | 37.9% | 103 | 11367 | 11.4 | histone cluster 2, H4b [Homo sapiens] |
| U | *gi|4504323|ref|NP\_003* | 5 | 22 | 37.9% | 103 | 11367 | 11.4 | histone cluster 2, H4a [Homo sapiens] |
| U | *gi|4504321|ref|NP\_003* | 5 | 22 | 37.9% | 103 | 11367 | 11.4 | histone cluster 1, H4i [Homo sapiens] |
| U | *gi|4504317|ref|NP\_003* | 5 | 22 | 37.9% | 103 | 11367 | 11.4 | histone cluster 1, H4l [Homo sapiens] |
| U | *gi|4504315|ref|NP\_003* | 5 | 22 | 37.9% | 103 | 11367 | 11.4 | histone cluster 1, H4e [Homo sapiens] |
| U | *gi|4504313|ref|NP\_003* | 5 | 22 | 37.9% | 103 | 11367 | 11.4 | histone cluster 1, H4b [Homo sapiens] |
| U | *gi|4504311|ref|NP\_003* | 5 | 22 | 37.9% | 103 | 11367 | 11.4 | histone cluster 1, H4h [Homo sapiens] |
| U | *gi|4504309|ref|NP\_003* | 5 | 22 | 37.9% | 103 | 11367 | 11.4 | histone cluster 1, H4c [Homo sapiens] |
| U | *gi|4504307|ref|NP\_003* | 5 | 22 | 37.9% | 103 | 11367 | 11.4 | histone cluster 1, H4k [Homo sapiens] |
| U | *gi|4504305|ref|NP\_003* | 5 | 22 | 37.9% | 103 | 11367 | 11.4 | histone cluster 1, H4f [Homo sapiens] |
| U | *gi|4504303|ref|NP\_003* | 5 | 22 | 37.9% | 103 | 11367 | 11.4 | histone cluster 1, H4d [Homo sapiens] |
| U | *gi|4504301|ref|NP\_003* | 5 | 22 | 37.9% | 103 | 11367 | 11.4 | histone cluster 1, H4a [Homo sapiens] |
| U | *gi|28173560|ref|NP\_77* | 5 | 22 | 37.9% | 103 | 11367 | 11.4 | histone cluster 4, H4 [Homo sapiens] |

| Filename XCorr DeltCN Conf% ObsM+H+ CalcM+H+ SpR ZScore Ion% # Sequence  | | | | | | | | | | | | |
| --- | --- | --- | --- | --- | --- | --- | --- | --- | --- | --- | --- | --- |
|  | Astrin\_STLCHLD\_tube2\_061314\_01.05330.05330.2 | 2.5801 | 0.1694 | 95.6% | 1337.8121 | 1337.5187 | 16 | 4.019 | 60.0% | 2 | K.RISGLIYEETR.G | 2 |
|  | Astrin\_STLCHLD\_tube2\_061314\_01.06653.06653.2 | 3.8506 | 0.3671 | 100.0% | 1181.3522 | 1181.3312 | 1 | 7.056 | 88.9% | 13 | R.ISGLIYEETR.G | 2 |
|  | Astrin\_STLCHLD\_tube2\_061314\_01.09551.09551.2 | 2.9102 | 0.1751 | 98.5% | 990.2522 | 990.19055 | 3 | 5.549 | 85.7% | 3 | K.VFLENVIR.D | 2 |
|  | Astrin\_STLCHLD\_061214\_01.08869.08869.2 | 3.1841 | 0.3866 | 100.0% | 1468.2322 | 1467.7667 | 1 | 6.593 | 66.7% | 2 | K.TVTAMDVVYALKR.Q | 2 |
|  | Astrin\_STLCHLD\_tube2\_061314\_01.09101.09101.1 | 1.8041 | 0.4889 | 100.0% | 714.44 | 714.796 | 1 | 8.303 | 75.0% | 2 | R.TLYGFGG.- | 1 |

---

|  |  |  |  |  |  |  |  |  |
| --- | --- | --- | --- | --- | --- | --- | --- | --- |
| U | *gi|4501885|ref|NP\_001* | 12 | 35 | 37.3% | 375 | 41737 | 5.5 | beta actin [Homo sapiens] |
| U | *gi|4501887|ref|NP\_001* | 12 | 35 | 37.3% | 375 | 41793 | 5.5 | actin, gamma 1 propeptide [Homo sapiens] |

| Filename XCorr DeltCN Conf% ObsM+H+ CalcM+H+ SpR ZScore Ion% # Sequence  | | | | | | | | | | | | |
| --- | --- | --- | --- | --- | --- | --- | --- | --- | --- | --- | --- | --- |
|  | Astrin\_STLCHLD\_061214\_01.05682.05682.2 | 2.7849 | 0.3968 | 100.0% | 1198.8322 | 1199.4415 | 2 | 6.403 | 70.0% | 3 | R.AVFPSIVGRPR.H | 22 |
|  | Astrin\_STLCHLD\_tube2\_061314\_01.05606.05606.2 | 2.8797 | 0.4114 | 100.0% | 1516.4521 | 1516.7019 | 2 | 6.49 | 65.0% | 2 | K.IWHHTFYNELR.V | 22 |
|  | Astrin\_STLCHLD\_tube2\_061314\_01.05597.05597.3 | 3.1756 | 0.156 | 96.2% | 1517.1843 | 1516.7019 | 1 | 5.007 | 60.0% | 3 | K.IWHHTFYNELR.V | 33 |
|  | Astrin\_STLCHLD\_tube2\_061314\_01.07787.07787.2 | 3.9621 | 0.3487 | 100.0% | 1954.7322 | 1955.2615 | 1 | 7.712 | 52.9% | 1 | R.VAPEEHPVLLTEAPLNPK.A | 2 |
|  | Astrin\_STLCHLD\_tube2\_061314\_01.11063.11063.3 | 6.1516 | 0.5743 | 100.0% | 3184.3743 | 3185.622 | 1 | 10.383 | 36.2% | 3 | R.TTGIVMDSGDGVTHTVPIYEGYALPHAILR.L | 3 |
|  | Astrin\_STLCHLD\_tube2\_061314\_01.10415.10415.2 | 1.9955 | 0.3931 | 98.1% | 999.03217 | 999.167 | 15 | 6.315 | 64.3% | 1 | R.DLTDYLMK.I | 222 |
|  | Astrin\_STLCHLD\_061214\_02.04627.04627.2 | 2.9147 | 0.4452 | 100.0% | 1133.2122 | 1133.2029 | 1 | 7.733 | 66.7% | 4 | R.GYSFTTTAER.E | 2 |
|  | Astrin\_STLCHLD\_tube2\_061314\_01.10440.10440.2 | 4.5438 | 0.3456 | 100.0% | 1792.6322 | 1791.9554 | 1 | 7.499 | 73.3% | 5 | K.SYELPDGQVITIGNER.F | 222 |
|  | Astrin\_STLCHLD\_061214\_02.07237.07237.3 | 5.3896 | 0.5494 | 100.0% | 2344.6443 | 2344.6448 | 1 | 8.789 | 39.3% | 1 | R.KDLYANTVLSGGTTMYPGIADR.M | 3 |
|  | Astrin\_STLCHLD\_tube2\_061314\_02.08176.08176.2 | 5.1728 | 0.6552 | 100.0% | 2215.5522 | 2216.4705 | 1 | 11.775 | 50.0% | 5 | K.DLYANTVLSGGTTMYPGIADR.M | 2 |
|  | Astrin\_STLCHLD\_061214\_01.05115.05115.3 | 3.7961 | 0.3358 | 100.0% | 1550.2144 | 1549.8843 | 10 | 6.274 | 44.2% | 1 | R.MQKEITALAPSTMK.I | 33 |
|  | Astrin\_STLCHLD\_tube2\_061314\_01.05532.05532.2 | 2.5818 | 0.3762 | 99.3% | 1162.1122 | 1162.3868 | 1 | 6.988 | 75.0% | 6 | K.EITALAPSTMK.I | 22 |

Similarities:
gi|4501881|ref|NP\_001(7:5)  
gi|63055057|ref|NP\_00(2:10)  

---

|  |  |  |  |  |  |  |  |  |
| --- | --- | --- | --- | --- | --- | --- | --- | --- |
| U | *gi|7669492|ref|NP\_002* | 7 | 23 | 36.4% | 335 | 36053 | 8.5 | glyceraldehyde-3-phosphate dehydrogenase [Homo sapiens] |

| Filename XCorr DeltCN Conf% ObsM+H+ CalcM+H+ SpR ZScore Ion% # Sequence  | | | | | | | | | | | | |
| --- | --- | --- | --- | --- | --- | --- | --- | --- | --- | --- | --- | --- |
| \* | Astrin\_STLCHLD\_tube2\_061314\_01.11910.11910.2 | 3.0361 | 0.3744 | 100.0% | 1614.7522 | 1614.8851 | 1 | 7.657 | 53.8% | 2 | K.LVINGNPITIFQER.D | 2 |
| \* | Astrin\_STLCHLD\_061214\_02.09089.09089.2 | 5.7349 | 0.588 | 100.0% | 2278.0122 | 2278.495 | 1 | 11.544 | 52.5% | 1 | K.WGDAGAEYVVESTGVFTTMEK.A | 2 |
| \* | Astrin\_STLCHLD\_tube2\_061314\_01.09300.09300.3 | 4.3177 | 0.3947 | 100.0% | 2370.5645 | 2370.79 | 1 | 6.841 | 40.5% | 1 | K.RVIISAPSADAPMFVMGVNHEK.Y | 3 |
| \* | Astrin\_STLCHLD\_tube2\_061314\_01.13621.13621.3 | 5.9896 | 0.5314 | 100.0% | 2597.3342 | 2597.0044 | 1 | 9.431 | 39.1% | 4 | K.VIHDNFGIVEGLMTTVHAITATQK.T | 3 |
| \* | Astrin\_STLCHLD\_tube2\_061314\_01.07740.07740.2 | 4.1922 | 0.3998 | 100.0% | 1412.5521 | 1412.6292 | 1 | 6.263 | 75.0% | 3 | R.GALQNIIPASTGAAK.A | 2 |
|  | Astrin\_STLCHLD\_061214\_01.09372.09372.2 | 4.4152 | 0.533 | 100.0% | 1764.3322 | 1764.8914 | 1 | 9.6 | 65.4% | 6 | K.LISWYDNEFGYSNR.V | 2 |
| \* | Astrin\_STLCHLD\_061214\_01.06181.06181.2 | 3.6269 | 0.4169 | 100.0% | 1331.5322 | 1331.5879 | 1 | 7.39 | 68.2% | 6 | R.VVDLMAHMASKE.- | 2 |

---

|  |  |  |  |  |  |  |  |  |
| --- | --- | --- | --- | --- | --- | --- | --- | --- |
| U | *gi|21396489|ref|NP\_00* | 21 | 50 | 35.9% | 959 | 106489 | 6.4 | mitochondrial lon peptidase 1 [Homo sapiens] |

| Filename XCorr DeltCN Conf% ObsM+H+ CalcM+H+ SpR ZScore Ion% # Sequence  | | | | | | | | | | | | |
| --- | --- | --- | --- | --- | --- | --- | --- | --- | --- | --- | --- | --- |
| \* | Astrin\_STLCHLD\_tube2\_061314\_01.10677.10677.2 | 2.165 | 0.2479 | 95.2% | 1235.5122 | 1235.512 | 124 | 4.808 | 55.0% | 1 | R.LAQPYVGVFLK.R | 2 |
| \* | Astrin\_STLCHLD\_tube2\_061314\_01.11201.11201.2 | 2.9426 | 0.4083 | 100.0% | 1188.3722 | 1188.4124 | 1 | 7.391 | 88.9% | 1 | R.DIIALNPLYR.E | 2 |
| \* | Astrin\_STLCHLD\_tube2\_061314\_01.09941.09941.2 | 3.0027 | 0.2231 | 98.2% | 1378.4122 | 1378.6044 | 1 | 5.955 | 63.6% | 2 | R.ESVLQMMQAGQR.V | 2 |
| \* | Astrin\_STLCHLD\_tube2\_061314\_01.13903.13903.3 | 4.9912 | 0.4308 | 100.0% | 3670.3743 | 3671.0674 | 1 | 7.028 | 28.0% | 1 | R.VVDNPIYLSDMGAALTGAESHELQDVLEETNIPK.R | 3 |
| \* | Astrin\_STLCHLD\_tube2\_061314\_01.07548.07548.2 | 3.9763 | 0.5798 | 100.0% | 1400.5922 | 1401.5745 | 1 | 10.055 | 72.7% | 2 | K.HVMDVVDEELSK.L | 2 |
| \* | Astrin\_STLCHLD\_061214\_01.07105.07105.2 | 3.718 | 0.4193 | 100.0% | 1702.4122 | 1702.8644 | 2 | 7.542 | 57.1% | 2 | K.LGLLDNHSSEFNVTR.N | 2 |
| \* | Astrin\_STLCHLD\_tube2\_061314\_02.06794.06794.3 | 4.5923 | 0.417 | 100.0% | 1704.5044 | 1702.8644 | 1 | 6.48 | 48.2% | 5 | K.LGLLDNHSSEFNVTR.N | 3 |
| \* | Astrin\_STLCHLD\_tube2\_061314\_01.14587.14587.2 | 4.003 | 0.4283 | 100.0% | 1594.2922 | 1593.8223 | 1 | 7.128 | 70.8% | 2 | R.NYLDWLTSIPWGK.Y | 2 |
| \* | Astrin\_STLCHLD\_061214\_02.05627.05627.2 | 3.9246 | 0.4418 | 100.0% | 1834.2322 | 1834.9954 | 1 | 8.452 | 60.0% | 1 | R.AQAVLEEDHYGMEDVK.K | 2 |
| \* | Astrin\_STLCHLD\_tube2\_061314\_02.08844.08844.2 | 3.3871 | 0.2801 | 100.0% | 1289.5721 | 1289.5608 | 2 | 5.854 | 75.0% | 3 | R.ILEFIAVSQLR.G | 2 |
| \* | Astrin\_STLCHLD\_061214\_01.07932.07932.2 | 3.6671 | 0.5042 | 100.0% | 1354.6522 | 1354.561 | 1 | 7.583 | 79.2% | 7 | R.FSVGGMTDVAEIK.G | 2 |
| \* | Astrin\_STLCHLD\_tube2\_061314\_01.12499.12499.2 | 2.7347 | 0.1668 | 95.2% | 1825.4521 | 1826.1002 | 1 | 5.461 | 46.7% | 1 | K.TENPLILIDEVDKIGR.G | 2 |
| \* | Astrin\_STLCHLD\_tube2\_061314\_01.15341.15341.3 | 5.0153 | 0.4565 | 100.0% | 3874.6443 | 3875.2373 | 1 | 8.058 | 22.8% | 3 | R.GYQGDPSSALLELLDPEQNANFLDHYLDVPVDLSK.V | 3 |
| \* | Astrin\_STLCHLD\_061214\_01.07819.07819.2 | 5.1839 | 0.5178 | 100.0% | 1600.3722 | 1599.8574 | 1 | 8.847 | 76.9% | 9 | R.MEMINVSGYVAQEK.L | 2 |
| \* | Astrin\_STLCHLD\_tube2\_061314\_02.08740.08740.3 | 2.9317 | 0.3292 | 98.7% | 3176.7544 | 3177.5352 | 1 | 5.765 | 23.2% | 1 | K.IVSGEAESVEVTPENLQDFVGKPVFTVER.M | 3 |
| \* | Astrin\_STLCHLD\_tube2\_061314\_01.18189.18189.2 | 5.6394 | 0.5801 | 100.0% | 3189.2322 | 3188.7422 | 1 | 10.784 | 39.7% | 1 | R.MYDVTPPGVVMGLAWTAMGGSTLFVETSLR.R | 2 |
| \* | Astrin\_STLCHLD\_tube2\_061314\_01.18186.18186.3 | 3.7519 | 0.3719 | 100.0% | 3189.7144 | 3188.7422 | 1 | 6.484 | 26.7% | 1 | R.MYDVTPPGVVMGLAWTAMGGSTLFVETSLR.R | 3 |
| \* | Astrin\_STLCHLD\_tube2\_061314\_02.06686.06686.3 | 3.6534 | 0.3158 | 99.8% | 1864.2843 | 1864.0778 | 1 | 5.324 | 44.1% | 1 | K.GDKDGSLEVTGQLGEVMK.E | 3 |
| \* | Astrin\_STLCHLD\_tube2\_061314\_02.06322.06322.2 | 3.4807 | 0.4713 | 100.0% | 1449.3522 | 1449.6624 | 1 | 7.907 | 73.1% | 4 | R.QNLAMTGEVSLTGK.I | 2 |
| \* | Astrin\_STLCHLD\_061214\_01.15104.15104.3 | 2.6191 | 0.2724 | 95.2% | 2674.6143 | 2672.956 | 1 | 4.576 | 32.1% | 1 | K.DFYDLAAFITEGLEVHFVEHYR.E | 3 |
| \* | Astrin\_STLCHLD\_tube2\_061314\_01.12708.12708.2 | 4.4113 | 0.3552 | 100.0% | 2165.4521 | 2164.3765 | 1 | 6.813 | 58.3% | 1 | R.EIFDIAFPDEQAEALAVER.- | 2 |

---

|  |  |  |  |  |  |  |  |  |
| --- | --- | --- | --- | --- | --- | --- | --- | --- |
| U | *gi|17921989|ref|NP\_00* | 20 | 91 | 35.7% | 448 | 49924 | 5.1 | tubulin, alpha 4a [Homo sapiens] |

| Filename XCorr DeltCN Conf% ObsM+H+ CalcM+H+ SpR ZScore Ion% # Sequence  | | | | | | | | | | | | |
| --- | --- | --- | --- | --- | --- | --- | --- | --- | --- | --- | --- | --- |
| \* | Astrin\_STLCHLD\_tube2\_061314\_01.12464.12464.2 | 2.9795 | 0.1508 | 95.8% | 1717.9722 | 1716.9719 | 1 | 4.562 | 60.7% | 2 | R.AVFVDLEPTVIDEIR.N | 2 |
|  | Astrin\_STLCHLD\_tube2\_061314\_01.08855.08855.2 | 1.7582 | 0.466 | 97.3% | 1411.6122 | 1411.6439 | 23 | 6.372 | 59.1% | 1 | R.QLFHPEQLITGK.E | 22 |
|  | Astrin\_STLCHLD\_tube2\_061314\_01.08195.08195.3 | 3.8919 | 0.4186 | 100.0% | 2417.5144 | 2416.6555 | 1 | 6.286 | 36.2% | 2 | R.QLFHPEQLITGKEDAANNYAR.G | 33 |
|  | Astrin\_STLCHLD\_tube2\_061314\_01.04847.04847.3 | 3.6369 | 0.3589 | 100.0% | 1876.6144 | 1876.0824 | 1 | 5.792 | 48.2% | 2 | R.RNLDIERPTYTNLNR.L | 33 |
|  | Astrin\_STLCHLD\_tube2\_061314\_01.06192.06192.3 | 2.5081 | 0.4178 | 99.8% | 1719.2644 | 1719.8949 | 13 | 5.415 | 46.2% | 2 | R.NLDIERPTYTNLNR.L | 33 |
|  | Astrin\_STLCHLD\_tube2\_061314\_01.06234.06234.2 | 3.2301 | 0.1671 | 97.4% | 1719.3722 | 1719.8949 | 2 | 5.169 | 57.7% | 2 | R.NLDIERPTYTNLNR.L | 22 |
|  | Astrin\_STLCHLD\_061214\_01.12381.12381.1 | 2.936 | 0.2665 | 100.0% | 1487.87 | 1488.7678 | 8 | 7.187 | 46.2% | 2 | R.LISQIVSSITASLR.F | 11 |
|  | Astrin\_STLCHLD\_061214\_02.10179.10179.2 | 4.4551 | 0.492 | 100.0% | 1489.4122 | 1488.7678 | 1 | 8.895 | 76.9% | 20 | R.LISQIVSSITASLR.F | 22 |
|  | Astrin\_STLCHLD\_tube2\_061314\_01.13637.13637.3 | 4.5167 | 0.4146 | 100.0% | 1489.6144 | 1488.7678 | 1 | 7.584 | 57.7% | 4 | R.LISQIVSSITASLR.F | 33 |
|  | Astrin\_STLCHLD\_tube2\_061314\_02.09638.09638.2 | 5.8424 | 0.5235 | 100.0% | 2410.7122 | 2410.6885 | 1 | 10.298 | 50.0% | 13 | R.FDGALNVDLTEFQTNLVPYPR.I | 22 |
|  | Astrin\_STLCHLD\_061214\_01.11526.11526.3 | 3.5553 | 0.4671 | 100.0% | 2410.7344 | 2410.6885 | 1 | 8.003 | 37.5% | 2 | R.FDGALNVDLTEFQTNLVPYPR.I | 33 |
|  | Astrin\_STLCHLD\_tube2\_061314\_01.10737.10737.2 | 4.0361 | 0.5047 | 100.0% | 1758.6522 | 1758.0703 | 1 | 8.332 | 73.3% | 6 | R.IHFPLATYAPVISAEK.A | 22 |
|  | Astrin\_STLCHLD\_tube2\_061314\_01.10739.10739.3 | 3.8365 | 0.3294 | 100.0% | 1759.0743 | 1758.0703 | 1 | 6.275 | 46.7% | 5 | R.IHFPLATYAPVISAEK.A | 33 |
|  | Astrin\_STLCHLD\_tube2\_061314\_01.08843.08843.2 | 4.4981 | 0.4675 | 100.0% | 1825.6721 | 1826.1027 | 1 | 7.437 | 67.6% | 5 | K.VGINYQPPTVVPGGDLAK.V | 22 |
|  | Astrin\_STLCHLD\_tube2\_061314\_01.06060.06060.3 | 4.3726 | 0.4192 | 100.0% | 1382.2144 | 1381.6324 | 1 | 6.674 | 60.0% | 5 | R.LDHKFDLMYAK.R | 33 |
|  | Astrin\_STLCHLD\_tube2\_061314\_01.08135.08135.2 | 2.4286 | 0.2419 | 98.2% | 887.8722 | 888.0692 | 1 | 4.937 | 91.7% | 1 | K.FDLMYAK.R | 22 |
|  | Astrin\_STLCHLD\_061214\_01.07863.07863.3 | 6.4401 | 0.5198 | 100.0% | 2488.7644 | 2487.7083 | 1 | 9.454 | 50.0% | 4 | K.RAFVHWYVGEGMEEGEFSEAR.E | 33 |
|  | Astrin\_STLCHLD\_tube2\_061314\_01.18852.18852.2 | 2.5758 | 0.2444 | 96.5% | 2331.2922 | 2331.5208 | 1 | 4.668 | 36.8% | 1 | R.AFVHWYVGEGMEEGEFSEAR.E | 22 |
|  | Astrin\_STLCHLD\_061214\_02.07921.07921.3 | 5.6027 | 0.4369 | 100.0% | 2332.3743 | 2331.5208 | 1 | 7.994 | 44.7% | 11 | R.AFVHWYVGEGMEEGEFSEAR.E | 33 |
|  | Astrin\_STLCHLD\_061214\_01.09956.09956.3 | 3.9499 | 0.2187 | 98.6% | 3220.7043 | 3219.524 | 1 | 4.394 | 28.7% | 1 | R.AFVHWYVGEGMEEGEFSEAREDMAALEK.D | 33 |

Similarities:
gi|57013276|ref|NP\_00(19:1)  

---

|  |  |  |  |  |  |  |  |  |
| --- | --- | --- | --- | --- | --- | --- | --- | --- |
| U | *contaminant\_gi|746301* | 9 | 76 | 35.7% | 269 | 27961 | 6.7 | lysyl endopeptidase (EC 3.4.21.50) - Lysobacter enzymogenes |

| Filename XCorr DeltCN Conf% ObsM+H+ CalcM+H+ SpR ZScore Ion% # Sequence  | | | | | | | | | | | | |
| --- | --- | --- | --- | --- | --- | --- | --- | --- | --- | --- | --- | --- |
| \* | Astrin\_STLCHLD\_tube2\_061314\_01.01418.01418.1 | 1.8205 | 0.267 | 100.0% | 725.64 | 725.8198 | 3 | 4.949 | 66.7% | 1 | R.SVAAYSK.Q | 1 |
| \* | Astrin\_STLCHLD\_061214\_01.04644.04644.2 | 6.1218 | 0.6276 | 100.0% | 2261.4722 | 2262.355 | 1 | 11.634 | 54.2% | 11 | R.APGSSSSGANGDGSLAQSQTGAVVR.A | 2 |
| \* | Astrin\_STLCHLD\_061214\_02.04609.04609.3 | 4.9943 | 0.4201 | 100.0% | 2261.7544 | 2262.355 | 1 | 7.234 | 37.5% | 11 | R.APGSSSSGANGDGSLAQSQTGAVVR.A | 3 |
| \* | Astrin\_STLCHLD\_061214\_01.14106.14106.3 | 4.5037 | 0.2996 | 100.0% | 3315.8943 | 3315.6257 | 1 | 5.0 | 22.4% | 3 | R.ATNAASDFTLLELNTAANPAYNLFWAGWDR.R | 3 |
| \* | Astrin\_STLCHLD\_tube2\_061314\_01.15306.15306.2 | 5.2982 | 0.478 | 100.0% | 3316.132 | 3315.6257 | 1 | 9.453 | 41.4% | 5 | R.ATNAASDFTLLELNTAANPAYNLFWAGWDR.R | 2 |
| \* | Astrin\_STLCHLD\_tube2\_061314\_01.14477.14477.3 | 6.2092 | 0.443 | 100.0% | 3472.2544 | 3471.813 | 1 | 9.607 | 30.8% | 13 | R.ATNAASDFTLLELNTAANPAYNLFWAGWDRR.D | 3 |
| \* | Astrin\_STLCHLD\_tube2\_061314\_01.04205.04205.3 | 3.9047 | 0.4734 | 100.0% | 2077.0444 | 2077.2668 | 1 | 7.108 | 34.7% | 2 | R.DQNFAGATAIHHPNVAEKR.I | 3 |
| \* | Astrin\_STLCHLD\_tube2\_061314\_01.06141.06141.1 | 1.8821 | 0.2444 | 96.7% | 1427.71 | 1428.5443 | 75 | 4.324 | 38.5% | 1 | R.VFTSWTGGGTSATR.L | 1 |
| \* | Astrin\_STLCHLD\_tube2\_061314\_02.06032.06032.2 | 4.8733 | 0.5492 | 100.0% | 1428.3121 | 1428.5443 | 1 | 9.16 | 73.1% | 29 | R.VFTSWTGGGTSATR.L | 2 |

---

|  |  |  |  |  |  |  |  |  |
| --- | --- | --- | --- | --- | --- | --- | --- | --- |
| U | *gi|4506645|ref|NP\_000* | 3 | 3 | 35.7% | 70 | 8218 | 10.1 | ribosomal protein L38 [Homo sapiens] |
| U | *gi|78214522|ref|NP\_00* | 3 | 3 | 35.7% | 70 | 8218 | 10.1 | ribosomal protein L38 [Homo sapiens] |

| Filename XCorr DeltCN Conf% ObsM+H+ CalcM+H+ SpR ZScore Ion% # Sequence  | | | | | | | | | | | | |
| --- | --- | --- | --- | --- | --- | --- | --- | --- | --- | --- | --- | --- |
|  | Astrin\_STLCHLD\_tube2\_061314\_01.09769.09769.2 | 2.6043 | 0.2502 | 97.1% | 1576.2722 | 1576.8766 | 13 | 4.903 | 50.0% | 1 | R.KIEEIKDFLLTAR.R | 2 |
|  | Astrin\_STLCHLD\_tube2\_061314\_01.09733.09733.3 | 3.3666 | 0.3504 | 100.0% | 1576.5543 | 1576.8766 | 13 | 6.159 | 43.8% | 1 | R.KIEEIKDFLLTAR.R | 3 |
|  | Astrin\_STLCHLD\_061214\_01.07384.07384.2 | 3.2532 | 0.2262 | 98.8% | 1486.6921 | 1486.7484 | 1 | 5.177 | 72.7% | 1 | R.YLYTLVITDKEK.A | 2 |

---

|  |  |  |  |  |  |  |  |  |
| --- | --- | --- | --- | --- | --- | --- | --- | --- |
| U | *gi|62414289|ref|NP\_00* | 18 | 27 | 35.6% | 466 | 53652 | 5.1 | vimentin [Homo sapiens] |

| Filename XCorr DeltCN Conf% ObsM+H+ CalcM+H+ SpR ZScore Ion% # Sequence  | | | | | | | | | | | | |
| --- | --- | --- | --- | --- | --- | --- | --- | --- | --- | --- | --- | --- |
| \* | Astrin\_STLCHLD\_061214\_01.04982.04982.2 | 3.1482 | 0.3793 | 100.0% | 1428.2322 | 1429.5724 | 1 | 6.614 | 69.2% | 2 | R.SLYASSPGGVYATR.S | 2 |
| \* | Astrin\_STLCHLD\_tube2\_061314\_01.06205.06205.2 | 3.1186 | 0.4148 | 100.0% | 1508.6921 | 1509.5724 | 1 | 6.684 | 65.4% | 2 | R.SLYASS\*PGGVYATR.S | 2 |
|  | Astrin\_STLCHLD\_tube2\_061314\_01.04639.04639.2 | 2.2121 | 0.2317 | 95.7% | 1116.7722 | 1116.2163 | 2 | 5.072 | 75.0% | 1 | K.VELQELNDR.F | 2 |
| \* | Astrin\_STLCHLD\_tube2\_061314\_01.04746.04746.2 | 2.524 | 0.2686 | 98.2% | 1126.0721 | 1126.3005 | 89 | 5.074 | 62.5% | 1 | R.FANYIDKVR.F | 2 |
| \* | Astrin\_STLCHLD\_tube2\_061314\_01.06923.06923.2 | 3.524 | 0.5082 | 100.0% | 1255.0922 | 1255.385 | 1 | 8.554 | 77.8% | 2 | R.LGDLYEEEMR.E | 2 |
| \* | Astrin\_STLCHLD\_tube2\_061314\_01.08217.08217.2 | 2.9911 | 0.2618 | 98.3% | 1689.2522 | 1689.881 | 2 | 5.306 | 57.7% | 1 | R.VEVERDNLAEDIMR.L | 2 |
| \* | Astrin\_STLCHLD\_tube2\_061314\_01.08243.08243.3 | 3.6392 | 0.2798 | 99.7% | 1690.5543 | 1689.881 | 1 | 5.576 | 48.1% | 2 | R.VEVERDNLAEDIMR.L | 3 |
| \* | Astrin\_STLCHLD\_tube2\_061314\_01.09476.09476.2 | 2.4164 | 0.2937 | 98.1% | 1077.1322 | 1077.1975 | 26 | 5.706 | 62.5% | 1 | R.DNLAEDIMR.L | 2 |
| \* | Astrin\_STLCHLD\_061214\_02.06980.06980.3 | 3.4785 | 0.3597 | 100.0% | 2351.1543 | 2352.581 | 9 | 5.691 | 33.3% | 2 | K.LQEEMLQREEAENTLQSFR.Q | 3 |
| \* | Astrin\_STLCHLD\_tube2\_061314\_01.05790.05790.2 | 2.7409 | 0.4147 | 100.0% | 1324.2322 | 1324.3898 | 1 | 6.362 | 65.0% | 2 | R.EEAENTLQSFR.Q | 2 |
| \* | Astrin\_STLCHLD\_tube2\_061314\_01.10518.10518.2 | 2.8212 | 0.2304 | 97.4% | 1534.0521 | 1534.793 | 57 | 4.855 | 50.0% | 1 | R.KVESLQEEIAFLK.K | 2 |
|  | Astrin\_STLCHLD\_tube2\_061314\_01.07835.07835.2 | 2.681 | 0.2074 | 97.4% | 1311.3722 | 1310.4056 | 1 | 4.323 | 77.8% | 1 | K.NLQEAEEWYK.S | 2 |
| \* | Astrin\_STLCHLD\_061214\_01.04651.04651.2 | 3.2951 | 0.457 | 100.0% | 1094.2922 | 1094.1692 | 1 | 8.283 | 83.3% | 1 | K.FADLSEAANR.N | 2 |
| \* | Astrin\_STLCHLD\_tube2\_061314\_02.07605.07605.3 | 3.8555 | 0.3695 | 100.0% | 2188.0144 | 2188.33 | 1 | 6.39 | 37.5% | 2 | R.EMEENFAVEAANYQDTIGR.L | 3 |
| \* | Astrin\_STLCHLD\_tube2\_061314\_02.07607.07607.2 | 5.978 | 0.5604 | 100.0% | 2188.372 | 2188.33 | 1 | 11.284 | 69.4% | 2 | R.EMEENFAVEAANYQDTIGR.L | 2 |
|  | Astrin\_STLCHLD\_tube2\_061314\_01.06375.06375.3 | 2.7562 | 0.2421 | 97.2% | 1528.7943 | 1528.7513 | 1 | 5.249 | 50.0% | 1 | R.HLREYQDLLNVK.M | 3 |
|  | Astrin\_STLCHLD\_tube2\_061314\_02.08062.08062.2 | 3.8579 | 0.3576 | 100.0% | 1296.7122 | 1296.5243 | 1 | 8.926 | 85.0% | 2 | K.MALDIEIATYR.K | 2 |
| \* | Astrin\_STLCHLD\_061214\_01.04346.04346.2 | 2.8138 | 0.235 | 97.2% | 1837.8121 | 1837.854 | 1 | 4.402 | 50.0% | 1 | R.DGQVINETSQHHDDLE.- | 2 |

---

|  |  |  |  |  |  |  |  |  |
| --- | --- | --- | --- | --- | --- | --- | --- | --- |
| U | *gi|218505827|ref|NP\_1* | 9 | 22 | 35.4% | 316 | 35438 | 6.3 | TRAF4 associated factor 1 isoform a [Homo sapiens] |
| U | *gi|218505831|ref|NP\_0* | 9 | 22 | 39.2% | 286 | 31880 | 7.1 | TRAF4 associated factor 1 isoform b [Homo sapiens] |

| Filename XCorr DeltCN Conf% ObsM+H+ CalcM+H+ SpR ZScore Ion% # Sequence  | | | | | | | | | | | | |
| --- | --- | --- | --- | --- | --- | --- | --- | --- | --- | --- | --- | --- |
|  | Astrin\_STLCHLD\_tube2\_061314\_01.07949.07949.2 | 5.4991 | 0.5401 | 100.0% | 2274.7122 | 2275.4802 | 1 | 8.789 | 61.9% | 4 | K.TVYSLQPPSALSGGQPADTQTR.A | 2 |
|  | Astrin\_STLCHLD\_tube2\_061314\_01.08022.08022.3 | 5.1071 | 0.432 | 100.0% | 2275.3442 | 2275.4802 | 1 | 8.103 | 40.5% | 2 | K.TVYSLQPPSALSGGQPADTQTR.A | 3 |
|  | Astrin\_STLCHLD\_tube2\_061314\_01.08015.08015.3 | 2.9256 | 0.2665 | 96.9% | 3411.6243 | 3408.7502 | 318 | 4.241 | 17.7% | 1 | K.TVYSLQPPSALSGGQPADTQTRATSKS\*LLPVR.S | 3 |
|  | Astrin\_STLCHLD\_tube2\_061314\_01.05448.05448.3 | 4.8994 | 0.4277 | 100.0% | 1987.8844 | 1988.2023 | 1 | 7.986 | 40.6% | 4 | K.SEEELKDKNQLLEAVNK.Q | 3 |
|  | Astrin\_STLCHLD\_tube2\_061314\_01.04971.04971.2 | 4.0646 | 0.4176 | 100.0% | 1604.5322 | 1604.7979 | 2 | 6.683 | 65.4% | 1 | K.LTETQGELKDLTQK.V | 2 |
|  | Astrin\_STLCHLD\_tube2\_061314\_01.10803.10803.3 | 4.4678 | 0.3836 | 100.0% | 2318.0645 | 2316.6543 | 1 | 5.951 | 38.2% | 1 | K.LTETQGELKDLTQKVELLEK.F | 3 |
|  | Astrin\_STLCHLD\_tube2\_061314\_01.07682.07682.2 | 3.4354 | 0.2252 | 99.5% | 1316.3121 | 1316.5376 | 1 | 5.453 | 75.0% | 2 | K.DLTQKVELLEK.F | 2 |
|  | Astrin\_STLCHLD\_tube2\_061314\_01.07062.07062.2 | 4.0669 | 0.5142 | 100.0% | 1388.8722 | 1387.5327 | 1 | 8.998 | 76.9% | 5 | K.GLDPALGSETLASR.Q | 2 |
|  | Astrin\_STLCHLD\_tube2\_061314\_01.14763.14763.3 | 5.6288 | 0.4502 | 100.0% | 3396.6543 | 3396.8062 | 1 | 7.548 | 28.6% | 2 | R.QESTTDHMDSMLLLETLQEELKLFNETAK.K | 3 |

---

|  |  |  |  |  |  |  |  |  |
| --- | --- | --- | --- | --- | --- | --- | --- | --- |
| U | *gi|30795231|ref|NP\_00* | 3 | 5 | 35.2% | 227 | 22693 | 4.6 | brain abundant, membrane attached signal protein 1 [Homo sapiens] |

| Filename XCorr DeltCN Conf% ObsM+H+ CalcM+H+ SpR ZScore Ion% # Sequence  | | | | | | | | | | | | |
| --- | --- | --- | --- | --- | --- | --- | --- | --- | --- | --- | --- | --- |
| \* | Astrin\_STLCHLD\_061214\_01.04158.04158.3 | 4.5362 | 0.403 | 100.0% | 2699.5444 | 2699.7986 | 1 | 7.035 | 32.7% | 1 | K.AEGAATEEEGTPKESEPQAAAEPAEAK.E | 3 |
| \* | Astrin\_STLCHLD\_061214\_01.04140.04140.3 | 3.7066 | 0.3118 | 99.7% | 2298.7744 | 2299.5022 | 1 | 4.808 | 43.5% | 2 | K.AEPPKAPEQEQAAPGPAAGGEAPK.A | 3 |
| \* | Astrin\_STLCHLD\_061214\_01.04627.04627.3 | 5.4847 | 0.4521 | 100.0% | 2894.6643 | 2894.1216 | 1 | 7.925 | 33.9% | 2 | K.AQGPAASAEEPKPVEAPAANSDQTVTVKE.- | 3 |

---

|  |  |  |  |  |  |  |  |  |
| --- | --- | --- | --- | --- | --- | --- | --- | --- |
| U | *gi|5902102|ref|NP\_008* | 2 | 3 | 34.5% | 119 | 13282 | 11.6 | small nuclear ribonucleoprotein D1 polypeptide 16kDa [Homo sapiens] |

| Filename XCorr DeltCN Conf% ObsM+H+ CalcM+H+ SpR ZScore Ion% # Sequence  | | | | | | | | | | | | |
| --- | --- | --- | --- | --- | --- | --- | --- | --- | --- | --- | --- | --- |
| \* | Astrin\_STLCHLD\_tube2\_061314\_02.05645.05645.3 | 3.617 | 0.1745 | 96.0% | 2210.4844 | 2210.47 | 458 | 4.906 | 26.2% | 1 | K.NGTQVHGTITGVDVSMNTHLK.A | 3 |
| \* | Astrin\_STLCHLD\_tube2\_061314\_01.15843.15843.2 | 4.4797 | 0.4172 | 100.0% | 2288.872 | 2288.6863 | 1 | 7.187 | 52.6% | 2 | R.YFILPDSLPLDTLLVDVEPK.V | 2 |

---

|  |  |  |  |  |  |  |  |  |
| --- | --- | --- | --- | --- | --- | --- | --- | --- |
| U | *gi|119395750|ref|NP\_0* | 14 | 47 | 34.2% | 644 | 66039 | 8.1 | keratin 1 [Homo sapiens] |

| Filename XCorr DeltCN Conf% ObsM+H+ CalcM+H+ SpR ZScore Ion% # Sequence  | | | | | | | | | | | | |
| --- | --- | --- | --- | --- | --- | --- | --- | --- | --- | --- | --- | --- |
| \* | Astrin\_STLCHLD\_061214\_02.06106.06106.2 | 4.4309 | 0.3988 | 100.0% | 1658.9521 | 1658.7678 | 1 | 6.986 | 56.2% | 6 | R.SGGGFSSGSAGIINYQR.R | 2 |
|  | Astrin\_STLCHLD\_tube2\_061314\_01.10411.10411.2 | 3.6496 | 0.4402 | 100.0% | 1385.4321 | 1384.5315 | 1 | 7.666 | 72.7% | 2 | K.SLNNQFASFIDK.V | 2 |
|  | Astrin\_STLCHLD\_tube2\_061314\_01.05226.05226.2 | 4.1811 | 0.1216 | 99.3% | 1476.2922 | 1476.6726 | 1 | 6.927 | 90.9% | 3 | R.FLEQQNQVLQTK.W | 22 |
|  | Astrin\_STLCHLD\_061214\_01.08490.08490.2 | 4.3907 | 0.5135 | 100.0% | 1476.5521 | 1476.6293 | 1 | 9.299 | 86.4% | 6 | K.WELLQQVDTSTR.T | 2 |
|  | Astrin\_STLCHLD\_tube2\_061314\_01.12770.12770.2 | 3.1021 | 0.314 | 98.9% | 1995.3922 | 1995.2017 | 1 | 6.362 | 56.7% | 1 | R.THNLEPYFESFINNLR.R | 2 |
| \* | Astrin\_STLCHLD\_tube2\_061314\_01.12637.12637.2 | 3.8541 | 0.4353 | 100.0% | 1303.2922 | 1303.4955 | 1 | 8.202 | 81.8% | 1 | R.SLDLDSIIAEVK.A | 2 |
|  | Astrin\_STLCHLD\_061214\_01.06446.06446.3 | 5.3788 | 0.4434 | 100.0% | 2502.7444 | 2502.7405 | 1 | 8.439 | 44.0% | 1 | K.SKAEAESLYQSKYEELQITAGR.H | 3 |
|  | Astrin\_STLCHLD\_061214\_01.06978.06978.3 | 3.8098 | 0.2572 | 99.6% | 2289.5044 | 2287.4883 | 2 | 5.515 | 32.9% | 1 | K.AEAESLYQSKYEELQITAGR.H | 3 |
|  | Astrin\_STLCHLD\_tube2\_061314\_01.05725.05725.2 | 3.887 | 0.3539 | 100.0% | 1181.3722 | 1180.303 | 2 | 6.792 | 83.3% | 16 | K.YEELQITAGR.H | 22 |
|  | Astrin\_STLCHLD\_tube2\_061314\_01.09176.09176.2 | 4.4087 | 0.3798 | 100.0% | 1601.5721 | 1600.769 | 1 | 6.672 | 80.8% | 1 | K.NKLNDLEDALQQAK.E | 2 |
| \* | Astrin\_STLCHLD\_tube2\_061314\_01.11840.11840.3 | 4.5283 | 0.48 | 100.0% | 2184.8943 | 2185.399 | 1 | 8.25 | 44.4% | 1 | K.NKLNDLEDALQQAKEDLAR.L | 3 |
|  | Astrin\_STLCHLD\_061214\_01.04202.04202.3 | 5.6466 | 0.5281 | 100.0% | 2385.0842 | 2385.298 | 1 | 9.58 | 30.0% | 1 | R.GGGGGGYGSGGSSYGSGGGSYGSGGGGGGGR.G | 3 |
| \* | Astrin\_STLCHLD\_061214\_01.04316.04316.3 | 5.6201 | 0.2052 | 99.7% | 3314.4844 | 3314.2085 | 1 | 8.476 | 22.4% | 4 | R.GSYGSGGSSYGSGGGSYGSGGGGGGHGSYGSGSSSGGYR.G | 3 |
| \* | Astrin\_STLCHLD\_tube2\_061314\_01.09537.09537.3 | 3.769 | 0.2214 | 97.9% | 2240.7844 | 2241.0396 | 5 | 4.793 | 30.6% | 3 | R.GGSGGGGGGS\*S\*GGRGSGGGSSGGSIGGR.G | 3 |

Similarities:
gi|47132620|ref|NP\_00(1:13)  
gi|119703753|ref|NP\_0(1:13)  

---

|  |  |  |  |  |  |  |  |  |
| --- | --- | --- | --- | --- | --- | --- | --- | --- |
| U | *gi|20127519|ref|NP\_03* | 32 | 79 | 33.6% | 747 | 85653 | 9.2 | TPX2, microtubule-associated protein homolog [Homo sapiens] |

| Filename XCorr DeltCN Conf% ObsM+H+ CalcM+H+ SpR ZScore Ion% # Sequence  | | | | | | | | | | | | |
| --- | --- | --- | --- | --- | --- | --- | --- | --- | --- | --- | --- | --- |
| \* | Astrin\_STLCHLD\_tube2\_061314\_01.09078.09078.3 | 4.0427 | 0.4612 | 100.0% | 2277.4443 | 2277.6255 | 1 | 7.374 | 32.9% | 2 | K.ANLQQAIVTPLKPVDNTYYK.E | 3 |
| \* | Astrin\_STLCHLD\_tube2\_061314\_01.08910.08910.3 | 3.4098 | 0.2828 | 99.3% | 2357.9644 | 2357.6255 | 2 | 5.779 | 28.9% | 1 | K.ANLQQAIVT#PLKPVDNTYYK.E | 3 |
| \* | Astrin\_STLCHLD\_tube2\_061314\_01.07550.07550.2 | 2.1733 | 0.3002 | 96.7% | 1066.8522 | 1067.317 | 245 | 6.611 | 50.0% | 2 | K.LALAGIGQPVK.K | 2 |
| \* | Astrin\_STLCHLD\_tube2\_061314\_01.05111.05111.2 | 3.2754 | 0.4251 | 100.0% | 1195.4922 | 1195.4911 | 18 | 7.187 | 59.1% | 2 | K.LALAGIGQPVKK.S | 2 |
| \* | Astrin\_STLCHLD\_tube2\_061314\_01.05285.05285.2 | 2.0477 | 0.282 | 97.0% | 908.21216 | 908.0043 | 199 | 5.611 | 66.7% | 2 | K.SVDFHFR.T | 2 |
| \* | Astrin\_STLCHLD\_061214\_01.06502.06502.2 | 4.948 | 0.4904 | 100.0% | 1886.4122 | 1887.013 | 1 | 9.182 | 82.1% | 3 | K.NQEEYKEVNFTSELR.K | 2 |
| \* | Astrin\_STLCHLD\_tube2\_061314\_01.07373.07373.3 | 4.6303 | 0.243 | 100.0% | 1889.3644 | 1887.013 | 7 | 5.489 | 41.1% | 8 | K.NQEEYKEVNFTSELR.K | 3 |
| \* | Astrin\_STLCHLD\_tube2\_061314\_01.07301.07301.2 | 2.3257 | 0.3335 | 98.4% | 1095.4521 | 1095.1974 | 4 | 5.959 | 68.8% | 1 | K.EVNFTSELR.K | 2 |
| \* | Astrin\_STLCHLD\_tube2\_061314\_01.10854.10854.3 | 2.9051 | 0.301 | 98.4% | 2612.5144 | 2611.8706 | 15 | 5.271 | 25.0% | 1 | R.TFDETVSTYVPLAQQVEDFHKR.T | 3 |
| \* | Astrin\_STLCHLD\_tube2\_061314\_01.05526.05526.2 | 3.5897 | 0.2513 | 100.0% | 1349.6721 | 1349.4344 | 1 | 5.703 | 81.8% | 7 | K.STAELEAEELEK.L | 2 |
| \* | Astrin\_STLCHLD\_tube2\_061314\_01.09596.09596.2 | 5.4151 | 0.337 | 100.0% | 2009.9321 | 2010.2053 | 1 | 7.987 | 65.6% | 1 | K.STAELEAEELEKLQQYK.F | 2 |
| \* | Astrin\_STLCHLD\_tube2\_061314\_01.07205.07205.2 | 3.3083 | 0.3095 | 100.0% | 1037.4321 | 1037.2877 | 1 | 5.99 | 77.8% | 5 | R.ILEGGPILPK.K | 2 |
| \* | Astrin\_STLCHLD\_tube2\_061314\_01.08814.08814.3 | 3.9812 | 0.4162 | 100.0% | 2136.1443 | 2135.5083 | 1 | 6.377 | 41.7% | 4 | K.KPPVKPPTEPIGFDLEIEK.R | 3 |
| \* | Astrin\_STLCHLD\_061214\_01.06651.06651.3 | 4.7489 | 0.467 | 100.0% | 2292.0544 | 2291.6958 | 1 | 7.636 | 39.5% | 2 | K.KPPVKPPTEPIGFDLEIEKR.I | 3 |
| \* | Astrin\_STLCHLD\_tube2\_061314\_01.07331.07331.2 | 3.3595 | 0.2939 | 100.0% | 1199.4722 | 1198.402 | 1 | 5.756 | 85.0% | 3 | K.ILEDVVGVPEK.K | 2 |
| \* | Astrin\_STLCHLD\_tube2\_061314\_01.05588.05588.2 | 3.4136 | 0.3166 | 100.0% | 1326.0122 | 1326.576 | 2 | 6.256 | 72.7% | 3 | K.ILEDVVGVPEKK.V | 2 |
| \* | Astrin\_STLCHLD\_tube2\_061314\_01.05573.05573.3 | 3.4615 | 0.4163 | 100.0% | 1326.8944 | 1326.576 | 85 | 6.214 | 45.5% | 3 | K.ILEDVVGVPEKK.V | 3 |
| \* | Astrin\_STLCHLD\_tube2\_061314\_01.10643.10643.2 | 3.7637 | 0.3957 | 100.0% | 1662.5521 | 1661.9823 | 1 | 6.783 | 67.9% | 2 | K.VLPITVPKS\*PAFALK.N | 2 |
| \* | Astrin\_STLCHLD\_tube2\_061314\_01.05364.05364.3 | 5.0164 | 0.304 | 100.0% | 2158.6443 | 2158.4285 | 1 | 7.045 | 38.2% | 2 | R.IRMPTKEDEEEDEPVVIK.A | 3 |
| \* | Astrin\_STLCHLD\_tube2\_061314\_01.04547.04547.2 | 5.0201 | 0.349 | 100.0% | 1888.6322 | 1889.0815 | 1 | 7.966 | 80.0% | 2 | R.MPTKEDEEEDEPVVIK.A | 2 |
| \* | Astrin\_STLCHLD\_tube2\_061314\_01.04595.04595.3 | 5.3209 | 0.2908 | 100.0% | 1890.2644 | 1889.0815 | 1 | 5.782 | 55.0% | 2 | R.MPTKEDEEEDEPVVIK.A | 3 |
| \* | Astrin\_STLCHLD\_tube2\_061314\_01.10759.10759.2 | 3.1638 | 0.3949 | 100.0% | 1706.5721 | 1705.9945 | 1 | 7.391 | 64.3% | 1 | K.ALPLPHFDTINLPEK.K | 2 |
| \* | Astrin\_STLCHLD\_tube2\_061314\_01.06275.06275.2 | 3.4662 | 0.3158 | 100.0% | 1683.7122 | 1683.9481 | 4 | 5.712 | 50.0% | 1 | K.ARPNTVISQEPFVPK.K | 2 |
| \* | Astrin\_STLCHLD\_tube2\_061314\_01.06384.06384.3 | 3.8837 | 0.2979 | 100.0% | 1684.6144 | 1683.9481 | 1 | 5.884 | 48.2% | 2 | K.ARPNTVISQEPFVPK.K | 3 |
| \* | Astrin\_STLCHLD\_tube2\_061314\_01.04688.04688.3 | 4.5754 | 0.4098 | 100.0% | 1812.5944 | 1812.1222 | 1 | 7.541 | 50.0% | 1 | K.ARPNTVISQEPFVPKK.E | 3 |
| \* | Astrin\_STLCHLD\_tube2\_061314\_01.09992.09992.3 | 3.7228 | 0.2685 | 99.3% | 2474.6042 | 2475.8044 | 1 | 5.538 | 30.7% | 1 | K.KSVAEGLSGSLVQEPFQLATEKR.A | 3 |
| \* | Astrin\_STLCHLD\_tube2\_061314\_01.10829.10829.3 | 4.4333 | 0.414 | 100.0% | 2347.7344 | 2347.6304 | 3 | 7.393 | 32.1% | 3 | K.SVAEGLSGSLVQEPFQLATEKR.A | 3 |
| \* | Astrin\_STLCHLD\_tube2\_061314\_01.02165.02165.2 | 2.7516 | 0.2427 | 98.9% | 906.21216 | 906.0417 | 26 | 5.246 | 78.6% | 5 | R.MAEVEAQK.A | 2 |
| \* | Astrin\_STLCHLD\_tube2\_061314\_01.04551.04551.2 | 4.7993 | 0.5038 | 100.0% | 1831.5721 | 1832.0386 | 1 | 9.04 | 70.0% | 1 | R.MAEVEAQKAQQLEEAR.L | 2 |
| \* | Astrin\_STLCHLD\_tube2\_061314\_01.04602.04602.3 | 3.7307 | 0.3479 | 100.0% | 1832.6643 | 1832.0386 | 1 | 6.185 | 46.7% | 1 | R.MAEVEAQKAQQLEEAR.L | 3 |
| \* | Astrin\_STLCHLD\_tube2\_061314\_01.02317.02317.2 | 2.5843 | 0.181 | 97.4% | 944.5722 | 945.02014 | 3 | 5.28 | 78.6% | 2 | K.AQQLEEAR.L | 2 |
| \* | Astrin\_STLCHLD\_tube2\_061314\_01.05825.05825.2 | 3.3936 | 0.3472 | 100.0% | 1435.4722 | 1435.5309 | 1 | 6.511 | 75.0% | 3 | K.SSDQPLTVPVS\*PK.F | 2 |

---

|  |  |  |  |  |  |  |  |  |
| --- | --- | --- | --- | --- | --- | --- | --- | --- |
| U | *gi|195927041|ref|NP\_0* | 7 | 13 | 33.3% | 297 | 34081 | 8.4 | cell division cycle 2 isoform 3 [Homo sapiens] |
| U | *gi|4502709|ref|NP\_001* | 7 | 13 | 33.3% | 297 | 34095 | 8.4 | cell division cycle 2 isoform 1 [Homo sapiens] |

| Filename XCorr DeltCN Conf% ObsM+H+ CalcM+H+ SpR ZScore Ion% # Sequence  | | | | | | | | | | | | |
| --- | --- | --- | --- | --- | --- | --- | --- | --- | --- | --- | --- | --- |
|  | Astrin\_STLCHLD\_tube2\_061314\_01.05425.05425.2 | 2.6379 | 0.3342 | 98.9% | 1185.4922 | 1186.3501 | 1 | 7.804 | 75.0% | 1 | K.IGEGTYGVVYK.G | 2 |
|  | Astrin\_STLCHLD\_tube2\_061314\_01.04585.04585.2 | 4.5977 | 0.4397 | 100.0% | 1518.4722 | 1517.6329 | 1 | 7.485 | 76.9% | 1 | R.LESEEEGVPSTAIR.E | 2 |
|  | Astrin\_STLCHLD\_061214\_01.06729.06729.3 | 3.324 | 0.2928 | 99.7% | 1813.5243 | 1812.1167 | 4 | 5.508 | 36.7% | 1 | R.DLKPQNLLIDDKGTIK.L | 3 |
|  | Astrin\_STLCHLD\_tube2\_061314\_01.05000.05000.2 | 2.9431 | 0.261 | 98.9% | 1031.0521 | 1029.1814 | 1 | 6.251 | 77.8% | 2 | R.SPEVLLGSAR.Y | 2 |
|  | Astrin\_STLCHLD\_tube2\_061314\_01.16923.16923.2 | 3.6713 | 0.3867 | 100.0% | 2213.9521 | 2213.5352 | 2 | 6.521 | 36.8% | 6 | R.YSTPVDIWSIGTIFAELATK.K | 2 |
|  | Astrin\_STLCHLD\_tube2\_061314\_01.09642.09642.2 | 3.3352 | 0.3511 | 100.0% | 1331.4722 | 1331.4656 | 1 | 7.089 | 68.2% | 1 | K.NLDENGLDLLSK.M | 2 |
|  | Astrin\_STLCHLD\_tube2\_061314\_01.08873.08873.3 | 4.6438 | 0.3492 | 100.0% | 1933.9744 | 1934.1768 | 1 | 6.298 | 55.0% | 1 | K.MALNHPYFNDLDNQIK.K | 3 |

---

|  |  |  |  |  |  |  |  |  |
| --- | --- | --- | --- | --- | --- | --- | --- | --- |
| U | *gi|20357599|ref|NP\_61* | 2 | 5 | 33.3% | 114 | 12146 | 10.5 | H2A histone family, member V isoform 2 [Homo sapiens] |
| U | *gi|6912616|ref|NP\_036* | 2 | 5 | 29.7% | 128 | 13509 | 10.6 | H2A histone family, member V isoform 1 [Homo sapiens] |
| U | *gi|4504255|ref|NP\_002* | 2 | 5 | 29.7% | 128 | 13553 | 10.6 | H2A histone family, member Z [Homo sapiens] |

| Filename XCorr DeltCN Conf% ObsM+H+ CalcM+H+ SpR ZScore Ion% # Sequence  | | | | | | | | | | | | |
| --- | --- | --- | --- | --- | --- | --- | --- | --- | --- | --- | --- | --- |
|  | Astrin\_STLCHLD\_tube2\_061314\_01.07517.07517.2 | 3.2091 | 0.3209 | 100.0% | 945.39215 | 945.1093 | 1 | 5.94 | 81.2% | 4 | R.AGLQFPVGR.I | 222 |
|  | Astrin\_STLCHLD\_tube2\_061314\_01.18247.18247.3 | 3.6918 | 0.3524 | 100.0% | 2897.0344 | 2897.2952 | 1 | 6.076 | 24.1% | 1 | R.VGATAAVYSAAILEYLTAEVLELAGNASK.D | 3 |

Similarities:
gi|10800130|ref|NP\_06(1:1)  
gi|106775678|ref|NP\_0(1:1)  

---

|  |  |  |  |  |  |  |  |  |
| --- | --- | --- | --- | --- | --- | --- | --- | --- |
| U | *gi|5174457|ref|NP\_006* | 18 | 49 | 32.1% | 642 | 73913 | 5.6 | kinetochore associated 2 [Homo sapiens] |

| Filename XCorr DeltCN Conf% ObsM+H+ CalcM+H+ SpR ZScore Ion% # Sequence  | | | | | | | | | | | | |
| --- | --- | --- | --- | --- | --- | --- | --- | --- | --- | --- | --- | --- |
| \* | Astrin\_STLCHLD\_tube2\_061314\_01.04851.04851.2 | 2.5709 | 0.1176 | 96.8% | 877.39215 | 877.0466 | 21 | 5.011 | 75.0% | 1 | R.LSMQELR.S | 2 |
| \* | Astrin\_STLCHLD\_tube2\_061314\_01.07167.07167.2 | 2.3108 | 0.3288 | 97.4% | 1296.3322 | 1297.4075 | 1 | 5.73 | 72.7% | 1 | R.NSQLGIFSSSEK.I | 2 |
| \* | Astrin\_STLCHLD\_tube2\_061314\_01.10514.10514.2 | 3.286 | 0.3752 | 100.0% | 1499.5322 | 1499.793 | 1 | 5.984 | 62.5% | 1 | R.IFKDLGYPFALSK.S | 2 |
| \* | Astrin\_STLCHLD\_tube2\_061314\_01.11455.11455.3 | 5.3241 | 0.4939 | 100.0% | 2081.9944 | 2081.4167 | 1 | 7.886 | 42.6% | 1 | K.LKDLFNVDAFKLESLEAK.N | 3 |
| \* | Astrin\_STLCHLD\_061214\_02.06601.06601.3 | 2.9025 | 0.3011 | 98.7% | 2099.8743 | 2099.3228 | 20 | 5.968 | 33.8% | 5 | K.YQAYMSNLESHSAILDQK.L | 3 |
| \* | Astrin\_STLCHLD\_tube2\_061314\_01.04769.04769.2 | 3.4722 | 0.3155 | 100.0% | 1129.4722 | 1129.2584 | 5 | 7.013 | 72.2% | 2 | K.LNGLNEEIAR.V | 2 |
| \* | Astrin\_STLCHLD\_tube2\_061314\_01.05046.05046.2 | 2.5349 | 0.3718 | 100.0% | 953.03217 | 953.03973 | 1 | 7.214 | 85.7% | 2 | K.YSVADIER.I | 2 |
| \* | Astrin\_STLCHLD\_061214\_01.04951.04951.3 | 4.3576 | 0.2393 | 100.0% | 1618.1044 | 1617.7991 | 1 | 7.381 | 46.2% | 1 | R.GKEAIETQLAEYHK.L | 3 |
| \* | Astrin\_STLCHLD\_tube2\_061314\_01.12735.12735.3 | 4.0502 | 0.4061 | 100.0% | 2360.3643 | 2360.6665 | 1 | 7.832 | 39.5% | 1 | R.AQVYVPLKELLNETEEEINK.A | 3 |
| \* | Astrin\_STLCHLD\_tube2\_061314\_01.06487.06487.2 | 3.6438 | 0.4177 | 100.0% | 1461.5922 | 1461.5658 | 1 | 7.267 | 77.3% | 2 | K.ELLNETEEEINK.A | 2 |
| \* | Astrin\_STLCHLD\_tube2\_061314\_01.14538.14538.2 | 4.1411 | 0.4555 | 100.0% | 2024.0521 | 2024.3088 | 1 | 8.266 | 52.9% | 1 | K.MGLEDTLEQLNAMITESK.R | 2 |
| \* | Astrin\_STLCHLD\_tube2\_061314\_01.13548.13548.3 | 4.0969 | 0.4342 | 100.0% | 2180.5444 | 2180.4963 | 1 | 6.878 | 45.8% | 2 | K.MGLEDTLEQLNAMITESKR.S | 3 |
| \* | Astrin\_STLCHLD\_tube2\_061314\_01.09534.09534.3 | 4.4893 | 0.4165 | 100.0% | 1979.6044 | 1979.2377 | 1 | 7.551 | 50.0% | 3 | R.TLKEEVQKLDDLYQQK.I | 3 |
| \* | Astrin\_STLCHLD\_tube2\_061314\_01.12535.12535.2 | 5.6523 | 0.6249 | 100.0% | 2554.8523 | 2555.8223 | 1 | 11.19 | 61.4% | 1 | K.HLLESTVNQGLSEAMNELDAVQR.E | 2 |
| \* | Astrin\_STLCHLD\_tube2\_061314\_02.05996.05996.2 | 4.2444 | 0.4498 | 100.0% | 1596.4521 | 1596.7344 | 1 | 8.659 | 79.2% | 14 | R.EYQLVVQTTTEER.R | 2 |
| \* | Astrin\_STLCHLD\_061214\_01.05467.05467.2 | 3.4773 | 0.3186 | 100.0% | 1752.5322 | 1752.9219 | 1 | 5.932 | 61.5% | 1 | R.EYQLVVQTTTEERR.K | 2 |
| \* | Astrin\_STLCHLD\_tube2\_061314\_01.07158.07158.2 | 4.3931 | 0.431 | 100.0% | 1513.3322 | 1513.7925 | 1 | 8.496 | 76.9% | 7 | R.LLEMVATHVGSVEK.H | 2 |
| \* | Astrin\_STLCHLD\_tube2\_061314\_01.07199.07199.3 | 2.3862 | 0.2998 | 96.9% | 1514.0343 | 1513.7925 | 1 | 5.462 | 44.2% | 3 | R.LLEMVATHVGSVEK.H | 3 |

---

|  |  |  |  |  |  |  |  |  |
| --- | --- | --- | --- | --- | --- | --- | --- | --- |
| U | *gi|4503571|ref|NP\_001* | 9 | 22 | 31.3% | 434 | 47169 | 7.4 | enolase 1 [Homo sapiens] |

| Filename XCorr DeltCN Conf% ObsM+H+ CalcM+H+ SpR ZScore Ion% # Sequence  | | | | | | | | | | | | |
| --- | --- | --- | --- | --- | --- | --- | --- | --- | --- | --- | --- | --- |
| \* | Astrin\_STLCHLD\_061214\_01.08010.08010.2 | 3.3018 | 0.338 | 100.0% | 1407.3121 | 1407.5634 | 2 | 7.839 | 62.5% | 5 | R.GNPTVEVDLFTSK.G | 2 |
|  | Astrin\_STLCHLD\_061214\_01.09480.09480.2 | 4.4819 | 0.4076 | 100.0% | 1806.5521 | 1806.0258 | 1 | 8.124 | 64.7% | 4 | R.AAVPSGASTGIYEALELR.D | 2 |
| \* | Astrin\_STLCHLD\_tube2\_061314\_01.06450.06450.2 | 3.0749 | 0.2313 | 98.7% | 1282.4122 | 1281.4817 | 10 | 6.125 | 65.0% | 2 | K.LMIEMDGTENK.S | 2 |
| \* | Astrin\_STLCHLD\_tube2\_061314\_01.11426.11426.3 | 5.4415 | 0.4333 | 100.0% | 3012.7144 | 3013.383 | 1 | 6.914 | 27.6% | 1 | R.HIADLAGNSEVILPVPAFNVINGGSHAGNK.L | 3 |
| \* | Astrin\_STLCHLD\_tube2\_061314\_01.13451.13451.2 | 4.3783 | 0.4917 | 100.0% | 1909.0521 | 1909.3148 | 1 | 8.88 | 62.5% | 2 | K.LAMQEFMILPVGAANFR.E | 2 |
| \* | Astrin\_STLCHLD\_tube2\_061314\_01.10637.10637.2 | 3.4224 | 0.4557 | 100.0% | 1426.4321 | 1426.6091 | 1 | 7.205 | 86.4% | 2 | R.YISPDQLADLYK.S | 2 |
| \* | Astrin\_STLCHLD\_061214\_02.07563.07563.2 | 5.2562 | 0.5188 | 100.0% | 2033.6522 | 2034.2737 | 1 | 10.48 | 52.6% | 1 | K.FTASAGIQVVGDDLTVTNPK.R | 2 |
| \* | Astrin\_STLCHLD\_061214\_02.07143.07143.3 | 4.0357 | 0.3012 | 99.8% | 2193.1443 | 2190.4612 | 2 | 6.161 | 36.2% | 3 | K.FTASAGIQVVGDDLTVTNPKR.I | 3 |
| \* | Astrin\_STLCHLD\_061214\_01.06408.06408.3 | 3.4126 | 0.2588 | 99.6% | 1528.0144 | 1526.7563 | 1 | 5.316 | 53.8% | 2 | K.LAQANGWGVMVSHR.S | 3 |

---

|  |  |  |  |  |  |  |  |  |
| --- | --- | --- | --- | --- | --- | --- | --- | --- |
| U | *gi|32455264|ref|NP\_85* | 6 | 11 | 31.2% | 199 | 22110 | 8.1 | peroxiredoxin 1 [Homo sapiens] |
| U | *gi|4505591|ref|NP\_002* | 6 | 11 | 31.2% | 199 | 22110 | 8.1 | peroxiredoxin 1 [Homo sapiens] |
| U | *gi|32455266|ref|NP\_85* | 6 | 11 | 31.2% | 199 | 22110 | 8.1 | peroxiredoxin 1 [Homo sapiens] |

| Filename XCorr DeltCN Conf% ObsM+H+ CalcM+H+ SpR ZScore Ion% # Sequence  | | | | | | | | | | | | |
| --- | --- | --- | --- | --- | --- | --- | --- | --- | --- | --- | --- | --- |
|  | Astrin\_STLCHLD\_tube2\_061314\_01.04662.04662.2 | 2.6098 | 0.4011 | 99.8% | 1165.4122 | 1165.3496 | 1 | 6.698 | 70.0% | 1 | K.ATAVMPDGQFK.D | 2 |
|  | Astrin\_STLCHLD\_tube2\_061314\_01.06503.06503.2 | 2.3462 | 0.3684 | 98.6% | 1107.5922 | 1108.2798 | 1 | 5.806 | 83.3% | 1 | R.TIAQDYGVLK.A | 2 |
|  | Astrin\_STLCHLD\_061214\_02.07447.07447.3 | 2.8758 | 0.4665 | 100.0% | 1984.4644 | 1984.2163 | 1 | 6.477 | 45.6% | 1 | R.TIAQDYGVLKADEGISFR.G | 3 |
|  | Astrin\_STLCHLD\_061214\_01.09338.09338.2 | 2.4777 | 0.2204 | 96.2% | 1362.0922 | 1360.6395 | 1 | 5.026 | 68.2% | 2 | R.GLFIIDDKGILR.Q | 2 |
|  | Astrin\_STLCHLD\_tube2\_061314\_01.07182.07182.2 | 2.561 | 0.3933 | 99.5% | 1211.8922 | 1212.3915 | 34 | 6.97 | 65.0% | 2 | R.QITVNDLPVGR.S | 22 |
|  | Astrin\_STLCHLD\_tube2\_061314\_01.08472.08472.2 | 3.3514 | 0.4978 | 100.0% | 1197.0122 | 1197.3763 | 1 | 7.848 | 88.9% | 4 | R.LVQAFQFTDK.H | 2 |

Similarities:
gi|32189392|ref|NP\_00(1:5)  

---

|  |  |  |  |  |  |  |  |  |
| --- | --- | --- | --- | --- | --- | --- | --- | --- |
| U | *gi|33286418|ref|NP\_00* | 10 | 17 | 30.7% | 531 | 57937 | 7.8 | pyruvate kinase, muscle isoform M2 [Homo sapiens] |

| Filename XCorr DeltCN Conf% ObsM+H+ CalcM+H+ SpR ZScore Ion% # Sequence  | | | | | | | | | | | | |
| --- | --- | --- | --- | --- | --- | --- | --- | --- | --- | --- | --- | --- |
|  | Astrin\_STLCHLD\_tube2\_061314\_01.06713.06713.2 | 3.0873 | 0.2172 | 98.5% | 1199.4321 | 1198.3617 | 8 | 5.464 | 70.0% | 3 | R.LDIDSPPITAR.N | 2 |
|  | Astrin\_STLCHLD\_061214\_01.04407.04407.3 | 2.9565 | 0.2356 | 96.9% | 1885.0443 | 1885.0458 | 1 | 4.555 | 36.7% | 1 | R.LNFSHGTHEYHAETIK.N | 3 |
|  | Astrin\_STLCHLD\_tube2\_061314\_01.11603.11603.3 | 3.9061 | 0.4711 | 100.0% | 2467.1943 | 2466.7937 | 1 | 6.88 | 30.7% | 1 | R.TATESFASDPILYRPVAVALDTK.G | 3 |
|  | Astrin\_STLCHLD\_tube2\_061314\_01.11132.11132.2 | 3.241 | 0.2975 | 99.5% | 1465.2722 | 1463.7142 | 1 | 5.342 | 66.7% | 1 | K.IYVDDGLISLQVK.Q | 2 |
|  | Astrin\_STLCHLD\_061214\_02.07936.07936.2 | 4.0569 | 0.4468 | 100.0% | 1780.8722 | 1780.9292 | 2 | 8.128 | 50.0% | 3 | K.GADFLVTEVENGGSLGSK.K | 2 |
|  | Astrin\_STLCHLD\_tube2\_061314\_01.15558.15558.2 | 4.088 | 0.5007 | 100.0% | 1860.2922 | 1861.1224 | 1 | 8.883 | 63.3% | 3 | K.FGVEQDVDMVFASFIR.K | 2 |
|  | Astrin\_STLCHLD\_tube2\_061314\_02.07996.07996.3 | 4.1481 | 0.3465 | 100.0% | 1823.9944 | 1823.0741 | 1 | 6.232 | 46.7% | 2 | R.RFDEILEASDGIMVAR.G | 3 |
|  | Astrin\_STLCHLD\_tube2\_061314\_01.07907.07907.2 | 2.807 | 0.3153 | 99.0% | 1141.7122 | 1142.2946 | 1 | 5.731 | 80.0% | 1 | R.GDLGIEIPAEK.V | 2 |
| \* | Astrin\_STLCHLD\_061214\_02.08114.08114.3 | 2.6294 | 0.2961 | 97.6% | 2088.5942 | 2089.3586 | 6 | 4.697 | 31.2% | 1 | R.EAEAAIYHLQLFEELRR.L | 3 |
| \* | Astrin\_STLCHLD\_061214\_02.08531.08531.2 | 3.9782 | 0.5102 | 100.0% | 2175.3323 | 2176.4282 | 1 | 9.047 | 50.0% | 1 | R.LAPITSDPTEATAVGAVEASFK.C | 2 |

---

|  |  |  |  |  |  |  |  |  |
| --- | --- | --- | --- | --- | --- | --- | --- | --- |
| U | *gi|150456457|ref|NP\_9* | 11 | 35 | 29.1% | 347 | 39929 | 5.6 | HMT1 hnRNP methyltransferase-like 2 isoform 2 [Homo sapiens] |
| U | *gi|154759421|ref|NP\_0* | 11 | 35 | 27.2% | 371 | 42462 | 5.3 | HMT1 hnRNP methyltransferase-like 2 isoform 1 [Homo sapiens] |
| U | *gi|151301219|ref|NP\_9* | 11 | 35 | 28.6% | 353 | 40548 | 5.5 | HMT1 hnRNP methyltransferase-like 2 isoform 3 [Homo sapiens] |

| Filename XCorr DeltCN Conf% ObsM+H+ CalcM+H+ SpR ZScore Ion% # Sequence  | | | | | | | | | | | | |
| --- | --- | --- | --- | --- | --- | --- | --- | --- | --- | --- | --- | --- |
|  | Astrin\_STLCHLD\_tube2\_061314\_01.04817.04817.3 | 4.4421 | 0.4346 | 100.0% | 1352.1543 | 1351.6322 | 1 | 7.842 | 54.5% | 2 | K.ANKLDHVVTIIK.G | 3 |
|  | Astrin\_STLCHLD\_tube2\_061314\_01.11397.11397.2 | 3.8739 | 0.4103 | 100.0% | 1643.5521 | 1643.8827 | 1 | 7.047 | 65.4% | 2 | R.DKWLAPDGLIFPDR.A | 2 |
|  | Astrin\_STLCHLD\_tube2\_061314\_01.11460.11460.3 | 3.0568 | 0.3727 | 100.0% | 1644.7144 | 1643.8827 | 5 | 5.962 | 44.2% | 1 | R.DKWLAPDGLIFPDR.A | 3 |
|  | Astrin\_STLCHLD\_061214\_01.06990.06990.2 | 3.6435 | 0.4859 | 100.0% | 1252.5721 | 1252.4099 | 2 | 8.25 | 70.0% | 16 | R.ATLYVTAIEDR.Q | 2 |
|  | Astrin\_STLCHLD\_tube2\_061314\_01.09152.09152.2 | 3.3368 | 0.3007 | 99.5% | 1637.6721 | 1637.914 | 1 | 6.241 | 60.7% | 1 | K.DVAIKEPLVDVVDPK.Q | 2 |
|  | Astrin\_STLCHLD\_tube2\_061314\_02.10048.10048.3 | 4.172 | 0.4212 | 100.0% | 2229.6843 | 2229.5027 | 1 | 7.832 | 39.7% | 2 | K.RNDYVHALVAYFNIEFTR.C | 3 |
|  | Astrin\_STLCHLD\_061214\_02.10687.10687.2 | 4.4908 | 0.5714 | 100.0% | 2072.672 | 2073.3152 | 1 | 9.607 | 53.1% | 1 | R.NDYVHALVAYFNIEFTR.C | 2 |
|  | Astrin\_STLCHLD\_tube2\_061314\_01.06572.06572.2 | 3.4415 | 0.3576 | 100.0% | 1726.4122 | 1725.8547 | 1 | 6.254 | 57.1% | 1 | R.TGFSTSPESPYTHWK.Q | 2 |
|  | Astrin\_STLCHLD\_tube2\_061314\_01.06606.06606.3 | 2.3517 | 0.3104 | 96.8% | 1727.0343 | 1725.8547 | 1 | 5.274 | 37.5% | 1 | R.TGFSTSPESPYTHWK.Q | 3 |
|  | Astrin\_STLCHLD\_tube2\_061314\_01.08448.08448.3 | 3.069 | 0.3324 | 99.7% | 1721.2144 | 1721.969 | 3 | 5.686 | 36.7% | 4 | K.TGEEIFGTIGMRPNAK.N | 3 |
|  | Astrin\_STLCHLD\_061214\_01.07171.07171.2 | 4.133 | 0.4976 | 100.0% | 1721.6122 | 1721.969 | 1 | 8.026 | 53.3% | 4 | K.TGEEIFGTIGMRPNAK.N | 2 |

---

|  |  |  |  |  |  |  |  |  |
| --- | --- | --- | --- | --- | --- | --- | --- | --- |
| U | *gi|50592996|ref|NP\_00* | 18 | 94 | 27.6% | 450 | 50433 | 4.9 | tubulin, beta, 4 [Homo sapiens] |

| Filename XCorr DeltCN Conf% ObsM+H+ CalcM+H+ SpR ZScore Ion% # Sequence  | | | | | | | | | | | | |
| --- | --- | --- | --- | --- | --- | --- | --- | --- | --- | --- | --- | --- |
|  | Astrin\_STLCHLD\_tube2\_061314\_01.10607.10607.2 | 4.4949 | 0.5136 | 100.0% | 1616.5521 | 1616.8701 | 1 | 8.677 | 71.4% | 9 | R.AILVDLEPGTMDSVR.S | 222 |
|  | Astrin\_STLCHLD\_061214\_01.11227.11227.2 | 6.4727 | 0.5531 | 100.0% | 1959.4521 | 1960.151 | 1 | 10.56 | 73.5% | 4 | K.GHYTEGAELVDSVLDVVR.K | 2222 |
|  | Astrin\_STLCHLD\_061214\_01.11221.11221.3 | 4.0137 | 0.3325 | 100.0% | 1961.2144 | 1960.151 | 1 | 6.21 | 44.1% | 5 | K.GHYTEGAELVDSVLDVVR.K | 3333 |
|  | Astrin\_STLCHLD\_tube2\_061314\_01.12007.12007.2 | 5.7392 | 0.5731 | 100.0% | 2087.912 | 2088.325 | 1 | 10.188 | 69.4% | 1 | K.GHYTEGAELVDSVLDVVRK.E | 2222 |
|  | Astrin\_STLCHLD\_tube2\_061314\_01.11982.11982.3 | 4.7827 | 0.4838 | 100.0% | 2088.1143 | 2088.325 | 1 | 8.148 | 44.4% | 7 | K.GHYTEGAELVDSVLDVVRK.E | 3333 |
|  | Astrin\_STLCHLD\_tube2\_061314\_01.08593.08593.1 | 1.9485 | 0.2768 | 100.0% | 1319.74 | 1320.5896 | 79 | 4.843 | 45.5% | 2 | R.IMNTFSVVPSPK.V | 111 |
|  | Astrin\_STLCHLD\_tube2\_061314\_01.08597.08597.2 | 4.5471 | 0.3836 | 100.0% | 1320.4122 | 1320.5896 | 1 | 7.86 | 77.3% | 9 | R.IMNTFSVVPSPK.V | 222 |
|  | Astrin\_STLCHLD\_tube2\_061314\_01.07361.07361.2 | 3.2733 | 0.2391 | 99.5% | 1132.2322 | 1131.2767 | 1 | 5.073 | 83.3% | 14 | R.FPGQLNADLR.K | 22222 |
|  | Astrin\_STLCHLD\_tube2\_061314\_01.04904.04904.2 | 2.5627 | 0.1707 | 95.5% | 1259.2922 | 1259.4508 | 1 | 4.474 | 70.0% | 2 | R.FPGQLNADLRK.L | 22222 |
|  | Astrin\_STLCHLD\_tube2\_061314\_01.04842.04842.3 | 2.8966 | 0.3686 | 100.0% | 1260.2043 | 1259.4508 | 14 | 5.742 | 42.5% | 5 | R.FPGQLNADLRK.L | 33333 |
|  | Astrin\_STLCHLD\_tube2\_061314\_01.08730.08730.2 | 3.8464 | 0.3753 | 100.0% | 1272.4122 | 1272.5945 | 1 | 7.898 | 75.0% | 4 | R.KLAVNMVPFPR.L | 22222 |
|  | Astrin\_STLCHLD\_tube2\_061314\_01.10148.10148.1 | 2.1143 | 0.2239 | 96.6% | 1143.61 | 1144.4204 | 18 | 6.431 | 61.1% | 1 | K.LAVNMVPFPR.L | 11111 |
|  | Astrin\_STLCHLD\_tube2\_061314\_01.10247.10247.2 | 3.6473 | 0.5079 | 100.0% | 1145.2722 | 1144.4204 | 1 | 8.426 | 94.4% | 4 | K.LAVNMVPFPR.L | 22222 |
|  | Astrin\_STLCHLD\_tube2\_061314\_01.12186.12186.2 | 4.648 | 0.4524 | 100.0% | 1692.6122 | 1692.9678 | 1 | 7.8 | 78.6% | 2 | R.ALTVPELTQQMFDAK.N | 22 |
|  | Astrin\_STLCHLD\_tube2\_061314\_01.11781.11781.2 | 4.295 | 0.2978 | 100.0% | 1698.5322 | 1697.8877 | 1 | 7.708 | 73.1% | 2 | K.NSSYFVEWIPNNVK.V | 22222 |
| \* | Astrin\_STLCHLD\_tube2\_061314\_02.09999.09999.2 | 3.2328 | 0.1169 | 96.4% | 1874.5521 | 1875.1469 | 5 | 3.593 | 46.9% | 1 | K.MSSTFIGNSTAIQELFK.R | 2 |
|  | Astrin\_STLCHLD\_061214\_01.07812.07812.2 | 3.8437 | 0.3944 | 100.0% | 1386.3522 | 1386.6116 | 1 | 7.545 | 80.0% | 6 | K.RISEQFTAMFR.R | 2222 |
|  | Astrin\_STLCHLD\_tube2\_061314\_02.07946.07946.2 | 3.9325 | 0.4891 | 100.0% | 1229.9321 | 1230.4241 | 1 | 8.487 | 94.4% | 16 | R.ISEQFTAMFR.R | 2222 |

Similarities:
gi|29788785|ref|NP\_82(16:2)  
gi|5174735|ref|NP\_006(16:2)  
gi|29788768|ref|NP\_82(14:4)  
gi|14210536|ref|NP\_11(7:11)  

---

|  |  |  |  |  |  |  |  |  |
| --- | --- | --- | --- | --- | --- | --- | --- | --- |
| U | *gi|4507357|ref|NP\_003* | 4 | 4 | 27.6% | 199 | 22391 | 8.2 | transgelin 2 [Homo sapiens] |

| Filename XCorr DeltCN Conf% ObsM+H+ CalcM+H+ SpR ZScore Ion% # Sequence  | | | | | | | | | | | | |
| --- | --- | --- | --- | --- | --- | --- | --- | --- | --- | --- | --- | --- |
| \* | Astrin\_STLCHLD\_tube2\_061314\_02.10361.10361.2 | 2.6382 | 0.3725 | 98.8% | 2101.2722 | 2101.3203 | 97 | 6.012 | 32.4% | 1 | R.YGINTTDIFQTVDLWEGK.N | 2 |
| \* | Astrin\_STLCHLD\_tube2\_061314\_01.10310.10310.2 | 2.0321 | 0.358 | 96.8% | 1216.1122 | 1216.4845 | 32 | 5.918 | 50.0% | 1 | R.TLMNLGGLAVAR.D | 2 |
| \* | Astrin\_STLCHLD\_tube2\_061314\_01.06549.06549.2 | 2.9452 | 0.4609 | 100.0% | 1203.6921 | 1203.402 | 1 | 8.668 | 85.0% | 1 | K.NVIGLQMGTNR.G | 2 |
|  | Astrin\_STLCHLD\_tube2\_061314\_01.04635.04635.2 | 3.4213 | 0.453 | 100.0% | 1383.9922 | 1384.5677 | 1 | 8.988 | 69.2% | 1 | R.GASQAGMTGYGMPR.Q | 2 |

---

|  |  |  |  |  |  |  |  |  |
| --- | --- | --- | --- | --- | --- | --- | --- | --- |
| U | *gi|20149594|ref|NP\_03* | 14 | 36 | 26.7% | 724 | 83264 | 5.0 | heat shock 90kDa protein 1, beta [Homo sapiens] |

| Filename XCorr DeltCN Conf% ObsM+H+ CalcM+H+ SpR ZScore Ion% # Sequence  | | | | | | | | | | | | |
| --- | --- | --- | --- | --- | --- | --- | --- | --- | --- | --- | --- | --- |
|  | Astrin\_STLCHLD\_tube2\_061314\_01.05813.05813.2 | 3.1355 | 0.4012 | 100.0% | 1276.3322 | 1276.3861 | 2 | 6.953 | 68.2% | 1 | R.ELISNASDALDK.I | 2 |
|  | Astrin\_STLCHLD\_tube2\_061314\_01.08535.08535.2 | 2.8014 | 0.2344 | 97.4% | 1546.7722 | 1545.733 | 1 | 5.396 | 61.5% | 1 | R.ELISNASDALDKIR.Y | 2 |
|  | Astrin\_STLCHLD\_tube2\_061314\_01.09271.09271.2 | 2.5815 | 0.1988 | 96.3% | 1242.3922 | 1243.4459 | 6 | 5.215 | 54.5% | 1 | K.ADLINNLGTIAK.S | 22 |
|  | Astrin\_STLCHLD\_061214\_02.06734.06734.3 | 3.3418 | 0.257 | 98.6% | 2258.3342 | 2257.294 | 1 | 5.261 | 35.5% | 2 | K.HNDDEQYAWESSAGGSFTVR.A | 33 |
|  | Astrin\_STLCHLD\_061214\_01.06097.06097.3 | 4.2275 | 0.4235 | 100.0% | 2016.4143 | 2016.2584 | 1 | 7.282 | 48.3% | 5 | K.VILHLKEDQTEYLEER.R | 33 |
| \* | Astrin\_STLCHLD\_tube2\_061314\_02.06786.06786.2 | 5.084 | 0.5321 | 100.0% | 1849.3722 | 1848.9171 | 1 | 9.586 | 82.1% | 4 | R.NPDDITQEEYGEFYK.S | 2 |
|  | Astrin\_STLCHLD\_061214\_01.07095.07095.2 | 4.0697 | 0.4045 | 100.0% | 1528.3322 | 1528.6616 | 1 | 7.91 | 75.0% | 2 | K.SLTNDWEDHLAVK.H | 22 |
|  | Astrin\_STLCHLD\_061214\_01.06870.06870.2 | 2.9414 | 0.4216 | 100.0% | 1349.4521 | 1349.4886 | 1 | 7.003 | 70.0% | 5 | K.HFSVEGQLEFR.A | 22 |
| \* | Astrin\_STLCHLD\_tube2\_061314\_01.10236.10236.2 | 1.9625 | 0.2378 | 95.2% | 830.3722 | 830.06104 | 1 | 4.869 | 83.3% | 1 | R.ALLFIPR.R | 2 |
| \* | Astrin\_STLCHLD\_tube2\_061314\_01.09371.09371.2 | 2.6882 | 0.1449 | 96.4% | 1237.1921 | 1237.4008 | 7 | 4.433 | 66.7% | 2 | R.RAPFDLFENK.K | 2 |
| \* | Astrin\_STLCHLD\_tube2\_061314\_02.06674.06674.3 | 4.6511 | 0.4153 | 100.0% | 2178.6843 | 2178.2915 | 1 | 7.014 | 38.9% | 7 | R.YHTSQSGDEMTSLSEYVSR.M | 3 |
| \* | Astrin\_STLCHLD\_tube2\_061314\_01.05965.05965.2 | 3.2708 | 0.2184 | 98.9% | 1250.4122 | 1249.4574 | 1 | 6.279 | 80.0% | 1 | R.DNSTMGYMMAK.K | 2 |
| \* | Astrin\_STLCHLD\_tube2\_061314\_01.06941.06941.3 | 3.9647 | 0.4814 | 100.0% | 1782.6543 | 1784.025 | 4 | 8.206 | 41.1% | 3 | K.HLEINPDHPIVETLR.Q | 3 |
| \* | Astrin\_STLCHLD\_tube2\_061314\_01.16956.16956.3 | 3.469 | 0.2518 | 98.3% | 3290.6643 | 3288.725 | 63 | 4.073 | 19.0% | 1 | K.AVKDLVVLLFETALLSSGFSLEDPQTHSNR.I | 3 |

Similarities:
gi|153792590|ref|NP\_0(5:9)  

---

|  |  |  |  |  |  |  |  |  |
| --- | --- | --- | --- | --- | --- | --- | --- | --- |
| U | *gi|12667788|ref|NP\_00* | 42 | 80 | 26.1% | 1960 | 226530 | 5.6 | myosin, heavy polypeptide 9, non-muscle [Homo sapiens] |

| Filename XCorr DeltCN Conf% ObsM+H+ CalcM+H+ SpR ZScore Ion% # Sequence  | | | | | | | | | | | | |
| --- | --- | --- | --- | --- | --- | --- | --- | --- | --- | --- | --- | --- |
| \* | Astrin\_STLCHLD\_tube2\_061314\_01.11199.11199.2 | 3.7482 | 0.2957 | 100.0% | 1673.3522 | 1673.8687 | 1 | 7.707 | 64.3% | 1 | K.NFINNPLAQADWAAK.K | 2 |
| \* | Astrin\_STLCHLD\_tube2\_061314\_01.05234.05234.2 | 2.3678 | 0.2485 | 97.2% | 1073.4722 | 1072.2926 | 265 | 5.022 | 50.0% | 1 | K.KLVWVPSDK.S | 2 |
| \* | Astrin\_STLCHLD\_tube2\_061314\_01.05205.05205.3 | 3.3482 | 0.3068 | 99.7% | 2072.5444 | 2072.3489 | 1 | 5.708 | 34.4% | 1 | K.RHEMPPHIYAITDTAYR.S | 3 |
| \* | Astrin\_STLCHLD\_tube2\_061314\_01.06187.06187.3 | 3.7096 | 0.3335 | 100.0% | 1917.2344 | 1916.1614 | 1 | 6.54 | 46.7% | 6 | R.HEMPPHIYAITDTAYR.S | 3 |
| \* | Astrin\_STLCHLD\_tube2\_061314\_01.07561.07561.3 | 2.5201 | 0.2632 | 96.3% | 1608.0243 | 1607.8931 | 2 | 4.974 | 42.3% | 1 | K.KVIQYLAYVASSHK.S | 3 |
| \* | Astrin\_STLCHLD\_061214\_01.07249.07249.2 | 3.9864 | 0.4528 | 100.0% | 1479.8322 | 1479.719 | 1 | 8.588 | 75.0% | 1 | K.VIQYLAYVASSHK.S | 2 |
| \* | Astrin\_STLCHLD\_061214\_01.07225.07225.3 | 2.4856 | 0.3792 | 99.7% | 1480.6144 | 1479.719 | 4 | 6.208 | 39.6% | 1 | K.VIQYLAYVASSHK.S | 3 |
| \* | Astrin\_STLCHLD\_tube2\_061314\_01.12587.12587.3 | 4.9721 | 0.3904 | 100.0% | 1997.6044 | 1997.3037 | 1 | 7.865 | 45.3% | 2 | R.TFHIFYYLLSGAGEHLK.T | 3 |
| \* | Astrin\_STLCHLD\_tube2\_061314\_01.11153.11153.2 | 3.6426 | 0.4331 | 100.0% | 1616.4722 | 1616.9313 | 1 | 7.978 | 73.1% | 3 | R.IMGIPEEEQMGLLR.V | 2 |
| \* | Astrin\_STLCHLD\_tube2\_061314\_01.10393.10393.2 | 3.0244 | 0.2968 | 98.8% | 1572.2522 | 1572.8044 | 1 | 6.636 | 57.7% | 1 | K.VSHLLGINVTDFTR.G | 2 |
| \* | Astrin\_STLCHLD\_tube2\_061314\_01.10433.10433.3 | 4.0122 | 0.263 | 100.0% | 1573.1044 | 1572.8044 | 3 | 4.832 | 51.9% | 1 | K.VSHLLGINVTDFTR.G | 3 |
|  | Astrin\_STLCHLD\_tube2\_061314\_01.11798.11798.3 | 3.5308 | 0.177 | 96.3% | 2468.6042 | 2468.7893 | 1 | 5.224 | 37.5% | 1 | K.LQQLFNHTMFILEQEEYQR.E | 33 |
|  | Astrin\_STLCHLD\_061214\_01.07250.07250.2 | 2.6822 | 0.2747 | 98.2% | 1398.6721 | 1398.6166 | 1 | 5.315 | 75.0% | 1 | K.VDYKADEWLMK.N | 22 |
| \* | Astrin\_STLCHLD\_tube2\_061314\_01.12537.12537.2 | 4.5639 | 0.2039 | 100.0% | 2019.9722 | 2019.3636 | 1 | 7.321 | 55.3% | 1 | R.IIGLDQVAGMSETALPGAFK.T | 2 |
| \* | Astrin\_STLCHLD\_tube2\_061314\_01.07224.07224.2 | 2.0908 | 0.4187 | 99.3% | 925.7322 | 925.07513 | 1 | 6.878 | 75.0% | 3 | R.VVFQEFR.Q | 2 |
| \* | Astrin\_STLCHLD\_tube2\_061314\_01.08136.08136.2 | 3.1863 | 0.4445 | 100.0% | 1194.2722 | 1194.33 | 1 | 7.748 | 77.8% | 3 | K.ALELDSNLYR.I | 2 |
|  | Astrin\_STLCHLD\_tube2\_061314\_01.05545.05545.2 | 3.5792 | 0.372 | 100.0% | 1225.5122 | 1224.3591 | 1 | 6.298 | 75.0% | 1 | R.AGVLAHLEEER.D | 22 |
| \* | Astrin\_STLCHLD\_061214\_02.07924.07924.2 | 4.4629 | 0.394 | 100.0% | 1752.4922 | 1753.0358 | 1 | 7.225 | 57.1% | 4 | R.LTEMETLQSQLMAEK.L | 2 |
| \* | Astrin\_STLCHLD\_tube2\_061314\_01.10902.10902.3 | 3.9911 | 0.3125 | 100.0% | 2335.3743 | 2334.4736 | 2 | 5.488 | 33.3% | 1 | K.MQQNIQELEEQLEEEESAR.Q | 3 |
| \* | Astrin\_STLCHLD\_061214\_01.06582.06582.2 | 4.7748 | 0.4945 | 100.0% | 1654.4321 | 1654.7681 | 1 | 8.072 | 73.1% | 6 | R.IAEFTTNLTEEEEK.S | 2 |
| \* | Astrin\_STLCHLD\_tube2\_061314\_01.11659.11659.2 | 3.2919 | 0.4572 | 100.0% | 2034.3722 | 2035.126 | 1 | 8.579 | 59.4% | 1 | R.ELESQISELQEDLESER.A | 2 |
| \* | Astrin\_STLCHLD\_tube2\_061314\_01.15641.15641.3 | 3.4249 | 0.1999 | 95.3% | 3018.2644 | 3019.2434 | 1 | 4.405 | 26.0% | 1 | R.DLGEELEALKTELEDTLDSTAAQQELR.S | 3 |
| \* | Astrin\_STLCHLD\_tube2\_061314\_01.15624.15624.2 | 4.9772 | 0.5491 | 100.0% | 3019.172 | 3019.2434 | 1 | 9.594 | 48.1% | 1 | R.DLGEELEALKTELEDTLDSTAAQQELR.S | 2 |
| \* | Astrin\_STLCHLD\_tube2\_061314\_01.06098.06098.3 | 3.599 | 0.2939 | 99.7% | 2045.8444 | 2044.2439 | 2 | 5.194 | 32.8% | 1 | K.TLEEEAKTHEAQIQEMR.Q | 3 |
| \* | Astrin\_STLCHLD\_tube2\_061314\_01.08315.08315.3 | 5.5822 | 0.4818 | 100.0% | 1997.0343 | 1997.1722 | 1 | 8.928 | 43.8% | 3 | K.HSQAVEELAEQLEQTKR.V | 3 |
| \* | Astrin\_STLCHLD\_tube2\_061314\_02.08471.08471.2 | 5.442 | 0.5601 | 100.0% | 1946.4521 | 1947.1498 | 1 | 9.768 | 73.5% | 3 | K.LQVELDNVTGLLSQSDSK.S | 2 |
| \* | Astrin\_STLCHLD\_tube2\_061314\_01.12135.12135.3 | 3.5296 | 0.3198 | 99.8% | 1951.8243 | 1951.1436 | 1 | 4.977 | 46.7% | 1 | R.LQQELDDLLVDLDHQR.Q | 3 |
|  | Astrin\_STLCHLD\_tube2\_061314\_01.05081.05081.2 | 2.9194 | 0.1221 | 96.9% | 1220.8522 | 1221.3959 | 9 | 4.855 | 66.7% | 1 | K.KFDQLLAEEK.T | 22 |
|  | Astrin\_STLCHLD\_tube2\_061314\_01.05537.05537.2 | 2.7381 | 0.2319 | 98.4% | 1093.4321 | 1093.2218 | 1 | 6.533 | 81.2% | 3 | K.FDQLLAEEK.T | 22 |
| \* | Astrin\_STLCHLD\_tube2\_061314\_02.05524.05524.2 | 4.0204 | 0.46 | 100.0% | 1647.4722 | 1647.8407 | 1 | 7.674 | 76.9% | 2 | R.ALEEAMEQKAELER.L | 2 |
| \* | Astrin\_STLCHLD\_061214\_02.05439.05439.3 | 4.0671 | 0.3888 | 100.0% | 1648.1044 | 1647.8407 | 1 | 6.816 | 57.7% | 3 | R.ALEEAMEQKAELER.L | 3 |
| \* | Astrin\_STLCHLD\_tube2\_061314\_01.13920.13920.3 | 3.3801 | 0.2322 | 96.9% | 3148.8843 | 3149.4048 | 1 | 4.022 | 23.1% | 1 | R.ALEQQVEEMKTQLEELEDELQATEDAK.L | 3 |
| \* | Astrin\_STLCHLD\_061214\_01.07334.07334.2 | 3.4795 | 0.1931 | 99.1% | 1315.2522 | 1315.6171 | 1 | 7.441 | 65.0% | 2 | K.LRLEVNLQAMK.A | 2 |
| \* | Astrin\_STLCHLD\_tube2\_061314\_01.07457.07457.2 | 2.408 | 0.2871 | 98.0% | 1045.8522 | 1046.2701 | 1 | 5.288 | 81.2% | 1 | R.LEVNLQAMK.A | 2 |
| \* | Astrin\_STLCHLD\_tube2\_061314\_01.12914.12914.2 | 2.7629 | 0.3596 | 98.9% | 2049.5923 | 2050.3064 | 1 | 6.523 | 52.9% | 1 | K.SMEAEMIQLQEELAAAER.A | 2 |
| \* | Astrin\_STLCHLD\_tube2\_061314\_01.05621.05621.3 | 3.9424 | 0.4664 | 100.0% | 2090.0645 | 2090.168 | 1 | 7.213 | 40.3% | 1 | R.QAQQERDELADEIANSSGK.G | 3 |
| \* | Astrin\_STLCHLD\_tube2\_061314\_02.07680.07680.2 | 6.3228 | 0.5145 | 100.0% | 2473.9722 | 2473.6099 | 1 | 9.283 | 62.5% | 2 | R.IAQLEEELEEEQGNTELINDR.L | 2 |
| \* | Astrin\_STLCHLD\_tube2\_061314\_02.07664.07664.3 | 4.7839 | 0.3452 | 100.0% | 2474.3643 | 2473.6099 | 1 | 6.612 | 37.5% | 2 | R.IAQLEEELEEEQGNTELINDR.L | 3 |
| \* | Astrin\_STLCHLD\_tube2\_061314\_01.10289.10289.2 | 5.3383 | 0.489 | 100.0% | 1870.6721 | 1871.0574 | 1 | 9.581 | 66.7% | 2 | K.ANLQIDQINTDLNLER.S | 2 |
| \* | Astrin\_STLCHLD\_tube2\_061314\_01.06338.06338.2 | 4.3734 | 0.4003 | 100.0% | 1531.3322 | 1531.6598 | 1 | 7.655 | 75.0% | 2 | K.IAQLEEQLDNETK.E | 2 |
| \* | Astrin\_STLCHLD\_tube2\_061314\_01.04549.04549.2 | 4.5441 | 0.5434 | 100.0% | 1566.3722 | 1566.6367 | 1 | 9.726 | 73.1% | 2 | R.ELEDATETADAMNR.E | 2 |
| \* | Astrin\_STLCHLD\_tube2\_061314\_01.07775.07775.2 | 3.8167 | 0.2489 | 100.0% | 1156.3322 | 1156.3732 | 1 | 6.143 | 88.9% | 4 | R.RGDLPFVVPR.R | 2 |

Similarities:
gi|41406064|ref|NP\_00(5:37)  

---

|  |  |  |  |  |  |  |  |  |
| --- | --- | --- | --- | --- | --- | --- | --- | --- |
| U | *gi|27436946|ref|NP\_73* | 16 | 52 | 25.6% | 664 | 74140 | 7.0 | lamin A/C isoform 1 precursor [Homo sapiens] |

| Filename XCorr DeltCN Conf% ObsM+H+ CalcM+H+ SpR ZScore Ion% # Sequence  | | | | | | | | | | | | |
| --- | --- | --- | --- | --- | --- | --- | --- | --- | --- | --- | --- | --- |
|  | Astrin\_STLCHLD\_061214\_01.04990.04990.2 | 4.3021 | 0.4597 | 100.0% | 1418.1921 | 1418.5901 | 1 | 8.438 | 72.7% | 2 | R.LRITESEEVVSR.E | 2 |
|  | Astrin\_STLCHLD\_tube2\_061314\_02.05014.05014.3 | 3.4976 | 0.2994 | 100.0% | 1418.6344 | 1418.5901 | 8 | 5.139 | 50.0% | 4 | R.LRITESEEVVSR.E | 3 |
|  | Astrin\_STLCHLD\_tube2\_061314\_01.09252.09252.2 | 3.2981 | 0.3392 | 100.0% | 1245.4122 | 1244.474 | 1 | 6.009 | 75.0% | 1 | R.LKDLEALLNSK.E | 2 |
|  | Astrin\_STLCHLD\_tube2\_061314\_01.06561.06561.2 | 2.5508 | 0.2355 | 97.4% | 1184.4521 | 1183.3066 | 149 | 4.89 | 61.1% | 1 | R.TLEGELHDLR.G | 2 |
|  | Astrin\_STLCHLD\_tube2\_061314\_01.07482.07482.2 | 3.6161 | 0.3066 | 100.0% | 1029.1921 | 1029.1814 | 1 | 5.98 | 87.5% | 3 | R.LADALQELR.A | 2 |
|  | Astrin\_STLCHLD\_061214\_01.05402.05402.2 | 4.7548 | 0.4962 | 100.0% | 1753.3121 | 1753.8693 | 1 | 8.557 | 70.0% | 4 | R.NSNLVGAAHEELQQSR.I | 2 |
|  | Astrin\_STLCHLD\_tube2\_061314\_01.09799.09799.2 | 4.0109 | 0.3893 | 100.0% | 1699.8922 | 1700.9762 | 1 | 7.137 | 67.9% | 1 | R.IRIDSLSAQLSQLQK.Q | 2 |
|  | Astrin\_STLCHLD\_tube2\_061314\_01.04782.04782.2 | 3.1849 | 0.3413 | 100.0% | 1188.3322 | 1188.3262 | 1 | 5.813 | 83.3% | 2 | K.LRDLEDSLAR.E | 2 |
|  | Astrin\_STLCHLD\_tube2\_061314\_01.04765.04765.3 | 3.3945 | 0.1629 | 98.2% | 1189.0743 | 1188.3262 | 1 | 4.491 | 52.8% | 1 | K.LRDLEDSLAR.E | 3 |
|  | Astrin\_STLCHLD\_tube2\_061314\_01.11615.11615.2 | 5.489 | 0.4506 | 100.0% | 1894.7322 | 1895.1346 | 1 | 8.371 | 75.0% | 1 | R.MQQQLDEYQELLDIK.L | 2 |
|  | Astrin\_STLCHLD\_061214\_01.05484.05484.2 | 4.0344 | 0.5406 | 100.0% | 1606.4521 | 1606.7728 | 1 | 8.972 | 73.1% | 4 | R.VAVEEVDEEGKFVR.L | 2 |
|  | Astrin\_STLCHLD\_061214\_01.05412.05412.3 | 2.9439 | 0.3267 | 99.7% | 1607.2144 | 1606.7728 | 5 | 5.539 | 38.5% | 5 | R.VAVEEVDEEGKFVR.L | 3 |
|  | Astrin\_STLCHLD\_tube2\_061314\_02.06002.06002.2 | 4.3827 | 0.613 | 100.0% | 1492.2122 | 1492.6874 | 1 | 9.872 | 69.2% | 11 | R.TALINSTGEEVAMR.K | 2 |
|  | Astrin\_STLCHLD\_tube2\_061314\_02.05033.05033.2 | 3.4177 | 0.5345 | 100.0% | 2365.7322 | 2366.504 | 1 | 9.42 | 36.5% | 1 | K.ASASGSGAQVGGPISSGSSASSVTVTR.S | 2 |
|  | Astrin\_STLCHLD\_tube2\_061314\_02.05010.05010.3 | 4.1628 | 0.4955 | 100.0% | 2366.3943 | 2366.504 | 1 | 7.772 | 34.6% | 2 | K.ASASGSGAQVGGPISSGSSASSVTVTR.S | 3 |
|  | Astrin\_STLCHLD\_061214\_01.06014.06014.2 | 4.4214 | 0.5221 | 100.0% | 1567.7122 | 1567.6555 | 1 | 9.825 | 53.1% | 9 | R.SVGGSGGGSFGDNLVTR.S | 2 |

---

|  |  |  |  |  |  |  |  |  |
| --- | --- | --- | --- | --- | --- | --- | --- | --- |
| U | *contaminant\_KERATIN02* | 10 | 27 | 24.6% | 622 | 61987 | 5.2 | no description |
| U | *gi|55956899|ref|NP\_00* | 10 | 27 | 24.6% | 623 | 62064 | 5.2 | keratin 9 [Homo sapiens] |

| Filename XCorr DeltCN Conf% ObsM+H+ CalcM+H+ SpR ZScore Ion% # Sequence  | | | | | | | | | | | | |
| --- | --- | --- | --- | --- | --- | --- | --- | --- | --- | --- | --- | --- |
|  | Astrin\_STLCHLD\_061214\_02.07521.07521.2 | 4.9205 | 0.4601 | 100.0% | 2705.8123 | 2706.7605 | 1 | 8.922 | 38.7% | 1 | R.GGGGSFGYSYGGGSGGGFSASSLGGGFGGGSR.G | 2 |
|  | Astrin\_STLCHLD\_061214\_02.07514.07514.3 | 4.0048 | 0.313 | 99.8% | 2706.5344 | 2706.7605 | 1 | 5.803 | 24.2% | 3 | R.GGGGSFGYSYGGGSGGGFSASSLGGGFGGGSR.G | 3 |
|  | Astrin\_STLCHLD\_tube2\_061314\_01.10086.10086.3 | 3.5259 | 0.1897 | 96.5% | 2379.0244 | 2378.5981 | 129 | 4.153 | 32.5% | 1 | R.LASYLDKVQALEEANNDLENK.I | 3 |
|  | Astrin\_STLCHLD\_tube2\_061314\_01.06869.06869.2 | 2.6971 | 0.3585 | 100.0% | 1061.4521 | 1061.1802 | 1 | 6.428 | 93.8% | 1 | K.TLLDIDNTR.M | 2 |
|  | Astrin\_STLCHLD\_tube2\_061314\_01.05373.05373.2 | 2.6456 | 0.189 | 96.7% | 1158.4722 | 1158.2566 | 2 | 4.833 | 80.0% | 2 | R.QGVDADINGLR.Q | 2 |
|  | Astrin\_STLCHLD\_061214\_02.07940.07940.3 | 4.2505 | 0.2979 | 100.0% | 1839.4143 | 1839.0557 | 1 | 5.863 | 41.7% | 2 | R.HGVQELEIELQSQLSK.K | 3 |
|  | Astrin\_STLCHLD\_061214\_02.07912.07912.2 | 3.0402 | 0.2119 | 97.4% | 1840.4922 | 1839.0557 | 1 | 5.058 | 60.0% | 1 | R.HGVQELEIELQSQLSK.K | 2 |
|  | Astrin\_STLCHLD\_061214\_01.08264.08264.3 | 5.2959 | 0.3595 | 100.0% | 1967.6643 | 1967.2297 | 1 | 6.466 | 46.9% | 5 | R.HGVQELEIELQSQLSKK.A | 3 |
|  | Astrin\_STLCHLD\_tube2\_061314\_02.07080.07080.3 | 5.2193 | 0.1107 | 97.8% | 2511.3542 | 2511.6177 | 1 | 6.408 | 37.5% | 4 | K.EIETYHNLLEGGQEDFESSGAGK.I | 3 |
|  | Astrin\_STLCHLD\_061214\_01.04327.04327.3 | 8.1976 | 0.6145 | 100.0% | 3224.5745 | 3225.1118 | 1 | 11.349 | 28.8% | 7 | R.GGSGGSHGGGSGFGGESGGSYGGGEEASGSGGGYGGGSGK.S | 3 |

---

|  |  |  |  |  |  |  |  |  |
| --- | --- | --- | --- | --- | --- | --- | --- | --- |
| U | *gi|4506901|ref|NP\_003* | 3 | 5 | 24.4% | 164 | 19330 | 11.6 | splicing factor, arginine/serine-rich 3 [Homo sapiens] |

| Filename XCorr DeltCN Conf% ObsM+H+ CalcM+H+ SpR ZScore Ion% # Sequence  | | | | | | | | | | | | |
| --- | --- | --- | --- | --- | --- | --- | --- | --- | --- | --- | --- | --- |
| \* | Astrin\_STLCHLD\_tube2\_061314\_01.04557.04557.2 | 4.6238 | 0.3445 | 100.0% | 1878.1921 | 1878.0519 | 1 | 7.16 | 65.6% | 1 | K.VYVGNLGNNGNKTELER.A | 2 |
| \* | Astrin\_STLCHLD\_tube2\_061314\_01.08489.08489.2 | 3.074 | 0.4513 | 100.0% | 1044.0922 | 1044.198 | 1 | 8.287 | 87.5% | 3 | R.AFGYYGPLR.S | 2 |
|  | Astrin\_STLCHLD\_tube2\_061314\_01.11862.11862.2 | 3.2875 | 0.4192 | 100.0% | 1622.4122 | 1622.7771 | 1 | 6.759 | 69.2% | 1 | R.NPPGFAFVEFEDPR.D | 22 |

Similarities:
gi|72534660|ref|NP\_00(1:2)  

---

|  |  |  |  |  |  |  |  |  |
| --- | --- | --- | --- | --- | --- | --- | --- | --- |
| U | *gi|10863927|ref|NP\_06* | 5 | 7 | 24.2% | 165 | 18012 | 7.8 | peptidylprolyl isomerase A [Homo sapiens] |
| U | *gi|169215435|ref|XP\_0* | 5 | 7 | 17.9% | 223 | 24376 | 6.9 | PREDICTED: similar to peptidylprolyl isomerase A-like [Homo sapiens] |

| Filename XCorr DeltCN Conf% ObsM+H+ CalcM+H+ SpR ZScore Ion% # Sequence  | | | | | | | | | | | | |
| --- | --- | --- | --- | --- | --- | --- | --- | --- | --- | --- | --- | --- |
|  | Astrin\_STLCHLD\_tube2\_061314\_01.10077.10077.2 | 3.548 | 0.3518 | 100.0% | 1380.0122 | 1380.6268 | 3 | 6.696 | 68.2% | 1 | R.VSFELFADKVPK.T | 2 |
|  | Astrin\_STLCHLD\_tube2\_061314\_01.10122.10122.3 | 2.3476 | 0.2969 | 97.0% | 1381.2843 | 1380.6268 | 159 | 5.769 | 38.6% | 1 | R.VSFELFADKVPK.T | 3 |
|  | Astrin\_STLCHLD\_061214\_01.08164.08164.2 | 4.3359 | 0.4478 | 100.0% | 1832.4122 | 1833.0477 | 1 | 7.476 | 64.3% | 2 | K.SIYGEKFEDENFILK.H | 2 |
|  | Astrin\_STLCHLD\_061214\_01.07093.07093.2 | 3.6308 | 0.0369 | 96.6% | 1506.5721 | 1506.7755 | 2 | 7.875 | 79.2% | 2 | K.VKEGMNIVEAMER.F | 2 |
|  | Astrin\_STLCHLD\_tube2\_061314\_01.09937.09937.2 | 2.8721 | 0.1741 | 97.3% | 1279.0521 | 1279.4689 | 2 | 6.871 | 70.0% | 1 | K.EGMNIVEAMER.F | 2 |

---

|  |  |  |  |  |  |  |  |  |
| --- | --- | --- | --- | --- | --- | --- | --- | --- |
| U | *gi|21464101|ref|NP\_03* | 4 | 6 | 23.9% | 247 | 28303 | 4.9 | tyrosine 3-monooxygenase/tryptophan 5-monooxygenase activation protein, gamma polypeptide [Homo sapiens] |

| Filename XCorr DeltCN Conf% ObsM+H+ CalcM+H+ SpR ZScore Ion% # Sequence  | | | | | | | | | | | | |
| --- | --- | --- | --- | --- | --- | --- | --- | --- | --- | --- | --- | --- |
| \* | Astrin\_STLCHLD\_tube2\_061314\_01.05457.05457.2 | 4.5282 | 0.4765 | 100.0% | 1644.4122 | 1644.7356 | 1 | 9.072 | 76.9% | 1 | K.NVTELNEPLSNEER.N | 2 |
| \* | Astrin\_STLCHLD\_tube2\_061314\_02.07689.07689.2 | 3.1832 | 0.1036 | 95.4% | 1798.2722 | 1798.8473 | 130 | 3.469 | 40.0% | 1 | R.VIS\*SIEQK@TSADGNEK.K | 2 |
| \* | Astrin\_STLCHLD\_tube2\_061314\_01.16157.16157.2 | 3.1102 | 0.1597 | 96.7% | 1798.6921 | 1798.8473 | 18 | 3.644 | 50.0% | 2 | R.VISS\*IEQK@TSADGNEK.K | 2 |
| \* | Astrin\_STLCHLD\_tube2\_061314\_01.16739.16739.3 | 4.4061 | 0.3479 | 100.0% | 3305.1243 | 3303.6626 | 1 | 5.484 | 24.1% | 2 | K.TAFDDAIAELDTLNEDSYKDSTLIMQLLR.D | 3 |

---

|  |  |  |  |  |  |  |  |  |
| --- | --- | --- | --- | --- | --- | --- | --- | --- |
| U | *TEV-Speptide* | 2 | 3 | 23.5% | 51 | 5423 | 9.4 | no description |

| Filename XCorr DeltCN Conf% ObsM+H+ CalcM+H+ SpR ZScore Ion% # Sequence  | | | | | | | | | | | | |
| --- | --- | --- | --- | --- | --- | --- | --- | --- | --- | --- | --- | --- |
| \* | Astrin\_STLCHLD\_061214\_01.04581.04581.2 | 3.8157 | 0.3807 | 100.0% | 1383.7722 | 1384.5345 | 1 | 6.937 | 72.7% | 2 | R.SRENLYFQGAAK.F | 2 |
| \* | Astrin\_STLCHLD\_061214\_02.04525.04525.3 | 2.8082 | 0.2921 | 99.0% | 1384.5243 | 1384.5345 | 1 | 5.137 | 54.5% | 1 | R.SRENLYFQGAAK.F | 3 |

---

|  |  |  |  |  |  |  |  |  |
| --- | --- | --- | --- | --- | --- | --- | --- | --- |
| U | *gi|224028244|ref|NP\_0* | 10 | 32 | 23.4% | 471 | 54232 | 8.9 | non-POU domain containing, octamer-binding isoform 1 [Homo sapiens] |
| U | *gi|34932414|ref|NP\_03* | 10 | 32 | 23.4% | 471 | 54232 | 8.9 | non-POU domain containing, octamer-binding isoform 1 [Homo sapiens] |
| U | *gi|224028246|ref|NP\_0* | 10 | 32 | 23.4% | 471 | 54232 | 8.9 | non-POU domain containing, octamer-binding isoform 1 [Homo sapiens] |

| Filename XCorr DeltCN Conf% ObsM+H+ CalcM+H+ SpR ZScore Ion% # Sequence  | | | | | | | | | | | | |
| --- | --- | --- | --- | --- | --- | --- | --- | --- | --- | --- | --- | --- |
|  | Astrin\_STLCHLD\_tube2\_061314\_01.10848.10848.2 | 3.1282 | 0.2917 | 98.8% | 1861.6522 | 1861.12 | 1 | 5.045 | 63.3% | 2 | R.LFVGNLPPDITEEEMR.K | 2 |
|  | Astrin\_STLCHLD\_tube2\_061314\_01.07147.07147.2 | 2.3552 | 0.3041 | 98.1% | 1086.8722 | 1087.2793 | 22 | 6.147 | 75.0% | 1 | K.VELDNMPLR.G | 2 |
|  | Astrin\_STLCHLD\_tube2\_061314\_01.16643.16643.3 | 3.8191 | 0.3489 | 100.0% | 2669.4844 | 2669.9507 | 1 | 5.77 | 33.0% | 2 | R.NLPQYVSNELLEEAFSVFGQVER.A | 3 |
|  | Astrin\_STLCHLD\_tube2\_061314\_01.16622.16622.2 | 5.2693 | 0.489 | 100.0% | 2670.0322 | 2669.9507 | 1 | 10.666 | 52.3% | 4 | R.NLPQYVSNELLEEAFSVFGQVER.A | 2 |
|  | Astrin\_STLCHLD\_tube2\_061314\_01.09333.09333.2 | 3.9971 | 0.5275 | 100.0% | 1696.3922 | 1696.8744 | 1 | 8.956 | 69.2% | 4 | R.FAQPGSFEYEYAMR.W | 2 |
|  | Astrin\_STLCHLD\_061214\_01.05223.05223.2 | 2.8488 | 0.3884 | 100.0% | 1337.2122 | 1337.5488 | 3 | 6.013 | 70.0% | 1 | R.EKLEMEMEAAR.H | 2 |
|  | Astrin\_STLCHLD\_061214\_01.06282.06282.2 | 4.4584 | 0.5135 | 100.0% | 1539.3722 | 1539.8441 | 1 | 8.657 | 67.9% | 13 | R.MGQMAMGGAMGINNR.G | 2 |
|  | Astrin\_STLCHLD\_061214\_02.07799.07799.2 | 4.5498 | 0.5297 | 100.0% | 2163.672 | 2164.4436 | 1 | 9.192 | 45.2% | 1 | R.FGQAATMEGIGAIGGTPPAFNR.A | 2 |
|  | Astrin\_STLCHLD\_061214\_02.07887.07887.2 | 3.6038 | 0.4767 | 100.0% | 2243.5923 | 2244.4436 | 1 | 7.949 | 45.2% | 1 | R.FGQAATMEGIGAIGGT#PPAFNR.A | 2 |
|  | Astrin\_STLCHLD\_061214\_02.07903.07903.3 | 3.9924 | 0.3208 | 100.0% | 2245.4944 | 2244.4436 | 1 | 5.663 | 42.9% | 3 | R.FGQAATMEGIGAIGGT#PPAFNR.A | 3 |

---

|  |  |  |  |  |  |  |  |  |
| --- | --- | --- | --- | --- | --- | --- | --- | --- |
| U | *gi|14043072|ref|NP\_11* | 7 | 13 | 23.2% | 353 | 37430 | 8.9 | heterogeneous nuclear ribonucleoprotein A2/B1 isoform B1 [Homo sapiens] |
| U | *gi|4504447|ref|NP\_002* | 7 | 13 | 24.0% | 341 | 36006 | 8.6 | heterogeneous nuclear ribonucleoprotein A2/B1 isoform A2 [Homo sapiens] |

| Filename XCorr DeltCN Conf% ObsM+H+ CalcM+H+ SpR ZScore Ion% # Sequence  | | | | | | | | | | | | |
| --- | --- | --- | --- | --- | --- | --- | --- | --- | --- | --- | --- | --- |
|  | Astrin\_STLCHLD\_061214\_01.09447.09447.3 | 2.6923 | 0.2783 | 97.1% | 1927.3444 | 1928.1925 | 65 | 4.503 | 29.7% | 1 | R.KLFIGGLSFETTEESLR.N | 3 |
|  | Astrin\_STLCHLD\_061214\_01.09438.09438.2 | 3.7127 | 0.3762 | 100.0% | 1927.6721 | 1928.1925 | 21 | 7.005 | 40.6% | 1 | R.KLFIGGLSFETTEESLR.N | 2 |
|  | Astrin\_STLCHLD\_061214\_01.10752.10752.2 | 4.6941 | 0.4342 | 100.0% | 1800.5922 | 1800.0184 | 1 | 7.449 | 70.0% | 4 | K.LFIGGLSFETTEESLR.N | 2 |
|  | Astrin\_STLCHLD\_tube2\_061314\_01.09365.09365.2 | 3.1639 | 0.4599 | 100.0% | 1188.6721 | 1189.3513 | 1 | 7.898 | 88.9% | 1 | K.IDTIEIITDR.Q | 2 |
|  | Astrin\_STLCHLD\_tube2\_061314\_01.10166.10166.2 | 2.4878 | 0.4319 | 98.9% | 1696.3121 | 1696.8132 | 1 | 7.145 | 50.0% | 1 | R.GFGFVTFDDHDPVDK.I | 2 |
|  | Astrin\_STLCHLD\_tube2\_061314\_01.04727.04727.2 | 3.0426 | 0.4014 | 100.0% | 1378.2922 | 1378.4465 | 1 | 6.705 | 57.1% | 3 | R.GGGGNFGPGPGSNFR.G | 2 |
|  | Astrin\_STLCHLD\_tube2\_061314\_01.04985.04985.2 | 6.2516 | 0.6399 | 100.0% | 2190.5122 | 2191.2554 | 1 | 12.21 | 56.2% | 2 | R.NMGGPYGGGNYGPGGSGGSGGYGGR.S | 2 |

---

|  |  |  |  |  |  |  |  |  |
| --- | --- | --- | --- | --- | --- | --- | --- | --- |
| U | *gi|226530908|ref|NP\_0* | 4 | 7 | 23.2% | 285 | 30315 | 7.5 | protein-L-isoaspartate (D-aspartate) O-methyltransferase [Homo sapiens] |

| Filename XCorr DeltCN Conf% ObsM+H+ CalcM+H+ SpR ZScore Ion% # Sequence  | | | | | | | | | | | | |
| --- | --- | --- | --- | --- | --- | --- | --- | --- | --- | --- | --- | --- |
| \* | Astrin\_STLCHLD\_061214\_01.07975.07975.2 | 3.8014 | 0.4828 | 100.0% | 1525.7322 | 1525.7601 | 1 | 9.122 | 70.8% | 3 | K.TDKVFEVMLATDR.S | 2 |
| \* | Astrin\_STLCHLD\_061214\_01.08007.08007.2 | 3.1814 | 0.5257 | 100.0% | 1181.4321 | 1181.3923 | 1 | 8.617 | 94.4% | 2 | K.VFEVMLATDR.S | 2 |
| \* | Astrin\_STLCHLD\_tube2\_061314\_01.11972.11972.3 | 6.1638 | 0.4286 | 100.0% | 3508.4043 | 3507.0015 | 1 | 6.539 | 23.5% | 1 | R.MGYAEEAPYDAIHVGAAAPVVPQALIDQLKPGGR.L | 3 |
| \* | Astrin\_STLCHLD\_tube2\_061314\_01.11163.11163.2 | 3.9321 | 0.3545 | 100.0% | 2044.7522 | 2044.3734 | 1 | 6.416 | 72.2% | 1 | R.LILPVGPAGGNQMLEQYDK.L | 2 |

---

|  |  |  |  |  |  |  |  |  |
| --- | --- | --- | --- | --- | --- | --- | --- | --- |
| U | *gi|67782365|ref|NP\_00* | 9 | 12 | 22.6% | 469 | 51386 | 5.5 | keratin 7 [Homo sapiens] |

| Filename XCorr DeltCN Conf% ObsM+H+ CalcM+H+ SpR ZScore Ion% # Sequence  | | | | | | | | | | | | |
| --- | --- | --- | --- | --- | --- | --- | --- | --- | --- | --- | --- | --- |
|  | Astrin\_STLCHLD\_061214\_01.04626.04626.2 | 2.7309 | 0.4487 | 100.0% | 1104.3922 | 1105.2388 | 1 | 7.206 | 68.2% | 1 | R.SAYGGPVGAGIR.E | 2 |
|  | Astrin\_STLCHLD\_tube2\_061314\_01.06264.06264.2 | 2.4085 | 0.2136 | 97.8% | 828.2922 | 827.95544 | 5 | 5.059 | 91.7% | 1 | K.FASFIDK.V | 2222 |
|  | Astrin\_STLCHLD\_tube2\_061314\_01.12363.12363.2 | 3.5418 | 0.4734 | 100.0% | 1443.6921 | 1443.686 | 1 | 8.906 | 75.0% | 1 | R.LPDIFEAQIAGLR.G | 2 |
|  | Astrin\_STLCHLD\_tube2\_061314\_01.05339.05339.2 | 3.5899 | 0.3893 | 100.0% | 1243.0521 | 1243.3622 | 1 | 7.451 | 77.3% | 1 | R.GQLEALQVDGGR.L | 2 |
| \* | Astrin\_STLCHLD\_tube2\_061314\_02.07842.07842.3 | 4.0299 | 0.4026 | 100.0% | 1956.8644 | 1955.1783 | 1 | 6.921 | 44.1% | 2 | R.GQLEALQVDGGRLEAELR.S | 3 |
|  | Astrin\_STLCHLD\_tube2\_061314\_01.11269.11269.2 | 3.9974 | 0.1518 | 99.3% | 1419.4922 | 1419.5773 | 1 | 7.823 | 72.7% | 1 | K.VDALNDEINFLR.T | 2 |
|  | Astrin\_STLCHLD\_061214\_02.07203.07203.3 | 4.2369 | 0.3193 | 100.0% | 2272.2544 | 2271.4912 | 1 | 6.402 | 34.2% | 1 | R.AEAEAWYQTKFETLQAQAGK.H | 3 |
|  | Astrin\_STLCHLD\_061214\_01.04774.04774.3 | 3.7922 | 0.1496 | 98.3% | 1386.9243 | 1386.548 | 15 | 4.826 | 45.5% | 1 | R.AKQEELEAALQR.G | 3 |
|  | Astrin\_STLCHLD\_061214\_01.07862.07862.2 | 3.174 | 0.25 | 99.0% | 1407.6322 | 1406.6653 | 1 | 4.86 | 77.3% | 3 | K.LALDIEIATYRK.L | 22 |

Similarities:
gi|4504919|ref|NP\_002(2:7)  
gi|47132620|ref|NP\_00(1:8)  
gi|119703753|ref|NP\_0(1:8)  

---

|  |  |  |  |  |  |  |  |  |
| --- | --- | --- | --- | --- | --- | --- | --- | --- |
| U | *gi|117968353|ref|NP\_1* | 9 | 14 | 22.2% | 464 | 54304 | 8.3 | NUF2, NDC80 kinetochore complex component [Homo sapiens] |
| U | *gi|117968420|ref|NP\_6* | 9 | 14 | 22.2% | 464 | 54304 | 8.3 | NUF2, NDC80 kinetochore complex component [Homo sapiens] |

| Filename XCorr DeltCN Conf% ObsM+H+ CalcM+H+ SpR ZScore Ion% # Sequence  | | | | | | | | | | | | |
| --- | --- | --- | --- | --- | --- | --- | --- | --- | --- | --- | --- | --- |
|  | Astrin\_STLCHLD\_061214\_01.08884.08884.2 | 3.3367 | 0.4544 | 100.0% | 1326.7522 | 1327.5693 | 1 | 8.546 | 85.0% | 5 | R.YNVAEIVIHIR.N | 2 |
|  | Astrin\_STLCHLD\_061214\_01.08881.08881.3 | 3.4198 | 0.4484 | 100.0% | 1327.9443 | 1327.5693 | 17 | 7.054 | 42.5% | 1 | R.YNVAEIVIHIR.N | 3 |
|  | Astrin\_STLCHLD\_tube2\_061314\_01.12852.12852.2 | 1.9637 | 0.3274 | 95.4% | 1464.8121 | 1464.7532 | 211 | 5.687 | 40.9% | 1 | R.FLSGIINFIHFR.E | 2 |
|  | Astrin\_STLCHLD\_tube2\_061314\_01.04559.04559.3 | 4.9594 | 0.4189 | 100.0% | 2102.1543 | 2102.3882 | 1 | 7.6 | 44.4% | 1 | K.SSADKMQQLNAAHQEALMK.L | 3 |
|  | Astrin\_STLCHLD\_tube2\_061314\_01.06446.06446.2 | 3.4662 | 0.376 | 100.0% | 1550.2722 | 1549.6746 | 1 | 7.091 | 79.2% | 2 | R.LDSVPVEEQEEFK.Q | 2 |
|  | Astrin\_STLCHLD\_tube2\_061314\_01.14311.14311.3 | 3.7437 | 0.2826 | 99.6% | 3886.4944 | 3888.193 | 1 | 4.553 | 24.2% | 1 | R.LDSVPVEEQEEFKQLSDGIQELQQSLNQDFHQK.T | 3 |
|  | Astrin\_STLCHLD\_tube2\_061314\_01.10806.10806.2 | 4.5361 | 0.4425 | 100.0% | 1978.4122 | 1979.0588 | 1 | 7.668 | 62.5% | 1 | K.ESLNLEDQIESDESELK.K | 2 |
|  | Astrin\_STLCHLD\_tube2\_061314\_01.08485.08485.2 | 2.6269 | 0.2207 | 97.2% | 1334.2522 | 1333.6139 | 2 | 5.136 | 65.0% | 1 | K.LKSQEIFLNLK.T | 2 |
|  | Astrin\_STLCHLD\_tube2\_061314\_01.09479.09479.2 | 2.5212 | 0.2554 | 98.0% | 1091.7722 | 1092.2804 | 1 | 6.561 | 87.5% | 1 | K.SQEIFLNLK.T | 2 |

---

|  |  |  |  |  |  |  |  |  |
| --- | --- | --- | --- | --- | --- | --- | --- | --- |
| U | *gi|222352151|ref|NP\_0* | 6 | 12 | 22.2% | 356 | 37498 | 7.1 | poly(rC) binding protein 1 [Homo sapiens] |

| Filename XCorr DeltCN Conf% ObsM+H+ CalcM+H+ SpR ZScore Ion% # Sequence  | | | | | | | | | | | | |
| --- | --- | --- | --- | --- | --- | --- | --- | --- | --- | --- | --- | --- |
| \* | Astrin\_STLCHLD\_tube2\_061314\_01.10992.10992.2 | 2.948 | 0.2687 | 98.5% | 1389.5721 | 1389.6781 | 1 | 6.513 | 62.5% | 1 | R.IITLTGPTNAIFK.A | 2 |
|  | Astrin\_STLCHLD\_061214\_01.06314.06314.2 | 5.6661 | 0.5681 | 100.0% | 2090.4521 | 2091.2573 | 1 | 9.651 | 60.5% | 6 | R.ESTGAQVQVAGDMLPNSTER.A | 22 |
|  | Astrin\_STLCHLD\_061214\_01.06312.06312.3 | 4.5802 | 0.3485 | 100.0% | 2091.4744 | 2091.2573 | 1 | 6.752 | 38.2% | 1 | R.ESTGAQVQVAGDMLPNSTER.A | 33 |
| \* | Astrin\_STLCHLD\_tube2\_061314\_02.06305.06305.3 | 4.1556 | 0.3742 | 100.0% | 2606.5745 | 2607.875 | 4 | 6.884 | 26.0% | 1 | R.QQSHFAMMHGGTGFAGIDSSSPEVK.G | 3 |
| \* | Astrin\_STLCHLD\_tube2\_061314\_02.06542.06542.3 | 3.5645 | 0.3096 | 99.7% | 2686.8542 | 2687.875 | 1 | 5.296 | 34.4% | 2 | R.QQSHFAMMHGGTGFAGIDSSS\*PEVK.G | 3 |
| \* | Astrin\_STLCHLD\_061214\_02.09502.09502.2 | 2.3759 | 0.2492 | 95.4% | 2177.2722 | 2178.4937 | 14 | 5.073 | 32.5% | 1 | R.QVTITGSAASISLAQYLINAR.L | 2 |

Similarities:
gi|14141166|ref|NP\_11(2:4)  

---

|  |  |  |  |  |  |  |  |  |
| --- | --- | --- | --- | --- | --- | --- | --- | --- |
| U | *gi|72534660|ref|NP\_00* | 4 | 7 | 21.4% | 238 | 27367 | 11.8 | splicing factor, arginine/serine-rich 7 [Homo sapiens] |

| Filename XCorr DeltCN Conf% ObsM+H+ CalcM+H+ SpR ZScore Ion% # Sequence  | | | | | | | | | | | | |
| --- | --- | --- | --- | --- | --- | --- | --- | --- | --- | --- | --- | --- |
| \* | Astrin\_STLCHLD\_tube2\_061314\_02.05361.05361.3 | 2.5843 | 0.3712 | 99.6% | 1721.2144 | 1720.923 | 22 | 6.482 | 34.4% | 2 | K.VYVGNLGTGAGKGELER.A | 3 |
| \* | Astrin\_STLCHLD\_tube2\_061314\_01.08327.08327.2 | 2.3844 | 0.3699 | 98.9% | 1073.8722 | 1074.2242 | 1 | 6.842 | 75.0% | 1 | R.AFSYYGPLR.T | 2 |
|  | Astrin\_STLCHLD\_tube2\_061314\_01.11862.11862.2 | 3.2875 | 0.4192 | 100.0% | 1622.4122 | 1622.7771 | 1 | 6.759 | 69.2% | 1 | R.NPPGFAFVEFEDPR.D | 22 |
| \* | Astrin\_STLCHLD\_tube2\_061314\_01.04813.04813.2 | 3.5702 | 0.3557 | 100.0% | 1245.3322 | 1245.4827 | 8 | 6.956 | 60.0% | 3 | R.VRVELSTGMPR.R | 2 |

Similarities:
gi|4506901|ref|NP\_003(1:3)  

---

|  |  |  |  |  |  |  |  |  |
| --- | --- | --- | --- | --- | --- | --- | --- | --- |
| U | *gi|15718687|ref|NP\_00* | 4 | 12 | 21.4% | 243 | 26688 | 9.7 | ribosomal protein S3 [Homo sapiens] |

| Filename XCorr DeltCN Conf% ObsM+H+ CalcM+H+ SpR ZScore Ion% # Sequence  | | | | | | | | | | | | |
| --- | --- | --- | --- | --- | --- | --- | --- | --- | --- | --- | --- | --- |
| \* | Astrin\_STLCHLD\_tube2\_061314\_01.08645.08645.2 | 2.3616 | 0.3021 | 98.1% | 1093.5521 | 1093.2249 | 1 | 5.858 | 75.0% | 2 | K.AELNEFLTR.E | 2 |
| \* | Astrin\_STLCHLD\_061214\_01.05522.05522.2 | 3.7935 | 0.4065 | 100.0% | 1424.2522 | 1424.5071 | 1 | 8.173 | 83.3% | 7 | R.ELAEDGYSGVEVR.V | 2 |
| \* | Astrin\_STLCHLD\_tube2\_061314\_01.10701.10701.2 | 2.6489 | 0.3679 | 98.8% | 1574.2522 | 1573.7423 | 1 | 5.934 | 53.8% | 2 | R.FGFPEGSVELYAEK.V | 2 |
| \* | Astrin\_STLCHLD\_tube2\_061314\_01.05691.05691.2 | 3.2282 | 0.3506 | 99.9% | 1574.4922 | 1574.8352 | 9 | 5.804 | 50.0% | 1 | K.GGKPEPPAMPQPVPTA.- | 2 |

---

|  |  |  |  |  |  |  |  |  |
| --- | --- | --- | --- | --- | --- | --- | --- | --- |
| U | *contaminant\_INT-STD1* | 13 | 47 | 21.3% | 607 | 69271 | 6.1 | BSA |

| Filename XCorr DeltCN Conf% ObsM+H+ CalcM+H+ SpR ZScore Ion% # Sequence  | | | | | | | | | | | | |
| --- | --- | --- | --- | --- | --- | --- | --- | --- | --- | --- | --- | --- |
| \* | Astrin\_STLCHLD\_tube2\_061314\_01.08933.08933.2 | 3.518 | 0.3173 | 100.0% | 1164.3522 | 1164.344 | 1 | 6.825 | 83.3% | 2 | K.LVNELTEFAK.T | 2 |
|  | Astrin\_STLCHLD\_tube2\_061314\_01.06131.06131.2 | 2.3824 | 0.1857 | 97.0% | 928.1722 | 928.0758 | 1 | 5.29 | 83.3% | 2 | K.YLYEIAR.R | 2 |
| \* | Astrin\_STLCHLD\_tube2\_061314\_01.10453.10453.3 | 3.5099 | 0.2808 | 99.7% | 2047.6444 | 2046.3354 | 1 | 5.016 | 43.3% | 2 | R.RHPYFYAPELLYYANK.Y | 3 |
| \* | Astrin\_STLCHLD\_061214\_02.09681.09681.2 | 4.6319 | 0.4808 | 100.0% | 1568.8322 | 1568.7258 | 1 | 8.632 | 79.2% | 4 | K.DAFLGSFLYEYSR.R | 2 |
| \* | Astrin\_STLCHLD\_061214\_01.06450.06450.2 | 3.1946 | 0.4217 | 100.0% | 1440.6322 | 1440.6884 | 11 | 7.078 | 59.1% | 3 | R.RHPEYAVSVLLR.L | 2 |
| \* | Astrin\_STLCHLD\_061214\_01.06522.06522.3 | 4.6296 | 0.3275 | 100.0% | 1441.4343 | 1440.6884 | 1 | 5.822 | 61.4% | 11 | R.RHPEYAVSVLLR.L | 3 |
| \* | Astrin\_STLCHLD\_tube2\_061314\_01.05489.05489.2 | 3.3287 | 0.4312 | 100.0% | 1306.4722 | 1306.5046 | 1 | 7.692 | 70.0% | 5 | K.HLVDEPQNLIK.Q | 2 |
| \* | Astrin\_STLCHLD\_tube2\_061314\_01.10727.10727.2 | 4.5152 | 0.4047 | 100.0% | 1480.6522 | 1480.7068 | 1 | 8.187 | 70.8% | 9 | K.LGEYGFQNALIVR.Y | 2 |
|  | Astrin\_STLCHLD\_tube2\_061314\_01.06455.06455.2 | 3.8101 | 0.4449 | 100.0% | 1640.5721 | 1640.9205 | 1 | 9.228 | 75.0% | 2 | R.KVPQVSTPTLVEVSR.S | 2 |
|  | Astrin\_STLCHLD\_tube2\_061314\_01.06413.06413.3 | 4.6097 | 0.445 | 100.0% | 1641.6543 | 1640.9205 | 3 | 7.85 | 46.4% | 3 | R.KVPQVSTPTLVEVSR.S | 3 |
| \* | Astrin\_STLCHLD\_tube2\_061314\_01.07841.07841.2 | 2.6125 | 0.1802 | 96.7% | 1143.0922 | 1143.4124 | 39 | 4.994 | 61.1% | 1 | K.KQTALVELLK.H | 2 |
| \* | Astrin\_STLCHLD\_tube2\_061314\_01.12469.12469.2 | 3.1803 | 0.5389 | 100.0% | 1399.9122 | 1400.6324 | 1 | 9.973 | 77.3% | 2 | K.TVMENFVAFVDK.C | 2 |
| \* | Astrin\_STLCHLD\_tube2\_061314\_02.06280.06280.2 | 2.2621 | 0.3725 | 98.4% | 1003.0522 | 1003.1839 | 33 | 6.225 | 61.1% | 1 | K.LVVSTQTALA.- | 2 |

---

|  |  |  |  |  |  |  |  |  |
| --- | --- | --- | --- | --- | --- | --- | --- | --- |
| U | *gi|14165435|ref|NP\_11* | 7 | 21 | 21.2% | 463 | 50976 | 5.5 | heterogeneous nuclear ribonucleoprotein K isoform b [Homo sapiens] |
| U | *gi|14165439|ref|NP\_00* | 7 | 21 | 21.1% | 464 | 51028 | 5.3 | heterogeneous nuclear ribonucleoprotein K isoform a [Homo sapiens] |
| U | *gi|14165437|ref|NP\_11* | 7 | 21 | 21.1% | 464 | 51028 | 5.3 | heterogeneous nuclear ribonucleoprotein K isoform a [Homo sapiens] |

| Filename XCorr DeltCN Conf% ObsM+H+ CalcM+H+ SpR ZScore Ion% # Sequence  | | | | | | | | | | | | |
| --- | --- | --- | --- | --- | --- | --- | --- | --- | --- | --- | --- | --- |
|  | Astrin\_STLCHLD\_tube2\_061314\_01.04679.04679.2 | 4.0426 | 0.5063 | 100.0% | 1781.4722 | 1781.8302 | 6 | 8.636 | 53.1% | 2 | R.TDYNASVSVPDSSGPER.I | 2 |
|  | Astrin\_STLCHLD\_tube2\_061314\_01.14988.14988.2 | 3.8625 | 0.4074 | 100.0% | 1715.9922 | 1716.0251 | 1 | 7.229 | 56.7% | 3 | R.ILSISADIETIGEILK.K | 2 |
|  | Astrin\_STLCHLD\_061214\_01.07743.07743.2 | 3.2626 | 0.4672 | 100.0% | 1519.0521 | 1519.8711 | 1 | 7.942 | 67.9% | 4 | R.LLIHQSLAGGIIGVK.G | 2 |
|  | Astrin\_STLCHLD\_061214\_02.07147.07147.3 | 4.9236 | 0.4824 | 100.0% | 1520.9343 | 1519.8711 | 1 | 8.001 | 53.6% | 5 | R.LLIHQSLAGGIIGVK.G | 3 |
|  | Astrin\_STLCHLD\_tube2\_061314\_01.12146.12146.2 | 3.6355 | 0.3902 | 100.0% | 1342.3121 | 1341.6311 | 1 | 6.924 | 72.7% | 1 | K.IILDLISESPIK.G | 2 |
|  | Astrin\_STLCHLD\_tube2\_061314\_01.10470.10470.2 | 4.6265 | 0.5417 | 100.0% | 1917.5521 | 1918.1974 | 1 | 10.008 | 52.8% | 2 | R.GSYGDLGGPIITTQVTIPK.D | 2 |
|  | Astrin\_STLCHLD\_tube2\_061314\_01.04938.04938.3 | 4.0036 | 0.3524 | 100.0% | 2070.4143 | 2070.1772 | 1 | 6.842 | 34.7% | 4 | R.HESGASIKIDEPLEGSEDR.I | 3 |

---

|  |  |  |  |  |  |  |  |  |
| --- | --- | --- | --- | --- | --- | --- | --- | --- |
| U | *gi|117190174|ref|NP\_0* | 4 | 14 | 21.2% | 293 | 32338 | 5.1 | heterogeneous nuclear ribonucleoprotein C isoform b [Homo sapiens] |
| U | *gi|117190254|ref|NP\_0* | 4 | 14 | 21.2% | 293 | 32338 | 5.1 | heterogeneous nuclear ribonucleoprotein C isoform b [Homo sapiens] |

| Filename XCorr DeltCN Conf% ObsM+H+ CalcM+H+ SpR ZScore Ion% # Sequence  | | | | | | | | | | | | |
| --- | --- | --- | --- | --- | --- | --- | --- | --- | --- | --- | --- | --- |
|  | Astrin\_STLCHLD\_061214\_01.09169.09169.2 | 3.8274 | 0.2744 | 100.0% | 1318.5521 | 1317.6145 | 1 | 6.335 | 81.8% | 3 | R.VFIGNLNTLVVK.K | 2 |
|  | Astrin\_STLCHLD\_061214\_01.08648.08648.2 | 3.4644 | 0.4933 | 100.0% | 1330.5322 | 1330.4857 | 1 | 7.822 | 75.0% | 4 | K.GFAFVQYVNER.N | 2 |
|  | Astrin\_STLCHLD\_061214\_02.08137.08137.2 | 5.1 | 0.481 | 100.0% | 1683.2922 | 1684.0038 | 1 | 8.259 | 73.3% | 6 | R.MIAGQVLDINLAAEPK.V | 2 |
|  | Astrin\_STLCHLD\_061214\_02.08278.08278.3 | 3.0199 | 0.241 | 96.2% | 2816.0044 | 2815.9404 | 1 | 5.096 | 27.3% | 1 | R.SAAEMYGSSFDLDYDFQRDYYDR.M | 3 |

---

|  |  |  |  |  |  |  |  |  |
| --- | --- | --- | --- | --- | --- | --- | --- | --- |
| U | *gi|32189392|ref|NP\_00* | 4 | 9 | 21.2% | 198 | 21892 | 6.0 | peroxiredoxin 2 isoform a [Homo sapiens] |

| Filename XCorr DeltCN Conf% ObsM+H+ CalcM+H+ SpR ZScore Ion% # Sequence  | | | | | | | | | | | | |
| --- | --- | --- | --- | --- | --- | --- | --- | --- | --- | --- | --- | --- |
|  | Astrin\_STLCHLD\_tube2\_061314\_01.04754.04754.2 | 2.2457 | 0.2683 | 95.4% | 1335.1122 | 1335.5431 | 86 | 5.211 | 50.0% | 1 | K.ATAVVDGAFKEVK.L | 2 |
| \* | Astrin\_STLCHLD\_061214\_01.06154.06154.3 | 4.0052 | 0.3487 | 100.0% | 2085.5942 | 2086.309 | 1 | 5.959 | 44.1% | 4 | R.RLSEDYGVLKTDEGIAYR.G | 3 |
| \* | Astrin\_STLCHLD\_061214\_01.06690.06690.3 | 3.3387 | 0.3875 | 100.0% | 1930.7344 | 1930.1217 | 1 | 6.052 | 37.5% | 2 | R.LSEDYGVLKTDEGIAYR.G | 3 |
|  | Astrin\_STLCHLD\_tube2\_061314\_01.07182.07182.2 | 2.561 | 0.3933 | 99.5% | 1211.8922 | 1212.3915 | 34 | 6.97 | 65.0% | 2 | R.QITVNDLPVGR.S | 22 |

Similarities:
gi|32455264|ref|NP\_85(1:3)  

---

|  |  |  |  |  |  |  |  |  |
| --- | --- | --- | --- | --- | --- | --- | --- | --- |
| U | *gi|5729877|ref|NP\_006* | 16 | 33 | 21.1% | 646 | 70898 | 5.5 | heat shock 70kDa protein 8 isoform 1 [Homo sapiens] |

| Filename XCorr DeltCN Conf% ObsM+H+ CalcM+H+ SpR ZScore Ion% # Sequence  | | | | | | | | | | | | |
| --- | --- | --- | --- | --- | --- | --- | --- | --- | --- | --- | --- | --- |
|  | Astrin\_STLCHLD\_tube2\_061314\_01.07280.07280.2 | 3.3247 | 0.4793 | 100.0% | 1488.3922 | 1488.5939 | 1 | 8.565 | 75.0% | 4 | R.TTPSYVAFTDTER.L | 222 |
|  | Astrin\_STLCHLD\_tube2\_061314\_01.07236.07236.2 | 4.4575 | 0.4745 | 100.0% | 1650.3121 | 1650.8468 | 1 | 9.859 | 78.6% | 2 | K.NQVAMNPTNTVFDAK.R | 2 |
|  | Astrin\_STLCHLD\_tube2\_061314\_01.08334.08334.3 | 3.7435 | 0.3487 | 100.0% | 1656.5343 | 1654.9298 | 1 | 6.032 | 46.2% | 2 | K.HWPFMVVNDAGRPK.V | 3 |
|  | Astrin\_STLCHLD\_tube2\_061314\_02.05702.05702.3 | 3.0767 | 0.3316 | 100.0% | 1253.3043 | 1253.4993 | 1 | 6.03 | 42.5% | 2 | K.MKEIAEAYLGK.T | 3 |
|  | Astrin\_STLCHLD\_tube2\_061314\_01.10183.10183.2 | 4.3082 | 0.3802 | 100.0% | 1662.4722 | 1660.9078 | 1 | 7.981 | 80.0% | 2 | R.IINEPTAAAIAYGLDK.K | 22 |
|  | Astrin\_STLCHLD\_tube2\_061314\_01.09043.09043.2 | 3.7373 | 0.3654 | 100.0% | 1788.4321 | 1789.0819 | 1 | 7.697 | 56.2% | 1 | R.IINEPTAAAIAYGLDKK.V | 2 |
|  | Astrin\_STLCHLD\_061214\_01.04423.04423.3 | 2.3236 | 0.3023 | 96.5% | 1692.8944 | 1692.6958 | 46 | 5.416 | 30.0% | 1 | K.STAGDTHLGGEDFDNR.M | 3 |
|  | Astrin\_STLCHLD\_061214\_01.06922.06922.3 | 3.2026 | 0.4045 | 100.0% | 1236.5044 | 1236.4741 | 1 | 6.894 | 50.0% | 2 | R.MVNHFIAEFK.R | 3 |
|  | Astrin\_STLCHLD\_tube2\_061314\_01.07791.07791.2 | 3.3058 | 0.4308 | 100.0% | 1237.4321 | 1236.4741 | 1 | 7.796 | 83.3% | 1 | R.MVNHFIAEFK.R | 2 |
|  | Astrin\_STLCHLD\_tube2\_061314\_01.09696.09696.2 | 3.311 | 0.3151 | 100.0% | 1481.0322 | 1481.6511 | 1 | 6.689 | 72.7% | 2 | R.ARFEELNADLFR.G | 2 |
|  | Astrin\_STLCHLD\_tube2\_061314\_01.09683.09683.3 | 3.7659 | 0.2928 | 100.0% | 1481.1543 | 1481.6511 | 1 | 5.359 | 54.5% | 1 | R.ARFEELNADLFR.G | 3 |
|  | Astrin\_STLCHLD\_tube2\_061314\_01.10777.10777.2 | 2.7357 | 0.3363 | 99.5% | 1255.7922 | 1254.3849 | 4 | 5.855 | 72.2% | 1 | R.FEELNADLFR.G | 2 |
|  | Astrin\_STLCHLD\_061214\_01.06200.06200.3 | 4.6652 | 0.3129 | 100.0% | 1839.3243 | 1839.1019 | 1 | 7.047 | 45.3% | 4 | K.LDKSQIHDIVLVGGSTR.I | 3 |
|  | Astrin\_STLCHLD\_tube2\_061314\_01.05916.05916.2 | 3.7545 | 0.4442 | 100.0% | 1482.4521 | 1482.6798 | 1 | 7.389 | 69.2% | 4 | K.SQIHDIVLVGGSTR.I | 2 |
|  | Astrin\_STLCHLD\_tube2\_061314\_02.05836.05836.3 | 3.4679 | 0.3067 | 99.8% | 1484.3344 | 1482.6798 | 2 | 5.69 | 44.2% | 2 | K.SQIHDIVLVGGSTR.I | 3 |
| \* | Astrin\_STLCHLD\_tube2\_061314\_01.09200.09200.2 | 3.3037 | 0.4607 | 100.0% | 1304.3522 | 1304.4602 | 3 | 7.468 | 70.0% | 2 | K.NSLESYAFNMK.A | 2 |

Similarities:
gi|167466173|ref|NP\_0(1:15)  
gi|124256496|ref|NP\_0(2:14)  

---

|  |  |  |  |  |  |  |  |  |
| --- | --- | --- | --- | --- | --- | --- | --- | --- |
| U | *gi|14210536|ref|NP\_11* | 12 | 39 | 20.4% | 446 | 49857 | 4.9 | tubulin, beta 6 [Homo sapiens] |

| Filename XCorr DeltCN Conf% ObsM+H+ CalcM+H+ SpR ZScore Ion% # Sequence  | | | | | | | | | | | | |
| --- | --- | --- | --- | --- | --- | --- | --- | --- | --- | --- | --- | --- |
| \* | Astrin\_STLCHLD\_tube2\_061314\_01.09413.09413.2 | 3.3723 | 0.3072 | 99.5% | 1574.4722 | 1574.7894 | 1 | 5.903 | 64.3% | 1 | R.AALVDLEPGTMDSVR.S | 2 |
|  | Astrin\_STLCHLD\_tube2\_061314\_01.07361.07361.2 | 3.2733 | 0.2391 | 99.5% | 1132.2322 | 1131.2767 | 1 | 5.073 | 83.3% | 14 | R.FPGQLNADLR.K | 22222 |
|  | Astrin\_STLCHLD\_tube2\_061314\_01.04904.04904.2 | 2.5627 | 0.1707 | 95.5% | 1259.2922 | 1259.4508 | 1 | 4.474 | 70.0% | 2 | R.FPGQLNADLRK.L | 22222 |
|  | Astrin\_STLCHLD\_tube2\_061314\_01.04842.04842.3 | 2.8966 | 0.3686 | 100.0% | 1260.2043 | 1259.4508 | 14 | 5.742 | 42.5% | 5 | R.FPGQLNADLRK.L | 33333 |
|  | Astrin\_STLCHLD\_tube2\_061314\_01.08730.08730.2 | 3.8464 | 0.3753 | 100.0% | 1272.4122 | 1272.5945 | 1 | 7.898 | 75.0% | 4 | R.KLAVNMVPFPR.L | 22222 |
|  | Astrin\_STLCHLD\_tube2\_061314\_01.10148.10148.1 | 2.1143 | 0.2239 | 96.6% | 1143.61 | 1144.4204 | 18 | 6.431 | 61.1% | 1 | K.LAVNMVPFPR.L | 11111 |
|  | Astrin\_STLCHLD\_tube2\_061314\_01.10247.10247.2 | 3.6473 | 0.5079 | 100.0% | 1145.2722 | 1144.4204 | 1 | 8.426 | 94.4% | 4 | K.LAVNMVPFPR.L | 22222 |
|  | Astrin\_STLCHLD\_tube2\_061314\_01.12143.12143.2 | 3.7666 | 0.4528 | 100.0% | 1621.8322 | 1621.9403 | 1 | 8.756 | 76.9% | 2 | R.LHFFMPGFAPLTSR.G | 2222 |
|  | Astrin\_STLCHLD\_tube2\_061314\_01.12204.12204.3 | 4.3867 | 0.3891 | 100.0% | 1622.3944 | 1621.9403 | 1 | 6.411 | 53.8% | 2 | R.LHFFMPGFAPLTSR.G | 3333 |
|  | Astrin\_STLCHLD\_tube2\_061314\_01.11781.11781.2 | 4.295 | 0.2978 | 100.0% | 1698.5322 | 1697.8877 | 1 | 7.708 | 73.1% | 2 | K.NSSYFVEWIPNNVK.V | 22222 |
| \* | Astrin\_STLCHLD\_061214\_01.11426.11426.2 | 3.8519 | 0.0023 | 95.4% | 1858.0721 | 1859.1475 | 1 | 8.136 | 62.5% | 1 | K.MASTFIGNSTAIQELFK.R | 2 |
| \* | Astrin\_STLCHLD\_061214\_01.08408.08408.2 | 2.4102 | 0.3327 | 98.4% | 1216.1721 | 1216.3972 | 3 | 5.814 | 72.2% | 1 | R.ISEQFSAMFR.R | 2 |

Similarities:
gi|29788785|ref|NP\_82(9:3)  
gi|5174735|ref|NP\_006(9:3)  
gi|29788768|ref|NP\_82(9:3)  
gi|50592996|ref|NP\_00(7:5)  

---

|  |  |  |  |  |  |  |  |  |
| --- | --- | --- | --- | --- | --- | --- | --- | --- |
| U | *gi|5031699|ref|NP\_005* | 6 | 13 | 20.4% | 427 | 47355 | 7.5 | flotillin 1 [Homo sapiens] |

| Filename XCorr DeltCN Conf% ObsM+H+ CalcM+H+ SpR ZScore Ion% # Sequence  | | | | | | | | | | | | |
| --- | --- | --- | --- | --- | --- | --- | --- | --- | --- | --- | --- | --- |
| \* | Astrin\_STLCHLD\_tube2\_061314\_01.11125.11125.3 | 3.7674 | 0.4133 | 100.0% | 2020.2843 | 2019.2217 | 1 | 7.211 | 42.6% | 1 | K.TEAEIAHIALETLEGHQR.A | 3 |
| \* | Astrin\_STLCHLD\_061214\_01.07519.07519.2 | 2.7884 | 0.3367 | 99.0% | 1468.3322 | 1469.693 | 8 | 5.637 | 54.2% | 3 | K.VSAQYLSEIEMAK.A | 2 |
| \* | Astrin\_STLCHLD\_061214\_02.06314.06314.2 | 4.0207 | 0.5207 | 100.0% | 1419.6122 | 1419.6206 | 1 | 9.193 | 70.8% | 4 | R.AQADLAYQLQVAK.T | 2 |
| \* | Astrin\_STLCHLD\_061214\_02.07493.07493.2 | 4.9822 | 0.544 | 100.0% | 1605.6322 | 1604.8187 | 1 | 9.528 | 71.4% | 1 | K.SQLIMQAEAEAASVR.M | 2 |
| \* | Astrin\_STLCHLD\_061214\_01.05671.05671.2 | 3.0559 | 0.2966 | 98.9% | 1379.2722 | 1379.5768 | 2 | 5.81 | 70.8% | 1 | R.MRGEAEAFAIGAR.A | 2 |
| \* | Astrin\_STLCHLD\_061214\_02.05374.05374.2 | 4.2052 | 0.4458 | 100.0% | 1380.1721 | 1380.5994 | 1 | 8.962 | 75.0% | 3 | K.ITLVSSGSGTMGAAK.V | 2 |

---

|  |  |  |  |  |  |  |  |  |
| --- | --- | --- | --- | --- | --- | --- | --- | --- |
| U | *gi|4501881|ref|NP\_001* | 8 | 22 | 20.2% | 377 | 42051 | 5.4 | actin, alpha 1, skeletal muscle [Homo sapiens] |
| U | *gi|4885049|ref|NP\_005* | 8 | 22 | 20.2% | 377 | 42019 | 5.4 | cardiac muscle alpha actin 1 proprotein [Homo sapiens] |

| Filename XCorr DeltCN Conf% ObsM+H+ CalcM+H+ SpR ZScore Ion% # Sequence  | | | | | | | | | | | | |
| --- | --- | --- | --- | --- | --- | --- | --- | --- | --- | --- | --- | --- |
|  | Astrin\_STLCHLD\_061214\_01.05682.05682.2 | 2.7849 | 0.3968 | 100.0% | 1198.8322 | 1199.4415 | 2 | 6.403 | 70.0% | 3 | R.AVFPSIVGRPR.H | 22 |
|  | Astrin\_STLCHLD\_tube2\_061314\_01.09287.09287.2 | 3.6396 | 0.3843 | 100.0% | 1961.6921 | 1962.1841 | 1 | 6.963 | 63.3% | 1 | K.YPIEHGIITNWDDMEK.I | 2 |
|  | Astrin\_STLCHLD\_tube2\_061314\_01.05606.05606.2 | 2.8797 | 0.4114 | 100.0% | 1516.4521 | 1516.7019 | 2 | 6.49 | 65.0% | 2 | K.IWHHTFYNELR.V | 22 |
|  | Astrin\_STLCHLD\_tube2\_061314\_01.05597.05597.3 | 3.1756 | 0.156 | 96.2% | 1517.1843 | 1516.7019 | 1 | 5.007 | 60.0% | 3 | K.IWHHTFYNELR.V | 33 |
|  | Astrin\_STLCHLD\_tube2\_061314\_01.10415.10415.2 | 1.9955 | 0.3931 | 98.1% | 999.03217 | 999.167 | 15 | 6.315 | 64.3% | 1 | R.DLTDYLMK.I | 222 |
|  | Astrin\_STLCHLD\_tube2\_061314\_01.10440.10440.2 | 4.5438 | 0.3456 | 100.0% | 1792.6322 | 1791.9554 | 1 | 7.499 | 73.3% | 5 | K.SYELPDGQVITIGNER.F | 222 |
|  | Astrin\_STLCHLD\_061214\_01.05115.05115.3 | 3.7961 | 0.3358 | 100.0% | 1550.2144 | 1549.8843 | 10 | 6.274 | 44.2% | 1 | R.MQKEITALAPSTMK.I | 33 |
|  | Astrin\_STLCHLD\_tube2\_061314\_01.05532.05532.2 | 2.5818 | 0.3762 | 99.3% | 1162.1122 | 1162.3868 | 1 | 6.988 | 75.0% | 6 | K.EITALAPSTMK.I | 22 |

Similarities:
gi|4501885|ref|NP\_001(7:1)  
gi|63055057|ref|NP\_00(2:6)  

---

|  |  |  |  |  |  |  |  |  |
| --- | --- | --- | --- | --- | --- | --- | --- | --- |
| U | *gi|208973238|ref|NP\_0* | 2 | 2 | 20.0% | 245 | 27745 | 4.8 | tyrosine 3/tryptophan 5 -monooxygenase activation protein, zeta polypeptide [Homo sapiens] |
| U | *gi|4507953|ref|NP\_003* | 2 | 2 | 20.0% | 245 | 27745 | 4.8 | tyrosine 3/tryptophan 5 -monooxygenase activation protein, zeta polypeptide [Homo sapiens] |
| U | *gi|21735625|ref|NP\_66* | 2 | 2 | 20.0% | 245 | 27745 | 4.8 | tyrosine 3/tryptophan 5 -monooxygenase activation protein, zeta polypeptide [Homo sapiens] |
| U | *gi|208973244|ref|NP\_0* | 2 | 2 | 20.0% | 245 | 27745 | 4.8 | tyrosine 3/tryptophan 5 -monooxygenase activation protein, zeta polypeptide [Homo sapiens] |
| U | *gi|208973242|ref|NP\_0* | 2 | 2 | 20.0% | 245 | 27745 | 4.8 | tyrosine 3/tryptophan 5 -monooxygenase activation protein, zeta polypeptide [Homo sapiens] |
| U | *gi|208973240|ref|NP\_0* | 2 | 2 | 20.0% | 245 | 27745 | 4.8 | tyrosine 3/tryptophan 5 -monooxygenase activation protein, zeta polypeptide [Homo sapiens] |

| Filename XCorr DeltCN Conf% ObsM+H+ CalcM+H+ SpR ZScore Ion% # Sequence  | | | | | | | | | | | | |
| --- | --- | --- | --- | --- | --- | --- | --- | --- | --- | --- | --- | --- |
|  | Astrin\_STLCHLD\_061214\_02.11572.11572.2 | 2.7554 | 0.3391 | 98.5% | 2318.5122 | 2318.6746 | 13 | 6.339 | 34.2% | 1 | R.LGLALNFSVFYYEILNSPEK.A | 22 |
|  | Astrin\_STLCHLD\_tube2\_061314\_01.18065.18065.3 | 5.6291 | 0.5467 | 100.0% | 3303.9844 | 3304.6907 | 1 | 10.021 | 30.4% | 1 | K.TAFDEAIAELDTLSEESYKDSTLIMQLLR.D | 3 |

Similarities:
gi|21328448|ref|NP\_64(1:1)  

---

|  |  |  |  |  |  |  |  |  |
| --- | --- | --- | --- | --- | --- | --- | --- | --- |
| U | *gi|21328448|ref|NP\_64* | 2 | 2 | 19.9% | 246 | 28082 | 4.8 | tyrosine 3-monooxygenase/tryptophan 5-monooxygenase activation protein, beta polypeptide [Homo sapiens] |
| U | *gi|4507949|ref|NP\_003* | 2 | 2 | 19.9% | 246 | 28082 | 4.8 | tyrosine 3-monooxygenase/tryptophan 5-monooxygenase activation protein, beta polypeptide [Homo sapiens] |

| Filename XCorr DeltCN Conf% ObsM+H+ CalcM+H+ SpR ZScore Ion% # Sequence  | | | | | | | | | | | | |
| --- | --- | --- | --- | --- | --- | --- | --- | --- | --- | --- | --- | --- |
|  | Astrin\_STLCHLD\_061214\_02.11572.11572.2 | 2.7554 | 0.3391 | 98.5% | 2318.5122 | 2318.6746 | 13 | 6.339 | 34.2% | 1 | R.LGLALNFSVFYYEILNSPEK.A | 22 |
|  | Astrin\_STLCHLD\_tube2\_061314\_01.17019.17019.3 | 3.3171 | 0.3453 | 99.7% | 3331.9744 | 3331.7163 | 2 | 5.351 | 22.3% | 1 | K.TAFDEAIAELDTLNEESYKDSTLIMQLLR.D | 3 |

Similarities:
gi|208973238|ref|NP\_0(1:1)  

---

|  |  |  |  |  |  |  |  |  |
| --- | --- | --- | --- | --- | --- | --- | --- | --- |
| U | *gi|5453555|ref|NP\_006* | 4 | 5 | 19.9% | 216 | 24423 | 7.5 | ras-related nuclear protein [Homo sapiens] |

| Filename XCorr DeltCN Conf% ObsM+H+ CalcM+H+ SpR ZScore Ion% # Sequence  | | | | | | | | | | | | |
| --- | --- | --- | --- | --- | --- | --- | --- | --- | --- | --- | --- | --- |
| \* | Astrin\_STLCHLD\_061214\_01.07653.07653.3 | 3.0796 | 0.4012 | 100.0% | 2054.3044 | 2053.3713 | 1 | 6.174 | 41.2% | 2 | K.YVATLGVEVHPLVFHTNR.G | 3 |
| \* | Astrin\_STLCHLD\_tube2\_061314\_01.07910.07910.2 | 2.3273 | 0.2387 | 96.0% | 1294.4722 | 1295.394 | 1 | 5.25 | 70.0% | 1 | K.FNVWDTAGQEK.F | 2 |
| \* | Astrin\_STLCHLD\_tube2\_061314\_01.12129.12129.2 | 3.8981 | 0.4643 | 100.0% | 1785.6721 | 1786.0427 | 1 | 8.081 | 61.5% | 1 | K.SNYNFEKPFLWLAR.K | 2 |
| \* | Astrin\_STLCHLD\_tube2\_061314\_01.12139.12139.3 | 4.5034 | 0.4328 | 100.0% | 1786.1344 | 1786.0427 | 1 | 6.926 | 51.9% | 1 | K.SNYNFEKPFLWLAR.K | 3 |

---

|  |  |  |  |  |  |  |  |  |
| --- | --- | --- | --- | --- | --- | --- | --- | --- |
| U | *gi|163965362|ref|NP\_0* | 3 | 5 | 19.5% | 215 | 23384 | 4.6 | nascent polypeptide-associated complex alpha subunit isoform b [Homo sapiens] |
| U | *gi|5031931|ref|NP\_005* | 3 | 5 | 19.5% | 215 | 23384 | 4.6 | nascent polypeptide-associated complex alpha subunit isoform b [Homo sapiens] |
| U | *gi|163965366|ref|NP\_0* | 3 | 5 | 2.0% | 2078 | 205419 | 9.6 | nascent polypeptide-associated complex alpha subunit isoform a [Homo sapiens] |
| U | *gi|163965364|ref|NP\_0* | 3 | 5 | 19.5% | 215 | 23384 | 4.6 | nascent polypeptide-associated complex alpha subunit isoform b [Homo sapiens] |

| Filename XCorr DeltCN Conf% ObsM+H+ CalcM+H+ SpR ZScore Ion% # Sequence  | | | | | | | | | | | | |
| --- | --- | --- | --- | --- | --- | --- | --- | --- | --- | --- | --- | --- |
|  | Astrin\_STLCHLD\_tube2\_061314\_01.09062.09062.2 | 3.1045 | 0.3867 | 100.0% | 1485.6122 | 1485.6335 | 10 | 7.771 | 50.0% | 1 | K.SPASDTYIVFGEAK.I | 2 |
|  | Astrin\_STLCHLD\_tube2\_061314\_02.05718.05718.2 | 3.7315 | 0.2581 | 99.7% | 1615.5122 | 1615.7808 | 1 | 6.847 | 60.7% | 3 | K.IEDLSQQAQLAAAEK.F | 2 |
|  | Astrin\_STLCHLD\_tube2\_061314\_02.07019.07019.2 | 2.4101 | 0.2547 | 96.4% | 1462.7322 | 1462.6611 | 1 | 5.293 | 58.3% | 1 | K.DIELVMSQANVSR.A | 2 |

---

|  |  |  |  |  |  |  |  |  |
| --- | --- | --- | --- | --- | --- | --- | --- | --- |
| U | *gi|4758086|ref|NP\_004* | 2 | 9 | 19.2% | 193 | 20567 | 8.6 | cysteine and glycine-rich protein 1 isoform 1 [Homo sapiens] |

| Filename XCorr DeltCN Conf% ObsM+H+ CalcM+H+ SpR ZScore Ion% # Sequence  | | | | | | | | | | | | |
| --- | --- | --- | --- | --- | --- | --- | --- | --- | --- | --- | --- | --- |
|  | Astrin\_STLCHLD\_061214\_02.05795.05795.3 | 3.0818 | 0.2823 | 98.2% | 2160.6543 | 2160.3452 | 3 | 4.231 | 31.0% | 2 | K.GYGYGQGAGTLSTDKGESLGIK.H | 3 |
| \* | Astrin\_STLCHLD\_061214\_01.07878.07878.2 | 3.9117 | 0.536 | 100.0% | 1433.7522 | 1434.551 | 1 | 9.286 | 57.1% | 7 | K.GFGFGQGAGALVHSE.- | 2 |

---

|  |  |  |  |  |  |  |  |  |
| --- | --- | --- | --- | --- | --- | --- | --- | --- |
| U | *gi|10800138|ref|NP\_06* | 2 | 7 | 19.0% | 126 | 13936 | 10.3 | histone cluster 1, H2bd [Homo sapiens] |
| U | *gi|66912162|ref|NP\_00* | 2 | 7 | 19.0% | 126 | 13920 | 10.3 | histone cluster 2, H2bf [Homo sapiens] |
| U | *gi|4504277|ref|NP\_003* | 2 | 7 | 19.0% | 126 | 13920 | 10.3 | histone cluster 2, H2be [Homo sapiens] |
| U | *gi|4504271|ref|NP\_003* | 2 | 7 | 19.0% | 126 | 13906 | 10.3 | histone cluster 1, H2bi [Homo sapiens] |
| U | *gi|4504269|ref|NP\_003* | 2 | 7 | 19.0% | 126 | 13892 | 10.3 | histone cluster 1, H2bh [Homo sapiens] |
| U | *gi|4504265|ref|NP\_003* | 2 | 7 | 19.0% | 126 | 13906 | 10.3 | histone cluster 1, H2bf [Homo sapiens] |
| U | *gi|4504263|ref|NP\_003* | 2 | 7 | 19.0% | 126 | 13989 | 10.3 | histone cluster 1, H2bm [Homo sapiens] |
| U | *gi|4504261|ref|NP\_003* | 2 | 7 | 19.0% | 126 | 13922 | 10.3 | histone cluster 1, H2bn [Homo sapiens] |
| U | *gi|4504259|ref|NP\_003* | 2 | 7 | 19.0% | 126 | 13952 | 10.3 | histone cluster 1, H2bl [Homo sapiens] |
| U | *gi|4504257|ref|NP\_003* | 2 | 7 | 19.0% | 126 | 13906 | 10.3 | histone cluster 1, H2bg [Homo sapiens] |
| U | *gi|28173554|ref|NP\_77* | 2 | 7 | 19.0% | 126 | 13908 | 10.3 | histone cluster 3, H2bb [Homo sapiens] |
| U | *gi|21396484|ref|NP\_00* | 2 | 7 | 19.0% | 126 | 13906 | 10.3 | histone cluster 1, H2be [Homo sapiens] |
| U | *gi|21166389|ref|NP\_00* | 2 | 7 | 19.0% | 126 | 13906 | 10.3 | histone cluster 1, H2bc [Homo sapiens] |
| U | *gi|20336754|ref|NP\_06* | 2 | 7 | 19.0% | 126 | 13904 | 10.3 | histone cluster 1, H2bj [Homo sapiens] |
| U | *gi|20336752|ref|NP\_61* | 2 | 7 | 19.0% | 126 | 13936 | 10.3 | histone cluster 1, H2bd [Homo sapiens] |
| U | *gi|18105048|ref|NP\_54* | 2 | 7 | 19.0% | 126 | 13890 | 10.3 | histone cluster 1, H2bk [Homo sapiens] |
| U | *gi|16306566|ref|NP\_00* | 2 | 7 | 19.0% | 126 | 13906 | 10.3 | histone cluster 1, H2bo [Homo sapiens] |
| U | *gi|10800140|ref|NP\_06* | 2 | 7 | 19.0% | 126 | 13950 | 10.3 | histone cluster 1, H2bb [Homo sapiens] |

| Filename XCorr DeltCN Conf% ObsM+H+ CalcM+H+ SpR ZScore Ion% # Sequence  | | | | | | | | | | | | |
| --- | --- | --- | --- | --- | --- | --- | --- | --- | --- | --- | --- | --- |
|  | Astrin\_STLCHLD\_tube2\_061314\_01.13745.13745.2 | 4.6238 | 0.385 | 100.0% | 1746.0122 | 1745.0211 | 1 | 7.927 | 78.6% | 6 | K.AMGIMNSFVNDIFER.I | 2 |
|  | Astrin\_STLCHLD\_tube2\_061314\_01.08891.08891.2 | 2.3749 | 0.1706 | 95.4% | 954.3122 | 954.19794 | 8 | 4.914 | 81.2% | 1 | R.LLLPGELAK.H | 2 |

---

|  |  |  |  |  |  |  |  |  |
| --- | --- | --- | --- | --- | --- | --- | --- | --- |
| U | *gi|4503483|ref|NP\_001* | 11 | 27 | 18.5% | 858 | 95338 | 6.8 | eukaryotic translation elongation factor 2 [Homo sapiens] |

| Filename XCorr DeltCN Conf% ObsM+H+ CalcM+H+ SpR ZScore Ion% # Sequence  | | | | | | | | | | | | |
| --- | --- | --- | --- | --- | --- | --- | --- | --- | --- | --- | --- | --- |
| \* | Astrin\_STLCHLD\_tube2\_061314\_01.14825.14825.2 | 3.4118 | 0.4039 | 100.0% | 2205.152 | 2205.4692 | 1 | 7.166 | 50.0% | 1 | K.STAISLFYELSENDLNFIK.Q | 2 |
| \* | Astrin\_STLCHLD\_tube2\_061314\_01.13338.13338.2 | 3.5745 | 0.2993 | 100.0% | 2222.172 | 2221.5151 | 1 | 5.011 | 52.9% | 1 | R.ALLELQLEPEELYQTFQR.I | 2 |
| \* | Astrin\_STLCHLD\_tube2\_061314\_01.14996.14996.2 | 3.348 | 0.419 | 100.0% | 2599.8523 | 2602.11 | 1 | 6.665 | 39.1% | 1 | R.WLPAGDALLQMITIHLPSPVTAQK.Y | 2 |
| \* | Astrin\_STLCHLD\_tube2\_061314\_01.09097.09097.2 | 2.9726 | 0.5209 | 100.0% | 1039.8522 | 1040.3241 | 1 | 8.097 | 75.0% | 1 | K.GPLMMYISK.M | 2 |
| \* | Astrin\_STLCHLD\_tube2\_061314\_01.08418.08418.2 | 3.1101 | 0.3736 | 100.0% | 1107.9722 | 1108.3231 | 1 | 6.948 | 75.0% | 3 | R.VFSGLVSTGLK.V | 2 |
| \* | Astrin\_STLCHLD\_tube2\_061314\_01.06324.06324.2 | 1.9443 | 0.294 | 96.6% | 822.27216 | 822.0717 | 118 | 5.174 | 66.7% | 1 | R.TILMMGR.Y | 2 |
| \* | Astrin\_STLCHLD\_tube2\_061314\_01.08550.08550.3 | 6.0248 | 0.4993 | 100.0% | 2144.6343 | 2144.3489 | 1 | 9.011 | 42.1% | 6 | K.ARPFPDGLAEDIDKGEVSAR.Q | 3 |
| \* | Astrin\_STLCHLD\_061214\_01.06889.06889.3 | 3.7198 | 0.46 | 100.0% | 1744.3143 | 1743.9133 | 1 | 7.574 | 55.8% | 4 | R.YLAEKYEWDVAEAR.K | 3 |
| \* | Astrin\_STLCHLD\_061214\_01.06904.06904.2 | 5.0188 | 0.4919 | 100.0% | 1744.4321 | 1743.9133 | 1 | 8.264 | 80.8% | 2 | R.YLAEKYEWDVAEAR.K | 2 |
| \* | Astrin\_STLCHLD\_061214\_02.10064.10064.3 | 4.1826 | 0.3202 | 100.0% | 2354.6343 | 2354.6677 | 41 | 5.617 | 27.5% | 1 | K.GVQYLNEIKDSVVAGFQWATK.E | 3 |
| \* | Astrin\_STLCHLD\_tube2\_061314\_02.08825.08825.2 | 4.2054 | 0.4747 | 100.0% | 1800.8722 | 1801.0087 | 1 | 7.689 | 60.0% | 6 | K.AYLPVNESFGFTADLR.S | 2 |

---

|  |  |  |  |  |  |  |  |  |
| --- | --- | --- | --- | --- | --- | --- | --- | --- |
| U | *gi|193794814|ref|NP\_0* | 4 | 7 | 18.4% | 364 | 39420 | 8.1 | fructose-bisphosphate aldolase A [Homo sapiens] |
| U | *gi|4557305|ref|NP\_000* | 4 | 7 | 18.4% | 364 | 39420 | 8.1 | fructose-bisphosphate aldolase A [Homo sapiens] |
| U | *gi|34577112|ref|NP\_90* | 4 | 7 | 18.4% | 364 | 39420 | 8.1 | fructose-bisphosphate aldolase A [Homo sapiens] |
| U | *gi|34577110|ref|NP\_90* | 4 | 7 | 18.4% | 364 | 39420 | 8.1 | fructose-bisphosphate aldolase A [Homo sapiens] |

| Filename XCorr DeltCN Conf% ObsM+H+ CalcM+H+ SpR ZScore Ion% # Sequence  | | | | | | | | | | | | |
| --- | --- | --- | --- | --- | --- | --- | --- | --- | --- | --- | --- | --- |
|  | Astrin\_STLCHLD\_tube2\_061314\_01.05120.05120.2 | 3.56 | 0.5292 | 100.0% | 1333.5721 | 1333.4814 | 1 | 8.922 | 73.1% | 4 | K.GILAADESTGSIAK.R | 2 |
|  | Astrin\_STLCHLD\_tube2\_061314\_01.11335.11335.3 | 3.4173 | 0.3614 | 100.0% | 2108.0344 | 2108.4204 | 46 | 5.396 | 27.6% | 1 | K.IGEHTPSALAIMENANVLAR.Y | 3 |
|  | Astrin\_STLCHLD\_061214\_02.07175.07175.3 | 3.966 | 0.3655 | 100.0% | 3343.9443 | 3343.6 | 188 | 6.459 | 16.4% | 1 | R.ALANSLACQGKYTPSGQAGAAASESLFVSNHAY.- | 3 |
|  | Astrin\_STLCHLD\_061214\_02.07175.07175.2 | 4.8085 | 0.4568 | 100.0% | 2229.632 | 2229.3672 | 1 | 8.447 | 42.9% | 1 | K.YTPSGQAGAAASESLFVSNHAY.- | 3 |

---

|  |  |  |  |  |  |  |  |  |
| --- | --- | --- | --- | --- | --- | --- | --- | --- |
| U | *gi|87196351|ref|NP\_00* | 9 | 19 | 18.0% | 662 | 73244 | 7.2 | DEAD/H (Asp-Glu-Ala-Asp/His) box polypeptide 3 [Homo sapiens] |

| Filename XCorr DeltCN Conf% ObsM+H+ CalcM+H+ SpR ZScore Ion% # Sequence  | | | | | | | | | | | | |
| --- | --- | --- | --- | --- | --- | --- | --- | --- | --- | --- | --- | --- |
| \* | Astrin\_STLCHLD\_tube2\_061314\_01.15355.15355.2 | 6.0037 | 0.4562 | 100.0% | 2334.0923 | 2333.6897 | 1 | 9.978 | 47.6% | 1 | K.TAAFLLPILSQIYSDGPGEALR.A | 2 |
|  | Astrin\_STLCHLD\_061214\_01.07375.07375.2 | 3.01 | 0.3707 | 100.0% | 1320.7522 | 1321.4729 | 3 | 6.571 | 75.0% | 5 | R.ELAVQIYEEAR.K | 2 |
|  | Astrin\_STLCHLD\_tube2\_061314\_01.06625.06625.2 | 2.6144 | 0.1698 | 96.9% | 1095.4122 | 1094.2096 | 1 | 4.529 | 87.5% | 1 | K.YLVLDEADR.M | 2 |
|  | Astrin\_STLCHLD\_tube2\_061314\_01.10391.10391.2 | 3.7307 | 0.5056 | 100.0% | 1337.5122 | 1337.5946 | 1 | 8.716 | 85.0% | 3 | R.MLDMGFEPQIR.R | 22 |
| \* | Astrin\_STLCHLD\_tube2\_061314\_01.13560.13560.2 | 3.7906 | 0.5065 | 100.0% | 1292.4722 | 1292.5181 | 1 | 9.424 | 77.3% | 1 | R.SFLLDLLNATGK.D | 2 |
| \* | Astrin\_STLCHLD\_tube2\_061314\_02.11962.11962.3 | 3.6676 | 0.3638 | 100.0% | 2525.9343 | 2525.945 | 60 | 6.256 | 26.1% | 1 | R.SFLLDLLNATGKDSLTLVFVETK.K | 3 |
|  | Astrin\_STLCHLD\_tube2\_061314\_02.06586.06586.2 | 3.6781 | 0.4136 | 100.0% | 1169.2922 | 1169.4099 | 1 | 8.206 | 72.7% | 3 | K.SPILVATAVAAR.G | 2 |
|  | Astrin\_STLCHLD\_tube2\_061314\_01.11528.11528.3 | 4.105 | 0.4628 | 100.0% | 2084.2444 | 2084.2957 | 1 | 7.349 | 50.0% | 2 | K.HVINFDLPSDIEEYVHR.I | 3 |
| \* | Astrin\_STLCHLD\_tube2\_061314\_01.11784.11784.2 | 3.8069 | 0.4963 | 100.0% | 1525.0521 | 1525.7043 | 1 | 9.242 | 76.9% | 2 | R.VGNLGLATSFFNER.N | 2 |

Similarities:
gi|4758138|ref|NP\_004(1:8)  

---

|  |  |  |  |  |  |  |  |  |
| --- | --- | --- | --- | --- | --- | --- | --- | --- |
| U | *gi|47132620|ref|NP\_00* | 9 | 19 | 17.7% | 639 | 65433 | 8.0 | keratin 2 [Homo sapiens] |

| Filename XCorr DeltCN Conf% ObsM+H+ CalcM+H+ SpR ZScore Ion% # Sequence  | | | | | | | | | | | | |
| --- | --- | --- | --- | --- | --- | --- | --- | --- | --- | --- | --- | --- |
|  | Astrin\_STLCHLD\_tube2\_061314\_02.07469.07469.2 | 3.7977 | 0.365 | 100.0% | 1840.3922 | 1840.0055 | 1 | 7.328 | 38.1% | 2 | K.SISISVAGGGGGFGAAGGFGGR.G | 2 |
| \* | Astrin\_STLCHLD\_061214\_02.07645.07645.2 | 5.8444 | 0.5738 | 100.0% | 2399.672 | 2400.4446 | 1 | 10.507 | 51.7% | 2 | R.GGGFGGGSSFGGGSGFSGGGFGGGGFGGGR.F | 2 |
| \* | Astrin\_STLCHLD\_061214\_02.07641.07641.3 | 5.5878 | 0.4765 | 100.0% | 2399.8442 | 2400.4446 | 1 | 9.353 | 49.1% | 1 | R.GGGFGGGSSFGGGSGFSGGGFGGGGFGGGR.F | 3 |
|  | Astrin\_STLCHLD\_tube2\_061314\_01.06264.06264.2 | 2.4085 | 0.2136 | 97.8% | 828.2922 | 827.95544 | 5 | 5.059 | 91.7% | 1 | K.FASFIDK.V | 2222 |
|  | Astrin\_STLCHLD\_tube2\_061314\_01.05226.05226.2 | 4.1811 | 0.1216 | 99.3% | 1476.2922 | 1476.6726 | 1 | 6.927 | 90.9% | 3 | R.FLEQQNQVLQTK.W | 22 |
|  | Astrin\_STLCHLD\_tube2\_061314\_01.05694.05694.2 | 2.6949 | 0.323 | 99.5% | 1038.3322 | 1038.1454 | 1 | 6.716 | 87.5% | 1 | R.YLDGLTAER.T | 2 |
|  | Astrin\_STLCHLD\_tube2\_061314\_01.12653.12653.2 | 2.489 | 0.2745 | 97.4% | 1331.5721 | 1330.5211 | 3 | 5.659 | 59.1% | 1 | R.NLDLDSIIAEVK.A | 22 |
|  | Astrin\_STLCHLD\_061214\_01.05516.05516.2 | 3.1494 | 0.4324 | 100.0% | 1194.3722 | 1194.33 | 1 | 8.999 | 72.2% | 5 | K.YEELQVTVGR.H | 2 |
|  | Astrin\_STLCHLD\_061214\_02.07769.07769.2 | 3.2347 | 0.4112 | 100.0% | 1264.5922 | 1264.4644 | 1 | 8.501 | 65.0% | 3 | K.LALDVEIATYR.K | 22 |

Similarities:
gi|4504919|ref|NP\_002(1:8)  
gi|119395750|ref|NP\_0(1:8)  
gi|67782365|ref|NP\_00(1:8)  
gi|119703753|ref|NP\_0(3:6)  

---

|  |  |  |  |  |  |  |  |  |
| --- | --- | --- | --- | --- | --- | --- | --- | --- |
| U | *gi|4503471|ref|NP\_001* | 9 | 52 | 17.7% | 462 | 50141 | 9.0 | eukaryotic translation elongation factor 1 alpha 1 [Homo sapiens] |

| Filename XCorr DeltCN Conf% ObsM+H+ CalcM+H+ SpR ZScore Ion% # Sequence  | | | | | | | | | | | | |
| --- | --- | --- | --- | --- | --- | --- | --- | --- | --- | --- | --- | --- |
|  | Astrin\_STLCHLD\_061214\_02.05785.05785.3 | 5.207 | 0.4985 | 100.0% | 1591.0144 | 1589.835 | 1 | 8.247 | 53.6% | 28 | K.THINIVVIGHVDSGK.S | 3 |
|  | Astrin\_STLCHLD\_061214\_01.05884.05884.2 | 3.7143 | 0.403 | 100.0% | 1591.2122 | 1589.835 | 1 | 7.705 | 71.4% | 2 | K.THINIVVIGHVDSGK.S | 2 |
| \* | Astrin\_STLCHLD\_tube2\_061314\_01.07055.07055.2 | 3.0583 | 0.4399 | 100.0% | 1405.4321 | 1405.5962 | 1 | 7.21 | 68.2% | 1 | K.YYVTIIDAPGHR.D | 2 |
| \* | Astrin\_STLCHLD\_tube2\_061314\_01.07037.07037.3 | 3.6552 | 0.3458 | 100.0% | 1407.1743 | 1405.5962 | 8 | 5.745 | 43.2% | 3 | K.YYVTIIDAPGHR.D | 3 |
|  | Astrin\_STLCHLD\_061214\_01.07464.07464.2 | 3.5198 | 0.4793 | 100.0% | 1315.5521 | 1315.5553 | 1 | 8.723 | 77.3% | 6 | R.EHALLAYTLGVK.Q | 2 |
|  | Astrin\_STLCHLD\_tube2\_061314\_02.07064.07064.3 | 4.1206 | 0.4223 | 100.0% | 1316.1244 | 1315.5553 | 2 | 7.496 | 56.8% | 2 | R.EHALLAYTLGVK.Q | 3 |
|  | Astrin\_STLCHLD\_tube2\_061314\_01.06557.06557.2 | 2.6251 | 0.1908 | 98.0% | 976.53217 | 976.1607 | 7 | 5.999 | 71.4% | 2 | R.LPLQDVYK.I | 2 |
|  | Astrin\_STLCHLD\_tube2\_061314\_01.05214.05214.2 | 3.399 | 0.3851 | 100.0% | 1026.3922 | 1026.2241 | 1 | 7.487 | 80.0% | 4 | K.IGGIGTVPVGR.V | 2 |
| \* | Astrin\_STLCHLD\_061214\_01.09192.09192.3 | 4.6953 | 0.3145 | 100.0% | 2519.0645 | 2516.999 | 1 | 5.331 | 35.9% | 4 | R.VETGVLKPGMVVTFAPVNVTTEVK.S | 3 |

---

|  |  |  |  |  |  |  |  |  |
| --- | --- | --- | --- | --- | --- | --- | --- | --- |
| U | *contaminant\_KERATIN12* | 8 | 20 | 17.6% | 431 | 47974 | 5.0 | no description |
| U | *gi|4557701|ref|NP\_000* | 8 | 20 | 17.6% | 432 | 48106 | 5.0 | keratin 17 [Homo sapiens] |

| Filename XCorr DeltCN Conf% ObsM+H+ CalcM+H+ SpR ZScore Ion% # Sequence  | | | | | | | | | | | | |
| --- | --- | --- | --- | --- | --- | --- | --- | --- | --- | --- | --- | --- |
|  | Astrin\_STLCHLD\_061214\_01.04909.04909.2 | 3.8958 | 0.4304 | 100.0% | 1347.1522 | 1346.4772 | 1 | 7.343 | 77.3% | 11 | R.ALEEANTELEVK.I | 2 |
|  | Astrin\_STLCHLD\_tube2\_061314\_01.08023.08023.2 | 3.2303 | 0.3839 | 100.0% | 1030.1122 | 1030.2096 | 2 | 6.85 | 81.2% | 2 | R.VLDELTLAR.A | 2222 |
|  | Astrin\_STLCHLD\_061214\_01.05173.05173.2 | 3.2804 | 0.4266 | 100.0% | 1404.7922 | 1404.4764 | 3 | 6.723 | 58.3% | 2 | K.ASLEGNLAETENR.Y | 2 |
|  | Astrin\_STLCHLD\_tube2\_061314\_01.05822.05822.2 | 3.2909 | 0.3194 | 100.0% | 1380.3722 | 1380.5437 | 1 | 5.474 | 70.0% | 1 | K.TRLEQEIATYR.R | 222 |
|  | Astrin\_STLCHLD\_tube2\_061314\_01.04778.04778.3 | 2.8055 | 0.2787 | 98.7% | 1537.4043 | 1536.7311 | 1 | 5.293 | 45.5% | 1 | K.TRLEQEIATYRR.L | 333 |
|  | Astrin\_STLCHLD\_tube2\_061314\_01.04573.04573.3 | 3.0426 | 0.2237 | 97.6% | 1674.7743 | 1673.8662 | 2 | 5.681 | 38.5% | 1 | R.RLLEGEDAHLTQYK.K | 3 |
|  | Astrin\_STLCHLD\_tube2\_061314\_01.04453.04453.3 | 3.4582 | 0.3532 | 100.0% | 2330.1843 | 2329.6152 | 1 | 5.641 | 34.2% | 1 | R.LLEGEDAHLTQYKKEPVTTR.Q | 3 |
|  | Astrin\_STLCHLD\_061214\_01.04531.04531.2 | 2.1646 | 0.3035 | 96.9% | 1117.3722 | 1118.2291 | 1 | 5.872 | 83.3% | 1 | R.TIVEEVQDGK.V | 2 |

Similarities:
contaminant\_KERATIN05(3:5)  
contaminant\_KERATIN08(3:5)  
contaminant\_KERATIN10(1:7)  

---

|  |  |  |  |  |  |  |  |  |
| --- | --- | --- | --- | --- | --- | --- | --- | --- |
| U | *gi|15809016|ref|NP\_29* | 3 | 5 | 17.4% | 172 | 19779 | 4.8 | myosin regulatory light chain MRCL2 isoform A [Homo sapiens] |
| U | *gi|5453740|ref|NP\_006* | 3 | 5 | 17.5% | 171 | 19794 | 4.8 | myosin, light chain 12A, regulatory, non-sarcomeric [Homo sapiens] |
| U | *gi|222144328|ref|NP\_0* | 3 | 5 | 19.5% | 154 | 17757 | 4.4 | myosin regulatory light chain MRCL2 isoform B [Homo sapiens] |
| U | *gi|222144326|ref|NP\_0* | 3 | 5 | 17.4% | 172 | 19779 | 4.8 | myosin regulatory light chain MRCL2 isoform A [Homo sapiens] |
| U | *gi|222144324|ref|NP\_0* | 3 | 5 | 17.4% | 172 | 19779 | 4.8 | myosin regulatory light chain MRCL2 isoform A [Homo sapiens] |

| Filename XCorr DeltCN Conf% ObsM+H+ CalcM+H+ SpR ZScore Ion% # Sequence  | | | | | | | | | | | | |
| --- | --- | --- | --- | --- | --- | --- | --- | --- | --- | --- | --- | --- |
|  | Astrin\_STLCHLD\_tube2\_061314\_01.11388.11388.3 | 3.4097 | 0.2013 | 96.9% | 2433.3542 | 2433.649 | 1 | 4.527 | 31.6% | 2 | R.ELLTTMGDRFTDEEVDELYR.E | 3 |
|  | Astrin\_STLCHLD\_tube2\_061314\_01.08257.08257.2 | 3.5653 | 0.4316 | 100.0% | 1417.6322 | 1416.4839 | 1 | 6.934 | 70.0% | 2 | R.FTDEEVDELYR.E | 2 |
|  | Astrin\_STLCHLD\_tube2\_061314\_01.09614.09614.2 | 3.1857 | 0.2754 | 99.9% | 1261.7322 | 1261.3794 | 1 | 8.008 | 72.2% | 1 | K.GNFNYIEFTR.I | 2 |

---

|  |  |  |  |  |  |  |  |  |
| --- | --- | --- | --- | --- | --- | --- | --- | --- |
| U | *contaminant\_KERATIN03* | 8 | 21 | 17.0% | 593 | 59519 | 5.2 | no description |
| U | *gi|195972866|ref|NP\_0* | 8 | 21 | 17.3% | 584 | 58801 | 5.2 | keratin 10 [Homo sapiens] |

| Filename XCorr DeltCN Conf% ObsM+H+ CalcM+H+ SpR ZScore Ion% # Sequence  | | | | | | | | | | | | |
| --- | --- | --- | --- | --- | --- | --- | --- | --- | --- | --- | --- | --- |
|  | Astrin\_STLCHLD\_061214\_02.06871.06871.2 | 5.5529 | 0.5159 | 100.0% | 1708.3722 | 1708.7844 | 1 | 8.976 | 63.9% | 8 | K.GSLGGGFSSGGFSGGSFSR.G | 2 |
|  | Astrin\_STLCHLD\_tube2\_061314\_01.04569.04569.2 | 3.5967 | 0.3869 | 100.0% | 1382.5122 | 1382.4668 | 1 | 7.842 | 68.2% | 2 | R.ALEESNYELEGK.I | 2 |
|  | Astrin\_STLCHLD\_tube2\_061314\_01.14066.14066.3 | 3.6898 | 0.2695 | 99.0% | 3054.2644 | 3054.4277 | 1 | 5.785 | 28.8% | 1 | K.TIDDLKNQILNLTTDNANILLQIDNAR.L | 3 |
|  | Astrin\_STLCHLD\_061214\_01.05007.05007.2 | 2.7783 | 0.3049 | 99.1% | 1235.4122 | 1235.4258 | 1 | 6.909 | 77.8% | 1 | R.LKYENEVALR.Q | 2 |
|  | Astrin\_STLCHLD\_061214\_01.05064.05064.3 | 3.5896 | 0.2666 | 100.0% | 1236.4744 | 1235.4258 | 1 | 5.47 | 55.6% | 2 | R.LKYENEVALR.Q | 3 |
|  | Astrin\_STLCHLD\_tube2\_061314\_01.07416.07416.2 | 3.0497 | 0.3998 | 100.0% | 1032.4521 | 1032.2224 | 1 | 7.0 | 75.0% | 1 | R.VLDELTLTK.A | 2 |
|  | Astrin\_STLCHLD\_061214\_01.06354.06354.2 | 3.6179 | 0.5389 | 100.0% | 1391.4122 | 1391.4778 | 1 | 9.1 | 70.8% | 4 | K.QSLEASLAETEGR.Y | 2 |
|  | Astrin\_STLCHLD\_061214\_01.05703.05703.2 | 3.6217 | 0.3459 | 100.0% | 1435.4521 | 1435.623 | 1 | 7.079 | 85.0% | 2 | K.IRLENEIQTYR.S | 2 |

---

|  |  |  |  |  |  |  |  |  |
| --- | --- | --- | --- | --- | --- | --- | --- | --- |
| U | *gi|108936958|ref|NP\_0* | 4 | 9 | 17.0% | 342 | 38926 | 5.5 | WD-repeat protein [Homo sapiens] |

| Filename XCorr DeltCN Conf% ObsM+H+ CalcM+H+ SpR ZScore Ion% # Sequence  | | | | | | | | | | | | |
| --- | --- | --- | --- | --- | --- | --- | --- | --- | --- | --- | --- | --- |
| \* | Astrin\_STLCHLD\_061214\_01.08654.08654.2 | 3.6461 | 0.4035 | 100.0% | 1485.0521 | 1484.6488 | 1 | 7.335 | 70.8% | 2 | R.LALGSFVEEYNNK.V | 2 |
| \* | Astrin\_STLCHLD\_tube2\_061314\_01.11642.11642.2 | 3.2005 | 0.5078 | 100.0% | 1640.8322 | 1640.8333 | 1 | 8.102 | 53.6% | 1 | K.GVYPDLLATSGDYLR.V | 2 |
| \* | Astrin\_STLCHLD\_tube2\_061314\_02.06758.06758.3 | 5.0382 | 0.3909 | 100.0% | 2007.2043 | 2007.2535 | 1 | 8.847 | 43.8% | 3 | K.TQLIAHDKEVYDIAFSR.A | 3 |
| \* | Astrin\_STLCHLD\_061214\_02.06560.06560.2 | 3.8348 | 0.4221 | 100.0% | 1312.5122 | 1312.44 | 1 | 8.892 | 75.0% | 3 | R.DMFASVGADGSVR.M | 2 |

---

|  |  |  |  |  |  |  |  |  |
| --- | --- | --- | --- | --- | --- | --- | --- | --- |
| U | *gi|10835063|ref|NP\_00* | 2 | 2 | 16.7% | 294 | 32575 | 4.8 | nucleophosmin 1 isoform 1 [Homo sapiens] |
| U | *gi|83641870|ref|NP\_00* | 2 | 2 | 18.9% | 259 | 28400 | 4.7 | nucleophosmin 1 isoform 3 [Homo sapiens] |
| U | *gi|40353734|ref|NP\_95* | 2 | 2 | 18.5% | 265 | 29465 | 4.6 | nucleophosmin 1 isoform 2 [Homo sapiens] |

| Filename XCorr DeltCN Conf% ObsM+H+ CalcM+H+ SpR ZScore Ion% # Sequence  | | | | | | | | | | | | |
| --- | --- | --- | --- | --- | --- | --- | --- | --- | --- | --- | --- | --- |
|  | Astrin\_STLCHLD\_tube2\_061314\_02.08484.08484.3 | 3.7747 | 0.39 | 100.0% | 2931.2944 | 2931.2874 | 3 | 5.921 | 23.1% | 1 | R.TVSLGAGAKDELHIVEAEAMNYEGSPIK.V | 3 |
|  | Astrin\_STLCHLD\_tube2\_061314\_01.12851.12851.2 | 3.2324 | 0.2992 | 99.2% | 2228.9922 | 2228.655 | 1 | 5.169 | 37.5% | 1 | K.MSVQPTVSLGGFEITPPVVLR.L | 2 |

---

|  |  |  |  |  |  |  |  |  |
| --- | --- | --- | --- | --- | --- | --- | --- | --- |
| U | *gi|5803225|ref|NP\_006* | 2 | 6 | 16.5% | 255 | 29174 | 4.7 | tyrosine 3/tryptophan 5 -monooxygenase activation protein, epsilon polypeptide [Homo sapiens] |

| Filename XCorr DeltCN Conf% ObsM+H+ CalcM+H+ SpR ZScore Ion% # Sequence  | | | | | | | | | | | | |
| --- | --- | --- | --- | --- | --- | --- | --- | --- | --- | --- | --- | --- |
| \* | Astrin\_STLCHLD\_061214\_02.06812.06812.2 | 3.7822 | 0.4089 | 100.0% | 1449.5122 | 1448.6312 | 1 | 8.472 | 62.5% | 5 | K.VAGMDVELTVEER.N | 2 |
| \* | Astrin\_STLCHLD\_tube2\_061314\_01.17676.17676.3 | 4.9018 | 0.4442 | 100.0% | 3260.1843 | 3260.6375 | 1 | 8.935 | 30.4% | 1 | K.AAFDDAIAELDTLSEESYKDSTLIMQLLR.D | 3 |

---

|  |  |  |  |  |  |  |  |  |
| --- | --- | --- | --- | --- | --- | --- | --- | --- |
| U | *GFP* | 4 | 6 | 16.4% | 238 | 26813 | 5.8 | no description |

| Filename XCorr DeltCN Conf% ObsM+H+ CalcM+H+ SpR ZScore Ion% # Sequence  | | | | | | | | | | | | |
| --- | --- | --- | --- | --- | --- | --- | --- | --- | --- | --- | --- | --- |
| \* | Astrin\_STLCHLD\_061214\_01.04634.04634.2 | 3.4954 | 0.5434 | 100.0% | 1504.5122 | 1504.5499 | 1 | 9.233 | 60.7% | 1 | K.FSVSGEGEGDATYGK.L | 2 |
| \* | Astrin\_STLCHLD\_061214\_01.04579.04579.2 | 2.5085 | 0.4252 | 99.9% | 1267.3121 | 1267.399 | 3 | 6.63 | 65.0% | 2 | K.SAMPEGYVQER.T | 2 |
| \* | Astrin\_STLCHLD\_061214\_01.05138.05138.3 | 2.4914 | 0.2501 | 95.3% | 1479.1144 | 1478.6451 | 29 | 4.788 | 35.4% | 1 | R.AEVKFEGDTLVNR.I | 3 |
| \* | Astrin\_STLCHLD\_061214\_01.04669.04669.2 | 2.9503 | 0.3904 | 100.0% | 1051.0521 | 1051.1442 | 1 | 6.439 | 87.5% | 2 | K.FEGDTLVNR.I | 2 |

---

|  |  |  |  |  |  |  |  |  |
| --- | --- | --- | --- | --- | --- | --- | --- | --- |
| U | *gi|38045913|ref|NP\_93* | 2 | 2 | 16.4% | 177 | 19654 | 5.6 | non-metastatic cells 1, protein (NM23A) expressed in isoform a [Homo sapiens] |
| U | *gi|66392227|ref|NP\_00* | 2 | 2 | 19.1% | 152 | 17298 | 8.4 | non-metastatic cells 2, protein (NM23B) expressed in [Homo sapiens] |
| U | *gi|66392205|ref|NP\_00* | 2 | 2 | 19.1% | 152 | 17298 | 8.4 | non-metastatic cells 2, protein (NM23B) expressed in [Homo sapiens] |
| U | *gi|66392203|ref|NP\_00* | 2 | 2 | 10.9% | 267 | 30137 | 8.9 | NME1-NME2 protein [Homo sapiens] |
| U | *gi|66392192|ref|NP\_00* | 2 | 2 | 19.1% | 152 | 17298 | 8.4 | non-metastatic cells 2, protein (NM23B) expressed in [Homo sapiens] |
| U | *gi|4557797|ref|NP\_000* | 2 | 2 | 19.1% | 152 | 17149 | 6.2 | non-metastatic cells 1, protein (NM23A) expressed in isoform b [Homo sapiens] |
| U | *gi|4505409|ref|NP\_002* | 2 | 2 | 19.1% | 152 | 17298 | 8.4 | non-metastatic cells 2, protein (NM23B) expressed in [Homo sapiens] |

| Filename XCorr DeltCN Conf% ObsM+H+ CalcM+H+ SpR ZScore Ion% # Sequence  | | | | | | | | | | | | |
| --- | --- | --- | --- | --- | --- | --- | --- | --- | --- | --- | --- | --- |
|  | Astrin\_STLCHLD\_tube2\_061314\_01.05387.05387.2 | 2.7698 | 0.1954 | 97.0% | 1345.5521 | 1345.5846 | 64 | 4.483 | 50.0% | 1 | R.TFIAIKPDGVQR.G | 2 |
|  | Astrin\_STLCHLD\_tube2\_061314\_01.04621.04621.2 | 4.4885 | 0.4123 | 100.0% | 1786.4321 | 1787.041 | 1 | 8.017 | 65.6% | 1 | R.VMLGETNPADSKPGTIR.G | 2 |

---

|  |  |  |  |  |  |  |  |  |
| --- | --- | --- | --- | --- | --- | --- | --- | --- |
| U | *gi|169201338|ref|XP\_0* | 3 | 8 | 16.2% | 160 | 18565 | 10.5 | PREDICTED: hypothetical protein [Homo sapiens] |
| U | *gi|89040203|ref|XP\_93* | 3 | 8 | 16.2% | 160 | 18593 | 10.5 | PREDICTED: hypothetical protein [Homo sapiens] |
| U | *gi|18104948|ref|NP\_00* | 3 | 8 | 16.2% | 160 | 18565 | 10.5 | ribosomal protein L21 [Homo sapiens] |
| U | *gi|169213854|ref|XP\_0* | 3 | 8 | 16.2% | 160 | 18790 | 10.3 | PREDICTED: hypothetical protein [Homo sapiens] |
| U | *gi|169210381|ref|XP\_0* | 3 | 8 | 16.2% | 160 | 18535 | 10.6 | PREDICTED: hypothetical protein isoform 2 [Homo sapiens] |
| U | *gi|169210379|ref|XP\_0* | 3 | 8 | 16.2% | 160 | 18535 | 10.6 | PREDICTED: hypothetical protein isoform 3 [Homo sapiens] |
| U | *gi|169210377|ref|XP\_0* | 3 | 8 | 16.2% | 160 | 18535 | 10.6 | PREDICTED: hypothetical protein isoform 1 [Homo sapiens] |
| U | *gi|169202779|ref|XP\_0* | 3 | 8 | 16.2% | 160 | 18521 | 10.5 | PREDICTED: similar to ribosomal protein L21 isoform 1 [Homo sapiens] |
| U | *gi|169202777|ref|XP\_0* | 3 | 8 | 16.2% | 160 | 18521 | 10.5 | PREDICTED: similar to ribosomal protein L21 isoform 2 [Homo sapiens] |
| U | *gi|169201750|ref|XP\_0* | 3 | 8 | 16.2% | 160 | 18550 | 10.5 | PREDICTED: hypothetical protein [Homo sapiens] |

| Filename XCorr DeltCN Conf% ObsM+H+ CalcM+H+ SpR ZScore Ion% # Sequence  | | | | | | | | | | | | |
| --- | --- | --- | --- | --- | --- | --- | --- | --- | --- | --- | --- | --- |
|  | Astrin\_STLCHLD\_tube2\_061314\_01.06972.06972.2 | 2.498 | 0.3414 | 98.5% | 1244.6721 | 1244.4973 | 2 | 6.461 | 65.0% | 2 | K.HGVVPLATYMR.I | 2 |
|  | Astrin\_STLCHLD\_061214\_01.05950.05950.2 | 4.4976 | 0.4473 | 100.0% | 1641.3922 | 1641.9108 | 1 | 8.306 | 85.7% | 1 | R.VYNVTQHAVGIVVNK.Q | 2 |
|  | Astrin\_STLCHLD\_061214\_01.05961.05961.3 | 4.2836 | 0.4717 | 100.0% | 1643.1843 | 1641.9108 | 1 | 8.316 | 50.0% | 5 | R.VYNVTQHAVGIVVNK.Q | 3 |

---

|  |  |  |  |  |  |  |  |  |
| --- | --- | --- | --- | --- | --- | --- | --- | --- |
| U | *gi|4502891|ref|NP\_001* | 2 | 2 | 16.0% | 237 | 26215 | 4.1 | chloride channel, nucleotide-sensitive, 1A [Homo sapiens] |

| Filename XCorr DeltCN Conf% ObsM+H+ CalcM+H+ SpR ZScore Ion% # Sequence  | | | | | | | | | | | | |
| --- | --- | --- | --- | --- | --- | --- | --- | --- | --- | --- | --- | --- |
| \* | Astrin\_STLCHLD\_tube2\_061314\_02.06694.06694.2 | 2.6882 | 0.3767 | 99.3% | 1337.3322 | 1338.5034 | 5 | 7.071 | 54.2% | 1 | K.GLGTGTLYIAESR.L | 2 |
| \* | Astrin\_STLCHLD\_061214\_01.12537.12537.3 | 3.0984 | 0.3012 | 98.7% | 2721.6243 | 2721.085 | 3 | 5.273 | 27.1% | 1 | R.LSWLDGSGLGFSLEYPTISLHALSR.D | 3 |

---

|  |  |  |  |  |  |  |  |  |
| --- | --- | --- | --- | --- | --- | --- | --- | --- |
| U | *gi|219555707|ref|NP\_0* | 2 | 2 | 15.8% | 184 | 20170 | 7.0 | eukaryotic translation initiation factor 5A isoform A [Homo sapiens] |
| U | *gi|4503545|ref|NP\_001* | 2 | 2 | 18.8% | 154 | 16832 | 5.2 | eukaryotic translation initiation factor 5A isoform B [Homo sapiens] |
| U | *gi|219555712|ref|NP\_0* | 2 | 2 | 18.8% | 154 | 16832 | 5.2 | eukaryotic translation initiation factor 5A isoform B [Homo sapiens] |
| U | *gi|219555710|ref|NP\_0* | 2 | 2 | 18.8% | 154 | 16832 | 5.2 | eukaryotic translation initiation factor 5A isoform B [Homo sapiens] |

| Filename XCorr DeltCN Conf% ObsM+H+ CalcM+H+ SpR ZScore Ion% # Sequence  | | | | | | | | | | | | |
| --- | --- | --- | --- | --- | --- | --- | --- | --- | --- | --- | --- | --- |
|  | Astrin\_STLCHLD\_tube2\_061314\_01.10879.10879.2 | 2.4334 | 0.5499 | 100.0% | 1298.5721 | 1299.5559 | 1 | 8.114 | 63.6% | 1 | K.VHLVGIDIFTGK.K | 2 |
|  | Astrin\_STLCHLD\_tube2\_061314\_01.07562.07562.3 | 3.654 | 0.3293 | 100.0% | 1970.6643 | 1970.187 | 7 | 5.956 | 35.9% | 1 | R.EDLRLPEGDLGKEIEQK.Y | 3 |

---

|  |  |  |  |  |  |  |  |  |
| --- | --- | --- | --- | --- | --- | --- | --- | --- |
| U | *gi|148470397|ref|NP\_0* | 3 | 5 | 15.7% | 415 | 45672 | 5.6 | heterogeneous nuclear ribonucleoprotein F [Homo sapiens] |
| U | *gi|4826760|ref|NP\_004* | 3 | 5 | 15.7% | 415 | 45672 | 5.6 | heterogeneous nuclear ribonucleoprotein F [Homo sapiens] |
| U | *gi|148470406|ref|NP\_0* | 3 | 5 | 15.7% | 415 | 45672 | 5.6 | heterogeneous nuclear ribonucleoprotein F [Homo sapiens] |
| U | *gi|148470404|ref|NP\_0* | 3 | 5 | 15.7% | 415 | 45672 | 5.6 | heterogeneous nuclear ribonucleoprotein F [Homo sapiens] |
| U | *gi|148470402|ref|NP\_0* | 3 | 5 | 15.7% | 415 | 45672 | 5.6 | heterogeneous nuclear ribonucleoprotein F [Homo sapiens] |
| U | *gi|148470400|ref|NP\_0* | 3 | 5 | 15.7% | 415 | 45672 | 5.6 | heterogeneous nuclear ribonucleoprotein F [Homo sapiens] |

| Filename XCorr DeltCN Conf% ObsM+H+ CalcM+H+ SpR ZScore Ion% # Sequence  | | | | | | | | | | | | |
| --- | --- | --- | --- | --- | --- | --- | --- | --- | --- | --- | --- | --- |
|  | Astrin\_STLCHLD\_061214\_02.06573.06573.2 | 2.8696 | 0.3734 | 99.3% | 1710.7322 | 1710.7919 | 1 | 6.02 | 40.0% | 2 | R.QSGEAFVELGSEDDVK.M | 2 |
|  | Astrin\_STLCHLD\_tube2\_061314\_01.12633.12633.2 | 3.7435 | 0.323 | 100.0% | 1868.7122 | 1869.0813 | 2 | 6.709 | 43.8% | 2 | K.ITGEAFVQFASQELAEK.A | 2 |
|  | Astrin\_STLCHLD\_tube2\_061314\_02.09105.09105.3 | 7.3535 | 0.5648 | 100.0% | 3476.5144 | 3476.7114 | 1 | 10.711 | 30.6% | 1 | R.MRPGAYSTGYGGYEEYSGLSDGYGFTTDLFGR.D | 3 |

---

|  |  |  |  |  |  |  |  |  |
| --- | --- | --- | --- | --- | --- | --- | --- | --- |
| U | *gi|36287110|ref|NP\_91* | 4 | 10 | 15.6% | 379 | 40907 | 4.6 | FGFR1 oncogene partner isoform b [Homo sapiens] |
| U | *gi|5901954|ref|NP\_008* | 4 | 10 | 14.8% | 399 | 43065 | 4.8 | FGFR1 oncogene partner isoform a [Homo sapiens] |

| Filename XCorr DeltCN Conf% ObsM+H+ CalcM+H+ SpR ZScore Ion% # Sequence  | | | | | | | | | | | | |
| --- | --- | --- | --- | --- | --- | --- | --- | --- | --- | --- | --- | --- |
|  | Astrin\_STLCHLD\_tube2\_061314\_01.14561.14561.2 | 4.6731 | 0.4372 | 100.0% | 2165.4922 | 2165.5352 | 1 | 7.414 | 52.5% | 4 | R.DLGIIEAEGTVGGPLLLEVIR.R | 2 |
|  | Astrin\_STLCHLD\_061214\_02.10101.10101.3 | 3.5067 | 0.3729 | 100.0% | 2322.6843 | 2321.7227 | 1 | 5.685 | 31.0% | 4 | R.DLGIIEAEGTVGGPLLLEVIRR.C | 3 |
|  | Astrin\_STLCHLD\_tube2\_061314\_01.07464.07464.2 | 3.2088 | 0.3307 | 100.0% | 1188.5122 | 1188.3666 | 4 | 6.69 | 66.7% | 1 | K.SGLSSLAGAPSLK.D | 2 |
|  | Astrin\_STLCHLD\_tube2\_061314\_01.09465.09465.3 | 3.5427 | 0.345 | 99.8% | 2571.2944 | 2571.63 | 23 | 5.424 | 23.9% | 1 | K.IGSLGLGTGEDDDYVDDFNSTSHR.S | 3 |

---

|  |  |  |  |  |  |  |  |  |
| --- | --- | --- | --- | --- | --- | --- | --- | --- |
| U | *gi|118582269|ref|NP\_0* | 3 | 10 | 15.4% | 201 | 22460 | 8.0 | splicing factor, arginine/serine-rich 1 isoform 2 [Homo sapiens] |
| U | *gi|5902076|ref|NP\_008* | 3 | 10 | 12.5% | 248 | 27745 | 10.4 | splicing factor, arginine/serine-rich 1 isoform 1 [Homo sapiens] |

| Filename XCorr DeltCN Conf% ObsM+H+ CalcM+H+ SpR ZScore Ion% # Sequence  | | | | | | | | | | | | |
| --- | --- | --- | --- | --- | --- | --- | --- | --- | --- | --- | --- | --- |
|  | Astrin\_STLCHLD\_tube2\_061314\_01.08237.08237.2 | 3.1598 | 0.4102 | 100.0% | 1257.5122 | 1257.4752 | 1 | 6.732 | 80.0% | 4 | R.IYVGNLPPDIR.T | 2 |
|  | Astrin\_STLCHLD\_tube2\_061314\_01.06794.06794.2 | 2.8784 | 0.3116 | 99.5% | 1258.9321 | 1258.4137 | 1 | 5.969 | 77.8% | 3 | R.TKDIEDVFYK.Y | 2 |
|  | Astrin\_STLCHLD\_tube2\_061314\_01.07433.07433.2 | 2.5246 | 0.2326 | 97.3% | 1079.1322 | 1079.198 | 1 | 5.09 | 83.3% | 3 | R.DGTGVVEFVR.K | 2 |

---

|  |  |  |  |  |  |  |  |  |
| --- | --- | --- | --- | --- | --- | --- | --- | --- |
| U | *gi|10190716|ref|NP\_06* | 2 | 4 | 15.2% | 224 | 26153 | 8.0 | spindle pole body component 25 [Homo sapiens] |

| Filename XCorr DeltCN Conf% ObsM+H+ CalcM+H+ SpR ZScore Ion% # Sequence  | | | | | | | | | | | | |
| --- | --- | --- | --- | --- | --- | --- | --- | --- | --- | --- | --- | --- |
| \* | Astrin\_STLCHLD\_061214\_02.07743.07743.3 | 4.1186 | 0.3642 | 100.0% | 2508.0544 | 2506.6445 | 1 | 6.281 | 35.7% | 3 | R.DYEVSDSAPHLEGLAEFQENVR.K | 3 |
| \* | Astrin\_STLCHLD\_tube2\_061314\_01.11307.11307.2 | 2.8965 | 0.2664 | 98.5% | 1354.2922 | 1354.5082 | 18 | 6.004 | 50.0% | 1 | K.TNNFSAFLANVR.K | 2 |

---

|  |  |  |  |  |  |  |  |  |
| --- | --- | --- | --- | --- | --- | --- | --- | --- |
| U | *gi|4757834|ref|NP\_004* | 2 | 5 | 15.2% | 211 | 23772 | 6.7 | BCL2-associated athanogene 2 [Homo sapiens] |

| Filename XCorr DeltCN Conf% ObsM+H+ CalcM+H+ SpR ZScore Ion% # Sequence  | | | | | | | | | | | | |
| --- | --- | --- | --- | --- | --- | --- | --- | --- | --- | --- | --- | --- |
| \* | Astrin\_STLCHLD\_tube2\_061314\_01.10953.10953.2 | 3.2304 | 0.4406 | 100.0% | 1329.3322 | 1329.5364 | 1 | 7.531 | 75.0% | 2 | R.LLESLDQLELR.V | 2 |
| \* | Astrin\_STLCHLD\_061214\_02.06589.06589.3 | 3.9064 | 0.3474 | 100.0% | 2400.1143 | 2400.6917 | 1 | 6.635 | 33.8% | 3 | R.TLTVEVSVETIRNPQQQESLK.H | 3 |

---

|  |  |  |  |  |  |  |  |  |
| --- | --- | --- | --- | --- | --- | --- | --- | --- |
| U | *gi|4504425|ref|NP\_002* | 2 | 2 | 14.9% | 215 | 24894 | 5.7 | high-mobility group box 1 [Homo sapiens] |

| Filename XCorr DeltCN Conf% ObsM+H+ CalcM+H+ SpR ZScore Ion% # Sequence  | | | | | | | | | | | | |
| --- | --- | --- | --- | --- | --- | --- | --- | --- | --- | --- | --- | --- |
| \* | Astrin\_STLCHLD\_tube2\_061314\_01.05023.05023.3 | 3.3207 | 0.2798 | 99.7% | 1594.5243 | 1593.7361 | 100 | 5.683 | 32.7% | 1 | K.KHPDASVNFSEFSK.K | 3 |
|  | Astrin\_STLCHLD\_tube2\_061314\_01.05919.05919.3 | 2.9443 | 0.2585 | 97.6% | 2109.5645 | 2111.2903 | 13 | 4.419 | 32.4% | 1 | K.LGEMWNNTAADDKQPYEK.K | 3 |

---

|  |  |  |  |  |  |  |  |  |
| --- | --- | --- | --- | --- | --- | --- | --- | --- |
| U | *gi|221316642|ref|NP\_0* | 4 | 15 | 14.3% | 335 | 37721 | 5.2 | nuclear distribution gene E homolog 1 [Homo sapiens] |
| U | *gi|8923110|ref|NP\_060* | 4 | 15 | 14.3% | 335 | 37721 | 5.2 | nuclear distribution gene E homolog 1 [Homo sapiens] |

| Filename XCorr DeltCN Conf% ObsM+H+ CalcM+H+ SpR ZScore Ion% # Sequence  | | | | | | | | | | | | |
| --- | --- | --- | --- | --- | --- | --- | --- | --- | --- | --- | --- | --- |
|  | Astrin\_STLCHLD\_061214\_01.04617.04617.1 | 1.6387 | 0.3483 | 100.0% | 841.57 | 841.99774 | 11 | 5.947 | 58.3% | 1 | K.DLAMTYK.Q | 1 |
|  | Astrin\_STLCHLD\_061214\_02.04267.04267.2 | 3.2686 | 0.3915 | 100.0% | 1332.2522 | 1332.3666 | 1 | 6.488 | 70.0% | 9 | R.ELEQANDDLER.A | 2 |
|  | Astrin\_STLCHLD\_tube2\_061314\_01.04158.04158.2 | 2.7047 | 0.3704 | 99.5% | 1204.0721 | 1204.3405 | 1 | 6.089 | 80.0% | 4 | R.TPMPSSVEAER.T | 2 |
|  | Astrin\_STLCHLD\_061214\_01.04582.04582.3 | 2.7514 | 0.3159 | 98.6% | 1909.8243 | 1910.0941 | 118 | 4.828 | 27.8% | 1 | R.TDTAVQATGSVPSTPIAHR.G | 3 |

---

|  |  |  |  |  |  |  |  |  |
| --- | --- | --- | --- | --- | --- | --- | --- | --- |
| U | *gi|9966881|ref|NP\_065* | 10 | 14 | 14.2% | 925 | 106374 | 5.4 | nucleoporin 107kDa [Homo sapiens] |

| Filename XCorr DeltCN Conf% ObsM+H+ CalcM+H+ SpR ZScore Ion% # Sequence  | | | | | | | | | | | | |
| --- | --- | --- | --- | --- | --- | --- | --- | --- | --- | --- | --- | --- |
| \* | Astrin\_STLCHLD\_tube2\_061314\_01.09123.09123.2 | 2.9207 | 0.2707 | 98.5% | 1329.5521 | 1329.4093 | 1 | 7.075 | 68.2% | 1 | R.SGFGEISS\*PVIR.E | 2 |
| \* | Astrin\_STLCHLD\_061214\_01.06166.06166.2 | 5.1874 | 0.5513 | 100.0% | 1890.7522 | 1891.0476 | 1 | 9.768 | 65.6% | 3 | R.VLLQASQDENFGNTTPR.N | 2 |
| \* | Astrin\_STLCHLD\_tube2\_061314\_02.06047.06047.3 | 3.2221 | 0.2003 | 96.5% | 1892.9343 | 1891.0476 | 19 | 4.937 | 35.9% | 1 | R.VLLQASQDENFGNTTPR.N | 3 |
| \* | Astrin\_STLCHLD\_tube2\_061314\_02.06180.06180.2 | 4.2771 | 0.4332 | 100.0% | 1971.3722 | 1971.0476 | 1 | 7.574 | 65.6% | 2 | R.VLLQASQDENFGNTT#PR.N | 2 |
| \* | Astrin\_STLCHLD\_tube2\_061314\_02.09011.09011.3 | 3.7417 | 0.2731 | 99.4% | 2619.3843 | 2619.9495 | 13 | 5.648 | 26.1% | 1 | R.SGLFTNTEPHSITEDVTISAVMLR.E | 3 |
| \* | Astrin\_STLCHLD\_tube2\_061314\_01.17149.17149.2 | 3.927 | 0.4548 | 100.0% | 2452.9922 | 2453.6975 | 1 | 8.194 | 42.9% | 1 | R.EDDPGEAASMSMFSDFLQSFLK.H | 2 |
| \* | Astrin\_STLCHLD\_tube2\_061314\_02.06593.06593.2 | 2.9983 | 0.3776 | 100.0% | 1121.0922 | 1121.3219 | 1 | 7.479 | 75.0% | 1 | R.AIYAALSGNLK.Q | 2 |
| \* | Astrin\_STLCHLD\_061214\_01.07918.07918.2 | 2.4933 | 0.2403 | 97.0% | 1324.3722 | 1324.476 | 1 | 5.539 | 75.0% | 2 | R.EYLGANWTLEK.V | 2 |
| \* | Astrin\_STLCHLD\_tube2\_061314\_01.14363.14363.2 | 2.9019 | 0.3503 | 99.3% | 1687.8722 | 1687.9359 | 4 | 5.762 | 42.3% | 1 | K.IDVIDWLVFDPAQR.A | 2 |
| \* | Astrin\_STLCHLD\_tube2\_061314\_01.06537.06537.3 | 2.8362 | 0.3135 | 98.7% | 2264.9043 | 2264.6477 | 45 | 4.933 | 27.6% | 1 | K.HMNSVPQKPALIPQPTFTEK.V | 3 |

---

|  |  |  |  |  |  |  |  |  |
| --- | --- | --- | --- | --- | --- | --- | --- | --- |
| U | *gi|14141152|ref|NP\_00* | 7 | 18 | 14.2% | 730 | 77516 | 8.7 | heterogeneous nuclear ribonucleoprotein M isoform a [Homo sapiens] |
| U | *gi|157412270|ref|NP\_1* | 7 | 18 | 15.1% | 691 | 73621 | 8.8 | heterogeneous nuclear ribonucleoprotein M isoform b [Homo sapiens] |

| Filename XCorr DeltCN Conf% ObsM+H+ CalcM+H+ SpR ZScore Ion% # Sequence  | | | | | | | | | | | | |
| --- | --- | --- | --- | --- | --- | --- | --- | --- | --- | --- | --- | --- |
|  | Astrin\_STLCHLD\_tube2\_061314\_02.07546.07546.2 | 3.4088 | 0.3706 | 100.0% | 1428.5521 | 1427.6403 | 3 | 6.169 | 50.0% | 1 | R.LGSTVFVANLDYK.V | 2 |
|  | Astrin\_STLCHLD\_tube2\_061314\_01.08963.08963.3 | 3.2537 | 0.5034 | 100.0% | 1615.0144 | 1614.875 | 2 | 7.572 | 44.6% | 1 | R.MGPLGLDHMASSIER.M | 3 |
|  | Astrin\_STLCHLD\_tube2\_061314\_02.06921.06921.2 | 3.4497 | 0.5585 | 100.0% | 1126.1921 | 1126.3337 | 1 | 8.747 | 75.0% | 3 | R.MGAGMGFGLER.M | 2 |
|  | Astrin\_STLCHLD\_tube2\_061314\_01.05765.05765.2 | 2.1349 | 0.3246 | 96.7% | 1189.8322 | 1189.4333 | 2 | 4.77 | 68.2% | 1 | R.MVPAGMGAGLER.M | 2 |
|  | Astrin\_STLCHLD\_tube2\_061314\_01.07993.07993.2 | 3.2002 | 0.3441 | 99.8% | 1428.4922 | 1428.7076 | 1 | 6.202 | 71.4% | 1 | R.MGPAMGPALGAGIER.M | 2 |
|  | Astrin\_STLCHLD\_061214\_02.06385.06385.2 | 3.9967 | 0.4418 | 100.0% | 1385.1921 | 1384.5677 | 1 | 8.495 | 75.0% | 8 | R.MGLAMGGGGGASFDR.A | 2 |
|  | Astrin\_STLCHLD\_061214\_01.06750.06750.3 | 3.3105 | 0.3856 | 100.0% | 2035.9143 | 2036.1735 | 1 | 6.717 | 30.7% | 3 | R.GNFGGSFAGSFGGAGGHAPGVAR.K | 3 |

---

|  |  |  |  |  |  |  |  |  |
| --- | --- | --- | --- | --- | --- | --- | --- | --- |
| U | *gi|14602427|ref|NP\_12* | 4 | 10 | 14.1% | 277 | 31293 | 5.2 | ZW10 interactor isoform a [Homo sapiens] |
| U | *gi|14602429|ref|NP\_00* | 4 | 10 | 14.1% | 277 | 31293 | 5.2 | ZW10 interactor isoform a [Homo sapiens] |

| Filename XCorr DeltCN Conf% ObsM+H+ CalcM+H+ SpR ZScore Ion% # Sequence  | | | | | | | | | | | | |
| --- | --- | --- | --- | --- | --- | --- | --- | --- | --- | --- | --- | --- |
|  | Astrin\_STLCHLD\_tube2\_061314\_01.05567.05567.2 | 2.1271 | 0.3828 | 97.7% | 1261.4521 | 1261.3312 | 1 | 6.23 | 63.6% | 1 | K.GLDPLASEDTSR.Q | 2 |
|  | Astrin\_STLCHLD\_061214\_01.04692.04692.3 | 4.5553 | 0.3499 | 100.0% | 1490.4243 | 1489.6743 | 1 | 7.644 | 56.2% | 7 | K.HLQHLAEVSAEVR.E | 3 |
|  | Astrin\_STLCHLD\_061214\_01.04648.04648.2 | 3.3933 | 0.464 | 100.0% | 1490.5322 | 1489.6743 | 1 | 7.956 | 66.7% | 1 | K.HLQHLAEVSAEVR.E | 2 |
|  | Astrin\_STLCHLD\_tube2\_061314\_01.14799.14799.2 | 3.7403 | 0.4996 | 100.0% | 1716.5322 | 1717.0178 | 1 | 9.878 | 65.4% | 1 | R.YQTFLQLLYTLQGK.L | 2 |

---

|  |  |  |  |  |  |  |  |  |
| --- | --- | --- | --- | --- | --- | --- | --- | --- |
| U | *gi|56699409|ref|NP\_00* | 4 | 14 | 13.8% | 391 | 42332 | 10.1 | RNA binding motif protein, X-linked [Homo sapiens] |

| Filename XCorr DeltCN Conf% ObsM+H+ CalcM+H+ SpR ZScore Ion% # Sequence  | | | | | | | | | | | | |
| --- | --- | --- | --- | --- | --- | --- | --- | --- | --- | --- | --- | --- |
|  | Astrin\_STLCHLD\_tube2\_061314\_01.08381.08381.2 | 3.7391 | 0.2809 | 100.0% | 1436.2922 | 1436.6049 | 3 | 6.444 | 70.8% | 4 | K.LFIGGLNTETNEK.A | 2 |
|  | Astrin\_STLCHLD\_tube2\_061314\_01.09717.09717.2 | 2.6119 | 0.1862 | 97.6% | 945.3722 | 945.2488 | 29 | 5.401 | 71.4% | 1 | R.IVEVLLMK.D | 2 |
|  | Astrin\_STLCHLD\_tube2\_061314\_01.11367.11367.2 | 2.945 | 0.3674 | 99.5% | 1487.5721 | 1487.6519 | 1 | 5.87 | 61.5% | 2 | R.GFAFVTFESPADAK.D | 2 |
|  | Astrin\_STLCHLD\_061214\_01.06366.06366.3 | 4.379 | 0.4033 | 100.0% | 2051.4243 | 2051.1873 | 1 | 7.636 | 41.7% | 7 | R.GGHMDDGGYSMNFNMSSSR.G | 3 |

---

|  |  |  |  |  |  |  |  |  |
| --- | --- | --- | --- | --- | --- | --- | --- | --- |
| U | *gi|4506607|ref|NP\_000* | 2 | 8 | 13.8% | 188 | 21634 | 11.7 | ribosomal protein L18 [Homo sapiens] |

| Filename XCorr DeltCN Conf% ObsM+H+ CalcM+H+ SpR ZScore Ion% # Sequence  | | | | | | | | | | | | |
| --- | --- | --- | --- | --- | --- | --- | --- | --- | --- | --- | --- | --- |
| \* | Astrin\_STLCHLD\_tube2\_061314\_02.06315.06315.2 | 3.7982 | 0.4291 | 100.0% | 1346.3322 | 1346.5236 | 1 | 8.39 | 79.2% | 7 | K.TAVVVGTITDDVR.V | 2 |
| \* | Astrin\_STLCHLD\_tube2\_061314\_01.11443.11443.2 | 3.4867 | 0.4319 | 100.0% | 1461.2722 | 1461.6982 | 1 | 7.513 | 70.8% | 1 | K.ILTFDQLALDSPK.G | 2 |

---

|  |  |  |  |  |  |  |  |  |
| --- | --- | --- | --- | --- | --- | --- | --- | --- |
| U | *gi|4506743|ref|NP\_001* | 3 | 6 | 13.5% | 208 | 24205 | 10.3 | ribosomal protein S8 [Homo sapiens] |

| Filename XCorr DeltCN Conf% ObsM+H+ CalcM+H+ SpR ZScore Ion% # Sequence  | | | | | | | | | | | | |
| --- | --- | --- | --- | --- | --- | --- | --- | --- | --- | --- | --- | --- |
| \* | Astrin\_STLCHLD\_061214\_01.07729.07729.2 | 3.2556 | 0.301 | 99.3% | 1719.3722 | 1719.9353 | 1 | 5.718 | 53.6% | 2 | R.IIDVVYNASNNELVR.T | 2 |
| \* | Astrin\_STLCHLD\_tube2\_061314\_02.07160.07160.3 | 4.3009 | 0.3264 | 100.0% | 1720.1643 | 1719.9353 | 3 | 5.993 | 44.6% | 2 | R.IIDVVYNASNNELVR.T | 3 |
| \* | Astrin\_STLCHLD\_tube2\_061314\_01.08540.08540.2 | 3.8376 | 0.4547 | 100.0% | 1507.4722 | 1507.6836 | 1 | 7.977 | 75.0% | 2 | K.ISSLLEEQFQQGK.L | 2 |

---

|  |  |  |  |  |  |  |  |  |
| --- | --- | --- | --- | --- | --- | --- | --- | --- |
| U | *gi|46367787|ref|NP\_00* | 5 | 7 | 13.1% | 636 | 70671 | 9.5 | poly(A) binding protein, cytoplasmic 1 [Homo sapiens] |

| Filename XCorr DeltCN Conf% ObsM+H+ CalcM+H+ SpR ZScore Ion% # Sequence  | | | | | | | | | | | | |
| --- | --- | --- | --- | --- | --- | --- | --- | --- | --- | --- | --- | --- |
|  | Astrin\_STLCHLD\_tube2\_061314\_02.07175.07175.2 | 3.5503 | 0.4429 | 100.0% | 1929.3322 | 1930.0837 | 1 | 7.947 | 50.0% | 1 | R.SLGYAYVNFQQPADAER.A | 2 |
|  | Astrin\_STLCHLD\_tube2\_061314\_01.10395.10395.2 | 2.2639 | 0.2756 | 96.7% | 1267.5122 | 1267.4828 | 1 | 5.803 | 75.0% | 1 | R.ALDTMNFDVIK.G | 2 |
| \* | Astrin\_STLCHLD\_061214\_01.06691.06691.3 | 2.8605 | 0.3629 | 99.7% | 1742.2144 | 1741.857 | 27 | 5.998 | 35.7% | 2 | K.GYGFVHFETQEAAER.A | 3 |
|  | Astrin\_STLCHLD\_tube2\_061314\_01.14111.14111.3 | 4.0269 | 0.3013 | 99.7% | 2742.5645 | 2742.175 | 1 | 6.054 | 33.7% | 2 | K.ITGMLLEIDNSELLHMLESPESLR.S | 3 |
|  | Astrin\_STLCHLD\_tube2\_061314\_01.05820.05820.3 | 5.0651 | 0.4396 | 100.0% | 1696.8544 | 1694.9285 | 1 | 7.856 | 51.7% | 1 | R.SKVDEAVAVLQAHQAK.E | 3 |

---

|  |  |  |  |  |  |  |  |  |
| --- | --- | --- | --- | --- | --- | --- | --- | --- |
| U | *gi|24234688|ref|NP\_00* | 7 | 10 | 13.0% | 679 | 73681 | 6.2 | heat shock 70kDa protein 9 precursor [Homo sapiens] |

| Filename XCorr DeltCN Conf% ObsM+H+ CalcM+H+ SpR ZScore Ion% # Sequence  | | | | | | | | | | | | |
| --- | --- | --- | --- | --- | --- | --- | --- | --- | --- | --- | --- | --- |
| \* | Astrin\_STLCHLD\_tube2\_061314\_01.07197.07197.2 | 3.8118 | 0.5575 | 100.0% | 1452.2922 | 1451.576 | 1 | 9.044 | 80.8% | 1 | R.TTPSVVAFTADGER.L | 2 |
| \* | Astrin\_STLCHLD\_tube2\_061314\_01.08904.08904.2 | 3.428 | 0.3479 | 100.0% | 1242.7322 | 1243.4056 | 1 | 7.085 | 81.8% | 1 | K.DAGQISGLNVLR.V | 2 |
| \* | Astrin\_STLCHLD\_tube2\_061314\_01.12121.12121.2 | 3.4345 | 0.3831 | 100.0% | 1363.7322 | 1362.5687 | 2 | 6.805 | 68.2% | 2 | R.AQFEGIVTDLIR.R | 2 |
| \* | Astrin\_STLCHLD\_tube2\_061314\_01.08613.08613.2 | 3.5423 | 0.2378 | 100.0% | 1291.4521 | 1291.4496 | 1 | 7.083 | 75.0% | 2 | K.VQQTVQDLFGR.A | 2 |
| \* | Astrin\_STLCHLD\_061214\_02.05889.05889.2 | 4.3979 | 0.4843 | 100.0% | 1809.4722 | 1809.9707 | 1 | 9.552 | 50.0% | 1 | K.SQVFSTAADGQTQVEIK.V | 2 |
| \* | Astrin\_STLCHLD\_tube2\_061314\_02.05240.05240.2 | 2.7093 | 0.4056 | 99.5% | 1474.2722 | 1474.6543 | 3 | 6.95 | 53.8% | 1 | R.EQQIVIQSSGGLSK.D | 2 |
| \* | Astrin\_STLCHLD\_tube2\_061314\_02.07038.07038.3 | 3.216 | 0.4148 | 100.0% | 2420.9043 | 2419.7095 | 9 | 5.78 | 27.4% | 2 | R.EQQIVIQSSGGLSKDDIENMVK.N | 3 |

---

|  |  |  |  |  |  |  |  |  |
| --- | --- | --- | --- | --- | --- | --- | --- | --- |
| U | *gi|16905517|ref|NP\_47* | 3 | 4 | 13.0% | 262 | 31301 | 11.3 | FUS interacting protein (serine-arginine rich) 1 isoform 2 [Homo sapiens] |
| U | *gi|5730079|ref|NP\_006* | 3 | 4 | 18.6% | 183 | 22222 | 10.3 | FUS interacting protein (serine-arginine rich) 1 isoform 1 [Homo sapiens] |
| U | *gi|169161980|ref|XP\_0* | 3 | 4 | 18.8% | 181 | 22022 | 10.3 | PREDICTED: hypothetical protein, partial [Homo sapiens] |
| U | *gi|169161109|ref|XP\_0* | 3 | 4 | 18.6% | 183 | 22222 | 10.3 | PREDICTED: hypothetical protein LOC642558 [Homo sapiens] |
| U | *gi|169161107|ref|XP\_0* | 3 | 4 | 13.0% | 262 | 31301 | 11.3 | PREDICTED: hypothetical protein LOC642558 [Homo sapiens] |

| Filename XCorr DeltCN Conf% ObsM+H+ CalcM+H+ SpR ZScore Ion% # Sequence  | | | | | | | | | | | | |
| --- | --- | --- | --- | --- | --- | --- | --- | --- | --- | --- | --- | --- |
|  | Astrin\_STLCHLD\_tube2\_061314\_01.08033.08033.3 | 3.8345 | 0.2548 | 99.8% | 1463.5144 | 1463.7227 | 1 | 5.336 | 61.4% | 2 | R.YLRPPNTSLFVR.N | 3 |
|  | Astrin\_STLCHLD\_061214\_01.08395.08395.2 | 3.1586 | 0.3172 | 100.0% | 1332.6721 | 1331.4705 | 1 | 5.889 | 70.0% | 1 | R.GFAYVQFEDVR.D | 2 |
|  | Astrin\_STLCHLD\_061214\_01.06560.06560.2 | 2.724 | 0.3071 | 98.8% | 1305.6322 | 1305.4331 | 7 | 5.62 | 65.0% | 1 | R.QIEIQFAQGDR.K | 2 |

---

|  |  |  |  |  |  |  |  |  |
| --- | --- | --- | --- | --- | --- | --- | --- | --- |
| U | *gi|14043070|ref|NP\_11* | 7 | 21 | 12.9% | 372 | 38747 | 9.1 | heterogeneous nuclear ribonucleoprotein A1 isoform b [Homo sapiens] |
| U | *gi|88958985|ref|XP\_94* | 7 | 21 | 15.9% | 301 | 32399 | 9.0 | PREDICTED: similar to heterogeneous nuclear ribonucleoprotein A1 [Homo sapiens] |
| U | *gi|88953883|ref|XP\_93* | 7 | 21 | 15.9% | 301 | 32380 | 8.9 | PREDICTED: similar to heterogeneous nuclear ribonucleoprotein A1 [Homo sapiens] |
| U | *gi|58761498|ref|NP\_00* | 7 | 21 | 15.0% | 320 | 34225 | 9.0 | heterogeneous nuclear ribonucleoprotein A1-like [Homo sapiens] |
| U | *gi|58761496|ref|NP\_00* | 7 | 21 | 15.0% | 320 | 34225 | 9.0 | heterogeneous nuclear ribonucleoprotein A1-like [Homo sapiens] |
| U | *gi|4504445|ref|NP\_002* | 7 | 21 | 15.0% | 320 | 34196 | 9.2 | heterogeneous nuclear ribonucleoprotein A1 isoform a [Homo sapiens] |
| U | *gi|169164476|ref|XP\_0* | 7 | 21 | 15.9% | 301 | 32399 | 9.0 | PREDICTED: similar to heterogeneous nuclear ribonucleoprotein A1 [Homo sapiens] |

| Filename XCorr DeltCN Conf% ObsM+H+ CalcM+H+ SpR ZScore Ion% # Sequence  | | | | | | | | | | | | |
| --- | --- | --- | --- | --- | --- | --- | --- | --- | --- | --- | --- | --- |
|  | Astrin\_STLCHLD\_061214\_01.09312.09312.2 | 4.4587 | 0.4494 | 100.0% | 1913.0322 | 1914.1656 | 3 | 7.125 | 53.1% | 3 | R.KLFIGGLSFETTDESLR.S | 2 |
|  | Astrin\_STLCHLD\_061214\_01.09344.09344.3 | 3.279 | 0.2071 | 96.9% | 1914.2043 | 1914.1656 | 1 | 4.76 | 42.2% | 1 | R.KLFIGGLSFETTDESLR.S | 3 |
|  | Astrin\_STLCHLD\_tube2\_061314\_02.09065.09065.2 | 4.3919 | 0.4126 | 100.0% | 1785.9122 | 1785.9916 | 1 | 7.905 | 63.3% | 6 | K.LFIGGLSFETTDESLR.S | 2 |
|  | Astrin\_STLCHLD\_tube2\_061314\_02.08051.08051.2 | 3.535 | 0.3695 | 100.0% | 1219.4722 | 1219.4387 | 1 | 7.055 | 88.9% | 4 | K.IEVIEIMTDR.G | 2 |
|  | Astrin\_STLCHLD\_061214\_01.07474.07474.3 | 4.5096 | 0.4498 | 100.0% | 1857.4143 | 1856.989 | 1 | 7.285 | 46.7% | 3 | K.RGFAFVTFDDHDSVDK.I | 3 |
|  | Astrin\_STLCHLD\_061214\_02.07643.07643.2 | 2.2313 | 0.3868 | 97.8% | 1701.6921 | 1700.8016 | 12 | 5.773 | 35.7% | 1 | R.GFAFVTFDDHDSVDK.I | 2 |
|  | Astrin\_STLCHLD\_tube2\_061314\_02.08037.08037.3 | 4.2373 | 0.3299 | 100.0% | 2282.9944 | 2282.5579 | 1 | 6.213 | 38.2% | 3 | R.GFAFVTFDDHDSVDKIVIQK.Y | 3 |

---

|  |  |  |  |  |  |  |  |  |
| --- | --- | --- | --- | --- | --- | --- | --- | --- |
| U | *gi|4504517|ref|NP\_001* | 2 | 3 | 12.7% | 205 | 22783 | 6.4 | heat shock protein beta-1 [Homo sapiens] |

| Filename XCorr DeltCN Conf% ObsM+H+ CalcM+H+ SpR ZScore Ion% # Sequence  | | | | | | | | | | | | |
| --- | --- | --- | --- | --- | --- | --- | --- | --- | --- | --- | --- | --- |
| \* | Astrin\_STLCHLD\_tube2\_061314\_01.10772.10772.2 | 3.2948 | 0.415 | 100.0% | 1163.9122 | 1164.3494 | 1 | 7.891 | 83.3% | 2 | R.LFDQAFGLPR.L | 2 |
| \* | Astrin\_STLCHLD\_tube2\_061314\_01.09518.09518.2 | 3.6372 | 0.4163 | 100.0% | 1784.5322 | 1785.0068 | 1 | 7.108 | 50.0% | 1 | R.VSLDVNHFAPDELTVK.T | 2 |

---

|  |  |  |  |  |  |  |  |  |
| --- | --- | --- | --- | --- | --- | --- | --- | --- |
| U | *gi|11136628|ref|NP\_06* | 2 | 2 | 12.4% | 225 | 24764 | 4.7 | eukaryotic translation elongation factor 1 beta 2 [Homo sapiens] |
| U | *gi|83376130|ref|NP\_00* | 2 | 2 | 12.4% | 225 | 24764 | 4.7 | eukaryotic translation elongation factor 1 beta 2 [Homo sapiens] |
| U | *gi|4503477|ref|NP\_001* | 2 | 2 | 12.4% | 225 | 24764 | 4.7 | eukaryotic translation elongation factor 1 beta 2 [Homo sapiens] |

| Filename XCorr DeltCN Conf% ObsM+H+ CalcM+H+ SpR ZScore Ion% # Sequence  | | | | | | | | | | | | |
| --- | --- | --- | --- | --- | --- | --- | --- | --- | --- | --- | --- | --- |
|  | Astrin\_STLCHLD\_tube2\_061314\_01.11736.11736.2 | 2.6641 | 0.3245 | 98.1% | 1603.5122 | 1604.8003 | 1 | 5.184 | 57.1% | 1 | K.SPAGLQVLNDYLADK.S | 2 |
|  | Astrin\_STLCHLD\_tube2\_061314\_02.06983.06983.2 | 2.4864 | 0.2609 | 96.9% | 1349.8121 | 1348.4985 | 1 | 4.869 | 62.5% | 1 | R.SIQADGLVWGSSK.L | 2 |

---

|  |  |  |  |  |  |  |  |  |
| --- | --- | --- | --- | --- | --- | --- | --- | --- |
| U | *gi|33469968|ref|NP\_00* | 6 | 12 | 12.0% | 719 | 81308 | 6.5 | minichromosome maintenance complex component 7 isoform 1 [Homo sapiens] |

| Filename XCorr DeltCN Conf% ObsM+H+ CalcM+H+ SpR ZScore Ion% # Sequence  | | | | | | | | | | | | |
| --- | --- | --- | --- | --- | --- | --- | --- | --- | --- | --- | --- | --- |
| \* | Astrin\_STLCHLD\_tube2\_061314\_02.06801.06801.3 | 3.5232 | 0.2499 | 99.4% | 1828.6743 | 1829.063 | 3 | 6.315 | 41.1% | 3 | R.EVVNKDVLDVYIEHR.L | 3 |
| \* | Astrin\_STLCHLD\_tube2\_061314\_01.06979.06979.3 | 3.3761 | 0.314 | 99.7% | 1827.1743 | 1827.0525 | 1 | 6.329 | 48.2% | 1 | R.RFELYFQGPSSNKPR.V | 3 |
|  | Astrin\_STLCHLD\_061214\_02.06095.06095.2 | 3.252 | 0.4325 | 100.0% | 1318.5122 | 1318.4697 | 1 | 7.122 | 68.2% | 2 | R.SITVLVEGENTR.I | 2 |
|  | Astrin\_STLCHLD\_061214\_01.06915.06915.2 | 2.523 | 0.2322 | 96.5% | 1188.4321 | 1188.3695 | 1 | 4.49 | 66.7% | 1 | R.GSSGVGLTAAVLR.D | 2 |
|  | Astrin\_STLCHLD\_tube2\_061314\_02.06051.06051.3 | 3.4119 | 0.2304 | 98.6% | 1800.5044 | 1800.0802 | 14 | 5.075 | 33.3% | 1 | R.TAIHEVMEQQTISIAK.A | 3 |
|  | Astrin\_STLCHLD\_tube2\_061314\_02.05663.05663.3 | 3.8392 | 0.3536 | 100.0% | 1747.8844 | 1746.9733 | 1 | 6.282 | 48.2% | 4 | R.MVDVVEKEDVNEAIR.L | 3 |

---

|  |  |  |  |  |  |  |  |  |
| --- | --- | --- | --- | --- | --- | --- | --- | --- |
| U | *gi|63055057|ref|NP\_00* | 3 | 7 | 12.0% | 376 | 42003 | 5.6 | actin, beta-like 2 [Homo sapiens] |

| Filename XCorr DeltCN Conf% ObsM+H+ CalcM+H+ SpR ZScore Ion% # Sequence  | | | | | | | | | | | | |
| --- | --- | --- | --- | --- | --- | --- | --- | --- | --- | --- | --- | --- |
| \* | Astrin\_STLCHLD\_tube2\_061314\_01.08363.08363.3 | 3.4293 | 0.19 | 95.9% | 2340.7444 | 2338.7122 | 2 | 4.628 | 36.2% | 1 | R.VAPDEHPILLTEAPLNPKINR.E | 3 |
|  | Astrin\_STLCHLD\_tube2\_061314\_01.10415.10415.2 | 1.9955 | 0.3931 | 98.1% | 999.03217 | 999.167 | 15 | 6.315 | 64.3% | 1 | R.DLTDYLMK.I | 222 |
|  | Astrin\_STLCHLD\_tube2\_061314\_01.10440.10440.2 | 4.5438 | 0.3456 | 100.0% | 1792.6322 | 1791.9554 | 1 | 7.499 | 73.3% | 5 | R.SYELPDGQVITIGNER.F | 222 |

Similarities:
gi|4501885|ref|NP\_001(2:1)  
gi|4501881|ref|NP\_001(2:1)  

---

|  |  |  |  |  |  |  |  |  |
| --- | --- | --- | --- | --- | --- | --- | --- | --- |
| U | *gi|94538362|ref|NP\_00* | 4 | 10 | 11.9% | 428 | 47064 | 5.3 | flotillin 2 [Homo sapiens] |

| Filename XCorr DeltCN Conf% ObsM+H+ CalcM+H+ SpR ZScore Ion% # Sequence  | | | | | | | | | | | | |
| --- | --- | --- | --- | --- | --- | --- | --- | --- | --- | --- | --- | --- |
| \* | Astrin\_STLCHLD\_061214\_01.04431.04431.2 | 2.6264 | 0.1767 | 96.4% | 1146.1721 | 1146.1992 | 2 | 4.19 | 70.0% | 1 | R.DADIGVAEAER.D | 2 |
| \* | Astrin\_STLCHLD\_061214\_01.07258.07258.2 | 4.3407 | 0.4123 | 100.0% | 1522.4122 | 1521.6702 | 1 | 7.272 | 76.9% | 6 | K.TAEAQLAYELQGAR.E | 2 |
| \* | Astrin\_STLCHLD\_tube2\_061314\_02.06045.06045.3 | 4.0117 | 0.2316 | 99.7% | 1641.9243 | 1641.865 | 7 | 5.614 | 47.9% | 2 | K.IRQEEIEIEVVQR.K | 3 |
| \* | Astrin\_STLCHLD\_tube2\_061314\_01.07033.07033.2 | 3.0038 | 0.3873 | 100.0% | 1375.2322 | 1375.5187 | 1 | 5.477 | 66.7% | 1 | K.VDEIVVLSGDNSK.V | 2 |

---

|  |  |  |  |  |  |  |  |  |
| --- | --- | --- | --- | --- | --- | --- | --- | --- |
| U | *gi|113412878|ref|XP\_0* | 2 | 2 | 11.9% | 293 | 31479 | 7.6 | PREDICTED: similar to voltage-dependent anion channel [Homo sapiens] |
| U | *gi|42476281|ref|NP\_00* | 2 | 2 | 11.9% | 294 | 31566 | 7.6 | voltage-dependent anion channel 2 [Homo sapiens] |
| U | *gi|169164151|ref|XP\_0* | 2 | 2 | 11.9% | 293 | 31445 | 7.6 | PREDICTED: similar to voltage-dependent anion channel [Homo sapiens] |

| Filename XCorr DeltCN Conf% ObsM+H+ CalcM+H+ SpR ZScore Ion% # Sequence  | | | | | | | | | | | | |
| --- | --- | --- | --- | --- | --- | --- | --- | --- | --- | --- | --- | --- |
|  | Astrin\_STLCHLD\_061214\_02.06687.06687.3 | 4.4368 | 0.3583 | 100.0% | 2529.4143 | 2529.682 | 1 | 6.064 | 34.1% | 1 | R.TGDFQLHTNVNDGTEFGGSIYQK.V | 3 |
|  | Astrin\_STLCHLD\_tube2\_061314\_01.06189.06189.2 | 2.1352 | 0.3968 | 98.0% | 1294.4122 | 1294.4473 | 8 | 6.066 | 54.5% | 1 | K.YQLDPTASISAK.V | 2 |

---

|  |  |  |  |  |  |  |  |  |
| --- | --- | --- | --- | --- | --- | --- | --- | --- |
| U | *gi|52632383|ref|NP\_00* | 5 | 6 | 11.7% | 589 | 64133 | 8.2 | heterogeneous nuclear ribonucleoprotein L isoform a [Homo sapiens] |

| Filename XCorr DeltCN Conf% ObsM+H+ CalcM+H+ SpR ZScore Ion% # Sequence  | | | | | | | | | | | | |
| --- | --- | --- | --- | --- | --- | --- | --- | --- | --- | --- | --- | --- |
| \* | Astrin\_STLCHLD\_tube2\_061314\_01.18157.18157.2 | 3.958 | 0.5343 | 100.0% | 3088.372 | 3089.6143 | 1 | 10.14 | 37.5% | 1 | R.GLIDGVVEADLVEALQEFGPISYVVVMPK.K | 2 |
| \* | Astrin\_STLCHLD\_061214\_01.16704.16704.3 | 3.6939 | 0.2656 | 98.9% | 3088.9143 | 3089.6143 | 83 | 5.602 | 22.3% | 1 | R.GLIDGVVEADLVEALQEFGPISYVVVMPK.K | 3 |
|  | Astrin\_STLCHLD\_061214\_02.06500.06500.3 | 3.1443 | 0.223 | 96.9% | 1869.4143 | 1869.1176 | 6 | 5.039 | 35.3% | 2 | K.SKPGAAMVEMADGYAVDR.A | 3 |
|  | Astrin\_STLCHLD\_tube2\_061314\_01.08552.08552.2 | 2.697 | 0.2563 | 98.0% | 1224.5721 | 1223.3251 | 7 | 5.096 | 60.0% | 1 | R.SSSGLLEWESK.S | 2 |
|  | Astrin\_STLCHLD\_tube2\_061314\_01.05477.05477.2 | 2.2782 | 0.3058 | 97.3% | 1264.4122 | 1264.4233 | 8 | 4.947 | 50.0% | 1 | K.NPNGPYPYTLK.L | 2 |

---

|  |  |  |  |  |  |  |  |  |
| --- | --- | --- | --- | --- | --- | --- | --- | --- |
| U | *contaminant\_KERATIN05* | 5 | 7 | 11.7% | 471 | 51531 | 5.2 | no description |
| U | *gi|15431310|ref|NP\_00* | 5 | 7 | 11.7% | 472 | 51622 | 5.2 | keratin 14 [Homo sapiens] |

| Filename XCorr DeltCN Conf% ObsM+H+ CalcM+H+ SpR ZScore Ion% # Sequence  | | | | | | | | | | | | |
| --- | --- | --- | --- | --- | --- | --- | --- | --- | --- | --- | --- | --- |
|  | Astrin\_STLCHLD\_061214\_01.05414.05414.2 | 3.1224 | 0.1417 | 97.3% | 1302.4722 | 1302.4241 | 1 | 5.667 | 72.7% | 1 | R.ALEEANADLEVK.I | 22 |
|  | Astrin\_STLCHLD\_tube2\_061314\_01.08023.08023.2 | 3.2303 | 0.3839 | 100.0% | 1030.1122 | 1030.2096 | 2 | 6.85 | 81.2% | 2 | R.VLDELTLAR.A | 2222 |
|  | Astrin\_STLCHLD\_tube2\_061314\_01.05822.05822.2 | 3.2909 | 0.3194 | 100.0% | 1380.3722 | 1380.5437 | 1 | 5.474 | 70.0% | 1 | K.TRLEQEIATYR.R | 222 |
|  | Astrin\_STLCHLD\_tube2\_061314\_01.04778.04778.3 | 2.8055 | 0.2787 | 98.7% | 1537.4043 | 1536.7311 | 1 | 5.293 | 45.5% | 1 | K.TRLEQEIATYRR.L | 333 |
|  | Astrin\_STLCHLD\_061214\_02.05348.05348.3 | 4.6262 | 0.3893 | 100.0% | 2310.8943 | 2310.396 | 1 | 7.152 | 34.5% | 2 | R.LLEGEDAHLSSSQFSSGSQSSR.D | 3 |

Similarities:
contaminant\_KERATIN12(3:2)  
contaminant\_KERATIN08(4:1)  
contaminant\_KERATIN10(1:4)  

---

|  |  |  |  |  |  |  |  |  |
| --- | --- | --- | --- | --- | --- | --- | --- | --- |
| U | *contaminant\_KERATIN08* | 5 | 6 | 11.7% | 469 | 50499 | 5.0 | no description |
| U | *gi|24430192|ref|NP\_00* | 5 | 6 | 11.6% | 473 | 51268 | 5.0 | keratin 16 [Homo sapiens] |

| Filename XCorr DeltCN Conf% ObsM+H+ CalcM+H+ SpR ZScore Ion% # Sequence  | | | | | | | | | | | | |
| --- | --- | --- | --- | --- | --- | --- | --- | --- | --- | --- | --- | --- |
|  | Astrin\_STLCHLD\_061214\_01.05414.05414.2 | 3.1224 | 0.1417 | 97.3% | 1302.4722 | 1302.4241 | 1 | 5.667 | 72.7% | 1 | R.ALEEANADLEVK.I | 22 |
|  | Astrin\_STLCHLD\_tube2\_061314\_01.08023.08023.2 | 3.2303 | 0.3839 | 100.0% | 1030.1122 | 1030.2096 | 2 | 6.85 | 81.2% | 2 | R.VLDELTLAR.T | 2222 |
|  | Astrin\_STLCHLD\_tube2\_061314\_01.05822.05822.2 | 3.2909 | 0.3194 | 100.0% | 1380.3722 | 1380.5437 | 1 | 5.474 | 70.0% | 1 | K.TRLEQEIATYR.R | 222 |
|  | Astrin\_STLCHLD\_tube2\_061314\_01.04778.04778.3 | 2.8055 | 0.2787 | 98.7% | 1537.4043 | 1536.7311 | 1 | 5.293 | 45.5% | 1 | K.TRLEQEIATYRR.L | 333 |
|  | Astrin\_STLCHLD\_061214\_01.04797.04797.3 | 3.7146 | 0.2813 | 99.6% | 2351.6643 | 2351.4485 | 1 | 5.436 | 29.8% | 1 | R.LLEGEDAHLSSQQASGQSYSSR.E | 3 |

Similarities:
contaminant\_KERATIN12(3:2)  
contaminant\_KERATIN05(4:1)  
contaminant\_KERATIN10(1:4)  

---

|  |  |  |  |  |  |  |  |  |
| --- | --- | --- | --- | --- | --- | --- | --- | --- |
| U | *gi|167466173|ref|NP\_0* | 5 | 11 | 11.4% | 641 | 70052 | 5.6 | heat shock 70kDa protein 1B [Homo sapiens] |
| U | *gi|194248072|ref|NP\_0* | 5 | 11 | 11.4% | 641 | 70052 | 5.6 | heat shock 70kDa protein 1A [Homo sapiens] |

| Filename XCorr DeltCN Conf% ObsM+H+ CalcM+H+ SpR ZScore Ion% # Sequence  | | | | | | | | | | | | |
| --- | --- | --- | --- | --- | --- | --- | --- | --- | --- | --- | --- | --- |
|  | Astrin\_STLCHLD\_tube2\_061314\_01.07280.07280.2 | 3.3247 | 0.4793 | 100.0% | 1488.3922 | 1488.5939 | 1 | 8.565 | 75.0% | 4 | R.TTPSYVAFTDTER.L | 222 |
|  | Astrin\_STLCHLD\_tube2\_061314\_01.07645.07645.2 | 4.3014 | 0.2728 | 100.0% | 1659.7122 | 1659.8394 | 1 | 6.949 | 78.6% | 1 | K.NQVALNPQNTVFDAK.R | 2 |
|  | Astrin\_STLCHLD\_061214\_01.09072.09072.2 | 3.199 | 0.3048 | 99.9% | 1199.4922 | 1198.408 | 1 | 6.267 | 77.3% | 4 | K.DAGVIAGLNVLR.I | 22 |
|  | Astrin\_STLCHLD\_tube2\_061314\_01.10472.10472.2 | 4.2547 | 0.5464 | 100.0% | 1688.5322 | 1688.9213 | 1 | 10.36 | 70.0% | 1 | R.IINEPTAAAIAYGLDR.T | 2 |
|  | Astrin\_STLCHLD\_tube2\_061314\_02.06341.06341.3 | 2.8417 | 0.2577 | 96.9% | 1822.3143 | 1823.1025 | 6 | 5.037 | 32.8% | 1 | K.LDKAQIHDLVLVGGSTR.I | 3 |

Similarities:
gi|5729877|ref|NP\_006(1:4)  
gi|124256496|ref|NP\_0(2:3)  

---

|  |  |  |  |  |  |  |  |  |
| --- | --- | --- | --- | --- | --- | --- | --- | --- |
| U | *gi|14249348|ref|NP\_11* | 2 | 9 | 11.4% | 123 | 13941 | 5.5 | thioredoxin-like 5 [Homo sapiens] |

| Filename XCorr DeltCN Conf% ObsM+H+ CalcM+H+ SpR ZScore Ion% # Sequence  | | | | | | | | | | | | |
| --- | --- | --- | --- | --- | --- | --- | --- | --- | --- | --- | --- | --- |
| \* | Astrin\_STLCHLD\_061214\_01.07267.07267.2 | 4.0604 | 0.5116 | 100.0% | 1715.3722 | 1715.8162 | 1 | 7.922 | 57.7% | 2 | R.YEEVSVSGFEEFHR.A | 2 |
| \* | Astrin\_STLCHLD\_061214\_02.06877.06877.3 | 3.1045 | 0.379 | 100.0% | 1716.6843 | 1715.8162 | 5 | 5.749 | 38.5% | 7 | R.YEEVSVSGFEEFHR.A | 3 |

---

|  |  |  |  |  |  |  |  |  |
| --- | --- | --- | --- | --- | --- | --- | --- | --- |
| U | *gi|47271443|ref|NP\_00* | 2 | 2 | 11.3% | 221 | 25476 | 11.9 | splicing factor, arginine/serine-rich 2 [Homo sapiens] |

| Filename XCorr DeltCN Conf% ObsM+H+ CalcM+H+ SpR ZScore Ion% # Sequence  | | | | | | | | | | | | |
| --- | --- | --- | --- | --- | --- | --- | --- | --- | --- | --- | --- | --- |
|  | Astrin\_STLCHLD\_tube2\_061314\_01.04889.04889.2 | 2.5146 | 0.2956 | 98.8% | 918.7522 | 919.0684 | 1 | 6.057 | 85.7% | 1 | R.VGDVYIPR.D | 2 |
| \* | Astrin\_STLCHLD\_tube2\_061314\_01.10668.10668.2 | 3.9648 | 0.4499 | 100.0% | 1752.5922 | 1752.8654 | 1 | 8.638 | 68.8% | 1 | R.DAEDAMDAMDGAVLDGR.E | 2 |

---

|  |  |  |  |  |  |  |  |  |
| --- | --- | --- | --- | --- | --- | --- | --- | --- |
| U | *gi|16579885|ref|NP\_00* | 3 | 4 | 11.2% | 427 | 47697 | 11.1 | ribosomal protein L4 [Homo sapiens] |

| Filename XCorr DeltCN Conf% ObsM+H+ CalcM+H+ SpR ZScore Ion% # Sequence  | | | | | | | | | | | | |
| --- | --- | --- | --- | --- | --- | --- | --- | --- | --- | --- | --- | --- |
| \* | Astrin\_STLCHLD\_tube2\_061314\_01.09404.09404.3 | 3.3119 | 0.3234 | 99.7% | 1864.1643 | 1863.1727 | 4 | 5.506 | 36.7% | 1 | K.APIRPDIVNFVHTNLR.K | 3 |
| \* | Astrin\_STLCHLD\_061214\_02.06275.06275.3 | 3.3518 | 0.3651 | 99.8% | 2333.4243 | 2333.479 | 1 | 5.664 | 28.6% | 1 | R.QPYAVSELAGHQTSAESWGTGR.A | 3 |
| \* | Astrin\_STLCHLD\_tube2\_061314\_01.07585.07585.2 | 3.3286 | 0.3132 | 100.0% | 1281.2922 | 1281.4539 | 1 | 5.543 | 83.3% | 2 | R.KLDELYGTWR.K | 2 |

---

|  |  |  |  |  |  |  |  |  |
| --- | --- | --- | --- | --- | --- | --- | --- | --- |
| U | *gi|14141161|ref|NP\_00* | 5 | 13 | 11.0% | 806 | 88980 | 5.8 | heterogeneous nuclear ribonucleoprotein U isoform b [Homo sapiens] |
| U | *gi|74136883|ref|NP\_11* | 5 | 13 | 10.8% | 825 | 90585 | 6.0 | heterogeneous nuclear ribonucleoprotein U isoform a [Homo sapiens] |

| Filename XCorr DeltCN Conf% ObsM+H+ CalcM+H+ SpR ZScore Ion% # Sequence  | | | | | | | | | | | | |
| --- | --- | --- | --- | --- | --- | --- | --- | --- | --- | --- | --- | --- |
|  | Astrin\_STLCHLD\_061214\_01.06674.06674.3 | 4.8842 | 0.3353 | 100.0% | 3129.6243 | 3128.311 | 1 | 5.521 | 25.8% | 3 | R.LQAALDDEEAGGRPAMEPGNGSLDLGGDSAGR.S | 3 |
|  | Astrin\_STLCHLD\_tube2\_061314\_01.12297.12297.3 | 3.084 | 0.2938 | 98.7% | 2725.9443 | 2726.0576 | 1 | 5.205 | 29.8% | 1 | K.EKPYFPIPEEYTFIQNVPLEDR.V | 3 |
|  | Astrin\_STLCHLD\_tube2\_061314\_01.06980.06980.3 | 3.5103 | 0.3474 | 100.0% | 2188.0745 | 2188.4631 | 1 | 5.847 | 35.5% | 1 | K.HAAENPGKYNILGTNTIMDK.M | 3 |
|  | Astrin\_STLCHLD\_tube2\_061314\_02.06768.06768.3 | 3.7312 | 0.2297 | 99.4% | 1648.5543 | 1648.816 | 1 | 5.248 | 44.6% | 2 | R.NFILDQTNVSAAAQR.R | 3 |
|  | Astrin\_STLCHLD\_061214\_01.07052.07052.2 | 5.1599 | 0.4221 | 100.0% | 1649.3322 | 1648.816 | 1 | 8.007 | 75.0% | 6 | R.NFILDQTNVSAAAQR.R | 2 |

---

|  |  |  |  |  |  |  |  |  |
| --- | --- | --- | --- | --- | --- | --- | --- | --- |
| U | *gi|4506411|ref|NP\_002* | 5 | 7 | 10.9% | 587 | 63542 | 4.7 | Ran GTPase activating protein 1 [Homo sapiens] |

| Filename XCorr DeltCN Conf% ObsM+H+ CalcM+H+ SpR ZScore Ion% # Sequence  | | | | | | | | | | | | |
| --- | --- | --- | --- | --- | --- | --- | --- | --- | --- | --- | --- | --- |
| \* | Astrin\_STLCHLD\_tube2\_061314\_01.04941.04941.2 | 2.3914 | 0.3199 | 98.0% | 1136.5922 | 1136.2932 | 2 | 6.273 | 70.0% | 1 | K.TQVAGGQLSFK.G | 2 |
| \* | Astrin\_STLCHLD\_tube2\_061314\_01.09662.09662.2 | 3.678 | 0.4273 | 100.0% | 1748.4122 | 1748.8931 | 1 | 7.074 | 63.3% | 1 | R.NRLENDGATALAEAFR.V | 2 |
| \* | Astrin\_STLCHLD\_tube2\_061314\_01.07281.07281.2 | 3.7272 | 0.3418 | 100.0% | 1409.3121 | 1408.551 | 1 | 6.439 | 77.3% | 2 | R.VINLNDNTFTEK.G | 2 |
| \* | Astrin\_STLCHLD\_tube2\_061314\_01.09579.09579.2 | 3.6185 | 0.4151 | 100.0% | 1693.3121 | 1693.8131 | 1 | 8.306 | 64.3% | 1 | K.AFNSSSFNSNTFLTR.L | 2 |
| \* | Astrin\_STLCHLD\_tube2\_061314\_01.09522.09522.2 | 3.0361 | 0.4371 | 100.0% | 1202.3322 | 1202.4392 | 2 | 7.639 | 72.2% | 2 | R.HSLLQTLYKV.- | 2 |

---

|  |  |  |  |  |  |  |  |  |
| --- | --- | --- | --- | --- | --- | --- | --- | --- |
| U | *gi|5031753|ref|NP\_005* | 3 | 7 | 10.7% | 449 | 49229 | 6.3 | heterogeneous nuclear ribonucleoprotein H1 [Homo sapiens] |

| Filename XCorr DeltCN Conf% ObsM+H+ CalcM+H+ SpR ZScore Ion% # Sequence  | | | | | | | | | | | | |
| --- | --- | --- | --- | --- | --- | --- | --- | --- | --- | --- | --- | --- |
| \* | Astrin\_STLCHLD\_061214\_01.08788.08788.2 | 2.4886 | 0.2565 | 97.3% | 1334.7522 | 1335.5176 | 5 | 5.408 | 55.0% | 2 | K.SNNVEMDWVLK.H | 2 |
|  | Astrin\_STLCHLD\_061214\_01.09620.09620.2 | 4.9781 | 0.5073 | 100.0% | 1842.5322 | 1843.0001 | 1 | 8.777 | 71.9% | 4 | R.STGEAFVQFASQEIAEK.A | 2 |
| \* | Astrin\_STLCHLD\_061214\_02.07219.07219.3 | 3.0033 | 0.3417 | 99.6% | 2143.3743 | 2143.32 | 8 | 6.264 | 30.3% | 1 | R.YVELFLNSTAGASGGAYEHR.Y | 3 |

---

|  |  |  |  |  |  |  |  |  |
| --- | --- | --- | --- | --- | --- | --- | --- | --- |
| U | *gi|116812577|ref|NP\_0* | 3 | 3 | 10.5% | 392 | 46514 | 10.0 | LUC7-like 2 [Homo sapiens] |

| Filename XCorr DeltCN Conf% ObsM+H+ CalcM+H+ SpR ZScore Ion% # Sequence  | | | | | | | | | | | | |
| --- | --- | --- | --- | --- | --- | --- | --- | --- | --- | --- | --- | --- |
| \* | Astrin\_STLCHLD\_tube2\_061314\_01.09623.09623.2 | 2.7211 | 0.4142 | 100.0% | 1223.9722 | 1223.448 | 1 | 7.196 | 80.0% | 1 | R.AMLDQLMGTSR.D | 2 |
| \* | Astrin\_STLCHLD\_tube2\_061314\_02.05567.05567.2 | 4.1297 | 0.4733 | 100.0% | 1589.4321 | 1589.7399 | 1 | 10.23 | 67.9% | 1 | R.LAETQEEISAEVAAK.A | 2 |
| \* | Astrin\_STLCHLD\_061214\_01.04453.04453.2 | 3.0374 | 0.4383 | 100.0% | 1617.5322 | 1617.71 | 3 | 7.271 | 50.0% | 1 | K.VEQLGAEGNVEESQK.V | 2 |

---

|  |  |  |  |  |  |  |  |  |
| --- | --- | --- | --- | --- | --- | --- | --- | --- |
| U | *gi|32483377|ref|NP\_05* | 2 | 3 | 10.5% | 238 | 25839 | 7.5 | peroxiredoxin 3 isoform b [Homo sapiens] |
| U | *gi|5802974|ref|NP\_006* | 2 | 3 | 9.8% | 256 | 27693 | 7.8 | peroxiredoxin 3 isoform a precursor [Homo sapiens] |

| Filename XCorr DeltCN Conf% ObsM+H+ CalcM+H+ SpR ZScore Ion% # Sequence  | | | | | | | | | | | | |
| --- | --- | --- | --- | --- | --- | --- | --- | --- | --- | --- | --- | --- |
|  | Astrin\_STLCHLD\_tube2\_061314\_02.08652.08652.2 | 3.8334 | 0.5327 | 100.0% | 1463.1522 | 1463.6738 | 1 | 8.727 | 73.1% | 2 | R.DYGVLLEGSGLALR.G | 2 |
|  | Astrin\_STLCHLD\_tube2\_061314\_01.05413.05413.2 | 3.0109 | 0.3107 | 99.6% | 1208.4922 | 1207.375 | 4 | 6.221 | 60.0% | 1 | K.HLSVNDLPVGR.S | 2 |

---

|  |  |  |  |  |  |  |  |  |
| --- | --- | --- | --- | --- | --- | --- | --- | --- |
| U | *gi|38327562|ref|NP\_00* | 3 | 3 | 10.4% | 403 | 45823 | 9.4 | serine/threonine protein kinase 6 [Homo sapiens] |
| U | *gi|38327572|ref|NP\_94* | 3 | 3 | 10.4% | 403 | 45823 | 9.4 | serine/threonine protein kinase 6 [Homo sapiens] |
| U | *gi|38327570|ref|NP\_94* | 3 | 3 | 10.4% | 403 | 45823 | 9.4 | serine/threonine protein kinase 6 [Homo sapiens] |
| U | *gi|38327568|ref|NP\_94* | 3 | 3 | 10.4% | 403 | 45823 | 9.4 | serine/threonine protein kinase 6 [Homo sapiens] |
| U | *gi|38327566|ref|NP\_94* | 3 | 3 | 10.4% | 403 | 45823 | 9.4 | serine/threonine protein kinase 6 [Homo sapiens] |
| U | *gi|38327564|ref|NP\_94* | 3 | 3 | 10.4% | 403 | 45823 | 9.4 | serine/threonine protein kinase 6 [Homo sapiens] |

| Filename XCorr DeltCN Conf% ObsM+H+ CalcM+H+ SpR ZScore Ion% # Sequence  | | | | | | | | | | | | |
| --- | --- | --- | --- | --- | --- | --- | --- | --- | --- | --- | --- | --- |
|  | Astrin\_STLCHLD\_tube2\_061314\_01.05076.05076.3 | 3.5725 | 0.2416 | 98.7% | 2166.5942 | 2166.3496 | 1 | 5.259 | 36.8% | 1 | K.SKQPLPSAPENNPEEELASK.Q | 3 |
|  | Astrin\_STLCHLD\_tube2\_061314\_01.05867.05867.2 | 2.4815 | 0.3598 | 99.5% | 940.6922 | 940.0898 | 1 | 5.6 | 85.7% | 1 | K.FGNVYLAR.E | 2 |
|  | Astrin\_STLCHLD\_tube2\_061314\_01.12517.12517.2 | 3.1054 | 0.3475 | 99.8% | 1615.8121 | 1615.7826 | 3 | 6.667 | 53.8% | 1 | R.VEFTFPDFVTEGAR.D | 2 |

---

|  |  |  |  |  |  |  |  |  |
| --- | --- | --- | --- | --- | --- | --- | --- | --- |
| U | *gi|25777713|ref|NP\_73* | 2 | 3 | 10.4% | 163 | 18658 | 4.5 | S-phase kinase-associated protein 1 isoform b [Homo sapiens] |

| Filename XCorr DeltCN Conf% ObsM+H+ CalcM+H+ SpR ZScore Ion% # Sequence  | | | | | | | | | | | | |
| --- | --- | --- | --- | --- | --- | --- | --- | --- | --- | --- | --- | --- |
| \* | Astrin\_STLCHLD\_tube2\_061314\_02.06736.06736.3 | 3.1055 | 0.2327 | 97.2% | 2072.0044 | 2071.2078 | 1 | 5.018 | 37.5% | 2 | K.TFNIKNDFTEEEEAQVR.K | 3 |
| \* | Astrin\_STLCHLD\_061214\_02.05174.05174.2 | 2.2523 | 0.243 | 95.3% | 1467.3121 | 1467.4888 | 7 | 5.217 | 54.5% | 1 | K.NDFTEEEEAQVR.K | 2 |

---

|  |  |  |  |  |  |  |  |  |
| --- | --- | --- | --- | --- | --- | --- | --- | --- |
| U | *gi|209862831|ref|NP\_0* | 2 | 6 | 10.3% | 339 | 38604 | 7.8 | annexin A2 isoform 2 [Homo sapiens] |
| U | *gi|50845388|ref|NP\_00* | 2 | 6 | 9.8% | 357 | 40411 | 8.4 | annexin A2 isoform 1 [Homo sapiens] |
| U | *gi|50845386|ref|NP\_00* | 2 | 6 | 10.3% | 339 | 38604 | 7.8 | annexin A2 isoform 2 [Homo sapiens] |
| U | *gi|4757756|ref|NP\_004* | 2 | 6 | 10.3% | 339 | 38604 | 7.8 | annexin A2 isoform 2 [Homo sapiens] |

| Filename XCorr DeltCN Conf% ObsM+H+ CalcM+H+ SpR ZScore Ion% # Sequence  | | | | | | | | | | | | |
| --- | --- | --- | --- | --- | --- | --- | --- | --- | --- | --- | --- | --- |
|  | Astrin\_STLCHLD\_tube2\_061314\_01.08599.08599.3 | 4.4844 | 0.4396 | 100.0% | 1941.5343 | 1941.102 | 1 | 7.797 | 53.1% | 2 | K.TDLEKDIISDTSGDFRK.L | 3 |
|  | Astrin\_STLCHLD\_tube2\_061314\_02.06976.06976.3 | 4.062 | 0.189 | 98.7% | 2065.4944 | 2066.1887 | 1 | 5.533 | 50.0% | 4 | R.RAEDGSVIDYELIDQDAR.D | 3 |

---

|  |  |  |  |  |  |  |  |  |
| --- | --- | --- | --- | --- | --- | --- | --- | --- |
| U | *gi|153792590|ref|NP\_0* | 6 | 16 | 10.2% | 854 | 98161 | 5.2 | heat shock 90kDa protein 1, alpha isoform 1 [Homo sapiens] |
| U | *gi|154146191|ref|NP\_0* | 6 | 16 | 11.9% | 732 | 84660 | 5.0 | heat shock 90kDa protein 1, alpha isoform 2 [Homo sapiens] |

| Filename XCorr DeltCN Conf% ObsM+H+ CalcM+H+ SpR ZScore Ion% # Sequence  | | | | | | | | | | | | |
| --- | --- | --- | --- | --- | --- | --- | --- | --- | --- | --- | --- | --- |
|  | Astrin\_STLCHLD\_tube2\_061314\_01.09271.09271.2 | 2.5815 | 0.1988 | 96.3% | 1242.3922 | 1243.4459 | 6 | 5.215 | 54.5% | 1 | K.ADLINNLGTIAK.S | 22 |
|  | Astrin\_STLCHLD\_061214\_02.06734.06734.3 | 3.3418 | 0.257 | 98.6% | 2258.3342 | 2257.294 | 1 | 5.261 | 35.5% | 2 | K.HNDDEQYAWESSAGGSFTVR.T | 33 |
|  | Astrin\_STLCHLD\_061214\_01.06097.06097.3 | 4.2275 | 0.4235 | 100.0% | 2016.4143 | 2016.2584 | 1 | 7.282 | 48.3% | 5 | K.VILHLKEDQTEYLEER.R | 33 |
|  | Astrin\_STLCHLD\_061214\_01.07095.07095.2 | 4.0697 | 0.4045 | 100.0% | 1528.3322 | 1528.6616 | 1 | 7.91 | 75.0% | 2 | K.SLTNDWEDHLAVK.H | 22 |
|  | Astrin\_STLCHLD\_061214\_01.06870.06870.2 | 2.9414 | 0.4216 | 100.0% | 1349.4521 | 1349.4886 | 1 | 7.003 | 70.0% | 5 | K.HFSVEGQLEFR.A | 22 |
|  | Astrin\_STLCHLD\_tube2\_061314\_01.08267.08267.3 | 3.3296 | 0.3321 | 99.8% | 1787.7244 | 1788.0134 | 1 | 6.469 | 41.1% | 1 | K.HLEINPDHSIIETLR.Q | 3 |

Similarities:
gi|20149594|ref|NP\_03(5:1)  

---

|  |  |  |  |  |  |  |  |  |
| --- | --- | --- | --- | --- | --- | --- | --- | --- |
| U | *gi|4758158|ref|NP\_004* | 2 | 5 | 10.2% | 361 | 41487 | 6.6 | septin 2 [Homo sapiens] |
| U | *gi|56549640|ref|NP\_00* | 2 | 5 | 10.2% | 361 | 41487 | 6.6 | septin 2 [Homo sapiens] |
| U | *gi|56549638|ref|NP\_00* | 2 | 5 | 10.2% | 361 | 41487 | 6.6 | septin 2 [Homo sapiens] |
| U | *gi|56549636|ref|NP\_00* | 2 | 5 | 10.2% | 361 | 41487 | 6.6 | septin 2 [Homo sapiens] |

| Filename XCorr DeltCN Conf% ObsM+H+ CalcM+H+ SpR ZScore Ion% # Sequence  | | | | | | | | | | | | |
| --- | --- | --- | --- | --- | --- | --- | --- | --- | --- | --- | --- | --- |
|  | Astrin\_STLCHLD\_tube2\_061314\_02.06158.06158.2 | 3.6538 | 0.44 | 100.0% | 1604.6322 | 1604.7545 | 4 | 7.42 | 61.5% | 2 | R.TVQIEASTVEIEER.G | 2 |
|  | Astrin\_STLCHLD\_061214\_01.05799.05799.3 | 5.1304 | 0.4226 | 100.0% | 2385.2644 | 2385.6675 | 1 | 7.032 | 44.3% | 3 | R.MQAQMQMQMQGGDGDGGALGHHV.- | 3 |

---

|  |  |  |  |  |  |  |  |  |
| --- | --- | --- | --- | --- | --- | --- | --- | --- |
| U | *gi|31542947|ref|NP\_00* | 3 | 4 | 10.1% | 573 | 61055 | 5.9 | chaperonin [Homo sapiens] |
| U | *gi|41399285|ref|NP\_95* | 3 | 4 | 10.1% | 573 | 61055 | 5.9 | chaperonin [Homo sapiens] |

| Filename XCorr DeltCN Conf% ObsM+H+ CalcM+H+ SpR ZScore Ion% # Sequence  | | | | | | | | | | | | |
| --- | --- | --- | --- | --- | --- | --- | --- | --- | --- | --- | --- | --- |
|  | Astrin\_STLCHLD\_tube2\_061314\_02.12651.12651.3 | 3.9517 | 0.3602 | 100.0% | 2114.8145 | 2114.5667 | 1 | 6.658 | 38.8% | 1 | R.ALMLQGVDLLADAVAVTMGPK.G | 3 |
|  | Astrin\_STLCHLD\_061214\_01.06684.06684.2 | 2.8353 | 0.221 | 97.7% | 1345.6322 | 1345.5382 | 1 | 5.117 | 68.2% | 1 | R.TVIIEQSWGSPK.V | 2 |
|  | Astrin\_STLCHLD\_061214\_02.05695.05695.3 | 3.2223 | 0.2879 | 98.6% | 2562.2043 | 2561.7222 | 159 | 5.71 | 24.0% | 2 | K.LVQDVANNTNEEAGDGTTTATVLAR.S | 3 |

---

|  |  |  |  |  |  |  |  |  |
| --- | --- | --- | --- | --- | --- | --- | --- | --- |
| U | *gi|156523260|ref|NP\_6* | 3 | 4 | 9.7% | 412 | 46373 | 5.1 | hypothetical protein LOC221150 [Homo sapiens] |

| Filename XCorr DeltCN Conf% ObsM+H+ CalcM+H+ SpR ZScore Ion% # Sequence  | | | | | | | | | | | | |
| --- | --- | --- | --- | --- | --- | --- | --- | --- | --- | --- | --- | --- |
| \* | Astrin\_STLCHLD\_tube2\_061314\_01.08611.08611.2 | 3.6867 | 0.5248 | 100.0% | 1774.5122 | 1774.853 | 1 | 8.99 | 57.1% | 1 | R.ALDGEESDFEDYPMR.I | 2 |
| \* | Astrin\_STLCHLD\_tube2\_061314\_01.08227.08227.2 | 3.4644 | 0.2803 | 100.0% | 1307.5322 | 1306.4583 | 1 | 5.515 | 80.0% | 2 | R.LENQEGIDFIK.A | 2 |
| \* | Astrin\_STLCHLD\_tube2\_061314\_01.08605.08605.2 | 3.3526 | 0.0912 | 96.5% | 1507.4922 | 1507.7269 | 4 | 6.221 | 61.5% | 1 | K.NSIALVSTNYPLSK.T | 2 |

---

|  |  |  |  |  |  |  |  |  |
| --- | --- | --- | --- | --- | --- | --- | --- | --- |
| U | *gi|224586882|ref|NP\_0* | 2 | 5 | 9.7% | 372 | 40090 | 9.8 | cold shock domain protein A isoform a [Homo sapiens] |
| U | *gi|224586884|ref|NP\_0* | 2 | 5 | 11.9% | 303 | 31947 | 9.7 | cold shock domain protein A isoform b [Homo sapiens] |

| Filename XCorr DeltCN Conf% ObsM+H+ CalcM+H+ SpR ZScore Ion% # Sequence  | | | | | | | | | | | | |
| --- | --- | --- | --- | --- | --- | --- | --- | --- | --- | --- | --- | --- |
|  | Astrin\_STLCHLD\_tube2\_061314\_02.06922.06922.2 | 4.5895 | 0.526 | 100.0% | 1796.5922 | 1796.8822 | 1 | 9.606 | 65.6% | 4 | R.SVGDGETVEFDVVEGEK.G | 2 |
|  | Astrin\_STLCHLD\_tube2\_061314\_01.04508.04508.2 | 3.9487 | 0.4397 | 100.0% | 1782.7122 | 1783.8925 | 4 | 8.084 | 38.9% | 1 | K.GAEAANVTGPDGVPVEGSR.Y | 2 |

---

|  |  |  |  |  |  |  |  |  |
| --- | --- | --- | --- | --- | --- | --- | --- | --- |
| U | *gi|55956788|ref|NP\_00* | 4 | 10 | 9.6% | 710 | 76615 | 4.7 | nucleolin [Homo sapiens] |

| Filename XCorr DeltCN Conf% ObsM+H+ CalcM+H+ SpR ZScore Ion% # Sequence  | | | | | | | | | | | | |
| --- | --- | --- | --- | --- | --- | --- | --- | --- | --- | --- | --- | --- |
| \* | Astrin\_STLCHLD\_061214\_01.09019.09019.2 | 4.0105 | 0.4612 | 100.0% | 1649.8121 | 1649.751 | 1 | 7.49 | 69.2% | 3 | K.FGYVDFESAEDLEK.A | 2 |
| \* | Astrin\_STLCHLD\_tube2\_061314\_01.04431.04431.3 | 2.9864 | 0.2486 | 96.9% | 2255.0645 | 2255.357 | 22 | 4.629 | 30.3% | 1 | K.TEADAEKTFEEKQGTEIDGR.S | 3 |
| \* | Astrin\_STLCHLD\_061214\_01.07454.07454.3 | 3.2974 | 0.3105 | 99.6% | 2201.9043 | 2201.3057 | 1 | 5.913 | 35.5% | 3 | K.GLSEDTTEETLKESFDGSVR.A | 3 |
| \* | Astrin\_STLCHLD\_061214\_01.09001.09001.2 | 4.3179 | 0.5486 | 100.0% | 1562.6122 | 1562.6323 | 1 | 8.678 | 61.5% | 3 | K.GFGFVDFNSEEDAK.A | 2 |

---

|  |  |  |  |  |  |  |  |  |
| --- | --- | --- | --- | --- | --- | --- | --- | --- |
| U | *gi|20357552|ref|NP\_00* | 3 | 3 | 9.6% | 550 | 61586 | 5.4 | cortactin isoform a [Homo sapiens] |
| U | *gi|20357556|ref|NP\_61* | 3 | 3 | 10.3% | 513 | 57467 | 5.3 | cortactin isoform b [Homo sapiens] |

| Filename XCorr DeltCN Conf% ObsM+H+ CalcM+H+ SpR ZScore Ion% # Sequence  | | | | | | | | | | | | |
| --- | --- | --- | --- | --- | --- | --- | --- | --- | --- | --- | --- | --- |
|  | Astrin\_STLCHLD\_tube2\_061314\_02.06260.06260.3 | 4.0399 | 0.3131 | 100.0% | 2251.5244 | 2250.442 | 3 | 6.127 | 32.9% | 1 | R.MDKNASTFEDVTQVSSAYQK.T | 3 |
|  | Astrin\_STLCHLD\_tube2\_061314\_01.08491.08491.2 | 4.6084 | 0.4749 | 100.0% | 2155.8523 | 2156.3284 | 1 | 9.019 | 55.3% | 1 | R.GPVSGTEPEPVYSMEAADYR.E | 2 |
|  | Astrin\_STLCHLD\_tube2\_061314\_01.11469.11469.2 | 3.1892 | 0.4546 | 100.0% | 1570.7722 | 1570.7881 | 1 | 8.016 | 79.2% | 1 | R.YGLFPANYVELRQ.- | 2 |

---

|  |  |  |  |  |  |  |  |  |
| --- | --- | --- | --- | --- | --- | --- | --- | --- |
| U | *gi|148277065|ref|NP\_0* | 2 | 2 | 9.6% | 551 | 60269 | 6.7 | thioredoxin reductase 1 isoform 1 [Homo sapiens] |
| U | *gi|33519430|ref|NP\_87* | 2 | 2 | 10.6% | 499 | 54604 | 6.5 | thioredoxin reductase 1 isoform 2 [Homo sapiens] |
| U | *gi|33519428|ref|NP\_87* | 2 | 2 | 10.6% | 499 | 54604 | 6.5 | thioredoxin reductase 1 isoform 2 [Homo sapiens] |
| U | *gi|33519426|ref|NP\_87* | 2 | 2 | 10.6% | 499 | 54604 | 6.5 | thioredoxin reductase 1 isoform 2 [Homo sapiens] |
| U | *gi|148277071|ref|NP\_0* | 2 | 2 | 8.2% | 649 | 70756 | 7.4 | thioredoxin reductase 1 isoform 3 [Homo sapiens] |

| Filename XCorr DeltCN Conf% ObsM+H+ CalcM+H+ SpR ZScore Ion% # Sequence  | | | | | | | | | | | | |
| --- | --- | --- | --- | --- | --- | --- | --- | --- | --- | --- | --- | --- |
|  | Astrin\_STLCHLD\_tube2\_061314\_02.07432.07432.3 | 3.8086 | 0.4003 | 100.0% | 1989.4443 | 1989.2592 | 5 | 6.222 | 39.1% | 1 | R.MIEAVQNHIGSLNWGYR.V | 3 |
|  | Astrin\_STLCHLD\_tube2\_061314\_01.14336.14336.3 | 4.0373 | 0.2395 | 98.9% | 3973.1042 | 3972.4814 | 27 | 4.906 | 16.4% | 1 | K.IPVTDEEQTNVPYIYAIGDILEDKVELTPVAIQAGR.L | 3 |

---

|  |  |  |  |  |  |  |  |  |
| --- | --- | --- | --- | --- | --- | --- | --- | --- |
| U | *gi|4557032|ref|NP\_002* | 2 | 3 | 9.3% | 334 | 36639 | 6.1 | L-lactate dehydrogenase B [Homo sapiens] |

| Filename XCorr DeltCN Conf% ObsM+H+ CalcM+H+ SpR ZScore Ion% # Sequence  | | | | | | | | | | | | |
| --- | --- | --- | --- | --- | --- | --- | --- | --- | --- | --- | --- | --- |
| \* | Astrin\_STLCHLD\_tube2\_061314\_02.06798.06798.2 | 2.57 | 0.3256 | 98.5% | 1267.6721 | 1268.5109 | 2 | 6.027 | 70.0% | 2 | K.MVVESAYEVIK.L | 2 |
| \* | Astrin\_STLCHLD\_tube2\_061314\_01.18115.18115.2 | 3.1912 | 0.2722 | 98.8% | 2182.0923 | 2182.5396 | 4 | 5.803 | 34.2% | 1 | K.GYTNWAIGLSVADLIESMLK.N | 2 |

---

|  |  |  |  |  |  |  |  |  |
| --- | --- | --- | --- | --- | --- | --- | --- | --- |
| U | *gi|4758138|ref|NP\_004* | 5 | 10 | 9.1% | 614 | 69148 | 8.9 | DEAD (Asp-Glu-Ala-Asp) box polypeptide 5 [Homo sapiens] |

| Filename XCorr DeltCN Conf% ObsM+H+ CalcM+H+ SpR ZScore Ion% # Sequence  | | | | | | | | | | | | |
| --- | --- | --- | --- | --- | --- | --- | --- | --- | --- | --- | --- | --- |
| \* | Astrin\_STLCHLD\_tube2\_061314\_01.08235.08235.2 | 3.4157 | 0.4725 | 100.0% | 1296.6522 | 1296.4198 | 1 | 8.344 | 75.0% | 2 | R.TTYLVLDEADR.M | 2 |
|  | Astrin\_STLCHLD\_tube2\_061314\_01.10391.10391.2 | 3.7307 | 0.5056 | 100.0% | 1337.5122 | 1337.5946 | 1 | 8.716 | 85.0% | 3 | R.MLDMGFEPQIR.K | 22 |
|  | Astrin\_STLCHLD\_tube2\_061314\_01.07739.07739.2 | 3.9151 | 0.2657 | 100.0% | 1227.1721 | 1227.4465 | 2 | 7.287 | 81.8% | 3 | K.APILIATDVASR.G | 2 |
| \* | Astrin\_STLCHLD\_tube2\_061314\_01.10038.10038.2 | 2.6361 | 0.2743 | 97.4% | 1575.0521 | 1575.7612 | 1 | 5.73 | 61.5% | 1 | K.TGTAYTFFTPNNIK.Q | 2 |
| \* | Astrin\_STLCHLD\_tube2\_061314\_01.07081.07081.2 | 2.4953 | 0.1827 | 96.9% | 985.6122 | 986.1564 | 1 | 4.904 | 85.7% | 1 | K.LLQLVEDR.G | 2 |

Similarities:
gi|87196351|ref|NP\_00(1:4)  

---

|  |  |  |  |  |  |  |  |  |
| --- | --- | --- | --- | --- | --- | --- | --- | --- |
| U | *gi|14141166|ref|NP\_11* | 3 | 9 | 9.1% | 362 | 38222 | 6.8 | poly(rC) binding protein 2 isoform b [Homo sapiens] |
| U | *gi|193083114|ref|NP\_0* | 3 | 9 | 10.4% | 318 | 33497 | 8.2 | poly(rC) binding protein 2 isoform g [Homo sapiens] |
| U | *gi|193083112|ref|NP\_0* | 3 | 9 | 9.9% | 335 | 35347 | 8.0 | poly(rC) binding protein 2 isoform f [Homo sapiens] |
| U | *gi|193083110|ref|NP\_0* | 3 | 9 | 9.1% | 361 | 38151 | 6.8 | poly(rC) binding protein 2 isoform e [Homo sapiens] |
| U | *gi|193083108|ref|NP\_0* | 3 | 9 | 9.0% | 365 | 38580 | 6.8 | poly(rC) binding protein 2 isoform d [Homo sapiens] |
| U | *gi|148833484|ref|NP\_0* | 3 | 9 | 10.0% | 331 | 34917 | 8.0 | poly(rC) binding protein 2 isoform c [Homo sapiens] |
| U | *gi|14141168|ref|NP\_00* | 3 | 9 | 9.0% | 366 | 38651 | 6.8 | poly(rC) binding protein 2 isoform a [Homo sapiens] |

| Filename XCorr DeltCN Conf% ObsM+H+ CalcM+H+ SpR ZScore Ion% # Sequence  | | | | | | | | | | | | |
| --- | --- | --- | --- | --- | --- | --- | --- | --- | --- | --- | --- | --- |
|  | Astrin\_STLCHLD\_tube2\_061314\_01.11003.11003.2 | 3.0738 | 0.311 | 99.3% | 1359.7122 | 1359.6519 | 2 | 5.614 | 54.2% | 2 | R.IITLAGPTNAIFK.A | 2 |
|  | Astrin\_STLCHLD\_061214\_01.06314.06314.2 | 5.6661 | 0.5681 | 100.0% | 2090.4521 | 2091.2573 | 1 | 9.651 | 60.5% | 6 | R.ESTGAQVQVAGDMLPNSTER.A | 22 |
|  | Astrin\_STLCHLD\_061214\_01.06312.06312.3 | 4.5802 | 0.3485 | 100.0% | 2091.4744 | 2091.2573 | 1 | 6.752 | 38.2% | 1 | R.ESTGAQVQVAGDMLPNSTER.A | 33 |

Similarities:
gi|222352151|ref|NP\_0(2:1)  

---

|  |  |  |  |  |  |  |  |  |
| --- | --- | --- | --- | --- | --- | --- | --- | --- |
| U | *gi|221307584|ref|NP\_0* | 2 | 3 | 9.0% | 299 | 33296 | 9.8 | prohibitin 2 isoform 1 [Homo sapiens] |
| U | *gi|6005854|ref|NP\_009* | 2 | 3 | 9.0% | 299 | 33296 | 9.8 | prohibitin 2 isoform 2 [Homo sapiens] |

| Filename XCorr DeltCN Conf% ObsM+H+ CalcM+H+ SpR ZScore Ion% # Sequence  | | | | | | | | | | | | |
| --- | --- | --- | --- | --- | --- | --- | --- | --- | --- | --- | --- | --- |
|  | Astrin\_STLCHLD\_tube2\_061314\_02.07955.07955.3 | 3.9957 | 0.4152 | 100.0% | 1855.4343 | 1855.1038 | 6 | 6.751 | 37.5% | 1 | R.IGGVQQDTILAEGLHFR.I | 3 |
|  | Astrin\_STLCHLD\_tube2\_061314\_01.07765.07765.2 | 2.6158 | 0.3122 | 98.8% | 1178.1522 | 1178.3335 | 3 | 5.684 | 66.7% | 2 | K.FNASQLITQR.A | 2 |

---

|  |  |  |  |  |  |  |  |  |
| --- | --- | --- | --- | --- | --- | --- | --- | --- |
| U | *gi|169212778|ref|XP\_0* | 2 | 3 | 9.0% | 266 | 30042 | 10.6 | PREDICTED: similar to ribosomal protein L7a [Homo sapiens] |
| U | *gi|4506661|ref|NP\_000* | 2 | 3 | 9.0% | 266 | 29996 | 10.6 | ribosomal protein L7a [Homo sapiens] |
| U | *gi|169213130|ref|XP\_0* | 2 | 3 | 9.0% | 266 | 30042 | 10.6 | PREDICTED: similar to ribosomal protein L7a [Homo sapiens] |
| U | *gi|169212940|ref|XP\_0* | 2 | 3 | 9.0% | 266 | 30028 | 10.6 | PREDICTED: similar to ribosomal protein L7a [Homo sapiens] |

| Filename XCorr DeltCN Conf% ObsM+H+ CalcM+H+ SpR ZScore Ion% # Sequence  | | | | | | | | | | | | |
| --- | --- | --- | --- | --- | --- | --- | --- | --- | --- | --- | --- | --- |
|  | Astrin\_STLCHLD\_tube2\_061314\_01.06563.06563.2 | 3.3234 | 0.3492 | 100.0% | 1217.3722 | 1217.3672 | 3 | 6.145 | 70.0% | 1 | K.NFGIGQDIQPK.R | 2 |
|  | Astrin\_STLCHLD\_tube2\_061314\_01.07009.07009.2 | 3.4189 | 0.4002 | 100.0% | 1346.2722 | 1346.5236 | 5 | 6.204 | 62.5% | 2 | R.AGVNTVTTLVENK.K | 2 |

---

|  |  |  |  |  |  |  |  |  |
| --- | --- | --- | --- | --- | --- | --- | --- | --- |
| U | *gi|4507877|ref|NP\_003* | 8 | 9 | 8.9% | 1066 | 116722 | 6.1 | vinculin isoform VCL [Homo sapiens] |
| U | *gi|7669550|ref|NP\_054* | 8 | 9 | 8.4% | 1134 | 123799 | 5.7 | vinculin isoform meta-VCL [Homo sapiens] |

| Filename XCorr DeltCN Conf% ObsM+H+ CalcM+H+ SpR ZScore Ion% # Sequence  | | | | | | | | | | | | |
| --- | --- | --- | --- | --- | --- | --- | --- | --- | --- | --- | --- | --- |
|  | Astrin\_STLCHLD\_tube2\_061314\_01.06576.06576.2 | 2.1688 | 0.2573 | 95.4% | 1237.4321 | 1236.5305 | 2 | 4.702 | 70.0% | 1 | R.VMLVNSMNTVK.E | 2 |
|  | Astrin\_STLCHLD\_061214\_01.07138.07138.2 | 2.9357 | 0.1839 | 98.0% | 1177.2722 | 1176.3734 | 5 | 5.52 | 77.8% | 1 | K.MSAEINEIIR.V | 2 |
|  | Astrin\_STLCHLD\_tube2\_061314\_01.08683.08683.2 | 2.935 | 0.2952 | 98.8% | 1478.3322 | 1478.7217 | 43 | 6.299 | 50.0% | 1 | K.MLGQMTDQVADLR.A | 2 |
|  | Astrin\_STLCHLD\_tube2\_061314\_01.07479.07479.2 | 2.7743 | 0.26 | 98.3% | 1106.1721 | 1106.2615 | 1 | 6.86 | 70.0% | 1 | R.SLGEISALTSK.L | 2 |
|  | Astrin\_STLCHLD\_061214\_01.04291.04291.3 | 2.8995 | 0.3773 | 100.0% | 1550.6643 | 1550.758 | 2 | 5.486 | 36.5% | 1 | K.AAVHLEGKIEQAQR.W | 3 |
|  | Astrin\_STLCHLD\_tube2\_061314\_01.05270.05270.2 | 2.6451 | 0.2633 | 98.2% | 1231.3722 | 1231.3073 | 1 | 5.569 | 83.3% | 1 | R.WIDNPTVDDR.G | 2 |
|  | Astrin\_STLCHLD\_061214\_01.06553.06553.2 | 2.7707 | 0.3946 | 100.0% | 1294.0922 | 1293.4749 | 1 | 7.213 | 68.2% | 2 | K.MTGLVDEAIDTK.S | 2 |
|  | Astrin\_STLCHLD\_tube2\_061314\_01.07580.07580.2 | 3.6445 | 0.3719 | 100.0% | 1519.1322 | 1519.8354 | 1 | 6.516 | 73.1% | 1 | K.AGEVINQPMMMAAR.Q | 2 |

---

|  |  |  |  |  |  |  |  |  |
| --- | --- | --- | --- | --- | --- | --- | --- | --- |
| U | *gi|4826998|ref|NP\_005* | 5 | 17 | 8.9% | 707 | 76150 | 9.4 | splicing factor proline/glutamine rich (polypyrimidine tract binding protein associated) [Homo sapiens] |

| Filename XCorr DeltCN Conf% ObsM+H+ CalcM+H+ SpR ZScore Ion% # Sequence  | | | | | | | | | | | | |
| --- | --- | --- | --- | --- | --- | --- | --- | --- | --- | --- | --- | --- |
| \* | Astrin\_STLCHLD\_061214\_01.07773.07773.3 | 3.5628 | 0.3054 | 99.8% | 1652.6643 | 1650.8723 | 2 | 5.453 | 41.1% | 2 | K.ISDSEGFKANLSLLR.R | 3 |
| \* | Astrin\_STLCHLD\_tube2\_061314\_01.06738.06738.2 | 2.255 | 0.3713 | 98.2% | 1252.4321 | 1253.3971 | 1 | 6.663 | 65.0% | 1 | K.YGEPGEVFINK.G | 2 |
| \* | Astrin\_STLCHLD\_tube2\_061314\_01.14268.14268.2 | 4.9294 | 0.5887 | 100.0% | 2640.9722 | 2640.9092 | 1 | 10.109 | 45.5% | 1 | R.NLSPYVSNELLEEAFSQFGPIER.A | 2 |
| \* | Astrin\_STLCHLD\_061214\_01.05383.05383.2 | 4.1947 | 0.4685 | 100.0% | 1763.3922 | 1763.8632 | 1 | 8.352 | 73.1% | 3 | R.FAQHGTFEYEYSQR.W | 2 |
| \* | Astrin\_STLCHLD\_061214\_01.05406.05406.3 | 4.6572 | 0.3743 | 100.0% | 1764.0844 | 1763.8632 | 1 | 6.417 | 46.2% | 10 | R.FAQHGTFEYEYSQR.W | 3 |

---

|  |  |  |  |  |  |  |  |  |
| --- | --- | --- | --- | --- | --- | --- | --- | --- |
| U | *gi|119703753|ref|NP\_0* | 5 | 22 | 8.7% | 564 | 60067 | 8.0 | keratin 6B [Homo sapiens] |

| Filename XCorr DeltCN Conf% ObsM+H+ CalcM+H+ SpR ZScore Ion% # Sequence  | | | | | | | | | | | | |
| --- | --- | --- | --- | --- | --- | --- | --- | --- | --- | --- | --- | --- |
|  | Astrin\_STLCHLD\_tube2\_061314\_01.06264.06264.2 | 2.4085 | 0.2136 | 97.8% | 828.2922 | 827.95544 | 5 | 5.059 | 91.7% | 1 | K.FASFIDK.V | 2222 |
|  | Astrin\_STLCHLD\_tube2\_061314\_01.12653.12653.2 | 2.489 | 0.2745 | 97.4% | 1331.5721 | 1330.5211 | 3 | 5.659 | 59.1% | 1 | R.NLDLDSIIAEVK.A | 22 |
|  | Astrin\_STLCHLD\_tube2\_061314\_01.05725.05725.2 | 3.887 | 0.3539 | 100.0% | 1181.3722 | 1180.303 | 2 | 6.792 | 83.3% | 16 | K.YEELQITAGR.H | 22 |
|  | Astrin\_STLCHLD\_tube2\_061314\_01.05844.05844.2 | 2.4271 | 0.225 | 97.0% | 1154.3322 | 1154.3234 | 78 | 6.209 | 56.2% | 1 | K.EYQELMNVK.L | 22 |
|  | Astrin\_STLCHLD\_061214\_02.07769.07769.2 | 3.2347 | 0.4112 | 100.0% | 1264.5922 | 1264.4644 | 1 | 8.501 | 65.0% | 3 | K.LALDVEIATYR.K | 22 |

Similarities:
gi|4504919|ref|NP\_002(2:3)  
gi|119395750|ref|NP\_0(1:4)  
gi|67782365|ref|NP\_00(1:4)  
gi|47132620|ref|NP\_00(3:2)  

---

|  |  |  |  |  |  |  |  |  |
| --- | --- | --- | --- | --- | --- | --- | --- | --- |
| U | *gi|223555975|ref|NP\_0* | 2 | 2 | 8.5% | 284 | 32723 | 4.7 | tropomyosin 4 isoform 1 [Homo sapiens] |
| U | *gi|4507651|ref|NP\_003* | 2 | 2 | 9.7% | 248 | 28522 | 4.7 | tropomyosin 4 isoform 2 [Homo sapiens] |

| Filename XCorr DeltCN Conf% ObsM+H+ CalcM+H+ SpR ZScore Ion% # Sequence  | | | | | | | | | | | | |
| --- | --- | --- | --- | --- | --- | --- | --- | --- | --- | --- | --- | --- |
|  | Astrin\_STLCHLD\_061214\_01.07005.07005.2 | 2.3665 | 0.2966 | 97.4% | 1298.6322 | 1299.5535 | 72 | 5.675 | 55.0% | 1 | R.KLVILEGELER.A | 2 |
|  | Astrin\_STLCHLD\_061214\_01.04944.04944.3 | 2.5759 | 0.2967 | 98.3% | 1493.2444 | 1492.7153 | 316 | 5.346 | 33.3% | 1 | R.AEFAERTVAKLEK.T | 3 |

---

|  |  |  |  |  |  |  |  |  |
| --- | --- | --- | --- | --- | --- | --- | --- | --- |
| U | *gi|20143967|ref|NP\_61* | 5 | 7 | 8.1% | 960 | 110059 | 8.5 | kinesin family member 23 isoform 1 [Homo sapiens] |
| U | *gi|6754472|ref|NP\_004* | 5 | 7 | 9.1% | 856 | 98105 | 8.5 | kinesin family member 23 isoform 2 [Homo sapiens] |

| Filename XCorr DeltCN Conf% ObsM+H+ CalcM+H+ SpR ZScore Ion% # Sequence  | | | | | | | | | | | | |
| --- | --- | --- | --- | --- | --- | --- | --- | --- | --- | --- | --- | --- |
|  | Astrin\_STLCHLD\_tube2\_061314\_02.07787.07787.2 | 2.6753 | 0.1816 | 95.4% | 1414.7922 | 1414.6012 | 1 | 4.963 | 57.7% | 1 | K.NGLLFTYGVTGSGK.T | 2 |
|  | Astrin\_STLCHLD\_tube2\_061314\_01.07068.07068.2 | 3.2793 | 0.4583 | 100.0% | 1497.3322 | 1497.6196 | 1 | 7.41 | 72.7% | 1 | K.AEDYEENLQVMR.F | 2 |
|  | Astrin\_STLCHLD\_tube2\_061314\_01.04641.04641.2 | 4.0945 | 0.209 | 100.0% | 1473.5721 | 1473.5828 | 1 | 5.948 | 72.7% | 1 | R.NLQQELETQNQK.L | 2 |
|  | Astrin\_STLCHLD\_tube2\_061314\_02.05673.05673.3 | 3.7785 | 0.3473 | 100.0% | 1965.2943 | 1966.17 | 1 | 5.956 | 43.8% | 2 | K.YMLTHQELASDGEIETK.L | 3 |
|  | Astrin\_STLCHLD\_061214\_01.06748.06748.3 | 3.7283 | 0.3367 | 99.8% | 2516.3943 | 2516.5974 | 2 | 5.353 | 28.4% | 2 | R.GGGQSVQFTDIETLKQES\*PNGSR.K | 3 |

---

|  |  |  |  |  |  |  |  |  |
| --- | --- | --- | --- | --- | --- | --- | --- | --- |
| U | *gi|4757810|ref|NP\_004* | 3 | 5 | 8.0% | 553 | 59751 | 9.1 | ATP synthase, H+ transporting, mitochondrial F1 complex, alpha subunit precursor [Homo sapiens] |
| U | *gi|50345984|ref|NP\_00* | 3 | 5 | 8.0% | 553 | 59751 | 9.1 | ATP synthase, H+ transporting, mitochondrial F1 complex, alpha subunit precursor [Homo sapiens] |

| Filename XCorr DeltCN Conf% ObsM+H+ CalcM+H+ SpR ZScore Ion% # Sequence  | | | | | | | | | | | | |
| --- | --- | --- | --- | --- | --- | --- | --- | --- | --- | --- | --- | --- |
|  | Astrin\_STLCHLD\_061214\_01.07119.07119.2 | 3.6652 | 0.359 | 100.0% | 1424.9521 | 1424.5659 | 1 | 7.172 | 62.5% | 1 | K.TGTAEMSSILEER.I | 2 |
|  | Astrin\_STLCHLD\_tube2\_061314\_02.06334.06334.2 | 4.7508 | 0.5135 | 100.0% | 1576.2922 | 1576.7007 | 1 | 9.499 | 60.7% | 3 | R.ILGADTSVDLEETGR.V | 2 |
|  | Astrin\_STLCHLD\_tube2\_061314\_01.11311.11311.2 | 2.646 | 0.2125 | 95.8% | 1625.6721 | 1625.8625 | 1 | 5.503 | 56.7% | 1 | R.TGAIVDVPVGEELLGR.V | 2 |

---

|  |  |  |  |  |  |  |  |  |
| --- | --- | --- | --- | --- | --- | --- | --- | --- |
| U | *gi|31543397|ref|NP\_62* | 3 | 7 | 7.9% | 417 | 44796 | 8.5 | phosphoglycerate kinase 2 [Homo sapiens] |
| U | *gi|4505763|ref|NP\_000* | 3 | 7 | 7.9% | 417 | 44615 | 8.1 | phosphoglycerate kinase 1 [Homo sapiens] |

| Filename XCorr DeltCN Conf% ObsM+H+ CalcM+H+ SpR ZScore Ion% # Sequence  | | | | | | | | | | | | |
| --- | --- | --- | --- | --- | --- | --- | --- | --- | --- | --- | --- | --- |
|  | Astrin\_STLCHLD\_061214\_01.06664.06664.2 | 3.7965 | 0.4767 | 100.0% | 1635.2722 | 1635.7764 | 1 | 8.029 | 64.3% | 1 | K.LGDVYVNDAFGTAHR.A | 2 |
|  | Astrin\_STLCHLD\_061214\_01.06636.06636.3 | 3.4489 | 0.4269 | 100.0% | 1636.3143 | 1635.7764 | 1 | 6.697 | 48.2% | 5 | K.LGDVYVNDAFGTAHR.A | 3 |
|  | Astrin\_STLCHLD\_tube2\_061314\_02.06428.06428.3 | 3.1951 | 0.2429 | 97.9% | 1742.1543 | 1741.939 | 4 | 5.013 | 35.3% | 1 | K.VSHVSTGGGASLELLEGK.I | 3 |

---

|  |  |  |  |  |  |  |  |  |
| --- | --- | --- | --- | --- | --- | --- | --- | --- |
| U | *gi|217272802|ref|NP\_0* | 3 | 3 | 7.7% | 725 | 84229 | 5.9 | hyaluronan-mediated motility receptor isoform a [Homo sapiens] |
| U | *gi|217416398|ref|NP\_0* | 3 | 3 | 7.9% | 709 | 82301 | 5.9 | hyaluronan-mediated motility receptor isoform c [Homo sapiens] |
| U | *gi|217416394|ref|NP\_0* | 3 | 3 | 7.7% | 724 | 84100 | 5.8 | hyaluronan-mediated motility receptor isoform b [Homo sapiens] |
| U | *gi|217272804|ref|NP\_0* | 3 | 3 | 8.8% | 638 | 74495 | 5.4 | hyaluronan-mediated motility receptor isoform d [Homo sapiens] |

| Filename XCorr DeltCN Conf% ObsM+H+ CalcM+H+ SpR ZScore Ion% # Sequence  | | | | | | | | | | | | |
| --- | --- | --- | --- | --- | --- | --- | --- | --- | --- | --- | --- | --- |
|  | Astrin\_STLCHLD\_061214\_02.05429.05429.3 | 3.3334 | 0.3717 | 100.0% | 1598.3644 | 1598.7966 | 1 | 6.27 | 42.9% | 1 | K.SSAAHTQATLLLQEK.Y | 3 |
|  | Astrin\_STLCHLD\_tube2\_061314\_02.06704.06704.3 | 2.7898 | 0.3414 | 99.2% | 2218.9744 | 2219.451 | 13 | 5.106 | 28.9% | 1 | K.ALTASEIEDLKLENSSLQEK.A | 3 |
|  | Astrin\_STLCHLD\_tube2\_061314\_02.06346.06346.3 | 3.8199 | 0.3259 | 100.0% | 2432.6343 | 2432.5657 | 3 | 5.431 | 31.2% | 1 | K.NAEDVQHQILATESSNQEYVR.M | 3 |

---

|  |  |  |  |  |  |  |  |  |
| --- | --- | --- | --- | --- | --- | --- | --- | --- |
| U | *gi|18375623|ref|NP\_54* | 2 | 3 | 7.7% | 428 | 48991 | 5.7 | HLA-B associated transcript 1 [Homo sapiens] |
| U | *gi|4758112|ref|NP\_004* | 2 | 3 | 7.7% | 428 | 48991 | 5.7 | HLA-B associated transcript 1 [Homo sapiens] |

| Filename XCorr DeltCN Conf% ObsM+H+ CalcM+H+ SpR ZScore Ion% # Sequence  | | | | | | | | | | | | |
| --- | --- | --- | --- | --- | --- | --- | --- | --- | --- | --- | --- | --- |
|  | Astrin\_STLCHLD\_tube2\_061314\_01.09990.09990.3 | 3.3618 | 0.3309 | 99.7% | 2301.5645 | 2301.492 | 1 | 5.673 | 33.3% | 1 | R.VNIAFNYDMPEDSDTYLHR.V | 3 |
|  | Astrin\_STLCHLD\_061214\_01.07771.07771.2 | 3.3911 | 0.3994 | 100.0% | 1480.3722 | 1480.6146 | 1 | 6.985 | 61.5% | 2 | K.GLAITFVSDENDAK.I | 2 |

---

|  |  |  |  |  |  |  |  |  |
| --- | --- | --- | --- | --- | --- | --- | --- | --- |
| U | *gi|19920317|ref|NP\_00* | 3 | 6 | 7.5% | 602 | 66023 | 5.9 | cytoskeleton-associated protein 4 [Homo sapiens] |

| Filename XCorr DeltCN Conf% ObsM+H+ CalcM+H+ SpR ZScore Ion% # Sequence  | | | | | | | | | | | | |
| --- | --- | --- | --- | --- | --- | --- | --- | --- | --- | --- | --- | --- |
| \* | Astrin\_STLCHLD\_tube2\_061314\_01.06837.06837.2 | 2.5929 | 0.1686 | 96.4% | 1294.3922 | 1293.44 | 1 | 4.146 | 72.2% | 1 | K.SREWDMEALR.S | 2 |
| \* | Astrin\_STLCHLD\_061214\_01.06379.06379.3 | 3.4953 | 0.3423 | 100.0% | 1841.1843 | 1841.0923 | 1 | 5.841 | 46.9% | 3 | R.LQHVEDGVLSMQVASAR.Q | 3 |
| \* | Astrin\_STLCHLD\_tube2\_061314\_01.04892.04892.3 | 4.0226 | 0.216 | 99.2% | 2023.0743 | 2023.2131 | 3 | 5.425 | 32.4% | 2 | K.VQEQVHTLLSQDQAQAAR.L | 32 |

---

|  |  |  |  |  |  |  |  |  |
| --- | --- | --- | --- | --- | --- | --- | --- | --- |
| U | *gi|23397427|ref|NP\_00* | 3 | 4 | 7.4% | 623 | 69633 | 8.6 | synaptotagmin binding, cytoplasmic RNA interacting protein [Homo sapiens] |

| Filename XCorr DeltCN Conf% ObsM+H+ CalcM+H+ SpR ZScore Ion% # Sequence  | | | | | | | | | | | | |
| --- | --- | --- | --- | --- | --- | --- | --- | --- | --- | --- | --- | --- |
| \* | Astrin\_STLCHLD\_tube2\_061314\_01.12799.12799.2 | 3.1591 | 0.3128 | 99.2% | 2336.0122 | 2335.6624 | 1 | 6.205 | 35.0% | 1 | R.AIEALKEFNEDGALAVLQQFK.D | 2 |
|  | Astrin\_STLCHLD\_tube2\_061314\_01.05269.05269.2 | 2.9066 | 0.3893 | 100.0% | 1312.3322 | 1312.4221 | 1 | 7.704 | 72.7% | 2 | R.TGYTLDVTTGQR.K | 2 |
| \* | Astrin\_STLCHLD\_tube2\_061314\_01.11427.11427.2 | 3.171 | 0.2588 | 98.8% | 1475.4722 | 1474.6512 | 4 | 6.384 | 50.0% | 1 | R.NLANTVTEEILEK.A | 2 |

---

|  |  |  |  |  |  |  |  |  |
| --- | --- | --- | --- | --- | --- | --- | --- | --- |
| U | *gi|5454064|ref|NP\_006* | 3 | 5 | 7.2% | 669 | 69492 | 9.7 | RNA binding motif protein 14 [Homo sapiens] |

| Filename XCorr DeltCN Conf% ObsM+H+ CalcM+H+ SpR ZScore Ion% # Sequence  | | | | | | | | | | | | |
| --- | --- | --- | --- | --- | --- | --- | --- | --- | --- | --- | --- | --- |
| \* | Astrin\_STLCHLD\_tube2\_061314\_01.06660.06660.2 | 3.1316 | 0.4487 | 100.0% | 1609.3522 | 1609.8223 | 1 | 7.87 | 67.9% | 1 | R.ASYVAPLTAQPATYR.A | 2 |
| \* | Astrin\_STLCHLD\_061214\_01.05262.05262.3 | 4.2278 | 0.5247 | 100.0% | 2465.5745 | 2466.6292 | 1 | 9.219 | 35.9% | 3 | R.TQSSASLAASYAAQQHPQAAASYR.G | 3 |
| \* | Astrin\_STLCHLD\_tube2\_061314\_01.06222.06222.2 | 2.3546 | 0.1893 | 95.7% | 1068.0922 | 1067.1869 | 2 | 4.121 | 75.0% | 1 | R.LSESQLSFR.R | 2 |

---

|  |  |  |  |  |  |  |  |  |
| --- | --- | --- | --- | --- | --- | --- | --- | --- |
| U | *Reverse\_gi|188595697|* | 2 | 3 | 7.0% | 299 | 32541 | 7.9 | sprouty homolog 4 isoform 2 [Homo sapiens] |
| U | *Reverse\_gi|23308574|r* | 2 | 3 | 6.5% | 322 | 34929 | 7.9 | sprouty homolog 4 isoform 1 [Homo sapiens] |

| Filename XCorr DeltCN Conf% ObsM+H+ CalcM+H+ SpR ZScore Ion% # Sequence  | | | | | | | | | | | | |
| --- | --- | --- | --- | --- | --- | --- | --- | --- | --- | --- | --- | --- |
|  | Astrin\_STLCHLD\_061214\_02.10287.10287.2 | 3.25 | 0.2925 | 98.9% | 2507.5923 | 2508.4355 | 4 | 4.325 | 32.5% | 1 | K.DPRS\*TKADGS\*AAK@CIVSNT#HK@.C | 2 |
|  | Astrin\_STLCHLD\_tube2\_061314\_01.13849.13849.2 | 3.163 | 0.1516 | 96.8% | 2509.0522 | 2508.4355 | 17 | 3.658 | 32.5% | 2 | K.DPRST#KADGS\*AAK@CIVSNT#HK@.C | 2 |

---

|  |  |  |  |  |  |  |  |  |
| --- | --- | --- | --- | --- | --- | --- | --- | --- |
| U | *gi|21327708|ref|NP\_63* | 2 | 2 | 6.9% | 391 | 45374 | 4.5 | nucleosome assembly protein 1-like 1 [Homo sapiens] |
| U | *gi|4758756|ref|NP\_004* | 2 | 2 | 6.9% | 391 | 45374 | 4.5 | nucleosome assembly protein 1-like 1 [Homo sapiens] |

| Filename XCorr DeltCN Conf% ObsM+H+ CalcM+H+ SpR ZScore Ion% # Sequence  | | | | | | | | | | | | |
| --- | --- | --- | --- | --- | --- | --- | --- | --- | --- | --- | --- | --- |
|  | Astrin\_STLCHLD\_tube2\_061314\_01.11431.11431.2 | 3.9645 | 0.275 | 100.0% | 1860.6522 | 1861.102 | 1 | 6.079 | 62.5% | 1 | R.LDGLVETPTGYIESLPR.V | 2 |
|  | Astrin\_STLCHLD\_tube2\_061314\_01.05163.05163.2 | 2.4907 | 0.3624 | 98.9% | 1337.4922 | 1337.4314 | 1 | 6.275 | 77.8% | 1 | K.FYEEVHDLER.K | 2 |

---

|  |  |  |  |  |  |  |  |  |
| --- | --- | --- | --- | --- | --- | --- | --- | --- |
| U | *gi|18765752|ref|NP\_56* | 3 | 3 | 6.8% | 754 | 84557 | 8.8 | dual-specificity tyrosine-(Y)-phosphorylation regulated kinase 1A isoform 2 [Homo sapiens] |

| Filename XCorr DeltCN Conf% ObsM+H+ CalcM+H+ SpR ZScore Ion% # Sequence  | | | | | | | | | | | | |
| --- | --- | --- | --- | --- | --- | --- | --- | --- | --- | --- | --- | --- |
| \* | Astrin\_STLCHLD\_tube2\_061314\_02.06530.06530.3 | 3.7786 | 0.3847 | 100.0% | 3115.2844 | 3116.3745 | 1 | 7.335 | 31.7% | 1 | R.RQPNISDQQVSALSYSDQIQQPLTNQR.R | 3 |
|  | Astrin\_STLCHLD\_tube2\_061314\_02.06629.06629.2 | 3.1246 | 0.2531 | 98.9% | 1288.6522 | 1289.477 | 14 | 6.707 | 60.0% | 1 | K.AFLNQAQIEVR.L | 2 |
|  | Astrin\_STLCHLD\_tube2\_061314\_01.04892.04892.2 | 2.8386 | 0.2349 | 97.7% | 1349.0521 | 1348.4552 | 1 | 4.784 | 66.7% | 1 | R.AGESGHTVADYLK.F | 32 |

---

|  |  |  |  |  |  |  |  |  |
| --- | --- | --- | --- | --- | --- | --- | --- | --- |
| U | *gi|5174447|ref|NP\_006* | 2 | 2 | 6.6% | 317 | 35077 | 7.7 | guanine nucleotide binding protein (G protein), beta polypeptide 2-like 1 [Homo sapiens] |

| Filename XCorr DeltCN Conf% ObsM+H+ CalcM+H+ SpR ZScore Ion% # Sequence  | | | | | | | | | | | | |
| --- | --- | --- | --- | --- | --- | --- | --- | --- | --- | --- | --- | --- |
| \* | Astrin\_STLCHLD\_tube2\_061314\_02.07385.07385.2 | 2.807 | 0.4918 | 100.0% | 1309.5122 | 1310.4062 | 2 | 8.848 | 63.6% | 1 | K.DVLSVAFSSDNR.Q | 2 |
| \* | Astrin\_STLCHLD\_tube2\_061314\_01.07752.07752.2 | 2.2105 | 0.2291 | 95.7% | 1060.5122 | 1060.2412 | 4 | 4.99 | 75.0% | 1 | R.VWQVTIGTR.- | 2 |

---

|  |  |  |  |  |  |  |  |  |
| --- | --- | --- | --- | --- | --- | --- | --- | --- |
| U | *gi|4506649|ref|NP\_000* | 3 | 3 | 6.5% | 403 | 46109 | 10.2 | ribosomal protein L3 isoform a [Homo sapiens] |
| U | *gi|76496472|ref|NP\_00* | 3 | 4 | 7.3% | 354 | 40152 | 10.2 | ribosomal protein L3 isoform b [Homo sapiens] |

| Filename XCorr DeltCN Conf% ObsM+H+ CalcM+H+ SpR ZScore Ion% # Sequence  | | | | | | | | | | | | |
| --- | --- | --- | --- | --- | --- | --- | --- | --- | --- | --- | --- | --- |
|  | Astrin\_STLCHLD\_tube2\_061314\_01.05739.05739.2 | 2.4959 | 0.3695 | 99.5% | 984.33215 | 984.14594 | 2 | 6.184 | 68.8% | 1 | R.HGSLGFLPR.K | 2 |
|  | Astrin\_STLCHLD\_tube2\_061314\_02.05261.05261.3 | 3.8295 | 0.1555 | 96.9% | 1826.3344 | 1826.1211 | 1 | 4.156 | 39.1% | 1 | K.KAHLMEIQVNGGTVAEK.L | 3 |
|  | Astrin\_STLCHLD\_tube2\_061314\_02.05807.05807.3 | 3.8163 | 0.2645 | 99.7% | 1698.3243 | 1697.947 | 1 | 6.196 | 41.7% | 1 | K.AHLMEIQVNGGTVAEK.L | 3 |

---

|  |  |  |  |  |  |  |  |  |
| --- | --- | --- | --- | --- | --- | --- | --- | --- |
| U | *gi|13376259|ref|NP\_07* | 3 | 4 | 6.4% | 656 | 75019 | 5.6 | nucleoporin 85 [Homo sapiens] |

| Filename XCorr DeltCN Conf% ObsM+H+ CalcM+H+ SpR ZScore Ion% # Sequence  | | | | | | | | | | | | |
| --- | --- | --- | --- | --- | --- | --- | --- | --- | --- | --- | --- | --- |
| \* | Astrin\_STLCHLD\_061214\_01.08316.08316.2 | 2.3653 | 0.3405 | 98.4% | 1090.3522 | 1090.2676 | 168 | 5.862 | 55.6% | 1 | R.LGSALSWSIR.A | 2 |
| \* | Astrin\_STLCHLD\_tube2\_061314\_01.15614.15614.2 | 2.2087 | 0.2899 | 95.4% | 1596.2322 | 1596.8822 | 144 | 5.533 | 35.7% | 1 | R.FADAASLLLSLMTSR.I | 2 |
| \* | Astrin\_STLCHLD\_tube2\_061314\_01.17495.17495.2 | 2.7984 | 0.4109 | 99.7% | 2006.9722 | 2007.3955 | 1 | 6.922 | 43.8% | 2 | R.SFWMTLLTDALPLLEQK.Q | 2 |

---

|  |  |  |  |  |  |  |  |  |
| --- | --- | --- | --- | --- | --- | --- | --- | --- |
| U | *gi|124256496|ref|NP\_0* | 3 | 10 | 6.4% | 641 | 70375 | 6.0 | heat shock 70kDa protein 1-like [Homo sapiens] |

| Filename XCorr DeltCN Conf% ObsM+H+ CalcM+H+ SpR ZScore Ion% # Sequence  | | | | | | | | | | | | |
| --- | --- | --- | --- | --- | --- | --- | --- | --- | --- | --- | --- | --- |
|  | Astrin\_STLCHLD\_tube2\_061314\_01.07280.07280.2 | 3.3247 | 0.4793 | 100.0% | 1488.3922 | 1488.5939 | 1 | 8.565 | 75.0% | 4 | R.TTPSYVAFTDTER.L | 222 |
|  | Astrin\_STLCHLD\_061214\_01.09072.09072.2 | 3.199 | 0.3048 | 99.9% | 1199.4922 | 1198.408 | 1 | 6.267 | 77.3% | 4 | K.DAGVIAGLNVLR.I | 22 |
|  | Astrin\_STLCHLD\_tube2\_061314\_01.10183.10183.2 | 4.3082 | 0.3802 | 100.0% | 1662.4722 | 1660.9078 | 1 | 7.981 | 80.0% | 2 | R.IINEPTAAAIAYGLDK.G | 22 |

Similarities:
gi|5729877|ref|NP\_006(2:1)  
gi|167466173|ref|NP\_0(2:1)  

---

|  |  |  |  |  |  |  |  |  |
| --- | --- | --- | --- | --- | --- | --- | --- | --- |
| U | *gi|205277463|ref|NP\_0* | 2 | 5 | 6.4% | 623 | 67878 | 7.7 | transketolase isoform 1 [Homo sapiens] |
| U | *gi|4507521|ref|NP\_001* | 2 | 5 | 6.4% | 623 | 67878 | 7.7 | transketolase isoform 1 [Homo sapiens] |
| U | *gi|205277465|ref|NP\_0* | 2 | 5 | 7.4% | 540 | 58982 | 7.7 | transketolase isoform 2 [Homo sapiens] |

| Filename XCorr DeltCN Conf% ObsM+H+ CalcM+H+ SpR ZScore Ion% # Sequence  | | | | | | | | | | | | |
| --- | --- | --- | --- | --- | --- | --- | --- | --- | --- | --- | --- | --- |
|  | Astrin\_STLCHLD\_tube2\_061314\_01.07737.07737.2 | 3.673 | 0.4458 | 100.0% | 1886.1322 | 1886.0656 | 1 | 9.009 | 55.9% | 1 | R.SVPTSTVFYPSDGVATEK.A | 2 |
|  | Astrin\_STLCHLD\_061214\_01.06104.06104.3 | 5.3385 | 0.4808 | 100.0% | 2508.5645 | 2509.6946 | 1 | 7.603 | 42.9% | 4 | R.TSRPENAIIYNNNEDFQVGQAK.V | 3 |

---

|  |  |  |  |  |  |  |  |  |
| --- | --- | --- | --- | --- | --- | --- | --- | --- |
| U | *gi|4507943|ref|NP\_003* | 4 | 6 | 6.3% | 1071 | 123386 | 6.1 | exportin 1 [Homo sapiens] |

| Filename XCorr DeltCN Conf% ObsM+H+ CalcM+H+ SpR ZScore Ion% # Sequence  | | | | | | | | | | | | |
| --- | --- | --- | --- | --- | --- | --- | --- | --- | --- | --- | --- | --- |
| \* | Astrin\_STLCHLD\_tube2\_061314\_02.06282.06282.3 | 4.4732 | 0.3326 | 100.0% | 2141.9644 | 2142.4321 | 1 | 6.783 | 38.9% | 2 | R.MAKPEEVLVVENDQGEVVR.E | 3 |
| \* | Astrin\_STLCHLD\_061214\_02.07779.07779.3 | 3.5941 | 0.4825 | 100.0% | 1968.3544 | 1968.1705 | 1 | 7.266 | 45.0% | 2 | R.ETLVYLTHLDYVDTER.I | 3 |
| \* | Astrin\_STLCHLD\_tube2\_061314\_01.14761.14761.2 | 4.1863 | 0.4813 | 100.0% | 2149.2922 | 2149.5127 | 1 | 8.635 | 61.8% | 1 | K.YMLLPNQVWDSIIQQATK.N | 2 |
| \* | Astrin\_STLCHLD\_061214\_02.07797.07797.2 | 3.9684 | 0.5001 | 100.0% | 1759.2122 | 1758.8358 | 1 | 8.844 | 60.7% | 1 | K.EFAGEDTSDLFLEER.E | 2 |

---

|  |  |  |  |  |  |  |  |  |
| --- | --- | --- | --- | --- | --- | --- | --- | --- |
| U | *gi|5032013|ref|NP\_005* | 5 | 6 | 6.3% | 890 | 100278 | 6.9 | kinesin family member 20A [Homo sapiens] |

| Filename XCorr DeltCN Conf% ObsM+H+ CalcM+H+ SpR ZScore Ion% # Sequence  | | | | | | | | | | | | |
| --- | --- | --- | --- | --- | --- | --- | --- | --- | --- | --- | --- | --- |
| \* | Astrin\_STLCHLD\_061214\_01.08796.08796.2 | 2.6589 | 0.2555 | 97.4% | 1454.1721 | 1454.7068 | 2 | 5.705 | 66.7% | 1 | R.IENVETLVLQAPK.D | 2 |
| \* | Astrin\_STLCHLD\_061214\_01.06226.06226.2 | 2.8039 | 0.3868 | 99.7% | 1419.1721 | 1419.6641 | 2 | 6.333 | 58.3% | 1 | R.ILHLQGEGDIVPK.I | 2 |
| \* | Astrin\_STLCHLD\_tube2\_061314\_02.06077.06077.3 | 2.881 | 0.2393 | 97.5% | 1420.3744 | 1419.6641 | 36 | 4.721 | 39.6% | 1 | R.ILHLQGEGDIVPK.I | 3 |
| \* | Astrin\_STLCHLD\_061214\_01.05143.05143.3 | 3.0159 | 0.2825 | 98.7% | 1724.1244 | 1724.9578 | 3 | 4.809 | 35.0% | 1 | R.LKEAGNINTSLHTLGR.C | 3 |
| \* | Astrin\_STLCHLD\_061214\_01.04822.04822.2 | 2.9404 | 0.306 | 98.8% | 1473.9321 | 1474.6543 | 2 | 6.091 | 57.7% | 2 | R.LAASASTQQLQEVK.A | 2 |

---

|  |  |  |  |  |  |  |  |  |
| --- | --- | --- | --- | --- | --- | --- | --- | --- |
| U | *gi|7661920|ref|NP\_055* | 3 | 4 | 6.3% | 411 | 46871 | 6.7 | eukaryotic translation initiation factor 4A, isoform 3 [Homo sapiens] |

| Filename XCorr DeltCN Conf% ObsM+H+ CalcM+H+ SpR ZScore Ion% # Sequence  | | | | | | | | | | | | |
| --- | --- | --- | --- | --- | --- | --- | --- | --- | --- | --- | --- | --- |
|  | Astrin\_STLCHLD\_tube2\_061314\_01.07218.07218.2 | 4.8055 | 0.5196 | 100.0% | 1828.6721 | 1829.0654 | 1 | 8.339 | 70.0% | 1 | R.GIYAYGFEKPSAIQQR.A | 22 |
|  | Astrin\_STLCHLD\_tube2\_061314\_01.07230.07230.3 | 3.6443 | 0.3709 | 100.0% | 1829.9944 | 1829.0654 | 1 | 6.418 | 45.0% | 2 | R.GIYAYGFEKPSAIQQR.A | 33 |
| \* | Astrin\_STLCHLD\_061214\_01.08088.08088.2 | 2.8816 | 0.343 | 100.0% | 1160.7322 | 1160.3585 | 1 | 7.225 | 77.8% | 1 | R.VLISTDVWAR.G | 2 |

Similarities:
gi|4503529|ref|NP\_001(2:1)  

---

|  |  |  |  |  |  |  |  |  |
| --- | --- | --- | --- | --- | --- | --- | --- | --- |
| U | *gi|150418007|ref|NP\_0* | 15 | 22 | 5.6% | 3224 | 358201 | 6.2 | RAN binding protein 2 [Homo sapiens] |

| Filename XCorr DeltCN Conf% ObsM+H+ CalcM+H+ SpR ZScore Ion% # Sequence  | | | | | | | | | | | | |
| --- | --- | --- | --- | --- | --- | --- | --- | --- | --- | --- | --- | --- |
|  | Astrin\_STLCHLD\_tube2\_061314\_02.13383.13383.3 | 3.8374 | 0.4215 | 100.0% | 2310.2043 | 2309.7266 | 1 | 7.3 | 37.5% | 1 | R.ATNTDLLLAYANLMLLTLSTR.D | 3 |
|  | Astrin\_STLCHLD\_tube2\_061314\_01.11995.11995.2 | 3.1312 | 0.4641 | 100.0% | 1564.6721 | 1565.7637 | 10 | 8.102 | 50.0% | 1 | R.ELLQSFDSALQSVK.S | 2 |
|  | Astrin\_STLCHLD\_061214\_02.06287.06287.3 | 3.9278 | 0.3988 | 100.0% | 1458.2644 | 1456.7031 | 32 | 6.212 | 37.5% | 5 | R.LSQSGHMLLNLSR.G | 3 |
|  | Astrin\_STLCHLD\_tube2\_061314\_01.05921.05921.3 | 2.8511 | 0.3626 | 100.0% | 1337.3944 | 1336.5773 | 1 | 5.543 | 45.0% | 1 | R.LLVQHEINTLR.A | 3 |
|  | Astrin\_STLCHLD\_tube2\_061314\_01.05900.05900.2 | 2.5073 | 0.2045 | 96.4% | 1337.4922 | 1336.5773 | 1 | 4.686 | 80.0% | 1 | R.LLVQHEINTLR.A | 2 |
|  | Astrin\_STLCHLD\_tube2\_061314\_01.06198.06198.2 | 2.7219 | 0.3036 | 98.5% | 1344.9321 | 1345.4111 | 1 | 5.586 | 63.6% | 1 | K.TGSGLNSFYDQR.E | 2 |
| \* | Astrin\_STLCHLD\_tube2\_061314\_02.06292.06292.2 | 4.4747 | 0.5511 | 100.0% | 1814.5521 | 1814.9647 | 1 | 9.903 | 62.5% | 2 | K.NVSGISFTENMGSSQQK.N | 2 |
| \* | Astrin\_STLCHLD\_tube2\_061314\_01.11979.11979.3 | 4.1932 | 0.2796 | 99.7% | 2600.1243 | 2599.9487 | 1 | 6.451 | 33.3% | 1 | R.SFVWHALDYADELPKPEQLAIR.F | 3 |
| \* | Astrin\_STLCHLD\_061214\_01.07940.07940.2 | 3.4417 | 0.2664 | 99.3% | 1540.4521 | 1540.6726 | 1 | 6.405 | 57.7% | 3 | K.SDAGNLNFEFQVAK.K | 2 |
| \* | Astrin\_STLCHLD\_tube2\_061314\_01.07724.07724.3 | 3.5595 | 0.507 | 100.0% | 1692.3243 | 1691.8839 | 1 | 8.615 | 44.6% | 1 | K.FGQGDLPKPINSDFR.S | 3 |
| \* | Astrin\_STLCHLD\_tube2\_061314\_01.08846.08846.2 | 2.268 | 0.3712 | 98.6% | 1004.1322 | 1004.14557 | 1 | 6.438 | 81.2% | 1 | K.SGFEGMFTK.K | 2 |
| \* | Astrin\_STLCHLD\_tube2\_061314\_01.06463.06463.2 | 2.5088 | 0.2883 | 98.1% | 1132.3322 | 1132.3197 | 1 | 5.592 | 72.2% | 1 | K.SGFEGMFTKK.E | 2 |
|  | Astrin\_STLCHLD\_tube2\_061314\_01.06740.06740.2 | 2.2832 | 0.1923 | 95.4% | 1110.7122 | 1110.2114 | 2 | 3.972 | 75.0% | 1 | R.FDAEVSQWK.E | 2 |
|  | Astrin\_STLCHLD\_tube2\_061314\_02.06616.06616.2 | 3.3175 | 0.3655 | 100.0% | 1202.1721 | 1202.3934 | 1 | 8.015 | 80.0% | 1 | K.IAVAVLEETTR.E | 2 |
| \* | Astrin\_STLCHLD\_061214\_02.05224.05224.2 | 2.2939 | 0.3681 | 98.3% | 1112.7122 | 1112.2712 | 1 | 6.235 | 65.0% | 1 | K.GHVSLAAELSK.E | 2 |

---

|  |  |  |  |  |  |  |  |  |
| --- | --- | --- | --- | --- | --- | --- | --- | --- |
| U | *gi|21264343|ref|NP\_00* | 3 | 4 | 5.6% | 915 | 102642 | 5.5 | scaffold attachment factor B [Homo sapiens] |

| Filename XCorr DeltCN Conf% ObsM+H+ CalcM+H+ SpR ZScore Ion% # Sequence  | | | | | | | | | | | | |
| --- | --- | --- | --- | --- | --- | --- | --- | --- | --- | --- | --- | --- |
|  | Astrin\_STLCHLD\_tube2\_061314\_01.07266.07266.3 | 4.3464 | 0.3011 | 100.0% | 2849.6643 | 2849.9788 | 1 | 6.104 | 39.0% | 1 | K.SEPVKEESSELEQPFAQDTSSVGPDR.K | 33 |
| \* | Astrin\_STLCHLD\_tube2\_061314\_01.09998.09998.2 | 2.7779 | 0.3777 | 99.7% | 1354.7722 | 1355.4929 | 1 | 5.867 | 72.7% | 2 | R.NFWVSGLSSTTR.A | 2 |
| \* | Astrin\_STLCHLD\_tube2\_061314\_01.05367.05367.2 | 3.5114 | 0.3736 | 100.0% | 1412.1921 | 1412.5348 | 1 | 7.226 | 75.0% | 1 | R.SWQGTADGGMMDR.D | 2 |

Similarities:
gi|7661936|ref|NP\_055(1:2)  

---

|  |  |  |  |  |  |  |  |  |
| --- | --- | --- | --- | --- | --- | --- | --- | --- |
| U | *gi|19923142|ref|NP\_00* | 3 | 3 | 5.4% | 876 | 97170 | 4.8 | karyopherin beta 1 [Homo sapiens] |

| Filename XCorr DeltCN Conf% ObsM+H+ CalcM+H+ SpR ZScore Ion% # Sequence  | | | | | | | | | | | | |
| --- | --- | --- | --- | --- | --- | --- | --- | --- | --- | --- | --- | --- |
| \* | Astrin\_STLCHLD\_tube2\_061314\_01.13527.13527.2 | 4.1396 | 0.3566 | 100.0% | 1659.5322 | 1659.9231 | 1 | 7.008 | 57.1% | 1 | R.AAVENLPTFLVELSR.V | 2 |
| \* | Astrin\_STLCHLD\_tube2\_061314\_01.12111.12111.2 | 3.9449 | 0.4534 | 100.0% | 1606.8922 | 1606.8595 | 1 | 7.554 | 53.6% | 1 | K.LAATNALLNSLEFTK.A | 2 |
| \* | Astrin\_STLCHLD\_tube2\_061314\_02.06056.06056.3 | 3.4741 | 0.2243 | 98.6% | 2001.3844 | 2001.2218 | 1 | 5.34 | 46.9% | 1 | R.LQQVLQMESHIQSTSDR.I | 3 |

---

|  |  |  |  |  |  |  |  |  |
| --- | --- | --- | --- | --- | --- | --- | --- | --- |
| U | *gi|4826686|ref|NP\_004* | 2 | 2 | 5.3% | 740 | 82432 | 7.2 | DEAD (Asp-Glu-Ala-Asp) box polypeptide 1 [Homo sapiens] |

| Filename XCorr DeltCN Conf% ObsM+H+ CalcM+H+ SpR ZScore Ion% # Sequence  | | | | | | | | | | | | |
| --- | --- | --- | --- | --- | --- | --- | --- | --- | --- | --- | --- | --- |
| \* | Astrin\_STLCHLD\_tube2\_061314\_01.14654.14654.2 | 3.5495 | 0.2645 | 99.3% | 2374.412 | 2373.6245 | 1 | 5.981 | 37.5% | 1 | R.FLVLDEADGLLSQGYSDFINR.M | 2 |
| \* | Astrin\_STLCHLD\_tube2\_061314\_01.12434.12434.2 | 2.0946 | 0.3201 | 95.4% | 2134.6921 | 2135.4302 | 27 | 4.999 | 29.4% | 1 | K.EAQTSFLHLGYLPNQLFR.T | 2 |

---

|  |  |  |  |  |  |  |  |  |
| --- | --- | --- | --- | --- | --- | --- | --- | --- |
| U | *gi|4503481|ref|NP\_001* | 2 | 5 | 5.3% | 437 | 50119 | 6.7 | eukaryotic translation elongation factor 1 gamma [Homo sapiens] |

| Filename XCorr DeltCN Conf% ObsM+H+ CalcM+H+ SpR ZScore Ion% # Sequence  | | | | | | | | | | | | |
| --- | --- | --- | --- | --- | --- | --- | --- | --- | --- | --- | --- | --- |
| \* | Astrin\_STLCHLD\_061214\_02.05633.05633.2 | 4.1236 | 0.4967 | 100.0% | 1348.4122 | 1348.5448 | 1 | 9.154 | 66.7% | 4 | K.ALIAAQYSGAQVR.V | 2 |
| \* | Astrin\_STLCHLD\_tube2\_061314\_01.08454.08454.2 | 2.2555 | 0.3304 | 98.0% | 1242.0122 | 1242.4172 | 1 | 7.182 | 72.2% | 1 | K.STFVLDEFKR.K | 2 |

---

|  |  |  |  |  |  |  |  |  |
| --- | --- | --- | --- | --- | --- | --- | --- | --- |
| U | *gi|41322908|ref|NP\_95* | 16 | 22 | 5.2% | 4525 | 513712 | 5.8 | plectin 1 isoform 3 [Homo sapiens] |
| U | *gi|47607492|ref|NP\_00* | 16 | 22 | 5.2% | 4574 | 518478 | 5.7 | plectin 1 isoform 1 [Homo sapiens] |
| U | *gi|41322923|ref|NP\_95* | 16 | 22 | 5.2% | 4547 | 516204 | 5.8 | plectin 1 isoform 11 [Homo sapiens] |
| U | *gi|41322919|ref|NP\_95* | 16 | 22 | 5.2% | 4547 | 516282 | 5.8 | plectin 1 isoform 8 [Homo sapiens] |
| U | *gi|41322916|ref|NP\_95* | 16 | 22 | 5.1% | 4684 | 531796 | 6.0 | plectin 1 isoform 6 [Homo sapiens] |
| U | *gi|41322914|ref|NP\_95* | 16 | 22 | 5.2% | 4551 | 516484 | 5.8 | plectin 1 isoform 10 [Homo sapiens] |
| U | *gi|41322912|ref|NP\_95* | 16 | 22 | 5.2% | 4533 | 514780 | 5.7 | plectin 1 isoform 2 [Homo sapiens] |
| U | *gi|41322910|ref|NP\_95* | 16 | 22 | 5.2% | 4515 | 512609 | 5.8 | plectin 1 isoform 7 [Homo sapiens] |

| Filename XCorr DeltCN Conf% ObsM+H+ CalcM+H+ SpR ZScore Ion% # Sequence  | | | | | | | | | | | | |
| --- | --- | --- | --- | --- | --- | --- | --- | --- | --- | --- | --- | --- |
|  | Astrin\_STLCHLD\_tube2\_061314\_01.09143.09143.2 | 2.5283 | 0.29 | 97.2% | 1532.5322 | 1532.8235 | 11 | 5.178 | 46.4% | 1 | K.VLALPEPSPAAPTLR.S | 2 |
|  | Astrin\_STLCHLD\_tube2\_061314\_01.07231.07231.2 | 2.8965 | 0.284 | 99.0% | 1288.8922 | 1287.4612 | 14 | 5.499 | 60.0% | 2 | R.WQAVLAQTDVR.Q | 2 |
|  | Astrin\_STLCHLD\_tube2\_061314\_02.08129.08129.2 | 3.058 | 0.3666 | 99.8% | 1810.2322 | 1809.9707 | 1 | 5.954 | 46.7% | 2 | K.VQSGSESVIQEYVDLR.T | 2 |
|  | Astrin\_STLCHLD\_tube2\_061314\_01.05250.05250.2 | 2.8466 | 0.1241 | 96.9% | 1043.6322 | 1043.2083 | 1 | 5.312 | 81.2% | 1 | R.LAAEQELIR.L | 2 |
|  | Astrin\_STLCHLD\_tube2\_061314\_01.08930.08930.2 | 3.0618 | 0.3801 | 100.0% | 1519.9521 | 1519.738 | 1 | 7.117 | 70.8% | 1 | K.AKLEQLFQDEVAK.A | 2 |
|  | Astrin\_STLCHLD\_061214\_01.05713.05713.3 | 3.8376 | 0.1846 | 98.9% | 1693.5844 | 1692.9156 | 6 | 4.596 | 50.0% | 1 | R.LREQLQLLEEQHR.A | 3 |
|  | Astrin\_STLCHLD\_061214\_02.04693.04693.3 | 2.826 | 0.2652 | 97.0% | 1783.6144 | 1784.964 | 1 | 5.148 | 36.8% | 1 | R.AALAHSEEVTASQVAATK.T | 3 |
|  | Astrin\_STLCHLD\_tube2\_061314\_02.06924.06924.2 | 4.8155 | 0.3755 | 100.0% | 1558.1122 | 1557.744 | 1 | 7.915 | 73.1% | 4 | R.LQEAGILSAEELQR.L | 2 |
|  | Astrin\_STLCHLD\_061214\_01.06595.06595.3 | 4.4241 | 0.4301 | 100.0% | 2014.4043 | 2014.292 | 1 | 6.563 | 37.5% | 1 | R.LLEAQIATGGVIDPVHSHR.V | 33 |
|  | Astrin\_STLCHLD\_tube2\_061314\_01.06297.06297.2 | 2.1372 | 0.3926 | 98.5% | 1161.0922 | 1161.2311 | 1 | 7.483 | 75.0% | 1 | R.GYFDEEMNR.V | 22 |
|  | Astrin\_STLCHLD\_061214\_02.06849.06849.2 | 3.8587 | 0.4379 | 100.0% | 1614.6921 | 1614.8363 | 1 | 7.688 | 56.7% | 1 | R.LLDAQLSTGGIVDPSK.S | 2 |
|  | Astrin\_STLCHLD\_tube2\_061314\_01.10922.10922.2 | 3.6225 | 0.5109 | 100.0% | 1566.7522 | 1567.8259 | 1 | 8.785 | 73.3% | 1 | R.APVPASELLASGVLSR.A | 2 |
|  | Astrin\_STLCHLD\_061214\_01.07137.07137.3 | 3.2706 | 0.211 | 96.8% | 2029.9443 | 2028.3188 | 54 | 4.583 | 27.8% | 1 | R.LLEAQIATGGIIDPVHSHR.V | 33 |
|  | Astrin\_STLCHLD\_061214\_02.07247.07247.2 | 2.1525 | 0.3034 | 95.4% | 1538.5122 | 1539.7721 | 1 | 5.697 | 53.6% | 1 | R.LLDAQLATGGIVDPR.L | 2 |
|  | Astrin\_STLCHLD\_tube2\_061314\_02.06843.06843.2 | 3.2366 | 0.2693 | 99.1% | 1462.6721 | 1462.6611 | 1 | 5.581 | 66.7% | 2 | R.SQVMDEATALQLR.E | 2 |
|  | Astrin\_STLCHLD\_tube2\_061314\_01.13937.13937.2 | 3.6794 | 0.4483 | 100.0% | 2115.912 | 2116.3533 | 1 | 8.447 | 40.0% | 1 | R.AGTLSITEFADMLSGNAGGFR.S | 2 |

Similarities:
gi|207452735|ref|NP\_1(3:13)  

---

|  |  |  |  |  |  |  |  |  |
| --- | --- | --- | --- | --- | --- | --- | --- | --- |
| U | *gi|221316723|ref|NP\_0* | 4 | 5 | 5.2% | 1025 | 115704 | 8.3 | N-acetyltransferase 10 isoform a [Homo sapiens] |
| U | *gi|221316741|ref|NP\_0* | 4 | 5 | 5.6% | 953 | 107271 | 7.0 | N-acetyltransferase 10 isoform b [Homo sapiens] |

| Filename XCorr DeltCN Conf% ObsM+H+ CalcM+H+ SpR ZScore Ion% # Sequence  | | | | | | | | | | | | |
| --- | --- | --- | --- | --- | --- | --- | --- | --- | --- | --- | --- | --- |
|  | Astrin\_STLCHLD\_061214\_01.05546.05546.2 | 2.5899 | 0.356 | 98.8% | 1411.8121 | 1412.5858 | 1 | 6.416 | 72.7% | 1 | R.TLHEVSLQESIR.Y | 2 |
|  | Astrin\_STLCHLD\_061214\_01.05515.05515.3 | 3.6768 | 0.294 | 100.0% | 1412.7244 | 1412.5858 | 11 | 5.142 | 45.5% | 2 | R.TLHEVSLQESIR.Y | 3 |
|  | Astrin\_STLCHLD\_tube2\_061314\_01.10653.10653.2 | 2.6059 | 0.306 | 98.0% | 1454.7922 | 1454.6659 | 1 | 5.419 | 58.3% | 1 | R.LDYLGVSYGLTPR.L | 2 |
|  | Astrin\_STLCHLD\_tube2\_061314\_01.18144.18144.3 | 4.4898 | 0.4008 | 100.0% | 2969.8145 | 2969.4973 | 1 | 6.779 | 28.7% | 1 | R.IYFLNQLGDLALSAAQSALLLGIGLQHK.S | 3 |

---

|  |  |  |  |  |  |  |  |  |
| --- | --- | --- | --- | --- | --- | --- | --- | --- |
| U | *gi|20127499|ref|NP\_00* | 2 | 3 | 5.2% | 344 | 39587 | 11.4 | arginine/serine-rich splicing factor 6 [Homo sapiens] |

| Filename XCorr DeltCN Conf% ObsM+H+ CalcM+H+ SpR ZScore Ion% # Sequence  | | | | | | | | | | | | |
| --- | --- | --- | --- | --- | --- | --- | --- | --- | --- | --- | --- | --- |
|  | Astrin\_STLCHLD\_tube2\_061314\_01.05262.05262.2 | 2.7427 | 0.2909 | 99.1% | 1031.3322 | 1031.1973 | 1 | 5.559 | 87.5% | 2 | R.LIVENLSSR.C | 2 |
| \* | Astrin\_STLCHLD\_tube2\_061314\_01.06018.06018.2 | 2.4406 | 0.2649 | 98.0% | 1064.6322 | 1065.171 | 1 | 6.412 | 81.2% | 1 | R.TNEGVIEFR.S | 2 |

---

|  |  |  |  |  |  |  |  |  |
| --- | --- | --- | --- | --- | --- | --- | --- | --- |
| U | *gi|74048514|ref|NP\_73* | 8 | 12 | 5.1% | 2342 | 265290 | 5.4 | cancer susceptibility candidate 5 isoform 1 [Homo sapiens] |
| U | *gi|74048554|ref|NP\_65* | 8 | 12 | 5.1% | 2316 | 262530 | 5.4 | cancer susceptibility candidate 5 isoform 2 [Homo sapiens] |

| Filename XCorr DeltCN Conf% ObsM+H+ CalcM+H+ SpR ZScore Ion% # Sequence  | | | | | | | | | | | | |
| --- | --- | --- | --- | --- | --- | --- | --- | --- | --- | --- | --- | --- |
|  | Astrin\_STLCHLD\_tube2\_061314\_01.07685.07685.2 | 2.2979 | 0.3658 | 98.5% | 1086.6322 | 1086.2303 | 7 | 6.277 | 61.1% | 1 | K.GLLDNPISEK.S | 2 |
|  | Astrin\_STLCHLD\_tube2\_061314\_01.11136.11136.2 | 2.5498 | 0.3184 | 98.4% | 1222.8522 | 1223.4117 | 1 | 5.996 | 65.0% | 1 | K.IDTTSFLANLK.L | 2 |
|  | Astrin\_STLCHLD\_061214\_01.05623.05623.2 | 4.0594 | 0.5134 | 100.0% | 1501.4122 | 1501.6484 | 1 | 8.447 | 87.5% | 4 | K.TIYSGEENMDITK.S | 2 |
|  | Astrin\_STLCHLD\_tube2\_061314\_01.10431.10431.2 | 3.5789 | 0.4193 | 100.0% | 1772.3922 | 1772.9963 | 1 | 8.164 | 56.7% | 1 | R.IQQSLSNPLSISLTDR.K | 2 |
|  | Astrin\_STLCHLD\_tube2\_061314\_01.09989.09989.3 | 3.58 | 0.2705 | 99.7% | 1964.2144 | 1964.1852 | 468 | 6.348 | 30.0% | 1 | K.NSLTDTWNKDKDWVLK.I | 3 |
|  | Astrin\_STLCHLD\_tube2\_061314\_01.13790.13790.2 | 3.3453 | 0.2561 | 98.8% | 2794.172 | 2793.2354 | 20 | 5.047 | 29.2% | 1 | K.TGEFLAFQTVHLPPLPEQLLELGNK.A | 2 |
|  | Astrin\_STLCHLD\_tube2\_061314\_01.06062.06062.2 | 2.6224 | 0.2231 | 97.5% | 1213.1721 | 1213.3508 | 1 | 4.522 | 83.3% | 1 | R.SSQMESQFLR.D | 2 |
|  | Astrin\_STLCHLD\_tube2\_061314\_01.08019.08019.3 | 4.5132 | 0.3964 | 100.0% | 2174.5444 | 2174.3696 | 1 | 8.018 | 38.2% | 2 | R.AAEKELEQLKTEEEELQR.N | 3 |

---

|  |  |  |  |  |  |  |  |  |
| --- | --- | --- | --- | --- | --- | --- | --- | --- |
| U | *gi|154800453|ref|NP\_0* | 2 | 4 | 4.9% | 778 | 83857 | 7.4 | tastin isoform 1 [Homo sapiens] |

| Filename XCorr DeltCN Conf% ObsM+H+ CalcM+H+ SpR ZScore Ion% # Sequence  | | | | | | | | | | | | |
| --- | --- | --- | --- | --- | --- | --- | --- | --- | --- | --- | --- | --- |
| \* | Astrin\_STLCHLD\_tube2\_061314\_02.05738.05738.3 | 3.6692 | 0.3616 | 100.0% | 1926.0543 | 1926.0935 | 4 | 6.032 | 36.1% | 2 | R.EVVTHSDEGGVASLGLAQR.V | 3 |
| \* | Astrin\_STLCHLD\_tube2\_061314\_02.05505.05505.3 | 4.3897 | 0.3999 | 100.0% | 1991.2144 | 1991.2113 | 1 | 7.184 | 33.3% | 2 | R.TLNATEHNSGTSHLPGLLK.H | 3 |

---

|  |  |  |  |  |  |  |  |  |
| --- | --- | --- | --- | --- | --- | --- | --- | --- |
| U | *gi|148612849|ref|NP\_0* | 2 | 2 | 4.7% | 744 | 84089 | 6.4 | kinesin heavy chain member 2 isoform 2 [Homo sapiens] |
| U | *gi|148612877|ref|NP\_0* | 2 | 2 | 5.0% | 706 | 79955 | 6.7 | kinesin heavy chain member 2 isoform 1 [Homo sapiens] |

| Filename XCorr DeltCN Conf% ObsM+H+ CalcM+H+ SpR ZScore Ion% # Sequence  | | | | | | | | | | | | |
| --- | --- | --- | --- | --- | --- | --- | --- | --- | --- | --- | --- | --- |
|  | Astrin\_STLCHLD\_tube2\_061314\_01.06677.06677.3 | 3.0464 | 0.2089 | 95.3% | 2057.5745 | 2057.2273 | 17 | 4.231 | 30.9% | 1 | R.GSLDYRPLTTADPIDEHR.I | 3 |
|  | Astrin\_STLCHLD\_tube2\_061314\_02.05458.05458.3 | 3.2472 | 0.2408 | 98.3% | 1925.7244 | 1926.1802 | 23 | 4.663 | 35.9% | 1 | R.VLEDGKQQVQVVGLQER.E | 3 |

---

|  |  |  |  |  |  |  |  |  |
| --- | --- | --- | --- | --- | --- | --- | --- | --- |
| U | *gi|167466272|ref|NP\_6* | 3 | 4 | 4.7% | 745 | 83587 | 9.8 | cytoskeleton associated protein 2-like [Homo sapiens] |

| Filename XCorr DeltCN Conf% ObsM+H+ CalcM+H+ SpR ZScore Ion% # Sequence  | | | | | | | | | | | | |
| --- | --- | --- | --- | --- | --- | --- | --- | --- | --- | --- | --- | --- |
| \* | Astrin\_STLCHLD\_tube2\_061314\_01.07921.07921.2 | 2.9724 | 0.2016 | 97.5% | 1556.3522 | 1555.6873 | 1 | 5.471 | 66.7% | 1 | R.TYPSLLQGEYNNR.H | 2 |
| \* | Astrin\_STLCHLD\_tube2\_061314\_01.08948.08948.2 | 3.5942 | 0.3351 | 100.0% | 1272.0521 | 1271.4594 | 1 | 6.967 | 70.0% | 2 | K.VVLNILQDSNR.T | 2 |
| \* | Astrin\_STLCHLD\_tube2\_061314\_01.06188.06188.2 | 2.8117 | 0.3226 | 99.3% | 1261.4722 | 1262.4817 | 6 | 5.33 | 60.0% | 1 | R.INGMPEVQDMK.F | 2 |

---

|  |  |  |  |  |  |  |  |  |
| --- | --- | --- | --- | --- | --- | --- | --- | --- |
| U | *contaminant\_KERATIN10* | 2 | 5 | 4.5% | 400 | 44106 | 5.1 | no description |
| U | *gi|24234699|ref|NP\_00* | 2 | 5 | 4.5% | 400 | 44106 | 5.1 | keratin 19 [Homo sapiens] |

| Filename XCorr DeltCN Conf% ObsM+H+ CalcM+H+ SpR ZScore Ion% # Sequence  | | | | | | | | | | | | |
| --- | --- | --- | --- | --- | --- | --- | --- | --- | --- | --- | --- | --- |
|  | Astrin\_STLCHLD\_tube2\_061314\_01.05939.05939.2 | 3.1982 | 0.1994 | 99.3% | 1042.1921 | 1042.2235 | 2 | 6.692 | 87.5% | 3 | R.IVLQIDNAR.L | 22 |
|  | Astrin\_STLCHLD\_tube2\_061314\_01.08023.08023.2 | 3.2303 | 0.3839 | 100.0% | 1030.1122 | 1030.2096 | 2 | 6.85 | 81.2% | 2 | R.VLDELTLAR.T | 2222 |

Similarities:
contaminant\_KERATIN09(1:1)  
contaminant\_KERATIN12(1:1)  
contaminant\_KERATIN05(1:1)  
contaminant\_KERATIN08(1:1)  

---

|  |  |  |  |  |  |  |  |  |
| --- | --- | --- | --- | --- | --- | --- | --- | --- |
| U | *gi|26051235|ref|NP\_06* | 3 | 3 | 4.4% | 1156 | 128979 | 5.1 | nucleoporin 133kDa [Homo sapiens] |

| Filename XCorr DeltCN Conf% ObsM+H+ CalcM+H+ SpR ZScore Ion% # Sequence  | | | | | | | | | | | | |
| --- | --- | --- | --- | --- | --- | --- | --- | --- | --- | --- | --- | --- |
| \* | Astrin\_STLCHLD\_061214\_01.05388.05388.3 | 3.8303 | 0.2393 | 99.6% | 1904.7843 | 1905.1351 | 1 | 5.744 | 48.3% | 1 | R.MFPHHSITESVNYDVK.T | 3 |
| \* | Astrin\_STLCHLD\_tube2\_061314\_01.07101.07101.3 | 3.1617 | 0.2942 | 99.2% | 1943.1244 | 1943.2773 | 1 | 6.066 | 38.2% | 1 | K.IHQHILPQGQGMLSGIGR.K | 3 |
| \* | Astrin\_STLCHLD\_tube2\_061314\_01.13999.13999.3 | 3.5499 | 0.4469 | 100.0% | 1975.9744 | 1976.3062 | 1 | 7.005 | 32.8% | 1 | K.AHSFLMDFIHQVGLFGR.L | 3 |

---

|  |  |  |  |  |  |  |  |  |
| --- | --- | --- | --- | --- | --- | --- | --- | --- |
| U | *gi|148664201|ref|NP\_0* | 2 | 2 | 4.4% | 683 | 76987 | 9.4 | cytoskeleton associated protein 2 isoform 2 [Homo sapiens] |
| U | *gi|148664244|ref|NP\_0* | 2 | 2 | 4.4% | 682 | 76900 | 9.4 | cytoskeleton associated protein 2 isoform 1 [Homo sapiens] |

| Filename XCorr DeltCN Conf% ObsM+H+ CalcM+H+ SpR ZScore Ion% # Sequence  | | | | | | | | | | | | |
| --- | --- | --- | --- | --- | --- | --- | --- | --- | --- | --- | --- | --- |
|  | Astrin\_STLCHLD\_tube2\_061314\_01.05699.05699.3 | 2.9053 | 0.322 | 99.4% | 1946.4543 | 1945.229 | 18 | 5.807 | 34.4% | 1 | K.FVSTTSQNTQLVRPPIR.S | 3 |
|  | Astrin\_STLCHLD\_tube2\_061314\_01.08071.08071.2 | 2.6407 | 0.3987 | 99.4% | 1398.7722 | 1399.6483 | 1 | 7.233 | 66.7% | 1 | K.AILAGAQPIEEMR.H | 2 |

---

|  |  |  |  |  |  |  |  |  |
| --- | --- | --- | --- | --- | --- | --- | --- | --- |
| U | *gi|38016914|ref|NP\_05* | 2 | 2 | 4.3% | 626 | 72201 | 7.1 | SAM domain- and HD domain-containing protein 1 [Homo sapiens] |

| Filename XCorr DeltCN Conf% ObsM+H+ CalcM+H+ SpR ZScore Ion% # Sequence  | | | | | | | | | | | | |
| --- | --- | --- | --- | --- | --- | --- | --- | --- | --- | --- | --- | --- |
| \* | Astrin\_STLCHLD\_tube2\_061314\_01.09325.09325.3 | 3.2544 | 0.406 | 100.0% | 1726.5243 | 1725.9165 | 1 | 6.851 | 44.2% | 1 | R.DKEVGNLYDMFHTR.N | 3 |
| \* | Astrin\_STLCHLD\_tube2\_061314\_01.08887.08887.2 | 2.6234 | 0.1986 | 96.1% | 1458.4722 | 1458.6233 | 5 | 4.201 | 54.2% | 1 | R.ISTAIDDMEAYTK.L | 2 |

---

|  |  |  |  |  |  |  |  |  |
| --- | --- | --- | --- | --- | --- | --- | --- | --- |
| U | *gi|7661936|ref|NP\_055* | 2 | 2 | 4.1% | 953 | 107474 | 6.2 | scaffold attachment factor B2 [Homo sapiens] |

| Filename XCorr DeltCN Conf% ObsM+H+ CalcM+H+ SpR ZScore Ion% # Sequence  | | | | | | | | | | | | |
| --- | --- | --- | --- | --- | --- | --- | --- | --- | --- | --- | --- | --- |
|  | Astrin\_STLCHLD\_tube2\_061314\_01.07266.07266.3 | 4.3464 | 0.3011 | 100.0% | 2849.6643 | 2849.9788 | 1 | 6.104 | 39.0% | 1 | K.SEPVKEESSELEQPFAQDTSSVGPDR.K | 33 |
| \* | Astrin\_STLCHLD\_061214\_01.05101.05101.2 | 2.1003 | 0.3149 | 95.8% | 1292.5521 | 1292.4119 | 10 | 5.275 | 58.3% | 1 | R.AWQGAMDAGAASR.E | 2 |

Similarities:
gi|21264343|ref|NP\_00(1:1)  

---

|  |  |  |  |  |  |  |  |  |
| --- | --- | --- | --- | --- | --- | --- | --- | --- |
| U | *gi|5032179|ref|NP\_005* | 2 | 2 | 4.0% | 835 | 88550 | 5.8 | tripartite motif-containing 28 protein [Homo sapiens] |

| Filename XCorr DeltCN Conf% ObsM+H+ CalcM+H+ SpR ZScore Ion% # Sequence  | | | | | | | | | | | | |
| --- | --- | --- | --- | --- | --- | --- | --- | --- | --- | --- | --- | --- |
| \* | Astrin\_STLCHLD\_061214\_02.08168.08168.2 | 2.9321 | 0.2998 | 98.5% | 1954.4521 | 1954.187 | 31 | 5.345 | 43.8% | 1 | K.VFPGSTTEDYNLIVIER.G | 2 |
| \* | Astrin\_STLCHLD\_061214\_02.06634.06634.3 | 3.209 | 0.2085 | 96.9% | 1786.4343 | 1787.0232 | 3 | 4.691 | 41.7% | 1 | K.LTEDKADVQSIIGLQR.F | 3 |

---

|  |  |  |  |  |  |  |  |  |
| --- | --- | --- | --- | --- | --- | --- | --- | --- |
| U | *gi|41406064|ref|NP\_00* | 7 | 10 | 3.8% | 1976 | 228997 | 5.5 | myosin, heavy polypeptide 10, non-muscle [Homo sapiens] |

| Filename XCorr DeltCN Conf% ObsM+H+ CalcM+H+ SpR ZScore Ion% # Sequence  | | | | | | | | | | | | |
| --- | --- | --- | --- | --- | --- | --- | --- | --- | --- | --- | --- | --- |
|  | Astrin\_STLCHLD\_tube2\_061314\_01.11798.11798.3 | 3.5308 | 0.177 | 96.3% | 2468.6042 | 2468.7893 | 1 | 5.224 | 37.5% | 1 | K.LQQLFNHTMFILEQEEYQR.E | 33 |
|  | Astrin\_STLCHLD\_061214\_01.07250.07250.2 | 2.6822 | 0.2747 | 98.2% | 1398.6721 | 1398.6166 | 1 | 5.315 | 75.0% | 1 | K.VDYKADEWLMK.N | 22 |
|  | Astrin\_STLCHLD\_tube2\_061314\_01.05545.05545.2 | 3.5792 | 0.372 | 100.0% | 1225.5122 | 1224.3591 | 1 | 6.298 | 75.0% | 1 | R.AGVLAHLEEER.D | 22 |
|  | Astrin\_STLCHLD\_tube2\_061314\_01.05081.05081.2 | 2.9194 | 0.1221 | 96.9% | 1220.8522 | 1221.3959 | 9 | 4.855 | 66.7% | 1 | K.KFDQLLAEEK.S | 22 |
|  | Astrin\_STLCHLD\_tube2\_061314\_01.05537.05537.2 | 2.7381 | 0.2319 | 98.4% | 1093.4321 | 1093.2218 | 1 | 6.533 | 81.2% | 3 | K.FDQLLAEEK.S | 22 |
| \* | Astrin\_STLCHLD\_tube2\_061314\_01.05569.05569.2 | 2.9142 | 0.2643 | 98.9% | 1233.6522 | 1233.382 | 1 | 5.757 | 77.8% | 2 | R.ALEQQVEEMR.T | 2 |
| \* | Astrin\_STLCHLD\_061214\_01.04448.04448.2 | 3.3835 | 0.3127 | 99.9% | 1520.8121 | 1520.5498 | 4 | 6.788 | 50.0% | 1 | R.ELDDATEANEGLSR.E | 2 |

Similarities:
gi|12667788|ref|NP\_00(5:2)  

---

|  |  |  |  |  |  |  |  |  |
| --- | --- | --- | --- | --- | --- | --- | --- | --- |
| U | *gi|194239723|ref|NP\_0* | 2 | 3 | 3.7% | 647 | 71408 | 6.4 | eukaryotic translation elongation factor 1 delta isoform 1 [Homo sapiens] |
| U | *gi|25453474|ref|NP\_11* | 2 | 3 | 3.7% | 647 | 71408 | 6.4 | eukaryotic translation elongation factor 1 delta isoform 1 [Homo sapiens] |
| U | *gi|25453472|ref|NP\_00* | 2 | 3 | 8.5% | 281 | 31122 | 5.0 | eukaryotic translation elongation factor 1 delta isoform 2 [Homo sapiens] |
| U | *gi|194239731|ref|NP\_0* | 2 | 3 | 8.5% | 281 | 31122 | 5.0 | eukaryotic translation elongation factor 1 delta isoform 2 [Homo sapiens] |
| U | *gi|194239729|ref|NP\_0* | 2 | 3 | 9.3% | 257 | 28558 | 4.9 | eukaryotic translation elongation factor 1 delta isoform 4 [Homo sapiens] |
| U | *gi|194239727|ref|NP\_0* | 2 | 3 | 8.5% | 281 | 31122 | 5.0 | eukaryotic translation elongation factor 1 delta isoform 2 [Homo sapiens] |
| U | *gi|194239725|ref|NP\_0* | 2 | 3 | 3.7% | 646 | 71266 | 6.4 | eukaryotic translation elongation factor 1 delta isoform 3 [Homo sapiens] |

| Filename XCorr DeltCN Conf% ObsM+H+ CalcM+H+ SpR ZScore Ion% # Sequence  | | | | | | | | | | | | |
| --- | --- | --- | --- | --- | --- | --- | --- | --- | --- | --- | --- | --- |
|  | Astrin\_STLCHLD\_tube2\_061314\_01.06039.06039.2 | 3.5467 | 0.4087 | 100.0% | 1359.6322 | 1359.5223 | 1 | 7.204 | 77.3% | 2 | R.IASLEVENQSLR.G | 2 |
|  | Astrin\_STLCHLD\_tube2\_061314\_01.11217.11217.2 | 3.0382 | 0.3657 | 100.0% | 1300.6522 | 1300.4978 | 1 | 5.958 | 68.2% | 1 | R.GVVQELQQAISK.L | 2 |

---

|  |  |  |  |  |  |  |  |  |
| --- | --- | --- | --- | --- | --- | --- | --- | --- |
| U | *gi|41872631|ref|NP\_00* | 7 | 8 | 3.6% | 2511 | 273424 | 6.4 | fatty acid synthase [Homo sapiens] |

| Filename XCorr DeltCN Conf% ObsM+H+ CalcM+H+ SpR ZScore Ion% # Sequence  | | | | | | | | | | | | |
| --- | --- | --- | --- | --- | --- | --- | --- | --- | --- | --- | --- | --- |
| \* | Astrin\_STLCHLD\_tube2\_061314\_01.09303.09303.2 | 2.2444 | 0.2349 | 95.4% | 1254.0322 | 1252.4148 | 1 | 5.232 | 65.0% | 1 | R.FDASFFGVHPK.Q | 2 |
| \* | Astrin\_STLCHLD\_061214\_01.05283.05283.3 | 4.2461 | 0.4295 | 100.0% | 1889.9944 | 1889.1215 | 1 | 7.727 | 38.9% | 1 | R.VTVAGGVHISGLHTESAPR.R | 3 |
| \* | Astrin\_STLCHLD\_tube2\_061314\_02.07386.07386.2 | 2.8324 | 0.2784 | 98.5% | 1407.5721 | 1407.6709 | 2 | 6.404 | 63.6% | 1 | K.VLQGDLVMNVYR.D | 2 |
| \* | Astrin\_STLCHLD\_tube2\_061314\_01.08576.08576.2 | 3.21 | 0.4082 | 100.0% | 1469.5922 | 1470.5815 | 1 | 6.755 | 75.0% | 2 | R.FPQLDSTSFANSR.D | 2 |
| \* | Astrin\_STLCHLD\_tube2\_061314\_01.09685.09685.2 | 2.7191 | 0.3129 | 98.4% | 1427.6921 | 1427.702 | 20 | 5.507 | 54.2% | 1 | R.SLLVNPEGPTLMR.L | 2 |
| \* | Astrin\_STLCHLD\_tube2\_061314\_01.15228.15228.2 | 3.988 | 0.3302 | 100.0% | 2424.4722 | 2423.769 | 5 | 6.899 | 31.8% | 1 | R.TLLEGSGLESIISIIHSSLAEPR.V | 2 |
| \* | Astrin\_STLCHLD\_tube2\_061314\_01.15209.15209.3 | 2.9741 | 0.236 | 95.2% | 2425.9744 | 2423.769 | 1 | 4.931 | 29.5% | 1 | R.TLLEGSGLESIISIIHSSLAEPR.V | 3 |

---

|  |  |  |  |  |  |  |  |  |
| --- | --- | --- | --- | --- | --- | --- | --- | --- |
| U | *gi|21361368|ref|NP\_00* | 2 | 2 | 3.6% | 795 | 87302 | 7.1 | pyrroline-5-carboxylate synthetase isoform 1 [Homo sapiens] |
| U | *gi|62912457|ref|NP\_00* | 2 | 2 | 3.7% | 793 | 87089 | 7.1 | pyrroline-5-carboxylate synthetase isoform 2 [Homo sapiens] |

| Filename XCorr DeltCN Conf% ObsM+H+ CalcM+H+ SpR ZScore Ion% # Sequence  | | | | | | | | | | | | |
| --- | --- | --- | --- | --- | --- | --- | --- | --- | --- | --- | --- | --- |
|  | Astrin\_STLCHLD\_061214\_01.07869.07869.2 | 2.3124 | 0.2803 | 95.8% | 1773.3722 | 1773.1289 | 165 | 5.256 | 36.7% | 1 | R.ILHLLTQEALSIHGVK.E | 2 |
|  | Astrin\_STLCHLD\_061214\_01.07273.07273.2 | 2.3377 | 0.3698 | 98.1% | 1294.5721 | 1294.4502 | 14 | 6.794 | 45.8% | 1 | R.FGLGAEVGISTSR.I | 2 |

---

|  |  |  |  |  |  |  |  |  |
| --- | --- | --- | --- | --- | --- | --- | --- | --- |
| U | *gi|154355000|ref|NP\_0* | 2 | 3 | 3.5% | 711 | 73115 | 7.3 | KH-type splicing regulatory protein (FUSE binding protein 2) [Homo sapiens] |

| Filename XCorr DeltCN Conf% ObsM+H+ CalcM+H+ SpR ZScore Ion% # Sequence  | | | | | | | | | | | | |
| --- | --- | --- | --- | --- | --- | --- | --- | --- | --- | --- | --- | --- |
| \* | Astrin\_STLCHLD\_tube2\_061314\_01.07135.07135.2 | 3.1252 | 0.4066 | 100.0% | 1080.3922 | 1080.2725 | 1 | 6.414 | 75.0% | 1 | R.IGGGIDVPVPR.H | 2 |
| \* | Astrin\_STLCHLD\_tube2\_061314\_02.06245.06245.2 | 4.2919 | 0.3272 | 100.0% | 1535.2322 | 1534.7123 | 1 | 7.321 | 69.2% | 2 | K.AINQQTGAFVEISR.Q | 2 |

---

|  |  |  |  |  |  |  |  |  |
| --- | --- | --- | --- | --- | --- | --- | --- | --- |
| U | *gi|116063573|ref|NP\_0* | 6 | 9 | 3.4% | 2639 | 280016 | 6.0 | filamin A, alpha isoform 1 [Homo sapiens] |
| U | *gi|160420317|ref|NP\_0* | 6 | 9 | 3.4% | 2647 | 280737 | 6.1 | filamin A, alpha isoform 2 [Homo sapiens] |

| Filename XCorr DeltCN Conf% ObsM+H+ CalcM+H+ SpR ZScore Ion% # Sequence  | | | | | | | | | | | | |
| --- | --- | --- | --- | --- | --- | --- | --- | --- | --- | --- | --- | --- |
|  | Astrin\_STLCHLD\_tube2\_061314\_01.09163.09163.2 | 2.9042 | 0.2753 | 98.4% | 1416.6921 | 1416.5742 | 2 | 6.087 | 66.7% | 1 | R.IANLQTDLSDGLR.L | 2 |
|  | Astrin\_STLCHLD\_tube2\_061314\_01.06254.06254.2 | 2.8363 | 0.3075 | 98.6% | 1435.5922 | 1435.5767 | 2 | 6.647 | 58.3% | 1 | R.ANLPQSFQVDTSK.A | 2 |
|  | Astrin\_STLCHLD\_061214\_01.07162.07162.2 | 2.8347 | 0.2991 | 98.3% | 1501.9321 | 1501.6335 | 1 | 5.864 | 60.0% | 1 | K.DAGEGGLSLAIEGPSK.A | 2 |
|  | Astrin\_STLCHLD\_tube2\_061314\_01.07536.07536.2 | 2.2581 | 0.2834 | 95.4% | 1504.3522 | 1503.6982 | 7 | 4.708 | 46.4% | 1 | K.VNQPASFAVSLNGAK.G | 2 |
|  | Astrin\_STLCHLD\_tube2\_061314\_01.06482.06482.3 | 2.1396 | 0.3454 | 97.6% | 1381.5844 | 1380.5455 | 19 | 5.431 | 37.5% | 1 | K.YGGPYHIGGSPFK.A | 3 |
|  | Astrin\_STLCHLD\_tube2\_061314\_02.06640.06640.3 | 4.3123 | 0.4186 | 100.0% | 2201.3643 | 2201.4412 | 1 | 6.77 | 32.9% | 4 | R.LVSNHSLHETSSVFVDSLTK.A | 3 |

---

|  |  |  |  |  |  |  |  |  |
| --- | --- | --- | --- | --- | --- | --- | --- | --- |
| U | *gi|42716280|ref|NP\_97* | 3 | 4 | 3.4% | 1268 | 141439 | 6.9 | high density lipoprotein binding protein [Homo sapiens] |
| U | *gi|4885409|ref|NP\_005* | 3 | 4 | 3.4% | 1268 | 141439 | 6.9 | high density lipoprotein binding protein [Homo sapiens] |

| Filename XCorr DeltCN Conf% ObsM+H+ CalcM+H+ SpR ZScore Ion% # Sequence  | | | | | | | | | | | | |
| --- | --- | --- | --- | --- | --- | --- | --- | --- | --- | --- | --- | --- |
|  | Astrin\_STLCHLD\_tube2\_061314\_02.05241.05241.2 | 2.5123 | 0.2401 | 96.6% | 1329.5322 | 1328.5516 | 2 | 4.471 | 58.3% | 1 | R.LQTQASATVAIPK.E | 2 |
|  | Astrin\_STLCHLD\_tube2\_061314\_01.06339.06339.2 | 2.8459 | 0.4514 | 100.0% | 1334.3922 | 1334.5304 | 1 | 7.135 | 59.1% | 1 | R.LVGEIMQETGTR.I | 2 |
|  | Astrin\_STLCHLD\_061214\_02.06536.06536.3 | 3.8566 | 0.3809 | 100.0% | 1990.9143 | 1991.2517 | 1 | 6.236 | 39.7% | 2 | R.TEIVFTGEKEQLAQAVAR.I | 3 |

---

|  |  |  |  |  |  |  |  |  |
| --- | --- | --- | --- | --- | --- | --- | --- | --- |
| U | *gi|4504505|ref|NP\_000* | 2 | 2 | 3.4% | 736 | 79686 | 8.8 | hydroxysteroid (17-beta) dehydrogenase 4 [Homo sapiens] |

| Filename XCorr DeltCN Conf% ObsM+H+ CalcM+H+ SpR ZScore Ion% # Sequence  | | | | | | | | | | | | |
| --- | --- | --- | --- | --- | --- | --- | --- | --- | --- | --- | --- | --- |
| \* | Astrin\_STLCHLD\_tube2\_061314\_02.06028.06028.2 | 2.7813 | 0.3857 | 99.5% | 1169.2722 | 1170.3976 | 1 | 7.074 | 66.7% | 1 | R.VVLVTGAGAGLGR.A | 2 |
| \* | Astrin\_STLCHLD\_061214\_01.07335.07335.2 | 2.2381 | 0.2423 | 95.2% | 1283.8522 | 1283.5137 | 177 | 4.964 | 54.5% | 1 | R.IDVVVNNAGILR.D | 2 |

---

|  |  |  |  |  |  |  |  |  |
| --- | --- | --- | --- | --- | --- | --- | --- | --- |
| U | *gi|17402900|ref|NP\_00* | 2 | 3 | 3.4% | 644 | 67560 | 7.6 | far upstream element-binding protein [Homo sapiens] |

| Filename XCorr DeltCN Conf% ObsM+H+ CalcM+H+ SpR ZScore Ion% # Sequence  | | | | | | | | | | | | |
| --- | --- | --- | --- | --- | --- | --- | --- | --- | --- | --- | --- | --- |
| \* | Astrin\_STLCHLD\_tube2\_061314\_01.05382.05382.2 | 2.3639 | 0.1747 | 95.4% | 1114.2522 | 1114.3304 | 8 | 4.411 | 62.5% | 1 | K.RLLDQIVEK.G | 2 |
| \* | Astrin\_STLCHLD\_tube2\_061314\_01.08293.08293.2 | 2.8322 | 0.2947 | 98.5% | 1337.6721 | 1337.5187 | 1 | 5.867 | 79.2% | 2 | R.IGGNEGIDVPIPR.F | 2 |

---

|  |  |  |  |  |  |  |  |  |
| --- | --- | --- | --- | --- | --- | --- | --- | --- |
| U | *gi|57164942|ref|NP\_00* | 4 | 4 | 2.9% | 2032 | 225493 | 7.8 | colonic and hepatic tumor over-expressed protein isoform a [Homo sapiens] |
| U | *gi|57222563|ref|NP\_05* | 4 | 4 | 3.0% | 1972 | 218524 | 8.1 | colonic and hepatic tumor over-expressed protein isoform b [Homo sapiens] |

| Filename XCorr DeltCN Conf% ObsM+H+ CalcM+H+ SpR ZScore Ion% # Sequence  | | | | | | | | | | | | |
| --- | --- | --- | --- | --- | --- | --- | --- | --- | --- | --- | --- | --- |
|  | Astrin\_STLCHLD\_tube2\_061314\_02.06154.06154.2 | 3.2142 | 0.2835 | 99.5% | 1321.5721 | 1321.5162 | 2 | 7.519 | 68.2% | 1 | K.FVTDSNAVVQLK.G | 2 |
|  | Astrin\_STLCHLD\_tube2\_061314\_01.08492.08492.3 | 2.8498 | 0.2852 | 98.6% | 1660.9143 | 1659.9255 | 11 | 5.327 | 33.9% | 1 | K.FGQYAGHVVPTILEK.F | 3 |
|  | Astrin\_STLCHLD\_tube2\_061314\_02.08097.08097.3 | 3.0748 | 0.3035 | 98.9% | 2204.8743 | 2204.4905 | 27 | 5.327 | 27.6% | 1 | R.NAALNTIVTVYNVHGDQVFK.L | 3 |
|  | Astrin\_STLCHLD\_tube2\_061314\_01.08826.08826.2 | 3.5576 | 0.3714 | 100.0% | 1492.5922 | 1492.5876 | 3 | 6.132 | 63.6% | 1 | K.NSSQFFQSYVER.G | 2 |

---

|  |  |  |  |  |  |  |  |  |
| --- | --- | --- | --- | --- | --- | --- | --- | --- |
| U | *gi|222136639|ref|NP\_0* | 2 | 2 | 2.9% | 935 | 101531 | 7.3 | methylenetetrahydrofolate dehydrogenase 1 [Homo sapiens] |

| Filename XCorr DeltCN Conf% ObsM+H+ CalcM+H+ SpR ZScore Ion% # Sequence  | | | | | | | | | | | | |
| --- | --- | --- | --- | --- | --- | --- | --- | --- | --- | --- | --- | --- |
| \* | Astrin\_STLCHLD\_tube2\_061314\_01.05667.05667.2 | 2.0754 | 0.4089 | 98.0% | 1200.4122 | 1200.3342 | 90 | 6.116 | 50.0% | 1 | K.TPVPSDIDISR.S | 2 |
| \* | Astrin\_STLCHLD\_tube2\_061314\_01.09098.09098.2 | 3.9213 | 0.4462 | 100.0% | 1632.5721 | 1631.9097 | 1 | 7.661 | 63.3% | 1 | K.YVVVTGITPTPLGEGK.S | 2 |

---

|  |  |  |  |  |  |  |  |  |
| --- | --- | --- | --- | --- | --- | --- | --- | --- |
| U | *gi|156523968|ref|NP\_0* | 2 | 4 | 2.8% | 1014 | 113084 | 8.9 | poly (ADP-ribose) polymerase family, member 1 [Homo sapiens] |

| Filename XCorr DeltCN Conf% ObsM+H+ CalcM+H+ SpR ZScore Ion% # Sequence  | | | | | | | | | | | | |
| --- | --- | --- | --- | --- | --- | --- | --- | --- | --- | --- | --- | --- |
| \* | Astrin\_STLCHLD\_tube2\_061314\_02.07617.07617.2 | 5.0401 | 0.5758 | 100.0% | 1626.2722 | 1625.7728 | 1 | 10.674 | 75.0% | 3 | R.VVSEDFLQDVSASTK.S | 2 |
| \* | Astrin\_STLCHLD\_tube2\_061314\_01.11403.11403.2 | 2.9007 | 0.51 | 100.0% | 1378.8121 | 1378.5712 | 3 | 7.917 | 54.2% | 1 | R.TTNFAGILSQGLR.I | 2 |

---

|  |  |  |  |  |  |  |  |  |
| --- | --- | --- | --- | --- | --- | --- | --- | --- |
| U | *gi|71361682|ref|NP\_00* | 4 | 5 | 2.7% | 2115 | 238257 | 5.8 | nuclear mitotic apparatus protein 1 [Homo sapiens] |

| Filename XCorr DeltCN Conf% ObsM+H+ CalcM+H+ SpR ZScore Ion% # Sequence  | | | | | | | | | | | | |
| --- | --- | --- | --- | --- | --- | --- | --- | --- | --- | --- | --- | --- |
| \* | Astrin\_STLCHLD\_061214\_01.06481.06481.2 | 2.9856 | 0.2609 | 98.3% | 1595.9922 | 1594.7185 | 1 | 5.348 | 65.4% | 1 | K.INQLSEENGDLSFK.L | 2 |
| \* | Astrin\_STLCHLD\_061214\_02.07565.07565.2 | 2.4025 | 0.2468 | 95.6% | 1561.1721 | 1561.6862 | 1 | 4.192 | 61.5% | 1 | R.DSALETLQGQLEEK.A | 2 |
| \* | Astrin\_STLCHLD\_tube2\_061314\_02.06730.06730.2 | 2.385 | 0.425 | 99.3% | 1264.7322 | 1264.5231 | 1 | 6.805 | 70.0% | 1 | R.TTQIINITMTK.K | 2 |
| \* | Astrin\_STLCHLD\_061214\_02.05564.05564.3 | 3.1904 | 0.3666 | 99.8% | 1796.7843 | 1796.9321 | 12 | 5.92 | 31.9% | 2 | R.IATTTASAATAAAIGAT#PR.A | 3 |

---

|  |  |  |  |  |  |  |  |  |
| --- | --- | --- | --- | --- | --- | --- | --- | --- |
| U | *gi|153792294|ref|NP\_1* | 2 | 2 | 2.7% | 1320 | 145257 | 6.8 | myopalladin [Homo sapiens] |

| Filename XCorr DeltCN Conf% ObsM+H+ CalcM+H+ SpR ZScore Ion% # Sequence  | | | | | | | | | | | | |
| --- | --- | --- | --- | --- | --- | --- | --- | --- | --- | --- | --- | --- |
| \* | Astrin\_STLCHLD\_tube2\_061314\_01.07881.07881.2 | 2.3056 | 0.2867 | 96.8% | 1379.4122 | 1379.4662 | 1 | 5.384 | 63.6% | 1 | R.EGTLIEDSPDFR.I | 2 |
| \* | Astrin\_STLCHLD\_061214\_01.05779.05779.3 | 4.5907 | 0.4913 | 100.0% | 2545.2844 | 2546.71 | 1 | 8.402 | 35.9% | 1 | R.VHFNLPEDDKGSEASSEAGVVTTR.Q | 3 |

---

|  |  |  |  |  |  |  |  |  |
| --- | --- | --- | --- | --- | --- | --- | --- | --- |
| U | *gi|38569421|ref|NP\_00* | 2 | 3 | 2.7% | 1101 | 120839 | 7.3 | ATP citrate lyase isoform 1 [Homo sapiens] |
| U | *gi|38569423|ref|NP\_94* | 2 | 3 | 2.7% | 1091 | 119772 | 7.3 | ATP citrate lyase isoform 2 [Homo sapiens] |

| Filename XCorr DeltCN Conf% ObsM+H+ CalcM+H+ SpR ZScore Ion% # Sequence  | | | | | | | | | | | | |
| --- | --- | --- | --- | --- | --- | --- | --- | --- | --- | --- | --- | --- |
|  | Astrin\_STLCHLD\_tube2\_061314\_02.06935.06935.2 | 5.0196 | 0.5221 | 100.0% | 1881.5322 | 1882.07 | 1 | 9.116 | 66.7% | 2 | R.SAYDSTMETMNYAQIR.T | 2 |
|  | Astrin\_STLCHLD\_tube2\_061314\_01.09246.09246.2 | 3.4484 | 0.3632 | 100.0% | 1492.5521 | 1492.647 | 1 | 5.726 | 69.2% | 1 | R.SGGMSNELNNIISR.T | 2 |

---

|  |  |  |  |  |  |  |  |  |
| --- | --- | --- | --- | --- | --- | --- | --- | --- |
| U | *gi|30089940|ref|NP\_00* | 2 | 2 | 2.1% | 1498 | 167354 | 5.4 | Golgi autoantigen, golgin subfamily a, 3 [Homo sapiens] |

| Filename XCorr DeltCN Conf% ObsM+H+ CalcM+H+ SpR ZScore Ion% # Sequence  | | | | | | | | | | | | |
| --- | --- | --- | --- | --- | --- | --- | --- | --- | --- | --- | --- | --- |
| \* | Astrin\_STLCHLD\_061214\_01.04250.04250.3 | 3.2344 | 0.3714 | 100.0% | 1751.6344 | 1751.8419 | 1 | 6.117 | 42.9% | 1 | R.RLEEGTEETSETLEK.L | 3 |
| \* | Astrin\_STLCHLD\_061214\_01.04466.04466.3 | 3.8392 | 0.2776 | 99.7% | 1767.9844 | 1767.9799 | 8 | 5.752 | 38.3% | 1 | K.SGQVEHLQQETAALKK.Q | 3 |

---

|  |  |  |  |  |  |  |  |  |
| --- | --- | --- | --- | --- | --- | --- | --- | --- |
| U | *gi|24430149|ref|NP\_70* | 2 | 3 | 2.1% | 1391 | 155199 | 6.2 | nucleoporin 155kDa isoform 1 [Homo sapiens] |
| U | *gi|4758844|ref|NP\_004* | 2 | 3 | 2.2% | 1332 | 149016 | 6.3 | nucleoporin 155kDa isoform 2 [Homo sapiens] |

| Filename XCorr DeltCN Conf% ObsM+H+ CalcM+H+ SpR ZScore Ion% # Sequence  | | | | | | | | | | | | |
| --- | --- | --- | --- | --- | --- | --- | --- | --- | --- | --- | --- | --- |
|  | Astrin\_STLCHLD\_tube2\_061314\_02.07002.07002.2 | 3.5693 | 0.3801 | 100.0% | 1729.4521 | 1728.9462 | 1 | 6.792 | 58.8% | 2 | R.VASVSQNAIVSAAGNIAR.T | 2 |
|  | Astrin\_STLCHLD\_tube2\_061314\_02.06754.06754.2 | 2.842 | 0.2253 | 98.0% | 1370.7522 | 1370.5919 | 2 | 5.628 | 70.0% | 1 | R.IQLQIQETLQR.Q | 2 |

---

|  |  |  |  |  |  |  |  |  |
| --- | --- | --- | --- | --- | --- | --- | --- | --- |
| U | *gi|4758012|ref|NP\_004* | 2 | 2 | 1.9% | 1675 | 191613 | 5.7 | clathrin heavy chain 1 [Homo sapiens] |

| Filename XCorr DeltCN Conf% ObsM+H+ CalcM+H+ SpR ZScore Ion% # Sequence  | | | | | | | | | | | | |
| --- | --- | --- | --- | --- | --- | --- | --- | --- | --- | --- | --- | --- |
|  | Astrin\_STLCHLD\_tube2\_061314\_01.08132.08132.2 | 2.289 | 0.3441 | 97.8% | 1338.4521 | 1338.5646 | 1 | 5.968 | 59.1% | 1 | R.VVGAMQLYSVDR.K | 2 |
| \* | Astrin\_STLCHLD\_tube2\_061314\_02.07029.07029.3 | 3.8798 | 0.3606 | 100.0% | 1972.5543 | 1972.2083 | 1 | 6.706 | 41.7% | 1 | R.LASTLVHLGEYQAAVDGAR.K | 3 |

---

|  |  |  |  |  |  |  |  |  |
| --- | --- | --- | --- | --- | --- | --- | --- | --- |
| U | *gi|207452735|ref|NP\_1* | 4 | 4 | 1.3% | 5090 | 555629 | 5.6 | epiplakin 1 [Homo sapiens] |

| Filename XCorr DeltCN Conf% ObsM+H+ CalcM+H+ SpR ZScore Ion% # Sequence  | | | | | | | | | | | | |
| --- | --- | --- | --- | --- | --- | --- | --- | --- | --- | --- | --- | --- |
|  | Astrin\_STLCHLD\_061214\_01.06595.06595.3 | 4.4241 | 0.4301 | 100.0% | 2014.4043 | 2014.292 | 1 | 6.563 | 37.5% | 1 | R.LLEAQIATGGVIDPVHSHR.V | 33 |
|  | Astrin\_STLCHLD\_061214\_01.07137.07137.3 | 3.2706 | 0.211 | 96.8% | 2029.9443 | 2028.3188 | 54 | 4.583 | 27.8% | 1 | R.LLEAQIATGGIIDPVHSHR.V | 33 |
|  | Astrin\_STLCHLD\_tube2\_061314\_01.06297.06297.2 | 2.1372 | 0.3926 | 98.5% | 1161.0922 | 1161.2311 | 1 | 7.483 | 75.0% | 1 | C.GYFDEEMNR.I | 22 |
| \* | Astrin\_STLCHLD\_tube2\_061314\_01.04417.04417.2 | 4.7695 | 0.5927 | 100.0% | 1788.5322 | 1788.8253 | 1 | 11.346 | 69.4% | 1 | R.EGQGEGETQEAAAAAAAAR.R | 2 |

Similarities:
gi|41322908|ref|NP\_95(3:1)  

---

|  |  |  |  |  |  |  |  |  |
| --- | --- | --- | --- | --- | --- | --- | --- | --- |
| U | *gi|55770834|ref|NP\_05* | 3 | 4 | 1.2% | 3114 | 357527 | 5.1 | centromere protein F [Homo sapiens] |

| Filename XCorr DeltCN Conf% ObsM+H+ CalcM+H+ SpR ZScore Ion% # Sequence  | | | | | | | | | | | | |
| --- | --- | --- | --- | --- | --- | --- | --- | --- | --- | --- | --- | --- |
| \* | Astrin\_STLCHLD\_tube2\_061314\_02.06526.06526.3 | 3.355 | 0.375 | 100.0% | 1706.3944 | 1705.9238 | 1 | 5.732 | 38.5% | 2 | R.TLEMDRENLSVEIR.N | 3 |
| \* | Astrin\_STLCHLD\_tube2\_061314\_01.08995.08995.2 | 2.7066 | 0.1233 | 95.6% | 1222.3121 | 1222.3403 | 1 | 4.239 | 77.8% | 1 | R.FLDVENELSR.I | 2 |
| \* | Astrin\_STLCHLD\_tube2\_061314\_01.05005.05005.2 | 2.7526 | 0.2581 | 98.0% | 1334.2922 | 1334.4229 | 414 | 5.249 | 45.5% | 1 | K.TAELQEELSGEK.N | 2 |

---

|  |  |  |  |  |  |  |  |  |
| --- | --- | --- | --- | --- | --- | --- | --- | --- |
| U | *gi|58530840|ref|NP\_00* | 2 | 2 | 0.7% | 2871 | 331774 | 6.8 | desmoplakin isoform I [Homo sapiens] |

| Filename XCorr DeltCN Conf% ObsM+H+ CalcM+H+ SpR ZScore Ion% # Sequence  | | | | | | | | | | | | |
| --- | --- | --- | --- | --- | --- | --- | --- | --- | --- | --- | --- | --- |
|  | Astrin\_STLCHLD\_tube2\_061314\_01.06636.06636.2 | 2.5085 | 0.2594 | 98.0% | 1159.3522 | 1159.3892 | 5 | 5.887 | 68.8% | 1 | R.LLQLQEQMR.A | 2 |
| \* | Astrin\_STLCHLD\_tube2\_061314\_01.06992.06992.2 | 2.4899 | 0.2467 | 96.9% | 1389.1322 | 1388.5205 | 1 | 4.739 | 68.2% | 1 | R.LNDSILQATEQR.R | 2 |

---

|  |  |  |  |  |  |  |  |  |
| --- | --- | --- | --- | --- | --- | --- | --- | --- |
| U | *Reverse\_gi|19115954|r* | 2 | 2 | 0.5% | 4624 | 529025 | 6.1 | dynein, axonemal, heavy chain 5 [Homo sapiens] |

| Filename XCorr DeltCN Conf% ObsM+H+ CalcM+H+ SpR ZScore Ion% # Sequence  | | | | | | | | | | | | |
| --- | --- | --- | --- | --- | --- | --- | --- | --- | --- | --- | --- | --- |
| \* | Astrin\_STLCHLD\_061214\_01.12404.12404.2 | 2.7133 | 0.1348 | 95.2% | 1491.7922 | 1490.6481 | 91 | 3.386 | 50.0% | 1 | K.VKFT#SGTK@IFNR.E | 2 |
| \* | Astrin\_STLCHLD\_061214\_01.04668.04668.3 | 3.3934 | 0.2019 | 98.4% | 1582.3744 | 1582.6696 | 37 | 4.216 | 41.7% | 1 | K.TQAAFEQSQETWR.E | 3 |

---

|  |  |  |  |  |  |  |  |  |
| --- | --- | --- | --- | --- | --- | --- | --- | --- |
| U | *Reverse\_gi|90903231|r* | 2 | 2 | 0.5% | 3144 | 347860 | 6.2 | huntingtin [Homo sapiens] |

| Filename XCorr DeltCN Conf% ObsM+H+ CalcM+H+ SpR ZScore Ion% # Sequence  | | | | | | | | | | | | |
| --- | --- | --- | --- | --- | --- | --- | --- | --- | --- | --- | --- | --- |
| \* | Astrin\_STLCHLD\_061214\_02.06392.06392.2 | 2.6499 | 0.2191 | 96.2% | 1912.4321 | 1914.0516 | 1 | 5.256 | 53.6% | 1 | R.S\*FCSKLYGLIEEVCK.G | 2 |
| \* | Astrin\_STLCHLD\_tube2\_061314\_02.06455.06455.2 | 2.6751 | 0.2486 | 96.9% | 1912.6122 | 1914.0516 | 1 | 4.574 | 53.6% | 1 | R.SFCS\*KLYGLIEEVCK.G | 2 |

|  |  |  |  |
| --- | --- | --- | --- |
|  | Proteins | Peptide IDs | Spectra |
| Unfiltered | 43282 | 87851 | 150607 |
| Filtered | 196 | 1064 | 2616 |
| Forward matches | 193 | 1058 | 2609 |
| Decoy matches | 3 | 6 | 7 |
| Forward FP rate | 1.55% | 0.57% | 0.27% |

  
/nfs/cheeseman\_massspec/David/Astrin\_STLCHLD2
