# Supplementary material for: Astrin-SKAP complex reconstitution reveals its kinetochore interaction with microtubule-bound Ndc80
Source: eLife. 2017 Aug 25;6:e26866. doi: 10.7554/eLife.26866 (PMC5602300; doi:10.7554/eLife.26866)
Supplement: Source data 1. — Complete mass spectrometry searches using methods described in (Washburn et al., 2001) for affinity purification/mass spectrometry data sets described in this paper (data from this study; [Kern et al., 2016] [Gascoigne et al., 2011]). Individual Astrin cross-linking immunoprecipitations are listed based on the order in Figure 4—figure supplement 1. These samples have not been pruned for common or antibody-specific contaminants. [file elife-26866-data1.zip › Astrin_Crosslinking#6.html]

D AstrinSTLCNLD
DTASelect v2.0.21  
/nfs/cheeseman\_massspec/David/AstrinSTLCNLD  
/nfs/cheeseman\_massspec/Databases/NCBI-RefSeq\_human\_na\_04-13-2009\_con\_reversed.fasta  
SEQUEST 3.0 in SQT format.  
-p 1  
 Jump  to the summary table.  
  
sequest.params modifications:

|  |  |  |
| --- | --- | --- |
| \* | S | 80.0 |
| # | T | 80.0 |
| @ | K | 12.0 |
| Static | C | 57.0 |

|  |  |
| --- | --- |
| true | Use criteria |
| 0.0 | Minimum peptide confidence |
| 0.05 | Peptide false positive rate |
| 0.0 | Minimum protein confidence |
| 1.0 | Protein false positive rate |
| 1 | Minimum charge state |
| 16 | Maximum charge state |
| 0.0 | Minimum ion proportion |
| 1000 | Maximum Sp rank |
| -1.0 | Minimum Sp score |
| Include | Modified peptide inclusion |
| Any | Tryptic status requirement |
| false | Multiple, ambiguous IDs allowed |
| Ignore | Peptide validation handling |
| XCorr | Purge duplicate peptides by protein |
| false | Include only loci with unique peptide |
| true | Remove subset proteins |
| Ignore | Locus validation handling |
| 0 | Minimum modified peptides per locus |
| 1000 | Minimum redundancy for low coverage loci |
| 1 | Minimum peptides per locus |

#### Locus Key:

|  |  |  |  |  |  |  |  |  |
| --- | --- | --- | --- | --- | --- | --- | --- | --- |
| Validation Status | Locus | Sequence Count | Spectrum Count | Sequence Coverage | Length | MolWt | pI | Descriptive Name |

#### Similarity Key:

|  |  |  |
| --- | --- | --- |
| Locus | # of identical peptides | # of differing peptides |

---

|  |  |  |  |  |  |  |  |  |
| --- | --- | --- | --- | --- | --- | --- | --- | --- |
| U | *contaminant\_KERATIN09* | 20 | 62 | 54.5% | 429 | 47927 | 5.5 | no description |
| U | *gi|4557888|ref|NP\_000* | 20 | 62 | 54.4% | 430 | 48058 | 5.5 | keratin 18 [Homo sapiens] |
| U | *gi|40354195|ref|NP\_95* | 20 | 62 | 54.4% | 430 | 48058 | 5.5 | keratin 18 [Homo sapiens] |

| Filename XCorr DeltCN Conf% ObsM+H+ CalcM+H+ SpR ZScore Ion% # Sequence  | | | | | | | | | | | | |
| --- | --- | --- | --- | --- | --- | --- | --- | --- | --- | --- | --- | --- |
|  | AstrinSTLCLD\_041714\_01.05952.05952.2 | 5.051 | 0.4354 | 100.0% | 2856.4922 | 2856.0813 | 1 | 8.143 | 33.3% | 1 | R.SLGSVQAPSYGARPVSSAASVYAGAGGSGSR.I | 2 |
|  | AstrinSTLCLD\_041714\_02.06050.06050.3 | 5.1099 | 0.4926 | 100.0% | 2857.1042 | 2856.0813 | 1 | 9.6 | 32.5% | 11 | R.SLGSVQAPSYGARPVSSAASVYAGAGGSGSR.I | 3 |
|  | AstrinSTLCLD\_041714\_02.08390.08390.3 | 6.2196 | 0.5712 | 100.0% | 3337.3145 | 3337.7224 | 1 | 10.171 | 27.9% | 5 | R.GGMGSGGLATGIAGGLAGMGGIQNEKETMQSLNDR.L | 3 |
|  | AstrinSTLCLD\_041714\_01.07136.07136.2 | 2.1891 | 0.2036 | 96.3% | 982.6922 | 983.0709 | 1 | 5.041 | 83.3% | 1 | R.DWSHYFK.I | 2 |
|  | AstrinSTLCLD\_041714\_01.05102.05102.2 | 2.0712 | 0.2926 | 97.3% | 1042.1522 | 1042.2235 | 1 | 5.281 | 75.0% | 1 | R.IVLQIDNAR.L | 22 |
|  | AstrinSTLCLD\_041714\_01.04719.04719.2 | 2.5019 | 0.3906 | 100.0% | 1239.8121 | 1240.4601 | 9 | 6.819 | 72.2% | 1 | R.VKYETELAMR.Q | 2 |
|  | AstrinSTLCLD\_041714\_01.13150.13150.2 | 5.7639 | 0.5625 | 100.0% | 2177.892 | 2178.589 | 1 | 10.376 | 58.8% | 1 | R.LQLETEIEALKEELLFMK.K | 2 |
|  | AstrinSTLCLD\_041714\_02.11436.11436.3 | 2.6902 | 0.2473 | 96.2% | 2178.2344 | 2178.589 | 189 | 4.45 | 30.9% | 3 | R.LQLETEIEALKEELLFMK.K | 3 |
|  | AstrinSTLCLD\_041714\_01.07850.07850.3 | 5.0859 | 0.4625 | 100.0% | 2750.1243 | 2751.0227 | 1 | 9.03 | 38.0% | 1 | K.NHEEEVKGLQAQIASSGLTVEVDAPK.S | 3 |
|  | AstrinSTLCLD\_041714\_01.08284.08284.2 | 3.9515 | 0.4646 | 100.0% | 1884.2122 | 1885.1246 | 1 | 7.567 | 61.1% | 1 | K.GLQAQIASSGLTVEVDAPK.S | 2 |
|  | AstrinSTLCLD\_041714\_01.09598.09598.1 | 2.0501 | 0.4188 | 100.0% | 1506.64 | 1507.699 | 2 | 6.326 | 54.2% | 1 | R.TVQSLEIDLDSMR.N | 1 |
|  | AstrinSTLCLD\_041714\_02.08151.08151.2 | 4.0833 | 0.5085 | 100.0% | 1508.3922 | 1507.699 | 1 | 9.756 | 75.0% | 6 | R.TVQSLEIDLDSMR.N | 2 |
|  | AstrinSTLCLD\_041714\_01.05887.05887.3 | 2.4075 | 0.2669 | 97.0% | 1475.0044 | 1474.6139 | 1 | 5.137 | 45.8% | 1 | K.ASLENSLREVEAR.Y | 3 |
|  | AstrinSTLCLD\_041714\_01.14072.14072.3 | 6.5086 | 0.523 | 100.0% | 2671.7944 | 2672.0715 | 1 | 9.15 | 46.6% | 14 | R.YALQMEQLNGILLHLESELAQTR.A | 3 |
|  | AstrinSTLCLD\_041714\_01.14060.14060.2 | 6.0115 | 0.4821 | 100.0% | 2672.0122 | 2672.0715 | 1 | 9.191 | 47.7% | 2 | R.YALQMEQLNGILLHLESELAQTR.A | 2 |
|  | AstrinSTLCLD\_041714\_01.08763.08763.2 | 3.6713 | 0.422 | 100.0% | 1420.4122 | 1420.6055 | 2 | 6.47 | 72.7% | 2 | R.QAQEYEALLNIK.V | 2 |
|  | AstrinSTLCLD\_041714\_02.06047.06047.2 | 3.1308 | 0.4022 | 100.0% | 1292.7122 | 1293.5059 | 1 | 6.963 | 85.0% | 6 | K.VKLEAEIATYR.R | 2 |
|  | AstrinSTLCLD\_041714\_01.09434.09434.3 | 4.2827 | 0.3186 | 100.0% | 2897.9644 | 2898.128 | 9 | 5.802 | 25.0% | 1 | R.RLLEDGEDFNLGDALDSSNSMQTIQK.T | 3 |
|  | AstrinSTLCLD\_041714\_01.10139.10139.2 | 5.8711 | 0.6523 | 100.0% | 2740.632 | 2741.9404 | 1 | 12.13 | 56.2% | 1 | R.LLEDGEDFNLGDALDSSNSMQTIQK.T | 2 |
|  | AstrinSTLCLD\_041714\_01.10133.10133.3 | 5.6454 | 0.5234 | 100.0% | 2741.9043 | 2741.9404 | 1 | 9.408 | 35.4% | 2 | R.LLEDGEDFNLGDALDSSNSMQTIQK.T | 3 |

Similarities:
contaminant\_KERATIN10(1:19)  

---

|  |  |  |  |  |  |  |  |  |
| --- | --- | --- | --- | --- | --- | --- | --- | --- |
| U | *gi|29788785|ref|NP\_82* | 21 | 51 | 52.3% | 444 | 49671 | 4.9 | tubulin, beta [Homo sapiens] |

| Filename XCorr DeltCN Conf% ObsM+H+ CalcM+H+ SpR ZScore Ion% # Sequence  | | | | | | | | | | | | |
| --- | --- | --- | --- | --- | --- | --- | --- | --- | --- | --- | --- | --- |
| \* | AstrinSTLCLD\_041714\_02.07954.07954.3 | 5.67 | 0.4131 | 100.0% | 3103.5244 | 3104.2725 | 1 | 8.372 | 29.8% | 2 | K.FWEVISDEHGIDPTGTYHGDSDLQLDR.I | 3 |
| \* | AstrinSTLCLD\_041714\_02.05325.05325.2 | 2.8414 | 0.3414 | 100.0% | 1302.3522 | 1302.4265 | 19 | 6.084 | 54.5% | 1 | R.ISVYYNEATGGK.Y | 2 |
| \* | AstrinSTLCLD\_041714\_02.05894.05894.3 | 2.6766 | 0.2563 | 97.4% | 1817.8744 | 1818.0392 | 371 | 5.391 | 28.3% | 1 | R.ISVYYNEATGGKYVPR.A | 3 |
|  | AstrinSTLCLD\_041714\_01.09356.09356.2 | 4.4304 | 0.4973 | 100.0% | 1617.3322 | 1616.8701 | 1 | 9.033 | 71.4% | 5 | R.AILVDLEPGTMDSVR.S | 22 |
|  | AstrinSTLCLD\_041714\_01.10952.10952.3 | 7.1011 | 0.4962 | 100.0% | 2799.1143 | 2800.0647 | 1 | 8.497 | 38.0% | 5 | R.SGPFGQIFRPDNFVFGQSGAGNNWAK.G | 33 |
|  | AstrinSTLCLD\_041714\_01.11397.11397.2 | 6.437 | 0.554 | 100.0% | 1959.8121 | 1960.151 | 1 | 10.53 | 79.4% | 1 | K.GHYTEGAELVDSVLDVVR.K | 222 |
|  | AstrinSTLCLD\_041714\_01.10706.10706.3 | 4.4871 | 0.4257 | 100.0% | 2088.8643 | 2088.325 | 1 | 7.56 | 40.3% | 2 | K.GHYTEGAELVDSVLDVVRK.E | 333 |
|  | AstrinSTLCLD\_041714\_01.07358.07358.2 | 4.3453 | 0.4254 | 100.0% | 1320.1322 | 1320.5896 | 1 | 8.343 | 72.7% | 6 | R.IMNTFSVVPSPK.V | 222 |
|  | AstrinSTLCLD\_041714\_01.06043.06043.2 | 2.8729 | 0.2906 | 100.0% | 1131.0922 | 1131.2767 | 9 | 5.247 | 77.8% | 7 | R.FPGQLNADLR.K | 222 |
|  | AstrinSTLCLD\_041714\_01.07593.07593.2 | 3.7915 | 0.3644 | 100.0% | 1272.4722 | 1272.5945 | 1 | 7.372 | 70.0% | 2 | R.KLAVNMVPFPR.L | 222 |
|  | AstrinSTLCLD\_041714\_01.08967.08967.2 | 3.7007 | 0.4846 | 100.0% | 1144.3922 | 1144.4204 | 1 | 9.08 | 94.4% | 2 | K.LAVNMVPFPR.L | 222 |
|  | AstrinSTLCLD\_041714\_01.10875.10875.3 | 4.0389 | 0.3577 | 100.0% | 1622.3043 | 1621.9403 | 1 | 6.217 | 51.9% | 2 | R.LHFFMPGFAPLTSR.G | 33 |
|  | AstrinSTLCLD\_041714\_01.10880.10880.2 | 3.6408 | 0.3737 | 100.0% | 1622.7122 | 1621.9403 | 1 | 7.77 | 73.1% | 2 | R.LHFFMPGFAPLTSR.G | 22 |
| \* | AstrinSTLCLD\_041714\_01.10652.10652.2 | 2.9198 | 0.4691 | 100.0% | 1660.3722 | 1660.9078 | 1 | 7.568 | 60.7% | 2 | R.ALTVPELTQQVFDAK.N | 2 |
|  | AstrinSTLCLD\_041714\_01.09495.09495.2 | 2.7652 | 0.2691 | 100.0% | 1041.4521 | 1040.2505 | 1 | 6.163 | 87.5% | 1 | R.YLTVAAVFR.G | 22 |
|  | AstrinSTLCLD\_041714\_02.06758.06758.3 | 3.9569 | 0.2413 | 100.0% | 1924.8544 | 1925.2405 | 7 | 5.619 | 41.7% | 2 | R.MSMKEVDEQMLNVQNK.N | 33 |
|  | AstrinSTLCLD\_041714\_01.10293.10293.2 | 2.9679 | 0.4318 | 100.0% | 1697.3121 | 1697.8877 | 1 | 7.31 | 57.7% | 1 | K.NSSYFVEWIPNNVK.T | 222 |
| \* | AstrinSTLCLD\_041714\_02.10392.10392.2 | 4.3811 | 0.4373 | 100.0% | 1870.8322 | 1871.2018 | 1 | 7.933 | 59.4% | 1 | K.MAVTFIGNSTAIQELFK.R | 2 |
| \* | AstrinSTLCLD\_041714\_01.11186.11186.3 | 3.5389 | 0.3944 | 100.0% | 2027.8744 | 2027.3893 | 2 | 6.924 | 35.3% | 2 | K.MAVTFIGNSTAIQELFKR.I | 3 |
|  | AstrinSTLCLD\_041714\_01.08030.08030.2 | 3.2012 | 0.3385 | 100.0% | 1385.6122 | 1386.6116 | 47 | 5.663 | 60.0% | 1 | K.RISEQFTAMFR.R | 222 |
|  | AstrinSTLCLD\_041714\_02.07989.07989.2 | 3.7427 | 0.5204 | 100.0% | 1229.6721 | 1230.4241 | 1 | 8.103 | 94.4% | 3 | R.ISEQFTAMFR.R | 222 |

Similarities:
gi|5174735|ref|NP\_006(14:7)  
gi|50592996|ref|NP\_00(10:11)  

---

|  |  |  |  |  |  |  |  |  |
| --- | --- | --- | --- | --- | --- | --- | --- | --- |
| U | *gi|73623035|ref|NP\_00* | 55 | 192 | 51.7% | 1193 | 134422 | 5.0 | sperm associated antigen 5 [Homo sapiens] |

| Filename XCorr DeltCN Conf% ObsM+H+ CalcM+H+ SpR ZScore Ion% # Sequence  | | | | | | | | | | | | |
| --- | --- | --- | --- | --- | --- | --- | --- | --- | --- | --- | --- | --- |
| \* | AstrinSTLCLD\_041714\_01.08306.08306.2 | 4.2511 | 0.3612 | 100.0% | 1653.2122 | 1653.8445 | 1 | 8.567 | 71.4% | 2 | K.TSEEAVDPLGNYMVK.T | 2 |
| \* | AstrinSTLCLD\_041714\_01.11240.11240.2 | 2.5701 | 0.4459 | 100.0% | 2322.4521 | 2323.6262 | 1 | 7.034 | 42.1% | 1 | K.TIVLVPS\*PLGQQQDMIFEAR.L | 2 |
| \* | AstrinSTLCLD\_041714\_01.08032.08032.2 | 5.2488 | 0.4901 | 100.0% | 1833.3121 | 1833.0668 | 1 | 8.481 | 65.6% | 5 | R.LDTMAETNSISLNGPLR.T | 2 |
| \* | AstrinSTLCLD\_041714\_02.07683.07683.3 | 4.0083 | 0.4268 | 100.0% | 2532.3843 | 2532.8286 | 1 | 7.446 | 35.2% | 2 | R.LDTMAETNSISLNGPLRTDDLVR.E | 3 |
| \* | AstrinSTLCLD\_041714\_01.12964.12964.3 | 5.8557 | 0.5502 | 100.0% | 3777.0842 | 3778.2102 | 1 | 8.519 | 27.9% | 1 | R.TEAVREDLVPSESNAFLPSSVLWLSPSTALAADFR.V | 3 |
| \* | AstrinSTLCLD\_041714\_01.12986.12986.3 | 5.5512 | 0.1572 | 100.0% | 3856.4644 | 3858.2102 | 1 | 7.059 | 36.0% | 6 | R.TEAVREDLVPSESNAFLPSSVLWLS\*PSTALAADFR.V | 3 |
| \* | AstrinSTLCLD\_041714\_01.05755.05755.3 | 5.2465 | 0.3419 | 100.0% | 2221.4343 | 2220.3752 | 1 | 5.991 | 40.3% | 11 | R.VNHVDPEEEIVEHGAMEER.E | 3 |
| \* | AstrinSTLCLD\_041714\_01.13520.13520.2 | 4.9362 | 0.5336 | 100.0% | 2063.4722 | 2064.3606 | 1 | 9.086 | 64.7% | 2 | R.ILGSDTESWMSPLAWLEK.G | 2 |
| \* | AstrinSTLCLD\_041714\_01.14030.14030.2 | 4.5714 | 0.4134 | 100.0% | 2144.7322 | 2144.3606 | 1 | 6.79 | 61.8% | 2 | R.ILGSDTESWMS\*PLAWLEK.G | 2 |
| \* | AstrinSTLCLD\_041714\_01.08012.08012.2 | 3.0586 | 0.2966 | 100.0% | 1332.6522 | 1333.5457 | 1 | 6.595 | 81.8% | 1 | K.GVNTSVMLENLR.Q | 2 |
| \* | AstrinSTLCLD\_041714\_01.08883.08883.2 | 2.3577 | 0.2212 | 96.4% | 1131.5521 | 1132.3635 | 415 | 5.743 | 55.6% | 1 | R.QSLSLPSMLR.D | 2 |
| \* | AstrinSTLCLD\_041714\_01.15311.15311.3 | 3.098 | 0.3 | 99.2% | 2165.9043 | 2166.4795 | 20 | 5.417 | 33.3% | 2 | R.HDLEDNLLSSLVILEVLSR.Q | 3 |
| \* | AstrinSTLCLD\_041714\_01.15182.15182.2 | 7.2986 | 0.6241 | 100.0% | 2166.632 | 2166.4795 | 1 | 10.15 | 72.2% | 10 | R.HDLEDNLLSSLVILEVLSR.Q | 2 |
| \* | AstrinSTLCLD\_041714\_01.04286.04286.3 | 5.4155 | 0.5229 | 100.0% | 2867.5745 | 2868.0 | 1 | 8.058 | 32.7% | 1 | K.SQLAVPHPETQDSSTQTDTSHSGITNK.L | 3 |
| \* | AstrinSTLCLD\_041714\_02.08594.08594.2 | 3.8977 | 0.4341 | 100.0% | 1305.3121 | 1305.578 | 1 | 7.428 | 75.0% | 3 | R.NVMQSWVLISK.E | 2 |
| \* | AstrinSTLCLD\_041714\_01.10932.10932.3 | 5.1613 | 0.4128 | 100.0% | 2893.1643 | 2892.2793 | 1 | 6.838 | 30.2% | 3 | K.ELISLLHLSLLHLEEDKTTVSQESR.R | 3 |
| \* | AstrinSTLCLD\_041714\_01.10332.10332.3 | 3.787 | 0.3171 | 100.0% | 2075.9644 | 2074.267 | 1 | 5.892 | 37.5% | 1 | R.GKDAAEIVLEAFCAHASQR.I | 3 |
| \* | AstrinSTLCLD\_041714\_01.11028.11028.2 | 4.3111 | 0.4346 | 100.0% | 1888.0521 | 1889.041 | 1 | 7.824 | 59.4% | 1 | K.DAAEIVLEAFCAHASQR.I | 2 |
| \* | AstrinSTLCLD\_041714\_01.08912.08912.2 | 3.7816 | 0.3764 | 100.0% | 1390.6721 | 1391.5823 | 1 | 8.066 | 68.2% | 4 | R.ISQLEQDLASMR.E | 2 |
| \* | AstrinSTLCLD\_041714\_01.09837.09837.3 | 2.852 | 0.2503 | 97.9% | 1823.8444 | 1824.0618 | 1 | 5.568 | 39.3% | 1 | R.ISQLEQDLASMREFR.G | 3 |
| \* | AstrinSTLCLD\_041714\_01.06846.06846.3 | 4.342 | 0.254 | 100.0% | 1693.5543 | 1692.9994 | 1 | 5.081 | 40.0% | 2 | R.GLLKDAQTQLVGLHAK.Q | 3 |
| \* | AstrinSTLCLD\_041714\_02.09617.09617.2 | 3.3235 | 0.467 | 100.0% | 2389.5923 | 2390.612 | 27 | 7.119 | 26.3% | 1 | K.QEELVQQTVSLTSTLQQDWR.S | 2 |
| \* | AstrinSTLCLD\_041714\_02.10195.10195.2 | 4.1076 | 0.2614 | 100.0% | 1787.9321 | 1787.0405 | 1 | 6.089 | 71.4% | 2 | R.SMQLDYTTWTALLSR.S | 2 |
| \* | AstrinSTLCLD\_041714\_01.04342.04342.3 | 5.4811 | 0.3753 | 100.0% | 2346.2344 | 2344.5437 | 1 | 6.218 | 43.4% | 1 | K.SQQALQERDVAIEEKQEVSR.V | 3 |
| \* | AstrinSTLCLD\_041714\_01.03830.03830.2 | 4.083 | 0.4469 | 100.0% | 1403.3121 | 1403.5321 | 1 | 7.329 | 72.7% | 4 | R.DVAIEEKQEVSR.V | 2 |
| \* | AstrinSTLCLD\_041714\_02.04298.04298.3 | 3.1579 | 0.3611 | 100.0% | 1404.1743 | 1403.5321 | 2 | 5.962 | 52.3% | 3 | R.DVAIEEKQEVSR.V | 3 |
| \* | AstrinSTLCLD\_041714\_02.05948.05948.2 | 4.5104 | 0.4241 | 100.0% | 1534.4722 | 1533.6849 | 1 | 8.436 | 70.8% | 1 | R.VLEQVSAQLEECK.G | 2 |
| \* | AstrinSTLCLD\_041714\_02.06807.06807.3 | 5.9366 | 0.4453 | 100.0% | 2919.8342 | 2919.1382 | 1 | 7.951 | 35.4% | 2 | R.VLEQVSAQLEECKGQTEQLELENSR.L | 3 |
| \* | AstrinSTLCLD\_041714\_01.09689.09689.2 | 5.2526 | 0.4386 | 100.0% | 1573.5521 | 1573.848 | 1 | 7.671 | 76.9% | 11 | R.AQLQILANMDSQLK.E | 2 |
| \* | AstrinSTLCLD\_041714\_02.05198.05198.3 | 5.0933 | 0.2955 | 100.0% | 1724.0643 | 1723.9879 | 1 | 5.988 | 51.8% | 13 | K.HMQAELQQQQAVLAK.E | 3 |
| \* | AstrinSTLCLD\_041714\_02.05133.05133.2 | 5.5331 | 0.5247 | 100.0% | 1724.3522 | 1723.9879 | 1 | 9.831 | 82.1% | 8 | K.HMQAELQQQQAVLAK.E | 2 |
| \* | AstrinSTLCLD\_041714\_02.09637.09637.3 | 6.1261 | 0.5123 | 100.0% | 3286.0745 | 3286.5862 | 1 | 8.948 | 38.0% | 2 | R.DLKETLEFADQENQVAHLELGQVECQLK.T | 3 |
| \* | AstrinSTLCLD\_041714\_01.07671.07671.3 | 4.3843 | 0.296 | 100.0% | 1964.4543 | 1964.1462 | 1 | 6.178 | 46.9% | 2 | R.SLQCENLKDTVENLTAK.L | 3 |
| \* | AstrinSTLCLD\_041714\_02.04995.04995.2 | 4.2536 | 0.3583 | 100.0% | 1675.0922 | 1675.7899 | 1 | 7.873 | 53.6% | 3 | K.LASTIADNQEQDLEK.T | 2 |
| \* | AstrinSTLCLD\_041714\_02.14805.14805.3 | 4.3697 | 0.3765 | 100.0% | 2762.0942 | 2762.1326 | 11 | 7.532 | 27.3% | 1 | R.QYS\*QKLGLLTEQLQSLTLFLQTK.L | 3 |
| \* | AstrinSTLCLD\_041714\_01.14318.14318.2 | 5.6953 | 0.512 | 100.0% | 2047.6721 | 2047.443 | 1 | 9.799 | 64.7% | 24 | K.LGLLTEQLQSLTLFLQTK.L | 2 |
| \* | AstrinSTLCLD\_041714\_02.12470.12470.3 | 5.3374 | 0.4204 | 100.0% | 2048.4243 | 2047.443 | 1 | 8.408 | 51.5% | 6 | K.LGLLTEQLQSLTLFLQTK.L | 3 |
| \* | AstrinSTLCLD\_041714\_01.13934.13934.3 | 6.3332 | 0.5378 | 100.0% | 2788.3442 | 2788.121 | 1 | 10.882 | 34.6% | 1 | R.TFLGSILTAVADEEPESTPVPLLGSDK.S | 3 |
| \* | AstrinSTLCLD\_041714\_01.14002.14002.2 | 5.1496 | 0.3525 | 100.0% | 2789.8123 | 2788.121 | 1 | 6.287 | 44.2% | 6 | R.TFLGSILTAVADEEPESTPVPLLGSDK.S | 2 |
| \* | AstrinSTLCLD\_041714\_01.14309.14309.2 | 4.1694 | 0.5129 | 100.0% | 2867.5723 | 2868.121 | 1 | 7.611 | 42.3% | 2 | R.TFLGSILTAVADEEPESTPVPLLGS\*DK.S | 2 |
| \* | AstrinSTLCLD\_041714\_01.13527.13527.3 | 4.2404 | 0.3381 | 100.0% | 3351.6243 | 3350.7473 | 4 | 5.782 | 21.0% | 2 | R.TFLGSILTAVADEEPESTPVPLLGSDKSAFTR.V | 3 |
| \* | AstrinSTLCLD\_041714\_01.13757.13757.3 | 4.926 | 0.3865 | 100.0% | 3430.5544 | 3430.7473 | 3 | 7.513 | 27.4% | 4 | R.TFLGSILTAVADEEPESTPVPLLGS\*DKSAFTR.V | 3 |
| \* | AstrinSTLCLD\_041714\_01.03441.03441.2 | 3.0517 | 0.3155 | 100.0% | 1594.6721 | 1595.7092 | 1 | 5.669 | 58.3% | 1 | R.LQAQEEQHQEVQK.A | 2 |
| \* | AstrinSTLCLD\_041714\_01.08064.08064.2 | 2.5985 | 0.3462 | 99.6% | 1672.4122 | 1673.873 | 2 | 6.038 | 50.0% | 1 | K.EADIEKLNQALCLR.Y | 2 |
| \* | AstrinSTLCLD\_041714\_01.09831.09831.3 | 4.8398 | 0.273 | 100.0% | 2679.9844 | 2681.0618 | 1 | 6.163 | 38.6% | 1 | K.ILEQIDKSGELISLREEVTHLTR.S | 3 |
| \* | AstrinSTLCLD\_041714\_01.08500.08500.2 | 3.8062 | 0.4109 | 100.0% | 1840.3922 | 1841.0745 | 1 | 6.082 | 63.3% | 2 | K.SGELISLREEVTHLTR.S | 2 |
| \* | AstrinSTLCLD\_041714\_01.08517.08517.3 | 5.2065 | 0.3873 | 100.0% | 1842.2344 | 1841.0745 | 1 | 7.062 | 51.7% | 10 | K.SGELISLREEVTHLTR.S | 3 |
| \* | AstrinSTLCLD\_041714\_01.07887.07887.2 | 3.019 | 0.2432 | 99.7% | 1373.3922 | 1373.595 | 1 | 5.414 | 85.0% | 2 | K.VWLSQEVDKLR.V | 2 |
| \* | AstrinSTLCLD\_041714\_01.08528.08528.2 | 2.8363 | 0.3369 | 100.0% | 898.15216 | 898.16644 | 1 | 7.131 | 91.7% | 2 | R.VMFLEMK.N | 2 |
| \* | AstrinSTLCLD\_041714\_01.08720.08720.3 | 5.5206 | 0.5253 | 100.0% | 2229.5344 | 2230.526 | 1 | 7.877 | 42.6% | 3 | R.RSDKELEKLDDIVQHIYK.T | 3 |
| \* | AstrinSTLCLD\_041714\_01.09536.09536.3 | 4.8524 | 0.2995 | 100.0% | 2074.6443 | 2074.3384 | 1 | 6.299 | 43.8% | 2 | R.SDKELEKLDDIVQHIYK.T | 3 |
| \* | AstrinSTLCLD\_041714\_01.09516.09516.2 | 4.5856 | 0.448 | 100.0% | 2075.2922 | 2074.3384 | 1 | 7.624 | 65.6% | 1 | R.SDKELEKLDDIVQHIYK.T | 2 |
| \* | AstrinSTLCLD\_041714\_01.09410.09410.2 | 2.6135 | 0.3196 | 100.0% | 1127.2122 | 1127.3696 | 4 | 5.586 | 77.8% | 3 | K.TLLSIPEVVR.G | 2 |
| \* | AstrinSTLCLD\_041714\_01.12556.12556.2 | 3.0008 | 0.4741 | 100.0% | 1494.6522 | 1494.6941 | 1 | 7.411 | 70.8% | 1 | R.GCKELQGLLEFLS.- | 2 |
| \* | AstrinSTLCLD\_041714\_01.13791.13791.1 | 1.7805 | 0.3401 | 100.0% | 1148.7 | 1149.3293 | 13 | 5.263 | 44.4% | 2 | K.ELQGLLEFLS.- | 1 |

---

|  |  |  |  |  |  |  |  |  |
| --- | --- | --- | --- | --- | --- | --- | --- | --- |
| U | *gi|106775678|ref|NP\_0* | 7 | 16 | 45.4% | 130 | 14095 | 10.9 | histone cluster 2, H2aa4 [Homo sapiens] |
| U | *gi|4504251|ref|NP\_003* | 7 | 16 | 45.4% | 130 | 14095 | 10.9 | histone cluster 2, H2aa3 [Homo sapiens] |
| U | *gi|24638446|ref|NP\_00* | 7 | 16 | 45.7% | 129 | 13988 | 10.9 | histone cluster 2, H2ac [Homo sapiens] |

| Filename XCorr DeltCN Conf% ObsM+H+ CalcM+H+ SpR ZScore Ion% # Sequence  | | | | | | | | | | | | |
| --- | --- | --- | --- | --- | --- | --- | --- | --- | --- | --- | --- | --- |
|  | AstrinSTLCLD\_041714\_01.06035.06035.2 | 3.1245 | 0.3446 | 100.0% | 944.7522 | 945.1093 | 2 | 6.049 | 81.2% | 5 | R.AGLQFPVGR.V | 222 |
|  | AstrinSTLCLD\_041714\_01.17692.17692.3 | 3.433 | 0.3386 | 100.0% | 2934.5344 | 2935.4082 | 1 | 5.684 | 23.2% | 1 | R.VGAGAPVYMAAVLEYLTAEILELAGNAAR.D | 3 |
|  | AstrinSTLCLD\_041714\_01.17631.17631.2 | 5.2869 | 0.5168 | 100.0% | 2936.1921 | 2935.4082 | 1 | 9.238 | 41.1% | 2 | R.VGAGAPVYMAAVLEYLTAEILELAGNAAR.D | 2 |
|  | AstrinSTLCLD\_041714\_01.17409.17409.3 | 3.5002 | 0.4151 | 100.0% | 3291.7444 | 3292.7747 | 1 | 6.34 | 21.8% | 1 | R.VGAGAPVYMAAVLEYLTAEILELAGNAARDNK.K | 3 |
|  | AstrinSTLCLD\_041714\_02.06002.06002.3 | 3.0348 | 0.3547 | 100.0% | 1695.1444 | 1693.9004 | 1 | 6.097 | 42.3% | 4 | R.HLQLAIRNDEELNK.L | 333 |
|  | AstrinSTLCLD\_041714\_01.08000.08000.3 | 3.993 | 0.3608 | 100.0% | 2106.3843 | 2105.4453 | 1 | 5.767 | 38.2% | 1 | R.HLQLAIRNDEELNKLLGK.V | 33 |
|  | AstrinSTLCLD\_041714\_01.06227.06227.2 | 3.5524 | 0.3511 | 100.0% | 1273.0521 | 1273.4288 | 12 | 6.622 | 65.0% | 2 | R.NDEELNKLLGK.V | 22 |

Similarities:
gi|10645195|ref|NP\_06(2:5)  
gi|10800130|ref|NP\_06(4:3)  

---

|  |  |  |  |  |  |  |  |  |
| --- | --- | --- | --- | --- | --- | --- | --- | --- |
| U | *gi|4504919|ref|NP\_002* | 22 | 50 | 45.1% | 483 | 53704 | 5.6 | keratin 8 [Homo sapiens] |

| Filename XCorr DeltCN Conf% ObsM+H+ CalcM+H+ SpR ZScore Ion% # Sequence  | | | | | | | | | | | | |
| --- | --- | --- | --- | --- | --- | --- | --- | --- | --- | --- | --- | --- |
| \* | AstrinSTLCLD\_041714\_02.11027.11027.3 | 3.5614 | 0.2104 | 97.8% | 3926.5144 | 3927.465 | 371 | 4.194 | 15.6% | 1 | R.GGLGGGYGGASGMGGITAVTVNQSLLSPLVLEVDPNIQAVR.T | 3 |
|  | AstrinSTLCLD\_041714\_01.06305.06305.2 | 3.32 | 0.1255 | 99.7% | 1030.8121 | 1031.1997 | 3 | 4.084 | 92.9% | 4 | K.WSLLQQQK.T | 2 |
|  | AstrinSTLCLD\_041714\_01.08998.08998.2 | 3.1834 | 0.448 | 100.0% | 1353.1322 | 1353.5732 | 1 | 7.267 | 75.0% | 4 | R.TEMENEFVLIK.K | 2 |
|  | AstrinSTLCLD\_041714\_01.06903.06903.2 | 3.5228 | 0.2248 | 100.0% | 1482.4321 | 1481.7473 | 8 | 5.243 | 59.1% | 2 | R.TEMENEFVLIKK.D | 2 |
|  | AstrinSTLCLD\_041714\_01.03918.03918.2 | 2.3238 | 0.4049 | 100.0% | 1085.0721 | 1085.1737 | 138 | 6.082 | 56.2% | 1 | K.DVDEAYMNK.V | 2 |
|  | AstrinSTLCLD\_041714\_01.05846.05846.2 | 4.289 | 0.484 | 100.0% | 1797.6522 | 1798.9623 | 1 | 7.892 | 67.9% | 2 | K.DVDEAYMNKVELESR.L | 2 |
|  | AstrinSTLCLD\_041714\_02.06098.06098.3 | 4.2727 | 0.2983 | 100.0% | 1799.4844 | 1798.9623 | 1 | 6.316 | 51.8% | 5 | K.DVDEAYMNKVELESR.L | 3 |
|  | AstrinSTLCLD\_041714\_02.10287.10287.3 | 4.334 | 0.4169 | 100.0% | 3198.7444 | 3200.5444 | 83 | 5.714 | 22.1% | 1 | K.DVDEAYMNKVELESRLEGLTDEINFLR.Q | 3 |
|  | AstrinSTLCLD\_041714\_01.10680.10680.2 | 4.1638 | 0.3658 | 100.0% | 1421.6522 | 1420.6055 | 1 | 7.205 | 90.9% | 5 | R.LEGLTDEINFLR.Q | 2 |
|  | AstrinSTLCLD\_041714\_02.07204.07204.2 | 5.6295 | 0.5674 | 100.0% | 2109.4321 | 2110.3008 | 1 | 9.671 | 77.8% | 2 | R.ELQSQISDTSVVLSMDNSR.S | 2 |
|  | AstrinSTLCLD\_041714\_01.10343.10343.2 | 4.1023 | 0.4135 | 100.0% | 1321.3922 | 1321.5286 | 1 | 8.367 | 77.3% | 2 | R.SLDMDSIIAEVK.A | 2 |
|  | AstrinSTLCLD\_041714\_01.03866.03866.2 | 2.6959 | 0.2317 | 99.2% | 1081.3922 | 1080.1423 | 1 | 5.818 | 87.5% | 1 | K.AQYEDIANR.S | 22 |
|  | AstrinSTLCLD\_041714\_01.07906.07906.3 | 5.0795 | 0.3705 | 100.0% | 2532.5645 | 2532.828 | 1 | 6.734 | 39.3% | 2 | R.SRAEAESMYQIKYEELQSLAGK.H | 3 |
|  | AstrinSTLCLD\_041714\_02.07606.07606.3 | 4.1576 | 0.3353 | 100.0% | 2289.1443 | 2289.5623 | 1 | 5.996 | 34.2% | 2 | R.AEAESMYQIKYEELQSLAGK.H | 3 |
|  | AstrinSTLCLD\_041714\_01.07997.07997.2 | 3.683 | 0.3898 | 100.0% | 1345.3121 | 1345.452 | 1 | 6.713 | 70.8% | 2 | R.ASLEAAIADAEQR.G | 2 |
|  | AstrinSTLCLD\_041714\_01.10182.10182.2 | 5.0648 | 0.4339 | 100.0% | 1956.5922 | 1957.1912 | 1 | 8.494 | 58.3% | 3 | R.ASLEAAIADAEQRGELAIK.D | 2 |
|  | AstrinSTLCLD\_041714\_01.10214.10214.3 | 3.7721 | 0.3542 | 100.0% | 1957.2544 | 1957.1912 | 1 | 6.306 | 41.7% | 2 | R.ASLEAAIADAEQRGELAIK.D | 3 |
|  | AstrinSTLCLD\_041714\_01.10449.10449.3 | 5.2998 | 0.4227 | 100.0% | 2455.6443 | 2456.7153 | 1 | 7.654 | 33.7% | 1 | R.ASLEAAIADAEQRGELAIKDANAK.L | 3 |
|  | AstrinSTLCLD\_041714\_01.06576.06576.2 | 4.1677 | 0.2247 | 100.0% | 1129.8322 | 1130.2865 | 1 | 5.977 | 77.8% | 4 | K.LSELEAALQR.A | 2 |
|  | AstrinSTLCLD\_041714\_01.06241.06241.2 | 3.5517 | 0.0751 | 97.8% | 1551.0521 | 1551.801 | 6 | 4.463 | 63.6% | 1 | R.QLREYQELMNVK.L | 2 |
|  | AstrinSTLCLD\_041714\_01.06799.06799.3 | 4.5873 | 0.3682 | 100.0% | 2518.5244 | 2518.8628 | 1 | 6.311 | 35.7% | 1 | R.KLLEGEESRLESGMQNMSIHTK.T | 3 |
|  | AstrinSTLCLD\_041714\_01.07408.07408.3 | 5.8143 | 0.4398 | 100.0% | 2390.7244 | 2390.6887 | 1 | 7.772 | 41.2% | 2 | K.LLEGEESRLESGMQNMSIHTK.T | 3 |

Similarities:
gi|153791158|ref|NP\_0(1:21)  

---

|  |  |  |  |  |  |  |  |  |
| --- | --- | --- | --- | --- | --- | --- | --- | --- |
| U | *gi|10645195|ref|NP\_06* | 5 | 13 | 43.1% | 130 | 14135 | 11.1 | histone cluster 1, H2ae [Homo sapiens] |
| U | *gi|19557656|ref|NP\_00* | 5 | 13 | 43.1% | 130 | 14135 | 11.1 | histone cluster 1, H2ab [Homo sapiens] |
| U | *gi|15617199|ref|NP\_25* | 5 | 13 | 43.1% | 130 | 14121 | 11.1 | histone cluster 3, H2a [Homo sapiens] |

| Filename XCorr DeltCN Conf% ObsM+H+ CalcM+H+ SpR ZScore Ion% # Sequence  | | | | | | | | | | | | |
| --- | --- | --- | --- | --- | --- | --- | --- | --- | --- | --- | --- | --- |
|  | AstrinSTLCLD\_041714\_01.06035.06035.2 | 3.1245 | 0.3446 | 100.0% | 944.7522 | 945.1093 | 2 | 6.049 | 81.2% | 5 | R.AGLQFPVGR.V | 222 |
|  | AstrinSTLCLD\_041714\_01.17776.17776.2 | 4.845 | 0.5749 | 100.0% | 2916.0923 | 2917.3752 | 1 | 10.395 | 42.9% | 2 | R.VGAGAPVYLAAVLEYLTAEILELAGNAAR.D | 22 |
|  | AstrinSTLCLD\_041714\_01.17772.17772.3 | 3.1971 | 0.3939 | 100.0% | 2916.1443 | 2917.3752 | 17 | 6.548 | 23.2% | 1 | R.VGAGAPVYLAAVLEYLTAEILELAGNAAR.D | 33 |
|  | AstrinSTLCLD\_041714\_02.06002.06002.3 | 3.0348 | 0.3547 | 100.0% | 1695.1444 | 1693.9004 | 1 | 6.097 | 42.3% | 4 | R.HLQLAIRNDEELNK.L | 333 |
|  | AstrinSTLCLD\_041714\_01.06855.06855.2 | 2.9694 | 0.3141 | 100.0% | 1300.5122 | 1301.4423 | 3 | 5.925 | 70.0% | 1 | R.NDEELNKLLGR.V | 2 |

Similarities:
gi|106775678|ref|NP\_0(2:3)  
gi|10800130|ref|NP\_06(4:1)  

---

|  |  |  |  |  |  |  |  |  |
| --- | --- | --- | --- | --- | --- | --- | --- | --- |
| U | *gi|10800130|ref|NP\_06* | 6 | 15 | 43.1% | 130 | 14107 | 10.9 | histone cluster 1, H2ad [Homo sapiens] |
| U | *gi|4504243|ref|NP\_003* | 6 | 15 | 43.1% | 130 | 14091 | 10.9 | histone cluster 1, H2al [Homo sapiens] |
| U | *gi|4504239|ref|NP\_003* | 6 | 15 | 43.1% | 130 | 14091 | 10.9 | histone cluster 1, H2ai [Homo sapiens] |
| U | *gi|18105045|ref|NP\_54* | 6 | 15 | 43.8% | 128 | 13906 | 10.9 | histone cluster 1, H2ah [Homo sapiens] |
| U | *gi|10800144|ref|NP\_06* | 6 | 15 | 43.8% | 128 | 13936 | 10.9 | histone cluster 1, H2aj [Homo sapiens] |
| U | *gi|10800132|ref|NP\_06* | 6 | 15 | 43.1% | 130 | 14091 | 10.9 | histone cluster 1, H2ag [Homo sapiens] |

| Filename XCorr DeltCN Conf% ObsM+H+ CalcM+H+ SpR ZScore Ion% # Sequence  | | | | | | | | | | | | |
| --- | --- | --- | --- | --- | --- | --- | --- | --- | --- | --- | --- | --- |
|  | AstrinSTLCLD\_041714\_01.06035.06035.2 | 3.1245 | 0.3446 | 100.0% | 944.7522 | 945.1093 | 2 | 6.049 | 81.2% | 5 | R.AGLQFPVGR.V | 222 |
|  | AstrinSTLCLD\_041714\_01.17776.17776.2 | 4.845 | 0.5749 | 100.0% | 2916.0923 | 2917.3752 | 1 | 10.395 | 42.9% | 2 | R.VGAGAPVYLAAVLEYLTAEILELAGNAAR.D | 22 |
|  | AstrinSTLCLD\_041714\_01.17772.17772.3 | 3.1971 | 0.3939 | 100.0% | 2916.1443 | 2917.3752 | 17 | 6.548 | 23.2% | 1 | R.VGAGAPVYLAAVLEYLTAEILELAGNAAR.D | 33 |
|  | AstrinSTLCLD\_041714\_02.06002.06002.3 | 3.0348 | 0.3547 | 100.0% | 1695.1444 | 1693.9004 | 1 | 6.097 | 42.3% | 4 | R.HLQLAIRNDEELNK.L | 333 |
|  | AstrinSTLCLD\_041714\_01.08000.08000.3 | 3.993 | 0.3608 | 100.0% | 2106.3843 | 2105.4453 | 1 | 5.767 | 38.2% | 1 | R.HLQLAIRNDEELNKLLGK.V | 33 |
|  | AstrinSTLCLD\_041714\_01.06227.06227.2 | 3.5524 | 0.3511 | 100.0% | 1273.0521 | 1273.4288 | 12 | 6.622 | 65.0% | 2 | R.NDEELNKLLGK.V | 22 |

Similarities:
gi|106775678|ref|NP\_0(4:2)  
gi|10645195|ref|NP\_06(4:2)  

---

|  |  |  |  |  |  |  |  |  |
| --- | --- | --- | --- | --- | --- | --- | --- | --- |
| U | *contaminant\_gi|746301* | 8 | 46 | 42.8% | 269 | 27961 | 6.7 | lysyl endopeptidase (EC 3.4.21.50) - Lysobacter enzymogenes |

| Filename XCorr DeltCN Conf% ObsM+H+ CalcM+H+ SpR ZScore Ion% # Sequence  | | | | | | | | | | | | |
| --- | --- | --- | --- | --- | --- | --- | --- | --- | --- | --- | --- | --- |
| \* | AstrinSTLCLD\_041714\_01.04203.04203.2 | 5.8542 | 0.5905 | 100.0% | 2261.672 | 2262.355 | 1 | 10.971 | 54.2% | 6 | R.APGSSSSGANGDGSLAQSQTGAVVR.A | 2 |
| \* | AstrinSTLCLD\_041714\_02.04598.04598.3 | 4.6375 | 0.4333 | 100.0% | 2262.0842 | 2262.355 | 1 | 7.151 | 40.6% | 9 | R.APGSSSSGANGDGSLAQSQTGAVVR.A | 3 |
| \* | AstrinSTLCLD\_041714\_01.14312.14312.3 | 4.4975 | 0.3064 | 100.0% | 3316.5244 | 3315.6257 | 1 | 5.314 | 28.4% | 2 | R.ATNAASDFTLLELNTAANPAYNLFWAGWDR.R | 3 |
| \* | AstrinSTLCLD\_041714\_01.14262.14262.2 | 4.7474 | 0.3008 | 100.0% | 3317.172 | 3315.6257 | 1 | 6.99 | 39.7% | 3 | R.ATNAASDFTLLELNTAANPAYNLFWAGWDR.R | 2 |
| \* | AstrinSTLCLD\_041714\_02.11630.11630.3 | 6.3666 | 0.5538 | 100.0% | 3470.0645 | 3471.813 | 1 | 11.057 | 27.5% | 12 | R.ATNAASDFTLLELNTAANPAYNLFWAGWDRR.D | 3 |
| \* | AstrinSTLCLD\_041714\_01.04124.04124.3 | 4.1545 | 0.4752 | 100.0% | 2077.4343 | 2077.2668 | 1 | 7.789 | 37.5% | 7 | R.DQNFAGATAIHHPNVAEKR.I | 3 |
| \* | AstrinSTLCLD\_041714\_02.06008.06008.2 | 4.2482 | 0.4935 | 100.0% | 1428.2522 | 1428.5443 | 1 | 8.211 | 69.2% | 6 | R.VFTSWTGGGTSATR.L | 2 |
| \* | AstrinSTLCLD\_041714\_01.12896.12896.2 | 4.2573 | 0.3892 | 100.0% | 2605.5723 | 2605.8174 | 1 | 8.313 | 32.0% | 1 | R.LSDWLDAAGTGAQFIDGLDSTGTPPV.- | 2 |

---

|  |  |  |  |  |  |  |  |  |
| --- | --- | --- | --- | --- | --- | --- | --- | --- |
| U | *gi|5174735|ref|NP\_006* | 17 | 41 | 41.3% | 445 | 49831 | 4.9 | tubulin, beta, 2 [Homo sapiens] |

| Filename XCorr DeltCN Conf% ObsM+H+ CalcM+H+ SpR ZScore Ion% # Sequence  | | | | | | | | | | | | |
| --- | --- | --- | --- | --- | --- | --- | --- | --- | --- | --- | --- | --- |
| \* | AstrinSTLCLD\_041714\_02.05303.05303.2 | 3.2846 | 0.3658 | 100.0% | 1330.4321 | 1329.4521 | 1 | 6.996 | 68.2% | 1 | R.INVYYNEATGGK.Y | 2 |
|  | AstrinSTLCLD\_041714\_01.08693.08693.2 | 4.1991 | 0.4374 | 100.0% | 1603.4521 | 1602.8431 | 1 | 8.651 | 78.6% | 2 | R.AVLVDLEPGTMDSVR.S | 2 |
|  | AstrinSTLCLD\_041714\_01.10952.10952.3 | 7.1011 | 0.4962 | 100.0% | 2799.1143 | 2800.0647 | 1 | 8.497 | 38.0% | 5 | R.SGPFGQIFRPDNFVFGQSGAGNNWAK.G | 33 |
|  | AstrinSTLCLD\_041714\_01.11397.11397.2 | 6.437 | 0.554 | 100.0% | 1959.8121 | 1960.151 | 1 | 10.53 | 79.4% | 1 | K.GHYTEGAELVDSVLDVVR.K | 222 |
|  | AstrinSTLCLD\_041714\_01.10706.10706.3 | 4.4871 | 0.4257 | 100.0% | 2088.8643 | 2088.325 | 1 | 7.56 | 40.3% | 2 | K.GHYTEGAELVDSVLDVVRK.E | 333 |
|  | AstrinSTLCLD\_041714\_01.07358.07358.2 | 4.3453 | 0.4254 | 100.0% | 1320.1322 | 1320.5896 | 1 | 8.343 | 72.7% | 6 | R.IMNTFSVVPSPK.V | 222 |
|  | AstrinSTLCLD\_041714\_01.06043.06043.2 | 2.8729 | 0.2906 | 100.0% | 1131.0922 | 1131.2767 | 9 | 5.247 | 77.8% | 7 | R.FPGQLNADLR.K | 222 |
|  | AstrinSTLCLD\_041714\_01.07593.07593.2 | 3.7915 | 0.3644 | 100.0% | 1272.4722 | 1272.5945 | 1 | 7.372 | 70.0% | 2 | R.KLAVNMVPFPR.L | 222 |
|  | AstrinSTLCLD\_041714\_01.08967.08967.2 | 3.7007 | 0.4846 | 100.0% | 1144.3922 | 1144.4204 | 1 | 9.08 | 94.4% | 2 | K.LAVNMVPFPR.L | 222 |
|  | AstrinSTLCLD\_041714\_01.10875.10875.3 | 4.0389 | 0.3577 | 100.0% | 1622.3043 | 1621.9403 | 1 | 6.217 | 51.9% | 2 | R.LHFFMPGFAPLTSR.G | 33 |
|  | AstrinSTLCLD\_041714\_01.10880.10880.2 | 3.6408 | 0.3737 | 100.0% | 1622.7122 | 1621.9403 | 1 | 7.77 | 73.1% | 2 | R.LHFFMPGFAPLTSR.G | 22 |
|  | AstrinSTLCLD\_041714\_01.10805.10805.2 | 3.6537 | 0.4002 | 100.0% | 1692.4321 | 1692.9678 | 1 | 8.342 | 71.4% | 1 | R.ALTVPELTQQMFDAK.N | 22 |
|  | AstrinSTLCLD\_041714\_01.09495.09495.2 | 2.7652 | 0.2691 | 100.0% | 1041.4521 | 1040.2505 | 1 | 6.163 | 87.5% | 1 | R.YLTVAAVFR.G | 22 |
|  | AstrinSTLCLD\_041714\_02.06758.06758.3 | 3.9569 | 0.2413 | 100.0% | 1924.8544 | 1925.2405 | 7 | 5.619 | 41.7% | 2 | R.MSMKEVDEQMLNVQNK.N | 33 |
|  | AstrinSTLCLD\_041714\_01.10293.10293.2 | 2.9679 | 0.4318 | 100.0% | 1697.3121 | 1697.8877 | 1 | 7.31 | 57.7% | 1 | K.NSSYFVEWIPNNVK.T | 222 |
|  | AstrinSTLCLD\_041714\_01.08030.08030.2 | 3.2012 | 0.3385 | 100.0% | 1385.6122 | 1386.6116 | 47 | 5.663 | 60.0% | 1 | K.RISEQFTAMFR.R | 222 |
|  | AstrinSTLCLD\_041714\_02.07989.07989.2 | 3.7427 | 0.5204 | 100.0% | 1229.6721 | 1230.4241 | 1 | 8.103 | 94.4% | 3 | R.ISEQFTAMFR.R | 222 |

Similarities:
gi|29788785|ref|NP\_82(14:3)  
gi|50592996|ref|NP\_00(10:7)  

---

|  |  |  |  |  |  |  |  |  |
| --- | --- | --- | --- | --- | --- | --- | --- | --- |
| U | *gi|11415030|ref|NP\_06* | 4 | 7 | 36.9% | 103 | 11367 | 11.4 | histone cluster 1, H4j [Homo sapiens] |
| U | *gi|77539758|ref|NP\_00* | 4 | 7 | 36.9% | 103 | 11367 | 11.4 | histone cluster 2, H4b [Homo sapiens] |
| U | *gi|4504323|ref|NP\_003* | 4 | 7 | 36.9% | 103 | 11367 | 11.4 | histone cluster 2, H4a [Homo sapiens] |
| U | *gi|4504321|ref|NP\_003* | 4 | 7 | 36.9% | 103 | 11367 | 11.4 | histone cluster 1, H4i [Homo sapiens] |
| U | *gi|4504317|ref|NP\_003* | 4 | 7 | 36.9% | 103 | 11367 | 11.4 | histone cluster 1, H4l [Homo sapiens] |
| U | *gi|4504315|ref|NP\_003* | 4 | 7 | 36.9% | 103 | 11367 | 11.4 | histone cluster 1, H4e [Homo sapiens] |
| U | *gi|4504313|ref|NP\_003* | 4 | 7 | 36.9% | 103 | 11367 | 11.4 | histone cluster 1, H4b [Homo sapiens] |
| U | *gi|4504311|ref|NP\_003* | 4 | 7 | 36.9% | 103 | 11367 | 11.4 | histone cluster 1, H4h [Homo sapiens] |
| U | *gi|4504309|ref|NP\_003* | 4 | 7 | 36.9% | 103 | 11367 | 11.4 | histone cluster 1, H4c [Homo sapiens] |
| U | *gi|4504307|ref|NP\_003* | 4 | 7 | 36.9% | 103 | 11367 | 11.4 | histone cluster 1, H4k [Homo sapiens] |
| U | *gi|4504305|ref|NP\_003* | 4 | 7 | 36.9% | 103 | 11367 | 11.4 | histone cluster 1, H4f [Homo sapiens] |
| U | *gi|4504303|ref|NP\_003* | 4 | 7 | 36.9% | 103 | 11367 | 11.4 | histone cluster 1, H4d [Homo sapiens] |
| U | *gi|4504301|ref|NP\_003* | 4 | 7 | 36.9% | 103 | 11367 | 11.4 | histone cluster 1, H4a [Homo sapiens] |
| U | *gi|28173560|ref|NP\_77* | 4 | 7 | 36.9% | 103 | 11367 | 11.4 | histone cluster 4, H4 [Homo sapiens] |

| Filename XCorr DeltCN Conf% ObsM+H+ CalcM+H+ SpR ZScore Ion% # Sequence  | | | | | | | | | | | | |
| --- | --- | --- | --- | --- | --- | --- | --- | --- | --- | --- | --- | --- |
|  | AstrinSTLCLD\_041714\_02.06238.06238.2 | 3.5854 | 0.3552 | 100.0% | 1182.1322 | 1181.3312 | 1 | 6.59 | 88.9% | 1 | R.ISGLIYEETR.G | 2 |
|  | AstrinSTLCLD\_041714\_01.08282.08282.2 | 3.0977 | 0.2474 | 100.0% | 990.53217 | 990.19055 | 3 | 5.676 | 85.7% | 3 | K.VFLENVIR.D | 2 |
|  | AstrinSTLCLD\_041714\_01.09314.09314.2 | 3.5126 | 0.4945 | 100.0% | 1467.5122 | 1467.7667 | 1 | 7.533 | 70.8% | 1 | K.TVTAMDVVYALKR.Q | 2 |
|  | AstrinSTLCLD\_041714\_01.07604.07604.1 | 1.7587 | 0.3983 | 100.0% | 714.25 | 714.796 | 1 | 7.293 | 75.0% | 2 | R.TLYGFGG.- | 1 |

---

|  |  |  |  |  |  |  |  |  |
| --- | --- | --- | --- | --- | --- | --- | --- | --- |
| U | *gi|14389309|ref|NP\_11* | 15 | 36 | 36.1% | 449 | 49895 | 5.1 | tubulin alpha 6 [Homo sapiens] |
| U | *gi|57013276|ref|NP\_00* | 15 | 36 | 35.9% | 451 | 50152 | 5.1 | tubulin, alpha, ubiquitous [Homo sapiens] |

| Filename XCorr DeltCN Conf% ObsM+H+ CalcM+H+ SpR ZScore Ion% # Sequence  | | | | | | | | | | | | |
| --- | --- | --- | --- | --- | --- | --- | --- | --- | --- | --- | --- | --- |
|  | AstrinSTLCLD\_041714\_01.09922.09922.2 | 5.5935 | 0.5825 | 100.0% | 2009.4321 | 2009.093 | 1 | 10.84 | 60.5% | 4 | K.TIGGGDDSFNTFFSETGAGK.H | 2 |
|  | AstrinSTLCLD\_041714\_01.10568.10568.2 | 4.6973 | 0.4621 | 100.0% | 1702.3722 | 1702.9451 | 1 | 7.878 | 78.6% | 4 | R.AVFVDLEPTVIDEVR.T | 2 |
|  | AstrinSTLCLD\_041714\_01.07219.07219.3 | 3.7043 | 0.3204 | 100.0% | 2417.2144 | 2416.6555 | 1 | 5.299 | 33.8% | 1 | R.QLFHPEQLITGKEDAANNYAR.G | 33 |
|  | AstrinSTLCLD\_041714\_01.11350.11350.2 | 4.3586 | 0.4869 | 100.0% | 1843.5122 | 1843.1332 | 1 | 8.224 | 66.7% | 1 | R.GHYTIGKEIIDLVLDR.I | 2 |
|  | AstrinSTLCLD\_041714\_01.11322.11322.3 | 3.6774 | 0.3693 | 100.0% | 1843.5543 | 1843.1332 | 1 | 7.584 | 48.3% | 1 | R.GHYTIGKEIIDLVLDR.I | 3 |
|  | AstrinSTLCLD\_041714\_01.10167.10167.2 | 2.226 | 0.3686 | 99.7% | 1085.8722 | 1086.2737 | 15 | 6.275 | 75.0% | 1 | K.EIIDLVLDR.I | 2 |
|  | AstrinSTLCLD\_041714\_02.10890.10890.2 | 4.8009 | 0.5004 | 100.0% | 1489.3522 | 1488.7678 | 1 | 9.204 | 80.8% | 6 | R.LISQIVSSITASLR.F | 22 |
|  | AstrinSTLCLD\_041714\_02.10868.10868.3 | 4.1356 | 0.3592 | 100.0% | 1489.3744 | 1488.7678 | 1 | 6.987 | 48.1% | 2 | R.LISQIVSSITASLR.F | 33 |
|  | AstrinSTLCLD\_041714\_02.10015.10015.2 | 5.2411 | 0.5596 | 100.0% | 2409.7322 | 2410.6885 | 1 | 10.253 | 50.0% | 3 | R.FDGALNVDLTEFQTNLVPYPR.I | 22 |
|  | AstrinSTLCLD\_041714\_02.10025.10025.3 | 3.4541 | 0.5091 | 100.0% | 2410.8542 | 2410.6885 | 3 | 7.371 | 31.2% | 1 | R.FDGALNVDLTEFQTNLVPYPR.I | 33 |
|  | AstrinSTLCLD\_041714\_01.09417.09417.2 | 4.1458 | 0.4952 | 100.0% | 1758.2722 | 1758.0703 | 1 | 8.517 | 63.3% | 4 | R.IHFPLATYAPVISAEK.A | 22 |
|  | AstrinSTLCLD\_041714\_01.09483.09483.3 | 3.8158 | 0.3589 | 100.0% | 1758.8344 | 1758.0703 | 2 | 6.303 | 51.7% | 2 | R.IHFPLATYAPVISAEK.A | 33 |
|  | AstrinSTLCLD\_041714\_01.07724.07724.2 | 3.9402 | 0.4208 | 100.0% | 1825.5721 | 1826.1027 | 1 | 7.072 | 61.8% | 3 | K.VGINYQPPTVVPGGDLAK.V | 22 |
|  | AstrinSTLCLD\_041714\_01.08327.08327.3 | 4.7328 | 0.4371 | 100.0% | 2488.2844 | 2487.7083 | 1 | 6.495 | 40.0% | 1 | K.RAFVHWYVGEGMEEGEFSEAR.E | 33 |
|  | AstrinSTLCLD\_041714\_02.08190.08190.3 | 3.9491 | 0.1957 | 98.9% | 2333.6343 | 2331.5208 | 1 | 4.754 | 40.8% | 2 | R.AFVHWYVGEGMEEGEFSEAR.E | 33 |

Similarities:
gi|17921989|ref|NP\_00(10:5)  

---

|  |  |  |  |  |  |  |  |  |
| --- | --- | --- | --- | --- | --- | --- | --- | --- |
| U | *gi|218505827|ref|NP\_1* | 5 | 9 | 30.1% | 316 | 35438 | 6.3 | TRAF4 associated factor 1 isoform a [Homo sapiens] |
| U | *gi|218505831|ref|NP\_0* | 5 | 9 | 33.2% | 286 | 31880 | 7.1 | TRAF4 associated factor 1 isoform b [Homo sapiens] |

| Filename XCorr DeltCN Conf% ObsM+H+ CalcM+H+ SpR ZScore Ion% # Sequence  | | | | | | | | | | | | |
| --- | --- | --- | --- | --- | --- | --- | --- | --- | --- | --- | --- | --- |
|  | AstrinSTLCLD\_041714\_01.06918.06918.2 | 5.4469 | 0.4993 | 100.0% | 2274.4922 | 2275.4802 | 1 | 8.592 | 59.5% | 2 | K.TVYSLQPPSALSGGQPADTQTR.A | 2 |
|  | AstrinSTLCLD\_041714\_01.09513.09513.3 | 5.0575 | 0.3191 | 100.0% | 2317.9744 | 2316.6543 | 1 | 6.15 | 40.8% | 1 | K.LTETQGELKDLTQKVELLEK.F | 3 |
|  | AstrinSTLCLD\_041714\_01.07162.07162.2 | 2.6584 | 0.1916 | 97.8% | 1163.5122 | 1163.2793 | 1 | 5.099 | 72.2% | 1 | R.DNCLAILESK.G | 2 |
|  | AstrinSTLCLD\_041714\_01.05921.05921.2 | 3.7009 | 0.5414 | 100.0% | 1387.6522 | 1387.5327 | 1 | 9.735 | 65.4% | 4 | K.GLDPALGSETLASR.Q | 2 |
|  | AstrinSTLCLD\_041714\_01.13595.13595.3 | 5.694 | 0.3451 | 100.0% | 3396.1143 | 3396.8062 | 20 | 6.508 | 21.4% | 1 | R.QESTTDHMDSMLLLETLQEELKLFNETAK.K | 3 |

---

|  |  |  |  |  |  |  |  |  |
| --- | --- | --- | --- | --- | --- | --- | --- | --- |
| U | *gi|57242777|ref|NP\_03* | 2 | 2 | 30.1% | 103 | 11967 | 5.9 | c-myc binding protein [Homo sapiens] |

| Filename XCorr DeltCN Conf% ObsM+H+ CalcM+H+ SpR ZScore Ion% # Sequence  | | | | | | | | | | | | |
| --- | --- | --- | --- | --- | --- | --- | --- | --- | --- | --- | --- | --- |
| \* | AstrinSTLCLD\_041714\_01.10350.10350.3 | 3.5673 | 0.4114 | 100.0% | 2276.6343 | 2276.6348 | 1 | 6.662 | 32.9% | 1 | K.VLVALYEEPEKPNSALDFLK.H | 3 |
| \* | AstrinSTLCLD\_041714\_01.03912.03912.2 | 2.8981 | 0.3091 | 100.0% | 1331.8922 | 1332.4528 | 5 | 5.217 | 60.0% | 1 | K.LAQYEPPQEEK.R | 2 |

---

|  |  |  |  |  |  |  |  |  |
| --- | --- | --- | --- | --- | --- | --- | --- | --- |
| U | *gi|62414289|ref|NP\_00* | 10 | 11 | 29.0% | 466 | 53652 | 5.1 | vimentin [Homo sapiens] |

| Filename XCorr DeltCN Conf% ObsM+H+ CalcM+H+ SpR ZScore Ion% # Sequence  | | | | | | | | | | | | |
| --- | --- | --- | --- | --- | --- | --- | --- | --- | --- | --- | --- | --- |
| \* | AstrinSTLCLD\_041714\_01.05410.05410.2 | 3.7666 | 0.4979 | 100.0% | 1254.6522 | 1255.385 | 1 | 8.427 | 77.8% | 1 | R.LGDLYEEEMR.E | 2 |
| \* | AstrinSTLCLD\_041714\_01.08100.08100.2 | 2.5353 | 0.1582 | 96.2% | 1076.2322 | 1077.1975 | 37 | 5.349 | 68.8% | 1 | R.DNLAEDIMR.L | 2 |
| \* | AstrinSTLCLD\_041714\_02.07134.07134.3 | 3.6943 | 0.3104 | 100.0% | 2351.3943 | 2352.581 | 5 | 5.732 | 30.6% | 1 | K.LQEEMLQREEAENTLQSFR.Q | 3 |
| \* | AstrinSTLCLD\_041714\_01.07792.07792.3 | 3.4682 | 0.2325 | 99.3% | 1662.9543 | 1662.967 | 3 | 5.157 | 36.5% | 1 | R.KVESLQEEIAFLKK.L | 3 |
|  | AstrinSTLCLD\_041714\_01.06568.06568.2 | 2.8232 | 0.1999 | 99.0% | 1311.9122 | 1310.4056 | 2 | 4.001 | 72.2% | 2 | K.NLQEAEEWYK.S | 2 |
| \* | AstrinSTLCLD\_041714\_02.07569.07569.2 | 5.8502 | 0.5495 | 100.0% | 2187.912 | 2188.33 | 1 | 10.889 | 63.9% | 1 | R.EMEENFAVEAANYQDTIGR.L | 2 |
| \* | AstrinSTLCLD\_041714\_01.05785.05785.2 | 4.582 | 0.4837 | 100.0% | 1735.4521 | 1735.9679 | 1 | 8.286 | 73.1% | 1 | R.LQDEIQNMKEEMAR.H | 2 |
|  | AstrinSTLCLD\_041714\_02.08124.08124.2 | 2.8872 | 0.2045 | 99.0% | 1296.3121 | 1296.5243 | 1 | 5.777 | 65.0% | 1 | K.MALDIEIATYR.K | 2 |
| \* | AstrinSTLCLD\_041714\_01.11576.11576.2 | 3.4267 | 0.4032 | 100.0% | 1571.4722 | 1571.8601 | 1 | 7.541 | 80.8% | 1 | R.ISLPLPNFSSLNLR.E | 2 |
| \* | AstrinSTLCLD\_041714\_01.06795.06795.2 | 2.806 | 0.1885 | 96.2% | 1670.4321 | 1669.829 | 1 | 4.818 | 57.1% | 1 | R.ETNLDSLPLVDTHSK.R | 2 |

---

|  |  |  |  |  |  |  |  |  |
| --- | --- | --- | --- | --- | --- | --- | --- | --- |
| U | *gi|4504517|ref|NP\_001* | 5 | 6 | 28.3% | 205 | 22783 | 6.4 | heat shock protein beta-1 [Homo sapiens] |

| Filename XCorr DeltCN Conf% ObsM+H+ CalcM+H+ SpR ZScore Ion% # Sequence  | | | | | | | | | | | | |
| --- | --- | --- | --- | --- | --- | --- | --- | --- | --- | --- | --- | --- |
| \* | AstrinSTLCLD\_041714\_01.09892.09892.3 | 2.5665 | 0.2543 | 96.8% | 1903.4944 | 1904.0537 | 3 | 5.194 | 35.7% | 1 | R.GPSWDPFRDWYPHSR.L | 3 |
| \* | AstrinSTLCLD\_041714\_01.09430.09430.2 | 3.4821 | 0.4166 | 100.0% | 1164.2322 | 1164.3494 | 1 | 7.513 | 83.3% | 1 | R.LFDQAFGLPR.L | 2 |
| \* | AstrinSTLCLD\_041714\_01.08274.08274.2 | 4.5367 | 0.5117 | 100.0% | 1784.2322 | 1785.0068 | 1 | 8.005 | 53.3% | 1 | R.VSLDVNHFAPDELTVK.T | 2 |
| \* | AstrinSTLCLD\_041714\_01.08324.08324.3 | 2.6847 | 0.2707 | 97.9% | 1784.7843 | 1785.0068 | 430 | 5.331 | 28.3% | 2 | R.VSLDVNHFAPDELTVK.T | 3 |
| \* | AstrinSTLCLD\_041714\_01.08888.08888.2 | 3.228 | 0.4372 | 100.0% | 1906.3322 | 1907.1307 | 7 | 6.68 | 40.6% | 1 | K.LATQSNEITIPVTFESR.A | 2 |

---

|  |  |  |  |  |  |  |  |  |
| --- | --- | --- | --- | --- | --- | --- | --- | --- |
| U | *gi|17921989|ref|NP\_00* | 11 | 26 | 28.1% | 448 | 49924 | 5.1 | tubulin, alpha 4a [Homo sapiens] |

| Filename XCorr DeltCN Conf% ObsM+H+ CalcM+H+ SpR ZScore Ion% # Sequence  | | | | | | | | | | | | |
| --- | --- | --- | --- | --- | --- | --- | --- | --- | --- | --- | --- | --- |
| \* | AstrinSTLCLD\_041714\_01.11122.11122.2 | 3.1182 | 0.2629 | 99.6% | 1716.6322 | 1716.9719 | 1 | 5.475 | 67.9% | 1 | R.AVFVDLEPTVIDEIR.N | 2 |
|  | AstrinSTLCLD\_041714\_01.07219.07219.3 | 3.7043 | 0.3204 | 100.0% | 2417.2144 | 2416.6555 | 1 | 5.299 | 33.8% | 1 | R.QLFHPEQLITGKEDAANNYAR.G | 33 |
|  | AstrinSTLCLD\_041714\_02.10890.10890.2 | 4.8009 | 0.5004 | 100.0% | 1489.3522 | 1488.7678 | 1 | 9.204 | 80.8% | 6 | R.LISQIVSSITASLR.F | 22 |
|  | AstrinSTLCLD\_041714\_02.10868.10868.3 | 4.1356 | 0.3592 | 100.0% | 1489.3744 | 1488.7678 | 1 | 6.987 | 48.1% | 2 | R.LISQIVSSITASLR.F | 33 |
|  | AstrinSTLCLD\_041714\_02.10015.10015.2 | 5.2411 | 0.5596 | 100.0% | 2409.7322 | 2410.6885 | 1 | 10.253 | 50.0% | 3 | R.FDGALNVDLTEFQTNLVPYPR.I | 22 |
|  | AstrinSTLCLD\_041714\_02.10025.10025.3 | 3.4541 | 0.5091 | 100.0% | 2410.8542 | 2410.6885 | 3 | 7.371 | 31.2% | 1 | R.FDGALNVDLTEFQTNLVPYPR.I | 33 |
|  | AstrinSTLCLD\_041714\_01.09417.09417.2 | 4.1458 | 0.4952 | 100.0% | 1758.2722 | 1758.0703 | 1 | 8.517 | 63.3% | 4 | R.IHFPLATYAPVISAEK.A | 22 |
|  | AstrinSTLCLD\_041714\_01.09483.09483.3 | 3.8158 | 0.3589 | 100.0% | 1758.8344 | 1758.0703 | 2 | 6.303 | 51.7% | 2 | R.IHFPLATYAPVISAEK.A | 33 |
|  | AstrinSTLCLD\_041714\_01.07724.07724.2 | 3.9402 | 0.4208 | 100.0% | 1825.5721 | 1826.1027 | 1 | 7.072 | 61.8% | 3 | K.VGINYQPPTVVPGGDLAK.V | 22 |
|  | AstrinSTLCLD\_041714\_01.08327.08327.3 | 4.7328 | 0.4371 | 100.0% | 2488.2844 | 2487.7083 | 1 | 6.495 | 40.0% | 1 | K.RAFVHWYVGEGMEEGEFSEAR.E | 33 |
|  | AstrinSTLCLD\_041714\_02.08190.08190.3 | 3.9491 | 0.1957 | 98.9% | 2333.6343 | 2331.5208 | 1 | 4.754 | 40.8% | 2 | R.AFVHWYVGEGMEEGEFSEAR.E | 33 |

Similarities:
gi|14389309|ref|NP\_11(10:1)  

---

|  |  |  |  |  |  |  |  |  |
| --- | --- | --- | --- | --- | --- | --- | --- | --- |
| U | *gi|14043072|ref|NP\_11* | 7 | 13 | 27.8% | 353 | 37430 | 8.9 | heterogeneous nuclear ribonucleoprotein A2/B1 isoform B1 [Homo sapiens] |
| U | *gi|4504447|ref|NP\_002* | 7 | 13 | 28.7% | 341 | 36006 | 8.6 | heterogeneous nuclear ribonucleoprotein A2/B1 isoform A2 [Homo sapiens] |

| Filename XCorr DeltCN Conf% ObsM+H+ CalcM+H+ SpR ZScore Ion% # Sequence  | | | | | | | | | | | | |
| --- | --- | --- | --- | --- | --- | --- | --- | --- | --- | --- | --- | --- |
|  | AstrinSTLCLD\_041714\_02.09491.09491.2 | 4.5858 | 0.3746 | 100.0% | 1800.8121 | 1800.0184 | 1 | 6.029 | 60.0% | 6 | K.LFIGGLSFETTEESLR.N | 2 |
|  | AstrinSTLCLD\_041714\_01.12291.12291.3 | 3.7523 | 0.2937 | 100.0% | 2868.2944 | 2869.1597 | 1 | 4.667 | 29.3% | 1 | K.LFIGGLSFETTEESLRNYYEQWGK.L | 3 |
|  | AstrinSTLCLD\_041714\_01.04703.04703.3 | 2.966 | 0.3477 | 100.0% | 1881.9543 | 1881.0984 | 7 | 5.717 | 35.0% | 1 | K.LFVGGIKEDTEEHHLR.D | 3 |
|  | AstrinSTLCLD\_041714\_01.08076.08076.2 | 3.1574 | 0.3984 | 100.0% | 1189.3722 | 1189.3513 | 8 | 7.427 | 77.8% | 2 | K.IDTIEIITDR.Q | 2 |
|  | AstrinSTLCLD\_041714\_01.08933.08933.2 | 3.1853 | 0.4102 | 100.0% | 1696.2522 | 1696.8132 | 1 | 7.603 | 60.7% | 1 | R.GFGFVTFDDHDPVDK.I | 2 |
|  | AstrinSTLCLD\_041714\_01.09966.09966.3 | 3.2684 | 0.3324 | 100.0% | 2278.4343 | 2278.5693 | 1 | 7.237 | 31.6% | 1 | R.GFGFVTFDDHDPVDKIVLQK.Y | 3 |
|  | AstrinSTLCLD\_041714\_01.06758.06758.3 | 3.2202 | 0.2169 | 96.2% | 2496.8643 | 2496.5303 | 339 | 4.776 | 23.1% | 1 | R.GFGDGYNGYGGGPGGGNFGGSPGYGGGR.G | 3 |

---

|  |  |  |  |  |  |  |  |  |
| --- | --- | --- | --- | --- | --- | --- | --- | --- |
| U | *gi|5902102|ref|NP\_008* | 2 | 5 | 27.7% | 119 | 13282 | 11.6 | small nuclear ribonucleoprotein D1 polypeptide 16kDa [Homo sapiens] |

| Filename XCorr DeltCN Conf% ObsM+H+ CalcM+H+ SpR ZScore Ion% # Sequence  | | | | | | | | | | | | |
| --- | --- | --- | --- | --- | --- | --- | --- | --- | --- | --- | --- | --- |
|  | AstrinSTLCLD\_041714\_01.06430.06430.2 | 3.4885 | 0.2627 | 100.0% | 1555.4922 | 1555.7745 | 1 | 5.393 | 62.5% | 1 | K.NREPVQLETLSIR.G | 2 |
| \* | AstrinSTLCLD\_041714\_01.14682.14682.2 | 3.4384 | 0.3992 | 100.0% | 2287.0522 | 2288.6863 | 1 | 7.083 | 42.1% | 4 | R.YFILPDSLPLDTLLVDVEPK.V | 2 |

---

|  |  |  |  |  |  |  |  |  |
| --- | --- | --- | --- | --- | --- | --- | --- | --- |
| U | *gi|224028244|ref|NP\_0* | 17 | 60 | 27.6% | 471 | 54232 | 8.9 | non-POU domain containing, octamer-binding isoform 1 [Homo sapiens] |
| U | *gi|34932414|ref|NP\_03* | 17 | 60 | 27.6% | 471 | 54232 | 8.9 | non-POU domain containing, octamer-binding isoform 1 [Homo sapiens] |
| U | *gi|224028246|ref|NP\_0* | 17 | 60 | 27.6% | 471 | 54232 | 8.9 | non-POU domain containing, octamer-binding isoform 1 [Homo sapiens] |

| Filename XCorr DeltCN Conf% ObsM+H+ CalcM+H+ SpR ZScore Ion% # Sequence  | | | | | | | | | | | | |
| --- | --- | --- | --- | --- | --- | --- | --- | --- | --- | --- | --- | --- |
|  | AstrinSTLCLD\_041714\_01.08770.08770.2 | 3.2564 | 0.2634 | 99.9% | 2103.7322 | 2104.3855 | 1 | 5.583 | 41.2% | 1 | R.SRLFVGNLPPDITEEEMR.K | 2 |
|  | AstrinSTLCLD\_041714\_01.08661.08661.3 | 2.8642 | 0.2483 | 97.8% | 2104.2544 | 2104.3855 | 71 | 4.4 | 30.9% | 1 | R.SRLFVGNLPPDITEEEMR.K | 3 |
|  | AstrinSTLCLD\_041714\_01.09486.09486.2 | 3.5371 | 0.4157 | 100.0% | 1860.4521 | 1861.12 | 2 | 6.675 | 60.0% | 6 | R.LFVGNLPPDITEEEMR.K | 2 |
|  | AstrinSTLCLD\_041714\_01.08336.08336.3 | 2.7817 | 0.2583 | 97.8% | 1989.1743 | 1989.2941 | 11 | 4.882 | 32.8% | 1 | R.LFVGNLPPDITEEEMRK.L | 3 |
|  | AstrinSTLCLD\_041714\_01.10480.10480.2 | 5.0197 | 0.5579 | 100.0% | 1813.6122 | 1814.1504 | 1 | 8.877 | 70.0% | 2 | R.TLAEIAKVELDNMPLR.G | 2 |
|  | AstrinSTLCLD\_041714\_01.10476.10476.3 | 5.5193 | 0.3973 | 100.0% | 1815.2043 | 1814.1504 | 1 | 6.982 | 48.3% | 1 | R.TLAEIAKVELDNMPLR.G | 3 |
|  | AstrinSTLCLD\_041714\_01.09563.09563.3 | 4.4853 | 0.4148 | 100.0% | 1999.1044 | 1999.3765 | 1 | 7.209 | 44.1% | 1 | R.TLAEIAKVELDNMPLRGK.Q | 3 |
|  | AstrinSTLCLD\_041714\_01.05718.05718.2 | 3.1946 | 0.2844 | 100.0% | 1087.1322 | 1087.2793 | 15 | 5.339 | 75.0% | 3 | K.VELDNMPLR.G | 2 |
|  | AstrinSTLCLD\_041714\_01.15770.15770.2 | 5.7209 | 0.5289 | 100.0% | 2668.892 | 2669.9507 | 1 | 10.765 | 54.5% | 8 | R.NLPQYVSNELLEEAFSVFGQVER.A | 2 |
|  | AstrinSTLCLD\_041714\_01.04070.04070.2 | 1.9543 | 0.2861 | 96.1% | 886.3522 | 887.0238 | 1 | 6.609 | 85.7% | 1 | R.AVVIVDDR.G | 22 |
|  | AstrinSTLCLD\_041714\_01.08198.08198.2 | 4.2867 | 0.5597 | 100.0% | 1696.3121 | 1696.8744 | 1 | 9.688 | 73.1% | 8 | R.FAQPGSFEYEYAMR.W | 2 |
|  | AstrinSTLCLD\_041714\_01.04833.04833.3 | 2.678 | 0.2991 | 99.3% | 1336.9744 | 1337.5488 | 1 | 5.823 | 55.0% | 1 | R.EKLEMEMEAAR.H | 3 |
|  | AstrinSTLCLD\_041714\_01.04892.04892.2 | 3.008 | 0.3712 | 100.0% | 1337.1122 | 1337.5488 | 1 | 6.229 | 75.0% | 5 | R.EKLEMEMEAAR.H | 2 |
|  | AstrinSTLCLD\_041714\_02.06176.06176.2 | 4.6157 | 0.5414 | 100.0% | 1539.0721 | 1539.8441 | 1 | 9.288 | 67.9% | 17 | R.MGQMAMGGAMGINNR.G | 2 |
|  | AstrinSTLCLD\_041714\_02.07829.07829.2 | 5.8222 | 0.5063 | 100.0% | 2163.5322 | 2164.4436 | 1 | 9.23 | 59.5% | 2 | R.FGQAATMEGIGAIGGTPPAFNR.A | 2 |
|  | AstrinSTLCLD\_041714\_02.07894.07894.2 | 4.8705 | 0.5504 | 100.0% | 2243.5522 | 2244.4436 | 1 | 10.905 | 61.9% | 1 | R.FGQAATMEGIGAIGGT#PPAFNR.A | 2 |
|  | AstrinSTLCLD\_041714\_02.07953.07953.3 | 4.1506 | 0.1994 | 99.1% | 2246.2144 | 2244.4436 | 1 | 4.841 | 42.9% | 1 | R.FGQAATMEGIGAIGGT#PPAFNR.A | 3 |

Similarities:
gi|4826998|ref|NP\_005(1:16)  

---

|  |  |  |  |  |  |  |  |  |
| --- | --- | --- | --- | --- | --- | --- | --- | --- |
| U | *gi|4501885|ref|NP\_001* | 6 | 11 | 26.7% | 375 | 41737 | 5.5 | beta actin [Homo sapiens] |
| U | *gi|4501887|ref|NP\_001* | 6 | 11 | 26.7% | 375 | 41793 | 5.5 | actin, gamma 1 propeptide [Homo sapiens] |

| Filename XCorr DeltCN Conf% ObsM+H+ CalcM+H+ SpR ZScore Ion% # Sequence  | | | | | | | | | | | | |
| --- | --- | --- | --- | --- | --- | --- | --- | --- | --- | --- | --- | --- |
|  | AstrinSTLCLD\_041714\_01.06840.06840.2 | 4.2922 | 0.2928 | 100.0% | 1955.6122 | 1955.2615 | 1 | 8.555 | 58.8% | 2 | R.VAPEEHPVLLTEAPLNPK.A | 2 |
|  | AstrinSTLCLD\_041714\_01.09806.09806.3 | 6.3385 | 0.4283 | 100.0% | 3186.8342 | 3185.622 | 1 | 7.101 | 31.9% | 3 | R.TTGIVMDSGDGVTHTVPIYEGYALPHAILR.L | 3 |
|  | AstrinSTLCLD\_041714\_01.09872.09872.2 | 2.8839 | 0.3323 | 100.0% | 1624.0521 | 1624.8927 | 1 | 6.051 | 57.7% | 1 | R.LDLAGRDLTDYLMK.I | 22 |
|  | AstrinSTLCLD\_041714\_01.09164.09164.2 | 4.0368 | 0.3695 | 100.0% | 1790.8522 | 1791.9554 | 1 | 6.586 | 70.0% | 2 | K.SYELPDGQVITIGNER.F | 22 |
|  | AstrinSTLCLD\_041714\_02.07364.07364.3 | 5.3662 | 0.4542 | 100.0% | 2345.6042 | 2344.6448 | 1 | 7.233 | 38.1% | 1 | R.KDLYANTVLSGGTTMYPGIADR.M | 3 |
|  | AstrinSTLCLD\_041714\_02.08261.08261.2 | 2.8281 | 0.4054 | 100.0% | 2215.5122 | 2216.4705 | 6 | 7.28 | 30.0% | 2 | K.DLYANTVLSGGTTMYPGIADR.M | 2 |

Similarities:
gi|213688375|ref|NP\_0(2:4)  

---

|  |  |  |  |  |  |  |  |  |
| --- | --- | --- | --- | --- | --- | --- | --- | --- |
| U | *gi|38016907|ref|NP\_93* | 2 | 2 | 26.0% | 123 | 13475 | 8.0 | stomatin isoform b [Homo sapiens] |
| U | *gi|38016911|ref|NP\_00* | 2 | 2 | 11.1% | 288 | 31731 | 7.9 | stomatin isoform a [Homo sapiens] |

| Filename XCorr DeltCN Conf% ObsM+H+ CalcM+H+ SpR ZScore Ion% # Sequence  | | | | | | | | | | | | |
| --- | --- | --- | --- | --- | --- | --- | --- | --- | --- | --- | --- | --- |
|  | AstrinSTLCLD\_041714\_02.04488.04488.2 | 3.4087 | 0.5396 | 100.0% | 1248.1122 | 1248.3966 | 1 | 8.695 | 68.2% | 1 | K.VIAAEGEMNASR.A | 2 |
|  | AstrinSTLCLD\_041714\_01.15232.15232.2 | 3.2749 | 0.3614 | 100.0% | 2128.5322 | 2128.5781 | 1 | 5.708 | 47.4% | 1 | K.NSTIVFPLPIDMLQGIIGAK.H | 2 |

---

|  |  |  |  |  |  |  |  |  |
| --- | --- | --- | --- | --- | --- | --- | --- | --- |
| U | *gi|150456457|ref|NP\_9* | 14 | 39 | 25.6% | 347 | 39929 | 5.6 | HMT1 hnRNP methyltransferase-like 2 isoform 2 [Homo sapiens] |
| U | *gi|154759421|ref|NP\_0* | 13 | 38 | 24.0% | 371 | 42462 | 5.3 | HMT1 hnRNP methyltransferase-like 2 isoform 1 [Homo sapiens] |
| U | *gi|151301219|ref|NP\_9* | 14 | 39 | 25.2% | 353 | 40548 | 5.5 | HMT1 hnRNP methyltransferase-like 2 isoform 3 [Homo sapiens] |

| Filename XCorr DeltCN Conf% ObsM+H+ CalcM+H+ SpR ZScore Ion% # Sequence  | | | | | | | | | | | | |
| --- | --- | --- | --- | --- | --- | --- | --- | --- | --- | --- | --- | --- |
|  | AstrinSTLCLD\_041714\_01.10055.10055.2 | 3.789 | 0.4035 | 100.0% | 1643.5521 | 1643.8827 | 1 | 6.848 | 69.2% | 2 | R.DKWLAPDGLIFPDR.A | 2 |
|  | AstrinSTLCLD\_041714\_01.10120.10120.3 | 3.5687 | 0.3996 | 100.0% | 1645.0443 | 1643.8827 | 3 | 5.894 | 44.2% | 2 | R.DKWLAPDGLIFPDR.A | 3 |
|  | AstrinSTLCLD\_041714\_01.11307.11307.3 | 3.0474 | 0.2305 | 96.2% | 2875.7644 | 2877.2695 | 173 | 3.895 | 22.9% | 1 | R.DKWLAPDGLIFPDRATLYVTAIEDR.Q | 3 |
|  | AstrinSTLCLD\_041714\_01.10824.10824.2 | 3.606 | 0.414 | 100.0% | 1400.5322 | 1400.6201 | 1 | 7.485 | 72.7% | 1 | K.WLAPDGLIFPDR.A | 2 |
|  | AstrinSTLCLD\_041714\_01.06858.06858.2 | 3.7416 | 0.4363 | 100.0% | 1253.0721 | 1252.4099 | 1 | 7.763 | 75.0% | 12 | R.ATLYVTAIEDR.Q | 2 |
|  | AstrinSTLCLD\_041714\_01.07918.07918.2 | 3.203 | 0.3474 | 100.0% | 1637.4122 | 1637.914 | 1 | 6.143 | 57.1% | 2 | K.DVAIKEPLVDVVDPK.Q | 2 |
|  | AstrinSTLCLD\_041714\_01.07971.07971.3 | 2.7304 | 0.2446 | 97.4% | 1637.9043 | 1637.914 | 261 | 5.186 | 28.6% | 1 | K.DVAIKEPLVDVVDPK.Q | 3 |
|  | AstrinSTLCLD\_041714\_02.10672.10672.3 | 4.3154 | 0.3444 | 100.0% | 2229.4143 | 2229.5027 | 1 | 7.422 | 38.2% | 1 | K.RNDYVHALVAYFNIEFTR.C | 3 |
|  | AstrinSTLCLD\_041714\_02.11467.11467.2 | 4.7025 | 0.5818 | 100.0% | 2072.2122 | 2073.3152 | 1 | 10.353 | 68.8% | 1 | R.NDYVHALVAYFNIEFTR.C | 2 |
|  | AstrinSTLCLD\_041714\_02.11428.11428.3 | 4.1302 | 0.3734 | 100.0% | 2073.3245 | 2073.3152 | 1 | 6.542 | 37.5% | 1 | R.NDYVHALVAYFNIEFTR.C | 3 |
|  | AstrinSTLCLD\_041714\_01.05537.05537.2 | 3.7268 | 0.379 | 100.0% | 1726.2322 | 1725.8547 | 1 | 8.132 | 67.9% | 3 | R.TGFSTSPESPYTHWK.Q | 2 |
|  | AstrinSTLCLD\_041714\_01.05639.05639.3 | 2.9364 | 0.3935 | 100.0% | 1726.2843 | 1725.8547 | 1 | 6.363 | 37.5% | 2 | R.TGFSTSPESPYTHWK.Q | 3 |
|  | AstrinSTLCLD\_041714\_01.07348.07348.2 | 4.2977 | 0.3936 | 100.0% | 1721.5322 | 1721.969 | 1 | 7.403 | 60.0% | 5 | K.TGEEIFGTIGMRPNAK.N | 2 |
|  | AstrinSTLCLD\_041714\_01.07328.07328.3 | 3.3474 | 0.3559 | 100.0% | 1722.7743 | 1721.969 | 1 | 6.219 | 40.0% | 5 | K.TGEEIFGTIGMRPNAK.N | 3 |

---

|  |  |  |  |  |  |  |  |  |
| --- | --- | --- | --- | --- | --- | --- | --- | --- |
| U | *gi|32455264|ref|NP\_85* | 4 | 5 | 25.6% | 199 | 22110 | 8.1 | peroxiredoxin 1 [Homo sapiens] |
| U | *gi|4505591|ref|NP\_002* | 4 | 5 | 25.6% | 199 | 22110 | 8.1 | peroxiredoxin 1 [Homo sapiens] |
| U | *gi|32455266|ref|NP\_85* | 4 | 5 | 25.6% | 199 | 22110 | 8.1 | peroxiredoxin 1 [Homo sapiens] |

| Filename XCorr DeltCN Conf% ObsM+H+ CalcM+H+ SpR ZScore Ion% # Sequence  | | | | | | | | | | | | |
| --- | --- | --- | --- | --- | --- | --- | --- | --- | --- | --- | --- | --- |
|  | AstrinSTLCLD\_041714\_02.07578.07578.3 | 3.2509 | 0.4742 | 100.0% | 1984.7344 | 1984.2163 | 1 | 6.772 | 36.8% | 1 | R.TIAQDYGVLKADEGISFR.G | 3 |
|  | AstrinSTLCLD\_041714\_01.09855.09855.2 | 3.1895 | 0.4009 | 100.0% | 1360.1522 | 1360.6395 | 1 | 7.454 | 77.3% | 1 | R.GLFIIDDKGILR.Q | 2 |
|  | AstrinSTLCLD\_041714\_01.05935.05935.2 | 2.6721 | 0.4061 | 100.0% | 1211.6921 | 1212.3915 | 21 | 7.244 | 65.0% | 2 | R.QITVNDLPVGR.S | 2 |
|  | AstrinSTLCLD\_041714\_01.07314.07314.2 | 2.6276 | 0.3846 | 100.0% | 1197.6721 | 1197.3763 | 1 | 6.408 | 77.8% | 1 | R.LVQAFQFTDK.H | 2 |

---

|  |  |  |  |  |  |  |  |  |
| --- | --- | --- | --- | --- | --- | --- | --- | --- |
| U | *Reverse\_gi|38788353|r* | 1 | 1 | 25.4% | 173 | 18158 | 5.5 | hepatitis B virus x-interacting protein [Homo sapiens] |

| Filename XCorr DeltCN Conf% ObsM+H+ CalcM+H+ SpR ZScore Ion% # Sequence  | | | | | | | | | | | | |
| --- | --- | --- | --- | --- | --- | --- | --- | --- | --- | --- | --- | --- |
| \* | AstrinSTLCLD\_041714\_01.16116.16116.3 | 3.8078 | 0.2655 | 99.7% | 4904.8145 | 4906.1807 | 79 | 4.449 | 15.1% | 1 | K.QIMINGNDSELCVVPIDT#PDST#LKAAQQALVS\*IVGAHEDSLTGR.C | 3 |

---

|  |  |  |  |  |  |  |  |  |
| --- | --- | --- | --- | --- | --- | --- | --- | --- |
| U | *gi|21396489|ref|NP\_00* | 14 | 21 | 25.2% | 959 | 106489 | 6.4 | mitochondrial lon peptidase 1 [Homo sapiens] |

| Filename XCorr DeltCN Conf% ObsM+H+ CalcM+H+ SpR ZScore Ion% # Sequence  | | | | | | | | | | | | |
| --- | --- | --- | --- | --- | --- | --- | --- | --- | --- | --- | --- | --- |
| \* | AstrinSTLCLD\_041714\_01.09452.09452.2 | 2.397 | 0.2554 | 97.6% | 1235.1522 | 1235.512 | 5 | 5.397 | 70.0% | 1 | R.LAQPYVGVFLK.R | 2 |
| \* | AstrinSTLCLD\_041714\_01.10228.10228.2 | 3.1443 | 0.4023 | 100.0% | 1558.4521 | 1558.8644 | 1 | 6.528 | 66.7% | 1 | K.TIRDIIALNPLYR.E | 2 |
| \* | AstrinSTLCLD\_041714\_01.08626.08626.2 | 2.84 | 0.2581 | 99.2% | 1378.4722 | 1378.6044 | 2 | 5.621 | 59.1% | 1 | R.ESVLQMMQAGQR.V | 2 |
| \* | AstrinSTLCLD\_041714\_01.12812.12812.3 | 4.6513 | 0.3051 | 100.0% | 3671.0645 | 3671.0674 | 1 | 5.757 | 31.1% | 1 | R.VVDNPIYLSDMGAALTGAESHELQDVLEETNIPK.R | 3 |
| \* | AstrinSTLCLD\_041714\_01.06361.06361.2 | 3.562 | 0.51 | 100.0% | 1401.4321 | 1401.5745 | 1 | 10.483 | 77.3% | 2 | K.HVMDVVDEELSK.L | 2 |
| \* | AstrinSTLCLD\_041714\_01.07209.07209.3 | 4.0502 | 0.4264 | 100.0% | 1703.6344 | 1702.8644 | 1 | 7.046 | 42.9% | 3 | K.LGLLDNHSSEFNVTR.N | 3 |
| \* | AstrinSTLCLD\_041714\_01.13431.13431.2 | 3.4703 | 0.4019 | 100.0% | 1593.4122 | 1593.8223 | 1 | 6.507 | 70.8% | 1 | R.NYLDWLTSIPWGK.Y | 2 |
| \* | AstrinSTLCLD\_041714\_01.10581.10581.2 | 3.528 | 0.2823 | 100.0% | 1289.5122 | 1289.5608 | 2 | 5.819 | 70.0% | 1 | R.ILEFIAVSQLR.G | 2 |
| \* | AstrinSTLCLD\_041714\_02.07341.07341.2 | 3.7787 | 0.4338 | 100.0% | 1354.1122 | 1354.561 | 1 | 7.596 | 75.0% | 2 | R.FSVGGMTDVAEIK.G | 2 |
| \* | AstrinSTLCLD\_041714\_01.11284.11284.2 | 2.9325 | 0.2349 | 98.7% | 1825.8322 | 1826.1002 | 1 | 5.055 | 46.7% | 1 | K.TENPLILIDEVDKIGR.G | 2 |
| \* | AstrinSTLCLD\_041714\_01.14264.14264.3 | 5.6227 | 0.4075 | 100.0% | 3874.1042 | 3875.2373 | 1 | 8.723 | 29.4% | 2 | R.GYQGDPSSALLELLDPEQNANFLDHYLDVPVDLSK.V | 3 |
| \* | AstrinSTLCLD\_041714\_02.07222.07222.2 | 4.9938 | 0.5195 | 100.0% | 1600.0721 | 1599.8574 | 1 | 8.9 | 76.9% | 2 | R.MEMINVSGYVAQEK.L | 2 |
| \* | AstrinSTLCLD\_041714\_02.08898.08898.3 | 3.4974 | 0.2561 | 99.1% | 3178.2844 | 3177.5352 | 5 | 4.78 | 23.2% | 2 | K.IVSGEAESVEVTPENLQDFVGKPVFTVER.M | 3 |
| \* | AstrinSTLCLD\_041714\_02.06316.06316.2 | 3.6425 | 0.4573 | 100.0% | 1449.2922 | 1449.6624 | 1 | 7.686 | 73.1% | 1 | R.QNLAMTGEVSLTGK.I | 2 |

---

|  |  |  |  |  |  |  |  |  |
| --- | --- | --- | --- | --- | --- | --- | --- | --- |
| U | *gi|27436946|ref|NP\_73* | 14 | 29 | 24.1% | 664 | 74140 | 7.0 | lamin A/C isoform 1 precursor [Homo sapiens] |

| Filename XCorr DeltCN Conf% ObsM+H+ CalcM+H+ SpR ZScore Ion% # Sequence  | | | | | | | | | | | | |
| --- | --- | --- | --- | --- | --- | --- | --- | --- | --- | --- | --- | --- |
|  | AstrinSTLCLD\_041714\_01.03987.03987.2 | 2.5226 | 0.3051 | 99.6% | 1089.5721 | 1090.1783 | 2 | 4.997 | 72.2% | 1 | R.SLETENAGLR.L | 2 |
|  | AstrinSTLCLD\_041714\_01.04622.04622.2 | 3.1989 | 0.3658 | 100.0% | 1418.0521 | 1418.5901 | 1 | 8.041 | 72.7% | 1 | R.LRITESEEVVSR.E | 2 |
|  | AstrinSTLCLD\_041714\_02.05163.05163.3 | 3.1173 | 0.2894 | 100.0% | 1419.3844 | 1418.5901 | 7 | 5.633 | 40.9% | 1 | R.LRITESEEVVSR.E | 3 |
|  | AstrinSTLCLD\_041714\_01.07995.07995.2 | 3.4017 | 0.3511 | 100.0% | 1244.5122 | 1244.474 | 2 | 6.483 | 75.0% | 1 | R.LKDLEALLNSK.E | 2 |
|  | AstrinSTLCLD\_041714\_01.06036.06036.2 | 3.7888 | 0.3389 | 100.0% | 1028.7122 | 1029.1814 | 1 | 6.384 | 87.5% | 3 | R.LADALQELR.A | 2 |
|  | AstrinSTLCLD\_041714\_01.05057.05057.2 | 4.4279 | 0.5087 | 100.0% | 1753.4321 | 1753.8693 | 1 | 7.966 | 50.0% | 1 | R.NSNLVGAAHEELQQSR.I | 2 |
|  | AstrinSTLCLD\_041714\_02.05540.05540.3 | 2.9818 | 0.3469 | 100.0% | 1754.0343 | 1753.8693 | 10 | 5.645 | 36.7% | 1 | R.NSNLVGAAHEELQQSR.I | 3 |
|  | AstrinSTLCLD\_041714\_01.08508.08508.2 | 4.4366 | 0.2393 | 100.0% | 1701.8722 | 1700.9762 | 1 | 5.712 | 75.0% | 1 | R.IRIDSLSAQLSQLQK.Q | 2 |
|  | AstrinSTLCLD\_041714\_01.10220.10220.2 | 3.4834 | 0.4212 | 100.0% | 1893.9321 | 1895.1346 | 1 | 7.113 | 57.1% | 2 | R.MQQQLDEYQELLDIK.L | 2 |
|  | AstrinSTLCLD\_041714\_01.05114.05114.2 | 4.0644 | 0.5467 | 100.0% | 1606.3121 | 1606.7728 | 1 | 9.245 | 73.1% | 2 | R.VAVEEVDEEGKFVR.L | 2 |
|  | AstrinSTLCLD\_041714\_02.05709.05709.3 | 2.9487 | 0.3528 | 100.0% | 1606.3444 | 1606.7728 | 7 | 5.342 | 38.5% | 4 | R.VAVEEVDEEGKFVR.L | 3 |
|  | AstrinSTLCLD\_041714\_02.05906.05906.2 | 4.2766 | 0.581 | 100.0% | 1492.0322 | 1492.6874 | 1 | 9.244 | 73.1% | 5 | R.TALINSTGEEVAMR.K | 2 |
|  | AstrinSTLCLD\_041714\_01.04649.04649.3 | 3.7665 | 0.3994 | 100.0% | 2367.3843 | 2366.504 | 1 | 7.465 | 32.7% | 3 | K.ASASGSGAQVGGPISSGSSASSVTVTR.S | 3 |
|  | AstrinSTLCLD\_041714\_02.05925.05925.2 | 4.6089 | 0.5472 | 100.0% | 1567.3322 | 1567.6555 | 1 | 9.689 | 62.5% | 3 | R.SVGGSGGGSFGDNLVTR.S | 2 |

---

|  |  |  |  |  |  |  |  |  |
| --- | --- | --- | --- | --- | --- | --- | --- | --- |
| U | *gi|4826998|ref|NP\_005* | 16 | 41 | 24.0% | 707 | 76150 | 9.4 | splicing factor proline/glutamine rich (polypyrimidine tract binding protein associated) [Homo sapiens] |

| Filename XCorr DeltCN Conf% ObsM+H+ CalcM+H+ SpR ZScore Ion% # Sequence  | | | | | | | | | | | | |
| --- | --- | --- | --- | --- | --- | --- | --- | --- | --- | --- | --- | --- |
| \* | AstrinSTLCLD\_041714\_02.07149.07149.3 | 4.0011 | 0.4068 | 100.0% | 2123.4844 | 2124.3555 | 1 | 6.611 | 37.5% | 2 | R.SEEKISDSEGFKANLSLLR.R | 3 |
| \* | AstrinSTLCLD\_041714\_02.07395.07395.3 | 4.5582 | 0.4672 | 100.0% | 1650.9543 | 1650.8723 | 1 | 7.328 | 44.6% | 3 | K.ISDSEGFKANLSLLR.R | 3 |
| \* | AstrinSTLCLD\_041714\_01.10634.10634.2 | 3.4062 | 0.4339 | 100.0% | 1808.4521 | 1809.0258 | 6 | 7.438 | 46.7% | 1 | R.LFVGNLPADITEDEFK.R | 2 |
| \* | AstrinSTLCLD\_041714\_01.09722.09722.2 | 4.4455 | 0.4718 | 100.0% | 1964.6322 | 1965.2133 | 1 | 8.156 | 59.4% | 2 | R.LFVGNLPADITEDEFKR.L | 2 |
| \* | AstrinSTLCLD\_041714\_01.09686.09686.3 | 3.0256 | 0.257 | 98.5% | 1966.5844 | 1965.2133 | 11 | 5.022 | 35.9% | 1 | R.LFVGNLPADITEDEFKR.L | 3 |
| \* | AstrinSTLCLD\_041714\_01.08013.08013.2 | 4.6661 | 0.5079 | 100.0% | 1743.7122 | 1745.0007 | 1 | 9.931 | 70.0% | 1 | R.ALAEIAKAELDDTPMR.G | 2 |
| \* | AstrinSTLCLD\_041714\_01.04214.04214.2 | 2.9274 | 0.4072 | 100.0% | 1143.8322 | 1144.3188 | 1 | 8.089 | 85.0% | 1 | R.FATHAAALSVR.N | 2 |
| \* | AstrinSTLCLD\_041714\_01.13115.13115.2 | 5.1552 | 0.56 | 100.0% | 2640.152 | 2640.9092 | 1 | 10.078 | 47.7% | 2 | R.NLSPYVSNELLEEAFSQFGPIER.A | 2 |
|  | AstrinSTLCLD\_041714\_01.04070.04070.2 | 1.9543 | 0.2861 | 96.1% | 886.3522 | 887.0238 | 1 | 6.609 | 85.7% | 1 | R.AVVIVDDR.G | 22 |
| \* | AstrinSTLCLD\_041714\_02.05571.05571.2 | 4.4321 | 0.5099 | 100.0% | 1763.1721 | 1763.8632 | 1 | 8.955 | 73.1% | 6 | R.FAQHGTFEYEYSQR.W | 2 |
| \* | AstrinSTLCLD\_041714\_02.05708.05708.3 | 4.8419 | 0.3406 | 100.0% | 1763.9043 | 1763.8632 | 1 | 6.861 | 46.2% | 15 | R.FAQHGTFEYEYSQR.W | 3 |
| \* | AstrinSTLCLD\_041714\_01.07941.07941.3 | 4.0775 | 0.2825 | 100.0% | 2744.7244 | 2743.9648 | 1 | 5.507 | 33.0% | 1 | K.DAKDKLESEMEDAYHEHQANLLR.Q | 3 |
| \* | AstrinSTLCLD\_041714\_01.07544.07544.3 | 5.7488 | 0.4493 | 100.0% | 2429.9343 | 2429.6233 | 1 | 7.26 | 43.4% | 2 | K.DKLESEMEDAYHEHQANLLR.Q | 3 |
| \* | AstrinSTLCLD\_041714\_01.03604.03604.3 | 2.9679 | 0.2426 | 99.1% | 1573.5243 | 1573.7821 | 2 | 5.17 | 40.9% | 1 | R.RMEELHNQEMQK.R | 3 |
| \* | AstrinSTLCLD\_041714\_01.03875.03875.2 | 3.9418 | 0.4968 | 100.0% | 1341.6522 | 1342.4569 | 1 | 8.576 | 78.6% | 1 | R.FGQGGAGPVGGQGPR.G | 2 |
| \* | AstrinSTLCLD\_041714\_01.03928.03928.2 | 2.7089 | 0.3704 | 100.0% | 1121.2522 | 1121.2561 | 124 | 5.82 | 54.5% | 1 | R.GMGPGTPAGYGR.G | 2 |

Similarities:
gi|224028244|ref|NP\_0(1:15)  

---

|  |  |  |  |  |  |  |  |  |
| --- | --- | --- | --- | --- | --- | --- | --- | --- |
| U | *gi|169164494|ref|XP\_0* | 2 | 2 | 24.0% | 100 | 11493 | 10.1 | PREDICTED: similar to ribosomal protein L10 [Homo sapiens] |
| U | *gi|41151097|ref|XP\_20* | 2 | 2 | 11.2% | 214 | 24627 | 10.1 | PREDICTED: similar to QM protein isoform 1 [Homo sapiens] |
| U | *gi|223890243|ref|NP\_0* | 2 | 2 | 11.2% | 214 | 24604 | 10.1 | ribosomal protein L10 [Homo sapiens] |
| U | *gi|169213734|ref|XP\_0* | 2 | 2 | 14.4% | 167 | 19409 | 9.9 | PREDICTED: similar to Q1Z 7F5 isoform 2 [Homo sapiens] |
| U | *gi|169213732|ref|XP\_0* | 2 | 2 | 11.2% | 214 | 24600 | 10.1 | PREDICTED: similar to Q1Z 7F5 isoform 1 [Homo sapiens] |
| U | *gi|169213538|ref|XP\_0* | 2 | 2 | 14.4% | 167 | 19436 | 9.9 | PREDICTED: similar to QM protein isoform 2 [Homo sapiens] |
| U | *gi|169213536|ref|XP\_0* | 2 | 2 | 11.2% | 214 | 24627 | 10.1 | PREDICTED: similar to QM protein isoform 1 [Homo sapiens] |

| Filename XCorr DeltCN Conf% ObsM+H+ CalcM+H+ SpR ZScore Ion% # Sequence  | | | | | | | | | | | | |
| --- | --- | --- | --- | --- | --- | --- | --- | --- | --- | --- | --- | --- |
|  | AstrinSTLCLD\_041714\_01.07714.07714.2 | 2.6241 | 0.4276 | 100.0% | 1252.8121 | 1253.5486 | 1 | 8.034 | 80.0% | 1 | R.VHIGQVIMSIR.T | 2 |
|  | AstrinSTLCLD\_041714\_01.09449.09449.2 | 3.4425 | 0.3069 | 100.0% | 1545.5721 | 1545.6606 | 1 | 6.216 | 62.5% | 1 | K.FNADEFEDMVAEK.W | 2 |

---

|  |  |  |  |  |  |  |  |  |
| --- | --- | --- | --- | --- | --- | --- | --- | --- |
| U | *gi|50592996|ref|NP\_00* | 11 | 31 | 23.8% | 450 | 50433 | 4.9 | tubulin, beta, 4 [Homo sapiens] |

| Filename XCorr DeltCN Conf% ObsM+H+ CalcM+H+ SpR ZScore Ion% # Sequence  | | | | | | | | | | | | |
| --- | --- | --- | --- | --- | --- | --- | --- | --- | --- | --- | --- | --- |
|  | AstrinSTLCLD\_041714\_01.09356.09356.2 | 4.4304 | 0.4973 | 100.0% | 1617.3322 | 1616.8701 | 1 | 9.033 | 71.4% | 5 | R.AILVDLEPGTMDSVR.S | 22 |
|  | AstrinSTLCLD\_041714\_01.11397.11397.2 | 6.437 | 0.554 | 100.0% | 1959.8121 | 1960.151 | 1 | 10.53 | 79.4% | 1 | K.GHYTEGAELVDSVLDVVR.K | 222 |
|  | AstrinSTLCLD\_041714\_01.10706.10706.3 | 4.4871 | 0.4257 | 100.0% | 2088.8643 | 2088.325 | 1 | 7.56 | 40.3% | 2 | K.GHYTEGAELVDSVLDVVRK.E | 333 |
|  | AstrinSTLCLD\_041714\_01.07358.07358.2 | 4.3453 | 0.4254 | 100.0% | 1320.1322 | 1320.5896 | 1 | 8.343 | 72.7% | 6 | R.IMNTFSVVPSPK.V | 222 |
|  | AstrinSTLCLD\_041714\_01.06043.06043.2 | 2.8729 | 0.2906 | 100.0% | 1131.0922 | 1131.2767 | 9 | 5.247 | 77.8% | 7 | R.FPGQLNADLR.K | 222 |
|  | AstrinSTLCLD\_041714\_01.07593.07593.2 | 3.7915 | 0.3644 | 100.0% | 1272.4722 | 1272.5945 | 1 | 7.372 | 70.0% | 2 | R.KLAVNMVPFPR.L | 222 |
|  | AstrinSTLCLD\_041714\_01.08967.08967.2 | 3.7007 | 0.4846 | 100.0% | 1144.3922 | 1144.4204 | 1 | 9.08 | 94.4% | 2 | K.LAVNMVPFPR.L | 222 |
|  | AstrinSTLCLD\_041714\_01.10805.10805.2 | 3.6537 | 0.4002 | 100.0% | 1692.4321 | 1692.9678 | 1 | 8.342 | 71.4% | 1 | R.ALTVPELTQQMFDAK.N | 22 |
|  | AstrinSTLCLD\_041714\_01.10293.10293.2 | 2.9679 | 0.4318 | 100.0% | 1697.3121 | 1697.8877 | 1 | 7.31 | 57.7% | 1 | K.NSSYFVEWIPNNVK.V | 222 |
|  | AstrinSTLCLD\_041714\_01.08030.08030.2 | 3.2012 | 0.3385 | 100.0% | 1385.6122 | 1386.6116 | 47 | 5.663 | 60.0% | 1 | K.RISEQFTAMFR.R | 222 |
|  | AstrinSTLCLD\_041714\_02.07989.07989.2 | 3.7427 | 0.5204 | 100.0% | 1229.6721 | 1230.4241 | 1 | 8.103 | 94.4% | 3 | R.ISEQFTAMFR.R | 222 |

Similarities:
gi|29788785|ref|NP\_82(10:1)  
gi|5174735|ref|NP\_006(10:1)  

---

|  |  |  |  |  |  |  |  |  |
| --- | --- | --- | --- | --- | --- | --- | --- | --- |
| U | *gi|5031753|ref|NP\_005* | 6 | 9 | 23.8% | 449 | 49229 | 6.3 | heterogeneous nuclear ribonucleoprotein H1 [Homo sapiens] |

| Filename XCorr DeltCN Conf% ObsM+H+ CalcM+H+ SpR ZScore Ion% # Sequence  | | | | | | | | | | | | |
| --- | --- | --- | --- | --- | --- | --- | --- | --- | --- | --- | --- | --- |
| \* | AstrinSTLCLD\_041714\_01.06399.06399.2 | 3.3519 | 0.3313 | 100.0% | 1505.4321 | 1505.5933 | 12 | 5.842 | 58.3% | 1 | R.GLPWSCSADEVQR.F | 2 |
| \* | AstrinSTLCLD\_041714\_02.06674.06674.3 | 3.6208 | 0.1504 | 95.2% | 2109.2644 | 2108.2231 | 1 | 4.34 | 38.9% | 1 | R.EGRPSGEAFVELESEDEVK.L | 3 |
|  | AstrinSTLCLD\_041714\_01.10112.10112.2 | 4.613 | 0.5272 | 100.0% | 1842.6721 | 1843.0001 | 1 | 9.872 | 71.9% | 4 | R.STGEAFVQFASQEIAEK.A | 22 |
|  | AstrinSTLCLD\_041714\_01.11628.11628.2 | 2.624 | 0.2571 | 97.8% | 1998.1522 | 1998.2023 | 161 | 5.285 | 34.4% | 1 | R.ATENDIYNFFSPLNPVR.V | 22 |
| \* | AstrinSTLCLD\_041714\_02.06668.06668.3 | 2.9494 | 0.3338 | 100.0% | 2178.4744 | 2179.363 | 1 | 5.381 | 32.5% | 1 | R.VTGEADVEFATHEDAVAAMSK.D | 3 |
| \* | AstrinSTLCLD\_041714\_02.07392.07392.3 | 3.077 | 0.2433 | 97.9% | 2142.3542 | 2143.32 | 5 | 5.075 | 34.2% | 1 | R.YVELFLNSTAGASGGAYEHR.Y | 3 |

Similarities:
gi|148470397|ref|NP\_0(1:5)  
gi|74099697|ref|NP\_00(1:5)  

---

|  |  |  |  |  |  |  |  |  |
| --- | --- | --- | --- | --- | --- | --- | --- | --- |
| U | *TEV-Speptide* | 1 | 1 | 23.5% | 51 | 5423 | 9.4 | no description |

| Filename XCorr DeltCN Conf% ObsM+H+ CalcM+H+ SpR ZScore Ion% # Sequence  | | | | | | | | | | | | |
| --- | --- | --- | --- | --- | --- | --- | --- | --- | --- | --- | --- | --- |
| \* | AstrinSTLCLD\_041714\_01.04359.04359.3 | 2.9748 | 0.21 | 97.9% | 1386.2644 | 1384.5345 | 25 | 4.832 | 38.6% | 1 | R.SRENLYFQGAAK.F | 3 |

---

|  |  |  |  |  |  |  |  |  |
| --- | --- | --- | --- | --- | --- | --- | --- | --- |
| U | *gi|16905517|ref|NP\_47* | 4 | 7 | 23.3% | 262 | 31301 | 11.3 | FUS interacting protein (serine-arginine rich) 1 isoform 2 [Homo sapiens] |
| U | *gi|5730079|ref|NP\_006* | 4 | 7 | 33.3% | 183 | 22222 | 10.3 | FUS interacting protein (serine-arginine rich) 1 isoform 1 [Homo sapiens] |
| U | *gi|169161980|ref|XP\_0* | 4 | 7 | 33.7% | 181 | 22022 | 10.3 | PREDICTED: hypothetical protein, partial [Homo sapiens] |
| U | *gi|169161109|ref|XP\_0* | 4 | 7 | 33.3% | 183 | 22222 | 10.3 | PREDICTED: hypothetical protein LOC642558 [Homo sapiens] |
| U | *gi|169161107|ref|XP\_0* | 4 | 7 | 23.3% | 262 | 31301 | 11.3 | PREDICTED: hypothetical protein LOC642558 [Homo sapiens] |

| Filename XCorr DeltCN Conf% ObsM+H+ CalcM+H+ SpR ZScore Ion% # Sequence  | | | | | | | | | | | | |
| --- | --- | --- | --- | --- | --- | --- | --- | --- | --- | --- | --- | --- |
|  | AstrinSTLCLD\_041714\_01.06966.06966.3 | 3.5377 | 0.1919 | 99.1% | 1464.1144 | 1463.7227 | 1 | 5.075 | 59.1% | 2 | R.YLRPPNTSLFVR.N | 3 |
|  | AstrinSTLCLD\_041714\_01.11934.11934.2 | 3.1055 | 0.1841 | 97.9% | 1919.1721 | 1918.1992 | 2 | 4.745 | 50.0% | 2 | R.YGPIVDVYVPLDFYTR.R | 2 |
|  | AstrinSTLCLD\_041714\_01.10626.10626.3 | 3.8139 | 0.2888 | 100.0% | 2581.1343 | 2581.7605 | 179 | 5.064 | 26.2% | 1 | R.GFAYVQFEDVRDAEDALHNLDR.K | 3 |
|  | AstrinSTLCLD\_041714\_01.05826.05826.2 | 2.3831 | 0.2618 | 97.8% | 1304.2922 | 1305.4331 | 71 | 4.433 | 60.0% | 2 | R.QIEIQFAQGDR.K | 2 |

---

|  |  |  |  |  |  |  |  |  |
| --- | --- | --- | --- | --- | --- | --- | --- | --- |
| U | *gi|14165435|ref|NP\_11* | 8 | 11 | 22.9% | 463 | 50976 | 5.5 | heterogeneous nuclear ribonucleoprotein K isoform b [Homo sapiens] |
| U | *gi|14165439|ref|NP\_00* | 8 | 11 | 22.8% | 464 | 51028 | 5.3 | heterogeneous nuclear ribonucleoprotein K isoform a [Homo sapiens] |
| U | *gi|14165437|ref|NP\_11* | 8 | 11 | 22.8% | 464 | 51028 | 5.3 | heterogeneous nuclear ribonucleoprotein K isoform a [Homo sapiens] |

| Filename XCorr DeltCN Conf% ObsM+H+ CalcM+H+ SpR ZScore Ion% # Sequence  | | | | | | | | | | | | |
| --- | --- | --- | --- | --- | --- | --- | --- | --- | --- | --- | --- | --- |
|  | AstrinSTLCLD\_041714\_01.13826.13826.2 | 2.8396 | 0.4618 | 100.0% | 1716.0521 | 1716.0251 | 1 | 7.606 | 46.7% | 1 | R.ILSISADIETIGEILK.K | 2 |
|  | AstrinSTLCLD\_041714\_01.07996.07996.3 | 5.0666 | 0.4418 | 100.0% | 1520.4844 | 1519.8711 | 1 | 7.212 | 48.2% | 2 | R.LLIHQSLAGGIIGVK.G | 3 |
|  | AstrinSTLCLD\_041714\_01.07991.07991.2 | 3.8982 | 0.3625 | 100.0% | 1520.5922 | 1519.8711 | 1 | 7.013 | 71.4% | 2 | R.LLIHQSLAGGIIGVK.G | 2 |
|  | AstrinSTLCLD\_041714\_01.10774.10774.2 | 3.5497 | 0.4455 | 100.0% | 1341.6721 | 1341.6311 | 1 | 7.512 | 72.7% | 1 | K.IILDLISESPIK.G | 2 |
|  | AstrinSTLCLD\_041714\_01.09504.09504.2 | 3.6935 | 0.3298 | 100.0% | 1554.9122 | 1554.8705 | 1 | 5.988 | 61.5% | 1 | K.IILDLISESPIKGR.A | 2 |
|  | AstrinSTLCLD\_041714\_01.09126.09126.2 | 4.0982 | 0.2912 | 100.0% | 1919.5322 | 1918.1974 | 1 | 6.416 | 52.8% | 2 | R.GSYGDLGGPIITTQVTIPK.D | 2 |
|  | AstrinSTLCLD\_041714\_01.04917.04917.3 | 4.0722 | 0.3832 | 100.0% | 2070.1143 | 2070.1772 | 1 | 6.602 | 38.9% | 1 | R.HESGASIKIDEPLEGSEDR.I | 3 |
|  | AstrinSTLCLD\_041714\_01.10714.10714.2 | 4.9892 | 0.4979 | 100.0% | 2589.872 | 2590.9365 | 1 | 8.661 | 47.7% | 1 | R.IITITGTQDQIQNAQYLLQNSVK.Q | 2 |

---

|  |  |  |  |  |  |  |  |  |
| --- | --- | --- | --- | --- | --- | --- | --- | --- |
| U | *gi|4503529|ref|NP\_001* | 6 | 8 | 22.4% | 406 | 46154 | 5.5 | eukaryotic translation initiation factor 4A isoform 1 [Homo sapiens] |

| Filename XCorr DeltCN Conf% ObsM+H+ CalcM+H+ SpR ZScore Ion% # Sequence  | | | | | | | | | | | | |
| --- | --- | --- | --- | --- | --- | --- | --- | --- | --- | --- | --- | --- |
| \* | AstrinSTLCLD\_041714\_01.14554.14554.3 | 4.52 | 0.2318 | 100.0% | 4170.7144 | 4169.451 | 1 | 4.312 | 22.9% | 1 | R.SRDNGPDGMEPEGVIESNWNEIVDSFDDMNLSESLLR.G | 3 |
|  | AstrinSTLCLD\_041714\_01.06209.06209.2 | 4.693 | 0.5396 | 100.0% | 1828.3121 | 1829.0654 | 1 | 8.803 | 73.3% | 1 | R.GIYAYGFEKPSAIQQR.A | 2 |
|  | AstrinSTLCLD\_041714\_01.06251.06251.3 | 3.4194 | 0.4001 | 100.0% | 1829.1244 | 1829.0654 | 1 | 6.999 | 43.3% | 1 | R.GIYAYGFEKPSAIQQR.A | 3 |
| \* | AstrinSTLCLD\_041714\_01.06794.06794.3 | 3.4092 | 0.3118 | 100.0% | 1618.9443 | 1619.9225 | 4 | 6.66 | 39.3% | 2 | K.LQMEAPHIIVGTPGR.V | 3 |
|  | AstrinSTLCLD\_041714\_01.11094.11094.2 | 4.1409 | 0.4418 | 100.0% | 1557.8121 | 1556.789 | 1 | 7.852 | 79.2% | 1 | K.MFVLDEADEMLSR.G | 2 |
|  | AstrinSTLCLD\_041714\_01.08974.08974.2 | 2.6082 | 0.3428 | 100.0% | 1115.4722 | 1115.3585 | 8 | 6.581 | 61.1% | 2 | R.VLITTDLLAR.G | 2 |

---

|  |  |  |  |  |  |  |  |  |
| --- | --- | --- | --- | --- | --- | --- | --- | --- |
| U | *gi|20127519|ref|NP\_03* | 15 | 26 | 22.1% | 747 | 85653 | 9.2 | TPX2, microtubule-associated protein homolog [Homo sapiens] |

| Filename XCorr DeltCN Conf% ObsM+H+ CalcM+H+ SpR ZScore Ion% # Sequence  | | | | | | | | | | | | |
| --- | --- | --- | --- | --- | --- | --- | --- | --- | --- | --- | --- | --- |
| \* | AstrinSTLCLD\_041714\_01.06693.06693.3 | 4.8189 | 0.397 | 100.0% | 2405.9644 | 2405.7996 | 1 | 7.15 | 40.0% | 1 | R.KANLQQAIVTPLKPVDNTYYK.E | 3 |
| \* | AstrinSTLCLD\_041714\_01.07868.07868.3 | 3.7535 | 0.3383 | 100.0% | 2277.6243 | 2277.6255 | 1 | 6.245 | 34.2% | 1 | K.ANLQQAIVTPLKPVDNTYYK.E | 3 |
| \* | AstrinSTLCLD\_041714\_01.06137.06137.2 | 3.2276 | 0.3745 | 100.0% | 1067.5122 | 1067.317 | 23 | 7.746 | 55.0% | 3 | K.LALAGIGQPVK.K | 2 |
| \* | AstrinSTLCLD\_041714\_01.06424.06424.2 | 4.9758 | 0.4689 | 100.0% | 1887.1322 | 1887.013 | 1 | 7.994 | 64.3% | 2 | K.NQEEYKEVNFTSELR.K | 2 |
| \* | AstrinSTLCLD\_041714\_01.06377.06377.3 | 4.5126 | 0.2568 | 100.0% | 1889.6044 | 1887.013 | 2 | 5.516 | 42.9% | 5 | K.NQEEYKEVNFTSELR.K | 3 |
| \* | AstrinSTLCLD\_041714\_02.05603.05603.2 | 3.54 | 0.2349 | 100.0% | 1349.9922 | 1349.4344 | 1 | 5.211 | 81.8% | 1 | K.STAELEAEELEK.L | 2 |
| \* | AstrinSTLCLD\_041714\_01.05814.05814.2 | 3.1719 | 0.3021 | 100.0% | 1036.8922 | 1037.2877 | 1 | 5.317 | 77.8% | 3 | R.ILEGGPILPK.K | 2 |
| \* | AstrinSTLCLD\_041714\_01.07649.07649.3 | 3.3171 | 0.3305 | 100.0% | 2135.7844 | 2135.5083 | 2 | 5.133 | 33.3% | 1 | K.KPPVKPPTEPIGFDLEIEK.R | 3 |
| \* | AstrinSTLCLD\_041714\_01.06723.06723.3 | 4.8175 | 0.5121 | 100.0% | 2290.4944 | 2291.6958 | 1 | 9.821 | 44.7% | 1 | K.KPPVKPPTEPIGFDLEIEKR.I | 3 |
| \* | AstrinSTLCLD\_041714\_01.05933.05933.2 | 3.2571 | 0.4344 | 100.0% | 1198.4321 | 1198.402 | 1 | 7.793 | 80.0% | 3 | K.ILEDVVGVPEK.K | 2 |
| \* | AstrinSTLCLD\_041714\_01.09332.09332.2 | 3.9978 | 0.4731 | 100.0% | 1661.4122 | 1661.9823 | 1 | 7.913 | 64.3% | 1 | K.VLPITVPKS\*PAFALK.N | 2 |
| \* | AstrinSTLCLD\_041714\_01.06577.06577.3 | 2.2573 | 0.3538 | 98.5% | 2131.7344 | 2132.473 | 100 | 5.177 | 26.4% | 1 | K.AQPVPHYGVPFKPQIPEAR.T | 3 |
| \* | AstrinSTLCLD\_041714\_01.03836.03836.2 | 2.388 | 0.2513 | 99.0% | 1053.4122 | 1054.1478 | 1 | 5.149 | 78.6% | 1 | K.HQLEEELR.Q | 2 |
| \* | AstrinSTLCLD\_041714\_01.08730.08730.3 | 3.7048 | 0.2157 | 98.3% | 2475.0842 | 2475.8044 | 13 | 5.237 | 27.3% | 1 | K.KSVAEGLSGSLVQEPFQLATEKR.A | 3 |
| \* | AstrinSTLCLD\_041714\_01.09489.09489.3 | 4.096 | 0.409 | 100.0% | 2347.1943 | 2347.6304 | 3 | 7.18 | 27.4% | 1 | K.SVAEGLSGSLVQEPFQLATEKR.A | 3 |

---

|  |  |  |  |  |  |  |  |  |
| --- | --- | --- | --- | --- | --- | --- | --- | --- |
| U | *gi|14043070|ref|NP\_11* | 5 | 13 | 21.5% | 372 | 38747 | 9.1 | heterogeneous nuclear ribonucleoprotein A1 isoform b [Homo sapiens] |

| Filename XCorr DeltCN Conf% ObsM+H+ CalcM+H+ SpR ZScore Ion% # Sequence  | | | | | | | | | | | | |
| --- | --- | --- | --- | --- | --- | --- | --- | --- | --- | --- | --- | --- |
|  | AstrinSTLCLD\_041714\_01.09846.09846.2 | 3.826 | 0.3895 | 100.0% | 1913.6522 | 1914.1656 | 3 | 6.581 | 43.8% | 1 | R.KLFIGGLSFETTDESLR.S | 2 |
|  | AstrinSTLCLD\_041714\_02.09290.09290.2 | 5.3461 | 0.5136 | 100.0% | 1785.6322 | 1785.9916 | 1 | 8.928 | 66.7% | 6 | K.LFIGGLSFETTDESLR.S | 2 |
|  | AstrinSTLCLD\_041714\_01.09416.09416.2 | 3.6877 | 0.4049 | 100.0% | 1219.3722 | 1219.4387 | 1 | 7.502 | 88.9% | 3 | K.IEVIEIMTDR.G | 2 |
|  | AstrinSTLCLD\_041714\_02.08295.08295.3 | 3.8188 | 0.2328 | 99.3% | 2283.3843 | 2282.5579 | 1 | 7.051 | 35.5% | 2 | R.GFAFVTFDDHDSVDKIVIQK.Y | 3 |
| \* | AstrinSTLCLD\_041714\_01.05237.05237.3 | 4.9259 | 0.4157 | 100.0% | 2875.2244 | 2875.8137 | 10 | 7.346 | 23.4% | 1 | R.GGGGYGGSGDGYNGFGNDGGYGGGGPGYSGGSR.G | 3 |

---

|  |  |  |  |  |  |  |  |  |
| --- | --- | --- | --- | --- | --- | --- | --- | --- |
| U | *gi|117190174|ref|NP\_0* | 4 | 7 | 21.2% | 293 | 32338 | 5.1 | heterogeneous nuclear ribonucleoprotein C isoform b [Homo sapiens] |
| U | *gi|117190254|ref|NP\_0* | 4 | 7 | 21.2% | 293 | 32338 | 5.1 | heterogeneous nuclear ribonucleoprotein C isoform b [Homo sapiens] |

| Filename XCorr DeltCN Conf% ObsM+H+ CalcM+H+ SpR ZScore Ion% # Sequence  | | | | | | | | | | | | |
| --- | --- | --- | --- | --- | --- | --- | --- | --- | --- | --- | --- | --- |
|  | AstrinSTLCLD\_041714\_02.08243.08243.2 | 3.5704 | 0.2712 | 100.0% | 1318.6122 | 1317.6145 | 1 | 5.878 | 81.8% | 2 | R.VFIGNLNTLVVK.K | 2 |
|  | AstrinSTLCLD\_041714\_01.09119.09119.2 | 3.9994 | 0.5109 | 100.0% | 1331.2722 | 1330.4857 | 1 | 8.415 | 80.0% | 1 | K.GFAFVQYVNER.N | 2 |
|  | AstrinSTLCLD\_041714\_02.08207.08207.2 | 4.7854 | 0.3993 | 100.0% | 1684.1522 | 1684.0038 | 1 | 8.49 | 83.3% | 3 | R.MIAGQVLDINLAAEPK.V | 2 |
|  | AstrinSTLCLD\_041714\_02.08518.08518.3 | 3.4815 | 0.3429 | 100.0% | 2814.9543 | 2815.9404 | 1 | 5.591 | 29.5% | 1 | R.SAAEMYGSSFDLDYDFQRDYYDR.M | 3 |

---

|  |  |  |  |  |  |  |  |  |
| --- | --- | --- | --- | --- | --- | --- | --- | --- |
| U | *gi|12667788|ref|NP\_00* | 26 | 42 | 21.1% | 1960 | 226530 | 5.6 | myosin, heavy polypeptide 9, non-muscle [Homo sapiens] |

| Filename XCorr DeltCN Conf% ObsM+H+ CalcM+H+ SpR ZScore Ion% # Sequence  | | | | | | | | | | | | |
| --- | --- | --- | --- | --- | --- | --- | --- | --- | --- | --- | --- | --- |
| \* | AstrinSTLCLD\_041714\_01.05701.05701.3 | 3.4482 | 0.3134 | 100.0% | 1916.9944 | 1916.1614 | 1 | 5.696 | 46.7% | 4 | R.HEMPPHIYAITDTAYR.S | 3 |
| \* | AstrinSTLCLD\_041714\_01.07325.07325.2 | 3.0484 | 0.3631 | 100.0% | 1479.1721 | 1479.719 | 1 | 7.124 | 75.0% | 1 | K.VIQYLAYVASSHK.S | 2 |
|  | AstrinSTLCLD\_041714\_01.11376.11376.2 | 2.6953 | 0.3247 | 99.5% | 1727.1921 | 1728.0012 | 1 | 5.79 | 43.3% | 1 | R.QLLQANPILEAFGNAK.T | 2 |
| \* | AstrinSTLCLD\_041714\_02.10257.10257.3 | 4.6563 | 0.431 | 100.0% | 1997.3344 | 1997.3037 | 1 | 8.365 | 43.8% | 2 | R.TFHIFYYLLSGAGEHLK.T | 3 |
| \* | AstrinSTLCLD\_041714\_01.09507.09507.3 | 5.1396 | 0.29 | 100.0% | 3012.5044 | 3012.4 | 1 | 6.342 | 32.0% | 1 | R.FLSNGHVTIPGQQDKDMFQETMEAMR.I | 3 |
| \* | AstrinSTLCLD\_041714\_01.09812.09812.2 | 3.4864 | 0.4262 | 100.0% | 1616.3922 | 1616.9313 | 1 | 7.291 | 80.8% | 2 | R.IMGIPEEEQMGLLR.V | 2 |
| \* | AstrinSTLCLD\_041714\_01.09167.09167.2 | 4.1154 | 0.4263 | 100.0% | 1573.2922 | 1572.8044 | 1 | 8.281 | 80.8% | 1 | K.VSHLLGINVTDFTR.G | 2 |
| \* | AstrinSTLCLD\_041714\_01.09160.09160.3 | 3.6637 | 0.3139 | 100.0% | 1573.7043 | 1572.8044 | 4 | 5.347 | 46.2% | 1 | K.VSHLLGINVTDFTR.G | 3 |
| \* | AstrinSTLCLD\_041714\_01.11171.11171.2 | 4.4854 | 0.5481 | 100.0% | 2018.3922 | 2019.3636 | 1 | 9.868 | 44.7% | 1 | R.IIGLDQVAGMSETALPGAFK.T | 2 |
| \* | AstrinSTLCLD\_041714\_01.06915.06915.2 | 2.6001 | 0.4065 | 100.0% | 1193.8922 | 1194.33 | 4 | 6.233 | 66.7% | 1 | K.ALELDSNLYR.I | 2 |
| \* | AstrinSTLCLD\_041714\_01.09459.09459.2 | 3.5121 | 0.3667 | 100.0% | 1752.2922 | 1753.0358 | 3 | 6.418 | 53.6% | 1 | R.LTEMETLQSQLMAEK.L | 2 |
| \* | AstrinSTLCLD\_041714\_01.09568.09568.3 | 2.8356 | 0.2263 | 95.2% | 2333.7244 | 2334.4736 | 38 | 4.586 | 27.8% | 1 | K.MQQNIQELEEQLEEEESAR.Q | 3 |
| \* | AstrinSTLCLD\_041714\_02.06414.06414.2 | 4.8103 | 0.437 | 100.0% | 1654.6122 | 1654.7681 | 1 | 7.801 | 73.1% | 3 | R.IAEFTTNLTEEEEK.S | 2 |
| \* | AstrinSTLCLD\_041714\_01.10071.10071.3 | 4.2247 | 0.3554 | 100.0% | 2304.2644 | 2304.473 | 1 | 5.6 | 41.7% | 1 | K.IRELESQISELQEDLESER.A | 3 |
| \* | AstrinSTLCLD\_041714\_01.14536.14536.2 | 4.0724 | 0.549 | 100.0% | 3017.4922 | 3019.2434 | 1 | 9.502 | 44.2% | 1 | R.DLGEELEALKTELEDTLDSTAAQQELR.S | 2 |
| \* | AstrinSTLCLD\_041714\_01.14552.14552.3 | 3.7242 | 0.3017 | 100.0% | 3019.3442 | 3019.2434 | 2 | 5.261 | 26.0% | 1 | R.DLGEELEALKTELEDTLDSTAAQQELR.S | 3 |
| \* | AstrinSTLCLD\_041714\_01.05392.05392.3 | 4.1503 | 0.4586 | 100.0% | 2043.7144 | 2044.2439 | 1 | 7.262 | 37.5% | 3 | K.TLEEEAKTHEAQIQEMR.Q | 3 |
| \* | AstrinSTLCLD\_041714\_01.07276.07276.3 | 5.5989 | 0.4917 | 100.0% | 1996.4043 | 1997.1722 | 1 | 9.025 | 45.3% | 2 | K.HSQAVEELAEQLEQTKR.V | 3 |
| \* | AstrinSTLCLD\_041714\_01.10230.10230.2 | 4.1034 | 0.4875 | 100.0% | 1945.7922 | 1947.1498 | 1 | 8.328 | 58.8% | 2 | K.LQVELDNVTGLLSQSDSK.S | 2 |
| \* | AstrinSTLCLD\_041714\_01.10772.10772.3 | 3.3087 | 0.2548 | 99.3% | 1951.7344 | 1951.1436 | 1 | 4.45 | 46.7% | 1 | R.LQQELDDLLVDLDHQR.Q | 3 |
| \* | AstrinSTLCLD\_041714\_02.05672.05672.3 | 3.5456 | 0.3217 | 100.0% | 1648.1643 | 1647.8407 | 1 | 6.175 | 51.9% | 3 | R.ALEEAMEQKAELER.L | 3 |
| \* | AstrinSTLCLD\_041714\_01.12772.12772.3 | 4.0295 | 0.3671 | 100.0% | 3147.7144 | 3149.4048 | 54 | 5.795 | 22.1% | 1 | R.ALEQQVEEMKTQLEELEDELQATEDAK.L | 3 |
| \* | AstrinSTLCLD\_041714\_01.11592.11592.2 | 3.7236 | 0.3665 | 100.0% | 2049.672 | 2050.3064 | 1 | 6.287 | 50.0% | 1 | K.SMEAEMIQLQEELAAAER.A | 2 |
| \* | AstrinSTLCLD\_041714\_01.08985.08985.2 | 5.474 | 0.4817 | 100.0% | 2472.5322 | 2473.6099 | 1 | 8.35 | 55.0% | 2 | R.IAQLEEELEEEQGNTELINDR.L | 2 |
| \* | AstrinSTLCLD\_041714\_01.09006.09006.2 | 5.5646 | 0.5191 | 100.0% | 1870.3922 | 1871.0574 | 1 | 9.585 | 66.7% | 2 | K.ANLQIDQINTDLNLER.S | 2 |
| \* | AstrinSTLCLD\_041714\_01.06571.06571.2 | 3.5186 | 0.2483 | 100.0% | 1156.1721 | 1156.3732 | 1 | 6.484 | 88.9% | 2 | R.RGDLPFVVPR.R | 2 |

---

|  |  |  |  |  |  |  |  |  |
| --- | --- | --- | --- | --- | --- | --- | --- | --- |
| U | *gi|72534660|ref|NP\_00* | 3 | 3 | 20.6% | 238 | 27367 | 11.8 | splicing factor, arginine/serine-rich 7 [Homo sapiens] |

| Filename XCorr DeltCN Conf% ObsM+H+ CalcM+H+ SpR ZScore Ion% # Sequence  | | | | | | | | | | | | |
| --- | --- | --- | --- | --- | --- | --- | --- | --- | --- | --- | --- | --- |
| \* | AstrinSTLCLD\_041714\_02.05469.05469.3 | 2.1603 | 0.32 | 97.0% | 1720.9744 | 1720.923 | 1 | 6.08 | 37.5% | 1 | K.VYVGNLGTGAGKGELER.A | 3 |
| \* | AstrinSTLCLD\_041714\_01.10203.10203.3 | 2.5362 | 0.2896 | 97.5% | 2379.2043 | 2379.5474 | 1 | 4.717 | 30.0% | 1 | R.NPPGFAFVEFEDPRDAEDAVR.G | 3 |
| \* | AstrinSTLCLD\_041714\_01.04913.04913.2 | 2.6989 | 0.1595 | 96.2% | 1245.3922 | 1245.4827 | 12 | 4.573 | 60.0% | 1 | R.VRVELSTGMPR.R | 2 |

---

|  |  |  |  |  |  |  |  |  |
| --- | --- | --- | --- | --- | --- | --- | --- | --- |
| U | *gi|14141152|ref|NP\_00* | 11 | 33 | 19.6% | 730 | 77516 | 8.7 | heterogeneous nuclear ribonucleoprotein M isoform a [Homo sapiens] |
| U | *gi|157412270|ref|NP\_1* | 11 | 33 | 20.7% | 691 | 73621 | 8.8 | heterogeneous nuclear ribonucleoprotein M isoform b [Homo sapiens] |

| Filename XCorr DeltCN Conf% ObsM+H+ CalcM+H+ SpR ZScore Ion% # Sequence  | | | | | | | | | | | | |
| --- | --- | --- | --- | --- | --- | --- | --- | --- | --- | --- | --- | --- |
|  | AstrinSTLCLD\_041714\_01.10131.10131.2 | 3.6249 | 0.4006 | 100.0% | 1265.2722 | 1265.4949 | 1 | 7.428 | 80.0% | 1 | R.AFITNIPFDVK.W | 2 |
|  | AstrinSTLCLD\_041714\_02.07600.07600.2 | 3.4985 | 0.3952 | 100.0% | 1428.4922 | 1427.6403 | 1 | 6.883 | 58.3% | 5 | R.LGSTVFVANLDYK.V | 2 |
|  | AstrinSTLCLD\_041714\_02.07530.07530.2 | 3.3381 | 0.3474 | 100.0% | 1435.1122 | 1435.768 | 1 | 6.456 | 75.0% | 1 | K.LKEVFSMAGVVVR.A | 2 |
|  | AstrinSTLCLD\_041714\_01.08471.08471.2 | 3.8125 | 0.4793 | 100.0% | 1716.3121 | 1715.9724 | 1 | 8.265 | 65.6% | 2 | K.MGGMEGPFGGGMENMGR.F | 2 |
|  | AstrinSTLCLD\_041714\_01.07021.07021.2 | 3.0141 | 0.3325 | 100.0% | 1114.7122 | 1115.3152 | 4 | 5.709 | 72.2% | 2 | R.INEILSNALK.R | 2 |
|  | AstrinSTLCLD\_041714\_01.07832.07832.2 | 4.4517 | 0.4865 | 100.0% | 1613.9922 | 1614.875 | 1 | 10.299 | 78.6% | 2 | R.MGPLGLDHMASSIER.M | 2 |
|  | AstrinSTLCLD\_041714\_01.07760.07760.3 | 3.3348 | 0.4568 | 100.0% | 1614.5643 | 1614.875 | 2 | 7.191 | 41.1% | 2 | R.MGPLGLDHMASSIER.M | 3 |
|  | AstrinSTLCLD\_041714\_02.06915.06915.2 | 3.6273 | 0.4618 | 100.0% | 1126.6721 | 1126.3337 | 1 | 9.119 | 80.0% | 6 | R.MGAGMGFGLER.M | 2 |
|  | AstrinSTLCLD\_041714\_01.06828.06828.2 | 3.5705 | 0.395 | 100.0% | 1428.3522 | 1428.7076 | 1 | 7.364 | 71.4% | 2 | R.MGPAMGPALGAGIER.M | 2 |
|  | AstrinSTLCLD\_041714\_02.06458.06458.2 | 4.2122 | 0.5395 | 100.0% | 1384.0721 | 1384.5677 | 1 | 10.435 | 82.1% | 6 | R.MGLAMGGGGGASFDR.A | 2 |
|  | AstrinSTLCLD\_041714\_02.06698.06698.3 | 3.7913 | 0.4534 | 100.0% | 2035.9143 | 2036.1735 | 1 | 7.338 | 36.4% | 4 | R.GNFGGSFAGSFGGAGGHAPGVAR.K | 3 |

---

|  |  |  |  |  |  |  |  |  |
| --- | --- | --- | --- | --- | --- | --- | --- | --- |
| U | *gi|7669492|ref|NP\_002* | 4 | 10 | 19.4% | 335 | 36053 | 8.5 | glyceraldehyde-3-phosphate dehydrogenase [Homo sapiens] |

| Filename XCorr DeltCN Conf% ObsM+H+ CalcM+H+ SpR ZScore Ion% # Sequence  | | | | | | | | | | | | |
| --- | --- | --- | --- | --- | --- | --- | --- | --- | --- | --- | --- | --- |
| \* | AstrinSTLCLD\_041714\_01.12592.12592.3 | 7.2002 | 0.5946 | 100.0% | 2597.2444 | 2597.0044 | 1 | 10.413 | 41.3% | 3 | K.VIHDNFGIVEGLMTTVHAITATQK.T | 3 |
| \* | AstrinSTLCLD\_041714\_01.06648.06648.2 | 4.0443 | 0.3539 | 100.0% | 1412.5322 | 1412.6292 | 1 | 6.303 | 71.4% | 3 | R.GALQNIIPASTGAAK.A | 2 |
|  | AstrinSTLCLD\_041714\_01.09784.09784.2 | 4.1864 | 0.543 | 100.0% | 1764.6721 | 1764.8914 | 1 | 8.729 | 57.7% | 2 | K.LISWYDNEFGYSNR.V | 2 |
| \* | AstrinSTLCLD\_041714\_02.06317.06317.2 | 3.0802 | 0.4438 | 100.0% | 1331.3722 | 1331.5879 | 1 | 7.024 | 68.2% | 2 | R.VVDLMAHMASKE.- | 2 |

---

|  |  |  |  |  |  |  |  |  |
| --- | --- | --- | --- | --- | --- | --- | --- | --- |
| U | *gi|118582269|ref|NP\_0* | 3 | 6 | 19.4% | 201 | 22460 | 8.0 | splicing factor, arginine/serine-rich 1 isoform 2 [Homo sapiens] |
| U | *gi|5902076|ref|NP\_008* | 3 | 6 | 15.7% | 248 | 27745 | 10.4 | splicing factor, arginine/serine-rich 1 isoform 1 [Homo sapiens] |

| Filename XCorr DeltCN Conf% ObsM+H+ CalcM+H+ SpR ZScore Ion% # Sequence  | | | | | | | | | | | | |
| --- | --- | --- | --- | --- | --- | --- | --- | --- | --- | --- | --- | --- |
|  | AstrinSTLCLD\_041714\_01.07120.07120.2 | 2.8731 | 0.4494 | 100.0% | 1257.3121 | 1257.4752 | 1 | 6.703 | 75.0% | 4 | R.IYVGNLPPDIR.T | 2 |
|  | AstrinSTLCLD\_041714\_01.05501.05501.2 | 2.5702 | 0.2749 | 99.2% | 1258.9321 | 1258.4137 | 1 | 4.974 | 72.2% | 1 | R.TKDIEDVFYK.Y | 2 |
|  | AstrinSTLCLD\_041714\_01.05206.05206.3 | 2.9665 | 0.3111 | 99.2% | 2101.7344 | 2101.1064 | 223 | 5.237 | 27.9% | 1 | R.DAEDAVYGRDGYDYDGYR.L | 3 |

---

|  |  |  |  |  |  |  |  |  |
| --- | --- | --- | --- | --- | --- | --- | --- | --- |
| U | *gi|4758792|ref|NP\_004* | 1 | 1 | 19.4% | 124 | 13712 | 8.3 | NADH dehydrogenase (ubiquinone) Fe-S protein 6, 13kDa (NADH-coenzyme Q reductase) [Homo sapiens] |

| Filename XCorr DeltCN Conf% ObsM+H+ CalcM+H+ SpR ZScore Ion% # Sequence  | | | | | | | | | | | | |
| --- | --- | --- | --- | --- | --- | --- | --- | --- | --- | --- | --- | --- |
| \* | AstrinSTLCLD\_041714\_01.10025.10025.3 | 4.2039 | 0.2238 | 99.3% | 2760.1443 | 2760.03 | 92 | 4.721 | 26.1% | 1 | R.QKEVNENFAIDLIAEQPVSEVETR.V | 3 |

---

|  |  |  |  |  |  |  |  |  |
| --- | --- | --- | --- | --- | --- | --- | --- | --- |
| U | *gi|119395750|ref|NP\_0* | 8 | 10 | 18.9% | 644 | 66039 | 8.1 | keratin 1 [Homo sapiens] |

| Filename XCorr DeltCN Conf% ObsM+H+ CalcM+H+ SpR ZScore Ion% # Sequence  | | | | | | | | | | | | |
| --- | --- | --- | --- | --- | --- | --- | --- | --- | --- | --- | --- | --- |
| \* | AstrinSTLCLD\_041714\_02.06090.06090.2 | 3.9734 | 0.4255 | 100.0% | 1658.6721 | 1658.7678 | 1 | 6.99 | 59.4% | 1 | R.SGGGFSSGSAGIINYQR.R | 2 |
|  | AstrinSTLCLD\_041714\_01.09156.09156.2 | 3.6516 | 0.4413 | 100.0% | 1385.3522 | 1384.5315 | 1 | 7.262 | 68.2% | 1 | K.SLNNQFASFIDK.V | 2 |
|  | AstrinSTLCLD\_041714\_02.07775.07775.2 | 4.2731 | 0.4497 | 100.0% | 1477.7122 | 1476.6293 | 1 | 7.676 | 86.4% | 2 | K.WELLQQVDTSTR.T | 2 |
| \* | AstrinSTLCLD\_041714\_01.11297.11297.2 | 4.1979 | 0.4951 | 100.0% | 1303.2322 | 1303.4955 | 1 | 9.01 | 81.8% | 1 | R.SLDLDSIIAEVK.A | 2 |
|  | AstrinSTLCLD\_041714\_02.05781.05781.2 | 2.7695 | 0.2987 | 100.0% | 1179.7922 | 1180.303 | 2 | 7.587 | 72.2% | 1 | K.YEELQITAGR.H | 22 |
| \* | AstrinSTLCLD\_041714\_01.10456.10456.3 | 4.4867 | 0.2967 | 100.0% | 2185.1643 | 2185.399 | 1 | 5.662 | 43.1% | 1 | K.NKLNDLEDALQQAKEDLAR.L | 3 |
|  | AstrinSTLCLD\_041714\_01.06374.06374.2 | 2.9557 | 0.2684 | 99.7% | 1524.4122 | 1524.7754 | 1 | 5.401 | 72.7% | 1 | R.LLRDYQELMNTK.L | 2 |
| \* | AstrinSTLCLD\_041714\_01.08276.08276.3 | 4.1298 | 0.2366 | 99.3% | 2242.1643 | 2241.0396 | 1 | 5.258 | 36.1% | 2 | R.GGSGGGGGGS\*S\*GGRGSGGGSSGGSIGGR.G | 3 |

Similarities:
gi|119703753|ref|NP\_0(1:7)  

---

|  |  |  |  |  |  |  |  |  |
| --- | --- | --- | --- | --- | --- | --- | --- | --- |
| U | *gi|4506901|ref|NP\_003* | 3 | 5 | 18.3% | 164 | 19330 | 11.6 | splicing factor, arginine/serine-rich 3 [Homo sapiens] |

| Filename XCorr DeltCN Conf% ObsM+H+ CalcM+H+ SpR ZScore Ion% # Sequence  | | | | | | | | | | | | |
| --- | --- | --- | --- | --- | --- | --- | --- | --- | --- | --- | --- | --- |
| \* | AstrinSTLCLD\_041714\_01.07211.07211.2 | 3.0321 | 0.4257 | 100.0% | 1044.6721 | 1044.198 | 1 | 7.701 | 81.2% | 3 | R.AFGYYGPLR.S | 2 |
| \* | AstrinSTLCLD\_041714\_01.10192.10192.2 | 3.0111 | 0.218 | 98.6% | 2320.7722 | 2321.5107 | 33 | 5.199 | 37.5% | 1 | R.NPPGFAFVEFEDPRDAADAVR.E | 2 |
| \* | AstrinSTLCLD\_041714\_01.10196.10196.3 | 2.8024 | 0.2484 | 96.5% | 2321.8145 | 2321.5107 | 1 | 4.896 | 30.0% | 1 | R.NPPGFAFVEFEDPRDAADAVR.E | 3 |

---

|  |  |  |  |  |  |  |  |  |
| --- | --- | --- | --- | --- | --- | --- | --- | --- |
| U | *gi|21464101|ref|NP\_03* | 2 | 2 | 18.2% | 247 | 28303 | 4.9 | tyrosine 3-monooxygenase/tryptophan 5-monooxygenase activation protein, gamma polypeptide [Homo sapiens] |

| Filename XCorr DeltCN Conf% ObsM+H+ CalcM+H+ SpR ZScore Ion% # Sequence  | | | | | | | | | | | | |
| --- | --- | --- | --- | --- | --- | --- | --- | --- | --- | --- | --- | --- |
| \* | AstrinSTLCLD\_041714\_01.17673.17673.2 | 3.2257 | 0.1229 | 96.2% | 1798.3922 | 1798.8473 | 59 | 3.452 | 43.3% | 1 | R.VISS\*IEQK@TSADGNEK.K | 2 |
| \* | AstrinSTLCLD\_041714\_01.15875.15875.3 | 4.5559 | 0.4132 | 100.0% | 3303.2944 | 3303.6626 | 1 | 6.41 | 26.8% | 1 | K.TAFDDAIAELDTLNEDSYKDSTLIMQLLR.D | 3 |

---

|  |  |  |  |  |  |  |  |  |
| --- | --- | --- | --- | --- | --- | --- | --- | --- |
| U | *gi|4758352|ref|NP\_004* | 1 | 1 | 17.9% | 184 | 19393 | 5.8 | ferredoxin 1 precursor [Homo sapiens] |

| Filename XCorr DeltCN Conf% ObsM+H+ CalcM+H+ SpR ZScore Ion% # Sequence  | | | | | | | | | | | | |
| --- | --- | --- | --- | --- | --- | --- | --- | --- | --- | --- | --- | --- |
| \* | AstrinSTLCLD\_041714\_01.17157.17157.3 | 3.2058 | 0.2234 | 96.8% | 3510.7744 | 3511.75 | 4 | 3.721 | 21.1% | 1 | R.GPGGSAEASRSLSVSARARS\*SSEDKITVHFINR.D | 3 |

---

|  |  |  |  |  |  |  |  |  |
| --- | --- | --- | --- | --- | --- | --- | --- | --- |
| U | *gi|15809016|ref|NP\_29* | 2 | 2 | 17.4% | 172 | 19779 | 4.8 | myosin regulatory light chain MRCL2 isoform A [Homo sapiens] |
| U | *gi|5453740|ref|NP\_006* | 2 | 2 | 17.5% | 171 | 19794 | 4.8 | myosin, light chain 12A, regulatory, non-sarcomeric [Homo sapiens] |
| U | *gi|222144328|ref|NP\_0* | 2 | 2 | 19.5% | 154 | 17757 | 4.4 | myosin regulatory light chain MRCL2 isoform B [Homo sapiens] |
| U | *gi|222144326|ref|NP\_0* | 2 | 2 | 17.4% | 172 | 19779 | 4.8 | myosin regulatory light chain MRCL2 isoform A [Homo sapiens] |
| U | *gi|222144324|ref|NP\_0* | 2 | 2 | 17.4% | 172 | 19779 | 4.8 | myosin regulatory light chain MRCL2 isoform A [Homo sapiens] |

| Filename XCorr DeltCN Conf% ObsM+H+ CalcM+H+ SpR ZScore Ion% # Sequence  | | | | | | | | | | | | |
| --- | --- | --- | --- | --- | --- | --- | --- | --- | --- | --- | --- | --- |
|  | AstrinSTLCLD\_041714\_02.08566.08566.3 | 3.8709 | 0.4427 | 100.0% | 2433.2944 | 2433.649 | 1 | 6.61 | 35.5% | 1 | R.ELLTTMGDRFTDEEVDELYR.E | 3 |
|  | AstrinSTLCLD\_041714\_01.08372.08372.2 | 2.7747 | 0.287 | 100.0% | 1261.2922 | 1261.3794 | 1 | 7.201 | 72.2% | 1 | K.GNFNYIEFTR.I | 2 |

---

|  |  |  |  |  |  |  |  |  |
| --- | --- | --- | --- | --- | --- | --- | --- | --- |
| U | *gi|87196351|ref|NP\_00* | 8 | 13 | 17.1% | 662 | 73244 | 7.2 | DEAD/H (Asp-Glu-Ala-Asp/His) box polypeptide 3 [Homo sapiens] |

| Filename XCorr DeltCN Conf% ObsM+H+ CalcM+H+ SpR ZScore Ion% # Sequence  | | | | | | | | | | | | |
| --- | --- | --- | --- | --- | --- | --- | --- | --- | --- | --- | --- | --- |
| \* | AstrinSTLCLD\_041714\_01.14244.14244.2 | 5.1076 | 0.5642 | 100.0% | 2333.672 | 2333.6897 | 1 | 10.007 | 52.4% | 1 | K.TAAFLLPILSQIYSDGPGEALR.A | 2 |
|  | AstrinSTLCLD\_041714\_01.06682.06682.3 | 3.5855 | 0.3023 | 100.0% | 1643.4844 | 1642.9852 | 21 | 5.327 | 40.4% | 1 | R.RKQYPISLVLAPTR.E | 3 |
|  | AstrinSTLCLD\_041714\_02.06957.06957.2 | 3.1518 | 0.4289 | 100.0% | 1321.0922 | 1321.4729 | 1 | 7.595 | 80.0% | 3 | R.ELAVQIYEEAR.K | 2 |
|  | AstrinSTLCLD\_041714\_01.09135.09135.2 | 4.0964 | 0.4602 | 100.0% | 1337.1721 | 1337.5946 | 1 | 8.584 | 85.0% | 3 | R.MLDMGFEPQIR.R | 222 |
| \* | AstrinSTLCLD\_041714\_01.12342.12342.2 | 2.9708 | 0.464 | 100.0% | 1292.4922 | 1292.5181 | 1 | 7.934 | 72.7% | 1 | R.SFLLDLLNATGK.D | 2 |
|  | AstrinSTLCLD\_041714\_01.06402.06402.2 | 2.5131 | 0.2208 | 96.7% | 1168.6721 | 1169.4099 | 1 | 5.336 | 77.3% | 2 | K.SPILVATAVAAR.G | 2 |
|  | AstrinSTLCLD\_041714\_01.10229.10229.3 | 5.2007 | 0.2375 | 100.0% | 2086.4944 | 2084.2957 | 1 | 6.789 | 48.4% | 1 | K.HVINFDLPSDIEEYVHR.I | 3 |
| \* | AstrinSTLCLD\_041714\_01.10419.10419.2 | 4.2903 | 0.4477 | 100.0% | 1526.0322 | 1525.7043 | 1 | 7.589 | 73.1% | 1 | R.VGNLGLATSFFNER.N | 2 |

Similarities:
gi|4758138|ref|NP\_004(1:7)  
gi|148613856|ref|NP\_0(1:7)  

---

|  |  |  |  |  |  |  |  |  |
| --- | --- | --- | --- | --- | --- | --- | --- | --- |
| U | *contaminant\_INT-STD1* | 11 | 22 | 16.6% | 607 | 69271 | 6.1 | BSA |

| Filename XCorr DeltCN Conf% ObsM+H+ CalcM+H+ SpR ZScore Ion% # Sequence  | | | | | | | | | | | | |
| --- | --- | --- | --- | --- | --- | --- | --- | --- | --- | --- | --- | --- |
| \* | AstrinSTLCLD\_041714\_01.07682.07682.2 | 3.2089 | 0.3687 | 100.0% | 1164.2522 | 1164.344 | 1 | 6.921 | 72.2% | 2 | K.LVNELTEFAK.T | 2 |
| \* | AstrinSTLCLD\_041714\_01.09273.09273.3 | 3.9908 | 0.3794 | 100.0% | 2046.4744 | 2046.3354 | 1 | 6.109 | 48.3% | 1 | R.RHPYFYAPELLYYANK.Y | 3 |
| \* | AstrinSTLCLD\_041714\_01.11769.11769.2 | 3.1329 | 0.4374 | 100.0% | 1569.3121 | 1568.7258 | 1 | 7.445 | 79.2% | 1 | K.DAFLGSFLYEYSR.R | 2 |
| \* | AstrinSTLCLD\_041714\_01.05904.05904.2 | 2.9384 | 0.4199 | 100.0% | 1440.6122 | 1440.6884 | 47 | 6.505 | 54.5% | 1 | R.RHPEYAVSVLLR.L | 2 |
| \* | AstrinSTLCLD\_041714\_01.05903.05903.3 | 4.6493 | 0.3335 | 100.0% | 1441.6444 | 1440.6884 | 1 | 5.781 | 61.4% | 4 | R.RHPEYAVSVLLR.L | 3 |
| \* | AstrinSTLCLD\_041714\_02.08091.08091.2 | 4.4326 | 0.4039 | 100.0% | 1481.1122 | 1480.7068 | 1 | 8.224 | 79.2% | 4 | K.LGEYGFQNALIVR.Y | 2 |
|  | AstrinSTLCLD\_041714\_01.05382.05382.2 | 3.7627 | 0.4462 | 100.0% | 1640.4922 | 1640.9205 | 1 | 8.536 | 57.1% | 2 | R.KVPQVSTPTLVEVSR.S | 2 |
|  | AstrinSTLCLD\_041714\_01.05428.05428.3 | 4.6485 | 0.4418 | 100.0% | 1642.0144 | 1640.9205 | 2 | 7.206 | 46.4% | 2 | R.KVPQVSTPTLVEVSR.S | 3 |
|  | AstrinSTLCLD\_041714\_01.06650.06650.2 | 2.439 | 0.4176 | 100.0% | 1512.7722 | 1512.7465 | 8 | 7.17 | 53.8% | 1 | K.VPQVSTPTLVEVSR.S | 2 |
| \* | AstrinSTLCLD\_041714\_01.11157.11157.2 | 3.3585 | 0.4707 | 100.0% | 1400.2922 | 1400.6324 | 1 | 8.087 | 77.3% | 3 | K.TVMENFVAFVDK.C | 2 |
| \* | AstrinSTLCLD\_041714\_02.06249.06249.2 | 2.8563 | 0.6059 | 100.0% | 1003.5522 | 1003.1839 | 2 | 8.592 | 66.7% | 1 | K.LVVSTQTALA.- | 2 |

---

|  |  |  |  |  |  |  |  |  |
| --- | --- | --- | --- | --- | --- | --- | --- | --- |
| U | *gi|169201338|ref|XP\_0* | 2 | 3 | 16.2% | 160 | 18565 | 10.5 | PREDICTED: hypothetical protein [Homo sapiens] |
| U | *gi|89040203|ref|XP\_93* | 2 | 3 | 16.2% | 160 | 18593 | 10.5 | PREDICTED: hypothetical protein [Homo sapiens] |
| U | *gi|18104948|ref|NP\_00* | 2 | 3 | 16.2% | 160 | 18565 | 10.5 | ribosomal protein L21 [Homo sapiens] |
| U | *gi|169213854|ref|XP\_0* | 2 | 3 | 16.2% | 160 | 18790 | 10.3 | PREDICTED: hypothetical protein [Homo sapiens] |
| U | *gi|169210381|ref|XP\_0* | 2 | 3 | 16.2% | 160 | 18535 | 10.6 | PREDICTED: hypothetical protein isoform 2 [Homo sapiens] |
| U | *gi|169210379|ref|XP\_0* | 2 | 3 | 16.2% | 160 | 18535 | 10.6 | PREDICTED: hypothetical protein isoform 3 [Homo sapiens] |
| U | *gi|169210377|ref|XP\_0* | 2 | 3 | 16.2% | 160 | 18535 | 10.6 | PREDICTED: hypothetical protein isoform 1 [Homo sapiens] |
| U | *gi|169202779|ref|XP\_0* | 2 | 3 | 16.2% | 160 | 18521 | 10.5 | PREDICTED: similar to ribosomal protein L21 isoform 1 [Homo sapiens] |
| U | *gi|169202777|ref|XP\_0* | 2 | 3 | 16.2% | 160 | 18521 | 10.5 | PREDICTED: similar to ribosomal protein L21 isoform 2 [Homo sapiens] |
| U | *gi|169201750|ref|XP\_0* | 2 | 3 | 16.2% | 160 | 18550 | 10.5 | PREDICTED: hypothetical protein [Homo sapiens] |

| Filename XCorr DeltCN Conf% ObsM+H+ CalcM+H+ SpR ZScore Ion% # Sequence  | | | | | | | | | | | | |
| --- | --- | --- | --- | --- | --- | --- | --- | --- | --- | --- | --- | --- |
|  | AstrinSTLCLD\_041714\_01.05744.05744.2 | 2.1339 | 0.4104 | 99.6% | 1243.3121 | 1244.4973 | 15 | 5.699 | 50.0% | 1 | K.HGVVPLATYMR.I | 2 |
|  | AstrinSTLCLD\_041714\_02.06081.06081.3 | 3.8468 | 0.4522 | 100.0% | 1642.2843 | 1641.9108 | 1 | 8.787 | 51.8% | 2 | R.VYNVTQHAVGIVVNK.Q | 3 |

---

|  |  |  |  |  |  |  |  |  |
| --- | --- | --- | --- | --- | --- | --- | --- | --- |
| U | *gi|148470397|ref|NP\_0* | 3 | 5 | 15.9% | 415 | 45672 | 5.6 | heterogeneous nuclear ribonucleoprotein F [Homo sapiens] |
| U | *gi|4826760|ref|NP\_004* | 3 | 5 | 15.9% | 415 | 45672 | 5.6 | heterogeneous nuclear ribonucleoprotein F [Homo sapiens] |
| U | *gi|148470406|ref|NP\_0* | 3 | 5 | 15.9% | 415 | 45672 | 5.6 | heterogeneous nuclear ribonucleoprotein F [Homo sapiens] |
| U | *gi|148470404|ref|NP\_0* | 3 | 5 | 15.9% | 415 | 45672 | 5.6 | heterogeneous nuclear ribonucleoprotein F [Homo sapiens] |
| U | *gi|148470402|ref|NP\_0* | 3 | 5 | 15.9% | 415 | 45672 | 5.6 | heterogeneous nuclear ribonucleoprotein F [Homo sapiens] |
| U | *gi|148470400|ref|NP\_0* | 3 | 5 | 15.9% | 415 | 45672 | 5.6 | heterogeneous nuclear ribonucleoprotein F [Homo sapiens] |

| Filename XCorr DeltCN Conf% ObsM+H+ CalcM+H+ SpR ZScore Ion% # Sequence  | | | | | | | | | | | | |
| --- | --- | --- | --- | --- | --- | --- | --- | --- | --- | --- | --- | --- |
|  | AstrinSTLCLD\_041714\_01.11273.11273.2 | 4.8819 | 0.4627 | 100.0% | 1868.4722 | 1869.0813 | 1 | 9.03 | 68.8% | 1 | K.ITGEAFVQFASQELAEK.A | 2 |
|  | AstrinSTLCLD\_041714\_02.09415.09415.3 | 7.0814 | 0.5707 | 100.0% | 3475.3743 | 3476.7114 | 1 | 11.266 | 34.7% | 3 | R.MRPGAYSTGYGGYEEYSGLSDGYGFTTDLFGR.D | 3 |
|  | AstrinSTLCLD\_041714\_01.11628.11628.2 | 2.624 | 0.2571 | 97.8% | 1998.1522 | 1998.2023 | 161 | 5.285 | 34.4% | 1 | K.ATENDIYNFFSPLNPVR.V | 22 |

Similarities:
gi|5031753|ref|NP\_005(1:2)  

---

|  |  |  |  |  |  |  |  |  |
| --- | --- | --- | --- | --- | --- | --- | --- | --- |
| U | *gi|4506903|ref|NP\_003* | 3 | 7 | 15.8% | 221 | 25542 | 8.6 | splicing factor, arginine/serine-rich 9 [Homo sapiens] |

| Filename XCorr DeltCN Conf% ObsM+H+ CalcM+H+ SpR ZScore Ion% # Sequence  | | | | | | | | | | | | |
| --- | --- | --- | --- | --- | --- | --- | --- | --- | --- | --- | --- | --- |
| \* | AstrinSTLCLD\_041714\_01.06395.06395.2 | 3.1851 | 0.3485 | 100.0% | 1247.4521 | 1247.4368 | 1 | 7.176 | 65.0% | 3 | R.IYVGNLPTDVR.E | 2 |
| \* | AstrinSTLCLD\_041714\_01.09191.09191.2 | 2.2977 | 0.2384 | 96.6% | 1143.7322 | 1143.3768 | 36 | 6.006 | 55.6% | 2 | R.HGLVPFAFVR.F | 2 |
| \* | AstrinSTLCLD\_041714\_01.06102.06102.3 | 3.3126 | 0.3423 | 100.0% | 1654.2843 | 1654.7332 | 1 | 7.54 | 46.2% | 2 | R.FEDPRDAEDAIYGR.N | 3 |

---

|  |  |  |  |  |  |  |  |  |
| --- | --- | --- | --- | --- | --- | --- | --- | --- |
| U | *gi|5031699|ref|NP\_005* | 5 | 6 | 15.7% | 427 | 47355 | 7.5 | flotillin 1 [Homo sapiens] |

| Filename XCorr DeltCN Conf% ObsM+H+ CalcM+H+ SpR ZScore Ion% # Sequence  | | | | | | | | | | | | |
| --- | --- | --- | --- | --- | --- | --- | --- | --- | --- | --- | --- | --- |
| \* | AstrinSTLCLD\_041714\_01.08879.08879.2 | 2.7339 | 0.3148 | 100.0% | 1216.4521 | 1216.4636 | 10 | 6.168 | 60.0% | 1 | R.ISLNTLTLNVK.S | 2 |
| \* | AstrinSTLCLD\_041714\_02.07024.07024.2 | 2.7236 | 0.3825 | 100.0% | 1468.6122 | 1469.693 | 3 | 6.936 | 58.3% | 1 | K.VSAQYLSEIEMAK.A | 2 |
| \* | AstrinSTLCLD\_041714\_02.06346.06346.2 | 4.3185 | 0.5263 | 100.0% | 1419.5521 | 1419.6206 | 1 | 9.555 | 75.0% | 1 | R.AQADLAYQLQVAK.T | 2 |
| \* | AstrinSTLCLD\_041714\_02.07557.07557.2 | 4.2661 | 0.5077 | 100.0% | 1605.2522 | 1604.8187 | 1 | 8.673 | 75.0% | 1 | K.SQLIMQAEAEAASVR.M | 2 |
| \* | AstrinSTLCLD\_041714\_02.05390.05390.2 | 4.3936 | 0.4895 | 100.0% | 1380.2922 | 1380.5994 | 1 | 8.576 | 82.1% | 2 | K.ITLVSSGSGTMGAAK.V | 2 |

---

|  |  |  |  |  |  |  |  |  |
| --- | --- | --- | --- | --- | --- | --- | --- | --- |
| U | *gi|15431295|ref|NP\_15* | 3 | 3 | 15.6% | 211 | 24261 | 11.7 | ribosomal protein L13 [Homo sapiens] |
| U | *gi|15431297|ref|NP\_00* | 3 | 3 | 15.6% | 211 | 24261 | 11.7 | ribosomal protein L13 [Homo sapiens] |

| Filename XCorr DeltCN Conf% ObsM+H+ CalcM+H+ SpR ZScore Ion% # Sequence  | | | | | | | | | | | | |
| --- | --- | --- | --- | --- | --- | --- | --- | --- | --- | --- | --- | --- |
|  | AstrinSTLCLD\_041714\_01.06013.06013.2 | 2.6617 | 0.3152 | 100.0% | 1190.3121 | 1190.3469 | 10 | 5.561 | 77.8% | 1 | R.VATWFNQPAR.K | 2 |
|  | AstrinSTLCLD\_041714\_01.08658.08658.3 | 3.2938 | 0.2738 | 99.1% | 2430.9844 | 2428.8064 | 1 | 5.354 | 31.8% | 1 | K.KGDSSAEELKLATQLTGPVMPVR.N | 3 |
|  | AstrinSTLCLD\_041714\_01.06890.06890.2 | 3.0773 | 0.4111 | 100.0% | 1383.2322 | 1383.6923 | 1 | 6.745 | 58.3% | 1 | K.LATQLTGPVMPVR.N | 2 |

---

|  |  |  |  |  |  |  |  |  |
| --- | --- | --- | --- | --- | --- | --- | --- | --- |
| U | *gi|169160598|ref|XP\_0* | 1 | 1 | 15.5% | 84 | 9461 | 9.5 | PREDICTED: similar to hCG1783679 [Homo sapiens] |
| U | *gi|7705706|ref|NP\_057* | 1 | 1 | 15.5% | 84 | 9477 | 9.5 | ribosomal protein S27-like [Homo sapiens] |
| U | *gi|4506711|ref|NP\_001* | 1 | 1 | 15.5% | 84 | 9461 | 9.5 | ribosomal protein S27 [Homo sapiens] |
| U | *gi|169214231|ref|XP\_0* | 1 | 1 | 15.7% | 83 | 9378 | 9.6 | PREDICTED: similar to hCG2027326 [Homo sapiens] |
| U | *gi|169213802|ref|XP\_0* | 1 | 1 | 15.7% | 83 | 9378 | 9.6 | PREDICTED: similar to hCG2027326 [Homo sapiens] |
| U | *gi|169213575|ref|XP\_0* | 1 | 1 | 15.7% | 83 | 9378 | 9.6 | PREDICTED: similar to hCG2027326 [Homo sapiens] |
| U | *gi|169166679|ref|XP\_0* | 1 | 1 | 15.5% | 84 | 9450 | 9.4 | PREDICTED: similar to metallopanstimulin [Homo sapiens] |
| U | *gi|169166621|ref|XP\_0* | 1 | 1 | 15.5% | 84 | 9465 | 9.5 | PREDICTED: similar to metallopanstimulin [Homo sapiens] |
| U | *gi|169166508|ref|XP\_0* | 1 | 1 | 15.5% | 84 | 9450 | 9.4 | PREDICTED: similar to metallopanstimulin [Homo sapiens] |
| U | *gi|169161552|ref|XP\_0* | 1 | 1 | 15.5% | 84 | 9461 | 9.5 | PREDICTED: similar to hCG1783679 [Homo sapiens] |
| U | *gi|169161255|ref|XP\_0* | 1 | 1 | 15.5% | 84 | 9461 | 9.5 | PREDICTED: hypothetical protein [Homo sapiens] |

| Filename XCorr DeltCN Conf% ObsM+H+ CalcM+H+ SpR ZScore Ion% # Sequence  | | | | | | | | | | | | |
| --- | --- | --- | --- | --- | --- | --- | --- | --- | --- | --- | --- | --- |
|  | AstrinSTLCLD\_041714\_01.08026.08026.2 | 3.5866 | 0.4314 | 100.0% | 1528.2922 | 1528.7632 | 1 | 6.769 | 79.2% | 1 | R.LVQSPNSYFMDVK.C | 2 |

---

|  |  |  |  |  |  |  |  |  |
| --- | --- | --- | --- | --- | --- | --- | --- | --- |
| U | *gi|4506623|ref|NP\_000* | 1 | 1 | 15.4% | 136 | 15798 | 10.6 | ribosomal protein L27 [Homo sapiens] |

| Filename XCorr DeltCN Conf% ObsM+H+ CalcM+H+ SpR ZScore Ion% # Sequence  | | | | | | | | | | | | |
| --- | --- | --- | --- | --- | --- | --- | --- | --- | --- | --- | --- | --- |
| \* | AstrinSTLCLD\_041714\_01.05309.05309.3 | 3.847 | 0.3575 | 100.0% | 2273.3342 | 2273.4238 | 1 | 6.294 | 33.8% | 1 | K.NIDDGTSDRPYSHALVAGIDR.Y | 3 |

---

|  |  |  |  |  |  |  |  |  |
| --- | --- | --- | --- | --- | --- | --- | --- | --- |
| U | *gi|5174457|ref|NP\_006* | 6 | 13 | 15.3% | 642 | 73913 | 5.6 | kinetochore associated 2 [Homo sapiens] |

| Filename XCorr DeltCN Conf% ObsM+H+ CalcM+H+ SpR ZScore Ion% # Sequence  | | | | | | | | | | | | |
| --- | --- | --- | --- | --- | --- | --- | --- | --- | --- | --- | --- | --- |
| \* | AstrinSTLCLD\_041714\_01.10168.10168.3 | 3.4733 | 0.2537 | 99.3% | 2081.3342 | 2081.4167 | 1 | 5.516 | 36.8% | 1 | K.LKDLFNVDAFKLESLEAK.N | 3 |
| \* | AstrinSTLCLD\_041714\_02.06824.06824.3 | 2.702 | 0.3363 | 99.3% | 2099.8145 | 2099.3228 | 15 | 4.856 | 32.4% | 2 | K.YQAYMSNLESHSAILDQK.L | 3 |
| \* | AstrinSTLCLD\_041714\_01.12413.12413.3 | 3.3151 | 0.3008 | 100.0% | 2179.7944 | 2180.4963 | 1 | 6.315 | 34.7% | 1 | K.MGLEDTLEQLNAMITESKR.S | 3 |
| \* | AstrinSTLCLD\_041714\_01.08240.08240.3 | 4.3547 | 0.3972 | 100.0% | 1978.8844 | 1979.2377 | 1 | 6.904 | 48.3% | 2 | R.TLKEEVQKLDDLYQQK.I | 3 |
| \* | AstrinSTLCLD\_041714\_02.05919.05919.2 | 4.4006 | 0.4926 | 100.0% | 1596.1921 | 1596.7344 | 1 | 8.744 | 79.2% | 3 | R.EYQLVVQTTTEER.R | 2 |
| \* | AstrinSTLCLD\_041714\_01.06087.06087.2 | 4.3732 | 0.3809 | 100.0% | 1513.4922 | 1513.7925 | 1 | 7.459 | 76.9% | 4 | R.LLEMVATHVGSVEK.H | 2 |

---

|  |  |  |  |  |  |  |  |  |
| --- | --- | --- | --- | --- | --- | --- | --- | --- |
| U | *gi|56699409|ref|NP\_00* | 5 | 10 | 15.3% | 391 | 42332 | 10.1 | RNA binding motif protein, X-linked [Homo sapiens] |

| Filename XCorr DeltCN Conf% ObsM+H+ CalcM+H+ SpR ZScore Ion% # Sequence  | | | | | | | | | | | | |
| --- | --- | --- | --- | --- | --- | --- | --- | --- | --- | --- | --- | --- |
|  | AstrinSTLCLD\_041714\_01.07118.07118.2 | 3.6997 | 0.3551 | 100.0% | 1435.6921 | 1436.6049 | 2 | 6.459 | 70.8% | 3 | K.LFIGGLNTETNEK.A | 2 |
|  | AstrinSTLCLD\_041714\_01.06536.06536.2 | 3.1471 | 0.3557 | 100.0% | 1216.3322 | 1216.5249 | 2 | 6.043 | 77.8% | 2 | R.IVEVLLMKDR.E | 2 |
|  | AstrinSTLCLD\_041714\_01.10034.10034.2 | 4.1677 | 0.4521 | 100.0% | 1487.2922 | 1487.6519 | 1 | 9.298 | 73.1% | 2 | R.GFAFVTFESPADAK.D | 2 |
|  | AstrinSTLCLD\_041714\_01.09080.09080.3 | 3.1051 | 0.2333 | 97.9% | 1902.4143 | 1901.0856 | 3 | 5.124 | 33.8% | 1 | R.GFAFVTFESPADAKDAAR.D | 3 |
|  | AstrinSTLCLD\_041714\_02.06326.06326.3 | 4.472 | 0.362 | 100.0% | 2050.9443 | 2051.1873 | 1 | 7.432 | 38.9% | 2 | R.GGHMDDGGYSMNFNMSSSR.G | 3 |

---

|  |  |  |  |  |  |  |  |  |
| --- | --- | --- | --- | --- | --- | --- | --- | --- |
| U | *gi|10863927|ref|NP\_06* | 2 | 3 | 15.2% | 165 | 18012 | 7.8 | peptidylprolyl isomerase A [Homo sapiens] |
| U | *gi|169215435|ref|XP\_0* | 2 | 3 | 11.2% | 223 | 24376 | 6.9 | PREDICTED: similar to peptidylprolyl isomerase A-like [Homo sapiens] |

| Filename XCorr DeltCN Conf% ObsM+H+ CalcM+H+ SpR ZScore Ion% # Sequence  | | | | | | | | | | | | |
| --- | --- | --- | --- | --- | --- | --- | --- | --- | --- | --- | --- | --- |
|  | AstrinSTLCLD\_041714\_01.08909.08909.2 | 3.079 | 0.2081 | 99.3% | 1380.5922 | 1380.6268 | 6 | 5.52 | 59.1% | 2 | R.VSFELFADKVPK.T | 2 |
|  | AstrinSTLCLD\_041714\_01.06999.06999.2 | 3.5591 | 0.0416 | 95.1% | 1505.9922 | 1506.7755 | 2 | 8.447 | 75.0% | 1 | K.VKEGMNIVEAMER.F | 2 |

---

|  |  |  |  |  |  |  |  |  |
| --- | --- | --- | --- | --- | --- | --- | --- | --- |
| U | *gi|5729877|ref|NP\_006* | 7 | 10 | 15.0% | 646 | 70898 | 5.5 | heat shock 70kDa protein 8 isoform 1 [Homo sapiens] |

| Filename XCorr DeltCN Conf% ObsM+H+ CalcM+H+ SpR ZScore Ion% # Sequence  | | | | | | | | | | | | |
| --- | --- | --- | --- | --- | --- | --- | --- | --- | --- | --- | --- | --- |
|  | AstrinSTLCLD\_041714\_01.05795.05795.2 | 3.5555 | 0.4534 | 100.0% | 1488.0721 | 1488.5939 | 1 | 8.647 | 75.0% | 3 | R.TTPSYVAFTDTER.L | 2 |
|  | AstrinSTLCLD\_041714\_01.08388.08388.2 | 2.6826 | 0.3481 | 99.7% | 1982.7522 | 1983.1882 | 4 | 6.582 | 38.2% | 1 | K.TVTNAVVTVPAYFNDSQR.Q | 2 |
|  | AstrinSTLCLD\_041714\_01.08927.08927.2 | 2.9462 | 0.3708 | 100.0% | 1659.8322 | 1660.9078 | 1 | 6.768 | 60.0% | 1 | R.IINEPTAAAIAYGLDK.K | 2 |
|  | AstrinSTLCLD\_041714\_01.06649.06649.2 | 2.5327 | 0.4504 | 100.0% | 1235.7522 | 1236.4741 | 1 | 6.654 | 77.8% | 1 | R.MVNHFIAEFK.R | 2 |
|  | AstrinSTLCLD\_041714\_01.08438.08438.2 | 3.5925 | 0.3673 | 100.0% | 1481.3322 | 1481.6511 | 1 | 6.385 | 77.3% | 1 | R.ARFEELNADLFR.G | 2 |
|  | AstrinSTLCLD\_041714\_01.05696.05696.3 | 3.1818 | 0.2493 | 99.1% | 1838.1244 | 1839.1019 | 66 | 4.954 | 29.7% | 2 | K.LDKSQIHDIVLVGGSTR.I | 3 |
| \* | AstrinSTLCLD\_041714\_01.07967.07967.2 | 2.526 | 0.3802 | 100.0% | 1304.1921 | 1304.4602 | 7 | 6.429 | 65.0% | 1 | K.NSLESYAFNMK.A | 2 |

---

|  |  |  |  |  |  |  |  |  |
| --- | --- | --- | --- | --- | --- | --- | --- | --- |
| U | *gi|67782365|ref|NP\_00* | 4 | 4 | 13.9% | 469 | 51386 | 5.5 | keratin 7 [Homo sapiens] |

| Filename XCorr DeltCN Conf% ObsM+H+ CalcM+H+ SpR ZScore Ion% # Sequence  | | | | | | | | | | | | |
| --- | --- | --- | --- | --- | --- | --- | --- | --- | --- | --- | --- | --- |
|  | AstrinSTLCLD\_041714\_01.10455.10455.3 | 2.4711 | 0.296 | 97.3% | 2450.2744 | 2450.7979 | 1 | 4.861 | 27.4% | 1 | R.EVTINQSLLAPLRLDADPSLQR.V | 3 |
|  | AstrinSTLCLD\_041714\_01.10994.10994.2 | 3.0786 | 0.441 | 100.0% | 1442.3922 | 1443.686 | 2 | 8.298 | 62.5% | 1 | R.LPDIFEAQIAGLR.G | 2 |
| \* | AstrinSTLCLD\_041714\_02.07908.07908.3 | 4.0864 | 0.4329 | 100.0% | 1954.7344 | 1955.1783 | 1 | 7.724 | 45.6% | 1 | R.GQLEALQVDGGRLEAELR.S | 3 |
|  | AstrinSTLCLD\_041714\_01.09920.09920.2 | 3.8722 | 0.1334 | 99.7% | 1419.0721 | 1419.5773 | 1 | 8.036 | 77.3% | 1 | K.VDALNDEINFLR.T | 2 |

---

|  |  |  |  |  |  |  |  |  |
| --- | --- | --- | --- | --- | --- | --- | --- | --- |
| U | *contaminant\_KERATIN12* | 5 | 11 | 13.9% | 431 | 47974 | 5.0 | no description |
| U | *gi|4557701|ref|NP\_000* | 5 | 11 | 13.9% | 432 | 48106 | 5.0 | keratin 17 [Homo sapiens] |

| Filename XCorr DeltCN Conf% ObsM+H+ CalcM+H+ SpR ZScore Ion% # Sequence  | | | | | | | | | | | | |
| --- | --- | --- | --- | --- | --- | --- | --- | --- | --- | --- | --- | --- |
|  | AstrinSTLCLD\_041714\_01.05007.05007.2 | 3.841 | 0.4007 | 100.0% | 1346.2122 | 1346.4772 | 2 | 7.338 | 63.6% | 6 | R.ALEEANTELEVK.I | 2 |
|  | AstrinSTLCLD\_041714\_01.07227.07227.2 | 3.2033 | 0.3687 | 100.0% | 1187.3121 | 1187.3384 | 1 | 6.94 | 80.0% | 1 | R.LSVEADINGLR.R | 2 |
|  | AstrinSTLCLD\_041714\_01.06680.06680.2 | 3.5857 | 0.3941 | 100.0% | 1029.6522 | 1030.2096 | 1 | 7.292 | 81.2% | 2 | R.VLDELTLAR.A | 22 |
|  | AstrinSTLCLD\_041714\_01.10018.10018.2 | 2.9703 | 0.3361 | 100.0% | 1887.4122 | 1888.0001 | 1 | 5.68 | 53.6% | 1 | K.DAEDWFFSKTEELNR.E | 2 |
|  | AstrinSTLCLD\_041714\_02.05181.05181.2 | 3.7212 | 0.4269 | 100.0% | 1404.5521 | 1404.4764 | 1 | 7.985 | 70.8% | 1 | K.ASLEGNLAETENR.Y | 2 |

Similarities:
contaminant\_KERATIN10(1:4)  

---

|  |  |  |  |  |  |  |  |  |
| --- | --- | --- | --- | --- | --- | --- | --- | --- |
| U | *gi|4506607|ref|NP\_000* | 2 | 4 | 13.8% | 188 | 21634 | 11.7 | ribosomal protein L18 [Homo sapiens] |

| Filename XCorr DeltCN Conf% ObsM+H+ CalcM+H+ SpR ZScore Ion% # Sequence  | | | | | | | | | | | | |
| --- | --- | --- | --- | --- | --- | --- | --- | --- | --- | --- | --- | --- |
| \* | AstrinSTLCLD\_041714\_02.06302.06302.2 | 4.0505 | 0.4089 | 100.0% | 1346.4321 | 1346.5236 | 1 | 8.841 | 83.3% | 3 | K.TAVVVGTITDDVR.V | 2 |
| \* | AstrinSTLCLD\_041714\_01.10092.10092.2 | 3.7549 | 0.4117 | 100.0% | 1461.4122 | 1461.6982 | 1 | 7.491 | 62.5% | 1 | K.ILTFDQLALDSPK.G | 2 |

---

|  |  |  |  |  |  |  |  |  |
| --- | --- | --- | --- | --- | --- | --- | --- | --- |
| U | *gi|4503471|ref|NP\_001* | 4 | 10 | 13.6% | 462 | 50141 | 9.0 | eukaryotic translation elongation factor 1 alpha 1 [Homo sapiens] |

| Filename XCorr DeltCN Conf% ObsM+H+ CalcM+H+ SpR ZScore Ion% # Sequence  | | | | | | | | | | | | |
| --- | --- | --- | --- | --- | --- | --- | --- | --- | --- | --- | --- | --- |
|  | AstrinSTLCLD\_041714\_02.06386.06386.3 | 4.8743 | 0.4336 | 100.0% | 1590.2644 | 1589.835 | 1 | 7.625 | 51.8% | 7 | K.THINIVVIGHVDSGK.S | 3 |
| \* | AstrinSTLCLD\_041714\_01.05838.05838.3 | 2.5322 | 0.2474 | 97.5% | 1406.1543 | 1405.5962 | 22 | 5.298 | 45.5% | 1 | K.YYVTIIDAPGHR.D | 3 |
|  | AstrinSTLCLD\_041714\_01.07623.07623.2 | 2.6677 | 0.285 | 99.3% | 1315.5122 | 1315.5553 | 1 | 6.427 | 68.2% | 1 | R.EHALLAYTLGVK.Q | 2 |
| \* | AstrinSTLCLD\_041714\_01.09705.09705.3 | 4.661 | 0.3507 | 100.0% | 2518.3145 | 2516.999 | 1 | 6.332 | 34.8% | 1 | R.VETGVLKPGMVVTFAPVNVTTEVK.S | 3 |

---

|  |  |  |  |  |  |  |  |  |
| --- | --- | --- | --- | --- | --- | --- | --- | --- |
| U | *gi|4506743|ref|NP\_001* | 2 | 4 | 13.5% | 208 | 24205 | 10.3 | ribosomal protein S8 [Homo sapiens] |

| Filename XCorr DeltCN Conf% ObsM+H+ CalcM+H+ SpR ZScore Ion% # Sequence  | | | | | | | | | | | | |
| --- | --- | --- | --- | --- | --- | --- | --- | --- | --- | --- | --- | --- |
| \* | AstrinSTLCLD\_041714\_02.07142.07142.2 | 4.0113 | 0.4765 | 100.0% | 1719.4321 | 1719.9353 | 1 | 8.998 | 78.6% | 2 | R.IIDVVYNASNNELVR.T | 2 |
| \* | AstrinSTLCLD\_041714\_01.07349.07349.2 | 3.8511 | 0.4035 | 100.0% | 1507.4722 | 1507.6836 | 1 | 7.087 | 70.8% | 2 | K.ISSLLEEQFQQGK.L | 2 |

---

|  |  |  |  |  |  |  |  |  |
| --- | --- | --- | --- | --- | --- | --- | --- | --- |
| U | *gi|4505813|ref|NP\_003* | 1 | 1 | 13.5% | 89 | 10366 | 7.4 | dynein light chain 1 [Homo sapiens] |
| U | *gi|83267868|ref|NP\_00* | 1 | 1 | 13.5% | 89 | 10366 | 7.4 | dynein light chain 1 [Homo sapiens] |
| U | *gi|83267866|ref|NP\_00* | 1 | 1 | 13.5% | 89 | 10366 | 7.4 | dynein light chain 1 [Homo sapiens] |

| Filename XCorr DeltCN Conf% ObsM+H+ CalcM+H+ SpR ZScore Ion% # Sequence  | | | | | | | | | | | | |
| --- | --- | --- | --- | --- | --- | --- | --- | --- | --- | --- | --- | --- |
|  | AstrinSTLCLD\_041714\_01.06481.06481.3 | 2.6007 | 0.2184 | 95.2% | 1415.7544 | 1415.6322 | 7 | 4.331 | 45.5% | 1 | K.YNIEKDIAAHIK.K | 3 |

---

|  |  |  |  |  |  |  |  |  |
| --- | --- | --- | --- | --- | --- | --- | --- | --- |
| U | *gi|5453555|ref|NP\_006* | 2 | 2 | 13.4% | 216 | 24423 | 7.5 | ras-related nuclear protein [Homo sapiens] |

| Filename XCorr DeltCN Conf% ObsM+H+ CalcM+H+ SpR ZScore Ion% # Sequence  | | | | | | | | | | | | |
| --- | --- | --- | --- | --- | --- | --- | --- | --- | --- | --- | --- | --- |
| \* | AstrinSTLCLD\_041714\_01.07788.07788.2 | 3.3234 | 0.4333 | 100.0% | 1689.5521 | 1690.8962 | 1 | 8.235 | 57.1% | 1 | R.GPIKFNVWDTAGQEK.F | 2 |
| \* | AstrinSTLCLD\_041714\_01.10791.10791.3 | 4.3221 | 0.5036 | 100.0% | 1786.5243 | 1786.0427 | 1 | 7.991 | 48.1% | 1 | K.SNYNFEKPFLWLAR.K | 3 |

---

|  |  |  |  |  |  |  |  |  |
| --- | --- | --- | --- | --- | --- | --- | --- | --- |
| U | *gi|94538362|ref|NP\_00* | 4 | 6 | 13.1% | 428 | 47064 | 5.3 | flotillin 2 [Homo sapiens] |

| Filename XCorr DeltCN Conf% ObsM+H+ CalcM+H+ SpR ZScore Ion% # Sequence  | | | | | | | | | | | | |
| --- | --- | --- | --- | --- | --- | --- | --- | --- | --- | --- | --- | --- |
| \* | AstrinSTLCLD\_041714\_02.05886.05886.2 | 2.3862 | 0.3371 | 99.6% | 1123.9922 | 1124.2358 | 3 | 6.709 | 72.2% | 1 | K.SAFSEEVNIK.T | 2 |
| \* | AstrinSTLCLD\_041714\_01.07571.07571.2 | 3.4997 | 0.2676 | 100.0% | 1522.3522 | 1521.6702 | 1 | 5.534 | 65.4% | 3 | K.TAEAQLAYELQGAR.E | 2 |
| \* | AstrinSTLCLD\_041714\_01.05490.05490.2 | 2.9223 | 0.171 | 97.5% | 1375.6721 | 1375.5187 | 1 | 4.569 | 70.8% | 1 | K.VDEIVVLSGDNSK.V | 2 |
| \* | AstrinSTLCLD\_041714\_01.10109.10109.2 | 3.8834 | 0.505 | 100.0% | 1934.5122 | 1935.2712 | 1 | 8.76 | 55.6% | 1 | R.LLAELPASVHALTGVDLSK.I | 2 |

---

|  |  |  |  |  |  |  |  |  |
| --- | --- | --- | --- | --- | --- | --- | --- | --- |
| U | *gi|17149836|ref|NP\_46* | 1 | 1 | 13.0% | 108 | 11951 | 8.2 | FK506 binding protein 1A, 12kDa [Homo sapiens] |
| U | *gi|4503725|ref|NP\_000* | 1 | 1 | 13.0% | 108 | 11951 | 8.2 | FK506 binding protein 1A, 12kDa [Homo sapiens] |

| Filename XCorr DeltCN Conf% ObsM+H+ CalcM+H+ SpR ZScore Ion% # Sequence  | | | | | | | | | | | | |
| --- | --- | --- | --- | --- | --- | --- | --- | --- | --- | --- | --- | --- |
|  | AstrinSTLCLD\_041714\_02.06688.06688.2 | 2.8748 | 0.264 | 99.3% | 1533.7322 | 1534.6868 | 1 | 6.355 | 53.8% | 1 | R.GWEEGVAQMSVGQR.A | 2 |

---

|  |  |  |  |  |  |  |  |  |
| --- | --- | --- | --- | --- | --- | --- | --- | --- |
| U | *gi|19920317|ref|NP\_00* | 5 | 9 | 12.8% | 602 | 66023 | 5.9 | cytoskeleton-associated protein 4 [Homo sapiens] |

| Filename XCorr DeltCN Conf% ObsM+H+ CalcM+H+ SpR ZScore Ion% # Sequence  | | | | | | | | | | | | |
| --- | --- | --- | --- | --- | --- | --- | --- | --- | --- | --- | --- | --- |
| \* | AstrinSTLCLD\_041714\_01.05648.05648.2 | 1.9248 | 0.3132 | 96.2% | 987.1922 | 987.1845 | 69 | 5.86 | 62.5% | 1 | R.LALQALTEK.L | 2 |
| \* | AstrinSTLCLD\_041714\_02.06411.06411.3 | 3.8716 | 0.3865 | 100.0% | 1840.7943 | 1841.0923 | 1 | 6.676 | 50.0% | 2 | R.LQHVEDGVLSMQVASAR.Q | 3 |
| \* | AstrinSTLCLD\_041714\_01.06113.06113.2 | 3.8461 | 0.4521 | 100.0% | 1904.4521 | 1906.0135 | 2 | 8.817 | 44.4% | 1 | R.LEGLGSSEADQDGLASTVR.S | 2 |
| \* | AstrinSTLCLD\_041714\_01.06405.06405.2 | 3.769 | 0.3413 | 100.0% | 1474.3121 | 1474.6512 | 1 | 6.716 | 73.1% | 2 | R.SVGELPSTVESLQK.V | 2 |
| \* | AstrinSTLCLD\_041714\_01.04857.04857.3 | 4.1843 | 0.2984 | 100.0% | 2023.3143 | 2023.2131 | 1 | 6.766 | 38.2% | 3 | K.VQEQVHTLLSQDQAQAAR.L | 3 |

---

|  |  |  |  |  |  |  |  |  |
| --- | --- | --- | --- | --- | --- | --- | --- | --- |
| U | *gi|14141161|ref|NP\_00* | 6 | 10 | 12.7% | 806 | 88980 | 5.8 | heterogeneous nuclear ribonucleoprotein U isoform b [Homo sapiens] |
| U | *gi|74136883|ref|NP\_11* | 6 | 10 | 12.4% | 825 | 90585 | 6.0 | heterogeneous nuclear ribonucleoprotein U isoform a [Homo sapiens] |

| Filename XCorr DeltCN Conf% ObsM+H+ CalcM+H+ SpR ZScore Ion% # Sequence  | | | | | | | | | | | | |
| --- | --- | --- | --- | --- | --- | --- | --- | --- | --- | --- | --- | --- |
|  | AstrinSTLCLD\_041714\_01.06810.06810.3 | 6.1163 | 0.507 | 100.0% | 3127.7944 | 3128.311 | 1 | 6.984 | 33.9% | 3 | R.LQAALDDEEAGGRPAMEPGNGSLDLGGDSAGR.S | 3 |
|  | AstrinSTLCLD\_041714\_01.07196.07196.2 | 3.8053 | 0.4667 | 100.0% | 1698.5122 | 1698.8291 | 1 | 7.467 | 75.0% | 1 | R.GYFEYIEENKYSR.A | 2 |
|  | AstrinSTLCLD\_041714\_01.10947.10947.3 | 3.8966 | 0.3099 | 100.0% | 2725.3442 | 2726.0576 | 2 | 5.363 | 31.0% | 1 | K.EKPYFPIPEEYTFIQNVPLEDR.V | 3 |
|  | AstrinSTLCLD\_041714\_01.06111.06111.3 | 3.8996 | 0.3694 | 100.0% | 2188.7644 | 2188.4631 | 32 | 5.37 | 34.2% | 2 | K.HAAENPGKYNILGTNTIMDK.M | 3 |
|  | AstrinSTLCLD\_041714\_02.06782.06782.2 | 5.1433 | 0.4611 | 100.0% | 1648.2722 | 1648.816 | 1 | 8.293 | 82.1% | 2 | R.NFILDQTNVSAAAQR.R | 2 |
|  | AstrinSTLCLD\_041714\_02.06737.06737.3 | 3.4435 | 0.1865 | 97.9% | 1648.5543 | 1648.816 | 1 | 5.327 | 51.8% | 1 | R.NFILDQTNVSAAAQR.R | 3 |

---

|  |  |  |  |  |  |  |  |  |
| --- | --- | --- | --- | --- | --- | --- | --- | --- |
| U | *gi|74099697|ref|NP\_00* | 3 | 6 | 12.2% | 449 | 49264 | 6.3 | heterogeneous nuclear ribonucleoprotein H2 [Homo sapiens] |
| U | *gi|9624998|ref|NP\_062* | 3 | 6 | 12.2% | 449 | 49264 | 6.3 | heterogeneous nuclear ribonucleoprotein H2 [Homo sapiens] |

| Filename XCorr DeltCN Conf% ObsM+H+ CalcM+H+ SpR ZScore Ion% # Sequence  | | | | | | | | | | | | |
| --- | --- | --- | --- | --- | --- | --- | --- | --- | --- | --- | --- | --- |
|  | AstrinSTLCLD\_041714\_01.10112.10112.2 | 4.613 | 0.5272 | 100.0% | 1842.6721 | 1843.0001 | 1 | 9.872 | 71.9% | 4 | R.STGEAFVQFASQEIAEK.A | 22 |
|  | AstrinSTLCLD\_041714\_01.12022.12022.2 | 3.1031 | 0.393 | 100.0% | 2029.6122 | 2030.2622 | 1 | 7.623 | 50.0% | 1 | R.ATENDIYNFFSPLNPMR.V | 2 |
|  | AstrinSTLCLD\_041714\_02.06759.06759.3 | 3.5013 | 0.2625 | 99.3% | 2162.9343 | 2163.3638 | 17 | 4.353 | 28.8% | 1 | R.VTGEADVEFATHEDAVAAMAK.D | 3 |

Similarities:
gi|5031753|ref|NP\_005(1:2)  

---

|  |  |  |  |  |  |  |  |  |
| --- | --- | --- | --- | --- | --- | --- | --- | --- |
| U | *gi|213688375|ref|NP\_0* | 4 | 5 | 12.2% | 377 | 42009 | 5.4 | alpha 2 actin [Homo sapiens] |
| U | *gi|4885049|ref|NP\_005* | 4 | 5 | 12.2% | 377 | 42019 | 5.4 | cardiac muscle alpha actin 1 proprotein [Homo sapiens] |
| U | *gi|4501889|ref|NP\_001* | 4 | 5 | 12.2% | 376 | 41877 | 5.5 | actin, gamma 2 propeptide [Homo sapiens] |
| U | *gi|4501883|ref|NP\_001* | 4 | 5 | 12.2% | 377 | 42009 | 5.4 | alpha 2 actin [Homo sapiens] |
| U | *gi|4501881|ref|NP\_001* | 4 | 5 | 12.2% | 377 | 42051 | 5.4 | actin, alpha 1, skeletal muscle [Homo sapiens] |

| Filename XCorr DeltCN Conf% ObsM+H+ CalcM+H+ SpR ZScore Ion% # Sequence  | | | | | | | | | | | | |
| --- | --- | --- | --- | --- | --- | --- | --- | --- | --- | --- | --- | --- |
|  | AstrinSTLCLD\_041714\_01.08008.08008.3 | 3.8408 | 0.2592 | 100.0% | 1961.6643 | 1962.1841 | 1 | 5.685 | 48.3% | 1 | K.YPIEHGIITNWDDMEK.I | 3 |
|  | AstrinSTLCLD\_041714\_01.08027.08027.2 | 3.9367 | 0.1965 | 100.0% | 1962.2922 | 1962.1841 | 1 | 5.934 | 60.0% | 1 | K.YPIEHGIITNWDDMEK.I | 2 |
|  | AstrinSTLCLD\_041714\_01.09872.09872.2 | 2.8839 | 0.3323 | 100.0% | 1624.0521 | 1624.8927 | 1 | 6.051 | 57.7% | 1 | R.LDLAGRDLTDYLMK.I | 22 |
|  | AstrinSTLCLD\_041714\_01.09164.09164.2 | 4.0368 | 0.3695 | 100.0% | 1790.8522 | 1791.9554 | 1 | 6.586 | 70.0% | 2 | K.SYELPDGQVITIGNER.F | 22 |

Similarities:
gi|4501885|ref|NP\_001(2:2)  

---

|  |  |  |  |  |  |  |  |  |
| --- | --- | --- | --- | --- | --- | --- | --- | --- |
| U | *gi|36287110|ref|NP\_91* | 3 | 5 | 12.1% | 379 | 40907 | 4.6 | FGFR1 oncogene partner isoform b [Homo sapiens] |
| U | *gi|5901954|ref|NP\_008* | 3 | 5 | 11.5% | 399 | 43065 | 4.8 | FGFR1 oncogene partner isoform a [Homo sapiens] |

| Filename XCorr DeltCN Conf% ObsM+H+ CalcM+H+ SpR ZScore Ion% # Sequence  | | | | | | | | | | | | |
| --- | --- | --- | --- | --- | --- | --- | --- | --- | --- | --- | --- | --- |
|  | AstrinSTLCLD\_041714\_01.13390.13390.2 | 3.9313 | 0.3457 | 100.0% | 2166.0322 | 2165.5352 | 1 | 6.66 | 42.5% | 2 | R.DLGIIEAEGTVGGPLLLEVIR.R | 2 |
|  | AstrinSTLCLD\_041714\_02.10690.10690.3 | 3.2721 | 0.2468 | 98.3% | 2321.2144 | 2321.7227 | 1 | 5.554 | 29.8% | 2 | R.DLGIIEAEGTVGGPLLLEVIRR.C | 3 |
|  | AstrinSTLCLD\_041714\_01.08247.08247.3 | 3.3331 | 0.2145 | 97.5% | 2570.3643 | 2571.63 | 287 | 4.738 | 22.8% | 1 | K.IGSLGLGTGEDDDYVDDFNSTSHR.S | 3 |

---

|  |  |  |  |  |  |  |  |  |
| --- | --- | --- | --- | --- | --- | --- | --- | --- |
| U | *gi|20149594|ref|NP\_03* | 6 | 8 | 11.9% | 724 | 83264 | 5.0 | heat shock 90kDa protein 1, beta [Homo sapiens] |

| Filename XCorr DeltCN Conf% ObsM+H+ CalcM+H+ SpR ZScore Ion% # Sequence  | | | | | | | | | | | | |
| --- | --- | --- | --- | --- | --- | --- | --- | --- | --- | --- | --- | --- |
|  | AstrinSTLCLD\_041714\_01.08062.08062.2 | 3.3951 | 0.2859 | 100.0% | 1243.1921 | 1243.4459 | 1 | 6.134 | 72.7% | 1 | K.ADLINNLGTIAK.S | 22 |
|  | AstrinSTLCLD\_041714\_02.06522.06522.3 | 3.5957 | 0.3866 | 100.0% | 2015.9944 | 2016.2584 | 1 | 6.42 | 41.7% | 2 | K.VILHLKEDQTEYLEER.R | 33 |
|  | AstrinSTLCLD\_041714\_01.07229.07229.2 | 3.4213 | 0.3608 | 100.0% | 1528.3722 | 1528.6616 | 1 | 6.434 | 62.5% | 1 | K.SLTNDWEDHLAVK.H | 22 |
|  | AstrinSTLCLD\_041714\_02.06833.06833.2 | 2.8835 | 0.4073 | 100.0% | 1348.6322 | 1349.4886 | 1 | 6.329 | 65.0% | 1 | K.HFSVEGQLEFR.A | 22 |
| \* | AstrinSTLCLD\_041714\_02.06735.06735.3 | 4.1954 | 0.3869 | 100.0% | 2178.0544 | 2178.2915 | 1 | 6.659 | 33.3% | 1 | R.YHTSQSGDEMTSLSEYVSR.M | 3 |
| \* | AstrinSTLCLD\_041714\_01.06143.06143.3 | 3.7771 | 0.4692 | 100.0% | 1783.8544 | 1784.025 | 1 | 7.415 | 46.4% | 2 | K.HLEINPDHPIVETLR.Q | 3 |

Similarities:
gi|153792590|ref|NP\_0(4:2)  

---

|  |  |  |  |  |  |  |  |  |
| --- | --- | --- | --- | --- | --- | --- | --- | --- |
| U | *gi|16579885|ref|NP\_00* | 3 | 3 | 11.9% | 427 | 47697 | 11.1 | ribosomal protein L4 [Homo sapiens] |

| Filename XCorr DeltCN Conf% ObsM+H+ CalcM+H+ SpR ZScore Ion% # Sequence  | | | | | | | | | | | | |
| --- | --- | --- | --- | --- | --- | --- | --- | --- | --- | --- | --- | --- |
| \* | AstrinSTLCLD\_041714\_01.08319.08319.3 | 3.4729 | 0.1554 | 96.2% | 1865.9343 | 1863.1727 | 1 | 3.87 | 43.3% | 1 | K.APIRPDIVNFVHTNLR.K | 3 |
| \* | AstrinSTLCLD\_041714\_01.05629.05629.3 | 4.715 | 0.4181 | 100.0% | 2716.2844 | 2717.874 | 1 | 7.123 | 34.4% | 1 | K.NNRQPYAVSELAGHQTSAESWGTGR.A | 3 |
| \* | AstrinSTLCLD\_041714\_01.06472.06472.2 | 3.0992 | 0.2193 | 100.0% | 1282.3922 | 1281.4539 | 119 | 4.768 | 55.6% | 1 | R.KLDELYGTWR.K | 2 |

---

|  |  |  |  |  |  |  |  |  |
| --- | --- | --- | --- | --- | --- | --- | --- | --- |
| U | *gi|10835063|ref|NP\_00* | 2 | 2 | 11.9% | 294 | 32575 | 4.8 | nucleophosmin 1 isoform 1 [Homo sapiens] |
| U | *gi|40353734|ref|NP\_95* | 2 | 2 | 13.2% | 265 | 29465 | 4.6 | nucleophosmin 1 isoform 2 [Homo sapiens] |

| Filename XCorr DeltCN Conf% ObsM+H+ CalcM+H+ SpR ZScore Ion% # Sequence  | | | | | | | | | | | | |
| --- | --- | --- | --- | --- | --- | --- | --- | --- | --- | --- | --- | --- |
|  | AstrinSTLCLD\_041714\_01.11564.11564.2 | 4.4451 | 0.5322 | 100.0% | 2227.6921 | 2228.655 | 1 | 9.386 | 50.0% | 1 | K.MSVQPTVSLGGFEITPPVVLR.L | 2 |
|  | AstrinSTLCLD\_041714\_01.11547.11547.2 | 3.6227 | 0.3295 | 100.0% | 1820.4321 | 1821.0172 | 1 | 6.25 | 57.7% | 1 | R.MTDQEAIQDLWQWR.K | 2 |

---

|  |  |  |  |  |  |  |  |  |
| --- | --- | --- | --- | --- | --- | --- | --- | --- |
| U | *gi|4506741|ref|NP\_001* | 1 | 1 | 11.9% | 194 | 22127 | 10.1 | ribosomal protein S7 [Homo sapiens] |

| Filename XCorr DeltCN Conf% ObsM+H+ CalcM+H+ SpR ZScore Ion% # Sequence  | | | | | | | | | | | | |
| --- | --- | --- | --- | --- | --- | --- | --- | --- | --- | --- | --- | --- |
| \* | AstrinSTLCLD\_041714\_01.11441.11441.3 | 5.1378 | 0.3342 | 100.0% | 2524.4043 | 2524.92 | 1 | 6.49 | 38.6% | 1 | R.TLTAVHDAILEDLVFPSEIVGKR.I | 3 |

---

|  |  |  |  |  |  |  |  |  |
| --- | --- | --- | --- | --- | --- | --- | --- | --- |
| U | *gi|10800138|ref|NP\_06* | 1 | 5 | 11.9% | 126 | 13936 | 10.3 | histone cluster 1, H2bd [Homo sapiens] |
| U | *gi|66912162|ref|NP\_00* | 1 | 5 | 11.9% | 126 | 13920 | 10.3 | histone cluster 2, H2bf [Homo sapiens] |
| U | *gi|4504277|ref|NP\_003* | 1 | 5 | 11.9% | 126 | 13920 | 10.3 | histone cluster 2, H2be [Homo sapiens] |
| U | *gi|4504271|ref|NP\_003* | 1 | 5 | 11.9% | 126 | 13906 | 10.3 | histone cluster 1, H2bi [Homo sapiens] |
| U | *gi|4504269|ref|NP\_003* | 1 | 5 | 11.9% | 126 | 13892 | 10.3 | histone cluster 1, H2bh [Homo sapiens] |
| U | *gi|4504265|ref|NP\_003* | 1 | 5 | 11.9% | 126 | 13906 | 10.3 | histone cluster 1, H2bf [Homo sapiens] |
| U | *gi|4504263|ref|NP\_003* | 1 | 5 | 11.9% | 126 | 13989 | 10.3 | histone cluster 1, H2bm [Homo sapiens] |
| U | *gi|4504261|ref|NP\_003* | 1 | 5 | 11.9% | 126 | 13922 | 10.3 | histone cluster 1, H2bn [Homo sapiens] |
| U | *gi|4504259|ref|NP\_003* | 1 | 5 | 11.9% | 126 | 13952 | 10.3 | histone cluster 1, H2bl [Homo sapiens] |
| U | *gi|4504257|ref|NP\_003* | 1 | 5 | 11.9% | 126 | 13906 | 10.3 | histone cluster 1, H2bg [Homo sapiens] |
| U | *gi|28173554|ref|NP\_77* | 1 | 5 | 11.9% | 126 | 13908 | 10.3 | histone cluster 3, H2bb [Homo sapiens] |
| U | *gi|21396484|ref|NP\_00* | 1 | 5 | 11.9% | 126 | 13906 | 10.3 | histone cluster 1, H2be [Homo sapiens] |
| U | *gi|21166389|ref|NP\_00* | 1 | 5 | 11.9% | 126 | 13906 | 10.3 | histone cluster 1, H2bc [Homo sapiens] |
| U | *gi|20336754|ref|NP\_06* | 1 | 5 | 11.9% | 126 | 13904 | 10.3 | histone cluster 1, H2bj [Homo sapiens] |
| U | *gi|20336752|ref|NP\_61* | 1 | 5 | 11.9% | 126 | 13936 | 10.3 | histone cluster 1, H2bd [Homo sapiens] |
| U | *gi|18105048|ref|NP\_54* | 1 | 5 | 11.9% | 126 | 13890 | 10.3 | histone cluster 1, H2bk [Homo sapiens] |
| U | *gi|16306566|ref|NP\_00* | 1 | 5 | 11.9% | 126 | 13906 | 10.3 | histone cluster 1, H2bo [Homo sapiens] |
| U | *gi|10800140|ref|NP\_06* | 1 | 5 | 11.9% | 126 | 13950 | 10.3 | histone cluster 1, H2bb [Homo sapiens] |

| Filename XCorr DeltCN Conf% ObsM+H+ CalcM+H+ SpR ZScore Ion% # Sequence  | | | | | | | | | | | | |
| --- | --- | --- | --- | --- | --- | --- | --- | --- | --- | --- | --- | --- |
|  | AstrinSTLCLD\_041714\_01.12596.12596.2 | 5.3752 | 0.4842 | 100.0% | 1744.4122 | 1745.0211 | 1 | 9.593 | 78.6% | 5 | K.AMGIMNSFVNDIFER.I | 2 |

---

|  |  |  |  |  |  |  |  |  |
| --- | --- | --- | --- | --- | --- | --- | --- | --- |
| U | *gi|5454064|ref|NP\_006* | 5 | 12 | 11.8% | 669 | 69492 | 9.7 | RNA binding motif protein 14 [Homo sapiens] |

| Filename XCorr DeltCN Conf% ObsM+H+ CalcM+H+ SpR ZScore Ion% # Sequence  | | | | | | | | | | | | |
| --- | --- | --- | --- | --- | --- | --- | --- | --- | --- | --- | --- | --- |
| \* | AstrinSTLCLD\_041714\_01.06636.06636.3 | 3.3714 | 0.3431 | 100.0% | 1898.5743 | 1898.2767 | 1 | 6.232 | 36.7% | 2 | R.ALVVEMSRPRPLNTWK.I | 3 |
| \* | AstrinSTLCLD\_041714\_01.05445.05445.2 | 3.2698 | 0.5027 | 100.0% | 1608.9722 | 1609.8223 | 1 | 8.262 | 71.4% | 2 | R.ASYVAPLTAQPATYR.A | 2 |
| \* | AstrinSTLCLD\_041714\_02.05522.05522.2 | 2.6501 | 0.4058 | 100.0% | 1220.2722 | 1220.3707 | 2 | 7.539 | 59.1% | 1 | R.AQPSVSLGAAYR.A | 2 |
| \* | AstrinSTLCLD\_041714\_01.03950.03950.2 | 2.6167 | 0.3747 | 100.0% | 1325.3322 | 1325.482 | 16 | 5.752 | 54.5% | 1 | R.TQPMTAQAASYR.A | 2 |
| \* | AstrinSTLCLD\_041714\_02.05487.05487.3 | 4.5486 | 0.4818 | 100.0% | 2467.0745 | 2466.6292 | 1 | 7.386 | 35.9% | 6 | R.TQSSASLAASYAAQQHPQAAASYR.G | 3 |

---

|  |  |  |  |  |  |  |  |  |
| --- | --- | --- | --- | --- | --- | --- | --- | --- |
| U | *gi|208973238|ref|NP\_0* | 1 | 1 | 11.8% | 245 | 27745 | 4.8 | tyrosine 3/tryptophan 5 -monooxygenase activation protein, zeta polypeptide [Homo sapiens] |
| U | *gi|4507953|ref|NP\_003* | 1 | 1 | 11.8% | 245 | 27745 | 4.8 | tyrosine 3/tryptophan 5 -monooxygenase activation protein, zeta polypeptide [Homo sapiens] |
| U | *gi|21735625|ref|NP\_66* | 1 | 1 | 11.8% | 245 | 27745 | 4.8 | tyrosine 3/tryptophan 5 -monooxygenase activation protein, zeta polypeptide [Homo sapiens] |
| U | *gi|208973244|ref|NP\_0* | 1 | 1 | 11.8% | 245 | 27745 | 4.8 | tyrosine 3/tryptophan 5 -monooxygenase activation protein, zeta polypeptide [Homo sapiens] |
| U | *gi|208973242|ref|NP\_0* | 1 | 1 | 11.8% | 245 | 27745 | 4.8 | tyrosine 3/tryptophan 5 -monooxygenase activation protein, zeta polypeptide [Homo sapiens] |
| U | *gi|208973240|ref|NP\_0* | 1 | 1 | 11.8% | 245 | 27745 | 4.8 | tyrosine 3/tryptophan 5 -monooxygenase activation protein, zeta polypeptide [Homo sapiens] |

| Filename XCorr DeltCN Conf% ObsM+H+ CalcM+H+ SpR ZScore Ion% # Sequence  | | | | | | | | | | | | |
| --- | --- | --- | --- | --- | --- | --- | --- | --- | --- | --- | --- | --- |
|  | AstrinSTLCLD\_041714\_01.17127.17127.3 | 4.1847 | 0.4273 | 100.0% | 3303.6543 | 3304.6907 | 1 | 6.381 | 25.9% | 1 | K.TAFDEAIAELDTLSEESYKDSTLIMQLLR.D | 3 |

---

|  |  |  |  |  |  |  |  |  |
| --- | --- | --- | --- | --- | --- | --- | --- | --- |
| U | *gi|5453898|ref|NP\_006* | 1 | 1 | 11.7% | 163 | 18243 | 8.8 | protein (peptidyl-prolyl cis/trans isomerase) NIMA-interacting 1 [Homo sapiens] |

| Filename XCorr DeltCN Conf% ObsM+H+ CalcM+H+ SpR ZScore Ion% # Sequence  | | | | | | | | | | | | |
| --- | --- | --- | --- | --- | --- | --- | --- | --- | --- | --- | --- | --- |
| \* | AstrinSTLCLD\_041714\_01.09929.09929.3 | 3.4944 | 0.3631 | 100.0% | 2031.6244 | 2031.3344 | 1 | 6.577 | 41.7% | 1 | R.TGEMSGPVFTDSGIHIILR.T | 3 |

---

|  |  |  |  |  |  |  |  |  |
| --- | --- | --- | --- | --- | --- | --- | --- | --- |
| U | *gi|214830438|ref|NP\_0* | 2 | 3 | 11.5% | 356 | 38629 | 5.5 | sequestosome 1 isoform 2 [Homo sapiens] |
| U | *gi|4505571|ref|NP\_003* | 2 | 3 | 9.3% | 440 | 47687 | 5.2 | sequestosome 1 isoform 1 [Homo sapiens] |
| U | *gi|214830451|ref|NP\_0* | 2 | 3 | 11.5% | 356 | 38629 | 5.5 | sequestosome 1 isoform 2 [Homo sapiens] |

| Filename XCorr DeltCN Conf% ObsM+H+ CalcM+H+ SpR ZScore Ion% # Sequence  | | | | | | | | | | | | |
| --- | --- | --- | --- | --- | --- | --- | --- | --- | --- | --- | --- | --- |
|  | AstrinSTLCLD\_041714\_01.06122.06122.3 | 5.1438 | 0.3057 | 100.0% | 2572.2844 | 2572.7478 | 1 | 7.661 | 34.0% | 2 | R.AGEARPGPTAESASGPSEDPSVNFLK.N | 3 |
|  | AstrinSTLCLD\_041714\_01.09443.09443.2 | 3.6681 | 0.53 | 100.0% | 1672.0922 | 1672.8322 | 1 | 8.905 | 75.0% | 1 | K.NYDIGAALDTIQYSK.H | 2 |

---

|  |  |  |  |  |  |  |  |  |
| --- | --- | --- | --- | --- | --- | --- | --- | --- |
| U | *gi|5803137|ref|NP\_006* | 1 | 1 | 11.5% | 157 | 17170 | 8.9 | RNA binding motif protein 3 [Homo sapiens] |

| Filename XCorr DeltCN Conf% ObsM+H+ CalcM+H+ SpR ZScore Ion% # Sequence  | | | | | | | | | | | | |
| --- | --- | --- | --- | --- | --- | --- | --- | --- | --- | --- | --- | --- |
| \* | AstrinSTLCLD\_041714\_01.09976.09976.3 | 3.1764 | 0.3094 | 100.0% | 1984.4343 | 1983.2518 | 1 | 5.321 | 44.1% | 1 | R.GFGFITFTNPEHASVAMR.A | 3 |

---

|  |  |  |  |  |  |  |  |  |
| --- | --- | --- | --- | --- | --- | --- | --- | --- |
| U | *gi|4757714|ref|NP\_004* | 1 | 1 | 11.4% | 158 | 18042 | 6.8 | acid phosphatase 1, soluble isoform c [Homo sapiens] |
| U | *gi|96304457|ref|NP\_00* | 1 | 1 | 16.1% | 112 | 12230 | 7.7 | acid phosphatase 1, soluble isoform d [Homo sapiens] |

| Filename XCorr DeltCN Conf% ObsM+H+ CalcM+H+ SpR ZScore Ion% # Sequence  | | | | | | | | | | | | |
| --- | --- | --- | --- | --- | --- | --- | --- | --- | --- | --- | --- | --- |
|  | AstrinSTLCLD\_041714\_01.05193.05193.2 | 2.6668 | 0.4047 | 100.0% | 1912.3522 | 1913.0074 | 1 | 7.604 | 47.1% | 1 | R.VDSAATSGYEIGNPPDYR.G | 2 |

---

|  |  |  |  |  |  |  |  |  |
| --- | --- | --- | --- | --- | --- | --- | --- | --- |
| U | *gi|4826898|ref|NP\_005* | 1 | 1 | 11.4% | 140 | 15054 | 8.3 | profilin 1 [Homo sapiens] |

| Filename XCorr DeltCN Conf% ObsM+H+ CalcM+H+ SpR ZScore Ion% # Sequence  | | | | | | | | | | | | |
| --- | --- | --- | --- | --- | --- | --- | --- | --- | --- | --- | --- | --- |
| \* | AstrinSTLCLD\_041714\_01.10710.10710.2 | 3.1449 | 0.2953 | 100.0% | 1644.6921 | 1644.9518 | 2 | 6.029 | 46.7% | 1 | K.TFVNITPAEVGVLVGK.D | 2 |

---

|  |  |  |  |  |  |  |  |  |
| --- | --- | --- | --- | --- | --- | --- | --- | --- |
| U | *gi|14249348|ref|NP\_11* | 2 | 5 | 11.4% | 123 | 13941 | 5.5 | thioredoxin-like 5 [Homo sapiens] |

| Filename XCorr DeltCN Conf% ObsM+H+ CalcM+H+ SpR ZScore Ion% # Sequence  | | | | | | | | | | | | |
| --- | --- | --- | --- | --- | --- | --- | --- | --- | --- | --- | --- | --- |
| \* | AstrinSTLCLD\_041714\_02.06941.06941.2 | 2.6131 | 0.3872 | 100.0% | 1715.3121 | 1715.8162 | 16 | 6.294 | 38.5% | 1 | R.YEEVSVSGFEEFHR.A | 2 |
| \* | AstrinSTLCLD\_041714\_01.07551.07551.3 | 3.1464 | 0.35 | 100.0% | 1717.1044 | 1715.8162 | 16 | 5.444 | 36.5% | 4 | R.YEEVSVSGFEEFHR.A | 3 |

---

|  |  |  |  |  |  |  |  |  |
| --- | --- | --- | --- | --- | --- | --- | --- | --- |
| U | *gi|169212778|ref|XP\_0* | 2 | 4 | 10.9% | 266 | 30042 | 10.6 | PREDICTED: similar to ribosomal protein L7a [Homo sapiens] |
| U | *gi|4506661|ref|NP\_000* | 2 | 4 | 10.9% | 266 | 29996 | 10.6 | ribosomal protein L7a [Homo sapiens] |
| U | *gi|169213130|ref|XP\_0* | 2 | 4 | 10.9% | 266 | 30042 | 10.6 | PREDICTED: similar to ribosomal protein L7a [Homo sapiens] |
| U | *gi|169212940|ref|XP\_0* | 2 | 4 | 10.9% | 266 | 30028 | 10.6 | PREDICTED: similar to ribosomal protein L7a [Homo sapiens] |

| Filename XCorr DeltCN Conf% ObsM+H+ CalcM+H+ SpR ZScore Ion% # Sequence  | | | | | | | | | | | | |
| --- | --- | --- | --- | --- | --- | --- | --- | --- | --- | --- | --- | --- |
|  | AstrinSTLCLD\_041714\_01.10019.10019.3 | 3.8106 | 0.2074 | 99.3% | 1811.9343 | 1812.1222 | 2 | 6.361 | 48.3% | 1 | R.LKVPPAINQFTQALDR.Q | 3 |
|  | AstrinSTLCLD\_041714\_01.05784.05784.2 | 3.4015 | 0.4093 | 100.0% | 1346.4521 | 1346.5236 | 5 | 7.276 | 62.5% | 3 | R.AGVNTVTTLVENK.K | 2 |

---

|  |  |  |  |  |  |  |  |  |
| --- | --- | --- | --- | --- | --- | --- | --- | --- |
| U | *gi|4502491|ref|NP\_001* | 1 | 1 | 10.6% | 282 | 31362 | 4.8 | complement component 1, q subcomponent binding protein precursor [Homo sapiens] |

| Filename XCorr DeltCN Conf% ObsM+H+ CalcM+H+ SpR ZScore Ion% # Sequence  | | | | | | | | | | | | |
| --- | --- | --- | --- | --- | --- | --- | --- | --- | --- | --- | --- | --- |
| \* | AstrinSTLCLD\_041714\_01.15996.15996.3 | 4.5044 | 0.3899 | 100.0% | 3440.8442 | 3441.77 | 87 | 6.352 | 20.7% | 1 | R.GVDNTFADELVELSTALEHQEYITFLEDLK.S | 3 |

---

|  |  |  |  |  |  |  |  |  |
| --- | --- | --- | --- | --- | --- | --- | --- | --- |
| U | *Reverse\_gi|17986258|r* | 1 | 12 | 10.6% | 151 | 16930 | 4.7 | myosin, light chain 6, alkali, smooth muscle and non-muscle isoform 1 [Homo sapiens] |
| U | *Reverse\_gi|88999583|r* | 1 | 12 | 10.6% | 151 | 16961 | 4.6 | myosin, light chain 6, alkali, smooth muscle and non-muscle isoform 2 [Homo sapiens] |

| Filename XCorr DeltCN Conf% ObsM+H+ CalcM+H+ SpR ZScore Ion% # Sequence  | | | | | | | | | | | | |
| --- | --- | --- | --- | --- | --- | --- | --- | --- | --- | --- | --- | --- |
|  | AstrinSTLCLD\_041714\_02.07726.07726.2 | 4.3021 | 0.1185 | 99.6% | 1801.2322 | 1800.9905 | 152 | 5.024 | 40.0% | 12 | R.MVDGCQSYLIKGDGTR.D | 2 |

---

|  |  |  |  |  |  |  |  |  |
| --- | --- | --- | --- | --- | --- | --- | --- | --- |
| U | *gi|25777713|ref|NP\_73* | 2 | 2 | 10.4% | 163 | 18658 | 4.5 | S-phase kinase-associated protein 1 isoform b [Homo sapiens] |

| Filename XCorr DeltCN Conf% ObsM+H+ CalcM+H+ SpR ZScore Ion% # Sequence  | | | | | | | | | | | | |
| --- | --- | --- | --- | --- | --- | --- | --- | --- | --- | --- | --- | --- |
| \* | AstrinSTLCLD\_041714\_02.06844.06844.3 | 3.3204 | 0.3114 | 100.0% | 2070.6543 | 2071.2078 | 1 | 5.841 | 42.2% | 1 | K.TFNIKNDFTEEEEAQVR.K | 3 |
| \* | AstrinSTLCLD\_041714\_02.05194.05194.2 | 3.8816 | 0.386 | 100.0% | 1467.4521 | 1467.4888 | 1 | 6.759 | 72.7% | 1 | K.NDFTEEEEAQVR.K | 2 |

---

|  |  |  |  |  |  |  |  |  |
| --- | --- | --- | --- | --- | --- | --- | --- | --- |
| U | *contaminant\_KERATIN03* | 4 | 6 | 10.3% | 593 | 59519 | 5.2 | no description |
| U | *gi|195972866|ref|NP\_0* | 4 | 6 | 10.4% | 584 | 58801 | 5.2 | keratin 10 [Homo sapiens] |

| Filename XCorr DeltCN Conf% ObsM+H+ CalcM+H+ SpR ZScore Ion% # Sequence  | | | | | | | | | | | | |
| --- | --- | --- | --- | --- | --- | --- | --- | --- | --- | --- | --- | --- |
|  | AstrinSTLCLD\_041714\_01.04792.04792.2 | 2.8182 | 0.3766 | 100.0% | 1382.1721 | 1382.4668 | 24 | 6.423 | 54.5% | 1 | R.ALEESNYELEGK.I | 2 |
|  | AstrinSTLCLD\_041714\_01.12957.12957.3 | 5.7925 | 0.4874 | 100.0% | 3053.6643 | 3054.4277 | 1 | 8.062 | 31.7% | 2 | K.TIDDLKNQILNLTTDNANILLQIDNAR.L | 3 |
|  | AstrinSTLCLD\_041714\_01.05993.05993.2 | 3.1 | 0.4514 | 100.0% | 1032.1322 | 1032.2224 | 2 | 7.474 | 75.0% | 2 | R.VLDELTLTK.A | 2 |
|  | AstrinSTLCLD\_041714\_01.05408.05408.2 | 3.7803 | 0.441 | 100.0% | 1391.2122 | 1391.4778 | 1 | 7.681 | 70.8% | 1 | K.QSLEASLAETEGR.Y | 2 |

---

|  |  |  |  |  |  |  |  |  |
| --- | --- | --- | --- | --- | --- | --- | --- | --- |
| U | *gi|209862831|ref|NP\_0* | 2 | 2 | 10.3% | 339 | 38604 | 7.8 | annexin A2 isoform 2 [Homo sapiens] |
| U | *gi|50845388|ref|NP\_00* | 2 | 2 | 9.8% | 357 | 40411 | 8.4 | annexin A2 isoform 1 [Homo sapiens] |
| U | *gi|50845386|ref|NP\_00* | 2 | 2 | 10.3% | 339 | 38604 | 7.8 | annexin A2 isoform 2 [Homo sapiens] |
| U | *gi|4757756|ref|NP\_004* | 2 | 2 | 10.3% | 339 | 38604 | 7.8 | annexin A2 isoform 2 [Homo sapiens] |

| Filename XCorr DeltCN Conf% ObsM+H+ CalcM+H+ SpR ZScore Ion% # Sequence  | | | | | | | | | | | | |
| --- | --- | --- | --- | --- | --- | --- | --- | --- | --- | --- | --- | --- |
|  | AstrinSTLCLD\_041714\_01.07437.07437.3 | 3.7253 | 0.3972 | 100.0% | 1941.1743 | 1941.102 | 43 | 6.403 | 35.9% | 1 | K.TDLEKDIISDTSGDFRK.L | 3 |
|  | AstrinSTLCLD\_041714\_01.07685.07685.3 | 3.5869 | 0.2618 | 100.0% | 2066.3943 | 2066.1887 | 1 | 5.496 | 47.1% | 1 | R.RAEDGSVIDYELIDQDAR.D | 3 |

---

|  |  |  |  |  |  |  |  |  |
| --- | --- | --- | --- | --- | --- | --- | --- | --- |
| U | *gi|4758158|ref|NP\_004* | 2 | 2 | 10.2% | 361 | 41487 | 6.6 | septin 2 [Homo sapiens] |
| U | *gi|56549640|ref|NP\_00* | 2 | 2 | 10.2% | 361 | 41487 | 6.6 | septin 2 [Homo sapiens] |
| U | *gi|56549638|ref|NP\_00* | 2 | 2 | 10.2% | 361 | 41487 | 6.6 | septin 2 [Homo sapiens] |
| U | *gi|56549636|ref|NP\_00* | 2 | 2 | 10.2% | 361 | 41487 | 6.6 | septin 2 [Homo sapiens] |

| Filename XCorr DeltCN Conf% ObsM+H+ CalcM+H+ SpR ZScore Ion% # Sequence  | | | | | | | | | | | | |
| --- | --- | --- | --- | --- | --- | --- | --- | --- | --- | --- | --- | --- |
|  | AstrinSTLCLD\_041714\_02.06142.06142.2 | 3.7051 | 0.4184 | 100.0% | 1604.2122 | 1604.7545 | 1 | 7.613 | 73.1% | 1 | R.TVQIEASTVEIEER.G | 2 |
|  | AstrinSTLCLD\_041714\_02.05865.05865.3 | 4.356 | 0.2967 | 100.0% | 2384.4844 | 2385.6675 | 1 | 6.931 | 38.6% | 1 | R.MQAQMQMQMQGGDGDGGALGHHV.- | 3 |

---

|  |  |  |  |  |  |  |  |  |
| --- | --- | --- | --- | --- | --- | --- | --- | --- |
| U | *gi|4503571|ref|NP\_001* | 3 | 3 | 10.1% | 434 | 47169 | 7.4 | enolase 1 [Homo sapiens] |

| Filename XCorr DeltCN Conf% ObsM+H+ CalcM+H+ SpR ZScore Ion% # Sequence  | | | | | | | | | | | | |
| --- | --- | --- | --- | --- | --- | --- | --- | --- | --- | --- | --- | --- |
|  | AstrinSTLCLD\_041714\_01.09978.09978.2 | 3.6044 | 0.3005 | 100.0% | 1806.2522 | 1806.0258 | 1 | 6.751 | 52.9% | 1 | R.AAVPSGASTGIYEALELR.D | 2 |
| \* | AstrinSTLCLD\_041714\_01.09333.09333.2 | 2.9602 | 0.4671 | 100.0% | 1426.2722 | 1426.6091 | 1 | 6.551 | 81.8% | 1 | R.YISPDQLADLYK.S | 2 |
| \* | AstrinSTLCLD\_041714\_02.06387.06387.3 | 2.9064 | 0.2146 | 97.5% | 1526.7244 | 1526.7563 | 1 | 4.548 | 51.9% | 1 | K.LAQANGWGVMVSHR.S | 3 |

---

|  |  |  |  |  |  |  |  |  |
| --- | --- | --- | --- | --- | --- | --- | --- | --- |
| U | *gi|221307584|ref|NP\_0* | 2 | 2 | 10.0% | 299 | 33296 | 9.8 | prohibitin 2 isoform 1 [Homo sapiens] |
| U | *gi|6005854|ref|NP\_009* | 2 | 2 | 10.0% | 299 | 33296 | 9.8 | prohibitin 2 isoform 2 [Homo sapiens] |

| Filename XCorr DeltCN Conf% ObsM+H+ CalcM+H+ SpR ZScore Ion% # Sequence  | | | | | | | | | | | | |
| --- | --- | --- | --- | --- | --- | --- | --- | --- | --- | --- | --- | --- |
|  | AstrinSTLCLD\_041714\_02.08111.08111.3 | 3.3644 | 0.4199 | 100.0% | 1855.4644 | 1855.1038 | 1 | 6.394 | 42.2% | 1 | R.IGGVQQDTILAEGLHFR.I | 3 |
|  | AstrinSTLCLD\_041714\_01.12801.12801.2 | 3.3912 | 0.4642 | 100.0% | 1725.1322 | 1725.0428 | 1 | 7.727 | 70.8% | 1 | R.IPWFQYPIIYDIR.A | 2 |

---

|  |  |  |  |  |  |  |  |  |
| --- | --- | --- | --- | --- | --- | --- | --- | --- |
| U | *gi|193794814|ref|NP\_0* | 2 | 2 | 9.9% | 364 | 39420 | 8.1 | fructose-bisphosphate aldolase A [Homo sapiens] |
| U | *gi|4557305|ref|NP\_000* | 2 | 2 | 9.9% | 364 | 39420 | 8.1 | fructose-bisphosphate aldolase A [Homo sapiens] |
| U | *gi|34577112|ref|NP\_90* | 2 | 2 | 9.9% | 364 | 39420 | 8.1 | fructose-bisphosphate aldolase A [Homo sapiens] |
| U | *gi|34577110|ref|NP\_90* | 2 | 2 | 9.9% | 364 | 39420 | 8.1 | fructose-bisphosphate aldolase A [Homo sapiens] |

| Filename XCorr DeltCN Conf% ObsM+H+ CalcM+H+ SpR ZScore Ion% # Sequence  | | | | | | | | | | | | |
| --- | --- | --- | --- | --- | --- | --- | --- | --- | --- | --- | --- | --- |
|  | AstrinSTLCLD\_041714\_02.05504.05504.2 | 3.2543 | 0.4416 | 100.0% | 1333.1122 | 1333.4814 | 1 | 7.754 | 73.1% | 1 | K.GILAADESTGSIAK.R | 2 |
|  | AstrinSTLCLD\_041714\_01.08050.08050.2 | 3.0931 | 0.3164 | 100.0% | 2229.3323 | 2229.3672 | 4 | 6.383 | 33.3% | 1 | K.YTPSGQAGAAASESLFVSNHAY.- | 23 |

---

|  |  |  |  |  |  |  |  |  |
| --- | --- | --- | --- | --- | --- | --- | --- | --- |
| U | *gi|15431303|ref|NP\_00* | 1 | 1 | 9.9% | 192 | 21863 | 10.0 | ribosomal protein L9 [Homo sapiens] |
| U | *gi|67944630|ref|NP\_00* | 1 | 1 | 9.9% | 192 | 21863 | 10.0 | ribosomal protein L9 [Homo sapiens] |

| Filename XCorr DeltCN Conf% ObsM+H+ CalcM+H+ SpR ZScore Ion% # Sequence  | | | | | | | | | | | | |
| --- | --- | --- | --- | --- | --- | --- | --- | --- | --- | --- | --- | --- |
|  | AstrinSTLCLD\_041714\_01.09695.09695.2 | 4.6227 | 0.49 | 100.0% | 2114.3123 | 2114.401 | 1 | 7.906 | 47.2% | 1 | K.TILSNQTVDIPENVDITLK.G | 2 |

---

|  |  |  |  |  |  |  |  |  |
| --- | --- | --- | --- | --- | --- | --- | --- | --- |
| U | *gi|21626466|ref|NP\_06* | 4 | 5 | 9.8% | 847 | 94623 | 6.3 | matrin 3 [Homo sapiens] |
| U | *gi|62750354|ref|NP\_95* | 4 | 5 | 9.8% | 847 | 94623 | 6.3 | matrin 3 [Homo sapiens] |

| Filename XCorr DeltCN Conf% ObsM+H+ CalcM+H+ SpR ZScore Ion% # Sequence  | | | | | | | | | | | | |
| --- | --- | --- | --- | --- | --- | --- | --- | --- | --- | --- | --- | --- |
|  | AstrinSTLCLD\_041714\_02.12037.12037.3 | 3.8223 | 0.275 | 100.0% | 2373.8342 | 2372.7424 | 3 | 5.735 | 28.1% | 1 | R.DLSAAGIGLLAAATQSLSMPASLGR.M | 3 |
|  | AstrinSTLCLD\_041714\_01.12962.12962.3 | 4.1897 | 0.3115 | 100.0% | 2439.8943 | 2439.9036 | 3 | 6.267 | 31.2% | 1 | R.YQLLQLVEPFGVISNHLILNK.I | 3 |
|  | AstrinSTLCLD\_041714\_01.06083.06083.3 | 3.4532 | 0.4658 | 100.0% | 2039.1543 | 2038.3109 | 1 | 7.017 | 34.7% | 2 | R.VIHLSNLPHSGYSDSAVLK.L | 3 |
|  | AstrinSTLCLD\_041714\_01.10193.10193.2 | 3.6701 | 0.4277 | 100.0% | 1969.4722 | 1970.319 | 1 | 6.635 | 55.9% | 1 | R.IGPYQPNVPVGIDYVIPK.T | 2 |

---

|  |  |  |  |  |  |  |  |  |
| --- | --- | --- | --- | --- | --- | --- | --- | --- |
| U | *gi|50409691|ref|NP\_00* | 1 | 1 | 9.5% | 338 | 36211 | 11.2 | SRp25 nuclear protein isoform 4 [Homo sapiens] |
| U | *gi|50409756|ref|NP\_06* | 1 | 1 | 8.9% | 360 | 38395 | 11.1 | SRp25 nuclear protein isoform 1 [Homo sapiens] |
| U | *gi|50409738|ref|NP\_00* | 1 | 1 | 9.1% | 352 | 37638 | 11.2 | SRp25 nuclear protein isoform 3 [Homo sapiens] |
| U | *gi|50409707|ref|NP\_05* | 1 | 1 | 9.4% | 341 | 36612 | 11.2 | SRp25 nuclear protein isoform 2 [Homo sapiens] |

| Filename XCorr DeltCN Conf% ObsM+H+ CalcM+H+ SpR ZScore Ion% # Sequence  | | | | | | | | | | | | |
| --- | --- | --- | --- | --- | --- | --- | --- | --- | --- | --- | --- | --- |
|  | AstrinSTLCLD\_041714\_01.18653.18653.3 | 2.7567 | 0.3687 | 100.0% | 3357.1743 | 3357.947 | 7 | 2.743 | 24.2% | 1 | R.T#RSSSSSS\*SSSSSSSS\*SSSSSSS\*SSSSDGRKK.R | 3 |

---

|  |  |  |  |  |  |  |  |  |
| --- | --- | --- | --- | --- | --- | --- | --- | --- |
| U | *gi|222352151|ref|NP\_0* | 3 | 5 | 9.3% | 356 | 37498 | 7.1 | poly(rC) binding protein 1 [Homo sapiens] |

| Filename XCorr DeltCN Conf% ObsM+H+ CalcM+H+ SpR ZScore Ion% # Sequence  | | | | | | | | | | | | |
| --- | --- | --- | --- | --- | --- | --- | --- | --- | --- | --- | --- | --- |
| \* | AstrinSTLCLD\_041714\_01.09696.09696.2 | 2.6841 | 0.2081 | 97.1% | 1390.3522 | 1389.6781 | 1 | 6.3 | 66.7% | 1 | R.IITLTGPTNAIFK.A | 2 |
|  | AstrinSTLCLD\_041714\_02.06086.06086.3 | 3.8238 | 0.3541 | 100.0% | 2090.5444 | 2091.2573 | 1 | 5.812 | 36.8% | 1 | R.ESTGAQVQVAGDMLPNSTER.A | 3 |
|  | AstrinSTLCLD\_041714\_02.06087.06087.2 | 5.2491 | 0.5364 | 100.0% | 2090.5723 | 2091.2573 | 1 | 9.725 | 55.3% | 3 | R.ESTGAQVQVAGDMLPNSTER.A | 2 |

---

|  |  |  |  |  |  |  |  |  |
| --- | --- | --- | --- | --- | --- | --- | --- | --- |
| U | *gi|52632383|ref|NP\_00* | 4 | 7 | 9.2% | 589 | 64133 | 8.2 | heterogeneous nuclear ribonucleoprotein L isoform a [Homo sapiens] |

| Filename XCorr DeltCN Conf% ObsM+H+ CalcM+H+ SpR ZScore Ion% # Sequence  | | | | | | | | | | | | |
| --- | --- | --- | --- | --- | --- | --- | --- | --- | --- | --- | --- | --- |
| \* | AstrinSTLCLD\_041714\_01.17175.17175.3 | 5.7375 | 0.5015 | 100.0% | 3089.4243 | 3089.6143 | 1 | 9.072 | 31.2% | 2 | R.GLIDGVVEADLVEALQEFGPISYVVVMPK.K | 3 |
|  | AstrinSTLCLD\_041714\_01.06908.06908.2 | 3.7668 | 0.3992 | 100.0% | 1635.8522 | 1635.881 | 1 | 6.722 | 65.4% | 1 | R.AITHLNNNFMFGQK.L | 2 |
|  | AstrinSTLCLD\_041714\_02.06872.06872.3 | 3.3354 | 0.1753 | 97.6% | 1637.5144 | 1635.881 | 11 | 4.034 | 38.5% | 2 | R.AITHLNNNFMFGQK.L | 3 |
|  | AstrinSTLCLD\_041714\_01.07239.07239.2 | 2.7012 | 0.3515 | 100.0% | 1223.3322 | 1223.3251 | 3 | 6.042 | 60.0% | 2 | R.SSSGLLEWESK.S | 2 |

---

|  |  |  |  |  |  |  |  |  |
| --- | --- | --- | --- | --- | --- | --- | --- | --- |
| U | *gi|20270311|ref|NP\_62* | 1 | 1 | 9.1% | 418 | 46174 | 7.4 | solute carrier family 25, member 46 [Homo sapiens] |

| Filename XCorr DeltCN Conf% ObsM+H+ CalcM+H+ SpR ZScore Ion% # Sequence  | | | | | | | | | | | | |
| --- | --- | --- | --- | --- | --- | --- | --- | --- | --- | --- | --- | --- |
| \* | AstrinSTLCLD\_041714\_02.12169.12169.3 | 2.9406 | 0.2415 | 96.2% | 4431.2944 | 4430.1187 | 175 | 4.008 | 15.5% | 1 | K.WSPKQIGEHLLLKS\*LTYVVAMPFYSASLIETVQSEIIR.D | 3 |

---

|  |  |  |  |  |  |  |  |  |
| --- | --- | --- | --- | --- | --- | --- | --- | --- |
| U | *gi|15718687|ref|NP\_00* | 2 | 5 | 9.1% | 243 | 26688 | 9.7 | ribosomal protein S3 [Homo sapiens] |

| Filename XCorr DeltCN Conf% ObsM+H+ CalcM+H+ SpR ZScore Ion% # Sequence  | | | | | | | | | | | | |
| --- | --- | --- | --- | --- | --- | --- | --- | --- | --- | --- | --- | --- |
| \* | AstrinSTLCLD\_041714\_01.07403.07403.2 | 2.0789 | 0.274 | 96.4% | 1093.2722 | 1093.2249 | 1 | 5.936 | 81.2% | 1 | K.AELNEFLTR.E | 2 |
| \* | AstrinSTLCLD\_041714\_02.05512.05512.2 | 3.9023 | 0.4016 | 100.0% | 1424.2722 | 1424.5071 | 1 | 8.343 | 83.3% | 4 | R.ELAEDGYSGVEVR.V | 2 |

---

|  |  |  |  |  |  |  |  |  |
| --- | --- | --- | --- | --- | --- | --- | --- | --- |
| U | *gi|113420883|ref|XP\_0* | 1 | 1 | 9.1% | 121 | 13997 | 6.0 | PREDICTED: similar to Spindle and kinetochore-associated protein 2 (Protein FAM33A) [Homo sapiens] |
| U | *gi|32699054|ref|NP\_87* | 1 | 1 | 9.1% | 121 | 14188 | 7.3 | spindle and KT associated 2 isoform 1 [Homo sapiens] |
| U | *gi|169178338|ref|XP\_0* | 1 | 1 | 9.1% | 121 | 13997 | 6.0 | PREDICTED: similar to Spindle and kinetochore-associated protein 2 (Protein FAM33A) [Homo sapiens] |
| U | *gi|113421249|ref|XP\_0* | 1 | 1 | 9.1% | 121 | 13997 | 6.0 | PREDICTED: similar to Spindle and kinetochore-associated protein 2 (Protein FAM33A) [Homo sapiens] |

| Filename XCorr DeltCN Conf% ObsM+H+ CalcM+H+ SpR ZScore Ion% # Sequence  | | | | | | | | | | | | |
| --- | --- | --- | --- | --- | --- | --- | --- | --- | --- | --- | --- | --- |
|  | AstrinSTLCLD\_041714\_01.06448.06448.2 | 2.6017 | 0.1958 | 97.1% | 1374.8722 | 1373.4619 | 3 | 5.039 | 65.0% | 1 | K.AESDLDYIQYR.L | 2 |

---

|  |  |  |  |  |  |  |  |  |
| --- | --- | --- | --- | --- | --- | --- | --- | --- |
| U | *gi|4507357|ref|NP\_003* | 1 | 1 | 9.0% | 199 | 22391 | 8.2 | transgelin 2 [Homo sapiens] |

| Filename XCorr DeltCN Conf% ObsM+H+ CalcM+H+ SpR ZScore Ion% # Sequence  | | | | | | | | | | | | |
| --- | --- | --- | --- | --- | --- | --- | --- | --- | --- | --- | --- | --- |
| \* | AstrinSTLCLD\_041714\_01.12489.12489.2 | 3.72 | 0.4983 | 100.0% | 2100.4922 | 2101.3203 | 1 | 7.908 | 52.9% | 1 | R.YGINTTDIFQTVDLWEGK.N | 2 |

---

|  |  |  |  |  |  |  |  |  |
| --- | --- | --- | --- | --- | --- | --- | --- | --- |
| U | *gi|8400715|ref|NP\_058* | 1 | 1 | 8.8% | 352 | 36760 | 9.4 | microtubule-associated protein tau isoform 4 [Homo sapiens] |

| Filename XCorr DeltCN Conf% ObsM+H+ CalcM+H+ SpR ZScore Ion% # Sequence  | | | | | | | | | | | | |
| --- | --- | --- | --- | --- | --- | --- | --- | --- | --- | --- | --- | --- |
| \* | AstrinSTLCLD\_041714\_01.17707.17707.3 | 3.5215 | 0.2193 | 97.7% | 3572.0044 | 3573.8337 | 120 | 3.752 | 19.2% | 1 | K.IGS\*T#ENLKHQPGGGKVQIVYK@PVDLSK@VTS\*K.C | 3 |

---

|  |  |  |  |  |  |  |  |  |
| --- | --- | --- | --- | --- | --- | --- | --- | --- |
| U | *gi|4506625|ref|NP\_000* | 1 | 2 | 8.8% | 148 | 16561 | 11.0 | ribosomal protein L27a [Homo sapiens] |

| Filename XCorr DeltCN Conf% ObsM+H+ CalcM+H+ SpR ZScore Ion% # Sequence  | | | | | | | | | | | | |
| --- | --- | --- | --- | --- | --- | --- | --- | --- | --- | --- | --- | --- |
| \* | AstrinSTLCLD\_041714\_01.05672.05672.3 | 2.7586 | 0.3151 | 100.0% | 1586.3644 | 1586.7899 | 73 | 5.967 | 35.4% | 2 | R.INFDKYHPGYFGK.V | 3 |

---

|  |  |  |  |  |  |  |  |  |
| --- | --- | --- | --- | --- | --- | --- | --- | --- |
| U | *gi|4506609|ref|NP\_000* | 1 | 1 | 8.7% | 196 | 23466 | 11.5 | ribosomal protein L19 [Homo sapiens] |

| Filename XCorr DeltCN Conf% ObsM+H+ CalcM+H+ SpR ZScore Ion% # Sequence  | | | | | | | | | | | | |
| --- | --- | --- | --- | --- | --- | --- | --- | --- | --- | --- | --- | --- |
| \* | AstrinSTLCLD\_041714\_01.07422.07422.2 | 5.1602 | 0.5578 | 100.0% | 1943.6122 | 1944.0679 | 1 | 10.467 | 68.8% | 1 | K.VWLDPNETNEIANANSR.Q | 2 |

---

|  |  |  |  |  |  |  |  |  |
| --- | --- | --- | --- | --- | --- | --- | --- | --- |
| U | *gi|48762932|ref|NP\_00* | 2 | 2 | 8.6% | 548 | 59621 | 5.6 | chaperonin containing TCP1, subunit 8 (theta) [Homo sapiens] |

| Filename XCorr DeltCN Conf% ObsM+H+ CalcM+H+ SpR ZScore Ion% # Sequence  | | | | | | | | | | | | |
| --- | --- | --- | --- | --- | --- | --- | --- | --- | --- | --- | --- | --- |
| \* | AstrinSTLCLD\_041714\_01.14307.14307.3 | 3.3217 | 0.2361 | 97.7% | 3572.9043 | 3572.7717 | 331 | 4.062 | 17.7% | 1 | K.ILGS\*GIS\*S\*SSVLHGMVFKK@ETEGDVTSVK@DAK.I | 3 |
| \* | AstrinSTLCLD\_041714\_02.05021.05021.2 | 3.3373 | 0.5666 | 100.0% | 1373.3121 | 1373.5492 | 5 | 9.601 | 50.0% | 1 | K.AIADTGANVVVTGGK.V | 2 |

---

|  |  |  |  |  |  |  |  |  |
| --- | --- | --- | --- | --- | --- | --- | --- | --- |
| U | *gi|37187860|ref|NP\_00* | 1 | 1 | 8.6% | 374 | 42494 | 9.0 | chemokine (C-C motif) receptor 6 [Homo sapiens] |
| U | *gi|37188165|ref|NP\_11* | 1 | 1 | 8.6% | 374 | 42494 | 9.0 | chemokine (C-C motif) receptor 6 [Homo sapiens] |

| Filename XCorr DeltCN Conf% ObsM+H+ CalcM+H+ SpR ZScore Ion% # Sequence  | | | | | | | | | | | | |
| --- | --- | --- | --- | --- | --- | --- | --- | --- | --- | --- | --- | --- |
|  | AstrinSTLCLD\_041714\_02.11080.11080.3 | 3.4304 | 0.19 | 95.2% | 3637.1343 | 3639.5496 | 25 | 3.716 | 18.5% | 1 | K.SSGFSCAGRYSENIS\*RQTSET#ADNDNASSFTM.- | 3 |

---

|  |  |  |  |  |  |  |  |  |
| --- | --- | --- | --- | --- | --- | --- | --- | --- |
| U | *gi|14141157|ref|NP\_03* | 2 | 4 | 8.4% | 346 | 36926 | 6.9 | heterogeneous nuclear ribonucleoprotein H3 isoform a [Homo sapiens] |
| U | *gi|14141159|ref|NP\_06* | 2 | 4 | 8.8% | 331 | 35239 | 6.9 | heterogeneous nuclear ribonucleoprotein H3 isoform b [Homo sapiens] |

| Filename XCorr DeltCN Conf% ObsM+H+ CalcM+H+ SpR ZScore Ion% # Sequence  | | | | | | | | | | | | |
| --- | --- | --- | --- | --- | --- | --- | --- | --- | --- | --- | --- | --- |
|  | AstrinSTLCLD\_041714\_02.06683.06683.2 | 3.1872 | 0.4051 | 100.0% | 1272.5721 | 1272.4001 | 1 | 7.479 | 77.3% | 3 | R.STGEAFVQFASK.E | 2 |
|  | AstrinSTLCLD\_041714\_01.11670.11670.2 | 2.9743 | 0.4126 | 100.0% | 1920.3922 | 1920.132 | 8 | 6.695 | 34.4% | 1 | R.ATENDIANFFSPLNPIR.V | 2 |

---

|  |  |  |  |  |  |  |  |  |
| --- | --- | --- | --- | --- | --- | --- | --- | --- |
| U | *Reverse\_gi|226437599|* | 1 | 1 | 8.4% | 285 | 30297 | 8.1 | V-set and transmembrane domain containing 2B [Homo sapiens] |

| Filename XCorr DeltCN Conf% ObsM+H+ CalcM+H+ SpR ZScore Ion% # Sequence  | | | | | | | | | | | | |
| --- | --- | --- | --- | --- | --- | --- | --- | --- | --- | --- | --- | --- |
| \* | AstrinSTLCLD\_041714\_02.09384.09384.3 | 3.1318 | 0.2232 | 96.1% | 2893.3743 | 2895.974 | 21 | 3.859 | 23.9% | 1 | R.HS\*IDNGQVRVT#S\*IKTADKNTVKSR.A | 3 |

---

|  |  |  |  |  |  |  |  |  |
| --- | --- | --- | --- | --- | --- | --- | --- | --- |
| U | *gi|40255109|ref|NP\_68* | 1 | 1 | 8.4% | 225 | 25431 | 7.7 | GrpE-like 2, mitochondrial [Homo sapiens] |

| Filename XCorr DeltCN Conf% ObsM+H+ CalcM+H+ SpR ZScore Ion% # Sequence  | | | | | | | | | | | | |
| --- | --- | --- | --- | --- | --- | --- | --- | --- | --- | --- | --- | --- |
| \* | AstrinSTLCLD\_041714\_01.16432.16432.2 | 4.0932 | 0.0259 | 96.9% | 2195.9521 | 2196.6418 | 227 | 4.349 | 36.1% | 1 | R.ALRVKAVKLEKEVQDLTVR.Y | 2 |

---

|  |  |  |  |  |  |  |  |  |
| --- | --- | --- | --- | --- | --- | --- | --- | --- |
| U | *gi|16306492|ref|NP\_20* | 1 | 1 | 8.3% | 240 | 27503 | 7.1 | cell division cycle 2 isoform 2 [Homo sapiens] |
| U | *gi|4502709|ref|NP\_001* | 1 | 1 | 6.7% | 297 | 34095 | 8.4 | cell division cycle 2 isoform 1 [Homo sapiens] |
| U | *gi|195927041|ref|NP\_0* | 1 | 1 | 6.7% | 297 | 34081 | 8.4 | cell division cycle 2 isoform 3 [Homo sapiens] |

| Filename XCorr DeltCN Conf% ObsM+H+ CalcM+H+ SpR ZScore Ion% # Sequence  | | | | | | | | | | | | |
| --- | --- | --- | --- | --- | --- | --- | --- | --- | --- | --- | --- | --- |
|  | AstrinSTLCLD\_041714\_01.15970.15970.2 | 2.8964 | 0.3444 | 100.0% | 2213.9521 | 2213.5352 | 1 | 5.75 | 39.5% | 1 | R.YSTPVDIWSIGTIFAELATK.K | 2 |

---

|  |  |  |  |  |  |  |  |  |
| --- | --- | --- | --- | --- | --- | --- | --- | --- |
| U | *gi|17105394|ref|NP\_00* | 1 | 2 | 8.3% | 156 | 17695 | 10.4 | ribosomal protein L23a [Homo sapiens] |

| Filename XCorr DeltCN Conf% ObsM+H+ CalcM+H+ SpR ZScore Ion% # Sequence  | | | | | | | | | | | | |
| --- | --- | --- | --- | --- | --- | --- | --- | --- | --- | --- | --- | --- |
| \* | AstrinSTLCLD\_041714\_01.06279.06279.2 | 3.4542 | 0.5375 | 100.0% | 1405.5721 | 1405.5474 | 1 | 8.208 | 66.7% | 2 | R.LAPDYDALDVANK.I | 2 |

---

|  |  |  |  |  |  |  |  |  |
| --- | --- | --- | --- | --- | --- | --- | --- | --- |
| U | *Reverse\_gi|185136049|* | 1 | 1 | 8.2% | 282 | 30798 | 7.4 | FSHD region gene 2 family, member C [Homo sapiens] |

| Filename XCorr DeltCN Conf% ObsM+H+ CalcM+H+ SpR ZScore Ion% # Sequence  | | | | | | | | | | | | |
| --- | --- | --- | --- | --- | --- | --- | --- | --- | --- | --- | --- | --- |
| \* | AstrinSTLCLD\_041714\_01.13694.13694.3 | 3.2664 | 0.2489 | 98.1% | 2695.6743 | 2697.6567 | 5 | 4.557 | 25.0% | 1 | K.NPNPDSGAQRQT#HK@ES\*SHSFATK.G | 3 |

---

|  |  |  |  |  |  |  |  |  |
| --- | --- | --- | --- | --- | --- | --- | --- | --- |
| U | *gi|169208824|ref|XP\_0* | 2 | 2 | 8.2% | 219 | 25125 | 6.7 | PREDICTED: hypothetical protein [Homo sapiens] |
| U | *gi|89038927|ref|XP\_94* | 2 | 2 | 7.4% | 243 | 27417 | 7.4 | PREDICTED: hypothetical protein [Homo sapiens] |
| U | *gi|89038281|ref|XP\_49* | 2 | 2 | 7.4% | 243 | 27340 | 7.4 | PREDICTED: hypothetical protein [Homo sapiens] |
| U | *gi|68800138|ref|NP\_00* | 2 | 2 | 10.8% | 167 | 19715 | 9.1 | U2 small nuclear RNA auxillary factor 1 isoform c [Homo sapiens] |
| U | *gi|68800128|ref|NP\_00* | 2 | 2 | 7.5% | 240 | 27882 | 8.7 | U2 small nuclear RNA auxillary factor 1 isoform b [Homo sapiens] |
| U | *gi|5803207|ref|NP\_006* | 2 | 2 | 7.5% | 240 | 27872 | 8.8 | U2 small nuclear RNA auxillary factor 1 isoform a [Homo sapiens] |

| Filename XCorr DeltCN Conf% ObsM+H+ CalcM+H+ SpR ZScore Ion% # Sequence  | | | | | | | | | | | | |
| --- | --- | --- | --- | --- | --- | --- | --- | --- | --- | --- | --- | --- |
|  | AstrinSTLCLD\_041714\_01.09395.09395.2 | 3.7518 | 0.366 | 100.0% | 2115.4922 | 2115.3555 | 1 | 7.472 | 50.0% | 1 | R.WFNGQPIHAELSPVTDFR.G | 2 |
|  | AstrinSTLCLD\_041714\_01.09399.09399.3 | 4.9124 | 0.4136 | 100.0% | 2116.2844 | 2115.3555 | 1 | 7.938 | 42.6% | 1 | R.WFNGQPIHAELSPVTDFR.G | 3 |

---

|  |  |  |  |  |  |  |  |  |
| --- | --- | --- | --- | --- | --- | --- | --- | --- |
| U | *gi|113429348|ref|XP\_3* | 1 | 1 | 8.2% | 171 | 19863 | 10.1 | PREDICTED: similar to ribosomal protein S10 [Homo sapiens] |
| U | *gi|4506679|ref|NP\_001* | 1 | 1 | 8.5% | 165 | 18898 | 10.2 | ribosomal protein S10 [Homo sapiens] |
| U | *gi|113429532|ref|XP\_9* | 1 | 1 | 8.2% | 171 | 19863 | 10.1 | PREDICTED: similar to ribosomal protein S10 [Homo sapiens] |

| Filename XCorr DeltCN Conf% ObsM+H+ CalcM+H+ SpR ZScore Ion% # Sequence  | | | | | | | | | | | | |
| --- | --- | --- | --- | --- | --- | --- | --- | --- | --- | --- | --- | --- |
|  | AstrinSTLCLD\_041714\_02.06246.06246.2 | 2.8867 | 0.2909 | 99.6% | 1442.3121 | 1442.5278 | 1 | 6.076 | 61.5% | 1 | K.AEAGAGSATEFQFR.G | 2 |

---

|  |  |  |  |  |  |  |  |  |
| --- | --- | --- | --- | --- | --- | --- | --- | --- |
| U | *gi|106049292|ref|NP\_0* | 6 | 8 | 8.1% | 1178 | 129634 | 6.8 | pyruvate carboxylase precursor [Homo sapiens] |
| U | *gi|106049528|ref|NP\_0* | 6 | 8 | 8.1% | 1178 | 129634 | 6.8 | pyruvate carboxylase precursor [Homo sapiens] |
| U | *gi|106049295|ref|NP\_0* | 6 | 8 | 8.1% | 1178 | 129634 | 6.8 | pyruvate carboxylase precursor [Homo sapiens] |

| Filename XCorr DeltCN Conf% ObsM+H+ CalcM+H+ SpR ZScore Ion% # Sequence  | | | | | | | | | | | | |
| --- | --- | --- | --- | --- | --- | --- | --- | --- | --- | --- | --- | --- |
|  | AstrinSTLCLD\_041714\_01.10336.10336.3 | 3.3041 | 0.3257 | 100.0% | 2410.7944 | 2411.7227 | 80 | 5.479 | 26.3% | 2 | R.HIEVQILGDQYGNILHLYER.D | 3 |
|  | AstrinSTLCLD\_041714\_01.09261.09261.3 | 3.564 | 0.3265 | 100.0% | 2346.3245 | 2346.645 | 1 | 5.333 | 38.1% | 1 | R.LDNASAFQGAVISPHYDSLLVK.V | 3 |
|  | AstrinSTLCLD\_041714\_01.09172.09172.2 | 3.242 | 0.3651 | 100.0% | 1522.7522 | 1522.6134 | 1 | 7.397 | 77.3% | 1 | R.VFDYSEYWEGAR.G | 2 |
|  | AstrinSTLCLD\_041714\_01.10853.10853.2 | 4.0076 | 0.434 | 100.0% | 1748.5521 | 1749.038 | 1 | 8.018 | 53.3% | 1 | K.IVGDLAQFMVQNGLSR.A | 2 |
|  | AstrinSTLCLD\_041714\_02.06435.06435.2 | 3.6083 | 0.4588 | 100.0% | 1548.2322 | 1548.6494 | 1 | 7.467 | 65.4% | 2 | R.AEAEAQAEELSFPR.S | 2 |
|  | AstrinSTLCLD\_041714\_02.07242.07242.2 | 3.1835 | 0.4257 | 100.0% | 1364.1721 | 1364.4949 | 1 | 7.426 | 75.0% | 1 | K.IAEEFEVELER.G | 2 |

---

|  |  |  |  |  |  |  |  |  |
| --- | --- | --- | --- | --- | --- | --- | --- | --- |
| U | *gi|55956919|ref|NP\_11* | 2 | 3 | 8.1% | 332 | 35968 | 6.9 | heterogeneous nuclear ribonucleoprotein A/B isoform a [Homo sapiens] |
| U | *gi|55956921|ref|NP\_00* | 2 | 3 | 9.5% | 285 | 30588 | 7.9 | heterogeneous nuclear ribonucleoprotein A/B isoform b [Homo sapiens] |

| Filename XCorr DeltCN Conf% ObsM+H+ CalcM+H+ SpR ZScore Ion% # Sequence  | | | | | | | | | | | | |
| --- | --- | --- | --- | --- | --- | --- | --- | --- | --- | --- | --- | --- |
|  | AstrinSTLCLD\_041714\_01.07883.07883.2 | 2.8169 | 0.3877 | 100.0% | 1456.4122 | 1456.6996 | 85 | 6.435 | 50.0% | 1 | K.MFVGGLSWDTSKK.D | 2 |
|  | AstrinSTLCLD\_041714\_01.06662.06662.2 | 3.9807 | 0.443 | 100.0% | 1504.6522 | 1504.6799 | 1 | 7.473 | 73.1% | 2 | K.IFVGGLNPEATEEK.I | 2 |

---

|  |  |  |  |  |  |  |  |  |
| --- | --- | --- | --- | --- | --- | --- | --- | --- |
| U | *gi|4507669|ref|NP\_003* | 1 | 1 | 8.1% | 172 | 19595 | 4.9 | tumor protein, translationally-controlled 1 [Homo sapiens] |

| Filename XCorr DeltCN Conf% ObsM+H+ CalcM+H+ SpR ZScore Ion% # Sequence  | | | | | | | | | | | | |
| --- | --- | --- | --- | --- | --- | --- | --- | --- | --- | --- | --- | --- |
| \* | AstrinSTLCLD\_041714\_01.09227.09227.2 | 2.4939 | 0.2495 | 96.9% | 1712.9722 | 1713.8994 | 154 | 5.101 | 42.3% | 1 | R.DLISHDEMFSDIYK.I | 2 |

---

|  |  |  |  |  |  |  |  |  |
| --- | --- | --- | --- | --- | --- | --- | --- | --- |
| U | *gi|110225358|ref|NP\_0* | 4 | 4 | 8.0% | 858 | 96185 | 6.4 | tetratricopeptide repeat domain 7A [Homo sapiens] |

| Filename XCorr DeltCN Conf% ObsM+H+ CalcM+H+ SpR ZScore Ion% # Sequence  | | | | | | | | | | | | |
| --- | --- | --- | --- | --- | --- | --- | --- | --- | --- | --- | --- | --- |
| \* | AstrinSTLCLD\_041714\_01.13510.13510.2 | 2.9583 | 0.3664 | 100.0% | 1662.2922 | 1662.9261 | 1 | 5.77 | 57.7% | 1 | R.ASWIAQVFLQELEK.T | 2 |
| \* | AstrinSTLCLD\_041714\_01.09587.09587.2 | 3.4622 | 0.3933 | 100.0% | 1765.5122 | 1766.075 | 1 | 5.618 | 50.0% | 1 | R.LEEAMSELTMPSSVLK.Q | 2 |
| \* | AstrinSTLCLD\_041714\_01.17301.17301.3 | 4.6674 | 0.435 | 100.0% | 3399.6243 | 3399.9502 | 1 | 7.494 | 26.9% | 1 | K.QGPMQLWTTLEQIWLQAAELFMEQQHLK.E | 3 |
| \* | AstrinSTLCLD\_041714\_01.07504.07504.2 | 2.8778 | 0.3468 | 100.0% | 1258.3522 | 1258.583 | 2 | 6.27 | 60.0% | 1 | R.IMHSLGLMLSR.L | 2 |

---

|  |  |  |  |  |  |  |  |  |
| --- | --- | --- | --- | --- | --- | --- | --- | --- |
| U | *gi|4503483|ref|NP\_001* | 5 | 10 | 8.0% | 858 | 95338 | 6.8 | eukaryotic translation elongation factor 2 [Homo sapiens] |

| Filename XCorr DeltCN Conf% ObsM+H+ CalcM+H+ SpR ZScore Ion% # Sequence  | | | | | | | | | | | | |
| --- | --- | --- | --- | --- | --- | --- | --- | --- | --- | --- | --- | --- |
| \* | AstrinSTLCLD\_041714\_01.13959.13959.3 | 3.1782 | 0.2838 | 99.1% | 2602.4343 | 2602.11 | 1 | 4.997 | 30.4% | 1 | R.WLPAGDALLQMITIHLPSPVTAQK.Y | 3 |
| \* | AstrinSTLCLD\_041714\_01.07231.07231.2 | 2.8236 | 0.2725 | 99.7% | 1108.2722 | 1108.3231 | 1 | 6.717 | 80.0% | 1 | R.VFSGLVSTGLK.V | 2 |
| \* | AstrinSTLCLD\_041714\_01.07301.07301.3 | 5.3389 | 0.4516 | 100.0% | 2144.3044 | 2144.3489 | 1 | 7.754 | 42.1% | 5 | K.ARPFPDGLAEDIDKGEVSAR.Q | 3 |
| \* | AstrinSTLCLD\_041714\_01.07066.07066.2 | 2.7015 | 0.3556 | 100.0% | 1743.4922 | 1743.9133 | 20 | 6.199 | 42.3% | 1 | R.YLAEKYEWDVAEAR.K | 2 |
| \* | AstrinSTLCLD\_041714\_02.06819.06819.3 | 3.3205 | 0.4251 | 100.0% | 1743.5944 | 1743.9133 | 1 | 6.66 | 46.2% | 2 | R.YLAEKYEWDVAEAR.K | 3 |

---

|  |  |  |  |  |  |  |  |  |
| --- | --- | --- | --- | --- | --- | --- | --- | --- |
| U | *gi|4758138|ref|NP\_004* | 5 | 10 | 8.0% | 614 | 69148 | 8.9 | DEAD (Asp-Glu-Ala-Asp) box polypeptide 5 [Homo sapiens] |

| Filename XCorr DeltCN Conf% ObsM+H+ CalcM+H+ SpR ZScore Ion% # Sequence  | | | | | | | | | | | | |
| --- | --- | --- | --- | --- | --- | --- | --- | --- | --- | --- | --- | --- |
| \* | AstrinSTLCLD\_041714\_01.06930.06930.2 | 3.7437 | 0.4786 | 100.0% | 1296.1522 | 1296.4198 | 1 | 9.355 | 80.0% | 2 | R.TTYLVLDEADR.M | 2 |
|  | AstrinSTLCLD\_041714\_01.09135.09135.2 | 4.0964 | 0.4602 | 100.0% | 1337.1721 | 1337.5946 | 1 | 8.584 | 85.0% | 3 | R.MLDMGFEPQIR.K | 222 |
|  | AstrinSTLCLD\_041714\_01.07284.07284.2 | 2.6786 | 0.2242 | 98.0% | 1465.5922 | 1465.7688 | 7 | 5.28 | 59.1% | 1 | R.MLDMGFEPQIRK.I | 22 |
|  | AstrinSTLCLD\_041714\_01.06420.06420.2 | 4.1554 | 0.2906 | 100.0% | 1227.4521 | 1227.4465 | 2 | 7.268 | 81.8% | 3 | K.APILIATDVASR.G | 22 |
| \* | AstrinSTLCLD\_041714\_01.08783.08783.2 | 2.6072 | 0.2925 | 99.0% | 1575.4122 | 1575.7612 | 1 | 5.798 | 53.8% | 1 | K.TGTAYTFFTPNNIK.Q | 2 |

Similarities:
gi|87196351|ref|NP\_00(1:4)  
gi|148613856|ref|NP\_0(3:2)  

---

|  |  |  |  |  |  |  |  |  |
| --- | --- | --- | --- | --- | --- | --- | --- | --- |
| U | *gi|20336746|ref|NP\_61* | 1 | 1 | 7.9% | 369 | 39183 | 9.8 | H2A histone family, member Y isoform 1 [Homo sapiens] |
| U | *gi|93141020|ref|NP\_00* | 1 | 1 | 7.8% | 371 | 39489 | 9.8 | H2A histone family, member Y isoform 2 [Homo sapiens] |
| U | *gi|93141018|ref|NP\_61* | 1 | 1 | 7.8% | 372 | 39617 | 9.8 | H2A histone family, member Y isoform 3 [Homo sapiens] |
| U | *gi|4758496|ref|NP\_004* | 1 | 1 | 7.8% | 371 | 39489 | 9.8 | H2A histone family, member Y isoform 2 [Homo sapiens] |

| Filename XCorr DeltCN Conf% ObsM+H+ CalcM+H+ SpR ZScore Ion% # Sequence  | | | | | | | | | | | | |
| --- | --- | --- | --- | --- | --- | --- | --- | --- | --- | --- | --- | --- |
|  | AstrinSTLCLD\_041714\_01.17751.17751.3 | 5.8516 | 0.2836 | 100.0% | 2977.7644 | 2977.489 | 1 | 8.049 | 33.0% | 1 | R.IGVGAPVYMAAVLEYLTAEILELAGNAAR.D | 3 |

---

|  |  |  |  |  |  |  |  |  |
| --- | --- | --- | --- | --- | --- | --- | --- | --- |
| U | *gi|5174447|ref|NP\_006* | 2 | 3 | 7.9% | 317 | 35077 | 7.7 | guanine nucleotide binding protein (G protein), beta polypeptide 2-like 1 [Homo sapiens] |

| Filename XCorr DeltCN Conf% ObsM+H+ CalcM+H+ SpR ZScore Ion% # Sequence  | | | | | | | | | | | | |
| --- | --- | --- | --- | --- | --- | --- | --- | --- | --- | --- | --- | --- |
| \* | AstrinSTLCLD\_041714\_01.07862.07862.2 | 3.8851 | 0.4869 | 100.0% | 1788.5922 | 1790.0642 | 1 | 8.409 | 60.0% | 1 | K.IIVDELKQEVISTSSK.A | 2 |
| \* | AstrinSTLCLD\_041714\_01.06416.06416.2 | 2.63 | 0.2746 | 99.7% | 1059.7122 | 1060.2412 | 1 | 5.562 | 87.5% | 2 | R.VWQVTIGTR.- | 2 |

---

|  |  |  |  |  |  |  |  |  |
| --- | --- | --- | --- | --- | --- | --- | --- | --- |
| U | *gi|5901926|ref|NP\_008* | 1 | 1 | 7.9% | 227 | 26227 | 8.8 | cleavage and polyadenylation specific factor 5 [Homo sapiens] |

| Filename XCorr DeltCN Conf% ObsM+H+ CalcM+H+ SpR ZScore Ion% # Sequence  | | | | | | | | | | | | |
| --- | --- | --- | --- | --- | --- | --- | --- | --- | --- | --- | --- | --- |
| \* | AstrinSTLCLD\_041714\_01.05730.05730.3 | 4.2412 | 0.3954 | 100.0% | 1910.0343 | 1910.0911 | 2 | 7.585 | 39.7% | 1 | K.LPGGELNPGEDEVEGLKR.L | 3 |

---

|  |  |  |  |  |  |  |  |  |
| --- | --- | --- | --- | --- | --- | --- | --- | --- |
| U | *gi|15431290|ref|NP\_00* | 1 | 1 | 7.9% | 178 | 20252 | 9.6 | ribosomal protein L11 [Homo sapiens] |

| Filename XCorr DeltCN Conf% ObsM+H+ CalcM+H+ SpR ZScore Ion% # Sequence  | | | | | | | | | | | | |
| --- | --- | --- | --- | --- | --- | --- | --- | --- | --- | --- | --- | --- |
| \* | AstrinSTLCLD\_041714\_01.07473.07473.2 | 3.4458 | 0.4833 | 100.0% | 1547.4722 | 1547.7917 | 1 | 8.384 | 61.5% | 1 | K.VLEQLTGQTPVFSK.A | 2 |

---

|  |  |  |  |  |  |  |  |  |
| --- | --- | --- | --- | --- | --- | --- | --- | --- |
| U | *gi|4759160|ref|NP\_004* | 1 | 2 | 7.9% | 126 | 13916 | 10.3 | small nuclear ribonucleoprotein polypeptide D3 [Homo sapiens] |

| Filename XCorr DeltCN Conf% ObsM+H+ CalcM+H+ SpR ZScore Ion% # Sequence  | | | | | | | | | | | | |
| --- | --- | --- | --- | --- | --- | --- | --- | --- | --- | --- | --- | --- |
| \* | AstrinSTLCLD\_041714\_01.05858.05858.2 | 3.0635 | 0.3067 | 100.0% | 1219.3121 | 1219.4264 | 1 | 6.311 | 77.8% | 2 | R.VAQLEQVYIR.G | 2 |

---

|  |  |  |  |  |  |  |  |  |
| --- | --- | --- | --- | --- | --- | --- | --- | --- |
| U | *gi|153792590|ref|NP\_0* | 5 | 6 | 7.8% | 854 | 98161 | 5.2 | heat shock 90kDa protein 1, alpha isoform 1 [Homo sapiens] |
| U | *gi|154146191|ref|NP\_0* | 5 | 6 | 9.2% | 732 | 84660 | 5.0 | heat shock 90kDa protein 1, alpha isoform 2 [Homo sapiens] |

| Filename XCorr DeltCN Conf% ObsM+H+ CalcM+H+ SpR ZScore Ion% # Sequence  | | | | | | | | | | | | |
| --- | --- | --- | --- | --- | --- | --- | --- | --- | --- | --- | --- | --- |
|  | AstrinSTLCLD\_041714\_01.08062.08062.2 | 3.3951 | 0.2859 | 100.0% | 1243.1921 | 1243.4459 | 1 | 6.134 | 72.7% | 1 | K.ADLINNLGTIAK.S | 22 |
|  | AstrinSTLCLD\_041714\_02.06522.06522.3 | 3.5957 | 0.3866 | 100.0% | 2015.9944 | 2016.2584 | 1 | 6.42 | 41.7% | 2 | K.VILHLKEDQTEYLEER.R | 33 |
|  | AstrinSTLCLD\_041714\_01.07229.07229.2 | 3.4213 | 0.3608 | 100.0% | 1528.3722 | 1528.6616 | 1 | 6.434 | 62.5% | 1 | K.SLTNDWEDHLAVK.H | 22 |
|  | AstrinSTLCLD\_041714\_02.06833.06833.2 | 2.8835 | 0.4073 | 100.0% | 1348.6322 | 1349.4886 | 1 | 6.329 | 65.0% | 1 | K.HFSVEGQLEFR.A | 22 |
|  | AstrinSTLCLD\_041714\_01.07269.07269.3 | 2.6023 | 0.2463 | 96.5% | 1787.9343 | 1788.0134 | 1 | 4.447 | 41.1% | 1 | K.HLEINPDHSIIETLR.Q | 3 |

Similarities:
gi|20149594|ref|NP\_03(4:1)  

---

|  |  |  |  |  |  |  |  |  |
| --- | --- | --- | --- | --- | --- | --- | --- | --- |
| U | *gi|4758086|ref|NP\_004* | 1 | 3 | 7.8% | 193 | 20567 | 8.6 | cysteine and glycine-rich protein 1 isoform 1 [Homo sapiens] |

| Filename XCorr DeltCN Conf% ObsM+H+ CalcM+H+ SpR ZScore Ion% # Sequence  | | | | | | | | | | | | |
| --- | --- | --- | --- | --- | --- | --- | --- | --- | --- | --- | --- | --- |
| \* | AstrinSTLCLD\_041714\_02.07295.07295.2 | 3.5921 | 0.5587 | 100.0% | 1434.3522 | 1434.551 | 3 | 9.154 | 50.0% | 3 | K.GFGFGQGAGALVHSE.- | 2 |

---

|  |  |  |  |  |  |  |  |  |
| --- | --- | --- | --- | --- | --- | --- | --- | --- |
| U | *gi|41393577|ref|NP\_07* | 1 | 1 | 7.8% | 167 | 18824 | 5.1 | gemin 6 [Homo sapiens] |

| Filename XCorr DeltCN Conf% ObsM+H+ CalcM+H+ SpR ZScore Ion% # Sequence  | | | | | | | | | | | | |
| --- | --- | --- | --- | --- | --- | --- | --- | --- | --- | --- | --- | --- |
| \* | AstrinSTLCLD\_041714\_02.06080.06080.2 | 3.3647 | 0.3646 | 100.0% | 1410.6921 | 1411.5547 | 1 | 7.756 | 75.0% | 1 | R.VQDLIEGHLTASQ.- | 2 |

---

|  |  |  |  |  |  |  |  |  |
| --- | --- | --- | --- | --- | --- | --- | --- | --- |
| U | *gi|153791632|ref|NP\_0* | 1 | 1 | 7.8% | 154 | 16773 | 5.0 | eukaryotic translation initiation factor 5A-like 1 [Homo sapiens] |
| U | *gi|9966867|ref|NP\_065* | 1 | 1 | 7.8% | 153 | 16793 | 5.6 | eIF-5A2 protein [Homo sapiens] |
| U | *gi|4503545|ref|NP\_001* | 1 | 1 | 7.8% | 154 | 16832 | 5.2 | eukaryotic translation initiation factor 5A isoform B [Homo sapiens] |
| U | *gi|219555712|ref|NP\_0* | 1 | 1 | 7.8% | 154 | 16832 | 5.2 | eukaryotic translation initiation factor 5A isoform B [Homo sapiens] |
| U | *gi|219555710|ref|NP\_0* | 1 | 1 | 7.8% | 154 | 16832 | 5.2 | eukaryotic translation initiation factor 5A isoform B [Homo sapiens] |
| U | *gi|219555707|ref|NP\_0* | 1 | 1 | 6.5% | 184 | 20170 | 7.0 | eukaryotic translation initiation factor 5A isoform A [Homo sapiens] |

| Filename XCorr DeltCN Conf% ObsM+H+ CalcM+H+ SpR ZScore Ion% # Sequence  | | | | | | | | | | | | |
| --- | --- | --- | --- | --- | --- | --- | --- | --- | --- | --- | --- | --- |
|  | AstrinSTLCLD\_041714\_01.09614.09614.2 | 3.3468 | 0.4539 | 100.0% | 1299.6721 | 1299.5559 | 1 | 8.271 | 81.8% | 1 | K.VHLVGIDIFTGK.K | 2 |

---

|  |  |  |  |  |  |  |  |  |
| --- | --- | --- | --- | --- | --- | --- | --- | --- |
| U | *gi|148613856|ref|NP\_0* | 5 | 9 | 7.7% | 731 | 80458 | 8.4 | DEAD box polypeptide 17 isoform 3 [Homo sapiens] |
| U | *gi|38201710|ref|NP\_00* | 5 | 9 | 7.7% | 729 | 80273 | 8.3 | DEAD box polypeptide 17 isoform 1 [Homo sapiens] |

| Filename XCorr DeltCN Conf% ObsM+H+ CalcM+H+ SpR ZScore Ion% # Sequence  | | | | | | | | | | | | |
| --- | --- | --- | --- | --- | --- | --- | --- | --- | --- | --- | --- | --- |
|  | AstrinSTLCLD\_041714\_01.07562.07562.2 | 4.1416 | 0.4534 | 100.0% | 1692.0322 | 1692.8229 | 1 | 7.452 | 64.3% | 1 | R.ELAQQVQQVADDYGK.C | 2 |
|  | AstrinSTLCLD\_041714\_01.09135.09135.2 | 4.0964 | 0.4602 | 100.0% | 1337.1721 | 1337.5946 | 1 | 8.584 | 85.0% | 3 | R.MLDMGFEPQIR.K | 222 |
|  | AstrinSTLCLD\_041714\_01.07284.07284.2 | 2.6786 | 0.2242 | 98.0% | 1465.5922 | 1465.7688 | 7 | 5.28 | 59.1% | 1 | R.MLDMGFEPQIRK.I | 22 |
|  | AstrinSTLCLD\_041714\_01.06420.06420.2 | 4.1554 | 0.2906 | 100.0% | 1227.4521 | 1227.4465 | 2 | 7.268 | 81.8% | 3 | K.APILIATDVASR.G | 22 |
|  | AstrinSTLCLD\_041714\_01.07431.07431.3 | 4.0359 | 0.2971 | 100.0% | 2118.7144 | 2119.2542 | 24 | 6.525 | 32.8% | 1 | K.FVINYDYPNSSEDYVHR.I | 3 |

Similarities:
gi|87196351|ref|NP\_00(1:4)  
gi|4758138|ref|NP\_004(3:2)  

---

|  |  |  |  |  |  |  |  |  |
| --- | --- | --- | --- | --- | --- | --- | --- | --- |
| U | *gi|47271443|ref|NP\_00* | 1 | 2 | 7.7% | 221 | 25476 | 11.9 | splicing factor, arginine/serine-rich 2 [Homo sapiens] |

| Filename XCorr DeltCN Conf% ObsM+H+ CalcM+H+ SpR ZScore Ion% # Sequence  | | | | | | | | | | | | |
| --- | --- | --- | --- | --- | --- | --- | --- | --- | --- | --- | --- | --- |
| \* | AstrinSTLCLD\_041714\_01.09327.09327.2 | 4.1878 | 0.4447 | 100.0% | 1752.2522 | 1752.8654 | 1 | 9.508 | 68.8% | 2 | R.DAEDAMDAMDGAVLDGR.E | 2 |

---

|  |  |  |  |  |  |  |  |  |
| --- | --- | --- | --- | --- | --- | --- | --- | --- |
| U | *gi|4557303|ref|NP\_000* | 3 | 3 | 7.6% | 485 | 54848 | 7.9 | aldehyde dehydrogenase 3A2 isoform 2 [Homo sapiens] |
| U | *gi|73466520|ref|NP\_00* | 3 | 3 | 7.3% | 508 | 57669 | 8.9 | aldehyde dehydrogenase 3A2 isoform 1 [Homo sapiens] |

| Filename XCorr DeltCN Conf% ObsM+H+ CalcM+H+ SpR ZScore Ion% # Sequence  | | | | | | | | | | | | |
| --- | --- | --- | --- | --- | --- | --- | --- | --- | --- | --- | --- | --- |
|  | AstrinSTLCLD\_041714\_02.05175.05175.2 | 2.6878 | 0.3297 | 100.0% | 1267.3322 | 1267.3379 | 2 | 6.497 | 68.2% | 1 | K.IAFGGETDEATR.Y | 2 |
|  | AstrinSTLCLD\_041714\_01.07811.07811.2 | 3.2418 | 0.3598 | 100.0% | 1433.3522 | 1432.6567 | 1 | 6.418 | 62.5% | 1 | R.YIAPTVLTDVDPK.T | 2 |
|  | AstrinSTLCLD\_041714\_01.08424.08424.2 | 3.9149 | 0.3752 | 100.0% | 1435.4521 | 1434.5486 | 1 | 7.977 | 77.3% | 1 | K.NVDEAINFINER.E | 2 |

---

|  |  |  |  |  |  |  |  |  |
| --- | --- | --- | --- | --- | --- | --- | --- | --- |
| U | *gi|108936958|ref|NP\_0* | 2 | 2 | 7.6% | 342 | 38926 | 5.5 | WD-repeat protein [Homo sapiens] |

| Filename XCorr DeltCN Conf% ObsM+H+ CalcM+H+ SpR ZScore Ion% # Sequence  | | | | | | | | | | | | |
| --- | --- | --- | --- | --- | --- | --- | --- | --- | --- | --- | --- | --- |
| \* | AstrinSTLCLD\_041714\_01.09116.09116.2 | 3.7137 | 0.4135 | 100.0% | 1484.0721 | 1484.6488 | 1 | 7.569 | 62.5% | 1 | R.LALGSFVEEYNNK.V | 2 |
| \* | AstrinSTLCLD\_041714\_02.06509.06509.2 | 2.5722 | 0.3024 | 99.1% | 1313.3522 | 1312.44 | 2 | 6.07 | 62.5% | 1 | R.DMFASVGADGSVR.M | 2 |

---

|  |  |  |  |  |  |  |  |  |
| --- | --- | --- | --- | --- | --- | --- | --- | --- |
| U | *gi|14165464|ref|NP\_11* | 2 | 2 | 7.5% | 550 | 59037 | 9.2 | polypyrimidine tract-binding protein 1 isoform b [Homo sapiens] |
| U | *gi|4506243|ref|NP\_002* | 2 | 2 | 7.4% | 557 | 59633 | 9.2 | polypyrimidine tract-binding protein 1 isoform a [Homo sapiens] |
| U | *gi|14165466|ref|NP\_11* | 2 | 2 | 7.7% | 531 | 57221 | 9.2 | polypyrimidine tract-binding protein 1 isoform c [Homo sapiens] |

| Filename XCorr DeltCN Conf% ObsM+H+ CalcM+H+ SpR ZScore Ion% # Sequence  | | | | | | | | | | | | |
| --- | --- | --- | --- | --- | --- | --- | --- | --- | --- | --- | --- | --- |
|  | AstrinSTLCLD\_041714\_02.09704.09704.2 | 4.2518 | 0.4793 | 100.0% | 2276.152 | 2276.6414 | 1 | 9.017 | 40.9% | 1 | R.IAIPGLAGAGNSVLLVSNLNPER.V | 2 |
|  | AstrinSTLCLD\_041714\_02.11644.11644.2 | 3.2977 | 0.4424 | 100.0% | 2040.2322 | 2040.3696 | 1 | 6.821 | 44.1% | 1 | R.VTPQSLFILFGVYGDVQR.V | 2 |

---

|  |  |  |  |  |  |  |  |  |
| --- | --- | --- | --- | --- | --- | --- | --- | --- |
| U | *gi|169161020|ref|XP\_0* | 1 | 1 | 7.5% | 146 | 16448 | 9.8 | PREDICTED: similar to Rps16 protein [Homo sapiens] |
| U | *gi|88946109|ref|XP\_94* | 1 | 1 | 7.5% | 146 | 16448 | 9.8 | PREDICTED: similar to Rps16 protein isoform 3 [Homo sapiens] |
| U | *gi|4506691|ref|NP\_001* | 1 | 1 | 7.5% | 146 | 16445 | 10.2 | ribosomal protein S16 [Homo sapiens] |

| Filename XCorr DeltCN Conf% ObsM+H+ CalcM+H+ SpR ZScore Ion% # Sequence  | | | | | | | | | | | | |
| --- | --- | --- | --- | --- | --- | --- | --- | --- | --- | --- | --- | --- |
|  | AstrinSTLCLD\_041714\_01.07728.07728.2 | 2.6914 | 0.3845 | 100.0% | 1187.9122 | 1188.372 | 1 | 6.799 | 60.0% | 1 | K.GPLQSVQVFGR.K | 2 |

---

|  |  |  |  |  |  |  |  |  |
| --- | --- | --- | --- | --- | --- | --- | --- | --- |
| U | *gi|4507879|ref|NP\_003* | 1 | 1 | 7.4% | 283 | 30773 | 8.5 | voltage-dependent anion channel 1 [Homo sapiens] |

| Filename XCorr DeltCN Conf% ObsM+H+ CalcM+H+ SpR ZScore Ion% # Sequence  | | | | | | | | | | | | |
| --- | --- | --- | --- | --- | --- | --- | --- | --- | --- | --- | --- | --- |
| \* | AstrinSTLCLD\_041714\_02.04846.04846.3 | 3.2758 | 0.2144 | 97.4% | 2191.7344 | 2190.2822 | 20 | 4.652 | 30.0% | 1 | K.TKSENGLEFTSSGSANTETTK.V | 3 |

---

|  |  |  |  |  |  |  |  |  |
| --- | --- | --- | --- | --- | --- | --- | --- | --- |
| U | *Reverse\_gi|169172022|* | 1 | 1 | 7.2% | 265 | 30612 | 7.7 | PREDICTED: similar to actin-related protein 3-beta [Homo sapiens] |
| U | *Reverse\_gi|9966913|re* | 1 | 1 | 4.5% | 418 | 47608 | 5.9 | actin-related protein 3-beta isoform 1 [Homo sapiens] |
| U | *Reverse\_gi|92373393|r* | 1 | 1 | 5.5% | 348 | 39698 | 5.1 | actin-related protein 3-beta isoform 2 [Homo sapiens] |

| Filename XCorr DeltCN Conf% ObsM+H+ CalcM+H+ SpR ZScore Ion% # Sequence  | | | | | | | | | | | | |
| --- | --- | --- | --- | --- | --- | --- | --- | --- | --- | --- | --- | --- |
|  | AstrinSTLCLD\_041714\_02.05366.05366.3 | 3.7038 | 0.2484 | 99.2% | 2097.1743 | 2096.3884 | 4 | 4.704 | 31.9% | 1 | K.EKIAKATELSQEPPIGVER.E | 3 |

---

|  |  |  |  |  |  |  |  |  |
| --- | --- | --- | --- | --- | --- | --- | --- | --- |
| U | *Reverse\_gi|194363768|* | 1 | 1 | 7.1% | 352 | 36495 | 9.3 | homeobox D9 [Homo sapiens] |

| Filename XCorr DeltCN Conf% ObsM+H+ CalcM+H+ SpR ZScore Ion% # Sequence  | | | | | | | | | | | | |
| --- | --- | --- | --- | --- | --- | --- | --- | --- | --- | --- | --- | --- |
| \* | AstrinSTLCLD\_041714\_02.10879.10879.2 | 2.4066 | 0.3206 | 98.5% | 2404.112 | 2404.4526 | 42 | 4.682 | 25.0% | 1 | R.K@SSSSLSTSSSS\*TTSAATAPAPAAR.T | 2 |

---

|  |  |  |  |  |  |  |  |  |
| --- | --- | --- | --- | --- | --- | --- | --- | --- |
| U | *Reverse\_gi|188595697|* | 1 | 2 | 7.0% | 299 | 32541 | 7.9 | sprouty homolog 4 isoform 2 [Homo sapiens] |
| U | *Reverse\_gi|23308574|r* | 1 | 2 | 6.5% | 322 | 34929 | 7.9 | sprouty homolog 4 isoform 1 [Homo sapiens] |

| Filename XCorr DeltCN Conf% ObsM+H+ CalcM+H+ SpR ZScore Ion% # Sequence  | | | | | | | | | | | | |
| --- | --- | --- | --- | --- | --- | --- | --- | --- | --- | --- | --- | --- |
|  | AstrinSTLCLD\_041714\_01.13444.13444.2 | 3.175 | 0.1354 | 96.3% | 2507.9521 | 2508.4355 | 42 | 3.633 | 32.5% | 2 | K.DPRS\*TKADGS\*AAK@CIVSNT#HK@.C | 2 |

---

|  |  |  |  |  |  |  |  |  |
| --- | --- | --- | --- | --- | --- | --- | --- | --- |
| U | *gi|56090146|ref|NP\_00* | 1 | 1 | 7.0% | 285 | 31689 | 7.5 | jumonji domain containing 8 [Homo sapiens] |

| Filename XCorr DeltCN Conf% ObsM+H+ CalcM+H+ SpR ZScore Ion% # Sequence  | | | | | | | | | | | | |
| --- | --- | --- | --- | --- | --- | --- | --- | --- | --- | --- | --- | --- |
| \* | AstrinSTLCLD\_041714\_02.08414.08414.3 | 2.9527 | 0.3203 | 99.2% | 2474.5444 | 2475.739 | 1 | 4.791 | 28.9% | 1 | R.FRALCSRDRLLAS\*FGDRVVR.L | 3 |

---

|  |  |  |  |  |  |  |  |  |
| --- | --- | --- | --- | --- | --- | --- | --- | --- |
| U | *gi|11024714|ref|NP\_06* | 1 | 1 | 7.0% | 229 | 25762 | 7.4 | ubiquitin B precursor [Homo sapiens] |
| U | *gi|77539055|ref|NP\_00* | 1 | 1 | 12.5% | 128 | 14728 | 9.8 | ubiquitin and ribosomal protein L40 precursor [Homo sapiens] |
| U | *gi|67191208|ref|NP\_06* | 1 | 1 | 2.3% | 685 | 77029 | 7.7 | ubiquitin C [Homo sapiens] |
| U | *gi|4507761|ref|NP\_003* | 1 | 1 | 12.5% | 128 | 14728 | 9.8 | ubiquitin and ribosomal protein L40 precursor [Homo sapiens] |
| U | *gi|4506713|ref|NP\_002* | 1 | 1 | 10.3% | 156 | 17965 | 9.6 | ubiquitin and ribosomal protein S27a precursor [Homo sapiens] |
| U | *gi|208022622|ref|NP\_0* | 1 | 1 | 10.3% | 156 | 17965 | 9.6 | ubiquitin and ribosomal protein S27a precursor [Homo sapiens] |

| Filename XCorr DeltCN Conf% ObsM+H+ CalcM+H+ SpR ZScore Ion% # Sequence  | | | | | | | | | | | | |
| --- | --- | --- | --- | --- | --- | --- | --- | --- | --- | --- | --- | --- |
|  | AstrinSTLCLD\_041714\_01.07928.07928.2 | 4.2859 | 0.4256 | 100.0% | 1788.4722 | 1788.9897 | 1 | 7.396 | 56.7% | 1 | K.TITLEVEPSDTIENVK.A | 2 |

---

|  |  |  |  |  |  |  |  |  |
| --- | --- | --- | --- | --- | --- | --- | --- | --- |
| U | *gi|34740329|ref|NP\_91* | 2 | 3 | 6.9% | 378 | 39595 | 9.0 | heterogeneous nuclear ribonucleoprotein A3 [Homo sapiens] |

| Filename XCorr DeltCN Conf% ObsM+H+ CalcM+H+ SpR ZScore Ion% # Sequence  | | | | | | | | | | | | |
| --- | --- | --- | --- | --- | --- | --- | --- | --- | --- | --- | --- | --- |
| \* | AstrinSTLCLD\_041714\_02.06699.06699.3 | 3.4067 | 0.3664 | 100.0% | 1883.5144 | 1884.096 | 1 | 5.959 | 40.0% | 1 | K.IFVGGIKEDTEEYNLR.D | 3 |
| \* | AstrinSTLCLD\_041714\_01.05736.05736.2 | 2.7876 | 0.4924 | 100.0% | 1234.4922 | 1235.3948 | 2 | 7.809 | 83.3% | 2 | K.IETIEVMEDR.Q | 2 |

---

|  |  |  |  |  |  |  |  |  |
| --- | --- | --- | --- | --- | --- | --- | --- | --- |
| U | *gi|15431293|ref|NP\_00* | 1 | 1 | 6.9% | 204 | 24146 | 11.6 | ribosomal protein L15 [Homo sapiens] |
| U | *gi|88998868|ref|XP\_94* | 1 | 1 | 6.9% | 204 | 24174 | 11.6 | PREDICTED: hypothetical protein isoform 4 [Homo sapiens] |
| U | *gi|88992455|ref|XP\_93* | 1 | 1 | 6.9% | 204 | 24174 | 11.6 | PREDICTED: hypothetical protein isoform 1 [Homo sapiens] |
| U | *gi|169169711|ref|XP\_0* | 1 | 1 | 6.9% | 204 | 24174 | 11.6 | PREDICTED: hypothetical protein [Homo sapiens] |

| Filename XCorr DeltCN Conf% ObsM+H+ CalcM+H+ SpR ZScore Ion% # Sequence  | | | | | | | | | | | | |
| --- | --- | --- | --- | --- | --- | --- | --- | --- | --- | --- | --- | --- |
|  | AstrinSTLCLD\_041714\_02.06990.06990.2 | 4.1561 | 0.5228 | 100.0% | 1661.3322 | 1661.8083 | 1 | 9.261 | 69.2% | 1 | R.VLNSYWVGEDSTYK.F | 2 |

---

|  |  |  |  |  |  |  |  |  |
| --- | --- | --- | --- | --- | --- | --- | --- | --- |
| U | *gi|149944593|ref|NP\_0* | 1 | 1 | 6.8% | 400 | 45192 | 5.7 | hypothetical protein LOC23349 [Homo sapiens] |

| Filename XCorr DeltCN Conf% ObsM+H+ CalcM+H+ SpR ZScore Ion% # Sequence  | | | | | | | | | | | | |
| --- | --- | --- | --- | --- | --- | --- | --- | --- | --- | --- | --- | --- |
| \* | AstrinSTLCLD\_041714\_01.11304.11304.3 | 2.9919 | 0.2955 | 98.9% | 3027.8643 | 3029.4697 | 263 | 4.67 | 23.1% | 1 | R.QTVEQVQKVSLAVSAFKDGLRDRPSIR.R | 3 |

---

|  |  |  |  |  |  |  |  |  |
| --- | --- | --- | --- | --- | --- | --- | --- | --- |
| U | *Reverse\_gi|194353946|* | 1 | 1 | 6.7% | 270 | 29031 | 9.8 | homeobox D12 [Homo sapiens] |

| Filename XCorr DeltCN Conf% ObsM+H+ CalcM+H+ SpR ZScore Ion% # Sequence  | | | | | | | | | | | | |
| --- | --- | --- | --- | --- | --- | --- | --- | --- | --- | --- | --- | --- |
| \* | AstrinSTLCLD\_041714\_02.08555.08555.3 | 3.2396 | 0.2361 | 98.2% | 2179.3145 | 2179.4343 | 1 | 4.227 | 38.2% | 1 | R.NIFENVLFENELEAIQQK.T | 3 |

---

|  |  |  |  |  |  |  |  |  |
| --- | --- | --- | --- | --- | --- | --- | --- | --- |
| U | *gi|116812577|ref|NP\_0* | 2 | 2 | 6.6% | 392 | 46514 | 10.0 | LUC7-like 2 [Homo sapiens] |

| Filename XCorr DeltCN Conf% ObsM+H+ CalcM+H+ SpR ZScore Ion% # Sequence  | | | | | | | | | | | | |
| --- | --- | --- | --- | --- | --- | --- | --- | --- | --- | --- | --- | --- |
| \* | AstrinSTLCLD\_041714\_01.08297.08297.2 | 2.8709 | 0.3835 | 100.0% | 1223.2322 | 1223.448 | 1 | 7.283 | 75.0% | 1 | R.AMLDQLMGTSR.D | 2 |
| \* | AstrinSTLCLD\_041714\_02.05518.05518.2 | 4.5527 | 0.4492 | 100.0% | 1589.4321 | 1589.7399 | 1 | 9.492 | 64.3% | 1 | R.LAETQEEISAEVAAK.A | 2 |

---

|  |  |  |  |  |  |  |  |  |
| --- | --- | --- | --- | --- | --- | --- | --- | --- |
| U | *gi|31542947|ref|NP\_00* | 2 | 2 | 6.5% | 573 | 61055 | 5.9 | chaperonin [Homo sapiens] |
| U | *gi|41399285|ref|NP\_95* | 2 | 2 | 6.5% | 573 | 61055 | 5.9 | chaperonin [Homo sapiens] |

| Filename XCorr DeltCN Conf% ObsM+H+ CalcM+H+ SpR ZScore Ion% # Sequence  | | | | | | | | | | | | |
| --- | --- | --- | --- | --- | --- | --- | --- | --- | --- | --- | --- | --- |
|  | AstrinSTLCLD\_041714\_02.05710.05710.3 | 3.8806 | 0.3838 | 100.0% | 2561.6042 | 2561.7222 | 1 | 6.405 | 30.2% | 1 | K.LVQDVANNTNEEAGDGTTTATVLAR.S | 3 |
|  | AstrinSTLCLD\_041714\_02.05705.05705.2 | 2.6736 | 0.2976 | 99.5% | 1215.8522 | 1216.377 | 1 | 6.313 | 63.6% | 1 | K.NAGVEGSLIVEK.I | 2 |

---

|  |  |  |  |  |  |  |  |  |
| --- | --- | --- | --- | --- | --- | --- | --- | --- |
| U | *gi|154355000|ref|NP\_0* | 3 | 3 | 6.3% | 711 | 73115 | 7.3 | KH-type splicing regulatory protein (FUSE binding protein 2) [Homo sapiens] |

| Filename XCorr DeltCN Conf% ObsM+H+ CalcM+H+ SpR ZScore Ion% # Sequence  | | | | | | | | | | | | |
| --- | --- | --- | --- | --- | --- | --- | --- | --- | --- | --- | --- | --- |
| \* | AstrinSTLCLD\_041714\_01.10654.10654.3 | 3.2919 | 0.3143 | 100.0% | 2225.0044 | 2225.583 | 1 | 5.04 | 32.9% | 1 | R.TSMTEEYRVPDGMVGLIIGR.G | 3 |
| \* | AstrinSTLCLD\_041714\_01.05928.05928.2 | 2.7954 | 0.1522 | 96.7% | 1079.4122 | 1080.2725 | 1 | 4.918 | 90.0% | 1 | R.IGGGIDVPVPR.H | 2 |
| \* | AstrinSTLCLD\_041714\_02.06212.06212.2 | 3.9019 | 0.3632 | 100.0% | 1534.2322 | 1534.7123 | 1 | 7.502 | 65.4% | 1 | K.AINQQTGAFVEISR.Q | 2 |

---

|  |  |  |  |  |  |  |  |  |
| --- | --- | --- | --- | --- | --- | --- | --- | --- |
| U | *gi|4826734|ref|NP\_004* | 2 | 3 | 6.3% | 526 | 53426 | 9.4 | fusion (involved in t(12;16) in malignant liposarcoma) [Homo sapiens] |

| Filename XCorr DeltCN Conf% ObsM+H+ CalcM+H+ SpR ZScore Ion% # Sequence  | | | | | | | | | | | | |
| --- | --- | --- | --- | --- | --- | --- | --- | --- | --- | --- | --- | --- |
| \* | AstrinSTLCLD\_041714\_02.05288.05288.3 | 4.1975 | 0.4374 | 100.0% | 1662.7444 | 1662.837 | 1 | 6.635 | 51.7% | 2 | K.LKGEATVSFDDPPSAK.A | 3 |
| \* | AstrinSTLCLD\_041714\_01.09874.09874.2 | 4.2857 | 0.4788 | 100.0% | 1895.4922 | 1896.1094 | 1 | 7.75 | 62.5% | 1 | K.AAIDWFDGKEFSGNPIK.V | 2 |

---

|  |  |  |  |  |  |  |  |  |
| --- | --- | --- | --- | --- | --- | --- | --- | --- |
| U | *Reverse\_gi|124249394|* | 1 | 1 | 6.1% | 244 | 27507 | 6.4 | glutathione S-transferase theta 2B [Homo sapiens] |
| U | *Reverse\_gi|4504187|re* | 1 | 1 | 6.1% | 244 | 27507 | 6.4 | glutathione S-transferase theta 2 [Homo sapiens] |

| Filename XCorr DeltCN Conf% ObsM+H+ CalcM+H+ SpR ZScore Ion% # Sequence  | | | | | | | | | | | | |
| --- | --- | --- | --- | --- | --- | --- | --- | --- | --- | --- | --- | --- |
|  | AstrinSTLCLD\_041714\_02.07514.07514.3 | 2.9435 | 0.2405 | 97.9% | 1782.2344 | 1782.0471 | 22 | 4.494 | 41.1% | 1 | K.LT#PLKGLSNIQLFEK.S | 3 |

---

|  |  |  |  |  |  |  |  |  |
| --- | --- | --- | --- | --- | --- | --- | --- | --- |
| U | *Reverse\_gi|22748649|r* | 1 | 1 | 6.0% | 480 | 55240 | 9.0 | acid phosphatase-like 2 [Homo sapiens] |
| U | *Reverse\_gi|81295410|r* | 1 | 1 | 6.0% | 480 | 55240 | 9.0 | acid phosphatase-like 2 [Homo sapiens] |

| Filename XCorr DeltCN Conf% ObsM+H+ CalcM+H+ SpR ZScore Ion% # Sequence  | | | | | | | | | | | | |
| --- | --- | --- | --- | --- | --- | --- | --- | --- | --- | --- | --- | --- |
|  | AstrinSTLCLD\_041714\_02.10245.10245.3 | 3.045 | 0.2263 | 95.3% | 3368.8743 | 3368.8162 | 263 | 4.346 | 20.5% | 1 | R.GETARQMRGITQNLIPHAGLLS\*YGFYLK@K@.E | 3 |

---

|  |  |  |  |  |  |  |  |  |
| --- | --- | --- | --- | --- | --- | --- | --- | --- |
| U | *gi|17158044|ref|NP\_00* | 1 | 1 | 6.0% | 249 | 28681 | 10.8 | ribosomal protein S6 [Homo sapiens] |

| Filename XCorr DeltCN Conf% ObsM+H+ CalcM+H+ SpR ZScore Ion% # Sequence  | | | | | | | | | | | | |
| --- | --- | --- | --- | --- | --- | --- | --- | --- | --- | --- | --- | --- |
| \* | AstrinSTLCLD\_041714\_02.07538.07538.2 | 3.2265 | 0.4469 | 100.0% | 1622.2722 | 1621.8022 | 1 | 6.438 | 67.9% | 1 | R.MATEVAADALGEEWK.G | 2 |

---

|  |  |  |  |  |  |  |  |  |
| --- | --- | --- | --- | --- | --- | --- | --- | --- |
| U | *gi|205277463|ref|NP\_0* | 2 | 3 | 5.8% | 623 | 67878 | 7.7 | transketolase isoform 1 [Homo sapiens] |
| U | *gi|4507521|ref|NP\_001* | 2 | 3 | 5.8% | 623 | 67878 | 7.7 | transketolase isoform 1 [Homo sapiens] |
| U | *gi|205277465|ref|NP\_0* | 2 | 3 | 6.7% | 540 | 58982 | 7.7 | transketolase isoform 2 [Homo sapiens] |

| Filename XCorr DeltCN Conf% ObsM+H+ CalcM+H+ SpR ZScore Ion% # Sequence  | | | | | | | | | | | | |
| --- | --- | --- | --- | --- | --- | --- | --- | --- | --- | --- | --- | --- |
|  | AstrinSTLCLD\_041714\_02.06051.06051.3 | 4.5323 | 0.383 | 100.0% | 2509.3442 | 2509.6946 | 1 | 6.04 | 40.5% | 2 | R.TSRPENAIIYNNNEDFQVGQAK.V | 3 |
|  | AstrinSTLCLD\_041714\_01.08291.08291.2 | 2.6107 | 0.2352 | 97.3% | 1563.4321 | 1563.815 | 1 | 4.693 | 61.5% | 1 | K.MFGIDRDAIAQAVR.G | 2 |

---

|  |  |  |  |  |  |  |  |  |
| --- | --- | --- | --- | --- | --- | --- | --- | --- |
| U | *gi|11136628|ref|NP\_06* | 1 | 1 | 5.8% | 225 | 24764 | 4.7 | eukaryotic translation elongation factor 1 beta 2 [Homo sapiens] |
| U | *gi|83376130|ref|NP\_00* | 1 | 1 | 5.8% | 225 | 24764 | 4.7 | eukaryotic translation elongation factor 1 beta 2 [Homo sapiens] |
| U | *gi|4503477|ref|NP\_001* | 1 | 1 | 5.8% | 225 | 24764 | 4.7 | eukaryotic translation elongation factor 1 beta 2 [Homo sapiens] |

| Filename XCorr DeltCN Conf% ObsM+H+ CalcM+H+ SpR ZScore Ion% # Sequence  | | | | | | | | | | | | |
| --- | --- | --- | --- | --- | --- | --- | --- | --- | --- | --- | --- | --- |
|  | AstrinSTLCLD\_041714\_02.06935.06935.2 | 3.379 | 0.4512 | 100.0% | 1348.2922 | 1348.4985 | 1 | 7.428 | 62.5% | 1 | R.SIQADGLVWGSSK.L | 2 |

---

|  |  |  |  |  |  |  |  |  |
| --- | --- | --- | --- | --- | --- | --- | --- | --- |
| U | *gi|14591909|ref|NP\_00* | 1 | 1 | 5.7% | 297 | 34363 | 9.7 | ribosomal protein L5 [Homo sapiens] |

| Filename XCorr DeltCN Conf% ObsM+H+ CalcM+H+ SpR ZScore Ion% # Sequence  | | | | | | | | | | | | |
| --- | --- | --- | --- | --- | --- | --- | --- | --- | --- | --- | --- | --- |
| \* | AstrinSTLCLD\_041714\_01.05266.05266.3 | 3.7191 | 0.2623 | 100.0% | 2012.7843 | 2013.1301 | 1 | 5.65 | 37.5% | 1 | R.FPGYDSESKEFNAEVHR.K | 3 |

---

|  |  |  |  |  |  |  |  |  |
| --- | --- | --- | --- | --- | --- | --- | --- | --- |
| U | *gi|117956403|ref|NP\_0* | 2 | 2 | 5.6% | 569 | 63543 | 4.8 | rabaptin, RAB GTPase binding effector protein 2 [Homo sapiens] |

| Filename XCorr DeltCN Conf% ObsM+H+ CalcM+H+ SpR ZScore Ion% # Sequence  | | | | | | | | | | | | |
| --- | --- | --- | --- | --- | --- | --- | --- | --- | --- | --- | --- | --- |
| \* | AstrinSTLCLD\_041714\_02.05250.05250.3 | 2.7068 | 0.359 | 100.0% | 1917.9844 | 1917.1265 | 1 | 5.404 | 36.1% | 1 | K.AVAEVSESTKAEAVAAVQR.Q | 3 |
|  | AstrinSTLCLD\_041714\_02.04890.04890.2 | 4.5191 | 0.245 | 100.0% | 1530.4521 | 1531.6628 | 1 | 8.595 | 70.8% | 1 | R.LQAELETSEQVQR.D | 2 |

---

|  |  |  |  |  |  |  |  |  |
| --- | --- | --- | --- | --- | --- | --- | --- | --- |
| U | *gi|169204156|ref|XP\_0* | 1 | 1 | 5.6% | 516 | 54130 | 11.5 | PREDICTED: hypothetical protein [Homo sapiens] |
| U | *gi|169205279|ref|XP\_0* | 1 | 1 | 6.1% | 479 | 49902 | 11.7 | PREDICTED: hypothetical protein [Homo sapiens] |
| U | *gi|169204716|ref|XP\_0* | 1 | 1 | 6.1% | 479 | 49892 | 11.7 | PREDICTED: hypothetical protein [Homo sapiens] |

| Filename XCorr DeltCN Conf% ObsM+H+ CalcM+H+ SpR ZScore Ion% # Sequence  | | | | | | | | | | | | |
| --- | --- | --- | --- | --- | --- | --- | --- | --- | --- | --- | --- | --- |
|  | AstrinSTLCLD\_041714\_02.10303.10303.3 | 3.4769 | 0.2857 | 99.3% | 3303.8643 | 3302.934 | 122 | 4.377 | 20.5% | 1 | R.T#S\*ASRPGPS\*PHLEAT#GT#HAGS\*GTCQGSRG.- | 3 |

---

|  |  |  |  |  |  |  |  |  |
| --- | --- | --- | --- | --- | --- | --- | --- | --- |
| U | *gi|4759098|ref|NP\_004* | 1 | 3 | 5.6% | 288 | 33666 | 11.2 | splicing factor, arginine/serine-rich 10 [Homo sapiens] |

| Filename XCorr DeltCN Conf% ObsM+H+ CalcM+H+ SpR ZScore Ion% # Sequence  | | | | | | | | | | | | |
| --- | --- | --- | --- | --- | --- | --- | --- | --- | --- | --- | --- | --- |
| \* | AstrinSTLCLD\_041714\_02.07533.07533.2 | 4.4654 | 0.3903 | 100.0% | 1812.1122 | 1811.989 | 1 | 7.347 | 60.0% | 3 | K.YGPIADVSIVYDQQSR.R | 2 |

---

|  |  |  |  |  |  |  |  |  |
| --- | --- | --- | --- | --- | --- | --- | --- | --- |
| U | *gi|78000181|ref|NP\_00* | 1 | 1 | 5.6% | 215 | 23432 | 10.9 | ribosomal protein L14 [Homo sapiens] |
| U | *gi|78000183|ref|NP\_00* | 1 | 1 | 5.6% | 215 | 23432 | 10.9 | ribosomal protein L14 [Homo sapiens] |

| Filename XCorr DeltCN Conf% ObsM+H+ CalcM+H+ SpR ZScore Ion% # Sequence  | | | | | | | | | | | | |
| --- | --- | --- | --- | --- | --- | --- | --- | --- | --- | --- | --- | --- |
|  | AstrinSTLCLD\_041714\_01.09611.09611.2 | 2.4518 | 0.3673 | 99.9% | 1355.1122 | 1355.5773 | 4 | 6.341 | 59.1% | 1 | K.LVAIVDVIDQNR.A | 2 |

---

|  |  |  |  |  |  |  |  |  |
| --- | --- | --- | --- | --- | --- | --- | --- | --- |
| U | *Reverse\_gi|16945972|r* | 1 | 1 | 5.5% | 382 | 43088 | 8.1 | kelch domain containing 3 [Homo sapiens] |

| Filename XCorr DeltCN Conf% ObsM+H+ CalcM+H+ SpR ZScore Ion% # Sequence  | | | | | | | | | | | | |
| --- | --- | --- | --- | --- | --- | --- | --- | --- | --- | --- | --- | --- |
| \* | AstrinSTLCLD\_041714\_02.05902.05902.3 | 3.0059 | 0.2838 | 98.9% | 2615.7244 | 2614.8127 | 21 | 4.474 | 26.2% | 1 | R.WRAPS\*GKT#CILTWTMTSTDLK.H | 3 |

---

|  |  |  |  |  |  |  |  |  |
| --- | --- | --- | --- | --- | --- | --- | --- | --- |
| U | *gi|38201714|ref|NP\_00* | 1 | 1 | 5.5% | 326 | 36092 | 9.2 | ELAV-like 1 [Homo sapiens] |

| Filename XCorr DeltCN Conf% ObsM+H+ CalcM+H+ SpR ZScore Ion% # Sequence  | | | | | | | | | | | | |
| --- | --- | --- | --- | --- | --- | --- | --- | --- | --- | --- | --- | --- |
| \* | AstrinSTLCLD\_041714\_01.10323.10323.2 | 2.6623 | 0.2525 | 97.8% | 2163.5723 | 2163.4534 | 153 | 4.452 | 32.4% | 1 | R.TNLIVNYLPQNMTQDELR.S | 2 |

---

|  |  |  |  |  |  |  |  |  |
| --- | --- | --- | --- | --- | --- | --- | --- | --- |
| U | *contaminant\_KERATIN22* | 3 | 4 | 5.3% | 645 | 65865 | 8.0 | no description |
| U | *gi|47132620|ref|NP\_00* | 3 | 4 | 5.3% | 639 | 65433 | 8.0 | keratin 2 [Homo sapiens] |

| Filename XCorr DeltCN Conf% ObsM+H+ CalcM+H+ SpR ZScore Ion% # Sequence  | | | | | | | | | | | | |
| --- | --- | --- | --- | --- | --- | --- | --- | --- | --- | --- | --- | --- |
|  | AstrinSTLCLD\_041714\_01.11080.11080.2 | 3.2621 | 0.3077 | 100.0% | 1461.7722 | 1461.6982 | 1 | 6.293 | 68.2% | 1 | K.VDLLNQEIEFLK.V | 2 |
|  | AstrinSTLCLD\_041714\_01.11253.11253.2 | 2.6041 | 0.2862 | 99.1% | 1330.4922 | 1330.5211 | 1 | 6.131 | 72.7% | 1 | R.NLDLDSIIAEVK.A | 2222 |
|  | AstrinSTLCLD\_041714\_02.05811.05811.2 | 2.9277 | 0.3584 | 100.0% | 1194.1322 | 1194.33 | 1 | 7.088 | 72.2% | 2 | K.YEELQVTVGR.H | 2 |

Similarities:
contaminant\_KERATIN17(1:2)  
gi|119703753|ref|NP\_0(1:2)  
gi|153791158|ref|NP\_0(1:2)  

---

|  |  |  |  |  |  |  |  |  |
| --- | --- | --- | --- | --- | --- | --- | --- | --- |
| U | *gi|221316723|ref|NP\_0* | 3 | 3 | 5.2% | 1025 | 115704 | 8.3 | N-acetyltransferase 10 isoform a [Homo sapiens] |
| U | *gi|221316741|ref|NP\_0* | 3 | 3 | 5.6% | 953 | 107271 | 7.0 | N-acetyltransferase 10 isoform b [Homo sapiens] |

| Filename XCorr DeltCN Conf% ObsM+H+ CalcM+H+ SpR ZScore Ion% # Sequence  | | | | | | | | | | | | |
| --- | --- | --- | --- | --- | --- | --- | --- | --- | --- | --- | --- | --- |
|  | AstrinSTLCLD\_041714\_01.05176.05176.2 | 2.1492 | 0.339 | 98.1% | 1412.2322 | 1412.5858 | 55 | 5.073 | 54.5% | 1 | R.TLHEVSLQESIR.Y | 2 |
|  | AstrinSTLCLD\_041714\_01.09387.09387.2 | 2.9984 | 0.3593 | 100.0% | 1454.4122 | 1454.6659 | 8 | 6.858 | 58.3% | 1 | R.LDYLGVSYGLTPR.L | 2 |
|  | AstrinSTLCLD\_041714\_01.17295.17295.3 | 5.1232 | 0.4487 | 100.0% | 2968.7344 | 2969.4973 | 1 | 8.202 | 33.3% | 1 | R.IYFLNQLGDLALSAAQSALLLGIGLQHK.S | 3 |

---

|  |  |  |  |  |  |  |  |  |
| --- | --- | --- | --- | --- | --- | --- | --- | --- |
| U | *gi|5803036|ref|NP\_006* | 1 | 3 | 5.2% | 305 | 30841 | 9.3 | heterogeneous nuclear ribonucleoprotein A0 [Homo sapiens] |

| Filename XCorr DeltCN Conf% ObsM+H+ CalcM+H+ SpR ZScore Ion% # Sequence  | | | | | | | | | | | | |
| --- | --- | --- | --- | --- | --- | --- | --- | --- | --- | --- | --- | --- |
| \* | AstrinSTLCLD\_041714\_02.08051.08051.2 | 5.1034 | 0.5475 | 100.0% | 1691.5521 | 1691.9248 | 1 | 9.488 | 80.0% | 3 | K.LFIGGLNVQTSESGLR.G | 2 |

---

|  |  |  |  |  |  |  |  |  |
| --- | --- | --- | --- | --- | --- | --- | --- | --- |
| U | *gi|6005926|ref|NP\_009* | 1 | 1 | 5.1% | 475 | 53501 | 9.1 | U2 (RNU2) small nuclear RNA auxiliary factor 2 isoform a [Homo sapiens] |
| U | *gi|60279268|ref|NP\_00* | 1 | 1 | 5.1% | 471 | 53121 | 9.1 | U2 (RNU2) small nuclear RNA auxiliary factor 2 isoform b [Homo sapiens] |

| Filename XCorr DeltCN Conf% ObsM+H+ CalcM+H+ SpR ZScore Ion% # Sequence  | | | | | | | | | | | | |
| --- | --- | --- | --- | --- | --- | --- | --- | --- | --- | --- | --- | --- |
|  | AstrinSTLCLD\_041714\_01.15754.15754.2 | 2.7303 | 0.3075 | 99.2% | 2795.2322 | 2796.246 | 1 | 5.442 | 34.8% | 1 | R.LYVGNIPFGITEEAMMDFFNAQMR.L | 2 |

---

|  |  |  |  |  |  |  |  |  |
| --- | --- | --- | --- | --- | --- | --- | --- | --- |
| U | *gi|222418639|ref|NP\_0* | 1 | 1 | 5.0% | 319 | 35277 | 8.8 | melanoma antigen family B, 2 [Homo sapiens] |

| Filename XCorr DeltCN Conf% ObsM+H+ CalcM+H+ SpR ZScore Ion% # Sequence  | | | | | | | | | | | | |
| --- | --- | --- | --- | --- | --- | --- | --- | --- | --- | --- | --- | --- |
| \* | AstrinSTLCLD\_041714\_01.07520.07520.3 | 2.8347 | 0.3673 | 100.0% | 1873.2544 | 1876.2322 | 263 | 5.105 | 30.0% | 1 | K.IK@KSVT#K@GEMLK@IVGK.R | 3 |

---

|  |  |  |  |  |  |  |  |  |
| --- | --- | --- | --- | --- | --- | --- | --- | --- |
| U | *gi|9558733|ref|NP\_037* | 1 | 2 | 5.0% | 282 | 32689 | 11.3 | transformer-2 alpha [Homo sapiens] |

| Filename XCorr DeltCN Conf% ObsM+H+ CalcM+H+ SpR ZScore Ion% # Sequence  | | | | | | | | | | | | |
| --- | --- | --- | --- | --- | --- | --- | --- | --- | --- | --- | --- | --- |
| \* | AstrinSTLCLD\_041714\_01.07900.07900.2 | 3.4906 | 0.3888 | 100.0% | 1567.1122 | 1567.7416 | 1 | 6.793 | 65.4% | 2 | R.YGPLSGVNVVYDQR.T | 2 |

---

|  |  |  |  |  |  |  |  |  |
| --- | --- | --- | --- | --- | --- | --- | --- | --- |
| U | *gi|156523260|ref|NP\_6* | 1 | 2 | 4.9% | 412 | 46373 | 5.1 | hypothetical protein LOC221150 [Homo sapiens] |

| Filename XCorr DeltCN Conf% ObsM+H+ CalcM+H+ SpR ZScore Ion% # Sequence  | | | | | | | | | | | | |
| --- | --- | --- | --- | --- | --- | --- | --- | --- | --- | --- | --- | --- |
| \* | AstrinSTLCLD\_041714\_02.05858.05858.3 | 2.9835 | 0.3156 | 99.2% | 2350.4343 | 2348.2942 | 6 | 4.769 | 27.6% | 2 | K.TDVK@DDLS\*DPPVASS\*CISEK@.S | 3 |

---

|  |  |  |  |  |  |  |  |  |
| --- | --- | --- | --- | --- | --- | --- | --- | --- |
| U | *gi|31542711|ref|NP\_06* | 1 | 1 | 4.9% | 371 | 40214 | 6.9 | hypothetical protein LOC54978 [Homo sapiens] |

| Filename XCorr DeltCN Conf% ObsM+H+ CalcM+H+ SpR ZScore Ion% # Sequence  | | | | | | | | | | | | |
| --- | --- | --- | --- | --- | --- | --- | --- | --- | --- | --- | --- | --- |
| \* | AstrinSTLCLD\_041714\_01.10107.10107.2 | 3.2002 | 0.1522 | 97.5% | 1924.3522 | 1925.3274 | 7 | 4.236 | 41.2% | 1 | R.GAVIIFTGLFSVAFLGRR.L | 2 |

---

|  |  |  |  |  |  |  |  |  |
| --- | --- | --- | --- | --- | --- | --- | --- | --- |
| U | *gi|20070302|ref|NP\_06* | 1 | 1 | 4.9% | 326 | 37028 | 8.1 | docking protein 4 [Homo sapiens] |

| Filename XCorr DeltCN Conf% ObsM+H+ CalcM+H+ SpR ZScore Ion% # Sequence  | | | | | | | | | | | | |
| --- | --- | --- | --- | --- | --- | --- | --- | --- | --- | --- | --- | --- |
| \* | AstrinSTLCLD\_041714\_01.06318.06318.3 | 3.2429 | 0.2607 | 99.3% | 2081.1843 | 2083.3562 | 1 | 4.419 | 40.0% | 1 | R.VK@LVSWPLCS\*LRRYGR.D | 3 |

---

|  |  |  |  |  |  |  |  |  |
| --- | --- | --- | --- | --- | --- | --- | --- | --- |
| U | *gi|4506725|ref|NP\_000* | 1 | 1 | 4.9% | 263 | 29598 | 10.2 | ribosomal protein S4, X-linked X isoform [Homo sapiens] |

| Filename XCorr DeltCN Conf% ObsM+H+ CalcM+H+ SpR ZScore Ion% # Sequence  | | | | | | | | | | | | |
| --- | --- | --- | --- | --- | --- | --- | --- | --- | --- | --- | --- | --- |
| \* | AstrinSTLCLD\_041714\_02.06650.06650.2 | 3.1217 | 0.3985 | 100.0% | 1445.7322 | 1446.5975 | 1 | 6.576 | 66.7% | 1 | K.VNDTIQIDLETGK.I | 2 |

---

|  |  |  |  |  |  |  |  |  |
| --- | --- | --- | --- | --- | --- | --- | --- | --- |
| U | *gi|109240550|ref|NP\_0* | 2 | 4 | 4.8% | 523 | 58744 | 6.7 | paraspeckle protein 1 [Homo sapiens] |

| Filename XCorr DeltCN Conf% ObsM+H+ CalcM+H+ SpR ZScore Ion% # Sequence  | | | | | | | | | | | | |
| --- | --- | --- | --- | --- | --- | --- | --- | --- | --- | --- | --- | --- |
| \* | AstrinSTLCLD\_041714\_01.05717.05717.2 | 3.7084 | 0.5102 | 100.0% | 1310.9722 | 1311.4368 | 1 | 8.377 | 70.0% | 3 | R.YGEPSEVFINR.D | 2 |
| \* | AstrinSTLCLD\_041714\_01.08410.08410.2 | 2.9658 | 0.3638 | 100.0% | 1650.4922 | 1650.7875 | 1 | 6.081 | 65.4% | 1 | R.FAQPGTFEFEYASR.W | 2 |

---

|  |  |  |  |  |  |  |  |  |
| --- | --- | --- | --- | --- | --- | --- | --- | --- |
| U | *gi|4758256|ref|NP\_004* | 1 | 1 | 4.8% | 315 | 36112 | 5.1 | eukaryotic translation initiation factor 2, subunit 1 alpha, 35kDa [Homo sapiens] |

| Filename XCorr DeltCN Conf% ObsM+H+ CalcM+H+ SpR ZScore Ion% # Sequence  | | | | | | | | | | | | |
| --- | --- | --- | --- | --- | --- | --- | --- | --- | --- | --- | --- | --- |
| \* | AstrinSTLCLD\_041714\_02.09937.09937.2 | 2.3069 | 0.2893 | 96.6% | 1548.6522 | 1547.8505 | 360 | 4.807 | 35.7% | 1 | R.TEGLSVLSQAMAVIK.E | 2 |

---

|  |  |  |  |  |  |  |  |  |
| --- | --- | --- | --- | --- | --- | --- | --- | --- |
| U | *Reverse\_gi|22219477|r* | 1 | 1 | 4.7% | 512 | 57468 | 6.4 | tigger transposable element derived 4 [Homo sapiens] |

| Filename XCorr DeltCN Conf% ObsM+H+ CalcM+H+ SpR ZScore Ion% # Sequence  | | | | | | | | | | | | |
| --- | --- | --- | --- | --- | --- | --- | --- | --- | --- | --- | --- | --- |
| \* | AstrinSTLCLD\_041714\_02.10609.10609.3 | 2.9502 | 0.3363 | 99.5% | 2857.2244 | 2859.1484 | 38 | 5.113 | 25.0% | 1 | R.K@K@GIVLLPLK@ES\*GDMNT#GVVLT#IR.D | 3 |

---

|  |  |  |  |  |  |  |  |  |
| --- | --- | --- | --- | --- | --- | --- | --- | --- |
| U | *gi|14602427|ref|NP\_12* | 1 | 1 | 4.7% | 277 | 31293 | 5.2 | ZW10 interactor isoform a [Homo sapiens] |
| U | *gi|14602429|ref|NP\_00* | 1 | 1 | 4.7% | 277 | 31293 | 5.2 | ZW10 interactor isoform a [Homo sapiens] |

| Filename XCorr DeltCN Conf% ObsM+H+ CalcM+H+ SpR ZScore Ion% # Sequence  | | | | | | | | | | | | |
| --- | --- | --- | --- | --- | --- | --- | --- | --- | --- | --- | --- | --- |
|  | AstrinSTLCLD\_041714\_01.04398.04398.3 | 3.3843 | 0.2722 | 100.0% | 1490.0643 | 1489.6743 | 1 | 5.39 | 39.6% | 1 | K.HLQHLAEVSAEVR.E | 3 |

---

|  |  |  |  |  |  |  |  |  |
| --- | --- | --- | --- | --- | --- | --- | --- | --- |
| U | *gi|14110414|ref|NP\_00* | 1 | 3 | 4.6% | 306 | 32835 | 8.2 | heterogeneous nuclear ribonucleoprotein D isoform c [Homo sapiens] |
| U | *gi|51477708|ref|NP\_00* | 1 | 3 | 4.9% | 287 | 30672 | 8.4 | heterogeneous nuclear ribonucleoprotein D isoform d [Homo sapiens] |
| U | *gi|14110420|ref|NP\_11* | 1 | 3 | 3.9% | 355 | 38434 | 7.8 | heterogeneous nuclear ribonucleoprotein D isoform a [Homo sapiens] |
| U | *gi|14110417|ref|NP\_11* | 1 | 3 | 4.2% | 336 | 36272 | 8.1 | heterogeneous nuclear ribonucleoprotein D isoform b [Homo sapiens] |

| Filename XCorr DeltCN Conf% ObsM+H+ CalcM+H+ SpR ZScore Ion% # Sequence  | | | | | | | | | | | | |
| --- | --- | --- | --- | --- | --- | --- | --- | --- | --- | --- | --- | --- |
|  | AstrinSTLCLD\_041714\_01.06441.06441.2 | 4.2134 | 0.3181 | 100.0% | 1490.4321 | 1489.6653 | 1 | 6.717 | 69.2% | 3 | K.IFVGGLSPDTPEEK.I | 2 |

---

|  |  |  |  |  |  |  |  |  |
| --- | --- | --- | --- | --- | --- | --- | --- | --- |
| U | *gi|156151392|ref|NP\_0* | 2 | 3 | 4.5% | 532 | 59682 | 9.2 | heterogeneous nuclear ribonucleoprotein R isoform 4 [Homo sapiens] |
| U | *gi|5031755|ref|NP\_005* | 2 | 3 | 3.8% | 633 | 70943 | 8.1 | heterogeneous nuclear ribonucleoprotein R isoform 2 [Homo sapiens] |
| U | *gi|156151396|ref|NP\_0* | 2 | 3 | 4.5% | 535 | 59953 | 9.2 | heterogeneous nuclear ribonucleoprotein R isoform 3 [Homo sapiens] |
| U | *gi|156151394|ref|NP\_0* | 2 | 3 | 3.8% | 636 | 71214 | 8.1 | heterogeneous nuclear ribonucleoprotein R isoform 1 [Homo sapiens] |

| Filename XCorr DeltCN Conf% ObsM+H+ CalcM+H+ SpR ZScore Ion% # Sequence  | | | | | | | | | | | | |
| --- | --- | --- | --- | --- | --- | --- | --- | --- | --- | --- | --- | --- |
|  | AstrinSTLCLD\_041714\_01.06564.06564.2 | 2.7706 | 0.2588 | 99.2% | 1262.1721 | 1262.4846 | 1 | 6.401 | 70.0% | 2 | R.LMMDPLSGQNR.G | 2 |
|  | AstrinSTLCLD\_041714\_01.11013.11013.2 | 3.3944 | 0.4343 | 100.0% | 1461.2722 | 1461.6525 | 1 | 6.843 | 70.8% | 1 | R.NLATTVTEEILEK.S | 2 |

---

|  |  |  |  |  |  |  |  |  |
| --- | --- | --- | --- | --- | --- | --- | --- | --- |
| U | *gi|114796640|ref|NP\_0* | 1 | 2 | 4.5% | 421 | 44969 | 7.5 | regulator of chromosome condensation 1 [Homo sapiens] |
| U | *gi|4502801|ref|NP\_001* | 1 | 2 | 4.5% | 421 | 44969 | 7.5 | regulator of chromosome condensation 1 isoform c [Homo sapiens] |
| U | *gi|114796648|ref|NP\_0* | 1 | 2 | 4.5% | 421 | 44969 | 7.5 | regulator of chromosome condensation 1 [Homo sapiens] |
| U | *gi|114796646|ref|NP\_0* | 1 | 2 | 4.3% | 438 | 46753 | 8.1 | regulator of chromosome condensation 1 isoform b [Homo sapiens] |
| U | *gi|114796644|ref|NP\_0* | 1 | 2 | 4.2% | 452 | 48146 | 8.2 | regulator of chromosome condensation 1 isoform a [Homo sapiens] |
| U | *gi|114796642|ref|NP\_0* | 1 | 2 | 4.5% | 421 | 44969 | 7.5 | regulator of chromosome condensation 1 [Homo sapiens] |

| Filename XCorr DeltCN Conf% ObsM+H+ CalcM+H+ SpR ZScore Ion% # Sequence  | | | | | | | | | | | | |
| --- | --- | --- | --- | --- | --- | --- | --- | --- | --- | --- | --- | --- |
|  | AstrinSTLCLD\_041714\_02.04754.04754.3 | 3.8905 | 0.3833 | 100.0% | 1901.0343 | 1900.0122 | 1 | 6.761 | 36.1% | 2 | K.VVQVSAGDSHTAALTDDGR.V | 3 |

---

|  |  |  |  |  |  |  |  |  |
| --- | --- | --- | --- | --- | --- | --- | --- | --- |
| U | *contaminant\_KERATIN10* | 2 | 3 | 4.5% | 400 | 44106 | 5.1 | no description |
| U | *gi|24234699|ref|NP\_00* | 2 | 3 | 4.5% | 400 | 44106 | 5.1 | keratin 19 [Homo sapiens] |

| Filename XCorr DeltCN Conf% ObsM+H+ CalcM+H+ SpR ZScore Ion% # Sequence  | | | | | | | | | | | | |
| --- | --- | --- | --- | --- | --- | --- | --- | --- | --- | --- | --- | --- |
|  | AstrinSTLCLD\_041714\_01.05102.05102.2 | 2.0712 | 0.2926 | 97.3% | 1042.1522 | 1042.2235 | 1 | 5.281 | 75.0% | 1 | R.IVLQIDNAR.L | 22 |
|  | AstrinSTLCLD\_041714\_01.06680.06680.2 | 3.5857 | 0.3941 | 100.0% | 1029.6522 | 1030.2096 | 1 | 7.292 | 81.2% | 2 | R.VLDELTLAR.T | 22 |

Similarities:
contaminant\_KERATIN09(1:1)  
contaminant\_KERATIN12(1:1)  

---

|  |  |  |  |  |  |  |  |  |
| --- | --- | --- | --- | --- | --- | --- | --- | --- |
| U | *Reverse\_gi|13786127|r* | 1 | 1 | 4.5% | 356 | 37980 | 5.2 | Cdc42 effector protein 4 [Homo sapiens] |

| Filename XCorr DeltCN Conf% ObsM+H+ CalcM+H+ SpR ZScore Ion% # Sequence  | | | | | | | | | | | | |
| --- | --- | --- | --- | --- | --- | --- | --- | --- | --- | --- | --- | --- |
| \* | AstrinSTLCLD\_041714\_01.09714.09714.3 | 2.5936 | 0.2595 | 97.1% | 1814.4844 | 1816.9524 | 60 | 4.627 | 30.0% | 1 | K.KVPSSSLS\*KPLKST#GK@.E | 3 |

---

|  |  |  |  |  |  |  |  |  |
| --- | --- | --- | --- | --- | --- | --- | --- | --- |
| U | *gi|4506003|ref|NP\_002* | 1 | 1 | 4.5% | 330 | 37512 | 6.3 | protein phosphatase 1, catalytic subunit, alpha isoform 1 [Homo sapiens] |
| U | *gi|56790945|ref|NP\_00* | 1 | 1 | 4.4% | 341 | 38631 | 6.6 | protein phosphatase 1, catalytic subunit, alpha isoform 3 [Homo sapiens] |
| U | *gi|46249376|ref|NP\_99* | 1 | 1 | 4.6% | 327 | 37187 | 6.2 | protein phosphatase 1, catalytic subunit, beta isoform 1 [Homo sapiens] |
| U | *gi|45827798|ref|NP\_99* | 1 | 1 | 5.2% | 286 | 32595 | 6.1 | protein phosphatase 1, catalytic subunit, alpha isoform 2 [Homo sapiens] |
| U | *gi|4506007|ref|NP\_002* | 1 | 1 | 4.6% | 323 | 36984 | 6.5 | protein phosphatase 1, catalytic subunit, gamma isoform [Homo sapiens] |
| U | *gi|4506005|ref|NP\_002* | 1 | 1 | 4.6% | 327 | 37187 | 6.2 | protein phosphatase 1, catalytic subunit, beta isoform 1 [Homo sapiens] |

| Filename XCorr DeltCN Conf% ObsM+H+ CalcM+H+ SpR ZScore Ion% # Sequence  | | | | | | | | | | | | |
| --- | --- | --- | --- | --- | --- | --- | --- | --- | --- | --- | --- | --- |
|  | AstrinSTLCLD\_041714\_01.06818.06818.3 | 3.3135 | 0.4576 | 100.0% | 1797.5044 | 1796.9799 | 1 | 7.3 | 41.1% | 1 | R.AHQVVEDGYEFFAKR.Q | 3 |

---

|  |  |  |  |  |  |  |  |  |
| --- | --- | --- | --- | --- | --- | --- | --- | --- |
| U | *Reverse\_gi|21686995|r* | 1 | 1 | 4.5% | 291 | 32135 | 8.6 | stomatin-like 3 isoform 1 [Homo sapiens] |

| Filename XCorr DeltCN Conf% ObsM+H+ CalcM+H+ SpR ZScore Ion% # Sequence  | | | | | | | | | | | | |
| --- | --- | --- | --- | --- | --- | --- | --- | --- | --- | --- | --- | --- |
| \* | AstrinSTLCLD\_041714\_01.08991.08991.2 | 2.4761 | 0.27 | 97.8% | 1393.9122 | 1393.5144 | 238 | 4.675 | 45.8% | 1 | R.AERTAEAEAAMSR.Q | 2 |

---

|  |  |  |  |  |  |  |  |  |
| --- | --- | --- | --- | --- | --- | --- | --- | --- |
| U | *gi|7657307|ref|NP\_055* | 2 | 2 | 4.4% | 676 | 72190 | 6.7 | LIM domains containing 1 [Homo sapiens] |

| Filename XCorr DeltCN Conf% ObsM+H+ CalcM+H+ SpR ZScore Ion% # Sequence  | | | | | | | | | | | | |
| --- | --- | --- | --- | --- | --- | --- | --- | --- | --- | --- | --- | --- |
| \* | AstrinSTLCLD\_041714\_02.07059.07059.2 | 3.0299 | 0.3431 | 100.0% | 1460.4722 | 1460.6416 | 1 | 5.944 | 72.7% | 1 | K.FIEDLNMYEASK.D | 2 |
| \* | AstrinSTLCLD\_041714\_01.06606.06606.3 | 4.2011 | 0.2676 | 100.0% | 2230.6743 | 2231.5168 | 14 | 5.35 | 32.4% | 1 | K.IHLQQQQQQLLQEETLPR.G | 3 |

---

|  |  |  |  |  |  |  |  |  |
| --- | --- | --- | --- | --- | --- | --- | --- | --- |
| U | *gi|93277122|ref|NP\_00* | 1 | 1 | 4.4% | 364 | 40314 | 7.1 | RNA binding motif protein 4 [Homo sapiens] |

| Filename XCorr DeltCN Conf% ObsM+H+ CalcM+H+ SpR ZScore Ion% # Sequence  | | | | | | | | | | | | |
| --- | --- | --- | --- | --- | --- | --- | --- | --- | --- | --- | --- | --- |
| \* | AstrinSTLCLD\_041714\_01.05303.05303.2 | 5.393 | 0.3533 | 100.0% | 1856.2322 | 1856.9866 | 1 | 9.459 | 66.7% | 1 | R.VADLTEQYNEQYGAVR.T | 2 |

---

|  |  |  |  |  |  |  |  |  |
| --- | --- | --- | --- | --- | --- | --- | --- | --- |
| U | *gi|15431301|ref|NP\_00* | 1 | 2 | 4.4% | 248 | 29226 | 10.7 | ribosomal protein L7 [Homo sapiens] |
| U | *gi|88988289|ref|XP\_94* | 1 | 2 | 4.2% | 259 | 30508 | 10.8 | PREDICTED: hypothetical protein LOC648000 isoform 3 [Homo sapiens] |
| U | *gi|169171881|ref|XP\_0* | 1 | 2 | 4.5% | 247 | 29037 | 10.7 | PREDICTED: hypothetical protein [Homo sapiens] |
| U | *gi|169171450|ref|XP\_0* | 1 | 2 | 4.5% | 247 | 28971 | 10.6 | PREDICTED: hypothetical protein [Homo sapiens] |
| U | *gi|169171114|ref|XP\_0* | 1 | 2 | 4.5% | 247 | 29037 | 10.7 | PREDICTED: hypothetical protein [Homo sapiens] |
| U | *gi|169170622|ref|XP\_0* | 1 | 2 | 4.5% | 247 | 29009 | 10.7 | PREDICTED: hypothetical protein [Homo sapiens] |
| U | *gi|169168181|ref|XP\_0* | 1 | 2 | 4.2% | 259 | 30508 | 10.8 | PREDICTED: hypothetical protein LOC648000 [Homo sapiens] |
| U | *gi|169167651|ref|XP\_0* | 1 | 2 | 4.2% | 259 | 30508 | 10.8 | PREDICTED: hypothetical protein LOC648000 [Homo sapiens] |

| Filename XCorr DeltCN Conf% ObsM+H+ CalcM+H+ SpR ZScore Ion% # Sequence  | | | | | | | | | | | | |
| --- | --- | --- | --- | --- | --- | --- | --- | --- | --- | --- | --- | --- |
|  | AstrinSTLCLD\_041714\_02.06821.06821.2 | 3.2598 | 0.3809 | 100.0% | 1171.1122 | 1171.3823 | 1 | 7.986 | 90.0% | 2 | R.IALTDNALIAR.S | 2 |

---

|  |  |  |  |  |  |  |  |  |
| --- | --- | --- | --- | --- | --- | --- | --- | --- |
| U | *gi|9966881|ref|NP\_065* | 4 | 5 | 4.3% | 925 | 106374 | 5.4 | nucleoporin 107kDa [Homo sapiens] |

| Filename XCorr DeltCN Conf% ObsM+H+ CalcM+H+ SpR ZScore Ion% # Sequence  | | | | | | | | | | | | |
| --- | --- | --- | --- | --- | --- | --- | --- | --- | --- | --- | --- | --- |
| \* | AstrinSTLCLD\_041714\_01.07800.07800.2 | 2.7029 | 0.2884 | 99.5% | 1329.1921 | 1329.4093 | 1 | 6.61 | 68.2% | 2 | R.SGFGEISS\*PVIR.E | 2 |
| \* | AstrinSTLCLD\_041714\_02.05991.05991.2 | 5.3457 | 0.4991 | 100.0% | 1890.2722 | 1891.0476 | 1 | 9.964 | 71.9% | 1 | R.VLLQASQDENFGNTTPR.N | 2 |
| \* | AstrinSTLCLD\_041714\_02.06122.06122.2 | 4.4396 | 0.4513 | 100.0% | 1970.2722 | 1971.0476 | 1 | 8.106 | 62.5% | 1 | R.VLLQASQDENFGNTT#PR.N | 2 |
| \* | AstrinSTLCLD\_041714\_02.06606.06606.2 | 2.891 | 0.3782 | 100.0% | 1121.1322 | 1121.3219 | 1 | 7.807 | 85.0% | 1 | R.AIYAALSGNLK.Q | 2 |

---

|  |  |  |  |  |  |  |  |  |
| --- | --- | --- | --- | --- | --- | --- | --- | --- |
| U | *gi|14150165|ref|NP\_11* | 1 | 1 | 4.3% | 488 | 57486 | 8.7 | coiled-coil domain containing 77 isoform a [Homo sapiens] |
| U | *gi|194306660|ref|NP\_0* | 1 | 1 | 4.6% | 456 | 53979 | 8.0 | coiled-coil domain containing 77 isoform b [Homo sapiens] |
| U | *gi|194306658|ref|NP\_0* | 1 | 1 | 4.6% | 456 | 53979 | 8.0 | coiled-coil domain containing 77 isoform b [Homo sapiens] |
| U | *gi|194306535|ref|NP\_0* | 1 | 1 | 4.6% | 456 | 53979 | 8.0 | coiled-coil domain containing 77 isoform b [Homo sapiens] |

| Filename XCorr DeltCN Conf% ObsM+H+ CalcM+H+ SpR ZScore Ion% # Sequence  | | | | | | | | | | | | |
| --- | --- | --- | --- | --- | --- | --- | --- | --- | --- | --- | --- | --- |
|  | AstrinSTLCLD\_041714\_01.14097.14097.2 | 2.3729 | 0.2998 | 97.5% | 2340.7322 | 2340.6794 | 1 | 4.934 | 35.0% | 1 | R.DIQTLILQVEALQAQLGEQTK.L | 2 |

---

|  |  |  |  |  |  |  |  |  |
| --- | --- | --- | --- | --- | --- | --- | --- | --- |
| U | *gi|94721342|ref|NP\_00* | 1 | 1 | 4.3% | 441 | 50141 | 7.2 | zinc finger and BTB domain containing 8A [Homo sapiens] |

| Filename XCorr DeltCN Conf% ObsM+H+ CalcM+H+ SpR ZScore Ion% # Sequence  | | | | | | | | | | | | |
| --- | --- | --- | --- | --- | --- | --- | --- | --- | --- | --- | --- | --- |
| \* | AstrinSTLCLD\_041714\_01.09206.09206.3 | 3.7465 | 0.2408 | 99.3% | 2399.8743 | 2401.4814 | 5 | 4.61 | 31.9% | 1 | K.CKRHVT#DLTGQVVQEGT#RR.Y | 3 |

---

|  |  |  |  |  |  |  |  |  |
| --- | --- | --- | --- | --- | --- | --- | --- | --- |
| U | *gi|4507877|ref|NP\_003* | 3 | 4 | 4.2% | 1066 | 116722 | 6.1 | vinculin isoform VCL [Homo sapiens] |
| U | *gi|7669550|ref|NP\_054* | 3 | 4 | 4.0% | 1134 | 123799 | 5.7 | vinculin isoform meta-VCL [Homo sapiens] |

| Filename XCorr DeltCN Conf% ObsM+H+ CalcM+H+ SpR ZScore Ion% # Sequence  | | | | | | | | | | | | |
| --- | --- | --- | --- | --- | --- | --- | --- | --- | --- | --- | --- | --- |
|  | AstrinSTLCLD\_041714\_01.05365.05365.3 | 3.5798 | 0.3554 | 100.0% | 1983.1444 | 1984.1356 | 1 | 6.339 | 33.3% | 1 | K.GWLRDPSASPGDAGEQAIR.Q | 3 |
|  | AstrinSTLCLD\_041714\_01.06177.06177.2 | 3.0502 | 0.2814 | 99.7% | 1458.6921 | 1458.6549 | 1 | 7.055 | 61.5% | 1 | K.AQQVSQGLDVLTAK.V | 2 |
|  | AstrinSTLCLD\_041714\_01.06171.06171.2 | 3.4619 | 0.4442 | 100.0% | 1294.1921 | 1293.4749 | 1 | 7.034 | 68.2% | 2 | K.MTGLVDEAIDTK.S | 2 |

---

|  |  |  |  |  |  |  |  |  |
| --- | --- | --- | --- | --- | --- | --- | --- | --- |
| U | *gi|21264343|ref|NP\_00* | 2 | 3 | 4.2% | 915 | 102642 | 5.5 | scaffold attachment factor B [Homo sapiens] |

| Filename XCorr DeltCN Conf% ObsM+H+ CalcM+H+ SpR ZScore Ion% # Sequence  | | | | | | | | | | | | |
| --- | --- | --- | --- | --- | --- | --- | --- | --- | --- | --- | --- | --- |
|  | AstrinSTLCLD\_041714\_01.06319.06319.3 | 5.2639 | 0.3544 | 100.0% | 2849.2144 | 2849.9788 | 1 | 6.982 | 38.0% | 1 | K.SEPVKEESSELEQPFAQDTSSVGPDR.K | 3 |
| \* | AstrinSTLCLD\_041714\_01.08738.08738.2 | 3.0318 | 0.4148 | 100.0% | 1355.0721 | 1355.4929 | 1 | 6.082 | 81.8% | 2 | R.NFWVSGLSSTTR.A | 2 |

---

|  |  |  |  |  |  |  |  |  |
| --- | --- | --- | --- | --- | --- | --- | --- | --- |
| U | *gi|33469968|ref|NP\_00* | 2 | 3 | 4.2% | 719 | 81308 | 6.5 | minichromosome maintenance complex component 7 isoform 1 [Homo sapiens] |

| Filename XCorr DeltCN Conf% ObsM+H+ CalcM+H+ SpR ZScore Ion% # Sequence  | | | | | | | | | | | | |
| --- | --- | --- | --- | --- | --- | --- | --- | --- | --- | --- | --- | --- |
| \* | AstrinSTLCLD\_041714\_02.07038.07038.3 | 3.0437 | 0.2139 | 97.8% | 1830.4443 | 1829.063 | 31 | 4.936 | 35.7% | 1 | R.EVVNKDVLDVYIEHR.L | 3 |
|  | AstrinSTLCLD\_041714\_02.05820.05820.3 | 2.9641 | 0.287 | 99.3% | 1746.7743 | 1746.9733 | 1 | 5.222 | 42.9% | 2 | R.MVDVVEKEDVNEAIR.L | 3 |

---

|  |  |  |  |  |  |  |  |  |
| --- | --- | --- | --- | --- | --- | --- | --- | --- |
| U | *gi|20357512|ref|NP\_61* | 1 | 1 | 4.2% | 451 | 49762 | 5.9 | macrophage scavenger receptor 1 isoform type 1 [Homo sapiens] |
| U | *gi|4505259|ref|NP\_002* | 1 | 1 | 5.3% | 358 | 39584 | 5.6 | macrophage scavenger receptor 1 isoform type 2 [Homo sapiens] |
| U | *gi|20357515|ref|NP\_61* | 1 | 1 | 4.9% | 388 | 42942 | 5.3 | macrophage scavenger receptor 1 isoform type 3 [Homo sapiens] |

| Filename XCorr DeltCN Conf% ObsM+H+ CalcM+H+ SpR ZScore Ion% # Sequence  | | | | | | | | | | | | |
| --- | --- | --- | --- | --- | --- | --- | --- | --- | --- | --- | --- | --- |
|  | AstrinSTLCLD\_041714\_01.16407.16407.2 | 2.5524 | 0.2471 | 96.9% | 2213.112 | 2213.3813 | 66 | 4.538 | 33.3% | 1 | R.S\*MT#ALLPPNPKNSPSLQEK.L | 2 |

---

|  |  |  |  |  |  |  |  |  |
| --- | --- | --- | --- | --- | --- | --- | --- | --- |
| U | *Reverse\_gi|10835242|r* | 1 | 1 | 4.1% | 686 | 77804 | 5.4 | protein kinase, cGMP-dependent, type I isoform 2 [Homo sapiens] |

| Filename XCorr DeltCN Conf% ObsM+H+ CalcM+H+ SpR ZScore Ion% # Sequence  | | | | | | | | | | | | |
| --- | --- | --- | --- | --- | --- | --- | --- | --- | --- | --- | --- | --- |
| \* | AstrinSTLCLD\_041714\_01.11456.11456.3 | 3.6165 | 0.1921 | 97.0% | 3357.8943 | 3355.576 | 7 | 3.822 | 22.2% | 1 | R.K@T#RPEGQLTS\*ASQK@QAQQTAPRIVSRYK@.D | 3 |

---

|  |  |  |  |  |  |  |  |  |
| --- | --- | --- | --- | --- | --- | --- | --- | --- |
| U | *contaminant\_KERATIN17* | 2 | 2 | 4.1% | 590 | 62461 | 8.1 | no description |
| U | *gi|119395754|ref|NP\_0* | 2 | 2 | 4.1% | 590 | 62378 | 7.8 | keratin 5 [Homo sapiens] |

| Filename XCorr DeltCN Conf% ObsM+H+ CalcM+H+ SpR ZScore Ion% # Sequence  | | | | | | | | | | | | |
| --- | --- | --- | --- | --- | --- | --- | --- | --- | --- | --- | --- | --- |
|  | AstrinSTLCLD\_041714\_01.11253.11253.2 | 2.6041 | 0.2862 | 99.1% | 1330.4922 | 1330.5211 | 1 | 6.131 | 72.7% | 1 | R.NLDLDSIIAEVK.A | 2222 |
|  | AstrinSTLCLD\_041714\_01.09393.09393.2 | 3.2216 | 0.1732 | 99.0% | 1538.4722 | 1538.8022 | 1 | 4.349 | 63.6% | 1 | R.LLREYQELMNTK.L | 2 |

Similarities:
contaminant\_KERATIN22(1:1)  
gi|119703753|ref|NP\_0(1:1)  
gi|153791158|ref|NP\_0(1:1)  

---

|  |  |  |  |  |  |  |  |  |
| --- | --- | --- | --- | --- | --- | --- | --- | --- |
| U | *Reverse\_gi|21361503|r* | 1 | 1 | 4.0% | 401 | 44593 | 9.2 | solute carrier family 35, member B3 [Homo sapiens] |
| U | *Reverse\_gi|216547942|* | 1 | 1 | 4.0% | 401 | 44593 | 9.2 | solute carrier family 35, member B3 [Homo sapiens] |
| U | *Reverse\_gi|216547920|* | 1 | 1 | 4.0% | 401 | 44593 | 9.2 | solute carrier family 35, member B3 [Homo sapiens] |

| Filename XCorr DeltCN Conf% ObsM+H+ CalcM+H+ SpR ZScore Ion% # Sequence  | | | | | | | | | | | | |
| --- | --- | --- | --- | --- | --- | --- | --- | --- | --- | --- | --- | --- |
|  | AstrinSTLCLD\_041714\_02.06917.06917.3 | 2.8083 | 0.2773 | 98.7% | 2014.3744 | 2014.3043 | 10 | 5.166 | 35.0% | 1 | K.NILDYLS\*PLRIKDMNK.S | 3 |

---

|  |  |  |  |  |  |  |  |  |
| --- | --- | --- | --- | --- | --- | --- | --- | --- |
| U | *gi|119703753|ref|NP\_0* | 2 | 2 | 3.9% | 564 | 60067 | 8.0 | keratin 6B [Homo sapiens] |

| Filename XCorr DeltCN Conf% ObsM+H+ CalcM+H+ SpR ZScore Ion% # Sequence  | | | | | | | | | | | | |
| --- | --- | --- | --- | --- | --- | --- | --- | --- | --- | --- | --- | --- |
|  | AstrinSTLCLD\_041714\_01.11253.11253.2 | 2.6041 | 0.2862 | 99.1% | 1330.4922 | 1330.5211 | 1 | 6.131 | 72.7% | 1 | R.NLDLDSIIAEVK.A | 2222 |
|  | AstrinSTLCLD\_041714\_02.05781.05781.2 | 2.7695 | 0.2987 | 100.0% | 1179.7922 | 1180.303 | 2 | 7.587 | 72.2% | 1 | K.YEELQITAGR.H | 22 |

Similarities:
gi|119395750|ref|NP\_0(1:1)  
contaminant\_KERATIN22(1:1)  
contaminant\_KERATIN17(1:1)  
gi|153791158|ref|NP\_0(1:1)  

---

|  |  |  |  |  |  |  |  |  |
| --- | --- | --- | --- | --- | --- | --- | --- | --- |
| U | *gi|66933016|ref|NP\_00* | 1 | 1 | 3.9% | 514 | 55805 | 6.9 | inosine monophosphate dehydrogenase 2 [Homo sapiens] |

| Filename XCorr DeltCN Conf% ObsM+H+ CalcM+H+ SpR ZScore Ion% # Sequence  | | | | | | | | | | | | |
| --- | --- | --- | --- | --- | --- | --- | --- | --- | --- | --- | --- | --- |
| \* | AstrinSTLCLD\_041714\_01.07686.07686.3 | 4.7594 | 0.2244 | 100.0% | 2050.3442 | 2049.3835 | 1 | 6.095 | 44.7% | 1 | R.RFGVPVIADGGIQNVGHIAK.A | 3 |

---

|  |  |  |  |  |  |  |  |  |
| --- | --- | --- | --- | --- | --- | --- | --- | --- |
| U | *Reverse\_gi|118498359|* | 1 | 1 | 3.9% | 490 | 54973 | 10.1 | ribosomal L1 domain containing 1 [Homo sapiens] |

| Filename XCorr DeltCN Conf% ObsM+H+ CalcM+H+ SpR ZScore Ion% # Sequence  | | | | | | | | | | | | |
| --- | --- | --- | --- | --- | --- | --- | --- | --- | --- | --- | --- | --- |
| \* | AstrinSTLCLD\_041714\_01.10187.10187.2 | 3.0876 | 0.1423 | 96.1% | 2230.5322 | 2231.294 | 8 | 3.945 | 44.4% | 1 | K.TAEDWNSVFS\*S\*FIPLAASK.E | 2 |

---

|  |  |  |  |  |  |  |  |  |
| --- | --- | --- | --- | --- | --- | --- | --- | --- |
| U | *gi|24234688|ref|NP\_00* | 2 | 3 | 3.8% | 679 | 73681 | 6.2 | heat shock 70kDa protein 9 precursor [Homo sapiens] |

| Filename XCorr DeltCN Conf% ObsM+H+ CalcM+H+ SpR ZScore Ion% # Sequence  | | | | | | | | | | | | |
| --- | --- | --- | --- | --- | --- | --- | --- | --- | --- | --- | --- | --- |
| \* | AstrinSTLCLD\_041714\_01.05816.05816.2 | 3.8232 | 0.502 | 100.0% | 1451.3522 | 1451.576 | 1 | 10.306 | 73.1% | 2 | R.TTPSVVAFTADGER.L | 2 |
| \* | AstrinSTLCLD\_041714\_01.10796.10796.2 | 2.9982 | 0.2754 | 100.0% | 1362.6122 | 1362.5687 | 9 | 6.143 | 63.6% | 1 | R.AQFEGIVTDLIR.R | 2 |

---

|  |  |  |  |  |  |  |  |  |
| --- | --- | --- | --- | --- | --- | --- | --- | --- |
| U | *gi|153791158|ref|NP\_0* | 2 | 2 | 3.8% | 551 | 59560 | 7.7 | keratin 75 [Homo sapiens] |

| Filename XCorr DeltCN Conf% ObsM+H+ CalcM+H+ SpR ZScore Ion% # Sequence  | | | | | | | | | | | | |
| --- | --- | --- | --- | --- | --- | --- | --- | --- | --- | --- | --- | --- |
|  | AstrinSTLCLD\_041714\_01.11253.11253.2 | 2.6041 | 0.2862 | 99.1% | 1330.4922 | 1330.5211 | 1 | 6.131 | 72.7% | 1 | R.NLDLDSIIAEVK.A | 2222 |
|  | AstrinSTLCLD\_041714\_01.03866.03866.2 | 2.6959 | 0.2317 | 99.2% | 1081.3922 | 1080.1423 | 1 | 5.818 | 87.5% | 1 | K.AQYEDIANR.S | 22 |

Similarities:
gi|4504919|ref|NP\_002(1:1)  
contaminant\_KERATIN22(1:1)  
contaminant\_KERATIN17(1:1)  
gi|119703753|ref|NP\_0(1:1)  

---

|  |  |  |  |  |  |  |  |  |
| --- | --- | --- | --- | --- | --- | --- | --- | --- |
| U | *GFP* | 1 | 1 | 3.8% | 238 | 26813 | 5.8 | no description |

| Filename XCorr DeltCN Conf% ObsM+H+ CalcM+H+ SpR ZScore Ion% # Sequence  | | | | | | | | | | | | |
| --- | --- | --- | --- | --- | --- | --- | --- | --- | --- | --- | --- | --- |
| \* | AstrinSTLCLD\_041714\_01.04395.04395.2 | 2.3652 | 0.2427 | 98.0% | 1051.4321 | 1051.1442 | 1 | 4.685 | 87.5% | 1 | K.FEGDTLVNR.I | 2 |

---

|  |  |  |  |  |  |  |  |  |
| --- | --- | --- | --- | --- | --- | --- | --- | --- |
| U | *gi|4885225|ref|NP\_005* | 1 | 1 | 3.7% | 656 | 68478 | 9.3 | Ewing sarcoma breakpoint region 1 isoform EWS [Homo sapiens] |

| Filename XCorr DeltCN Conf% ObsM+H+ CalcM+H+ SpR ZScore Ion% # Sequence  | | | | | | | | | | | | |
| --- | --- | --- | --- | --- | --- | --- | --- | --- | --- | --- | --- | --- |
| \* | AstrinSTLCLD\_041714\_02.05450.05450.3 | 5.0648 | 0.4548 | 100.0% | 2481.2644 | 2481.572 | 1 | 7.867 | 35.9% | 1 | R.QDHPSSMGVYGQESGGFSGPGENR.S | 3 |

---

|  |  |  |  |  |  |  |  |  |
| --- | --- | --- | --- | --- | --- | --- | --- | --- |
| U | *gi|169168574|ref|XP\_0* | 1 | 1 | 3.7% | 354 | 37950 | 11.9 | PREDICTED: hypothetical protein [Homo sapiens] |

| Filename XCorr DeltCN Conf% ObsM+H+ CalcM+H+ SpR ZScore Ion% # Sequence  | | | | | | | | | | | | |
| --- | --- | --- | --- | --- | --- | --- | --- | --- | --- | --- | --- | --- |
| \* | AstrinSTLCLD\_041714\_01.04200.04200.3 | 3.1159 | 0.2726 | 100.0% | 1696.9143 | 1699.6945 | 1 | 4.671 | 41.7% | 1 | R.T#RDRTSSRGT#VFR.A | 3 |

---

|  |  |  |  |  |  |  |  |  |
| --- | --- | --- | --- | --- | --- | --- | --- | --- |
| U | *gi|42518078|ref|NP\_96* | 1 | 1 | 3.7% | 269 | 30175 | 9.8 | transcription factor 7 (T-cell specific, HMG-box) isoform 2 [Homo sapiens] |
| U | *gi|47419938|ref|NP\_99* | 1 | 1 | 3.7% | 269 | 30175 | 9.8 | transcription factor 7 (T-cell specific, HMG-box) isoform 2 [Homo sapiens] |
| U | *gi|42518080|ref|NP\_00* | 1 | 1 | 2.6% | 384 | 41552 | 7.5 | transcription factor 7 (T-cell specific, HMG-box) isoform 1 [Homo sapiens] |

| Filename XCorr DeltCN Conf% ObsM+H+ CalcM+H+ SpR ZScore Ion% # Sequence  | | | | | | | | | | | | |
| --- | --- | --- | --- | --- | --- | --- | --- | --- | --- | --- | --- | --- |
|  | AstrinSTLCLD\_041714\_01.04023.04023.2 | 2.061 | 0.3025 | 96.9% | 1181.9122 | 1181.1643 | 26 | 4.597 | 55.6% | 1 | K.HQESTT#GGKR.N | 2 |

---

|  |  |  |  |  |  |  |  |  |
| --- | --- | --- | --- | --- | --- | --- | --- | --- |
| U | *gi|18765752|ref|NP\_56* | 1 | 1 | 3.6% | 754 | 84557 | 8.8 | dual-specificity tyrosine-(Y)-phosphorylation regulated kinase 1A isoform 2 [Homo sapiens] |

| Filename XCorr DeltCN Conf% ObsM+H+ CalcM+H+ SpR ZScore Ion% # Sequence  | | | | | | | | | | | | |
| --- | --- | --- | --- | --- | --- | --- | --- | --- | --- | --- | --- | --- |
| \* | AstrinSTLCLD\_041714\_02.06564.06564.3 | 4.0921 | 0.4834 | 100.0% | 3115.1042 | 3116.3745 | 1 | 7.017 | 27.9% | 1 | R.RQPNISDQQVSALSYSDQIQQPLTNQR.R | 3 |

---

|  |  |  |  |  |  |  |  |  |
| --- | --- | --- | --- | --- | --- | --- | --- | --- |
| U | *gi|49472841|ref|NP\_05* | 1 | 1 | 3.6% | 646 | 71649 | 5.1 | A kinase (PRKA) anchor protein 8-like [Homo sapiens] |

| Filename XCorr DeltCN Conf% ObsM+H+ CalcM+H+ SpR ZScore Ion% # Sequence  | | | | | | | | | | | | |
| --- | --- | --- | --- | --- | --- | --- | --- | --- | --- | --- | --- | --- |
| \* | AstrinSTLCLD\_041714\_01.09249.09249.2 | 2.7832 | 0.3736 | 100.0% | 2480.2322 | 2480.7878 | 103 | 6.028 | 22.7% | 1 | R.LDMVPHLETDMMQGGVYGSGGER.Y | 2 |

---

|  |  |  |  |  |  |  |  |  |
| --- | --- | --- | --- | --- | --- | --- | --- | --- |
| U | *gi|5031877|ref|NP\_005* | 1 | 1 | 3.6% | 586 | 66408 | 5.2 | lamin B1 [Homo sapiens] |

| Filename XCorr DeltCN Conf% ObsM+H+ CalcM+H+ SpR ZScore Ion% # Sequence  | | | | | | | | | | | | |
| --- | --- | --- | --- | --- | --- | --- | --- | --- | --- | --- | --- | --- |
| \* | AstrinSTLCLD\_041714\_01.07361.07361.3 | 3.2951 | 0.2066 | 97.5% | 2372.2744 | 2372.5842 | 1 | 5.147 | 36.2% | 1 | R.LSSEMNTSTVNSAREELMESR.M | 3 |

---

|  |  |  |  |  |  |  |  |  |
| --- | --- | --- | --- | --- | --- | --- | --- | --- |
| U | *gi|20357552|ref|NP\_00* | 1 | 2 | 3.6% | 550 | 61586 | 5.4 | cortactin isoform a [Homo sapiens] |
| U | *gi|20357556|ref|NP\_61* | 1 | 2 | 3.9% | 513 | 57467 | 5.3 | cortactin isoform b [Homo sapiens] |

| Filename XCorr DeltCN Conf% ObsM+H+ CalcM+H+ SpR ZScore Ion% # Sequence  | | | | | | | | | | | | |
| --- | --- | --- | --- | --- | --- | --- | --- | --- | --- | --- | --- | --- |
|  | AstrinSTLCLD\_041714\_02.06268.06268.3 | 3.7043 | 0.3396 | 100.0% | 2250.0244 | 2250.442 | 1 | 5.558 | 32.9% | 2 | R.MDKNASTFEDVTQVSSAYQK.T | 3 |

---

|  |  |  |  |  |  |  |  |  |
| --- | --- | --- | --- | --- | --- | --- | --- | --- |
| U | *gi|160707950|ref|NP\_0* | 1 | 1 | 3.6% | 498 | 53323 | 9.9 | methyl CpG binding protein 2 isoform 2 [Homo sapiens] |
| U | *gi|4826830|ref|NP\_004* | 1 | 1 | 3.7% | 486 | 52441 | 9.9 | methyl CpG binding protein 2 isoform 1 [Homo sapiens] |

| Filename XCorr DeltCN Conf% ObsM+H+ CalcM+H+ SpR ZScore Ion% # Sequence  | | | | | | | | | | | | |
| --- | --- | --- | --- | --- | --- | --- | --- | --- | --- | --- | --- | --- |
|  | AstrinSTLCLD\_041714\_01.07180.07180.3 | 2.9808 | 0.2215 | 97.0% | 1955.5443 | 1956.2185 | 46 | 4.656 | 32.4% | 1 | K.KPKS\*PKAPGTGRGRGRPK.G | 3 |

---

|  |  |  |  |  |  |  |  |  |
| --- | --- | --- | --- | --- | --- | --- | --- | --- |
| U | *gi|87578255|ref|NP\_11* | 1 | 1 | 3.6% | 502 | 52980 | 9.6 | microtubule-associated protein 2 isoform 4 [Homo sapiens] |
| U | *gi|87578394|ref|NP\_00* | 1 | 1 | 3.2% | 559 | 58954 | 9.7 | microtubule-associated protein 2 isoform 5 [Homo sapiens] |

| Filename XCorr DeltCN Conf% ObsM+H+ CalcM+H+ SpR ZScore Ion% # Sequence  | | | | | | | | | | | | |
| --- | --- | --- | --- | --- | --- | --- | --- | --- | --- | --- | --- | --- |
|  | AstrinSTLCLD\_041714\_02.06728.06728.3 | 3.1488 | 0.2287 | 97.9% | 2189.3943 | 2191.372 | 5 | 4.472 | 30.9% | 1 | K.KIDFSKVQSRCGS\*KDNIK.H | 3 |

---

|  |  |  |  |  |  |  |  |  |
| --- | --- | --- | --- | --- | --- | --- | --- | --- |
| U | *gi|31543397|ref|NP\_62* | 2 | 2 | 3.6% | 417 | 44796 | 8.5 | phosphoglycerate kinase 2 [Homo sapiens] |
| U | *gi|4505763|ref|NP\_000* | 2 | 2 | 3.6% | 417 | 44615 | 8.1 | phosphoglycerate kinase 1 [Homo sapiens] |

| Filename XCorr DeltCN Conf% ObsM+H+ CalcM+H+ SpR ZScore Ion% # Sequence  | | | | | | | | | | | | |
| --- | --- | --- | --- | --- | --- | --- | --- | --- | --- | --- | --- | --- |
|  | AstrinSTLCLD\_041714\_01.06463.06463.2 | 2.9895 | 0.466 | 100.0% | 1633.9521 | 1635.7764 | 1 | 7.424 | 57.1% | 1 | K.LGDVYVNDAFGTAHR.A | 2 |
|  | AstrinSTLCLD\_041714\_01.06457.06457.3 | 3.1338 | 0.2985 | 100.0% | 1635.8344 | 1635.7764 | 1 | 5.192 | 42.9% | 1 | K.LGDVYVNDAFGTAHR.A | 3 |

---

|  |  |  |  |  |  |  |  |  |
| --- | --- | --- | --- | --- | --- | --- | --- | --- |
| U | *gi|5032013|ref|NP\_005* | 2 | 2 | 3.4% | 890 | 100278 | 6.9 | kinesin family member 20A [Homo sapiens] |

| Filename XCorr DeltCN Conf% ObsM+H+ CalcM+H+ SpR ZScore Ion% # Sequence  | | | | | | | | | | | | |
| --- | --- | --- | --- | --- | --- | --- | --- | --- | --- | --- | --- | --- |
| \* | AstrinSTLCLD\_041714\_01.04778.04778.3 | 3.119 | 0.2246 | 97.9% | 1724.3043 | 1724.9578 | 19 | 4.717 | 33.3% | 1 | R.LKEAGNINTSLHTLGR.C | 3 |
| \* | AstrinSTLCLD\_041714\_02.04809.04809.2 | 3.2817 | 0.4251 | 100.0% | 1474.0721 | 1474.6543 | 1 | 6.413 | 53.8% | 1 | R.LAASASTQQLQEVK.A | 2 |

---

|  |  |  |  |  |  |  |  |  |
| --- | --- | --- | --- | --- | --- | --- | --- | --- |
| U | *Reverse\_gi|87578394|r* | 1 | 1 | 3.4% | 559 | 58954 | 9.7 | microtubule-associated protein 2 isoform 5 [Homo sapiens] |

| Filename XCorr DeltCN Conf% ObsM+H+ CalcM+H+ SpR ZScore Ion% # Sequence  | | | | | | | | | | | | |
| --- | --- | --- | --- | --- | --- | --- | --- | --- | --- | --- | --- | --- |
| \* | AstrinSTLCLD\_041714\_01.15138.15138.2 | 3.4192 | 0.1319 | 97.7% | 2207.172 | 2208.1277 | 131 | 4.021 | 30.6% | 1 | R.DT#SGASTPQILLSDS\*FT#AR.K | 2 |

---

|  |  |  |  |  |  |  |  |  |
| --- | --- | --- | --- | --- | --- | --- | --- | --- |
| U | *Reverse\_gi|24307887|r* | 1 | 1 | 3.4% | 465 | 51023 | 9.7 | one cut homeobox 1 [Homo sapiens] |

| Filename XCorr DeltCN Conf% ObsM+H+ CalcM+H+ SpR ZScore Ion% # Sequence  | | | | | | | | | | | | |
| --- | --- | --- | --- | --- | --- | --- | --- | --- | --- | --- | --- | --- |
| \* | AstrinSTLCLD\_041714\_02.06399.06399.2 | 2.8132 | 0.1802 | 95.2% | 1754.8922 | 1753.9708 | 3 | 4.767 | 43.3% | 1 | R.EDRMLTFSGSVNGALR.Q | 2 |

---

|  |  |  |  |  |  |  |  |  |
| --- | --- | --- | --- | --- | --- | --- | --- | --- |
| U | *gi|154800487|ref|NP\_0* | 1 | 2 | 3.4% | 348 | 39171 | 7.9 | ER lipid raft associated 1 [Homo sapiens] |
| U | *gi|6005721|ref|NP\_009* | 1 | 2 | 3.5% | 339 | 37840 | 5.6 | ER lipid raft associated 2 isoform 1 [Homo sapiens] |
| U | *gi|154800489|ref|NP\_0* | 1 | 2 | 3.4% | 348 | 39171 | 7.9 | ER lipid raft associated 1 [Homo sapiens] |

| Filename XCorr DeltCN Conf% ObsM+H+ CalcM+H+ SpR ZScore Ion% # Sequence  | | | | | | | | | | | | |
| --- | --- | --- | --- | --- | --- | --- | --- | --- | --- | --- | --- | --- |
|  | AstrinSTLCLD\_041714\_02.07468.07468.2 | 3.3397 | 0.4526 | 100.0% | 1335.3922 | 1335.4998 | 1 | 8.418 | 81.8% | 2 | R.ISEIEDAAFLAR.E | 2 |

---

|  |  |  |  |  |  |  |  |  |
| --- | --- | --- | --- | --- | --- | --- | --- | --- |
| U | *gi|153792294|ref|NP\_1* | 2 | 2 | 3.3% | 1320 | 145257 | 6.8 | myopalladin [Homo sapiens] |

| Filename XCorr DeltCN Conf% ObsM+H+ CalcM+H+ SpR ZScore Ion% # Sequence  | | | | | | | | | | | | |
| --- | --- | --- | --- | --- | --- | --- | --- | --- | --- | --- | --- | --- |
| \* | AstrinSTLCLD\_041714\_01.05294.05294.3 | 4.6266 | 0.4202 | 100.0% | 2546.0645 | 2546.71 | 1 | 7.215 | 33.7% | 1 | R.VHFNLPEDDKGSEASSEAGVVTTR.Q | 3 |
| \* | AstrinSTLCLD\_041714\_01.07511.07511.3 | 3.1255 | 0.3121 | 100.0% | 2211.2644 | 2213.5242 | 32 | 4.706 | 33.3% | 1 | R.FFRPHFLQAPGDMVAHEGR.L | 3 |

---

|  |  |  |  |  |  |  |  |  |
| --- | --- | --- | --- | --- | --- | --- | --- | --- |
| U | *gi|29826282|ref|NP\_81* | 1 | 1 | 3.3% | 546 | 59272 | 4.4 | protein phosphatase 1G [Homo sapiens] |
| U | *gi|4505999|ref|NP\_002* | 1 | 1 | 3.3% | 546 | 59272 | 4.4 | protein phosphatase 1G [Homo sapiens] |

| Filename XCorr DeltCN Conf% ObsM+H+ CalcM+H+ SpR ZScore Ion% # Sequence  | | | | | | | | | | | | |
| --- | --- | --- | --- | --- | --- | --- | --- | --- | --- | --- | --- | --- |
|  | AstrinSTLCLD\_041714\_01.05297.05297.3 | 3.2688 | 0.2847 | 100.0% | 2119.4944 | 2119.3105 | 1 | 5.604 | 36.8% | 1 | K.ALDMSYDHKPEDEVELAR.I | 3 |

---

|  |  |  |  |  |  |  |  |  |
| --- | --- | --- | --- | --- | --- | --- | --- | --- |
| U | *gi|18375623|ref|NP\_54* | 1 | 1 | 3.3% | 428 | 48991 | 5.7 | HLA-B associated transcript 1 [Homo sapiens] |
| U | *gi|4758112|ref|NP\_004* | 1 | 1 | 3.3% | 428 | 48991 | 5.7 | HLA-B associated transcript 1 [Homo sapiens] |
| U | *gi|21040371|ref|NP\_00* | 1 | 1 | 3.3% | 427 | 49130 | 5.7 | DEAD (Asp-Glu-Ala-Asp) box polypeptide 39 [Homo sapiens] |

| Filename XCorr DeltCN Conf% ObsM+H+ CalcM+H+ SpR ZScore Ion% # Sequence  | | | | | | | | | | | | |
| --- | --- | --- | --- | --- | --- | --- | --- | --- | --- | --- | --- | --- |
|  | AstrinSTLCLD\_041714\_02.07214.07214.2 | 3.1625 | 0.3576 | 100.0% | 1480.1721 | 1480.6146 | 1 | 6.06 | 69.2% | 1 | K.GLAITFVSDENDAK.I | 2 |

---

|  |  |  |  |  |  |  |  |  |
| --- | --- | --- | --- | --- | --- | --- | --- | --- |
| U | *gi|224451077|ref|NP\_0* | 1 | 1 | 3.3% | 399 | 44762 | 5.2 | HBV PreS1-transactivated protein 3 isoform b [Homo sapiens] |
| U | *gi|93102389|ref|NP\_00* | 1 | 1 | 3.3% | 390 | 44140 | 5.2 | HBV PreS1-transactivated protein 3 isoform a [Homo sapiens] |

| Filename XCorr DeltCN Conf% ObsM+H+ CalcM+H+ SpR ZScore Ion% # Sequence  | | | | | | | | | | | | |
| --- | --- | --- | --- | --- | --- | --- | --- | --- | --- | --- | --- | --- |
|  | AstrinSTLCLD\_041714\_02.06094.06094.2 | 3.3273 | 0.4585 | 100.0% | 1274.4922 | 1274.4166 | 1 | 7.421 | 62.5% | 1 | K.ISAEAVGVDISGR.F | 2 |

---

|  |  |  |  |  |  |  |  |  |
| --- | --- | --- | --- | --- | --- | --- | --- | --- |
| U | *gi|19923485|ref|NP\_05* | 1 | 1 | 3.2% | 432 | 51466 | 9.8 | cisplatin resistance-associated overexpressed protein [Homo sapiens] |
| U | *gi|52426743|ref|NP\_00* | 1 | 1 | 3.2% | 432 | 51466 | 9.8 | cisplatin resistance-associated overexpressed protein [Homo sapiens] |

| Filename XCorr DeltCN Conf% ObsM+H+ CalcM+H+ SpR ZScore Ion% # Sequence  | | | | | | | | | | | | |
| --- | --- | --- | --- | --- | --- | --- | --- | --- | --- | --- | --- | --- |
|  | AstrinSTLCLD\_041714\_01.06287.06287.2 | 2.9679 | 0.2592 | 99.2% | 1500.0521 | 1500.6024 | 9 | 4.964 | 53.8% | 1 | R.STTSTIESFAAQEK.Q | 2 |

---

|  |  |  |  |  |  |  |  |  |
| --- | --- | --- | --- | --- | --- | --- | --- | --- |
| U | *Reverse\_gi|89111953|r* | 1 | 1 | 3.1% | 1078 | 119476 | 4.7 | transmembrane protein 132B [Homo sapiens] |

| Filename XCorr DeltCN Conf% ObsM+H+ CalcM+H+ SpR ZScore Ion% # Sequence  | | | | | | | | | | | | |
| --- | --- | --- | --- | --- | --- | --- | --- | --- | --- | --- | --- | --- |
| \* | AstrinSTLCLD\_041714\_01.17033.17033.3 | 2.9286 | 0.2358 | 95.0% | 3580.4944 | 3577.743 | 71 | 3.988 | 17.2% | 1 | R.ST#VTLDSPNNPDPSK@GQT#PFSTFADPGGSKLLK.N | 3 |

---

|  |  |  |  |  |  |  |  |  |
| --- | --- | --- | --- | --- | --- | --- | --- | --- |
| U | *gi|28195388|ref|NP\_11* | 1 | 1 | 3.1% | 637 | 71928 | 4.9 | RAB11 family interacting protein 4 (class II) [Homo sapiens] |

| Filename XCorr DeltCN Conf% ObsM+H+ CalcM+H+ SpR ZScore Ion% # Sequence  | | | | | | | | | | | | |
| --- | --- | --- | --- | --- | --- | --- | --- | --- | --- | --- | --- | --- |
| \* | AstrinSTLCLD\_041714\_01.20707.20707.3 | 2.9879 | 0.2428 | 97.8% | 2171.4844 | 2174.113 | 304 | 4.278 | 26.3% | 1 | R.TNVYSDLGSS\*VSSSAGQT#PR.K | 3 |

---

|  |  |  |  |  |  |  |  |  |
| --- | --- | --- | --- | --- | --- | --- | --- | --- |
| U | *gi|112420960|ref|NP\_9* | 1 | 1 | 3.0% | 1197 | 135786 | 8.0 | hypothetical protein LOC374467 [Homo sapiens] |

| Filename XCorr DeltCN Conf% ObsM+H+ CalcM+H+ SpR ZScore Ion% # Sequence  | | | | | | | | | | | | |
| --- | --- | --- | --- | --- | --- | --- | --- | --- | --- | --- | --- | --- |
| \* | AstrinSTLCLD\_041714\_02.10989.10989.3 | 3.374 | 0.2451 | 98.1% | 4172.8145 | 4175.7236 | 172 | 3.841 | 16.4% | 1 | R.LQLAAVALQRHRAAYS\*AAIVFSTLTLLQDS\*K@LFEKK.V | 3 |

---

|  |  |  |  |  |  |  |  |  |
| --- | --- | --- | --- | --- | --- | --- | --- | --- |
| U | *gi|155030232|ref|NP\_0* | 2 | 2 | 3.0% | 1130 | 119700 | 4.3 | proline, glutamic acid and leucine rich protein 1 [Homo sapiens] |

| Filename XCorr DeltCN Conf% ObsM+H+ CalcM+H+ SpR ZScore Ion% # Sequence  | | | | | | | | | | | | |
| --- | --- | --- | --- | --- | --- | --- | --- | --- | --- | --- | --- | --- |
| \* | AstrinSTLCLD\_041714\_01.08391.08391.2 | 3.2104 | 0.4393 | 100.0% | 1275.6322 | 1275.4906 | 1 | 7.677 | 62.5% | 1 | R.LPSLGAGFSQGLK.H | 2 |
| \* | AstrinSTLCLD\_041714\_01.06235.06235.2 | 2.6792 | 0.2654 | 98.2% | 2108.3123 | 2110.2854 | 15 | 5.574 | 30.0% | 1 | R.GADTAPTLAPEALPSQGEVER.E | 2 |

---

|  |  |  |  |  |  |  |  |  |
| --- | --- | --- | --- | --- | --- | --- | --- | --- |
| U | *Reverse\_gi|7657265|re* | 1 | 1 | 3.0% | 627 | 70264 | 6.6 | fem-1 homolog b [Homo sapiens] |

| Filename XCorr DeltCN Conf% ObsM+H+ CalcM+H+ SpR ZScore Ion% # Sequence  | | | | | | | | | | | | |
| --- | --- | --- | --- | --- | --- | --- | --- | --- | --- | --- | --- | --- |
| \* | AstrinSTLCLD\_041714\_01.08332.08332.2 | 3.5607 | 0.1207 | 97.9% | 2239.5322 | 2240.4783 | 14 | 4.412 | 47.2% | 1 | K.NAIS\*INANNEVLYKVIDLR.G | 2 |

---

|  |  |  |  |  |  |  |  |  |
| --- | --- | --- | --- | --- | --- | --- | --- | --- |
| U | *gi|4503481|ref|NP\_001* | 1 | 1 | 3.0% | 437 | 50119 | 6.7 | eukaryotic translation elongation factor 1 gamma [Homo sapiens] |

| Filename XCorr DeltCN Conf% ObsM+H+ CalcM+H+ SpR ZScore Ion% # Sequence  | | | | | | | | | | | | |
| --- | --- | --- | --- | --- | --- | --- | --- | --- | --- | --- | --- | --- |
| \* | AstrinSTLCLD\_041714\_02.05668.05668.2 | 3.9142 | 0.4232 | 100.0% | 1348.3322 | 1348.5448 | 1 | 8.158 | 66.7% | 1 | K.ALIAAQYSGAQVR.V | 2 |

---

|  |  |  |  |  |  |  |  |  |
| --- | --- | --- | --- | --- | --- | --- | --- | --- |
| U | *gi|5453660|ref|NP\_006* | 1 | 1 | 2.9% | 907 | 103571 | 8.1 | tubulin, gamma complex associated protein 3 [Homo sapiens] |

| Filename XCorr DeltCN Conf% ObsM+H+ CalcM+H+ SpR ZScore Ion% # Sequence  | | | | | | | | | | | | |
| --- | --- | --- | --- | --- | --- | --- | --- | --- | --- | --- | --- | --- |
| \* | AstrinSTLCLD\_041714\_02.09194.09194.3 | 3.2396 | 0.2379 | 97.8% | 3208.8245 | 3211.5913 | 112 | 4.571 | 23.0% | 1 | R.K@LHSQGVLKNKWSILYLLLS\*LS\*EDPR.R | 3 |

---

|  |  |  |  |  |  |  |  |  |
| --- | --- | --- | --- | --- | --- | --- | --- | --- |
| U | *gi|42560244|ref|NP\_00* | 1 | 1 | 2.9% | 754 | 88617 | 10.3 | peptidylprolyl isomerase G [Homo sapiens] |

| Filename XCorr DeltCN Conf% ObsM+H+ CalcM+H+ SpR ZScore Ion% # Sequence  | | | | | | | | | | | | |
| --- | --- | --- | --- | --- | --- | --- | --- | --- | --- | --- | --- | --- |
| \* | AstrinSTLCLD\_041714\_01.14052.14052.3 | 2.8867 | 0.2338 | 95.5% | 2875.4343 | 2872.8835 | 4 | 4.124 | 25.0% | 1 | K.QS\*S\*QDNELKS\*SMLK@NK@EDEKIR.S | 3 |

---

|  |  |  |  |  |  |  |  |  |
| --- | --- | --- | --- | --- | --- | --- | --- | --- |
| U | *gi|217272802|ref|NP\_0* | 1 | 1 | 2.9% | 725 | 84229 | 5.9 | hyaluronan-mediated motility receptor isoform a [Homo sapiens] |
| U | *gi|217416398|ref|NP\_0* | 1 | 1 | 3.0% | 709 | 82301 | 5.9 | hyaluronan-mediated motility receptor isoform c [Homo sapiens] |
| U | *gi|217416394|ref|NP\_0* | 1 | 1 | 2.9% | 724 | 84100 | 5.8 | hyaluronan-mediated motility receptor isoform b [Homo sapiens] |
| U | *gi|217272804|ref|NP\_0* | 1 | 1 | 3.3% | 638 | 74495 | 5.4 | hyaluronan-mediated motility receptor isoform d [Homo sapiens] |

| Filename XCorr DeltCN Conf% ObsM+H+ CalcM+H+ SpR ZScore Ion% # Sequence  | | | | | | | | | | | | |
| --- | --- | --- | --- | --- | --- | --- | --- | --- | --- | --- | --- | --- |
|  | AstrinSTLCLD\_041714\_01.06596.06596.3 | 2.7949 | 0.3269 | 99.3% | 2432.8743 | 2432.5657 | 1 | 5.087 | 30.0% | 1 | K.NAEDVQHQILATESSNQEYVR.M | 3 |

---

|  |  |  |  |  |  |  |  |  |
| --- | --- | --- | --- | --- | --- | --- | --- | --- |
| U | *Reverse\_gi|28827807|r* | 1 | 3 | 2.9% | 655 | 75032 | 7.2 | NLR family, pyrin domain containing 10 [Homo sapiens] |

| Filename XCorr DeltCN Conf% ObsM+H+ CalcM+H+ SpR ZScore Ion% # Sequence  | | | | | | | | | | | | |
| --- | --- | --- | --- | --- | --- | --- | --- | --- | --- | --- | --- | --- |
| \* | AstrinSTLCLD\_041714\_01.08219.08219.2 | 4.2482 | 0.0276 | 97.6% | 2215.2322 | 2215.2212 | 73 | 3.834 | 38.9% | 3 | K.QT#GAINDKGEKQGHVS\*PCK.Q | 2 |

---

|  |  |  |  |  |  |  |  |  |
| --- | --- | --- | --- | --- | --- | --- | --- | --- |
| U | *gi|21327701|ref|NP\_63* | 1 | 1 | 2.9% | 592 | 61830 | 8.0 | TBP-associated factor 15 isoform 1 [Homo sapiens] |
| U | *gi|4507353|ref|NP\_003* | 1 | 1 | 2.9% | 589 | 61558 | 8.0 | TBP-associated factor 15 isoform 2 [Homo sapiens] |

| Filename XCorr DeltCN Conf% ObsM+H+ CalcM+H+ SpR ZScore Ion% # Sequence  | | | | | | | | | | | | |
| --- | --- | --- | --- | --- | --- | --- | --- | --- | --- | --- | --- | --- |
|  | AstrinSTLCLD\_041714\_01.09287.09287.3 | 3.2738 | 0.2764 | 99.2% | 1962.4744 | 1962.2151 | 12 | 5.454 | 35.9% | 1 | K.AAIDWFDGKEFHGNIIK.V | 3 |

---

|  |  |  |  |  |  |  |  |  |
| --- | --- | --- | --- | --- | --- | --- | --- | --- |
| U | *Reverse\_gi|17975768|r* | 1 | 1 | 2.8% | 998 | 110330 | 6.3 | ephrin receptor EphB3 precursor [Homo sapiens] |

| Filename XCorr DeltCN Conf% ObsM+H+ CalcM+H+ SpR ZScore Ion% # Sequence  | | | | | | | | | | | | |
| --- | --- | --- | --- | --- | --- | --- | --- | --- | --- | --- | --- | --- |
| \* | AstrinSTLCLD\_041714\_02.00038.00038.3 | 3.2228 | 0.2681 | 98.5% | 3336.4744 | 3337.797 | 98 | 4.715 | 20.4% | 1 | R.LFSDLACNEMFETLIMVPRS\*KTVVGELR.I | 3 |

---

|  |  |  |  |  |  |  |  |  |
| --- | --- | --- | --- | --- | --- | --- | --- | --- |
| U | *gi|109948283|ref|NP\_0* | 1 | 1 | 2.8% | 648 | 73584 | 9.6 | KIAA0020 protein [Homo sapiens] |

| Filename XCorr DeltCN Conf% ObsM+H+ CalcM+H+ SpR ZScore Ion% # Sequence  | | | | | | | | | | | | |
| --- | --- | --- | --- | --- | --- | --- | --- | --- | --- | --- | --- | --- |
| \* | AstrinSTLCLD\_041714\_02.07370.07370.3 | 3.1429 | 0.3605 | 100.0% | 2318.5144 | 2319.2283 | 4 | 4.214 | 33.8% | 1 | K.NRFHKNS\*DSGS\*S\*K@TFPTR.K | 3 |

---

|  |  |  |  |  |  |  |  |  |
| --- | --- | --- | --- | --- | --- | --- | --- | --- |
| U | *gi|5453952|ref|NP\_006* | 1 | 1 | 2.8% | 497 | 57393 | 6.7 | beta isoform of regulatory subunit B56, protein phosphatase 2A [Homo sapiens] |

| Filename XCorr DeltCN Conf% ObsM+H+ CalcM+H+ SpR ZScore Ion% # Sequence  | | | | | | | | | | | | |
| --- | --- | --- | --- | --- | --- | --- | --- | --- | --- | --- | --- | --- |
| \* | AstrinSTLCLD\_041714\_01.10188.10188.2 | 2.3125 | 0.2826 | 96.7% | 1671.4922 | 1674.045 | 92 | 4.866 | 34.6% | 1 | K.TILHRVYGKFLGLR.A | 2 |

---

|  |  |  |  |  |  |  |  |  |
| --- | --- | --- | --- | --- | --- | --- | --- | --- |
| U | *gi|32189394|ref|NP\_00* | 1 | 1 | 2.8% | 529 | 56560 | 5.4 | mitochondrial ATP synthase beta subunit precursor [Homo sapiens] |

| Filename XCorr DeltCN Conf% ObsM+H+ CalcM+H+ SpR ZScore Ion% # Sequence  | | | | | | | | | | | | |
| --- | --- | --- | --- | --- | --- | --- | --- | --- | --- | --- | --- | --- |
| \* | AstrinSTLCLD\_041714\_02.07193.07193.2 | 2.8681 | 0.3608 | 100.0% | 1651.0322 | 1651.9034 | 1 | 5.39 | 57.1% | 1 | R.LVLEVAQHLGESTVR.T | 2 |

---

|  |  |  |  |  |  |  |  |  |
| --- | --- | --- | --- | --- | --- | --- | --- | --- |
| U | *gi|163792208|ref|NP\_0* | 1 | 1 | 2.7% | 709 | 80105 | 7.4 | Rho guanine exchange factor 16 [Homo sapiens] |

| Filename XCorr DeltCN Conf% ObsM+H+ CalcM+H+ SpR ZScore Ion% # Sequence  | | | | | | | | | | | | |
| --- | --- | --- | --- | --- | --- | --- | --- | --- | --- | --- | --- | --- |
| \* | AstrinSTLCLD\_041714\_01.09756.09756.2 | 3.3211 | 0.169 | 98.6% | 2287.5723 | 2288.3796 | 37 | 3.937 | 33.3% | 1 | R.DPK@LLPAPS\*FS\*LDDMDVDK@.D | 2 |

---

|  |  |  |  |  |  |  |  |  |
| --- | --- | --- | --- | --- | --- | --- | --- | --- |
| U | *gi|4757810|ref|NP\_004* | 1 | 2 | 2.7% | 553 | 59751 | 9.1 | ATP synthase, H+ transporting, mitochondrial F1 complex, alpha subunit precursor [Homo sapiens] |
| U | *gi|50345984|ref|NP\_00* | 1 | 2 | 2.7% | 553 | 59751 | 9.1 | ATP synthase, H+ transporting, mitochondrial F1 complex, alpha subunit precursor [Homo sapiens] |

| Filename XCorr DeltCN Conf% ObsM+H+ CalcM+H+ SpR ZScore Ion% # Sequence  | | | | | | | | | | | | |
| --- | --- | --- | --- | --- | --- | --- | --- | --- | --- | --- | --- | --- |
|  | AstrinSTLCLD\_041714\_02.06318.06318.2 | 4.4969 | 0.5878 | 100.0% | 1577.3322 | 1576.7007 | 1 | 9.934 | 78.6% | 2 | R.ILGADTSVDLEETGR.V | 2 |

---

|  |  |  |  |  |  |  |  |  |
| --- | --- | --- | --- | --- | --- | --- | --- | --- |
| U | *gi|41872631|ref|NP\_00* | 3 | 3 | 2.6% | 2511 | 273424 | 6.4 | fatty acid synthase [Homo sapiens] |

| Filename XCorr DeltCN Conf% ObsM+H+ CalcM+H+ SpR ZScore Ion% # Sequence  | | | | | | | | | | | | |
| --- | --- | --- | --- | --- | --- | --- | --- | --- | --- | --- | --- | --- |
| \* | AstrinSTLCLD\_041714\_01.14656.14656.3 | 4.6205 | 0.3649 | 100.0% | 3165.1743 | 3165.415 | 1 | 7.043 | 26.9% | 1 | K.LPESENLQEFWDNLIGGVDMVTDDDRR.W | 3 |
| \* | AstrinSTLCLD\_041714\_01.14403.14403.3 | 3.096 | 0.2404 | 97.5% | 2787.6543 | 2787.3152 | 1 | 4.645 | 29.0% | 1 | R.ALGLGVEQLPVVFEDVVLHQATILPK.T | 3 |
| \* | AstrinSTLCLD\_041714\_01.07372.07372.2 | 3.7585 | 0.4582 | 100.0% | 1469.9922 | 1470.5815 | 1 | 7.341 | 79.2% | 1 | R.FPQLDSTSFANSR.D | 2 |

---

|  |  |  |  |  |  |  |  |  |
| --- | --- | --- | --- | --- | --- | --- | --- | --- |
| U | *Reverse\_gi|21536466|r* | 1 | 1 | 2.6% | 894 | 98337 | 5.4 | AXL receptor tyrosine kinase isoform 1 [Homo sapiens] |
| U | *Reverse\_gi|21536468|r* | 1 | 1 | 2.6% | 885 | 97378 | 5.4 | AXL receptor tyrosine kinase isoform 2 [Homo sapiens] |

| Filename XCorr DeltCN Conf% ObsM+H+ CalcM+H+ SpR ZScore Ion% # Sequence  | | | | | | | | | | | | |
| --- | --- | --- | --- | --- | --- | --- | --- | --- | --- | --- | --- | --- |
|  | AstrinSTLCLD\_041714\_02.08349.08349.3 | 3.8963 | 0.1605 | 96.8% | 2772.7144 | 2773.8845 | 3 | 3.597 | 31.8% | 1 | K.EKLEES\*IGLSNLTAETTRRS\*YSK.R | 3 |

---

|  |  |  |  |  |  |  |  |  |
| --- | --- | --- | --- | --- | --- | --- | --- | --- |
| U | *Reverse\_gi|93102364|r* | 1 | 1 | 2.6% | 585 | 68998 | 5.9 | SWAP-70 protein [Homo sapiens] |

| Filename XCorr DeltCN Conf% ObsM+H+ CalcM+H+ SpR ZScore Ion% # Sequence  | | | | | | | | | | | | |
| --- | --- | --- | --- | --- | --- | --- | --- | --- | --- | --- | --- | --- |
| \* | AstrinSTLCLD\_041714\_01.06248.06248.3 | 2.2776 | 0.2846 | 96.0% | 1924.8243 | 1925.1339 | 272 | 4.598 | 32.1% | 1 | K.DKWSK@TK@NT#AMELK@K.K | 3 |

---

|  |  |  |  |  |  |  |  |  |
| --- | --- | --- | --- | --- | --- | --- | --- | --- |
| U | *gi|55956899|ref|NP\_00* | 1 | 1 | 2.6% | 623 | 62064 | 5.2 | keratin 9 [Homo sapiens] |

| Filename XCorr DeltCN Conf% ObsM+H+ CalcM+H+ SpR ZScore Ion% # Sequence  | | | | | | | | | | | | |
| --- | --- | --- | --- | --- | --- | --- | --- | --- | --- | --- | --- | --- |
| \* | AstrinSTLCLD\_041714\_01.03717.03717.2 | 2.5725 | 0.2555 | 97.5% | 1233.3522 | 1233.2833 | 6 | 5.992 | 46.7% | 1 | R.SGGGGGGGLGSGGSIR.S | 2 |

---

|  |  |  |  |  |  |  |  |  |
| --- | --- | --- | --- | --- | --- | --- | --- | --- |
| U | *gi|100913206|ref|NP\_0* | 3 | 3 | 2.5% | 1270 | 140958 | 6.8 | DEAH (Asp-Glu-Ala-His) box polypeptide 9 [Homo sapiens] |

| Filename XCorr DeltCN Conf% ObsM+H+ CalcM+H+ SpR ZScore Ion% # Sequence  | | | | | | | | | | | | |
| --- | --- | --- | --- | --- | --- | --- | --- | --- | --- | --- | --- | --- |
| \* | AstrinSTLCLD\_041714\_01.05621.05621.2 | 2.2762 | 0.2958 | 98.1% | 1392.3922 | 1392.6307 | 1 | 6.218 | 65.0% | 1 | R.LETHMTPEMFR.T | 2 |
| \* | AstrinSTLCLD\_041714\_01.06675.06675.2 | 2.2437 | 0.2629 | 96.4% | 1223.4521 | 1222.4703 | 152 | 5.404 | 55.0% | 1 | R.TPLHEIALSIK.L | 2 |
| \* | AstrinSTLCLD\_041714\_01.08489.08489.2 | 2.6768 | 0.3338 | 100.0% | 1004.1922 | 1004.21747 | 1 | 6.372 | 83.3% | 1 | R.LGGIGQFLAK.A | 2 |

---

|  |  |  |  |  |  |  |  |  |
| --- | --- | --- | --- | --- | --- | --- | --- | --- |
| U | *Reverse\_gi|117606347|* | 1 | 1 | 2.5% | 674 | 74448 | 8.2 | F-box protein 43 isoform a [Homo sapiens] |
| U | *Reverse\_gi|117606351|* | 1 | 1 | 2.4% | 708 | 78402 | 8.1 | F-box protein 43 isoform b [Homo sapiens] |

| Filename XCorr DeltCN Conf% ObsM+H+ CalcM+H+ SpR ZScore Ion% # Sequence  | | | | | | | | | | | | |
| --- | --- | --- | --- | --- | --- | --- | --- | --- | --- | --- | --- | --- |
|  | AstrinSTLCLD\_041714\_01.10925.10925.3 | 2.3842 | 0.2912 | 97.3% | 2260.6143 | 2258.25 | 2 | 4.49 | 31.2% | 1 | K.EK@K@GLYEK@DINDFS\*CS\*K.L | 3 |

---

|  |  |  |  |  |  |  |  |  |
| --- | --- | --- | --- | --- | --- | --- | --- | --- |
| U | *gi|186910300|ref|NP\_0* | 1 | 1 | 2.5% | 632 | 71027 | 8.9 | Rac GTPase activating protein 1 [Homo sapiens] |
| U | *gi|21361397|ref|NP\_03* | 1 | 1 | 2.5% | 632 | 71027 | 8.9 | Rac GTPase activating protein 1 [Homo sapiens] |
| U | *gi|186910302|ref|NP\_0* | 1 | 1 | 2.5% | 632 | 71027 | 8.9 | Rac GTPase activating protein 1 [Homo sapiens] |

| Filename XCorr DeltCN Conf% ObsM+H+ CalcM+H+ SpR ZScore Ion% # Sequence  | | | | | | | | | | | | |
| --- | --- | --- | --- | --- | --- | --- | --- | --- | --- | --- | --- | --- |
|  | AstrinSTLCLD\_041714\_02.05249.05249.2 | 2.5788 | 0.282 | 98.1% | 1576.7722 | 1575.716 | 1 | 5.636 | 50.0% | 1 | R.SIGSAVDQGNESIVAK.T | 2 |

---

|  |  |  |  |  |  |  |  |  |
| --- | --- | --- | --- | --- | --- | --- | --- | --- |
| U | *Reverse\_gi|7705915|re* | 1 | 1 | 2.5% | 475 | 52932 | 6.6 | tubulin, epsilon 1 [Homo sapiens] |

| Filename XCorr DeltCN Conf% ObsM+H+ CalcM+H+ SpR ZScore Ion% # Sequence  | | | | | | | | | | | | |
| --- | --- | --- | --- | --- | --- | --- | --- | --- | --- | --- | --- | --- |
| \* | AstrinSTLCLD\_041714\_01.12854.12854.2 | 2.1689 | 0.3037 | 97.1% | 1649.7322 | 1649.9093 | 1 | 5.636 | 50.0% | 1 | R.MFREK@LEMFT#PK.V | 2 |

---

|  |  |  |  |  |  |  |  |  |
| --- | --- | --- | --- | --- | --- | --- | --- | --- |
| U | *gi|41322908|ref|NP\_95* | 7 | 10 | 2.4% | 4525 | 513712 | 5.8 | plectin 1 isoform 3 [Homo sapiens] |
| U | *gi|47607492|ref|NP\_00* | 7 | 10 | 2.4% | 4574 | 518478 | 5.7 | plectin 1 isoform 1 [Homo sapiens] |
| U | *gi|41322923|ref|NP\_95* | 7 | 10 | 2.4% | 4547 | 516204 | 5.8 | plectin 1 isoform 11 [Homo sapiens] |
| U | *gi|41322919|ref|NP\_95* | 7 | 10 | 2.4% | 4547 | 516282 | 5.8 | plectin 1 isoform 8 [Homo sapiens] |
| U | *gi|41322916|ref|NP\_95* | 7 | 10 | 2.3% | 4684 | 531796 | 6.0 | plectin 1 isoform 6 [Homo sapiens] |
| U | *gi|41322914|ref|NP\_95* | 7 | 10 | 2.4% | 4551 | 516484 | 5.8 | plectin 1 isoform 10 [Homo sapiens] |
| U | *gi|41322912|ref|NP\_95* | 7 | 10 | 2.4% | 4533 | 514780 | 5.7 | plectin 1 isoform 2 [Homo sapiens] |
| U | *gi|41322910|ref|NP\_95* | 7 | 10 | 2.4% | 4515 | 512609 | 5.8 | plectin 1 isoform 7 [Homo sapiens] |

| Filename XCorr DeltCN Conf% ObsM+H+ CalcM+H+ SpR ZScore Ion% # Sequence  | | | | | | | | | | | | |
| --- | --- | --- | --- | --- | --- | --- | --- | --- | --- | --- | --- | --- |
|  | AstrinSTLCLD\_041714\_01.10769.10769.2 | 2.7756 | 0.3823 | 100.0% | 1529.7522 | 1529.6494 | 1 | 6.992 | 53.8% | 1 | R.ESADPLGAWLQDAR.R | 2 |
|  | AstrinSTLCLD\_041714\_01.07708.07708.2 | 2.8668 | 0.3441 | 100.0% | 1520.6322 | 1519.738 | 1 | 6.309 | 58.3% | 1 | K.AKLEQLFQDEVAK.A | 2 |
|  | AstrinSTLCLD\_041714\_02.04880.04880.3 | 3.3562 | 0.1925 | 97.5% | 1784.6943 | 1784.964 | 1 | 5.379 | 36.8% | 2 | R.AALAHSEEVTASQVAATK.T | 3 |
|  | AstrinSTLCLD\_041714\_02.06912.06912.2 | 4.2775 | 0.4132 | 100.0% | 1556.9321 | 1557.744 | 1 | 7.117 | 84.6% | 3 | R.LQEAGILSAEELQR.L | 2 |
|  | AstrinSTLCLD\_041714\_02.06845.06845.2 | 4.275 | 0.536 | 100.0% | 1614.2722 | 1614.8363 | 1 | 8.91 | 70.0% | 1 | R.LLDAQLSTGGIVDPSK.S | 2 |
|  | AstrinSTLCLD\_041714\_02.06815.06815.2 | 3.0406 | 0.2374 | 99.2% | 1463.4922 | 1462.6611 | 2 | 4.349 | 62.5% | 1 | R.SQVMDEATALQLR.E | 2 |
|  | AstrinSTLCLD\_041714\_01.12840.12840.2 | 4.9975 | 0.4823 | 100.0% | 2115.5322 | 2116.3533 | 1 | 8.937 | 37.5% | 1 | R.AGTLSITEFADMLSGNAGGFR.S | 2 |

---

|  |  |  |  |  |  |  |  |  |
| --- | --- | --- | --- | --- | --- | --- | --- | --- |
| U | *gi|44955929|ref|NP\_98* | 1 | 1 | 2.4% | 1478 | 166731 | 6.7 | calmodulin regulated spectrin-associated protein 1-like 1 [Homo sapiens] |

| Filename XCorr DeltCN Conf% ObsM+H+ CalcM+H+ SpR ZScore Ion% # Sequence  | | | | | | | | | | | | |
| --- | --- | --- | --- | --- | --- | --- | --- | --- | --- | --- | --- | --- |
| \* | AstrinSTLCLD\_041714\_01.14885.14885.3 | 3.5746 | 0.27 | 99.3% | 4170.5645 | 4169.1523 | 22 | 4.074 | 19.3% | 1 | R.DHIES\*PK@T#PIKGPPVS\*SLSLASLNT#GDNESVHS\*GKR.T | 3 |

---

|  |  |  |  |  |  |  |  |  |
| --- | --- | --- | --- | --- | --- | --- | --- | --- |
| U | *Reverse\_gi|224458284|* | 1 | 1 | 2.4% | 1397 | 151775 | 9.2 | Rho GTPase activating protein 23 [Homo sapiens] |

| Filename XCorr DeltCN Conf% ObsM+H+ CalcM+H+ SpR ZScore Ion% # Sequence  | | | | | | | | | | | | |
| --- | --- | --- | --- | --- | --- | --- | --- | --- | --- | --- | --- | --- |
| \* | AstrinSTLCLD\_041714\_01.13146.13146.3 | 3.4888 | 0.2231 | 97.7% | 3419.4243 | 3416.6995 | 101 | 3.728 | 18.8% | 1 | R.AAS\*QK@LFESK@LGGLGRSGKPSSDAK@PGSSHSVK.R | 3 |

---

|  |  |  |  |  |  |  |  |  |
| --- | --- | --- | --- | --- | --- | --- | --- | --- |
| U | *Reverse\_gi|34328899|r* | 1 | 1 | 2.4% | 1187 | 135261 | 8.3 | protein tyrosine phosphatase, non-receptor type 14 [Homo sapiens] |

| Filename XCorr DeltCN Conf% ObsM+H+ CalcM+H+ SpR ZScore Ion% # Sequence  | | | | | | | | | | | | |
| --- | --- | --- | --- | --- | --- | --- | --- | --- | --- | --- | --- | --- |
| \* | AstrinSTLCLD\_041714\_01.16893.16893.3 | 3.4287 | 0.2187 | 97.6% | 3582.3245 | 3580.5317 | 165 | 3.764 | 19.4% | 1 | K.LGT#T#AYCVSDTRFKTTVK@FKGYT#AS\*S\*HK@.S | 3 |

---

|  |  |  |  |  |  |  |  |  |
| --- | --- | --- | --- | --- | --- | --- | --- | --- |
| U | *gi|193083125|ref|NP\_0* | 1 | 1 | 2.4% | 753 | 84489 | 9.6 | MAP/microtubule affinity-regulating kinase 3 isoform a [Homo sapiens] |
| U | *gi|193083131|ref|NP\_0* | 1 | 1 | 2.7% | 659 | 74151 | 9.8 | MAP/microtubule affinity-regulating kinase 3 isoform e [Homo sapiens] |
| U | *gi|193083127|ref|NP\_0* | 1 | 1 | 2.4% | 744 | 83308 | 9.5 | MAP/microtubule affinity-regulating kinase 3 isoform b [Homo sapiens] |

| Filename XCorr DeltCN Conf% ObsM+H+ CalcM+H+ SpR ZScore Ion% # Sequence  | | | | | | | | | | | | |
| --- | --- | --- | --- | --- | --- | --- | --- | --- | --- | --- | --- | --- |
|  | AstrinSTLCLD\_041714\_02.06279.06279.3 | 2.8131 | 0.2403 | 97.0% | 2384.2744 | 2385.3726 | 2 | 4.353 | 35.3% | 1 | R.SRGSTNLFS\*KLT#S\*K@LT#RR.N | 3 |

---

|  |  |  |  |  |  |  |  |  |
| --- | --- | --- | --- | --- | --- | --- | --- | --- |
| U | *Reverse\_gi|118150654|* | 1 | 1 | 2.4% | 673 | 75111 | 5.6 | non-protein coding RNA 153 [Homo sapiens] |

| Filename XCorr DeltCN Conf% ObsM+H+ CalcM+H+ SpR ZScore Ion% # Sequence  | | | | | | | | | | | | |
| --- | --- | --- | --- | --- | --- | --- | --- | --- | --- | --- | --- | --- |
| \* | AstrinSTLCLD\_041714\_01.08364.08364.2 | 3.4337 | 0.0892 | 95.7% | 1907.3722 | 1909.0226 | 1 | 3.666 | 56.7% | 1 | K.QFRNETQNTNDQLTAK.K | 2 |

---

|  |  |  |  |  |  |  |  |  |
| --- | --- | --- | --- | --- | --- | --- | --- | --- |
| U | *gi|4557445|ref|NP\_001* | 1 | 1 | 2.4% | 551 | 60315 | 5.5 | regulator of chromosome condensation and BTB domain containing protein 2 [Homo sapiens] |

| Filename XCorr DeltCN Conf% ObsM+H+ CalcM+H+ SpR ZScore Ion% # Sequence  | | | | | | | | | | | | |
| --- | --- | --- | --- | --- | --- | --- | --- | --- | --- | --- | --- | --- |
| \* | AstrinSTLCLD\_041714\_01.11158.11158.2 | 3.0175 | 0.1734 | 97.9% | 1530.5721 | 1530.5884 | 8 | 4.472 | 54.2% | 1 | K.SNQS\*YPTPVTVEK.D | 2 |

---

|  |  |  |  |  |  |  |  |  |
| --- | --- | --- | --- | --- | --- | --- | --- | --- |
| U | *gi|4502643|ref|NP\_001* | 1 | 1 | 2.4% | 531 | 58024 | 6.7 | chaperonin containing TCP1, subunit 6A isoform a [Homo sapiens] |
| U | *gi|58331171|ref|NP\_00* | 1 | 1 | 2.7% | 486 | 53289 | 7.3 | chaperonin containing TCP1, subunit 6A isoform b [Homo sapiens] |

| Filename XCorr DeltCN Conf% ObsM+H+ CalcM+H+ SpR ZScore Ion% # Sequence  | | | | | | | | | | | | |
| --- | --- | --- | --- | --- | --- | --- | --- | --- | --- | --- | --- | --- |
|  | AstrinSTLCLD\_041714\_02.05921.05921.2 | 2.9206 | 0.3167 | 100.0% | 1256.9321 | 1256.4478 | 1 | 6.34 | 70.8% | 1 | R.AQAALAVNISAAR.G | 2 |

---

|  |  |  |  |  |  |  |  |  |
| --- | --- | --- | --- | --- | --- | --- | --- | --- |
| U | *gi|117968353|ref|NP\_1* | 1 | 2 | 2.4% | 464 | 54304 | 8.3 | NUF2, NDC80 kinetochore complex component [Homo sapiens] |
| U | *gi|117968420|ref|NP\_6* | 1 | 2 | 2.4% | 464 | 54304 | 8.3 | NUF2, NDC80 kinetochore complex component [Homo sapiens] |

| Filename XCorr DeltCN Conf% ObsM+H+ CalcM+H+ SpR ZScore Ion% # Sequence  | | | | | | | | | | | | |
| --- | --- | --- | --- | --- | --- | --- | --- | --- | --- | --- | --- | --- |
|  | AstrinSTLCLD\_041714\_01.09279.09279.2 | 2.3887 | 0.4393 | 100.0% | 1327.5122 | 1327.5693 | 1 | 6.687 | 55.0% | 2 | R.YNVAEIVIHIR.N | 2 |

---

|  |  |  |  |  |  |  |  |  |
| --- | --- | --- | --- | --- | --- | --- | --- | --- |
| U | *gi|164420685|ref|NP\_0* | 1 | 1 | 2.3% | 1198 | 130965 | 8.4 | homeodomain interacting protein kinase 2 isoform 1 [Homo sapiens] |
| U | *gi|164420691|ref|NP\_0* | 1 | 1 | 2.4% | 1171 | 128158 | 8.5 | homeodomain interacting protein kinase 2 isoform 2 [Homo sapiens] |

| Filename XCorr DeltCN Conf% ObsM+H+ CalcM+H+ SpR ZScore Ion% # Sequence  | | | | | | | | | | | | |
| --- | --- | --- | --- | --- | --- | --- | --- | --- | --- | --- | --- | --- |
|  | AstrinSTLCLD\_041714\_01.08050.08050.3 | 2.7885 | 0.2629 | 96.7% | 3343.4944 | 3344.3298 | 219 | 4.009 | 21.3% | 1 | R.KS\*KQHQSS\*VRNVS\*TCEVSSSQAISSPQR.S | 23 |

---

|  |  |  |  |  |  |  |  |  |
| --- | --- | --- | --- | --- | --- | --- | --- | --- |
| U | *gi|116235478|ref|NP\_4* | 1 | 1 | 2.3% | 840 | 94983 | 9.2 | establishment of cohesion 1 homolog 1 [Homo sapiens] |

| Filename XCorr DeltCN Conf% ObsM+H+ CalcM+H+ SpR ZScore Ion% # Sequence  | | | | | | | | | | | | |
| --- | --- | --- | --- | --- | --- | --- | --- | --- | --- | --- | --- | --- |
| \* | AstrinSTLCLD\_041714\_02.15059.15059.3 | 2.8877 | 0.2336 | 96.8% | 2405.1843 | 2403.2488 | 131 | 4.574 | 27.8% | 1 | K.S\*DDK@NS\*ETEIQDS\*QKNLAK.K | 3 |

---

|  |  |  |  |  |  |  |  |  |
| --- | --- | --- | --- | --- | --- | --- | --- | --- |
| U | *gi|13376259|ref|NP\_07* | 1 | 2 | 2.3% | 656 | 75019 | 5.6 | nucleoporin 85 [Homo sapiens] |

| Filename XCorr DeltCN Conf% ObsM+H+ CalcM+H+ SpR ZScore Ion% # Sequence  | | | | | | | | | | | | |
| --- | --- | --- | --- | --- | --- | --- | --- | --- | --- | --- | --- | --- |
| \* | AstrinSTLCLD\_041714\_01.14528.14528.2 | 3.8416 | 0.4552 | 100.0% | 1596.7722 | 1596.8822 | 1 | 8.15 | 71.4% | 2 | R.FADAASLLLSLMTSR.I | 2 |

---

|  |  |  |  |  |  |  |  |  |
| --- | --- | --- | --- | --- | --- | --- | --- | --- |
| U | *gi|27436951|ref|NP\_11* | 1 | 1 | 2.3% | 600 | 67689 | 5.3 | lamin B2 [Homo sapiens] |

| Filename XCorr DeltCN Conf% ObsM+H+ CalcM+H+ SpR ZScore Ion% # Sequence  | | | | | | | | | | | | |
| --- | --- | --- | --- | --- | --- | --- | --- | --- | --- | --- | --- | --- |
| \* | AstrinSTLCLD\_041714\_02.05910.05910.2 | 2.8267 | 0.3361 | 100.0% | 1506.2122 | 1504.6984 | 1 | 6.197 | 65.4% | 1 | R.TVLVNADGEEVAMR.T | 2 |

---

|  |  |  |  |  |  |  |  |  |
| --- | --- | --- | --- | --- | --- | --- | --- | --- |
| U | *gi|21265037|ref|NP\_05* | 2 | 2 | 2.2% | 1205 | 135575 | 7.1 | ADAM metallopeptidase with thrombospondin type 1 motif, 3 proprotein [Homo sapiens] |

| Filename XCorr DeltCN Conf% ObsM+H+ CalcM+H+ SpR ZScore Ion% # Sequence  | | | | | | | | | | | | |
| --- | --- | --- | --- | --- | --- | --- | --- | --- | --- | --- | --- | --- |
| \* | AstrinSTLCLD\_041714\_01.14043.14043.3 | 4.2732 | 0.1406 | 96.8% | 3379.0144 | 3381.4216 | 45 | 3.834 | 24.0% | 1 | R.EYELVT#PVS\*T#NLEGRYLSHTLS\*ASHKK.R | 3 |
| \* | AstrinSTLCLD\_041714\_01.14108.14108.3 | 4.3823 | 0.1555 | 97.7% | 3379.3442 | 3381.4216 | 14 | 3.917 | 24.0% | 1 | R.EYELVT#PVS\*T#NLEGRYLSHT#LSASHKK.R | 3 |

---

|  |  |  |  |  |  |  |  |  |
| --- | --- | --- | --- | --- | --- | --- | --- | --- |
| U | *gi|33859670|ref|NP\_05* | 1 | 1 | 2.2% | 728 | 80885 | 5.9 | leucine-rich repeats and calponin homology (CH) domain containing 1 [Homo sapiens] |

| Filename XCorr DeltCN Conf% ObsM+H+ CalcM+H+ SpR ZScore Ion% # Sequence  | | | | | | | | | | | | |
| --- | --- | --- | --- | --- | --- | --- | --- | --- | --- | --- | --- | --- |
| \* | AstrinSTLCLD\_041714\_02.05772.05772.2 | 3.7287 | 0.4526 | 100.0% | 1573.3922 | 1573.7031 | 1 | 7.62 | 56.7% | 1 | R.ALEEAANSGGLNLSAR.K | 2 |

---

|  |  |  |  |  |  |  |  |  |
| --- | --- | --- | --- | --- | --- | --- | --- | --- |
| U | *gi|119220550|ref|NP\_0* | 1 | 1 | 2.2% | 679 | 74385 | 6.0 | sidekick 1 isoform 2 [Homo sapiens] |
| U | *gi|119220552|ref|NP\_6* | 1 | 1 | 0.7% | 2213 | 242109 | 6.4 | sidekick 1 isoform 1 [Homo sapiens] |

| Filename XCorr DeltCN Conf% ObsM+H+ CalcM+H+ SpR ZScore Ion% # Sequence  | | | | | | | | | | | | |
| --- | --- | --- | --- | --- | --- | --- | --- | --- | --- | --- | --- | --- |
|  | AstrinSTLCLD\_041714\_02.07738.07738.2 | 2.6879 | 0.199 | 95.0% | 1821.5521 | 1821.9688 | 148 | 3.872 | 39.3% | 1 | R.HLNVK@ST#FSKK@NGTR.S | 2 |

---

|  |  |  |  |  |  |  |  |  |
| --- | --- | --- | --- | --- | --- | --- | --- | --- |
| U | *gi|32483374|ref|NP\_00* | 1 | 1 | 2.2% | 594 | 66050 | 9.2 | nucleolar protein 5A [Homo sapiens] |

| Filename XCorr DeltCN Conf% ObsM+H+ CalcM+H+ SpR ZScore Ion% # Sequence  | | | | | | | | | | | | |
| --- | --- | --- | --- | --- | --- | --- | --- | --- | --- | --- | --- | --- |
| \* | AstrinSTLCLD\_041714\_01.05963.05963.2 | 3.128 | 0.395 | 100.0% | 1377.8322 | 1377.5803 | 1 | 6.314 | 70.8% | 1 | K.YPASTVQILGAEK.A | 2 |

---

|  |  |  |  |  |  |  |  |  |
| --- | --- | --- | --- | --- | --- | --- | --- | --- |
| U | *Reverse\_gi|122937213|* | 1 | 1 | 2.1% | 1036 | 120095 | 9.3 | zinc finger protein 99 [Homo sapiens] |

| Filename XCorr DeltCN Conf% ObsM+H+ CalcM+H+ SpR ZScore Ion% # Sequence  | | | | | | | | | | | | |
| --- | --- | --- | --- | --- | --- | --- | --- | --- | --- | --- | --- | --- |
| \* | AstrinSTLCLD\_041714\_02.10693.10693.3 | 2.4474 | 0.2836 | 95.5% | 2812.2544 | 2813.86 | 1 | 4.315 | 28.6% | 1 | K.YSK@EGT#HIIEHKRLTS\*S\*NNFAK.G | 3 |

---

|  |  |  |  |  |  |  |  |  |
| --- | --- | --- | --- | --- | --- | --- | --- | --- |
| U | *Reverse\_gi|188497756|* | 1 | 1 | 2.1% | 983 | 103930 | 7.0 | ubiquitin associated protein 2-like isoform b [Homo sapiens] |
| U | *Reverse\_gi|188497758|* | 1 | 1 | 1.9% | 1087 | 114534 | 7.1 | ubiquitin associated protein 2-like isoform a [Homo sapiens] |

| Filename XCorr DeltCN Conf% ObsM+H+ CalcM+H+ SpR ZScore Ion% # Sequence  | | | | | | | | | | | | |
| --- | --- | --- | --- | --- | --- | --- | --- | --- | --- | --- | --- | --- |
|  | AstrinSTLCLD\_041714\_01.17728.17728.2 | 2.136 | 0.325 | 96.4% | 2056.0322 | 2053.2224 | 6 | 5.205 | 32.5% | 1 | K.SPLPS\*SPPPPAATSTAVAPSK@.E | 2 |

---

|  |  |  |  |  |  |  |  |  |
| --- | --- | --- | --- | --- | --- | --- | --- | --- |
| U | *gi|156416003|ref|NP\_0* | 1 | 1 | 2.1% | 664 | 72692 | 7.4 | succinate dehydrogenase complex, subunit A, flavoprotein precursor [Homo sapiens] |

| Filename XCorr DeltCN Conf% ObsM+H+ CalcM+H+ SpR ZScore Ion% # Sequence  | | | | | | | | | | | | |
| --- | --- | --- | --- | --- | --- | --- | --- | --- | --- | --- | --- | --- |
| \* | AstrinSTLCLD\_041714\_01.13230.13230.2 | 2.8241 | 0.4465 | 100.0% | 1474.6522 | 1474.7434 | 2 | 7.182 | 46.2% | 1 | R.LGANSLLDLVVFGR.A | 2 |

---

|  |  |  |  |  |  |  |  |  |
| --- | --- | --- | --- | --- | --- | --- | --- | --- |
| U | *gi|23397427|ref|NP\_00* | 1 | 1 | 2.1% | 623 | 69633 | 8.6 | synaptotagmin binding, cytoplasmic RNA interacting protein [Homo sapiens] |

| Filename XCorr DeltCN Conf% ObsM+H+ CalcM+H+ SpR ZScore Ion% # Sequence  | | | | | | | | | | | | |
| --- | --- | --- | --- | --- | --- | --- | --- | --- | --- | --- | --- | --- |
| \* | AstrinSTLCLD\_041714\_01.10090.10090.2 | 3.3101 | 0.3418 | 100.0% | 1474.4321 | 1474.6512 | 1 | 6.356 | 70.8% | 1 | R.NLANTVTEEILEK.A | 2 |

---

|  |  |  |  |  |  |  |  |  |
| --- | --- | --- | --- | --- | --- | --- | --- | --- |
| U | *Reverse\_gi|224451032|* | 1 | 1 | 2.0% | 1367 | 147265 | 10.2 | KIAA1683 isoform a [Homo sapiens] |
| U | *Reverse\_gi|54606862|r* | 1 | 1 | 2.3% | 1180 | 127693 | 10.2 | KIAA1683 isoform b [Homo sapiens] |
| U | *Reverse\_gi|224451036|* | 1 | 1 | 2.4% | 1134 | 122900 | 10.2 | KIAA1683 isoform c [Homo sapiens] |

| Filename XCorr DeltCN Conf% ObsM+H+ CalcM+H+ SpR ZScore Ion% # Sequence  | | | | | | | | | | | | |
| --- | --- | --- | --- | --- | --- | --- | --- | --- | --- | --- | --- | --- |
|  | AstrinSTLCLD\_041714\_02.10802.10802.3 | 3.2265 | 0.2093 | 95.2% | 3029.0645 | 3031.3833 | 1 | 4.021 | 25.0% | 1 | R.VGAQIT#IVALIRIEEAAQMSVMPEGS\*R.R | 3 |

---

|  |  |  |  |  |  |  |  |  |
| --- | --- | --- | --- | --- | --- | --- | --- | --- |
| U | *gi|14670350|ref|NP\_12* | 1 | 1 | 2.0% | 998 | 112416 | 6.4 | general transcription factor II, i isoform 1 [Homo sapiens] |
| U | *gi|169881252|ref|NP\_0* | 1 | 1 | 2.1% | 957 | 107970 | 7.9 | general transcription factor II, i isoform 4 [Homo sapiens] |
| U | *gi|14670354|ref|NP\_12* | 1 | 1 | 2.0% | 977 | 110106 | 7.4 | general transcription factor II, i isoform 3 [Homo sapiens] |
| U | *gi|14670352|ref|NP\_12* | 1 | 1 | 2.0% | 978 | 110280 | 6.7 | general transcription factor II, i isoform 2 [Homo sapiens] |

| Filename XCorr DeltCN Conf% ObsM+H+ CalcM+H+ SpR ZScore Ion% # Sequence  | | | | | | | | | | | | |
| --- | --- | --- | --- | --- | --- | --- | --- | --- | --- | --- | --- | --- |
|  | AstrinSTLCLD\_041714\_01.09934.09934.2 | 2.4811 | 0.2589 | 96.8% | 2357.5122 | 2358.6152 | 6 | 5.209 | 36.8% | 1 | K.FEAHPNDLYVEGLPENIPFR.S | 2 |

---

|  |  |  |  |  |  |  |  |  |
| --- | --- | --- | --- | --- | --- | --- | --- | --- |
| U | *gi|21536320|ref|NP\_65* | 1 | 1 | 2.0% | 756 | 84794 | 8.8 | heterogeneous nuclear ribonucleoprotein U-like 1 isoform d [Homo sapiens] |
| U | *gi|21536326|ref|NP\_00* | 1 | 1 | 1.8% | 856 | 95739 | 6.9 | heterogeneous nuclear ribonucleoprotein U-like 1 isoform a [Homo sapiens] |

| Filename XCorr DeltCN Conf% ObsM+H+ CalcM+H+ SpR ZScore Ion% # Sequence  | | | | | | | | | | | | |
| --- | --- | --- | --- | --- | --- | --- | --- | --- | --- | --- | --- | --- |
|  | AstrinSTLCLD\_041714\_02.06417.06417.2 | 5.3118 | 0.5049 | 100.0% | 1742.0322 | 1742.8857 | 1 | 9.152 | 85.7% | 1 | R.NYILDQTNVYGSAQR.R | 2 |

---

|  |  |  |  |  |  |  |  |  |
| --- | --- | --- | --- | --- | --- | --- | --- | --- |
| U | *gi|55956788|ref|NP\_00* | 1 | 1 | 2.0% | 710 | 76615 | 4.7 | nucleolin [Homo sapiens] |

| Filename XCorr DeltCN Conf% ObsM+H+ CalcM+H+ SpR ZScore Ion% # Sequence  | | | | | | | | | | | | |
| --- | --- | --- | --- | --- | --- | --- | --- | --- | --- | --- | --- | --- |
| \* | AstrinSTLCLD\_041714\_02.08100.08100.2 | 3.8808 | 0.4921 | 100.0% | 1563.4122 | 1562.6323 | 1 | 7.965 | 65.4% | 1 | K.GFGFVDFNSEEDAK.A | 2 |

---

|  |  |  |  |  |  |  |  |  |
| --- | --- | --- | --- | --- | --- | --- | --- | --- |
| U | *gi|17402900|ref|NP\_00* | 1 | 2 | 2.0% | 644 | 67560 | 7.6 | far upstream element-binding protein [Homo sapiens] |

| Filename XCorr DeltCN Conf% ObsM+H+ CalcM+H+ SpR ZScore Ion% # Sequence  | | | | | | | | | | | | |
| --- | --- | --- | --- | --- | --- | --- | --- | --- | --- | --- | --- | --- |
| \* | AstrinSTLCLD\_041714\_01.07155.07155.2 | 2.7804 | 0.4406 | 100.0% | 1336.4722 | 1337.5187 | 1 | 6.98 | 62.5% | 2 | R.IGGNEGIDVPIPR.F | 2 |

---

|  |  |  |  |  |  |  |  |  |
| --- | --- | --- | --- | --- | --- | --- | --- | --- |
| U | *gi|4506411|ref|NP\_002* | 1 | 2 | 2.0% | 587 | 63542 | 4.7 | Ran GTPase activating protein 1 [Homo sapiens] |

| Filename XCorr DeltCN Conf% ObsM+H+ CalcM+H+ SpR ZScore Ion% # Sequence  | | | | | | | | | | | | |
| --- | --- | --- | --- | --- | --- | --- | --- | --- | --- | --- | --- | --- |
| \* | AstrinSTLCLD\_041714\_01.06026.06026.2 | 2.4993 | 0.2825 | 98.7% | 1408.3922 | 1408.551 | 32 | 4.975 | 59.1% | 2 | R.VINLNDNTFTEK.G | 2 |

---

|  |  |  |  |  |  |  |  |  |
| --- | --- | --- | --- | --- | --- | --- | --- | --- |
| U | *gi|45243534|ref|NP\_98* | 1 | 1 | 1.9% | 1237 | 136064 | 5.5 | protocadherin 9 isoform 1 precursor [Homo sapiens] |
| U | *gi|9966883|ref|NP\_065* | 1 | 1 | 1.9% | 1203 | 132251 | 5.5 | protocadherin 9 isoform 2 precursor [Homo sapiens] |

| Filename XCorr DeltCN Conf% ObsM+H+ CalcM+H+ SpR ZScore Ion% # Sequence  | | | | | | | | | | | | |
| --- | --- | --- | --- | --- | --- | --- | --- | --- | --- | --- | --- | --- |
|  | AstrinSTLCLD\_041714\_01.18737.18737.3 | 3.0911 | 0.2667 | 98.3% | 2700.8342 | 2699.9734 | 2 | 4.758 | 27.3% | 1 | K.IALIT#VSDKDTDVNGK@VICFIER.E | 3 |

---

|  |  |  |  |  |  |  |  |  |
| --- | --- | --- | --- | --- | --- | --- | --- | --- |
| U | *gi|52630449|ref|NP\_11* | 1 | 1 | 1.9% | 1035 | 119032 | 6.1 | ubiquitin specific protease 48 isoform a [Homo sapiens] |
| U | *gi|76257392|ref|NP\_00* | 1 | 1 | 4.1% | 485 | 56151 | 6.4 | ubiquitin specific protease 48 isoform b [Homo sapiens] |

| Filename XCorr DeltCN Conf% ObsM+H+ CalcM+H+ SpR ZScore Ion% # Sequence  | | | | | | | | | | | | |
| --- | --- | --- | --- | --- | --- | --- | --- | --- | --- | --- | --- | --- |
|  | AstrinSTLCLD\_041714\_01.05622.05622.3 | 2.5165 | 0.3103 | 97.9% | 2431.2844 | 2431.6238 | 76 | 5.603 | 31.6% | 1 | R.WAETVRPEEVSQEHIETAYR.I | 3 |

---

|  |  |  |  |  |  |  |  |  |
| --- | --- | --- | --- | --- | --- | --- | --- | --- |
| U | *gi|225637473|ref|NP\_1* | 1 | 1 | 1.9% | 848 | 91864 | 7.7 | solute carrier organic anion transporter family, member 5A1 isoform 1 [Homo sapiens] |
| U | *gi|225637477|ref|NP\_0* | 1 | 1 | 2.3% | 687 | 73843 | 8.1 | solute carrier organic anion transporter family, member 5A1 isoform 2 [Homo sapiens] |
| U | *gi|225637475|ref|NP\_0* | 1 | 1 | 2.0% | 793 | 85850 | 7.7 | solute carrier organic anion transporter family, member 5A1 isoform 3 [Homo sapiens] |

| Filename XCorr DeltCN Conf% ObsM+H+ CalcM+H+ SpR ZScore Ion% # Sequence  | | | | | | | | | | | | |
| --- | --- | --- | --- | --- | --- | --- | --- | --- | --- | --- | --- | --- |
|  | AstrinSTLCLD\_041714\_01.06882.06882.3 | 2.7798 | 0.2902 | 99.1% | 1889.7544 | 1887.0428 | 115 | 4.623 | 31.7% | 1 | K.KKKFS\*VDAVSDDDVLK@.E | 3 |

---

|  |  |  |  |  |  |  |  |  |
| --- | --- | --- | --- | --- | --- | --- | --- | --- |
| U | *gi|150456415|ref|NP\_0* | 1 | 1 | 1.8% | 1753 | 190542 | 6.3 | HEAT repeat containing 5A [Homo sapiens] |

| Filename XCorr DeltCN Conf% ObsM+H+ CalcM+H+ SpR ZScore Ion% # Sequence  | | | | | | | | | | | | |
| --- | --- | --- | --- | --- | --- | --- | --- | --- | --- | --- | --- | --- |
| \* | AstrinSTLCLD\_041714\_01.16434.16434.3 | 3.4715 | 0.2007 | 96.8% | 3329.3044 | 3330.7625 | 47 | 4.153 | 18.5% | 1 | K.LPGGQLSS\*TVAASLQALK@GILSSPMARAEK@SR.T | 3 |

---

|  |  |  |  |  |  |  |  |  |
| --- | --- | --- | --- | --- | --- | --- | --- | --- |
| U | *gi|148491088|ref|NP\_0* | 2 | 2 | 1.8% | 1066 | 118870 | 8.3 | zinc finger protein 295 isoform L [Homo sapiens] |
| U | *gi|50345873|ref|NP\_06* | 2 | 2 | 1.8% | 1066 | 118870 | 8.3 | zinc finger protein 295 isoform L [Homo sapiens] |
| U | *gi|148491106|ref|NP\_0* | 2 | 2 | 2.2% | 865 | 95807 | 6.5 | zinc finger protein 295 isoform S [Homo sapiens] |

| Filename XCorr DeltCN Conf% ObsM+H+ CalcM+H+ SpR ZScore Ion% # Sequence  | | | | | | | | | | | | |
| --- | --- | --- | --- | --- | --- | --- | --- | --- | --- | --- | --- | --- |
|  | AstrinSTLCLD\_041714\_02.05164.05164.3 | 3.0691 | 0.3446 | 100.0% | 1753.3143 | 1752.876 | 15 | 5.572 | 33.3% | 1 | R.VTVGDAATTAAASSSSVTR.D | 3 |
|  | AstrinSTLCLD\_041714\_02.05192.05192.2 | 5.1464 | 0.5472 | 100.0% | 1753.8722 | 1752.876 | 1 | 8.974 | 52.8% | 1 | R.VTVGDAATTAAASSSSVTR.D | 2 |

---

|  |  |  |  |  |  |  |  |  |
| --- | --- | --- | --- | --- | --- | --- | --- | --- |
| U | *gi|20143967|ref|NP\_61* | 1 | 1 | 1.8% | 960 | 110059 | 8.5 | kinesin family member 23 isoform 1 [Homo sapiens] |
| U | *gi|6754472|ref|NP\_004* | 1 | 1 | 2.0% | 856 | 98105 | 8.5 | kinesin family member 23 isoform 2 [Homo sapiens] |

| Filename XCorr DeltCN Conf% ObsM+H+ CalcM+H+ SpR ZScore Ion% # Sequence  | | | | | | | | | | | | |
| --- | --- | --- | --- | --- | --- | --- | --- | --- | --- | --- | --- | --- |
|  | AstrinSTLCLD\_041714\_02.05792.05792.3 | 3.5433 | 0.3273 | 100.0% | 1965.4443 | 1966.17 | 3 | 5.653 | 32.8% | 1 | K.YMLTHQELASDGEIETK.L | 3 |

---

|  |  |  |  |  |  |  |  |  |
| --- | --- | --- | --- | --- | --- | --- | --- | --- |
| U | *gi|4505343|ref|NP\_002* | 1 | 1 | 1.8% | 790 | 91839 | 6.4 | nuclear cap binding protein subunit 1, 80kDa [Homo sapiens] |

| Filename XCorr DeltCN Conf% ObsM+H+ CalcM+H+ SpR ZScore Ion% # Sequence  | | | | | | | | | | | | |
| --- | --- | --- | --- | --- | --- | --- | --- | --- | --- | --- | --- | --- |
| \* | AstrinSTLCLD\_041714\_02.10402.10402.2 | 2.561 | 0.2568 | 97.7% | 1788.5521 | 1788.8662 | 5 | 5.134 | 50.0% | 1 | K.LT#IYT#T#LVGLLNAR.N | 2 |

---

|  |  |  |  |  |  |  |  |  |
| --- | --- | --- | --- | --- | --- | --- | --- | --- |
| U | *gi|4505749|ref|NP\_000* | 1 | 1 | 1.8% | 780 | 85182 | 8.0 | phosphofructokinase, muscle [Homo sapiens] |

| Filename XCorr DeltCN Conf% ObsM+H+ CalcM+H+ SpR ZScore Ion% # Sequence  | | | | | | | | | | | | |
| --- | --- | --- | --- | --- | --- | --- | --- | --- | --- | --- | --- | --- |
| \* | AstrinSTLCLD\_041714\_01.12180.12180.2 | 3.1674 | 0.2067 | 99.0% | 1674.8722 | 1675.9315 | 89 | 4.46 | 50.0% | 1 | R.EREGRLRAAYNLVK.R | 2 |

---

|  |  |  |  |  |  |  |  |  |
| --- | --- | --- | --- | --- | --- | --- | --- | --- |
| U | *gi|23510448|ref|NP\_00* | 1 | 1 | 1.8% | 734 | 82286 | 8.4 | minichromosome maintenance complex component 5 [Homo sapiens] |

| Filename XCorr DeltCN Conf% ObsM+H+ CalcM+H+ SpR ZScore Ion% # Sequence  | | | | | | | | | | | | |
| --- | --- | --- | --- | --- | --- | --- | --- | --- | --- | --- | --- | --- |
| \* | AstrinSTLCLD\_041714\_02.06064.06064.2 | 2.4555 | 0.3318 | 99.3% | 1316.9321 | 1317.4417 | 5 | 6.643 | 62.5% | 1 | R.VLGIQVDTDGSGR.S | 2 |

---

|  |  |  |  |  |  |  |  |  |
| --- | --- | --- | --- | --- | --- | --- | --- | --- |
| U | *Reverse\_gi|110347443|* | 1 | 1 | 1.7% | 1093 | 122842 | 4.9 | TATA element modulatory factor 1 [Homo sapiens] |

| Filename XCorr DeltCN Conf% ObsM+H+ CalcM+H+ SpR ZScore Ion% # Sequence  | | | | | | | | | | | | |
| --- | --- | --- | --- | --- | --- | --- | --- | --- | --- | --- | --- | --- |
| \* | AstrinSTLCLD\_041714\_01.09868.09868.2 | 2.5629 | 0.2857 | 98.1% | 2226.2722 | 2228.3752 | 142 | 5.015 | 25.0% | 1 | K.EKLLDATESSNLRT#ALEEK.I | 2 |

---

|  |  |  |  |  |  |  |  |  |
| --- | --- | --- | --- | --- | --- | --- | --- | --- |
| U | *Reverse\_gi|4506467|re* | 1 | 1 | 1.7% | 583 | 68564 | 6.4 | radixin [Homo sapiens] |

| Filename XCorr DeltCN Conf% ObsM+H+ CalcM+H+ SpR ZScore Ion% # Sequence  | | | | | | | | | | | | |
| --- | --- | --- | --- | --- | --- | --- | --- | --- | --- | --- | --- | --- |
| \* | AstrinSTLCLD\_041714\_01.06666.06666.2 | 2.4452 | 0.2681 | 98.8% | 1142.6322 | 1143.4124 | 75 | 5.176 | 55.6% | 1 | K.KKAEELLAIK.A | 2 |

---

|  |  |  |  |  |  |  |  |  |
| --- | --- | --- | --- | --- | --- | --- | --- | --- |
| U | *Reverse\_gi|71725360|r* | 1 | 1 | 1.6% | 1411 | 151190 | 8.0 | zinc finger protein 609 [Homo sapiens] |

| Filename XCorr DeltCN Conf% ObsM+H+ CalcM+H+ SpR ZScore Ion% # Sequence  | | | | | | | | | | | | |
| --- | --- | --- | --- | --- | --- | --- | --- | --- | --- | --- | --- | --- |
| \* | AstrinSTLCLD\_041714\_01.17020.17020.2 | 2.3072 | 0.3002 | 97.2% | 2380.5723 | 2381.4163 | 134 | 4.672 | 26.2% | 1 | R.GCEAGTK@AESAEK@SLHS\*ADSLK.V | 2 |

---

|  |  |  |  |  |  |  |  |  |
| --- | --- | --- | --- | --- | --- | --- | --- | --- |
| U | *gi|113420243|ref|XP\_9* | 1 | 1 | 1.6% | 1218 | 134310 | 6.9 | PREDICTED: hypothetical protein LOC377711 isoform 5 [Homo sapiens] |
| U | *gi|150010654|ref|NP\_1* | 1 | 1 | 1.2% | 1641 | 181276 | 6.9 | HEAT repeat containing 7A isoform 1 [Homo sapiens] |

| Filename XCorr DeltCN Conf% ObsM+H+ CalcM+H+ SpR ZScore Ion% # Sequence  | | | | | | | | | | | | |
| --- | --- | --- | --- | --- | --- | --- | --- | --- | --- | --- | --- | --- |
|  | AstrinSTLCLD\_041714\_01.10919.10919.2 | 2.5107 | 0.3312 | 99.0% | 2317.372 | 2314.739 | 2 | 5.807 | 31.6% | 1 | R.LVHLVESWDLRSGLLHVAIR.I | 2 |

---

|  |  |  |  |  |  |  |  |  |
| --- | --- | --- | --- | --- | --- | --- | --- | --- |
| U | *gi|21237725|ref|NP\_00* | 1 | 1 | 1.6% | 1102 | 126454 | 7.5 | phosphoinositide-3-kinase, catalytic, gamma polypeptide [Homo sapiens] |

| Filename XCorr DeltCN Conf% ObsM+H+ CalcM+H+ SpR ZScore Ion% # Sequence  | | | | | | | | | | | | |
| --- | --- | --- | --- | --- | --- | --- | --- | --- | --- | --- | --- | --- |
| \* | AstrinSTLCLD\_041714\_01.08112.08112.2 | 4.0496 | 0.0168 | 96.2% | 2216.612 | 2215.3857 | 3 | 3.493 | 50.0% | 1 | K.K@S\*LMDIPESQSEQDFVLR.V | 2 |

---

|  |  |  |  |  |  |  |  |  |
| --- | --- | --- | --- | --- | --- | --- | --- | --- |
| U | *gi|54234034|ref|NP\_05* | 1 | 3 | 1.6% | 977 | 110589 | 6.6 | vacuolar protein sorting 54 isoform 1 [Homo sapiens] |
| U | *gi|54234040|ref|NP\_00* | 1 | 3 | 1.7% | 965 | 109361 | 6.6 | vacuolar protein sorting 54 isoform 2 [Homo sapiens] |

| Filename XCorr DeltCN Conf% ObsM+H+ CalcM+H+ SpR ZScore Ion% # Sequence  | | | | | | | | | | | | |
| --- | --- | --- | --- | --- | --- | --- | --- | --- | --- | --- | --- | --- |
|  | AstrinSTLCLD\_041714\_01.08873.08873.2 | 3.0428 | 0.1744 | 97.5% | 1841.0922 | 1841.2218 | 3 | 4.398 | 46.7% | 3 | R.AVKFLMSRAKDGFLEK.L | 2 |

---

|  |  |  |  |  |  |  |  |  |
| --- | --- | --- | --- | --- | --- | --- | --- | --- |
| U | *Reverse\_gi|119226260|* | 1 | 1 | 1.6% | 916 | 103702 | 9.0 | calcium homeostasis endoplasmic reticulum protein [Homo sapiens] |

| Filename XCorr DeltCN Conf% ObsM+H+ CalcM+H+ SpR ZScore Ion% # Sequence  | | | | | | | | | | | | |
| --- | --- | --- | --- | --- | --- | --- | --- | --- | --- | --- | --- | --- |
| \* | AstrinSTLCLD\_041714\_02.07990.07990.2 | 3.1417 | 0.149 | 96.8% | 1801.2922 | 1799.7286 | 2 | 3.795 | 46.4% | 1 | K.S\*S\*RSNSRSSSRGRSK.S | 2 |

---

|  |  |  |  |  |  |  |  |  |
| --- | --- | --- | --- | --- | --- | --- | --- | --- |
| U | *Reverse\_gi|45238858|r* | 1 | 1 | 1.6% | 913 | 101366 | 5.5 | WD repeat domain 44 protein [Homo sapiens] |

| Filename XCorr DeltCN Conf% ObsM+H+ CalcM+H+ SpR ZScore Ion% # Sequence  | | | | | | | | | | | | |
| --- | --- | --- | --- | --- | --- | --- | --- | --- | --- | --- | --- | --- |
| \* | AstrinSTLCLD\_041714\_01.09839.09839.2 | 2.7218 | 0.2802 | 98.9% | 1825.8722 | 1826.9493 | 1 | 6.074 | 50.0% | 1 | K.S\*WSLDLLDATHGKYK@.C | 2 |

---

|  |  |  |  |  |  |  |  |  |
| --- | --- | --- | --- | --- | --- | --- | --- | --- |
| U | *gi|167466272|ref|NP\_6* | 2 | 2 | 1.6% | 745 | 83587 | 9.8 | cytoskeleton associated protein 2-like [Homo sapiens] |

| Filename XCorr DeltCN Conf% ObsM+H+ CalcM+H+ SpR ZScore Ion% # Sequence  | | | | | | | | | | | | |
| --- | --- | --- | --- | --- | --- | --- | --- | --- | --- | --- | --- | --- |
| \* | AstrinSTLCLD\_041714\_01.05839.05839.2 | 3.412 | 0.4265 | 100.0% | 1399.2322 | 1399.6334 | 4 | 6.777 | 68.2% | 1 | R.KVVLNILQDSNR.T | 2 |
| \* | AstrinSTLCLD\_041714\_01.07701.07701.2 | 3.3505 | 0.3581 | 100.0% | 1271.3522 | 1271.4594 | 1 | 6.789 | 75.0% | 1 | K.VVLNILQDSNR.T | 2 |

---

|  |  |  |  |  |  |  |  |  |
| --- | --- | --- | --- | --- | --- | --- | --- | --- |
| U | *gi|4507555|ref|NP\_003* | 1 | 1 | 1.6% | 694 | 75492 | 7.7 | thymopoietin isoform alpha [Homo sapiens] |

| Filename XCorr DeltCN Conf% ObsM+H+ CalcM+H+ SpR ZScore Ion% # Sequence  | | | | | | | | | | | | |
| --- | --- | --- | --- | --- | --- | --- | --- | --- | --- | --- | --- | --- |
| \* | AstrinSTLCLD\_041714\_01.05336.05336.2 | 3.5549 | 0.4566 | 100.0% | 1431.1122 | 1431.5449 | 1 | 7.713 | 85.0% | 1 | K.VIEEEWQQVDR.Q | 2 |

---

|  |  |  |  |  |  |  |  |  |
| --- | --- | --- | --- | --- | --- | --- | --- | --- |
| U | *gi|31652242|ref|NP\_85* | 1 | 1 | 1.5% | 1938 | 209652 | 9.1 | transcription factor 20 isoform 2 [Homo sapiens] |
| U | *gi|31652244|ref|NP\_00* | 1 | 1 | 1.5% | 1960 | 211769 | 9.0 | transcription factor 20 isoform 1 [Homo sapiens] |

| Filename XCorr DeltCN Conf% ObsM+H+ CalcM+H+ SpR ZScore Ion% # Sequence  | | | | | | | | | | | | |
| --- | --- | --- | --- | --- | --- | --- | --- | --- | --- | --- | --- | --- |
|  | AstrinSTLCLD\_041714\_01.12761.12761.3 | 3.5094 | 0.2375 | 98.3% | 3573.7144 | 3574.6938 | 139 | 4.526 | 18.1% | 1 | R.LLHSSKEGADKAFNS\*YAHLS\*HSQDIK@S\*IPK@.R | 3 |

---

|  |  |  |  |  |  |  |  |  |
| --- | --- | --- | --- | --- | --- | --- | --- | --- |
| U | *gi|30348954|ref|NP\_06* | 1 | 1 | 1.5% | 1006 | 110136 | 6.9 | mindbomb homolog 1 [Homo sapiens] |

| Filename XCorr DeltCN Conf% ObsM+H+ CalcM+H+ SpR ZScore Ion% # Sequence  | | | | | | | | | | | | |
| --- | --- | --- | --- | --- | --- | --- | --- | --- | --- | --- | --- | --- |
| \* | AstrinSTLCLD\_041714\_01.15387.15387.2 | 2.0994 | 0.3218 | 95.9% | 1800.6122 | 1800.9393 | 1 | 5.432 | 46.4% | 1 | R.K@S\*K@K@IT#ARGIFAGAR.V | 2 |

---

|  |  |  |  |  |  |  |  |  |
| --- | --- | --- | --- | --- | --- | --- | --- | --- |
| U | *gi|166795250|ref|NP\_0* | 1 | 1 | 1.5% | 725 | 81313 | 7.8 | kinesin family member 2C [Homo sapiens] |

| Filename XCorr DeltCN Conf% ObsM+H+ CalcM+H+ SpR ZScore Ion% # Sequence  | | | | | | | | | | | | |
| --- | --- | --- | --- | --- | --- | --- | --- | --- | --- | --- | --- | --- |
| \* | AstrinSTLCLD\_041714\_02.05572.05572.2 | 3.1968 | 0.3796 | 100.0% | 1275.5122 | 1276.4503 | 2 | 7.547 | 70.0% | 1 | R.LAMQLEEQASR.Q | 2 |

---

|  |  |  |  |  |  |  |  |  |
| --- | --- | --- | --- | --- | --- | --- | --- | --- |
| U | *gi|32481206|ref|NP\_00* | 1 | 1 | 1.4% | 1927 | 218585 | 6.3 | lactase-phlorizin hydrolase preproprotein [Homo sapiens] |

| Filename XCorr DeltCN Conf% ObsM+H+ CalcM+H+ SpR ZScore Ion% # Sequence  | | | | | | | | | | | | |
| --- | --- | --- | --- | --- | --- | --- | --- | --- | --- | --- | --- | --- |
| \* | AstrinSTLCLD\_041714\_01.11720.11720.3 | 2.851 | 0.2464 | 95.5% | 3327.7744 | 3325.5017 | 22 | 5.065 | 21.2% | 1 | R.GVASIADRS\*WPDSGS\*FWLK@MT#PFGFRR.I | 3 |

---

|  |  |  |  |  |  |  |  |  |
| --- | --- | --- | --- | --- | --- | --- | --- | --- |
| U | *Reverse\_gi|11968023|r* | 1 | 1 | 1.4% | 1883 | 208882 | 7.2 | zinc finger protein 106 homolog [Homo sapiens] |

| Filename XCorr DeltCN Conf% ObsM+H+ CalcM+H+ SpR ZScore Ion% # Sequence  | | | | | | | | | | | | |
| --- | --- | --- | --- | --- | --- | --- | --- | --- | --- | --- | --- | --- |
| \* | AstrinSTLCLD\_041714\_02.09022.09022.3 | 3.9943 | 0.1586 | 96.7% | 3064.4944 | 3066.2964 | 4 | 3.5 | 26.0% | 1 | K.TNKSINK@PDQK@QSLS\*KTAPCPLLPS\*K.L | 3 |

---

|  |  |  |  |  |  |  |  |  |
| --- | --- | --- | --- | --- | --- | --- | --- | --- |
| U | *Reverse\_gi|21264337|r* | 1 | 1 | 1.4% | 1544 | 169843 | 7.1 | ArfGAP with RhoGAP domain, ankyrin repeat and PH domain 3 [Homo sapiens] |

| Filename XCorr DeltCN Conf% ObsM+H+ CalcM+H+ SpR ZScore Ion% # Sequence  | | | | | | | | | | | | |
| --- | --- | --- | --- | --- | --- | --- | --- | --- | --- | --- | --- | --- |
| \* | AstrinSTLCLD\_041714\_02.12063.12063.2 | 2.4427 | 0.3688 | 99.4% | 2225.4321 | 2224.4355 | 2 | 5.0 | 31.0% | 1 | R.LAPTPASIQSSDPAKDQAAQAR.G | 2 |

---

|  |  |  |  |  |  |  |  |  |
| --- | --- | --- | --- | --- | --- | --- | --- | --- |
| U | *gi|26051235|ref|NP\_06* | 1 | 1 | 1.4% | 1156 | 128979 | 5.1 | nucleoporin 133kDa [Homo sapiens] |

| Filename XCorr DeltCN Conf% ObsM+H+ CalcM+H+ SpR ZScore Ion% # Sequence  | | | | | | | | | | | | |
| --- | --- | --- | --- | --- | --- | --- | --- | --- | --- | --- | --- | --- |
| \* | AstrinSTLCLD\_041714\_01.09429.09429.2 | 3.2507 | 0.391 | 100.0% | 1909.3522 | 1910.2206 | 1 | 6.814 | 46.7% | 1 | R.FLLHQETLPEQLLAEK.Q | 2 |

---

|  |  |  |  |  |  |  |  |  |
| --- | --- | --- | --- | --- | --- | --- | --- | --- |
| U | *gi|77404397|ref|NP\_05* | 1 | 1 | 1.4% | 910 | 101997 | 7.2 | staphylococcal nuclease domain containing 1 [Homo sapiens] |

| Filename XCorr DeltCN Conf% ObsM+H+ CalcM+H+ SpR ZScore Ion% # Sequence  | | | | | | | | | | | | |
| --- | --- | --- | --- | --- | --- | --- | --- | --- | --- | --- | --- | --- |
| \* | AstrinSTLCLD\_041714\_02.05844.05844.2 | 3.4952 | 0.4815 | 100.0% | 1466.4321 | 1466.6311 | 1 | 8.191 | 66.7% | 1 | K.VITEYLNAQESAK.S | 2 |

---

|  |  |  |  |  |  |  |  |  |
| --- | --- | --- | --- | --- | --- | --- | --- | --- |
| U | *gi|54859722|ref|NP\_05* | 1 | 1 | 1.3% | 1436 | 162121 | 5.5 | nucleoporin 160kDa [Homo sapiens] |

| Filename XCorr DeltCN Conf% ObsM+H+ CalcM+H+ SpR ZScore Ion% # Sequence  | | | | | | | | | | | | |
| --- | --- | --- | --- | --- | --- | --- | --- | --- | --- | --- | --- | --- |
| \* | AstrinSTLCLD\_041714\_01.15864.15864.2 | 2.7077 | 0.2888 | 99.0% | 2098.392 | 2098.4062 | 2 | 5.511 | 35.3% | 1 | R.FVSSPQTIVELFFQEVAR.K | 2 |

---

|  |  |  |  |  |  |  |  |  |
| --- | --- | --- | --- | --- | --- | --- | --- | --- |
| U | *gi|205360932|ref|NP\_0* | 1 | 1 | 1.3% | 1204 | 132888 | 5.9 | fibronectin type III domain containing 3B [Homo sapiens] |
| U | *gi|205360934|ref|NP\_0* | 1 | 1 | 1.3% | 1204 | 132888 | 5.9 | fibronectin type III domain containing 3B [Homo sapiens] |

| Filename XCorr DeltCN Conf% ObsM+H+ CalcM+H+ SpR ZScore Ion% # Sequence  | | | | | | | | | | | | |
| --- | --- | --- | --- | --- | --- | --- | --- | --- | --- | --- | --- | --- |
|  | AstrinSTLCLD\_041714\_01.09077.09077.3 | 2.9534 | 0.2024 | 95.5% | 1912.5543 | 1911.9713 | 50 | 4.404 | 33.3% | 1 | K.GPVTS\*HGFS\*VKWDPPK@.D | 3 |

---

|  |  |  |  |  |  |  |  |  |
| --- | --- | --- | --- | --- | --- | --- | --- | --- |
| U | *gi|38569421|ref|NP\_00* | 1 | 1 | 1.3% | 1101 | 120839 | 7.3 | ATP citrate lyase isoform 1 [Homo sapiens] |
| U | *gi|38569423|ref|NP\_94* | 1 | 1 | 1.3% | 1091 | 119772 | 7.3 | ATP citrate lyase isoform 2 [Homo sapiens] |

| Filename XCorr DeltCN Conf% ObsM+H+ CalcM+H+ SpR ZScore Ion% # Sequence  | | | | | | | | | | | | |
| --- | --- | --- | --- | --- | --- | --- | --- | --- | --- | --- | --- | --- |
|  | AstrinSTLCLD\_041714\_01.08009.08009.2 | 3.3607 | 0.2882 | 100.0% | 1493.2322 | 1492.647 | 1 | 5.952 | 65.4% | 1 | R.SGGMSNELNNIISR.T | 2 |

---

|  |  |  |  |  |  |  |  |  |
| --- | --- | --- | --- | --- | --- | --- | --- | --- |
| U | *gi|34878777|ref|NP\_06* | 1 | 1 | 1.3% | 975 | 113662 | 5.9 | ring finger protein 20 [Homo sapiens] |

| Filename XCorr DeltCN Conf% ObsM+H+ CalcM+H+ SpR ZScore Ion% # Sequence  | | | | | | | | | | | | |
| --- | --- | --- | --- | --- | --- | --- | --- | --- | --- | --- | --- | --- |
| \* | AstrinSTLCLD\_041714\_02.06214.06214.2 | 2.3309 | 0.3455 | 99.0% | 1376.2322 | 1376.5065 | 16 | 5.859 | 50.0% | 1 | K.LGGVSSTEELDIR.T | 2 |

---

|  |  |  |  |  |  |  |  |  |
| --- | --- | --- | --- | --- | --- | --- | --- | --- |
| U | *gi|156523968|ref|NP\_0* | 1 | 1 | 1.3% | 1014 | 113084 | 8.9 | poly (ADP-ribose) polymerase family, member 1 [Homo sapiens] |

| Filename XCorr DeltCN Conf% ObsM+H+ CalcM+H+ SpR ZScore Ion% # Sequence  | | | | | | | | | | | | |
| --- | --- | --- | --- | --- | --- | --- | --- | --- | --- | --- | --- | --- |
| \* | AstrinSTLCLD\_041714\_01.10070.10070.2 | 3.7048 | 0.5588 | 100.0% | 1378.6921 | 1378.5712 | 1 | 9.132 | 70.8% | 1 | R.TTNFAGILSQGLR.I | 2 |

---

|  |  |  |  |  |  |  |  |  |
| --- | --- | --- | --- | --- | --- | --- | --- | --- |
| U | *gi|171184451|ref|NP\_0* | 2 | 2 | 1.2% | 3117 | 350931 | 6.3 | centrosome-associated protein 350 [Homo sapiens] |

| Filename XCorr DeltCN Conf% ObsM+H+ CalcM+H+ SpR ZScore Ion% # Sequence  | | | | | | | | | | | | |
| --- | --- | --- | --- | --- | --- | --- | --- | --- | --- | --- | --- | --- |
| \* | AstrinSTLCLD\_041714\_01.08058.08058.2 | 2.248 | 0.3553 | 99.0% | 1279.1921 | 1279.5686 | 2 | 6.47 | 63.6% | 1 | R.VLIGNVQPGILR.F | 2 |
| \* | AstrinSTLCLD\_041714\_01.17415.17415.3 | 3.3053 | 0.3223 | 100.0% | 2835.6543 | 2836.2703 | 1 | 5.526 | 25.0% | 1 | K.MQLADGIFETLIKDTIDVLNQISEK.Q | 3 |

---

|  |  |  |  |  |  |  |  |  |
| --- | --- | --- | --- | --- | --- | --- | --- | --- |
| U | *gi|117553586|ref|NP\_0* | 1 | 1 | 1.2% | 1870 | 215307 | 8.0 | dedicator of cytokinesis 5 [Homo sapiens] |

| Filename XCorr DeltCN Conf% ObsM+H+ CalcM+H+ SpR ZScore Ion% # Sequence  | | | | | | | | | | | | |
| --- | --- | --- | --- | --- | --- | --- | --- | --- | --- | --- | --- | --- |
| \* | AstrinSTLCLD\_041714\_01.15639.15639.3 | 2.7031 | 0.2553 | 95.2% | 2705.5144 | 2702.7104 | 102 | 4.232 | 25.0% | 1 | R.LS\*PFHGSSPPQS\*T#PLS\*PPPLTPK@.A | 3 |

---

|  |  |  |  |  |  |  |  |  |
| --- | --- | --- | --- | --- | --- | --- | --- | --- |
| U | *gi|222537754|ref|NP\_0* | 1 | 1 | 1.2% | 1692 | 190926 | 7.9 | hypothetical protein LOC375337 [Homo sapiens] |

| Filename XCorr DeltCN Conf% ObsM+H+ CalcM+H+ SpR ZScore Ion% # Sequence  | | | | | | | | | | | | |
| --- | --- | --- | --- | --- | --- | --- | --- | --- | --- | --- | --- | --- |
| \* | AstrinSTLCLD\_041714\_01.09408.09408.3 | 3.0139 | 0.2541 | 97.9% | 2519.9043 | 2519.5586 | 143 | 4.252 | 25.0% | 1 | R.KESLTSSES\*FQT#VECLQSLGK.E | 3 |

---

|  |  |  |  |  |  |  |  |  |
| --- | --- | --- | --- | --- | --- | --- | --- | --- |
| U | *Reverse\_gi|55770888|r* | 1 | 1 | 1.2% | 1411 | 162465 | 5.7 | early endosome antigen 1, 162kD [Homo sapiens] |

| Filename XCorr DeltCN Conf% ObsM+H+ CalcM+H+ SpR ZScore Ion% # Sequence  | | | | | | | | | | | | |
| --- | --- | --- | --- | --- | --- | --- | --- | --- | --- | --- | --- | --- |
| \* | AstrinSTLCLD\_041714\_01.04612.04612.3 | 3.7461 | 0.2135 | 99.1% | 2148.9543 | 2150.4338 | 3 | 4.079 | 46.9% | 1 | K.EIFEKKLEEEEKKVQDK.L | 3 |

---

|  |  |  |  |  |  |  |  |  |
| --- | --- | --- | --- | --- | --- | --- | --- | --- |
| U | *Reverse\_gi|7661952|re* | 1 | 1 | 1.2% | 963 | 109935 | 5.6 | squamous cell carcinoma antigen recognized by T cells 3 [Homo sapiens] |

| Filename XCorr DeltCN Conf% ObsM+H+ CalcM+H+ SpR ZScore Ion% # Sequence  | | | | | | | | | | | | |
| --- | --- | --- | --- | --- | --- | --- | --- | --- | --- | --- | --- | --- |
| \* | AstrinSTLCLD\_041714\_02.06105.06105.2 | 2.5746 | 0.1915 | 95.4% | 1445.3522 | 1445.6188 | 18 | 4.194 | 59.1% | 1 | R.IARNHVS\*LVLDK.V | 2 |

---

|  |  |  |  |  |  |  |  |  |
| --- | --- | --- | --- | --- | --- | --- | --- | --- |
| U | *gi|115298682|ref|NP\_0* | 1 | 1 | 1.1% | 2817 | 308606 | 9.1 | HBxAg transactivated protein 2 [Homo sapiens] |

| Filename XCorr DeltCN Conf% ObsM+H+ CalcM+H+ SpR ZScore Ion% # Sequence  | | | | | | | | | | | | |
| --- | --- | --- | --- | --- | --- | --- | --- | --- | --- | --- | --- | --- |
| \* | AstrinSTLCLD\_041714\_01.05130.05130.3 | 4.9053 | 0.4213 | 100.0% | 3343.6443 | 3344.3513 | 3 | 6.063 | 29.8% | 1 | K.AAGS\*PSSSDQDEKLPGQDESTAGTSEQNDILK.V | 3 |

---

|  |  |  |  |  |  |  |  |  |
| --- | --- | --- | --- | --- | --- | --- | --- | --- |
| U | *gi|21536376|ref|NP\_00* | 1 | 1 | 1.1% | 2261 | 254300 | 6.9 | ATP-binding cassette, sub-family A member 1 [Homo sapiens] |

| Filename XCorr DeltCN Conf% ObsM+H+ CalcM+H+ SpR ZScore Ion% # Sequence  | | | | | | | | | | | | |
| --- | --- | --- | --- | --- | --- | --- | --- | --- | --- | --- | --- | --- |
| \* | AstrinSTLCLD\_041714\_01.06234.06234.3 | 3.5145 | 0.2406 | 98.5% | 3100.0144 | 3100.3555 | 28 | 4.164 | 21.0% | 1 | K.DLS\*LHKNQTVVDVAVLTS\*FLQDEK@VK.E | 3 |

---

|  |  |  |  |  |  |  |  |  |
| --- | --- | --- | --- | --- | --- | --- | --- | --- |
| U | *Reverse\_gi|209969819|* | 1 | 1 | 1.1% | 2151 | 237297 | 6.3 | hypothetical protein LOC253143 [Homo sapiens] |

| Filename XCorr DeltCN Conf% ObsM+H+ CalcM+H+ SpR ZScore Ion% # Sequence  | | | | | | | | | | | | |
| --- | --- | --- | --- | --- | --- | --- | --- | --- | --- | --- | --- | --- |
| \* | AstrinSTLCLD\_041714\_02.09417.09417.3 | 2.6129 | 0.289 | 97.4% | 2923.1643 | 2922.047 | 189 | 4.7 | 23.9% | 1 | K.QQS\*ISILTGNREK@PEEFIT#ELCR.N | 3 |

---

|  |  |  |  |  |  |  |  |  |
| --- | --- | --- | --- | --- | --- | --- | --- | --- |
| U | *Reverse\_gi|221316699|* | 1 | 1 | 1.1% | 1857 | 209851 | 5.5 | maltase-glucoamylase [Homo sapiens] |

| Filename XCorr DeltCN Conf% ObsM+H+ CalcM+H+ SpR ZScore Ion% # Sequence  | | | | | | | | | | | | |
| --- | --- | --- | --- | --- | --- | --- | --- | --- | --- | --- | --- | --- |
| \* | AstrinSTLCLD\_041714\_02.13678.13678.2 | 3.0237 | 0.1868 | 97.5% | 2331.7122 | 2333.4302 | 43 | 3.786 | 31.6% | 1 | K.NAVTDKT#EGNDWFLEGKAEK.N | 2 |

---

|  |  |  |  |  |  |  |  |  |
| --- | --- | --- | --- | --- | --- | --- | --- | --- |
| U | *gi|21071077|ref|NP\_05* | 1 | 1 | 1.1% | 1078 | 123473 | 7.2 | dishevelled-associated activator of morphogenesis 1 [Homo sapiens] |

| Filename XCorr DeltCN Conf% ObsM+H+ CalcM+H+ SpR ZScore Ion% # Sequence  | | | | | | | | | | | | |
| --- | --- | --- | --- | --- | --- | --- | --- | --- | --- | --- | --- | --- |
| \* | AstrinSTLCLD\_041714\_01.10077.10077.2 | 2.8437 | 0.1518 | 96.3% | 1598.6122 | 1599.598 | 5 | 4.475 | 54.5% | 1 | R.S\*K@TIES\*LKT#ALR.T | 2 |

---

|  |  |  |  |  |  |  |  |  |
| --- | --- | --- | --- | --- | --- | --- | --- | --- |
| U | *gi|21626468|ref|NP\_05* | 1 | 1 | 1.0% | 1978 | 220623 | 6.4 | zinc finger protein 638 [Homo sapiens] |
| U | *gi|62526045|ref|NP\_00* | 1 | 1 | 1.0% | 1978 | 220623 | 6.4 | zinc finger protein 638 [Homo sapiens] |

| Filename XCorr DeltCN Conf% ObsM+H+ CalcM+H+ SpR ZScore Ion% # Sequence  | | | | | | | | | | | | |
| --- | --- | --- | --- | --- | --- | --- | --- | --- | --- | --- | --- | --- |
|  | AstrinSTLCLD\_041714\_01.11146.11146.2 | 2.7358 | 0.2657 | 98.6% | 2130.0923 | 2129.4329 | 36 | 5.224 | 30.6% | 1 | K.AILQLDSPESAQSMYSFLK.Q | 2 |

---

|  |  |  |  |  |  |  |  |  |
| --- | --- | --- | --- | --- | --- | --- | --- | --- |
| U | *Reverse\_gi|33620716|r* | 1 | 3 | 1.0% | 1758 | 197286 | 9.6 | retinoblastoma-binding protein 6 isoform 2 [Homo sapiens] |
| U | *Reverse\_gi|33620769|r* | 1 | 3 | 0.9% | 1792 | 201563 | 9.6 | retinoblastoma-binding protein 6 isoform 1 [Homo sapiens] |

| Filename XCorr DeltCN Conf% ObsM+H+ CalcM+H+ SpR ZScore Ion% # Sequence  | | | | | | | | | | | | |
| --- | --- | --- | --- | --- | --- | --- | --- | --- | --- | --- | --- | --- |
|  | AstrinSTLCLD\_041714\_01.08151.08151.2 | 3.4132 | 0.1189 | 97.4% | 2212.7122 | 2213.2517 | 51 | 4.029 | 40.6% | 3 | K.EEKT#T#LKESKVDK@SYDK@.K | 2 |

---

|  |  |  |  |  |  |  |  |  |
| --- | --- | --- | --- | --- | --- | --- | --- | --- |
| U | *gi|42716280|ref|NP\_97* | 1 | 1 | 1.0% | 1268 | 141439 | 6.9 | high density lipoprotein binding protein [Homo sapiens] |
| U | *gi|4885409|ref|NP\_005* | 1 | 1 | 1.0% | 1268 | 141439 | 6.9 | high density lipoprotein binding protein [Homo sapiens] |

| Filename XCorr DeltCN Conf% ObsM+H+ CalcM+H+ SpR ZScore Ion% # Sequence  | | | | | | | | | | | | |
| --- | --- | --- | --- | --- | --- | --- | --- | --- | --- | --- | --- | --- |
|  | AstrinSTLCLD\_041714\_02.05236.05236.2 | 3.6846 | 0.3444 | 100.0% | 1328.5922 | 1328.5516 | 1 | 6.543 | 66.7% | 1 | R.LQTQASATVAIPK.E | 2 |

---

|  |  |  |  |  |  |  |  |  |
| --- | --- | --- | --- | --- | --- | --- | --- | --- |
| U | *gi|4502523|ref|NP\_000* | 1 | 1 | 0.9% | 2339 | 262494 | 8.5 | calcium channel, voltage-dependent, N type, alpha 1B subunit [Homo sapiens] |

| Filename XCorr DeltCN Conf% ObsM+H+ CalcM+H+ SpR ZScore Ion% # Sequence  | | | | | | | | | | | | |
| --- | --- | --- | --- | --- | --- | --- | --- | --- | --- | --- | --- | --- |
| \* | AstrinSTLCLD\_041714\_01.10540.10540.3 | 3.3152 | 0.1991 | 97.0% | 2509.9744 | 2511.8015 | 295 | 3.984 | 25.0% | 1 | K.VT#KYWSSLRNLVVS\*LLNSMK@.S | 3 |

---

|  |  |  |  |  |  |  |  |  |
| --- | --- | --- | --- | --- | --- | --- | --- | --- |
| U | *Reverse\_gi|207113160|* | 1 | 1 | 0.9% | 1488 | 152106 | 9.0 | Treacher Collins-Franceschetti syndrome 1 isoform d [Homo sapiens] |
| U | *Reverse\_gi|57164979|r* | 1 | 1 | 1.4% | 958 | 96757 | 5.6 | Treacher Collins-Franceschetti syndrome 1 isoform c [Homo sapiens] |
| U | *Reverse\_gi|57164977|r* | 1 | 1 | 0.9% | 1450 | 148253 | 8.9 | Treacher Collins-Franceschetti syndrome 1 isoform a [Homo sapiens] |
| U | *Reverse\_gi|57164975|r* | 1 | 1 | 0.9% | 1411 | 144314 | 9.0 | Treacher Collins-Franceschetti syndrome 1 isoform b [Homo sapiens] |
| U | *Reverse\_gi|207113164|* | 1 | 1 | 0.9% | 1412 | 144413 | 9.0 | Treacher Collins-Franceschetti syndrome 1 isoform f [Homo sapiens] |
| U | *Reverse\_gi|207113162|* | 1 | 1 | 0.9% | 1451 | 148352 | 8.9 | Treacher Collins-Franceschetti syndrome 1 isoform e [Homo sapiens] |

| Filename XCorr DeltCN Conf% ObsM+H+ CalcM+H+ SpR ZScore Ion% # Sequence  | | | | | | | | | | | | |
| --- | --- | --- | --- | --- | --- | --- | --- | --- | --- | --- | --- | --- |
|  | AstrinSTLCLD\_041714\_01.04485.04485.3 | 2.3185 | 0.2673 | 95.7% | 1496.5144 | 1495.5754 | 32 | 4.569 | 37.5% | 1 | R.T#KK@AQLAADEEAK.R | 3 |

---

|  |  |  |  |  |  |  |  |  |
| --- | --- | --- | --- | --- | --- | --- | --- | --- |
| U | *gi|118722349|ref|NP\_9* | 1 | 1 | 0.9% | 1001 | 118103 | 6.8 | RNA binding motif protein 12B [Homo sapiens] |

| Filename XCorr DeltCN Conf% ObsM+H+ CalcM+H+ SpR ZScore Ion% # Sequence  | | | | | | | | | | | | |
| --- | --- | --- | --- | --- | --- | --- | --- | --- | --- | --- | --- | --- |
| \* | AstrinSTLCLD\_041714\_01.09238.09238.2 | 2.4535 | 0.2827 | 99.2% | 1048.8522 | 1048.2706 | 4 | 5.35 | 81.2% | 1 | R.FLGTEVLLR.L | 2 |

---

|  |  |  |  |  |  |  |  |  |
| --- | --- | --- | --- | --- | --- | --- | --- | --- |
| U | *gi|153945728|ref|NP\_0* | 1 | 1 | 0.8% | 2468 | 270632 | 4.8 | microtubule-associated protein 1B [Homo sapiens] |

| Filename XCorr DeltCN Conf% ObsM+H+ CalcM+H+ SpR ZScore Ion% # Sequence  | | | | | | | | | | | | |
| --- | --- | --- | --- | --- | --- | --- | --- | --- | --- | --- | --- | --- |
| \* | AstrinSTLCLD\_041714\_02.07431.07431.3 | 3.4462 | 0.1767 | 96.5% | 2225.6643 | 2226.3074 | 1 | 4.025 | 40.3% | 1 | R.KLGDVS\*PTQIDVSQFGS\*FK@.E | 3 |

---

|  |  |  |  |  |  |  |  |  |
| --- | --- | --- | --- | --- | --- | --- | --- | --- |
| U | *Reverse\_gi|22538461|r* | 1 | 1 | 0.8% | 2440 | 270207 | 7.1 | nuclear receptor co-repressor 1 [Homo sapiens] |

| Filename XCorr DeltCN Conf% ObsM+H+ CalcM+H+ SpR ZScore Ion% # Sequence  | | | | | | | | | | | | |
| --- | --- | --- | --- | --- | --- | --- | --- | --- | --- | --- | --- | --- |
| \* | AstrinSTLCLD\_041714\_02.11251.11251.2 | 2.536 | 0.2298 | 95.3% | 2333.7922 | 2336.5154 | 2 | 4.874 | 33.3% | 1 | K.GRT#RLEEEYRSK@PPPGKDK.G | 2 |

---

|  |  |  |  |  |  |  |  |  |
| --- | --- | --- | --- | --- | --- | --- | --- | --- |
| U | *gi|21040314|ref|NP\_11* | 1 | 1 | 0.8% | 2303 | 250386 | 5.6 | SON DNA-binding protein isoform B [Homo sapiens] |
| U | *gi|21040326|ref|NP\_62* | 1 | 1 | 0.7% | 2426 | 263827 | 5.6 | SON DNA-binding protein isoform F [Homo sapiens] |

| Filename XCorr DeltCN Conf% ObsM+H+ CalcM+H+ SpR ZScore Ion% # Sequence  | | | | | | | | | | | | |
| --- | --- | --- | --- | --- | --- | --- | --- | --- | --- | --- | --- | --- |
|  | AstrinSTLCLD\_041714\_01.09741.09741.3 | 3.6023 | 0.1826 | 97.7% | 2273.1243 | 2274.1326 | 5 | 4.374 | 38.2% | 1 | K.T#KS\*HDDGNIDLES\*DSFLK@.F | 3 |

---

|  |  |  |  |  |  |  |  |  |
| --- | --- | --- | --- | --- | --- | --- | --- | --- |
| U | *Reverse\_gi|213972634|* | 1 | 1 | 0.8% | 1975 | 213987 | 4.7 | tumor protein p53 binding protein 1 isoform 2 [Homo sapiens] |
| U | *Reverse\_gi|5032189|re* | 1 | 1 | 0.8% | 1972 | 213573 | 4.7 | tumor protein p53 binding protein 1 isoform 3 [Homo sapiens] |
| U | *Reverse\_gi|213972636|* | 1 | 1 | 0.8% | 1977 | 214115 | 4.7 | tumor protein p53 binding protein 1 isoform 1 [Homo sapiens] |

| Filename XCorr DeltCN Conf% ObsM+H+ CalcM+H+ SpR ZScore Ion% # Sequence  | | | | | | | | | | | | |
| --- | --- | --- | --- | --- | --- | --- | --- | --- | --- | --- | --- | --- |
|  | AstrinSTLCLD\_041714\_01.08609.08609.2 | 2.6505 | 0.2106 | 95.5% | 1781.3722 | 1781.8983 | 120 | 3.918 | 33.3% | 1 | K.SCEVTLSSSHMDGDKK.G | 2 |

---

|  |  |  |  |  |  |  |  |  |
| --- | --- | --- | --- | --- | --- | --- | --- | --- |
| U | *gi|169218225|ref|XP\_9* | 1 | 1 | 0.8% | 1700 | 194322 | 6.7 | PREDICTED: hypothetical protein, partial [Homo sapiens] |
| U | *gi|40217847|ref|NP\_05* | 1 | 1 | 0.6% | 2136 | 244505 | 6.1 | activating signal cointegrator 1 complex subunit 3-like 1 [Homo sapiens] |

| Filename XCorr DeltCN Conf% ObsM+H+ CalcM+H+ SpR ZScore Ion% # Sequence  | | | | | | | | | | | | |
| --- | --- | --- | --- | --- | --- | --- | --- | --- | --- | --- | --- | --- |
|  | AstrinSTLCLD\_041714\_02.06470.06470.2 | 2.7038 | 0.3428 | 100.0% | 1376.5322 | 1376.5902 | 2 | 6.494 | 58.3% | 1 | K.VVLLTGETSTDLK.L | 2 |

---

|  |  |  |  |  |  |  |  |  |
| --- | --- | --- | --- | --- | --- | --- | --- | --- |
| U | *Reverse\_gi|221139764|* | 1 | 1 | 0.8% | 1648 | 178578 | 8.9 | PHD and ring finger domains 1 [Homo sapiens] |

| Filename XCorr DeltCN Conf% ObsM+H+ CalcM+H+ SpR ZScore Ion% # Sequence  | | | | | | | | | | | | |
| --- | --- | --- | --- | --- | --- | --- | --- | --- | --- | --- | --- | --- |
| \* | AstrinSTLCLD\_041714\_01.04154.04154.3 | 2.8213 | 0.2069 | 96.3% | 1315.8544 | 1314.2651 | 14 | 3.998 | 41.7% | 1 | R.ET#DSAGEAAVAGR.L | 3 |

---

|  |  |  |  |  |  |  |  |  |
| --- | --- | --- | --- | --- | --- | --- | --- | --- |
| U | *gi|24430149|ref|NP\_70* | 1 | 1 | 0.8% | 1391 | 155199 | 6.2 | nucleoporin 155kDa isoform 1 [Homo sapiens] |
| U | *gi|4758844|ref|NP\_004* | 1 | 1 | 0.8% | 1332 | 149016 | 6.3 | nucleoporin 155kDa isoform 2 [Homo sapiens] |

| Filename XCorr DeltCN Conf% ObsM+H+ CalcM+H+ SpR ZScore Ion% # Sequence  | | | | | | | | | | | | |
| --- | --- | --- | --- | --- | --- | --- | --- | --- | --- | --- | --- | --- |
|  | AstrinSTLCLD\_041714\_02.06761.06761.2 | 2.8011 | 0.1665 | 97.5% | 1370.4521 | 1370.5919 | 1 | 4.996 | 70.0% | 1 | R.IQLQIQETLQR.Q | 2 |

---

|  |  |  |  |  |  |  |  |  |
| --- | --- | --- | --- | --- | --- | --- | --- | --- |
| U | *Reverse\_gi|118442837|* | 1 | 1 | 0.8% | 1324 | 140405 | 7.0 | sal-like 1 isoform a [Homo sapiens] |
| U | *Reverse\_gi|189217876|* | 1 | 1 | 0.9% | 1227 | 129571 | 7.0 | sal-like 1 isoform b [Homo sapiens] |

| Filename XCorr DeltCN Conf% ObsM+H+ CalcM+H+ SpR ZScore Ion% # Sequence  | | | | | | | | | | | | |
| --- | --- | --- | --- | --- | --- | --- | --- | --- | --- | --- | --- | --- |
|  | AstrinSTLCLD\_041714\_01.06059.06059.2 | 2.6309 | 0.27 | 99.1% | 1444.5721 | 1444.4606 | 49 | 4.489 | 50.0% | 1 | R.HVS\*YHT#KLNGK.T | 2 |

---

|  |  |  |  |  |  |  |  |  |
| --- | --- | --- | --- | --- | --- | --- | --- | --- |
| U | *gi|105990514|ref|NP\_0* | 1 | 1 | 0.7% | 2602 | 278162 | 5.7 | filamin B, beta (actin binding protein 278) [Homo sapiens] |

| Filename XCorr DeltCN Conf% ObsM+H+ CalcM+H+ SpR ZScore Ion% # Sequence  | | | | | | | | | | | | |
| --- | --- | --- | --- | --- | --- | --- | --- | --- | --- | --- | --- | --- |
| \* | AstrinSTLCLD\_041714\_02.07019.07019.2 | 2.3899 | 0.3298 | 98.7% | 1630.3522 | 1629.8076 | 10 | 5.241 | 38.2% | 1 | R.GAGIGGLGITVEGPSESK.I | 2 |

---

|  |  |  |  |  |  |  |  |  |
| --- | --- | --- | --- | --- | --- | --- | --- | --- |
| U | *gi|112382250|ref|NP\_0* | 1 | 1 | 0.7% | 2364 | 274608 | 5.6 | spectrin, beta, non-erythrocytic 1 isoform 1 [Homo sapiens] |
| U | *gi|112382252|ref|NP\_8* | 1 | 1 | 0.8% | 2155 | 251395 | 5.5 | spectrin, beta, non-erythrocytic 1 isoform 2 [Homo sapiens] |

| Filename XCorr DeltCN Conf% ObsM+H+ CalcM+H+ SpR ZScore Ion% # Sequence  | | | | | | | | | | | | |
| --- | --- | --- | --- | --- | --- | --- | --- | --- | --- | --- | --- | --- |
|  | AstrinSTLCLD\_041714\_02.06472.06472.2 | 2.5462 | 0.3344 | 99.3% | 1778.0322 | 1778.914 | 1 | 5.447 | 46.9% | 1 | R.SQNIVTDSSSLSAEAIR.Q | 2 |

---

|  |  |  |  |  |  |  |  |  |
| --- | --- | --- | --- | --- | --- | --- | --- | --- |
| U | *gi|21264365|ref|NP\_05* | 1 | 1 | 0.7% | 1800 | 195816 | 6.4 | nucleoporin 98kD isoform 1 [Homo sapiens] |
| U | *gi|56549645|ref|NP\_62* | 1 | 1 | 0.8% | 1726 | 187198 | 6.5 | nucleoporin 98kD isoform 4 [Homo sapiens] |

| Filename XCorr DeltCN Conf% ObsM+H+ CalcM+H+ SpR ZScore Ion% # Sequence  | | | | | | | | | | | | |
| --- | --- | --- | --- | --- | --- | --- | --- | --- | --- | --- | --- | --- |
|  | AstrinSTLCLD\_041714\_01.09396.09396.2 | 2.3885 | 0.2391 | 95.2% | 1580.1322 | 1580.811 | 2 | 5.698 | 62.5% | 1 | R.LPMPEDYAMDELR.S | 2 |

---

|  |  |  |  |  |  |  |  |  |
| --- | --- | --- | --- | --- | --- | --- | --- | --- |
| U | *gi|74048514|ref|NP\_73* | 1 | 1 | 0.6% | 2342 | 265290 | 5.4 | cancer susceptibility candidate 5 isoform 1 [Homo sapiens] |
| U | *gi|74048554|ref|NP\_65* | 1 | 1 | 0.6% | 2316 | 262530 | 5.4 | cancer susceptibility candidate 5 isoform 2 [Homo sapiens] |

| Filename XCorr DeltCN Conf% ObsM+H+ CalcM+H+ SpR ZScore Ion% # Sequence  | | | | | | | | | | | | |
| --- | --- | --- | --- | --- | --- | --- | --- | --- | --- | --- | --- | --- |
|  | AstrinSTLCLD\_041714\_02.05555.05555.2 | 3.6514 | 0.5239 | 100.0% | 1501.1921 | 1501.6484 | 1 | 8.188 | 79.2% | 1 | K.TIYSGEENMDITK.S | 2 |

---

|  |  |  |  |  |  |  |  |  |
| --- | --- | --- | --- | --- | --- | --- | --- | --- |
| U | *Reverse\_gi|169218268|* | 1 | 4 | 0.5% | 2934 | 336126 | 6.5 | PREDICTED: similar to mutated in ataxia telangiectasia, partial [Homo sapiens] |

| Filename XCorr DeltCN Conf% ObsM+H+ CalcM+H+ SpR ZScore Ion% # Sequence  | | | | | | | | | | | | |
| --- | --- | --- | --- | --- | --- | --- | --- | --- | --- | --- | --- | --- |
| \* | AstrinSTLCLD\_041714\_01.08961.08961.2 | 2.7849 | 0.2836 | 99.0% | 1770.4722 | 1771.0046 | 12 | 5.001 | 46.7% | 4 | K.FLTISEGACQMLSNAK.Q | 2 |

---

|  |  |  |  |  |  |  |  |  |
| --- | --- | --- | --- | --- | --- | --- | --- | --- |
| U | *gi|49640009|ref|NP\_00* | 1 | 1 | 0.5% | 2025 | 229867 | 7.5 | tetratricopeptide repeat domain 3 [Homo sapiens] |
| U | *gi|49640011|ref|NP\_00* | 1 | 1 | 0.5% | 2025 | 229867 | 7.5 | tetratricopeptide repeat domain 3 [Homo sapiens] |

| Filename XCorr DeltCN Conf% ObsM+H+ CalcM+H+ SpR ZScore Ion% # Sequence  | | | | | | | | | | | | |
| --- | --- | --- | --- | --- | --- | --- | --- | --- | --- | --- | --- | --- |
|  | AstrinSTLCLD\_041714\_02.04790.04790.2 | 3.0683 | 0.1087 | 96.9% | 1448.2722 | 1447.5084 | 4 | 4.44 | 65.0% | 1 | K.MK@GNEEFS\*KER.F | 2 |

---

|  |  |  |  |  |  |  |  |  |
| --- | --- | --- | --- | --- | --- | --- | --- | --- |
| U | *gi|150378498|ref|NP\_0* | 1 | 1 | 0.4% | 5005 | 555490 | 6.6 | fragile site-associated protein [Homo sapiens] |

| Filename XCorr DeltCN Conf% ObsM+H+ CalcM+H+ SpR ZScore Ion% # Sequence  | | | | | | | | | | | | |
| --- | --- | --- | --- | --- | --- | --- | --- | --- | --- | --- | --- | --- |
| \* | AstrinSTLCLD\_041714\_01.07382.07382.3 | 2.5062 | 0.2873 | 97.6% | 2057.8743 | 2059.2158 | 139 | 4.821 | 26.5% | 1 | R.DREIS\*MSVGLGRSQLDSK.G | 3 |

---

|  |  |  |  |  |  |  |  |  |
| --- | --- | --- | --- | --- | --- | --- | --- | --- |
| U | *gi|126012562|ref|NP\_0* | 1 | 1 | 0.4% | 4544 | 504610 | 5.4 | low density lipoprotein-related protein 1 [Homo sapiens] |

| Filename XCorr DeltCN Conf% ObsM+H+ CalcM+H+ SpR ZScore Ion% # Sequence  | | | | | | | | | | | | |
| --- | --- | --- | --- | --- | --- | --- | --- | --- | --- | --- | --- | --- |
| \* | AstrinSTLCLD\_041714\_01.09074.09074.3 | 3.084 | 0.2595 | 98.2% | 2397.9543 | 2399.5422 | 1 | 4.677 | 32.9% | 1 | R.TNT#LAKANKWTGHNVT#VVQR.T | 3 |

---

|  |  |  |  |  |  |  |  |  |
| --- | --- | --- | --- | --- | --- | --- | --- | --- |
| U | *Reverse\_gi|157885806|* | 1 | 1 | 0.4% | 3996 | 439346 | 6.2 | chromosome 12 open reading frame 51 [Homo sapiens] |

| Filename XCorr DeltCN Conf% ObsM+H+ CalcM+H+ SpR ZScore Ion% # Sequence  | | | | | | | | | | | | |
| --- | --- | --- | --- | --- | --- | --- | --- | --- | --- | --- | --- | --- |
| \* | AstrinSTLCLD\_041714\_01.10666.10666.2 | 2.3391 | 0.2711 | 96.0% | 2042.1322 | 2043.1222 | 33 | 4.618 | 34.4% | 1 | K.K@ES\*LATRSVS\*NLVAK@DK@.A | 2 |

---

|  |  |  |  |  |  |  |  |  |
| --- | --- | --- | --- | --- | --- | --- | --- | --- |
| U | *gi|55770834|ref|NP\_05* | 1 | 1 | 0.4% | 3114 | 357527 | 5.1 | centromere protein F [Homo sapiens] |

| Filename XCorr DeltCN Conf% ObsM+H+ CalcM+H+ SpR ZScore Ion% # Sequence  | | | | | | | | | | | | |
| --- | --- | --- | --- | --- | --- | --- | --- | --- | --- | --- | --- | --- |
| \* | AstrinSTLCLD\_041714\_02.06610.06610.3 | 2.6982 | 0.2194 | 95.2% | 1705.6144 | 1705.9238 | 38 | 5.006 | 30.8% | 1 | R.TLEMDRENLSVEIR.N | 3 |

---

|  |  |  |  |  |  |  |  |  |
| --- | --- | --- | --- | --- | --- | --- | --- | --- |
| U | *gi|58530840|ref|NP\_00* | 1 | 1 | 0.4% | 2871 | 331774 | 6.8 | desmoplakin isoform I [Homo sapiens] |

| Filename XCorr DeltCN Conf% ObsM+H+ CalcM+H+ SpR ZScore Ion% # Sequence  | | | | | | | | | | | | |
| --- | --- | --- | --- | --- | --- | --- | --- | --- | --- | --- | --- | --- |
| \* | AstrinSTLCLD\_041714\_01.05588.05588.2 | 2.7727 | 0.3645 | 100.0% | 1388.1522 | 1388.5205 | 1 | 6.308 | 68.2% | 1 | R.LNDSILQATEQR.R | 2 |

---

|  |  |  |  |  |  |  |  |  |
| --- | --- | --- | --- | --- | --- | --- | --- | --- |
| U | *Reverse\_gi|83367077|r* | 2 | 2 | 0.3% | 14507 | 1519247 | 5.3 | mucin 16 [Homo sapiens] |

| Filename XCorr DeltCN Conf% ObsM+H+ CalcM+H+ SpR ZScore Ion% # Sequence  | | | | | | | | | | | | |
| --- | --- | --- | --- | --- | --- | --- | --- | --- | --- | --- | --- | --- |
| \* | AstrinSTLCLD\_041714\_01.06733.06733.2 | 2.5877 | 0.1909 | 96.6% | 1404.0922 | 1403.3617 | 96 | 4.262 | 50.0% | 1 | K.EKENWS\*DSTAR.A | 2 |
| \* | AstrinSTLCLD\_041714\_02.11417.11417.3 | 2.6677 | 0.2677 | 95.8% | 3370.2544 | 3369.0688 | 138 | 2.774 | 18.5% | 1 | K.T#VDMTSS\*AIST#NILDS\*S\*SSSDSPLENT#K.R | 3 |

---

|  |  |  |  |  |  |  |  |  |
| --- | --- | --- | --- | --- | --- | --- | --- | --- |
| U | *gi|207452735|ref|NP\_1* | 1 | 1 | 0.3% | 5090 | 555629 | 5.6 | epiplakin 1 [Homo sapiens] |

| Filename XCorr DeltCN Conf% ObsM+H+ CalcM+H+ SpR ZScore Ion% # Sequence  | | | | | | | | | | | | |
| --- | --- | --- | --- | --- | --- | --- | --- | --- | --- | --- | --- | --- |
| \* | AstrinSTLCLD\_041714\_02.06335.06335.2 | 2.8804 | 0.3068 | 100.0% | 1414.9922 | 1415.5864 | 1 | 6.698 | 62.5% | 1 | R.ALEAEQVEITVGR.F | 2 |

|  |  |  |  |
| --- | --- | --- | --- |
|  | Proteins | Peptide IDs | Spectra |
| Unfiltered | 31484 | 44501 | 75879 |
| Filtered | 347 | 861 | 1562 |
| Forward matches | 294 | 807 | 1489 |
| Decoy matches | 53 | 54 | 73 |
| Forward FP rate | 18.03% | 6.69% | 4.9% |

  
/nfs/cheeseman\_massspec/David/AstrinSTLCNLD
