# Supplementary material for: Astrin-SKAP complex reconstitution reveals its kinetochore interaction with microtubule-bound Ndc80
Source: eLife. 2017 Aug 25;6:e26866. doi: 10.7554/eLife.26866 (PMC5602300; doi:10.7554/eLife.26866)
Supplement: Source data 1. — Complete mass spectrometry searches using methods described in (Washburn et al., 2001) for affinity purification/mass spectrometry data sets described in this paper (data from this study; [Kern et al., 2016] [Gascoigne et al., 2011]). Individual Astrin cross-linking immunoprecipitations are listed based on the order in Figure 4—figure supplement 1. These samples have not been pruned for common or antibody-specific contaminants. [file elife-26866-data1.zip › Astrin_Crosslinking_Combined.html]

D PooledAstrinAbCAKEpreps
DTASelect v2.0.21  
/lab/cheeseman\_lab/David/ASKinetochorePaper/PooledAstrinAbCAKEpreps  
/nfs/cheeseman\_massspec/Databases/NCBI-RefSeq\_human\_na\_04-13-2009\_con\_reversed.fasta  
SEQUEST 3.0 in SQT format.  
  
 Jump  to the summary table.  
  
sequest.params modifications:

|  |  |  |
| --- | --- | --- |
| \* | S | 80.0 |
| # | T | 80.0 |
| @ | K | 12.0 |
| Static | C | 57.0 |

|  |  |
| --- | --- |
| true | Use criteria |
| 0.0 | Minimum peptide confidence |
| 0.05 | Peptide false positive rate |
| 0.0 | Minimum protein confidence |
| 1.0 | Protein false positive rate |
| 1 | Minimum charge state |
| 16 | Maximum charge state |
| 0.0 | Minimum ion proportion |
| 1000 | Maximum Sp rank |
| -1.0 | Minimum Sp score |
| Include | Modified peptide inclusion |
| Any | Tryptic status requirement |
| false | Multiple, ambiguous IDs allowed |
| Ignore | Peptide validation handling |
| XCorr | Purge duplicate peptides by protein |
| false | Include only loci with unique peptide |
| true | Remove subset proteins |
| Ignore | Locus validation handling |
| 0 | Minimum modified peptides per locus |
| 1000 | Minimum redundancy for low coverage loci |
| 2 | Minimum peptides per locus |

#### Locus Key:

|  |  |  |  |  |  |  |  |  |
| --- | --- | --- | --- | --- | --- | --- | --- | --- |
| Validation Status | Locus | Sequence Count | Spectrum Count | Sequence Coverage | Length | MolWt | pI | Descriptive Name |

#### Similarity Key:

|  |  |  |
| --- | --- | --- |
| Locus | # of identical peptides | # of differing peptides |

---

|  |  |  |  |  |  |  |  |  |
| --- | --- | --- | --- | --- | --- | --- | --- | --- |
| U | *gi|154800483|ref|NP\_0* | 59 | 570 | 94.1% | 339 | 39541 | 9.1 | centromere protein N isoform 2 [Homo sapiens] |

| Filename XCorr DeltCN Conf% ObsM+H+ CalcM+H+ SpR ZScore Ion% # Sequence  | | | | | | | | | | | | |
| --- | --- | --- | --- | --- | --- | --- | --- | --- | --- | --- | --- | --- |
|  | Astrin\_NLD\_STLC\_tube2\_021014\_01.09740.09740.2 | 3.6546 | 0.4522 | 100.0% | 1182.9722 | 1183.3618 | 1 | 8.176 | 77.8% | 6 | -.MDETVAEFIK.R | 22 |
|  | Astrin\_NLD\_STLC\_031014\_02.06572.06572.3 | 2.4158 | 0.2871 | 98.1% | 1338.5643 | 1339.5493 | 477 | 5.488 | 32.5% | 1 | -.MDETVAEFIKR.T | 3 |
|  | Astrin\_NLD\_STLC\_tube2\_021014\_01.08414.08414.2 | 3.2945 | 0.3467 | 100.0% | 1338.9321 | 1339.5493 | 1 | 6.946 | 75.0% | 13 | -.MDETVAEFIKR.T | 22 |
|  | Astrin\_NLD\_STLC\_tube2\_021014\_01.12992.12992.2 | 3.4768 | 0.2917 | 99.9% | 1728.4922 | 1729.1719 | 2 | 5.034 | 60.7% | 7 | R.TILKIPMNELTTILK.A | 22 |
|  | Astrin\_NLD\_STLC\_031014\_01.11240.11240.3 | 4.4357 | 0.4426 | 100.0% | 1729.9143 | 1729.1719 | 14 | 6.628 | 46.4% | 8 | R.TILKIPMNELTTILK.A | 33 |
|  | Astrin\_NLD\_STLC\_tube2\_021014\_01.17877.17877.3 | 6.2108 | 0.4843 | 100.0% | 3677.9644 | 3679.3123 | 1 | 8.134 | 35.0% | 1 | R.TILKIPMNELTTILKAWDFLSENQLQTVNFR.Q | 33 |
|  | Astrin\_NLD\_STLC\_tube2\_021014\_01.11900.11900.2 | 3.6942 | 0.417 | 100.0% | 1273.2922 | 1273.5737 | 1 | 7.132 | 85.0% | 29 | K.IPMNELTTILK.A | 22 |
|  | Astrin\_NLD\_STLC\_tube2\_021014\_01.12981.12981.1 | 2.1951 | 0.5538 | 100.0% | 1967.88 | 1969.1637 | 3 | 8.043 | 40.0% | 1 | K.AWDFLSENQLQTVNFR.Q | 11 |
|  | Astrin\_NLD\_STLC\_tube2\_021014\_02.09398.09398.2 | 5.0365 | 0.5022 | 100.0% | 1968.2722 | 1969.1637 | 1 | 8.886 | 70.0% | 44 | K.AWDFLSENQLQTVNFR.Q | 22 |
|  | Astrin\_NLD\_STLC\_tube2\_021014\_02.09404.09404.3 | 5.1607 | 0.5166 | 100.0% | 1969.5243 | 1969.1637 | 1 | 9.199 | 41.7% | 13 | K.AWDFLSENQLQTVNFR.Q | 33 |
|  | Astrin\_NLD\_STLC\_031014\_01.09758.09758.3 | 3.732 | 0.2028 | 99.3% | 1849.5844 | 1850.0912 | 1 | 5.154 | 42.9% | 1 | R.KESVVQHLIHLCEEK.R | 33 |
|  | Astrin\_NLD\_STLC\_031014\_01.10187.10187.2 | 3.5328 | 0.3523 | 100.0% | 1720.7922 | 1721.9171 | 1 | 6.518 | 57.7% | 1 | K.ESVVQHLIHLCEEK.R | 22 |
|  | Astrin\_NLD\_STLC\_tube2\_021014\_01.12916.12916.3 | 6.8447 | 0.4011 | 100.0% | 2586.2344 | 2586.9714 | 1 | 8.212 | 38.1% | 2 | K.RASISDAALLDIIYMQFHQHQK.V | 33 |
|  | Astrin\_NLD\_STLC\_tube2\_021014\_01.13785.13785.2 | 4.7865 | 0.4513 | 100.0% | 2429.652 | 2430.784 | 1 | 8.408 | 50.0% | 1 | R.ASISDAALLDIIYMQFHQHQK.V | 22 |
|  | Astrin\_NLD\_STLC\_tube2\_021014\_01.13844.13844.3 | 3.1585 | 0.266 | 98.6% | 2429.9944 | 2430.784 | 1 | 5.157 | 35.0% | 3 | R.ASISDAALLDIIYMQFHQHQK.V | 33 |
|  | Astrin\_NLD\_STLC\_tube2\_021014\_01.15286.15286.3 | 4.5068 | 0.415 | 100.0% | 3551.3342 | 3552.103 | 1 | 6.204 | 26.7% | 1 | R.ASISDAALLDIIYMQFHQHQKVWDVFQMSK.G | 33 |
|  | Astrin\_NLD\_STLC\_tube2\_021014\_01.11547.11547.1 | 2.0847 | 0.3667 | 96.5% | 1139.49 | 1140.3423 | 1 | 6.837 | 75.0% | 2 | K.VWDVFQMSK.G | 11 |
|  | Astrin\_NLD\_STLC\_tube2\_021014\_01.11516.11516.2 | 3.399 | 0.3196 | 100.0% | 1140.0521 | 1140.3423 | 1 | 6.892 | 81.2% | 6 | K.VWDVFQMSK.G | 22 |
|  | Astrin\_NLD\_STLC\_tube2\_021014\_01.10488.10488.1 | 2.6404 | 0.387 | 100.0% | 1322.45 | 1323.4602 | 2 | 8.362 | 59.1% | 1 | K.GPGEDVDLFDMK.Q | 11 |
|  | Astrin\_NLD\_STLC\_tube2\_021014\_01.10502.10502.2 | 4.1308 | 0.5977 | 100.0% | 1323.0122 | 1323.4602 | 1 | 10.286 | 68.2% | 22 | K.GPGEDVDLFDMK.Q | 22 |
|  | Astrin\_NLD\_STLC\_tube2\_021014\_01.10685.10685.2 | 4.5421 | 0.4915 | 100.0% | 1726.1921 | 1726.9415 | 1 | 7.831 | 64.3% | 1 | K.GPGEDVDLFDMKQFK.N | 22 |
|  | Astrin\_NLD\_STLC\_tube2\_021014\_01.10688.10688.3 | 3.4703 | 0.5038 | 100.0% | 1726.8544 | 1726.9415 | 2 | 7.142 | 44.6% | 1 | K.GPGEDVDLFDMKQFK.N | 33 |
|  | Astrin\_NLD\_STLC\_tube2\_021014\_01.06052.06052.2 | 3.5049 | 0.3337 | 100.0% | 1135.4521 | 1135.3518 | 5 | 7.113 | 77.8% | 2 | R.ALKNVTVSFR.E | 22 |
|  | Astrin\_NLD\_STLC\_tube2\_021014\_01.07694.07694.1 | 1.8497 | 0.2567 | 96.9% | 1246.58 | 1247.3501 | 230 | 6.095 | 50.0% | 1 | R.ETEENAVWIR.I | 11 |
|  | Astrin\_NLD\_STLC\_tube2\_021014\_01.07724.07724.2 | 2.8236 | 0.3418 | 100.0% | 1247.0122 | 1247.3501 | 7 | 5.842 | 77.8% | 5 | R.ETEENAVWIR.I | 22 |
|  | Astrin\_NLD\_STLC\_tube2\_021014\_01.11631.11631.3 | 5.959 | 0.4887 | 100.0% | 4141.9443 | 4142.675 | 1 | 7.631 | 27.2% | 3 | R.IAWGTQYTKPNQYKPTYVVYYSQTPYAFTSSSMLR.R | 33 |
|  | Astrin\_NLD\_STLC\_tube2\_021014\_01.09380.09380.2 | 4.4996 | 0.4962 | 100.0% | 1583.2722 | 1583.8717 | 1 | 8.02 | 71.4% | 11 | R.RNTPLLGQALTIASK.H | 22 |
|  | Astrin\_NLD\_STLC\_tube2\_021014\_01.09268.09268.3 | 4.5168 | 0.4501 | 100.0% | 1584.4744 | 1583.8717 | 1 | 7.835 | 44.6% | 4 | R.RNTPLLGQALTIASK.H | 33 |
|  | Astrin\_NLD\_STLC\_tube2\_021014\_01.10896.10896.1 | 2.4538 | 0.2844 | 97.6% | 1426.73 | 1427.6842 | 1 | 5.212 | 57.7% | 4 | R.NTPLLGQALTIASK.H | 11 |
|  | Astrin\_NLD\_STLC\_tube2\_021014\_01.11392.11392.2 | 4.5659 | 0.5253 | 100.0% | 1428.4922 | 1427.6842 | 1 | 8.647 | 80.8% | 53 | R.NTPLLGQALTIASK.H | 22 |
|  | Astrin\_NLD\_STLC\_tube2\_021014\_01.10924.10924.3 | 3.4434 | 0.2154 | 99.1% | 1429.0443 | 1427.6842 | 1 | 5.448 | 51.9% | 1 | R.NTPLLGQALTIASK.H | 33 |
|  | Astrin\_NLD\_STLC\_tube2\_021014\_01.04931.04931.3 | 2.7735 | 0.1862 | 95.3% | 1277.5144 | 1277.5303 | 1 | 4.804 | 52.8% | 2 | K.HHQIVKMDLR.S | 33 |
|  | Astrin\_NLD\_STLC\_031014\_01.09153.09153.2 | 2.7419 | 0.1846 | 99.1% | 981.9922 | 982.12463 | 2 | 4.094 | 85.7% | 3 | R.SRYLDSLK.A | 22 |
|  | Astrin\_NLD\_STLC\_031014\_01.09605.09605.3 | 5.1117 | 0.4057 | 100.0% | 2753.2444 | 2754.0312 | 1 | 6.858 | 34.1% | 2 | K.AIVFKQYNQTFETHNSTTPLQER.S | 33 |
|  | Astrin\_NLD\_STLC\_tube2\_021014\_01.05517.05517.2 | 3.3667 | 0.5209 | 100.0% | 2194.392 | 2195.3098 | 1 | 7.519 | 50.0% | 4 | K.QYNQTFETHNSTTPLQER.S | 22 |
|  | Astrin\_NLD\_STLC\_tube2\_021014\_01.05397.05397.3 | 3.7725 | 0.399 | 99.9% | 2194.6743 | 2195.3098 | 2 | 6.537 | 35.3% | 4 | K.QYNQTFETHNSTTPLQER.S | 33 |
|  | Astrin\_NLD\_STLC\_tube2\_021014\_01.09226.09226.1 | 2.5736 | 0.5097 | 100.0% | 1220.53 | 1221.371 | 1 | 7.395 | 70.0% | 8 | R.SLGLDINMDSR.I | 11 |
|  | Astrin\_NLD\_STLC\_tube2\_021014\_01.09284.09284.2 | 4.1376 | 0.5111 | 100.0% | 1221.1721 | 1221.371 | 1 | 8.089 | 90.0% | 142 | R.SLGLDINMDSR.I | 22 |
|  | Astrin\_NLD\_STLC\_031014\_01.08974.08974.1 | 2.3599 | 0.2128 | 97.5% | 1094.56 | 1095.284 | 2 | 5.322 | 68.8% | 1 | R.IIHENIVEK.E | 11 |
|  | Astrin\_NLD\_STLC\_031014\_01.08972.08972.2 | 2.4044 | 0.2891 | 99.1% | 1095.2122 | 1095.284 | 1 | 5.446 | 87.5% | 3 | R.IIHENIVEK.E | 22 |
|  | Astrin\_NLD\_STLC\_031014\_01.09148.09148.3 | 2.9882 | 0.3121 | 99.8% | 1380.0543 | 1380.587 | 1 | 5.512 | 45.0% | 5 | R.IIHENIVEKER.V | 33 |
|  | Astrin\_STLCLD20\_112214\_tube2\_01.06152.06152.2 | 3.6573 | 0.3712 | 100.0% | 1380.3722 | 1380.587 | 1 | 7.537 | 85.0% | 5 | R.IIHENIVEKER.V | 22 |
|  | Astrin\_NLD\_STLC\_031014\_01.10464.10464.2 | 5.8669 | 0.5523 | 100.0% | 2403.912 | 2404.638 | 1 | 10.859 | 65.8% | 22 | R.ITQETFGDYPQPQLEFAQYK.L | 22 |
|  | Astrin\_NLD\_STLC\_031014\_01.10479.10479.3 | 5.0854 | 0.4505 | 100.0% | 2406.0842 | 2404.638 | 1 | 7.92 | 43.4% | 8 | R.ITQETFGDYPQPQLEFAQYK.L | 33 |
|  | Astrin\_NLD\_STLC\_031014\_01.10454.10454.2 | 4.886 | 0.5122 | 100.0% | 2875.5923 | 2876.192 | 1 | 9.971 | 43.5% | 3 | R.ITQETFGDYPQPQLEFAQYKLETK.F | 22 |
|  | Astrin\_NLD\_STLC\_031014\_01.10466.10466.3 | 6.1483 | 0.5449 | 100.0% | 2876.0344 | 2876.192 | 1 | 8.838 | 42.4% | 12 | R.ITQETFGDYPQPQLEFAQYKLETK.F | 33 |
|  | Astrin\_NLD\_STLC\_tube2\_021014\_02.05792.05792.2 | 3.464 | 0.3926 | 100.0% | 1391.8322 | 1392.598 | 1 | 6.903 | 79.2% | 4 | K.FKSGLNGSILAER.E | 22 |
|  | Astrin\_NLD\_STLC\_031014\_02.05832.05832.3 | 4.0889 | 0.3354 | 99.9% | 1392.0243 | 1392.598 | 1 | 6.271 | 52.1% | 2 | K.FKSGLNGSILAER.E | 33 |
|  | Astrin\_NLD\_STLC\_031014\_01.09611.09611.2 | 3.5811 | 0.235 | 99.7% | 2016.6522 | 2017.2926 | 80 | 5.4 | 35.3% | 1 | K.FKSGLNGSILAEREEPLR.C | 22 |
|  | Astrin\_NLD\_STLC\_tube2\_021014\_01.07227.07227.1 | 2.0669 | 0.2647 | 97.0% | 1116.69 | 1117.2474 | 157 | 4.789 | 45.0% | 1 | K.SGLNGSILAER.E | 11 |
|  | Astrin\_NLD\_STLC\_tube2\_021014\_01.07274.07274.2 | 3.4635 | 0.3447 | 100.0% | 1117.1322 | 1117.2474 | 1 | 7.452 | 85.0% | 23 | K.SGLNGSILAER.E | 22 |
|  | Astrin\_NLD\_STLC\_tube2\_021014\_01.07886.07886.2 | 3.1578 | 0.2282 | 99.1% | 1741.3522 | 1741.9419 | 69 | 4.684 | 40.0% | 11 | K.SGLNGSILAEREEPLR.C | 22 |
|  | Astrin\_NLD\_STLC\_031014\_01.09680.09680.3 | 3.267 | 0.2989 | 99.8% | 1742.0343 | 1741.9419 | 1 | 5.25 | 40.0% | 14 | K.SGLNGSILAEREEPLR.C | 33 |
|  | Astrin\_NLD\_STLC\_tube2\_021014\_01.08934.08934.1 | 2.2654 | 0.2482 | 97.4% | 1241.62 | 1242.4606 | 1 | 4.955 | 55.0% | 3 | K.FSSPHLLEALK.S | 11 |
|  | Astrin\_NLD\_STLC\_tube2\_021014\_01.09356.09356.2 | 3.2793 | 0.3937 | 100.0% | 1242.3522 | 1242.4606 | 1 | 6.693 | 85.0% | 34 | K.FSSPHLLEALK.S | 22 |
|  | Astrin\_NLD\_STLC\_tube2\_021014\_01.09339.09339.3 | 2.2147 | 0.2802 | 95.6% | 1242.4744 | 1242.4606 | 2 | 5.171 | 47.5% | 1 | K.FSSPHLLEALK.S | 33 |
| \* | Astrin\_NLD\_STLC\_031014\_01.10766.10766.3 | 2.5487 | 0.2867 | 95.8% | 2375.0044 | 2376.768 | 4 | 5.563 | 29.5% | 1 | K.SLAPAGIADAPLSPLLTCIPNKR.M | 3 |
| \* | Astrin\_NLD\_STLC\_031014\_01.10755.10755.2 | 4.4336 | 0.5067 | 100.0% | 2375.7122 | 2376.768 | 1 | 9.105 | 43.2% | 1 | K.SLAPAGIADAPLSPLLTCIPNKR.M | 2 |
| \* | Astrin\_NLD\_STLC\_tube2\_021014\_01.08979.08979.2 | 2.1014 | 0.2217 | 97.0% | 972.27216 | 972.1931 | 45 | 5.363 | 66.7% | 1 | R.MNYFKIR.D | 2 |

Similarities:
gi|154800485|ref|NP\_0(55:4)  

---

|  |  |  |  |  |  |  |  |  |
| --- | --- | --- | --- | --- | --- | --- | --- | --- |
| U | *gi|154800485|ref|NP\_0* | 57 | 572 | 81.9% | 353 | 41180 | 8.9 | centromere protein N isoform 1 [Homo sapiens] |

| Filename XCorr DeltCN Conf% ObsM+H+ CalcM+H+ SpR ZScore Ion% # Sequence  | | | | | | | | | | | | |
| --- | --- | --- | --- | --- | --- | --- | --- | --- | --- | --- | --- | --- |
|  | Astrin\_NLD\_STLC\_tube2\_021014\_01.09740.09740.2 | 3.6546 | 0.4522 | 100.0% | 1182.9722 | 1183.3618 | 1 | 8.176 | 77.8% | 6 | -.MDETVAEFIK.R | 22 |
|  | Astrin\_NLD\_STLC\_tube2\_021014\_01.08414.08414.2 | 3.2945 | 0.3467 | 100.0% | 1338.9321 | 1339.5493 | 1 | 6.946 | 75.0% | 13 | -.MDETVAEFIKR.T | 22 |
|  | Astrin\_NLD\_STLC\_tube2\_021014\_01.08673.08673.3 | 2.5121 | 0.3029 | 99.2% | 1340.2743 | 1339.5493 | 177 | 5.187 | 37.5% | 4 | -.MDETVAEFIKR.T | 3 |
|  | Astrin\_NLD\_STLC\_tube2\_021014\_01.12992.12992.2 | 3.4768 | 0.2917 | 99.9% | 1728.4922 | 1729.1719 | 2 | 5.034 | 60.7% | 7 | R.TILKIPMNELTTILK.A | 22 |
|  | Astrin\_NLD\_STLC\_031014\_01.11240.11240.3 | 4.4357 | 0.4426 | 100.0% | 1729.9143 | 1729.1719 | 14 | 6.628 | 46.4% | 8 | R.TILKIPMNELTTILK.A | 33 |
|  | Astrin\_NLD\_STLC\_tube2\_021014\_01.17877.17877.3 | 6.2108 | 0.4843 | 100.0% | 3677.9644 | 3679.3123 | 1 | 8.134 | 35.0% | 1 | R.TILKIPMNELTTILKAWDFLSENQLQTVNFR.Q | 33 |
|  | Astrin\_NLD\_STLC\_tube2\_021014\_01.11900.11900.2 | 3.6942 | 0.417 | 100.0% | 1273.2922 | 1273.5737 | 1 | 7.132 | 85.0% | 29 | K.IPMNELTTILK.A | 22 |
|  | Astrin\_NLD\_STLC\_tube2\_021014\_01.12981.12981.1 | 2.1951 | 0.5538 | 100.0% | 1967.88 | 1969.1637 | 3 | 8.043 | 40.0% | 1 | K.AWDFLSENQLQTVNFR.Q | 11 |
|  | Astrin\_NLD\_STLC\_tube2\_021014\_02.09398.09398.2 | 5.0365 | 0.5022 | 100.0% | 1968.2722 | 1969.1637 | 1 | 8.886 | 70.0% | 44 | K.AWDFLSENQLQTVNFR.Q | 22 |
|  | Astrin\_NLD\_STLC\_tube2\_021014\_02.09404.09404.3 | 5.1607 | 0.5166 | 100.0% | 1969.5243 | 1969.1637 | 1 | 9.199 | 41.7% | 13 | K.AWDFLSENQLQTVNFR.Q | 33 |
|  | Astrin\_NLD\_STLC\_031014\_01.09758.09758.3 | 3.732 | 0.2028 | 99.3% | 1849.5844 | 1850.0912 | 1 | 5.154 | 42.9% | 1 | R.KESVVQHLIHLCEEK.R | 33 |
|  | Astrin\_NLD\_STLC\_031014\_01.10187.10187.2 | 3.5328 | 0.3523 | 100.0% | 1720.7922 | 1721.9171 | 1 | 6.518 | 57.7% | 1 | K.ESVVQHLIHLCEEK.R | 22 |
|  | Astrin\_NLD\_STLC\_tube2\_021014\_01.12916.12916.3 | 6.8447 | 0.4011 | 100.0% | 2586.2344 | 2586.9714 | 1 | 8.212 | 38.1% | 2 | K.RASISDAALLDIIYMQFHQHQK.V | 33 |
|  | Astrin\_NLD\_STLC\_tube2\_021014\_01.13785.13785.2 | 4.7865 | 0.4513 | 100.0% | 2429.652 | 2430.784 | 1 | 8.408 | 50.0% | 1 | R.ASISDAALLDIIYMQFHQHQK.V | 22 |
|  | Astrin\_NLD\_STLC\_tube2\_021014\_01.13844.13844.3 | 3.1585 | 0.266 | 98.6% | 2429.9944 | 2430.784 | 1 | 5.157 | 35.0% | 3 | R.ASISDAALLDIIYMQFHQHQK.V | 33 |
|  | Astrin\_NLD\_STLC\_tube2\_021014\_01.15286.15286.3 | 4.5068 | 0.415 | 100.0% | 3551.3342 | 3552.103 | 1 | 6.204 | 26.7% | 1 | R.ASISDAALLDIIYMQFHQHQKVWDVFQMSK.G | 33 |
|  | Astrin\_NLD\_STLC\_tube2\_021014\_01.11547.11547.1 | 2.0847 | 0.3667 | 96.5% | 1139.49 | 1140.3423 | 1 | 6.837 | 75.0% | 2 | K.VWDVFQMSK.G | 11 |
|  | Astrin\_NLD\_STLC\_tube2\_021014\_01.11516.11516.2 | 3.399 | 0.3196 | 100.0% | 1140.0521 | 1140.3423 | 1 | 6.892 | 81.2% | 6 | K.VWDVFQMSK.G | 22 |
|  | Astrin\_NLD\_STLC\_tube2\_021014\_01.10488.10488.1 | 2.6404 | 0.387 | 100.0% | 1322.45 | 1323.4602 | 2 | 8.362 | 59.1% | 1 | K.GPGEDVDLFDMK.Q | 11 |
|  | Astrin\_NLD\_STLC\_tube2\_021014\_01.10502.10502.2 | 4.1308 | 0.5977 | 100.0% | 1323.0122 | 1323.4602 | 1 | 10.286 | 68.2% | 22 | K.GPGEDVDLFDMK.Q | 22 |
|  | Astrin\_NLD\_STLC\_tube2\_021014\_01.10685.10685.2 | 4.5421 | 0.4915 | 100.0% | 1726.1921 | 1726.9415 | 1 | 7.831 | 64.3% | 1 | K.GPGEDVDLFDMKQFK.N | 22 |
|  | Astrin\_NLD\_STLC\_tube2\_021014\_01.10688.10688.3 | 3.4703 | 0.5038 | 100.0% | 1726.8544 | 1726.9415 | 2 | 7.142 | 44.6% | 1 | K.GPGEDVDLFDMKQFK.N | 33 |
|  | Astrin\_NLD\_STLC\_tube2\_021014\_01.06052.06052.2 | 3.5049 | 0.3337 | 100.0% | 1135.4521 | 1135.3518 | 5 | 7.113 | 77.8% | 2 | R.ALKNVTVSFR.E | 22 |
|  | Astrin\_NLD\_STLC\_tube2\_021014\_01.07694.07694.1 | 1.8497 | 0.2567 | 96.9% | 1246.58 | 1247.3501 | 230 | 6.095 | 50.0% | 1 | R.ETEENAVWIR.I | 11 |
|  | Astrin\_NLD\_STLC\_tube2\_021014\_01.07724.07724.2 | 2.8236 | 0.3418 | 100.0% | 1247.0122 | 1247.3501 | 7 | 5.842 | 77.8% | 5 | R.ETEENAVWIR.I | 22 |
|  | Astrin\_NLD\_STLC\_tube2\_021014\_01.11631.11631.3 | 5.959 | 0.4887 | 100.0% | 4141.9443 | 4142.675 | 1 | 7.631 | 27.2% | 3 | R.IAWGTQYTKPNQYKPTYVVYYSQTPYAFTSSSMLR.R | 33 |
|  | Astrin\_NLD\_STLC\_tube2\_021014\_01.09380.09380.2 | 4.4996 | 0.4962 | 100.0% | 1583.2722 | 1583.8717 | 1 | 8.02 | 71.4% | 11 | R.RNTPLLGQALTIASK.H | 22 |
|  | Astrin\_NLD\_STLC\_tube2\_021014\_01.09268.09268.3 | 4.5168 | 0.4501 | 100.0% | 1584.4744 | 1583.8717 | 1 | 7.835 | 44.6% | 4 | R.RNTPLLGQALTIASK.H | 33 |
|  | Astrin\_NLD\_STLC\_tube2\_021014\_01.10896.10896.1 | 2.4538 | 0.2844 | 97.6% | 1426.73 | 1427.6842 | 1 | 5.212 | 57.7% | 4 | R.NTPLLGQALTIASK.H | 11 |
|  | Astrin\_NLD\_STLC\_tube2\_021014\_01.11392.11392.2 | 4.5659 | 0.5253 | 100.0% | 1428.4922 | 1427.6842 | 1 | 8.647 | 80.8% | 53 | R.NTPLLGQALTIASK.H | 22 |
|  | Astrin\_NLD\_STLC\_tube2\_021014\_01.10924.10924.3 | 3.4434 | 0.2154 | 99.1% | 1429.0443 | 1427.6842 | 1 | 5.448 | 51.9% | 1 | R.NTPLLGQALTIASK.H | 33 |
|  | Astrin\_NLD\_STLC\_tube2\_021014\_01.04931.04931.3 | 2.7735 | 0.1862 | 95.3% | 1277.5144 | 1277.5303 | 1 | 4.804 | 52.8% | 2 | K.HHQIVKMDLR.S | 33 |
|  | Astrin\_NLD\_STLC\_031014\_01.09153.09153.2 | 2.7419 | 0.1846 | 99.1% | 981.9922 | 982.12463 | 2 | 4.094 | 85.7% | 3 | R.SRYLDSLK.A | 22 |
|  | Astrin\_NLD\_STLC\_031014\_01.09605.09605.3 | 5.1117 | 0.4057 | 100.0% | 2753.2444 | 2754.0312 | 1 | 6.858 | 34.1% | 2 | K.AIVFKQYNQTFETHNSTTPLQER.S | 33 |
|  | Astrin\_NLD\_STLC\_tube2\_021014\_01.05517.05517.2 | 3.3667 | 0.5209 | 100.0% | 2194.392 | 2195.3098 | 1 | 7.519 | 50.0% | 4 | K.QYNQTFETHNSTTPLQER.S | 22 |
|  | Astrin\_NLD\_STLC\_tube2\_021014\_01.05397.05397.3 | 3.7725 | 0.399 | 99.9% | 2194.6743 | 2195.3098 | 2 | 6.537 | 35.3% | 4 | K.QYNQTFETHNSTTPLQER.S | 33 |
|  | Astrin\_NLD\_STLC\_tube2\_021014\_01.09226.09226.1 | 2.5736 | 0.5097 | 100.0% | 1220.53 | 1221.371 | 1 | 7.395 | 70.0% | 8 | R.SLGLDINMDSR.I | 11 |
|  | Astrin\_NLD\_STLC\_tube2\_021014\_01.09284.09284.2 | 4.1376 | 0.5111 | 100.0% | 1221.1721 | 1221.371 | 1 | 8.089 | 90.0% | 142 | R.SLGLDINMDSR.I | 22 |
|  | Astrin\_NLD\_STLC\_031014\_01.08974.08974.1 | 2.3599 | 0.2128 | 97.5% | 1094.56 | 1095.284 | 2 | 5.322 | 68.8% | 1 | R.IIHENIVEK.E | 11 |
|  | Astrin\_NLD\_STLC\_031014\_01.08972.08972.2 | 2.4044 | 0.2891 | 99.1% | 1095.2122 | 1095.284 | 1 | 5.446 | 87.5% | 3 | R.IIHENIVEK.E | 22 |
|  | Astrin\_NLD\_STLC\_031014\_01.09148.09148.3 | 2.9882 | 0.3121 | 99.8% | 1380.0543 | 1380.587 | 1 | 5.512 | 45.0% | 5 | R.IIHENIVEKER.V | 33 |
|  | Astrin\_STLCLD20\_112214\_tube2\_01.06152.06152.2 | 3.6573 | 0.3712 | 100.0% | 1380.3722 | 1380.587 | 1 | 7.537 | 85.0% | 5 | R.IIHENIVEKER.V | 22 |
|  | Astrin\_NLD\_STLC\_031014\_01.10464.10464.2 | 5.8669 | 0.5523 | 100.0% | 2403.912 | 2404.638 | 1 | 10.859 | 65.8% | 22 | R.ITQETFGDYPQPQLEFAQYK.L | 22 |
|  | Astrin\_NLD\_STLC\_031014\_01.10479.10479.3 | 5.0854 | 0.4505 | 100.0% | 2406.0842 | 2404.638 | 1 | 7.92 | 43.4% | 8 | R.ITQETFGDYPQPQLEFAQYK.L | 33 |
|  | Astrin\_NLD\_STLC\_031014\_01.10454.10454.2 | 4.886 | 0.5122 | 100.0% | 2875.5923 | 2876.192 | 1 | 9.971 | 43.5% | 3 | R.ITQETFGDYPQPQLEFAQYKLETK.F | 22 |
|  | Astrin\_NLD\_STLC\_031014\_01.10466.10466.3 | 6.1483 | 0.5449 | 100.0% | 2876.0344 | 2876.192 | 1 | 8.838 | 42.4% | 12 | R.ITQETFGDYPQPQLEFAQYKLETK.F | 33 |
|  | Astrin\_NLD\_STLC\_tube2\_021014\_02.05792.05792.2 | 3.464 | 0.3926 | 100.0% | 1391.8322 | 1392.598 | 1 | 6.903 | 79.2% | 4 | K.FKSGLNGSILAER.E | 22 |
|  | Astrin\_NLD\_STLC\_031014\_02.05832.05832.3 | 4.0889 | 0.3354 | 99.9% | 1392.0243 | 1392.598 | 1 | 6.271 | 52.1% | 2 | K.FKSGLNGSILAER.E | 33 |
|  | Astrin\_NLD\_STLC\_031014\_01.09611.09611.2 | 3.5811 | 0.235 | 99.7% | 2016.6522 | 2017.2926 | 80 | 5.4 | 35.3% | 1 | K.FKSGLNGSILAEREEPLR.C | 22 |
| \* | Astrin\_NLD\_STLC\_031014\_02.06338.06338.3 | 2.7321 | 0.263 | 96.9% | 2017.7943 | 2017.2926 | 181 | 4.364 | 27.9% | 2 | K.FKSGLNGSILAEREEPLR.C | 3 |
|  | Astrin\_NLD\_STLC\_tube2\_021014\_01.07227.07227.1 | 2.0669 | 0.2647 | 97.0% | 1116.69 | 1117.2474 | 157 | 4.789 | 45.0% | 1 | K.SGLNGSILAER.E | 11 |
|  | Astrin\_NLD\_STLC\_tube2\_021014\_01.07274.07274.2 | 3.4635 | 0.3447 | 100.0% | 1117.1322 | 1117.2474 | 1 | 7.452 | 85.0% | 23 | K.SGLNGSILAER.E | 22 |
|  | Astrin\_NLD\_STLC\_tube2\_021014\_01.07886.07886.2 | 3.1578 | 0.2282 | 99.1% | 1741.3522 | 1741.9419 | 69 | 4.684 | 40.0% | 11 | K.SGLNGSILAEREEPLR.C | 22 |
|  | Astrin\_NLD\_STLC\_031014\_01.09680.09680.3 | 3.267 | 0.2989 | 99.8% | 1742.0343 | 1741.9419 | 1 | 5.25 | 40.0% | 14 | K.SGLNGSILAEREEPLR.C | 33 |
|  | Astrin\_NLD\_STLC\_tube2\_021014\_01.08934.08934.1 | 2.2654 | 0.2482 | 97.4% | 1241.62 | 1242.4606 | 1 | 4.955 | 55.0% | 3 | K.FSSPHLLEALK.S | 11 |
|  | Astrin\_NLD\_STLC\_tube2\_021014\_01.09356.09356.2 | 3.2793 | 0.3937 | 100.0% | 1242.3522 | 1242.4606 | 1 | 6.693 | 85.0% | 34 | K.FSSPHLLEALK.S | 22 |
|  | Astrin\_NLD\_STLC\_tube2\_021014\_01.09339.09339.3 | 2.2147 | 0.2802 | 95.6% | 1242.4744 | 1242.4606 | 2 | 5.171 | 47.5% | 1 | K.FSSPHLLEALK.S | 33 |

Similarities:
gi|154800483|ref|NP\_0(55:2)  

---

|  |  |  |  |  |  |  |  |  |
| --- | --- | --- | --- | --- | --- | --- | --- | --- |
| U | *gi|40354195|ref|NP\_95* | 44 | 550 | 71.6% | 430 | 48058 | 5.5 | keratin 18 [Homo sapiens] |
| U | *gi|4557888|ref|NP\_000* | 44 | 550 | 71.6% | 430 | 48058 | 5.5 | keratin 18 [Homo sapiens] |

| Filename XCorr DeltCN Conf% ObsM+H+ CalcM+H+ SpR ZScore Ion% # Sequence  | | | | | | | | | | | | |
| --- | --- | --- | --- | --- | --- | --- | --- | --- | --- | --- | --- | --- |
|  | Astrin\_STLCHLD\_050114\_01.05293.05293.2 | 1.8227 | 0.3647 | 98.1% | 975.9922 | 976.0336 | 1 | 6.297 | 78.6% | 3 | R.STFSTNYR.S | 2 |
|  | Astrin\_NLD\_STLC\_031014\_02.06081.06081.3 | 5.207 | 0.5785 | 100.0% | 2855.3943 | 2856.0813 | 1 | 9.903 | 29.2% | 58 | R.SLGSVQAPSYGARPVSSAASVYAGAGGSGSR.I | 3 |
|  | Astrin\_STLCLD20\_112214\_tube2\_01.09368.09368.2 | 5.1021 | 0.501 | 100.0% | 2855.8523 | 2856.0813 | 1 | 8.057 | 30.0% | 7 | R.SLGSVQAPSYGARPVSSAASVYAGAGGSGSR.I | 2 |
|  | Astrin\_STLCLD20\_112214\_02.09957.09957.3 | 3.8771 | 0.3909 | 99.9% | 2935.5244 | 2936.0813 | 2 | 5.917 | 26.7% | 1 | R.SLGSVQAPSYGARPVSSAASVYAGAGGS\*GSR.I | 3 |
|  | Astrin\_STLCHLD\_tube2\_050114\_02.08289.08289.2 | 5.9754 | 0.5229 | 100.0% | 2262.7322 | 2262.561 | 1 | 8.534 | 52.0% | 6 | R.GGMGSGGLATGIAGGLAGMGGIQNEK.E | 2 |
|  | Astrin\_STLCLD20\_112214\_tube2\_02.08814.08814.3 | 4.6793 | 0.31 | 99.9% | 2265.1443 | 2262.561 | 1 | 6.114 | 38.0% | 6 | R.GGMGSGGLATGIAGGLAGMGGIQNEK.E | 3 |
|  | AstrinSTLCLD\_041714\_02.08390.08390.3 | 6.2196 | 0.5712 | 100.0% | 3337.3145 | 3337.7224 | 1 | 10.171 | 27.9% | 25 | R.GGMGSGGLATGIAGGLAGMGGIQNEKETMQSLNDR.L | 3 |
|  | Astrin\_NLD\_STLC\_tube2\_021014\_01.05040.05040.2 | 1.9022 | 0.2598 | 95.9% | 837.2322 | 837.9511 | 20 | 4.708 | 83.3% | 5 | R.LASYLDR.V | 2 |
|  | Astrin\_STLCLD20\_112214\_01.10024.10024.1 | 1.7507 | 0.3024 | 98.1% | 982.5 | 983.0709 | 5 | 5.045 | 58.3% | 2 | R.DWSHYFK.I | 11 |
|  | AstrinSTLCLD\_041714\_01.07136.07136.2 | 2.1891 | 0.2036 | 97.3% | 982.6922 | 983.0709 | 1 | 5.041 | 83.3% | 1 | R.DWSHYFK.I | 22 |
|  | Astrin\_STLCLD20\_112214\_02.12161.12161.3 | 3.8851 | 0.3999 | 99.9% | 2059.4944 | 2060.3176 | 4 | 6.977 | 36.8% | 1 | K.IIEDLRAQIFANTVDNAR.I | 3 |
|  | Astrin\_NLD\_STLC\_tube2\_021014\_01.06450.06450.1 | 2.2261 | 0.4378 | 100.0% | 1319.41 | 1320.4478 | 15 | 6.781 | 45.5% | 3 | R.AQIFANTVDNAR.I | 1 |
|  | Astrin\_STLCLD20\_112214\_01.07189.07189.2 | 4.1827 | 0.4578 | 100.0% | 1320.3121 | 1320.4478 | 1 | 8.414 | 77.3% | 39 | R.AQIFANTVDNAR.I | 22 |
|  | Astrin\_STLCLD20\_112214\_tube2\_01.09495.09495.1 | 2.6834 | 0.3062 | 96.7% | 1041.62 | 1042.2235 | 1 | 7.063 | 68.8% | 7 | R.IVLQIDNAR.L | 11 |
|  | Astrin\_NLD\_STLC\_tube2\_021014\_01.07100.07100.2 | 3.2063 | 0.1355 | 99.2% | 1042.1122 | 1042.2235 | 1 | 6.273 | 87.5% | 20 | R.IVLQIDNAR.L | 22 |
|  | Astrin\_STLCHLD\_050114\_01.06505.06505.2 | 2.4865 | 0.2457 | 99.3% | 808.09216 | 807.8815 | 46 | 6.454 | 66.7% | 16 | R.LAADDFR.V | 222222 |
|  | Astrin\_STLCLD20\_112214\_01.05707.05707.2 | 3.073 | 0.4746 | 100.0% | 1240.3121 | 1240.4601 | 16 | 7.561 | 72.2% | 23 | R.VKYETELAMR.Q | 2 |
|  | Astrin\_NLD\_STLC\_tube2\_021014\_02.04946.04946.3 | 3.329 | 0.1842 | 99.0% | 1240.3744 | 1240.4601 | 43 | 4.76 | 50.0% | 4 | R.VKYETELAMR.Q | 3 |
|  | Astrin\_STLCHLD\_050114\_01.08619.08619.2 | 1.8966 | 0.5107 | 99.9% | 1013.6722 | 1013.1535 | 40 | 6.936 | 71.4% | 1 | K.YETELAMR.Q | 2 |
|  | Astrin\_NLD\_STLC\_031014\_01.08912.08912.2 | 3.0829 | 0.1829 | 99.2% | 1176.2522 | 1175.3274 | 6 | 5.49 | 72.2% | 4 | R.KVIDDTNITR.L | 2 |
|  | Astrin\_STLCLD20\_112214\_tube2\_01.06578.06578.2 | 2.3695 | 0.3345 | 99.5% | 1046.4722 | 1047.1533 | 6 | 7.068 | 68.8% | 1 | K.VIDDTNITR.L | 2 |
|  | Astrin\_STLCLD20\_112214\_tube2\_01.06584.06584.1 | 1.8182 | 0.3081 | 98.2% | 1046.56 | 1047.1533 | 2 | 5.245 | 62.5% | 1 | K.VIDDTNITR.L | 1 |
|  | Astrin\_STLCLD20\_112214\_01.15206.15206.2 | 6.4218 | 0.5637 | 100.0% | 2177.7322 | 2178.589 | 1 | 10.167 | 67.6% | 17 | R.LQLETEIEALKEELLFMK.K | 22 |
|  | Astrin\_STLCLD20\_112214\_tube2\_02.11539.11539.3 | 3.2119 | 0.4364 | 99.9% | 2178.1143 | 2178.589 | 67 | 6.355 | 33.8% | 13 | R.LQLETEIEALKEELLFMK.K | 33 |
|  | Astrin\_STLCHLD\_tube2\_050114\_01.08716.08716.3 | 5.8449 | 0.5194 | 100.0% | 2750.7544 | 2751.0227 | 1 | 9.699 | 35.0% | 15 | K.NHEEEVKGLQAQIASSGLTVEVDAPK.S | 3 |
|  | Astrin\_STLCLD20\_112214\_tube2\_01.11565.11565.2 | 6.4403 | 0.5816 | 100.0% | 2750.872 | 2751.0227 | 1 | 11.094 | 56.0% | 1 | K.NHEEEVKGLQAQIASSGLTVEVDAPK.S | 2 |
|  | Astrin\_STLCLD20\_112214\_tube2\_01.12294.12294.2 | 4.8914 | 0.519 | 100.0% | 1884.5922 | 1885.1246 | 1 | 9.854 | 66.7% | 10 | K.GLQAQIASSGLTVEVDAPK.S | 2 |
|  | Astrin\_STLCLD20\_112214\_tube2\_01.06986.06986.1 | 1.9887 | 0.2102 | 96.0% | 965.61 | 966.0385 | 63 | 5.014 | 50.0% | 5 | R.AQYDELAR.K | 1 |
|  | Astrin\_STLCLD20\_112214\_tube2\_01.12106.12106.2 | 3.5869 | 0.4313 | 100.0% | 1664.3722 | 1663.8865 | 1 | 7.071 | 73.1% | 3 | R.RTVQSLEIDLDSMR.N | 2 |
|  | Astrin\_STLCHLD\_tube2\_050114\_01.10480.10480.1 | 2.3465 | 0.4692 | 100.0% | 1506.68 | 1507.699 | 1 | 7.556 | 62.5% | 4 | R.TVQSLEIDLDSMR.N | 1 |
|  | Astrin\_STLCLD20\_112214\_01.11641.11641.2 | 4.4726 | 0.494 | 100.0% | 1508.5322 | 1507.699 | 1 | 9.071 | 75.0% | 45 | R.TVQSLEIDLDSMR.N | 2 |
|  | Astrin\_STLCHLD\_050114\_01.05607.05607.2 | 2.6608 | 0.2448 | 99.5% | 891.1122 | 889.9841 | 107 | 5.434 | 71.4% | 6 | K.ASLENSLR.E | 2 |
|  | Astrin\_STLCLD20\_112214\_01.08857.08857.2 | 2.7572 | 0.1465 | 95.9% | 1473.8722 | 1474.6139 | 1 | 4.465 | 58.3% | 1 | K.ASLENSLREVEAR.Y | 2 |
|  | Astrin\_STLCLD20\_112214\_tube2\_01.09820.09820.3 | 2.6679 | 0.2342 | 96.2% | 1475.4543 | 1474.6139 | 27 | 5.197 | 37.5% | 3 | K.ASLENSLREVEAR.Y | 3 |
|  | Astrin\_STLCLD20\_112214\_tube2\_02.12146.12146.2 | 6.175 | 0.4456 | 100.0% | 2671.7122 | 2672.0715 | 1 | 9.736 | 54.5% | 17 | R.YALQMEQLNGILLHLESELAQTR.A | 2 |
|  | Astrin\_STLCLD20\_112214\_02.17163.17163.3 | 6.6694 | 0.512 | 100.0% | 2672.5444 | 2672.0715 | 1 | 9.274 | 46.6% | 96 | R.YALQMEQLNGILLHLESELAQTR.A | 3 |
|  | AstrinSTLCLD\_041714\_01.08763.08763.2 | 3.6713 | 0.422 | 100.0% | 1420.4122 | 1420.6055 | 2 | 6.47 | 72.7% | 18 | R.QAQEYEALLNIK.V | 2 |
|  | Astrin\_STLCHLD\_050114\_02.06194.06194.2 | 3.7113 | 0.3085 | 100.0% | 1293.7522 | 1293.5059 | 1 | 6.541 | 75.0% | 27 | K.VKLEAEIATYR.R | 2 |
|  | Astrin\_STLCLD20\_112214\_tube2\_01.08554.08554.1 | 2.3173 | 0.3228 | 96.9% | 1065.55 | 1066.1992 | 72 | 5.913 | 56.2% | 2 | K.LEAEIATYR.R | 1 |
|  | Astrin\_STLCLD20\_112214\_01.06829.06829.2 | 3.5529 | 0.3712 | 100.0% | 1066.2722 | 1066.1992 | 1 | 6.82 | 87.5% | 15 | K.LEAEIATYR.R | 2 |
|  | Astrin\_STLCLD20\_112214\_02.12562.12562.3 | 5.335 | 0.3869 | 100.0% | 2897.6643 | 2898.128 | 1 | 6.627 | 29.0% | 7 | R.RLLEDGEDFNLGDALDSSNSMQTIQK.T | 3 |
|  | Astrin\_STLCLD20\_112214\_tube2\_01.14951.14951.2 | 6.0933 | 0.6171 | 100.0% | 2740.652 | 2741.9404 | 1 | 12.384 | 52.1% | 5 | R.LLEDGEDFNLGDALDSSNSMQTIQK.T | 2 |
|  | AstrinSTLCLD\_041714\_01.10133.10133.3 | 5.6454 | 0.5234 | 100.0% | 2741.9043 | 2741.9404 | 1 | 9.408 | 35.4% | 3 | R.LLEDGEDFNLGDALDSSNSMQTIQK.T | 3 |
|  | Astrin\_NLD\_STLC\_031014\_01.06272.06272.3 | 3.1775 | 0.3293 | 99.8% | 1661.7544 | 1661.8528 | 1 | 6.689 | 42.9% | 3 | R.RIVDGKVVSETNDTK.V | 3 |

Similarities:
gi|4557701|ref|NP\_000(1:43)  
gi|15431310|ref|NP\_00(1:43)  
contaminant\_KERATIN03(1:43)  
gi|24430192|ref|NP\_00(1:43)  
gi|24234699|ref|NP\_00(3:41)  
gi|169164037|ref|XP\_0(5:39)  

---

|  |  |  |  |  |  |  |  |  |
| --- | --- | --- | --- | --- | --- | --- | --- | --- |
| U | *gi|57013276|ref|NP\_00* | 39 | 487 | 69.8% | 451 | 50152 | 5.1 | tubulin, alpha, ubiquitous [Homo sapiens] |

| Filename XCorr DeltCN Conf% ObsM+H+ CalcM+H+ SpR ZScore Ion% # Sequence  | | | | | | | | | | | | |
| --- | --- | --- | --- | --- | --- | --- | --- | --- | --- | --- | --- | --- |
|  | Astrin\_STLCHLD\_061214\_01.09384.09384.2 | 6.1825 | 0.6572 | 100.0% | 2008.3922 | 2009.093 | 1 | 11.538 | 63.2% | 37 | K.TIGGGDDSFNTFFSETGAGK.H | 22 |
|  | Astrin\_STLCHLD\_050114\_02.08865.08865.3 | 3.8391 | 0.2974 | 99.8% | 2009.6044 | 2009.093 | 12 | 5.573 | 31.6% | 1 | K.TIGGGDDSFNTFFSETGAGK.H | 33 |
|  | Astrin\_STLCHLD\_tube2\_061314\_01.11935.11935.1 | 3.3623 | 0.5322 | 100.0% | 1702.95 | 1702.9451 | 1 | 8.203 | 57.1% | 7 | R.AVFVDLEPTVIDEVR.T | 11 |
|  | Astrin\_STLCHLD\_061214\_02.08717.08717.2 | 5.1706 | 0.51 | 100.0% | 1703.3522 | 1702.9451 | 1 | 8.59 | 78.6% | 47 | R.AVFVDLEPTVIDEVR.T | 22 |
|  | Astrin\_STLCHLD\_tube2\_050114\_01.11676.11676.3 | 4.6957 | 0.4534 | 100.0% | 1704.2943 | 1702.9451 | 1 | 7.889 | 51.8% | 6 | R.AVFVDLEPTVIDEVR.T | 33 |
|  | Astrin\_STLCLD20\_112214\_01.10293.10293.2 | 1.9386 | 0.3075 | 95.5% | 1411.5521 | 1411.6439 | 367 | 5.745 | 50.0% | 4 | R.QLFHPEQLITGK.E | 222 |
|  | Astrin\_STLCLD20\_112214\_tube2\_01.10802.10802.3 | 3.9221 | 0.4709 | 100.0% | 2416.4644 | 2416.6555 | 2 | 7.043 | 30.0% | 13 | R.QLFHPEQLITGKEDAANNYAR.G | 333 |
|  | Astrin\_STLCLD20\_112214\_tube2\_01.10767.10767.2 | 2.6947 | 0.3834 | 99.8% | 2416.5723 | 2416.6555 | 6 | 5.895 | 37.5% | 2 | R.QLFHPEQLITGKEDAANNYAR.G | 222 |
|  | Astrin\_STLCLD20\_112214\_01.13269.13269.3 | 4.8135 | 0.4526 | 100.0% | 1843.2544 | 1843.1332 | 2 | 7.617 | 43.3% | 6 | R.GHYTIGKEIIDLVLDR.I | 33 |
|  | Astrin\_STLCHLD\_tube2\_050114\_01.12647.12647.2 | 5.0104 | 0.5165 | 100.0% | 1843.7322 | 1843.1332 | 1 | 8.617 | 70.0% | 6 | R.GHYTIGKEIIDLVLDR.I | 22 |
|  | Astrin\_STLCLD20\_112214\_tube2\_01.15296.15296.1 | 2.1784 | 0.3293 | 97.1% | 1085.77 | 1086.2737 | 2 | 6.033 | 62.5% | 5 | K.EIIDLVLDR.I | 11 |
|  | Astrin\_STLCHLD\_tube2\_061314\_01.11543.11543.2 | 3.0514 | 0.3519 | 100.0% | 1086.4722 | 1086.2737 | 9 | 6.025 | 81.2% | 8 | K.EIIDLVLDR.I | 22 |
|  | Astrin\_STLCHLD\_tube2\_050114\_02.11004.11004.3 | 3.9515 | 0.2893 | 99.8% | 3392.8145 | 3392.7769 | 1 | 5.149 | 23.4% | 1 | K.LADQCTGLQGFLVFHSFGGGTGSGFTSLLMER.L | 33 |
|  | Astrin\_STLCHLD\_tube2\_061314\_01.04847.04847.3 | 3.6369 | 0.3589 | 99.9% | 1876.6144 | 1876.0824 | 1 | 5.792 | 48.2% | 6 | R.RNLDIERPTYTNLNR.L | 333 |
|  | Astrin\_STLCHLD\_050114\_01.11147.11147.2 | 3.4174 | 0.0864 | 97.3% | 1719.6322 | 1719.8949 | 1 | 5.134 | 61.5% | 7 | R.NLDIERPTYTNLNR.L | 222 |
|  | Astrin\_STLCHLD\_tube2\_050114\_01.06465.06465.3 | 2.9056 | 0.311 | 99.8% | 1721.3644 | 1719.8949 | 2 | 5.652 | 46.2% | 9 | R.NLDIERPTYTNLNR.L | 333 |
|  | Astrin\_STLCHLD\_050114\_01.13856.13856.1 | 3.7074 | 0.3194 | 100.0% | 1488.91 | 1488.7678 | 3 | 6.528 | 50.0% | 9 | R.LISQIVSSITASLR.F | 111 |
|  | Astrin\_STLCHLD\_tube2\_050114\_02.10976.10976.2 | 4.8703 | 0.4751 | 100.0% | 1490.2522 | 1488.7678 | 1 | 9.358 | 73.1% | 71 | R.LISQIVSSITASLR.F | 222 |
|  | Astrin\_STLCHLD\_tube2\_050114\_01.13991.13991.3 | 4.7196 | 0.3289 | 99.9% | 1490.3344 | 1488.7678 | 2 | 6.09 | 50.0% | 15 | R.LISQIVSSITASLR.F | 333 |
|  | Astrin\_STLCHLD\_tube2\_061314\_02.09638.09638.2 | 5.8424 | 0.5235 | 100.0% | 2410.7122 | 2410.6885 | 1 | 10.298 | 50.0% | 50 | R.FDGALNVDLTEFQTNLVPYPR.I | 222 |
|  | Astrin\_STLCLD20\_112214\_tube2\_02.10416.10416.3 | 3.7941 | 0.4445 | 99.9% | 2410.8245 | 2410.6885 | 1 | 7.732 | 36.2% | 7 | R.FDGALNVDLTEFQTNLVPYPR.I | 333 |
|  | Astrin\_STLCHLD\_tube2\_050114\_01.10237.10237.2 | 4.4468 | 0.5586 | 100.0% | 1757.4722 | 1758.0703 | 1 | 9.549 | 76.7% | 35 | R.IHFPLATYAPVISAEK.A | 222 |
|  | Astrin\_STLCHLD\_050114\_01.12271.12271.3 | 4.3329 | 0.4459 | 100.0% | 1757.9644 | 1758.0703 | 1 | 7.63 | 51.7% | 19 | R.IHFPLATYAPVISAEK.A | 333 |
|  | Astrin\_STLCHLD\_050114\_02.08102.08102.3 | 4.2224 | 0.3505 | 99.9% | 2751.6543 | 2752.0369 | 5 | 6.013 | 27.2% | 1 | K.AYHEQLSVAEITNACFEPANQMVK.C | 33 |
|  | Astrin\_STLCHLD\_050114\_01.12021.12021.2 | 2.5571 | 0.4159 | 100.0% | 1250.3722 | 1250.4304 | 1 | 6.932 | 75.0% | 3 | K.YMACCLLYR.G | 222 |
|  | Astrin\_STLCHLD\_tube2\_050114\_01.08558.08558.2 | 2.9016 | 0.2756 | 99.2% | 1611.1322 | 1611.879 | 15 | 5.949 | 46.7% | 1 | R.GDVVPKDVNAAIATIK.T | 22 |
|  | Astrin\_STLCHLD\_tube2\_061314\_01.06093.06093.1 | 1.9394 | 0.2937 | 98.3% | 1015.66 | 1016.1827 | 1 | 5.809 | 77.8% | 10 | K.DVNAAIATIK.T | 11 |
|  | Astrin\_STLCHLD\_tube2\_061314\_01.06251.06251.2 | 3.4569 | 0.4018 | 100.0% | 1016.1922 | 1016.1827 | 1 | 7.757 | 88.9% | 15 | K.DVNAAIATIK.T | 22 |
|  | Astrin\_STLCHLD\_tube2\_061314\_01.08843.08843.2 | 4.4981 | 0.4675 | 100.0% | 1825.6721 | 1826.1027 | 1 | 7.437 | 67.6% | 25 | K.VGINYQPPTVVPGGDLAK.V | 222 |
|  | Astrin\_STLCHLD\_050114\_02.09635.09635.2 | 4.2429 | 0.3899 | 100.0% | 1865.7122 | 1866.1084 | 1 | 8.618 | 56.2% | 2 | R.AVCMLSNTTAIAEAWAR.L | 22 |
|  | Astrin\_STLCLD20\_112214\_01.08110.08110.2 | 3.5435 | 0.367 | 100.0% | 1382.0521 | 1381.6324 | 1 | 6.667 | 65.0% | 2 | R.LDHKFDLMYAK.R | 222 |
|  | Astrin\_STLCHLD\_tube2\_061314\_01.06060.06060.3 | 4.3726 | 0.4192 | 100.0% | 1382.2144 | 1381.6324 | 1 | 6.674 | 60.0% | 13 | R.LDHKFDLMYAK.R | 333 |
|  | Astrin\_STLCHLD\_050114\_01.09772.09772.3 | 2.6871 | 0.2432 | 97.3% | 1538.2444 | 1537.82 | 69 | 4.407 | 40.9% | 1 | R.LDHKFDLMYAKR.A | 333 |
|  | Astrin\_STLCHLD\_tube2\_061314\_01.08135.08135.2 | 2.4286 | 0.2419 | 99.2% | 887.8722 | 888.0692 | 1 | 4.937 | 91.7% | 1 | K.FDLMYAK.R | 222 |
|  | Astrin\_STLCHLD\_050114\_02.07700.07700.3 | 6.6327 | 0.4886 | 100.0% | 2487.6843 | 2487.7083 | 1 | 9.145 | 48.8% | 10 | K.RAFVHWYVGEGMEEGEFSEAR.E | 333 |
|  | Astrin\_STLCHLD\_050114\_02.08531.08531.2 | 5.3032 | 0.4677 | 100.0% | 2331.412 | 2331.5208 | 1 | 8.46 | 65.8% | 2 | R.AFVHWYVGEGMEEGEFSEAR.E | 222 |
|  | Astrin\_STLCHLD\_061214\_02.07921.07921.3 | 5.6027 | 0.4369 | 100.0% | 2332.3743 | 2331.5208 | 1 | 7.994 | 44.7% | 24 | R.AFVHWYVGEGMEEGEFSEAR.E | 333 |
|  | Astrin\_STLCHLD\_tube2\_050114\_01.11503.11503.3 | 4.258 | 0.3976 | 99.9% | 3219.3843 | 3219.524 | 1 | 7.266 | 31.5% | 3 | R.AFVHWYVGEGMEEGEFSEAREDMAALEK.D | 333 |
|  | Astrin\_STLCHLD\_tube2\_061314\_02.06330.06330.2 | 3.6552 | 0.5707 | 100.0% | 2349.612 | 2350.2751 | 1 | 10.384 | 52.5% | 3 | K.DYEEVGVDSVEGEGEEEGEEY.- | 2 |

Similarities:
gi|14389309|ref|NP\_11(36:3)  
gi|17921989|ref|NP\_00(25:14)  

---

|  |  |  |  |  |  |  |  |  |
| --- | --- | --- | --- | --- | --- | --- | --- | --- |
| U | *gi|29788785|ref|NP\_82* | 45 | 634 | 66.0% | 444 | 49671 | 4.9 | tubulin, beta [Homo sapiens] |

| Filename XCorr DeltCN Conf% ObsM+H+ CalcM+H+ SpR ZScore Ion% # Sequence  | | | | | | | | | | | | |
| --- | --- | --- | --- | --- | --- | --- | --- | --- | --- | --- | --- | --- |
| \* | Astrin\_STLCHLD\_050114\_02.08224.08224.3 | 6.5782 | 0.409 | 100.0% | 3103.5842 | 3104.2725 | 1 | 9.131 | 36.5% | 23 | K.FWEVISDEHGIDPTGTYHGDSDLQLDR.I | 3 |
| \* | Astrin\_STLCHLD\_tube2\_050114\_01.05498.05498.2 | 3.9324 | 0.5962 | 100.0% | 1302.2122 | 1302.4265 | 1 | 9.872 | 86.4% | 44 | R.ISVYYNEATGGK.Y | 2 |
| \* | Astrin\_STLCHLD\_tube2\_061314\_01.04659.04659.1 | 2.4724 | 0.2415 | 97.3% | 1303.66 | 1302.4265 | 6 | 5.053 | 50.0% | 5 | R.ISVYYNEATGGK.Y | 1 |
| \* | Astrin\_STLCHLD\_061214\_01.05823.05823.2 | 4.198 | 0.518 | 100.0% | 1817.2722 | 1818.0392 | 1 | 9.098 | 70.0% | 2 | R.ISVYYNEATGGKYVPR.A | 2 |
| \* | AstrinSTLCLD\_041714\_02.05894.05894.3 | 2.6766 | 0.2563 | 96.6% | 1817.8744 | 1818.0392 | 371 | 5.391 | 28.3% | 3 | R.ISVYYNEATGGKYVPR.A | 3 |
|  | Astrin\_STLCHLD\_tube2\_050114\_01.10300.10300.1 | 1.9796 | 0.4452 | 100.0% | 1615.8 | 1616.8701 | 25 | 6.88 | 42.9% | 1 | R.AILVDLEPGTMDSVR.S | 111 |
|  | Astrin\_STLCLD20\_112214\_01.11522.11522.2 | 4.511 | 0.4938 | 100.0% | 1617.3121 | 1616.8701 | 1 | 8.254 | 64.3% | 40 | R.AILVDLEPGTMDSVR.S | 222 |
|  | Astrin\_STLCHLD\_tube2\_050114\_01.12338.12338.2 | 5.6846 | 0.5176 | 100.0% | 2798.8523 | 2800.0647 | 1 | 8.445 | 40.0% | 14 | R.SGPFGQIFRPDNFVFGQSGAGNNWAK.G | 2222 |
|  | AstrinSTLCLD\_041714\_01.10952.10952.3 | 7.1011 | 0.4962 | 100.0% | 2799.1143 | 2800.0647 | 1 | 8.497 | 38.0% | 33 | R.SGPFGQIFRPDNFVFGQSGAGNNWAK.G | 3333 |
|  | Astrin\_STLCHLD\_tube2\_050114\_01.12857.12857.2 | 7.2534 | 0.4819 | 100.0% | 1960.4722 | 1960.151 | 1 | 9.656 | 79.4% | 13 | K.GHYTEGAELVDSVLDVVR.K | 2222 |
|  | Astrin\_STLCHLD\_tube2\_050114\_01.12839.12839.3 | 4.3447 | 0.3812 | 99.9% | 1960.7644 | 1960.151 | 1 | 7.142 | 45.6% | 12 | K.GHYTEGAELVDSVLDVVR.K | 3333 |
|  | Astrin\_STLCHLD\_tube2\_061314\_01.11982.11982.3 | 4.7827 | 0.4838 | 100.0% | 2088.1143 | 2088.325 | 1 | 8.148 | 44.4% | 19 | K.GHYTEGAELVDSVLDVVRK.E | 3333 |
|  | Astrin\_STLCHLD\_050114\_01.12405.12405.2 | 6.3217 | 0.4514 | 100.0% | 2088.9321 | 2088.325 | 1 | 8.891 | 66.7% | 6 | K.GHYTEGAELVDSVLDVVRK.E | 2222 |
|  | Astrin\_STLCHLD\_050114\_01.04328.04328.2 | 2.6855 | 0.1744 | 98.8% | 1078.0122 | 1078.1698 | 9 | 4.186 | 78.6% | 2 | K.IREEYPDR.I | 222 |
|  | Astrin\_STLCHLD\_050114\_01.11995.11995.1 | 2.1993 | 0.2639 | 97.3% | 1319.69 | 1320.5896 | 245 | 5.053 | 40.9% | 6 | R.IMNTFSVVPSPK.V | 1111 |
|  | Astrin\_STLCHLD\_tube2\_061314\_01.08597.08597.2 | 4.5471 | 0.3836 | 100.0% | 1320.4122 | 1320.5896 | 1 | 7.86 | 77.3% | 40 | R.IMNTFSVVPSPK.V | 2222 |
|  | Astrin\_STLCHLD\_050114\_01.12590.12590.3 | 4.1941 | 0.3268 | 100.0% | 2710.8843 | 2710.0405 | 1 | 6.714 | 30.2% | 1 | K.LTTPTYGDLNHLVSATMSGVTTCLR.F | 3333 |
|  | Astrin\_STLCHLD\_tube2\_061314\_01.07361.07361.2 | 3.2733 | 0.2391 | 99.9% | 1132.2322 | 1131.2767 | 1 | 5.073 | 83.3% | 60 | R.FPGQLNADLR.K | 222222 |
|  | Astrin\_STLCHLD\_tube2\_050114\_01.05601.05601.2 | 2.7143 | 0.2756 | 99.3% | 1258.5521 | 1259.4508 | 11 | 5.358 | 65.0% | 14 | R.FPGQLNADLRK.L | 222222 |
|  | Astrin\_STLCHLD\_tube2\_050114\_01.05583.05583.3 | 3.152 | 0.3056 | 99.8% | 1259.8744 | 1259.4508 | 8 | 5.284 | 45.0% | 17 | R.FPGQLNADLRK.L | 333333 |
|  | Astrin\_STLCHLD\_tube2\_061314\_01.08730.08730.2 | 3.8464 | 0.3753 | 100.0% | 1272.4122 | 1272.5945 | 1 | 7.898 | 75.0% | 16 | R.KLAVNMVPFPR.L | 222222 |
|  | Astrin\_STLCLD20\_112214\_01.11192.11192.1 | 2.2399 | 0.2147 | 96.5% | 1143.63 | 1144.4204 | 6 | 6.378 | 61.1% | 4 | K.LAVNMVPFPR.L | 111111 |
|  | Astrin\_STLCHLD\_tube2\_050114\_01.09839.09839.2 | 3.9889 | 0.4725 | 100.0% | 1144.4122 | 1144.4204 | 1 | 8.55 | 94.4% | 18 | K.LAVNMVPFPR.L | 222222 |
|  | Astrin\_STLCHLD\_tube2\_061314\_01.12143.12143.2 | 3.7666 | 0.4528 | 100.0% | 1621.8322 | 1621.9403 | 1 | 8.756 | 76.9% | 17 | R.LHFFMPGFAPLTSR.G | 22222 |
|  | Astrin\_STLCHLD\_tube2\_061314\_01.12204.12204.3 | 4.3867 | 0.3891 | 100.0% | 1622.3944 | 1621.9403 | 1 | 6.411 | 53.8% | 14 | R.LHFFMPGFAPLTSR.G | 33333 |
| \* | Astrin\_STLCHLD\_tube2\_061314\_01.11378.11378.3 | 2.9228 | 0.2689 | 97.7% | 2381.3044 | 2380.6628 | 1 | 4.399 | 30.0% | 1 | R.GSQQYRALTVPELTQQVFDAK.N | 3 |
| \* | Astrin\_STLCHLD\_tube2\_050114\_01.11701.11701.2 | 4.2031 | 0.5417 | 100.0% | 1660.4321 | 1660.9078 | 1 | 9.309 | 67.9% | 18 | R.ALTVPELTQQVFDAK.N | 2 |
| \* | Astrin\_STLCHLD\_tube2\_050114\_01.11774.11774.3 | 3.158 | 0.3724 | 100.0% | 1660.8243 | 1660.9078 | 1 | 6.247 | 39.3% | 1 | R.ALTVPELTQQVFDAK.N | 3 |
|  | Astrin\_STLCHLD\_050114\_01.04952.04952.2 | 2.8672 | 0.3941 | 100.0% | 1066.2322 | 1066.2013 | 2 | 7.174 | 68.8% | 2 | K.NMMAACDPR.H | 222222 |
|  | Astrin\_STLCHLD\_050114\_02.06747.06747.3 | 4.3076 | 0.2581 | 99.9% | 1391.1244 | 1390.631 | 1 | 7.084 | 47.7% | 4 | R.HGRYLTVAAVFR.G | 333 |
|  | Astrin\_STLCHLD\_tube2\_050114\_01.10402.10402.1 | 1.7815 | 0.4511 | 100.0% | 1039.71 | 1040.2505 | 1 | 6.99 | 81.2% | 15 | R.YLTVAAVFR.G | 111 |
|  | Astrin\_STLCHLD\_tube2\_050114\_01.10269.10269.2 | 3.417 | 0.3601 | 100.0% | 1041.3322 | 1040.2505 | 1 | 6.884 | 87.5% | 30 | R.YLTVAAVFR.G | 222 |
|  | Astrin\_STLCHLD\_tube2\_050114\_02.06679.06679.2 | 5.6297 | 0.5004 | 100.0% | 1924.5322 | 1925.2405 | 1 | 8.712 | 56.7% | 4 | R.MSMKEVDEQMLNVQNK.N | 222 |
|  | Astrin\_STLCHLD\_tube2\_061314\_02.06675.06675.3 | 4.9405 | 0.1867 | 99.8% | 1926.6843 | 1925.2405 | 1 | 5.512 | 51.7% | 15 | R.MSMKEVDEQMLNVQNK.N | 333 |
|  | Astrin\_STLCHLD\_tube2\_050114\_01.05776.05776.1 | 2.9126 | 0.2315 | 98.0% | 1446.63 | 1447.6031 | 1 | 5.343 | 68.2% | 1 | K.EVDEQMLNVQNK.N | 111 |
|  | Astrin\_NLD\_STLC\_tube2\_021014\_01.06237.06237.2 | 4.3052 | 0.2375 | 100.0% | 1448.2522 | 1447.6031 | 6 | 5.817 | 63.6% | 16 | K.EVDEQMLNVQNK.N | 222 |
|  | Astrin\_STLCHLD\_tube2\_061314\_01.11781.11781.2 | 4.295 | 0.2978 | 100.0% | 1698.5322 | 1697.8877 | 1 | 7.708 | 73.1% | 8 | K.NSSYFVEWIPNNVK.T | 222222 |
|  | Astrin\_STLCHLD\_050114\_01.05029.05029.2 | 2.2588 | 0.224 | 97.2% | 1029.1921 | 1029.1473 | 9 | 4.44 | 62.5% | 1 | K.TAVCDIPPR.G | 2222 |
| \* | Astrin\_STLCHLD\_tube2\_061314\_02.09998.09998.2 | 5.7383 | 0.5106 | 100.0% | 1870.6122 | 1871.2018 | 1 | 9.377 | 71.9% | 11 | K.MAVTFIGNSTAIQELFK.R | 2 |
| \* | Astrin\_STLCLD20\_112214\_tube2\_01.16989.16989.2 | 5.2038 | 0.5156 | 100.0% | 2026.4521 | 2027.3893 | 1 | 8.599 | 70.6% | 3 | K.MAVTFIGNSTAIQELFKR.I | 2 |
| \* | Astrin\_STLCHLD\_061214\_02.09223.09223.3 | 3.6251 | 0.3561 | 99.9% | 2028.0543 | 2027.3893 | 1 | 6.713 | 41.2% | 16 | K.MAVTFIGNSTAIQELFKR.I | 3 |
|  | Astrin\_STLCHLD\_061214\_01.07812.07812.2 | 3.8437 | 0.3944 | 100.0% | 1386.3522 | 1386.6116 | 1 | 7.545 | 80.0% | 16 | K.RISEQFTAMFR.R | 22222 |
|  | Astrin\_STLCHLD\_tube2\_050114\_02.06787.06787.3 | 3.1948 | 0.3082 | 99.8% | 1543.3143 | 1542.7991 | 1 | 5.726 | 52.3% | 1 | K.RISEQFTAMFRR.K | 33333 |
|  | Astrin\_STLCLD20\_112214\_tube2\_01.13623.13623.1 | 1.9755 | 0.2993 | 98.1% | 1229.5 | 1230.4241 | 1 | 5.342 | 61.1% | 1 | R.ISEQFTAMFR.R | 11111 |
|  | Astrin\_STLCHLD\_tube2\_050114\_02.08042.08042.2 | 4.0657 | 0.4262 | 100.0% | 1231.3121 | 1230.4241 | 1 | 6.725 | 94.4% | 46 | R.ISEQFTAMFR.R | 22222 |

Similarities:
gi|5174735|ref|NP\_006(32:13)  
gi|29788768|ref|NP\_82(29:16)  
gi|21361322|ref|NP\_00(23:22)  
gi|50592996|ref|NP\_00(20:25)  
gi|14210536|ref|NP\_11(10:35)  

---

|  |  |  |  |  |  |  |  |  |
| --- | --- | --- | --- | --- | --- | --- | --- | --- |
| U | *gi|4504919|ref|NP\_002* | 52 | 502 | 64.8% | 483 | 53704 | 5.6 | keratin 8 [Homo sapiens] |

| Filename XCorr DeltCN Conf% ObsM+H+ CalcM+H+ SpR ZScore Ion% # Sequence  | | | | | | | | | | | | |
| --- | --- | --- | --- | --- | --- | --- | --- | --- | --- | --- | --- | --- |
|  | Astrin\_STLCHLD\_050114\_01.10456.10456.2 | 2.4721 | 0.2629 | 97.5% | 1618.0521 | 1618.7465 | 73 | 4.409 | 39.3% | 1 | R.ISSSSFSRVGSSNFR.G | 2 |
| \* | Astrin\_NLD\_STLC\_tube2\_021014\_01.15436.15436.3 | 4.9228 | 0.3462 | 99.9% | 3926.5444 | 3927.465 | 1 | 6.555 | 21.2% | 4 | R.GGLGGGYGGASGMGGITAVTVNQSLLSPLVLEVDPNIQAVR.T | 3 |
|  | Astrin\_STLCHLD\_tube2\_061314\_01.06264.06264.2 | 2.4085 | 0.2136 | 98.8% | 828.2922 | 827.95544 | 5 | 5.059 | 91.7% | 3 | K.FASFIDK.V | 22222222 |
|  | Astrin\_NLD\_STLC\_tube2\_021014\_01.07623.07623.2 | 2.9741 | 0.1656 | 99.1% | 1082.8121 | 1083.2755 | 7 | 7.028 | 75.0% | 6 | K.FASFIDKVR.F | 2222222 |
|  | AstrinSTLCLD\_041714\_01.06305.06305.2 | 3.32 | 0.1255 | 99.6% | 1030.8121 | 1031.1997 | 3 | 4.084 | 92.9% | 12 | K.WSLLQQQK.T | 2 |
|  | Astrin\_NLD\_STLC\_tube2\_021014\_01.13679.13679.2 | 3.7561 | 0.3979 | 100.0% | 1849.3322 | 1849.0431 | 1 | 6.537 | 50.0% | 3 | R.SNMDNMFESYINNLR.R | 2 |
|  | Astrin\_STLCHLD\_tube2\_061314\_01.12083.12083.3 | 2.3488 | 0.3489 | 99.0% | 2005.2244 | 2005.2306 | 6 | 5.196 | 30.0% | 1 | R.SNMDNMFESYINNLRR.Q | 3 |
|  | Astrin\_STLCLD20\_112214\_tube2\_01.16650.16650.2 | 5.033 | 0.3575 | 100.0% | 2034.4922 | 2035.363 | 1 | 9.481 | 61.8% | 1 | K.LKLEAELGNMQGLVEDFK.N | 2 |
|  | Astrin\_STLCHLD\_050114\_02.09917.09917.3 | 4.9914 | 0.2895 | 99.9% | 2036.6044 | 2035.363 | 1 | 7.594 | 45.6% | 8 | K.LKLEAELGNMQGLVEDFK.N | 3 |
|  | Astrin\_STLCLD20\_112214\_tube2\_01.16520.16520.2 | 3.9658 | 0.2823 | 100.0% | 1793.9922 | 1794.0295 | 2 | 8.639 | 50.0% | 5 | K.LEAELGNMQGLVEDFK.N | 2 |
|  | Astrin\_STLCLD20\_112214\_tube2\_01.13413.13413.2 | 3.5674 | 0.4238 | 100.0% | 1353.4122 | 1353.5732 | 1 | 7.877 | 85.0% | 25 | R.TEMENEFVLIK.K | 2 |
|  | Astrin\_NLD\_STLC\_tube2\_021014\_02.06556.06556.2 | 3.7042 | 0.3079 | 100.0% | 1481.2522 | 1481.7473 | 1 | 5.659 | 72.7% | 20 | R.TEMENEFVLIKK.D | 2 |
|  | Astrin\_STLCHLD\_tube2\_050114\_01.07946.07946.3 | 2.3312 | 0.3006 | 97.7% | 1483.3744 | 1481.7473 | 16 | 4.946 | 40.9% | 2 | R.TEMENEFVLIKK.D | 3 |
|  | Astrin\_STLCHLD\_050114\_02.05722.05722.3 | 3.982 | 0.4171 | 100.0% | 1926.6244 | 1927.1365 | 1 | 6.668 | 36.7% | 6 | K.KDVDEAYMNKVELESR.L | 3 |
|  | AstrinSTLCLD\_041714\_01.03918.03918.2 | 2.3238 | 0.4049 | 99.9% | 1085.0721 | 1085.1737 | 138 | 6.082 | 56.2% | 2 | K.DVDEAYMNK.V | 2 |
|  | Astrin\_STLCHLD\_tube2\_050114\_01.07050.07050.2 | 4.4449 | 0.5029 | 100.0% | 1798.2922 | 1798.9623 | 1 | 8.594 | 75.0% | 21 | K.DVDEAYMNKVELESR.L | 2 |
|  | Astrin\_STLCLD20\_112214\_tube2\_02.06816.06816.3 | 4.5861 | 0.3449 | 99.9% | 1799.6643 | 1798.9623 | 1 | 6.319 | 55.4% | 26 | K.DVDEAYMNKVELESR.L | 3 |
|  | AstrinSTLCLD\_041714\_02.10287.10287.3 | 4.334 | 0.4169 | 99.9% | 3198.7444 | 3200.5444 | 83 | 5.714 | 22.1% | 2 | K.DVDEAYMNKVELESRLEGLTDEINFLR.Q | 3 |
|  | Astrin\_STLCLD20\_112214\_01.12742.12742.1 | 2.9453 | 0.2941 | 96.7% | 1419.6 | 1420.6055 | 1 | 5.852 | 63.6% | 3 | R.LEGLTDEINFLR.Q | 1 |
|  | AstrinSTLCLD\_041714\_01.10680.10680.2 | 4.1638 | 0.3658 | 100.0% | 1421.6522 | 1420.6055 | 1 | 7.205 | 90.9% | 29 | R.LEGLTDEINFLR.Q | 2 |
|  | Astrin\_STLCHLD\_050114\_02.07643.07643.2 | 5.8514 | 0.5637 | 100.0% | 2109.6921 | 2110.3008 | 1 | 10.001 | 72.2% | 9 | R.ELQSQISDTSVVLSMDNSR.S | 2 |
|  | Astrin\_STLCLD20\_112214\_tube2\_01.15461.15461.1 | 2.5272 | 0.3514 | 95.6% | 1320.64 | 1321.5286 | 4 | 6.314 | 59.1% | 5 | R.SLDMDSIIAEVK.A | 1 |
|  | Astrin\_STLCHLD\_tube2\_061314\_01.11803.11803.2 | 4.4966 | 0.4215 | 100.0% | 1321.4922 | 1321.5286 | 1 | 8.047 | 72.7% | 17 | R.SLDMDSIIAEVK.A | 2 |
|  | Astrin\_STLCHLD\_tube2\_050114\_01.14632.14632.2 | 3.4678 | 0.4072 | 100.0% | 2381.9321 | 2382.6477 | 1 | 6.067 | 40.0% | 3 | R.SLDMDSIIAEVKAQYEDIANR.S | 2 |
|  | Astrin\_NLD\_STLC\_tube2\_021014\_01.04317.04317.1 | 1.9579 | 0.2777 | 97.3% | 1079.45 | 1080.1423 | 4 | 5.773 | 56.2% | 4 | K.AQYEDIANR.S | 11 |
|  | Astrin\_NLD\_STLC\_031014\_01.09014.09014.2 | 3.2681 | 0.2878 | 100.0% | 1079.9722 | 1080.1423 | 2 | 6.731 | 75.0% | 14 | K.AQYEDIANR.S | 22 |
|  | Astrin\_STLCHLD\_050114\_01.06423.06423.2 | 3.5727 | 0.2997 | 100.0% | 1412.6721 | 1413.5884 | 1 | 6.14 | 81.8% | 8 | R.SRAEAESMYQIK.Y | 2 |
|  | Astrin\_STLCHLD\_050114\_02.07454.07454.3 | 6.3252 | 0.4019 | 100.0% | 2533.1343 | 2532.828 | 1 | 7.97 | 38.1% | 17 | R.SRAEAESMYQIKYEELQSLAGK.H | 3 |
|  | Astrin\_NLD\_STLC\_tube2\_021014\_01.05788.05788.2 | 3.391 | 0.4615 | 100.0% | 1170.0521 | 1170.3228 | 1 | 7.159 | 77.8% | 9 | R.AEAESMYQIK.Y | 2 |
|  | Astrin\_STLCLD20\_112214\_02.11686.11686.3 | 5.1538 | 0.4307 | 100.0% | 2289.2344 | 2289.5623 | 1 | 8.344 | 34.2% | 16 | R.AEAESMYQIKYEELQSLAGK.H | 3 |
|  | Astrin\_NLD\_STLC\_tube2\_021014\_01.06752.06752.2 | 3.8055 | 0.0737 | 99.3% | 1138.1122 | 1138.2627 | 2 | 7.092 | 77.8% | 20 | K.YEELQSLAGK.H | 2 |
|  | Astrin\_STLCLD20\_112214\_tube2\_01.08957.08957.1 | 2.0941 | 0.2302 | 96.8% | 1000.5 | 1001.168 | 10 | 5.469 | 62.5% | 2 | R.LQAEIEGLK.G | 1 |
|  | Astrin\_STLCLD20\_112214\_tube2\_01.08900.08900.2 | 3.2154 | 0.2175 | 99.9% | 1001.3122 | 1001.168 | 3 | 5.211 | 87.5% | 14 | R.LQAEIEGLK.G | 2 |
|  | Astrin\_STLCLD20\_112214\_01.05091.05091.2 | 3.6977 | 0.2385 | 100.0% | 1342.2922 | 1342.5381 | 1 | 6.616 | 77.3% | 13 | R.LQAEIEGLKGQR.A | 2 |
|  | Astrin\_STLCLD20\_112214\_tube2\_01.12674.12674.3 | 4.1554 | 0.4053 | 99.9% | 2669.7544 | 2668.967 | 1 | 7.6 | 31.2% | 1 | R.LQAEIEGLKGQRASLEAAIADAEQR.G | 3 |
|  | Astrin\_STLCLD20\_112214\_01.10636.10636.1 | 2.8357 | 0.5447 | 100.0% | 1344.6 | 1345.452 | 1 | 8.051 | 62.5% | 1 | R.ASLEAAIADAEQR.G | 1 |
|  | Astrin\_STLCLD20\_112214\_01.10616.10616.2 | 4.3054 | 0.4007 | 100.0% | 1346.3522 | 1345.452 | 1 | 7.313 | 70.8% | 18 | R.ASLEAAIADAEQR.G | 2 |
|  | Astrin\_NLD\_STLC\_tube2\_021014\_02.08550.08550.3 | 3.8611 | 0.4245 | 99.9% | 1956.2943 | 1957.1912 | 7 | 6.411 | 37.5% | 9 | R.ASLEAAIADAEQRGELAIK.D | 3 |
|  | Astrin\_NLD\_STLC\_tube2\_021014\_01.11879.11879.2 | 5.4067 | 0.423 | 100.0% | 1957.4521 | 1957.1912 | 1 | 7.653 | 55.6% | 14 | R.ASLEAAIADAEQRGELAIK.D | 2 |
|  | Astrin\_STLCLD20\_112214\_tube2\_01.15478.15478.3 | 5.8266 | 0.4404 | 100.0% | 2456.6343 | 2456.7153 | 1 | 7.699 | 33.7% | 13 | R.ASLEAAIADAEQRGELAIKDANAK.L | 3 |
|  | Astrin\_NLD\_STLC\_031014\_01.09888.09888.1 | 2.1905 | 0.296 | 97.8% | 1129.57 | 1130.2865 | 1 | 5.118 | 55.6% | 6 | K.LSELEAALQR.A | 1 |
|  | Astrin\_NLD\_STLC\_tube2\_021014\_01.08426.08426.2 | 4.343 | 0.2502 | 100.0% | 1130.1921 | 1130.2865 | 32 | 6.039 | 72.2% | 23 | K.LSELEAALQR.A | 2 |
|  | Astrin\_STLCLD20\_112214\_tube2\_01.10042.10042.2 | 3.7422 | 0.2042 | 99.9% | 1551.3121 | 1551.801 | 1 | 5.206 | 72.7% | 4 | R.QLREYQELMNVK.L | 2 |
|  | Astrin\_STLCLD20\_112214\_tube2\_01.09255.09255.1 | 2.466 | 0.2121 | 97.3% | 1153.62 | 1154.3234 | 15 | 5.518 | 68.8% | 3 | R.EYQELMNVK.L | 11 |
|  | Astrin\_STLCHLD\_050114\_01.11141.11141.2 | 2.8705 | 0.2502 | 99.6% | 1154.1322 | 1154.3234 | 15 | 6.116 | 62.5% | 5 | R.EYQELMNVK.L | 22 |
|  | Astrin\_STLCHLD\_061214\_01.07862.07862.2 | 3.174 | 0.25 | 99.6% | 1407.6322 | 1406.6653 | 1 | 4.86 | 77.3% | 9 | K.LALDIEIATYRK.L | 222 |
|  | Astrin\_NLD\_STLC\_tube2\_021014\_01.08200.08200.3 | 5.273 | 0.4907 | 100.0% | 2518.2844 | 2518.8628 | 1 | 7.947 | 38.1% | 5 | R.KLLEGEESRLESGMQNMSIHTK.T | 3 |
|  | AstrinSTLCLD\_041714\_01.07408.07408.3 | 5.8143 | 0.4398 | 100.0% | 2390.7244 | 2390.6887 | 1 | 7.772 | 41.2% | 7 | K.LLEGEESRLESGMQNMSIHTK.T | 3 |
|  | Astrin\_NLD\_STLC\_031014\_01.09158.09158.2 | 4.0999 | 0.48 | 100.0% | 1476.2322 | 1476.7058 | 1 | 8.072 | 83.3% | 8 | R.LESGMQNMSIHTK.T | 2 |
|  | Astrin\_NLD\_STLC\_tube2\_021014\_01.05038.05038.3 | 3.4427 | 0.3061 | 99.9% | 1478.5443 | 1476.7058 | 4 | 4.801 | 41.7% | 8 | R.LESGMQNMSIHTK.T | 3 |
|  | Astrin\_NLD\_STLC\_tube2\_021014\_01.06256.06256.2 | 3.7761 | 0.3793 | 100.0% | 1474.1921 | 1474.6512 | 1 | 7.267 | 65.4% | 6 | R.DGKLVSESSDVLPK.- | 2 |
|  | Astrin\_STLCHLD\_tube2\_050114\_01.05480.05480.2 | 3.5787 | 0.4495 | 100.0% | 1175.1721 | 1174.3367 | 1 | 7.381 | 75.0% | 31 | K.LVSESSDVLPK.- | 2 |

Similarities:
gi|67782365|ref|NP\_00(3:49)  
gi|47132620|ref|NP\_00(2:50)  
gi|119703753|ref|NP\_0(4:48)  
gi|32567786|ref|NP\_78(2:50)  
gi|119395754|ref|NP\_0(2:50)  
gi|153791158|ref|NP\_0(4:48)  
gi|109255249|ref|NP\_0(2:50)  

---

|  |  |  |  |  |  |  |  |  |
| --- | --- | --- | --- | --- | --- | --- | --- | --- |
| U | *gi|5174735|ref|NP\_006* | 39 | 529 | 63.6% | 445 | 49831 | 4.9 | tubulin, beta, 2 [Homo sapiens] |

| Filename XCorr DeltCN Conf% ObsM+H+ CalcM+H+ SpR ZScore Ion% # Sequence  | | | | | | | | | | | | |
| --- | --- | --- | --- | --- | --- | --- | --- | --- | --- | --- | --- | --- |
|  | Astrin\_STLCHLD\_050114\_02.08218.08218.3 | 6.3129 | 0.4919 | 100.0% | 3117.2344 | 3118.2996 | 1 | 8.572 | 35.6% | 9 | K.FWEVISDEHGIDPTGTYHGDSDLQLER.I | 33 |
| \* | Astrin\_STLCHLD\_050114\_01.09653.09653.1 | 2.0865 | 0.2983 | 97.9% | 1328.61 | 1329.4521 | 1 | 6.346 | 54.5% | 1 | R.INVYYNEATGGK.Y | 1 |
| \* | Astrin\_STLCHLD\_tube2\_061314\_01.04739.04739.2 | 4.0385 | 0.3962 | 100.0% | 1328.9722 | 1329.4521 | 1 | 8.671 | 81.8% | 16 | R.INVYYNEATGGK.Y | 2 |
|  | Astrin\_STLCLD20\_112214\_tube2\_01.12946.12946.2 | 4.312 | 0.3215 | 100.0% | 1604.5322 | 1602.8431 | 1 | 6.404 | 67.9% | 22 | R.AVLVDLEPGTMDSVR.S | 22 |
|  | Astrin\_STLCHLD\_tube2\_050114\_01.12338.12338.2 | 5.6846 | 0.5176 | 100.0% | 2798.8523 | 2800.0647 | 1 | 8.445 | 40.0% | 14 | R.SGPFGQIFRPDNFVFGQSGAGNNWAK.G | 2222 |
|  | AstrinSTLCLD\_041714\_01.10952.10952.3 | 7.1011 | 0.4962 | 100.0% | 2799.1143 | 2800.0647 | 1 | 8.497 | 38.0% | 33 | R.SGPFGQIFRPDNFVFGQSGAGNNWAK.G | 3333 |
|  | Astrin\_STLCHLD\_tube2\_050114\_01.12857.12857.2 | 7.2534 | 0.4819 | 100.0% | 1960.4722 | 1960.151 | 1 | 9.656 | 79.4% | 13 | K.GHYTEGAELVDSVLDVVR.K | 2222 |
|  | Astrin\_STLCHLD\_tube2\_050114\_01.12839.12839.3 | 4.3447 | 0.3812 | 99.9% | 1960.7644 | 1960.151 | 1 | 7.142 | 45.6% | 12 | K.GHYTEGAELVDSVLDVVR.K | 3333 |
|  | Astrin\_STLCHLD\_tube2\_061314\_01.11982.11982.3 | 4.7827 | 0.4838 | 100.0% | 2088.1143 | 2088.325 | 1 | 8.148 | 44.4% | 19 | K.GHYTEGAELVDSVLDVVRK.E | 3333 |
|  | Astrin\_STLCHLD\_050114\_01.12405.12405.2 | 6.3217 | 0.4514 | 100.0% | 2088.9321 | 2088.325 | 1 | 8.891 | 66.7% | 6 | K.GHYTEGAELVDSVLDVVRK.E | 2222 |
|  | Astrin\_STLCHLD\_050114\_01.04328.04328.2 | 2.6855 | 0.1744 | 98.8% | 1078.0122 | 1078.1698 | 9 | 4.186 | 78.6% | 2 | K.IREEYPDR.I | 222 |
|  | Astrin\_STLCHLD\_050114\_01.11995.11995.1 | 2.1993 | 0.2639 | 97.3% | 1319.69 | 1320.5896 | 245 | 5.053 | 40.9% | 6 | R.IMNTFSVVPSPK.V | 1111 |
|  | Astrin\_STLCHLD\_tube2\_061314\_01.08597.08597.2 | 4.5471 | 0.3836 | 100.0% | 1320.4122 | 1320.5896 | 1 | 7.86 | 77.3% | 41 | R.IMNTFSVVPSPK.V | 2222 |
|  | Astrin\_STLCHLD\_050114\_01.12590.12590.3 | 4.1941 | 0.3268 | 100.0% | 2710.8843 | 2710.0405 | 1 | 6.714 | 30.2% | 1 | K.LTTPTYGDLNHLVSATMSGVTTCLR.F | 3333 |
|  | Astrin\_STLCHLD\_tube2\_061314\_01.07361.07361.2 | 3.2733 | 0.2391 | 99.9% | 1132.2322 | 1131.2767 | 1 | 5.073 | 83.3% | 60 | R.FPGQLNADLR.K | 222222 |
|  | Astrin\_STLCHLD\_tube2\_050114\_01.05601.05601.2 | 2.7143 | 0.2756 | 99.3% | 1258.5521 | 1259.4508 | 11 | 5.358 | 65.0% | 14 | R.FPGQLNADLRK.L | 222222 |
|  | Astrin\_STLCHLD\_tube2\_050114\_01.05583.05583.3 | 3.152 | 0.3056 | 99.8% | 1259.8744 | 1259.4508 | 8 | 5.284 | 45.0% | 17 | R.FPGQLNADLRK.L | 333333 |
|  | Astrin\_STLCHLD\_tube2\_061314\_01.08730.08730.2 | 3.8464 | 0.3753 | 100.0% | 1272.4122 | 1272.5945 | 1 | 7.898 | 75.0% | 16 | R.KLAVNMVPFPR.L | 222222 |
|  | Astrin\_STLCLD20\_112214\_01.11192.11192.1 | 2.2399 | 0.2147 | 96.5% | 1143.63 | 1144.4204 | 6 | 6.378 | 61.1% | 4 | K.LAVNMVPFPR.L | 111111 |
|  | Astrin\_STLCHLD\_tube2\_050114\_01.09839.09839.2 | 3.9889 | 0.4725 | 100.0% | 1144.4122 | 1144.4204 | 1 | 8.55 | 94.4% | 18 | K.LAVNMVPFPR.L | 222222 |
|  | Astrin\_STLCHLD\_tube2\_061314\_01.12143.12143.2 | 3.7666 | 0.4528 | 100.0% | 1621.8322 | 1621.9403 | 1 | 8.756 | 76.9% | 17 | R.LHFFMPGFAPLTSR.G | 22222 |
|  | Astrin\_STLCHLD\_tube2\_061314\_01.12204.12204.3 | 4.3867 | 0.3891 | 100.0% | 1622.3944 | 1621.9403 | 1 | 6.411 | 53.8% | 14 | R.LHFFMPGFAPLTSR.G | 33333 |
|  | Astrin\_STLCHLD\_tube2\_061314\_01.12186.12186.2 | 4.648 | 0.4524 | 100.0% | 1692.6122 | 1692.9678 | 1 | 7.8 | 78.6% | 12 | R.ALTVPELTQQMFDAK.N | 222 |
|  | Astrin\_STLCHLD\_050114\_01.04952.04952.2 | 2.8672 | 0.3941 | 100.0% | 1066.2322 | 1066.2013 | 2 | 7.174 | 68.8% | 2 | K.NMMAACDPR.H | 222222 |
|  | Astrin\_STLCHLD\_050114\_02.06747.06747.3 | 4.3076 | 0.2581 | 99.9% | 1391.1244 | 1390.631 | 1 | 7.084 | 47.7% | 4 | R.HGRYLTVAAVFR.G | 333 |
|  | Astrin\_STLCHLD\_tube2\_050114\_01.10402.10402.1 | 1.7815 | 0.4511 | 100.0% | 1039.71 | 1040.2505 | 1 | 6.99 | 81.2% | 15 | R.YLTVAAVFR.G | 111 |
|  | Astrin\_STLCHLD\_tube2\_050114\_01.10269.10269.2 | 3.417 | 0.3601 | 100.0% | 1041.3322 | 1040.2505 | 1 | 6.884 | 87.5% | 30 | R.YLTVAAVFR.G | 222 |
|  | Astrin\_STLCHLD\_tube2\_050114\_02.06679.06679.2 | 5.6297 | 0.5004 | 100.0% | 1924.5322 | 1925.2405 | 1 | 8.712 | 56.7% | 4 | R.MSMKEVDEQMLNVQNK.N | 222 |
|  | Astrin\_STLCHLD\_tube2\_061314\_02.06675.06675.3 | 4.9405 | 0.1867 | 99.8% | 1926.6843 | 1925.2405 | 1 | 5.512 | 51.7% | 15 | R.MSMKEVDEQMLNVQNK.N | 333 |
|  | Astrin\_STLCHLD\_tube2\_050114\_01.05776.05776.1 | 2.9126 | 0.2315 | 98.0% | 1446.63 | 1447.6031 | 1 | 5.343 | 68.2% | 1 | K.EVDEQMLNVQNK.N | 111 |
|  | Astrin\_NLD\_STLC\_tube2\_021014\_01.06237.06237.2 | 4.3052 | 0.2375 | 100.0% | 1448.2522 | 1447.6031 | 6 | 5.817 | 63.6% | 16 | K.EVDEQMLNVQNK.N | 222 |
|  | Astrin\_STLCHLD\_tube2\_061314\_01.11781.11781.2 | 4.295 | 0.2978 | 100.0% | 1698.5322 | 1697.8877 | 1 | 7.708 | 73.1% | 8 | K.NSSYFVEWIPNNVK.T | 222222 |
|  | Astrin\_STLCHLD\_050114\_01.05029.05029.2 | 2.2588 | 0.224 | 97.2% | 1029.1921 | 1029.1473 | 9 | 4.44 | 62.5% | 1 | K.TAVCDIPPR.G | 2222 |
|  | Astrin\_STLCHLD\_tube2\_061314\_02.09608.09608.2 | 3.6979 | 0.0138 | 95.3% | 1859.8522 | 1859.1475 | 1 | 5.87 | 50.0% | 1 | K.MSATFIGNSTAIQELFK.R | 22 |
|  | Astrin\_STLCLD20\_112214\_02.14525.14525.3 | 3.4924 | 0.1623 | 95.1% | 2016.4443 | 2015.335 | 1 | 5.966 | 32.4% | 1 | K.MSATFIGNSTAIQELFKR.I | 33 |
|  | Astrin\_STLCHLD\_061214\_01.07812.07812.2 | 3.8437 | 0.3944 | 100.0% | 1386.3522 | 1386.6116 | 1 | 7.545 | 80.0% | 16 | K.RISEQFTAMFR.R | 22222 |
|  | Astrin\_STLCHLD\_tube2\_050114\_02.06787.06787.3 | 3.1948 | 0.3082 | 99.8% | 1543.3143 | 1542.7991 | 1 | 5.726 | 52.3% | 1 | K.RISEQFTAMFRR.K | 33333 |
|  | Astrin\_STLCLD20\_112214\_tube2\_01.13623.13623.1 | 1.9755 | 0.2993 | 98.1% | 1229.5 | 1230.4241 | 1 | 5.342 | 61.1% | 1 | R.ISEQFTAMFR.R | 11111 |
|  | Astrin\_STLCHLD\_tube2\_050114\_02.08042.08042.2 | 4.0657 | 0.4262 | 100.0% | 1231.3121 | 1230.4241 | 1 | 6.725 | 94.4% | 46 | R.ISEQFTAMFR.R | 22222 |

Similarities:
gi|29788785|ref|NP\_82(32:7)  
gi|29788768|ref|NP\_82(29:10)  
gi|21361322|ref|NP\_00(26:13)  
gi|50592996|ref|NP\_00(19:20)  
gi|14210536|ref|NP\_11(10:29)  

---

|  |  |  |  |  |  |  |  |  |
| --- | --- | --- | --- | --- | --- | --- | --- | --- |
| U | *gi|106775678|ref|NP\_0* | 12 | 100 | 62.3% | 130 | 14095 | 10.9 | histone cluster 2, H2aa4 [Homo sapiens] |
| U | *gi|4504251|ref|NP\_003* | 12 | 100 | 62.3% | 130 | 14095 | 10.9 | histone cluster 2, H2aa3 [Homo sapiens] |
| U | *gi|24638446|ref|NP\_00* | 12 | 100 | 62.8% | 129 | 13988 | 10.9 | histone cluster 2, H2ac [Homo sapiens] |

| Filename XCorr DeltCN Conf% ObsM+H+ CalcM+H+ SpR ZScore Ion% # Sequence  | | | | | | | | | | | | |
| --- | --- | --- | --- | --- | --- | --- | --- | --- | --- | --- | --- | --- |
|  | Astrin\_STLCHLD\_050114\_01.10552.10552.2 | 2.9962 | 0.2759 | 99.6% | 1276.0322 | 1275.4531 | 1 | 5.752 | 77.3% | 5 | R.SSRAGLQFPVGR.V | 222 |
|  | AstrinSTLCLD\_041714\_01.06063.06063.1 | 1.9612 | 0.2801 | 97.2% | 944.67 | 945.1093 | 4 | 5.26 | 62.5% | 2 | R.AGLQFPVGR.V | 1111 |
|  | Astrin\_STLCHLD\_tube2\_061314\_01.07517.07517.2 | 3.2091 | 0.3209 | 100.0% | 945.39215 | 945.1093 | 1 | 5.94 | 81.2% | 21 | R.AGLQFPVGR.V | 2222 |
|  | Astrin\_STLCLD20\_112214\_tube2\_02.00464.00464.3 | 4.7352 | 0.3446 | 99.9% | 2935.4644 | 2935.4082 | 1 | 7.289 | 30.4% | 6 | R.VGAGAPVYMAAVLEYLTAEILELAGNAAR.D | 3 |
|  | Astrin\_NLD\_STLC\_tube2\_021014\_01.20505.20505.2 | 5.5694 | 0.5404 | 100.0% | 2935.7722 | 2935.4082 | 1 | 9.66 | 44.6% | 12 | R.VGAGAPVYMAAVLEYLTAEILELAGNAAR.D | 2 |
|  | Astrin\_STLCLD20\_112214\_tube2\_02.00326.00326.3 | 3.9845 | 0.3733 | 99.9% | 3293.1243 | 3292.7747 | 1 | 6.832 | 25.8% | 2 | R.VGAGAPVYMAAVLEYLTAEILELAGNAARDNK.K | 3 |
|  | Astrin\_STLCLD20\_112214\_tube2\_01.07832.07832.2 | 2.6508 | 0.2524 | 99.7% | 851.2922 | 851.0396 | 4 | 6.019 | 83.3% | 5 | R.HLQLAIR.N | 2222 |
|  | Astrin\_STLCHLD\_tube2\_061314\_02.05192.05192.3 | 3.2474 | 0.3885 | 99.9% | 1694.3344 | 1693.9004 | 1 | 6.694 | 44.2% | 17 | R.HLQLAIRNDEELNK.L | 333 |
|  | Astrin\_STLCLD20\_112214\_tube2\_01.11649.11649.3 | 4.8389 | 0.5071 | 100.0% | 2106.0244 | 2105.4453 | 1 | 7.497 | 39.7% | 9 | R.HLQLAIRNDEELNKLLGK.V | 33 |
|  | Astrin\_STLCHLD\_tube2\_061314\_01.07421.07421.2 | 3.6 | 0.3554 | 100.0% | 1273.4122 | 1273.4288 | 1 | 6.312 | 75.0% | 13 | R.NDEELNKLLGK.V | 22 |
|  | Astrin\_STLCLD20\_112214\_tube2\_01.17512.17512.1 | 2.388 | 0.2221 | 97.4% | 1931.12 | 1932.3573 | 40 | 5.511 | 33.3% | 1 | K.VTIAQGGVLPNIQAVLLPK.K | 111 |
|  | Astrin\_NLD\_STLC\_tube2\_021014\_01.13730.13730.2 | 4.9626 | 0.4911 | 100.0% | 1931.8522 | 1932.3573 | 1 | 8.543 | 66.7% | 7 | K.VTIAQGGVLPNIQAVLLPK.K | 222 |

Similarities:
gi|10645195|ref|NP\_06(7:5)  
gi|10800130|ref|NP\_06(9:3)  
gi|20357599|ref|NP\_61(3:9)  

---

|  |  |  |  |  |  |  |  |  |
| --- | --- | --- | --- | --- | --- | --- | --- | --- |
| U | *gi|14389309|ref|NP\_11* | 37 | 482 | 61.7% | 449 | 49895 | 5.1 | tubulin alpha 6 [Homo sapiens] |

| Filename XCorr DeltCN Conf% ObsM+H+ CalcM+H+ SpR ZScore Ion% # Sequence  | | | | | | | | | | | | |
| --- | --- | --- | --- | --- | --- | --- | --- | --- | --- | --- | --- | --- |
|  | Astrin\_STLCHLD\_061214\_01.09384.09384.2 | 6.1825 | 0.6572 | 100.0% | 2008.3922 | 2009.093 | 1 | 11.538 | 63.2% | 37 | K.TIGGGDDSFNTFFSETGAGK.H | 22 |
|  | Astrin\_STLCHLD\_050114\_02.08865.08865.3 | 3.8391 | 0.2974 | 99.8% | 2009.6044 | 2009.093 | 12 | 5.573 | 31.6% | 1 | K.TIGGGDDSFNTFFSETGAGK.H | 33 |
|  | Astrin\_STLCHLD\_tube2\_061314\_01.11935.11935.1 | 3.3623 | 0.5322 | 100.0% | 1702.95 | 1702.9451 | 1 | 8.203 | 57.1% | 7 | R.AVFVDLEPTVIDEVR.T | 11 |
|  | Astrin\_STLCHLD\_061214\_02.08717.08717.2 | 5.1706 | 0.51 | 100.0% | 1703.3522 | 1702.9451 | 1 | 8.59 | 78.6% | 47 | R.AVFVDLEPTVIDEVR.T | 22 |
|  | Astrin\_STLCHLD\_tube2\_050114\_01.11676.11676.3 | 4.6957 | 0.4534 | 100.0% | 1704.2943 | 1702.9451 | 1 | 7.889 | 51.8% | 6 | R.AVFVDLEPTVIDEVR.T | 33 |
|  | Astrin\_STLCLD20\_112214\_01.10293.10293.2 | 1.9386 | 0.3075 | 95.5% | 1411.5521 | 1411.6439 | 367 | 5.745 | 50.0% | 4 | R.QLFHPEQLITGK.E | 222 |
|  | Astrin\_STLCLD20\_112214\_tube2\_01.10802.10802.3 | 3.9221 | 0.4709 | 100.0% | 2416.4644 | 2416.6555 | 2 | 7.043 | 30.0% | 13 | R.QLFHPEQLITGKEDAANNYAR.G | 333 |
|  | Astrin\_STLCLD20\_112214\_tube2\_01.10767.10767.2 | 2.6947 | 0.3834 | 99.8% | 2416.5723 | 2416.6555 | 6 | 5.895 | 37.5% | 2 | R.QLFHPEQLITGKEDAANNYAR.G | 222 |
|  | Astrin\_STLCLD20\_112214\_01.13269.13269.3 | 4.8135 | 0.4526 | 100.0% | 1843.2544 | 1843.1332 | 2 | 7.617 | 43.3% | 6 | R.GHYTIGKEIIDLVLDR.I | 33 |
|  | Astrin\_STLCHLD\_tube2\_050114\_01.12647.12647.2 | 5.0104 | 0.5165 | 100.0% | 1843.7322 | 1843.1332 | 1 | 8.617 | 70.0% | 6 | R.GHYTIGKEIIDLVLDR.I | 22 |
|  | Astrin\_STLCLD20\_112214\_tube2\_01.15296.15296.1 | 2.1784 | 0.3293 | 97.1% | 1085.77 | 1086.2737 | 2 | 6.033 | 62.5% | 5 | K.EIIDLVLDR.I | 11 |
|  | Astrin\_STLCHLD\_tube2\_061314\_01.11543.11543.2 | 3.0514 | 0.3519 | 100.0% | 1086.4722 | 1086.2737 | 9 | 6.025 | 81.2% | 8 | K.EIIDLVLDR.I | 22 |
|  | Astrin\_STLCHLD\_tube2\_050114\_02.11004.11004.3 | 3.9515 | 0.2893 | 99.8% | 3392.8145 | 3392.7769 | 1 | 5.149 | 23.4% | 1 | K.LADQCTGLQGFLVFHSFGGGTGSGFTSLLMER.L | 33 |
|  | Astrin\_STLCHLD\_tube2\_061314\_01.04847.04847.3 | 3.6369 | 0.3589 | 99.9% | 1876.6144 | 1876.0824 | 1 | 5.792 | 48.2% | 6 | R.RNLDIERPTYTNLNR.L | 333 |
|  | Astrin\_STLCHLD\_050114\_01.11147.11147.2 | 3.4174 | 0.0864 | 97.3% | 1719.6322 | 1719.8949 | 1 | 5.134 | 61.5% | 7 | R.NLDIERPTYTNLNR.L | 222 |
|  | Astrin\_STLCHLD\_tube2\_050114\_01.06465.06465.3 | 2.9056 | 0.311 | 99.8% | 1721.3644 | 1719.8949 | 2 | 5.652 | 46.2% | 9 | R.NLDIERPTYTNLNR.L | 333 |
|  | Astrin\_STLCHLD\_050114\_01.13856.13856.1 | 3.7074 | 0.3194 | 100.0% | 1488.91 | 1488.7678 | 3 | 6.528 | 50.0% | 9 | R.LISQIVSSITASLR.F | 111 |
|  | Astrin\_STLCHLD\_tube2\_050114\_02.10976.10976.2 | 4.8703 | 0.4751 | 100.0% | 1490.2522 | 1488.7678 | 1 | 9.358 | 73.1% | 71 | R.LISQIVSSITASLR.F | 222 |
|  | Astrin\_STLCHLD\_tube2\_050114\_01.13991.13991.3 | 4.7196 | 0.3289 | 99.9% | 1490.3344 | 1488.7678 | 2 | 6.09 | 50.0% | 15 | R.LISQIVSSITASLR.F | 333 |
|  | Astrin\_STLCHLD\_tube2\_061314\_02.09638.09638.2 | 5.8424 | 0.5235 | 100.0% | 2410.7122 | 2410.6885 | 1 | 10.298 | 50.0% | 50 | R.FDGALNVDLTEFQTNLVPYPR.I | 222 |
|  | Astrin\_STLCLD20\_112214\_tube2\_02.10416.10416.3 | 3.7941 | 0.4445 | 99.9% | 2410.8245 | 2410.6885 | 1 | 7.732 | 36.2% | 7 | R.FDGALNVDLTEFQTNLVPYPR.I | 333 |
|  | Astrin\_STLCHLD\_tube2\_050114\_01.10237.10237.2 | 4.4468 | 0.5586 | 100.0% | 1757.4722 | 1758.0703 | 1 | 9.549 | 76.7% | 35 | R.IHFPLATYAPVISAEK.A | 222 |
|  | Astrin\_STLCHLD\_050114\_01.12271.12271.3 | 4.3329 | 0.4459 | 100.0% | 1757.9644 | 1758.0703 | 1 | 7.63 | 51.7% | 19 | R.IHFPLATYAPVISAEK.A | 333 |
| \* | Astrin\_STLCHLD\_050114\_02.08192.08192.3 | 3.8453 | 0.2905 | 99.8% | 2765.9944 | 2766.064 | 343 | 5.097 | 23.9% | 1 | K.AYHEQLTVAEITNACFEPANQMVK.C | 3 |
|  | Astrin\_STLCHLD\_050114\_01.12021.12021.2 | 2.5571 | 0.4159 | 100.0% | 1250.3722 | 1250.4304 | 1 | 6.932 | 75.0% | 3 | K.YMACCLLYR.G | 222 |
|  | Astrin\_STLCHLD\_tube2\_050114\_01.08558.08558.2 | 2.9016 | 0.2756 | 99.2% | 1611.1322 | 1611.879 | 15 | 5.949 | 46.7% | 1 | R.GDVVPKDVNAAIATIK.T | 22 |
|  | Astrin\_STLCHLD\_tube2\_061314\_01.06093.06093.1 | 1.9394 | 0.2937 | 98.3% | 1015.66 | 1016.1827 | 1 | 5.809 | 77.8% | 10 | K.DVNAAIATIK.T | 11 |
|  | Astrin\_STLCHLD\_tube2\_061314\_01.06251.06251.2 | 3.4569 | 0.4018 | 100.0% | 1016.1922 | 1016.1827 | 1 | 7.757 | 88.9% | 15 | K.DVNAAIATIK.T | 22 |
|  | Astrin\_STLCHLD\_tube2\_061314\_01.08843.08843.2 | 4.4981 | 0.4675 | 100.0% | 1825.6721 | 1826.1027 | 1 | 7.437 | 67.6% | 25 | K.VGINYQPPTVVPGGDLAK.V | 222 |
|  | Astrin\_STLCLD20\_112214\_01.08110.08110.2 | 3.5435 | 0.367 | 100.0% | 1382.0521 | 1381.6324 | 1 | 6.667 | 65.0% | 2 | R.LDHKFDLMYAK.R | 222 |
|  | Astrin\_STLCHLD\_tube2\_061314\_01.06060.06060.3 | 4.3726 | 0.4192 | 100.0% | 1382.2144 | 1381.6324 | 1 | 6.674 | 60.0% | 13 | R.LDHKFDLMYAK.R | 333 |
|  | Astrin\_STLCHLD\_050114\_01.09772.09772.3 | 2.6871 | 0.2432 | 97.3% | 1538.2444 | 1537.82 | 69 | 4.407 | 40.9% | 1 | R.LDHKFDLMYAKR.A | 333 |
|  | Astrin\_STLCHLD\_tube2\_061314\_01.08135.08135.2 | 2.4286 | 0.2419 | 99.2% | 887.8722 | 888.0692 | 1 | 4.937 | 91.7% | 1 | K.FDLMYAK.R | 222 |
|  | Astrin\_STLCHLD\_050114\_02.07700.07700.3 | 6.6327 | 0.4886 | 100.0% | 2487.6843 | 2487.7083 | 1 | 9.145 | 48.8% | 10 | K.RAFVHWYVGEGMEEGEFSEAR.E | 333 |
|  | Astrin\_STLCHLD\_050114\_02.08531.08531.2 | 5.3032 | 0.4677 | 100.0% | 2331.412 | 2331.5208 | 1 | 8.46 | 65.8% | 2 | R.AFVHWYVGEGMEEGEFSEAR.E | 222 |
|  | Astrin\_STLCHLD\_061214\_02.07921.07921.3 | 5.6027 | 0.4369 | 100.0% | 2332.3743 | 2331.5208 | 1 | 7.994 | 44.7% | 24 | R.AFVHWYVGEGMEEGEFSEAR.E | 333 |
|  | Astrin\_STLCHLD\_tube2\_050114\_01.11503.11503.3 | 4.258 | 0.3976 | 99.9% | 3219.3843 | 3219.524 | 1 | 7.266 | 31.5% | 3 | R.AFVHWYVGEGMEEGEFSEAREDMAALEK.D | 333 |

Similarities:
gi|57013276|ref|NP\_00(36:1)  
gi|17921989|ref|NP\_00(23:14)  

---

|  |  |  |  |  |  |  |  |  |
| --- | --- | --- | --- | --- | --- | --- | --- | --- |
| U | *gi|11415030|ref|NP\_06* | 11 | 114 | 60.2% | 103 | 11367 | 11.4 | histone cluster 1, H4j [Homo sapiens] |
| U | *gi|77539758|ref|NP\_00* | 11 | 114 | 60.2% | 103 | 11367 | 11.4 | histone cluster 2, H4b [Homo sapiens] |
| U | *gi|4504323|ref|NP\_003* | 10 | 112 | 60.2% | 103 | 11367 | 11.4 | histone cluster 2, H4a [Homo sapiens] |
| U | *gi|4504321|ref|NP\_003* | 11 | 114 | 60.2% | 103 | 11367 | 11.4 | histone cluster 1, H4i [Homo sapiens] |
| U | *gi|4504317|ref|NP\_003* | 11 | 114 | 60.2% | 103 | 11367 | 11.4 | histone cluster 1, H4l [Homo sapiens] |
| U | *gi|4504315|ref|NP\_003* | 11 | 114 | 60.2% | 103 | 11367 | 11.4 | histone cluster 1, H4e [Homo sapiens] |
| U | *gi|4504313|ref|NP\_003* | 11 | 114 | 60.2% | 103 | 11367 | 11.4 | histone cluster 1, H4b [Homo sapiens] |
| U | *gi|4504311|ref|NP\_003* | 11 | 114 | 60.2% | 103 | 11367 | 11.4 | histone cluster 1, H4h [Homo sapiens] |
| U | *gi|4504309|ref|NP\_003* | 11 | 114 | 60.2% | 103 | 11367 | 11.4 | histone cluster 1, H4c [Homo sapiens] |
| U | *gi|4504307|ref|NP\_003* | 11 | 114 | 60.2% | 103 | 11367 | 11.4 | histone cluster 1, H4k [Homo sapiens] |
| U | *gi|4504305|ref|NP\_003* | 11 | 114 | 60.2% | 103 | 11367 | 11.4 | histone cluster 1, H4f [Homo sapiens] |
| U | *gi|4504303|ref|NP\_003* | 11 | 114 | 60.2% | 103 | 11367 | 11.4 | histone cluster 1, H4d [Homo sapiens] |
| U | *gi|4504301|ref|NP\_003* | 11 | 114 | 60.2% | 103 | 11367 | 11.4 | histone cluster 1, H4a [Homo sapiens] |
| U | *gi|28173560|ref|NP\_77* | 11 | 114 | 60.2% | 103 | 11367 | 11.4 | histone cluster 4, H4 [Homo sapiens] |

| Filename XCorr DeltCN Conf% ObsM+H+ CalcM+H+ SpR ZScore Ion% # Sequence  | | | | | | | | | | | | |
| --- | --- | --- | --- | --- | --- | --- | --- | --- | --- | --- | --- | --- |
|  | Astrin\_STLCHLD\_050114\_01.08137.08137.2 | 3.6336 | 0.2083 | 99.8% | 1326.2122 | 1326.5387 | 1 | 6.833 | 81.8% | 20 | R.DNIQGITKPAIR.R | 2 |
|  | Astrin\_STLCHLD\_tube2\_050114\_01.05855.05855.2 | 2.9559 | 0.2856 | 99.7% | 1337.2922 | 1337.5187 | 13 | 5.64 | 60.0% | 3 | K.RISGLIYEETR.G | 2 |
|  | Astrin\_STLCLD20\_112214\_01.08653.08653.1 | 2.6057 | 0.2463 | 98.1% | 1180.57 | 1181.3312 | 3 | 5.159 | 66.7% | 5 | R.ISGLIYEETR.G | 1 |
|  | Astrin\_STLCLD20\_112214\_tube2\_01.10004.10004.2 | 3.9194 | 0.3998 | 100.0% | 1181.0521 | 1181.3312 | 1 | 7.38 | 88.9% | 40 | R.ISGLIYEETR.G | 2 |
|  | Astrin\_STLCLD20\_112214\_01.10903.10903.1 | 2.0827 | 0.3308 | 97.1% | 989.64 | 990.19055 | 8 | 6.091 | 64.3% | 2 | K.VFLENVIR.D | 1 |
|  | AstrinSTLCLD\_041714\_01.08282.08282.2 | 3.0977 | 0.2474 | 100.0% | 990.53217 | 990.19055 | 3 | 5.676 | 85.7% | 16 | K.VFLENVIR.D | 2 |
|  | Astrin\_STLCHLD\_tube2\_050114\_01.13015.13015.3 | 4.7061 | 0.4251 | 100.0% | 2106.6543 | 2106.386 | 1 | 7.019 | 44.1% | 1 | K.VFLENVIRDAVTYTEHAK.R | 3 |
|  | Astrin\_STLCHLD\_tube2\_050114\_01.08799.08799.3 | 3.8216 | 0.4229 | 100.0% | 1595.3944 | 1595.9409 | 5 | 5.954 | 38.5% | 3 | R.KTVTAMDVVYALKR.Q | 3 |
|  | Astrin\_NLD\_STLC\_tube2\_021014\_01.12010.12010.2 | 3.3679 | 0.375 | 100.0% | 1311.8522 | 1311.5793 | 1 | 6.509 | 72.7% | 1 | K.TVTAMDVVYALK.R | 2 |
|  | Astrin\_STLCHLD\_050114\_01.12252.12252.2 | 3.6021 | 0.4683 | 100.0% | 1467.4922 | 1467.7667 | 1 | 7.764 | 62.5% | 13 | K.TVTAMDVVYALKR.Q | 2 |
|  | Astrin\_STLCHLD\_tube2\_061314\_01.09101.09101.1 | 1.8041 | 0.4889 | 100.0% | 714.44 | 714.796 | 1 | 8.303 | 75.0% | 10 | R.TLYGFGG.- | 1 |

---

|  |  |  |  |  |  |  |  |  |
| --- | --- | --- | --- | --- | --- | --- | --- | --- |
| U | *gi|10645195|ref|NP\_06* | 11 | 81 | 60.0% | 130 | 14135 | 11.1 | histone cluster 1, H2ae [Homo sapiens] |
| U | *gi|4504245|ref|NP\_003* | 9 | 58 | 60.0% | 130 | 14105 | 11.1 | histone cluster 1, H2ac [Homo sapiens] |
| U | *gi|19557656|ref|NP\_00* | 11 | 81 | 60.0% | 130 | 14135 | 11.1 | histone cluster 1, H2ab [Homo sapiens] |
| U | *gi|15617199|ref|NP\_25* | 11 | 81 | 60.0% | 130 | 14121 | 11.1 | histone cluster 3, H2a [Homo sapiens] |

| Filename XCorr DeltCN Conf% ObsM+H+ CalcM+H+ SpR ZScore Ion% # Sequence  | | | | | | | | | | | | |
| --- | --- | --- | --- | --- | --- | --- | --- | --- | --- | --- | --- | --- |
|  | Astrin\_STLCHLD\_050114\_01.10552.10552.2 | 2.9962 | 0.2759 | 99.6% | 1276.0322 | 1275.4531 | 1 | 5.752 | 77.3% | 5 | R.SSRAGLQFPVGR.V | 222 |
|  | AstrinSTLCLD\_041714\_01.06063.06063.1 | 1.9612 | 0.2801 | 97.2% | 944.67 | 945.1093 | 4 | 5.26 | 62.5% | 2 | R.AGLQFPVGR.V | 1111 |
|  | Astrin\_STLCHLD\_tube2\_061314\_01.07517.07517.2 | 3.2091 | 0.3209 | 100.0% | 945.39215 | 945.1093 | 1 | 5.94 | 81.2% | 21 | R.AGLQFPVGR.V | 2222 |
|  | Astrin\_STLCHLD\_tube2\_061314\_01.18624.18624.2 | 5.7651 | 0.5322 | 100.0% | 2917.172 | 2917.3752 | 1 | 11.331 | 44.6% | 11 | R.VGAGAPVYLAAVLEYLTAEILELAGNAAR.D | 22 |
|  | Astrin\_STLCLD20\_112214\_tube2\_02.00560.00560.3 | 6.2608 | 0.5052 | 100.0% | 2917.9143 | 2917.3752 | 1 | 8.493 | 33.0% | 7 | R.VGAGAPVYLAAVLEYLTAEILELAGNAAR.D | 33 |
|  | Astrin\_STLCLD20\_112214\_tube2\_01.07832.07832.2 | 2.6508 | 0.2524 | 99.7% | 851.2922 | 851.0396 | 4 | 6.019 | 83.3% | 5 | R.HLQLAIR.N | 2222 |
|  | Astrin\_STLCHLD\_tube2\_061314\_02.05192.05192.3 | 3.2474 | 0.3885 | 99.9% | 1694.3344 | 1693.9004 | 1 | 6.694 | 44.2% | 17 | R.HLQLAIRNDEELNK.L | 333 |
|  | Astrin\_STLCLD20\_112214\_01.10678.10678.3 | 4.1655 | 0.4581 | 100.0% | 2132.6643 | 2133.4587 | 1 | 7.186 | 39.7% | 2 | R.HLQLAIRNDEELNKLLGR.V | 3 |
|  | Astrin\_STLCHLD\_tube2\_050114\_01.07926.07926.2 | 3.3565 | 0.3178 | 100.0% | 1301.4321 | 1301.4423 | 2 | 6.011 | 70.0% | 3 | R.NDEELNKLLGR.V | 2 |
|  | Astrin\_STLCLD20\_112214\_tube2\_01.17512.17512.1 | 2.388 | 0.2221 | 97.4% | 1931.12 | 1932.3573 | 40 | 5.511 | 33.3% | 1 | R.VTIAQGGVLPNIQAVLLPK.K | 111 |
|  | Astrin\_NLD\_STLC\_tube2\_021014\_01.13730.13730.2 | 4.9626 | 0.4911 | 100.0% | 1931.8522 | 1932.3573 | 1 | 8.543 | 66.7% | 7 | R.VTIAQGGVLPNIQAVLLPK.K | 222 |

Similarities:
gi|106775678|ref|NP\_0(7:4)  
gi|10800130|ref|NP\_06(9:2)  
gi|20357599|ref|NP\_61(3:8)  

---

|  |  |  |  |  |  |  |  |  |
| --- | --- | --- | --- | --- | --- | --- | --- | --- |
| U | *gi|10800130|ref|NP\_06* | 11 | 98 | 60.0% | 130 | 14107 | 10.9 | histone cluster 1, H2ad [Homo sapiens] |
| U | *gi|4504249|ref|NP\_003* | 9 | 75 | 60.0% | 130 | 14091 | 10.9 | histone cluster 1, H2am [Homo sapiens] |
| U | *gi|4504243|ref|NP\_003* | 11 | 98 | 60.0% | 130 | 14091 | 10.9 | histone cluster 1, H2al [Homo sapiens] |
| U | *gi|4504241|ref|NP\_003* | 8 | 70 | 60.0% | 130 | 14091 | 10.9 | histone cluster 1, H2ak [Homo sapiens] |
| U | *gi|4504239|ref|NP\_003* | 11 | 98 | 60.0% | 130 | 14091 | 10.9 | histone cluster 1, H2ai [Homo sapiens] |
| U | *gi|29553970|ref|NP\_80* | 8 | 70 | 60.5% | 129 | 14019 | 10.9 | H2A histone family, member J [Homo sapiens] |
| U | *gi|18105045|ref|NP\_54* | 11 | 98 | 60.9% | 128 | 13906 | 10.9 | histone cluster 1, H2ah [Homo sapiens] |
| U | *gi|10800144|ref|NP\_06* | 11 | 98 | 60.9% | 128 | 13936 | 10.9 | histone cluster 1, H2aj [Homo sapiens] |
| U | *gi|10800132|ref|NP\_06* | 11 | 98 | 60.0% | 130 | 14091 | 10.9 | histone cluster 1, H2ag [Homo sapiens] |

| Filename XCorr DeltCN Conf% ObsM+H+ CalcM+H+ SpR ZScore Ion% # Sequence  | | | | | | | | | | | | |
| --- | --- | --- | --- | --- | --- | --- | --- | --- | --- | --- | --- | --- |
|  | Astrin\_STLCHLD\_050114\_01.10552.10552.2 | 2.9962 | 0.2759 | 99.6% | 1276.0322 | 1275.4531 | 1 | 5.752 | 77.3% | 5 | R.SSRAGLQFPVGR.V | 222 |
|  | AstrinSTLCLD\_041714\_01.06063.06063.1 | 1.9612 | 0.2801 | 97.2% | 944.67 | 945.1093 | 4 | 5.26 | 62.5% | 2 | R.AGLQFPVGR.V | 1111 |
|  | Astrin\_STLCHLD\_tube2\_061314\_01.07517.07517.2 | 3.2091 | 0.3209 | 100.0% | 945.39215 | 945.1093 | 1 | 5.94 | 81.2% | 21 | R.AGLQFPVGR.V | 2222 |
|  | Astrin\_STLCHLD\_tube2\_061314\_01.18624.18624.2 | 5.7651 | 0.5322 | 100.0% | 2917.172 | 2917.3752 | 1 | 11.331 | 44.6% | 11 | R.VGAGAPVYLAAVLEYLTAEILELAGNAAR.D | 22 |
|  | Astrin\_STLCLD20\_112214\_tube2\_02.00560.00560.3 | 6.2608 | 0.5052 | 100.0% | 2917.9143 | 2917.3752 | 1 | 8.493 | 33.0% | 7 | R.VGAGAPVYLAAVLEYLTAEILELAGNAAR.D | 33 |
|  | Astrin\_STLCLD20\_112214\_tube2\_01.07832.07832.2 | 2.6508 | 0.2524 | 99.7% | 851.2922 | 851.0396 | 4 | 6.019 | 83.3% | 5 | R.HLQLAIR.N | 2222 |
|  | Astrin\_STLCHLD\_tube2\_061314\_02.05192.05192.3 | 3.2474 | 0.3885 | 99.9% | 1694.3344 | 1693.9004 | 1 | 6.694 | 44.2% | 17 | R.HLQLAIRNDEELNK.L | 333 |
|  | Astrin\_STLCLD20\_112214\_tube2\_01.11649.11649.3 | 4.8389 | 0.5071 | 100.0% | 2106.0244 | 2105.4453 | 1 | 7.497 | 39.7% | 9 | R.HLQLAIRNDEELNKLLGK.V | 33 |
|  | Astrin\_STLCHLD\_tube2\_061314\_01.07421.07421.2 | 3.6 | 0.3554 | 100.0% | 1273.4122 | 1273.4288 | 1 | 6.312 | 75.0% | 13 | R.NDEELNKLLGK.V | 22 |
|  | Astrin\_STLCLD20\_112214\_tube2\_01.17512.17512.1 | 2.388 | 0.2221 | 97.4% | 1931.12 | 1932.3573 | 40 | 5.511 | 33.3% | 1 | K.VTIAQGGVLPNIQAVLLPK.K | 111 |
|  | Astrin\_NLD\_STLC\_tube2\_021014\_01.13730.13730.2 | 4.9626 | 0.4911 | 100.0% | 1931.8522 | 1932.3573 | 1 | 8.543 | 66.7% | 7 | K.VTIAQGGVLPNIQAVLLPK.K | 222 |

Similarities:
gi|106775678|ref|NP\_0(9:2)  
gi|10645195|ref|NP\_06(9:2)  
gi|20357599|ref|NP\_61(3:8)  

---

|  |  |  |  |  |  |  |  |  |
| --- | --- | --- | --- | --- | --- | --- | --- | --- |
| U | *gi|62414289|ref|NP\_00* | 35 | 120 | 59.2% | 466 | 53652 | 5.1 | vimentin [Homo sapiens] |

| Filename XCorr DeltCN Conf% ObsM+H+ CalcM+H+ SpR ZScore Ion% # Sequence  | | | | | | | | | | | | |
| --- | --- | --- | --- | --- | --- | --- | --- | --- | --- | --- | --- | --- |
| \* | Astrin\_STLCLD20\_112214\_tube2\_01.06398.06398.2 | 2.6618 | 0.2317 | 97.7% | 1496.2522 | 1495.6531 | 199 | 4.474 | 39.3% | 1 | R.MFGGPGTASRPSSSR.S | 2 |
| \* | Astrin\_STLCLD20\_112214\_tube2\_01.08566.08566.3 | 2.6309 | 0.3632 | 99.8% | 1496.6344 | 1496.6633 | 4 | 5.97 | 36.5% | 1 | R.TYSLGSALRPSTSR.S | 3 |
| \* | Astrin\_STLCLD20\_112214\_01.07441.07441.2 | 2.2012 | 0.3149 | 97.7% | 1497.3322 | 1496.6633 | 99 | 5.096 | 42.3% | 1 | R.TYSLGSALRPSTSR.S | 2 |
| \* | Astrin\_STLCLD20\_112214\_tube2\_01.08585.08585.2 | 3.4496 | 0.2935 | 100.0% | 1429.2522 | 1429.5724 | 1 | 5.546 | 69.2% | 4 | R.SLYASSPGGVYATR.S | 2 |
| \* | Astrin\_STLCHLD\_tube2\_050114\_01.06697.06697.2 | 3.9865 | 0.3548 | 100.0% | 1509.4321 | 1509.5724 | 1 | 6.371 | 61.5% | 8 | R.SLYASS\*PGGVYATR.S | 2 |
|  | Astrin\_STLCHLD\_tube2\_050114\_01.05117.05117.2 | 4.1023 | 0.4856 | 100.0% | 1588.2722 | 1588.7147 | 1 | 7.801 | 79.2% | 2 | R.TNEKVELQELNDR.F | 2 |
|  | Astrin\_STLCHLD\_tube2\_050114\_01.05114.05114.3 | 3.677 | 0.3006 | 100.0% | 1590.0543 | 1588.7147 | 1 | 5.749 | 47.9% | 3 | R.TNEKVELQELNDR.F | 3 |
|  | Astrin\_STLCHLD\_tube2\_061314\_01.04639.04639.2 | 2.2121 | 0.2317 | 97.0% | 1116.7722 | 1116.2163 | 2 | 5.072 | 75.0% | 1 | K.VELQELNDR.F | 2 |
| \* | Astrin\_STLCHLD\_050114\_01.07387.07387.2 | 2.1513 | 0.285 | 98.8% | 871.2922 | 870.9805 | 3 | 4.865 | 91.7% | 1 | R.FANYIDK.V | 2 |
| \* | Astrin\_STLCHLD\_tube2\_050114\_01.05457.05457.2 | 2.9656 | 0.3507 | 100.0% | 1125.3121 | 1126.3005 | 2 | 6.675 | 75.0% | 3 | R.FANYIDKVR.F | 2 |
| \* | Astrin\_NLD\_STLC\_tube2\_021014\_01.11856.11856.2 | 2.6046 | 0.3192 | 99.6% | 1170.1721 | 1170.4349 | 5 | 6.205 | 66.7% | 1 | K.ILLAELEQLK.G | 2 |
| \* | Astrin\_STLCLD20\_112214\_01.10841.10841.2 | 3.3857 | 0.4495 | 100.0% | 1539.9321 | 1540.8436 | 1 | 8.963 | 65.4% | 3 | K.ILLAELEQLKGQGK.S | 2 |
| \* | Astrin\_STLCLD20\_112214\_tube2\_01.10439.10439.2 | 3.0329 | 0.365 | 100.0% | 1498.3322 | 1498.6508 | 1 | 6.624 | 77.3% | 1 | K.SRLGDLYEEEMR.E | 2 |
| \* | AstrinSTLCLD\_041714\_01.05410.05410.2 | 3.7666 | 0.4979 | 100.0% | 1254.6522 | 1255.385 | 1 | 8.427 | 77.8% | 10 | R.LGDLYEEEMR.E | 2 |
| \* | Astrin\_STLCHLD\_tube2\_050114\_01.08030.08030.2 | 3.2495 | 0.2607 | 99.6% | 1690.4321 | 1689.881 | 1 | 5.238 | 53.8% | 2 | R.VEVERDNLAEDIMR.L | 2 |
| \* | Astrin\_STLCHLD\_tube2\_061314\_01.08243.08243.3 | 3.6392 | 0.2798 | 99.8% | 1690.5543 | 1689.881 | 1 | 5.576 | 48.1% | 3 | R.VEVERDNLAEDIMR.L | 3 |
| \* | AstrinSTLCLD\_041714\_01.08100.08100.2 | 2.5353 | 0.1582 | 97.1% | 1076.2322 | 1077.1975 | 37 | 5.349 | 68.8% | 2 | R.DNLAEDIMR.L | 2 |
| \* | Astrin\_NLD\_STLC\_tube2\_021014\_02.06952.06952.3 | 4.1475 | 0.258 | 99.8% | 2352.9243 | 2352.581 | 9 | 5.515 | 33.3% | 6 | K.LQEEMLQREEAENTLQSFR.Q | 3 |
| \* | Astrin\_STLCHLD\_tube2\_061314\_01.05790.05790.2 | 2.7409 | 0.4147 | 100.0% | 1324.2322 | 1324.3898 | 1 | 6.362 | 65.0% | 5 | R.EEAENTLQSFR.Q | 2 |
| \* | Astrin\_STLCLD20\_112214\_tube2\_01.12725.12725.3 | 3.0053 | 0.2617 | 97.8% | 2394.2644 | 2394.5168 | 4 | 5.627 | 30.0% | 1 | R.EEAENTLQSFRQDVDNASLAR.L | 3 |
| \* | Astrin\_STLCLD20\_112214\_tube2\_01.13305.13305.2 | 4.6213 | 0.4259 | 100.0% | 1535.5922 | 1534.793 | 1 | 8.079 | 70.8% | 7 | R.KVESLQEEIAFLK.K | 2 |
| \* | Astrin\_STLCLD20\_112214\_01.10371.10371.3 | 3.5289 | 0.278 | 99.8% | 1663.2843 | 1662.967 | 4 | 5.53 | 40.4% | 2 | R.KVESLQEEIAFLKK.L | 3 |
|  | Astrin\_STLCHLD\_tube2\_050114\_01.07782.07782.2 | 3.1061 | 0.2515 | 99.8% | 1311.0322 | 1310.4056 | 2 | 4.977 | 77.8% | 8 | K.NLQEAEEWYK.S | 2 |
| \* | Astrin\_STLCHLD\_tube2\_050114\_01.05064.05064.2 | 3.4768 | 0.4107 | 100.0% | 1095.2922 | 1094.1692 | 1 | 7.784 | 77.8% | 8 | K.FADLSEAANR.N | 2 |
| \* | Astrin\_NLD\_STLC\_tube2\_021014\_01.06321.06321.3 | 2.7323 | 0.2722 | 98.0% | 1777.5243 | 1777.8912 | 4 | 5.449 | 35.0% | 1 | K.FADLSEAANRNNDALR.Q | 3 |
| \* | Astrin\_STLCHLD\_050114\_02.08058.08058.2 | 6.0084 | 0.5746 | 100.0% | 2187.8323 | 2188.33 | 1 | 11.216 | 66.7% | 6 | R.EMEENFAVEAANYQDTIGR.L | 2 |
| \* | Astrin\_STLCHLD\_tube2\_061314\_02.07605.07605.3 | 3.8555 | 0.3695 | 99.9% | 2188.0144 | 2188.33 | 1 | 6.39 | 37.5% | 3 | R.EMEENFAVEAANYQDTIGR.L | 3 |
| \* | Astrin\_STLCHLD\_tube2\_050114\_01.06983.06983.2 | 4.6638 | 0.5108 | 100.0% | 1734.6122 | 1735.9679 | 1 | 8.873 | 76.9% | 6 | R.LQDEIQNMKEEMAR.H | 2 |
|  | Astrin\_STLCLD20\_112214\_01.08361.08361.2 | 3.0768 | 0.2236 | 99.3% | 1528.5922 | 1528.7513 | 1 | 4.891 | 68.2% | 1 | R.HLREYQDLLNVK.M | 2 |
|  | Astrin\_STLCLD20\_112214\_01.08344.08344.3 | 3.5739 | 0.3364 | 99.9% | 1529.4844 | 1528.7513 | 2 | 5.877 | 54.5% | 4 | R.HLREYQDLLNVK.M | 3 |
|  | Astrin\_STLCHLD\_050114\_02.08578.08578.2 | 3.9851 | 0.4267 | 100.0% | 1296.1921 | 1296.5243 | 1 | 9.369 | 85.0% | 6 | K.MALDIEIATYR.K | 2 |
|  | Astrin\_STLCLD20\_112214\_tube2\_02.07794.07794.3 | 2.8535 | 0.2242 | 97.4% | 1424.8143 | 1424.6984 | 1 | 5.103 | 45.5% | 1 | K.MALDIEIATYRK.L | 3 |
| \* | AstrinSTLCLD\_041714\_01.11576.11576.2 | 3.4267 | 0.4032 | 100.0% | 1571.4722 | 1571.8601 | 1 | 7.541 | 80.8% | 2 | R.ISLPLPNFSSLNLR.E | 2 |
| \* | AstrinSTLCLD\_041714\_01.06795.06795.2 | 2.806 | 0.1885 | 97.1% | 1670.4321 | 1669.829 | 1 | 4.818 | 57.1% | 1 | R.ETNLDSLPLVDTHSK.R | 2 |
| \* | Astrin\_STLCHLD\_050114\_01.06021.06021.2 | 3.8523 | 0.4076 | 100.0% | 1837.2922 | 1837.854 | 1 | 7.163 | 56.7% | 5 | R.DGQVINETSQHHDDLE.- | 2 |

---

|  |  |  |  |  |  |  |  |  |
| --- | --- | --- | --- | --- | --- | --- | --- | --- |
| U | *gi|73623035|ref|NP\_00* | 103 | 1263 | 58.8% | 1193 | 134422 | 5.0 | sperm associated antigen 5 [Homo sapiens] |

| Filename XCorr DeltCN Conf% ObsM+H+ CalcM+H+ SpR ZScore Ion% # Sequence  | | | | | | | | | | | | |
| --- | --- | --- | --- | --- | --- | --- | --- | --- | --- | --- | --- | --- |
| \* | Astrin\_NLD\_STLC\_tube2\_021014\_01.03771.03771.3 | 3.3991 | 0.2235 | 99.3% | 1618.5243 | 1618.7031 | 106 | 5.29 | 32.7% | 1 | K.RTDLSSEHFSHSSK.W | 3 |
| \* | Astrin\_NLD\_STLC\_tube2\_021014\_01.03894.03894.2 | 3.8054 | 0.3962 | 100.0% | 1463.0122 | 1462.5156 | 1 | 6.235 | 70.8% | 2 | R.TDLSSEHFSHSSK.W | 2 |
| \* | Astrin\_STLCHLD\_tube2\_061314\_01.09504.09504.2 | 4.3024 | 0.3726 | 100.0% | 1653.4321 | 1653.8445 | 1 | 9.01 | 71.4% | 9 | K.TSEEAVDPLGNYMVK.T | 2 |
| \* | Astrin\_STLCHLD\_tube2\_050114\_01.12828.12828.2 | 3.4052 | 0.502 | 100.0% | 2323.2922 | 2323.6262 | 1 | 8.634 | 52.6% | 6 | K.TIVLVPS\*PLGQQQDMIFEAR.L | 2 |
| \* | AstrinSTLCLD\_041714\_01.08032.08032.2 | 5.2488 | 0.4901 | 100.0% | 1833.3121 | 1833.0668 | 1 | 8.481 | 65.6% | 23 | R.LDTMAETNSISLNGPLR.T | 2 |
| \* | Astrin\_NLD\_STLC\_tube2\_021014\_02.07582.07582.3 | 4.2406 | 0.4176 | 99.9% | 2532.9243 | 2532.8286 | 6 | 7.222 | 27.3% | 14 | R.LDTMAETNSISLNGPLRTDDLVR.E | 3 |
| \* | Astrin\_STLCLD20\_112214\_tube2\_01.19553.19553.3 | 6.1013 | 0.4712 | 100.0% | 3778.6443 | 3778.2102 | 1 | 7.846 | 23.5% | 2 | R.TEAVREDLVPSESNAFLPSSVLWLSPSTALAADFR.V | 3 |
| \* | Astrin\_STLCLD20\_112214\_tube2\_02.11436.11436.3 | 4.6436 | 0.2379 | 99.8% | 3857.0344 | 3858.2102 | 2 | 5.468 | 22.8% | 1 | R.TEAVREDLVPSESNAFLPSSVLWLSPST#ALAADFR.V | 3 |
| \* | Astrin\_STLCLD20\_112214\_01.15097.15097.3 | 5.6025 | 0.2446 | 100.0% | 3858.0842 | 3858.2102 | 2 | 6.411 | 27.9% | 1 | R.TEAVREDLVPSESNAFLPSSVLWLSPS\*TALAADFR.V | 3 |
| \* | Astrin\_NLD\_STLC\_tube2\_021014\_01.15512.15512.3 | 6.1907 | 0.2218 | 100.0% | 3858.8044 | 3858.2102 | 1 | 6.438 | 28.7% | 24 | R.TEAVREDLVPSESNAFLPSSVLWLS\*PSTALAADFR.V | 3 |
| \* | Astrin\_STLCLD20\_112214\_tube2\_01.09050.09050.2 | 5.8438 | 0.5651 | 100.0% | 2219.6921 | 2220.3752 | 1 | 9.622 | 72.2% | 4 | R.VNHVDPEEEIVEHGAMEER.E | 2 |
| \* | AstrinSTLCLD\_041714\_01.05755.05755.3 | 5.2465 | 0.3419 | 100.0% | 2221.4343 | 2220.3752 | 1 | 5.991 | 40.3% | 47 | R.VNHVDPEEEIVEHGAMEER.E | 3 |
| \* | Astrin\_STLCHLD\_tube2\_061314\_01.14687.14687.2 | 5.7115 | 0.5692 | 100.0% | 2063.9521 | 2064.3606 | 1 | 9.506 | 67.6% | 11 | R.ILGSDTESWMSPLAWLEK.G | 2 |
| \* | Astrin\_STLCLD20\_112214\_tube2\_01.20486.20486.2 | 4.7872 | 0.5115 | 100.0% | 2143.392 | 2144.3606 | 1 | 8.46 | 61.8% | 10 | R.ILGSDTESWMS\*PLAWLEK.G | 2 |
| \* | Astrin\_STLCHLD\_tube2\_061314\_01.09263.09263.2 | 3.4886 | 0.3311 | 100.0% | 1334.6721 | 1333.5457 | 1 | 6.338 | 81.8% | 7 | K.GVNTSVMLENLR.Q | 2 |
| \* | AstrinSTLCLD\_041714\_01.08883.08883.2 | 2.3577 | 0.2212 | 97.3% | 1131.5521 | 1132.3635 | 415 | 5.743 | 55.6% | 2 | R.QSLSLPSMLR.D | 2 |
| \* | Astrin\_NLD\_STLC\_tube2\_021014\_01.03957.03957.2 | 3.6103 | 0.5046 | 100.0% | 1294.4122 | 1294.4044 | 1 | 8.093 | 70.8% | 1 | K.STNTSQTGLVGTK.H | 2 |
| \* | Astrin\_STLCLD20\_112214\_tube2\_01.00602.00602.2 | 7.5875 | 0.6387 | 100.0% | 2165.7722 | 2166.4795 | 1 | 11.53 | 72.2% | 62 | R.HDLEDNLLSSLVILEVLSR.Q | 2 |
| \* | Astrin\_STLCHLD\_061214\_01.14881.14881.3 | 3.6229 | 0.3024 | 99.8% | 2166.3843 | 2166.4795 | 7 | 5.81 | 33.3% | 13 | R.HDLEDNLLSSLVILEVLSR.Q | 3 |
| \* | AstrinSTLCLD\_041714\_01.04286.04286.3 | 5.4155 | 0.5229 | 100.0% | 2867.5745 | 2868.0 | 1 | 8.058 | 32.7% | 11 | K.SQLAVPHPETQDSSTQTDTSHSGITNK.L | 3 |
| \* | Astrin\_STLCHLD\_tube2\_050114\_01.04396.04396.2 | 5.4276 | 0.4906 | 100.0% | 2105.5522 | 2105.3794 | 1 | 8.697 | 70.6% | 1 | K.LQHLKESHEMGQALQQAR.N | 2 |
| \* | Astrin\_STLCHLD\_tube2\_050114\_01.04389.04389.3 | 5.4181 | 0.3977 | 100.0% | 2106.5942 | 2105.3794 | 1 | 6.693 | 47.1% | 2 | K.LQHLKESHEMGQALQQAR.N | 3 |
| \* | Astrin\_NLD\_STLC\_031014\_01.08975.08975.2 | 3.9642 | 0.402 | 100.0% | 1485.1322 | 1485.6146 | 1 | 6.432 | 62.5% | 6 | K.ESHEMGQALQQAR.N | 2 |
| \* | Astrin\_STLCHLD\_050114\_01.04885.04885.3 | 4.0989 | 0.0781 | 95.5% | 1486.5243 | 1485.6146 | 13 | 4.774 | 47.9% | 1 | K.ESHEMGQALQQAR.N | 3 |
| \* | Astrin\_STLCHLD\_tube2\_061314\_01.11369.11369.2 | 4.1386 | 0.4382 | 100.0% | 1305.5922 | 1305.578 | 1 | 7.437 | 80.0% | 19 | R.NVMQSWVLISK.E | 2 |
| \* | Astrin\_STLCHLD\_050114\_01.12426.12426.3 | 6.364 | 0.5078 | 100.0% | 2891.5144 | 2892.2793 | 1 | 7.949 | 35.4% | 16 | K.ELISLLHLSLLHLEEDKTTVSQESR.R | 3 |
| \* | AstrinSTLCLD\_041714\_01.10332.10332.3 | 3.787 | 0.3171 | 100.0% | 2075.9644 | 2074.267 | 1 | 5.892 | 37.5% | 4 | R.GKDAAEIVLEAFCAHASQR.I | 3 |
| \* | AstrinSTLCLD\_041714\_01.11028.11028.2 | 4.3111 | 0.4346 | 100.0% | 1888.0521 | 1889.041 | 1 | 7.824 | 59.4% | 2 | K.DAAEIVLEAFCAHASQR.I | 2 |
| \* | Astrin\_STLCLD20\_112214\_tube2\_01.13140.13140.1 | 2.4811 | 0.4938 | 100.0% | 1390.58 | 1391.5823 | 4 | 7.486 | 54.5% | 2 | R.ISQLEQDLASMR.E | 1 |
| \* | Astrin\_STLCLD20\_112214\_tube2\_01.13196.13196.2 | 3.8244 | 0.3755 | 100.0% | 1391.2522 | 1391.5823 | 1 | 8.253 | 68.2% | 19 | R.ISQLEQDLASMR.E | 2 |
| \* | AstrinSTLCLD\_041714\_01.09837.09837.3 | 2.852 | 0.2503 | 97.7% | 1823.8444 | 1824.0618 | 1 | 5.568 | 39.3% | 2 | R.ISQLEQDLASMREFR.G | 3 |
| \* | Astrin\_STLCLD20\_112214\_tube2\_01.11734.11734.3 | 4.8788 | 0.5072 | 100.0% | 2124.8643 | 2125.4788 | 1 | 8.156 | 40.3% | 6 | R.EFRGLLKDAQTQLVGLHAK.Q | 3 |
| \* | Astrin\_STLCLD20\_112214\_01.09409.09409.2 | 3.886 | 0.3525 | 100.0% | 1692.7522 | 1692.9994 | 1 | 6.81 | 60.0% | 1 | R.GLLKDAQTQLVGLHAK.Q | 2 |
| \* | AstrinSTLCLD\_041714\_01.06846.06846.3 | 4.342 | 0.254 | 99.9% | 1693.5543 | 1692.9994 | 1 | 5.081 | 40.0% | 6 | R.GLLKDAQTQLVGLHAK.Q | 3 |
| \* | Astrin\_STLCLD20\_112214\_tube2\_01.07730.07730.2 | 3.6744 | 0.4439 | 100.0% | 1280.6921 | 1281.4545 | 1 | 7.255 | 68.2% | 7 | K.DAQTQLVGLHAK.Q | 2 |
| \* | Astrin\_STLCLD20\_112214\_tube2\_01.17201.17201.2 | 5.0867 | 0.5326 | 100.0% | 2390.6921 | 2390.612 | 1 | 8.921 | 50.0% | 10 | K.QEELVQQTVSLTSTLQQDWR.S | 2 |
| \* | Astrin\_STLCLD20\_112214\_02.15251.15251.3 | 4.0911 | 0.4276 | 100.0% | 2391.3245 | 2390.612 | 1 | 6.242 | 35.5% | 4 | K.QEELVQQTVSLTSTLQQDWR.S | 3 |
| \* | Astrin\_STLCHLD\_061214\_02.09740.09740.2 | 5.4525 | 0.4219 | 100.0% | 1786.7922 | 1787.0405 | 1 | 8.603 | 75.0% | 20 | R.SMQLDYTTWTALLSR.S | 2 |
| \* | Astrin\_STLCHLD\_tube2\_061314\_01.01591.01591.2 | 2.7269 | 0.1876 | 99.1% | 959.77216 | 960.0348 | 30 | 5.025 | 71.4% | 3 | K.SQQALQER.D | 2 |
| \* | AstrinSTLCLD\_041714\_01.04342.04342.3 | 5.4811 | 0.3753 | 100.0% | 2346.2344 | 2344.5437 | 1 | 6.218 | 43.4% | 7 | K.SQQALQERDVAIEEKQEVSR.V | 3 |
| \* | AstrinSTLCLD\_041714\_01.03830.03830.2 | 4.083 | 0.4469 | 100.0% | 1403.3121 | 1403.5321 | 1 | 7.329 | 72.7% | 31 | R.DVAIEEKQEVSR.V | 2 |
| \* | AstrinSTLCLD\_041714\_02.04298.04298.3 | 3.1579 | 0.3611 | 99.9% | 1404.1743 | 1403.5321 | 2 | 5.962 | 52.3% | 10 | R.DVAIEEKQEVSR.V | 3 |
| \* | AstrinSTLCLD\_041714\_02.05948.05948.2 | 4.5104 | 0.4241 | 100.0% | 1534.4722 | 1533.6849 | 1 | 8.436 | 70.8% | 7 | R.VLEQVSAQLEECK.G | 2 |
| \* | AstrinSTLCLD\_041714\_02.06807.06807.3 | 5.9366 | 0.4453 | 100.0% | 2919.8342 | 2919.1382 | 1 | 7.951 | 35.4% | 7 | R.VLEQVSAQLEECKGQTEQLELENSR.L | 3 |
| \* | Astrin\_STLCHLD\_050114\_01.06979.06979.2 | 4.3543 | 0.4622 | 100.0% | 1404.3121 | 1404.4764 | 1 | 8.036 | 68.2% | 12 | K.GQTEQLELENSR.L | 2 |
| \* | Astrin\_STLCLD20\_112214\_tube2\_01.14447.14447.2 | 5.6941 | 0.3751 | 100.0% | 1574.5521 | 1573.848 | 1 | 7.272 | 76.9% | 68 | R.AQLQILANMDSQLK.E | 2 |
| \* | Astrin\_STLCHLD\_050114\_02.08750.08750.3 | 5.3272 | 0.3177 | 100.0% | 1574.6044 | 1573.848 | 1 | 6.912 | 51.9% | 5 | R.AQLQILANMDSQLK.E | 3 |
| \* | Astrin\_STLCHLD\_tube2\_050114\_01.04895.04895.2 | 5.6943 | 0.5635 | 100.0% | 1722.7122 | 1723.9879 | 1 | 10.394 | 82.1% | 48 | K.HMQAELQQQQAVLAK.E | 2 |
| \* | Astrin\_STLCHLD\_050114\_02.05204.05204.3 | 5.1866 | 0.3005 | 99.9% | 1724.5443 | 1723.9879 | 1 | 5.834 | 50.0% | 59 | K.HMQAELQQQQAVLAK.E | 3 |
| \* | Astrin\_STLCHLD\_050114\_02.05957.05957.3 | 4.9848 | 0.4934 | 100.0% | 2107.4644 | 2108.4233 | 1 | 8.772 | 47.1% | 6 | K.HMQAELQQQQAVLAKEVR.D | 3 |
| \* | AstrinSTLCLD\_041714\_02.09637.09637.3 | 6.1261 | 0.5123 | 100.0% | 3286.0745 | 3286.5862 | 1 | 8.948 | 38.0% | 5 | R.DLKETLEFADQENQVAHLELGQVECQLK.T | 3 |
| \* | Astrin\_STLCHLD\_050114\_02.08849.08849.3 | 3.2023 | 0.2962 | 99.3% | 2931.1443 | 2930.1638 | 16 | 4.866 | 25.0% | 1 | K.ETLEFADQENQVAHLELGQVECQLK.T | 3 |
| \* | Astrin\_STLCHLD\_050114\_01.09823.09823.2 | 2.2633 | 0.2319 | 98.4% | 832.47217 | 831.9878 | 21 | 5.008 | 83.3% | 4 | K.TTLEVLR.E | 2 |
| \* | Astrin\_STLCLD20\_112214\_tube2\_01.11487.11487.2 | 4.8751 | 0.4855 | 100.0% | 1963.7522 | 1964.1462 | 1 | 8.859 | 68.8% | 1 | R.SLQCENLKDTVENLTAK.L | 2 |
| \* | AstrinSTLCLD\_041714\_01.07671.07671.3 | 4.3843 | 0.296 | 99.9% | 1964.4543 | 1964.1462 | 1 | 6.178 | 46.9% | 6 | R.SLQCENLKDTVENLTAK.L | 3 |
| \* | Astrin\_STLCLD20\_112214\_tube2\_01.07992.07992.1 | 2.6097 | 0.3571 | 95.2% | 1674.85 | 1675.7899 | 1 | 6.876 | 53.6% | 1 | K.LASTIADNQEQDLEK.T | 1 |
| \* | Astrin\_NLD\_STLC\_tube2\_021014\_01.05678.05678.2 | 5.0084 | 0.4301 | 100.0% | 1675.3522 | 1675.7899 | 1 | 7.362 | 71.4% | 40 | K.LASTIADNQEQDLEK.T | 2 |
| \* | Astrin\_STLCLD20\_112214\_tube2\_01.07742.07742.2 | 4.3765 | 0.5366 | 100.0% | 1932.5922 | 1933.0825 | 1 | 8.354 | 62.5% | 6 | K.LASTIADNQEQDLEKTR.Q | 2 |
| \* | Astrin\_STLCHLD\_tube2\_050114\_01.05090.05090.3 | 2.3525 | 0.2975 | 96.3% | 1933.4944 | 1933.0825 | 140 | 4.854 | 32.8% | 1 | K.LASTIADNQEQDLEKTR.Q | 3 |
| \* | AstrinSTLCLD\_041714\_02.14805.14805.3 | 4.3697 | 0.3765 | 99.9% | 2762.0942 | 2762.1326 | 11 | 7.532 | 27.3% | 4 | R.QYS\*QKLGLLTEQLQSLTLFLQTK.L | 3 |
| \* | Astrin\_STLCHLD\_050114\_02.12775.12775.2 | 6.2077 | 0.513 | 100.0% | 2046.7922 | 2047.443 | 1 | 9.979 | 67.6% | 117 | K.LGLLTEQLQSLTLFLQTK.L | 2 |
| \* | Astrin\_STLCHLD\_tube2\_061314\_01.15384.15384.3 | 5.5133 | 0.4316 | 100.0% | 2048.3044 | 2047.443 | 1 | 7.514 | 51.5% | 33 | K.LGLLTEQLQSLTLFLQTK.L | 3 |
| \* | Astrin\_STLCHLD\_tube2\_050114\_01.15850.15850.2 | 5.4955 | 0.4967 | 100.0% | 2787.6921 | 2788.121 | 1 | 9.007 | 42.3% | 41 | R.TFLGSILTAVADEEPESTPVPLLGSDK.S | 2 |
| \* | AstrinSTLCLD\_041714\_01.13934.13934.3 | 6.3332 | 0.5378 | 100.0% | 2788.3442 | 2788.121 | 1 | 10.882 | 34.6% | 5 | R.TFLGSILTAVADEEPESTPVPLLGSDK.S | 3 |
| \* | Astrin\_STLCHLD\_tube2\_061314\_01.15537.15537.2 | 4.841 | 0.5327 | 100.0% | 2868.4521 | 2868.121 | 1 | 8.704 | 44.2% | 11 | R.TFLGSILTAVADEEPESTPVPLLGS\*DK.S | 2 |
| \* | Astrin\_STLCHLD\_tube2\_050114\_01.15226.15226.3 | 4.5468 | 0.3527 | 99.9% | 3350.5745 | 3350.7473 | 1 | 5.944 | 23.4% | 5 | R.TFLGSILTAVADEEPESTPVPLLGSDKSAFTR.V | 3 |
| \* | Astrin\_NLD\_STLC\_tube2\_021014\_01.16511.16511.3 | 4.8387 | 0.4261 | 100.0% | 3429.0544 | 3430.7473 | 1 | 5.944 | 28.2% | 2 | R.TFLGSILTAVADEEPESTPVPLLGSDKSAFT#R.V | 3 |
| \* | Astrin\_STLCLD20\_112214\_tube2\_01.20102.20102.3 | 4.9757 | 0.4221 | 100.0% | 3429.3542 | 3430.7473 | 2 | 7.39 | 28.2% | 7 | R.TFLGSILTAVADEEPESTPVPLLGSDKS\*AFTR.V | 3 |
| \* | Astrin\_STLCHLD\_tube2\_061314\_01.14916.14916.3 | 5.2701 | 0.325 | 99.9% | 3430.3743 | 3430.7473 | 3 | 6.639 | 26.6% | 12 | R.TFLGSILTAVADEEPESTPVPLLGS\*DKSAFTR.V | 3 |
| \* | Astrin\_STLCLD20\_112214\_tube2\_01.05642.05642.2 | 4.6908 | 0.413 | 100.0% | 1595.3922 | 1595.7092 | 1 | 7.726 | 83.3% | 7 | R.LQAQEEQHQEVQK.A | 2 |
| \* | AstrinSTLCLD\_041714\_01.08064.08064.2 | 2.5985 | 0.3462 | 99.4% | 1672.4122 | 1673.873 | 2 | 6.038 | 50.0% | 1 | K.EADIEKLNQALCLR.Y | 2 |
| \* | Astrin\_STLCHLD\_050114\_01.09140.09140.2 | 2.4527 | 0.2997 | 99.5% | 988.27216 | 988.1412 | 1 | 5.219 | 92.9% | 2 | K.LNQALCLR.Y | 2 |
| \* | Astrin\_NLD\_STLC\_tube2\_021014\_01.05634.05634.2 | 6.3816 | 0.3913 | 100.0% | 2148.7922 | 2149.3652 | 1 | 7.744 | 68.8% | 6 | R.YKNEKELQEVIQQQNEK.I | 2 |
| \* | Astrin\_STLCHLD\_tube2\_050114\_01.05098.05098.3 | 6.2748 | 0.2843 | 100.0% | 2149.7344 | 2149.3652 | 1 | 5.996 | 51.6% | 12 | R.YKNEKELQEVIQQQNEK.I | 3 |
| \* | Astrin\_STLCLD20\_112214\_01.05057.05057.1 | 3.3999 | 0.2453 | 97.1% | 1485.72 | 1486.622 | 1 | 6.034 | 72.7% | 1 | K.ELQEVIQQQNEK.I | 1 |
| \* | Astrin\_NLD\_STLC\_tube2\_021014\_01.05085.05085.2 | 4.8324 | 0.2354 | 100.0% | 1486.3121 | 1486.622 | 1 | 6.368 | 86.4% | 27 | K.ELQEVIQQQNEK.I | 2 |
| \* | Astrin\_STLCHLD\_050114\_01.05869.05869.2 | 2.7162 | 0.0506 | 96.4% | 859.03217 | 859.01044 | 15 | 3.939 | 83.3% | 2 | K.ILEQIDK.S | 2 |
| \* | Astrin\_STLCHLD\_tube2\_061314\_01.09344.09344.2 | 4.2731 | 0.4963 | 100.0% | 1714.9321 | 1715.0 | 1 | 8.609 | 67.9% | 3 | K.ILEQIDKSGELISLR.E | 2 |
| \* | Astrin\_STLCHLD\_tube2\_061314\_02.07263.07263.3 | 4.1239 | 0.361 | 99.9% | 1715.2444 | 1715.0 | 14 | 7.42 | 39.3% | 4 | K.ILEQIDKSGELISLR.E | 3 |
| \* | Astrin\_STLCLD20\_112214\_tube2\_01.13996.13996.3 | 5.0916 | 0.2947 | 99.9% | 2680.3743 | 2681.0618 | 1 | 6.413 | 40.9% | 11 | K.ILEQIDKSGELISLREEVTHLTR.S | 3 |
| \* | Astrin\_STLCHLD\_tube2\_061314\_01.06269.06269.2 | 3.1318 | 0.1818 | 99.7% | 875.0522 | 875.0128 | 36 | 4.94 | 78.6% | 5 | K.SGELISLR.E | 2 |
| \* | Astrin\_STLCLD20\_112214\_tube2\_01.12387.12387.2 | 4.1255 | 0.4074 | 100.0% | 1840.6721 | 1841.0745 | 1 | 6.812 | 56.7% | 17 | K.SGELISLREEVTHLTR.S | 2 |
| \* | Astrin\_STLCHLD\_tube2\_061314\_02.07526.07526.3 | 5.2215 | 0.3986 | 100.0% | 1841.5743 | 1841.0745 | 1 | 7.207 | 53.3% | 56 | K.SGELISLREEVTHLTR.S | 3 |
| \* | Astrin\_STLCLD20\_112214\_tube2\_01.09807.09807.2 | 2.8019 | 0.2287 | 99.3% | 1104.1721 | 1104.248 | 16 | 5.894 | 75.0% | 6 | K.VWLSQEVDK.L | 2 |
| \* | Astrin\_STLCHLD\_tube2\_061314\_01.09041.09041.2 | 3.6669 | 0.3763 | 100.0% | 1373.5322 | 1373.595 | 1 | 6.866 | 85.0% | 19 | K.VWLSQEVDKLR.V | 2 |
| \* | Astrin\_STLCHLD\_050114\_01.12266.12266.1 | 2.3236 | 0.186 | 96.8% | 897.65 | 898.16644 | 1 | 4.715 | 75.0% | 1 | R.VMFLEMK.N | 1 |
| \* | AstrinSTLCLD\_041714\_01.08528.08528.2 | 2.8363 | 0.3369 | 100.0% | 898.15216 | 898.16644 | 1 | 7.131 | 91.7% | 12 | R.VMFLEMK.N | 2 |
| \* | Astrin\_STLCHLD\_tube2\_061314\_01.06813.06813.2 | 2.9476 | 0.376 | 100.0% | 1270.5322 | 1269.5598 | 1 | 6.797 | 83.3% | 10 | R.VMFLEMKNEK.E | 2 |
| \* | Astrin\_STLCHLD\_tube2\_050114\_01.06875.06875.1 | 2.5079 | 0.2754 | 97.4% | 1000.5 | 1001.1277 | 4 | 4.839 | 71.4% | 16 | R.NILEENLR.R | 1 |
| \* | Astrin\_STLCLD20\_112214\_01.08473.08473.2 | 2.7832 | 0.041 | 95.3% | 1001.1122 | 1001.1277 | 2 | 4.345 | 85.7% | 1 | R.NILEENLR.R | 2 |
| \* | Astrin\_STLCLD20\_112214\_01.10858.10858.2 | 5.4045 | 0.4677 | 100.0% | 2229.9922 | 2230.526 | 1 | 8.234 | 70.6% | 2 | R.RSDKELEKLDDIVQHIYK.T | 2 |
| \* | Astrin\_STLCHLD\_tube2\_050114\_01.09328.09328.3 | 5.8334 | 0.4605 | 100.0% | 2231.3342 | 2230.526 | 1 | 8.389 | 51.5% | 16 | R.RSDKELEKLDDIVQHIYK.T | 3 |
| \* | Astrin\_STLCLD20\_112214\_tube2\_01.13635.13635.3 | 5.3612 | 0.3134 | 99.9% | 2074.1643 | 2074.3384 | 1 | 6.803 | 48.4% | 13 | R.SDKELEKLDDIVQHIYK.T | 3 |
| \* | Astrin\_STLCLD20\_112214\_01.11344.11344.2 | 5.2585 | 0.535 | 100.0% | 2074.632 | 2074.3384 | 1 | 9.406 | 68.8% | 3 | R.SDKELEKLDDIVQHIYK.T | 2 |
| \* | Astrin\_NLD\_STLC\_tube2\_021014\_01.10367.10367.3 | 3.1497 | 0.26 | 99.4% | 1743.8344 | 1743.9977 | 1 | 5.596 | 44.2% | 3 | K.ELEKLDDIVQHIYK.T | 3 |
| \* | Astrin\_STLCHLD\_tube2\_050114\_01.09691.09691.2 | 2.7641 | 0.1917 | 97.3% | 1744.0122 | 1743.9977 | 2 | 4.947 | 50.0% | 2 | K.ELEKLDDIVQHIYK.T | 2 |
| \* | Astrin\_STLCLD20\_112214\_01.08659.08659.2 | 3.4257 | 0.3789 | 100.0% | 1244.5122 | 1244.4331 | 44 | 6.931 | 61.1% | 11 | K.LDDIVQHIYK.T | 2 |
| \* | Astrin\_STLCHLD\_050114\_01.11483.11483.3 | 2.5959 | 0.2643 | 98.5% | 1244.6943 | 1244.4331 | 60 | 4.98 | 44.4% | 1 | K.LDDIVQHIYK.T | 3 |
| \* | Astrin\_STLCHLD\_050114\_01.12307.12307.1 | 1.8279 | 0.2628 | 96.7% | 1126.71 | 1127.3696 | 7 | 5.51 | 55.6% | 3 | K.TLLSIPEVVR.G | 1 |
| \* | Astrin\_STLCHLD\_tube2\_061314\_01.10589.10589.2 | 2.8975 | 0.4307 | 100.0% | 1127.5521 | 1127.3696 | 3 | 6.64 | 83.3% | 21 | K.TLLSIPEVVR.G | 2 |
| \* | AstrinSTLCLD\_041714\_01.12556.12556.2 | 3.0008 | 0.4741 | 100.0% | 1494.6522 | 1494.6941 | 1 | 7.411 | 70.8% | 2 | R.GCKELQGLLEFLS.- | 2 |
| \* | Astrin\_NLD\_STLC\_031014\_01.13590.13590.1 | 1.9685 | 0.3639 | 96.9% | 1148.57 | 1149.3293 | 1 | 5.763 | 61.1% | 21 | K.ELQGLLEFLS.- | 1 |
| \* | Astrin\_STLCHLD\_tube2\_050114\_01.15586.15586.2 | 1.9953 | 0.2828 | 96.0% | 1148.8922 | 1149.3293 | 1 | 5.429 | 66.7% | 2 | K.ELQGLLEFLS.- | 2 |

---

|  |  |  |  |  |  |  |  |  |
| --- | --- | --- | --- | --- | --- | --- | --- | --- |
| U | *gi|4501885|ref|NP\_001* | 22 | 196 | 58.7% | 375 | 41737 | 5.5 | beta actin [Homo sapiens] |
| U | *gi|4501887|ref|NP\_001* | 22 | 196 | 58.7% | 375 | 41793 | 5.5 | actin, gamma 1 propeptide [Homo sapiens] |

| Filename XCorr DeltCN Conf% ObsM+H+ CalcM+H+ SpR ZScore Ion% # Sequence  | | | | | | | | | | | | |
| --- | --- | --- | --- | --- | --- | --- | --- | --- | --- | --- | --- | --- |
|  | Astrin\_NLD\_STLC\_031014\_01.06884.06884.2 | 3.2662 | 0.4547 | 100.0% | 976.33215 | 977.02136 | 2 | 7.839 | 72.2% | 30 | K.AGFAGDDAPR.A | 22 |
|  | Astrin\_STLCLD20\_112214\_01.08519.08519.2 | 2.8688 | 0.378 | 100.0% | 1199.3522 | 1199.4415 | 19 | 6.226 | 60.0% | 12 | R.AVFPSIVGRPR.H | 22 |
|  | Astrin\_STLCLD20\_112214\_tube2\_01.06642.06642.1 | 2.0003 | 0.2433 | 96.8% | 1171.74 | 1172.4058 | 1 | 5.709 | 65.0% | 1 | R.HQGVMVGMGQK.D | 111 |
|  | AstrinSTLCLD\_041714\_01.03582.03582.2 | 2.8958 | 0.0922 | 95.9% | 1199.4922 | 1199.2163 | 1 | 7.356 | 65.0% | 1 | K.DSYVGDEAQSK.R | 22 |
|  | Astrin\_STLCHLD\_tube2\_050114\_01.05846.05846.3 | 3.4498 | 0.3159 | 100.0% | 1515.5643 | 1516.7019 | 1 | 6.429 | 55.0% | 9 | K.IWHHTFYNELR.V | 33 |
|  | Astrin\_STLCHLD\_tube2\_050114\_01.05795.05795.2 | 3.3616 | 0.4634 | 100.0% | 1516.2722 | 1516.7019 | 1 | 7.494 | 80.0% | 9 | K.IWHHTFYNELR.V | 22 |
|  | Astrin\_STLCLD20\_112214\_tube2\_01.10730.10730.3 | 4.2988 | 0.0975 | 95.2% | 1954.4944 | 1955.2615 | 2 | 7.035 | 39.7% | 1 | R.VAPEEHPVLLTEAPLNPK.A | 3 |
|  | Astrin\_STLCHLD\_tube2\_050114\_01.07740.07740.2 | 4.5586 | 0.3648 | 100.0% | 1954.7122 | 1955.2615 | 1 | 7.903 | 61.8% | 13 | R.VAPEEHPVLLTEAPLNPK.A | 2 |
|  | Astrin\_STLCHLD\_050114\_02.15382.15382.3 | 3.6962 | 0.1804 | 95.6% | 3257.0044 | 3255.8325 | 1 | 4.968 | 22.3% | 1 | K.MTQIMFETFNTPAMYVAIQAVLSLYASGR.T | 3 |
|  | Astrin\_STLCLD20\_112214\_tube2\_01.14018.14018.3 | 6.925 | 0.4464 | 100.0% | 3186.7744 | 3185.622 | 1 | 7.981 | 33.6% | 19 | R.TTGIVMDSGDGVTHTVPIYEGYALPHAILR.L | 3 |
|  | Astrin\_NLD\_STLC\_tube2\_021014\_01.11475.11475.2 | 2.9524 | 0.2059 | 98.4% | 1624.5322 | 1624.8927 | 2 | 5.245 | 57.7% | 4 | R.LDLAGRDLTDYLMK.I | 222 |
|  | AstrinSTLCLD\_041714\_01.09070.09070.1 | 2.0807 | 0.1982 | 96.1% | 998.79 | 999.167 | 3 | 4.182 | 71.4% | 3 | R.DLTDYLMK.I | 111 |
|  | Astrin\_STLCLD20\_112214\_tube2\_01.13726.13726.2 | 2.0606 | 0.342 | 98.9% | 999.21216 | 999.167 | 16 | 5.639 | 71.4% | 2 | R.DLTDYLMK.I | 222 |
|  | Astrin\_STLCHLD\_050114\_01.08528.08528.2 | 3.1329 | 0.5222 | 100.0% | 1132.9722 | 1133.2029 | 1 | 8.979 | 77.8% | 26 | R.GYSFTTTAER.E | 2 |
|  | Astrin\_NLD\_STLC\_tube2\_021014\_01.10676.10676.2 | 4.8426 | 0.3035 | 100.0% | 1791.2922 | 1791.9554 | 1 | 8.531 | 83.3% | 21 | K.SYELPDGQVITIGNER.F | 222 |
|  | Astrin\_STLCHLD\_061214\_02.07237.07237.3 | 5.3896 | 0.5494 | 100.0% | 2344.6443 | 2344.6448 | 1 | 8.789 | 39.3% | 8 | R.KDLYANTVLSGGTTMYPGIADR.M | 3 |
|  | Astrin\_STLCHLD\_tube2\_061314\_02.08176.08176.2 | 5.1728 | 0.6552 | 100.0% | 2215.5522 | 2216.4705 | 1 | 11.775 | 50.0% | 12 | K.DLYANTVLSGGTTMYPGIADR.M | 2 |
|  | Astrin\_STLCLD20\_112214\_02.13135.13135.3 | 3.9604 | 0.4186 | 99.9% | 2216.3943 | 2216.4705 | 1 | 6.825 | 41.2% | 1 | K.DLYANTVLSGGTTMYPGIADR.M | 3 |
|  | Astrin\_STLCHLD\_061214\_01.05115.05115.3 | 3.7961 | 0.3358 | 99.9% | 1550.2144 | 1549.8843 | 10 | 6.274 | 44.2% | 1 | R.MQKEITALAPSTMK.I | 33 |
|  | Astrin\_NLD\_STLC\_tube2\_021014\_01.06791.06791.1 | 2.5336 | 0.4765 | 100.0% | 1161.6 | 1162.3868 | 1 | 7.532 | 55.0% | 2 | K.EITALAPSTMK.I | 11 |
|  | Astrin\_STLCLD20\_112214\_tube2\_01.09039.09039.2 | 2.8112 | 0.3091 | 99.7% | 1162.4122 | 1162.3868 | 13 | 5.941 | 55.0% | 19 | K.EITALAPSTMK.I | 22 |
|  | Astrin\_STLCHLD\_tube2\_050114\_01.04500.04500.3 | 2.417 | 0.3928 | 99.8% | 1517.7244 | 1517.595 | 1 | 6.263 | 43.8% | 1 | K.QEYDESGPSIVHR.K | 3 |

Similarities:
gi|4501881|ref|NP\_001(13:9)  
gi|63055057|ref|NP\_00(5:17)  

---

|  |  |  |  |  |  |  |  |  |
| --- | --- | --- | --- | --- | --- | --- | --- | --- |
| U | *gi|8923110|ref|NP\_060* | 18 | 126 | 58.5% | 335 | 37721 | 5.2 | nuclear distribution gene E homolog 1 [Homo sapiens] |

| Filename XCorr DeltCN Conf% ObsM+H+ CalcM+H+ SpR ZScore Ion% # Sequence  | | | | | | | | | | | | |
| --- | --- | --- | --- | --- | --- | --- | --- | --- | --- | --- | --- | --- |
|  | Astrin\_STLCHLD\_061214\_01.04617.04617.1 | 1.6387 | 0.3483 | 97.5% | 841.57 | 841.99774 | 11 | 5.947 | 58.3% | 1 | K.DLAMTYK.Q | 1 |
|  | Astrin\_STLCHLD\_050114\_02.05486.05486.3 | 2.9283 | 0.2483 | 97.9% | 1923.3844 | 1923.9908 | 1 | 5.254 | 40.0% | 1 | R.AENTQEELREFQEGSR.E | 3 |
| \* | Astrin\_STLCHLD\_050114\_01.05239.05239.3 | 3.1744 | 0.2708 | 99.8% | 1501.8544 | 1500.6578 | 225 | 4.789 | 38.6% | 1 | R.NRDLLSENNRLR.M | 3 |
|  | Astrin\_STLCHLD\_050114\_02.04517.04517.2 | 3.1405 | 0.3403 | 100.0% | 1251.6721 | 1252.3286 | 1 | 5.421 | 72.2% | 1 | K.FEVQHSEGYR.Q | 2 |
|  | Astrin\_STLCHLD\_050114\_01.11316.11316.2 | 3.6075 | 0.1861 | 99.5% | 1434.3922 | 1432.5707 | 1 | 6.027 | 66.7% | 3 | R.QISALEDDLAQTK.A | 2 |
|  | Astrin\_STLCHLD\_050114\_02.05610.05610.3 | 3.5797 | 0.2107 | 99.3% | 1763.5443 | 1764.8894 | 5 | 5.143 | 42.3% | 1 | K.YIRELEQANDDLER.A | 3 |
|  | Astrin\_STLCHLD\_050114\_02.04664.04664.2 | 3.3547 | 0.381 | 100.0% | 1332.0922 | 1332.3666 | 1 | 6.314 | 75.0% | 15 | R.ELEQANDDLER.A | 2 |
|  | Astrin\_STLCHLD\_050114\_02.08097.08097.3 | 4.5724 | 0.3229 | 99.9% | 2364.7444 | 2364.5713 | 1 | 7.224 | 38.2% | 2 | R.NAFLESELDEKENLLESVQR.L | 3 |
|  | Astrin\_STLCHLD\_050114\_01.07874.07874.2 | 2.801 | 0.1268 | 96.5% | 1328.0922 | 1328.5112 | 1 | 4.319 | 70.0% | 3 | R.DLRQELAVQQK.Q | 2 |
|  | Astrin\_STLCHLD\_050114\_02.05216.05216.3 | 3.9148 | 0.1358 | 99.0% | 1329.2943 | 1328.5112 | 7 | 5.119 | 52.5% | 1 | R.DLRQELAVQQK.Q | 3 |
|  | Astrin\_STLCHLD\_tube2\_061314\_01.04158.04158.2 | 2.7047 | 0.3704 | 100.0% | 1204.0721 | 1204.3405 | 1 | 6.089 | 80.0% | 4 | R.TPMPSSVEAER.T | 2 |
|  | Astrin\_STLCHLD\_050114\_01.10937.10937.3 | 4.4531 | 0.3325 | 99.9% | 3095.0344 | 3095.4114 | 1 | 6.548 | 30.2% | 1 | R.TPMPSSVEAERTDTAVQATGSVPSTPIAHR.G | 3 |
|  | Astrin\_STLCHLD\_tube2\_050114\_02.04502.04502.3 | 3.213 | 0.3653 | 99.9% | 1911.0844 | 1910.0941 | 1 | 6.378 | 38.9% | 3 | R.TDTAVQATGSVPSTPIAHR.G | 3 |
|  | Astrin\_STLCHLD\_tube2\_050114\_02.04648.04648.2 | 3.3929 | 0.4412 | 100.0% | 1307.3322 | 1307.4056 | 1 | 8.037 | 58.3% | 4 | R.GPSSSLNTPGSFR.R | 2 |
|  | Astrin\_STLCHLD\_050114\_01.07725.07725.2 | 4.603 | 0.504 | 100.0% | 1529.8121 | 1529.647 | 1 | 9.106 | 63.3% | 6 | R.GLDDSTGGTPLTPAAR.I | 2 |
|  | Astrin\_STLCHLD\_050114\_01.13333.13333.2 | 4.1749 | 0.42 | 100.0% | 1284.0521 | 1284.5419 | 1 | 7.891 | 90.9% | 2 | R.ISALNIVGDLLR.K | 2 |
|  | Astrin\_STLCHLD\_tube2\_050114\_02.18050.18050.2 | 2.9916 | 0.3024 | 100.0% | 1206.1721 | 1206.3005 | 13 | 5.784 | 66.7% | 75 | R.NLVYDQSPNR.T | 2 |
|  | Astrin\_STLCHLD\_050114\_02.04400.04400.2 | 2.64 | 0.3593 | 99.7% | 1244.2122 | 1244.3904 | 1 | 6.649 | 68.2% | 2 | R.RPSSTSVPLGDK.G | 2 |

---

|  |  |  |  |  |  |  |  |  |
| --- | --- | --- | --- | --- | --- | --- | --- | --- |
| U | *gi|58743363|ref|NP\_20* | 29 | 200 | 56.4% | 344 | 38998 | 6.5 | centromere protein L isoform 2 [Homo sapiens] |

| Filename XCorr DeltCN Conf% ObsM+H+ CalcM+H+ SpR ZScore Ion% # Sequence  | | | | | | | | | | | | |
| --- | --- | --- | --- | --- | --- | --- | --- | --- | --- | --- | --- | --- |
|  | Astrin\_NLD\_STLC\_tube2\_021014\_01.09915.09915.3 | 5.4419 | 0.4444 | 100.0% | 3133.1042 | 3134.398 | 1 | 7.751 | 33.9% | 14 | -.MDSYSAPESTPSASSRPEDYFIGATPLQK.R | 3 |
|  | Astrin\_NLD\_STLC\_tube2\_021014\_01.09735.09735.2 | 4.0764 | 0.5197 | 100.0% | 3134.372 | 3134.398 | 1 | 7.389 | 37.5% | 4 | -.MDSYSAPESTPSASSRPEDYFIGATPLQK.R | 2 |
|  | Astrin\_NLD\_STLC\_tube2\_021014\_01.09057.09057.3 | 5.0803 | 0.3926 | 100.0% | 3288.8342 | 3290.5854 | 1 | 6.645 | 25.9% | 3 | -.MDSYSAPESTPSASSRPEDYFIGATPLQKR.L | 3 |
|  | Astrin\_NLD\_STLC\_031014\_01.09603.09603.2 | 3.2777 | 0.3596 | 100.0% | 1274.3522 | 1274.5057 | 1 | 6.008 | 70.0% | 17 | R.KQSSFILTPPR.R | 2 |
|  | Astrin\_NLD\_STLC\_tube2\_021014\_01.05284.05284.2 | 2.5993 | 0.1621 | 95.8% | 1430.3922 | 1430.6932 | 1 | 4.544 | 63.6% | 2 | R.KQSSFILTPPRR.K | 2 |
|  | Astrin\_NLD\_STLC\_031014\_01.09220.09220.3 | 3.0635 | 0.2543 | 99.4% | 1431.1444 | 1430.6932 | 19 | 5.743 | 38.6% | 5 | R.KQSSFILTPPRR.K | 3 |
|  | Astrin\_NLD\_STLC\_031014\_01.09938.09938.2 | 3.0631 | 0.3633 | 100.0% | 1146.1322 | 1146.3317 | 1 | 6.183 | 83.3% | 13 | K.QSSFILTPPR.R | 2 |
|  | Astrin\_NLD\_STLC\_031014\_01.09381.09381.2 | 3.9284 | 0.2973 | 100.0% | 1785.6122 | 1785.9147 | 1 | 6.447 | 75.0% | 1 | K.IPQCSQLQEDVDPQK.V | 2 |
|  | Astrin\_NLD\_STLC\_tube2\_021014\_01.06339.06339.2 | 2.2896 | 0.1549 | 96.4% | 827.7522 | 828.04517 | 3 | 5.234 | 83.3% | 3 | K.VAFLLHK.Q | 2 |
|  | Astrin\_NLD\_STLC\_tube2\_021014\_01.12443.12443.3 | 3.7774 | 0.3228 | 100.0% | 2322.0842 | 2322.7983 | 1 | 5.579 | 31.9% | 2 | K.VAFLLHKQWTLYSLTPLYK.F | 3 |
|  | Astrin\_NLD\_STLC\_tube2\_021014\_01.13494.13494.1 | 2.1704 | 0.4412 | 100.0% | 1512.59 | 1513.7765 | 1 | 6.647 | 54.5% | 1 | K.QWTLYSLTPLYK.F | 1 |
|  | Astrin\_NLD\_STLC\_031014\_01.11434.11434.2 | 3.3237 | 0.3971 | 100.0% | 1513.3522 | 1513.7765 | 1 | 6.924 | 72.7% | 5 | K.QWTLYSLTPLYK.F | 2 |
|  | Astrin\_NLD\_STLC\_tube2\_021014\_01.06632.06632.2 | 3.5919 | 0.4094 | 100.0% | 1394.1921 | 1394.5266 | 1 | 7.537 | 75.0% | 4 | K.FSYSNLKEYSR.L | 2 |
|  | Astrin\_NLD\_STLC\_031014\_01.10449.10449.1 | 2.1006 | 0.2964 | 97.9% | 1117.6 | 1118.3617 | 3 | 5.399 | 66.7% | 3 | R.LLNAFIVAEK.Q | 1 |
|  | Astrin\_NLD\_STLC\_031014\_02.07809.07809.2 | 3.3264 | 0.3709 | 100.0% | 1118.3322 | 1118.3617 | 2 | 6.753 | 72.2% | 33 | R.LLNAFIVAEK.Q | 2 |
|  | Astrin\_NLD\_STLC\_tube2\_021014\_01.08601.08601.2 | 3.3731 | 0.316 | 100.0% | 1374.3121 | 1374.6665 | 1 | 6.565 | 72.7% | 1 | R.LLNAFIVAEKQK.G | 2 |
|  | Astrin\_NLD\_STLC\_031014\_02.06982.06982.3 | 3.5939 | 0.1954 | 98.6% | 1647.4143 | 1647.8687 | 206 | 4.864 | 37.5% | 2 | K.QKGLAVEVGEDFNIK.V | 3 |
|  | Astrin\_NLD\_STLC\_tube2\_021014\_02.07719.07719.2 | 4.755 | 0.4584 | 100.0% | 1391.4321 | 1391.5638 | 1 | 8.37 | 75.0% | 28 | K.GLAVEVGEDFNIK.V | 2 |
|  | Astrin\_NLD\_STLC\_tube2\_021014\_02.12936.12936.3 | 3.55 | 0.1914 | 96.3% | 2482.5544 | 2481.9531 | 84 | 4.623 | 25.0% | 1 | K.GLAVEVGEDFNIKVIFSTLLGMK.G | 3 |
|  | Astrin\_NLD\_STLC\_tube2\_021014\_01.12419.12419.1 | 1.7295 | 0.3539 | 97.6% | 1108.62 | 1109.4125 | 7 | 6.016 | 50.0% | 1 | K.VIFSTLLGMK.G | 1 |
|  | Astrin\_NLD\_STLC\_031014\_01.11093.11093.2 | 3.3838 | 0.4607 | 100.0% | 1109.5322 | 1109.4125 | 1 | 6.813 | 77.8% | 7 | K.VIFSTLLGMK.G | 2 |
| \* | Astrin\_NLD\_STLC\_031014\_01.10732.10732.2 | 4.9511 | 0.3349 | 100.0% | 1788.4521 | 1789.0415 | 1 | 7.185 | 66.7% | 5 | K.GTQRDPEAFLVQIVSK.S | 2 |
| \* | Astrin\_NLD\_STLC\_tube2\_021014\_01.11750.11750.3 | 4.9766 | 0.2946 | 99.9% | 1788.6244 | 1789.0415 | 1 | 6.901 | 50.0% | 6 | K.GTQRDPEAFLVQIVSK.S | 3 |
| \* | Astrin\_NLD\_STLC\_tube2\_021014\_01.13637.13637.1 | 2.7251 | 0.3344 | 95.8% | 1345.51 | 1346.5663 | 2 | 5.679 | 68.2% | 3 | R.DPEAFLVQIVSK.S | 1 |
| \* | Astrin\_NLD\_STLC\_tube2\_021014\_01.13706.13706.2 | 4.4481 | 0.403 | 100.0% | 1346.3722 | 1346.5663 | 1 | 8.3 | 68.2% | 21 | R.DPEAFLVQIVSK.S | 2 |
|  | Astrin\_NLD\_STLC\_tube2\_021014\_01.06338.06338.2 | 2.4255 | 0.2335 | 98.7% | 956.0522 | 956.0892 | 4 | 4.616 | 78.6% | 4 | K.ALWDSVHK.T | 2 |
|  | Astrin\_NLD\_STLC\_031014\_01.12364.12364.3 | 4.7712 | 0.3817 | 99.9% | 2813.2444 | 2812.0486 | 1 | 6.051 | 39.8% | 2 | K.TPGEVTQEEVDLFMDCLYSHFHR.H | 3 |
|  | Astrin\_NLD\_STLC\_tube2\_021014\_02.05048.05048.3 | 3.4761 | 0.4254 | 100.0% | 1210.8243 | 1210.4244 | 3 | 7.284 | 50.0% | 2 | R.HFKIHLSATR.L | 3 |
|  | Astrin\_NLD\_STLC\_031014\_01.06423.06423.2 | 3.5559 | 0.3853 | 100.0% | 1501.2722 | 1501.6799 | 1 | 6.597 | 53.6% | 7 | R.VSTSVASAHTDGKIK.I | 2 |

---

|  |  |  |  |  |  |  |  |  |
| --- | --- | --- | --- | --- | --- | --- | --- | --- |
| U | *gi|29788768|ref|NP\_82* | 33 | 416 | 56.0% | 445 | 49953 | 4.9 | tubulin, beta 2B [Homo sapiens] |
| U | *gi|4507729|ref|NP\_001* | 33 | 418 | 56.0% | 445 | 49907 | 4.9 | tubulin, beta 2 [Homo sapiens] |

| Filename XCorr DeltCN Conf% ObsM+H+ CalcM+H+ SpR ZScore Ion% # Sequence  | | | | | | | | | | | | |
| --- | --- | --- | --- | --- | --- | --- | --- | --- | --- | --- | --- | --- |
|  | Astrin\_STLCHLD\_tube2\_050114\_01.10300.10300.1 | 1.9796 | 0.4452 | 100.0% | 1615.8 | 1616.8701 | 25 | 6.88 | 42.9% | 1 | R.AILVDLEPGTMDSVR.S | 111 |
|  | Astrin\_STLCLD20\_112214\_01.11522.11522.2 | 4.511 | 0.4938 | 100.0% | 1617.3121 | 1616.8701 | 1 | 8.254 | 64.3% | 40 | R.AILVDLEPGTMDSVR.S | 222 |
|  | Astrin\_STLCHLD\_tube2\_050114\_01.12338.12338.2 | 5.6846 | 0.5176 | 100.0% | 2798.8523 | 2800.0647 | 1 | 8.445 | 40.0% | 14 | R.SGPFGQIFRPDNFVFGQSGAGNNWAK.G | 2222 |
|  | AstrinSTLCLD\_041714\_01.10952.10952.3 | 7.1011 | 0.4962 | 100.0% | 2799.1143 | 2800.0647 | 1 | 8.497 | 38.0% | 33 | R.SGPFGQIFRPDNFVFGQSGAGNNWAK.G | 3333 |
|  | Astrin\_STLCHLD\_tube2\_050114\_01.12857.12857.2 | 7.2534 | 0.4819 | 100.0% | 1960.4722 | 1960.151 | 1 | 9.656 | 79.4% | 13 | K.GHYTEGAELVDSVLDVVR.K | 2222 |
|  | Astrin\_STLCHLD\_tube2\_050114\_01.12839.12839.3 | 4.3447 | 0.3812 | 99.9% | 1960.7644 | 1960.151 | 1 | 7.142 | 45.6% | 12 | K.GHYTEGAELVDSVLDVVR.K | 3333 |
|  | Astrin\_STLCHLD\_tube2\_061314\_01.11982.11982.3 | 4.7827 | 0.4838 | 100.0% | 2088.1143 | 2088.325 | 1 | 8.148 | 44.4% | 19 | K.GHYTEGAELVDSVLDVVRK.E | 3333 |
|  | Astrin\_STLCHLD\_050114\_01.12405.12405.2 | 6.3217 | 0.4514 | 100.0% | 2088.9321 | 2088.325 | 1 | 8.891 | 66.7% | 6 | K.GHYTEGAELVDSVLDVVRK.E | 2222 |
|  | Astrin\_STLCHLD\_061214\_01.09505.09505.3 | 5.6867 | 0.3777 | 100.0% | 3327.7744 | 3329.5925 | 1 | 7.024 | 29.8% | 1 | K.ESESCDCLQGFQLTHSLGGGTGSGMGTLLISK.I | 3 |
|  | Astrin\_STLCHLD\_050114\_01.04328.04328.2 | 2.6855 | 0.1744 | 98.8% | 1078.0122 | 1078.1698 | 9 | 4.186 | 78.6% | 2 | K.IREEYPDR.I | 222 |
|  | Astrin\_STLCLD20\_112214\_01.10486.10486.2 | 3.3502 | 0.4172 | 100.0% | 1352.5322 | 1352.6497 | 1 | 6.771 | 63.6% | 1 | R.IMNTFSVMPSPK.V | 22 |
|  | Astrin\_STLCHLD\_050114\_01.12590.12590.3 | 4.1941 | 0.3268 | 100.0% | 2710.8843 | 2710.0405 | 1 | 6.714 | 30.2% | 1 | K.LTTPTYGDLNHLVSATMSGVTTCLR.F | 3333 |
|  | Astrin\_STLCHLD\_tube2\_061314\_01.07361.07361.2 | 3.2733 | 0.2391 | 99.9% | 1132.2322 | 1131.2767 | 1 | 5.073 | 83.3% | 60 | R.FPGQLNADLR.K | 222222 |
|  | Astrin\_STLCHLD\_tube2\_050114\_01.05601.05601.2 | 2.7143 | 0.2756 | 99.3% | 1258.5521 | 1259.4508 | 11 | 5.358 | 65.0% | 14 | R.FPGQLNADLRK.L | 222222 |
|  | Astrin\_STLCHLD\_tube2\_050114\_01.05583.05583.3 | 3.152 | 0.3056 | 99.8% | 1259.8744 | 1259.4508 | 8 | 5.284 | 45.0% | 17 | R.FPGQLNADLRK.L | 333333 |
|  | Astrin\_STLCHLD\_tube2\_061314\_01.08730.08730.2 | 3.8464 | 0.3753 | 100.0% | 1272.4122 | 1272.5945 | 1 | 7.898 | 75.0% | 16 | R.KLAVNMVPFPR.L | 222222 |
|  | Astrin\_STLCLD20\_112214\_01.11192.11192.1 | 2.2399 | 0.2147 | 96.5% | 1143.63 | 1144.4204 | 6 | 6.378 | 61.1% | 4 | K.LAVNMVPFPR.L | 111111 |
|  | Astrin\_STLCHLD\_tube2\_050114\_01.09839.09839.2 | 3.9889 | 0.4725 | 100.0% | 1144.4122 | 1144.4204 | 1 | 8.55 | 94.4% | 18 | K.LAVNMVPFPR.L | 222222 |
|  | Astrin\_STLCHLD\_tube2\_061314\_01.12143.12143.2 | 3.7666 | 0.4528 | 100.0% | 1621.8322 | 1621.9403 | 1 | 8.756 | 76.9% | 17 | R.LHFFMPGFAPLTSR.G | 22222 |
|  | Astrin\_STLCHLD\_tube2\_061314\_01.12204.12204.3 | 4.3867 | 0.3891 | 100.0% | 1622.3944 | 1621.9403 | 1 | 6.411 | 53.8% | 14 | R.LHFFMPGFAPLTSR.G | 33333 |
|  | Astrin\_STLCHLD\_050114\_01.04952.04952.2 | 2.8672 | 0.3941 | 100.0% | 1066.2322 | 1066.2013 | 2 | 7.174 | 68.8% | 2 | K.NMMAACDPR.H | 222222 |
|  | Astrin\_STLCHLD\_tube2\_050114\_02.06679.06679.2 | 5.6297 | 0.5004 | 100.0% | 1924.5322 | 1925.2405 | 1 | 8.712 | 56.7% | 4 | R.MSMKEVDEQMLNVQNK.N | 222 |
|  | Astrin\_STLCHLD\_tube2\_061314\_02.06675.06675.3 | 4.9405 | 0.1867 | 99.8% | 1926.6843 | 1925.2405 | 1 | 5.512 | 51.7% | 15 | R.MSMKEVDEQMLNVQNK.N | 333 |
|  | Astrin\_STLCHLD\_tube2\_050114\_01.05776.05776.1 | 2.9126 | 0.2315 | 98.0% | 1446.63 | 1447.6031 | 1 | 5.343 | 68.2% | 1 | K.EVDEQMLNVQNK.N | 111 |
|  | Astrin\_NLD\_STLC\_tube2\_021014\_01.06237.06237.2 | 4.3052 | 0.2375 | 100.0% | 1448.2522 | 1447.6031 | 6 | 5.817 | 63.6% | 16 | K.EVDEQMLNVQNK.N | 222 |
|  | Astrin\_STLCHLD\_tube2\_061314\_01.11781.11781.2 | 4.295 | 0.2978 | 100.0% | 1698.5322 | 1697.8877 | 1 | 7.708 | 73.1% | 8 | K.NSSYFVEWIPNNVK.T | 222222 |
|  | Astrin\_STLCHLD\_050114\_01.05029.05029.2 | 2.2588 | 0.224 | 97.2% | 1029.1921 | 1029.1473 | 9 | 4.44 | 62.5% | 1 | K.TAVCDIPPR.G | 2222 |
|  | Astrin\_STLCHLD\_tube2\_061314\_02.09608.09608.2 | 3.6979 | 0.0138 | 95.3% | 1859.8522 | 1859.1475 | 1 | 5.87 | 50.0% | 1 | K.MSATFIGNSTAIQELFK.R | 22 |
|  | Astrin\_STLCLD20\_112214\_02.14525.14525.3 | 3.4924 | 0.1623 | 95.1% | 2016.4443 | 2015.335 | 1 | 5.966 | 32.4% | 1 | K.MSATFIGNSTAIQELFKR.I | 33 |
|  | Astrin\_STLCHLD\_061214\_01.07812.07812.2 | 3.8437 | 0.3944 | 100.0% | 1386.3522 | 1386.6116 | 1 | 7.545 | 80.0% | 16 | K.RISEQFTAMFR.R | 22222 |
|  | Astrin\_STLCHLD\_tube2\_050114\_02.06787.06787.3 | 3.1948 | 0.3082 | 99.8% | 1543.3143 | 1542.7991 | 1 | 5.726 | 52.3% | 1 | K.RISEQFTAMFRR.K | 33333 |
|  | Astrin\_STLCLD20\_112214\_tube2\_01.13623.13623.1 | 1.9755 | 0.2993 | 98.1% | 1229.5 | 1230.4241 | 1 | 5.342 | 61.1% | 1 | R.ISEQFTAMFR.R | 11111 |
|  | Astrin\_STLCHLD\_tube2\_050114\_02.08042.08042.2 | 4.0657 | 0.4262 | 100.0% | 1231.3121 | 1230.4241 | 1 | 6.725 | 94.4% | 46 | R.ISEQFTAMFR.R | 22222 |

Similarities:
gi|29788785|ref|NP\_82(29:4)  
gi|5174735|ref|NP\_006(29:4)  
gi|21361322|ref|NP\_00(18:15)  
gi|50592996|ref|NP\_00(18:15)  
gi|14210536|ref|NP\_11(11:22)  

---

|  |  |  |  |  |  |  |  |  |
| --- | --- | --- | --- | --- | --- | --- | --- | --- |
| U | *gi|57242777|ref|NP\_03* | 7 | 20 | 55.3% | 103 | 11967 | 5.9 | c-myc binding protein [Homo sapiens] |

| Filename XCorr DeltCN Conf% ObsM+H+ CalcM+H+ SpR ZScore Ion% # Sequence  | | | | | | | | | | | | |
| --- | --- | --- | --- | --- | --- | --- | --- | --- | --- | --- | --- | --- |
| \* | Astrin\_STLCHLD\_tube2\_061314\_01.06689.06689.2 | 2.5818 | 0.2999 | 99.6% | 933.7522 | 934.07764 | 3 | 5.869 | 75.0% | 1 | K.SGVLDTLTK.V | 2 |
| \* | Astrin\_STLCHLD\_tube2\_061314\_01.16490.16490.3 | 4.8728 | 0.3685 | 99.9% | 3191.3342 | 3191.6892 | 1 | 5.676 | 23.2% | 1 | K.SGVLDTLTKVLVALYEEPEKPNSALDFLK.H | 3 |
| \* | Astrin\_STLCLD20\_112214\_01.12430.12430.2 | 4.5601 | 0.4139 | 100.0% | 2275.6921 | 2276.6348 | 1 | 7.695 | 44.7% | 2 | K.VLVALYEEPEKPNSALDFLK.H | 2 |
| \* | Astrin\_STLCHLD\_tube2\_050114\_01.11423.11423.3 | 4.7952 | 0.4795 | 100.0% | 2276.6343 | 2276.6348 | 1 | 7.923 | 39.5% | 4 | K.VLVALYEEPEKPNSALDFLK.H | 3 |
| \* | Astrin\_STLCHLD\_tube2\_061314\_01.07005.07005.3 | 4.0617 | 0.3109 | 99.9% | 1896.8043 | 1898.1289 | 67 | 5.527 | 37.5% | 4 | K.HHLGAATPENPEIELLR.L | 3 |
| \* | Astrin\_STLCLD20\_112214\_tube2\_01.09737.09737.2 | 4.217 | 0.4143 | 100.0% | 1897.2522 | 1898.1289 | 1 | 7.352 | 71.9% | 2 | K.HHLGAATPENPEIELLR.L | 2 |
| \* | Astrin\_STLCHLD\_050114\_01.05491.05491.2 | 3.1578 | 0.2815 | 99.9% | 1333.0521 | 1332.4528 | 76 | 5.237 | 55.0% | 6 | K.LAQYEPPQEEK.R | 2 |

---

|  |  |  |  |  |  |  |  |  |
| --- | --- | --- | --- | --- | --- | --- | --- | --- |
| U | *gi|223890147|ref|NP\_0* | 24 | 92 | 54.2% | 356 | 40067 | 7.0 | DSN1, MIND kinetochore complex component, homolog isoform 1 [Homo sapiens] |
| U | *gi|223972618|ref|NP\_0* | 24 | 92 | 54.2% | 356 | 40067 | 7.0 | DSN1, MIND kinetochore complex component, homolog isoform 1 [Homo sapiens] |
| U | *gi|223890149|ref|NP\_0* | 24 | 92 | 54.2% | 356 | 40067 | 7.0 | DSN1, MIND kinetochore complex component, homolog isoform 1 [Homo sapiens] |

| Filename XCorr DeltCN Conf% ObsM+H+ CalcM+H+ SpR ZScore Ion% # Sequence  | | | | | | | | | | | | |
| --- | --- | --- | --- | --- | --- | --- | --- | --- | --- | --- | --- | --- |
|  | Astrin\_STLCHLD\_tube2\_050114\_01.04899.04899.2 | 4.2495 | 0.3785 | 100.0% | 1433.4521 | 1433.6599 | 1 | 7.196 | 75.0% | 8 | R.SEIIDEKGPVMSK.T | 2 |
|  | Astrin\_STLCHLD\_tube2\_050114\_01.08214.08214.2 | 6.5535 | 0.6047 | 100.0% | 2024.6522 | 2025.2255 | 1 | 11.316 | 79.4% | 3 | K.THDHQLESSLSPVEVFAK.T | 2 |
|  | Astrin\_STLCHLD\_tube2\_050114\_01.08202.08202.3 | 5.4903 | 0.3636 | 100.0% | 2025.8344 | 2025.2255 | 1 | 7.09 | 47.1% | 4 | K.THDHQLESSLSPVEVFAK.T | 3 |
|  | Astrin\_STLCHLD\_050114\_02.05258.05258.2 | 4.7966 | 0.4593 | 100.0% | 1638.2722 | 1638.7467 | 1 | 8.859 | 57.1% | 12 | K.TSASLEMNQGVSEER.I | 2 |
|  | Astrin\_STLCHLD\_tube2\_050114\_01.04714.04714.2 | 5.4626 | 0.5507 | 100.0% | 1846.5922 | 1846.9512 | 1 | 9.901 | 66.7% | 2 | K.SLHLSPQEQSASYQDR.R | 2 |
|  | Astrin\_STLCHLD\_050114\_01.07135.07135.3 | 4.6132 | 0.3599 | 100.0% | 1847.3944 | 1846.9512 | 3 | 6.422 | 41.7% | 5 | K.SLHLSPQEQSASYQDR.R | 3 |
|  | Astrin\_STLCHLD\_tube2\_050114\_01.04385.04385.2 | 3.9021 | 0.3923 | 100.0% | 2002.4122 | 2003.1387 | 1 | 6.383 | 56.2% | 1 | K.SLHLSPQEQSASYQDRR.Q | 2 |
|  | Astrin\_STLCHLD\_050114\_01.05332.05332.3 | 3.7003 | 0.4868 | 100.0% | 1717.2843 | 1716.981 | 1 | 7.415 | 46.4% | 1 | R.KSLHPIHQGITELSR.S | 3 |
|  | Astrin\_STLCHLD\_tube2\_050114\_01.04896.04896.2 | 3.8435 | 0.4583 | 100.0% | 1588.6322 | 1588.8069 | 1 | 7.934 | 84.6% | 4 | K.SLHPIHQGITELSR.S | 2 |
|  | Astrin\_STLCHLD\_tube2\_050114\_01.04892.04892.3 | 4.1868 | 0.5459 | 100.0% | 1589.5443 | 1588.8069 | 1 | 8.734 | 51.9% | 13 | K.SLHPIHQGITELSR.S | 3 |
|  | Astrin\_STLCHLD\_050114\_01.09977.09977.2 | 2.4377 | 0.3293 | 99.4% | 1049.6522 | 1049.1663 | 1 | 6.253 | 77.8% | 2 | R.SISVDLAESK.R | 2 |
|  | Astrin\_STLCHLD\_050114\_01.07346.07346.2 | 3.1436 | 0.3608 | 100.0% | 1204.9122 | 1205.3538 | 1 | 6.463 | 80.0% | 8 | R.SISVDLAESKR.L | 2 |
|  | Astrin\_STLCHLD\_tube2\_050114\_01.09538.09538.1 | 1.7298 | 0.3129 | 98.1% | 942.45 | 943.04706 | 99 | 5.318 | 50.0% | 2 | K.GFSLESFR.A | 1 |
|  | Astrin\_STLCHLD\_tube2\_050114\_01.09502.09502.2 | 2.5362 | 0.4913 | 100.0% | 943.09216 | 943.04706 | 2 | 7.305 | 71.4% | 2 | K.GFSLESFR.A | 2 |
|  | Astrin\_STLCHLD\_050114\_01.06038.06038.2 | 2.7127 | 0.3453 | 100.0% | 964.1922 | 964.06055 | 304 | 6.882 | 56.2% | 2 | K.ASSLSEELK.H | 2 |
|  | Astrin\_STLCHLD\_tube2\_050114\_01.11721.11721.3 | 6.1593 | 0.4367 | 100.0% | 2477.7244 | 2477.6873 | 1 | 8.469 | 39.8% | 2 | K.ASSLSEELKHFADGLETDGTLQK.C | 3 |
|  | Astrin\_STLCHLD\_050114\_01.09850.09850.2 | 4.0074 | 0.5199 | 100.0% | 1532.3121 | 1532.65 | 1 | 8.582 | 80.8% | 4 | K.HFADGLETDGTLQK.C | 2 |
|  | Astrin\_STLCHLD\_tube2\_050114\_02.07744.07744.2 | 4.6703 | 0.4649 | 100.0% | 1486.6921 | 1485.6489 | 1 | 8.56 | 73.1% | 3 | K.ASDFSLEASVAEMK.E | 2 |
|  | Astrin\_STLCHLD\_tube2\_050114\_01.12679.12679.2 | 4.8989 | 0.4961 | 100.0% | 2120.5122 | 2120.379 | 1 | 9.053 | 52.8% | 1 | K.ASDFSLEASVAEMKEYITK.F | 2 |
|  | Astrin\_STLCHLD\_tube2\_050114\_02.09718.09718.3 | 3.2296 | 0.272 | 99.3% | 2120.9644 | 2120.379 | 6 | 5.504 | 33.3% | 1 | K.ASDFSLEASVAEMKEYITK.F | 3 |
|  | Astrin\_STLCHLD\_tube2\_050114\_01.10517.10517.2 | 2.444 | 0.2969 | 98.2% | 1901.7722 | 1902.1167 | 210 | 4.394 | 39.3% | 1 | R.QTWDQLLLHYQQEAK.E | 2 |
|  | Astrin\_STLCHLD\_tube2\_050114\_01.08664.08664.3 | 6.7309 | 0.4988 | 100.0% | 3212.9043 | 3213.627 | 1 | 8.034 | 36.1% | 1 | K.ITEVKVEPMTYLGSSQNEVLNTKPDYQK.I | 3 |
|  | Astrin\_STLCHLD\_tube2\_050114\_01.08302.08302.3 | 2.1374 | 0.3443 | 96.3% | 2641.7944 | 2642.9404 | 1 | 4.781 | 30.7% | 1 | K.VEPMTYLGSSQNEVLNTKPDYQK.I | 3 |
|  | Astrin\_STLCHLD\_050114\_01.05955.05955.2 | 4.1475 | 0.3478 | 100.0% | 1230.0922 | 1230.3813 | 1 | 6.874 | 85.0% | 9 | R.SMQQLDPSPAR.K | 2 |

---

|  |  |  |  |  |  |  |  |  |
| --- | --- | --- | --- | --- | --- | --- | --- | --- |
| U | *gi|5174457|ref|NP\_006* | 43 | 229 | 53.4% | 642 | 73913 | 5.6 | kinetochore associated 2 [Homo sapiens] |

| Filename XCorr DeltCN Conf% ObsM+H+ CalcM+H+ SpR ZScore Ion% # Sequence  | | | | | | | | | | | | |
| --- | --- | --- | --- | --- | --- | --- | --- | --- | --- | --- | --- | --- |
| \* | Astrin\_STLCHLD\_tube2\_050114\_02.05643.05643.3 | 3.333 | 0.2712 | 99.6% | 1803.2043 | 1802.914 | 1 | 5.096 | 39.1% | 1 | R.SSVSSGGAGRLS\*MQELR.S | 3 |
| \* | Astrin\_STLCHLD\_tube2\_061314\_01.04851.04851.2 | 2.5709 | 0.1176 | 97.7% | 877.39215 | 877.0466 | 21 | 5.011 | 75.0% | 1 | R.LSMQELR.S | 2 |
| \* | Astrin\_NLD\_STLC\_031014\_01.08957.08957.2 | 4.0437 | 0.4579 | 100.0% | 1707.4122 | 1707.8809 | 1 | 8.281 | 57.1% | 1 | R.SQDVNKQGLYTPQTK.E | 2 |
| \* | Astrin\_NLD\_STLC\_031014\_01.08648.08648.2 | 2.5354 | 0.2615 | 98.9% | 1145.4321 | 1145.3011 | 1 | 5.272 | 77.8% | 3 | K.LSINKPTSER.K | 2 |
| \* | Astrin\_NLD\_STLC\_tube2\_021014\_01.08037.08037.2 | 2.8764 | 0.1961 | 98.4% | 1299.1322 | 1297.4075 | 5 | 4.813 | 63.6% | 5 | R.NSQLGIFSSSEK.I | 2 |
| \* | Astrin\_STLCHLD\_050114\_01.05834.05834.2 | 2.2876 | 0.3155 | 99.5% | 906.4122 | 905.9829 | 1 | 4.853 | 83.3% | 2 | K.FEEEVPR.I | 2 |
| \* | Astrin\_NLD\_STLC\_tube2\_021014\_01.10764.10764.2 | 4.2097 | 0.4127 | 100.0% | 1499.5721 | 1499.793 | 1 | 6.872 | 79.2% | 4 | R.IFKDLGYPFALSK.S | 2 |
| \* | Astrin\_NLD\_STLC\_tube2\_021014\_01.10739.10739.2 | 2.6376 | 0.2237 | 99.2% | 1013.03217 | 1013.2218 | 1 | 5.192 | 85.7% | 2 | K.LFLDYTIK.C | 2 |
| \* | Astrin\_STLCHLD\_tube2\_061314\_01.11455.11455.3 | 5.3241 | 0.4939 | 100.0% | 2081.9944 | 2081.4167 | 1 | 7.886 | 42.6% | 7 | K.LKDLFNVDAFKLESLEAK.N | 3 |
| \* | Astrin\_NLD\_STLC\_tube2\_021014\_01.12731.12731.2 | 3.3875 | 0.3629 | 100.0% | 1840.1322 | 1840.0831 | 8 | 5.711 | 43.3% | 2 | K.DLFNVDAFKLESLEAK.N | 2 |
| \* | Astrin\_NLD\_STLC\_tube2\_021014\_01.04272.04272.3 | 3.1118 | 0.2613 | 99.1% | 2026.8544 | 2027.2449 | 63 | 5.201 | 30.0% | 2 | R.LEQEREKEPNRLESLR.K | 3 |
| \* | Astrin\_NLD\_STLC\_031014\_01.08961.08961.2 | 2.6053 | 0.204 | 97.9% | 1372.2522 | 1371.5363 | 35 | 4.513 | 60.0% | 1 | R.EKEPNRLESLR.K | 2 |
| \* | Astrin\_STLCHLD\_tube2\_050114\_01.07991.07991.2 | 5.5815 | 0.506 | 100.0% | 2098.5122 | 2099.3228 | 1 | 9.397 | 64.7% | 3 | K.YQAYMSNLESHSAILDQK.L | 2 |
| \* | Astrin\_STLCHLD\_050114\_02.07010.07010.3 | 3.4858 | 0.4813 | 100.0% | 2099.5444 | 2099.3228 | 3 | 7.011 | 33.8% | 17 | K.YQAYMSNLESHSAILDQK.L | 3 |
| \* | Astrin\_STLCHLD\_tube2\_061314\_01.04769.04769.2 | 3.4722 | 0.3155 | 100.0% | 1129.4722 | 1129.2584 | 5 | 7.013 | 72.2% | 13 | K.LNGLNEEIAR.V | 2 |
| \* | Astrin\_NLD\_STLC\_tube2\_021014\_01.04790.04790.2 | 3.1566 | 0.1868 | 99.6% | 1086.2122 | 1086.2334 | 7 | 4.71 | 75.0% | 7 | R.LQNIIDNQK.Y | 2 |
| \* | Astrin\_STLCHLD\_tube2\_050114\_01.07915.07915.3 | 3.5864 | 0.3533 | 99.9% | 2021.2144 | 2020.2499 | 1 | 5.623 | 39.1% | 1 | R.LQNIIDNQKYSVADIER.I | 3 |
| \* | Astrin\_STLCHLD\_050114\_01.10153.10153.2 | 2.7647 | 0.4499 | 100.0% | 952.4922 | 953.03973 | 1 | 7.426 | 85.7% | 12 | K.YSVADIER.I | 2 |
| \* | Astrin\_STLCHLD\_050114\_01.04592.04592.3 | 4.565 | 0.3836 | 100.0% | 1738.1943 | 1737.9132 | 1 | 5.965 | 51.9% | 2 | R.INHERNELQQTINK.L | 3 |
| \* | Astrin\_NLD\_STLC\_031014\_01.08922.08922.2 | 4.3474 | 0.3365 | 100.0% | 1738.5122 | 1737.9132 | 1 | 6.252 | 73.1% | 2 | R.INHERNELQQTINK.L | 2 |
| \* | Astrin\_NLD\_STLC\_tube2\_021014\_01.07492.07492.3 | 3.9761 | 0.3568 | 99.9% | 2078.9944 | 2080.3518 | 1 | 6.359 | 37.5% | 1 | R.INHERNELQQTINKLTK.D | 3 |
| \* | Astrin\_STLCHLD\_050114\_01.05066.05066.2 | 2.9083 | 0.1383 | 98.5% | 1088.2322 | 1088.2058 | 1 | 5.085 | 81.2% | 1 | R.NELQQTINK.L | 2 |
| \* | Astrin\_NLD\_STLC\_tube2\_021014\_01.09846.09846.3 | 2.9182 | 0.2661 | 98.7% | 1874.3644 | 1874.0575 | 104 | 4.54 | 32.1% | 1 | K.DLEAEQQKLWNEELK.Y | 3 |
| \* | Astrin\_NLD\_STLC\_tube2\_021014\_01.07142.07142.2 | 2.3551 | 0.1244 | 95.9% | 931.9922 | 932.0642 | 44 | 4.142 | 75.0% | 1 | K.LWNEELK.Y | 2 |
| \* | Astrin\_STLCHLD\_tube2\_050114\_01.04944.04944.2 | 4.1119 | 0.4631 | 100.0% | 1617.5122 | 1617.7991 | 2 | 8.165 | 61.5% | 2 | R.GKEAIETQLAEYHK.L | 2 |
| \* | Astrin\_NLD\_STLC\_tube2\_021014\_02.04805.04805.3 | 4.4636 | 0.4011 | 100.0% | 1618.6743 | 1617.7991 | 4 | 6.702 | 44.2% | 10 | R.GKEAIETQLAEYHK.L | 3 |
| \* | Astrin\_STLCHLD\_050114\_01.11235.11235.2 | 2.1734 | 0.26 | 96.0% | 1320.2722 | 1320.454 | 1 | 4.664 | 77.3% | 1 | K.FNPEAGANCLVK.Y | 2 |
| \* | Astrin\_NLD\_STLC\_tube2\_021014\_01.13724.13724.2 | 5.2272 | 0.4431 | 100.0% | 2359.5322 | 2360.6665 | 1 | 7.884 | 57.9% | 4 | R.AQVYVPLKELLNETEEEINK.A | 2 |
| \* | Astrin\_NLD\_STLC\_031014\_01.11481.11481.3 | 4.7675 | 0.4288 | 100.0% | 2360.7844 | 2360.6665 | 1 | 6.717 | 36.8% | 5 | R.AQVYVPLKELLNETEEEINK.A | 3 |
| \* | Astrin\_NLD\_STLC\_tube2\_021014\_01.07271.07271.2 | 3.6965 | 0.3624 | 100.0% | 1461.5122 | 1461.5658 | 1 | 6.505 | 77.3% | 5 | K.ELLNETEEEINK.A | 2 |
| \* | Astrin\_STLCHLD\_050114\_01.15012.15012.2 | 4.8879 | 0.4708 | 100.0% | 2024.8121 | 2024.3088 | 1 | 8.436 | 64.7% | 4 | K.MGLEDTLEQLNAMITESK.R | 2 |
| \* | Astrin\_STLCHLD\_tube2\_050114\_01.14125.14125.2 | 5.0525 | 0.5754 | 100.0% | 2179.7922 | 2180.4963 | 1 | 9.591 | 63.9% | 2 | K.MGLEDTLEQLNAMITESKR.S | 2 |
| \* | Astrin\_STLCLD20\_112214\_tube2\_01.19070.19070.3 | 4.84 | 0.4471 | 100.0% | 2180.0044 | 2180.4963 | 1 | 7.939 | 43.1% | 11 | K.MGLEDTLEQLNAMITESKR.S | 3 |
| \* | Astrin\_STLCLD20\_112214\_tube2\_01.12110.12110.3 | 4.7894 | 0.3146 | 99.9% | 1979.1843 | 1979.2377 | 1 | 7.301 | 45.0% | 11 | R.TLKEEVQKLDDLYQQK.I | 3 |
| \* | Astrin\_STLCHLD\_tube2\_050114\_01.08922.08922.2 | 4.6593 | 0.4127 | 100.0% | 1979.6721 | 1979.2377 | 1 | 8.128 | 63.3% | 1 | R.TLKEEVQKLDDLYQQK.I | 2 |
| \* | Astrin\_STLCHLD\_050114\_01.05660.05660.2 | 2.7565 | 0.33 | 100.0% | 1023.4322 | 1023.1308 | 1 | 5.677 | 85.7% | 1 | K.LDDLYQQK.I | 2 |
| \* | Astrin\_STLCLD20\_112214\_tube2\_01.16977.16977.2 | 5.7535 | 0.5461 | 100.0% | 2554.9321 | 2555.8223 | 1 | 10.084 | 59.1% | 2 | K.HLLESTVNQGLSEAMNELDAVQR.E | 2 |
| \* | Astrin\_STLCLD20\_112214\_tube2\_01.16974.16974.3 | 3.2034 | 0.3282 | 99.8% | 2555.3943 | 2555.8223 | 4 | 5.668 | 27.3% | 1 | K.HLLESTVNQGLSEAMNELDAVQR.E | 3 |
| \* | Astrin\_STLCHLD\_tube2\_050114\_02.06003.06003.2 | 4.6226 | 0.4061 | 100.0% | 1597.4922 | 1596.7344 | 1 | 8.874 | 79.2% | 38 | R.EYQLVVQTTTEER.R | 2 |
| \* | Astrin\_STLCHLD\_061214\_01.05467.05467.2 | 3.4773 | 0.3186 | 100.0% | 1752.5322 | 1752.9219 | 1 | 5.932 | 61.5% | 3 | R.EYQLVVQTTTEERR.K | 2 |
| \* | Astrin\_STLCHLD\_tube2\_061314\_01.07158.07158.2 | 4.3931 | 0.431 | 100.0% | 1513.3322 | 1513.7925 | 1 | 8.496 | 76.9% | 26 | R.LLEMVATHVGSVEK.H | 2 |
| \* | Astrin\_STLCHLD\_tube2\_050114\_02.06402.06402.3 | 3.6309 | 0.38 | 99.9% | 1514.6044 | 1513.7925 | 1 | 6.536 | 44.2% | 9 | R.LLEMVATHVGSVEK.H | 3 |
| \* | Astrin\_STLCHLD\_050114\_02.06885.06885.3 | 3.3791 | 0.3574 | 100.0% | 2245.9744 | 2247.3584 | 1 | 5.933 | 45.6% | 1 | K.VDREYEECMSEDLSENIK.E | 3 |

---

|  |  |  |  |  |  |  |  |  |
| --- | --- | --- | --- | --- | --- | --- | --- | --- |
| U | *gi|13129022|ref|NP\_07* | 8 | 37 | 53.3% | 180 | 19737 | 7.2 | centromere protein M isoform a [Homo sapiens] |

| Filename XCorr DeltCN Conf% ObsM+H+ CalcM+H+ SpR ZScore Ion% # Sequence  | | | | | | | | | | | | |
| --- | --- | --- | --- | --- | --- | --- | --- | --- | --- | --- | --- | --- |
|  | Astrin\_NLD\_STLC\_031014\_01.16166.16166.3 | 4.3415 | 0.3774 | 99.9% | 2939.4844 | 2940.4658 | 1 | 7.293 | 27.8% | 2 | K.LPGLNTATILLVGTEDALLQQLADSMLK.E | 3 |
|  | Astrin\_NLD\_STLC\_031014\_01.09248.09248.2 | 2.9914 | 0.3336 | 100.0% | 1323.2122 | 1323.5381 | 2 | 5.333 | 63.6% | 4 | K.SLPLPSSVNRPR.I | 2 |
|  | Astrin\_NLD\_STLC\_031014\_02.10204.10204.3 | 3.8269 | 0.2228 | 99.8% | 1497.9543 | 1497.821 | 18 | 5.576 | 37.5% | 5 | R.IDLIVFVVNLHSK.Y | 3 |
|  | Astrin\_NLD\_STLC\_tube2\_021014\_02.09977.09977.2 | 4.273 | 0.4635 | 100.0% | 1497.9722 | 1497.821 | 1 | 8.493 | 75.0% | 6 | R.IDLIVFVVNLHSK.Y | 2 |
|  | Astrin\_NLD\_STLC\_031014\_01.09479.09479.2 | 3.2708 | 0.2929 | 100.0% | 1339.9922 | 1340.4325 | 1 | 8.37 | 75.0% | 4 | K.YSLQNTEESLR.H | 2 |
|  | Astrin\_NLD\_STLC\_tube2\_021014\_01.08948.08948.2 | 3.3721 | 0.5493 | 100.0% | 1121.1522 | 1121.281 | 1 | 9.061 | 72.2% | 13 | R.HVDASFFLGK.V | 2 |
|  | Astrin\_NLD\_STLC\_031014\_01.12580.12580.2 | 3.4366 | 0.4169 | 100.0% | 2330.4722 | 2331.7734 | 1 | 7.419 | 40.5% | 1 | R.VLQICAGHVPGVSALNLLSLLR.S | 2 |
|  | Astrin\_NLD\_STLC\_031014\_01.12596.12596.3 | 4.3254 | 0.3384 | 99.9% | 2331.5942 | 2331.7734 | 1 | 6.964 | 39.3% | 2 | R.VLQICAGHVPGVSALNLLSLLR.S | 3 |

---

|  |  |  |  |  |  |  |  |  |
| --- | --- | --- | --- | --- | --- | --- | --- | --- |
| U | *gi|7669492|ref|NP\_002* | 12 | 103 | 52.5% | 335 | 36053 | 8.5 | glyceraldehyde-3-phosphate dehydrogenase [Homo sapiens] |

| Filename XCorr DeltCN Conf% ObsM+H+ CalcM+H+ SpR ZScore Ion% # Sequence  | | | | | | | | | | | | |
| --- | --- | --- | --- | --- | --- | --- | --- | --- | --- | --- | --- | --- |
| \* | Astrin\_STLCHLD\_050114\_01.06145.06145.2 | 2.424 | 0.2581 | 99.1% | 806.27216 | 805.912 | 15 | 5.995 | 71.4% | 3 | K.VGVNGFGR.I | 2 |
| \* | Astrin\_STLCHLD\_050114\_02.13705.13705.3 | 4.2906 | 0.4006 | 99.9% | 3310.0444 | 3310.7634 | 7 | 6.463 | 24.1% | 4 | K.VDIVAINDPFIDLNYMVYMFQYDSTHGK.F | 3 |
| \* | Astrin\_STLCLD20\_112214\_tube2\_01.15888.15888.2 | 3.632 | 0.4113 | 100.0% | 1614.8322 | 1614.8851 | 1 | 7.927 | 61.5% | 6 | K.LVINGNPITIFQER.D | 2 |
| \* | Astrin\_STLCLD20\_112214\_01.11543.11543.2 | 2.9456 | 0.3341 | 99.7% | 2042.4521 | 2042.3427 | 86 | 5.955 | 29.4% | 1 | K.LVINGNPITIFQERDPSK.I | 2 |
| \* | Astrin\_STLCHLD\_061214\_02.09089.09089.2 | 5.7349 | 0.588 | 100.0% | 2278.0122 | 2278.495 | 1 | 11.544 | 52.5% | 1 | K.WGDAGAEYVVESTGVFTTMEK.A | 2 |
| \* | Astrin\_STLCLD20\_112214\_01.10694.10694.3 | 4.9672 | 0.3163 | 99.9% | 2371.5544 | 2370.79 | 1 | 6.111 | 40.5% | 3 | K.RVIISAPSADAPMFVMGVNHEK.Y | 3 |
| \* | AstrinSTLCLD\_041714\_01.12592.12592.3 | 7.2002 | 0.5946 | 100.0% | 2597.2444 | 2597.0044 | 1 | 10.413 | 41.3% | 20 | K.VIHDNFGIVEGLMTTVHAITATQK.T | 3 |
| \* | Astrin\_STLCLD20\_112214\_tube2\_01.19043.19043.2 | 5.9089 | 0.509 | 100.0% | 2597.3123 | 2597.0044 | 1 | 9.752 | 47.8% | 2 | K.VIHDNFGIVEGLMTTVHAITATQK.T | 2 |
| \* | Astrin\_STLCHLD\_tube2\_061314\_01.07740.07740.2 | 4.1922 | 0.3998 | 100.0% | 1412.5521 | 1412.6292 | 1 | 6.263 | 75.0% | 19 | R.GALQNIIPASTGAAK.A | 2 |
| \* | Astrin\_STLCLD20\_112214\_01.09829.09829.2 | 3.2203 | 0.368 | 100.0% | 1531.7122 | 1531.7155 | 13 | 6.808 | 50.0% | 3 | R.VPTANVSVVDLTCR.L | 2 |
|  | Astrin\_NLD\_STLC\_tube2\_021014\_02.08255.08255.2 | 4.5351 | 0.5625 | 100.0% | 1764.3722 | 1764.8914 | 1 | 9.95 | 65.4% | 18 | K.LISWYDNEFGYSNR.V | 2 |
| \* | Astrin\_STLCHLD\_tube2\_050114\_01.06777.06777.2 | 4.0811 | 0.4896 | 100.0% | 1331.4321 | 1331.5879 | 1 | 8.771 | 77.3% | 23 | R.VVDLMAHMASKE.- | 2 |

---

|  |  |  |  |  |  |  |  |  |
| --- | --- | --- | --- | --- | --- | --- | --- | --- |
| U | *contaminant\_gi|746301* | 18 | 537 | 52.4% | 269 | 27961 | 6.7 | lysyl endopeptidase (EC 3.4.21.50) - Lysobacter enzymogenes |

| Filename XCorr DeltCN Conf% ObsM+H+ CalcM+H+ SpR ZScore Ion% # Sequence  | | | | | | | | | | | | |
| --- | --- | --- | --- | --- | --- | --- | --- | --- | --- | --- | --- | --- |
| \* | Astrin\_NLD\_STLC\_031014\_01.03735.03735.1 | 1.9528 | 0.257 | 97.4% | 725.86 | 725.8198 | 1 | 5.169 | 66.7% | 3 | R.SVAAYSK.Q | 1 |
| \* | Astrin\_STLCHLD\_050114\_01.06523.06523.2 | 6.3901 | 0.6156 | 100.0% | 2261.4922 | 2262.355 | 1 | 11.178 | 56.2% | 84 | R.APGSSSSGANGDGSLAQSQTGAVVR.A | 2 |
| \* | Astrin\_NLD\_STLC\_031014\_01.09083.09083.3 | 5.0972 | 0.4292 | 100.0% | 2262.1443 | 2262.355 | 1 | 7.495 | 37.5% | 63 | R.APGSSSSGANGDGSLAQSQTGAVVR.A | 3 |
| \* | Astrin\_NLD\_STLC\_031014\_02.12422.12422.3 | 6.4532 | 0.4802 | 100.0% | 3315.7144 | 3315.6257 | 1 | 7.903 | 27.6% | 18 | R.ATNAASDFTLLELNTAANPAYNLFWAGWDR.R | 3 |
| \* | Astrin\_STLCHLD\_050114\_02.12788.12788.2 | 5.4228 | 0.4616 | 100.0% | 3316.2722 | 3315.6257 | 1 | 8.99 | 39.7% | 26 | R.ATNAASDFTLLELNTAANPAYNLFWAGWDR.R | 2 |
| \* | Astrin\_NLD\_STLC\_tube2\_021014\_01.15998.15998.3 | 6.7063 | 0.4961 | 100.0% | 3470.8442 | 3471.813 | 1 | 9.888 | 29.2% | 103 | R.ATNAASDFTLLELNTAANPAYNLFWAGWDRR.D | 3 |
| \* | Astrin\_STLCHLD\_tube2\_050114\_01.04346.04346.3 | 4.7789 | 0.313 | 99.9% | 2077.1042 | 2077.2668 | 1 | 6.482 | 47.2% | 8 | R.RDQNFAGATAIHHPNVAEK.R | 3 |
| \* | Astrin\_NLD\_STLC\_031014\_01.08907.08907.3 | 4.8561 | 0.3694 | 100.0% | 2233.4644 | 2233.4543 | 1 | 6.291 | 39.5% | 6 | R.RDQNFAGATAIHHPNVAEKR.I | 3 |
| \* | Astrin\_NLD\_STLC\_tube2\_021014\_01.04192.04192.2 | 5.9375 | 0.5266 | 100.0% | 2233.5522 | 2233.4543 | 1 | 9.318 | 60.5% | 2 | R.RDQNFAGATAIHHPNVAEKR.I | 2 |
| \* | Astrin\_STLCHLD\_tube2\_050114\_01.04670.04670.2 | 4.9285 | 0.5221 | 100.0% | 1920.7522 | 1921.0793 | 1 | 8.428 | 58.8% | 14 | R.DQNFAGATAIHHPNVAEK.R | 2 |
| \* | Astrin\_STLCHLD\_050114\_01.07117.07117.3 | 3.0045 | 0.3658 | 99.8% | 1921.3744 | 1921.0793 | 1 | 5.83 | 38.2% | 5 | R.DQNFAGATAIHHPNVAEK.R | 3 |
| \* | Astrin\_STLCHLD\_tube2\_050114\_01.04448.04448.2 | 5.5433 | 0.4268 | 100.0% | 2076.5322 | 2077.2668 | 1 | 7.603 | 55.6% | 13 | R.DQNFAGATAIHHPNVAEKR.I | 2 |
| \* | Astrin\_STLCHLD\_tube2\_050114\_01.04412.04412.3 | 4.5726 | 0.4477 | 100.0% | 2078.3044 | 2077.2668 | 1 | 8.101 | 43.1% | 35 | R.DQNFAGATAIHHPNVAEKR.I | 3 |
| \* | Astrin\_NLD\_STLC\_031014\_01.09015.09015.2 | 4.8879 | 0.4086 | 100.0% | 1870.0322 | 1870.983 | 1 | 7.99 | 66.7% | 3 | R.VLGQLHGGPSSCSATGADR.S | 2 |
| \* | Astrin\_NLD\_STLC\_031014\_01.09009.09009.3 | 4.41 | 0.4726 | 100.0% | 1870.4043 | 1870.983 | 1 | 7.682 | 38.9% | 5 | R.VLGQLHGGPSSCSATGADR.S | 3 |
| \* | Astrin\_NLD\_STLC\_tube2\_021014\_01.07809.07809.2 | 5.1869 | 0.4699 | 100.0% | 1429.4321 | 1428.5443 | 1 | 8.767 | 73.1% | 139 | R.VFTSWTGGGTSATR.L | 2 |
| \* | Astrin\_STLCLD20\_112214\_tube2\_01.09540.09540.1 | 2.6307 | 0.1733 | 96.0% | 1429.62 | 1428.5443 | 23 | 4.616 | 38.5% | 9 | R.VFTSWTGGGTSATR.L | 1 |
| \* | AstrinSTLCLD\_041714\_01.12896.12896.2 | 4.2573 | 0.3892 | 100.0% | 2605.5723 | 2605.8174 | 1 | 8.313 | 32.0% | 1 | R.LSDWLDAAGTGAQFIDGLDSTGTPPV.- | 2 |

---

|  |  |  |  |  |  |  |  |  |
| --- | --- | --- | --- | --- | --- | --- | --- | --- |
| U | *gi|21361322|ref|NP\_00* | 27 | 422 | 51.1% | 444 | 49586 | 4.9 | tubulin, beta 4 [Homo sapiens] |

| Filename XCorr DeltCN Conf% ObsM+H+ CalcM+H+ SpR ZScore Ion% # Sequence  | | | | | | | | | | | | |
| --- | --- | --- | --- | --- | --- | --- | --- | --- | --- | --- | --- | --- |
|  | Astrin\_STLCHLD\_050114\_02.08218.08218.3 | 6.3129 | 0.4919 | 100.0% | 3117.2344 | 3118.2996 | 1 | 8.572 | 35.6% | 9 | K.FWEVISDEHGIDPTGTYHGDSDLQLER.I | 33 |
| \* | Astrin\_STLCHLD\_050114\_02.06892.06892.3 | 2.6021 | 0.361 | 99.8% | 1909.8544 | 1910.9945 | 2 | 5.146 | 33.3% | 1 | R.INVYYNEAT#GGNYVPR.A | 3 |
|  | Astrin\_STLCLD20\_112214\_tube2\_01.12946.12946.2 | 4.312 | 0.3215 | 100.0% | 1604.5322 | 1602.8431 | 1 | 6.404 | 67.9% | 22 | R.AVLVDLEPGTMDSVR.S | 22 |
|  | Astrin\_STLCHLD\_tube2\_050114\_01.12338.12338.2 | 5.6846 | 0.5176 | 100.0% | 2798.8523 | 2800.0647 | 1 | 8.445 | 40.0% | 14 | R.SGPFGQIFRPDNFVFGQSGAGNNWAK.G | 2222 |
|  | AstrinSTLCLD\_041714\_01.10952.10952.3 | 7.1011 | 0.4962 | 100.0% | 2799.1143 | 2800.0647 | 1 | 8.497 | 38.0% | 33 | R.SGPFGQIFRPDNFVFGQSGAGNNWAK.G | 3333 |
|  | Astrin\_STLCHLD\_050114\_01.11995.11995.1 | 2.1993 | 0.2639 | 97.3% | 1319.69 | 1320.5896 | 245 | 5.053 | 40.9% | 6 | R.IMNTFSVVPSPK.V | 1111 |
|  | Astrin\_STLCHLD\_tube2\_061314\_01.08597.08597.2 | 4.5471 | 0.3836 | 100.0% | 1320.4122 | 1320.5896 | 1 | 7.86 | 77.3% | 40 | R.IMNTFSVVPSPK.V | 2222 |
|  | Astrin\_STLCHLD\_050114\_01.12590.12590.3 | 4.1941 | 0.3268 | 100.0% | 2710.8843 | 2710.0405 | 1 | 6.714 | 30.2% | 1 | K.LTTPTYGDLNHLVSATMSGVTTCLR.F | 3333 |
|  | Astrin\_STLCHLD\_tube2\_061314\_01.07361.07361.2 | 3.2733 | 0.2391 | 99.9% | 1132.2322 | 1131.2767 | 1 | 5.073 | 83.3% | 60 | R.FPGQLNADLR.K | 222222 |
|  | Astrin\_STLCHLD\_tube2\_050114\_01.05601.05601.2 | 2.7143 | 0.2756 | 99.3% | 1258.5521 | 1259.4508 | 11 | 5.358 | 65.0% | 14 | R.FPGQLNADLRK.L | 222222 |
|  | Astrin\_STLCHLD\_tube2\_050114\_01.05583.05583.3 | 3.152 | 0.3056 | 99.8% | 1259.8744 | 1259.4508 | 8 | 5.284 | 45.0% | 17 | R.FPGQLNADLRK.L | 333333 |
|  | Astrin\_STLCHLD\_tube2\_061314\_01.08730.08730.2 | 3.8464 | 0.3753 | 100.0% | 1272.4122 | 1272.5945 | 1 | 7.898 | 75.0% | 16 | R.KLAVNMVPFPR.L | 222222 |
|  | Astrin\_STLCLD20\_112214\_01.11192.11192.1 | 2.2399 | 0.2147 | 96.5% | 1143.63 | 1144.4204 | 6 | 6.378 | 61.1% | 4 | K.LAVNMVPFPR.L | 111111 |
|  | Astrin\_STLCHLD\_tube2\_050114\_01.09839.09839.2 | 3.9889 | 0.4725 | 100.0% | 1144.4122 | 1144.4204 | 1 | 8.55 | 94.4% | 18 | K.LAVNMVPFPR.L | 222222 |
|  | Astrin\_STLCHLD\_tube2\_061314\_01.12143.12143.2 | 3.7666 | 0.4528 | 100.0% | 1621.8322 | 1621.9403 | 1 | 8.756 | 76.9% | 17 | R.LHFFMPGFAPLTSR.G | 22222 |
|  | Astrin\_STLCHLD\_tube2\_061314\_01.12204.12204.3 | 4.3867 | 0.3891 | 100.0% | 1622.3944 | 1621.9403 | 1 | 6.411 | 53.8% | 14 | R.LHFFMPGFAPLTSR.G | 33333 |
|  | Astrin\_STLCHLD\_tube2\_061314\_01.12186.12186.2 | 4.648 | 0.4524 | 100.0% | 1692.6122 | 1692.9678 | 1 | 7.8 | 78.6% | 12 | R.ALTVPELTQQMFDAK.N | 222 |
|  | Astrin\_STLCHLD\_050114\_01.04952.04952.2 | 2.8672 | 0.3941 | 100.0% | 1066.2322 | 1066.2013 | 2 | 7.174 | 68.8% | 2 | K.NMMAACDPR.H | 222222 |
|  | Astrin\_STLCHLD\_050114\_02.06747.06747.3 | 4.3076 | 0.2581 | 99.9% | 1391.1244 | 1390.631 | 1 | 7.084 | 47.7% | 4 | R.HGRYLTVAAVFR.G | 333 |
|  | Astrin\_STLCHLD\_tube2\_050114\_01.10402.10402.1 | 1.7815 | 0.4511 | 100.0% | 1039.71 | 1040.2505 | 1 | 6.99 | 81.2% | 15 | R.YLTVAAVFR.G | 111 |
|  | Astrin\_STLCHLD\_tube2\_050114\_01.10269.10269.2 | 3.417 | 0.3601 | 100.0% | 1041.3322 | 1040.2505 | 1 | 6.884 | 87.5% | 30 | R.YLTVAAVFR.G | 222 |
|  | Astrin\_STLCHLD\_tube2\_061314\_01.11781.11781.2 | 4.295 | 0.2978 | 100.0% | 1698.5322 | 1697.8877 | 1 | 7.708 | 73.1% | 8 | K.NSSYFVEWIPNNVK.T | 222222 |
|  | Astrin\_STLCHLD\_050114\_01.05029.05029.2 | 2.2588 | 0.224 | 97.2% | 1029.1921 | 1029.1473 | 9 | 4.44 | 62.5% | 1 | K.TAVCDIPPR.G | 2222 |
|  | Astrin\_STLCHLD\_061214\_01.07812.07812.2 | 3.8437 | 0.3944 | 100.0% | 1386.3522 | 1386.6116 | 1 | 7.545 | 80.0% | 16 | K.RISEQFTAMFR.R | 22222 |
|  | Astrin\_STLCHLD\_tube2\_050114\_02.06787.06787.3 | 3.1948 | 0.3082 | 99.8% | 1543.3143 | 1542.7991 | 1 | 5.726 | 52.3% | 1 | K.RISEQFTAMFRR.K | 33333 |
|  | Astrin\_STLCLD20\_112214\_tube2\_01.13623.13623.1 | 1.9755 | 0.2993 | 98.1% | 1229.5 | 1230.4241 | 1 | 5.342 | 61.1% | 1 | R.ISEQFTAMFR.R | 11111 |
|  | Astrin\_STLCHLD\_tube2\_050114\_02.08042.08042.2 | 4.0657 | 0.4262 | 100.0% | 1231.3121 | 1230.4241 | 1 | 6.725 | 94.4% | 46 | R.ISEQFTAMFR.R | 22222 |

Similarities:
gi|29788785|ref|NP\_82(23:4)  
gi|5174735|ref|NP\_006(26:1)  
gi|29788768|ref|NP\_82(18:9)  
gi|50592996|ref|NP\_00(15:12)  
gi|14210536|ref|NP\_11(10:17)  

---

|  |  |  |  |  |  |  |  |  |
| --- | --- | --- | --- | --- | --- | --- | --- | --- |
| U | *gi|150456457|ref|NP\_9* | 24 | 219 | 50.4% | 347 | 39929 | 5.6 | HMT1 hnRNP methyltransferase-like 2 isoform 2 [Homo sapiens] |
| U | *gi|154759421|ref|NP\_0* | 23 | 218 | 47.2% | 371 | 42462 | 5.3 | HMT1 hnRNP methyltransferase-like 2 isoform 1 [Homo sapiens] |
| U | *gi|151301219|ref|NP\_9* | 24 | 219 | 49.6% | 353 | 40548 | 5.5 | HMT1 hnRNP methyltransferase-like 2 isoform 3 [Homo sapiens] |

| Filename XCorr DeltCN Conf% ObsM+H+ CalcM+H+ SpR ZScore Ion% # Sequence  | | | | | | | | | | | | |
| --- | --- | --- | --- | --- | --- | --- | --- | --- | --- | --- | --- | --- |
|  | Astrin\_STLCHLD\_050114\_02.08259.08259.3 | 4.9772 | 0.4767 | 100.0% | 2764.9443 | 2766.0132 | 1 | 8.839 | 33.3% | 2 | K.DYYFDSYAHFGIHEEMLKDEVR.T | 3 |
|  | Astrin\_STLCHLD\_tube2\_050114\_01.05380.05380.2 | 3.4675 | 0.2305 | 99.8% | 1351.5521 | 1351.6322 | 4 | 5.821 | 68.2% | 9 | K.ANKLDHVVTIIK.G | 2 |
|  | Astrin\_STLCHLD\_tube2\_061314\_01.04817.04817.3 | 4.4421 | 0.4346 | 100.0% | 1352.1543 | 1351.6322 | 1 | 7.842 | 54.5% | 10 | K.ANKLDHVVTIIK.G | 3 |
|  | Astrin\_STLCHLD\_050114\_01.09831.09831.2 | 3.3185 | 0.3983 | 100.0% | 1356.1322 | 1356.559 | 1 | 6.365 | 72.7% | 2 | K.GKVEEVELPVEK.V | 2 |
|  | Astrin\_STLCHLD\_tube2\_050114\_01.06750.06750.2 | 2.4186 | 0.2674 | 98.6% | 1171.3522 | 1171.333 | 100 | 5.696 | 55.6% | 1 | K.VEEVELPVEK.V | 2 |
|  | Astrin\_STLCLD20\_112214\_tube2\_01.14894.14894.2 | 3.9991 | 0.4029 | 100.0% | 1643.5521 | 1643.8827 | 1 | 7.156 | 73.1% | 17 | R.DKWLAPDGLIFPDR.A | 2 |
|  | Astrin\_STLCHLD\_tube2\_050114\_01.11119.11119.3 | 3.9731 | 0.4103 | 100.0% | 1644.1444 | 1643.8827 | 2 | 6.113 | 46.2% | 4 | R.DKWLAPDGLIFPDR.A | 3 |
|  | AstrinSTLCLD\_041714\_01.11307.11307.3 | 3.0474 | 0.2305 | 95.1% | 2875.7644 | 2877.2695 | 173 | 3.895 | 22.9% | 1 | R.DKWLAPDGLIFPDRATLYVTAIEDR.Q | 3 |
|  | AstrinSTLCLD\_041714\_01.10824.10824.2 | 3.606 | 0.414 | 100.0% | 1400.5322 | 1400.6201 | 1 | 7.485 | 72.7% | 5 | K.WLAPDGLIFPDR.A | 2 |
|  | Astrin\_STLCLD20\_112214\_tube2\_01.11110.11110.1 | 2.0704 | 0.3927 | 95.5% | 1251.55 | 1252.4099 | 2 | 6.621 | 60.0% | 2 | R.ATLYVTAIEDR.Q | 1 |
|  | Astrin\_STLCHLD\_050114\_02.07118.07118.2 | 3.9077 | 0.4948 | 100.0% | 1252.0521 | 1252.4099 | 1 | 8.151 | 75.0% | 67 | R.ATLYVTAIEDR.Q | 2 |
|  | Astrin\_NLD\_STLC\_tube2\_021014\_01.09382.09382.2 | 3.9554 | 0.36 | 100.0% | 1637.4122 | 1637.914 | 1 | 7.031 | 71.4% | 10 | K.DVAIKEPLVDVVDPK.Q | 2 |
|  | AstrinSTLCLD\_041714\_01.07971.07971.3 | 2.7304 | 0.2446 | 96.6% | 1637.9043 | 1637.914 | 261 | 5.186 | 28.6% | 1 | K.DVAIKEPLVDVVDPK.Q | 3 |
|  | Astrin\_STLCLD20\_112214\_tube2\_01.18464.18464.2 | 3.1424 | 0.3674 | 100.0% | 2229.6921 | 2229.5027 | 4 | 5.42 | 41.2% | 2 | K.RNDYVHALVAYFNIEFTR.C | 2 |
|  | Astrin\_STLCLD20\_112214\_02.15965.15965.3 | 5.1918 | 0.3604 | 100.0% | 2230.1643 | 2229.5027 | 1 | 8.991 | 50.0% | 9 | K.RNDYVHALVAYFNIEFTR.C | 3 |
|  | Astrin\_STLCLD20\_112214\_tube2\_01.19751.19751.2 | 5.6507 | 0.5334 | 100.0% | 2072.9321 | 2073.3152 | 1 | 9.27 | 65.6% | 9 | R.NDYVHALVAYFNIEFTR.C | 2 |
|  | Astrin\_STLCHLD\_050114\_02.11816.11816.3 | 4.5928 | 0.3813 | 100.0% | 2073.4744 | 2073.3152 | 1 | 6.78 | 40.6% | 3 | R.NDYVHALVAYFNIEFTR.C | 3 |
|  | AstrinSTLCLD\_041714\_01.05537.05537.2 | 3.7268 | 0.379 | 100.0% | 1726.2322 | 1725.8547 | 1 | 8.132 | 67.9% | 14 | R.TGFSTSPESPYTHWK.Q | 2 |
|  | AstrinSTLCLD\_041714\_01.05639.05639.3 | 2.9364 | 0.3935 | 100.0% | 1726.2843 | 1725.8547 | 1 | 6.363 | 37.5% | 5 | R.TGFSTSPESPYTHWK.Q | 3 |
|  | Astrin\_STLCLD20\_112214\_02.14441.14441.2 | 4.2178 | 0.4677 | 100.0% | 1638.5521 | 1637.8878 | 1 | 8.309 | 75.0% | 4 | K.QTVFYMEDYLTVK.T | 2 |
|  | AstrinSTLCLD\_041714\_01.07348.07348.2 | 4.2977 | 0.3936 | 100.0% | 1721.5322 | 1721.969 | 1 | 7.403 | 60.0% | 21 | K.TGEEIFGTIGMRPNAK.N | 2 |
|  | AstrinSTLCLD\_041714\_01.07328.07328.3 | 3.3474 | 0.3559 | 100.0% | 1722.7743 | 1721.969 | 1 | 6.219 | 40.0% | 19 | K.TGEEIFGTIGMRPNAK.N | 3 |
|  | AstrinSTLCLD\_041714\_02.08664.08664.2 | 2.1576 | 0.2707 | 95.5% | 1726.9922 | 1726.8834 | 40 | 4.812 | 34.6% | 1 | K.NNRDLDFTIDLDFK.G | 2 |
|  | Astrin\_STLCLD20\_112214\_tube2\_01.08688.08688.2 | 3.5922 | 0.3292 | 100.0% | 1590.1921 | 1589.6313 | 1 | 6.455 | 62.5% | 1 | K.GQLCELSCSTDYR.M | 2 |

---

|  |  |  |  |  |  |  |  |  |
| --- | --- | --- | --- | --- | --- | --- | --- | --- |
| U | *GFP* | 15 | 231 | 50.0% | 238 | 26813 | 5.8 | no description |

| Filename XCorr DeltCN Conf% ObsM+H+ CalcM+H+ SpR ZScore Ion% # Sequence  | | | | | | | | | | | | |
| --- | --- | --- | --- | --- | --- | --- | --- | --- | --- | --- | --- | --- |
| \* | Astrin\_STLCHLD\_tube2\_050114\_01.00077.00077.3 | 4.5694 | 0.4756 | 100.0% | 3922.7644 | 3924.2666 | 1 | 8.431 | 21.6% | 1 | K.GEELFTGVVPILVELDGDVNGHKFSVSGEGEGDATYGK.L | 3 |
| \* | Astrin\_STLCHLD\_tube2\_050114\_02.04556.04556.2 | 4.3666 | 0.5894 | 100.0% | 1504.3922 | 1504.5499 | 1 | 9.676 | 71.4% | 46 | K.FSVSGEGEGDATYGK.L | 2 |
| \* | Astrin\_STLCHLD\_050114\_02.04848.04848.3 | 2.775 | 0.2722 | 99.1% | 1593.2943 | 1593.7998 | 1 | 4.311 | 52.3% | 1 | R.YPDHMKQHDFFK.S | 3 |
| \* | Astrin\_STLCHLD\_tube2\_050114\_02.04526.04526.2 | 3.1874 | 0.4521 | 100.0% | 1267.1122 | 1267.399 | 1 | 7.704 | 80.0% | 60 | K.SAMPEGYVQER.T | 2 |
| \* | Astrin\_STLCHLD\_050114\_02.05606.05606.2 | 3.4397 | 0.4891 | 100.0% | 1348.3522 | 1348.4979 | 1 | 7.845 | 85.0% | 20 | R.TIFFKDDGNYK.T | 2 |
| \* | Astrin\_STLCHLD\_tube2\_050114\_02.04785.04785.3 | 2.6865 | 0.2446 | 97.1% | 1606.6444 | 1605.7905 | 1 | 4.649 | 41.7% | 3 | R.TIFFKDDGNYKTR.A | 3 |
| \* | Astrin\_STLCHLD\_050114\_02.05099.05099.3 | 3.0692 | 0.2348 | 98.2% | 1736.4543 | 1735.9376 | 106 | 4.237 | 32.1% | 1 | K.TRAEVKFEGDTLVNR.I | 3 |
| \* | Astrin\_STLCHLD\_050114\_02.05481.05481.2 | 4.2464 | 0.4888 | 100.0% | 1478.3322 | 1478.6451 | 1 | 8.587 | 75.0% | 8 | R.AEVKFEGDTLVNR.I | 2 |
| \* | Astrin\_STLCHLD\_050114\_02.05518.05518.3 | 3.0655 | 0.3424 | 99.9% | 1479.9844 | 1478.6451 | 2 | 5.668 | 41.7% | 9 | R.AEVKFEGDTLVNR.I | 3 |
| \* | Astrin\_STLCHLD\_050114\_02.05006.05006.2 | 3.4221 | 0.4217 | 100.0% | 1051.2322 | 1051.1442 | 1 | 7.609 | 87.5% | 55 | K.FEGDTLVNR.I | 2 |
| \* | Astrin\_STLCHLD\_050114\_01.10953.10953.2 | 4.0302 | 0.3871 | 100.0% | 1543.4521 | 1543.7196 | 2 | 6.937 | 65.4% | 3 | K.GIDFKEDGNILGHK.L | 2 |
| \* | Astrin\_STLCHLD\_tube2\_050114\_02.05504.05504.3 | 4.3541 | 0.3373 | 99.9% | 1544.3644 | 1543.7196 | 12 | 5.868 | 42.3% | 13 | K.GIDFKEDGNILGHK.L | 3 |
| \* | Astrin\_STLCHLD\_tube2\_050114\_02.05732.05732.2 | 4.9532 | 0.502 | 100.0% | 1974.5721 | 1975.1829 | 1 | 9.76 | 70.0% | 2 | K.LEYNYNSHNVYIMADK.Q | 2 |
| \* | Astrin\_STLCHLD\_050114\_02.06298.06298.3 | 3.0052 | 0.2915 | 99.4% | 1974.8944 | 1975.1829 | 32 | 5.373 | 30.0% | 7 | K.LEYNYNSHNVYIMADK.Q | 3 |
| \* | Astrin\_STLCHLD\_050114\_02.05604.05604.3 | 4.4255 | 0.4436 | 100.0% | 2233.3442 | 2231.4875 | 19 | 6.853 | 32.4% | 2 | K.LEYNYNSHNVYIMADKQK.N | 3 |

---

|  |  |  |  |  |  |  |  |  |
| --- | --- | --- | --- | --- | --- | --- | --- | --- |
| U | *gi|4503529|ref|NP\_001* | 17 | 62 | 49.3% | 406 | 46154 | 5.5 | eukaryotic translation initiation factor 4A isoform 1 [Homo sapiens] |

| Filename XCorr DeltCN Conf% ObsM+H+ CalcM+H+ SpR ZScore Ion% # Sequence  | | | | | | | | | | | | |
| --- | --- | --- | --- | --- | --- | --- | --- | --- | --- | --- | --- | --- |
| \* | Astrin\_STLCLD20\_112214\_tube2\_01.20542.20542.3 | 4.9976 | 0.4117 | 100.0% | 4169.994 | 4169.451 | 1 | 6.889 | 22.2% | 7 | R.SRDNGPDGMEPEGVIESNWNEIVDSFDDMNLSESLLR.G | 3 |
|  | Astrin\_STLCHLD\_tube2\_061314\_01.07218.07218.2 | 4.8055 | 0.5196 | 100.0% | 1828.6721 | 1829.0654 | 1 | 8.339 | 70.0% | 8 | R.GIYAYGFEKPSAIQQR.A | 22 |
|  | Astrin\_STLCHLD\_tube2\_061314\_01.07230.07230.3 | 3.6443 | 0.3709 | 99.9% | 1829.9944 | 1829.0654 | 1 | 6.418 | 45.0% | 6 | R.GIYAYGFEKPSAIQQR.A | 33 |
|  | Astrin\_STLCHLD\_tube2\_050114\_01.05626.05626.2 | 4.2106 | 0.5396 | 100.0% | 1395.3121 | 1395.512 | 1 | 9.128 | 76.9% | 4 | K.GYDVIAQAQSGTGK.T | 2 |
| \* | Astrin\_STLCLD20\_112214\_01.09521.09521.2 | 3.7186 | 0.3161 | 100.0% | 1619.5521 | 1619.9225 | 2 | 7.215 | 64.3% | 2 | K.LQMEAPHIIVGTPGR.V | 2 |
| \* | Astrin\_STLCHLD\_tube2\_061314\_01.07853.07853.3 | 3.59 | 0.3972 | 99.9% | 1620.5044 | 1619.9225 | 1 | 6.559 | 48.2% | 6 | K.LQMEAPHIIVGTPGR.V | 3 |
|  | Astrin\_STLCLD20\_112214\_tube2\_01.17031.17031.2 | 5.1714 | 0.5104 | 100.0% | 1557.3922 | 1556.789 | 1 | 9.616 | 79.2% | 5 | K.MFVLDEADEMLSR.G | 2 |
| \* | Astrin\_STLCHLD\_tube2\_050114\_01.10464.10464.2 | 3.7722 | 0.2826 | 100.0% | 1501.8322 | 1502.71 | 1 | 6.134 | 68.2% | 5 | R.GFKDQIYDIFQK.L | 2 |
| \* | Astrin\_STLCHLD\_050114\_01.11173.11173.2 | 2.5097 | 0.247 | 98.6% | 1188.3522 | 1188.3666 | 1 | 4.66 | 83.3% | 1 | K.KEELTLEGIR.Q | 2 |
|  | Astrin\_STLCHLD\_tube2\_050114\_01.06438.06438.2 | 3.0458 | 0.2519 | 99.2% | 1581.9922 | 1582.7432 | 1 | 5.715 | 65.4% | 1 | R.DFTVSAMHGDMDQK.E | 2 |
|  | Astrin\_STLCHLD\_tube2\_061314\_01.05057.05057.3 | 2.4889 | 0.284 | 96.8% | 1867.4343 | 1868.0461 | 1 | 4.89 | 40.0% | 1 | R.DFTVSAMHGDMDQKER.D | 3 |
|  | Astrin\_STLCLD20\_112214\_tube2\_01.13348.13348.1 | 1.6928 | 0.2508 | 95.8% | 1114.59 | 1115.3585 | 7 | 5.286 | 55.6% | 1 | R.VLITTDLLAR.G | 1 |
|  | Astrin\_STLCHLD\_tube2\_050114\_01.09870.09870.2 | 2.8784 | 0.407 | 100.0% | 1115.7722 | 1115.3585 | 1 | 7.634 | 83.3% | 6 | R.VLITTDLLAR.G | 2 |
|  | Astrin\_NLD\_STLC\_tube2\_021014\_02.08946.08946.2 | 2.4874 | 0.235 | 96.6% | 2145.1921 | 2145.4204 | 3 | 4.308 | 36.1% | 1 | R.GIDVQQVSLVINYDLPTNR.E | 2 |
| \* | Astrin\_STLCLD20\_112214\_tube2\_01.07329.07329.3 | 3.7809 | 0.3615 | 99.9% | 1591.5243 | 1590.8352 | 1 | 6.213 | 44.2% | 5 | R.KGVAINMVTEEDKR.T | 3 |
|  | Astrin\_STLCHLD\_tube2\_050114\_01.05526.05526.2 | 2.517 | 0.1776 | 95.2% | 1463.7322 | 1462.6611 | 12 | 4.242 | 62.5% | 1 | K.GVAINMVTEEDKR.T | 2 |
| \* | Astrin\_STLCHLD\_tube2\_061314\_01.15633.15633.2 | 3.9342 | 0.477 | 100.0% | 2799.132 | 2799.1653 | 1 | 8.452 | 47.8% | 2 | R.TLRDIETFYNTSIEEMPLNVADLI.- | 2 |

Similarities:
gi|7661920|ref|NP\_055(2:15)  

---

|  |  |  |  |  |  |  |  |  |
| --- | --- | --- | --- | --- | --- | --- | --- | --- |
| U | *gi|10800140|ref|NP\_06* | 7 | 58 | 48.4% | 126 | 13950 | 10.3 | histone cluster 1, H2bb [Homo sapiens] |
| U | *gi|4504277|ref|NP\_003* | 7 | 58 | 48.4% | 126 | 13920 | 10.3 | histone cluster 2, H2be [Homo sapiens] |
| U | *gi|20336754|ref|NP\_06* | 7 | 58 | 48.4% | 126 | 13904 | 10.3 | histone cluster 1, H2bj [Homo sapiens] |
| U | *gi|16306566|ref|NP\_00* | 7 | 58 | 48.4% | 126 | 13906 | 10.3 | histone cluster 1, H2bo [Homo sapiens] |

| Filename XCorr DeltCN Conf% ObsM+H+ CalcM+H+ SpR ZScore Ion% # Sequence  | | | | | | | | | | | | |
| --- | --- | --- | --- | --- | --- | --- | --- | --- | --- | --- | --- | --- |
|  | Astrin\_STLCLD20\_112214\_tube2\_01.08565.08565.2 | 2.3869 | 0.2779 | 98.7% | 1280.0521 | 1280.4631 | 18 | 5.608 | 61.1% | 1 | R.KESYSIYVYK.V | 2 |
|  | Astrin\_STLCLD20\_112214\_tube2\_01.19358.19358.2 | 5.4029 | 0.5019 | 100.0% | 1744.1721 | 1745.0211 | 1 | 9.93 | 82.1% | 36 | K.AMGIMNSFVNDIFER.I | 22 |
|  | Astrin\_NLD\_STLC\_tube2\_021014\_01.17006.17006.3 | 3.2816 | 0.2993 | 99.6% | 2430.8342 | 2429.7712 | 3 | 5.063 | 32.1% | 1 | K.AMGIMNSFVNDIFERIAGEASR.L | 33 |
|  | Astrin\_NLD\_STLC\_031014\_01.08852.08852.3 | 3.7836 | 0.348 | 99.9% | 1587.0243 | 1586.7941 | 1 | 6.046 | 46.2% | 3 | R.IAGEASRLAHYNKR.S | 33 |
|  | Astrin\_STLCLD20\_112214\_tube2\_01.07574.07574.2 | 3.8004 | 0.4522 | 100.0% | 1462.4321 | 1462.6462 | 1 | 7.074 | 66.7% | 9 | R.STITSREIQTAVR.L | 22 |
|  | Astrin\_STLCHLD\_tube2\_050114\_01.04809.04809.3 | 3.6418 | 0.3964 | 99.9% | 1463.3644 | 1462.6462 | 86 | 6.688 | 37.5% | 6 | R.STITSREIQTAVR.L | 33 |
|  | Astrin\_STLCLD20\_112214\_tube2\_01.12044.12044.2 | 2.5857 | 0.152 | 97.3% | 954.27216 | 954.19794 | 17 | 4.077 | 68.8% | 2 | R.LLLPGELAK.H | 22 |

Similarities:
gi|10800138|ref|NP\_06(6:1)  

---

|  |  |  |  |  |  |  |  |  |
| --- | --- | --- | --- | --- | --- | --- | --- | --- |
| U | *gi|10800138|ref|NP\_06* | 7 | 62 | 48.4% | 126 | 13936 | 10.3 | histone cluster 1, H2bd [Homo sapiens] |
| U | *gi|66912162|ref|NP\_00* | 7 | 62 | 48.4% | 126 | 13920 | 10.3 | histone cluster 2, H2bf [Homo sapiens] |
| U | *gi|4504271|ref|NP\_003* | 7 | 62 | 48.4% | 126 | 13906 | 10.3 | histone cluster 1, H2bi [Homo sapiens] |
| U | *gi|4504269|ref|NP\_003* | 7 | 62 | 48.4% | 126 | 13892 | 10.3 | histone cluster 1, H2bh [Homo sapiens] |
| U | *gi|4504265|ref|NP\_003* | 7 | 62 | 48.4% | 126 | 13906 | 10.3 | histone cluster 1, H2bf [Homo sapiens] |
| U | *gi|4504263|ref|NP\_003* | 7 | 62 | 48.4% | 126 | 13989 | 10.3 | histone cluster 1, H2bm [Homo sapiens] |
| U | *gi|4504261|ref|NP\_003* | 7 | 62 | 48.4% | 126 | 13922 | 10.3 | histone cluster 1, H2bn [Homo sapiens] |
| U | *gi|4504257|ref|NP\_003* | 7 | 62 | 48.4% | 126 | 13906 | 10.3 | histone cluster 1, H2bg [Homo sapiens] |
| U | *gi|21396484|ref|NP\_00* | 7 | 62 | 48.4% | 126 | 13906 | 10.3 | histone cluster 1, H2be [Homo sapiens] |
| U | *gi|21166389|ref|NP\_00* | 7 | 62 | 48.4% | 126 | 13906 | 10.3 | histone cluster 1, H2bc [Homo sapiens] |
| U | *gi|20336752|ref|NP\_61* | 7 | 62 | 48.4% | 126 | 13936 | 10.3 | histone cluster 1, H2bd [Homo sapiens] |
| U | *gi|18105048|ref|NP\_54* | 7 | 62 | 48.4% | 126 | 13890 | 10.3 | histone cluster 1, H2bk [Homo sapiens] |

| Filename XCorr DeltCN Conf% ObsM+H+ CalcM+H+ SpR ZScore Ion% # Sequence  | | | | | | | | | | | | |
| --- | --- | --- | --- | --- | --- | --- | --- | --- | --- | --- | --- | --- |
|  | Astrin\_STLCHLD\_tube2\_050114\_01.05016.05016.2 | 3.2571 | 0.3992 | 100.0% | 1266.4321 | 1266.4363 | 1 | 6.337 | 77.8% | 5 | R.KESYSVYVYK.V | 2 |
|  | Astrin\_STLCLD20\_112214\_tube2\_01.19358.19358.2 | 5.4029 | 0.5019 | 100.0% | 1744.1721 | 1745.0211 | 1 | 9.93 | 82.1% | 36 | K.AMGIMNSFVNDIFER.I | 22 |
|  | Astrin\_NLD\_STLC\_tube2\_021014\_01.17006.17006.3 | 3.2816 | 0.2993 | 99.6% | 2430.8342 | 2429.7712 | 3 | 5.063 | 32.1% | 1 | K.AMGIMNSFVNDIFERIAGEASR.L | 33 |
|  | Astrin\_NLD\_STLC\_031014\_01.08852.08852.3 | 3.7836 | 0.348 | 99.9% | 1587.0243 | 1586.7941 | 1 | 6.046 | 46.2% | 3 | R.IAGEASRLAHYNKR.S | 33 |
|  | Astrin\_STLCLD20\_112214\_tube2\_01.07574.07574.2 | 3.8004 | 0.4522 | 100.0% | 1462.4321 | 1462.6462 | 1 | 7.074 | 66.7% | 9 | R.STITSREIQTAVR.L | 22 |
|  | Astrin\_STLCHLD\_tube2\_050114\_01.04809.04809.3 | 3.6418 | 0.3964 | 99.9% | 1463.3644 | 1462.6462 | 86 | 6.688 | 37.5% | 6 | R.STITSREIQTAVR.L | 33 |
|  | Astrin\_STLCLD20\_112214\_tube2\_01.12044.12044.2 | 2.5857 | 0.152 | 97.3% | 954.27216 | 954.19794 | 17 | 4.077 | 68.8% | 2 | R.LLLPGELAK.H | 22 |

Similarities:
gi|10800140|ref|NP\_06(6:1)  

---

|  |  |  |  |  |  |  |  |  |
| --- | --- | --- | --- | --- | --- | --- | --- | --- |
| U | *gi|20127519|ref|NP\_03* | 52 | 367 | 47.8% | 747 | 85653 | 9.2 | TPX2, microtubule-associated protein homolog [Homo sapiens] |

| Filename XCorr DeltCN Conf% ObsM+H+ CalcM+H+ SpR ZScore Ion% # Sequence  | | | | | | | | | | | | |
| --- | --- | --- | --- | --- | --- | --- | --- | --- | --- | --- | --- | --- |
| \* | AstrinSTLCLD\_041714\_01.06693.06693.3 | 4.8189 | 0.397 | 100.0% | 2405.9644 | 2405.7996 | 1 | 7.15 | 40.0% | 4 | R.KANLQQAIVTPLKPVDNTYYK.E | 3 |
| \* | Astrin\_STLCHLD\_tube2\_061314\_01.09078.09078.3 | 4.0427 | 0.4612 | 100.0% | 2277.4443 | 2277.6255 | 1 | 7.374 | 32.9% | 8 | K.ANLQQAIVTPLKPVDNTYYK.E | 3 |
| \* | Astrin\_STLCHLD\_tube2\_061314\_01.08910.08910.3 | 3.4098 | 0.2828 | 99.7% | 2357.9644 | 2357.6255 | 2 | 5.779 | 28.9% | 1 | K.ANLQQAIVT#PLKPVDNTYYK.E | 3 |
| \* | Astrin\_STLCHLD\_050114\_01.07153.07153.2 | 3.5532 | 0.4055 | 100.0% | 1150.1721 | 1150.3534 | 1 | 7.323 | 81.2% | 11 | K.MQQEVVEMR.K | 2 |
| \* | Astrin\_STLCHLD\_tube2\_050114\_01.07642.07642.2 | 3.3083 | 0.4009 | 100.0% | 1067.2522 | 1067.317 | 1 | 8.242 | 65.0% | 11 | K.LALAGIGQPVK.K | 2 |
| \* | Astrin\_STLCHLD\_050114\_01.10574.10574.2 | 3.3019 | 0.3182 | 100.0% | 1195.4321 | 1195.4911 | 18 | 6.89 | 59.1% | 14 | K.LALAGIGQPVKK.S | 2 |
| \* | Astrin\_STLCLD20\_112214\_01.07591.07591.2 | 2.1992 | 0.3647 | 99.7% | 908.0722 | 908.0043 | 3 | 6.183 | 83.3% | 3 | K.SVDFHFR.T | 2 |
| \* | Astrin\_STLCHLD\_tube2\_050114\_01.07379.07379.2 | 5.1042 | 0.4608 | 100.0% | 1887.4521 | 1887.013 | 1 | 7.895 | 67.9% | 12 | K.NQEEYKEVNFTSELR.K | 2 |
| \* | Astrin\_STLCHLD\_tube2\_061314\_01.07373.07373.3 | 4.6303 | 0.243 | 100.0% | 1889.3644 | 1887.013 | 7 | 5.489 | 41.1% | 30 | K.NQEEYKEVNFTSELR.K | 3 |
| \* | Astrin\_STLCHLD\_050114\_01.11039.11039.3 | 4.1704 | 0.4179 | 100.0% | 2015.5144 | 2015.187 | 1 | 6.159 | 38.3% | 2 | K.NQEEYKEVNFTSELRK.H | 3 |
| \* | Astrin\_STLCHLD\_tube2\_061314\_01.07301.07301.2 | 2.3257 | 0.3335 | 99.3% | 1095.4521 | 1095.1974 | 4 | 5.959 | 68.8% | 2 | K.EVNFTSELR.K | 2 |
| \* | Astrin\_STLCHLD\_tube2\_050114\_01.10518.10518.3 | 3.5177 | 0.3841 | 99.9% | 2611.2844 | 2611.8706 | 1 | 5.681 | 31.0% | 2 | R.TFDETVSTYVPLAQQVEDFHKR.T | 3 |
| \* | Astrin\_STLCLD20\_112214\_tube2\_01.07426.07426.2 | 2.9998 | 0.1647 | 98.3% | 1358.4321 | 1358.5779 | 2 | 5.294 | 72.7% | 1 | R.SKKDDINLLPSK.S | 2 |
| \* | Astrin\_STLCHLD\_050114\_01.06237.06237.3 | 3.6722 | 0.1461 | 98.0% | 1359.8644 | 1358.5779 | 159 | 4.557 | 43.2% | 3 | R.SKKDDINLLPSK.S | 3 |
| \* | Astrin\_STLCHLD\_050114\_01.05060.05060.2 | 2.3717 | 0.188 | 96.0% | 1207.2122 | 1207.2828 | 1 | 5.109 | 77.8% | 1 | R.DPQT#PVLQTK.H | 2 |
| \* | Astrin\_STLCLD20\_112214\_01.07561.07561.2 | 3.8276 | 0.2713 | 100.0% | 1349.5922 | 1349.4344 | 1 | 5.811 | 81.8% | 20 | K.STAELEAEELEK.L | 2 |
| \* | Astrin\_STLCHLD\_tube2\_061314\_01.09596.09596.2 | 5.4151 | 0.337 | 100.0% | 2009.9321 | 2010.2053 | 1 | 7.987 | 65.6% | 3 | K.STAELEAEELEKLQQYK.F | 2 |
| \* | Astrin\_STLCLD20\_112214\_tube2\_01.12225.12225.3 | 3.0024 | 0.2708 | 98.9% | 2010.4143 | 2010.2053 | 11 | 4.979 | 34.4% | 4 | K.STAELEAEELEKLQQYK.F | 3 |
| \* | Astrin\_STLCHLD\_tube2\_050114\_01.07461.07461.2 | 3.4002 | 0.3369 | 100.0% | 1037.3722 | 1037.2877 | 1 | 5.997 | 72.2% | 20 | R.ILEGGPILPK.K | 2 |
| \* | Astrin\_NLD\_STLC\_tube2\_021014\_01.09058.09058.3 | 4.1786 | 0.3902 | 99.9% | 2135.3044 | 2135.5083 | 1 | 6.469 | 37.5% | 13 | K.KPPVKPPTEPIGFDLEIEK.R | 3 |
| \* | Astrin\_STLCHLD\_050114\_01.11684.11684.3 | 5.2407 | 0.5365 | 100.0% | 2291.7544 | 2291.6958 | 1 | 8.875 | 39.5% | 8 | K.KPPVKPPTEPIGFDLEIEKR.I | 3 |
| \* | Astrin\_NLD\_STLC\_031014\_01.08832.08832.3 | 3.4451 | 0.2687 | 99.7% | 2273.7544 | 2274.4639 | 3 | 4.872 | 35.3% | 1 | K.KKTEDEHFEFHSRPCPTK.I | 3 |
| \* | Astrin\_STLCLD20\_112214\_tube2\_01.10466.10466.2 | 3.5848 | 0.3396 | 100.0% | 1198.4122 | 1198.402 | 1 | 7.719 | 80.0% | 14 | K.ILEDVVGVPEK.K | 2 |
| \* | Astrin\_STLCHLD\_tube2\_050114\_01.06140.06140.2 | 3.8866 | 0.3407 | 100.0% | 1326.3322 | 1326.576 | 2 | 6.104 | 72.7% | 14 | K.ILEDVVGVPEKK.V | 2 |
| \* | Astrin\_NLD\_STLC\_tube2\_021014\_01.06698.06698.3 | 3.5301 | 0.3012 | 100.0% | 1327.0144 | 1326.576 | 116 | 5.112 | 40.9% | 7 | K.ILEDVVGVPEKK.V | 3 |
| \* | Astrin\_STLCHLD\_tube2\_050114\_01.08911.08911.3 | 3.1289 | 0.2999 | 99.8% | 1790.6643 | 1790.1564 | 4 | 5.418 | 38.3% | 1 | K.KVLPITVPKS\*PAFALK.N | 3 |
| \* | AstrinSTLCLD\_041714\_01.09332.09332.2 | 3.9978 | 0.4731 | 100.0% | 1661.4122 | 1661.9823 | 1 | 7.913 | 64.3% | 12 | K.VLPITVPKS\*PAFALK.N | 2 |
| \* | Astrin\_STLCHLD\_tube2\_061314\_01.05364.05364.3 | 5.0164 | 0.304 | 99.9% | 2158.6443 | 2158.4285 | 1 | 7.045 | 38.2% | 9 | R.IRMPTKEDEEEDEPVVIK.A | 3 |
| \* | Astrin\_STLCLD20\_112214\_tube2\_01.08044.08044.2 | 5.2133 | 0.3839 | 100.0% | 1889.1122 | 1889.0815 | 1 | 7.553 | 83.3% | 12 | R.MPTKEDEEEDEPVVIK.A | 2 |
| \* | Astrin\_STLCLD20\_112214\_01.05767.05767.3 | 5.5648 | 0.2988 | 100.0% | 1889.5743 | 1889.0815 | 1 | 6.346 | 56.7% | 20 | R.MPTKEDEEEDEPVVIK.A | 3 |
| \* | Astrin\_STLCLD20\_112214\_01.09100.09100.2 | 3.7769 | 0.4376 | 100.0% | 2131.8323 | 2132.473 | 1 | 8.115 | 61.1% | 2 | K.AQPVPHYGVPFKPQIPEAR.T | 2 |
| \* | Astrin\_STLCLD20\_112214\_01.09170.09170.3 | 3.046 | 0.355 | 99.8% | 2132.8145 | 2132.473 | 1 | 5.925 | 31.9% | 9 | K.AQPVPHYGVPFKPQIPEAR.T | 3 |
| \* | Astrin\_STLCLD20\_112214\_tube2\_01.13964.13964.2 | 2.9604 | 0.3961 | 100.0% | 1458.2922 | 1458.5769 | 1 | 6.818 | 77.3% | 3 | R.TVEICPFSFDSR.D | 2 |
| \* | Astrin\_STLCHLD\_tube2\_050114\_01.10361.10361.2 | 3.4232 | 0.3986 | 100.0% | 1705.1721 | 1705.9945 | 1 | 7.85 | 67.9% | 4 | K.ALPLPHFDTINLPEK.K | 2 |
| \* | Astrin\_STLCHLD\_tube2\_050114\_01.09246.09246.2 | 3.2188 | 0.4602 | 100.0% | 1833.5922 | 1834.1686 | 3 | 7.08 | 46.7% | 1 | K.ALPLPHFDTINLPEKK.V | 2 |
| \* | Astrin\_NLD\_STLC\_031014\_01.08010.08010.2 | 2.7091 | 0.2531 | 99.6% | 1053.5521 | 1054.1478 | 2 | 5.117 | 78.6% | 8 | K.HQLEEELR.Q | 2 |
| \* | Astrin\_STLCHLD\_tube2\_061314\_01.06275.06275.2 | 3.4662 | 0.3158 | 100.0% | 1683.7122 | 1683.9481 | 4 | 5.712 | 50.0% | 7 | K.ARPNTVISQEPFVPK.K | 2 |
| \* | Astrin\_STLCHLD\_tube2\_061314\_01.06384.06384.3 | 3.8837 | 0.2979 | 100.0% | 1684.6144 | 1683.9481 | 1 | 5.884 | 48.2% | 7 | K.ARPNTVISQEPFVPK.K | 3 |
| \* | Astrin\_STLCHLD\_tube2\_061314\_01.04688.04688.3 | 4.5754 | 0.4098 | 100.0% | 1812.5944 | 1812.1222 | 1 | 7.541 | 50.0% | 10 | K.ARPNTVISQEPFVPKK.E | 3 |
| \* | Astrin\_STLCLD20\_112214\_tube2\_01.07376.07376.3 | 3.4547 | 0.2565 | 99.4% | 2069.3044 | 2069.4116 | 154 | 5.326 | 30.9% | 1 | K.ARPNTVISQEPFVPKKEK.K | 3 |
| \* | Astrin\_STLCHLD\_050114\_01.12111.12111.3 | 4.7169 | 0.3978 | 100.0% | 2475.4143 | 2475.8044 | 1 | 6.377 | 37.5% | 6 | K.KSVAEGLSGSLVQEPFQLATEKR.A | 3 |
| \* | Astrin\_STLCLD20\_112214\_tube2\_01.15162.15162.2 | 4.59 | 0.5379 | 100.0% | 2190.3123 | 2191.4429 | 1 | 9.331 | 47.5% | 1 | K.SVAEGLSGSLVQEPFQLATEK.R | 2 |
| \* | Astrin\_STLCHLD\_tube2\_050114\_01.10427.10427.2 | 4.9567 | 0.4993 | 100.0% | 2347.0322 | 2347.6304 | 1 | 9.444 | 45.2% | 2 | K.SVAEGLSGSLVQEPFQLATEKR.A | 2 |
| \* | Astrin\_STLCHLD\_tube2\_061314\_01.10829.10829.3 | 4.4333 | 0.414 | 100.0% | 2347.7344 | 2347.6304 | 3 | 7.393 | 32.1% | 8 | K.SVAEGLSGSLVQEPFQLATEKR.A | 3 |
| \* | Astrin\_STLCHLD\_tube2\_050114\_01.10978.10978.3 | 3.9127 | 0.5041 | 100.0% | 2427.3542 | 2427.6304 | 1 | 7.625 | 39.3% | 2 | K.SVAEGLSGS\*LVQEPFQLATEKR.A | 3 |
| \* | Astrin\_STLCHLD\_tube2\_061314\_01.02165.02165.2 | 2.7516 | 0.2427 | 99.6% | 906.21216 | 906.0417 | 26 | 5.246 | 78.6% | 5 | R.MAEVEAQK.A | 2 |
| \* | Astrin\_STLCHLD\_tube2\_050114\_01.05018.05018.2 | 5.2937 | 0.5472 | 100.0% | 1831.5322 | 1832.0386 | 1 | 9.62 | 73.3% | 2 | R.MAEVEAQKAQQLEEAR.L | 2 |
| \* | Astrin\_STLCHLD\_tube2\_050114\_01.05015.05015.3 | 5.1008 | 0.4613 | 100.0% | 1832.2144 | 1832.0386 | 1 | 8.668 | 48.3% | 6 | R.MAEVEAQKAQQLEEAR.L | 3 |
| \* | Astrin\_STLCHLD\_050114\_01.03613.03613.2 | 2.6639 | 0.1714 | 98.6% | 944.7322 | 945.02014 | 4 | 4.723 | 78.6% | 4 | K.AQQLEEAR.L | 2 |
| \* | Astrin\_STLCLD20\_112214\_tube2\_01.05727.05727.2 | 5.1049 | 0.3282 | 100.0% | 1630.2722 | 1630.7954 | 1 | 8.296 | 79.2% | 2 | R.LQEEEQKKEELAR.L | 2 |
| \* | Astrin\_STLCHLD\_050114\_01.04216.04216.3 | 3.2147 | 0.2639 | 99.8% | 1630.9143 | 1630.7954 | 1 | 5.53 | 47.9% | 3 | R.LQEEEQKKEELAR.L | 3 |
| \* | Astrin\_STLCHLD\_tube2\_050114\_01.06617.06617.2 | 3.6328 | 0.4166 | 100.0% | 1436.4122 | 1435.5309 | 1 | 6.327 | 79.2% | 11 | K.SSDQPLTVPVS\*PK.F | 2 |

---

|  |  |  |  |  |  |  |  |  |
| --- | --- | --- | --- | --- | --- | --- | --- | --- |
| U | *gi|17921989|ref|NP\_00* | 26 | 339 | 46.9% | 448 | 49924 | 5.1 | tubulin, alpha 4a [Homo sapiens] |

| Filename XCorr DeltCN Conf% ObsM+H+ CalcM+H+ SpR ZScore Ion% # Sequence  | | | | | | | | | | | | |
| --- | --- | --- | --- | --- | --- | --- | --- | --- | --- | --- | --- | --- |
| \* | Astrin\_STLCHLD\_tube2\_050114\_01.12483.12483.2 | 3.1905 | 0.287 | 99.6% | 1716.6921 | 1716.9719 | 1 | 6.486 | 75.0% | 5 | R.AVFVDLEPTVIDEIR.N | 2 |
|  | Astrin\_STLCLD20\_112214\_01.10293.10293.2 | 1.9386 | 0.3075 | 95.5% | 1411.5521 | 1411.6439 | 367 | 5.745 | 50.0% | 4 | R.QLFHPEQLITGK.E | 222 |
|  | Astrin\_STLCLD20\_112214\_tube2\_01.10802.10802.3 | 3.9221 | 0.4709 | 100.0% | 2416.4644 | 2416.6555 | 2 | 7.043 | 30.0% | 13 | R.QLFHPEQLITGKEDAANNYAR.G | 333 |
|  | Astrin\_STLCLD20\_112214\_tube2\_01.10767.10767.2 | 2.6947 | 0.3834 | 99.8% | 2416.5723 | 2416.6555 | 6 | 5.895 | 37.5% | 2 | R.QLFHPEQLITGKEDAANNYAR.G | 222 |
|  | Astrin\_STLCHLD\_tube2\_061314\_01.04847.04847.3 | 3.6369 | 0.3589 | 99.9% | 1876.6144 | 1876.0824 | 1 | 5.792 | 48.2% | 6 | R.RNLDIERPTYTNLNR.L | 333 |
|  | Astrin\_STLCHLD\_050114\_01.11147.11147.2 | 3.4174 | 0.0864 | 97.3% | 1719.6322 | 1719.8949 | 1 | 5.134 | 61.5% | 7 | R.NLDIERPTYTNLNR.L | 222 |
|  | Astrin\_STLCHLD\_tube2\_050114\_01.06465.06465.3 | 2.9056 | 0.311 | 99.8% | 1721.3644 | 1719.8949 | 2 | 5.652 | 46.2% | 9 | R.NLDIERPTYTNLNR.L | 333 |
|  | Astrin\_STLCHLD\_050114\_01.13856.13856.1 | 3.7074 | 0.3194 | 100.0% | 1488.91 | 1488.7678 | 3 | 6.528 | 50.0% | 9 | R.LISQIVSSITASLR.F | 111 |
|  | Astrin\_STLCHLD\_tube2\_050114\_02.10976.10976.2 | 4.8703 | 0.4751 | 100.0% | 1490.2522 | 1488.7678 | 1 | 9.358 | 73.1% | 71 | R.LISQIVSSITASLR.F | 222 |
|  | Astrin\_STLCHLD\_tube2\_050114\_01.13991.13991.3 | 4.7196 | 0.3289 | 99.9% | 1490.3344 | 1488.7678 | 2 | 6.09 | 50.0% | 15 | R.LISQIVSSITASLR.F | 333 |
|  | Astrin\_STLCHLD\_tube2\_061314\_02.09638.09638.2 | 5.8424 | 0.5235 | 100.0% | 2410.7122 | 2410.6885 | 1 | 10.298 | 50.0% | 50 | R.FDGALNVDLTEFQTNLVPYPR.I | 222 |
|  | Astrin\_STLCLD20\_112214\_tube2\_02.10416.10416.3 | 3.7941 | 0.4445 | 99.9% | 2410.8245 | 2410.6885 | 1 | 7.732 | 36.2% | 7 | R.FDGALNVDLTEFQTNLVPYPR.I | 333 |
|  | Astrin\_STLCHLD\_tube2\_050114\_01.10237.10237.2 | 4.4468 | 0.5586 | 100.0% | 1757.4722 | 1758.0703 | 1 | 9.549 | 76.7% | 35 | R.IHFPLATYAPVISAEK.A | 222 |
|  | Astrin\_STLCHLD\_050114\_01.12271.12271.3 | 4.3329 | 0.4459 | 100.0% | 1757.9644 | 1758.0703 | 1 | 7.63 | 51.7% | 19 | R.IHFPLATYAPVISAEK.A | 333 |
|  | Astrin\_STLCHLD\_050114\_02.08102.08102.3 | 4.2224 | 0.3505 | 99.9% | 2751.6543 | 2752.0369 | 5 | 6.013 | 27.2% | 1 | K.AYHEQLSVAEITNACFEPANQMVK.C | 33 |
|  | Astrin\_STLCHLD\_050114\_01.12021.12021.2 | 2.5571 | 0.4159 | 100.0% | 1250.3722 | 1250.4304 | 1 | 6.932 | 75.0% | 3 | K.YMACCLLYR.G | 222 |
|  | Astrin\_STLCHLD\_tube2\_061314\_01.08843.08843.2 | 4.4981 | 0.4675 | 100.0% | 1825.6721 | 1826.1027 | 1 | 7.437 | 67.6% | 25 | K.VGINYQPPTVVPGGDLAK.V | 222 |
|  | Astrin\_STLCHLD\_050114\_02.09635.09635.2 | 4.2429 | 0.3899 | 100.0% | 1865.7122 | 1866.1084 | 1 | 8.618 | 56.2% | 2 | R.AVCMLSNTTAIAEAWAR.L | 22 |
|  | Astrin\_STLCLD20\_112214\_01.08110.08110.2 | 3.5435 | 0.367 | 100.0% | 1382.0521 | 1381.6324 | 1 | 6.667 | 65.0% | 2 | R.LDHKFDLMYAK.R | 222 |
|  | Astrin\_STLCHLD\_tube2\_061314\_01.06060.06060.3 | 4.3726 | 0.4192 | 100.0% | 1382.2144 | 1381.6324 | 1 | 6.674 | 60.0% | 13 | R.LDHKFDLMYAK.R | 333 |
|  | Astrin\_STLCHLD\_050114\_01.09772.09772.3 | 2.6871 | 0.2432 | 97.3% | 1538.2444 | 1537.82 | 69 | 4.407 | 40.9% | 1 | R.LDHKFDLMYAKR.A | 333 |
|  | Astrin\_STLCHLD\_tube2\_061314\_01.08135.08135.2 | 2.4286 | 0.2419 | 99.2% | 887.8722 | 888.0692 | 1 | 4.937 | 91.7% | 1 | K.FDLMYAK.R | 222 |
|  | Astrin\_STLCHLD\_050114\_02.07700.07700.3 | 6.6327 | 0.4886 | 100.0% | 2487.6843 | 2487.7083 | 1 | 9.145 | 48.8% | 10 | K.RAFVHWYVGEGMEEGEFSEAR.E | 333 |
|  | Astrin\_STLCHLD\_050114\_02.08531.08531.2 | 5.3032 | 0.4677 | 100.0% | 2331.412 | 2331.5208 | 1 | 8.46 | 65.8% | 2 | R.AFVHWYVGEGMEEGEFSEAR.E | 222 |
|  | Astrin\_STLCHLD\_061214\_02.07921.07921.3 | 5.6027 | 0.4369 | 100.0% | 2332.3743 | 2331.5208 | 1 | 7.994 | 44.7% | 24 | R.AFVHWYVGEGMEEGEFSEAR.E | 333 |
|  | Astrin\_STLCHLD\_tube2\_050114\_01.11503.11503.3 | 4.258 | 0.3976 | 99.9% | 3219.3843 | 3219.524 | 1 | 7.266 | 31.5% | 3 | R.AFVHWYVGEGMEEGEFSEAREDMAALEK.D | 333 |

Similarities:
gi|57013276|ref|NP\_00(25:1)  
gi|14389309|ref|NP\_11(23:3)  

---

|  |  |  |  |  |  |  |  |  |
| --- | --- | --- | --- | --- | --- | --- | --- | --- |
| U | *gi|4505813|ref|NP\_003* | 5 | 15 | 46.1% | 89 | 10366 | 7.4 | dynein light chain 1 [Homo sapiens] |
| U | *gi|83267868|ref|NP\_00* | 5 | 15 | 46.1% | 89 | 10366 | 7.4 | dynein light chain 1 [Homo sapiens] |
| U | *gi|83267866|ref|NP\_00* | 5 | 15 | 46.1% | 89 | 10366 | 7.4 | dynein light chain 1 [Homo sapiens] |

| Filename XCorr DeltCN Conf% ObsM+H+ CalcM+H+ SpR ZScore Ion% # Sequence  | | | | | | | | | | | | |
| --- | --- | --- | --- | --- | --- | --- | --- | --- | --- | --- | --- | --- |
|  | Astrin\_STLCHLD\_tube2\_050114\_01.07279.07279.2 | 3.1321 | 0.3495 | 100.0% | 1415.8922 | 1415.6322 | 1 | 6.407 | 68.2% | 1 | K.YNIEKDIAAHIK.K | 2 |
|  | Astrin\_STLCHLD\_tube2\_061314\_01.07322.07322.3 | 3.1616 | 0.3401 | 100.0% | 1417.3143 | 1415.6322 | 10 | 5.803 | 45.5% | 3 | K.YNIEKDIAAHIK.K | 3 |
|  | Astrin\_STLCHLD\_050114\_01.06503.06503.2 | 3.232 | 0.3623 | 100.0% | 1283.2922 | 1283.383 | 8 | 6.356 | 60.0% | 4 | R.NFGSYVTHETK.H | 2 |
|  | Astrin\_STLCHLD\_050114\_01.16517.16517.3 | 3.9645 | 0.2972 | 99.8% | 3237.4443 | 3237.771 | 1 | 5.752 | 28.8% | 4 | R.NFGSYVTHETKHFIYFYLGQVAILLFK.S | 3 |
|  | Astrin\_STLCLD20\_112214\_tube2\_01.20860.20860.3 | 4.2952 | 0.1552 | 97.1% | 3381.0544 | 3381.9011 | 1 | 5.102 | 25.9% | 3 | R.NFGSYVTHETKHFIYFYLGQVAILLFKSG.- | 3 |

---

|  |  |  |  |  |  |  |  |  |
| --- | --- | --- | --- | --- | --- | --- | --- | --- |
| U | *gi|14043072|ref|NP\_11* | 16 | 82 | 45.9% | 353 | 37430 | 8.9 | heterogeneous nuclear ribonucleoprotein A2/B1 isoform B1 [Homo sapiens] |
| U | *gi|4504447|ref|NP\_002* | 16 | 81 | 47.5% | 341 | 36006 | 8.6 | heterogeneous nuclear ribonucleoprotein A2/B1 isoform A2 [Homo sapiens] |

| Filename XCorr DeltCN Conf% ObsM+H+ CalcM+H+ SpR ZScore Ion% # Sequence  | | | | | | | | | | | | |
| --- | --- | --- | --- | --- | --- | --- | --- | --- | --- | --- | --- | --- |
|  | Astrin\_STLCHLD\_061214\_01.09447.09447.3 | 2.6923 | 0.2783 | 97.7% | 1927.3444 | 1928.1925 | 65 | 4.503 | 29.7% | 1 | R.KLFIGGLSFETTEESLR.N | 3 |
|  | Astrin\_STLCHLD\_tube2\_050114\_01.10899.10899.2 | 3.9211 | 0.2938 | 100.0% | 1928.0322 | 1928.1925 | 1 | 5.951 | 59.4% | 7 | R.KLFIGGLSFETTEESLR.N | 2 |
|  | Astrin\_STLCHLD\_061214\_01.10752.10752.2 | 4.6941 | 0.4342 | 100.0% | 1800.5922 | 1800.0184 | 1 | 7.449 | 70.0% | 19 | K.LFIGGLSFETTEESLR.N | 2 |
|  | AstrinSTLCLD\_041714\_01.12291.12291.3 | 3.7523 | 0.2937 | 99.8% | 2868.2944 | 2869.1597 | 1 | 4.667 | 29.3% | 1 | K.LFIGGLSFETTEESLRNYYEQWGK.L | 3 |
|  | Astrin\_STLCHLD\_050114\_01.10760.10760.2 | 2.4711 | 0.3508 | 99.9% | 1088.0721 | 1088.1644 | 1 | 5.901 | 85.7% | 2 | R.NYYEQWGK.L | 2 |
|  | AstrinSTLCLD\_041714\_01.04703.04703.3 | 2.966 | 0.3477 | 99.8% | 1881.9543 | 1881.0984 | 7 | 5.717 | 35.0% | 1 | K.LFVGGIKEDTEEHHLR.D | 3 |
|  | Astrin\_STLCLD20\_112214\_tube2\_01.12449.12449.2 | 3.3941 | 0.4952 | 100.0% | 1189.3322 | 1189.3513 | 4 | 7.966 | 77.8% | 5 | K.IDTIEIITDR.Q | 2 |
|  | Astrin\_STLCLD20\_112214\_01.11173.11173.2 | 3.6407 | 0.4217 | 100.0% | 1696.4321 | 1696.8132 | 1 | 8.234 | 64.3% | 3 | R.GFGFVTFDDHDPVDK.I | 2 |
|  | Astrin\_STLCHLD\_tube2\_050114\_01.10969.10969.3 | 4.3152 | 0.4963 | 100.0% | 2278.1343 | 2278.5693 | 1 | 7.586 | 39.5% | 8 | R.GFGFVTFDDHDPVDKIVLQK.Y | 3 |
|  | Astrin\_STLCLD20\_112214\_tube2\_01.05752.05752.2 | 3.4351 | 0.3521 | 100.0% | 1411.4922 | 1411.5198 | 1 | 7.011 | 81.8% | 1 | K.YHTINGHNAEVR.K | 2 |
|  | Astrin\_STLCLD20\_112214\_tube2\_01.05622.05622.2 | 2.7917 | 0.1615 | 96.7% | 1540.4122 | 1539.6938 | 1 | 4.982 | 66.7% | 1 | K.YHTINGHNAEVRK.A | 2 |
|  | Astrin\_STLCLD20\_112214\_tube2\_01.08646.08646.2 | 2.4727 | 0.1657 | 96.0% | 1014.1922 | 1014.0421 | 2 | 4.934 | 72.2% | 1 | R.GGNFGFGDSR.G | 2 |
|  | Astrin\_STLCLD20\_112214\_tube2\_01.08471.08471.2 | 3.1269 | 0.4721 | 100.0% | 1378.0922 | 1378.4465 | 1 | 6.934 | 53.6% | 18 | R.GGGGNFGPGPGSNFR.G | 2 |
|  | AstrinSTLCLD\_041714\_01.06758.06758.3 | 3.2202 | 0.2169 | 95.3% | 2496.8643 | 2496.5303 | 339 | 4.776 | 23.1% | 1 | R.GFGDGYNGYGGGPGGGNFGGSPGYGGGR.G | 3 |
|  | Astrin\_STLCHLD\_tube2\_061314\_01.04985.04985.2 | 6.2516 | 0.6399 | 100.0% | 2190.5122 | 2191.2554 | 1 | 12.21 | 56.2% | 10 | R.NMGGPYGGGNYGPGGSGGSGGYGGR.S | 2 |
|  | Astrin\_STLCLD20\_112214\_01.06530.06530.3 | 4.9634 | 0.5095 | 100.0% | 2190.7144 | 2191.2554 | 1 | 8.872 | 36.5% | 3 | R.NMGGPYGGGNYGPGGSGGSGGYGGR.S | 3 |

---

|  |  |  |  |  |  |  |  |  |
| --- | --- | --- | --- | --- | --- | --- | --- | --- |
| U | *gi|218505827|ref|NP\_1* | 15 | 80 | 45.9% | 316 | 35438 | 6.3 | TRAF4 associated factor 1 isoform a [Homo sapiens] |

| Filename XCorr DeltCN Conf% ObsM+H+ CalcM+H+ SpR ZScore Ion% # Sequence  | | | | | | | | | | | | |
| --- | --- | --- | --- | --- | --- | --- | --- | --- | --- | --- | --- | --- |
|  | Astrin\_STLCLD20\_112214\_tube2\_01.10773.10773.2 | 6.1531 | 0.5839 | 100.0% | 2274.7522 | 2275.4802 | 1 | 9.807 | 61.9% | 14 | K.TVYSLQPPSALSGGQPADTQTR.A | 2 |
|  | Astrin\_STLCHLD\_tube2\_061314\_01.08022.08022.3 | 5.1071 | 0.432 | 100.0% | 2275.3442 | 2275.4802 | 1 | 8.103 | 40.5% | 4 | K.TVYSLQPPSALSGGQPADTQTR.A | 3 |
|  | Astrin\_STLCLD20\_112214\_01.09561.09561.3 | 3.4029 | 0.2939 | 99.5% | 3411.6243 | 3408.7502 | 26 | 4.306 | 21.0% | 2 | K.TVYSLQPPSALSGGQPADTQTRATSKS\*LLPVR.S | 3 |
|  | Astrin\_NLD\_STLC\_tube2\_021014\_01.03730.03730.2 | 2.166 | 0.4906 | 99.9% | 1382.1122 | 1382.4728 | 4 | 7.844 | 58.3% | 1 | K.QLHSGGPENDVTK.I | 2 |
|  | Astrin\_STLCHLD\_tube2\_050114\_01.05848.05848.3 | 4.9753 | 0.4362 | 100.0% | 1989.5044 | 1988.2023 | 1 | 6.989 | 45.3% | 8 | K.SEEELKDKNQLLEAVNK.Q | 3 |
|  | Astrin\_STLCHLD\_tube2\_061314\_01.04971.04971.2 | 4.0646 | 0.4176 | 100.0% | 1604.5322 | 1604.7979 | 2 | 6.683 | 65.4% | 3 | K.LTETQGELKDLTQK.V | 2 |
|  | Astrin\_STLCLD20\_112214\_tube2\_01.13703.13703.2 | 5.4086 | 0.4003 | 100.0% | 2316.912 | 2316.6543 | 1 | 7.418 | 60.5% | 1 | K.LTETQGELKDLTQKVELLEK.F | 2 |
|  | AstrinSTLCLD\_041714\_01.09513.09513.3 | 5.0575 | 0.3191 | 99.9% | 2317.9744 | 2316.6543 | 1 | 6.15 | 40.8% | 7 | K.LTETQGELKDLTQKVELLEK.F | 3 |
|  | Astrin\_STLCHLD\_tube2\_061314\_01.07682.07682.2 | 3.4354 | 0.2252 | 99.9% | 1316.3121 | 1316.5376 | 1 | 5.453 | 75.0% | 2 | K.DLTQKVELLEK.F | 2 |
|  | AstrinSTLCLD\_041714\_01.07132.07132.1 | 2.5017 | 0.2369 | 97.4% | 1163.18 | 1163.2793 | 19 | 4.95 | 55.6% | 1 | R.DNCLAILESK.G | 1 |
|  | AstrinSTLCLD\_041714\_01.07162.07162.2 | 2.6584 | 0.1916 | 98.2% | 1163.5122 | 1163.2793 | 1 | 5.099 | 72.2% | 1 | R.DNCLAILESK.G | 2 |
|  | Astrin\_STLCLD20\_112214\_01.08995.08995.2 | 4.3186 | 0.5406 | 100.0% | 1387.2722 | 1387.5327 | 1 | 9.581 | 65.4% | 25 | K.GLDPALGSETLASR.Q | 2 |
|  | Astrin\_STLCHLD\_050114\_01.13533.13533.2 | 3.2031 | 0.1119 | 96.5% | 2593.7922 | 2592.8928 | 2 | 3.906 | 33.3% | 1 | R.QESTTDHMDSMLLLETLQEELK.L | 2 |
|  | AstrinSTLCLD\_041714\_01.13595.13595.3 | 5.694 | 0.3451 | 100.0% | 3396.1143 | 3396.8062 | 20 | 6.508 | 21.4% | 9 | R.QESTTDHMDSMLLLETLQEELKLFNETAK.K | 3 |
| \* | Astrin\_STLCHLD\_050114\_01.07264.07264.2 | 3.0147 | 0.2102 | 99.3% | 1217.7522 | 1218.454 | 35 | 4.894 | 66.7% | 1 | K.KQMEELQALK.V | 2 |

---

|  |  |  |  |  |  |  |  |  |
| --- | --- | --- | --- | --- | --- | --- | --- | --- |
| U | *gi|183076548|ref|NP\_0* | 5 | 16 | 45.6% | 136 | 15388 | 11.3 | histone cluster 2, H3d [Homo sapiens] |
| U | *gi|53793688|ref|NP\_00* | 5 | 16 | 45.6% | 136 | 15388 | 11.3 | histone cluster 2, H3a [Homo sapiens] |
| U | *gi|31742503|ref|NP\_06* | 5 | 16 | 45.6% | 136 | 15388 | 11.3 | histone cluster 2, H3c [Homo sapiens] |

| Filename XCorr DeltCN Conf% ObsM+H+ CalcM+H+ SpR ZScore Ion% # Sequence  | | | | | | | | | | | | |
| --- | --- | --- | --- | --- | --- | --- | --- | --- | --- | --- | --- | --- |
|  | Astrin\_STLCLD20\_112214\_tube2\_01.06626.06626.2 | 2.3346 | 0.2004 | 96.9% | 1032.9321 | 1033.2186 | 18 | 4.911 | 68.8% | 4 | R.YRPGTVALR.E | 2 |
|  | Astrin\_STLCLD20\_112214\_01.05207.05207.2 | 2.2973 | 0.1987 | 95.7% | 1252.0521 | 1251.4685 | 1 | 5.567 | 88.9% | 1 | R.YQKSTELLIR.K | 2 |
|  | Astrin\_STLCLD20\_112214\_tube2\_01.08714.08714.2 | 3.0084 | 0.3456 | 100.0% | 1336.4922 | 1336.4875 | 1 | 5.672 | 80.0% | 9 | R.EIAQDFKTDLR.F | 2 |
|  | Astrin\_STLCHLD\_tube2\_050114\_01.16857.16857.3 | 3.4445 | 0.3219 | 99.8% | 3516.1143 | 3515.9258 | 2 | 4.604 | 21.0% | 1 | R.FQSSAVMALQEASEAYLVGLFEDTNLCAIHAK.R | 3 |
|  | Astrin\_NLD\_STLC\_tube2\_021014\_02.14273.14273.3 | 3.5615 | 0.2719 | 99.4% | 3674.0645 | 3675.9258 | 1 | 3.879 | 20.2% | 1 | R.FQSSAVMALQEAS\*EAYLVGLFEDT#NLCAIHAK.R | 3 |

---

|  |  |  |  |  |  |  |  |  |
| --- | --- | --- | --- | --- | --- | --- | --- | --- |
| U | *gi|5902102|ref|NP\_008* | 3 | 18 | 45.4% | 119 | 13282 | 11.6 | small nuclear ribonucleoprotein D1 polypeptide 16kDa [Homo sapiens] |

| Filename XCorr DeltCN Conf% ObsM+H+ CalcM+H+ SpR ZScore Ion% # Sequence  | | | | | | | | | | | | |
| --- | --- | --- | --- | --- | --- | --- | --- | --- | --- | --- | --- | --- |
| \* | Astrin\_STLCHLD\_050114\_02.05975.05975.3 | 4.6454 | 0.3889 | 100.0% | 2211.0244 | 2210.47 | 1 | 7.581 | 38.8% | 2 | K.NGTQVHGTITGVDVSMNTHLK.A | 3 |
|  | Astrin\_STLCLD20\_112214\_01.09279.09279.2 | 3.6488 | 0.3364 | 100.0% | 1556.3922 | 1555.7745 | 10 | 6.072 | 58.3% | 2 | K.NREPVQLETLSIR.G | 2 |
| \* | Astrin\_STLCLD20\_112214\_tube2\_01.20769.20769.2 | 4.7631 | 0.5653 | 100.0% | 2287.652 | 2288.6863 | 1 | 8.697 | 63.2% | 14 | R.YFILPDSLPLDTLLVDVEPK.V | 2 |

---

|  |  |  |  |  |  |  |  |  |
| --- | --- | --- | --- | --- | --- | --- | --- | --- |
| U | *gi|118582269|ref|NP\_0* | 9 | 44 | 45.3% | 201 | 22460 | 8.0 | splicing factor, arginine/serine-rich 1 isoform 2 [Homo sapiens] |
| U | *gi|5902076|ref|NP\_008* | 9 | 44 | 36.7% | 248 | 27745 | 10.4 | splicing factor, arginine/serine-rich 1 isoform 1 [Homo sapiens] |

| Filename XCorr DeltCN Conf% ObsM+H+ CalcM+H+ SpR ZScore Ion% # Sequence  | | | | | | | | | | | | |
| --- | --- | --- | --- | --- | --- | --- | --- | --- | --- | --- | --- | --- |
|  | Astrin\_STLCHLD\_tube2\_061314\_01.08237.08237.2 | 3.1598 | 0.4102 | 100.0% | 1257.5122 | 1257.4752 | 1 | 6.732 | 80.0% | 20 | R.IYVGNLPPDIR.T | 2 |
|  | Astrin\_STLCLD20\_112214\_01.08852.08852.2 | 3.4968 | 0.3227 | 100.0% | 1259.3121 | 1258.4137 | 1 | 5.93 | 83.3% | 10 | R.TKDIEDVFYK.Y | 2 |
|  | Astrin\_STLCLD20\_112214\_tube2\_01.16248.16248.2 | 3.1318 | 0.1409 | 97.3% | 2541.7522 | 2542.7234 | 1 | 4.28 | 38.6% | 2 | R.GGPPFAFVEFEDPRDAEDAVYGR.D | 2 |
|  | AstrinSTLCLD\_041714\_01.05206.05206.3 | 2.9665 | 0.3111 | 99.5% | 2101.7344 | 2101.1064 | 223 | 5.237 | 27.9% | 1 | R.DAEDAVYGRDGYDYDGYR.L | 3 |
|  | Astrin\_STLCHLD\_050114\_01.08651.08651.2 | 2.5253 | 0.3205 | 99.6% | 1123.9521 | 1124.1083 | 1 | 5.309 | 75.0% | 2 | R.DGYDYDGYR.L | 2 |
|  | Astrin\_STLCLD20\_112214\_01.07489.07489.2 | 2.3638 | 0.1102 | 95.3% | 917.47217 | 917.0989 | 2 | 4.174 | 83.3% | 2 | R.LRVEFPR.S | 22 |
|  | Astrin\_STLCHLD\_tube2\_050114\_01.06284.06284.2 | 2.5582 | 0.2258 | 97.9% | 1418.7922 | 1418.4688 | 1 | 4.303 | 68.2% | 2 | R.EAGDVCYADVYR.D | 2 |
|  | Astrin\_STLCHLD\_tube2\_061314\_01.07433.07433.2 | 2.5246 | 0.2326 | 98.4% | 1079.1322 | 1079.198 | 1 | 5.09 | 83.3% | 4 | R.DGTGVVEFVR.K | 2 |
|  | Astrin\_STLCHLD\_tube2\_050114\_02.07461.07461.3 | 3.5217 | 0.3421 | 100.0% | 2173.1943 | 2173.4485 | 1 | 5.329 | 36.1% | 1 | R.DGTGVVEFVRKEDMTYAVR.K | 3 |

Similarities:
gi|4506903|ref|NP\_003(1:8)  

---

|  |  |  |  |  |  |  |  |  |
| --- | --- | --- | --- | --- | --- | --- | --- | --- |
| U | *TEV-Speptide* | 5 | 125 | 45.1% | 51 | 5423 | 9.4 | no description |

| Filename XCorr DeltCN Conf% ObsM+H+ CalcM+H+ SpR ZScore Ion% # Sequence  | | | | | | | | | | | | |
| --- | --- | --- | --- | --- | --- | --- | --- | --- | --- | --- | --- | --- |
| \* | Astrin\_STLCHLD\_tube2\_050114\_02.00065.00065.2 | 4.3888 | 0.3797 | 100.0% | 1384.8121 | 1384.5345 | 1 | 7.387 | 68.2% | 59 | R.SRENLYFQGAAK.F | 2 |
| \* | Astrin\_STLCHLD\_050114\_01.05803.05803.3 | 3.9215 | 0.3863 | 100.0% | 1385.1244 | 1384.5345 | 1 | 6.95 | 52.3% | 54 | R.SRENLYFQGAAK.F | 3 |
| \* | Astrin\_STLCHLD\_050114\_02.05378.05378.2 | 3.0698 | 0.261 | 99.8% | 1140.6522 | 1141.2688 | 3 | 5.398 | 72.2% | 10 | R.ENLYFQGAAK.F | 2 |
| \* | Astrin\_STLCHLD\_050114\_02.04496.04496.2 | 2.85 | 0.3403 | 100.0% | 1299.1721 | 1298.4844 | 1 | 6.368 | 70.0% | 1 | K.FKETAAAKFER.Q | 2 |
| \* | Astrin\_STLCHLD\_050114\_01.04651.04651.3 | 3.5284 | 0.2032 | 99.5% | 1299.6244 | 1298.4844 | 1 | 5.632 | 55.0% | 1 | K.FKETAAAKFER.Q | 3 |

---

|  |  |  |  |  |  |  |  |  |
| --- | --- | --- | --- | --- | --- | --- | --- | --- |
| U | *gi|67782365|ref|NP\_00* | 20 | 82 | 44.3% | 469 | 51386 | 5.5 | keratin 7 [Homo sapiens] |

| Filename XCorr DeltCN Conf% ObsM+H+ CalcM+H+ SpR ZScore Ion% # Sequence  | | | | | | | | | | | | |
| --- | --- | --- | --- | --- | --- | --- | --- | --- | --- | --- | --- | --- |
|  | Astrin\_STLCLD20\_112214\_01.08062.08062.3 | 4.4152 | 0.4135 | 100.0% | 2249.2144 | 2247.519 | 4 | 7.024 | 30.7% | 6 | R.LSSARPGGLGSSSLYGLGASRPR.V | 3 |
|  | Astrin\_STLCHLD\_tube2\_050114\_01.04937.04937.2 | 3.4126 | 0.4558 | 100.0% | 1106.3121 | 1105.2388 | 1 | 6.855 | 86.4% | 11 | R.SAYGGPVGAGIR.E | 2 |
|  | Astrin\_STLCLD20\_112214\_tube2\_01.15480.15480.3 | 3.0959 | 0.2473 | 97.3% | 2451.3843 | 2450.7979 | 3 | 4.596 | 28.6% | 2 | R.EVTINQSLLAPLRLDADPSLQR.V | 3 |
|  | Astrin\_STLCHLD\_tube2\_061314\_01.06264.06264.2 | 2.4085 | 0.2136 | 98.8% | 828.2922 | 827.95544 | 5 | 5.059 | 91.7% | 3 | K.FASFIDK.V | 22222222 |
|  | Astrin\_NLD\_STLC\_tube2\_021014\_01.07623.07623.2 | 2.9741 | 0.1656 | 99.1% | 1082.8121 | 1083.2755 | 7 | 7.028 | 75.0% | 6 | K.FASFIDKVR.F | 2222222 |
|  | Astrin\_STLCHLD\_tube2\_050114\_01.12328.12328.2 | 4.4723 | 0.4584 | 100.0% | 1443.2522 | 1443.686 | 2 | 8.868 | 62.5% | 7 | R.LPDIFEAQIAGLR.G | 2 |
|  | Astrin\_STLCHLD\_tube2\_061314\_01.05339.05339.2 | 3.5899 | 0.3893 | 100.0% | 1243.0521 | 1243.3622 | 1 | 7.451 | 77.3% | 2 | R.GQLEALQVDGGR.L | 2 |
| \* | Astrin\_STLCLD20\_112214\_tube2\_01.13338.13338.2 | 3.2423 | 0.1977 | 98.9% | 1954.5721 | 1955.1783 | 2 | 5.197 | 41.2% | 2 | R.GQLEALQVDGGRLEAELR.S | 2 |
| \* | AstrinSTLCLD\_041714\_02.07908.07908.3 | 4.0864 | 0.4329 | 100.0% | 1954.7344 | 1955.1783 | 1 | 7.724 | 45.6% | 10 | R.GQLEALQVDGGRLEAELR.S | 3 |
|  | Astrin\_STLCHLD\_tube2\_061314\_01.11269.11269.2 | 3.9974 | 0.1518 | 99.8% | 1419.4922 | 1419.5773 | 1 | 7.823 | 72.7% | 7 | K.VDALNDEINFLR.T | 2 |
|  | Astrin\_STLCLD20\_112214\_tube2\_01.16433.16433.2 | 3.3848 | 0.4027 | 100.0% | 1273.4722 | 1273.4692 | 1 | 8.166 | 72.7% | 1 | R.SLDLDGIIAEVK.A | 2 |
|  | Astrin\_STLCLD20\_112214\_01.06345.06345.2 | 2.3708 | 0.1992 | 96.4% | 1196.4122 | 1197.2897 | 6 | 5.586 | 66.7% | 1 | R.AEAEAWYQTK.F | 22 |
|  | Astrin\_STLCHLD\_061214\_02.07203.07203.3 | 4.2369 | 0.3193 | 99.9% | 2272.2544 | 2271.4912 | 1 | 6.402 | 34.2% | 2 | R.AEAEAWYQTKFETLQAQAGK.H | 3 |
|  | Astrin\_STLCHLD\_050114\_01.05710.05710.2 | 2.015 | 0.2999 | 97.0% | 1092.9122 | 1093.2249 | 1 | 5.15 | 72.2% | 1 | K.FETLQAQAGK.H | 2 |
|  | Astrin\_STLCHLD\_tube2\_050114\_01.04607.04607.3 | 2.3556 | 0.2927 | 97.5% | 1442.6943 | 1442.6151 | 4 | 5.166 | 40.9% | 1 | R.LQAEIDNIKNQR.A | 3 |
| \* | Astrin\_NLD\_STLC\_tube2\_021014\_02.07823.07823.3 | 3.8712 | 0.298 | 99.8% | 2014.0144 | 2013.2987 | 1 | 5.268 | 43.1% | 2 | R.AKLEAAIAEAEERGELALK.D | 3 |
|  | Astrin\_STLCLD20\_112214\_01.05824.05824.2 | 4.1189 | 0.3595 | 100.0% | 1386.2522 | 1386.548 | 1 | 7.488 | 72.7% | 7 | R.AKQEELEAALQR.G | 2 |
|  | Astrin\_STLCHLD\_061214\_01.04774.04774.3 | 3.7922 | 0.1496 | 98.6% | 1386.9243 | 1386.548 | 15 | 4.826 | 45.5% | 1 | R.AKQEELEAALQR.G | 3 |
|  | Astrin\_STLCLD20\_112214\_01.09243.09243.2 | 2.7639 | 0.2529 | 99.1% | 1524.2522 | 1524.7754 | 24 | 5.185 | 59.1% | 1 | R.QLREYQELMSVK.L | 2 |
|  | Astrin\_STLCHLD\_061214\_01.07862.07862.2 | 3.174 | 0.25 | 99.6% | 1407.6322 | 1406.6653 | 1 | 4.86 | 77.3% | 9 | K.LALDIEIATYRK.L | 222 |

Similarities:
gi|4504919|ref|NP\_002(3:17)  
gi|47132620|ref|NP\_00(2:18)  
gi|119703753|ref|NP\_0(2:18)  
gi|32567786|ref|NP\_78(3:17)  
gi|119395754|ref|NP\_0(2:18)  
gi|153791158|ref|NP\_0(2:18)  
gi|109255249|ref|NP\_0(2:18)  

---

|  |  |  |  |  |  |  |  |  |
| --- | --- | --- | --- | --- | --- | --- | --- | --- |
| U | *gi|224028244|ref|NP\_0* | 31 | 252 | 44.2% | 471 | 54232 | 8.9 | non-POU domain containing, octamer-binding isoform 1 [Homo sapiens] |
| U | *gi|34932414|ref|NP\_03* | 31 | 252 | 44.2% | 471 | 54232 | 8.9 | non-POU domain containing, octamer-binding isoform 1 [Homo sapiens] |
| U | *gi|224028246|ref|NP\_0* | 31 | 252 | 44.2% | 471 | 54232 | 8.9 | non-POU domain containing, octamer-binding isoform 1 [Homo sapiens] |

| Filename XCorr DeltCN Conf% ObsM+H+ CalcM+H+ SpR ZScore Ion% # Sequence  | | | | | | | | | | | | |
| --- | --- | --- | --- | --- | --- | --- | --- | --- | --- | --- | --- | --- |
|  | AstrinSTLCLD\_041714\_01.08770.08770.2 | 3.2564 | 0.2634 | 99.6% | 2103.7322 | 2104.3855 | 1 | 5.583 | 41.2% | 1 | R.SRLFVGNLPPDITEEEMR.K | 2 |
|  | AstrinSTLCLD\_041714\_01.08661.08661.3 | 2.8642 | 0.2483 | 96.9% | 2104.2544 | 2104.3855 | 71 | 4.4 | 30.9% | 1 | R.SRLFVGNLPPDITEEEMR.K | 3 |
|  | Astrin\_STLCLD20\_112214\_01.11551.11551.2 | 3.8157 | 0.5133 | 100.0% | 1860.3922 | 1861.12 | 1 | 8.105 | 70.0% | 23 | R.LFVGNLPPDITEEEMR.K | 2 |
|  | AstrinSTLCLD\_041714\_01.08336.08336.3 | 2.7817 | 0.2583 | 97.2% | 1989.1743 | 1989.2941 | 11 | 4.882 | 32.8% | 1 | R.LFVGNLPPDITEEEMRK.L | 3 |
|  | Astrin\_STLCHLD\_tube2\_050114\_01.11539.11539.2 | 5.1585 | 0.5425 | 100.0% | 1813.0122 | 1814.1504 | 1 | 9.765 | 73.3% | 7 | R.TLAEIAKVELDNMPLR.G | 2 |
|  | AstrinSTLCLD\_041714\_01.10476.10476.3 | 5.5193 | 0.3973 | 100.0% | 1815.2043 | 1814.1504 | 1 | 6.982 | 48.3% | 4 | R.TLAEIAKVELDNMPLR.G | 3 |
|  | AstrinSTLCLD\_041714\_01.09563.09563.3 | 4.4853 | 0.4148 | 100.0% | 1999.1044 | 1999.3765 | 1 | 7.209 | 44.1% | 3 | R.TLAEIAKVELDNMPLRGK.Q | 3 |
|  | AstrinSTLCLD\_041714\_01.05718.05718.2 | 3.1946 | 0.2844 | 100.0% | 1087.1322 | 1087.2793 | 15 | 5.339 | 75.0% | 11 | K.VELDNMPLR.G | 2 |
|  | Astrin\_STLCHLD\_tube2\_050114\_01.05652.05652.2 | 2.7312 | 0.1537 | 97.2% | 1271.8922 | 1272.5052 | 1 | 4.174 | 80.0% | 2 | K.VELDNMPLRGK.Q | 2 |
|  | Astrin\_STLCHLD\_050114\_01.05543.05543.3 | 2.9639 | 0.2589 | 99.4% | 1250.0044 | 1249.3782 | 13 | 5.138 | 42.5% | 1 | R.FACHSASLTVR.N | 3 |
|  | AstrinSTLCLD\_041714\_01.15770.15770.2 | 5.7209 | 0.5289 | 100.0% | 2668.892 | 2669.9507 | 1 | 10.765 | 54.5% | 35 | R.NLPQYVSNELLEEAFSVFGQVER.A | 2 |
|  | Astrin\_STLCLD20\_112214\_01.17785.17785.3 | 5.0303 | 0.3864 | 100.0% | 2670.5344 | 2669.9507 | 1 | 8.075 | 39.8% | 11 | R.NLPQYVSNELLEEAFSVFGQVER.A | 3 |
|  | Astrin\_NLD\_STLC\_tube2\_021014\_01.04706.04706.2 | 2.3101 | 0.2583 | 98.6% | 886.47217 | 887.0238 | 1 | 6.189 | 78.6% | 5 | R.AVVIVDDR.G | 22 |
|  | Astrin\_NLD\_STLC\_031014\_01.09174.09174.1 | 2.1813 | 0.3489 | 96.4% | 886.54 | 887.0238 | 12 | 6.823 | 64.3% | 5 | R.AVVIVDDR.G | 11 |
|  | Astrin\_NLD\_STLC\_tube2\_021014\_01.04250.04250.3 | 3.0065 | 0.2286 | 96.6% | 1816.6743 | 1815.0854 | 40 | 4.659 | 29.4% | 1 | R.GRPSGKGIVEFSGKPAAR.K | 3 |
|  | Astrin\_STLCLD20\_112214\_tube2\_01.07612.07612.2 | 3.0678 | 0.3722 | 100.0% | 1232.1921 | 1232.4252 | 1 | 7.863 | 72.7% | 7 | K.GIVEFSGKPAAR.K | 2 |
|  | Astrin\_STLCLD20\_112214\_tube2\_01.12293.12293.2 | 4.6923 | 0.5562 | 100.0% | 1696.5122 | 1696.8744 | 1 | 10.467 | 76.9% | 31 | R.FAQPGSFEYEYAMR.W | 2 |
|  | Astrin\_STLCHLD\_tube2\_050114\_01.05164.05164.3 | 2.7696 | 0.3303 | 99.8% | 1832.2144 | 1832.0386 | 1 | 4.799 | 35.7% | 1 | K.ALIEMEKQQQDQVDR.N | 3 |
|  | AstrinSTLCLD\_041714\_01.04833.04833.3 | 2.678 | 0.2991 | 99.4% | 1336.9744 | 1337.5488 | 1 | 5.823 | 55.0% | 1 | R.EKLEMEMEAAR.H | 3 |
|  | Astrin\_STLCHLD\_tube2\_050114\_01.05237.05237.2 | 3.1846 | 0.4268 | 100.0% | 1337.3322 | 1337.5488 | 1 | 7.137 | 80.0% | 18 | R.EKLEMEMEAAR.H | 2 |
|  | Astrin\_STLCHLD\_tube2\_050114\_02.06202.06202.3 | 4.2419 | 0.2807 | 99.9% | 2499.3245 | 2499.942 | 1 | 5.713 | 32.9% | 1 | R.EKLEMEMEAARHEHQVMLMR.Q | 3 |
|  | Astrin\_STLCLD20\_112214\_tube2\_01.06486.06486.2 | 2.2656 | 0.2388 | 97.6% | 1180.6522 | 1181.4161 | 1 | 5.523 | 68.8% | 1 | R.HEHQVMLMR.Q | 2 |
|  | Astrin\_STLCLD20\_112214\_tube2\_01.05771.05771.2 | 3.9157 | 0.3321 | 100.0% | 1541.2122 | 1541.7222 | 1 | 6.254 | 72.7% | 1 | R.RMEELHNQEVQK.R | 2 |
|  | Astrin\_STLCLD20\_112214\_tube2\_01.05681.05681.2 | 4.6847 | 0.2587 | 100.0% | 1697.3522 | 1697.9097 | 1 | 5.697 | 70.8% | 1 | R.RMEELHNQEVQKR.K | 2 |
|  | Astrin\_STLCHLD\_050114\_01.04185.04185.3 | 3.651 | 0.1176 | 95.4% | 1697.8143 | 1697.9097 | 1 | 4.722 | 54.2% | 1 | R.RMEELHNQEVQKR.K | 3 |
|  | AstrinSTLCLD\_041714\_02.06176.06176.2 | 4.6157 | 0.5414 | 100.0% | 1539.0721 | 1539.8441 | 1 | 9.288 | 67.9% | 56 | R.MGQMAMGGAMGINNR.G | 2 |
|  | Astrin\_STLCHLD\_050114\_02.06501.06501.3 | 3.353 | 0.3897 | 99.9% | 1539.5343 | 1539.8441 | 2 | 5.802 | 42.9% | 2 | R.MGQMAMGGAMGINNR.G | 3 |
|  | AstrinSTLCLD\_041714\_02.07829.07829.2 | 5.8222 | 0.5063 | 100.0% | 2163.5322 | 2164.4436 | 1 | 9.23 | 59.5% | 3 | R.FGQAATMEGIGAIGGTPPAFNR.A | 2 |
|  | AstrinSTLCLD\_041714\_02.07894.07894.2 | 4.8705 | 0.5504 | 100.0% | 2243.5522 | 2244.4436 | 1 | 10.905 | 61.9% | 4 | R.FGQAATMEGIGAIGGT#PPAFNR.A | 2 |
|  | Astrin\_STLCLD20\_112214\_tube2\_02.08617.08617.3 | 4.2158 | 0.4155 | 99.9% | 2243.9343 | 2244.4436 | 1 | 6.861 | 44.0% | 10 | R.FGQAATMEGIGAIGGT#PPAFNR.A | 3 |
|  | Astrin\_STLCLD20\_112214\_tube2\_01.06364.06364.2 | 2.2854 | 0.2556 | 97.0% | 1229.2322 | 1229.3811 | 1 | 4.6 | 72.7% | 3 | R.AAPGAEFAPNKR.R | 2 |

Similarities:
gi|4826998|ref|NP\_005(2:29)  

---

|  |  |  |  |  |  |  |  |  |
| --- | --- | --- | --- | --- | --- | --- | --- | --- |
| U | *gi|12667788|ref|NP\_00* | 87 | 447 | 44.0% | 1960 | 226530 | 5.6 | myosin, heavy polypeptide 9, non-muscle [Homo sapiens] |

| Filename XCorr DeltCN Conf% ObsM+H+ CalcM+H+ SpR ZScore Ion% # Sequence  | | | | | | | | | | | | |
| --- | --- | --- | --- | --- | --- | --- | --- | --- | --- | --- | --- | --- |
| \* | Astrin\_STLCLD20\_112214\_tube2\_01.14602.14602.2 | 3.9986 | 0.2871 | 100.0% | 1673.5122 | 1673.8687 | 1 | 6.584 | 71.4% | 7 | K.NFINNPLAQADWAAK.K | 2 |
| \* | Astrin\_STLCHLD\_tube2\_061314\_01.05234.05234.2 | 2.3678 | 0.2485 | 98.4% | 1073.4722 | 1072.2926 | 265 | 5.022 | 50.0% | 4 | K.KLVWVPSDK.S | 2 |
| \* | Astrin\_STLCLD20\_112214\_tube2\_01.15735.15735.2 | 2.4809 | 0.2311 | 96.7% | 1728.6921 | 1728.9978 | 11 | 5.623 | 42.3% | 1 | K.NLPIYSEEIVEMYK.G | 2 |
| \* | Astrin\_STLCLD20\_112214\_tube2\_01.08433.08433.3 | 4.3809 | 0.3901 | 100.0% | 2072.3342 | 2072.3489 | 1 | 7.218 | 45.3% | 3 | K.RHEMPPHIYAITDTAYR.S | 3 |
| \* | Astrin\_STLCLD20\_112214\_01.08240.08240.3 | 4.3084 | 0.4868 | 100.0% | 1916.1543 | 1916.1614 | 1 | 8.118 | 53.3% | 32 | R.HEMPPHIYAITDTAYR.S | 3 |
| \* | Astrin\_NLD\_STLC\_tube2\_021014\_01.08230.08230.3 | 3.0737 | 0.3262 | 99.8% | 1609.5844 | 1607.8931 | 1 | 5.092 | 44.2% | 2 | K.KVIQYLAYVASSHK.S | 3 |
| \* | Astrin\_STLCHLD\_061214\_01.07249.07249.2 | 3.9864 | 0.4528 | 100.0% | 1479.8322 | 1479.719 | 1 | 8.588 | 75.0% | 5 | K.VIQYLAYVASSHK.S | 2 |
| \* | Astrin\_STLCHLD\_061214\_01.07225.07225.3 | 2.4856 | 0.3792 | 99.8% | 1480.6144 | 1479.719 | 4 | 6.208 | 39.6% | 4 | K.VIQYLAYVASSHK.S | 3 |
|  | Astrin\_STLCLD20\_112214\_01.13430.13430.2 | 4.9502 | 0.4604 | 100.0% | 1727.9321 | 1728.0012 | 1 | 9.572 | 63.3% | 4 | R.QLLQANPILEAFGNAK.T | 22 |
| \* | Astrin\_STLCHLD\_tube2\_050114\_02.11081.11081.2 | 4.1881 | 0.4969 | 100.0% | 2385.9321 | 2386.7068 | 1 | 9.329 | 42.5% | 1 | R.INFDVNGYIVGANIETYLLEK.S | 2 |
| \* | Astrin\_STLCHLD\_050114\_02.10203.10203.3 | 5.0084 | 0.4593 | 100.0% | 1997.3944 | 1997.3037 | 1 | 8.252 | 45.3% | 10 | R.TFHIFYYLLSGAGEHLK.T | 3 |
| \* | Astrin\_STLCLD20\_112214\_tube2\_01.17004.17004.2 | 4.3074 | 0.3687 | 100.0% | 1997.8522 | 1997.3037 | 1 | 7.166 | 59.4% | 1 | R.TFHIFYYLLSGAGEHLK.T | 2 |
| \* | Astrin\_STLCHLD\_tube2\_061314\_01.08497.08497.2 | 2.1138 | 0.2417 | 95.6% | 1205.7522 | 1206.3812 | 1 | 4.568 | 72.2% | 1 | K.TDLLLEPYNK.Y | 2 |
| \* | AstrinSTLCLD\_041714\_01.09507.09507.3 | 5.1396 | 0.29 | 99.9% | 3012.5044 | 3012.4 | 1 | 6.342 | 32.0% | 2 | R.FLSNGHVTIPGQQDKDMFQETMEAMR.I | 3 |
| \* | Astrin\_STLCLD20\_112214\_01.11827.11827.2 | 4.0814 | 0.4199 | 100.0% | 1616.5122 | 1616.9313 | 1 | 8.078 | 80.8% | 16 | R.IMGIPEEEQMGLLR.V | 2 |
| \* | Astrin\_STLCHLD\_050114\_01.12674.12674.2 | 4.0689 | 0.5232 | 100.0% | 1487.7522 | 1487.8259 | 1 | 9.075 | 69.2% | 2 | R.VISGVLQLGNIVFK.K | 2 |
| \* | Astrin\_STLCLD20\_112214\_tube2\_01.15566.15566.2 | 3.8874 | 0.2548 | 100.0% | 1616.1522 | 1616.0 | 1 | 5.467 | 67.9% | 3 | R.VISGVLQLGNIVFKK.E | 2 |
|  | Astrin\_NLD\_STLC\_031014\_01.05930.05930.2 | 5.1525 | 0.4536 | 100.0% | 1593.3322 | 1592.6776 | 1 | 7.792 | 75.0% | 18 | R.NTDQASMPDNTAAQK.V | 2 |
| \* | Astrin\_STLCHLD\_tube2\_061314\_01.10433.10433.3 | 4.0122 | 0.263 | 99.9% | 1573.1044 | 1572.8044 | 3 | 4.832 | 51.9% | 4 | K.VSHLLGINVTDFTR.G | 3 |
| \* | Astrin\_NLD\_STLC\_tube2\_021014\_01.10668.10668.2 | 4.2189 | 0.4152 | 100.0% | 1573.2522 | 1572.8044 | 1 | 7.856 | 76.9% | 9 | K.VSHLLGINVTDFTR.G | 2 |
|  | Astrin\_STLCHLD\_tube2\_061314\_01.11798.11798.3 | 3.5308 | 0.177 | 96.3% | 2468.6042 | 2468.7893 | 1 | 5.224 | 37.5% | 1 | K.LQQLFNHTMFILEQEEYQR.E | 33 |
|  | Astrin\_STLCHLD\_061214\_01.07250.07250.2 | 2.6822 | 0.2747 | 99.2% | 1398.6721 | 1398.6166 | 1 | 5.315 | 75.0% | 1 | K.VDYKADEWLMK.N | 22 |
| \* | Astrin\_STLCHLD\_tube2\_050114\_01.12703.12703.2 | 4.6844 | 0.4506 | 100.0% | 2018.6921 | 2019.3636 | 1 | 9.411 | 57.9% | 7 | R.IIGLDQVAGMSETALPGAFK.T | 2 |
| \* | Astrin\_STLCLD20\_112214\_tube2\_01.07731.07731.2 | 2.9455 | 0.2813 | 99.6% | 1378.1721 | 1378.6115 | 8 | 5.025 | 54.5% | 2 | R.TVGQLYKEQLAK.L | 2 |
|  | Astrin\_STLCLD20\_112214\_tube2\_01.12982.12982.2 | 2.3095 | 0.3423 | 99.1% | 1319.8722 | 1319.5468 | 23 | 5.288 | 60.0% | 2 | K.LDPHLVLDQLR.C | 22 |
| \* | Astrin\_STLCHLD\_050114\_01.11817.11817.2 | 2.3691 | 0.3028 | 99.6% | 925.5722 | 925.07513 | 1 | 6.503 | 75.0% | 6 | R.VVFQEFR.Q | 2 |
| \* | Astrin\_STLCLD20\_112214\_tube2\_01.11349.11349.2 | 2.7434 | 0.2948 | 99.5% | 1275.4521 | 1275.4875 | 1 | 6.763 | 65.0% | 3 | R.YEILTPNSIPK.G | 2 |
| \* | Astrin\_STLCHLD\_tube2\_061314\_01.08136.08136.2 | 3.1863 | 0.4445 | 100.0% | 1194.2722 | 1194.33 | 1 | 7.748 | 77.8% | 10 | K.ALELDSNLYR.I | 2 |
|  | Astrin\_STLCHLD\_tube2\_061314\_01.05545.05545.2 | 3.5792 | 0.372 | 100.0% | 1225.5122 | 1224.3591 | 1 | 6.298 | 75.0% | 6 | R.AGVLAHLEEER.D | 22 |
| \* | Astrin\_STLCHLD\_tube2\_050114\_02.08132.08132.2 | 5.0968 | 0.4607 | 100.0% | 1752.4722 | 1753.0358 | 1 | 8.123 | 67.9% | 13 | R.LTEMETLQSQLMAEK.L | 2 |
| \* | Astrin\_STLCHLD\_tube2\_050114\_01.10626.10626.2 | 6.3834 | 0.4357 | 100.0% | 2333.7522 | 2334.4736 | 1 | 9.573 | 66.7% | 2 | K.MQQNIQELEEQLEEEESAR.Q | 2 |
| \* | Astrin\_STLCHLD\_tube2\_061314\_01.10902.10902.3 | 3.9911 | 0.3125 | 100.0% | 2335.3743 | 2334.4736 | 2 | 5.488 | 33.3% | 1 | K.MQQNIQELEEQLEEEESAR.Q | 3 |
| \* | Astrin\_STLCHLD\_050114\_02.06750.06750.2 | 4.8512 | 0.4747 | 100.0% | 1654.3722 | 1654.7681 | 1 | 8.353 | 73.1% | 20 | R.IAEFTTNLTEEEEK.S | 2 |
| \* | Astrin\_STLCLD20\_112214\_tube2\_01.09180.09180.2 | 3.9685 | 0.3047 | 100.0% | 1870.5122 | 1870.0203 | 1 | 5.918 | 53.3% | 1 | R.IAEFTTNLTEEEEKSK.S | 2 |
| \* | Astrin\_STLCHLD\_tube2\_050114\_01.04702.04702.3 | 3.2573 | 0.352 | 100.0% | 1828.0743 | 1828.0936 | 11 | 5.453 | 37.5% | 1 | K.LKNKHEAMITDLEER.L | 3 |
| \* | Astrin\_STLCLD20\_112214\_tube2\_01.07839.07839.2 | 3.1375 | 0.1804 | 98.7% | 1587.5721 | 1586.76 | 2 | 6.838 | 62.5% | 1 | K.NKHEAMITDLEER.L | 2 |
| \* | Astrin\_STLCLD20\_112214\_01.07474.07474.2 | 2.6176 | 0.4724 | 100.0% | 1343.9521 | 1344.4822 | 1 | 7.711 | 75.0% | 2 | K.HEAMITDLEER.L | 2 |
|  | Astrin\_STLCLD20\_112214\_01.05162.05162.2 | 3.2255 | 0.3083 | 100.0% | 1258.1921 | 1258.4172 | 5 | 5.34 | 75.0% | 3 | K.KEEELQAALAR.V | 22 |
| \* | Astrin\_STLCLD20\_112214\_01.11944.11944.3 | 5.011 | 0.3452 | 99.9% | 2305.1343 | 2304.473 | 1 | 6.165 | 38.9% | 3 | K.IRELESQISELQEDLESER.A | 3 |
| \* | Astrin\_STLCLD20\_112214\_tube2\_01.15195.15195.2 | 3.9789 | 0.4771 | 100.0% | 2034.2122 | 2035.126 | 1 | 8.323 | 59.4% | 2 | R.ELESQISELQEDLESER.A | 2 |
| \* | Astrin\_STLCLD20\_112214\_01.16693.16693.3 | 4.1211 | 0.3383 | 100.0% | 3018.2944 | 3019.2434 | 1 | 6.406 | 35.6% | 4 | R.DLGEELEALKTELEDTLDSTAAQQELR.S | 3 |
| \* | Astrin\_STLCLD20\_112214\_01.16679.16679.2 | 5.1368 | 0.5418 | 100.0% | 3019.152 | 3019.2434 | 1 | 10.356 | 44.2% | 5 | R.DLGEELEALKTELEDTLDSTAAQQELR.S | 2 |
| \* | AstrinSTLCLD\_041714\_01.05392.05392.3 | 4.1503 | 0.4586 | 100.0% | 2043.7144 | 2044.2439 | 1 | 7.262 | 37.5% | 7 | K.TLEEEAKTHEAQIQEMR.Q | 3 |
| \* | AstrinSTLCLD\_041714\_01.07276.07276.3 | 5.5989 | 0.4917 | 100.0% | 1996.4043 | 1997.1722 | 1 | 9.025 | 45.3% | 14 | K.HSQAVEELAEQLEQTKR.V | 3 |
| \* | Astrin\_STLCLD20\_112214\_01.04990.04990.3 | 3.7062 | 0.2806 | 99.8% | 1930.3444 | 1930.1252 | 1 | 4.772 | 42.2% | 3 | K.AKQTLENERGELANEVK.V | 3 |
| \* | Astrin\_STLCHLD\_050114\_01.07318.07318.3 | 3.1659 | 0.2247 | 98.6% | 1542.1444 | 1541.8314 | 197 | 4.784 | 35.4% | 2 | R.KKVEAQLQELQVK.F | 3 |
| \* | Astrin\_STLCLD20\_112214\_01.06953.06953.2 | 3.9367 | 0.3241 | 100.0% | 1413.8922 | 1413.6573 | 1 | 6.179 | 72.7% | 2 | K.KVEAQLQELQVK.F | 2 |
| \* | Astrin\_STLCLD20\_112214\_01.08309.08309.2 | 3.5521 | 0.3183 | 100.0% | 1286.0322 | 1285.4833 | 1 | 6.283 | 85.0% | 1 | K.VEAQLQELQVK.F | 2 |
| \* | Astrin\_STLCLD20\_112214\_tube2\_02.09156.09156.2 | 5.6644 | 0.5434 | 100.0% | 1946.8121 | 1947.1498 | 1 | 10.073 | 73.5% | 10 | K.LQVELDNVTGLLSQSDSK.S | 2 |
| \* | Astrin\_NLD\_STLC\_031014\_01.08837.08837.2 | 3.0217 | 0.2137 | 99.1% | 1492.3722 | 1493.6598 | 1 | 4.968 | 72.7% | 2 | K.LKQVEDEKNSFR.E | 2 |
| \* | Astrin\_STLCHLD\_050114\_01.04478.04478.3 | 3.0958 | 0.3149 | 99.8% | 1493.6943 | 1493.6598 | 14 | 5.673 | 40.9% | 2 | K.LKQVEDEKNSFR.E | 3 |
| \* | Astrin\_STLCLD20\_112214\_01.12899.12899.2 | 5.0288 | 0.4855 | 100.0% | 1950.3322 | 1951.1436 | 1 | 9.727 | 76.7% | 3 | R.LQQELDDLLVDLDHQR.Q | 2 |
| \* | Astrin\_STLCLD20\_112214\_tube2\_01.16215.16215.3 | 3.564 | 0.2624 | 99.8% | 1951.5844 | 1951.1436 | 1 | 4.912 | 48.3% | 7 | R.LQQELDDLLVDLDHQR.Q | 3 |
|  | Astrin\_STLCHLD\_tube2\_061314\_01.05081.05081.2 | 2.9194 | 0.1221 | 97.8% | 1220.8522 | 1221.3959 | 9 | 4.855 | 66.7% | 6 | K.KFDQLLAEEK.T | 222 |
|  | Astrin\_NLD\_STLC\_tube2\_021014\_01.06868.06868.2 | 3.1864 | 0.2186 | 99.8% | 1093.1122 | 1093.2218 | 1 | 5.928 | 81.2% | 6 | K.FDQLLAEEK.T | 222 |
| \* | Astrin\_STLCHLD\_tube2\_050114\_02.05512.05512.2 | 4.4595 | 0.4129 | 100.0% | 1647.0322 | 1647.8407 | 1 | 7.937 | 76.9% | 9 | R.ALEEAMEQKAELER.L | 2 |
| \* | Astrin\_STLCHLD\_061214\_02.05439.05439.3 | 4.0671 | 0.3888 | 100.0% | 1648.1044 | 1647.8407 | 1 | 6.816 | 57.7% | 12 | R.ALEEAMEQKAELER.L | 3 |
| \* | Astrin\_NLD\_STLC\_tube2\_021014\_01.07383.07383.2 | 2.3517 | 0.266 | 98.3% | 1170.7722 | 1171.3229 | 18 | 6.377 | 61.1% | 1 | R.TEMEDLMSSK.D | 2 |
| \* | Astrin\_STLCHLD\_tube2\_050114\_01.06575.06575.2 | 3.8653 | 0.5138 | 100.0% | 1685.0521 | 1685.8586 | 1 | 8.499 | 67.9% | 3 | R.TEMEDLMSSKDDVGK.S | 2 |
| \* | Astrin\_STLCHLD\_tube2\_050114\_01.05654.05654.2 | 3.4135 | 0.4385 | 100.0% | 1204.9722 | 1205.3685 | 4 | 7.91 | 66.7% | 9 | R.ALEQQVEEMK.T | 2 |
| \* | Astrin\_NLD\_STLC\_tube2\_021014\_01.15297.15297.3 | 4.6822 | 0.407 | 100.0% | 3148.1343 | 3149.4048 | 2 | 7.685 | 26.0% | 5 | R.ALEQQVEEMKTQLEELEDELQATEDAK.L | 3 |
| \* | Astrin\_STLCHLD\_061214\_01.07334.07334.2 | 3.4795 | 0.1931 | 99.7% | 1315.2522 | 1315.6171 | 1 | 7.441 | 65.0% | 8 | K.LRLEVNLQAMK.A | 2 |
| \* | Astrin\_STLCHLD\_tube2\_061314\_01.07457.07457.2 | 2.408 | 0.2871 | 99.1% | 1045.8522 | 1046.2701 | 1 | 5.288 | 81.2% | 2 | R.LEVNLQAMK.A | 2 |
| \* | Astrin\_STLCLD20\_112214\_tube2\_01.07773.07773.2 | 2.61 | 0.3203 | 99.6% | 1251.1721 | 1251.3075 | 1 | 6.21 | 72.2% | 5 | R.EMEAELEDER.K | 2 |
| \* | Astrin\_STLCLD20\_112214\_tube2\_01.06603.06603.2 | 2.334 | 0.1879 | 95.0% | 1380.3722 | 1379.4816 | 18 | 4.015 | 55.0% | 1 | R.EMEAELEDERK.Q | 2 |
| \* | Astrin\_NLD\_STLC\_tube2\_021014\_01.04332.04332.2 | 3.4196 | 0.4899 | 100.0% | 1212.3121 | 1213.2896 | 1 | 9.3 | 75.0% | 2 | K.DLEAHIDSANK.N | 2 |
| \* | Astrin\_STLCLD20\_112214\_tube2\_01.06416.06416.2 | 2.9415 | 0.1937 | 98.9% | 1216.1921 | 1216.3799 | 2 | 4.959 | 70.0% | 1 | R.ASREEILAQAK.E | 2 |
| \* | Astrin\_STLCHLD\_050114\_01.04815.04815.3 | 2.9432 | 0.2167 | 98.1% | 1217.2444 | 1216.3799 | 180 | 4.533 | 35.0% | 1 | R.ASREEILAQAK.E | 3 |
| \* | Astrin\_STLCLD20\_112214\_tube2\_01.06051.06051.3 | 3.573 | 0.2372 | 99.4% | 1844.4844 | 1845.0629 | 12 | 4.739 | 33.3% | 1 | R.ASREEILAQAKENEKK.L | 3 |
| \* | Astrin\_NLD\_STLC\_tube2\_021014\_01.13919.13919.2 | 4.1472 | 0.4239 | 100.0% | 2049.0923 | 2050.3064 | 1 | 7.744 | 50.0% | 4 | K.SMEAEMIQLQEELAAAER.A | 2 |
| \* | Astrin\_STLCHLD\_tube2\_061314\_01.05621.05621.3 | 3.9424 | 0.4664 | 100.0% | 2090.0645 | 2090.168 | 1 | 7.213 | 40.3% | 3 | R.QAQQERDELADEIANSSGK.G | 3 |
| \* | Astrin\_STLCHLD\_tube2\_061314\_02.07680.07680.2 | 6.3228 | 0.5145 | 100.0% | 2473.9722 | 2473.6099 | 1 | 9.283 | 62.5% | 6 | R.IAQLEEELEEEQGNTELINDR.L | 2 |
| \* | Astrin\_STLCHLD\_tube2\_061314\_02.07664.07664.3 | 4.7839 | 0.3452 | 99.9% | 2474.3643 | 2473.6099 | 1 | 6.612 | 37.5% | 11 | R.IAQLEEELEEEQGNTELINDR.L | 3 |
| \* | Astrin\_NLD\_STLC\_tube2\_021014\_01.09255.09255.3 | 3.4457 | 0.2572 | 99.5% | 1999.1643 | 1999.2314 | 3 | 4.868 | 34.4% | 1 | K.KANLQIDQINTDLNLER.S | 3 |
| \* | AstrinSTLCLD\_041714\_01.09006.09006.2 | 5.5646 | 0.5191 | 100.0% | 1870.3922 | 1871.0574 | 1 | 9.585 | 66.7% | 13 | K.ANLQIDQINTDLNLER.S | 2 |
| \* | Astrin\_NLD\_STLC\_tube2\_021014\_01.05201.05201.2 | 2.4213 | 0.3631 | 99.8% | 904.0722 | 904.05133 | 1 | 6.72 | 68.8% | 1 | K.ASITALEAK.I | 2 |
| \* | Astrin\_STLCHLD\_050114\_01.11329.11329.2 | 4.6611 | 0.4513 | 100.0% | 1531.4122 | 1531.6598 | 1 | 8.23 | 79.2% | 7 | K.IAQLEEQLDNETK.E | 2 |
| \* | Astrin\_STLCLD20\_112214\_01.07952.07952.2 | 4.9376 | 0.505 | 100.0% | 1816.4922 | 1816.9628 | 1 | 8.37 | 78.6% | 2 | K.IAQLEEQLDNETKER.Q | 2 |
| \* | Astrin\_STLCHLD\_tube2\_050114\_01.06314.06314.3 | 2.9142 | 0.2397 | 97.6% | 1817.4543 | 1816.9628 | 132 | 5.35 | 32.1% | 2 | K.IAQLEEQLDNETKER.Q | 3 |
| \* | Astrin\_STLCHLD\_tube2\_050114\_01.05897.05897.3 | 2.99 | 0.2733 | 99.4% | 1601.0944 | 1599.8278 | 1 | 5.898 | 50.0% | 1 | K.LKDVLLQVDDERR.N | 3 |
| \* | Astrin\_NLD\_STLC\_031014\_01.05478.05478.2 | 3.0491 | 0.3724 | 100.0% | 1725.1721 | 1725.8125 | 7 | 7.401 | 46.4% | 1 | R.NAEQYKDQADKASTR.L | 2 |
| \* | Astrin\_NLD\_STLC\_031014\_01.05498.05498.3 | 3.9237 | 0.4273 | 100.0% | 1726.1044 | 1725.8125 | 8 | 7.033 | 39.3% | 9 | R.NAEQYKDQADKASTR.L | 3 |
| \* | Astrin\_STLCHLD\_050114\_01.04462.04462.2 | 4.4929 | 0.3654 | 100.0% | 1488.2722 | 1488.5541 | 1 | 6.954 | 81.8% | 3 | K.RQLEEAEEEAQR.A | 2 |
| \* | Astrin\_STLCLD20\_112214\_tube2\_01.06171.06171.2 | 3.5123 | 0.342 | 100.0% | 1333.3322 | 1332.3666 | 1 | 6.31 | 80.0% | 2 | R.QLEEAEEEAQR.A | 2 |
| \* | Astrin\_NLD\_STLC\_tube2\_021014\_01.05723.05723.2 | 4.5793 | 0.5497 | 100.0% | 1566.1122 | 1566.6367 | 1 | 9.208 | 65.4% | 10 | R.ELEDATETADAMNR.E | 2 |
| \* | Astrin\_STLCHLD\_tube2\_061314\_01.07775.07775.2 | 3.8167 | 0.2489 | 100.0% | 1156.3322 | 1156.3732 | 1 | 6.143 | 88.9% | 18 | R.RGDLPFVVPR.R | 2 |
| \* | Astrin\_STLCLD20\_112214\_tube2\_01.05817.05817.3 | 4.7448 | 0.362 | 99.9% | 2384.5745 | 2384.3428 | 1 | 7.269 | 33.7% | 3 | R.KGAGDGS\*DEEVDGKADGAEAKPAE.- | 3 |

Similarities:
gi|41406064|ref|NP\_00(6:81)  
gi|116284394|ref|NP\_0(4:83)  

---

|  |  |  |  |  |  |  |  |  |
| --- | --- | --- | --- | --- | --- | --- | --- | --- |
| U | *gi|14043070|ref|NP\_11* | 13 | 104 | 43.8% | 372 | 38747 | 9.1 | heterogeneous nuclear ribonucleoprotein A1 isoform b [Homo sapiens] |

| Filename XCorr DeltCN Conf% ObsM+H+ CalcM+H+ SpR ZScore Ion% # Sequence  | | | | | | | | | | | | |
| --- | --- | --- | --- | --- | --- | --- | --- | --- | --- | --- | --- | --- |
|  | Astrin\_NLD\_STLC\_031014\_01.06768.06768.2 | 2.52 | 0.2405 | 98.3% | 1300.3121 | 1300.4111 | 3 | 4.823 | 65.0% | 5 | K.SESPKEPEQLR.K | 2 |
|  | Astrin\_NLD\_STLC\_tube2\_021014\_02.08289.08289.3 | 4.0072 | 0.4087 | 99.9% | 1913.6344 | 1914.1656 | 1 | 6.271 | 39.1% | 5 | R.KLFIGGLSFETTDESLR.S | 3 |
|  | Astrin\_STLCLD20\_112214\_tube2\_01.14348.14348.2 | 4.7721 | 0.4437 | 100.0% | 1913.6522 | 1914.1656 | 1 | 7.581 | 59.4% | 9 | R.KLFIGGLSFETTDESLR.S | 2 |
|  | AstrinSTLCLD\_041714\_02.09290.09290.2 | 5.3461 | 0.5136 | 100.0% | 1785.6322 | 1785.9916 | 1 | 8.928 | 66.7% | 27 | K.LFIGGLSFETTDESLR.S | 2 |
|  | Astrin\_STLCHLD\_tube2\_050114\_02.09551.09551.3 | 2.863 | 0.2574 | 96.3% | 2511.9243 | 2511.8145 | 7 | 5.066 | 28.4% | 1 | R.GFGFVTYATVEEVDAAMNARPHK.V | 3 |
|  | Astrin\_STLCLD20\_112214\_tube2\_01.05668.05668.2 | 2.7633 | 0.2421 | 98.4% | 1566.5521 | 1566.7574 | 9 | 4.408 | 53.8% | 1 | R.EDSQRPGAHLTVKK.I | 2 |
|  | AstrinSTLCLD\_041714\_01.09416.09416.2 | 3.6877 | 0.4049 | 100.0% | 1219.3722 | 1219.4387 | 1 | 7.502 | 88.9% | 17 | K.IEVIEIMTDR.G | 2 |
|  | Astrin\_STLCHLD\_061214\_01.07474.07474.3 | 4.5096 | 0.4498 | 100.0% | 1857.4143 | 1856.989 | 1 | 7.285 | 46.7% | 6 | K.RGFAFVTFDDHDSVDK.I | 3 |
|  | Astrin\_STLCLD20\_112214\_tube2\_01.13103.13103.2 | 3.5452 | 0.2203 | 99.5% | 1701.5322 | 1700.8016 | 1 | 5.102 | 57.1% | 2 | R.GFAFVTFDDHDSVDK.I | 2 |
|  | Astrin\_STLCHLD\_050114\_02.08540.08540.3 | 4.2942 | 0.261 | 99.8% | 2283.3542 | 2282.5579 | 1 | 6.841 | 35.5% | 11 | R.GFAFVTFDDHDSVDKIVIQK.Y | 3 |
| \* | AstrinSTLCLD\_041714\_01.05237.05237.3 | 4.9259 | 0.4157 | 100.0% | 2875.2244 | 2875.8137 | 10 | 7.346 | 23.4% | 1 | R.GGGGYGGSGDGYNGFGNDGGYGGGGPGYSGGSR.G | 3 |
|  | Astrin\_STLCHLD\_050114\_01.08305.08305.2 | 4.1816 | 0.5067 | 100.0% | 1628.7922 | 1629.7721 | 1 | 8.198 | 66.7% | 12 | R.SSGPYGGGGQYFAKPR.N | 2 |
|  | Astrin\_NLD\_STLC\_031014\_01.05216.05216.2 | 4.87 | 0.5279 | 100.0% | 1695.3322 | 1695.6561 | 1 | 10.254 | 64.7% | 7 | R.NQGGYGGSSSSSSYGSGR.R | 2 |

---

|  |  |  |  |  |  |  |  |  |
| --- | --- | --- | --- | --- | --- | --- | --- | --- |
| U | *gi|4502709|ref|NP\_001* | 10 | 31 | 42.4% | 297 | 34095 | 8.4 | cell division cycle 2 isoform 1 [Homo sapiens] |

| Filename XCorr DeltCN Conf% ObsM+H+ CalcM+H+ SpR ZScore Ion% # Sequence  | | | | | | | | | | | | |
| --- | --- | --- | --- | --- | --- | --- | --- | --- | --- | --- | --- | --- |
|  | Astrin\_STLCHLD\_050114\_01.10755.10755.2 | 3.1102 | 0.4672 | 100.0% | 1186.4321 | 1186.3501 | 1 | 8.533 | 70.0% | 4 | K.IGEGTYGVVYK.G | 2 |
|  | Astrin\_STLCHLD\_tube2\_061314\_01.04585.04585.2 | 4.5977 | 0.4397 | 100.0% | 1518.4722 | 1517.6329 | 1 | 7.485 | 76.9% | 1 | R.LESEEEGVPSTAIR.E | 2 |
|  | Astrin\_STLCHLD\_061214\_01.06729.06729.3 | 3.324 | 0.2928 | 99.8% | 1813.5243 | 1812.1167 | 4 | 5.508 | 36.7% | 1 | R.DLKPQNLLIDDKGTIK.L | 3 |
| \* | Astrin\_STLCHLD\_050114\_02.07818.07818.3 | 3.9341 | 0.2219 | 99.8% | 1567.1344 | 1566.7997 | 3 | 5.963 | 50.0% | 1 | R.VYTHEVVTLWYR.S | 3 |
|  | Astrin\_STLCHLD\_050114\_01.10261.10261.2 | 2.9548 | 0.3379 | 100.0% | 1029.1721 | 1029.1814 | 1 | 6.28 | 83.3% | 9 | R.SPEVLLGSAR.Y | 2 |
|  | Astrin\_STLCHLD\_tube2\_061314\_01.16923.16923.2 | 3.6713 | 0.3867 | 100.0% | 2213.9521 | 2213.5352 | 2 | 6.521 | 36.8% | 9 | R.YSTPVDIWSIGTIFAELATK.K | 2 |
|  | Astrin\_STLCHLD\_050114\_01.12117.12117.2 | 3.1535 | 0.2461 | 99.2% | 1802.6322 | 1803.0275 | 1 | 5.522 | 57.1% | 1 | K.KPLFHGDSEIDQLFR.I | 2 |
|  | Astrin\_STLCHLD\_tube2\_050114\_01.09313.09313.3 | 4.0191 | 0.2843 | 100.0% | 1803.3544 | 1803.0275 | 1 | 5.901 | 46.4% | 2 | K.KPLFHGDSEIDQLFR.I | 3 |
|  | Astrin\_STLCHLD\_tube2\_061314\_01.09642.09642.2 | 3.3352 | 0.3511 | 100.0% | 1331.4722 | 1331.4656 | 1 | 7.089 | 68.2% | 2 | K.NLDENGLDLLSK.M | 2 |
|  | Astrin\_STLCHLD\_tube2\_061314\_01.08873.08873.3 | 4.6438 | 0.3492 | 99.9% | 1933.9744 | 1934.1768 | 1 | 6.298 | 55.0% | 1 | K.MALNHPYFNDLDNQIK.K | 3 |

---

|  |  |  |  |  |  |  |  |  |
| --- | --- | --- | --- | --- | --- | --- | --- | --- |
| U | *gi|27436946|ref|NP\_73* | 32 | 213 | 41.6% | 664 | 74140 | 7.0 | lamin A/C isoform 1 precursor [Homo sapiens] |

| Filename XCorr DeltCN Conf% ObsM+H+ CalcM+H+ SpR ZScore Ion% # Sequence  | | | | | | | | | | | | |
| --- | --- | --- | --- | --- | --- | --- | --- | --- | --- | --- | --- | --- |
|  | Astrin\_NLD\_STLC\_031014\_01.08848.08848.2 | 3.6563 | 0.4903 | 100.0% | 1361.2922 | 1360.4667 | 1 | 8.105 | 57.7% | 1 | R.SGAQASSTPLSPTR.I | 2 |
|  | Astrin\_STLCLD20\_112214\_tube2\_01.07804.07804.3 | 3.6249 | 0.301 | 99.8% | 2001.7144 | 2001.204 | 1 | 5.341 | 38.3% | 1 | R.ITRLQEKEDLQELNDR.L | 3 |
|  | Astrin\_STLCHLD\_tube2\_050114\_01.04823.04823.3 | 4.0271 | 0.236 | 99.8% | 1630.5543 | 1630.7521 | 28 | 4.825 | 47.9% | 4 | R.LQEKEDLQELNDR.L | 3 |
|  | Astrin\_STLCHLD\_tube2\_050114\_01.04829.04829.2 | 4.7687 | 0.3346 | 100.0% | 1630.5721 | 1630.7521 | 1 | 7.693 | 75.0% | 3 | R.LQEKEDLQELNDR.L | 2 |
|  | Astrin\_STLCHLD\_050114\_01.05409.05409.2 | 2.9416 | 0.2321 | 99.4% | 1091.3121 | 1090.1783 | 1 | 4.863 | 77.8% | 5 | R.SLETENAGLR.L | 2 |
|  | Astrin\_STLCHLD\_061214\_01.04990.04990.2 | 4.3021 | 0.4597 | 100.0% | 1418.1921 | 1418.5901 | 1 | 8.438 | 72.7% | 5 | R.LRITESEEVVSR.E | 2 |
|  | Astrin\_NLD\_STLC\_tube2\_021014\_02.04823.04823.3 | 3.74 | 0.2033 | 99.8% | 1418.3344 | 1418.5901 | 19 | 4.984 | 45.5% | 8 | R.LRITESEEVVSR.E | 3 |
|  | Astrin\_STLCHLD\_050114\_01.04789.04789.2 | 3.471 | 0.4691 | 100.0% | 1149.0721 | 1149.2432 | 2 | 8.35 | 77.8% | 4 | R.ITESEEVVSR.E | 2 |
|  | Astrin\_STLCHLD\_050114\_01.07574.07574.2 | 3.406 | 0.4553 | 100.0% | 1165.9722 | 1166.2328 | 1 | 7.373 | 85.0% | 7 | K.AAYEAELGDAR.K | 2 |
|  | Astrin\_STLCHLD\_050114\_01.05674.05674.2 | 2.9068 | 0.3053 | 99.9% | 1044.2322 | 1044.1527 | 16 | 5.992 | 66.7% | 2 | K.EGDLIAAQAR.L | 2 |
|  | Astrin\_STLCLD20\_112214\_tube2\_01.12057.12057.2 | 3.4708 | 0.3768 | 100.0% | 1244.4122 | 1244.474 | 1 | 6.959 | 80.0% | 5 | R.LKDLEALLNSK.E | 2 |
|  | Astrin\_STLCLD20\_112214\_01.08570.08570.2 | 3.0528 | 0.2967 | 100.0% | 1183.5922 | 1183.3066 | 6 | 6.442 | 77.8% | 7 | R.TLEGELHDLR.G | 2 |
|  | Astrin\_STLCHLD\_tube2\_050114\_01.06528.06528.2 | 3.8853 | 0.3583 | 100.0% | 1510.2722 | 1510.7455 | 3 | 6.884 | 63.6% | 1 | R.LQTMKEELDFQK.N | 2 |
|  | Astrin\_STLCHLD\_tube2\_050114\_01.04920.04920.2 | 2.6856 | 0.2747 | 99.2% | 1381.5521 | 1382.5132 | 63 | 5.129 | 55.0% | 1 | K.NIYSEELRETK.R | 2 |
|  | AstrinSTLCLD\_041714\_01.06036.06036.2 | 3.7888 | 0.3389 | 100.0% | 1028.7122 | 1029.1814 | 1 | 6.384 | 87.5% | 13 | R.LADALQELR.A | 2 |
|  | Astrin\_NLD\_STLC\_tube2\_021014\_01.06210.06210.2 | 5.0424 | 0.5227 | 100.0% | 1753.1921 | 1753.8693 | 1 | 8.622 | 70.0% | 13 | R.NSNLVGAAHEELQQSR.I | 2 |
|  | AstrinSTLCLD\_041714\_02.05540.05540.3 | 2.9818 | 0.3469 | 99.8% | 1754.0343 | 1753.8693 | 10 | 5.645 | 36.7% | 2 | R.NSNLVGAAHEELQQSR.I | 3 |
|  | Astrin\_STLCHLD\_050114\_01.12115.12115.2 | 4.6168 | 0.4308 | 100.0% | 1700.6522 | 1700.9762 | 1 | 7.861 | 71.4% | 4 | R.IRIDSLSAQLSQLQK.Q | 2 |
|  | Astrin\_STLCLD20\_112214\_01.10593.10593.2 | 2.6175 | 0.1904 | 96.6% | 1431.1322 | 1431.6293 | 4 | 4.084 | 62.5% | 1 | R.IDSLSAQLSQLQK.Q | 2 |
|  | Astrin\_STLCHLD\_tube2\_061314\_01.04782.04782.2 | 3.1849 | 0.3413 | 100.0% | 1188.3322 | 1188.3262 | 1 | 5.813 | 83.3% | 11 | K.LRDLEDSLAR.E | 2 |
|  | Astrin\_STLCHLD\_tube2\_061314\_01.04765.04765.3 | 3.3945 | 0.1629 | 98.4% | 1189.0743 | 1188.3262 | 1 | 4.491 | 52.8% | 1 | K.LRDLEDSLAR.E | 3 |
|  | Astrin\_STLCHLD\_tube2\_061314\_01.11615.11615.2 | 5.489 | 0.4506 | 100.0% | 1894.7322 | 1895.1346 | 1 | 8.371 | 75.0% | 7 | R.MQQQLDEYQELLDIK.L | 2 |
|  | Astrin\_STLCHLD\_050114\_02.07234.07234.2 | 2.6902 | 0.4308 | 100.0% | 1332.2922 | 1332.5603 | 1 | 7.215 | 55.0% | 1 | K.LALDMEIHAYR.K | 2 |
|  | Astrin\_STLCHLD\_050114\_01.06712.06712.2 | 2.4118 | 0.2949 | 98.8% | 1402.1921 | 1402.4208 | 45 | 4.984 | 60.0% | 2 | R.LRLS\*PSPT#SQR.S | 2 |
|  | Astrin\_STLCHLD\_tube2\_050114\_01.04672.04672.2 | 2.8028 | 0.3221 | 99.8% | 1402.2322 | 1402.4208 | 12 | 5.193 | 65.0% | 2 | R.LRLS\*PS\*PTSQR.S | 2 |
|  | Astrin\_STLCHLD\_050114\_01.04970.04970.2 | 2.8172 | 0.4066 | 100.0% | 1204.1921 | 1204.2762 | 1 | 7.56 | 75.0% | 1 | R.VAVEEVDEEGK.F | 2 |
|  | AstrinSTLCLD\_041714\_02.05709.05709.3 | 2.9487 | 0.3528 | 99.8% | 1606.3444 | 1606.7728 | 7 | 5.342 | 38.5% | 18 | R.VAVEEVDEEGKFVR.L | 3 |
|  | Astrin\_STLCHLD\_tube2\_050114\_01.05812.05812.2 | 4.3557 | 0.5296 | 100.0% | 1606.3522 | 1606.7728 | 1 | 9.048 | 73.1% | 17 | R.VAVEEVDEEGKFVR.L | 2 |
|  | Astrin\_STLCHLD\_tube2\_061314\_02.06002.06002.2 | 4.3827 | 0.613 | 100.0% | 1492.2122 | 1492.6874 | 1 | 9.872 | 69.2% | 32 | R.TALINSTGEEVAMR.K | 2 |
|  | Astrin\_STLCHLD\_tube2\_061314\_02.05033.05033.2 | 3.4177 | 0.5345 | 100.0% | 2365.7322 | 2366.504 | 1 | 9.42 | 36.5% | 1 | K.ASASGSGAQVGGPISSGSSASSVTVTR.S | 2 |
|  | Astrin\_NLD\_STLC\_tube2\_021014\_02.04828.04828.3 | 4.6464 | 0.4856 | 100.0% | 2366.4243 | 2366.504 | 1 | 8.809 | 35.6% | 9 | K.ASASGSGAQVGGPISSGSSASSVTVTR.S | 3 |
|  | AstrinSTLCLD\_041714\_02.05925.05925.2 | 4.6089 | 0.5472 | 100.0% | 1567.3322 | 1567.6555 | 1 | 9.689 | 62.5% | 24 | R.SVGGSGGGSFGDNLVTR.S | 2 |

---

|  |  |  |  |  |  |  |  |  |
| --- | --- | --- | --- | --- | --- | --- | --- | --- |
| U | *gi|4826898|ref|NP\_005* | 4 | 14 | 41.4% | 140 | 15054 | 8.3 | profilin 1 [Homo sapiens] |

| Filename XCorr DeltCN Conf% ObsM+H+ CalcM+H+ SpR ZScore Ion% # Sequence  | | | | | | | | | | | | |
| --- | --- | --- | --- | --- | --- | --- | --- | --- | --- | --- | --- | --- |
| \* | Astrin\_STLCLD20\_112214\_tube2\_01.16176.16176.2 | 3.8876 | 0.3527 | 100.0% | 1644.4521 | 1644.9518 | 1 | 7.431 | 66.7% | 4 | K.TFVNITPAEVGVLVGK.D | 2 |
| \* | Astrin\_STLCLD20\_112214\_tube2\_01.12378.12378.2 | 3.9425 | 0.5238 | 100.0% | 1471.5322 | 1471.6531 | 1 | 8.483 | 73.1% | 6 | R.SSFYVNGLTLGGQK.C | 2 |
| \* | Astrin\_STLCHLD\_tube2\_061314\_02.08597.08597.2 | 4.3052 | 0.4348 | 100.0% | 1628.0521 | 1626.7784 | 1 | 6.972 | 69.2% | 3 | R.DSLLQDGEFSMDLR.T | 2 |
| \* | Astrin\_STLCHLD\_tube2\_061314\_01.05958.05958.2 | 3.452 | 0.4581 | 100.0% | 1379.9922 | 1380.5406 | 1 | 7.537 | 69.2% | 1 | K.STGGAPTFNVTVTK.T | 2 |

---

|  |  |  |  |  |  |  |  |  |
| --- | --- | --- | --- | --- | --- | --- | --- | --- |
| U | *gi|21396489|ref|NP\_00* | 30 | 206 | 40.8% | 959 | 106489 | 6.4 | mitochondrial lon peptidase 1 [Homo sapiens] |

| Filename XCorr DeltCN Conf% ObsM+H+ CalcM+H+ SpR ZScore Ion% # Sequence  | | | | | | | | | | | | |
| --- | --- | --- | --- | --- | --- | --- | --- | --- | --- | --- | --- | --- |
| \* | Astrin\_STLCLD20\_112214\_tube2\_01.13929.13929.2 | 3.1352 | 0.4476 | 100.0% | 1235.7122 | 1235.512 | 1 | 8.055 | 75.0% | 5 | R.LAQPYVGVFLK.R | 2 |
| \* | AstrinSTLCLD\_041714\_01.09776.09776.2 | 3.0185 | 0.1084 | 95.1% | 1797.2522 | 1796.8809 | 17 | 3.463 | 39.3% | 8 | K.S\*K@RGKKEAEDELSAR.H | 2 |
| \* | Astrin\_STLCLD20\_112214\_01.12195.12195.2 | 3.4342 | 0.2748 | 100.0% | 1558.9122 | 1558.8644 | 2 | 5.71 | 58.3% | 5 | K.TIRDIIALNPLYR.E | 2 |
| \* | Astrin\_STLCHLD\_tube2\_050114\_01.10954.10954.1 | 1.9918 | 0.2879 | 97.1% | 1187.74 | 1188.4124 | 90 | 5.389 | 50.0% | 1 | R.DIIALNPLYR.E | 1 |
| \* | Astrin\_STLCHLD\_tube2\_061314\_01.11201.11201.2 | 2.9426 | 0.4083 | 100.0% | 1188.3722 | 1188.4124 | 1 | 7.391 | 88.9% | 2 | R.DIIALNPLYR.E | 2 |
| \* | Astrin\_STLCHLD\_tube2\_061314\_01.09941.09941.2 | 3.0027 | 0.2231 | 99.2% | 1378.4122 | 1378.6044 | 1 | 5.955 | 63.6% | 5 | R.ESVLQMMQAGQR.V | 2 |
| \* | Astrin\_STLCLD20\_112214\_01.14803.14803.3 | 5.0179 | 0.467 | 100.0% | 3670.6443 | 3671.0674 | 1 | 8.375 | 28.0% | 8 | R.VVDNPIYLSDMGAALTGAESHELQDVLEETNIPK.R | 3 |
| \* | Astrin\_STLCHLD\_050114\_01.06242.06242.2 | 2.5661 | 0.2257 | 99.1% | 926.0122 | 926.18756 | 3 | 4.45 | 85.7% | 1 | R.LKELVVPK.H | 2 |
| \* | Astrin\_STLCHLD\_tube2\_061314\_01.07548.07548.2 | 3.9763 | 0.5798 | 100.0% | 1400.5922 | 1401.5745 | 1 | 10.055 | 72.7% | 9 | K.HVMDVVDEELSK.L | 2 |
| \* | Astrin\_STLCHLD\_tube2\_050114\_01.13058.13058.3 | 6.0468 | 0.4902 | 100.0% | 3085.5842 | 3085.4155 | 1 | 8.446 | 38.5% | 1 | K.HVMDVVDEELSKLGLLDNHSSEFNVTR.N | 3 |
| \* | Astrin\_STLCHLD\_050114\_02.07166.07166.2 | 4.1233 | 0.3618 | 100.0% | 1702.2922 | 1702.8644 | 6 | 7.708 | 50.0% | 7 | K.LGLLDNHSSEFNVTR.N | 2 |
| \* | Astrin\_NLD\_STLC\_tube2\_021014\_02.06656.06656.3 | 4.7187 | 0.4163 | 100.0% | 1703.2444 | 1702.8644 | 1 | 7.051 | 42.9% | 19 | K.LGLLDNHSSEFNVTR.N | 3 |
| \* | Astrin\_STLCHLD\_tube2\_050114\_01.15217.15217.2 | 4.7028 | 0.407 | 100.0% | 1594.6721 | 1593.8223 | 1 | 7.016 | 70.8% | 12 | R.NYLDWLTSIPWGK.Y | 2 |
| \* | Astrin\_STLCHLD\_tube2\_050114\_01.05217.05217.2 | 3.3792 | 0.3859 | 100.0% | 1195.3322 | 1195.2743 | 1 | 7.069 | 77.8% | 13 | K.YSNENLDLAR.A | 2 |
| \* | Astrin\_STLCHLD\_050114\_01.11054.11054.2 | 5.1178 | 0.5263 | 100.0% | 1834.3722 | 1834.9954 | 1 | 10.475 | 70.0% | 4 | R.AQAVLEEDHYGMEDVK.K | 2 |
| \* | Astrin\_STLCHLD\_tube2\_050114\_01.11606.11606.2 | 3.6998 | 0.2623 | 100.0% | 1289.3922 | 1289.5608 | 1 | 5.776 | 75.0% | 13 | R.ILEFIAVSQLR.G | 2 |
| \* | AstrinSTLCLD\_041714\_02.07341.07341.2 | 3.7787 | 0.4338 | 100.0% | 1354.1122 | 1354.561 | 1 | 7.596 | 75.0% | 19 | R.FSVGGMTDVAEIK.G | 2 |
| \* | Astrin\_STLCHLD\_050114\_01.05191.05191.2 | 2.2434 | 0.4689 | 100.0% | 923.5522 | 924.1028 | 19 | 7.648 | 68.8% | 1 | R.TYVGAMPGK.I | 2 |
| \* | Astrin\_STLCHLD\_tube2\_050114\_01.11238.11238.3 | 4.4153 | 0.3296 | 99.9% | 2055.1743 | 2055.3794 | 1 | 6.054 | 42.6% | 2 | K.TKTENPLILIDEVDKIGR.G | 3 |
| \* | Astrin\_STLCLD20\_112214\_01.13279.13279.2 | 4.3277 | 0.4636 | 100.0% | 1825.1921 | 1826.1002 | 1 | 8.541 | 66.7% | 8 | K.TENPLILIDEVDKIGR.G | 2 |
| \* | Astrin\_STLCHLD\_050114\_01.15733.15733.3 | 6.0052 | 0.4575 | 100.0% | 3875.6643 | 3875.2373 | 1 | 8.951 | 30.1% | 13 | R.GYQGDPSSALLELLDPEQNANFLDHYLDVPVDLSK.V | 3 |
| \* | Astrin\_STLCHLD\_061214\_01.07819.07819.2 | 5.1839 | 0.5178 | 100.0% | 1600.3722 | 1599.8574 | 1 | 8.847 | 76.9% | 20 | R.MEMINVSGYVAQEK.L | 2 |
| \* | Astrin\_STLCHLD\_tube2\_050114\_01.11674.11674.3 | 4.729 | 0.5291 | 100.0% | 3177.1743 | 3177.5352 | 1 | 8.586 | 25.9% | 8 | K.IVSGEAESVEVTPENLQDFVGKPVFTVER.M | 3 |
| \* | Astrin\_STLCHLD\_tube2\_061314\_01.18189.18189.2 | 5.6394 | 0.5801 | 100.0% | 3189.2322 | 3188.7422 | 1 | 10.784 | 39.7% | 2 | R.MYDVTPPGVVMGLAWTAMGGSTLFVETSLR.R | 2 |
| \* | Astrin\_STLCHLD\_tube2\_061314\_01.18186.18186.3 | 3.7519 | 0.3719 | 99.9% | 3189.7144 | 3188.7422 | 1 | 6.484 | 26.7% | 1 | R.MYDVTPPGVVMGLAWTAMGGSTLFVETSLR.R | 3 |
| \* | Astrin\_STLCHLD\_tube2\_061314\_02.06686.06686.3 | 3.6534 | 0.3158 | 99.9% | 1864.2843 | 1864.0778 | 1 | 5.324 | 44.1% | 3 | K.GDKDGSLEVTGQLGEVMK.E | 3 |
| \* | Astrin\_STLCHLD\_050114\_02.07766.07766.2 | 2.6133 | 0.2707 | 98.3% | 1564.5322 | 1563.7632 | 26 | 5.122 | 46.4% | 1 | K.DGSLEVTGQLGEVMK.E | 2 |
| \* | Astrin\_STLCLD20\_112214\_tube2\_02.07028.07028.2 | 3.9176 | 0.4712 | 100.0% | 1450.2922 | 1449.6624 | 3 | 8.12 | 57.7% | 10 | R.QNLAMTGEVSLTGK.I | 2 |
| \* | Astrin\_STLCLD20\_112214\_tube2\_01.00634.00634.3 | 3.3213 | 0.2739 | 99.3% | 2671.7644 | 2672.956 | 12 | 4.922 | 27.4% | 3 | K.DFYDLAAFITEGLEVHFVEHYR.E | 3 |
| \* | Astrin\_STLCHLD\_tube2\_061314\_01.12708.12708.2 | 4.4113 | 0.3552 | 100.0% | 2165.4521 | 2164.3765 | 1 | 6.813 | 58.3% | 2 | R.EIFDIAFPDEQAEALAVER.- | 2 |

---

|  |  |  |  |  |  |  |  |  |
| --- | --- | --- | --- | --- | --- | --- | --- | --- |
| U | *gi|119395750|ref|NP\_0* | 23 | 104 | 40.8% | 644 | 66039 | 8.1 | keratin 1 [Homo sapiens] |

| Filename XCorr DeltCN Conf% ObsM+H+ CalcM+H+ SpR ZScore Ion% # Sequence  | | | | | | | | | | | | |
| --- | --- | --- | --- | --- | --- | --- | --- | --- | --- | --- | --- | --- |
| \* | Astrin\_STLCHLD\_061214\_02.06106.06106.2 | 4.4309 | 0.3988 | 100.0% | 1658.9521 | 1658.7678 | 1 | 6.986 | 56.2% | 10 | R.SGGGFSSGSAGIINYQR.R | 2 |
|  | AstrinSTLCLD\_041714\_01.09156.09156.2 | 3.6516 | 0.4413 | 100.0% | 1385.3522 | 1384.5315 | 1 | 7.262 | 68.2% | 5 | K.SLNNQFASFIDK.V | 2 |
|  | Astrin\_STLCLD20\_112214\_01.11787.11787.2 | 2.6999 | 0.3446 | 99.6% | 1640.3322 | 1639.8516 | 8 | 4.891 | 42.3% | 2 | K.SLNNQFASFIDKVR.F | 2 |
|  | Astrin\_STLCLD20\_112214\_01.07333.07333.2 | 4.5527 | 0.0972 | 99.9% | 1476.4922 | 1476.6726 | 1 | 7.289 | 90.9% | 10 | R.FLEQQNQVLQTK.W | 22 |
|  | Astrin\_STLCHLD\_061214\_01.08490.08490.2 | 4.3907 | 0.5135 | 100.0% | 1476.5521 | 1476.6293 | 1 | 9.299 | 86.4% | 12 | K.WELLQQVDTSTR.T | 2 |
|  | Astrin\_NLD\_STLC\_tube2\_021014\_01.13704.13704.2 | 3.2918 | 0.3599 | 100.0% | 1994.2122 | 1995.2017 | 1 | 5.808 | 56.7% | 3 | R.THNLEPYFESFINNLR.R | 2 |
|  | Astrin\_NLD\_STLC\_tube2\_021014\_01.13762.13762.3 | 3.6231 | 0.1519 | 96.3% | 1995.4143 | 1995.2017 | 2 | 5.064 | 41.7% | 1 | R.THNLEPYFESFINNLR.R | 3 |
|  | Astrin\_NLD\_STLC\_tube2\_021014\_01.07334.07334.2 | 2.9649 | 0.275 | 99.7% | 1266.1921 | 1266.3934 | 1 | 5.904 | 75.0% | 2 | R.TNAENEFVTIK.K | 2 |
|  | Astrin\_STLCHLD\_tube2\_050114\_01.04962.04962.2 | 2.9535 | 0.2244 | 99.1% | 1394.8522 | 1394.5675 | 4 | 5.232 | 63.6% | 2 | R.TNAENEFVTIKK.D | 2 |
| \* | AstrinSTLCLD\_041714\_01.11297.11297.2 | 4.1979 | 0.4951 | 100.0% | 1303.2322 | 1303.4955 | 1 | 9.01 | 81.8% | 5 | R.SLDLDSIIAEVK.A | 2 |
|  | Astrin\_STLCHLD\_061214\_01.06446.06446.3 | 5.3788 | 0.4434 | 100.0% | 2502.7444 | 2502.7405 | 1 | 8.439 | 44.0% | 1 | K.SKAEAESLYQSKYEELQITAGR.H | 3 |
|  | Astrin\_STLCHLD\_061214\_01.06978.06978.3 | 3.8098 | 0.2572 | 99.8% | 2289.5044 | 2287.4883 | 2 | 5.515 | 32.9% | 1 | K.AEAESLYQSKYEELQITAGR.H | 3 |
|  | Astrin\_STLCHLD\_tube2\_061314\_01.05725.05725.2 | 3.887 | 0.3539 | 100.0% | 1181.3722 | 1180.303 | 2 | 6.792 | 83.3% | 26 | K.YEELQITAGR.H | 22 |
|  | Astrin\_NLD\_STLC\_tube2\_021014\_01.06564.06564.2 | 2.9459 | 0.0753 | 98.0% | 974.1322 | 974.102 | 175 | 4.257 | 64.3% | 1 | K.IEISELNR.V | 22 |
|  | Astrin\_STLCHLD\_tube2\_061314\_01.09176.09176.2 | 4.4087 | 0.3798 | 100.0% | 1601.5721 | 1600.769 | 1 | 6.672 | 80.8% | 1 | K.NKLNDLEDALQQAK.E | 2 |
| \* | Astrin\_NLD\_STLC\_tube2\_021014\_01.12202.12202.3 | 4.8778 | 0.4232 | 100.0% | 2185.1643 | 2185.399 | 1 | 6.791 | 48.6% | 4 | K.NKLNDLEDALQQAKEDLAR.L | 3 |
|  | Astrin\_NLD\_STLC\_tube2\_021014\_01.08040.08040.3 | 2.8193 | 0.2094 | 96.3% | 1524.2344 | 1524.7754 | 66 | 4.582 | 34.1% | 1 | R.LLRDYQELMNTK.L | 3 |
|  | AstrinSTLCLD\_041714\_01.06374.06374.2 | 2.9557 | 0.2684 | 99.5% | 1524.4122 | 1524.7754 | 1 | 5.401 | 72.7% | 1 | R.LLRDYQELMNTK.L | 2 |
|  | Astrin\_STLCHLD\_050114\_01.05411.05411.2 | 2.2176 | 0.202 | 95.7% | 1034.3522 | 1034.1112 | 67 | 5.011 | 62.5% | 1 | R.TLLEGEESR.M | 2 |
|  | Astrin\_NLD\_STLC\_tube2\_021014\_01.04131.04131.2 | 6.1204 | 0.5987 | 100.0% | 2384.2122 | 2385.298 | 1 | 11.749 | 41.7% | 1 | R.GGGGGGYGSGGSSYGSGGGSYGSGGGGGGGR.G | 2 |
|  | Astrin\_STLCHLD\_061214\_01.04202.04202.3 | 5.6466 | 0.5281 | 100.0% | 2385.0842 | 2385.298 | 1 | 9.58 | 30.0% | 1 | R.GGGGGGYGSGGSSYGSGGGSYGSGGGGGGGR.G | 3 |
| \* | Astrin\_STLCHLD\_061214\_01.04316.04316.3 | 5.6201 | 0.2052 | 99.8% | 3314.4844 | 3314.2085 | 1 | 8.476 | 22.4% | 5 | R.GSYGSGGSSYGSGGGSYGSGGGGGGHGSYGSGSSSGGYR.G | 3 |
| \* | AstrinSTLCLD\_041714\_01.08276.08276.3 | 4.1298 | 0.2366 | 99.5% | 2242.1643 | 2241.0396 | 1 | 5.258 | 36.1% | 8 | R.GGSGGGGGGS\*S\*GGRGSGGGSSGGSIGGR.G | 3 |

Similarities:
gi|47132620|ref|NP\_00(2:21)  
gi|119703753|ref|NP\_0(1:22)  

---

|  |  |  |  |  |  |  |  |  |
| --- | --- | --- | --- | --- | --- | --- | --- | --- |
| U | *gi|4557701|ref|NP\_000* | 21 | 133 | 39.8% | 432 | 48106 | 5.0 | keratin 17 [Homo sapiens] |

| Filename XCorr DeltCN Conf% ObsM+H+ CalcM+H+ SpR ZScore Ion% # Sequence  | | | | | | | | | | | | |
| --- | --- | --- | --- | --- | --- | --- | --- | --- | --- | --- | --- | --- |
|  | Astrin\_STLCHLD\_tube2\_050114\_01.05063.05063.2 | 3.0476 | 0.142 | 99.0% | 1064.9722 | 1065.2578 | 35 | 6.057 | 62.5% | 4 | R.LASYLDKVR.A | 22222 |
|  | Astrin\_NLD\_STLC\_tube2\_021014\_02.05198.05198.2 | 4.0318 | 0.4298 | 100.0% | 1346.4122 | 1346.4772 | 1 | 7.983 | 72.7% | 36 | R.ALEEANTELEVK.I | 2 |
|  | Astrin\_STLCLD20\_112214\_02.14227.14227.3 | 4.6244 | 0.3541 | 99.9% | 2070.4443 | 2069.366 | 1 | 6.58 | 44.4% | 1 | K.ILTATVDNANILLQIDNAR.L | 3 |
|  | Astrin\_STLCHLD\_050114\_01.06505.06505.2 | 2.4865 | 0.2457 | 99.3% | 808.09216 | 807.8815 | 46 | 6.454 | 66.7% | 16 | R.LAADDFR.T | 222222 |
|  | Astrin\_NLD\_STLC\_tube2\_021014\_01.04334.04334.2 | 2.6976 | 0.3418 | 99.9% | 1223.0122 | 1223.3715 | 19 | 5.786 | 66.7% | 2 | R.TKFETEQALR.L | 22 |
|  | Astrin\_NLD\_STLC\_tube2\_021014\_02.06634.06634.2 | 3.2897 | 0.3272 | 100.0% | 1187.2922 | 1187.3384 | 1 | 7.29 | 80.0% | 4 | R.LSVEADINGLR.R | 2 |
|  | Astrin\_STLCLD20\_112214\_01.08975.08975.2 | 2.9226 | 0.3172 | 100.0% | 1186.2522 | 1186.397 | 1 | 5.85 | 83.3% | 4 | R.RVLDELTLAR.A | 2222 |
|  | Astrin\_STLCLD20\_112214\_01.09782.09782.1 | 2.1585 | 0.3224 | 97.2% | 1029.49 | 1030.2096 | 10 | 5.815 | 56.2% | 1 | R.VLDELTLAR.A | 1111 |
|  | Astrin\_STLCHLD\_tube2\_050114\_01.08094.08094.2 | 3.8958 | 0.4282 | 100.0% | 1030.5721 | 1030.2096 | 1 | 7.786 | 93.8% | 11 | R.VLDELTLAR.A | 2222 |
|  | Astrin\_STLCLD20\_112214\_01.05006.05006.2 | 3.4896 | 0.2654 | 100.0% | 1439.7322 | 1439.6263 | 1 | 5.072 | 80.0% | 5 | R.ILNEMRDQYEK.M | 22 |
|  | Astrin\_STLCHLD\_tube2\_050114\_01.11020.11020.2 | 3.7708 | 0.4345 | 100.0% | 1887.4722 | 1888.0001 | 1 | 6.784 | 53.6% | 4 | K.DAEDWFFSKTEELNR.E | 2 |
|  | Astrin\_STLCLD20\_112214\_01.06820.06820.3 | 3.5479 | 0.1718 | 95.9% | 2105.6042 | 2105.2664 | 54 | 4.942 | 30.6% | 2 | K.TEELNREVATNSELVQSGK.S | 33 |
|  | Astrin\_STLCHLD\_050114\_01.04933.04933.2 | 4.0231 | 0.5067 | 100.0% | 1362.1122 | 1362.4796 | 1 | 9.592 | 75.0% | 4 | R.EVATNSELVQSGK.S | 22 |
|  | Astrin\_NLD\_STLC\_tube2\_021014\_01.05760.05760.2 | 4.0567 | 0.4237 | 100.0% | 1405.0922 | 1404.4764 | 1 | 7.95 | 70.8% | 10 | K.ASLEGNLAETENR.Y | 2 |
|  | Astrin\_STLCHLD\_tube2\_050114\_01.06167.06167.2 | 3.6142 | 0.3306 | 100.0% | 1381.3722 | 1380.5437 | 4 | 5.939 | 65.0% | 7 | K.TRLEQEIATYR.R | 222 |
|  | Astrin\_NLD\_STLC\_tube2\_021014\_01.05813.05813.3 | 3.1264 | 0.2733 | 99.8% | 1536.1743 | 1536.7311 | 2 | 5.027 | 38.6% | 4 | K.TRLEQEIATYRR.L | 333 |
|  | Astrin\_STLCLD20\_112214\_01.05125.05125.2 | 3.1188 | 0.3235 | 100.0% | 1123.3922 | 1123.2511 | 2 | 6.193 | 81.2% | 5 | R.LEQEIATYR.R | 2222 |
|  | Astrin\_STLCHLD\_tube2\_061314\_01.04573.04573.3 | 3.0426 | 0.2237 | 97.9% | 1674.7743 | 1673.8662 | 2 | 5.681 | 38.5% | 1 | R.RLLEGEDAHLTQYK.K | 3 |
|  | Astrin\_STLCLD20\_112214\_01.06881.06881.2 | 4.3342 | 0.3729 | 100.0% | 1518.7922 | 1517.6787 | 1 | 7.328 | 79.2% | 3 | R.LLEGEDAHLTQYK.K | 2 |
|  | Astrin\_NLD\_STLC\_tube2\_021014\_01.05164.05164.3 | 4.5016 | 0.3981 | 100.0% | 2329.1042 | 2329.6152 | 1 | 7.25 | 39.5% | 3 | R.LLEGEDAHLTQYKKEPVTTR.Q | 3 |
|  | Astrin\_NLD\_STLC\_tube2\_021014\_01.05175.05175.2 | 2.7251 | 0.2799 | 99.5% | 1118.2722 | 1118.2291 | 1 | 5.73 | 72.2% | 6 | R.TIVEEVQDGK.V | 2 |

Similarities:
gi|40354195|ref|NP\_95(1:20)  
gi|15431310|ref|NP\_00(11:10)  
contaminant\_KERATIN03(2:19)  
gi|24430192|ref|NP\_00(8:13)  
gi|24234699|ref|NP\_00(7:14)  

---

|  |  |  |  |  |  |  |  |  |
| --- | --- | --- | --- | --- | --- | --- | --- | --- |
| U | *gi|4758792|ref|NP\_004* | 5 | 10 | 39.5% | 124 | 13712 | 8.3 | NADH dehydrogenase (ubiquinone) Fe-S protein 6, 13kDa (NADH-coenzyme Q reductase) [Homo sapiens] |

| Filename XCorr DeltCN Conf% ObsM+H+ CalcM+H+ SpR ZScore Ion% # Sequence  | | | | | | | | | | | | |
| --- | --- | --- | --- | --- | --- | --- | --- | --- | --- | --- | --- | --- |
| \* | Astrin\_STLCLD20\_112214\_tube2\_01.05955.05955.3 | 3.2878 | 0.3325 | 100.0% | 1697.9944 | 1697.8015 | 13 | 5.01 | 36.5% | 1 | K.VTHTGQVYDDKDYR.R | 3 |
| \* | Astrin\_STLCLD20\_112214\_tube2\_01.05781.05781.2 | 3.2194 | 0.3586 | 100.0% | 1853.2522 | 1853.989 | 1 | 6.006 | 60.7% | 1 | K.VTHTGQVYDDKDYRR.I | 2 |
| \* | Astrin\_STLCLD20\_112214\_tube2\_01.05777.05777.3 | 2.8601 | 0.4235 | 99.9% | 1853.6044 | 1853.989 | 1 | 6.026 | 41.1% | 4 | K.VTHTGQVYDDKDYRR.I | 3 |
| \* | AstrinSTLCLD\_041714\_01.10025.10025.3 | 4.2039 | 0.2238 | 99.4% | 2760.1443 | 2760.03 | 92 | 4.721 | 26.1% | 1 | R.QKEVNENFAIDLIAEQPVSEVETR.V | 3 |
| \* | Astrin\_STLCHLD\_050114\_01.07499.07499.2 | 3.1894 | 0.2778 | 100.0% | 1222.9521 | 1223.4117 | 1 | 5.99 | 77.8% | 3 | K.VYINLDKETK.T | 2 |

---

|  |  |  |  |  |  |  |  |  |
| --- | --- | --- | --- | --- | --- | --- | --- | --- |
| U | *gi|20357599|ref|NP\_61* | 5 | 30 | 39.5% | 114 | 12146 | 10.5 | H2A histone family, member V isoform 2 [Homo sapiens] |
| U | *gi|6912616|ref|NP\_036* | 5 | 30 | 35.2% | 128 | 13509 | 10.6 | H2A histone family, member V isoform 1 [Homo sapiens] |
| U | *gi|4504255|ref|NP\_002* | 5 | 30 | 35.2% | 128 | 13553 | 10.6 | H2A histone family, member Z [Homo sapiens] |

| Filename XCorr DeltCN Conf% ObsM+H+ CalcM+H+ SpR ZScore Ion% # Sequence  | | | | | | | | | | | | |
| --- | --- | --- | --- | --- | --- | --- | --- | --- | --- | --- | --- | --- |
|  | AstrinSTLCLD\_041714\_01.06063.06063.1 | 1.9612 | 0.2801 | 97.2% | 944.67 | 945.1093 | 4 | 5.26 | 62.5% | 2 | R.AGLQFPVGR.I | 1111 |
|  | Astrin\_STLCHLD\_tube2\_061314\_01.07517.07517.2 | 3.2091 | 0.3209 | 100.0% | 945.39215 | 945.1093 | 1 | 5.94 | 81.2% | 21 | R.AGLQFPVGR.I | 2222 |
|  | Astrin\_STLCLD20\_112214\_01.19147.19147.2 | 2.8134 | 0.2206 | 98.1% | 2896.0723 | 2897.2952 | 72 | 4.788 | 23.2% | 1 | R.VGATAAVYSAAILEYLTAEVLELAGNASK.D | 2 |
|  | Astrin\_STLCHLD\_tube2\_061314\_01.18247.18247.3 | 3.6918 | 0.3524 | 100.0% | 2897.0344 | 2897.2952 | 1 | 6.076 | 24.1% | 1 | R.VGATAAVYSAAILEYLTAEVLELAGNASK.D | 3 |
|  | Astrin\_STLCLD20\_112214\_tube2\_01.07832.07832.2 | 2.6508 | 0.2524 | 99.7% | 851.2922 | 851.0396 | 4 | 6.019 | 83.3% | 5 | R.HLQLAIR.G | 2222 |

Similarities:
gi|106775678|ref|NP\_0(3:2)  
gi|10645195|ref|NP\_06(3:2)  
gi|10800130|ref|NP\_06(3:2)  

---

|  |  |  |  |  |  |  |  |  |
| --- | --- | --- | --- | --- | --- | --- | --- | --- |
| U | *GST* | 11 | 28 | 39.3% | 244 | 28430 | 6.2 | no description |

| Filename XCorr DeltCN Conf% ObsM+H+ CalcM+H+ SpR ZScore Ion% # Sequence  | | | | | | | | | | | | |
| --- | --- | --- | --- | --- | --- | --- | --- | --- | --- | --- | --- | --- |
| \* | Astrin\_STLCLD20\_112214\_tube2\_01.16154.16154.3 | 4.356 | 0.2701 | 100.0% | 2270.4844 | 2270.5437 | 1 | 7.169 | 39.1% | 3 | R.LLLEYLEEKYEEHLYER.D | 3 |
| \* | Astrin\_STLCLD20\_112214\_01.12117.12117.3 | 4.5262 | 0.4061 | 99.9% | 3156.9243 | 3157.4631 | 2 | 6.517 | 28.3% | 2 | R.LLLEYLEEKYEEHLYERDEGDKWR.N | 3 |
| \* | Astrin\_STLCLD20\_112214\_01.12859.12859.3 | 4.5631 | 0.3678 | 99.9% | 2601.1743 | 2600.9739 | 1 | 6.711 | 41.7% | 1 | R.NKKFELGLEFPNLPYYIDGDVK.L | 3 |
| \* | Astrin\_STLCLD20\_112214\_01.14750.14750.2 | 4.5319 | 0.3891 | 100.0% | 2230.632 | 2230.522 | 1 | 7.242 | 50.0% | 3 | K.FELGLEFPNLPYYIDGDVK.L | 2 |
| \* | Astrin\_STLCLD20\_112214\_tube2\_01.09198.09198.1 | 1.8273 | 0.2961 | 97.2% | 1032.51 | 1033.2744 | 21 | 5.316 | 56.2% | 1 | K.LTQSMAIIR.Y | 1 |
| \* | Astrin\_STLCLD20\_112214\_tube2\_01.09266.09266.2 | 3.1021 | 0.228 | 99.8% | 1033.1921 | 1033.2744 | 2 | 5.747 | 81.2% | 4 | K.LTQSMAIIR.Y | 2 |
| \* | Astrin\_STLCLD20\_112214\_tube2\_01.17315.17315.2 | 4.8964 | 0.3311 | 100.0% | 1518.5322 | 1517.7809 | 1 | 6.924 | 73.1% | 5 | R.AEISMLEGAVLDIR.Y | 2 |
| \* | Astrin\_STLCLD20\_112214\_tube2\_01.13656.13656.3 | 3.6225 | 0.3785 | 99.9% | 2005.7043 | 2005.3184 | 1 | 5.965 | 34.4% | 2 | R.IAYSKDFETLKVDFLSK.L | 3 |
| \* | Astrin\_STLCLD20\_112214\_01.11925.11925.2 | 3.6655 | 0.3331 | 100.0% | 1443.6122 | 1442.6519 | 1 | 6.182 | 72.7% | 2 | K.DFETLKVDFLSK.L | 2 |
| \* | Astrin\_STLCLD20\_112214\_01.06206.06206.2 | 2.475 | 0.223 | 98.0% | 1182.6522 | 1183.3934 | 1 | 4.599 | 83.3% | 3 | K.RIEAIPQIDK.Y | 2 |
| \* | Astrin\_STLCLD20\_112214\_01.07862.07862.2 | 2.425 | 0.143 | 95.3% | 1027.3922 | 1027.2059 | 50 | 4.547 | 56.2% | 2 | R.IEAIPQIDK.Y | 2 |

---

|  |  |  |  |  |  |  |  |  |
| --- | --- | --- | --- | --- | --- | --- | --- | --- |
| U | *gi|5031699|ref|NP\_005* | 14 | 59 | 39.1% | 427 | 47355 | 7.5 | flotillin 1 [Homo sapiens] |

| Filename XCorr DeltCN Conf% ObsM+H+ CalcM+H+ SpR ZScore Ion% # Sequence  | | | | | | | | | | | | |
| --- | --- | --- | --- | --- | --- | --- | --- | --- | --- | --- | --- | --- |
| \* | Astrin\_STLCHLD\_tube2\_050114\_01.09747.09747.2 | 3.1121 | 0.3557 | 100.0% | 1216.5521 | 1216.4636 | 2 | 7.288 | 70.0% | 3 | R.ISLNTLTLNVK.S | 2 |
| \* | Astrin\_STLCHLD\_tube2\_050114\_01.06985.06985.2 | 2.8035 | 0.4339 | 100.0% | 1406.2722 | 1406.6682 | 1 | 6.761 | 61.5% | 1 | R.HGVPISVTGIAQVK.I | 2 |
| \* | Astrin\_STLCHLD\_tube2\_050114\_01.10692.10692.3 | 4.3487 | 0.4111 | 100.0% | 2018.2444 | 2019.2217 | 1 | 8.424 | 45.6% | 3 | K.TEAEIAHIALETLEGHQR.A | 3 |
| \* | Astrin\_STLCLD20\_112214\_02.10325.10325.3 | 3.1934 | 0.3426 | 99.9% | 1808.0643 | 1808.1206 | 1 | 5.613 | 41.1% | 2 | R.AIMAHMTVEEIYKDR.Q | 3 |
| \* | Astrin\_STLCHLD\_050114\_02.07443.07443.2 | 4.0736 | 0.4621 | 100.0% | 1470.0721 | 1469.693 | 2 | 8.311 | 62.5% | 7 | K.VSAQYLSEIEMAK.A | 2 |
| \* | Astrin\_STLCHLD\_tube2\_050114\_02.05820.05820.3 | 3.0215 | 0.309 | 99.8% | 1576.4944 | 1575.8081 | 1 | 5.381 | 44.2% | 1 | R.RAQADLAYQLQVAK.T | 3 |
| \* | AstrinSTLCLD\_041714\_02.06346.06346.2 | 4.3185 | 0.5263 | 100.0% | 1419.5521 | 1419.6206 | 1 | 9.555 | 75.0% | 8 | R.AQADLAYQLQVAK.T | 2 |
| \* | Astrin\_STLCHLD\_050114\_01.06520.06520.2 | 4.7613 | 0.4519 | 100.0% | 1470.3121 | 1470.6255 | 1 | 7.908 | 70.8% | 2 | R.AQQVAVQEQEIAR.R | 2 |
| \* | Astrin\_STLCHLD\_tube2\_050114\_02.07635.07635.2 | 5.3854 | 0.5966 | 100.0% | 1604.4722 | 1604.8187 | 1 | 10.241 | 71.4% | 7 | K.SQLIMQAEAEAASVR.M | 2 |
| \* | Astrin\_STLCHLD\_tube2\_050114\_01.05790.05790.2 | 3.8045 | 0.3974 | 100.0% | 1379.5521 | 1379.5768 | 1 | 6.886 | 79.2% | 8 | R.MRGEAEAFAIGAR.A | 2 |
| \* | Astrin\_STLCHLD\_050114\_02.05997.05997.3 | 2.9975 | 0.2396 | 98.6% | 1379.8444 | 1379.5768 | 2 | 5.171 | 47.9% | 1 | R.MRGEAEAFAIGAR.A | 3 |
| \* | Astrin\_STLCHLD\_tube2\_050114\_02.05433.05433.2 | 4.7378 | 0.5073 | 100.0% | 1380.4722 | 1380.5994 | 1 | 8.571 | 82.1% | 10 | K.ITLVSSGSGTMGAAK.V | 2 |
| \* | Astrin\_STLCHLD\_tube2\_050114\_01.10553.10553.2 | 3.8674 | 0.3758 | 100.0% | 1216.2522 | 1216.4203 | 1 | 6.928 | 80.0% | 4 | K.VTGEVLDILTR.L | 2 |
| \* | Astrin\_STLCLD20\_112214\_tube2\_01.07527.07527.2 | 3.1161 | 0.2975 | 99.6% | 1649.9722 | 1649.934 | 1 | 5.777 | 53.6% | 2 | R.LTGVSISQVNHKPLR.T | 2 |

---

|  |  |  |  |  |  |  |  |  |
| --- | --- | --- | --- | --- | --- | --- | --- | --- |
| U | *gi|4503571|ref|NP\_001* | 12 | 59 | 37.3% | 434 | 47169 | 7.4 | enolase 1 [Homo sapiens] |

| Filename XCorr DeltCN Conf% ObsM+H+ CalcM+H+ SpR ZScore Ion% # Sequence  | | | | | | | | | | | | |
| --- | --- | --- | --- | --- | --- | --- | --- | --- | --- | --- | --- | --- |
| \* | Astrin\_STLCHLD\_061214\_01.08010.08010.2 | 3.3018 | 0.338 | 100.0% | 1407.3121 | 1407.5634 | 2 | 7.839 | 62.5% | 8 | R.GNPTVEVDLFTSK.G | 2 |
|  | Astrin\_STLCHLD\_061214\_01.09480.09480.2 | 4.4819 | 0.4076 | 100.0% | 1806.5521 | 1806.0258 | 1 | 8.124 | 64.7% | 12 | R.AAVPSGASTGIYEALELR.D | 2 |
| \* | Astrin\_STLCHLD\_tube2\_061314\_01.06450.06450.2 | 3.0749 | 0.2313 | 99.5% | 1282.4122 | 1281.4817 | 10 | 6.125 | 65.0% | 3 | K.LMIEMDGTENK.S | 2 |
| \* | Astrin\_STLCHLD\_tube2\_061314\_01.11426.11426.3 | 5.4415 | 0.4333 | 100.0% | 3012.7144 | 3013.383 | 1 | 6.914 | 27.6% | 4 | R.HIADLAGNSEVILPVPAFNVINGGSHAGNK.L | 3 |
| \* | Astrin\_STLCLD20\_112214\_01.14282.14282.2 | 4.5006 | 0.4715 | 100.0% | 1909.5721 | 1909.3148 | 1 | 7.834 | 62.5% | 3 | K.LAMQEFMILPVGAANFR.E | 2 |
| \* | Astrin\_STLCHLD\_050114\_01.06059.06059.2 | 2.888 | 0.2163 | 99.2% | 1145.3922 | 1144.3158 | 4 | 4.703 | 72.2% | 2 | R.IGAEVYHNLK.N | 2 |
| \* | Astrin\_STLCLD20\_112214\_01.06779.06779.3 | 3.4238 | 0.3141 | 99.8% | 1829.4243 | 1827.9451 | 1 | 5.181 | 40.0% | 3 | R.SGKYDLDFKSPDDPSR.Y | 3 |
| \* | Astrin\_STLCHLD\_tube2\_050114\_01.10250.10250.2 | 3.7409 | 0.4546 | 100.0% | 1426.4722 | 1426.6091 | 2 | 7.537 | 77.3% | 10 | R.YISPDQLADLYK.S | 2 |
| \* | Astrin\_STLCHLD\_061214\_02.07563.07563.2 | 5.2562 | 0.5188 | 100.0% | 2033.6522 | 2034.2737 | 1 | 10.48 | 52.6% | 1 | K.FTASAGIQVVGDDLTVTNPK.R | 2 |
| \* | Astrin\_STLCLD20\_112214\_tube2\_02.07890.07890.3 | 4.326 | 0.3663 | 99.9% | 2191.7043 | 2190.4612 | 2 | 6.524 | 32.5% | 7 | K.FTASAGIQVVGDDLTVTNPKR.I | 3 |
| \* | Astrin\_STLCLD20\_112214\_01.08806.08806.2 | 2.9646 | 0.2833 | 99.4% | 1526.2522 | 1526.7563 | 1 | 4.914 | 65.4% | 1 | K.LAQANGWGVMVSHR.S | 2 |
| \* | Astrin\_STLCHLD\_061214\_01.06408.06408.3 | 3.4126 | 0.2588 | 99.8% | 1528.0144 | 1526.7563 | 1 | 5.316 | 53.8% | 5 | K.LAQANGWGVMVSHR.S | 3 |

---

|  |  |  |  |  |  |  |  |  |
| --- | --- | --- | --- | --- | --- | --- | --- | --- |
| U | *gi|4506901|ref|NP\_003* | 7 | 29 | 37.2% | 164 | 19330 | 11.6 | splicing factor, arginine/serine-rich 3 [Homo sapiens] |

| Filename XCorr DeltCN Conf% ObsM+H+ CalcM+H+ SpR ZScore Ion% # Sequence  | | | | | | | | | | | | |
| --- | --- | --- | --- | --- | --- | --- | --- | --- | --- | --- | --- | --- |
| \* | Astrin\_STLCHLD\_tube2\_061314\_01.04557.04557.2 | 4.6238 | 0.3445 | 100.0% | 1878.1921 | 1878.0519 | 1 | 7.16 | 65.6% | 5 | K.VYVGNLGNNGNKTELER.A | 2 |
| \* | Astrin\_NLD\_STLC\_tube2\_021014\_01.08889.08889.1 | 1.8643 | 0.2613 | 97.5% | 1043.56 | 1044.198 | 93 | 5.102 | 43.8% | 1 | R.AFGYYGPLR.S | 1 |
| \* | Astrin\_STLCHLD\_tube2\_061314\_01.08489.08489.2 | 3.074 | 0.4513 | 100.0% | 1044.0922 | 1044.198 | 1 | 8.287 | 87.5% | 12 | R.AFGYYGPLR.S | 2 |
|  | Astrin\_STLCLD20\_112214\_tube2\_01.15758.15758.2 | 3.3718 | 0.37 | 100.0% | 1622.3522 | 1622.7771 | 1 | 6.459 | 65.4% | 4 | R.NPPGFAFVEFEDPR.D | 22 |
| \* | Astrin\_STLCLD20\_112214\_tube2\_01.14967.14967.2 | 3.4948 | 0.4018 | 100.0% | 2320.652 | 2321.5107 | 11 | 6.421 | 40.0% | 2 | R.NPPGFAFVEFEDPRDAADAVR.E | 2 |
| \* | Astrin\_STLCLD20\_112214\_01.12193.12193.3 | 2.8981 | 0.3195 | 99.4% | 2322.1443 | 2321.5107 | 1 | 5.096 | 33.8% | 4 | R.NPPGFAFVEFEDPRDAADAVR.E | 3 |
| \* | Astrin\_NLD\_STLC\_031014\_02.06932.06932.3 | 3.1176 | 0.1909 | 96.4% | 1835.7544 | 1832.9338 | 2 | 4.831 | 38.5% | 1 | R.ERSLS\*RERNHKPSR.S | 3 |

Similarities:
gi|72534660|ref|NP\_00(1:6)  

---

|  |  |  |  |  |  |  |  |  |
| --- | --- | --- | --- | --- | --- | --- | --- | --- |
| U | *gi|4504517|ref|NP\_001* | 6 | 26 | 36.6% | 205 | 22783 | 6.4 | heat shock protein beta-1 [Homo sapiens] |

| Filename XCorr DeltCN Conf% ObsM+H+ CalcM+H+ SpR ZScore Ion% # Sequence  | | | | | | | | | | | | |
| --- | --- | --- | --- | --- | --- | --- | --- | --- | --- | --- | --- | --- |
| \* | Astrin\_STLCHLD\_tube2\_050114\_01.10764.10764.3 | 3.3775 | 0.2942 | 99.8% | 1904.8744 | 1904.0537 | 1 | 5.649 | 41.1% | 6 | R.GPSWDPFRDWYPHSR.L | 3 |
| \* | Astrin\_STLCHLD\_tube2\_050114\_01.10418.10418.2 | 4.0182 | 0.4491 | 100.0% | 1164.1322 | 1164.3494 | 1 | 8.048 | 83.3% | 9 | R.LFDQAFGLPR.L | 2 |
| \* | Astrin\_STLCLD20\_112214\_tube2\_01.12213.12213.2 | 4.8386 | 0.4427 | 100.0% | 1784.5922 | 1785.0068 | 1 | 8.434 | 60.0% | 5 | R.VSLDVNHFAPDELTVK.T | 2 |
| \* | Astrin\_STLCLD20\_112214\_tube2\_01.12236.12236.3 | 2.9535 | 0.369 | 99.8% | 1784.8143 | 1785.0068 | 4 | 6.029 | 35.0% | 2 | R.VSLDVNHFAPDELTVK.T | 3 |
| \* | Astrin\_STLCLD20\_112214\_01.11115.11115.2 | 3.7385 | 0.527 | 100.0% | 1906.5922 | 1907.1307 | 1 | 8.434 | 50.0% | 3 | K.LATQSNEITIPVTFESR.A | 2 |
| \* | Astrin\_NLD\_STLC\_031014\_01.07510.07510.2 | 3.3963 | 0.5073 | 100.0% | 1644.2122 | 1644.7789 | 1 | 9.311 | 62.5% | 1 | R.AQLGGPEAAKSDETAAK.- | 2 |

---

|  |  |  |  |  |  |  |  |  |
| --- | --- | --- | --- | --- | --- | --- | --- | --- |
| U | *gi|4826998|ref|NP\_005* | 36 | 219 | 36.4% | 707 | 76150 | 9.4 | splicing factor proline/glutamine rich (polypyrimidine tract binding protein associated) [Homo sapiens] |

| Filename XCorr DeltCN Conf% ObsM+H+ CalcM+H+ SpR ZScore Ion% # Sequence  | | | | | | | | | | | | |
| --- | --- | --- | --- | --- | --- | --- | --- | --- | --- | --- | --- | --- |
| \* | Astrin\_STLCLD20\_112214\_tube2\_01.06933.06933.2 | 2.2186 | 0.3038 | 98.0% | 1268.1921 | 1268.4332 | 3 | 6.656 | 59.1% | 2 | R.SPPPGMGLNQNR.G | 2 |
| \* | Astrin\_NLD\_STLC\_031014\_01.08885.08885.3 | 4.5679 | 0.3873 | 99.9% | 2371.2844 | 2371.725 | 1 | 6.95 | 34.4% | 1 | K.MPGGPKPGGGPGLSTPGGHPKPPHR.G | 3 |
| \* | Astrin\_STLCHLD\_tube2\_050114\_01.04173.04173.3 | 3.1936 | 0.2827 | 99.0% | 2451.6843 | 2451.725 | 7 | 4.652 | 28.1% | 1 | K.MPGGPKPGGGPGLS\*TPGGHPKPPHR.G | 3 |
| \* | Astrin\_STLCLD20\_112214\_tube2\_01.05523.05523.3 | 3.3624 | 0.4263 | 99.9% | 2404.1343 | 2404.5747 | 1 | 6.416 | 34.5% | 2 | R.QHHPPYHQQHHQGPPPGGPGGR.S | 3 |
| \* | Astrin\_STLCHLD\_050114\_01.04865.04865.3 | 3.0292 | 0.3215 | 99.8% | 1358.0343 | 1356.429 | 2 | 5.852 | 38.6% | 1 | R.SEEKISDSEGFK.A | 3 |
| \* | AstrinSTLCLD\_041714\_02.07149.07149.3 | 4.0011 | 0.4068 | 99.9% | 2123.4844 | 2124.3555 | 1 | 6.611 | 37.5% | 3 | R.SEEKISDSEGFKANLSLLR.R | 3 |
| \* | Astrin\_STLCHLD\_tube2\_050114\_01.08873.08873.2 | 3.4427 | 0.282 | 99.8% | 1649.7922 | 1650.8723 | 2 | 5.022 | 57.1% | 1 | K.ISDSEGFKANLSLLR.R | 2 |
| \* | AstrinSTLCLD\_041714\_02.07395.07395.3 | 4.5582 | 0.4672 | 100.0% | 1650.9543 | 1650.8723 | 1 | 7.328 | 44.6% | 14 | K.ISDSEGFKANLSLLR.R | 3 |
| \* | Astrin\_STLCLD20\_112214\_tube2\_01.16137.16137.2 | 3.5934 | 0.3997 | 100.0% | 1808.7122 | 1809.0258 | 1 | 6.208 | 53.3% | 6 | R.LFVGNLPADITEDEFK.R | 2 |
| \* | Astrin\_STLCLD20\_112214\_tube2\_01.14229.14229.2 | 4.4517 | 0.5094 | 100.0% | 1964.5922 | 1965.2133 | 1 | 9.002 | 65.6% | 6 | R.LFVGNLPADITEDEFKR.L | 2 |
| \* | AstrinSTLCLD\_041714\_01.09686.09686.3 | 3.0256 | 0.257 | 98.4% | 1966.5844 | 1965.2133 | 11 | 5.022 | 35.9% | 5 | R.LFVGNLPADITEDEFKR.L | 3 |
| \* | Astrin\_NLD\_STLC\_tube2\_021014\_01.07648.07648.2 | 3.5824 | 0.4683 | 100.0% | 1254.2122 | 1253.3971 | 5 | 7.353 | 60.0% | 12 | K.YGEPGEVFINK.G | 2 |
| \* | AstrinSTLCLD\_041714\_01.08013.08013.2 | 4.6661 | 0.5079 | 100.0% | 1743.7122 | 1745.0007 | 1 | 9.931 | 70.0% | 2 | R.ALAEIAKAELDDTPMR.G | 2 |
| \* | Astrin\_NLD\_STLC\_tube2\_021014\_01.09422.09422.3 | 3.4497 | 0.3101 | 99.8% | 1744.5844 | 1745.0007 | 2 | 5.824 | 43.3% | 1 | R.ALAEIAKAELDDTPMR.G | 3 |
| \* | Astrin\_NLD\_STLC\_tube2\_021014\_01.04878.04878.1 | 1.5919 | 0.2516 | 95.2% | 1047.52 | 1048.1559 | 46 | 3.923 | 43.8% | 1 | K.AELDDTPMR.G | 1 |
| \* | Astrin\_STLCHLD\_050114\_01.06235.06235.2 | 2.5667 | 0.2665 | 99.2% | 1048.1122 | 1048.1559 | 1 | 5.81 | 81.2% | 3 | K.AELDDTPMR.G | 2 |
| \* | Astrin\_NLD\_STLC\_031014\_01.09147.09147.1 | 2.2164 | 0.4345 | 100.0% | 1143.61 | 1144.3188 | 1 | 6.516 | 60.0% | 3 | R.FATHAAALSVR.N | 1 |
| \* | Astrin\_STLCHLD\_050114\_01.06320.06320.2 | 3.4433 | 0.3813 | 100.0% | 1144.4922 | 1144.3188 | 1 | 7.598 | 90.0% | 10 | R.FATHAAALSVR.N | 2 |
| \* | Astrin\_STLCLD20\_112214\_tube2\_01.18612.18612.3 | 5.5508 | 0.3735 | 100.0% | 3767.8145 | 3766.2046 | 1 | 6.603 | 25.8% | 1 | R.FATHAAALSVRNLSPYVSNELLEEAFSQFGPIER.A | 3 |
| \* | Astrin\_STLCHLD\_tube2\_050114\_01.14975.14975.3 | 3.6456 | 0.4038 | 99.9% | 2640.3245 | 2640.9092 | 1 | 6.545 | 30.7% | 1 | R.NLSPYVSNELLEEAFSQFGPIER.A | 3 |
| \* | Astrin\_STLCLD20\_112214\_01.15302.15302.2 | 5.2212 | 0.5868 | 100.0% | 2640.5522 | 2640.9092 | 1 | 9.563 | 47.7% | 13 | R.NLSPYVSNELLEEAFSQFGPIER.A | 2 |
|  | Astrin\_NLD\_STLC\_tube2\_021014\_01.04706.04706.2 | 2.3101 | 0.2583 | 98.6% | 886.47217 | 887.0238 | 1 | 6.189 | 78.6% | 5 | R.AVVIVDDR.G | 22 |
|  | Astrin\_NLD\_STLC\_031014\_01.09174.09174.1 | 2.1813 | 0.3489 | 96.4% | 886.54 | 887.0238 | 12 | 6.823 | 64.3% | 5 | R.AVVIVDDR.G | 11 |
| \* | Astrin\_NLD\_STLC\_031014\_01.09130.09130.3 | 2.8129 | 0.3365 | 99.8% | 1619.1543 | 1619.8613 | 1 | 5.606 | 38.3% | 1 | R.STGKGIVEFASKPAAR.K | 3 |
| \* | Astrin\_NLD\_STLC\_031014\_01.09250.09250.2 | 2.8399 | 0.3557 | 100.0% | 1246.1322 | 1246.452 | 1 | 6.958 | 72.7% | 10 | K.GIVEFASKPAAR.K | 2 |
| \* | Astrin\_NLD\_STLC\_tube2\_021014\_02.05219.05219.2 | 4.6092 | 0.4974 | 100.0% | 1762.5721 | 1763.8632 | 1 | 8.028 | 69.2% | 17 | R.FAQHGTFEYEYSQR.W | 2 |
| \* | AstrinSTLCLD\_041714\_02.05708.05708.3 | 4.8419 | 0.3406 | 100.0% | 1763.9043 | 1763.8632 | 1 | 6.861 | 46.2% | 53 | R.FAQHGTFEYEYSQR.W | 3 |
| \* | AstrinSTLCLD\_041714\_01.07941.07941.3 | 4.0775 | 0.2825 | 99.8% | 2744.7244 | 2743.9648 | 1 | 5.507 | 33.0% | 2 | K.DAKDKLESEMEDAYHEHQANLLR.Q | 3 |
| \* | Astrin\_NLD\_STLC\_031014\_01.09880.09880.3 | 5.9824 | 0.4465 | 100.0% | 2429.4844 | 2429.6233 | 1 | 8.472 | 42.1% | 10 | K.DKLESEMEDAYHEHQANLLR.Q | 3 |
| \* | Astrin\_STLCLD20\_112214\_tube2\_01.05859.05859.2 | 3.5096 | 0.1883 | 99.6% | 1573.4922 | 1573.7821 | 1 | 5.32 | 72.7% | 1 | R.RMEELHNQEMQK.R | 2 |
| \* | AstrinSTLCLD\_041714\_01.03604.03604.3 | 2.9679 | 0.2426 | 98.8% | 1573.5243 | 1573.7821 | 2 | 5.17 | 40.9% | 1 | R.RMEELHNQEMQK.R | 3 |
| \* | Astrin\_STLCHLD\_050114\_01.04232.04232.3 | 3.8661 | 0.1659 | 99.0% | 1730.0044 | 1729.9696 | 2 | 4.784 | 41.7% | 1 | R.RMEELHNQEMQKR.K | 3 |
| \* | Astrin\_NLD\_STLC\_031014\_01.08504.08504.2 | 3.9628 | 0.352 | 100.0% | 1417.2522 | 1417.5946 | 1 | 6.181 | 85.0% | 3 | R.MEELHNQEMQK.R | 2 |
| \* | Astrin\_NLD\_STLC\_031014\_01.08526.08526.3 | 2.598 | 0.2336 | 96.4% | 1418.1843 | 1417.5946 | 7 | 4.588 | 47.5% | 1 | R.MEELHNQEMQK.R | 3 |
| \* | Astrin\_STLCHLD\_050114\_01.05311.05311.2 | 4.2654 | 0.571 | 100.0% | 1342.3522 | 1342.4569 | 1 | 9.217 | 78.6% | 16 | R.FGQGGAGPVGGQGPR.G | 2 |
| \* | Astrin\_NLD\_STLC\_tube2\_021014\_01.04508.04508.2 | 2.9289 | 0.3445 | 100.0% | 1120.8322 | 1121.2561 | 10 | 7.923 | 63.6% | 4 | R.GMGPGTPAGYGR.G | 2 |

Similarities:
gi|224028244|ref|NP\_0(2:34)  

---

|  |  |  |  |  |  |  |  |  |
| --- | --- | --- | --- | --- | --- | --- | --- | --- |
| U | *gi|33286418|ref|NP\_00* | 13 | 40 | 36.3% | 531 | 57937 | 7.8 | pyruvate kinase, muscle isoform M2 [Homo sapiens] |

| Filename XCorr DeltCN Conf% ObsM+H+ CalcM+H+ SpR ZScore Ion% # Sequence  | | | | | | | | | | | | |
| --- | --- | --- | --- | --- | --- | --- | --- | --- | --- | --- | --- | --- |
|  | Astrin\_STLCLD20\_112214\_tube2\_01.09890.09890.2 | 3.3459 | 0.3238 | 100.0% | 1198.4722 | 1198.3617 | 2 | 6.845 | 80.0% | 9 | R.LDIDSPPITAR.N | 2 |
|  | Astrin\_STLCHLD\_tube2\_050114\_01.04382.04382.3 | 4.0463 | 0.357 | 99.9% | 1885.6144 | 1885.0458 | 1 | 7.06 | 45.0% | 4 | R.LNFSHGTHEYHAETIK.N | 3 |
|  | Astrin\_STLCHLD\_tube2\_061314\_01.11603.11603.3 | 3.9061 | 0.4711 | 100.0% | 2467.1943 | 2466.7937 | 1 | 6.88 | 30.7% | 2 | R.TATESFASDPILYRPVAVALDTK.G | 3 |
|  | Astrin\_STLCHLD\_tube2\_061314\_01.11132.11132.2 | 3.241 | 0.2975 | 99.9% | 1465.2722 | 1463.7142 | 1 | 5.342 | 66.7% | 2 | K.IYVDDGLISLQVK.Q | 2 |
|  | Astrin\_STLCHLD\_061214\_02.07936.07936.2 | 4.0569 | 0.4468 | 100.0% | 1780.8722 | 1780.9292 | 2 | 8.128 | 50.0% | 5 | K.GADFLVTEVENGGSLGSK.K | 2 |
|  | Astrin\_STLCHLD\_tube2\_061314\_01.15558.15558.2 | 4.088 | 0.5007 | 100.0% | 1860.2922 | 1861.1224 | 1 | 8.883 | 63.3% | 8 | K.FGVEQDVDMVFASFIR.K | 2 |
|  | Astrin\_STLCLD20\_112214\_01.11403.11403.2 | 2.8914 | 0.1979 | 97.9% | 1823.9521 | 1823.0741 | 1 | 4.637 | 53.3% | 1 | R.RFDEILEASDGIMVAR.G | 2 |
|  | Astrin\_STLCHLD\_tube2\_061314\_02.07996.07996.3 | 4.1481 | 0.3465 | 99.9% | 1823.9944 | 1823.0741 | 1 | 6.232 | 46.7% | 3 | R.RFDEILEASDGIMVAR.G | 3 |
|  | Astrin\_STLCHLD\_tube2\_061314\_01.07907.07907.2 | 2.807 | 0.3153 | 99.8% | 1141.7122 | 1142.2946 | 1 | 5.731 | 80.0% | 1 | R.GDLGIEIPAEK.V | 2 |
| \* | Astrin\_STLCHLD\_061214\_02.08114.08114.3 | 2.6294 | 0.2961 | 98.2% | 2088.5942 | 2089.3586 | 6 | 4.697 | 31.2% | 2 | R.EAEAAIYHLQLFEELRR.L | 3 |
| \* | Astrin\_STLCHLD\_061214\_02.08531.08531.2 | 3.9782 | 0.5102 | 100.0% | 2175.3323 | 2176.4282 | 1 | 9.047 | 50.0% | 1 | R.LAPITSDPTEATAVGAVEASFK.C | 2 |
|  | Astrin\_NLD\_STLC\_tube2\_021014\_01.05291.05291.1 | 1.734 | 0.3165 | 98.0% | 840.5 | 841.0415 | 27 | 5.158 | 57.1% | 1 | R.APIIAVTR.N | 1 |
|  | Astrin\_STLCLD20\_112214\_tube2\_01.13672.13672.3 | 3.7788 | 0.1958 | 97.9% | 2392.7043 | 2392.7815 | 1 | 4.149 | 36.9% | 1 | K.KGDVVIVLTGWRPGSGFTNTMR.V | 3 |

---

|  |  |  |  |  |  |  |  |  |
| --- | --- | --- | --- | --- | --- | --- | --- | --- |
| U | *gi|4506623|ref|NP\_000* | 5 | 9 | 36.0% | 136 | 15798 | 10.6 | ribosomal protein L27 [Homo sapiens] |

| Filename XCorr DeltCN Conf% ObsM+H+ CalcM+H+ SpR ZScore Ion% # Sequence  | | | | | | | | | | | | |
| --- | --- | --- | --- | --- | --- | --- | --- | --- | --- | --- | --- | --- |
| \* | Astrin\_STLCLD20\_112214\_01.08674.08674.1 | 1.8258 | 0.2726 | 97.1% | 826.56 | 827.058 | 1 | 5.575 | 71.4% | 1 | K.VVLVLAGR.Y | 1 |
| \* | AstrinSTLCLD\_041714\_01.05309.05309.3 | 3.847 | 0.3575 | 99.9% | 2273.3342 | 2273.4238 | 1 | 6.294 | 33.8% | 1 | K.NIDDGTSDRPYSHALVAGIDR.Y | 3 |
|  | Astrin\_STLCLD20\_112214\_tube2\_01.08604.08604.2 | 3.2147 | 0.3582 | 100.0% | 1408.1522 | 1408.6177 | 1 | 7.302 | 75.0% | 3 | K.VYNYNHLMPTR.Y | 2 |
|  | Astrin\_STLCHLD\_tube2\_061314\_01.05534.05534.3 | 2.3343 | 0.3236 | 99.0% | 1409.4243 | 1408.6177 | 26 | 4.966 | 47.5% | 2 | K.VYNYNHLMPTR.Y | 3 |
| \* | Astrin\_NLD\_STLC\_tube2\_021014\_01.08306.08306.2 | 2.2155 | 0.3333 | 99.1% | 1049.7522 | 1050.1968 | 42 | 6.058 | 50.0% | 2 | R.YSVDIPLDK.T | 2 |

---

|  |  |  |  |  |  |  |  |  |
| --- | --- | --- | --- | --- | --- | --- | --- | --- |
| U | *gi|4506645|ref|NP\_000* | 3 | 4 | 35.7% | 70 | 8218 | 10.1 | ribosomal protein L38 [Homo sapiens] |
| U | *gi|78214522|ref|NP\_00* | 3 | 4 | 35.7% | 70 | 8218 | 10.1 | ribosomal protein L38 [Homo sapiens] |

| Filename XCorr DeltCN Conf% ObsM+H+ CalcM+H+ SpR ZScore Ion% # Sequence  | | | | | | | | | | | | |
| --- | --- | --- | --- | --- | --- | --- | --- | --- | --- | --- | --- | --- |
|  | Astrin\_STLCHLD\_tube2\_061314\_01.09769.09769.2 | 2.6043 | 0.2502 | 98.3% | 1576.2722 | 1576.8766 | 13 | 4.903 | 50.0% | 1 | R.KIEEIKDFLLTAR.R | 2 |
|  | Astrin\_STLCHLD\_tube2\_050114\_01.09299.09299.3 | 3.589 | 0.297 | 100.0% | 1576.5243 | 1576.8766 | 1 | 5.515 | 47.9% | 2 | R.KIEEIKDFLLTAR.R | 3 |
|  | Astrin\_STLCHLD\_061214\_01.07384.07384.2 | 3.2532 | 0.2262 | 99.6% | 1486.6921 | 1486.7484 | 1 | 5.177 | 72.7% | 1 | R.YLYTLVITDKEK.A | 2 |

---

|  |  |  |  |  |  |  |  |  |
| --- | --- | --- | --- | --- | --- | --- | --- | --- |
| U | *gi|34098946|ref|NP\_00* | 6 | 19 | 35.5% | 324 | 35924 | 9.9 | nuclease sensitive element binding protein 1 [Homo sapiens] |

| Filename XCorr DeltCN Conf% ObsM+H+ CalcM+H+ SpR ZScore Ion% # Sequence  | | | | | | | | | | | | |
| --- | --- | --- | --- | --- | --- | --- | --- | --- | --- | --- | --- | --- |
|  | Astrin\_STLCHLD\_tube2\_050114\_01.04841.04841.3 | 3.4665 | 0.2201 | 99.1% | 1745.1843 | 1745.9298 | 1 | 5.08 | 44.6% | 3 | R.NDTKEDVFVHQTAIK.K | 33 |
|  | Astrin\_STLCHLD\_tube2\_061314\_02.06922.06922.2 | 4.5895 | 0.526 | 100.0% | 1796.5922 | 1796.8822 | 1 | 9.606 | 65.6% | 7 | R.SVGDGETVEFDVVEGEK.G | 22 |
| \* | Astrin\_STLCHLD\_050114\_01.04435.04435.3 | 6.1553 | 0.5007 | 100.0% | 3258.5344 | 3259.2566 | 1 | 9.553 | 33.9% | 3 | R.NYQQNYQNSESGEKNEGSESAPEGQAQQR.R | 3 |
| \* | Astrin\_STLCLD20\_112214\_tube2\_01.07647.07647.3 | 5.3307 | 0.3882 | 100.0% | 3224.4844 | 3225.4795 | 1 | 6.441 | 27.6% | 4 | R.RPQYSNPPVQGEVMEGADNQGAGEQGRPVR.Q | 3 |
| \* | Astrin\_NLD\_STLC\_031014\_01.05030.05030.3 | 4.1415 | 0.3514 | 99.9% | 2628.9243 | 2629.5835 | 1 | 6.334 | 33.0% | 1 | R.EDGNEEDKENQGDETQGQQPPQR.R | 3 |
| \* | Astrin\_STLCLD20\_112214\_tube2\_01.05595.05595.3 | 3.0194 | 0.3757 | 99.8% | 2785.0444 | 2785.771 | 1 | 5.514 | 26.1% | 1 | R.EDGNEEDKENQGDETQGQQPPQRR.Y | 3 |

Similarities:
gi|224586882|ref|NP\_0(2:4)  

---

|  |  |  |  |  |  |  |  |  |
| --- | --- | --- | --- | --- | --- | --- | --- | --- |
| U | *gi|209862831|ref|NP\_0* | 13 | 49 | 35.4% | 339 | 38604 | 7.8 | annexin A2 isoform 2 [Homo sapiens] |
| U | *gi|50845388|ref|NP\_00* | 13 | 49 | 33.6% | 357 | 40411 | 8.4 | annexin A2 isoform 1 [Homo sapiens] |
| U | *gi|50845386|ref|NP\_00* | 13 | 49 | 35.4% | 339 | 38604 | 7.8 | annexin A2 isoform 2 [Homo sapiens] |
| U | *gi|4757756|ref|NP\_004* | 13 | 49 | 35.4% | 339 | 38604 | 7.8 | annexin A2 isoform 2 [Homo sapiens] |

| Filename XCorr DeltCN Conf% ObsM+H+ CalcM+H+ SpR ZScore Ion% # Sequence  | | | | | | | | | | | | |
| --- | --- | --- | --- | --- | --- | --- | --- | --- | --- | --- | --- | --- |
|  | Astrin\_STLCLD20\_112214\_01.13384.13384.2 | 3.7393 | 0.4068 | 100.0% | 1544.0122 | 1543.7605 | 1 | 7.363 | 57.7% | 3 | K.GVDEVTIVNILTNR.S | 2 |
|  | Astrin\_STLCHLD\_050114\_01.11177.11177.2 | 2.8515 | 0.2602 | 99.7% | 1112.1322 | 1112.2303 | 1 | 4.998 | 68.8% | 2 | R.QDIAFAYQR.R | 2 |
|  | Astrin\_STLCLD20\_112214\_tube2\_01.19079.19079.2 | 4.9535 | 0.4297 | 100.0% | 1651.6721 | 1651.9872 | 1 | 8.778 | 70.0% | 6 | K.SALSGHLETVILGLLK.T | 2 |
|  | Astrin\_STLCHLD\_050114\_01.13731.13731.3 | 4.2852 | 0.309 | 99.9% | 1651.9744 | 1651.9872 | 23 | 5.874 | 41.7% | 2 | K.SALSGHLETVILGLLK.T | 3 |
|  | Astrin\_STLCHLD\_050114\_01.06446.06446.2 | 3.6633 | 0.4393 | 100.0% | 1223.4521 | 1223.3251 | 1 | 7.66 | 80.0% | 6 | K.TPAQYDASELK.A | 2 |
|  | Astrin\_STLCHLD\_050114\_01.05768.05768.2 | 3.1872 | 0.2786 | 100.0% | 1245.0122 | 1245.3347 | 1 | 6.28 | 83.3% | 6 | R.TNQELQEINR.V | 2 |
|  | Astrin\_STLCHLD\_tube2\_050114\_01.09366.09366.2 | 3.5243 | 0.4317 | 100.0% | 1812.5521 | 1812.928 | 1 | 7.877 | 56.7% | 1 | K.TDLEKDIISDTSGDFR.K | 2 |
|  | Astrin\_STLCHLD\_tube2\_061314\_01.08599.08599.3 | 4.4844 | 0.4396 | 100.0% | 1941.5343 | 1941.102 | 1 | 7.797 | 53.1% | 9 | K.TDLEKDIISDTSGDFRK.L | 3 |
|  | Astrin\_STLCLD20\_112214\_01.10295.10295.3 | 4.504 | 0.3014 | 99.9% | 2066.6943 | 2066.1887 | 1 | 5.951 | 52.9% | 10 | R.RAEDGSVIDYELIDQDAR.D | 3 |
|  | Astrin\_STLCHLD\_tube2\_050114\_01.09590.09590.2 | 3.0368 | 0.4258 | 100.0% | 1909.6522 | 1910.0013 | 2 | 6.959 | 46.9% | 1 | R.AEDGSVIDYELIDQDAR.D | 2 |
|  | Astrin\_STLCHLD\_050114\_01.05119.05119.2 | 1.9942 | 0.2843 | 96.6% | 1037.0721 | 1037.1606 | 156 | 4.812 | 62.5% | 1 | R.DLYDAGVKR.K | 2 |
|  | Astrin\_STLCLD20\_112214\_tube2\_01.11715.11715.2 | 2.5552 | 0.277 | 99.0% | 1422.9321 | 1422.5774 | 1 | 5.066 | 75.0% | 1 | K.SLYYYIQQDTK.G | 2 |
|  | Astrin\_STLCHLD\_061214\_02.06698.06698.3 | 2.7229 | 0.2328 | 95.2% | 2014.4343 | 2014.1986 | 13 | 4.227 | 31.7% | 1 | K.SLYYYIQQDTKGDYQK.A | 3 |

---

|  |  |  |  |  |  |  |  |  |
| --- | --- | --- | --- | --- | --- | --- | --- | --- |
| U | *gi|30795231|ref|NP\_00* | 3 | 5 | 35.2% | 227 | 22693 | 4.6 | brain abundant, membrane attached signal protein 1 [Homo sapiens] |

| Filename XCorr DeltCN Conf% ObsM+H+ CalcM+H+ SpR ZScore Ion% # Sequence  | | | | | | | | | | | | |
| --- | --- | --- | --- | --- | --- | --- | --- | --- | --- | --- | --- | --- |
| \* | Astrin\_STLCHLD\_061214\_01.04158.04158.3 | 4.5362 | 0.403 | 99.9% | 2699.5444 | 2699.7986 | 1 | 7.035 | 32.7% | 1 | K.AEGAATEEEGTPKESEPQAAAEPAEAK.E | 3 |
| \* | Astrin\_STLCHLD\_061214\_01.04140.04140.3 | 3.7066 | 0.3118 | 99.8% | 2298.7744 | 2299.5022 | 1 | 4.808 | 43.5% | 2 | K.AEPPKAPEQEQAAPGPAAGGEAPK.A | 3 |
| \* | Astrin\_STLCHLD\_061214\_01.04627.04627.3 | 5.4847 | 0.4521 | 100.0% | 2894.6643 | 2894.1216 | 1 | 7.925 | 33.9% | 2 | K.AQGPAASAEEPKPVEAPAANSDQTVTVKE.- | 3 |

---

|  |  |  |  |  |  |  |  |  |
| --- | --- | --- | --- | --- | --- | --- | --- | --- |
| U | *gi|4501881|ref|NP\_001* | 17 | 128 | 35.0% | 377 | 42051 | 5.4 | actin, alpha 1, skeletal muscle [Homo sapiens] |
| U | *gi|4885049|ref|NP\_005* | 17 | 128 | 35.0% | 377 | 42019 | 5.4 | cardiac muscle alpha actin 1 proprotein [Homo sapiens] |

| Filename XCorr DeltCN Conf% ObsM+H+ CalcM+H+ SpR ZScore Ion% # Sequence  | | | | | | | | | | | | |
| --- | --- | --- | --- | --- | --- | --- | --- | --- | --- | --- | --- | --- |
|  | Astrin\_NLD\_STLC\_031014\_01.06884.06884.2 | 3.2662 | 0.4547 | 100.0% | 976.33215 | 977.02136 | 2 | 7.839 | 72.2% | 30 | K.AGFAGDDAPR.A | 22 |
|  | Astrin\_STLCLD20\_112214\_01.08519.08519.2 | 2.8688 | 0.378 | 100.0% | 1199.3522 | 1199.4415 | 19 | 6.226 | 60.0% | 12 | R.AVFPSIVGRPR.H | 22 |
|  | Astrin\_STLCLD20\_112214\_tube2\_01.06642.06642.1 | 2.0003 | 0.2433 | 96.8% | 1171.74 | 1172.4058 | 1 | 5.709 | 65.0% | 1 | R.HQGVMVGMGQK.D | 111 |
|  | AstrinSTLCLD\_041714\_01.03582.03582.2 | 2.8958 | 0.0922 | 95.9% | 1199.4922 | 1199.2163 | 1 | 7.356 | 65.0% | 1 | K.DSYVGDEAQSK.R | 22 |
|  | Astrin\_STLCLD20\_112214\_tube2\_01.12063.12063.2 | 4.5689 | 0.4291 | 100.0% | 1961.2522 | 1962.1841 | 1 | 7.923 | 63.3% | 7 | K.YPIEHGIITNWDDMEK.I | 2 |
|  | Astrin\_NLD\_STLC\_tube2\_021014\_01.09402.09402.3 | 4.3625 | 0.2297 | 99.8% | 1962.5944 | 1962.1841 | 52 | 4.981 | 40.0% | 4 | K.YPIEHGIITNWDDMEK.I | 3 |
|  | Astrin\_STLCLD20\_112214\_01.13180.13180.3 | 5.0812 | 0.3412 | 99.9% | 3459.3843 | 3459.8628 | 1 | 5.511 | 32.7% | 2 | K.YPIEHGIITNWDDMEKIWHHTFYNELR.V | 3 |
|  | Astrin\_STLCHLD\_tube2\_050114\_01.05846.05846.3 | 3.4498 | 0.3159 | 100.0% | 1515.5643 | 1516.7019 | 1 | 6.429 | 55.0% | 9 | K.IWHHTFYNELR.V | 33 |
|  | Astrin\_STLCHLD\_tube2\_050114\_01.05795.05795.2 | 3.3616 | 0.4634 | 100.0% | 1516.2722 | 1516.7019 | 1 | 7.494 | 80.0% | 9 | K.IWHHTFYNELR.V | 22 |
|  | Astrin\_STLCLD20\_112214\_01.09520.09520.2 | 4.9868 | 0.4013 | 100.0% | 1957.7122 | 1957.234 | 1 | 7.383 | 64.7% | 1 | R.VAPEEHPTLLTEAPLNPK.A | 2 |
|  | Astrin\_NLD\_STLC\_tube2\_021014\_01.11475.11475.2 | 2.9524 | 0.2059 | 98.4% | 1624.5322 | 1624.8927 | 2 | 5.245 | 57.7% | 4 | R.LDLAGRDLTDYLMK.I | 222 |
|  | AstrinSTLCLD\_041714\_01.09070.09070.1 | 2.0807 | 0.1982 | 96.1% | 998.79 | 999.167 | 3 | 4.182 | 71.4% | 3 | R.DLTDYLMK.I | 111 |
|  | Astrin\_STLCLD20\_112214\_tube2\_01.13726.13726.2 | 2.0606 | 0.342 | 98.9% | 999.21216 | 999.167 | 16 | 5.639 | 71.4% | 2 | R.DLTDYLMK.I | 222 |
|  | Astrin\_NLD\_STLC\_tube2\_021014\_01.10676.10676.2 | 4.8426 | 0.3035 | 100.0% | 1791.2922 | 1791.9554 | 1 | 8.531 | 83.3% | 21 | K.SYELPDGQVITIGNER.F | 222 |
|  | Astrin\_STLCHLD\_061214\_01.05115.05115.3 | 3.7961 | 0.3358 | 99.9% | 1550.2144 | 1549.8843 | 10 | 6.274 | 44.2% | 1 | R.MQKEITALAPSTMK.I | 33 |
|  | Astrin\_NLD\_STLC\_tube2\_021014\_01.06791.06791.1 | 2.5336 | 0.4765 | 100.0% | 1161.6 | 1162.3868 | 1 | 7.532 | 55.0% | 2 | K.EITALAPSTMK.I | 11 |
|  | Astrin\_STLCLD20\_112214\_tube2\_01.09039.09039.2 | 2.8112 | 0.3091 | 99.7% | 1162.4122 | 1162.3868 | 13 | 5.941 | 55.0% | 19 | K.EITALAPSTMK.I | 22 |

Similarities:
gi|4501885|ref|NP\_001(13:4)  
gi|63055057|ref|NP\_00(5:12)  

---

|  |  |  |  |  |  |  |  |  |
| --- | --- | --- | --- | --- | --- | --- | --- | --- |
| U | *gi|20149594|ref|NP\_03* | 20 | 110 | 34.7% | 724 | 83264 | 5.0 | heat shock 90kDa protein 1, beta [Homo sapiens] |

| Filename XCorr DeltCN Conf% ObsM+H+ CalcM+H+ SpR ZScore Ion% # Sequence  | | | | | | | | | | | | |
| --- | --- | --- | --- | --- | --- | --- | --- | --- | --- | --- | --- | --- |
|  | Astrin\_STLCHLD\_tube2\_061314\_01.05813.05813.2 | 3.1355 | 0.4012 | 100.0% | 1276.3322 | 1276.3861 | 2 | 6.953 | 68.2% | 2 | R.ELISNASDALDK.I | 2 |
|  | Astrin\_STLCLD20\_112214\_tube2\_01.11159.11159.2 | 3.7146 | 0.4163 | 100.0% | 1545.5721 | 1545.733 | 1 | 6.528 | 73.1% | 4 | R.ELISNASDALDKIR.Y | 2 |
| \* | Astrin\_STLCLD20\_112214\_01.10736.10736.2 | 2.144 | 0.3129 | 97.5% | 1350.3322 | 1350.6135 | 181 | 5.701 | 41.7% | 1 | R.TLTLVDTGIGMTK.A | 2 |
|  | Astrin\_STLCLD20\_112214\_tube2\_01.12254.12254.2 | 3.4834 | 0.4327 | 100.0% | 1243.4521 | 1243.4459 | 1 | 7.724 | 81.8% | 6 | K.ADLINNLGTIAK.S | 22 |
|  | Astrin\_STLCHLD\_061214\_02.06734.06734.3 | 3.3418 | 0.257 | 99.0% | 2258.3342 | 2257.294 | 1 | 5.261 | 35.5% | 2 | K.HNDDEQYAWESSAGGSFTVR.A | 33 |
|  | Astrin\_STLCHLD\_050114\_02.06302.06302.3 | 4.6555 | 0.5081 | 100.0% | 2016.2344 | 2016.2584 | 1 | 8.269 | 48.3% | 16 | K.VILHLKEDQTEYLEER.R | 33 |
| \* | Astrin\_STLCLD20\_112214\_tube2\_01.15354.15354.2 | 4.5798 | 0.4949 | 100.0% | 1810.2322 | 1810.1027 | 1 | 8.36 | 71.4% | 1 | K.HSQFIGYPITLYLEK.E | 2 |
|  | Astrin\_STLCHLD\_050114\_01.05627.05627.2 | 2.9558 | 0.3011 | 100.0% | 1152.4722 | 1152.2462 | 1 | 5.822 | 81.2% | 4 | K.YIDQEELNK.T | 22 |
| \* | Astrin\_STLCHLD\_tube2\_061314\_02.06786.06786.2 | 5.084 | 0.5321 | 100.0% | 1849.3722 | 1848.9171 | 1 | 9.586 | 82.1% | 7 | R.NPDDITQEEYGEFYK.S | 2 |
|  | Astrin\_STLCLD20\_112214\_01.09962.09962.2 | 4.3602 | 0.4022 | 100.0% | 1528.3922 | 1528.6616 | 1 | 7.855 | 70.8% | 7 | K.SLTNDWEDHLAVK.H | 22 |
|  | Astrin\_STLCHLD\_050114\_02.06964.06964.3 | 2.8425 | 0.2263 | 98.0% | 1349.2444 | 1349.4886 | 308 | 5.284 | 40.0% | 2 | K.HFSVEGQLEFR.A | 33 |
|  | Astrin\_NLD\_STLC\_tube2\_021014\_02.06472.06472.2 | 3.0916 | 0.4043 | 100.0% | 1349.2722 | 1349.4886 | 1 | 6.76 | 65.0% | 18 | K.HFSVEGQLEFR.A | 22 |
| \* | Astrin\_STLCHLD\_tube2\_061314\_01.10236.10236.2 | 1.9625 | 0.2378 | 95.9% | 830.3722 | 830.06104 | 1 | 4.869 | 83.3% | 1 | R.ALLFIPR.R | 2 |
| \* | Astrin\_STLCLD20\_112214\_tube2\_01.12466.12466.2 | 2.7135 | 0.2089 | 98.7% | 1237.3922 | 1237.4008 | 3 | 4.694 | 72.2% | 3 | R.RAPFDLFENK.K | 2 |
| \* | Astrin\_STLCHLD\_tube2\_061314\_02.06674.06674.3 | 4.6511 | 0.4153 | 100.0% | 2178.6843 | 2178.2915 | 1 | 7.014 | 38.9% | 17 | R.YHTSQSGDEMTSLSEYVSR.M | 3 |
| \* | Astrin\_NLD\_STLC\_tube2\_021014\_01.06894.06894.2 | 2.1488 | 0.3197 | 98.4% | 1161.9922 | 1161.297 | 29 | 5.324 | 55.6% | 1 | K.SIYYITGESK.E | 2 |
| \* | Astrin\_STLCHLD\_050114\_01.07091.07091.2 | 2.6319 | 0.3153 | 99.5% | 1250.3922 | 1250.3538 | 245 | 7.101 | 50.0% | 3 | K.EQVANSAFVER.V | 2 |
| \* | Astrin\_STLCHLD\_tube2\_061314\_01.05965.05965.2 | 3.2708 | 0.2184 | 99.6% | 1250.4122 | 1249.4574 | 1 | 6.279 | 80.0% | 1 | R.DNSTMGYMMAK.K | 2 |
| \* | Astrin\_STLCHLD\_tube2\_050114\_01.07024.07024.3 | 4.1236 | 0.5214 | 100.0% | 1783.4944 | 1784.025 | 1 | 8.673 | 46.4% | 13 | K.HLEINPDHPIVETLR.Q | 3 |
| \* | Astrin\_STLCHLD\_tube2\_061314\_01.16956.16956.3 | 3.469 | 0.2518 | 98.6% | 3290.6643 | 3288.725 | 63 | 4.073 | 19.0% | 1 | K.AVKDLVVLLFETALLSSGFSLEDPQTHSNR.I | 3 |

Similarities:
gi|153792590|ref|NP\_0(7:13)  

---

|  |  |  |  |  |  |  |  |  |
| --- | --- | --- | --- | --- | --- | --- | --- | --- |
| U | *gi|4506699|ref|NP\_001* | 2 | 7 | 33.7% | 83 | 9111 | 8.5 | ribosomal protein S21 [Homo sapiens] |

| Filename XCorr DeltCN Conf% ObsM+H+ CalcM+H+ SpR ZScore Ion% # Sequence  | | | | | | | | | | | | |
| --- | --- | --- | --- | --- | --- | --- | --- | --- | --- | --- | --- | --- |
| \* | Astrin\_STLCHLD\_tube2\_050114\_02.06229.06229.3 | 4.4081 | 0.473 | 100.0% | 1971.3844 | 1971.1956 | 1 | 9.273 | 38.2% | 6 | K.DHASIQMNVAEVDKVTGR.F | 3 |
| \* | Astrin\_STLCLD20\_112214\_01.06173.06173.2 | 2.8323 | 0.3965 | 100.0% | 1122.2522 | 1123.2231 | 8 | 6.288 | 66.7% | 1 | R.MGESDDSILR.L | 2 |

---

|  |  |  |  |  |  |  |  |  |
| --- | --- | --- | --- | --- | --- | --- | --- | --- |
| U | *gi|14165435|ref|NP\_11* | 14 | 88 | 32.2% | 463 | 50976 | 5.5 | heterogeneous nuclear ribonucleoprotein K isoform b [Homo sapiens] |
| U | *gi|14165439|ref|NP\_00* | 14 | 88 | 32.1% | 464 | 51028 | 5.3 | heterogeneous nuclear ribonucleoprotein K isoform a [Homo sapiens] |
| U | *gi|14165437|ref|NP\_11* | 14 | 88 | 32.1% | 464 | 51028 | 5.3 | heterogeneous nuclear ribonucleoprotein K isoform a [Homo sapiens] |

| Filename XCorr DeltCN Conf% ObsM+H+ CalcM+H+ SpR ZScore Ion% # Sequence  | | | | | | | | | | | | |
| --- | --- | --- | --- | --- | --- | --- | --- | --- | --- | --- | --- | --- |
|  | Astrin\_STLCLD20\_112214\_tube2\_01.06615.06615.3 | 4.0547 | 0.3536 | 99.9% | 1736.7244 | 1736.8969 | 1 | 5.851 | 46.2% | 4 | K.RPAEDMEEEQAFKR.S | 3 |
|  | Astrin\_STLCLD20\_112214\_tube2\_01.07581.07581.2 | 3.3204 | 0.3255 | 100.0% | 1350.2522 | 1350.4894 | 1 | 5.87 | 80.0% | 1 | R.SRNTDEMVELR.I | 2 |
|  | Astrin\_STLCLD20\_112214\_01.06733.06733.2 | 2.8448 | 0.3794 | 100.0% | 1107.3922 | 1107.2238 | 1 | 6.833 | 81.2% | 4 | R.NTDEMVELR.I | 2 |
|  | Astrin\_STLCLD20\_112214\_01.05876.05876.2 | 4.1582 | 0.5045 | 100.0% | 1781.2322 | 1781.8302 | 3 | 8.669 | 56.2% | 9 | R.TDYNASVSVPDSSGPER.I | 2 |
|  | Astrin\_STLCLD20\_112214\_02.17237.17237.2 | 4.3464 | 0.5109 | 100.0% | 1716.0922 | 1716.0251 | 1 | 9.141 | 60.0% | 9 | R.ILSISADIETIGEILK.K | 2 |
|  | Astrin\_STLCLD20\_112214\_tube2\_01.19446.19446.2 | 3.2268 | 0.3788 | 100.0% | 1843.3722 | 1844.1992 | 6 | 6.48 | 37.5% | 2 | R.ILSISADIETIGEILKK.I | 2 |
|  | Astrin\_STLCHLD\_tube2\_050114\_02.07330.07330.2 | 3.9984 | 0.4974 | 100.0% | 1519.9122 | 1519.8711 | 1 | 8.471 | 71.4% | 13 | R.LLIHQSLAGGIIGVK.G | 2 |
|  | AstrinSTLCLD\_041714\_01.07996.07996.3 | 5.0666 | 0.4418 | 100.0% | 1520.4844 | 1519.8711 | 1 | 7.212 | 48.2% | 14 | R.LLIHQSLAGGIIGVK.G | 3 |
|  | Astrin\_STLCHLD\_tube2\_061314\_01.12146.12146.2 | 3.6355 | 0.3902 | 100.0% | 1342.3121 | 1341.6311 | 1 | 6.924 | 72.7% | 6 | K.IILDLISESPIK.G | 2 |
|  | AstrinSTLCLD\_041714\_01.09504.09504.2 | 3.6935 | 0.3298 | 100.0% | 1554.9122 | 1554.8705 | 1 | 5.988 | 61.5% | 3 | K.IILDLISESPIKGR.A | 2 |
|  | Astrin\_NLD\_STLC\_tube2\_021014\_01.10666.10666.2 | 5.3361 | 0.552 | 100.0% | 1917.5922 | 1918.1974 | 1 | 9.927 | 61.1% | 10 | R.GSYGDLGGPIITTQVTIPK.D | 2 |
|  | AstrinSTLCLD\_041714\_01.04917.04917.3 | 4.0722 | 0.3832 | 99.9% | 2070.1143 | 2070.1772 | 1 | 6.602 | 38.9% | 8 | R.HESGASIKIDEPLEGSEDR.I | 3 |
|  | Astrin\_STLCHLD\_050114\_01.06543.06543.2 | 3.3909 | 0.4183 | 100.0% | 1260.1122 | 1260.3 | 1 | 6.946 | 85.0% | 3 | K.IDEPLEGSEDR.I | 2 |
|  | Astrin\_STLCLD20\_112214\_tube2\_01.16102.16102.2 | 5.6302 | 0.537 | 100.0% | 2590.9922 | 2590.9365 | 1 | 9.445 | 52.3% | 2 | R.IITITGTQDQIQNAQYLLQNSVK.Q | 2 |

---

|  |  |  |  |  |  |  |  |  |
| --- | --- | --- | --- | --- | --- | --- | --- | --- |
| U | *gi|94538362|ref|NP\_00* | 11 | 48 | 32.2% | 428 | 47064 | 5.3 | flotillin 2 [Homo sapiens] |

| Filename XCorr DeltCN Conf% ObsM+H+ CalcM+H+ SpR ZScore Ion% # Sequence  | | | | | | | | | | | | |
| --- | --- | --- | --- | --- | --- | --- | --- | --- | --- | --- | --- | --- |
| \* | Astrin\_STLCLD20\_112214\_tube2\_01.12927.12927.2 | 3.1387 | 0.4533 | 100.0% | 1378.8922 | 1379.6023 | 1 | 7.786 | 77.3% | 2 | K.NVVLQTLEGHLR.S | 2 |
| \* | Astrin\_STLCHLD\_tube2\_050114\_01.15887.15887.3 | 5.476 | 0.5188 | 100.0% | 2835.2344 | 2836.313 | 3 | 8.944 | 29.2% | 1 | R.MGIEILSFTIKDVYDKVDYLSSLGK.T | 3 |
| \* | Astrin\_STLCHLD\_050114\_01.06652.06652.2 | 2.9759 | 0.3144 | 100.0% | 1145.7122 | 1146.1992 | 1 | 5.146 | 80.0% | 2 | R.DADIGVAEAER.D | 2 |
| \* | Astrin\_STLCHLD\_050114\_02.06252.06252.2 | 2.4332 | 0.2805 | 98.9% | 1124.6322 | 1124.2358 | 1 | 5.793 | 72.2% | 4 | K.SAFSEEVNIK.T | 2 |
| \* | Astrin\_STLCHLD\_tube2\_050114\_02.06907.06907.2 | 4.6022 | 0.4009 | 100.0% | 1521.5322 | 1521.6702 | 1 | 7.677 | 76.9% | 19 | K.TAEAQLAYELQGAR.E | 2 |
| \* | Astrin\_STLCHLD\_050114\_02.07300.07300.3 | 3.7467 | 0.2962 | 100.0% | 1523.0343 | 1521.6702 | 1 | 5.213 | 59.6% | 2 | K.TAEAQLAYELQGAR.E | 3 |
| \* | Astrin\_STLCHLD\_tube2\_061314\_02.06045.06045.3 | 4.0117 | 0.2316 | 99.8% | 1641.9243 | 1641.865 | 7 | 5.614 | 47.9% | 5 | K.IRQEEIEIEVVQR.K | 3 |
| \* | Astrin\_STLCLD20\_112214\_02.11136.11136.2 | 2.105 | 0.2651 | 96.0% | 1270.3922 | 1270.4716 | 273 | 4.573 | 55.0% | 1 | K.QIAVEAQEILR.T | 2 |
| \* | Astrin\_STLCHLD\_050114\_01.05918.05918.2 | 2.3794 | 0.2066 | 96.9% | 1146.1721 | 1146.3292 | 13 | 4.303 | 66.7% | 1 | R.TDKELIATVR.R | 2 |
| \* | Astrin\_STLCLD20\_112214\_01.09003.09003.2 | 3.5627 | 0.4266 | 100.0% | 1376.3522 | 1375.5187 | 1 | 7.56 | 75.0% | 6 | K.VDEIVVLSGDNSK.V | 2 |
| \* | Astrin\_STLCLD20\_112214\_01.11996.11996.2 | 4.1248 | 0.3682 | 100.0% | 1935.7522 | 1935.2712 | 1 | 7.175 | 58.3% | 5 | R.LLAELPASVHALTGVDLSK.I | 2 |

---

|  |  |  |  |  |  |  |  |  |
| --- | --- | --- | --- | --- | --- | --- | --- | --- |
| U | *gi|5031753|ref|NP\_005* | 10 | 54 | 32.1% | 449 | 49229 | 6.3 | heterogeneous nuclear ribonucleoprotein H1 [Homo sapiens] |

| Filename XCorr DeltCN Conf% ObsM+H+ CalcM+H+ SpR ZScore Ion% # Sequence  | | | | | | | | | | | | |
| --- | --- | --- | --- | --- | --- | --- | --- | --- | --- | --- | --- | --- |
| \* | AstrinSTLCLD\_041714\_01.06399.06399.2 | 3.3519 | 0.3313 | 100.0% | 1505.4321 | 1505.5933 | 12 | 5.842 | 58.3% | 1 | R.GLPWSCSADEVQR.F | 2 |
| \* | Astrin\_NLD\_STLC\_tube2\_021014\_02.06448.06448.3 | 3.6979 | 0.1852 | 97.6% | 2109.2043 | 2108.2231 | 2 | 4.056 | 37.5% | 1 | R.EGRPSGEAFVELESEDEVK.L | 3 |
| \* | Astrin\_NLD\_STLC\_tube2\_021014\_02.07875.07875.2 | 3.2962 | 0.3272 | 100.0% | 1334.7522 | 1335.5176 | 2 | 6.311 | 70.0% | 7 | K.SNNVEMDWVLK.H | 2 |
|  | Astrin\_STLCHLD\_050114\_01.05415.05415.2 | 4.1137 | 0.511 | 100.0% | 1685.3722 | 1685.7501 | 1 | 8.232 | 70.0% | 3 | K.HTGPNSPDTANDGFVR.L | 22 |
|  | Astrin\_STLCHLD\_050114\_01.05353.05353.3 | 3.9638 | 0.2195 | 99.8% | 1686.9543 | 1685.7501 | 2 | 4.836 | 48.3% | 2 | K.HTGPNSPDTANDGFVR.L | 33 |
|  | Astrin\_NLD\_STLC\_tube2\_021014\_01.11806.11806.2 | 5.1994 | 0.4738 | 100.0% | 1842.3121 | 1843.0001 | 1 | 9.375 | 68.8% | 18 | R.STGEAFVQFASQEIAEK.A | 22 |
|  | Astrin\_STLCHLD\_050114\_01.12769.12769.2 | 4.1076 | 0.4915 | 100.0% | 1997.4521 | 1998.2023 | 1 | 7.45 | 59.4% | 7 | R.ATENDIYNFFSPLNPVR.V | 22 |
|  | Astrin\_STLCLD20\_112214\_tube2\_01.07737.07737.2 | 3.2067 | 0.508 | 100.0% | 1093.4122 | 1093.2278 | 1 | 8.327 | 83.3% | 9 | R.VHIEIGPDGR.V | 222 |
| \* | AstrinSTLCLD\_041714\_02.06668.06668.3 | 2.9494 | 0.3338 | 99.8% | 2178.4744 | 2179.363 | 1 | 5.381 | 32.5% | 3 | R.VTGEADVEFATHEDAVAAMSK.D | 3 |
| \* | Astrin\_STLCHLD\_050114\_02.07676.07676.3 | 3.2425 | 0.3422 | 99.8% | 2144.0645 | 2143.32 | 1 | 6.494 | 36.8% | 3 | R.YVELFLNSTAGASGGAYEHR.Y | 3 |

Similarities:
gi|148470397|ref|NP\_0(2:8)  
gi|74099697|ref|NP\_00(4:6)  

---

|  |  |  |  |  |  |  |  |  |
| --- | --- | --- | --- | --- | --- | --- | --- | --- |
| U | *gi|117190174|ref|NP\_0* | 6 | 49 | 31.4% | 293 | 32338 | 5.1 | heterogeneous nuclear ribonucleoprotein C isoform b [Homo sapiens] |
| U | *gi|117190254|ref|NP\_0* | 6 | 49 | 31.4% | 293 | 32338 | 5.1 | heterogeneous nuclear ribonucleoprotein C isoform b [Homo sapiens] |

| Filename XCorr DeltCN Conf% ObsM+H+ CalcM+H+ SpR ZScore Ion% # Sequence  | | | | | | | | | | | | |
| --- | --- | --- | --- | --- | --- | --- | --- | --- | --- | --- | --- | --- |
|  | Astrin\_STLCHLD\_061214\_01.09169.09169.2 | 3.8274 | 0.2744 | 100.0% | 1318.5521 | 1317.6145 | 1 | 6.335 | 81.8% | 14 | R.VFIGNLNTLVVK.K | 2 |
|  | AstrinSTLCLD\_041714\_01.09119.09119.2 | 3.9994 | 0.5109 | 100.0% | 1331.2722 | 1330.4857 | 1 | 8.415 | 80.0% | 12 | K.GFAFVQYVNER.N | 2 |
|  | Astrin\_STLCHLD\_061214\_02.08137.08137.2 | 5.1 | 0.481 | 100.0% | 1683.2922 | 1684.0038 | 1 | 8.259 | 73.3% | 19 | R.MIAGQVLDINLAAEPK.V | 2 |
|  | AstrinSTLCLD\_041714\_02.08518.08518.3 | 3.4815 | 0.3429 | 99.8% | 2814.9543 | 2815.9404 | 1 | 5.591 | 29.5% | 2 | R.SAAEMYGSSFDLDYDFQRDYYDR.M | 3 |
|  | Astrin\_STLCHLD\_050114\_01.06009.06009.2 | 2.1321 | 0.3654 | 99.2% | 943.7922 | 944.1649 | 3 | 7.008 | 68.8% | 1 | R.VPPPPPIAR.A | 2 |
|  | Astrin\_STLCLD20\_112214\_tube2\_01.05634.05634.3 | 4.0924 | 0.343 | 99.9% | 2369.3943 | 2369.4583 | 1 | 5.555 | 45.0% | 1 | K.NDKSEEEQSSSSVKKDETNVK.M | 3 |

---

|  |  |  |  |  |  |  |  |  |
| --- | --- | --- | --- | --- | --- | --- | --- | --- |
| U | *gi|32455264|ref|NP\_85* | 7 | 44 | 31.2% | 199 | 22110 | 8.1 | peroxiredoxin 1 [Homo sapiens] |
| U | *gi|4505591|ref|NP\_002* | 7 | 44 | 31.2% | 199 | 22110 | 8.1 | peroxiredoxin 1 [Homo sapiens] |
| U | *gi|32455266|ref|NP\_85* | 7 | 44 | 31.2% | 199 | 22110 | 8.1 | peroxiredoxin 1 [Homo sapiens] |

| Filename XCorr DeltCN Conf% ObsM+H+ CalcM+H+ SpR ZScore Ion% # Sequence  | | | | | | | | | | | | |
| --- | --- | --- | --- | --- | --- | --- | --- | --- | --- | --- | --- | --- |
|  | Astrin\_STLCHLD\_tube2\_061314\_01.04662.04662.2 | 2.6098 | 0.4011 | 100.0% | 1165.4122 | 1165.3496 | 1 | 6.698 | 70.0% | 2 | K.ATAVMPDGQFK.D | 2 |
|  | Astrin\_STLCLD20\_112214\_tube2\_01.09722.09722.2 | 3.0434 | 0.2851 | 100.0% | 1108.1921 | 1108.2798 | 1 | 6.364 | 83.3% | 5 | R.TIAQDYGVLK.A | 2 |
|  | Astrin\_NLD\_STLC\_tube2\_021014\_02.07452.07452.3 | 3.7651 | 0.3529 | 99.9% | 1985.3644 | 1984.2163 | 1 | 6.946 | 39.7% | 7 | R.TIAQDYGVLKADEGISFR.G | 3 |
|  | Astrin\_STLCHLD\_tube2\_061314\_01.09906.09906.2 | 1.918 | 0.2523 | 95.0% | 920.83215 | 921.0813 | 12 | 5.812 | 71.4% | 1 | R.GLFIIDDK.G | 22 |
|  | Astrin\_STLCHLD\_tube2\_050114\_01.10778.10778.2 | 3.2519 | 0.2212 | 99.5% | 1361.9122 | 1360.6395 | 1 | 5.696 | 68.2% | 5 | R.GLFIIDDKGILR.Q | 22 |
|  | Astrin\_STLCLD20\_112214\_tube2\_01.10262.10262.2 | 2.9791 | 0.2073 | 99.1% | 1212.5122 | 1212.3915 | 3 | 6.522 | 75.0% | 13 | R.QITVNDLPVGR.S | 22 |
|  | Astrin\_STLCHLD\_tube2\_061314\_01.08472.08472.2 | 3.3514 | 0.4978 | 100.0% | 1197.0122 | 1197.3763 | 1 | 7.848 | 88.9% | 11 | R.LVQAFQFTDK.H | 2 |

Similarities:
gi|32189392|ref|NP\_00(1:6)  
gi|5453549|ref|NP\_006(2:5)  

---

|  |  |  |  |  |  |  |  |  |
| --- | --- | --- | --- | --- | --- | --- | --- | --- |
| U | *gi|222352151|ref|NP\_0* | 8 | 36 | 30.9% | 356 | 37498 | 7.1 | poly(rC) binding protein 1 [Homo sapiens] |

| Filename XCorr DeltCN Conf% ObsM+H+ CalcM+H+ SpR ZScore Ion% # Sequence  | | | | | | | | | | | | |
| --- | --- | --- | --- | --- | --- | --- | --- | --- | --- | --- | --- | --- |
| \* | Astrin\_STLCLD20\_112214\_tube2\_01.14316.14316.2 | 3.5479 | 0.3875 | 100.0% | 1390.4722 | 1389.6781 | 2 | 6.884 | 66.7% | 5 | R.IITLTGPTNAIFK.A | 2 |
| \* | Astrin\_STLCHLD\_tube2\_050114\_01.13743.13743.3 | 4.7225 | 0.3663 | 99.9% | 3380.4543 | 3380.8562 | 1 | 6.483 | 25.8% | 4 | K.AFAMIIDKLEEDINSSMTNSTAASRPPVTLR.L | 3 |
|  | Astrin\_STLCHLD\_061214\_01.06314.06314.2 | 5.6661 | 0.5681 | 100.0% | 2090.4521 | 2091.2573 | 1 | 9.651 | 60.5% | 15 | R.ESTGAQVQVAGDMLPNSTER.A | 22 |
|  | Astrin\_STLCHLD\_061214\_01.06312.06312.3 | 4.5802 | 0.3485 | 99.9% | 2091.4744 | 2091.2573 | 1 | 6.752 | 38.2% | 5 | R.ESTGAQVQVAGDMLPNSTER.A | 33 |
| \* | Astrin\_STLCHLD\_tube2\_061314\_02.06305.06305.3 | 4.1556 | 0.3742 | 99.9% | 2606.5745 | 2607.875 | 4 | 6.884 | 26.0% | 2 | R.QQSHFAMMHGGTGFAGIDSSSPEVK.G | 3 |
| \* | Astrin\_STLCHLD\_050114\_02.06918.06918.3 | 3.6103 | 0.239 | 98.6% | 2687.6042 | 2687.875 | 1 | 6.076 | 30.2% | 3 | R.QQSHFAMMHGGTGFAGIDSSS\*PEVK.G | 3 |
| \* | Astrin\_STLCLD20\_112214\_tube2\_02.07244.07244.3 | 2.9638 | 0.2691 | 97.3% | 2688.1743 | 2687.875 | 2 | 4.541 | 29.2% | 1 | R.QQSHFAMMHGGTGFAGIDSS\*SPEVK.G | 3 |
| \* | Astrin\_STLCHLD\_061214\_02.09502.09502.2 | 2.3759 | 0.2492 | 96.3% | 2177.2722 | 2178.4937 | 14 | 5.073 | 32.5% | 1 | R.QVTITGSAASISLAQYLINAR.L | 2 |

Similarities:
gi|14141166|ref|NP\_11(2:6)  

---

|  |  |  |  |  |  |  |  |  |
| --- | --- | --- | --- | --- | --- | --- | --- | --- |
| U | *gi|221307584|ref|NP\_0* | 8 | 36 | 30.8% | 299 | 33296 | 9.8 | prohibitin 2 isoform 1 [Homo sapiens] |
| U | *gi|6005854|ref|NP\_009* | 8 | 36 | 30.8% | 299 | 33296 | 9.8 | prohibitin 2 isoform 2 [Homo sapiens] |

| Filename XCorr DeltCN Conf% ObsM+H+ CalcM+H+ SpR ZScore Ion% # Sequence  | | | | | | | | | | | | |
| --- | --- | --- | --- | --- | --- | --- | --- | --- | --- | --- | --- | --- |
|  | Astrin\_NLD\_STLC\_tube2\_021014\_02.07409.07409.2 | 3.3729 | 0.5669 | 100.0% | 1260.2322 | 1260.5222 | 1 | 10.015 | 70.8% | 1 | K.LLLGAGAVAYGVR.E | 2 |
|  | Astrin\_STLCLD20\_112214\_01.11302.11302.2 | 3.0964 | 0.2653 | 99.3% | 1854.8722 | 1855.1038 | 12 | 5.06 | 40.6% | 2 | R.IGGVQQDTILAEGLHFR.I | 2 |
|  | Astrin\_STLCHLD\_050114\_02.08445.08445.3 | 4.5089 | 0.5137 | 100.0% | 1855.5543 | 1855.1038 | 1 | 8.476 | 50.0% | 12 | R.IGGVQQDTILAEGLHFR.I | 3 |
|  | Astrin\_STLCHLD\_050114\_01.14243.14243.2 | 4.167 | 0.4088 | 100.0% | 1724.5122 | 1725.0428 | 1 | 7.427 | 66.7% | 5 | R.IPWFQYPIIYDIR.A | 2 |
|  | Astrin\_NLD\_STLC\_tube2\_021014\_01.06501.06501.2 | 2.2541 | 0.3451 | 99.5% | 995.3122 | 995.077 | 1 | 6.764 | 85.7% | 2 | R.LGLDYEER.V | 2 |
|  | Astrin\_STLCHLD\_tube2\_061314\_01.07765.07765.2 | 2.6158 | 0.3122 | 99.6% | 1178.1522 | 1178.3335 | 3 | 5.684 | 66.7% | 2 | K.FNASQLITQR.A | 2 |
|  | Astrin\_NLD\_STLC\_031014\_01.06956.06956.2 | 3.2321 | 0.416 | 100.0% | 1215.4321 | 1216.3336 | 1 | 8.011 | 68.2% | 11 | K.IVQAEGEAEAAK.M | 2 |
|  | Astrin\_STLCHLD\_tube2\_050114\_02.09942.09942.2 | 2.554 | 0.2094 | 96.0% | 2226.3123 | 2226.4912 | 6 | 4.795 | 38.9% | 1 | R.IYLTADNLVLNLQDESFTR.G | 2 |

---

|  |  |  |  |  |  |  |  |  |
| --- | --- | --- | --- | --- | --- | --- | --- | --- |
| U | *gi|15431295|ref|NP\_15* | 8 | 23 | 30.8% | 211 | 24261 | 11.7 | ribosomal protein L13 [Homo sapiens] |
| U | *gi|15431297|ref|NP\_00* | 8 | 23 | 30.8% | 211 | 24261 | 11.7 | ribosomal protein L13 [Homo sapiens] |

| Filename XCorr DeltCN Conf% ObsM+H+ CalcM+H+ SpR ZScore Ion% # Sequence  | | | | | | | | | | | | |
| --- | --- | --- | --- | --- | --- | --- | --- | --- | --- | --- | --- | --- |
|  | Astrin\_STLCHLD\_050114\_01.11000.11000.2 | 2.5357 | 0.1586 | 95.7% | 1347.3121 | 1346.5344 | 2 | 4.1 | 70.0% | 1 | R.RVATWFNQPAR.K | 2 |
|  | Astrin\_STLCHLD\_050114\_01.11911.11911.2 | 3.1878 | 0.2922 | 100.0% | 1190.2722 | 1190.3469 | 45 | 6.134 | 66.7% | 5 | R.VATWFNQPAR.K | 2 |
|  | Astrin\_STLCLD20\_112214\_tube2\_01.12533.12533.2 | 2.2876 | 0.3844 | 99.8% | 951.3522 | 951.0672 | 2 | 6.824 | 78.6% | 1 | R.GFSLEELR.V | 2 |
|  | Astrin\_NLD\_STLC\_031014\_01.08555.08555.2 | 2.3169 | 0.3225 | 98.5% | 1475.0322 | 1475.6017 | 14 | 5.987 | 50.0% | 1 | R.NKSTESLQANVQR.L | 2 |
|  | Astrin\_STLCHLD\_050114\_01.04715.04715.2 | 3.8243 | 0.3532 | 100.0% | 1234.4521 | 1233.3237 | 1 | 5.771 | 80.0% | 3 | K.STESLQANVQR.L | 2 |
|  | AstrinSTLCLD\_041714\_01.08658.08658.3 | 3.2938 | 0.2738 | 99.1% | 2430.9844 | 2428.8064 | 1 | 5.354 | 31.8% | 1 | K.KGDSSAEELKLATQLTGPVMPVR.N | 3 |
|  | Astrin\_STLCHLD\_tube2\_061314\_01.07994.07994.2 | 3.1064 | 0.3873 | 100.0% | 1383.5521 | 1383.6923 | 1 | 7.39 | 70.8% | 9 | K.LATQLTGPVMPVR.N | 2 |
|  | Astrin\_STLCHLD\_050114\_01.04858.04858.2 | 2.5146 | 0.258 | 98.8% | 1238.0721 | 1237.3953 | 19 | 4.469 | 61.1% | 2 | R.VITEEEKNFK.A | 2 |

---

|  |  |  |  |  |  |  |  |  |
| --- | --- | --- | --- | --- | --- | --- | --- | --- |
| U | *gi|117968353|ref|NP\_1* | 14 | 46 | 30.4% | 464 | 54304 | 8.3 | NUF2, NDC80 kinetochore complex component [Homo sapiens] |
| U | *gi|117968420|ref|NP\_6* | 14 | 46 | 30.4% | 464 | 54304 | 8.3 | NUF2, NDC80 kinetochore complex component [Homo sapiens] |

| Filename XCorr DeltCN Conf% ObsM+H+ CalcM+H+ SpR ZScore Ion% # Sequence  | | | | | | | | | | | | |
| --- | --- | --- | --- | --- | --- | --- | --- | --- | --- | --- | --- | --- |
|  | Astrin\_STLCHLD\_050114\_02.08458.08458.2 | 3.7181 | 0.4326 | 100.0% | 1327.1522 | 1327.5693 | 1 | 9.25 | 85.0% | 15 | R.YNVAEIVIHIR.N | 2 |
|  | Astrin\_STLCHLD\_061214\_01.08881.08881.3 | 3.4198 | 0.4484 | 100.0% | 1327.9443 | 1327.5693 | 17 | 7.054 | 42.5% | 4 | R.YNVAEIVIHIR.N | 3 |
|  | Astrin\_STLCHLD\_050114\_01.12172.12172.3 | 3.0581 | 0.3165 | 99.8% | 2231.3643 | 2231.6355 | 3 | 5.377 | 32.4% | 1 | K.NDLYPNPKPEVLHMIYMR.A | 3 |
|  | Astrin\_STLCHLD\_tube2\_050114\_01.08644.08644.2 | 2.4365 | 0.289 | 99.2% | 1033.5122 | 1033.259 | 3 | 5.793 | 75.0% | 1 | R.ALQIVYGIR.L | 2 |
|  | Astrin\_STLCHLD\_050114\_01.12633.12633.2 | 4.2653 | 0.4713 | 100.0% | 1465.3522 | 1464.7532 | 1 | 9.118 | 68.2% | 3 | R.FLSGIINFIHFR.E | 2 |
|  | Astrin\_STLCHLD\_tube2\_061314\_01.04559.04559.3 | 4.9594 | 0.4189 | 100.0% | 2102.1543 | 2102.3882 | 1 | 7.6 | 44.4% | 1 | K.SSADKMQQLNAAHQEALMK.L | 3 |
|  | Astrin\_STLCHLD\_tube2\_050114\_01.04935.04935.2 | 4.475 | 0.4713 | 100.0% | 1612.7122 | 1613.8903 | 1 | 8.087 | 76.9% | 2 | K.MQQLNAAHQEALMK.L | 2 |
|  | Astrin\_STLCHLD\_tube2\_061314\_01.06446.06446.2 | 3.4662 | 0.376 | 100.0% | 1550.2722 | 1549.6746 | 1 | 7.091 | 79.2% | 5 | R.LDSVPVEEQEEFK.Q | 2 |
|  | Astrin\_STLCHLD\_tube2\_061314\_01.14311.14311.3 | 3.7437 | 0.2826 | 99.8% | 3886.4944 | 3888.193 | 1 | 4.553 | 24.2% | 2 | R.LDSVPVEEQEEFKQLSDGIQELQQSLNQDFHQK.T | 3 |
|  | Astrin\_STLCHLD\_tube2\_061314\_01.10806.10806.2 | 4.5361 | 0.4425 | 100.0% | 1978.4122 | 1979.0588 | 1 | 7.668 | 62.5% | 2 | K.ESLNLEDQIESDESELK.K | 2 |
|  | Astrin\_STLCHLD\_tube2\_050114\_01.09516.09516.2 | 3.4745 | 0.3725 | 100.0% | 2106.3523 | 2107.233 | 1 | 6.249 | 41.2% | 1 | K.ESLNLEDQIESDESELKK.L | 2 |
|  | Astrin\_STLCHLD\_050114\_01.05665.05665.2 | 3.4211 | 0.4159 | 100.0% | 1174.1921 | 1174.3396 | 1 | 6.642 | 77.8% | 6 | R.VTTINQEIQK.I | 2 |
|  | Astrin\_STLCHLD\_tube2\_061314\_01.08485.08485.2 | 2.6269 | 0.2207 | 98.3% | 1334.2522 | 1333.6139 | 2 | 5.136 | 65.0% | 1 | K.LKSQEIFLNLK.T | 2 |
|  | Astrin\_STLCHLD\_tube2\_061314\_01.09479.09479.2 | 2.5212 | 0.2554 | 99.1% | 1091.7722 | 1092.2804 | 1 | 6.561 | 87.5% | 2 | K.SQEIFLNLK.T | 2 |

---

|  |  |  |  |  |  |  |  |  |
| --- | --- | --- | --- | --- | --- | --- | --- | --- |
| U | *gi|32189392|ref|NP\_00* | 5 | 27 | 30.3% | 198 | 21892 | 6.0 | peroxiredoxin 2 isoform a [Homo sapiens] |

| Filename XCorr DeltCN Conf% ObsM+H+ CalcM+H+ SpR ZScore Ion% # Sequence  | | | | | | | | | | | | |
| --- | --- | --- | --- | --- | --- | --- | --- | --- | --- | --- | --- | --- |
|  | Astrin\_STLCHLD\_tube2\_061314\_01.04754.04754.2 | 2.2457 | 0.2683 | 96.6% | 1335.1122 | 1335.5431 | 86 | 5.211 | 50.0% | 1 | K.ATAVVDGAFKEVK.L | 2 |
| \* | Astrin\_STLCLD20\_112214\_tube2\_01.17223.17223.2 | 4.3767 | 0.5141 | 100.0% | 1863.9922 | 1864.1954 | 1 | 8.409 | 58.8% | 3 | R.KEGGLGPLNIPLLADVTR.R | 2 |
| \* | Astrin\_STLCHLD\_tube2\_050114\_02.06111.06111.3 | 4.2187 | 0.3145 | 99.9% | 2086.1943 | 2086.309 | 1 | 5.88 | 36.8% | 6 | R.RLSEDYGVLKTDEGIAYR.G | 3 |
| \* | Astrin\_STLCHLD\_tube2\_050114\_02.06492.06492.3 | 3.9077 | 0.4421 | 100.0% | 1930.4043 | 1930.1217 | 1 | 8.124 | 37.5% | 4 | R.LSEDYGVLKTDEGIAYR.G | 3 |
|  | Astrin\_STLCLD20\_112214\_tube2\_01.10262.10262.2 | 2.9791 | 0.2073 | 99.1% | 1212.5122 | 1212.3915 | 3 | 6.522 | 75.0% | 13 | R.QITVNDLPVGR.S | 22 |

Similarities:
gi|32455264|ref|NP\_85(1:4)  

---

|  |  |  |  |  |  |  |  |  |
| --- | --- | --- | --- | --- | --- | --- | --- | --- |
| U | *gi|50592996|ref|NP\_00* | 22 | 353 | 29.8% | 450 | 50433 | 4.9 | tubulin, beta, 4 [Homo sapiens] |

| Filename XCorr DeltCN Conf% ObsM+H+ CalcM+H+ SpR ZScore Ion% # Sequence  | | | | | | | | | | | | |
| --- | --- | --- | --- | --- | --- | --- | --- | --- | --- | --- | --- | --- |
|  | Astrin\_STLCHLD\_tube2\_050114\_01.10300.10300.1 | 1.9796 | 0.4452 | 100.0% | 1615.8 | 1616.8701 | 25 | 6.88 | 42.9% | 1 | R.AILVDLEPGTMDSVR.S | 111 |
|  | Astrin\_STLCLD20\_112214\_01.11522.11522.2 | 4.511 | 0.4938 | 100.0% | 1617.3121 | 1616.8701 | 1 | 8.254 | 64.3% | 40 | R.AILVDLEPGTMDSVR.S | 222 |
|  | Astrin\_STLCHLD\_tube2\_050114\_01.12857.12857.2 | 7.2534 | 0.4819 | 100.0% | 1960.4722 | 1960.151 | 1 | 9.656 | 79.4% | 13 | K.GHYTEGAELVDSVLDVVR.K | 2222 |
|  | Astrin\_STLCHLD\_tube2\_050114\_01.12839.12839.3 | 4.3447 | 0.3812 | 99.9% | 1960.7644 | 1960.151 | 1 | 7.142 | 45.6% | 12 | K.GHYTEGAELVDSVLDVVR.K | 3333 |
|  | Astrin\_STLCHLD\_tube2\_061314\_01.11982.11982.3 | 4.7827 | 0.4838 | 100.0% | 2088.1143 | 2088.325 | 1 | 8.148 | 44.4% | 19 | K.GHYTEGAELVDSVLDVVRK.E | 3333 |
|  | Astrin\_STLCHLD\_050114\_01.12405.12405.2 | 6.3217 | 0.4514 | 100.0% | 2088.9321 | 2088.325 | 1 | 8.891 | 66.7% | 6 | K.GHYTEGAELVDSVLDVVRK.E | 2222 |
|  | Astrin\_STLCHLD\_050114\_01.11995.11995.1 | 2.1993 | 0.2639 | 97.3% | 1319.69 | 1320.5896 | 245 | 5.053 | 40.9% | 6 | R.IMNTFSVVPSPK.V | 1111 |
|  | Astrin\_STLCHLD\_tube2\_061314\_01.08597.08597.2 | 4.5471 | 0.3836 | 100.0% | 1320.4122 | 1320.5896 | 1 | 7.86 | 77.3% | 41 | R.IMNTFSVVPSPK.V | 2222 |
|  | Astrin\_STLCHLD\_tube2\_061314\_01.07361.07361.2 | 3.2733 | 0.2391 | 99.9% | 1132.2322 | 1131.2767 | 1 | 5.073 | 83.3% | 60 | R.FPGQLNADLR.K | 222222 |
|  | Astrin\_STLCHLD\_tube2\_050114\_01.05601.05601.2 | 2.7143 | 0.2756 | 99.3% | 1258.5521 | 1259.4508 | 11 | 5.358 | 65.0% | 14 | R.FPGQLNADLRK.L | 222222 |
|  | Astrin\_STLCHLD\_tube2\_050114\_01.05583.05583.3 | 3.152 | 0.3056 | 99.8% | 1259.8744 | 1259.4508 | 8 | 5.284 | 45.0% | 17 | R.FPGQLNADLRK.L | 333333 |
|  | Astrin\_STLCHLD\_tube2\_061314\_01.08730.08730.2 | 3.8464 | 0.3753 | 100.0% | 1272.4122 | 1272.5945 | 1 | 7.898 | 75.0% | 16 | R.KLAVNMVPFPR.L | 222222 |
|  | Astrin\_STLCLD20\_112214\_01.11192.11192.1 | 2.2399 | 0.2147 | 96.5% | 1143.63 | 1144.4204 | 6 | 6.378 | 61.1% | 4 | K.LAVNMVPFPR.L | 111111 |
|  | Astrin\_STLCHLD\_tube2\_050114\_01.09839.09839.2 | 3.9889 | 0.4725 | 100.0% | 1144.4122 | 1144.4204 | 1 | 8.55 | 94.4% | 18 | K.LAVNMVPFPR.L | 222222 |
|  | Astrin\_STLCHLD\_tube2\_061314\_01.12186.12186.2 | 4.648 | 0.4524 | 100.0% | 1692.6122 | 1692.9678 | 1 | 7.8 | 78.6% | 12 | R.ALTVPELTQQMFDAK.N | 222 |
|  | Astrin\_STLCHLD\_050114\_01.04952.04952.2 | 2.8672 | 0.3941 | 100.0% | 1066.2322 | 1066.2013 | 2 | 7.174 | 68.8% | 2 | K.NMMAACDPR.H | 222222 |
|  | Astrin\_STLCHLD\_tube2\_061314\_01.11781.11781.2 | 4.295 | 0.2978 | 100.0% | 1698.5322 | 1697.8877 | 1 | 7.708 | 73.1% | 8 | K.NSSYFVEWIPNNVK.V | 222222 |
| \* | Astrin\_STLCHLD\_tube2\_061314\_02.09999.09999.2 | 3.2328 | 0.1169 | 97.0% | 1874.5521 | 1875.1469 | 5 | 3.593 | 46.9% | 1 | K.MSSTFIGNSTAIQELFK.R | 2 |
|  | Astrin\_STLCHLD\_061214\_01.07812.07812.2 | 3.8437 | 0.3944 | 100.0% | 1386.3522 | 1386.6116 | 1 | 7.545 | 80.0% | 15 | K.RISEQFTAMFR.R | 22222 |
|  | Astrin\_STLCHLD\_tube2\_050114\_02.06787.06787.3 | 3.1948 | 0.3082 | 99.8% | 1543.3143 | 1542.7991 | 1 | 5.726 | 52.3% | 1 | K.RISEQFTAMFRR.K | 33333 |
|  | Astrin\_STLCLD20\_112214\_tube2\_01.13623.13623.1 | 1.9755 | 0.2993 | 98.1% | 1229.5 | 1230.4241 | 1 | 5.342 | 61.1% | 1 | R.ISEQFTAMFR.R | 11111 |
|  | Astrin\_STLCHLD\_tube2\_050114\_02.08042.08042.2 | 4.0657 | 0.4262 | 100.0% | 1231.3121 | 1230.4241 | 1 | 6.725 | 94.4% | 46 | R.ISEQFTAMFR.R | 22222 |

Similarities:
gi|29788785|ref|NP\_82(20:2)  
gi|5174735|ref|NP\_006(19:3)  
gi|29788768|ref|NP\_82(18:4)  
gi|21361322|ref|NP\_00(15:7)  
gi|14210536|ref|NP\_11(8:14)  

---

|  |  |  |  |  |  |  |  |  |
| --- | --- | --- | --- | --- | --- | --- | --- | --- |
| U | *gi|5032051|ref|NP\_005* | 3 | 8 | 29.8% | 151 | 16273 | 10.1 | ribosomal protein S14 [Homo sapiens] |
| U | *gi|68160922|ref|NP\_00* | 3 | 8 | 29.8% | 151 | 16273 | 10.1 | ribosomal protein S14 [Homo sapiens] |
| U | *gi|68160915|ref|NP\_00* | 3 | 8 | 29.8% | 151 | 16273 | 10.1 | ribosomal protein S14 [Homo sapiens] |

| Filename XCorr DeltCN Conf% ObsM+H+ CalcM+H+ SpR ZScore Ion% # Sequence  | | | | | | | | | | | | |
| --- | --- | --- | --- | --- | --- | --- | --- | --- | --- | --- | --- | --- |
|  | Astrin\_STLCLD20\_112214\_tube2\_01.11889.11889.3 | 3.6945 | 0.3807 | 99.9% | 2266.6443 | 2266.4473 | 1 | 5.984 | 41.2% | 1 | K.ADRDESSPYAAMLAAQDVAQR.C | 3 |
|  | Astrin\_NLD\_STLC\_031014\_01.08787.08787.2 | 3.1492 | 0.3511 | 100.0% | 1055.3722 | 1055.179 | 2 | 7.726 | 65.0% | 2 | K.TPGPGAQSALR.A | 2 |
|  | Astrin\_STLCLD20\_112214\_01.06518.06518.2 | 3.8788 | 0.4443 | 100.0% | 1430.4122 | 1430.5547 | 1 | 8.188 | 75.0% | 5 | R.IEDVTPIPSDSTR.R | 2 |

---

|  |  |  |  |  |  |  |  |  |
| --- | --- | --- | --- | --- | --- | --- | --- | --- |
| U | *gi|5729877|ref|NP\_006* | 22 | 104 | 29.7% | 646 | 70898 | 5.5 | heat shock 70kDa protein 8 isoform 1 [Homo sapiens] |

| Filename XCorr DeltCN Conf% ObsM+H+ CalcM+H+ SpR ZScore Ion% # Sequence  | | | | | | | | | | | | |
| --- | --- | --- | --- | --- | --- | --- | --- | --- | --- | --- | --- | --- |
|  | Astrin\_STLCLD20\_112214\_01.09043.09043.2 | 3.6782 | 0.4657 | 100.0% | 1488.2922 | 1488.5939 | 1 | 9.028 | 75.0% | 19 | R.TTPSYVAFTDTER.L | 222 |
|  | Astrin\_STLCHLD\_tube2\_061314\_01.07236.07236.2 | 4.4575 | 0.4745 | 100.0% | 1650.3121 | 1650.8468 | 1 | 9.859 | 78.6% | 7 | K.NQVAMNPTNTVFDAK.R | 2 |
|  | Astrin\_STLCHLD\_050114\_01.08992.08992.2 | 2.9837 | 0.3636 | 100.0% | 1410.8121 | 1411.5725 | 1 | 6.38 | 72.7% | 3 | R.RFDDAVVQSDMK.H | 2 |
|  | Astrin\_STLCHLD\_tube2\_061314\_01.08334.08334.3 | 3.7435 | 0.3487 | 99.9% | 1656.5343 | 1654.9298 | 1 | 6.032 | 46.2% | 3 | K.HWPFMVVNDAGRPK.V | 3 |
|  | Astrin\_NLD\_STLC\_tube2\_021014\_01.03838.03838.2 | 2.3256 | 0.2652 | 98.2% | 1181.0721 | 1181.3312 | 71 | 4.615 | 61.1% | 1 | K.VQVEYKGETK.S | 2 |
|  | Astrin\_STLCHLD\_tube2\_061314\_02.05702.05702.3 | 3.0767 | 0.3316 | 100.0% | 1253.3043 | 1253.4993 | 1 | 6.03 | 42.5% | 3 | K.MKEIAEAYLGK.T | 3 |
|  | Astrin\_STLCHLD\_tube2\_050114\_01.05915.05915.2 | 3.0165 | 0.1928 | 99.1% | 1254.2922 | 1253.4993 | 1 | 4.57 | 80.0% | 1 | K.MKEIAEAYLGK.T | 2 |
|  | Astrin\_STLCHLD\_tube2\_050114\_01.09337.09337.2 | 3.0032 | 0.3503 | 99.9% | 1983.3522 | 1983.1882 | 3 | 5.942 | 44.1% | 3 | K.TVTNAVVTVPAYFNDSQR.Q | 2 |
|  | Astrin\_NLD\_STLC\_tube2\_021014\_01.10414.10414.2 | 4.7786 | 0.4625 | 100.0% | 1661.2522 | 1660.9078 | 1 | 8.509 | 73.3% | 8 | R.IINEPTAAAIAYGLDK.K | 222 |
|  | Astrin\_STLCHLD\_tube2\_061314\_01.09043.09043.2 | 3.7373 | 0.3654 | 100.0% | 1788.4321 | 1789.0819 | 1 | 7.697 | 56.2% | 1 | R.IINEPTAAAIAYGLDKK.V | 2 |
|  | Astrin\_STLCHLD\_tube2\_050114\_01.04599.04599.2 | 4.3472 | 0.4848 | 100.0% | 1692.2522 | 1692.6958 | 1 | 9.085 | 63.3% | 4 | K.STAGDTHLGGEDFDNR.M | 2 |
|  | Astrin\_STLCHLD\_050114\_01.06475.06475.3 | 2.9132 | 0.4213 | 99.9% | 1692.3544 | 1692.6958 | 2 | 6.602 | 36.7% | 4 | K.STAGDTHLGGEDFDNR.M | 3 |
|  | Astrin\_STLCHLD\_061214\_01.06922.06922.3 | 3.2026 | 0.4045 | 99.9% | 1236.5044 | 1236.4741 | 1 | 6.894 | 50.0% | 2 | R.MVNHFIAEFK.R | 3 |
|  | Astrin\_STLCHLD\_tube2\_061314\_01.07791.07791.2 | 3.3058 | 0.4308 | 100.0% | 1237.4321 | 1236.4741 | 1 | 7.796 | 83.3% | 4 | R.MVNHFIAEFK.R | 2 |
|  | Astrin\_STLCHLD\_tube2\_061314\_01.09683.09683.3 | 3.7659 | 0.2928 | 100.0% | 1481.1543 | 1481.6511 | 1 | 5.359 | 54.5% | 1 | R.ARFEELNADLFR.G | 3 |
|  | Astrin\_STLCHLD\_tube2\_050114\_01.09239.09239.2 | 3.6423 | 0.337 | 100.0% | 1482.3922 | 1481.6511 | 1 | 6.341 | 77.3% | 10 | R.ARFEELNADLFR.G | 2 |
|  | Astrin\_STLCHLD\_tube2\_061314\_01.10777.10777.2 | 2.7357 | 0.3363 | 99.9% | 1255.7922 | 1254.3849 | 4 | 5.855 | 72.2% | 2 | R.FEELNADLFR.G | 2 |
|  | Astrin\_STLCHLD\_061214\_01.06200.06200.3 | 4.6652 | 0.3129 | 99.9% | 1839.3243 | 1839.1019 | 1 | 7.047 | 45.3% | 10 | K.LDKSQIHDIVLVGGSTR.I | 3 |
|  | Astrin\_STLCHLD\_tube2\_061314\_01.05916.05916.2 | 3.7545 | 0.4442 | 100.0% | 1482.4521 | 1482.6798 | 1 | 7.389 | 69.2% | 8 | K.SQIHDIVLVGGSTR.I | 2 |
|  | Astrin\_STLCHLD\_tube2\_061314\_02.05836.05836.3 | 3.4679 | 0.3067 | 99.9% | 1484.3344 | 1482.6798 | 2 | 5.69 | 44.2% | 2 | K.SQIHDIVLVGGSTR.I | 3 |
| \* | Astrin\_STLCLD20\_112214\_tube2\_01.05751.05751.3 | 4.0546 | 0.3064 | 99.9% | 1983.3544 | 1983.2036 | 3 | 6.357 | 40.0% | 2 | R.MVQEAEKYKAEDEKQR.D | 3 |
| \* | Astrin\_STLCLD20\_112214\_tube2\_01.12108.12108.2 | 3.8702 | 0.4388 | 100.0% | 1305.2922 | 1304.4602 | 1 | 6.787 | 85.0% | 6 | K.NSLESYAFNMK.A | 2 |

Similarities:
gi|167466173|ref|NP\_0(1:21)  
gi|124256496|ref|NP\_0(2:20)  
contaminant\_GR78\_HUMA(1:21)  

---

|  |  |  |  |  |  |  |  |  |
| --- | --- | --- | --- | --- | --- | --- | --- | --- |
| U | *gi|47132620|ref|NP\_00* | 17 | 55 | 29.6% | 639 | 65433 | 8.0 | keratin 2 [Homo sapiens] |

| Filename XCorr DeltCN Conf% ObsM+H+ CalcM+H+ SpR ZScore Ion% # Sequence  | | | | | | | | | | | | |
| --- | --- | --- | --- | --- | --- | --- | --- | --- | --- | --- | --- | --- |
|  | Astrin\_NLD\_STLC\_tube2\_021014\_01.04533.04533.2 | 3.3624 | 0.406 | 100.0% | 1256.4122 | 1255.3298 | 1 | 7.47 | 69.2% | 1 | R.GFSSGSAVVSGGSR.R | 2 |
|  | Astrin\_STLCHLD\_tube2\_061314\_02.07469.07469.2 | 3.7977 | 0.365 | 100.0% | 1840.3922 | 1840.0055 | 1 | 7.328 | 38.1% | 2 | K.SISISVAGGGGGFGAAGGFGGR.G | 2 |
| \* | Astrin\_STLCHLD\_061214\_02.07645.07645.2 | 5.8444 | 0.5738 | 100.0% | 2399.672 | 2400.4446 | 1 | 10.507 | 51.7% | 2 | R.GGGFGGGSSFGGGSGFSGGGFGGGGFGGGR.F | 2 |
| \* | Astrin\_STLCHLD\_061214\_02.07641.07641.3 | 5.5878 | 0.4765 | 100.0% | 2399.8442 | 2400.4446 | 1 | 9.353 | 49.1% | 2 | R.GGGFGGGSSFGGGSGFSGGGFGGGGFGGGR.F | 3 |
|  | Astrin\_STLCHLD\_tube2\_061314\_01.06264.06264.2 | 2.4085 | 0.2136 | 98.8% | 828.2922 | 827.95544 | 5 | 5.059 | 91.7% | 3 | K.FASFIDK.V | 22222222 |
|  | Astrin\_NLD\_STLC\_tube2\_021014\_01.07623.07623.2 | 2.9741 | 0.1656 | 99.1% | 1082.8121 | 1083.2755 | 7 | 7.028 | 75.0% | 6 | K.FASFIDKVR.F | 2222222 |
|  | Astrin\_STLCLD20\_112214\_01.07333.07333.2 | 4.5527 | 0.0972 | 99.9% | 1476.4922 | 1476.6726 | 1 | 7.289 | 90.9% | 10 | R.FLEQQNQVLQTK.W | 22 |
|  | Astrin\_NLD\_STLC\_tube2\_021014\_01.06866.06866.2 | 2.9544 | 0.3384 | 100.0% | 1038.1322 | 1038.1454 | 1 | 6.526 | 87.5% | 4 | R.YLDGLTAER.T | 2 |
|  | Astrin\_NLD\_STLC\_tube2\_021014\_01.07499.07499.2 | 2.4179 | 0.2734 | 98.4% | 1209.3121 | 1209.3416 | 4 | 5.422 | 70.0% | 1 | R.TAAENDFVTLK.K | 2 |
|  | Astrin\_NLD\_STLC\_tube2\_021014\_01.12972.12972.2 | 3.7648 | 0.3089 | 100.0% | 1461.3522 | 1461.6982 | 2 | 7.072 | 72.7% | 2 | K.VDLLNQEIEFLK.V | 2 |
|  | Astrin\_NLD\_STLC\_tube2\_021014\_01.13338.13338.2 | 4.1957 | 0.3905 | 100.0% | 1331.0922 | 1330.5211 | 1 | 7.868 | 86.4% | 3 | R.NLDLDSIIAEVK.A | 22222 |
|  | Astrin\_NLD\_STLC\_tube2\_021014\_01.04328.04328.2 | 3.2426 | 0.0852 | 98.6% | 1108.1122 | 1108.196 | 2 | 6.567 | 75.0% | 1 | K.AQYEEIAQR.S | 222 |
|  | Astrin\_STLCHLD\_061214\_01.05516.05516.2 | 3.1494 | 0.4324 | 100.0% | 1194.3722 | 1194.33 | 1 | 8.999 | 72.2% | 11 | K.YEELQVTVGR.H | 2 |
|  | Astrin\_NLD\_STLC\_tube2\_021014\_01.06564.06564.2 | 2.9459 | 0.0753 | 98.0% | 974.1322 | 974.102 | 175 | 4.257 | 64.3% | 1 | K.IEISELNR.V | 22 |
|  | Astrin\_STLCLD20\_112214\_tube2\_01.15761.15761.3 | 4.4833 | 0.3719 | 99.9% | 2199.5344 | 2199.4258 | 2 | 6.021 | 36.1% | 1 | R.NKLNDLEEALQQAKEDLAR.L | 3 |
|  | Astrin\_STLCHLD\_061214\_02.07769.07769.2 | 3.2347 | 0.4112 | 100.0% | 1264.5922 | 1264.4644 | 1 | 8.501 | 65.0% | 4 | K.LALDVEIATYR.K | 22222 |
|  | Astrin\_STLCHLD\_061214\_01.07207.07207.2 | 2.6141 | 0.1428 | 95.1% | 1392.5322 | 1392.6384 | 23 | 4.252 | 59.1% | 1 | K.LALDVEIATYRK.L | 22222 |

Similarities:
gi|4504919|ref|NP\_002(2:15)  
gi|67782365|ref|NP\_00(2:15)  
gi|119395750|ref|NP\_0(2:15)  
gi|119703753|ref|NP\_0(6:11)  
gi|32567786|ref|NP\_78(5:12)  
gi|119395754|ref|NP\_0(5:12)  
gi|153791158|ref|NP\_0(5:12)  
gi|109255249|ref|NP\_0(2:15)  

---

|  |  |  |  |  |  |  |  |  |
| --- | --- | --- | --- | --- | --- | --- | --- | --- |
| U | *gi|17986258|ref|NP\_06* | 3 | 9 | 29.1% | 151 | 16930 | 4.7 | myosin, light chain 6, alkali, smooth muscle and non-muscle isoform 1 [Homo sapiens] |
| U | *gi|88999583|ref|NP\_52* | 3 | 9 | 29.1% | 151 | 16961 | 4.6 | myosin, light chain 6, alkali, smooth muscle and non-muscle isoform 2 [Homo sapiens] |

| Filename XCorr DeltCN Conf% ObsM+H+ CalcM+H+ SpR ZScore Ion% # Sequence  | | | | | | | | | | | | |
| --- | --- | --- | --- | --- | --- | --- | --- | --- | --- | --- | --- | --- |
|  | Astrin\_NLD\_STLC\_tube2\_021014\_01.05705.05705.2 | 3.6548 | 0.3657 | 100.0% | 1355.4122 | 1355.5339 | 1 | 6.243 | 66.7% | 6 | R.ALGQNPTNAEVLK.V | 2 |
|  | Astrin\_STLCLD20\_112214\_01.14357.14357.2 | 3.2261 | 0.3214 | 99.9% | 1889.8121 | 1889.2628 | 1 | 6.422 | 50.0% | 2 | K.VLDFEHFLPMLQTVAK.N | 2 |
|  | Astrin\_STLCHLD\_tube2\_061314\_01.07496.07496.3 | 3.511 | 0.3983 | 99.9% | 1787.7843 | 1787.8804 | 1 | 6.141 | 50.0% | 1 | K.NKDQGTYEDYVEGLR.V | 3 |

---

|  |  |  |  |  |  |  |  |  |
| --- | --- | --- | --- | --- | --- | --- | --- | --- |
| U | *gi|55956899|ref|NP\_00* | 12 | 35 | 28.7% | 623 | 62064 | 5.2 | keratin 9 [Homo sapiens] |

| Filename XCorr DeltCN Conf% ObsM+H+ CalcM+H+ SpR ZScore Ion% # Sequence  | | | | | | | | | | | | |
| --- | --- | --- | --- | --- | --- | --- | --- | --- | --- | --- | --- | --- |
| \* | AstrinSTLCLD\_041714\_01.03717.03717.2 | 2.5725 | 0.2555 | 97.9% | 1233.3522 | 1233.2833 | 6 | 5.992 | 46.7% | 1 | R.SGGGGGGGLGSGGSIR.S | 2 |
|  | Astrin\_STLCHLD\_061214\_02.07521.07521.2 | 4.9205 | 0.4601 | 100.0% | 2705.8123 | 2706.7605 | 1 | 8.922 | 38.7% | 1 | R.GGGGSFGYSYGGGSGGGFSASSLGGGFGGGSR.G | 2 |
|  | Astrin\_STLCHLD\_061214\_02.07514.07514.3 | 4.0048 | 0.313 | 99.8% | 2706.5344 | 2706.7605 | 1 | 5.803 | 24.2% | 3 | R.GGGGSFGYSYGGGSGGGFSASSLGGGFGGGSR.G | 3 |
|  | Astrin\_STLCHLD\_tube2\_061314\_01.10086.10086.3 | 3.5259 | 0.1897 | 96.4% | 2379.0244 | 2378.5981 | 129 | 4.153 | 32.5% | 1 | R.LASYLDKVQALEEANNDLENK.I | 3 |
|  | Astrin\_STLCHLD\_tube2\_061314\_01.06869.06869.2 | 2.6971 | 0.3585 | 100.0% | 1061.4521 | 1061.1802 | 1 | 6.428 | 93.8% | 2 | K.TLLDIDNTR.M | 2 |
|  | Astrin\_STLCHLD\_061214\_01.06081.06081.2 | 2.0882 | 0.2367 | 95.1% | 1308.5721 | 1308.5383 | 9 | 5.014 | 61.1% | 1 | R.IKFEMEQNLR.Q | 2 |
|  | Astrin\_STLCLD20\_112214\_01.07406.07406.2 | 3.0642 | 0.3213 | 100.0% | 1158.4922 | 1158.2566 | 8 | 5.376 | 65.0% | 4 | R.QGVDADINGLR.Q | 2 |
|  | Astrin\_STLCHLD\_061214\_02.07940.07940.3 | 4.2505 | 0.2979 | 99.9% | 1839.4143 | 1839.0557 | 1 | 5.863 | 41.7% | 2 | R.HGVQELEIELQSQLSK.K | 3 |
|  | Astrin\_STLCHLD\_061214\_02.07912.07912.2 | 3.0402 | 0.2119 | 98.5% | 1840.4922 | 1839.0557 | 1 | 5.058 | 60.0% | 2 | R.HGVQELEIELQSQLSK.K | 2 |
|  | Astrin\_STLCLD20\_112214\_02.11724.11724.3 | 5.6819 | 0.4051 | 100.0% | 1967.4243 | 1967.2297 | 1 | 7.486 | 48.4% | 7 | R.HGVQELEIELQSQLSKK.A | 3 |
|  | Astrin\_STLCHLD\_tube2\_061314\_02.07080.07080.3 | 5.2193 | 0.1107 | 98.2% | 2511.3542 | 2511.6177 | 1 | 6.408 | 37.5% | 4 | K.EIETYHNLLEGGQEDFESSGAGK.I | 3 |
|  | Astrin\_STLCHLD\_061214\_01.04327.04327.3 | 8.1976 | 0.6145 | 100.0% | 3224.5745 | 3225.1118 | 1 | 11.349 | 28.8% | 7 | R.GGSGGSHGGGSGFGGESGGSYGGGEEASGSGGGYGGGSGK.S | 3 |

---

|  |  |  |  |  |  |  |  |  |
| --- | --- | --- | --- | --- | --- | --- | --- | --- |
| U | *gi|10835063|ref|NP\_00* | 4 | 14 | 28.6% | 294 | 32575 | 4.8 | nucleophosmin 1 isoform 1 [Homo sapiens] |
| U | *gi|40353734|ref|NP\_95* | 4 | 14 | 31.7% | 265 | 29465 | 4.6 | nucleophosmin 1 isoform 2 [Homo sapiens] |

| Filename XCorr DeltCN Conf% ObsM+H+ CalcM+H+ SpR ZScore Ion% # Sequence  | | | | | | | | | | | | |
| --- | --- | --- | --- | --- | --- | --- | --- | --- | --- | --- | --- | --- |
|  | Astrin\_STLCLD20\_112214\_01.05853.05853.3 | 3.9254 | 0.224 | 99.4% | 2573.9343 | 2574.7258 | 21 | 5.307 | 28.8% | 1 | K.ADKDYHFKVDNDENEHQLSLR.T | 3 |
|  | Astrin\_NLD\_STLC\_tube2\_021014\_01.11933.11933.3 | 5.4874 | 0.4468 | 100.0% | 2931.1443 | 2931.2874 | 1 | 7.843 | 32.4% | 5 | R.TVSLGAGAKDELHIVEAEAMNYEGSPIK.V | 3 |
|  | Astrin\_STLCHLD\_tube2\_050114\_01.13190.13190.2 | 4.6233 | 0.5332 | 100.0% | 2229.112 | 2228.655 | 1 | 8.229 | 52.5% | 5 | K.MSVQPTVSLGGFEITPPVVLR.L | 2 |
|  | Astrin\_STLCHLD\_050114\_01.12661.12661.2 | 3.6322 | 0.2054 | 99.6% | 1820.6322 | 1821.0172 | 1 | 5.438 | 53.8% | 3 | R.MTDQEAIQDLWQWR.K | 2 |

---

|  |  |  |  |  |  |  |  |  |
| --- | --- | --- | --- | --- | --- | --- | --- | --- |
| U | *gi|219555707|ref|NP\_0* | 3 | 11 | 28.3% | 184 | 20170 | 7.0 | eukaryotic translation initiation factor 5A isoform A [Homo sapiens] |
| U | *gi|4503545|ref|NP\_001* | 3 | 11 | 33.8% | 154 | 16832 | 5.2 | eukaryotic translation initiation factor 5A isoform B [Homo sapiens] |
| U | *gi|219555712|ref|NP\_0* | 3 | 11 | 33.8% | 154 | 16832 | 5.2 | eukaryotic translation initiation factor 5A isoform B [Homo sapiens] |
| U | *gi|219555710|ref|NP\_0* | 3 | 11 | 33.8% | 154 | 16832 | 5.2 | eukaryotic translation initiation factor 5A isoform B [Homo sapiens] |

| Filename XCorr DeltCN Conf% ObsM+H+ CalcM+H+ SpR ZScore Ion% # Sequence  | | | | | | | | | | | | |
| --- | --- | --- | --- | --- | --- | --- | --- | --- | --- | --- | --- | --- |
|  | AstrinSTLCLD\_041714\_01.09614.09614.2 | 3.3468 | 0.4539 | 100.0% | 1299.6721 | 1299.5559 | 1 | 8.271 | 81.8% | 9 | K.VHLVGIDIFTGK.K | 2 |
|  | Astrin\_STLCHLD\_050114\_02.11271.11271.2 | 5.0697 | 0.4686 | 100.0% | 2581.8323 | 2581.8418 | 1 | 9.659 | 47.7% | 1 | R.NDFQLIGIQDGYLSLLQDSGEVR.E | 2 |
|  | Astrin\_STLCHLD\_tube2\_061314\_01.07562.07562.3 | 3.654 | 0.3293 | 100.0% | 1970.6643 | 1970.187 | 7 | 5.956 | 35.9% | 1 | R.EDLRLPEGDLGKEIEQK.Y | 3 |

---

|  |  |  |  |  |  |  |  |  |
| --- | --- | --- | --- | --- | --- | --- | --- | --- |
| U | *gi|4506687|ref|NP\_001* | 2 | 4 | 28.3% | 145 | 17040 | 10.4 | ribosomal protein S15 [Homo sapiens] |

| Filename XCorr DeltCN Conf% ObsM+H+ CalcM+H+ SpR ZScore Ion% # Sequence  | | | | | | | | | | | | |
| --- | --- | --- | --- | --- | --- | --- | --- | --- | --- | --- | --- | --- |
|  | Astrin\_STLCLD20\_112214\_01.17566.17566.2 | 3.8631 | 0.5116 | 100.0% | 2588.5522 | 2589.938 | 1 | 8.707 | 45.2% | 3 | R.GVDLDQLLDMSYEQLMQLYSAR.Q | 2 |
| \* | Astrin\_STLCLD20\_112214\_01.15038.15038.2 | 2.9751 | 0.4218 | 100.0% | 2054.1921 | 2054.4856 | 1 | 6.719 | 47.2% | 1 | R.DMIILPEMVGSMVGVYNGK.T | 2 |

---

|  |  |  |  |  |  |  |  |  |
| --- | --- | --- | --- | --- | --- | --- | --- | --- |
| U | *gi|4758086|ref|NP\_004* | 3 | 26 | 28.0% | 193 | 20567 | 8.6 | cysteine and glycine-rich protein 1 isoform 1 [Homo sapiens] |

| Filename XCorr DeltCN Conf% ObsM+H+ CalcM+H+ SpR ZScore Ion% # Sequence  | | | | | | | | | | | | |
| --- | --- | --- | --- | --- | --- | --- | --- | --- | --- | --- | --- | --- |
|  | Astrin\_STLCHLD\_061214\_02.05795.05795.3 | 3.0818 | 0.2823 | 98.8% | 2160.6543 | 2160.3452 | 3 | 4.231 | 31.0% | 4 | K.GYGYGQGAGTLSTDKGESLGIK.H | 3 |
|  | Astrin\_STLCLD20\_112214\_tube2\_01.05512.05512.2 | 3.3109 | 0.4094 | 100.0% | 1842.8322 | 1843.9535 | 1 | 6.957 | 59.4% | 1 | K.HEEAPGHRPTTNPNASK.F | 2 |
| \* | Astrin\_STLCHLD\_061214\_01.07878.07878.2 | 3.9117 | 0.536 | 100.0% | 1433.7522 | 1434.551 | 1 | 9.286 | 57.1% | 21 | K.GFGFGQGAGALVHSE.- | 2 |

---

|  |  |  |  |  |  |  |  |  |
| --- | --- | --- | --- | --- | --- | --- | --- | --- |
| U | *gi|4507357|ref|NP\_003* | 4 | 10 | 27.6% | 199 | 22391 | 8.2 | transgelin 2 [Homo sapiens] |

| Filename XCorr DeltCN Conf% ObsM+H+ CalcM+H+ SpR ZScore Ion% # Sequence  | | | | | | | | | | | | |
| --- | --- | --- | --- | --- | --- | --- | --- | --- | --- | --- | --- | --- |
| \* | AstrinSTLCLD\_041714\_01.12489.12489.2 | 3.72 | 0.4983 | 100.0% | 2100.4922 | 2101.3203 | 1 | 7.908 | 52.9% | 3 | R.YGINTTDIFQTVDLWEGK.N | 2 |
| \* | Astrin\_NLD\_STLC\_tube2\_021014\_01.10605.10605.2 | 3.1005 | 0.3311 | 100.0% | 1216.7322 | 1216.4845 | 1 | 6.984 | 72.7% | 3 | R.TLMNLGGLAVAR.D | 2 |
| \* | Astrin\_STLCHLD\_tube2\_061314\_01.06549.06549.2 | 2.9452 | 0.4609 | 100.0% | 1203.6921 | 1203.402 | 1 | 8.668 | 85.0% | 1 | K.NVIGLQMGTNR.G | 2 |
|  | Astrin\_STLCHLD\_tube2\_061314\_01.04635.04635.2 | 3.4213 | 0.453 | 100.0% | 1383.9922 | 1384.5677 | 1 | 8.988 | 69.2% | 3 | R.GASQAGMTGYGMPR.Q | 2 |

---

|  |  |  |  |  |  |  |  |  |
| --- | --- | --- | --- | --- | --- | --- | --- | --- |
| U | *gi|14141152|ref|NP\_00* | 16 | 136 | 27.5% | 730 | 77516 | 8.7 | heterogeneous nuclear ribonucleoprotein M isoform a [Homo sapiens] |
| U | *gi|157412270|ref|NP\_1* | 16 | 136 | 29.1% | 691 | 73621 | 8.8 | heterogeneous nuclear ribonucleoprotein M isoform b [Homo sapiens] |

| Filename XCorr DeltCN Conf% ObsM+H+ CalcM+H+ SpR ZScore Ion% # Sequence  | | | | | | | | | | | | |
| --- | --- | --- | --- | --- | --- | --- | --- | --- | --- | --- | --- | --- |
|  | AstrinSTLCLD\_041714\_01.10131.10131.2 | 3.6249 | 0.4006 | 100.0% | 1265.2722 | 1265.4949 | 1 | 7.428 | 80.0% | 1 | R.AFITNIPFDVK.W | 2 |
|  | Astrin\_STLCLD20\_112214\_02.12070.12070.2 | 4.079 | 0.4854 | 100.0% | 1427.5721 | 1427.6403 | 1 | 8.279 | 58.3% | 10 | R.LGSTVFVANLDYK.V | 2 |
|  | AstrinSTLCLD\_041714\_02.07530.07530.2 | 3.3381 | 0.3474 | 100.0% | 1435.1122 | 1435.768 | 1 | 6.456 | 75.0% | 2 | K.LKEVFSMAGVVVR.A | 2 |
|  | Astrin\_STLCLD20\_112214\_02.14731.14731.2 | 2.7006 | 0.3273 | 99.3% | 2179.9922 | 2179.5752 | 1 | 5.832 | 31.8% | 1 | K.GIGMGNIGPAGMGMEGIGFGINK.M | 2 |
|  | AstrinSTLCLD\_041714\_01.08471.08471.2 | 3.8125 | 0.4793 | 100.0% | 1716.3121 | 1715.9724 | 1 | 8.265 | 65.6% | 4 | K.MGGMEGPFGGGMENMGR.F | 2 |
|  | Astrin\_STLCHLD\_050114\_01.06481.06481.2 | 2.0128 | 0.3171 | 98.0% | 957.21216 | 957.11017 | 5 | 6.066 | 75.0% | 2 | R.FGSGMNMGR.I | 2 |
|  | AstrinSTLCLD\_041714\_01.07021.07021.2 | 3.0141 | 0.3325 | 100.0% | 1114.7122 | 1115.3152 | 4 | 5.709 | 72.2% | 5 | R.INEILSNALK.R | 2 |
|  | Astrin\_STLCHLD\_050114\_01.09317.09317.2 | 2.3259 | 0.3439 | 99.8% | 822.1722 | 822.0283 | 2 | 6.503 | 83.3% | 1 | R.MGLVMDR.M | 2 |
|  | Astrin\_STLCLD20\_112214\_01.10383.10383.2 | 4.732 | 0.4895 | 100.0% | 1614.3722 | 1614.875 | 1 | 9.627 | 75.0% | 6 | R.MGPLGLDHMASSIER.M | 2 |
|  | Astrin\_NLD\_STLC\_tube2\_021014\_01.09274.09274.3 | 3.9711 | 0.4338 | 100.0% | 1614.5643 | 1614.875 | 1 | 7.76 | 42.9% | 10 | R.MGPLGLDHMASSIER.M | 3 |
|  | Astrin\_STLCHLD\_050114\_02.07302.07302.2 | 3.6724 | 0.4778 | 100.0% | 1126.3322 | 1126.3337 | 1 | 9.623 | 75.0% | 23 | R.MGAGMGFGLER.M | 2 |
|  | Astrin\_STLCHLD\_050114\_01.07514.07514.2 | 2.2174 | 0.2001 | 97.4% | 825.2322 | 824.00085 | 1 | 6.176 | 66.7% | 2 | R.MGLSMER.M | 2 |
|  | Astrin\_NLD\_STLC\_tube2\_021014\_01.06839.06839.2 | 3.0605 | 0.4796 | 100.0% | 1189.3322 | 1189.4333 | 1 | 7.712 | 77.3% | 8 | R.MVPAGMGAGLER.M | 2 |
|  | AstrinSTLCLD\_041714\_01.06828.06828.2 | 3.5705 | 0.395 | 100.0% | 1428.3522 | 1428.7076 | 1 | 7.364 | 71.4% | 10 | R.MGPAMGPALGAGIER.M | 2 |
|  | AstrinSTLCLD\_041714\_02.06458.06458.2 | 4.2122 | 0.5395 | 100.0% | 1384.0721 | 1384.5677 | 1 | 10.435 | 82.1% | 33 | R.MGLAMGGGGGASFDR.A | 2 |
|  | Astrin\_NLD\_STLC\_tube2\_021014\_02.06404.06404.3 | 3.9794 | 0.4499 | 100.0% | 2035.4043 | 2036.1735 | 1 | 7.122 | 34.1% | 18 | R.GNFGGSFAGSFGGAGGHAPGVAR.K | 3 |

---

|  |  |  |  |  |  |  |  |  |
| --- | --- | --- | --- | --- | --- | --- | --- | --- |
| U | *gi|21464101|ref|NP\_03* | 5 | 16 | 27.5% | 247 | 28303 | 4.9 | tyrosine 3-monooxygenase/tryptophan 5-monooxygenase activation protein, gamma polypeptide [Homo sapiens] |

| Filename XCorr DeltCN Conf% ObsM+H+ CalcM+H+ SpR ZScore Ion% # Sequence  | | | | | | | | | | | | |
| --- | --- | --- | --- | --- | --- | --- | --- | --- | --- | --- | --- | --- |
|  | Astrin\_STLCHLD\_050114\_01.06965.06965.2 | 2.0972 | 0.249 | 96.4% | 1016.2522 | 1016.172 | 1 | 4.78 | 68.8% | 2 | R.YDDMAAAMK.N | 22 |
| \* | Astrin\_STLCHLD\_tube2\_061314\_01.05457.05457.2 | 4.5282 | 0.4765 | 100.0% | 1644.4122 | 1644.7356 | 1 | 9.072 | 76.9% | 2 | K.NVTELNEPLSNEER.N | 2 |
| \* | Astrin\_STLCHLD\_tube2\_061314\_02.07689.07689.2 | 3.1832 | 0.1036 | 96.0% | 1798.2722 | 1798.8473 | 130 | 3.469 | 40.0% | 1 | R.VIS\*SIEQK@TSADGNEK.K | 2 |
| \* | AstrinSTLCLD\_041714\_01.17673.17673.2 | 3.2257 | 0.1229 | 97.2% | 1798.3922 | 1798.8473 | 59 | 3.452 | 43.3% | 6 | R.VISS\*IEQK@TSADGNEK.K | 2 |
| \* | AstrinSTLCLD\_041714\_01.15875.15875.3 | 4.5559 | 0.4132 | 100.0% | 3303.2944 | 3303.6626 | 1 | 6.41 | 26.8% | 5 | K.TAFDDAIAELDTLNEDSYKDSTLIMQLLR.D | 3 |

Similarities:
gi|21328448|ref|NP\_64(1:4)  

---

|  |  |  |  |  |  |  |  |  |
| --- | --- | --- | --- | --- | --- | --- | --- | --- |
| U | *gi|15718687|ref|NP\_00* | 5 | 38 | 27.2% | 243 | 26688 | 9.7 | ribosomal protein S3 [Homo sapiens] |

| Filename XCorr DeltCN Conf% ObsM+H+ CalcM+H+ SpR ZScore Ion% # Sequence  | | | | | | | | | | | | |
| --- | --- | --- | --- | --- | --- | --- | --- | --- | --- | --- | --- | --- |
| \* | Astrin\_STLCHLD\_tube2\_061314\_01.08645.08645.2 | 2.3616 | 0.3021 | 99.2% | 1093.5521 | 1093.2249 | 1 | 5.858 | 75.0% | 3 | K.AELNEFLTR.E | 2 |
| \* | AstrinSTLCLD\_041714\_02.05512.05512.2 | 3.9023 | 0.4016 | 100.0% | 1424.2722 | 1424.5071 | 1 | 8.343 | 83.3% | 24 | R.ELAEDGYSGVEVR.V | 2 |
| \* | Astrin\_NLD\_STLC\_tube2\_021014\_01.08898.08898.3 | 3.0347 | 0.2674 | 99.4% | 1585.3744 | 1584.8998 | 1 | 5.697 | 51.9% | 2 | R.VTPTRTEIIILATR.T | 3 |
| \* | Astrin\_STLCHLD\_tube2\_050114\_02.08135.08135.2 | 2.7249 | 0.2792 | 98.9% | 1574.7922 | 1573.7423 | 1 | 5.115 | 50.0% | 5 | R.FGFPEGSVELYAEK.V | 2 |
| \* | Astrin\_NLD\_STLC\_tube2\_021014\_01.06700.06700.2 | 3.4169 | 0.262 | 99.7% | 1574.5322 | 1574.8352 | 4 | 5.478 | 53.3% | 4 | K.GGKPEPPAMPQPVPTA.- | 2 |

---

|  |  |  |  |  |  |  |  |  |
| --- | --- | --- | --- | --- | --- | --- | --- | --- |
| U | *gi|32698866|ref|NP\_87* | 3 | 7 | 26.9% | 197 | 22443 | 4.7 | spindle pole body component 24 homolog [Homo sapiens] |

| Filename XCorr DeltCN Conf% ObsM+H+ CalcM+H+ SpR ZScore Ion% # Sequence  | | | | | | | | | | | | |
| --- | --- | --- | --- | --- | --- | --- | --- | --- | --- | --- | --- | --- |
| \* | Astrin\_STLCHLD\_tube2\_050114\_01.15665.15665.2 | 3.1429 | 0.3664 | 100.0% | 1814.7522 | 1815.0336 | 4 | 6.692 | 53.1% | 2 | R.DIEEVSQGLLSLLGANR.A | 2 |
| \* | Astrin\_STLCHLD\_tube2\_061314\_01.04831.04831.3 | 4.7433 | 0.4166 | 100.0% | 2251.8843 | 2251.51 | 1 | 7.284 | 36.2% | 1 | K.GIHHGPSVAQPIHLDSTQLSR.K | 3 |
| \* | Astrin\_STLCHLD\_050114\_02.13037.13037.2 | 3.3941 | 0.4218 | 100.0% | 1903.3322 | 1903.1412 | 1 | 6.95 | 50.0% | 4 | R.KFISDYLWSLVDTEW.- | 2 |

---

|  |  |  |  |  |  |  |  |  |
| --- | --- | --- | --- | --- | --- | --- | --- | --- |
| U | *gi|15431310|ref|NP\_00* | 15 | 72 | 26.7% | 472 | 51622 | 5.2 | keratin 14 [Homo sapiens] |

| Filename XCorr DeltCN Conf% ObsM+H+ CalcM+H+ SpR ZScore Ion% # Sequence  | | | | | | | | | | | | |
| --- | --- | --- | --- | --- | --- | --- | --- | --- | --- | --- | --- | --- |
| \* | Astrin\_STLCHLD\_tube2\_050114\_01.04863.04863.2 | 2.1145 | 0.3273 | 97.2% | 1426.0521 | 1426.526 | 28 | 5.388 | 39.3% | 1 | R.APSTYGGGLSVSSSR.F | 2 |
|  | Astrin\_NLD\_STLC\_tube2\_021014\_01.04526.04526.2 | 2.8206 | 0.328 | 100.0% | 1090.9922 | 1091.2273 | 46 | 6.066 | 68.8% | 1 | K.VTMQNLNDR.L | 222 |
|  | Astrin\_STLCHLD\_tube2\_050114\_01.05063.05063.2 | 3.0476 | 0.142 | 99.0% | 1064.9722 | 1065.2578 | 35 | 6.057 | 62.5% | 4 | R.LASYLDKVR.A | 22222 |
|  | Astrin\_NLD\_STLC\_tube2\_021014\_02.05150.05150.2 | 3.5297 | 0.3564 | 100.0% | 1301.4521 | 1302.4241 | 2 | 7.873 | 63.6% | 4 | R.ALEEANADLEVK.I | 22 |
|  | Astrin\_STLCHLD\_050114\_01.06505.06505.2 | 2.4865 | 0.2457 | 99.3% | 808.09216 | 807.8815 | 46 | 6.454 | 66.7% | 16 | R.LAADDFR.T | 222222 |
|  | Astrin\_STLCLD20\_112214\_01.08975.08975.2 | 2.9226 | 0.3172 | 100.0% | 1186.2522 | 1186.397 | 1 | 5.85 | 83.3% | 4 | R.RVLDELTLAR.A | 2222 |
|  | Astrin\_STLCLD20\_112214\_01.09782.09782.1 | 2.1585 | 0.3224 | 97.2% | 1029.49 | 1030.2096 | 10 | 5.815 | 56.2% | 1 | R.VLDELTLAR.A | 1111 |
|  | Astrin\_STLCHLD\_tube2\_050114\_01.08094.08094.2 | 3.8958 | 0.4282 | 100.0% | 1030.5721 | 1030.2096 | 1 | 7.786 | 93.8% | 11 | R.VLDELTLAR.A | 2222 |
|  | Astrin\_STLCLD20\_112214\_01.05006.05006.2 | 3.4896 | 0.2654 | 100.0% | 1439.7322 | 1439.6263 | 1 | 5.072 | 80.0% | 5 | R.ILNEMRDQYEK.M | 22 |
|  | Astrin\_STLCLD20\_112214\_01.06820.06820.3 | 3.5479 | 0.1718 | 95.9% | 2105.6042 | 2105.2664 | 54 | 4.942 | 30.6% | 2 | K.TEELNREVATNSELVQSGK.S | 33 |
|  | Astrin\_STLCHLD\_050114\_01.04933.04933.2 | 4.0231 | 0.5067 | 100.0% | 1362.1122 | 1362.4796 | 1 | 9.592 | 75.0% | 4 | R.EVATNSELVQSGK.S | 22 |
|  | Astrin\_STLCHLD\_tube2\_050114\_01.06167.06167.2 | 3.6142 | 0.3306 | 100.0% | 1381.3722 | 1380.5437 | 4 | 5.939 | 65.0% | 7 | K.TRLEQEIATYR.R | 222 |
|  | Astrin\_NLD\_STLC\_tube2\_021014\_01.05813.05813.3 | 3.1264 | 0.2733 | 99.8% | 1536.1743 | 1536.7311 | 2 | 5.027 | 38.6% | 4 | K.TRLEQEIATYRR.L | 333 |
|  | Astrin\_STLCLD20\_112214\_01.05125.05125.2 | 3.1188 | 0.3235 | 100.0% | 1123.3922 | 1123.2511 | 2 | 6.193 | 81.2% | 5 | R.LEQEIATYR.R | 2222 |
|  | Astrin\_NLD\_STLC\_tube2\_021014\_02.05234.05234.3 | 5.64 | 0.5171 | 100.0% | 2310.4143 | 2310.396 | 1 | 7.992 | 39.3% | 3 | R.LLEGEDAHLSSSQFSSGSQSSR.D | 3 |

Similarities:
gi|40354195|ref|NP\_95(1:14)  
gi|4557701|ref|NP\_000(11:4)  
contaminant\_KERATIN03(3:12)  
gi|24430192|ref|NP\_00(10:5)  
gi|24234699|ref|NP\_00(6:9)  

---

|  |  |  |  |  |  |  |  |  |
| --- | --- | --- | --- | --- | --- | --- | --- | --- |
| U | *gi|87196351|ref|NP\_00* | 14 | 77 | 26.0% | 662 | 73244 | 7.2 | DEAD/H (Asp-Glu-Ala-Asp/His) box polypeptide 3 [Homo sapiens] |

| Filename XCorr DeltCN Conf% ObsM+H+ CalcM+H+ SpR ZScore Ion% # Sequence  | | | | | | | | | | | | |
| --- | --- | --- | --- | --- | --- | --- | --- | --- | --- | --- | --- | --- |
| \* | Astrin\_STLCLD20\_112214\_tube2\_01.20415.20415.3 | 3.8626 | 0.2726 | 99.8% | 2333.6643 | 2333.6897 | 5 | 5.303 | 29.8% | 1 | K.TAAFLLPILSQIYSDGPGEALR.A | 3 |
| \* | Astrin\_STLCHLD\_tube2\_061314\_01.15355.15355.2 | 6.0037 | 0.4562 | 100.0% | 2334.0923 | 2333.6897 | 1 | 9.978 | 47.6% | 7 | K.TAAFLLPILSQIYSDGPGEALR.A | 2 |
|  | AstrinSTLCLD\_041714\_01.06682.06682.3 | 3.5855 | 0.3023 | 99.9% | 1643.4844 | 1642.9852 | 21 | 5.327 | 40.4% | 1 | R.RKQYPISLVLAPTR.E | 3 |
|  | Astrin\_STLCLD20\_112214\_tube2\_02.07622.07622.2 | 3.2353 | 0.4493 | 100.0% | 1321.3722 | 1321.4729 | 1 | 7.761 | 80.0% | 14 | R.ELAVQIYEEAR.K | 2 |
|  | Astrin\_STLCHLD\_tube2\_061314\_01.06625.06625.2 | 2.6144 | 0.1698 | 98.0% | 1095.4122 | 1094.2096 | 1 | 4.529 | 87.5% | 2 | K.YLVLDEADR.M | 2 |
|  | Astrin\_STLCLD20\_112214\_01.11336.11336.2 | 4.1408 | 0.4316 | 100.0% | 1338.4122 | 1337.5946 | 1 | 7.877 | 85.0% | 14 | R.MLDMGFEPQIR.R | 222 |
|  | Astrin\_STLCHLD\_tube2\_050114\_02.11896.11896.2 | 2.7157 | 0.3014 | 99.3% | 1558.2122 | 1558.774 | 10 | 6.473 | 54.2% | 2 | R.DFLDEYIFLAVGR.V | 2 |
| \* | Astrin\_STLCHLD\_tube2\_061314\_01.13560.13560.2 | 3.7906 | 0.5065 | 100.0% | 1292.4722 | 1292.5181 | 1 | 9.424 | 77.3% | 3 | R.SFLLDLLNATGK.D | 2 |
| \* | Astrin\_STLCHLD\_tube2\_061314\_02.11962.11962.3 | 3.6676 | 0.3638 | 100.0% | 2525.9343 | 2525.945 | 60 | 6.256 | 26.1% | 1 | R.SFLLDLLNATGKDSLTLVFVETK.K | 3 |
|  | Astrin\_STLCHLD\_050114\_01.05005.05005.3 | 2.9733 | 0.164 | 95.6% | 1302.2043 | 1301.4043 | 1 | 4.155 | 47.2% | 1 | R.DREEALHQFR.S | 3 |
|  | Astrin\_STLCHLD\_050114\_02.06951.06951.2 | 3.8789 | 0.5069 | 100.0% | 1169.4321 | 1169.4099 | 1 | 7.497 | 81.8% | 14 | K.SPILVATAVAAR.G | 2 |
|  | AstrinSTLCLD\_041714\_01.10229.10229.3 | 5.2007 | 0.2375 | 99.9% | 2086.4944 | 2084.2957 | 1 | 6.789 | 48.4% | 8 | K.HVINFDLPSDIEEYVHR.I | 3 |
| \* | AstrinSTLCLD\_041714\_01.10419.10419.2 | 4.2903 | 0.4477 | 100.0% | 1526.0322 | 1525.7043 | 1 | 7.589 | 73.1% | 8 | R.VGNLGLATSFFNER.N | 2 |
| \* | Astrin\_STLCLD20\_112214\_tube2\_01.19626.19626.2 | 2.9849 | 0.4816 | 100.0% | 1812.8322 | 1813.1448 | 6 | 8.139 | 46.7% | 1 | R.NINITKDLLDLLVEAK.Q | 2 |

Similarities:
gi|4758138|ref|NP\_004(1:13)  
gi|148613856|ref|NP\_0(1:13)  

---

|  |  |  |  |  |  |  |  |  |
| --- | --- | --- | --- | --- | --- | --- | --- | --- |
| U | *gi|148470397|ref|NP\_0* | 6 | 41 | 26.0% | 415 | 45672 | 5.6 | heterogeneous nuclear ribonucleoprotein F [Homo sapiens] |
| U | *gi|4826760|ref|NP\_004* | 6 | 41 | 26.0% | 415 | 45672 | 5.6 | heterogeneous nuclear ribonucleoprotein F [Homo sapiens] |
| U | *gi|148470406|ref|NP\_0* | 6 | 41 | 26.0% | 415 | 45672 | 5.6 | heterogeneous nuclear ribonucleoprotein F [Homo sapiens] |
| U | *gi|148470404|ref|NP\_0* | 6 | 41 | 26.0% | 415 | 45672 | 5.6 | heterogeneous nuclear ribonucleoprotein F [Homo sapiens] |
| U | *gi|148470402|ref|NP\_0* | 6 | 41 | 26.0% | 415 | 45672 | 5.6 | heterogeneous nuclear ribonucleoprotein F [Homo sapiens] |
| U | *gi|148470400|ref|NP\_0* | 6 | 41 | 26.0% | 415 | 45672 | 5.6 | heterogeneous nuclear ribonucleoprotein F [Homo sapiens] |

| Filename XCorr DeltCN Conf% ObsM+H+ CalcM+H+ SpR ZScore Ion% # Sequence  | | | | | | | | | | | | |
| --- | --- | --- | --- | --- | --- | --- | --- | --- | --- | --- | --- | --- |
|  | Astrin\_STLCHLD\_050114\_02.07005.07005.2 | 3.4015 | 0.3848 | 100.0% | 1710.2722 | 1710.7919 | 1 | 6.499 | 53.3% | 3 | R.QSGEAFVELGSEDDVK.M | 2 |
|  | Astrin\_STLCHLD\_050114\_01.04711.04711.3 | 3.0961 | 0.2811 | 99.4% | 1631.4543 | 1631.6584 | 332 | 4.94 | 30.0% | 1 | K.HSGPNSADSANDGFVR.L | 3 |
|  | Astrin\_STLCLD20\_112214\_tube2\_01.17202.17202.2 | 5.5366 | 0.3923 | 100.0% | 1869.4922 | 1869.0813 | 1 | 8.299 | 62.5% | 11 | K.ITGEAFVQFASQELAEK.A | 2 |
|  | Astrin\_STLCHLD\_tube2\_061314\_02.09105.09105.3 | 7.3535 | 0.5648 | 100.0% | 3476.5144 | 3476.7114 | 1 | 10.711 | 30.6% | 10 | R.MRPGAYSTGYGGYEEYSGLSDGYGFTTDLFGR.D | 3 |
|  | Astrin\_STLCHLD\_050114\_01.12769.12769.2 | 4.1076 | 0.4915 | 100.0% | 1997.4521 | 1998.2023 | 1 | 7.45 | 59.4% | 7 | K.ATENDIYNFFSPLNPVR.V | 22 |
|  | Astrin\_STLCLD20\_112214\_tube2\_01.07737.07737.2 | 3.2067 | 0.508 | 100.0% | 1093.4122 | 1093.2278 | 1 | 8.327 | 83.3% | 9 | R.VHIEIGPDGR.V | 222 |

Similarities:
gi|5031753|ref|NP\_005(2:4)  
gi|74099697|ref|NP\_00(1:5)  

---

|  |  |  |  |  |  |  |  |  |
| --- | --- | --- | --- | --- | --- | --- | --- | --- |
| U | *gi|19920317|ref|NP\_00* | 11 | 38 | 25.9% | 602 | 66023 | 5.9 | cytoskeleton-associated protein 4 [Homo sapiens] |

| Filename XCorr DeltCN Conf% ObsM+H+ CalcM+H+ SpR ZScore Ion% # Sequence  | | | | | | | | | | | | |
| --- | --- | --- | --- | --- | --- | --- | --- | --- | --- | --- | --- | --- |
| \* | Astrin\_STLCLD20\_112214\_tube2\_01.19055.19055.2 | 3.7145 | 0.4323 | 100.0% | 1797.7922 | 1798.049 | 1 | 7.181 | 53.3% | 1 | K.VQSLQATFGTFESILR.S | 2 |
| \* | Astrin\_STLCHLD\_tube2\_050114\_01.07062.07062.2 | 2.6292 | 0.188 | 98.0% | 1293.1522 | 1293.44 | 3 | 4.677 | 66.7% | 2 | K.SREWDMEALR.S | 2 |
| \* | AstrinSTLCLD\_041714\_01.05648.05648.2 | 1.9248 | 0.3132 | 97.1% | 987.1922 | 987.1845 | 69 | 5.86 | 62.5% | 1 | R.LALQALTEK.L | 2 |
| \* | Astrin\_STLCLD20\_112214\_tube2\_01.07522.07522.2 | 2.9519 | 0.3464 | 100.0% | 1288.0521 | 1288.4026 | 31 | 5.914 | 65.0% | 1 | R.HSEAFEALQQK.S | 2 |
| \* | AstrinSTLCLD\_041714\_02.06411.06411.3 | 3.8716 | 0.3865 | 99.9% | 1840.7943 | 1841.0923 | 1 | 6.676 | 50.0% | 10 | R.LQHVEDGVLSMQVASAR.Q | 3 |
| \* | AstrinSTLCLD\_041714\_01.06113.06113.2 | 3.8461 | 0.4521 | 100.0% | 1904.4521 | 1906.0135 | 2 | 8.817 | 44.4% | 2 | R.LEGLGSSEADQDGLASTVR.S | 2 |
| \* | Astrin\_STLCLD20\_112214\_tube2\_02.08778.08778.3 | 2.9238 | 0.2936 | 99.2% | 2051.4844 | 2050.3164 | 10 | 4.949 | 30.9% | 2 | R.SLGETQLVLYGDVEELKR.S | 3 |
| \* | AstrinSTLCLD\_041714\_01.06405.06405.2 | 3.769 | 0.3413 | 100.0% | 1474.3121 | 1474.6512 | 1 | 6.716 | 73.1% | 5 | R.SVGELPSTVESLQK.V | 2 |
| \* | Astrin\_NLD\_STLC\_tube2\_021014\_02.05081.05081.3 | 5.3313 | 0.4634 | 100.0% | 2022.2043 | 2023.2131 | 1 | 8.503 | 38.2% | 12 | K.VQEQVHTLLSQDQAQAAR.L | 3 |
| \* | Astrin\_STLCHLD\_tube2\_050114\_02.06931.06931.2 | 2.1003 | 0.3091 | 97.3% | 1253.5122 | 1253.4381 | 5 | 5.727 | 59.1% | 1 | R.TAVDSLVAYSVK.I | 2 |
| \* | Astrin\_STLCHLD\_050114\_01.04658.04658.2 | 3.2078 | 0.4433 | 100.0% | 1361.8121 | 1362.4363 | 1 | 7.454 | 72.7% | 1 | K.IETNENNLESAK.G | 2 |

---

|  |  |  |  |  |  |  |  |  |
| --- | --- | --- | --- | --- | --- | --- | --- | --- |
| U | *gi|16753227|ref|NP\_00* | 6 | 14 | 25.7% | 288 | 32728 | 10.6 | ribosomal protein L6 [Homo sapiens] |
| U | *gi|67189747|ref|NP\_00* | 6 | 14 | 25.7% | 288 | 32728 | 10.6 | ribosomal protein L6 [Homo sapiens] |

| Filename XCorr DeltCN Conf% ObsM+H+ CalcM+H+ SpR ZScore Ion% # Sequence  | | | | | | | | | | | | |
| --- | --- | --- | --- | --- | --- | --- | --- | --- | --- | --- | --- | --- |
|  | Astrin\_NLD\_STLC\_031014\_01.08940.08940.2 | 2.5387 | 0.3982 | 99.8% | 1284.3722 | 1285.5266 | 9 | 6.593 | 54.2% | 1 | K.VLATVTKPVGGDK.N | 2 |
|  | Astrin\_NLD\_STLC\_031014\_01.08584.08584.3 | 2.9628 | 0.2181 | 95.5% | 1771.2244 | 1771.0269 | 6 | 5.192 | 33.8% | 1 | K.VLATVTKPVGGDKNGGTR.V | 3 |
|  | Astrin\_STLCHLD\_050114\_01.15124.15124.2 | 3.4033 | 0.5136 | 100.0% | 1526.4922 | 1526.8601 | 1 | 9.07 | 75.0% | 2 | R.ASITPGTILIILTGR.H | 2 |
|  | Astrin\_STLCLD20\_112214\_01.05191.05191.2 | 2.5442 | 0.3402 | 99.9% | 995.15216 | 995.1228 | 1 | 6.838 | 71.4% | 3 | K.HLTDAYFK.K | 2 |
|  | Astrin\_STLCHLD\_tube2\_050114\_02.05508.05508.3 | 4.4273 | 0.4352 | 100.0% | 2510.2744 | 2510.6763 | 1 | 7.25 | 42.1% | 4 | R.HQEGEIFDTEKEKYEITEQR.K | 3 |
|  | Astrin\_STLCHLD\_tube2\_050114\_01.08895.08895.2 | 3.1901 | 0.3874 | 100.0% | 1447.0922 | 1447.6769 | 4 | 7.303 | 54.2% | 3 | R.SVFALTNGIYPHK.L | 2 |

---

|  |  |  |  |  |  |  |  |  |
| --- | --- | --- | --- | --- | --- | --- | --- | --- |
| U | *gi|4505409|ref|NP\_002* | 4 | 6 | 25.7% | 152 | 17298 | 8.4 | non-metastatic cells 2, protein (NM23B) expressed in [Homo sapiens] |
| U | *gi|66392227|ref|NP\_00* | 4 | 6 | 25.7% | 152 | 17298 | 8.4 | non-metastatic cells 2, protein (NM23B) expressed in [Homo sapiens] |
| U | *gi|66392205|ref|NP\_00* | 4 | 6 | 25.7% | 152 | 17298 | 8.4 | non-metastatic cells 2, protein (NM23B) expressed in [Homo sapiens] |
| U | *gi|66392203|ref|NP\_00* | 4 | 6 | 14.6% | 267 | 30137 | 8.9 | NME1-NME2 protein [Homo sapiens] |
| U | *gi|66392192|ref|NP\_00* | 4 | 6 | 25.7% | 152 | 17298 | 8.4 | non-metastatic cells 2, protein (NM23B) expressed in [Homo sapiens] |

| Filename XCorr DeltCN Conf% ObsM+H+ CalcM+H+ SpR ZScore Ion% # Sequence  | | | | | | | | | | | | |
| --- | --- | --- | --- | --- | --- | --- | --- | --- | --- | --- | --- | --- |
|  | Astrin\_STLCHLD\_050114\_01.10834.10834.2 | 3.0239 | 0.1826 | 98.6% | 1346.1322 | 1345.5846 | 5 | 4.767 | 63.6% | 3 | R.TFIAIKPDGVQR.G | 2 |
|  | Astrin\_STLCHLD\_tube2\_061314\_01.09228.09228.2 | 2.0916 | 0.243 | 95.4% | 1176.5322 | 1176.4038 | 7 | 5.048 | 61.1% | 1 | K.DRPFFPGLVK.Y | 2 |
|  | Astrin\_STLCHLD\_tube2\_061314\_01.04621.04621.2 | 4.4885 | 0.4123 | 100.0% | 1786.4321 | 1787.041 | 1 | 8.017 | 65.6% | 1 | R.VMLGETNPADSKPGTIR.G | 2 |
|  | Astrin\_STLCHLD\_tube2\_061314\_01.04655.04655.3 | 2.5464 | 0.2619 | 95.4% | 1787.0343 | 1787.041 | 7 | 5.506 | 31.2% | 1 | R.VMLGETNPADSKPGTIR.G | 3 |

---

|  |  |  |  |  |  |  |  |  |
| --- | --- | --- | --- | --- | --- | --- | --- | --- |
| U | *gi|14210536|ref|NP\_11* | 14 | 179 | 25.1% | 446 | 49857 | 4.9 | tubulin, beta 6 [Homo sapiens] |

| Filename XCorr DeltCN Conf% ObsM+H+ CalcM+H+ SpR ZScore Ion% # Sequence  | | | | | | | | | | | | |
| --- | --- | --- | --- | --- | --- | --- | --- | --- | --- | --- | --- | --- |
| \* | Astrin\_STLCHLD\_tube2\_050114\_01.09148.09148.2 | 3.6548 | 0.403 | 100.0% | 1575.3121 | 1574.7894 | 1 | 6.653 | 60.7% | 5 | R.AALVDLEPGTMDSVR.S | 2 |
|  | Astrin\_STLCLD20\_112214\_01.10486.10486.2 | 3.3502 | 0.4172 | 100.0% | 1352.5322 | 1352.6497 | 1 | 6.771 | 63.6% | 1 | R.IMNTFSVMPSPK.V | 22 |
|  | Astrin\_STLCHLD\_tube2\_061314\_01.07361.07361.2 | 3.2733 | 0.2391 | 99.9% | 1132.2322 | 1131.2767 | 1 | 5.073 | 83.3% | 60 | R.FPGQLNADLR.K | 222222 |
|  | Astrin\_STLCHLD\_tube2\_050114\_01.05601.05601.2 | 2.7143 | 0.2756 | 99.3% | 1258.5521 | 1259.4508 | 11 | 5.358 | 65.0% | 14 | R.FPGQLNADLRK.L | 222222 |
|  | Astrin\_STLCHLD\_tube2\_050114\_01.05583.05583.3 | 3.152 | 0.3056 | 99.8% | 1259.8744 | 1259.4508 | 8 | 5.284 | 45.0% | 17 | R.FPGQLNADLRK.L | 333333 |
|  | Astrin\_STLCHLD\_tube2\_061314\_01.08730.08730.2 | 3.8464 | 0.3753 | 100.0% | 1272.4122 | 1272.5945 | 1 | 7.898 | 75.0% | 16 | R.KLAVNMVPFPR.L | 222222 |
|  | Astrin\_STLCLD20\_112214\_01.11192.11192.1 | 2.2399 | 0.2147 | 96.5% | 1143.63 | 1144.4204 | 6 | 6.378 | 61.1% | 4 | K.LAVNMVPFPR.L | 111111 |
|  | Astrin\_STLCHLD\_tube2\_050114\_01.09839.09839.2 | 3.9889 | 0.4725 | 100.0% | 1144.4122 | 1144.4204 | 1 | 8.55 | 94.4% | 18 | K.LAVNMVPFPR.L | 222222 |
|  | Astrin\_STLCHLD\_tube2\_061314\_01.12143.12143.2 | 3.7666 | 0.4528 | 100.0% | 1621.8322 | 1621.9403 | 1 | 8.756 | 76.9% | 17 | R.LHFFMPGFAPLTSR.G | 22222 |
|  | Astrin\_STLCHLD\_tube2\_061314\_01.12204.12204.3 | 4.3867 | 0.3891 | 100.0% | 1622.3944 | 1621.9403 | 1 | 6.411 | 53.8% | 14 | R.LHFFMPGFAPLTSR.G | 33333 |
|  | Astrin\_STLCHLD\_050114\_01.04952.04952.2 | 2.8672 | 0.3941 | 100.0% | 1066.2322 | 1066.2013 | 2 | 7.174 | 68.8% | 2 | R.NMMAACDPR.H | 222222 |
|  | Astrin\_STLCHLD\_tube2\_061314\_01.11781.11781.2 | 4.295 | 0.2978 | 100.0% | 1698.5322 | 1697.8877 | 1 | 7.708 | 73.1% | 8 | K.NSSYFVEWIPNNVK.V | 222222 |
| \* | Astrin\_STLCHLD\_061214\_01.11426.11426.2 | 3.8519 | 0.0023 | 95.7% | 1858.0721 | 1859.1475 | 1 | 8.136 | 62.5% | 1 | K.MASTFIGNSTAIQELFK.R | 2 |
| \* | Astrin\_STLCHLD\_tube2\_050114\_01.09654.09654.2 | 2.4815 | 0.2046 | 97.6% | 1215.9922 | 1216.3972 | 7 | 4.948 | 66.7% | 2 | R.ISEQFSAMFR.R | 2 |

Similarities:
gi|29788785|ref|NP\_82(10:4)  
gi|5174735|ref|NP\_006(10:4)  
gi|29788768|ref|NP\_82(11:3)  
gi|21361322|ref|NP\_00(10:4)  
gi|50592996|ref|NP\_00(8:6)  

---

|  |  |  |  |  |  |  |  |  |
| --- | --- | --- | --- | --- | --- | --- | --- | --- |
| U | *gi|56699409|ref|NP\_00* | 9 | 58 | 25.1% | 391 | 42332 | 10.1 | RNA binding motif protein, X-linked [Homo sapiens] |

| Filename XCorr DeltCN Conf% ObsM+H+ CalcM+H+ SpR ZScore Ion% # Sequence  | | | | | | | | | | | | |
| --- | --- | --- | --- | --- | --- | --- | --- | --- | --- | --- | --- | --- |
|  | Astrin\_STLCHLD\_tube2\_061314\_01.08381.08381.2 | 3.7391 | 0.2809 | 100.0% | 1436.2922 | 1436.6049 | 3 | 6.444 | 70.8% | 16 | K.LFIGGLNTETNEK.A | 2 |
|  | Astrin\_STLCHLD\_tube2\_061314\_01.09717.09717.2 | 2.6119 | 0.1862 | 98.6% | 945.3722 | 945.2488 | 29 | 5.401 | 71.4% | 2 | R.IVEVLLMK.D | 2 |
|  | AstrinSTLCLD\_041714\_01.06536.06536.2 | 3.1471 | 0.3557 | 100.0% | 1216.3322 | 1216.5249 | 2 | 6.043 | 77.8% | 3 | R.IVEVLLMKDR.E | 2 |
|  | Astrin\_STLCHLD\_tube2\_050114\_01.11028.11028.2 | 4.438 | 0.4571 | 100.0% | 1487.3121 | 1487.6519 | 1 | 8.812 | 73.1% | 7 | R.GFAFVTFESPADAK.D | 2 |
|  | AstrinSTLCLD\_041714\_01.09080.09080.3 | 3.1051 | 0.2333 | 97.5% | 1902.4143 | 1901.0856 | 3 | 5.124 | 33.8% | 1 | R.GFAFVTFESPADAKDAAR.D | 3 |
| \* | Astrin\_NLD\_STLC\_031014\_01.09039.09039.3 | 3.7804 | 0.2563 | 99.8% | 1749.6244 | 1748.9768 | 1 | 6.693 | 41.7% | 3 | K.AIKVEQATKPSFESGR.R | 3 |
|  | AstrinSTLCLD\_041714\_02.06326.06326.3 | 4.472 | 0.362 | 99.9% | 2050.9443 | 2051.1873 | 1 | 7.432 | 38.9% | 18 | R.GGHMDDGGYSMNFNMSSSR.G | 3 |
|  | Astrin\_STLCHLD\_tube2\_050114\_01.04769.04769.2 | 2.9954 | 0.4334 | 100.0% | 1514.3322 | 1514.6372 | 35 | 6.897 | 54.5% | 1 | R.DYAPPPRDYTYR.D | 2 |
|  | Astrin\_NLD\_STLC\_031014\_01.06534.06534.2 | 2.8932 | 0.3918 | 100.0% | 1178.1522 | 1178.1571 | 1 | 5.897 | 72.2% | 7 | R.DSYESYGNSR.S | 2 |

---

|  |  |  |  |  |  |  |  |  |
| --- | --- | --- | --- | --- | --- | --- | --- | --- |
| U | *contaminant\_KERATIN03* | 16 | 73 | 25.0% | 593 | 59519 | 5.2 | no description |
| U | *gi|195972866|ref|NP\_0* | 16 | 76 | 25.3% | 584 | 58801 | 5.2 | keratin 10 [Homo sapiens] |

| Filename XCorr DeltCN Conf% ObsM+H+ CalcM+H+ SpR ZScore Ion% # Sequence  | | | | | | | | | | | | |
| --- | --- | --- | --- | --- | --- | --- | --- | --- | --- | --- | --- | --- |
|  | Astrin\_STLCHLD\_061214\_02.06871.06871.2 | 5.5529 | 0.5159 | 100.0% | 1708.3722 | 1708.7844 | 1 | 8.976 | 63.9% | 11 | K.GSLGGGFSSGGFSGGSFSR.G | 2 |
|  | Astrin\_NLD\_STLC\_tube2\_021014\_01.04526.04526.2 | 2.8206 | 0.328 | 100.0% | 1090.9922 | 1091.2273 | 46 | 6.066 | 68.8% | 1 | K.VTMQNLNDR.L | 222 |
|  | Astrin\_STLCHLD\_tube2\_050114\_01.05063.05063.2 | 3.0476 | 0.142 | 99.0% | 1064.9722 | 1065.2578 | 35 | 6.057 | 62.5% | 4 | R.LASYLDKVR.A | 22222 |
|  | Astrin\_STLCLD20\_112214\_01.06026.06026.2 | 3.6248 | 0.46 | 100.0% | 1382.3522 | 1382.4668 | 1 | 7.97 | 63.6% | 7 | R.ALEESNYELEGK.I | 2 |
|  | AstrinSTLCLD\_041714\_01.12957.12957.3 | 5.7925 | 0.4874 | 100.0% | 3053.6643 | 3054.4277 | 1 | 8.062 | 31.7% | 5 | K.TIDDLKNQILNLTTDNANILLQIDNAR.L | 3 |
|  | Astrin\_STLCHLD\_050114\_01.06505.06505.2 | 2.4865 | 0.2457 | 99.3% | 808.09216 | 807.8815 | 46 | 6.454 | 66.7% | 13 | R.LAADDFR.L | 222222 |
|  | Astrin\_STLCLD20\_112214\_01.05560.05560.2 | 2.8917 | 0.3716 | 100.0% | 1235.7922 | 1235.4258 | 6 | 6.199 | 72.2% | 6 | R.LKYENEVALR.Q | 2 |
|  | Astrin\_STLCHLD\_061214\_01.05064.05064.3 | 3.5896 | 0.2666 | 100.0% | 1236.4744 | 1235.4258 | 1 | 5.47 | 55.6% | 2 | R.LKYENEVALR.Q | 3 |
|  | Astrin\_NLD\_STLC\_tube2\_021014\_01.07449.07449.2 | 2.6343 | 0.2881 | 99.4% | 1188.2722 | 1188.4099 | 1 | 5.736 | 77.8% | 1 | R.RVLDELTLTK.A | 2 |
|  | AstrinSTLCLD\_041714\_01.05993.05993.2 | 3.1 | 0.4514 | 100.0% | 1032.1322 | 1032.2224 | 2 | 7.474 | 75.0% | 5 | R.VLDELTLTK.A | 2 |
|  | Astrin\_NLD\_STLC\_tube2\_021014\_01.04660.04660.2 | 3.9455 | 0.3756 | 100.0% | 1366.2122 | 1366.43 | 6 | 6.829 | 65.0% | 1 | R.SQYEQLAEQNR.K | 2 |
|  | Astrin\_STLCLD20\_112214\_tube2\_01.06234.06234.2 | 3.0523 | 0.2143 | 99.2% | 1495.4922 | 1494.6041 | 1 | 5.032 | 68.2% | 1 | R.SQYEQLAEQNRK.D | 2 |
|  | Astrin\_NLD\_STLC\_tube2\_021014\_01.08760.08760.2 | 2.793 | 0.1416 | 98.1% | 1111.2322 | 1110.1681 | 2 | 5.579 | 75.0% | 1 | K.DAEAWFNEK.S | 2 |
|  | AstrinSTLCLD\_041714\_01.05408.05408.2 | 3.7803 | 0.441 | 100.0% | 1391.2122 | 1391.4778 | 1 | 7.681 | 70.8% | 8 | K.QSLEASLAETEGR.Y | 2 |
|  | Astrin\_STLCHLD\_061214\_01.05703.05703.2 | 3.6217 | 0.3459 | 100.0% | 1435.4521 | 1435.623 | 1 | 7.079 | 85.0% | 4 | K.IRLENEIQTYR.S | 2 |
|  | Astrin\_STLCHLD\_tube2\_050114\_01.04778.04778.2 | 3.44 | 0.0158 | 98.0% | 1167.3722 | 1166.2761 | 1 | 4.539 | 87.5% | 3 | R.LENEIQTYR.S | 2 |

Similarities:
gi|40354195|ref|NP\_95(1:15)  
gi|4557701|ref|NP\_000(2:14)  
gi|15431310|ref|NP\_00(3:13)  
gi|24430192|ref|NP\_00(3:13)  
gi|24234699|ref|NP\_00(2:14)  

---

|  |  |  |  |  |  |  |  |  |
| --- | --- | --- | --- | --- | --- | --- | --- | --- |
| U | *gi|208973238|ref|NP\_0* | 4 | 6 | 24.9% | 245 | 27745 | 4.8 | tyrosine 3/tryptophan 5 -monooxygenase activation protein, zeta polypeptide [Homo sapiens] |
| U | *gi|4507953|ref|NP\_003* | 4 | 6 | 24.9% | 245 | 27745 | 4.8 | tyrosine 3/tryptophan 5 -monooxygenase activation protein, zeta polypeptide [Homo sapiens] |
| U | *gi|21735625|ref|NP\_66* | 4 | 6 | 24.9% | 245 | 27745 | 4.8 | tyrosine 3/tryptophan 5 -monooxygenase activation protein, zeta polypeptide [Homo sapiens] |
| U | *gi|208973244|ref|NP\_0* | 4 | 6 | 24.9% | 245 | 27745 | 4.8 | tyrosine 3/tryptophan 5 -monooxygenase activation protein, zeta polypeptide [Homo sapiens] |
| U | *gi|208973242|ref|NP\_0* | 4 | 6 | 24.9% | 245 | 27745 | 4.8 | tyrosine 3/tryptophan 5 -monooxygenase activation protein, zeta polypeptide [Homo sapiens] |
| U | *gi|208973240|ref|NP\_0* | 4 | 6 | 24.9% | 245 | 27745 | 4.8 | tyrosine 3/tryptophan 5 -monooxygenase activation protein, zeta polypeptide [Homo sapiens] |

| Filename XCorr DeltCN Conf% ObsM+H+ CalcM+H+ SpR ZScore Ion% # Sequence  | | | | | | | | | | | | |
| --- | --- | --- | --- | --- | --- | --- | --- | --- | --- | --- | --- | --- |
|  | Astrin\_STLCHLD\_050114\_01.04787.04787.2 | 2.3869 | 0.2659 | 98.0% | 1280.2922 | 1280.4203 | 1 | 5.456 | 68.2% | 1 | R.YLAEVAAGDDKK.G | 2 |
|  | Astrin\_STLCHLD\_061214\_02.11572.11572.2 | 2.7554 | 0.3391 | 99.5% | 2318.5122 | 2318.6746 | 13 | 6.339 | 34.2% | 1 | R.LGLALNFSVFYYEILNSPEK.A | 22 |
|  | Astrin\_STLCHLD\_tube2\_061314\_01.18065.18065.3 | 5.6291 | 0.5467 | 100.0% | 3303.9844 | 3304.6907 | 1 | 10.021 | 30.4% | 3 | K.TAFDEAIAELDTLSEESYKDSTLIMQLLR.D | 3 |
|  | Astrin\_STLCHLD\_050114\_01.18307.18307.2 | 3.6207 | 0.4753 | 100.0% | 3305.0723 | 3304.6907 | 1 | 8.951 | 28.6% | 1 | K.TAFDEAIAELDTLSEESYKDSTLIMQLLR.D | 2 |

Similarities:
gi|21328448|ref|NP\_64(1:3)  

---

|  |  |  |  |  |  |  |  |  |
| --- | --- | --- | --- | --- | --- | --- | --- | --- |
| U | *gi|72534660|ref|NP\_00* | 7 | 29 | 24.4% | 238 | 27367 | 11.8 | splicing factor, arginine/serine-rich 7 [Homo sapiens] |

| Filename XCorr DeltCN Conf% ObsM+H+ CalcM+H+ SpR ZScore Ion% # Sequence  | | | | | | | | | | | | |
| --- | --- | --- | --- | --- | --- | --- | --- | --- | --- | --- | --- | --- |
| \* | Astrin\_STLCLD20\_112214\_01.06891.06891.2 | 3.8301 | 0.4885 | 100.0% | 1720.4922 | 1720.923 | 1 | 8.375 | 68.8% | 2 | K.VYVGNLGTGAGKGELER.A | 2 |
| \* | Astrin\_STLCHLD\_tube2\_061314\_02.05361.05361.3 | 2.5843 | 0.3712 | 99.8% | 1721.2144 | 1720.923 | 22 | 6.482 | 34.4% | 6 | K.VYVGNLGTGAGKGELER.A | 3 |
| \* | Astrin\_STLCLD20\_112214\_tube2\_01.11373.11373.2 | 2.5498 | 0.3219 | 99.7% | 1074.2522 | 1074.2242 | 19 | 6.915 | 68.8% | 5 | R.AFSYYGPLR.T | 2 |
|  | Astrin\_STLCLD20\_112214\_tube2\_01.15758.15758.2 | 3.3718 | 0.37 | 100.0% | 1622.3522 | 1622.7771 | 1 | 6.459 | 65.4% | 4 | R.NPPGFAFVEFEDPR.D | 22 |
| \* | AstrinSTLCLD\_041714\_01.10203.10203.3 | 2.5362 | 0.2896 | 96.4% | 2379.2043 | 2379.5474 | 1 | 4.717 | 30.0% | 1 | R.NPPGFAFVEFEDPRDAEDAVR.G | 3 |
| \* | Astrin\_STLCHLD\_tube2\_061314\_01.04813.04813.2 | 3.5702 | 0.3557 | 100.0% | 1245.3322 | 1245.4827 | 8 | 6.956 | 60.0% | 10 | R.VRVELSTGMPR.R | 2 |
| \* | Astrin\_STLCHLD\_050114\_01.08723.08723.2 | 2.1797 | 0.2127 | 95.8% | 991.0522 | 990.1626 | 88 | 3.732 | 68.8% | 1 | R.VELSTGMPR.R | 2 |

Similarities:
gi|4506901|ref|NP\_003(1:6)  

---

|  |  |  |  |  |  |  |  |  |
| --- | --- | --- | --- | --- | --- | --- | --- | --- |
| U | *gi|10863927|ref|NP\_06* | 5 | 27 | 24.2% | 165 | 18012 | 7.8 | peptidylprolyl isomerase A [Homo sapiens] |
| U | *gi|169215435|ref|XP\_0* | 5 | 27 | 17.9% | 223 | 24376 | 6.9 | PREDICTED: similar to peptidylprolyl isomerase A-like [Homo sapiens] |

| Filename XCorr DeltCN Conf% ObsM+H+ CalcM+H+ SpR ZScore Ion% # Sequence  | | | | | | | | | | | | |
| --- | --- | --- | --- | --- | --- | --- | --- | --- | --- | --- | --- | --- |
|  | Astrin\_STLCHLD\_tube2\_061314\_01.10077.10077.2 | 3.548 | 0.3518 | 100.0% | 1380.0122 | 1380.6268 | 3 | 6.696 | 68.2% | 9 | R.VSFELFADKVPK.T | 2 |
|  | Astrin\_NLD\_STLC\_tube2\_021014\_01.10332.10332.3 | 2.7666 | 0.3171 | 99.8% | 1380.0844 | 1380.6268 | 53 | 5.737 | 38.6% | 2 | R.VSFELFADKVPK.T | 3 |
|  | Astrin\_STLCLD20\_112214\_01.10942.10942.2 | 4.4902 | 0.3905 | 100.0% | 1832.7322 | 1833.0477 | 1 | 6.635 | 60.7% | 5 | K.SIYGEKFEDENFILK.H | 2 |
|  | Astrin\_STLCLD20\_112214\_01.09839.09839.2 | 3.8121 | 0.0891 | 98.8% | 1506.2522 | 1506.7755 | 2 | 8.373 | 75.0% | 5 | K.VKEGMNIVEAMER.F | 2 |
|  | Astrin\_STLCHLD\_tube2\_061314\_01.09937.09937.2 | 2.8721 | 0.1741 | 98.3% | 1279.0521 | 1279.4689 | 2 | 6.871 | 70.0% | 6 | K.EGMNIVEAMER.F | 2 |

---

|  |  |  |  |  |  |  |  |  |
| --- | --- | --- | --- | --- | --- | --- | --- | --- |
| U | *gi|4506743|ref|NP\_001* | 5 | 31 | 24.0% | 208 | 24205 | 10.3 | ribosomal protein S8 [Homo sapiens] |

| Filename XCorr DeltCN Conf% ObsM+H+ CalcM+H+ SpR ZScore Ion% # Sequence  | | | | | | | | | | | | |
| --- | --- | --- | --- | --- | --- | --- | --- | --- | --- | --- | --- | --- |
| \* | Astrin\_NLD\_STLC\_031014\_01.08871.08871.2 | 2.6286 | 0.3809 | 100.0% | 1220.0122 | 1220.3707 | 1 | 6.0 | 75.0% | 1 | K.YELGRPAANTK.I | 2 |
| \* | AstrinSTLCLD\_041714\_02.07142.07142.2 | 4.0113 | 0.4765 | 100.0% | 1719.4321 | 1719.9353 | 1 | 8.998 | 78.6% | 9 | R.IIDVVYNASNNELVR.T | 2 |
| \* | Astrin\_STLCHLD\_tube2\_061314\_02.07160.07160.3 | 4.3009 | 0.3264 | 99.9% | 1720.1643 | 1719.9353 | 3 | 5.993 | 44.6% | 4 | R.IIDVVYNASNNELVR.T | 3 |
|  | Astrin\_STLCLD20\_112214\_01.08348.08348.2 | 2.5987 | 0.3235 | 99.5% | 1315.2522 | 1315.4631 | 2 | 5.185 | 65.0% | 2 | K.LTPEEEEILNK.K | 2 |
| \* | Astrin\_NLD\_STLC\_tube2\_021014\_01.08871.08871.2 | 4.2275 | 0.4411 | 100.0% | 1507.6721 | 1507.6836 | 1 | 7.435 | 79.2% | 15 | K.ISSLLEEQFQQGK.L | 2 |

---

|  |  |  |  |  |  |  |  |  |
| --- | --- | --- | --- | --- | --- | --- | --- | --- |
| U | *gi|169164494|ref|XP\_0* | 2 | 8 | 24.0% | 100 | 11493 | 10.1 | PREDICTED: similar to ribosomal protein L10 [Homo sapiens] |
| U | *gi|41151097|ref|XP\_20* | 2 | 8 | 11.2% | 214 | 24627 | 10.1 | PREDICTED: similar to QM protein isoform 1 [Homo sapiens] |
| U | *gi|223890243|ref|NP\_0* | 2 | 8 | 11.2% | 214 | 24604 | 10.1 | ribosomal protein L10 [Homo sapiens] |
| U | *gi|169213734|ref|XP\_0* | 2 | 8 | 14.4% | 167 | 19409 | 9.9 | PREDICTED: similar to Q1Z 7F5 isoform 2 [Homo sapiens] |
| U | *gi|169213732|ref|XP\_0* | 2 | 8 | 11.2% | 214 | 24600 | 10.1 | PREDICTED: similar to Q1Z 7F5 isoform 1 [Homo sapiens] |
| U | *gi|169213538|ref|XP\_0* | 2 | 8 | 14.4% | 167 | 19436 | 9.9 | PREDICTED: similar to QM protein isoform 2 [Homo sapiens] |
| U | *gi|169213536|ref|XP\_0* | 2 | 8 | 11.2% | 214 | 24627 | 10.1 | PREDICTED: similar to QM protein isoform 1 [Homo sapiens] |

| Filename XCorr DeltCN Conf% ObsM+H+ CalcM+H+ SpR ZScore Ion% # Sequence  | | | | | | | | | | | | |
| --- | --- | --- | --- | --- | --- | --- | --- | --- | --- | --- | --- | --- |
|  | Astrin\_STLCLD20\_112214\_01.10352.10352.2 | 3.2788 | 0.5335 | 100.0% | 1253.4722 | 1253.5486 | 2 | 9.089 | 65.0% | 6 | R.VHIGQVIMSIR.T | 2 |
|  | AstrinSTLCLD\_041714\_01.09449.09449.2 | 3.4425 | 0.3069 | 100.0% | 1545.5721 | 1545.6606 | 1 | 6.216 | 62.5% | 2 | K.FNADEFEDMVAEK.W | 2 |

---

|  |  |  |  |  |  |  |  |  |
| --- | --- | --- | --- | --- | --- | --- | --- | --- |
| U | *gi|15809016|ref|NP\_29* | 4 | 16 | 23.8% | 172 | 19779 | 4.8 | myosin regulatory light chain MRCL2 isoform A [Homo sapiens] |
| U | *gi|5453740|ref|NP\_006* | 4 | 16 | 24.0% | 171 | 19794 | 4.8 | myosin, light chain 12A, regulatory, non-sarcomeric [Homo sapiens] |
| U | *gi|222144328|ref|NP\_0* | 4 | 16 | 26.6% | 154 | 17757 | 4.4 | myosin regulatory light chain MRCL2 isoform B [Homo sapiens] |
| U | *gi|222144326|ref|NP\_0* | 4 | 16 | 23.8% | 172 | 19779 | 4.8 | myosin regulatory light chain MRCL2 isoform A [Homo sapiens] |
| U | *gi|222144324|ref|NP\_0* | 4 | 16 | 23.8% | 172 | 19779 | 4.8 | myosin regulatory light chain MRCL2 isoform A [Homo sapiens] |

| Filename XCorr DeltCN Conf% ObsM+H+ CalcM+H+ SpR ZScore Ion% # Sequence  | | | | | | | | | | | | |
| --- | --- | --- | --- | --- | --- | --- | --- | --- | --- | --- | --- | --- |
|  | Astrin\_STLCLD20\_112214\_tube2\_01.08741.08741.2 | 2.8659 | 0.2328 | 99.2% | 1229.5922 | 1229.3324 | 6 | 5.195 | 65.0% | 1 | K.LNGTDPEDVIR.N | 2 |
|  | Astrin\_STLCLD20\_112214\_tube2\_01.14668.14668.3 | 4.4127 | 0.3918 | 99.9% | 2433.4744 | 2433.649 | 1 | 6.504 | 38.2% | 8 | R.ELLTTMGDRFTDEEVDELYR.E | 3 |
|  | Astrin\_STLCHLD\_tube2\_061314\_01.08257.08257.2 | 3.5653 | 0.4316 | 100.0% | 1417.6322 | 1416.4839 | 1 | 6.934 | 70.0% | 4 | R.FTDEEVDELYR.E | 2 |
|  | Astrin\_STLCHLD\_tube2\_061314\_01.09614.09614.2 | 3.1857 | 0.2754 | 100.0% | 1261.7322 | 1261.3794 | 1 | 8.008 | 72.2% | 3 | K.GNFNYIEFTR.I | 2 |

---

|  |  |  |  |  |  |  |  |  |
| --- | --- | --- | --- | --- | --- | --- | --- | --- |
| U | *gi|16905517|ref|NP\_47* | 7 | 31 | 23.7% | 262 | 31301 | 11.3 | FUS interacting protein (serine-arginine rich) 1 isoform 2 [Homo sapiens] |
| U | *gi|5730079|ref|NP\_006* | 7 | 31 | 33.9% | 183 | 22222 | 10.3 | FUS interacting protein (serine-arginine rich) 1 isoform 1 [Homo sapiens] |
| U | *gi|169161980|ref|XP\_0* | 7 | 31 | 34.3% | 181 | 22022 | 10.3 | PREDICTED: hypothetical protein, partial [Homo sapiens] |
| U | *gi|169161109|ref|XP\_0* | 7 | 31 | 33.9% | 183 | 22222 | 10.3 | PREDICTED: hypothetical protein LOC642558 [Homo sapiens] |
| U | *gi|169161107|ref|XP\_0* | 7 | 31 | 23.7% | 262 | 31301 | 11.3 | PREDICTED: hypothetical protein LOC642558 [Homo sapiens] |

| Filename XCorr DeltCN Conf% ObsM+H+ CalcM+H+ SpR ZScore Ion% # Sequence  | | | | | | | | | | | | |
| --- | --- | --- | --- | --- | --- | --- | --- | --- | --- | --- | --- | --- |
|  | Astrin\_STLCHLD\_tube2\_061314\_01.08033.08033.3 | 3.8345 | 0.2548 | 99.9% | 1463.5144 | 1463.7227 | 1 | 5.336 | 61.4% | 9 | R.YLRPPNTSLFVR.N | 3 |
|  | Astrin\_STLCLD20\_112214\_tube2\_01.18545.18545.2 | 4.3356 | 0.5495 | 100.0% | 1918.0922 | 1918.1992 | 1 | 9.21 | 60.0% | 7 | R.YGPIVDVYVPLDFYTR.R | 2 |
|  | Astrin\_STLCHLD\_061214\_01.08395.08395.2 | 3.1586 | 0.3172 | 100.0% | 1332.6721 | 1331.4705 | 1 | 5.889 | 70.0% | 5 | R.GFAYVQFEDVR.D | 2 |
|  | Astrin\_STLCLD20\_112214\_tube2\_01.15809.15809.3 | 4.1756 | 0.4364 | 100.0% | 2580.5942 | 2581.7605 | 1 | 7.225 | 34.5% | 2 | R.GFAYVQFEDVRDAEDALHNLDR.K | 3 |
|  | Astrin\_STLCLD20\_112214\_tube2\_01.14609.14609.3 | 4.1533 | 0.3468 | 99.9% | 2709.5942 | 2709.9346 | 1 | 6.223 | 37.5% | 2 | R.GFAYVQFEDVRDAEDALHNLDRK.W | 3 |
|  | Astrin\_STLCLD20\_112214\_01.07516.07516.2 | 2.1582 | 0.3058 | 98.0% | 1269.2922 | 1269.3134 | 1 | 4.903 | 75.0% | 1 | R.DAEDALHNLDR.K | 2 |
|  | Astrin\_STLCHLD\_061214\_01.06560.06560.2 | 2.724 | 0.3071 | 99.6% | 1305.6322 | 1305.4331 | 7 | 5.62 | 65.0% | 5 | R.QIEIQFAQGDR.K | 2 |

---

|  |  |  |  |  |  |  |  |  |
| --- | --- | --- | --- | --- | --- | --- | --- | --- |
| U | *gi|21328448|ref|NP\_64* | 3 | 4 | 23.6% | 246 | 28082 | 4.8 | tyrosine 3-monooxygenase/tryptophan 5-monooxygenase activation protein, beta polypeptide [Homo sapiens] |
| U | *gi|4507949|ref|NP\_003* | 3 | 4 | 23.6% | 246 | 28082 | 4.8 | tyrosine 3-monooxygenase/tryptophan 5-monooxygenase activation protein, beta polypeptide [Homo sapiens] |

| Filename XCorr DeltCN Conf% ObsM+H+ CalcM+H+ SpR ZScore Ion% # Sequence  | | | | | | | | | | | | |
| --- | --- | --- | --- | --- | --- | --- | --- | --- | --- | --- | --- | --- |
|  | Astrin\_STLCHLD\_050114\_01.06965.06965.2 | 2.0972 | 0.249 | 96.4% | 1016.2522 | 1016.172 | 1 | 4.78 | 68.8% | 2 | R.YDDMAAAMK.A | 22 |
|  | Astrin\_STLCHLD\_061214\_02.11572.11572.2 | 2.7554 | 0.3391 | 99.5% | 2318.5122 | 2318.6746 | 13 | 6.339 | 34.2% | 1 | R.LGLALNFSVFYYEILNSPEK.A | 22 |
|  | Astrin\_STLCHLD\_tube2\_061314\_01.17019.17019.3 | 3.3171 | 0.3453 | 99.8% | 3331.9744 | 3331.7163 | 2 | 5.351 | 22.3% | 1 | K.TAFDEAIAELDTLNEESYKDSTLIMQLLR.D | 3 |

Similarities:
gi|21464101|ref|NP\_03(1:2)  
gi|208973238|ref|NP\_0(1:2)  

---

|  |  |  |  |  |  |  |  |  |
| --- | --- | --- | --- | --- | --- | --- | --- | --- |
| U | *gi|4503483|ref|NP\_001* | 20 | 80 | 23.5% | 858 | 95338 | 6.8 | eukaryotic translation elongation factor 2 [Homo sapiens] |

| Filename XCorr DeltCN Conf% ObsM+H+ CalcM+H+ SpR ZScore Ion% # Sequence  | | | | | | | | | | | | |
| --- | --- | --- | --- | --- | --- | --- | --- | --- | --- | --- | --- | --- |
| \* | Astrin\_STLCLD20\_112214\_tube2\_01.06542.06542.2 | 2.6912 | 0.309 | 99.4% | 1307.3922 | 1308.4979 | 133 | 6.142 | 54.5% | 1 | R.NMSVIAHVDHGK.S | 2 |
| \* | Astrin\_STLCHLD\_tube2\_061314\_01.14825.14825.2 | 3.4118 | 0.4039 | 100.0% | 2205.152 | 2205.4692 | 1 | 7.166 | 50.0% | 1 | K.STAISLFYELSENDLNFIK.Q | 2 |
| \* | Astrin\_STLCHLD\_050114\_01.13058.13058.2 | 4.0366 | 0.3752 | 100.0% | 2221.612 | 2221.5151 | 1 | 5.773 | 50.0% | 5 | R.ALLELQLEPEELYQTFQR.I | 2 |
| \* | Astrin\_STLCLD20\_112214\_tube2\_01.19284.19284.3 | 5.1017 | 0.3406 | 99.9% | 2757.9243 | 2758.2976 | 1 | 6.244 | 36.5% | 1 | R.RWLPAGDALLQMITIHLPSPVTAQK.Y | 3 |
| \* | Astrin\_STLCHLD\_050114\_01.15437.15437.2 | 4.8207 | 0.4994 | 100.0% | 2602.2522 | 2602.11 | 1 | 8.118 | 47.8% | 2 | R.WLPAGDALLQMITIHLPSPVTAQK.Y | 2 |
| \* | AstrinSTLCLD\_041714\_01.13959.13959.3 | 3.1782 | 0.2838 | 99.0% | 2602.4343 | 2602.11 | 1 | 4.997 | 30.4% | 1 | R.WLPAGDALLQMITIHLPSPVTAQK.Y | 3 |
| \* | Astrin\_STLCHLD\_tube2\_061314\_01.09097.09097.2 | 2.9726 | 0.5209 | 100.0% | 1039.8522 | 1040.3241 | 1 | 8.097 | 75.0% | 3 | K.GPLMMYISK.M | 2 |
| \* | Astrin\_STLCHLD\_tube2\_061314\_01.08418.08418.2 | 3.1101 | 0.3736 | 100.0% | 1107.9722 | 1108.3231 | 1 | 6.948 | 75.0% | 10 | R.VFSGLVSTGLK.V | 2 |
| \* | Astrin\_STLCHLD\_tube2\_061314\_01.06324.06324.2 | 1.9443 | 0.294 | 97.8% | 822.27216 | 822.0717 | 118 | 5.174 | 66.7% | 1 | R.TILMMGR.Y | 2 |
| \* | Astrin\_STLCLD20\_112214\_tube2\_01.07274.07274.2 | 4.2458 | 0.5054 | 100.0% | 1616.2122 | 1616.7917 | 1 | 8.957 | 57.7% | 1 | K.TGTITTFEHAHNMR.V | 2 |
| \* | Astrin\_STLCHLD\_tube2\_061314\_01.08550.08550.3 | 6.0248 | 0.4993 | 100.0% | 2144.6343 | 2144.3489 | 1 | 9.011 | 42.1% | 24 | K.ARPFPDGLAEDIDKGEVSAR.Q | 3 |
| \* | Astrin\_STLCLD20\_112214\_02.10126.10126.3 | 3.2024 | 0.2954 | 99.8% | 1969.4343 | 1971.1796 | 57 | 4.538 | 33.3% | 1 | R.ARYLAEKYEWDVAEAR.K | 3 |
| \* | Astrin\_STLCHLD\_061214\_01.06904.06904.2 | 5.0188 | 0.4919 | 100.0% | 1744.4321 | 1743.9133 | 1 | 8.264 | 80.8% | 4 | R.YLAEKYEWDVAEAR.K | 2 |
| \* | Astrin\_STLCHLD\_050114\_02.07028.07028.3 | 3.7729 | 0.4121 | 100.0% | 1744.7043 | 1743.9133 | 1 | 7.107 | 51.9% | 10 | R.YLAEKYEWDVAEAR.K | 3 |
| \* | Astrin\_STLCHLD\_tube2\_050114\_02.06065.06065.3 | 3.0634 | 0.2002 | 96.2% | 1872.7444 | 1872.0874 | 1 | 4.844 | 42.9% | 1 | R.YLAEKYEWDVAEARK.I | 3 |
| \* | Astrin\_STLCLD20\_112214\_tube2\_01.10478.10478.2 | 1.9764 | 0.3184 | 97.7% | 1139.0122 | 1139.2096 | 7 | 5.832 | 75.0% | 1 | K.YEWDVAEAR.K | 2 |
| \* | Astrin\_STLCHLD\_tube2\_050114\_01.13914.13914.2 | 4.7915 | 0.5382 | 100.0% | 2353.912 | 2354.6677 | 1 | 9.87 | 52.5% | 1 | K.GVQYLNEIKDSVVAGFQWATK.E | 2 |
| \* | Astrin\_STLCHLD\_061214\_02.10064.10064.3 | 4.1826 | 0.3202 | 100.0% | 2354.6343 | 2354.6677 | 41 | 5.617 | 27.5% | 1 | K.GVQYLNEIKDSVVAGFQWATK.E | 3 |
| \* | Astrin\_STLCHLD\_tube2\_061314\_02.08825.08825.2 | 4.2054 | 0.4747 | 100.0% | 1800.8722 | 1801.0087 | 1 | 7.689 | 60.0% | 9 | K.AYLPVNESFGFTADLR.S | 2 |
| \* | Astrin\_STLCLD20\_112214\_tube2\_01.19799.19799.2 | 3.3217 | 0.4781 | 100.0% | 1446.6522 | 1445.6555 | 1 | 7.089 | 62.5% | 2 | K.EGIPALDNFLDKL.- | 2 |

---

|  |  |  |  |  |  |  |  |  |
| --- | --- | --- | --- | --- | --- | --- | --- | --- |
| U | *gi|4506903|ref|NP\_003* | 5 | 20 | 23.5% | 221 | 25542 | 8.6 | splicing factor, arginine/serine-rich 9 [Homo sapiens] |

| Filename XCorr DeltCN Conf% ObsM+H+ CalcM+H+ SpR ZScore Ion% # Sequence  | | | | | | | | | | | | |
| --- | --- | --- | --- | --- | --- | --- | --- | --- | --- | --- | --- | --- |
| \* | Astrin\_NLD\_STLC\_tube2\_021014\_01.08199.08199.2 | 3.2839 | 0.2534 | 99.9% | 1247.2922 | 1247.4368 | 20 | 5.623 | 65.0% | 10 | R.IYVGNLPTDVR.E | 2 |
| \* | Astrin\_STLCLD20\_112214\_tube2\_01.12360.12360.2 | 2.6465 | 0.2784 | 99.3% | 1300.1322 | 1300.4509 | 82 | 5.778 | 55.6% | 1 | R.EKDLEDLFYK.Y | 2 |
| \* | Astrin\_STLCHLD\_tube2\_050114\_01.09905.09905.2 | 2.7046 | 0.4609 | 100.0% | 1143.9722 | 1143.3768 | 1 | 7.781 | 72.2% | 5 | R.HGLVPFAFVR.F | 2 |
| \* | AstrinSTLCLD\_041714\_01.06102.06102.3 | 3.3126 | 0.3423 | 100.0% | 1654.2843 | 1654.7332 | 1 | 7.54 | 46.2% | 2 | R.FEDPRDAEDAIYGR.N | 3 |
|  | Astrin\_STLCLD20\_112214\_01.07489.07489.2 | 2.3638 | 0.1102 | 95.3% | 917.47217 | 917.0989 | 2 | 4.174 | 83.3% | 2 | R.LRVEFPR.T | 22 |

Similarities:
gi|118582269|ref|NP\_0(1:4)  

---

|  |  |  |  |  |  |  |  |  |
| --- | --- | --- | --- | --- | --- | --- | --- | --- |
| U | *gi|226530908|ref|NP\_0* | 4 | 7 | 23.2% | 285 | 30315 | 7.5 | protein-L-isoaspartate (D-aspartate) O-methyltransferase [Homo sapiens] |

| Filename XCorr DeltCN Conf% ObsM+H+ CalcM+H+ SpR ZScore Ion% # Sequence  | | | | | | | | | | | | |
| --- | --- | --- | --- | --- | --- | --- | --- | --- | --- | --- | --- | --- |
| \* | Astrin\_STLCHLD\_061214\_01.07975.07975.2 | 3.8014 | 0.4828 | 100.0% | 1525.7322 | 1525.7601 | 1 | 9.122 | 70.8% | 3 | K.TDKVFEVMLATDR.S | 2 |
| \* | Astrin\_STLCHLD\_061214\_01.08007.08007.2 | 3.1814 | 0.5257 | 100.0% | 1181.4321 | 1181.3923 | 1 | 8.617 | 94.4% | 2 | K.VFEVMLATDR.S | 2 |
| \* | Astrin\_STLCHLD\_tube2\_061314\_01.11972.11972.3 | 6.1638 | 0.4286 | 100.0% | 3508.4043 | 3507.0015 | 1 | 6.539 | 23.5% | 1 | R.MGYAEEAPYDAIHVGAAAPVVPQALIDQLKPGGR.L | 3 |
| \* | Astrin\_STLCHLD\_tube2\_061314\_01.11163.11163.2 | 3.9321 | 0.3545 | 100.0% | 2044.7522 | 2044.3734 | 1 | 6.416 | 72.2% | 1 | R.LILPVGPAGGNQMLEQYDK.L | 2 |

---

|  |  |  |  |  |  |  |  |  |
| --- | --- | --- | --- | --- | --- | --- | --- | --- |
| U | *gi|38201714|ref|NP\_00* | 5 | 6 | 23.0% | 326 | 36092 | 9.2 | ELAV-like 1 [Homo sapiens] |

| Filename XCorr DeltCN Conf% ObsM+H+ CalcM+H+ SpR ZScore Ion% # Sequence  | | | | | | | | | | | | |
| --- | --- | --- | --- | --- | --- | --- | --- | --- | --- | --- | --- | --- |
| \* | Astrin\_STLCLD20\_112214\_01.12364.12364.2 | 3.2114 | 0.4459 | 100.0% | 2162.9521 | 2163.4534 | 105 | 6.497 | 32.4% | 2 | R.TNLIVNYLPQNMTQDELR.S | 2 |
| \* | Astrin\_NLD\_STLC\_tube2\_021014\_01.10607.10607.2 | 3.1163 | 0.3502 | 100.0% | 1354.6322 | 1354.4998 | 7 | 6.241 | 54.2% | 1 | R.SLFSSIGEVESAK.L | 2 |
| \* | Astrin\_STLCLD20\_112214\_02.11121.11121.3 | 3.3174 | 0.2756 | 99.4% | 2027.8444 | 2027.2871 | 1 | 5.14 | 37.5% | 1 | R.DKVAGHSLGYGFVNYVTAK.D | 3 |
| \* | Astrin\_NLD\_STLC\_tube2\_021014\_01.05542.05542.2 | 2.1989 | 0.281 | 97.5% | 1189.2522 | 1189.3542 | 2 | 5.121 | 60.0% | 1 | R.VLVDQTTGLSR.G | 2 |
| \* | Astrin\_STLCLD20\_112214\_tube2\_01.12957.12957.2 | 2.6 | 0.1932 | 96.1% | 1649.8121 | 1649.8038 | 1 | 4.904 | 57.7% | 1 | K.NVALLSQLYHS\*PAR.R | 2 |

---

|  |  |  |  |  |  |  |  |  |
| --- | --- | --- | --- | --- | --- | --- | --- | --- |
| U | *gi|31542947|ref|NP\_00* | 9 | 19 | 22.9% | 573 | 61055 | 5.9 | chaperonin [Homo sapiens] |
| U | *gi|41399285|ref|NP\_95* | 9 | 19 | 22.9% | 573 | 61055 | 5.9 | chaperonin [Homo sapiens] |

| Filename XCorr DeltCN Conf% ObsM+H+ CalcM+H+ SpR ZScore Ion% # Sequence  | | | | | | | | | | | | |
| --- | --- | --- | --- | --- | --- | --- | --- | --- | --- | --- | --- | --- |
|  | Astrin\_STLCHLD\_tube2\_061314\_02.12651.12651.3 | 3.9517 | 0.3602 | 99.9% | 2114.8145 | 2114.5667 | 1 | 6.658 | 38.8% | 1 | R.ALMLQGVDLLADAVAVTMGPK.G | 3 |
|  | Astrin\_STLCLD20\_112214\_01.17625.17625.2 | 2.8206 | 0.2404 | 98.4% | 2116.0122 | 2114.5667 | 12 | 4.907 | 32.5% | 2 | R.ALMLQGVDLLADAVAVTMGPK.G | 2 |
|  | Astrin\_STLCHLD\_061214\_01.06684.06684.2 | 2.8353 | 0.221 | 98.8% | 1345.6322 | 1345.5382 | 1 | 5.117 | 68.2% | 1 | R.TVIIEQSWGSPK.V | 2 |
|  | AstrinSTLCLD\_041714\_02.05710.05710.3 | 3.8806 | 0.3838 | 99.9% | 2561.6042 | 2561.7222 | 1 | 6.405 | 30.2% | 4 | K.LVQDVANNTNEEAGDGTTTATVLAR.S | 3 |
|  | Astrin\_STLCLD20\_112214\_tube2\_01.18668.18668.2 | 2.2203 | 0.2822 | 96.3% | 1557.6522 | 1557.9324 | 2 | 5.215 | 46.4% | 1 | R.GVMLAVDAVIAELKK.Q | 2 |
|  | Astrin\_STLCLD20\_112214\_tube2\_02.07460.07460.3 | 3.2314 | 0.3878 | 99.9% | 1631.9644 | 1631.9684 | 2 | 6.293 | 41.1% | 3 | K.VGEVIVTKDDAMLLK.G | 3 |
|  | Astrin\_STLCLD20\_112214\_tube2\_02.08629.08629.3 | 3.5924 | 0.2646 | 99.8% | 2297.6042 | 2296.5334 | 445 | 4.324 | 33.3% | 1 | R.IQEIIEQLDVTTSEYEKEK.L | 3 |
|  | Astrin\_NLD\_STLC\_031014\_01.05726.05726.2 | 2.6436 | 0.4257 | 100.0% | 1234.3522 | 1234.3055 | 12 | 6.724 | 59.1% | 3 | K.VGGTSDVEVNEK.K | 2 |
|  | Astrin\_STLCLD20\_112214\_02.09691.09691.2 | 2.9257 | 0.3545 | 100.0% | 1216.2722 | 1216.377 | 1 | 6.806 | 77.3% | 3 | K.NAGVEGSLIVEK.I | 2 |

---

|  |  |  |  |  |  |  |  |  |
| --- | --- | --- | --- | --- | --- | --- | --- | --- |
| U | *gi|50592994|ref|NP\_00* | 2 | 2 | 22.9% | 105 | 11737 | 4.9 | thioredoxin [Homo sapiens] |

| Filename XCorr DeltCN Conf% ObsM+H+ CalcM+H+ SpR ZScore Ion% # Sequence  | | | | | | | | | | | | |
| --- | --- | --- | --- | --- | --- | --- | --- | --- | --- | --- | --- | --- |
| \* | Astrin\_STLCHLD\_tube2\_061314\_01.08496.08496.2 | 2.8603 | 0.2661 | 99.2% | 1337.5322 | 1337.429 | 1 | 5.101 | 70.8% | 1 | K.TAFQEALDAAGDK.L | 2 |
| \* | Astrin\_STLCHLD\_tube2\_061314\_01.10400.10400.2 | 2.0578 | 0.2633 | 95.3% | 1259.7922 | 1259.4424 | 2 | 5.495 | 65.0% | 1 | K.EKLEATINELV.- | 2 |

---

|  |  |  |  |  |  |  |  |  |
| --- | --- | --- | --- | --- | --- | --- | --- | --- |
| U | *gi|193794814|ref|NP\_0* | 5 | 14 | 22.8% | 364 | 39420 | 8.1 | fructose-bisphosphate aldolase A [Homo sapiens] |
| U | *gi|4557305|ref|NP\_000* | 5 | 14 | 22.8% | 364 | 39420 | 8.1 | fructose-bisphosphate aldolase A [Homo sapiens] |
| U | *gi|34577112|ref|NP\_90* | 5 | 14 | 22.8% | 364 | 39420 | 8.1 | fructose-bisphosphate aldolase A [Homo sapiens] |

| Filename XCorr DeltCN Conf% ObsM+H+ CalcM+H+ SpR ZScore Ion% # Sequence  | | | | | | | | | | | | |
| --- | --- | --- | --- | --- | --- | --- | --- | --- | --- | --- | --- | --- |
|  | Astrin\_STLCHLD\_tube2\_061314\_01.05120.05120.2 | 3.56 | 0.5292 | 100.0% | 1333.5721 | 1333.4814 | 1 | 8.922 | 73.1% | 9 | K.GILAADESTGSIAK.R | 2 |
|  | Astrin\_STLCHLD\_tube2\_061314\_01.11335.11335.3 | 3.4173 | 0.3614 | 100.0% | 2108.0344 | 2108.4204 | 46 | 5.396 | 27.6% | 1 | K.IGEHTPSALAIMENANVLAR.Y | 3 |
|  | Astrin\_STLCHLD\_061214\_01.07200.07200.3 | 2.1684 | 0.3087 | 95.3% | 1833.7743 | 1833.1156 | 4 | 4.987 | 38.3% | 1 | K.FSHEEIAMATVTALRR.T | 3 |
|  | Astrin\_STLCHLD\_061214\_02.07175.07175.3 | 3.966 | 0.3655 | 99.9% | 3343.9443 | 3343.6 | 188 | 6.459 | 16.4% | 1 | R.ALANSLACQGKYTPSGQAGAAASESLFVSNHAY.- | 3 |
|  | Astrin\_STLCHLD\_061214\_02.07175.07175.2 | 4.8085 | 0.4568 | 100.0% | 2229.632 | 2229.3672 | 1 | 8.447 | 42.9% | 2 | K.YTPSGQAGAAASESLFVSNHAY.- | 3 |

---

|  |  |  |  |  |  |  |  |  |
| --- | --- | --- | --- | --- | --- | --- | --- | --- |
| U | *gi|4503471|ref|NP\_001* | 13 | 163 | 22.7% | 462 | 50141 | 9.0 | eukaryotic translation elongation factor 1 alpha 1 [Homo sapiens] |

| Filename XCorr DeltCN Conf% ObsM+H+ CalcM+H+ SpR ZScore Ion% # Sequence  | | | | | | | | | | | | |
| --- | --- | --- | --- | --- | --- | --- | --- | --- | --- | --- | --- | --- |
|  | Astrin\_STLCHLD\_tube2\_050114\_02.05919.05919.2 | 4.232 | 0.5034 | 100.0% | 1590.5521 | 1589.835 | 1 | 8.679 | 71.4% | 6 | K.THINIVVIGHVDSGK.S | 2 |
|  | Astrin\_STLCHLD\_061214\_02.05785.05785.3 | 5.207 | 0.4985 | 100.0% | 1591.0144 | 1589.835 | 1 | 8.247 | 53.6% | 74 | K.THINIVVIGHVDSGK.S | 3 |
|  | Astrin\_STLCLD20\_112214\_01.07457.07457.3 | 2.8286 | 0.3334 | 99.8% | 1613.3043 | 1611.8506 | 14 | 5.88 | 34.6% | 1 | R.TIEKFEKEAAEMGK.G | 3 |
| \* | Astrin\_STLCHLD\_tube2\_050114\_01.07277.07277.2 | 3.2302 | 0.4753 | 100.0% | 1405.5322 | 1405.5962 | 1 | 8.302 | 72.7% | 7 | K.YYVTIIDAPGHR.D | 2 |
| \* | Astrin\_STLCHLD\_tube2\_061314\_01.07037.07037.3 | 3.6552 | 0.3458 | 99.9% | 1407.1743 | 1405.5962 | 8 | 5.745 | 43.2% | 5 | K.YYVTIIDAPGHR.D | 3 |
|  | Astrin\_STLCHLD\_061214\_01.07464.07464.2 | 3.5198 | 0.4793 | 100.0% | 1315.5521 | 1315.5553 | 1 | 8.723 | 77.3% | 17 | R.EHALLAYTLGVK.Q | 2 |
|  | Astrin\_STLCHLD\_tube2\_061314\_02.07064.07064.3 | 4.1206 | 0.4223 | 100.0% | 1316.1244 | 1315.5553 | 2 | 7.496 | 56.8% | 4 | R.EHALLAYTLGVK.Q | 3 |
|  | Astrin\_STLCHLD\_tube2\_061314\_01.06557.06557.2 | 2.6251 | 0.1908 | 98.8% | 976.53217 | 976.1607 | 7 | 5.999 | 71.4% | 4 | R.LPLQDVYK.I | 2 |
|  | Astrin\_STLCLD20\_112214\_tube2\_01.08742.08742.1 | 1.7253 | 0.3275 | 98.0% | 1025.64 | 1026.2241 | 3 | 5.554 | 55.0% | 3 | K.IGGIGTVPVGR.V | 1 |
|  | Astrin\_STLCLD20\_112214\_01.07327.07327.2 | 3.7298 | 0.3353 | 100.0% | 1027.1921 | 1026.2241 | 1 | 6.092 | 80.0% | 23 | K.IGGIGTVPVGR.V | 2 |
| \* | Astrin\_STLCLD20\_112214\_tube2\_01.14084.14084.3 | 4.7835 | 0.4081 | 100.0% | 2517.2644 | 2516.999 | 1 | 6.311 | 35.9% | 14 | R.VETGVLKPGMVVTFAPVNVTTEVK.S | 3 |
| \* | Astrin\_STLCHLD\_tube2\_050114\_01.10679.10679.2 | 5.2973 | 0.4452 | 100.0% | 2517.4521 | 2516.999 | 1 | 7.528 | 47.8% | 4 | R.VETGVLKPGMVVTFAPVNVTTEVK.S | 2 |
|  | Astrin\_STLCLD20\_112214\_tube2\_01.07851.07851.1 | 1.9061 | 0.3959 | 95.9% | 914.56 | 915.1209 | 60 | 5.532 | 56.2% | 1 | R.QTVAVGVIK.A | 1 |

---

|  |  |  |  |  |  |  |  |  |
| --- | --- | --- | --- | --- | --- | --- | --- | --- |
| U | *contaminant\_INT-STD1* | 16 | 200 | 22.6% | 607 | 69271 | 6.1 | BSA |

| Filename XCorr DeltCN Conf% ObsM+H+ CalcM+H+ SpR ZScore Ion% # Sequence  | | | | | | | | | | | | |
| --- | --- | --- | --- | --- | --- | --- | --- | --- | --- | --- | --- | --- |
| \* | Astrin\_STLCHLD\_tube2\_050114\_01.08720.08720.2 | 3.5324 | 0.3784 | 100.0% | 1164.1721 | 1164.344 | 3 | 6.967 | 77.8% | 13 | K.LVNELTEFAK.T | 2 |
|  | Astrin\_STLCHLD\_tube2\_050114\_01.06729.06729.2 | 2.4104 | 0.2791 | 99.5% | 927.5722 | 928.0758 | 1 | 5.881 | 83.3% | 4 | K.YLYEIAR.R | 2 |
| \* | AstrinSTLCLD\_041714\_01.09273.09273.3 | 3.9908 | 0.3794 | 99.9% | 2046.4744 | 2046.3354 | 1 | 6.109 | 48.3% | 7 | R.RHPYFYAPELLYYANK.Y | 3 |
| \* | Astrin\_NLD\_STLC\_tube2\_021014\_01.05134.05134.1 | 1.9382 | 0.2146 | 96.1% | 922.46 | 923.05383 | 10 | 4.51 | 57.1% | 1 | K.AEFVEVTK.L | 1 |
| \* | Astrin\_NLD\_STLC\_tube2\_021014\_01.05169.05169.2 | 2.3412 | 0.2107 | 98.0% | 922.5522 | 923.05383 | 1 | 5.35 | 85.7% | 1 | K.AEFVEVTK.L | 2 |
| \* | Astrin\_NLD\_STLC\_tube2\_021014\_02.10065.10065.2 | 4.6769 | 0.5065 | 100.0% | 1568.8121 | 1568.7258 | 1 | 9.045 | 79.2% | 13 | K.DAFLGSFLYEYSR.R | 2 |
| \* | Astrin\_STLCHLD\_tube2\_050114\_01.07049.07049.2 | 3.2494 | 0.3408 | 100.0% | 1441.3722 | 1440.6884 | 1 | 6.315 | 68.2% | 13 | R.RHPEYAVSVLLR.L | 2 |
| \* | AstrinSTLCLD\_041714\_01.05903.05903.3 | 4.6493 | 0.3335 | 100.0% | 1441.6444 | 1440.6884 | 1 | 5.781 | 61.4% | 40 | R.RHPEYAVSVLLR.L | 3 |
| \* | Astrin\_STLCHLD\_tube2\_050114\_01.05978.05978.2 | 3.3761 | 0.413 | 100.0% | 1306.3522 | 1306.5046 | 1 | 7.769 | 70.0% | 16 | K.HLVDEPQNLIK.Q | 2 |
| \* | Astrin\_STLCHLD\_tube2\_050114\_02.08219.08219.2 | 4.7237 | 0.3588 | 100.0% | 1482.0322 | 1480.7068 | 1 | 7.228 | 75.0% | 41 | K.LGEYGFQNALIVR.Y | 2 |
|  | Astrin\_STLCHLD\_tube2\_050114\_01.06773.06773.2 | 4.0709 | 0.3843 | 100.0% | 1641.4922 | 1640.9205 | 1 | 8.109 | 60.7% | 15 | R.KVPQVSTPTLVEVSR.S | 2 |
|  | AstrinSTLCLD\_041714\_01.05428.05428.3 | 4.6485 | 0.4418 | 100.0% | 1642.0144 | 1640.9205 | 2 | 7.206 | 46.4% | 11 | R.KVPQVSTPTLVEVSR.S | 3 |
|  | Astrin\_NLD\_STLC\_tube2\_021014\_01.08284.08284.2 | 2.9449 | 0.4363 | 100.0% | 1512.0721 | 1512.7465 | 1 | 8.143 | 61.5% | 2 | K.VPQVSTPTLVEVSR.S | 2 |
| \* | Astrin\_STLCHLD\_tube2\_050114\_01.07832.07832.2 | 2.7361 | 0.3021 | 99.7% | 1143.4521 | 1143.4124 | 1 | 6.076 | 77.8% | 3 | K.KQTALVELLK.H | 2 |
| \* | Astrin\_STLCHLD\_tube2\_050114\_01.12457.12457.2 | 4.0676 | 0.5221 | 100.0% | 1401.2722 | 1400.6324 | 1 | 8.136 | 77.3% | 15 | K.TVMENFVAFVDK.C | 2 |
| \* | AstrinSTLCLD\_041714\_02.06249.06249.2 | 2.8563 | 0.6059 | 100.0% | 1003.5522 | 1003.1839 | 2 | 8.592 | 66.7% | 5 | K.LVVSTQTALA.- | 2 |

---

|  |  |  |  |  |  |  |  |  |
| --- | --- | --- | --- | --- | --- | --- | --- | --- |
| U | *gi|24234688|ref|NP\_00* | 12 | 42 | 22.4% | 679 | 73681 | 6.2 | heat shock 70kDa protein 9 precursor [Homo sapiens] |

| Filename XCorr DeltCN Conf% ObsM+H+ CalcM+H+ SpR ZScore Ion% # Sequence  | | | | | | | | | | | | |
| --- | --- | --- | --- | --- | --- | --- | --- | --- | --- | --- | --- | --- |
| \* | AstrinSTLCLD\_041714\_01.05816.05816.2 | 3.8232 | 0.502 | 100.0% | 1451.3522 | 1451.576 | 1 | 10.306 | 73.1% | 8 | R.TTPSVVAFTADGER.L | 2 |
| \* | Astrin\_STLCLD20\_112214\_tube2\_01.17133.17133.2 | 2.6871 | 0.2419 | 98.2% | 1554.1522 | 1554.8878 | 1 | 5.253 | 65.4% | 2 | K.LYSPSQIGAFVLMK.M | 2 |
| \* | Astrin\_STLCHLD\_tube2\_050114\_01.09437.09437.2 | 2.9344 | 0.3783 | 99.9% | 1696.4722 | 1695.8723 | 1 | 5.875 | 60.7% | 1 | K.NAVITVPAYFNDSQR.Q | 2 |
| \* | Astrin\_STLCHLD\_tube2\_050114\_01.08744.08744.2 | 3.4737 | 0.3321 | 100.0% | 1243.5922 | 1243.4056 | 1 | 6.368 | 77.3% | 3 | K.DAGQISGLNVLR.V | 2 |
| \* | Astrin\_STLCLD20\_112214\_tube2\_01.15821.15821.2 | 4.3515 | 0.4823 | 100.0% | 2057.0522 | 2057.181 | 1 | 7.872 | 47.2% | 1 | K.STNGDTFLGGEDFDQALLR.H | 2 |
| \* | Astrin\_STLCHLD\_050114\_01.11035.11035.2 | 2.336 | 0.2327 | 95.2% | 1692.5721 | 1691.8969 | 2 | 4.51 | 57.1% | 1 | R.ETGVDLTKDNMALQR.V | 2 |
| \* | Astrin\_STLCLD20\_112214\_tube2\_01.16337.16337.2 | 4.0023 | 0.4104 | 100.0% | 1362.5322 | 1362.5687 | 1 | 8.152 | 63.6% | 13 | R.AQFEGIVTDLIR.R | 2 |
| \* | Astrin\_STLCHLD\_tube2\_061314\_01.08613.08613.2 | 3.5423 | 0.2378 | 100.0% | 1291.4521 | 1291.4496 | 1 | 7.083 | 75.0% | 5 | K.VQQTVQDLFGR.A | 2 |
| \* | Astrin\_STLCHLD\_050114\_02.05636.05636.3 | 4.162 | 0.2243 | 99.8% | 1939.2544 | 1938.1448 | 7 | 5.177 | 36.8% | 1 | K.KSQVFSTAADGQTQVEIK.V | 3 |
| \* | Astrin\_STLCHLD\_061214\_02.05889.05889.2 | 4.3979 | 0.4843 | 100.0% | 1809.4722 | 1809.9707 | 1 | 9.552 | 50.0% | 2 | K.SQVFSTAADGQTQVEIK.V | 2 |
| \* | Astrin\_STLCHLD\_tube2\_061314\_02.05240.05240.2 | 2.7093 | 0.4056 | 100.0% | 1474.2722 | 1474.6543 | 3 | 6.95 | 53.8% | 1 | R.EQQIVIQSSGGLSK.D | 2 |
| \* | Astrin\_STLCHLD\_tube2\_061314\_02.07038.07038.3 | 3.216 | 0.4148 | 99.9% | 2420.9043 | 2419.7095 | 9 | 5.78 | 27.4% | 4 | R.EQQIVIQSSGGLSKDDIENMVK.N | 3 |

---

|  |  |  |  |  |  |  |  |  |
| --- | --- | --- | --- | --- | --- | --- | --- | --- |
| U | *gi|5454064|ref|NP\_006* | 11 | 67 | 22.0% | 669 | 69492 | 9.7 | RNA binding motif protein 14 [Homo sapiens] |

| Filename XCorr DeltCN Conf% ObsM+H+ CalcM+H+ SpR ZScore Ion% # Sequence  | | | | | | | | | | | | |
| --- | --- | --- | --- | --- | --- | --- | --- | --- | --- | --- | --- | --- |
| \* | Astrin\_STLCLD20\_112214\_tube2\_01.06362.06362.3 | 3.5703 | 0.4672 | 100.0% | 1557.5643 | 1556.7677 | 1 | 7.219 | 44.2% | 4 | R.AIEALHGHELRPGR.A | 3 |
| \* | AstrinSTLCLD\_041714\_01.06636.06636.3 | 3.3714 | 0.3431 | 100.0% | 1898.5743 | 1898.2767 | 1 | 6.232 | 36.7% | 3 | R.ALVVEMSRPRPLNTWK.I | 3 |
| \* | Astrin\_NLD\_STLC\_tube2\_021014\_01.07517.07517.2 | 4.0231 | 0.4514 | 100.0% | 1610.4122 | 1609.8223 | 1 | 7.418 | 71.4% | 9 | R.ASYVAPLTAQPATYR.A | 2 |
| \* | AstrinSTLCLD\_041714\_02.05522.05522.2 | 2.6501 | 0.4058 | 100.0% | 1220.2722 | 1220.3707 | 2 | 7.539 | 59.1% | 3 | R.AQPSVSLGAAYR.A | 2 |
| \* | Astrin\_STLCHLD\_tube2\_050114\_01.05150.05150.2 | 2.5876 | 0.3317 | 99.4% | 1206.2322 | 1206.3439 | 1 | 6.208 | 68.2% | 1 | R.AQPSASLGVGYR.T | 2 |
| \* | Astrin\_NLD\_STLC\_tube2\_021014\_01.04527.04527.2 | 2.9166 | 0.4039 | 100.0% | 1324.9521 | 1325.482 | 1 | 6.819 | 72.7% | 5 | R.TQPMTAQAASYR.A | 2 |
| \* | Astrin\_STLCHLD\_tube2\_050114\_01.05991.05991.2 | 2.624 | 0.3236 | 99.4% | 1246.1322 | 1246.4087 | 1 | 5.455 | 63.6% | 4 | R.AQPSVSLGAPYR.G | 2 |
| \* | AstrinSTLCLD\_041714\_02.05487.05487.3 | 4.5486 | 0.4818 | 100.0% | 2467.0745 | 2466.6292 | 1 | 7.386 | 35.9% | 27 | R.TQSSASLAASYAAQQHPQAAASYR.G | 3 |
| \* | Astrin\_STLCHLD\_tube2\_050114\_01.06705.06705.2 | 2.9759 | 0.3875 | 100.0% | 1068.2922 | 1067.1869 | 1 | 6.177 | 81.2% | 4 | R.LSESQLSFR.R | 2 |
| \* | Astrin\_STLCLD20\_112214\_tube2\_01.05927.05927.2 | 2.3917 | 0.2693 | 98.3% | 1300.5922 | 1301.4043 | 22 | 5.101 | 55.0% | 1 | R.RLPDAHSDYAR.Y | 2 |
| \* | Astrin\_STLCHLD\_050114\_01.11120.11120.2 | 3.1048 | 0.3939 | 100.0% | 1237.8322 | 1238.2988 | 1 | 7.871 | 83.3% | 6 | R.YSGSYNDYLR.A | 2 |

---

|  |  |  |  |  |  |  |  |  |
| --- | --- | --- | --- | --- | --- | --- | --- | --- |
| U | *gi|36287110|ref|NP\_91* | 6 | 29 | 21.9% | 379 | 40907 | 4.6 | FGFR1 oncogene partner isoform b [Homo sapiens] |
| U | *gi|5901954|ref|NP\_008* | 6 | 29 | 20.8% | 399 | 43065 | 4.8 | FGFR1 oncogene partner isoform a [Homo sapiens] |

| Filename XCorr DeltCN Conf% ObsM+H+ CalcM+H+ SpR ZScore Ion% # Sequence  | | | | | | | | | | | | |
| --- | --- | --- | --- | --- | --- | --- | --- | --- | --- | --- | --- | --- |
|  | Astrin\_STLCHLD\_tube2\_061314\_01.14561.14561.2 | 4.6731 | 0.4372 | 100.0% | 2165.4922 | 2165.5352 | 1 | 7.414 | 52.5% | 10 | R.DLGIIEAEGTVGGPLLLEVIR.R | 2 |
|  | Astrin\_STLCHLD\_061214\_02.10101.10101.3 | 3.5067 | 0.3729 | 100.0% | 2322.6843 | 2321.7227 | 1 | 5.685 | 31.0% | 11 | R.DLGIIEAEGTVGGPLLLEVIRR.C | 3 |
|  | Astrin\_NLD\_STLC\_tube2\_021014\_01.06860.06860.2 | 2.2177 | 0.3726 | 99.6% | 894.1722 | 894.0183 | 33 | 6.733 | 64.3% | 1 | K.IGSFLSNR.T | 2 |
|  | Astrin\_STLCLD20\_112214\_01.08091.08091.2 | 3.2469 | 0.2453 | 99.3% | 1584.1921 | 1583.8253 | 1 | 5.232 | 56.7% | 2 | R.KQAGSLASLSDAPPLK.S | 2 |
|  | Astrin\_STLCHLD\_tube2\_061314\_01.07464.07464.2 | 3.2088 | 0.3307 | 100.0% | 1188.5122 | 1188.3666 | 4 | 6.69 | 66.7% | 2 | K.SGLSSLAGAPSLK.D | 2 |
|  | Astrin\_STLCHLD\_tube2\_050114\_01.09113.09113.3 | 4.1855 | 0.3134 | 100.0% | 2571.3542 | 2571.63 | 10 | 5.463 | 26.1% | 3 | K.IGSLGLGTGEDDDYVDDFNSTSHR.S | 3 |

---

|  |  |  |  |  |  |  |  |  |
| --- | --- | --- | --- | --- | --- | --- | --- | --- |
| U | *gi|38016911|ref|NP\_00* | 4 | 5 | 21.9% | 288 | 31731 | 7.9 | stomatin isoform a [Homo sapiens] |

| Filename XCorr DeltCN Conf% ObsM+H+ CalcM+H+ SpR ZScore Ion% # Sequence  | | | | | | | | | | | | |
| --- | --- | --- | --- | --- | --- | --- | --- | --- | --- | --- | --- | --- |
| \* | Astrin\_STLCLD20\_112214\_02.10439.10439.2 | 5.2381 | 0.3371 | 100.0% | 1932.1921 | 1931.113 | 1 | 9.317 | 55.6% | 1 | R.VQNATLAVANITNADSATR.L | 2 |
|  | AstrinSTLCLD\_041714\_02.04488.04488.2 | 3.4087 | 0.5396 | 100.0% | 1248.1122 | 1248.3966 | 1 | 8.695 | 68.2% | 1 | K.VIAAEGEMNASR.A | 2 |
|  | Astrin\_STLCLD20\_112214\_02.10869.10869.2 | 2.4659 | 0.2881 | 98.6% | 1352.5521 | 1352.5707 | 5 | 5.679 | 54.5% | 1 | R.YLQTLTTIAAEK.N | 2 |
|  | AstrinSTLCLD\_041714\_01.15232.15232.2 | 3.2749 | 0.3614 | 100.0% | 2128.5322 | 2128.5781 | 1 | 5.708 | 47.4% | 2 | K.NSTIVFPLPIDMLQGIIGAK.H | 2 |

---

|  |  |  |  |  |  |  |  |  |
| --- | --- | --- | --- | --- | --- | --- | --- | --- |
| U | *gi|4504425|ref|NP\_002* | 3 | 3 | 21.9% | 215 | 24894 | 5.7 | high-mobility group box 1 [Homo sapiens] |

| Filename XCorr DeltCN Conf% ObsM+H+ CalcM+H+ SpR ZScore Ion% # Sequence  | | | | | | | | | | | | |
| --- | --- | --- | --- | --- | --- | --- | --- | --- | --- | --- | --- | --- |
| \* | Astrin\_STLCHLD\_tube2\_061314\_01.05023.05023.3 | 3.3207 | 0.2798 | 99.8% | 1594.5243 | 1593.7361 | 100 | 5.683 | 32.7% | 1 | K.KHPDASVNFSEFSK.K | 3 |
|  | Astrin\_STLCHLD\_tube2\_050114\_01.04911.04911.3 | 3.1148 | 0.3237 | 99.8% | 1522.1044 | 1521.7568 | 1 | 6.162 | 37.5% | 1 | K.IKGEHPGLSIGDVAK.K | 3 |
|  | Astrin\_STLCHLD\_tube2\_061314\_01.05919.05919.3 | 2.9443 | 0.2585 | 98.0% | 2109.5645 | 2111.2903 | 13 | 4.419 | 32.4% | 1 | K.LGEMWNNTAADDKQPYEK.K | 3 |

---

|  |  |  |  |  |  |  |  |  |
| --- | --- | --- | --- | --- | --- | --- | --- | --- |
| U | *gi|5453555|ref|NP\_006* | 5 | 15 | 21.8% | 216 | 24423 | 7.5 | ras-related nuclear protein [Homo sapiens] |

| Filename XCorr DeltCN Conf% ObsM+H+ CalcM+H+ SpR ZScore Ion% # Sequence  | | | | | | | | | | | | |
| --- | --- | --- | --- | --- | --- | --- | --- | --- | --- | --- | --- | --- |
| \* | Astrin\_STLCHLD\_061214\_01.07653.07653.3 | 3.0796 | 0.4012 | 100.0% | 2054.3044 | 2053.3713 | 1 | 6.174 | 41.2% | 2 | K.YVATLGVEVHPLVFHTNR.G | 3 |
| \* | Astrin\_STLCLD20\_112214\_01.10414.10414.2 | 3.3742 | 0.3505 | 100.0% | 1690.1522 | 1690.8962 | 1 | 5.903 | 60.7% | 2 | R.GPIKFNVWDTAGQEK.F | 2 |
| \* | Astrin\_STLCLD20\_112214\_01.09711.09711.2 | 2.8096 | 0.3328 | 99.9% | 1295.9321 | 1295.394 | 1 | 5.766 | 75.0% | 2 | K.FNVWDTAGQEK.F | 2 |
| \* | Astrin\_STLCHLD\_tube2\_061314\_01.12129.12129.2 | 3.8981 | 0.4643 | 100.0% | 1785.6721 | 1786.0427 | 1 | 8.081 | 61.5% | 5 | K.SNYNFEKPFLWLAR.K | 2 |
| \* | Astrin\_STLCHLD\_tube2\_061314\_01.12139.12139.3 | 4.5034 | 0.4328 | 100.0% | 1786.1344 | 1786.0427 | 1 | 6.926 | 51.9% | 4 | K.SNYNFEKPFLWLAR.K | 3 |

---

|  |  |  |  |  |  |  |  |  |
| --- | --- | --- | --- | --- | --- | --- | --- | --- |
| U | *gi|4757714|ref|NP\_004* | 2 | 2 | 21.5% | 158 | 18042 | 6.8 | acid phosphatase 1, soluble isoform c [Homo sapiens] |

| Filename XCorr DeltCN Conf% ObsM+H+ CalcM+H+ SpR ZScore Ion% # Sequence  | | | | | | | | | | | | |
| --- | --- | --- | --- | --- | --- | --- | --- | --- | --- | --- | --- | --- |
|  | AstrinSTLCLD\_041714\_01.05193.05193.2 | 2.6668 | 0.4047 | 99.8% | 1912.3522 | 1913.0074 | 1 | 7.604 | 47.1% | 1 | R.VDSAATSGYEIGNPPDYR.G | 2 |
|  | Astrin\_NLD\_STLC\_tube2\_021014\_01.10988.10988.3 | 3.095 | 0.3034 | 99.8% | 1851.6843 | 1852.104 | 117 | 4.878 | 31.7% | 1 | K.TCKAKIELLGSYDPQK.Q | 3 |

---

|  |  |  |  |  |  |  |  |  |
| --- | --- | --- | --- | --- | --- | --- | --- | --- |
| U | *gi|15431303|ref|NP\_00* | 2 | 4 | 21.4% | 192 | 21863 | 10.0 | ribosomal protein L9 [Homo sapiens] |
| U | *gi|67944630|ref|NP\_00* | 2 | 4 | 21.4% | 192 | 21863 | 10.0 | ribosomal protein L9 [Homo sapiens] |

| Filename XCorr DeltCN Conf% ObsM+H+ CalcM+H+ SpR ZScore Ion% # Sequence  | | | | | | | | | | | | |
| --- | --- | --- | --- | --- | --- | --- | --- | --- | --- | --- | --- | --- |
|  | AstrinSTLCLD\_041714\_01.09695.09695.2 | 4.6227 | 0.49 | 100.0% | 2114.3123 | 2114.401 | 1 | 7.906 | 47.2% | 3 | K.TILSNQTVDIPENVDITLK.G | 2 |
|  | Astrin\_NLD\_STLC\_tube2\_021014\_02.09149.09149.3 | 4.028 | 0.3414 | 99.9% | 2486.4243 | 2485.8455 | 3 | 6.567 | 32.1% | 1 | R.SVYAHFPINVVIQENGSLVEIR.N | 3 |

---

|  |  |  |  |  |  |  |  |  |
| --- | --- | --- | --- | --- | --- | --- | --- | --- |
| U | *gi|4506607|ref|NP\_000* | 3 | 33 | 21.3% | 188 | 21634 | 11.7 | ribosomal protein L18 [Homo sapiens] |

| Filename XCorr DeltCN Conf% ObsM+H+ CalcM+H+ SpR ZScore Ion% # Sequence  | | | | | | | | | | | | |
| --- | --- | --- | --- | --- | --- | --- | --- | --- | --- | --- | --- | --- |
| \* | Astrin\_STLCLD20\_112214\_tube2\_02.07076.07076.2 | 4.1965 | 0.4117 | 100.0% | 1347.6322 | 1346.5236 | 1 | 7.494 | 83.3% | 26 | K.TAVVVGTITDDVR.V | 2 |
| \* | AstrinSTLCLD\_041714\_01.10092.10092.2 | 3.7549 | 0.4117 | 100.0% | 1461.4122 | 1461.6982 | 1 | 7.491 | 62.5% | 6 | K.ILTFDQLALDSPK.G | 2 |
| \* | Astrin\_STLCLD20\_112214\_tube2\_01.05580.05580.3 | 3.0457 | 0.3925 | 99.9% | 1549.9143 | 1548.7446 | 1 | 6.13 | 46.2% | 1 | K.APGTPHSHTKPYVR.S | 3 |

---

|  |  |  |  |  |  |  |  |  |
| --- | --- | --- | --- | --- | --- | --- | --- | --- |
| U | *gi|5803225|ref|NP\_006* | 4 | 10 | 21.2% | 255 | 29174 | 4.7 | tyrosine 3/tryptophan 5 -monooxygenase activation protein, epsilon polypeptide [Homo sapiens] |

| Filename XCorr DeltCN Conf% ObsM+H+ CalcM+H+ SpR ZScore Ion% # Sequence  | | | | | | | | | | | | |
| --- | --- | --- | --- | --- | --- | --- | --- | --- | --- | --- | --- | --- |
| \* | Astrin\_STLCHLD\_061214\_02.06812.06812.2 | 3.7822 | 0.4089 | 100.0% | 1449.5122 | 1448.6312 | 1 | 8.472 | 62.5% | 6 | K.VAGMDVELTVEER.N | 2 |
| \* | Astrin\_STLCHLD\_tube2\_061314\_01.06038.06038.2 | 2.0438 | 0.2589 | 95.1% | 1257.4321 | 1257.3452 | 1 | 4.588 | 70.0% | 1 | R.YLAEFATGNDR.K | 2 |
| \* | Astrin\_STLCHLD\_tube2\_050114\_01.05014.05014.2 | 2.493 | 0.1668 | 95.1% | 1384.9521 | 1385.5193 | 100 | 5.271 | 50.0% | 1 | R.YLAEFATGNDRK.E | 2 |
| \* | Astrin\_STLCHLD\_tube2\_061314\_01.17676.17676.3 | 4.9018 | 0.4442 | 100.0% | 3260.1843 | 3260.6375 | 1 | 8.935 | 30.4% | 2 | K.AAFDDAIAELDTLSEESYKDSTLIMQLLR.D | 3 |

---

|  |  |  |  |  |  |  |  |  |
| --- | --- | --- | --- | --- | --- | --- | --- | --- |
| U | *gi|5803137|ref|NP\_006* | 2 | 2 | 21.0% | 157 | 17170 | 8.9 | RNA binding motif protein 3 [Homo sapiens] |

| Filename XCorr DeltCN Conf% ObsM+H+ CalcM+H+ SpR ZScore Ion% # Sequence  | | | | | | | | | | | | |
| --- | --- | --- | --- | --- | --- | --- | --- | --- | --- | --- | --- | --- |
| \* | AstrinSTLCLD\_041714\_01.09976.09976.3 | 3.1764 | 0.3094 | 99.8% | 1984.4343 | 1983.2518 | 1 | 5.321 | 44.1% | 1 | R.GFGFITFTNPEHASVAMR.A | 3 |
| \* | Astrin\_STLCHLD\_tube2\_061314\_01.04497.04497.3 | 3.1247 | 0.3931 | 99.9% | 1730.3944 | 1731.821 | 6 | 6.331 | 35.7% | 1 | R.YYDSRPGGYGYGYGR.S | 3 |

---

|  |  |  |  |  |  |  |  |  |
| --- | --- | --- | --- | --- | --- | --- | --- | --- |
| U | *gi|15431293|ref|NP\_00* | 3 | 7 | 20.6% | 204 | 24146 | 11.6 | ribosomal protein L15 [Homo sapiens] |
| U | *gi|88998868|ref|XP\_94* | 3 | 7 | 20.6% | 204 | 24174 | 11.6 | PREDICTED: hypothetical protein isoform 4 [Homo sapiens] |
| U | *gi|88992455|ref|XP\_93* | 3 | 7 | 20.6% | 204 | 24174 | 11.6 | PREDICTED: hypothetical protein isoform 1 [Homo sapiens] |
| U | *gi|169169711|ref|XP\_0* | 3 | 7 | 20.6% | 204 | 24174 | 11.6 | PREDICTED: hypothetical protein [Homo sapiens] |

| Filename XCorr DeltCN Conf% ObsM+H+ CalcM+H+ SpR ZScore Ion% # Sequence  | | | | | | | | | | | | |
| --- | --- | --- | --- | --- | --- | --- | --- | --- | --- | --- | --- | --- |
|  | Astrin\_STLCHLD\_050114\_01.04201.04201.3 | 3.2348 | 0.3619 | 100.0% | 1706.7544 | 1706.945 | 3 | 6.462 | 40.0% | 2 | K.GATYGKPVHHGVNQLK.F | 3 |
|  | AstrinSTLCLD\_041714\_02.06990.06990.2 | 4.1561 | 0.5228 | 100.0% | 1661.3322 | 1661.8083 | 1 | 9.261 | 69.2% | 4 | R.VLNSYWVGEDSTYK.F | 2 |
|  | Astrin\_STLCHLD\_050114\_01.12909.12909.2 | 2.7043 | 0.2142 | 98.2% | 1506.8322 | 1505.7997 | 14 | 4.569 | 50.0% | 1 | K.FFEVILIDPFHK.A | 2 |

---

|  |  |  |  |  |  |  |  |  |
| --- | --- | --- | --- | --- | --- | --- | --- | --- |
| U | *gi|11024714|ref|NP\_06* | 5 | 31 | 20.5% | 229 | 25762 | 7.4 | ubiquitin B precursor [Homo sapiens] |
| U | *gi|77539055|ref|NP\_00* | 5 | 31 | 36.7% | 128 | 14728 | 9.8 | ubiquitin and ribosomal protein L40 precursor [Homo sapiens] |
| U | *gi|67191208|ref|NP\_06* | 5 | 31 | 6.9% | 685 | 77029 | 7.7 | ubiquitin C [Homo sapiens] |
| U | *gi|4507761|ref|NP\_003* | 5 | 31 | 36.7% | 128 | 14728 | 9.8 | ubiquitin and ribosomal protein L40 precursor [Homo sapiens] |
| U | *gi|4506713|ref|NP\_002* | 5 | 31 | 30.1% | 156 | 17965 | 9.6 | ubiquitin and ribosomal protein S27a precursor [Homo sapiens] |
| U | *gi|208022622|ref|NP\_0* | 5 | 31 | 30.1% | 156 | 17965 | 9.6 | ubiquitin and ribosomal protein S27a precursor [Homo sapiens] |

| Filename XCorr DeltCN Conf% ObsM+H+ CalcM+H+ SpR ZScore Ion% # Sequence  | | | | | | | | | | | | |
| --- | --- | --- | --- | --- | --- | --- | --- | --- | --- | --- | --- | --- |
|  | Astrin\_NLD\_STLC\_tube2\_021014\_01.09353.09353.2 | 4.3008 | 0.4693 | 100.0% | 1789.1522 | 1788.9897 | 1 | 8.187 | 70.0% | 17 | K.TITLEVEPSDTIENVK.A | 22 |
|  | Astrin\_STLCLD20\_112214\_tube2\_01.05832.05832.2 | 3.587 | 0.3876 | 100.0% | 1524.4122 | 1524.6738 | 1 | 7.514 | 79.2% | 3 | K.IQDKEGIPPDQQR.L | 22 |
|  | Astrin\_STLCHLD\_050114\_01.06895.06895.2 | 2.5347 | 0.2384 | 98.9% | 1082.3722 | 1082.1986 | 2 | 5.538 | 75.0% | 5 | R.TLSDYNIQK.E | 22 |
|  | Astrin\_STLCHLD\_tube2\_050114\_02.07117.07117.3 | 3.373 | 0.449 | 99.9% | 2130.3542 | 2131.4368 | 1 | 6.513 | 38.2% | 3 | R.TLSDYNIQKESTLHLVLR.L | 33 |
|  | Astrin\_STLCLD20\_112214\_tube2\_01.09656.09656.2 | 2.9144 | 0.3629 | 100.0% | 1068.2522 | 1068.2615 | 2 | 5.969 | 87.5% | 3 | K.ESTLHLVLR.L | 2 |

Similarities:
contaminant\_UBIQUITIN(4:1)  

---

|  |  |  |  |  |  |  |  |  |
| --- | --- | --- | --- | --- | --- | --- | --- | --- |
| U | *gi|38327562|ref|NP\_00* | 7 | 13 | 20.3% | 403 | 45823 | 9.4 | serine/threonine protein kinase 6 [Homo sapiens] |
| U | *gi|38327572|ref|NP\_94* | 7 | 14 | 20.3% | 403 | 45823 | 9.4 | serine/threonine protein kinase 6 [Homo sapiens] |
| U | *gi|38327570|ref|NP\_94* | 7 | 14 | 20.3% | 403 | 45823 | 9.4 | serine/threonine protein kinase 6 [Homo sapiens] |
| U | *gi|38327568|ref|NP\_94* | 7 | 14 | 20.3% | 403 | 45823 | 9.4 | serine/threonine protein kinase 6 [Homo sapiens] |
| U | *gi|38327566|ref|NP\_94* | 7 | 14 | 20.3% | 403 | 45823 | 9.4 | serine/threonine protein kinase 6 [Homo sapiens] |
| U | *gi|38327564|ref|NP\_94* | 7 | 14 | 20.3% | 403 | 45823 | 9.4 | serine/threonine protein kinase 6 [Homo sapiens] |

| Filename XCorr DeltCN Conf% ObsM+H+ CalcM+H+ SpR ZScore Ion% # Sequence  | | | | | | | | | | | | |
| --- | --- | --- | --- | --- | --- | --- | --- | --- | --- | --- | --- | --- |
|  | Astrin\_STLCHLD\_050114\_01.10582.10582.3 | 3.9156 | 0.3095 | 99.9% | 2167.4343 | 2166.3496 | 3 | 5.137 | 32.9% | 2 | K.SKQPLPSAPENNPEEELASK.Q | 3 |
|  | Astrin\_STLCHLD\_tube2\_061314\_01.05867.05867.2 | 2.4815 | 0.3598 | 99.9% | 940.6922 | 940.0898 | 1 | 5.6 | 85.7% | 1 | K.FGNVYLAR.E | 2 |
|  | Astrin\_STLCHLD\_050114\_01.10382.10382.2 | 2.768 | 0.362 | 100.0% | 1243.3322 | 1243.3641 | 1 | 6.331 | 83.3% | 2 | R.LYGYFHDATR.V | 2 |
|  | Astrin\_STLCHLD\_tube2\_050114\_01.10298.10298.2 | 2.6322 | 0.2373 | 97.7% | 1696.8522 | 1697.9695 | 1 | 6.419 | 50.0% | 1 | R.DIKPENLLLGSAGELK.I | 2 |
|  | Astrin\_STLCHLD\_tube2\_050114\_01.08101.08101.3 | 2.047 | 0.4221 | 99.7% | 1530.4744 | 1530.683 | 2 | 5.998 | 38.5% | 1 | K.IADFGWSVHAPSSR.R | 3 |
|  | Astrin\_STLCHLD\_tube2\_050114\_01.08085.08085.2 | 3.1272 | 0.3426 | 100.0% | 1531.8722 | 1530.683 | 3 | 5.443 | 53.8% | 1 | K.IADFGWSVHAPSSR.R | 2 |
|  | Astrin\_STLCHLD\_tube2\_061314\_01.12517.12517.2 | 3.1054 | 0.3475 | 100.0% | 1615.8121 | 1615.7826 | 3 | 6.667 | 53.8% | 5 | R.VEFTFPDFVTEGAR.D | 2 |

---

|  |  |  |  |  |  |  |  |  |
| --- | --- | --- | --- | --- | --- | --- | --- | --- |
| U | *gi|4758138|ref|NP\_004* | 12 | 64 | 20.2% | 614 | 69148 | 8.9 | DEAD (Asp-Glu-Ala-Asp) box polypeptide 5 [Homo sapiens] |

| Filename XCorr DeltCN Conf% ObsM+H+ CalcM+H+ SpR ZScore Ion% # Sequence  | | | | | | | | | | | | |
| --- | --- | --- | --- | --- | --- | --- | --- | --- | --- | --- | --- | --- |
| \* | Astrin\_STLCHLD\_050114\_01.08955.08955.2 | 3.4659 | 0.412 | 100.0% | 1390.3121 | 1390.4978 | 1 | 7.145 | 65.0% | 2 | K.NFYQEHPDLAR.R | 2 |
| \* | Astrin\_STLCLD20\_112214\_tube2\_01.05714.05714.3 | 2.7174 | 0.2256 | 97.1% | 1410.2644 | 1409.5448 | 4 | 5.271 | 37.5% | 1 | R.RTAQEVETYRR.S | 3 |
| \* | AstrinSTLCLD\_041714\_01.06930.06930.2 | 3.7437 | 0.4786 | 100.0% | 1296.1522 | 1296.4198 | 1 | 9.355 | 80.0% | 12 | R.TTYLVLDEADR.M | 2 |
|  | Astrin\_STLCLD20\_112214\_01.11336.11336.2 | 4.1408 | 0.4316 | 100.0% | 1338.4122 | 1337.5946 | 1 | 7.877 | 85.0% | 14 | R.MLDMGFEPQIR.K | 222 |
|  | AstrinSTLCLD\_041714\_01.07284.07284.2 | 2.6786 | 0.2242 | 98.3% | 1465.5922 | 1465.7688 | 7 | 5.28 | 59.1% | 2 | R.MLDMGFEPQIRK.I | 22 |
|  | Astrin\_STLCLD20\_112214\_01.11839.11839.2 | 2.3141 | 0.2075 | 95.7% | 1350.0122 | 1349.5902 | 128 | 4.927 | 50.0% | 1 | R.QTLMWSATWPK.E | 22 |
| \* | Astrin\_NLD\_STLC\_tube2\_021014\_01.05285.05285.2 | 2.1695 | 0.2641 | 97.7% | 1093.1921 | 1093.3115 | 382 | 4.668 | 56.2% | 1 | K.TIVFVETKR.R | 2 |
|  | AstrinSTLCLD\_041714\_01.06420.06420.2 | 4.1554 | 0.2906 | 100.0% | 1227.4521 | 1227.4465 | 2 | 7.268 | 81.8% | 18 | K.APILIATDVASR.G | 22 |
|  | Astrin\_NLD\_STLC\_tube2\_021014\_01.05238.05238.1 | 1.9714 | 0.2367 | 97.1% | 874.51 | 874.96643 | 3 | 5.852 | 71.4% | 1 | R.GLDVEDVK.F | 11 |
| \* | Astrin\_STLCLD20\_112214\_01.10442.10442.3 | 3.3089 | 0.1841 | 96.0% | 2133.0544 | 2133.281 | 194 | 4.553 | 29.7% | 1 | K.FVINYDYPNSSEDYIHR.I | 3 |
| \* | Astrin\_STLCHLD\_tube2\_050114\_01.09655.09655.2 | 3.4412 | 0.3084 | 100.0% | 1575.6122 | 1575.7612 | 1 | 6.327 | 69.2% | 5 | K.TGTAYTFFTPNNIK.Q | 2 |
| \* | Astrin\_NLD\_STLC\_tube2\_021014\_01.07962.07962.2 | 2.7053 | 0.2163 | 99.3% | 986.2522 | 986.1564 | 1 | 5.774 | 85.7% | 6 | K.LLQLVEDR.G | 2 |

Similarities:
gi|87196351|ref|NP\_00(1:11)  
gi|148613856|ref|NP\_0(5:7)  

---

|  |  |  |  |  |  |  |  |  |
| --- | --- | --- | --- | --- | --- | --- | --- | --- |
| U | *gi|14141157|ref|NP\_03* | 4 | 17 | 19.9% | 346 | 36926 | 6.9 | heterogeneous nuclear ribonucleoprotein H3 isoform a [Homo sapiens] |
| U | *gi|14141159|ref|NP\_06* | 4 | 17 | 20.8% | 331 | 35239 | 6.9 | heterogeneous nuclear ribonucleoprotein H3 isoform b [Homo sapiens] |

| Filename XCorr DeltCN Conf% ObsM+H+ CalcM+H+ SpR ZScore Ion% # Sequence  | | | | | | | | | | | | |
| --- | --- | --- | --- | --- | --- | --- | --- | --- | --- | --- | --- | --- |
|  | Astrin\_STLCLD20\_112214\_02.10767.10767.2 | 3.3936 | 0.429 | 100.0% | 1272.5721 | 1272.4001 | 1 | 7.799 | 81.8% | 13 | R.STGEAFVQFASK.E | 2 |
|  | Astrin\_STLCLD20\_112214\_01.05368.05368.3 | 5.0538 | 0.5125 | 100.0% | 2544.5645 | 2544.7437 | 1 | 8.377 | 38.0% | 1 | R.GMGGHGYGGAGDASSGFHGGHFVHMR.G | 3 |
|  | AstrinSTLCLD\_041714\_01.11670.11670.2 | 2.9743 | 0.4126 | 100.0% | 1920.3922 | 1920.132 | 8 | 6.695 | 34.4% | 1 | R.ATENDIANFFSPLNPIR.V | 2 |
|  | Astrin\_STLCHLD\_050114\_01.05698.05698.2 | 2.9896 | 0.3715 | 100.0% | 1412.3121 | 1413.4613 | 1 | 6.639 | 76.9% | 2 | R.DGMDNQGGYGSVGR.M | 2 |

---

|  |  |  |  |  |  |  |  |  |
| --- | --- | --- | --- | --- | --- | --- | --- | --- |
| U | *gi|169212778|ref|XP\_0* | 8 | 25 | 19.9% | 266 | 30042 | 10.6 | PREDICTED: similar to ribosomal protein L7a [Homo sapiens] |
| U | *gi|4506661|ref|NP\_000* | 8 | 25 | 19.9% | 266 | 29996 | 10.6 | ribosomal protein L7a [Homo sapiens] |
| U | *gi|169213130|ref|XP\_0* | 8 | 25 | 19.9% | 266 | 30042 | 10.6 | PREDICTED: similar to ribosomal protein L7a [Homo sapiens] |
| U | *gi|169212940|ref|XP\_0* | 8 | 25 | 19.9% | 266 | 30028 | 10.6 | PREDICTED: similar to ribosomal protein L7a [Homo sapiens] |

| Filename XCorr DeltCN Conf% ObsM+H+ CalcM+H+ SpR ZScore Ion% # Sequence  | | | | | | | | | | | | |
| --- | --- | --- | --- | --- | --- | --- | --- | --- | --- | --- | --- | --- |
|  | Astrin\_STLCHLD\_tube2\_061314\_01.06563.06563.2 | 3.3234 | 0.3492 | 100.0% | 1217.3722 | 1217.3672 | 3 | 6.145 | 70.0% | 5 | K.NFGIGQDIQPK.R | 2 |
|  | Astrin\_STLCHLD\_tube2\_050114\_01.10915.10915.2 | 2.5138 | 0.3246 | 98.9% | 1812.1522 | 1812.1222 | 12 | 4.965 | 46.7% | 1 | R.LKVPPAINQFTQALDR.Q | 2 |
|  | Astrin\_STLCHLD\_tube2\_050114\_01.10929.10929.3 | 5.2131 | 0.3967 | 100.0% | 1812.3243 | 1812.1222 | 1 | 7.107 | 46.7% | 3 | R.LKVPPAINQFTQALDR.Q | 3 |
|  | Astrin\_STLCHLD\_tube2\_050114\_01.10555.10555.2 | 2.2917 | 0.2614 | 96.4% | 1571.6522 | 1570.7886 | 34 | 5.075 | 42.3% | 1 | K.VPPAINQFTQALDR.Q | 2 |
|  | Astrin\_STLCHLD\_050114\_01.11635.11635.2 | 3.5757 | 0.3672 | 100.0% | 1346.2522 | 1346.5236 | 1 | 6.062 | 66.7% | 12 | R.AGVNTVTTLVENK.K | 2 |
|  | Astrin\_STLCLD20\_112214\_01.07407.07407.2 | 2.8089 | 0.2659 | 99.0% | 1474.5322 | 1474.6976 | 24 | 4.827 | 53.8% | 1 | R.AGVNTVTTLVENKK.A | 2 |
|  | Astrin\_STLCLD20\_112214\_tube2\_01.08536.08536.3 | 2.8712 | 0.3074 | 99.7% | 1475.3644 | 1474.6976 | 15 | 5.931 | 32.7% | 1 | R.AGVNTVTTLVENKK.A | 3 |
|  | Astrin\_NLD\_STLC\_tube2\_021014\_01.04221.04221.3 | 3.4287 | 0.159 | 97.3% | 1615.7043 | 1615.7025 | 15 | 5.1 | 45.5% | 1 | R.TNYNDRYDEIRR.H | 3 |

---

|  |  |  |  |  |  |  |  |  |
| --- | --- | --- | --- | --- | --- | --- | --- | --- |
| U | *gi|9966881|ref|NP\_065* | 15 | 41 | 19.7% | 925 | 106374 | 5.4 | nucleoporin 107kDa [Homo sapiens] |

| Filename XCorr DeltCN Conf% ObsM+H+ CalcM+H+ SpR ZScore Ion% # Sequence  | | | | | | | | | | | | |
| --- | --- | --- | --- | --- | --- | --- | --- | --- | --- | --- | --- | --- |
| \* | Astrin\_STLCHLD\_tube2\_061314\_01.09123.09123.2 | 2.9207 | 0.2707 | 99.5% | 1329.5521 | 1329.4093 | 1 | 7.075 | 68.2% | 6 | R.SGFGEISS\*PVIR.E | 2 |
| \* | Astrin\_STLCHLD\_050114\_02.06380.06380.2 | 5.4062 | 0.5284 | 100.0% | 1890.3722 | 1891.0476 | 1 | 10.531 | 68.8% | 10 | R.VLLQASQDENFGNTTPR.N | 2 |
| \* | Astrin\_STLCHLD\_tube2\_061314\_02.06047.06047.3 | 3.2221 | 0.2003 | 96.4% | 1892.9343 | 1891.0476 | 19 | 4.937 | 35.9% | 1 | R.VLLQASQDENFGNTTPR.N | 3 |
| \* | Astrin\_STLCLD20\_112214\_tube2\_02.06870.06870.3 | 3.6004 | 0.319 | 99.9% | 1971.2344 | 1971.0476 | 1 | 5.472 | 37.5% | 1 | R.VLLQASQDENFGNTT#PR.N | 3 |
| \* | Astrin\_STLCLD20\_112214\_02.10230.10230.2 | 4.674 | 0.4057 | 100.0% | 1971.4122 | 1971.0476 | 1 | 7.124 | 62.5% | 5 | R.VLLQASQDENFGNTT#PR.N | 2 |
| \* | Astrin\_STLCHLD\_tube2\_050114\_01.05448.05448.3 | 3.0298 | 0.4213 | 99.9% | 1611.4744 | 1609.7819 | 7 | 7.096 | 38.5% | 1 | R.TPSSFRQPFTPTSR.S | 3 |
| \* | Astrin\_STLCHLD\_tube2\_061314\_02.09011.09011.3 | 3.7417 | 0.2731 | 99.8% | 2619.3843 | 2619.9495 | 13 | 5.648 | 26.1% | 1 | R.SGLFTNTEPHSITEDVTISAVMLR.E | 3 |
| \* | Astrin\_STLCHLD\_tube2\_061314\_01.17149.17149.2 | 3.927 | 0.4548 | 100.0% | 2452.9922 | 2453.6975 | 1 | 8.194 | 42.9% | 1 | R.EDDPGEAASMSMFSDFLQSFLK.H | 2 |
| \* | Astrin\_STLCHLD\_tube2\_050114\_01.09373.09373.2 | 2.307 | 0.3188 | 99.2% | 1063.1522 | 1063.242 | 2 | 5.813 | 68.8% | 1 | K.TVVEALFQR.D | 2 |
| \* | Astrin\_STLCHLD\_tube2\_050114\_01.08294.08294.2 | 3.4707 | 0.4093 | 100.0% | 1491.3922 | 1491.6866 | 1 | 6.697 | 63.6% | 1 | K.SVYWENTLHTLK.Q | 2 |
| \* | Astrin\_STLCHLD\_050114\_02.06957.06957.2 | 3.4525 | 0.3563 | 100.0% | 1122.5721 | 1121.3219 | 1 | 6.986 | 75.0% | 6 | R.AIYAALSGNLK.Q | 2 |
| \* | Astrin\_STLCHLD\_050114\_02.07763.07763.2 | 2.546 | 0.222 | 98.0% | 1325.2322 | 1324.476 | 29 | 5.292 | 55.0% | 3 | R.EYLGANWTLEK.V | 2 |
| \* | Astrin\_STLCHLD\_tube2\_050114\_01.16037.16037.2 | 4.7782 | 0.4857 | 100.0% | 1814.6322 | 1814.1035 | 1 | 8.768 | 70.0% | 2 | K.FLILGDIDGLMDEFSK.W | 2 |
| \* | Astrin\_STLCHLD\_tube2\_061314\_01.14363.14363.2 | 2.9019 | 0.3503 | 99.8% | 1687.8722 | 1687.9359 | 4 | 5.762 | 42.3% | 1 | K.IDVIDWLVFDPAQR.A | 2 |
| \* | Astrin\_STLCHLD\_tube2\_061314\_01.06537.06537.3 | 2.8362 | 0.3135 | 99.3% | 2264.9043 | 2264.6477 | 45 | 4.933 | 27.6% | 1 | K.HMNSVPQKPALIPQPTFTEK.V | 3 |

---

|  |  |  |  |  |  |  |  |  |
| --- | --- | --- | --- | --- | --- | --- | --- | --- |
| U | *gi|14249348|ref|NP\_11* | 3 | 26 | 19.5% | 123 | 13941 | 5.5 | thioredoxin-like 5 [Homo sapiens] |

| Filename XCorr DeltCN Conf% ObsM+H+ CalcM+H+ SpR ZScore Ion% # Sequence  | | | | | | | | | | | | |
| --- | --- | --- | --- | --- | --- | --- | --- | --- | --- | --- | --- | --- |
| \* | Astrin\_STLCHLD\_061214\_01.07267.07267.2 | 4.0604 | 0.5116 | 100.0% | 1715.3722 | 1715.8162 | 1 | 7.922 | 57.7% | 5 | R.YEEVSVSGFEEFHR.A | 2 |
| \* | Astrin\_STLCLD20\_112214\_tube2\_01.11408.11408.3 | 3.1867 | 0.4485 | 99.9% | 1716.2344 | 1715.8162 | 1 | 7.349 | 46.2% | 20 | R.YEEVSVSGFEEFHR.A | 3 |
| \* | Astrin\_NLD\_STLC\_tube2\_021014\_01.11482.11482.2 | 1.9471 | 0.3741 | 98.4% | 1135.1721 | 1135.3049 | 30 | 5.877 | 50.0% | 1 | K.TIFAYFTGSK.D | 2 |

---

|  |  |  |  |  |  |  |  |  |
| --- | --- | --- | --- | --- | --- | --- | --- | --- |
| U | *gi|169212979|ref|XP\_0* | 3 | 4 | 19.0% | 184 | 21397 | 10.2 | PREDICTED: hypothetical protein [Homo sapiens] |
| U | *gi|78000186|ref|NP\_00* | 3 | 4 | 19.0% | 184 | 21397 | 10.2 | ribosomal protein L17 [Homo sapiens] |
| U | *gi|4506617|ref|NP\_000* | 3 | 4 | 19.0% | 184 | 21397 | 10.2 | ribosomal protein L17 [Homo sapiens] |

| Filename XCorr DeltCN Conf% ObsM+H+ CalcM+H+ SpR ZScore Ion% # Sequence  | | | | | | | | | | | | |
| --- | --- | --- | --- | --- | --- | --- | --- | --- | --- | --- | --- | --- |
|  | Astrin\_STLCLD20\_112214\_01.06526.06526.2 | 2.1912 | 0.2103 | 95.1% | 1164.2122 | 1164.2572 | 2 | 6.247 | 55.6% | 2 | R.YSLDPENPTK.S | 2 |
|  | Astrin\_STLCHLD\_tube2\_050114\_01.08530.08530.2 | 2.8896 | 0.2493 | 99.3% | 1316.5721 | 1317.6323 | 430 | 5.091 | 45.0% | 1 | K.KSAEFLLHMLK.N | 2 |
|  | Astrin\_STLCLD20\_112214\_tube2\_01.07060.07060.2 | 3.2596 | 0.2644 | 99.6% | 1624.0922 | 1624.8314 | 1 | 5.722 | 57.7% | 1 | K.EQIVPKPEEEVAQK.K | 2 |

---

|  |  |  |  |  |  |  |  |  |
| --- | --- | --- | --- | --- | --- | --- | --- | --- |
| U | *gi|4757880|ref|NP\_004* | 4 | 9 | 18.9% | 328 | 37155 | 6.8 | budding uninhibited by benzimidazoles 3 isoform a [Homo sapiens] |
| U | *gi|56550081|ref|NP\_00* | 4 | 9 | 19.0% | 326 | 36955 | 6.8 | budding uninhibited by benzimidazoles 3 isoform b [Homo sapiens] |

| Filename XCorr DeltCN Conf% ObsM+H+ CalcM+H+ SpR ZScore Ion% # Sequence  | | | | | | | | | | | | |
| --- | --- | --- | --- | --- | --- | --- | --- | --- | --- | --- | --- | --- |
|  | Astrin\_STLCHLD\_050114\_01.07263.07263.2 | 3.0556 | 0.2433 | 99.3% | 1384.6522 | 1384.529 | 27 | 5.344 | 54.2% | 2 | K.LNQPPEDGISSVK.F | 2 |
|  | Astrin\_STLCHLD\_tube2\_050114\_02.09785.09785.2 | 3.5043 | 0.4444 | 100.0% | 2172.9321 | 2172.402 | 1 | 7.047 | 44.4% | 1 | K.FSPNTSQFLLVSSWDTSVR.L | 2 |
|  | Astrin\_STLCHLD\_tube2\_050114\_01.05711.05711.2 | 2.4974 | 0.2568 | 98.7% | 1166.1721 | 1166.3373 | 17 | 4.866 | 66.7% | 1 | R.LYDVPANSMR.L | 2 |
|  | Astrin\_STLCHLD\_050114\_01.10786.10786.3 | 5.11 | 0.4458 | 100.0% | 2276.2144 | 2277.4736 | 1 | 8.026 | 42.1% | 5 | K.MHDLNTDQENLVGTHDAPIR.C | 3 |

---

|  |  |  |  |  |  |  |  |  |
| --- | --- | --- | --- | --- | --- | --- | --- | --- |
| U | *gi|4505773|ref|NP\_002* | 4 | 5 | 18.8% | 272 | 29804 | 5.8 | prohibitin [Homo sapiens] |

| Filename XCorr DeltCN Conf% ObsM+H+ CalcM+H+ SpR ZScore Ion% # Sequence  | | | | | | | | | | | | |
| --- | --- | --- | --- | --- | --- | --- | --- | --- | --- | --- | --- | --- |
| \* | Astrin\_STLCLD20\_112214\_01.09515.09515.2 | 2.8121 | 0.2586 | 99.2% | 1398.0521 | 1397.7067 | 4 | 5.089 | 63.6% | 1 | R.ILFRPVASQLPR.I | 2 |
| \* | Astrin\_STLCLD20\_112214\_01.09895.09895.2 | 2.6741 | 0.2631 | 99.2% | 1150.6522 | 1150.2767 | 7 | 6.162 | 66.7% | 2 | R.FDAGELITQR.E | 2 |
| \* | Astrin\_STLCHLD\_050114\_02.11112.11112.2 | 2.3246 | 0.241 | 95.4% | 2119.632 | 2120.4534 | 216 | 4.99 | 26.3% | 1 | R.AATFGLILDDVSLTHLTFGK.E | 2 |
| \* | Astrin\_STLCLD20\_112214\_tube2\_02.11209.11209.3 | 3.8672 | 0.2809 | 99.8% | 3126.6843 | 3125.5457 | 2 | 4.698 | 25.0% | 1 | R.AATFGLILDDVSLTHLTFGKEFTEAVEAK.Q | 3 |

---

|  |  |  |  |  |  |  |  |  |
| --- | --- | --- | --- | --- | --- | --- | --- | --- |
| U | *gi|4506613|ref|NP\_000* | 2 | 12 | 18.8% | 128 | 14787 | 9.2 | ribosomal protein L22 proprotein [Homo sapiens] |

| Filename XCorr DeltCN Conf% ObsM+H+ CalcM+H+ SpR ZScore Ion% # Sequence  | | | | | | | | | | | | |
| --- | --- | --- | --- | --- | --- | --- | --- | --- | --- | --- | --- | --- |
| \* | Astrin\_STLCLD20\_112214\_01.07748.07748.2 | 3.503 | 0.4599 | 100.0% | 1243.4922 | 1243.4056 | 1 | 7.109 | 70.8% | 10 | K.AGNLGGGVVTIER.S | 2 |
| \* | Astrin\_STLCHLD\_tube2\_050114\_01.07525.07525.2 | 2.4014 | 0.3155 | 99.1% | 1208.3922 | 1208.3971 | 14 | 5.563 | 65.0% | 2 | K.ITVTSEVPFSK.R | 2 |

---

|  |  |  |  |  |  |  |  |  |
| --- | --- | --- | --- | --- | --- | --- | --- | --- |
| U | *gi|4506741|ref|NP\_001* | 2 | 4 | 18.6% | 194 | 22127 | 10.1 | ribosomal protein S7 [Homo sapiens] |

| Filename XCorr DeltCN Conf% ObsM+H+ CalcM+H+ SpR ZScore Ion% # Sequence  | | | | | | | | | | | | |
| --- | --- | --- | --- | --- | --- | --- | --- | --- | --- | --- | --- | --- |
| \* | Astrin\_STLCLD20\_112214\_tube2\_01.14750.14750.2 | 2.8793 | 0.1971 | 98.2% | 1466.1522 | 1466.8937 | 2 | 5.165 | 58.3% | 1 | R.KAIIIFVPVPQLK.S | 2 |
| \* | AstrinSTLCLD\_041714\_01.11441.11441.3 | 5.1378 | 0.3342 | 99.9% | 2524.4043 | 2524.92 | 1 | 6.49 | 38.6% | 3 | R.TLTAVHDAILEDLVFPSEIVGKR.I | 3 |

---

|  |  |  |  |  |  |  |  |  |
| --- | --- | --- | --- | --- | --- | --- | --- | --- |
| U | *gi|74099697|ref|NP\_00* | 6 | 34 | 18.0% | 449 | 49264 | 6.3 | heterogeneous nuclear ribonucleoprotein H2 [Homo sapiens] |
| U | *gi|9624998|ref|NP\_062* | 6 | 34 | 18.0% | 449 | 49264 | 6.3 | heterogeneous nuclear ribonucleoprotein H2 [Homo sapiens] |

| Filename XCorr DeltCN Conf% ObsM+H+ CalcM+H+ SpR ZScore Ion% # Sequence  | | | | | | | | | | | | |
| --- | --- | --- | --- | --- | --- | --- | --- | --- | --- | --- | --- | --- |
|  | Astrin\_STLCHLD\_050114\_01.05415.05415.2 | 4.1137 | 0.511 | 100.0% | 1685.3722 | 1685.7501 | 1 | 8.232 | 70.0% | 3 | K.HTGPNSPDTANDGFVR.L | 22 |
|  | Astrin\_STLCHLD\_050114\_01.05353.05353.3 | 3.9638 | 0.2195 | 99.8% | 1686.9543 | 1685.7501 | 2 | 4.836 | 48.3% | 2 | K.HTGPNSPDTANDGFVR.L | 33 |
|  | Astrin\_NLD\_STLC\_tube2\_021014\_01.11806.11806.2 | 5.1994 | 0.4738 | 100.0% | 1842.3121 | 1843.0001 | 1 | 9.375 | 68.8% | 18 | R.STGEAFVQFASQEIAEK.A | 22 |
|  | AstrinSTLCLD\_041714\_01.12022.12022.2 | 3.1031 | 0.393 | 100.0% | 2029.6122 | 2030.2622 | 1 | 7.623 | 50.0% | 1 | R.ATENDIYNFFSPLNPMR.V | 2 |
|  | Astrin\_STLCLD20\_112214\_tube2\_01.07737.07737.2 | 3.2067 | 0.508 | 100.0% | 1093.4122 | 1093.2278 | 1 | 8.327 | 83.3% | 9 | R.VHIEIGPDGR.V | 222 |
|  | AstrinSTLCLD\_041714\_02.06759.06759.3 | 3.5013 | 0.2625 | 99.4% | 2162.9343 | 2163.3638 | 17 | 4.353 | 28.8% | 1 | R.VTGEADVEFATHEDAVAAMAK.D | 3 |

Similarities:
gi|5031753|ref|NP\_005(4:2)  
gi|148470397|ref|NP\_0(1:5)  

---

|  |  |  |  |  |  |  |  |  |
| --- | --- | --- | --- | --- | --- | --- | --- | --- |
| U | *gi|4885375|ref|NP\_005* | 7 | 22 | 17.8% | 213 | 21365 | 10.9 | histone cluster 1, H1c [Homo sapiens] |
| U | *gi|4885379|ref|NP\_005* | 7 | 22 | 17.4% | 219 | 21865 | 11.0 | histone cluster 1, H1e [Homo sapiens] |
| U | *gi|4885377|ref|NP\_005* | 7 | 22 | 17.2% | 221 | 22350 | 11.0 | histone cluster 1, H1d [Homo sapiens] |

| Filename XCorr DeltCN Conf% ObsM+H+ CalcM+H+ SpR ZScore Ion% # Sequence  | | | | | | | | | | | | |
| --- | --- | --- | --- | --- | --- | --- | --- | --- | --- | --- | --- | --- |
|  | Astrin\_STLCLD20\_112214\_tube2\_01.08475.08475.2 | 3.6798 | 0.5046 | 100.0% | 1327.4922 | 1327.5638 | 2 | 7.608 | 58.3% | 7 | R.KASGPPVSELITK.A | 2 |
|  | Astrin\_STLCHLD\_tube2\_050114\_01.05486.05486.3 | 2.8438 | 0.2643 | 98.8% | 1328.1843 | 1327.5638 | 1 | 4.67 | 47.9% | 1 | R.KASGPPVSELITK.A | 3 |
|  | Astrin\_STLCHLD\_050114\_01.11658.11658.2 | 2.7397 | 0.3717 | 99.9% | 1199.2122 | 1199.3898 | 1 | 6.5 | 68.2% | 3 | K.ASGPPVSELITK.A | 2 |
|  | Astrin\_STLCHLD\_050114\_01.07187.07187.2 | 2.9073 | 0.1523 | 98.3% | 974.4322 | 974.1887 | 6 | 4.872 | 77.8% | 1 | R.SGVSLAALKK.A | 2 |
|  | Astrin\_STLCLD20\_112214\_tube2\_01.07986.07986.1 | 2.6116 | 0.4079 | 100.0% | 1108.09 | 1108.2365 | 217 | 6.902 | 45.0% | 2 | K.ALAAAGYDVEK.N | 1 |
|  | Astrin\_STLCHLD\_tube2\_050114\_01.04839.04839.2 | 3.213 | 0.3866 | 100.0% | 1109.2722 | 1108.2365 | 3 | 6.976 | 70.0% | 5 | K.ALAAAGYDVEK.N | 2 |
|  | Astrin\_NLD\_STLC\_tube2\_021014\_01.04505.04505.2 | 4.0855 | 0.4439 | 100.0% | 1579.3922 | 1579.7098 | 1 | 8.835 | 75.0% | 3 | K.ALAAAGYDVEKNNSR.I | 2 |

---

|  |  |  |  |  |  |  |  |  |
| --- | --- | --- | --- | --- | --- | --- | --- | --- |
| U | *gi|214830438|ref|NP\_0* | 3 | 10 | 17.7% | 356 | 38629 | 5.5 | sequestosome 1 isoform 2 [Homo sapiens] |
| U | *gi|4505571|ref|NP\_003* | 3 | 10 | 14.3% | 440 | 47687 | 5.2 | sequestosome 1 isoform 1 [Homo sapiens] |
| U | *gi|214830451|ref|NP\_0* | 3 | 10 | 17.7% | 356 | 38629 | 5.5 | sequestosome 1 isoform 2 [Homo sapiens] |

| Filename XCorr DeltCN Conf% ObsM+H+ CalcM+H+ SpR ZScore Ion% # Sequence  | | | | | | | | | | | | |
| --- | --- | --- | --- | --- | --- | --- | --- | --- | --- | --- | --- | --- |
|  | AstrinSTLCLD\_041714\_01.06122.06122.3 | 5.1438 | 0.3057 | 99.9% | 2572.2844 | 2572.7478 | 1 | 7.661 | 34.0% | 5 | R.AGEARPGPTAESASGPSEDPSVNFLK.N | 3 |
|  | Astrin\_STLCLD20\_112214\_tube2\_02.12048.12048.2 | 2.7959 | 0.3386 | 99.6% | 2458.1921 | 2458.8066 | 1 | 5.108 | 38.1% | 1 | R.LIESLSQMLSMGFSDEGGWLTR.L | 2 |
|  | Astrin\_STLCHLD\_tube2\_050114\_02.08111.08111.2 | 4.0816 | 0.5202 | 100.0% | 1672.5521 | 1672.8322 | 1 | 8.035 | 64.3% | 4 | K.NYDIGAALDTIQYSK.H | 2 |

---

|  |  |  |  |  |  |  |  |  |
| --- | --- | --- | --- | --- | --- | --- | --- | --- |
| U | *gi|14602427|ref|NP\_12* | 5 | 21 | 17.7% | 277 | 31293 | 5.2 | ZW10 interactor isoform a [Homo sapiens] |
| U | *gi|14602429|ref|NP\_00* | 5 | 21 | 17.7% | 277 | 31293 | 5.2 | ZW10 interactor isoform a [Homo sapiens] |

| Filename XCorr DeltCN Conf% ObsM+H+ CalcM+H+ SpR ZScore Ion% # Sequence  | | | | | | | | | | | | |
| --- | --- | --- | --- | --- | --- | --- | --- | --- | --- | --- | --- | --- |
|  | Astrin\_STLCHLD\_tube2\_061314\_01.05567.05567.2 | 2.1271 | 0.3828 | 98.9% | 1261.4521 | 1261.3312 | 1 | 6.23 | 63.6% | 1 | K.GLDPLASEDTSR.Q | 2 |
|  | Astrin\_STLCHLD\_050114\_01.05097.05097.2 | 2.6717 | 0.2842 | 99.4% | 1177.4722 | 1177.3177 | 2 | 5.649 | 77.8% | 1 | K.ALTQMEEAQR.K | 2 |
|  | Astrin\_STLCHLD\_061214\_01.04692.04692.3 | 4.5553 | 0.3499 | 100.0% | 1490.4243 | 1489.6743 | 1 | 7.644 | 56.2% | 14 | K.HLQHLAEVSAEVR.E | 3 |
|  | Astrin\_STLCHLD\_061214\_01.04648.04648.2 | 3.3933 | 0.464 | 100.0% | 1490.5322 | 1489.6743 | 1 | 7.956 | 66.7% | 1 | K.HLQHLAEVSAEVR.E | 2 |
|  | Astrin\_STLCHLD\_tube2\_061314\_01.14799.14799.2 | 3.7403 | 0.4996 | 100.0% | 1716.5322 | 1717.0178 | 1 | 9.878 | 65.4% | 4 | R.YQTFLQLLYTLQGK.L | 2 |

---

|  |  |  |  |  |  |  |  |  |
| --- | --- | --- | --- | --- | --- | --- | --- | --- |
| U | *gi|14165469|ref|NP\_00* | 2 | 4 | 17.7% | 130 | 14839 | 10.1 | ribosomal protein S15a [Homo sapiens] |
| U | *gi|71772415|ref|NP\_00* | 2 | 4 | 17.7% | 130 | 14839 | 10.1 | ribosomal protein S15a [Homo sapiens] |

| Filename XCorr DeltCN Conf% ObsM+H+ CalcM+H+ SpR ZScore Ion% # Sequence  | | | | | | | | | | | | |
| --- | --- | --- | --- | --- | --- | --- | --- | --- | --- | --- | --- | --- |
|  | Astrin\_STLCLD20\_112214\_01.09970.09970.3 | 3.7859 | 0.3596 | 99.9% | 1702.6743 | 1701.8357 | 5 | 5.966 | 48.1% | 3 | K.HGYIGEFEIIDDHR.A | 3 |
|  | Astrin\_STLCHLD\_tube2\_061314\_01.07063.07063.2 | 2.687 | 0.2332 | 99.2% | 1128.0721 | 1128.276 | 3 | 4.829 | 87.5% | 1 | K.WQNNLLPSR.Q | 2 |

---

|  |  |  |  |  |  |  |  |  |
| --- | --- | --- | --- | --- | --- | --- | --- | --- |
| U | *gi|156523260|ref|NP\_6* | 5 | 7 | 17.5% | 412 | 46373 | 5.1 | hypothetical protein LOC221150 [Homo sapiens] |

| Filename XCorr DeltCN Conf% ObsM+H+ CalcM+H+ SpR ZScore Ion% # Sequence  | | | | | | | | | | | | |
| --- | --- | --- | --- | --- | --- | --- | --- | --- | --- | --- | --- | --- |
| \* | Astrin\_STLCHLD\_tube2\_061314\_01.08611.08611.2 | 3.6867 | 0.5248 | 100.0% | 1774.5122 | 1774.853 | 1 | 8.99 | 57.1% | 1 | R.ALDGEESDFEDYPMR.I | 2 |
| \* | Astrin\_STLCHLD\_tube2\_061314\_01.08227.08227.2 | 3.4644 | 0.2803 | 100.0% | 1307.5322 | 1306.4583 | 1 | 5.515 | 80.0% | 3 | R.LENQEGIDFIK.A | 2 |
| \* | AstrinSTLCLD\_041714\_02.05858.05858.3 | 2.9835 | 0.3156 | 99.4% | 2350.4343 | 2348.2942 | 6 | 4.769 | 27.6% | 1 | K.TDVK@DDLS\*DPPVASS\*CISEK@.S | 3 |
| \* | Astrin\_STLCHLD\_tube2\_061314\_01.08605.08605.2 | 3.3526 | 0.0912 | 97.2% | 1507.4922 | 1507.7269 | 4 | 6.221 | 61.5% | 1 | K.NSIALVSTNYPLSK.T | 2 |
| \* | Astrin\_STLCHLD\_tube2\_050114\_01.07468.07468.2 | 2.6748 | 0.131 | 95.1% | 1386.5322 | 1385.5168 | 3 | 4.016 | 68.2% | 1 | K.YNSNLAT#PIAIK.A | 2 |

---

|  |  |  |  |  |  |  |  |  |
| --- | --- | --- | --- | --- | --- | --- | --- | --- |
| U | *gi|153792590|ref|NP\_0* | 12 | 63 | 17.4% | 854 | 98161 | 5.2 | heat shock 90kDa protein 1, alpha isoform 1 [Homo sapiens] |
| U | *gi|154146191|ref|NP\_0* | 12 | 63 | 20.4% | 732 | 84660 | 5.0 | heat shock 90kDa protein 1, alpha isoform 2 [Homo sapiens] |

| Filename XCorr DeltCN Conf% ObsM+H+ CalcM+H+ SpR ZScore Ion% # Sequence  | | | | | | | | | | | | |
| --- | --- | --- | --- | --- | --- | --- | --- | --- | --- | --- | --- | --- |
|  | Astrin\_STLCLD20\_112214\_tube2\_01.12254.12254.2 | 3.4834 | 0.4327 | 100.0% | 1243.4521 | 1243.4459 | 1 | 7.724 | 81.8% | 6 | K.ADLINNLGTIAK.S | 22 |
|  | Astrin\_STLCHLD\_061214\_02.06734.06734.3 | 3.3418 | 0.257 | 99.0% | 2258.3342 | 2257.294 | 1 | 5.261 | 35.5% | 2 | K.HNDDEQYAWESSAGGSFTVR.T | 33 |
|  | Astrin\_STLCHLD\_050114\_02.06302.06302.3 | 4.6555 | 0.5081 | 100.0% | 2016.2344 | 2016.2584 | 1 | 8.269 | 48.3% | 16 | K.VILHLKEDQTEYLEER.R | 33 |
|  | Astrin\_STLCLD20\_112214\_01.11969.11969.3 | 4.4434 | 0.3415 | 99.9% | 2066.8442 | 2065.3794 | 1 | 6.369 | 37.5% | 1 | K.HSQFIGYPITLFVEKER.D | 3 |
|  | Astrin\_STLCHLD\_050114\_01.05627.05627.2 | 2.9558 | 0.3011 | 100.0% | 1152.4722 | 1152.2462 | 1 | 5.822 | 81.2% | 4 | K.YIDQEELNK.T | 22 |
|  | Astrin\_STLCHLD\_050114\_02.07073.07073.2 | 2.7322 | 0.3456 | 99.5% | 1833.3322 | 1834.8903 | 12 | 6.582 | 42.9% | 1 | R.NPDDITNEEYGEFYK.S | 2 |
|  | Astrin\_STLCLD20\_112214\_01.09962.09962.2 | 4.3602 | 0.4022 | 100.0% | 1528.3922 | 1528.6616 | 1 | 7.855 | 70.8% | 7 | K.SLTNDWEDHLAVK.H | 22 |
|  | Astrin\_STLCHLD\_050114\_02.06964.06964.3 | 2.8425 | 0.2263 | 98.0% | 1349.2444 | 1349.4886 | 308 | 5.284 | 40.0% | 2 | K.HFSVEGQLEFR.A | 33 |
|  | Astrin\_NLD\_STLC\_tube2\_021014\_02.06472.06472.2 | 3.0916 | 0.4043 | 100.0% | 1349.2722 | 1349.4886 | 1 | 6.76 | 65.0% | 18 | K.HFSVEGQLEFR.A | 22 |
|  | Astrin\_STLCLD20\_112214\_tube2\_01.12694.12694.2 | 2.1497 | 0.3204 | 98.4% | 1265.7322 | 1265.4142 | 5 | 5.612 | 61.1% | 1 | R.RAPFDLFENR.K | 2 |
|  | Astrin\_STLCLD20\_112214\_01.05137.05137.2 | 2.5728 | 0.2789 | 99.1% | 1236.1122 | 1236.3268 | 1 | 5.31 | 75.0% | 1 | K.DQVANSAFVER.L | 2 |
|  | Astrin\_STLCHLD\_tube2\_061314\_01.08267.08267.3 | 3.3296 | 0.3321 | 99.9% | 1787.7244 | 1788.0134 | 1 | 6.469 | 41.1% | 4 | K.HLEINPDHSIIETLR.Q | 3 |

Similarities:
gi|20149594|ref|NP\_03(7:5)  

---

|  |  |  |  |  |  |  |  |  |
| --- | --- | --- | --- | --- | --- | --- | --- | --- |
| U | *gi|34740329|ref|NP\_91* | 5 | 14 | 17.2% | 378 | 39595 | 9.0 | heterogeneous nuclear ribonucleoprotein A3 [Homo sapiens] |

| Filename XCorr DeltCN Conf% ObsM+H+ CalcM+H+ SpR ZScore Ion% # Sequence  | | | | | | | | | | | | |
| --- | --- | --- | --- | --- | --- | --- | --- | --- | --- | --- | --- | --- |
| \* | Astrin\_STLCLD20\_112214\_02.15077.15077.2 | 2.5633 | 0.3066 | 98.8% | 1772.1921 | 1771.9646 | 326 | 5.41 | 33.3% | 1 | K.LFIGGLSFETTDDSLR.E | 2 |
| \* | Astrin\_NLD\_STLC\_tube2\_021014\_02.06459.06459.3 | 4.0223 | 0.3032 | 100.0% | 1884.7743 | 1884.096 | 1 | 6.375 | 41.7% | 3 | K.IFVGGIKEDTEEYNLR.D | 3 |
| \* | Astrin\_STLCLD20\_112214\_01.08883.08883.2 | 3.0923 | 0.3813 | 100.0% | 1583.4321 | 1583.7968 | 1 | 5.927 | 70.8% | 1 | K.YGKIETIEVMEDR.Q | 2 |
| \* | Astrin\_NLD\_STLC\_tube2\_021014\_01.07974.07974.2 | 2.937 | 0.362 | 100.0% | 1234.9722 | 1235.3948 | 1 | 6.683 | 77.8% | 6 | K.IETIEVMEDR.Q | 2 |
[truncated: 264,628 more chars]
