# Supplementary material for: Astrin-SKAP complex reconstitution reveals its kinetochore interaction with microtubule-bound Ndc80
Source: eLife. 2017 Aug 25;6:e26866. doi: 10.7554/eLife.26866 (PMC5602300; doi:10.7554/eLife.26866)
Supplement: Source data 1. — Complete mass spectrometry searches using methods described in (Washburn et al., 2001) for affinity purification/mass spectrometry data sets described in this paper (data from this study; [Kern et al., 2016] [Gascoigne et al., 2011]). Individual Astrin cross-linking immunoprecipitations are listed based on the order in Figure 4—figure supplement 1. These samples have not been pruned for common or antibody-specific contaminants. [file elife-26866-data1.zip › Astrin_NocodazoleIP_Kern2016.html]

D AstrinIP
DTASelect v2.0.21  
/nfs/cheeseman\_massspec/David/AstrinIP  
/nfs/cheeseman\_massspec/Databases/NCBI-RefSeq\_human\_na\_04-13-2009\_con\_reversed.fasta  
SEQUEST 3.0 in SQT format.  
  
 Jump  to the summary table.  
  
sequest.params modifications:

|  |  |  |
| --- | --- | --- |
| \* | S | 80.0 |
| # | T | 80.0 |
| @ | Y | 80.0 |
| Static | C | 57.0 |

|  |  |
| --- | --- |
| true | Use criteria |
| 0.0 | Minimum peptide confidence |
| 0.05 | Peptide false positive rate |
| 0.0 | Minimum protein confidence |
| 1.0 | Protein false positive rate |
| 1 | Minimum charge state |
| 16 | Maximum charge state |
| 0.0 | Minimum ion proportion |
| 1000 | Maximum Sp rank |
| -1.0 | Minimum Sp score |
| Include | Modified peptide inclusion |
| Any | Tryptic status requirement |
| false | Multiple, ambiguous IDs allowed |
| Ignore | Peptide validation handling |
| XCorr | Purge duplicate peptides by protein |
| false | Include only loci with unique peptide |
| true | Remove subset proteins |
| Ignore | Locus validation handling |
| 0 | Minimum modified peptides per locus |
| 1000 | Minimum redundancy for low coverage loci |
| 2 | Minimum peptides per locus |

#### Locus Key:

|  |  |  |  |  |  |  |  |  |
| --- | --- | --- | --- | --- | --- | --- | --- | --- |
| Validation Status | Locus | Sequence Count | Spectrum Count | Sequence Coverage | Length | MolWt | pI | Descriptive Name |

#### Similarity Key:

|  |  |  |
| --- | --- | --- |
| Locus | # of identical peptides | # of differing peptides |

---

|  |  |  |  |  |  |  |  |  |
| --- | --- | --- | --- | --- | --- | --- | --- | --- |
| U | *gi|73623035|ref|NP\_00* | 223 | 2314 | 89.6% | 1193 | 134422 | 5.0 | sperm associated antigen 5 [Homo sapiens] |

| Filename XCorr DeltCN Conf% ObsM+H+ CalcM+H+ SpR ZScore Ion% # Sequence  | | | | | | | | | | | | |
| --- | --- | --- | --- | --- | --- | --- | --- | --- | --- | --- | --- | --- |
| \* | AstrinIP\_MS1\_022614\_01.05800.05800.3 | 4.8096 | 0.4338 | 100.0% | 1915.2544 | 1915.2585 | 1 | 7.531 | 41.2% | 3 | K.KLSLSLSPSPQTGKPSMR.T | 3 |
| \* | AstrinIP\_MS1\_022614\_01.05782.05782.2 | 4.2795 | 0.4528 | 100.0% | 1915.3522 | 1915.2585 | 1 | 6.872 | 61.8% | 2 | K.KLSLSLSPSPQTGKPSMR.T | 2 |
| \* | AstrinIP\_MS2\_022614\_01.07025.07025.3 | 3.1631 | 0.3421 | 99.8% | 1995.4744 | 1995.2585 | 27 | 5.443 | 32.4% | 1 | K.KLS\*LSLSPSPQTGKPSMR.T | 3 |
| \* | AstrinIP\_MS1\_022614\_01.07020.07020.3 | 2.7341 | 0.3242 | 99.2% | 2073.4744 | 2075.2585 | 58 | 4.879 | 32.4% | 1 | K.KLSLS\*LSPS\*PQTGKPSMR.T | 3 |
| \* | AstrinIP\_MS2\_022614\_01.07172.07172.2 | 3.7599 | 0.4311 | 100.0% | 1785.6921 | 1787.0845 | 1 | 7.574 | 53.1% | 6 | K.LSLSLSPSPQTGKPSMR.T | 2 |
| \* | AstrinIP\_MS1\_022614\_01.06891.06891.3 | 2.5804 | 0.2732 | 96.9% | 1787.0944 | 1787.0845 | 1 | 4.982 | 39.1% | 1 | K.LSLSLSPSPQTGKPSMR.T | 3 |
| \* | AstrinIP\_MS2\_022614\_01.08526.08526.2 | 5.399 | 0.5254 | 100.0% | 1896.8922 | 1897.182 | 1 | 9.587 | 64.7% | 1 | R.TPLRELTLQPGALTNSGK.R | 2 |
| \* | AstrinIP\_MS1\_022614\_01.07140.07140.3 | 4.2258 | 0.2291 | 99.4% | 2054.3943 | 2053.3694 | 2 | 5.285 | 37.5% | 2 | R.TPLRELTLQPGALTNSGKR.S | 3 |
| \* | AstrinIP\_MS1\_022614\_01.07388.07388.2 | 3.8288 | 0.3591 | 100.0% | 1429.2322 | 1429.6133 | 1 | 7.364 | 80.8% | 11 | R.ELTLQPGALTNSGK.R | 2 |
| \* | AstrinIP\_MS2\_022614\_01.07793.07793.1 | 3.349 | 0.4471 | 100.0% | 1429.68 | 1429.6133 | 1 | 8.373 | 61.5% | 6 | R.ELTLQPGALTNSGK.R | 1 |
| \* | AstrinIP\_MS2\_022614\_01.08207.08207.2 | 2.9224 | 0.2636 | 99.5% | 1509.2322 | 1509.6133 | 1 | 5.252 | 69.2% | 1 | R.ELTLQPGALTNS\*GK.R | 2 |
| \* | AstrinIP\_MS2\_022614\_01.06632.06632.2 | 3.3299 | 0.3738 | 100.0% | 1585.4922 | 1585.8008 | 3 | 6.599 | 57.1% | 4 | R.ELTLQPGALTNSGKR.S | 2 |
| \* | AstrinIP\_MS1\_022614\_01.05100.05100.2 | 2.2895 | 0.3336 | 98.7% | 1643.7722 | 1644.7113 | 1 | 5.335 | 65.4% | 1 | K.RS\*PACSSLTPSLCK.L | 2 |
| \* | AstrinIP\_MS1\_022614\_01.05463.05463.2 | 3.5969 | 0.4869 | 100.0% | 1407.6322 | 1408.5238 | 1 | 8.354 | 66.7% | 2 | R.SPACSSLTPSLCK.L | 2 |
| \* | AstrinIP\_MS1\_022614\_01.08343.08343.2 | 5.4967 | 0.5416 | 100.0% | 2019.4122 | 2020.1631 | 1 | 9.432 | 66.7% | 4 | K.LGLQEGSNNSSPVDFVNNK.R | 2 |
| \* | AstrinIP\_MS2\_022614\_01.09896.09896.2 | 4.5442 | 0.5172 | 100.0% | 2099.5923 | 2100.163 | 1 | 7.477 | 58.3% | 2 | K.LGLQEGS\*NNSSPVDFVNNK.R | 2 |
| \* | AstrinIP\_MS2\_022614\_01.07917.07917.2 | 3.9025 | 0.5698 | 100.0% | 2175.3123 | 2176.3506 | 1 | 9.45 | 47.4% | 5 | K.LGLQEGSNNSSPVDFVNNKR.T | 2 |
| \* | AstrinIP\_MS2\_022614\_01.07862.07862.3 | 4.2494 | 0.4112 | 100.0% | 2177.0044 | 2176.3506 | 1 | 7.204 | 36.8% | 9 | K.LGLQEGSNNSSPVDFVNNKR.T | 3 |
| \* | AstrinIP\_MS1\_022614\_01.09509.09509.2 | 4.1979 | 0.5317 | 100.0% | 2179.0122 | 2180.163 | 1 | 7.249 | 58.3% | 1 | K.LGLQEGS\*NNS\*SPVDFVNNK.R | 2 |
| \* | AstrinIP\_MS2\_022614\_01.09977.09977.2 | 4.5047 | 0.5245 | 100.0% | 2179.2722 | 2180.163 | 1 | 7.247 | 61.1% | 2 | K.LGLQEGS\*NNSS\*PVDFVNNK.R | 2 |
| \* | AstrinIP\_MS1\_022614\_01.07667.07667.2 | 3.4257 | 0.2772 | 99.8% | 2255.152 | 2256.3506 | 2 | 4.919 | 39.5% | 1 | K.LGLQEGSNNSS\*PVDFVNNKR.T | 2 |
| \* | AstrinIP\_MS2\_022614\_01.08055.08055.2 | 3.1136 | 0.3941 | 100.0% | 2255.4321 | 2256.3506 | 1 | 6.82 | 42.1% | 1 | K.LGLQEGSNNS\*SPVDFVNNKR.T | 2 |
| \* | AstrinIP\_MS1\_022614\_01.08332.08332.3 | 3.5211 | 0.4047 | 100.0% | 2255.6042 | 2256.3506 | 14 | 6.041 | 32.9% | 5 | K.LGLQEGSNNS\*SPVDFVNNKR.T | 3 |
| \* | AstrinIP\_MS2\_022614\_01.09014.09014.3 | 2.7406 | 0.2613 | 96.5% | 2335.5244 | 2336.3506 | 12 | 4.48 | 28.9% | 1 | K.LGLQEGS\*NNSS\*PVDFVNNKR.T | 3 |
| \* | AstrinIP\_MS2\_022614\_01.08948.08948.3 | 3.0926 | 0.3932 | 100.0% | 2335.8843 | 2336.3506 | 2 | 5.343 | 31.6% | 3 | K.LGLQEGSNNS\*S\*PVDFVNNKR.T | 3 |
| \* | AstrinIP\_MS2\_022614\_01.03495.03495.2 | 3.5685 | 0.3901 | 100.0% | 1618.0922 | 1618.7031 | 1 | 6.906 | 65.4% | 2 | K.RTDLSSEHFSHSSK.W | 2 |
| \* | AstrinIP\_MS1\_022614\_01.03536.03536.3 | 3.1344 | 0.2793 | 99.3% | 1619.1543 | 1618.7031 | 2 | 4.984 | 42.3% | 2 | K.RTDLSSEHFSHSSK.W | 3 |
| \* | AstrinIP\_MS2\_022614\_01.03586.03586.1 | 2.4768 | 0.5135 | 100.0% | 1461.57 | 1462.5156 | 1 | 8.196 | 50.0% | 1 | R.TDLSSEHFSHSSK.W | 1 |
| \* | AstrinIP\_MS2\_022614\_01.03578.03578.2 | 4.1954 | 0.5028 | 100.0% | 1462.2122 | 1462.5156 | 1 | 7.745 | 79.2% | 1 | R.TDLSSEHFSHSSK.W | 2 |
| \* | AstrinIP\_MS2\_022614\_01.03579.03579.3 | 2.1094 | 0.3215 | 97.3% | 1462.9143 | 1462.5156 | 72 | 5.323 | 35.4% | 1 | R.TDLSSEHFSHSSK.W | 3 |
| \* | AstrinIP\_MS1\_022614\_01.09358.09358.3 | 4.9889 | 0.422 | 100.0% | 2936.3643 | 2937.1555 | 1 | 6.947 | 38.5% | 4 | K.WLETCQHESDEQPLDPIPQISSTPK.T | 3 |
| \* | AstrinIP\_MS1\_022614\_01.09384.09384.2 | 4.0326 | 0.4516 | 100.0% | 2936.7922 | 2937.1555 | 1 | 7.482 | 43.8% | 1 | K.WLETCQHESDEQPLDPIPQISSTPK.T | 2 |
| \* | AstrinIP\_MS1\_022614\_02.06375.06375.3 | 4.2486 | 0.275 | 99.8% | 3016.1943 | 3017.1555 | 1 | 5.415 | 38.5% | 1 | K.WLETCQHESDEQPLDPIPQISST#PK.T | 3 |
| \* | AstrinIP\_MS1\_022614\_01.09970.09970.2 | 3.2596 | 0.2857 | 99.8% | 3017.412 | 3017.1555 | 2 | 5.321 | 33.3% | 1 | K.WLETCQHESDEQPLDPIPQIS\*STPK.T | 2 |
| \* | AstrinIP\_MS2\_022614\_01.09662.09662.2 | 4.5944 | 0.4373 | 100.0% | 1653.1721 | 1653.8445 | 1 | 9.167 | 71.4% | 13 | K.TSEEAVDPLGNYMVK.T | 2 |
| \* | AstrinIP\_MS1\_022614\_01.09051.09051.1 | 3.6463 | 0.4038 | 100.0% | 1653.72 | 1653.8445 | 1 | 7.996 | 60.7% | 9 | K.TSEEAVDPLGNYMVK.T | 1 |
| \* | AstrinIP\_MS2\_022614\_01.13029.13029.3 | 3.4101 | 0.4163 | 100.0% | 2243.0942 | 2243.6262 | 1 | 5.775 | 36.8% | 4 | K.TIVLVPSPLGQQQDMIFEAR.L | 3 |
| \* | AstrinIP\_MS2\_022614\_01.13024.13024.2 | 4.4861 | 0.5096 | 100.0% | 2243.372 | 2243.6262 | 1 | 8.728 | 63.2% | 5 | K.TIVLVPSPLGQQQDMIFEAR.L | 2 |
| \* | AstrinIP\_MS1\_022614\_01.12884.12884.3 | 3.2797 | 0.4519 | 100.0% | 2323.1042 | 2323.6262 | 1 | 7.818 | 40.8% | 6 | K.TIVLVPS\*PLGQQQDMIFEAR.L | 3 |
| \* | AstrinIP\_MS1\_022614\_01.12878.12878.2 | 4.1129 | 0.5463 | 100.0% | 2323.672 | 2323.6262 | 1 | 8.499 | 60.5% | 12 | K.TIVLVPS\*PLGQQQDMIFEAR.L | 2 |
| \* | AstrinIP\_MS2\_022614\_02.06374.06374.3 | 3.4187 | 0.3039 | 99.8% | 1832.9043 | 1833.0668 | 4 | 5.184 | 39.1% | 2 | R.LDTMAETNSISLNGPLR.T | 3 |
| \* | AstrinIP\_MS1\_022614\_01.08961.08961.2 | 5.3921 | 0.4718 | 100.0% | 1833.1322 | 1833.0668 | 1 | 8.587 | 68.8% | 25 | R.LDTMAETNSISLNGPLR.T | 2 |
| \* | AstrinIP\_MS2\_022614\_01.10202.10202.2 | 5.016 | 0.4416 | 100.0% | 1913.2522 | 1913.0668 | 1 | 7.129 | 59.4% | 10 | R.LDTMAETNSIS\*LNGPLR.T | 2 |
| \* | AstrinIP\_MS2\_022614\_01.10037.10037.2 | 3.6717 | 0.2334 | 99.8% | 1913.5122 | 1913.0668 | 2 | 4.984 | 53.1% | 2 | R.LDTMAETNS\*ISLNGPLR.T | 2 |
| \* | AstrinIP\_MS1\_022614\_01.09806.09806.3 | 4.7475 | 0.4664 | 100.0% | 2531.5444 | 2532.8286 | 1 | 8.649 | 35.2% | 13 | R.LDTMAETNSISLNGPLRTDDLVR.E | 3 |
| \* | AstrinIP\_MS1\_022614\_01.10205.10205.3 | 3.1319 | 0.2866 | 99.0% | 2612.2744 | 2612.8286 | 8 | 4.489 | 26.1% | 1 | R.LDTMAETNS\*ISLNGPLRTDDLVR.E | 3 |
| \* | AstrinIP\_MS1\_022614\_01.10430.10430.3 | 4.0321 | 0.2371 | 99.2% | 2614.6743 | 2612.8286 | 2 | 4.265 | 27.3% | 4 | R.LDTMAETNSIS\*LNGPLRTDDLVR.E | 3 |
| \* | AstrinIP\_MS1\_022614\_01.06414.06414.2 | 2.7172 | 0.3211 | 99.6% | 1862.9521 | 1864.0033 | 1 | 5.316 | 50.0% | 1 | R.TDDLVREEVAPCMGDR.F | 2 |
| \* | AstrinIP\_MS1\_022614\_01.04096.04096.2 | 2.6123 | 0.3161 | 99.8% | 1164.0322 | 1164.2416 | 3 | 5.344 | 72.2% | 2 | R.EEVAPCMGDR.F | 2 |
| \* | AstrinIP\_MS2\_022614\_02.10586.10586.3 | 6.3482 | 0.5315 | 100.0% | 3778.1943 | 3778.2102 | 1 | 8.16 | 28.7% | 6 | R.TEAVREDLVPSESNAFLPSSVLWLSPSTALAADFR.V | 3 |
| \* | AstrinIP\_MS2\_022614\_02.10510.10510.3 | 6.4255 | 0.2115 | 100.0% | 3857.8743 | 3858.2102 | 1 | 7.501 | 32.4% | 14 | R.TEAVREDLVPSESNAFLPSSVLWLS\*PSTALAADFR.V | 3 |
| \* | AstrinIP\_MS1\_022614\_01.15592.15592.3 | 4.9313 | 0.1078 | 95.4% | 3937.1643 | 3938.2102 | 2 | 5.439 | 21.3% | 1 | R.TEAVREDLVPS\*ESNAFLPSSVLWLS\*PSTALAADFR.V | 3 |
| \* | AstrinIP\_MS2\_022614\_01.16437.16437.2 | 4.1564 | 0.5354 | 100.0% | 3221.0322 | 3221.5908 | 1 | 8.807 | 36.2% | 2 | R.EDLVPSESNAFLPSSVLWLSPSTALAADFR.V | 2 |
| \* | AstrinIP\_MS1\_022614\_01.16130.16130.2 | 3.9306 | 0.5132 | 100.0% | 3301.412 | 3301.5908 | 1 | 6.883 | 34.5% | 5 | R.EDLVPSESNAFLPSSVLWLS\*PSTALAADFR.V | 2 |
| \* | AstrinIP\_MS1\_022614\_01.06591.06591.2 | 5.988 | 0.5626 | 100.0% | 2219.5322 | 2220.3752 | 1 | 10.714 | 72.2% | 15 | R.VNHVDPEEEIVEHGAMEER.E | 2 |
| \* | AstrinIP\_MS2\_022614\_01.06854.06854.3 | 5.3732 | 0.4456 | 100.0% | 2219.5745 | 2220.3752 | 1 | 7.418 | 44.4% | 82 | R.VNHVDPEEEIVEHGAMEER.E | 3 |
| \* | AstrinIP\_MS1\_022614\_01.16982.16982.3 | 5.1975 | 0.5728 | 100.0% | 4634.574 | 4633.9536 | 1 | 8.461 | 20.8% | 1 | K.ESETEDQALVSSVEDILSTCLTPNLVEMESQEAPGPAVEDVGR.I | 3 |
| \* | AstrinIP\_MS1\_022614\_01.15536.15536.2 | 6.1691 | 0.5527 | 100.0% | 2063.5122 | 2064.3606 | 1 | 9.235 | 67.6% | 14 | R.ILGSDTESWMSPLAWLEK.G | 2 |
| \* | AstrinIP\_MS2\_022614\_01.16112.16112.3 | 4.8082 | 0.4108 | 100.0% | 2065.2544 | 2064.3606 | 1 | 7.147 | 41.2% | 6 | R.ILGSDTESWMSPLAWLEK.G | 3 |
| \* | AstrinIP\_MS2\_022614\_01.17375.17375.2 | 4.0788 | 0.4755 | 100.0% | 2143.3323 | 2144.3606 | 2 | 7.183 | 50.0% | 2 | R.ILGSDT#ESWMSPLAWLEK.G | 2 |
| \* | AstrinIP\_MS2\_022614\_01.16677.16677.2 | 5.1468 | 0.5054 | 100.0% | 2143.4922 | 2144.3606 | 1 | 8.898 | 67.6% | 11 | R.ILGSDTESWMS\*PLAWLEK.G | 2 |
| \* | AstrinIP\_MS1\_022614\_01.16862.16862.2 | 3.9634 | 0.4914 | 100.0% | 2143.5723 | 2144.3606 | 1 | 7.765 | 52.9% | 2 | R.ILGS\*DTESWMSPLAWLEK.G | 2 |
| \* | AstrinIP\_MS2\_022614\_01.16673.16673.3 | 4.6072 | 0.489 | 100.0% | 2143.9443 | 2144.3606 | 1 | 7.817 | 42.6% | 3 | R.ILGSDTESWMS\*PLAWLEK.G | 3 |
| \* | AstrinIP\_MS2\_022614\_01.17200.17200.2 | 3.8022 | 0.436 | 100.0% | 2144.3323 | 2144.3606 | 2 | 6.004 | 44.1% | 2 | R.ILGSDTES\*WMSPLAWLEK.G | 2 |
| \* | AstrinIP\_MS1\_022614\_01.17181.17181.2 | 3.9689 | 0.2116 | 99.8% | 2223.872 | 2224.3606 | 1 | 6.127 | 55.9% | 2 | R.ILGS\*DTESWMS\*PLAWLEK.G | 2 |
| \* | AstrinIP\_MS2\_022614\_01.17648.17648.2 | 3.8999 | 0.2026 | 99.8% | 2224.4321 | 2224.3606 | 2 | 5.808 | 50.0% | 5 | R.ILGSDT#ESWMS\*PLAWLEK.G | 2 |
| \* | AstrinIP\_MS2\_022614\_01.09345.09345.1 | 2.5466 | 0.3536 | 100.0% | 1332.6 | 1333.5457 | 1 | 5.431 | 54.5% | 4 | K.GVNTSVMLENLR.Q | 1 |
| \* | AstrinIP\_MS2\_022614\_01.09404.09404.2 | 3.9957 | 0.3982 | 100.0% | 1333.1322 | 1333.5457 | 1 | 7.47 | 81.8% | 8 | K.GVNTSVMLENLR.Q | 2 |
| \* | AstrinIP\_MS1\_022614\_01.09999.09999.1 | 1.4677 | 0.4635 | 100.0% | 1412.5 | 1413.5457 | 248 | 6.537 | 36.4% | 2 | K.GVNT#SVMLENLR.Q | 1 |
| \* | AstrinIP\_MS1\_022614\_01.09934.09934.1 | 1.3523 | 0.3386 | 99.0% | 1412.52 | 1413.5457 | 240 | 6.654 | 40.9% | 1 | K.GVNTS\*VMLENLR.Q | 1 |
| \* | AstrinIP\_MS2\_022614\_01.10556.10556.2 | 3.9941 | 0.4087 | 100.0% | 1413.1522 | 1413.5457 | 1 | 6.552 | 77.3% | 6 | K.GVNTS\*VMLENLR.Q | 2 |
| \* | AstrinIP\_MS1\_022614\_02.09175.09175.3 | 2.9217 | 0.267 | 97.9% | 2606.0942 | 2606.886 | 1 | 4.596 | 29.8% | 2 | K.GVNTS\*VMLENLRQS\*LSLPSMLR.D | 3 |
| \* | AstrinIP\_MS2\_022614\_01.10346.10346.1 | 1.2377 | 0.2982 | 97.4% | 1131.5 | 1132.3635 | 137 | 5.224 | 50.0% | 1 | R.QSLSLPSMLR.D | 1 |
| \* | AstrinIP\_MS1\_022614\_02.06207.06207.2 | 2.3303 | 0.2343 | 98.1% | 1132.1322 | 1132.3635 | 99 | 5.396 | 61.1% | 4 | R.QSLSLPSMLR.D | 2 |
| \* | AstrinIP\_MS1\_022614\_01.12208.12208.2 | 4.5907 | 0.5097 | 100.0% | 2757.4521 | 2757.9795 | 1 | 9.706 | 38.0% | 4 | R.DAAIGTTPFSTCSVGTWFTPSAPQEK.S | 2 |
| \* | AstrinIP\_MS2\_022614\_01.03752.03752.2 | 3.6058 | 0.464 | 100.0% | 1294.2722 | 1294.4044 | 1 | 8.287 | 75.0% | 5 | K.STNTSQTGLVGTK.H | 2 |
| \* | AstrinIP\_MS1\_022614\_01.03873.03873.1 | 1.6752 | 0.2154 | 95.7% | 1373.57 | 1374.4044 | 18 | 4.782 | 45.8% | 2 | K.STNT#SQTGLVGTK.H | 1 |
| \* | AstrinIP\_MS2\_022614\_01.04100.04100.2 | 3.0381 | 0.1163 | 97.7% | 1373.9521 | 1374.4044 | 11 | 4.62 | 54.2% | 5 | K.STNT#SQTGLVGTK.H | 2 |
| \* | AstrinIP\_MS1\_022614\_01.08602.08602.2 | 3.2977 | 0.4914 | 100.0% | 2468.612 | 2469.68 | 1 | 7.422 | 40.5% | 3 | K.HSTSETEQLLCGRPPDLTALSR.H | 2 |
| \* | AstrinIP\_MS1\_022614\_01.08702.08702.3 | 3.4855 | 0.3019 | 99.6% | 2469.4443 | 2469.68 | 1 | 6.185 | 39.3% | 3 | K.HSTSETEQLLCGRPPDLTALSR.H | 3 |
| \* | AstrinIP\_MS2\_022614\_01.18152.18152.2 | 7.6774 | 0.6838 | 100.0% | 2165.5923 | 2166.4795 | 1 | 11.942 | 83.3% | 127 | R.HDLEDNLLSSLVILEVLSR.Q | 2 |
| \* | AstrinIP\_MS2\_022614\_01.18032.18032.3 | 3.7674 | 0.2801 | 99.7% | 2166.8044 | 2166.4795 | 1 | 5.837 | 41.7% | 23 | R.HDLEDNLLSSLVILEVLSR.Q | 3 |
| \* | AstrinIP\_MS1\_022614\_01.04246.04246.2 | 4.4118 | 0.4895 | 100.0% | 2867.5923 | 2868.0 | 1 | 7.831 | 42.3% | 7 | K.SQLAVPHPETQDSSTQTDTSHSGITNK.L | 2 |
| \* | AstrinIP\_MS1\_022614\_01.04395.04395.3 | 5.3117 | 0.541 | 100.0% | 2867.7244 | 2868.0 | 2 | 8.99 | 32.7% | 21 | K.SQLAVPHPETQDSSTQTDTSHSGITNK.L | 3 |
| \* | AstrinIP\_MS1\_022614\_01.04749.04749.3 | 4.084 | 0.1638 | 96.0% | 2947.7644 | 2948.0 | 5 | 5.366 | 30.8% | 1 | K.SQLAVPHPETQDSSTQTDTS\*HSGITNK.L | 3 |
| \* | AstrinIP\_MS1\_022614\_01.03996.03996.2 | 6.1689 | 0.5315 | 100.0% | 2104.2722 | 2105.3794 | 1 | 10.664 | 70.6% | 5 | K.LQHLKESHEMGQALQQAR.N | 2 |
| \* | AstrinIP\_MS2\_022614\_01.04130.04130.3 | 5.4292 | 0.456 | 100.0% | 2106.2944 | 2105.3794 | 2 | 7.873 | 48.5% | 6 | K.LQHLKESHEMGQALQQAR.N | 3 |
| \* | AstrinIP\_MS1\_022614\_02.03379.03379.2 | 3.9053 | 0.3512 | 100.0% | 1484.4321 | 1485.6146 | 1 | 6.82 | 75.0% | 5 | K.ESHEMGQALQQAR.N | 2 |
| \* | AstrinIP\_MS2\_022614\_01.03944.03944.3 | 3.7867 | 0.1339 | 96.9% | 1485.7144 | 1485.6146 | 11 | 4.946 | 45.8% | 1 | K.ESHEMGQALQQAR.N | 3 |
| \* | AstrinIP\_MS1\_022614\_01.03860.03860.1 | 3.5338 | 0.1684 | 99.0% | 1486.58 | 1485.6146 | 1 | 5.349 | 66.7% | 8 | K.ESHEMGQALQQAR.N | 1 |
| \* | AstrinIP\_MS2\_022614\_01.11589.11589.2 | 4.2993 | 0.3328 | 100.0% | 1306.2922 | 1305.578 | 1 | 6.76 | 80.0% | 24 | R.NVMQSWVLISK.E | 2 |
| \* | AstrinIP\_MS2\_022614\_01.19937.19937.3 | 7.2736 | 0.5772 | 100.0% | 3288.5645 | 3289.9011 | 1 | 9.618 | 32.4% | 1 | R.NVMQSWVLISKELISLLHLSLLHLEEDK.T | 3 |
| \* | AstrinIP\_MS2\_022614\_01.19653.19653.3 | 7.3529 | 0.5958 | 100.0% | 4177.344 | 4178.834 | 1 | 9.486 | 25.7% | 2 | R.NVMQSWVLISKELISLLHLSLLHLEEDKTTVSQESR.R | 3 |
| \* | AstrinIP\_MS2\_022614\_01.12981.12981.3 | 4.2115 | 0.2478 | 99.3% | 4337.514 | 4338.834 | 1 | 4.94 | 20.0% | 2 | R.NVMQS\*WVLIS\*KELISLLHLSLLHLEEDKTTVSQESR.R | 3 |
| \* | AstrinIP\_MS2\_022614\_01.13869.13869.2 | 4.9283 | 0.44 | 100.0% | 2003.4922 | 2003.3464 | 1 | 8.669 | 56.2% | 3 | K.ELISLLHLSLLHLEEDK.T | 2 |
| \* | AstrinIP\_MS2\_022614\_01.13862.13862.3 | 4.2828 | 0.4175 | 100.0% | 2003.5443 | 2003.3464 | 1 | 7.235 | 42.2% | 7 | K.ELISLLHLSLLHLEEDK.T | 3 |
| \* | AstrinIP\_MS2\_022614\_01.12981.12981.2 | 5.6176 | 0.4711 | 100.0% | 2892.0122 | 2892.2793 | 1 | 9.277 | 41.7% | 5 | K.ELISLLHLSLLHLEEDKTTVSQESR.R | 3 |
| \* | AstrinIP\_MS1\_022614\_01.12320.12320.3 | 7.682 | 0.432 | 100.0% | 2892.0842 | 2892.2793 | 1 | 7.99 | 37.5% | 147 | K.ELISLLHLSLLHLEEDKTTVSQESR.R | 3 |
| \* | AstrinIP\_MS1\_022614\_01.09844.09844.2 | 4.0451 | 0.378 | 100.0% | 1785.3522 | 1785.9766 | 1 | 7.205 | 76.9% | 4 | R.RAETLVCCCFDLLK.K | 2 |
| \* | AstrinIP\_MS1\_022614\_01.08475.08475.3 | 4.3325 | 0.4436 | 100.0% | 1913.3944 | 1914.1506 | 1 | 6.828 | 44.6% | 3 | R.RAETLVCCCFDLLKK.L | 3 |
| \* | AstrinIP\_MS1\_022614\_01.08481.08481.2 | 3.4297 | 0.2285 | 99.7% | 1913.5322 | 1914.1506 | 1 | 4.974 | 67.9% | 1 | R.RAETLVCCCFDLLKK.L | 2 |
| \* | AstrinIP\_MS1\_022614\_02.07071.07071.2 | 3.9651 | 0.4831 | 100.0% | 1629.1921 | 1629.7891 | 1 | 8.232 | 62.5% | 6 | R.AETLVCCCFDLLK.K | 2 |
| \* | AstrinIP\_MS1\_022614\_01.09621.09621.2 | 3.9803 | 0.3261 | 100.0% | 1756.9521 | 1757.9631 | 1 | 6.653 | 61.5% | 2 | R.AETLVCCCFDLLKK.L | 2 |
| \* | AstrinIP\_MS2\_022614\_01.03498.03498.2 | 2.7853 | 0.1187 | 96.3% | 1430.1122 | 1430.6041 | 1 | 5.043 | 72.7% | 1 | K.LQSLKAEREEAR.H | 2 |
| \* | AstrinIP\_MS1\_022614\_01.11522.11522.2 | 6.7759 | 0.5354 | 100.0% | 2073.412 | 2074.267 | 1 | 10.369 | 75.0% | 2 | R.GKDAAEIVLEAFCAHASQR.I | 2 |
| \* | AstrinIP\_MS1\_022614\_02.08119.08119.3 | 5.3076 | 0.5281 | 100.0% | 2074.4343 | 2074.267 | 1 | 7.964 | 44.4% | 8 | R.GKDAAEIVLEAFCAHASQR.I | 3 |
| \* | AstrinIP\_MS1\_022614\_01.12567.12567.3 | 4.0364 | 0.4531 | 100.0% | 1888.5844 | 1889.041 | 1 | 8.202 | 46.9% | 8 | K.DAAEIVLEAFCAHASQR.I | 3 |
| \* | AstrinIP\_MS1\_022614\_01.12371.12371.2 | 5.6245 | 0.4487 | 100.0% | 1889.1122 | 1889.041 | 1 | 8.65 | 71.9% | 21 | K.DAAEIVLEAFCAHASQR.I | 2 |
| \* | AstrinIP\_MS1\_022614\_01.09854.09854.2 | 4.0999 | 0.4427 | 100.0% | 1390.8522 | 1391.5823 | 1 | 8.669 | 68.2% | 20 | R.ISQLEQDLASMR.E | 2 |
| \* | AstrinIP\_MS1\_022614\_01.09878.09878.1 | 2.8029 | 0.3355 | 100.0% | 1392.59 | 1391.5823 | 1 | 5.413 | 59.1% | 13 | R.ISQLEQDLASMR.E | 1 |
| \* | AstrinIP\_MS1\_022614\_01.10863.10863.2 | 3.097 | 0.1785 | 98.6% | 1823.4722 | 1824.0618 | 1 | 4.69 | 46.4% | 4 | R.ISQLEQDLASMREFR.G | 2 |
| \* | AstrinIP\_MS2\_022614\_01.11494.11494.3 | 3.3642 | 0.3995 | 100.0% | 1824.2644 | 1824.0618 | 2 | 6.028 | 42.9% | 4 | R.ISQLEQDLASMREFR.G | 3 |
| \* | AstrinIP\_MS1\_022614\_01.07828.07828.2 | 4.6942 | 0.574 | 100.0% | 1691.7122 | 1692.9994 | 1 | 9.46 | 63.3% | 5 | R.GLLKDAQTQLVGLHAK.Q | 2 |
| \* | AstrinIP\_MS2\_022614\_01.08228.08228.3 | 4.7497 | 0.3302 | 100.0% | 1693.2544 | 1692.9994 | 1 | 6.756 | 45.0% | 7 | R.GLLKDAQTQLVGLHAK.Q | 3 |
| \* | AstrinIP\_MS1\_022614\_01.14457.14457.3 | 5.8738 | 0.5677 | 100.0% | 4062.9844 | 4064.5884 | 1 | 10.339 | 27.1% | 1 | R.GLLKDAQTQLVGLHAKQEELVQQTVSLTSTLQQDWR.S | 3 |
| \* | AstrinIP\_MS2\_022614\_01.04802.04802.2 | 4.0782 | 0.4115 | 100.0% | 1281.2522 | 1281.4545 | 1 | 7.213 | 77.3% | 14 | K.DAQTQLVGLHAK.Q | 2 |
| \* | AstrinIP\_MS2\_022614\_01.04844.04844.3 | 2.7765 | 0.2679 | 98.9% | 1281.8644 | 1281.4545 | 1 | 5.526 | 45.5% | 1 | K.DAQTQLVGLHAK.Q | 3 |
| \* | AstrinIP\_MS2\_022614\_01.04817.04817.1 | 3.1433 | 0.2695 | 100.0% | 1282.54 | 1281.4545 | 1 | 5.076 | 63.6% | 7 | K.DAQTQLVGLHAK.Q | 1 |
| \* | AstrinIP\_MS2\_022614\_01.13670.13670.2 | 5.9251 | 0.6546 | 100.0% | 2390.0322 | 2390.612 | 1 | 12.065 | 65.8% | 57 | K.QEELVQQTVSLTSTLQQDWR.S | 2 |
| \* | AstrinIP\_MS2\_022614\_01.13634.13634.3 | 5.4577 | 0.5491 | 100.0% | 2390.0344 | 2390.612 | 1 | 9.885 | 39.5% | 20 | K.QEELVQQTVSLTSTLQQDWR.S | 3 |
| \* | AstrinIP\_MS2\_022614\_02.13348.13348.3 | 5.3831 | 0.5287 | 100.0% | 4157.784 | 4158.6294 | 1 | 8.929 | 25.0% | 3 | K.QEELVQQTVSLTSTLQQDWRSMQLDYTTWTALLSR.S | 3 |
| \* | AstrinIP\_MS1\_022614\_02.09469.09469.2 | 5.6297 | 0.4615 | 100.0% | 1786.2522 | 1787.0405 | 1 | 9.749 | 82.1% | 83 | R.SMQLDYTTWTALLSR.S | 2 |
| \* | AstrinIP\_MS1\_022614\_01.13574.13574.3 | 3.8896 | 0.4119 | 100.0% | 1786.7043 | 1787.0405 | 1 | 7.018 | 50.0% | 2 | R.SMQLDYTTWTALLSR.S | 3 |
| \* | AstrinIP\_MS2\_022614\_01.14061.14061.1 | 3.3013 | 0.4595 | 100.0% | 1786.81 | 1787.0405 | 1 | 6.97 | 50.0% | 4 | R.SMQLDYTTWTALLSR.S | 1 |
| \* | AstrinIP\_MS2\_022614\_01.03840.03840.3 | 3.5759 | 0.1729 | 98.5% | 1303.5243 | 1303.5448 | 37 | 3.818 | 45.0% | 1 | R.SRQLTEKLTVK.S | 3 |
| \* | AstrinIP\_MS2\_022614\_01.03834.03834.2 | 3.65 | 0.1581 | 99.8% | 1303.6522 | 1303.5448 | 1 | 5.621 | 80.0% | 1 | R.SRQLTEKLTVK.S | 2 |
| \* | AstrinIP\_MS2\_022614\_01.03304.03304.2 | 2.5927 | 0.1366 | 98.2% | 959.9522 | 960.0348 | 1 | 4.693 | 85.7% | 2 | K.SQQALQER.D | 2 |
| \* | AstrinIP\_MS2\_022614\_01.04430.04430.2 | 3.4367 | 0.3757 | 100.0% | 1744.3922 | 1744.8992 | 1 | 6.603 | 67.9% | 1 | K.SQQALQERDVAIEEK.Q | 2 |
| \* | AstrinIP\_MS1\_022614\_01.04419.04419.3 | 5.5505 | 0.3881 | 100.0% | 2345.0044 | 2344.5437 | 1 | 7.1 | 46.1% | 6 | K.SQQALQERDVAIEEKQEVSR.V | 3 |
| \* | AstrinIP\_MS1\_022614\_01.03827.03827.1 | 3.0295 | 0.4037 | 100.0% | 1402.71 | 1403.5321 | 1 | 7.4 | 63.6% | 2 | R.DVAIEEKQEVSR.V | 1 |
| \* | AstrinIP\_MS2\_022614\_02.03413.03413.2 | 3.8463 | 0.3634 | 100.0% | 1402.7722 | 1403.5321 | 1 | 7.486 | 86.4% | 25 | R.DVAIEEKQEVSR.V | 2 |
| \* | AstrinIP\_MS2\_022614\_01.03914.03914.3 | 2.9556 | 0.4247 | 100.0% | 1403.6643 | 1403.5321 | 1 | 6.803 | 47.7% | 2 | R.DVAIEEKQEVSR.V | 3 |
| \* | AstrinIP\_MS1\_022614\_01.07025.07025.1 | 3.6356 | 0.4606 | 100.0% | 1532.55 | 1533.6849 | 1 | 9.183 | 54.2% | 1 | R.VLEQVSAQLEECK.G | 1 |
| \* | AstrinIP\_MS1\_022614\_01.07040.07040.2 | 4.9305 | 0.4726 | 100.0% | 1533.1322 | 1533.6849 | 1 | 9.052 | 70.8% | 23 | R.VLEQVSAQLEECK.G | 2 |
| \* | AstrinIP\_MS1\_022614\_02.06085.06085.3 | 6.2673 | 0.4923 | 100.0% | 2918.1543 | 2919.1382 | 1 | 8.253 | 36.5% | 6 | R.VLEQVSAQLEECKGQTEQLELENSR.L | 3 |
| \* | AstrinIP\_MS1\_022614\_01.08493.08493.2 | 5.0393 | 0.5318 | 100.0% | 2918.2122 | 2919.1382 | 1 | 9.126 | 41.7% | 3 | R.VLEQVSAQLEECKGQTEQLELENSR.L | 2 |
| \* | AstrinIP\_MS1\_022614\_01.04659.04659.2 | 4.4176 | 0.4966 | 100.0% | 1404.0521 | 1404.4764 | 1 | 7.631 | 81.8% | 12 | K.GQTEQLELENSR.L | 2 |
| \* | AstrinIP\_MS2\_022614\_01.04784.04784.1 | 3.0534 | 0.4097 | 100.0% | 1404.68 | 1404.4764 | 1 | 6.26 | 54.5% | 6 | K.GQTEQLELENSR.L | 1 |
| \* | AstrinIP\_MS1\_022614\_01.10761.10761.1 | 3.8457 | 0.5052 | 100.0% | 1572.68 | 1573.848 | 1 | 8.588 | 65.4% | 10 | R.AQLQILANMDSQLK.E | 1 |
| \* | AstrinIP\_MS1\_022614\_02.07501.07501.2 | 5.719 | 0.4314 | 100.0% | 1573.3121 | 1573.848 | 1 | 7.883 | 80.8% | 156 | R.AQLQILANMDSQLK.E | 2 |
| \* | AstrinIP\_MS2\_022614\_02.07478.07478.3 | 4.4801 | 0.357 | 100.0% | 1573.9443 | 1573.848 | 3 | 5.959 | 48.1% | 2 | R.AQLQILANMDSQLK.E | 3 |
| \* | AstrinIP\_MS1\_022614\_01.03758.03758.2 | 3.9237 | 0.2675 | 100.0% | 1897.2122 | 1898.0668 | 1 | 7.604 | 60.0% | 1 | K.ELQSQHTHCAQDLAMK.D | 2 |
| \* | AstrinIP\_MS1\_022614\_01.09574.09574.3 | 6.3411 | 0.5332 | 100.0% | 4298.3945 | 4299.6045 | 1 | 10.187 | 25.0% | 2 | K.ELQSQHTHCAQDLAMKDELLCQLTQSNEEQAAQWQK.E | 3 |
| \* | AstrinIP\_MS2\_022614\_02.07248.07248.3 | 4.2459 | 0.3902 | 100.0% | 2418.0244 | 2420.561 | 1 | 6.494 | 36.8% | 2 | K.DELLCQLTQSNEEQAAQWQK.E | 3 |
| \* | AstrinIP\_MS1\_022614\_01.10371.10371.2 | 5.9619 | 0.5426 | 100.0% | 2419.612 | 2420.561 | 1 | 10.069 | 60.5% | 1 | K.DELLCQLTQSNEEQAAQWQK.E | 2 |
| \* | AstrinIP\_MS2\_022614\_01.04895.04895.2 | 5.7627 | 0.5758 | 100.0% | 1723.4521 | 1723.9879 | 1 | 10.581 | 85.7% | 97 | K.HMQAELQQQQAVLAK.E | 2 |
| \* | AstrinIP\_MS2\_022614\_01.04641.04641.1 | 3.9418 | 0.4241 | 100.0% | 1723.85 | 1723.9879 | 1 | 7.183 | 60.7% | 5 | K.HMQAELQQQQAVLAK.E | 1 |
| \* | AstrinIP\_MS1\_022614\_01.04664.04664.3 | 4.8619 | 0.3728 | 100.0% | 1724.1244 | 1723.9879 | 1 | 6.744 | 51.8% | 14 | K.HMQAELQQQQAVLAK.E | 3 |
| \* | AstrinIP\_MS1\_022614\_02.08822.08822.3 | 7.0438 | 0.5196 | 100.0% | 3286.7344 | 3286.5862 | 1 | 8.212 | 34.3% | 17 | R.DLKETLEFADQENQVAHLELGQVECQLK.T | 3 |
| \* | AstrinIP\_MS1\_022614\_01.11154.11154.2 | 4.9416 | 0.438 | 100.0% | 2929.9321 | 2930.1638 | 1 | 7.623 | 39.6% | 3 | K.ETLEFADQENQVAHLELGQVECQLK.T | 2 |
| \* | AstrinIP\_MS1\_022614\_02.07651.07651.3 | 6.0255 | 0.5251 | 100.0% | 2930.3342 | 2930.1638 | 1 | 9.119 | 37.5% | 9 | K.ETLEFADQENQVAHLELGQVECQLK.T | 3 |
| \* | AstrinIP\_MS2\_022614\_01.06356.06356.1 | 1.629 | 0.2368 | 97.3% | 831.47 | 831.9878 | 43 | 4.993 | 58.3% | 2 | K.TTLEVLR.E | 1 |
| \* | AstrinIP\_MS1\_022614\_01.06082.06082.2 | 2.3545 | 0.1823 | 98.5% | 831.9922 | 831.9878 | 6 | 4.868 | 83.3% | 6 | K.TTLEVLR.E | 2 |
| \* | AstrinIP\_MS1\_022614\_01.07887.07887.3 | 3.3816 | 0.3084 | 99.7% | 2249.3943 | 2249.4492 | 1 | 5.094 | 37.5% | 1 | R.ERSLQCENLKDTVENLTAK.L | 3 |
| \* | AstrinIP\_MS1\_022614\_01.08564.08564.2 | 5.6114 | 0.5769 | 100.0% | 1963.4722 | 1964.1462 | 1 | 10.064 | 68.8% | 4 | R.SLQCENLKDTVENLTAK.L | 2 |
| \* | AstrinIP\_MS1\_022614\_01.08558.08558.3 | 4.5941 | 0.3348 | 100.0% | 1964.5743 | 1964.1462 | 1 | 6.28 | 46.9% | 4 | R.SLQCENLKDTVENLTAK.L | 3 |
| \* | AstrinIP\_MS1\_022614\_01.04083.04083.1 | 2.023 | 0.4048 | 100.0% | 990.49 | 991.0862 | 1 | 6.146 | 62.5% | 5 | K.DTVENLTAK.L | 1 |
| \* | AstrinIP\_MS2\_022614\_01.04425.04425.2 | 2.2792 | 0.3219 | 99.3% | 990.5122 | 991.0862 | 1 | 5.22 | 87.5% | 3 | K.DTVENLTAK.L | 2 |
| \* | AstrinIP\_MS1\_022614\_01.05264.05264.2 | 4.9408 | 0.4957 | 100.0% | 1675.3121 | 1675.7899 | 1 | 7.906 | 75.0% | 56 | K.LASTIADNQEQDLEK.T | 2 |
| \* | AstrinIP\_MS1\_022614\_01.05140.05140.1 | 3.467 | 0.4086 | 100.0% | 1675.83 | 1675.7899 | 1 | 6.475 | 53.6% | 9 | K.LASTIADNQEQDLEK.T | 1 |
| \* | AstrinIP\_MS1\_022614\_01.05301.05301.3 | 4.4723 | 0.2692 | 100.0% | 1676.1543 | 1675.7899 | 1 | 6.169 | 48.2% | 6 | K.LASTIADNQEQDLEK.T | 3 |
| \* | AstrinIP\_MS2\_022614\_01.05151.05151.2 | 4.2953 | 0.485 | 100.0% | 1931.8722 | 1933.0825 | 3 | 8.399 | 50.0% | 7 | K.LASTIADNQEQDLEKTR.Q | 2 |
| \* | AstrinIP\_MS1\_022614\_01.20891.20891.2 | 6.044 | 0.5345 | 100.0% | 2046.8922 | 2047.443 | 1 | 10.065 | 64.7% | 240 | K.LGLLTEQLQSLTLFLQTK.L | 2 |
| \* | AstrinIP\_MS2\_022614\_01.17492.17492.3 | 5.4208 | 0.3955 | 100.0% | 2047.6444 | 2047.443 | 1 | 8.631 | 48.5% | 88 | K.LGLLTEQLQSLTLFLQTK.L | 3 |
| \* | AstrinIP\_MS1\_022614\_01.07575.07575.3 | 4.5102 | 0.3809 | 100.0% | 3005.4844 | 3006.2622 | 1 | 6.655 | 28.0% | 2 | K.EKTEQETLLLSTACPPTQEHPLPNDR.T | 3 |
| \* | AstrinIP\_MS1\_022614\_01.08174.08174.2 | 4.1464 | 0.5364 | 100.0% | 2747.5522 | 2748.9727 | 1 | 9.043 | 39.1% | 5 | K.TEQETLLLSTACPPTQEHPLPNDR.T | 2 |
| \* | AstrinIP\_MS1\_022614\_01.08246.08246.3 | 3.5343 | 0.3261 | 99.8% | 2748.9543 | 2748.9727 | 1 | 5.699 | 31.5% | 4 | K.TEQETLLLSTACPPTQEHPLPNDR.T | 3 |
| \* | AstrinIP\_MS2\_022614\_01.16496.16496.3 | 6.6632 | 0.6039 | 100.0% | 2788.4644 | 2788.121 | 1 | 10.343 | 34.6% | 10 | R.TFLGSILTAVADEEPESTPVPLLGSDK.S | 3 |
| \* | AstrinIP\_MS2\_022614\_02.10655.10655.2 | 5.2321 | 0.4264 | 100.0% | 2788.652 | 2788.121 | 1 | 8.417 | 42.3% | 25 | R.TFLGSILTAVADEEPESTPVPLLGSDK.S | 2 |
| \* | AstrinIP\_MS1\_022614\_01.16653.16653.2 | 4.9802 | 0.5714 | 100.0% | 2867.652 | 2868.121 | 1 | 9.226 | 44.2% | 4 | R.TFLGSILTAVADEEPESTPVPLLGS\*DK.S | 2 |
| \* | AstrinIP\_MS1\_022614\_01.15518.15518.3 | 5.3221 | 0.5709 | 100.0% | 3349.6143 | 3350.7473 | 1 | 9.497 | 27.4% | 3 | R.TFLGSILTAVADEEPESTPVPLLGSDKSAFTR.V | 3 |
| \* | AstrinIP\_MS1\_022614\_01.15938.15938.3 | 4.4494 | 0.4114 | 100.0% | 3429.3542 | 3430.7473 | 2 | 7.043 | 29.0% | 2 | R.TFLGSILTAVADEEPESTPVPLLGS\*DKSAFTR.V | 3 |
| \* | AstrinIP\_MS2\_022614\_01.16389.16389.3 | 5.7185 | 0.3671 | 100.0% | 3429.5645 | 3430.7473 | 2 | 8.674 | 25.8% | 3 | R.TFLGSILTAVADEEPESTPVPLLGSDKS\*AFTR.V | 3 |
| \* | AstrinIP\_MS1\_022614\_02.12577.12577.3 | 5.7757 | 0.4825 | 100.0% | 4358.8745 | 4358.957 | 1 | 8.346 | 24.4% | 6 | R.VASMVSLQPAETPGMEESLAEMSIMTTELQSLCSLLQESK.E | 3 |
| \* | AstrinIP\_MS1\_022614\_01.17558.17558.3 | 4.3492 | 0.3111 | 100.0% | 4956.5645 | 4957.614 | 1 | 5.567 | 19.9% | 2 | R.VASMVSLQPAETPGMEESLAEMSIMTTELQSLCSLLQESKEEAIR.T | 3 |
| \* | AstrinIP\_MS1\_022614\_01.03462.03462.1 | 2.32 | 0.294 | 100.0% | 1594.71 | 1595.7092 | 18 | 4.755 | 45.8% | 1 | R.LQAQEEQHQEVQK.A | 1 |
| \* | AstrinIP\_MS2\_022614\_01.03398.03398.2 | 4.7416 | 0.4304 | 100.0% | 1595.3522 | 1595.7092 | 1 | 7.711 | 83.3% | 4 | R.LQAQEEQHQEVQK.A | 2 |
| \* | AstrinIP\_MS1\_022614\_01.07908.07908.3 | 4.3105 | 0.3694 | 100.0% | 1872.8344 | 1873.126 | 1 | 6.893 | 45.0% | 1 | K.AKEADIEKLNQALCLR.Y | 3 |
| \* | AstrinIP\_MS2\_022614\_01.06875.06875.3 | 3.2499 | 0.2766 | 99.2% | 2162.9644 | 2164.476 | 1 | 5.012 | 30.9% | 1 | K.AKEADIEKLNQALCLRYK.N | 3 |
| \* | AstrinIP\_MS1\_022614\_01.08900.08900.2 | 3.7949 | 0.4342 | 100.0% | 1673.3922 | 1673.873 | 1 | 7.779 | 76.9% | 1 | K.EADIEKLNQALCLR.Y | 2 |
| \* | AstrinIP\_MS1\_022614\_01.05570.05570.1 | 1.7867 | 0.2321 | 98.0% | 987.43 | 988.1412 | 4 | 5.139 | 57.1% | 2 | K.LNQALCLR.Y | 1 |
| \* | AstrinIP\_MS1\_022614\_01.05582.05582.2 | 3.1789 | 0.3339 | 100.0% | 987.97217 | 988.1412 | 1 | 5.524 | 85.7% | 3 | K.LNQALCLR.Y | 2 |
| \* | AstrinIP\_MS2\_022614\_01.05144.05144.2 | 6.3385 | 0.4233 | 100.0% | 2148.5322 | 2149.3652 | 1 | 8.339 | 68.8% | 7 | R.YKNEKELQEVIQQQNEK.I | 2 |
| \* | AstrinIP\_MS1\_022614\_01.05019.05019.3 | 6.2624 | 0.2569 | 100.0% | 2149.3743 | 2149.3652 | 1 | 6.048 | 51.6% | 8 | R.YKNEKELQEVIQQQNEK.I | 3 |
| \* | AstrinIP\_MS1\_022614\_01.04736.04736.1 | 3.5839 | 0.2473 | 100.0% | 1485.68 | 1486.622 | 1 | 6.463 | 72.7% | 5 | K.ELQEVIQQQNEK.I | 1 |
| \* | AstrinIP\_MS1\_022614\_01.04826.04826.2 | 4.7936 | 0.2338 | 100.0% | 1486.1721 | 1486.622 | 1 | 6.568 | 86.4% | 22 | K.ELQEVIQQQNEK.I | 2 |
| \* | AstrinIP\_MS1\_022614\_01.13077.13077.3 | 3.5247 | 0.2058 | 96.4% | 3181.3442 | 3182.5986 | 277 | 4.059 | 20.2% | 1 | K.ELQEVIQQQNEKILEQIDKSGELISLR.E | 3 |
| \* | AstrinIP\_MS2\_022614\_01.04676.04676.2 | 2.8845 | 0.0503 | 98.5% | 859.2322 | 859.01044 | 13 | 3.951 | 83.3% | 4 | K.ILEQIDK.S | 2 |
| \* | AstrinIP\_MS1\_022614\_01.09056.09056.2 | 4.8472 | 0.4363 | 100.0% | 1714.2322 | 1715.0 | 1 | 7.499 | 78.6% | 6 | K.ILEQIDKSGELISLR.E | 2 |
| \* | AstrinIP\_MS1\_022614\_01.08990.08990.3 | 4.864 | 0.3845 | 100.0% | 1715.1244 | 1715.0 | 1 | 7.188 | 57.1% | 6 | K.ILEQIDKSGELISLR.E | 3 |
| \* | AstrinIP\_MS2\_022614\_01.11559.11559.3 | 5.7572 | 0.3338 | 100.0% | 2680.8843 | 2681.0618 | 1 | 6.959 | 40.9% | 10 | K.ILEQIDKSGELISLREEVTHLTR.S | 3 |
| \* | AstrinIP\_MS1\_022614\_01.06956.06956.1 | 2.0036 | 0.1888 | 97.1% | 874.39 | 875.0128 | 88 | 4.993 | 57.1% | 4 | K.SGELISLR.E | 1 |
| \* | AstrinIP\_MS2\_022614\_01.07298.07298.2 | 3.1831 | 0.1752 | 99.8% | 874.9922 | 875.0128 | 2 | 5.294 | 85.7% | 7 | K.SGELISLR.E | 2 |
| \* | AstrinIP\_MS2\_022614\_01.10100.10100.2 | 4.1592 | 0.3926 | 100.0% | 1840.4922 | 1841.0745 | 1 | 6.516 | 60.0% | 14 | K.SGELISLREEVTHLTR.S | 2 |
| \* | AstrinIP\_MS2\_022614\_01.09950.09950.3 | 5.243 | 0.4348 | 100.0% | 1841.6044 | 1841.0745 | 1 | 7.777 | 53.3% | 33 | K.SGELISLREEVTHLTR.S | 3 |
| \* | AstrinIP\_MS1\_022614\_01.10754.10754.3 | 6.1017 | 0.5778 | 100.0% | 2845.3145 | 2846.1506 | 1 | 11.176 | 40.6% | 4 | K.VLQEALAGQLDSNCQPMATNWIQEK.V | 3 |
| \* | AstrinIP\_MS1\_022614\_01.10644.10644.2 | 6.0058 | 0.6553 | 100.0% | 2846.112 | 2846.1506 | 1 | 11.496 | 50.0% | 4 | K.VLQEALAGQLDSNCQPMATNWIQEK.V | 2 |
| \* | AstrinIP\_MS2\_022614\_01.07324.07324.1 | 1.8376 | 0.3668 | 100.0% | 1103.52 | 1104.248 | 110 | 5.328 | 50.0% | 2 | K.VWLSQEVDK.L | 1 |
| \* | AstrinIP\_MS2\_022614\_01.07244.07244.2 | 2.8842 | 0.3217 | 100.0% | 1104.0721 | 1104.248 | 1 | 5.995 | 87.5% | 10 | K.VWLSQEVDK.L | 2 |
| \* | AstrinIP\_MS1\_022614\_01.08694.08694.1 | 2.7102 | 0.3291 | 100.0% | 1372.61 | 1373.595 | 1 | 6.502 | 70.0% | 4 | K.VWLSQEVDKLR.V | 1 |
| \* | AstrinIP\_MS2\_022614\_01.09164.09164.2 | 3.6908 | 0.3736 | 100.0% | 1373.3121 | 1373.595 | 1 | 7.077 | 90.0% | 11 | K.VWLSQEVDKLR.V | 2 |
| \* | AstrinIP\_MS1\_022614\_01.09423.09423.2 | 2.8747 | 0.2493 | 99.9% | 898.1922 | 898.16644 | 1 | 7.002 | 83.3% | 8 | R.VMFLEMK.N | 2 |
| \* | AstrinIP\_MS1\_022614\_01.07124.07124.2 | 3.0715 | 0.4444 | 100.0% | 1268.9922 | 1269.5598 | 1 | 7.659 | 77.8% | 5 | R.VMFLEMKNEK.E | 2 |
| \* | AstrinIP\_MS1\_022614\_01.05804.05804.2 | 3.6483 | 0.2082 | 99.8% | 1526.3121 | 1526.8494 | 1 | 5.07 | 81.8% | 5 | R.VMFLEMKNEKEK.L | 2 |
| \* | AstrinIP\_MS2\_022614\_01.06146.06146.3 | 2.596 | 0.3913 | 99.9% | 1526.6943 | 1526.8494 | 11 | 5.675 | 36.4% | 3 | R.VMFLEMKNEKEK.L | 3 |
| \* | AstrinIP\_MS1\_022614\_01.07755.07755.3 | 3.5078 | 0.3246 | 99.9% | 2012.5144 | 2012.5349 | 1 | 5.324 | 43.3% | 2 | R.VMFLEMKNEKEKLMIK.F | 3 |
| \* | AstrinIP\_MS1\_022614\_01.06926.06926.1 | 2.3856 | 0.2487 | 100.0% | 1000.43 | 1001.1277 | 1 | 4.657 | 78.6% | 8 | R.NILEENLR.R | 1 |
| \* | AstrinIP\_MS1\_022614\_01.06938.06938.2 | 2.7489 | 0.0354 | 95.6% | 1001.0722 | 1001.1277 | 7 | 4.254 | 85.7% | 1 | R.NILEENLR.R | 2 |
| \* | AstrinIP\_MS2\_022614\_01.10179.10179.3 | 5.9837 | 0.4937 | 100.0% | 2229.8342 | 2230.526 | 1 | 9.389 | 48.5% | 14 | R.RSDKELEKLDDIVQHIYK.T | 3 |
| \* | AstrinIP\_MS1\_022614\_01.09371.09371.2 | 5.6666 | 0.4707 | 100.0% | 2230.4521 | 2230.526 | 1 | 8.37 | 67.6% | 7 | R.RSDKELEKLDDIVQHIYK.T | 2 |
| \* | AstrinIP\_MS2\_022614\_01.11139.11139.2 | 5.6193 | 0.5337 | 100.0% | 2073.4321 | 2074.3384 | 1 | 10.503 | 65.6% | 4 | R.SDKELEKLDDIVQHIYK.T | 2 |
| \* | AstrinIP\_MS1\_022614\_01.10418.10418.3 | 5.4829 | 0.3307 | 100.0% | 2074.3442 | 2074.3384 | 1 | 6.766 | 45.3% | 7 | R.SDKELEKLDDIVQHIYK.T | 3 |
| \* | AstrinIP\_MS1\_022614\_01.17732.17732.3 | 4.2141 | 0.2909 | 99.8% | 3181.0444 | 3182.6848 | 14 | 5.709 | 22.1% | 1 | R.SDKELEKLDDIVQHIYKTLLSIPEVVR.G | 3 |
| \* | AstrinIP\_MS1\_022614\_01.09848.09848.2 | 3.8856 | 0.4612 | 100.0% | 1743.4922 | 1743.9977 | 1 | 7.385 | 73.1% | 6 | K.ELEKLDDIVQHIYK.T | 2 |
| \* | AstrinIP\_MS2\_022614\_01.10436.10436.3 | 3.8692 | 0.4427 | 100.0% | 1743.8944 | 1743.9977 | 1 | 7.103 | 50.0% | 8 | K.ELEKLDDIVQHIYK.T | 3 |
| \* | AstrinIP\_MS1\_022614\_01.07084.07084.1 | 1.8541 | 0.2864 | 99.0% | 1243.54 | 1244.4331 | 1 | 5.086 | 55.6% | 3 | K.LDDIVQHIYK.T | 1 |
| \* | AstrinIP\_MS1\_022614\_01.07100.07100.2 | 3.6514 | 0.3931 | 100.0% | 1243.7122 | 1244.4331 | 1 | 7.542 | 83.3% | 6 | K.LDDIVQHIYK.T | 2 |
| \* | AstrinIP\_MS2\_022614\_01.07413.07413.3 | 3.1639 | 0.2336 | 99.1% | 1244.6044 | 1244.4331 | 7 | 5.11 | 55.6% | 1 | K.LDDIVQHIYK.T | 3 |
| \* | AstrinIP\_MS1\_022614\_01.10322.10322.2 | 2.8109 | 0.3081 | 99.8% | 1127.1522 | 1127.3696 | 1 | 5.729 | 83.3% | 9 | K.TLLSIPEVVR.G | 2 |
| \* | AstrinIP\_MS1\_022614\_01.10158.10158.1 | 1.9365 | 0.1954 | 96.3% | 1128.62 | 1127.3696 | 36 | 3.825 | 50.0% | 4 | K.TLLSIPEVVR.G | 1 |
| \* | AstrinIP\_MS1\_022614\_01.14505.14505.2 | 3.2348 | 0.4491 | 100.0% | 1494.2722 | 1494.6941 | 1 | 8.006 | 75.0% | 2 | R.GCKELQGLLEFLS.- | 2 |
| \* | AstrinIP\_MS1\_022614\_01.15854.15854.1 | 2.0011 | 0.3561 | 100.0% | 1148.52 | 1149.3293 | 1 | 5.876 | 61.1% | 8 | K.ELQGLLEFLS.- | 1 |
| \* | AstrinIP\_MS2\_022614\_01.16244.16244.2 | 2.6394 | 0.409 | 100.0% | 1149.1322 | 1149.3293 | 1 | 6.738 | 66.7% | 2 | K.ELQGLLEFLS.- | 2 |

---

|  |  |  |  |  |  |  |  |  |
| --- | --- | --- | --- | --- | --- | --- | --- | --- |
| U | *gi|4505813|ref|NP\_003* | 11 | 66 | 84.3% | 89 | 10366 | 7.4 | dynein light chain 1 [Homo sapiens] |
| U | *gi|83267868|ref|NP\_00* | 11 | 66 | 84.3% | 89 | 10366 | 7.4 | dynein light chain 1 [Homo sapiens] |
| U | *gi|83267866|ref|NP\_00* | 11 | 66 | 84.3% | 89 | 10366 | 7.4 | dynein light chain 1 [Homo sapiens] |

| Filename XCorr DeltCN Conf% ObsM+H+ CalcM+H+ SpR ZScore Ion% # Sequence  | | | | | | | | | | | | |
| --- | --- | --- | --- | --- | --- | --- | --- | --- | --- | --- | --- | --- |
|  | AstrinIP\_MS1\_022614\_01.09394.09394.2 | 5.9069 | 0.5232 | 100.0% | 2514.3123 | 2515.6462 | 1 | 11.407 | 59.5% | 3 | K.NADMSEEMQQDSVECATQALEK.Y | 2 |
|  | AstrinIP\_MS1\_022614\_02.06595.06595.3 | 6.0162 | 0.4855 | 100.0% | 2515.0444 | 2515.6462 | 1 | 9.507 | 45.2% | 4 | K.NADMSEEMQQDSVECATQALEK.Y | 3 |
|  | AstrinIP\_MS1\_022614\_01.07503.07503.2 | 4.255 | 0.3243 | 100.0% | 1414.8922 | 1415.6322 | 1 | 6.89 | 81.8% | 5 | K.YNIEKDIAAHIK.K | 2 |
|  | AstrinIP\_MS2\_022614\_01.07920.07920.3 | 3.2896 | 0.2877 | 99.8% | 1415.3644 | 1415.6322 | 2 | 5.042 | 56.8% | 1 | K.YNIEKDIAAHIK.K | 3 |
|  | AstrinIP\_MS2\_022614\_01.06728.06728.2 | 4.2899 | 0.4176 | 100.0% | 1543.3121 | 1543.8064 | 1 | 7.272 | 75.0% | 2 | K.YNIEKDIAAHIKK.E | 2 |
|  | AstrinIP\_MS2\_022614\_01.06723.06723.3 | 4.3648 | 0.3515 | 100.0% | 1543.5844 | 1543.8064 | 1 | 6.284 | 47.9% | 5 | K.YNIEKDIAAHIKK.E | 3 |
|  | AstrinIP\_MS1\_022614\_01.06758.06758.2 | 3.4596 | 0.4052 | 100.0% | 1404.1122 | 1403.5493 | 1 | 7.521 | 75.0% | 2 | K.YNPTWHCIVGR.N | 22 |
|  | AstrinIP\_MS2\_022614\_01.04534.04534.2 | 3.008 | 0.2354 | 99.7% | 1285.1921 | 1283.383 | 1 | 5.574 | 70.0% | 3 | R.NFGSYVTHETK.H | 22 |
|  | AstrinIP\_MS2\_022614\_01.18886.18886.3 | 5.6956 | 0.5088 | 100.0% | 3236.8442 | 3237.771 | 1 | 8.289 | 36.5% | 23 | R.NFGSYVTHETKHFIYFYLGQVAILLFK.S | 33 |
|  | AstrinIP\_MS1\_022614\_02.12331.12331.3 | 4.9276 | 0.3904 | 100.0% | 3381.8044 | 3381.9011 | 1 | 5.718 | 31.2% | 12 | R.NFGSYVTHETKHFIYFYLGQVAILLFKSG.- | 33 |
|  | AstrinIP\_MS2\_022614\_01.18827.18827.2 | 4.6597 | 0.6023 | 100.0% | 1972.6122 | 1973.4111 | 1 | 10.006 | 56.7% | 6 | K.HFIYFYLGQVAILLFK.S | 22 |

Similarities:
gi|18087855|ref|NP\_54(5:6)  

---

|  |  |  |  |  |  |  |  |  |
| --- | --- | --- | --- | --- | --- | --- | --- | --- |
| U | *gi|57242777|ref|NP\_03* | 11 | 38 | 73.8% | 103 | 11967 | 5.9 | c-myc binding protein [Homo sapiens] |

| Filename XCorr DeltCN Conf% ObsM+H+ CalcM+H+ SpR ZScore Ion% # Sequence  | | | | | | | | | | | | |
| --- | --- | --- | --- | --- | --- | --- | --- | --- | --- | --- | --- | --- |
| \* | AstrinIP\_MS1\_022614\_01.07202.07202.1 | 1.6463 | 0.2788 | 99.1% | 933.4 | 934.07764 | 11 | 5.122 | 56.2% | 2 | K.SGVLDTLTK.V | 1 |
| \* | AstrinIP\_MS2\_022614\_01.07502.07502.2 | 2.8219 | 0.2671 | 99.8% | 934.0122 | 934.07764 | 3 | 5.043 | 75.0% | 4 | K.SGVLDTLTK.V | 2 |
| \* | AstrinIP\_MS1\_022614\_01.11618.11618.2 | 4.8539 | 0.482 | 100.0% | 2275.5723 | 2276.6348 | 1 | 8.978 | 57.9% | 4 | K.VLVALYEEPEKPNSALDFLK.H | 2 |
| \* | AstrinIP\_MS2\_022614\_02.07395.07395.3 | 4.7956 | 0.5037 | 100.0% | 2276.3342 | 2276.6348 | 1 | 8.236 | 38.2% | 8 | K.VLVALYEEPEKPNSALDFLK.H | 3 |
| \* | AstrinIP\_MS1\_022614\_01.07118.07118.3 | 3.9762 | 0.2908 | 99.9% | 1897.7943 | 1898.1289 | 4 | 5.478 | 42.2% | 3 | K.HHLGAATPENPEIELLR.L | 3 |
| \* | AstrinIP\_MS2\_022614\_01.07563.07563.2 | 4.3343 | 0.3195 | 100.0% | 1898.3922 | 1898.1289 | 1 | 6.821 | 71.9% | 4 | K.HHLGAATPENPEIELLR.L | 2 |
| \* | AstrinIP\_MS1\_022614\_01.11397.11397.3 | 3.1022 | 0.2673 | 98.2% | 2711.3643 | 2713.124 | 1 | 4.689 | 28.3% | 1 | K.HHLGAATPENPEIELLRLELAEMK.E | 3 |
| \* | AstrinIP\_MS2\_022614\_02.06255.06255.3 | 4.6175 | 0.346 | 100.0% | 2295.4443 | 2295.6533 | 1 | 5.472 | 40.3% | 3 | R.LELAEMKEKYEAIVEENKK.L | 3 |
| \* | AstrinIP\_MS1\_022614\_01.03794.03794.2 | 3.856 | 0.4097 | 100.0% | 1480.2322 | 1480.658 | 6 | 6.881 | 63.6% | 2 | K.EKYEAIVEENKK.L | 2 |
| \* | AstrinIP\_MS2\_022614\_01.03710.03710.2 | 3.0158 | 0.2761 | 99.8% | 1224.1322 | 1223.3684 | 1 | 5.907 | 88.9% | 1 | K.YEAIVEENKK.L | 2 |
| \* | AstrinIP\_MS1\_022614\_01.04118.04118.2 | 3.3912 | 0.2433 | 99.8% | 1332.0322 | 1332.4528 | 3 | 5.665 | 65.0% | 6 | K.LAQYEPPQEEK.R | 2 |

---

|  |  |  |  |  |  |  |  |  |
| --- | --- | --- | --- | --- | --- | --- | --- | --- |
| U | *gi|150456457|ref|NP\_9* | 34 | 157 | 70.3% | 347 | 39929 | 5.6 | HMT1 hnRNP methyltransferase-like 2 isoform 2 [Homo sapiens] |
| U | *gi|154759421|ref|NP\_0* | 34 | 157 | 65.8% | 371 | 42462 | 5.3 | HMT1 hnRNP methyltransferase-like 2 isoform 1 [Homo sapiens] |
| U | *gi|151301219|ref|NP\_9* | 34 | 157 | 69.1% | 353 | 40548 | 5.5 | HMT1 hnRNP methyltransferase-like 2 isoform 3 [Homo sapiens] |

| Filename XCorr DeltCN Conf% ObsM+H+ CalcM+H+ SpR ZScore Ion% # Sequence  | | | | | | | | | | | | |
| --- | --- | --- | --- | --- | --- | --- | --- | --- | --- | --- | --- | --- |
|  | AstrinIP\_MS2\_022614\_02.07336.07336.3 | 3.9822 | 0.4443 | 100.0% | 2266.4343 | 2266.489 | 1 | 6.553 | 38.2% | 2 | K.DYYFDSYAHFGIHEEMLK.D | 3 |
|  | AstrinIP\_MS2\_022614\_01.10786.10786.3 | 5.7399 | 0.4798 | 100.0% | 2765.0942 | 2766.0132 | 1 | 9.358 | 42.9% | 4 | K.DYYFDSYAHFGIHEEMLKDEVR.T | 3 |
|  | AstrinIP\_MS1\_022614\_02.09387.09387.2 | 5.599 | 0.3636 | 100.0% | 1838.3522 | 1839.1665 | 1 | 9.781 | 76.5% | 4 | K.VVLDVGSGTGILCMFAAK.A | 2 |
|  | AstrinIP\_MS1\_022614\_02.05624.05624.2 | 4.0734 | 0.4925 | 100.0% | 1769.1522 | 1769.999 | 1 | 7.745 | 53.3% | 2 | R.KVIGIECSSISDYAVK.I | 2 |
|  | AstrinIP\_MS1\_022614\_01.08768.08768.2 | 4.7529 | 0.583 | 100.0% | 1641.0721 | 1641.825 | 1 | 10.547 | 78.6% | 5 | K.VIGIECSSISDYAVK.I | 2 |
|  | AstrinIP\_MS1\_022614\_01.05465.05465.1 | 2.4014 | 0.3473 | 100.0% | 1350.75 | 1351.6322 | 10 | 7.099 | 54.5% | 2 | K.ANKLDHVVTIIK.G | 1 |
|  | AstrinIP\_MS2\_022614\_01.05487.05487.2 | 3.5365 | 0.3062 | 100.0% | 1350.9122 | 1351.6322 | 1 | 6.432 | 68.2% | 8 | K.ANKLDHVVTIIK.G | 2 |
|  | AstrinIP\_MS2\_022614\_01.05522.05522.3 | 4.7075 | 0.4344 | 100.0% | 1351.6144 | 1351.6322 | 1 | 7.951 | 61.4% | 9 | K.ANKLDHVVTIIK.G | 3 |
|  | AstrinIP\_MS1\_022614\_01.05514.05514.1 | 2.1191 | 0.2518 | 99.0% | 1037.6 | 1038.2755 | 4 | 6.453 | 62.5% | 3 | K.LDHVVTIIK.G | 1 |
|  | AstrinIP\_MS2\_022614\_01.05690.05690.2 | 2.7381 | 0.2985 | 99.8% | 1038.1721 | 1038.2755 | 1 | 5.333 | 87.5% | 4 | K.LDHVVTIIK.G | 2 |
|  | AstrinIP\_MS2\_022614\_01.05769.05769.2 | 4.0198 | 0.4017 | 100.0% | 1356.0521 | 1356.559 | 1 | 7.664 | 72.7% | 6 | K.GKVEEVELPVEK.V | 2 |
|  | AstrinIP\_MS2\_022614\_01.11870.11870.2 | 3.9373 | 0.4127 | 100.0% | 1643.1721 | 1643.8827 | 1 | 6.68 | 76.9% | 5 | R.DKWLAPDGLIFPDR.A | 2 |
|  | AstrinIP\_MS2\_022614\_01.11859.11859.3 | 3.335 | 0.2613 | 99.3% | 1644.5044 | 1643.8827 | 3 | 4.589 | 44.2% | 1 | R.DKWLAPDGLIFPDR.A | 3 |
|  | AstrinIP\_MS2\_022614\_01.13599.13599.3 | 4.0914 | 0.4927 | 100.0% | 2876.7544 | 2877.2695 | 1 | 7.246 | 30.2% | 3 | R.DKWLAPDGLIFPDRATLYVTAIEDR.Q | 3 |
|  | AstrinIP\_MS1\_022614\_01.12065.12065.2 | 3.3967 | 0.4255 | 100.0% | 1401.0322 | 1400.6201 | 1 | 6.398 | 77.3% | 3 | K.WLAPDGLIFPDR.A | 2 |
|  | AstrinIP\_MS1\_022614\_01.13560.13560.3 | 3.1355 | 0.2383 | 96.9% | 2633.6042 | 2634.0068 | 1 | 4.662 | 29.5% | 1 | K.WLAPDGLIFPDRATLYVTAIEDR.Q | 32 |
|  | AstrinIP\_MS2\_022614\_01.08541.08541.1 | 2.4844 | 0.4572 | 100.0% | 1251.61 | 1252.4099 | 1 | 7.745 | 60.0% | 4 | R.ATLYVTAIEDR.Q | 1 |
|  | AstrinIP\_MS2\_022614\_01.08606.08606.2 | 3.698 | 0.499 | 100.0% | 1252.3522 | 1252.4099 | 1 | 8.595 | 70.0% | 14 | R.ATLYVTAIEDR.Q | 2 |
|  | AstrinIP\_MS1\_022614\_01.12456.12456.2 | 4.8219 | 0.56 | 100.0% | 2085.4722 | 2086.3381 | 1 | 9.468 | 76.7% | 1 | K.IHWWENVYGFDMSCIK.D | 2 |
|  | AstrinIP\_MS2\_022614\_01.09356.09356.2 | 4.0252 | 0.4007 | 100.0% | 1637.5122 | 1637.914 | 1 | 7.433 | 75.0% | 4 | K.DVAIKEPLVDVVDPK.Q | 2 |
|  | AstrinIP\_MS1\_022614\_01.08808.08808.3 | 4.0459 | 0.379 | 100.0% | 1637.5443 | 1637.914 | 1 | 6.235 | 42.9% | 2 | K.DVAIKEPLVDVVDPK.Q | 3 |
|  | AstrinIP\_MS1\_022614\_01.06593.06593.2 | 2.2905 | 0.2908 | 98.8% | 1159.6122 | 1160.3655 | 44 | 6.22 | 61.1% | 1 | K.QLVTNACLIK.E | 2 |
|  | AstrinIP\_MS1\_022614\_01.06090.06090.1 | 1.9368 | 0.2407 | 99.1% | 966.52 | 967.107 | 35 | 4.763 | 57.1% | 2 | K.EVDIYTVK.V | 1 |
|  | AstrinIP\_MS1\_022614\_01.12130.12130.2 | 3.9424 | 0.4397 | 100.0% | 1784.3322 | 1785.0131 | 1 | 7.458 | 57.1% | 2 | K.VEDLTFTSPFCLQVK.R | 2 |
|  | AstrinIP\_MS1\_022614\_01.13992.13992.2 | 5.506 | 0.5518 | 100.0% | 2228.9521 | 2229.5027 | 1 | 9.625 | 67.6% | 5 | K.RNDYVHALVAYFNIEFTR.C | 2 |
|  | AstrinIP\_MS2\_022614\_02.09932.09932.3 | 5.6392 | 0.3744 | 100.0% | 2230.0745 | 2229.5027 | 1 | 9.184 | 51.5% | 10 | K.RNDYVHALVAYFNIEFTR.C | 3 |
|  | AstrinIP\_MS2\_022614\_02.10772.10772.3 | 3.8466 | 0.4324 | 100.0% | 2072.4543 | 2073.3152 | 1 | 7.045 | 43.8% | 2 | R.NDYVHALVAYFNIEFTR.C | 3 |
|  | AstrinIP\_MS2\_022614\_01.15808.15808.2 | 5.5933 | 0.6045 | 100.0% | 2072.5122 | 2073.3152 | 1 | 10.304 | 75.0% | 4 | R.NDYVHALVAYFNIEFTR.C | 2 |
|  | AstrinIP\_MS2\_022614\_01.07292.07292.2 | 3.6137 | 0.4566 | 100.0% | 1725.1721 | 1725.8547 | 1 | 7.163 | 67.9% | 5 | R.TGFSTSPESPYTHWK.Q | 2 |
|  | AstrinIP\_MS2\_022614\_01.07263.07263.3 | 2.7425 | 0.4543 | 100.0% | 1725.7144 | 1725.8547 | 13 | 6.343 | 33.9% | 3 | R.TGFSTSPESPYTHWK.Q | 3 |
|  | AstrinIP\_MS1\_022614\_02.08221.08221.2 | 4.0451 | 0.4192 | 100.0% | 1637.1921 | 1637.8878 | 1 | 8.282 | 70.8% | 20 | K.QTVFYMEDYLTVK.T | 2 |
|  | AstrinIP\_MS2\_022614\_02.06036.06036.2 | 4.1698 | 0.3586 | 100.0% | 1721.3522 | 1721.969 | 1 | 7.226 | 56.7% | 6 | K.TGEEIFGTIGMRPNAK.N | 2 |
|  | AstrinIP\_MS2\_022614\_02.06056.06056.3 | 3.3341 | 0.3725 | 100.0% | 1721.8744 | 1721.969 | 15 | 6.365 | 35.0% | 6 | K.TGEEIFGTIGMRPNAK.N | 3 |
|  | AstrinIP\_MS1\_022614\_01.06135.06135.2 | 4.1438 | 0.5344 | 100.0% | 1589.1122 | 1589.6313 | 1 | 9.203 | 75.0% | 4 | K.GQLCELSCSTDYR.M | 2 |

---

|  |  |  |  |  |  |  |  |  |
| --- | --- | --- | --- | --- | --- | --- | --- | --- |
| U | *gi|218505827|ref|NP\_1* | 46 | 281 | 63.6% | 316 | 35438 | 6.3 | TRAF4 associated factor 1 isoform a [Homo sapiens] |

| Filename XCorr DeltCN Conf% ObsM+H+ CalcM+H+ SpR ZScore Ion% # Sequence  | | | | | | | | | | | | |
| --- | --- | --- | --- | --- | --- | --- | --- | --- | --- | --- | --- | --- |
|  | AstrinIP\_MS2\_022614\_01.08552.08552.2 | 5.624 | 0.531 | 100.0% | 2274.672 | 2275.4802 | 1 | 9.591 | 64.3% | 26 | K.TVYSLQPPSALSGGQPADTQTR.A | 22 |
|  | AstrinIP\_MS1\_022614\_02.05275.05275.3 | 5.516 | 0.5174 | 100.0% | 2275.4343 | 2275.4802 | 1 | 8.435 | 51.2% | 18 | K.TVYSLQPPSALSGGQPADTQTR.A | 33 |
|  | AstrinIP\_MS2\_022614\_01.08288.08288.3 | 3.0087 | 0.3374 | 99.3% | 3411.1143 | 3408.7502 | 134 | 4.647 | 17.7% | 1 | K.TVYSLQPPSALSGGQPADTQTRATSKS\*LLPVR.S | 33 |
|  | AstrinIP\_MS2\_022614\_01.08486.08486.3 | 3.003 | 0.2502 | 96.5% | 3411.2644 | 3408.7502 | 265 | 4.86 | 18.5% | 1 | K.TVYSLQPPSALSGGQPADTQT#RATSKSLLPVR.S | 33 |
|  | AstrinIP\_MS1\_022614\_01.03945.03945.3 | 3.6499 | 0.2294 | 98.7% | 2255.7844 | 2255.4465 | 1 | 4.705 | 36.2% | 1 | R.SKEVDVSKQLHSGGPENDVTK.I | 33 |
|  | AstrinIP\_MS2\_022614\_01.03465.03465.2 | 2.2072 | 0.3877 | 99.3% | 1382.1322 | 1382.4728 | 1 | 7.573 | 75.0% | 2 | K.QLHSGGPENDVTK.I | 22 |
|  | AstrinIP\_MS2\_022614\_01.03467.03467.3 | 2.5001 | 0.2515 | 95.6% | 1382.4543 | 1382.4728 | 212 | 4.778 | 41.7% | 1 | K.QLHSGGPENDVTK.I | 33 |
|  | AstrinIP\_MS1\_022614\_01.05002.05002.2 | 3.8213 | 0.4185 | 100.0% | 2243.7922 | 2244.507 | 2 | 6.577 | 41.7% | 5 | K.QKSEEELKDKNQLLEAVNK.Q | 22 |
|  | AstrinIP\_MS2\_022614\_01.04965.04965.3 | 3.5796 | 0.3012 | 99.8% | 2244.2944 | 2244.507 | 5 | 6.245 | 31.9% | 12 | K.QKSEEELKDKNQLLEAVNK.Q | 33 |
|  | AstrinIP\_MS2\_022614\_01.06431.06431.2 | 5.2517 | 0.447 | 100.0% | 1987.4922 | 1988.2023 | 1 | 9.133 | 65.6% | 8 | K.SEEELKDKNQLLEAVNK.Q | 22 |
|  | AstrinIP\_MS1\_022614\_02.04897.04897.3 | 5.2827 | 0.5339 | 100.0% | 1987.7344 | 1988.2023 | 1 | 8.958 | 40.6% | 12 | K.SEEELKDKNQLLEAVNK.Q | 33 |
|  | AstrinIP\_MS1\_022614\_01.05512.05512.1 | 2.1761 | 0.2415 | 99.1% | 1271.52 | 1272.4441 | 1 | 5.11 | 65.0% | 1 | K.DKNQLLEAVNK.Q | 11 |
|  | AstrinIP\_MS2\_022614\_01.05732.05732.2 | 3.8153 | 0.2607 | 100.0% | 1272.3121 | 1272.4441 | 1 | 5.812 | 85.0% | 5 | K.DKNQLLEAVNK.Q | 22 |
|  | AstrinIP\_MS2\_022614\_01.05985.05985.1 | 2.1877 | 0.2289 | 99.1% | 1028.53 | 1029.1814 | 20 | 4.994 | 62.5% | 7 | K.NQLLEAVNK.Q | 11 |
|  | AstrinIP\_MS1\_022614\_01.05679.05679.2 | 3.2837 | 0.2346 | 99.9% | 1029.0322 | 1029.1814 | 2 | 4.95 | 87.5% | 8 | K.NQLLEAVNK.Q | 22 |
|  | AstrinIP\_MS1\_022614\_01.07854.07854.2 | 2.608 | 0.2416 | 98.4% | 1663.7922 | 1663.9175 | 1 | 5.219 | 53.8% | 1 | K.NQLLEAVNKQLHQK.L | 22 |
|  | AstrinIP\_MS2\_022614\_01.03794.03794.2 | 2.799 | 0.2947 | 99.8% | 1019.1122 | 1019.13995 | 1 | 6.817 | 87.5% | 2 | K.LTETQGELK.D | 22 |
|  | AstrinIP\_MS1\_022614\_01.05594.05594.2 | 4.388 | 0.4022 | 100.0% | 1604.4321 | 1604.7979 | 1 | 7.253 | 65.4% | 9 | K.LTETQGELKDLTQK.V | 22 |
|  | AstrinIP\_MS2\_022614\_01.05880.05880.1 | 3.5506 | 0.3994 | 100.0% | 1604.72 | 1604.7979 | 1 | 6.394 | 53.8% | 2 | K.LTETQGELKDLTQK.V | 11 |
|  | AstrinIP\_MS2\_022614\_01.05810.05810.3 | 1.9233 | 0.341 | 96.0% | 1605.0243 | 1604.7979 | 3 | 4.811 | 42.3% | 1 | K.LTETQGELKDLTQK.V | 33 |
|  | AstrinIP\_MS2\_022614\_01.11206.11206.2 | 5.9733 | 0.5075 | 100.0% | 2315.5723 | 2316.6543 | 1 | 8.935 | 55.3% | 7 | K.LTETQGELKDLTQKVELLEK.F | 22 |
|  | AstrinIP\_MS2\_022614\_01.11204.11204.3 | 5.1978 | 0.3235 | 100.0% | 2317.1943 | 2316.6543 | 1 | 7.157 | 48.7% | 10 | K.LTETQGELKDLTQKVELLEK.F | 33 |
|  | AstrinIP\_MS2\_022614\_01.08172.08172.3 | 2.6367 | 0.2285 | 96.4% | 1316.7244 | 1316.5376 | 5 | 4.985 | 50.0% | 1 | K.DLTQKVELLEK.F | 33 |
|  | AstrinIP\_MS1\_022614\_01.07719.07719.2 | 4.0724 | 0.2096 | 100.0% | 1317.2922 | 1316.5376 | 1 | 5.055 | 75.0% | 12 | K.DLTQKVELLEK.F | 22 |
|  | AstrinIP\_MS1\_022614\_01.07949.07949.3 | 3.1825 | 0.2123 | 98.3% | 1466.3644 | 1466.6434 | 36 | 4.837 | 43.2% | 1 | K.FRDNCLAILESK.G | 33 |
|  | AstrinIP\_MS1\_022614\_01.07928.07928.2 | 3.4832 | 0.3777 | 100.0% | 1466.3922 | 1466.6434 | 2 | 6.384 | 72.7% | 5 | K.FRDNCLAILESK.G | 22 |
|  | AstrinIP\_MS1\_022614\_01.08420.08420.2 | 3.7674 | 0.2248 | 100.0% | 1162.7122 | 1163.2793 | 1 | 6.503 | 77.8% | 3 | R.DNCLAILESK.G | 22 |
|  | AstrinIP\_MS1\_022614\_01.08374.08374.1 | 2.3816 | 0.1485 | 95.1% | 1164.43 | 1163.2793 | 1 | 3.52 | 66.7% | 3 | R.DNCLAILESK.G | 11 |
|  | AstrinIP\_MS1\_022614\_01.07517.07517.1 | 1.5464 | 0.3522 | 100.0% | 1386.65 | 1387.5327 | 4 | 6.414 | 50.0% | 1 | K.GLDPALGSETLASR.Q | 11 |
|  | AstrinIP\_MS1\_022614\_01.07436.07436.2 | 4.3619 | 0.6123 | 100.0% | 1387.0521 | 1387.5327 | 1 | 10.485 | 84.6% | 49 | K.GLDPALGSETLASR.Q | 22 |
|  | AstrinIP\_MS1\_022614\_01.07611.07611.2 | 2.4204 | 0.2862 | 98.5% | 1466.7322 | 1467.5327 | 3 | 5.526 | 57.7% | 1 | K.GLDPALGS\*ETLASR.Q | 22 |
|  | AstrinIP\_MS2\_022614\_01.07966.07966.2 | 2.9295 | 0.4656 | 100.0% | 1467.3522 | 1467.5327 | 1 | 6.645 | 69.2% | 1 | K.GLDPALGSET#LASR.Q | 22 |
|  | AstrinIP\_MS2\_022614\_01.14264.14264.3 | 3.9413 | 0.1871 | 97.9% | 2592.8044 | 2592.8928 | 83 | 4.273 | 28.6% | 2 | R.QESTTDHMDSMLLLETLQEELK.L | 33 |
|  | AstrinIP\_MS2\_022614\_01.14471.14471.2 | 5.1585 | 0.3552 | 100.0% | 2592.8323 | 2592.8928 | 1 | 6.259 | 45.2% | 17 | R.QESTTDHMDSMLLLETLQEELK.L | 22 |
|  | AstrinIP\_MS2\_022614\_01.16311.16311.3 | 6.0367 | 0.459 | 100.0% | 3395.1843 | 3396.8062 | 1 | 7.35 | 26.8% | 14 | R.QESTTDHMDSMLLLETLQEELKLFNETAK.K | 33 |
|  | AstrinIP\_MS2\_022614\_01.15146.15146.3 | 3.574 | 0.3752 | 100.0% | 3523.7344 | 3524.9802 | 39 | 6.169 | 19.8% | 2 | R.QESTTDHMDSMLLLETLQEELKLFNETAKK.Q | 3 |
|  | AstrinIP\_MS2\_022614\_01.03956.03956.1 | 1.8115 | 0.2288 | 98.3% | 822.35 | 822.9365 | 23 | 4.387 | 66.7% | 2 | K.LFNETAK.K | 11 |
| \* | AstrinIP\_MS1\_022614\_01.04671.04671.1 | 2.8004 | 0.1353 | 96.5% | 1217.55 | 1218.454 | 235 | 4.561 | 55.6% | 1 | K.KQMEELQALK.V | 1 |
| \* | AstrinIP\_MS1\_022614\_02.03829.03829.2 | 3.4477 | 0.2583 | 100.0% | 1218.1522 | 1218.454 | 27 | 4.944 | 72.2% | 9 | K.KQMEELQALK.V | 2 |
| \* | AstrinIP\_MS2\_022614\_01.03627.03627.1 | 2.1887 | 0.216 | 98.7% | 1161.52 | 1162.3899 | 6 | 5.022 | 68.8% | 1 | K.VKLEMKEER.V | 1 |
| \* | AstrinIP\_MS2\_022614\_01.03620.03620.2 | 3.3233 | 0.3162 | 100.0% | 1162.2322 | 1162.3899 | 1 | 6.477 | 93.8% | 1 | K.VKLEMKEER.V | 2 |
| \* | AstrinIP\_MS1\_022614\_02.07067.07067.3 | 3.479 | 0.3323 | 99.8% | 3525.2944 | 3522.9648 | 3 | 4.898 | 19.6% | 1 | K.LEMKEERVRFLEQQTLCNNQVNDLTTALK.E | 3 |
| \* | AstrinIP\_MS1\_022614\_01.17858.17858.3 | 3.5297 | 0.263 | 99.0% | 3612.1143 | 3611.086 | 4 | 4.339 | 21.6% | 1 | R.VRFLEQQTLCNNQVNDLTTALKEMEQLLEM.- | 3 |
| \* | AstrinIP\_MS1\_022614\_01.10166.10166.2 | 6.323 | 0.5723 | 100.0% | 2350.5522 | 2351.5847 | 1 | 10.59 | 63.2% | 7 | R.FLEQQTLCNNQVNDLTTALK.E | 2 |
| \* | AstrinIP\_MS1\_022614\_01.10130.10130.3 | 6.1915 | 0.4025 | 100.0% | 2351.8743 | 2351.5847 | 1 | 7.753 | 57.9% | 4 | R.FLEQQTLCNNQVNDLTTALK.E | 3 |
| \* | AstrinIP\_MS1\_022614\_01.19169.19169.3 | 3.6619 | 0.292 | 99.5% | 3356.2444 | 3355.7659 | 8 | 5.333 | 23.1% | 2 | R.FLEQQTLCNNQVNDLTTALKEMEQLLEM.- | 3 |

Similarities:
gi|218505831|ref|NP\_0(36:10)  

---

|  |  |  |  |  |  |  |  |  |
| --- | --- | --- | --- | --- | --- | --- | --- | --- |
| U | *gi|18087855|ref|NP\_54* | 7 | 50 | 59.6% | 89 | 10350 | 7.4 | dynein, light chain, LC8-type 2 [Homo sapiens] |

| Filename XCorr DeltCN Conf% ObsM+H+ CalcM+H+ SpR ZScore Ion% # Sequence  | | | | | | | | | | | | |
| --- | --- | --- | --- | --- | --- | --- | --- | --- | --- | --- | --- | --- |
| \* | AstrinIP\_MS2\_022614\_01.09329.09329.3 | 3.8132 | 0.3818 | 100.0% | 1569.6244 | 1569.8412 | 1 | 6.938 | 56.2% | 1 | K.YNIEKDIAAYIKK.E | 3 |
| \* | AstrinIP\_MS2\_022614\_01.07606.07606.1 | 1.9958 | 0.2366 | 99.0% | 793.5 | 793.93835 | 3 | 6.283 | 75.0% | 3 | K.DIAAYIK.K | 1 |
|  | AstrinIP\_MS1\_022614\_01.06758.06758.2 | 3.4596 | 0.4052 | 100.0% | 1404.1122 | 1403.5493 | 1 | 7.521 | 75.0% | 2 | K.YNPTWHCIVGR.N | 22 |
|  | AstrinIP\_MS2\_022614\_01.04534.04534.2 | 3.008 | 0.2354 | 99.7% | 1285.1921 | 1283.383 | 1 | 5.574 | 70.0% | 3 | R.NFGSYVTHETK.H | 22 |
|  | AstrinIP\_MS2\_022614\_01.18886.18886.3 | 5.6956 | 0.5088 | 100.0% | 3236.8442 | 3237.771 | 1 | 8.289 | 36.5% | 23 | R.NFGSYVTHETKHFIYFYLGQVAILLFK.S | 33 |
|  | AstrinIP\_MS1\_022614\_02.12331.12331.3 | 4.9276 | 0.3904 | 100.0% | 3381.8044 | 3381.9011 | 1 | 5.718 | 31.2% | 12 | R.NFGSYVTHETKHFIYFYLGQVAILLFKSG.- | 33 |
|  | AstrinIP\_MS2\_022614\_01.18827.18827.2 | 4.6597 | 0.6023 | 100.0% | 1972.6122 | 1973.4111 | 1 | 10.006 | 56.7% | 6 | K.HFIYFYLGQVAILLFK.S | 22 |

Similarities:
gi|4505813|ref|NP\_003(5:2)  

---

|  |  |  |  |  |  |  |  |  |
| --- | --- | --- | --- | --- | --- | --- | --- | --- |
| U | *gi|4501885|ref|NP\_001* | 21 | 46 | 56.5% | 375 | 41737 | 5.5 | beta actin [Homo sapiens] |
| U | *gi|4501887|ref|NP\_001* | 21 | 46 | 56.5% | 375 | 41793 | 5.5 | actin, gamma 1 propeptide [Homo sapiens] |

| Filename XCorr DeltCN Conf% ObsM+H+ CalcM+H+ SpR ZScore Ion% # Sequence  | | | | | | | | | | | | |
| --- | --- | --- | --- | --- | --- | --- | --- | --- | --- | --- | --- | --- |
|  | AstrinIP\_MS2\_022614\_01.04052.04052.2 | 3.1372 | 0.3107 | 100.0% | 976.3522 | 977.02136 | 1 | 6.669 | 77.8% | 1 | K.AGFAGDDAPR.A | 22 |
|  | AstrinIP\_MS2\_022614\_01.04036.04036.1 | 1.9667 | 0.3834 | 100.0% | 976.48 | 977.02136 | 6 | 5.951 | 55.6% | 1 | K.AGFAGDDAPR.A | 11 |
|  | AstrinIP\_MS1\_022614\_01.06808.06808.2 | 2.6818 | 0.321 | 99.8% | 1199.3922 | 1199.4415 | 6 | 5.253 | 60.0% | 4 | R.AVFPSIVGRPR.H | 22 |
|  | AstrinIP\_MS2\_022614\_01.03922.03922.2 | 2.2208 | 0.3178 | 98.7% | 1170.5922 | 1172.4058 | 1 | 6.061 | 70.0% | 1 | R.HQGVMVGMGQK.D | 22 |
|  | AstrinIP\_MS2\_022614\_01.03903.03903.1 | 2.5908 | 0.3969 | 100.0% | 1171.48 | 1172.4058 | 1 | 7.283 | 65.0% | 1 | R.HQGVMVGMGQK.D | 11 |
|  | AstrinIP\_MS2\_022614\_01.03548.03548.2 | 2.8969 | 0.1276 | 97.7% | 1355.0122 | 1355.4038 | 1 | 7.345 | 77.3% | 1 | K.DSYVGDEAQSKR.G | 22 |
|  | AstrinIP\_MS1\_022614\_01.06177.06177.2 | 3.2759 | 0.338 | 100.0% | 1516.3322 | 1516.7019 | 1 | 5.686 | 75.0% | 4 | K.IWHHTFYNELR.V | 22 |
|  | AstrinIP\_MS2\_022614\_01.06611.06611.3 | 2.7762 | 0.2022 | 95.4% | 1517.3944 | 1516.7019 | 10 | 5.19 | 50.0% | 1 | K.IWHHTFYNELR.V | 33 |
|  | AstrinIP\_MS1\_022614\_01.07862.07862.2 | 4.3889 | 0.3567 | 100.0% | 1954.5922 | 1955.2615 | 1 | 8.857 | 61.8% | 4 | R.VAPEEHPVLLTEAPLNPK.A | 2 |
|  | AstrinIP\_MS1\_022614\_01.10785.10785.3 | 6.4363 | 0.5656 | 100.0% | 3184.2244 | 3185.622 | 1 | 9.596 | 37.9% | 4 | R.TTGIVMDSGDGVTHTVPIYEGYALPHAILR.L | 3 |
|  | AstrinIP\_MS2\_022614\_01.11638.11638.2 | 2.5561 | 0.2653 | 98.6% | 1624.1522 | 1624.8927 | 15 | 4.862 | 42.3% | 1 | R.LDLAGRDLTDYLMK.I | 22 |
|  | AstrinIP\_MS1\_022614\_01.10119.10119.1 | 2.0248 | 0.2294 | 99.1% | 998.3 | 999.167 | 2 | 4.596 | 71.4% | 2 | R.DLTDYLMK.I | 11 |
|  | AstrinIP\_MS2\_022614\_01.05397.05397.2 | 2.9985 | 0.4844 | 100.0% | 1132.9122 | 1133.2029 | 1 | 8.627 | 88.9% | 2 | R.GYSFTTTAER.E | 2 |
|  | AstrinIP\_MS2\_022614\_01.10803.10803.2 | 4.4281 | 0.2928 | 100.0% | 1791.3722 | 1791.9554 | 2 | 8.716 | 73.3% | 6 | K.SYELPDGQVITIGNER.F | 22 |
|  | AstrinIP\_MS2\_022614\_02.06500.06500.3 | 3.2525 | 0.3108 | 99.3% | 2342.7244 | 2344.6448 | 1 | 5.081 | 28.6% | 2 | R.KDLYANTVLSGGTTMYPGIADR.M | 3 |
|  | AstrinIP\_MS2\_022614\_01.11591.11591.2 | 4.1166 | 0.4827 | 100.0% | 2215.5322 | 2216.4705 | 2 | 8.412 | 47.5% | 2 | K.DLYANTVLSGGTTMYPGIADR.M | 2 |
|  | AstrinIP\_MS2\_022614\_02.07455.07455.3 | 3.6566 | 0.3295 | 99.9% | 2216.9644 | 2216.4705 | 7 | 5.714 | 28.8% | 1 | K.DLYANTVLSGGTTMYPGIADR.M | 3 |
|  | AstrinIP\_MS1\_022614\_01.06375.06375.1 | 2.52 | 0.4797 | 100.0% | 1161.52 | 1162.3868 | 1 | 7.293 | 65.0% | 3 | K.EITALAPSTMK.I | 11 |
|  | AstrinIP\_MS2\_022614\_01.06662.06662.2 | 2.8359 | 0.3956 | 100.0% | 1162.0721 | 1162.3868 | 1 | 6.257 | 75.0% | 3 | K.EITALAPSTMK.I | 22 |
|  | AstrinIP\_MS2\_022614\_01.17754.17754.3 | 4.7034 | 0.3123 | 100.0% | 4102.6743 | 4102.6104 | 1 | 5.368 | 22.1% | 1 | K.YSVWIGGSILASLSTFQQMWISKQEYDESGPSIVHR.K | 3 |
|  | AstrinIP\_MS2\_022614\_01.04419.04419.2 | 1.9518 | 0.3269 | 96.6% | 1517.1921 | 1517.595 | 1 | 5.751 | 66.7% | 1 | K.QEYDESGPSIVHR.K | 2 |

Similarities:
gi|4501881|ref|NP\_001(13:8)  

---

|  |  |  |  |  |  |  |  |  |
| --- | --- | --- | --- | --- | --- | --- | --- | --- |
| U | *gi|20127519|ref|NP\_03* | 70 | 227 | 56.2% | 747 | 85653 | 9.2 | TPX2, microtubule-associated protein homolog [Homo sapiens] |

| Filename XCorr DeltCN Conf% ObsM+H+ CalcM+H+ SpR ZScore Ion% # Sequence  | | | | | | | | | | | | |
| --- | --- | --- | --- | --- | --- | --- | --- | --- | --- | --- | --- | --- |
| \* | AstrinIP\_MS2\_022614\_02.10014.10014.3 | 4.6398 | 0.391 | 100.0% | 3811.4944 | 3810.8867 | 1 | 6.615 | 22.7% | 2 | K.SSYSYDAPSDFINFSSLDDEGDTQNIDSWFEEK.A | 3 |
| \* | AstrinIP\_MS1\_022614\_01.07678.07678.2 | 4.7396 | 0.4451 | 100.0% | 2404.4521 | 2405.7996 | 1 | 8.689 | 52.5% | 1 | R.KANLQQAIVTPLKPVDNTYYK.E | 2 |
| \* | AstrinIP\_MS1\_022614\_01.07695.07695.3 | 5.3433 | 0.4179 | 100.0% | 2405.2444 | 2405.7996 | 1 | 8.823 | 47.5% | 5 | R.KANLQQAIVTPLKPVDNTYYK.E | 3 |
| \* | AstrinIP\_MS1\_022614\_01.08775.08775.2 | 4.8721 | 0.5495 | 100.0% | 2276.4722 | 2277.6255 | 1 | 9.633 | 52.6% | 3 | K.ANLQQAIVTPLKPVDNTYYK.E | 2 |
| \* | AstrinIP\_MS1\_022614\_01.08776.08776.3 | 4.1651 | 0.4447 | 100.0% | 2278.8843 | 2277.6255 | 1 | 7.064 | 32.9% | 5 | K.ANLQQAIVTPLKPVDNTYYK.E | 3 |
| \* | AstrinIP\_MS2\_022614\_01.03599.03599.1 | 1.9249 | 0.2718 | 99.0% | 1092.46 | 1093.1327 | 47 | 4.774 | 56.2% | 1 | K.STEEQELEK.S | 1 |
| \* | AstrinIP\_MS2\_022614\_01.04857.04857.1 | 2.2504 | 0.2856 | 100.0% | 1149.56 | 1150.3534 | 5 | 5.457 | 68.8% | 1 | K.MQQEVVEMR.K | 1 |
| \* | AstrinIP\_MS2\_022614\_01.04893.04893.2 | 3.4648 | 0.4406 | 100.0% | 1150.0322 | 1150.3534 | 1 | 7.802 | 87.5% | 4 | K.MQQEVVEMR.K | 2 |
| \* | AstrinIP\_MS2\_022614\_01.04607.04607.2 | 2.8108 | 0.2506 | 99.2% | 1324.1721 | 1323.6652 | 1 | 4.823 | 66.7% | 1 | K.KLALAGIGQPVKK.S | 2 |
| \* | AstrinIP\_MS2\_022614\_01.08036.08036.2 | 3.0987 | 0.4169 | 100.0% | 1066.5521 | 1067.317 | 1 | 7.858 | 85.0% | 2 | K.LALAGIGQPVK.K | 2 |
| \* | AstrinIP\_MS2\_022614\_01.06171.06171.1 | 2.2437 | 0.4259 | 100.0% | 1194.61 | 1195.4911 | 5 | 6.679 | 54.5% | 2 | K.LALAGIGQPVKK.S | 1 |
| \* | AstrinIP\_MS1\_022614\_01.05871.05871.2 | 3.3658 | 0.3305 | 100.0% | 1196.1322 | 1195.4911 | 2 | 6.345 | 63.6% | 7 | K.LALAGIGQPVKK.S | 2 |
| \* | AstrinIP\_MS1\_022614\_01.06246.06246.2 | 1.9826 | 0.3226 | 98.8% | 908.0122 | 908.0043 | 16 | 6.299 | 66.7% | 2 | K.SVDFHFR.T | 2 |
| \* | AstrinIP\_MS2\_022614\_01.07900.07900.2 | 5.078 | 0.4497 | 100.0% | 1887.3522 | 1887.013 | 1 | 7.971 | 75.0% | 4 | K.NQEEYKEVNFTSELR.K | 2 |
| \* | AstrinIP\_MS1\_022614\_01.07551.07551.3 | 4.4822 | 0.3629 | 100.0% | 1887.6543 | 1887.013 | 1 | 6.578 | 50.0% | 8 | K.NQEEYKEVNFTSELR.K | 3 |
| \* | AstrinIP\_MS2\_022614\_01.06656.06656.3 | 3.4628 | 0.298 | 99.8% | 2014.8544 | 2015.187 | 3 | 5.03 | 41.7% | 2 | K.NQEEYKEVNFTSELRK.H | 3 |
| \* | AstrinIP\_MS1\_022614\_01.07589.07589.2 | 2.1579 | 0.1991 | 95.1% | 1095.2122 | 1095.1974 | 5 | 5.079 | 68.8% | 2 | K.EVNFTSELR.K | 2 |
| \* | AstrinIP\_MS1\_022614\_01.06651.06651.2 | 3.5916 | 0.4332 | 100.0% | 1548.6322 | 1549.7765 | 1 | 7.155 | 69.2% | 2 | K.GCTIVKPFNLSQGK.K | 2 |
| \* | AstrinIP\_MS2\_022614\_01.12090.12090.3 | 3.7636 | 0.4099 | 100.0% | 2454.8342 | 2455.683 | 1 | 6.58 | 36.2% | 1 | R.TFDETVSTYVPLAQQVEDFHK.R | 3 |
| \* | AstrinIP\_MS1\_022614\_01.10625.10625.2 | 3.7433 | 0.519 | 100.0% | 2611.0923 | 2611.8706 | 1 | 8.422 | 40.5% | 4 | R.TFDETVSTYVPLAQQVEDFHKR.T | 2 |
| \* | AstrinIP\_MS1\_022614\_01.10629.10629.3 | 3.8858 | 0.4129 | 100.0% | 2611.9143 | 2611.8706 | 1 | 6.213 | 39.3% | 2 | R.TFDETVSTYVPLAQQVEDFHKR.T | 3 |
| \* | AstrinIP\_MS1\_022614\_01.04384.04384.2 | 3.9178 | 0.2449 | 100.0% | 1357.8722 | 1358.5779 | 1 | 6.529 | 77.3% | 4 | R.SKKDDINLLPSK.S | 2 |
| \* | AstrinIP\_MS1\_022614\_01.04332.04332.3 | 3.7321 | 0.156 | 98.2% | 1359.1444 | 1358.5779 | 3 | 4.356 | 52.3% | 2 | R.SKKDDINLLPSK.S | 3 |
| \* | AstrinIP\_MS2\_022614\_01.05835.05835.2 | 2.9136 | 0.2637 | 99.8% | 1143.4922 | 1143.3256 | 1 | 4.949 | 83.3% | 4 | K.KDDINLLPSK.S | 2 |
| \* | AstrinIP\_MS1\_022614\_01.04049.04049.2 | 3.7012 | 0.3636 | 100.0% | 1556.4922 | 1556.7686 | 1 | 6.449 | 70.8% | 2 | K.ICRDPQTPVLQTK.H | 2 |
| \* | AstrinIP\_MS2\_022614\_01.06484.06484.1 | 2.5128 | 0.2801 | 100.0% | 1348.45 | 1349.4344 | 12 | 5.488 | 54.5% | 2 | K.STAELEAEELEK.L | 1 |
| \* | AstrinIP\_MS2\_022614\_01.06518.06518.2 | 4.0747 | 0.2758 | 100.0% | 1349.0322 | 1349.4344 | 2 | 5.96 | 77.3% | 7 | K.STAELEAEELEK.L | 2 |
| \* | AstrinIP\_MS1\_022614\_01.09256.09256.2 | 5.6275 | 0.5352 | 100.0% | 2009.4122 | 2010.2053 | 1 | 9.407 | 65.6% | 4 | K.STAELEAEELEKLQQYK.F | 2 |
| \* | AstrinIP\_MS2\_022614\_02.06526.06526.3 | 3.5092 | 0.3244 | 99.9% | 2010.8344 | 2010.2053 | 1 | 7.29 | 35.9% | 1 | K.STAELEAEELEKLQQYK.F | 3 |
| \* | AstrinIP\_MS2\_022614\_01.07756.07756.2 | 3.489 | 0.318 | 100.0% | 1037.2522 | 1037.2877 | 1 | 6.113 | 83.3% | 8 | R.ILEGGPILPK.K | 2 |
| \* | AstrinIP\_MS2\_022614\_01.08992.08992.2 | 4.2063 | 0.5033 | 100.0% | 2134.872 | 2135.5083 | 1 | 9.203 | 61.1% | 2 | K.KPPVKPPTEPIGFDLEIEK.R | 2 |
| \* | AstrinIP\_MS2\_022614\_01.09056.09056.3 | 4.2665 | 0.4667 | 100.0% | 2135.4243 | 2135.5083 | 1 | 6.66 | 50.0% | 8 | K.KPPVKPPTEPIGFDLEIEK.R | 3 |
| \* | AstrinIP\_MS1\_022614\_01.07666.07666.2 | 3.7128 | 0.4866 | 100.0% | 2290.392 | 2291.6958 | 1 | 8.732 | 50.0% | 1 | K.KPPVKPPTEPIGFDLEIEKR.I | 2 |
| \* | AstrinIP\_MS1\_022614\_01.07665.07665.3 | 5.4295 | 0.5133 | 100.0% | 2291.2144 | 2291.6958 | 1 | 8.591 | 44.7% | 7 | K.KPPVKPPTEPIGFDLEIEKR.I | 3 |
| \* | AstrinIP\_MS1\_022614\_01.03658.03658.2 | 4.7578 | 0.4487 | 100.0% | 2273.5522 | 2274.4639 | 1 | 9.119 | 55.9% | 1 | K.KKTEDEHFEFHSRPCPTK.I | 2 |
| \* | AstrinIP\_MS2\_022614\_01.07923.07923.2 | 3.735 | 0.4049 | 100.0% | 1198.5521 | 1198.402 | 1 | 7.21 | 85.0% | 4 | K.ILEDVVGVPEK.K | 2 |
| \* | AstrinIP\_MS2\_022614\_01.06477.06477.2 | 3.7808 | 0.2957 | 100.0% | 1326.2322 | 1326.576 | 1 | 5.976 | 77.3% | 8 | K.ILEDVVGVPEKK.V | 2 |
| \* | AstrinIP\_MS2\_022614\_01.06548.06548.3 | 3.2353 | 0.3375 | 100.0% | 1327.1344 | 1326.576 | 2 | 5.792 | 47.7% | 3 | K.ILEDVVGVPEKK.V | 3 |
| \* | AstrinIP\_MS1\_022614\_01.10329.10329.2 | 3.9888 | 0.5225 | 100.0% | 1661.4122 | 1661.9823 | 1 | 8.015 | 67.9% | 3 | K.VLPITVPKS\*PAFALK.N | 2 |
| \* | AstrinIP\_MS2\_022614\_01.05678.05678.1 | 1.8089 | 0.2566 | 99.0% | 733.39 | 733.8858 | 4 | 5.162 | 75.0% | 2 | K.SPAFALK.N | 1 |
| \* | AstrinIP\_MS1\_022614\_01.05934.05934.2 | 4.212 | 0.4857 | 100.0% | 2157.412 | 2158.4285 | 1 | 8.128 | 55.9% | 1 | R.IRMPTKEDEEEDEPVVIK.A | 2 |
| \* | AstrinIP\_MS2\_022614\_01.06305.06305.3 | 5.1504 | 0.2843 | 100.0% | 2157.7144 | 2158.4285 | 1 | 7.081 | 42.6% | 7 | R.IRMPTKEDEEEDEPVVIK.A | 3 |
| \* | AstrinIP\_MS2\_022614\_01.05117.05117.2 | 5.3279 | 0.4055 | 100.0% | 1888.5322 | 1889.0815 | 1 | 8.47 | 80.0% | 6 | R.MPTKEDEEEDEPVVIK.A | 2 |
| \* | AstrinIP\_MS2\_022614\_01.05150.05150.3 | 5.6037 | 0.3985 | 100.0% | 1888.6144 | 1889.0815 | 1 | 7.054 | 53.3% | 9 | R.MPTKEDEEEDEPVVIK.A | 3 |
| \* | AstrinIP\_MS2\_022614\_01.05668.05668.2 | 2.9808 | 0.3253 | 99.8% | 1431.0922 | 1431.4932 | 1 | 5.642 | 68.2% | 1 | K.EDEEEDEPVVIK.A | 2 |
| \* | AstrinIP\_MS1\_022614\_01.07510.07510.2 | 4.2334 | 0.4404 | 100.0% | 2132.5923 | 2132.473 | 1 | 8.547 | 58.3% | 4 | K.AQPVPHYGVPFKPQIPEAR.T | 2 |
| \* | AstrinIP\_MS2\_022614\_01.07887.07887.3 | 3.2943 | 0.4056 | 100.0% | 2133.5942 | 2132.473 | 5 | 6.794 | 34.7% | 4 | K.AQPVPHYGVPFKPQIPEAR.T | 3 |
| \* | AstrinIP\_MS1\_022614\_01.10346.10346.2 | 3.2327 | 0.4748 | 100.0% | 1458.1921 | 1458.5769 | 1 | 7.282 | 77.3% | 3 | R.TVEICPFSFDSR.D | 2 |
| \* | AstrinIP\_MS2\_022614\_01.11105.11105.2 | 2.9747 | 0.4068 | 100.0% | 1705.3322 | 1705.9945 | 1 | 7.301 | 67.9% | 1 | K.ALPLPHFDTINLPEK.K | 2 |
| \* | AstrinIP\_MS1\_022614\_01.09232.09232.2 | 3.6338 | 0.3049 | 100.0% | 1832.6322 | 1834.1686 | 1 | 6.883 | 56.7% | 2 | K.ALPLPHFDTINLPEKK.V | 2 |
| \* | AstrinIP\_MS2\_022614\_01.03942.03942.2 | 3.1103 | 0.2243 | 99.9% | 1054.1122 | 1054.1478 | 3 | 5.625 | 78.6% | 2 | K.HQLEEELR.Q | 2 |
| \* | AstrinIP\_MS1\_022614\_01.03719.03719.2 | 3.7585 | 0.3689 | 100.0% | 1438.2722 | 1438.5834 | 1 | 6.763 | 85.0% | 2 | K.HQLEEELRQQK.E | 2 |
| \* | AstrinIP\_MS1\_022614\_01.03722.03722.3 | 3.0048 | 0.2653 | 99.1% | 1438.3444 | 1438.5834 | 5 | 5.19 | 42.5% | 1 | K.HQLEEELRQQK.E | 3 |
| \* | AstrinIP\_MS1\_022614\_01.06712.06712.2 | 3.7302 | 0.2801 | 99.9% | 1683.3722 | 1683.9481 | 3 | 5.743 | 57.1% | 6 | K.ARPNTVISQEPFVPK.K | 2 |
| \* | AstrinIP\_MS1\_022614\_01.05106.05106.3 | 4.6843 | 0.3798 | 100.0% | 1811.5443 | 1812.1222 | 1 | 7.108 | 48.3% | 8 | K.ARPNTVISQEPFVPKK.E | 3 |
| \* | AstrinIP\_MS1\_022614\_01.05154.05154.2 | 2.5724 | 0.3831 | 99.8% | 1811.8121 | 1812.1222 | 1 | 6.193 | 53.3% | 1 | K.ARPNTVISQEPFVPKK.E | 2 |
| \* | AstrinIP\_MS2\_022614\_01.04455.04455.3 | 3.9697 | 0.263 | 99.7% | 2069.6943 | 2069.4116 | 6 | 4.528 | 38.2% | 2 | K.ARPNTVISQEPFVPKKEK.K | 3 |
| \* | AstrinIP\_MS2\_022614\_01.11092.11092.2 | 6.1658 | 0.5663 | 100.0% | 2318.632 | 2319.617 | 1 | 10.498 | 64.3% | 2 | K.KSVAEGLSGSLVQEPFQLATEK.R | 2 |
| \* | AstrinIP\_MS2\_022614\_01.11076.11076.3 | 5.8678 | 0.3715 | 100.0% | 2320.1343 | 2319.617 | 1 | 6.762 | 44.0% | 2 | K.KSVAEGLSGSLVQEPFQLATEK.R | 3 |
| \* | AstrinIP\_MS1\_022614\_01.09674.09674.3 | 5.8494 | 0.4898 | 100.0% | 2475.6843 | 2475.8044 | 1 | 8.474 | 46.6% | 3 | K.KSVAEGLSGSLVQEPFQLATEKR.A | 3 |
| \* | AstrinIP\_MS1\_022614\_01.11435.11435.2 | 6.1693 | 0.5699 | 100.0% | 2190.5522 | 2191.4429 | 1 | 11.006 | 57.5% | 3 | K.SVAEGLSGSLVQEPFQLATEK.R | 2 |
| \* | AstrinIP\_MS1\_022614\_01.11436.11436.3 | 4.5058 | 0.337 | 100.0% | 2192.9043 | 2191.4429 | 1 | 6.443 | 37.5% | 4 | K.SVAEGLSGSLVQEPFQLATEK.R | 3 |
| \* | AstrinIP\_MS2\_022614\_01.11193.11193.2 | 4.838 | 0.5717 | 100.0% | 2347.632 | 2347.6304 | 1 | 9.513 | 47.6% | 2 | K.SVAEGLSGSLVQEPFQLATEKR.A | 2 |
| \* | AstrinIP\_MS1\_022614\_01.10526.10526.3 | 4.4897 | 0.3939 | 100.0% | 2348.0344 | 2347.6304 | 1 | 7.532 | 32.1% | 4 | K.SVAEGLSGSLVQEPFQLATEKR.A | 3 |
| \* | AstrinIP\_MS1\_022614\_01.04932.04932.3 | 4.1403 | 0.3928 | 100.0% | 1832.4543 | 1832.0386 | 1 | 6.608 | 41.7% | 3 | R.MAEVEAQKAQQLEEAR.L | 3 |
| \* | AstrinIP\_MS2\_022614\_01.03476.03476.2 | 5.3049 | 0.3426 | 100.0% | 1630.2922 | 1630.7954 | 1 | 7.907 | 83.3% | 1 | R.LQEEEQKKEELAR.L | 2 |
| \* | AstrinIP\_MS2\_022614\_01.03464.03464.3 | 3.5398 | 0.2529 | 99.6% | 1631.1843 | 1630.7954 | 1 | 5.467 | 52.1% | 1 | R.LQEEEQKKEELAR.L | 3 |
| \* | AstrinIP\_MS2\_022614\_01.06144.06144.2 | 3.3468 | 0.122 | 98.8% | 1356.5922 | 1355.5309 | 1 | 5.002 | 66.7% | 2 | K.SSDQPLTVPVSPK.F | 2 |
| \* | AstrinIP\_MS1\_022614\_01.06477.06477.1 | 1.5295 | 0.2481 | 96.8% | 1434.49 | 1435.5309 | 2 | 4.049 | 58.3% | 1 | K.SSDQPLTVPVS\*PK.F | 1 |
| \* | AstrinIP\_MS1\_022614\_01.06464.06464.2 | 3.3637 | 0.341 | 100.0% | 1434.8922 | 1435.5309 | 1 | 6.42 | 75.0% | 5 | K.SSDQPLTVPVS\*PK.F | 2 |

---

|  |  |  |  |  |  |  |  |  |
| --- | --- | --- | --- | --- | --- | --- | --- | --- |
| U | *gi|226530908|ref|NP\_0* | 23 | 87 | 54.4% | 285 | 30315 | 7.5 | protein-L-isoaspartate (D-aspartate) O-methyltransferase [Homo sapiens] |

| Filename XCorr DeltCN Conf% ObsM+H+ CalcM+H+ SpR ZScore Ion% # Sequence  | | | | | | | | | | | | |
| --- | --- | --- | --- | --- | --- | --- | --- | --- | --- | --- | --- | --- |
| \* | AstrinIP\_MS1\_022614\_01.03951.03951.2 | 4.0384 | 0.3212 | 100.0% | 1478.3322 | 1478.6078 | 1 | 8.033 | 65.4% | 3 | K.SGGASHSELIHNLR.K | 2 |
| \* | AstrinIP\_MS2\_022614\_01.04047.04047.3 | 4.1764 | 0.3037 | 100.0% | 1480.1344 | 1478.6078 | 2 | 6.427 | 46.2% | 3 | K.SGGASHSELIHNLR.K | 3 |
| \* | AstrinIP\_MS2\_022614\_01.03665.03665.2 | 4.0636 | 0.4843 | 100.0% | 1605.5922 | 1606.7819 | 1 | 7.813 | 57.1% | 1 | K.SGGASHSELIHNLRK.N | 2 |
| \* | AstrinIP\_MS2\_022614\_01.03662.03662.3 | 3.888 | 0.3738 | 100.0% | 1607.6643 | 1606.7819 | 1 | 6.65 | 42.9% | 1 | K.SGGASHSELIHNLRK.N | 3 |
| \* | AstrinIP\_MS2\_022614\_01.10792.10792.3 | 4.1461 | 0.4345 | 100.0% | 2050.7344 | 2051.409 | 1 | 7.545 | 33.8% | 4 | K.NGIIKTDKVFEVMLATDR.S | 3 |
| \* | AstrinIP\_MS2\_022614\_01.09483.09483.3 | 3.7277 | 0.3612 | 100.0% | 1525.7344 | 1525.7601 | 1 | 6.603 | 52.1% | 1 | K.TDKVFEVMLATDR.S | 3 |
| \* | AstrinIP\_MS1\_022614\_01.08930.08930.2 | 4.7151 | 0.5257 | 100.0% | 1526.0521 | 1525.7601 | 1 | 9.142 | 83.3% | 9 | K.TDKVFEVMLATDR.S | 2 |
| \* | AstrinIP\_MS1\_022614\_01.09096.09096.1 | 2.4875 | 0.4445 | 100.0% | 1180.6 | 1181.3923 | 5 | 6.713 | 61.1% | 2 | K.VFEVMLATDR.S | 1 |
| \* | AstrinIP\_MS1\_022614\_01.09050.09050.2 | 3.9686 | 0.4535 | 100.0% | 1182.2322 | 1181.3923 | 1 | 7.553 | 94.4% | 10 | K.VFEVMLATDR.S | 2 |
| \* | AstrinIP\_MS1\_022614\_02.07321.07321.2 | 5.8616 | 0.6678 | 100.0% | 1695.1921 | 1695.8792 | 1 | 12.258 | 81.2% | 5 | K.ALDVGSGSGILTACFAR.M | 2 |
| \* | AstrinIP\_MS1\_022614\_01.04582.04582.1 | 1.5228 | 0.355 | 100.0% | 894.48 | 895.0898 | 113 | 5.584 | 57.1% | 2 | K.VIGIDHIK.E | 1 |
| \* | AstrinIP\_MS1\_022614\_01.04660.04660.2 | 2.6567 | 0.3848 | 100.0% | 895.1922 | 895.0898 | 1 | 6.796 | 85.7% | 4 | K.VIGIDHIK.E | 2 |
| \* | AstrinIP\_MS2\_022614\_01.04185.04185.1 | 1.6336 | 0.4457 | 100.0% | 1188.55 | 1189.3109 | 2 | 6.803 | 50.0% | 2 | R.KDDPTLLSSGR.V | 1 |
| \* | AstrinIP\_MS2\_022614\_01.04160.04160.2 | 3.0462 | 0.4016 | 100.0% | 1188.6721 | 1189.3109 | 1 | 6.999 | 75.0% | 3 | R.KDDPTLLSSGR.V | 2 |
| \* | AstrinIP\_MS1\_022614\_01.04959.04959.1 | 2.1963 | 0.4219 | 100.0% | 942.49 | 943.091 | 1 | 8.35 | 68.8% | 3 | R.VQLVVGDGR.M | 1 |
| \* | AstrinIP\_MS1\_022614\_01.04946.04946.2 | 2.5711 | 0.3038 | 99.8% | 943.3522 | 943.091 | 1 | 6.201 | 87.5% | 1 | R.VQLVVGDGR.M | 2 |
| \* | AstrinIP\_MS2\_022614\_01.12686.12686.3 | 6.8534 | 0.6375 | 100.0% | 3505.3743 | 3507.0015 | 1 | 11.561 | 34.8% | 5 | R.MGYAEEAPYDAIHVGAAAPVVPQALIDQLKPGGR.L | 3 |
| \* | AstrinIP\_MS2\_022614\_01.11588.11588.2 | 5.0422 | 0.5428 | 100.0% | 2043.4321 | 2044.3734 | 1 | 9.614 | 80.6% | 7 | R.LILPVGPAGGNQMLEQYDK.L | 2 |
| \* | AstrinIP\_MS1\_022614\_01.10929.10929.3 | 3.4857 | 0.4517 | 100.0% | 2043.9543 | 2044.3734 | 1 | 6.309 | 38.9% | 3 | R.LILPVGPAGGNQMLEQYDK.L | 3 |
| \* | AstrinIP\_MS1\_022614\_01.11471.11471.2 | 4.8964 | 0.507 | 100.0% | 2785.2122 | 2786.2158 | 1 | 8.933 | 40.0% | 3 | R.LILPVGPAGGNQMLEQYDKLQDGSIK.M | 2 |
| \* | AstrinIP\_MS2\_022614\_01.11631.11631.2 | 4.4542 | 0.4427 | 100.0% | 1706.3922 | 1706.1549 | 1 | 7.488 | 78.6% | 6 | K.MKPLMGVIYVPLTDK.E | 2 |
| \* | AstrinIP\_MS1\_022614\_01.09602.09602.2 | 4.9935 | 0.6105 | 100.0% | 1962.5322 | 1963.4445 | 1 | 10.072 | 71.9% | 4 | K.MKPLMGVIYVPLTDKEK.Q | 2 |
| \* | AstrinIP\_MS1\_022614\_01.09584.09584.3 | 3.7517 | 0.3265 | 100.0% | 1962.5944 | 1963.4445 | 1 | 6.311 | 42.2% | 5 | K.MKPLMGVIYVPLTDKEK.Q | 3 |

---

|  |  |  |  |  |  |  |  |  |
| --- | --- | --- | --- | --- | --- | --- | --- | --- |
| U | *gi|62414289|ref|NP\_00* | 27 | 44 | 53.9% | 466 | 53652 | 5.1 | vimentin [Homo sapiens] |

| Filename XCorr DeltCN Conf% ObsM+H+ CalcM+H+ SpR ZScore Ion% # Sequence  | | | | | | | | | | | | |
| --- | --- | --- | --- | --- | --- | --- | --- | --- | --- | --- | --- | --- |
| \* | AstrinIP\_MS1\_022614\_01.05944.05944.2 | 2.3335 | 0.315 | 98.6% | 1496.2322 | 1496.6633 | 9 | 5.283 | 46.2% | 2 | R.TYSLGSALRPSTSR.S | 2 |
| \* | AstrinIP\_MS1\_022614\_01.05879.05879.2 | 3.8337 | 0.2296 | 99.8% | 1430.1522 | 1429.5724 | 1 | 5.507 | 73.1% | 2 | R.SLYASSPGGVYATR.S | 2 |
| \* | AstrinIP\_MS1\_022614\_01.06736.06736.2 | 3.2702 | 0.2897 | 99.8% | 1509.2122 | 1509.5724 | 1 | 5.694 | 61.5% | 2 | R.SLYASS\*PGGVYATR.S | 2 |
|  | AstrinIP\_MS2\_022614\_01.05860.05860.2 | 2.7755 | 0.1175 | 98.2% | 1115.6522 | 1116.2163 | 151 | 4.423 | 62.5% | 1 | K.VELQELNDR.F | 2 |
| \* | AstrinIP\_MS2\_022614\_01.05675.05675.2 | 2.1107 | 0.3309 | 98.9% | 1126.1322 | 1126.3005 | 1 | 5.91 | 81.2% | 1 | R.FANYIDKVR.F | 2 |
| \* | AstrinIP\_MS2\_022614\_01.12065.12065.2 | 3.6176 | 0.2915 | 99.9% | 1170.0122 | 1170.4349 | 1 | 7.296 | 83.3% | 1 | K.ILLAELEQLK.G | 2 |
| \* | AstrinIP\_MS2\_022614\_01.09660.09660.2 | 3.471 | 0.3585 | 100.0% | 1540.7722 | 1540.8436 | 1 | 6.343 | 61.5% | 1 | K.ILLAELEQLKGQGK.S | 2 |
| \* | AstrinIP\_MS2\_022614\_01.08141.08141.2 | 3.378 | 0.3579 | 100.0% | 1499.1322 | 1498.6508 | 1 | 6.643 | 72.7% | 1 | K.SRLGDLYEEEMR.E | 2 |
| \* | AstrinIP\_MS2\_022614\_01.07562.07562.2 | 3.5871 | 0.4279 | 100.0% | 1255.0322 | 1255.385 | 1 | 7.83 | 77.8% | 3 | R.LGDLYEEEMR.E | 2 |
| \* | AstrinIP\_MS2\_022614\_01.08666.08666.2 | 2.7987 | 0.2065 | 98.4% | 1689.4321 | 1689.881 | 2 | 4.523 | 53.8% | 1 | R.VEVERDNLAEDIMR.L | 2 |
| \* | AstrinIP\_MS2\_022614\_01.08642.08642.3 | 2.9432 | 0.2809 | 99.2% | 1690.5844 | 1689.881 | 1 | 4.83 | 40.4% | 2 | R.VEVERDNLAEDIMR.L | 3 |
| \* | AstrinIP\_MS2\_022614\_01.09248.09248.3 | 3.0543 | 0.3797 | 100.0% | 2352.3542 | 2352.581 | 4 | 5.95 | 31.9% | 2 | K.LQEEMLQREEAENTLQSFR.Q | 3 |
| \* | AstrinIP\_MS1\_022614\_01.06406.06406.2 | 2.8655 | 0.4084 | 100.0% | 1324.0122 | 1324.3898 | 1 | 6.284 | 65.0% | 2 | R.EEAENTLQSFR.Q | 2 |
| \* | AstrinIP\_MS2\_022614\_01.03807.03807.2 | 2.2988 | 0.3922 | 99.8% | 1088.4122 | 1089.1503 | 1 | 5.964 | 88.9% | 1 | R.QDVDNASLAR.L | 2 |
| \* | AstrinIP\_MS2\_022614\_01.09126.09126.2 | 5.1348 | 0.4893 | 100.0% | 1662.4521 | 1662.967 | 1 | 8.701 | 84.6% | 2 | R.KVESLQEEIAFLKK.L | 2 |
| \* | AstrinIP\_MS1\_022614\_01.08548.08548.3 | 4.1132 | 0.2748 | 100.0% | 1663.2843 | 1662.967 | 2 | 5.574 | 42.3% | 2 | R.KVESLQEEIAFLKK.L | 3 |
| \* | AstrinIP\_MS2\_022614\_01.12185.12185.2 | 2.6305 | 0.3162 | 99.7% | 1405.8522 | 1406.6189 | 1 | 5.63 | 72.7% | 1 | K.VESLQEEIAFLK.K | 2 |
|  | AstrinIP\_MS2\_022614\_01.08343.08343.2 | 2.783 | 0.3387 | 99.9% | 1309.5922 | 1310.4056 | 1 | 6.028 | 77.8% | 4 | K.NLQEAEEWYK.S | 2 |
| \* | AstrinIP\_MS2\_022614\_01.05409.05409.2 | 3.507 | 0.4347 | 100.0% | 1093.9122 | 1094.1692 | 1 | 7.662 | 94.4% | 3 | K.FADLSEAANR.N | 2 |
| \* | AstrinIP\_MS2\_022614\_02.06828.06828.3 | 4.2674 | 0.3896 | 100.0% | 2187.8044 | 2188.33 | 1 | 6.919 | 45.8% | 1 | R.EMEENFAVEAANYQDTIGR.L | 3 |
| \* | AstrinIP\_MS2\_022614\_01.07420.07420.2 | 4.4792 | 0.4368 | 100.0% | 1735.2722 | 1735.9679 | 1 | 7.972 | 76.9% | 2 | R.LQDEIQNMKEEMAR.H | 2 |
|  | AstrinIP\_MS2\_022614\_01.07086.07086.2 | 2.6857 | 0.274 | 99.3% | 1527.9521 | 1528.7513 | 1 | 4.647 | 68.2% | 1 | R.HLREYQDLLNVK.M | 2 |
|  | AstrinIP\_MS2\_022614\_01.07080.07080.3 | 3.6459 | 0.3194 | 100.0% | 1528.9443 | 1528.7513 | 1 | 5.76 | 52.3% | 1 | R.HLREYQDLLNVK.M | 3 |
|  | AstrinIP\_MS2\_022614\_02.07295.07295.2 | 2.663 | 0.4732 | 100.0% | 1295.5521 | 1296.5243 | 1 | 7.325 | 70.0% | 1 | K.MALDIEIATYR.K | 2 |
| \* | AstrinIP\_MS1\_022614\_01.13340.13340.2 | 3.0288 | 0.2198 | 99.2% | 1572.4521 | 1571.8601 | 2 | 6.023 | 69.2% | 2 | R.ISLPLPNFSSLNLR.E | 2 |
| \* | AstrinIP\_MS1\_022614\_01.06784.06784.2 | 3.1586 | 0.3494 | 99.9% | 1825.8722 | 1826.0165 | 43 | 5.675 | 36.7% | 1 | R.ETNLDSLPLVDTHSKR.T | 2 |
| \* | AstrinIP\_MS2\_022614\_01.04401.04401.2 | 3.5776 | 0.2899 | 99.9% | 1837.0721 | 1837.854 | 1 | 6.317 | 56.7% | 1 | R.DGQVINETSQHHDDLE.- | 2 |

---

|  |  |  |  |  |  |  |  |  |
| --- | --- | --- | --- | --- | --- | --- | --- | --- |
| U | *gi|218505831|ref|NP\_0* | 37 | 255 | 53.5% | 286 | 31880 | 7.1 | TRAF4 associated factor 1 isoform b [Homo sapiens] |

| Filename XCorr DeltCN Conf% ObsM+H+ CalcM+H+ SpR ZScore Ion% # Sequence  | | | | | | | | | | | | |
| --- | --- | --- | --- | --- | --- | --- | --- | --- | --- | --- | --- | --- |
|  | AstrinIP\_MS2\_022614\_01.08552.08552.2 | 5.624 | 0.531 | 100.0% | 2274.672 | 2275.4802 | 1 | 9.591 | 64.3% | 26 | K.TVYSLQPPSALSGGQPADTQTR.A | 22 |
|  | AstrinIP\_MS1\_022614\_02.05275.05275.3 | 5.516 | 0.5174 | 100.0% | 2275.4343 | 2275.4802 | 1 | 8.435 | 51.2% | 18 | K.TVYSLQPPSALSGGQPADTQTR.A | 33 |
|  | AstrinIP\_MS2\_022614\_01.08288.08288.3 | 3.0087 | 0.3374 | 99.3% | 3411.1143 | 3408.7502 | 134 | 4.647 | 17.7% | 1 | K.TVYSLQPPSALSGGQPADTQTRATSKS\*LLPVR.S | 33 |
|  | AstrinIP\_MS2\_022614\_01.08486.08486.3 | 3.003 | 0.2502 | 96.5% | 3411.2644 | 3408.7502 | 265 | 4.86 | 18.5% | 1 | K.TVYSLQPPSALSGGQPADTQT#RATSKSLLPVR.S | 33 |
|  | AstrinIP\_MS1\_022614\_01.03945.03945.3 | 3.6499 | 0.2294 | 98.7% | 2255.7844 | 2255.4465 | 1 | 4.705 | 36.2% | 1 | R.SKEVDVSKQLHSGGPENDVTK.I | 33 |
|  | AstrinIP\_MS2\_022614\_01.03465.03465.2 | 2.2072 | 0.3877 | 99.3% | 1382.1322 | 1382.4728 | 1 | 7.573 | 75.0% | 2 | K.QLHSGGPENDVTK.I | 22 |
|  | AstrinIP\_MS2\_022614\_01.03467.03467.3 | 2.5001 | 0.2515 | 95.6% | 1382.4543 | 1382.4728 | 212 | 4.778 | 41.7% | 1 | K.QLHSGGPENDVTK.I | 33 |
|  | AstrinIP\_MS1\_022614\_01.05002.05002.2 | 3.8213 | 0.4185 | 100.0% | 2243.7922 | 2244.507 | 2 | 6.577 | 41.7% | 5 | K.QKSEEELKDKNQLLEAVNK.Q | 22 |
|  | AstrinIP\_MS2\_022614\_01.04965.04965.3 | 3.5796 | 0.3012 | 99.8% | 2244.2944 | 2244.507 | 5 | 6.245 | 31.9% | 12 | K.QKSEEELKDKNQLLEAVNK.Q | 33 |
|  | AstrinIP\_MS2\_022614\_01.06431.06431.2 | 5.2517 | 0.447 | 100.0% | 1987.4922 | 1988.2023 | 1 | 9.133 | 65.6% | 8 | K.SEEELKDKNQLLEAVNK.Q | 22 |
|  | AstrinIP\_MS1\_022614\_02.04897.04897.3 | 5.2827 | 0.5339 | 100.0% | 1987.7344 | 1988.2023 | 1 | 8.958 | 40.6% | 12 | K.SEEELKDKNQLLEAVNK.Q | 33 |
|  | AstrinIP\_MS1\_022614\_01.05512.05512.1 | 2.1761 | 0.2415 | 99.1% | 1271.52 | 1272.4441 | 1 | 5.11 | 65.0% | 1 | K.DKNQLLEAVNK.Q | 11 |
|  | AstrinIP\_MS2\_022614\_01.05732.05732.2 | 3.8153 | 0.2607 | 100.0% | 1272.3121 | 1272.4441 | 1 | 5.812 | 85.0% | 5 | K.DKNQLLEAVNK.Q | 22 |
|  | AstrinIP\_MS2\_022614\_01.05985.05985.1 | 2.1877 | 0.2289 | 99.1% | 1028.53 | 1029.1814 | 20 | 4.994 | 62.5% | 7 | K.NQLLEAVNK.Q | 11 |
|  | AstrinIP\_MS1\_022614\_01.05679.05679.2 | 3.2837 | 0.2346 | 99.9% | 1029.0322 | 1029.1814 | 2 | 4.95 | 87.5% | 8 | K.NQLLEAVNK.Q | 22 |
|  | AstrinIP\_MS1\_022614\_01.07854.07854.2 | 2.608 | 0.2416 | 98.4% | 1663.7922 | 1663.9175 | 1 | 5.219 | 53.8% | 1 | K.NQLLEAVNKQLHQK.L | 22 |
|  | AstrinIP\_MS2\_022614\_01.03794.03794.2 | 2.799 | 0.2947 | 99.8% | 1019.1122 | 1019.13995 | 1 | 6.817 | 87.5% | 2 | K.LTETQGELK.D | 22 |
|  | AstrinIP\_MS1\_022614\_01.05594.05594.2 | 4.388 | 0.4022 | 100.0% | 1604.4321 | 1604.7979 | 1 | 7.253 | 65.4% | 9 | K.LTETQGELKDLTQK.V | 22 |
|  | AstrinIP\_MS2\_022614\_01.05880.05880.1 | 3.5506 | 0.3994 | 100.0% | 1604.72 | 1604.7979 | 1 | 6.394 | 53.8% | 2 | K.LTETQGELKDLTQK.V | 11 |
|  | AstrinIP\_MS2\_022614\_01.05810.05810.3 | 1.9233 | 0.341 | 96.0% | 1605.0243 | 1604.7979 | 3 | 4.811 | 42.3% | 1 | K.LTETQGELKDLTQK.V | 33 |
|  | AstrinIP\_MS2\_022614\_01.11206.11206.2 | 5.9733 | 0.5075 | 100.0% | 2315.5723 | 2316.6543 | 1 | 8.935 | 55.3% | 7 | K.LTETQGELKDLTQKVELLEK.F | 22 |
|  | AstrinIP\_MS2\_022614\_01.11204.11204.3 | 5.1978 | 0.3235 | 100.0% | 2317.1943 | 2316.6543 | 1 | 7.157 | 48.7% | 10 | K.LTETQGELKDLTQKVELLEK.F | 33 |
|  | AstrinIP\_MS2\_022614\_01.08172.08172.3 | 2.6367 | 0.2285 | 96.4% | 1316.7244 | 1316.5376 | 5 | 4.985 | 50.0% | 1 | K.DLTQKVELLEK.F | 33 |
|  | AstrinIP\_MS1\_022614\_01.07719.07719.2 | 4.0724 | 0.2096 | 100.0% | 1317.2922 | 1316.5376 | 1 | 5.055 | 75.0% | 12 | K.DLTQKVELLEK.F | 22 |
|  | AstrinIP\_MS1\_022614\_01.07949.07949.3 | 3.1825 | 0.2123 | 98.3% | 1466.3644 | 1466.6434 | 36 | 4.837 | 43.2% | 1 | K.FRDNCLAILESK.G | 33 |
|  | AstrinIP\_MS1\_022614\_01.07928.07928.2 | 3.4832 | 0.3777 | 100.0% | 1466.3922 | 1466.6434 | 2 | 6.384 | 72.7% | 5 | K.FRDNCLAILESK.G | 22 |
|  | AstrinIP\_MS1\_022614\_01.08420.08420.2 | 3.7674 | 0.2248 | 100.0% | 1162.7122 | 1163.2793 | 1 | 6.503 | 77.8% | 3 | R.DNCLAILESK.G | 22 |
|  | AstrinIP\_MS1\_022614\_01.08374.08374.1 | 2.3816 | 0.1485 | 95.1% | 1164.43 | 1163.2793 | 1 | 3.52 | 66.7% | 3 | R.DNCLAILESK.G | 11 |
|  | AstrinIP\_MS1\_022614\_01.07517.07517.1 | 1.5464 | 0.3522 | 100.0% | 1386.65 | 1387.5327 | 4 | 6.414 | 50.0% | 1 | K.GLDPALGSETLASR.Q | 11 |
|  | AstrinIP\_MS1\_022614\_01.07436.07436.2 | 4.3619 | 0.6123 | 100.0% | 1387.0521 | 1387.5327 | 1 | 10.485 | 84.6% | 49 | K.GLDPALGSETLASR.Q | 22 |
|  | AstrinIP\_MS1\_022614\_01.07611.07611.2 | 2.4204 | 0.2862 | 98.5% | 1466.7322 | 1467.5327 | 3 | 5.526 | 57.7% | 1 | K.GLDPALGS\*ETLASR.Q | 22 |
|  | AstrinIP\_MS2\_022614\_01.07966.07966.2 | 2.9295 | 0.4656 | 100.0% | 1467.3522 | 1467.5327 | 1 | 6.645 | 69.2% | 1 | K.GLDPALGSET#LASR.Q | 22 |
|  | AstrinIP\_MS2\_022614\_01.14264.14264.3 | 3.9413 | 0.1871 | 97.9% | 2592.8044 | 2592.8928 | 83 | 4.273 | 28.6% | 2 | R.QESTTDHMDSMLLLETLQEELK.L | 33 |
|  | AstrinIP\_MS2\_022614\_01.14471.14471.2 | 5.1585 | 0.3552 | 100.0% | 2592.8323 | 2592.8928 | 1 | 6.259 | 45.2% | 17 | R.QESTTDHMDSMLLLETLQEELK.L | 22 |
|  | AstrinIP\_MS2\_022614\_01.16311.16311.3 | 6.0367 | 0.459 | 100.0% | 3395.1843 | 3396.8062 | 1 | 7.35 | 26.8% | 14 | R.QESTTDHMDSMLLLETLQEELKLFNETAK.K | 33 |
| \* | AstrinIP\_MS1\_022614\_01.14559.14559.3 | 4.0725 | 0.3894 | 100.0% | 3523.9143 | 3524.9802 | 35 | 7.072 | 19.0% | 3 | R.QESTTDHMDSMLLLETLQEELKLFNETAKK.Q | 3 |
|  | AstrinIP\_MS2\_022614\_01.03956.03956.1 | 1.8115 | 0.2288 | 98.3% | 822.35 | 822.9365 | 23 | 4.387 | 66.7% | 2 | K.LFNETAK.K | 11 |

Similarities:
gi|218505827|ref|NP\_1(36:1)  

---

|  |  |  |  |  |  |  |  |  |
| --- | --- | --- | --- | --- | --- | --- | --- | --- |
| U | *gi|4504517|ref|NP\_001* | 7 | 12 | 52.2% | 205 | 22783 | 6.4 | heat shock protein beta-1 [Homo sapiens] |

| Filename XCorr DeltCN Conf% ObsM+H+ CalcM+H+ SpR ZScore Ion% # Sequence  | | | | | | | | | | | | |
| --- | --- | --- | --- | --- | --- | --- | --- | --- | --- | --- | --- | --- |
| \* | AstrinIP\_MS2\_022614\_01.11550.11550.3 | 3.11 | 0.2727 | 99.2% | 1903.7943 | 1904.0537 | 1 | 5.364 | 42.9% | 1 | R.GPSWDPFRDWYPHSR.L | 3 |
| \* | AstrinIP\_MS2\_022614\_01.11070.11070.2 | 3.4941 | 0.3516 | 100.0% | 1164.2722 | 1164.3494 | 1 | 7.105 | 88.9% | 3 | R.LFDQAFGLPR.L | 2 |
| \* | AstrinIP\_MS2\_022614\_01.14052.14052.3 | 6.9159 | 0.5602 | 100.0% | 4094.9043 | 4095.606 | 1 | 9.656 | 33.1% | 2 | R.LPEEWSQWLGGSSWPGYVRPLPPAAIESPAVAAPAYSR.A | 3 |
| \* | AstrinIP\_MS2\_022614\_01.09747.09747.2 | 5.0939 | 0.5101 | 100.0% | 1784.1921 | 1785.0068 | 1 | 8.618 | 63.3% | 2 | R.VSLDVNHFAPDELTVK.T | 2 |
| \* | AstrinIP\_MS1\_022614\_01.09172.09172.3 | 3.3216 | 0.374 | 100.0% | 1784.9644 | 1785.0068 | 40 | 6.88 | 30.0% | 2 | R.VSLDVNHFAPDELTVK.T | 3 |
| \* | AstrinIP\_MS2\_022614\_01.03936.03936.2 | 2.5763 | 0.2376 | 98.8% | 1147.4321 | 1147.314 | 1 | 4.931 | 75.0% | 1 | K.TKDGVVEITGK.H | 2 |
| \* | AstrinIP\_MS2\_022614\_01.10508.10508.2 | 2.991 | 0.3838 | 99.9% | 1906.8722 | 1907.1307 | 1 | 6.391 | 40.6% | 1 | K.LATQSNEITIPVTFESR.A | 2 |

---

|  |  |  |  |  |  |  |  |  |
| --- | --- | --- | --- | --- | --- | --- | --- | --- |
| U | *gi|5729877|ref|NP\_006* | 51 | 180 | 51.1% | 646 | 70898 | 5.5 | heat shock 70kDa protein 8 isoform 1 [Homo sapiens] |

| Filename XCorr DeltCN Conf% ObsM+H+ CalcM+H+ SpR ZScore Ion% # Sequence  | | | | | | | | | | | | |
| --- | --- | --- | --- | --- | --- | --- | --- | --- | --- | --- | --- | --- |
|  | AstrinIP\_MS1\_022614\_02.06931.06931.3 | 3.9696 | 0.4267 | 100.0% | 2266.0144 | 2264.509 | 1 | 7.14 | 36.9% | 4 | K.GPAVGIDLGTTYSCVGVFQHGK.V | 3 |
|  | AstrinIP\_MS2\_022614\_01.07734.07734.1 | 2.2714 | 0.4224 | 100.0% | 1487.62 | 1488.5939 | 1 | 6.368 | 50.0% | 5 | R.TTPSYVAFTDTER.L | 11111 |
|  | AstrinIP\_MS1\_022614\_02.04868.04868.2 | 3.849 | 0.5598 | 100.0% | 1489.2922 | 1488.5939 | 1 | 9.39 | 79.2% | 10 | R.TTPSYVAFTDTER.L | 22222 |
|  | AstrinIP\_MS1\_022614\_01.07349.07349.2 | 5.1458 | 0.5614 | 100.0% | 1650.2122 | 1650.8468 | 1 | 10.415 | 82.1% | 10 | K.NQVAMNPTNTVFDAK.R | 2 |
|  | AstrinIP\_MS2\_022614\_01.06663.06663.2 | 4.6058 | 0.3665 | 100.0% | 1806.3522 | 1807.0343 | 1 | 7.549 | 60.0% | 2 | K.NQVAMNPTNTVFDAKR.L | 2 |
|  | AstrinIP\_MS1\_022614\_01.05186.05186.2 | 3.6406 | 0.4442 | 100.0% | 1411.1721 | 1411.5725 | 1 | 7.27 | 77.3% | 5 | R.RFDDAVVQSDMK.H | 2 |
|  | AstrinIP\_MS1\_022614\_01.10060.10060.3 | 4.197 | 0.2697 | 99.7% | 3048.1443 | 3047.4792 | 1 | 5.376 | 31.0% | 1 | R.RFDDAVVQSDMKHWPFMVVNDAGRPK.V | 3 |
|  | AstrinIP\_MS2\_022614\_01.06290.06290.2 | 3.6817 | 0.5225 | 100.0% | 1254.7522 | 1255.385 | 1 | 8.408 | 90.0% | 2 | R.FDDAVVQSDMK.H | 2 |
|  | AstrinIP\_MS2\_022614\_01.08706.08706.2 | 3.2984 | 0.5125 | 100.0% | 1654.0922 | 1654.9298 | 1 | 7.831 | 65.4% | 5 | K.HWPFMVVNDAGRPK.V | 2 |
|  | AstrinIP\_MS2\_022614\_01.08798.08798.3 | 4.2556 | 0.4165 | 100.0% | 1654.7943 | 1654.9298 | 1 | 7.254 | 42.3% | 5 | K.HWPFMVVNDAGRPK.V | 3 |
|  | AstrinIP\_MS2\_022614\_01.03561.03561.1 | 2.2284 | 0.2726 | 99.4% | 1180.55 | 1181.3312 | 1 | 5.221 | 66.7% | 1 | K.VQVEYKGETK.S | 11 |
|  | AstrinIP\_MS2\_022614\_01.03554.03554.2 | 3.2438 | 0.3839 | 100.0% | 1181.1721 | 1181.3312 | 1 | 6.258 | 77.8% | 1 | K.VQVEYKGETK.S | 22 |
|  | AstrinIP\_MS1\_022614\_01.10876.10876.1 | 2.7527 | 0.5396 | 100.0% | 1616.56 | 1617.8542 | 1 | 8.889 | 53.8% | 2 | K.SFYPEEVSSMVLTK.M | 1 |
|  | AstrinIP\_MS2\_022614\_01.11504.11504.2 | 4.4845 | 0.4942 | 100.0% | 1618.2722 | 1617.8542 | 1 | 8.668 | 80.8% | 7 | K.SFYPEEVSSMVLTK.M | 2 |
|  | AstrinIP\_MS2\_022614\_01.06528.06528.1 | 2.5105 | 0.2494 | 99.4% | 1252.53 | 1253.4993 | 1 | 6.909 | 70.0% | 2 | K.MKEIAEAYLGK.T | 1 |
|  | AstrinIP\_MS2\_022614\_01.06512.06512.2 | 4.0128 | 0.2762 | 100.0% | 1253.2322 | 1253.4993 | 1 | 6.222 | 90.0% | 8 | K.MKEIAEAYLGK.T | 2 |
|  | AstrinIP\_MS2\_022614\_01.06263.06263.1 | 2.1455 | 0.3922 | 100.0% | 993.36 | 994.1326 | 8 | 6.49 | 56.2% | 1 | K.EIAEAYLGK.T | 1 |
|  | AstrinIP\_MS2\_022614\_01.09857.09857.2 | 4.0008 | 0.4473 | 100.0% | 1982.3722 | 1983.1882 | 1 | 7.404 | 61.8% | 7 | K.TVTNAVVTVPAYFNDSQR.Q | 2 |
|  | AstrinIP\_MS2\_022614\_01.09929.09929.3 | 4.5313 | 0.3892 | 100.0% | 1983.5643 | 1983.1882 | 1 | 7.124 | 45.6% | 4 | K.TVTNAVVTVPAYFNDSQR.Q | 3 |
|  | AstrinIP\_MS1\_022614\_01.09921.09921.2 | 5.232 | 0.5436 | 100.0% | 1660.4922 | 1660.9078 | 1 | 10.069 | 80.0% | 8 | R.IINEPTAAAIAYGLDK.K | 2222 |
|  | AstrinIP\_MS2\_022614\_01.09232.09232.2 | 4.6507 | 0.5188 | 100.0% | 1788.4922 | 1789.0819 | 1 | 9.486 | 68.8% | 5 | R.IINEPTAAAIAYGLDKK.V | 22 |
|  | AstrinIP\_MS2\_022614\_01.04514.04514.2 | 4.3459 | 0.4677 | 100.0% | 1692.2122 | 1692.6958 | 1 | 7.869 | 63.3% | 3 | K.STAGDTHLGGEDFDNR.M | 22 |
|  | AstrinIP\_MS1\_022614\_01.04403.04403.3 | 2.4102 | 0.2905 | 97.0% | 1693.8844 | 1692.6958 | 28 | 4.578 | 28.3% | 1 | K.STAGDTHLGGEDFDNR.M | 33 |
|  | AstrinIP\_MS2\_022614\_01.08270.08270.2 | 3.5175 | 0.547 | 100.0% | 1235.9922 | 1236.4741 | 1 | 9.183 | 88.9% | 6 | R.MVNHFIAEFK.R | 2 |
|  | AstrinIP\_MS2\_022614\_01.08230.08230.3 | 3.4825 | 0.4315 | 100.0% | 1237.7344 | 1236.4741 | 1 | 6.984 | 58.3% | 1 | R.MVNHFIAEFK.R | 3 |
|  | AstrinIP\_MS1\_022614\_01.06455.06455.2 | 3.0328 | 0.4531 | 100.0% | 1391.9922 | 1392.6616 | 1 | 7.251 | 80.0% | 2 | R.MVNHFIAEFKR.K | 2 |
|  | AstrinIP\_MS2\_022614\_01.06804.06804.3 | 3.3963 | 0.3889 | 100.0% | 1392.2043 | 1392.6616 | 1 | 6.624 | 50.0% | 1 | R.MVNHFIAEFKR.K | 3 |
|  | AstrinIP\_MS1\_022614\_01.09297.09297.2 | 3.3038 | 0.4288 | 100.0% | 1480.5122 | 1481.6511 | 1 | 6.801 | 77.3% | 3 | R.ARFEELNADLFR.G | 22 |
|  | AstrinIP\_MS2\_022614\_01.09827.09827.3 | 4.0793 | 0.3183 | 100.0% | 1481.5743 | 1481.6511 | 1 | 5.772 | 54.5% | 1 | R.ARFEELNADLFR.G | 33 |
|  | AstrinIP\_MS2\_022614\_01.11079.11079.1 | 2.2045 | 0.4317 | 100.0% | 1253.55 | 1254.3849 | 5 | 6.105 | 50.0% | 1 | R.FEELNADLFR.G | 11 |
|  | AstrinIP\_MS1\_022614\_01.10436.10436.2 | 3.4707 | 0.3983 | 100.0% | 1253.9321 | 1254.3849 | 1 | 7.554 | 72.2% | 4 | R.FEELNADLFR.G | 22 |
|  | AstrinIP\_MS2\_022614\_01.07304.07304.2 | 4.6159 | 0.4454 | 100.0% | 1838.3722 | 1839.1019 | 1 | 9.304 | 65.6% | 2 | K.LDKSQIHDIVLVGGSTR.I | 2 |
|  | AstrinIP\_MS2\_022614\_01.07347.07347.3 | 5.3986 | 0.346 | 100.0% | 1840.2544 | 1839.1019 | 1 | 8.405 | 46.9% | 6 | K.LDKSQIHDIVLVGGSTR.I | 3 |
|  | AstrinIP\_MS1\_022614\_01.06417.06417.1 | 3.6222 | 0.5524 | 100.0% | 1481.62 | 1482.6798 | 1 | 10.256 | 69.2% | 2 | K.SQIHDIVLVGGSTR.I | 1 |
|  | AstrinIP\_MS1\_022614\_01.06458.06458.2 | 4.6554 | 0.6045 | 100.0% | 1481.8121 | 1482.6798 | 1 | 10.418 | 84.6% | 9 | K.SQIHDIVLVGGSTR.I | 2 |
|  | AstrinIP\_MS1\_022614\_01.09870.09870.1 | 2.2447 | 0.2928 | 100.0% | 1081.36 | 1082.2444 | 1 | 4.936 | 75.0% | 4 | K.LLQDFFNGK.E | 111 |
|  | AstrinIP\_MS2\_022614\_01.10502.10502.2 | 3.0573 | 0.3647 | 99.9% | 1082.0922 | 1082.2444 | 1 | 5.829 | 81.2% | 5 | K.LLQDFFNGK.E | 222 |
|  | AstrinIP\_MS2\_022614\_01.10005.10005.2 | 3.8674 | 0.1637 | 99.7% | 1567.3722 | 1566.7972 | 1 | 4.208 | 70.8% | 5 | K.LLQDFFNGKELNK.S | 222 |
|  | AstrinIP\_MS2\_022614\_01.13952.13952.3 | 5.3519 | 0.5915 | 100.0% | 2261.2144 | 2261.4937 | 1 | 9.58 | 37.5% | 2 | K.SINPDEAVAYGAAVQAAILSGDK.S | 3 |
|  | AstrinIP\_MS2\_022614\_02.09334.09334.2 | 4.7431 | 0.5447 | 100.0% | 2262.4521 | 2261.4937 | 1 | 9.524 | 47.7% | 4 | K.SINPDEAVAYGAAVQAAILSGDK.S | 2 |
|  | AstrinIP\_MS1\_022614\_01.19506.19506.2 | 4.2739 | 0.4207 | 100.0% | 3239.9722 | 3240.8206 | 3 | 7.115 | 25.0% | 1 | K.SENVQDLLLLDVTPLSLGIETAGGVMTVLIK.R | 2 |
|  | AstrinIP\_MS2\_022614\_01.20078.20078.3 | 3.5807 | 0.3187 | 99.7% | 3239.9944 | 3240.8206 | 1 | 5.513 | 28.3% | 1 | K.SENVQDLLLLDVTPLSLGIETAGGVMTVLIK.R | 3 |
|  | AstrinIP\_MS1\_022614\_02.13573.13573.3 | 3.9493 | 0.3836 | 100.0% | 3396.4443 | 3397.008 | 2 | 6.673 | 21.0% | 1 | K.SENVQDLLLLDVTPLSLGIETAGGVMTVLIKR.N | 3 |
|  | AstrinIP\_MS2\_022614\_01.11600.11600.2 | 4.7896 | 0.4483 | 100.0% | 2774.8523 | 2775.9885 | 1 | 10.122 | 45.7% | 5 | K.QTQTFTTYSDNQPGVLIQVYEGER.A | 2 |
| \* | AstrinIP\_MS2\_022614\_01.03588.03588.2 | 2.5772 | 0.1194 | 97.8% | 989.6122 | 990.10144 | 5 | 4.764 | 85.7% | 1 | R.LSKEDIER.M | 2 |
|  | AstrinIP\_MS2\_022614\_01.03545.03545.2 | 1.9886 | 0.2475 | 95.1% | 1126.0122 | 1126.313 | 7 | 5.056 | 68.8% | 1 | R.MVQEAEKYK.A | 22 |
| \* | AstrinIP\_MS2\_022614\_01.03512.03512.2 | 5.1515 | 0.4402 | 100.0% | 1982.3121 | 1983.2036 | 1 | 6.723 | 73.3% | 1 | R.MVQEAEKYKAEDEKQR.D | 2 |
| \* | AstrinIP\_MS2\_022614\_01.03506.03506.3 | 4.7211 | 0.4352 | 100.0% | 1983.1444 | 1983.2036 | 1 | 7.301 | 50.0% | 2 | R.MVQEAEKYKAEDEKQR.D | 3 |
| \* | AstrinIP\_MS2\_022614\_01.03509.03509.2 | 2.0975 | 0.2922 | 98.3% | 1168.3322 | 1167.2639 | 62 | 4.777 | 62.5% | 1 | K.YKAEDEKQR.D | 2 |
| \* | AstrinIP\_MS2\_022614\_01.09354.09354.1 | 2.9995 | 0.4368 | 100.0% | 1303.43 | 1304.4602 | 1 | 6.957 | 65.0% | 2 | K.NSLESYAFNMK.A | 1 |
| \* | AstrinIP\_MS2\_022614\_01.09348.09348.2 | 3.6475 | 0.2717 | 100.0% | 1306.2322 | 1304.4602 | 1 | 5.676 | 80.0% | 7 | K.NSLESYAFNMK.A | 2 |

Similarities:
gi|16507237|ref|NP\_00(1:50)  
gi|167466173|ref|NP\_0(3:48)  
gi|124256496|ref|NP\_0(3:48)  
gi|13676857|ref|NP\_06(15:36)  
gi|34419635|ref|NP\_00(5:46)  

---

|  |  |  |  |  |  |  |  |  |
| --- | --- | --- | --- | --- | --- | --- | --- | --- |
| U | *gi|4504919|ref|NP\_002* | 32 | 67 | 51.1% | 483 | 53704 | 5.6 | keratin 8 [Homo sapiens] |

| Filename XCorr DeltCN Conf% ObsM+H+ CalcM+H+ SpR ZScore Ion% # Sequence  | | | | | | | | | | | | |
| --- | --- | --- | --- | --- | --- | --- | --- | --- | --- | --- | --- | --- |
|  | AstrinIP\_MS1\_022614\_01.07103.07103.2 | 2.5876 | 0.1854 | 98.7% | 1083.1322 | 1083.2755 | 2 | 6.016 | 75.0% | 2 | K.FASFIDKVR.F | 2222 |
|  | AstrinIP\_MS2\_022614\_01.08363.08363.2 | 2.9633 | 0.1309 | 99.3% | 1031.1322 | 1031.1997 | 1 | 3.806 | 92.9% | 3 | K.WSLLQQQK.T | 2 |
|  | AstrinIP\_MS1\_022614\_01.13210.13210.2 | 3.8475 | 0.3847 | 100.0% | 1849.3722 | 1849.0431 | 1 | 6.435 | 67.9% | 3 | R.SNMDNMFESYINNLR.R | 2 |
|  | AstrinIP\_MS2\_022614\_01.13238.13238.3 | 5.1883 | 0.2568 | 100.0% | 2035.4343 | 2035.363 | 1 | 8.321 | 47.1% | 4 | K.LKLEAELGNMQGLVEDFK.N | 3 |
|  | AstrinIP\_MS2\_022614\_01.12912.12912.2 | 2.9436 | 0.355 | 99.8% | 1792.8922 | 1794.0295 | 1 | 6.496 | 46.7% | 2 | K.LEAELGNMQGLVEDFK.N | 2 |
|  | AstrinIP\_MS1\_022614\_01.03650.03650.2 | 2.8304 | 0.2913 | 99.8% | 1309.3522 | 1309.4215 | 161 | 5.387 | 55.6% | 2 | K.NKYEDEINKR.T | 222 |
|  | AstrinIP\_MS2\_022614\_01.10557.10557.2 | 3.2453 | 0.4839 | 100.0% | 1353.2322 | 1353.5732 | 1 | 7.311 | 75.0% | 2 | R.TEMENEFVLIK.K | 2 |
|  | AstrinIP\_MS2\_022614\_01.08462.08462.2 | 2.459 | 0.1788 | 95.9% | 1480.5922 | 1481.7473 | 1 | 4.548 | 59.1% | 1 | R.TEMENEFVLIKK.D | 2 |
|  | AstrinIP\_MS1\_022614\_02.05385.05385.3 | 3.8787 | 0.3457 | 100.0% | 1798.9143 | 1798.9623 | 1 | 5.515 | 44.6% | 2 | K.DVDEAYMNKVELESR.L | 3 |
|  | AstrinIP\_MS2\_022614\_01.07468.07468.2 | 3.3952 | 0.3456 | 100.0% | 1799.7122 | 1798.9623 | 1 | 5.621 | 57.1% | 1 | K.DVDEAYMNKVELESR.L | 2 |
|  | AstrinIP\_MS1\_022614\_01.11865.11865.2 | 3.9647 | 0.4499 | 100.0% | 1421.1522 | 1420.6055 | 1 | 8.176 | 90.9% | 4 | R.LEGLTDEINFLR.Q | 2 |
|  | AstrinIP\_MS1\_022614\_01.05772.05772.2 | 2.3103 | 0.1976 | 98.1% | 1081.8522 | 1080.1827 | 1 | 4.224 | 78.6% | 1 | R.QLYEEEIR.E | 2 |
|  | AstrinIP\_MS2\_022614\_01.12279.12279.2 | 4.224 | 0.4437 | 100.0% | 1321.3722 | 1321.5286 | 1 | 8.286 | 77.3% | 2 | R.SLDMDSIIAEVK.A | 2 |
|  | AstrinIP\_MS2\_022614\_01.04235.04235.1 | 1.9188 | 0.3079 | 100.0% | 1079.25 | 1080.1423 | 62 | 5.673 | 56.2% | 1 | K.AQYEDIANR.S | 1 |
|  | AstrinIP\_MS2\_022614\_01.09298.09298.3 | 5.4037 | 0.4769 | 100.0% | 2531.5144 | 2532.828 | 1 | 7.376 | 42.9% | 1 | R.SRAEAESMYQIKYEELQSLAGK.H | 3 |
|  | AstrinIP\_MS2\_022614\_01.05518.05518.2 | 3.4187 | 0.4835 | 100.0% | 1170.0322 | 1170.3228 | 1 | 6.91 | 83.3% | 1 | R.AEAESMYQIK.Y | 2 |
|  | AstrinIP\_MS2\_022614\_01.10236.10236.3 | 3.0702 | 0.3018 | 99.2% | 2288.3643 | 2289.5623 | 4 | 5.076 | 26.3% | 1 | R.AEAESMYQIKYEELQSLAGK.H | 3 |
|  | AstrinIP\_MS1\_022614\_01.06303.06303.2 | 3.329 | 0.0745 | 98.8% | 1138.1122 | 1138.2627 | 1 | 6.978 | 83.3% | 2 | K.YEELQSLAGK.H | 2 |
|  | AstrinIP\_MS2\_022614\_01.03615.03615.2 | 3.2173 | 0.4397 | 100.0% | 1210.0122 | 1209.36 | 1 | 6.93 | 88.9% | 1 | R.TKTEISEMNR.N | 2 |
|  | AstrinIP\_MS2\_022614\_01.06599.06599.1 | 2.1469 | 0.191 | 97.4% | 1000.6 | 1001.168 | 5 | 5.746 | 68.8% | 2 | R.LQAEIEGLK.G | 1 |
|  | AstrinIP\_MS2\_022614\_01.04728.04728.2 | 3.1028 | 0.2975 | 99.8% | 1342.2122 | 1342.5381 | 1 | 7.822 | 72.7% | 2 | R.LQAEIEGLKGQR.A | 2 |
|  | AstrinIP\_MS1\_022614\_01.08805.08805.2 | 4.1103 | 0.3698 | 100.0% | 1345.8522 | 1345.452 | 1 | 6.879 | 66.7% | 3 | R.ASLEAAIADAEQR.G | 2 |
|  | AstrinIP\_MS1\_022614\_01.11399.11399.2 | 4.6087 | 0.4962 | 100.0% | 1956.7122 | 1957.1912 | 1 | 8.891 | 58.3% | 3 | R.ASLEAAIADAEQRGELAIK.D | 2 |
|  | AstrinIP\_MS2\_022614\_01.12456.12456.3 | 4.4153 | 0.4112 | 100.0% | 2456.9644 | 2456.7153 | 1 | 5.963 | 34.8% | 2 | R.ASLEAAIADAEQRGELAIKDANAK.L | 3 |
|  | AstrinIP\_MS2\_022614\_01.03687.03687.2 | 3.2598 | 0.3466 | 100.0% | 1130.1522 | 1130.2865 | 3 | 6.237 | 70.0% | 1 | R.GELAIKDANAK.L | 2 |
|  | AstrinIP\_MS2\_022614\_01.08400.08400.1 | 2.2917 | 0.2629 | 99.4% | 1129.59 | 1130.2865 | 4 | 5.525 | 55.6% | 2 | K.LSELEAALQR.A | 1 |
|  | AstrinIP\_MS1\_022614\_01.08002.08002.2 | 3.9923 | 0.247 | 100.0% | 1130.0721 | 1130.2865 | 1 | 6.065 | 83.3% | 5 | K.LSELEAALQR.A | 2 |
|  | AstrinIP\_MS2\_022614\_01.06780.06780.1 | 2.5379 | 0.2335 | 99.5% | 1153.47 | 1154.3234 | 178 | 5.811 | 56.2% | 1 | R.EYQELMNVK.L | 11 |
|  | AstrinIP\_MS2\_022614\_01.08886.08886.3 | 4.572 | 0.3562 | 100.0% | 2390.0942 | 2390.6887 | 1 | 5.849 | 36.2% | 1 | K.LLEGEESRLESGMQNMSIHTK.T | 3 |
|  | AstrinIP\_MS1\_022614\_01.04522.04522.2 | 3.7835 | 0.383 | 100.0% | 1476.0521 | 1476.7058 | 1 | 7.102 | 75.0% | 1 | R.LESGMQNMSIHTK.T | 2 |
|  | AstrinIP\_MS2\_022614\_01.04589.04589.3 | 3.1487 | 0.2583 | 99.2% | 1477.1643 | 1476.7058 | 14 | 4.931 | 39.6% | 1 | R.LESGMQNMSIHTK.T | 3 |
|  | AstrinIP\_MS2\_022614\_01.05703.05703.2 | 3.2637 | 0.3927 | 100.0% | 1174.2122 | 1174.3367 | 1 | 7.751 | 75.0% | 7 | K.LVSESSDVLPK.- | 2 |

Similarities:
gi|47132620|ref|NP\_00(1:31)  
gi|67782365|ref|NP\_00(1:31)  
gi|119395750|ref|NP\_0(1:31)  
gi|119703753|ref|NP\_0(3:29)  

---

|  |  |  |  |  |  |  |  |  |
| --- | --- | --- | --- | --- | --- | --- | --- | --- |
| U | *gi|4758792|ref|NP\_004* | 4 | 5 | 50.8% | 124 | 13712 | 8.3 | NADH dehydrogenase (ubiquinone) Fe-S protein 6, 13kDa (NADH-coenzyme Q reductase) [Homo sapiens] |

| Filename XCorr DeltCN Conf% ObsM+H+ CalcM+H+ SpR ZScore Ion% # Sequence  | | | | | | | | | | | | |
| --- | --- | --- | --- | --- | --- | --- | --- | --- | --- | --- | --- | --- |
| \* | AstrinIP\_MS1\_022614\_01.03639.03639.3 | 3.1152 | 0.3403 | 99.9% | 1698.1444 | 1697.8015 | 1 | 5.367 | 38.5% | 1 | K.VTHTGQVYDDKDYR.R | 3 |
| \* | AstrinIP\_MS2\_022614\_01.11836.11836.3 | 3.7494 | 0.2252 | 98.4% | 2759.8743 | 2760.03 | 11 | 4.406 | 25.0% | 1 | R.QKEVNENFAIDLIAEQPVSEVETR.V | 3 |
| \* | AstrinIP\_MS1\_022614\_01.03749.03749.2 | 3.4197 | 0.4126 | 100.0% | 1409.4922 | 1409.5511 | 1 | 7.125 | 71.4% | 1 | R.VIACDGGGGALGHPK.V | 2 |
| \* | AstrinIP\_MS1\_022614\_01.04727.04727.2 | 3.0558 | 0.216 | 99.8% | 1223.4722 | 1223.4117 | 4 | 4.995 | 66.7% | 2 | K.VYINLDKETK.T | 2 |

---

|  |  |  |  |  |  |  |  |  |
| --- | --- | --- | --- | --- | --- | --- | --- | --- |
| U | *contaminant\_KERATIN09* | 17 | 48 | 50.6% | 429 | 47927 | 5.5 | no description |
| U | *gi|4557888|ref|NP\_000* | 17 | 48 | 50.5% | 430 | 48058 | 5.5 | keratin 18 [Homo sapiens] |
| U | *gi|40354195|ref|NP\_95* | 17 | 48 | 50.5% | 430 | 48058 | 5.5 | keratin 18 [Homo sapiens] |

| Filename XCorr DeltCN Conf% ObsM+H+ CalcM+H+ SpR ZScore Ion% # Sequence  | | | | | | | | | | | | |
| --- | --- | --- | --- | --- | --- | --- | --- | --- | --- | --- | --- | --- |
|  | AstrinIP\_MS1\_022614\_02.05305.05305.3 | 5.0831 | 0.5919 | 100.0% | 2854.9744 | 2856.0813 | 1 | 9.384 | 30.0% | 3 | R.SLGSVQAPSYGARPVSSAASVYAGAGGSGSR.I | 3 |
|  | AstrinIP\_MS2\_022614\_01.11666.11666.3 | 4.4651 | 0.4406 | 100.0% | 3336.4443 | 3337.7224 | 1 | 6.878 | 24.3% | 1 | R.GGMGSGGLATGIAGGLAGMGGIQNEKETMQSLNDR.L | 3 |
|  | AstrinIP\_MS2\_022614\_01.08771.08771.2 | 2.0623 | 0.2236 | 97.4% | 983.03217 | 983.0709 | 22 | 4.665 | 75.0% | 1 | R.DWSHYFK.I | 2 |
|  | AstrinIP\_MS2\_022614\_01.06284.06284.2 | 3.8334 | 0.4126 | 100.0% | 1320.2722 | 1320.4478 | 1 | 8.184 | 81.8% | 5 | R.AQIFANTVDNAR.I | 2 |
|  | AstrinIP\_MS2\_022614\_01.06832.06832.2 | 2.8567 | 0.2333 | 99.8% | 1041.4521 | 1042.2235 | 1 | 6.542 | 87.5% | 2 | R.IVLQIDNAR.L | 22 |
|  | AstrinIP\_MS2\_022614\_01.06845.06845.1 | 2.5181 | 0.3095 | 100.0% | 1041.62 | 1042.2235 | 1 | 6.391 | 68.8% | 1 | R.IVLQIDNAR.L | 11 |
|  | AstrinIP\_MS2\_022614\_01.05006.05006.2 | 2.8702 | 0.2739 | 99.8% | 1241.4922 | 1240.4601 | 12 | 5.592 | 72.2% | 2 | R.VKYETELAMR.Q | 2 |
|  | AstrinIP\_MS1\_022614\_01.03784.03784.2 | 2.8204 | 0.1634 | 98.7% | 1176.0521 | 1175.3274 | 5 | 5.449 | 72.2% | 1 | R.KVIDDTNITR.L | 2 |
|  | AstrinIP\_MS2\_022614\_01.15755.15755.2 | 6.05 | 0.5596 | 100.0% | 2177.892 | 2178.589 | 1 | 9.837 | 52.9% | 2 | R.LQLETEIEALKEELLFMK.K | 2 |
|  | AstrinIP\_MS2\_022614\_02.10737.10737.3 | 2.7454 | 0.3115 | 99.1% | 2178.5645 | 2178.589 | 130 | 5.098 | 27.9% | 3 | R.LQLETEIEALKEELLFMK.K | 3 |
|  | AstrinIP\_MS2\_022614\_01.09275.09275.3 | 5.1529 | 0.4941 | 100.0% | 2750.3342 | 2751.0227 | 1 | 7.508 | 38.0% | 3 | K.NHEEEVKGLQAQIASSGLTVEVDAPK.S | 3 |
|  | AstrinIP\_MS2\_022614\_01.11246.11246.2 | 4.0233 | 0.534 | 100.0% | 1507.1721 | 1507.699 | 1 | 9.19 | 75.0% | 6 | R.TVQSLEIDLDSMR.N | 2 |
|  | AstrinIP\_MS2\_022614\_01.16784.16784.2 | 5.6445 | 0.5358 | 100.0% | 2671.1921 | 2672.0715 | 1 | 9.644 | 56.8% | 2 | R.YALQMEQLNGILLHLESELAQTR.A | 2 |
|  | AstrinIP\_MS2\_022614\_01.16778.16778.3 | 6.084 | 0.5023 | 100.0% | 2672.3044 | 2672.0715 | 1 | 8.614 | 45.5% | 10 | R.YALQMEQLNGILLHLESELAQTR.A | 3 |
|  | AstrinIP\_MS2\_022614\_01.10239.10239.2 | 3.3575 | 0.4186 | 100.0% | 1420.2122 | 1420.6055 | 2 | 6.871 | 72.7% | 2 | R.QAQEYEALLNIK.V | 2 |
|  | AstrinIP\_MS2\_022614\_02.05049.05049.2 | 2.9922 | 0.3591 | 100.0% | 1292.8322 | 1293.5059 | 1 | 7.031 | 80.0% | 3 | K.VKLEAEIATYR.R | 2 |
|  | AstrinIP\_MS2\_022614\_01.06021.06021.2 | 2.4758 | 0.3038 | 99.7% | 1066.2122 | 1066.1992 | 1 | 5.703 | 87.5% | 1 | K.LEAEIATYR.R | 2 |

Similarities:
contaminant\_KERATIN10(2:15)  

---

|  |  |  |  |  |  |  |  |  |
| --- | --- | --- | --- | --- | --- | --- | --- | --- |
| U | *contaminant\_gi|746301* | 17 | 96 | 49.8% | 269 | 27961 | 6.7 | lysyl endopeptidase (EC 3.4.21.50) - Lysobacter enzymogenes |

| Filename XCorr DeltCN Conf% ObsM+H+ CalcM+H+ SpR ZScore Ion% # Sequence  | | | | | | | | | | | | |
| --- | --- | --- | --- | --- | --- | --- | --- | --- | --- | --- | --- | --- |
| \* | AstrinIP\_MS1\_022614\_01.04352.04352.2 | 6.0946 | 0.5861 | 100.0% | 2261.5122 | 2262.355 | 1 | 11.048 | 50.0% | 14 | R.APGSSSSGANGDGSLAQSQTGAVVR.A | 2 |
| \* | AstrinIP\_MS2\_022614\_01.04539.04539.3 | 4.9517 | 0.4334 | 100.0% | 2262.0544 | 2262.355 | 1 | 7.946 | 38.5% | 8 | R.APGSSSSGANGDGSLAQSQTGAVVR.A | 3 |
| \* | AstrinIP\_MS2\_022614\_02.11607.11607.3 | 5.1952 | 0.3654 | 100.0% | 3314.9043 | 3315.6257 | 1 | 5.297 | 25.0% | 5 | R.ATNAASDFTLLELNTAANPAYNLFWAGWDR.R | 3 |
| \* | AstrinIP\_MS2\_022614\_01.17097.17097.2 | 5.4131 | 0.5201 | 100.0% | 3315.9521 | 3315.6257 | 1 | 9.768 | 41.4% | 10 | R.ATNAASDFTLLELNTAANPAYNLFWAGWDR.R | 2 |
| \* | AstrinIP\_MS1\_022614\_01.15476.15476.3 | 6.1847 | 0.534 | 100.0% | 3472.8843 | 3471.813 | 1 | 9.0 | 28.3% | 10 | R.ATNAASDFTLLELNTAANPAYNLFWAGWDRR.D | 3 |
| \* | AstrinIP\_MS2\_022614\_01.15992.15992.3 | 4.3121 | 0.2183 | 99.2% | 3475.2544 | 3475.6257 | 40 | 4.07 | 19.8% | 1 | R.ATNAASDFTLLELNT#AANPAY@NLFWAGWDR.R | 3 |
| \* | AstrinIP\_MS1\_022614\_01.03960.03960.2 | 4.0013 | 0.44 | 100.0% | 2076.2522 | 2077.2668 | 1 | 7.221 | 47.2% | 2 | R.RDQNFAGATAIHHPNVAEK.R | 2 |
| \* | AstrinIP\_MS1\_022614\_01.03936.03936.3 | 4.6527 | 0.3842 | 100.0% | 2076.4443 | 2077.2668 | 1 | 6.592 | 43.1% | 2 | R.RDQNFAGATAIHHPNVAEK.R | 3 |
| \* | AstrinIP\_MS1\_022614\_01.04468.04468.2 | 4.5806 | 0.4778 | 100.0% | 1921.2122 | 1921.0793 | 1 | 7.836 | 55.9% | 5 | R.DQNFAGATAIHHPNVAEK.R | 2 |
| \* | AstrinIP\_MS2\_022614\_02.03508.03508.3 | 2.9129 | 0.306 | 99.2% | 1921.8243 | 1921.0793 | 2 | 5.756 | 36.8% | 2 | R.DQNFAGATAIHHPNVAEK.R | 3 |
| \* | AstrinIP\_MS2\_022614\_01.04144.04144.2 | 5.4298 | 0.4281 | 100.0% | 2076.4722 | 2077.2668 | 1 | 7.697 | 52.8% | 3 | R.DQNFAGATAIHHPNVAEKR.I | 2 |
| \* | AstrinIP\_MS2\_022614\_01.04190.04190.3 | 3.9421 | 0.4536 | 100.0% | 2077.0444 | 2077.2668 | 1 | 7.302 | 37.5% | 6 | R.DQNFAGATAIHHPNVAEKR.I | 3 |
| \* | AstrinIP\_MS1\_022614\_01.04006.04006.3 | 3.9199 | 0.5078 | 100.0% | 1870.2544 | 1870.983 | 1 | 8.364 | 41.7% | 2 | R.VLGQLHGGPSSCSATGADR.S | 3 |
| \* | AstrinIP\_MS1\_022614\_01.04001.04001.2 | 4.4505 | 0.4055 | 100.0% | 1870.4321 | 1870.983 | 1 | 7.003 | 63.9% | 2 | R.VLGQLHGGPSSCSATGADR.S | 2 |
| \* | AstrinIP\_MS2\_022614\_01.06857.06857.2 | 4.7236 | 0.5269 | 100.0% | 1427.8922 | 1428.5443 | 1 | 8.482 | 76.9% | 16 | R.VFTSWTGGGTSATR.L | 2 |
| \* | AstrinIP\_MS2\_022614\_01.06909.06909.1 | 2.3525 | 0.2784 | 100.0% | 1429.63 | 1428.5443 | 1 | 4.877 | 53.8% | 7 | R.VFTSWTGGGTSATR.L | 1 |
| \* | AstrinIP\_MS2\_022614\_01.15401.15401.2 | 4.0227 | 0.5483 | 100.0% | 2604.7322 | 2605.8174 | 1 | 9.123 | 38.0% | 1 | R.LSDWLDAAGTGAQFIDGLDSTGTPPV.- | 2 |

---

|  |  |  |  |  |  |  |  |  |
| --- | --- | --- | --- | --- | --- | --- | --- | --- |
| U | *gi|16507237|ref|NP\_00* | 40 | 129 | 49.4% | 654 | 72333 | 5.2 | heat shock 70kDa protein 5 [Homo sapiens] |

| Filename XCorr DeltCN Conf% ObsM+H+ CalcM+H+ SpR ZScore Ion% # Sequence  | | | | | | | | | | | | |
| --- | --- | --- | --- | --- | --- | --- | --- | --- | --- | --- | --- | --- |
|  | AstrinIP\_MS1\_022614\_01.03897.03897.2 | 3.2626 | 0.2866 | 99.8% | 1556.4521 | 1556.6786 | 1 | 5.499 | 69.2% | 2 | K.NGRVEIIANDQGNR.I | 2 |
|  | AstrinIP\_MS1\_022614\_01.08324.08324.2 | 3.7808 | 0.4627 | 100.0% | 1568.1522 | 1567.7386 | 1 | 9.238 | 69.2% | 6 | R.ITPSYVAFTPEGER.L | 2 |
|  | AstrinIP\_MS1\_022614\_01.07148.07148.2 | 4.7206 | 0.4906 | 100.0% | 1679.3522 | 1678.796 | 1 | 8.943 | 78.6% | 6 | K.NQLTSNPENTVFDAK.R | 2 |
|  | AstrinIP\_MS2\_022614\_01.06448.06448.2 | 4.4444 | 0.4873 | 100.0% | 1834.4922 | 1834.9835 | 1 | 8.799 | 70.0% | 2 | K.NQLTSNPENTVFDAKR.L | 2 |
|  | AstrinIP\_MS1\_022614\_01.06189.06189.2 | 3.8106 | 0.3422 | 100.0% | 1432.1322 | 1431.5449 | 1 | 6.931 | 81.8% | 6 | R.TWNDPSVQQDIK.F | 2 |
|  | AstrinIP\_MS2\_022614\_01.06405.06405.2 | 4.2859 | 0.523 | 100.0% | 1605.3922 | 1605.8314 | 1 | 8.831 | 89.3% | 9 | K.TKPYIQVDIGGGQTK.T | 2 |
|  | AstrinIP\_MS2\_022614\_01.12558.12558.1 | 2.4231 | 0.4899 | 100.0% | 1536.62 | 1537.8114 | 11 | 7.966 | 42.3% | 1 | K.TFAPEEISAMVLTK.M | 1 |
|  | AstrinIP\_MS2\_022614\_01.12578.12578.2 | 4.6116 | 0.5278 | 100.0% | 1538.2522 | 1537.8114 | 1 | 9.013 | 84.6% | 5 | K.TFAPEEISAMVLTK.M | 2 |
|  | AstrinIP\_MS2\_022614\_01.04500.04500.2 | 3.384 | 0.3427 | 100.0% | 1240.5122 | 1241.445 | 1 | 6.647 | 80.0% | 2 | K.MKETAEAYLGK.K | 2 |
|  | AstrinIP\_MS2\_022614\_01.03926.03926.2 | 3.7121 | 0.3075 | 100.0% | 1369.0721 | 1369.619 | 1 | 6.34 | 77.3% | 1 | K.MKETAEAYLGKK.V | 2 |
|  | AstrinIP\_MS2\_022614\_01.03928.03928.3 | 3.9427 | 0.3935 | 100.0% | 1371.1144 | 1369.619 | 1 | 6.312 | 50.0% | 1 | K.MKETAEAYLGKK.V | 3 |
|  | AstrinIP\_MS1\_022614\_01.07880.07880.2 | 4.6234 | 0.507 | 100.0% | 1888.5521 | 1889.121 | 1 | 9.523 | 71.9% | 4 | K.VTHAVVTVPAYFNDAQR.Q | 2 |
|  | AstrinIP\_MS1\_022614\_01.07916.07916.3 | 4.4212 | 0.4155 | 100.0% | 1888.7043 | 1889.121 | 1 | 7.325 | 40.6% | 3 | K.VTHAVVTVPAYFNDAQR.Q | 3 |
|  | AstrinIP\_MS1\_022614\_01.08560.08560.1 | 2.3738 | 0.408 | 100.0% | 1217.44 | 1218.4137 | 154 | 6.54 | 50.0% | 1 | K.DAGTIAGLNVMR.I | 1 |
|  | AstrinIP\_MS1\_022614\_01.08596.08596.2 | 3.8689 | 0.4284 | 100.0% | 1218.1522 | 1218.4137 | 1 | 7.466 | 86.4% | 4 | K.DAGTIAGLNVMR.I | 2 |
|  | AstrinIP\_MS1\_022614\_01.09921.09921.2 | 5.232 | 0.5436 | 100.0% | 1660.4922 | 1660.9078 | 1 | 10.069 | 80.0% | 8 | R.IINEPTAAAIAYGLDK.R | 2222 |
|  | AstrinIP\_MS1\_022614\_01.08842.08842.2 | 5.0342 | 0.4454 | 100.0% | 1815.7122 | 1817.0953 | 1 | 7.836 | 81.2% | 2 | R.IINEPTAAAIAYGLDKR.E | 2 |
|  | AstrinIP\_MS1\_022614\_01.08889.08889.3 | 2.7471 | 0.2552 | 97.0% | 1817.0643 | 1817.0953 | 92 | 5.521 | 26.6% | 1 | R.IINEPTAAAIAYGLDKR.E | 3 |
|  | AstrinIP\_MS2\_022614\_02.08158.08158.2 | 5.9482 | 0.603 | 100.0% | 2165.5522 | 2166.3025 | 1 | 11.014 | 73.5% | 3 | R.IEIESFYEGEDFSETLTR.A | 2 |
|  | AstrinIP\_MS2\_022614\_01.11256.11256.3 | 4.5178 | 0.3106 | 100.0% | 1514.0343 | 1513.7516 | 1 | 5.471 | 56.8% | 1 | R.AKFEELNMDLFR.S | 3 |
|  | AstrinIP\_MS1\_022614\_01.10524.10524.2 | 3.934 | 0.4777 | 100.0% | 1514.6522 | 1513.7516 | 1 | 7.471 | 68.2% | 3 | R.AKFEELNMDLFR.S | 2 |
|  | AstrinIP\_MS2\_022614\_01.12604.12604.2 | 3.5805 | 0.4315 | 100.0% | 1314.6322 | 1314.4987 | 1 | 7.81 | 77.8% | 2 | K.FEELNMDLFR.S | 2 |
|  | AstrinIP\_MS1\_022614\_01.03948.03948.1 | 1.9054 | 0.2407 | 99.1% | 918.34 | 919.0196 | 2 | 5.479 | 71.4% | 2 | K.VLEDSDLK.K | 1 |
|  | AstrinIP\_MS2\_022614\_01.08481.08481.2 | 4.1273 | 0.2897 | 100.0% | 1589.9922 | 1589.7863 | 1 | 7.463 | 71.4% | 6 | K.KSDIDEIVLVGGSTR.I | 2 |
|  | AstrinIP\_MS1\_022614\_01.07550.07550.2 | 5.2088 | 0.5282 | 100.0% | 1837.4122 | 1838.0245 | 1 | 9.838 | 75.0% | 8 | K.SQIFSTASDNQPTVTIK.V | 22 |
|  | AstrinIP\_MS2\_022614\_01.03674.03674.2 | 2.4981 | 0.3322 | 99.8% | 1192.0721 | 1192.3574 | 1 | 6.147 | 72.2% | 1 | K.VYEGERPLTK.D | 22 |
|  | AstrinIP\_MS1\_022614\_01.11088.11088.2 | 4.1652 | 0.564 | 100.0% | 1934.5122 | 1935.19 | 1 | 9.377 | 61.8% | 4 | K.DNHLLGTFDLTGIPPAPR.G | 22 |
|  | AstrinIP\_MS2\_022614\_01.03741.03741.2 | 2.5616 | 0.2861 | 99.7% | 1075.0322 | 1075.1667 | 5 | 6.002 | 75.0% | 1 | K.ITITNDQNR.L | 22 |
|  | AstrinIP\_MS1\_022614\_01.08865.08865.2 | 2.7128 | 0.3476 | 99.7% | 1801.4722 | 1802.9788 | 1 | 5.941 | 60.7% | 1 | R.IDTRNELESYAYSLK.N | 2 |
|  | AstrinIP\_MS2\_022614\_01.09395.09395.3 | 3.8743 | 0.2334 | 99.5% | 1804.1344 | 1802.9788 | 1 | 4.226 | 46.4% | 1 | R.IDTRNELESYAYSLK.N | 3 |
|  | AstrinIP\_MS2\_022614\_01.09075.09075.1 | 2.4132 | 0.2165 | 99.1% | 1316.6 | 1317.4381 | 1 | 4.461 | 65.0% | 1 | R.NELESYAYSLK.N | 1 |
|  | AstrinIP\_MS2\_022614\_01.09066.09066.2 | 3.2504 | 0.1992 | 99.8% | 1318.9722 | 1317.4381 | 1 | 4.631 | 90.0% | 4 | R.NELESYAYSLK.N | 2 |
|  | AstrinIP\_MS2\_022614\_02.06641.06641.3 | 4.3207 | 0.3775 | 100.0% | 2532.9543 | 2532.723 | 1 | 6.091 | 36.2% | 4 | K.AVEEKIEWLESHQDADIEDFK.A | 3 |
|  | AstrinIP\_MS2\_022614\_01.10173.10173.2 | 5.1961 | 0.3803 | 100.0% | 1975.4122 | 1976.1064 | 1 | 8.593 | 66.7% | 2 | K.IEWLESHQDADIEDFK.A | 2 |
|  | AstrinIP\_MS2\_022614\_01.10150.10150.3 | 4.4153 | 0.3649 | 100.0% | 1976.3944 | 1976.1064 | 1 | 6.41 | 45.0% | 4 | K.IEWLESHQDADIEDFK.A | 3 |
|  | AstrinIP\_MS1\_022614\_02.06199.06199.3 | 4.7665 | 0.3464 | 100.0% | 2175.7144 | 2175.3594 | 1 | 6.433 | 38.2% | 2 | K.IEWLESHQDADIEDFKAK.K | 3 |
|  | AstrinIP\_MS1\_022614\_01.07157.07157.2 | 5.0975 | 0.3443 | 100.0% | 1654.4521 | 1654.9878 | 1 | 7.274 | 84.6% | 4 | K.KKELEEIVQPIISK.L | 2 |
|  | AstrinIP\_MS1\_022614\_01.07156.07156.3 | 3.4863 | 0.1946 | 98.3% | 1654.7644 | 1654.9878 | 16 | 4.607 | 38.5% | 1 | K.KKELEEIVQPIISK.L | 3 |
|  | AstrinIP\_MS1\_022614\_01.09304.09304.2 | 3.9748 | 0.4283 | 100.0% | 1398.3522 | 1398.6396 | 1 | 7.223 | 81.8% | 4 | K.ELEEIVQPIISK.L | 2 |
|  | AstrinIP\_MS1\_022614\_01.06797.06797.2 | 5.8034 | 0.6523 | 100.0% | 2177.5522 | 2177.283 | 1 | 11.211 | 57.5% | 6 | K.LYGSAGPPPTGEEDTAEKDEL.- | 2 |

Similarities:
gi|5729877|ref|NP\_006(1:39)  
contaminant\_GR78\_PIG(4:36)  
gi|124256496|ref|NP\_0(1:39)  
gi|13676857|ref|NP\_06(1:39)  

---

|  |  |  |  |  |  |  |  |  |
| --- | --- | --- | --- | --- | --- | --- | --- | --- |
| U | *gi|14389309|ref|NP\_11* | 19 | 58 | 47.7% | 449 | 49895 | 5.1 | tubulin alpha 6 [Homo sapiens] |
| U | *gi|57013276|ref|NP\_00* | 19 | 58 | 47.5% | 451 | 50152 | 5.1 | tubulin, alpha, ubiquitous [Homo sapiens] |

| Filename XCorr DeltCN Conf% ObsM+H+ CalcM+H+ SpR ZScore Ion% # Sequence  | | | | | | | | | | | | |
| --- | --- | --- | --- | --- | --- | --- | --- | --- | --- | --- | --- | --- |
|  | AstrinIP\_MS2\_022614\_01.11679.11679.2 | 5.387 | 0.6075 | 100.0% | 2008.4922 | 2009.093 | 1 | 11.234 | 57.9% | 6 | K.TIGGGDDSFNTFFSETGAGK.H | 2 |
|  | AstrinIP\_MS2\_022614\_02.07323.07323.2 | 3.8151 | 0.5117 | 100.0% | 1702.6522 | 1702.9451 | 2 | 8.268 | 53.6% | 7 | R.AVFVDLEPTVIDEVR.T | 2 |
|  | AstrinIP\_MS2\_022614\_01.09048.09048.2 | 2.6573 | 0.4517 | 100.0% | 1410.3121 | 1411.6439 | 51 | 6.74 | 54.5% | 2 | R.QLFHPEQLITGK.E | 2 |
|  | AstrinIP\_MS1\_022614\_01.08129.08129.3 | 3.9292 | 0.4711 | 100.0% | 2415.5645 | 2416.6555 | 1 | 7.419 | 31.2% | 3 | R.QLFHPEQLITGKEDAANNYAR.G | 3 |
|  | AstrinIP\_MS2\_022614\_01.13612.13612.2 | 3.6378 | 0.4056 | 100.0% | 1843.5721 | 1843.1332 | 1 | 7.241 | 56.7% | 1 | R.GHYTIGKEIIDLVLDR.I | 2 |
|  | AstrinIP\_MS2\_022614\_01.11937.11937.2 | 2.2201 | 0.3385 | 99.4% | 1086.2922 | 1086.2737 | 1 | 6.305 | 81.2% | 1 | K.EIIDLVLDR.I | 2 |
|  | AstrinIP\_MS2\_022614\_01.03693.03693.2 | 2.3076 | 0.2225 | 98.5% | 909.9922 | 910.05804 | 2 | 5.244 | 78.6% | 1 | R.LSVDYGKK.S | 2 |
|  | AstrinIP\_MS2\_022614\_01.05537.05537.3 | 3.1267 | 0.2098 | 97.2% | 1876.0443 | 1876.0824 | 1 | 5.415 | 41.1% | 1 | R.RNLDIERPTYTNLNR.L | 3 |
|  | AstrinIP\_MS1\_022614\_01.06474.06474.2 | 3.1464 | 0.1804 | 98.9% | 1719.8922 | 1719.8949 | 1 | 4.53 | 53.8% | 3 | R.NLDIERPTYTNLNR.L | 2 |
|  | AstrinIP\_MS2\_022614\_01.14877.14877.2 | 4.5225 | 0.4952 | 100.0% | 1489.4521 | 1488.7678 | 1 | 9.541 | 80.8% | 6 | R.LISQIVSSITASLR.F | 2 |
|  | AstrinIP\_MS1\_022614\_01.13464.13464.2 | 4.7644 | 0.4704 | 100.0% | 2411.2522 | 2410.6885 | 1 | 8.273 | 47.5% | 2 | R.FDGALNVDLTEFQTNLVPYPR.I | 2 |
|  | AstrinIP\_MS2\_022614\_01.11182.11182.2 | 4.2471 | 0.5532 | 100.0% | 1758.3722 | 1758.0703 | 1 | 9.202 | 66.7% | 7 | R.IHFPLATYAPVISAEK.A | 2 |
|  | AstrinIP\_MS1\_022614\_01.08429.08429.2 | 2.7335 | 0.3316 | 99.9% | 1249.9922 | 1250.4304 | 1 | 6.82 | 81.2% | 1 | K.YMACCLLYR.G | 2 |
|  | AstrinIP\_MS1\_022614\_01.06694.06694.1 | 1.7775 | 0.417 | 100.0% | 1015.52 | 1016.1827 | 2 | 6.128 | 61.1% | 4 | K.DVNAAIATIK.T | 1 |
|  | AstrinIP\_MS2\_022614\_01.09147.09147.2 | 4.5299 | 0.4948 | 100.0% | 1825.4722 | 1826.1027 | 1 | 7.664 | 61.8% | 7 | K.VGINYQPPTVVPGGDLAK.V | 2 |
|  | AstrinIP\_MS2\_022614\_01.06821.06821.2 | 2.9495 | 0.3954 | 100.0% | 1380.5322 | 1381.6324 | 1 | 6.968 | 75.0% | 1 | R.LDHKFDLMYAK.R | 2 |
|  | AstrinIP\_MS2\_022614\_01.06819.06819.3 | 4.0314 | 0.3662 | 100.0% | 1381.5243 | 1381.6324 | 1 | 6.356 | 60.0% | 2 | R.LDHKFDLMYAK.R | 3 |
|  | AstrinIP\_MS2\_022614\_01.06849.06849.1 | 2.8794 | 0.1799 | 99.1% | 1382.19 | 1381.6324 | 1 | 4.024 | 65.0% | 1 | R.LDHKFDLMYAK.R | 1 |
|  | AstrinIP\_MS2\_022614\_01.11064.11064.3 | 3.8386 | 0.3568 | 100.0% | 2330.6343 | 2331.5208 | 1 | 6.258 | 35.5% | 2 | R.AFVHWYVGEGMEEGEFSEAR.E | 3 |

---

|  |  |  |  |  |  |  |  |  |
| --- | --- | --- | --- | --- | --- | --- | --- | --- |
| U | *gi|167466173|ref|NP\_0* | 28 | 84 | 46.0% | 641 | 70052 | 5.6 | heat shock 70kDa protein 1B [Homo sapiens] |
| U | *gi|194248072|ref|NP\_0* | 28 | 84 | 46.0% | 641 | 70052 | 5.6 | heat shock 70kDa protein 1A [Homo sapiens] |

| Filename XCorr DeltCN Conf% ObsM+H+ CalcM+H+ SpR ZScore Ion% # Sequence  | | | | | | | | | | | | |
| --- | --- | --- | --- | --- | --- | --- | --- | --- | --- | --- | --- | --- |
|  | AstrinIP\_MS2\_022614\_01.07734.07734.1 | 2.2714 | 0.4224 | 100.0% | 1487.62 | 1488.5939 | 1 | 6.368 | 50.0% | 5 | R.TTPSYVAFTDTER.L | 11111 |
|  | AstrinIP\_MS1\_022614\_02.04868.04868.2 | 3.849 | 0.5598 | 100.0% | 1489.2922 | 1488.5939 | 1 | 9.39 | 79.2% | 10 | R.TTPSYVAFTDTER.L | 22222 |
|  | AstrinIP\_MS2\_022614\_01.08174.08174.2 | 5.0676 | 0.42 | 100.0% | 1659.4122 | 1659.8394 | 1 | 8.71 | 82.1% | 5 | K.NQVALNPQNTVFDAK.R | 2 |
|  | AstrinIP\_MS1\_022614\_01.04853.04853.2 | 2.7714 | 0.3221 | 99.8% | 1350.6721 | 1351.5603 | 1 | 6.154 | 63.6% | 2 | R.KFGDPVVQSDMK.H | 2 |
|  | AstrinIP\_MS2\_022614\_01.06262.06262.2 | 2.8727 | 0.4972 | 100.0% | 1222.4122 | 1223.3862 | 1 | 8.266 | 70.0% | 4 | K.FGDPVVQSDMK.H | 2 |
|  | AstrinIP\_MS2\_022614\_01.08219.08219.2 | 4.0316 | 0.4162 | 100.0% | 1681.5322 | 1681.8912 | 1 | 7.259 | 73.1% | 4 | K.HWPFQVINDGDKPK.V | 2 |
|  | AstrinIP\_MS1\_022614\_01.07809.07809.3 | 4.2975 | 0.3162 | 100.0% | 1682.0343 | 1681.8912 | 1 | 6.003 | 51.9% | 5 | K.HWPFQVINDGDKPK.V | 3 |
|  | AstrinIP\_MS1\_022614\_01.11918.11918.2 | 4.8916 | 0.5504 | 100.0% | 1616.3722 | 1615.8817 | 1 | 8.96 | 84.6% | 7 | K.AFYPEEISSMVLTK.M | 22 |
|  | AstrinIP\_MS2\_022614\_01.13391.13391.3 | 2.8753 | 0.2499 | 95.3% | 3262.8245 | 3262.7046 | 1 | 5.318 | 23.2% | 1 | K.MKEIAEAYLGYPVTNAVITVPAYFNDSQR.Q | 3 |
|  | AstrinIP\_MS1\_022614\_01.10547.10547.1 | 2.3999 | 0.437 | 100.0% | 1197.55 | 1198.408 | 8 | 5.848 | 50.0% | 2 | K.DAGVIAGLNVLR.I | 11 |
|  | AstrinIP\_MS2\_022614\_01.11162.11162.2 | 3.7606 | 0.2878 | 100.0% | 1198.1322 | 1198.408 | 1 | 7.4 | 86.4% | 3 | K.DAGVIAGLNVLR.I | 22 |
|  | AstrinIP\_MS2\_022614\_01.10839.10839.2 | 5.2925 | 0.516 | 100.0% | 1688.1122 | 1688.9213 | 1 | 10.5 | 83.3% | 6 | R.IINEPTAAAIAYGLDR.T | 22 |
|  | AstrinIP\_MS2\_022614\_01.04504.04504.2 | 4.2557 | 0.5152 | 100.0% | 1676.1522 | 1676.6964 | 1 | 8.323 | 66.7% | 3 | K.ATAGDTHLGGEDFDNR.L | 222 |
|  | AstrinIP\_MS1\_022614\_01.07200.07200.2 | 3.2548 | 0.3946 | 100.0% | 1261.6721 | 1262.4508 | 1 | 8.051 | 88.9% | 4 | R.LVNHFVEEFK.R | 2 |
|  | AstrinIP\_MS2\_022614\_01.06551.06551.2 | 2.9311 | 0.3484 | 100.0% | 1418.2322 | 1418.6383 | 4 | 6.379 | 60.0% | 2 | R.LVNHFVEEFKR.K | 2 |
|  | AstrinIP\_MS2\_022614\_01.06556.06556.3 | 2.727 | 0.3683 | 99.9% | 1418.5443 | 1418.6383 | 1 | 6.037 | 45.0% | 1 | R.LVNHFVEEFKR.K | 3 |
|  | AstrinIP\_MS1\_022614\_01.09669.09669.2 | 2.8156 | 0.2188 | 99.1% | 1544.7722 | 1543.6855 | 1 | 4.313 | 63.6% | 1 | R.ARFEELCSDLFR.S | 22 |
|  | AstrinIP\_MS1\_022614\_01.10366.10366.2 | 3.1778 | 0.4564 | 100.0% | 1316.2522 | 1316.4193 | 1 | 6.925 | 83.3% | 1 | R.FEELCSDLFR.S | 22 |
|  | AstrinIP\_MS2\_022614\_02.05492.05492.3 | 4.6614 | 0.3166 | 100.0% | 1822.6743 | 1823.1025 | 1 | 5.977 | 42.2% | 2 | K.LDKAQIHDLVLVGGSTR.I | 3 |
|  | AstrinIP\_MS2\_022614\_01.10762.10762.1 | 2.0368 | 0.176 | 95.7% | 1109.5 | 1110.2578 | 3 | 4.061 | 56.2% | 1 | K.LLQDFFNGR.D | 1 |
|  | AstrinIP\_MS1\_022614\_01.10118.10118.2 | 2.8908 | 0.33 | 100.0% | 1110.0721 | 1110.2578 | 1 | 6.487 | 87.5% | 4 | K.LLQDFFNGR.D | 2 |
|  | AstrinIP\_MS2\_022614\_02.09924.09924.3 | 3.7164 | 0.4297 | 100.0% | 2305.0444 | 2305.608 | 1 | 6.568 | 33.0% | 1 | K.SINPDEAVAYGAAVQAAILMGDK.S | 33 |
|  | AstrinIP\_MS1\_022614\_01.19496.19496.3 | 3.3496 | 0.2583 | 98.4% | 3181.4644 | 3182.7407 | 2 | 4.758 | 21.7% | 1 | K.SENVQDLLLLDVAPLSLGLETAGGVMTALIK.R | 3 |
|  | AstrinIP\_MS1\_022614\_01.19508.19508.2 | 5.0635 | 0.4581 | 100.0% | 3182.0322 | 3182.7407 | 1 | 7.491 | 35.0% | 1 | K.SENVQDLLLLDVAPLSLGLETAGGVMTALIK.R | 2 |
|  | AstrinIP\_MS1\_022614\_01.11703.11703.2 | 4.922 | 0.5638 | 100.0% | 2787.7122 | 2788.043 | 1 | 10.506 | 41.3% | 2 | K.QTQIFTTYSDNQPGVLIQVYEGER.A | 22 |
|  | AstrinIP\_MS2\_022614\_01.03545.03545.2 | 1.9886 | 0.2475 | 95.1% | 1126.0122 | 1126.313 | 7 | 5.056 | 68.8% | 1 | R.MVQEAEKYK.A | 22 |
|  | AstrinIP\_MS2\_022614\_01.03896.03896.3 | 3.6747 | 0.3151 | 100.0% | 1954.4343 | 1954.1621 | 1 | 5.786 | 48.3% | 2 | R.MVQEAEKYKAEDEVQR.E | 3 |
|  | AstrinIP\_MS2\_022614\_01.09441.09441.2 | 3.7744 | 0.3869 | 100.0% | 1289.1322 | 1288.4608 | 1 | 6.901 | 80.0% | 3 | K.NALESYAFNMK.S | 22 |

Similarities:
gi|5729877|ref|NP\_006(3:25)  
gi|124256496|ref|NP\_0(9:19)  
gi|13676857|ref|NP\_06(2:26)  
gi|34419635|ref|NP\_00(6:22)  

---

|  |  |  |  |  |  |  |  |  |
| --- | --- | --- | --- | --- | --- | --- | --- | --- |
| U | *gi|10800130|ref|NP\_06* | 3 | 5 | 45.4% | 130 | 14107 | 10.9 | histone cluster 1, H2ad [Homo sapiens] |
| U | *gi|4504249|ref|NP\_003* | 3 | 5 | 45.4% | 130 | 14091 | 10.9 | histone cluster 1, H2am [Homo sapiens] |
| U | *gi|4504243|ref|NP\_003* | 3 | 5 | 45.4% | 130 | 14091 | 10.9 | histone cluster 1, H2al [Homo sapiens] |
| U | *gi|4504241|ref|NP\_003* | 3 | 5 | 45.4% | 130 | 14091 | 10.9 | histone cluster 1, H2ak [Homo sapiens] |
| U | *gi|4504239|ref|NP\_003* | 3 | 5 | 45.4% | 130 | 14091 | 10.9 | histone cluster 1, H2ai [Homo sapiens] |
| U | *gi|29553970|ref|NP\_80* | 3 | 5 | 45.7% | 129 | 14019 | 10.9 | H2A histone family, member J [Homo sapiens] |
| U | *gi|18105045|ref|NP\_54* | 3 | 5 | 46.1% | 128 | 13906 | 10.9 | histone cluster 1, H2ah [Homo sapiens] |
| U | *gi|10800144|ref|NP\_06* | 3 | 5 | 46.1% | 128 | 13936 | 10.9 | histone cluster 1, H2aj [Homo sapiens] |
| U | *gi|10800132|ref|NP\_06* | 3 | 5 | 45.4% | 130 | 14091 | 10.9 | histone cluster 1, H2ag [Homo sapiens] |

| Filename XCorr DeltCN Conf% ObsM+H+ CalcM+H+ SpR ZScore Ion% # Sequence  | | | | | | | | | | | | |
| --- | --- | --- | --- | --- | --- | --- | --- | --- | --- | --- | --- | --- |
|  | AstrinIP\_MS2\_022614\_01.20630.20630.3 | 4.8361 | 0.442 | 100.0% | 2917.7944 | 2917.3752 | 1 | 7.078 | 32.1% | 1 | R.VGAGAPVYLAAVLEYLTAEILELAGNAAR.D | 3 |
|  | AstrinIP\_MS1\_022614\_01.07631.07631.2 | 3.006 | 0.4194 | 100.0% | 1273.3522 | 1273.4288 | 3 | 6.156 | 65.0% | 1 | R.NDEELNKLLGK.V | 2 |
|  | AstrinIP\_MS1\_022614\_01.13294.13294.2 | 4.339 | 0.5193 | 100.0% | 1931.5721 | 1932.3573 | 1 | 7.819 | 52.8% | 3 | K.VTIAQGGVLPNIQAVLLPK.K | 2 |

---

|  |  |  |  |  |  |  |  |  |
| --- | --- | --- | --- | --- | --- | --- | --- | --- |
| U | *gi|5032161|ref|NP\_005* | 4 | 16 | 42.0% | 112 | 12473 | 4.8 | elongin C [Homo sapiens] |

| Filename XCorr DeltCN Conf% ObsM+H+ CalcM+H+ SpR ZScore Ion% # Sequence  | | | | | | | | | | | | |
| --- | --- | --- | --- | --- | --- | --- | --- | --- | --- | --- | --- | --- |
| \* | AstrinIP\_MS1\_022614\_01.05957.05957.2 | 4.2378 | 0.492 | 100.0% | 1549.3121 | 1548.6736 | 1 | 7.748 | 76.9% | 1 | K.TYGGCEGPDAMYVK.L | 2 |
|  | AstrinIP\_MS1\_022614\_01.06428.06428.2 | 4.0218 | 0.4237 | 100.0% | 1344.4722 | 1345.5382 | 1 | 8.504 | 81.8% | 2 | K.LISSDGHEFIVK.R | 2 |
|  | AstrinIP\_MS2\_022614\_01.05021.05021.2 | 3.3651 | 0.373 | 100.0% | 1501.3522 | 1501.7257 | 1 | 5.956 | 75.0% | 1 | K.LISSDGHEFIVKR.E | 2 |
| \* | AstrinIP\_MS1\_022614\_01.09605.09605.2 | 6.5462 | 0.5265 | 100.0% | 2212.132 | 2212.3984 | 1 | 10.18 | 60.5% | 12 | K.AMLSGPGQFAENETNEVNFR.E | 2 |

---

|  |  |  |  |  |  |  |  |  |
| --- | --- | --- | --- | --- | --- | --- | --- | --- |
| U | *gi|29788785|ref|NP\_82* | 14 | 31 | 40.1% | 444 | 49671 | 4.9 | tubulin, beta [Homo sapiens] |

| Filename XCorr DeltCN Conf% ObsM+H+ CalcM+H+ SpR ZScore Ion% # Sequence  | | | | | | | | | | | | |
| --- | --- | --- | --- | --- | --- | --- | --- | --- | --- | --- | --- | --- |
| \* | AstrinIP\_MS2\_022614\_01.05794.05794.2 | 3.2499 | 0.5557 | 100.0% | 1302.1322 | 1302.4265 | 1 | 8.899 | 81.8% | 4 | R.ISVYYNEATGGK.Y | 2 |
|  | AstrinIP\_MS2\_022614\_01.11037.11037.2 | 4.1941 | 0.4786 | 100.0% | 1617.1721 | 1616.8701 | 1 | 8.153 | 75.0% | 4 | R.AILVDLEPGTMDSVR.S | 2 |
|  | AstrinIP\_MS1\_022614\_01.12682.12682.3 | 5.7439 | 0.4835 | 100.0% | 2798.9343 | 2800.0647 | 1 | 7.897 | 31.0% | 4 | R.SGPFGQIFRPDNFVFGQSGAGNNWAK.G | 3 |
|  | AstrinIP\_MS2\_022614\_01.13616.13616.2 | 6.3235 | 0.5614 | 100.0% | 1959.3322 | 1960.151 | 1 | 10.147 | 76.5% | 2 | K.GHYTEGAELVDSVLDVVR.K | 2 |
|  | AstrinIP\_MS1\_022614\_01.11945.11945.3 | 2.2622 | 0.3446 | 98.3% | 2088.5645 | 2088.325 | 4 | 5.668 | 33.3% | 1 | K.GHYTEGAELVDSVLDVVRK.E | 3 |
|  | AstrinIP\_MS2\_022614\_01.08927.08927.2 | 4.1111 | 0.4049 | 100.0% | 1319.7722 | 1320.5896 | 1 | 7.548 | 81.8% | 4 | R.IMNTFSVVPSPK.V | 2 |
|  | AstrinIP\_MS2\_022614\_01.08963.08963.2 | 2.653 | 0.3897 | 99.9% | 1271.3322 | 1272.5945 | 1 | 5.814 | 75.0% | 1 | R.KLAVNMVPFPR.L | 2 |
|  | AstrinIP\_MS2\_022614\_01.10388.10388.2 | 2.8081 | 0.456 | 100.0% | 1144.2122 | 1144.4204 | 8 | 6.971 | 66.7% | 1 | K.LAVNMVPFPR.L | 2 |
|  | AstrinIP\_MS1\_022614\_01.12106.12106.2 | 3.4086 | 0.3149 | 100.0% | 1622.7322 | 1621.9403 | 1 | 6.665 | 65.4% | 2 | R.LHFFMPGFAPLTSR.G | 2 |
| \* | AstrinIP\_MS1\_022614\_01.11870.11870.2 | 2.6759 | 0.2572 | 98.6% | 1661.4722 | 1660.9078 | 44 | 5.463 | 42.9% | 1 | R.ALTVPELTQQVFDAK.N | 2 |
|  | AstrinIP\_MS1\_022614\_01.05895.05895.2 | 3.1192 | 0.2974 | 99.8% | 1446.4321 | 1447.6031 | 1 | 6.017 | 72.7% | 3 | K.EVDEQMLNVQNK.N | 2 |
|  | AstrinIP\_MS1\_022614\_01.11489.11489.2 | 2.6661 | 0.3758 | 99.8% | 1696.3322 | 1697.8877 | 2 | 5.691 | 42.3% | 1 | K.NSSYFVEWIPNNVK.T | 2 |
| \* | AstrinIP\_MS2\_022614\_01.13418.13418.3 | 3.3821 | 0.4401 | 100.0% | 2027.5743 | 2027.3893 | 4 | 7.025 | 33.8% | 1 | K.MAVTFIGNSTAIQELFKR.I | 3 |
|  | AstrinIP\_MS2\_022614\_01.10769.10769.2 | 3.5506 | 0.408 | 100.0% | 1231.1721 | 1230.4241 | 1 | 6.579 | 94.4% | 2 | R.ISEQFTAMFR.R | 2 |

---

|  |  |  |  |  |  |  |  |  |
| --- | --- | --- | --- | --- | --- | --- | --- | --- |
| U | *gi|5901954|ref|NP\_008* | 17 | 32 | 38.6% | 399 | 43065 | 4.8 | FGFR1 oncogene partner isoform a [Homo sapiens] |

| Filename XCorr DeltCN Conf% ObsM+H+ CalcM+H+ SpR ZScore Ion% # Sequence  | | | | | | | | | | | | |
| --- | --- | --- | --- | --- | --- | --- | --- | --- | --- | --- | --- | --- |
|  | AstrinIP\_MS2\_022614\_01.13464.13464.2 | 3.3614 | 0.392 | 100.0% | 1670.7122 | 1671.8912 | 1 | 7.436 | 67.9% | 2 | R.DLLVQTLENSGVLNR.I | 2 |
|  | AstrinIP\_MS2\_022614\_01.05314.05314.2 | 2.1399 | 0.2681 | 98.1% | 1000.9922 | 1001.168 | 2 | 5.398 | 81.2% | 1 | K.TPLVNESLK.K | 2 |
|  | AstrinIP\_MS1\_022614\_01.03994.03994.2 | 3.1243 | 0.3525 | 100.0% | 1129.0922 | 1129.342 | 25 | 6.856 | 61.1% | 1 | K.TPLVNESLKK.F | 2 |
|  | AstrinIP\_MS2\_022614\_02.10848.10848.2 | 5.8663 | 0.5327 | 100.0% | 2164.892 | 2165.5352 | 1 | 9.264 | 60.0% | 4 | R.DLGIIEAEGTVGGPLLLEVIR.R | 2 |
|  | AstrinIP\_MS2\_022614\_02.10822.10822.3 | 3.5605 | 0.2783 | 99.3% | 2165.3342 | 2165.5352 | 1 | 6.081 | 36.2% | 1 | R.DLGIIEAEGTVGGPLLLEVIR.R | 3 |
|  | AstrinIP\_MS1\_022614\_01.14310.14310.3 | 3.1726 | 0.2731 | 98.7% | 2321.3342 | 2321.7227 | 1 | 5.315 | 32.1% | 5 | R.DLGIIEAEGTVGGPLLLEVIRR.C | 3 |
| \* | AstrinIP\_MS2\_022614\_01.03698.03698.2 | 2.4479 | 0.1799 | 95.7% | 1367.6322 | 1367.4587 | 10 | 4.01 | 54.5% | 1 | K.TSAQTT#PSKIPR.Y | 2 |
|  | AstrinIP\_MS2\_022614\_01.04622.04622.2 | 4.9678 | 0.4871 | 100.0% | 1892.4922 | 1892.928 | 1 | 8.328 | 61.8% | 2 | K.ANDEANQSDTSVSLSEPK.S | 2 |
|  | AstrinIP\_MS2\_022614\_01.03968.03968.2 | 3.4427 | 0.3701 | 100.0% | 1252.1322 | 1252.413 | 2 | 6.209 | 65.0% | 1 | K.SSLHLLSHETK.I | 2 |
|  | AstrinIP\_MS2\_022614\_01.03970.03970.3 | 3.0787 | 0.3701 | 100.0% | 1253.0044 | 1252.413 | 9 | 7.867 | 45.0% | 1 | K.SSLHLLSHETK.I | 3 |
|  | AstrinIP\_MS2\_022614\_01.06730.06730.1 | 2.0375 | 0.3349 | 100.0% | 893.54 | 894.0183 | 1 | 4.348 | 64.3% | 2 | K.IGSFLSNR.T | 1 |
|  | AstrinIP\_MS2\_022614\_01.06740.06740.2 | 2.5024 | 0.4694 | 100.0% | 893.9122 | 894.0183 | 1 | 7.739 | 78.6% | 1 | K.IGSFLSNR.T | 2 |
|  | AstrinIP\_MS1\_022614\_01.06492.06492.2 | 4.1754 | 0.4034 | 100.0% | 1583.3922 | 1583.8253 | 1 | 7.473 | 73.3% | 4 | R.KQAGSLASLSDAPPLK.S | 2 |
|  | AstrinIP\_MS2\_022614\_01.08086.08086.2 | 2.9056 | 0.3525 | 99.8% | 1188.2522 | 1188.3666 | 1 | 6.046 | 70.8% | 1 | K.SGLSSLAGAPSLK.D | 2 |
|  | AstrinIP\_MS1\_022614\_01.06381.06381.2 | 3.5907 | 0.4896 | 100.0% | 1734.3722 | 1734.9011 | 1 | 7.881 | 55.9% | 2 | K.SGLSSLAGAPSLKDSESK.R | 2 |
|  | AstrinIP\_MS2\_022614\_01.06699.06699.3 | 3.2412 | 0.2922 | 99.3% | 1734.8944 | 1734.9011 | 2 | 5.271 | 35.3% | 1 | K.SGLSSLAGAPSLKDSESK.R | 3 |
|  | AstrinIP\_MS1\_022614\_02.06001.06001.3 | 3.5326 | 0.2591 | 99.0% | 2571.2043 | 2571.63 | 2 | 4.236 | 27.2% | 2 | K.IGSLGLGTGEDDDYVDDFNSTSHR.S | 3 |

---

|  |  |  |  |  |  |  |  |  |
| --- | --- | --- | --- | --- | --- | --- | --- | --- |
| U | *gi|25777713|ref|NP\_73* | 4 | 4 | 37.4% | 163 | 18658 | 4.5 | S-phase kinase-associated protein 1 isoform b [Homo sapiens] |

| Filename XCorr DeltCN Conf% ObsM+H+ CalcM+H+ SpR ZScore Ion% # Sequence  | | | | | | | | | | | | |
| --- | --- | --- | --- | --- | --- | --- | --- | --- | --- | --- | --- | --- |
|  | AstrinIP\_MS2\_022614\_01.12743.12743.3 | 3.838 | 0.3449 | 100.0% | 3126.3245 | 3127.5056 | 1 | 7.514 | 23.2% | 1 | K.TMLEDLGMDDEGDDDPVPLPNVNAAILKK.V | 3 |
|  | AstrinIP\_MS2\_022614\_01.11214.11214.2 | 3.5726 | 0.487 | 100.0% | 1762.3121 | 1762.9597 | 1 | 7.531 | 61.5% | 1 | K.RTDDIPVWDQEFLK.V | 2 |
| \* | AstrinIP\_MS2\_022614\_02.05898.05898.3 | 3.215 | 0.3002 | 99.5% | 2069.8743 | 2071.2078 | 1 | 5.167 | 35.9% | 1 | K.TFNIKNDFTEEEEAQVR.K | 3 |
| \* | AstrinIP\_MS2\_022614\_02.05417.05417.3 | 3.4187 | 0.2581 | 99.2% | 2199.8643 | 2199.3818 | 22 | 5.722 | 32.4% | 1 | K.TFNIKNDFTEEEEAQVRK.E | 3 |

---

|  |  |  |  |  |  |  |  |  |
| --- | --- | --- | --- | --- | --- | --- | --- | --- |
| U | *gi|7669492|ref|NP\_002* | 7 | 10 | 36.1% | 335 | 36053 | 8.5 | glyceraldehyde-3-phosphate dehydrogenase [Homo sapiens] |

| Filename XCorr DeltCN Conf% ObsM+H+ CalcM+H+ SpR ZScore Ion% # Sequence  | | | | | | | | | | | | |
| --- | --- | --- | --- | --- | --- | --- | --- | --- | --- | --- | --- | --- |
| \* | AstrinIP\_MS2\_022614\_01.18441.18441.3 | 3.264 | 0.2576 | 98.2% | 3309.9243 | 3310.7634 | 21 | 4.512 | 20.4% | 1 | K.VDIVAINDPFIDLNYMVYMFQYDSTHGK.F | 3 |
| \* | AstrinIP\_MS1\_022614\_01.11776.11776.2 | 3.3021 | 0.3396 | 100.0% | 1614.3522 | 1614.8851 | 1 | 7.692 | 65.4% | 1 | K.LVINGNPITIFQER.D | 2 |
| \* | AstrinIP\_MS2\_022614\_01.15058.15058.3 | 5.3863 | 0.5188 | 100.0% | 2596.0144 | 2597.0044 | 1 | 8.726 | 38.0% | 3 | K.VIHDNFGIVEGLMTTVHAITATQK.T | 3 |
| \* | AstrinIP\_MS1\_022614\_01.07911.07911.2 | 2.9914 | 0.1659 | 98.1% | 1412.2122 | 1412.6292 | 6 | 4.808 | 53.6% | 2 | R.GALQNIIPASTGAAK.A | 2 |
| \* | AstrinIP\_MS1\_022614\_01.08200.08200.2 | 2.8188 | 0.375 | 99.8% | 1532.3322 | 1531.7155 | 1 | 6.09 | 57.7% | 1 | R.VPTANVSVVDLTCR.L | 2 |
|  | AstrinIP\_MS2\_022614\_01.11506.11506.2 | 3.9215 | 0.523 | 100.0% | 1764.2922 | 1764.8914 | 1 | 8.681 | 61.5% | 1 | K.LISWYDNEFGYSNR.V | 2 |
| \* | AstrinIP\_MS2\_022614\_01.07092.07092.2 | 2.9041 | 0.4028 | 100.0% | 1331.1122 | 1331.5879 | 8 | 6.555 | 54.5% | 1 | R.VVDLMAHMASKE.- | 2 |

---

|  |  |  |  |  |  |  |  |  |
| --- | --- | --- | --- | --- | --- | --- | --- | --- |
| U | *gi|38016907|ref|NP\_93* | 3 | 5 | 35.8% | 123 | 13475 | 8.0 | stomatin isoform b [Homo sapiens] |
| U | *gi|38016911|ref|NP\_00* | 3 | 5 | 15.3% | 288 | 31731 | 7.9 | stomatin isoform a [Homo sapiens] |

| Filename XCorr DeltCN Conf% ObsM+H+ CalcM+H+ SpR ZScore Ion% # Sequence  | | | | | | | | | | | | |
| --- | --- | --- | --- | --- | --- | --- | --- | --- | --- | --- | --- | --- |
|  | AstrinIP\_MS2\_022614\_01.04348.04348.2 | 3.1016 | 0.5037 | 100.0% | 1248.0122 | 1248.3966 | 1 | 8.285 | 77.3% | 1 | K.VIAAEGEMNASR.A | 2 |
|  | AstrinIP\_MS2\_022614\_01.08603.08603.2 | 2.8995 | 0.4205 | 100.0% | 1352.4521 | 1352.5707 | 1 | 6.773 | 81.8% | 3 | R.YLQTLTTIAAEK.N | 2 |
|  | AstrinIP\_MS2\_022614\_01.18197.18197.2 | 3.7587 | 0.5091 | 100.0% | 2127.4321 | 2128.5781 | 1 | 7.425 | 55.3% | 1 | K.NSTIVFPLPIDMLQGIIGAK.H | 2 |

---

|  |  |  |  |  |  |  |  |  |
| --- | --- | --- | --- | --- | --- | --- | --- | --- |
| U | *gi|4826998|ref|NP\_005* | 20 | 49 | 34.7% | 707 | 76150 | 9.4 | splicing factor proline/glutamine rich (polypyrimidine tract binding protein associated) [Homo sapiens] |

| Filename XCorr DeltCN Conf% ObsM+H+ CalcM+H+ SpR ZScore Ion% # Sequence  | | | | | | | | | | | | |
| --- | --- | --- | --- | --- | --- | --- | --- | --- | --- | --- | --- | --- |
| \* | AstrinIP\_MS2\_022614\_01.04414.04414.2 | 2.3595 | 0.4165 | 99.8% | 1268.1921 | 1268.4332 | 26 | 7.387 | 54.5% | 1 | R.SPPPGMGLNQNR.G | 2 |
| \* | AstrinIP\_MS1\_022614\_01.03741.03741.3 | 4.9435 | 0.5096 | 100.0% | 2371.6143 | 2371.725 | 1 | 7.846 | 37.5% | 1 | K.MPGGPKPGGGPGLSTPGGHPKPPHR.G | 3 |
| \* | AstrinIP\_MS2\_022614\_01.03341.03341.3 | 2.8458 | 0.4286 | 100.0% | 2404.4944 | 2404.5747 | 1 | 6.313 | 32.1% | 1 | R.QHHPPYHQQHHQGPPPGGPGGR.S | 3 |
| \* | AstrinIP\_MS2\_022614\_02.06473.06473.3 | 3.7218 | 0.3832 | 100.0% | 1650.6843 | 1650.8723 | 1 | 6.385 | 41.1% | 1 | K.ISDSEGFKANLSLLR.R | 3 |
| \* | AstrinIP\_MS1\_022614\_02.06985.06985.3 | 2.6789 | 0.2589 | 96.6% | 1964.7843 | 1965.2133 | 89 | 4.554 | 29.7% | 2 | R.LFVGNLPADITEDEFKR.L | 3 |
| \* | AstrinIP\_MS2\_022614\_01.11478.11478.2 | 3.8537 | 0.4401 | 100.0% | 1965.0721 | 1965.2133 | 1 | 6.749 | 59.4% | 3 | R.LFVGNLPADITEDEFKR.L | 2 |
| \* | AstrinIP\_MS2\_022614\_01.07458.07458.1 | 2.6355 | 0.4499 | 100.0% | 1252.61 | 1253.3971 | 1 | 7.65 | 65.0% | 1 | K.YGEPGEVFINK.G | 1 |
| \* | AstrinIP\_MS1\_022614\_01.07136.07136.2 | 3.0641 | 0.4327 | 100.0% | 1252.7722 | 1253.3971 | 1 | 7.044 | 70.0% | 5 | K.YGEPGEVFINK.G | 2 |
| \* | AstrinIP\_MS2\_022614\_01.04448.04448.1 | 2.05 | 0.417 | 100.0% | 1143.55 | 1144.3188 | 1 | 7.162 | 60.0% | 2 | R.FATHAAALSVR.N | 1 |
| \* | AstrinIP\_MS1\_022614\_01.04331.04331.2 | 3.3617 | 0.4896 | 100.0% | 1144.1721 | 1144.3188 | 1 | 7.808 | 85.0% | 3 | R.FATHAAALSVR.N | 2 |
| \* | AstrinIP\_MS2\_022614\_01.15740.15740.2 | 4.7135 | 0.5878 | 100.0% | 2639.5522 | 2640.9092 | 1 | 10.495 | 52.3% | 4 | R.NLSPYVSNELLEEAFSQFGPIER.A | 2 |
|  | AstrinIP\_MS1\_022614\_01.04426.04426.1 | 2.0327 | 0.335 | 100.0% | 886.5 | 887.0238 | 6 | 6.694 | 71.4% | 2 | R.AVVIVDDR.G | 11 |
| \* | AstrinIP\_MS2\_022614\_01.05026.05026.2 | 3.0553 | 0.4237 | 100.0% | 1246.2722 | 1246.452 | 1 | 8.188 | 68.2% | 5 | K.GIVEFASKPAAR.K | 2 |
| \* | AstrinIP\_MS2\_022614\_01.03587.03587.2 | 2.6914 | 0.3454 | 99.8% | 1220.5122 | 1221.4601 | 30 | 5.786 | 61.1% | 1 | K.LAQKNPMYQK.E | 2 |
| \* | AstrinIP\_MS2\_022614\_01.05963.05963.2 | 4.3517 | 0.5201 | 100.0% | 1763.2922 | 1763.8632 | 1 | 9.028 | 73.1% | 3 | R.FAQHGTFEYEYSQR.W | 2 |
| \* | AstrinIP\_MS2\_022614\_02.04584.04584.3 | 4.2266 | 0.2357 | 99.8% | 1763.6044 | 1763.8632 | 1 | 5.918 | 44.2% | 4 | R.FAQHGTFEYEYSQR.W | 3 |
| \* | AstrinIP\_MS2\_022614\_01.08918.08918.3 | 5.3916 | 0.4706 | 100.0% | 2430.0244 | 2429.6233 | 1 | 7.906 | 42.1% | 3 | K.DKLESEMEDAYHEHQANLLR.Q | 3 |
| \* | AstrinIP\_MS2\_022614\_01.03706.03706.2 | 4.0362 | 0.3972 | 100.0% | 1417.0922 | 1417.5946 | 1 | 6.936 | 85.0% | 1 | R.MEELHNQEMQK.R | 2 |
| \* | AstrinIP\_MS2\_022614\_01.06015.06015.2 | 4.3398 | 0.5033 | 100.0% | 1772.3922 | 1772.9631 | 1 | 9.195 | 55.6% | 1 | R.MGGGGAMNMGDPYGSGGQK.F | 2 |
| \* | AstrinIP\_MS1\_022614\_01.04089.04089.2 | 3.8913 | 0.5179 | 100.0% | 1341.8522 | 1342.4569 | 1 | 8.294 | 78.6% | 5 | R.FGQGGAGPVGGQGPR.G | 2 |

Similarities:
gi|224028244|ref|NP\_0(1:19)  

---

|  |  |  |  |  |  |  |  |  |
| --- | --- | --- | --- | --- | --- | --- | --- | --- |
| U | *gi|46276893|ref|NP\_99* | 5 | 10 | 34.2% | 161 | 17911 | 6.1 | elongin B isoform b [Homo sapiens] |
| U | *gi|6005890|ref|NP\_009* | 5 | 10 | 46.6% | 118 | 13133 | 4.9 | elongin B isoform a [Homo sapiens] |

| Filename XCorr DeltCN Conf% ObsM+H+ CalcM+H+ SpR ZScore Ion% # Sequence  | | | | | | | | | | | | |
| --- | --- | --- | --- | --- | --- | --- | --- | --- | --- | --- | --- | --- |
|  | AstrinIP\_MS2\_022614\_01.05045.05045.1 | 1.6956 | 0.2562 | 98.3% | 896.43 | 897.0159 | 1 | 5.211 | 71.4% | 1 | K.TTIFTDAK.E | 1 |
|  | AstrinIP\_MS2\_022614\_02.06203.06203.3 | 3.714 | 0.3809 | 100.0% | 2074.2244 | 2074.3384 | 4 | 5.767 | 30.9% | 1 | K.TTIFTDAKESSTVFELKR.I | 3 |
|  | AstrinIP\_MS2\_022614\_01.05560.05560.2 | 2.4997 | 0.3524 | 99.7% | 1196.1122 | 1196.3458 | 31 | 5.185 | 61.1% | 3 | K.ESSTVFELKR.I | 2 |
|  | AstrinIP\_MS1\_022614\_01.05268.05268.2 | 4.0885 | 0.3871 | 100.0% | 1424.4122 | 1423.5627 | 1 | 7.557 | 81.8% | 4 | R.LYKDDQLLDDGK.T | 2 |
|  | AstrinIP\_MS1\_022614\_01.08825.08825.3 | 4.6263 | 0.4788 | 100.0% | 2637.3245 | 2637.9214 | 1 | 7.949 | 34.4% | 1 | K.TLGECGFTSQTARPQAPATVGLAFR.A | 3 |

---

|  |  |  |  |  |  |  |  |  |
| --- | --- | --- | --- | --- | --- | --- | --- | --- |
| U | *gi|24234688|ref|NP\_00* | 20 | 54 | 34.0% | 679 | 73681 | 6.2 | heat shock 70kDa protein 9 precursor [Homo sapiens] |

| Filename XCorr DeltCN Conf% ObsM+H+ CalcM+H+ SpR ZScore Ion% # Sequence  | | | | | | | | | | | | |
| --- | --- | --- | --- | --- | --- | --- | --- | --- | --- | --- | --- | --- |
| \* | AstrinIP\_MS2\_022614\_01.07731.07731.2 | 3.734 | 0.5042 | 100.0% | 1451.2522 | 1451.576 | 1 | 8.165 | 65.4% | 5 | R.TTPSVVAFTADGER.L | 2 |
| \* | AstrinIP\_MS2\_022614\_01.05134.05134.2 | 3.3752 | 0.4509 | 100.0% | 1569.3322 | 1569.7141 | 2 | 7.486 | 65.4% | 4 | R.QAVTNPNNTFYATK.R | 2 |
| \* | AstrinIP\_MS2\_022614\_01.13407.13407.2 | 4.1916 | 0.4909 | 100.0% | 1554.4722 | 1554.8878 | 1 | 8.014 | 76.9% | 5 | K.LYSPSQIGAFVLMK.M | 2 |
| \* | AstrinIP\_MS2\_022614\_01.03989.03989.2 | 4.4674 | 0.4431 | 100.0% | 1593.3522 | 1593.7949 | 1 | 7.979 | 76.9% | 2 | K.MKETAENYLGHTAK.N | 2 |
| \* | AstrinIP\_MS2\_022614\_01.09942.09942.2 | 2.8678 | 0.3214 | 99.7% | 1695.1122 | 1695.8723 | 1 | 6.185 | 64.3% | 1 | K.NAVITVPAYFNDSQR.Q | 2 |
| \* | AstrinIP\_MS2\_022614\_01.09132.09132.1 | 2.625 | 0.3212 | 100.0% | 1242.56 | 1243.4056 | 1 | 5.397 | 63.6% | 1 | K.DAGQISGLNVLR.V | 1 |
| \* | AstrinIP\_MS2\_022614\_01.09116.09116.2 | 3.6252 | 0.3272 | 100.0% | 1242.6522 | 1243.4056 | 1 | 6.555 | 81.8% | 3 | K.DAGQISGLNVLR.V | 2 |
| \* | AstrinIP\_MS2\_022614\_01.12464.12464.2 | 5.5675 | 0.491 | 100.0% | 2056.2522 | 2057.181 | 1 | 8.618 | 66.7% | 2 | K.STNGDTFLGGEDFDQALLR.H | 2 |
| \* | AstrinIP\_MS1\_022614\_01.06286.06286.2 | 3.2144 | 0.2262 | 99.5% | 1692.2722 | 1691.8969 | 1 | 5.608 | 60.7% | 2 | R.ETGVDLTKDNMALQR.V | 2 |
| \* | AstrinIP\_MS1\_022614\_01.12039.12039.2 | 3.6923 | 0.3654 | 100.0% | 1362.0721 | 1362.5687 | 1 | 7.961 | 77.3% | 4 | R.AQFEGIVTDLIR.R | 2 |
| \* | AstrinIP\_MS2\_022614\_02.07242.07242.3 | 3.4233 | 0.198 | 95.4% | 2408.8145 | 2407.7595 | 1 | 5.281 | 35.2% | 1 | K.AMQDAEVSKSDIGEVILVGGMTR.M | 3 |
| \* | AstrinIP\_MS2\_022614\_02.07440.07440.2 | 3.5425 | 0.4224 | 100.0% | 1447.3722 | 1447.6898 | 1 | 7.886 | 69.2% | 1 | K.SDIGEVILVGGMTR.M | 2 |
| \* | AstrinIP\_MS2\_022614\_01.08984.08984.2 | 3.8971 | 0.1755 | 99.9% | 1292.3322 | 1291.4496 | 1 | 6.618 | 80.0% | 5 | K.VQQTVQDLFGR.A | 2 |
| \* | AstrinIP\_MS2\_022614\_01.13326.13326.2 | 2.8724 | 0.3982 | 99.9% | 1593.9521 | 1593.9529 | 9 | 5.944 | 50.0% | 2 | K.LLGQFTLIGIPPAPR.G | 2 |
| \* | AstrinIP\_MS1\_022614\_02.04556.04556.2 | 3.4427 | 0.3671 | 100.0% | 1473.9321 | 1474.6543 | 1 | 7.447 | 69.2% | 2 | R.EQQIVIQSSGGLSK.D | 2 |
| \* | AstrinIP\_MS2\_022614\_02.06212.06212.3 | 3.6321 | 0.4908 | 100.0% | 2420.3044 | 2419.7095 | 1 | 7.418 | 36.9% | 3 | R.EQQIVIQSSGGLSKDDIENMVK.N | 3 |
| \* | AstrinIP\_MS2\_022614\_01.10199.10199.3 | 5.1597 | 0.4101 | 100.0% | 2142.4443 | 2143.3765 | 1 | 7.527 | 47.2% | 2 | K.ERVEAVNMAEGIIHDTETK.M | 3 |
| \* | AstrinIP\_MS1\_022614\_01.10040.10040.3 | 3.4367 | 0.3716 | 100.0% | 1857.8944 | 1858.0735 | 3 | 6.613 | 40.6% | 3 | R.VEAVNMAEGIIHDTETK.M | 3 |
| \* | AstrinIP\_MS2\_022614\_01.10781.10781.2 | 4.8579 | 0.4808 | 100.0% | 1858.2722 | 1858.0735 | 1 | 8.011 | 75.0% | 2 | R.VEAVNMAEGIIHDTETK.M | 2 |
| \* | AstrinIP\_MS2\_022614\_01.04209.04209.2 | 3.4237 | 0.4272 | 100.0% | 1232.0521 | 1232.3794 | 1 | 7.26 | 81.8% | 4 | R.QAASSLQQASLK.L | 2 |

---

|  |  |  |  |  |  |  |  |  |
| --- | --- | --- | --- | --- | --- | --- | --- | --- |
| U | *contaminant\_GR78\_PIG* | 5 | 15 | 33.5% | 200 | 21709 | 5.0 | owl|P34935| 78 KD GLUCOSE REGULATED PROTEIN (GRP 78) (IMMUNOGLOBULIN HEAVY... |

| Filename XCorr DeltCN Conf% ObsM+H+ CalcM+H+ SpR ZScore Ion% # Sequence  | | | | | | | | | | | | |
| --- | --- | --- | --- | --- | --- | --- | --- | --- | --- | --- | --- | --- |
| \* | AstrinIP\_MS1\_022614\_01.17182.17182.2 | 3.1606 | 0.1676 | 98.9% | 1450.1122 | 1449.6475 | 12 | 4.0 | 58.3% | 1 | -.EIVLVGGST#RIPK.I | 2 |
|  | AstrinIP\_MS1\_022614\_01.07550.07550.2 | 5.2088 | 0.5282 | 100.0% | 1837.4122 | 1838.0245 | 1 | 9.838 | 75.0% | 8 | K.SQIFSTASDNQPTVTIK.V | 22 |
|  | AstrinIP\_MS2\_022614\_01.03674.03674.2 | 2.4981 | 0.3322 | 99.8% | 1192.0721 | 1192.3574 | 1 | 6.147 | 72.2% | 1 | K.VYEGERPLTK.D | 22 |
|  | AstrinIP\_MS1\_022614\_01.11088.11088.2 | 4.1652 | 0.564 | 100.0% | 1934.5122 | 1935.19 | 1 | 9.377 | 61.8% | 4 | K.DNHLLGTFDLTGIPPAPR.G | 22 |
|  | AstrinIP\_MS2\_022614\_01.03741.03741.2 | 2.5616 | 0.2861 | 99.7% | 1075.0322 | 1075.1667 | 5 | 6.002 | 75.0% | 1 | K.ITITNDQNR.L | 22 |

Similarities:
gi|16507237|ref|NP\_00(4:1)  

---

|  |  |  |  |  |  |  |  |  |
| --- | --- | --- | --- | --- | --- | --- | --- | --- |
| U | *gi|7657307|ref|NP\_055* | 13 | 38 | 31.1% | 676 | 72190 | 6.7 | LIM domains containing 1 [Homo sapiens] |

| Filename XCorr DeltCN Conf% ObsM+H+ CalcM+H+ SpR ZScore Ion% # Sequence  | | | | | | | | | | | | |
| --- | --- | --- | --- | --- | --- | --- | --- | --- | --- | --- | --- | --- |
| \* | AstrinIP\_MS2\_022614\_01.09290.09290.2 | 3.6628 | 0.4343 | 100.0% | 1460.0122 | 1460.6416 | 1 | 6.867 | 77.3% | 7 | K.FIEDLNMYEASK.D | 2 |
| \* | AstrinIP\_MS1\_022614\_01.07659.07659.2 | 6.7768 | 0.5464 | 100.0% | 2230.612 | 2231.5168 | 1 | 10.619 | 76.5% | 4 | K.IHLQQQQQQLLQEETLPR.G | 2 |
| \* | AstrinIP\_MS1\_022614\_01.07701.07701.3 | 4.7575 | 0.2998 | 100.0% | 2231.3342 | 2231.5168 | 1 | 5.929 | 41.2% | 5 | K.IHLQQQQQQLLQEETLPR.G | 3 |
| \* | AstrinIP\_MS1\_022614\_01.04923.04923.1 | 1.7101 | 0.3829 | 100.0% | 804.4 | 804.9212 | 9 | 6.432 | 58.3% | 2 | R.WEVVGSK.L | 1 |
| \* | AstrinIP\_MS2\_022614\_01.04008.04008.2 | 4.354 | 0.3165 | 100.0% | 1464.1921 | 1463.5039 | 1 | 7.837 | 70.0% | 5 | R.SSEGSLGGQNSGIGGR.S | 2 |
| \* | AstrinIP\_MS2\_022614\_01.05302.05302.2 | 4.1403 | 0.3174 | 100.0% | 1722.3722 | 1722.8522 | 1 | 8.669 | 76.7% | 2 | R.SSEKPTGLWSTASSQR.V | 2 |
| \* | AstrinIP\_MS2\_022614\_01.08896.08896.2 | 3.6113 | 0.3114 | 100.0% | 2255.652 | 2254.551 | 1 | 5.866 | 47.7% | 2 | R.VSPGLPSPNLENGAPAVGPVQPR.T | 2 |
| \* | AstrinIP\_MS1\_022614\_01.07690.07690.2 | 3.3861 | 0.4958 | 100.0% | 1456.1322 | 1456.6483 | 1 | 7.705 | 69.2% | 2 | R.TPSVSAPLALSCPR.Q | 2 |
| \* | AstrinIP\_MS2\_022614\_02.06948.06948.3 | 5.4041 | 0.3635 | 100.0% | 3015.1743 | 3016.3118 | 1 | 6.781 | 31.2% | 4 | R.SNSGLGGEVSGVMSKPNVDPQPWFQDGPK.S | 3 |
| \* | AstrinIP\_MS2\_022614\_01.12975.12975.2 | 4.4517 | 0.5244 | 100.0% | 2400.4722 | 2401.592 | 1 | 9.761 | 47.8% | 1 | K.EGPLGWSSDGSLGSVLLDSPSSPR.V | 2 |
| \* | AstrinIP\_MS1\_022614\_01.10542.10542.3 | 3.1035 | 0.2876 | 98.7% | 3110.7844 | 3112.2646 | 1 | 4.734 | 25.0% | 1 | K.GVFGAGQACQAMGNLYHDTCFTCAACSR.K | 3 |
| \* | AstrinIP\_MS2\_022614\_01.08181.08181.1 | 2.3634 | 0.3823 | 100.0% | 945.61 | 946.0935 | 1 | 6.827 | 64.3% | 2 | K.AFYFVNGK.V | 1 |
| \* | AstrinIP\_MS1\_022614\_01.03596.03596.3 | 3.2862 | 0.2905 | 99.6% | 1789.1643 | 1789.0066 | 1 | 5.022 | 37.5% | 1 | R.LEKRPSSTALHQHHF.- | 3 |

---

|  |  |  |  |  |  |  |  |  |
| --- | --- | --- | --- | --- | --- | --- | --- | --- |
| U | *gi|4501881|ref|NP\_001* | 15 | 33 | 29.7% | 377 | 42051 | 5.4 | actin, alpha 1, skeletal muscle [Homo sapiens] |
| U | *gi|4885049|ref|NP\_005* | 15 | 33 | 29.7% | 377 | 42019 | 5.4 | cardiac muscle alpha actin 1 proprotein [Homo sapiens] |

| Filename XCorr DeltCN Conf% ObsM+H+ CalcM+H+ SpR ZScore Ion% # Sequence  | | | | | | | | | | | | |
| --- | --- | --- | --- | --- | --- | --- | --- | --- | --- | --- | --- | --- |
|  | AstrinIP\_MS2\_022614\_01.04052.04052.2 | 3.1372 | 0.3107 | 100.0% | 976.3522 | 977.02136 | 1 | 6.669 | 77.8% | 1 | K.AGFAGDDAPR.A | 22 |
|  | AstrinIP\_MS2\_022614\_01.04036.04036.1 | 1.9667 | 0.3834 | 100.0% | 976.48 | 977.02136 | 6 | 5.951 | 55.6% | 1 | K.AGFAGDDAPR.A | 11 |
|  | AstrinIP\_MS1\_022614\_01.06808.06808.2 | 2.6818 | 0.321 | 99.8% | 1199.3922 | 1199.4415 | 6 | 5.253 | 60.0% | 4 | R.AVFPSIVGRPR.H | 22 |
|  | AstrinIP\_MS2\_022614\_01.03922.03922.2 | 2.2208 | 0.3178 | 98.7% | 1170.5922 | 1172.4058 | 1 | 6.061 | 70.0% | 1 | R.HQGVMVGMGQK.D | 22 |
|  | AstrinIP\_MS2\_022614\_01.03903.03903.1 | 2.5908 | 0.3969 | 100.0% | 1171.48 | 1172.4058 | 1 | 7.283 | 65.0% | 1 | R.HQGVMVGMGQK.D | 11 |
|  | AstrinIP\_MS2\_022614\_01.03548.03548.2 | 2.8969 | 0.1276 | 97.7% | 1355.0122 | 1355.4038 | 1 | 7.345 | 77.3% | 1 | K.DSYVGDEAQSKR.G | 22 |
|  | AstrinIP\_MS1\_022614\_02.05980.05980.3 | 3.9274 | 0.1879 | 98.9% | 1961.6943 | 1962.1841 | 3 | 4.724 | 41.7% | 2 | K.YPIEHGIITNWDDMEK.I | 3 |
|  | AstrinIP\_MS1\_022614\_01.08859.08859.2 | 4.082 | 0.3739 | 100.0% | 1962.2122 | 1962.1841 | 1 | 6.733 | 63.3% | 2 | K.YPIEHGIITNWDDMEK.I | 2 |
|  | AstrinIP\_MS1\_022614\_01.06177.06177.2 | 3.2759 | 0.338 | 100.0% | 1516.3322 | 1516.7019 | 1 | 5.686 | 75.0% | 4 | K.IWHHTFYNELR.V | 22 |
|  | AstrinIP\_MS2\_022614\_01.06611.06611.3 | 2.7762 | 0.2022 | 95.4% | 1517.3944 | 1516.7019 | 10 | 5.19 | 50.0% | 1 | K.IWHHTFYNELR.V | 33 |
|  | AstrinIP\_MS2\_022614\_01.11638.11638.2 | 2.5561 | 0.2653 | 98.6% | 1624.1522 | 1624.8927 | 15 | 4.862 | 42.3% | 1 | R.LDLAGRDLTDYLMK.I | 22 |
|  | AstrinIP\_MS1\_022614\_01.10119.10119.1 | 2.0248 | 0.2294 | 99.1% | 998.3 | 999.167 | 2 | 4.596 | 71.4% | 2 | R.DLTDYLMK.I | 11 |
|  | AstrinIP\_MS2\_022614\_01.10803.10803.2 | 4.4281 | 0.2928 | 100.0% | 1791.3722 | 1791.9554 | 2 | 8.716 | 73.3% | 6 | K.SYELPDGQVITIGNER.F | 22 |
|  | AstrinIP\_MS1\_022614\_01.06375.06375.1 | 2.52 | 0.4797 | 100.0% | 1161.52 | 1162.3868 | 1 | 7.293 | 65.0% | 3 | K.EITALAPSTMK.I | 11 |
|  | AstrinIP\_MS2\_022614\_01.06662.06662.2 | 2.8359 | 0.3956 | 100.0% | 1162.0721 | 1162.3868 | 1 | 6.257 | 75.0% | 3 | K.EITALAPSTMK.I | 22 |

Similarities:
gi|4501885|ref|NP\_001(13:2)  

---

|  |  |  |  |  |  |  |  |  |
| --- | --- | --- | --- | --- | --- | --- | --- | --- |
| U | *gi|224028244|ref|NP\_0* | 16 | 38 | 29.3% | 471 | 54232 | 8.9 | non-POU domain containing, octamer-binding isoform 1 [Homo sapiens] |
| U | *gi|34932414|ref|NP\_03* | 16 | 38 | 29.3% | 471 | 54232 | 8.9 | non-POU domain containing, octamer-binding isoform 1 [Homo sapiens] |
| U | *gi|224028246|ref|NP\_0* | 16 | 38 | 29.3% | 471 | 54232 | 8.9 | non-POU domain containing, octamer-binding isoform 1 [Homo sapiens] |

| Filename XCorr DeltCN Conf% ObsM+H+ CalcM+H+ SpR ZScore Ion% # Sequence  | | | | | | | | | | | | |
| --- | --- | --- | --- | --- | --- | --- | --- | --- | --- | --- | --- | --- |
|  | AstrinIP\_MS1\_022614\_01.10516.10516.2 | 3.6222 | 0.5635 | 100.0% | 1860.2522 | 1861.12 | 2 | 8.874 | 56.7% | 3 | R.LFVGNLPPDITEEEMR.K | 2 |
|  | AstrinIP\_MS1\_022614\_01.11774.11774.3 | 2.8229 | 0.2829 | 98.7% | 1814.4243 | 1814.1504 | 1 | 5.018 | 36.7% | 1 | R.TLAEIAKVELDNMPLR.G | 3 |
|  | AstrinIP\_MS2\_022614\_01.11238.11238.3 | 3.4407 | 0.3263 | 99.9% | 2000.0343 | 1999.3765 | 1 | 5.493 | 32.4% | 1 | R.TLAEIAKVELDNMPLRGK.Q | 3 |
|  | AstrinIP\_MS2\_022614\_01.06026.06026.2 | 3.0743 | 0.3093 | 99.9% | 1272.2322 | 1272.5052 | 1 | 5.205 | 80.0% | 2 | K.VELDNMPLRGK.Q | 2 |
|  | AstrinIP\_MS1\_022614\_01.18044.18044.3 | 3.316 | 0.3194 | 99.6% | 2669.1543 | 2669.9507 | 1 | 5.746 | 31.8% | 1 | R.NLPQYVSNELLEEAFSVFGQVER.A | 3 |
|  | AstrinIP\_MS2\_022614\_01.18765.18765.2 | 5.5821 | 0.5114 | 100.0% | 2669.5723 | 2669.9507 | 1 | 10.946 | 50.0% | 6 | R.NLPQYVSNELLEEAFSVFGQVER.A | 2 |
|  | AstrinIP\_MS1\_022614\_01.04426.04426.1 | 2.0327 | 0.335 | 100.0% | 886.5 | 887.0238 | 6 | 6.694 | 71.4% | 2 | R.AVVIVDDR.G | 11 |
|  | AstrinIP\_MS1\_022614\_01.04617.04617.2 | 3.0912 | 0.3977 | 100.0% | 1232.0521 | 1232.4252 | 1 | 7.289 | 72.7% | 3 | K.GIVEFSGKPAAR.K | 2 |
|  | AstrinIP\_MS1\_022614\_01.08936.08936.2 | 3.4962 | 0.542 | 100.0% | 1696.0322 | 1696.8744 | 1 | 8.809 | 65.4% | 7 | R.FAQPGSFEYEYAMR.W | 2 |
|  | AstrinIP\_MS2\_022614\_01.05406.05406.2 | 3.143 | 0.4328 | 100.0% | 1337.3322 | 1337.5488 | 1 | 7.788 | 75.0% | 2 | R.EKLEMEMEAAR.H | 2 |
|  | AstrinIP\_MS2\_022614\_01.06038.06038.2 | 2.3641 | 0.2069 | 98.1% | 1079.9122 | 1080.2593 | 1 | 4.334 | 81.2% | 1 | K.LEMEMEAAR.H | 2 |
|  | AstrinIP\_MS2\_022614\_01.03874.03874.2 | 2.5108 | 0.3314 | 99.8% | 1181.2522 | 1181.4161 | 1 | 6.357 | 75.0% | 1 | R.HEHQVMLMR.Q | 2 |
|  | AstrinIP\_MS2\_022614\_01.03494.03494.2 | 3.6586 | 0.2075 | 99.8% | 1541.1721 | 1541.7222 | 1 | 5.503 | 86.4% | 1 | R.RMEELHNQEVQK.R | 2 |
|  | AstrinIP\_MS2\_022614\_01.03500.03500.3 | 3.433 | 0.2745 | 99.8% | 1541.3944 | 1541.7222 | 1 | 5.419 | 56.8% | 1 | R.RMEELHNQEVQK.R | 3 |
|  | AstrinIP\_MS2\_022614\_01.03603.03603.2 | 3.0982 | 0.2113 | 99.7% | 1384.2522 | 1385.5347 | 1 | 4.831 | 80.0% | 1 | R.MEELHNQEVQK.R | 2 |
|  | AstrinIP\_MS2\_022614\_02.05289.05289.2 | 4.0777 | 0.4806 | 100.0% | 1538.7722 | 1539.8441 | 1 | 8.303 | 60.7% | 5 | R.MGQMAMGGAMGINNR.G | 2 |

Similarities:
gi|4826998|ref|NP\_005(1:15)  

---

|  |  |  |  |  |  |  |  |  |
| --- | --- | --- | --- | --- | --- | --- | --- | --- |
| U | *gi|14141152|ref|NP\_00* | 15 | 23 | 28.6% | 730 | 77516 | 8.7 | heterogeneous nuclear ribonucleoprotein M isoform a [Homo sapiens] |
| U | *gi|157412270|ref|NP\_1* | 15 | 23 | 30.2% | 691 | 73621 | 8.8 | heterogeneous nuclear ribonucleoprotein M isoform b [Homo sapiens] |

| Filename XCorr DeltCN Conf% ObsM+H+ CalcM+H+ SpR ZScore Ion% # Sequence  | | | | | | | | | | | | |
| --- | --- | --- | --- | --- | --- | --- | --- | --- | --- | --- | --- | --- |
|  | AstrinIP\_MS2\_022614\_01.11895.11895.2 | 2.4014 | 0.2706 | 98.7% | 1267.3522 | 1265.4949 | 13 | 5.094 | 60.0% | 1 | R.AFITNIPFDVK.W | 2 |
|  | AstrinIP\_MS2\_022614\_02.09210.09210.2 | 4.187 | 0.5367 | 100.0% | 1753.8522 | 1754.0051 | 1 | 9.838 | 53.3% | 3 | K.VGEVTYVELLMDAEGK.S | 2 |
|  | AstrinIP\_MS1\_022614\_02.06793.06793.2 | 3.2004 | 0.4422 | 100.0% | 1427.5322 | 1427.6403 | 1 | 7.704 | 62.5% | 1 | R.LGSTVFVANLDYK.V | 2 |
|  | AstrinIP\_MS2\_022614\_02.06585.06585.2 | 2.4879 | 0.1885 | 96.2% | 1435.5322 | 1435.768 | 2 | 4.392 | 58.3% | 1 | K.LKEVFSMAGVVVR.A | 2 |
|  | AstrinIP\_MS2\_022614\_01.12892.12892.2 | 4.0363 | 0.4122 | 100.0% | 2179.3523 | 2179.5752 | 1 | 7.832 | 45.5% | 1 | K.GIGMGNIGPAGMGMEGIGFGINK.M | 2 |
|  | AstrinIP\_MS1\_022614\_01.09429.09429.2 | 2.5024 | 0.3018 | 98.8% | 1714.3522 | 1715.9724 | 1 | 5.332 | 50.0% | 1 | K.MGGMEGPFGGGMENMGR.F | 2 |
|  | AstrinIP\_MS1\_022614\_01.08220.08220.2 | 2.5209 | 0.3428 | 99.8% | 1115.1721 | 1115.3152 | 1 | 5.586 | 72.2% | 1 | R.INEILSNALK.R | 2 |
|  | AstrinIP\_MS1\_022614\_01.03816.03816.2 | 3.0412 | 0.2784 | 99.8% | 1103.2922 | 1102.2714 | 1 | 5.285 | 95.0% | 1 | R.MGAGLGHGMDR.V | 2 |
|  | AstrinIP\_MS2\_022614\_01.09137.09137.3 | 3.468 | 0.4849 | 100.0% | 1614.5643 | 1614.875 | 1 | 8.429 | 50.0% | 1 | R.MGPLGLDHMASSIER.M | 3 |
|  | AstrinIP\_MS2\_022614\_01.08783.08783.2 | 3.2741 | 0.5125 | 100.0% | 1126.2522 | 1126.3337 | 1 | 9.561 | 80.0% | 3 | R.MGAGMGFGLER.M | 2 |
|  | AstrinIP\_MS2\_022614\_01.03695.03695.2 | 2.5944 | 0.3216 | 99.8% | 905.0722 | 905.0166 | 5 | 5.566 | 78.6% | 1 | R.MGANNLER.M | 2 |
|  | AstrinIP\_MS2\_022614\_01.03837.03837.1 | 1.9184 | 0.2917 | 100.0% | 877.53 | 877.99097 | 4 | 5.198 | 64.3% | 1 | R.MGANSLER.M | 1 |
|  | AstrinIP\_MS1\_022614\_01.08019.08019.2 | 3.2475 | 0.3292 | 99.8% | 1428.3322 | 1428.7076 | 1 | 6.169 | 60.7% | 3 | R.MGPAMGPALGAGIER.M | 2 |
|  | AstrinIP\_MS2\_022614\_02.05579.05579.2 | 3.5288 | 0.4237 | 100.0% | 1384.1322 | 1384.5677 | 1 | 7.872 | 67.9% | 2 | R.MGLAMGGGGGASFDR.A | 2 |
|  | AstrinIP\_MS2\_022614\_02.05720.05720.3 | 3.3969 | 0.4193 | 100.0% | 2036.0044 | 2036.1735 | 1 | 6.289 | 34.1% | 2 | R.GNFGGSFAGSFGGAGGHAPGVAR.K | 3 |

---

|  |  |  |  |  |  |  |  |  |
| --- | --- | --- | --- | --- | --- | --- | --- | --- |
| U | *contaminant\_INT-STD1* | 22 | 71 | 27.8% | 607 | 69271 | 6.1 | BSA |

| Filename XCorr DeltCN Conf% ObsM+H+ CalcM+H+ SpR ZScore Ion% # Sequence  | | | | | | | | | | | | |
| --- | --- | --- | --- | --- | --- | --- | --- | --- | --- | --- | --- | --- |
| \* | AstrinIP\_MS1\_022614\_01.08655.08655.2 | 3.3594 | 0.3748 | 100.0% | 1164.3722 | 1164.344 | 1 | 6.876 | 77.8% | 5 | K.LVNELTEFAK.T | 2 |
|  | AstrinIP\_MS1\_022614\_01.06783.06783.1 | 1.844 | 0.1904 | 96.5% | 927.4 | 928.0758 | 78 | 4.429 | 66.7% | 2 | K.YLYEIAR.R | 1 |
|  | AstrinIP\_MS2\_022614\_01.07061.07061.2 | 2.3428 | 0.1896 | 98.6% | 928.39215 | 928.0758 | 1 | 5.542 | 91.7% | 1 | K.YLYEIAR.R | 2 |
| \* | AstrinIP\_MS2\_022614\_01.10791.10791.2 | 5.0127 | 0.5382 | 100.0% | 2045.5122 | 2046.3354 | 1 | 9.051 | 70.0% | 3 | R.RHPYFYAPELLYYANK.Y | 2 |
| \* | AstrinIP\_MS1\_022614\_01.10024.10024.3 | 3.6863 | 0.3362 | 100.0% | 2046.0543 | 2046.3354 | 1 | 6.227 | 50.0% | 2 | R.RHPYFYAPELLYYANK.Y | 3 |
| \* | AstrinIP\_MS1\_022614\_01.11342.11342.2 | 2.2468 | 0.4155 | 99.5% | 1888.7722 | 1890.148 | 68 | 6.345 | 35.7% | 1 | R.HPYFYAPELLYYANK.Y | 2 |
| \* | AstrinIP\_MS1\_022614\_01.04864.04864.1 | 2.0635 | 0.1948 | 97.3% | 922.39 | 923.05383 | 2 | 4.717 | 57.1% | 3 | K.AEFVEVTK.L | 1 |
| \* | AstrinIP\_MS1\_022614\_01.03786.03786.2 | 4.0132 | 0.2878 | 100.0% | 1533.3121 | 1533.7377 | 2 | 5.666 | 72.7% | 1 | K.LKECCDKPLLEK.S | 2 |
| \* | AstrinIP\_MS1\_022614\_01.03788.03788.3 | 3.6604 | 0.1826 | 99.0% | 1533.8644 | 1533.7377 | 2 | 5.324 | 50.0% | 1 | K.LKECCDKPLLEK.S | 3 |
| \* | AstrinIP\_MS1\_022614\_01.13518.13518.2 | 4.7989 | 0.4472 | 100.0% | 1569.3121 | 1568.7258 | 1 | 7.379 | 79.2% | 6 | K.DAFLGSFLYEYSR.R | 2 |
| \* | AstrinIP\_MS2\_022614\_02.05540.05540.3 | 4.3123 | 0.3463 | 100.0% | 1440.9844 | 1440.6884 | 1 | 5.738 | 56.8% | 1 | R.RHPEYAVSVLLR.L | 3 |
| \* | AstrinIP\_MS1\_022614\_01.06966.06966.2 | 3.1988 | 0.2983 | 99.9% | 1441.5922 | 1440.6884 | 1 | 6.184 | 63.6% | 5 | R.RHPEYAVSVLLR.L | 2 |
| \* | AstrinIP\_MS1\_022614\_01.08373.08373.2 | 2.2624 | 0.2617 | 98.0% | 1284.3522 | 1284.5009 | 188 | 4.48 | 50.0% | 1 | R.HPEYAVSVLLR.L | 2 |
| \* | AstrinIP\_MS2\_022614\_01.06444.06444.2 | 3.2744 | 0.4169 | 100.0% | 1306.3121 | 1306.5046 | 2 | 7.532 | 65.0% | 7 | K.HLVDEPQNLIK.Q | 2 |
| \* | AstrinIP\_MS1\_022614\_01.10412.10412.2 | 4.1745 | 0.3967 | 100.0% | 1480.1122 | 1480.7068 | 1 | 8.012 | 75.0% | 11 | K.LGEYGFQNALIVR.Y | 2 |
|  | AstrinIP\_MS2\_022614\_01.07082.07082.2 | 3.9051 | 0.4476 | 100.0% | 1640.5322 | 1640.9205 | 1 | 9.045 | 71.4% | 6 | R.KVPQVSTPTLVEVSR.S | 2 |
|  | AstrinIP\_MS1\_022614\_01.06791.06791.3 | 3.3201 | 0.2978 | 99.7% | 1640.5743 | 1640.9205 | 1 | 6.108 | 42.9% | 2 | R.KVPQVSTPTLVEVSR.S | 3 |
|  | AstrinIP\_MS1\_022614\_01.07838.07838.2 | 2.8698 | 0.3908 | 99.9% | 1512.1322 | 1512.7465 | 1 | 8.011 | 69.2% | 1 | K.VPQVSTPTLVEVSR.S | 2 |
| \* | AstrinIP\_MS1\_022614\_01.13383.13383.2 | 4.772 | 0.4807 | 100.0% | 1726.2122 | 1725.9636 | 1 | 8.457 | 69.2% | 1 | R.MPCTEDYLSLILNR.L | 2 |
| \* | AstrinIP\_MS1\_022614\_01.07810.07810.2 | 3.223 | 0.44 | 100.0% | 1881.4122 | 1882.0894 | 1 | 6.37 | 50.0% | 1 | R.RPCFSALTPDETYVPK.A | 2 |
| \* | AstrinIP\_MS2\_022614\_01.08326.08326.2 | 3.2228 | 0.3499 | 100.0% | 1143.2722 | 1143.4124 | 1 | 6.331 | 72.2% | 4 | K.KQTALVELLK.H | 2 |
| \* | AstrinIP\_MS2\_022614\_01.13246.13246.2 | 4.0485 | 0.4795 | 100.0% | 1401.2922 | 1400.6324 | 1 | 8.118 | 86.4% | 6 | K.TVMENFVAFVDK.C | 2 |

---

|  |  |  |  |  |  |  |  |  |
| --- | --- | --- | --- | --- | --- | --- | --- | --- |
| U | *gi|190360566|ref|NP\_4* | 10 | 20 | 27.5% | 357 | 40675 | 5.7 | protein-L-isoaspartate (D-aspartate) O-methyltransferase domain containing 1 [Homo sapiens] |

| Filename XCorr DeltCN Conf% ObsM+H+ CalcM+H+ SpR ZScore Ion% # Sequence  | | | | | | | | | | | | |
| --- | --- | --- | --- | --- | --- | --- | --- | --- | --- | --- | --- | --- |
| \* | AstrinIP\_MS1\_022614\_01.19708.19708.2 | 3.2563 | 0.3239 | 99.8% | 2014.4321 | 2015.0698 | 1 | 6.455 | 41.7% | 2 | -.MGGAVS\*AGEDNDDLIDNLK.E | 2 |
| \* | AstrinIP\_MS2\_022614\_01.20004.20004.2 | 4.0547 | 0.452 | 100.0% | 2774.7522 | 2775.9177 | 1 | 8.345 | 39.6% | 2 | -.MGGAVS\*AGEDNDDLIDNLKEAQYIR.T | 2 |
| \* | AstrinIP\_MS2\_022614\_01.09737.09737.3 | 3.6135 | 0.2268 | 98.3% | 2797.0444 | 2797.0557 | 1 | 4.34 | 33.0% | 1 | R.AIDRGDYYLEGYRDNAYKDLAWK.H | 3 |
| \* | AstrinIP\_MS2\_022614\_01.09966.09966.3 | 3.49 | 0.2159 | 98.2% | 2341.7043 | 2341.5413 | 8 | 4.943 | 27.8% | 1 | R.GDYYLEGYRDNAYKDLAWK.H | 3 |
| \* | AstrinIP\_MS2\_022614\_01.06888.06888.2 | 2.3238 | 0.237 | 98.1% | 1225.4922 | 1224.3586 | 19 | 4.513 | 72.2% | 1 | R.DNAYKDLAWK.H | 2 |
| \* | AstrinIP\_MS2\_022614\_01.15206.15206.2 | 4.5029 | 0.6022 | 100.0% | 2013.3722 | 2014.4492 | 1 | 10.539 | 58.8% | 5 | K.VGGILVMPIEDQLTQIMR.T | 2 |
| \* | AstrinIP\_MS1\_022614\_01.14698.14698.3 | 2.6518 | 0.2799 | 97.8% | 2016.0243 | 2014.4492 | 122 | 5.205 | 26.5% | 1 | K.VGGILVMPIEDQLTQIMR.T | 3 |
| \* | AstrinIP\_MS2\_022614\_01.12819.12819.2 | 3.6369 | 0.4182 | 100.0% | 1584.7322 | 1584.8992 | 2 | 6.896 | 60.7% | 3 | K.NILAVSFAPLVQPSK.N | 2 |
| \* | AstrinIP\_MS1\_022614\_02.04606.04606.2 | 2.586 | 0.236 | 99.1% | 1209.4722 | 1210.3472 | 9 | 4.618 | 72.2% | 3 | R.NFINDEMQAK.G | 2 |
| \* | AstrinIP\_MS2\_022614\_01.09015.09015.1 | 1.6176 | 0.3238 | 100.0% | 933.51 | 934.0825 | 173 | 5.085 | 50.0% | 1 | K.AYLTYFR.D | 1 |

---

|  |  |  |  |  |  |  |  |  |
| --- | --- | --- | --- | --- | --- | --- | --- | --- |
| U | *gi|34098946|ref|NP\_00* | 7 | 10 | 26.9% | 324 | 35924 | 9.9 | nuclease sensitive element binding protein 1 [Homo sapiens] |

| Filename XCorr DeltCN Conf% ObsM+H+ CalcM+H+ SpR ZScore Ion% # Sequence  | | | | | | | | | | | | |
| --- | --- | --- | --- | --- | --- | --- | --- | --- | --- | --- | --- | --- |
|  | AstrinIP\_MS2\_022614\_01.04626.04626.3 | 3.3734 | 0.3198 | 99.9% | 1745.8744 | 1745.9298 | 1 | 5.888 | 42.9% | 2 | R.NDTKEDVFVHQTAIK.K | 3 |
|  | AstrinIP\_MS1\_022614\_01.03978.03978.2 | 4.2667 | 0.3315 | 100.0% | 1873.4722 | 1874.1039 | 1 | 6.283 | 70.0% | 1 | R.NDTKEDVFVHQTAIKK.N | 2 |
|  | AstrinIP\_MS2\_022614\_01.04049.04049.3 | 3.9789 | 0.3503 | 100.0% | 1873.9443 | 1874.1039 | 1 | 6.543 | 48.3% | 2 | R.NDTKEDVFVHQTAIKK.N | 3 |
|  | AstrinIP\_MS2\_022614\_02.06077.06077.2 | 4.7515 | 0.4461 | 100.0% | 1796.4521 | 1796.8822 | 1 | 9.364 | 68.8% | 1 | R.SVGDGETVEFDVVEGEK.G | 2 |
| \* | AstrinIP\_MS2\_022614\_01.04947.04947.3 | 5.2235 | 0.3894 | 100.0% | 3225.7144 | 3225.4795 | 1 | 6.828 | 26.7% | 2 | R.RPQYSNPPVQGEVMEGADNQGAGEQGRPVR.Q | 3 |
| \* | AstrinIP\_MS2\_022614\_01.03422.03422.3 | 4.9179 | 0.3841 | 100.0% | 2629.8542 | 2629.5835 | 1 | 7.317 | 42.0% | 1 | R.EDGNEEDKENQGDETQGQQPPQR.R | 3 |
| \* | AstrinIP\_MS2\_022614\_01.03368.03368.3 | 3.1245 | 0.3993 | 100.0% | 2784.8342 | 2785.771 | 1 | 6.639 | 28.3% | 1 | R.EDGNEEDKENQGDETQGQQPPQRR.Y | 3 |

---

|  |  |  |  |  |  |  |  |  |
| --- | --- | --- | --- | --- | --- | --- | --- | --- |
| U | *gi|117189975|ref|NP\_1* | 6 | 11 | 26.8% | 306 | 33670 | 5.1 | heterogeneous nuclear ribonucleoprotein C isoform a [Homo sapiens] |
| U | *gi|117190254|ref|NP\_0* | 6 | 11 | 28.0% | 293 | 32338 | 5.1 | heterogeneous nuclear ribonucleoprotein C isoform b [Homo sapiens] |
| U | *gi|117190192|ref|NP\_0* | 6 | 11 | 26.8% | 306 | 33670 | 5.1 | heterogeneous nuclear ribonucleoprotein C isoform a [Homo sapiens] |
| U | *gi|117190174|ref|NP\_0* | 6 | 11 | 28.0% | 293 | 32338 | 5.1 | heterogeneous nuclear ribonucleoprotein C isoform b [Homo sapiens] |

| Filename XCorr DeltCN Conf% ObsM+H+ CalcM+H+ SpR ZScore Ion% # Sequence  | | | | | | | | | | | | |
| --- | --- | --- | --- | --- | --- | --- | --- | --- | --- | --- | --- | --- |
|  | AstrinIP\_MS2\_022614\_02.07448.07448.2 | 3.8045 | 0.2987 | 100.0% | 1317.2922 | 1317.6145 | 1 | 7.071 | 86.4% | 2 | R.VFIGNLNTLVVK.K | 2 |
|  | AstrinIP\_MS1\_022614\_01.06326.06326.2 | 2.7944 | 0.3876 | 100.0% | 1124.1721 | 1124.2792 | 1 | 6.144 | 72.2% | 2 | K.KSDVEAIFSK.Y | 2 |
|  | AstrinIP\_MS1\_022614\_01.10008.10008.2 | 3.4887 | 0.4576 | 100.0% | 1329.9321 | 1330.4857 | 1 | 9.012 | 80.0% | 2 | K.GFAFVQYVNER.N | 2 |
|  | AstrinIP\_MS1\_022614\_01.10602.10602.2 | 4.8158 | 0.4242 | 100.0% | 1683.2922 | 1684.0038 | 1 | 7.211 | 83.3% | 3 | R.MIAGQVLDINLAAEPK.V | 2 |
|  | AstrinIP\_MS2\_022614\_01.08973.08973.2 | 2.1364 | 0.3571 | 98.8% | 1416.1522 | 1416.6146 | 3 | 5.578 | 72.7% | 1 | K.QKVDSLLENLEK.I | 2 |
|  | AstrinIP\_MS2\_022614\_01.03416.03416.3 | 4.2168 | 0.4106 | 100.0% | 2368.6443 | 2369.4583 | 1 | 6.234 | 41.2% | 1 | K.NDKSEEEQSSSSVKKDETNVK.M | 3 |

---

|  |  |  |  |  |  |  |  |  |
| --- | --- | --- | --- | --- | --- | --- | --- | --- |
| U | *gi|28875797|ref|NP\_05* | 5 | 12 | 25.8% | 248 | 26397 | 12.2 | hypothetical protein LOC26097 [Homo sapiens] |

| Filename XCorr DeltCN Conf% ObsM+H+ CalcM+H+ SpR ZScore Ion% # Sequence  | | | | | | | | | | | | |
| --- | --- | --- | --- | --- | --- | --- | --- | --- | --- | --- | --- | --- |
| \* | AstrinIP\_MS2\_022614\_01.03872.03872.2 | 4.1474 | 0.4282 | 100.0% | 1447.1921 | 1447.6091 | 3 | 7.903 | 62.5% | 2 | R.ASMQQQQQLASAR.N | 2 |
| \* | AstrinIP\_MS2\_022614\_01.04930.04930.2 | 4.4525 | 0.3008 | 100.0% | 1784.3322 | 1785.0715 | 1 | 6.254 | 60.0% | 2 | R.LAQQMENRPSVQAALK.L | 2 |
| \* | AstrinIP\_MS2\_022614\_01.04905.04905.3 | 2.8744 | 0.3716 | 99.9% | 1784.8143 | 1785.0715 | 2 | 5.709 | 33.3% | 2 | R.LAQQMENRPSVQAALK.L | 3 |
| \* | AstrinIP\_MS2\_022614\_02.06020.06020.2 | 3.7207 | 0.5112 | 100.0% | 1555.1522 | 1555.6997 | 1 | 8.323 | 62.5% | 5 | K.EQLDNQLDAYMSK.T | 2 |
| \* | AstrinIP\_MS2\_022614\_02.06147.06147.3 | 3.289 | 0.3097 | 99.3% | 2435.5144 | 2436.566 | 1 | 5.118 | 32.1% | 1 | K.TKGHLDAELDAYMAQTDPETND.- | 3 |

---

|  |  |  |  |  |  |  |  |  |
| --- | --- | --- | --- | --- | --- | --- | --- | --- |
| U | *gi|27436946|ref|NP\_73* | 13 | 20 | 24.5% | 664 | 74140 | 7.0 | lamin A/C isoform 1 precursor [Homo sapiens] |

| Filename XCorr DeltCN Conf% ObsM+H+ CalcM+H+ SpR ZScore Ion% # Sequence  | | | | | | | | | | | | |
| --- | --- | --- | --- | --- | --- | --- | --- | --- | --- | --- | --- | --- |
|  | AstrinIP\_MS2\_022614\_01.04773.04773.3 | 3.4582 | 0.2061 | 98.9% | 1631.5443 | 1630.7521 | 4 | 5.312 | 52.1% | 1 | R.LQEKEDLQELNDR.L | 3 |
|  | AstrinIP\_MS2\_022614\_01.04002.04002.2 | 3.1654 | 0.3149 | 100.0% | 1149.9521 | 1149.2432 | 1 | 6.559 | 88.9% | 1 | R.ITESEEVVSR.E | 2 |
|  | AstrinIP\_MS2\_022614\_01.05034.05034.2 | 2.8395 | 0.3765 | 100.0% | 1166.3121 | 1166.2328 | 1 | 6.529 | 75.0% | 1 | K.AAYEAELGDAR.K | 2 |
|  | AstrinIP\_MS2\_022614\_01.13389.13389.3 | 2.935 | 0.2319 | 95.7% | 2346.2344 | 2346.56 | 4 | 3.875 | 29.2% | 1 | R.KT#LDSVAKERARLQLELS\*K.V | 3 |
|  | AstrinIP\_MS1\_022614\_01.08828.08828.2 | 3.2431 | 0.2425 | 99.8% | 1244.4321 | 1244.474 | 1 | 5.301 | 80.0% | 2 | R.LKDLEALLNSK.E | 2 |
|  | AstrinIP\_MS2\_022614\_01.06834.06834.2 | 3.4274 | 0.3856 | 100.0% | 1510.0521 | 1510.7455 | 1 | 6.295 | 68.2% | 1 | R.LQTMKEELDFQK.N | 2 |
|  | AstrinIP\_MS2\_022614\_01.06042.06042.2 | 4.4861 | 0.4965 | 100.0% | 1754.2922 | 1753.8693 | 1 | 8.783 | 66.7% | 2 | R.NSNLVGAAHEELQQSR.I | 2 |
|  | AstrinIP\_MS2\_022614\_01.10114.10114.2 | 4.475 | 0.4506 | 100.0% | 1700.4122 | 1700.9762 | 1 | 7.274 | 64.3% | 1 | R.IRIDSLSAQLSQLQK.Q | 2 |
|  | AstrinIP\_MS2\_022614\_01.09323.09323.2 | 3.4185 | 0.2502 | 99.8% | 1431.5721 | 1431.6293 | 1 | 4.902 | 70.8% | 1 | R.IDSLSAQLSQLQK.Q | 2 |
|  | AstrinIP\_MS1\_022614\_01.05673.05673.2 | 2.5387 | 0.2712 | 99.3% | 1188.5122 | 1188.3262 | 1 | 5.34 | 72.2% | 3 | K.LRDLEDSLAR.E | 2 |
|  | AstrinIP\_MS1\_022614\_01.11444.11444.2 | 4.1786 | 0.3975 | 100.0% | 1894.2722 | 1895.1346 | 1 | 7.198 | 71.4% | 1 | R.MQQQLDEYQELLDIK.L | 2 |
|  | AstrinIP\_MS2\_022614\_02.04379.04379.2 | 3.5819 | 0.438 | 100.0% | 1491.9521 | 1492.6874 | 1 | 7.425 | 69.2% | 4 | R.TALINSTGEEVAMR.K | 2 |
|  | AstrinIP\_MS2\_022614\_01.06874.06874.2 | 3.9533 | 0.5018 | 100.0% | 1567.0322 | 1567.6555 | 1 | 8.058 | 53.1% | 1 | R.SVGGSGGGSFGDNLVTR.S | 2 |

---

|  |  |  |  |  |  |  |  |  |
| --- | --- | --- | --- | --- | --- | --- | --- | --- |
| U | *gi|20149594|ref|NP\_03* | 14 | 30 | 24.4% | 724 | 83264 | 5.0 | heat shock 90kDa protein 1, beta [Homo sapiens] |

| Filename XCorr DeltCN Conf% ObsM+H+ CalcM+H+ SpR ZScore Ion% # Sequence  | | | | | | | | | | | | |
| --- | --- | --- | --- | --- | --- | --- | --- | --- | --- | --- | --- | --- |
|  | AstrinIP\_MS2\_022614\_01.06681.06681.2 | 2.9726 | 0.3961 | 100.0% | 1275.7522 | 1276.3861 | 1 | 6.52 | 68.2% | 2 | R.ELISNASDALDK.I | 2 |
|  | AstrinIP\_MS1\_022614\_01.08340.08340.2 | 3.1905 | 0.4368 | 100.0% | 1544.2922 | 1545.733 | 1 | 7.258 | 61.5% | 1 | R.ELISNASDALDKIR.Y | 2 |
|  | AstrinIP\_MS1\_022614\_01.08895.08895.2 | 3.6434 | 0.4326 | 100.0% | 1243.4722 | 1243.4459 | 1 | 7.604 | 77.3% | 3 | K.ADLINNLGTIAK.S | 22 |
|  | AstrinIP\_MS2\_022614\_02.05165.05165.3 | 4.0265 | 0.4323 | 100.0% | 2015.5443 | 2016.2584 | 1 | 7.512 | 41.7% | 5 | K.VILHLKEDQTEYLEER.R | 33 |
| \* | AstrinIP\_MS1\_022614\_01.11604.11604.2 | 4.6682 | 0.5387 | 100.0% | 1809.5322 | 1810.1027 | 1 | 9.377 | 71.4% | 3 | K.HSQFIGYPITLYLEK.E | 2 |
| \* | AstrinIP\_MS2\_022614\_01.03515.03515.3 | 3.5796 | 0.2691 | 99.5% | 1946.0944 | 1946.8871 | 15 | 5.377 | 35.9% | 1 | K.IEDVGS\*DEEDDSGKDKK.K | 3 |
|  | AstrinIP\_MS2\_022614\_01.04424.04424.2 | 2.9971 | 0.2018 | 99.8% | 1151.9922 | 1152.2462 | 2 | 5.324 | 81.2% | 1 | K.YIDQEELNK.T | 22 |
| \* | AstrinIP\_MS2\_022614\_01.08842.08842.2 | 4.3865 | 0.5275 | 100.0% | 1848.3522 | 1848.9171 | 1 | 8.873 | 71.4% | 2 | R.NPDDITQEEYGEFYK.S | 2 |
|  | AstrinIP\_MS2\_022614\_01.08724.08724.2 | 4.0147 | 0.4128 | 100.0% | 1528.5122 | 1528.6616 | 5 | 7.355 | 62.5% | 3 | K.SLTNDWEDHLAVK.H | 22 |
|  | AstrinIP\_MS2\_022614\_02.05787.05787.2 | 3.0185 | 0.4175 | 100.0% | 1349.5521 | 1349.4886 | 2 | 6.35 | 60.0% | 3 | K.HFSVEGQLEFR.A | 22 |
| \* | AstrinIP\_MS2\_022614\_01.03560.03560.2 | 2.7415 | 0.3823 | 100.0% | 1141.9722 | 1142.2137 | 1 | 6.489 | 72.2% | 1 | K.LGIHEDSTNR.R | 2 |
| \* | AstrinIP\_MS2\_022614\_02.05834.05834.3 | 4.1481 | 0.4423 | 100.0% | 2177.5745 | 2178.2915 | 1 | 8.02 | 41.7% | 2 | R.YHTSQSGDEMTSLSEYVSR.M | 3 |
| \* | AstrinIP\_MS2\_022614\_01.04797.04797.2 | 2.0918 | 0.3515 | 98.7% | 1249.9122 | 1250.3538 | 42 | 5.522 | 50.0% | 1 | K.EQVANSAFVER.V | 2 |
| \* | AstrinIP\_MS1\_022614\_01.07120.07120.3 | 3.8629 | 0.5004 | 100.0% | 1783.7344 | 1784.025 | 1 | 7.642 | 50.0% | 2 | K.HLEINPDHPIVETLR.Q | 3 |

Similarities:
gi|153792590|ref|NP\_0(5:9)  

---

|  |  |  |  |  |  |  |  |  |
| --- | --- | --- | --- | --- | --- | --- | --- | --- |
| U | *gi|31542947|ref|NP\_00* | 9 | 12 | 23.4% | 573 | 61055 | 5.9 | chaperonin [Homo sapiens] |
| U | *gi|41399285|ref|NP\_95* | 9 | 12 | 23.4% | 573 | 61055 | 5.9 | chaperonin [Homo sapiens] |

| Filename XCorr DeltCN Conf% ObsM+H+ CalcM+H+ SpR ZScore Ion% # Sequence  | | | | | | | | | | | | |
| --- | --- | --- | --- | --- | --- | --- | --- | --- | --- | --- | --- | --- |
|  | AstrinIP\_MS2\_022614\_02.12828.12828.3 | 4.3966 | 0.4135 | 100.0% | 2114.3943 | 2114.5667 | 1 | 7.396 | 43.8% | 2 | R.ALMLQGVDLLADAVAVTMGPK.G | 3 |
|  | AstrinIP\_MS2\_022614\_01.18516.18516.2 | 4.6211 | 0.4826 | 100.0% | 2114.9722 | 2114.5667 | 1 | 9.137 | 55.0% | 2 | R.ALMLQGVDLLADAVAVTMGPK.G | 2 |
|  | AstrinIP\_MS1\_022614\_01.07689.07689.2 | 2.6942 | 0.1869 | 98.1% | 1345.4722 | 1345.5382 | 1 | 5.288 | 68.2% | 2 | R.TVIIEQSWGSPK.V | 2 |
|  | AstrinIP\_MS1\_022614\_02.05073.05073.3 | 3.1001 | 0.2946 | 99.0% | 2561.0645 | 2561.7222 | 159 | 6.706 | 24.0% | 1 | K.LVQDVANNTNEEAGDGTTTATVLAR.S | 3 |
|  | AstrinIP\_MS2\_022614\_01.14631.14631.2 | 3.4502 | 0.2587 | 99.8% | 1557.5922 | 1557.9324 | 1 | 6.647 | 71.4% | 1 | R.GVMLAVDAVIAELKK.Q | 2 |
|  | AstrinIP\_MS2\_022614\_01.08709.08709.3 | 2.6979 | 0.3159 | 99.2% | 1632.3844 | 1631.9684 | 8 | 4.977 | 35.7% | 1 | K.VGEVIVTKDDAMLLK.G | 3 |
|  | AstrinIP\_MS2\_022614\_01.03869.03869.2 | 2.3616 | 0.2843 | 99.2% | 961.1322 | 961.0629 | 5 | 5.228 | 75.0% | 1 | R.VTDALNATR.A | 2 |
|  | AstrinIP\_MS1\_022614\_02.04947.04947.2 | 2.7513 | 0.3829 | 99.9% | 1215.3121 | 1216.377 | 1 | 6.717 | 72.7% | 1 | K.NAGVEGSLIVEK.I | 2 |
|  | AstrinIP\_MS1\_022614\_02.13645.13645.3 | 4.5074 | 0.2664 | 99.8% | 2483.8743 | 2483.9055 | 1 | 6.588 | 36.5% | 1 | R.TALLDAAGVASLLTTAEVVVTEIPK.E | 3 |

---

|  |  |  |  |  |  |  |  |  |
| --- | --- | --- | --- | --- | --- | --- | --- | --- |
| U | *gi|157388995|ref|NP\_0* | 5 | 5 | 22.2% | 361 | 41072 | 6.1 | protein-L-isoaspartate (D-aspartate) O-methyltransferase domain containing 2 isoform 1 [Homo sapiens] |

| Filename XCorr DeltCN Conf% ObsM+H+ CalcM+H+ SpR ZScore Ion% # Sequence  | | | | | | | | | | | | |
| --- | --- | --- | --- | --- | --- | --- | --- | --- | --- | --- | --- | --- |
|  | AstrinIP\_MS2\_022614\_01.19984.19984.2 | 3.4035 | 0.5052 | 100.0% | 2788.0122 | 2789.9446 | 1 | 8.125 | 31.2% | 1 | -.MGGAVS\*AGEDNDELIDNLKEAQYIR.T | 2 |
|  | AstrinIP\_MS2\_022614\_01.07530.07530.2 | 2.934 | 0.3668 | 100.0% | 1093.1122 | 1093.2249 | 2 | 6.305 | 75.0% | 1 | R.TELVEQAFR.A | 2 |
| \* | AstrinIP\_MS2\_022614\_01.11085.11085.2 | 3.7262 | 0.3989 | 100.0% | 1285.2522 | 1285.5848 | 1 | 7.253 | 77.3% | 1 | K.VGGILVMPLEEK.L | 2 |
|  | AstrinIP\_MS2\_022614\_02.07475.07475.3 | 5.0494 | 0.4681 | 100.0% | 1942.1643 | 1942.2836 | 1 | 8.643 | 51.7% | 1 | R.RMETIVFLDKEVFASR.I | 3 |
|  | AstrinIP\_MS1\_022614\_01.15308.15308.2 | 2.2142 | 0.2724 | 96.4% | 2225.152 | 2227.6946 | 56 | 4.123 | 29.4% | 1 | K.VLSLPLPDPLKYYLLYYR.E | 2 |

---

|  |  |  |  |  |  |  |  |  |
| --- | --- | --- | --- | --- | --- | --- | --- | --- |
| U | *gi|14141161|ref|NP\_00* | 14 | 28 | 22.1% | 806 | 88980 | 5.8 | heterogeneous nuclear ribonucleoprotein U isoform b [Homo sapiens] |
| U | *gi|74136883|ref|NP\_11* | 14 | 28 | 21.6% | 825 | 90585 | 6.0 | heterogeneous nuclear ribonucleoprotein U isoform a [Homo sapiens] |

| Filename XCorr DeltCN Conf% ObsM+H+ CalcM+H+ SpR ZScore Ion% # Sequence  | | | | | | | | | | | | |
| --- | --- | --- | --- | --- | --- | --- | --- | --- | --- | --- | --- | --- |
|  | AstrinIP\_MS1\_022614\_01.03868.03868.2 | 3.0743 | 0.1905 | 99.8% | 1074.9321 | 1075.2474 | 3 | 5.989 | 81.2% | 2 | K.VSELKEELK.K | 2 |
|  | AstrinIP\_MS2\_022614\_01.03622.03622.2 | 2.5742 | 0.1432 | 97.0% | 1202.3322 | 1203.4215 | 1 | 4.74 | 77.8% | 1 | K.VSELKEELKK.R | 2 |
|  | AstrinIP\_MS2\_022614\_01.08302.08302.3 | 4.9793 | 0.4099 | 100.0% | 3128.0044 | 3128.311 | 1 | 7.536 | 30.6% | 1 | R.LQAALDDEEAGGRPAMEPGNGSLDLGGDSAGR.S | 3 |
|  | AstrinIP\_MS1\_022614\_01.08208.08208.2 | 3.8009 | 0.4694 | 100.0% | 1698.2922 | 1698.8291 | 1 | 7.659 | 75.0% | 2 | R.GYFEYIEENKYSR.A | 2 |
|  | AstrinIP\_MS2\_022614\_01.03718.03718.2 | 2.1573 | 0.2697 | 98.6% | 942.09216 | 942.1466 | 1 | 5.13 | 78.6% | 1 | K.VTEKIPVR.H | 2 |
|  | AstrinIP\_MS1\_022614\_01.04535.04535.2 | 2.3908 | 0.1763 | 98.1% | 996.47217 | 997.0959 | 4 | 5.783 | 78.6% | 2 | K.DIDIHEVR.I | 2 |
|  | AstrinIP\_MS2\_022614\_01.06650.06650.2 | 2.9742 | 0.2707 | 99.8% | 1050.2922 | 1049.1716 | 1 | 6.604 | 72.2% | 1 | K.NGQDLGVAFK.I | 2 |
|  | AstrinIP\_MS2\_022614\_01.13113.13113.3 | 4.4151 | 0.2918 | 100.0% | 2725.9744 | 2726.0576 | 2 | 5.063 | 31.0% | 2 | K.EKPYFPIPEEYTFIQNVPLEDR.V | 3 |
|  | AstrinIP\_MS2\_022614\_01.07498.07498.3 | 4.7894 | 0.4901 | 100.0% | 2188.7043 | 2188.4631 | 1 | 8.784 | 36.8% | 2 | K.HAAENPGKYNILGTNTIMDK.M | 3 |
|  | AstrinIP\_MS2\_022614\_02.05646.05646.2 | 3.0709 | 0.2583 | 99.8% | 1384.3121 | 1383.6025 | 2 | 5.726 | 63.6% | 3 | K.YNILGTNTIMDK.M | 2 |
|  | AstrinIP\_MS1\_022614\_01.08262.08262.2 | 4.8242 | 0.4183 | 100.0% | 1649.2522 | 1648.816 | 1 | 7.718 | 78.6% | 8 | R.NFILDQTNVSAAAQR.R | 2 |
|  | AstrinIP\_MS1\_022614\_01.03858.03858.2 | 2.9825 | 0.222 | 99.7% | 1266.6322 | 1267.4216 | 1 | 5.078 | 77.8% | 1 | K.LLEQYKEESK.K | 2 |
|  | AstrinIP\_MS2\_022614\_01.03651.03651.2 | 3.6406 | 0.418 | 100.0% | 1395.0521 | 1395.5957 | 1 | 6.979 | 80.0% | 1 | K.LLEQYKEESKK.A | 2 |
|  | AstrinIP\_MS1\_022614\_01.09477.09477.3 | 3.7428 | 0.3429 | 99.9% | 3659.9043 | 3660.8645 | 1 | 4.446 | 25.0% | 1 | K.NQSQGYNQWQQGQFWGQKPWSQHYHQGYY.- | 3 |

---

|  |  |  |  |  |  |  |  |  |
| --- | --- | --- | --- | --- | --- | --- | --- | --- |
| U | *gi|11415030|ref|NP\_06* | 2 | 2 | 21.4% | 103 | 11367 | 11.4 | histone cluster 1, H4j [Homo sapiens] |
| U | *gi|77539758|ref|NP\_00* | 2 | 2 | 21.4% | 103 | 11367 | 11.4 | histone cluster 2, H4b [Homo sapiens] |
| U | *gi|4504323|ref|NP\_003* | 2 | 2 | 21.4% | 103 | 11367 | 11.4 | histone cluster 2, H4a [Homo sapiens] |
| U | *gi|4504321|ref|NP\_003* | 2 | 2 | 21.4% | 103 | 11367 | 11.4 | histone cluster 1, H4i [Homo sapiens] |
| U | *gi|4504317|ref|NP\_003* | 2 | 2 | 21.4% | 103 | 11367 | 11.4 | histone cluster 1, H4l [Homo sapiens] |
| U | *gi|4504315|ref|NP\_003* | 2 | 2 | 21.4% | 103 | 11367 | 11.4 | histone cluster 1, H4e [Homo sapiens] |
| U | *gi|4504313|ref|NP\_003* | 2 | 2 | 21.4% | 103 | 11367 | 11.4 | histone cluster 1, H4b [Homo sapiens] |
| U | *gi|4504311|ref|NP\_003* | 2 | 2 | 21.4% | 103 | 11367 | 11.4 | histone cluster 1, H4h [Homo sapiens] |
| U | *gi|4504309|ref|NP\_003* | 2 | 2 | 21.4% | 103 | 11367 | 11.4 | histone cluster 1, H4c [Homo sapiens] |
| U | *gi|4504307|ref|NP\_003* | 2 | 2 | 21.4% | 103 | 11367 | 11.4 | histone cluster 1, H4k [Homo sapiens] |
| U | *gi|4504305|ref|NP\_003* | 2 | 2 | 21.4% | 103 | 11367 | 11.4 | histone cluster 1, H4f [Homo sapiens] |
| U | *gi|4504303|ref|NP\_003* | 2 | 2 | 21.4% | 103 | 11367 | 11.4 | histone cluster 1, H4d [Homo sapiens] |
| U | *gi|4504301|ref|NP\_003* | 2 | 2 | 21.4% | 103 | 11367 | 11.4 | histone cluster 1, H4a [Homo sapiens] |
| U | *gi|28173560|ref|NP\_77* | 2 | 2 | 21.4% | 103 | 11367 | 11.4 | histone cluster 4, H4 [Homo sapiens] |

| Filename XCorr DeltCN Conf% ObsM+H+ CalcM+H+ SpR ZScore Ion% # Sequence  | | | | | | | | | | | | |
| --- | --- | --- | --- | --- | --- | --- | --- | --- | --- | --- | --- | --- |
|  | AstrinIP\_MS1\_022614\_01.04852.04852.2 | 3.1008 | 0.21 | 99.5% | 1326.1322 | 1326.5387 | 1 | 6.228 | 68.2% | 1 | R.DNIQGITKPAIR.R | 2 |
|  | AstrinIP\_MS2\_022614\_01.07444.07444.2 | 3.1958 | 0.3698 | 100.0% | 1181.5122 | 1181.3312 | 1 | 6.346 | 88.9% | 1 | R.ISGLIYEETR.G | 2 |

---

|  |  |  |  |  |  |  |  |  |
| --- | --- | --- | --- | --- | --- | --- | --- | --- |
| U | *gi|38569421|ref|NP\_00* | 14 | 22 | 21.3% | 1101 | 120839 | 7.3 | ATP citrate lyase isoform 1 [Homo sapiens] |
| U | *gi|38569423|ref|NP\_94* | 14 | 22 | 21.5% | 1091 | 119772 | 7.3 | ATP citrate lyase isoform 2 [Homo sapiens] |

| Filename XCorr DeltCN Conf% ObsM+H+ CalcM+H+ SpR ZScore Ion% # Sequence  | | | | | | | | | | | | |
| --- | --- | --- | --- | --- | --- | --- | --- | --- | --- | --- | --- | --- |
|  | AstrinIP\_MS2\_022614\_01.12281.12281.3 | 5.5438 | 0.4875 | 100.0% | 2597.9043 | 2599.0916 | 1 | 8.55 | 40.5% | 2 | R.LLQDHPWLLSQNLVVKPDQLIK.R | 3 |
|  | AstrinIP\_MS2\_022614\_01.12389.12389.2 | 4.3965 | 0.4909 | 100.0% | 1399.4321 | 1398.686 | 1 | 8.356 | 73.1% | 2 | K.LGLVGVNLTLDGVK.S | 2 |
|  | AstrinIP\_MS2\_022614\_02.05602.05602.3 | 3.3244 | 0.287 | 99.1% | 2258.3342 | 2259.3936 | 48 | 4.806 | 26.2% | 1 | R.EGDYVLFHHEGGVDVGDVDAK.A | 3 |
|  | AstrinIP\_MS1\_022614\_01.08320.08320.3 | 2.8099 | 0.2459 | 97.0% | 1932.5044 | 1932.1863 | 1 | 4.165 | 38.3% | 1 | R.DYQGPLKEHEVTIFVR.R | 3 |
|  | AstrinIP\_MS2\_022614\_01.03825.03825.2 | 2.9857 | 0.3611 | 100.0% | 1246.4321 | 1247.3561 | 1 | 5.821 | 70.0% | 1 | R.RGGPNYQEGLR.V | 2 |
|  | AstrinIP\_MS2\_022614\_01.12677.12677.2 | 3.2603 | 0.3019 | 99.8% | 1568.0122 | 1568.854 | 1 | 6.967 | 60.7% | 1 | R.TIAIIAEGIPEALTR.K | 2 |
|  | AstrinIP\_MS1\_022614\_01.10485.10485.2 | 4.7948 | 0.4318 | 100.0% | 1504.1721 | 1504.7417 | 1 | 9.146 | 71.4% | 3 | K.IGNTGGMLDNILASK.L | 2 |
|  | AstrinIP\_MS2\_022614\_01.04788.04788.2 | 2.749 | 0.3033 | 99.7% | 1368.1921 | 1368.5785 | 1 | 5.133 | 68.2% | 2 | K.LYRPGSVAYVSR.S | 2 |
|  | AstrinIP\_MS1\_022614\_01.08867.08867.2 | 3.5684 | 0.2478 | 99.8% | 1493.3722 | 1492.647 | 2 | 5.883 | 61.5% | 2 | R.SGGMSNELNNIISR.T | 2 |
|  | AstrinIP\_MS1\_022614\_02.12952.12952.3 | 3.7707 | 0.3645 | 100.0% | 3555.2644 | 3556.2153 | 1 | 5.356 | 21.8% | 2 | R.GQELIYAGMPITEVFKEEMGIGGVLGLLWFQK.R | 3 |
|  | AstrinIP\_MS2\_022614\_01.15993.15993.2 | 3.8581 | 0.4569 | 100.0% | 1647.7322 | 1647.8662 | 1 | 8.977 | 70.0% | 2 | K.DLVSSLTSGLLTIGDR.F | 2 |
|  | AstrinIP\_MS2\_022614\_01.12522.12522.2 | 3.5523 | 0.3924 | 100.0% | 1567.6721 | 1568.8279 | 3 | 6.662 | 57.7% | 1 | K.AFDSGIIPMEFVNK.M | 2 |
|  | AstrinIP\_MS1\_022614\_01.17864.17864.2 | 3.6674 | 0.5058 | 100.0% | 2296.912 | 2298.7925 | 1 | 7.849 | 40.0% | 1 | K.KPNLILNVDGLIGVAFVDMLR.N | 2 |
|  | AstrinIP\_MS1\_022614\_01.08847.08847.2 | 3.0899 | 0.2642 | 99.8% | 1395.9922 | 1396.6038 | 1 | 5.805 | 72.7% | 1 | R.SMGFIGHYLDQK.R | 2 |

---

|  |  |  |  |  |  |  |  |  |
| --- | --- | --- | --- | --- | --- | --- | --- | --- |
| U | *gi|12667788|ref|NP\_00* | 29 | 47 | 20.9% | 1960 | 226530 | 5.6 | myosin, heavy polypeptide 9, non-muscle [Homo sapiens] |

| Filename XCorr DeltCN Conf% ObsM+H+ CalcM+H+ SpR ZScore Ion% # Sequence  | | | | | | | | | | | | |
| --- | --- | --- | --- | --- | --- | --- | --- | --- | --- | --- | --- | --- |
| \* | AstrinIP\_MS2\_022614\_01.11561.11561.2 | 3.666 | 0.2327 | 99.8% | 1675.3522 | 1673.8687 | 1 | 5.111 | 67.9% | 5 | K.NFINNPLAQADWAAK.K | 2 |
| \* | AstrinIP\_MS2\_022614\_01.12297.12297.3 | 4.6612 | 0.4537 | 100.0% | 2789.5144 | 2790.0967 | 1 | 6.951 | 33.0% | 1 | K.SGFEPASLKEEVGEEAIVELVENGKK.V | 3 |
| \* | AstrinIP\_MS1\_022614\_01.11765.11765.2 | 2.8181 | 0.3392 | 99.8% | 1728.4722 | 1728.9978 | 1 | 6.882 | 50.0% | 2 | K.NLPIYSEEIVEMYK.G | 2 |
| \* | AstrinIP\_MS2\_022614\_01.06828.06828.3 | 2.7663 | 0.2968 | 99.0% | 1916.5443 | 1916.1614 | 1 | 5.131 | 41.7% | 1 | R.HEMPPHIYAITDTAYR.S | 3 |
| \* | AstrinIP\_MS2\_022614\_01.08817.08817.2 | 2.8669 | 0.3854 | 99.9% | 1479.6522 | 1479.719 | 1 | 6.546 | 66.7% | 1 | K.VIQYLAYVASSHK.S | 2 |
|  | AstrinIP\_MS1\_022614\_01.13002.13002.2 | 4.0727 | 0.5241 | 100.0% | 1727.5521 | 1728.0012 | 1 | 8.472 | 70.0% | 3 | R.QLLQANPILEAFGNAK.T | 22 |
| \* | AstrinIP\_MS1\_022614\_01.13022.13022.2 | 3.3609 | 0.2764 | 99.8% | 1995.7322 | 1997.3037 | 1 | 6.535 | 56.2% | 1 | R.TFHIFYYLLSGAGEHLK.T | 2 |
| \* | AstrinIP\_MS1\_022614\_01.10774.10774.2 | 2.4893 | 0.1873 | 95.2% | 1616.4922 | 1616.9313 | 2 | 5.347 | 57.7% | 1 | R.IMGIPEEEQMGLLR.V | 2 |
| \* | AstrinIP\_MS2\_022614\_01.13913.13913.2 | 2.574 | 0.4231 | 99.8% | 1486.5322 | 1487.8259 | 1 | 6.699 | 57.7% | 1 | R.VISGVLQLGNIVFK.K | 2 |
| \* | AstrinIP\_MS2\_022614\_01.12459.12459.2 | 2.9286 | 0.1827 | 98.2% | 1614.7722 | 1616.0 | 13 | 4.388 | 53.6% | 1 | R.VISGVLQLGNIVFKK.E | 2 |
| \* | AstrinIP\_MS1\_022614\_01.10042.10042.2 | 3.9715 | 0.2775 | 100.0% | 1573.4922 | 1572.8044 | 1 | 7.643 | 76.9% | 1 | K.VSHLLGINVTDFTR.G | 2 |
| \* | AstrinIP\_MS1\_022614\_01.12801.12801.2 | 3.185 | 0.3534 | 99.9% | 2018.2922 | 2019.3636 | 2 | 5.323 | 44.7% | 1 | R.IIGLDQVAGMSETALPGAFK.T | 2 |
|  | AstrinIP\_MS2\_022614\_01.10344.10344.2 | 2.6066 | 0.1937 | 98.2% | 1319.6122 | 1319.5468 | 37 | 5.417 | 65.0% | 1 | K.LDPHLVLDQLR.C | 22 |
| \* | AstrinIP\_MS2\_022614\_01.08788.08788.2 | 2.4705 | 0.2422 | 98.6% | 1275.1921 | 1275.4875 | 8 | 4.904 | 55.0% | 1 | R.YEILTPNSIPK.G | 2 |
| \* | AstrinIP\_MS2\_022614\_01.08638.08638.2 | 2.7514 | 0.3699 | 100.0% | 1193.6721 | 1194.33 | 1 | 6.842 | 77.8% | 3 | K.ALELDSNLYR.I | 2 |
|  | AstrinIP\_MS2\_022614\_01.06628.06628.2 | 3.2823 | 0.4021 | 100.0% | 1223.9722 | 1224.3591 | 1 | 7.157 | 75.0% | 2 | R.AGVLAHLEEER.D | 22 |
| \* | AstrinIP\_MS2\_022614\_01.11073.11073.2 | 4.0798 | 0.4418 | 100.0% | 1752.3322 | 1753.0358 | 1 | 7.711 | 60.7% | 1 | R.LTEMETLQSQLMAEK.L | 2 |
| \* | AstrinIP\_MS1\_022614\_02.05696.05696.2 | 4.1937 | 0.422 | 100.0% | 1654.4321 | 1654.7681 | 1 | 7.318 | 73.1% | 4 | R.IAEFTTNLTEEEEK.S | 2 |
|  | AstrinIP\_MS1\_022614\_01.04691.04691.2 | 2.5804 | 0.283 | 99.3% | 1257.8322 | 1258.4172 | 210 | 4.791 | 60.0% | 2 | K.KEEELQAALAR.V | 22 |
| \* | AstrinIP\_MS1\_022614\_01.16818.16818.3 | 3.4471 | 0.2784 | 99.2% | 3018.8643 | 3019.2434 | 2 | 5.465 | 24.0% | 1 | R.DLGEELEALKTELEDTLDSTAAQQELR.S | 3 |
| \* | AstrinIP\_MS2\_022614\_01.08714.08714.3 | 4.3587 | 0.4618 | 100.0% | 1996.7644 | 1997.1722 | 1 | 7.029 | 37.5% | 2 | K.HSQAVEELAEQLEQTKR.V | 3 |
| \* | AstrinIP\_MS2\_022614\_01.03580.03580.2 | 2.9476 | 0.1511 | 98.6% | 1260.2922 | 1260.4764 | 3 | 4.161 | 70.0% | 1 | R.VRTELADKVTK.L | 2 |
| \* | AstrinIP\_MS2\_022614\_01.12070.12070.2 | 2.935 | 0.4096 | 100.0% | 1946.1522 | 1947.1498 | 40 | 6.907 | 32.4% | 1 | K.LQVELDNVTGLLSQSDSK.S | 2 |
| \* | AstrinIP\_MS1\_022614\_01.03700.03700.2 | 3.3318 | 0.1315 | 99.1% | 1493.3722 | 1493.6598 | 16 | 4.207 | 59.1% | 1 | K.LKQVEDEKNSFR.E | 2 |
| \* | AstrinIP\_MS2\_022614\_01.12849.12849.3 | 3.2444 | 0.1875 | 95.5% | 1950.8944 | 1951.1436 | 2 | 4.761 | 41.7% | 2 | R.LQQELDDLLVDLDHQR.Q | 3 |
| \* | AstrinIP\_MS2\_022614\_01.06057.06057.2 | 2.2257 | 0.3078 | 98.8% | 1205.5322 | 1205.3685 | 2 | 5.927 | 66.7% | 1 | R.ALEQQVEEMK.T | 2 |
| \* | AstrinIP\_MS2\_022614\_01.03708.03708.2 | 3.5905 | 0.2596 | 100.0% | 1487.8121 | 1488.5541 | 1 | 6.269 | 72.7% | 1 | K.RQLEEAEEEAQR.A | 2 |
| \* | AstrinIP\_MS2\_022614\_01.05550.05550.2 | 4.1871 | 0.5174 | 100.0% | 1565.9521 | 1566.6367 | 1 | 9.23 | 73.1% | 2 | R.ELEDATETADAMNR.E | 2 |
| \* | AstrinIP\_MS2\_022614\_01.08268.08268.2 | 3.1746 | 0.2214 | 99.8% | 1156.1322 | 1156.3732 | 1 | 4.913 | 88.9% | 2 | R.RGDLPFVVPR.R | 2 |

Similarities:
gi|116284394|ref|NP\_0(2:27)  
gi|41406064|ref|NP\_00(2:27)  

---

|  |  |  |  |  |  |  |  |  |
| --- | --- | --- | --- | --- | --- | --- | --- | --- |
| U | *gi|21626466|ref|NP\_06* | 12 | 17 | 20.7% | 847 | 94623 | 6.3 | matrin 3 [Homo sapiens] |
| U | *gi|62750354|ref|NP\_95* | 12 | 17 | 20.7% | 847 | 94623 | 6.3 | matrin 3 [Homo sapiens] |

| Filename XCorr DeltCN Conf% ObsM+H+ CalcM+H+ SpR ZScore Ion% # Sequence  | | | | | | | | | | | | |
| --- | --- | --- | --- | --- | --- | --- | --- | --- | --- | --- | --- | --- |
|  | AstrinIP\_MS2\_022614\_01.04056.04056.2 | 2.1591 | 0.2744 | 98.3% | 1039.4922 | 1040.121 | 2 | 4.751 | 75.0% | 1 | K.SFQQSSLSR.D | 2 |
|  | AstrinIP\_MS2\_022614\_01.16764.16764.2 | 3.3395 | 0.4131 | 100.0% | 2371.8323 | 2372.7424 | 1 | 7.347 | 37.5% | 1 | R.DLSAAGIGLLAAATQSLSMPASLGR.M | 2 |
|  | AstrinIP\_MS1\_022614\_01.12958.12958.2 | 3.8091 | 0.4651 | 100.0% | 1793.8922 | 1793.931 | 1 | 7.939 | 47.1% | 1 | R.GDADQASNILASFGLSAR.D | 2 |
|  | AstrinIP\_MS1\_022614\_01.14050.14050.2 | 2.6527 | 0.1946 | 97.5% | 1620.7322 | 1620.9731 | 2 | 4.995 | 57.7% | 1 | K.ITPENLPQILLQLK.R | 2 |
|  | AstrinIP\_MS2\_022614\_01.04989.04989.2 | 2.5235 | 0.3243 | 99.7% | 1210.2122 | 1210.2859 | 1 | 5.861 | 70.0% | 1 | R.TEEGPTLSYGR.D | 2 |
|  | AstrinIP\_MS2\_022614\_01.08777.08777.3 | 3.7213 | 0.2264 | 98.7% | 2361.7444 | 2363.462 | 189 | 5.23 | 26.2% | 1 | R.DSFDDRGPSLNPVLDYDHGSR.S | 3 |
|  | AstrinIP\_MS2\_022614\_01.07118.07118.2 | 2.3326 | 0.1949 | 97.5% | 1145.6322 | 1145.3647 | 1 | 5.183 | 75.0% | 1 | R.VVHIMDFQR.G | 2 |
|  | AstrinIP\_MS1\_022614\_01.15009.15009.3 | 4.5169 | 0.3641 | 100.0% | 2439.1443 | 2439.9036 | 9 | 6.846 | 27.5% | 3 | R.YQLLQLVEPFGVISNHLILNK.I | 3 |
|  | AstrinIP\_MS1\_022614\_01.14974.14974.2 | 5.2786 | 0.4219 | 100.0% | 2440.0522 | 2439.9036 | 1 | 6.944 | 50.0% | 1 | R.YQLLQLVEPFGVISNHLILNK.I | 2 |
|  | AstrinIP\_MS2\_022614\_01.07402.07402.3 | 2.7148 | 0.2612 | 96.5% | 2039.9043 | 2038.3109 | 1 | 5.053 | 34.7% | 1 | R.VIHLSNLPHSGYSDSAVLK.L | 3 |
|  | AstrinIP\_MS2\_022614\_01.07370.07370.2 | 2.9157 | 0.2614 | 99.8% | 1212.2722 | 1212.363 | 1 | 5.399 | 77.8% | 1 | K.SQAFIEMETR.E | 2 |
|  | AstrinIP\_MS2\_022614\_01.12048.12048.2 | 4.058 | 0.4795 | 100.0% | 1969.2722 | 1970.319 | 1 | 8.815 | 52.9% | 4 | R.IGPYQPNVPVGIDYVIPK.T | 2 |

---

|  |  |  |  |  |  |  |  |  |
| --- | --- | --- | --- | --- | --- | --- | --- | --- |
| U | *gi|11024714|ref|NP\_06* | 5 | 13 | 20.5% | 229 | 25762 | 7.4 | ubiquitin B precursor [Homo sapiens] |
| U | *gi|77539055|ref|NP\_00* | 5 | 13 | 36.7% | 128 | 14728 | 9.8 | ubiquitin and ribosomal protein L40 precursor [Homo sapiens] |
| U | *gi|67191208|ref|NP\_06* | 5 | 13 | 6.9% | 685 | 77029 | 7.7 | ubiquitin C [Homo sapiens] |
| U | *gi|4507761|ref|NP\_003* | 5 | 13 | 36.7% | 128 | 14728 | 9.8 | ubiquitin and ribosomal protein L40 precursor [Homo sapiens] |
| U | *gi|4506713|ref|NP\_002* | 5 | 13 | 30.1% | 156 | 17965 | 9.6 | ubiquitin and ribosomal protein S27a precursor [Homo sapiens] |
| U | *gi|208022622|ref|NP\_0* | 5 | 13 | 30.1% | 156 | 17965 | 9.6 | ubiquitin and ribosomal protein S27a precursor [Homo sapiens] |

| Filename XCorr DeltCN Conf% ObsM+H+ CalcM+H+ SpR ZScore Ion% # Sequence  | | | | | | | | | | | | |
| --- | --- | --- | --- | --- | --- | --- | --- | --- | --- | --- | --- | --- |
|  | AstrinIP\_MS1\_022614\_01.08810.08810.2 | 4.3752 | 0.522 | 100.0% | 1788.3322 | 1788.9897 | 1 | 8.632 | 70.0% | 6 | K.TITLEVEPSDTIENVK.A | 2 |
|  | AstrinIP\_MS1\_022614\_01.03566.03566.2 | 3.7891 | 0.3517 | 100.0% | 1524.1921 | 1524.6738 | 1 | 7.134 | 79.2% | 1 | K.IQDKEGIPPDQQR.L | 2 |
|  | AstrinIP\_MS2\_022614\_01.04787.04787.1 | 1.5881 | 0.2186 | 95.0% | 1081.46 | 1082.1986 | 12 | 4.61 | 56.2% | 1 | R.TLSDYNIQK.E | 1 |
|  | AstrinIP\_MS2\_022614\_01.04767.04767.2 | 2.1425 | 0.3873 | 99.7% | 1082.0922 | 1082.1986 | 1 | 5.832 | 87.5% | 1 | R.TLSDYNIQK.E | 2 |
|  | AstrinIP\_MS1\_022614\_01.06720.06720.2 | 2.6645 | 0.2867 | 99.8% | 1068.2322 | 1068.2615 | 2 | 4.612 | 81.2% | 4 | K.ESTLHLVLR.L | 2 |

---

|  |  |  |  |  |  |  |  |  |
| --- | --- | --- | --- | --- | --- | --- | --- | --- |
| U | *contaminant\_KERATIN03* | 9 | 12 | 20.4% | 593 | 59519 | 5.2 | no description |
| U | *gi|195972866|ref|NP\_0* | 9 | 12 | 20.7% | 584 | 58801 | 5.2 | keratin 10 [Homo sapiens] |

| Filename XCorr DeltCN Conf% ObsM+H+ CalcM+H+ SpR ZScore Ion% # Sequence  | | | | | | | | | | | | |
| --- | --- | --- | --- | --- | --- | --- | --- | --- | --- | --- | --- | --- |
|  | AstrinIP\_MS1\_022614\_02.06229.06229.2 | 4.6396 | 0.5518 | 100.0% | 1708.1921 | 1708.7844 | 1 | 9.744 | 61.1% | 1 | K.GSLGGGFSSGGFSGGSFSR.G | 2 |
|  | AstrinIP\_MS1\_022614\_01.04173.04173.2 | 2.1506 | 0.2051 | 95.4% | 1091.5322 | 1091.2273 | 78 | 4.696 | 62.5% | 1 | K.VTMQNLNDR.L | 22 |
|  | AstrinIP\_MS1\_022614\_01.05027.05027.2 | 2.3369 | 0.1774 | 96.8% | 1065.8322 | 1065.2578 | 35 | 4.948 | 62.5% | 1 | R.LASYLDKVR.A | 2222 |
|  | AstrinIP\_MS1\_022614\_01.05417.05417.2 | 3.8136 | 0.4851 | 100.0% | 1381.8322 | 1382.4668 | 1 | 9.425 | 72.7% | 3 | R.ALEESNYELEGK.I | 2 |
|  | AstrinIP\_MS1\_022614\_01.15044.15044.3 | 5.3797 | 0.4548 | 100.0% | 3053.2744 | 3054.4277 | 1 | 7.523 | 27.9% | 2 | K.TIDDLKNQILNLTTDNANILLQIDNAR.L | 3 |
|  | AstrinIP\_MS1\_022614\_01.04851.04851.2 | 3.2675 | 0.3532 | 100.0% | 1234.5521 | 1235.4258 | 1 | 7.168 | 83.3% | 1 | R.LKYENEVALR.Q | 2 |
|  | AstrinIP\_MS2\_022614\_01.04432.04432.2 | 3.0074 | 0.1571 | 98.8% | 1366.3322 | 1366.43 | 91 | 4.833 | 60.0% | 1 | R.SQYEQLAEQNR.K | 2 |
|  | AstrinIP\_MS1\_022614\_01.07204.07204.2 | 3.4375 | 0.5087 | 100.0% | 1391.2322 | 1391.4778 | 1 | 7.847 | 70.8% | 1 | K.QSLEASLAETEGR.Y | 2 |
|  | AstrinIP\_MS1\_022614\_01.06332.06332.2 | 2.9036 | 0.279 | 99.8% | 1434.3322 | 1435.623 | 2 | 5.614 | 65.0% | 1 | K.IRLENEIQTYR.S | 2 |

Similarities:
contaminant\_KERATIN12(1:8)  
contaminant\_KERATIN05(2:7)  
contaminant\_KERATIN10(1:8)  

---

|  |  |  |  |  |  |  |  |  |
| --- | --- | --- | --- | --- | --- | --- | --- | --- |
| U | *gi|124256496|ref|NP\_0* | 10 | 44 | 20.1% | 641 | 70375 | 6.0 | heat shock 70kDa protein 1-like [Homo sapiens] |

| Filename XCorr DeltCN Conf% ObsM+H+ CalcM+H+ SpR ZScore Ion% # Sequence  | | | | | | | | | | | | |
| --- | --- | --- | --- | --- | --- | --- | --- | --- | --- | --- | --- | --- |
|  | AstrinIP\_MS2\_022614\_01.07734.07734.1 | 2.2714 | 0.4224 | 100.0% | 1487.62 | 1488.5939 | 1 | 6.368 | 50.0% | 5 | R.TTPSYVAFTDTER.L | 11111 |
|  | AstrinIP\_MS1\_022614\_02.04868.04868.2 | 3.849 | 0.5598 | 100.0% | 1489.2922 | 1488.5939 | 1 | 9.39 | 79.2% | 10 | R.TTPSYVAFTDTER.L | 22222 |
|  | AstrinIP\_MS1\_022614\_01.11918.11918.2 | 4.8916 | 0.5504 | 100.0% | 1616.3722 | 1615.8817 | 1 | 8.96 | 84.6% | 7 | K.AFYPEEISSMVLTK.L | 22 |
|  | AstrinIP\_MS1\_022614\_01.10547.10547.1 | 2.3999 | 0.437 | 100.0% | 1197.55 | 1198.408 | 8 | 5.848 | 50.0% | 2 | K.DAGVIAGLNVLR.I | 11 |
|  | AstrinIP\_MS2\_022614\_01.11162.11162.2 | 3.7606 | 0.2878 | 100.0% | 1198.1322 | 1198.408 | 1 | 7.4 | 86.4% | 3 | K.DAGVIAGLNVLR.I | 22 |
|  | AstrinIP\_MS1\_022614\_01.09921.09921.2 | 5.232 | 0.5436 | 100.0% | 1660.4922 | 1660.9078 | 1 | 10.069 | 80.0% | 8 | R.IINEPTAAAIAYGLDK.G | 2222 |
|  | AstrinIP\_MS2\_022614\_01.04504.04504.2 | 4.2557 | 0.5152 | 100.0% | 1676.1522 | 1676.6964 | 1 | 8.323 | 66.7% | 3 | K.ATAGDTHLGGEDFDNR.L | 222 |
|  | AstrinIP\_MS2\_022614\_02.09924.09924.3 | 3.7164 | 0.4297 | 100.0% | 2305.0444 | 2305.608 | 1 | 6.568 | 33.0% | 1 | K.SINPDEAVAYGAAVQAAILMGDK.S | 33 |
|  | AstrinIP\_MS1\_022614\_01.11703.11703.2 | 4.922 | 0.5638 | 100.0% | 2787.7122 | 2788.043 | 1 | 10.506 | 41.3% | 2 | K.QTQIFTTYSDNQPGVLIQVYEGER.A | 22 |
|  | AstrinIP\_MS2\_022614\_01.09441.09441.2 | 3.7744 | 0.3869 | 100.0% | 1289.1322 | 1288.4608 | 1 | 6.901 | 80.0% | 3 | K.NALESYAFNMK.S | 22 |

Similarities:
gi|5729877|ref|NP\_006(3:7)  
gi|16507237|ref|NP\_00(1:9)  
gi|167466173|ref|NP\_0(9:1)  
gi|13676857|ref|NP\_06(3:7)  
gi|34419635|ref|NP\_00(3:7)  

---

|  |  |  |  |  |  |  |  |  |
| --- | --- | --- | --- | --- | --- | --- | --- | --- |
| U | *contaminant\_KERATIN12* | 8 | 10 | 20.0% | 431 | 47974 | 5.0 | no description |
| U | *gi|4557701|ref|NP\_000* | 8 | 10 | 19.9% | 432 | 48106 | 5.0 | keratin 17 [Homo sapiens] |

| Filename XCorr DeltCN Conf% ObsM+H+ CalcM+H+ SpR ZScore Ion% # Sequence  | | | | | | | | | | | | |
| --- | --- | --- | --- | --- | --- | --- | --- | --- | --- | --- | --- | --- |
|  | AstrinIP\_MS1\_022614\_01.05027.05027.2 | 2.3369 | 0.1774 | 96.8% | 1065.8322 | 1065.2578 | 35 | 4.948 | 62.5% | 1 | R.LASYLDKVR.A | 2222 |
|  | AstrinIP\_MS1\_022614\_02.04731.04731.2 | 3.1222 | 0.349 | 100.0% | 1345.8322 | 1346.4772 | 1 | 5.929 | 63.6% | 2 | R.ALEEANTELEVK.I | 2 |
|  | AstrinIP\_MS2\_022614\_01.08524.08524.2 | 2.4955 | 0.3861 | 99.9% | 1030.1522 | 1030.2096 | 1 | 7.085 | 81.2% | 1 | R.VLDELTLAR.A | 222 |
|  | AstrinIP\_MS2\_022614\_01.12924.12924.3 | 3.3493 | 0.2961 | 99.5% | 2278.5244 | 2279.6538 | 1 | 5.58 | 36.1% | 1 | R.ADLEMQIENLKEELAYLKK.N | 3 |
|  | AstrinIP\_MS2\_022614\_01.05519.05519.1 | 2.3703 | 0.2345 | 99.0% | 1404.15 | 1404.4764 | 10 | 4.841 | 45.8% | 1 | K.ASLEGNLAETENR.Y | 1 |
|  | AstrinIP\_MS2\_022614\_01.05506.05506.2 | 2.9053 | 0.1674 | 98.3% | 1404.2522 | 1404.4764 | 1 | 5.087 | 62.5% | 1 | K.ASLEGNLAETENR.Y | 2 |
|  | AstrinIP\_MS1\_022614\_01.06353.06353.2 | 2.9214 | 0.3246 | 99.8% | 1380.4321 | 1380.5437 | 1 | 5.656 | 75.0% | 2 | K.TRLEQEIATYR.R | 22 |
|  | AstrinIP\_MS2\_022614\_01.05939.05939.2 | 3.8318 | 0.3322 | 100.0% | 1516.9922 | 1517.6787 | 1 | 7.084 | 66.7% | 1 | R.LLEGEDAHLTQYK.K | 2 |

Similarities:
contaminant\_KERATIN03(1:7)  
contaminant\_KERATIN05(3:5)  
contaminant\_KERATIN10(2:6)  

---

|  |  |  |  |  |  |  |  |  |
| --- | --- | --- | --- | --- | --- | --- | --- | --- |
| U | *gi|21359873|ref|NP\_00* | 9 | 21 | 19.7% | 603 | 68255 | 8.9 | polo-like kinase [Homo sapiens] |

| Filename XCorr DeltCN Conf% ObsM+H+ CalcM+H+ SpR ZScore Ion% # Sequence  | | | | | | | | | | | | |
| --- | --- | --- | --- | --- | --- | --- | --- | --- | --- | --- | --- | --- |
| \* | AstrinIP\_MS2\_022614\_01.05296.05296.2 | 4.1062 | 0.4857 | 100.0% | 1570.2922 | 1570.832 | 1 | 8.164 | 75.0% | 4 | K.AGVPGVAAPGAPAAAPPAK.E | 2 |
| \* | AstrinIP\_MS1\_022614\_01.11195.11195.2 | 3.8071 | 0.3994 | 100.0% | 1504.0521 | 1504.7234 | 1 | 7.123 | 66.7% | 4 | K.LGNLFLNEDLEVK.I | 2 |
| \* | AstrinIP\_MS2\_022614\_01.03670.03670.2 | 2.4609 | 0.1858 | 97.6% | 1220.5122 | 1220.4545 | 83 | 3.876 | 55.6% | 1 | R.IKKNEYSIPK.H | 2 |
| \* | AstrinIP\_MS1\_022614\_01.06044.06044.2 | 3.2245 | 0.3885 | 100.0% | 1291.2122 | 1291.5363 | 1 | 6.854 | 81.8% | 4 | K.HINPVAASLIQK.M | 2 |
| \* | AstrinIP\_MS2\_022614\_01.08757.08757.2 | 3.1337 | 0.3722 | 100.0% | 1391.1921 | 1391.5236 | 2 | 6.495 | 66.7% | 2 | R.FSIAPSSLDPSNR.K | 2 |
| \* | AstrinIP\_MS2\_022614\_01.09032.09032.3 | 3.1032 | 0.2733 | 98.6% | 2285.5444 | 2285.6487 | 1 | 5.707 | 35.0% | 1 | R.FSIAPSSLDPSNRKPLTVLNK.G | 3 |
| \* | AstrinIP\_MS2\_022614\_01.04932.04932.2 | 2.3561 | 0.2789 | 98.6% | 1277.7122 | 1278.4539 | 1 | 4.81 | 65.0% | 1 | K.GLENPLPERPR.E | 2 |
| \* | AstrinIP\_MS2\_022614\_01.10883.10883.2 | 4.2036 | 0.4621 | 100.0% | 1812.5922 | 1813.0172 | 1 | 8.935 | 71.4% | 3 | R.LILYNDGDSLQYIER.D | 2 |
| \* | AstrinIP\_MS2\_022614\_01.08025.08025.2 | 4.592 | 0.475 | 100.0% | 1966.4922 | 1967.1578 | 2 | 8.172 | 50.0% | 1 | R.DGTESYLTVSSHPNSLMK.K | 2 |

---

|  |  |  |  |  |  |  |  |  |
| --- | --- | --- | --- | --- | --- | --- | --- | --- |
| U | *gi|17986258|ref|NP\_06* | 2 | 6 | 19.2% | 151 | 16930 | 4.7 | myosin, light chain 6, alkali, smooth muscle and non-muscle isoform 1 [Homo sapiens] |
| U | *gi|88999583|ref|NP\_52* | 2 | 6 | 19.2% | 151 | 16961 | 4.6 | myosin, light chain 6, alkali, smooth muscle and non-muscle isoform 2 [Homo sapiens] |

| Filename XCorr DeltCN Conf% ObsM+H+ CalcM+H+ SpR ZScore Ion% # Sequence  | | | | | | | | | | | | |
| --- | --- | --- | --- | --- | --- | --- | --- | --- | --- | --- | --- | --- |
|  | AstrinIP\_MS2\_022614\_01.05387.05387.2 | 3.1909 | 0.2648 | 99.8% | 1355.3922 | 1355.5339 | 2 | 5.515 | 62.5% | 4 | R.ALGQNPTNAEVLK.V | 2 |
|  | AstrinIP\_MS2\_022614\_01.14862.14862.2 | 3.705 | 0.4201 | 100.0% | 1888.2722 | 1889.2628 | 1 | 7.094 | 56.7% | 2 | K.VLDFEHFLPMLQTVAK.N | 2 |

---

|  |  |  |  |  |  |  |  |  |
| --- | --- | --- | --- | --- | --- | --- | --- | --- |
| U | *gi|47132620|ref|NP\_00* | 8 | 11 | 18.6% | 639 | 65433 | 8.0 | keratin 2 [Homo sapiens] |

| Filename XCorr DeltCN Conf% ObsM+H+ CalcM+H+ SpR ZScore Ion% # Sequence  | | | | | | | | | | | | |
| --- | --- | --- | --- | --- | --- | --- | --- | --- | --- | --- | --- | --- |
|  | AstrinIP\_MS2\_022614\_01.04353.04353.2 | 2.4278 | 0.2567 | 98.0% | 1255.5122 | 1255.3298 | 1 | 4.975 | 65.4% | 1 | R.GFSSGSAVVSGGSR.R | 2 |
|  | AstrinIP\_MS1\_022614\_01.04997.04997.1 | 1.5068 | 0.2943 | 99.1% | 831.42 | 831.9878 | 3 | 5.18 | 50.0% | 1 | R.SLVGLGGTK.S | 1 |
|  | AstrinIP\_MS1\_022614\_01.12798.12798.3 | 5.8301 | 0.4425 | 100.0% | 4094.9043 | 4094.5786 | 1 | 8.393 | 20.3% | 1 | R.FGGFGGPGGVGGLGGPGGFGPGGYPGGIHEVSVNQSLLQPLNVK.V | 3 |
|  | AstrinIP\_MS1\_022614\_01.07103.07103.2 | 2.5876 | 0.1854 | 98.7% | 1083.1322 | 1083.2755 | 2 | 6.016 | 75.0% | 2 | K.FASFIDKVR.F | 2222 |
|  | AstrinIP\_MS1\_022614\_01.05997.05997.2 | 3.9133 | 0.0682 | 99.3% | 1475.9122 | 1476.6726 | 1 | 6.651 | 81.8% | 3 | R.FLEQQNQVLQTK.W | 22 |
|  | AstrinIP\_MS1\_022614\_01.12636.12636.2 | 2.7149 | 0.2381 | 99.1% | 1460.3121 | 1461.6982 | 1 | 5.462 | 68.2% | 1 | K.VDLLNQEIEFLK.V | 2 |
|  | AstrinIP\_MS1\_022614\_01.09212.09212.2 | 3.1819 | 0.3522 | 100.0% | 1614.6522 | 1614.796 | 1 | 6.342 | 61.5% | 1 | R.NKLNDLEEALQQAK.E | 2 |
|  | AstrinIP\_MS1\_022614\_01.11903.11903.3 | 4.2765 | 0.3747 | 100.0% | 2199.0544 | 2199.4258 | 1 | 6.889 | 40.3% | 1 | R.NKLNDLEEALQQAKEDLAR.L | 3 |

Similarities:
gi|4504919|ref|NP\_002(1:7)  
gi|67782365|ref|NP\_00(1:7)  
gi|119395750|ref|NP\_0(1:7)  
gi|119703753|ref|NP\_0(1:7)  

---

|  |  |  |  |  |  |  |  |  |
| --- | --- | --- | --- | --- | --- | --- | --- | --- |
| U | *gi|67782365|ref|NP\_00* | 7 | 8 | 18.6% | 469 | 51386 | 5.5 | keratin 7 [Homo sapiens] |

| Filename XCorr DeltCN Conf% ObsM+H+ CalcM+H+ SpR ZScore Ion% # Sequence  | | | | | | | | | | | | |
| --- | --- | --- | --- | --- | --- | --- | --- | --- | --- | --- | --- | --- |
|  | AstrinIP\_MS1\_022614\_01.06466.06466.3 | 3.8047 | 0.2341 | 99.1% | 2246.4243 | 2247.519 | 28 | 5.83 | 25.0% | 1 | R.LSSARPGGLGSSSLYGLGASRPR.V | 3 |
|  | AstrinIP\_MS1\_022614\_01.07103.07103.2 | 2.5876 | 0.1854 | 98.7% | 1083.1322 | 1083.2755 | 2 | 6.016 | 75.0% | 2 | K.FASFIDKVR.F | 2222 |
|  | AstrinIP\_MS2\_022614\_01.13040.13040.2 | 2.3293 | 0.2673 | 97.9% | 1441.4521 | 1443.686 | 4 | 5.874 | 58.3% | 1 | R.LPDIFEAQIAGLR.G | 2 |
|  | AstrinIP\_MS2\_022614\_01.06519.06519.1 | 2.2516 | 0.2656 | 99.5% | 1242.63 | 1243.3622 | 2 | 5.261 | 54.5% | 1 | R.GQLEALQVDGGR.L | 1 |
| \* | AstrinIP\_MS2\_022614\_02.07110.07110.3 | 3.0528 | 0.3337 | 99.6% | 1955.6943 | 1955.1783 | 1 | 5.559 | 30.9% | 1 | R.GQLEALQVDGGRLEAELR.S | 3 |
|  | AstrinIP\_MS1\_022614\_01.11008.11008.2 | 2.6027 | 0.1709 | 97.1% | 1419.5721 | 1419.5773 | 1 | 5.958 | 63.6% | 1 | K.VDALNDEINFLR.T | 2 |
|  | AstrinIP\_MS2\_022614\_01.05238.05238.2 | 3.0922 | 0.3307 | 99.9% | 1386.1322 | 1386.548 | 2 | 6.797 | 68.2% | 1 | R.AKQEELEAALQR.G | 2 |

Similarities:
gi|4504919|ref|NP\_002(1:6)  
gi|47132620|ref|NP\_00(1:6)  
gi|119703753|ref|NP\_0(1:6)  

---

|  |  |  |  |  |  |  |  |  |
| --- | --- | --- | --- | --- | --- | --- | --- | --- |
| U | *gi|4885399|ref|NP\_005* | 16 | 21 | 18.3% | 1217 | 141541 | 7.2 | structural maintenance of chromosomes 3 [Homo sapiens] |

| Filename XCorr DeltCN Conf% ObsM+H+ CalcM+H+ SpR ZScore Ion% # Sequence  | | | | | | | | | | | | |
| --- | --- | --- | --- | --- | --- | --- | --- | --- | --- | --- | --- | --- |
| \* | AstrinIP\_MS2\_022614\_01.16882.16882.3 | 3.994 | 0.3985 | 100.0% | 2832.1743 | 2832.1472 | 1 | 7.131 | 30.7% | 1 | K.SNFFYAIQFVLSDEFSHLRPEQR.L | 3 |
| \* | AstrinIP\_MS2\_022614\_01.04954.04954.2 | 2.7538 | 0.3439 | 99.8% | 1165.6122 | 1164.35 | 1 | 5.852 | 80.0% | 1 | R.LALLHEGTGPR.V | 2 |
| \* | AstrinIP\_MS2\_022614\_01.13638.13638.2 | 2.4214 | 0.2097 | 95.9% | 1552.1322 | 1553.7301 | 2 | 4.968 | 57.7% | 1 | K.NDVMNLLESAGFSR.S | 2 |
| \* | AstrinIP\_MS1\_022614\_01.03694.03694.2 | 3.6233 | 0.3399 | 100.0% | 1332.0721 | 1332.4741 | 1 | 6.439 | 81.8% | 2 | K.INQMATAPDSQR.L | 2 |
| \* | AstrinIP\_MS1\_022614\_01.06448.06448.2 | 4.9592 | 0.523 | 100.0% | 1988.2922 | 1989.1895 | 1 | 8.469 | 66.7% | 1 | R.LHTLEEEKEELAQYQK.W | 2 |
| \* | AstrinIP\_MS2\_022614\_01.08418.08418.3 | 3.568 | 0.2572 | 99.3% | 1964.1543 | 1964.1393 | 1 | 5.01 | 38.3% | 1 | K.NLEQYNKLDQDLNEVK.A | 3 |
| \* | AstrinIP\_MS2\_022614\_01.04271.04271.2 | 2.7094 | 0.3485 | 99.8% | 1302.0122 | 1302.3837 | 3 | 6.848 | 68.2% | 2 | R.EENAEQQALAAK.R | 2 |
| \* | AstrinIP\_MS2\_022614\_02.05524.05524.3 | 2.9656 | 0.4399 | 100.0% | 1652.4844 | 1653.8291 | 1 | 6.54 | 44.2% | 1 | R.LFYHIVDSDEVSTK.I | 3 |
| \* | AstrinIP\_MS1\_022614\_01.04673.04673.2 | 3.2547 | 0.2407 | 99.8% | 1346.1122 | 1346.4772 | 2 | 6.363 | 68.2% | 3 | R.KAEEELGELEAK.L | 2 |
| \* | AstrinIP\_MS1\_022614\_01.05403.05403.2 | 2.123 | 0.2087 | 95.1% | 1045.0322 | 1045.1375 | 7 | 4.851 | 68.8% | 1 | R.VDALNDEIR.Q | 2 |
| \* | AstrinIP\_MS2\_022614\_01.07107.07107.2 | 2.3433 | 0.1787 | 96.9% | 1043.2122 | 1043.295 | 28 | 4.856 | 75.0% | 1 | R.IKLEGIITR.V | 2 |
| \* | AstrinIP\_MS2\_022614\_01.06814.06814.2 | 2.8118 | 0.3586 | 100.0% | 1251.3922 | 1251.3818 | 1 | 6.851 | 83.3% | 1 | R.VETYLNENLR.K | 2 |
| \* | AstrinIP\_MS1\_022614\_01.08318.08318.3 | 3.6702 | 0.251 | 99.2% | 2234.8145 | 2234.422 | 1 | 5.546 | 39.5% | 2 | R.SEDLDNSIDKTEAGIKELQK.S | 3 |
| \* | AstrinIP\_MS2\_022614\_01.08117.08117.2 | 2.5912 | 0.2088 | 97.6% | 1618.5922 | 1617.8424 | 1 | 4.074 | 53.8% | 1 | K.IRELGSLPQEAFEK.Y | 2 |
| \* | AstrinIP\_MS2\_022614\_01.08164.08164.2 | 2.9952 | 0.3014 | 99.9% | 1241.3922 | 1241.4729 | 1 | 5.936 | 88.9% | 1 | R.KYEAIQLTFK.Q | 2 |
| \* | AstrinIP\_MS2\_022614\_01.03371.03371.3 | 4.3841 | 0.2947 | 100.0% | 2168.2744 | 2168.1057 | 1 | 5.645 | 35.0% | 1 | K.KGDVEGSQSQDEGEGSGESER.G | 3 |

---

|  |  |  |  |  |  |  |  |  |
| --- | --- | --- | --- | --- | --- | --- | --- | --- |
| U | *gi|4503471|ref|NP\_001* | 7 | 16 | 18.0% | 462 | 50141 | 9.0 | eukaryotic translation elongation factor 1 alpha 1 [Homo sapiens] |

| Filename XCorr DeltCN Conf% ObsM+H+ CalcM+H+ SpR ZScore Ion% # Sequence  | | | | | | | | | | | | |
| --- | --- | --- | --- | --- | --- | --- | --- | --- | --- | --- | --- | --- |
|  | AstrinIP\_MS2\_022614\_02.05022.05022.2 | 3.6199 | 0.4601 | 100.0% | 1588.4321 | 1589.835 | 1 | 7.601 | 64.3% | 2 | K.THINIVVIGHVDSGK.S | 2 |
|  | AstrinIP\_MS2\_022614\_02.04997.04997.3 | 4.2437 | 0.3898 | 100.0% | 1589.7244 | 1589.835 | 1 | 7.029 | 46.4% | 5 | K.THINIVVIGHVDSGK.S | 3 |
| \* | AstrinIP\_MS2\_022614\_01.07587.07587.2 | 2.3114 | 0.2463 | 97.5% | 1405.1122 | 1405.5962 | 1 | 4.566 | 68.2% | 1 | K.YYVTIIDAPGHR.D | 2 |
|  | AstrinIP\_MS1\_022614\_01.08501.08501.2 | 3.5256 | 0.4125 | 100.0% | 1315.9722 | 1315.5553 | 1 | 6.863 | 72.7% | 4 | R.EHALLAYTLGVK.Q | 2 |
|  | AstrinIP\_MS2\_022614\_01.06399.06399.2 | 3.4518 | 0.393 | 100.0% | 1026.0922 | 1026.2241 | 1 | 7.518 | 85.0% | 1 | K.IGGIGTVPVGR.V | 2 |
| \* | AstrinIP\_MS2\_022614\_01.11476.11476.3 | 3.9387 | 0.2187 | 98.6% | 2518.2844 | 2516.999 | 1 | 5.093 | 31.5% | 2 | R.VETGVLKPGMVVTFAPVNVTTEVK.S | 3 |
|  | AstrinIP\_MS1\_022614\_01.04901.04901.1 | 2.0299 | 0.2803 | 99.4% | 914.56 | 915.1209 | 4 | 5.144 | 68.8% | 1 | R.QTVAVGVIK.A | 1 |

---

|  |  |  |  |  |  |  |  |  |
| --- | --- | --- | --- | --- | --- | --- | --- | --- |
| U | *gi|119395750|ref|NP\_0* | 10 | 17 | 17.9% | 644 | 66039 | 8.1 | keratin 1 [Homo sapiens] |

| Filename XCorr DeltCN Conf% ObsM+H+ CalcM+H+ SpR ZScore Ion% # Sequence  | | | | | | | | | | | | |
| --- | --- | --- | --- | --- | --- | --- | --- | --- | --- | --- | --- | --- |
|  | AstrinIP\_MS1\_022614\_01.10035.10035.2 | 3.2188 | 0.3563 | 100.0% | 1384.2122 | 1384.5315 | 3 | 6.359 | 59.1% | 2 | K.SLNNQFASFIDK.V | 2 |
|  | AstrinIP\_MS1\_022614\_01.05997.05997.2 | 3.9133 | 0.0682 | 99.3% | 1475.9122 | 1476.6726 | 1 | 6.651 | 81.8% | 3 | R.FLEQQNQVLQTK.W | 22 |
|  | AstrinIP\_MS1\_022614\_02.06991.06991.2 | 2.7932 | 0.3252 | 99.8% | 1475.5721 | 1476.6293 | 3 | 5.825 | 59.1% | 1 | K.WELLQQVDTSTR.T | 2 |
|  | AstrinIP\_MS1\_022614\_01.13319.13319.2 | 3.827 | 0.4795 | 100.0% | 1994.6122 | 1995.2017 | 1 | 6.942 | 56.7% | 1 | R.THNLEPYFESFINNLR.R | 2 |
|  | AstrinIP\_MS1\_022614\_01.03650.03650.2 | 2.8304 | 0.2913 | 99.8% | 1309.3522 | 1309.4215 | 161 | 5.387 | 55.6% | 2 | R.NKYEDEINKR.T | 222 |
|  | AstrinIP\_MS1\_022614\_01.04863.04863.2 | 3.3345 | 0.3468 | 100.0% | 1394.2722 | 1394.5675 | 1 | 6.817 | 72.7% | 1 | R.TNAENEFVTIKK.D | 2 |
| \* | AstrinIP\_MS1\_022614\_01.12891.12891.2 | 4.3086 | 0.4959 | 100.0% | 1303.5521 | 1303.4955 | 1 | 8.79 | 81.8% | 2 | R.SLDLDSIIAEVK.A | 2 |
|  | AstrinIP\_MS1\_022614\_01.06365.06365.2 | 3.2145 | 0.3532 | 100.0% | 1181.2922 | 1180.303 | 1 | 6.204 | 88.9% | 3 | K.YEELQITAGR.H | 22 |
| \* | AstrinIP\_MS1\_022614\_01.11741.11741.3 | 5.3615 | 0.407 | 100.0% | 2185.5842 | 2185.399 | 1 | 6.809 | 43.1% | 1 | K.NKLNDLEDALQQAKEDLAR.L | 3 |
|  | AstrinIP\_MS1\_022614\_01.08430.08430.2 | 3.6926 | 0.3227 | 99.9% | 1358.2922 | 1358.4912 | 1 | 6.566 | 81.8% | 1 | K.LNDLEDALQQAK.E | 2 |

Similarities:
gi|4504919|ref|NP\_002(1:9)  
gi|47132620|ref|NP\_00(1:9)  
gi|119703753|ref|NP\_0(2:8)  

---

|  |  |  |  |  |  |  |  |  |
| --- | --- | --- | --- | --- | --- | --- | --- | --- |
| U | *gi|117956403|ref|NP\_0* | 8 | 13 | 17.8% | 569 | 63543 | 4.8 | rabaptin, RAB GTPase binding effector protein 2 [Homo sapiens] |

| Filename XCorr DeltCN Conf% ObsM+H+ CalcM+H+ SpR ZScore Ion% # Sequence  | | | | | | | | | | | | |
| --- | --- | --- | --- | --- | --- | --- | --- | --- | --- | --- | --- | --- |
| \* | AstrinIP\_MS1\_022614\_01.03695.03695.2 | 4.5729 | 0.5215 | 100.0% | 1621.2122 | 1621.6146 | 1 | 8.274 | 66.7% | 2 | R.SQEGANGEAESGELSR.L | 2 |
| \* | AstrinIP\_MS2\_022614\_02.04313.04313.3 | 2.9207 | 0.3027 | 99.2% | 1917.0243 | 1917.1265 | 1 | 5.235 | 34.7% | 1 | K.AVAEVSESTKAEAVAAVQR.Q | 3 |
| \* | AstrinIP\_MS1\_022614\_01.03772.03772.2 | 2.3011 | 0.2052 | 96.1% | 1401.1721 | 1401.5222 | 63 | 3.901 | 55.0% | 1 | R.ERDELQEGLRR.S | 2 |
| \* | AstrinIP\_MS1\_022614\_01.08578.08578.2 | 3.0281 | 0.2657 | 99.8% | 1113.1522 | 1113.389 | 1 | 5.634 | 87.5% | 2 | R.LRIEIVTLR.E | 2 |
| \* | AstrinIP\_MS1\_022614\_01.04274.04274.2 | 2.5287 | 0.1779 | 97.9% | 1147.2122 | 1147.2273 | 24 | 4.259 | 66.7% | 1 | R.EALEEETVAR.A | 2 |
| \* | AstrinIP\_MS2\_022614\_01.09206.09206.2 | 2.7041 | 0.3373 | 99.8% | 1270.1522 | 1270.4282 | 1 | 6.52 | 65.0% | 2 | K.AQLPDLLSEQR.A | 2 |
|  | AstrinIP\_MS2\_022614\_02.04098.04098.2 | 4.7919 | 0.3195 | 100.0% | 1531.1721 | 1531.6628 | 1 | 8.861 | 83.3% | 3 | R.LQAELETSEQVQR.D | 2 |
|  | AstrinIP\_MS2\_022614\_01.09081.09081.2 | 2.6399 | 0.2417 | 98.8% | 1348.0721 | 1347.5261 | 1 | 4.646 | 63.6% | 1 | R.SIMDEAPLTDVR.D | 2 |

---

|  |  |  |  |  |  |  |  |  |
| --- | --- | --- | --- | --- | --- | --- | --- | --- |
| U | *gi|14141166|ref|NP\_11* | 3 | 3 | 17.7% | 362 | 38222 | 6.8 | poly(rC) binding protein 2 isoform b [Homo sapiens] |
| U | *gi|193083114|ref|NP\_0* | 3 | 3 | 20.1% | 318 | 33497 | 8.2 | poly(rC) binding protein 2 isoform g [Homo sapiens] |
| U | *gi|193083112|ref|NP\_0* | 3 | 3 | 19.1% | 335 | 35347 | 8.0 | poly(rC) binding protein 2 isoform f [Homo sapiens] |
| U | *gi|193083110|ref|NP\_0* | 3 | 3 | 17.7% | 361 | 38151 | 6.8 | poly(rC) binding protein 2 isoform e [Homo sapiens] |
| U | *gi|193083108|ref|NP\_0* | 3 | 3 | 17.5% | 365 | 38580 | 6.8 | poly(rC) binding protein 2 isoform d [Homo sapiens] |
| U | *gi|148833484|ref|NP\_0* | 3 | 3 | 19.3% | 331 | 34917 | 8.0 | poly(rC) binding protein 2 isoform c [Homo sapiens] |
| U | *gi|14141168|ref|NP\_00* | 3 | 3 | 17.5% | 366 | 38651 | 6.8 | poly(rC) binding protein 2 isoform a [Homo sapiens] |

| Filename XCorr DeltCN Conf% ObsM+H+ CalcM+H+ SpR ZScore Ion% # Sequence  | | | | | | | | | | | | |
| --- | --- | --- | --- | --- | --- | --- | --- | --- | --- | --- | --- | --- |
|  | AstrinIP\_MS2\_022614\_01.11334.11334.2 | 3.3119 | 0.4479 | 100.0% | 1360.4521 | 1359.6519 | 1 | 8.419 | 66.7% | 1 | R.IITLAGPTNAIFK.A | 2 |
|  | AstrinIP\_MS1\_022614\_01.14804.14804.3 | 3.2538 | 0.2215 | 95.5% | 3354.4143 | 3353.8306 | 1 | 4.183 | 25.0% | 1 | K.AFAMIIDKLEEDISSSMTNSTAASRPPVTLR.L | 3 |
|  | AstrinIP\_MS2\_022614\_02.05321.05321.3 | 3.2617 | 0.2337 | 97.9% | 2092.1042 | 2091.2573 | 1 | 3.903 | 36.8% | 1 | R.ESTGAQVQVAGDMLPNSTER.A | 33 |

Similarities:
gi|222352151|ref|NP\_0(1:2)  

---

|  |  |  |  |  |  |  |  |  |
| --- | --- | --- | --- | --- | --- | --- | --- | --- |
| U | *Reverse\_gi|4507723|re* | 2 | 2 | 16.9% | 278 | 31750 | 7.7 | tocopherol (alpha) transfer protein [Homo sapiens] |

| Filename XCorr DeltCN Conf% ObsM+H+ CalcM+H+ SpR ZScore Ion% # Sequence  | | | | | | | | | | | | |
| --- | --- | --- | --- | --- | --- | --- | --- | --- | --- | --- | --- | --- |
| \* | AstrinIP\_MS1\_022614\_02.05626.05626.3 | 2.7701 | 0.276 | 98.5% | 1826.5144 | 1826.6929 | 12 | 4.129 | 34.6% | 1 | -.QISES\*IS\*S\*LYDESK.M | 3 |
| \* | AstrinIP\_MS2\_022614\_01.07307.07307.3 | 3.1077 | 0.238 | 96.0% | 3467.1543 | 3464.9707 | 14 | 4.497 | 18.8% | 1 | R.RRLAALGPQLLPSHDPLANLQPGASPQSRAEAM.- | 3 |

---

|  |  |  |  |  |  |  |  |  |
| --- | --- | --- | --- | --- | --- | --- | --- | --- |
| U | *gi|10835063|ref|NP\_00* | 2 | 2 | 16.7% | 294 | 32575 | 4.8 | nucleophosmin 1 isoform 1 [Homo sapiens] |
| U | *gi|83641870|ref|NP\_00* | 2 | 2 | 18.9% | 259 | 28400 | 4.7 | nucleophosmin 1 isoform 3 [Homo sapiens] |
| U | *gi|40353734|ref|NP\_95* | 2 | 2 | 18.5% | 265 | 29465 | 4.6 | nucleophosmin 1 isoform 2 [Homo sapiens] |

| Filename XCorr DeltCN Conf% ObsM+H+ CalcM+H+ SpR ZScore Ion% # Sequence  | | | | | | | | | | | | |
| --- | --- | --- | --- | --- | --- | --- | --- | --- | --- | --- | --- | --- |
|  | AstrinIP\_MS2\_022614\_02.07899.07899.3 | 3.7027 | 0.1914 | 96.1% | 2931.1743 | 2931.2874 | 1 | 4.376 | 26.9% | 1 | R.TVSLGAGAKDELHIVEAEAMNYEGSPIK.V | 3 |
|  | AstrinIP\_MS2\_022614\_01.13940.13940.2 | 2.1794 | 0.3 | 97.3% | 2227.412 | 2228.655 | 1 | 4.856 | 35.0% | 1 | K.MSVQPTVSLGGFEITPPVVLR.L | 2 |

---

|  |  |  |  |  |  |  |  |  |
| --- | --- | --- | --- | --- | --- | --- | --- | --- |
| U | *gi|33350932|ref|NP\_00* | 53 | 80 | 16.5% | 4646 | 532412 | 6.4 | cytoplasmic dynein 1 heavy chain 1 [Homo sapiens] |

| Filename XCorr DeltCN Conf% ObsM+H+ CalcM+H+ SpR ZScore Ion% # Sequence  | | | | | | | | | | | | |
| --- | --- | --- | --- | --- | --- | --- | --- | --- | --- | --- | --- | --- |
| \* | AstrinIP\_MS2\_022614\_01.13786.13786.3 | 4.8238 | 0.3721 | 100.0% | 2477.8743 | 2477.8577 | 1 | 7.316 | 35.9% | 1 | R.KLVPLLLEDGGEAPAALEAALEEK.S | 3 |
| \* | AstrinIP\_MS2\_022614\_01.14901.14901.3 | 3.8516 | 0.3804 | 100.0% | 2348.6943 | 2349.6836 | 1 | 6.308 | 33.0% | 1 | K.LVPLLLEDGGEAPAALEAALEEK.S | 3 |
| \* | AstrinIP\_MS2\_022614\_01.07965.07965.2 | 3.5312 | 0.4177 | 100.0% | 1640.2522 | 1640.8796 | 1 | 6.851 | 73.1% | 2 | K.FLSDPQVHTVLVER.S | 2 |
| \* | AstrinIP\_MS1\_022614\_01.05482.05482.2 | 4.4798 | 0.4657 | 100.0% | 1626.2122 | 1626.8503 | 1 | 8.578 | 67.9% | 2 | R.TPVIDADKPVSSQLR.V | 2 |
| \* | AstrinIP\_MS1\_022614\_01.05446.05446.3 | 3.4418 | 0.3092 | 99.9% | 1627.3744 | 1626.8503 | 15 | 6.706 | 32.1% | 3 | R.TPVIDADKPVSSQLR.V | 3 |
| \* | AstrinIP\_MS1\_022614\_01.17709.17709.3 | 4.3816 | 0.4703 | 100.0% | 2913.2944 | 2914.2842 | 1 | 7.171 | 30.0% | 3 | R.VLTLSEDSPYETLHSFISNAVAPFFK.S | 3 |
| \* | AstrinIP\_MS1\_022614\_01.10265.10265.3 | 4.0561 | 0.3337 | 100.0% | 2579.8743 | 2580.8137 | 1 | 6.043 | 31.8% | 1 | K.VTDFGDKVEDPTFLNQLQSGVNR.W | 3 |
| \* | AstrinIP\_MS2\_022614\_01.06768.06768.3 | 2.7117 | 0.3303 | 99.0% | 2663.1543 | 2664.0085 | 3 | 5.671 | 28.3% | 1 | R.VLRPQVTAVAQQNQGEVPEPQDMK.V | 3 |
| \* | AstrinIP\_MS2\_022614\_01.12492.12492.2 | 3.1521 | 0.482 | 100.0% | 1507.4722 | 1508.6305 | 1 | 7.288 | 68.2% | 2 | K.LNTQEIFDDWAR.K | 2 |
| \* | AstrinIP\_MS1\_022614\_01.12347.12347.2 | 3.363 | 0.348 | 100.0% | 1616.1721 | 1615.997 | 1 | 6.456 | 69.2% | 1 | K.LKVNFLPEIITLSK.E | 2 |
| \* | AstrinIP\_MS2\_022614\_01.13781.13781.2 | 2.8225 | 0.1259 | 97.0% | 1374.2522 | 1374.6635 | 6 | 5.11 | 59.1% | 1 | K.VNFLPEIITLSK.E | 2 |
| \* | AstrinIP\_MS1\_022614\_01.10078.10078.2 | 2.648 | 0.2475 | 98.7% | 1459.3922 | 1459.6421 | 1 | 5.163 | 66.7% | 1 | R.AWTQVLLGQAEDK.A | 2 |
| \* | AstrinIP\_MS2\_022614\_01.20013.20013.3 | 4.1852 | 0.4156 | 100.0% | 2928.9243 | 2929.3267 | 21 | 6.479 | 23.1% | 1 | R.MPDGPVALEESYSAVMGIVSEVEQYVK.V | 3 |
| \* | AstrinIP\_MS1\_022614\_01.12804.12804.2 | 3.2214 | 0.3797 | 100.0% | 1896.2322 | 1897.2278 | 1 | 5.765 | 56.7% | 1 | R.LGEDLNKWQALLVQIR.K | 2 |
| \* | AstrinIP\_MS2\_022614\_01.08962.08962.2 | 3.8925 | 0.4262 | 100.0% | 1574.3722 | 1574.7925 | 1 | 7.564 | 73.1% | 2 | K.DSAIQQQVANLQMK.I | 2 |
| \* | AstrinIP\_MS2\_022614\_02.06222.06222.2 | 2.8214 | 0.1297 | 97.2% | 1386.3922 | 1385.6006 | 6 | 4.413 | 59.1% | 1 | R.VQVALEELQDLK.G | 2 |
| \* | AstrinIP\_MS2\_022614\_02.10175.10175.3 | 3.8621 | 0.308 | 99.9% | 2273.1843 | 2272.6035 | 1 | 6.079 | 35.5% | 1 | R.VQVALEELQDLKGVWSELSK.V | 3 |
| \* | AstrinIP\_MS2\_022614\_01.10559.10559.3 | 3.1387 | 0.2263 | 97.0% | 2383.5842 | 2384.7148 | 4 | 4.067 | 31.9% | 1 | K.VWEQIDQMKEQPWVSVQPR.K | 3 |
| \* | AstrinIP\_MS2\_022614\_01.11873.11873.2 | 3.1536 | 0.2204 | 99.8% | 1270.3722 | 1270.4716 | 1 | 4.844 | 75.0% | 1 | R.QNLDALLNQLK.S | 2 |
| \* | AstrinIP\_MS2\_022614\_01.06576.06576.2 | 3.0449 | 0.3323 | 99.9% | 1561.0521 | 1560.7526 | 72 | 5.627 | 54.5% | 1 | R.LRQYASYEFVQR.L | 2 |
| \* | AstrinIP\_MS1\_022614\_01.14801.14801.2 | 4.5565 | 0.5748 | 100.0% | 1958.9321 | 1960.1908 | 1 | 10.339 | 68.8% | 4 | R.FYFVGDEDLLEIIGNSK.N | 2 |
| \* | AstrinIP\_MS2\_022614\_01.08206.08206.3 | 3.7742 | 0.3215 | 100.0% | 1616.5443 | 1616.904 | 1 | 6.102 | 47.9% | 2 | R.KLEHLITELVHQR.D | 3 |
| \* | AstrinIP\_MS2\_022614\_01.12378.12378.2 | 3.023 | 0.3765 | 99.9% | 1788.2922 | 1789.0569 | 1 | 5.813 | 46.7% | 1 | K.QTDVLQQLSIQMANAK.F | 2 |
| \* | AstrinIP\_MS2\_022614\_01.11222.11222.2 | 3.7995 | 0.3032 | 100.0% | 1580.2122 | 1580.7368 | 1 | 6.748 | 87.5% | 1 | K.FNYGFEYLGVQDK.L | 2 |
| \* | AstrinIP\_MS1\_022614\_01.05717.05717.2 | 2.7036 | 0.196 | 98.1% | 1145.9122 | 1146.2883 | 174 | 4.827 | 50.0% | 2 | R.LGGSPFGPAGTGK.T | 2 |
| \* | AstrinIP\_MS2\_022614\_01.13383.13383.2 | 4.2772 | 0.4827 | 100.0% | 1654.8121 | 1654.9678 | 1 | 9.189 | 69.2% | 2 | R.QLIAQVMLYSQGFR.T | 2 |
| \* | AstrinIP\_MS1\_022614\_01.03834.03834.2 | 2.456 | 0.2064 | 97.8% | 1130.2122 | 1130.3329 | 4 | 5.209 | 65.0% | 1 | K.SVLVSAGNVKR.E | 2 |
| \* | AstrinIP\_MS2\_022614\_01.19805.19805.2 | 4.9666 | 0.6011 | 100.0% | 2729.172 | 2730.179 | 1 | 10.405 | 45.7% | 1 | K.LVAEDIPLLFSLLSDVFPGVQYHR.G | 2 |
| \* | AstrinIP\_MS1\_022614\_01.11213.11213.2 | 2.8897 | 0.2179 | 99.2% | 1475.3121 | 1474.6592 | 5 | 4.825 | 59.1% | 1 | R.EWTDGLFTHVLR.K | 2 |
| \* | AstrinIP\_MS2\_022614\_01.16328.16328.3 | 4.978 | 0.4469 | 100.0% | 3941.2444 | 3942.3306 | 1 | 7.916 | 22.0% | 1 | R.QWIVFDGDVDPEWVENLNSVLDDNKLLTLPNGER.L | 3 |
| \* | AstrinIP\_MS2\_022614\_01.11688.11688.2 | 2.8299 | 0.3905 | 99.8% | 1956.1921 | 1958.2366 | 1 | 6.929 | 44.1% | 1 | R.DAATIMQPYFTSNGLVTK.A | 2 |
| \* | AstrinIP\_MS1\_022614\_01.09546.09546.2 | 3.0393 | 0.3369 | 99.8% | 1552.4521 | 1552.8113 | 1 | 7.246 | 53.6% | 1 | K.VAAPDVVVPTLDTVR.H | 2 |
| \* | AstrinIP\_MS2\_022614\_01.08824.08824.2 | 3.1148 | 0.2982 | 99.8% | 1393.1322 | 1393.6696 | 1 | 6.162 | 65.4% | 2 | R.TPNGVVLAPVQLGK.W | 2 |
| \* | AstrinIP\_MS2\_022614\_01.15730.15730.2 | 5.2076 | 0.5393 | 100.0% | 2340.2122 | 2339.64 | 1 | 10.226 | 55.3% | 1 | R.TYAEPLTAAMVEFYTMSQER.F | 2 |
| \* | AstrinIP\_MS2\_022614\_01.15717.15717.3 | 3.2891 | 0.4491 | 100.0% | 2340.5942 | 2339.64 | 1 | 6.703 | 32.9% | 1 | R.TYAEPLTAAMVEFYTMSQER.F | 3 |
| \* | AstrinIP\_MS1\_022614\_01.14590.14590.2 | 3.7099 | 0.4421 | 100.0% | 2122.4521 | 2124.5315 | 1 | 8.069 | 50.0% | 2 | R.GIFEALRPLETLPVEGLIR.I | 2 |
| \* | AstrinIP\_MS2\_022614\_02.05874.05874.2 | 2.8797 | 0.3588 | 99.9% | 1405.2722 | 1405.5474 | 1 | 6.178 | 72.7% | 4 | R.WTDENIDTVALK.H | 2 |
| \* | AstrinIP\_MS2\_022614\_01.19835.19835.3 | 7.4381 | 0.5404 | 100.0% | 3030.4143 | 3030.534 | 1 | 10.276 | 38.5% | 2 | R.LKVFYEEELDVPLVLFNEVLDHVLR.I | 3 |
| \* | AstrinIP\_MS2\_022614\_02.08969.08969.2 | 2.5881 | 0.3407 | 99.6% | 1657.3121 | 1656.983 | 2 | 5.413 | 50.0% | 1 | R.FVAWMNGLSVYQIK.V | 2 |
| \* | AstrinIP\_MS1\_022614\_01.07709.07709.2 | 2.5028 | 0.2097 | 97.3% | 1565.2122 | 1564.7504 | 178 | 4.41 | 41.7% | 1 | K.EGLMLDSHEELYK.W | 2 |
| \* | AstrinIP\_MS2\_022614\_01.05328.05328.2 | 3.6867 | 0.3285 | 100.0% | 1471.5122 | 1470.6837 | 1 | 7.032 | 77.3% | 1 | K.VMSQEIQEQLHK.Q | 2 |
| \* | AstrinIP\_MS2\_022614\_01.08150.08150.2 | 5.2728 | 0.5517 | 100.0% | 1864.2322 | 1865.0271 | 1 | 9.76 | 85.7% | 1 | K.NYMSNPSYNYEIVNR.A | 2 |
| \* | AstrinIP\_MS1\_022614\_01.12662.12662.2 | 3.7858 | 0.2302 | 99.9% | 1538.6522 | 1537.8169 | 1 | 6.27 | 79.2% | 2 | K.WAIAQLNYADMLK.R | 2 |
| \* | AstrinIP\_MS2\_022614\_01.06725.06725.2 | 2.567 | 0.1532 | 98.0% | 1090.3522 | 1090.2396 | 15 | 4.47 | 68.8% | 1 | K.ANEVEQMIR.D | 2 |
| \* | AstrinIP\_MS2\_022614\_01.10889.10889.2 | 3.9484 | 0.4577 | 100.0% | 1855.4122 | 1856.126 | 1 | 8.056 | 73.3% | 1 | R.YKEEYAVLISEAQAIK.A | 2 |
| \* | AstrinIP\_MS2\_022614\_01.08872.08872.2 | 2.9102 | 0.1865 | 99.1% | 1200.2722 | 1200.3367 | 1 | 5.988 | 77.8% | 1 | R.TSFLDDAFRK.N | 2 |
| \* | AstrinIP\_MS1\_022614\_01.07772.07772.2 | 2.6593 | 0.1797 | 97.4% | 1505.6721 | 1506.6624 | 372 | 4.156 | 41.7% | 1 | R.SSLQSQCLNEVLK.A | 2 |
| \* | AstrinIP\_MS2\_022614\_01.10695.10695.2 | 2.7904 | 0.2381 | 99.6% | 1115.8722 | 1115.3152 | 3 | 4.991 | 72.2% | 1 | K.SLLQALNEVK.G | 2 |
| \* | AstrinIP\_MS2\_022614\_01.09239.09239.2 | 4.432 | 0.4047 | 100.0% | 1513.2722 | 1513.7343 | 1 | 9.259 | 88.5% | 5 | R.IQGLTVEQAEAVVR.L | 2 |
| \* | AstrinIP\_MS1\_022614\_02.04604.04604.2 | 2.38 | 0.3237 | 99.3% | 1196.8922 | 1197.3948 | 11 | 5.381 | 60.0% | 1 | K.TLMAQSIYGGR.V | 2 |
| \* | AstrinIP\_MS2\_022614\_01.09378.09378.2 | 2.4151 | 0.2845 | 99.6% | 1006.1722 | 1006.19 | 3 | 5.467 | 85.7% | 1 | R.LLNTFLER.L | 2 |
| \* | AstrinIP\_MS2\_022614\_01.09292.09292.2 | 4.3277 | 0.5 | 100.0% | 1405.4722 | 1405.6929 | 1 | 8.954 | 79.2% | 3 | R.VLLTTQGVDMISK.M | 2 |
| \* | AstrinIP\_MS2\_022614\_01.15420.15420.3 | 3.1426 | 0.2614 | 98.2% | 2683.7644 | 2684.9856 | 30 | 5.097 | 25.0% | 1 | R.SWSHYTVPAGMTVIQWVSDFSER.I | 3 |

---

|  |  |  |  |  |  |  |  |  |
| --- | --- | --- | --- | --- | --- | --- | --- | --- |
| U | *gi|4502491|ref|NP\_001* | 2 | 5 | 16.0% | 282 | 31362 | 4.8 | complement component 1, q subcomponent binding protein precursor [Homo sapiens] |

| Filename XCorr DeltCN Conf% ObsM+H+ CalcM+H+ SpR ZScore Ion% # Sequence  | | | | | | | | | | | | |
| --- | --- | --- | --- | --- | --- | --- | --- | --- | --- | --- | --- | --- |
| \* | AstrinIP\_MS1\_022614\_02.06695.06695.2 | 2.6548 | 0.3509 | 99.7% | 1621.4722 | 1622.79 | 26 | 5.603 | 42.9% | 1 | K.MSGGWELELNGTEAK.L | 2 |
| \* | AstrinIP\_MS2\_022614\_01.18992.18992.3 | 4.9884 | 0.4352 | 100.0% | 3441.5044 | 3441.77 | 1 | 6.272 | 26.7% | 4 | R.GVDNTFADELVELSTALEHQEYITFLEDLK.S | 3 |

---

|  |  |  |  |  |  |  |  |  |
| --- | --- | --- | --- | --- | --- | --- | --- | --- |
| U | *gi|13676857|ref|NP\_06* | 16 | 58 | 14.9% | 639 | 70021 | 5.7 | heat shock 70kDa protein 2 [Homo sapiens] |

| Filename XCorr DeltCN Conf% ObsM+H+ CalcM+H+ SpR ZScore Ion% # Sequence  | | | | | | | | | | | | |
| --- | --- | --- | --- | --- | --- | --- | --- | --- | --- | --- | --- | --- |
|  | AstrinIP\_MS2\_022614\_01.07734.07734.1 | 2.2714 | 0.4224 | 100.0% | 1487.62 | 1488.5939 | 1 | 6.368 | 50.0% | 5 | R.TTPSYVAFTDTER.L | 11111 |
|  | AstrinIP\_MS1\_022614\_02.04868.04868.2 | 3.849 | 0.5598 | 100.0% | 1489.2922 | 1488.5939 | 1 | 9.39 | 79.2% | 10 | R.TTPSYVAFTDTER.L | 22222 |
|  | AstrinIP\_MS2\_022614\_01.03561.03561.1 | 2.2284 | 0.2726 | 99.4% | 1180.55 | 1181.3312 | 1 | 5.221 | 66.7% | 1 | K.VQVEYKGETK.T | 11 |
|  | AstrinIP\_MS2\_022614\_01.03554.03554.2 | 3.2438 | 0.3839 | 100.0% | 1181.1721 | 1181.3312 | 1 | 6.258 | 77.8% | 1 | K.VQVEYKGETK.T | 22 |
| \* | AstrinIP\_MS2\_022614\_01.13734.13734.2 | 2.5795 | 0.3671 | 99.8% | 1630.0521 | 1629.9086 | 1 | 5.69 | 73.1% | 1 | K.TFFPEEISSMVLTK.M | 2 |
|  | AstrinIP\_MS1\_022614\_01.09921.09921.2 | 5.232 | 0.5436 | 100.0% | 1660.4922 | 1660.9078 | 1 | 10.069 | 80.0% | 8 | R.IINEPTAAAIAYGLDK.K | 2222 |
|  | AstrinIP\_MS2\_022614\_01.09232.09232.2 | 4.6507 | 0.5188 | 100.0% | 1788.4922 | 1789.0819 | 1 | 9.486 | 68.8% | 5 | R.IINEPTAAAIAYGLDKK.G | 22 |
|  | AstrinIP\_MS2\_022614\_01.04514.04514.2 | 4.3459 | 0.4677 | 100.0% | 1692.2122 | 1692.6958 | 1 | 7.869 | 63.3% | 3 | K.STAGDTHLGGEDFDNR.M | 22 |
|  | AstrinIP\_MS1\_022614\_01.04403.04403.3 | 2.4102 | 0.2905 | 97.0% | 1693.8844 | 1692.6958 | 28 | 4.578 | 28.3% | 1 | K.STAGDTHLGGEDFDNR.M | 33 |
|  | AstrinIP\_MS1\_022614\_01.09297.09297.2 | 3.3038 | 0.4288 | 100.0% | 1480.5122 | 1481.6511 | 1 | 6.801 | 77.3% | 3 | R.ARFEELNADLFR.G | 22 |
|  | AstrinIP\_MS2\_022614\_01.09827.09827.3 | 4.0793 | 0.3183 | 100.0% | 1481.5743 | 1481.6511 | 1 | 5.772 | 54.5% | 1 | R.ARFEELNADLFR.G | 33 |
|  | AstrinIP\_MS2\_022614\_01.11079.11079.1 | 2.2045 | 0.4317 | 100.0% | 1253.55 | 1254.3849 | 5 | 6.105 | 50.0% | 1 | R.FEELNADLFR.G | 11 |
|  | AstrinIP\_MS1\_022614\_01.10436.10436.2 | 3.4707 | 0.3983 | 100.0% | 1253.9321 | 1254.3849 | 1 | 7.554 | 72.2% | 4 | R.FEELNADLFR.G | 22 |
|  | AstrinIP\_MS1\_022614\_01.09870.09870.1 | 2.2447 | 0.2928 | 100.0% | 1081.36 | 1082.2444 | 1 | 4.936 | 75.0% | 4 | K.LLQDFFNGK.E | 111 |
|  | AstrinIP\_MS2\_022614\_01.10502.10502.2 | 3.0573 | 0.3647 | 99.9% | 1082.0922 | 1082.2444 | 1 | 5.829 | 81.2% | 5 | K.LLQDFFNGK.E | 222 |
|  | AstrinIP\_MS2\_022614\_01.10005.10005.2 | 3.8674 | 0.1637 | 99.7% | 1567.3722 | 1566.7972 | 1 | 4.208 | 70.8% | 5 | K.LLQDFFNGKELNK.S | 222 |

Similarities:
gi|5729877|ref|NP\_006(15:1)  
gi|16507237|ref|NP\_00(1:15)  
gi|167466173|ref|NP\_0(2:14)  
gi|124256496|ref|NP\_0(3:13)  
gi|34419635|ref|NP\_00(5:11)  

---

|  |  |  |  |  |  |  |  |  |
| --- | --- | --- | --- | --- | --- | --- | --- | --- |
| U | *gi|148470397|ref|NP\_0* | 4 | 7 | 14.7% | 415 | 45672 | 5.6 | heterogeneous nuclear ribonucleoprotein F [Homo sapiens] |
| U | *gi|4826760|ref|NP\_004* | 4 | 7 | 14.7% | 415 | 45672 | 5.6 | heterogeneous nuclear ribonucleoprotein F [Homo sapiens] |
| U | *gi|148470406|ref|NP\_0* | 4 | 7 | 14.7% | 415 | 45672 | 5.6 | heterogeneous nuclear ribonucleoprotein F [Homo sapiens] |
| U | *gi|148470404|ref|NP\_0* | 4 | 7 | 14.7% | 415 | 45672 | 5.6 | heterogeneous nuclear ribonucleoprotein F [Homo sapiens] |
| U | *gi|148470402|ref|NP\_0* | 4 | 7 | 14.7% | 415 | 45672 | 5.6 | heterogeneous nuclear ribonucleoprotein F [Homo sapiens] |
| U | *gi|148470400|ref|NP\_0* | 4 | 7 | 14.7% | 415 | 45672 | 5.6 | heterogeneous nuclear ribonucleoprotein F [Homo sapiens] |

| Filename XCorr DeltCN Conf% ObsM+H+ CalcM+H+ SpR ZScore Ion% # Sequence  | | | | | | | | | | | | |
| --- | --- | --- | --- | --- | --- | --- | --- | --- | --- | --- | --- | --- |
|  | AstrinIP\_MS2\_022614\_01.13479.13479.2 | 5.5778 | 0.4468 | 100.0% | 1868.8722 | 1869.0813 | 1 | 8.802 | 68.8% | 2 | K.ITGEAFVQFASQELAEK.A | 2 |
|  | AstrinIP\_MS1\_022614\_01.04413.04413.3 | 3.0391 | 0.2555 | 98.3% | 1936.1943 | 1936.1986 | 1 | 5.027 | 42.2% | 1 | K.FMSVQRPGPYDRPGTAR.R | 3 |
|  | AstrinIP\_MS2\_022614\_01.14010.14010.2 | 4.2566 | 0.4097 | 100.0% | 1997.5922 | 1998.2023 | 1 | 7.754 | 59.4% | 2 | K.ATENDIYNFFSPLNPVR.V | 22 |
|  | AstrinIP\_MS1\_022614\_01.04682.04682.2 | 2.7054 | 0.408 | 100.0% | 1094.5122 | 1093.2278 | 56 | 7.129 | 61.1% | 2 | R.VHIEIGPDGR.V | 22 |

Similarities:
gi|5031753|ref|NP\_005(2:2)  

---

|  |  |  |  |  |  |  |  |  |
| --- | --- | --- | --- | --- | --- | --- | --- | --- |
| U | *gi|4503513|ref|NP\_003* | 3 | 3 | 14.5% | 325 | 36502 | 5.6 | eukaryotic translation initiation factor 3, subunit 2 beta, 36kDa [Homo sapiens] |

| Filename XCorr DeltCN Conf% ObsM+H+ CalcM+H+ SpR ZScore Ion% # Sequence  | | | | | | | | | | | | |
| --- | --- | --- | --- | --- | --- | --- | --- | --- | --- | --- | --- | --- |
| \* | AstrinIP\_MS2\_022614\_01.14741.14741.3 | 4.261 | 0.4492 | 100.0% | 2724.0544 | 2723.0576 | 1 | 6.518 | 32.6% | 1 | R.EGDLLFTVAKDPIVNVWYSVNGER.L | 3 |
| \* | AstrinIP\_MS2\_022614\_01.05363.05363.2 | 2.948 | 0.2749 | 99.8% | 1319.3121 | 1319.457 | 6 | 5.58 | 65.0% | 1 | K.LFDSTTLEHQK.T | 2 |
| \* | AstrinIP\_MS2\_022614\_01.12167.12167.2 | 3.1912 | 0.358 | 100.0% | 1530.1322 | 1529.6946 | 1 | 6.448 | 68.2% | 1 | R.FFHLAFEEEFGR.V | 2 |

---

|  |  |  |  |  |  |  |  |  |
| --- | --- | --- | --- | --- | --- | --- | --- | --- |
| U | *gi|56682959|ref|NP\_00* | 2 | 5 | 14.2% | 183 | 21226 | 5.6 | ferritin, heavy polypeptide 1 [Homo sapiens] |

| Filename XCorr DeltCN Conf% ObsM+H+ CalcM+H+ SpR ZScore Ion% # Sequence  | | | | | | | | | | | | |
| --- | --- | --- | --- | --- | --- | --- | --- | --- | --- | --- | --- | --- |
| \* | AstrinIP\_MS2\_022614\_01.06578.06578.2 | 3.6698 | 0.3123 | 100.0% | 1296.3522 | 1295.4813 | 2 | 5.879 | 70.0% | 2 | K.NVNQSLLELHK.L | 2 |
| \* | AstrinIP\_MS1\_022614\_01.11999.11999.2 | 3.1846 | 0.3346 | 99.8% | 1627.8722 | 1628.8372 | 1 | 5.179 | 50.0% | 3 | K.MGAPESGLAEYLFDK.H | 2 |

---

|  |  |  |  |  |  |  |  |  |
| --- | --- | --- | --- | --- | --- | --- | --- | --- |
| U | *gi|4758012|ref|NP\_004* | 16 | 30 | 14.1% | 1675 | 191613 | 5.7 | clathrin heavy chain 1 [Homo sapiens] |

| Filename XCorr DeltCN Conf% ObsM+H+ CalcM+H+ SpR ZScore Ion% # Sequence  | | | | | | | | | | | | |
| --- | --- | --- | --- | --- | --- | --- | --- | --- | --- | --- | --- | --- |
| \* | AstrinIP\_MS2\_022614\_02.10718.10718.3 | 3.3135 | 0.3325 | 99.7% | 3175.0745 | 3176.5708 | 23 | 5.089 | 19.4% | 1 | K.WISLNTVALVTDNAVYHWSMEGESQPVK.M | 3 |
|  | AstrinIP\_MS2\_022614\_01.08555.08555.2 | 3.6264 | 0.4937 | 100.0% | 1338.3722 | 1338.5646 | 1 | 8.776 | 77.3% | 4 | R.VVGAMQLYSVDR.K | 2 |
| \* | AstrinIP\_MS2\_022614\_01.08813.08813.2 | 3.1627 | 0.3278 | 99.8% | 1946.1122 | 1946.2566 | 1 | 5.786 | 52.9% | 2 | K.LHIIEVGTPPTGNQPFPK.K | 2 |
| \* | AstrinIP\_MS2\_022614\_01.08178.08178.2 | 2.1725 | 0.3554 | 99.5% | 1071.9321 | 1072.2926 | 5 | 6.506 | 68.8% | 1 | K.HDVVFLITK.Y | 2 |
| \* | AstrinIP\_MS2\_022614\_01.10702.10702.3 | 4.462 | 0.3863 | 100.0% | 2354.2744 | 2354.6682 | 1 | 8.01 | 35.2% | 2 | R.ISGETIFVTAPHEATAGIIGVNR.K | 3 |
| \* | AstrinIP\_MS2\_022614\_01.09826.09826.2 | 4.0539 | 0.4243 | 100.0% | 1305.1721 | 1305.4331 | 1 | 7.501 | 86.4% | 1 | R.NNLAGAEELFAR.K | 2 |
| \* | AstrinIP\_MS1\_022614\_01.07242.07242.2 | 4.962 | 0.4949 | 100.0% | 1759.3322 | 1759.9591 | 1 | 8.142 | 66.7% | 1 | R.KFNALFAQGNYSEAAK.V | 2 |
| \* | AstrinIP\_MS2\_022614\_01.12928.12928.2 | 2.9119 | 0.4704 | 100.0% | 1435.0322 | 1434.6757 | 1 | 6.952 | 70.8% | 2 | K.SVDPTLALSVYLR.A | 2 |
|  | AstrinIP\_MS1\_022614\_01.13179.13179.2 | 3.3631 | 0.3583 | 100.0% | 1608.4722 | 1608.9237 | 1 | 7.761 | 66.7% | 1 | K.KVGYTPDWIFLLR.N | 2 |
|  | AstrinIP\_MS1\_022614\_01.14830.14830.2 | 2.7051 | 0.3312 | 99.8% | 1480.2722 | 1480.7496 | 1 | 6.417 | 68.2% | 2 | K.VGYTPDWIFLLR.N | 2 |
| \* | AstrinIP\_MS2\_022614\_01.08396.08396.3 | 2.6979 | 0.2283 | 95.6% | 1622.3043 | 1621.8333 | 4 | 5.333 | 39.6% | 1 | R.ALEHFTDLYDIKR.A | 3 |
| \* | AstrinIP\_MS2\_022614\_01.04433.04433.2 | 2.8419 | 0.2599 | 99.7% | 1335.0322 | 1335.416 | 2 | 6.385 | 70.0% | 1 | K.IYIDSNNNPER.F | 2 |
|  | AstrinIP\_MS1\_022614\_01.16599.16599.2 | 3.5841 | 0.4368 | 100.0% | 1947.3322 | 1948.2819 | 5 | 7.324 | 43.8% | 4 | K.AFMTADLPNELIELLEK.I | 2 |
| \* | AstrinIP\_MS1\_022614\_01.13528.13528.3 | 6.2202 | 0.4895 | 100.0% | 2369.0942 | 2369.6829 | 1 | 7.781 | 38.8% | 2 | R.KFDVNTSAVQVLIEHIGNLDR.A | 3 |
| \* | AstrinIP\_MS1\_022614\_01.07952.07952.2 | 3.455 | 0.4863 | 100.0% | 1297.0922 | 1297.4563 | 1 | 7.867 | 75.0% | 3 | K.LLYNNVSNFGR.L | 2 |
| \* | AstrinIP\_MS2\_022614\_02.06202.06202.3 | 4.0274 | 0.5044 | 100.0% | 1972.2843 | 1972.2083 | 1 | 8.17 | 41.7% | 2 | R.LASTLVHLGEYQAAVDGAR.K | 3 |

---

|  |  |  |  |  |  |  |  |  |
| --- | --- | --- | --- | --- | --- | --- | --- | --- |
| U | *gi|30581135|ref|NP\_00* | 12 | 15 | 14.1% | 1233 | 143233 | 7.6 | structural maintenance of chromosomes 1A [Homo sapiens] |

| Filename XCorr DeltCN Conf% ObsM+H+ CalcM+H+ SpR ZScore Ion% # Sequence  | | | | | | | | | | | | |
| --- | --- | --- | --- | --- | --- | --- | --- | --- | --- | --- | --- | --- |
| \* | AstrinIP\_MS1\_022614\_01.15882.15882.2 | 4.6222 | 0.397 | 100.0% | 1525.6122 | 1524.7723 | 1 | 6.971 | 80.8% | 2 | K.SNLMDAISFVLGEK.T | 2 |
| \* | AstrinIP\_MS2\_022614\_01.04326.04326.3 | 2.7684 | 0.2733 | 98.3% | 1517.4543 | 1516.7433 | 165 | 5.325 | 28.6% | 1 | R.DLIHGAPVGKPAANR.A | 3 |
| \* | AstrinIP\_MS1\_022614\_02.08386.08386.3 | 3.6707 | 0.2716 | 99.5% | 2241.8643 | 2241.633 | 1 | 4.996 | 34.7% | 1 | K.VVQLHEYSEELEKLGILIK.A | 3 |
| \* | AstrinIP\_MS2\_022614\_02.04980.04980.3 | 3.6855 | 0.2809 | 99.8% | 1762.1943 | 1763.0161 | 14 | 5.735 | 33.9% | 1 | K.KLEGELTEEVEMAKR.R | 3 |
| \* | AstrinIP\_MS1\_022614\_01.06173.06173.2 | 2.8309 | 0.0912 | 97.1% | 1216.1522 | 1216.3862 | 3 | 4.485 | 77.8% | 1 | R.LIDLCQPTQK.K | 2 |
| \* | AstrinIP\_MS2\_022614\_01.04258.04258.2 | 2.4981 | 0.4167 | 99.9% | 1248.5122 | 1249.4651 | 126 | 6.683 | 50.0% | 1 | R.LTEELKEQMK.A | 2 |
| \* | AstrinIP\_MS2\_022614\_01.03798.03798.2 | 2.6623 | 0.1968 | 98.2% | 1383.4321 | 1383.5681 | 1 | 4.429 | 72.7% | 1 | R.QVQSQAHGLQMR.L | 2 |
| \* | AstrinIP\_MS2\_022614\_01.04818.04818.2 | 2.5764 | 0.3242 | 99.8% | 1065.9922 | 1066.2455 | 1 | 6.471 | 87.5% | 1 | R.HLALNLQEK.S | 2 |
| \* | AstrinIP\_MS1\_022614\_01.08309.08309.2 | 3.5075 | 0.4145 | 100.0% | 1448.1122 | 1448.6188 | 1 | 7.69 | 70.8% | 3 | K.SKLESELANFGPR.I | 2 |
| \* | AstrinIP\_MS1\_022614\_01.06462.06462.3 | 4.2727 | 0.3861 | 100.0% | 2257.0144 | 2257.5261 | 1 | 6.215 | 38.2% | 1 | K.NQLKEDQDKVHMWEQTVK.K | 3 |
| \* | AstrinIP\_MS2\_022614\_01.18083.18083.3 | 3.6287 | 0.2101 | 97.3% | 3346.1343 | 3346.1694 | 9 | 3.834 | 20.5% | 1 | K.LPLS\*KGT#MDDIS\*QEEGSS\*QGEDSVSGSQR.I | 3 |
| \* | AstrinIP\_MS2\_022614\_01.04395.04395.2 | 2.9629 | 0.2862 | 99.8% | 1215.5122 | 1215.3518 | 1 | 5.248 | 83.3% | 1 | K.LNEQQSVLQR.I | 2 |

---

|  |  |  |  |  |  |  |  |  |
| --- | --- | --- | --- | --- | --- | --- | --- | --- |
| U | *gi|38327562|ref|NP\_00* | 3 | 4 | 13.9% | 403 | 45823 | 9.4 | serine/threonine protein kinase 6 [Homo sapiens] |
| U | *gi|38327572|ref|NP\_94* | 3 | 4 | 13.9% | 403 | 45823 | 9.4 | serine/threonine protein kinase 6 [Homo sapiens] |
| U | *gi|38327570|ref|NP\_94* | 3 | 4 | 13.9% | 403 | 45823 | 9.4 | serine/threonine protein kinase 6 [Homo sapiens] |
| U | *gi|38327568|ref|NP\_94* | 3 | 4 | 13.9% | 403 | 45823 | 9.4 | serine/threonine protein kinase 6 [Homo sapiens] |
| U | *gi|38327566|ref|NP\_94* | 3 | 4 | 13.9% | 403 | 45823 | 9.4 | serine/threonine protein kinase 6 [Homo sapiens] |
| U | *gi|38327564|ref|NP\_94* | 3 | 4 | 13.9% | 403 | 45823 | 9.4 | serine/threonine protein kinase 6 [Homo sapiens] |

| Filename XCorr DeltCN Conf% ObsM+H+ CalcM+H+ SpR ZScore Ion% # Sequence  | | | | | | | | | | | | |
| --- | --- | --- | --- | --- | --- | --- | --- | --- | --- | --- | --- | --- |
|  | AstrinIP\_MS1\_022614\_01.04209.04209.3 | 2.8893 | 0.3025 | 99.1% | 2216.9644 | 2216.5051 | 229 | 5.709 | 22.4% | 1 | K.QLQATSVPHPVSRPLNNTQK.S | 3 |
|  | AstrinIP\_MS1\_022614\_01.05692.05692.3 | 4.6552 | 0.436 | 100.0% | 2165.8743 | 2166.3496 | 1 | 7.186 | 39.5% | 2 | K.SKQPLPSAPENNPEEELASK.Q | 3 |
|  | AstrinIP\_MS1\_022614\_01.10331.10331.2 | 3.412 | 0.3279 | 100.0% | 1697.4922 | 1697.9695 | 1 | 6.25 | 56.7% | 1 | R.DIKPENLLLGSAGELK.I | 2 |

---

|  |  |  |  |  |  |  |  |  |
| --- | --- | --- | --- | --- | --- | --- | --- | --- |
| U | *gi|32698730|ref|NP\_06* | 6 | 11 | 13.5% | 695 | 76121 | 8.7 | nuclear fragile X mental retardation protein interacting protein 2 [Homo sapiens] |

| Filename XCorr DeltCN Conf% ObsM+H+ CalcM+H+ SpR ZScore Ion% # Sequence  | | | | | | | | | | | | |
| --- | --- | --- | --- | --- | --- | --- | --- | --- | --- | --- | --- | --- |
| \* | AstrinIP\_MS1\_022614\_01.04146.04146.2 | 2.188 | 0.2479 | 95.4% | 1338.0521 | 1338.3763 | 36 | 5.122 | 50.0% | 1 | K.TGYGELNGNAGER.E | 2 |
| \* | AstrinIP\_MS2\_022614\_01.05734.05734.2 | 3.891 | 0.5572 | 100.0% | 1515.5521 | 1515.7068 | 1 | 9.154 | 76.9% | 4 | R.VLNGNQQVVDTSLK.Q | 2 |
| \* | AstrinIP\_MS2\_022614\_01.06189.06189.2 | 3.6615 | 0.3702 | 100.0% | 1376.3522 | 1375.6233 | 1 | 5.936 | 81.8% | 3 | K.IMQQETSVPTLK.Q | 2 |
| \* | AstrinIP\_MS1\_022614\_01.06496.06496.2 | 2.605 | 0.1511 | 97.6% | 1074.3121 | 1074.3239 | 88 | 4.038 | 61.1% | 1 | R.LSQVPMSALK.S | 2 |
| \* | AstrinIP\_MS2\_022614\_01.13031.13031.3 | 4.2093 | 0.4148 | 100.0% | 3559.8843 | 3561.0437 | 4 | 6.527 | 20.3% | 1 | K.VMEVTFQGEYPATLVSQGAEIIPSGTEHPVFPK.A | 3 |
| \* | AstrinIP\_MS2\_022614\_01.09027.09027.2 | 2.473 | 0.1988 | 97.1% | 1379.0122 | 1379.5565 | 1 | 4.504 | 68.2% | 1 | K.RTS\*PQVLGSILK.S | 2 |

---

|  |  |  |  |  |  |  |  |  |
| --- | --- | --- | --- | --- | --- | --- | --- | --- |
| U | *gi|5453994|ref|NP\_006* | 6 | 9 | 13.5% | 631 | 71690 | 4.7 | RAD21 homolog [Homo sapiens] |

| Filename XCorr DeltCN Conf% ObsM+H+ CalcM+H+ SpR ZScore Ion% # Sequence  | | | | | | | | | | | | |
| --- | --- | --- | --- | --- | --- | --- | --- | --- | --- | --- | --- | --- |
|  | AstrinIP\_MS2\_022614\_01.07199.07199.2 | 2.6243 | 0.3176 | 99.7% | 1152.1721 | 1152.3823 | 1 | 5.897 | 75.0% | 1 | R.TSGHLLLGVVR.I | 2 |
| \* | AstrinIP\_MS2\_022614\_01.08532.08532.3 | 3.8482 | 0.237 | 99.5% | 1731.3544 | 1730.9794 | 9 | 4.598 | 42.9% | 1 | K.MAFRPGVVDLPEENR.E | 3 |
| \* | AstrinIP\_MS2\_022614\_01.12444.12444.2 | 3.4809 | 0.3009 | 100.0% | 1556.3121 | 1556.8075 | 1 | 7.196 | 70.8% | 2 | K.LFSLPAQPLWNNR.L | 2 |
| \* | AstrinIP\_MS2\_022614\_01.08723.08723.2 | 3.9221 | 0.4304 | 100.0% | 1512.1522 | 1512.6567 | 1 | 7.476 | 79.2% | 2 | R.DVIDEPIIEEPSR.L | 2 |
| \* | AstrinIP\_MS1\_022614\_01.03513.03513.3 | 5.7814 | 0.4667 | 100.0% | 2683.8843 | 2684.478 | 1 | 7.914 | 43.2% | 1 | K.EKEDDEEEEDEDASGGDQDQEER.R | 3 |
| \* | AstrinIP\_MS1\_022614\_01.04220.04220.2 | 2.6532 | 0.1966 | 98.6% | 1212.0521 | 1212.4124 | 14 | 4.501 | 72.2% | 2 | R.TQQMLHGLQR.A | 2 |

---

|  |  |  |  |  |  |  |  |  |
| --- | --- | --- | --- | --- | --- | --- | --- | --- |
| U | *gi|108936958|ref|NP\_0* | 3 | 4 | 13.2% | 342 | 38926 | 5.5 | WD-repeat protein [Homo sapiens] |

| Filename XCorr DeltCN Conf% ObsM+H+ CalcM+H+ SpR ZScore Ion% # Sequence  | | | | | | | | | | | | |
| --- | --- | --- | --- | --- | --- | --- | --- | --- | --- | --- | --- | --- |
| \* | AstrinIP\_MS2\_022614\_01.10679.10679.2 | 2.7882 | 0.3434 | 99.8% | 1485.7722 | 1484.6488 | 1 | 5.437 | 62.5% | 1 | R.LALGSFVEEYNNK.V | 2 |
| \* | AstrinIP\_MS1\_022614\_01.11452.11452.2 | 2.7373 | 0.301 | 99.3% | 1640.2522 | 1640.8333 | 1 | 6.028 | 50.0% | 2 | K.GVYPDLLATSGDYLR.V | 2 |
| \* | AstrinIP\_MS2\_022614\_02.05920.05920.3 | 3.4218 | 0.2848 | 99.6% | 2009.3043 | 2007.2535 | 1 | 6.111 | 39.1% | 1 | K.TQLIAHDKEVYDIAFSR.A | 3 |

---

|  |  |  |  |  |  |  |  |  |
| --- | --- | --- | --- | --- | --- | --- | --- | --- |
| U | *gi|6678271|ref|NP\_031* | 3 | 4 | 12.6% | 414 | 44740 | 6.2 | TAR DNA binding protein [Homo sapiens] |

| Filename XCorr DeltCN Conf% ObsM+H+ CalcM+H+ SpR ZScore Ion% # Sequence  | | | | | | | | | | | | |
| --- | --- | --- | --- | --- | --- | --- | --- | --- | --- | --- | --- | --- |
| \* | AstrinIP\_MS2\_022614\_01.03533.03533.2 | 3.7433 | 0.364 | 100.0% | 1282.3322 | 1282.4082 | 1 | 6.96 | 72.7% | 1 | R.KMDETDASSAVK.V | 2 |
| \* | AstrinIP\_MS1\_022614\_01.14837.14837.2 | 4.1535 | 0.3386 | 100.0% | 2592.612 | 2593.9514 | 1 | 7.384 | 47.6% | 1 | K.TTEQDLKEYFSTFGEVLMVQVK.K | 2 |
| \* | AstrinIP\_MS2\_022614\_01.07161.07161.2 | 4.3392 | 0.4953 | 100.0% | 1727.2322 | 1727.7928 | 1 | 7.669 | 73.5% | 2 | R.FGGNPGGFGNQGGFGNSR.G | 2 |

---

|  |  |  |  |  |  |  |  |  |
| --- | --- | --- | --- | --- | --- | --- | --- | --- |
| U | *gi|167234419|ref|NP\_0* | 10 | 18 | 12.4% | 955 | 108666 | 10.2 | thyroid hormone receptor associated protein 3 [Homo sapiens] |

| Filename XCorr DeltCN Conf% ObsM+H+ CalcM+H+ SpR ZScore Ion% # Sequence  | | | | | | | | | | | | |
| --- | --- | --- | --- | --- | --- | --- | --- | --- | --- | --- | --- | --- |
| \* | AstrinIP\_MS2\_022614\_01.09034.09034.2 | 2.9236 | 0.2719 | 99.8% | 1217.1122 | 1217.4099 | 2 | 5.969 | 70.0% | 1 | K.WEGLVYAPPGK.E | 2 |
| \* | AstrinIP\_MS1\_022614\_01.05121.05121.2 | 3.1206 | 0.4316 | 100.0% | 1623.8922 | 1623.7136 | 1 | 7.067 | 58.3% | 1 | R.KTEELEEESFPER.S | 2 |
| \* | AstrinIP\_MS2\_022614\_01.06642.06642.2 | 2.1089 | 0.301 | 97.7% | 1494.0521 | 1495.5396 | 1 | 5.849 | 72.7% | 1 | K.TEELEEESFPER.S | 2 |
| \* | AstrinIP\_MS1\_022614\_01.09563.09563.2 | 2.653 | 0.2884 | 99.1% | 2150.9722 | 2151.3557 | 4 | 4.811 | 41.7% | 1 | R.MDSFDEDLARPSGLLAQER.K | 2 |
| \* | AstrinIP\_MS2\_022614\_01.10174.10174.3 | 3.5003 | 0.344 | 100.0% | 2150.9944 | 2151.3557 | 6 | 5.705 | 29.2% | 2 | R.MDSFDEDLARPSGLLAQER.K | 3 |
| \* | AstrinIP\_MS1\_022614\_01.05577.05577.2 | 4.0094 | 0.4091 | 100.0% | 1530.2922 | 1530.6835 | 1 | 7.36 | 75.0% | 4 | R.SIFQHIQSAQSQR.S | 2 |
| \* | AstrinIP\_MS2\_022614\_01.10126.10126.3 | 5.008 | 0.5101 | 100.0% | 2043.6843 | 2043.3762 | 1 | 8.441 | 42.6% | 4 | R.SPSELFAQHIVTIVHHVK.E | 3 |
| \* | AstrinIP\_MS2\_022614\_01.03819.03819.2 | 3.6265 | 0.4557 | 100.0% | 1625.1522 | 1625.7588 | 1 | 8.464 | 61.5% | 2 | K.EHHFGSSGMTLHER.F | 2 |
| \* | AstrinIP\_MS1\_022614\_01.03630.03630.3 | 2.6599 | 0.2707 | 97.4% | 2033.6044 | 2033.1827 | 77 | 4.173 | 29.7% | 1 | K.HGLAHDEMKS\*PREPGYK.A | 3 |
| \* | AstrinIP\_MS2\_022614\_01.05992.05992.2 | 3.2942 | 0.3594 | 100.0% | 1693.0322 | 1693.7667 | 1 | 7.12 | 62.5% | 1 | R.NREEEWDPEYTPK.S | 2 |

---

|  |  |  |  |  |  |  |  |  |
| --- | --- | --- | --- | --- | --- | --- | --- | --- |
| U | *gi|11136628|ref|NP\_06* | 2 | 3 | 12.4% | 225 | 24764 | 4.7 | eukaryotic translation elongation factor 1 beta 2 [Homo sapiens] |
| U | *gi|83376130|ref|NP\_00* | 2 | 3 | 12.4% | 225 | 24764 | 4.7 | eukaryotic translation elongation factor 1 beta 2 [Homo sapiens] |
| U | *gi|4503477|ref|NP\_001* | 2 | 3 | 12.4% | 225 | 24764 | 4.7 | eukaryotic translation elongation factor 1 beta 2 [Homo sapiens] |

| Filename XCorr DeltCN Conf% ObsM+H+ CalcM+H+ SpR ZScore Ion% # Sequence  | | | | | | | | | | | | |
| --- | --- | --- | --- | --- | --- | --- | --- | --- | --- | --- | --- | --- |
|  | AstrinIP\_MS1\_022614\_01.11495.11495.2 | 3.893 | 0.3835 | 100.0% | 1604.6721 | 1604.8003 | 1 | 7.31 | 60.7% | 1 | K.SPAGLQVLNDYLADK.S | 2 |
|  | AstrinIP\_MS2\_022614\_02.06106.06106.2 | 3.1831 | 0.4547 | 100.0% | 1347.8522 | 1348.4985 | 1 | 7.126 | 66.7% | 2 | R.SIQADGLVWGSSK.L | 2 |

---

|  |  |  |  |  |  |  |  |  |
| --- | --- | --- | --- | --- | --- | --- | --- | --- |
| U | *gi|6715600|ref|NP\_002* | 21 | 35 | 12.3% | 2230 | 261137 | 5.4 | golgi autoantigen, golgin subfamily a, 4 [Homo sapiens] |

| Filename XCorr DeltCN Conf% ObsM+H+ CalcM+H+ SpR ZScore Ion% # Sequence  | | | | | | | | | | | | |
| --- | --- | --- | --- | --- | --- | --- | --- | --- | --- | --- | --- | --- |
| \* | AstrinIP\_MS2\_022614\_01.08705.08705.2 | 3.0561 | 0.233 | 99.8% | 1161.1921 | 1161.3379 | 1 | 5.636 | 83.3% | 1 | R.ILELESSLEK.S | 2 |
| \* | AstrinIP\_MS2\_022614\_01.13490.13490.2 | 4.3668 | 0.356 | 100.0% | 1639.7722 | 1639.9762 | 1 | 6.833 | 80.8% | 2 | K.ALKDQINQLELLLK.E | 2 |
| \* | AstrinIP\_MS2\_022614\_01.06682.06682.2 | 2.5753 | 0.2249 | 98.4% | 1448.4722 | 1449.6036 | 8 | 4.211 | 54.5% | 1 | K.AYEEQLAQLQQK.L | 2 |
| \* | AstrinIP\_MS1\_022614\_02.05332.05332.3 | 2.9716 | 0.2689 | 98.4% | 2120.4844 | 2120.3208 | 31 | 5.024 | 30.9% | 1 | K.LSAKEDSIHILNEEYETK.F | 3 |
| \* | AstrinIP\_MS2\_022614\_01.07236.07236.2 | 3.4497 | 0.3351 | 100.0% | 1375.0521 | 1375.5187 | 3 | 5.969 | 68.2% | 1 | K.ELENTALELSQK.E | 2 |
| \* | AstrinIP\_MS2\_022614\_01.06748.06748.2 | 3.2666 | 0.3389 | 100.0% | 1206.3722 | 1206.3385 | 1 | 5.861 | 80.0% | 2 | K.TNELINISSSK.T | 2 |
| \* | AstrinIP\_MS2\_022614\_01.08282.08282.2 | 2.334 | 0.235 | 98.1% | 1105.3922 | 1105.2303 | 378 | 4.65 | 50.0% | 1 | K.ADIESLVTEK.E | 2 |
| \* | AstrinIP\_MS1\_022614\_01.05811.05811.2 | 3.4135 | 0.311 | 100.0% | 1357.2922 | 1357.5646 | 1 | 5.451 | 80.0% | 3 | R.IMELEDHITQK.T | 2 |
| \* | AstrinIP\_MS2\_022614\_01.11535.11535.2 | 3.7969 | 0.3461 | 100.0% | 1388.2122 | 1388.6012 | 1 | 7.897 | 81.8% | 3 | K.TIEIESLNEVLK.N | 2 |
| \* | AstrinIP\_MS2\_022614\_01.09461.09461.2 | 3.645 | 0.424 | 100.0% | 1439.9922 | 1439.71 | 1 | 6.825 | 81.8% | 4 | K.ILTLENQVYSMK.A | 2 |
| \* | AstrinIP\_MS2\_022614\_01.03881.03881.2 | 3.7315 | 0.3453 | 100.0% | 1315.9521 | 1316.4105 | 2 | 7.101 | 68.2% | 1 | K.GTESHLSELNTK.L | 2 |
| \* | AstrinIP\_MS2\_022614\_01.04569.04569.3 | 3.574 | 0.3094 | 99.9% | 1894.3143 | 1894.0662 | 1 | 5.008 | 45.0% | 1 | R.VGQEKEETVSSHFEMR.C | 3 |
| \* | AstrinIP\_MS1\_022614\_01.05734.05734.2 | 2.672 | 0.2099 | 98.6% | 1287.3922 | 1287.5016 | 1 | 5.482 | 65.0% | 2 | K.YSLIVAQHVEK.E | 2 |
| \* | AstrinIP\_MS2\_022614\_01.09432.09432.2 | 3.7051 | 0.3954 | 100.0% | 1449.1921 | 1449.5602 | 1 | 6.457 | 77.3% | 1 | K.QNLENVFDDVQK.T | 2 |
| \* | AstrinIP\_MS2\_022614\_01.05579.05579.2 | 2.8027 | 0.2722 | 99.8% | 1160.0122 | 1160.3336 | 1 | 6.515 | 88.9% | 2 | K.LQALQQMDGR.N | 2 |
| \* | AstrinIP\_MS1\_022614\_01.04734.04734.2 | 3.6259 | 0.3027 | 100.0% | 1673.8121 | 1674.8021 | 1 | 6.742 | 84.6% | 1 | R.NKPTELLEENTEEK.S | 2 |
| \* | AstrinIP\_MS1\_022614\_01.04284.04284.2 | 3.3055 | 0.1097 | 98.9% | 1396.2322 | 1395.5986 | 3 | 4.125 | 70.0% | 1 | K.EHQQELEILKK.E | 2 |
| \* | AstrinIP\_MS2\_022614\_01.04368.04368.2 | 3.3644 | 0.1084 | 99.1% | 1372.4521 | 1373.5461 | 28 | 4.59 | 60.0% | 2 | K.IKQEQEDLELK.H | 2 |
| \* | AstrinIP\_MS2\_022614\_01.09270.09270.3 | 4.0047 | 0.3236 | 100.0% | 2438.3044 | 2439.6384 | 1 | 6.71 | 37.5% | 3 | K.AQEVEAELLESHQEETNQLLK.K | 3 |
| \* | AstrinIP\_MS2\_022614\_01.10337.10337.2 | 3.3436 | 0.1485 | 99.5% | 1345.8121 | 1345.4924 | 1 | 4.875 | 80.0% | 1 | R.DLQTQLEELQK.K | 2 |
| \* | AstrinIP\_MS2\_022614\_01.11483.11483.3 | 4.2838 | 0.3092 | 100.0% | 2673.5044 | 2673.9414 | 1 | 5.178 | 30.7% | 1 | K.GGNLYHTDVSLFGEPTEFEYLRK.V | 3 |

---

|  |  |  |  |  |  |  |  |  |
| --- | --- | --- | --- | --- | --- | --- | --- | --- |
| U | *gi|40254446|ref|NP\_00* | 7 | 9 | 12.3% | 780 | 90955 | 8.0 | Vasopressin-activated calcium-mobilizing receptor-1 [Homo sapiens] |

| Filename XCorr DeltCN Conf% ObsM+H+ CalcM+H+ SpR ZScore Ion% # Sequence  | | | | | | | | | | | | |
| --- | --- | --- | --- | --- | --- | --- | --- | --- | --- | --- | --- | --- |
| \* | AstrinIP\_MS1\_022614\_01.11626.11626.3 | 3.4206 | 0.187 | 96.0% | 2211.1143 | 2210.5989 | 3 | 4.427 | 36.8% | 1 | K.GSLQFEDKWDFMRPIVLK.L | 3 |
| \* | AstrinIP\_MS1\_022614\_01.10134.10134.3 | 3.2866 | 0.4335 | 100.0% | 1697.4844 | 1698.0153 | 4 | 6.984 | 38.5% | 1 | K.IHQALKEDILEFIK.Q | 3 |
| \* | AstrinIP\_MS1\_022614\_01.04566.04566.2 | 3.3269 | 0.3391 | 100.0% | 1340.0922 | 1340.5193 | 1 | 6.308 | 72.7% | 1 | R.VLSHQDDTALLK.A | 2 |
| \* | AstrinIP\_MS1\_022614\_01.13449.13449.2 | 3.2219 | 0.3803 | 100.0% | 1811.8322 | 1812.0906 | 1 | 6.619 | 57.1% | 2 | K.LMLDTWNESIFSNIK.N | 2 |
| \* | AstrinIP\_MS2\_022614\_02.07332.07332.2 | 3.0909 | 0.3652 | 100.0% | 1504.6721 | 1504.7263 | 1 | 6.346 | 61.5% | 1 | R.LGEAFDSQLVIGVR.E | 2 |
| \* | AstrinIP\_MS1\_022614\_01.07724.07724.2 | 3.0455 | 0.3224 | 99.8% | 1515.4122 | 1515.7496 | 1 | 6.787 | 79.2% | 2 | R.QVLLYEPQVNSPK.D | 2 |
| \* | AstrinIP\_MS2\_022614\_01.09060.09060.2 | 2.5302 | 0.258 | 99.2% | 1175.7122 | 1175.429 | 3 | 5.23 | 72.2% | 1 | R.TQEAIIQIMK.M | 2 |

---

|  |  |  |  |  |  |  |  |  |
| --- | --- | --- | --- | --- | --- | --- | --- | --- |
| U | *gi|15809016|ref|NP\_29* | 2 | 3 | 12.2% | 172 | 19779 | 4.8 | myosin regulatory light chain MRCL2 isoform A [Homo sapiens] |
| U | *gi|5453740|ref|NP\_006* | 2 | 3 | 12.3% | 171 | 19794 | 4.8 | myosin, light chain 12A, regulatory, non-sarcomeric [Homo sapiens] |
| U | *gi|222144328|ref|NP\_0* | 2 | 3 | 13.6% | 154 | 17757 | 4.4 | myosin regulatory light chain MRCL2 isoform B [Homo sapiens] |
| U | *gi|222144326|ref|NP\_0* | 2 | 3 | 12.2% | 172 | 19779 | 4.8 | myosin regulatory light chain MRCL2 isoform A [Homo sapiens] |
| U | *gi|222144324|ref|NP\_0* | 2 | 3 | 12.2% | 172 | 19779 | 4.8 | myosin regulatory light chain MRCL2 isoform A [Homo sapiens] |

| Filename XCorr DeltCN Conf% ObsM+H+ CalcM+H+ SpR ZScore Ion% # Sequence  | | | | | | | | | | | | |
| --- | --- | --- | --- | --- | --- | --- | --- | --- | --- | --- | --- | --- |
|  | AstrinIP\_MS2\_022614\_01.08699.08699.2 | 3.0903 | 0.3516 | 100.0% | 1417.2522 | 1416.4839 | 1 | 6.42 | 80.0% | 1 | R.FTDEEVDELYR.E | 2 |
|  | AstrinIP\_MS2\_022614\_01.09800.09800.2 | 2.9643 | 0.2305 | 99.7% | 1261.2322 | 1261.3794 | 1 | 6.833 | 77.8% | 2 | K.GNFNYIEFTR.I | 2 |

---

|  |  |  |  |  |  |  |  |  |
| --- | --- | --- | --- | --- | --- | --- | --- | --- |
| U | *gi|157266292|ref|NP\_0* | 5 | 9 | 11.9% | 528 | 56812 | 5.9 | intestinal alkaline phosphatase precursor [Homo sapiens] |

| Filename XCorr DeltCN Conf% ObsM+H+ CalcM+H+ SpR ZScore Ion% # Sequence  | | | | | | | | | | | | |
| --- | --- | --- | --- | --- | --- | --- | --- | --- | --- | --- | --- | --- |
| \* | AstrinIP\_MS2\_022614\_01.14844.14844.2 | 3.9496 | 0.5171 | 100.0% | 1957.4521 | 1958.3085 | 1 | 8.725 | 52.8% | 1 | K.NLILFLGDGLGVPTVTATR.I | 2 |
| \* | AstrinIP\_MS1\_022614\_01.07722.07722.2 | 3.3045 | 0.3756 | 100.0% | 1321.5922 | 1320.4911 | 1 | 6.143 | 75.0% | 4 | K.ANFQTIGLSAAAR.F | 2 |
|  | AstrinIP\_MS2\_022614\_01.03719.03719.1 | 1.804 | 0.2646 | 99.1% | 919.53 | 920.0537 | 5 | 5.674 | 56.2% | 1 | K.SVGVVTTTR.V | 1 |
| \* | AstrinIP\_MS2\_022614\_01.07953.07953.2 | 3.5495 | 0.5264 | 100.0% | 1484.2122 | 1484.5829 | 1 | 8.532 | 75.0% | 2 | R.NWYSDADMPASAR.Q | 2 |
|  | AstrinIP\_MS2\_022614\_01.12192.12192.2 | 2.1268 | 0.3616 | 99.3% | 1101.2322 | 1101.2908 | 1 | 5.512 | 81.2% | 1 | K.NLVQEWLAK.H | 2 |

---

|  |  |  |  |  |  |  |  |  |
| --- | --- | --- | --- | --- | --- | --- | --- | --- |
| U | *gi|16753203|ref|NP\_03* | 5 | 7 | 11.7% | 589 | 62519 | 5.1 | ubiquilin 1 isoform 1 [Homo sapiens] |
| U | *gi|16753205|ref|NP\_44* | 5 | 7 | 12.3% | 561 | 59220 | 5.1 | ubiquilin 1 isoform 2 [Homo sapiens] |

| Filename XCorr DeltCN Conf% ObsM+H+ CalcM+H+ SpR ZScore Ion% # Sequence  | | | | | | | | | | | | |
| --- | --- | --- | --- | --- | --- | --- | --- | --- | --- | --- | --- | --- |
|  | AstrinIP\_MS2\_022614\_01.10722.10722.2 | 2.575 | 0.3377 | 99.7% | 1429.3322 | 1429.6592 | 11 | 6.391 | 54.2% | 1 | K.SHTDQLVLIFAGK.I | 2 |
|  | AstrinIP\_MS1\_022614\_01.10032.10032.2 | 4.012 | 0.355 | 100.0% | 1812.4321 | 1813.1865 | 2 | 6.119 | 60.7% | 2 | R.QLIMANPQMQQLIQR.N | 2 |
|  | AstrinIP\_MS2\_022614\_01.08799.08799.3 | 3.152 | 0.2059 | 97.0% | 1781.5443 | 1782.0424 | 115 | 3.907 | 37.5% | 1 | R.NPEISHMLNNPDIMR.Q | 3 |
|  | AstrinIP\_MS2\_022614\_01.08043.08043.2 | 3.0479 | 0.5082 | 100.0% | 1239.0922 | 1239.5265 | 1 | 8.692 | 77.8% | 1 | R.NPAMMQEMMR.N | 2 |
|  | AstrinIP\_MS2\_022614\_01.12603.12603.2 | 3.8881 | 0.4594 | 100.0% | 1910.6522 | 1911.1858 | 1 | 7.456 | 53.3% | 2 | R.FQQQLEQLSAMGFLNR.E | 2 |

---

|  |  |  |  |  |  |  |  |  |
| --- | --- | --- | --- | --- | --- | --- | --- | --- |
| U | *gi|157384956|ref|NP\_0* | 4 | 7 | 11.6% | 319 | 34083 | 9.0 | nucleotide binding protein-like [Homo sapiens] |

| Filename XCorr DeltCN Conf% ObsM+H+ CalcM+H+ SpR ZScore Ion% # Sequence  | | | | | | | | | | | | |
| --- | --- | --- | --- | --- | --- | --- | --- | --- | --- | --- | --- | --- |
| \* | AstrinIP\_MS1\_022614\_01.11578.11578.2 | 4.0832 | 0.5135 | 100.0% | 1643.4722 | 1643.9207 | 1 | 9.03 | 70.0% | 3 | K.AIGLLDVDVYGPSVPK.M | 2 |
| \* | AstrinIP\_MS1\_022614\_01.13358.13358.3 | 5.4334 | 0.4216 | 100.0% | 2314.6143 | 2314.777 | 1 | 8.22 | 46.2% | 2 | R.KLAQTLGLEVLGDIPLHLNIR.E | 3 |
| \* | AstrinIP\_MS2\_022614\_01.14799.14799.2 | 4.3382 | 0.527 | 100.0% | 2186.372 | 2186.6028 | 1 | 8.393 | 50.0% | 1 | K.LAQTLGLEVLGDIPLHLNIR.E | 2 |
| \* | AstrinIP\_MS1\_022614\_01.14272.14272.3 | 3.9183 | 0.2754 | 99.7% | 2186.7844 | 2186.6028 | 1 | 6.316 | 36.8% | 1 | K.LAQTLGLEVLGDIPLHLNIR.E | 3 |

---

|  |  |  |  |  |  |  |  |  |
| --- | --- | --- | --- | --- | --- | --- | --- | --- |
| U | *gi|18087819|ref|NP\_54* | 2 | 2 | 11.3% | 275 | 32363 | 8.4 | hypothetical protein LOC91283 [Homo sapiens] |

| Filename XCorr DeltCN Conf% ObsM+H+ CalcM+H+ SpR ZScore Ion% # Sequence  | | | | | | | | | | | | |
| --- | --- | --- | --- | --- | --- | --- | --- | --- | --- | --- | --- | --- |
| \* | AstrinIP\_MS2\_022614\_01.08920.08920.3 | 3.5661 | 0.197 | 97.9% | 2087.3943 | 2088.287 | 5 | 4.847 | 38.2% | 1 | R.TWQALAHEYNSQPSVSLR.D | 3 |
| \* | AstrinIP\_MS1\_022614\_01.08547.08547.2 | 2.4526 | 0.3326 | 99.3% | 1418.3522 | 1418.5901 | 2 | 6.754 | 62.5% | 1 | R.SVS\*PLLSTHVLGK.E | 2 |

---

|  |  |  |  |  |  |  |  |  |
| --- | --- | --- | --- | --- | --- | --- | --- | --- |
| U | *gi|116063573|ref|NP\_0* | 22 | 28 | 11.0% | 2639 | 280016 | 6.0 | filamin A, alpha isoform 1 [Homo sapiens] |
| U | *gi|160420317|ref|NP\_0* | 22 | 29 | 10.9% | 2647 | 280737 | 6.1 | filamin A, alpha isoform 2 [Homo sapiens] |

| Filename XCorr DeltCN Conf% ObsM+H+ CalcM+H+ SpR ZScore Ion% # Sequence  | | | | | | | | | | | | |
| --- | --- | --- | --- | --- | --- | --- | --- | --- | --- | --- | --- | --- |
|  | AstrinIP\_MS1\_022614\_01.08790.08790.2 | 2.9289 | 0.3585 | 99.8% | 1416.1122 | 1416.5742 | 1 | 6.888 | 79.2% | 2 | R.IANLQTDLSDGLR.L | 2 |
|  | AstrinIP\_MS2\_022614\_01.10980.10980.2 | 2.7839 | 0.3021 | 99.8% | 1286.4922 | 1286.5167 | 11 | 5.586 | 70.0% | 1 | K.LPQLPITNFSR.D | 2 |
|  | AstrinIP\_MS1\_022614\_01.03918.03918.2 | 2.4458 | 0.16 | 97.3% | 1109.2122 | 1109.2272 | 1 | 5.051 | 75.0% | 1 | K.RAEFTVETR.S | 2 |
|  | AstrinIP\_MS2\_022614\_01.06900.06900.2 | 2.5403 | 0.2421 | 98.6% | 1283.7922 | 1284.5443 | 1 | 4.908 | 59.1% | 1 | K.VTVLFAGQHIAK.S | 2 |
|  | AstrinIP\_MS2\_022614\_01.08183.08183.2 | 2.321 | 0.3294 | 99.6% | 1084.3722 | 1084.2139 | 5 | 6.311 | 68.8% | 1 | K.SPFEVYVDK.S | 2 |
|  | AstrinIP\_MS1\_022614\_01.04782.04782.2 | 5.0365 | 0.535 | 100.0% | 1653.5721 | 1653.8326 | 1 | 9.767 | 71.9% | 2 | K.VTAQGPGLEPSGNIANK.T | 2 |
|  | AstrinIP\_MS2\_022614\_01.05751.05751.3 | 2.1644 | 0.3564 | 98.5% | 1646.4844 | 1647.8687 | 1 | 5.065 | 36.7% | 1 | K.TGVAVNKPAEFTVDAK.H | 3 |
|  | AstrinIP\_MS2\_022614\_01.05758.05758.2 | 4.1904 | 0.4709 | 100.0% | 1648.3322 | 1647.8687 | 1 | 7.886 | 70.0% | 1 | K.TGVAVNKPAEFTVDAK.H | 2 |
|  | AstrinIP\_MS2\_022614\_01.04971.04971.3 | 3.7162 | 0.336 | 100.0% | 1700.2743 | 1699.9475 | 4 | 6.202 | 35.0% | 1 | R.TGVELGKPTHFTVNAK.A | 3 |
|  | AstrinIP\_MS2\_022614\_01.05974.05974.2 | 3.3079 | 0.4338 | 100.0% | 1785.1322 | 1785.9078 | 1 | 6.396 | 57.1% | 1 | R.DVDIIDHHDNTYTVK.Y | 2 |
|  | AstrinIP\_MS2\_022614\_01.08997.08997.2 | 4.8457 | 0.6099 | 100.0% | 2286.5923 | 2287.5774 | 1 | 10.03 | 57.1% | 1 | K.YTPVQQGPVGVNVTYGGDPIPK.S | 2 |
|  | AstrinIP\_MS2\_022614\_01.05609.05609.2 | 2.8304 | 0.4268 | 100.0% | 1225.8722 | 1226.2854 | 4 | 6.745 | 65.0% | 1 | R.EATTEFSVDAR.A | 2 |
|  | AstrinIP\_MS2\_022614\_01.06573.06573.2 | 4.9325 | 0.5335 | 100.0% | 1764.5721 | 1764.8894 | 1 | 8.807 | 66.7% | 2 | R.VANPSGNLTETYVQDR.G | 2 |
|  | AstrinIP\_MS2\_022614\_02.05205.05205.2 | 3.5389 | 0.4303 | 100.0% | 1570.3722 | 1571.7275 | 5 | 7.341 | 38.2% | 1 | R.GAGTGGLGLAVEGPSEAK.M | 2 |
|  | AstrinIP\_MS2\_022614\_01.06959.06959.2 | 2.276 | 0.388 | 99.5% | 1435.9321 | 1435.5767 | 1 | 6.927 | 70.8% | 2 | R.ANLPQSFQVDTSK.A | 2 |
|  | AstrinIP\_MS2\_022614\_01.05934.05934.2 | 2.9533 | 0.2664 | 99.5% | 1503.6921 | 1503.7565 | 1 | 4.702 | 61.5% | 1 | R.FVPAEMGTHTVSVK.Y | 2 |
|  | AstrinIP\_MS2\_022614\_01.12044.12044.2 | 2.4929 | 0.2934 | 98.8% | 1533.1322 | 1534.7117 | 49 | 4.988 | 46.2% | 1 | R.AEAGVPAEFSIWTR.E | 2 |
|  | AstrinIP\_MS2\_022614\_02.05300.05300.2 | 3.8351 | 0.4228 | 100.0% | 1427.0521 | 1427.5974 | 1 | 6.895 | 56.7% | 2 | R.EAGAGGLAIAVEGPSK.A | 2 |
|  | AstrinIP\_MS1\_022614\_01.06779.06779.2 | 2.9034 | 0.3725 | 99.9% | 1380.0322 | 1380.5455 | 1 | 5.361 | 58.3% | 1 | K.YGGPYHIGGSPFK.A | 2 |
|  | AstrinIP\_MS2\_022614\_02.05852.05852.3 | 3.3391 | 0.3306 | 99.8% | 2200.7644 | 2201.4412 | 1 | 5.497 | 35.5% | 2 | R.LVSNHSLHETSSVFVDSLTK.A | 3 |
|  | AstrinIP\_MS2\_022614\_01.07121.07121.2 | 2.292 | 0.2908 | 98.1% | 1435.4722 | 1435.6873 | 1 | 5.7 | 53.8% | 1 | K.AGNNMLLVGVHGPR.T | 2 |
|  | AstrinIP\_MS2\_022614\_01.07116.07116.3 | 2.3732 | 0.2738 | 95.7% | 1435.5543 | 1435.6873 | 1 | 5.736 | 40.4% | 1 | K.AGNNMLLVGVHGPR.T | 3 |

---

|  |  |  |  |  |  |  |  |  |
| --- | --- | --- | --- | --- | --- | --- | --- | --- |
| U | *gi|4503481|ref|NP\_001* | 4 | 7 | 11.0% | 437 | 50119 | 6.7 | eukaryotic translation elongation factor 1 gamma [Homo sapiens] |

| Filename XCorr DeltCN Conf% ObsM+H+ CalcM+H+ SpR ZScore Ion% # Sequence  | | | | | | | | | | | | |
| --- | --- | --- | --- | --- | --- | --- | --- | --- | --- | --- | --- | --- |
| \* | AstrinIP\_MS2\_022614\_01.06585.06585.2 | 4.1658 | 0.5029 | 100.0% | 1348.3121 | 1348.5448 | 1 | 8.592 | 75.0% | 3 | K.ALIAAQYSGAQVR.V | 2 |
| \* | AstrinIP\_MS1\_022614\_01.11775.11775.2 | 2.9904 | 0.305 | 99.8% | 1610.4122 | 1610.8218 | 1 | 5.26 | 68.2% | 1 | R.WFLTCINQPQFR.A | 2 |
| \* | AstrinIP\_MS2\_022614\_01.08891.08891.2 | 2.4378 | 0.397 | 99.8% | 1242.0322 | 1242.4172 | 1 | 7.771 | 61.1% | 2 | K.STFVLDEFKR.K | 2 |
| \* | AstrinIP\_MS2\_022614\_01.06008.06008.2 | 3.0159 | 0.3776 | 100.0% | 1445.1122 | 1445.5693 | 4 | 6.425 | 62.5% | 1 | K.LDPGSEETQTLVR.E | 2 |

---

|  |  |  |  |  |  |  |  |  |
| --- | --- | --- | --- | --- | --- | --- | --- | --- |
| U | *gi|16933546|ref|NP\_44* | 3 | 4 | 11.0% | 317 | 34274 | 6.0 | ribosomal protein P0 [Homo sapiens] |
| U | *gi|4506667|ref|NP\_000* | 3 | 4 | 11.0% | 317 | 34274 | 6.0 | ribosomal protein P0 [Homo sapiens] |

| Filename XCorr DeltCN Conf% ObsM+H+ CalcM+H+ SpR ZScore Ion% # Sequence  | | | | | | | | | | | | |
| --- | --- | --- | --- | --- | --- | --- | --- | --- | --- | --- | --- | --- |
|  | AstrinIP\_MS2\_022614\_01.10248.10248.2 | 3.1396 | 0.3185 | 100.0% | 1217.6721 | 1218.4357 | 1 | 6.862 | 88.9% | 1 | K.IIQLLDDYPK.C | 2 |
|  | AstrinIP\_MS2\_022614\_01.06737.06737.2 | 3.1286 | 0.3164 | 99.8% | 1369.7722 | 1370.6531 | 2 | 5.176 | 66.7% | 2 | R.DMLLANKVPAAAR.A | 2 |
|  | AstrinIP\_MS2\_022614\_01.13092.13092.2 | 3.2187 | 0.3696 | 100.0% | 1314.4122 | 1314.5242 | 4 | 7.799 | 59.1% | 1 | K.TSFFQALGITTK.I | 2 |

---

|  |  |  |  |  |  |  |  |  |
| --- | --- | --- | --- | --- | --- | --- | --- | --- |
| U | *gi|34419635|ref|NP\_00* | 9 | 40 | 10.9% | 643 | 71028 | 6.1 | heat shock 70kDa protein 6 (HSP70B') [Homo sapiens] |

| Filename XCorr DeltCN Conf% ObsM+H+ CalcM+H+ SpR ZScore Ion% # Sequence  | | | | | | | | | | | | |
| --- | --- | --- | --- | --- | --- | --- | --- | --- | --- | --- | --- | --- |
|  | AstrinIP\_MS2\_022614\_01.07734.07734.1 | 2.2714 | 0.4224 | 100.0% | 1487.62 | 1488.5939 | 1 | 6.368 | 50.0% | 5 | R.TTPSYVAFTDTER.L | 11111 |
|  | AstrinIP\_MS1\_022614\_02.04868.04868.2 | 3.849 | 0.5598 | 100.0% | 1489.2922 | 1488.5939 | 1 | 9.39 | 79.2% | 10 | R.TTPSYVAFTDTER.L | 22222 |
|  | AstrinIP\_MS2\_022614\_01.10839.10839.2 | 5.2925 | 0.516 | 100.0% | 1688.1122 | 1688.9213 | 1 | 10.5 | 83.3% | 6 | R.IINEPTAAAIAYGLDR.R | 22 |
|  | AstrinIP\_MS2\_022614\_01.04504.04504.2 | 4.2557 | 0.5152 | 100.0% | 1676.1522 | 1676.6964 | 1 | 8.323 | 66.7% | 3 | K.ATAGDTHLGGEDFDNR.L | 222 |
|  | AstrinIP\_MS1\_022614\_01.09669.09669.2 | 2.8156 | 0.2188 | 99.1% | 1544.7722 | 1543.6855 | 1 | 4.313 | 63.6% | 1 | R.ARFEELCSDLFR.S | 22 |
|  | AstrinIP\_MS1\_022614\_01.10366.10366.2 | 3.1778 | 0.4564 | 100.0% | 1316.2522 | 1316.4193 | 1 | 6.925 | 83.3% | 1 | R.FEELCSDLFR.S | 22 |
|  | AstrinIP\_MS1\_022614\_01.09870.09870.1 | 2.2447 | 0.2928 | 100.0% | 1081.36 | 1082.2444 | 1 | 4.936 | 75.0% | 4 | K.LLQDFFNGK.E | 111 |
|  | AstrinIP\_MS2\_022614\_01.10502.10502.2 | 3.0573 | 0.3647 | 99.9% | 1082.0922 | 1082.2444 | 1 | 5.829 | 81.2% | 5 | K.LLQDFFNGK.E | 222 |
|  | AstrinIP\_MS2\_022614\_01.10005.10005.2 | 3.8674 | 0.1637 | 99.7% | 1567.3722 | 1566.7972 | 1 | 4.208 | 70.8% | 5 | K.LLQDFFNGKELNK.S | 222 |

Similarities:
gi|5729877|ref|NP\_006(5:4)  
gi|167466173|ref|NP\_0(6:3)  
gi|124256496|ref|NP\_0(3:6)  
gi|13676857|ref|NP\_06(5:4)  

---

|  |  |  |  |  |  |  |  |  |
| --- | --- | --- | --- | --- | --- | --- | --- | --- |
| U | *gi|55770834|ref|NP\_05* | 23 | 27 | 10.6% | 3114 | 357527 | 5.1 | centromere protein F [Homo sapiens] |

| Filename XCorr DeltCN Conf% ObsM+H+ CalcM+H+ SpR ZScore Ion% # Sequence  | | | | | | | | | | | | |
| --- | --- | --- | --- | --- | --- | --- | --- | --- | --- | --- | --- | --- |
| \* | AstrinIP\_MS2\_022614\_01.12219.12219.2 | 3.9069 | 0.2971 | 100.0% | 1491.3121 | 1491.6842 | 1 | 5.876 | 79.2% | 1 | R.QFQLDSLEAALQK.Q | 2 |
| \* | AstrinIP\_MS2\_022614\_01.06796.06796.2 | 4.8066 | 0.487 | 100.0% | 1649.2922 | 1649.7599 | 1 | 8.415 | 80.8% | 1 | R.HQASSSVFSWQQEK.T | 2 |
| \* | AstrinIP\_MS2\_022614\_01.04968.04968.2 | 2.386 | 0.1921 | 97.9% | 1129.2522 | 1129.3018 | 4 | 5.015 | 75.0% | 1 | R.NKINELELR.L | 2 |
| \* | AstrinIP\_MS2\_022614\_01.04799.04799.2 | 3.5876 | 0.3746 | 100.0% | 1416.9922 | 1417.559 | 1 | 6.586 | 81.8% | 1 | K.INQQENSLTLEK.L | 2 |
| \* | AstrinIP\_MS2\_022614\_02.05666.05666.3 | 3.1101 | 0.3602 | 100.0% | 1706.6044 | 1705.9238 | 3 | 5.297 | 36.5% | 1 | R.TLEMDRENLSVEIR.N | 3 |
| \* | AstrinIP\_MS2\_022614\_01.06034.06034.2 | 3.486 | 0.2567 | 99.8% | 1585.6721 | 1585.711 | 1 | 6.736 | 65.4% | 1 | K.TSQLTGQVEDLEHK.L | 2 |
| \* | AstrinIP\_MS2\_022614\_01.03504.03504.3 | 3.2259 | 0.3995 | 100.0% | 1601.3344 | 1600.6854 | 1 | 6.382 | 46.2% | 1 | K.SKDASLVTNEDHQR.S | 3 |
| \* | AstrinIP\_MS2\_022614\_02.06120.06120.2 | 2.6045 | 0.3687 | 99.8% | 1382.2122 | 1382.5101 | 2 | 5.452 | 63.6% | 1 | R.VDSLEFSLESQK.Q | 2 |
| \* | AstrinIP\_MS2\_022614\_01.09201.09201.3 | 5.044 | 0.4236 | 100.0% | 2498.5144 | 2499.821 | 1 | 6.888 | 32.5% | 1 | K.ELQLLNDKVETEQAEIQELKK.S | 3 |
| \* | AstrinIP\_MS2\_022614\_01.16414.16414.3 | 3.5923 | 0.299 | 99.6% | 2641.9143 | 2640.991 | 1 | 5.404 | 28.4% | 1 | K.SNHLLEDSLKELQLLSETLSLEK.K | 3 |
| \* | AstrinIP\_MS1\_022614\_01.13834.13834.2 | 2.8033 | 0.1754 | 97.4% | 1900.6322 | 1901.1882 | 17 | 4.645 | 43.3% | 1 | R.NQNLMLELETVQQALR.S | 2 |
| \* | AstrinIP\_MS1\_022614\_01.09215.09215.2 | 4.4002 | 0.4159 | 100.0% | 1703.7722 | 1703.9049 | 1 | 7.688 | 73.1% | 2 | K.EVNDLLQENEQLMK.V | 2 |
| \* | AstrinIP\_MS2\_022614\_02.10229.10229.2 | 2.8436 | 0.226 | 98.6% | 2195.0522 | 2194.5078 | 9 | 5.813 | 33.3% | 1 | K.EISLDSYNAQLVQLEAMLR.N | 2 |
| \* | AstrinIP\_MS1\_022614\_01.10536.10536.2 | 3.4556 | 0.2576 | 99.9% | 1415.2722 | 1414.6135 | 1 | 5.887 | 77.3% | 2 | K.MSELQTYVDSLK.A | 2 |
| \* | AstrinIP\_MS2\_022614\_01.06882.06882.2 | 2.5738 | 0.394 | 99.9% | 1190.9922 | 1191.4283 | 6 | 6.722 | 55.6% | 1 | R.KVESLLNEMK.E | 2 |
| \* | AstrinIP\_MS2\_022614\_02.04900.04900.3 | 4.0269 | 0.3905 | 100.0% | 2132.2144 | 2132.3728 | 1 | 6.096 | 33.8% | 1 | K.SLLEEKEQAEIQIKEESK.T | 3 |
| \* | AstrinIP\_MS2\_022614\_01.08626.08626.2 | 2.735 | 0.3264 | 99.8% | 1275.1721 | 1275.506 | 1 | 6.075 | 70.0% | 1 | K.TAVEMLQNQLK.E | 2 |
| \* | AstrinIP\_MS2\_022614\_01.04439.04439.3 | 5.3937 | 0.4838 | 100.0% | 2298.1743 | 2299.4563 | 1 | 8.019 | 43.8% | 2 | R.TNQEHAALEAENSKGEVETLK.A | 3 |
| \* | AstrinIP\_MS2\_022614\_01.06033.06033.2 | 3.3816 | 0.3624 | 100.0% | 1454.2322 | 1454.6255 | 1 | 6.02 | 68.2% | 1 | K.LSQVEGEHQLWK.E | 2 |
| \* | AstrinIP\_MS2\_022614\_01.12488.12488.2 | 3.9586 | 0.3975 | 100.0% | 1797.4122 | 1796.9719 | 1 | 6.722 | 56.7% | 1 | K.NASLQDTLEVLQSSYK.N | 2 |
| \* | AstrinIP\_MS2\_022614\_01.06186.06186.2 | 2.9869 | 0.4137 | 100.0% | 1334.3121 | 1334.4229 | 1 | 6.673 | 68.2% | 1 | K.TAELQEELSGEK.N | 2 |
| \* | AstrinIP\_MS2\_022614\_02.10282.10282.2 | 4.0089 | 0.2197 | 100.0% | 1470.0922 | 1469.7618 | 1 | 6.674 | 75.0% | 2 | R.LAGELQLLLEEIK.S | 2 |
| \* | AstrinIP\_MS1\_022614\_01.04268.04268.2 | 2.1603 | 0.2467 | 96.5% | 1244.6921 | 1245.3751 | 221 | 4.307 | 55.0% | 1 | K.ELSQLQAAQEK.Q | 2 |

---

|  |  |  |  |  |  |  |  |  |
| --- | --- | --- | --- | --- | --- | --- | --- | --- |
| U | *gi|4504511|ref|NP\_001* | 3 | 5 | 10.3% | 397 | 44868 | 7.1 | DnaJ (Hsp40) homolog, subfamily A, member 1 [Homo sapiens] |

| Filename XCorr DeltCN Conf% ObsM+H+ CalcM+H+ SpR ZScore Ion% # Sequence  | | | | | | | | | | | | |
| --- | --- | --- | --- | --- | --- | --- | --- | --- | --- | --- | --- | --- |
| \* | AstrinIP\_MS1\_022614\_01.07874.07874.2 | 2.7106 | 0.3834 | 99.8% | 1451.8722 | 1452.6042 | 1 | 6.218 | 70.8% | 2 | K.QISQAYEVLSDAK.K | 2 |
| \* | AstrinIP\_MS2\_022614\_01.05369.05369.2 | 3.2895 | 0.3606 | 100.0% | 1393.2922 | 1393.6696 | 2 | 6.736 | 62.5% | 2 | R.TIVITSHPGQIVK.H | 2 |
| \* | AstrinIP\_MS2\_022614\_01.03443.03443.3 | 3.7538 | 0.3388 | 100.0% | 1871.3043 | 1869.8607 | 2 | 5.785 | 39.3% | 1 | R.HYNGEAYEDDEHHPR.G | 3 |

---

|  |  |  |  |  |  |  |  |  |
| --- | --- | --- | --- | --- | --- | --- | --- | --- |
| U | *gi|16905517|ref|NP\_47* | 2 | 2 | 10.3% | 262 | 31301 | 11.3 | FUS interacting protein (serine-arginine rich) 1 isoform 2 [Homo sapiens] |
| U | *gi|5730079|ref|NP\_006* | 2 | 2 | 14.8% | 183 | 22222 | 10.3 | FUS interacting protein (serine-arginine rich) 1 isoform 1 [Homo sapiens] |
| U | *gi|169161980|ref|XP\_0* | 2 | 2 | 14.9% | 181 | 22022 | 10.3 | PREDICTED: hypothetical protein, partial [Homo sapiens] |
| U | *gi|169161109|ref|XP\_0* | 2 | 2 | 14.8% | 183 | 22222 | 10.3 | PREDICTED: hypothetical protein LOC642558 [Homo sapiens] |
| U | *gi|169161107|ref|XP\_0* | 2 | 2 | 10.3% | 262 | 31301 | 11.3 | PREDICTED: hypothetical protein LOC642558 [Homo sapiens] |

| Filename XCorr DeltCN Conf% ObsM+H+ CalcM+H+ SpR ZScore Ion% # Sequence  | | | | | | | | | | | | |
| --- | --- | --- | --- | --- | --- | --- | --- | --- | --- | --- | --- | --- |
|  | AstrinIP\_MS2\_022614\_01.14265.14265.2 | 3.5672 | 0.3256 | 100.0% | 1918.7922 | 1918.1992 | 1 | 5.901 | 50.0% | 1 | R.YGPIVDVYVPLDFYTR.R | 2 |
|  | AstrinIP\_MS2\_022614\_01.10325.10325.2 | 2.9723 | 0.353 | 100.0% | 1331.8121 | 1331.4705 | 1 | 5.667 | 65.0% | 1 | R.GFAYVQFEDVR.D | 2 |

---

|  |  |  |  |  |  |  |  |  |
| --- | --- | --- | --- | --- | --- | --- | --- | --- |
| U | *gi|5031877|ref|NP\_005* | 4 | 4 | 10.2% | 586 | 66408 | 5.2 | lamin B1 [Homo sapiens] |

| Filename XCorr DeltCN Conf% ObsM+H+ CalcM+H+ SpR ZScore Ion% # Sequence  | | | | | | | | | | | | |
| --- | --- | --- | --- | --- | --- | --- | --- | --- | --- | --- | --- | --- |
| \* | AstrinIP\_MS2\_022614\_01.07238.07238.2 | 2.562 | 0.2144 | 98.4% | 1252.1322 | 1252.3666 | 6 | 5.07 | 65.0% | 1 | K.ALYETELADAR.R | 2 |
| \* | AstrinIP\_MS2\_022614\_01.04943.04943.2 | 3.0175 | 0.2215 | 99.5% | 1174.0922 | 1174.3396 | 1 | 5.579 | 77.3% | 1 | K.DAALATALGDKK.S | 2 |
| \* | AstrinIP\_MS1\_022614\_01.16781.16781.3 | 4.0051 | 0.4342 | 100.0% | 2528.5444 | 2529.804 | 1 | 6.387 | 27.2% | 1 | K.SLEGDLEDLKDQIAQLEASLAAAK.K | 3 |
| \* | AstrinIP\_MS2\_022614\_01.08865.08865.2 | 3.0333 | 0.2827 | 99.8% | 1446.3522 | 1447.6287 | 1 | 5.653 | 66.7% | 1 | R.IESLSSQLSNLQK.E | 2 |

---

|  |  |  |  |  |  |  |  |  |
| --- | --- | --- | --- | --- | --- | --- | --- | --- |
| U | *gi|46367787|ref|NP\_00* | 4 | 8 | 10.1% | 636 | 70671 | 9.5 | poly(A) binding protein, cytoplasmic 1 [Homo sapiens] |

| Filename XCorr DeltCN Conf% ObsM+H+ CalcM+H+ SpR ZScore Ion% # Sequence  | | | | | | | | | | | | |
| --- | --- | --- | --- | --- | --- | --- | --- | --- | --- | --- | --- | --- |
|  | AstrinIP\_MS2\_022614\_01.10727.10727.2 | 3.323 | 0.5001 | 100.0% | 1267.1122 | 1267.4828 | 1 | 7.854 | 80.0% | 1 | R.ALDTMNFDVIK.G | 2 |
| \* | AstrinIP\_MS1\_022614\_01.07774.07774.2 | 2.97 | 0.3567 | 99.9% | 1413.3922 | 1413.6134 | 3 | 5.186 | 58.3% | 1 | R.KEFSPFGTITSAK.V | 2 |
|  | AstrinIP\_MS2\_022614\_01.15636.15636.3 | 4.0994 | 0.3497 | 100.0% | 2741.6042 | 2742.175 | 1 | 5.693 | 32.6% | 3 | K.ITGMLLEIDNSELLHMLESPESLR.S | 3 |
|  | AstrinIP\_MS1\_022614\_01.06351.06351.3 | 5.0081 | 0.4436 | 100.0% | 1695.2344 | 1694.9285 | 1 | 8.105 | 55.0% | 3 | R.SKVDEAVAVLQAHQAK.E | 3 |

---

|  |  |  |  |  |  |  |  |  |
| --- | --- | --- | --- | --- | --- | --- | --- | --- |
| U | *gi|5031753|ref|NP\_005* | 3 | 7 | 9.8% | 449 | 49229 | 6.3 | heterogeneous nuclear ribonucleoprotein H1 [Homo sapiens] |

| Filename XCorr DeltCN Conf% ObsM+H+ CalcM+H+ SpR ZScore Ion% # Sequence  | | | | | | | | | | | | |
| --- | --- | --- | --- | --- | --- | --- | --- | --- | --- | --- | --- | --- |
|  | AstrinIP\_MS2\_022614\_01.12017.12017.2 | 4.8574 | 0.5612 | 100.0% | 1842.4722 | 1843.0001 | 1 | 9.917 | 68.8% | 3 | R.STGEAFVQFASQEIAEK.A | 2 |
|  | AstrinIP\_MS2\_022614\_01.14010.14010.2 | 4.2566 | 0.4097 | 100.0% | 1997.5922 | 1998.2023 | 1 | 7.754 | 59.4% | 2 | R.ATENDIYNFFSPLNPVR.V | 22 |
|  | AstrinIP\_MS1\_022614\_01.04682.04682.2 | 2.7054 | 0.408 | 100.0% | 1094.5122 | 1093.2278 | 56 | 7.129 | 61.1% | 2 | R.VHIEIGPDGR.V | 22 |

Similarities:
gi|148470397|ref|NP\_0(2:1)  

---

|  |  |  |  |  |  |  |  |  |
| --- | --- | --- | --- | --- | --- | --- | --- | --- |
| U | *gi|224451077|ref|NP\_0* | 3 | 7 | 9.8% | 399 | 44762 | 5.2 | HBV PreS1-transactivated protein 3 isoform b [Homo sapiens] |
| U | *gi|93102389|ref|NP\_00* | 3 | 7 | 10.0% | 390 | 44140 | 5.2 | HBV PreS1-transactivated protein 3 isoform a [Homo sapiens] |

| Filename XCorr DeltCN Conf% ObsM+H+ CalcM+H+ SpR ZScore Ion% # Sequence  | | | | | | | | | | | | |
| --- | --- | --- | --- | --- | --- | --- | --- | --- | --- | --- | --- | --- |
|  | AstrinIP\_MS2\_022614\_01.07490.07490.2 | 3.8358 | 0.5381 | 100.0% | 1274.2122 | 1274.4166 | 1 | 8.981 | 70.8% | 3 | K.ISAEAVGVDISGR.F | 2 |
|  | AstrinIP\_MS1\_022614\_01.11837.11837.2 | 3.624 | 0.3485 | 100.0% | 1678.4521 | 1678.9006 | 1 | 6.254 | 61.5% | 2 | K.VMVDENTWTNLLSR.V | 2 |
|  | AstrinIP\_MS1\_022614\_01.11592.11592.2 | 2.9591 | 0.3575 | 99.9% | 1377.1522 | 1377.6794 | 1 | 6.357 | 72.7% | 2 | R.VTLLETEMLLSK.K | 2 |

---

|  |  |  |  |  |  |  |  |  |
| --- | --- | --- | --- | --- | --- | --- | --- | --- |
| U | *gi|4506457|ref|NP\_002* | 2 | 2 | 9.8% | 317 | 36876 | 4.4 | reticulocalbin 2 precursor [Homo sapiens] |

| Filename XCorr DeltCN Conf% ObsM+H+ CalcM+H+ SpR ZScore Ion% # Sequence  | | | | | | | | | | | | |
| --- | --- | --- | --- | --- | --- | --- | --- | --- | --- | --- | --- | --- |
| \* | AstrinIP\_MS2\_022614\_02.07194.07194.2 | 4.1806 | 0.4421 | 100.0% | 1707.0922 | 1707.8749 | 1 | 7.714 | 60.7% | 1 | R.EALLGVQEDVDEYVK.L | 2 |
| \* | AstrinIP\_MS2\_022614\_01.12377.12377.2 | 3.8455 | 0.4278 | 100.0% | 1941.8322 | 1943.1199 | 1 | 7.106 | 60.0% | 1 | R.WDPTANEDPEWILVEK.D | 2 |

---

|  |  |  |  |  |  |  |  |  |
| --- | --- | --- | --- | --- | --- | --- | --- | --- |
| U | *gi|9966881|ref|NP\_065* | 5 | 5 | 9.6% | 925 | 106374 | 5.4 | nucleoporin 107kDa [Homo sapiens] |

| Filename XCorr DeltCN Conf% ObsM+H+ CalcM+H+ SpR ZScore Ion% # Sequence  | | | | | | | | | | | | |
| --- | --- | --- | --- | --- | --- | --- | --- | --- | --- | --- | --- | --- |
| \* | AstrinIP\_MS2\_022614\_01.19281.19281.2 | 2.886 | 0.3115 | 99.7% | 2454.2522 | 2453.6975 | 1 | 5.683 | 38.1% | 1 | R.EDDPGEAASMSMFSDFLQSFLK.H | 2 |
| \* | AstrinIP\_MS1\_022614\_01.18009.18009.3 | 3.8837 | 0.334 | 100.0% | 3375.2043 | 3375.7131 | 1 | 5.084 | 23.2% | 1 | R.QSQLVVDWLESIAKDEIGEFSDNIEFYAK.S | 3 |
| \* | AstrinIP\_MS2\_022614\_01.08933.08933.2 | 2.9812 | 0.2341 | 99.5% | 1491.8922 | 1491.6866 | 1 | 4.617 | 63.6% | 1 | K.SVYWENTLHTLK.Q | 2 |
| \* | AstrinIP\_MS2\_022614\_01.06155.06155.2 | 2.7695 | 0.4043 | 100.0% | 1180.5922 | 1180.3 | 1 | 6.824 | 83.3% | 1 | K.VFEELQATDK.K | 2 |
| \* | AstrinIP\_MS2\_022614\_01.16895.16895.2 | 4.296 | 0.5672 | 100.0% | 1812.6921 | 1814.1035 | 1 | 9.429 | 73.3% | 1 | K.FLILGDIDGLMDEFSK.W | 2 |

---

|  |  |  |  |  |  |  |  |  |
| --- | --- | --- | --- | --- | --- | --- | --- | --- |
| U | *gi|32483374|ref|NP\_00* | 3 | 4 | 9.6% | 594 | 66050 | 9.2 | nucleolar protein 5A [Homo sapiens] |

| Filename XCorr DeltCN Conf% ObsM+H+ CalcM+H+ SpR ZScore Ion% # Sequence  | | | | | | | | | | | | |
| --- | --- | --- | --- | --- | --- | --- | --- | --- | --- | --- | --- | --- |
| \* | AstrinIP\_MS2\_022614\_01.19617.19617.3 | 4.2954 | 0.3745 | 100.0% | 2910.9243 | 2911.3584 | 1 | 7.108 | 29.2% | 2 | R.VDNMIIQSISLLDQLDKDINTFSMR.V | 3 |
| \* | AstrinIP\_MS1\_022614\_01.13917.13917.2 | 3.4811 | 0.3888 | 100.0% | 1858.1322 | 1858.1632 | 1 | 6.238 | 52.8% | 1 | K.MSQVAPSLSALIGEAVGAR.L | 2 |
| \* | AstrinIP\_MS2\_022614\_01.07841.07841.2 | 2.9607 | 0.441 | 100.0% | 1376.6522 | 1377.5803 | 1 | 7.103 | 66.7% | 1 | K.YPASTVQILGAEK.A | 2 |

---

|  |  |  |  |  |  |  |  |  |
| --- | --- | --- | --- | --- | --- | --- | --- | --- |
| U | *gi|222352151|ref|NP\_0* | 2 | 3 | 9.3% | 356 | 37498 | 7.1 | poly(rC) binding protein 1 [Homo sapiens] |

| Filename XCorr DeltCN Conf% ObsM+H+ CalcM+H+ SpR ZScore Ion% # Sequence  | | | | | | | | | | | | |
| --- | --- | --- | --- | --- | --- | --- | --- | --- | --- | --- | --- | --- |
| \* | AstrinIP\_MS1\_022614\_01.10600.10600.2 | 3.5785 | 0.396 | 100.0% | 1389.6721 | 1389.6781 | 1 | 8.084 | 62.5% | 2 | R.IITLTGPTNAIFK.A | 2 |
|  | AstrinIP\_MS2\_022614\_02.05321.05321.3 | 3.2617 | 0.2337 | 97.9% | 2092.1042 | 2091.2573 | 1 | 3.903 | 36.8% | 1 | R.ESTGAQVQVAGDMLPNSTER.A | 33 |

Similarities:
gi|14141166|ref|NP\_11(1:1)  

---

|  |  |  |  |  |  |  |  |  |
| --- | --- | --- | --- | --- | --- | --- | --- | --- |
| U | *gi|38679912|ref|NP\_05* | 3 | 3 | 9.0% | 376 | 42009 | 7.4 | hypothetical protein LOC51490 [Homo sapiens] |

| Filename XCorr DeltCN Conf% ObsM+H+ CalcM+H+ SpR ZScore Ion% # Sequence  | | | | | | | | | | | | |
| --- | --- | --- | --- | --- | --- | --- | --- | --- | --- | --- | --- | --- |
| \* | AstrinIP\_MS1\_022614\_01.03666.03666.3 | 4.2116 | 0.3694 | 100.0% | 1675.8243 | 1675.7496 | 1 | 7.021 | 42.3% | 1 | K.RLEEEEAAAEKEDR.G | 3 |
| \* | AstrinIP\_MS1\_022614\_01.03612.03612.2 | 3.4681 | 0.3183 | 100.0% | 1518.8722 | 1519.5621 | 1 | 6.613 | 70.8% | 1 | R.LEEEEAAAEKEDR.G | 2 |
| \* | AstrinIP\_MS2\_022614\_01.09839.09839.3 | 4.0029 | 0.2658 | 99.6% | 2328.4443 | 2327.6255 | 1 | 5.296 | 36.8% | 1 | K.HQDLQFAGLLNPLDSPHHMR.Q | 3 |

---

|  |  |  |  |  |  |  |  |  |
| --- | --- | --- | --- | --- | --- | --- | --- | --- |
| U | *gi|153792590|ref|NP\_0* | 6 | 16 | 8.9% | 854 | 98161 | 5.2 | heat shock 90kDa protein 1, alpha isoform 1 [Homo sapiens] |
| U | *gi|154146191|ref|NP\_0* | 6 | 16 | 10.4% | 732 | 84660 | 5.0 | heat shock 90kDa protein 1, alpha isoform 2 [Homo sapiens] |

| Filename XCorr DeltCN Conf% ObsM+H+ CalcM+H+ SpR ZScore Ion% # Sequence  | | | | | | | | | | | | |
| --- | --- | --- | --- | --- | --- | --- | --- | --- | --- | --- | --- | --- |
|  | AstrinIP\_MS1\_022614\_01.08895.08895.2 | 3.6434 | 0.4326 | 100.0% | 1243.4722 | 1243.4459 | 1 | 7.604 | 77.3% | 3 | K.ADLINNLGTIAK.S | 22 |
|  | AstrinIP\_MS2\_022614\_02.05165.05165.3 | 4.0265 | 0.4323 | 100.0% | 2015.5443 | 2016.2584 | 1 | 7.512 | 41.7% | 5 | K.VILHLKEDQTEYLEER.R | 33 |
|  | AstrinIP\_MS2\_022614\_01.04424.04424.2 | 2.9971 | 0.2018 | 99.8% | 1151.9922 | 1152.2462 | 2 | 5.324 | 81.2% | 1 | K.YIDQEELNK.T | 22 |
|  | AstrinIP\_MS2\_022614\_01.08724.08724.2 | 4.0147 | 0.4128 | 100.0% | 1528.5122 | 1528.6616 | 5 | 7.355 | 62.5% | 3 | K.SLTNDWEDHLAVK.H | 22 |
|  | AstrinIP\_MS2\_022614\_02.05787.05787.2 | 3.0185 | 0.4175 | 100.0% | 1349.5521 | 1349.4886 | 2 | 6.35 | 60.0% | 3 | K.HFSVEGQLEFR.A | 22 |
|  | AstrinIP\_MS2\_022614\_01.08718.08718.3 | 3.1593 | 0.4034 | 100.0% | 1787.6943 | 1788.0134 | 1 | 6.429 | 46.4% | 1 | K.HLEINPDHSIIETLR.Q | 3 |

Similarities:
gi|20149594|ref|NP\_03(5:1)  

---

|  |  |  |  |  |  |  |  |  |
| --- | --- | --- | --- | --- | --- | --- | --- | --- |
| U | *gi|114155142|ref|NP\_0* | 15 | 16 | 8.8% | 2363 | 267290 | 5.0 | nuclear pore complex-associated protein TPR [Homo sapiens] |

| Filename XCorr DeltCN Conf% ObsM+H+ CalcM+H+ SpR ZScore Ion% # Sequence  | | | | | | | | | | | | |
| --- | --- | --- | --- | --- | --- | --- | --- | --- | --- | --- | --- | --- |
| \* | AstrinIP\_MS1\_022614\_01.07680.07680.2 | 3.4423 | 0.4416 | 100.0% | 1463.9521 | 1464.6152 | 1 | 7.252 | 62.5% | 1 | K.FLADQQSEIDGLK.G | 2 |
| \* | AstrinIP\_MS2\_022614\_01.10636.10636.2 | 2.9227 | 0.1868 | 98.6% | 1568.1122 | 1567.7332 | 3 | 4.192 | 54.2% | 1 | R.LSQELEYLTEDVK.R | 2 |
| \* | AstrinIP\_MS2\_022614\_01.06352.06352.2 | 2.3501 | 0.2109 | 96.3% | 1305.5122 | 1304.44 | 1 | 4.612 | 63.6% | 1 | K.LDELQASDVSVK.Y | 2 |
| \* | AstrinIP\_MS1\_022614\_01.10077.10077.2 | 3.3485 | 0.296 | 100.0% | 1204.6721 | 1203.4393 | 1 | 6.659 | 88.9% | 1 | R.VLLMELEEAR.G | 2 |
| \* | AstrinIP\_MS2\_022614\_01.11652.11652.3 | 4.3224 | 0.2914 | 99.9% | 2835.0842 | 2836.2554 | 2 | 6.037 | 23.1% | 2 | R.ILLSQTTGVAIPLHASSLDDVSLASTPK.R | 3 |
| \* | AstrinIP\_MS1\_022614\_01.08446.08446.3 | 4.211 | 0.3108 | 99.9% | 2696.1243 | 2697.0146 | 1 | 6.404 | 34.0% | 1 | K.RPSTSQTVSTPAPVPVIESTEAIEAK.A | 3 |
| \* | AstrinIP\_MS2\_022614\_01.05012.05012.2 | 3.2683 | 0.2938 | 100.0% | 1101.9521 | 1102.2328 | 1 | 5.544 | 87.5% | 1 | K.LQEQVTDLR.S | 2 |
| \* | AstrinIP\_MS1\_022614\_01.15959.15959.2 | 4.2877 | 0.4459 | 100.0% | 2024.2122 | 2025.3561 | 1 | 8.587 | 50.0% | 1 | R.GQNLLLTNLQTIQGILER.S | 2 |
| \* | AstrinIP\_MS1\_022614\_01.03728.03728.2 | 2.4469 | 0.2728 | 98.8% | 1320.7722 | 1321.4758 | 4 | 5.516 | 60.0% | 1 | K.IAVEAQNKYER.E | 2 |
| \* | AstrinIP\_MS2\_022614\_02.06104.06104.2 | 3.2845 | 0.3795 | 100.0% | 1710.8121 | 1710.9861 | 1 | 6.46 | 50.0% | 1 | R.ELMLHAADVEALQAAK.E | 2 |
| \* | AstrinIP\_MS2\_022614\_01.08655.08655.2 | 2.9361 | 0.255 | 99.8% | 1178.1921 | 1178.3304 | 1 | 5.594 | 83.3% | 1 | R.FEVAQVESLR.Y | 2 |
| \* | AstrinIP\_MS2\_022614\_01.08032.08032.2 | 3.5789 | 0.4114 | 100.0% | 1545.1921 | 1545.6464 | 1 | 7.226 | 75.0% | 1 | R.ELQELQDSLNAER.E | 2 |
| \* | AstrinIP\_MS1\_022614\_01.03549.03549.3 | 3.8097 | 0.363 | 100.0% | 2216.2744 | 2216.372 | 1 | 6.402 | 45.6% | 1 | R.NQHLVSQQKDPDTEEYRK.L | 3 |
| \* | AstrinIP\_MS1\_022614\_01.03557.03557.2 | 5.0267 | 0.4971 | 100.0% | 2216.372 | 2216.372 | 1 | 9.659 | 67.6% | 1 | R.NQHLVSQQKDPDTEEYRK.L | 2 |
| \* | AstrinIP\_MS2\_022614\_01.06266.06266.2 | 2.0733 | 0.2423 | 95.4% | 1186.3722 | 1187.3384 | 164 | 4.468 | 50.0% | 1 | R.IQQLTEEIGR.L | 2 |

---

|  |  |  |  |  |  |  |  |  |
| --- | --- | --- | --- | --- | --- | --- | --- | --- |
| U | *gi|169211155|ref|XP\_0* | 3 | 4 | 8.7% | 529 | 57862 | 5.4 | PREDICTED: similar to Karyopherin alpha 2 (RAG cohort 1, importin alpha 1) isoform 1 [Homo sapiens] |
| U | *gi|4504897|ref|NP\_002* | 3 | 4 | 8.7% | 529 | 57862 | 5.4 | karyopherin alpha 2 [Homo sapiens] |
| U | *gi|169211157|ref|XP\_0* | 3 | 4 | 8.7% | 529 | 57862 | 5.4 | PREDICTED: similar to Karyopherin alpha 2 (RAG cohort 1, importin alpha 1) isoform 2 [Homo sapiens] |

| Filename XCorr DeltCN Conf% ObsM+H+ CalcM+H+ SpR ZScore Ion% # Sequence  | | | | | | | | | | | | |
| --- | --- | --- | --- | --- | --- | --- | --- | --- | --- | --- | --- | --- |
|  | AstrinIP\_MS2\_022614\_01.06623.06623.2 | 4.9347 | 0.3625 | 100.0% | 1901.4321 | 1902.0311 | 1 | 8.571 | 55.9% | 1 | K.GINSSNVENQLQATQAAR.K | 2 |
|  | AstrinIP\_MS1\_022614\_01.11465.11465.2 | 3.9848 | 0.4548 | 100.0% | 1550.3522 | 1550.8821 | 13 | 7.825 | 53.6% | 2 | K.LLGASELPIVTPALR.A | 2 |
|  | AstrinIP\_MS2\_022614\_01.08954.08954.2 | 2.4423 | 0.2612 | 98.3% | 1437.7922 | 1438.5981 | 2 | 5.098 | 62.5% | 1 | K.EATWTMSNITAGR.Q | 2 |

---

|  |  |  |  |  |  |  |  |  |
| --- | --- | --- | --- | --- | --- | --- | --- | --- |
| U | *gi|71361682|ref|NP\_00* | 13 | 21 | 8.6% | 2115 | 238257 | 5.8 | nuclear mitotic apparatus protein 1 [Homo sapiens] |

| Filename XCorr DeltCN Conf% ObsM+H+ CalcM+H+ SpR ZScore Ion% # Sequence  | | | | | | | | | | | | |
| --- | --- | --- | --- | --- | --- | --- | --- | --- | --- | --- | --- | --- |
| \* | AstrinIP\_MS2\_022614\_01.04097.04097.2 | 2.8448 | 0.434 | 100.0% | 1256.0322 | 1256.4014 | 1 | 6.453 | 70.0% | 1 | R.HQVEQLSSSLK.Q | 2 |
| \* | AstrinIP\_MS2\_022614\_01.06604.06604.2 | 2.9824 | 0.2453 | 99.7% | 1233.3322 | 1233.3611 | 1 | 5.507 | 85.0% | 2 | K.LADDLSTLQEK.M | 2 |
| \* | AstrinIP\_MS1\_022614\_01.03807.03807.2 | 4.8231 | 0.5571 | 100.0% | 1654.4321 | 1654.78 | 1 | 8.523 | 70.0% | 1 | K.AQELGHSQSALASAQR.E | 2 |
| \* | AstrinIP\_MS1\_022614\_01.03771.03771.2 | 4.0517 | 0.4612 | 100.0% | 1261.1322 | 1261.3774 | 1 | 8.675 | 81.8% | 2 | R.LLQAETASNSAR.A | 2 |
| \* | AstrinIP\_MS1\_022614\_01.04224.04224.2 | 3.0703 | 0.4192 | 100.0% | 1725.6122 | 1725.8651 | 1 | 7.004 | 71.4% | 1 | K.HLCQQLQAEQAAAEK.R | 2 |
| \* | AstrinIP\_MS1\_022614\_01.08892.08892.2 | 4.8395 | 0.4998 | 100.0% | 1712.1721 | 1712.9437 | 1 | 8.929 | 75.0% | 2 | R.LQAQLNELQAQLSQK.E | 2 |
| \* | AstrinIP\_MS2\_022614\_01.03934.03934.2 | 4.0424 | 0.1725 | 99.9% | 1542.4722 | 1542.692 | 1 | 6.044 | 90.9% | 2 | K.KQQNQELQEQLR.S | 2 |
| \* | AstrinIP\_MS2\_022614\_01.04252.04252.2 | 2.625 | 0.2797 | 99.5% | 1195.3722 | 1194.3762 | 1 | 4.991 | 70.0% | 2 | R.LGHELQQAGLK.T | 2 |
| \* | AstrinIP\_MS2\_022614\_01.06754.06754.2 | 3.7899 | 0.4825 | 100.0% | 1566.2122 | 1566.7141 | 1 | 8.401 | 57.7% | 2 | R.SLEAQVAHADQQLR.D | 2 |
| \* | AstrinIP\_MS1\_022614\_01.14189.14189.2 | 3.6173 | 0.2533 | 99.8% | 2193.2122 | 2194.3599 | 3 | 6.845 | 32.5% | 2 | R.SQAPLESSLDSLGDVFLDSGR.K | 2 |
| \* | AstrinIP\_MS2\_022614\_01.08535.08535.2 | 1.8105 | 0.3515 | 96.7% | 1264.5922 | 1264.5231 | 1 | 5.5 | 70.0% | 1 | R.TTQIINITMTK.K | 2 |
| \* | AstrinIP\_MS1\_022614\_01.08718.08718.2 | 2.6943 | 0.409 | 99.8% | 1774.4321 | 1775.03 | 1 | 6.008 | 53.1% | 2 | R.ASMQPIQIAEGTGITTR.Q | 2 |
| \* | AstrinIP\_MS1\_022614\_01.03821.03821.2 | 3.573 | 0.308 | 100.0% | 1562.9321 | 1563.7074 | 1 | 6.279 | 60.7% | 1 | R.VSLEPHQGPGTPESK.K | 2 |

---

|  |  |  |  |  |  |  |  |  |
| --- | --- | --- | --- | --- | --- | --- | --- | --- |
| U | *gi|4757834|ref|NP\_004* | 2 | 2 | 8.5% | 211 | 23772 | 6.7 | BCL2-associated athanogene 2 [Homo sapiens] |

| Filename XCorr DeltCN Conf% ObsM+H+ CalcM+H+ SpR ZScore Ion% # Sequence  | | | | | | | | | | | | |
| --- | --- | --- | --- | --- | --- | --- | --- | --- | --- | --- | --- | --- |
| \* | AstrinIP\_MS2\_022614\_01.11292.11292.2 | 2.8941 | 0.3091 | 99.8% | 1329.0521 | 1329.5364 | 1 | 5.95 | 75.0% | 1 | R.LLESLDQLELR.V | 2 |
| \* | AstrinIP\_MS2\_022614\_01.04924.04924.2 | 2.5074 | 0.1312 | 98.3% | 901.15216 | 901.09705 | 53 | 4.043 | 75.0% | 1 | R.RLETLLR.N | 2 |

---

|  |  |  |  |  |  |  |  |  |
| --- | --- | --- | --- | --- | --- | --- | --- | --- |
| U | *gi|33239445|ref|NP\_00* | 4 | 5 | 8.4% | 814 | 92482 | 5.0 | eukaryotic translation initiation factor 3, subunit 9 eta, 116kDa [Homo sapiens] |
| U | *gi|83367072|ref|NP\_00* | 4 | 5 | 8.4% | 814 | 92482 | 5.0 | eukaryotic translation initiation factor 3, subunit 9 eta, 116kDa [Homo sapiens] |

| Filename XCorr DeltCN Conf% ObsM+H+ CalcM+H+ SpR ZScore Ion% # Sequence  | | | | | | | | | | | | |
| --- | --- | --- | --- | --- | --- | --- | --- | --- | --- | --- | --- | --- |
|  | AstrinIP\_MS1\_022614\_01.06330.06330.2 | 2.5064 | 0.319 | 99.4% | 1428.1122 | 1428.495 | 1 | 4.93 | 68.2% | 1 | K.ITNDFYPEEDGK.T | 2 |
|  | AstrinIP\_MS2\_022614\_01.11262.11262.3 | 2.7271 | 0.3545 | 99.6% | 1951.9443 | 1952.2169 | 339 | 5.554 | 25.0% | 1 | K.GYIFLEYASPAHAVDAVK.N | 3 |
|  | AstrinIP\_MS2\_022614\_01.17594.17594.3 | 3.759 | 0.3363 | 99.9% | 3231.4443 | 3232.7188 | 63 | 5.441 | 20.4% | 2 | R.YLVTFSPLMDTQDDPQAIIIWDILTGHK.K | 3 |
|  | AstrinIP\_MS2\_022614\_01.05844.05844.2 | 2.2504 | 0.2529 | 98.1% | 1271.0721 | 1271.489 | 4 | 4.531 | 72.2% | 1 | K.MAQELYMEQK.N | 2 |

---

|  |  |  |  |  |  |  |  |  |
| --- | --- | --- | --- | --- | --- | --- | --- | --- |
| U | *gi|41322908|ref|NP\_95* | 26 | 34 | 8.2% | 4525 | 513712 | 5.8 | plectin 1 isoform 3 [Homo sapiens] |
| U | *gi|47607492|ref|NP\_00* | 26 | 34 | 8.2% | 4574 | 518478 | 5.7 | plectin 1 isoform 1 [Homo sapiens] |
| U | *gi|41322923|ref|NP\_95* | 26 | 34 | 8.2% | 4547 | 516204 | 5.8 | plectin 1 isoform 11 [Homo sapiens] |
| U | *gi|41322919|ref|NP\_95* | 26 | 34 | 8.2% | 4547 | 516282 | 5.8 | plectin 1 isoform 8 [Homo sapiens] |
| U | *gi|41322916|ref|NP\_95* | 26 | 34 | 8.0% | 4684 | 531796 | 6.0 | plectin 1 isoform 6 [Homo sapiens] |
| U | *gi|41322914|ref|NP\_95* | 26 | 34 | 8.2% | 4551 | 516484 | 5.8 | plectin 1 isoform 10 [Homo sapiens] |
| U | *gi|41322912|ref|NP\_95* | 26 | 34 | 8.2% | 4533 | 514780 | 5.7 | plectin 1 isoform 2 [Homo sapiens] |
| U | *gi|41322910|ref|NP\_95* | 26 | 34 | 8.3% | 4515 | 512609 | 5.8 | plectin 1 isoform 7 [Homo sapiens] |

| Filename XCorr DeltCN Conf% ObsM+H+ CalcM+H+ SpR ZScore Ion% # Sequence  | | | | | | | | | | | | |
| --- | --- | --- | --- | --- | --- | --- | --- | --- | --- | --- | --- | --- |
|  | AstrinIP\_MS1\_022614\_01.16266.16266.3 | 5.7895 | 0.4548 | 100.0% | 3280.2844 | 3278.6462 | 1 | 8.353 | 30.4% | 2 | R.HISDLYEDLRDGHNLISLLEVLSGDSLPR.E | 3 |
|  | AstrinIP\_MS2\_022614\_01.11975.11975.2 | 2.5611 | 0.2123 | 98.1% | 1446.2522 | 1446.6897 | 1 | 5.663 | 63.6% | 1 | K.LQNVQIALDYLR.H | 2 |
|  | AstrinIP\_MS1\_022614\_01.10404.10404.2 | 3.1039 | 0.362 | 99.9% | 1836.3322 | 1835.9683 | 1 | 6.696 | 46.7% | 1 | R.QTNLENLDQAFSVAER.D | 2 |
|  | AstrinIP\_MS2\_022614\_01.17811.17811.2 | 4.7557 | 0.5015 | 100.0% | 2256.7122 | 2257.5938 | 1 | 8.912 | 55.9% | 2 | R.RFPSSFEEIEILWSQFLK.F | 2 |
|  | AstrinIP\_MS2\_022614\_01.11212.11212.2 | 4.0611 | 0.399 | 100.0% | 1663.0521 | 1662.8833 | 1 | 6.968 | 63.3% | 1 | K.GIYQSLEGAVQAGQLK.V | 2 |
|  | AstrinIP\_MS1\_022614\_01.04047.04047.2 | 2.3941 | 0.1593 | 95.4% | 1215.9521 | 1216.3365 | 3 | 4.193 | 66.7% | 1 | R.RPELEDSTLR.Y | 2 |
|  | AstrinIP\_MS2\_022614\_01.13851.13851.2 | 4.0875 | 0.4932 | 100.0% | 1914.2122 | 1915.1155 | 1 | 8.092 | 64.3% | 2 | R.YLQDLLAWVEENQHR.V | 2 |
|  | AstrinIP\_MS1\_022614\_01.05402.05402.2 | 3.27 | 0.3634 | 100.0% | 1216.3121 | 1216.339 | 1 | 6.141 | 83.3% | 1 | R.GLHQSIEEFR.A | 2 |
|  | AstrinIP\_MS2\_022614\_02.07661.07661.3 | 3.5067 | 0.3145 | 99.8% | 2397.7444 | 2398.788 | 8 | 5.469 | 30.3% | 1 | R.LEAQHQALVTLWHQLHVDMK.S | 3 |
|  | AstrinIP\_MS2\_022614\_01.09327.09327.2 | 2.9628 | 0.2836 | 99.6% | 1533.3922 | 1532.8235 | 1 | 5.634 | 60.7% | 1 | K.VLALPEPSPAAPTLR.S | 2 |
|  | AstrinIP\_MS2\_022614\_01.07782.07782.2 | 2.6715 | 0.2299 | 99.0% | 1287.2922 | 1287.4612 | 1 | 4.912 | 75.0% | 1 | R.WQAVLAQTDVR.Q | 2 |
|  | AstrinIP\_MS2\_022614\_01.12803.12803.2 | 3.0453 | 0.4562 | 100.0% | 1529.5922 | 1529.6494 | 1 | 6.558 | 53.8% | 1 | R.ESADPLGAWLQDAR.R | 2 |
|  | AstrinIP\_MS1\_022614\_02.07430.07430.2 | 2.6802 | 0.2973 | 99.2% | 1808.7522 | 1809.9707 | 1 | 5.949 | 43.3% | 1 | K.VQSGSESVIQEYVDLR.T | 2 |
|  | AstrinIP\_MS1\_022614\_01.07161.07161.2 | 2.2666 | 0.2098 | 95.8% | 1288.5922 | 1288.4435 | 4 | 4.378 | 65.0% | 1 | K.AQVEQELTTLR.L | 2 |
|  | AstrinIP\_MS1\_022614\_01.08561.08561.2 | 3.0385 | 0.3587 | 99.9% | 1519.5122 | 1519.738 | 1 | 6.129 | 70.8% | 2 | K.AKLEQLFQDEVAK.A | 2 |
|  | AstrinIP\_MS2\_022614\_01.08448.08448.2 | 2.7334 | 0.1184 | 96.5% | 1320.0922 | 1320.4851 | 1 | 4.144 | 75.0% | 1 | K.LEQLFQDEVAK.A | 2 |
|  | AstrinIP\_MS1\_022614\_02.06177.06177.2 | 2.8626 | 0.228 | 98.9% | 1557.4321 | 1557.744 | 17 | 5.752 | 50.0% | 2 | R.LQEAGILSAEELQR.L | 2 |
|  | AstrinIP\_MS1\_022614\_01.04192.04192.2 | 2.9331 | 0.32 | 99.8% | 1411.1122 | 1411.5577 | 1 | 5.082 | 70.8% | 1 | R.LAQGHTTVDELAR.R | 2 |
|  | AstrinIP\_MS2\_022614\_01.08033.08033.3 | 4.0343 | 0.3821 | 100.0% | 2013.9844 | 2014.292 | 1 | 5.925 | 38.9% | 3 | R.LLEAQIATGGVIDPVHSHR.V | 33 |
|  | AstrinIP\_MS2\_022614\_01.07104.07104.2 | 2.1779 | 0.2594 | 98.1% | 1161.1322 | 1161.2311 | 1 | 5.235 | 75.0% | 1 | R.GYFDEEMNR.V | 22 |
|  | AstrinIP\_MS1\_022614\_02.06170.06170.2 | 2.6646 | 0.2855 | 99.1% | 1615.6921 | 1614.8363 | 1 | 5.043 | 53.3% | 1 | R.LLDAQLSTGGIVDPSK.S | 2 |
|  | AstrinIP\_MS2\_022614\_01.07635.07635.2 | 2.2414 | 0.2724 | 98.1% | 1130.0922 | 1130.3293 | 13 | 5.837 | 60.0% | 1 | K.TPVEVPVGGFK.G | 2 |
|  | AstrinIP\_MS2\_022614\_01.11248.11248.2 | 3.8077 | 0.4851 | 100.0% | 1568.1921 | 1567.8259 | 1 | 8.967 | 76.7% | 1 | R.APVPASELLASGVLSR.A | 2 |
|  | AstrinIP\_MS2\_022614\_01.07906.07906.2 | 3.383 | 0.3291 | 99.9% | 1778.0322 | 1778.0153 | 2 | 5.963 | 50.0% | 1 | K.AGVVGPELHEQLLSAEK.A | 2 |
|  | AstrinIP\_MS2\_022614\_01.08807.08807.3 | 3.1153 | 0.2831 | 99.2% | 2028.6244 | 2028.3188 | 95 | 5.473 | 29.2% | 1 | R.LLEAQIATGGIIDPVHSHR.V | 33 |
|  | AstrinIP\_MS2\_022614\_01.08880.08880.2 | 3.3342 | 0.4012 | 100.0% | 1462.0521 | 1462.6611 | 1 | 6.877 | 66.7% | 2 | R.SQVMDEATALQLR.E | 2 |

Similarities:
gi|207452735|ref|NP\_1(3:23)  

---

|  |  |  |  |  |  |  |  |  |
| --- | --- | --- | --- | --- | --- | --- | --- | --- |
| U | *gi|40789249|ref|NP\_06* | 3 | 4 | 8.2% | 645 | 73563 | 8.0 | aspartyl-tRNA synthetase 2, mitochondrial [Homo sapiens] |

| Filename XCorr DeltCN Conf% ObsM+H+ CalcM+H+ SpR ZScore Ion% # Sequence  | | | | | | | | | | | | |
| --- | --- | --- | --- | --- | --- | --- | --- | --- | --- | --- | --- | --- |
| \* | AstrinIP\_MS1\_022614\_01.12298.12298.2 | 2.4383 | 0.2182 | 96.1% | 1794.3322 | 1793.9946 | 341 | 4.717 | 32.1% | 2 | R.NWNSPVANFIMESQR.L | 2 |
| \* | AstrinIP\_MS1\_022614\_02.06449.06449.3 | 2.7358 | 0.2606 | 96.6% | 2157.3843 | 2157.4004 | 78 | 4.572 | 30.6% | 1 | R.LMETQEEDVVLLTAGEHNK.A | 3 |
| \* | AstrinIP\_MS2\_022614\_02.05958.05958.3 | 3.4546 | 0.2618 | 99.2% | 2028.8644 | 2030.2046 | 72 | 4.731 | 25.0% | 1 | R.SQHYDLVLNGNEIGGGSIR.I | 3 |

---

|  |  |  |  |  |  |  |  |  |
| --- | --- | --- | --- | --- | --- | --- | --- | --- |
| U | *gi|87196351|ref|NP\_00* | 4 | 8 | 8.2% | 662 | 73244 | 7.2 | DEAD/H (Asp-Glu-Ala-Asp/His) box polypeptide 3 [Homo sapiens] |

| Filename XCorr DeltCN Conf% ObsM+H+ CalcM+H+ SpR ZScore Ion% # Sequence  | | | | | | | | | | | | |
| --- | --- | --- | --- | --- | --- | --- | --- | --- | --- | --- | --- | --- |
|  | AstrinIP\_MS2\_022614\_01.10708.10708.2 | 3.6869 | 0.4418 | 100.0% | 1337.3922 | 1337.5946 | 1 | 8.364 | 85.0% | 2 | R.MLDMGFEPQIR.R | 22 |
|  | AstrinIP\_MS2\_022614\_01.08211.08211.2 | 3.1488 | 0.3395 | 100.0% | 1169.5922 | 1169.4099 | 1 | 7.082 | 77.3% | 3 | K.SPILVATAVAAR.G | 2 |
|  | AstrinIP\_MS2\_022614\_01.12126.12126.3 | 4.5699 | 0.4741 | 100.0% | 2084.3643 | 2084.2957 | 1 | 8.239 | 42.2% | 2 | K.HVINFDLPSDIEEYVHR.I | 3 |
| \* | AstrinIP\_MS1\_022614\_01.11558.11558.2 | 3.0626 | 0.4022 | 100.0% | 1525.8522 | 1525.7043 | 2 | 6.491 | 57.7% | 1 | R.VGNLGLATSFFNER.N | 2 |

Similarities:
gi|4758138|ref|NP\_004(1:3)  

---

|  |  |  |  |  |  |  |  |  |
| --- | --- | --- | --- | --- | --- | --- | --- | --- |
| U | *gi|113408840|ref|XP\_0* | 2 | 2 | 8.2% | 294 | 32601 | 4.8 | PREDICTED: similar to 40S ribosomal protein SA (p40) (34/67 kDa laminin receptor) (Colon carcinoma laminin-binding protein) (NEM/1CHD4) (Multidrug resistance-associated protein MGr1-Ag) isoform 1 [Homo sapiens] |
| U | *gi|9845502|ref|NP\_002* | 2 | 2 | 8.1% | 295 | 32854 | 4.9 | ribosomal protein SA [Homo sapiens] |
| U | *gi|88951511|ref|XP\_94* | 2 | 2 | 8.2% | 294 | 32601 | 4.8 | PREDICTED: similar to 40S ribosomal protein SA (p40) (34/67 kDa laminin receptor) (Colon carcinoma laminin-binding protein) (NEM/1CHD4) (Multidrug resistance-associated protein MGr1-Ag) isoform 4 [Homo sapiens] |
| U | *gi|59859885|ref|NP\_00* | 2 | 2 | 8.1% | 295 | 32854 | 4.9 | ribosomal protein SA [Homo sapiens] |
| U | *gi|41201737|ref|XP\_37* | 2 | 2 | 8.1% | 295 | 32744 | 4.5 | PREDICTED: similar to 40S ribosomal protein SA (p40) (34/67 kDa laminin receptor) (Colon carcinoma laminin-binding protein) (NEM/1CHD4) (Multidrug resistance-associated protein MGr1-Ag) isoform 3 [Homo sapiens] |
| U | *gi|169205444|ref|XP\_0* | 2 | 2 | 8.1% | 295 | 32744 | 4.5 | PREDICTED: similar to 40S ribosomal protein SA (p40) (34/67 kDa laminin receptor) (Colon carcinoma laminin-binding protein) (NEM/1CHD4) (Multidrug resistance-associated protein MGr1-Ag) isoform 1 [Homo sapiens] |
| U | *gi|169205442|ref|XP\_0* | 2 | 2 | 8.1% | 295 | 32744 | 4.5 | PREDICTED: similar to 40S ribosomal protein SA (p40) (34/67 kDa laminin receptor) (Colon carcinoma laminin-binding protein) (NEM/1CHD4) (Multidrug resistance-associated protein MGr1-Ag) isoform 3 [Homo sapiens] |
| U | *gi|169205440|ref|XP\_0* | 2 | 2 | 8.1% | 295 | 32744 | 4.5 | PREDICTED: similar to 40S ribosomal protein SA (p40) (34/67 kDa laminin receptor) (Colon carcinoma laminin-binding protein) (NEM/1CHD4) (Multidrug resistance-associated protein MGr1-Ag) isoform 2 [Homo sapiens] |
| U | *gi|169204812|ref|XP\_0* | 2 | 2 | 8.1% | 295 | 32774 | 4.5 | PREDICTED: similar to Ribosomal protein SA pseudogene isoform 1 [Homo sapiens] |
| U | *gi|169204810|ref|XP\_0* | 2 | 2 | 8.1% | 295 | 32774 | 4.5 | PREDICTED: similar to Ribosomal protein SA pseudogene isoform 3 [Homo sapiens] |
| U | *gi|169204808|ref|XP\_0* | 2 | 2 | 8.1% | 295 | 32774 | 4.5 | PREDICTED: similar to Ribosomal protein SA pseudogene isoform 2 [Homo sapiens] |
| U | *gi|169204115|ref|XP\_0* | 2 | 2 | 8.1% | 295 | 32744 | 4.5 | PREDICTED: similar to 40S ribosomal protein SA (p40) (34/67 kDa laminin receptor) (Colon carcinoma laminin-binding protein) (NEM/1CHD4) (Multidrug resistance-associated protein MGr1-Ag) isoform 1 [Homo sapiens] |
| U | *gi|169204113|ref|XP\_0* | 2 | 2 | 8.1% | 295 | 32744 | 4.5 | PREDICTED: similar to 40S ribosomal protein SA (p40) (34/67 kDa laminin receptor) (Colon carcinoma laminin-binding protein) (NEM/1CHD4) (Multidrug resistance-associated protein MGr1-Ag) isoform 2 [Homo sapiens] |
| U | *gi|169160827|ref|XP\_0* | 2 | 2 | 8.2% | 294 | 32601 | 4.8 | PREDICTED: similar to 40S ribosomal protein SA (p40) (34/67 kDa laminin receptor) (Colon carcinoma laminin-binding protein) (NEM/1CHD4) (Multidrug resistance-associated protein MGr1-Ag) [Homo sapiens] |

| Filename XCorr DeltCN Conf% ObsM+H+ CalcM+H+ SpR ZScore Ion% # Sequence  | | | | | | | | | | | | |
| --- | --- | --- | --- | --- | --- | --- | --- | --- | --- | --- | --- | --- |
|  | AstrinIP\_MS2\_022614\_01.08856.08856.2 | 2.7585 | 0.1609 | 98.2% | 1263.9321 | 1264.5077 | 1 | 5.339 | 80.0% | 1 | R.KSDGIYIINLK.R | 2 |
|  | AstrinIP\_MS2\_022614\_01.04880.04880.2 | 2.9081 | 0.2364 | 99.1% | 1203.9521 | 1204.3713 | 1 | 6.122 | 75.0% | 1 | K.FAAATGATPIAGR.F | 2 |

---

|  |  |  |  |  |  |  |  |  |
| --- | --- | --- | --- | --- | --- | --- | --- | --- |
| U | *contaminant\_KERATIN05* | 4 | 5 | 8.1% | 471 | 51531 | 5.2 | no description |
| U | *gi|24430192|ref|NP\_00* | 4 | 5 | 8.0% | 473 | 51268 | 5.0 | keratin 16 [Homo sapiens] |
| U | *gi|15431310|ref|NP\_00* | 4 | 5 | 8.1% | 472 | 51622 | 5.2 | keratin 14 [Homo sapiens] |
| U | *contaminant\_KERATIN08* | 4 | 5 | 8.1% | 469 | 50499 | 5.0 | no description |

| Filename XCorr DeltCN Conf% ObsM+H+ CalcM+H+ SpR ZScore Ion% # Sequence  | | | | | | | | | | | | |
| --- | --- | --- | --- | --- | --- | --- | --- | --- | --- | --- | --- | --- |
|  | AstrinIP\_MS1\_022614\_01.04173.04173.2 | 2.1506 | 0.2051 | 95.4% | 1091.5322 | 1091.2273 | 78 | 4.696 | 62.5% | 1 | K.VTMQNLNDR.L | 22 |
|  | AstrinIP\_MS1\_022614\_01.05027.05027.2 | 2.3369 | 0.1774 | 96.8% | 1065.8322 | 1065.2578 | 35 | 4.948 | 62.5% | 1 | R.LASYLDKVR.A | 2222 |
|  | AstrinIP\_MS2\_022614\_01.08524.08524.2 | 2.4955 | 0.3861 | 99.9% | 1030.1522 | 1030.2096 | 1 | 7.085 | 81.2% | 1 | R.VLDELTLAR.A | 222 |
|  | AstrinIP\_MS1\_022614\_01.06353.06353.2 | 2.9214 | 0.3246 | 99.8% | 1380.4321 | 1380.5437 | 1 | 5.656 | 75.0% | 2 | K.TRLEQEIATYR.R | 22 |

Similarities:
contaminant\_KERATIN03(2:2)  
contaminant\_KERATIN12(3:1)  
contaminant\_KERATIN10(2:2)  

---

|  |  |  |  |  |  |  |  |  |
| --- | --- | --- | --- | --- | --- | --- | --- | --- |
| U | *Reverse\_gi|28827807|r* | 2 | 9 | 7.8% | 655 | 75032 | 7.2 | NLR family, pyrin domain containing 10 [Homo sapiens] |

| Filename XCorr DeltCN Conf% ObsM+H+ CalcM+H+ SpR ZScore Ion% # Sequence  | | | | | | | | | | | | |
| --- | --- | --- | --- | --- | --- | --- | --- | --- | --- | --- | --- | --- |
| \* | AstrinIP\_MS2\_022614\_01.09682.09682.2 | 4.1985 | 0.0328 | 98.7% | 2215.4521 | 2215.2212 | 29 | 4.428 | 38.9% | 8 | K.QT#GAINDKGEKQGHVS\*PCK.Q | 2 |
| \* | AstrinIP\_MS1\_022614\_01.11645.11645.3 | 3.0914 | 0.3413 | 99.5% | 3577.9443 | 3577.711 | 43 | 4.484 | 17.7% | 1 | K.EGSDFLAEVTVSELEQEPFPCALS\*EPSESSPK.A | 3 |

---

|  |  |  |  |  |  |  |  |  |
| --- | --- | --- | --- | --- | --- | --- | --- | --- |
| U | *gi|4758138|ref|NP\_004* | 4 | 7 | 7.8% | 614 | 69148 | 8.9 | DEAD (Asp-Glu-Ala-Asp) box polypeptide 5 [Homo sapiens] |

| Filename XCorr DeltCN Conf% ObsM+H+ CalcM+H+ SpR ZScore Ion% # Sequence  | | | | | | | | | | | | |
| --- | --- | --- | --- | --- | --- | --- | --- | --- | --- | --- | --- | --- |
| \* | AstrinIP\_MS2\_022614\_01.08633.08633.2 | 2.4143 | 0.3064 | 99.2% | 1296.3922 | 1296.4198 | 1 | 5.671 | 65.0% | 1 | R.TTYLVLDEADR.M | 2 |
|  | AstrinIP\_MS2\_022614\_01.10708.10708.2 | 3.6869 | 0.4418 | 100.0% | 1337.3922 | 1337.5946 | 1 | 8.364 | 85.0% | 2 | R.MLDMGFEPQIR.K | 22 |
|  | AstrinIP\_MS2\_022614\_01.08246.08246.2 | 3.5346 | 0.2644 | 100.0% | 1227.0922 | 1227.4465 | 1 | 7.552 | 81.8% | 3 | K.APILIATDVASR.G | 2 |
| \* | AstrinIP\_MS2\_022614\_01.10254.10254.2 | 3.3599 | 0.2888 | 99.8% | 1576.3922 | 1575.7612 | 1 | 5.169 | 65.4% | 1 | K.TGTAYTFFTPNNIK.Q | 2 |

Similarities:
gi|87196351|ref|NP\_00(1:3)  

---

|  |  |  |  |  |  |  |  |  |
| --- | --- | --- | --- | --- | --- | --- | --- | --- |
| U | *gi|156071459|ref|NP\_0* | 2 | 3 | 7.7% | 298 | 32852 | 9.7 | solute carrier family 25, member 5 [Homo sapiens] |

| Filename XCorr DeltCN Conf% ObsM+H+ CalcM+H+ SpR ZScore Ion% # Sequence  | | | | | | | | | | | | |
| --- | --- | --- | --- | --- | --- | --- | --- | --- | --- | --- | --- | --- |
| \* | AstrinIP\_MS2\_022614\_02.07012.07012.2 | 2.7634 | 0.4505 | 100.0% | 1219.7522 | 1220.4111 | 1 | 8.043 | 70.8% | 1 | K.DFLAGGVAAAISK.T | 2 |
|  | AstrinIP\_MS2\_022614\_01.04590.04590.2 | 2.901 | 0.3314 | 99.9% | 1136.4722 | 1137.3677 | 3 | 6.137 | 66.7% | 2 | K.LLLQVQHASK.Q | 2 |

---

|  |  |  |  |  |  |  |  |  |
| --- | --- | --- | --- | --- | --- | --- | --- | --- |
| U | *gi|19923229|ref|NP\_00* | 5 | 6 | 7.5% | 1150 | 130278 | 5.2 | protein phosphatase 2, regulatory subunit B'', alpha isoform 1 [Homo sapiens] |

| Filename XCorr DeltCN Conf% ObsM+H+ CalcM+H+ SpR ZScore Ion% # Sequence  | | | | | | | | | | | | |
| --- | --- | --- | --- | --- | --- | --- | --- | --- | --- | --- | --- | --- |
| \* | AstrinIP\_MS2\_022614\_02.05519.05519.3 | 2.6203 | 0.3313 | 99.2% | 1788.4744 | 1789.0415 | 9 | 5.4 | 35.0% | 1 | R.LVVSTVNHYSSVVIDR.R | 3 |
| \* | AstrinIP\_MS2\_022614\_02.06954.06954.3 | 3.6521 | 0.2011 | 97.4% | 2443.0745 | 2443.6758 | 1 | 4.688 | 38.1% | 1 | K.NSNFLNSHSQLTGQTLVDLEPK.S | 3 |
|  | AstrinIP\_MS2\_022614\_02.07310.07310.3 | 3.6207 | 0.2947 | 99.7% | 2363.5144 | 2362.6719 | 1 | 6.559 | 32.9% | 2 | R.IETAFMDIEEQKADIYEMGK.I | 3 |
|  | AstrinIP\_MS2\_022614\_02.10102.10102.2 | 2.359 | 0.222 | 95.8% | 1616.8722 | 1615.8901 | 1 | 5.442 | 50.0% | 1 | K.TGFVTAQSFIAMWR.K | 2 |
|  | AstrinIP\_MS1\_022614\_01.12248.12248.2 | 2.3105 | 0.2715 | 97.7% | 1775.1721 | 1777.0472 | 13 | 4.791 | 46.2% | 1 | R.MAHIFYDTFFNLEK.Y | 2 |

---

|  |  |  |  |  |  |  |  |  |
| --- | --- | --- | --- | --- | --- | --- | --- | --- |
| U | *gi|4503483|ref|NP\_001* | 3 | 3 | 7.3% | 858 | 95338 | 6.8 | eukaryotic translation elongation factor 2 [Homo sapiens] |

| Filename XCorr DeltCN Conf% ObsM+H+ CalcM+H+ SpR ZScore Ion% # Sequence  | | | | | | | | | | | | |
| --- | --- | --- | --- | --- | --- | --- | --- | --- | --- | --- | --- | --- |
| \* | AstrinIP\_MS2\_022614\_01.16374.16374.2 | 2.7746 | 0.2939 | 99.3% | 2205.412 | 2205.4692 | 1 | 4.911 | 38.9% | 1 | K.STAISLFYELSENDLNFIK.Q | 2 |
| \* | AstrinIP\_MS2\_022614\_01.16534.16534.2 | 3.2913 | 0.4373 | 100.0% | 2600.9321 | 2602.11 | 2 | 7.311 | 32.6% | 1 | R.WLPAGDALLQMITIHLPSPVTAQK.Y | 2 |
| \* | AstrinIP\_MS1\_022614\_01.08370.08370.3 | 4.0301 | 0.3129 | 100.0% | 2144.2744 | 2144.3489 | 27 | 5.024 | 30.3% | 1 | K.ARPFPDGLAEDIDKGEVSAR.Q | 3 |

---

|  |  |  |  |  |  |  |  |  |
| --- | --- | --- | --- | --- | --- | --- | --- | --- |
| U | *gi|27477138|ref|NP\_07* | 3 | 3 | 7.2% | 699 | 77903 | 8.4 | zinc finger antiviral protein isoform 2 [Homo sapiens] |

| Filename XCorr DeltCN Conf% ObsM+H+ CalcM+H+ SpR ZScore Ion% # Sequence  | | | | | | | | | | | | |
| --- | --- | --- | --- | --- | --- | --- | --- | --- | --- | --- | --- | --- |
|  | AstrinIP\_MS2\_022614\_02.05919.05919.2 | 2.5016 | 0.317 | 99.2% | 1448.8121 | 1449.647 | 6 | 5.145 | 53.8% | 1 | R.FVVLETGGEAGITR.S | 2 |
|  | AstrinIP\_MS1\_022614\_01.13797.13797.2 | 2.7879 | 0.1725 | 97.2% | 2783.4521 | 2785.9714 | 1 | 4.784 | 31.8% | 1 | R.EHGLNPDVVQNIQDICNS\*KHMQK.N | 2 |
| \* | AstrinIP\_MS2\_022614\_01.07985.07985.2 | 2.4859 | 0.3212 | 99.2% | 1402.2922 | 1402.501 | 301 | 5.117 | 37.5% | 1 | R.ASLEDAPVDDLTR.K | 2 |

---

|  |  |  |  |  |  |  |  |  |
| --- | --- | --- | --- | --- | --- | --- | --- | --- |
| U | *gi|21361809|ref|NP\_06* | 2 | 3 | 6.9% | 390 | 42142 | 9.9 | kynurenine aminotransferase III isoform 3 [Homo sapiens] |
| U | *gi|56699409|ref|NP\_00* | 2 | 3 | 6.9% | 391 | 42332 | 10.1 | RNA binding motif protein, X-linked [Homo sapiens] |

| Filename XCorr DeltCN Conf% ObsM+H+ CalcM+H+ SpR ZScore Ion% # Sequence  | | | | | | | | | | | | |
| --- | --- | --- | --- | --- | --- | --- | --- | --- | --- | --- | --- | --- |
|  | AstrinIP\_MS2\_022614\_01.08781.08781.2 | 3.4591 | 0.2735 | 99.9% | 1436.2922 | 1436.6049 | 1 | 5.673 | 79.2% | 1 | K.LFIGGLNTETNEK.A | 2 |
|  | AstrinIP\_MS1\_022614\_01.11134.11134.2 | 3.8892 | 0.4034 | 100.0% | 1488.3922 | 1487.6519 | 1 | 7.882 | 73.1% | 2 | R.GFAFVTFESPADAK.D | 2 |

---

|  |  |  |  |  |  |  |  |  |
| --- | --- | --- | --- | --- | --- | --- | --- | --- |
| U | *contaminant\_KERATIN10* | 4 | 5 | 6.8% | 400 | 44106 | 5.1 | no description |
| U | *gi|24234699|ref|NP\_00* | 4 | 5 | 6.8% | 400 | 44106 | 5.1 | keratin 19 [Homo sapiens] |

| Filename XCorr DeltCN Conf% ObsM+H+ CalcM+H+ SpR ZScore Ion% # Sequence  | | | | | | | | | | | | |
| --- | --- | --- | --- | --- | --- | --- | --- | --- | --- | --- | --- | --- |
|  | AstrinIP\_MS1\_022614\_01.05027.05027.2 | 2.3369 | 0.1774 | 96.8% | 1065.8322 | 1065.2578 | 35 | 4.948 | 62.5% | 1 | R.LASYLDKVR.A | 2222 |
|  | AstrinIP\_MS2\_022614\_01.06832.06832.2 | 2.8567 | 0.2333 | 99.8% | 1041.4521 | 1042.2235 | 1 | 6.542 | 87.5% | 2 | R.IVLQIDNAR.L | 22 |
|  | AstrinIP\_MS2\_022614\_01.06845.06845.1 | 2.5181 | 0.3095 | 100.0% | 1041.62 | 1042.2235 | 1 | 6.391 | 68.8% | 1 | R.IVLQIDNAR.L | 11 |
|  | AstrinIP\_MS2\_022614\_01.08524.08524.2 | 2.4955 | 0.3861 | 99.9% | 1030.1522 | 1030.2096 | 1 | 7.085 | 81.2% | 1 | R.VLDELTLAR.T | 222 |

Similarities:
contaminant\_KERATIN09(2:2)  
contaminant\_KERATIN03(1:3)  
contaminant\_KERATIN12(2:2)  
contaminant\_KERATIN05(2:2)  

---

|  |  |  |  |  |  |  |  |  |
| --- | --- | --- | --- | --- | --- | --- | --- | --- |
| U | *gi|119703753|ref|NP\_0* | 4 | 8 | 6.7% | 564 | 60067 | 8.0 | keratin 6B [Homo sapiens] |

| Filename XCorr DeltCN Conf% ObsM+H+ CalcM+H+ SpR ZScore Ion% # Sequence  | | | | | | | | | | | | |
| --- | --- | --- | --- | --- | --- | --- | --- | --- | --- | --- | --- | --- |
|  | AstrinIP\_MS1\_022614\_01.07103.07103.2 | 2.5876 | 0.1854 | 98.7% | 1083.1322 | 1083.2755 | 2 | 6.016 | 75.0% | 2 | K.FASFIDKVR.F | 2222 |
|  | AstrinIP\_MS1\_022614\_01.03650.03650.2 | 2.8304 | 0.2913 | 99.8% | 1309.3522 | 1309.4215 | 161 | 5.387 | 55.6% | 2 | K.NKYEDEINKR.T | 222 |
|  | AstrinIP\_MS1\_022614\_01.06365.06365.2 | 3.2145 | 0.3532 | 100.0% | 1181.2922 | 1180.303 | 1 | 6.204 | 88.9% | 3 | K.YEELQITAGR.H | 22 |
|  | AstrinIP\_MS2\_022614\_01.06780.06780.1 | 2.5379 | 0.2335 | 99.5% | 1153.47 | 1154.3234 | 178 | 5.811 | 56.2% | 1 | K.EYQELMNVK.L | 11 |

Similarities:
gi|4504919|ref|NP\_002(3:1)  
gi|47132620|ref|NP\_00(1:3)  
gi|67782365|ref|NP\_00(1:3)  
gi|119395750|ref|NP\_0(2:2)  

---

|  |  |  |  |  |  |  |  |  |
| --- | --- | --- | --- | --- | --- | --- | --- | --- |
| U | *gi|154759259|ref|NP\_0* | 10 | 11 | 6.6% | 2472 | 284538 | 5.3 | spectrin, alpha, non-erythrocytic 1 (alpha-fodrin) isoform 2 [Homo sapiens] |
| U | *gi|194595509|ref|NP\_0* | 10 | 11 | 6.6% | 2477 | 285093 | 5.4 | spectrin, alpha, non-erythrocytic 1 (alpha-fodrin) isoform 1 [Homo sapiens] |

| Filename XCorr DeltCN Conf% ObsM+H+ CalcM+H+ SpR ZScore Ion% # Sequence  | | | | | | | | | | | | |
| --- | --- | --- | --- | --- | --- | --- | --- | --- | --- | --- | --- | --- |
|  | AstrinIP\_MS1\_022614\_01.04811.04811.2 | 2.3898 | 0.3274 | 99.3% | 1303.6721 | 1303.4117 | 1 | 5.143 | 75.0% | 1 | K.VLETAEDIQER.R | 2 |
|  | AstrinIP\_MS2\_022614\_01.15764.15764.2 | 4.8194 | 0.4573 | 100.0% | 2128.172 | 2128.344 | 1 | 9.139 | 57.5% | 1 | K.ALINADELASDVAGAEALLDR.H | 2 |
|  | AstrinIP\_MS2\_022614\_01.08693.08693.2 | 2.3796 | 0.1674 | 95.8% | 1292.5122 | 1292.3446 | 4 | 4.233 | 61.1% | 1 | R.DVEDEETWIR.E | 2 |
|  | AstrinIP\_MS1\_022614\_01.11861.11861.3 | 3.4325 | 0.4336 | 100.0% | 2033.9343 | 2033.3348 | 1 | 6.418 | 39.1% | 1 | K.KGDILTLLNSTNKDWWK.V | 3 |
|  | AstrinIP\_MS1\_022614\_01.03981.03981.2 | 3.0169 | 0.249 | 99.7% | 1324.9521 | 1325.4673 | 1 | 5.457 | 68.2% | 1 | R.SQLLGSAHEVQR.F | 2 |
|  | AstrinIP\_MS1\_022614\_01.13647.13647.3 | 2.68 | 0.2961 | 98.3% | 1973.4243 | 1973.2822 | 10 | 4.521 | 29.2% | 1 | K.IAALQAFADQLIAAGHYAK.G | 3 |
|  | AstrinIP\_MS2\_022614\_02.04161.04161.3 | 3.6657 | 0.33 | 100.0% | 1634.6943 | 1634.7458 | 34 | 7.027 | 38.5% | 1 | K.HQLLEADISAHEDR.L | 3 |
|  | AstrinIP\_MS2\_022614\_01.16240.16240.3 | 5.41 | 0.5176 | 100.0% | 3234.1143 | 3234.6335 | 1 | 8.249 | 27.6% | 2 | K.QETFDAGLQAFQQEGIANITALKDQLLAAK.H | 3 |
|  | AstrinIP\_MS2\_022614\_01.11584.11584.2 | 3.8068 | 0.4893 | 100.0% | 1311.4122 | 1311.5638 | 1 | 7.927 | 85.0% | 1 | R.KVEDLFLTFAK.K | 2 |
|  | AstrinIP\_MS1\_022614\_01.11043.11043.2 | 2.337 | 0.2346 | 95.6% | 1955.0521 | 1953.0728 | 2 | 4.765 | 35.3% | 1 | R.SSLSSAQADFNQLAELDR.Q | 2 |

---

|  |  |  |  |  |  |  |  |  |
| --- | --- | --- | --- | --- | --- | --- | --- | --- |
| U | *gi|19923142|ref|NP\_00* | 4 | 6 | 6.6% | 876 | 97170 | 4.8 | karyopherin beta 1 [Homo sapiens] |

| Filename XCorr DeltCN Conf% ObsM+H+ CalcM+H+ SpR ZScore Ion% # Sequence  | | | | | | | | | | | | |
| --- | --- | --- | --- | --- | --- | --- | --- | --- | --- | --- | --- | --- |
| \* | AstrinIP\_MS2\_022614\_01.14733.14733.2 | 4.3367 | 0.4433 | 100.0% | 1659.4122 | 1659.9231 | 1 | 7.612 | 71.4% | 2 | R.AAVENLPTFLVELSR.V | 2 |
| \* | AstrinIP\_MS1\_022614\_01.03732.03732.2 | 2.9965 | 0.473 | 100.0% | 1226.0322 | 1226.378 | 1 | 7.715 | 68.2% | 1 | R.VLANPGNSQVAR.V | 2 |
| \* | AstrinIP\_MS2\_022614\_01.12717.12717.2 | 3.4298 | 0.3994 | 100.0% | 1606.4122 | 1606.8595 | 1 | 7.097 | 60.7% | 2 | K.LAATNALLNSLEFTK.A | 2 |
| \* | AstrinIP\_MS1\_022614\_01.03833.03833.3 | 2.9948 | 0.278 | 99.1% | 1880.8143 | 1880.0275 | 1 | 5.424 | 40.0% | 1 | K.LLETTDRPDGHQNNLR.S | 3 |

---

|  |  |  |  |  |  |  |  |  |
| --- | --- | --- | --- | --- | --- | --- | --- | --- |
| U | *gi|117938251|ref|NP\_0* | 5 | 7 | 6.4% | 869 | 100232 | 10.0 | BCL2-associated transcription factor 1 isoform 2 [Homo sapiens] |
| U | *gi|7661958|ref|NP\_055* | 5 | 7 | 6.1% | 920 | 106122 | 10.0 | BCL2-associated transcription factor 1 isoform 1 [Homo sapiens] |

| Filename XCorr DeltCN Conf% ObsM+H+ CalcM+H+ SpR ZScore Ion% # Sequence  | | | | | | | | | | | | |
| --- | --- | --- | --- | --- | --- | --- | --- | --- | --- | --- | --- | --- |
|  | AstrinIP\_MS1\_022614\_01.03850.03850.2 | 4.3615 | 0.4656 | 100.0% | 1708.5721 | 1708.9524 | 1 | 8.225 | 64.3% | 1 | K.LKETGYVVERPSTTK.D | 2 |
|  | AstrinIP\_MS1\_022614\_01.03828.03828.2 | 2.9704 | 0.4269 | 100.0% | 1467.4321 | 1467.6189 | 3 | 6.429 | 54.2% | 1 | K.ETGYVVERPSTTK.D | 2 |
|  | AstrinIP\_MS2\_022614\_01.03502.03502.2 | 3.2397 | 0.3113 | 100.0% | 1370.1122 | 1370.5664 | 1 | 5.722 | 68.2% | 1 | K.MIASDSHRPEVK.L | 2 |
|  | AstrinIP\_MS1\_022614\_01.06507.06507.2 | 3.457 | 0.3411 | 100.0% | 1168.3922 | 1168.4221 | 1 | 6.952 | 80.0% | 2 | R.LLASTLVHSVK.K | 2 |
|  | AstrinIP\_MS1\_022614\_01.11986.11986.3 | 3.6215 | 0.3569 | 100.0% | 2049.3843 | 2049.3372 | 2 | 6.276 | 41.2% | 2 | K.STSESFIQHIVSLVHHVK.E | 3 |

---

|  |  |  |  |  |  |  |  |  |
| --- | --- | --- | --- | --- | --- | --- | --- | --- |
| U | *gi|14165435|ref|NP\_11* | 2 | 2 | 6.3% | 463 | 50976 | 5.5 | heterogeneous nuclear ribonucleoprotein K isoform b [Homo sapiens] |
| U | *gi|14165439|ref|NP\_00* | 2 | 2 | 6.2% | 464 | 51028 | 5.3 | heterogeneous nuclear ribonucleoprotein K isoform a [Homo sapiens] |
| U | *gi|14165437|ref|NP\_11* | 2 | 2 | 6.2% | 464 | 51028 | 5.3 | heterogeneous nuclear ribonucleoprotein K isoform a [Homo sapiens] |

| Filename XCorr DeltCN Conf% ObsM+H+ CalcM+H+ SpR ZScore Ion% # Sequence  | | | | | | | | | | | | |
| --- | --- | --- | --- | --- | --- | --- | --- | --- | --- | --- | --- | --- |
|  | AstrinIP\_MS2\_022614\_01.15350.15350.2 | 2.9748 | 0.3619 | 99.8% | 1844.2122 | 1844.1992 | 83 | 6.721 | 28.1% | 1 | R.ILSISADIETIGEILKK.I | 2 |
|  | AstrinIP\_MS2\_022614\_01.12761.12761.2 | 3.21 | 0.3226 | 100.0% | 1342.0721 | 1341.6311 | 1 | 5.415 | 77.3% | 1 | K.IILDLISESPIK.G | 2 |

---

|  |  |  |  |  |  |  |  |  |
| --- | --- | --- | --- | --- | --- | --- | --- | --- |
| U | *gi|5031699|ref|NP\_005* | 2 | 3 | 6.1% | 427 | 47355 | 7.5 | flotillin 1 [Homo sapiens] |

| Filename XCorr DeltCN Conf% ObsM+H+ CalcM+H+ SpR ZScore Ion% # Sequence  | | | | | | | | | | | | |
| --- | --- | --- | --- | --- | --- | --- | --- | --- | --- | --- | --- | --- |
| \* | AstrinIP\_MS1\_022614\_02.05651.05651.2 | 2.8939 | 0.3995 | 100.0% | 1420.2722 | 1419.6206 | 1 | 6.329 | 62.5% | 2 | R.AQADLAYQLQVAK.T | 2 |
| \* | AstrinIP\_MS2\_022614\_01.06273.06273.2 | 2.3743 | 0.2452 | 97.6% | 1379.5322 | 1379.5768 | 1 | 4.516 | 58.3% | 1 | R.MRGEAEAFAIGAR.A | 2 |

---

|  |  |  |  |  |  |  |  |  |
| --- | --- | --- | --- | --- | --- | --- | --- | --- |
| U | *gi|115298682|ref|NP\_0* | 13 | 18 | 6.0% | 2817 | 308606 | 9.1 | HBxAg transactivated protein 2 [Homo sapiens] |

| Filename XCorr DeltCN Conf% ObsM+H+ CalcM+H+ SpR ZScore Ion% # Sequence  | | | | | | | | | | | | |
| --- | --- | --- | --- | --- | --- | --- | --- | --- | --- | --- | --- | --- |
| \* | AstrinIP\_MS1\_022614\_01.10646.10646.2 | 2.9047 | 0.2455 | 99.7% | 1322.4122 | 1321.5156 | 1 | 5.717 | 70.0% | 1 | K.YATLSLFNTYK.G | 2 |
|  | AstrinIP\_MS1\_022614\_01.06160.06160.2 | 2.5862 | 0.2671 | 99.1% | 1322.3722 | 1321.6238 | 1 | 4.814 | 63.6% | 2 | R.RMPPPANLPSLK.A | 2 |
| \* | AstrinIP\_MS2\_022614\_01.04949.04949.2 | 4.3261 | 0.4596 | 100.0% | 1420.2522 | 1420.5681 | 1 | 8.875 | 75.0% | 1 | K.LNGQQAALASQYR.A | 2 |
| \* | AstrinIP\_MS2\_022614\_01.03585.03585.2 | 3.3232 | 0.3865 | 100.0% | 1118.1921 | 1118.278 | 1 | 6.497 | 70.0% | 1 | R.GTSSHLPPPPK.L | 2 |
| \* | AstrinIP\_MS2\_022614\_01.03569.03569.2 | 2.9622 | 0.3362 | 99.9% | 1272.0521 | 1272.4496 | 1 | 5.359 | 65.0% | 1 | K.LLAQQHPPPDR.Q | 2 |
| \* | AstrinIP\_MS2\_022614\_01.04278.04278.2 | 2.5062 | 0.3047 | 99.1% | 1337.6921 | 1338.5521 | 31 | 5.706 | 54.2% | 1 | R.QAVPGRPGPFPSK.Q | 2 |
| \* | AstrinIP\_MS2\_022614\_01.07467.07467.2 | 2.8217 | 0.3352 | 99.8% | 1361.2922 | 1361.4509 | 9 | 5.931 | 55.0% | 1 | K.QQVADEDEIWK.Q | 2 |
| \* | AstrinIP\_MS1\_022614\_01.04269.04269.2 | 2.6332 | 0.2596 | 99.1% | 1217.6721 | 1218.3091 | 14 | 4.993 | 70.0% | 2 | R.QQSEISAAVER.A | 2 |
| \* | AstrinIP\_MS2\_022614\_01.08290.08290.3 | 3.4638 | 0.3043 | 99.3% | 2913.7144 | 2914.2012 | 1 | 4.922 | 26.0% | 1 | K.EATPVVHETEPESGSQPRPAVLSGYFK.Q | 3 |
| \* | AstrinIP\_MS2\_022614\_01.12159.12159.2 | 3.281 | 0.4713 | 100.0% | 1457.9122 | 1458.7512 | 1 | 7.802 | 80.0% | 3 | R.WLMMQSYMDPR.M | 2 |
| \* | AstrinIP\_MS2\_022614\_01.05631.05631.3 | 3.6692 | 0.2671 | 99.3% | 2257.4944 | 2258.2976 | 15 | 5.244 | 33.3% | 1 | R.RDQMEGS\*PNSSESFEHIAR.S | 3 |
| \* | AstrinIP\_MS2\_022614\_01.04426.04426.2 | 2.9697 | 0.3964 | 100.0% | 1124.7322 | 1125.2266 | 1 | 8.007 | 88.9% | 2 | R.DHAISLSEPR.M | 2 |
| \* | AstrinIP\_MS2\_022614\_01.03558.03558.2 | 3.1623 | 0.3604 | 100.0% | 1073.9922 | 1074.179 | 1 | 6.876 | 83.3% | 1 | R.ISAVESQPSR.K | 2 |

---

|  |  |  |  |  |  |  |  |  |
| --- | --- | --- | --- | --- | --- | --- | --- | --- |
| U | *gi|54607053|ref|NP\_00* | 10 | 13 | 5.7% | 2671 | 292708 | 7.4 | GCN1 general control of amino-acid synthesis 1-like 1 [Homo sapiens] |

| Filename XCorr DeltCN Conf% ObsM+H+ CalcM+H+ SpR ZScore Ion% # Sequence  | | | | | | | | | | | | |
| --- | --- | --- | --- | --- | --- | --- | --- | --- | --- | --- | --- | --- |
| \* | AstrinIP\_MS2\_022614\_01.07630.07630.2 | 2.8976 | 0.3753 | 99.8% | 1440.4321 | 1441.6273 | 1 | 5.846 | 69.2% | 2 | K.NLLHSLQSSGIGSK.A | 2 |
| \* | AstrinIP\_MS1\_022614\_02.13677.13677.2 | 4.1667 | 0.4409 | 100.0% | 2237.2522 | 2238.627 | 1 | 9.264 | 50.0% | 2 | R.LQELDGELEAALGLLDIILAK.N | 2 |
| \* | AstrinIP\_MS2\_022614\_01.20030.20030.3 | 4.8077 | 0.3701 | 100.0% | 2238.0244 | 2238.627 | 1 | 8.061 | 48.8% | 1 | R.LQELDGELEAALGLLDIILAK.N | 3 |
| \* | AstrinIP\_MS1\_022614\_01.07790.07790.2 | 2.8862 | 0.3979 | 100.0% | 1267.9722 | 1267.5143 | 1 | 5.772 | 59.1% | 1 | K.ALGTLVSHVTLR.L | 2 |
| \* | AstrinIP\_MS1\_022614\_01.06460.06460.2 | 2.4171 | 0.2566 | 98.1% | 1513.9122 | 1514.6348 | 12 | 4.309 | 54.2% | 1 | R.VISESPPDQWEAR.C | 2 |
| \* | AstrinIP\_MS2\_022614\_01.16356.16356.3 | 3.4789 | 0.2721 | 99.2% | 2390.1843 | 2388.8154 | 2 | 4.327 | 32.1% | 1 | R.IRFSSVQLLGDLLFHISGVTGK.M | 3 |
| \* | AstrinIP\_MS1\_022614\_01.15261.15261.2 | 2.8938 | 0.3125 | 99.8% | 1605.3121 | 1605.9585 | 1 | 5.64 | 57.7% | 2 | K.ILPEIIPILEEGLR.S | 2 |
| \* | AstrinIP\_MS2\_022614\_01.17820.17820.2 | 5.5085 | 0.4977 | 100.0% | 2028.6122 | 2028.2878 | 1 | 8.995 | 64.7% | 1 | K.NIVSLLLSMLGHDEDNTR.I | 2 |
| \* | AstrinIP\_MS1\_022614\_01.12125.12125.3 | 3.1529 | 0.279 | 98.5% | 2767.8843 | 2767.1267 | 1 | 4.45 | 24.0% | 1 | R.YSSDVQEMILSSATADRIPIAVSGVR.G | 3 |
| \* | AstrinIP\_MS2\_022614\_01.11765.11765.2 | 3.1301 | 0.3008 | 99.8% | 1335.2722 | 1335.5431 | 3 | 5.742 | 63.6% | 1 | R.AYSDQAIVNLLK.M | 2 |

---

|  |  |  |  |  |  |  |  |  |
| --- | --- | --- | --- | --- | --- | --- | --- | --- |
| U | *gi|42740897|ref|NP\_00* | 3 | 4 | 5.6% | 804 | 90996 | 5.2 | CTAGE family, member 5 isoform 1 [Homo sapiens] |
| U | *gi|42740905|ref|NP\_97* | 3 | 4 | 5.7% | 792 | 89647 | 5.2 | CTAGE family, member 5 isoform 2 [Homo sapiens] |
| U | *gi|42740901|ref|NP\_97* | 3 | 4 | 5.8% | 775 | 87837 | 5.3 | CTAGE family, member 5 isoform 4 [Homo sapiens] |
| U | *gi|42740899|ref|NP\_97* | 3 | 4 | 5.9% | 761 | 86532 | 5.2 | CTAGE family, member 5 isoform 3 [Homo sapiens] |

| Filename XCorr DeltCN Conf% ObsM+H+ CalcM+H+ SpR ZScore Ion% # Sequence  | | | | | | | | | | | | |
| --- | --- | --- | --- | --- | --- | --- | --- | --- | --- | --- | --- | --- |
|  | AstrinIP\_MS2\_022614\_02.04845.04845.3 | 3.2562 | 0.2777 | 99.1% | 2030.7544 | 2030.2426 | 36 | 4.826 | 30.9% | 2 | K.IAIKDALNENSQLQESQK.Q | 3 |
|  | AstrinIP\_MS1\_022614\_01.03753.03753.3 | 2.7716 | 0.3406 | 99.6% | 1747.8544 | 1747.9486 | 1 | 6.36 | 39.3% | 1 | K.VHAEQVLNDKESHIK.T | 3 |
|  | AstrinIP\_MS1\_022614\_01.06748.06748.2 | 2.6281 | 0.1538 | 96.5% | 1515.9722 | 1515.7365 | 7 | 4.515 | 54.5% | 1 | K.VMTELYQENEMK.L | 2 |

---

|  |  |  |  |  |  |  |  |  |
| --- | --- | --- | --- | --- | --- | --- | --- | --- |
| U | *gi|145309326|ref|NP\_0* | 6 | 6 | 5.5% | 1609 | 177602 | 5.1 | laminin, gamma 1 precursor [Homo sapiens] |

| Filename XCorr DeltCN Conf% ObsM+H+ CalcM+H+ SpR ZScore Ion% # Sequence  | | | | | | | | | | | | |
| --- | --- | --- | --- | --- | --- | --- | --- | --- | --- | --- | --- | --- |
| \* | AstrinIP\_MS1\_022614\_01.11717.11717.2 | 2.9835 | 0.214 | 99.0% | 1444.6921 | 1443.6403 | 1 | 5.552 | 65.4% | 1 | R.LSAEDLVLEGAGLR.V | 2 |
| \* | AstrinIP\_MS2\_022614\_01.05698.05698.2 | 4.5142 | 0.2517 | 99.9% | 1547.3922 | 1546.6342 | 1 | 6.716 | 76.9% | 1 | R.NTIEETGNLAEQAR.A | 2 |
| \* | AstrinIP\_MS1\_022614\_01.10342.10342.2 | 3.3596 | 0.1865 | 99.6% | 1562.0322 | 1561.7473 | 3 | 5.116 | 70.8% | 1 | K.MEAENLEQLIDQK.L | 2 |
| \* | AstrinIP\_MS2\_022614\_01.12786.12786.2 | 2.5141 | 0.1815 | 95.2% | 1601.8722 | 1601.7539 | 1 | 3.92 | 61.5% | 1 | R.DTLQEANDILNNLK.D | 2 |
| \* | AstrinIP\_MS2\_022614\_01.05074.05074.2 | 4.7373 | 0.5155 | 100.0% | 1632.1122 | 1632.7245 | 1 | 9.791 | 78.1% | 1 | R.EAQQALGSAAADATEAK.N | 2 |
| \* | AstrinIP\_MS1\_022614\_01.12850.12850.2 | 3.4307 | 0.2919 | 99.8% | 1954.8322 | 1954.159 | 4 | 5.247 | 40.6% | 1 | R.TFAEVTDLDNEVNNMLK.Q | 2 |

---

|  |  |  |  |  |  |  |  |  |
| --- | --- | --- | --- | --- | --- | --- | --- | --- |
| U | *gi|112382250|ref|NP\_0* | 8 | 9 | 5.1% | 2364 | 274608 | 5.6 | spectrin, beta, non-erythrocytic 1 isoform 1 [Homo sapiens] |
| U | *gi|112382252|ref|NP\_8* | 8 | 9 | 5.6% | 2155 | 251395 | 5.5 | spectrin, beta, non-erythrocytic 1 isoform 2 [Homo sapiens] |

| Filename XCorr DeltCN Conf% ObsM+H+ CalcM+H+ SpR ZScore Ion% # Sequence  | | | | | | | | | | | | |
| --- | --- | --- | --- | --- | --- | --- | --- | --- | --- | --- | --- | --- |
|  | AstrinIP\_MS2\_022614\_01.12248.12248.3 | 6.0072 | 0.4692 | 100.0% | 2484.4744 | 2484.7336 | 1 | 8.301 | 44.0% | 2 | K.SNAHYNLQNAFNLAEQHLGLTK.L | 3 |
|  | AstrinIP\_MS2\_022614\_01.17388.17388.2 | 2.9929 | 0.2228 | 99.7% | 1348.3722 | 1348.628 | 5 | 6.589 | 66.7% | 1 | R.LWEYLLELLR.A | 2 |
|  | AstrinIP\_MS2\_022614\_01.09182.09182.2 | 3.0571 | 0.3865 | 100.0% | 1799.9922 | 1801.0216 | 1 | 6.968 | 53.3% | 1 | K.ILSSDDYGKDLTSVMR.L | 2 |
|  | AstrinIP\_MS2\_022614\_01.19750.19750.3 | 3.3794 | 0.2742 | 99.0% | 3001.6443 | 3002.3655 | 1 | 5.422 | 27.0% | 1 | R.LEEASLLHQFQADADDIDAWMLDILK.I | 3 |
|  | AstrinIP\_MS2\_022614\_01.13248.13248.2 | 2.5392 | 0.2193 | 98.4% | 1382.7722 | 1382.4723 | 1 | 4.753 | 70.0% | 1 | R.DLDDFQSWLSR.T | 2 |
|  | AstrinIP\_MS1\_022614\_01.08044.08044.2 | 3.1189 | 0.183 | 99.1% | 1525.3522 | 1525.7043 | 1 | 5.154 | 75.0% | 1 | R.LQALDTGWNELHK.M | 2 |
|  | AstrinIP\_MS2\_022614\_01.12196.12196.2 | 2.4581 | 0.2505 | 98.6% | 1391.6122 | 1391.6251 | 1 | 5.536 | 75.0% | 1 | K.FMELLEPLNER.K | 2 |
|  | AstrinIP\_MS2\_022614\_01.06713.06713.2 | 2.9922 | 0.3881 | 100.0% | 1374.0122 | 1374.5797 | 1 | 5.788 | 72.7% | 1 | R.TQILAASYELHK.F | 2 |

---

|  |  |  |  |  |  |  |  |  |
| --- | --- | --- | --- | --- | --- | --- | --- | --- |
| U | *gi|50659095|ref|NP\_00* | 2 | 2 | 5.1% | 783 | 87344 | 9.3 | DEAD (Asp-Glu-Ala-Asp) box polypeptide 21 [Homo sapiens] |

| Filename XCorr DeltCN Conf% ObsM+H+ CalcM+H+ SpR ZScore Ion% # Sequence  | | | | | | | | | | | | |
| --- | --- | --- | --- | --- | --- | --- | --- | --- | --- | --- | --- | --- |
| \* | AstrinIP\_MS2\_022614\_01.08064.08064.2 | 2.5337 | 0.1894 | 97.8% | 1165.1122 | 1165.4215 | 2 | 4.594 | 65.0% | 1 | R.APQVLVLAPTR.E | 2 |
| \* | AstrinIP\_MS2\_022614\_01.17766.17766.3 | 3.2292 | 0.2191 | 95.3% | 3322.1343 | 3323.8008 | 67 | 3.8 | 20.5% | 1 | K.HVVLDEVDQMLDMGFADQVEEILSVAYKK.D | 3 |

---

|  |  |  |  |  |  |  |  |  |
| --- | --- | --- | --- | --- | --- | --- | --- | --- |
| U | *gi|4503571|ref|NP\_001* | 2 | 2 | 5.1% | 434 | 47169 | 7.4 | enolase 1 [Homo sapiens] |

| Filename XCorr DeltCN Conf% ObsM+H+ CalcM+H+ SpR ZScore Ion% # Sequence  | | | | | | | | | | | | |
| --- | --- | --- | --- | --- | --- | --- | --- | --- | --- | --- | --- | --- |
| \* | AstrinIP\_MS2\_022614\_01.04391.04391.2 | 2.5927 | 0.1088 | 95.1% | 1145.1921 | 1144.3158 | 4 | 3.717 | 72.2% | 1 | R.IGAEVYHNLK.N | 2 |
| \* | AstrinIP\_MS2\_022614\_01.10974.10974.2 | 2.6301 | 0.1425 | 95.9% | 1426.0922 | 1426.6091 | 1 | 5.546 | 68.2% | 1 | R.YISPDQLADLYK.S | 2 |

---

|  |  |  |  |  |  |  |  |  |
| --- | --- | --- | --- | --- | --- | --- | --- | --- |
| U | *gi|154800453|ref|NP\_0* | 2 | 2 | 5.0% | 778 | 83857 | 7.4 | tastin isoform 1 [Homo sapiens] |

| Filename XCorr DeltCN Conf% ObsM+H+ CalcM+H+ SpR ZScore Ion% # Sequence  | | | | | | | | | | | | |
| --- | --- | --- | --- | --- | --- | --- | --- | --- | --- | --- | --- | --- |
| \* | AstrinIP\_MS1\_022614\_01.03734.03734.3 | 3.2227 | 0.304 | 99.3% | 1975.7644 | 1976.1564 | 1 | 5.888 | 38.2% | 1 | R.SLEGSGKPPVATPSGPHSNR.T | 3 |
| \* | AstrinIP\_MS1\_022614\_01.05789.05789.3 | 2.9291 | 0.2688 | 98.2% | 1991.6344 | 1991.2113 | 13 | 5.012 | 29.2% | 1 | R.TLNATEHNSGTSHLPGLLK.H | 3 |

---

|  |  |  |  |  |  |  |  |  |
| --- | --- | --- | --- | --- | --- | --- | --- | --- |
| U | *gi|4506411|ref|NP\_002* | 2 | 2 | 4.9% | 587 | 63542 | 4.7 | Ran GTPase activating protein 1 [Homo sapiens] |

| Filename XCorr DeltCN Conf% ObsM+H+ CalcM+H+ SpR ZScore Ion% # Sequence  | | | | | | | | | | | | |
| --- | --- | --- | --- | --- | --- | --- | --- | --- | --- | --- | --- | --- |
| \* | AstrinIP\_MS2\_022614\_01.09814.09814.2 | 3.6456 | 0.3255 | 100.0% | 1748.4521 | 1748.8931 | 1 | 6.35 | 53.3% | 1 | R.NRLENDGATALAEAFR.V | 2 |
| \* | AstrinIP\_MS2\_022614\_01.12174.12174.2 | 2.4488 | 0.3428 | 99.4% | 1419.8722 | 1420.682 | 1 | 5.615 | 58.3% | 1 | R.MAVQDAVDALMQK.A | 2 |

---

|  |  |  |  |  |  |  |  |  |
| --- | --- | --- | --- | --- | --- | --- | --- | --- |
| U | *gi|206597472|ref|NP\_0* | 2 | 2 | 4.9% | 534 | 61139 | 7.3 | zinc finger protein 397 isoform 1 [Homo sapiens] |

| Filename XCorr DeltCN Conf% ObsM+H+ CalcM+H+ SpR ZScore Ion% # Sequence  | | | | | | | | | | | | |
| --- | --- | --- | --- | --- | --- | --- | --- | --- | --- | --- | --- | --- |
|  | AstrinIP\_MS1\_022614\_01.05706.05706.2 | 2.9593 | 0.3838 | 99.9% | 1567.8922 | 1567.7391 | 69 | 6.026 | 42.3% | 1 | R.ASQESTDIHLQPLK.T | 2 |
| \* | AstrinIP\_MS1\_022614\_01.06219.06219.2 | 2.6953 | 0.2803 | 99.5% | 1399.3121 | 1399.5461 | 8 | 5.121 | 50.0% | 1 | R.GISEHESNLVWK.Q | 2 |

---

|  |  |  |  |  |  |  |  |  |
| --- | --- | --- | --- | --- | --- | --- | --- | --- |
| U | *gi|119395758|ref|NP\_0* | 3 | 4 | 4.8% | 1272 | 141347 | 5.4 | diaphanous 1 isoform 1 [Homo sapiens] |
| U | *gi|119395760|ref|NP\_0* | 3 | 4 | 4.8% | 1263 | 140289 | 5.4 | diaphanous 1 isoform 2 [Homo sapiens] |

| Filename XCorr DeltCN Conf% ObsM+H+ CalcM+H+ SpR ZScore Ion% # Sequence  | | | | | | | | | | | | |
| --- | --- | --- | --- | --- | --- | --- | --- | --- | --- | --- | --- | --- |
|  | AstrinIP\_MS2\_022614\_01.20001.20001.3 | 3.959 | 0.4704 | 100.0% | 3043.1343 | 3043.493 | 1 | 7.923 | 30.6% | 2 | R.VSLNNNPVSWVQTFGAEGLASLLDILKR.L | 3 |
|  | AstrinIP\_MS2\_022614\_01.03455.03455.3 | 3.9153 | 0.4132 | 100.0% | 1737.0844 | 1737.7771 | 1 | 7.297 | 44.6% | 1 | R.LHDEKEETAGSYDSR.N | 3 |
|  | AstrinIP\_MS2\_022614\_01.19095.19095.2 | 3.2701 | 0.3382 | 99.9% | 2185.3523 | 2187.5305 | 159 | 5.353 | 32.4% | 1 | R.MEMDDFNEVFQILLNTVK.D | 2 |

---

|  |  |  |  |  |  |  |  |  |
| --- | --- | --- | --- | --- | --- | --- | --- | --- |
| U | *gi|4507555|ref|NP\_003* | 2 | 2 | 4.8% | 694 | 75492 | 7.7 | thymopoietin isoform alpha [Homo sapiens] |

| Filename XCorr DeltCN Conf% ObsM+H+ CalcM+H+ SpR ZScore Ion% # Sequence  | | | | | | | | | | | | |
| --- | --- | --- | --- | --- | --- | --- | --- | --- | --- | --- | --- | --- |
|  | AstrinIP\_MS2\_022614\_01.06616.06616.2 | 2.3029 | 0.2827 | 98.1% | 1331.2522 | 1331.5143 | 1 | 4.565 | 66.7% | 1 | K.YGVNPGPIVGTTR.K | 2 |
| \* | AstrinIP\_MS2\_022614\_02.07642.07642.3 | 3.0283 | 0.3113 | 99.1% | 2034.6843 | 2035.3073 | 2 | 5.632 | 32.9% | 1 | R.AYEAAASALQIATHTAFVAK.A | 3 |

---

|  |  |  |  |  |  |  |  |  |
| --- | --- | --- | --- | --- | --- | --- | --- | --- |
| U | *gi|62241042|ref|NP\_00* | 5 | 6 | 4.5% | 1512 | 170590 | 7.3 | glutamyl-prolyl tRNA synthetase [Homo sapiens] |

| Filename XCorr DeltCN Conf% ObsM+H+ CalcM+H+ SpR ZScore Ion% # Sequence  | | | | | | | | | | | | |
| --- | --- | --- | --- | --- | --- | --- | --- | --- | --- | --- | --- | --- |
| \* | AstrinIP\_MS1\_022614\_02.05681.05681.2 | 2.2126 | 0.3066 | 98.2% | 1546.2522 | 1546.769 | 2 | 4.988 | 50.0% | 1 | K.AALLNQHYQVNFK.G | 2 |
| \* | AstrinIP\_MS1\_022614\_01.04614.04614.2 | 2.2955 | 0.2186 | 97.9% | 1071.4321 | 1071.265 | 9 | 5.186 | 68.8% | 1 | K.KGDIIQLQR.R | 2 |
| \* | AstrinIP\_MS2\_022614\_01.10911.10911.2 | 3.5342 | 0.3727 | 100.0% | 1700.2722 | 1699.9065 | 1 | 8.782 | 57.1% | 2 | K.THVADFAPEVAWVTR.S | 2 |
| \* | AstrinIP\_MS1\_022614\_01.03699.03699.2 | 4.344 | 0.5118 | 100.0% | 1683.0721 | 1682.8362 | 1 | 8.434 | 60.0% | 1 | R.AIQGGTSHHLGQNFSK.M | 2 |
| \* | AstrinIP\_MS1\_022614\_02.10403.10403.2 | 3.9033 | 0.529 | 100.0% | 1760.6921 | 1761.0715 | 1 | 9.561 | 67.9% | 1 | K.LQAILEDIQVTLFTR.A | 2 |

---

|  |  |  |  |  |  |  |  |  |
| --- | --- | --- | --- | --- | --- | --- | --- | --- |
| U | *gi|148536853|ref|NP\_0* | 4 | 5 | 4.5% | 1224 | 138345 | 7.7 | coatomer protein complex, subunit alpha isoform 2 [Homo sapiens] |
| U | *gi|148536855|ref|NP\_0* | 4 | 5 | 4.5% | 1233 | 139324 | 7.6 | coatomer protein complex, subunit alpha isoform 1 [Homo sapiens] |

| Filename XCorr DeltCN Conf% ObsM+H+ CalcM+H+ SpR ZScore Ion% # Sequence  | | | | | | | | | | | | |
| --- | --- | --- | --- | --- | --- | --- | --- | --- | --- | --- | --- | --- |
|  | AstrinIP\_MS2\_022614\_02.07641.07641.2 | 3.3987 | 0.4367 | 100.0% | 1593.8121 | 1593.8174 | 1 | 6.964 | 60.0% | 1 | R.GITGVDLFGTTDAVVK.H | 2 |
|  | AstrinIP\_MS1\_022614\_01.05475.05475.2 | 2.2003 | 0.2249 | 95.6% | 1277.2322 | 1276.3861 | 45 | 4.449 | 55.0% | 1 | R.QELILSNSEDK.S | 2 |
|  | AstrinIP\_MS1\_022614\_01.15002.15002.2 | 2.682 | 0.2221 | 98.5% | 1512.1721 | 1511.8015 | 1 | 4.952 | 54.2% | 1 | K.LSFLYLITGNLEK.L | 2 |
|  | AstrinIP\_MS2\_022614\_01.10264.10264.2 | 2.9444 | 0.1256 | 96.3% | 1601.2922 | 1601.8839 | 5 | 5.914 | 46.4% | 2 | R.VTTVTEIGKDVIGLR.I | 2 |

---

|  |  |  |  |  |  |  |  |  |
| --- | --- | --- | --- | --- | --- | --- | --- | --- |
| U | *gi|57164942|ref|NP\_00* | 7 | 10 | 4.2% | 2032 | 225493 | 7.8 | colonic and hepatic tumor over-expressed protein isoform a [Homo sapiens] |
| U | *gi|57222563|ref|NP\_05* | 7 | 10 | 4.3% | 1972 | 218524 | 8.1 | colonic and hepatic tumor over-expressed protein isoform b [Homo sapiens] |

| Filename XCorr DeltCN Conf% ObsM+H+ CalcM+H+ SpR ZScore Ion% # Sequence  | | | | | | | | | | | | |
| --- | --- | --- | --- | --- | --- | --- | --- | --- | --- | --- | --- | --- |
|  | AstrinIP\_MS2\_022614\_02.05331.05331.2 | 2.5702 | 0.3503 | 99.8% | 1320.7522 | 1321.5162 | 4 | 5.665 | 59.1% | 1 | K.FVTDSNAVVQLK.G | 2 |
|  | AstrinIP\_MS2\_022614\_02.06870.06870.2 | 2.8368 | 0.318 | 99.8% | 1345.6921 | 1345.5358 | 2 | 6.329 | 62.5% | 1 | K.TSAQVVLDGLVDK.I | 2 |
|  | AstrinIP\_MS2\_022614\_02.05775.05775.2 | 2.9073 | 0.3282 | 99.8% | 1549.4722 | 1550.7466 | 1 | 6.26 | 57.7% | 2 | R.TEISDKITSELVSK.I | 2 |
|  | AstrinIP\_MS2\_022614\_01.18812.18812.3 | 4.4293 | 0.3284 | 100.0% | 2402.6343 | 2401.842 | 1 | 6.432 | 32.1% | 2 | R.EASTGVLKDLMHGLITLMLDSR.I | 3 |
|  | AstrinIP\_MS2\_022614\_01.16995.16995.2 | 3.2554 | 0.3947 | 100.0% | 1614.8722 | 1615.9465 | 1 | 6.433 | 69.2% | 1 | K.DLMHGLITLMLDSR.I | 2 |
|  | AstrinIP\_MS1\_022614\_01.06453.06453.2 | 3.1183 | 0.2093 | 99.5% | 1429.4321 | 1429.57 | 3 | 4.98 | 68.2% | 1 | R.IEDLEEGQQVIR.S | 2 |
|  | AstrinIP\_MS2\_022614\_01.09089.09089.2 | 3.3067 | 0.3428 | 100.0% | 1492.2922 | 1492.5876 | 6 | 6.397 | 59.1% | 2 | K.NSSQFFQSYVER.G | 2 |

---

|  |  |  |  |  |  |  |  |  |
| --- | --- | --- | --- | --- | --- | --- | --- | --- |
| U | *gi|26051235|ref|NP\_06* | 4 | 4 | 4.2% | 1156 | 128979 | 5.1 | nucleoporin 133kDa [Homo sapiens] |

| Filename XCorr DeltCN Conf% ObsM+H+ CalcM+H+ SpR ZScore Ion% # Sequence  | | | | | | | | | | | | |
| --- | --- | --- | --- | --- | --- | --- | --- | --- | --- | --- | --- | --- |
| \* | AstrinIP\_MS2\_022614\_01.15586.15586.2 | 2.6948 | 0.2549 | 98.6% | 1975.3322 | 1976.3062 | 1 | 5.375 | 46.9% | 1 | K.AHSFLMDFIHQVGLFGR.L | 2 |
| \* | AstrinIP\_MS1\_022614\_01.14980.14980.3 | 2.783 | 0.35 | 99.6% | 1976.2743 | 1976.3062 | 1 | 5.623 | 35.9% | 1 | K.AHSFLMDFIHQVGLFGR.L | 3 |
| \* | AstrinIP\_MS1\_022614\_01.19301.19301.3 | 3.9109 | 0.4139 | 100.0% | 3544.2544 | 3545.0222 | 1 | 5.65 | 20.8% | 1 | R.DAPMDSIEWAEVVINVNNILKDMLQAASHYR.Q | 3 |
| \* | AstrinIP\_MS2\_022614\_01.06342.06342.2 | 2.1242 | 0.2944 | 98.2% | 1192.6921 | 1192.3348 | 62 | 5.014 | 55.6% | 1 | K.DMLQAASHYR.Q | 2 |

---

|  |  |  |  |  |  |  |  |  |
| --- | --- | --- | --- | --- | --- | --- | --- | --- |
| U | *gi|7657381|ref|NP\_055* | 2 | 4 | 4.2% | 504 | 55181 | 6.6 | PRP19/PSO4 pre-mRNA processing factor 19 homolog [Homo sapiens] |

| Filename XCorr DeltCN Conf% ObsM+H+ CalcM+H+ SpR ZScore Ion% # Sequence  | | | | | | | | | | | | |
| --- | --- | --- | --- | --- | --- | --- | --- | --- | --- | --- | --- | --- |
| \* | AstrinIP\_MS2\_022614\_01.08002.08002.2 | 2.8748 | 0.2833 | 99.8% | 1090.8922 | 1090.2621 | 2 | 5.294 | 72.2% | 1 | K.SSEQILATLK.G | 2 |
| \* | AstrinIP\_MS2\_022614\_01.08646.08646.2 | 2.7191 | 0.3315 | 99.8% | 1321.0521 | 1321.4729 | 9 | 5.421 | 60.0% | 3 | K.TLQLDNNFEVK.S | 2 |

---

|  |  |  |  |  |  |  |  |  |
| --- | --- | --- | --- | --- | --- | --- | --- | --- |
| U | *gi|4506787|ref|NP\_003* | 3 | 4 | 4.1% | 1657 | 189251 | 6.5 | IQ motif containing GTPase activating protein 1 [Homo sapiens] |

| Filename XCorr DeltCN Conf% ObsM+H+ CalcM+H+ SpR ZScore Ion% # Sequence  | | | | | | | | | | | | |
| --- | --- | --- | --- | --- | --- | --- | --- | --- | --- | --- | --- | --- |
| \* | AstrinIP\_MS1\_022614\_01.14279.14279.2 | 4.8362 | 0.5117 | 100.0% | 2195.4722 | 2195.498 | 1 | 8.577 | 52.8% | 1 | R.HTDNVIQWLNAMDEIGLPK.I | 2 |
| \* | AstrinIP\_MS1\_022614\_01.18959.18959.3 | 4.0769 | 0.2668 | 99.5% | 2960.9043 | 2960.3574 | 1 | 4.646 | 25.9% | 2 | K.IGGILANELSVDEAALHAAVIAINEAIDR.R | 3 |
| \* | AstrinIP\_MS2\_022614\_01.07830.07830.3 | 3.9337 | 0.2988 | 99.9% | 2285.2744 | 2284.5713 | 1 | 4.976 | 36.8% | 1 | K.VLKDSLHEKFPDAGEDELLK.I | 3 |

---

|  |  |  |  |  |  |  |  |  |
| --- | --- | --- | --- | --- | --- | --- | --- | --- |
| U | *gi|118498359|ref|NP\_0* | 2 | 2 | 4.1% | 490 | 54973 | 10.1 | ribosomal L1 domain containing 1 [Homo sapiens] |

| Filename XCorr DeltCN Conf% ObsM+H+ CalcM+H+ SpR ZScore Ion% # Sequence  | | | | | | | | | | | | |
| --- | --- | --- | --- | --- | --- | --- | --- | --- | --- | --- | --- | --- |
| \* | AstrinIP\_MS2\_022614\_01.15815.15815.2 | 3.1745 | 0.5161 | 100.0% | 2200.7722 | 2202.62 | 1 | 7.818 | 42.1% | 1 | R.IGHVGMQIEHIIENIVAVTK.G | 2 |
| \* | AstrinIP\_MS2\_022614\_01.15784.15784.3 | 3.4731 | 0.4618 | 100.0% | 2201.9343 | 2202.62 | 1 | 7.164 | 36.8% | 1 | R.IGHVGMQIEHIIENIVAVTK.G | 3 |

---

|  |  |  |  |  |  |  |  |  |
| --- | --- | --- | --- | --- | --- | --- | --- | --- |
| U | *gi|14251212|ref|NP\_00* | 2 | 2 | 4.0% | 824 | 92213 | 6.9 | DEAD (Asp-Glu-Ala-Asp) box polypeptide 20 [Homo sapiens] |

| Filename XCorr DeltCN Conf% ObsM+H+ CalcM+H+ SpR ZScore Ion% # Sequence  | | | | | | | | | | | | |
| --- | --- | --- | --- | --- | --- | --- | --- | --- | --- | --- | --- | --- |
| \* | AstrinIP\_MS2\_022614\_01.10674.10674.3 | 3.6882 | 0.416 | 100.0% | 1744.4343 | 1744.0061 | 1 | 7.729 | 48.2% | 1 | R.IPFNQALVFSNLHSR.A | 3 |
| \* | AstrinIP\_MS1\_022614\_01.03627.03627.2 | 3.6494 | 0.3748 | 100.0% | 1997.4122 | 1998.1167 | 2 | 6.763 | 44.1% | 1 | K.NSVQTPVENSTNSQHQVK.E | 2 |

---

|  |  |  |  |  |  |  |  |  |
| --- | --- | --- | --- | --- | --- | --- | --- | --- |
| U | *gi|4503509|ref|NP\_003* | 4 | 5 | 3.8% | 1382 | 166569 | 6.8 | eukaryotic translation initiation factor 3, subunit 10 theta, 150/170kDa [Homo sapiens] |

| Filename XCorr DeltCN Conf% ObsM+H+ CalcM+H+ SpR ZScore Ion% # Sequence  | | | | | | | | | | | | |
| --- | --- | --- | --- | --- | --- | --- | --- | --- | --- | --- | --- | --- |
| \* | AstrinIP\_MS1\_022614\_01.10964.10964.2 | 2.4229 | 0.2471 | 98.0% | 1520.5322 | 1520.8273 | 13 | 4.491 | 41.7% | 1 | K.KQPALDVLYDVMK.S | 2 |
| \* | AstrinIP\_MS2\_022614\_01.13554.13554.2 | 2.6799 | 0.2816 | 99.4% | 1435.3121 | 1435.7062 | 2 | 5.39 | 68.2% | 1 | R.FNVLQYVVPEVK.D | 2 |
| \* | AstrinIP\_MS1\_022614\_01.14422.14422.2 | 3.3872 | 0.386 | 100.0% | 1735.3322 | 1736.0209 | 1 | 6.567 | 53.6% | 2 | R.LTSLVPFVDAFQLER.A | 2 |
| \* | AstrinIP\_MS1\_022614\_02.06387.06387.2 | 3.0015 | 0.4492 | 100.0% | 1445.5721 | 1445.5718 | 1 | 7.302 | 62.5% | 1 | R.TLSFGSDLNYATR.E | 2 |

---

|  |  |  |  |  |  |  |  |  |
| --- | --- | --- | --- | --- | --- | --- | --- | --- |
| U | *gi|54859722|ref|NP\_05* | 3 | 3 | 3.8% | 1436 | 162121 | 5.5 | nucleoporin 160kDa [Homo sapiens] |

| Filename XCorr DeltCN Conf% ObsM+H+ CalcM+H+ SpR ZScore Ion% # Sequence  | | | | | | | | | | | | |
| --- | --- | --- | --- | --- | --- | --- | --- | --- | --- | --- | --- | --- |
| \* | AstrinIP\_MS2\_022614\_01.18844.18844.2 | 3.3573 | 0.5103 | 100.0% | 2097.412 | 2098.4062 | 1 | 7.917 | 47.1% | 1 | R.FVSSPQTIVELFFQEVAR.K | 2 |
| \* | AstrinIP\_MS2\_022614\_01.04319.04319.2 | 2.81 | 0.329 | 99.8% | 1189.9321 | 1190.2523 | 18 | 6.2 | 65.0% | 1 | R.SEDGEIVSTPR.L | 2 |
| \* | AstrinIP\_MS2\_022614\_01.15821.15821.3 | 3.3477 | 0.3034 | 99.2% | 2899.6443 | 2900.261 | 26 | 4.881 | 24.0% | 1 | R.SQLQDLVEFPYVNLHNEVVGIIESR.A | 3 |

---

|  |  |  |  |  |  |  |  |  |
| --- | --- | --- | --- | --- | --- | --- | --- | --- |
| U | *gi|194239723|ref|NP\_0* | 2 | 3 | 3.7% | 647 | 71408 | 6.4 | eukaryotic translation elongation factor 1 delta isoform 1 [Homo sapiens] |
| U | *gi|25453474|ref|NP\_11* | 2 | 3 | 3.7% | 647 | 71408 | 6.4 | eukaryotic translation elongation factor 1 delta isoform 1 [Homo sapiens] |
| U | *gi|25453472|ref|NP\_00* | 2 | 3 | 8.5% | 281 | 31122 | 5.0 | eukaryotic translation elongation factor 1 delta isoform 2 [Homo sapiens] |
| U | *gi|194239731|ref|NP\_0* | 2 | 3 | 8.5% | 281 | 31122 | 5.0 | eukaryotic translation elongation factor 1 delta isoform 2 [Homo sapiens] |
| U | *gi|194239729|ref|NP\_0* | 2 | 3 | 9.3% | 257 | 28558 | 4.9 | eukaryotic translation elongation factor 1 delta isoform 4 [Homo sapiens] |
| U | *gi|194239727|ref|NP\_0* | 2 | 3 | 8.5% | 281 | 31122 | 5.0 | eukaryotic translation elongation factor 1 delta isoform 2 [Homo sapiens] |
| U | *gi|194239725|ref|NP\_0* | 2 | 3 | 3.7% | 646 | 71266 | 6.4 | eukaryotic translation elongation factor 1 delta isoform 3 [Homo sapiens] |

| Filename XCorr DeltCN Conf% ObsM+H+ CalcM+H+ SpR ZScore Ion% # Sequence  | | | | | | | | | | | | |
| --- | --- | --- | --- | --- | --- | --- | --- | --- | --- | --- | --- | --- |
|  | AstrinIP\_MS1\_022614\_01.06549.06549.2 | 3.3102 | 0.3505 | 100.0% | 1359.9922 | 1359.5223 | 1 | 6.325 | 77.3% | 2 | R.IASLEVENQSLR.G | 2 |
|  | AstrinIP\_MS2\_022614\_01.11532.11532.2 | 2.7847 | 0.333 | 99.8% | 1300.6522 | 1300.4978 | 1 | 6.059 | 63.6% | 1 | R.GVVQELQQAISK.L | 2 |

---

|  |  |  |  |  |  |  |  |  |
| --- | --- | --- | --- | --- | --- | --- | --- | --- |
| U | *gi|207452735|ref|NP\_1* | 11 | 15 | 3.6% | 5090 | 555629 | 5.6 | epiplakin 1 [Homo sapiens] |

| Filename XCorr DeltCN Conf% ObsM+H+ CalcM+H+ SpR ZScore Ion% # Sequence  | | | | | | | | | | | | |
| --- | --- | --- | --- | --- | --- | --- | --- | --- | --- | --- | --- | --- |
| \* | AstrinIP\_MS2\_022614\_02.04121.04121.2 | 2.5065 | 0.4404 | 99.8% | 1334.3722 | 1334.5126 | 7 | 7.219 | 50.0% | 1 | R.QALSTATATVSVGK.F | 2 |
| \* | AstrinIP\_MS2\_022614\_01.15946.15946.2 | 2.8241 | 0.213 | 98.4% | 2305.3523 | 2305.5957 | 1 | 5.087 | 34.2% | 1 | R.GRPVSLWELLFSEAISSEQR.A | 2 |
| \* | AstrinIP\_MS1\_022614\_02.05525.05525.2 | 2.8879 | 0.4483 | 100.0% | 1796.1522 | 1797.0332 | 2 | 6.819 | 46.7% | 1 | R.AMLAQQYQEGTLSVEK.L | 2 |
|  | AstrinIP\_MS2\_022614\_01.08033.08033.3 | 4.0343 | 0.3821 | 100.0% | 2013.9844 | 2014.292 | 1 | 5.925 | 38.9% | 3 | R.LLEAQIATGGVIDPVHSHR.V | 33 |
|  | AstrinIP\_MS2\_022614\_01.08807.08807.3 | 3.1153 | 0.2831 | 99.2% | 2028.6244 | 2028.3188 | 95 | 5.473 | 29.2% | 1 | R.LLEAQIATGGIIDPVHSHR.V | 33 |
|  | AstrinIP\_MS2\_022614\_01.07104.07104.2 | 2.1779 | 0.2594 | 98.1% | 1161.1322 | 1161.2311 | 1 | 5.235 | 75.0% | 1 | C.GYFDEEMNR.I | 22 |
| \* | AstrinIP\_MS1\_022614\_01.15365.15365.2 | 4.9216 | 0.5308 | 100.0% | 2395.0522 | 2395.7202 | 1 | 8.407 | 54.8% | 1 | R.VTPGSGALQGQSVSVWELLFYR.E | 2 |
| \* | AstrinIP\_MS2\_022614\_01.17794.17794.2 | 3.6598 | 0.4512 | 100.0% | 2513.672 | 2514.8386 | 3 | 6.817 | 31.2% | 1 | R.AGTLTVEELGATLTSLLAQAQAQAR.A | 2 |
| \* | AstrinIP\_MS1\_022614\_01.17162.17162.3 | 4.7464 | 0.4638 | 100.0% | 2514.2344 | 2514.8386 | 1 | 7.554 | 35.4% | 3 | R.AGTLTVEELGATLTSLLAQAQAQAR.A | 3 |
| \* | AstrinIP\_MS2\_022614\_01.14537.14537.3 | 3.3464 | 0.2713 | 99.0% | 2500.8843 | 2500.904 | 8 | 4.832 | 27.2% | 1 | R.RDELLAQHAAGALGLPDLVAVLTR.V | 32 |
| \* | AstrinIP\_MS2\_022614\_01.05837.05837.2 | 2.2933 | 0.292 | 98.2% | 1535.8322 | 1536.7406 | 1 | 5.87 | 58.3% | 1 | K.TLQEVTEMDSVKR.Y | 2 |

Similarities:
gi|41322908|ref|NP\_95(3:8)  

---

|  |  |  |  |  |  |  |  |  |
| --- | --- | --- | --- | --- | --- | --- | --- | --- |
| U | *gi|41872631|ref|NP\_00* | 5 | 6 | 3.5% | 2511 | 273424 | 6.4 | fatty acid synthase [Homo sapiens] |

| Filename XCorr DeltCN Conf% ObsM+H+ CalcM+H+ SpR ZScore Ion% # Sequence  | | | | | | | | | | | | |
| --- | --- | --- | --- | --- | --- | --- | --- | --- | --- | --- | --- | --- |
| \* | AstrinIP\_MS2\_022614\_01.05739.05739.2 | 2.5365 | 0.4211 | 99.8% | 1298.2922 | 1299.4264 | 3 | 6.742 | 68.2% | 1 | K.VGDPQELNGITR.A | 2 |
| \* | AstrinIP\_MS2\_022614\_01.15078.15078.2 | 2.9085 | 0.3572 | 99.8% | 1595.2722 | 1594.9371 | 1 | 6.217 | 69.2% | 1 | R.VLFPATGYLSIVWK.T | 2 |
| \* | AstrinIP\_MS2\_022614\_01.17266.17266.3 | 3.7143 | 0.2243 | 98.2% | 2787.5344 | 2787.3152 | 1 | 4.926 | 29.0% | 2 | R.ALGLGVEQLPVVFEDVVLHQATILPK.T | 3 |
| \* | AstrinIP\_MS2\_022614\_01.08908.08908.2 | 2.6945 | 0.2588 | 99.1% | 1470.0721 | 1470.5815 | 1 | 5.42 | 75.0% | 1 | R.FPQLDSTSFANSR.D | 2 |
| \* | AstrinIP\_MS2\_022614\_01.16923.16923.3 | 2.7597 | 0.2968 | 98.2% | 2423.2444 | 2423.769 | 262 | 4.52 | 21.6% | 1 | R.TLLEGSGLESIISIIHSSLAEPR.V | 3 |

---

|  |  |  |  |  |  |  |  |  |
| --- | --- | --- | --- | --- | --- | --- | --- | --- |
| U | *gi|33946280|ref|NP\_89* | 2 | 2 | 3.4% | 1147 | 128734 | 4.6 | hypothetical protein LOC25962 isoform 2 [Homo sapiens] |
| U | *gi|33946282|ref|NP\_05* | 2 | 2 | 2.2% | 1812 | 202023 | 5.0 | hypothetical protein LOC25962 isoform 1 [Homo sapiens] |

| Filename XCorr DeltCN Conf% ObsM+H+ CalcM+H+ SpR ZScore Ion% # Sequence  | | | | | | | | | | | | |
| --- | --- | --- | --- | --- | --- | --- | --- | --- | --- | --- | --- | --- |
|  | AstrinIP\_MS2\_022614\_01.15172.15172.2 | 2.8898 | 0.2969 | 99.7% | 1612.5122 | 1612.95 | 1 | 4.842 | 57.7% | 1 | K.WVTALEEIPSLIIK.G | 2 |
|  | AstrinIP\_MS1\_022614\_01.17178.17178.3 | 5.7088 | 0.4219 | 100.0% | 2934.3843 | 2933.4968 | 1 | 7.744 | 35.4% | 1 | R.LINLLEEVFHLMETAPHTMIQQPVK.S | 3 |

---

|  |  |  |  |  |  |  |  |  |
| --- | --- | --- | --- | --- | --- | --- | --- | --- |
| U | *gi|171184451|ref|NP\_0* | 6 | 9 | 3.3% | 3117 | 350931 | 6.3 | centrosome-associated protein 350 [Homo sapiens] |

| Filename XCorr DeltCN Conf% ObsM+H+ CalcM+H+ SpR ZScore Ion% # Sequence  | | | | | | | | | | | | |
| --- | --- | --- | --- | --- | --- | --- | --- | --- | --- | --- | --- | --- |
| \* | AstrinIP\_MS2\_022614\_01.11068.11068.3 | 5.0792 | 0.4443 | 100.0% | 2335.5244 | 2335.625 | 1 | 7.496 | 38.1% | 2 | R.THISDAVVASGAPLAILYDHQR.Q | 3 |
| \* | AstrinIP\_MS2\_022614\_01.04301.04301.2 | 2.6991 | 0.1718 | 98.5% | 1201.6921 | 1202.3097 | 1 | 4.33 | 83.3% | 1 | R.RLDAEEAEIR.Q | 2 |
| \* | AstrinIP\_MS2\_022614\_02.04871.04871.3 | 3.4097 | 0.3131 | 99.7% | 2267.0044 | 2267.4087 | 25 | 5.139 | 27.6% | 1 | K.TEAELSQDLETS\*PTAKPQIK.T | 3 |
| \* | AstrinIP\_MS2\_022614\_02.05543.05543.2 | 3.6289 | 0.4439 | 100.0% | 1518.0922 | 1518.7045 | 1 | 7.458 | 69.2% | 1 | K.LVLEQGDSSEILSK.K | 2 |
| \* | AstrinIP\_MS1\_022614\_01.08915.08915.2 | 3.3021 | 0.3814 | 100.0% | 1279.4521 | 1279.5686 | 1 | 7.988 | 72.7% | 2 | R.VLIGNVQPGILR.F | 2 |
| \* | AstrinIP\_MS2\_022614\_01.20122.20122.3 | 5.5009 | 0.4239 | 100.0% | 2836.6143 | 2836.2703 | 1 | 7.337 | 35.4% | 2 | K.MQLADGIFETLIKDTIDVLNQISEK.Q | 3 |

---

|  |  |  |  |  |  |  |  |  |
| --- | --- | --- | --- | --- | --- | --- | --- | --- |
| U | *gi|21493022|ref|NP\_00* | 3 | 3 | 3.3% | 1782 | 191480 | 4.4 | A kinase (PRKA) anchor protein 12 isoform 1 [Homo sapiens] |
| U | *gi|21493024|ref|NP\_65* | 3 | 3 | 3.4% | 1684 | 181688 | 4.4 | A kinase (PRKA) anchor protein 12 isoform 2 [Homo sapiens] |

| Filename XCorr DeltCN Conf% ObsM+H+ CalcM+H+ SpR ZScore Ion% # Sequence  | | | | | | | | | | | | |
| --- | --- | --- | --- | --- | --- | --- | --- | --- | --- | --- | --- | --- |
|  | AstrinIP\_MS1\_022614\_01.03519.03519.3 | 5.4344 | 0.4182 | 100.0% | 2604.6243 | 2604.7844 | 1 | 7.074 | 34.1% | 1 | K.KKEQEPEKVDTEEDGKAEVASEK.L | 3 |
|  | AstrinIP\_MS1\_022614\_01.03602.03602.3 | 3.2925 | 0.3602 | 99.9% | 2186.0044 | 2186.3025 | 1 | 5.924 | 34.2% | 1 | K.LTASEQAHPQEPAESAHEPR.L | 3 |
|  | AstrinIP\_MS2\_022614\_01.15582.15582.2 | 2.7084 | 0.3509 | 99.7% | 1746.3522 | 1745.0317 | 46 | 5.148 | 35.7% | 1 | K.LVQNIIQTAVDQFVR.T | 2 |

---

|  |  |  |  |  |  |  |  |  |
| --- | --- | --- | --- | --- | --- | --- | --- | --- |
| U | *gi|62243696|ref|NP\_00* | 3 | 4 | 3.2% | 1258 | 144427 | 5.6 | stromal antigen 1 [Homo sapiens] |

| Filename XCorr DeltCN Conf% ObsM+H+ CalcM+H+ SpR ZScore Ion% # Sequence  | | | | | | | | | | | | |
| --- | --- | --- | --- | --- | --- | --- | --- | --- | --- | --- | --- | --- |
|  | AstrinIP\_MS2\_022614\_01.11075.11075.2 | 4.2029 | 0.4809 | 100.0% | 1567.7722 | 1567.7534 | 1 | 7.886 | 79.2% | 2 | K.MYSDAFLNDSYLK.Y | 22 |
| \* | AstrinIP\_MS1\_022614\_01.13560.13560.2 | 2.7543 | 0.3903 | 99.8% | 1756.0721 | 1757.1847 | 1 | 7.298 | 46.4% | 1 | K.LTEHFIITLPMLLSK.Y | 32 |
| \* | AstrinIP\_MS2\_022614\_01.12534.12534.2 | 2.8716 | 0.155 | 98.2% | 1546.6522 | 1547.7971 | 3 | 4.78 | 63.6% | 1 | R.REDVWLPLISYR.N | 2 |

Similarities:
gi|112789526|ref|NP\_0(1:2)  

---

|  |  |  |  |  |  |  |  |  |
| --- | --- | --- | --- | --- | --- | --- | --- | --- |
| U | *gi|30089926|ref|NP\_07* | 2 | 2 | 3.2% | 843 | 94056 | 6.9 | F-box only protein 11 isoform 1 [Homo sapiens] |

| Filename XCorr DeltCN Conf% ObsM+H+ CalcM+H+ SpR ZScore Ion% # Sequence  | | | | | | | | | | | | |
| --- | --- | --- | --- | --- | --- | --- | --- | --- | --- | --- | --- | --- |
| \* | AstrinIP\_MS2\_022614\_01.10787.10787.2 | 2.4032 | 0.2653 | 98.8% | 1205.5521 | 1206.341 | 2 | 5.213 | 72.2% | 1 | R.GLLEENDIFR.N | 2 |
| \* | AstrinIP\_MS2\_022614\_02.05259.05259.3 | 2.5476 | 0.2571 | 95.0% | 1793.1244 | 1792.0482 | 2 | 4.883 | 35.9% | 1 | R.NAQAGVLISTNSHPILR.K | 3 |

---

|  |  |  |  |  |  |  |  |  |
| --- | --- | --- | --- | --- | --- | --- | --- | --- |
| U | *gi|27262645|ref|NP\_66* | 2 | 2 | 3.1% | 1062 | 111920 | 8.7 | ataxin 2 related protein isoform B [Homo sapiens] |
| U | *gi|27262653|ref|NP\_68* | 2 | 2 | 3.2% | 1044 | 110143 | 8.8 | ataxin 2 related protein isoform E [Homo sapiens] |
| U | *gi|27262651|ref|NP\_68* | 2 | 2 | 3.2% | 1044 | 110326 | 8.9 | ataxin 2 related protein isoform D [Homo sapiens] |
| U | *gi|27262649|ref|NP\_68* | 2 | 2 | 3.0% | 1097 | 115582 | 8.7 | ataxin 2 related protein isoform C [Homo sapiens] |
| U | *gi|27262647|ref|NP\_00* | 2 | 2 | 3.1% | 1075 | 113374 | 8.6 | ataxin 2 related protein isoform A [Homo sapiens] |

| Filename XCorr DeltCN Conf% ObsM+H+ CalcM+H+ SpR ZScore Ion% # Sequence  | | | | | | | | | | | | |
| --- | --- | --- | --- | --- | --- | --- | --- | --- | --- | --- | --- | --- |
|  | AstrinIP\_MS1\_022614\_01.05435.05435.2 | 2.5055 | 0.341 | 99.5% | 1427.8922 | 1428.6001 | 1 | 4.849 | 70.8% | 1 | K.DKFTDSAIAMNSK.V | 2 |
|  | AstrinIP\_MS1\_022614\_01.03591.03591.3 | 4.2608 | 0.3266 | 100.0% | 2315.4543 | 2316.4644 | 1 | 6.228 | 34.2% | 1 | R.IAMENDDGRTEEEKHSAVQR.Q | 3 |

---

|  |  |  |  |  |  |  |  |  |
| --- | --- | --- | --- | --- | --- | --- | --- | --- |
| U | *gi|6005757|ref|NP\_009* | 2 | 3 | 3.0% | 1047 | 119914 | 5.7 | chromatin-specific transcription elongation factor large subunit [Homo sapiens] |

| Filename XCorr DeltCN Conf% ObsM+H+ CalcM+H+ SpR ZScore Ion% # Sequence  | | | | | | | | | | | | |
| --- | --- | --- | --- | --- | --- | --- | --- | --- | --- | --- | --- | --- |
| \* | AstrinIP\_MS2\_022614\_01.12258.12258.2 | 3.6531 | 0.4444 | 100.0% | 2153.4321 | 2154.338 | 1 | 7.666 | 38.9% | 2 | R.HTDVQFYTEVGEITTDLGK.H | 2 |
| \* | AstrinIP\_MS2\_022614\_01.08195.08195.2 | 2.9081 | 0.3268 | 99.8% | 1426.1721 | 1426.5687 | 1 | 6.364 | 59.1% | 1 | K.YTEGVQSLNWTK.I | 2 |

---

|  |  |  |  |  |  |  |  |  |
| --- | --- | --- | --- | --- | --- | --- | --- | --- |
| U | *gi|214010173|ref|NP\_0* | 3 | 4 | 2.8% | 1479 | 162914 | 8.8 | CLIP-associating protein 1 isoform 2 [Homo sapiens] |
| U | *gi|31563537|ref|NP\_05* | 3 | 4 | 2.7% | 1538 | 169450 | 9.0 | CLIP-associating protein 1 isoform 1 [Homo sapiens] |
| U | *gi|214010175|ref|NP\_0* | 3 | 4 | 2.8% | 1471 | 162110 | 8.8 | CLIP-associating protein 1 isoform 3 [Homo sapiens] |

| Filename XCorr DeltCN Conf% ObsM+H+ CalcM+H+ SpR ZScore Ion% # Sequence  | | | | | | | | | | | | |
| --- | --- | --- | --- | --- | --- | --- | --- | --- | --- | --- | --- | --- |
|  | AstrinIP\_MS2\_022614\_01.13836.13836.2 | 3.7777 | 0.3141 | 100.0% | 1655.6522 | 1655.845 | 1 | 7.578 | 69.2% | 2 | R.LQVGQELIDYFSDK.Q | 2 |
|  | AstrinIP\_MS2\_022614\_01.07079.07079.2 | 2.8153 | 0.2754 | 99.1% | 1381.9521 | 1382.5162 | 2 | 5.23 | 53.6% | 1 | K.LLGSGYGGLTGGSSR.G | 2 |
|  | AstrinIP\_MS1\_022614\_01.10614.10614.2 | 2.4306 | 0.2373 | 98.1% | 1326.2322 | 1326.6224 | 1 | 5.051 | 77.3% | 1 | R.KEGLLGLQNLLK.S | 22 |

Similarities:
gi|57863301|ref|NP\_05(1:2)  

---

|  |  |  |  |  |  |  |  |  |
| --- | --- | --- | --- | --- | --- | --- | --- | --- |
| U | *gi|112789526|ref|NP\_0* | 2 | 3 | 2.8% | 1268 | 145751 | 5.5 | stromal antigen 2 isoform a [Homo sapiens] |
| U | *gi|31563531|ref|NP\_00* | 2 | 3 | 2.9% | 1231 | 141326 | 5.4 | stromal antigen 2 isoform b [Homo sapiens] |
| U | *gi|112789530|ref|NP\_0* | 2 | 3 | 2.9% | 1231 | 141326 | 5.4 | stromal antigen 2 isoform b [Homo sapiens] |
| U | *gi|112789528|ref|NP\_0* | 2 | 3 | 2.8% | 1268 | 145751 | 5.5 | stromal antigen 2 isoform a [Homo sapiens] |

| Filename XCorr DeltCN Conf% ObsM+H+ CalcM+H+ SpR ZScore Ion% # Sequence  | | | | | | | | | | | | |
| --- | --- | --- | --- | --- | --- | --- | --- | --- | --- | --- | --- | --- |
|  | AstrinIP\_MS2\_022614\_01.11075.11075.2 | 4.2029 | 0.4809 | 100.0% | 1567.7722 | 1567.7534 | 1 | 7.886 | 79.2% | 2 | K.MYSDAFLNDSYLK.Y | 22 |
|  | AstrinIP\_MS1\_022614\_01.18008.18008.3 | 3.9316 | 0.3673 | 100.0% | 2788.0444 | 2788.1475 | 1 | 6.065 | 30.7% | 1 | K.TLILSLQQLFNEMIQENGYNFDR.S | 3 |

Similarities:
gi|62243696|ref|NP\_00(1:1)  

---

|  |  |  |  |  |  |  |  |  |
| --- | --- | --- | --- | --- | --- | --- | --- | --- |
| U | *gi|153792294|ref|NP\_1* | 2 | 5 | 2.8% | 1320 | 145257 | 6.8 | myopalladin [Homo sapiens] |

| Filename XCorr DeltCN Conf% ObsM+H+ CalcM+H+ SpR ZScore Ion% # Sequence  | | | | | | | | | | | | |
| --- | --- | --- | --- | --- | --- | --- | --- | --- | --- | --- | --- | --- |
| \* | AstrinIP\_MS2\_022614\_01.17595.17595.2 | 3.5006 | 0.4924 | 100.0% | 1470.3722 | 1470.6624 | 1 | 7.758 | 75.0% | 4 | K.AADFIEELSSLFK.S | 2 |
| \* | AstrinIP\_MS1\_022614\_02.05078.05078.3 | 3.3371 | 0.3531 | 99.9% | 2545.4043 | 2546.71 | 1 | 5.314 | 29.3% | 1 | R.VHFNLPEDDKGSEASSEAGVVTTR.Q | 3 |

---

|  |  |  |  |  |  |  |  |  |
| --- | --- | --- | --- | --- | --- | --- | --- | --- |
| U | *gi|158420731|ref|NP\_0* | 3 | 5 | 2.7% | 2059 | 233035 | 6.2 | chromodomain helicase DNA binding protein 3 isoform 3 [Homo sapiens] |
| U | *gi|52630326|ref|NP\_00* | 3 | 5 | 2.8% | 2000 | 226590 | 7.3 | chromodomain helicase DNA binding protein 3 isoform 1 [Homo sapiens] |
| U | *gi|52630322|ref|NP\_00* | 3 | 5 | 2.8% | 1966 | 222859 | 7.5 | chromodomain helicase DNA binding protein 3 isoform 2 [Homo sapiens] |
| U | *gi|51599156|ref|NP\_00* | 3 | 4 | 2.9% | 1912 | 218003 | 5.9 | chromodomain helicase DNA binding protein 4 [Homo sapiens] |

| Filename XCorr DeltCN Conf% ObsM+H+ CalcM+H+ SpR ZScore Ion% # Sequence  | | | | | | | | | | | | |
| --- | --- | --- | --- | --- | --- | --- | --- | --- | --- | --- | --- | --- |
|  | AstrinIP\_MS2\_022614\_01.15375.15375.2 | 3.3241 | 0.4506 | 100.0% | 1900.7322 | 1901.2157 | 1 | 7.351 | 53.1% | 3 | K.GPFLVSAPLSTIINWER.E | 2 |
|  | AstrinIP\_MS2\_022614\_01.19886.19886.3 | 3.1578 | 0.3185 | 99.2% | 3036.4443 | 3037.529 | 1 | 6.289 | 29.0% | 1 | K.LLLTGTPLQNNLEELFHLLNFLTPER.F | 3 |
|  | AstrinIP\_MS2\_022614\_01.03468.03468.2 | 3.8337 | 0.4406 | 100.0% | 1554.1921 | 1554.6188 | 1 | 7.586 | 77.3% | 1 | R.HHYEQQQEDLAR.N | 2 |

---

|  |  |  |  |  |  |  |  |  |
| --- | --- | --- | --- | --- | --- | --- | --- | --- |
| U | *gi|149158692|ref|NP\_0* | 2 | 2 | 2.7% | 1132 | 119409 | 5.6 | HLA-B associated transcript-3 isoform a [Homo sapiens] |
| U | *gi|18375632|ref|NP\_54* | 2 | 2 | 2.7% | 1126 | 118693 | 5.6 | HLA-B associated transcript-3 isoform b [Homo sapiens] |
| U | *gi|18375630|ref|NP\_54* | 2 | 2 | 2.7% | 1126 | 118693 | 5.6 | HLA-B associated transcript-3 isoform b [Homo sapiens] |
| U | *gi|149158696|ref|NP\_0* | 2 | 2 | 2.7% | 1126 | 118693 | 5.6 | HLA-B associated transcript-3 isoform b [Homo sapiens] |

| Filename XCorr DeltCN Conf% ObsM+H+ CalcM+H+ SpR ZScore Ion% # Sequence  | | | | | | | | | | | | |
| --- | --- | --- | --- | --- | --- | --- | --- | --- | --- | --- | --- | --- |
|  | AstrinIP\_MS2\_022614\_01.12812.12812.2 | 2.6929 | 0.2884 | 99.3% | 1420.5521 | 1419.6641 | 1 | 5.416 | 66.7% | 1 | R.LLGNTFVALSDLR.C | 2 |
|  | AstrinIP\_MS2\_022614\_01.11519.11519.2 | 3.1801 | 0.3087 | 99.8% | 1886.9922 | 1888.2787 | 35 | 6.157 | 37.5% | 1 | R.LQVVLEHMPVGPDAILR.Y | 2 |

---

|  |  |  |  |  |  |  |  |  |
| --- | --- | --- | --- | --- | --- | --- | --- | --- |
| U | *gi|12025678|ref|NP\_00* | 2 | 3 | 2.6% | 911 | 104854 | 5.4 | actinin, alpha 4 [Homo sapiens] |
| U | *gi|4501891|ref|NP\_001* | 2 | 3 | 2.7% | 892 | 103058 | 5.4 | actinin, alpha 1 isoform b [Homo sapiens] |
| U | *gi|194097352|ref|NP\_0* | 2 | 3 | 2.7% | 887 | 102709 | 5.5 | actinin, alpha 1 isoform c [Homo sapiens] |
| U | *gi|194097350|ref|NP\_0* | 2 | 3 | 2.6% | 914 | 105568 | 5.4 | actinin, alpha 1 isoform a [Homo sapiens] |

| Filename XCorr DeltCN Conf% ObsM+H+ CalcM+H+ SpR ZScore Ion% # Sequence  | | | | | | | | | | | | |
| --- | --- | --- | --- | --- | --- | --- | --- | --- | --- | --- | --- | --- |
|  | AstrinIP\_MS2\_022614\_01.14610.14610.2 | 2.1901 | 0.2853 | 97.9% | 1387.0721 | 1387.6218 | 16 | 5.286 | 50.0% | 1 | R.VGWEQLLTTIAR.T | 2 |
|  | AstrinIP\_MS2\_022614\_01.08758.08758.2 | 2.8804 | 0.2503 | 99.5% | 1429.9321 | 1430.6011 | 204 | 5.392 | 50.0% | 2 | R.TINEVENQILTR.D | 2 |

---

|  |  |  |  |  |  |  |  |  |
| --- | --- | --- | --- | --- | --- | --- | --- | --- |
| U | *gi|21264365|ref|NP\_05* | 3 | 4 | 2.5% | 1800 | 195816 | 6.4 | nucleoporin 98kD isoform 1 [Homo sapiens] |

| Filename XCorr DeltCN Conf% ObsM+H+ CalcM+H+ SpR ZScore Ion% # Sequence  | | | | | | | | | | | | |
| --- | --- | --- | --- | --- | --- | --- | --- | --- | --- | --- | --- | --- |
|  | AstrinIP\_MS2\_022614\_01.14537.14537.2 | 2.2131 | 0.3207 | 98.1% | 1667.5922 | 1668.89 | 1 | 5.464 | 46.4% | 1 | K.NSPVEAVFSYLTGKR.I | 32 |
| \* | AstrinIP\_MS1\_022614\_01.08838.08838.2 | 2.8485 | 0.1658 | 98.6% | 1399.3722 | 1398.5614 | 1 | 5.331 | 75.0% | 2 | R.HYDLNQLLEPR.S | 2 |
|  | AstrinIP\_MS2\_022614\_02.11627.11627.2 | 2.9142 | 0.3443 | 99.8% | 2260.5322 | 2259.5205 | 2 | 5.077 | 33.3% | 1 | R.SSLIQDWETSGLVYLDYIR.V | 2 |

---

|  |  |  |  |  |  |  |  |  |
| --- | --- | --- | --- | --- | --- | --- | --- | --- |
| U | *Reverse\_gi|56550047|r* | 2 | 3 | 2.4% | 1403 | 161510 | 6.9 | uveal autoantigen with coiled-coil domains and ankyrin repeats isoform 2 [Homo sapiens] |
| U | *Reverse\_gi|59850762|r* | 2 | 3 | 2.4% | 1416 | 162505 | 7.0 | uveal autoantigen with coiled-coil domains and ankyrin repeats isoform 1 [Homo sapiens] |

| Filename XCorr DeltCN Conf% ObsM+H+ CalcM+H+ SpR ZScore Ion% # Sequence  | | | | | | | | | | | | |
| --- | --- | --- | --- | --- | --- | --- | --- | --- | --- | --- | --- | --- |
|  | AstrinIP\_MS1\_022614\_01.19794.19794.2 | 2.6359 | 0.1753 | 97.0% | 1550.0922 | 1550.793 | 49 | 4.168 | 45.8% | 1 | K.LEVINS\*KLAIIEK.E | 2 |
|  | AstrinIP\_MS1\_022614\_01.14889.14889.2 | 4.1577 | 0.1624 | 99.8% | 2578.5723 | 2579.8848 | 20 | 3.901 | 37.5% | 2 | K.NEEILKGEDRKFEEVIMENQK.I | 2 |

---

|  |  |  |  |  |  |  |  |  |
| --- | --- | --- | --- | --- | --- | --- | --- | --- |
| U | *gi|42734325|ref|NP\_05* | 2 | 3 | 2.4% | 1190 | 132945 | 5.4 | wings apart-like homolog [Homo sapiens] |

| Filename XCorr DeltCN Conf% ObsM+H+ CalcM+H+ SpR ZScore Ion% # Sequence  | | | | | | | | | | | | |
| --- | --- | --- | --- | --- | --- | --- | --- | --- | --- | --- | --- | --- |
| \* | AstrinIP\_MS1\_022614\_01.06491.06491.2 | 2.5698 | 0.181 | 97.2% | 1305.4122 | 1304.44 | 159 | 4.5 | 50.0% | 2 | R.LLELEQDASSAK.L | 2 |
| \* | AstrinIP\_MS2\_022614\_02.13234.13234.2 | 1.9387 | 0.325 | 95.3% | 1731.1122 | 1731.0886 | 1 | 5.342 | 43.3% | 1 | R.VLGLGLLINLVEYSAR.N | 2 |

---

|  |  |  |  |  |  |  |  |  |
| --- | --- | --- | --- | --- | --- | --- | --- | --- |
| U | *gi|13259508|ref|NP\_07* | 2 | 2 | 2.4% | 1144 | 127404 | 5.4 | dynactin 1 isoform 2 [Homo sapiens] |
| U | *gi|205277396|ref|NP\_0* | 2 | 2 | 2.5% | 1139 | 126733 | 5.4 | dynactin 1 isoform 4 [Homo sapiens] |
| U | *gi|205277392|ref|NP\_0* | 2 | 2 | 2.2% | 1253 | 138750 | 5.5 | dynactin 1 isoform 3 [Homo sapiens] |
| U | *gi|13259510|ref|NP\_00* | 2 | 2 | 2.2% | 1278 | 141694 | 5.8 | dynactin 1 isoform 1 [Homo sapiens] |

| Filename XCorr DeltCN Conf% ObsM+H+ CalcM+H+ SpR ZScore Ion% # Sequence  | | | | | | | | | | | | |
| --- | --- | --- | --- | --- | --- | --- | --- | --- | --- | --- | --- | --- |
|  | AstrinIP\_MS2\_022614\_01.10689.10689.2 | 2.9705 | 0.3876 | 100.0% | 1529.5322 | 1530.7797 | 1 | 6.878 | 57.7% | 1 | K.SPSAQLMEQVAQLK.S | 2 |
|  | AstrinIP\_MS2\_022614\_01.05308.05308.2 | 2.5019 | 0.2667 | 98.5% | 1703.2522 | 1702.9541 | 3 | 6.384 | 50.0% | 1 | R.LVLTQEQLHQLHSR.L | 2 |

---

|  |  |  |  |  |  |  |  |  |
| --- | --- | --- | --- | --- | --- | --- | --- | --- |
| U | *gi|116284394|ref|NP\_0* | 3 | 6 | 2.2% | 1995 | 227868 | 5.6 | myosin, heavy chain 14 isoform 2 [Homo sapiens] |
| U | *gi|224831241|ref|NP\_0* | 3 | 6 | 2.2% | 2036 | 232009 | 5.6 | myosin, heavy chain 14 isoform 3 [Homo sapiens] |
| U | *gi|116284396|ref|NP\_0* | 3 | 6 | 2.2% | 2003 | 228663 | 5.6 | myosin, heavy chain 14 isoform 1 [Homo sapiens] |

| Filename XCorr DeltCN Conf% ObsM+H+ CalcM+H+ SpR ZScore Ion% # Sequence  | | | | | | | | | | | | |
| --- | --- | --- | --- | --- | --- | --- | --- | --- | --- | --- | --- | --- |
|  | AstrinIP\_MS1\_022614\_01.13002.13002.2 | 4.0727 | 0.5241 | 100.0% | 1727.5521 | 1728.0012 | 1 | 8.472 | 70.0% | 3 | R.QLLQANPILEAFGNAK.T | 22 |
|  | AstrinIP\_MS1\_022614\_01.16533.16533.3 | 2.8624 | 0.2648 | 98.2% | 2069.6343 | 2070.1807 | 29 | 4.393 | 34.4% | 1 | R.LLGLGVT#DFS\*RALLT#PR.I | 3 |
|  | AstrinIP\_MS1\_022614\_01.04691.04691.2 | 2.5804 | 0.283 | 99.3% | 1257.8322 | 1258.4172 | 210 | 4.791 | 60.0% | 2 | R.KEEELQAALAR.A | 22 |

Similarities:
gi|12667788|ref|NP\_00(2:1)  

---

|  |  |  |  |  |  |  |  |  |
| --- | --- | --- | --- | --- | --- | --- | --- | --- |
| U | *Reverse\_gi|31652242|r* | 2 | 2 | 2.2% | 1938 | 209652 | 9.1 | transcription factor 20 isoform 2 [Homo sapiens] |
| U | *Reverse\_gi|31652244|r* | 2 | 2 | 2.1% | 1960 | 211769 | 9.0 | transcription factor 20 isoform 1 [Homo sapiens] |

| Filename XCorr DeltCN Conf% ObsM+H+ CalcM+H+ SpR ZScore Ion% # Sequence  | | | | | | | | | | | | |
| --- | --- | --- | --- | --- | --- | --- | --- | --- | --- | --- | --- | --- |
|  | AstrinIP\_MS2\_022614\_01.08854.08854.2 | 2.5117 | 0.1888 | 95.7% | 1712.4722 | 1712.6384 | 1 | 4.158 | 50.0% | 1 | K.GAT#VTEAHT#ET#KVK.D | 2 |
|  | AstrinIP\_MS2\_022614\_02.08397.08397.3 | 3.2634 | 0.2542 | 98.1% | 3102.5942 | 3103.137 | 6 | 4.298 | 22.2% | 1 | R.RMPTPRS\*DQPGYDSLSDHTAAGPS\*ANGR.Y | 3 |

---

|  |  |  |  |  |  |  |  |  |
| --- | --- | --- | --- | --- | --- | --- | --- | --- |
| U | *Reverse\_gi|40255119|r* | 2 | 2 | 2.2% | 740 | 82879 | 5.1 | RAS and EF-hand domain containing [Homo sapiens] |

| Filename XCorr DeltCN Conf% ObsM+H+ CalcM+H+ SpR ZScore Ion% # Sequence  | | | | | | | | | | | | |
| --- | --- | --- | --- | --- | --- | --- | --- | --- | --- | --- | --- | --- |
| \* | AstrinIP\_MS1\_022614\_01.10318.10318.2 | 3.0614 | 0.1368 | 97.6% | 1764.4122 | 1765.7452 | 17 | 3.958 | 46.7% | 1 | K.RSSS\*VISGES\*VSGQPK.W | 2 |
| \* | AstrinIP\_MS1\_022614\_01.10253.10253.2 | 2.8735 | 0.1589 | 97.3% | 1764.4521 | 1765.7452 | 98 | 3.896 | 36.7% | 1 | K.RSSSVIS\*GES\*VSGQPK.W | 2 |

---

|  |  |  |  |  |  |  |  |  |
| --- | --- | --- | --- | --- | --- | --- | --- | --- |
| U | *gi|57863301|ref|NP\_05* | 2 | 2 | 2.1% | 1506 | 164886 | 8.2 | CLIP-associating protein 2 [Homo sapiens] |

| Filename XCorr DeltCN Conf% ObsM+H+ CalcM+H+ SpR ZScore Ion% # Sequence  | | | | | | | | | | | | |
| --- | --- | --- | --- | --- | --- | --- | --- | --- | --- | --- | --- | --- |
| \* | AstrinIP\_MS1\_022614\_01.17102.17102.3 | 3.7121 | 0.4276 | 100.0% | 2391.9243 | 2392.6733 | 1 | 6.808 | 36.1% | 1 | R.SFEFLDLLLQEWQTHSLER.H | 3 |
|  | AstrinIP\_MS1\_022614\_01.10614.10614.2 | 2.4306 | 0.2373 | 98.1% | 1326.2322 | 1326.6224 | 1 | 5.051 | 77.3% | 1 | R.KEGLLGLQNLLK.N | 22 |

Similarities:
gi|214010173|ref|NP\_0(1:1)  

---

|  |  |  |  |  |  |  |  |  |
| --- | --- | --- | --- | --- | --- | --- | --- | --- |
| U | *gi|4505257|ref|NP\_002* | 2 | 2 | 2.1% | 577 | 67820 | 6.4 | moesin [Homo sapiens] |

| Filename XCorr DeltCN Conf% ObsM+H+ CalcM+H+ SpR ZScore Ion% # Sequence  | | | | | | | | | | | | |
| --- | --- | --- | --- | --- | --- | --- | --- | --- | --- | --- | --- | --- |
| \* | AstrinIP\_MS2\_022614\_01.05334.05334.1 | 2.2161 | 0.2193 | 98.3% | 1190.63 | 1191.3417 | 198 | 4.304 | 50.0% | 1 | K.AQMVQEDLEK.T | 1 |
| \* | AstrinIP\_MS1\_022614\_01.05015.05015.2 | 2.6024 | 0.2786 | 99.2% | 1447.1322 | 1448.6343 | 3 | 5.142 | 63.6% | 1 | K.AQMVQEDLEKTR.A | 2 |

---

|  |  |  |  |  |  |  |  |  |
| --- | --- | --- | --- | --- | --- | --- | --- | --- |
| U | *gi|105990514|ref|NP\_0* | 4 | 6 | 2.0% | 2602 | 278162 | 5.7 | filamin B, beta (actin binding protein 278) [Homo sapiens] |

| Filename XCorr DeltCN Conf% ObsM+H+ CalcM+H+ SpR ZScore Ion% # Sequence  | | | | | | | | | | | | |
| --- | --- | --- | --- | --- | --- | --- | --- | --- | --- | --- | --- | --- |
| \* | AstrinIP\_MS2\_022614\_01.09104.09104.2 | 2.6714 | 0.1987 | 98.1% | 1402.4122 | 1402.5474 | 13 | 4.81 | 58.3% | 1 | R.IGNLQTDLSDGLR.L | 2 |
| \* | AstrinIP\_MS1\_022614\_01.06513.06513.2 | 3.0018 | 0.3488 | 99.9% | 1300.3722 | 1300.5437 | 2 | 5.981 | 63.6% | 1 | K.VTVLFAGQHISK.S | 2 |
| \* | AstrinIP\_MS1\_022614\_01.06630.06630.2 | 2.1432 | 0.3468 | 98.6% | 1278.0122 | 1277.4716 | 38 | 5.93 | 50.0% | 2 | R.AWGPGLHGGIVGR.S | 2 |
| \* | AstrinIP\_MS1\_022614\_01.07012.07012.2 | 2.9003 | 0.3299 | 99.8% | 1413.8522 | 1414.5988 | 1 | 5.384 | 57.1% | 2 | R.AGPGTLSVTIEGPSK.V | 2 |

---

|  |  |  |  |  |  |  |  |  |
| --- | --- | --- | --- | --- | --- | --- | --- | --- |
| U | *gi|32698688|ref|NP\_00* | 3 | 4 | 2.0% | 2027 | 231429 | 6.6 | citron [Homo sapiens] |

| Filename XCorr DeltCN Conf% ObsM+H+ CalcM+H+ SpR ZScore Ion% # Sequence  | | | | | | | | | | | | |
| --- | --- | --- | --- | --- | --- | --- | --- | --- | --- | --- | --- | --- |
| \* | AstrinIP\_MS2\_022614\_01.06076.06076.2 | 4.0676 | 0.4211 | 100.0% | 1382.1522 | 1382.5162 | 1 | 8.119 | 73.1% | 2 | R.NPLDAGAAEPIASR.A | 2 |
| \* | AstrinIP\_MS2\_022614\_01.12726.12726.2 | 3.2984 | 0.3026 | 99.8% | 1649.7122 | 1649.9178 | 1 | 6.394 | 69.2% | 1 | R.LMMNQLEEDLVSAR.R | 2 |
| \* | AstrinIP\_MS2\_022614\_01.09813.09813.2 | 3.0509 | 0.3293 | 99.8% | 1445.9722 | 1446.6005 | 1 | 5.832 | 66.7% | 1 | K.ASTEATELLQNIR.Q | 2 |

---

|  |  |  |  |  |  |  |  |  |
| --- | --- | --- | --- | --- | --- | --- | --- | --- |
| U | *gi|113430175|ref|XP\_0* | 2 | 2 | 1.9% | 1934 | 204058 | 10.7 | PREDICTED: similar to Predicted gene, OTTMUSG00000017677 [Homo sapiens] |
| U | *gi|169217315|ref|XP\_0* | 2 | 2 | 1.8% | 2061 | 217745 | 10.9 | PREDICTED: similar to Predicted gene, OTTMUSG00000017677 [Homo sapiens] |
| U | *gi|113430659|ref|XP\_0* | 2 | 2 | 1.8% | 2061 | 217774 | 10.9 | PREDICTED: similar to Predicted gene, OTTMUSG00000017677 [Homo sapiens] |
| U | *gi|113430177|ref|XP\_0* | 2 | 2 | 1.8% | 2028 | 214266 | 10.9 | PREDICTED: similar to Predicted gene, OTTMUSG00000017677 [Homo sapiens] |

| Filename XCorr DeltCN Conf% ObsM+H+ CalcM+H+ SpR ZScore Ion% # Sequence  | | | | | | | | | | | | |
| --- | --- | --- | --- | --- | --- | --- | --- | --- | --- | --- | --- | --- |
|  | AstrinIP\_MS2\_022614\_01.17086.17086.2 | 2.3103 | 0.2592 | 96.8% | 1853.5521 | 1856.0458 | 9 | 4.579 | 34.4% | 1 | R.ENPSSPTVPSESKRALR.D | 2 |
|  | AstrinIP\_MS1\_022614\_01.15303.15303.3 | 2.5703 | 0.304 | 98.1% | 2127.1743 | 2124.3677 | 177 | 4.659 | 25.0% | 1 | R.VGTGIALPAGALHRDRS\*PVR.R | 3 |

---

|  |  |  |  |  |  |  |  |  |
| --- | --- | --- | --- | --- | --- | --- | --- | --- |
| U | *gi|87162455|ref|NP\_05* | 2 | 3 | 1.9% | 1309 | 146669 | 6.1 | hypothetical protein LOC23277 [Homo sapiens] |

| Filename XCorr DeltCN Conf% ObsM+H+ CalcM+H+ SpR ZScore Ion% # Sequence  | | | | | | | | | | | | |
| --- | --- | --- | --- | --- | --- | --- | --- | --- | --- | --- | --- | --- |
| \* | AstrinIP\_MS2\_022614\_01.06800.06800.2 | 3.3086 | 0.4857 | 100.0% | 1274.1921 | 1274.4166 | 1 | 7.609 | 77.3% | 1 | R.SVEGLQEGSVLR.V | 2 |
| \* | AstrinIP\_MS2\_022614\_01.09090.09090.2 | 3.2437 | 0.4542 | 100.0% | 1324.0922 | 1324.4764 | 1 | 7.316 | 70.8% | 2 | K.AVGSISSTAFDIR.F | 2 |

---

|  |  |  |  |  |  |  |  |  |
| --- | --- | --- | --- | --- | --- | --- | --- | --- |
| U | *Reverse\_gi|26986534|r* | 2 | 12 | 1.9% | 846 | 96254 | 7.6 | Rho GTPase activating protein 12 [Homo sapiens] |

| Filename XCorr DeltCN Conf% ObsM+H+ CalcM+H+ SpR ZScore Ion% # Sequence  | | | | | | | | | | | | |
| --- | --- | --- | --- | --- | --- | --- | --- | --- | --- | --- | --- | --- |
| \* | AstrinIP\_MS1\_022614\_02.08179.08179.3 | 3.096 | 0.2575 | 98.7% | 2016.6843 | 2018.9597 | 15 | 4.66 | 36.7% | 3 | K.GFS\*S\*LEPLKNVNETS\*R.Q | 3 |
| \* | AstrinIP\_MS1\_022614\_01.12554.12554.3 | 3.5549 | 0.3016 | 99.8% | 2017.4944 | 2018.9597 | 2 | 4.707 | 43.3% | 9 | K.GFS\*S\*LEPLKNVNET#SR.Q | 3 |

---

|  |  |  |  |  |  |  |  |  |
| --- | --- | --- | --- | --- | --- | --- | --- | --- |
| U | *gi|41406064|ref|NP\_00* | 3 | 4 | 1.8% | 1976 | 228997 | 5.5 | myosin, heavy polypeptide 10, non-muscle [Homo sapiens] |

| Filename XCorr DeltCN Conf% ObsM+H+ CalcM+H+ SpR ZScore Ion% # Sequence  | | | | | | | | | | | | |
| --- | --- | --- | --- | --- | --- | --- | --- | --- | --- | --- | --- | --- |
|  | AstrinIP\_MS2\_022614\_01.10344.10344.2 | 2.6066 | 0.1937 | 98.2% | 1319.6122 | 1319.5468 | 37 | 5.417 | 65.0% | 1 | K.LDPHLVLDQLR.C | 22 |
|  | AstrinIP\_MS2\_022614\_01.06628.06628.2 | 3.2823 | 0.4021 | 100.0% | 1223.9722 | 1224.3591 | 1 | 7.157 | 75.0% | 2 | R.AGVLAHLEEER.D | 22 |
| \* | AstrinIP\_MS2\_022614\_01.08238.08238.2 | 2.6552 | 0.192 | 97.9% | 1516.5322 | 1515.6604 | 2 | 5.058 | 54.2% | 1 | K.IGQLEEQLEQEAK.E | 2 |

Similarities:
gi|12667788|ref|NP\_00(2:1)  

---

|  |  |  |  |  |  |  |  |  |
| --- | --- | --- | --- | --- | --- | --- | --- | --- |
| U | *gi|225543461|ref|NP\_2* | 2 | 3 | 1.8% | 1861 | 202218 | 8.3 | tetratricopeptide repeat, ankyrin repeat and coiled-coil containing 1 isoform 1 [Homo sapiens] |
| U | *gi|225543463|ref|NP\_0* | 2 | 3 | 2.4% | 1390 | 152183 | 8.3 | tetratricopeptide repeat, ankyrin repeat and coiled-coil containing 1 isoform 2 [Homo sapiens] |

| Filename XCorr DeltCN Conf% ObsM+H+ CalcM+H+ SpR ZScore Ion% # Sequence  | | | | | | | | | | | | |
| --- | --- | --- | --- | --- | --- | --- | --- | --- | --- | --- | --- | --- |
|  | AstrinIP\_MS2\_022614\_01.13601.13601.2 | 2.4188 | 0.2997 | 99.0% | 1600.4521 | 1600.7739 | 1 | 4.933 | 59.1% | 1 | R.DWLFHQIEENLR.N | 2 |
|  | AstrinIP\_MS2\_022614\_01.18527.18527.2 | 3.1259 | 0.4729 | 100.0% | 2328.5122 | 2329.652 | 1 | 6.488 | 35.0% | 2 | R.TPLDLAAFYGDAETVLYLVEK.G | 2 |

---

|  |  |  |  |  |  |  |  |  |
| --- | --- | --- | --- | --- | --- | --- | --- | --- |
| U | *gi|148612801|ref|NP\_0* | 2 | 3 | 1.8% | 1403 | 161942 | 5.5 | KIAA1009 protein [Homo sapiens] |

| Filename XCorr DeltCN Conf% ObsM+H+ CalcM+H+ SpR ZScore Ion% # Sequence  | | | | | | | | | | | | |
| --- | --- | --- | --- | --- | --- | --- | --- | --- | --- | --- | --- | --- |
| \* | AstrinIP\_MS2\_022614\_01.05466.05466.2 | 2.8277 | 0.4261 | 100.0% | 1452.1122 | 1452.5763 | 4 | 7.087 | 50.0% | 2 | R.SQLSSEEEGAVMGK.Q | 2 |
| \* | AstrinIP\_MS1\_022614\_01.06387.06387.2 | 2.875 | 0.2301 | 99.5% | 1320.6122 | 1320.4882 | 1 | 5.285 | 75.0% | 1 | K.LNQDNYILQAK.L | 2 |

---

|  |  |  |  |  |  |  |  |  |
| --- | --- | --- | --- | --- | --- | --- | --- | --- |
| U | *gi|19913406|ref|NP\_00* | 2 | 2 | 1.7% | 1531 | 174384 | 8.7 | DNA topoisomerase II, alpha isozyme [Homo sapiens] |
| U | *gi|19913408|ref|NP\_00* | 2 | 2 | 1.6% | 1621 | 182661 | 8.1 | DNA topoisomerase II, beta isozyme [Homo sapiens] |

| Filename XCorr DeltCN Conf% ObsM+H+ CalcM+H+ SpR ZScore Ion% # Sequence  | | | | | | | | | | | | |
| --- | --- | --- | --- | --- | --- | --- | --- | --- | --- | --- | --- | --- |
|  | AstrinIP\_MS2\_022614\_01.10648.10648.2 | 3.0855 | 0.1549 | 98.6% | 1462.4321 | 1462.6427 | 1 | 5.352 | 79.2% | 1 | K.IFDEILVNAADNK.Q | 2 |
|  | AstrinIP\_MS2\_022614\_01.09418.09418.2 | 3.5682 | 0.2095 | 99.8% | 1530.3121 | 1530.7797 | 1 | 4.604 | 75.0% | 1 | K.QIMENAEINNIIK.I | 2 |

---

|  |  |  |  |  |  |  |  |  |
| --- | --- | --- | --- | --- | --- | --- | --- | --- |
| U | *gi|150418007|ref|NP\_0* | 4 | 4 | 1.6% | 3224 | 358201 | 6.2 | RAN binding protein 2 [Homo sapiens] |

| Filename XCorr DeltCN Conf% ObsM+H+ CalcM+H+ SpR ZScore Ion% # Sequence  | | | | | | | | | | | | |
| --- | --- | --- | --- | --- | --- | --- | --- | --- | --- | --- | --- | --- |
|  | AstrinIP\_MS2\_022614\_01.06742.06742.2 | 2.979 | 0.4515 | 100.0% | 1336.0521 | 1336.5773 | 1 | 7.157 | 80.0% | 1 | R.LLVQHEINTLR.A | 22 |
| \* | AstrinIP\_MS2\_022614\_01.04392.04392.2 | 2.8535 | 0.3308 | 99.8% | 1486.3522 | 1487.6738 | 11 | 6.776 | 50.0% | 1 | K.APGTNVAMASNQAVR.I | 2 |
| \* | AstrinIP\_MS2\_022614\_02.06552.06552.2 | 2.8929 | 0.1072 | 95.0% | 1540.8722 | 1540.6726 | 1 | 3.699 | 53.8% | 1 | K.SDAGNLNFEFQVAK.K | 2 |
|  | AstrinIP\_MS2\_022614\_02.05770.05770.2 | 2.5299 | 0.3004 | 99.5% | 1202.5521 | 1202.3934 | 1 | 5.001 | 80.0% | 1 | K.IAVAVLEETTR.E | 2 |

Similarities:
gi|169162953|ref|XP\_0(1:3)  

---

|  |  |  |  |  |  |  |  |  |
| --- | --- | --- | --- | --- | --- | --- | --- | --- |
| U | *gi|27477134|ref|NP\_07* | 2 | 3 | 1.5% | 1887 | 205109 | 6.8 | nucleoporin 210 [Homo sapiens] |

| Filename XCorr DeltCN Conf% ObsM+H+ CalcM+H+ SpR ZScore Ion% # Sequence  | | | | | | | | | | | | |
| --- | --- | --- | --- | --- | --- | --- | --- | --- | --- | --- | --- | --- |
| \* | AstrinIP\_MS2\_022614\_01.12144.12144.2 | 3.6275 | 0.5145 | 100.0% | 1610.3922 | 1610.8712 | 1 | 8.071 | 69.2% | 2 | R.LPSQYNFAMNVLGR.V | 2 |
| \* | AstrinIP\_MS2\_022614\_01.08154.08154.2 | 2.8699 | 0.3474 | 99.8% | 1447.8722 | 1448.6188 | 1 | 7.132 | 73.1% | 1 | K.AVDPTSGQLYGLAR.E | 2 |

---

|  |  |  |  |  |  |  |  |  |
| --- | --- | --- | --- | --- | --- | --- | --- | --- |
| U | *gi|169218225|ref|XP\_9* | 2 | 2 | 1.5% | 1700 | 194322 | 6.7 | PREDICTED: hypothetical protein, partial [Homo sapiens] |
| U | *gi|40217847|ref|NP\_05* | 2 | 2 | 1.2% | 2136 | 244505 | 6.1 | activating signal cointegrator 1 complex subunit 3-like 1 [Homo sapiens] |

| Filename XCorr DeltCN Conf% ObsM+H+ CalcM+H+ SpR ZScore Ion% # Sequence  | | | | | | | | | | | | |
| --- | --- | --- | --- | --- | --- | --- | --- | --- | --- | --- | --- | --- |
|  | AstrinIP\_MS2\_022614\_01.10338.10338.2 | 2.9027 | 0.1256 | 96.5% | 1699.8121 | 1699.904 | 1 | 5.423 | 65.4% | 1 | R.LYDLNHNEIGELIR.M | 2 |
|  | AstrinIP\_MS2\_022614\_01.06564.06564.2 | 2.4199 | 0.2683 | 98.6% | 1189.9922 | 1190.3824 | 185 | 5.054 | 50.0% | 1 | R.IVALSSSLSNAK.D | 2 |

---

|  |  |  |  |  |  |  |  |  |
| --- | --- | --- | --- | --- | --- | --- | --- | --- |
| U | *gi|38679960|ref|NP\_94* | 2 | 2 | 1.4% | 2383 | 269997 | 6.5 | acetyl-Coenzyme A carboxylase alpha isoform 1 [Homo sapiens] |
| U | *gi|38679977|ref|NP\_94* | 2 | 2 | 1.4% | 2346 | 265551 | 6.4 | acetyl-Coenzyme A carboxylase alpha isoform 2 [Homo sapiens] |
| U | *gi|38679974|ref|NP\_94* | 2 | 2 | 1.5% | 2268 | 257236 | 6.6 | acetyl-Coenzyme A carboxylase alpha isoform 4 [Homo sapiens] |
| U | *gi|38679971|ref|NP\_94* | 2 | 2 | 1.4% | 2288 | 259684 | 6.6 | acetyl-Coenzyme A carboxylase alpha isoform 3 [Homo sapiens] |
| U | *gi|38679967|ref|NP\_94* | 2 | 2 | 1.4% | 2346 | 265551 | 6.4 | acetyl-Coenzyme A carboxylase alpha isoform 2 [Homo sapiens] |

| Filename XCorr DeltCN Conf% ObsM+H+ CalcM+H+ SpR ZScore Ion% # Sequence  | | | | | | | | | | | | |
| --- | --- | --- | --- | --- | --- | --- | --- | --- | --- | --- | --- | --- |
|  | AstrinIP\_MS2\_022614\_01.13534.13534.2 | 2.8732 | 0.3683 | 99.8% | 1591.9922 | 1592.8351 | 1 | 6.462 | 57.7% | 1 | R.IGSFGPQEDLLFLR.A | 2 |
|  | AstrinIP\_MS2\_022614\_02.05984.05984.3 | 3.0704 | 0.2873 | 99.2% | 1979.9644 | 1980.2719 | 13 | 4.43 | 30.6% | 1 | R.TIQVENSHLILTGAGALNK.V | 3 |

---

|  |  |  |  |  |  |  |  |  |
| --- | --- | --- | --- | --- | --- | --- | --- | --- |
| U | *gi|134142826|ref|NP\_0* | 3 | 3 | 1.4% | 2024 | 228531 | 5.0 | pericentriolar material 1 [Homo sapiens] |

| Filename XCorr DeltCN Conf% ObsM+H+ CalcM+H+ SpR ZScore Ion% # Sequence  | | | | | | | | | | | | |
| --- | --- | --- | --- | --- | --- | --- | --- | --- | --- | --- | --- | --- |
| \* | AstrinIP\_MS2\_022614\_01.10632.10632.2 | 3.2767 | 0.3235 | 99.8% | 2092.8123 | 2094.3716 | 3 | 5.252 | 43.8% | 1 | R.TPWLYEQEGEVEKPFIK.T | 2 |
| \* | AstrinIP\_MS2\_022614\_01.10641.10641.3 | 4.1525 | 0.4461 | 100.0% | 2093.2744 | 2094.3716 | 1 | 6.682 | 40.6% | 1 | R.TPWLYEQEGEVEKPFIK.T | 3 |
| \* | AstrinIP\_MS2\_022614\_01.10800.10800.2 | 3.2111 | 0.4332 | 100.0% | 1249.1122 | 1249.4526 | 1 | 7.519 | 80.0% | 1 | R.ALYALQDIVSR.H | 2 |

---

|  |  |  |  |  |  |  |  |  |
| --- | --- | --- | --- | --- | --- | --- | --- | --- |
| U | *gi|169162953|ref|XP\_0* | 2 | 2 | 1.3% | 1765 | 198991 | 6.5 | PREDICTED: hypothetical protein [Homo sapiens] |
| U | *gi|83267877|ref|NP\_00* | 2 | 2 | 1.3% | 1765 | 198922 | 6.4 | RANBP2-like and GRIP domain containing 5 isoform 1 [Homo sapiens] |
| U | *gi|182765478|ref|NP\_0* | 2 | 2 | 1.3% | 1765 | 198922 | 6.4 | RANBP2-like and GRIP domain containing 6 [Homo sapiens] |

| Filename XCorr DeltCN Conf% ObsM+H+ CalcM+H+ SpR ZScore Ion% # Sequence  | | | | | | | | | | | | |
| --- | --- | --- | --- | --- | --- | --- | --- | --- | --- | --- | --- | --- |
|  | AstrinIP\_MS2\_022614\_01.06742.06742.2 | 2.979 | 0.4515 | 100.0% | 1336.0521 | 1336.5773 | 1 | 7.157 | 80.0% | 1 | R.LLVQHEINTLR.A | 22 |
|  | AstrinIP\_MS2\_022614\_01.06976.06976.2 | 2.8645 | 0.0964 | 95.9% | 1431.9722 | 1432.6604 | 1 | 4.152 | 63.6% | 1 | K.EELVQKLRSTTK.S | 2 |

Similarities:
gi|150418007|ref|NP\_0(1:1)  

---

|  |  |  |  |  |  |  |  |  |
| --- | --- | --- | --- | --- | --- | --- | --- | --- |
| U | *gi|54607139|ref|NP\_05* | 2 | 2 | 1.1% | 4388 | 491920 | 6.6 | vacuolar protein sorting 13D isoform 1 [Homo sapiens] |
| U | *gi|54607141|ref|NP\_06* | 2 | 2 | 1.1% | 4363 | 489065 | 6.6 | vacuolar protein sorting 13D isoform 2 [Homo sapiens] |

| Filename XCorr DeltCN Conf% ObsM+H+ CalcM+H+ SpR ZScore Ion% # Sequence  | | | | | | | | | | | | |
| --- | --- | --- | --- | --- | --- | --- | --- | --- | --- | --- | --- | --- |
|  | AstrinIP\_MS2\_022614\_01.12509.12509.3 | 4.4488 | 0.1476 | 96.6% | 3329.2144 | 3328.5896 | 18 | 3.856 | 22.0% | 1 | R.SRHS\*PRIDCDIQLETIPLKLS\*QLQYR.Q | 3 |
|  | AstrinIP\_MS1\_022614\_01.19612.19612.3 | 2.9009 | 0.2457 | 95.7% | 2408.7544 | 2410.8655 | 106 | 4.055 | 25.0% | 1 | R.ALKVIQQGNRPGLIYNIGIDVK.K | 3 |

---

|  |  |  |  |  |  |  |  |  |
| --- | --- | --- | --- | --- | --- | --- | --- | --- |
| U | *gi|194248068|ref|NP\_0* | 2 | 4 | 1.1% | 1343 | 148284 | 9.0 | synaptic Ras GTPase activating protein 1 [Homo sapiens] |

| Filename XCorr DeltCN Conf% ObsM+H+ CalcM+H+ SpR ZScore Ion% # Sequence  | | | | | | | | | | | | |
| --- | --- | --- | --- | --- | --- | --- | --- | --- | --- | --- | --- | --- |
| \* | AstrinIP\_MS1\_022614\_01.07988.07988.2 | 2.3111 | 0.3211 | 98.6% | 1582.5922 | 1583.5841 | 4 | 4.83 | 39.3% | 1 | R.LS\*QGSGS\*SITAAGMR.L | 2 |
| \* | AstrinIP\_MS1\_022614\_01.08054.08054.2 | 2.487 | 0.3513 | 99.3% | 1582.7122 | 1583.5841 | 1 | 4.86 | 46.4% | 3 | R.LS\*QGSGSSIT#AAGMR.L | 2 |

---

|  |  |  |  |  |  |  |  |  |
| --- | --- | --- | --- | --- | --- | --- | --- | --- |
| U | *gi|212549532|ref|NP\_0* | 2 | 9 | 0.9% | 1982 | 222656 | 6.6 | caspase 8 associated protein 2 [Homo sapiens] |
| U | *gi|6912288|ref|NP\_036* | 2 | 9 | 0.9% | 1982 | 222656 | 6.6 | caspase 8 associated protein 2 [Homo sapiens] |
| U | *gi|212549782|ref|NP\_0* | 2 | 9 | 0.9% | 1982 | 222656 | 6.6 | caspase 8 associated protein 2 [Homo sapiens] |

| Filename XCorr DeltCN Conf% ObsM+H+ CalcM+H+ SpR ZScore Ion% # Sequence  | | | | | | | | | | | | |
| --- | --- | --- | --- | --- | --- | --- | --- | --- | --- | --- | --- | --- |
|  | AstrinIP\_MS1\_022614\_01.17316.17316.2 | 3.7772 | 0.0896 | 98.7% | 2169.5322 | 2171.4192 | 3 | 3.406 | 41.2% | 2 | K.S\*VTTLQKNLCDIIESKLK.Q | 2 |
|  | AstrinIP\_MS2\_022614\_01.18042.18042.2 | 4.4069 | 0.2032 | 100.0% | 2170.0923 | 2171.4192 | 5 | 4.505 | 41.2% | 7 | K.SVT#TLQKNLCDIIESKLK.Q | 2 |

---

|  |  |  |  |  |  |  |  |  |
| --- | --- | --- | --- | --- | --- | --- | --- | --- |
| U | *gi|154277116|ref|NP\_8* | 2 | 2 | 0.5% | 8797 | 1011050 | 5.5 | spectrin repeat containing, nuclear envelope 1 isoform 1 [Homo sapiens] |
| U | *gi|23097308|ref|NP\_14* | 2 | 2 | 0.5% | 8749 | 1005204 | 5.5 | spectrin repeat containing, nuclear envelope 1 isoform 2 [Homo sapiens] |

| Filename XCorr DeltCN Conf% ObsM+H+ CalcM+H+ SpR ZScore Ion% # Sequence  | | | | | | | | | | | | |
| --- | --- | --- | --- | --- | --- | --- | --- | --- | --- | --- | --- | --- |
|  | AstrinIP\_MS2\_022614\_01.07335.07335.2 | 2.6548 | 0.1143 | 95.2% | 1410.1921 | 1410.5388 | 54 | 3.572 | 55.0% | 1 | K.SQLNELCRFSR.D | 2 |
|  | AstrinIP\_MS2\_022614\_01.19964.19964.3 | 3.5223 | 0.2165 | 97.2% | 3293.5745 | 3295.375 | 16 | 3.632 | 21.4% | 1 | R.SADSLLSPQPSS\*NLS\*LSLAQPLRS\*ERSGR.D | 3 |

|  |  |  |  |
| --- | --- | --- | --- |
|  | Proteins | Peptide IDs | Spectra |
| Unfiltered | 47977 | 99928 | 166803 |
| Filtered | 193 | 1645 | 5495 |
| Forward matches | 187 | 1633 | 5465 |
| Decoy matches | 6 | 12 | 30 |
| Forward FP rate | 3.21% | 0.73% | 0.55% |

  
/nfs/cheeseman\_massspec/David/AstrinIP
