# Supplementary material for: Astrin-SKAP complex reconstitution reveals its kinetochore interaction with microtubule-bound Ndc80
Source: eLife. 2017 Aug 25;6:e26866. doi: 10.7554/eLife.26866 (PMC5602300; doi:10.7554/eLife.26866)
Supplement: Source data 1. — Complete mass spectrometry searches using methods described in (Washburn et al., 2001) for affinity purification/mass spectrometry data sets described in this paper (data from this study; [Kern et al., 2016] [Gascoigne et al., 2011]). Individual Astrin cross-linking immunoprecipitations are listed based on the order in Figure 4—figure supplement 1. These samples have not been pruned for common or antibody-specific contaminants. [file elife-26866-data1.zip › Astrin_STLCIP.html]

D Astrin\_STLC\_IP
DTASelect v2.0.21  
/nfs/cheeseman\_massspec/David/Astrin\_STLC\_IP  
/nfs/cheeseman\_massspec/Databases/HumanRefSeq\_20160627\_reversed\_small.fasta  
SEQUEST 3.0 in SQT format.  
  
 Jump  to the summary table.  
  
sequest.params modifications:

|  |  |  |
| --- | --- | --- |
| \* | S | 80.0 |
| # | T | 80.0 |
| @ | K | 12.0 |
| Static | C | 57.0 |

|  |  |
| --- | --- |
| true | Use criteria |
| 0.0 | Minimum peptide confidence |
| 0.05 | Peptide false positive rate |
| 0.0 | Minimum protein confidence |
| 1.0 | Protein false positive rate |
| 1 | Minimum charge state |
| 16 | Maximum charge state |
| 0.0 | Minimum ion proportion |
| 1000 | Maximum Sp rank |
| -1.0 | Minimum Sp score |
| Include | Modified peptide inclusion |
| Any | Tryptic status requirement |
| false | Multiple, ambiguous IDs allowed |
| Ignore | Peptide validation handling |
| XCorr | Purge duplicate peptides by protein |
| false | Include only loci with unique peptide |
| true | Remove subset proteins |
| Ignore | Locus validation handling |
| 0 | Minimum modified peptides per locus |
| 1000 | Minimum redundancy for low coverage loci |
| 2 | Minimum peptides per locus |

#### Locus Key:

|  |  |  |  |  |  |  |  |  |
| --- | --- | --- | --- | --- | --- | --- | --- | --- |
| Validation Status | Locus | Sequence Count | Spectrum Count | Sequence Coverage | Length | MolWt | pI | Descriptive Name |

#### Similarity Key:

|  |  |  |
| --- | --- | --- |
| Locus | # of identical peptides | # of differing peptides |

---

|  |  |  |  |  |  |  |  |  |
| --- | --- | --- | --- | --- | --- | --- | --- | --- |
| U | *gi|73623035|ref|NP\_00* | 100 | 646 | 66.1% | 1193 | 134422 | 5.0 | sperm-associated antigen 5 [Homo sapiens] &IC Astrin |

| Filename XCorr DeltCN Conf% ObsM+H+ CalcM+H+ SpR ZScore Ion% # Sequence  | | | | | | | | | | | | |
| --- | --- | --- | --- | --- | --- | --- | --- | --- | --- | --- | --- | --- |
| \* | Astrin\_STLC\_112116\_01.05859.05859.3 | 3.0089 | 0.2756 | 98.6% | 1916.2444 | 1915.2585 | 36 | 5.22 | 32.4% | 1 | K.KLSLSLSPSPQTGKPSMR.T | 3 |
| \* | Astrin\_STLC\_112116\_tube2\_01.07193.07193.3 | 4.154 | 0.4036 | 100.0% | 2051.8743 | 2053.3694 | 1 | 6.517 | 37.5% | 1 | R.TPLRELTLQPGALTNSGKR.S | 3 |
| \* | Astrin\_STLC\_112116\_01.05963.05963.2 | 2.8392 | 0.1764 | 96.8% | 1585.3922 | 1585.8008 | 13 | 3.819 | 53.6% | 1 | R.ELTLQPGALTNSGKR.S | 2 |
| \* | Astrin\_STLC\_112116\_tube2\_01.07353.07353.3 | 3.7813 | 0.449 | 100.0% | 2175.4443 | 2176.3506 | 1 | 7.009 | 34.2% | 2 | K.LGLQEGSNNSSPVDFVNNKR.T | 3 |
| \* | Astrin\_STLC\_112116\_tube2\_01.07973.07973.3 | 3.4695 | 0.3525 | 99.8% | 2255.1243 | 2256.3506 | 2 | 6.235 | 36.8% | 1 | K.LGLQEGSNNS\*SPVDFVNNKR.T | 3 |
| \* | Astrin\_STLC\_112116\_tube2\_01.03588.03588.3 | 3.0187 | 0.2361 | 97.8% | 1618.8243 | 1618.7031 | 41 | 5.298 | 36.5% | 2 | K.RTDLSSEHFSHSSK.W | 3 |
| \* | Astrin\_STLC\_112116\_tube2\_01.08544.08544.2 | 4.2979 | 0.4512 | 100.0% | 1652.9521 | 1653.8445 | 1 | 9.778 | 71.4% | 9 | K.TSEEAVDPLGNYMVK.T | 2 |
| \* | Astrin\_STLC\_112116\_01.09658.09658.2 | 2.606 | 0.3835 | 100.0% | 2242.2922 | 2243.6262 | 1 | 6.377 | 50.0% | 2 | K.TIVLVPSPLGQQQDMIFEAR.L | 2 |
| \* | Astrin\_STLC\_112116\_01.10808.10808.2 | 3.8297 | 0.5385 | 100.0% | 2322.4321 | 2323.6262 | 1 | 9.388 | 60.5% | 10 | K.TIVLVPS\*PLGQQQDMIFEAR.L | 2 |
| \* | Astrin\_STLC\_112116\_01.10827.10827.3 | 2.8667 | 0.3465 | 99.4% | 2322.6543 | 2323.6262 | 1 | 5.882 | 34.2% | 1 | K.TIVLVPS\*PLGQQQDMIFEAR.L | 3 |
| \* | Astrin\_STLC\_112116\_tube2\_01.08548.08548.2 | 4.8869 | 0.4207 | 100.0% | 1833.2522 | 1833.0668 | 1 | 7.825 | 53.1% | 11 | R.LDTMAETNSISLNGPLR.T | 2 |
| \* | Astrin\_STLC\_112116\_02.07865.07865.3 | 4.7325 | 0.5057 | 100.0% | 2533.0444 | 2532.8286 | 1 | 8.975 | 35.2% | 6 | R.LDTMAETNSISLNGPLRTDDLVR.E | 3 |
| \* | Astrin\_STLC\_112116\_tube2\_02.09087.09087.3 | 3.8049 | 0.3603 | 99.8% | 2612.0044 | 2612.8286 | 7 | 5.467 | 31.8% | 2 | R.LDTMAETNSIS\*LNGPLRTDDLVR.E | 3 |
| \* | Astrin\_STLC\_112116\_02.11191.11191.3 | 4.795 | 0.3747 | 100.0% | 3777.9844 | 3778.2102 | 1 | 6.378 | 25.0% | 2 | R.TEAVREDLVPSESNAFLPSSVLWLSPSTALAADFR.V | 3 |
| \* | Astrin\_STLC\_112116\_01.12635.12635.3 | 4.2819 | 0.2341 | 99.5% | 3856.2844 | 3858.2102 | 1 | 6.917 | 26.5% | 2 | R.TEAVREDLVPSESNAFLPSSVLWLSPS\*TALAADFR.V | 3 |
| \* | Astrin\_STLC\_112116\_tube2\_01.13508.13508.3 | 6.1518 | 0.1564 | 99.4% | 3856.5244 | 3858.2102 | 1 | 7.473 | 35.3% | 14 | R.TEAVREDLVPSESNAFLPSSVLWLS\*PSTALAADFR.V | 3 |
| \* | Astrin\_STLC\_112116\_01.05948.05948.2 | 5.8236 | 0.5825 | 100.0% | 2219.652 | 2220.3752 | 1 | 10.103 | 72.2% | 2 | R.VNHVDPEEEIVEHGAMEER.E | 2 |
| \* | Astrin\_STLC\_112116\_tube2\_01.06741.06741.3 | 5.407 | 0.4059 | 100.0% | 2220.2944 | 2220.3752 | 1 | 7.575 | 51.4% | 10 | R.VNHVDPEEEIVEHGAMEER.E | 3 |
| \* | Astrin\_STLC\_112116\_01.12764.12764.3 | 4.4252 | 0.3948 | 100.0% | 2063.4844 | 2064.3606 | 1 | 6.391 | 38.2% | 1 | R.ILGSDTESWMSPLAWLEK.G | 3 |
| \* | Astrin\_STLC\_112116\_01.12957.12957.2 | 5.8659 | 0.533 | 100.0% | 2063.612 | 2064.3606 | 1 | 9.42 | 73.5% | 10 | R.ILGSDTESWMSPLAWLEK.G | 2 |
| \* | Astrin\_STLC\_112116\_01.13022.13022.2 | 4.9076 | 0.4856 | 100.0% | 2144.1921 | 2144.3606 | 1 | 7.374 | 64.7% | 11 | R.ILGSDTESWMS\*PLAWLEK.G | 2 |
| \* | Astrin\_STLC\_112116\_01.13659.13659.2 | 4.0931 | 0.3975 | 100.0% | 2144.2922 | 2144.3606 | 2 | 6.705 | 52.9% | 3 | R.ILGSDT#ESWMSPLAWLEK.G | 2 |
| \* | Astrin\_STLC\_112116\_01.14558.14558.2 | 3.2578 | 0.2691 | 99.9% | 2223.672 | 2224.3606 | 1 | 6.364 | 47.1% | 3 | R.ILGS\*DTESWMS\*PLAWLEK.G | 2 |
| \* | Astrin\_STLC\_112116\_01.14492.14492.2 | 3.1433 | 0.2208 | 99.3% | 2224.672 | 2224.3606 | 2 | 5.512 | 38.2% | 1 | R.ILGSDT#ESWMS\*PLAWLEK.G | 2 |
| \* | Astrin\_STLC\_112116\_01.07070.07070.2 | 3.8705 | 0.4278 | 100.0% | 1333.7722 | 1333.5457 | 1 | 7.251 | 68.2% | 3 | K.GVNTSVMLENLR.Q | 2 |
| \* | Astrin\_STLC\_112116\_tube2\_01.09204.09204.2 | 3.3151 | 0.254 | 100.0% | 1414.1522 | 1413.5457 | 1 | 4.968 | 68.2% | 2 | K.GVNTS\*VMLENLR.Q | 2 |
| \* | Astrin\_STLC\_112116\_01.07394.07394.2 | 2.5833 | 0.2024 | 98.5% | 1132.1122 | 1132.3635 | 367 | 5.273 | 55.6% | 6 | R.QSLSLPSMLR.D | 2 |
| \* | Astrin\_STLC\_112116\_01.04160.04160.2 | 2.8325 | 0.1301 | 95.7% | 1373.8322 | 1374.4044 | 1 | 5.362 | 75.0% | 2 | K.STNTS\*QTGLVGTK.H | 2 |
| \* | Astrin\_STLC\_112116\_01.06994.06994.3 | 3.4494 | 0.3931 | 99.8% | 2468.8145 | 2469.68 | 1 | 6.647 | 41.7% | 1 | K.HSTSETEQLLCGRPPDLTALSR.H | 3 |
| \* | Astrin\_STLC\_112116\_tube2\_01.15143.15143.2 | 7.635 | 0.6419 | 100.0% | 2165.152 | 2166.4795 | 1 | 10.63 | 69.4% | 28 | R.HDLEDNLLSSLVILEVLSR.Q | 2 |
| \* | Astrin\_STLC\_112116\_01.14378.14378.3 | 3.9194 | 0.4078 | 100.0% | 2166.6843 | 2166.4795 | 2 | 6.425 | 37.5% | 6 | R.HDLEDNLLSSLVILEVLSR.Q | 3 |
| \* | Astrin\_STLC\_112116\_01.05612.05612.3 | 5.4382 | 0.4639 | 100.0% | 2867.3044 | 2868.0 | 1 | 8.755 | 33.7% | 5 | K.SQLAVPHPETQDSSTQTDTSHSGITNK.L | 3 |
| \* | Astrin\_STLC\_112116\_tube2\_01.04381.04381.3 | 5.334 | 0.4515 | 100.0% | 2103.7144 | 2105.3794 | 1 | 8.657 | 45.6% | 5 | K.LQHLKESHEMGQALQQAR.N | 3 |
| \* | Astrin\_STLC\_112116\_01.04502.04502.2 | 3.5875 | 0.3894 | 100.0% | 1484.3121 | 1485.6146 | 3 | 7.557 | 62.5% | 2 | K.ESHEMGQALQQAR.N | 2 |
| \* | Astrin\_STLC\_112116\_01.04496.04496.3 | 3.5839 | 0.1532 | 96.9% | 1485.3243 | 1485.6146 | 18 | 5.292 | 47.9% | 1 | K.ESHEMGQALQQAR.N | 3 |
| \* | Astrin\_STLC\_112116\_01.09200.09200.2 | 3.9507 | 0.464 | 100.0% | 1305.3722 | 1305.578 | 1 | 7.878 | 90.0% | 9 | R.NVMQSWVLISK.E | 2 |
| \* | Astrin\_STLC\_112116\_01.09408.09408.3 | 6.5163 | 0.4741 | 100.0% | 2892.9543 | 2892.2793 | 1 | 7.646 | 30.2% | 11 | K.ELISLLHLSLLHLEEDKTTVSQESR.R | 3 |
| \* | Astrin\_STLC\_112116\_tube2\_01.03632.03632.2 | 2.6299 | 0.1528 | 98.5% | 1041.6921 | 1042.201 | 7 | 4.976 | 71.4% | 3 | R.HREEMALR.G | 2 |
| \* | Astrin\_STLC\_112116\_01.09208.09208.3 | 4.9728 | 0.4063 | 100.0% | 2074.8245 | 2074.267 | 1 | 7.266 | 41.7% | 1 | R.GKDAAEIVLEAFCAHASQR.I | 3 |
| \* | Astrin\_STLC\_112116\_tube2\_01.09165.09165.2 | 4.0121 | 0.4098 | 100.0% | 1391.0922 | 1391.5823 | 1 | 8.376 | 72.7% | 29 | R.ISQLEQDLASMR.E | 2 |
| \* | Astrin\_STLC\_112116\_01.09120.09120.2 | 2.5177 | 0.1969 | 95.4% | 1823.0521 | 1824.0618 | 1 | 5.006 | 57.1% | 1 | R.ISQLEQDLASMREFR.G | 2 |
| \* | Astrin\_STLC\_112116\_tube2\_01.07645.07645.2 | 3.2491 | 0.3737 | 100.0% | 1692.0521 | 1692.9994 | 1 | 6.649 | 53.3% | 1 | R.GLLKDAQTQLVGLHAK.Q | 2 |
| \* | Astrin\_STLC\_112116\_tube2\_01.07665.07665.3 | 4.3914 | 0.3072 | 99.8% | 1692.7144 | 1692.9994 | 11 | 5.792 | 38.3% | 6 | R.GLLKDAQTQLVGLHAK.Q | 3 |
| \* | Astrin\_STLC\_112116\_01.05714.05714.2 | 3.8497 | 0.4654 | 100.0% | 1280.8722 | 1281.4545 | 3 | 7.593 | 63.6% | 3 | K.DAQTQLVGLHAK.Q | 2 |
| \* | Astrin\_STLC\_112116\_tube2\_01.04909.04909.3 | 2.6525 | 0.2885 | 98.8% | 1281.5044 | 1281.4545 | 13 | 5.118 | 38.6% | 1 | K.DAQTQLVGLHAK.Q | 3 |
| \* | Astrin\_STLC\_112116\_02.09150.09150.3 | 4.3575 | 0.3968 | 100.0% | 2389.2244 | 2390.612 | 1 | 7.671 | 35.5% | 7 | K.QEELVQQTVSLTSTLQQDWR.S | 3 |
| \* | Astrin\_STLC\_112116\_01.10857.10857.2 | 5.4723 | 0.6417 | 100.0% | 2390.2922 | 2390.612 | 1 | 11.337 | 55.3% | 8 | K.QEELVQQTVSLTSTLQQDWR.S | 2 |
| \* | Astrin\_STLC\_112116\_tube2\_01.12035.12035.2 | 5.0454 | 0.4599 | 100.0% | 1785.7522 | 1787.0405 | 1 | 9.506 | 78.6% | 19 | R.SMQLDYTTWTALLSR.S | 2 |
| \* | Astrin\_STLC\_112116\_02.10163.10163.3 | 3.8402 | 0.2999 | 99.8% | 1786.6444 | 1787.0405 | 1 | 6.255 | 48.2% | 1 | R.SMQLDYTTWTALLSR.S | 3 |
| \* | Astrin\_STLC\_112116\_tube2\_01.03456.03456.2 | 2.4955 | 0.1339 | 96.3% | 959.89215 | 960.0348 | 2 | 3.925 | 78.6% | 1 | K.SQQALQER.D | 2 |
| \* | Astrin\_STLC\_112116\_01.05621.05621.3 | 5.8116 | 0.3669 | 100.0% | 2344.9744 | 2344.5437 | 1 | 6.511 | 44.7% | 2 | K.SQQALQERDVAIEEKQEVSR.V | 3 |
| \* | Astrin\_STLC\_112116\_tube2\_01.04066.04066.2 | 3.7861 | 0.386 | 100.0% | 1403.0122 | 1403.5321 | 1 | 6.843 | 77.3% | 4 | R.DVAIEEKQEVSR.V | 2 |
| \* | Astrin\_STLC\_112116\_01.04406.04406.3 | 2.7089 | 0.3169 | 99.5% | 1403.5144 | 1403.5321 | 1 | 4.94 | 40.9% | 2 | R.DVAIEEKQEVSR.V | 3 |
| \* | Astrin\_STLC\_112116\_02.04540.04540.2 | 3.0954 | 0.3508 | 100.0% | 1532.8322 | 1533.6849 | 1 | 7.332 | 62.5% | 1 | R.VLEQVSAQLEECK.G | 2 |
| \* | Astrin\_STLC\_112116\_02.07157.07157.3 | 6.6616 | 0.5775 | 100.0% | 2918.8743 | 2919.1382 | 1 | 10.053 | 41.7% | 5 | R.VLEQVSAQLEECKGQTEQLELENSR.L | 3 |
| \* | Astrin\_STLC\_112116\_tube2\_01.04741.04741.2 | 4.0096 | 0.4791 | 100.0% | 1403.8922 | 1404.4764 | 1 | 7.821 | 77.3% | 2 | K.GQTEQLELENSR.L | 2 |
| \* | Astrin\_STLC\_112116\_tube2\_01.09845.09845.2 | 5.6983 | 0.4025 | 100.0% | 1573.1322 | 1573.848 | 1 | 7.042 | 73.1% | 24 | R.AQLQILANMDSQLK.E | 2 |
| \* | Astrin\_STLC\_112116\_tube2\_01.05036.05036.2 | 5.5109 | 0.5724 | 100.0% | 1722.9922 | 1723.9879 | 1 | 10.3 | 82.1% | 6 | K.HMQAELQQQQAVLAK.E | 2 |
| \* | Astrin\_STLC\_112116\_tube2\_01.04939.04939.3 | 5.2754 | 0.3481 | 100.0% | 1723.2544 | 1723.9879 | 1 | 7.1 | 51.8% | 6 | K.HMQAELQQQQAVLAK.E | 3 |
| \* | Astrin\_STLC\_112116\_02.09011.09011.3 | 6.5016 | 0.5388 | 100.0% | 3285.8943 | 3286.5862 | 1 | 9.594 | 33.3% | 6 | R.DLKETLEFADQENQVAHLELGQVECQLK.T | 3 |
| \* | Astrin\_STLC\_112116\_01.06002.06002.2 | 2.5069 | 0.2018 | 99.3% | 833.1922 | 831.9878 | 7 | 4.532 | 83.3% | 1 | K.TTLEVLR.E | 2 |
| \* | Astrin\_STLC\_112116\_01.06995.06995.2 | 5.2401 | 0.5878 | 100.0% | 1963.1921 | 1964.1462 | 1 | 9.995 | 62.5% | 2 | R.SLQCENLKDTVENLTAK.L | 2 |
| \* | Astrin\_STLC\_112116\_tube2\_01.05455.05455.2 | 4.9086 | 0.4225 | 100.0% | 1675.0122 | 1675.7899 | 1 | 7.805 | 75.0% | 20 | K.LASTIADNQEQDLEK.T | 2 |
| \* | Astrin\_STLC\_112116\_01.05728.05728.2 | 4.533 | 0.4656 | 100.0% | 1932.2922 | 1933.0825 | 1 | 8.042 | 56.2% | 2 | K.LASTIADNQEQDLEKTR.Q | 2 |
| \* | Astrin\_STLC\_112116\_01.05726.05726.3 | 2.6032 | 0.3208 | 99.0% | 1932.8944 | 1933.0825 | 2 | 5.37 | 35.9% | 1 | K.LASTIADNQEQDLEKTR.Q | 3 |
| \* | Astrin\_STLC\_112116\_tube2\_01.13568.13568.3 | 4.2396 | 0.4861 | 100.0% | 2681.7244 | 2682.1326 | 1 | 8.242 | 35.2% | 3 | R.QYSQKLGLLTEQLQSLTLFLQTK.L | 3 |
| \* | Astrin\_STLC\_112116\_tube2\_01.14681.14681.2 | 6.002 | 0.5464 | 100.0% | 2046.9722 | 2047.443 | 1 | 9.742 | 67.6% | 76 | K.LGLLTEQLQSLTLFLQTK.L | 2 |
| \* | Astrin\_STLC\_112116\_tube2\_02.12564.12564.3 | 5.3375 | 0.4744 | 100.0% | 2047.4644 | 2047.443 | 1 | 8.099 | 51.5% | 40 | K.LGLLTEQLQSLTLFLQTK.L | 3 |
| \* | Astrin\_STLC\_112116\_01.12879.12879.3 | 5.6727 | 0.5213 | 100.0% | 2789.3044 | 2788.121 | 1 | 8.225 | 30.8% | 12 | R.TFLGSILTAVADEEPESTPVPLLGSDK.S | 3 |
| \* | Astrin\_STLC\_112116\_tube2\_01.14262.14262.2 | 4.6007 | 0.3364 | 100.0% | 2789.892 | 2788.121 | 1 | 5.594 | 44.2% | 25 | R.TFLGSILTAVADEEPESTPVPLLGSDK.S | 2 |
| \* | Astrin\_STLC\_112116\_tube2\_01.14638.14638.2 | 3.0239 | 0.5307 | 100.0% | 2867.612 | 2868.121 | 1 | 7.638 | 46.2% | 5 | R.TFLGSILTAVADEEPESTPVPLLGS\*DK.S | 2 |
| \* | Astrin\_STLC\_112116\_01.13532.13532.3 | 3.8892 | 0.3486 | 99.8% | 2867.7544 | 2868.121 | 1 | 4.99 | 28.8% | 3 | R.TFLGSILTAVADEEPESTPVPLLGS\*DK.S | 3 |
| \* | Astrin\_STLC\_112116\_tube2\_01.13679.13679.3 | 5.0369 | 0.481 | 100.0% | 3349.7043 | 3350.7473 | 1 | 7.976 | 26.6% | 1 | R.TFLGSILTAVADEEPESTPVPLLGSDKSAFTR.V | 3 |
| \* | Astrin\_STLC\_112116\_01.14057.14057.3 | 3.6764 | 0.3166 | 99.6% | 3429.9543 | 3430.7473 | 1 | 5.351 | 21.8% | 1 | R.TFLGSILTAVADEEPESTPVPLLGSDKSAFT#R.V | 3 |
| \* | Astrin\_STLC\_112116\_01.12842.12842.3 | 5.4293 | 0.3459 | 100.0% | 3430.2244 | 3430.7473 | 3 | 6.859 | 28.2% | 9 | R.TFLGSILTAVADEEPESTPVPLLGS\*DKSAFTR.V | 3 |
| \* | Astrin\_STLC\_112116\_01.12780.12780.3 | 4.8138 | 0.365 | 100.0% | 3430.8245 | 3430.7473 | 2 | 7.522 | 31.5% | 9 | R.TFLGSILTAVADEEPESTPVPLLGSDKS\*AFTR.V | 3 |
| \* | Astrin\_STLC\_112116\_tube2\_01.03398.03398.2 | 4.5594 | 0.3998 | 100.0% | 1595.8922 | 1595.7092 | 1 | 7.837 | 83.3% | 1 | R.LQAQEEQHQEVQK.A | 2 |
| \* | Astrin\_STLC\_112116\_01.05736.05736.2 | 5.7952 | 0.3833 | 100.0% | 2148.5322 | 2149.3652 | 1 | 8.039 | 68.8% | 1 | R.YKNEKELQEVIQQQNEK.I | 2 |
| \* | Astrin\_STLC\_112116\_tube2\_01.05473.05473.3 | 6.7369 | 0.2223 | 99.8% | 2149.6143 | 2149.3652 | 1 | 5.793 | 56.2% | 4 | R.YKNEKELQEVIQQQNEK.I | 3 |
| \* | Astrin\_STLC\_112116\_tube2\_01.04831.04831.2 | 4.653 | 0.2459 | 100.0% | 1486.0122 | 1486.622 | 1 | 6.272 | 86.4% | 5 | K.ELQEVIQQQNEK.I | 2 |
| \* | Astrin\_STLC\_112116\_01.05270.05270.2 | 2.6404 | 0.0679 | 95.9% | 858.77216 | 859.01044 | 6 | 3.821 | 91.7% | 1 | K.ILEQIDK.S | 2 |
| \* | Astrin\_STLC\_112116\_01.07060.07060.2 | 4.0759 | 0.3141 | 100.0% | 1714.2522 | 1715.0 | 1 | 6.356 | 71.4% | 2 | K.ILEQIDKSGELISLR.E | 2 |
| \* | Astrin\_STLC\_112116\_tube2\_01.08509.08509.3 | 4.0695 | 0.3623 | 100.0% | 1714.8243 | 1715.0 | 8 | 6.06 | 41.1% | 1 | K.ILEQIDKSGELISLR.E | 3 |
| \* | Astrin\_STLC\_112116\_tube2\_01.09957.09957.3 | 5.2684 | 0.3723 | 100.0% | 2680.3145 | 2681.0618 | 1 | 6.731 | 38.6% | 3 | K.ILEQIDKSGELISLREEVTHLTR.S | 3 |
| \* | Astrin\_STLC\_112116\_tube2\_01.08778.08778.2 | 3.6561 | 0.3503 | 100.0% | 1840.4722 | 1841.0745 | 1 | 6.065 | 56.7% | 3 | K.SGELISLREEVTHLTR.S | 2 |
| \* | Astrin\_STLC\_112116\_tube2\_01.08732.08732.3 | 5.1014 | 0.4033 | 100.0% | 1841.8744 | 1841.0745 | 1 | 7.418 | 46.7% | 29 | K.SGELISLREEVTHLTR.S | 3 |
| \* | Astrin\_STLC\_112116\_02.08160.08160.3 | 6.1671 | 0.4922 | 100.0% | 2846.5444 | 2846.1506 | 1 | 9.214 | 37.5% | 1 | K.VLQEALAGQLDSNCQPMATNWIQEK.V | 3 |
| \* | Astrin\_STLC\_112116\_tube2\_01.06861.06861.2 | 2.7529 | 0.3267 | 100.0% | 1104.8522 | 1104.248 | 2 | 5.775 | 81.2% | 2 | K.VWLSQEVDK.L | 2 |
| \* | Astrin\_STLC\_112116\_tube2\_01.08267.08267.2 | 3.3756 | 0.4039 | 100.0% | 1372.3121 | 1373.595 | 1 | 7.314 | 85.0% | 6 | K.VWLSQEVDKLR.V | 2 |
| \* | Astrin\_STLC\_112116\_tube2\_01.08842.08842.2 | 2.1922 | 0.374 | 100.0% | 897.8522 | 898.16644 | 6 | 7.319 | 75.0% | 4 | R.VMFLEMK.N | 2 |
| \* | Astrin\_STLC\_112116\_tube2\_01.07110.07110.2 | 2.7392 | 0.2682 | 99.8% | 1269.0922 | 1269.5598 | 1 | 5.452 | 72.2% | 1 | R.VMFLEMKNEK.E | 2 |
| \* | Astrin\_STLC\_112116\_01.07092.07092.2 | 4.0749 | 0.3908 | 100.0% | 2229.4321 | 2230.526 | 1 | 6.717 | 52.9% | 1 | R.RSDKELEKLDDIVQHIYK.T | 2 |
| \* | Astrin\_STLC\_112116\_tube2\_01.08871.08871.3 | 5.3417 | 0.3681 | 100.0% | 2230.3442 | 2230.526 | 1 | 7.014 | 41.2% | 6 | R.RSDKELEKLDDIVQHIYK.T | 3 |
| \* | Astrin\_STLC\_112116\_tube2\_01.09658.09658.3 | 5.0276 | 0.3758 | 100.0% | 2075.6042 | 2074.3384 | 1 | 7.289 | 42.2% | 9 | R.SDKELEKLDDIVQHIYK.T | 3 |
| \* | Astrin\_STLC\_112116\_tube2\_01.09120.09120.3 | 3.3404 | 0.3233 | 99.8% | 1743.8644 | 1743.9977 | 1 | 6.193 | 48.1% | 3 | K.ELEKLDDIVQHIYK.T | 3 |
| \* | Astrin\_STLC\_112116\_tube2\_01.07006.07006.2 | 3.492 | 0.2711 | 100.0% | 1244.1522 | 1244.4331 | 12 | 5.942 | 66.7% | 2 | K.LDDIVQHIYK.T | 2 |
| \* | Astrin\_STLC\_112116\_01.08552.08552.2 | 2.7929 | 0.2488 | 99.8% | 1127.0922 | 1127.3696 | 1 | 4.84 | 83.3% | 19 | K.TLLSIPEVVR.G | 2 |
| \* | Astrin\_STLC\_112116\_01.12230.12230.2 | 3.3937 | 0.3784 | 100.0% | 1494.1122 | 1494.6941 | 1 | 6.369 | 58.3% | 1 | R.GCKELQGLLEFLS.- | 2 |
| \* | Astrin\_STLC\_112116\_tube2\_01.14002.14002.1 | 1.9369 | 0.4085 | 100.0% | 1148.43 | 1149.3293 | 19 | 5.951 | 44.4% | 1 | K.ELQGLLEFLS.- | 1 |
| \* | Astrin\_STLC\_112116\_01.12854.12854.2 | 2.3063 | 0.3966 | 100.0% | 1148.9922 | 1149.3293 | 2 | 7.019 | 66.7% | 2 | K.ELQGLLEFLS.- | 2 |

---

|  |  |  |  |  |  |  |  |  |
| --- | --- | --- | --- | --- | --- | --- | --- | --- |
| U | *gi|83267868|ref|NP\_00* | 8 | 18 | 59.6% | 89 | 10366 | 7.4 | dynein light chain 1, cytoplasmic [Homo sapiens] &IC LC8-type 1 |

| Filename XCorr DeltCN Conf% ObsM+H+ CalcM+H+ SpR ZScore Ion% # Sequence  | | | | | | | | | | | | |
| --- | --- | --- | --- | --- | --- | --- | --- | --- | --- | --- | --- | --- |
| \* | Astrin\_STLC\_112116\_tube2\_01.07411.07411.2 | 3.3208 | 0.3042 | 100.0% | 1415.1122 | 1415.6322 | 2 | 6.564 | 68.2% | 1 | K.YNIEKDIAAHIK.K | 2 |
| \* | Astrin\_STLC\_112116\_tube2\_01.07367.07367.3 | 3.0692 | 0.3069 | 99.6% | 1415.4543 | 1415.6322 | 63 | 5.216 | 43.2% | 2 | K.YNIEKDIAAHIK.K | 3 |
| \* | Astrin\_STLC\_112116\_tube2\_01.06610.06610.2 | 2.9763 | 0.3137 | 100.0% | 1543.1721 | 1543.8064 | 1 | 6.453 | 58.3% | 1 | K.YNIEKDIAAHIKK.E | 2 |
| \* | Astrin\_STLC\_112116\_tube2\_01.06604.06604.3 | 3.2011 | 0.3332 | 99.8% | 1543.4944 | 1543.8064 | 1 | 5.275 | 50.0% | 1 | K.YNIEKDIAAHIKK.E | 3 |
|  | Astrin\_STLC\_112116\_01.06027.06027.2 | 2.561 | 0.3843 | 100.0% | 1402.8121 | 1403.5493 | 3 | 5.913 | 65.0% | 1 | K.YNPTWHCIVGR.N | 22 |
|  | Astrin\_STLC\_112116\_tube2\_01.04588.04588.2 | 3.1401 | 0.4414 | 100.0% | 1282.9122 | 1283.383 | 1 | 7.567 | 75.0% | 2 | R.NFGSYVTHETK.H | 22 |
|  | Astrin\_STLC\_112116\_tube2\_01.15164.15164.3 | 5.9399 | 0.494 | 100.0% | 3236.6943 | 3237.771 | 1 | 9.774 | 37.5% | 6 | R.NFGSYVTHETKHFIYFYLGQVAILLFK.S | 33 |
|  | Astrin\_STLC\_112116\_tube2\_02.13478.13478.3 | 4.4142 | 0.4062 | 100.0% | 3380.4543 | 3381.9011 | 2 | 6.475 | 25.0% | 4 | R.NFGSYVTHETKHFIYFYLGQVAILLFKSG.- | 33 |

Similarities:
gi|18087855|ref|NP\_54(4:4)  

---

|  |  |  |  |  |  |  |  |  |
| --- | --- | --- | --- | --- | --- | --- | --- | --- |
| U | *gi|18087855|ref|NP\_54* | 5 | 14 | 59.6% | 89 | 10350 | 7.4 | dynein light chain 2, cytoplasmic [Homo sapiens] &IC LC8-type 2 |

| Filename XCorr DeltCN Conf% ObsM+H+ CalcM+H+ SpR ZScore Ion% # Sequence  | | | | | | | | | | | | |
| --- | --- | --- | --- | --- | --- | --- | --- | --- | --- | --- | --- | --- |
| \* | Astrin\_STLC\_112116\_tube2\_01.08310.08310.3 | 3.7441 | 0.3469 | 99.8% | 1570.1344 | 1569.8412 | 1 | 6.229 | 47.9% | 1 | K.YNIEKDIAAYIKK.E | 3 |
|  | Astrin\_STLC\_112116\_01.06027.06027.2 | 2.561 | 0.3843 | 100.0% | 1402.8121 | 1403.5493 | 3 | 5.913 | 65.0% | 1 | K.YNPTWHCIVGR.N | 22 |
|  | Astrin\_STLC\_112116\_tube2\_01.04588.04588.2 | 3.1401 | 0.4414 | 100.0% | 1282.9122 | 1283.383 | 1 | 7.567 | 75.0% | 2 | R.NFGSYVTHETK.H | 22 |
|  | Astrin\_STLC\_112116\_tube2\_01.15164.15164.3 | 5.9399 | 0.494 | 100.0% | 3236.6943 | 3237.771 | 1 | 9.774 | 37.5% | 6 | R.NFGSYVTHETKHFIYFYLGQVAILLFK.S | 33 |
|  | Astrin\_STLC\_112116\_tube2\_02.13478.13478.3 | 4.4142 | 0.4062 | 100.0% | 3380.4543 | 3381.9011 | 2 | 6.475 | 25.0% | 4 | R.NFGSYVTHETKHFIYFYLGQVAILLFKSG.- | 33 |

Similarities:
gi|83267868|ref|NP\_00(4:1)  

---

|  |  |  |  |  |  |  |  |  |
| --- | --- | --- | --- | --- | --- | --- | --- | --- |
| U | *contaminant\_KERATIN09* | 18 | 56 | 55.5% | 429 | 47927 | 5.5 | no description |

| Filename XCorr DeltCN Conf% ObsM+H+ CalcM+H+ SpR ZScore Ion% # Sequence  | | | | | | | | | | | | |
| --- | --- | --- | --- | --- | --- | --- | --- | --- | --- | --- | --- | --- |
|  | Astrin\_STLC\_112116\_tube2\_02.07872.07872.3 | 5.3719 | 0.5618 | 100.0% | 2856.4443 | 2856.0813 | 1 | 10.201 | 35.8% | 8 | R.SLGSVQAPSYGARPVSSAASVYAGAGGSGSR.I | 3 |
|  | Astrin\_STLC\_112116\_02.08356.08356.3 | 5.8972 | 0.5333 | 100.0% | 3336.8643 | 3337.7224 | 1 | 8.991 | 28.7% | 4 | R.GGMGSGGLATGIAGGLAGMGGIQNEKETMQSLNDR.L | 3 |
|  | Astrin\_STLC\_112116\_tube2\_01.07919.07919.2 | 2.0934 | 0.2072 | 96.2% | 982.9522 | 983.0709 | 3 | 5.09 | 83.3% | 1 | R.DWSHYFK.I | 2 |
|  | Astrin\_STLC\_112116\_tube2\_01.06111.06111.2 | 3.8607 | 0.3853 | 100.0% | 1320.0521 | 1320.4478 | 1 | 8.22 | 77.3% | 3 | R.AQIFANTVDNAR.I | 2 |
|  | Astrin\_STLC\_112116\_tube2\_01.06544.06544.2 | 2.8305 | 0.168 | 99.0% | 1041.6322 | 1042.2235 | 1 | 5.465 | 93.8% | 2 | R.IVLQIDNAR.L | 22 |
|  | Astrin\_STLC\_112116\_01.05584.05584.2 | 1.8322 | 0.255 | 95.2% | 807.8722 | 807.8815 | 186 | 5.3 | 66.7% | 1 | R.LAADDFR.V | 2222222 |
|  | Astrin\_STLC\_112116\_01.05772.05772.2 | 2.9101 | 0.3808 | 100.0% | 1240.1921 | 1240.4601 | 2 | 6.741 | 72.2% | 2 | R.VKYETELAMR.Q | 2 |
|  | Astrin\_STLC\_112116\_tube2\_01.05345.05345.2 | 2.3032 | 0.3424 | 99.9% | 1013.4122 | 1013.1535 | 40 | 5.931 | 64.3% | 1 | K.YETELAMR.Q | 2 |
|  | Astrin\_STLC\_112116\_01.04258.04258.2 | 3.1021 | 0.2629 | 100.0% | 1175.1721 | 1175.3274 | 2 | 6.164 | 77.8% | 1 | R.KVIDDTNITR.L | 2 |
|  | Astrin\_STLC\_112116\_01.12654.12654.2 | 6.1114 | 0.5007 | 100.0% | 2178.0522 | 2178.589 | 1 | 9.624 | 73.5% | 1 | R.LQLETEIEALKEELLFMK.K | 2 |
|  | Astrin\_STLC\_112116\_01.12650.12650.3 | 2.6851 | 0.2615 | 96.9% | 2179.1643 | 2178.589 | 4 | 4.574 | 41.2% | 2 | R.LQLETEIEALKEELLFMK.K | 3 |
|  | Astrin\_STLC\_112116\_02.07435.07435.3 | 6.1484 | 0.5026 | 100.0% | 2750.5745 | 2751.0227 | 1 | 8.821 | 39.0% | 2 | K.NHEEEVKGLQAQIASSGLTVEVDAPK.S | 3 |
|  | Astrin\_STLC\_112116\_tube2\_01.08606.08606.2 | 5.6205 | 0.5922 | 100.0% | 1884.2522 | 1885.1246 | 1 | 10.559 | 66.7% | 3 | K.GLQAQIASSGLTVEVDAPK.S | 2 |
|  | Astrin\_STLC\_112116\_tube2\_01.09722.09722.2 | 3.9248 | 0.4104 | 100.0% | 1508.0922 | 1507.699 | 1 | 8.302 | 70.8% | 9 | R.TVQSLEIDLDSMR.N | 2 |
|  | Astrin\_STLC\_112116\_tube2\_01.14173.14173.2 | 3.5179 | 0.4641 | 100.0% | 2670.5122 | 2672.0715 | 1 | 9.133 | 40.9% | 1 | R.YALQMEQLNGILLHLESELAQTR.A | 2 |
|  | Astrin\_STLC\_112116\_01.12813.12813.3 | 6.2942 | 0.4782 | 100.0% | 2670.9543 | 2672.0715 | 1 | 7.91 | 45.5% | 12 | R.YALQMEQLNGILLHLESELAQTR.A | 3 |
|  | Astrin\_STLC\_112116\_tube2\_01.08950.08950.2 | 3.5206 | 0.3808 | 100.0% | 1420.3322 | 1420.6055 | 2 | 5.868 | 68.2% | 2 | R.QAQEYEALLNIK.V | 2 |
|  | Astrin\_STLC\_112116\_tube2\_02.09384.09384.3 | 3.916 | 0.3478 | 99.8% | 2742.8044 | 2741.9404 | 1 | 5.899 | 32.3% | 1 | R.LLEDGEDFNLGDALDSSNSMQTIQK.T | 3 |

Similarities:
gi|24430192|ref|NP\_00(1:17)  
gi|15431310|ref|NP\_00(1:17)  
contaminant\_KERATIN12(1:17)  
contaminant\_KERATIN03(1:17)  
contaminant\_KERATIN07(1:17)  
contaminant\_KERATIN10(2:16)  

---

|  |  |  |  |  |  |  |  |  |
| --- | --- | --- | --- | --- | --- | --- | --- | --- |
| U | *gi|226530908|ref|NP\_0* | 18 | 87 | 51.9% | 285 | 30315 | 7.5 | protein-L-isoaspartate(D-aspartate) O-methyltransferase isoform 1 [Homo sapiens] |
| U | *gi|354983493|ref|NP\_0* | 18 | 87 | 51.7% | 286 | 30358 | 6.7 | protein-L-isoaspartate(D-aspartate) O-methyltransferase isoform 2 [Homo sapiens] |

| Filename XCorr DeltCN Conf% ObsM+H+ CalcM+H+ SpR ZScore Ion% # Sequence  | | | | | | | | | | | | |
| --- | --- | --- | --- | --- | --- | --- | --- | --- | --- | --- | --- | --- |
|  | Astrin\_STLC\_112116\_01.04652.04652.2 | 4.2935 | 0.4039 | 100.0% | 1478.2122 | 1478.6078 | 1 | 7.962 | 65.4% | 1 | K.SGGASHSELIHNLR.K | 2 |
|  | Astrin\_STLC\_112116\_01.04778.04778.3 | 3.884 | 0.3538 | 99.8% | 1478.4543 | 1478.6078 | 1 | 6.526 | 51.9% | 9 | K.SGGASHSELIHNLR.K | 3 |
|  | Astrin\_STLC\_112116\_01.04199.04199.2 | 4.1221 | 0.2432 | 100.0% | 1606.3121 | 1606.7819 | 6 | 6.173 | 53.6% | 2 | K.SGGASHSELIHNLRK.N | 2 |
|  | Astrin\_STLC\_112116\_01.04220.04220.3 | 3.489 | 0.2395 | 99.4% | 1606.6743 | 1606.7819 | 14 | 5.808 | 37.5% | 5 | K.SGGASHSELIHNLRK.N | 3 |
|  | Astrin\_STLC\_112116\_tube2\_02.09061.09061.3 | 4.3646 | 0.4953 | 100.0% | 2052.2944 | 2051.409 | 1 | 7.828 | 36.8% | 6 | K.NGIIKTDKVFEVMLATDR.S | 3 |
|  | Astrin\_STLC\_112116\_01.07098.07098.3 | 3.6877 | 0.4122 | 100.0% | 1524.9543 | 1525.7601 | 12 | 7.108 | 41.7% | 2 | K.TDKVFEVMLATDR.S | 3 |
|  | Astrin\_STLC\_112116\_01.07088.07088.2 | 4.2139 | 0.4546 | 100.0% | 1526.0721 | 1525.7601 | 1 | 8.674 | 79.2% | 9 | K.TDKVFEVMLATDR.S | 2 |
|  | Astrin\_STLC\_112116\_tube2\_01.08582.08582.2 | 3.5303 | 0.3829 | 100.0% | 1180.7722 | 1181.3923 | 1 | 8.594 | 83.3% | 18 | K.VFEVMLATDR.S | 2 |
|  | Astrin\_STLC\_112116\_02.08087.08087.2 | 5.2524 | 0.4954 | 100.0% | 1696.2322 | 1695.8792 | 1 | 9.158 | 75.0% | 3 | K.ALDVGSGSGILTACFAR.M | 2 |
|  | Astrin\_STLC\_112116\_tube2\_01.04580.04580.2 | 2.6507 | 0.314 | 100.0% | 894.4322 | 895.0898 | 1 | 5.274 | 85.7% | 2 | K.VIGIDHIK.E | 2 |
|  | Astrin\_STLC\_112116\_01.04466.04466.2 | 2.6585 | 0.322 | 100.0% | 1188.9521 | 1189.3109 | 3 | 5.526 | 65.0% | 2 | R.KDDPTLLSSGR.V | 2 |
|  | Astrin\_STLC\_112116\_tube2\_01.04578.04578.2 | 3.1806 | 0.333 | 100.0% | 942.9922 | 943.091 | 1 | 6.407 | 81.2% | 4 | R.VQLVVGDGR.M | 2 |
|  | Astrin\_STLC\_112116\_tube2\_01.10776.10776.3 | 7.5261 | 0.5558 | 100.0% | 3506.6643 | 3507.0015 | 1 | 10.435 | 34.8% | 9 | R.MGYAEEAPYDAIHVGAAAPVVPQALIDQLKPGGR.L | 3 |
|  | Astrin\_STLC\_112116\_01.09160.09160.2 | 4.7142 | 0.5143 | 100.0% | 2043.4122 | 2044.3734 | 1 | 9.419 | 72.2% | 4 | R.LILPVGPAGGNQMLEQYDK.L | 2 |
|  | Astrin\_STLC\_112116\_tube2\_01.10150.10150.2 | 4.0354 | 0.4511 | 100.0% | 1705.1122 | 1706.1549 | 1 | 7.392 | 78.6% | 2 | K.MKPLMGVIYVPLTDK.E | 2 |
|  | Astrin\_STLC\_112116\_tube2\_01.10121.10121.3 | 3.3251 | 0.2463 | 99.4% | 1706.1843 | 1706.1549 | 1 | 5.151 | 48.2% | 1 | K.MKPLMGVIYVPLTDK.E | 3 |
|  | Astrin\_STLC\_112116\_tube2\_01.08980.08980.2 | 4.8495 | 0.5119 | 100.0% | 1962.1522 | 1963.4445 | 1 | 9.08 | 59.4% | 3 | K.MKPLMGVIYVPLTDKEK.Q | 2 |
|  | Astrin\_STLC\_112116\_tube2\_01.09021.09021.3 | 3.5096 | 0.4265 | 100.0% | 1964.2444 | 1963.4445 | 18 | 7.016 | 39.1% | 5 | K.MKPLMGVIYVPLTDKEK.Q | 3 |

---

|  |  |  |  |  |  |  |  |  |
| --- | --- | --- | --- | --- | --- | --- | --- | --- |
| U | *contaminant\_KERATIN21* | 14 | 21 | 48.2% | 357 | 39219 | 5.2 | no description |

| Filename XCorr DeltCN Conf% ObsM+H+ CalcM+H+ SpR ZScore Ion% # Sequence  | | | | | | | | | | | | |
| --- | --- | --- | --- | --- | --- | --- | --- | --- | --- | --- | --- | --- |
|  | Astrin\_STLC\_112116\_tube2\_01.10060.10060.3 | 4.2038 | 0.3861 | 100.0% | 2471.8442 | 2472.732 | 3 | 6.193 | 31.6% | 1 | R.GMQDLVEDFKNKYEDEINKR.T | 33 |
|  | Astrin\_STLC\_112116\_tube2\_01.05492.05492.2 | 3.3041 | 0.3582 | 100.0% | 1351.4321 | 1351.5425 | 1 | 6.676 | 77.3% | 1 | R.TAAENEFVTLKK.D | 22222 |
|  | Astrin\_STLC\_112116\_tube2\_01.06933.06933.2 | 3.9229 | 0.1151 | 99.1% | 1695.1921 | 1695.9281 | 1 | 7.073 | 75.0% | 1 | K.DVDAAYMNKVELQAK.A | 2222 |
|  | Astrin\_STLC\_112116\_tube2\_01.10418.10418.2 | 3.1329 | 0.2102 | 99.6% | 1410.2522 | 1408.551 | 1 | 4.322 | 72.7% | 2 | K.ADTLTDEINFLR.A | 2222 |
|  | Astrin\_STLC\_112116\_tube2\_02.09447.09447.3 | 3.5552 | 0.2195 | 97.7% | 3040.3743 | 3040.3467 | 184 | 4.203 | 22.1% | 1 | R.ALYDAELSQMQTHISDTSVVLSMDNNR.N | 333 |
|  | Astrin\_STLC\_112116\_tube2\_01.11450.11450.2 | 4.018 | 0.3758 | 100.0% | 1329.9722 | 1330.5211 | 1 | 7.458 | 86.4% | 3 | R.NLDLDSIIAEVK.A | 22222222 |
|  | Astrin\_STLC\_112116\_tube2\_01.04497.04497.2 | 3.6681 | 0.3308 | 100.0% | 1456.0721 | 1456.5547 | 1 | 6.189 | 68.2% | 1 | R.SRAEAESWYQTK.Y | 22222 |
|  | Astrin\_STLC\_112116\_tube2\_01.05288.05288.2 | 3.4785 | 0.2191 | 100.0% | 1165.9722 | 1166.2761 | 1 | 6.834 | 88.9% | 1 | K.YEELQVTAGR.H | 2222 |
|  | Astrin\_STLC\_112116\_tube2\_01.07167.07167.2 | 3.8773 | 0.3103 | 100.0% | 1359.1721 | 1358.5345 | 1 | 5.962 | 81.8% | 2 | K.NKLEGLEDALQK.A | 2222 |
|  | Astrin\_STLC\_112116\_tube2\_01.08126.08126.3 | 2.8284 | 0.1968 | 95.4% | 1508.1244 | 1508.8163 | 1 | 5.635 | 54.5% | 1 | R.LLKEYQELMNVK.L | 3333 |
|  | Astrin\_STLC\_112116\_tube2\_01.08129.08129.2 | 3.9836 | 0.4378 | 100.0% | 1508.5521 | 1508.8163 | 1 | 7.33 | 81.8% | 1 | R.LLKEYQELMNVK.L | 2222 |
|  | Astrin\_STLC\_112116\_tube2\_01.06513.06513.2 | 2.4157 | 0.1475 | 95.4% | 1153.7722 | 1154.3234 | 1 | 4.653 | 75.0% | 1 | K.EYQELMNVK.L | 22222 |
|  | Astrin\_STLC\_112116\_tube2\_01.09346.09346.2 | 3.4818 | 0.346 | 100.0% | 1264.4122 | 1264.4644 | 1 | 7.748 | 80.0% | 3 | K.LALDVEIATYR.K | 222222222 |
|  | Astrin\_STLC\_112116\_tube2\_01.06151.06151.2 | 4.9435 | 0.5404 | 100.0% | 1448.1921 | 1448.6163 | 1 | 9.166 | 75.0% | 2 | R.AIGGGLSSVGGGSSTIK.Y | 22 |

Similarities:
contaminant\_KERATIN20(1:13)  
gi|119703753|ref|NP\_0(11:3)  
contaminant\_KERATIN18(12:2)  
gi|5031839|ref|NP\_005(13:1)  
contaminant\_KERATIN22(2:12)  
gi|119395754|ref|NP\_0(2:12)  
gi|153791158|ref|NP\_0(4:10)  
gi|109148552|ref|NP\_4(2:12)  
gi|32567786|ref|NP\_78(2:12)  

---

|  |  |  |  |  |  |  |  |  |
| --- | --- | --- | --- | --- | --- | --- | --- | --- |
| U | *gi|119395750|ref|NP\_0* | 40 | 126 | 48.1% | 644 | 66039 | 8.1 | keratin, type II cytoskeletal 1 [Homo sapiens] |

| Filename XCorr DeltCN Conf% ObsM+H+ CalcM+H+ SpR ZScore Ion% # Sequence  | | | | | | | | | | | | |
| --- | --- | --- | --- | --- | --- | --- | --- | --- | --- | --- | --- | --- |
| \* | Astrin\_STLC\_112116\_tube2\_01.07013.07013.2 | 4.1616 | 0.4638 | 100.0% | 1657.8922 | 1658.7678 | 1 | 7.637 | 62.5% | 5 | R.SGGGFSSGSAGIINYQR.R | 2 |
|  | Astrin\_STLC\_112116\_tube2\_01.09442.09442.2 | 3.7161 | 0.4548 | 100.0% | 1384.0521 | 1384.5315 | 1 | 7.922 | 81.8% | 8 | K.SLNNQFASFIDK.V | 22 |
|  | Astrin\_STLC\_112116\_tube2\_01.09971.09971.2 | 3.8673 | 0.4538 | 100.0% | 1639.1921 | 1639.8516 | 1 | 7.901 | 69.2% | 4 | K.SLNNQFASFIDKVR.F | 22 |
|  | Astrin\_STLC\_112116\_tube2\_01.06129.06129.2 | 4.5739 | 0.073 | 100.0% | 1475.7922 | 1476.6726 | 2 | 7.553 | 86.4% | 9 | R.FLEQQNQVLQTK.W | 222 |
|  | Astrin\_STLC\_112116\_tube2\_01.10258.10258.3 | 5.2837 | 0.4703 | 100.0% | 2932.7344 | 2934.2786 | 1 | 7.453 | 35.9% | 2 | R.FLEQQNQVLQTKWELLQQVDTSTR.T | 33 |
|  | Astrin\_STLC\_112116\_tube2\_01.09129.09129.2 | 4.2322 | 0.4964 | 100.0% | 1475.7922 | 1476.6293 | 1 | 8.564 | 81.8% | 15 | K.WELLQQVDTSTR.T | 22 |
|  | Astrin\_STLC\_112116\_tube2\_01.11817.11817.2 | 4.3979 | 0.5187 | 100.0% | 1994.3322 | 1995.2017 | 1 | 9.062 | 76.7% | 3 | R.THNLEPYFESFINNLR.R | 22 |
|  | Astrin\_STLC\_112116\_tube2\_01.11847.11847.3 | 4.9294 | 0.3858 | 100.0% | 1995.5044 | 1995.2017 | 1 | 6.843 | 56.7% | 3 | R.THNLEPYFESFINNLR.R | 33 |
|  | Astrin\_STLC\_112116\_tube2\_01.11117.11117.2 | 2.8864 | 0.2933 | 99.8% | 2150.172 | 2151.3892 | 1 | 5.344 | 53.1% | 1 | R.THNLEPYFESFINNLRR.R | 22 |
|  | Astrin\_STLC\_112116\_tube2\_01.11064.11064.3 | 3.4024 | 0.2482 | 99.4% | 2151.3542 | 2151.3892 | 4 | 5.721 | 35.9% | 1 | R.THNLEPYFESFINNLRR.R | 33 |
|  | Astrin\_STLC\_112116\_tube2\_01.07335.07335.2 | 3.2605 | 0.404 | 100.0% | 1300.4521 | 1301.4316 | 1 | 7.365 | 77.8% | 1 | K.NMQDMVEDYR.N | 22 |
|  | Astrin\_STLC\_112116\_tube2\_01.06784.06784.2 | 3.4701 | 0.4576 | 100.0% | 1265.9321 | 1266.3934 | 1 | 7.541 | 85.0% | 5 | R.TNAENEFVTIK.K | 22 |
|  | Astrin\_STLC\_112116\_tube2\_01.05125.05125.3 | 3.0508 | 0.3458 | 99.8% | 1393.8243 | 1394.5675 | 1 | 5.669 | 43.2% | 2 | R.TNAENEFVTIKK.D | 33 |
|  | Astrin\_STLC\_112116\_tube2\_01.05197.05197.2 | 3.5145 | 0.2883 | 100.0% | 1395.0922 | 1394.5675 | 3 | 5.84 | 68.2% | 3 | R.TNAENEFVTIKK.D | 22 |
| \* | Astrin\_STLC\_112116\_01.10761.10761.2 | 4.7686 | 0.4575 | 100.0% | 1304.1122 | 1303.4955 | 1 | 8.429 | 86.4% | 7 | R.SLDLDSIIAEVK.A | 2 |
| \* | Astrin\_STLC\_112116\_tube2\_01.13642.13642.2 | 3.3244 | 0.4177 | 100.0% | 2349.2722 | 2350.6282 | 1 | 7.054 | 37.5% | 1 | R.SLDLDSIIAEVKAQYEDIAQK.S | 2 |
| \* | Astrin\_STLC\_112116\_tube2\_01.13629.13629.3 | 4.1078 | 0.4102 | 100.0% | 2349.5645 | 2350.6282 | 1 | 7.674 | 36.2% | 1 | R.SLDLDSIIAEVKAQYEDIAQK.S | 3 |
| \* | Astrin\_STLC\_112116\_01.04304.04304.2 | 2.7505 | 0.3239 | 100.0% | 1066.0521 | 1066.1558 | 1 | 6.058 | 81.2% | 1 | K.AQYEDIAQK.S | 2 |
|  | Astrin\_STLC\_112116\_01.04029.04029.2 | 3.21 | 0.4634 | 100.0% | 1340.4321 | 1341.4607 | 1 | 7.831 | 86.4% | 1 | K.SKAEAESLYQSK.Y | 22 |
|  | Astrin\_STLC\_112116\_tube2\_02.07969.07969.3 | 5.8989 | 0.4718 | 100.0% | 2501.0645 | 2502.7405 | 1 | 8.807 | 44.0% | 3 | K.SKAEAESLYQSKYEELQITAGR.H | 33 |
|  | Astrin\_STLC\_112116\_01.04421.04421.2 | 2.9471 | 0.4197 | 100.0% | 1126.1322 | 1126.2084 | 1 | 6.888 | 83.3% | 2 | K.AEAESLYQSK.Y | 22 |
|  | Astrin\_STLC\_112116\_tube2\_01.06387.06387.2 | 3.8574 | 0.3511 | 100.0% | 1181.6322 | 1180.303 | 1 | 6.597 | 83.3% | 9 | K.YEELQITAGR.H | 222 |
|  | Astrin\_STLC\_112116\_tube2\_01.05228.05228.2 | 3.4414 | 0.3673 | 100.0% | 1302.8522 | 1303.4581 | 1 | 6.323 | 80.0% | 2 | R.NSKIEISELNR.V | 22 |
|  | Astrin\_STLC\_112116\_tube2\_01.06183.06183.2 | 2.735 | 0.1721 | 99.1% | 973.8722 | 974.102 | 1 | 4.527 | 85.7% | 2 | K.IEISELNR.V | 222 |
|  | Astrin\_STLC\_112116\_tube2\_01.07315.07315.2 | 3.8389 | 0.5429 | 100.0% | 1716.2722 | 1717.8333 | 1 | 9.232 | 67.9% | 1 | K.QISNLQQSISDAEQR.G | 22 |
|  | Astrin\_STLC\_112116\_tube2\_01.09578.09578.3 | 3.7777 | 0.4415 | 100.0% | 2643.2644 | 2644.8582 | 1 | 7.523 | 32.6% | 1 | K.QISNLQQSISDAEQRGENALKDAK.N | 33 |
|  | Astrin\_STLC\_112116\_tube2\_01.08272.08272.2 | 4.8409 | 0.476 | 100.0% | 1600.2122 | 1600.769 | 1 | 8.096 | 80.8% | 2 | K.NKLNDLEDALQQAK.E | 22 |
|  | Astrin\_STLC\_112116\_tube2\_01.08279.08279.3 | 4.3061 | 0.312 | 99.8% | 1600.7943 | 1600.769 | 1 | 6.056 | 53.8% | 1 | K.NKLNDLEDALQQAK.E | 33 |
| \* | Astrin\_STLC\_112116\_tube2\_01.10626.10626.3 | 5.3224 | 0.4195 | 100.0% | 2185.6443 | 2185.399 | 1 | 7.807 | 47.2% | 8 | K.NKLNDLEDALQQAKEDLAR.L | 3 |
|  | Astrin\_STLC\_112116\_tube2\_01.08034.08034.2 | 3.9419 | 0.2882 | 100.0% | 1359.0122 | 1358.4912 | 1 | 6.729 | 86.4% | 1 | K.LNDLEDALQQAK.E | 22 |
| \* | Astrin\_STLC\_112116\_tube2\_01.10693.10693.3 | 4.2882 | 0.3261 | 99.8% | 1943.0944 | 1943.121 | 1 | 6.285 | 48.4% | 1 | K.LNDLEDALQQAKEDLAR.L | 3 |
|  | Astrin\_STLC\_112116\_tube2\_01.07372.07372.2 | 3.7594 | 0.3157 | 100.0% | 1523.9922 | 1524.7754 | 1 | 5.893 | 77.3% | 2 | R.LLRDYQELMNTK.L | 22 |
|  | Astrin\_STLC\_112116\_tube2\_01.07431.07431.3 | 3.7261 | 0.2294 | 99.6% | 1524.6843 | 1524.7754 | 10 | 5.487 | 45.5% | 3 | R.LLRDYQELMNTK.L | 33 |
|  | Astrin\_STLC\_112116\_tube2\_01.06219.06219.2 | 2.6926 | 0.4107 | 100.0% | 1141.7722 | 1142.2689 | 7 | 7.095 | 68.8% | 1 | R.DYQELMNTK.L | 22 |
|  | Astrin\_STLC\_112116\_01.04743.04743.1 | 1.9357 | 0.3919 | 100.0% | 1033.26 | 1034.1112 | 96 | 6.034 | 50.0% | 1 | R.TLLEGEESR.M | 11 |
|  | Astrin\_STLC\_112116\_01.04742.04742.2 | 2.4529 | 0.2423 | 99.0% | 1033.9321 | 1034.1112 | 3 | 4.968 | 68.8% | 1 | R.TLLEGEESR.M | 22 |
|  | Astrin\_STLC\_112116\_tube2\_01.03948.03948.2 | 5.2399 | 0.5939 | 100.0% | 2384.1921 | 2385.298 | 1 | 11.598 | 41.7% | 5 | R.GGGGGGYGSGGSSYGSGGGSYGSGGGGGGGR.G | 22 |
|  | Astrin\_STLC\_112116\_tube2\_01.03942.03942.3 | 7.741 | 0.5156 | 100.0% | 2385.1443 | 2385.298 | 1 | 11.851 | 35.8% | 4 | R.GGGGGGYGSGGSSYGSGGGSYGSGGGGGGGR.G | 33 |
| \* | Astrin\_STLC\_112116\_tube2\_01.04344.04344.3 | 7.4421 | 0.1908 | 99.8% | 3313.2244 | 3314.2085 | 1 | 10.207 | 26.3% | 2 | R.GSYGSGGSSYGSGGGSYGSGGGGGGHGSYGSGSSSGGYR.G | 3 |
| \* | Astrin\_STLC\_112116\_tube2\_01.08552.08552.3 | 3.9672 | 0.2501 | 99.4% | 2240.5745 | 2241.0396 | 1 | 5.162 | 34.3% | 1 | R.GGSGGGGGGS\*S\*GGRGSGGGSSGGSIGGR.G | 3 |

Similarities:
gi|119703753|ref|NP\_0(1:39)  
contaminant\_KERATIN13(31:9)  
contaminant\_KERATIN22(2:38)  

---

|  |  |  |  |  |  |  |  |  |
| --- | --- | --- | --- | --- | --- | --- | --- | --- |
| U | *gi|29788785|ref|NP\_82* | 15 | 83 | 48.0% | 444 | 49671 | 4.9 | tubulin beta chain isoform b [Homo sapiens] |
| U | *gi|645912968|ref|NP\_0* | 15 | 83 | 45.9% | 464 | 52018 | 5.0 | tubulin beta chain isoform a [Homo sapiens] |

| Filename XCorr DeltCN Conf% ObsM+H+ CalcM+H+ SpR ZScore Ion% # Sequence  | | | | | | | | | | | | |
| --- | --- | --- | --- | --- | --- | --- | --- | --- | --- | --- | --- | --- |
|  | Astrin\_STLC\_112116\_tube2\_01.09428.09428.3 | 5.5691 | 0.4387 | 100.0% | 3102.7444 | 3104.2725 | 1 | 9.445 | 33.7% | 1 | K.FWEVISDEHGIDPTGTYHGDSDLQLDR.I | 3 |
|  | Astrin\_STLC\_112116\_tube2\_01.05738.05738.2 | 3.7169 | 0.5554 | 100.0% | 1301.6122 | 1302.4265 | 1 | 9.521 | 81.8% | 3 | R.ISVYYNEATGGK.Y | 2 |
|  | Astrin\_STLC\_112116\_tube2\_01.09574.09574.2 | 4.4914 | 0.5146 | 100.0% | 1617.2122 | 1616.8701 | 1 | 7.705 | 71.4% | 20 | R.AILVDLEPGTMDSVR.S | 2 |
|  | Astrin\_STLC\_112116\_01.09959.09959.3 | 7.2157 | 0.4557 | 100.0% | 2799.9543 | 2800.0647 | 1 | 8.259 | 44.0% | 16 | R.SGPFGQIFRPDNFVFGQSGAGNNWAK.G | 33 |
|  | Astrin\_STLC\_112116\_01.09293.09293.3 | 3.3494 | 0.3601 | 99.8% | 2087.9644 | 2088.325 | 2 | 6.356 | 36.1% | 3 | K.GHYTEGAELVDSVLDVVRK.E | 33 |
|  | Astrin\_STLC\_112116\_01.07034.07034.2 | 4.1256 | 0.3836 | 100.0% | 1320.3722 | 1320.5896 | 1 | 7.509 | 72.7% | 4 | R.IMNTFSVVPSPK.V | 22 |
|  | Astrin\_STLC\_112116\_01.07324.07324.2 | 3.8024 | 0.4427 | 100.0% | 1145.4321 | 1144.4204 | 1 | 7.812 | 83.3% | 6 | K.LAVNMVPFPR.L | 22 |
|  | Astrin\_STLC\_112116\_tube2\_01.10873.10873.2 | 4.1615 | 0.3869 | 100.0% | 1622.2122 | 1621.9403 | 1 | 7.766 | 80.8% | 5 | R.LHFFMPGFAPLTSR.G | 22 |
|  | Astrin\_STLC\_112116\_01.09405.09405.2 | 2.7134 | 0.3012 | 99.5% | 1660.3922 | 1660.9078 | 2 | 5.413 | 50.0% | 3 | R.ALTVPELTQQVFDAK.N | 2 |
|  | Astrin\_STLC\_112116\_01.08746.08746.2 | 2.2652 | 0.271 | 98.9% | 1041.2322 | 1040.2505 | 18 | 5.083 | 68.8% | 2 | R.YLTVAAVFR.G | 22 |
|  | Astrin\_STLC\_112116\_tube2\_01.05990.05990.2 | 4.3025 | 0.3035 | 100.0% | 1446.7722 | 1447.6031 | 9 | 6.202 | 68.2% | 2 | K.EVDEQMLNVQNK.N | 22 |
|  | Astrin\_STLC\_112116\_01.09240.09240.2 | 4.4601 | 0.4452 | 100.0% | 1698.1122 | 1697.8877 | 1 | 8.384 | 73.1% | 2 | K.NSSYFVEWIPNNVK.T | 22 |
|  | Astrin\_STLC\_112116\_tube2\_02.10952.10952.2 | 4.5781 | 0.3578 | 100.0% | 1871.6721 | 1871.2018 | 1 | 6.933 | 68.8% | 4 | K.MAVTFIGNSTAIQELFK.R | 2 |
|  | Astrin\_STLC\_112116\_01.09968.09968.3 | 3.5852 | 0.3887 | 99.8% | 2027.2444 | 2027.3893 | 2 | 6.412 | 39.7% | 6 | K.MAVTFIGNSTAIQELFKR.I | 3 |
|  | Astrin\_STLC\_112116\_tube2\_01.09413.09413.2 | 3.5833 | 0.3694 | 100.0% | 1231.1122 | 1230.4241 | 1 | 5.862 | 83.3% | 6 | R.ISEQFTAMFR.R | 22 |

Similarities:
gi|5174735|ref|NP\_006(9:6)  

---

|  |  |  |  |  |  |  |  |  |
| --- | --- | --- | --- | --- | --- | --- | --- | --- |
| U | *contaminant\_KERATIN20* | 26 | 58 | 47.6% | 483 | 53748 | 5.4 | no description |

| Filename XCorr DeltCN Conf% ObsM+H+ CalcM+H+ SpR ZScore Ion% # Sequence  | | | | | | | | | | | | |
| --- | --- | --- | --- | --- | --- | --- | --- | --- | --- | --- | --- | --- |
|  | Astrin\_STLC\_112116\_01.06138.06138.2 | 2.671 | 0.1258 | 96.8% | 1082.1522 | 1083.2755 | 1 | 5.557 | 81.2% | 1 | K.FASFIDKVR.F | 22222222 |
|  | Astrin\_STLC\_112116\_tube2\_01.07708.07708.2 | 2.7258 | 0.165 | 99.0% | 1030.1721 | 1031.1997 | 4 | 3.905 | 92.9% | 2 | K.WSLLQQQK.T | 2 |
|  | Astrin\_STLC\_112116\_tube2\_01.11776.11776.2 | 4.0885 | 0.4255 | 100.0% | 1848.8522 | 1849.0431 | 1 | 6.465 | 60.7% | 2 | R.SNMDNMFESYINNLR.R | 2 |
|  | Astrin\_STLC\_112116\_02.08689.08689.3 | 5.4198 | 0.4543 | 100.0% | 2035.8844 | 2035.363 | 1 | 9.558 | 48.5% | 3 | K.LKLEAELGNMQGLVEDFK.N | 3 |
|  | Astrin\_STLC\_112116\_01.09486.09486.2 | 2.6345 | 0.2882 | 99.2% | 1792.4922 | 1794.0295 | 1 | 5.266 | 46.7% | 1 | K.LEAELGNMQGLVEDFK.N | 2 |
|  | Astrin\_STLC\_112116\_01.07419.07419.2 | 2.4552 | 0.3456 | 99.9% | 1353.4321 | 1353.5732 | 1 | 6.534 | 65.0% | 1 | R.TEMENEFVLIK.K | 2 |
|  | Astrin\_STLC\_112116\_tube2\_01.07776.07776.2 | 3.5607 | 0.2561 | 100.0% | 1481.3722 | 1481.7473 | 1 | 5.483 | 72.7% | 1 | R.TEMENEFVLIKK.D | 2 |
|  | Astrin\_STLC\_112116\_tube2\_01.07109.07109.2 | 3.7519 | 0.528 | 100.0% | 1798.0322 | 1798.9623 | 1 | 8.717 | 75.0% | 1 | K.DVDEAYMNKVELESR.L | 2 |
|  | Astrin\_STLC\_112116\_01.06051.06051.3 | 3.28 | 0.3126 | 99.7% | 1798.9443 | 1798.9623 | 3 | 5.283 | 41.1% | 1 | K.DVDEAYMNKVELESR.L | 3 |
|  | Astrin\_STLC\_112116\_01.09351.09351.2 | 3.9158 | 0.4679 | 100.0% | 1420.2922 | 1420.6055 | 1 | 8.445 | 90.9% | 18 | R.LEGLTDEINFLR.Q | 2 |
|  | Astrin\_STLC\_112116\_tube2\_02.08701.08701.2 | 5.551 | 0.5754 | 100.0% | 2109.3323 | 2110.3008 | 1 | 10.863 | 63.9% | 2 | R.ELQSQISDTSVVLSMDNSR.S | 2 |
|  | Astrin\_STLC\_112116\_tube2\_01.10489.10489.2 | 4.0915 | 0.4751 | 100.0% | 1320.9521 | 1321.5286 | 1 | 8.821 | 81.8% | 2 | R.SLDMDSIIAEVK.A | 2 |
|  | Astrin\_STLC\_112116\_01.04611.04611.2 | 2.7736 | 0.209 | 99.5% | 1081.0721 | 1080.1423 | 1 | 5.464 | 81.2% | 3 | K.AQYEDIANR.S | 22 |
|  | Astrin\_STLC\_112116\_01.05570.05570.2 | 3.2757 | 0.2772 | 100.0% | 1412.9122 | 1413.5884 | 1 | 6.153 | 77.3% | 2 | R.SRAEAESMYQIK.Y | 2 |
|  | Astrin\_STLC\_112116\_tube2\_02.08593.08593.3 | 4.8522 | 0.228 | 99.6% | 2532.7144 | 2532.828 | 2 | 5.278 | 34.5% | 2 | R.SRAEAESMYQIKYEELQSLAGK.H | 3 |
|  | Astrin\_STLC\_112116\_01.05843.05843.2 | 2.3749 | 0.2292 | 98.0% | 1169.7522 | 1170.3228 | 5 | 4.639 | 66.7% | 1 | R.AEAESMYQIK.Y | 2 |
|  | Astrin\_STLC\_112116\_02.07812.07812.3 | 5.3911 | 0.4484 | 100.0% | 2288.9343 | 2289.5623 | 1 | 8.889 | 35.5% | 1 | R.AEAESMYQIKYEELQSLAGK.H | 3 |
|  | Astrin\_STLC\_112116\_tube2\_01.06245.06245.2 | 3.3714 | 0.0618 | 98.5% | 1138.0521 | 1138.2627 | 2 | 6.924 | 77.8% | 2 | K.YEELQSLAGK.H | 2 |
|  | Astrin\_STLC\_112116\_01.05704.05704.2 | 3.4343 | 0.268 | 100.0% | 1342.2522 | 1342.5381 | 1 | 7.174 | 77.3% | 2 | R.LQAEIEGLKGQR.A | 2 |
|  | Astrin\_STLC\_112116\_tube2\_01.08334.08334.2 | 2.7918 | 0.3727 | 100.0% | 1344.1721 | 1345.452 | 1 | 6.349 | 66.7% | 1 | R.ASLEAAIADAEQR.G | 2 |
|  | Astrin\_STLC\_112116\_01.09225.09225.3 | 4.6069 | 0.4533 | 100.0% | 2456.5745 | 2456.7153 | 1 | 7.952 | 32.6% | 1 | R.ASLEAAIADAEQRGELAIKDANAK.L | 3 |
|  | Astrin\_STLC\_112116\_tube2\_01.07731.07731.2 | 4.0915 | 0.2687 | 100.0% | 1130.0721 | 1130.2865 | 1 | 6.245 | 77.8% | 2 | K.LSELEAALQR.A | 2 |
|  | Astrin\_STLC\_112116\_tube2\_01.06513.06513.2 | 2.4157 | 0.1475 | 95.4% | 1153.7722 | 1154.3234 | 1 | 4.653 | 75.0% | 1 | R.EYQELMNVK.L | 22222 |
|  | Astrin\_STLC\_112116\_tube2\_01.04749.04749.2 | 3.0405 | 0.3387 | 100.0% | 1475.6921 | 1476.7058 | 1 | 6.924 | 70.8% | 1 | R.LESGMQNMSIHTK.T | 2 |
|  | Astrin\_STLC\_112116\_tube2\_01.04728.04728.3 | 2.7619 | 0.3466 | 99.6% | 1475.9343 | 1476.7058 | 1 | 6.974 | 43.8% | 1 | R.LESGMQNMSIHTK.T | 3 |
|  | Astrin\_STLC\_112116\_tube2\_01.05696.05696.2 | 3.0834 | 0.4089 | 100.0% | 1174.0122 | 1174.3367 | 1 | 7.266 | 75.0% | 3 | K.LVSESSDVLPK.- | 2 |

Similarities:
contaminant\_KERATIN21(1:25)  
gi|119703753|ref|NP\_0(2:24)  
contaminant\_KERATIN18(2:24)  
gi|5031839|ref|NP\_005(2:24)  
contaminant\_KERATIN22(1:25)  
gi|119395754|ref|NP\_0(1:25)  
contaminant\_KERATIN19(1:25)  
gi|153791158|ref|NP\_0(1:25)  
gi|109148552|ref|NP\_4(1:25)  

---

|  |  |  |  |  |  |  |  |  |
| --- | --- | --- | --- | --- | --- | --- | --- | --- |
| U | *gi|24430192|ref|NP\_00* | 21 | 47 | 46.1% | 473 | 51268 | 5.0 | keratin, type I cytoskeletal 16 [Homo sapiens] |

| Filename XCorr DeltCN Conf% ObsM+H+ CalcM+H+ SpR ZScore Ion% # Sequence  | | | | | | | | | | | | |
| --- | --- | --- | --- | --- | --- | --- | --- | --- | --- | --- | --- | --- |
| \* | Astrin\_STLC\_112116\_tube2\_01.05072.05072.2 | 3.6631 | 0.4631 | 100.0% | 1338.8722 | 1339.4478 | 1 | 9.803 | 76.9% | 4 | R.APSTYGGGLSVSSR.F | 2 |
|  | Astrin\_STLC\_112116\_tube2\_01.04204.04204.2 | 2.311 | 0.2065 | 96.9% | 1091.1322 | 1091.2273 | 68 | 4.975 | 62.5% | 1 | K.VTMQNLNDR.L | 222 |
|  | Astrin\_STLC\_112116\_tube2\_01.05299.05299.2 | 3.2103 | 0.1537 | 99.8% | 1064.5721 | 1065.2578 | 1 | 6.052 | 81.2% | 2 | R.LASYLDKVR.A | 222222 |
|  | Astrin\_STLC\_112116\_tube2\_02.07110.07110.2 | 4.4264 | 0.3632 | 100.0% | 1303.0322 | 1302.4241 | 1 | 7.671 | 81.8% | 6 | R.ALEEANADLEVK.I | 222 |
|  | Astrin\_STLC\_112116\_tube2\_01.06328.06328.3 | 3.7332 | 0.2635 | 99.6% | 1758.2943 | 1758.9713 | 27 | 5.33 | 38.5% | 1 | R.QRPSEIKDYSPYFK.T | 3 |
|  | Astrin\_STLC\_112116\_tube2\_01.09660.09660.2 | 5.1278 | 0.5192 | 100.0% | 2064.372 | 2065.3774 | 1 | 10.376 | 61.1% | 2 | K.IIAATIENAQPILQIDNAR.L | 22 |
|  | Astrin\_STLC\_112116\_tube2\_01.09652.09652.3 | 5.1324 | 0.4697 | 100.0% | 2064.5044 | 2065.3774 | 1 | 7.651 | 50.0% | 3 | K.IIAATIENAQPILQIDNAR.L | 33 |
|  | Astrin\_STLC\_112116\_01.05584.05584.2 | 1.8322 | 0.255 | 95.2% | 807.8722 | 807.8815 | 186 | 5.3 | 66.7% | 1 | R.LAADDFR.T | 2222222 |
|  | Astrin\_STLC\_112116\_tube2\_01.05831.05831.2 | 3.2025 | 0.2785 | 100.0% | 1202.1522 | 1202.3097 | 1 | 5.458 | 80.0% | 2 | R.QTVEADVNGLR.R | 2 |
|  | Astrin\_STLC\_112116\_tube2\_01.07209.07209.2 | 3.3704 | 0.3259 | 100.0% | 1186.0521 | 1186.397 | 1 | 6.037 | 83.3% | 1 | R.RVLDELTLAR.T | 2222 |
|  | Astrin\_STLC\_112116\_tube2\_01.07839.07839.2 | 3.2486 | 0.3989 | 100.0% | 1030.0122 | 1030.2096 | 1 | 7.534 | 87.5% | 4 | R.VLDELTLAR.T | 22222 |
|  | Astrin\_STLC\_112116\_tube2\_01.11418.11418.3 | 4.0859 | 0.468 | 100.0% | 2279.8442 | 2280.6416 | 3 | 7.585 | 34.7% | 1 | R.TDLEMQIEGLKEELAYLRK.N | 3 |
|  | Astrin\_STLC\_112116\_tube2\_01.07702.07702.2 | 5.5604 | 0.4896 | 100.0% | 2088.152 | 2089.2415 | 1 | 9.547 | 52.5% | 2 | R.GQTGGDVNVEMDAAPGVDLSR.I | 2 |
|  | Astrin\_STLC\_112116\_tube2\_01.09539.09539.2 | 3.3813 | 0.3778 | 100.0% | 1096.7922 | 1097.2126 | 1 | 6.496 | 81.2% | 1 | R.DAETWFLSK.T | 2 |
|  | Astrin\_STLC\_112116\_tube2\_01.05919.05919.2 | 5.3468 | 0.5761 | 100.0% | 2120.2922 | 2121.2659 | 1 | 10.22 | 63.9% | 1 | K.TEELNKEVASNSELVQSSR.S | 2 |
|  | Astrin\_STLC\_112116\_tube2\_01.05942.05942.3 | 4.4421 | 0.4545 | 100.0% | 2120.5444 | 2121.2659 | 1 | 8.105 | 45.8% | 2 | K.TEELNKEVASNSELVQSSR.S | 3 |
|  | Astrin\_STLC\_112116\_tube2\_01.05697.05697.2 | 3.1611 | 0.2971 | 100.0% | 1221.6721 | 1221.3068 | 1 | 6.8 | 80.0% | 3 | K.ASLENSLEETK.G | 222 |
|  | Astrin\_STLC\_112116\_tube2\_01.06467.06467.2 | 3.5426 | 0.3417 | 100.0% | 1381.0922 | 1380.5437 | 2 | 6.215 | 75.0% | 3 | K.TRLEQEIATYR.R | 2222 |
|  | Astrin\_STLC\_112116\_tube2\_01.05638.05638.3 | 3.1897 | 0.3263 | 99.8% | 1535.1543 | 1536.7311 | 1 | 5.56 | 45.5% | 3 | K.TRLEQEIATYRR.L | 3333 |
|  | Astrin\_STLC\_112116\_tube2\_01.04954.04954.2 | 2.7318 | 0.326 | 100.0% | 1122.9922 | 1123.2511 | 1 | 5.932 | 81.2% | 1 | R.LEQEIATYR.R | 22222 |
|  | Astrin\_STLC\_112116\_tube2\_01.05096.05096.3 | 5.5329 | 0.5566 | 100.0% | 2350.2244 | 2351.4485 | 1 | 9.653 | 41.7% | 3 | R.LLEGEDAHLSSQQASGQSYSSR.E | 3 |

Similarities:
contaminant\_KERATIN09(1:20)  
gi|15431310|ref|NP\_00(10:11)  
contaminant\_KERATIN12(7:14)  
contaminant\_KERATIN03(3:18)  
contaminant\_KERATIN07(10:11)  
contaminant\_KERATIN10(5:16)  

---

|  |  |  |  |  |  |  |  |  |
| --- | --- | --- | --- | --- | --- | --- | --- | --- |
| U | *contaminant\_KERATIN02* | 23 | 71 | 45.5% | 622 | 61987 | 5.2 | no description |
| U | *gi|55956899|ref|NP\_00* | 23 | 71 | 45.4% | 623 | 62064 | 5.2 | keratin, type I cytoskeletal 9 [Homo sapiens] |

| Filename XCorr DeltCN Conf% ObsM+H+ CalcM+H+ SpR ZScore Ion% # Sequence  | | | | | | | | | | | | |
| --- | --- | --- | --- | --- | --- | --- | --- | --- | --- | --- | --- | --- |
|  | Astrin\_STLC\_112116\_tube2\_01.03625.03625.2 | 2.5263 | 0.3137 | 99.5% | 1235.9922 | 1236.2401 | 1 | 6.821 | 62.5% | 1 | R.FSSSSGYGGGSSR.V | 2 |
|  | Astrin\_STLC\_112116\_tube2\_01.09272.09272.2 | 4.8124 | 0.5551 | 100.0% | 2706.2922 | 2706.7605 | 1 | 11.019 | 40.3% | 3 | R.GGGGSFGYSYGGGSGGGFSASSLGGGFGGGSR.G | 2 |
|  | Astrin\_STLC\_112116\_tube2\_02.08874.08874.3 | 5.3445 | 0.4557 | 100.0% | 2707.4043 | 2706.7605 | 1 | 8.041 | 27.4% | 6 | R.GGGGSFGYSYGGGSGGGFSASSLGGGFGGGSR.G | 3 |
|  | Astrin\_STLC\_112116\_tube2\_01.09171.09171.3 | 4.0682 | 0.2471 | 99.4% | 2378.3643 | 2378.5981 | 1 | 5.682 | 37.5% | 3 | R.LASYLDKVQALEEANNDLENK.I | 3 |
|  | Astrin\_STLC\_112116\_tube2\_01.11542.11542.3 | 4.4456 | 0.3547 | 99.8% | 3326.5745 | 3327.6287 | 1 | 6.055 | 25.9% | 1 | R.LASYLDKVQALEEANNDLENKIQDWYDK.K | 3 |
|  | Astrin\_STLC\_112116\_tube2\_01.05913.05913.2 | 5.1221 | 0.469 | 100.0% | 1587.8922 | 1587.6836 | 1 | 9.485 | 80.8% | 1 | K.VQALEEANNDLENK.I | 2 |
|  | Astrin\_STLC\_112116\_tube2\_01.09959.09959.3 | 3.8878 | 0.4345 | 100.0% | 2536.1343 | 2536.714 | 19 | 6.29 | 28.8% | 1 | K.VQALEEANNDLENKIQDWYDK.K | 3 |
|  | Astrin\_STLC\_112116\_tube2\_01.09625.09625.3 | 4.4931 | 0.3473 | 99.8% | 2664.2043 | 2664.8882 | 3 | 5.817 | 32.1% | 1 | K.VQALEEANNDLENKIQDWYDKK.G | 3 |
|  | Astrin\_STLC\_112116\_tube2\_01.05343.05343.3 | 4.0855 | 0.2664 | 99.7% | 1761.4143 | 1762.0183 | 1 | 5.258 | 50.0% | 1 | K.IQDWYDKKGPAAIQK.N | 3 |
|  | Astrin\_STLC\_112116\_tube2\_01.12300.12300.3 | 5.8503 | 0.5741 | 100.0% | 2902.7644 | 2904.1597 | 1 | 9.449 | 40.6% | 6 | K.NYSPYYNTIDDLKDQIVDLTVGNNK.T | 3 |
|  | Astrin\_STLC\_112116\_tube2\_01.07137.07137.2 | 2.7392 | 0.375 | 100.0% | 1060.8121 | 1061.1802 | 1 | 6.402 | 93.8% | 4 | K.TLLDIDNTR.M | 2 |
|  | Astrin\_STLC\_112116\_tube2\_01.06791.06791.2 | 2.4575 | 0.2933 | 99.5% | 1307.1122 | 1308.5383 | 6 | 5.514 | 66.7% | 1 | R.IKFEMEQNLR.Q | 2 |
|  | Astrin\_STLC\_112116\_tube2\_01.06106.06106.2 | 3.1686 | 0.3802 | 100.0% | 1157.9521 | 1158.2566 | 1 | 6.397 | 80.0% | 5 | R.QGVDADINGLR.Q | 2 |
|  | Astrin\_STLC\_112116\_tube2\_01.09330.09330.2 | 3.1129 | 0.2386 | 99.5% | 1851.9722 | 1853.1003 | 1 | 4.803 | 46.4% | 2 | K.TLNDMRQEYEQLIAK.N | 2 |
|  | Astrin\_STLC\_112116\_tube2\_01.09352.09352.3 | 3.5789 | 0.3729 | 99.8% | 1852.3143 | 1853.1003 | 1 | 6.08 | 42.9% | 2 | K.TLNDMRQEYEQLIAK.N | 3 |
|  | Astrin\_STLC\_112116\_tube2\_01.10795.10795.3 | 6.3437 | 0.551 | 100.0% | 3264.5344 | 3266.413 | 1 | 10.71 | 32.1% | 4 | K.DIENQYETQITQIEHEVSSSGQEVQSSAK.E | 3 |
|  | Astrin\_STLC\_112116\_tube2\_01.09610.09610.2 | 6.6467 | 0.5759 | 100.0% | 1838.3322 | 1839.0557 | 1 | 10.215 | 80.0% | 4 | R.HGVQELEIELQSQLSK.K | 2 |
|  | Astrin\_STLC\_112116\_tube2\_01.09583.09583.3 | 3.4754 | 0.2523 | 99.5% | 1838.5443 | 1839.0557 | 1 | 5.545 | 46.7% | 2 | R.HGVQELEIELQSQLSK.K | 3 |
|  | Astrin\_STLC\_112116\_01.07107.07107.3 | 6.2005 | 0.3296 | 100.0% | 1967.2743 | 1967.2297 | 1 | 6.943 | 53.1% | 9 | R.HGVQELEIELQSQLSKK.A | 3 |
|  | Astrin\_STLC\_112116\_tube2\_01.08207.08207.2 | 4.6762 | 0.3776 | 100.0% | 2512.5522 | 2511.6177 | 1 | 7.213 | 52.3% | 2 | K.EIETYHNLLEGGQEDFESSGAGK.I | 2 |
|  | Astrin\_STLC\_112116\_01.07013.07013.3 | 5.7589 | 0.2657 | 99.8% | 2512.8843 | 2511.6177 | 1 | 6.239 | 37.5% | 6 | K.EIETYHNLLEGGQEDFESSGAGK.I | 3 |
|  | Astrin\_STLC\_112116\_tube2\_01.03510.03510.2 | 5.3653 | 0.4955 | 100.0% | 1791.8922 | 1792.7324 | 1 | 12.425 | 47.7% | 2 | R.GGSGGSYGGGGSGGGYGGGSGSR.G | 2 |
|  | Astrin\_STLC\_112116\_tube2\_01.04338.04338.3 | 9.0148 | 0.6165 | 100.0% | 3224.1243 | 3225.1118 | 1 | 12.333 | 32.7% | 4 | R.GGSGGSHGGGSGFGGESGGSYGGGEEASGSGGGYGGGSGK.S | 3 |

---

|  |  |  |  |  |  |  |  |  |
| --- | --- | --- | --- | --- | --- | --- | --- | --- |
| U | *gi|10800130|ref|NP\_06* | 4 | 11 | 43.8% | 130 | 14107 | 10.9 | histone H2A type 1-D [Homo sapiens] |
| U | *gi|4504249|ref|NP\_003* | 4 | 11 | 43.8% | 130 | 14091 | 10.9 | histone H2A type 1 [Homo sapiens] |
| U | *gi|29553970|ref|NP\_80* | 4 | 11 | 44.2% | 129 | 14019 | 10.9 | histone H2A.J [Homo sapiens] |
| U | *gi|18105045|ref|NP\_54* | 4 | 11 | 44.5% | 128 | 13906 | 10.9 | histone H2A type 1-H [Homo sapiens] |
| U | *gi|10800144|ref|NP\_06* | 4 | 11 | 44.5% | 128 | 13936 | 10.9 | histone cluster 1, H2aj [Homo sapiens] |

| Filename XCorr DeltCN Conf% ObsM+H+ CalcM+H+ SpR ZScore Ion% # Sequence  | | | | | | | | | | | | |
| --- | --- | --- | --- | --- | --- | --- | --- | --- | --- | --- | --- | --- |
|  | Astrin\_STLC\_112116\_tube2\_01.07633.07633.2 | 2.54 | 0.345 | 100.0% | 945.33215 | 945.1093 | 131 | 6.211 | 62.5% | 2 | R.AGLQFPVGR.V | 222 |
|  | Astrin\_STLC\_112116\_01.15848.15848.3 | 6.0609 | 0.5426 | 100.0% | 2917.6143 | 2917.3752 | 1 | 9.275 | 33.9% | 3 | R.VGAGAPVYLAAVLEYLTAEILELAGNAAR.D | 3 |
|  | Astrin\_STLC\_112116\_01.15850.15850.2 | 4.316 | 0.4432 | 100.0% | 2918.0923 | 2917.3752 | 1 | 6.692 | 37.5% | 2 | R.VGAGAPVYLAAVLEYLTAEILELAGNAAR.D | 2 |
|  | Astrin\_STLC\_112116\_tube2\_01.11897.11897.2 | 4.762 | 0.4629 | 100.0% | 1932.4321 | 1932.3573 | 1 | 8.027 | 58.3% | 4 | K.VTIAQGGVLPNIQAVLLPK.K | 22 |

Similarities:
gi|106775678|ref|NP\_0(2:2)  
gi|20357599|ref|NP\_61(1:3)  

---

|  |  |  |  |  |  |  |  |  |
| --- | --- | --- | --- | --- | --- | --- | --- | --- |
| U | *gi|106775678|ref|NP\_0* | 4 | 11 | 43.8% | 130 | 14095 | 10.9 | histone H2A type 2-A [Homo sapiens] |
| U | *gi|24638446|ref|NP\_00* | 4 | 11 | 44.2% | 129 | 13988 | 10.9 | histone H2A type 2-C [Homo sapiens] |

| Filename XCorr DeltCN Conf% ObsM+H+ CalcM+H+ SpR ZScore Ion% # Sequence  | | | | | | | | | | | | |
| --- | --- | --- | --- | --- | --- | --- | --- | --- | --- | --- | --- | --- |
|  | Astrin\_STLC\_112116\_tube2\_01.07633.07633.2 | 2.54 | 0.345 | 100.0% | 945.33215 | 945.1093 | 131 | 6.211 | 62.5% | 2 | R.AGLQFPVGR.V | 222 |
|  | Astrin\_STLC\_112116\_tube2\_01.16255.16255.2 | 3.4191 | 0.5492 | 100.0% | 2933.7922 | 2935.4082 | 1 | 8.478 | 41.1% | 2 | R.VGAGAPVYMAAVLEYLTAEILELAGNAAR.D | 2 |
|  | Astrin\_STLC\_112116\_01.15752.15752.3 | 4.1268 | 0.3966 | 99.8% | 2934.0544 | 2935.4082 | 3 | 7.152 | 26.8% | 3 | R.VGAGAPVYMAAVLEYLTAEILELAGNAAR.D | 3 |
|  | Astrin\_STLC\_112116\_tube2\_01.11897.11897.2 | 4.762 | 0.4629 | 100.0% | 1932.4321 | 1932.3573 | 1 | 8.027 | 58.3% | 4 | K.VTIAQGGVLPNIQAVLLPK.K | 22 |

Similarities:
gi|10800130|ref|NP\_06(2:2)  
gi|20357599|ref|NP\_61(1:3)  

---

|  |  |  |  |  |  |  |  |  |
| --- | --- | --- | --- | --- | --- | --- | --- | --- |
| U | *gi|16507237|ref|NP\_00* | 21 | 45 | 43.1% | 654 | 72333 | 5.2 | 78 kDa glucose-regulated protein precursor [Homo sapiens] |

| Filename XCorr DeltCN Conf% ObsM+H+ CalcM+H+ SpR ZScore Ion% # Sequence  | | | | | | | | | | | | |
| --- | --- | --- | --- | --- | --- | --- | --- | --- | --- | --- | --- | --- |
|  | Astrin\_STLC\_112116\_tube2\_01.07912.07912.2 | 3.6535 | 0.3858 | 100.0% | 1568.3922 | 1567.7386 | 2 | 7.364 | 46.2% | 3 | R.ITPSYVAFTPEGER.L | 2 |
|  | Astrin\_STLC\_112116\_01.06107.06107.2 | 4.3367 | 0.4826 | 100.0% | 1679.0122 | 1678.796 | 1 | 7.592 | 71.4% | 3 | K.NQLTSNPENTVFDAK.R | 2 |
|  | Astrin\_STLC\_112116\_tube2\_01.06249.06249.2 | 3.8516 | 0.2833 | 100.0% | 1431.9922 | 1431.5449 | 1 | 5.905 | 81.8% | 3 | R.TWNDPSVQQDIK.F | 2 |
|  | Astrin\_STLC\_112116\_01.05916.05916.2 | 4.5147 | 0.4897 | 100.0% | 1606.0721 | 1605.8314 | 1 | 8.22 | 82.1% | 1 | K.TKPYIQVDIGGGQTK.T | 2 |
|  | Astrin\_STLC\_112116\_01.09342.09342.2 | 3.7621 | 0.4269 | 100.0% | 1537.2122 | 1537.8114 | 1 | 8.135 | 69.2% | 2 | K.TFAPEEISAMVLTK.M | 2 |
|  | Astrin\_STLC\_112116\_01.07056.07056.2 | 3.0595 | 0.3798 | 100.0% | 1218.0122 | 1218.4137 | 1 | 6.598 | 81.8% | 4 | K.DAGTIAGLNVMR.I | 2 |
|  | Astrin\_STLC\_112116\_tube2\_01.09259.09259.2 | 3.9717 | 0.4271 | 100.0% | 1661.6322 | 1660.9078 | 1 | 7.009 | 73.3% | 2 | R.IINEPTAAAIAYGLDK.R | 222 |
|  | Astrin\_STLC\_112116\_02.08504.08504.2 | 5.2098 | 0.6411 | 100.0% | 2165.4722 | 2166.3025 | 1 | 11.466 | 79.4% | 3 | R.IEIESFYEGEDFSETLTR.A | 2 |
|  | Astrin\_STLC\_112116\_tube2\_01.09773.09773.2 | 3.4486 | 0.5357 | 100.0% | 1512.5922 | 1513.7516 | 1 | 8.425 | 81.8% | 4 | R.AKFEELNMDLFR.S | 2 |
|  | Astrin\_STLC\_112116\_01.08717.08717.3 | 3.5215 | 0.4023 | 100.0% | 1513.8844 | 1513.7516 | 1 | 6.167 | 50.0% | 1 | R.AKFEELNMDLFR.S | 3 |
|  | Astrin\_STLC\_112116\_01.06936.06936.2 | 3.8512 | 0.1994 | 100.0% | 1589.4922 | 1589.7863 | 1 | 8.046 | 57.1% | 1 | K.KSDIDEIVLVGGSTR.I | 2 |
|  | Astrin\_STLC\_112116\_tube2\_01.04350.04350.2 | 2.8494 | 0.1826 | 99.0% | 1211.0322 | 1211.3195 | 2 | 4.669 | 72.2% | 1 | K.EFFNGKEPSR.G | 2 |
|  | Astrin\_STLC\_112116\_tube2\_01.07355.07355.2 | 5.1196 | 0.5702 | 100.0% | 1837.5521 | 1838.0245 | 1 | 9.68 | 78.1% | 4 | K.SQIFSTASDNQPTVTIK.V | 2 |
|  | Astrin\_STLC\_112116\_01.03946.03946.2 | 2.6467 | 0.3868 | 100.0% | 1192.8722 | 1192.3574 | 3 | 6.816 | 61.1% | 1 | K.VYEGERPLTK.D | 2 |
|  | Astrin\_STLC\_112116\_tube2\_01.10217.10217.2 | 3.0182 | 0.3481 | 100.0% | 1935.5922 | 1935.19 | 1 | 5.506 | 50.0% | 1 | K.DNHLLGTFDLTGIPPAPR.G | 2 |
|  | Astrin\_STLC\_112116\_tube2\_02.13359.13359.2 | 2.2111 | 0.2497 | 95.2% | 2000.1522 | 2000.3024 | 24 | 4.448 | 32.4% | 1 | R.GVPQIEVTFEIDVNGILR.V | 2 |
|  | Astrin\_STLC\_112116\_tube2\_01.08369.08369.3 | 3.3676 | 0.3237 | 99.7% | 1801.8544 | 1802.9788 | 1 | 5.335 | 44.6% | 1 | R.IDTRNELESYAYSLK.N | 3 |
|  | Astrin\_STLC\_112116\_02.07810.07810.3 | 4.0034 | 0.3758 | 100.0% | 1975.7043 | 1976.1064 | 37 | 6.307 | 36.7% | 2 | K.IEWLESHQDADIEDFK.A | 3 |
|  | Astrin\_STLC\_112116\_tube2\_02.08444.08444.3 | 3.8516 | 0.2496 | 99.4% | 2174.9043 | 2175.3594 | 1 | 5.977 | 39.7% | 2 | K.IEWLESHQDADIEDFKAK.K | 3 |
|  | Astrin\_STLC\_112116\_01.07149.07149.2 | 2.7115 | 0.3402 | 100.0% | 1398.0521 | 1398.6396 | 2 | 6.694 | 63.6% | 2 | K.ELEEIVQPIISK.L | 2 |
|  | Astrin\_STLC\_112116\_tube2\_01.06754.06754.2 | 5.5906 | 0.5956 | 100.0% | 2176.2322 | 2177.283 | 1 | 10.515 | 50.0% | 3 | K.LYGSAGPPPTGEEDTAEKDEL.- | 2 |

Similarities:
gi|5729877|ref|NP\_006(1:20)  
gi|124256496|ref|NP\_0(1:20)  

---

|  |  |  |  |  |  |  |  |  |
| --- | --- | --- | --- | --- | --- | --- | --- | --- |
| U | *gi|57013276|ref|NP\_00* | 16 | 58 | 42.8% | 451 | 50152 | 5.1 | tubulin alpha-1B chain [Homo sapiens] |

| Filename XCorr DeltCN Conf% ObsM+H+ CalcM+H+ SpR ZScore Ion% # Sequence  | | | | | | | | | | | | |
| --- | --- | --- | --- | --- | --- | --- | --- | --- | --- | --- | --- | --- |
|  | Astrin\_STLC\_112116\_tube2\_02.09308.09308.2 | 5.6232 | 0.5753 | 100.0% | 2009.0322 | 2009.093 | 1 | 10.785 | 52.6% | 5 | K.TIGGGDDSFNTFFSETGAGK.H | 222 |
|  | Astrin\_STLC\_112116\_tube2\_01.10687.10687.2 | 5.1392 | 0.5068 | 100.0% | 1703.0521 | 1702.9451 | 1 | 8.957 | 78.6% | 22 | R.AVFVDLEPTVIDEVR.T | 22 |
|  | Astrin\_STLC\_112116\_tube2\_01.07799.07799.3 | 3.6569 | 0.3544 | 99.8% | 2415.8044 | 2416.6555 | 1 | 6.145 | 31.2% | 2 | R.QLFHPEQLITGKEDAANNYAR.G | 3333 |
|  | Astrin\_STLC\_112116\_01.10010.10010.3 | 4.7103 | 0.4547 | 100.0% | 1843.1044 | 1843.1332 | 2 | 7.318 | 43.3% | 1 | R.GHYTIGKEIIDLVLDR.I | 33 |
|  | Astrin\_STLC\_112116\_tube2\_01.10239.10239.2 | 2.656 | 0.3391 | 100.0% | 1086.1322 | 1086.2737 | 1 | 5.902 | 81.2% | 1 | K.EIIDLVLDR.I | 22 |
|  | Astrin\_STLC\_112116\_01.05955.05955.2 | 3.0877 | 0.1237 | 96.6% | 1719.1921 | 1719.8949 | 10 | 4.661 | 53.8% | 1 | R.NLDIERPTYTNLNR.L | 2222 |
|  | Astrin\_STLC\_112116\_tube2\_01.12602.12602.2 | 4.4411 | 0.4757 | 100.0% | 1489.1921 | 1488.7678 | 1 | 9.088 | 65.4% | 6 | R.LISQIVSSITASLR.F | 222 |
|  | Astrin\_STLC\_112116\_01.11304.11304.2 | 5.0812 | 0.5156 | 100.0% | 2410.672 | 2410.6885 | 1 | 10.197 | 50.0% | 4 | R.FDGALNVDLTEFQTNLVPYPR.I | 2222 |
|  | Astrin\_STLC\_112116\_tube2\_01.09711.09711.2 | 3.8528 | 0.4144 | 100.0% | 1758.1921 | 1758.0703 | 1 | 8.032 | 73.3% | 2 | R.IHFPLATYAPVISAEK.A | 2222 |
|  | Astrin\_STLC\_112116\_01.08092.08092.3 | 3.5914 | 0.2803 | 99.6% | 1759.1344 | 1758.0703 | 12 | 5.388 | 38.3% | 3 | R.IHFPLATYAPVISAEK.A | 3333 |
|  | Astrin\_STLC\_112116\_01.06065.06065.1 | 1.6791 | 0.3806 | 100.0% | 1015.39 | 1016.1827 | 49 | 5.524 | 50.0% | 1 | K.DVNAAIATIK.T | 111 |
|  | Astrin\_STLC\_112116\_tube2\_01.06683.06683.2 | 2.5719 | 0.3262 | 100.0% | 1015.4522 | 1016.1827 | 1 | 6.838 | 72.2% | 3 | K.DVNAAIATIK.T | 222 |
|  | Astrin\_STLC\_112116\_01.07012.07012.2 | 4.5739 | 0.4617 | 100.0% | 1825.3722 | 1826.1027 | 1 | 7.363 | 64.7% | 3 | K.VGINYQPPTVVPGGDLAK.V | 2222 |
|  | Astrin\_STLC\_112116\_02.08574.08574.2 | 2.6993 | 0.2984 | 99.5% | 1865.4321 | 1866.1084 | 2 | 5.253 | 43.8% | 1 | R.AVCMLSNTTAIAEAWAR.L | 222 |
|  | Astrin\_STLC\_112116\_01.05996.05996.2 | 2.5896 | 0.4144 | 100.0% | 1380.8522 | 1381.6324 | 270 | 6.049 | 50.0% | 1 | R.LDHKFDLMYAK.R | 2222 |
|  | Astrin\_STLC\_112116\_01.05997.05997.3 | 3.9875 | 0.2967 | 99.8% | 1382.9043 | 1381.6324 | 4 | 6.138 | 55.0% | 2 | R.LDHKFDLMYAK.R | 3333 |

Similarities:
gi|14389309|ref|NP\_11(15:1)  
gi|156564363|ref|NP\_5(12:4)  
gi|17921989|ref|NP\_00(10:6)  

---

|  |  |  |  |  |  |  |  |  |
| --- | --- | --- | --- | --- | --- | --- | --- | --- |
| U | *gi|20127519|ref|NP\_03* | 34 | 78 | 42.2% | 747 | 85653 | 9.2 | targeting protein for Xklp2 [Homo sapiens] |

| Filename XCorr DeltCN Conf% ObsM+H+ CalcM+H+ SpR ZScore Ion% # Sequence  | | | | | | | | | | | | |
| --- | --- | --- | --- | --- | --- | --- | --- | --- | --- | --- | --- | --- |
| \* | Astrin\_STLC\_112116\_01.12281.12281.3 | 3.2837 | 0.3049 | 99.5% | 3809.9944 | 3810.8867 | 50 | 5.16 | 18.0% | 1 | K.SSYSYDAPSDFINFSSLDDEGDTQNIDSWFEEK.A | 3 |
| \* | Astrin\_STLC\_112116\_tube2\_01.07559.07559.3 | 4.8692 | 0.4027 | 100.0% | 2405.7544 | 2405.7996 | 1 | 7.4 | 40.0% | 1 | R.KANLQQAIVTPLKPVDNTYYK.E | 3 |
| \* | Astrin\_STLC\_112116\_tube2\_01.08292.08292.3 | 3.2686 | 0.352 | 99.7% | 2276.2144 | 2277.6255 | 1 | 5.544 | 31.6% | 1 | K.ANLQQAIVTPLKPVDNTYYK.E | 3 |
| \* | Astrin\_STLC\_112116\_tube2\_01.04729.04729.2 | 3.4758 | 0.3756 | 100.0% | 1150.8322 | 1150.3534 | 1 | 7.317 | 81.2% | 3 | K.MQQEVVEMR.K | 2 |
| \* | Astrin\_STLC\_112116\_01.05924.05924.2 | 3.1183 | 0.3461 | 100.0% | 1195.0521 | 1195.4911 | 41 | 6.509 | 54.5% | 2 | K.LALAGIGQPVKK.S | 2 |
| \* | Astrin\_STLC\_112116\_tube2\_01.07383.07383.2 | 4.9125 | 0.5165 | 100.0% | 1885.9521 | 1887.013 | 1 | 8.458 | 78.6% | 1 | K.NQEEYKEVNFTSELR.K | 2 |
| \* | Astrin\_STLC\_112116\_tube2\_01.07401.07401.3 | 4.0379 | 0.4365 | 100.0% | 1886.7544 | 1887.013 | 1 | 7.792 | 55.4% | 4 | K.NQEEYKEVNFTSELR.K | 3 |
| \* | Astrin\_STLC\_112116\_tube2\_01.06414.06414.3 | 3.2044 | 0.4365 | 99.7% | 2014.9443 | 2015.187 | 1 | 5.974 | 40.0% | 1 | K.NQEEYKEVNFTSELRK.H | 3 |
| \* | Astrin\_STLC\_112116\_tube2\_01.04500.04500.3 | 3.9394 | 0.1835 | 99.4% | 1358.2144 | 1358.5779 | 9 | 4.936 | 52.3% | 2 | R.SKKDDINLLPSK.S | 3 |
| \* | Astrin\_STLC\_112116\_01.05604.05604.2 | 3.7077 | 0.2592 | 100.0% | 1358.2522 | 1358.5779 | 1 | 6.038 | 81.8% | 1 | R.SKKDDINLLPSK.S | 2 |
| \* | Astrin\_STLC\_112116\_tube2\_01.06269.06269.2 | 3.3965 | 0.1565 | 99.5% | 1349.7922 | 1349.4344 | 1 | 4.731 | 81.8% | 2 | K.STAELEAEELEK.L | 2 |
| \* | Astrin\_STLC\_112116\_tube2\_01.08595.08595.2 | 5.3471 | 0.4274 | 100.0% | 2009.0122 | 2010.2053 | 1 | 8.491 | 62.5% | 2 | K.STAELEAEELEKLQQYK.F | 2 |
| \* | Astrin\_STLC\_112116\_tube2\_01.08636.08636.3 | 2.8119 | 0.439 | 99.8% | 2009.4543 | 2010.2053 | 235 | 6.135 | 26.6% | 1 | K.STAELEAEELEKLQQYK.F | 3 |
| \* | Astrin\_STLC\_112116\_01.06435.06435.2 | 3.0629 | 0.2106 | 99.8% | 1036.5322 | 1037.2877 | 2 | 4.64 | 72.2% | 6 | R.ILEGGPILPK.K | 2 |
| \* | Astrin\_STLC\_112116\_01.06992.06992.3 | 3.9212 | 0.4022 | 100.0% | 2135.9644 | 2135.5083 | 1 | 6.937 | 37.5% | 2 | K.KPPVKPPTEPIGFDLEIEK.R | 3 |
| \* | Astrin\_STLC\_112116\_tube2\_01.07578.07578.3 | 5.0389 | 0.4801 | 100.0% | 2292.4443 | 2291.6958 | 1 | 8.38 | 38.2% | 3 | K.KPPVKPPTEPIGFDLEIEKR.I | 3 |
| \* | Astrin\_STLC\_112116\_tube2\_01.07333.07333.2 | 3.1914 | 0.2328 | 100.0% | 1198.7122 | 1198.402 | 10 | 5.765 | 65.0% | 4 | K.ILEDVVGVPEK.K | 2 |
| \* | Astrin\_STLC\_112116\_tube2\_01.06327.06327.3 | 3.8925 | 0.3786 | 100.0% | 1326.5343 | 1326.576 | 197 | 6.32 | 43.2% | 2 | K.ILEDVVGVPEKK.V | 3 |
| \* | Astrin\_STLC\_112116\_tube2\_01.06298.06298.2 | 3.3998 | 0.0816 | 98.0% | 1328.4521 | 1326.576 | 3 | 4.163 | 72.7% | 4 | K.ILEDVVGVPEKK.V | 2 |
| \* | Astrin\_STLC\_112116\_tube2\_01.09619.09619.2 | 3.1909 | 0.3942 | 100.0% | 1661.3322 | 1661.9823 | 1 | 7.433 | 46.4% | 1 | K.VLPITVPKS\*PAFALK.N | 2 |
| \* | Astrin\_STLC\_112116\_tube2\_01.06149.06149.3 | 5.0065 | 0.5154 | 100.0% | 2157.5942 | 2158.4285 | 1 | 9.199 | 44.1% | 2 | R.IRMPTKEDEEEDEPVVIK.A | 3 |
| \* | Astrin\_STLC\_112116\_01.05756.05756.2 | 5.1006 | 0.392 | 100.0% | 1888.2122 | 1889.0815 | 1 | 8.153 | 83.3% | 1 | R.MPTKEDEEEDEPVVIK.A | 2 |
| \* | Astrin\_STLC\_112116\_tube2\_01.05401.05401.3 | 5.8379 | 0.3301 | 100.0% | 1889.0343 | 1889.0815 | 1 | 7.049 | 58.3% | 3 | R.MPTKEDEEEDEPVVIK.A | 3 |
| \* | Astrin\_STLC\_112116\_01.06897.06897.3 | 3.1348 | 0.3486 | 99.7% | 2131.8542 | 2132.473 | 230 | 6.611 | 29.2% | 1 | K.AQPVPHYGVPFKPQIPEAR.T | 3 |
| \* | Astrin\_STLC\_112116\_01.08111.08111.2 | 2.6855 | 0.3796 | 100.0% | 1458.1122 | 1458.5769 | 16 | 6.951 | 54.5% | 1 | R.TVEICPFSFDSR.D | 2 |
| \* | Astrin\_STLC\_112116\_01.04232.04232.2 | 2.7592 | 0.2964 | 100.0% | 1054.0721 | 1054.1478 | 2 | 5.328 | 78.6% | 3 | K.HQLEEELR.Q | 2 |
| \* | Astrin\_STLC\_112116\_tube2\_01.05659.05659.3 | 4.4082 | 0.3241 | 99.8% | 1812.2043 | 1812.1222 | 1 | 7.276 | 48.3% | 4 | K.ARPNTVISQEPFVPKK.E | 3 |
| \* | Astrin\_STLC\_112116\_01.07127.07127.3 | 5.1089 | 0.421 | 100.0% | 2474.4844 | 2475.8044 | 1 | 8.205 | 35.2% | 3 | K.KSVAEGLSGSLVQEPFQLATEKR.A | 3 |
| \* | Astrin\_STLC\_112116\_tube2\_01.10429.10429.2 | 4.7516 | 0.5749 | 100.0% | 2190.3123 | 2191.4429 | 1 | 9.982 | 57.5% | 1 | K.SVAEGLSGSLVQEPFQLATEK.R | 2 |
| \* | Astrin\_STLC\_112116\_01.08252.08252.3 | 4.404 | 0.3467 | 99.8% | 2347.3442 | 2347.6304 | 2 | 6.891 | 28.6% | 7 | K.SVAEGLSGSLVQEPFQLATEKR.A | 3 |
| \* | Astrin\_STLC\_112116\_tube2\_01.05297.05297.3 | 3.9235 | 0.2828 | 99.7% | 1831.2843 | 1832.0386 | 1 | 6.155 | 41.7% | 3 | R.MAEVEAQKAQQLEEAR.L | 3 |
| \* | Astrin\_STLC\_112116\_01.03698.03698.3 | 3.6718 | 0.2996 | 99.8% | 1630.2544 | 1630.7954 | 3 | 5.686 | 50.0% | 1 | R.LQEEEQKKEELAR.L | 3 |
| \* | Astrin\_STLC\_112116\_01.05909.05909.2 | 3.0899 | 0.3144 | 100.0% | 1355.1522 | 1355.5309 | 1 | 6.704 | 66.7% | 2 | K.SSDQPLTVPVSPK.F | 2 |
| \* | Astrin\_STLC\_112116\_tube2\_01.06459.06459.2 | 3.3675 | 0.4176 | 100.0% | 1434.3322 | 1435.5309 | 1 | 7.85 | 83.3% | 2 | K.SSDQPLTVPVS\*PK.F | 2 |

---

|  |  |  |  |  |  |  |  |  |
| --- | --- | --- | --- | --- | --- | --- | --- | --- |
| U | *gi|15431310|ref|NP\_00* | 21 | 38 | 42.2% | 472 | 51622 | 5.2 | keratin, type I cytoskeletal 14 [Homo sapiens] |

| Filename XCorr DeltCN Conf% ObsM+H+ CalcM+H+ SpR ZScore Ion% # Sequence  | | | | | | | | | | | | |
| --- | --- | --- | --- | --- | --- | --- | --- | --- | --- | --- | --- | --- |
| \* | Astrin\_STLC\_112116\_tube2\_01.04894.04894.2 | 3.8583 | 0.5548 | 100.0% | 1425.8922 | 1426.526 | 2 | 9.77 | 57.1% | 2 | R.APSTYGGGLSVSSSR.F | 2 |
|  | Astrin\_STLC\_112116\_tube2\_01.04204.04204.2 | 2.311 | 0.2065 | 96.9% | 1091.1322 | 1091.2273 | 68 | 4.975 | 62.5% | 1 | K.VTMQNLNDR.L | 222 |
|  | Astrin\_STLC\_112116\_tube2\_01.05299.05299.2 | 3.2103 | 0.1537 | 99.8% | 1064.5721 | 1065.2578 | 1 | 6.052 | 81.2% | 2 | R.LASYLDKVR.A | 222222 |
|  | Astrin\_STLC\_112116\_tube2\_02.07110.07110.2 | 4.4264 | 0.3632 | 100.0% | 1303.0322 | 1302.4241 | 1 | 7.671 | 81.8% | 6 | R.ALEEANADLEVK.I | 222 |
|  | Astrin\_STLC\_112116\_tube2\_01.07473.07473.3 | 3.5531 | 0.2747 | 99.6% | 1741.7644 | 1742.9719 | 3 | 5.098 | 40.4% | 2 | R.QRPAEIKDYSPYFK.T | 3 |
|  | Astrin\_STLC\_112116\_tube2\_02.09363.09363.2 | 5.2892 | 0.4468 | 100.0% | 2054.4521 | 2055.339 | 1 | 8.606 | 61.1% | 1 | K.ILTATVDNANVLLQIDNAR.L | 2 |
|  | Astrin\_STLC\_112116\_tube2\_02.09357.09357.3 | 5.7126 | 0.4408 | 100.0% | 2055.0544 | 2055.339 | 1 | 7.381 | 47.2% | 1 | K.ILTATVDNANVLLQIDNAR.L | 3 |
|  | Astrin\_STLC\_112116\_01.05584.05584.2 | 1.8322 | 0.255 | 95.2% | 807.8722 | 807.8815 | 186 | 5.3 | 66.7% | 1 | R.LAADDFR.T | 2222222 |
|  | Astrin\_STLC\_112116\_tube2\_01.05387.05387.2 | 2.3548 | 0.2289 | 97.8% | 1266.9321 | 1267.4246 | 1 | 4.953 | 72.2% | 1 | R.TKYETELNLR.M | 2 |
|  | Astrin\_STLC\_112116\_tube2\_01.07209.07209.2 | 3.3704 | 0.3259 | 100.0% | 1186.0521 | 1186.397 | 1 | 6.037 | 83.3% | 1 | R.RVLDELTLAR.A | 2222 |
|  | Astrin\_STLC\_112116\_tube2\_01.07839.07839.2 | 3.2486 | 0.3989 | 100.0% | 1030.0122 | 1030.2096 | 1 | 7.534 | 87.5% | 4 | R.VLDELTLAR.A | 22222 |
|  | Astrin\_STLC\_112116\_01.04197.04197.2 | 2.5639 | 0.369 | 100.0% | 1242.9922 | 1243.3367 | 8 | 6.59 | 66.7% | 1 | K.NHEEEMNALR.G | 22 |
|  | Astrin\_STLC\_112116\_tube2\_01.04705.04705.2 | 2.6519 | 0.2422 | 99.2% | 1438.2122 | 1439.6263 | 15 | 4.49 | 65.0% | 1 | R.ILNEMRDQYEK.M | 22 |
|  | Astrin\_STLC\_112116\_tube2\_01.10174.10174.2 | 3.5347 | 0.4118 | 100.0% | 1916.3722 | 1916.0538 | 1 | 7.108 | 57.1% | 1 | K.DAEEWFFTKTEELNR.E | 2 |
|  | Astrin\_STLC\_112116\_tube2\_01.05884.05884.3 | 4.6205 | 0.4462 | 100.0% | 2104.1943 | 2105.2664 | 1 | 7.923 | 43.1% | 1 | K.TEELNREVATNSELVQSGK.S | 33 |
|  | Astrin\_STLC\_112116\_01.04469.04469.2 | 4.0278 | 0.5084 | 100.0% | 1361.9922 | 1362.4796 | 1 | 9.172 | 75.0% | 1 | R.EVATNSELVQSGK.S | 22 |
|  | Astrin\_STLC\_112116\_tube2\_01.05697.05697.2 | 3.1611 | 0.2971 | 100.0% | 1221.6721 | 1221.3068 | 1 | 6.8 | 80.0% | 3 | K.ASLENSLEETK.G | 222 |
|  | Astrin\_STLC\_112116\_tube2\_01.06467.06467.2 | 3.5426 | 0.3417 | 100.0% | 1381.0922 | 1380.5437 | 2 | 6.215 | 75.0% | 3 | K.TRLEQEIATYR.R | 2222 |
|  | Astrin\_STLC\_112116\_tube2\_01.05638.05638.3 | 3.1897 | 0.3263 | 99.8% | 1535.1543 | 1536.7311 | 1 | 5.56 | 45.5% | 3 | K.TRLEQEIATYRR.L | 3333 |
|  | Astrin\_STLC\_112116\_tube2\_01.04954.04954.2 | 2.7318 | 0.326 | 100.0% | 1122.9922 | 1123.2511 | 1 | 5.932 | 81.2% | 1 | R.LEQEIATYR.R | 22222 |
|  | Astrin\_STLC\_112116\_tube2\_01.06072.06072.3 | 5.8051 | 0.5734 | 100.0% | 2309.4844 | 2310.396 | 1 | 9.99 | 47.6% | 1 | R.LLEGEDAHLSSSQFSSGSQSSR.D | 3 |

Similarities:
contaminant\_KERATIN09(1:20)  
gi|24430192|ref|NP\_00(10:11)  
contaminant\_KERATIN12(11:10)  
contaminant\_KERATIN03(3:18)  
contaminant\_KERATIN07(8:13)  
contaminant\_KERATIN10(5:16)  

---

|  |  |  |  |  |  |  |  |  |
| --- | --- | --- | --- | --- | --- | --- | --- | --- |
| U | *gi|57242777|ref|NP\_03* | 5 | 12 | 41.7% | 103 | 11967 | 5.9 | C-Myc-binding protein [Homo sapiens] &IC MYCBP |

| Filename XCorr DeltCN Conf% ObsM+H+ CalcM+H+ SpR ZScore Ion% # Sequence  | | | | | | | | | | | | |
| --- | --- | --- | --- | --- | --- | --- | --- | --- | --- | --- | --- | --- |
| \* | Astrin\_STLC\_112116\_tube2\_01.06940.06940.2 | 2.775 | 0.3415 | 100.0% | 934.0122 | 934.07764 | 6 | 5.819 | 68.8% | 2 | K.SGVLDTLTK.V | 2 |
| \* | Astrin\_STLC\_112116\_tube2\_01.10514.10514.2 | 4.7076 | 0.4352 | 100.0% | 2276.3323 | 2276.6348 | 1 | 7.043 | 47.4% | 2 | K.VLVALYEEPEKPNSALDFLK.H | 2 |
| \* | Astrin\_STLC\_112116\_tube2\_01.10502.10502.3 | 4.3278 | 0.4241 | 100.0% | 2276.8442 | 2276.6348 | 1 | 7.193 | 36.8% | 2 | K.VLVALYEEPEKPNSALDFLK.H | 3 |
| \* | Astrin\_STLC\_112116\_01.04863.04863.2 | 3.3519 | 0.307 | 100.0% | 1331.7922 | 1332.4528 | 5 | 5.136 | 65.0% | 5 | K.LAQYEPPQEEK.R | 2 |
| \* | Astrin\_STLC\_112116\_tube2\_01.04074.04074.2 | 3.268 | 0.2793 | 100.0% | 1687.9521 | 1688.8345 | 1 | 5.922 | 61.5% | 1 | K.LAQYEPPQEEKRAE.- | 2 |

---

|  |  |  |  |  |  |  |  |  |
| --- | --- | --- | --- | --- | --- | --- | --- | --- |
| U | *contaminant\_gi|746301* | 10 | 115 | 40.1% | 269 | 27961 | 6.7 | lysyl endopeptidase (EC 3.4.21.50) - Lysobacter enzymogenes &IC Lys-C |

| Filename XCorr DeltCN Conf% ObsM+H+ CalcM+H+ SpR ZScore Ion% # Sequence  | | | | | | | | | | | | |
| --- | --- | --- | --- | --- | --- | --- | --- | --- | --- | --- | --- | --- |
| \* | Astrin\_STLC\_112116\_tube2\_01.04585.04585.2 | 6.6164 | 0.6116 | 100.0% | 2261.2722 | 2262.355 | 1 | 11.435 | 58.3% | 10 | R.APGSSSSGANGDGSLAQSQTGAVVR.A | 2 |
| \* | Astrin\_STLC\_112116\_tube2\_01.04582.04582.3 | 5.051 | 0.4037 | 100.0% | 2261.6343 | 2262.355 | 1 | 7.837 | 36.5% | 7 | R.APGSSSSGANGDGSLAQSQTGAVVR.A | 3 |
| \* | Astrin\_STLC\_112116\_01.13028.13028.3 | 6.9738 | 0.5183 | 100.0% | 3316.5544 | 3315.6257 | 1 | 8.596 | 32.8% | 13 | R.ATNAASDFTLLELNTAANPAYNLFWAGWDR.R | 3 |
| \* | Astrin\_STLC\_112116\_tube2\_01.13773.13773.3 | 6.3333 | 0.4224 | 100.0% | 3472.4043 | 3471.813 | 1 | 9.554 | 26.7% | 44 | R.ATNAASDFTLLELNTAANPAYNLFWAGWDRR.D | 3 |
| \* | Astrin\_STLC\_112116\_tube2\_01.04334.04334.3 | 4.4178 | 0.2899 | 99.8% | 2077.7944 | 2077.2668 | 1 | 5.766 | 40.3% | 1 | R.RDQNFAGATAIHHPNVAEK.R | 3 |
| \* | Astrin\_STLC\_112116\_01.05608.05608.2 | 4.874 | 0.5229 | 100.0% | 1920.1322 | 1921.0793 | 1 | 8.923 | 58.8% | 1 | R.DQNFAGATAIHHPNVAEK.R | 2 |
| \* | Astrin\_STLC\_112116\_tube2\_01.04692.04692.3 | 2.7629 | 0.3799 | 99.7% | 1920.5944 | 1921.0793 | 1 | 5.8 | 38.2% | 2 | R.DQNFAGATAIHHPNVAEK.R | 3 |
| \* | Astrin\_STLC\_112116\_tube2\_01.04398.04398.3 | 3.9413 | 0.489 | 100.0% | 2077.7344 | 2077.2668 | 1 | 7.782 | 40.3% | 5 | R.DQNFAGATAIHHPNVAEKR.I | 3 |
| \* | Astrin\_STLC\_112116\_01.04820.04820.3 | 3.3109 | 0.4172 | 99.8% | 1872.1444 | 1870.983 | 1 | 6.179 | 37.5% | 1 | R.VLGQLHGGPSSCSATGADR.S | 3 |
| \* | Astrin\_STLC\_112116\_tube2\_01.06747.06747.2 | 4.7645 | 0.4647 | 100.0% | 1428.8121 | 1428.5443 | 1 | 8.467 | 69.2% | 31 | R.VFTSWTGGGTSATR.L | 2 |

---

|  |  |  |  |  |  |  |  |  |
| --- | --- | --- | --- | --- | --- | --- | --- | --- |
| U | *gi|14389309|ref|NP\_11* | 16 | 58 | 39.2% | 449 | 49895 | 5.1 | tubulin alpha-1C chain isoform c [Homo sapiens] |
| U | *gi|733606247|ref|NP\_0* | 16 | 58 | 42.5% | 414 | 46057 | 5.1 | tubulin alpha-1C chain isoform b [Homo sapiens] |
| U | *gi|733605926|ref|NP\_0* | 16 | 60 | 33.9% | 519 | 57730 | 5.1 | tubulin alpha-1C chain isoform a [Homo sapiens] |

| Filename XCorr DeltCN Conf% ObsM+H+ CalcM+H+ SpR ZScore Ion% # Sequence  | | | | | | | | | | | | |
| --- | --- | --- | --- | --- | --- | --- | --- | --- | --- | --- | --- | --- |
|  | Astrin\_STLC\_112116\_tube2\_02.09308.09308.2 | 5.6232 | 0.5753 | 100.0% | 2009.0322 | 2009.093 | 1 | 10.785 | 52.6% | 5 | K.TIGGGDDSFNTFFSETGAGK.H | 222 |
|  | Astrin\_STLC\_112116\_tube2\_01.10687.10687.2 | 5.1392 | 0.5068 | 100.0% | 1703.0521 | 1702.9451 | 1 | 8.957 | 78.6% | 22 | R.AVFVDLEPTVIDEVR.T | 22 |
|  | Astrin\_STLC\_112116\_tube2\_01.08150.08150.2 | 1.8772 | 0.3713 | 98.0% | 1411.0721 | 1411.6439 | 279 | 6.036 | 45.5% | 1 | R.QLFHPEQLITGK.E | 222 |
|  | Astrin\_STLC\_112116\_tube2\_01.07799.07799.3 | 3.6569 | 0.3544 | 99.8% | 2415.8044 | 2416.6555 | 1 | 6.145 | 31.2% | 2 | R.QLFHPEQLITGKEDAANNYAR.G | 3333 |
|  | Astrin\_STLC\_112116\_01.10010.10010.3 | 4.7103 | 0.4547 | 100.0% | 1843.1044 | 1843.1332 | 2 | 7.318 | 43.3% | 1 | R.GHYTIGKEIIDLVLDR.I | 33 |
|  | Astrin\_STLC\_112116\_tube2\_01.10239.10239.2 | 2.656 | 0.3391 | 100.0% | 1086.1322 | 1086.2737 | 1 | 5.902 | 81.2% | 1 | K.EIIDLVLDR.I | 22 |
|  | Astrin\_STLC\_112116\_01.05955.05955.2 | 3.0877 | 0.1237 | 96.6% | 1719.1921 | 1719.8949 | 10 | 4.661 | 53.8% | 1 | R.NLDIERPTYTNLNR.L | 2222 |
|  | Astrin\_STLC\_112116\_tube2\_01.12602.12602.2 | 4.4411 | 0.4757 | 100.0% | 1489.1921 | 1488.7678 | 1 | 9.088 | 65.4% | 6 | R.LISQIVSSITASLR.F | 222 |
|  | Astrin\_STLC\_112116\_01.11304.11304.2 | 5.0812 | 0.5156 | 100.0% | 2410.672 | 2410.6885 | 1 | 10.197 | 50.0% | 4 | R.FDGALNVDLTEFQTNLVPYPR.I | 2222 |
|  | Astrin\_STLC\_112116\_tube2\_01.09711.09711.2 | 3.8528 | 0.4144 | 100.0% | 1758.1921 | 1758.0703 | 1 | 8.032 | 73.3% | 2 | R.IHFPLATYAPVISAEK.A | 2222 |
|  | Astrin\_STLC\_112116\_01.08092.08092.3 | 3.5914 | 0.2803 | 99.6% | 1759.1344 | 1758.0703 | 12 | 5.388 | 38.3% | 3 | R.IHFPLATYAPVISAEK.A | 3333 |
|  | Astrin\_STLC\_112116\_01.06065.06065.1 | 1.6791 | 0.3806 | 100.0% | 1015.39 | 1016.1827 | 49 | 5.524 | 50.0% | 1 | K.DVNAAIATIK.T | 111 |
|  | Astrin\_STLC\_112116\_tube2\_01.06683.06683.2 | 2.5719 | 0.3262 | 100.0% | 1015.4522 | 1016.1827 | 1 | 6.838 | 72.2% | 3 | K.DVNAAIATIK.T | 222 |
|  | Astrin\_STLC\_112116\_01.07012.07012.2 | 4.5739 | 0.4617 | 100.0% | 1825.3722 | 1826.1027 | 1 | 7.363 | 64.7% | 3 | K.VGINYQPPTVVPGGDLAK.V | 2222 |
|  | Astrin\_STLC\_112116\_01.05996.05996.2 | 2.5896 | 0.4144 | 100.0% | 1380.8522 | 1381.6324 | 270 | 6.049 | 50.0% | 1 | R.LDHKFDLMYAK.R | 2222 |
|  | Astrin\_STLC\_112116\_01.05997.05997.3 | 3.9875 | 0.2967 | 99.8% | 1382.9043 | 1381.6324 | 4 | 6.138 | 55.0% | 2 | R.LDHKFDLMYAK.R | 3333 |

Similarities:
gi|57013276|ref|NP\_00(15:1)  
gi|156564363|ref|NP\_5(12:4)  
gi|17921989|ref|NP\_00(10:6)  

---

|  |  |  |  |  |  |  |  |  |
| --- | --- | --- | --- | --- | --- | --- | --- | --- |
| U | *gi|25777713|ref|NP\_73* | 4 | 6 | 38.7% | 163 | 18658 | 4.5 | S-phase kinase-associated protein 1 isoform b [Homo sapiens] &IC Skp1A |

| Filename XCorr DeltCN Conf% ObsM+H+ CalcM+H+ SpR ZScore Ion% # Sequence  | | | | | | | | | | | | |
| --- | --- | --- | --- | --- | --- | --- | --- | --- | --- | --- | --- | --- |
|  | Astrin\_STLC\_112116\_tube2\_02.09296.09296.2 | 3.3066 | 0.3792 | 100.0% | 1880.3922 | 1880.0588 | 1 | 6.813 | 53.1% | 1 | K.LQSSDGEIFEVDVEIAK.Q | 2 |
|  | Astrin\_STLC\_112116\_tube2\_01.10840.10840.3 | 4.2498 | 0.4347 | 100.0% | 3126.8643 | 3127.5056 | 1 | 7.224 | 29.5% | 2 | K.TMLEDLGMDDEGDDDPVPLPNVNAAILKK.V | 3 |
| \* | Astrin\_STLC\_112116\_tube2\_02.08269.08269.3 | 4.6736 | 0.3849 | 100.0% | 2071.4644 | 2071.2078 | 1 | 7.304 | 53.1% | 2 | K.TFNIKNDFTEEEEAQVR.K | 3 |
| \* | Astrin\_STLC\_112116\_tube2\_01.05715.05715.2 | 2.6896 | 0.3594 | 100.0% | 1466.8722 | 1467.4888 | 3 | 6.357 | 59.1% | 1 | K.NDFTEEEEAQVR.K | 2 |

---

|  |  |  |  |  |  |  |  |  |
| --- | --- | --- | --- | --- | --- | --- | --- | --- |
| U | *gi|4758792|ref|NP\_004* | 3 | 3 | 38.7% | 124 | 13712 | 8.3 | NADH dehydrogenase [ubiquinone] iron-sulfur protein 6, mitochondrial precursor [Homo sapiens] |

| Filename XCorr DeltCN Conf% ObsM+H+ CalcM+H+ SpR ZScore Ion% # Sequence  | | | | | | | | | | | | |
| --- | --- | --- | --- | --- | --- | --- | --- | --- | --- | --- | --- | --- |
| \* | Astrin\_STLC\_112116\_01.04005.04005.3 | 2.9186 | 0.2666 | 98.6% | 1697.3944 | 1697.8015 | 1 | 4.727 | 40.4% | 1 | K.VTHTGQVYDDKDYR.R | 3 |
| \* | Astrin\_STLC\_112116\_tube2\_02.09346.09346.3 | 4.5837 | 0.3804 | 100.0% | 2759.3643 | 2760.03 | 7 | 5.626 | 29.3% | 1 | R.QKEVNENFAIDLIAEQPVSEVETR.V | 3 |
| \* | Astrin\_STLC\_112116\_tube2\_01.04892.04892.2 | 2.9405 | 0.1095 | 97.6% | 1222.8922 | 1223.4117 | 1 | 5.359 | 88.9% | 1 | K.VYINLDKETK.T | 2 |

---

|  |  |  |  |  |  |  |  |  |
| --- | --- | --- | --- | --- | --- | --- | --- | --- |
| U | *gi|316659409|ref|NP\_0* | 9 | 22 | 36.8% | 375 | 41793 | 5.5 | actin, cytoplasmic 2 [Homo sapiens] |
| U | *gi|4501885|ref|NP\_001* | 9 | 22 | 36.8% | 375 | 41737 | 5.5 | actin, cytoplasmic 1 [Homo sapiens] |

| Filename XCorr DeltCN Conf% ObsM+H+ CalcM+H+ SpR ZScore Ion% # Sequence  | | | | | | | | | | | | |
| --- | --- | --- | --- | --- | --- | --- | --- | --- | --- | --- | --- | --- |
|  | Astrin\_STLC\_112116\_01.04121.04121.2 | 3.4288 | 0.4105 | 100.0% | 976.9122 | 977.02136 | 1 | 7.125 | 88.9% | 1 | K.AGFAGDDAPR.A | 22 |
|  | Astrin\_STLC\_112116\_01.06117.06117.2 | 2.8748 | 0.4046 | 100.0% | 1198.4922 | 1199.4415 | 1 | 6.426 | 75.0% | 2 | R.AVFPSIVGRPR.H | 22 |
|  | Astrin\_STLC\_112116\_01.05896.05896.3 | 3.0002 | 0.2301 | 98.6% | 1516.4343 | 1516.7019 | 5 | 5.183 | 45.0% | 2 | K.IWHHTFYNELR.V | 33 |
|  | Astrin\_STLC\_112116\_tube2\_01.07719.07719.2 | 4.4263 | 0.3482 | 100.0% | 1954.3922 | 1955.2615 | 1 | 8.813 | 64.7% | 1 | R.VAPEEHPVLLTEAPLNPK.A | 2 |
|  | Astrin\_STLC\_112116\_01.09093.09093.3 | 6.0445 | 0.5065 | 100.0% | 3184.7944 | 3185.622 | 1 | 6.472 | 34.5% | 2 | R.TTGIVMDSGDGVTHTVPIYEGYALPHAILR.L | 3 |
|  | Astrin\_STLC\_112116\_tube2\_01.05276.05276.2 | 3.0256 | 0.4835 | 100.0% | 1133.0122 | 1133.2029 | 1 | 7.302 | 77.8% | 4 | R.GYSFTTTAER.E | 2 |
|  | Astrin\_STLC\_112116\_tube2\_01.09467.09467.2 | 4.7773 | 0.4402 | 100.0% | 1792.1322 | 1791.9554 | 1 | 8.114 | 80.0% | 7 | K.SYELPDGQVITIGNER.F | 222 |
|  | Astrin\_STLC\_112116\_tube2\_02.09255.09255.2 | 3.8437 | 0.5455 | 100.0% | 2215.2522 | 2216.4705 | 1 | 9.367 | 47.5% | 2 | K.DLYANTVLSGGTTMYPGIADR.M | 2 |
|  | Astrin\_STLC\_112116\_tube2\_01.06340.06340.1 | 2.3171 | 0.4487 | 100.0% | 1161.5 | 1162.3868 | 22 | 7.087 | 55.0% | 1 | K.EITALAPSTMK.I | 11 |

Similarities:
gi|4501881|ref|NP\_001(5:4)  
gi|63055057|ref|NP\_00(1:8)  

---

|  |  |  |  |  |  |  |  |  |
| --- | --- | --- | --- | --- | --- | --- | --- | --- |
| U | *gi|5729877|ref|NP\_006* | 24 | 51 | 36.1% | 646 | 70898 | 5.5 | heat shock cognate 71 kDa protein isoform 1 [Homo sapiens] |

| Filename XCorr DeltCN Conf% ObsM+H+ CalcM+H+ SpR ZScore Ion% # Sequence  | | | | | | | | | | | | |
| --- | --- | --- | --- | --- | --- | --- | --- | --- | --- | --- | --- | --- |
|  | Astrin\_STLC\_112116\_tube2\_01.07222.07222.2 | 3.2644 | 0.4305 | 100.0% | 1488.9722 | 1488.5939 | 1 | 8.322 | 79.2% | 5 | R.TTPSYVAFTDTER.L | 2222 |
|  | Astrin\_STLC\_112116\_01.06333.06333.2 | 4.5037 | 0.5677 | 100.0% | 1649.9722 | 1650.8468 | 1 | 9.747 | 75.0% | 5 | K.NQVAMNPTNTVFDAK.R | 2 |
|  | Astrin\_STLC\_112116\_tube2\_01.05422.05422.2 | 3.3865 | 0.4261 | 100.0% | 1411.1322 | 1411.5725 | 1 | 7.646 | 86.4% | 2 | R.RFDDAVVQSDMK.H | 2 |
|  | Astrin\_STLC\_112116\_tube2\_01.06090.06090.2 | 3.0324 | 0.4271 | 100.0% | 1255.0521 | 1255.385 | 1 | 7.464 | 80.0% | 1 | R.FDDAVVQSDMK.H | 2 |
|  | Astrin\_STLC\_112116\_01.06993.06993.3 | 3.8494 | 0.341 | 99.8% | 1654.7043 | 1654.9298 | 10 | 6.059 | 40.4% | 2 | K.HWPFMVVNDAGRPK.V | 3 |
|  | Astrin\_STLC\_112116\_tube2\_01.09975.09975.2 | 3.2198 | 0.339 | 100.0% | 1616.2522 | 1617.8542 | 1 | 7.127 | 80.8% | 2 | K.SFYPEEVSSMVLTK.M | 2 |
|  | Astrin\_STLC\_112116\_01.05938.05938.2 | 3.6744 | 0.2846 | 100.0% | 1254.1322 | 1253.4993 | 1 | 5.407 | 85.0% | 3 | K.MKEIAEAYLGK.T | 2 |
|  | Astrin\_STLC\_112116\_tube2\_01.08694.08694.3 | 3.3966 | 0.3811 | 99.8% | 1982.3043 | 1983.1882 | 12 | 6.022 | 32.4% | 1 | K.TVTNAVVTVPAYFNDSQR.Q | 3 |
|  | Astrin\_STLC\_112116\_tube2\_01.08735.08735.2 | 4.0724 | 0.4155 | 100.0% | 1983.7122 | 1983.1882 | 1 | 7.21 | 55.9% | 3 | K.TVTNAVVTVPAYFNDSQR.Q | 2 |
|  | Astrin\_STLC\_112116\_tube2\_01.09259.09259.2 | 3.9717 | 0.4271 | 100.0% | 1661.6322 | 1660.9078 | 1 | 7.009 | 73.3% | 2 | R.IINEPTAAAIAYGLDK.K | 222 |
|  | Astrin\_STLC\_112116\_tube2\_01.08274.08274.2 | 4.3007 | 0.4262 | 100.0% | 1788.2322 | 1789.0819 | 1 | 7.569 | 56.2% | 1 | R.IINEPTAAAIAYGLDKK.V | 2 |
|  | Astrin\_STLC\_112116\_01.05542.05542.2 | 3.8999 | 0.5028 | 100.0% | 1692.0922 | 1692.6958 | 1 | 8.734 | 63.3% | 1 | K.STAGDTHLGGEDFDNR.M | 2 |
|  | Astrin\_STLC\_112116\_01.05529.05529.3 | 2.9354 | 0.4569 | 99.8% | 1692.1144 | 1692.6958 | 3 | 7.052 | 31.7% | 1 | K.STAGDTHLGGEDFDNR.M | 3 |
|  | Astrin\_STLC\_112116\_01.06924.06924.2 | 2.7279 | 0.3959 | 100.0% | 1236.1921 | 1236.4741 | 1 | 6.912 | 66.7% | 2 | R.MVNHFIAEFK.R | 2 |
|  | Astrin\_STLC\_112116\_01.07118.07118.2 | 3.368 | 0.3762 | 100.0% | 1480.9722 | 1481.6511 | 1 | 6.694 | 77.3% | 2 | R.ARFEELNADLFR.G | 2 |
|  | Astrin\_STLC\_112116\_tube2\_01.09636.09636.2 | 3.0623 | 0.3987 | 100.0% | 1254.3121 | 1254.3849 | 1 | 7.082 | 83.3% | 4 | R.FEELNADLFR.G | 2 |
|  | Astrin\_STLC\_112116\_01.06040.06040.2 | 4.3265 | 0.4667 | 100.0% | 1838.3121 | 1839.1019 | 2 | 9.93 | 59.4% | 1 | K.LDKSQIHDIVLVGGSTR.I | 2 |
|  | Astrin\_STLC\_112116\_01.06029.06029.3 | 4.8997 | 0.393 | 100.0% | 1838.6643 | 1839.1019 | 2 | 7.55 | 43.8% | 2 | K.LDKSQIHDIVLVGGSTR.I | 3 |
|  | Astrin\_STLC\_112116\_tube2\_01.06528.06528.2 | 4.8724 | 0.5614 | 100.0% | 1482.1122 | 1482.6798 | 1 | 9.499 | 84.6% | 1 | K.SQIHDIVLVGGSTR.I | 2 |
|  | Astrin\_STLC\_112116\_01.07436.07436.2 | 2.5451 | 0.3283 | 100.0% | 1081.7722 | 1082.2444 | 1 | 5.828 | 81.2% | 4 | K.LLQDFFNGK.E | 22 |
|  | Astrin\_STLC\_112116\_01.07146.07146.2 | 3.6893 | 0.1587 | 99.8% | 1567.1122 | 1566.7972 | 1 | 4.243 | 75.0% | 1 | K.LLQDFFNGKELNK.S | 22 |
|  | Astrin\_STLC\_112116\_tube2\_01.10033.10033.3 | 5.1002 | 0.5418 | 100.0% | 2775.0544 | 2775.9885 | 1 | 9.764 | 31.5% | 1 | K.QTQTFTTYSDNQPGVLIQVYEGER.A | 3 |
| \* | Astrin\_STLC\_112116\_tube2\_01.03580.03580.3 | 3.896 | 0.4066 | 100.0% | 1982.2743 | 1983.2036 | 5 | 6.615 | 38.3% | 2 | R.MVQEAEKYKAEDEKQR.D | 3 |
| \* | Astrin\_STLC\_112116\_01.07067.07067.2 | 3.6718 | 0.4091 | 100.0% | 1304.3522 | 1304.4602 | 4 | 7.39 | 70.0% | 2 | K.NSLESYAFNMK.A | 2 |

Similarities:
gi|16507237|ref|NP\_00(1:23)  
gi|167466173|ref|NP\_0(1:23)  
gi|124256496|ref|NP\_0(2:22)  
gi|34419635|ref|NP\_00(3:21)  

---

|  |  |  |  |  |  |  |  |  |
| --- | --- | --- | --- | --- | --- | --- | --- | --- |
| U | *gi|218505827|ref|NP\_1* | 14 | 61 | 36.1% | 316 | 35438 | 6.3 | small kinetochore-associated protein isoform a [Homo sapiens] &IC SKAP |

| Filename XCorr DeltCN Conf% ObsM+H+ CalcM+H+ SpR ZScore Ion% # Sequence  | | | | | | | | | | | | |
| --- | --- | --- | --- | --- | --- | --- | --- | --- | --- | --- | --- | --- |
|  | Astrin\_STLC\_112116\_tube2\_01.07728.07728.2 | 5.3413 | 0.5068 | 100.0% | 2274.372 | 2275.4802 | 1 | 9.047 | 69.0% | 4 | K.TVYSLQPPSALSGGQPADTQTR.A | 2 |
|  | Astrin\_STLC\_112116\_tube2\_01.07881.07881.3 | 4.2257 | 0.2413 | 99.4% | 2275.5544 | 2275.4802 | 1 | 6.286 | 36.9% | 3 | K.TVYSLQPPSALSGGQPADTQTR.A | 3 |
|  | Astrin\_STLC\_112116\_01.05740.05740.2 | 3.8732 | 0.3104 | 100.0% | 2243.5923 | 2244.507 | 1 | 7.207 | 52.8% | 1 | K.QKSEEELKDKNQLLEAVNK.Q | 2 |
|  | Astrin\_STLC\_112116\_01.05738.05738.3 | 3.4307 | 0.3015 | 99.6% | 2244.0544 | 2244.507 | 6 | 6.0 | 34.7% | 1 | K.QKSEEELKDKNQLLEAVNK.Q | 3 |
|  | Astrin\_STLC\_112116\_01.05874.05874.2 | 5.1781 | 0.5108 | 100.0% | 1986.7122 | 1988.2023 | 1 | 9.019 | 71.9% | 1 | K.SEEELKDKNQLLEAVNK.Q | 2 |
|  | Astrin\_STLC\_112116\_01.05867.05867.3 | 4.5757 | 0.4399 | 100.0% | 1988.0643 | 1988.2023 | 1 | 7.645 | 39.1% | 3 | K.SEEELKDKNQLLEAVNK.Q | 3 |
|  | Astrin\_STLC\_112116\_tube2\_01.05895.05895.2 | 4.0743 | 0.3268 | 100.0% | 1604.9722 | 1604.7979 | 1 | 5.863 | 65.4% | 2 | K.LTETQGELKDLTQK.V | 2 |
|  | Astrin\_STLC\_112116\_tube2\_01.09710.09710.2 | 5.1283 | 0.4236 | 100.0% | 2315.4722 | 2316.6543 | 1 | 7.992 | 52.6% | 1 | K.LTETQGELKDLTQKVELLEK.F | 2 |
|  | Astrin\_STLC\_112116\_01.08216.08216.3 | 5.4307 | 0.3701 | 100.0% | 2316.0842 | 2316.6543 | 1 | 6.742 | 44.7% | 19 | K.LTETQGELKDLTQKVELLEK.F | 3 |
|  | Astrin\_STLC\_112116\_tube2\_01.07608.07608.2 | 3.4211 | 0.2508 | 100.0% | 1316.2522 | 1316.5376 | 1 | 5.621 | 80.0% | 2 | K.DLTQKVELLEK.F | 2 |
|  | Astrin\_STLC\_112116\_01.06921.06921.2 | 4.2552 | 0.5717 | 100.0% | 1387.1522 | 1387.5327 | 1 | 10.032 | 76.9% | 19 | K.GLDPALGSETLASR.Q | 2 |
|  | Astrin\_STLC\_112116\_01.11474.11474.2 | 3.3123 | 0.5208 | 100.0% | 2591.3523 | 2592.8928 | 1 | 8.214 | 38.1% | 1 | R.QESTTDHMDSMLLLETLQEELK.L | 2 |
|  | Astrin\_STLC\_112116\_tube2\_02.11896.11896.3 | 4.5252 | 0.3349 | 99.8% | 3395.3943 | 3396.8062 | 1 | 6.404 | 27.7% | 2 | R.QESTTDHMDSMLLLETLQEELKLFNETAK.K | 3 |
| \* | Astrin\_STLC\_112116\_01.05715.05715.2 | 3.3695 | 0.2565 | 100.0% | 1218.0922 | 1218.454 | 10 | 4.939 | 72.2% | 2 | K.KQMEELQALK.V | 2 |

---

|  |  |  |  |  |  |  |  |  |
| --- | --- | --- | --- | --- | --- | --- | --- | --- |
| U | *gi|38016907|ref|NP\_93* | 3 | 9 | 35.8% | 123 | 13475 | 8.0 | erythrocyte band 7 integral membrane protein isoform b [Homo sapiens] |
| U | *gi|38016911|ref|NP\_00* | 3 | 9 | 15.3% | 288 | 31731 | 7.9 | erythrocyte band 7 integral membrane protein isoform a [Homo sapiens] |

| Filename XCorr DeltCN Conf% ObsM+H+ CalcM+H+ SpR ZScore Ion% # Sequence  | | | | | | | | | | | | |
| --- | --- | --- | --- | --- | --- | --- | --- | --- | --- | --- | --- | --- |
|  | Astrin\_STLC\_112116\_01.04952.04952.2 | 3.3167 | 0.422 | 100.0% | 1247.9722 | 1248.3966 | 1 | 7.422 | 77.3% | 4 | K.VIAAEGEMNASR.A | 2 |
|  | Astrin\_STLC\_112116\_tube2\_01.07894.07894.2 | 2.3124 | 0.3449 | 99.5% | 1351.9722 | 1352.5707 | 10 | 4.837 | 59.1% | 1 | R.YLQTLTTIAAEK.N | 2 |
|  | Astrin\_STLC\_112116\_tube2\_01.15144.15144.2 | 3.6184 | 0.4188 | 100.0% | 2127.5322 | 2128.5781 | 1 | 6.774 | 50.0% | 4 | K.NSTIVFPLPIDMLQGIIGAK.H | 2 |

---

|  |  |  |  |  |  |  |  |  |
| --- | --- | --- | --- | --- | --- | --- | --- | --- |
| U | *gi|119703753|ref|NP\_0* | 17 | 32 | 35.6% | 564 | 60067 | 8.0 | keratin, type II cytoskeletal 6B [Homo sapiens] |

| Filename XCorr DeltCN Conf% ObsM+H+ CalcM+H+ SpR ZScore Ion% # Sequence  | | | | | | | | | | | | |
| --- | --- | --- | --- | --- | --- | --- | --- | --- | --- | --- | --- | --- |
|  | Astrin\_STLC\_112116\_tube2\_01.06037.06037.2 | 2.3806 | 0.3142 | 99.6% | 1027.0122 | 1027.1222 | 2 | 5.743 | 66.7% | 1 | R.SGFSSISVSR.S | 22 |
|  | Astrin\_STLC\_112116\_01.06138.06138.2 | 2.671 | 0.1258 | 96.8% | 1082.1522 | 1083.2755 | 1 | 5.557 | 81.2% | 1 | K.FASFIDKVR.F | 22222222 |
|  | Astrin\_STLC\_112116\_tube2\_01.07809.07809.2 | 3.0691 | 0.323 | 100.0% | 1203.3121 | 1204.3684 | 1 | 6.71 | 77.8% | 2 | K.WTLLQEQGTK.T | 2222 |
| \* | Astrin\_STLC\_112116\_tube2\_01.10420.10420.3 | 4.3946 | 0.3851 | 100.0% | 2496.5645 | 2495.7668 | 1 | 6.191 | 32.9% | 1 | R.NMQDLVEDLKNKYEDEINKR.T | 3 |
|  | Astrin\_STLC\_112116\_tube2\_01.05492.05492.2 | 3.3041 | 0.3582 | 100.0% | 1351.4321 | 1351.5425 | 1 | 6.676 | 77.3% | 1 | R.TAAENEFVTLKK.D | 22222 |
|  | Astrin\_STLC\_112116\_tube2\_01.06933.06933.2 | 3.9229 | 0.1151 | 99.1% | 1695.1921 | 1695.9281 | 1 | 7.073 | 75.0% | 1 | K.DVDAAYMNKVELQAK.A | 2222 |
|  | Astrin\_STLC\_112116\_tube2\_01.10418.10418.2 | 3.1329 | 0.2102 | 99.6% | 1410.2522 | 1408.551 | 1 | 4.322 | 72.7% | 2 | K.ADTLTDEINFLR.A | 2222 |
|  | Astrin\_STLC\_112116\_tube2\_02.09447.09447.3 | 3.5552 | 0.2195 | 97.7% | 3040.3743 | 3040.3467 | 184 | 4.203 | 22.1% | 1 | R.ALYDAELSQMQTHISDTSVVLSMDNNR.N | 333 |
|  | Astrin\_STLC\_112116\_tube2\_01.11450.11450.2 | 4.018 | 0.3758 | 100.0% | 1329.9722 | 1330.5211 | 1 | 7.458 | 86.4% | 3 | R.NLDLDSIIAEVK.A | 22222222 |
|  | Astrin\_STLC\_112116\_tube2\_01.04497.04497.2 | 3.6681 | 0.3308 | 100.0% | 1456.0721 | 1456.5547 | 1 | 6.189 | 68.2% | 1 | R.SRAEAESWYQTK.Y | 22222 |
|  | Astrin\_STLC\_112116\_tube2\_01.06387.06387.2 | 3.8574 | 0.3511 | 100.0% | 1181.6322 | 1180.303 | 1 | 6.597 | 83.3% | 9 | K.YEELQITAGR.H | 222 |
|  | Astrin\_STLC\_112116\_tube2\_01.07167.07167.2 | 3.8773 | 0.3103 | 100.0% | 1359.1721 | 1358.5345 | 1 | 5.962 | 81.8% | 2 | K.NKLEGLEDALQK.A | 2222 |
|  | Astrin\_STLC\_112116\_tube2\_01.08126.08126.3 | 2.8284 | 0.1968 | 95.4% | 1508.1244 | 1508.8163 | 1 | 5.635 | 54.5% | 1 | R.LLKEYQELMNVK.L | 3333 |
|  | Astrin\_STLC\_112116\_tube2\_01.08129.08129.2 | 3.9836 | 0.4378 | 100.0% | 1508.5521 | 1508.8163 | 1 | 7.33 | 81.8% | 1 | R.LLKEYQELMNVK.L | 2222 |
|  | Astrin\_STLC\_112116\_tube2\_01.06513.06513.2 | 2.4157 | 0.1475 | 95.4% | 1153.7722 | 1154.3234 | 1 | 4.653 | 75.0% | 1 | K.EYQELMNVK.L | 22222 |
|  | Astrin\_STLC\_112116\_tube2\_01.09346.09346.2 | 3.4818 | 0.346 | 100.0% | 1264.4122 | 1264.4644 | 1 | 7.748 | 80.0% | 3 | K.LALDVEIATYR.K | 222222222 |
|  | Astrin\_STLC\_112116\_tube2\_01.04602.04602.2 | 3.9986 | 0.5265 | 100.0% | 1437.0521 | 1436.562 | 1 | 8.543 | 78.1% | 1 | R.ATGGGLSSVGGGSSTIK.Y | 22 |

Similarities:
contaminant\_KERATIN21(11:6)  
gi|119395750|ref|NP\_0(1:16)  
contaminant\_KERATIN20(2:15)  
contaminant\_KERATIN13(1:16)  
contaminant\_KERATIN18(15:2)  
gi|5031839|ref|NP\_005(12:5)  
contaminant\_KERATIN22(3:14)  
gi|119395754|ref|NP\_0(4:13)  
contaminant\_KERATIN19(1:16)  
gi|153791158|ref|NP\_0(3:14)  
gi|109148552|ref|NP\_4(3:14)  
gi|32567786|ref|NP\_78(2:15)  

---

|  |  |  |  |  |  |  |  |  |
| --- | --- | --- | --- | --- | --- | --- | --- | --- |
| U | *contaminant\_KERATIN13* | 32 | 100 | 34.5% | 643 | 65494 | 6.6 | no description |

| Filename XCorr DeltCN Conf% ObsM+H+ CalcM+H+ SpR ZScore Ion% # Sequence  | | | | | | | | | | | | |
| --- | --- | --- | --- | --- | --- | --- | --- | --- | --- | --- | --- | --- |
|  | Astrin\_STLC\_112116\_tube2\_01.09442.09442.2 | 3.7161 | 0.4548 | 100.0% | 1384.0521 | 1384.5315 | 1 | 7.922 | 81.8% | 8 | K.SLNNQFASFIDK.V | 22 |
|  | Astrin\_STLC\_112116\_tube2\_01.09971.09971.2 | 3.8673 | 0.4538 | 100.0% | 1639.1921 | 1639.8516 | 1 | 7.901 | 69.2% | 4 | K.SLNNQFASFIDKVR.F | 22 |
|  | Astrin\_STLC\_112116\_tube2\_01.06129.06129.2 | 4.5739 | 0.073 | 100.0% | 1475.7922 | 1476.6726 | 2 | 7.553 | 86.4% | 9 | R.FLEQQNQVLQTK.W | 222 |
|  | Astrin\_STLC\_112116\_tube2\_01.10258.10258.3 | 5.2837 | 0.4703 | 100.0% | 2932.7344 | 2934.2786 | 1 | 7.453 | 35.9% | 2 | R.FLEQQNQVLQTKWELLQQVDTSTR.T | 33 |
|  | Astrin\_STLC\_112116\_tube2\_01.09129.09129.2 | 4.2322 | 0.4964 | 100.0% | 1475.7922 | 1476.6293 | 1 | 8.564 | 81.8% | 15 | K.WELLQQVDTSTR.T | 22 |
|  | Astrin\_STLC\_112116\_tube2\_01.11817.11817.2 | 4.3979 | 0.5187 | 100.0% | 1994.3322 | 1995.2017 | 1 | 9.062 | 76.7% | 3 | R.THNLEPYFESFINNLR.R | 22 |
|  | Astrin\_STLC\_112116\_tube2\_01.11847.11847.3 | 4.9294 | 0.3858 | 100.0% | 1995.5044 | 1995.2017 | 1 | 6.843 | 56.7% | 3 | R.THNLEPYFESFINNLR.R | 33 |
|  | Astrin\_STLC\_112116\_tube2\_01.11117.11117.2 | 2.8864 | 0.2933 | 99.8% | 2150.172 | 2151.3892 | 1 | 5.344 | 53.1% | 1 | R.THNLEPYFESFINNLRR.G | 22 |
|  | Astrin\_STLC\_112116\_tube2\_01.11064.11064.3 | 3.4024 | 0.2482 | 99.4% | 2151.3542 | 2151.3892 | 4 | 5.721 | 35.9% | 1 | R.THNLEPYFESFINNLRR.G | 33 |
|  | Astrin\_STLC\_112116\_tube2\_01.07335.07335.2 | 3.2605 | 0.404 | 100.0% | 1300.4521 | 1301.4316 | 1 | 7.365 | 77.8% | 1 | K.NMQDMVEDYR.N | 22 |
|  | Astrin\_STLC\_112116\_tube2\_01.06784.06784.2 | 3.4701 | 0.4576 | 100.0% | 1265.9321 | 1266.3934 | 1 | 7.541 | 85.0% | 5 | R.TNAENEFVTIK.K | 22 |
|  | Astrin\_STLC\_112116\_tube2\_01.05125.05125.3 | 3.0508 | 0.3458 | 99.8% | 1393.8243 | 1394.5675 | 1 | 5.669 | 43.2% | 2 | R.TNAENEFVTIKK.D | 33 |
|  | Astrin\_STLC\_112116\_tube2\_01.05197.05197.2 | 3.5145 | 0.2883 | 100.0% | 1395.0922 | 1394.5675 | 3 | 5.84 | 68.2% | 3 | R.TNAENEFVTIKK.D | 22 |
|  | Astrin\_STLC\_112116\_01.04029.04029.2 | 3.21 | 0.4634 | 100.0% | 1340.4321 | 1341.4607 | 1 | 7.831 | 86.4% | 1 | K.SKAEAESLYQSK.Y | 22 |
|  | Astrin\_STLC\_112116\_tube2\_02.07969.07969.3 | 5.8989 | 0.4718 | 100.0% | 2501.0645 | 2502.7405 | 1 | 8.807 | 44.0% | 3 | K.SKAEAESLYQSKYEELQITAGR.H | 33 |
|  | Astrin\_STLC\_112116\_01.04421.04421.2 | 2.9471 | 0.4197 | 100.0% | 1126.1322 | 1126.2084 | 1 | 6.888 | 83.3% | 2 | K.AEAESLYQSK.Y | 22 |
|  | Astrin\_STLC\_112116\_tube2\_01.06387.06387.2 | 3.8574 | 0.3511 | 100.0% | 1181.6322 | 1180.303 | 1 | 6.597 | 83.3% | 9 | K.YEELQITAGR.H | 222 |
|  | Astrin\_STLC\_112116\_tube2\_01.05228.05228.2 | 3.4414 | 0.3673 | 100.0% | 1302.8522 | 1303.4581 | 1 | 6.323 | 80.0% | 2 | R.NSKIEISELNR.V | 22 |
|  | Astrin\_STLC\_112116\_tube2\_01.06183.06183.2 | 2.735 | 0.1721 | 99.1% | 973.8722 | 974.102 | 1 | 4.527 | 85.7% | 2 | K.IEISELNR.V | 222 |
|  | Astrin\_STLC\_112116\_tube2\_01.07315.07315.2 | 3.8389 | 0.5429 | 100.0% | 1716.2722 | 1717.8333 | 1 | 9.232 | 67.9% | 1 | K.QISNLQQSISDAEQR.G | 22 |
|  | Astrin\_STLC\_112116\_tube2\_01.09578.09578.3 | 3.7777 | 0.4415 | 100.0% | 2643.2644 | 2644.8582 | 1 | 7.523 | 32.6% | 1 | K.QISNLQQSISDAEQRGENALKDAK.N | 33 |
|  | Astrin\_STLC\_112116\_tube2\_01.08272.08272.2 | 4.8409 | 0.476 | 100.0% | 1600.2122 | 1600.769 | 1 | 8.096 | 80.8% | 2 | K.NKLNDLEDALQQAK.E | 22 |
|  | Astrin\_STLC\_112116\_tube2\_01.08279.08279.3 | 4.3061 | 0.312 | 99.8% | 1600.7943 | 1600.769 | 1 | 6.056 | 53.8% | 1 | K.NKLNDLEDALQQAK.E | 33 |
|  | Astrin\_STLC\_112116\_tube2\_01.08034.08034.2 | 3.9419 | 0.2882 | 100.0% | 1359.0122 | 1358.4912 | 1 | 6.729 | 86.4% | 1 | K.LNDLEDALQQAK.E | 22 |
|  | Astrin\_STLC\_112116\_tube2\_01.07372.07372.2 | 3.7594 | 0.3157 | 100.0% | 1523.9922 | 1524.7754 | 1 | 5.893 | 77.3% | 2 | R.LLRDYQELMNTK.L | 22 |
|  | Astrin\_STLC\_112116\_tube2\_01.07431.07431.3 | 3.7261 | 0.2294 | 99.6% | 1524.6843 | 1524.7754 | 10 | 5.487 | 45.5% | 3 | R.LLRDYQELMNTK.L | 33 |
|  | Astrin\_STLC\_112116\_tube2\_01.06219.06219.2 | 2.6926 | 0.4107 | 100.0% | 1141.7722 | 1142.2689 | 7 | 7.095 | 68.8% | 1 | R.DYQELMNTK.L | 22 |
|  | Astrin\_STLC\_112116\_01.04743.04743.1 | 1.9357 | 0.3919 | 100.0% | 1033.26 | 1034.1112 | 96 | 6.034 | 50.0% | 1 | R.TLLEGEESR.M | 11 |
|  | Astrin\_STLC\_112116\_01.04742.04742.2 | 2.4529 | 0.2423 | 99.0% | 1033.9321 | 1034.1112 | 3 | 4.968 | 68.8% | 1 | R.TLLEGEESR.M | 22 |
|  | Astrin\_STLC\_112116\_tube2\_01.03948.03948.2 | 5.2399 | 0.5939 | 100.0% | 2384.1921 | 2385.298 | 1 | 11.598 | 41.7% | 5 | R.GGGGGGYGSGGSSYGSGGGSYGSGGGGGGGR.G | 22 |
|  | Astrin\_STLC\_112116\_tube2\_01.03942.03942.3 | 7.741 | 0.5156 | 100.0% | 2385.1443 | 2385.298 | 1 | 11.851 | 35.8% | 4 | R.GGGGGGYGSGGSSYGSGGGSYGSGGGGGGGR.G | 33 |
| \* | Astrin\_STLC\_112116\_tube2\_02.09055.09055.2 | 2.6637 | 0.1638 | 95.1% | 1819.1322 | 1818.7141 | 2 | 4.056 | 38.1% | 1 | R.GSGGGSSGGSSGGRGS\*SSGGVK@.S | 2 |

Similarities:
gi|119395750|ref|NP\_0(31:1)  
gi|119703753|ref|NP\_0(1:31)  
contaminant\_KERATIN22(2:30)  

---

|  |  |  |  |  |  |  |  |  |
| --- | --- | --- | --- | --- | --- | --- | --- | --- |
| U | *gi|5174735|ref|NP\_006* | 11 | 49 | 34.4% | 445 | 49831 | 4.9 | tubulin beta-4B chain [Homo sapiens] |

| Filename XCorr DeltCN Conf% ObsM+H+ CalcM+H+ SpR ZScore Ion% # Sequence  | | | | | | | | | | | | |
| --- | --- | --- | --- | --- | --- | --- | --- | --- | --- | --- | --- | --- |
| \* | Astrin\_STLC\_112116\_tube2\_01.05820.05820.2 | 2.7052 | 0.2283 | 98.9% | 1329.3121 | 1329.4521 | 1 | 5.071 | 68.2% | 1 | R.INVYYNEATGGK.Y | 2 |
|  | Astrin\_STLC\_112116\_tube2\_01.08958.08958.2 | 4.0146 | 0.4372 | 100.0% | 1602.1721 | 1602.8431 | 1 | 8.501 | 64.3% | 2 | R.AVLVDLEPGTMDSVR.S | 2 |
|  | Astrin\_STLC\_112116\_01.09959.09959.3 | 7.2157 | 0.4557 | 100.0% | 2799.9543 | 2800.0647 | 1 | 8.259 | 44.0% | 16 | R.SGPFGQIFRPDNFVFGQSGAGNNWAK.G | 33 |
|  | Astrin\_STLC\_112116\_01.09293.09293.3 | 3.3494 | 0.3601 | 99.8% | 2087.9644 | 2088.325 | 2 | 6.356 | 36.1% | 3 | K.GHYTEGAELVDSVLDVVRK.E | 33 |
|  | Astrin\_STLC\_112116\_01.07034.07034.2 | 4.1256 | 0.3836 | 100.0% | 1320.3722 | 1320.5896 | 1 | 7.509 | 72.7% | 4 | R.IMNTFSVVPSPK.V | 22 |
|  | Astrin\_STLC\_112116\_01.07324.07324.2 | 3.8024 | 0.4427 | 100.0% | 1145.4321 | 1144.4204 | 1 | 7.812 | 83.3% | 6 | K.LAVNMVPFPR.L | 22 |
|  | Astrin\_STLC\_112116\_tube2\_01.10873.10873.2 | 4.1615 | 0.3869 | 100.0% | 1622.2122 | 1621.9403 | 1 | 7.766 | 80.8% | 5 | R.LHFFMPGFAPLTSR.G | 22 |
|  | Astrin\_STLC\_112116\_01.08746.08746.2 | 2.2652 | 0.271 | 98.9% | 1041.2322 | 1040.2505 | 18 | 5.083 | 68.8% | 2 | R.YLTVAAVFR.G | 22 |
|  | Astrin\_STLC\_112116\_tube2\_01.05990.05990.2 | 4.3025 | 0.3035 | 100.0% | 1446.7722 | 1447.6031 | 9 | 6.202 | 68.2% | 2 | K.EVDEQMLNVQNK.N | 22 |
|  | Astrin\_STLC\_112116\_01.09240.09240.2 | 4.4601 | 0.4452 | 100.0% | 1698.1122 | 1697.8877 | 1 | 8.384 | 73.1% | 2 | K.NSSYFVEWIPNNVK.T | 22 |
|  | Astrin\_STLC\_112116\_tube2\_01.09413.09413.2 | 3.5833 | 0.3694 | 100.0% | 1231.1122 | 1230.4241 | 1 | 5.862 | 83.3% | 6 | R.ISEQFTAMFR.R | 22 |

Similarities:
gi|29788785|ref|NP\_82(9:2)  

---

|  |  |  |  |  |  |  |  |  |
| --- | --- | --- | --- | --- | --- | --- | --- | --- |
| U | *gi|34098946|ref|NP\_00* | 7 | 13 | 33.6% | 324 | 35924 | 9.9 | nuclease-sensitive element-binding protein 1 [Homo sapiens] |

| Filename XCorr DeltCN Conf% ObsM+H+ CalcM+H+ SpR ZScore Ion% # Sequence  | | | | | | | | | | | | |
| --- | --- | --- | --- | --- | --- | --- | --- | --- | --- | --- | --- | --- |
|  | Astrin\_STLC\_112116\_02.07159.07159.2 | 4.7364 | 0.5548 | 100.0% | 1797.1522 | 1796.8822 | 1 | 9.886 | 68.8% | 2 | R.SVGDGETVEFDVVEGEK.G | 2 |
| \* | Astrin\_STLC\_112116\_02.07579.07579.3 | 5.0202 | 0.5018 | 100.0% | 3473.6042 | 3474.7168 | 1 | 8.439 | 22.9% | 1 | R.SVGDGETVEFDVVEGEKGAEAANVTGPGGVPVQGSK.Y | 3 |
| \* | Astrin\_STLC\_112116\_tube2\_01.04560.04560.2 | 4.8595 | 0.5046 | 100.0% | 1696.0521 | 1696.8577 | 1 | 9.394 | 61.1% | 2 | K.GAEAANVTGPGGVPVQGSK.Y | 2 |
| \* | Astrin\_STLC\_112116\_01.04114.04114.3 | 6.0986 | 0.5093 | 100.0% | 3258.1743 | 3259.2566 | 1 | 9.02 | 33.9% | 2 | R.NYQQNYQNSESGEKNEGSESAPEGQAQQR.R | 3 |
| \* | Astrin\_STLC\_112116\_01.03654.03654.3 | 4.7891 | 0.3581 | 100.0% | 2628.6243 | 2629.5835 | 1 | 6.824 | 38.6% | 1 | R.EDGNEEDKENQGDETQGQQPPQR.R | 3 |
| \* | Astrin\_STLC\_112116\_01.03594.03594.3 | 2.8056 | 0.408 | 99.7% | 2784.4744 | 2785.771 | 1 | 5.874 | 29.3% | 2 | R.EDGNEEDKENQGDETQGQQPPQRR.Y | 3 |
| \* | Astrin\_STLC\_112116\_01.04265.04265.2 | 3.4456 | 0.5384 | 100.0% | 1898.0721 | 1898.8914 | 10 | 8.864 | 39.5% | 3 | K.AADPPAENSSAPEAEQGGAE.- | 2 |

---

|  |  |  |  |  |  |  |  |  |
| --- | --- | --- | --- | --- | --- | --- | --- | --- |
| U | *gi|62414289|ref|NP\_00* | 15 | 16 | 33.3% | 466 | 53652 | 5.1 | vimentin [Homo sapiens] |

| Filename XCorr DeltCN Conf% ObsM+H+ CalcM+H+ SpR ZScore Ion% # Sequence  | | | | | | | | | | | | |
| --- | --- | --- | --- | --- | --- | --- | --- | --- | --- | --- | --- | --- |
| \* | Astrin\_STLC\_112116\_tube2\_01.06057.06057.2 | 3.9442 | 0.5227 | 100.0% | 1428.9922 | 1429.5724 | 1 | 8.576 | 76.9% | 1 | R.SLYASSPGGVYATR.S | 2 |
| \* | Astrin\_STLC\_112116\_02.10193.10193.2 | 3.2113 | 0.4291 | 100.0% | 2126.7922 | 2127.3557 | 16 | 6.693 | 33.3% | 1 | R.LLQDSVDFSLADAINTEFK.N | 2 |
|  | Astrin\_STLC\_112116\_01.05765.05765.2 | 3.6259 | 0.3997 | 100.0% | 1588.1322 | 1588.7147 | 1 | 6.857 | 79.2% | 1 | R.TNEKVELQELNDR.F | 2 |
|  | Astrin\_STLC\_112116\_tube2\_01.05355.05355.3 | 3.4698 | 0.3155 | 99.8% | 1589.0944 | 1588.7147 | 4 | 5.532 | 43.8% | 1 | R.TNEKVELQELNDR.F | 3 |
| \* | Astrin\_STLC\_112116\_tube2\_01.05799.05799.2 | 1.9794 | 0.2436 | 95.1% | 1126.2122 | 1126.3005 | 260 | 4.418 | 56.2% | 1 | R.FANYIDKVR.F | 2 |
| \* | Astrin\_STLC\_112116\_tube2\_01.08598.08598.2 | 2.8143 | 0.3746 | 100.0% | 1539.4521 | 1540.8436 | 1 | 6.528 | 69.2% | 1 | K.ILLAELEQLKGQGK.S | 2 |
| \* | Astrin\_STLC\_112116\_tube2\_01.07511.07511.2 | 2.7063 | 0.3327 | 100.0% | 1496.9122 | 1498.6508 | 1 | 5.319 | 68.2% | 1 | K.SRLGDLYEEEMR.E | 2 |
| \* | Astrin\_STLC\_112116\_tube2\_01.07081.07081.2 | 2.4909 | 0.3682 | 100.0% | 1255.9922 | 1255.385 | 1 | 5.675 | 72.2% | 1 | R.LGDLYEEEMR.E | 2 |
| \* | Astrin\_STLC\_112116\_tube2\_01.04687.04687.2 | 2.3454 | 0.1487 | 95.7% | 1046.7722 | 1047.2146 | 55 | 3.894 | 64.3% | 1 | K.LQEEMLQR.E | 2 |
| \* | Astrin\_STLC\_112116\_01.07378.07378.2 | 2.5241 | 0.2029 | 96.3% | 1535.2722 | 1534.793 | 60 | 3.524 | 50.0% | 2 | R.KVESLQEEIAFLK.K | 2 |
|  | Astrin\_STLC\_112116\_tube2\_01.07637.07637.2 | 2.3463 | 0.2721 | 98.9% | 1310.1522 | 1310.4056 | 1 | 4.572 | 77.8% | 1 | K.NLQEAEEWYK.S | 2 |
| \* | Astrin\_STLC\_112116\_tube2\_01.05168.05168.2 | 3.3194 | 0.4138 | 100.0% | 1093.6322 | 1094.1692 | 1 | 8.453 | 83.3% | 1 | K.FADLSEAANR.N | 2 |
| \* | Astrin\_STLC\_112116\_tube2\_02.08902.08902.3 | 3.2374 | 0.2188 | 96.8% | 2186.9043 | 2188.33 | 2 | 5.033 | 36.1% | 1 | R.EMEENFAVEAANYQDTIGR.L | 3 |
| \* | Astrin\_STLC\_112116\_02.07873.07873.2 | 5.777 | 0.5783 | 100.0% | 2187.5923 | 2188.33 | 1 | 11.403 | 66.7% | 1 | R.EMEENFAVEAANYQDTIGR.L | 2 |
| \* | Astrin\_STLC\_112116\_01.11214.11214.2 | 2.325 | 0.3213 | 98.9% | 1571.1721 | 1571.8601 | 6 | 5.264 | 53.8% | 1 | R.ISLPLPNFSSLNLR.E | 2 |

---

|  |  |  |  |  |  |  |  |  |
| --- | --- | --- | --- | --- | --- | --- | --- | --- |
| U | *gi|20357599|ref|NP\_61* | 2 | 4 | 33.3% | 114 | 12146 | 10.5 | histone H2A.V isoform 2 [Homo sapiens] |
| U | *gi|6912616|ref|NP\_036* | 2 | 4 | 29.7% | 128 | 13509 | 10.6 | histone H2A.V isoform 1 [Homo sapiens] |
| U | *gi|4504255|ref|NP\_002* | 2 | 4 | 29.7% | 128 | 13553 | 10.6 | histone H2A.Z [Homo sapiens] |

| Filename XCorr DeltCN Conf% ObsM+H+ CalcM+H+ SpR ZScore Ion% # Sequence  | | | | | | | | | | | | |
| --- | --- | --- | --- | --- | --- | --- | --- | --- | --- | --- | --- | --- |
|  | Astrin\_STLC\_112116\_tube2\_01.07633.07633.2 | 2.54 | 0.345 | 100.0% | 945.33215 | 945.1093 | 131 | 6.211 | 62.5% | 2 | R.AGLQFPVGR.I | 222 |
|  | Astrin\_STLC\_112116\_01.15428.15428.3 | 5.8982 | 0.5232 | 100.0% | 2896.9443 | 2897.2952 | 5 | 9.75 | 25.9% | 2 | R.VGATAAVYSAAILEYLTAEVLELAGNASK.D | 3 |

Similarities:
gi|10800130|ref|NP\_06(1:1)  
gi|106775678|ref|NP\_0(1:1)  

---

|  |  |  |  |  |  |  |  |  |
| --- | --- | --- | --- | --- | --- | --- | --- | --- |
| U | *contaminant\_KERATIN12* | 16 | 24 | 33.2% | 431 | 47974 | 5.0 | no description |

| Filename XCorr DeltCN Conf% ObsM+H+ CalcM+H+ SpR ZScore Ion% # Sequence  | | | | | | | | | | | | |
| --- | --- | --- | --- | --- | --- | --- | --- | --- | --- | --- | --- | --- |
|  | Astrin\_STLC\_112116\_tube2\_01.05299.05299.2 | 3.2103 | 0.1537 | 99.8% | 1064.5721 | 1065.2578 | 1 | 6.052 | 81.2% | 2 | R.LASYLDKVR.A | 222222 |
|  | Astrin\_STLC\_112116\_tube2\_01.05958.05958.2 | 3.5941 | 0.2573 | 100.0% | 1348.0521 | 1346.4772 | 1 | 5.765 | 77.3% | 1 | R.ALEEANTELEVK.I | 2 |
|  | Astrin\_STLC\_112116\_tube2\_01.10623.10623.2 | 4.6582 | 0.4216 | 100.0% | 2069.132 | 2069.366 | 1 | 7.546 | 55.6% | 1 | K.ILTATVDNANILLQIDNAR.L | 2 |
|  | Astrin\_STLC\_112116\_01.05584.05584.2 | 1.8322 | 0.255 | 95.2% | 807.8722 | 807.8815 | 186 | 5.3 | 66.7% | 1 | R.LAADDFR.T | 2222222 |
|  | Astrin\_STLC\_112116\_tube2\_01.07936.07936.2 | 2.4289 | 0.2874 | 99.2% | 1185.8322 | 1187.3384 | 6 | 4.904 | 65.0% | 1 | R.LSVEADINGLR.R | 2 |
|  | Astrin\_STLC\_112116\_tube2\_01.07209.07209.2 | 3.3704 | 0.3259 | 100.0% | 1186.0521 | 1186.397 | 1 | 6.037 | 83.3% | 1 | R.RVLDELTLAR.A | 2222 |
|  | Astrin\_STLC\_112116\_tube2\_01.07839.07839.2 | 3.2486 | 0.3989 | 100.0% | 1030.0122 | 1030.2096 | 1 | 7.534 | 87.5% | 4 | R.VLDELTLAR.A | 22222 |
|  | Astrin\_STLC\_112116\_01.04197.04197.2 | 2.5639 | 0.369 | 100.0% | 1242.9922 | 1243.3367 | 8 | 6.59 | 66.7% | 1 | K.NHEEEMNALR.G | 22 |
|  | Astrin\_STLC\_112116\_tube2\_01.04705.04705.2 | 2.6519 | 0.2422 | 99.2% | 1438.2122 | 1439.6263 | 15 | 4.49 | 65.0% | 1 | R.ILNEMRDQYEK.M | 22 |
|  | Astrin\_STLC\_112116\_tube2\_01.05884.05884.3 | 4.6205 | 0.4462 | 100.0% | 2104.1943 | 2105.2664 | 1 | 7.923 | 43.1% | 1 | K.TEELNREVATNSELVQSGK.S | 33 |
|  | Astrin\_STLC\_112116\_01.04469.04469.2 | 4.0278 | 0.5084 | 100.0% | 1361.9922 | 1362.4796 | 1 | 9.172 | 75.0% | 1 | R.EVATNSELVQSGK.S | 22 |
|  | Astrin\_STLC\_112116\_tube2\_01.05476.05476.2 | 3.2672 | 0.4215 | 100.0% | 1403.9122 | 1404.4764 | 1 | 6.882 | 62.5% | 1 | K.ASLEGNLAETENR.Y | 2 |
|  | Astrin\_STLC\_112116\_tube2\_01.06467.06467.2 | 3.5426 | 0.3417 | 100.0% | 1381.0922 | 1380.5437 | 2 | 6.215 | 75.0% | 3 | K.TRLEQEIATYR.R | 2222 |
|  | Astrin\_STLC\_112116\_tube2\_01.05638.05638.3 | 3.1897 | 0.3263 | 99.8% | 1535.1543 | 1536.7311 | 1 | 5.56 | 45.5% | 3 | K.TRLEQEIATYRR.L | 3333 |
|  | Astrin\_STLC\_112116\_tube2\_01.04954.04954.2 | 2.7318 | 0.326 | 100.0% | 1122.9922 | 1123.2511 | 1 | 5.932 | 81.2% | 1 | R.LEQEIATYR.R | 22222 |
|  | Astrin\_STLC\_112116\_01.05721.05721.2 | 2.1664 | 0.2992 | 98.6% | 1117.6921 | 1118.2291 | 2 | 5.998 | 66.7% | 1 | R.TIVEEVQDGK.V | 2 |

Similarities:
contaminant\_KERATIN09(1:15)  
gi|24430192|ref|NP\_00(7:9)  
gi|15431310|ref|NP\_00(11:5)  
contaminant\_KERATIN03(2:14)  
contaminant\_KERATIN07(6:10)  
contaminant\_KERATIN10(5:11)  

---

|  |  |  |  |  |  |  |  |  |
| --- | --- | --- | --- | --- | --- | --- | --- | --- |
| U | *gi|156564363|ref|NP\_5* | 13 | 29 | 32.9% | 450 | 49960 | 5.1 | tubulin alpha-3C/D chain [Homo sapiens] |

| Filename XCorr DeltCN Conf% ObsM+H+ CalcM+H+ SpR ZScore Ion% # Sequence  | | | | | | | | | | | | |
| --- | --- | --- | --- | --- | --- | --- | --- | --- | --- | --- | --- | --- |
|  | Astrin\_STLC\_112116\_tube2\_02.09308.09308.2 | 5.6232 | 0.5753 | 100.0% | 2009.0322 | 2009.093 | 1 | 10.785 | 52.6% | 5 | K.TIGGGDDSFNTFFSETGAGK.H | 222 |
|  | Astrin\_STLC\_112116\_tube2\_01.08150.08150.2 | 1.8772 | 0.3713 | 98.0% | 1411.0721 | 1411.6439 | 279 | 6.036 | 45.5% | 1 | R.QLFHPEQLITGK.E | 222 |
|  | Astrin\_STLC\_112116\_tube2\_01.07799.07799.3 | 3.6569 | 0.3544 | 99.8% | 2415.8044 | 2416.6555 | 1 | 6.145 | 31.2% | 2 | R.QLFHPEQLITGKEDAANNYAR.G | 3333 |
|  | Astrin\_STLC\_112116\_01.05955.05955.2 | 3.0877 | 0.1237 | 96.6% | 1719.1921 | 1719.8949 | 10 | 4.661 | 53.8% | 1 | R.NLDIERPTYTNLNR.L | 2222 |
|  | Astrin\_STLC\_112116\_01.11304.11304.2 | 5.0812 | 0.5156 | 100.0% | 2410.672 | 2410.6885 | 1 | 10.197 | 50.0% | 4 | R.FDGALNVDLTEFQTNLVPYPR.I | 2222 |
|  | Astrin\_STLC\_112116\_tube2\_01.09711.09711.2 | 3.8528 | 0.4144 | 100.0% | 1758.1921 | 1758.0703 | 1 | 8.032 | 73.3% | 2 | R.IHFPLATYAPVISAEK.A | 2222 |
|  | Astrin\_STLC\_112116\_01.08092.08092.3 | 3.5914 | 0.2803 | 99.6% | 1759.1344 | 1758.0703 | 12 | 5.388 | 38.3% | 3 | R.IHFPLATYAPVISAEK.A | 3333 |
|  | Astrin\_STLC\_112116\_01.06065.06065.1 | 1.6791 | 0.3806 | 100.0% | 1015.39 | 1016.1827 | 49 | 5.524 | 50.0% | 1 | K.DVNAAIATIK.T | 111 |
|  | Astrin\_STLC\_112116\_tube2\_01.06683.06683.2 | 2.5719 | 0.3262 | 100.0% | 1015.4522 | 1016.1827 | 1 | 6.838 | 72.2% | 3 | K.DVNAAIATIK.T | 222 |
|  | Astrin\_STLC\_112116\_01.07012.07012.2 | 4.5739 | 0.4617 | 100.0% | 1825.3722 | 1826.1027 | 1 | 7.363 | 64.7% | 3 | K.VGINYQPPTVVPGGDLAK.V | 2222 |
|  | Astrin\_STLC\_112116\_02.08574.08574.2 | 2.6993 | 0.2984 | 99.5% | 1865.4321 | 1866.1084 | 2 | 5.253 | 43.8% | 1 | R.AVCMLSNTTAIAEAWAR.L | 222 |
|  | Astrin\_STLC\_112116\_01.05996.05996.2 | 2.5896 | 0.4144 | 100.0% | 1380.8522 | 1381.6324 | 270 | 6.049 | 50.0% | 1 | R.LDHKFDLMYAK.R | 2222 |
|  | Astrin\_STLC\_112116\_01.05997.05997.3 | 3.9875 | 0.2967 | 99.8% | 1382.9043 | 1381.6324 | 4 | 6.138 | 55.0% | 2 | R.LDHKFDLMYAK.R | 3333 |

Similarities:
gi|57013276|ref|NP\_00(12:1)  
gi|14389309|ref|NP\_11(12:1)  
gi|17921989|ref|NP\_00(10:3)  

---

|  |  |  |  |  |  |  |  |  |
| --- | --- | --- | --- | --- | --- | --- | --- | --- |
| U | *contaminant\_KERATIN18* | 16 | 23 | 32.2% | 562 | 59822 | 8.0 | no description |

| Filename XCorr DeltCN Conf% ObsM+H+ CalcM+H+ SpR ZScore Ion% # Sequence  | | | | | | | | | | | | |
| --- | --- | --- | --- | --- | --- | --- | --- | --- | --- | --- | --- | --- |
|  | Astrin\_STLC\_112116\_tube2\_01.06037.06037.2 | 2.3806 | 0.3142 | 99.6% | 1027.0122 | 1027.1222 | 2 | 5.743 | 66.7% | 1 | R.SGFSSISVSR.S | 22 |
|  | Astrin\_STLC\_112116\_01.06138.06138.2 | 2.671 | 0.1258 | 96.8% | 1082.1522 | 1083.2755 | 1 | 5.557 | 81.2% | 1 | K.FASFIDKVR.F | 22222222 |
|  | Astrin\_STLC\_112116\_tube2\_01.07809.07809.2 | 3.0691 | 0.323 | 100.0% | 1203.3121 | 1204.3684 | 1 | 6.71 | 77.8% | 2 | K.WTLLQEQGTK.T | 2222 |
|  | Astrin\_STLC\_112116\_tube2\_01.05492.05492.2 | 3.3041 | 0.3582 | 100.0% | 1351.4321 | 1351.5425 | 1 | 6.676 | 77.3% | 1 | R.TAAENEFVTLKK.D | 22222 |
|  | Astrin\_STLC\_112116\_tube2\_01.06933.06933.2 | 3.9229 | 0.1151 | 99.1% | 1695.1921 | 1695.9281 | 1 | 7.073 | 75.0% | 1 | K.DVDAAYMNKVELQAK.A | 2222 |
|  | Astrin\_STLC\_112116\_tube2\_01.10418.10418.2 | 3.1329 | 0.2102 | 99.6% | 1410.2522 | 1408.551 | 1 | 4.322 | 72.7% | 2 | K.ADTLTDEINFLR.A | 2222 |
|  | Astrin\_STLC\_112116\_tube2\_02.09447.09447.3 | 3.5552 | 0.2195 | 97.7% | 3040.3743 | 3040.3467 | 184 | 4.203 | 22.1% | 1 | R.ALYDAELSQMQTHISDTSVVLSMDNNR.N | 333 |
|  | Astrin\_STLC\_112116\_tube2\_01.11450.11450.2 | 4.018 | 0.3758 | 100.0% | 1329.9722 | 1330.5211 | 1 | 7.458 | 86.4% | 3 | R.NLDLDSIIAEVK.A | 22222222 |
|  | Astrin\_STLC\_112116\_tube2\_01.04497.04497.2 | 3.6681 | 0.3308 | 100.0% | 1456.0721 | 1456.5547 | 1 | 6.189 | 68.2% | 1 | R.SRAEAESWYQTK.Y | 22222 |
|  | Astrin\_STLC\_112116\_tube2\_01.05288.05288.2 | 3.4785 | 0.2191 | 100.0% | 1165.9722 | 1166.2761 | 1 | 6.834 | 88.9% | 1 | K.YEELQVTAGR.H | 2222 |
|  | Astrin\_STLC\_112116\_tube2\_01.07167.07167.2 | 3.8773 | 0.3103 | 100.0% | 1359.1721 | 1358.5345 | 1 | 5.962 | 81.8% | 2 | K.NKLEGLEDALQK.A | 2222 |
|  | Astrin\_STLC\_112116\_tube2\_01.08126.08126.3 | 2.8284 | 0.1968 | 95.4% | 1508.1244 | 1508.8163 | 1 | 5.635 | 54.5% | 1 | R.LLKEYQELMNVK.L | 3333 |
|  | Astrin\_STLC\_112116\_tube2\_01.08129.08129.2 | 3.9836 | 0.4378 | 100.0% | 1508.5521 | 1508.8163 | 1 | 7.33 | 81.8% | 1 | R.LLKEYQELMNVK.L | 2222 |
|  | Astrin\_STLC\_112116\_tube2\_01.06513.06513.2 | 2.4157 | 0.1475 | 95.4% | 1153.7722 | 1154.3234 | 1 | 4.653 | 75.0% | 1 | K.EYQELMNVK.L | 22222 |
|  | Astrin\_STLC\_112116\_tube2\_01.09346.09346.2 | 3.4818 | 0.346 | 100.0% | 1264.4122 | 1264.4644 | 1 | 7.748 | 80.0% | 3 | K.LALDVEIATYR.K | 222222222 |
|  | Astrin\_STLC\_112116\_tube2\_01.04602.04602.2 | 3.9986 | 0.5265 | 100.0% | 1437.0521 | 1436.562 | 1 | 8.543 | 78.1% | 1 | R.ATGGGLSSVGGGSSTIK.Y | 22 |

Similarities:
contaminant\_KERATIN21(12:4)  
contaminant\_KERATIN20(2:14)  
gi|119703753|ref|NP\_0(15:1)  
gi|5031839|ref|NP\_005(13:3)  
contaminant\_KERATIN22(3:13)  
gi|119395754|ref|NP\_0(4:12)  
contaminant\_KERATIN19(1:15)  
gi|153791158|ref|NP\_0(4:12)  
gi|109148552|ref|NP\_4(3:13)  
gi|32567786|ref|NP\_78(2:14)  

---

|  |  |  |  |  |  |  |  |  |
| --- | --- | --- | --- | --- | --- | --- | --- | --- |
| U | *gi|34932414|ref|NP\_03* | 13 | 28 | 32.1% | 471 | 54232 | 8.9 | non-POU domain-containing octamer-binding protein isoform 1 [Homo sapiens] |

| Filename XCorr DeltCN Conf% ObsM+H+ CalcM+H+ SpR ZScore Ion% # Sequence  | | | | | | | | | | | | |
| --- | --- | --- | --- | --- | --- | --- | --- | --- | --- | --- | --- | --- |
| \* | Astrin\_STLC\_112116\_tube2\_01.09779.09779.2 | 3.4822 | 0.4231 | 100.0% | 1859.9521 | 1861.12 | 1 | 6.559 | 60.0% | 1 | R.LFVGNLPPDITEEEMR.K | 2 |
|  | Astrin\_STLC\_112116\_tube2\_01.09737.09737.3 | 4.2329 | 0.3981 | 100.0% | 1999.4944 | 1999.3765 | 1 | 6.626 | 42.6% | 1 | R.TLAEIAKVELDNMPLRGK.Q | 3 |
|  | Astrin\_STLC\_112116\_tube2\_01.07290.07290.2 | 2.5147 | 0.2926 | 99.8% | 1086.8722 | 1087.2793 | 3 | 5.494 | 81.2% | 2 | K.VELDNMPLR.G | 2 |
|  | Astrin\_STLC\_112116\_01.14882.14882.3 | 4.6589 | 0.4574 | 100.0% | 2669.8743 | 2669.9507 | 1 | 7.296 | 39.8% | 5 | R.NLPQYVSNELLEEAFSVFGQVER.A | 3 |
|  | Astrin\_STLC\_112116\_tube2\_01.15475.15475.2 | 5.6082 | 0.467 | 100.0% | 2670.2322 | 2669.9507 | 1 | 10.347 | 61.4% | 6 | R.NLPQYVSNELLEEAFSVFGQVER.A | 2 |
|  | Astrin\_STLC\_112116\_01.04916.04916.2 | 2.0201 | 0.2196 | 95.3% | 887.47217 | 887.0238 | 1 | 6.866 | 78.6% | 1 | R.AVVIVDDR.G | 22 |
|  | Astrin\_STLC\_112116\_01.05699.05699.2 | 2.9827 | 0.2386 | 99.7% | 1232.0521 | 1232.4252 | 2 | 5.704 | 68.2% | 2 | K.GIVEFSGKPAAR.K | 2 |
|  | Astrin\_STLC\_112116\_tube2\_01.08530.08530.2 | 4.1814 | 0.4996 | 100.0% | 1697.1322 | 1696.8744 | 1 | 8.939 | 65.4% | 3 | R.FAQPGSFEYEYAMR.W | 2 |
|  | Astrin\_STLC\_112116\_01.05819.05819.2 | 3.0309 | 0.3936 | 100.0% | 1337.1322 | 1337.5488 | 1 | 6.718 | 75.0% | 1 | R.EKLEMEMEAAR.H | 2 |
|  | Astrin\_STLC\_112116\_tube2\_01.06981.06981.2 | 3.7337 | 0.5031 | 100.0% | 1539.0122 | 1539.8441 | 1 | 7.942 | 64.3% | 2 | R.MGQMAMGGAMGINNR.G | 2 |
|  | Astrin\_STLC\_112116\_02.07988.07988.2 | 5.1373 | 0.5257 | 100.0% | 2163.4521 | 2164.4436 | 1 | 9.676 | 52.4% | 1 | R.FGQAATMEGIGAIGGTPPAFNR.A | 2 |
|  | Astrin\_STLC\_112116\_tube2\_02.09043.09043.3 | 2.9984 | 0.2438 | 96.9% | 2163.5645 | 2164.4436 | 58 | 4.9 | 26.2% | 2 | R.FGQAATMEGIGAIGGTPPAFNR.A | 3 |
|  | Astrin\_STLC\_112116\_01.04319.04319.2 | 2.2273 | 0.2536 | 96.3% | 1229.1921 | 1229.3811 | 5 | 5.451 | 59.1% | 1 | R.AAPGAEFAPNKR.R | 2 |

Similarities:
gi|4826998|ref|NP\_005(1:12)  

---

|  |  |  |  |  |  |  |  |  |
| --- | --- | --- | --- | --- | --- | --- | --- | --- |
| U | *gi|5031839|ref|NP\_005* | 16 | 25 | 30.9% | 564 | 60045 | 8.0 | keratin, type II cytoskeletal 6C [Homo sapiens] |

| Filename XCorr DeltCN Conf% ObsM+H+ CalcM+H+ SpR ZScore Ion% # Sequence  | | | | | | | | | | | | |
| --- | --- | --- | --- | --- | --- | --- | --- | --- | --- | --- | --- | --- |
| \* | Astrin\_STLC\_112116\_tube2\_01.04573.04573.2 | 2.6197 | 0.3552 | 100.0% | 1012.3722 | 1013.0953 | 1 | 5.925 | 77.8% | 2 | R.SGFSSVSVSR.S | 2 |
|  | Astrin\_STLC\_112116\_01.06138.06138.2 | 2.671 | 0.1258 | 96.8% | 1082.1522 | 1083.2755 | 1 | 5.557 | 81.2% | 1 | K.FASFIDKVR.F | 22222222 |
|  | Astrin\_STLC\_112116\_tube2\_01.07809.07809.2 | 3.0691 | 0.323 | 100.0% | 1203.3121 | 1204.3684 | 1 | 6.71 | 77.8% | 2 | K.WTLLQEQGTK.T | 2222 |
|  | Astrin\_STLC\_112116\_tube2\_01.10060.10060.3 | 4.2038 | 0.3861 | 100.0% | 2471.8442 | 2472.732 | 3 | 6.193 | 31.6% | 1 | R.GMQDLVEDFKNKYEDEINKR.T | 33 |
|  | Astrin\_STLC\_112116\_tube2\_01.05492.05492.2 | 3.3041 | 0.3582 | 100.0% | 1351.4321 | 1351.5425 | 1 | 6.676 | 77.3% | 1 | R.TAAENEFVTLKK.D | 22222 |
|  | Astrin\_STLC\_112116\_tube2\_01.06933.06933.2 | 3.9229 | 0.1151 | 99.1% | 1695.1921 | 1695.9281 | 1 | 7.073 | 75.0% | 1 | K.DVDAAYMNKVELQAK.A | 2222 |
|  | Astrin\_STLC\_112116\_tube2\_01.10418.10418.2 | 3.1329 | 0.2102 | 99.6% | 1410.2522 | 1408.551 | 1 | 4.322 | 72.7% | 2 | K.ADTLTDEINFLR.A | 2222 |
|  | Astrin\_STLC\_112116\_tube2\_01.11450.11450.2 | 4.018 | 0.3758 | 100.0% | 1329.9722 | 1330.5211 | 1 | 7.458 | 86.4% | 3 | R.NLDLDSIIAEVK.A | 22222222 |
|  | Astrin\_STLC\_112116\_tube2\_01.04497.04497.2 | 3.6681 | 0.3308 | 100.0% | 1456.0721 | 1456.5547 | 1 | 6.189 | 68.2% | 1 | R.SRAEAESWYQTK.Y | 22222 |
|  | Astrin\_STLC\_112116\_tube2\_01.05288.05288.2 | 3.4785 | 0.2191 | 100.0% | 1165.9722 | 1166.2761 | 1 | 6.834 | 88.9% | 1 | K.YEELQVTAGR.H | 2222 |
|  | Astrin\_STLC\_112116\_tube2\_01.07167.07167.2 | 3.8773 | 0.3103 | 100.0% | 1359.1721 | 1358.5345 | 1 | 5.962 | 81.8% | 2 | K.NKLEGLEDALQK.A | 2222 |
|  | Astrin\_STLC\_112116\_tube2\_01.08126.08126.3 | 2.8284 | 0.1968 | 95.4% | 1508.1244 | 1508.8163 | 1 | 5.635 | 54.5% | 1 | R.LLKEYQELMNVK.L | 3333 |
|  | Astrin\_STLC\_112116\_tube2\_01.08129.08129.2 | 3.9836 | 0.4378 | 100.0% | 1508.5521 | 1508.8163 | 1 | 7.33 | 81.8% | 1 | R.LLKEYQELMNVK.L | 2222 |
|  | Astrin\_STLC\_112116\_tube2\_01.06513.06513.2 | 2.4157 | 0.1475 | 95.4% | 1153.7722 | 1154.3234 | 1 | 4.653 | 75.0% | 1 | K.EYQELMNVK.L | 22222 |
|  | Astrin\_STLC\_112116\_tube2\_01.09346.09346.2 | 3.4818 | 0.346 | 100.0% | 1264.4122 | 1264.4644 | 1 | 7.748 | 80.0% | 3 | K.LALDVEIATYR.K | 222222222 |
|  | Astrin\_STLC\_112116\_tube2\_01.06151.06151.2 | 4.9435 | 0.5404 | 100.0% | 1448.1921 | 1448.6163 | 1 | 9.166 | 75.0% | 2 | R.AIGGGLSSVGGGSSTIK.Y | 22 |

Similarities:
contaminant\_KERATIN21(13:3)  
contaminant\_KERATIN20(2:14)  
gi|119703753|ref|NP\_0(12:4)  
contaminant\_KERATIN18(13:3)  
contaminant\_KERATIN22(3:13)  
gi|119395754|ref|NP\_0(4:12)  
contaminant\_KERATIN19(1:15)  
gi|153791158|ref|NP\_0(4:12)  
gi|109148552|ref|NP\_4(3:13)  
gi|32567786|ref|NP\_78(2:14)  

---

|  |  |  |  |  |  |  |  |  |
| --- | --- | --- | --- | --- | --- | --- | --- | --- |
| U | *gi|222352151|ref|NP\_0* | 7 | 10 | 30.1% | 356 | 37498 | 7.1 | poly(rC)-binding protein 1 [Homo sapiens] |

| Filename XCorr DeltCN Conf% ObsM+H+ CalcM+H+ SpR ZScore Ion% # Sequence  | | | | | | | | | | | | |
| --- | --- | --- | --- | --- | --- | --- | --- | --- | --- | --- | --- | --- |
|  | Astrin\_STLC\_112116\_01.04785.04785.2 | 2.6149 | 0.3304 | 100.0% | 1288.6921 | 1289.3538 | 1 | 6.269 | 70.0% | 1 | R.INISEGNCPER.I | 22 |
| \* | Astrin\_STLC\_112116\_01.08892.08892.2 | 3.2241 | 0.3189 | 100.0% | 1390.4722 | 1389.6781 | 1 | 6.69 | 70.8% | 2 | R.IITLTGPTNAIFK.A | 2 |
| \* | Astrin\_STLC\_112116\_01.11927.11927.3 | 4.3826 | 0.3701 | 99.8% | 3379.5244 | 3380.8562 | 1 | 6.072 | 34.2% | 1 | K.AFAMIIDKLEEDINSSMTNSTAASRPPVTLR.L | 3 |
|  | Astrin\_STLC\_112116\_tube2\_01.07200.07200.2 | 5.4171 | 0.5695 | 100.0% | 2089.9722 | 2091.2573 | 1 | 9.631 | 57.9% | 1 | R.ESTGAQVQVAGDMLPNSTER.A | 22 |
| \* | Astrin\_STLC\_112116\_01.04191.04191.2 | 2.4349 | 0.3014 | 99.3% | 1088.2122 | 1087.1777 | 7 | 4.999 | 65.0% | 1 | K.IANPVEGSSGR.Q | 2 |
| \* | Astrin\_STLC\_112116\_02.09976.09976.3 | 4.8351 | 0.3444 | 99.7% | 2178.1443 | 2178.4937 | 1 | 7.211 | 36.2% | 2 | R.QVTITGSAASISLAQYLINAR.L | 3 |
| \* | Astrin\_STLC\_112116\_02.09935.09935.2 | 5.8485 | 0.5371 | 100.0% | 2178.5122 | 2178.4937 | 1 | 10.526 | 62.5% | 2 | R.QVTITGSAASISLAQYLINAR.L | 2 |

Similarities:
gi|14141166|ref|NP\_11(2:5)  

---

|  |  |  |  |  |  |  |  |  |
| --- | --- | --- | --- | --- | --- | --- | --- | --- |
| U | *gi|167466173|ref|NP\_0* | 15 | 34 | 30.0% | 641 | 70052 | 5.6 | heat shock 70 kDa protein 1B [Homo sapiens] |
| U | *gi|194248072|ref|NP\_0* | 15 | 34 | 30.0% | 641 | 70052 | 5.6 | heat shock 70 kDa protein 1A [Homo sapiens] |

| Filename XCorr DeltCN Conf% ObsM+H+ CalcM+H+ SpR ZScore Ion% # Sequence  | | | | | | | | | | | | |
| --- | --- | --- | --- | --- | --- | --- | --- | --- | --- | --- | --- | --- |
|  | Astrin\_STLC\_112116\_tube2\_01.07222.07222.2 | 3.2644 | 0.4305 | 100.0% | 1488.9722 | 1488.5939 | 1 | 8.322 | 79.2% | 5 | R.TTPSYVAFTDTER.L | 2222 |
|  | Astrin\_STLC\_112116\_tube2\_01.07560.07560.2 | 3.5872 | 0.3447 | 100.0% | 1658.6522 | 1659.8394 | 1 | 7.166 | 71.4% | 1 | K.NQVALNPQNTVFDAK.R | 2 |
|  | Astrin\_STLC\_112116\_tube2\_01.07666.07666.3 | 3.4948 | 0.2066 | 98.6% | 1681.9744 | 1681.8912 | 4 | 4.61 | 38.5% | 1 | K.HWPFQVINDGDKPK.V | 3 |
|  | Astrin\_STLC\_112116\_01.09358.09358.2 | 3.1419 | 0.4076 | 100.0% | 1615.4122 | 1615.8817 | 1 | 6.598 | 73.1% | 3 | K.AFYPEEISSMVLTK.M | 22 |
|  | Astrin\_STLC\_112116\_01.09969.09969.3 | 4.194 | 0.4036 | 100.0% | 3261.6243 | 3262.7046 | 1 | 7.035 | 28.6% | 2 | K.MKEIAEAYLGYPVTNAVITVPAYFNDSQR.Q | 3 |
|  | Astrin\_STLC\_112116\_tube2\_01.09728.09728.2 | 3.5325 | 0.3274 | 100.0% | 1198.2322 | 1198.408 | 1 | 6.836 | 86.4% | 6 | K.DAGVIAGLNVLR.I | 22 |
|  | Astrin\_STLC\_112116\_tube2\_01.09474.09474.3 | 4.1847 | 0.3057 | 99.8% | 1688.9343 | 1688.9213 | 2 | 6.013 | 46.7% | 1 | R.IINEPTAAAIAYGLDR.T | 33 |
|  | Astrin\_STLC\_112116\_tube2\_01.09456.09456.2 | 5.2341 | 0.5236 | 100.0% | 1689.0322 | 1688.9213 | 1 | 9.8 | 80.0% | 6 | R.IINEPTAAAIAYGLDR.T | 22 |
|  | Astrin\_STLC\_112116\_01.05559.05559.2 | 3.8525 | 0.3976 | 100.0% | 1675.8522 | 1676.6964 | 1 | 8.003 | 56.7% | 1 | K.ATAGDTHLGGEDFDNR.L | 222 |
|  | Astrin\_STLC\_112116\_tube2\_01.04581.04581.3 | 3.1587 | 0.2499 | 98.6% | 1676.1543 | 1676.6964 | 126 | 5.668 | 31.7% | 1 | K.ATAGDTHLGGEDFDNR.L | 333 |
|  | Astrin\_STLC\_112116\_tube2\_01.07206.07206.2 | 2.7006 | 0.3866 | 100.0% | 1262.0322 | 1262.4508 | 1 | 6.337 | 66.7% | 2 | R.LVNHFVEEFK.R | 2 |
|  | Astrin\_STLC\_112116\_tube2\_01.07357.07357.3 | 2.7617 | 0.3656 | 99.6% | 1823.0343 | 1823.1025 | 38 | 5.749 | 31.2% | 1 | K.LDKAQIHDLVLVGGSTR.I | 3 |
|  | Astrin\_STLC\_112116\_tube2\_01.09374.09374.2 | 2.8119 | 0.3267 | 100.0% | 1109.8522 | 1110.2578 | 1 | 5.618 | 81.2% | 2 | K.LLQDFFNGR.D | 2 |
|  | Astrin\_STLC\_112116\_01.04437.04437.3 | 3.7834 | 0.3377 | 99.8% | 1953.7144 | 1954.1621 | 1 | 6.536 | 43.3% | 1 | R.MVQEAEKYKAEDEVQR.E | 3 |
|  | Astrin\_STLC\_112116\_tube2\_01.08427.08427.2 | 3.1583 | 0.3764 | 100.0% | 1288.0922 | 1288.4608 | 1 | 6.75 | 80.0% | 1 | K.NALESYAFNMK.S | 22 |

Similarities:
gi|5729877|ref|NP\_006(1:14)  
gi|124256496|ref|NP\_0(6:9)  
gi|34419635|ref|NP\_00(5:10)  

---

|  |  |  |  |  |  |  |  |  |
| --- | --- | --- | --- | --- | --- | --- | --- | --- |
| U | *gi|17921989|ref|NP\_00* | 11 | 26 | 29.5% | 448 | 49924 | 5.1 | tubulin alpha-4A chain isoform 1 [Homo sapiens] |
| U | *gi|514052659|ref|NP\_0* | 11 | 26 | 30.5% | 433 | 48329 | 5.0 | tubulin alpha-4A chain isoform 2 [Homo sapiens] |

| Filename XCorr DeltCN Conf% ObsM+H+ CalcM+H+ SpR ZScore Ion% # Sequence  | | | | | | | | | | | | |
| --- | --- | --- | --- | --- | --- | --- | --- | --- | --- | --- | --- | --- |
|  | Astrin\_STLC\_112116\_tube2\_01.08150.08150.2 | 1.8772 | 0.3713 | 98.0% | 1411.0721 | 1411.6439 | 279 | 6.036 | 45.5% | 1 | R.QLFHPEQLITGK.E | 222 |
|  | Astrin\_STLC\_112116\_tube2\_01.07799.07799.3 | 3.6569 | 0.3544 | 99.8% | 2415.8044 | 2416.6555 | 1 | 6.145 | 31.2% | 2 | R.QLFHPEQLITGKEDAANNYAR.G | 3333 |
|  | Astrin\_STLC\_112116\_01.05955.05955.2 | 3.0877 | 0.1237 | 96.6% | 1719.1921 | 1719.8949 | 10 | 4.661 | 53.8% | 1 | R.NLDIERPTYTNLNR.L | 2222 |
|  | Astrin\_STLC\_112116\_tube2\_01.12602.12602.2 | 4.4411 | 0.4757 | 100.0% | 1489.1921 | 1488.7678 | 1 | 9.088 | 65.4% | 6 | R.LISQIVSSITASLR.F | 222 |
|  | Astrin\_STLC\_112116\_01.11304.11304.2 | 5.0812 | 0.5156 | 100.0% | 2410.672 | 2410.6885 | 1 | 10.197 | 50.0% | 4 | R.FDGALNVDLTEFQTNLVPYPR.I | 2222 |
|  | Astrin\_STLC\_112116\_tube2\_01.09711.09711.2 | 3.8528 | 0.4144 | 100.0% | 1758.1921 | 1758.0703 | 1 | 8.032 | 73.3% | 2 | R.IHFPLATYAPVISAEK.A | 2222 |
|  | Astrin\_STLC\_112116\_01.08092.08092.3 | 3.5914 | 0.2803 | 99.6% | 1759.1344 | 1758.0703 | 12 | 5.388 | 38.3% | 3 | R.IHFPLATYAPVISAEK.A | 3333 |
|  | Astrin\_STLC\_112116\_01.07012.07012.2 | 4.5739 | 0.4617 | 100.0% | 1825.3722 | 1826.1027 | 1 | 7.363 | 64.7% | 3 | K.VGINYQPPTVVPGGDLAK.V | 2222 |
|  | Astrin\_STLC\_112116\_02.08574.08574.2 | 2.6993 | 0.2984 | 99.5% | 1865.4321 | 1866.1084 | 2 | 5.253 | 43.8% | 1 | R.AVCMLSNTTAIAEAWAR.L | 222 |
|  | Astrin\_STLC\_112116\_01.05996.05996.2 | 2.5896 | 0.4144 | 100.0% | 1380.8522 | 1381.6324 | 270 | 6.049 | 50.0% | 1 | R.LDHKFDLMYAK.R | 2222 |
|  | Astrin\_STLC\_112116\_01.05997.05997.3 | 3.9875 | 0.2967 | 99.8% | 1382.9043 | 1381.6324 | 4 | 6.138 | 55.0% | 2 | R.LDHKFDLMYAK.R | 3333 |

Similarities:
gi|57013276|ref|NP\_00(10:1)  
gi|14389309|ref|NP\_11(10:1)  
gi|156564363|ref|NP\_5(10:1)  

---

|  |  |  |  |  |  |  |  |  |
| --- | --- | --- | --- | --- | --- | --- | --- | --- |
| U | *gi|4504301|ref|NP\_003* | 3 | 8 | 29.1% | 103 | 11367 | 11.4 | histone H4 [Homo sapiens] |

| Filename XCorr DeltCN Conf% ObsM+H+ CalcM+H+ SpR ZScore Ion% # Sequence  | | | | | | | | | | | | |
| --- | --- | --- | --- | --- | --- | --- | --- | --- | --- | --- | --- | --- |
| \* | Astrin\_STLC\_112116\_tube2\_01.05146.05146.2 | 2.8649 | 0.2403 | 99.5% | 1325.9321 | 1326.5387 | 1 | 4.99 | 77.3% | 1 | R.DNIQGITKPAIR.R | 2 |
| \* | Astrin\_STLC\_112116\_tube2\_01.07029.07029.2 | 3.7081 | 0.39 | 100.0% | 1181.0922 | 1181.3312 | 1 | 6.606 | 77.8% | 6 | R.ISGLIYEETR.G | 2 |
| \* | Astrin\_STLC\_112116\_tube2\_01.08642.08642.2 | 2.994 | 0.2987 | 100.0% | 989.9122 | 990.19055 | 2 | 5.09 | 85.7% | 1 | K.VFLENVIR.D | 2 |

---

|  |  |  |  |  |  |  |  |  |
| --- | --- | --- | --- | --- | --- | --- | --- | --- |
| U | *gi|36287110|ref|NP\_91* | 9 | 18 | 29.0% | 379 | 40907 | 4.6 | FGFR1 oncogene partner isoform b [Homo sapiens] |
| U | *gi|5901954|ref|NP\_008* | 9 | 18 | 27.6% | 399 | 43065 | 4.8 | FGFR1 oncogene partner isoform a [Homo sapiens] |

| Filename XCorr DeltCN Conf% ObsM+H+ CalcM+H+ SpR ZScore Ion% # Sequence  | | | | | | | | | | | | |
| --- | --- | --- | --- | --- | --- | --- | --- | --- | --- | --- | --- | --- |
|  | Astrin\_STLC\_112116\_02.11623.11623.3 | 3.1922 | 0.2729 | 98.6% | 2164.7944 | 2165.5352 | 2 | 4.678 | 32.5% | 1 | R.DLGIIEAEGTVGGPLLLEVIR.R | 3 |
|  | Astrin\_STLC\_112116\_tube2\_01.13648.13648.2 | 5.8398 | 0.5166 | 100.0% | 2164.8323 | 2165.5352 | 1 | 9.018 | 55.0% | 5 | R.DLGIIEAEGTVGGPLLLEVIR.R | 2 |
|  | Astrin\_STLC\_112116\_01.12158.12158.3 | 3.5473 | 0.3582 | 99.8% | 2321.0942 | 2321.7227 | 1 | 6.763 | 32.1% | 5 | R.DLGIIEAEGTVGGPLLLEVIRR.C | 3 |
|  | Astrin\_STLC\_112116\_tube2\_01.06523.06523.3 | 3.6866 | 0.3549 | 99.8% | 2536.3145 | 2537.57 | 1 | 6.284 | 32.6% | 1 | K.GPTTGEGALDLSDVHS\*PPKS\*PEGK.T | 3 |
|  | Astrin\_STLC\_112116\_tube2\_01.04254.04254.3 | 3.7027 | 0.242 | 99.4% | 2021.0044 | 2021.102 | 1 | 5.174 | 41.7% | 1 | K.KANDEANQSDTSVSLSEPK.S | 3 |
|  | Astrin\_STLC\_112116\_01.05566.05566.2 | 5.1032 | 0.5812 | 100.0% | 1892.0521 | 1892.928 | 1 | 10.153 | 64.7% | 2 | K.ANDEANQSDTSVSLSEPK.S | 2 |
|  | Astrin\_STLC\_112116\_tube2\_01.06143.06143.2 | 2.0325 | 0.4608 | 100.0% | 894.03217 | 894.0183 | 36 | 7.065 | 64.3% | 1 | K.IGSFLSNR.T | 2 |
|  | Astrin\_STLC\_112116\_01.06534.06534.2 | 2.6074 | 0.1726 | 95.7% | 1188.4321 | 1188.3666 | 3 | 4.72 | 58.3% | 1 | K.SGLSSLAGAPSLK.D | 2 |
|  | Astrin\_STLC\_112116\_tube2\_01.08535.08535.3 | 4.1196 | 0.329 | 99.8% | 2570.7844 | 2571.63 | 1 | 5.831 | 28.3% | 1 | K.IGSLGLGTGEDDDYVDDFNSTSHR.S | 3 |

---

|  |  |  |  |  |  |  |  |  |
| --- | --- | --- | --- | --- | --- | --- | --- | --- |
| U | *gi|4758302|ref|NP\_004* | 3 | 4 | 28.8% | 104 | 12259 | 5.9 | enhancer of rudimentary homolog [Homo sapiens] |

| Filename XCorr DeltCN Conf% ObsM+H+ CalcM+H+ SpR ZScore Ion% # Sequence  | | | | | | | | | | | | |
| --- | --- | --- | --- | --- | --- | --- | --- | --- | --- | --- | --- | --- |
| \* | Astrin\_STLC\_112116\_tube2\_01.03598.03598.2 | 2.6215 | 0.2434 | 99.8% | 1105.7922 | 1106.2848 | 38 | 5.019 | 71.4% | 1 | K.MYEEHLKR.M | 2 |
| \* | Astrin\_STLC\_112116\_01.06022.06022.2 | 3.688 | 0.3632 | 100.0% | 1871.2722 | 1872.0441 | 2 | 6.082 | 50.0% | 2 | R.ADTQTYQPYNKDWIK.E | 2 |
| \* | Astrin\_STLC\_112116\_tube2\_01.06198.06198.2 | 2.2091 | 0.1617 | 95.7% | 932.3722 | 933.185 | 161 | 4.604 | 66.7% | 1 | K.IYVLLRR.Q | 2 |

---

|  |  |  |  |  |  |  |  |  |
| --- | --- | --- | --- | --- | --- | --- | --- | --- |
| U | *gi|31542947|ref|NP\_00* | 12 | 25 | 28.4% | 573 | 61055 | 5.9 | 60 kDa heat shock protein, mitochondrial [Homo sapiens] |

| Filename XCorr DeltCN Conf% ObsM+H+ CalcM+H+ SpR ZScore Ion% # Sequence  | | | | | | | | | | | | |
| --- | --- | --- | --- | --- | --- | --- | --- | --- | --- | --- | --- | --- |
| \* | Astrin\_STLC\_112116\_tube2\_01.15256.15256.2 | 5.5343 | 0.6168 | 100.0% | 2114.112 | 2114.5667 | 1 | 11.52 | 62.5% | 6 | R.ALMLQGVDLLADAVAVTMGPK.G | 2 |
| \* | Astrin\_STLC\_112116\_01.14642.14642.3 | 5.4619 | 0.4337 | 100.0% | 2114.6042 | 2114.5667 | 1 | 8.164 | 43.8% | 3 | R.ALMLQGVDLLADAVAVTMGPK.G | 3 |
| \* | Astrin\_STLC\_112116\_tube2\_01.06664.06664.3 | 4.5359 | 0.4983 | 100.0% | 2561.2144 | 2561.7222 | 1 | 8.252 | 33.3% | 3 | K.LVQDVANNTNEEAGDGTTTATVLAR.S | 3 |
| \* | Astrin\_STLC\_112116\_tube2\_01.10684.10684.2 | 2.2548 | 0.3255 | 98.9% | 1504.8121 | 1505.7235 | 5 | 5.204 | 50.0% | 1 | K.TLNDELEIIEGMK.F | 2 |
| \* | Astrin\_STLC\_112116\_tube2\_01.10825.10825.2 | 2.8491 | 0.3803 | 100.0% | 1922.8722 | 1924.1761 | 7 | 6.152 | 36.7% | 2 | K.TLNDELEIIEGMKFDR.G | 2 |
| \* | Astrin\_STLC\_112116\_tube2\_02.09698.09698.3 | 2.6446 | 0.2731 | 97.7% | 1923.8043 | 1924.1761 | 23 | 4.548 | 35.0% | 2 | K.TLNDELEIIEGMKFDR.G | 3 |
| \* | Astrin\_STLC\_112116\_tube2\_01.08488.08488.2 | 2.7148 | 0.4892 | 100.0% | 1390.1921 | 1390.5786 | 1 | 7.372 | 63.6% | 1 | R.GYISPYFINTSK.G | 2 |
| \* | Astrin\_STLC\_112116\_01.07242.07242.3 | 3.2768 | 0.2196 | 97.7% | 2048.2144 | 2048.3933 | 1 | 4.562 | 33.3% | 1 | K.KISSIQSIVPALEIANAHR.K | 3 |
| \* | Astrin\_STLC\_112116\_01.06984.06984.2 | 3.9542 | 0.3225 | 100.0% | 1632.2522 | 1631.9684 | 1 | 6.93 | 64.3% | 1 | K.VGEVIVTKDDAMLLK.G | 2 |
| \* | Astrin\_STLC\_112116\_tube2\_02.09274.09274.3 | 3.3402 | 0.2581 | 98.7% | 2450.9644 | 2452.721 | 144 | 4.267 | 26.3% | 1 | K.RIQEIIEQLDVTTSEYEKEK.L | 3 |
| \* | Astrin\_STLC\_112116\_tube2\_01.06166.06166.2 | 2.7897 | 0.367 | 100.0% | 1215.4321 | 1216.377 | 2 | 6.788 | 68.2% | 2 | K.NAGVEGSLIVEK.I | 2 |
| \* | Astrin\_STLC\_112116\_02.09059.09059.3 | 3.7441 | 0.3583 | 99.8% | 2509.1042 | 2509.8235 | 1 | 6.416 | 31.8% | 2 | K.IMQSSSEVGYDAMAGDFVNMVEK.G | 3 |

---

|  |  |  |  |  |  |  |  |  |
| --- | --- | --- | --- | --- | --- | --- | --- | --- |
| U | *gi|20149594|ref|NP\_03* | 15 | 31 | 27.6% | 724 | 83264 | 5.0 | heat shock protein HSP 90-beta isoform a [Homo sapiens] &IC Hsp90 |
| U | *gi|431822408|ref|NP\_0* | 15 | 31 | 28.0% | 714 | 82320 | 5.1 | heat shock protein HSP 90-beta isoform c [Homo sapiens] &IC Hsp90 |

| Filename XCorr DeltCN Conf% ObsM+H+ CalcM+H+ SpR ZScore Ion% # Sequence  | | | | | | | | | | | | |
| --- | --- | --- | --- | --- | --- | --- | --- | --- | --- | --- | --- | --- |
|  | Astrin\_STLC\_112116\_tube2\_01.06413.06413.2 | 2.4029 | 0.2186 | 96.4% | 1277.1322 | 1276.3861 | 13 | 4.594 | 54.5% | 1 | R.ELISNASDALDK.I | 22 |
|  | Astrin\_STLC\_112116\_tube2\_01.07956.07956.2 | 3.3531 | 0.2598 | 100.0% | 1545.4321 | 1545.733 | 1 | 5.563 | 65.4% | 1 | R.ELISNASDALDKIR.Y | 22 |
|  | Astrin\_STLC\_112116\_tube2\_01.08407.08407.2 | 3.4125 | 0.4213 | 100.0% | 1243.0322 | 1243.4459 | 1 | 6.622 | 72.7% | 2 | K.ADLINNLGTIAK.S | 22 |
|  | Astrin\_STLC\_112116\_02.04492.04492.3 | 4.9004 | 0.4837 | 100.0% | 2015.3644 | 2016.2584 | 1 | 8.339 | 45.0% | 5 | K.VILHLKEDQTEYLEER.R | 33 |
|  | Astrin\_STLC\_112116\_01.08555.08555.3 | 4.19 | 0.4108 | 100.0% | 2094.8044 | 2095.4058 | 1 | 7.087 | 42.2% | 2 | K.HSQFIGYPITLYLEKER.E | 3 |
|  | Astrin\_STLC\_112116\_01.05163.05163.2 | 3.0953 | 0.2673 | 100.0% | 1151.8922 | 1152.2462 | 2 | 5.479 | 75.0% | 4 | K.YIDQEELNK.T | 22 |
|  | Astrin\_STLC\_112116\_tube2\_01.07958.07958.2 | 5.0956 | 0.5175 | 100.0% | 1848.0721 | 1848.9171 | 1 | 9.314 | 82.1% | 1 | R.NPDDITQEEYGEFYK.S | 2 |
|  | Astrin\_STLC\_112116\_tube2\_01.07911.07911.2 | 4.0786 | 0.414 | 100.0% | 1528.5721 | 1528.6616 | 1 | 7.285 | 75.0% | 2 | K.SLTNDWEDHLAVK.H | 22 |
|  | Astrin\_STLC\_112116\_tube2\_02.08226.08226.2 | 3.5056 | 0.4499 | 100.0% | 1348.9922 | 1349.4886 | 1 | 7.643 | 80.0% | 5 | K.HFSVEGQLEFR.A | 22 |
|  | Astrin\_STLC\_112116\_02.06906.06906.3 | 3.6515 | 0.3161 | 99.7% | 2178.7444 | 2178.2915 | 22 | 5.077 | 30.6% | 1 | R.YHTSQSGDEMTSLSEYVSR.M | 3 |
|  | Astrin\_STLC\_112116\_02.07022.07022.3 | 3.8812 | 0.2203 | 98.7% | 2394.2344 | 2392.6274 | 1 | 5.218 | 40.0% | 1 | K.SIYYITGESKEQVANSAFVER.V | 3 |
|  | Astrin\_STLC\_112116\_01.05685.05685.2 | 2.7025 | 0.4574 | 100.0% | 1250.0521 | 1250.3538 | 21 | 7.336 | 55.0% | 2 | K.EQVANSAFVER.V | 2 |
|  | Astrin\_STLC\_112116\_01.06087.06087.2 | 2.1188 | 0.331 | 98.9% | 1248.4521 | 1249.4574 | 14 | 4.77 | 55.0% | 1 | R.DNSTMGYMMAK.K | 2 |
|  | Astrin\_STLC\_112116\_tube2\_01.07326.07326.3 | 2.9455 | 0.3097 | 99.5% | 1784.4844 | 1784.025 | 34 | 5.619 | 32.1% | 1 | K.HLEINPDHPIVETLR.Q | 3 |
|  | Astrin\_STLC\_112116\_01.15183.15183.3 | 4.3018 | 0.5036 | 100.0% | 2989.2544 | 2990.3398 | 1 | 8.889 | 28.8% | 2 | K.DLVVLLFETALLSSGFSLEDPQTHSNR.I | 3 |

Similarities:
gi|153792590|ref|NP\_0(5:10)  
gi|4507677|ref|NP\_003(2:13)  

---

|  |  |  |  |  |  |  |  |  |
| --- | --- | --- | --- | --- | --- | --- | --- | --- |
| U | *contaminant\_KERATIN03* | 15 | 22 | 27.2% | 593 | 59519 | 5.2 | no description |
| U | *gi|195972866|ref|NP\_0* | 15 | 22 | 27.6% | 584 | 58801 | 5.2 | keratin, type I cytoskeletal 10 [Homo sapiens] |

| Filename XCorr DeltCN Conf% ObsM+H+ CalcM+H+ SpR ZScore Ion% # Sequence  | | | | | | | | | | | | |
| --- | --- | --- | --- | --- | --- | --- | --- | --- | --- | --- | --- | --- |
|  | Astrin\_STLC\_112116\_tube2\_02.08484.08484.2 | 4.9595 | 0.5093 | 100.0% | 1708.1322 | 1708.7844 | 1 | 8.836 | 61.1% | 4 | K.GSLGGGFSSGGFSGGSFSR.G | 2 |
|  | Astrin\_STLC\_112116\_tube2\_01.04204.04204.2 | 2.311 | 0.2065 | 96.9% | 1091.1322 | 1091.2273 | 68 | 4.975 | 62.5% | 1 | K.VTMQNLNDR.L | 222 |
|  | Astrin\_STLC\_112116\_tube2\_01.05299.05299.2 | 3.2103 | 0.1537 | 99.8% | 1064.5721 | 1065.2578 | 1 | 6.052 | 81.2% | 2 | R.LASYLDKVR.A | 222222 |
|  | Astrin\_STLC\_112116\_tube2\_01.05546.05546.2 | 4.1293 | 0.3925 | 100.0% | 1381.7722 | 1382.4668 | 1 | 7.173 | 68.2% | 2 | R.ALEESNYELEGK.I | 2 |
|  | Astrin\_STLC\_112116\_01.12621.12621.3 | 5.6896 | 0.4886 | 100.0% | 3054.4143 | 3054.4277 | 1 | 8.271 | 33.7% | 3 | K.TIDDLKNQILNLTTDNANILLQIDNAR.L | 3 |
|  | Astrin\_STLC\_112116\_tube2\_02.10411.10411.3 | 5.4564 | 0.3309 | 100.0% | 2368.3145 | 2368.6523 | 1 | 6.519 | 45.0% | 1 | K.NQILNLTTDNANILLQIDNAR.L | 3 |
|  | Astrin\_STLC\_112116\_01.05584.05584.2 | 1.8322 | 0.255 | 95.2% | 807.8722 | 807.8815 | 186 | 5.3 | 66.7% | 1 | R.LAADDFR.L | 2222222 |
|  | Astrin\_STLC\_112116\_tube2\_01.05259.05259.2 | 2.3092 | 0.2619 | 98.7% | 1235.9321 | 1235.4258 | 1 | 4.502 | 83.3% | 1 | R.LKYENEVALR.Q | 2 |
|  | Astrin\_STLC\_112116\_tube2\_01.07450.07450.2 | 2.5043 | 0.2303 | 99.0% | 1032.0521 | 1032.2224 | 2 | 4.985 | 75.0% | 1 | R.VLDELTLTK.A | 2 |
|  | Astrin\_STLC\_112116\_01.04343.04343.2 | 2.9297 | 0.2275 | 99.5% | 1493.9722 | 1494.6041 | 2 | 5.292 | 63.6% | 1 | R.SQYEQLAEQNRK.D | 2 |
|  | Astrin\_STLC\_112116\_tube2\_01.07876.07876.2 | 2.7711 | 0.1186 | 97.5% | 1110.9922 | 1110.1681 | 1 | 5.317 | 75.0% | 1 | K.DAEAWFNEK.S | 2 |
|  | Astrin\_STLC\_112116\_tube2\_01.07063.07063.2 | 3.8322 | 0.4808 | 100.0% | 1391.0922 | 1391.4778 | 1 | 8.071 | 66.7% | 1 | K.QSLEASLAETEGR.Y | 2 |
|  | Astrin\_STLC\_112116\_tube2\_01.06394.06394.2 | 2.6306 | 0.2067 | 98.5% | 1434.7522 | 1435.623 | 2 | 4.491 | 65.0% | 1 | K.IRLENEIQTYR.S | 2 |
|  | Astrin\_STLC\_112116\_tube2\_01.04780.04780.2 | 3.088 | 0.0995 | 98.8% | 1165.7522 | 1166.2761 | 2 | 5.708 | 87.5% | 1 | R.LENEIQTYR.S | 2 |
|  | Astrin\_STLC\_112116\_01.04649.04649.2 | 3.3049 | 0.4075 | 100.0% | 1263.1322 | 1263.3066 | 1 | 7.991 | 69.2% | 1 | R.SLLEGEGSSGGGGR.G | 2 |

Similarities:
contaminant\_KERATIN09(1:14)  
gi|24430192|ref|NP\_00(3:12)  
gi|15431310|ref|NP\_00(3:12)  
contaminant\_KERATIN12(2:13)  
contaminant\_KERATIN07(2:13)  
contaminant\_KERATIN10(2:13)  

---

|  |  |  |  |  |  |  |  |  |
| --- | --- | --- | --- | --- | --- | --- | --- | --- |
| U | *gi|4506671|ref|NP\_000* | 2 | 11 | 27.0% | 115 | 11665 | 4.5 | 60S acidic ribosomal protein P2 [Homo sapiens] |

| Filename XCorr DeltCN Conf% ObsM+H+ CalcM+H+ SpR ZScore Ion% # Sequence  | | | | | | | | | | | | |
| --- | --- | --- | --- | --- | --- | --- | --- | --- | --- | --- | --- | --- |
| \* | Astrin\_STLC\_112116\_tube2\_01.11055.11055.2 | 4.7409 | 0.3875 | 100.0% | 1870.3121 | 1870.1124 | 1 | 8.689 | 58.3% | 2 | R.YVASYLLAALGGNSSPSAK.D | 2 |
| \* | Astrin\_STLC\_112116\_tube2\_01.09552.09552.2 | 3.6703 | 0.5248 | 100.0% | 1256.9122 | 1257.4294 | 1 | 8.821 | 81.8% | 9 | K.NIEDVIAQGIGK.L | 2 |

---

|  |  |  |  |  |  |  |  |  |
| --- | --- | --- | --- | --- | --- | --- | --- | --- |
| U | *gi|5901926|ref|NP\_008* | 3 | 7 | 26.0% | 227 | 26227 | 8.8 | cleavage and polyadenylation specificity factor subunit 5 [Homo sapiens] |

| Filename XCorr DeltCN Conf% ObsM+H+ CalcM+H+ SpR ZScore Ion% # Sequence  | | | | | | | | | | | | |
| --- | --- | --- | --- | --- | --- | --- | --- | --- | --- | --- | --- | --- |
| \* | Astrin\_STLC\_112116\_tube2\_01.05289.05289.3 | 2.8896 | 0.212 | 96.9% | 1490.4243 | 1490.7428 | 9 | 4.835 | 43.2% | 1 | K.YIQQTKPLTLER.T | 3 |
| \* | Astrin\_STLC\_112116\_tube2\_01.06951.06951.3 | 3.1163 | 0.2758 | 99.3% | 1909.6144 | 1910.0911 | 1 | 5.059 | 35.3% | 1 | K.LPGGELNPGEDEVEGLKR.L | 3 |
| \* | Astrin\_STLC\_112116\_tube2\_01.14884.14884.3 | 5.1223 | 0.4806 | 100.0% | 3115.7344 | 3116.6274 | 1 | 8.505 | 33.9% | 5 | K.LVAAPLFELYDNAPGYGPIISSLPQLLSR.F | 3 |

---

|  |  |  |  |  |  |  |  |  |
| --- | --- | --- | --- | --- | --- | --- | --- | --- |
| U | *gi|4504517|ref|NP\_001* | 4 | 10 | 25.9% | 205 | 22783 | 6.4 | heat shock protein beta-1 [Homo sapiens] |

| Filename XCorr DeltCN Conf% ObsM+H+ CalcM+H+ SpR ZScore Ion% # Sequence  | | | | | | | | | | | | |
| --- | --- | --- | --- | --- | --- | --- | --- | --- | --- | --- | --- | --- |
| \* | Astrin\_STLC\_112116\_tube2\_01.09676.09676.2 | 3.4257 | 0.3748 | 100.0% | 1164.0922 | 1164.3494 | 1 | 6.876 | 94.4% | 6 | R.LFDQAFGLPR.L | 2 |
| \* | Astrin\_STLC\_112116\_01.05586.05586.2 | 2.2872 | 0.2001 | 95.7% | 1076.1322 | 1076.1948 | 8 | 4.768 | 61.1% | 1 | R.QLSSGVSEIR.H | 2 |
| \* | Astrin\_STLC\_112116\_tube2\_01.08547.08547.3 | 3.0488 | 0.2585 | 98.6% | 1786.0144 | 1785.0068 | 117 | 5.214 | 31.7% | 1 | R.VSLDVNHFAPDELTVK.T | 3 |
| \* | Astrin\_STLC\_112116\_01.07258.07258.2 | 4.527 | 0.4529 | 100.0% | 1907.6522 | 1907.1307 | 1 | 8.394 | 46.9% | 2 | K.LATQSNEITIPVTFESR.A | 2 |

---

|  |  |  |  |  |  |  |  |  |
| --- | --- | --- | --- | --- | --- | --- | --- | --- |
| U | *gi|28875797|ref|NP\_05* | 4 | 5 | 25.8% | 248 | 26397 | 12.2 | chromatin target of PRMT1 protein isoform 1 [Homo sapiens] |
| U | *gi|951880973|ref|NP\_0* | 4 | 5 | 31.7% | 202 | 21918 | 12.0 | chromatin target of PRMT1 protein isoform 4 [Homo sapiens] |
| U | *gi|331028739|ref|NP\_0* | 4 | 5 | 25.7% | 249 | 26525 | 12.2 | chromatin target of PRMT1 protein isoform 2 [Homo sapiens] |

| Filename XCorr DeltCN Conf% ObsM+H+ CalcM+H+ SpR ZScore Ion% # Sequence  | | | | | | | | | | | | |
| --- | --- | --- | --- | --- | --- | --- | --- | --- | --- | --- | --- | --- |
|  | Astrin\_STLC\_112116\_01.04268.04268.2 | 4.0976 | 0.3904 | 100.0% | 1446.8121 | 1447.6091 | 1 | 7.7 | 70.8% | 1 | R.ASMQQQQQLASAR.N | 2 |
|  | Astrin\_STLC\_112116\_tube2\_01.05287.05287.3 | 3.3332 | 0.3772 | 99.8% | 1784.9644 | 1785.0715 | 1 | 6.336 | 40.0% | 2 | R.LAQQMENRPSVQAALK.L | 3 |
|  | Astrin\_STLC\_112116\_tube2\_01.08006.08006.2 | 3.3069 | 0.3878 | 100.0% | 1555.2122 | 1555.6997 | 1 | 6.327 | 62.5% | 1 | K.EQLDNQLDAYMSK.T | 2 |
|  | Astrin\_STLC\_112116\_tube2\_02.08518.08518.3 | 2.979 | 0.2965 | 98.8% | 2436.7744 | 2436.566 | 2 | 4.515 | 28.6% | 1 | K.TKGHLDAELDAYMAQTDPETND.- | 3 |

---

|  |  |  |  |  |  |  |  |  |
| --- | --- | --- | --- | --- | --- | --- | --- | --- |
| U | *gi|12667788|ref|NP\_00* | 36 | 56 | 25.4% | 1960 | 226530 | 5.6 | myosin-9 [Homo sapiens] |

| Filename XCorr DeltCN Conf% ObsM+H+ CalcM+H+ SpR ZScore Ion% # Sequence  | | | | | | | | | | | | |
| --- | --- | --- | --- | --- | --- | --- | --- | --- | --- | --- | --- | --- |
| \* | Astrin\_STLC\_112116\_tube2\_01.06702.06702.3 | 2.9942 | 0.3465 | 99.7% | 1915.5243 | 1916.1614 | 1 | 5.672 | 40.0% | 1 | R.HEMPPHIYAITDTAYR.S | 3 |
|  | Astrin\_STLC\_112116\_01.10971.10971.2 | 4.7517 | 0.4498 | 100.0% | 1727.3322 | 1728.0012 | 1 | 8.484 | 66.7% | 3 | R.QLLQANPILEAFGNAK.T | 2 |
| \* | Astrin\_STLC\_112116\_01.09150.09150.2 | 2.7222 | 0.2139 | 98.0% | 1616.6522 | 1616.9313 | 1 | 5.624 | 69.2% | 2 | R.IMGIPEEEQMGLLR.V | 2 |
|  | Astrin\_STLC\_112116\_01.03836.03836.2 | 4.2795 | 0.5178 | 100.0% | 1591.5322 | 1592.6776 | 1 | 7.321 | 78.6% | 1 | R.NTDQASMPDNTAAQK.V | 2 |
| \* | Astrin\_STLC\_112116\_01.07342.07342.3 | 3.5362 | 0.3315 | 99.8% | 1572.7144 | 1572.8044 | 3 | 5.186 | 48.1% | 2 | K.VSHLLGINVTDFTR.G | 3 |
| \* | Astrin\_STLC\_112116\_tube2\_01.11470.11470.2 | 5.0603 | 0.5503 | 100.0% | 2018.4722 | 2019.3636 | 1 | 10.264 | 60.5% | 4 | R.IIGLDQVAGMSETALPGAFK.T | 2 |
| \* | Astrin\_STLC\_112116\_tube2\_01.07828.07828.2 | 2.8418 | 0.4562 | 100.0% | 1193.7722 | 1194.33 | 1 | 7.672 | 77.8% | 1 | K.ALELDSNLYR.I | 2 |
| \* | Astrin\_STLC\_112116\_02.08077.08077.2 | 3.7622 | 0.3261 | 100.0% | 1753.5721 | 1753.0358 | 1 | 6.32 | 57.1% | 2 | R.LTEMETLQSQLMAEK.L | 2 |
| \* | Astrin\_STLC\_112116\_tube2\_01.07457.07457.2 | 4.3408 | 0.4482 | 100.0% | 1653.9722 | 1654.7681 | 1 | 7.958 | 73.1% | 2 | R.IAEFTTNLTEEEEK.S | 2 |
|  | Astrin\_STLC\_112116\_tube2\_01.04870.04870.2 | 3.0321 | 0.316 | 100.0% | 1258.0721 | 1258.4172 | 6 | 5.139 | 75.0% | 1 | K.KEEELQAALAR.V | 2 |
| \* | Astrin\_STLC\_112116\_tube2\_01.10261.10261.3 | 4.7398 | 0.3474 | 100.0% | 2304.7744 | 2304.473 | 2 | 6.263 | 36.1% | 1 | K.IRELESQISELQEDLESER.A | 3 |
| \* | Astrin\_STLC\_112116\_01.13401.13401.3 | 4.1534 | 0.4484 | 100.0% | 3019.2244 | 3019.2434 | 1 | 7.149 | 33.7% | 4 | R.DLGEELEALKTELEDTLDSTAAQQELR.S | 3 |
| \* | Astrin\_STLC\_112116\_tube2\_01.06589.06589.3 | 3.4234 | 0.3854 | 99.8% | 2043.9543 | 2044.2439 | 1 | 6.142 | 37.5% | 1 | K.TLEEEAKTHEAQIQEMR.Q | 3 |
| \* | Astrin\_STLC\_112116\_01.06950.06950.3 | 4.9445 | 0.5031 | 100.0% | 1996.4043 | 1997.1722 | 1 | 8.027 | 43.8% | 2 | K.HSQAVEELAEQLEQTKR.V | 3 |
| \* | Astrin\_STLC\_112116\_tube2\_01.06024.06024.2 | 3.5147 | 0.2033 | 100.0% | 1413.8322 | 1413.6573 | 57 | 4.434 | 59.1% | 1 | K.KVEAQLQELQVK.F | 2 |
| \* | Astrin\_STLC\_112116\_tube2\_01.06775.06775.2 | 3.5944 | 0.3976 | 100.0% | 1285.1522 | 1285.4833 | 1 | 6.437 | 90.0% | 1 | K.VEAQLQELQVK.F | 2 |
| \* | Astrin\_STLC\_112116\_tube2\_01.10408.10408.2 | 5.5145 | 0.5758 | 100.0% | 1946.2122 | 1947.1498 | 1 | 9.871 | 73.5% | 3 | K.LQVELDNVTGLLSQSDSK.S | 2 |
| \* | Astrin\_STLC\_112116\_01.11254.11254.3 | 5.3924 | 0.461 | 100.0% | 2494.8542 | 2494.631 | 1 | 8.18 | 37.5% | 1 | K.DFSALESQLQDTQELLQEENR.Q | 3 |
| \* | Astrin\_STLC\_112116\_01.04107.04107.3 | 3.0622 | 0.2968 | 99.6% | 1495.6144 | 1493.6598 | 82 | 5.115 | 36.4% | 1 | K.LKQVEDEKNSFR.E | 3 |
| \* | Astrin\_STLC\_112116\_01.09413.09413.3 | 3.179 | 0.2959 | 99.4% | 1950.8043 | 1951.1436 | 1 | 5.267 | 48.3% | 1 | R.LQQELDDLLVDLDHQR.Q | 3 |
| \* | Astrin\_STLC\_112116\_01.09416.09416.2 | 3.9382 | 0.3813 | 100.0% | 1951.8722 | 1951.1436 | 1 | 6.782 | 63.3% | 1 | R.LQQELDDLLVDLDHQR.Q | 2 |
|  | Astrin\_STLC\_112116\_tube2\_01.06060.06060.2 | 2.9889 | 0.1978 | 99.6% | 1222.1122 | 1221.3959 | 13 | 4.489 | 66.7% | 1 | K.KFDQLLAEEK.T | 22 |
|  | Astrin\_STLC\_112116\_tube2\_01.06341.06341.2 | 2.6047 | 0.1138 | 95.7% | 1093.1322 | 1093.2218 | 1 | 5.265 | 87.5% | 1 | K.FDQLLAEEK.T | 22 |
| \* | Astrin\_STLC\_112116\_01.04376.04376.2 | 2.2754 | 0.2189 | 97.2% | 1049.0322 | 1049.1841 | 8 | 5.507 | 68.8% | 1 | R.ALEEAMEQK.A | 2 |
| \* | Astrin\_STLC\_112116\_tube2\_01.13337.13337.3 | 3.1025 | 0.2865 | 98.6% | 3147.6843 | 3149.4048 | 34 | 4.76 | 21.2% | 1 | R.ALEQQVEEMKTQLEELEDELQATEDAK.L | 3 |
| \* | Astrin\_STLC\_112116\_tube2\_01.06229.06229.3 | 4.8478 | 0.4719 | 100.0% | 2089.7043 | 2090.168 | 1 | 8.683 | 43.1% | 1 | R.QAQQERDELADEIANSSGK.G | 3 |
| \* | Astrin\_STLC\_112116\_tube2\_01.06559.06559.2 | 2.7386 | 0.267 | 99.3% | 1348.4922 | 1349.394 | 4 | 4.465 | 62.5% | 1 | R.DELADEIANSSGK.G | 2 |
| \* | Astrin\_STLC\_112116\_tube2\_01.09311.09311.3 | 4.173 | 0.2313 | 99.5% | 2474.1243 | 2473.6099 | 1 | 6.605 | 37.5% | 1 | R.IAQLEEELEEEQGNTELINDR.L | 3 |
| \* | Astrin\_STLC\_112116\_tube2\_01.09320.09320.2 | 4.7587 | 0.447 | 100.0% | 1871.4722 | 1871.0574 | 1 | 7.815 | 60.0% | 2 | K.ANLQIDQINTDLNLER.S | 2 |
| \* | Astrin\_STLC\_112116\_tube2\_01.06711.06711.2 | 4.5646 | 0.3335 | 100.0% | 1531.0721 | 1531.6598 | 1 | 6.94 | 79.2% | 2 | K.IAQLEEQLDNETK.E | 2 |
| \* | Astrin\_STLC\_112116\_01.03794.03794.3 | 2.9138 | 0.2567 | 98.3% | 1724.2743 | 1725.8125 | 16 | 4.848 | 33.9% | 1 | R.NAEQYKDQADKASTR.L | 3 |
| \* | Astrin\_STLC\_112116\_01.03774.03774.2 | 3.3479 | 0.4581 | 100.0% | 1725.1921 | 1725.8125 | 3 | 7.35 | 50.0% | 1 | R.NAEQYKDQADKASTR.L | 2 |
| \* | Astrin\_STLC\_112116\_01.05474.05474.3 | 2.772 | 0.3493 | 99.6% | 1830.9543 | 1831.8937 | 145 | 5.312 | 31.7% | 2 | R.QLEEAEEEAQRANASR.R | 3 |
| \* | Astrin\_STLC\_112116\_01.05793.05793.2 | 3.735 | 0.5506 | 100.0% | 1565.9521 | 1566.6367 | 1 | 8.915 | 69.2% | 2 | R.ELEDATETADAMNR.E | 2 |
| \* | Astrin\_STLC\_112116\_tube2\_01.07721.07721.2 | 2.9655 | 0.332 | 100.0% | 1155.7522 | 1156.3732 | 3 | 5.899 | 72.2% | 1 | R.RGDLPFVVPR.R | 2 |
| \* | Astrin\_STLC\_112116\_01.03982.03982.3 | 4.5164 | 0.2999 | 99.8% | 2384.6042 | 2384.3428 | 1 | 6.541 | 34.8% | 2 | R.KGAGDGS\*DEEVDGKADGAEAKPAE.- | 3 |

Similarities:
gi|365192532|ref|NP\_0(2:34)  

---

|  |  |  |  |  |  |  |  |  |
| --- | --- | --- | --- | --- | --- | --- | --- | --- |
| U | *gi|17986258|ref|NP\_06* | 4 | 10 | 25.2% | 151 | 16930 | 4.7 | myosin light polypeptide 6 isoform 1 [Homo sapiens] |
| U | *gi|88999583|ref|NP\_52* | 4 | 10 | 25.2% | 151 | 16961 | 4.6 | myosin light polypeptide 6 isoform 2 [Homo sapiens] |

| Filename XCorr DeltCN Conf% ObsM+H+ CalcM+H+ SpR ZScore Ion% # Sequence  | | | | | | | | | | | | |
| --- | --- | --- | --- | --- | --- | --- | --- | --- | --- | --- | --- | --- |
|  | Astrin\_STLC\_112116\_01.05804.05804.2 | 3.6882 | 0.2481 | 100.0% | 1354.8922 | 1355.5339 | 12 | 6.046 | 58.3% | 3 | R.ALGQNPTNAEVLK.V | 2 |
|  | Astrin\_STLC\_112116\_01.12162.12162.3 | 3.9575 | 0.392 | 100.0% | 1888.4043 | 1889.2628 | 1 | 6.776 | 46.7% | 3 | K.VLDFEHFLPMLQTVAK.N | 3 |
|  | Astrin\_STLC\_112116\_tube2\_01.12664.12664.2 | 4.0536 | 0.306 | 100.0% | 1889.3722 | 1889.2628 | 1 | 6.438 | 56.7% | 2 | K.VLDFEHFLPMLQTVAK.N | 2 |
|  | Astrin\_STLC\_112116\_01.05576.05576.2 | 2.1228 | 0.2127 | 95.3% | 996.1322 | 996.1949 | 134 | 6.122 | 50.0% | 2 | R.HVLVTLGEK.M | 2 |

---

|  |  |  |  |  |  |  |  |  |
| --- | --- | --- | --- | --- | --- | --- | --- | --- |
| U | *gi|7657307|ref|NP\_055* | 12 | 21 | 24.7% | 676 | 72190 | 6.7 | LIM domain-containing protein 1 [Homo sapiens] |

| Filename XCorr DeltCN Conf% ObsM+H+ CalcM+H+ SpR ZScore Ion% # Sequence  | | | | | | | | | | | | |
| --- | --- | --- | --- | --- | --- | --- | --- | --- | --- | --- | --- | --- |
| \* | Astrin\_STLC\_112116\_tube2\_01.08293.08293.2 | 3.6569 | 0.3158 | 100.0% | 1459.9321 | 1460.6416 | 1 | 5.943 | 77.3% | 2 | K.FIEDLNMYEASK.D | 2 |
| \* | Astrin\_STLC\_112116\_02.08369.08369.3 | 2.5416 | 0.3506 | 99.5% | 2048.5144 | 2049.3057 | 3 | 5.512 | 37.5% | 1 | K.FIEDLNMYEASKDGLFR.V | 3 |
| \* | Astrin\_STLC\_112116\_01.04418.04418.2 | 2.8468 | 0.3631 | 100.0% | 1321.2722 | 1321.3458 | 8 | 5.551 | 63.6% | 1 | K.GAGNNPEFEETR.R | 2 |
| \* | Astrin\_STLC\_112116\_tube2\_01.07545.07545.2 | 6.6568 | 0.5193 | 100.0% | 2230.2522 | 2231.5168 | 1 | 9.977 | 73.5% | 1 | K.IHLQQQQQQLLQEETLPR.G | 2 |
| \* | Astrin\_STLC\_112116\_tube2\_01.07599.07599.3 | 4.6022 | 0.2553 | 99.8% | 2230.7043 | 2231.5168 | 2 | 5.196 | 36.8% | 2 | K.IHLQQQQQQLLQEETLPR.G | 3 |
| \* | Astrin\_STLC\_112116\_01.04436.04436.2 | 4.1575 | 0.3288 | 100.0% | 1462.6522 | 1463.5039 | 1 | 8.123 | 60.0% | 4 | R.SSEGSLGGQNSGIGGR.S | 2 |
| \* | Astrin\_STLC\_112116\_01.05817.05817.2 | 3.9277 | 0.163 | 99.8% | 1723.1522 | 1722.8522 | 1 | 7.36 | 70.0% | 1 | R.SSEKPTGLWSTASSQR.V | 2 |
| \* | Astrin\_STLC\_112116\_tube2\_01.08051.08051.2 | 3.9522 | 0.4134 | 100.0% | 2253.392 | 2254.551 | 1 | 7.525 | 59.1% | 1 | R.VSPGLPSPNLENGAPAVGPVQPR.T | 2 |
| \* | Astrin\_STLC\_112116\_tube2\_01.08040.08040.3 | 4.016 | 0.2337 | 99.4% | 2255.7244 | 2254.551 | 1 | 5.36 | 35.2% | 1 | R.VSPGLPSPNLENGAPAVGPVQPR.T | 3 |
| \* | Astrin\_STLC\_112116\_tube2\_02.08943.08943.3 | 5.8715 | 0.4296 | 100.0% | 3016.2244 | 3016.3118 | 1 | 8.049 | 32.1% | 4 | R.SNSGLGGEVSGVMSKPNVDPQPWFQDGPK.S | 3 |
| \* | Astrin\_STLC\_112116\_tube2\_01.11079.11079.2 | 5.3663 | 0.5828 | 100.0% | 2401.2722 | 2401.592 | 1 | 10.789 | 58.7% | 1 | K.EGPLGWSSDGSLGSVLLDSPSSPR.V | 2 |
| \* | Astrin\_STLC\_112116\_01.03711.03711.3 | 3.2926 | 0.2486 | 99.5% | 1418.4543 | 1418.5576 | 29 | 5.784 | 38.6% | 2 | K.RPSSTALHQHHF.- | 3 |

---

|  |  |  |  |  |  |  |  |  |
| --- | --- | --- | --- | --- | --- | --- | --- | --- |
| U | *gi|576583519|ref|NP\_0* | 4 | 4 | 24.2% | 335 | 36053 | 8.5 | glyceraldehyde-3-phosphate dehydrogenase isoform 1 [Homo sapiens] |

| Filename XCorr DeltCN Conf% ObsM+H+ CalcM+H+ SpR ZScore Ion% # Sequence  | | | | | | | | | | | | |
| --- | --- | --- | --- | --- | --- | --- | --- | --- | --- | --- | --- | --- |
| \* | Astrin\_STLC\_112116\_01.14630.14630.3 | 4.2261 | 0.2374 | 99.5% | 3311.2144 | 3310.7634 | 1 | 4.275 | 24.1% | 1 | K.VDIVAINDPFIDLNYMVYMFQYDSTHGK.F | 3 |
|  | Astrin\_STLC\_112116\_tube2\_01.12987.12987.3 | 4.9972 | 0.4317 | 100.0% | 2595.4143 | 2597.0044 | 1 | 7.559 | 34.8% | 1 | K.VIHDNFGIVEGLMTTVHAITATQK.T | 3 |
|  | Astrin\_STLC\_112116\_tube2\_01.07683.07683.2 | 3.1592 | 0.3571 | 100.0% | 1411.7522 | 1412.6292 | 52 | 6.113 | 46.4% | 1 | R.GALQNIIPASTGAAK.A | 2 |
|  | Astrin\_STLC\_112116\_tube2\_01.09912.09912.2 | 2.4271 | 0.2311 | 96.3% | 1764.0922 | 1764.8914 | 41 | 5.283 | 42.3% | 1 | K.LISWYDNEFGYSNR.V | 2 |

---

|  |  |  |  |  |  |  |  |  |
| --- | --- | --- | --- | --- | --- | --- | --- | --- |
| U | *gi|13654278|ref|NP\_11* | 2 | 2 | 23.9% | 109 | 12349 | 10.2 | SRA stem-loop-interacting RNA-binding protein, mitochondrial isoform 1 precursor [Homo sapiens] |
| U | *gi|392583865|ref|NP\_0* | 2 | 2 | 24.3% | 107 | 12122 | 10.2 | SRA stem-loop-interacting RNA-binding protein, mitochondrial isoform 2 precursor [Homo sapiens] |

| Filename XCorr DeltCN Conf% ObsM+H+ CalcM+H+ SpR ZScore Ion% # Sequence  | | | | | | | | | | | | |
| --- | --- | --- | --- | --- | --- | --- | --- | --- | --- | --- | --- | --- |
|  | Astrin\_STLC\_112116\_tube2\_02.09381.09381.2 | 2.8297 | 0.3103 | 99.9% | 1566.5922 | 1565.7257 | 56 | 5.053 | 46.2% | 1 | R.GLGWVQFSSEEGLR.N | 2 |
|  | Astrin\_STLC\_112116\_tube2\_01.04165.04165.3 | 2.6514 | 0.3633 | 99.7% | 1423.1643 | 1423.5193 | 14 | 5.572 | 40.9% | 1 | K.LPQTSDDEKKDF.- | 3 |

---

|  |  |  |  |  |  |  |  |  |
| --- | --- | --- | --- | --- | --- | --- | --- | --- |
| U | *gi|4826998|ref|NP\_005* | 13 | 32 | 23.2% | 707 | 76150 | 9.4 | splicing factor, proline- and glutamine-rich [Homo sapiens] |

| Filename XCorr DeltCN Conf% ObsM+H+ CalcM+H+ SpR ZScore Ion% # Sequence  | | | | | | | | | | | | |
| --- | --- | --- | --- | --- | --- | --- | --- | --- | --- | --- | --- | --- |
| \* | Astrin\_STLC\_112116\_02.07614.07614.3 | 3.0623 | 0.2889 | 99.5% | 1650.7144 | 1650.8723 | 16 | 5.528 | 37.5% | 2 | K.ISDSEGFKANLSLLR.R | 3 |
| \* | Astrin\_STLC\_112116\_01.09389.09389.2 | 3.2122 | 0.321 | 100.0% | 1808.2922 | 1809.0258 | 16 | 5.655 | 43.3% | 1 | R.LFVGNLPADITEDEFK.R | 2 |
| \* | Astrin\_STLC\_112116\_01.06178.06178.2 | 2.9859 | 0.4767 | 100.0% | 1252.5521 | 1253.3971 | 1 | 7.429 | 65.0% | 3 | K.YGEPGEVFINK.G | 2 |
| \* | Astrin\_STLC\_112116\_01.05438.05438.2 | 2.4808 | 0.2816 | 99.6% | 1047.9321 | 1048.1559 | 1 | 5.821 | 81.2% | 1 | K.AELDDTPMR.G | 2 |
| \* | Astrin\_STLC\_112116\_tube2\_01.04562.04562.2 | 3.101 | 0.3448 | 100.0% | 1144.1322 | 1144.3188 | 1 | 6.462 | 95.0% | 3 | R.FATHAAALSVR.N | 2 |
| \* | Astrin\_STLC\_112116\_01.12669.12669.2 | 4.2397 | 0.6089 | 100.0% | 2639.5522 | 2640.9092 | 1 | 12.06 | 59.1% | 3 | R.NLSPYVSNELLEEAFSQFGPIER.A | 2 |
| \* | Astrin\_STLC\_112116\_01.12668.12668.3 | 3.7798 | 0.4035 | 99.8% | 2640.4443 | 2640.9092 | 1 | 7.943 | 30.7% | 1 | R.NLSPYVSNELLEEAFSQFGPIER.A | 3 |
|  | Astrin\_STLC\_112116\_01.04916.04916.2 | 2.0201 | 0.2196 | 95.3% | 887.47217 | 887.0238 | 1 | 6.866 | 78.6% | 1 | R.AVVIVDDR.G | 22 |
| \* | Astrin\_STLC\_112116\_tube2\_01.05365.05365.2 | 2.8391 | 0.4043 | 100.0% | 1246.0721 | 1246.452 | 7 | 6.655 | 63.6% | 2 | K.GIVEFASKPAAR.K | 2 |
| \* | Astrin\_STLC\_112116\_tube2\_01.07992.07992.3 | 4.3943 | 0.2437 | 99.6% | 2429.6042 | 2429.6233 | 1 | 5.306 | 35.5% | 1 | K.DKLESEMEDAYHEHQANLLR.Q | 3 |
| \* | Astrin\_STLC\_112116\_01.03880.03880.3 | 3.1104 | 0.2084 | 97.9% | 1574.3043 | 1573.7821 | 2 | 4.983 | 47.7% | 2 | R.RMEELHNQEMQK.R | 3 |
| \* | Astrin\_STLC\_112116\_01.04730.04730.2 | 3.8965 | 0.5177 | 100.0% | 1342.0922 | 1342.4569 | 1 | 9.397 | 78.6% | 8 | R.FGQGGAGPVGGQGPR.G | 2 |
| \* | Astrin\_STLC\_112116\_01.05013.05013.2 | 2.5674 | 0.4673 | 100.0% | 1120.6721 | 1121.2561 | 10 | 6.59 | 59.1% | 4 | R.GMGPGTPAGYGR.G | 2 |

Similarities:
gi|34932414|ref|NP\_03(1:12)  

---

|  |  |  |  |  |  |  |  |  |
| --- | --- | --- | --- | --- | --- | --- | --- | --- |
| U | *gi|4757834|ref|NP\_004* | 3 | 4 | 23.2% | 211 | 23772 | 6.7 | BAG family molecular chaperone regulator 2 [Homo sapiens] |

| Filename XCorr DeltCN Conf% ObsM+H+ CalcM+H+ SpR ZScore Ion% # Sequence  | | | | | | | | | | | | |
| --- | --- | --- | --- | --- | --- | --- | --- | --- | --- | --- | --- | --- |
| \* | Astrin\_STLC\_112116\_tube2\_01.09834.09834.2 | 3.1991 | 0.2995 | 100.0% | 1329.1122 | 1329.5364 | 3 | 6.93 | 65.0% | 1 | R.LLESLDQLELR.V | 2 |
| \* | Astrin\_STLC\_112116\_tube2\_02.08245.08245.3 | 3.5019 | 0.2906 | 99.4% | 2400.5344 | 2400.6917 | 1 | 5.336 | 38.8% | 2 | R.TLTVEVSVETIRNPQQQESLK.H | 3 |
| \* | Astrin\_STLC\_112116\_tube2\_01.11814.11814.3 | 3.718 | 0.3578 | 99.8% | 1904.0944 | 1904.1705 | 1 | 6.578 | 40.6% | 1 | R.IIDEVVNKFLDDLGNAK.S | 3 |

---

|  |  |  |  |  |  |  |  |  |
| --- | --- | --- | --- | --- | --- | --- | --- | --- |
| U | *gi|14141152|ref|NP\_00* | 12 | 19 | 22.9% | 730 | 77516 | 8.7 | heterogeneous nuclear ribonucleoprotein M isoform a [Homo sapiens] |
| U | *gi|157412270|ref|NP\_1* | 12 | 19 | 24.2% | 691 | 73621 | 8.8 | heterogeneous nuclear ribonucleoprotein M isoform b [Homo sapiens] |

| Filename XCorr DeltCN Conf% ObsM+H+ CalcM+H+ SpR ZScore Ion% # Sequence  | | | | | | | | | | | | |
| --- | --- | --- | --- | --- | --- | --- | --- | --- | --- | --- | --- | --- |
|  | Astrin\_STLC\_112116\_tube2\_01.10287.10287.2 | 3.2183 | 0.3502 | 100.0% | 1264.9722 | 1265.4949 | 1 | 6.169 | 70.0% | 1 | R.AFITNIPFDVK.W | 2 |
|  | Astrin\_STLC\_112116\_01.11116.11116.2 | 3.999 | 0.5506 | 100.0% | 1753.6721 | 1754.0051 | 1 | 8.503 | 63.3% | 2 | K.VGEVTYVELLMDAEGK.S | 2 |
|  | Astrin\_STLC\_112116\_tube2\_01.08788.08788.2 | 2.6236 | 0.2599 | 98.9% | 1427.4321 | 1427.6403 | 1 | 4.936 | 62.5% | 1 | R.LGSTVFVANLDYK.V | 2 |
|  | Astrin\_STLC\_112116\_tube2\_01.07902.07902.2 | 3.0191 | 0.251 | 100.0% | 1115.9122 | 1115.3152 | 2 | 5.006 | 77.8% | 1 | R.INEILSNALK.R | 2 |
|  | Astrin\_STLC\_112116\_01.04600.04600.2 | 3.2498 | 0.4932 | 100.0% | 1285.1322 | 1285.3591 | 3 | 8.015 | 60.7% | 1 | K.QGGGGGGGSVPGIER.M | 2 |
|  | Astrin\_STLC\_112116\_01.04308.04308.2 | 2.5103 | 0.1773 | 96.1% | 1103.3922 | 1102.2714 | 368 | 4.146 | 50.0% | 1 | R.MGAGLGHGMDR.V | 2 |
|  | Astrin\_STLC\_112116\_01.07026.07026.3 | 3.2523 | 0.3772 | 99.8% | 1615.2244 | 1614.875 | 5 | 6.766 | 37.5% | 1 | R.MGPLGLDHMASSIER.M | 3 |
|  | Astrin\_STLC\_112116\_tube2\_01.07954.07954.2 | 3.5842 | 0.4959 | 100.0% | 1125.8922 | 1126.3337 | 1 | 9.319 | 80.0% | 1 | R.MGAGMGFGLER.M | 2 |
|  | Astrin\_STLC\_112116\_tube2\_01.06395.06395.2 | 2.8238 | 0.3909 | 100.0% | 1190.0521 | 1189.4333 | 1 | 7.091 | 68.2% | 2 | R.MVPAGMGAGLER.M | 2 |
|  | Astrin\_STLC\_112116\_01.06982.06982.2 | 3.027 | 0.3066 | 100.0% | 1428.1522 | 1428.7076 | 1 | 5.514 | 64.3% | 2 | R.MGPAMGPALGAGIER.M | 2 |
|  | Astrin\_STLC\_112116\_tube2\_02.08154.08154.2 | 3.5446 | 0.4728 | 100.0% | 1383.9521 | 1384.5677 | 1 | 8.892 | 64.3% | 3 | R.MGLAMGGGGGASFDR.A | 2 |
|  | Astrin\_STLC\_112116\_tube2\_02.08130.08130.3 | 3.3584 | 0.4688 | 100.0% | 2035.5543 | 2036.1735 | 256 | 7.401 | 21.6% | 3 | R.GNFGGSFAGSFGGAGGHAPGVAR.K | 3 |

---

|  |  |  |  |  |  |  |  |  |
| --- | --- | --- | --- | --- | --- | --- | --- | --- |
| U | *gi|1005261228|ref|NP\_* | 2 | 3 | 21.5% | 135 | 15516 | 9.8 | ubiquitin-60S ribosomal protein L40 isoform 2 [Homo sapiens] |
| U | *gi|601984520|ref|NP\_0* | 2 | 3 | 4.2% | 685 | 77039 | 7.7 | polyubiquitin-C [Homo sapiens] |
| U | *gi|528524471|ref|NP\_0* | 2 | 3 | 12.7% | 229 | 25762 | 7.4 | polyubiquitin-B precursor [Homo sapiens] |
| U | *gi|4507761|ref|NP\_003* | 2 | 3 | 22.7% | 128 | 14728 | 9.8 | ubiquitin-60S ribosomal protein L40 isoform 1 precursor [Homo sapiens] |
| U | *gi|294459921|ref|NP\_0* | 2 | 3 | 18.6% | 156 | 17965 | 9.6 | ubiquitin-40S ribosomal protein S27a precursor [Homo sapiens] |

| Filename XCorr DeltCN Conf% ObsM+H+ CalcM+H+ SpR ZScore Ion% # Sequence  | | | | | | | | | | | | |
| --- | --- | --- | --- | --- | --- | --- | --- | --- | --- | --- | --- | --- |
|  | Astrin\_STLC\_112116\_01.07041.07041.2 | 4.0022 | 0.4799 | 100.0% | 1788.1921 | 1788.9897 | 1 | 8.466 | 70.0% | 2 | K.TITLEVEPSDTIENVK.A | 2 |
|  | Astrin\_STLC\_112116\_tube2\_01.03603.03603.2 | 3.2005 | 0.3226 | 100.0% | 1524.0122 | 1524.6738 | 1 | 6.826 | 58.3% | 1 | K.IQDKEGIPPDQQR.L | 2 |

---

|  |  |  |  |  |  |  |  |  |
| --- | --- | --- | --- | --- | --- | --- | --- | --- |
| U | *gi|32698730|ref|NP\_06* | 7 | 21 | 21.3% | 695 | 76121 | 8.7 | nuclear fragile X mental retardation-interacting protein 2 [Homo sapiens] |

| Filename XCorr DeltCN Conf% ObsM+H+ CalcM+H+ SpR ZScore Ion% # Sequence  | | | | | | | | | | | | |
| --- | --- | --- | --- | --- | --- | --- | --- | --- | --- | --- | --- | --- |
| \* | Astrin\_STLC\_112116\_01.04832.04832.2 | 3.45 | 0.383 | 100.0% | 1337.9321 | 1338.3763 | 1 | 7.122 | 75.0% | 8 | K.TGYGELNGNAGER.E | 2 |
| \* | Astrin\_STLC\_112116\_tube2\_01.04873.04873.2 | 4.0454 | 0.3919 | 100.0% | 1404.2722 | 1404.4764 | 1 | 8.559 | 66.7% | 3 | K.NLSSDEATNPISR.V | 2 |
| \* | Astrin\_STLC\_112116\_tube2\_01.05805.05805.2 | 3.9026 | 0.4989 | 100.0% | 1515.3522 | 1515.7068 | 1 | 8.204 | 73.1% | 4 | R.VLNGNQQVVDTSLK.Q | 2 |
| \* | Astrin\_STLC\_112116\_02.07720.07720.3 | 3.5311 | 0.3258 | 99.6% | 3557.5745 | 3558.8555 | 1 | 4.707 | 22.7% | 1 | K.SGENQSVDKSDTIPIPNGVVTNNSGYITNGYMGK.G | 3 |
| \* | Astrin\_STLC\_112116\_01.05915.05915.2 | 3.4145 | 0.3147 | 100.0% | 1375.3722 | 1375.6233 | 5 | 5.166 | 68.2% | 3 | K.IMQQETSVPTLK.Q | 2 |
| \* | Astrin\_STLC\_112116\_tube2\_01.03686.03686.3 | 5.7114 | 0.0944 | 97.7% | 2973.0842 | 2973.903 | 3 | 5.638 | 39.3% | 1 | K.TIQNSSVSPT#SSSSSSSSTGETQTQSSSR.L | 3 |
| \* | Astrin\_STLC\_112116\_tube2\_01.11122.11122.3 | 4.2462 | 0.3851 | 99.8% | 3560.9343 | 3561.0437 | 4 | 5.593 | 20.3% | 1 | K.VMEVTFQGEYPATLVSQGAEIIPSGTEHPVFPK.A | 3 |

---

|  |  |  |  |  |  |  |  |  |
| --- | --- | --- | --- | --- | --- | --- | --- | --- |
| U | *gi|14141166|ref|NP\_11* | 5 | 6 | 21.0% | 362 | 38222 | 6.8 | poly(rC)-binding protein 2 isoform b [Homo sapiens] |
| U | *gi|193083114|ref|NP\_0* | 5 | 6 | 23.9% | 318 | 33497 | 8.2 | poly(rC)-binding protein 2 isoform g [Homo sapiens] |
| U | *gi|193083112|ref|NP\_0* | 5 | 6 | 22.7% | 335 | 35347 | 8.0 | poly(rC)-binding protein 2 isoform f [Homo sapiens] |
| U | *gi|193083110|ref|NP\_0* | 5 | 6 | 21.1% | 361 | 38151 | 6.8 | poly(rC)-binding protein 2 isoform e [Homo sapiens] |
| U | *gi|193083108|ref|NP\_0* | 5 | 6 | 20.8% | 365 | 38580 | 6.8 | poly(rC)-binding protein 2 isoform d [Homo sapiens] |
| U | *gi|148833484|ref|NP\_0* | 5 | 6 | 23.0% | 331 | 34917 | 8.0 | poly(rC)-binding protein 2 isoform c [Homo sapiens] |
| U | *gi|14141168|ref|NP\_00* | 5 | 6 | 20.8% | 366 | 38651 | 6.8 | poly(rC)-binding protein 2 isoform a [Homo sapiens] |

| Filename XCorr DeltCN Conf% ObsM+H+ CalcM+H+ SpR ZScore Ion% # Sequence  | | | | | | | | | | | | |
| --- | --- | --- | --- | --- | --- | --- | --- | --- | --- | --- | --- | --- |
|  | Astrin\_STLC\_112116\_01.04785.04785.2 | 2.6149 | 0.3304 | 100.0% | 1288.6921 | 1289.3538 | 1 | 6.269 | 70.0% | 1 | R.INISEGNCPER.I | 22 |
|  | Astrin\_STLC\_112116\_tube2\_01.09897.09897.2 | 2.6707 | 0.4497 | 100.0% | 1358.4122 | 1359.6519 | 6 | 6.877 | 54.2% | 2 | R.IITLAGPTNAIFK.A | 2 |
|  | Astrin\_STLC\_112116\_tube2\_01.07200.07200.2 | 5.4171 | 0.5695 | 100.0% | 2089.9722 | 2091.2573 | 1 | 9.631 | 57.9% | 1 | R.ESTGAQVQVAGDMLPNSTER.A | 22 |
|  | Astrin\_STLC\_112116\_01.04576.04576.2 | 2.4173 | 0.3959 | 100.0% | 1158.4521 | 1159.2413 | 1 | 6.628 | 65.0% | 1 | K.IANPVEGSTDR.Q | 2 |
|  | Astrin\_STLC\_112116\_02.10206.10206.3 | 4.2965 | 0.419 | 100.0% | 2205.9543 | 2206.5474 | 3 | 7.842 | 32.5% | 1 | R.QVTITGSAASISLAQYLINVR.L | 3 |

Similarities:
gi|222352151|ref|NP\_0(2:3)  

---

|  |  |  |  |  |  |  |  |  |
| --- | --- | --- | --- | --- | --- | --- | --- | --- |
| U | *contaminant\_KERATIN07* | 11 | 30 | 20.5% | 473 | 50915 | 5.5 | no description |

| Filename XCorr DeltCN Conf% ObsM+H+ CalcM+H+ SpR ZScore Ion% # Sequence  | | | | | | | | | | | | |
| --- | --- | --- | --- | --- | --- | --- | --- | --- | --- | --- | --- | --- |
|  | Astrin\_STLC\_112116\_tube2\_01.05299.05299.2 | 3.2103 | 0.1537 | 99.8% | 1064.5721 | 1065.2578 | 1 | 6.052 | 81.2% | 2 | R.LASYLDKVR.A | 222222 |
|  | Astrin\_STLC\_112116\_tube2\_02.07110.07110.2 | 4.4264 | 0.3632 | 100.0% | 1303.0322 | 1302.4241 | 1 | 7.671 | 81.8% | 6 | R.ALEEANADLEVK.I | 222 |
|  | Astrin\_STLC\_112116\_tube2\_01.09660.09660.2 | 5.1278 | 0.5192 | 100.0% | 2064.372 | 2065.3774 | 1 | 10.376 | 61.1% | 2 | K.IIAATIENAQPILQIDNAR.L | 22 |
|  | Astrin\_STLC\_112116\_tube2\_01.09652.09652.3 | 5.1324 | 0.4697 | 100.0% | 2064.5044 | 2065.3774 | 1 | 7.651 | 50.0% | 3 | K.IIAATIENAQPILQIDNAR.L | 33 |
|  | Astrin\_STLC\_112116\_01.05584.05584.2 | 1.8322 | 0.255 | 95.2% | 807.8722 | 807.8815 | 186 | 5.3 | 66.7% | 1 | R.LAADDFR.T | 2222222 |
|  | Astrin\_STLC\_112116\_tube2\_01.07839.07839.2 | 3.2486 | 0.3989 | 100.0% | 1030.0122 | 1030.2096 | 1 | 7.534 | 87.5% | 4 | R.VLDELTLAR.T | 22222 |
| \* | Astrin\_STLC\_112116\_tube2\_01.12263.12263.3 | 3.5927 | 0.1854 | 96.9% | 2169.4143 | 2170.5007 | 1 | 5.886 | 39.7% | 2 | R.TDLEMQMEGLKEELAYLR.K | 3 |
|  | Astrin\_STLC\_112116\_tube2\_01.05697.05697.2 | 3.1611 | 0.2971 | 100.0% | 1221.6721 | 1221.3068 | 1 | 6.8 | 80.0% | 3 | K.ASLENSLEETK.G | 222 |
|  | Astrin\_STLC\_112116\_tube2\_01.06467.06467.2 | 3.5426 | 0.3417 | 100.0% | 1381.0922 | 1380.5437 | 2 | 6.215 | 75.0% | 3 | K.TRLEQEIATYR.R | 2222 |
|  | Astrin\_STLC\_112116\_tube2\_01.05638.05638.3 | 3.1897 | 0.3263 | 99.8% | 1535.1543 | 1536.7311 | 1 | 5.56 | 45.5% | 3 | K.TRLEQEIATYRR.L | 3333 |
|  | Astrin\_STLC\_112116\_tube2\_01.04954.04954.2 | 2.7318 | 0.326 | 100.0% | 1122.9922 | 1123.2511 | 1 | 5.932 | 81.2% | 1 | R.LEQEIATYR.R | 22222 |

Similarities:
contaminant\_KERATIN09(1:10)  
gi|24430192|ref|NP\_00(10:1)  
gi|15431310|ref|NP\_00(8:3)  
contaminant\_KERATIN12(6:5)  
contaminant\_KERATIN03(2:9)  
contaminant\_KERATIN10(4:7)  

---

|  |  |  |  |  |  |  |  |  |
| --- | --- | --- | --- | --- | --- | --- | --- | --- |
| U | *gi|4501881|ref|NP\_001* | 6 | 14 | 19.9% | 377 | 42051 | 5.4 | actin, alpha skeletal muscle [Homo sapiens] |
| U | *gi|4885049|ref|NP\_005* | 6 | 14 | 19.9% | 377 | 42019 | 5.4 | actin, alpha cardiac muscle 1 precursor [Homo sapiens] |

| Filename XCorr DeltCN Conf% ObsM+H+ CalcM+H+ SpR ZScore Ion% # Sequence  | | | | | | | | | | | | |
| --- | --- | --- | --- | --- | --- | --- | --- | --- | --- | --- | --- | --- |
|  | Astrin\_STLC\_112116\_01.04121.04121.2 | 3.4288 | 0.4105 | 100.0% | 976.9122 | 977.02136 | 1 | 7.125 | 88.9% | 1 | K.AGFAGDDAPR.A | 22 |
|  | Astrin\_STLC\_112116\_01.06117.06117.2 | 2.8748 | 0.4046 | 100.0% | 1198.4922 | 1199.4415 | 1 | 6.426 | 75.0% | 2 | R.AVFPSIVGRPR.H | 22 |
|  | Astrin\_STLC\_112116\_tube2\_01.08398.08398.2 | 3.9523 | 0.2748 | 100.0% | 1961.9922 | 1962.1841 | 2 | 6.652 | 53.3% | 1 | K.YPIEHGIITNWDDMEK.I | 2 |
|  | Astrin\_STLC\_112116\_01.05896.05896.3 | 3.0002 | 0.2301 | 98.6% | 1516.4343 | 1516.7019 | 5 | 5.183 | 45.0% | 2 | K.IWHHTFYNELR.V | 33 |
|  | Astrin\_STLC\_112116\_tube2\_01.09467.09467.2 | 4.7773 | 0.4402 | 100.0% | 1792.1322 | 1791.9554 | 1 | 8.114 | 80.0% | 7 | K.SYELPDGQVITIGNER.F | 222 |
|  | Astrin\_STLC\_112116\_tube2\_01.06340.06340.1 | 2.3171 | 0.4487 | 100.0% | 1161.5 | 1162.3868 | 22 | 7.087 | 55.0% | 1 | K.EITALAPSTMK.I | 11 |

Similarities:
gi|316659409|ref|NP\_0(5:1)  
gi|63055057|ref|NP\_00(1:5)  

---

|  |  |  |  |  |  |  |  |  |
| --- | --- | --- | --- | --- | --- | --- | --- | --- |
| U | *gi|4505591|ref|NP\_002* | 3 | 4 | 19.6% | 199 | 22110 | 8.1 | peroxiredoxin-1 [Homo sapiens] |

| Filename XCorr DeltCN Conf% ObsM+H+ CalcM+H+ SpR ZScore Ion% # Sequence  | | | | | | | | | | | | |
| --- | --- | --- | --- | --- | --- | --- | --- | --- | --- | --- | --- | --- |
| \* | Astrin\_STLC\_112116\_tube2\_02.08834.08834.3 | 2.7159 | 0.3404 | 99.5% | 1984.0443 | 1984.2163 | 1 | 5.156 | 41.2% | 1 | R.TIAQDYGVLKADEGISFR.G | 3 |
|  | Astrin\_STLC\_112116\_01.06268.06268.2 | 2.5742 | 0.3079 | 99.8% | 1211.4321 | 1212.3915 | 155 | 5.375 | 55.0% | 2 | R.QITVNDLPVGR.S | 2 |
| \* | Astrin\_STLC\_112116\_tube2\_01.08045.08045.2 | 2.596 | 0.395 | 100.0% | 1196.8322 | 1197.3763 | 1 | 6.474 | 72.2% | 1 | R.LVQAFQFTDK.H | 2 |

---

|  |  |  |  |  |  |  |  |  |
| --- | --- | --- | --- | --- | --- | --- | --- | --- |
| U | *gi|22748747|ref|NP\_68* | 2 | 7 | 19.0% | 195 | 21701 | 7.8 | protein LSM12 homolog [Homo sapiens] |

| Filename XCorr DeltCN Conf% ObsM+H+ CalcM+H+ SpR ZScore Ion% # Sequence  | | | | | | | | | | | | |
| --- | --- | --- | --- | --- | --- | --- | --- | --- | --- | --- | --- | --- |
| \* | Astrin\_STLC\_112116\_02.06703.06703.2 | 4.2584 | 0.4837 | 100.0% | 1483.9722 | 1484.6488 | 1 | 9.269 | 75.0% | 3 | R.LQGEVVAFDYQSK.M | 2 |
| \* | Astrin\_STLC\_112116\_02.08539.08539.3 | 4.3583 | 0.4094 | 100.0% | 2589.9844 | 2590.939 | 1 | 7.511 | 33.7% | 4 | K.LSQAYAISAGVSLEGQQLFQTIHK.T | 3 |

---

|  |  |  |  |  |  |  |  |  |
| --- | --- | --- | --- | --- | --- | --- | --- | --- |
| U | *gi|21464101|ref|NP\_03* | 2 | 2 | 18.2% | 247 | 28303 | 4.9 | 14-3-3 protein gamma [Homo sapiens] |

| Filename XCorr DeltCN Conf% ObsM+H+ CalcM+H+ SpR ZScore Ion% # Sequence  | | | | | | | | | | | | |
| --- | --- | --- | --- | --- | --- | --- | --- | --- | --- | --- | --- | --- |
| \* | Astrin\_STLC\_112116\_tube2\_01.09189.09189.2 | 3.1391 | 0.1066 | 95.7% | 1799.1122 | 1798.8473 | 48 | 3.321 | 46.7% | 1 | R.VISS\*IEQK@TSADGNEK.K | 2 |
| \* | Astrin\_STLC\_112116\_tube2\_01.15540.15540.3 | 5.4392 | 0.5124 | 100.0% | 3303.4744 | 3303.6626 | 1 | 9.121 | 29.5% | 1 | K.TAFDDAIAELDTLNEDSYKDSTLIMQLLR.D | 3 |

---

|  |  |  |  |  |  |  |  |  |
| --- | --- | --- | --- | --- | --- | --- | --- | --- |
| U | *gi|21626466|ref|NP\_06* | 10 | 12 | 17.0% | 847 | 94623 | 6.3 | matrin-3 isoform a [Homo sapiens] |

| Filename XCorr DeltCN Conf% ObsM+H+ CalcM+H+ SpR ZScore Ion% # Sequence  | | | | | | | | | | | | |
| --- | --- | --- | --- | --- | --- | --- | --- | --- | --- | --- | --- | --- |
| \* | Astrin\_STLC\_112116\_01.04239.04239.2 | 2.3345 | 0.2874 | 99.3% | 1039.8922 | 1040.121 | 5 | 4.994 | 68.8% | 1 | K.SFQQSSLSR.D | 2 |
| \* | Astrin\_STLC\_112116\_tube2\_01.14306.14306.2 | 2.604 | 0.3496 | 99.8% | 2371.4521 | 2372.7424 | 192 | 6.247 | 27.1% | 1 | R.DLSAAGIGLLAAATQSLSMPASLGR.M | 2 |
| \* | Astrin\_STLC\_112116\_tube2\_02.12262.12262.3 | 4.457 | 0.4453 | 100.0% | 2371.6443 | 2372.7424 | 4 | 7.772 | 29.2% | 1 | R.DLSAAGIGLLAAATQSLSMPASLGR.M | 3 |
| \* | Astrin\_STLC\_112116\_tube2\_01.11575.11575.2 | 4.608 | 0.5151 | 100.0% | 1794.1122 | 1793.931 | 1 | 9.46 | 61.8% | 1 | R.GDADQASNILASFGLSAR.D | 2 |
| \* | Astrin\_STLC\_112116\_tube2\_01.04849.04849.2 | 2.2985 | 0.2367 | 96.8% | 1209.6522 | 1210.2859 | 6 | 4.363 | 60.0% | 2 | R.TEEGPTLSYGR.D | 2 |
|  | Astrin\_STLC\_112116\_01.04601.04601.2 | 2.5557 | 0.1824 | 95.4% | 1325.0721 | 1325.4269 | 1 | 5.772 | 76.9% | 1 | R.GNLGAGNGNLQGPR.H | 2 |
|  | Astrin\_STLC\_112116\_01.06084.06084.2 | 2.5213 | 0.2156 | 98.9% | 1146.0122 | 1145.3647 | 3 | 4.817 | 68.8% | 1 | R.VVHIMDFQR.G | 2 |
|  | Astrin\_STLC\_112116\_tube2\_01.13396.13396.3 | 5.1278 | 0.4535 | 100.0% | 2438.9644 | 2439.9036 | 8 | 7.409 | 31.2% | 2 | R.YQLLQLVEPFGVISNHLILNK.I | 3 |
|  | Astrin\_STLC\_112116\_tube2\_01.07103.07103.3 | 3.9983 | 0.4446 | 100.0% | 2036.8444 | 2038.3109 | 1 | 7.608 | 40.3% | 1 | R.VIHLSNLPHSGYSDSAVLK.L | 3 |
|  | Astrin\_STLC\_112116\_tube2\_01.10406.10406.2 | 3.8402 | 0.4386 | 100.0% | 1969.1721 | 1970.319 | 1 | 7.803 | 50.0% | 1 | R.IGPYQPNVPVGIDYVIPK.T | 2 |

---

|  |  |  |  |  |  |  |  |  |
| --- | --- | --- | --- | --- | --- | --- | --- | --- |
| U | *gi|117956403|ref|NP\_0* | 8 | 15 | 16.9% | 569 | 63543 | 4.8 | rab GTPase-binding effector protein 2 [Homo sapiens] |

| Filename XCorr DeltCN Conf% ObsM+H+ CalcM+H+ SpR ZScore Ion% # Sequence  | | | | | | | | | | | | |
| --- | --- | --- | --- | --- | --- | --- | --- | --- | --- | --- | --- | --- |
| \* | Astrin\_STLC\_112116\_01.04074.04074.2 | 4.8189 | 0.4987 | 100.0% | 1621.8522 | 1621.6146 | 1 | 8.071 | 66.7% | 1 | R.SQEGANGEAESGELSR.L | 2 |
| \* | Astrin\_STLC\_112116\_tube2\_01.06825.06825.3 | 3.581 | 0.3941 | 99.8% | 1715.3043 | 1715.9498 | 1 | 6.416 | 40.0% | 2 | R.HAPSLHGSTELLPLSR.D | 3 |
| \* | Astrin\_STLC\_112116\_01.04616.04616.2 | 3.6294 | 0.3859 | 100.0% | 1317.9722 | 1318.4294 | 1 | 6.618 | 77.3% | 4 | R.TLQGTVSQAQER.V | 2 |
| \* | Astrin\_STLC\_112116\_01.05060.05060.2 | 2.553 | 0.2882 | 99.7% | 1147.1921 | 1147.2273 | 43 | 5.331 | 66.7% | 1 | R.EALEEETVAR.A | 2 |
| \* | Astrin\_STLC\_112116\_02.04330.04330.2 | 4.5728 | 0.3164 | 100.0% | 1531.0922 | 1531.6628 | 1 | 8.424 | 79.2% | 3 | R.LQAELETSEQVQR.D | 2 |
| \* | Astrin\_STLC\_112116\_01.04898.04898.2 | 2.3781 | 0.2794 | 99.6% | 915.59216 | 915.08057 | 2 | 5.173 | 78.6% | 2 | R.LSQALQVR.L | 2 |
| \* | Astrin\_STLC\_112116\_01.04494.04494.2 | 2.2295 | 0.2546 | 98.2% | 1073.9321 | 1074.179 | 1 | 4.977 | 81.2% | 1 | R.QAETLEQVR.S | 2 |
| \* | Astrin\_STLC\_112116\_tube2\_01.08200.08200.2 | 2.1169 | 0.2812 | 96.5% | 1346.2722 | 1347.5261 | 14 | 4.927 | 54.5% | 1 | R.SIMDEAPLTDVR.D | 2 |

---

|  |  |  |  |  |  |  |  |  |
| --- | --- | --- | --- | --- | --- | --- | --- | --- |
| U | *gi|117190174|ref|NP\_0* | 4 | 10 | 16.7% | 293 | 32338 | 5.1 | heterogeneous nuclear ribonucleoproteins C1/C2 isoform b [Homo sapiens] |
| U | *gi|117190192|ref|NP\_0* | 4 | 10 | 16.0% | 306 | 33670 | 5.1 | heterogeneous nuclear ribonucleoproteins C1/C2 isoform a [Homo sapiens] |

| Filename XCorr DeltCN Conf% ObsM+H+ CalcM+H+ SpR ZScore Ion% # Sequence  | | | | | | | | | | | | |
| --- | --- | --- | --- | --- | --- | --- | --- | --- | --- | --- | --- | --- |
|  | Astrin\_STLC\_112116\_02.08268.08268.2 | 2.9808 | 0.2844 | 100.0% | 1316.8322 | 1317.6145 | 1 | 5.377 | 63.6% | 4 | R.VFIGNLNTLVVK.K | 2 |
|  | Astrin\_STLC\_112116\_tube2\_01.06247.06247.2 | 2.7967 | 0.2648 | 99.9% | 1123.9122 | 1124.2792 | 1 | 4.963 | 66.7% | 1 | K.KSDVEAIFSK.Y | 2 |
|  | Astrin\_STLC\_112116\_tube2\_01.09366.09366.2 | 3.3442 | 0.5625 | 100.0% | 1329.6322 | 1330.4857 | 1 | 9.379 | 80.0% | 2 | K.GFAFVQYVNER.N | 2 |
|  | Astrin\_STLC\_112116\_02.08272.08272.2 | 4.9223 | 0.4107 | 100.0% | 1683.9722 | 1684.0038 | 1 | 8.186 | 80.0% | 3 | R.MIAGQVLDINLAAEPK.V | 2 |

---

|  |  |  |  |  |  |  |  |  |
| --- | --- | --- | --- | --- | --- | --- | --- | --- |
| U | *gi|27436946|ref|NP\_73* | 9 | 14 | 16.3% | 664 | 74140 | 7.0 | lamin isoform A [Homo sapiens] |
| U | *gi|544063468|ref|NP\_0* | 9 | 14 | 17.6% | 614 | 69249 | 6.6 | lamin isoform A-delta50 [Homo sapiens] |
| U | *gi|5031875|ref|NP\_005* | 9 | 14 | 18.9% | 572 | 65135 | 6.8 | lamin isoform C [Homo sapiens] |

| Filename XCorr DeltCN Conf% ObsM+H+ CalcM+H+ SpR ZScore Ion% # Sequence  | | | | | | | | | | | | |
| --- | --- | --- | --- | --- | --- | --- | --- | --- | --- | --- | --- | --- |
|  | Astrin\_STLC\_112116\_01.04204.04204.2 | 2.9518 | 0.4097 | 100.0% | 1359.8322 | 1360.4667 | 41 | 6.866 | 46.2% | 1 | R.SGAQASSTPLSPTR.I | 2 |
|  | Astrin\_STLC\_112116\_tube2\_01.04885.04885.3 | 3.321 | 0.2604 | 99.5% | 1631.4844 | 1630.7521 | 18 | 4.746 | 41.7% | 1 | R.LQEKEDLQELNDR.L | 3 |
|  | Astrin\_STLC\_112116\_01.04979.04979.2 | 2.8266 | 0.3071 | 100.0% | 1089.5521 | 1090.1783 | 1 | 6.077 | 77.8% | 4 | R.SLETENAGLR.L | 2 |
|  | Astrin\_STLC\_112116\_01.04365.04365.2 | 2.5706 | 0.3927 | 100.0% | 1148.2922 | 1149.2432 | 2 | 7.192 | 72.2% | 2 | R.ITESEEVVSR.E | 2 |
|  | Astrin\_STLC\_112116\_tube2\_01.05009.05009.2 | 1.9496 | 0.2827 | 95.4% | 1165.9521 | 1166.2328 | 7 | 4.963 | 60.0% | 1 | K.AAYEAELGDAR.K | 2 |
|  | Astrin\_STLC\_112116\_tube2\_01.06636.06636.2 | 3.2398 | 0.2981 | 100.0% | 1510.6721 | 1510.7455 | 2 | 6.107 | 54.5% | 1 | R.LQTMKEELDFQK.N | 2 |
|  | Astrin\_STLC\_112116\_tube2\_01.07480.07480.2 | 2.2404 | 0.2554 | 98.4% | 1028.0322 | 1029.1814 | 1 | 5.565 | 87.5% | 1 | R.LADALQELR.A | 2 |
|  | Astrin\_STLC\_112116\_tube2\_01.10415.10415.2 | 2.5936 | 0.2654 | 98.6% | 1893.2322 | 1895.1346 | 119 | 5.503 | 39.3% | 1 | R.MQQQLDEYQELLDIK.L | 2 |
|  | Astrin\_STLC\_112116\_tube2\_01.06763.06763.2 | 3.5916 | 0.4932 | 100.0% | 1491.6322 | 1492.6874 | 1 | 7.902 | 73.1% | 2 | R.TALINSTGEEVAMR.K | 2 |

---

|  |  |  |  |  |  |  |  |  |
| --- | --- | --- | --- | --- | --- | --- | --- | --- |
| U | *contaminant\_KERATIN22* | 8 | 21 | 16.3% | 645 | 65865 | 8.0 | no description |
| U | *gi|47132620|ref|NP\_00* | 8 | 21 | 16.4% | 639 | 65433 | 8.0 | keratin, type II cytoskeletal 2 epidermal [Homo sapiens] |

| Filename XCorr DeltCN Conf% ObsM+H+ CalcM+H+ SpR ZScore Ion% # Sequence  | | | | | | | | | | | | |
| --- | --- | --- | --- | --- | --- | --- | --- | --- | --- | --- | --- | --- |
|  | Astrin\_STLC\_112116\_tube2\_02.08821.08821.2 | 4.0914 | 0.379 | 100.0% | 1840.0322 | 1840.0055 | 1 | 6.582 | 42.9% | 1 | K.SISISVAGGGGGFGAAGGFGGR.G | 2 |
|  | Astrin\_STLC\_112116\_01.06138.06138.2 | 2.671 | 0.1258 | 96.8% | 1082.1522 | 1083.2755 | 1 | 5.557 | 81.2% | 1 | K.FASFIDKVR.F | 22222222 |
|  | Astrin\_STLC\_112116\_tube2\_01.06129.06129.2 | 4.5739 | 0.073 | 100.0% | 1475.7922 | 1476.6726 | 2 | 7.553 | 86.4% | 9 | R.FLEQQNQVLQTK.W | 222 |
|  | Astrin\_STLC\_112116\_tube2\_01.11175.11175.2 | 3.1902 | 0.4265 | 100.0% | 1461.1122 | 1461.6982 | 1 | 6.942 | 68.2% | 1 | K.VDLLNQEIEFLK.V | 2 |
|  | Astrin\_STLC\_112116\_tube2\_01.11450.11450.2 | 4.018 | 0.3758 | 100.0% | 1329.9722 | 1330.5211 | 1 | 7.458 | 86.4% | 3 | R.NLDLDSIIAEVK.A | 22222222 |
|  | Astrin\_STLC\_112116\_tube2\_01.06183.06183.2 | 2.735 | 0.1721 | 99.1% | 973.8722 | 974.102 | 1 | 4.527 | 85.7% | 2 | K.IEISELNR.V | 222 |
|  | Astrin\_STLC\_112116\_tube2\_01.10694.10694.3 | 3.2647 | 0.288 | 99.5% | 2198.3643 | 2199.4258 | 2 | 5.483 | 37.5% | 1 | R.NKLNDLEEALQQAKEDLAR.L | 3 |
|  | Astrin\_STLC\_112116\_tube2\_01.09346.09346.2 | 3.4818 | 0.346 | 100.0% | 1264.4122 | 1264.4644 | 1 | 7.748 | 80.0% | 3 | K.LALDVEIATYR.K | 222222222 |

Similarities:
contaminant\_KERATIN21(2:6)  
gi|119395750|ref|NP\_0(2:6)  
contaminant\_KERATIN20(1:7)  
gi|119703753|ref|NP\_0(3:5)  
contaminant\_KERATIN13(2:6)  
contaminant\_KERATIN18(3:5)  
gi|5031839|ref|NP\_005(3:5)  
gi|119395754|ref|NP\_0(3:5)  
contaminant\_KERATIN19(1:7)  
gi|153791158|ref|NP\_0(2:6)  
gi|109148552|ref|NP\_4(2:6)  
gi|32567786|ref|NP\_78(2:6)  

---

|  |  |  |  |  |  |  |  |  |
| --- | --- | --- | --- | --- | --- | --- | --- | --- |
| U | *gi|119395754|ref|NP\_0* | 9 | 14 | 16.3% | 590 | 62378 | 7.8 | keratin, type II cytoskeletal 5 [Homo sapiens] |

| Filename XCorr DeltCN Conf% ObsM+H+ CalcM+H+ SpR ZScore Ion% # Sequence  | | | | | | | | | | | | |
| --- | --- | --- | --- | --- | --- | --- | --- | --- | --- | --- | --- | --- |
|  | Astrin\_STLC\_112116\_tube2\_01.06355.06355.2 | 2.5697 | 0.2089 | 96.6% | 1410.0322 | 1411.5547 | 1 | 4.93 | 61.5% | 1 | R.SFSTASAITPSVSR.T | 2 |
| \* | Astrin\_STLC\_112116\_tube2\_01.06227.06227.2 | 2.5039 | 0.2368 | 98.7% | 1111.9521 | 1112.2279 | 1 | 5.246 | 65.0% | 1 | R.ISISTSGGSFR.N | 2 |
|  | Astrin\_STLC\_112116\_01.06138.06138.2 | 2.671 | 0.1258 | 96.8% | 1082.1522 | 1083.2755 | 1 | 5.557 | 81.2% | 1 | K.FASFIDKVR.F | 22222222 |
|  | Astrin\_STLC\_112116\_tube2\_01.07809.07809.2 | 3.0691 | 0.323 | 100.0% | 1203.3121 | 1204.3684 | 1 | 6.71 | 77.8% | 2 | K.WTLLQEQGTK.T | 2222 |
|  | Astrin\_STLC\_112116\_tube2\_01.11450.11450.2 | 4.018 | 0.3758 | 100.0% | 1329.9722 | 1330.5211 | 1 | 7.458 | 86.4% | 3 | R.NLDLDSIIAEVK.A | 22222222 |
|  | Astrin\_STLC\_112116\_tube2\_01.08339.08339.2 | 2.7226 | 0.2099 | 98.6% | 1386.2922 | 1386.5883 | 7 | 4.299 | 63.6% | 1 | R.NKLAELEEALQK.A | 2 |
|  | Astrin\_STLC\_112116\_tube2\_01.07385.07385.2 | 2.734 | 0.2216 | 99.3% | 1143.9722 | 1144.3104 | 1 | 5.131 | 88.9% | 1 | K.LAELEEALQK.A | 2 |
|  | Astrin\_STLC\_112116\_tube2\_01.09346.09346.2 | 3.4818 | 0.346 | 100.0% | 1264.4122 | 1264.4644 | 1 | 7.748 | 80.0% | 3 | K.LALDVEIATYR.K | 222222222 |
|  | Astrin\_STLC\_112116\_tube2\_01.06252.06252.2 | 2.9438 | 0.3186 | 100.0% | 1439.7322 | 1440.5529 | 2 | 6.006 | 43.8% | 1 | R.GLGVGFGSGGGSSSSVK.F | 2 |

Similarities:
contaminant\_KERATIN21(2:7)  
contaminant\_KERATIN20(1:8)  
gi|119703753|ref|NP\_0(4:5)  
contaminant\_KERATIN18(4:5)  
gi|5031839|ref|NP\_005(4:5)  
contaminant\_KERATIN22(3:6)  
contaminant\_KERATIN19(1:8)  
gi|153791158|ref|NP\_0(2:7)  
gi|109148552|ref|NP\_4(2:7)  
gi|32567786|ref|NP\_78(2:7)  

---

|  |  |  |  |  |  |  |  |  |
| --- | --- | --- | --- | --- | --- | --- | --- | --- |
| U | *gi|4502491|ref|NP\_001* | 2 | 3 | 16.0% | 282 | 31362 | 4.8 | complement component 1 Q subcomponent-binding protein, mitochondrial precursor [Homo sapiens] |

| Filename XCorr DeltCN Conf% ObsM+H+ CalcM+H+ SpR ZScore Ion% # Sequence  | | | | | | | | | | | | |
| --- | --- | --- | --- | --- | --- | --- | --- | --- | --- | --- | --- | --- |
| \* | Astrin\_STLC\_112116\_tube2\_02.08800.08800.2 | 3.5255 | 0.4435 | 100.0% | 1621.7722 | 1622.79 | 1 | 7.162 | 60.7% | 2 | K.MSGGWELELNGTEAK.L | 2 |
| \* | Astrin\_STLC\_112116\_01.15045.15045.3 | 4.1454 | 0.2841 | 99.6% | 3440.5144 | 3441.77 | 1 | 5.983 | 23.3% | 1 | R.GVDNTFADELVELSTALEHQEYITFLEDLK.S | 3 |

---

|  |  |  |  |  |  |  |  |  |
| --- | --- | --- | --- | --- | --- | --- | --- | --- |
| U | *gi|345197228|ref|NP\_0* | 2 | 2 | 16.0% | 188 | 21935 | 10.2 | transformer-2 protein homolog beta isoform 2 [Homo sapiens] |
| U | *gi|4759098|ref|NP\_004* | 2 | 2 | 10.4% | 288 | 33666 | 11.2 | transformer-2 protein homolog beta isoform 1 [Homo sapiens] |

| Filename XCorr DeltCN Conf% ObsM+H+ CalcM+H+ SpR ZScore Ion% # Sequence  | | | | | | | | | | | | |
| --- | --- | --- | --- | --- | --- | --- | --- | --- | --- | --- | --- | --- |
|  | Astrin\_STLC\_112116\_tube2\_01.08873.08873.2 | 4.3464 | 0.4103 | 100.0% | 1812.0322 | 1811.989 | 1 | 8.716 | 63.3% | 1 | K.YGPIADVSIVYDQQSR.R | 2 |
|  | Astrin\_STLC\_112116\_02.08694.08694.2 | 3.0936 | 0.3167 | 100.0% | 1621.9722 | 1622.774 | 1 | 6.194 | 57.7% | 1 | R.GFAFVYFENVDDAK.E | 2 |

---

|  |  |  |  |  |  |  |  |  |
| --- | --- | --- | --- | --- | --- | --- | --- | --- |
| U | *gi|24234688|ref|NP\_00* | 8 | 10 | 15.9% | 679 | 73681 | 6.2 | stress-70 protein, mitochondrial precursor [Homo sapiens] |

| Filename XCorr DeltCN Conf% ObsM+H+ CalcM+H+ SpR ZScore Ion% # Sequence  | | | | | | | | | | | | |
| --- | --- | --- | --- | --- | --- | --- | --- | --- | --- | --- | --- | --- |
| \* | Astrin\_STLC\_112116\_01.06359.06359.2 | 2.4017 | 0.2438 | 96.7% | 1451.8522 | 1451.576 | 1 | 5.28 | 61.5% | 1 | R.TTPSVVAFTADGER.L | 2 |
| \* | Astrin\_STLC\_112116\_01.04500.04500.2 | 3.0303 | 0.2 | 99.2% | 1343.4922 | 1342.4105 | 49 | 5.151 | 54.2% | 1 | R.ASNGDAWVEAHGK.L | 2 |
| \* | Astrin\_STLC\_112116\_01.10181.10181.2 | 2.6987 | 0.2438 | 98.8% | 1554.0322 | 1554.8878 | 1 | 5.098 | 69.2% | 2 | K.LYSPSQIGAFVLMK.M | 2 |
| \* | Astrin\_STLC\_112116\_tube2\_01.08739.08739.2 | 2.494 | 0.2247 | 96.2% | 1695.2322 | 1695.8723 | 1 | 5.303 | 53.6% | 1 | K.NAVITVPAYFNDSQR.Q | 2 |
| \* | Astrin\_STLC\_112116\_tube2\_01.08201.08201.2 | 3.5531 | 0.2783 | 100.0% | 1242.9722 | 1243.4056 | 1 | 6.28 | 77.3% | 1 | K.DAGQISGLNVLR.V | 2 |
| \* | Astrin\_STLC\_112116\_01.09460.09460.2 | 2.9253 | 0.3252 | 100.0% | 1361.9122 | 1362.5687 | 1 | 7.17 | 63.6% | 1 | R.AQFEGIVTDLIR.R | 2 |
| \* | Astrin\_STLC\_112116\_tube2\_01.08102.08102.2 | 3.4569 | 0.2544 | 100.0% | 1292.1322 | 1291.4496 | 1 | 7.269 | 80.0% | 2 | K.VQQTVQDLFGR.A | 2 |
| \* | Astrin\_STLC\_112116\_tube2\_02.07888.07888.2 | 4.7192 | 0.5154 | 100.0% | 1809.1522 | 1809.9707 | 1 | 9.101 | 50.0% | 1 | K.SQVFSTAADGQTQVEIK.V | 2 |

---

|  |  |  |  |  |  |  |  |  |
| --- | --- | --- | --- | --- | --- | --- | --- | --- |
| U | *gi|157388995|ref|NP\_0* | 4 | 5 | 15.5% | 361 | 41072 | 6.1 | protein-L-isoaspartate O-methyltransferase domain-containing protein 2 isoform 1 [Homo sapiens] |

| Filename XCorr DeltCN Conf% ObsM+H+ CalcM+H+ SpR ZScore Ion% # Sequence  | | | | | | | | | | | | |
| --- | --- | --- | --- | --- | --- | --- | --- | --- | --- | --- | --- | --- |
|  | Astrin\_STLC\_112116\_tube2\_01.07045.07045.2 | 2.5742 | 0.1147 | 95.5% | 1093.9922 | 1093.2249 | 6 | 5.035 | 75.0% | 1 | R.TELVEQAFR.A | 2 |
|  | Astrin\_STLC\_112116\_tube2\_01.10493.10493.3 | 3.6113 | 0.237 | 99.1% | 2396.5144 | 2397.6458 | 1 | 5.117 | 30.6% | 1 | R.ADYYLEEFKENAYKDLAWK.H | 3 |
| \* | Astrin\_STLC\_112116\_01.08535.08535.2 | 2.4057 | 0.3015 | 99.2% | 1285.2722 | 1285.5848 | 57 | 4.935 | 50.0% | 2 | K.VGGILVMPLEEK.L | 2 |
|  | Astrin\_STLC\_112116\_tube2\_01.09682.09682.3 | 4.3729 | 0.3551 | 100.0% | 1941.2043 | 1942.2836 | 1 | 6.302 | 38.3% | 1 | R.RMETIVFLDKEVFASR.I | 3 |

---

|  |  |  |  |  |  |  |  |  |
| --- | --- | --- | --- | --- | --- | --- | --- | --- |
| U | *contaminant\_KERATIN19* | 5 | 6 | 15.2% | 468 | 51203 | 5.5 | no description |
| U | *gi|67782365|ref|NP\_00* | 5 | 6 | 15.1% | 469 | 51386 | 5.5 | keratin, type II cytoskeletal 7 [Homo sapiens] |

| Filename XCorr DeltCN Conf% ObsM+H+ CalcM+H+ SpR ZScore Ion% # Sequence  | | | | | | | | | | | | |
| --- | --- | --- | --- | --- | --- | --- | --- | --- | --- | --- | --- | --- |
|  | Astrin\_STLC\_112116\_tube2\_01.05073.05073.2 | 3.3562 | 0.4523 | 100.0% | 1105.1322 | 1105.2388 | 2 | 7.126 | 68.2% | 2 | R.SAYGGPVGAGIR.E | 2 |
|  | Astrin\_STLC\_112116\_01.06138.06138.2 | 2.671 | 0.1258 | 96.8% | 1082.1522 | 1083.2755 | 1 | 5.557 | 81.2% | 1 | K.FASFIDKVR.F | 22222222 |
|  | Astrin\_STLC\_112116\_tube2\_01.11061.11061.2 | 3.3565 | 0.4236 | 100.0% | 1443.1721 | 1443.686 | 1 | 7.82 | 66.7% | 1 | R.LPDIFEAQIAGLR.G | 2 |
|  | Astrin\_STLC\_112116\_02.10114.10114.3 | 4.001 | 0.1555 | 95.2% | 3170.2744 | 3172.2698 | 1 | 5.794 | 28.8% | 1 | R.T#LNET#ELTELQSQISDTSVVLSMDNSR.S | 3 |
|  | Astrin\_STLC\_112116\_tube2\_01.05594.05594.2 | 3.1639 | 0.2805 | 100.0% | 1197.0122 | 1197.2897 | 1 | 6.472 | 83.3% | 1 | R.AEAEAWYQTK.F | 22 |

Similarities:
contaminant\_KERATIN20(1:4)  
gi|119703753|ref|NP\_0(1:4)  
contaminant\_KERATIN18(1:4)  
gi|5031839|ref|NP\_005(1:4)  
contaminant\_KERATIN22(1:4)  
gi|119395754|ref|NP\_0(1:4)  
gi|109148552|ref|NP\_4(1:4)  
gi|32567786|ref|NP\_78(1:4)  

---

|  |  |  |  |  |  |  |  |  |
| --- | --- | --- | --- | --- | --- | --- | --- | --- |
| U | *gi|4506687|ref|NP\_001* | 2 | 2 | 15.2% | 145 | 17040 | 10.4 | 40S ribosomal protein S15 isoform 2 [Homo sapiens] |
| U | *gi|815891093|ref|NP\_0* | 2 | 2 | 14.5% | 152 | 17723 | 10.4 | 40S ribosomal protein S15 isoform 1 [Homo sapiens] |

| Filename XCorr DeltCN Conf% ObsM+H+ CalcM+H+ SpR ZScore Ion% # Sequence  | | | | | | | | | | | | |
| --- | --- | --- | --- | --- | --- | --- | --- | --- | --- | --- | --- | --- |
|  | Astrin\_STLC\_112116\_01.14703.14703.2 | 5.3901 | 0.5562 | 100.0% | 2588.892 | 2589.938 | 1 | 9.497 | 50.0% | 1 | R.GVDLDQLLDMSYEQLMQLYSAR.Q | 2 |
|  | Astrin\_STLC\_112116\_01.14693.14693.3 | 3.2868 | 0.3327 | 99.6% | 2589.0544 | 2589.938 | 24 | 5.573 | 26.2% | 1 | R.GVDLDQLLDMSYEQLMQLYSAR.Q | 3 |

---

|  |  |  |  |  |  |  |  |  |
| --- | --- | --- | --- | --- | --- | --- | --- | --- |
| U | *gi|190360566|ref|NP\_4* | 4 | 8 | 14.8% | 357 | 40675 | 5.7 | protein-L-isoaspartate O-methyltransferase domain-containing protein 1 isoform 1 [Homo sapiens] |

| Filename XCorr DeltCN Conf% ObsM+H+ CalcM+H+ SpR ZScore Ion% # Sequence  | | | | | | | | | | | | |
| --- | --- | --- | --- | --- | --- | --- | --- | --- | --- | --- | --- | --- |
| \* | Astrin\_STLC\_112116\_tube2\_01.16254.16254.2 | 3.6795 | 0.4196 | 100.0% | 2014.0922 | 2015.0698 | 1 | 6.777 | 58.3% | 2 | -.MGGAVS\*AGEDNDDLIDNLK.E | 2 |
| \* | Astrin\_STLC\_112116\_tube2\_01.15956.15956.2 | 3.1365 | 0.5759 | 100.0% | 2774.372 | 2775.9177 | 1 | 9.268 | 35.4% | 3 | -.MGGAVS\*AGEDNDDLIDNLKEAQYIR.T | 2 |
|  | Astrin\_STLC\_112116\_tube2\_01.13167.13167.2 | 4.8931 | 0.5768 | 100.0% | 2013.4722 | 2014.4492 | 1 | 10.526 | 67.6% | 1 | K.VGGILVMPIEDQLTQIMR.T | 2 |
|  | Astrin\_STLC\_112116\_tube2\_01.06891.06891.2 | 2.7392 | 0.1168 | 95.9% | 1209.9122 | 1210.3472 | 10 | 4.147 | 72.2% | 2 | R.NFINDEMQAK.G | 2 |

---

|  |  |  |  |  |  |  |  |  |
| --- | --- | --- | --- | --- | --- | --- | --- | --- |
| U | *gi|198041662|ref|NP\_0* | 3 | 3 | 14.3% | 286 | 29893 | 8.1 | pyrroline-5-carboxylate reductase 3 [Homo sapiens] |

| Filename XCorr DeltCN Conf% ObsM+H+ CalcM+H+ SpR ZScore Ion% # Sequence  | | | | | | | | | | | | |
| --- | --- | --- | --- | --- | --- | --- | --- | --- | --- | --- | --- | --- |
| \* | Astrin\_STLC\_112116\_tube2\_01.07614.07614.2 | 2.8052 | 0.3913 | 100.0% | 1100.1721 | 1101.3525 | 2 | 6.674 | 75.0% | 1 | R.MAGAIAQGLIR.A | 2 |
| \* | Astrin\_STLC\_112116\_tube2\_01.04608.04608.3 | 3.141 | 0.207 | 96.5% | 1764.5643 | 1764.9791 | 5 | 4.376 | 35.9% | 1 | R.AGKVEAQHILASAPTDR.N | 3 |
| \* | Astrin\_STLC\_112116\_tube2\_01.04384.04384.3 | 2.8977 | 0.2201 | 96.8% | 1531.4644 | 1530.7881 | 107 | 4.241 | 35.4% | 1 | K.MLLHEGQHPAQLR.S | 3 |

---

|  |  |  |  |  |  |  |  |  |
| --- | --- | --- | --- | --- | --- | --- | --- | --- |
| U | *gi|221307584|ref|NP\_0* | 3 | 3 | 14.0% | 299 | 33296 | 9.8 | prohibitin-2 isoform 1 [Homo sapiens] |

| Filename XCorr DeltCN Conf% ObsM+H+ CalcM+H+ SpR ZScore Ion% # Sequence  | | | | | | | | | | | | |
| --- | --- | --- | --- | --- | --- | --- | --- | --- | --- | --- | --- | --- |
|  | Astrin\_STLC\_112116\_tube2\_01.08728.08728.2 | 2.6107 | 0.29 | 99.3% | 1260.6522 | 1260.5222 | 1 | 6.401 | 62.5% | 1 | K.LLLGAGAVAYGVR.E | 2 |
|  | Astrin\_STLC\_112116\_tube2\_02.09115.09115.3 | 2.5832 | 0.2729 | 96.9% | 1854.5343 | 1855.1038 | 1 | 4.771 | 37.5% | 1 | R.IGGVQQDTILAEGLHFR.I | 3 |
| \* | Astrin\_STLC\_112116\_01.03916.03916.2 | 3.8409 | 0.4618 | 100.0% | 1216.0122 | 1216.3336 | 1 | 8.733 | 81.8% | 1 | K.IVQAEGEAEAAK.M | 2 |

---

|  |  |  |  |  |  |  |  |  |
| --- | --- | --- | --- | --- | --- | --- | --- | --- |
| U | *gi|4506619|ref|NP\_000* | 2 | 3 | 14.0% | 157 | 17779 | 11.3 | 60S ribosomal protein L24 [Homo sapiens] |

| Filename XCorr DeltCN Conf% ObsM+H+ CalcM+H+ SpR ZScore Ion% # Sequence  | | | | | | | | | | | | |
| --- | --- | --- | --- | --- | --- | --- | --- | --- | --- | --- | --- | --- |
| \* | Astrin\_STLC\_112116\_tube2\_01.09795.09795.2 | 2.3784 | 0.2813 | 99.3% | 1193.3522 | 1193.391 | 4 | 4.721 | 68.8% | 1 | R.QINWTVLYR.R | 2 |
| \* | Astrin\_STLC\_112116\_01.07278.07278.2 | 3.4967 | 0.4401 | 100.0% | 1262.0122 | 1262.5072 | 1 | 9.269 | 75.0% | 2 | R.AITGASLADIMAK.R | 2 |

---

|  |  |  |  |  |  |  |  |  |
| --- | --- | --- | --- | --- | --- | --- | --- | --- |
| U | *gi|4502303|ref|NP\_001* | 2 | 2 | 13.1% | 213 | 23277 | 10.0 | ATP synthase subunit O, mitochondrial precursor [Homo sapiens] |

| Filename XCorr DeltCN Conf% ObsM+H+ CalcM+H+ SpR ZScore Ion% # Sequence  | | | | | | | | | | | | |
| --- | --- | --- | --- | --- | --- | --- | --- | --- | --- | --- | --- | --- |
| \* | Astrin\_STLC\_112116\_tube2\_01.07123.07123.2 | 2.788 | 0.4408 | 100.0% | 1161.2122 | 1161.3867 | 1 | 6.59 | 75.0% | 1 | K.VAASVLNPYVK.R | 2 |
| \* | Astrin\_STLC\_112116\_tube2\_01.12709.12709.2 | 3.382 | 0.4355 | 100.0% | 1873.5922 | 1874.1472 | 3 | 7.455 | 46.9% | 1 | R.FSPLTTNLINLLAENGR.L | 2 |

---

|  |  |  |  |  |  |  |  |  |
| --- | --- | --- | --- | --- | --- | --- | --- | --- |
| U | *gi|4885399|ref|NP\_005* | 11 | 13 | 12.9% | 1217 | 141541 | 7.2 | structural maintenance of chromosomes protein 3 [Homo sapiens] &IC SMC3 |

| Filename XCorr DeltCN Conf% ObsM+H+ CalcM+H+ SpR ZScore Ion% # Sequence  | | | | | | | | | | | | |
| --- | --- | --- | --- | --- | --- | --- | --- | --- | --- | --- | --- | --- |
| \* | Astrin\_STLC\_112116\_tube2\_02.12320.12320.3 | 3.7025 | 0.3585 | 99.8% | 2832.7144 | 2832.1472 | 4 | 5.562 | 25.0% | 2 | K.SNFFYAIQFVLSDEFSHLRPEQR.L | 3 |
| \* | Astrin\_STLC\_112116\_tube2\_01.11656.11656.2 | 3.958 | 0.412 | 100.0% | 1553.5721 | 1553.7301 | 1 | 9.089 | 73.1% | 1 | K.NDVMNLLESAGFSR.S | 2 |
| \* | Astrin\_STLC\_112116\_01.04070.04070.2 | 3.6782 | 0.3056 | 100.0% | 1332.0521 | 1332.4741 | 1 | 6.473 | 81.8% | 1 | K.INQMATAPDSQR.L | 2 |
| \* | Astrin\_STLC\_112116\_tube2\_01.04160.04160.3 | 3.5569 | 0.3448 | 99.8% | 1749.6843 | 1749.9769 | 26 | 5.974 | 35.7% | 1 | K.ISAMKEEKEQLSAER.Q | 3 |
| \* | Astrin\_STLC\_112116\_tube2\_01.07720.07720.3 | 3.0653 | 0.2891 | 99.5% | 1964.0944 | 1964.1393 | 1 | 5.569 | 43.3% | 1 | K.NLEQYNKLDQDLNEVK.A | 3 |
| \* | Astrin\_STLC\_112116\_tube2\_01.05204.05204.2 | 2.7123 | 0.1888 | 98.4% | 1173.5122 | 1174.2554 | 5 | 4.558 | 70.0% | 1 | R.GALTGGYYDTR.K | 2 |
| \* | Astrin\_STLC\_112116\_tube2\_01.04856.04856.2 | 3.0857 | 0.325 | 100.0% | 1345.4321 | 1346.4772 | 4 | 6.374 | 63.6% | 1 | R.KAEEELGELEAK.L | 2 |
| \* | Astrin\_STLC\_112116\_tube2\_01.05296.05296.2 | 2.3817 | 0.2644 | 99.2% | 1043.8522 | 1045.1375 | 10 | 5.455 | 68.8% | 2 | R.VDALNDEIR.Q | 2 |
| \* | Astrin\_STLC\_112116\_tube2\_01.06571.06571.2 | 2.5763 | 0.162 | 96.7% | 1251.8922 | 1251.3818 | 1 | 4.536 | 72.2% | 1 | R.VETYLNENLR.K | 2 |
| \* | Astrin\_STLC\_112116\_tube2\_01.07920.07920.3 | 4.0224 | 0.3973 | 100.0% | 2233.2544 | 2234.422 | 17 | 6.266 | 34.2% | 1 | R.SEDLDNSIDKTEAGIKELQK.S | 3 |
| \* | Astrin\_STLC\_112116\_tube2\_01.06108.06108.3 | 2.6807 | 0.2402 | 96.4% | 1655.1543 | 1656.9379 | 29 | 4.563 | 35.7% | 1 | R.NKVSHIDVITAEMAK.D | 3 |

---

|  |  |  |  |  |  |  |  |  |
| --- | --- | --- | --- | --- | --- | --- | --- | --- |
| U | *gi|124256496|ref|NP\_0* | 7 | 19 | 12.8% | 641 | 70375 | 6.0 | heat shock 70 kDa protein 1-like [Homo sapiens] |

| Filename XCorr DeltCN Conf% ObsM+H+ CalcM+H+ SpR ZScore Ion% # Sequence  | | | | | | | | | | | | |
| --- | --- | --- | --- | --- | --- | --- | --- | --- | --- | --- | --- | --- |
|  | Astrin\_STLC\_112116\_tube2\_01.07222.07222.2 | 3.2644 | 0.4305 | 100.0% | 1488.9722 | 1488.5939 | 1 | 8.322 | 79.2% | 5 | R.TTPSYVAFTDTER.L | 2222 |
|  | Astrin\_STLC\_112116\_01.09358.09358.2 | 3.1419 | 0.4076 | 100.0% | 1615.4122 | 1615.8817 | 1 | 6.598 | 73.1% | 3 | K.AFYPEEISSMVLTK.L | 22 |
|  | Astrin\_STLC\_112116\_tube2\_01.09728.09728.2 | 3.5325 | 0.3274 | 100.0% | 1198.2322 | 1198.408 | 1 | 6.836 | 86.4% | 6 | K.DAGVIAGLNVLR.I | 22 |
|  | Astrin\_STLC\_112116\_tube2\_01.09259.09259.2 | 3.9717 | 0.4271 | 100.0% | 1661.6322 | 1660.9078 | 1 | 7.009 | 73.3% | 2 | R.IINEPTAAAIAYGLDK.G | 222 |
|  | Astrin\_STLC\_112116\_01.05559.05559.2 | 3.8525 | 0.3976 | 100.0% | 1675.8522 | 1676.6964 | 1 | 8.003 | 56.7% | 1 | K.ATAGDTHLGGEDFDNR.L | 222 |
|  | Astrin\_STLC\_112116\_tube2\_01.04581.04581.3 | 3.1587 | 0.2499 | 98.6% | 1676.1543 | 1676.6964 | 126 | 5.668 | 31.7% | 1 | K.ATAGDTHLGGEDFDNR.L | 333 |
|  | Astrin\_STLC\_112116\_tube2\_01.08427.08427.2 | 3.1583 | 0.3764 | 100.0% | 1288.0922 | 1288.4608 | 1 | 6.75 | 80.0% | 1 | K.NALESYAFNMK.S | 22 |

Similarities:
gi|16507237|ref|NP\_00(1:6)  
gi|5729877|ref|NP\_006(2:5)  
gi|167466173|ref|NP\_0(6:1)  
gi|34419635|ref|NP\_00(3:4)  

---

|  |  |  |  |  |  |  |  |  |
| --- | --- | --- | --- | --- | --- | --- | --- | --- |
| U | *gi|24307939|ref|NP\_03* | 4 | 6 | 12.8% | 541 | 59671 | 5.6 | T-complex protein 1 subunit epsilon isoform a [Homo sapiens] |
| U | *gi|807066366|ref|NP\_0* | 4 | 6 | 13.7% | 503 | 55349 | 5.5 | T-complex protein 1 subunit epsilon isoform e [Homo sapiens] |
| U | *gi|807066360|ref|NP\_0* | 4 | 6 | 13.3% | 520 | 57144 | 5.6 | T-complex protein 1 subunit epsilon isoform b [Homo sapiens] |

| Filename XCorr DeltCN Conf% ObsM+H+ CalcM+H+ SpR ZScore Ion% # Sequence  | | | | | | | | | | | | |
| --- | --- | --- | --- | --- | --- | --- | --- | --- | --- | --- | --- | --- |
|  | Astrin\_STLC\_112116\_01.15586.15586.3 | 3.3306 | 0.2331 | 97.4% | 3116.0044 | 3115.3752 | 4 | 4.271 | 21.6% | 1 | K.SQDDEIGDGTTGVVVLAGALLEEAEQLLDR.G | 3 |
|  | Astrin\_STLC\_112116\_01.04145.04145.2 | 2.5882 | 0.3716 | 100.0% | 1093.7922 | 1094.1692 | 1 | 6.164 | 77.8% | 1 | R.IADGYEQAAR.V | 2 |
|  | Astrin\_STLC\_112116\_tube2\_01.08564.08564.2 | 2.6565 | 0.2766 | 99.5% | 1391.6921 | 1392.592 | 1 | 5.162 | 68.2% | 1 | R.DVDFELIKVEGK.V | 2 |
|  | Astrin\_STLC\_112116\_tube2\_01.13162.13162.2 | 3.2017 | 0.3197 | 100.0% | 1740.2522 | 1740.0122 | 1 | 6.511 | 46.9% | 3 | R.WVGGPEIELIAIATGGR.I | 2 |

---

|  |  |  |  |  |  |  |  |  |
| --- | --- | --- | --- | --- | --- | --- | --- | --- |
| U | *gi|21359873|ref|NP\_00* | 4 | 10 | 12.4% | 603 | 68255 | 8.9 | serine/threonine-protein kinase PLK1 [Homo sapiens] &IC PLK1 |

| Filename XCorr DeltCN Conf% ObsM+H+ CalcM+H+ SpR ZScore Ion% # Sequence  | | | | | | | | | | | | |
| --- | --- | --- | --- | --- | --- | --- | --- | --- | --- | --- | --- | --- |
| \* | Astrin\_STLC\_112116\_tube2\_01.05579.05579.2 | 3.0923 | 0.3758 | 100.0% | 1570.1322 | 1570.832 | 1 | 7.282 | 63.9% | 1 | K.AGVPGVAAPGAPAAAPPAK.E | 2 |
| \* | Astrin\_STLC\_112116\_tube2\_01.06259.06259.2 | 2.2583 | 0.299 | 98.7% | 1291.2722 | 1291.5363 | 1 | 5.927 | 68.2% | 1 | K.HINPVAASLIQK.M | 2 |
| \* | Astrin\_STLC\_112116\_01.11223.11223.3 | 4.3752 | 0.3923 | 100.0% | 3311.3943 | 3312.7217 | 1 | 6.753 | 28.6% | 1 | K.MLQTDPTARPTINELLNDEFFTSGYIPAR.L | 3 |
| \* | Astrin\_STLC\_112116\_tube2\_01.09510.09510.2 | 4.3528 | 0.4026 | 100.0% | 1812.8922 | 1813.0172 | 1 | 7.557 | 67.9% | 7 | R.LILYNDGDSLQYIER.D | 2 |

---

|  |  |  |  |  |  |  |  |  |
| --- | --- | --- | --- | --- | --- | --- | --- | --- |
| U | *gi|6678271|ref|NP\_031* | 3 | 3 | 12.3% | 414 | 44740 | 6.2 | TAR DNA-binding protein 43 [Homo sapiens] |

| Filename XCorr DeltCN Conf% ObsM+H+ CalcM+H+ SpR ZScore Ion% # Sequence  | | | | | | | | | | | | |
| --- | --- | --- | --- | --- | --- | --- | --- | --- | --- | --- | --- | --- |
| \* | Astrin\_STLC\_112116\_tube2\_01.11716.11716.3 | 4.3398 | 0.4667 | 100.0% | 2625.0244 | 2626.03 | 2 | 7.546 | 29.3% | 1 | R.LVEGILHAPDAGWGNLVYVVNYPK.D | 3 |
| \* | Astrin\_STLC\_112116\_01.05464.05464.2 | 2.0996 | 0.3495 | 99.3% | 1144.2322 | 1145.2543 | 3 | 6.041 | 68.8% | 1 | R.FTEYETQVK.V | 2 |
| \* | Astrin\_STLC\_112116\_tube2\_01.06810.06810.2 | 4.7236 | 0.5177 | 100.0% | 1726.9922 | 1727.7928 | 1 | 8.302 | 79.4% | 1 | R.FGGNPGGFGNQGGFGNSR.G | 2 |

---

|  |  |  |  |  |  |  |  |  |
| --- | --- | --- | --- | --- | --- | --- | --- | --- |
| U | *gi|290656936|ref|NP\_0* | 3 | 3 | 12.1% | 265 | 31005 | 8.4 | thymidine kinase 2, mitochondrial isoform 1 precursor [Homo sapiens] |
| U | *gi|429836851|ref|NP\_0* | 3 | 3 | 14.8% | 216 | 25486 | 6.7 | thymidine kinase 2 isoform 5 [Homo sapiens] |
| U | *gi|290657146|ref|NP\_0* | 3 | 3 | 13.3% | 240 | 28374 | 9.2 | thymidine kinase 2, mitochondrial isoform 3 precursor [Homo sapiens] |
| U | *gi|290656975|ref|NP\_0* | 3 | 3 | 13.7% | 234 | 27562 | 6.8 | thymidine kinase 2 isoform 2 [Homo sapiens] |

| Filename XCorr DeltCN Conf% ObsM+H+ CalcM+H+ SpR ZScore Ion% # Sequence  | | | | | | | | | | | | |
| --- | --- | --- | --- | --- | --- | --- | --- | --- | --- | --- | --- | --- |
|  | Astrin\_STLC\_112116\_tube2\_01.06323.06323.3 | 2.5067 | 0.2687 | 96.9% | 1568.1843 | 1568.7502 | 15 | 5.906 | 28.8% | 1 | R.GHNPLGLMYHDASR.W | 3 |
|  | Astrin\_STLC\_112116\_tube2\_01.09554.09554.2 | 2.3635 | 0.3271 | 99.8% | 1216.8322 | 1217.4099 | 1 | 5.749 | 75.0% | 1 | R.YIFVENLYR.S | 2 |
|  | Astrin\_STLC\_112116\_tube2\_01.08868.08868.2 | 2.4927 | 0.3023 | 99.8% | 1179.8121 | 1180.3643 | 19 | 5.577 | 75.0% | 1 | R.MLELFEQNR.D | 2 |

---

|  |  |  |  |  |  |  |  |  |
| --- | --- | --- | --- | --- | --- | --- | --- | --- |
| U | *contaminant\_INT-STD1* | 7 | 11 | 12.0% | 607 | 69271 | 6.1 | BSA |

| Filename XCorr DeltCN Conf% ObsM+H+ CalcM+H+ SpR ZScore Ion% # Sequence  | | | | | | | | | | | | |
| --- | --- | --- | --- | --- | --- | --- | --- | --- | --- | --- | --- | --- |
| \* | Astrin\_STLC\_112116\_tube2\_01.08216.08216.2 | 2.7137 | 0.3802 | 100.0% | 1163.2722 | 1164.344 | 2 | 6.945 | 77.8% | 1 | K.LVNELTEFAK.T | 2 |
| \* | Astrin\_STLC\_112116\_tube2\_01.07150.07150.3 | 4.1847 | 0.3467 | 100.0% | 1440.6543 | 1440.6884 | 1 | 6.157 | 56.8% | 1 | R.RHPEYAVSVLLR.L | 3 |
| \* | Astrin\_STLC\_112116\_tube2\_01.06250.06250.2 | 2.6665 | 0.4137 | 100.0% | 1306.0521 | 1306.5046 | 1 | 7.644 | 70.0% | 1 | K.HLVDEPQNLIK.Q | 2 |
| \* | Astrin\_STLC\_112116\_02.08136.08136.2 | 3.9229 | 0.3685 | 100.0% | 1480.0122 | 1480.7068 | 1 | 7.708 | 66.7% | 4 | K.LGEYGFQNALIVR.Y | 2 |
|  | Astrin\_STLC\_112116\_tube2\_01.06873.06873.3 | 3.6048 | 0.3644 | 99.8% | 1640.2444 | 1640.9205 | 66 | 6.637 | 37.5% | 1 | R.KVPQVSTPTLVEVSR.S | 3 |
|  | Astrin\_STLC\_112116\_tube2\_01.06883.06883.2 | 2.852 | 0.2246 | 98.6% | 1641.0521 | 1640.9205 | 43 | 5.168 | 42.9% | 1 | R.KVPQVSTPTLVEVSR.S | 2 |
| \* | Astrin\_STLC\_112116\_tube2\_01.11150.11150.2 | 3.6025 | 0.5011 | 100.0% | 1400.1721 | 1400.6324 | 1 | 9.953 | 72.7% | 2 | K.TVMENFVAFVDK.C | 2 |

---

|  |  |  |  |  |  |  |  |  |
| --- | --- | --- | --- | --- | --- | --- | --- | --- |
| U | *gi|289577134|ref|NP\_0* | 21 | 29 | 11.6% | 2243 | 262325 | 5.4 | golgin subfamily A member 4 isoform 1 [Homo sapiens] |
| U | *gi|6715600|ref|NP\_002* | 21 | 29 | 11.7% | 2230 | 261137 | 5.4 | golgin subfamily A member 4 isoform 2 [Homo sapiens] |

| Filename XCorr DeltCN Conf% ObsM+H+ CalcM+H+ SpR ZScore Ion% # Sequence  | | | | | | | | | | | | |
| --- | --- | --- | --- | --- | --- | --- | --- | --- | --- | --- | --- | --- |
|  | Astrin\_STLC\_112116\_tube2\_01.07909.07909.2 | 2.8406 | 0.2563 | 99.9% | 1162.0721 | 1161.3379 | 2 | 4.851 | 72.2% | 1 | R.ILELESSLEK.S | 2 |
|  | Astrin\_STLC\_112116\_tube2\_01.06443.06443.2 | 3.0534 | 0.3196 | 100.0% | 1449.0922 | 1449.6036 | 1 | 5.143 | 68.2% | 1 | K.AYEEQLAQLQQK.L | 2 |
|  | Astrin\_STLC\_112116\_tube2\_01.10354.10354.2 | 2.697 | 0.1977 | 98.0% | 1473.2122 | 1473.7277 | 1 | 4.66 | 63.6% | 1 | K.IQVQDLMQQLEK.Q | 2 |
|  | Astrin\_STLC\_112116\_tube2\_01.04309.04309.3 | 3.2737 | 0.1928 | 96.9% | 1641.9543 | 1641.8217 | 19 | 4.458 | 32.1% | 1 | K.SAHVNSLAQDETKLK.A | 3 |
|  | Astrin\_STLC\_112116\_tube2\_01.09368.09368.3 | 4.0133 | 0.3418 | 99.8% | 1919.9944 | 1920.2133 | 2 | 5.586 | 35.0% | 1 | K.SLKENTFLQEQLVELK.M | 3 |
|  | Astrin\_STLC\_112116\_tube2\_01.05505.05505.2 | 2.953 | 0.3517 | 100.0% | 1197.7722 | 1198.272 | 7 | 7.263 | 66.7% | 1 | K.TTDEEFQSLK.S | 2 |
|  | Astrin\_STLC\_112116\_tube2\_01.06383.06383.2 | 2.3738 | 0.3119 | 99.3% | 1205.2322 | 1206.3385 | 1 | 7.444 | 80.0% | 1 | K.TNELINISSSK.T | 2 |
|  | Astrin\_STLC\_112116\_01.05883.05883.2 | 3.3554 | 0.2379 | 100.0% | 1358.1522 | 1357.5646 | 11 | 4.999 | 70.0% | 1 | R.IMELEDHITQK.T | 2 |
|  | Astrin\_STLC\_112116\_tube2\_01.10030.10030.2 | 3.3101 | 0.2141 | 99.9% | 1388.3722 | 1388.6012 | 5 | 5.681 | 59.1% | 1 | K.TIEIESLNEVLK.N | 2 |
|  | Astrin\_STLC\_112116\_01.05088.05088.3 | 2.8687 | 0.2603 | 98.5% | 1744.1044 | 1743.8735 | 49 | 4.692 | 32.7% | 2 | K.LQHFQELGEEKDNR.V | 3 |
|  | Astrin\_STLC\_112116\_tube2\_01.08508.08508.2 | 4.0443 | 0.4625 | 100.0% | 1440.0122 | 1439.71 | 1 | 7.778 | 81.8% | 3 | K.ILTLENQVYSMK.A | 2 |
|  | Astrin\_STLC\_112116\_01.05506.05506.2 | 2.6455 | 0.2792 | 99.3% | 1418.2722 | 1419.5315 | 1 | 5.767 | 54.2% | 2 | K.ALEDRLESESAAK.L | 2 |
|  | Astrin\_STLC\_112116\_01.04247.04247.2 | 3.7296 | 0.4778 | 100.0% | 1315.9722 | 1316.4105 | 1 | 7.592 | 72.7% | 1 | K.GTESHLSELNTK.L | 2 |
|  | Astrin\_STLC\_112116\_01.05654.05654.3 | 3.8986 | 0.4108 | 100.0% | 1892.9944 | 1894.0662 | 1 | 6.848 | 45.0% | 2 | R.VGQEKEETVSSHFEMR.C | 3 |
|  | Astrin\_STLC\_112116\_tube2\_01.06015.06015.2 | 1.8821 | 0.3458 | 97.5% | 1287.2322 | 1287.5016 | 17 | 5.166 | 50.0% | 1 | K.YSLIVAQHVEK.E | 2 |
|  | Astrin\_STLC\_112116\_tube2\_01.08414.08414.2 | 3.025 | 0.3965 | 100.0% | 1448.2922 | 1449.5602 | 1 | 6.958 | 72.7% | 1 | K.QNLENVFDDVQK.T | 2 |
|  | Astrin\_STLC\_112116\_tube2\_01.05487.05487.2 | 3.128 | 0.3239 | 100.0% | 1159.9521 | 1160.3336 | 1 | 7.155 | 88.9% | 3 | K.LQALQQMDGR.N | 2 |
|  | Astrin\_STLC\_112116\_tube2\_01.04943.04943.2 | 3.0045 | 0.18 | 98.5% | 1675.5721 | 1674.8021 | 1 | 4.292 | 69.2% | 1 | R.NKPTELLEENTEEK.S | 2 |
|  | Astrin\_STLC\_112116\_01.05297.05297.3 | 3.6511 | 0.2618 | 99.4% | 1889.2743 | 1890.0544 | 88 | 5.254 | 35.0% | 1 | R.NKPTELLEENTEEKSK.S | 3 |
|  | Astrin\_STLC\_112116\_tube2\_02.08580.08580.3 | 4.6369 | 0.4139 | 100.0% | 2438.4844 | 2439.6384 | 1 | 7.444 | 37.5% | 2 | K.AQEVEAELLESHQEETNQLLK.K | 3 |
|  | Astrin\_STLC\_112116\_01.06972.06972.2 | 2.0915 | 0.3235 | 97.2% | 1732.2722 | 1734.0024 | 138 | 6.173 | 39.3% | 1 | K.VITTVLKFPDDQTQK.I | 2 |

---

|  |  |  |  |  |  |  |  |  |
| --- | --- | --- | --- | --- | --- | --- | --- | --- |
| U | *gi|153792590|ref|NP\_0* | 8 | 22 | 11.6% | 854 | 98161 | 5.2 | heat shock protein HSP 90-alpha isoform 1 [Homo sapiens] &IC Hsp90 |
| U | *gi|767980445|ref|XP\_0* | 8 | 22 | 11.6% | 853 | 98074 | 5.2 | PREDICTED: heat shock protein HSP 90-alpha isoform X1 [Homo sapiens] &IC Hsp90 |
| U | *gi|154146191|ref|NP\_0* | 8 | 22 | 13.5% | 732 | 84660 | 5.0 | heat shock protein HSP 90-alpha isoform 2 [Homo sapiens] &IC Hsp90 |

| Filename XCorr DeltCN Conf% ObsM+H+ CalcM+H+ SpR ZScore Ion% # Sequence  | | | | | | | | | | | | |
| --- | --- | --- | --- | --- | --- | --- | --- | --- | --- | --- | --- | --- |
|  | Astrin\_STLC\_112116\_tube2\_01.08407.08407.2 | 3.4125 | 0.4213 | 100.0% | 1243.0322 | 1243.4459 | 1 | 6.622 | 72.7% | 2 | K.ADLINNLGTIAK.S | 22 |
|  | Astrin\_STLC\_112116\_02.04492.04492.3 | 4.9004 | 0.4837 | 100.0% | 2015.3644 | 2016.2584 | 1 | 8.339 | 45.0% | 5 | K.VILHLKEDQTEYLEER.R | 33 |
|  | Astrin\_STLC\_112116\_tube2\_01.10168.10168.3 | 3.266 | 0.2517 | 98.7% | 2064.7144 | 2065.3794 | 4 | 5.243 | 34.4% | 1 | K.HSQFIGYPITLFVEKER.D | 3 |
|  | Astrin\_STLC\_112116\_01.05163.05163.2 | 3.0953 | 0.2673 | 100.0% | 1151.8922 | 1152.2462 | 2 | 5.479 | 75.0% | 4 | K.YIDQEELNK.T | 22 |
|  | Astrin\_STLC\_112116\_tube2\_01.07911.07911.2 | 4.0786 | 0.414 | 100.0% | 1528.5721 | 1528.6616 | 1 | 7.285 | 75.0% | 2 | K.SLTNDWEDHLAVK.H | 22 |
|  | Astrin\_STLC\_112116\_tube2\_02.08226.08226.2 | 3.5056 | 0.4499 | 100.0% | 1348.9922 | 1349.4886 | 1 | 7.643 | 80.0% | 5 | K.HFSVEGQLEFR.A | 22 |
|  | Astrin\_STLC\_112116\_01.05684.05684.2 | 2.5022 | 0.212 | 98.3% | 1224.9922 | 1225.3867 | 1 | 5.829 | 77.8% | 2 | K.HIYYITGETK.D | 2 |
|  | Astrin\_STLC\_112116\_tube2\_02.07900.07900.3 | 3.6627 | 0.3155 | 99.7% | 2442.8342 | 2442.6904 | 194 | 6.004 | 27.5% | 1 | K.HIYYITGETKDQVANSAFVER.L | 3 |

Similarities:
gi|20149594|ref|NP\_03(5:3)  

---

|  |  |  |  |  |  |  |  |  |
| --- | --- | --- | --- | --- | --- | --- | --- | --- |
| U | *gi|162329583|ref|NP\_0* | 5 | 6 | 11.6% | 551 | 59210 | 7.2 | cleavage and polyadenylation specificity factor subunit 6 isoform 1 [Homo sapiens] |
| U | *gi|665821177|ref|NP\_0* | 5 | 6 | 10.9% | 588 | 63471 | 7.7 | cleavage and polyadenylation specificity factor subunit 6 isoform 2 [Homo sapiens] |

| Filename XCorr DeltCN Conf% ObsM+H+ CalcM+H+ SpR ZScore Ion% # Sequence  | | | | | | | | | | | | |
| --- | --- | --- | --- | --- | --- | --- | --- | --- | --- | --- | --- | --- |
|  | Astrin\_STLC\_112116\_tube2\_01.07786.07786.2 | 3.6878 | 0.3941 | 100.0% | 1309.1921 | 1309.4619 | 1 | 6.066 | 76.9% | 1 | K.GFALVGVGSEASSK.K | 2 |
|  | Astrin\_STLC\_112116\_tube2\_01.08622.08622.2 | 3.1625 | 0.2621 | 100.0% | 1402.1322 | 1401.5798 | 2 | 5.036 | 70.0% | 1 | K.QFLSQFEMQSR.K | 2 |
|  | Astrin\_STLC\_112116\_tube2\_01.10008.10008.2 | 5.0384 | 0.5818 | 100.0% | 1665.8722 | 1666.8433 | 1 | 10.035 | 80.8% | 1 | R.TPLSEAEFEEIMNR.N | 2 |
|  | Astrin\_STLC\_112116\_01.14870.14870.3 | 4.1663 | 0.4356 | 100.0% | 2453.2144 | 2453.749 | 1 | 7.59 | 39.6% | 1 | R.AVSDASAGDYGSAIETLVTAISLIK.Q | 3 |
|  | Astrin\_STLC\_112116\_01.14876.14876.2 | 6.1134 | 0.5576 | 100.0% | 2453.8323 | 2453.749 | 1 | 11.511 | 58.3% | 2 | R.AVSDASAGDYGSAIETLVTAISLIK.Q | 2 |

---

|  |  |  |  |  |  |  |  |  |
| --- | --- | --- | --- | --- | --- | --- | --- | --- |
| U | *gi|15809016|ref|NP\_29* | 2 | 4 | 11.6% | 172 | 19779 | 4.8 | myosin regulatory light chain 12B [Homo sapiens] |
| U | *gi|740087210|ref|NP\_0* | 2 | 4 | 11.3% | 177 | 20457 | 4.8 | myosin regulatory light chain 12A isoform 2 [Homo sapiens] |
| U | *gi|740087161|ref|NP\_0* | 2 | 4 | 11.7% | 171 | 19794 | 4.8 | myosin regulatory light chain 12A isoform 1 [Homo sapiens] |

| Filename XCorr DeltCN Conf% ObsM+H+ CalcM+H+ SpR ZScore Ion% # Sequence  | | | | | | | | | | | | |
| --- | --- | --- | --- | --- | --- | --- | --- | --- | --- | --- | --- | --- |
|  | Astrin\_STLC\_112116\_tube2\_02.09338.09338.3 | 3.5627 | 0.3819 | 99.8% | 2432.0645 | 2433.649 | 2 | 5.896 | 34.2% | 2 | R.ELLTTMGDRFTDEEVDELYR.E | 3 |
|  | Astrin\_STLC\_112116\_tube2\_01.07882.07882.2 | 3.3647 | 0.4492 | 100.0% | 1415.7722 | 1416.4839 | 1 | 8.394 | 90.0% | 2 | R.FTDEEVDELYR.E | 2 |

---

|  |  |  |  |  |  |  |  |  |
| --- | --- | --- | --- | --- | --- | --- | --- | --- |
| U | *gi|15431297|ref|NP\_00* | 2 | 2 | 11.4% | 211 | 24261 | 11.7 | 60S ribosomal protein L13 isoform 1 [Homo sapiens] |
| U | *gi|341604768|ref|NP\_0* | 2 | 2 | 12.5% | 192 | 22184 | 11.6 | 60S ribosomal protein L13 isoform 2 [Homo sapiens] |

| Filename XCorr DeltCN Conf% ObsM+H+ CalcM+H+ SpR ZScore Ion% # Sequence  | | | | | | | | | | | | |
| --- | --- | --- | --- | --- | --- | --- | --- | --- | --- | --- | --- | --- |
|  | Astrin\_STLC\_112116\_01.04331.04331.2 | 2.4384 | 0.2629 | 98.9% | 1232.2522 | 1233.3237 | 4 | 5.529 | 65.0% | 1 | K.STESLQANVQR.L | 2 |
|  | Astrin\_STLC\_112116\_tube2\_01.07823.07823.2 | 2.7609 | 0.2165 | 98.6% | 1384.2522 | 1383.6923 | 1 | 4.773 | 66.7% | 1 | K.LATQLTGPVMPVR.N | 2 |

---

|  |  |  |  |  |  |  |  |  |
| --- | --- | --- | --- | --- | --- | --- | --- | --- |
| U | *gi|5453994|ref|NP\_006* | 4 | 5 | 11.1% | 631 | 71690 | 4.7 | double-strand-break repair protein rad21 homolog [Homo sapiens] &IC Rad21 |

| Filename XCorr DeltCN Conf% ObsM+H+ CalcM+H+ SpR ZScore Ion% # Sequence  | | | | | | | | | | | | |
| --- | --- | --- | --- | --- | --- | --- | --- | --- | --- | --- | --- | --- |
| \* | Astrin\_STLC\_112116\_tube2\_01.10319.10319.2 | 3.3043 | 0.325 | 100.0% | 2278.5122 | 2277.5767 | 2 | 6.383 | 40.0% | 1 | R.AQLSDYSDIVTTLDLAPPTKK.L | 2 |
| \* | Astrin\_STLC\_112116\_tube2\_01.10605.10605.2 | 2.9078 | 0.3999 | 100.0% | 1555.5922 | 1556.8075 | 1 | 6.996 | 66.7% | 2 | K.LFSLPAQPLWNNR.L | 2 |
| \* | Astrin\_STLC\_112116\_tube2\_01.07915.07915.2 | 3.2459 | 0.4251 | 100.0% | 1512.0322 | 1512.6567 | 1 | 7.624 | 62.5% | 1 | R.DVIDEPIIEEPSR.L | 2 |
| \* | Astrin\_STLC\_112116\_01.03782.03782.3 | 5.5434 | 0.4273 | 100.0% | 2683.7043 | 2684.478 | 1 | 7.278 | 43.2% | 1 | K.EKEDDEEEEDEDASGGDQDQEER.R | 3 |

---

|  |  |  |  |  |  |  |  |  |
| --- | --- | --- | --- | --- | --- | --- | --- | --- |
| U | *contaminant\_KERATIN10* | 6 | 11 | 11.0% | 400 | 44106 | 5.1 | no description |

| Filename XCorr DeltCN Conf% ObsM+H+ CalcM+H+ SpR ZScore Ion% # Sequence  | | | | | | | | | | | | |
| --- | --- | --- | --- | --- | --- | --- | --- | --- | --- | --- | --- | --- |
|  | Astrin\_STLC\_112116\_tube2\_01.05299.05299.2 | 3.2103 | 0.1537 | 99.8% | 1064.5721 | 1065.2578 | 1 | 6.052 | 81.2% | 2 | R.LASYLDKVR.A | 222222 |
|  | Astrin\_STLC\_112116\_tube2\_01.06544.06544.2 | 2.8305 | 0.168 | 99.0% | 1041.6322 | 1042.2235 | 1 | 5.465 | 93.8% | 2 | R.IVLQIDNAR.L | 22 |
|  | Astrin\_STLC\_112116\_01.05584.05584.2 | 1.8322 | 0.255 | 95.2% | 807.8722 | 807.8815 | 186 | 5.3 | 66.7% | 1 | R.LAADDFR.T | 2222222 |
|  | Astrin\_STLC\_112116\_tube2\_01.07209.07209.2 | 3.3704 | 0.3259 | 100.0% | 1186.0521 | 1186.397 | 1 | 6.037 | 83.3% | 1 | R.RVLDELTLAR.T | 2222 |
|  | Astrin\_STLC\_112116\_tube2\_01.07839.07839.2 | 3.2486 | 0.3989 | 100.0% | 1030.0122 | 1030.2096 | 1 | 7.534 | 87.5% | 4 | R.VLDELTLAR.T | 22222 |
|  | Astrin\_STLC\_112116\_tube2\_01.04954.04954.2 | 2.7318 | 0.326 | 100.0% | 1122.9922 | 1123.2511 | 1 | 5.932 | 81.2% | 1 | R.LEQEIATYR.S | 22222 |

Similarities:
contaminant\_KERATIN09(2:4)  
gi|24430192|ref|NP\_00(5:1)  
gi|15431310|ref|NP\_00(5:1)  
contaminant\_KERATIN12(5:1)  
contaminant\_KERATIN03(2:4)  
contaminant\_KERATIN07(4:2)  

---

|  |  |  |  |  |  |  |  |  |
| --- | --- | --- | --- | --- | --- | --- | --- | --- |
| U | *gi|50845386|ref|NP\_00* | 4 | 5 | 10.9% | 339 | 38604 | 7.8 | annexin A2 isoform 2 [Homo sapiens] |
| U | *gi|50845388|ref|NP\_00* | 4 | 5 | 10.4% | 357 | 40411 | 8.4 | annexin A2 isoform 1 [Homo sapiens] |

| Filename XCorr DeltCN Conf% ObsM+H+ CalcM+H+ SpR ZScore Ion% # Sequence  | | | | | | | | | | | | |
| --- | --- | --- | --- | --- | --- | --- | --- | --- | --- | --- | --- | --- |
|  | Astrin\_STLC\_112116\_tube2\_01.12758.12758.2 | 2.4285 | 0.2709 | 97.9% | 1649.9922 | 1651.9872 | 1 | 5.509 | 50.0% | 1 | K.SALSGHLETVILGLLK.T | 2 |
|  | Astrin\_STLC\_112116\_tube2\_01.12712.12712.3 | 4.4865 | 0.1835 | 99.5% | 1651.9744 | 1651.9872 | 5 | 5.939 | 43.3% | 1 | K.SALSGHLETVILGLLK.T | 3 |
|  | Astrin\_STLC\_112116\_tube2\_01.04527.04527.2 | 3.2621 | 0.3162 | 100.0% | 1224.2722 | 1223.3251 | 1 | 6.046 | 75.0% | 2 | K.TPAQYDASELK.A | 2 |
|  | Astrin\_STLC\_112116\_01.05152.05152.2 | 3.156 | 0.2851 | 100.0% | 1244.8722 | 1245.3347 | 6 | 5.874 | 72.2% | 1 | R.TNQELQEINR.V | 2 |

---

|  |  |  |  |  |  |  |  |  |
| --- | --- | --- | --- | --- | --- | --- | --- | --- |
| U | *gi|4506399|ref|NP\_003* | 2 | 3 | 10.6% | 368 | 40968 | 7.8 | mRNA export factor [Homo sapiens] |

| Filename XCorr DeltCN Conf% ObsM+H+ CalcM+H+ SpR ZScore Ion% # Sequence  | | | | | | | | | | | | |
| --- | --- | --- | --- | --- | --- | --- | --- | --- | --- | --- | --- | --- |
| \* | Astrin\_STLC\_112116\_02.07897.07897.3 | 4.1791 | 0.4259 | 100.0% | 2252.5444 | 2253.5376 | 2 | 6.506 | 32.9% | 2 | K.MWDLSSNQAIQIAQHDAPVK.T | 3 |
| \* | Astrin\_STLC\_112116\_01.06948.06948.3 | 3.5449 | 0.1694 | 95.5% | 2187.5645 | 2187.5059 | 1 | 4.776 | 31.9% | 1 | R.VAIHYINPPNPAKDNFTFK.C | 3 |

---

|  |  |  |  |  |  |  |  |  |
| --- | --- | --- | --- | --- | --- | --- | --- | --- |
| U | *gi|151301219|ref|NP\_9* | 3 | 3 | 10.5% | 353 | 40548 | 5.5 | protein arginine N-methyltransferase 1 isoform 3 [Homo sapiens] |
| U | *gi|154759421|ref|NP\_0* | 3 | 3 | 10.0% | 371 | 42462 | 5.3 | protein arginine N-methyltransferase 1 isoform 1 [Homo sapiens] |

| Filename XCorr DeltCN Conf% ObsM+H+ CalcM+H+ SpR ZScore Ion% # Sequence  | | | | | | | | | | | | |
| --- | --- | --- | --- | --- | --- | --- | --- | --- | --- | --- | --- | --- |
|  | Astrin\_STLC\_112116\_01.05832.05832.3 | 2.6643 | 0.2342 | 96.9% | 1351.1044 | 1351.6322 | 44 | 4.348 | 43.2% | 1 | K.ANKLDHVVTIIK.G | 3 |
|  | Astrin\_STLC\_112116\_tube2\_01.10225.10225.2 | 3.3941 | 0.3301 | 100.0% | 1643.5721 | 1643.8827 | 1 | 6.084 | 65.4% | 1 | R.DKWLAPDGLIFPDR.A | 2 |
|  | Astrin\_STLC\_112116\_02.06949.06949.2 | 2.2171 | 0.3224 | 99.0% | 1252.7522 | 1252.4099 | 2 | 5.927 | 60.0% | 1 | R.ATLYVTAIEDR.Q | 2 |

---

|  |  |  |  |  |  |  |  |  |
| --- | --- | --- | --- | --- | --- | --- | --- | --- |
| U | *gi|4507513|ref|NP\_000* | 2 | 2 | 10.0% | 211 | 24145 | 8.7 | metalloproteinase inhibitor 3 precursor [Homo sapiens] |

| Filename XCorr DeltCN Conf% ObsM+H+ CalcM+H+ SpR ZScore Ion% # Sequence  | | | | | | | | | | | | |
| --- | --- | --- | --- | --- | --- | --- | --- | --- | --- | --- | --- | --- |
| \* | Astrin\_STLC\_112116\_tube2\_01.10145.10145.2 | 2.3509 | 0.3919 | 99.9% | 1324.8322 | 1325.5474 | 1 | 6.147 | 54.5% | 1 | K.EGPFGTLVYTIK.Q | 2 |
| \* | Astrin\_STLC\_112116\_tube2\_01.07125.07125.2 | 2.9934 | 0.2848 | 100.0% | 1147.2322 | 1147.2761 | 1 | 5.144 | 93.8% | 1 | R.WDQLTLSQR.K | 2 |

---

|  |  |  |  |  |  |  |  |  |
| --- | --- | --- | --- | --- | --- | --- | --- | --- |
| U | *gi|153791158|ref|NP\_0* | 5 | 11 | 9.8% | 551 | 59560 | 7.7 | keratin, type II cytoskeletal 75 [Homo sapiens] |

| Filename XCorr DeltCN Conf% ObsM+H+ CalcM+H+ SpR ZScore Ion% # Sequence  | | | | | | | | | | | | |
| --- | --- | --- | --- | --- | --- | --- | --- | --- | --- | --- | --- | --- |
|  | Astrin\_STLC\_112116\_tube2\_01.11450.11450.2 | 4.018 | 0.3758 | 100.0% | 1329.9722 | 1330.5211 | 1 | 7.458 | 86.4% | 3 | R.NLDLDSIIAEVK.A | 22222222 |
|  | Astrin\_STLC\_112116\_01.04611.04611.2 | 2.7736 | 0.209 | 99.5% | 1081.0721 | 1080.1423 | 1 | 5.464 | 81.2% | 3 | K.AQYEDIANR.S | 22 |
|  | Astrin\_STLC\_112116\_tube2\_01.04497.04497.2 | 3.6681 | 0.3308 | 100.0% | 1456.0721 | 1456.5547 | 1 | 6.189 | 68.2% | 1 | R.SRAEAESWYQTK.Y | 22222 |
|  | Astrin\_STLC\_112116\_tube2\_01.05288.05288.2 | 3.4785 | 0.2191 | 100.0% | 1165.9722 | 1166.2761 | 1 | 6.834 | 88.9% | 1 | K.YEELQVTAGR.H | 2222 |
|  | Astrin\_STLC\_112116\_tube2\_01.09346.09346.2 | 3.4818 | 0.346 | 100.0% | 1264.4122 | 1264.4644 | 1 | 7.748 | 80.0% | 3 | K.LALDVEIATYR.K | 222222222 |

Similarities:
contaminant\_KERATIN21(4:1)  
contaminant\_KERATIN20(1:4)  
gi|119703753|ref|NP\_0(3:2)  
contaminant\_KERATIN18(4:1)  
gi|5031839|ref|NP\_005(4:1)  
contaminant\_KERATIN22(2:3)  
gi|119395754|ref|NP\_0(2:3)  
gi|109148552|ref|NP\_4(1:4)  
gi|32567786|ref|NP\_78(2:3)  

---

|  |  |  |  |  |  |  |  |  |
| --- | --- | --- | --- | --- | --- | --- | --- | --- |
| U | *gi|109148552|ref|NP\_4* | 4 | 6 | 9.4% | 628 | 64417 | 6.5 | keratin, type II cytoskeletal 3 [Homo sapiens] |

| Filename XCorr DeltCN Conf% ObsM+H+ CalcM+H+ SpR ZScore Ion% # Sequence  | | | | | | | | | | | | |
| --- | --- | --- | --- | --- | --- | --- | --- | --- | --- | --- | --- | --- |
|  | Astrin\_STLC\_112116\_01.06138.06138.2 | 2.671 | 0.1258 | 96.8% | 1082.1522 | 1083.2755 | 1 | 5.557 | 81.2% | 1 | K.FASFIDKVR.F | 22222222 |
|  | Astrin\_STLC\_112116\_tube2\_01.05492.05492.2 | 3.3041 | 0.3582 | 100.0% | 1351.4321 | 1351.5425 | 1 | 6.676 | 77.3% | 1 | R.TAAENEFVTLKK.D | 22222 |
|  | Astrin\_STLC\_112116\_tube2\_02.08776.08776.3 | 3.8367 | 0.2205 | 98.3% | 3055.1943 | 3056.3462 | 70 | 5.955 | 20.2% | 1 | R.TLYDAELSQMQSHISDTSVVLSMDNNR.S | 3 |
|  | Astrin\_STLC\_112116\_tube2\_01.09346.09346.2 | 3.4818 | 0.346 | 100.0% | 1264.4122 | 1264.4644 | 1 | 7.748 | 80.0% | 3 | K.LALDVEIATYR.K | 222222222 |

Similarities:
contaminant\_KERATIN21(2:2)  
contaminant\_KERATIN20(1:3)  
gi|119703753|ref|NP\_0(3:1)  
contaminant\_KERATIN18(3:1)  
gi|5031839|ref|NP\_005(3:1)  
contaminant\_KERATIN22(2:2)  
gi|119395754|ref|NP\_0(2:2)  
contaminant\_KERATIN19(1:3)  
gi|153791158|ref|NP\_0(1:3)  
gi|32567786|ref|NP\_78(1:3)  

---

|  |  |  |  |  |  |  |  |  |
| --- | --- | --- | --- | --- | --- | --- | --- | --- |
| U | *gi|194239729|ref|NP\_0* | 2 | 3 | 9.3% | 257 | 28558 | 4.9 | elongation factor 1-delta isoform 4 [Homo sapiens] |
| U | *gi|304555583|ref|NP\_0* | 2 | 3 | 3.7% | 647 | 71422 | 6.4 | elongation factor 1-delta isoform 1 [Homo sapiens] |
| U | *gi|194239731|ref|NP\_0* | 2 | 3 | 8.5% | 281 | 31122 | 5.0 | elongation factor 1-delta isoform 2 [Homo sapiens] |

| Filename XCorr DeltCN Conf% ObsM+H+ CalcM+H+ SpR ZScore Ion% # Sequence  | | | | | | | | | | | | |
| --- | --- | --- | --- | --- | --- | --- | --- | --- | --- | --- | --- | --- |
|  | Astrin\_STLC\_112116\_tube2\_01.06605.06605.2 | 3.5718 | 0.4487 | 100.0% | 1358.8322 | 1359.5223 | 1 | 7.534 | 81.8% | 2 | R.IASLEVENQSLR.G | 2 |
|  | Astrin\_STLC\_112116\_tube2\_01.09972.09972.2 | 3.3291 | 0.2751 | 100.0% | 1301.1721 | 1300.4978 | 1 | 5.314 | 81.8% | 1 | R.GVVQELQQAISK.L | 2 |

---

|  |  |  |  |  |  |  |  |  |
| --- | --- | --- | --- | --- | --- | --- | --- | --- |
| U | *gi|157266292|ref|NP\_0* | 3 | 3 | 9.1% | 528 | 56812 | 5.9 | intestinal-type alkaline phosphatase precursor [Homo sapiens] |

| Filename XCorr DeltCN Conf% ObsM+H+ CalcM+H+ SpR ZScore Ion% # Sequence  | | | | | | | | | | | | |
| --- | --- | --- | --- | --- | --- | --- | --- | --- | --- | --- | --- | --- |
| \* | Astrin\_STLC\_112116\_01.12041.12041.2 | 3.3045 | 0.5426 | 100.0% | 1957.4521 | 1958.3085 | 1 | 9.223 | 61.1% | 1 | K.NLILFLGDGLGVPTVTATR.I | 2 |
|  | Astrin\_STLC\_112116\_01.03993.03993.3 | 2.8831 | 0.408 | 99.8% | 1710.0844 | 1709.8618 | 289 | 5.781 | 28.3% | 1 | R.VQHASPAGTYAHTVNR.N | 3 |
| \* | Astrin\_STLC\_112116\_tube2\_01.07339.07339.2 | 2.1794 | 0.2444 | 95.3% | 1485.6522 | 1484.5829 | 2 | 5.158 | 50.0% | 1 | R.NWYSDADMPASAR.Q | 2 |

---

|  |  |  |  |  |  |  |  |  |
| --- | --- | --- | --- | --- | --- | --- | --- | --- |
| U | *gi|34419635|ref|NP\_00* | 7 | 19 | 9.0% | 643 | 71028 | 6.1 | heat shock 70 kDa protein 6 [Homo sapiens] |

| Filename XCorr DeltCN Conf% ObsM+H+ CalcM+H+ SpR ZScore Ion% # Sequence  | | | | | | | | | | | | |
| --- | --- | --- | --- | --- | --- | --- | --- | --- | --- | --- | --- | --- |
|  | Astrin\_STLC\_112116\_tube2\_01.07222.07222.2 | 3.2644 | 0.4305 | 100.0% | 1488.9722 | 1488.5939 | 1 | 8.322 | 79.2% | 5 | R.TTPSYVAFTDTER.L | 2222 |
|  | Astrin\_STLC\_112116\_tube2\_01.09474.09474.3 | 4.1847 | 0.3057 | 99.8% | 1688.9343 | 1688.9213 | 2 | 6.013 | 46.7% | 1 | R.IINEPTAAAIAYGLDR.R | 33 |
|  | Astrin\_STLC\_112116\_tube2\_01.09456.09456.2 | 5.2341 | 0.5236 | 100.0% | 1689.0322 | 1688.9213 | 1 | 9.8 | 80.0% | 6 | R.IINEPTAAAIAYGLDR.R | 22 |
|  | Astrin\_STLC\_112116\_01.05559.05559.2 | 3.8525 | 0.3976 | 100.0% | 1675.8522 | 1676.6964 | 1 | 8.003 | 56.7% | 1 | K.ATAGDTHLGGEDFDNR.L | 222 |
|  | Astrin\_STLC\_112116\_tube2\_01.04581.04581.3 | 3.1587 | 0.2499 | 98.6% | 1676.1543 | 1676.6964 | 126 | 5.668 | 31.7% | 1 | K.ATAGDTHLGGEDFDNR.L | 333 |
|  | Astrin\_STLC\_112116\_01.07436.07436.2 | 2.5451 | 0.3283 | 100.0% | 1081.7722 | 1082.2444 | 1 | 5.828 | 81.2% | 4 | K.LLQDFFNGK.E | 22 |
|  | Astrin\_STLC\_112116\_01.07146.07146.2 | 3.6893 | 0.1587 | 99.8% | 1567.1122 | 1566.7972 | 1 | 4.243 | 75.0% | 1 | K.LLQDFFNGKELNK.S | 22 |

Similarities:
gi|5729877|ref|NP\_006(3:4)  
gi|167466173|ref|NP\_0(5:2)  
gi|124256496|ref|NP\_0(3:4)  

---

|  |  |  |  |  |  |  |  |  |
| --- | --- | --- | --- | --- | --- | --- | --- | --- |
| U | *gi|63055057|ref|NP\_00* | 2 | 8 | 9.0% | 376 | 42003 | 5.6 | beta-actin-like protein 2 [Homo sapiens] |

| Filename XCorr DeltCN Conf% ObsM+H+ CalcM+H+ SpR ZScore Ion% # Sequence  | | | | | | | | | | | | |
| --- | --- | --- | --- | --- | --- | --- | --- | --- | --- | --- | --- | --- |
| \* | Astrin\_STLC\_112116\_tube2\_01.07774.07774.3 | 3.5802 | 0.3173 | 99.7% | 1955.0343 | 1955.2615 | 10 | 5.664 | 33.8% | 1 | R.VAPDEHPILLTEAPLNPK.I | 3 |
|  | Astrin\_STLC\_112116\_tube2\_01.09467.09467.2 | 4.7773 | 0.4402 | 100.0% | 1792.1322 | 1791.9554 | 1 | 8.114 | 80.0% | 7 | R.SYELPDGQVITIGNER.F | 222 |

Similarities:
gi|316659409|ref|NP\_0(1:1)  
gi|4501881|ref|NP\_001(1:1)  

---

|  |  |  |  |  |  |  |  |  |
| --- | --- | --- | --- | --- | --- | --- | --- | --- |
| U | *gi|4506661|ref|NP\_000* | 2 | 2 | 9.0% | 266 | 29996 | 10.6 | 60S ribosomal protein L7a [Homo sapiens] |

| Filename XCorr DeltCN Conf% ObsM+H+ CalcM+H+ SpR ZScore Ion% # Sequence  | | | | | | | | | | | | |
| --- | --- | --- | --- | --- | --- | --- | --- | --- | --- | --- | --- | --- |
| \* | Astrin\_STLC\_112116\_tube2\_01.06942.06942.2 | 2.6034 | 0.2278 | 98.9% | 1217.6522 | 1217.3672 | 2 | 5.463 | 70.0% | 1 | K.NFGIGQDIQPK.R | 2 |
| \* | Astrin\_STLC\_112116\_tube2\_01.07171.07171.2 | 3.4442 | 0.0948 | 98.2% | 1346.8522 | 1346.5236 | 63 | 5.078 | 50.0% | 1 | R.AGVNTVTTLVENK.K | 2 |

---

|  |  |  |  |  |  |  |  |  |
| --- | --- | --- | --- | --- | --- | --- | --- | --- |
| U | *gi|94721250|ref|NP\_00* | 2 | 4 | 8.8% | 294 | 32614 | 8.9 | vesicle-associated membrane protein-associated protein A isoform 1 [Homo sapiens] |
| U | *gi|94721252|ref|NP\_91* | 2 | 4 | 10.4% | 249 | 27893 | 8.6 | vesicle-associated membrane protein-associated protein A isoform 2 [Homo sapiens] |

| Filename XCorr DeltCN Conf% ObsM+H+ CalcM+H+ SpR ZScore Ion% # Sequence  | | | | | | | | | | | | |
| --- | --- | --- | --- | --- | --- | --- | --- | --- | --- | --- | --- | --- |
|  | Astrin\_STLC\_112116\_tube2\_01.08329.08329.2 | 3.963 | 0.4148 | 100.0% | 1617.9122 | 1618.8705 | 1 | 7.315 | 69.2% | 2 | K.HEQILVLDPPTDLK.F | 2 |
|  | Astrin\_STLC\_112116\_tube2\_01.08240.08240.2 | 3.1828 | 0.289 | 100.0% | 1291.9922 | 1292.4747 | 17 | 6.544 | 54.5% | 2 | K.GPFTDVVTTNLK.L | 2 |

---

|  |  |  |  |  |  |  |  |  |
| --- | --- | --- | --- | --- | --- | --- | --- | --- |
| U | *gi|195947382|ref|NP\_8* | 2 | 3 | 8.7% | 403 | 47108 | 5.2 | RILP-like protein 1 isoform 1 [Homo sapiens] |
| U | *gi|984880766|ref|NP\_0* | 2 | 3 | 9.1% | 386 | 44766 | 5.1 | RILP-like protein 1 isoform 2 [Homo sapiens] |

| Filename XCorr DeltCN Conf% ObsM+H+ CalcM+H+ SpR ZScore Ion% # Sequence  | | | | | | | | | | | | |
| --- | --- | --- | --- | --- | --- | --- | --- | --- | --- | --- | --- | --- |
|  | Astrin\_STLC\_112116\_01.15130.15130.3 | 2.9415 | 0.2754 | 97.7% | 3911.6042 | 3909.173 | 4 | 3.986 | 19.1% | 1 | R.GS\*ALAAES\*ALEK@NVAELTVMDVYDIASLVGHEFER.V | 3 |
|  | Astrin\_STLC\_112116\_01.15130.15130.2 | 3.8769 | 0.4761 | 100.0% | 2608.0723 | 2608.9258 | 1 | 7.316 | 38.6% | 2 | K.NVAELTVMDVYDIASLVGHEFER.V | 3 |

---

|  |  |  |  |  |  |  |  |  |
| --- | --- | --- | --- | --- | --- | --- | --- | --- |
| U | *gi|14141161|ref|NP\_00* | 4 | 7 | 8.4% | 806 | 88980 | 5.8 | heterogeneous nuclear ribonucleoprotein U isoform b [Homo sapiens] |
| U | *gi|74136883|ref|NP\_11* | 4 | 7 | 8.2% | 825 | 90585 | 6.0 | heterogeneous nuclear ribonucleoprotein U isoform a [Homo sapiens] |

| Filename XCorr DeltCN Conf% ObsM+H+ CalcM+H+ SpR ZScore Ion% # Sequence  | | | | | | | | | | | | |
| --- | --- | --- | --- | --- | --- | --- | --- | --- | --- | --- | --- | --- |
|  | Astrin\_STLC\_112116\_tube2\_01.09606.09606.2 | 2.3466 | 0.2613 | 96.7% | 1715.3722 | 1715.9469 | 19 | 5.214 | 38.2% | 1 | K.SSGPTSLFAVTVAPPGAR.Q | 2 |
|  | Astrin\_STLC\_112116\_tube2\_01.07871.07871.2 | 3.2726 | 0.4675 | 100.0% | 1698.0322 | 1698.8291 | 1 | 7.699 | 58.3% | 2 | R.GYFEYIEENKYSR.A | 2 |
|  | Astrin\_STLC\_112116\_tube2\_01.11181.11181.3 | 4.5807 | 0.4411 | 100.0% | 2724.3245 | 2726.0576 | 1 | 6.353 | 38.1% | 1 | K.EKPYFPIPEEYTFIQNVPLEDR.V | 3 |
|  | Astrin\_STLC\_112116\_02.06988.06988.2 | 4.2999 | 0.4798 | 100.0% | 1648.3121 | 1648.816 | 1 | 8.18 | 75.0% | 3 | R.NFILDQTNVSAAAQR.R | 2 |

---

|  |  |  |  |  |  |  |  |  |
| --- | --- | --- | --- | --- | --- | --- | --- | --- |
| U | *gi|4504811|ref|NP\_002* | 4 | 4 | 8.2% | 745 | 81745 | 6.1 | junction plakoglobin [Homo sapiens] |

| Filename XCorr DeltCN Conf% ObsM+H+ CalcM+H+ SpR ZScore Ion% # Sequence  | | | | | | | | | | | | |
| --- | --- | --- | --- | --- | --- | --- | --- | --- | --- | --- | --- | --- |
| \* | Astrin\_STLC\_112116\_tube2\_02.08599.08599.3 | 4.7921 | 0.4607 | 100.0% | 2030.3344 | 2030.245 | 1 | 7.737 | 41.2% | 1 | K.SAIVHLINYQDDAELATR.A | 3 |
| \* | Astrin\_STLC\_112116\_tube2\_01.06561.06561.2 | 3.4126 | 0.4129 | 100.0% | 1342.0122 | 1342.5321 | 1 | 8.19 | 77.3% | 1 | K.LLNDEDPVVVTK.A | 2 |
| \* | Astrin\_STLC\_112116\_tube2\_01.15636.15636.3 | 3.3937 | 0.3533 | 99.8% | 2348.7544 | 2348.7478 | 1 | 5.818 | 32.9% | 1 | R.LNTIPLFVQLLYSSVENIQR.V | 3 |
| \* | Astrin\_STLC\_112116\_tube2\_01.09221.09221.2 | 2.1973 | 0.3212 | 98.9% | 1236.1721 | 1237.4387 | 365 | 5.275 | 45.0% | 1 | R.VSVELTNSLFK.H | 2 |

---

|  |  |  |  |  |  |  |  |  |
| --- | --- | --- | --- | --- | --- | --- | --- | --- |
| U | *gi|40254446|ref|NP\_00* | 5 | 6 | 8.1% | 780 | 90955 | 8.0 | cullin-5 [Homo sapiens] |

| Filename XCorr DeltCN Conf% ObsM+H+ CalcM+H+ SpR ZScore Ion% # Sequence  | | | | | | | | | | | | |
| --- | --- | --- | --- | --- | --- | --- | --- | --- | --- | --- | --- | --- |
| \* | Astrin\_STLC\_112116\_tube2\_01.11922.11922.2 | 3.7458 | 0.4723 | 100.0% | 1812.4122 | 1812.0906 | 1 | 7.762 | 46.4% | 2 | K.LMLDTWNESIFSNIK.N | 2 |
| \* | Astrin\_STLC\_112116\_02.08156.08156.2 | 4.0507 | 0.3852 | 100.0% | 1504.0721 | 1504.7263 | 1 | 8.045 | 57.7% | 1 | R.LGEAFDSQLVIGVR.E | 2 |
| \* | Astrin\_STLC\_112116\_tube2\_01.06538.06538.2 | 2.4587 | 0.2501 | 99.1% | 987.8722 | 988.13434 | 68 | 4.615 | 56.2% | 1 | K.ILNAGAWSR.S | 2 |
| \* | Astrin\_STLC\_112116\_tube2\_01.08160.08160.2 | 2.292 | 0.2429 | 97.8% | 1175.1921 | 1175.429 | 1 | 4.39 | 72.2% | 1 | R.TQEAIIQIMK.M | 2 |
| \* | Astrin\_STLC\_112116\_01.11176.11176.2 | 3.0683 | 0.357 | 100.0% | 1699.3522 | 1699.9854 | 55 | 6.59 | 42.9% | 1 | K.ISNAQLQTELVEILK.N | 2 |

---

|  |  |  |  |  |  |  |  |  |
| --- | --- | --- | --- | --- | --- | --- | --- | --- |
| U | *gi|1020738480|ref|NP\_* | 2 | 3 | 8.0% | 498 | 56190 | 10.1 | RNA-binding protein 39 isoform e [Homo sapiens] |
| U | *gi|4757926|ref|NP\_004* | 2 | 3 | 7.6% | 524 | 58657 | 10.1 | RNA-binding protein 39 isoform b [Homo sapiens] |
| U | *gi|35493811|ref|NP\_90* | 2 | 3 | 7.5% | 530 | 59380 | 10.1 | RNA-binding protein 39 isoform a [Homo sapiens] |
| U | *gi|336176066|ref|NP\_0* | 2 | 3 | 8.0% | 502 | 56367 | 10.0 | RNA-binding protein 39 isoform d [Homo sapiens] |
| U | *gi|336176064|ref|NP\_0* | 2 | 3 | 7.9% | 508 | 57090 | 10.0 | RNA-binding protein 39 isoform c [Homo sapiens] |
| U | *gi|1020738594|ref|NP\_* | 2 | 3 | 7.6% | 529 | 59293 | 10.1 | RNA-binding protein 39 isoform g [Homo sapiens] |
| U | *gi|1020738482|ref|NP\_* | 2 | 3 | 10.7% | 373 | 40541 | 6.3 | RNA-binding protein 39 isoform f [Homo sapiens] |

| Filename XCorr DeltCN Conf% ObsM+H+ CalcM+H+ SpR ZScore Ion% # Sequence  | | | | | | | | | | | | |
| --- | --- | --- | --- | --- | --- | --- | --- | --- | --- | --- | --- | --- |
|  | Astrin\_STLC\_112116\_02.10027.10027.3 | 3.8906 | 0.3315 | 99.8% | 2608.9143 | 2608.0098 | 6 | 5.848 | 27.1% | 1 | R.SKGIAYVEFVDVSSVPLAIGLTGQR.V | 3 |
|  | Astrin\_STLC\_112116\_tube2\_01.09472.09472.2 | 2.4647 | 0.3048 | 98.9% | 1551.9321 | 1552.8546 | 2 | 4.739 | 53.6% | 2 | R.VLGVPIIVQASQAEK.N | 2 |

---

|  |  |  |  |  |  |  |  |  |
| --- | --- | --- | --- | --- | --- | --- | --- | --- |
| U | *gi|4503471|ref|NP\_001* | 4 | 11 | 8.0% | 462 | 50141 | 9.0 | elongation factor 1-alpha 1 [Homo sapiens] |

| Filename XCorr DeltCN Conf% ObsM+H+ CalcM+H+ SpR ZScore Ion% # Sequence  | | | | | | | | | | | | |
| --- | --- | --- | --- | --- | --- | --- | --- | --- | --- | --- | --- | --- |
|  | Astrin\_STLC\_112116\_tube2\_02.07851.07851.2 | 2.929 | 0.498 | 100.0% | 1589.3922 | 1589.835 | 1 | 7.613 | 64.3% | 1 | K.THINIVVIGHVDSGK.S | 2 |
|  | Astrin\_STLC\_112116\_02.04456.04456.3 | 4.6557 | 0.4769 | 100.0% | 1589.8744 | 1589.835 | 1 | 8.198 | 55.4% | 6 | K.THINIVVIGHVDSGK.S | 3 |
| \* | Astrin\_STLC\_112116\_01.04186.04186.2 | 2.5589 | 0.2576 | 99.2% | 1283.1322 | 1283.3955 | 24 | 4.449 | 50.0% | 1 | K.MDSTEPPYSQK.R | 2 |
|  | Astrin\_STLC\_112116\_tube2\_01.06157.06157.2 | 3.234 | 0.348 | 100.0% | 1025.9521 | 1026.2241 | 3 | 6.616 | 75.0% | 3 | K.IGGIGTVPVGR.V | 2 |

---

|  |  |  |  |  |  |  |  |  |
| --- | --- | --- | --- | --- | --- | --- | --- | --- |
| U | *gi|41322908|ref|NP\_95* | 26 | 30 | 7.9% | 4525 | 513712 | 5.8 | plectin isoform 1e [Homo sapiens] |
| U | *gi|47607492|ref|NP\_00* | 26 | 30 | 7.8% | 4574 | 518478 | 5.7 | plectin isoform 1c [Homo sapiens] |
| U | *gi|41322923|ref|NP\_95* | 26 | 30 | 7.9% | 4547 | 516204 | 5.8 | plectin isoform 1a [Homo sapiens] |
| U | *gi|41322919|ref|NP\_95* | 26 | 30 | 7.9% | 4547 | 516282 | 5.8 | plectin isoform 1b [Homo sapiens] |
| U | *gi|41322916|ref|NP\_95* | 26 | 30 | 7.6% | 4684 | 531796 | 6.0 | plectin isoform 1 [Homo sapiens] |
| U | *gi|41322914|ref|NP\_95* | 26 | 30 | 7.9% | 4551 | 516484 | 5.8 | plectin isoform 1g [Homo sapiens] |
| U | *gi|41322912|ref|NP\_95* | 26 | 30 | 7.9% | 4533 | 514780 | 5.7 | plectin isoform 1f [Homo sapiens] |
| U | *gi|41322910|ref|NP\_95* | 26 | 30 | 7.9% | 4515 | 512609 | 5.8 | plectin isoform 1d [Homo sapiens] |

| Filename XCorr DeltCN Conf% ObsM+H+ CalcM+H+ SpR ZScore Ion% # Sequence  | | | | | | | | | | | | |
| --- | --- | --- | --- | --- | --- | --- | --- | --- | --- | --- | --- | --- |
|  | Astrin\_STLC\_112116\_tube2\_01.10271.10271.2 | 3.4164 | 0.2647 | 100.0% | 1445.9922 | 1446.6897 | 1 | 6.793 | 68.2% | 1 | K.LQNVQIALDYLR.H | 2 |
|  | Astrin\_STLC\_112116\_tube2\_01.07756.07756.2 | 3.9003 | 0.3462 | 100.0% | 1710.0922 | 1709.8474 | 1 | 6.584 | 64.3% | 1 | R.LLDPEDVDVPQPDEK.S | 2 |
|  | Astrin\_STLC\_112116\_tube2\_01.14804.14804.2 | 2.8791 | 0.2633 | 99.3% | 2256.5122 | 2257.5938 | 1 | 4.751 | 44.1% | 1 | R.RFPSSFEEIEILWSQFLK.F | 2 |
|  | Astrin\_STLC\_112116\_tube2\_01.08814.08814.2 | 2.8642 | 0.2148 | 99.5% | 1192.2322 | 1191.413 | 1 | 4.779 | 77.8% | 2 | R.LLFNDVQTLK.D | 2 |
|  | Astrin\_STLC\_112116\_01.04896.04896.2 | 2.7616 | 0.3255 | 100.0% | 1216.1522 | 1216.3365 | 18 | 5.55 | 61.1% | 1 | R.RPELEDSTLR.Y | 2 |
|  | Astrin\_STLC\_112116\_01.11181.11181.3 | 2.5602 | 0.2834 | 97.8% | 1914.8043 | 1915.1155 | 1 | 5.204 | 35.7% | 1 | R.YLQDLLAWVEENQHR.V | 3 |
|  | Astrin\_STLC\_112116\_01.05133.05133.2 | 2.6162 | 0.2195 | 98.0% | 1350.4321 | 1351.3287 | 1 | 4.537 | 66.7% | 1 | R.DSQDAGGFGPEDR.L | 2 |
|  | Astrin\_STLC\_112116\_01.07055.07055.2 | 2.2457 | 0.3449 | 98.9% | 1532.1322 | 1532.8235 | 9 | 5.621 | 46.4% | 1 | K.VLALPEPSPAAPTLR.S | 2 |
|  | Astrin\_STLC\_112116\_01.06388.06388.2 | 2.8177 | 0.2844 | 99.9% | 1288.1122 | 1287.4612 | 1 | 4.66 | 80.0% | 1 | R.WQAVLAQTDVR.Q | 2 |
|  | Astrin\_STLC\_112116\_tube2\_01.10816.10816.2 | 3.2358 | 0.4349 | 100.0% | 1530.0521 | 1529.6494 | 1 | 7.095 | 53.8% | 2 | R.ESADPLGAWLQDAR.R | 2 |
|  | Astrin\_STLC\_112116\_02.08231.08231.2 | 4.485 | 0.4918 | 100.0% | 1808.6322 | 1809.9707 | 1 | 8.727 | 63.3% | 2 | K.VQSGSESVIQEYVDLR.T | 2 |
|  | Astrin\_STLC\_112116\_tube2\_01.06322.06322.2 | 2.9294 | 0.3667 | 100.0% | 1073.0721 | 1073.2316 | 1 | 6.963 | 77.8% | 1 | R.LAEVEAALEK.Q | 2 |
|  | Astrin\_STLC\_112116\_tube2\_01.08004.08004.2 | 2.618 | 0.285 | 99.9% | 1043.1522 | 1043.2083 | 1 | 5.732 | 87.5% | 1 | R.ALQALEELR.L | 2 |
|  | Astrin\_STLC\_112116\_tube2\_01.03752.03752.3 | 3.5429 | 0.2528 | 99.5% | 1819.2544 | 1817.9506 | 13 | 5.032 | 38.3% | 1 | K.SLAQAEAEKQKEEAER.E | 3 |
|  | Astrin\_STLC\_112116\_tube2\_01.08628.08628.2 | 2.4794 | 0.3616 | 100.0% | 1323.1921 | 1323.4454 | 1 | 6.38 | 70.0% | 1 | R.SQVEEELFSVR.V | 2 |
|  | Astrin\_STLC\_112116\_tube2\_01.06300.06300.3 | 3.4831 | 0.2701 | 99.6% | 1690.8544 | 1692.9156 | 2 | 5.129 | 41.7% | 1 | R.LREQLQLLEEQHR.A | 3 |
|  | Astrin\_STLC\_112116\_tube2\_01.04771.04771.3 | 3.1031 | 0.3046 | 99.5% | 1784.7544 | 1784.964 | 1 | 5.424 | 39.7% | 1 | R.AALAHSEEVTASQVAATK.T | 3 |
|  | Astrin\_STLC\_112116\_tube2\_01.08080.08080.2 | 3.3048 | 0.2751 | 100.0% | 1558.2122 | 1557.744 | 1 | 5.391 | 61.5% | 1 | R.LQEAGILSAEELQR.L | 2 |
|  | Astrin\_STLC\_112116\_tube2\_01.04323.04323.3 | 3.9675 | 0.4418 | 100.0% | 1412.7843 | 1411.5577 | 1 | 6.976 | 50.0% | 2 | R.LAQGHTTVDELAR.R | 3 |
|  | Astrin\_STLC\_112116\_01.15173.15173.2 | 2.8756 | 0.4505 | 100.0% | 2693.5522 | 2695.1748 | 1 | 7.237 | 30.0% | 1 | R.QLLSPGTALILLEAQAASGFLLDPVR.N | 2 |
|  | Astrin\_STLC\_112116\_02.13241.13241.3 | 3.1255 | 0.3104 | 99.4% | 2694.2644 | 2695.1748 | 23 | 5.184 | 24.0% | 1 | R.QLLSPGTALILLEAQAASGFLLDPVR.N | 3 |
|  | Astrin\_STLC\_112116\_tube2\_01.06648.06648.2 | 1.9692 | 0.3195 | 98.2% | 1162.2122 | 1161.2311 | 3 | 5.458 | 68.8% | 1 | R.GYFDEEMNR.V | 22 |
|  | Astrin\_STLC\_112116\_02.07145.07145.2 | 3.6463 | 0.4526 | 100.0% | 1614.3322 | 1614.8363 | 1 | 8.909 | 63.3% | 1 | R.LLDAQLSTGGIVDPSK.S | 2 |
|  | Astrin\_STLC\_112116\_02.08628.08628.3 | 3.5561 | 0.4623 | 100.0% | 2141.5444 | 2142.5034 | 1 | 6.757 | 34.2% | 1 | R.LLLEAQAATGFLLDPVKGER.L | 3 |
|  | Astrin\_STLC\_112116\_tube2\_01.08003.08003.2 | 2.5916 | 0.3088 | 99.5% | 1462.0322 | 1462.6611 | 1 | 5.129 | 66.7% | 1 | R.SQVMDEATALQLR.E | 2 |
|  | Astrin\_STLC\_112116\_01.12603.12603.2 | 5.0976 | 0.5433 | 100.0% | 2116.152 | 2116.3533 | 1 | 9.676 | 57.5% | 1 | R.AGTLSITEFADMLSGNAGGFR.S | 2 |

Similarities:
gi|525507390|ref|NP\_1(1:25)  

---

|  |  |  |  |  |  |  |  |  |
| --- | --- | --- | --- | --- | --- | --- | --- | --- |
| U | *gi|30581135|ref|NP\_00* | 8 | 11 | 7.8% | 1233 | 143233 | 7.6 | structural maintenance of chromosomes protein 1A isoform 1 [Homo sapiens] &IC SMC1A |
| U | *gi|527317371|ref|NP\_0* | 8 | 11 | 7.9% | 1211 | 140859 | 7.4 | structural maintenance of chromosomes protein 1A isoform 2 [Homo sapiens] &IC SMC1A |

| Filename XCorr DeltCN Conf% ObsM+H+ CalcM+H+ SpR ZScore Ion% # Sequence  | | | | | | | | | | | | |
| --- | --- | --- | --- | --- | --- | --- | --- | --- | --- | --- | --- | --- |
|  | Astrin\_STLC\_112116\_01.12837.12837.2 | 4.0018 | 0.4071 | 100.0% | 1523.7722 | 1524.7723 | 1 | 7.51 | 65.4% | 2 | K.SNLMDAISFVLGEK.T | 2 |
|  | Astrin\_STLC\_112116\_02.09918.09918.2 | 3.3837 | 0.4107 | 100.0% | 1653.3722 | 1654.9647 | 1 | 7.813 | 57.1% | 1 | R.NFLVFQGAVESIAMK.N | 2 |
|  | Astrin\_STLC\_112116\_tube2\_01.06822.06822.3 | 3.5234 | 0.2281 | 99.5% | 1454.0944 | 1454.6604 | 1 | 5.142 | 50.0% | 1 | R.IEKLEEYITTSK.Q | 3 |
|  | Astrin\_STLC\_112116\_01.05420.05420.2 | 2.2875 | 0.2918 | 98.7% | 1091.2322 | 1091.2065 | 1 | 4.893 | 72.7% | 1 | K.SGVISGGASDLK.A | 2 |
|  | Astrin\_STLC\_112116\_01.04109.04109.2 | 2.3378 | 0.3722 | 99.8% | 1383.3722 | 1383.5681 | 2 | 6.037 | 68.2% | 1 | R.QVQSQAHGLQMR.L | 2 |
|  | Astrin\_STLC\_112116\_tube2\_01.04270.04270.3 | 3.0542 | 0.3201 | 99.7% | 1439.8143 | 1440.5933 | 1 | 5.778 | 47.7% | 1 | R.LKYSQSDLEQTK.T | 3 |
|  | Astrin\_STLC\_112116\_tube2\_01.04880.04880.2 | 2.4637 | 0.2974 | 99.8% | 1065.8322 | 1066.2455 | 2 | 6.936 | 68.8% | 1 | R.HLALNLQEK.S | 2 |
|  | Astrin\_STLC\_112116\_01.05116.05116.2 | 3.1528 | 0.2518 | 100.0% | 1216.1322 | 1215.3518 | 1 | 5.09 | 88.9% | 3 | K.LNEQQSVLQR.I | 2 |

---

|  |  |  |  |  |  |  |  |  |
| --- | --- | --- | --- | --- | --- | --- | --- | --- |
| U | *gi|164419758|ref|NP\_0* | 2 | 2 | 7.6% | 801 | 88061 | 9.1 | bromodomain-containing protein 2 isoform 1 [Homo sapiens] |
| U | *gi|634743319|ref|NP\_0* | 2 | 2 | 9.0% | 681 | 74881 | 8.7 | bromodomain-containing protein 2 isoform 4 [Homo sapiens] |
| U | *gi|313747419|ref|NP\_0* | 2 | 2 | 8.1% | 754 | 83151 | 9.1 | bromodomain-containing protein 2 isoform 3 [Homo sapiens] |
| U | *gi|313747417|ref|NP\_0* | 2 | 2 | 7.3% | 836 | 92033 | 9.1 | bromodomain-containing protein 2 isoform 2 [Homo sapiens] |

| Filename XCorr DeltCN Conf% ObsM+H+ CalcM+H+ SpR ZScore Ion% # Sequence  | | | | | | | | | | | | |
| --- | --- | --- | --- | --- | --- | --- | --- | --- | --- | --- | --- | --- |
|  | Astrin\_STLC\_112116\_tube2\_01.14634.14634.3 | 3.2912 | 0.2423 | 97.7% | 3327.2944 | 3328.5957 | 92 | 4.713 | 18.5% | 1 | K.GVK@RKADT#T#TPTPTAILAPGSPASPPGSLEPK@.A | 3 |
|  | Astrin\_STLC\_112116\_tube2\_02.16940.16940.3 | 2.801 | 0.2681 | 96.7% | 2599.1042 | 2596.697 | 4 | 4.206 | 24.1% | 1 | K.K@SKK@ASGSGGGSAALGPS\*GFGPSGGSGTK@.L | 3 |

---

|  |  |  |  |  |  |  |  |  |
| --- | --- | --- | --- | --- | --- | --- | --- | --- |
| U | *gi|29171705|ref|NP\_80* | 2 | 2 | 7.6% | 606 | 64954 | 9.3 | melanoma-associated antigen D2 [Homo sapiens] |

| Filename XCorr DeltCN Conf% ObsM+H+ CalcM+H+ SpR ZScore Ion% # Sequence  | | | | | | | | | | | | |
| --- | --- | --- | --- | --- | --- | --- | --- | --- | --- | --- | --- | --- |
| \* | Astrin\_STLC\_112116\_tube2\_01.06922.06922.2 | 2.8845 | 0.2058 | 98.4% | 2478.7522 | 2476.6567 | 7 | 4.234 | 30.4% | 1 | R.EAPATQASSTTQLTDTQVLAAENK.S | 2 |
| \* | Astrin\_STLC\_112116\_01.03632.03632.3 | 3.1293 | 0.3712 | 99.7% | 2191.0745 | 2192.1309 | 1 | 5.974 | 33.3% | 1 | K.HLDGEEDGSSDQSQASGTTGGR.R | 3 |

---

|  |  |  |  |  |  |  |  |  |
| --- | --- | --- | --- | --- | --- | --- | --- | --- |
| U | *gi|381342476|ref|NP\_0* | 2 | 3 | 7.6% | 449 | 49229 | 6.3 | heterogeneous nuclear ribonucleoprotein H [Homo sapiens] |

| Filename XCorr DeltCN Conf% ObsM+H+ CalcM+H+ SpR ZScore Ion% # Sequence  | | | | | | | | | | | | |
| --- | --- | --- | --- | --- | --- | --- | --- | --- | --- | --- | --- | --- |
|  | Astrin\_STLC\_112116\_tube2\_02.09416.09416.2 | 4.6949 | 0.4769 | 100.0% | 1842.0322 | 1843.0001 | 1 | 9.444 | 65.6% | 2 | R.STGEAFVQFASQEIAEK.A | 2 |
|  | Astrin\_STLC\_112116\_tube2\_01.11976.11976.2 | 4.062 | 0.4605 | 100.0% | 1998.2722 | 1998.2023 | 1 | 7.713 | 59.4% | 1 | R.ATENDIYNFFSPLNPVR.V | 2 |

---

|  |  |  |  |  |  |  |  |  |
| --- | --- | --- | --- | --- | --- | --- | --- | --- |
| U | *gi|38201714|ref|NP\_00* | 2 | 2 | 7.4% | 326 | 36092 | 9.2 | ELAV-like protein 1 [Homo sapiens] |

| Filename XCorr DeltCN Conf% ObsM+H+ CalcM+H+ SpR ZScore Ion% # Sequence  | | | | | | | | | | | | |
| --- | --- | --- | --- | --- | --- | --- | --- | --- | --- | --- | --- | --- |
| \* | Astrin\_STLC\_112116\_tube2\_01.09348.09348.2 | 2.4227 | 0.2825 | 98.8% | 1355.5922 | 1354.4998 | 1 | 4.371 | 62.5% | 1 | R.SLFSSIGEVESAK.L | 2 |
| \* | Astrin\_STLC\_112116\_tube2\_01.05222.05222.2 | 2.3559 | 0.3135 | 99.3% | 1188.5122 | 1189.3542 | 3 | 5.947 | 75.0% | 1 | R.VLVDQTTGLSR.G | 2 |

---

|  |  |  |  |  |  |  |  |  |
| --- | --- | --- | --- | --- | --- | --- | --- | --- |
| U | *gi|14165435|ref|NP\_11* | 2 | 2 | 7.1% | 463 | 50976 | 5.5 | heterogeneous nuclear ribonucleoprotein K isoform b [Homo sapiens] |
| U | *gi|970949444|ref|NP\_0* | 2 | 2 | 7.5% | 440 | 48562 | 5.5 | heterogeneous nuclear ribonucleoprotein K isoform d [Homo sapiens] |
| U | *gi|970598247|ref|NP\_0* | 2 | 2 | 7.5% | 439 | 48511 | 5.9 | heterogeneous nuclear ribonucleoprotein K isoform c [Homo sapiens] |
| U | *gi|14165439|ref|NP\_00* | 2 | 2 | 7.1% | 464 | 51028 | 5.3 | heterogeneous nuclear ribonucleoprotein K isoform a [Homo sapiens] |

| Filename XCorr DeltCN Conf% ObsM+H+ CalcM+H+ SpR ZScore Ion% # Sequence  | | | | | | | | | | | | |
| --- | --- | --- | --- | --- | --- | --- | --- | --- | --- | --- | --- | --- |
|  | Astrin\_STLC\_112116\_tube2\_01.04322.04322.3 | 3.7733 | 0.3442 | 99.8% | 1735.8544 | 1736.8969 | 1 | 5.935 | 42.3% | 1 | K.RPAEDMEEEQAFKR.S | 3 |
|  | Astrin\_STLC\_112116\_tube2\_01.09482.09482.2 | 4.3224 | 0.5024 | 100.0% | 1917.2722 | 1918.1974 | 1 | 9.151 | 61.1% | 1 | R.GSYGDLGGPIITTQVTIPK.D | 2 |

---

|  |  |  |  |  |  |  |  |  |
| --- | --- | --- | --- | --- | --- | --- | --- | --- |
| U | *gi|16753207|ref|NP\_03* | 3 | 6 | 6.6% | 624 | 65696 | 5.2 | ubiquilin-2 [Homo sapiens] |

| Filename XCorr DeltCN Conf% ObsM+H+ CalcM+H+ SpR ZScore Ion% # Sequence  | | | | | | | | | | | | |
| --- | --- | --- | --- | --- | --- | --- | --- | --- | --- | --- | --- | --- |
| \* | Astrin\_STLC\_112116\_01.03842.03842.2 | 3.7042 | 0.5133 | 100.0% | 1393.4122 | 1394.5272 | 1 | 9.35 | 70.0% | 1 | R.GPAAAQGSAAAPAEPK.I | 2 |
|  | Astrin\_STLC\_112116\_01.07331.07331.2 | 4.3527 | 0.3428 | 100.0% | 1812.8722 | 1813.1865 | 1 | 6.904 | 75.0% | 3 | R.QLIMANPQMQQLIQR.N | 2 |
|  | Astrin\_STLC\_112116\_tube2\_01.07459.07459.2 | 2.4541 | 0.4833 | 100.0% | 1238.0721 | 1239.5265 | 3 | 8.271 | 61.1% | 2 | R.NPAMMQEMMR.N | 2 |

---

|  |  |  |  |  |  |  |  |  |
| --- | --- | --- | --- | --- | --- | --- | --- | --- |
| U | *Reverse\_gi|21536349|r* | 2 | 2 | 6.6% | 528 | 59910 | 5.7 | acid-sensing ion channel 1 isoform b [Homo sapiens] |
| U | *Reverse\_gi|378744190|* | 2 | 2 | 6.2% | 562 | 62700 | 5.1 | acid-sensing ion channel 1 isoform c [Homo sapiens] |
| U | *Reverse\_gi|21536351|r* | 2 | 2 | 6.1% | 574 | 64783 | 6.1 | acid-sensing ion channel 1 isoform a [Homo sapiens] |

| Filename XCorr DeltCN Conf% ObsM+H+ CalcM+H+ SpR ZScore Ion% # Sequence  | | | | | | | | | | | | |
| --- | --- | --- | --- | --- | --- | --- | --- | --- | --- | --- | --- | --- |
|  | Astrin\_STLC\_112116\_02.11792.11792.2 | 2.0812 | 0.2785 | 95.2% | 1882.7122 | 1882.0269 | 2 | 5.223 | 37.5% | 1 | R.KVDDLS\*LAVGK@DASSRK.A | 2 |
|  | Astrin\_STLC\_112116\_01.12006.12006.2 | 3.7255 | 0.1503 | 99.4% | 2080.372 | 2081.402 | 1 | 4.11 | 47.1% | 1 | K.ALYK@ASAK@SPIK@VMS\*LEK.G | 2 |

---

|  |  |  |  |  |  |  |  |  |
| --- | --- | --- | --- | --- | --- | --- | --- | --- |
| U | *gi|115298682|ref|NP\_0* | 11 | 17 | 6.5% | 2817 | 308606 | 9.1 | protein PRRC2C [Homo sapiens] |

| Filename XCorr DeltCN Conf% ObsM+H+ CalcM+H+ SpR ZScore Ion% # Sequence  | | | | | | | | | | | | |
| --- | --- | --- | --- | --- | --- | --- | --- | --- | --- | --- | --- | --- |
| \* | Astrin\_STLC\_112116\_01.05177.05177.3 | 3.603 | 0.3235 | 99.8% | 1611.0543 | 1609.7794 | 84 | 5.101 | 35.7% | 2 | K.AENKGNDPNVNIVPK.D | 3 |
| \* | Astrin\_STLC\_112116\_02.07596.07596.3 | 4.1066 | 0.3021 | 99.7% | 3219.9844 | 3221.424 | 3 | 5.861 | 22.4% | 1 | K.QGGQGDGIQVNSQFQQEFPSLQAAGDQEKK.E | 3 |
| \* | Astrin\_STLC\_112116\_tube2\_01.06178.06178.3 | 5.8688 | 0.5251 | 100.0% | 3263.1243 | 3264.3513 | 1 | 9.259 | 29.8% | 1 | K.AAGSPSSSDQDEKLPGQDESTAGTSEQNDILK.V | 3 |
| \* | Astrin\_STLC\_112116\_tube2\_01.05078.05078.2 | 3.7867 | 0.4342 | 100.0% | 1419.8522 | 1420.5681 | 1 | 8.163 | 62.5% | 1 | K.LNGQQAALASQYR.A | 2 |
| \* | Astrin\_STLC\_112116\_01.04594.04594.2 | 2.7644 | 0.2017 | 98.2% | 1300.9722 | 1300.4545 | 86 | 4.493 | 54.2% | 4 | K.SSSQIPAQPSVAK.V | 2 |
| \* | Astrin\_STLC\_112116\_tube2\_01.07018.07018.2 | 2.4249 | 0.4014 | 100.0% | 1360.9722 | 1361.4509 | 5 | 6.606 | 60.0% | 1 | K.QQVADEDEIWK.Q | 2 |
| \* | Astrin\_STLC\_112116\_01.05320.05320.2 | 2.3359 | 0.3138 | 99.1% | 1217.9122 | 1218.3091 | 3 | 5.454 | 70.0% | 2 | R.QQSEISAAVER.A | 2 |
| \* | Astrin\_STLC\_112116\_tube2\_01.10366.10366.2 | 2.8977 | 0.3036 | 100.0% | 1458.0922 | 1458.7512 | 2 | 6.634 | 60.0% | 1 | R.WLMMQSYMDPR.M | 2 |
| \* | Astrin\_STLC\_112116\_01.05302.05302.2 | 3.1749 | 0.3754 | 100.0% | 1124.9722 | 1125.2266 | 2 | 6.645 | 77.8% | 2 | R.DHAISLSEPR.M | 2 |
| \* | Astrin\_STLC\_112116\_tube2\_01.07570.07570.3 | 3.5475 | 0.3824 | 99.8% | 2253.9844 | 2255.5098 | 1 | 6.068 | 35.5% | 1 | R.MLWGSDPYPHAEPQQATTPK.A | 3 |
| \* | Astrin\_STLC\_112116\_tube2\_01.04453.04453.3 | 2.3106 | 0.3053 | 97.3% | 1773.4443 | 1774.0424 | 90 | 4.965 | 28.3% | 1 | K.TVNQQTMAAPVVKEEK.Q | 3 |

---

|  |  |  |  |  |  |  |  |  |
| --- | --- | --- | --- | --- | --- | --- | --- | --- |
| U | *gi|4758012|ref|NP\_004* | 8 | 12 | 6.4% | 1675 | 191613 | 5.7 | clathrin heavy chain 1 isoform 1 [Homo sapiens] |
| U | *gi|568815719|ref|NP\_0* | 8 | 12 | 6.4% | 1679 | 192057 | 5.7 | clathrin heavy chain 1 isoform 2 [Homo sapiens] |

| Filename XCorr DeltCN Conf% ObsM+H+ CalcM+H+ SpR ZScore Ion% # Sequence  | | | | | | | | | | | | |
| --- | --- | --- | --- | --- | --- | --- | --- | --- | --- | --- | --- | --- |
|  | Astrin\_STLC\_112116\_tube2\_01.07853.07853.2 | 2.4226 | 0.2954 | 99.2% | 1337.9521 | 1338.5646 | 1 | 5.663 | 63.6% | 1 | R.VVGAMQLYSVDR.K | 2 |
|  | Astrin\_STLC\_112116\_01.07161.07161.2 | 3.3941 | 0.4674 | 100.0% | 1304.0721 | 1305.4331 | 1 | 7.655 | 68.2% | 2 | R.NNLAGAEELFAR.K | 2 |
|  | Astrin\_STLC\_112116\_01.09629.09629.2 | 1.9147 | 0.3058 | 95.3% | 1433.9321 | 1434.6757 | 13 | 4.964 | 45.8% | 1 | K.SVDPTLALSVYLR.A | 2 |
|  | Astrin\_STLC\_112116\_tube2\_01.07711.07711.3 | 2.7755 | 0.2864 | 99.4% | 1621.8243 | 1621.8333 | 18 | 5.071 | 39.6% | 1 | R.ALEHFTDLYDIKR.A | 3 |
|  | Astrin\_STLC\_112116\_tube2\_01.04287.04287.2 | 2.5983 | 0.2632 | 99.3% | 1334.4122 | 1335.416 | 3 | 6.759 | 70.0% | 2 | K.IYIDSNNNPER.F | 2 |
|  | Astrin\_STLC\_112116\_01.13156.13156.2 | 3.6595 | 0.3709 | 100.0% | 1947.2522 | 1948.2819 | 1 | 6.597 | 56.2% | 2 | K.AFMTADLPNELIELLEK.I | 2 |
|  | Astrin\_STLC\_112116\_tube2\_01.07713.07713.2 | 2.5189 | 0.3572 | 100.0% | 1296.3522 | 1297.4563 | 1 | 7.573 | 70.0% | 2 | K.LLYNNVSNFGR.L | 2 |
|  | Astrin\_STLC\_112116\_tube2\_01.08184.08184.3 | 3.6143 | 0.3117 | 99.7% | 1972.4343 | 1972.2083 | 1 | 5.868 | 37.5% | 1 | R.LASTLVHLGEYQAAVDGAR.K | 3 |

---

|  |  |  |  |  |  |  |  |  |
| --- | --- | --- | --- | --- | --- | --- | --- | --- |
| U | *gi|46367787|ref|NP\_00* | 2 | 4 | 6.3% | 636 | 70671 | 9.5 | polyadenylate-binding protein 1 [Homo sapiens] |

| Filename XCorr DeltCN Conf% ObsM+H+ CalcM+H+ SpR ZScore Ion% # Sequence  | | | | | | | | | | | | |
| --- | --- | --- | --- | --- | --- | --- | --- | --- | --- | --- | --- | --- |
|  | Astrin\_STLC\_112116\_tube2\_01.13450.13450.3 | 5.0966 | 0.4015 | 100.0% | 2741.6643 | 2742.175 | 1 | 7.354 | 37.0% | 2 | K.ITGMLLEIDNSELLHMLESPESLR.S | 3 |
|  | Astrin\_STLC\_112116\_tube2\_01.06430.06430.3 | 4.4118 | 0.4509 | 100.0% | 1694.4243 | 1694.9285 | 1 | 7.238 | 48.3% | 2 | R.SKVDEAVAVLQAHQAK.E | 3 |

---

|  |  |  |  |  |  |  |  |  |
| --- | --- | --- | --- | --- | --- | --- | --- | --- |
| U | *gi|32567786|ref|NP\_78* | 3 | 7 | 6.2% | 535 | 57836 | 7.2 | keratin, type II cytoskeletal 79 [Homo sapiens] |

| Filename XCorr DeltCN Conf% ObsM+H+ CalcM+H+ SpR ZScore Ion% # Sequence  | | | | | | | | | | | | |
| --- | --- | --- | --- | --- | --- | --- | --- | --- | --- | --- | --- | --- |
|  | Astrin\_STLC\_112116\_tube2\_01.11450.11450.2 | 4.018 | 0.3758 | 100.0% | 1329.9722 | 1330.5211 | 1 | 7.458 | 86.4% | 3 | R.NLDLDSIIAEVK.A | 22222222 |
|  | Astrin\_STLC\_112116\_tube2\_01.05594.05594.2 | 3.1639 | 0.2805 | 100.0% | 1197.0122 | 1197.2897 | 1 | 6.472 | 83.3% | 1 | R.AEAEAWYQTK.Y | 22 |
|  | Astrin\_STLC\_112116\_tube2\_01.09346.09346.2 | 3.4818 | 0.346 | 100.0% | 1264.4122 | 1264.4644 | 1 | 7.748 | 80.0% | 3 | K.LALDVEIATYR.K | 222222222 |

Similarities:
contaminant\_KERATIN21(2:1)  
gi|119703753|ref|NP\_0(2:1)  
contaminant\_KERATIN18(2:1)  
gi|5031839|ref|NP\_005(2:1)  
contaminant\_KERATIN22(2:1)  
gi|119395754|ref|NP\_0(2:1)  
contaminant\_KERATIN19(1:2)  
gi|153791158|ref|NP\_0(2:1)  
gi|109148552|ref|NP\_4(1:2)  

---

|  |  |  |  |  |  |  |  |  |
| --- | --- | --- | --- | --- | --- | --- | --- | --- |
| U | *gi|301171467|ref|NP\_0* | 3 | 4 | 5.9% | 661 | 73156 | 7.2 | ATP-dependent RNA helicase DDX3X isoform 2 [Homo sapiens] |
| U | *gi|87196351|ref|NP\_00* | 3 | 4 | 5.9% | 662 | 73244 | 7.2 | ATP-dependent RNA helicase DDX3X isoform 1 [Homo sapiens] |
| U | *gi|301171475|ref|NP\_0* | 3 | 4 | 6.0% | 646 | 71355 | 6.6 | ATP-dependent RNA helicase DDX3X isoform 3 [Homo sapiens] |

| Filename XCorr DeltCN Conf% ObsM+H+ CalcM+H+ SpR ZScore Ion% # Sequence  | | | | | | | | | | | | |
| --- | --- | --- | --- | --- | --- | --- | --- | --- | --- | --- | --- | --- |
|  | Astrin\_STLC\_112116\_tube2\_02.08493.08493.2 | 2.2027 | 0.3607 | 99.5% | 1321.5922 | 1321.4729 | 20 | 5.689 | 55.0% | 1 | R.ELAVQIYEEAR.K | 2 |
|  | Astrin\_STLC\_112116\_tube2\_01.09379.09379.2 | 3.221 | 0.4391 | 100.0% | 1337.3922 | 1337.5946 | 1 | 8.296 | 90.0% | 2 | R.MLDMGFEPQIR.R | 2 |
|  | Astrin\_STLC\_112116\_tube2\_01.10373.10373.3 | 3.9418 | 0.281 | 99.7% | 2083.6443 | 2084.2957 | 1 | 5.545 | 42.2% | 1 | K.HVINFDLPSDIEEYVHR.I | 3 |

---

|  |  |  |  |  |  |  |  |  |
| --- | --- | --- | --- | --- | --- | --- | --- | --- |
| U | *gi|4503529|ref|NP\_001* | 2 | 2 | 5.9% | 406 | 46154 | 5.5 | eukaryotic initiation factor 4A-I isoform 1 [Homo sapiens] |

| Filename XCorr DeltCN Conf% ObsM+H+ CalcM+H+ SpR ZScore Ion% # Sequence  | | | | | | | | | | | | |
| --- | --- | --- | --- | --- | --- | --- | --- | --- | --- | --- | --- | --- |
|  | Astrin\_STLC\_112116\_01.07460.07460.2 | 2.5025 | 0.2745 | 99.4% | 1115.6522 | 1115.3585 | 236 | 5.18 | 55.6% | 1 | R.VLITTDLLAR.G | 2 |
| \* | Astrin\_STLC\_112116\_01.05607.05607.3 | 3.7702 | 0.4216 | 100.0% | 1589.9644 | 1590.8352 | 1 | 7.034 | 46.2% | 1 | R.KGVAINMVTEEDKR.T | 3 |

---

|  |  |  |  |  |  |  |  |  |
| --- | --- | --- | --- | --- | --- | --- | --- | --- |
| U | *gi|114155142|ref|NP\_0* | 9 | 12 | 5.5% | 2363 | 267290 | 5.0 | nucleoprotein TPR [Homo sapiens] &IC TPR |
| U | *gi|767910362|ref|XP\_0* | 9 | 12 | 5.4% | 2368 | 267872 | 5.0 | PREDICTED: nucleoprotein TPR isoform X1 [Homo sapiens] &IC TPR |

| Filename XCorr DeltCN Conf% ObsM+H+ CalcM+H+ SpR ZScore Ion% # Sequence  | | | | | | | | | | | | |
| --- | --- | --- | --- | --- | --- | --- | --- | --- | --- | --- | --- | --- |
|  | Astrin\_STLC\_112116\_tube2\_01.09412.09412.2 | 3.7635 | 0.4595 | 100.0% | 1202.9521 | 1203.4393 | 1 | 8.222 | 88.9% | 2 | R.VLLMELEEAR.G | 2 |
|  | Astrin\_STLC\_112116\_02.08372.08372.3 | 5.3644 | 0.5286 | 100.0% | 2836.2844 | 2836.2554 | 1 | 9.189 | 36.1% | 2 | R.ILLSQTTGVAIPLHASSLDDVSLASTPK.R | 3 |
|  | Astrin\_STLC\_112116\_tube2\_01.08070.08070.3 | 4.2181 | 0.398 | 99.7% | 2695.7644 | 2697.0146 | 3 | 5.402 | 28.0% | 1 | K.RPSTSQTVSTPAPVPVIESTEAIEAK.A | 3 |
|  | Astrin\_STLC\_112116\_tube2\_01.04979.04979.2 | 2.7451 | 0.1094 | 96.7% | 1103.4321 | 1102.2328 | 19 | 5.204 | 68.8% | 1 | K.LQEQVTDLR.S | 2 |
|  | Astrin\_STLC\_112116\_01.12884.12884.3 | 3.7782 | 0.3272 | 99.8% | 2024.6943 | 2025.3561 | 15 | 4.833 | 36.8% | 1 | R.GQNLLLTNLQTIQGILER.S | 3 |
|  | Astrin\_STLC\_112116\_tube2\_01.14128.14128.2 | 5.1926 | 0.524 | 100.0% | 2025.4122 | 2025.3561 | 1 | 9.146 | 61.8% | 2 | R.GQNLLLTNLQTIQGILER.S | 2 |
|  | Astrin\_STLC\_112116\_tube2\_01.09401.09401.2 | 4.4664 | 0.2941 | 100.0% | 1703.2322 | 1702.8619 | 1 | 6.55 | 64.3% | 1 | K.TLSSVQNEVQEALQR.A | 2 |
|  | Astrin\_STLC\_112116\_tube2\_01.05982.05982.2 | 2.2201 | 0.3773 | 99.6% | 1397.3121 | 1397.6014 | 1 | 5.826 | 54.5% | 1 | K.TETMNVVMETNK.M | 2 |
|  | Astrin\_STLC\_112116\_tube2\_01.07145.07145.2 | 2.1959 | 0.217 | 95.2% | 1274.0122 | 1275.4014 | 9 | 4.799 | 60.0% | 1 | K.SLESQVENLQK.T | 2 |

---

|  |  |  |  |  |  |  |  |  |
| --- | --- | --- | --- | --- | --- | --- | --- | --- |
| U | *gi|50345982|ref|NP\_00* | 2 | 2 | 5.4% | 503 | 54494 | 8.2 | ATP synthase subunit alpha, mitochondrial isoform c [Homo sapiens] |
| U | *gi|50345984|ref|NP\_00* | 2 | 2 | 4.9% | 553 | 59751 | 9.1 | ATP synthase subunit alpha, mitochondrial isoform a precursor [Homo sapiens] |

| Filename XCorr DeltCN Conf% ObsM+H+ CalcM+H+ SpR ZScore Ion% # Sequence  | | | | | | | | | | | | |
| --- | --- | --- | --- | --- | --- | --- | --- | --- | --- | --- | --- | --- |
|  | Astrin\_STLC\_112116\_tube2\_01.10157.10157.2 | 3.2955 | 0.3221 | 100.0% | 1625.0721 | 1625.8625 | 1 | 6.186 | 60.0% | 1 | R.TGAIVDVPVGEELLGR.V | 2 |
|  | Astrin\_STLC\_112116\_tube2\_01.07619.07619.2 | 2.6717 | 0.3529 | 100.0% | 1288.9122 | 1288.4863 | 1 | 7.164 | 75.0% | 1 | K.HALIIYDDLSK.Q | 2 |

---

|  |  |  |  |  |  |  |  |  |
| --- | --- | --- | --- | --- | --- | --- | --- | --- |
| U | *gi|21361399|ref|NP\_05* | 2 | 2 | 5.1% | 589 | 65309 | 5.1 | serine/threonine-protein phosphatase 2A 65 kDa regulatory subunit A alpha isoform [Homo sapiens] |

| Filename XCorr DeltCN Conf% ObsM+H+ CalcM+H+ SpR ZScore Ion% # Sequence  | | | | | | | | | | | | |
| --- | --- | --- | --- | --- | --- | --- | --- | --- | --- | --- | --- | --- |
| \* | Astrin\_STLC\_112116\_tube2\_01.08330.08330.2 | 2.1714 | 0.2156 | 95.4% | 1110.2122 | 1110.2145 | 1 | 3.983 | 83.3% | 1 | R.LAGGDWFTSR.T | 2 |
| \* | Astrin\_STLC\_112116\_tube2\_01.09718.09718.3 | 2.6786 | 0.2899 | 97.8% | 2194.4944 | 2195.5615 | 160 | 4.538 | 27.6% | 1 | K.IGPILDNSTLQSEVKPILEK.L | 3 |

---

|  |  |  |  |  |  |  |  |  |
| --- | --- | --- | --- | --- | --- | --- | --- | --- |
| U | *gi|48762932|ref|NP\_00* | 2 | 2 | 4.9% | 548 | 59621 | 5.6 | T-complex protein 1 subunit theta isoform 1 [Homo sapiens] |
| U | *gi|544711070|ref|NP\_0* | 2 | 2 | 5.4% | 497 | 54106 | 5.3 | T-complex protein 1 subunit theta isoform 3 [Homo sapiens] |
| U | *gi|544711041|ref|NP\_0* | 2 | 2 | 5.1% | 529 | 57645 | 5.4 | T-complex protein 1 subunit theta isoform 2 [Homo sapiens] |

| Filename XCorr DeltCN Conf% ObsM+H+ CalcM+H+ SpR ZScore Ion% # Sequence  | | | | | | | | | | | | |
| --- | --- | --- | --- | --- | --- | --- | --- | --- | --- | --- | --- | --- |
|  | Astrin\_STLC\_112116\_tube2\_01.09252.09252.2 | 2.2518 | 0.2366 | 95.7% | 1334.2722 | 1334.5583 | 4 | 4.336 | 54.5% | 1 | K.LFVTNDAATILR.E | 2 |
|  | Astrin\_STLC\_112116\_tube2\_01.05379.05379.2 | 4.2609 | 0.6114 | 100.0% | 1373.4521 | 1373.5492 | 1 | 11.09 | 75.0% | 1 | K.AIADTGANVVVTGGK.V | 2 |

---

|  |  |  |  |  |  |  |  |  |
| --- | --- | --- | --- | --- | --- | --- | --- | --- |
| U | *gi|19923142|ref|NP\_00* | 3 | 6 | 4.8% | 876 | 97170 | 4.8 | importin subunit beta-1 isoform 1 [Homo sapiens] |

| Filename XCorr DeltCN Conf% ObsM+H+ CalcM+H+ SpR ZScore Ion% # Sequence  | | | | | | | | | | | | |
| --- | --- | --- | --- | --- | --- | --- | --- | --- | --- | --- | --- | --- |
| \* | Astrin\_STLC\_112116\_tube2\_01.12495.12495.2 | 3.0492 | 0.3278 | 100.0% | 1659.2922 | 1659.9231 | 2 | 5.982 | 53.6% | 1 | R.AAVENLPTFLVELSR.V | 2 |
| \* | Astrin\_STLC\_112116\_01.04164.04164.2 | 3.2859 | 0.488 | 100.0% | 1226.0122 | 1226.378 | 1 | 8.069 | 77.3% | 2 | R.VLANPGNSQVAR.V | 2 |
|  | Astrin\_STLC\_112116\_tube2\_01.10779.10779.2 | 3.9453 | 0.398 | 100.0% | 1607.2122 | 1606.8595 | 1 | 7.282 | 75.0% | 3 | K.LAATNALLNSLEFTK.A | 2 |

---

|  |  |  |  |  |  |  |  |  |
| --- | --- | --- | --- | --- | --- | --- | --- | --- |
| U | *gi|109240550|ref|NP\_0* | 2 | 2 | 4.8% | 523 | 58744 | 6.7 | paraspeckle component 1 [Homo sapiens] |

| Filename XCorr DeltCN Conf% ObsM+H+ CalcM+H+ SpR ZScore Ion% # Sequence  | | | | | | | | | | | | |
| --- | --- | --- | --- | --- | --- | --- | --- | --- | --- | --- | --- | --- |
| \* | Astrin\_STLC\_112116\_tube2\_01.07217.07217.2 | 2.4311 | 0.3062 | 99.5% | 1312.3922 | 1311.4368 | 1 | 5.516 | 80.0% | 1 | R.YGEPSEVFINR.D | 2 |
| \* | Astrin\_STLC\_112116\_tube2\_01.08750.08750.2 | 2.5839 | 0.1871 | 95.7% | 1649.6921 | 1650.7875 | 48 | 4.209 | 46.2% | 1 | R.FAQPGTFEFEYASR.W | 2 |

---

|  |  |  |  |  |  |  |  |  |
| --- | --- | --- | --- | --- | --- | --- | --- | --- |
| U | *gi|38569421|ref|NP\_00* | 4 | 6 | 4.7% | 1101 | 120839 | 7.3 | ATP-citrate synthase isoform 1 [Homo sapiens] |
| U | *gi|740086852|ref|NP\_0* | 4 | 6 | 4.5% | 1145 | 125151 | 8.2 | ATP-citrate synthase isoform 4 [Homo sapiens] |
| U | *gi|740086846|ref|NP\_0* | 4 | 6 | 4.5% | 1155 | 126218 | 8.2 | ATP-citrate synthase isoform 3 [Homo sapiens] |
| U | *gi|38569423|ref|NP\_94* | 4 | 6 | 4.8% | 1091 | 119772 | 7.3 | ATP-citrate synthase isoform 2 [Homo sapiens] |

| Filename XCorr DeltCN Conf% ObsM+H+ CalcM+H+ SpR ZScore Ion% # Sequence  | | | | | | | | | | | | |
| --- | --- | --- | --- | --- | --- | --- | --- | --- | --- | --- | --- | --- |
|  | Astrin\_STLC\_112116\_01.04157.04157.2 | 3.1199 | 0.3601 | 100.0% | 1248.2322 | 1247.3561 | 1 | 5.636 | 65.0% | 1 | R.RGGPNYQEGLR.V | 2 |
|  | Astrin\_STLC\_112116\_tube2\_01.09688.09688.2 | 3.4673 | 0.2527 | 100.0% | 1504.2522 | 1504.7417 | 1 | 6.899 | 60.7% | 3 | K.IGNTGGMLDNILASK.L | 2 |
|  | Astrin\_STLC\_112116\_01.05730.05730.2 | 2.6768 | 0.279 | 99.5% | 1368.1322 | 1368.5785 | 1 | 4.657 | 59.1% | 1 | K.LYRPGSVAYVSR.S | 2 |
|  | Astrin\_STLC\_112116\_tube2\_01.08381.08381.2 | 3.008 | 0.3584 | 100.0% | 1492.2122 | 1492.647 | 4 | 5.962 | 57.7% | 1 | R.SGGMSNELNNIISR.T | 2 |

---

|  |  |  |  |  |  |  |  |  |
| --- | --- | --- | --- | --- | --- | --- | --- | --- |
| U | *gi|586798161|ref|NP\_0* | 3 | 4 | 4.7% | 962 | 107895 | 4.9 | general vesicular transport factor p115 isoform 2 [Homo sapiens] |
| U | *gi|586798211|ref|NP\_0* | 3 | 4 | 4.6% | 973 | 109194 | 4.9 | general vesicular transport factor p115 isoform 1 [Homo sapiens] |

| Filename XCorr DeltCN Conf% ObsM+H+ CalcM+H+ SpR ZScore Ion% # Sequence  | | | | | | | | | | | | |
| --- | --- | --- | --- | --- | --- | --- | --- | --- | --- | --- | --- | --- |
|  | Astrin\_STLC\_112116\_tube2\_01.04261.04261.3 | 3.2639 | 0.2998 | 99.5% | 2026.7943 | 2027.2164 | 1 | 4.923 | 39.5% | 1 | R.GVMGGQSAGPQHTEAETIQK.L | 3 |
|  | Astrin\_STLC\_112116\_tube2\_01.04273.04273.3 | 3.2745 | 0.2324 | 99.1% | 1597.9744 | 1598.7556 | 1 | 5.906 | 47.9% | 1 | K.TLEQHDNIVTHYK.N | 3 |
|  | Astrin\_STLC\_112116\_01.05572.05572.2 | 3.3059 | 0.47 | 100.0% | 1333.8722 | 1334.4692 | 1 | 8.185 | 72.7% | 2 | K.SQLNSQSVEITK.L | 2 |

---

|  |  |  |  |  |  |  |  |  |
| --- | --- | --- | --- | --- | --- | --- | --- | --- |
| U | *gi|4507677|ref|NP\_003* | 4 | 4 | 4.6% | 803 | 92469 | 4.8 | endoplasmin precursor [Homo sapiens] |

| Filename XCorr DeltCN Conf% ObsM+H+ CalcM+H+ SpR ZScore Ion% # Sequence  | | | | | | | | | | | | |
| --- | --- | --- | --- | --- | --- | --- | --- | --- | --- | --- | --- | --- |
| \* | Astrin\_STLC\_112116\_tube2\_01.06912.06912.2 | 2.3994 | 0.2223 | 98.5% | 1081.7122 | 1082.204 | 3 | 5.429 | 68.8% | 1 | K.FAFQAEVNR.M | 2 |
|  | Astrin\_STLC\_112116\_tube2\_01.06413.06413.2 | 2.4029 | 0.2186 | 96.4% | 1277.1322 | 1276.3861 | 13 | 4.594 | 54.5% | 1 | R.ELISNASDALDK.I | 22 |
|  | Astrin\_STLC\_112116\_tube2\_01.07956.07956.2 | 3.3531 | 0.2598 | 100.0% | 1545.4321 | 1545.733 | 1 | 5.563 | 65.4% | 1 | R.ELISNASDALDKIR.L | 22 |
| \* | Astrin\_STLC\_112116\_tube2\_01.07908.07908.2 | 3.5147 | 0.4972 | 100.0% | 1486.1122 | 1486.622 | 8 | 7.518 | 53.8% | 1 | K.GVVDSDDLPLNVSR.E | 2 |

Similarities:
gi|20149594|ref|NP\_03(2:2)  

---

|  |  |  |  |  |  |  |  |  |
| --- | --- | --- | --- | --- | --- | --- | --- | --- |
| U | *gi|1002095850|ref|NP\_* | 2 | 2 | 4.6% | 759 | 86101 | 7.5 | scaffold attachment factor B1 isoform 5 [Homo sapiens] |
| U | *gi|321267473|ref|NP\_0* | 2 | 2 | 4.1% | 848 | 95181 | 6.5 | scaffold attachment factor B1 isoform 4 [Homo sapiens] |
| U | *gi|321267471|ref|NP\_0* | 2 | 2 | 3.8% | 916 | 102768 | 5.5 | scaffold attachment factor B1 isoform 2 [Homo sapiens] |
| U | *gi|321267469|ref|NP\_0* | 2 | 2 | 3.8% | 917 | 102855 | 5.5 | scaffold attachment factor B1 isoform 1 [Homo sapiens] |
| U | *gi|21264343|ref|NP\_00* | 2 | 2 | 3.8% | 915 | 102642 | 5.5 | scaffold attachment factor B1 isoform 3 [Homo sapiens] |
| U | *gi|1002095852|ref|NP\_* | 2 | 2 | 3.8% | 914 | 102555 | 5.5 | scaffold attachment factor B1 isoform 6 [Homo sapiens] |

| Filename XCorr DeltCN Conf% ObsM+H+ CalcM+H+ SpR ZScore Ion% # Sequence  | | | | | | | | | | | | |
| --- | --- | --- | --- | --- | --- | --- | --- | --- | --- | --- | --- | --- |
|  | Astrin\_STLC\_112116\_01.03845.03845.3 | 2.6355 | 0.3414 | 99.4% | 2647.8542 | 2648.5098 | 5 | 5.151 | 30.7% | 1 | K.ESSTSEGADQKMS\*S\*PEDDSDTKR.L | 3 |
|  | Astrin\_STLC\_112116\_tube2\_01.08939.08939.2 | 3.2507 | 0.4489 | 100.0% | 1355.5521 | 1355.4929 | 1 | 6.963 | 77.3% | 1 | R.NFWVSGLSSTTR.A | 2 |

---

|  |  |  |  |  |  |  |  |  |
| --- | --- | --- | --- | --- | --- | --- | --- | --- |
| U | *gi|751557636|ref|NP\_0* | 3 | 3 | 4.6% | 718 | 83067 | 5.9 | mitotic spindle assembly checkpoint protein MAD1 isoform a [Homo sapiens] &IC Mad1 |
| U | *gi|751557638|ref|NP\_0* | 3 | 3 | 5.3% | 626 | 72287 | 6.8 | mitotic spindle assembly checkpoint protein MAD1 isoform b precursor [Homo sapiens] &IC Mad1 |

| Filename XCorr DeltCN Conf% ObsM+H+ CalcM+H+ SpR ZScore Ion% # Sequence  | | | | | | | | | | | | |
| --- | --- | --- | --- | --- | --- | --- | --- | --- | --- | --- | --- | --- |
|  | Astrin\_STLC\_112116\_01.04446.04446.2 | 3.6617 | 0.2498 | 100.0% | 1273.2922 | 1273.3885 | 1 | 6.301 | 80.0% | 1 | K.IQELQASQEAR.A | 2 |
|  | Astrin\_STLC\_112116\_tube2\_01.06738.06738.2 | 3.0775 | 0.3827 | 100.0% | 1315.3121 | 1315.5095 | 1 | 7.106 | 77.3% | 1 | K.LSLQEQDAAIVK.N | 2 |
|  | Astrin\_STLC\_112116\_tube2\_01.07379.07379.2 | 2.9834 | 0.2663 | 100.0% | 1134.1122 | 1134.336 | 1 | 6.02 | 77.8% | 1 | R.LDQTMGLSIR.T | 2 |

---

|  |  |  |  |  |  |  |  |  |
| --- | --- | --- | --- | --- | --- | --- | --- | --- |
| U | *gi|119395758|ref|NP\_0* | 3 | 4 | 4.5% | 1272 | 141347 | 5.4 | protein diaphanous homolog 1 isoform 1 [Homo sapiens] |
| U | *gi|929981605|ref|NP\_0* | 3 | 4 | 4.6% | 1250 | 139413 | 5.3 | protein diaphanous homolog 1 isoform 3 [Homo sapiens] |
| U | *gi|119395760|ref|NP\_0* | 3 | 4 | 4.5% | 1263 | 140289 | 5.4 | protein diaphanous homolog 1 isoform 2 [Homo sapiens] |

| Filename XCorr DeltCN Conf% ObsM+H+ CalcM+H+ SpR ZScore Ion% # Sequence  | | | | | | | | | | | | |
| --- | --- | --- | --- | --- | --- | --- | --- | --- | --- | --- | --- | --- |
|  | Astrin\_STLC\_112116\_tube2\_01.15902.15902.3 | 3.877 | 0.3607 | 99.8% | 3043.4343 | 3043.493 | 1 | 5.981 | 28.7% | 2 | R.VSLNNNPVSWVQTFGAEGLASLLDILKR.L | 3 |
|  | Astrin\_STLC\_112116\_02.10327.10327.2 | 3.4601 | 0.365 | 100.0% | 1617.5122 | 1617.9415 | 3 | 5.99 | 53.8% | 1 | K.TMLETEEGILLLVR.A | 2 |
|  | Astrin\_STLC\_112116\_tube2\_01.11645.11645.2 | 2.6677 | 0.3279 | 99.7% | 1911.5322 | 1913.2012 | 1 | 5.097 | 42.9% | 1 | K.KLSVEEFFMDLHNFR.N | 2 |

---

|  |  |  |  |  |  |  |  |  |
| --- | --- | --- | --- | --- | --- | --- | --- | --- |
| U | *gi|116063573|ref|NP\_0* | 8 | 9 | 4.4% | 2639 | 280016 | 6.0 | filamin-A isoform 1 [Homo sapiens] |
| U | *gi|160420317|ref|NP\_0* | 8 | 9 | 4.3% | 2647 | 280737 | 6.1 | filamin-A isoform 2 [Homo sapiens] |

| Filename XCorr DeltCN Conf% ObsM+H+ CalcM+H+ SpR ZScore Ion% # Sequence  | | | | | | | | | | | | |
| --- | --- | --- | --- | --- | --- | --- | --- | --- | --- | --- | --- | --- |
|  | Astrin\_STLC\_112116\_01.07889.07889.2 | 2.2887 | 0.2349 | 96.7% | 1285.4521 | 1286.5167 | 3 | 4.852 | 75.0% | 1 | K.LPQLPITNFSR.D | 2 |
|  | Astrin\_STLC\_112116\_tube2\_01.05918.05918.3 | 3.7892 | 0.3671 | 99.8% | 1648.8544 | 1647.8687 | 1 | 6.327 | 40.0% | 1 | K.TGVAVNKPAEFTVDAK.H | 3 |
|  | Astrin\_STLC\_112116\_tube2\_01.05381.05381.2 | 2.9087 | 0.4938 | 100.0% | 1429.9321 | 1430.5632 | 1 | 8.232 | 63.3% | 1 | K.AFGPGLQGGSAGSPAR.F | 2 |
|  | Astrin\_STLC\_112116\_tube2\_01.05433.05433.2 | 2.966 | 0.3832 | 100.0% | 1225.7122 | 1226.2854 | 5 | 7.002 | 65.0% | 1 | R.EATTEFSVDAR.A | 2 |
|  | Astrin\_STLC\_112116\_tube2\_01.06871.06871.2 | 4.0499 | 0.541 | 100.0% | 1571.0122 | 1571.7275 | 1 | 10.053 | 55.9% | 1 | R.GAGTGGLGLAVEGPSEAK.M | 2 |
|  | Astrin\_STLC\_112116\_tube2\_01.06748.06748.2 | 2.3631 | 0.3207 | 99.2% | 1435.3522 | 1435.5767 | 1 | 6.033 | 58.3% | 1 | R.ANLPQSFQVDTSK.A | 2 |
|  | Astrin\_STLC\_112116\_02.06922.06922.3 | 3.8928 | 0.3404 | 99.8% | 2201.0645 | 2201.4412 | 1 | 6.341 | 35.5% | 2 | R.LVSNHSLHETSSVFVDSLTK.A | 3 |
|  | Astrin\_STLC\_112116\_01.05795.05795.2 | 2.3884 | 0.2441 | 98.5% | 1151.9722 | 1152.333 | 2 | 4.884 | 66.7% | 1 | K.DKGEYTLVVK.W | 2 |

---

|  |  |  |  |  |  |  |  |  |
| --- | --- | --- | --- | --- | --- | --- | --- | --- |
| U | *gi|4506787|ref|NP\_003* | 5 | 6 | 4.3% | 1657 | 189251 | 6.5 | ras GTPase-activating-like protein IQGAP1 [Homo sapiens] |

| Filename XCorr DeltCN Conf% ObsM+H+ CalcM+H+ SpR ZScore Ion% # Sequence  | | | | | | | | | | | | |
| --- | --- | --- | --- | --- | --- | --- | --- | --- | --- | --- | --- | --- |
| \* | Astrin\_STLC\_112116\_01.04524.04524.2 | 2.573 | 0.1987 | 98.8% | 1093.4521 | 1094.1816 | 9 | 5.868 | 75.0% | 1 | R.LTAEEMDER.R | 2 |
| \* | Astrin\_STLC\_112116\_01.15135.15135.3 | 5.9072 | 0.5382 | 100.0% | 2959.3743 | 2960.3574 | 1 | 8.866 | 33.9% | 1 | K.IGGILANELSVDEAALHAAVIAINEAIDR.R | 3 |
| \* | Astrin\_STLC\_112116\_tube2\_01.09991.09991.2 | 2.7637 | 0.3071 | 99.8% | 1543.7122 | 1543.763 | 6 | 6.949 | 62.5% | 1 | R.EQLWLANEGLITR.L | 2 |
| \* | Astrin\_STLC\_112116\_tube2\_01.05157.05157.2 | 2.4692 | 0.2134 | 98.0% | 1073.7122 | 1073.2316 | 2 | 5.386 | 72.2% | 1 | K.LTELGTVDPK.N | 2 |
| \* | Astrin\_STLC\_112116\_tube2\_01.04486.04486.2 | 3.1387 | 0.4591 | 100.0% | 1237.1122 | 1237.3983 | 1 | 7.697 | 75.0% | 2 | K.LQQTYAALNSK.A | 2 |

---

|  |  |  |  |  |  |  |  |  |
| --- | --- | --- | --- | --- | --- | --- | --- | --- |
| U | *gi|154759259|ref|NP\_0* | 6 | 8 | 3.8% | 2472 | 284538 | 5.3 | spectrin alpha chain, non-erythrocytic 1 isoform 2 [Homo sapiens] |
| U | *gi|306966132|ref|NP\_0* | 6 | 8 | 3.8% | 2452 | 282280 | 5.3 | spectrin alpha chain, non-erythrocytic 1 isoform 3 [Homo sapiens] |

| Filename XCorr DeltCN Conf% ObsM+H+ CalcM+H+ SpR ZScore Ion% # Sequence  | | | | | | | | | | | | |
| --- | --- | --- | --- | --- | --- | --- | --- | --- | --- | --- | --- | --- |
|  | Astrin\_STLC\_112116\_tube2\_01.04859.04859.2 | 2.9585 | 0.2861 | 100.0% | 1302.1322 | 1303.4117 | 4 | 5.781 | 70.0% | 1 | K.VLETAEDIQER.R | 2 |
|  | Astrin\_STLC\_112116\_tube2\_01.13522.13522.2 | 2.9373 | 0.3547 | 100.0% | 2128.2322 | 2128.344 | 1 | 7.079 | 35.0% | 1 | K.ALINADELASDVAGAEALLDR.H | 2 |
|  | Astrin\_STLC\_112116\_tube2\_01.10658.10658.3 | 3.0272 | 0.3221 | 99.4% | 2035.4944 | 2033.3348 | 5 | 5.795 | 34.4% | 1 | K.KGDILTLLNSTNKDWWK.V | 3 |
|  | Astrin\_STLC\_112116\_tube2\_01.06652.06652.2 | 3.2447 | 0.4539 | 100.0% | 1394.2922 | 1394.5272 | 1 | 7.596 | 63.6% | 1 | K.LGESQTLQQFSR.D | 2 |
|  | Astrin\_STLC\_112116\_tube2\_01.04404.04404.3 | 3.1959 | 0.3349 | 99.7% | 2026.2544 | 2026.1644 | 1 | 5.249 | 47.1% | 1 | K.LQTASDESYKDPTNIQSK.H | 3 |
|  | Astrin\_STLC\_112116\_tube2\_01.11005.11005.2 | 4.5899 | 0.4379 | 100.0% | 1631.1921 | 1631.915 | 1 | 8.481 | 73.1% | 3 | R.LAALADQWQFLVQK.S | 2 |

---

|  |  |  |  |  |  |  |  |  |
| --- | --- | --- | --- | --- | --- | --- | --- | --- |
| U | *gi|112382250|ref|NP\_0* | 5 | 5 | 3.6% | 2364 | 274608 | 5.6 | spectrin beta chain, non-erythrocytic 1 isoform 1 [Homo sapiens] |

| Filename XCorr DeltCN Conf% ObsM+H+ CalcM+H+ SpR ZScore Ion% # Sequence  | | | | | | | | | | | | |
| --- | --- | --- | --- | --- | --- | --- | --- | --- | --- | --- | --- | --- |
|  | Astrin\_STLC\_112116\_01.15191.15191.3 | 4.5431 | 0.3874 | 100.0% | 3001.3743 | 3002.3655 | 1 | 7.629 | 32.0% | 1 | R.LEEASLLHQFQADADDIDAWMLDILK.I | 3 |
|  | Astrin\_STLC\_112116\_01.10152.10152.2 | 2.5655 | 0.1953 | 97.6% | 1383.2522 | 1382.4723 | 1 | 5.317 | 70.0% | 1 | R.DLDDFQSWLSR.T | 2 |
|  | Astrin\_STLC\_112116\_01.14943.14943.2 | 3.0258 | 0.2556 | 99.5% | 1944.5922 | 1945.1364 | 1 | 5.063 | 46.9% | 1 | K.DGLNEAWADLLELIDTR.T | 2 |
|  | Astrin\_STLC\_112116\_tube2\_01.03908.03908.3 | 3.3061 | 0.288 | 99.4% | 1764.8644 | 1763.9481 | 1 | 4.695 | 40.0% | 1 | R.LQAAYAGDKADDIQKR.E | 3 |
| \* | Astrin\_STLC\_112116\_tube2\_01.05759.05759.2 | 2.6018 | 0.2366 | 98.0% | 1368.0721 | 1368.576 | 6 | 5.089 | 57.7% | 1 | K.TALPAQSAATLPAR.T | 2 |

---

|  |  |  |  |  |  |  |  |  |
| --- | --- | --- | --- | --- | --- | --- | --- | --- |
| U | *gi|33350932|ref|NP\_00* | 9 | 12 | 3.5% | 4646 | 532412 | 6.4 | cytoplasmic dynein 1 heavy chain 1 [Homo sapiens] &IC dynein heavy chain |

| Filename XCorr DeltCN Conf% ObsM+H+ CalcM+H+ SpR ZScore Ion% # Sequence  | | | | | | | | | | | | |
| --- | --- | --- | --- | --- | --- | --- | --- | --- | --- | --- | --- | --- |
| \* | Astrin\_STLC\_112116\_01.11159.11159.3 | 4.3101 | 0.292 | 99.7% | 2478.8044 | 2477.8577 | 1 | 5.069 | 38.0% | 1 | R.KLVPLLLEDGGEAPAALEAALEEK.S | 3 |
| \* | Astrin\_STLC\_112116\_tube2\_01.05824.05824.3 | 3.21 | 0.402 | 99.8% | 1625.7244 | 1626.8503 | 1 | 7.001 | 46.4% | 1 | R.TPVIDADKPVSSQLR.V | 3 |
| \* | Astrin\_STLC\_112116\_01.14463.14463.3 | 4.0522 | 0.4223 | 100.0% | 2914.3145 | 2914.2842 | 9 | 7.435 | 25.0% | 3 | R.VLTLSEDSPYETLHSFISNAVAPFFK.S | 3 |
| \* | Astrin\_STLC\_112116\_tube2\_01.09517.09517.3 | 3.9219 | 0.3338 | 99.8% | 2579.8442 | 2580.8137 | 1 | 5.884 | 31.8% | 1 | K.VTDFGDKVEDPTFLNQLQSGVNR.W | 3 |
| \* | Astrin\_STLC\_112116\_01.14817.14817.3 | 4.558 | 0.4181 | 100.0% | 2893.0745 | 2892.3245 | 1 | 6.927 | 33.3% | 1 | K.EVQALIAEGIALVWESYKLDPYVQR.L | 3 |
| \* | Astrin\_STLC\_112116\_02.09080.09080.2 | 2.3469 | 0.2176 | 95.3% | 1655.0122 | 1654.9678 | 2 | 4.886 | 50.0% | 1 | R.QLIAQVMLYSQGFR.T | 2 |
| \* | Astrin\_STLC\_112116\_01.04630.04630.2 | 2.2643 | 0.3042 | 99.2% | 1198.2322 | 1198.2316 | 59 | 6.184 | 50.0% | 1 | R.TEYLSNADER.L | 2 |
| \* | Astrin\_STLC\_112116\_02.07363.07363.2 | 4.4464 | 0.4997 | 100.0% | 1513.3722 | 1513.7343 | 1 | 9.69 | 80.8% | 2 | R.IQGLTVEQAEAVVR.L | 2 |
| \* | Astrin\_STLC\_112116\_tube2\_01.08281.08281.2 | 3.8027 | 0.485 | 100.0% | 1405.1122 | 1405.6929 | 1 | 8.779 | 75.0% | 1 | R.VLLTTQGVDMISK.M | 2 |

---

|  |  |  |  |  |  |  |  |  |
| --- | --- | --- | --- | --- | --- | --- | --- | --- |
| U | *gi|13376798|ref|NP\_07* | 2 | 2 | 3.5% | 721 | 79136 | 6.7 | WD repeat and coiled-coil-containing protein C2orf44 isoform 1 [Homo sapiens] |

| Filename XCorr DeltCN Conf% ObsM+H+ CalcM+H+ SpR ZScore Ion% # Sequence  | | | | | | | | | | | | |
| --- | --- | --- | --- | --- | --- | --- | --- | --- | --- | --- | --- | --- |
|  | Astrin\_STLC\_112116\_01.07518.07518.2 | 2.3317 | 0.2245 | 96.6% | 1266.1921 | 1265.452 | 42 | 4.487 | 55.0% | 1 | K.SDQYAISLIVR.E | 2 |
| \* | Astrin\_STLC\_112116\_tube2\_01.10299.10299.2 | 2.6597 | 0.1701 | 95.7% | 1499.4321 | 1499.5339 | 10 | 4.586 | 46.2% | 1 | R.DSFS\*HSPGAVSSLK.V | 2 |

---

|  |  |  |  |  |  |  |  |  |
| --- | --- | --- | --- | --- | --- | --- | --- | --- |
| U | *gi|194578921|ref|NP\_0* | 3 | 3 | 3.2% | 1311 | 145135 | 5.2 | C-Jun-amino-terminal kinase-interacting protein 4 isoform 2 [Homo sapiens] |
| U | *gi|354681995|ref|NP\_0* | 3 | 3 | 3.6% | 1177 | 128607 | 5.4 | C-Jun-amino-terminal kinase-interacting protein 4 isoform 4 [Homo sapiens] |
| U | *gi|27436920|ref|NP\_00* | 3 | 3 | 3.2% | 1307 | 144681 | 5.2 | C-Jun-amino-terminal kinase-interacting protein 4 isoform 3 [Homo sapiens] |
| U | *gi|194578923|ref|NP\_0* | 3 | 3 | 3.2% | 1321 | 146205 | 5.1 | C-Jun-amino-terminal kinase-interacting protein 4 isoform 1 [Homo sapiens] |

| Filename XCorr DeltCN Conf% ObsM+H+ CalcM+H+ SpR ZScore Ion% # Sequence  | | | | | | | | | | | | |
| --- | --- | --- | --- | --- | --- | --- | --- | --- | --- | --- | --- | --- |
|  | Astrin\_STLC\_112116\_01.04553.04553.2 | 2.4651 | 0.3719 | 100.0% | 1309.6721 | 1310.4526 | 1 | 7.445 | 60.0% | 1 | K.HIEVQVAQETR.N | 2 |
|  | Astrin\_STLC\_112116\_tube2\_02.09724.09724.2 | 2.3596 | 0.2908 | 98.1% | 1887.2322 | 1887.1376 | 21 | 4.98 | 40.0% | 1 | R.EVENLILENTQLLETK.N | 2 |
|  | Astrin\_STLC\_112116\_tube2\_01.04064.04064.2 | 3.4415 | 0.3344 | 100.0% | 1467.8121 | 1468.6066 | 195 | 5.901 | 39.3% | 1 | K.AGPSAQEPGSQTPLK.S | 2 |

---

|  |  |  |  |  |  |  |  |  |
| --- | --- | --- | --- | --- | --- | --- | --- | --- |
| U | *gi|557440899|ref|NP\_0* | 4 | 4 | 2.6% | 2101 | 236513 | 5.8 | nuclear mitotic apparatus protein 1 isoform 2 [Homo sapiens] &IC NuMA |
| U | *gi|71361682|ref|NP\_00* | 4 | 4 | 2.6% | 2115 | 238257 | 5.8 | nuclear mitotic apparatus protein 1 isoform 1 [Homo sapiens] &IC NuMA |

| Filename XCorr DeltCN Conf% ObsM+H+ CalcM+H+ SpR ZScore Ion% # Sequence  | | | | | | | | | | | | |
| --- | --- | --- | --- | --- | --- | --- | --- | --- | --- | --- | --- | --- |
|  | Astrin\_STLC\_112116\_01.04330.04330.2 | 3.6971 | 0.2577 | 100.0% | 1604.9722 | 1605.7197 | 1 | 6.079 | 69.2% | 1 | R.AALMESQGQQQEER.G | 2 |
|  | Astrin\_STLC\_112116\_01.04178.04178.2 | 3.9371 | 0.3999 | 100.0% | 1260.9922 | 1261.3774 | 1 | 7.938 | 72.7% | 1 | R.LLQAETASNSAR.A | 2 |
|  | Astrin\_STLC\_112116\_01.03692.03692.3 | 2.7008 | 0.2411 | 95.8% | 1829.0044 | 1828.9797 | 3 | 4.503 | 35.9% | 1 | K.LKAVQAQGGESQQEAQR.L | 3 |
|  | Astrin\_STLC\_112116\_01.05022.05022.2 | 1.8778 | 0.311 | 95.7% | 1193.0521 | 1194.3762 | 42 | 4.565 | 55.0% | 1 | R.LGHELQQAGLK.T | 2 |

---

|  |  |  |  |  |  |  |  |  |
| --- | --- | --- | --- | --- | --- | --- | --- | --- |
| U | *gi|54607053|ref|NP\_00* | 4 | 4 | 2.5% | 2671 | 292708 | 7.4 | eIF-2-alpha kinase activator GCN1 [Homo sapiens] |

| Filename XCorr DeltCN Conf% ObsM+H+ CalcM+H+ SpR ZScore Ion% # Sequence  | | | | | | | | | | | | |
| --- | --- | --- | --- | --- | --- | --- | --- | --- | --- | --- | --- | --- |
| \* | Astrin\_STLC\_112116\_tube2\_01.04934.04934.2 | 2.0734 | 0.2787 | 98.2% | 992.2522 | 992.1661 | 18 | 5.415 | 64.3% | 1 | R.HLDQIIPR.M | 2 |
| \* | Astrin\_STLC\_112116\_01.15404.15404.2 | 2.7719 | 0.2847 | 99.4% | 2237.1921 | 2238.627 | 1 | 6.426 | 40.0% | 1 | R.LQELDGELEAALGLLDIILAK.N | 2 |
| \* | Astrin\_STLC\_112116\_tube2\_01.15782.15782.2 | 3.7276 | 0.3765 | 100.0% | 2314.9922 | 2315.7583 | 1 | 7.24 | 40.0% | 1 | K.NPSGLTQYIPVLVDSFLPLLK.S | 2 |
| \* | Astrin\_STLC\_112116\_01.12922.12922.2 | 3.4635 | 0.4457 | 100.0% | 1826.3922 | 1827.1307 | 1 | 7.119 | 63.3% | 1 | K.LVLPSLLAALEEESWR.T | 2 |

---

|  |  |  |  |  |  |  |  |  |
| --- | --- | --- | --- | --- | --- | --- | --- | --- |
| U | *gi|62241042|ref|NP\_00* | 3 | 3 | 2.5% | 1512 | 170590 | 7.3 | bifunctional glutamate/proline--tRNA ligase [Homo sapiens] |

| Filename XCorr DeltCN Conf% ObsM+H+ CalcM+H+ SpR ZScore Ion% # Sequence  | | | | | | | | | | | | |
| --- | --- | --- | --- | --- | --- | --- | --- | --- | --- | --- | --- | --- |
| \* | Astrin\_STLC\_112116\_02.10184.10184.2 | 4.6942 | 0.3298 | 100.0% | 1945.5322 | 1945.1846 | 1 | 7.235 | 56.2% | 1 | R.WFGFLEAQQAFQSVGTK.W | 2 |
| \* | Astrin\_STLC\_112116\_01.06096.06096.2 | 2.1915 | 0.2188 | 95.7% | 1115.9321 | 1116.303 | 151 | 4.374 | 50.0% | 1 | R.LNLNNTVLSK.R | 2 |
| \* | Astrin\_STLC\_112116\_01.04228.04228.2 | 2.8086 | 0.3734 | 100.0% | 1204.6921 | 1205.3074 | 1 | 6.89 | 75.0% | 1 | K.LTVAENEAETK.L | 2 |

---

|  |  |  |  |  |  |  |  |  |
| --- | --- | --- | --- | --- | --- | --- | --- | --- |
| U | *gi|31621305|ref|NP\_57* | 2 | 2 | 2.3% | 1394 | 157904 | 6.1 | leucine-rich PPR motif-containing protein, mitochondrial precursor [Homo sapiens] |

| Filename XCorr DeltCN Conf% ObsM+H+ CalcM+H+ SpR ZScore Ion% # Sequence  | | | | | | | | | | | | |
| --- | --- | --- | --- | --- | --- | --- | --- | --- | --- | --- | --- | --- |
| \* | Astrin\_STLC\_112116\_02.11149.11149.2 | 2.1354 | 0.3035 | 96.6% | 1725.5322 | 1725.939 | 104 | 5.633 | 33.3% | 1 | R.SEAANGNLDFVLSFLK.S | 2 |
| \* | Astrin\_STLC\_112116\_tube2\_01.15008.15008.2 | 4.3934 | 0.5149 | 100.0% | 1954.5521 | 1955.2764 | 1 | 8.848 | 50.0% | 1 | R.SMNINLWSEITELLYK.D | 2 |

---

|  |  |  |  |  |  |  |  |  |
| --- | --- | --- | --- | --- | --- | --- | --- | --- |
| U | *gi|153792294|ref|NP\_1* | 2 | 5 | 2.3% | 1320 | 145257 | 6.8 | myopalladin isoform a [Homo sapiens] |

| Filename XCorr DeltCN Conf% ObsM+H+ CalcM+H+ SpR ZScore Ion% # Sequence  | | | | | | | | | | | | |
| --- | --- | --- | --- | --- | --- | --- | --- | --- | --- | --- | --- | --- |
| \* | Astrin\_STLC\_112116\_tube2\_01.07829.07829.3 | 3.432 | 0.3818 | 99.8% | 1947.9243 | 1948.1399 | 1 | 5.902 | 35.9% | 1 | R.LAINYDPLEKADETQAR.K | 3 |
| \* | Astrin\_STLC\_112116\_01.13574.13574.2 | 4.2978 | 0.4976 | 100.0% | 1470.2122 | 1470.6624 | 1 | 8.279 | 79.2% | 4 | K.AADFIEELSSLFK.S | 2 |

---

|  |  |  |  |  |  |  |  |  |
| --- | --- | --- | --- | --- | --- | --- | --- | --- |
| U | *gi|54859722|ref|NP\_05* | 2 | 2 | 2.0% | 1436 | 162121 | 5.5 | nuclear pore complex protein Nup160 isoform 1 [Homo sapiens] &IC Nup160 |

| Filename XCorr DeltCN Conf% ObsM+H+ CalcM+H+ SpR ZScore Ion% # Sequence  | | | | | | | | | | | | |
| --- | --- | --- | --- | --- | --- | --- | --- | --- | --- | --- | --- | --- |
| \* | Astrin\_STLC\_112116\_01.14832.14832.2 | 3.9425 | 0.4802 | 100.0% | 2097.7122 | 2098.4062 | 1 | 9.172 | 44.1% | 1 | R.FVSSPQTIVELFFQEVAR.K | 2 |
| \* | Astrin\_STLC\_112116\_01.04732.04732.2 | 2.5214 | 0.3074 | 99.7% | 1191.1522 | 1190.2523 | 12 | 5.342 | 60.0% | 1 | R.SEDGEIVSTPR.L | 2 |

---

|  |  |  |  |  |  |  |  |  |
| --- | --- | --- | --- | --- | --- | --- | --- | --- |
| U | *gi|365192532|ref|NP\_0* | 4 | 4 | 1.9% | 2007 | 232526 | 5.6 | myosin-10 isoform 1 [Homo sapiens] |
| U | *gi|367460090|ref|NP\_0* | 4 | 4 | 1.9% | 1985 | 230028 | 5.6 | myosin-10 isoform 3 [Homo sapiens] |
| U | *gi|367460087|ref|NP\_0* | 4 | 4 | 1.9% | 1976 | 228997 | 5.5 | myosin-10 isoform 2 [Homo sapiens] |

| Filename XCorr DeltCN Conf% ObsM+H+ CalcM+H+ SpR ZScore Ion% # Sequence  | | | | | | | | | | | | |
| --- | --- | --- | --- | --- | --- | --- | --- | --- | --- | --- | --- | --- |
|  | Astrin\_STLC\_112116\_01.06986.06986.2 | 2.3058 | 0.2699 | 96.7% | 1649.6921 | 1649.8436 | 77 | 4.566 | 46.4% | 1 | R.AVIYNPATQADWTAK.K | 2 |
|  | Astrin\_STLC\_112116\_tube2\_01.06060.06060.2 | 2.9889 | 0.1978 | 99.6% | 1222.1122 | 1221.3959 | 13 | 4.489 | 66.7% | 1 | K.KFDQLLAEEK.S | 22 |
|  | Astrin\_STLC\_112116\_tube2\_01.06341.06341.2 | 2.6047 | 0.1138 | 95.7% | 1093.1322 | 1093.2218 | 1 | 5.265 | 87.5% | 1 | K.FDQLLAEEK.S | 22 |
|  | Astrin\_STLC\_112116\_tube2\_01.07603.07603.2 | 2.9021 | 0.2302 | 99.1% | 1516.3922 | 1515.6604 | 1 | 4.474 | 66.7% | 1 | K.IGQLEEQLEQEAK.E | 2 |

Similarities:
gi|12667788|ref|NP\_00(2:2)  

---

|  |  |  |  |  |  |  |  |  |
| --- | --- | --- | --- | --- | --- | --- | --- | --- |
| U | *gi|62243696|ref|NP\_00* | 2 | 2 | 1.8% | 1258 | 144427 | 5.6 | cohesin subunit SA-1 [Homo sapiens] &IC SCC3 |
| U | *gi|767925496|ref|XP\_0* | 2 | 2 | 2.2% | 1032 | 119002 | 5.5 | PREDICTED: cohesin subunit SA-1 isoform X2 [Homo sapiens] &IC SCC3 |
| U | *gi|767925492|ref|XP\_0* | 2 | 2 | 2.1% | 1121 | 129413 | 5.4 | PREDICTED: cohesin subunit SA-1 isoform X1 [Homo sapiens] &IC SCC3 |

| Filename XCorr DeltCN Conf% ObsM+H+ CalcM+H+ SpR ZScore Ion% # Sequence  | | | | | | | | | | | | |
| --- | --- | --- | --- | --- | --- | --- | --- | --- | --- | --- | --- | --- |
|  | Astrin\_STLC\_112116\_01.14646.14646.2 | 3.696 | 0.2383 | 100.0% | 2670.3523 | 2670.0378 | 1 | 5.014 | 40.9% | 1 | K.TLILSLQQLFNELVQEQGPNLDR.T | 2 |
|  | Astrin\_STLC\_112116\_01.14634.14634.3 | 4.5808 | 0.3311 | 99.8% | 2671.1343 | 2670.0378 | 7 | 6.287 | 29.5% | 1 | K.TLILSLQQLFNELVQEQGPNLDR.T | 3 |

---

|  |  |  |  |  |  |  |  |  |
| --- | --- | --- | --- | --- | --- | --- | --- | --- |
| U | *gi|58530840|ref|NP\_00* | 4 | 4 | 1.6% | 2871 | 331774 | 6.8 | desmoplakin isoform I [Homo sapiens] |
| U | *gi|975830145|ref|NP\_0* | 4 | 4 | 1.9% | 2428 | 278914 | 7.0 | desmoplakin isoform Ia [Homo sapiens] |

| Filename XCorr DeltCN Conf% ObsM+H+ CalcM+H+ SpR ZScore Ion% # Sequence  | | | | | | | | | | | | |
| --- | --- | --- | --- | --- | --- | --- | --- | --- | --- | --- | --- | --- |
|  | Astrin\_STLC\_112116\_tube2\_01.07000.07000.2 | 2.6896 | 0.3081 | 100.0% | 1158.3522 | 1159.3892 | 1 | 5.655 | 87.5% | 1 | R.LLQLQEQMR.A | 2 |
|  | Astrin\_STLC\_112116\_tube2\_01.06787.06787.2 | 2.7651 | 0.232 | 99.3% | 1271.7922 | 1272.4471 | 10 | 4.98 | 60.0% | 1 | R.QLQNIIQATSR.E | 2 |
|  | Astrin\_STLC\_112116\_tube2\_01.07163.07163.2 | 3.0494 | 0.4288 | 100.0% | 1388.0521 | 1388.5205 | 1 | 6.571 | 77.3% | 1 | R.LNDSILQATEQR.R | 2 |
|  | Astrin\_STLC\_112116\_tube2\_02.08794.08794.2 | 3.0284 | 0.3827 | 100.0% | 1538.8121 | 1539.8125 | 1 | 6.598 | 60.7% | 1 | R.LLEAQIATGGIIDPK.E | 2 |

---

|  |  |  |  |  |  |  |  |  |
| --- | --- | --- | --- | --- | --- | --- | --- | --- |
| U | *gi|40217847|ref|NP\_05* | 2 | 2 | 1.6% | 2136 | 244505 | 6.1 | U5 small nuclear ribonucleoprotein 200 kDa helicase [Homo sapiens] |

| Filename XCorr DeltCN Conf% ObsM+H+ CalcM+H+ SpR ZScore Ion% # Sequence  | | | | | | | | | | | | |
| --- | --- | --- | --- | --- | --- | --- | --- | --- | --- | --- | --- | --- |
| \* | Astrin\_STLC\_112116\_01.14600.14600.3 | 3.7239 | 0.4431 | 100.0% | 2151.6843 | 2151.424 | 1 | 7.019 | 36.1% | 1 | R.ETYEVLLSFIQAALGDQPR.D | 3 |
| \* | Astrin\_STLC\_112116\_01.12915.12915.2 | 2.6676 | 0.2509 | 98.7% | 1715.4922 | 1717.0361 | 2 | 5.304 | 53.6% | 1 | R.WTELGALDILQMLGR.A | 2 |

---

|  |  |  |  |  |  |  |  |  |
| --- | --- | --- | --- | --- | --- | --- | --- | --- |
| U | *gi|525507390|ref|NP\_1* | 2 | 3 | 0.7% | 5088 | 555666 | 5.6 | epiplakin [Homo sapiens] |

| Filename XCorr DeltCN Conf% ObsM+H+ CalcM+H+ SpR ZScore Ion% # Sequence  | | | | | | | | | | | | |
| --- | --- | --- | --- | --- | --- | --- | --- | --- | --- | --- | --- | --- |
|  | Astrin\_STLC\_112116\_tube2\_01.06648.06648.2 | 1.9692 | 0.3195 | 98.2% | 1162.2122 | 1161.2311 | 3 | 5.458 | 68.8% | 1 | C.GYFDEEMNR.I | 22 |
| \* | Astrin\_STLC\_112116\_01.14277.14277.3 | 4.8782 | 0.4053 | 100.0% | 2515.4043 | 2514.8386 | 1 | 7.229 | 36.5% | 2 | R.AGTLTVEELGATLTSLLAQAQAQAR.A | 3 |

Similarities:
gi|41322908|ref|NP\_95(1:1)  


|  |  |  |  |
| --- | --- | --- | --- |
|  | Proteins | Peptide IDs | Spectra |
| Unfiltered | 44638 | 75168 | 154835 |
| Filtered | 160 | 1050 | 2627 |
| Forward matches | 159 | 1048 | 2625 |
| Decoy matches | 1 | 2 | 2 |
| Forward FP rate | 0.63% | 0.19% | 0.08% |

  
/nfs/cheeseman\_massspec/David/Astrin\_STLC\_IP
