# Supplementary material for: Astrin-SKAP complex reconstitution reveals its kinetochore interaction with microtubule-bound Ndc80
Source: eLife. 2017 Aug 25;6:e26866. doi: 10.7554/eLife.26866 (PMC5602300; doi:10.7554/eLife.26866)
Supplement: Source data 1. — Complete mass spectrometry searches using methods described in (Washburn et al., 2001) for affinity purification/mass spectrometry data sets described in this paper (data from this study; [Kern et al., 2016] [Gascoigne et al., 2011]). Individual Astrin cross-linking immunoprecipitations are listed based on the order in Figure 4—figure supplement 1. These samples have not been pruned for common or antibody-specific contaminants. [file elife-26866-data1.zip › CENPL_CrosslinkingIP.html]

D CENPL\_STLCLD
DTASelect v2.0.21  
/nfs/cheeseman\_massspec/David/CENPL\_STLCLD  
/nfs/cheeseman\_massspec/Databases/NCBI-RefSeq\_human\_na\_04-13-2009\_con\_reversed.fasta  
SEQUEST 3.0 in SQT format.  
  
 Jump  to the summary table.  
  
sequest.params modifications:

|  |  |  |
| --- | --- | --- |
| \* | S | 80.0 |
| # | T | 80.0 |
| @ | K | 12.0 |
| Static | C | 57.0 |

|  |  |
| --- | --- |
| true | Use criteria |
| 0.0 | Minimum peptide confidence |
| 0.05 | Peptide false positive rate |
| 0.0 | Minimum protein confidence |
| 1.0 | Protein false positive rate |
| 1 | Minimum charge state |
| 16 | Maximum charge state |
| 0.0 | Minimum ion proportion |
| 1000 | Maximum Sp rank |
| -1.0 | Minimum Sp score |
| Include | Modified peptide inclusion |
| Any | Tryptic status requirement |
| false | Multiple, ambiguous IDs allowed |
| Ignore | Peptide validation handling |
| XCorr | Purge duplicate peptides by protein |
| false | Include only loci with unique peptide |
| true | Remove subset proteins |
| Ignore | Locus validation handling |
| 0 | Minimum modified peptides per locus |
| 1000 | Minimum redundancy for low coverage loci |
| 2 | Minimum peptides per locus |

#### Locus Key:

|  |  |  |  |  |  |  |  |  |
| --- | --- | --- | --- | --- | --- | --- | --- | --- |
| Validation Status | Locus | Sequence Count | Spectrum Count | Sequence Coverage | Length | MolWt | pI | Descriptive Name |

#### Similarity Key:

|  |  |  |
| --- | --- | --- |
| Locus | # of identical peptides | # of differing peptides |

---

|  |  |  |  |  |  |  |  |  |
| --- | --- | --- | --- | --- | --- | --- | --- | --- |
| U | *gi|209862831|ref|NP\_0* | 27 | 96 | 66.1% | 339 | 38604 | 7.8 | annexin A2 isoform 2 [Homo sapiens] |
| U | *gi|50845388|ref|NP\_00* | 27 | 96 | 62.7% | 357 | 40411 | 8.4 | annexin A2 isoform 1 [Homo sapiens] |
| U | *gi|50845386|ref|NP\_00* | 27 | 96 | 66.1% | 339 | 38604 | 7.8 | annexin A2 isoform 2 [Homo sapiens] |
| U | *gi|4757756|ref|NP\_004* | 27 | 96 | 66.1% | 339 | 38604 | 7.8 | annexin A2 isoform 2 [Homo sapiens] |

| Filename XCorr DeltCN Conf% ObsM+H+ CalcM+H+ SpR ZScore Ion% # Sequence  | | | | | | | | | | | | |
| --- | --- | --- | --- | --- | --- | --- | --- | --- | --- | --- | --- | --- |
|  | CENPL\_stlcld\_122314\_01.11070.11070.2 | 4.9426 | 0.4202 | 100.0% | 1845.9521 | 1846.0038 | 1 | 9.231 | 52.9% | 4 | K.LSLEGDHSTPPSAYGSVK.A | 2 |
|  | CENPL\_stlcld\_tube2\_122314\_01.09339.09339.2 | 2.5776 | 0.4068 | 99.9% | 1087.2322 | 1087.1338 | 12 | 6.528 | 68.8% | 4 | K.AYTNFDAER.D | 2 |
|  | CENPL\_stlcld\_122314\_01.16877.16877.2 | 4.9197 | 0.5026 | 100.0% | 2155.5122 | 2156.357 | 1 | 9.042 | 52.8% | 3 | K.AYTNFDAERDALNIETAIK.T | 2 |
|  | CENPL\_stlcld\_tube2\_122314\_02.15260.15260.3 | 4.0062 | 0.2334 | 99.9% | 2157.5344 | 2156.357 | 1 | 5.205 | 44.4% | 6 | K.AYTNFDAERDALNIETAIK.T | 3 |
|  | CENPL\_stlcld\_122314\_01.14180.14180.2 | 2.6536 | 0.2657 | 99.4% | 1088.3522 | 1088.2462 | 1 | 6.282 | 77.8% | 1 | R.DALNIETAIK.T | 2 |
|  | CENPL\_stlcld\_122314\_01.18851.18851.3 | 3.8519 | 0.3232 | 100.0% | 1773.2344 | 1773.0397 | 1 | 5.759 | 48.3% | 1 | K.TKGVDEVTIVNILTNR.S | 3 |
|  | CENPL\_stlcld\_tube2\_122314\_02.16725.16725.2 | 3.9307 | 0.464 | 100.0% | 1543.4722 | 1543.7605 | 1 | 7.987 | 65.4% | 5 | K.GVDEVTIVNILTNR.S | 2 |
|  | CENPL\_stlcld\_122314\_01.12131.12131.2 | 2.4976 | 0.2605 | 99.1% | 1111.5922 | 1112.2303 | 1 | 6.368 | 87.5% | 2 | R.QDIAFAYQR.R | 2 |
|  | CENPL\_stlcld\_tube2\_122314\_01.21018.21018.3 | 4.7532 | 0.3102 | 100.0% | 1652.2743 | 1651.9872 | 14 | 5.87 | 41.7% | 4 | K.SALSGHLETVILGLLK.T | 3 |
|  | CENPL\_stlcld\_122314\_01.21815.21815.2 | 5.2862 | 0.4294 | 100.0% | 1652.7522 | 1651.9872 | 1 | 8.721 | 63.3% | 4 | K.SALSGHLETVILGLLK.T | 2 |
|  | CENPL\_stlcld\_tube2\_122314\_01.09134.09134.2 | 3.9521 | 0.3899 | 100.0% | 1223.2722 | 1223.3251 | 1 | 7.846 | 80.0% | 9 | K.TPAQYDASELK.A | 2 |
|  | CENPL\_stlcld\_tube2\_122314\_01.10323.10323.3 | 3.4976 | 0.3096 | 99.9% | 1641.6843 | 1640.8488 | 1 | 5.104 | 48.2% | 1 | K.TPAQYDASELKASMK.G | 3 |
|  | CENPL\_stlcld\_tube2\_122314\_01.19925.19925.2 | 2.506 | 0.1765 | 95.3% | 1779.6122 | 1778.9202 | 162 | 5.251 | 36.7% | 1 | K.GLGTDEDSLIEIICSR.T | 2 |
|  | CENPL\_stlcld\_tube2\_122314\_01.08811.08811.2 | 3.508 | 0.3328 | 100.0% | 1245.2322 | 1245.3347 | 1 | 6.628 | 88.9% | 7 | R.TNQELQEINR.V | 2 |
|  | CENPL\_stlcld\_122314\_01.15431.15431.2 | 3.9629 | 0.4561 | 100.0% | 1812.4722 | 1812.928 | 1 | 8.372 | 63.3% | 3 | K.TDLEKDIISDTSGDFR.K | 2 |
|  | CENPL\_stlcld\_tube2\_122314\_01.13646.13646.3 | 5.0532 | 0.5143 | 100.0% | 1940.6943 | 1941.102 | 1 | 8.281 | 51.6% | 6 | K.TDLEKDIISDTSGDFRK.L | 3 |
|  | CENPL\_stlcld\_tube2\_122314\_01.14249.14249.2 | 5.2083 | 0.4074 | 100.0% | 2065.4722 | 2066.1887 | 1 | 7.045 | 70.6% | 2 | R.RAEDGSVIDYELIDQDAR.D | 2 |
|  | CENPL\_stlcld\_tube2\_122314\_02.13682.13682.3 | 4.2136 | 0.2706 | 99.9% | 2067.3542 | 2066.1887 | 1 | 5.366 | 48.5% | 6 | R.RAEDGSVIDYELIDQDAR.D | 3 |
|  | CENPL\_stlcld\_122314\_01.16050.16050.2 | 4.1314 | 0.5311 | 100.0% | 1909.0922 | 1910.0013 | 4 | 8.992 | 46.9% | 5 | R.AEDGSVIDYELIDQDAR.D | 2 |
|  | CENPL\_stlcld\_122314\_01.08751.08751.2 | 2.2058 | 0.2418 | 97.9% | 1038.5322 | 1037.1606 | 118 | 4.75 | 62.5% | 2 | R.DLYDAGVKR.K | 2 |
|  | CENPL\_stlcld\_122314\_01.15616.15616.1 | 1.8927 | 0.3032 | 95.7% | 1035.61 | 1036.2341 | 12 | 4.6 | 64.3% | 1 | K.WISIMTER.S | 1 |
|  | CENPL\_stlcld\_tube2\_122314\_01.15692.15692.2 | 2.5797 | 0.3241 | 99.9% | 1036.4122 | 1036.2341 | 467 | 6.197 | 64.3% | 7 | K.WISIMTER.S | 2 |
|  | CENPL\_stlcld\_122314\_01.19725.19725.2 | 2.1973 | 0.2898 | 98.1% | 1461.2722 | 1461.6294 | 3 | 5.391 | 50.0% | 1 | K.SYSPYDMLESIR.K | 2 |
|  | CENPL\_stlcld\_122314\_01.16899.16899.2 | 3.5066 | 0.436 | 100.0% | 1589.8522 | 1589.8035 | 1 | 6.725 | 62.5% | 5 | K.SYSPYDMLESIRK.E | 2 |
|  | CENPL\_stlcld\_tube2\_122314\_02.00524.00524.3 | 4.8514 | 0.3147 | 100.0% | 2840.3643 | 2840.1743 | 1 | 6.683 | 30.4% | 1 | K.GDLENAFLNLVQCIQNKPLYFADR.L | 3 |
|  | CENPL\_stlcld\_tube2\_122314\_01.14404.14404.2 | 3.1848 | 0.2855 | 99.9% | 1422.8322 | 1422.5774 | 1 | 5.885 | 75.0% | 3 | K.SLYYYIQQDTK.G | 2 |
|  | CENPL\_stlcld\_tube2\_122314\_02.13293.13293.3 | 2.6482 | 0.2567 | 97.8% | 2013.8944 | 2014.1986 | 12 | 4.275 | 28.3% | 2 | K.SLYYYIQQDTKGDYQK.A | 3 |

---

|  |  |  |  |  |  |  |  |  |
| --- | --- | --- | --- | --- | --- | --- | --- | --- |
| U | *gi|40354195|ref|NP\_95* | 30 | 133 | 64.4% | 430 | 48058 | 5.5 | keratin 18 [Homo sapiens] |
| U | *gi|4557888|ref|NP\_000* | 30 | 133 | 64.4% | 430 | 48058 | 5.5 | keratin 18 [Homo sapiens] |

| Filename XCorr DeltCN Conf% ObsM+H+ CalcM+H+ SpR ZScore Ion% # Sequence  | | | | | | | | | | | | |
| --- | --- | --- | --- | --- | --- | --- | --- | --- | --- | --- | --- | --- |
|  | CENPL\_stlcld\_122314\_01.12092.12092.2 | 4.7052 | 0.528 | 100.0% | 2854.912 | 2856.0813 | 1 | 8.213 | 31.7% | 1 | R.SLGSVQAPSYGARPVSSAASVYAGAGGSGSR.I | 2 |
|  | CENPL\_stlcld\_tube2\_122314\_02.12044.12044.3 | 5.5704 | 0.5948 | 100.0% | 2856.3245 | 2856.0813 | 1 | 9.508 | 32.5% | 11 | R.SLGSVQAPSYGARPVSSAASVYAGAGGSGSR.I | 3 |
|  | CENPL\_stlcld\_tube2\_122314\_01.17652.17652.2 | 5.4891 | 0.5076 | 100.0% | 2262.7122 | 2262.561 | 1 | 9.381 | 52.0% | 5 | R.GGMGSGGLATGIAGGLAGMGGIQNEK.E | 2 |
|  | CENPL\_stlcld\_tube2\_122314\_02.15899.15899.3 | 4.3097 | 0.3906 | 100.0% | 2263.0745 | 2262.561 | 1 | 6.871 | 30.0% | 2 | R.GGMGSGGLATGIAGGLAGMGGIQNEK.E | 3 |
|  | CENPL\_stlcld\_122314\_02.14570.14570.3 | 5.9591 | 0.5083 | 100.0% | 3336.9543 | 3337.7224 | 1 | 8.437 | 28.7% | 11 | R.GGMGSGGLATGIAGGLAGMGGIQNEKETMQSLNDR.L | 3 |
|  | CENPL\_stlcld\_122314\_01.09825.09825.2 | 2.1269 | 0.2993 | 99.0% | 839.15216 | 837.9511 | 105 | 5.554 | 66.7% | 2 | R.LASYLDR.V | 2 |
|  | CENPL\_stlcld\_tube2\_122314\_02.15120.15120.3 | 4.0429 | 0.4379 | 100.0% | 2060.9944 | 2060.3176 | 1 | 6.664 | 38.2% | 1 | K.IIEDLRAQIFANTVDNAR.I | 3 |
|  | CENPL\_stlcld\_tube2\_122314\_01.11222.11222.2 | 3.9503 | 0.4826 | 100.0% | 1320.3522 | 1320.4478 | 1 | 8.732 | 72.7% | 13 | R.AQIFANTVDNAR.I | 2 |
|  | CENPL\_stlcld\_tube2\_122314\_01.11966.11966.2 | 3.0615 | 0.1615 | 99.4% | 1042.3722 | 1042.2235 | 3 | 6.157 | 87.5% | 6 | R.IVLQIDNAR.L | 22 |
|  | CENPL\_stlcld\_122314\_01.09788.09788.2 | 2.5662 | 0.2183 | 99.5% | 808.39215 | 807.8815 | 68 | 6.201 | 66.7% | 8 | R.LAADDFR.V | 22222 |
|  | CENPL\_stlcld\_122314\_02.09080.09080.3 | 2.8588 | 0.198 | 98.2% | 1240.8544 | 1240.4601 | 19 | 4.209 | 50.0% | 1 | R.VKYETELAMR.Q | 3 |
|  | CENPL\_stlcld\_tube2\_122314\_01.09735.09735.2 | 2.7647 | 0.3984 | 99.9% | 1241.3722 | 1240.4601 | 29 | 6.73 | 66.7% | 6 | R.VKYETELAMR.Q | 2 |
|  | CENPL\_stlcld\_122314\_01.08792.08792.2 | 2.3214 | 0.2539 | 98.5% | 1046.5922 | 1047.1533 | 21 | 5.956 | 62.5% | 1 | K.VIDDTNITR.L | 2 |
|  | CENPL\_stlcld\_tube2\_122314\_01.21706.21706.2 | 5.8878 | 0.5311 | 100.0% | 2177.5923 | 2178.589 | 1 | 10.29 | 64.7% | 4 | R.LQLETEIEALKEELLFMK.K | 2 |
|  | CENPL\_stlcld\_122314\_01.22586.22586.3 | 2.8797 | 0.314 | 99.9% | 2178.5044 | 2178.589 | 9 | 5.204 | 41.2% | 4 | R.LQLETEIEALKEELLFMK.K | 3 |
|  | CENPL\_stlcld\_122314\_01.14351.14351.3 | 5.3608 | 0.4507 | 100.0% | 2750.7244 | 2751.0227 | 1 | 8.144 | 38.0% | 3 | K.NHEEEVKGLQAQIASSGLTVEVDAPK.S | 3 |
|  | CENPL\_stlcld\_122314\_02.13044.13044.2 | 3.7959 | 0.4517 | 100.0% | 1884.4521 | 1885.1246 | 1 | 8.399 | 50.0% | 1 | K.GLQAQIASSGLTVEVDAPK.S | 2 |
|  | CENPL\_stlcld\_122314\_01.09090.09090.1 | 1.7846 | 0.2719 | 95.8% | 965.62 | 966.0385 | 7 | 5.278 | 57.1% | 1 | R.AQYDELAR.K | 1 |
|  | CENPL\_stlcld\_122314\_01.14919.14919.2 | 3.5895 | 0.4232 | 100.0% | 1664.5322 | 1663.8865 | 1 | 6.774 | 80.8% | 1 | R.RTVQSLEIDLDSMR.N | 2 |
|  | CENPL\_stlcld\_tube2\_122314\_01.17525.17525.1 | 2.181 | 0.3102 | 95.2% | 1506.66 | 1507.699 | 1 | 7.103 | 62.5% | 1 | R.TVQSLEIDLDSMR.N | 1 |
|  | CENPL\_stlcld\_122314\_01.17870.17870.2 | 4.2355 | 0.4915 | 100.0% | 1508.6122 | 1507.699 | 1 | 9.45 | 75.0% | 16 | R.TVQSLEIDLDSMR.N | 2 |
|  | CENPL\_stlcld\_tube2\_122314\_01.08674.08674.2 | 2.5468 | 0.2456 | 99.4% | 890.1922 | 889.9841 | 78 | 5.056 | 71.4% | 2 | K.ASLENSLR.E | 2 |
|  | CENPL\_stlcld\_tube2\_122314\_01.12178.12178.3 | 2.9071 | 0.3049 | 99.9% | 1475.6643 | 1474.6139 | 3 | 5.554 | 41.7% | 1 | K.ASLENSLREVEAR.Y | 3 |
|  | CENPL\_stlcld\_122314\_02.00249.00249.2 | 5.664 | 0.4547 | 100.0% | 2671.9321 | 2672.0715 | 1 | 10.041 | 52.3% | 4 | R.YALQMEQLNGILLHLESELAQTR.A | 2 |
|  | CENPL\_stlcld\_tube2\_122314\_02.00596.00596.3 | 6.3537 | 0.4071 | 100.0% | 2672.7844 | 2672.0715 | 1 | 7.911 | 44.3% | 8 | R.YALQMEQLNGILLHLESELAQTR.A | 3 |
|  | CENPL\_stlcld\_tube2\_122314\_01.16130.16130.2 | 3.1869 | 0.4214 | 100.0% | 1420.2922 | 1420.6055 | 58 | 6.318 | 59.1% | 6 | R.QAQEYEALLNIK.V | 2 |
|  | CENPL\_stlcld\_122314\_02.11114.11114.2 | 3.5905 | 0.3397 | 100.0% | 1293.5521 | 1293.5059 | 2 | 7.144 | 75.0% | 5 | K.VKLEAEIATYR.R | 2 |
|  | CENPL\_stlcld\_tube2\_122314\_01.10935.10935.2 | 2.8851 | 0.3641 | 99.9% | 1065.7722 | 1066.1992 | 1 | 5.595 | 87.5% | 4 | K.LEAEIATYR.R | 2 |
|  | CENPL\_stlcld\_122314\_02.14177.14177.3 | 4.7654 | 0.4486 | 100.0% | 2897.3643 | 2898.128 | 1 | 8.235 | 29.0% | 1 | R.RLLEDGEDFNLGDALDSSNSMQTIQK.T | 3 |
|  | CENPL\_stlcld\_122314\_01.19064.19064.2 | 6.3667 | 0.5766 | 100.0% | 2742.0723 | 2741.9404 | 1 | 10.81 | 52.1% | 3 | R.LLEDGEDFNLGDALDSSNSMQTIQK.T | 2 |

Similarities:
gi|4557701|ref|NP\_000(1:29)  
contaminant\_KERATIN03(1:29)  
contaminant\_KERATIN05(1:29)  
gi|24234699|ref|NP\_00(2:28)  

---

|  |  |  |  |  |  |  |  |  |
| --- | --- | --- | --- | --- | --- | --- | --- | --- |
| U | *gi|4501885|ref|NP\_001* | 30 | 140 | 63.7% | 375 | 41737 | 5.5 | beta actin [Homo sapiens] |
| U | *gi|4501887|ref|NP\_001* | 30 | 138 | 63.7% | 375 | 41793 | 5.5 | actin, gamma 1 propeptide [Homo sapiens] |

| Filename XCorr DeltCN Conf% ObsM+H+ CalcM+H+ SpR ZScore Ion% # Sequence  | | | | | | | | | | | | |
| --- | --- | --- | --- | --- | --- | --- | --- | --- | --- | --- | --- | --- |
|  | CENPL\_stlcld\_122314\_01.14021.14021.3 | 4.3293 | 0.48 | 100.0% | 2157.5645 | 2157.4397 | 2 | 8.358 | 33.8% | 2 | K.AGFAGDDAPRAVFPSIVGRPR.H | 33 |
|  | CENPL\_stlcld\_122314\_01.12266.12266.2 | 2.7778 | 0.3415 | 99.9% | 1199.4321 | 1199.4415 | 52 | 5.739 | 55.0% | 6 | R.AVFPSIVGRPR.H | 22 |
|  | CENPL\_stlcld\_122314\_01.11708.11708.2 | 3.3771 | 0.4633 | 100.0% | 1516.3322 | 1516.7019 | 1 | 6.87 | 80.0% | 6 | K.IWHHTFYNELR.V | 22 |
|  | CENPL\_stlcld\_122314\_01.11684.11684.3 | 3.1925 | 0.1607 | 97.9% | 1517.3644 | 1516.7019 | 1 | 4.968 | 55.0% | 6 | K.IWHHTFYNELR.V | 33 |
|  | CENPL\_stlcld\_tube2\_122314\_01.13322.13322.2 | 4.6571 | 0.4017 | 100.0% | 1954.6122 | 1955.2615 | 1 | 8.11 | 61.8% | 6 | R.VAPEEHPVLLTEAPLNPK.A | 2 |
|  | CENPL\_stlcld\_122314\_01.12105.12105.3 | 3.456 | 0.2392 | 99.1% | 2298.2344 | 2296.6316 | 125 | 4.787 | 25.0% | 1 | R.VAPEEHPVLLTEAPLNPKANR.E | 3 |
|  | CENPL\_stlcld\_tube2\_122314\_02.02096.02096.2 | 4.4413 | 0.1721 | 99.9% | 3255.392 | 3255.8325 | 1 | 10.154 | 39.3% | 2 | K.MTQIMFETFNTPAMYVAIQAVLSLYASGR.T | 2 |
|  | CENPL\_stlcld\_tube2\_122314\_01.17448.17448.2 | 4.7278 | 0.4606 | 100.0% | 3186.2522 | 3185.622 | 1 | 7.912 | 37.9% | 1 | R.TTGIVMDSGDGVTHTVPIYEGYALPHAILR.L | 2 |
|  | CENPL\_stlcld\_122314\_01.17780.17780.3 | 6.659 | 0.4542 | 100.0% | 3187.0444 | 3185.622 | 1 | 8.604 | 32.8% | 12 | R.TTGIVMDSGDGVTHTVPIYEGYALPHAILR.L | 3 |
|  | CENPL\_stlcld\_tube2\_122314\_01.17931.17931.2 | 3.3436 | 0.3565 | 99.9% | 1624.4521 | 1624.8927 | 1 | 6.039 | 61.5% | 3 | R.LDLAGRDLTDYLMK.I | 22 |
|  | CENPL\_stlcld\_tube2\_122314\_01.17907.17907.3 | 3.4423 | 0.4035 | 100.0% | 1625.8744 | 1624.8927 | 1 | 7.333 | 48.1% | 2 | R.LDLAGRDLTDYLMK.I | 33 |
|  | CENPL\_stlcld\_tube2\_122314\_01.21777.21777.3 | 5.0553 | 0.4004 | 100.0% | 2237.9343 | 2237.6196 | 1 | 7.674 | 43.1% | 2 | R.LDLAGRDLTDYLMKILTER.G | 33 |
|  | CENPL\_stlcld\_tube2\_122314\_01.17259.17259.1 | 2.0501 | 0.2549 | 96.8% | 998.57 | 999.167 | 4 | 5.345 | 71.4% | 4 | R.DLTDYLMK.I | 11 |
|  | CENPL\_stlcld\_122314\_01.17540.17540.2 | 2.3473 | 0.3499 | 99.8% | 998.9122 | 999.167 | 2 | 5.813 | 78.6% | 5 | R.DLTDYLMK.I | 22 |
|  | CENPL\_stlcld\_tube2\_122314\_02.00425.00425.2 | 2.7976 | 0.4206 | 99.9% | 1610.9521 | 1611.8939 | 4 | 6.881 | 54.2% | 1 | R.DLTDYLMKILTER.G | 22 |
|  | CENPL\_stlcld\_122314\_01.10626.10626.1 | 1.9059 | 0.2752 | 97.0% | 1132.52 | 1133.2029 | 24 | 4.252 | 50.0% | 1 | R.GYSFTTTAER.E | 1 |
|  | CENPL\_stlcld\_tube2\_122314\_01.10227.10227.2 | 3.3975 | 0.5255 | 100.0% | 1133.0721 | 1133.2029 | 1 | 8.56 | 77.8% | 20 | R.GYSFTTTAER.E | 2 |
|  | CENPL\_stlcld\_tube2\_122314\_01.16760.16760.2 | 4.594 | 0.3377 | 100.0% | 1792.2322 | 1791.9554 | 1 | 8.241 | 80.0% | 12 | K.SYELPDGQVITIGNER.F | 22 |
|  | CENPL\_stlcld\_122314\_01.15137.15137.2 | 6.2289 | 0.6046 | 100.0% | 2343.6921 | 2344.6448 | 1 | 11.296 | 66.7% | 2 | R.KDLYANTVLSGGTTMYPGIADR.M | 2 |
|  | CENPL\_stlcld\_tube2\_122314\_02.14258.14258.3 | 5.7907 | 0.552 | 100.0% | 2344.3442 | 2344.6448 | 1 | 9.522 | 39.3% | 12 | R.KDLYANTVLSGGTTMYPGIADR.M | 3 |
|  | CENPL\_stlcld\_122314\_02.14769.14769.2 | 5.236 | 0.6322 | 100.0% | 2215.672 | 2216.4705 | 1 | 10.711 | 60.0% | 17 | K.DLYANTVLSGGTTMYPGIADR.M | 2 |
|  | CENPL\_stlcld\_tube2\_122314\_02.16140.16140.3 | 3.8876 | 0.341 | 100.0% | 2216.7244 | 2216.4705 | 28 | 6.145 | 30.0% | 2 | K.DLYANTVLSGGTTMYPGIADR.M | 3 |
|  | CENPL\_stlcld\_tube2\_122314\_01.10899.10899.3 | 3.6397 | 0.2696 | 99.9% | 1550.0643 | 1549.8843 | 2 | 5.869 | 46.2% | 1 | R.MQKEITALAPSTMK.I | 33 |
|  | CENPL\_stlcld\_tube2\_122314\_01.11684.11684.1 | 2.5482 | 0.4302 | 100.0% | 1161.6 | 1162.3868 | 1 | 7.63 | 65.0% | 2 | K.EITALAPSTMK.I | 11 |
|  | CENPL\_stlcld\_122314\_02.09992.09992.2 | 2.6978 | 0.3741 | 99.9% | 1162.6721 | 1162.3868 | 2 | 6.662 | 60.0% | 5 | K.EITALAPSTMK.I | 22 |
|  | CENPL\_stlcld\_tube2\_122314\_01.09464.09464.2 | 2.3837 | 0.2092 | 98.3% | 1037.0721 | 1037.2908 | 1 | 4.75 | 75.0% | 1 | K.IKIIAPPER.K | 22 |
|  | CENPL\_stlcld\_tube2\_122314\_01.00675.00675.2 | 4.6438 | 0.4966 | 100.0% | 2730.8323 | 2732.213 | 1 | 7.45 | 52.2% | 1 | R.KYSVWIGGSILASLSTFQQMWISK.Q | 22 |
|  | CENPL\_stlcld\_tube2\_122314\_01.00293.00293.3 | 4.2968 | 0.3776 | 100.0% | 4100.9346 | 4102.6104 | 1 | 6.455 | 20.0% | 2 | K.YSVWIGGSILASLSTFQQMWISKQEYDESGPSIVHR.K | 3 |
|  | CENPL\_stlcld\_tube2\_122314\_01.08752.08752.2 | 1.8996 | 0.3427 | 97.5% | 1517.3121 | 1517.595 | 3 | 5.264 | 50.0% | 1 | K.QEYDESGPSIVHR.K | 2 |
|  | CENPL\_stlcld\_tube2\_122314\_01.08812.08812.3 | 2.5028 | 0.2847 | 98.6% | 1518.1743 | 1517.595 | 34 | 5.048 | 35.4% | 2 | K.QEYDESGPSIVHR.K | 3 |

Similarities:
gi|4885049|ref|NP\_005(16:14)  

---

|  |  |  |  |  |  |  |  |  |
| --- | --- | --- | --- | --- | --- | --- | --- | --- |
| U | *gi|10645195|ref|NP\_06* | 11 | 43 | 63.1% | 130 | 14135 | 11.1 | histone cluster 1, H2ae [Homo sapiens] |
| U | *gi|4504245|ref|NP\_003* | 10 | 33 | 63.1% | 130 | 14105 | 11.1 | histone cluster 1, H2ac [Homo sapiens] |
| U | *gi|19557656|ref|NP\_00* | 11 | 43 | 63.1% | 130 | 14135 | 11.1 | histone cluster 1, H2ab [Homo sapiens] |
| U | *gi|15617199|ref|NP\_25* | 11 | 43 | 63.1% | 130 | 14121 | 11.1 | histone cluster 3, H2a [Homo sapiens] |

| Filename XCorr DeltCN Conf% ObsM+H+ CalcM+H+ SpR ZScore Ion% # Sequence  | | | | | | | | | | | | |
| --- | --- | --- | --- | --- | --- | --- | --- | --- | --- | --- | --- | --- |
|  | CENPL\_stlcld\_122314\_01.11778.11778.2 | 2.6166 | 0.1881 | 97.8% | 1274.6921 | 1275.4531 | 1 | 4.59 | 77.3% | 2 | R.SSRAGLQFPVGR.V | 222 |
|  | CENPL\_stlcld\_122314\_01.13670.13670.2 | 3.2544 | 0.3534 | 100.0% | 945.1922 | 945.1093 | 3 | 6.291 | 81.2% | 10 | R.AGLQFPVGR.V | 2222 |
|  | CENPL\_stlcld\_tube2\_122314\_02.02384.02384.2 | 4.9732 | 0.5685 | 100.0% | 2916.5122 | 2917.3752 | 1 | 12.217 | 46.4% | 4 | R.VGAGAPVYLAAVLEYLTAEILELAGNAAR.D | 22 |
|  | CENPL\_stlcld\_122314\_02.02374.02374.3 | 4.9621 | 0.4378 | 100.0% | 2918.4844 | 2917.3752 | 1 | 8.203 | 28.6% | 1 | R.VGAGAPVYLAAVLEYLTAEILELAGNAAR.D | 33 |
|  | CENPL\_stlcld\_tube2\_122314\_02.02223.02223.3 | 4.5206 | 0.3937 | 100.0% | 3274.5244 | 3274.7417 | 1 | 6.418 | 31.5% | 2 | R.VGAGAPVYLAAVLEYLTAEILELAGNAARDNK.K | 33 |
|  | CENPL\_stlcld\_tube2\_122314\_02.02133.02133.3 | 3.9901 | 0.3106 | 99.9% | 3402.1143 | 3402.9158 | 1 | 6.402 | 25.8% | 1 | R.VGAGAPVYLAAVLEYLTAEILELAGNAARDNKK.T | 33 |
|  | CENPL\_stlcld\_122314\_01.09950.09950.2 | 2.5538 | 0.2207 | 99.5% | 851.0722 | 851.0396 | 4 | 5.466 | 83.3% | 7 | R.HLQLAIR.N | 2222 |
|  | CENPL\_stlcld\_tube2\_122314\_02.10960.10960.2 | 2.74 | 0.1373 | 96.0% | 1694.0521 | 1693.9004 | 2 | 4.347 | 46.2% | 1 | R.HLQLAIRNDEELNK.L | 222 |
|  | CENPL\_stlcld\_122314\_02.10160.10160.3 | 3.1474 | 0.3441 | 99.9% | 1694.3043 | 1693.9004 | 1 | 6.006 | 44.2% | 9 | R.HLQLAIRNDEELNK.L | 333 |
|  | CENPL\_stlcld\_122314\_01.13682.13682.2 | 3.3542 | 0.1596 | 99.4% | 1302.6921 | 1301.4423 | 3 | 4.71 | 70.0% | 2 | R.NDEELNKLLGR.V | 2 |
|  | CENPL\_stlcld\_122314\_01.20973.20973.2 | 3.494 | 0.4099 | 100.0% | 1931.9922 | 1932.3573 | 1 | 7.17 | 50.0% | 4 | R.VTIAQGGVLPNIQAVLLPK.K | 222 |

Similarities:
gi|10800130|ref|NP\_06(10:1)  
gi|106775678|ref|NP\_0(6:5)  
gi|20357599|ref|NP\_61(2:9)  

---

|  |  |  |  |  |  |  |  |  |
| --- | --- | --- | --- | --- | --- | --- | --- | --- |
| U | *gi|10800130|ref|NP\_06* | 12 | 48 | 63.1% | 130 | 14107 | 10.9 | histone cluster 1, H2ad [Homo sapiens] |
| U | *gi|4504249|ref|NP\_003* | 11 | 38 | 63.1% | 130 | 14091 | 10.9 | histone cluster 1, H2am [Homo sapiens] |
| U | *gi|4504243|ref|NP\_003* | 12 | 48 | 63.1% | 130 | 14091 | 10.9 | histone cluster 1, H2al [Homo sapiens] |
| U | *gi|4504241|ref|NP\_003* | 10 | 31 | 63.1% | 130 | 14091 | 10.9 | histone cluster 1, H2ak [Homo sapiens] |
| U | *gi|4504239|ref|NP\_003* | 12 | 48 | 63.1% | 130 | 14091 | 10.9 | histone cluster 1, H2ai [Homo sapiens] |
| U | *gi|29553970|ref|NP\_80* | 10 | 31 | 63.6% | 129 | 14019 | 10.9 | H2A histone family, member J [Homo sapiens] |
| U | *gi|18105045|ref|NP\_54* | 12 | 48 | 64.1% | 128 | 13906 | 10.9 | histone cluster 1, H2ah [Homo sapiens] |
| U | *gi|10800144|ref|NP\_06* | 12 | 48 | 64.1% | 128 | 13936 | 10.9 | histone cluster 1, H2aj [Homo sapiens] |
| U | *gi|10800132|ref|NP\_06* | 12 | 48 | 63.1% | 130 | 14091 | 10.9 | histone cluster 1, H2ag [Homo sapiens] |

| Filename XCorr DeltCN Conf% ObsM+H+ CalcM+H+ SpR ZScore Ion% # Sequence  | | | | | | | | | | | | |
| --- | --- | --- | --- | --- | --- | --- | --- | --- | --- | --- | --- | --- |
|  | CENPL\_stlcld\_122314\_01.11778.11778.2 | 2.6166 | 0.1881 | 97.8% | 1274.6921 | 1275.4531 | 1 | 4.59 | 77.3% | 2 | R.SSRAGLQFPVGR.V | 222 |
|  | CENPL\_stlcld\_122314\_01.13670.13670.2 | 3.2544 | 0.3534 | 100.0% | 945.1922 | 945.1093 | 3 | 6.291 | 81.2% | 10 | R.AGLQFPVGR.V | 2222 |
|  | CENPL\_stlcld\_tube2\_122314\_02.02384.02384.2 | 4.9732 | 0.5685 | 100.0% | 2916.5122 | 2917.3752 | 1 | 12.217 | 46.4% | 4 | R.VGAGAPVYLAAVLEYLTAEILELAGNAAR.D | 22 |
|  | CENPL\_stlcld\_122314\_02.02374.02374.3 | 4.9621 | 0.4378 | 100.0% | 2918.4844 | 2917.3752 | 1 | 8.203 | 28.6% | 1 | R.VGAGAPVYLAAVLEYLTAEILELAGNAAR.D | 33 |
|  | CENPL\_stlcld\_tube2\_122314\_02.02223.02223.3 | 4.5206 | 0.3937 | 100.0% | 3274.5244 | 3274.7417 | 1 | 6.418 | 31.5% | 2 | R.VGAGAPVYLAAVLEYLTAEILELAGNAARDNK.K | 33 |
|  | CENPL\_stlcld\_tube2\_122314\_02.02133.02133.3 | 3.9901 | 0.3106 | 99.9% | 3402.1143 | 3402.9158 | 1 | 6.402 | 25.8% | 1 | R.VGAGAPVYLAAVLEYLTAEILELAGNAARDNKK.T | 33 |
|  | CENPL\_stlcld\_122314\_01.09950.09950.2 | 2.5538 | 0.2207 | 99.5% | 851.0722 | 851.0396 | 4 | 5.466 | 83.3% | 7 | R.HLQLAIR.N | 2222 |
|  | CENPL\_stlcld\_tube2\_122314\_02.10960.10960.2 | 2.74 | 0.1373 | 96.0% | 1694.0521 | 1693.9004 | 2 | 4.347 | 46.2% | 1 | R.HLQLAIRNDEELNK.L | 222 |
|  | CENPL\_stlcld\_122314\_02.10160.10160.3 | 3.1474 | 0.3441 | 99.9% | 1694.3043 | 1693.9004 | 1 | 6.006 | 44.2% | 9 | R.HLQLAIRNDEELNK.L | 333 |
|  | CENPL\_stlcld\_122314\_01.14446.14446.3 | 4.3328 | 0.499 | 100.0% | 2105.6943 | 2105.4453 | 1 | 7.87 | 39.7% | 1 | R.HLQLAIRNDEELNKLLGK.V | 33 |
|  | CENPL\_stlcld\_122314\_01.13104.13104.2 | 3.8023 | 0.3492 | 100.0% | 1274.4321 | 1273.4288 | 1 | 5.513 | 75.0% | 6 | R.NDEELNKLLGK.V | 22 |
|  | CENPL\_stlcld\_122314\_01.20973.20973.2 | 3.494 | 0.4099 | 100.0% | 1931.9922 | 1932.3573 | 1 | 7.17 | 50.0% | 4 | K.VTIAQGGVLPNIQAVLLPK.K | 222 |

Similarities:
gi|10645195|ref|NP\_06(10:2)  
gi|106775678|ref|NP\_0(8:4)  
gi|20357599|ref|NP\_61(2:10)  

---

|  |  |  |  |  |  |  |  |  |
| --- | --- | --- | --- | --- | --- | --- | --- | --- |
| U | *gi|106775678|ref|NP\_0* | 11 | 44 | 63.1% | 130 | 14095 | 10.9 | histone cluster 2, H2aa4 [Homo sapiens] |
| U | *gi|4504251|ref|NP\_003* | 11 | 44 | 63.1% | 130 | 14095 | 10.9 | histone cluster 2, H2aa3 [Homo sapiens] |
| U | *gi|24638446|ref|NP\_00* | 11 | 44 | 63.6% | 129 | 13988 | 10.9 | histone cluster 2, H2ac [Homo sapiens] |

| Filename XCorr DeltCN Conf% ObsM+H+ CalcM+H+ SpR ZScore Ion% # Sequence  | | | | | | | | | | | | |
| --- | --- | --- | --- | --- | --- | --- | --- | --- | --- | --- | --- | --- |
|  | CENPL\_stlcld\_122314\_01.11778.11778.2 | 2.6166 | 0.1881 | 97.8% | 1274.6921 | 1275.4531 | 1 | 4.59 | 77.3% | 2 | R.SSRAGLQFPVGR.V | 222 |
|  | CENPL\_stlcld\_122314\_01.13670.13670.2 | 3.2544 | 0.3534 | 100.0% | 945.1922 | 945.1093 | 3 | 6.291 | 81.2% | 10 | R.AGLQFPVGR.V | 2222 |
|  | CENPL\_stlcld\_122314\_02.02277.02277.2 | 5.1231 | 0.5694 | 100.0% | 2935.132 | 2935.4082 | 1 | 10.185 | 42.9% | 2 | R.VGAGAPVYMAAVLEYLTAEILELAGNAAR.D | 2 |
|  | CENPL\_stlcld\_122314\_02.02276.02276.3 | 5.2178 | 0.4144 | 100.0% | 2935.8542 | 2935.4082 | 1 | 7.172 | 31.2% | 1 | R.VGAGAPVYMAAVLEYLTAEILELAGNAAR.D | 3 |
|  | CENPL\_stlcld\_tube2\_122314\_02.02091.02091.3 | 4.1799 | 0.2714 | 99.9% | 3421.9443 | 3420.949 | 22 | 4.789 | 19.5% | 1 | R.VGAGAPVYMAAVLEYLTAEILELAGNAARDNKK.T | 3 |
|  | CENPL\_stlcld\_122314\_01.09950.09950.2 | 2.5538 | 0.2207 | 99.5% | 851.0722 | 851.0396 | 4 | 5.466 | 83.3% | 7 | R.HLQLAIR.N | 2222 |
|  | CENPL\_stlcld\_tube2\_122314\_02.10960.10960.2 | 2.74 | 0.1373 | 96.0% | 1694.0521 | 1693.9004 | 2 | 4.347 | 46.2% | 1 | R.HLQLAIRNDEELNK.L | 222 |
|  | CENPL\_stlcld\_122314\_02.10160.10160.3 | 3.1474 | 0.3441 | 99.9% | 1694.3043 | 1693.9004 | 1 | 6.006 | 44.2% | 9 | R.HLQLAIRNDEELNK.L | 333 |
|  | CENPL\_stlcld\_122314\_01.14446.14446.3 | 4.3328 | 0.499 | 100.0% | 2105.6943 | 2105.4453 | 1 | 7.87 | 39.7% | 1 | R.HLQLAIRNDEELNKLLGK.V | 33 |
|  | CENPL\_stlcld\_122314\_01.13104.13104.2 | 3.8023 | 0.3492 | 100.0% | 1274.4321 | 1273.4288 | 1 | 5.513 | 75.0% | 6 | R.NDEELNKLLGK.V | 22 |
|  | CENPL\_stlcld\_122314\_01.20973.20973.2 | 3.494 | 0.4099 | 100.0% | 1931.9922 | 1932.3573 | 1 | 7.17 | 50.0% | 4 | K.VTIAQGGVLPNIQAVLLPK.K | 222 |

Similarities:
gi|10645195|ref|NP\_06(6:5)  
gi|10800130|ref|NP\_06(8:3)  
gi|20357599|ref|NP\_61(2:9)  

---

|  |  |  |  |  |  |  |  |  |
| --- | --- | --- | --- | --- | --- | --- | --- | --- |
| U | *gi|11415030|ref|NP\_06* | 11 | 71 | 59.2% | 103 | 11367 | 11.4 | histone cluster 1, H4j [Homo sapiens] |
| U | *gi|77539758|ref|NP\_00* | 11 | 71 | 59.2% | 103 | 11367 | 11.4 | histone cluster 2, H4b [Homo sapiens] |
| U | *gi|4504323|ref|NP\_003* | 10 | 69 | 59.2% | 103 | 11367 | 11.4 | histone cluster 2, H4a [Homo sapiens] |
| U | *gi|4504321|ref|NP\_003* | 11 | 71 | 59.2% | 103 | 11367 | 11.4 | histone cluster 1, H4i [Homo sapiens] |
| U | *gi|4504317|ref|NP\_003* | 11 | 71 | 59.2% | 103 | 11367 | 11.4 | histone cluster 1, H4l [Homo sapiens] |
| U | *gi|4504315|ref|NP\_003* | 11 | 71 | 59.2% | 103 | 11367 | 11.4 | histone cluster 1, H4e [Homo sapiens] |
| U | *gi|4504313|ref|NP\_003* | 11 | 71 | 59.2% | 103 | 11367 | 11.4 | histone cluster 1, H4b [Homo sapiens] |
| U | *gi|4504311|ref|NP\_003* | 11 | 71 | 59.2% | 103 | 11367 | 11.4 | histone cluster 1, H4h [Homo sapiens] |
| U | *gi|4504309|ref|NP\_003* | 11 | 71 | 59.2% | 103 | 11367 | 11.4 | histone cluster 1, H4c [Homo sapiens] |
| U | *gi|4504307|ref|NP\_003* | 11 | 71 | 59.2% | 103 | 11367 | 11.4 | histone cluster 1, H4k [Homo sapiens] |
| U | *gi|4504305|ref|NP\_003* | 11 | 71 | 59.2% | 103 | 11367 | 11.4 | histone cluster 1, H4f [Homo sapiens] |
| U | *gi|4504303|ref|NP\_003* | 11 | 71 | 59.2% | 103 | 11367 | 11.4 | histone cluster 1, H4d [Homo sapiens] |
| U | *gi|4504301|ref|NP\_003* | 11 | 71 | 59.2% | 103 | 11367 | 11.4 | histone cluster 1, H4a [Homo sapiens] |
| U | *gi|28173560|ref|NP\_77* | 11 | 71 | 59.2% | 103 | 11367 | 11.4 | histone cluster 4, H4 [Homo sapiens] |

| Filename XCorr DeltCN Conf% ObsM+H+ CalcM+H+ SpR ZScore Ion% # Sequence  | | | | | | | | | | | | |
| --- | --- | --- | --- | --- | --- | --- | --- | --- | --- | --- | --- | --- |
|  | CENPL\_stlcld\_tube2\_122314\_01.09596.09596.2 | 3.4676 | 0.2001 | 99.7% | 1326.0922 | 1326.5387 | 1 | 6.544 | 81.8% | 11 | R.DNIQGITKPAIR.R | 2 |
|  | CENPL\_stlcld\_tube2\_122314\_01.12508.12508.1 | 2.4096 | 0.2223 | 95.9% | 1180.62 | 1181.3312 | 3 | 4.757 | 66.7% | 2 | R.ISGLIYEETR.G | 1 |
|  | CENPL\_stlcld\_122314\_01.12644.12644.2 | 3.8212 | 0.386 | 100.0% | 1181.4321 | 1181.3312 | 1 | 7.301 | 88.9% | 18 | R.ISGLIYEETR.G | 2 |
|  | CENPL\_stlcld\_tube2\_122314\_01.15843.15843.1 | 1.8858 | 0.2757 | 96.7% | 989.73 | 990.19055 | 34 | 5.265 | 57.1% | 2 | K.VFLENVIR.D | 1 |
|  | CENPL\_stlcld\_122314\_01.15728.15728.2 | 3.0974 | 0.2608 | 99.9% | 989.97217 | 990.19055 | 1 | 5.644 | 85.7% | 11 | K.VFLENVIR.D | 2 |
|  | CENPL\_stlcld\_tube2\_122314\_01.19950.19950.3 | 5.0362 | 0.4197 | 100.0% | 2106.2344 | 2106.386 | 1 | 7.255 | 44.1% | 2 | K.VFLENVIRDAVTYTEHAK.R | 3 |
|  | CENPL\_stlcld\_tube2\_122314\_01.16396.16396.2 | 3.7416 | 0.4016 | 100.0% | 1440.2122 | 1439.7534 | 1 | 6.137 | 83.3% | 2 | R.KTVTAMDVVYALK.R | 2 |
|  | CENPL\_stlcld\_tube2\_122314\_01.14666.14666.3 | 4.4416 | 0.4106 | 100.0% | 1595.4543 | 1595.9409 | 1 | 6.145 | 48.1% | 5 | R.KTVTAMDVVYALKR.Q | 3 |
|  | CENPL\_stlcld\_tube2\_122314\_01.18710.18710.2 | 3.5276 | 0.501 | 100.0% | 1311.4321 | 1311.5793 | 1 | 8.936 | 81.8% | 3 | K.TVTAMDVVYALK.R | 2 |
|  | CENPL\_stlcld\_tube2\_122314\_01.16829.16829.2 | 3.6389 | 0.4746 | 100.0% | 1467.3722 | 1467.7667 | 1 | 7.785 | 70.8% | 10 | K.TVTAMDVVYALKR.Q | 2 |
|  | CENPL\_stlcld\_tube2\_122314\_01.15974.15974.1 | 2.2499 | 0.5402 | 100.0% | 715.48 | 714.796 | 1 | 7.354 | 66.7% | 5 | R.TLYGFGG.- | 1 |

---

|  |  |  |  |  |  |  |  |  |
| --- | --- | --- | --- | --- | --- | --- | --- | --- |
| U | *gi|14165270|ref|NP\_05* | 11 | 31 | 52.8% | 178 | 20692 | 9.1 | mitochondrial ribosomal protein L13 [Homo sapiens] |

| Filename XCorr DeltCN Conf% ObsM+H+ CalcM+H+ SpR ZScore Ion% # Sequence  | | | | | | | | | | | | |
| --- | --- | --- | --- | --- | --- | --- | --- | --- | --- | --- | --- | --- |
| \* | CENPL\_stlcld\_tube2\_122314\_01.13160.13160.2 | 3.6094 | 0.3625 | 100.0% | 1177.4122 | 1176.3201 | 1 | 6.735 | 88.9% | 7 | R.APQQWATFAR.I | 2 |
| \* | CENPL\_stlcld\_tube2\_122314\_01.08738.08738.3 | 2.7866 | 0.29 | 99.9% | 1446.0543 | 1445.6206 | 22 | 4.946 | 38.6% | 1 | R.HIAFSGNKWEQK.V | 3 |
| \* | CENPL\_stlcld\_122314\_01.09546.09546.2 | 3.6336 | 0.4192 | 100.0% | 1428.4521 | 1428.5468 | 1 | 8.046 | 66.7% | 2 | K.VYSSHTGYPGGFR.Q | 2 |
| \* | CENPL\_stlcld\_tube2\_122314\_01.09110.09110.3 | 3.1169 | 0.3288 | 99.9% | 1429.1943 | 1428.5468 | 6 | 5.614 | 35.4% | 3 | K.VYSSHTGYPGGFR.Q | 3 |
| \* | CENPL\_stlcld\_122314\_01.17019.17019.2 | 2.5082 | 0.3908 | 99.9% | 1006.21216 | 1006.29156 | 1 | 6.512 | 75.0% | 1 | K.LAIYGMLPK.N | 2 |
| \* | CENPL\_stlcld\_122314\_01.20294.20294.2 | 4.1079 | 0.4438 | 100.0% | 1843.7522 | 1843.1296 | 1 | 7.359 | 75.0% | 2 | R.LHLFPDEYIPEDILK.N | 2 |
| \* | CENPL\_stlcld\_tube2\_122314\_02.00491.00491.3 | 4.856 | 0.526 | 100.0% | 3018.4143 | 3019.4675 | 1 | 8.283 | 36.5% | 1 | R.LHLFPDEYIPEDILKNLVEELPQPR.K | 3 |
| \* | CENPL\_stlcld\_tube2\_122314\_01.13042.13042.2 | 3.037 | 0.1689 | 99.1% | 1196.4122 | 1195.361 | 7 | 4.555 | 83.3% | 6 | K.NLVEELPQPR.K | 2 |
| \* | CENPL\_stlcld\_122314\_01.14100.14100.2 | 4.3708 | 0.4565 | 100.0% | 1882.6322 | 1883.0245 | 1 | 7.895 | 85.7% | 2 | K.RLDEYTQEEIDAFPR.L | 2 |
| \* | CENPL\_stlcld\_tube2\_122314\_02.13484.13484.3 | 3.1276 | 0.3327 | 99.9% | 1882.9744 | 1883.0245 | 1 | 5.622 | 42.9% | 5 | K.RLDEYTQEEIDAFPR.L | 3 |
| \* | CENPL\_stlcld\_122314\_01.17457.17457.2 | 2.0019 | 0.3073 | 97.8% | 1290.4321 | 1290.4613 | 104 | 4.98 | 55.6% | 1 | R.LWTPPEDYRL.- | 2 |

---

|  |  |  |  |  |  |  |  |  |
| --- | --- | --- | --- | --- | --- | --- | --- | --- |
| U | *gi|29788785|ref|NP\_82* | 28 | 127 | 52.0% | 444 | 49671 | 4.9 | tubulin, beta [Homo sapiens] |

| Filename XCorr DeltCN Conf% ObsM+H+ CalcM+H+ SpR ZScore Ion% # Sequence  | | | | | | | | | | | | |
| --- | --- | --- | --- | --- | --- | --- | --- | --- | --- | --- | --- | --- |
| \* | CENPL\_stlcld\_tube2\_122314\_01.16468.16468.3 | 5.9362 | 0.4844 | 100.0% | 3103.8843 | 3104.2725 | 1 | 8.662 | 36.5% | 5 | K.FWEVISDEHGIDPTGTYHGDSDLQLDR.I | 3 |
| \* | CENPL\_stlcld\_tube2\_122314\_01.10557.10557.2 | 3.6054 | 0.4988 | 100.0% | 1302.1122 | 1302.4265 | 1 | 8.899 | 77.3% | 10 | R.ISVYYNEATGGK.Y | 2 |
|  | CENPL\_stlcld\_122314\_02.13131.13131.2 | 4.2785 | 0.369 | 100.0% | 1617.9722 | 1616.8701 | 1 | 6.59 | 67.9% | 11 | R.AILVDLEPGTMDSVR.S | 22 |
|  | CENPL\_stlcld\_122314\_01.20337.20337.2 | 5.5537 | 0.4908 | 100.0% | 2799.9722 | 2800.0647 | 1 | 7.549 | 40.0% | 3 | R.SGPFGQIFRPDNFVFGQSGAGNNWAK.G | 22 |
|  | CENPL\_stlcld\_122314\_01.20366.20366.3 | 6.7253 | 0.4336 | 100.0% | 2800.8542 | 2800.0647 | 1 | 7.18 | 34.0% | 4 | R.SGPFGQIFRPDNFVFGQSGAGNNWAK.G | 33 |
|  | CENPL\_stlcld\_122314\_01.20825.20825.2 | 5.647 | 0.559 | 100.0% | 1959.0322 | 1960.151 | 1 | 11.798 | 79.4% | 2 | K.GHYTEGAELVDSVLDVVR.K | 222 |
|  | CENPL\_stlcld\_122314\_01.20822.20822.3 | 2.9108 | 0.2585 | 98.5% | 1959.7743 | 1960.151 | 7 | 4.629 | 32.4% | 1 | K.GHYTEGAELVDSVLDVVR.K | 333 |
|  | CENPL\_stlcld\_tube2\_122314\_01.19052.19052.3 | 5.1569 | 0.4766 | 100.0% | 2087.9644 | 2088.325 | 1 | 8.639 | 44.4% | 6 | K.GHYTEGAELVDSVLDVVRK.E | 333 |
|  | CENPL\_stlcld\_tube2\_122314\_01.19009.19009.2 | 6.3337 | 0.4925 | 100.0% | 2088.612 | 2088.325 | 1 | 9.687 | 66.7% | 3 | K.GHYTEGAELVDSVLDVVRK.E | 222 |
|  | CENPL\_stlcld\_tube2\_122314\_01.14282.14282.2 | 4.3911 | 0.4008 | 100.0% | 1320.4922 | 1320.5896 | 1 | 7.658 | 81.8% | 10 | R.IMNTFSVVPSPK.V | 222 |
|  | CENPL\_stlcld\_122314\_01.13025.13025.2 | 3.0807 | 0.3262 | 99.9% | 1130.8322 | 1131.2767 | 1 | 5.245 | 83.3% | 8 | R.FPGQLNADLR.K | 2222 |
|  | CENPL\_stlcld\_122314\_01.11442.11442.3 | 2.9929 | 0.3054 | 99.9% | 1259.5443 | 1259.4508 | 9 | 5.658 | 42.5% | 1 | R.FPGQLNADLRK.L | 3333 |
|  | CENPL\_stlcld\_tube2\_122314\_01.10454.10454.2 | 2.6368 | 0.2303 | 98.6% | 1259.7322 | 1259.4508 | 17 | 4.582 | 60.0% | 5 | R.FPGQLNADLRK.L | 2222 |
|  | CENPL\_stlcld\_tube2\_122314\_01.14181.14181.2 | 3.8965 | 0.404 | 100.0% | 1271.9922 | 1272.5945 | 1 | 7.764 | 75.0% | 4 | R.KLAVNMVPFPR.L | 2222 |
|  | CENPL\_stlcld\_122314\_01.16748.16748.2 | 3.646 | 0.4855 | 100.0% | 1143.9922 | 1144.4204 | 1 | 8.724 | 94.4% | 8 | K.LAVNMVPFPR.L | 2222 |
|  | CENPL\_stlcld\_122314\_01.20176.20176.3 | 4.2322 | 0.3658 | 100.0% | 1622.3644 | 1621.9403 | 1 | 6.191 | 55.8% | 3 | R.LHFFMPGFAPLTSR.G | 333 |
|  | CENPL\_stlcld\_122314\_01.20175.20175.2 | 3.8813 | 0.426 | 100.0% | 1622.5721 | 1621.9403 | 1 | 8.381 | 73.1% | 3 | R.LHFFMPGFAPLTSR.G | 222 |
| \* | CENPL\_stlcld\_tube2\_122314\_01.19195.19195.2 | 3.9944 | 0.4918 | 100.0% | 1660.4922 | 1660.9078 | 1 | 8.068 | 71.4% | 7 | R.ALTVPELTQQVFDAK.N | 2 |
|  | CENPL\_stlcld\_tube2\_122314\_01.12296.12296.3 | 2.2853 | 0.2706 | 96.2% | 1391.1543 | 1390.631 | 4 | 5.015 | 45.5% | 1 | R.HGRYLTVAAVFR.G | 33 |
|  | CENPL\_stlcld\_122314\_01.17753.17753.1 | 1.5504 | 0.3447 | 96.0% | 1039.71 | 1040.2505 | 1 | 5.617 | 68.8% | 2 | R.YLTVAAVFR.G | 11 |
|  | CENPL\_stlcld\_122314\_02.14903.14903.2 | 2.8302 | 0.4423 | 100.0% | 1040.4122 | 1040.2505 | 1 | 7.611 | 93.8% | 5 | R.YLTVAAVFR.G | 22 |
|  | CENPL\_stlcld\_tube2\_122314\_02.13035.13035.3 | 3.8475 | 0.2619 | 99.9% | 1925.4844 | 1925.2405 | 1 | 5.282 | 43.3% | 2 | R.MSMKEVDEQMLNVQNK.N | 33 |
|  | CENPL\_stlcld\_tube2\_122314\_01.10952.10952.2 | 4.3318 | 0.2038 | 99.9% | 1448.3322 | 1447.6031 | 1 | 5.927 | 72.7% | 6 | K.EVDEQMLNVQNK.N | 22 |
|  | CENPL\_stlcld\_122314\_01.19247.19247.2 | 3.5195 | 0.346 | 99.9% | 1698.4321 | 1697.8877 | 3 | 5.888 | 53.8% | 4 | K.NSSYFVEWIPNNVK.T | 2222 |
| \* | CENPL\_stlcld\_122314\_02.17240.17240.2 | 3.7909 | 0.1929 | 99.7% | 1872.6122 | 1871.2018 | 1 | 5.392 | 65.6% | 1 | K.MAVTFIGNSTAIQELFK.R | 2 |
| \* | CENPL\_stlcld\_tube2\_122314\_01.19733.19733.2 | 3.7875 | 0.4513 | 100.0% | 2027.3121 | 2027.3893 | 2 | 7.5 | 38.2% | 1 | K.MAVTFIGNSTAIQELFKR.I | 2 |
| \* | CENPL\_stlcld\_122314\_02.16545.16545.3 | 3.5835 | 0.2182 | 99.3% | 2029.5543 | 2027.3893 | 5 | 4.601 | 36.8% | 2 | K.MAVTFIGNSTAIQELFKR.I | 3 |
|  | CENPL\_stlcld\_122314\_02.14308.14308.2 | 3.8472 | 0.492 | 100.0% | 1230.5122 | 1230.4241 | 1 | 7.151 | 94.4% | 9 | R.ISEQFTAMFR.R | 222 |

Similarities:
gi|5174735|ref|NP\_006(21:7)  
gi|50592996|ref|NP\_00(13:15)  
gi|14210536|ref|NP\_11(8:20)  

---

|  |  |  |  |  |  |  |  |  |
| --- | --- | --- | --- | --- | --- | --- | --- | --- |
| U | *gi|4504919|ref|NP\_002* | 42 | 160 | 48.9% | 483 | 53704 | 5.6 | keratin 8 [Homo sapiens] |

| Filename XCorr DeltCN Conf% ObsM+H+ CalcM+H+ SpR ZScore Ion% # Sequence  | | | | | | | | | | | | |
| --- | --- | --- | --- | --- | --- | --- | --- | --- | --- | --- | --- | --- |
|  | CENPL\_stlcld\_tube2\_122314\_01.12512.12512.2 | 2.417 | 0.2122 | 98.9% | 828.09216 | 827.95544 | 5 | 4.999 | 91.7% | 4 | K.FASFIDK.V | 222222222 |
|  | CENPL\_stlcld\_122314\_01.12293.12293.2 | 2.815 | 0.2809 | 99.9% | 1082.9922 | 1083.2755 | 3 | 6.695 | 75.0% | 2 | K.FASFIDKVR.F | 222322223 |
|  | CENPL\_stlcld\_tube2\_122314\_02.11972.11972.2 | 2.8575 | 0.1847 | 99.5% | 1031.1322 | 1031.1997 | 2 | 4.271 | 92.9% | 6 | K.WSLLQQQK.T | 2 |
|  | CENPL\_stlcld\_tube2\_122314\_02.17030.17030.3 | 4.932 | 0.1662 | 99.9% | 2034.9844 | 2035.363 | 50 | 8.027 | 35.3% | 4 | K.LKLEAELGNMQGLVEDFK.N | 3 |
|  | CENPL\_stlcld\_122314\_02.16259.16259.2 | 5.143 | 0.2868 | 100.0% | 2035.4321 | 2035.363 | 2 | 9.57 | 52.9% | 1 | K.LKLEAELGNMQGLVEDFK.N | 2 |
|  | CENPL\_stlcld\_122314\_02.11433.11433.3 | 4.4899 | 0.2242 | 99.9% | 1639.4043 | 1637.9348 | 1 | 5.901 | 50.0% | 1 | K.RTEMENEFVLIKK.D | 3 |
|  | CENPL\_stlcld\_122314\_01.16916.16916.2 | 3.3951 | 0.4469 | 100.0% | 1353.4521 | 1353.5732 | 1 | 7.144 | 75.0% | 6 | R.TEMENEFVLIK.K | 2 |
|  | CENPL\_stlcld\_122314\_01.13659.13659.2 | 3.3697 | 0.2821 | 99.9% | 1481.2722 | 1481.7473 | 1 | 5.021 | 72.7% | 6 | R.TEMENEFVLIKK.D | 2 |
|  | CENPL\_stlcld\_tube2\_122314\_02.11224.11224.3 | 2.5093 | 0.2703 | 97.4% | 1928.2144 | 1927.1365 | 30 | 4.549 | 31.7% | 1 | K.KDVDEAYMNKVELESR.L | 3 |
|  | CENPL\_stlcld\_tube2\_122314\_01.12164.12164.2 | 4.606 | 0.4942 | 100.0% | 1798.4521 | 1798.9623 | 1 | 8.4 | 67.9% | 7 | K.DVDEAYMNKVELESR.L | 2 |
|  | CENPL\_stlcld\_tube2\_122314\_02.12248.12248.3 | 3.9884 | 0.4291 | 100.0% | 1798.5844 | 1798.9623 | 2 | 6.784 | 50.0% | 3 | K.DVDEAYMNKVELESR.L | 3 |
|  | CENPL\_stlcld\_tube2\_122314\_01.19289.19289.1 | 3.0156 | 0.39 | 100.0% | 1420.02 | 1420.6055 | 1 | 6.509 | 68.2% | 2 | R.LEGLTDEINFLR.Q | 1 |
|  | CENPL\_stlcld\_122314\_01.19928.19928.2 | 4.1183 | 0.5206 | 100.0% | 1420.7122 | 1420.6055 | 1 | 9.052 | 86.4% | 7 | R.LEGLTDEINFLR.Q | 2 |
|  | CENPL\_stlcld\_122314\_02.13020.13020.2 | 4.5122 | 0.585 | 100.0% | 2109.5522 | 2110.3008 | 1 | 10.696 | 69.4% | 1 | R.ELQSQISDTSVVLSMDNSR.S | 2 |
|  | CENPL\_stlcld\_tube2\_122314\_01.18877.18877.1 | 2.279 | 0.2777 | 95.9% | 1320.68 | 1321.5286 | 10 | 5.868 | 54.5% | 2 | R.SLDMDSIIAEVK.A | 1 |
|  | CENPL\_stlcld\_tube2\_122314\_01.18889.18889.2 | 4.5604 | 0.451 | 100.0% | 1321.7122 | 1321.5286 | 1 | 8.028 | 77.3% | 7 | R.SLDMDSIIAEVK.A | 2 |
|  | CENPL\_stlcld\_tube2\_122314\_01.21450.21450.2 | 3.1829 | 0.4261 | 100.0% | 2381.3123 | 2382.6477 | 1 | 6.87 | 37.5% | 1 | R.SLDMDSIIAEVKAQYEDIANR.S | 2 |
|  | CENPL\_stlcld\_tube2\_122314\_01.08468.08468.2 | 2.9007 | 0.2842 | 99.9% | 1080.2122 | 1080.1423 | 1 | 6.836 | 87.5% | 8 | K.AQYEDIANR.S | 22 |
|  | CENPL\_stlcld\_122314\_01.09435.09435.2 | 3.4971 | 0.2925 | 99.9% | 1413.0721 | 1413.5884 | 1 | 5.997 | 86.4% | 4 | R.SRAEAESMYQIK.Y | 2 |
|  | CENPL\_stlcld\_tube2\_122314\_01.08963.08963.3 | 3.4759 | 0.2262 | 99.9% | 1413.6244 | 1413.5884 | 5 | 5.011 | 50.0% | 2 | R.SRAEAESMYQIK.Y | 3 |
|  | CENPL\_stlcld\_122314\_01.14399.14399.3 | 5.7641 | 0.3614 | 100.0% | 2532.6843 | 2532.828 | 1 | 7.045 | 35.7% | 5 | R.SRAEAESMYQIKYEELQSLAGK.H | 3 |
|  | CENPL\_stlcld\_tube2\_122314\_01.10384.10384.2 | 3.1251 | 0.3506 | 99.9% | 1170.3121 | 1170.3228 | 1 | 7.149 | 77.8% | 7 | R.AEAESMYQIK.Y | 2 |
|  | CENPL\_stlcld\_122314\_02.13246.13246.3 | 5.2371 | 0.398 | 100.0% | 2289.6543 | 2289.5623 | 1 | 8.327 | 39.5% | 5 | R.AEAESMYQIKYEELQSLAGK.H | 3 |
|  | CENPL\_stlcld\_tube2\_122314\_01.15651.15651.2 | 3.2671 | 0.2775 | 99.7% | 2289.7322 | 2289.5623 | 19 | 5.75 | 34.2% | 1 | R.AEAESMYQIKYEELQSLAGK.H | 2 |
|  | CENPL\_stlcld\_tube2\_122314\_01.11492.11492.2 | 3.6968 | 0.0726 | 99.3% | 1138.5122 | 1138.2627 | 2 | 6.757 | 77.8% | 5 | K.YEELQSLAGK.H | 2 |
|  | CENPL\_stlcld\_122314\_02.09869.09869.2 | 2.734 | 0.1902 | 98.9% | 1001.5522 | 1001.168 | 278 | 4.754 | 68.8% | 4 | R.LQAEIEGLK.G | 2 |
|  | CENPL\_stlcld\_tube2\_122314\_01.09416.09416.2 | 3.3998 | 0.2642 | 99.9% | 1342.4521 | 1342.5381 | 1 | 6.334 | 68.2% | 4 | R.LQAEIEGLKGQR.A | 2 |
|  | CENPL\_stlcld\_122314\_01.15683.15683.3 | 4.3519 | 0.3317 | 100.0% | 2669.8743 | 2668.967 | 1 | 7.474 | 27.1% | 1 | R.LQAEIEGLKGQRASLEAAIADAEQR.G | 3 |
|  | CENPL\_stlcld\_122314\_01.14816.14816.2 | 4.2948 | 0.3991 | 100.0% | 1346.5521 | 1345.452 | 1 | 6.421 | 66.7% | 7 | R.ASLEAAIADAEQR.G | 2 |
|  | CENPL\_stlcld\_122314\_01.18677.18677.3 | 3.8204 | 0.3135 | 99.9% | 1958.4243 | 1957.1912 | 1 | 5.786 | 41.7% | 2 | R.ASLEAAIADAEQRGELAIK.D | 3 |
|  | CENPL\_stlcld\_122314\_01.18687.18687.2 | 4.6439 | 0.2805 | 100.0% | 1958.4521 | 1957.1912 | 1 | 6.218 | 63.9% | 2 | R.ASLEAAIADAEQRGELAIK.D | 2 |
|  | CENPL\_stlcld\_tube2\_122314\_01.18554.18554.3 | 5.6018 | 0.4621 | 100.0% | 2456.2744 | 2456.7153 | 1 | 7.584 | 32.6% | 5 | R.ASLEAAIADAEQRGELAIKDANAK.L | 3 |
|  | CENPL\_stlcld\_122314\_02.00114.00114.3 | 4.9644 | 0.3575 | 100.0% | 3567.3843 | 3567.9785 | 1 | 7.022 | 23.5% | 1 | R.ASLEAAIADAEQRGELAIKDANAKLSELEAALQR.A | 3 |
|  | CENPL\_stlcld\_122314\_01.13804.13804.1 | 2.2779 | 0.2419 | 97.0% | 1129.75 | 1130.2865 | 2 | 5.318 | 61.1% | 2 | K.LSELEAALQR.A | 1 |
|  | CENPL\_stlcld\_tube2\_122314\_01.13682.13682.2 | 4.2383 | 0.2402 | 100.0% | 1130.3922 | 1130.2865 | 1 | 6.399 | 83.3% | 7 | K.LSELEAALQR.A | 2 |
|  | CENPL\_stlcld\_122314\_01.12065.12065.1 | 2.418 | 0.2607 | 95.8% | 1153.63 | 1154.3234 | 450 | 5.874 | 50.0% | 1 | R.EYQELMNVK.L | 11 |
|  | CENPL\_stlcld\_122314\_01.12050.12050.2 | 2.7001 | 0.2224 | 99.2% | 1154.3121 | 1154.3234 | 9 | 6.13 | 68.8% | 3 | R.EYQELMNVK.L | 22 |
|  | CENPL\_stlcld\_122314\_02.13122.13122.2 | 3.3979 | 0.3814 | 100.0% | 1407.4122 | 1406.6653 | 2 | 6.236 | 68.2% | 2 | K.LALDIEIATYRK.L | 22222 |
|  | CENPL\_stlcld\_tube2\_122314\_01.12468.12468.3 | 5.3892 | 0.4464 | 100.0% | 2518.2544 | 2518.8628 | 1 | 7.439 | 40.5% | 2 | R.KLLEGEESRLESGMQNMSIHTK.T | 3 |
|  | CENPL\_stlcld\_tube2\_122314\_01.09164.09164.2 | 4.5351 | 0.4685 | 100.0% | 1476.3322 | 1476.7058 | 1 | 8.046 | 83.3% | 3 | R.LESGMQNMSIHTK.T | 2 |
|  | CENPL\_stlcld\_tube2\_122314\_02.09382.09382.3 | 3.2422 | 0.4298 | 100.0% | 1476.4143 | 1476.7058 | 4 | 6.376 | 45.8% | 4 | R.LESGMQNMSIHTK.T | 3 |
|  | CENPL\_stlcld\_122314\_01.10982.10982.2 | 3.3701 | 0.4946 | 100.0% | 1174.6921 | 1174.3367 | 1 | 7.746 | 80.0% | 12 | K.LVSESSDVLPK.- | 2 |

Similarities:
gi|47132620|ref|NP\_00(2:40)  
gi|67782365|ref|NP\_00(3:39)  
contaminant\_KERATIN19(1:41)  
gi|119703753|ref|NP\_0(4:38)  
gi|32567786|ref|NP\_78(2:40)  
gi|119395754|ref|NP\_0(2:40)  
contaminant\_KERATIN16(1:41)  
gi|153791158|ref|NP\_0(3:39)  
gi|109255249|ref|NP\_0(2:40)  
gi|15618995|ref|NP\_25(2:40)  

---

|  |  |  |  |  |  |  |  |  |
| --- | --- | --- | --- | --- | --- | --- | --- | --- |
| U | *gi|10800140|ref|NP\_06* | 10 | 26 | 48.4% | 126 | 13950 | 10.3 | histone cluster 1, H2bb [Homo sapiens] |
| U | *gi|4504277|ref|NP\_003* | 10 | 26 | 48.4% | 126 | 13920 | 10.3 | histone cluster 2, H2be [Homo sapiens] |
| U | *gi|20336754|ref|NP\_06* | 10 | 26 | 48.4% | 126 | 13904 | 10.3 | histone cluster 1, H2bj [Homo sapiens] |
| U | *gi|16306566|ref|NP\_00* | 10 | 26 | 48.4% | 126 | 13906 | 10.3 | histone cluster 1, H2bo [Homo sapiens] |

| Filename XCorr DeltCN Conf% ObsM+H+ CalcM+H+ SpR ZScore Ion% # Sequence  | | | | | | | | | | | | |
| --- | --- | --- | --- | --- | --- | --- | --- | --- | --- | --- | --- | --- |
|  | CENPL\_stlcld\_tube2\_122314\_01.10893.10893.2 | 2.9689 | 0.3626 | 99.9% | 1280.4321 | 1280.4631 | 4 | 5.99 | 66.7% | 2 | R.KESYSIYVYK.V | 2 |
|  | CENPL\_stlcld\_122314\_01.22058.22058.2 | 5.206 | 0.4897 | 100.0% | 1744.6522 | 1745.0211 | 1 | 9.43 | 78.6% | 6 | K.AMGIMNSFVNDIFER.I | 22 |
|  | CENPL\_stlcld\_122314\_02.00518.00518.3 | 3.8884 | 0.3461 | 100.0% | 2429.3943 | 2429.7712 | 7 | 6.545 | 31.0% | 2 | K.AMGIMNSFVNDIFERIAGEASR.L | 33 |
|  | CENPL\_stlcld\_122314\_01.08313.08313.2 | 3.1276 | 0.2408 | 99.5% | 1430.1322 | 1430.6066 | 2 | 5.4 | 70.8% | 1 | R.IAGEASRLAHYNK.R | 22 |
|  | CENPL\_stlcld\_122314\_01.08043.08043.2 | 2.6634 | 0.1536 | 96.1% | 1586.4922 | 1586.7941 | 4 | 3.489 | 57.7% | 1 | R.IAGEASRLAHYNKR.S | 22 |
|  | CENPL\_stlcld\_122314\_01.08066.08066.3 | 3.8171 | 0.3115 | 100.0% | 1586.9944 | 1586.7941 | 1 | 6.756 | 46.2% | 2 | R.IAGEASRLAHYNKR.S | 33 |
|  | CENPL\_stlcld\_122314\_01.09783.09783.2 | 3.7671 | 0.4336 | 100.0% | 1462.4722 | 1462.6462 | 1 | 6.99 | 66.7% | 4 | R.STITSREIQTAVR.L | 22 |
|  | CENPL\_stlcld\_tube2\_122314\_01.09272.09272.3 | 3.5752 | 0.4352 | 100.0% | 1463.4243 | 1462.6462 | 24 | 6.204 | 39.6% | 6 | R.STITSREIQTAVR.L | 33 |
|  | CENPL\_stlcld\_tube2\_122314\_01.19854.19854.3 | 2.9285 | 0.3178 | 99.9% | 2398.4944 | 2397.821 | 1 | 6.042 | 29.8% | 1 | R.STITSREIQTAVRLLLPGELAK.H | 33 |
|  | CENPL\_stlcld\_tube2\_122314\_01.14882.14882.2 | 2.2749 | 0.2131 | 97.7% | 954.3722 | 954.19794 | 6 | 4.41 | 87.5% | 1 | R.LLLPGELAK.H | 22 |

Similarities:
gi|10800138|ref|NP\_06(9:1)  

---

|  |  |  |  |  |  |  |  |  |
| --- | --- | --- | --- | --- | --- | --- | --- | --- |
| U | *gi|10800138|ref|NP\_06* | 12 | 28 | 48.4% | 126 | 13936 | 10.3 | histone cluster 1, H2bd [Homo sapiens] |
| U | *gi|66912162|ref|NP\_00* | 12 | 28 | 48.4% | 126 | 13920 | 10.3 | histone cluster 2, H2bf [Homo sapiens] |
| U | *gi|4504271|ref|NP\_003* | 12 | 28 | 48.4% | 126 | 13906 | 10.3 | histone cluster 1, H2bi [Homo sapiens] |
| U | *gi|4504269|ref|NP\_003* | 12 | 28 | 48.4% | 126 | 13892 | 10.3 | histone cluster 1, H2bh [Homo sapiens] |
| U | *gi|4504265|ref|NP\_003* | 12 | 28 | 48.4% | 126 | 13906 | 10.3 | histone cluster 1, H2bf [Homo sapiens] |
| U | *gi|4504263|ref|NP\_003* | 12 | 28 | 48.4% | 126 | 13989 | 10.3 | histone cluster 1, H2bm [Homo sapiens] |
| U | *gi|4504261|ref|NP\_003* | 12 | 28 | 48.4% | 126 | 13922 | 10.3 | histone cluster 1, H2bn [Homo sapiens] |
| U | *gi|4504257|ref|NP\_003* | 12 | 28 | 48.4% | 126 | 13906 | 10.3 | histone cluster 1, H2bg [Homo sapiens] |
| U | *gi|21396484|ref|NP\_00* | 12 | 28 | 48.4% | 126 | 13906 | 10.3 | histone cluster 1, H2be [Homo sapiens] |
| U | *gi|21166389|ref|NP\_00* | 12 | 28 | 48.4% | 126 | 13906 | 10.3 | histone cluster 1, H2bc [Homo sapiens] |
| U | *gi|20336752|ref|NP\_61* | 12 | 28 | 48.4% | 126 | 13936 | 10.3 | histone cluster 1, H2bd [Homo sapiens] |
| U | *gi|18105048|ref|NP\_54* | 12 | 28 | 48.4% | 126 | 13890 | 10.3 | histone cluster 1, H2bk [Homo sapiens] |

| Filename XCorr DeltCN Conf% ObsM+H+ CalcM+H+ SpR ZScore Ion% # Sequence  | | | | | | | | | | | | |
| --- | --- | --- | --- | --- | --- | --- | --- | --- | --- | --- | --- | --- |
|  | CENPL\_stlcld\_tube2\_122314\_01.09656.09656.2 | 3.0043 | 0.3552 | 99.9% | 1266.5521 | 1266.4363 | 1 | 6.211 | 77.8% | 2 | R.KESYSVYVYK.V | 2 |
|  | CENPL\_stlcld\_tube2\_122314\_01.11789.11789.1 | 1.9516 | 0.3182 | 95.3% | 1137.62 | 1138.2622 | 1 | 5.521 | 68.8% | 1 | K.ESYSVYVYK.V | 1 |
|  | CENPL\_stlcld\_tube2\_122314\_01.11756.11756.2 | 2.2693 | 0.4 | 99.9% | 1137.9722 | 1138.2622 | 1 | 6.436 | 68.8% | 1 | K.ESYSVYVYK.V | 2 |
|  | CENPL\_stlcld\_122314\_01.22058.22058.2 | 5.206 | 0.4897 | 100.0% | 1744.6522 | 1745.0211 | 1 | 9.43 | 78.6% | 6 | K.AMGIMNSFVNDIFER.I | 22 |
|  | CENPL\_stlcld\_122314\_02.00518.00518.3 | 3.8884 | 0.3461 | 100.0% | 2429.3943 | 2429.7712 | 7 | 6.545 | 31.0% | 2 | K.AMGIMNSFVNDIFERIAGEASR.L | 33 |
|  | CENPL\_stlcld\_122314\_01.08313.08313.2 | 3.1276 | 0.2408 | 99.5% | 1430.1322 | 1430.6066 | 2 | 5.4 | 70.8% | 1 | R.IAGEASRLAHYNK.R | 22 |
|  | CENPL\_stlcld\_122314\_01.08043.08043.2 | 2.6634 | 0.1536 | 96.1% | 1586.4922 | 1586.7941 | 4 | 3.489 | 57.7% | 1 | R.IAGEASRLAHYNKR.S | 22 |
|  | CENPL\_stlcld\_122314\_01.08066.08066.3 | 3.8171 | 0.3115 | 100.0% | 1586.9944 | 1586.7941 | 1 | 6.756 | 46.2% | 2 | R.IAGEASRLAHYNKR.S | 33 |
|  | CENPL\_stlcld\_122314\_01.09783.09783.2 | 3.7671 | 0.4336 | 100.0% | 1462.4722 | 1462.6462 | 1 | 6.99 | 66.7% | 4 | R.STITSREIQTAVR.L | 22 |
|  | CENPL\_stlcld\_tube2\_122314\_01.09272.09272.3 | 3.5752 | 0.4352 | 100.0% | 1463.4243 | 1462.6462 | 24 | 6.204 | 39.6% | 6 | R.STITSREIQTAVR.L | 33 |
|  | CENPL\_stlcld\_tube2\_122314\_01.19854.19854.3 | 2.9285 | 0.3178 | 99.9% | 2398.4944 | 2397.821 | 1 | 6.042 | 29.8% | 1 | R.STITSREIQTAVRLLLPGELAK.H | 33 |
|  | CENPL\_stlcld\_tube2\_122314\_01.14882.14882.2 | 2.2749 | 0.2131 | 97.7% | 954.3722 | 954.19794 | 6 | 4.41 | 87.5% | 1 | R.LLLPGELAK.H | 22 |

Similarities:
gi|10800140|ref|NP\_06(9:3)  

---

|  |  |  |  |  |  |  |  |  |
| --- | --- | --- | --- | --- | --- | --- | --- | --- |
| U | *gi|5174735|ref|NP\_006* | 25 | 105 | 47.9% | 445 | 49831 | 4.9 | tubulin, beta, 2 [Homo sapiens] |

| Filename XCorr DeltCN Conf% ObsM+H+ CalcM+H+ SpR ZScore Ion% # Sequence  | | | | | | | | | | | | |
| --- | --- | --- | --- | --- | --- | --- | --- | --- | --- | --- | --- | --- |
|  | CENPL\_stlcld\_122314\_02.13738.13738.3 | 5.4922 | 0.3582 | 100.0% | 3118.5244 | 3118.2996 | 1 | 6.464 | 31.7% | 1 | K.FWEVISDEHGIDPTGTYHGDSDLQLER.I | 3 |
| \* | CENPL\_stlcld\_122314\_01.11054.11054.2 | 3.4343 | 0.3581 | 100.0% | 1329.4321 | 1329.4521 | 1 | 6.874 | 81.8% | 6 | R.INVYYNEATGGK.Y | 2 |
|  | CENPL\_stlcld\_tube2\_122314\_02.13715.13715.2 | 3.735 | 0.4647 | 100.0% | 1602.6721 | 1602.8431 | 2 | 8.059 | 57.1% | 6 | R.AVLVDLEPGTMDSVR.S | 2 |
|  | CENPL\_stlcld\_122314\_01.20337.20337.2 | 5.5537 | 0.4908 | 100.0% | 2799.9722 | 2800.0647 | 1 | 7.549 | 40.0% | 3 | R.SGPFGQIFRPDNFVFGQSGAGNNWAK.G | 22 |
|  | CENPL\_stlcld\_122314\_01.20366.20366.3 | 6.7253 | 0.4336 | 100.0% | 2800.8542 | 2800.0647 | 1 | 7.18 | 34.0% | 4 | R.SGPFGQIFRPDNFVFGQSGAGNNWAK.G | 33 |
|  | CENPL\_stlcld\_122314\_01.20825.20825.2 | 5.647 | 0.559 | 100.0% | 1959.0322 | 1960.151 | 1 | 11.798 | 79.4% | 2 | K.GHYTEGAELVDSVLDVVR.K | 222 |
|  | CENPL\_stlcld\_122314\_01.20822.20822.3 | 2.9108 | 0.2585 | 98.5% | 1959.7743 | 1960.151 | 7 | 4.629 | 32.4% | 1 | K.GHYTEGAELVDSVLDVVR.K | 333 |
|  | CENPL\_stlcld\_tube2\_122314\_01.19052.19052.3 | 5.1569 | 0.4766 | 100.0% | 2087.9644 | 2088.325 | 1 | 8.639 | 44.4% | 6 | K.GHYTEGAELVDSVLDVVRK.E | 333 |
|  | CENPL\_stlcld\_tube2\_122314\_01.19009.19009.2 | 6.3337 | 0.4925 | 100.0% | 2088.612 | 2088.325 | 1 | 9.687 | 66.7% | 3 | K.GHYTEGAELVDSVLDVVRK.E | 222 |
|  | CENPL\_stlcld\_tube2\_122314\_01.14282.14282.2 | 4.3911 | 0.4008 | 100.0% | 1320.4922 | 1320.5896 | 1 | 7.658 | 81.8% | 10 | R.IMNTFSVVPSPK.V | 222 |
|  | CENPL\_stlcld\_122314\_01.13025.13025.2 | 3.0807 | 0.3262 | 99.9% | 1130.8322 | 1131.2767 | 1 | 5.245 | 83.3% | 8 | R.FPGQLNADLR.K | 2222 |
|  | CENPL\_stlcld\_122314\_01.11442.11442.3 | 2.9929 | 0.3054 | 99.9% | 1259.5443 | 1259.4508 | 9 | 5.658 | 42.5% | 1 | R.FPGQLNADLRK.L | 3333 |
|  | CENPL\_stlcld\_tube2\_122314\_01.10454.10454.2 | 2.6368 | 0.2303 | 98.6% | 1259.7322 | 1259.4508 | 17 | 4.582 | 60.0% | 5 | R.FPGQLNADLRK.L | 2222 |
|  | CENPL\_stlcld\_tube2\_122314\_01.14181.14181.2 | 3.8965 | 0.404 | 100.0% | 1271.9922 | 1272.5945 | 1 | 7.764 | 75.0% | 4 | R.KLAVNMVPFPR.L | 2222 |
|  | CENPL\_stlcld\_122314\_01.16748.16748.2 | 3.646 | 0.4855 | 100.0% | 1143.9922 | 1144.4204 | 1 | 8.724 | 94.4% | 8 | K.LAVNMVPFPR.L | 2222 |
|  | CENPL\_stlcld\_122314\_01.20176.20176.3 | 4.2322 | 0.3658 | 100.0% | 1622.3644 | 1621.9403 | 1 | 6.191 | 55.8% | 3 | R.LHFFMPGFAPLTSR.G | 333 |
|  | CENPL\_stlcld\_122314\_01.20175.20175.2 | 3.8813 | 0.426 | 100.0% | 1622.5721 | 1621.9403 | 1 | 8.381 | 73.1% | 3 | R.LHFFMPGFAPLTSR.G | 222 |
|  | CENPL\_stlcld\_122314\_01.20271.20271.2 | 3.407 | 0.4681 | 100.0% | 1692.8322 | 1692.9678 | 1 | 7.635 | 75.0% | 2 | R.ALTVPELTQQMFDAK.N | 22 |
|  | CENPL\_stlcld\_tube2\_122314\_01.12296.12296.3 | 2.2853 | 0.2706 | 96.2% | 1391.1543 | 1390.631 | 4 | 5.015 | 45.5% | 1 | R.HGRYLTVAAVFR.G | 33 |
|  | CENPL\_stlcld\_122314\_01.17753.17753.1 | 1.5504 | 0.3447 | 96.0% | 1039.71 | 1040.2505 | 1 | 5.617 | 68.8% | 2 | R.YLTVAAVFR.G | 11 |
|  | CENPL\_stlcld\_122314\_02.14903.14903.2 | 2.8302 | 0.4423 | 100.0% | 1040.4122 | 1040.2505 | 1 | 7.611 | 93.8% | 5 | R.YLTVAAVFR.G | 22 |
|  | CENPL\_stlcld\_tube2\_122314\_02.13035.13035.3 | 3.8475 | 0.2619 | 99.9% | 1925.4844 | 1925.2405 | 1 | 5.282 | 43.3% | 2 | R.MSMKEVDEQMLNVQNK.N | 33 |
|  | CENPL\_stlcld\_tube2\_122314\_01.10952.10952.2 | 4.3318 | 0.2038 | 99.9% | 1448.3322 | 1447.6031 | 1 | 5.927 | 72.7% | 6 | K.EVDEQMLNVQNK.N | 22 |
|  | CENPL\_stlcld\_122314\_01.19247.19247.2 | 3.5195 | 0.346 | 99.9% | 1698.4321 | 1697.8877 | 3 | 5.888 | 53.8% | 4 | K.NSSYFVEWIPNNVK.T | 2222 |
|  | CENPL\_stlcld\_122314\_02.14308.14308.2 | 3.8472 | 0.492 | 100.0% | 1230.5122 | 1230.4241 | 1 | 7.151 | 94.4% | 9 | R.ISEQFTAMFR.R | 222 |

Similarities:
gi|29788785|ref|NP\_82(21:4)  
gi|50592996|ref|NP\_00(13:12)  
gi|14210536|ref|NP\_11(8:17)  

---

|  |  |  |  |  |  |  |  |  |
| --- | --- | --- | --- | --- | --- | --- | --- | --- |
| U | *gi|7669492|ref|NP\_002* | 15 | 62 | 46.3% | 335 | 36053 | 8.5 | glyceraldehyde-3-phosphate dehydrogenase [Homo sapiens] |

| Filename XCorr DeltCN Conf% ObsM+H+ CalcM+H+ SpR ZScore Ion% # Sequence  | | | | | | | | | | | | |
| --- | --- | --- | --- | --- | --- | --- | --- | --- | --- | --- | --- | --- |
| \* | CENPL\_stlcld\_122314\_01.09591.09591.2 | 2.1658 | 0.2708 | 98.5% | 805.8722 | 805.912 | 70 | 6.693 | 64.3% | 3 | K.VGVNGFGR.I | 2 |
| \* | CENPL\_stlcld\_tube2\_122314\_01.19157.19157.2 | 3.7717 | 0.4407 | 100.0% | 1614.6122 | 1614.8851 | 1 | 8.102 | 57.7% | 3 | K.LVINGNPITIFQER.D | 2 |
| \* | CENPL\_stlcld\_tube2\_122314\_01.17453.17453.2 | 3.196 | 0.3565 | 99.9% | 2041.4321 | 2042.3427 | 1 | 6.496 | 44.1% | 1 | K.LVINGNPITIFQERDPSK.I | 2 |
| \* | CENPL\_stlcld\_122314\_01.20592.20592.2 | 5.4097 | 0.5992 | 100.0% | 2277.9521 | 2278.495 | 1 | 11.487 | 60.0% | 6 | K.WGDAGAEYVVESTGVFTTMEK.A | 2 |
| \* | CENPL\_stlcld\_122314\_02.16320.16320.3 | 3.4759 | 0.3891 | 100.0% | 2278.1343 | 2278.495 | 4 | 6.555 | 33.8% | 1 | K.WGDAGAEYVVESTGVFTTMEK.A | 3 |
| \* | CENPL\_stlcld\_122314\_01.14848.14848.3 | 5.6019 | 0.3762 | 100.0% | 2371.5842 | 2370.79 | 1 | 7.538 | 46.4% | 4 | K.RVIISAPSADAPMFVMGVNHEK.Y | 3 |
| \* | CENPL\_stlcld\_122314\_01.16749.16749.2 | 4.1121 | 0.4996 | 100.0% | 2214.412 | 2214.6025 | 1 | 8.36 | 50.0% | 2 | R.VIISAPSADAPMFVMGVNHEK.Y | 2 |
| \* | CENPL\_stlcld\_tube2\_122314\_01.21029.21029.2 | 5.5256 | 0.5757 | 100.0% | 2596.152 | 2597.0044 | 1 | 9.862 | 50.0% | 1 | K.VIHDNFGIVEGLMTTVHAITATQK.T | 2 |
| \* | CENPL\_stlcld\_122314\_01.21795.21795.3 | 6.6623 | 0.5639 | 100.0% | 2596.8843 | 2597.0044 | 1 | 9.418 | 33.7% | 5 | K.VIHDNFGIVEGLMTTVHAITATQK.T | 3 |
| \* | CENPL\_stlcld\_122314\_01.13742.13742.2 | 4.2332 | 0.3517 | 100.0% | 1413.7322 | 1412.6292 | 1 | 6.228 | 71.4% | 9 | R.GALQNIIPASTGAAK.A | 2 |
|  | CENPL\_stlcld\_tube2\_122314\_01.11391.11391.2 | 1.9936 | 0.3592 | 99.2% | 796.15216 | 795.97504 | 3 | 5.463 | 75.0% | 4 | K.LTGMAFR.V | 2 |
| \* | CENPL\_stlcld\_tube2\_122314\_01.13798.13798.2 | 3.2914 | 0.4994 | 100.0% | 1531.8722 | 1531.7155 | 3 | 8.344 | 57.7% | 2 | R.VPTANVSVVDLTCR.L | 2 |
|  | CENPL\_stlcld\_tube2\_122314\_01.17895.17895.2 | 4.5914 | 0.5448 | 100.0% | 1763.9321 | 1764.8914 | 1 | 9.27 | 65.4% | 13 | K.LISWYDNEFGYSNR.V | 2 |
| \* | CENPL\_stlcld\_122314\_01.12072.12072.2 | 3.483 | 0.447 | 100.0% | 1202.2722 | 1202.4724 | 1 | 7.843 | 85.0% | 1 | R.VVDLMAHMASK.E | 2 |
| \* | CENPL\_stlcld\_122314\_01.12143.12143.2 | 3.7504 | 0.4082 | 100.0% | 1331.4321 | 1331.5879 | 1 | 7.408 | 77.3% | 7 | R.VVDLMAHMASKE.- | 2 |

---

|  |  |  |  |  |  |  |  |  |
| --- | --- | --- | --- | --- | --- | --- | --- | --- |
| U | *gi|57013276|ref|NP\_00* | 21 | 83 | 45.9% | 451 | 50152 | 5.1 | tubulin, alpha, ubiquitous [Homo sapiens] |

| Filename XCorr DeltCN Conf% ObsM+H+ CalcM+H+ SpR ZScore Ion% # Sequence  | | | | | | | | | | | | |
| --- | --- | --- | --- | --- | --- | --- | --- | --- | --- | --- | --- | --- |
|  | CENPL\_stlcld\_122314\_01.18522.18522.2 | 5.5595 | 0.6546 | 100.0% | 2008.5922 | 2009.093 | 1 | 11.034 | 55.3% | 8 | K.TIGGGDDSFNTFFSETGAGK.H | 2 |
|  | CENPL\_stlcld\_122314\_01.19886.19886.2 | 4.4254 | 0.4643 | 100.0% | 1702.2722 | 1702.9451 | 1 | 9.192 | 78.6% | 5 | R.AVFVDLEPTVIDEVR.T | 2 |
|  | CENPL\_stlcld\_122314\_01.20664.20664.2 | 2.8601 | 0.2828 | 99.2% | 1841.6322 | 1843.1332 | 1 | 6.084 | 50.0% | 1 | R.GHYTIGKEIIDLVLDR.I | 2 |
|  | CENPL\_stlcld\_tube2\_122314\_01.19756.19756.3 | 4.5293 | 0.318 | 100.0% | 1843.1344 | 1843.1332 | 3 | 7.075 | 41.7% | 1 | R.GHYTIGKEIIDLVLDR.I | 3 |
|  | CENPL\_stlcld\_122314\_01.10546.10546.3 | 3.3396 | 0.203 | 98.8% | 1876.7943 | 1876.0824 | 1 | 5.292 | 39.3% | 2 | R.RNLDIERPTYTNLNR.L | 3 |
|  | CENPL\_stlcld\_tube2\_122314\_01.11552.11552.3 | 2.8493 | 0.3367 | 99.9% | 1721.3344 | 1719.8949 | 35 | 5.436 | 40.4% | 3 | R.NLDIERPTYTNLNR.L | 3 |
|  | CENPL\_stlcld\_tube2\_122314\_01.21188.21188.2 | 4.585 | 0.5339 | 100.0% | 1488.5322 | 1488.7678 | 1 | 9.488 | 73.1% | 9 | R.LISQIVSSITASLR.F | 2 |
|  | CENPL\_stlcld\_122314\_02.16884.16884.2 | 5.2781 | 0.5702 | 100.0% | 2410.8123 | 2410.6885 | 1 | 9.508 | 52.5% | 3 | R.FDGALNVDLTEFQTNLVPYPR.I | 2 |
|  | CENPL\_stlcld\_122314\_01.17512.17512.2 | 4.1634 | 0.5041 | 100.0% | 1758.4722 | 1758.0703 | 1 | 9.224 | 70.0% | 12 | R.IHFPLATYAPVISAEK.A | 2 |
|  | CENPL\_stlcld\_122314\_01.17438.17438.3 | 3.8493 | 0.3983 | 100.0% | 1758.5643 | 1758.0703 | 1 | 6.51 | 46.7% | 6 | R.IHFPLATYAPVISAEK.A | 3 |
|  | CENPL\_stlcld\_tube2\_122314\_01.14284.14284.2 | 2.5958 | 0.36 | 99.9% | 1250.6122 | 1250.4304 | 2 | 5.693 | 68.8% | 1 | K.YMACCLLYR.G | 2 |
|  | CENPL\_stlcld\_tube2\_122314\_01.12174.12174.1 | 1.6628 | 0.3496 | 95.5% | 1015.56 | 1016.1827 | 3 | 6.023 | 66.7% | 3 | K.DVNAAIATIK.T | 1 |
|  | CENPL\_stlcld\_122314\_01.12338.12338.2 | 3.3775 | 0.412 | 100.0% | 1017.4522 | 1016.1827 | 1 | 6.347 | 83.3% | 4 | K.DVNAAIATIK.T | 2 |
|  | CENPL\_stlcld\_122314\_01.19634.19634.2 | 2.8515 | 0.3566 | 99.9% | 1587.5922 | 1585.7656 | 1 | 5.974 | 58.3% | 2 | R.SIQFVDWCPTGFK.V | 2 |
|  | CENPL\_stlcld\_122314\_01.14600.14600.2 | 4.292 | 0.436 | 100.0% | 1825.6522 | 1826.1027 | 1 | 8.234 | 55.9% | 8 | K.VGINYQPPTVVPGGDLAK.V | 2 |
|  | CENPL\_stlcld\_tube2\_122314\_01.11698.11698.2 | 2.6983 | 0.3117 | 99.7% | 1382.3322 | 1381.6324 | 7 | 5.509 | 60.0% | 2 | R.LDHKFDLMYAK.R | 2 |
|  | CENPL\_stlcld\_tube2\_122314\_01.11625.11625.3 | 4.2112 | 0.3754 | 100.0% | 1382.9043 | 1381.6324 | 1 | 6.418 | 62.5% | 5 | R.LDHKFDLMYAK.R | 3 |
|  | CENPL\_stlcld\_122314\_01.14206.14206.2 | 2.1423 | 0.1839 | 97.2% | 888.1722 | 888.0692 | 2 | 4.788 | 83.3% | 1 | K.FDLMYAK.R | 2 |
|  | CENPL\_stlcld\_122314\_02.12904.12904.3 | 4.3076 | 0.4028 | 100.0% | 2487.2944 | 2487.7083 | 1 | 6.693 | 40.0% | 1 | K.RAFVHWYVGEGMEEGEFSEAR.E | 3 |
|  | CENPL\_stlcld\_122314\_02.14294.14294.3 | 4.2457 | 0.3191 | 100.0% | 2332.8245 | 2331.5208 | 1 | 5.351 | 40.8% | 5 | R.AFVHWYVGEGMEEGEFSEAR.E | 3 |
|  | CENPL\_stlcld\_122314\_01.19128.19128.3 | 4.4033 | 0.3336 | 100.0% | 3219.5344 | 3219.524 | 1 | 6.563 | 30.6% | 1 | R.AFVHWYVGEGMEEGEFSEAREDMAALEK.D | 3 |

---

|  |  |  |  |  |  |  |  |  |
| --- | --- | --- | --- | --- | --- | --- | --- | --- |
| U | *gi|10440560|ref|NP\_06* | 5 | 12 | 45.6% | 136 | 15404 | 11.1 | histone cluster 1, H3f [Homo sapiens] |
| U | *gi|4504297|ref|NP\_003* | 5 | 12 | 45.6% | 136 | 15404 | 11.1 | histone cluster 1, H3b [Homo sapiens] |
| U | *gi|4504295|ref|NP\_003* | 5 | 11 | 45.6% | 136 | 15404 | 11.1 | histone cluster 1, H3h [Homo sapiens] |
| U | *gi|4504293|ref|NP\_003* | 5 | 12 | 45.6% | 136 | 15404 | 11.1 | histone cluster 1, H3j [Homo sapiens] |
| U | *gi|4504291|ref|NP\_003* | 5 | 11 | 45.6% | 136 | 15404 | 11.1 | H3 histone family, member H [Homo sapiens] |
| U | *gi|4504289|ref|NP\_003* | 5 | 12 | 45.6% | 136 | 15404 | 11.1 | histone cluster 1, H3i [Homo sapiens] |
| U | *gi|4504287|ref|NP\_003* | 5 | 12 | 45.6% | 136 | 15404 | 11.1 | histone cluster 1, H3e [Homo sapiens] |
| U | *gi|4504285|ref|NP\_003* | 5 | 12 | 45.6% | 136 | 15404 | 11.1 | histone cluster 1, H3c [Homo sapiens] |
| U | *gi|4504281|ref|NP\_003* | 5 | 12 | 45.6% | 136 | 15404 | 11.1 | histone cluster 1, H3a [Homo sapiens] |
| U | *gi|21071021|ref|NP\_00* | 5 | 11 | 45.6% | 136 | 15404 | 11.1 | histone cluster 1, H3d [Homo sapiens] |

| Filename XCorr DeltCN Conf% ObsM+H+ CalcM+H+ SpR ZScore Ion% # Sequence  | | | | | | | | | | | | |
| --- | --- | --- | --- | --- | --- | --- | --- | --- | --- | --- | --- | --- |
|  | CENPL\_stlcld\_tube2\_122314\_01.08144.08144.2 | 2.3132 | 0.217 | 98.0% | 1034.5521 | 1033.2186 | 68 | 4.191 | 56.2% | 2 | R.YRPGTVALR.E | 22 |
|  | CENPL\_stlcld\_tube2\_122314\_01.09884.09884.3 | 2.879 | 0.2216 | 98.7% | 1251.9543 | 1251.4685 | 9 | 4.886 | 47.2% | 1 | R.YQKSTELLIR.K | 33 |
|  | CENPL\_stlcld\_122314\_01.11798.11798.2 | 2.3428 | 0.1646 | 98.1% | 832.1922 | 831.9878 | 1 | 4.539 | 83.3% | 5 | K.STELLIR.K | 22 |
|  | CENPL\_stlcld\_tube2\_122314\_01.11175.11175.2 | 3.0683 | 0.408 | 100.0% | 1336.4321 | 1336.4875 | 4 | 6.435 | 65.0% | 3 | R.EIAQDFKTDLR.F | 22 |
|  | CENPL\_stlcld\_tube2\_122314\_02.00546.00546.3 | 5.2786 | 0.4499 | 100.0% | 3588.5344 | 3588.9866 | 1 | 7.833 | 28.2% | 1 | R.FQSSAVMALQEACEAYLVGLFEDTNLCAIHAK.R | 3 |

Similarities:
gi|4504279|ref|NP\_002(4:1)  

---

|  |  |  |  |  |  |  |  |  |
| --- | --- | --- | --- | --- | --- | --- | --- | --- |
| U | *gi|4504279|ref|NP\_002* | 5 | 12 | 45.6% | 136 | 15328 | 11.3 | H3 histone, family 3A [Homo sapiens] |
| U | *gi|4885385|ref|NP\_005* | 5 | 12 | 45.6% | 136 | 15328 | 11.3 | H3 histone, family 3B [Homo sapiens] |

| Filename XCorr DeltCN Conf% ObsM+H+ CalcM+H+ SpR ZScore Ion% # Sequence  | | | | | | | | | | | | |
| --- | --- | --- | --- | --- | --- | --- | --- | --- | --- | --- | --- | --- |
|  | CENPL\_stlcld\_tube2\_122314\_01.08144.08144.2 | 2.3132 | 0.217 | 98.0% | 1034.5521 | 1033.2186 | 68 | 4.191 | 56.2% | 2 | R.YRPGTVALR.E | 22 |
|  | CENPL\_stlcld\_tube2\_122314\_01.09884.09884.3 | 2.879 | 0.2216 | 98.7% | 1251.9543 | 1251.4685 | 9 | 4.886 | 47.2% | 1 | R.YQKSTELLIR.K | 33 |
|  | CENPL\_stlcld\_122314\_01.11798.11798.2 | 2.3428 | 0.1646 | 98.1% | 832.1922 | 831.9878 | 1 | 4.539 | 83.3% | 5 | K.STELLIR.K | 22 |
|  | CENPL\_stlcld\_tube2\_122314\_01.11175.11175.2 | 3.0683 | 0.408 | 100.0% | 1336.4321 | 1336.4875 | 4 | 6.435 | 65.0% | 3 | R.EIAQDFKTDLR.F | 22 |
|  | CENPL\_stlcld\_tube2\_122314\_02.01005.01005.3 | 4.1717 | 0.396 | 100.0% | 3440.0645 | 3439.8127 | 1 | 5.765 | 23.4% | 1 | R.FQSAAIGALQEASEAYLVGLFEDTNLCAIHAK.R | 3 |

Similarities:
gi|10440560|ref|NP\_06(4:1)  

---

|  |  |  |  |  |  |  |  |  |
| --- | --- | --- | --- | --- | --- | --- | --- | --- |
| U | *gi|5902102|ref|NP\_008* | 3 | 5 | 45.4% | 119 | 13282 | 11.6 | small nuclear ribonucleoprotein D1 polypeptide 16kDa [Homo sapiens] |

| Filename XCorr DeltCN Conf% ObsM+H+ CalcM+H+ SpR ZScore Ion% # Sequence  | | | | | | | | | | | | |
| --- | --- | --- | --- | --- | --- | --- | --- | --- | --- | --- | --- | --- |
| \* | CENPL\_stlcld\_tube2\_122314\_02.11640.11640.3 | 3.8529 | 0.3392 | 100.0% | 2211.1143 | 2210.47 | 3 | 5.211 | 33.8% | 1 | K.NGTQVHGTITGVDVSMNTHLK.A | 3 |
|  | CENPL\_stlcld\_122314\_01.13006.13006.2 | 3.9897 | 0.4453 | 100.0% | 1555.7722 | 1555.7745 | 1 | 7.119 | 62.5% | 2 | K.NREPVQLETLSIR.G | 2 |
| \* | CENPL\_stlcld\_tube2\_122314\_02.01521.01521.2 | 4.3686 | 0.42 | 100.0% | 2289.2322 | 2288.6863 | 1 | 6.878 | 65.8% | 2 | R.YFILPDSLPLDTLLVDVEPK.V | 2 |

---

|  |  |  |  |  |  |  |  |  |
| --- | --- | --- | --- | --- | --- | --- | --- | --- |
| U | *gi|4506749|ref|NP\_001* | 30 | 132 | 44.1% | 792 | 90070 | 7.1 | ribonucleoside-diphosphate reductase M1 chain [Homo sapiens] |

| Filename XCorr DeltCN Conf% ObsM+H+ CalcM+H+ SpR ZScore Ion% # Sequence  | | | | | | | | | | | | |
| --- | --- | --- | --- | --- | --- | --- | --- | --- | --- | --- | --- | --- |
| \* | CENPL\_stlcld\_122314\_01.23037.23037.2 | 5.7775 | 0.5678 | 100.0% | 2868.5723 | 2868.2512 | 1 | 10.563 | 38.9% | 2 | K.VIQGLYSGVTTVELDTLAAETAATLTTK.H | 2 |
| \* | CENPL\_stlcld\_122314\_01.22114.22114.3 | 5.8481 | 0.4877 | 100.0% | 3975.9243 | 3976.5164 | 1 | 7.527 | 23.0% | 2 | K.VIQGLYSGVTTVELDTLAAETAATLTTKHPDYAILAAR.I | 3 |
| \* | CENPL\_stlcld\_tube2\_122314\_01.11045.11045.2 | 2.4157 | 0.3242 | 99.5% | 1127.2522 | 1127.2883 | 7 | 7.028 | 66.7% | 4 | K.HPDYAILAAR.I | 2 |
| \* | CENPL\_stlcld\_122314\_01.20864.20864.3 | 4.5024 | 0.5124 | 100.0% | 2383.7644 | 2384.6685 | 1 | 7.652 | 38.2% | 2 | K.KVFSDVMEDLYNYINPHNGK.H | 3 |
| \* | CENPL\_stlcld\_122314\_01.21575.21575.3 | 3.1415 | 0.3563 | 99.9% | 2254.9443 | 2256.4944 | 4 | 5.278 | 33.3% | 1 | K.VFSDVMEDLYNYINPHNGK.H | 3 |
| \* | CENPL\_stlcld\_122314\_01.12333.12333.2 | 3.0816 | 0.3068 | 99.9% | 1345.5521 | 1345.5388 | 1 | 5.94 | 77.3% | 7 | K.STLDIVLANKDR.L | 2 |
| \* | CENPL\_stlcld\_122314\_02.09815.09815.2 | 2.8354 | 0.2302 | 99.5% | 1065.3322 | 1065.2145 | 1 | 4.824 | 87.5% | 4 | R.LNSAIIYDR.D | 2 |
| \* | CENPL\_stlcld\_tube2\_122314\_02.16766.16766.3 | 5.289 | 0.421 | 100.0% | 2335.6743 | 2334.5928 | 1 | 7.08 | 43.1% | 5 | R.LNSAIIYDRDFSYNYFGFK.T | 3 |
| \* | CENPL\_stlcld\_122314\_01.08392.08392.2 | 2.1755 | 0.2017 | 95.1% | 1368.3322 | 1368.6581 | 55 | 4.658 | 50.0% | 1 | K.VAERPQHMLMR.V | 2 |
| \* | CENPL\_stlcld\_122314\_01.08444.08444.3 | 3.8077 | 0.4314 | 100.0% | 1369.4644 | 1368.6581 | 1 | 6.906 | 55.0% | 1 | K.VAERPQHMLMR.V | 3 |
| \* | CENPL\_stlcld\_122314\_01.18957.18957.2 | 4.4763 | 0.5075 | 100.0% | 2572.9321 | 2573.8625 | 1 | 9.088 | 38.6% | 1 | R.VSVGIHKEDIDAAIETYNLLSER.W | 2 |
| \* | CENPL\_stlcld\_122314\_02.15137.15137.3 | 3.8475 | 0.3665 | 100.0% | 2574.8643 | 2573.8625 | 1 | 6.184 | 29.5% | 3 | R.VSVGIHKEDIDAAIETYNLLSER.W | 3 |
| \* | CENPL\_stlcld\_tube2\_122314\_01.12305.12305.2 | 2.1358 | 0.3504 | 98.5% | 1247.6921 | 1247.4033 | 1 | 5.737 | 62.5% | 1 | K.SAGGIGVAVSCIR.A | 2 |
| \* | CENPL\_stlcld\_122314\_01.16294.16294.2 | 4.1437 | 0.4681 | 100.0% | 2093.632 | 2094.35 | 1 | 7.475 | 47.5% | 5 | R.ATGSYIAGTNGNSNGLVPMLR.V | 2 |
| \* | CENPL\_stlcld\_tube2\_122314\_02.01545.01545.2 | 4.4247 | 0.3655 | 100.0% | 1983.6322 | 1984.4094 | 1 | 8.216 | 73.3% | 2 | R.ARDLFFALWIPDLFMK.R | 2 |
| \* | CENPL\_stlcld\_122314\_02.02073.02073.1 | 3.6569 | 0.3802 | 100.0% | 1757.15 | 1757.1432 | 1 | 7.172 | 61.5% | 2 | R.DLFFALWIPDLFMK.R | 1 |
| \* | CENPL\_stlcld\_122314\_02.16637.16637.3 | 5.4185 | 0.4905 | 100.0% | 2066.0344 | 2065.4875 | 1 | 9.256 | 41.7% | 7 | R.HRPIGIGVQGLADAFILMR.Y | 3 |
| \* | CENPL\_stlcld\_122314\_01.16450.16450.2 | 4.1997 | 0.3306 | 100.0% | 1510.3922 | 1510.6866 | 1 | 6.997 | 91.7% | 20 | R.YPFESAEAQLLNK.Q | 2 |
| \* | CENPL\_stlcld\_tube2\_122314\_01.09861.09861.2 | 4.1032 | 0.4225 | 100.0% | 1671.3522 | 1671.7576 | 1 | 7.497 | 78.6% | 5 | K.EQGPYETYEGSPVSK.G | 2 |
| \* | CENPL\_stlcld\_tube2\_122314\_02.00440.00440.2 | 5.8789 | 0.5522 | 100.0% | 2382.7722 | 2382.6953 | 1 | 10.089 | 61.1% | 6 | K.GILQYDMWNVTPTDLWDWK.V | 2 |
| \* | CENPL\_stlcld\_122314\_01.20649.20649.2 | 2.4798 | 0.44 | 99.9% | 3580.912 | 3582.0198 | 1 | 8.519 | 21.9% | 1 | R.NSLLIAPMPTASTAQILGNNESIEPYTSNIYTR.R | 2 |
| \* | CENPL\_stlcld\_tube2\_122314\_01.14355.14355.3 | 5.8516 | 0.3831 | 100.0% | 1851.5643 | 1851.2021 | 1 | 6.905 | 56.7% | 9 | R.RVLSGEFQIVNPHLLK.D | 3 |
| \* | CENPL\_stlcld\_tube2\_122314\_01.16184.16184.3 | 3.6021 | 0.5094 | 100.0% | 1694.3644 | 1695.0146 | 3 | 7.17 | 46.4% | 6 | R.VLSGEFQIVNPHLLK.D | 3 |
| \* | CENPL\_stlcld\_tube2\_122314\_01.16226.16226.2 | 4.8139 | 0.3902 | 100.0% | 1695.6721 | 1695.0146 | 1 | 7.165 | 71.4% | 6 | R.VLSGEFQIVNPHLLK.D | 2 |
| \* | CENPL\_stlcld\_122314\_01.09983.09983.2 | 2.7765 | 0.3128 | 99.9% | 1030.4722 | 1030.1864 | 1 | 6.78 | 85.7% | 4 | R.GLWHEEMK.N | 2 |
| \* | CENPL\_stlcld\_tube2\_122314\_01.11852.11852.2 | 1.9787 | 0.2657 | 97.5% | 990.8122 | 991.13196 | 1 | 5.159 | 85.7% | 1 | K.TVWEISQK.T | 2 |
| \* | CENPL\_stlcld\_122314\_01.14955.14955.3 | 4.3335 | 0.3758 | 100.0% | 2303.5144 | 2303.5364 | 1 | 6.538 | 35.0% | 10 | R.GAFIDQSQSLNIHIAEPNYGK.L | 3 |
| \* | CENPL\_stlcld\_tube2\_122314\_01.14030.14030.2 | 2.9852 | 0.4089 | 100.0% | 1270.2122 | 1270.4913 | 1 | 6.692 | 83.3% | 4 | K.LTSMHFYGWK.Q | 2 |
| \* | CENPL\_stlcld\_122314\_01.12593.12593.2 | 4.6417 | 0.418 | 100.0% | 1572.2322 | 1571.8197 | 1 | 7.642 | 65.4% | 8 | R.TRPAANPIQFTLNK.E | 2 |
| \* | CENPL\_stlcld\_122314\_01.12646.12646.3 | 3.7276 | 0.3611 | 100.0% | 1573.6743 | 1571.8197 | 2 | 5.366 | 46.2% | 2 | R.TRPAANPIQFTLNK.E | 3 |

---

|  |  |  |  |  |  |  |  |  |
| --- | --- | --- | --- | --- | --- | --- | --- | --- |
| U | *gi|4505409|ref|NP\_002* | 6 | 17 | 44.1% | 152 | 17298 | 8.4 | non-metastatic cells 2, protein (NM23B) expressed in [Homo sapiens] |
| U | *gi|66392227|ref|NP\_00* | 6 | 17 | 44.1% | 152 | 17298 | 8.4 | non-metastatic cells 2, protein (NM23B) expressed in [Homo sapiens] |
| U | *gi|66392205|ref|NP\_00* | 6 | 17 | 44.1% | 152 | 17298 | 8.4 | non-metastatic cells 2, protein (NM23B) expressed in [Homo sapiens] |
| U | *gi|66392203|ref|NP\_00* | 6 | 17 | 25.1% | 267 | 30137 | 8.9 | NME1-NME2 protein [Homo sapiens] |
| U | *gi|66392192|ref|NP\_00* | 6 | 17 | 44.1% | 152 | 17298 | 8.4 | non-metastatic cells 2, protein (NM23B) expressed in [Homo sapiens] |

| Filename XCorr DeltCN Conf% ObsM+H+ CalcM+H+ SpR ZScore Ion% # Sequence  | | | | | | | | | | | | |
| --- | --- | --- | --- | --- | --- | --- | --- | --- | --- | --- | --- | --- |
|  | CENPL\_stlcld\_tube2\_122314\_01.11264.11264.2 | 2.9804 | 0.315 | 99.9% | 1345.5521 | 1345.5846 | 2 | 6.332 | 63.6% | 3 | R.TFIAIKPDGVQR.G | 22 |
|  | CENPL\_stlcld\_122314\_01.12003.12003.2 | 2.3734 | 0.2936 | 99.2% | 985.0522 | 985.21497 | 4 | 6.27 | 75.0% | 2 | R.GLVGEIIKR.F | 22 |
|  | CENPL\_stlcld\_122314\_01.15166.15166.2 | 2.3715 | 0.1792 | 97.0% | 1177.8522 | 1176.4038 | 10 | 4.625 | 61.1% | 3 | K.DRPFFPGLVK.Y | 2 |
|  | CENPL\_stlcld\_tube2\_122314\_02.00260.00260.2 | 4.9966 | 0.413 | 100.0% | 2094.9321 | 2094.4944 | 1 | 7.484 | 52.8% | 4 | K.YMNSGPVVAMVWEGLNVVK.T | 2 |
|  | CENPL\_stlcld\_tube2\_122314\_01.09785.09785.2 | 4.5302 | 0.4022 | 100.0% | 1787.5122 | 1787.041 | 1 | 7.109 | 71.9% | 3 | R.VMLGETNPADSKPGTIR.G | 22 |
|  | CENPL\_stlcld\_tube2\_122314\_01.09743.09743.3 | 2.3591 | 0.3203 | 98.3% | 1787.7544 | 1787.041 | 1 | 5.302 | 39.1% | 2 | R.VMLGETNPADSKPGTIR.G | 33 |

Similarities:
gi|38045913|ref|NP\_93(4:2)  

---

|  |  |  |  |  |  |  |  |  |
| --- | --- | --- | --- | --- | --- | --- | --- | --- |
| U | *gi|4885049|ref|NP\_005* | 21 | 77 | 43.0% | 377 | 42019 | 5.4 | cardiac muscle alpha actin 1 proprotein [Homo sapiens] |

| Filename XCorr DeltCN Conf% ObsM+H+ CalcM+H+ SpR ZScore Ion% # Sequence  | | | | | | | | | | | | |
| --- | --- | --- | --- | --- | --- | --- | --- | --- | --- | --- | --- | --- |
|  | CENPL\_stlcld\_122314\_01.14021.14021.3 | 4.3293 | 0.48 | 100.0% | 2157.5645 | 2157.4397 | 2 | 8.358 | 33.8% | 2 | K.AGFAGDDAPRAVFPSIVGRPR.H | 33 |
|  | CENPL\_stlcld\_122314\_01.12266.12266.2 | 2.7778 | 0.3415 | 99.9% | 1199.4321 | 1199.4415 | 52 | 5.739 | 55.0% | 6 | R.AVFPSIVGRPR.H | 22 |
|  | CENPL\_stlcld\_tube2\_122314\_01.14823.14823.2 | 4.4646 | 0.3111 | 100.0% | 1962.0521 | 1962.1841 | 1 | 6.523 | 63.3% | 5 | K.YPIEHGIITNWDDMEK.I | 2 |
|  | CENPL\_stlcld\_tube2\_122314\_01.14822.14822.3 | 4.2395 | 0.1002 | 97.8% | 1962.8644 | 1962.1841 | 20 | 4.627 | 41.7% | 6 | K.YPIEHGIITNWDDMEK.I | 3 |
|  | CENPL\_stlcld\_122314\_01.20463.20463.3 | 5.3074 | 0.3864 | 100.0% | 3458.8145 | 3459.8628 | 1 | 6.775 | 33.7% | 3 | K.YPIEHGIITNWDDMEKIWHHTFYNELR.V | 3 |
|  | CENPL\_stlcld\_122314\_01.11708.11708.2 | 3.3771 | 0.4633 | 100.0% | 1516.3322 | 1516.7019 | 1 | 6.87 | 80.0% | 6 | K.IWHHTFYNELR.V | 22 |
|  | CENPL\_stlcld\_122314\_01.11684.11684.3 | 3.1925 | 0.1607 | 97.9% | 1517.3644 | 1516.7019 | 1 | 4.968 | 55.0% | 6 | K.IWHHTFYNELR.V | 33 |
|  | CENPL\_stlcld\_tube2\_122314\_01.13294.13294.2 | 4.7866 | 0.4103 | 100.0% | 1957.6322 | 1957.234 | 1 | 6.416 | 55.9% | 2 | R.VAPEEHPTLLTEAPLNPK.A | 2 |
|  | CENPL\_stlcld\_tube2\_122314\_01.17931.17931.2 | 3.3436 | 0.3565 | 99.9% | 1624.4521 | 1624.8927 | 1 | 6.039 | 61.5% | 3 | R.LDLAGRDLTDYLMK.I | 22 |
|  | CENPL\_stlcld\_tube2\_122314\_01.17907.17907.3 | 3.4423 | 0.4035 | 100.0% | 1625.8744 | 1624.8927 | 1 | 7.333 | 48.1% | 2 | R.LDLAGRDLTDYLMK.I | 33 |
|  | CENPL\_stlcld\_tube2\_122314\_01.21777.21777.3 | 5.0553 | 0.4004 | 100.0% | 2237.9343 | 2237.6196 | 1 | 7.674 | 43.1% | 2 | R.LDLAGRDLTDYLMKILTER.G | 33 |
|  | CENPL\_stlcld\_tube2\_122314\_01.17259.17259.1 | 2.0501 | 0.2549 | 96.8% | 998.57 | 999.167 | 4 | 5.345 | 71.4% | 4 | R.DLTDYLMK.I | 11 |
|  | CENPL\_stlcld\_122314\_01.17540.17540.2 | 2.3473 | 0.3499 | 99.8% | 998.9122 | 999.167 | 2 | 5.813 | 78.6% | 5 | R.DLTDYLMK.I | 22 |
|  | CENPL\_stlcld\_tube2\_122314\_02.00425.00425.2 | 2.7976 | 0.4206 | 99.9% | 1610.9521 | 1611.8939 | 4 | 6.881 | 54.2% | 1 | R.DLTDYLMKILTER.G | 22 |
|  | CENPL\_stlcld\_tube2\_122314\_01.16760.16760.2 | 4.594 | 0.3377 | 100.0% | 1792.2322 | 1791.9554 | 1 | 8.241 | 80.0% | 12 | K.SYELPDGQVITIGNER.F | 22 |
|  | CENPL\_stlcld\_tube2\_122314\_01.10899.10899.3 | 3.6397 | 0.2696 | 99.9% | 1550.0643 | 1549.8843 | 2 | 5.869 | 46.2% | 1 | R.MQKEITALAPSTMK.I | 33 |
|  | CENPL\_stlcld\_tube2\_122314\_01.11684.11684.1 | 2.5482 | 0.4302 | 100.0% | 1161.6 | 1162.3868 | 1 | 7.63 | 65.0% | 2 | K.EITALAPSTMK.I | 11 |
|  | CENPL\_stlcld\_122314\_02.09992.09992.2 | 2.6978 | 0.3741 | 99.9% | 1162.6721 | 1162.3868 | 2 | 6.662 | 60.0% | 5 | K.EITALAPSTMK.I | 22 |
|  | CENPL\_stlcld\_tube2\_122314\_01.09464.09464.2 | 2.3837 | 0.2092 | 98.3% | 1037.0721 | 1037.2908 | 1 | 4.75 | 75.0% | 1 | K.IKIIAPPER.K | 22 |
|  | CENPL\_stlcld\_tube2\_122314\_01.00675.00675.2 | 4.6438 | 0.4966 | 100.0% | 2730.8323 | 2732.213 | 1 | 7.45 | 52.2% | 1 | R.KYSVWIGGSILASLSTFQQMWISK.Q | 22 |
|  | CENPL\_stlcld\_tube2\_122314\_01.08897.08897.2 | 2.9299 | 0.2134 | 98.6% | 1628.6322 | 1629.7697 | 2 | 4.885 | 53.8% | 2 | K.QEYDEAGPSIVHRK.C | 2 |

Similarities:
gi|4501885|ref|NP\_001(16:5)  

---

|  |  |  |  |  |  |  |  |  |
| --- | --- | --- | --- | --- | --- | --- | --- | --- |
| U | *contaminant\_gi|746301* | 15 | 124 | 42.8% | 269 | 27961 | 6.7 | lysyl endopeptidase (EC 3.4.21.50) - Lysobacter enzymogenes |

| Filename XCorr DeltCN Conf% ObsM+H+ CalcM+H+ SpR ZScore Ion% # Sequence  | | | | | | | | | | | | |
| --- | --- | --- | --- | --- | --- | --- | --- | --- | --- | --- | --- | --- |
| \* | CENPL\_stlcld\_122314\_01.06581.06581.1 | 1.7724 | 0.2515 | 96.0% | 725.54 | 725.8198 | 3 | 4.445 | 66.7% | 4 | R.SVAAYSK.Q | 1 |
| \* | CENPL\_stlcld\_tube2\_122314\_01.09020.09020.2 | 6.3724 | 0.5995 | 100.0% | 2261.672 | 2262.355 | 1 | 11.331 | 54.2% | 23 | R.APGSSSSGANGDGSLAQSQTGAVVR.A | 2 |
| \* | CENPL\_stlcld\_tube2\_122314\_01.08955.08955.3 | 4.5551 | 0.41 | 100.0% | 2261.9644 | 2262.355 | 1 | 7.024 | 39.6% | 13 | R.APGSSSSGANGDGSLAQSQTGAVVR.A | 3 |
| \* | CENPL\_stlcld\_tube2\_122314\_02.01058.01058.3 | 4.9942 | 0.3901 | 100.0% | 3316.2844 | 3315.6257 | 1 | 6.123 | 27.6% | 2 | R.ATNAASDFTLLELNTAANPAYNLFWAGWDR.R | 3 |
| \* | CENPL\_stlcld\_122314\_02.00656.00656.2 | 5.4248 | 0.4918 | 100.0% | 3316.5122 | 3315.6257 | 1 | 9.779 | 43.1% | 7 | R.ATNAASDFTLLELNTAANPAYNLFWAGWDR.R | 2 |
| \* | CENPL\_stlcld\_tube2\_122314\_02.00002.00002.3 | 6.3968 | 0.5164 | 100.0% | 3471.5344 | 3471.813 | 1 | 9.17 | 29.2% | 7 | R.ATNAASDFTLLELNTAANPAYNLFWAGWDRR.D | 3 |
| \* | CENPL\_stlcld\_122314\_01.08566.08566.3 | 3.9842 | 0.3943 | 100.0% | 2076.2043 | 2077.2668 | 1 | 7.013 | 41.7% | 2 | R.RDQNFAGATAIHHPNVAEK.R | 3 |
| \* | CENPL\_stlcld\_tube2\_122314\_01.07838.07838.3 | 3.822 | 0.2129 | 99.4% | 2233.4944 | 2233.4543 | 1 | 5.259 | 35.5% | 1 | R.RDQNFAGATAIHHPNVAEKR.I | 3 |
| \* | CENPL\_stlcld\_tube2\_122314\_01.08906.08906.2 | 4.7187 | 0.513 | 100.0% | 1920.6122 | 1921.0793 | 1 | 7.784 | 52.9% | 3 | R.DQNFAGATAIHHPNVAEK.R | 2 |
| \* | CENPL\_stlcld\_122314\_01.09405.09405.3 | 2.6161 | 0.2697 | 97.8% | 1921.2843 | 1921.0793 | 5 | 4.547 | 29.4% | 2 | R.DQNFAGATAIHHPNVAEK.R | 3 |
| \* | CENPL\_stlcld\_122314\_01.08870.08870.2 | 5.3838 | 0.4794 | 100.0% | 2076.5522 | 2077.2668 | 1 | 8.118 | 58.3% | 4 | R.DQNFAGATAIHHPNVAEKR.I | 2 |
| \* | CENPL\_stlcld\_122314\_01.08846.08846.3 | 4.375 | 0.4392 | 100.0% | 2078.3643 | 2077.2668 | 1 | 8.205 | 40.3% | 10 | R.DQNFAGATAIHHPNVAEKR.I | 3 |
| \* | CENPL\_stlcld\_tube2\_122314\_01.08363.08363.3 | 4.0983 | 0.3161 | 100.0% | 1871.1843 | 1870.983 | 2 | 6.578 | 36.1% | 3 | R.VLGQLHGGPSSCSATGADR.S | 3 |
| \* | CENPL\_stlcld\_tube2\_122314\_01.12104.12104.2 | 4.9355 | 0.5223 | 100.0% | 1428.1921 | 1428.5443 | 1 | 8.684 | 69.2% | 41 | R.VFTSWTGGGTSATR.L | 2 |
| \* | CENPL\_stlcld\_122314\_01.12155.12155.1 | 2.2399 | 0.2666 | 96.4% | 1429.83 | 1428.5443 | 147 | 4.272 | 34.6% | 2 | R.VFTSWTGGGTSATR.L | 1 |

---

|  |  |  |  |  |  |  |  |  |
| --- | --- | --- | --- | --- | --- | --- | --- | --- |
| U | *gi|33286418|ref|NP\_00* | 15 | 36 | 42.2% | 531 | 57937 | 7.8 | pyruvate kinase, muscle isoform M2 [Homo sapiens] |

| Filename XCorr DeltCN Conf% ObsM+H+ CalcM+H+ SpR ZScore Ion% # Sequence  | | | | | | | | | | | | |
| --- | --- | --- | --- | --- | --- | --- | --- | --- | --- | --- | --- | --- |
|  | CENPL\_stlcld\_122314\_01.12752.12752.2 | 3.4324 | 0.2783 | 99.9% | 1199.4321 | 1198.3617 | 2 | 6.001 | 80.0% | 5 | R.LDIDSPPITAR.N | 2 |
|  | CENPL\_stlcld\_122314\_01.12120.12120.2 | 2.5081 | 0.1396 | 96.6% | 1179.5521 | 1178.4296 | 28 | 4.969 | 55.6% | 2 | R.SVETLKEMIK.S | 2 |
|  | CENPL\_stlcld\_122314\_01.08715.08715.3 | 3.863 | 0.3461 | 100.0% | 1885.7043 | 1885.0458 | 1 | 6.3 | 43.3% | 3 | R.LNFSHGTHEYHAETIK.N | 3 |
|  | CENPL\_stlcld\_tube2\_122314\_01.17854.17854.3 | 6.0999 | 0.5387 | 100.0% | 3019.3442 | 3019.4246 | 1 | 8.16 | 30.6% | 1 | R.TATESFASDPILYRPVAVALDTKGPEIR.T | 3 |
|  | CENPL\_stlcld\_122314\_01.18550.18550.2 | 3.0604 | 0.3691 | 99.9% | 1463.4722 | 1463.7142 | 1 | 6.378 | 62.5% | 1 | K.IYVDDGLISLQVK.Q | 2 |
|  | CENPL\_stlcld\_122314\_01.17514.17514.2 | 3.1689 | 0.1999 | 98.8% | 1782.4922 | 1780.9292 | 6 | 4.948 | 41.2% | 1 | K.GADFLVTEVENGGSLGSK.K | 2 |
|  | CENPL\_stlcld\_tube2\_122314\_01.13625.13625.2 | 2.7174 | 0.1858 | 97.5% | 1766.7722 | 1766.0476 | 2 | 4.489 | 44.1% | 1 | K.KGVNLPGAAVDLPAVSEK.D | 2 |
|  | CENPL\_stlcld\_tube2\_122314\_01.15998.15998.3 | 6.926 | 0.5075 | 100.0% | 2479.3442 | 2478.8486 | 1 | 8.951 | 45.7% | 3 | K.KGVNLPGAAVDLPAVSEKDIQDLK.F | 3 |
|  | CENPL\_stlcld\_tube2\_122314\_01.00203.00203.2 | 4.96 | 0.6335 | 100.0% | 1860.5721 | 1861.1224 | 1 | 10.869 | 70.0% | 3 | K.FGVEQDVDMVFASFIR.K | 2 |
|  | CENPL\_stlcld\_tube2\_122314\_01.17182.17182.2 | 4.5381 | 0.4882 | 100.0% | 1822.5521 | 1823.0741 | 1 | 6.989 | 66.7% | 4 | R.RFDEILEASDGIMVAR.G | 2 |
|  | CENPL\_stlcld\_tube2\_122314\_02.15596.15596.3 | 3.0006 | 0.3103 | 99.9% | 1823.1843 | 1823.0741 | 1 | 5.764 | 40.0% | 1 | R.RFDEILEASDGIMVAR.G | 3 |
|  | CENPL\_stlcld\_tube2\_122314\_01.13701.13701.2 | 2.7955 | 0.2335 | 99.1% | 1142.5122 | 1142.2946 | 3 | 4.665 | 70.0% | 2 | R.GDLGIEIPAEK.V | 2 |
| \* | CENPL\_stlcld\_tube2\_122314\_01.17417.17417.3 | 2.9224 | 0.3613 | 99.9% | 2090.2444 | 2089.3586 | 65 | 5.715 | 31.2% | 4 | R.EAEAAIYHLQLFEELRR.L | 3 |
| \* | CENPL\_stlcld\_122314\_02.15384.15384.2 | 5.0655 | 0.5645 | 100.0% | 2176.412 | 2176.4282 | 1 | 9.891 | 54.8% | 2 | R.LAPITSDPTEATAVGAVEASFK.C | 2 |
|  | CENPL\_stlcld\_tube2\_122314\_01.17033.17033.3 | 3.9021 | 0.3535 | 100.0% | 2392.9143 | 2392.7815 | 1 | 5.969 | 35.7% | 3 | K.KGDVVIVLTGWRPGSGFTNTMR.V | 3 |

---

|  |  |  |  |  |  |  |  |  |
| --- | --- | --- | --- | --- | --- | --- | --- | --- |
| U | *gi|4503529|ref|NP\_001* | 14 | 49 | 42.1% | 406 | 46154 | 5.5 | eukaryotic translation initiation factor 4A isoform 1 [Homo sapiens] |

| Filename XCorr DeltCN Conf% ObsM+H+ CalcM+H+ SpR ZScore Ion% # Sequence  | | | | | | | | | | | | |
| --- | --- | --- | --- | --- | --- | --- | --- | --- | --- | --- | --- | --- |
| \* | CENPL\_stlcld\_tube2\_122314\_02.01070.01070.3 | 6.8139 | 0.5501 | 100.0% | 4168.8843 | 4169.451 | 1 | 7.748 | 34.7% | 3 | R.SRDNGPDGMEPEGVIESNWNEIVDSFDDMNLSESLLR.G | 3 |
|  | CENPL\_stlcld\_122314\_01.12581.12581.2 | 5.1051 | 0.5494 | 100.0% | 1828.8121 | 1829.0654 | 1 | 9.214 | 76.7% | 4 | R.GIYAYGFEKPSAIQQR.A | 2 |
|  | CENPL\_stlcld\_tube2\_122314\_01.12398.12398.3 | 3.4574 | 0.4445 | 100.0% | 1829.6943 | 1829.0654 | 1 | 5.77 | 38.3% | 3 | R.GIYAYGFEKPSAIQQR.A | 3 |
|  | CENPL\_stlcld\_tube2\_122314\_01.10623.10623.2 | 4.4162 | 0.4629 | 100.0% | 1395.4122 | 1395.512 | 1 | 8.784 | 80.8% | 3 | K.GYDVIAQAQSGTGK.T | 2 |
| \* | CENPL\_stlcld\_tube2\_122314\_01.13187.13187.2 | 4.0906 | 0.3342 | 100.0% | 1619.6721 | 1619.9225 | 3 | 7.39 | 57.1% | 4 | K.LQMEAPHIIVGTPGR.V | 2 |
| \* | CENPL\_stlcld\_tube2\_122314\_01.13238.13238.3 | 3.944 | 0.455 | 100.0% | 1621.2244 | 1619.9225 | 1 | 7.582 | 51.8% | 6 | K.LQMEAPHIIVGTPGR.V | 3 |
|  | CENPL\_stlcld\_tube2\_122314\_01.19790.19790.2 | 5.0995 | 0.5669 | 100.0% | 1556.3322 | 1556.789 | 1 | 9.976 | 79.2% | 3 | K.MFVLDEADEMLSR.G | 2 |
| \* | CENPL\_stlcld\_tube2\_122314\_01.17315.17315.2 | 3.6756 | 0.3967 | 100.0% | 1502.9321 | 1502.71 | 1 | 6.706 | 63.6% | 4 | R.GFKDQIYDIFQK.L | 2 |
|  | CENPL\_stlcld\_tube2\_122314\_01.10504.10504.3 | 2.966 | 0.2521 | 98.7% | 1867.5844 | 1868.0461 | 18 | 5.074 | 35.0% | 3 | R.DFTVSAMHGDMDQKER.D | 3 |
|  | CENPL\_stlcld\_tube2\_122314\_01.16832.16832.1 | 1.7303 | 0.267 | 96.2% | 1114.64 | 1115.3585 | 72 | 4.619 | 50.0% | 1 | R.VLITTDLLAR.G | 1 |
|  | CENPL\_stlcld\_tube2\_122314\_01.16881.16881.2 | 3.2158 | 0.4515 | 100.0% | 1115.4521 | 1115.3585 | 1 | 7.069 | 88.9% | 7 | R.VLITTDLLAR.G | 2 |
| \* | CENPL\_stlcld\_122314\_01.09455.09455.2 | 3.9373 | 0.4374 | 100.0% | 1590.6322 | 1590.8352 | 1 | 6.914 | 80.8% | 1 | R.KGVAINMVTEEDKR.T | 2 |
| \* | CENPL\_stlcld\_122314\_01.09423.09423.3 | 4.4661 | 0.3818 | 100.0% | 1591.4644 | 1590.8352 | 1 | 6.697 | 55.8% | 5 | R.KGVAINMVTEEDKR.T | 3 |
| \* | CENPL\_stlcld\_tube2\_122314\_02.01234.01234.2 | 3.4703 | 0.5267 | 100.0% | 2798.172 | 2799.1653 | 1 | 8.464 | 45.7% | 2 | R.TLRDIETFYNTSIEEMPLNVADLI.- | 2 |

---

|  |  |  |  |  |  |  |  |  |
| --- | --- | --- | --- | --- | --- | --- | --- | --- |
| U | *gi|4506671|ref|NP\_000* | 3 | 4 | 41.7% | 115 | 11665 | 4.5 | ribosomal protein P2 [Homo sapiens] |

| Filename XCorr DeltCN Conf% ObsM+H+ CalcM+H+ SpR ZScore Ion% # Sequence  | | | | | | | | | | | | |
| --- | --- | --- | --- | --- | --- | --- | --- | --- | --- | --- | --- | --- |
| \* | CENPL\_stlcld\_122314\_01.20502.20502.2 | 4.1284 | 0.4116 | 100.0% | 1869.8522 | 1870.1124 | 1 | 7.704 | 50.0% | 1 | R.YVASYLLAALGGNSSPSAK.D | 2 |
| \* | CENPL\_stlcld\_tube2\_122314\_01.17312.17312.2 | 3.4217 | 0.4695 | 100.0% | 1257.7722 | 1257.4294 | 1 | 7.304 | 77.3% | 2 | K.NIEDVIAQGIGK.L | 2 |
|  | CENPL\_stlcld\_122314\_02.16038.16038.2 | 2.0596 | 0.3179 | 97.5% | 1950.0922 | 1950.979 | 1 | 5.478 | 40.6% | 1 | K.KEESEESDDDMGFGLFD.- | 2 |

---

|  |  |  |  |  |  |  |  |  |
| --- | --- | --- | --- | --- | --- | --- | --- | --- |
| U | *gi|114155144|ref|NP\_0* | 8 | 15 | 41.5% | 248 | 28870 | 4.8 | tropomyosin 3 isoform 4 [Homo sapiens] |
| U | *gi|24119203|ref|NP\_70* | 8 | 15 | 41.5% | 248 | 29033 | 4.8 | tropomyosin 3 isoform 2 [Homo sapiens] |
| U | *gi|114155148|ref|NP\_0* | 8 | 15 | 41.7% | 247 | 28793 | 4.8 | tropomyosin 3 isoform 5 [Homo sapiens] |
| U | *gi|114155146|ref|NP\_0* | 8 | 15 | 41.7% | 247 | 28955 | 4.8 | tropomyosin 3 isoform 3 [Homo sapiens] |

| Filename XCorr DeltCN Conf% ObsM+H+ CalcM+H+ SpR ZScore Ion% # Sequence  | | | | | | | | | | | | |
| --- | --- | --- | --- | --- | --- | --- | --- | --- | --- | --- | --- | --- |
|  | CENPL\_stlcld\_tube2\_122314\_01.10272.10272.2 | 3.5009 | 0.2425 | 99.9% | 1645.1322 | 1643.7507 | 1 | 6.013 | 57.7% | 1 | K.IQVLQQQADDAEER.A | 23 |
|  | CENPL\_stlcld\_tube2\_122314\_01.08280.08280.3 | 3.8999 | 0.3282 | 100.0% | 1699.7644 | 1700.852 | 1 | 6.395 | 44.6% | 2 | R.AREQAEAEVASLNRR.I | 3 |
|  | CENPL\_stlcld\_tube2\_122314\_01.13634.13634.2 | 3.3821 | 0.2919 | 99.9% | 1244.1921 | 1244.3873 | 2 | 5.902 | 77.8% | 3 | R.IQLVEEELDR.A | 22 |
|  | CENPL\_stlcld\_122314\_01.12767.12767.2 | 3.0824 | 0.2575 | 99.6% | 1445.2922 | 1444.6683 | 1 | 5.012 | 70.8% | 1 | R.LATALQKLEEAEK.A | 22 |
|  | CENPL\_stlcld\_tube2\_122314\_01.13997.13997.3 | 3.2887 | 0.3151 | 99.9% | 2203.6443 | 2203.4111 | 1 | 5.586 | 31.6% | 2 | R.LATALQKLEEAEKAADESER.G | 33 |
|  | CENPL\_stlcld\_tube2\_122314\_02.12768.12768.3 | 3.8207 | 0.1822 | 99.2% | 1945.9143 | 1946.2665 | 6 | 4.465 | 38.3% | 4 | R.ALKDEEKMELQEIQLK.E | 3 |
|  | CENPL\_stlcld\_122314\_02.11206.11206.3 | 2.3833 | 0.283 | 97.5% | 1801.5543 | 1801.05 | 43 | 5.057 | 33.9% | 1 | R.KLVIIEGDLERTEER.A | 3 |
|  | CENPL\_stlcld\_122314\_02.09128.09128.3 | 2.9786 | 0.3096 | 99.9% | 1478.9343 | 1478.6885 | 2 | 5.599 | 43.8% | 1 | R.AEFAERSVAKLEK.T | 3 |

Similarities:
gi|4507651|ref|NP\_003(3:5)  

---

|  |  |  |  |  |  |  |  |  |
| --- | --- | --- | --- | --- | --- | --- | --- | --- |
| U | *gi|4826898|ref|NP\_005* | 4 | 7 | 41.4% | 140 | 15054 | 8.3 | profilin 1 [Homo sapiens] |

| Filename XCorr DeltCN Conf% ObsM+H+ CalcM+H+ SpR ZScore Ion% # Sequence  | | | | | | | | | | | | |
| --- | --- | --- | --- | --- | --- | --- | --- | --- | --- | --- | --- | --- |
| \* | CENPL\_stlcld\_122314\_01.20076.20076.2 | 2.9744 | 0.2286 | 98.8% | 1644.2322 | 1644.9518 | 6 | 5.765 | 46.7% | 1 | K.TFVNITPAEVGVLVGK.D | 2 |
| \* | CENPL\_stlcld\_tube2\_122314\_01.15352.15352.2 | 2.9435 | 0.3453 | 99.9% | 1471.3722 | 1471.6531 | 1 | 6.371 | 61.5% | 2 | R.SSFYVNGLTLGGQK.C | 2 |
| \* | CENPL\_stlcld\_tube2\_122314\_02.16655.16655.2 | 3.3041 | 0.4308 | 100.0% | 1626.4922 | 1626.7784 | 12 | 6.805 | 50.0% | 3 | R.DSLLQDGEFSMDLR.T | 2 |
| \* | CENPL\_stlcld\_tube2\_122314\_01.11762.11762.2 | 2.8631 | 0.177 | 98.1% | 1380.2722 | 1380.5406 | 1 | 4.709 | 61.5% | 1 | K.STGGAPTFNVTVTK.T | 2 |

---

|  |  |  |  |  |  |  |  |  |
| --- | --- | --- | --- | --- | --- | --- | --- | --- |
| U | *gi|15718687|ref|NP\_00* | 7 | 25 | 40.7% | 243 | 26688 | 9.7 | ribosomal protein S3 [Homo sapiens] |

| Filename XCorr DeltCN Conf% ObsM+H+ CalcM+H+ SpR ZScore Ion% # Sequence  | | | | | | | | | | | | |
| --- | --- | --- | --- | --- | --- | --- | --- | --- | --- | --- | --- | --- |
| \* | CENPL\_stlcld\_tube2\_122314\_01.14510.14510.2 | 2.849 | 0.3523 | 99.9% | 1093.4321 | 1093.2249 | 1 | 6.258 | 81.2% | 5 | K.AELNEFLTR.E | 2 |
| \* | CENPL\_stlcld\_tube2\_122314\_02.11555.11555.2 | 4.099 | 0.4057 | 100.0% | 1424.8121 | 1424.5071 | 1 | 8.208 | 83.3% | 9 | R.ELAEDGYSGVEVR.V | 2 |
| \* | CENPL\_stlcld\_122314\_01.14123.14123.2 | 3.1218 | 0.2425 | 99.4% | 1585.0122 | 1584.8998 | 1 | 4.736 | 57.7% | 2 | R.VTPTRTEIIILATR.T | 2 |
| \* | CENPL\_stlcld\_122314\_01.08753.08753.3 | 2.9986 | 0.1974 | 98.4% | 1314.4143 | 1313.5863 | 16 | 5.287 | 47.5% | 1 | R.IRELTAVVQKR.F | 3 |
| \* | CENPL\_stlcld\_122314\_02.14439.14439.2 | 3.0278 | 0.4926 | 100.0% | 1573.9722 | 1573.7423 | 1 | 7.395 | 73.1% | 4 | R.FGFPEGSVELYAEK.V | 2 |
| \* | CENPL\_stlcld\_tube2\_122314\_01.17831.17831.3 | 4.1715 | 0.4013 | 100.0% | 2468.8743 | 2469.7742 | 19 | 6.664 | 31.0% | 2 | K.FVDGLMIHSGDPVNYYVDTAVR.H | 3 |
| \* | CENPL\_stlcld\_tube2\_122314\_01.11333.11333.2 | 3.3359 | 0.3436 | 99.9% | 1574.3722 | 1574.8352 | 4 | 6.216 | 50.0% | 2 | K.GGKPEPPAMPQPVPTA.- | 2 |

---

|  |  |  |  |  |  |  |  |  |
| --- | --- | --- | --- | --- | --- | --- | --- | --- |
| U | *gi|224028244|ref|NP\_0* | 24 | 104 | 40.6% | 471 | 54232 | 8.9 | non-POU domain containing, octamer-binding isoform 1 [Homo sapiens] |
| U | *gi|34932414|ref|NP\_03* | 24 | 104 | 40.6% | 471 | 54232 | 8.9 | non-POU domain containing, octamer-binding isoform 1 [Homo sapiens] |
| U | *gi|224028246|ref|NP\_0* | 24 | 104 | 40.6% | 471 | 54232 | 8.9 | non-POU domain containing, octamer-binding isoform 1 [Homo sapiens] |

| Filename XCorr DeltCN Conf% ObsM+H+ CalcM+H+ SpR ZScore Ion% # Sequence  | | | | | | | | | | | | |
| --- | --- | --- | --- | --- | --- | --- | --- | --- | --- | --- | --- | --- |
|  | CENPL\_stlcld\_122314\_01.18086.18086.2 | 4.0782 | 0.4465 | 100.0% | 1860.7322 | 1861.12 | 1 | 8.4 | 66.7% | 15 | R.LFVGNLPPDITEEEMR.K | 2 |
|  | CENPL\_stlcld\_tube2\_122314\_01.18870.18870.2 | 5.4607 | 0.5258 | 100.0% | 1814.0922 | 1814.1504 | 1 | 9.077 | 73.3% | 5 | R.TLAEIAKVELDNMPLR.G | 2 |
|  | CENPL\_stlcld\_tube2\_122314\_01.18859.18859.3 | 2.8732 | 0.3412 | 99.9% | 1814.6643 | 1814.1504 | 1 | 5.522 | 41.7% | 2 | R.TLAEIAKVELDNMPLR.G | 3 |
|  | CENPL\_stlcld\_122314\_01.17302.17302.3 | 4.6589 | 0.4407 | 100.0% | 2000.2444 | 1999.3765 | 1 | 7.728 | 44.1% | 3 | R.TLAEIAKVELDNMPLRGK.Q | 3 |
|  | CENPL\_stlcld\_122314\_01.13221.13221.2 | 3.0608 | 0.3576 | 100.0% | 1087.3322 | 1087.2793 | 52 | 5.827 | 68.8% | 6 | K.VELDNMPLR.G | 2 |
|  | CENPL\_stlcld\_tube2\_122314\_01.08459.08459.2 | 2.5175 | 0.4994 | 100.0% | 1247.8922 | 1249.3782 | 1 | 7.258 | 65.0% | 2 | R.FACHSASLTVR.N | 2 |
|  | CENPL\_stlcld\_tube2\_122314\_01.08451.08451.3 | 2.8891 | 0.3226 | 99.9% | 1249.6144 | 1249.3782 | 2 | 5.906 | 50.0% | 1 | R.FACHSASLTVR.N | 3 |
|  | CENPL\_stlcld\_122314\_02.01790.01790.3 | 4.3514 | 0.338 | 100.0% | 2670.6843 | 2669.9507 | 1 | 5.786 | 37.5% | 1 | R.NLPQYVSNELLEEAFSVFGQVER.A | 3 |
|  | CENPL\_stlcld\_122314\_02.01763.01763.2 | 4.9772 | 0.3724 | 100.0% | 2671.5923 | 2669.9507 | 1 | 9.014 | 54.5% | 3 | R.NLPQYVSNELLEEAFSVFGQVER.A | 2 |
|  | CENPL\_stlcld\_tube2\_122314\_01.09248.09248.2 | 2.399 | 0.2054 | 98.4% | 887.3722 | 887.0238 | 1 | 6.834 | 85.7% | 2 | R.AVVIVDDR.G | 22 |
|  | CENPL\_stlcld\_122314\_01.08357.08357.3 | 3.7454 | 0.3149 | 99.9% | 1816.0443 | 1815.0854 | 7 | 6.261 | 35.3% | 1 | R.GRPSGKGIVEFSGKPAAR.K | 3 |
|  | CENPL\_stlcld\_122314\_02.08858.08858.3 | 2.0818 | 0.3513 | 98.7% | 1232.9644 | 1232.4252 | 3 | 5.105 | 40.9% | 2 | K.GIVEFSGKPAAR.K | 3 |
|  | CENPL\_stlcld\_122314\_01.09796.09796.2 | 3.2672 | 0.4202 | 100.0% | 1233.5922 | 1232.4252 | 1 | 7.133 | 81.8% | 6 | K.GIVEFSGKPAAR.K | 2 |
|  | CENPL\_stlcld\_122314\_01.15218.15218.2 | 4.4328 | 0.5093 | 100.0% | 1696.5721 | 1696.8744 | 1 | 9.881 | 73.1% | 12 | R.FAQPGSFEYEYAMR.W | 2 |
|  | CENPL\_stlcld\_tube2\_122314\_01.10796.10796.2 | 2.1935 | 0.1686 | 97.2% | 834.0722 | 834.0185 | 4 | 5.377 | 83.3% | 1 | K.ALIEMEK.Q | 2 |
|  | CENPL\_stlcld\_tube2\_122314\_01.09744.09744.3 | 3.3972 | 0.2755 | 99.9% | 1831.9143 | 1832.0386 | 1 | 5.998 | 41.1% | 1 | K.ALIEMEKQQQDQVDR.N | 3 |
|  | CENPL\_stlcld\_tube2\_122314\_01.10146.10146.2 | 3.0723 | 0.3796 | 99.9% | 1338.3121 | 1337.5488 | 1 | 6.59 | 75.0% | 14 | R.EKLEMEMEAAR.H | 2 |
|  | CENPL\_stlcld\_122314\_01.07626.07626.2 | 2.4853 | 0.2171 | 98.8% | 1109.2722 | 1108.2758 | 7 | 4.787 | 71.4% | 1 | R.RQQEEMMR.R | 2 |
|  | CENPL\_stlcld\_122314\_02.11546.11546.2 | 4.7804 | 0.5446 | 100.0% | 1539.2722 | 1539.8441 | 1 | 9.559 | 67.9% | 14 | R.MGQMAMGGAMGINNR.G | 2 |
|  | CENPL\_stlcld\_122314\_02.14087.14087.2 | 5.4758 | 0.4844 | 100.0% | 2164.5522 | 2164.4436 | 1 | 8.749 | 57.1% | 3 | R.FGQAATMEGIGAIGGTPPAFNR.A | 2 |
|  | CENPL\_stlcld\_tube2\_122314\_02.15420.15420.3 | 3.0157 | 0.3946 | 99.9% | 2164.8843 | 2164.4436 | 1 | 5.845 | 31.0% | 1 | R.FGQAATMEGIGAIGGTPPAFNR.A | 3 |
|  | CENPL\_stlcld\_122314\_02.14426.14426.2 | 5.6188 | 0.4903 | 100.0% | 2243.5322 | 2244.4436 | 1 | 10.757 | 59.5% | 3 | R.FGQAATMEGIGAIGGT#PPAFNR.A | 2 |
|  | CENPL\_stlcld\_122314\_02.14396.14396.3 | 3.7556 | 0.3277 | 99.9% | 2244.5344 | 2244.4436 | 1 | 6.396 | 44.0% | 4 | R.FGQAATMEGIGAIGGT#PPAFNR.A | 3 |
|  | CENPL\_stlcld\_122314\_01.08403.08403.2 | 2.4924 | 0.4756 | 99.9% | 1229.4122 | 1229.3811 | 1 | 7.254 | 72.7% | 1 | R.AAPGAEFAPNKR.R | 2 |

Similarities:
gi|4826998|ref|NP\_005(1:23)  

---

|  |  |  |  |  |  |  |  |  |
| --- | --- | --- | --- | --- | --- | --- | --- | --- |
| U | *gi|119395750|ref|NP\_0* | 30 | 99 | 39.6% | 644 | 66039 | 8.1 | keratin 1 [Homo sapiens] |

| Filename XCorr DeltCN Conf% ObsM+H+ CalcM+H+ SpR ZScore Ion% # Sequence  | | | | | | | | | | | | |
| --- | --- | --- | --- | --- | --- | --- | --- | --- | --- | --- | --- | --- |
| \* | CENPL\_stlcld\_122314\_02.11470.11470.2 | 4.3659 | 0.4388 | 100.0% | 1658.4521 | 1658.7678 | 1 | 6.849 | 65.6% | 4 | R.SGGGFSSGSAGIINYQR.R | 2 |
| \* | CENPL\_stlcld\_tube2\_122314\_01.10529.10529.1 | 1.5762 | 0.3375 | 96.2% | 874.67 | 875.0128 | 49 | 5.32 | 50.0% | 1 | R.SLVNLGGSK.S | 1 |
| \* | CENPL\_stlcld\_tube2\_122314\_02.10002.10002.2 | 2.4088 | 0.2166 | 98.4% | 875.1122 | 875.0128 | 34 | 4.461 | 62.5% | 3 | R.SLVNLGGSK.S | 2 |
| \* | CENPL\_stlcld\_tube2\_122314\_01.10372.10372.2 | 2.0041 | 0.226 | 96.1% | 833.15216 | 832.9755 | 1 | 4.79 | 78.6% | 1 | K.SISISVAR.G | 2 |
|  | CENPL\_stlcld\_122314\_01.16935.16935.2 | 3.9569 | 0.3476 | 100.0% | 1385.5122 | 1384.5315 | 1 | 6.057 | 72.7% | 9 | K.SLNNQFASFIDK.V | 2 |
|  | CENPL\_stlcld\_122314\_01.17976.17976.2 | 3.5764 | 0.3849 | 100.0% | 1639.8522 | 1639.8516 | 1 | 6.104 | 61.5% | 4 | K.SLNNQFASFIDKVR.F | 2 |
|  | CENPL\_stlcld\_tube2\_122314\_01.11162.11162.2 | 4.5228 | 0.0843 | 99.9% | 1476.4521 | 1476.6726 | 1 | 6.928 | 90.9% | 6 | R.FLEQQNQVLQTK.W | 22 |
|  | CENPL\_stlcld\_tube2\_122314\_01.16557.16557.2 | 4.2638 | 0.4896 | 100.0% | 1476.3522 | 1476.6293 | 1 | 9.112 | 86.4% | 20 | K.WELLQQVDTSTR.T | 2 |
|  | CENPL\_stlcld\_tube2\_122314\_01.19997.19997.2 | 4.2698 | 0.4572 | 100.0% | 1994.8522 | 1995.2017 | 1 | 7.874 | 63.3% | 1 | R.THNLEPYFESFINNLR.R | 2 |
|  | CENPL\_stlcld\_tube2\_122314\_02.17052.17052.3 | 4.1496 | 0.332 | 100.0% | 1995.3544 | 1995.2017 | 1 | 6.815 | 50.0% | 1 | R.THNLEPYFESFINNLR.R | 3 |
|  | CENPL\_stlcld\_tube2\_122314\_01.12140.12140.2 | 3.4077 | 0.3459 | 100.0% | 1266.3722 | 1266.3934 | 1 | 6.827 | 75.0% | 2 | R.TNAENEFVTIK.K | 2 |
|  | CENPL\_stlcld\_122314\_01.09999.09999.2 | 3.314 | 0.3223 | 99.9% | 1394.2722 | 1394.5675 | 4 | 6.053 | 59.1% | 5 | R.TNAENEFVTIKK.D | 2 |
|  | CENPL\_stlcld\_tube2\_122314\_01.09614.09614.3 | 2.3246 | 0.262 | 95.9% | 1395.0543 | 1394.5675 | 172 | 4.612 | 38.6% | 1 | R.TNAENEFVTIKK.D | 3 |
| \* | CENPL\_stlcld\_tube2\_122314\_01.19958.19958.1 | 2.4287 | 0.2628 | 96.1% | 1302.95 | 1303.4955 | 5 | 5.729 | 59.1% | 1 | R.SLDLDSIIAEVK.A | 1 |
| \* | CENPL\_stlcld\_tube2\_122314\_01.19934.19934.2 | 4.3788 | 0.5158 | 100.0% | 1303.6921 | 1303.4955 | 1 | 9.161 | 86.4% | 2 | R.SLDLDSIIAEVK.A | 2 |
|  | CENPL\_stlcld\_tube2\_122314\_01.12324.12324.3 | 4.7078 | 0.466 | 100.0% | 2502.0544 | 2502.7405 | 1 | 8.295 | 36.9% | 2 | K.SKAEAESLYQSKYEELQITAGR.H | 3 |
|  | CENPL\_stlcld\_tube2\_122314\_02.10658.10658.2 | 4.0519 | 0.3315 | 100.0% | 1181.4122 | 1180.303 | 2 | 6.856 | 83.3% | 7 | K.YEELQITAGR.H | 22 |
|  | CENPL\_stlcld\_tube2\_122314\_01.09705.09705.2 | 3.4258 | 0.3497 | 100.0% | 1303.5922 | 1303.4581 | 1 | 6.327 | 80.0% | 2 | R.NSKIEISELNR.V | 2 |
|  | CENPL\_stlcld\_tube2\_122314\_01.11376.11376.1 | 1.8862 | 0.2617 | 97.1% | 973.58 | 974.102 | 7 | 4.27 | 64.3% | 1 | K.IEISELNR.V | 11 |
|  | CENPL\_stlcld\_tube2\_122314\_01.11366.11366.2 | 3.0241 | 0.1669 | 99.6% | 974.4522 | 974.102 | 11 | 4.866 | 78.6% | 4 | K.IEISELNR.V | 22 |
|  | CENPL\_stlcld\_122314\_01.14625.14625.2 | 4.774 | 0.428 | 100.0% | 1600.6322 | 1600.769 | 1 | 7.866 | 80.8% | 4 | K.NKLNDLEDALQQAK.E | 2 |
|  | CENPL\_stlcld\_tube2\_122314\_01.14535.14535.3 | 3.7583 | 0.324 | 100.0% | 1601.9944 | 1600.769 | 2 | 5.696 | 48.1% | 1 | K.NKLNDLEDALQQAK.E | 3 |
| \* | CENPL\_stlcld\_tube2\_122314\_01.18561.18561.2 | 5.6233 | 0.4774 | 100.0% | 2185.1921 | 2185.399 | 1 | 8.458 | 75.0% | 1 | K.NKLNDLEDALQQAKEDLAR.L | 2 |
| \* | CENPL\_stlcld\_tube2\_122314\_01.18594.18594.3 | 5.7578 | 0.4136 | 100.0% | 2185.5842 | 2185.399 | 1 | 8.34 | 48.6% | 5 | K.NKLNDLEDALQQAKEDLAR.L | 3 |
|  | CENPL\_stlcld\_tube2\_122314\_01.14178.14178.2 | 3.2286 | 0.3768 | 100.0% | 1357.8322 | 1358.4912 | 1 | 6.479 | 63.6% | 1 | K.LNDLEDALQQAK.E | 2 |
| \* | CENPL\_stlcld\_122314\_01.19704.19704.2 | 3.4437 | 0.3506 | 99.9% | 1942.3322 | 1943.121 | 1 | 6.196 | 59.4% | 1 | K.LNDLEDALQQAKEDLAR.L | 2 |
|  | CENPL\_stlcld\_tube2\_122314\_01.12600.12600.2 | 3.39 | 0.3155 | 99.9% | 1524.5322 | 1524.7754 | 1 | 5.653 | 68.2% | 2 | R.LLRDYQELMNTK.L | 2 |
|  | CENPL\_stlcld\_tube2\_122314\_01.08583.08583.2 | 2.2292 | 0.2313 | 97.8% | 1035.5122 | 1034.1112 | 4 | 4.723 | 75.0% | 4 | R.TLLEGEESR.M | 2 |
|  | CENPL\_stlcld\_tube2\_122314\_01.07912.07912.2 | 5.5618 | 0.6213 | 100.0% | 2384.672 | 2385.298 | 1 | 12.217 | 41.7% | 1 | R.GGGGGGYGSGGSSYGSGGGSYGSGGGGGGGR.G | 2 |
| \* | CENPL\_stlcld\_tube2\_122314\_02.09119.09119.3 | 4.3554 | 0.2797 | 99.9% | 3314.4543 | 3314.2085 | 1 | 7.615 | 21.1% | 2 | R.GSYGSGGSSYGSGGGSYGSGGGGGGHGSYGSGSSSGGYR.G | 3 |

Similarities:
gi|47132620|ref|NP\_00(3:27)  
gi|119703753|ref|NP\_0(1:29)  

---

|  |  |  |  |  |  |  |  |  |
| --- | --- | --- | --- | --- | --- | --- | --- | --- |
| U | *gi|20357599|ref|NP\_61* | 3 | 19 | 39.5% | 114 | 12146 | 10.5 | H2A histone family, member V isoform 2 [Homo sapiens] |
| U | *gi|6912616|ref|NP\_036* | 3 | 19 | 35.2% | 128 | 13509 | 10.6 | H2A histone family, member V isoform 1 [Homo sapiens] |
| U | *gi|4504255|ref|NP\_002* | 3 | 19 | 35.2% | 128 | 13553 | 10.6 | H2A histone family, member Z [Homo sapiens] |

| Filename XCorr DeltCN Conf% ObsM+H+ CalcM+H+ SpR ZScore Ion% # Sequence  | | | | | | | | | | | | |
| --- | --- | --- | --- | --- | --- | --- | --- | --- | --- | --- | --- | --- |
|  | CENPL\_stlcld\_122314\_01.13670.13670.2 | 3.2544 | 0.3534 | 100.0% | 945.1922 | 945.1093 | 3 | 6.291 | 81.2% | 10 | R.AGLQFPVGR.I | 2222 |
|  | CENPL\_stlcld\_122314\_02.02105.02105.2 | 5.4585 | 0.4837 | 100.0% | 2898.4922 | 2897.2952 | 1 | 9.004 | 41.1% | 2 | R.VGATAAVYSAAILEYLTAEVLELAGNASK.D | 2 |
|  | CENPL\_stlcld\_122314\_01.09950.09950.2 | 2.5538 | 0.2207 | 99.5% | 851.0722 | 851.0396 | 4 | 5.466 | 83.3% | 7 | R.HLQLAIR.G | 2222 |

Similarities:
gi|10645195|ref|NP\_06(2:1)  
gi|10800130|ref|NP\_06(2:1)  
gi|106775678|ref|NP\_0(2:1)  

---

|  |  |  |  |  |  |  |  |  |
| --- | --- | --- | --- | --- | --- | --- | --- | --- |
| U | *gi|4557701|ref|NP\_000* | 16 | 48 | 39.1% | 432 | 48106 | 5.0 | keratin 17 [Homo sapiens] |

| Filename XCorr DeltCN Conf% ObsM+H+ CalcM+H+ SpR ZScore Ion% # Sequence  | | | | | | | | | | | | |
| --- | --- | --- | --- | --- | --- | --- | --- | --- | --- | --- | --- | --- |
|  | CENPL\_stlcld\_tube2\_122314\_01.09831.09831.2 | 3.0041 | 0.1699 | 99.3% | 1065.3322 | 1065.2578 | 54 | 5.893 | 62.5% | 1 | R.LASYLDKVR.A | 2222 |
|  | CENPL\_stlcld\_tube2\_122314\_02.11498.11498.2 | 3.6861 | 0.429 | 100.0% | 1346.4722 | 1346.4772 | 1 | 7.631 | 77.3% | 10 | R.ALEEANTELEVK.I | 2 |
|  | CENPL\_stlcld\_122314\_01.09504.09504.2 | 2.335 | 0.0893 | 95.2% | 1037.4922 | 1037.1661 | 21 | 4.386 | 75.0% | 1 | K.IRDWYQR.Q | 22 |
|  | CENPL\_stlcld\_122314\_01.09788.09788.2 | 2.5662 | 0.2183 | 99.5% | 808.39215 | 807.8815 | 68 | 6.201 | 66.7% | 8 | R.LAADDFR.T | 22222 |
|  | CENPL\_stlcld\_122314\_01.08733.08733.2 | 2.3452 | 0.299 | 99.0% | 1223.3522 | 1223.3715 | 7 | 5.286 | 77.8% | 1 | R.TKFETEQALR.L | 22 |
|  | CENPL\_stlcld\_122314\_01.14091.14091.2 | 3.0726 | 0.4118 | 100.0% | 1030.3522 | 1030.2096 | 3 | 6.871 | 81.2% | 5 | R.VLDELTLAR.A | 222 |
|  | CENPL\_stlcld\_tube2\_122314\_01.19296.19296.3 | 3.5397 | 0.3449 | 99.9% | 2279.8743 | 2279.6538 | 5 | 5.296 | 33.3% | 2 | R.ADLEMQIENLKEELAYLKK.N | 3 |
|  | CENPL\_stlcld\_122314\_02.13168.13168.2 | 2.3634 | 0.3033 | 98.4% | 2114.612 | 2115.3228 | 224 | 4.871 | 27.5% | 1 | R.GQVGGEINVEMDAAPGVDLSR.I | 2 |
|  | CENPL\_stlcld\_122314\_01.18683.18683.2 | 3.5274 | 0.4162 | 100.0% | 1887.2722 | 1888.0001 | 2 | 6.722 | 50.0% | 1 | K.DAEDWFFSKTEELNR.E | 2 |
|  | CENPL\_stlcld\_122314\_02.10301.10301.3 | 3.6063 | 0.3568 | 100.0% | 2106.0244 | 2105.2664 | 8 | 5.981 | 34.7% | 1 | K.TEELNREVATNSELVQSGK.S | 33 |
|  | CENPL\_stlcld\_122314\_01.08586.08586.2 | 3.5852 | 0.459 | 100.0% | 1362.2322 | 1362.4796 | 1 | 8.37 | 66.7% | 4 | R.EVATNSELVQSGK.S | 22 |
|  | CENPL\_stlcld\_tube2\_122314\_01.10187.10187.2 | 4.1407 | 0.5011 | 100.0% | 1404.5721 | 1404.4764 | 1 | 8.622 | 66.7% | 4 | K.ASLEGNLAETENR.Y | 2 |
|  | CENPL\_stlcld\_tube2\_122314\_01.11194.11194.2 | 3.5553 | 0.293 | 99.9% | 1380.4722 | 1380.5437 | 5 | 5.961 | 65.0% | 4 | K.TRLEQEIATYR.R | 22 |
|  | CENPL\_stlcld\_tube2\_122314\_01.09641.09641.2 | 2.9939 | 0.3424 | 99.9% | 1123.2722 | 1123.2511 | 2 | 6.024 | 81.2% | 1 | R.LEQEIATYR.R | 222 |
|  | CENPL\_stlcld\_122314\_01.11153.11153.2 | 2.7516 | 0.3183 | 99.6% | 1516.4521 | 1517.6787 | 8 | 5.957 | 54.2% | 1 | R.LLEGEDAHLTQYK.K | 2 |
|  | CENPL\_stlcld\_tube2\_122314\_01.09624.09624.2 | 2.6365 | 0.3602 | 99.9% | 1119.4922 | 1118.2291 | 1 | 6.883 | 77.8% | 3 | R.TIVEEVQDGK.V | 2 |

Similarities:
gi|40354195|ref|NP\_95(1:15)  
contaminant\_KERATIN03(2:14)  
contaminant\_KERATIN05(8:8)  
gi|24234699|ref|NP\_00(5:11)  

---

|  |  |  |  |  |  |  |  |  |
| --- | --- | --- | --- | --- | --- | --- | --- | --- |
| U | *gi|20149594|ref|NP\_03* | 27 | 78 | 38.1% | 724 | 83264 | 5.0 | heat shock 90kDa protein 1, beta [Homo sapiens] |

| Filename XCorr DeltCN Conf% ObsM+H+ CalcM+H+ SpR ZScore Ion% # Sequence  | | | | | | | | | | | | |
| --- | --- | --- | --- | --- | --- | --- | --- | --- | --- | --- | --- | --- |
|  | CENPL\_stlcld\_tube2\_122314\_01.11607.11607.2 | 3.4954 | 0.4147 | 100.0% | 1276.4321 | 1276.3861 | 2 | 7.643 | 63.6% | 1 | R.ELISNASDALDK.I | 2 |
|  | CENPL\_stlcld\_122314\_01.13920.13920.2 | 3.6254 | 0.4439 | 100.0% | 1545.1322 | 1545.733 | 1 | 7.464 | 73.1% | 3 | R.ELISNASDALDKIR.Y | 2 |
| \* | CENPL\_stlcld\_tube2\_122314\_01.10329.10329.3 | 3.4661 | 0.3832 | 100.0% | 1910.5743 | 1911.1162 | 5 | 6.544 | 35.9% | 1 | R.YESLTDPSKLDSGKELK.I | 3 |
| \* | CENPL\_stlcld\_122314\_02.12257.12257.2 | 2.3837 | 0.2888 | 98.5% | 1350.3522 | 1350.6135 | 19 | 5.849 | 54.2% | 1 | R.TLTLVDTGIGMTK.A | 2 |
|  | CENPL\_stlcld\_tube2\_122314\_01.15220.15220.2 | 3.8553 | 0.3359 | 100.0% | 1244.5521 | 1243.4459 | 1 | 7.087 | 72.7% | 5 | K.ADLINNLGTIAK.S | 22 |
|  | CENPL\_stlcld\_tube2\_122314\_02.13241.13241.3 | 3.0099 | 0.3033 | 99.9% | 2256.3843 | 2257.294 | 1 | 4.591 | 32.9% | 1 | K.HNDDEQYAWESSAGGSFTVR.A | 33 |
|  | CENPL\_stlcld\_tube2\_122314\_02.12039.12039.3 | 4.507 | 0.4049 | 100.0% | 2017.6743 | 2016.2584 | 1 | 7.479 | 48.3% | 6 | K.VILHLKEDQTEYLEER.R | 33 |
|  | CENPL\_stlcld\_122314\_02.10739.10739.3 | 3.6436 | 0.2957 | 99.9% | 2172.2644 | 2172.446 | 1 | 6.397 | 34.4% | 1 | K.VILHLKEDQTEYLEERR.V | 33 |
| \* | CENPL\_stlcld\_tube2\_122314\_01.18610.18610.2 | 4.7209 | 0.5292 | 100.0% | 1809.6522 | 1810.1027 | 1 | 9.108 | 71.4% | 3 | K.HSQFIGYPITLYLEK.E | 2 |
| \* | CENPL\_stlcld\_tube2\_122314\_01.17214.17214.2 | 3.176 | 0.2543 | 99.5% | 2094.7722 | 2095.4058 | 1 | 6.56 | 43.8% | 1 | K.HSQFIGYPITLYLEKER.E | 2 |
| \* | CENPL\_stlcld\_122314\_01.17481.17481.3 | 4.8987 | 0.4969 | 100.0% | 2095.3743 | 2095.4058 | 1 | 8.103 | 40.6% | 3 | K.HSQFIGYPITLYLEKER.E | 3 |
|  | CENPL\_stlcld\_122314\_01.09152.09152.2 | 3.4786 | 0.3417 | 100.0% | 1152.4521 | 1152.2462 | 1 | 5.806 | 87.5% | 5 | K.YIDQEELNK.T | 22 |
| \* | CENPL\_stlcld\_tube2\_122314\_01.13929.13929.2 | 5.0316 | 0.5061 | 100.0% | 1848.5122 | 1848.9171 | 1 | 10.012 | 75.0% | 5 | R.NPDDITQEEYGEFYK.S | 2 |
|  | CENPL\_stlcld\_tube2\_122314\_01.13653.13653.2 | 4.5186 | 0.4666 | 100.0% | 1528.1322 | 1528.6616 | 1 | 8.221 | 70.8% | 3 | K.SLTNDWEDHLAVK.H | 22 |
|  | CENPL\_stlcld\_122314\_01.13418.13418.2 | 3.3434 | 0.4267 | 100.0% | 1349.7322 | 1349.4886 | 3 | 7.174 | 65.0% | 10 | K.HFSVEGQLEFR.A | 22 |
|  | CENPL\_stlcld\_tube2\_122314\_02.13062.13062.3 | 2.6349 | 0.2058 | 95.2% | 1349.9043 | 1349.4886 | 279 | 4.978 | 40.0% | 1 | K.HFSVEGQLEFR.A | 33 |
| \* | CENPL\_stlcld\_122314\_01.15456.15456.2 | 3.123 | 0.2724 | 99.9% | 1237.4521 | 1237.4008 | 1 | 5.727 | 77.8% | 4 | R.RAPFDLFENK.K | 2 |
| \* | CENPL\_stlcld\_tube2\_122314\_01.11385.11385.2 | 2.0393 | 0.3434 | 99.2% | 892.33215 | 891.99884 | 4 | 5.509 | 83.3% | 1 | K.FYEAFSK.N | 2 |
| \* | CENPL\_stlcld\_tube2\_122314\_01.09776.09776.2 | 2.7208 | 0.0658 | 97.9% | 887.3122 | 887.0702 | 159 | 4.062 | 75.0% | 1 | R.RLSELLR.Y | 2 |
| \* | CENPL\_stlcld\_tube2\_122314\_02.12998.12998.3 | 4.6844 | 0.3876 | 100.0% | 2178.6843 | 2178.2915 | 1 | 6.72 | 40.3% | 8 | R.YHTSQSGDEMTSLSEYVSR.M | 3 |
| \* | CENPL\_stlcld\_122314\_02.09927.09927.2 | 2.8151 | 0.3921 | 99.9% | 1161.7122 | 1161.297 | 1 | 6.636 | 72.2% | 3 | K.SIYYITGESK.E | 2 |
| \* | CENPL\_stlcld\_122314\_01.09879.09879.2 | 2.829 | 0.4224 | 99.9% | 1250.2722 | 1250.3538 | 22 | 6.85 | 55.0% | 1 | K.EQVANSAFVER.V | 2 |
| \* | CENPL\_stlcld\_122314\_01.12256.12256.2 | 2.4705 | 0.4435 | 99.9% | 1248.8121 | 1249.4574 | 1 | 7.48 | 60.0% | 2 | R.DNSTMGYMMAK.K | 2 |
| \* | CENPL\_stlcld\_122314\_01.11572.11572.3 | 2.5568 | 0.2495 | 95.4% | 1911.4744 | 1912.1991 | 137 | 4.555 | 30.0% | 1 | K.KHLEINPDHPIVETLR.Q | 3 |
| \* | CENPL\_stlcld\_tube2\_122314\_01.12192.12192.2 | 4.4182 | 0.3118 | 100.0% | 1784.4722 | 1784.025 | 1 | 7.785 | 78.6% | 2 | K.HLEINPDHPIVETLR.Q | 2 |
| \* | CENPL\_stlcld\_tube2\_122314\_01.12182.12182.3 | 4.4621 | 0.4887 | 100.0% | 1785.4443 | 1784.025 | 1 | 8.375 | 46.4% | 4 | K.HLEINPDHPIVETLR.Q | 3 |
| \* | CENPL\_stlcld\_tube2\_122314\_02.02044.02044.3 | 4.3665 | 0.3947 | 100.0% | 2990.5144 | 2990.3398 | 1 | 7.311 | 33.7% | 1 | K.DLVVLLFETALLSSGFSLEDPQTHSNR.I | 3 |

Similarities:
gi|153792590|ref|NP\_0(8:19)  

---

|  |  |  |  |  |  |  |  |  |
| --- | --- | --- | --- | --- | --- | --- | --- | --- |
| U | *gi|47132620|ref|NP\_00* | 20 | 45 | 36.6% | 639 | 65433 | 8.0 | keratin 2 [Homo sapiens] |

| Filename XCorr DeltCN Conf% ObsM+H+ CalcM+H+ SpR ZScore Ion% # Sequence  | | | | | | | | | | | | |
| --- | --- | --- | --- | --- | --- | --- | --- | --- | --- | --- | --- | --- |
|  | CENPL\_stlcld\_tube2\_122314\_01.08702.08702.2 | 4.0594 | 0.429 | 100.0% | 1255.5322 | 1255.3298 | 1 | 8.32 | 76.9% | 3 | R.GFSSGSAVVSGGSR.R | 2 |
|  | CENPL\_stlcld\_122314\_02.13476.13476.2 | 3.5153 | 0.3402 | 99.9% | 1839.3121 | 1840.0055 | 2 | 7.863 | 40.5% | 1 | K.SISISVAGGGGGFGAAGGFGGR.G | 2 |
| \* | CENPL\_stlcld\_tube2\_122314\_02.15011.15011.3 | 4.6889 | 0.3547 | 100.0% | 2400.5942 | 2400.4446 | 1 | 8.533 | 40.5% | 2 | R.GGGFGGGSSFGGGSGFSGGGFGGGGFGGGR.F | 3 |
|  | CENPL\_stlcld\_tube2\_122314\_01.19682.19682.3 | 5.2436 | 0.456 | 100.0% | 4093.8245 | 4094.5786 | 1 | 7.852 | 22.7% | 1 | R.FGGFGGPGGVGGLGGPGGFGPGGYPGGIHEVSVNQSLLQPLNVK.V | 3 |
|  | CENPL\_stlcld\_tube2\_122314\_01.12512.12512.2 | 2.417 | 0.2122 | 98.9% | 828.09216 | 827.95544 | 5 | 4.999 | 91.7% | 4 | K.FASFIDK.V | 222222222 |
|  | CENPL\_stlcld\_122314\_01.12293.12293.2 | 2.815 | 0.2809 | 99.9% | 1082.9922 | 1083.2755 | 3 | 6.695 | 75.0% | 2 | K.FASFIDKVR.F | 222322223 |
|  | CENPL\_stlcld\_tube2\_122314\_01.11162.11162.2 | 4.5228 | 0.0843 | 99.9% | 1476.4521 | 1476.6726 | 1 | 6.928 | 90.9% | 6 | R.FLEQQNQVLQTK.W | 22 |
|  | CENPL\_stlcld\_tube2\_122314\_01.11662.11662.2 | 3.0741 | 0.3973 | 100.0% | 1038.3522 | 1038.1454 | 1 | 6.443 | 87.5% | 3 | R.YLDGLTAER.T | 2 |
|  | CENPL\_stlcld\_tube2\_122314\_01.12383.12383.2 | 2.4922 | 0.2797 | 98.9% | 1209.9321 | 1209.3416 | 5 | 5.613 | 65.0% | 1 | R.TAAENDFVTLK.K | 2 |
|  | CENPL\_stlcld\_tube2\_122314\_01.09773.09773.2 | 3.48 | 0.3618 | 100.0% | 1337.5922 | 1337.5156 | 1 | 5.666 | 81.8% | 1 | R.TAAENDFVTLKK.D | 2 |
|  | CENPL\_stlcld\_122314\_01.20684.20684.2 | 3.9417 | 0.3391 | 100.0% | 1461.8121 | 1461.6982 | 1 | 6.864 | 72.7% | 2 | K.VDLLNQEIEFLK.V | 2 |
|  | CENPL\_stlcld\_tube2\_122314\_01.19944.19944.1 | 2.8562 | 0.3917 | 100.0% | 1329.95 | 1330.5211 | 1 | 6.619 | 72.7% | 1 | R.NLDLDSIIAEVK.A | 11111 |
|  | CENPL\_stlcld\_122314\_01.20872.20872.2 | 3.7762 | 0.4242 | 100.0% | 1330.7522 | 1330.5211 | 1 | 7.499 | 81.8% | 2 | R.NLDLDSIIAEVK.A | 22222 |
|  | CENPL\_stlcld\_122314\_01.08782.08782.2 | 2.6273 | 0.1146 | 97.5% | 1108.5721 | 1108.196 | 60 | 6.069 | 62.5% | 2 | K.AQYEEIAQR.S | 2222 |
|  | CENPL\_stlcld\_tube2\_122314\_01.11808.11808.2 | 3.0504 | 0.1151 | 98.5% | 1195.6522 | 1194.33 | 1 | 6.613 | 77.8% | 4 | K.YEELQVTVGR.H | 2 |
|  | CENPL\_stlcld\_tube2\_122314\_01.11376.11376.1 | 1.8862 | 0.2617 | 97.1% | 973.58 | 974.102 | 7 | 4.27 | 64.3% | 1 | K.IEISELNR.V | 11 |
|  | CENPL\_stlcld\_tube2\_122314\_01.11366.11366.2 | 3.0241 | 0.1669 | 99.6% | 974.4522 | 974.102 | 11 | 4.866 | 78.6% | 4 | K.IEISELNR.V | 22 |
|  | CENPL\_stlcld\_tube2\_122314\_01.15156.15156.2 | 4.2003 | 0.3405 | 100.0% | 1614.6721 | 1614.796 | 1 | 7.303 | 73.1% | 1 | R.NKLNDLEEALQQAK.E | 2 |
|  | CENPL\_stlcld\_tube2\_122314\_01.18941.18941.3 | 5.0315 | 0.3874 | 100.0% | 2199.2644 | 2199.4258 | 1 | 7.307 | 45.8% | 3 | R.NKLNDLEEALQQAKEDLAR.L | 3 |
|  | CENPL\_stlcld\_tube2\_122314\_01.13841.13841.2 | 2.9177 | 0.2074 | 98.9% | 1522.5322 | 1522.8029 | 2 | 5.216 | 63.6% | 1 | R.LLRDYQELMNVK.L | 22 |

Similarities:
gi|4504919|ref|NP\_002(2:18)  
gi|119395750|ref|NP\_0(3:17)  
gi|67782365|ref|NP\_00(2:18)  
gi|119703753|ref|NP\_0(5:15)  
gi|32567786|ref|NP\_78(5:15)  
gi|119395754|ref|NP\_0(4:16)  
contaminant\_KERATIN16(1:19)  
gi|153791158|ref|NP\_0(4:16)  
gi|109255249|ref|NP\_0(2:18)  
gi|15618995|ref|NP\_25(2:18)  

---

|  |  |  |  |  |  |  |  |  |
| --- | --- | --- | --- | --- | --- | --- | --- | --- |
| U | *gi|31542947|ref|NP\_00* | 21 | 52 | 36.6% | 573 | 61055 | 5.9 | chaperonin [Homo sapiens] |
| U | *gi|41399285|ref|NP\_95* | 21 | 52 | 36.6% | 573 | 61055 | 5.9 | chaperonin [Homo sapiens] |

| Filename XCorr DeltCN Conf% ObsM+H+ CalcM+H+ SpR ZScore Ion% # Sequence  | | | | | | | | | | | | |
| --- | --- | --- | --- | --- | --- | --- | --- | --- | --- | --- | --- | --- |
|  | CENPL\_stlcld\_tube2\_122314\_02.01931.01931.2 | 3.9224 | 0.5996 | 100.0% | 2113.892 | 2114.5667 | 1 | 10.471 | 50.0% | 1 | R.ALMLQGVDLLADAVAVTMGPK.G | 2 |
|  | CENPL\_stlcld\_tube2\_122314\_02.01943.01943.3 | 3.3319 | 0.322 | 99.9% | 2114.6042 | 2114.5667 | 52 | 5.65 | 27.5% | 1 | R.ALMLQGVDLLADAVAVTMGPK.G | 3 |
|  | CENPL\_stlcld\_122314\_02.00429.00429.3 | 3.0049 | 0.24 | 97.5% | 2326.7644 | 2327.806 | 83 | 4.806 | 28.4% | 1 | R.ALMLQGVDLLADAVAVTMGPKGR.T | 3 |
|  | CENPL\_stlcld\_122314\_02.00430.00430.2 | 4.4505 | 0.4592 | 100.0% | 2327.4922 | 2327.806 | 1 | 8.338 | 52.3% | 1 | R.ALMLQGVDLLADAVAVTMGPKGR.T | 2 |
|  | CENPL\_stlcld\_122314\_01.13359.13359.2 | 3.2316 | 0.3368 | 99.9% | 1345.5521 | 1345.5382 | 1 | 6.302 | 72.7% | 8 | R.TVIIEQSWGSPK.V | 2 |
|  | CENPL\_stlcld\_122314\_02.11055.11055.2 | 5.9696 | 0.6457 | 100.0% | 2560.8523 | 2561.7222 | 1 | 13.394 | 58.3% | 1 | K.LVQDVANNTNEEAGDGTTTATVLAR.S | 2 |
|  | CENPL\_stlcld\_122314\_01.20072.20072.3 | 5.4662 | 0.3593 | 100.0% | 1715.6044 | 1714.1199 | 3 | 7.06 | 48.3% | 3 | R.RGVMLAVDAVIAELKK.Q | 3 |
|  | CENPL\_stlcld\_tube2\_122314\_01.20718.20718.2 | 4.433 | 0.4234 | 100.0% | 1558.7722 | 1557.9324 | 1 | 7.706 | 67.9% | 3 | R.GVMLAVDAVIAELKK.Q | 2 |
|  | CENPL\_stlcld\_122314\_01.19872.19872.2 | 4.5639 | 0.3027 | 100.0% | 1506.5922 | 1505.7235 | 1 | 7.897 | 70.8% | 3 | K.TLNDELEIIEGMK.F | 2 |
|  | CENPL\_stlcld\_tube2\_122314\_01.15333.15333.2 | 2.9115 | 0.4502 | 100.0% | 1390.7322 | 1390.5786 | 1 | 7.855 | 68.2% | 3 | R.GYISPYFINTSK.G | 2 |
|  | CENPL\_stlcld\_tube2\_122314\_01.16095.16095.3 | 5.1983 | 0.4304 | 100.0% | 2048.3943 | 2048.3933 | 1 | 7.278 | 43.1% | 3 | K.KISSIQSIVPALEIANAHR.K | 3 |
|  | CENPL\_stlcld\_tube2\_122314\_01.18065.18065.2 | 3.6936 | 0.4386 | 100.0% | 1919.9922 | 1920.2192 | 20 | 7.283 | 32.4% | 1 | K.ISSIQSIVPALEIANAHR.K | 2 |
|  | CENPL\_stlcld\_122314\_01.18640.18640.3 | 3.6429 | 0.2819 | 99.9% | 1920.6244 | 1920.2192 | 9 | 6.949 | 33.8% | 3 | K.ISSIQSIVPALEIANAHR.K | 3 |
|  | CENPL\_stlcld\_122314\_02.11597.11597.2 | 2.3404 | 0.2448 | 98.2% | 1154.7922 | 1154.4819 | 1 | 5.53 | 70.0% | 2 | R.LKVGLQVVAVK.A | 2 |
|  | CENPL\_stlcld\_tube2\_122314\_01.11951.11951.2 | 2.9309 | 0.2998 | 99.9% | 913.5522 | 913.14844 | 1 | 7.12 | 87.5% | 1 | K.VGLQVVAVK.A | 2 |
|  | CENPL\_stlcld\_122314\_01.13882.13882.2 | 4.4425 | 0.4674 | 100.0% | 1631.5922 | 1631.9684 | 1 | 8.225 | 60.7% | 1 | K.VGEVIVTKDDAMLLK.G | 2 |
|  | CENPL\_stlcld\_122314\_01.13862.13862.3 | 3.7945 | 0.453 | 100.0% | 1631.9644 | 1631.9684 | 3 | 7.188 | 41.1% | 4 | K.VGEVIVTKDDAMLLK.G | 3 |
|  | CENPL\_stlcld\_122314\_01.17704.17704.3 | 4.1918 | 0.1409 | 98.2% | 2454.5044 | 2452.721 | 1 | 4.624 | 32.9% | 1 | K.RIQEIIEQLDVTTSEYEKEK.L | 3 |
|  | CENPL\_stlcld\_122314\_02.09466.09466.2 | 2.5466 | 0.1941 | 98.5% | 902.3522 | 902.0788 | 9 | 6.234 | 75.0% | 2 | K.LSDGVAVLK.V | 2 |
|  | CENPL\_stlcld\_122314\_02.11043.11043.2 | 3.3456 | 0.3272 | 99.9% | 1216.1122 | 1216.377 | 1 | 7.136 | 68.2% | 8 | K.NAGVEGSLIVEK.I | 2 |
|  | CENPL\_stlcld\_122314\_02.16504.16504.3 | 5.3821 | 0.4211 | 100.0% | 2509.4944 | 2509.8235 | 1 | 7.577 | 37.5% | 1 | K.IMQSSSEVGYDAMAGDFVNMVEK.G | 3 |

---

|  |  |  |  |  |  |  |  |  |
| --- | --- | --- | --- | --- | --- | --- | --- | --- |
| U | *gi|14043072|ref|NP\_11* | 12 | 40 | 36.5% | 353 | 37430 | 8.9 | heterogeneous nuclear ribonucleoprotein A2/B1 isoform B1 [Homo sapiens] |
| U | *gi|4504447|ref|NP\_002* | 12 | 41 | 37.8% | 341 | 36006 | 8.6 | heterogeneous nuclear ribonucleoprotein A2/B1 isoform A2 [Homo sapiens] |

| Filename XCorr DeltCN Conf% ObsM+H+ CalcM+H+ SpR ZScore Ion% # Sequence  | | | | | | | | | | | | |
| --- | --- | --- | --- | --- | --- | --- | --- | --- | --- | --- | --- | --- |
|  | CENPL\_stlcld\_122314\_01.18336.18336.2 | 5.4376 | 0.4564 | 100.0% | 1928.3322 | 1928.1925 | 1 | 8.039 | 65.6% | 3 | R.KLFIGGLSFETTEESLR.N | 2 |
|  | CENPL\_stlcld\_122314\_02.16484.16484.2 | 4.9444 | 0.404 | 100.0% | 1800.7722 | 1800.0184 | 1 | 7.205 | 76.7% | 7 | K.LFIGGLSFETTEESLR.N | 2 |
|  | CENPL\_stlcld\_tube2\_122314\_01.11360.11360.2 | 2.6927 | 0.3746 | 99.9% | 1088.4122 | 1088.1644 | 72 | 5.833 | 78.6% | 3 | R.NYYEQWGK.L | 2 |
|  | CENPL\_stlcld\_122314\_02.09794.09794.3 | 3.1238 | 0.2951 | 99.9% | 1882.4343 | 1881.0984 | 79 | 6.306 | 33.3% | 1 | K.LFVGGIKEDTEEHHLR.D | 3 |
|  | CENPL\_stlcld\_tube2\_122314\_01.14079.14079.3 | 5.8679 | 0.1444 | 99.9% | 2911.7644 | 2913.1724 | 1 | 8.221 | 38.0% | 1 | K.LFVGGIKEDTEEHHLRDYFEEYGK.I | 3 |
|  | CENPL\_stlcld\_122314\_01.15506.15506.2 | 3.5188 | 0.4453 | 100.0% | 1189.1921 | 1189.3513 | 2 | 6.901 | 77.8% | 2 | K.IDTIEIITDR.Q | 2 |
|  | CENPL\_stlcld\_122314\_01.16455.16455.2 | 4.1486 | 0.4359 | 100.0% | 1697.4521 | 1696.8132 | 1 | 7.577 | 75.0% | 1 | R.GFGFVTFDDHDPVDK.I | 2 |
|  | CENPL\_stlcld\_tube2\_122314\_01.17946.17946.2 | 4.8466 | 0.5307 | 100.0% | 2278.0923 | 2278.5693 | 1 | 8.967 | 57.9% | 1 | R.GFGFVTFDDHDPVDKIVLQK.Y | 2 |
|  | CENPL\_stlcld\_tube2\_122314\_01.17910.17910.3 | 3.8539 | 0.4729 | 100.0% | 2278.6743 | 2278.5693 | 1 | 7.461 | 38.2% | 5 | R.GFGFVTFDDHDPVDKIVLQK.Y | 3 |
|  | CENPL\_stlcld\_122314\_02.09394.09394.2 | 3.0248 | 0.215 | 99.5% | 1014.3122 | 1014.0421 | 2 | 5.526 | 72.2% | 4 | R.GGNFGFGDSR.G | 2 |
|  | CENPL\_stlcld\_tube2\_122314\_01.10568.10568.2 | 3.1929 | 0.4653 | 100.0% | 1378.3522 | 1378.4465 | 5 | 6.845 | 53.6% | 8 | R.GGGGNFGPGPGSNFR.G | 2 |
|  | CENPL\_stlcld\_tube2\_122314\_01.10274.10274.2 | 6.2091 | 0.6294 | 100.0% | 2190.5322 | 2191.2554 | 1 | 11.768 | 52.1% | 4 | R.NMGGPYGGGNYGPGGSGGSGGYGGR.S | 2 |

---

|  |  |  |  |  |  |  |  |  |
| --- | --- | --- | --- | --- | --- | --- | --- | --- |
| U | *gi|14165435|ref|NP\_11* | 16 | 52 | 36.3% | 463 | 50976 | 5.5 | heterogeneous nuclear ribonucleoprotein K isoform b [Homo sapiens] |
| U | *gi|14165439|ref|NP\_00* | 16 | 54 | 36.2% | 464 | 51028 | 5.3 | heterogeneous nuclear ribonucleoprotein K isoform a [Homo sapiens] |

| Filename XCorr DeltCN Conf% ObsM+H+ CalcM+H+ SpR ZScore Ion% # Sequence  | | | | | | | | | | | | |
| --- | --- | --- | --- | --- | --- | --- | --- | --- | --- | --- | --- | --- |
|  | CENPL\_stlcld\_tube2\_122314\_01.08265.08265.3 | 4.502 | 0.2998 | 100.0% | 1737.0543 | 1736.8969 | 1 | 5.911 | 53.8% | 3 | K.RPAEDMEEEQAFKR.S | 3 |
|  | CENPL\_stlcld\_122314\_01.09736.09736.2 | 3.5457 | 0.3849 | 100.0% | 1350.5521 | 1350.4894 | 1 | 6.22 | 75.0% | 2 | R.SRNTDEMVELR.I | 2 |
|  | CENPL\_stlcld\_tube2\_122314\_01.11176.11176.2 | 3.1257 | 0.3414 | 100.0% | 1107.3722 | 1107.2238 | 1 | 5.972 | 81.2% | 4 | R.NTDEMVELR.I | 2 |
|  | CENPL\_stlcld\_122314\_01.10439.10439.2 | 3.5746 | 0.5319 | 100.0% | 1781.4722 | 1781.8302 | 381 | 8.888 | 37.5% | 6 | R.TDYNASVSVPDSSGPER.I | 2 |
|  | CENPL\_stlcld\_tube2\_122314\_01.21444.21444.2 | 3.9002 | 0.5282 | 100.0% | 1843.8322 | 1844.1992 | 1 | 8.968 | 53.1% | 2 | R.ILSISADIETIGEILKK.I | 2 |
|  | CENPL\_stlcld\_tube2\_122314\_01.10636.10636.2 | 2.2208 | 0.1587 | 95.0% | 1100.3322 | 1099.1083 | 40 | 4.583 | 62.5% | 1 | K.GSDFDCELR.L | 2 |
|  | CENPL\_stlcld\_122314\_01.14769.14769.2 | 4.196 | 0.4718 | 100.0% | 1519.5122 | 1519.8711 | 1 | 8.158 | 75.0% | 8 | R.LLIHQSLAGGIIGVK.G | 2 |
|  | CENPL\_stlcld\_tube2\_122314\_02.14157.14157.3 | 5.11 | 0.489 | 100.0% | 1520.8744 | 1519.8711 | 1 | 7.901 | 55.4% | 8 | R.LLIHQSLAGGIIGVK.G | 3 |
|  | CENPL\_stlcld\_122314\_01.08844.08844.2 | 2.4154 | 0.2389 | 98.4% | 1054.2722 | 1054.278 | 17 | 5.457 | 66.7% | 1 | R.VVLIGGKPDR.V | 2 |
|  | CENPL\_stlcld\_tube2\_122314\_01.19575.19575.2 | 3.9189 | 0.4621 | 100.0% | 1341.6921 | 1341.6311 | 1 | 7.31 | 81.8% | 4 | K.IILDLISESPIK.G | 2 |
|  | CENPL\_stlcld\_tube2\_122314\_01.17379.17379.2 | 3.712 | 0.3748 | 100.0% | 1554.5122 | 1554.8705 | 1 | 6.941 | 65.4% | 3 | K.IILDLISESPIKGR.A | 2 |
|  | CENPL\_stlcld\_tube2\_122314\_01.17387.17387.3 | 2.4528 | 0.3418 | 99.9% | 1555.2244 | 1554.8705 | 1 | 4.985 | 51.9% | 2 | K.IILDLISESPIKGR.A | 3 |
|  | CENPL\_stlcld\_tube2\_122314\_01.16910.16910.2 | 5.2348 | 0.5045 | 100.0% | 1917.4722 | 1918.1974 | 1 | 9.794 | 52.8% | 4 | R.GSYGDLGGPIITTQVTIPK.D | 2 |
|  | CENPL\_stlcld\_122314\_02.10367.10367.3 | 3.3039 | 0.3274 | 99.9% | 2070.5645 | 2070.1772 | 7 | 5.0 | 31.9% | 1 | R.HESGASIKIDEPLEGSEDR.I | 3 |
|  | CENPL\_stlcld\_tube2\_122314\_01.19268.19268.2 | 5.4066 | 0.4506 | 100.0% | 2591.112 | 2590.9365 | 1 | 9.548 | 52.3% | 2 | R.IITITGTQDQIQNAQYLLQNSVK.Q | 2 |
|  | CENPL\_stlcld\_122314\_01.19984.19984.3 | 5.5971 | 0.5321 | 100.0% | 2592.0842 | 2590.9365 | 1 | 9.46 | 42.0% | 1 | R.IITITGTQDQIQNAQYLLQNSVK.Q | 3 |

---

|  |  |  |  |  |  |  |  |  |
| --- | --- | --- | --- | --- | --- | --- | --- | --- |
| U | *gi|5031753|ref|NP\_005* | 11 | 36 | 36.1% | 449 | 49229 | 6.3 | heterogeneous nuclear ribonucleoprotein H1 [Homo sapiens] |

| Filename XCorr DeltCN Conf% ObsM+H+ CalcM+H+ SpR ZScore Ion% # Sequence  | | | | | | | | | | | | |
| --- | --- | --- | --- | --- | --- | --- | --- | --- | --- | --- | --- | --- |
| \* | CENPL\_stlcld\_tube2\_122314\_01.13290.13290.2 | 2.2128 | 0.2594 | 97.2% | 1505.6122 | 1505.5933 | 11 | 4.95 | 54.2% | 1 | R.GLPWSCSADEVQR.F | 2 |
| \* | CENPL\_stlcld\_122314\_02.11997.11997.3 | 3.7229 | 0.2107 | 99.3% | 2109.5645 | 2108.2231 | 5 | 4.574 | 33.3% | 1 | R.EGRPSGEAFVELESEDEVK.L | 3 |
| \* | CENPL\_stlcld\_122314\_02.14357.14357.2 | 3.5887 | 0.3899 | 100.0% | 1335.3121 | 1335.5176 | 1 | 7.699 | 70.0% | 4 | K.SNNVEMDWVLK.H | 2 |
|  | CENPL\_stlcld\_tube2\_122314\_01.08404.08404.2 | 4.4003 | 0.5584 | 100.0% | 1685.1921 | 1685.7501 | 1 | 8.836 | 73.3% | 3 | K.HTGPNSPDTANDGFVR.L | 2 |
|  | CENPL\_stlcld\_tube2\_122314\_01.08420.08420.3 | 4.3259 | 0.2674 | 100.0% | 1686.2043 | 1685.7501 | 3 | 5.634 | 43.3% | 3 | K.HTGPNSPDTANDGFVR.L | 3 |
| \* | CENPL\_stlcld\_tube2\_122314\_02.00776.00776.2 | 4.2551 | 0.5098 | 100.0% | 2906.2122 | 2906.3079 | 1 | 9.301 | 38.0% | 1 | K.EEIVQFFSGLEIVPNGITLPVDFQGR.S | 2 |
|  | CENPL\_stlcld\_122314\_02.15058.15058.2 | 4.9944 | 0.4903 | 100.0% | 1843.5122 | 1843.0001 | 1 | 8.935 | 68.8% | 9 | R.STGEAFVQFASQEIAEK.A | 2 |
|  | CENPL\_stlcld\_tube2\_122314\_01.12652.12652.2 | 2.8409 | 0.3124 | 99.7% | 1602.3121 | 1602.6844 | 3 | 5.598 | 58.3% | 1 | R.DLNYCFSGMSDHR.Y | 2 |
|  | CENPL\_stlcld\_tube2\_122314\_01.20397.20397.2 | 4.5599 | 0.463 | 100.0% | 1997.8121 | 1998.2023 | 1 | 7.917 | 59.4% | 4 | R.ATENDIYNFFSPLNPVR.V | 22 |
|  | CENPL\_stlcld\_tube2\_122314\_01.09572.09572.2 | 3.1384 | 0.502 | 100.0% | 1093.4922 | 1093.2278 | 1 | 8.474 | 83.3% | 7 | R.VHIEIGPDGR.V | 22 |
| \* | CENPL\_stlcld\_tube2\_122314\_02.14424.14424.3 | 3.0717 | 0.3037 | 99.9% | 2142.9844 | 2143.32 | 1 | 6.535 | 39.5% | 2 | R.YVELFLNSTAGASGGAYEHR.Y | 3 |

Similarities:
gi|148470397|ref|NP\_0(2:9)  

---

|  |  |  |  |  |  |  |  |  |
| --- | --- | --- | --- | --- | --- | --- | --- | --- |
| U | *gi|4506645|ref|NP\_000* | 3 | 3 | 35.7% | 70 | 8218 | 10.1 | ribosomal protein L38 [Homo sapiens] |
| U | *gi|78214522|ref|NP\_00* | 3 | 3 | 35.7% | 70 | 8218 | 10.1 | ribosomal protein L38 [Homo sapiens] |

| Filename XCorr DeltCN Conf% ObsM+H+ CalcM+H+ SpR ZScore Ion% # Sequence  | | | | | | | | | | | | |
| --- | --- | --- | --- | --- | --- | --- | --- | --- | --- | --- | --- | --- |
|  | CENPL\_stlcld\_122314\_01.15430.15430.2 | 3.9729 | 0.2003 | 99.9% | 1577.6322 | 1576.8766 | 1 | 5.099 | 75.0% | 1 | R.KIEEIKDFLLTAR.R | 2 |
|  | CENPL\_stlcld\_tube2\_122314\_01.15443.15443.3 | 3.5924 | 0.192 | 99.5% | 1578.2943 | 1576.8766 | 1 | 4.736 | 52.1% | 1 | R.KIEEIKDFLLTAR.R | 3 |
|  | CENPL\_stlcld\_122314\_01.14279.14279.2 | 3.315 | 0.2314 | 99.7% | 1486.5521 | 1486.7484 | 1 | 6.104 | 72.7% | 1 | R.YLYTLVITDKEK.A | 2 |

---

|  |  |  |  |  |  |  |  |  |
| --- | --- | --- | --- | --- | --- | --- | --- | --- |
| U | *gi|27735067|ref|NP\_77* | 18 | 42 | 33.4% | 679 | 75357 | 6.8 | hypothetical protein LOC126353 [Homo sapiens] |

| Filename XCorr DeltCN Conf% ObsM+H+ CalcM+H+ SpR ZScore Ion% # Sequence  | | | | | | | | | | | | |
| --- | --- | --- | --- | --- | --- | --- | --- | --- | --- | --- | --- | --- |
| \* | CENPL\_stlcld\_122314\_01.13180.13180.2 | 2.3633 | 0.2418 | 98.2% | 1264.7922 | 1265.5009 | 1 | 5.423 | 70.0% | 1 | R.YPILGIPQAHR.G | 2 |
| \* | CENPL\_stlcld\_122314\_01.13276.13276.3 | 3.0713 | 0.3666 | 100.0% | 1266.1743 | 1265.5009 | 2 | 5.996 | 45.0% | 4 | R.YPILGIPQAHR.G | 3 |
| \* | CENPL\_stlcld\_tube2\_122314\_02.10522.10522.3 | 2.9247 | 0.2308 | 97.8% | 1834.5844 | 1834.986 | 9 | 5.072 | 35.9% | 1 | R.QGVSYSVHAYTGQPSPR.G | 3 |
| \* | CENPL\_stlcld\_122314\_02.10203.10203.3 | 3.251 | 0.374 | 100.0% | 1976.7843 | 1976.072 | 1 | 6.305 | 38.3% | 5 | R.GLHSENREDEGWQVYR.L | 3 |
| \* | CENPL\_stlcld\_tube2\_122314\_01.12152.12152.2 | 3.3883 | 0.3002 | 99.9% | 1128.0122 | 1128.3195 | 1 | 6.212 | 88.9% | 4 | R.WAVIQGQAVR.K | 2 |
| \* | CENPL\_stlcld\_tube2\_122314\_01.09749.09749.2 | 2.3919 | 0.169 | 95.9% | 1256.2322 | 1256.4935 | 91 | 3.67 | 55.0% | 1 | R.WAVIQGQAVRK.S | 2 |
| \* | CENPL\_stlcld\_122314\_01.16780.16780.2 | 2.9871 | 0.3396 | 99.9% | 2302.7522 | 2303.534 | 1 | 6.348 | 42.1% | 2 | R.STPLEENVVDREQIDFLAAR.Q | 2 |
| \* | CENPL\_stlcld\_tube2\_122314\_01.16677.16677.3 | 3.6002 | 0.3329 | 99.9% | 2303.7544 | 2303.534 | 1 | 6.632 | 38.2% | 7 | R.STPLEENVVDREQIDFLAAR.Q | 3 |
| \* | CENPL\_stlcld\_tube2\_122314\_01.12830.12830.2 | 2.7957 | 0.3611 | 99.9% | 1306.6322 | 1306.4612 | 5 | 5.929 | 75.0% | 2 | R.QQFLSLEQANK.G | 2 |
| \* | CENPL\_stlcld\_122314\_01.07787.07787.3 | 3.0224 | 0.2447 | 97.8% | 2363.3643 | 2362.3916 | 138 | 4.407 | 25.0% | 1 | K.GAPHSS\*PARGT#PAGTTPGASQAPK.A | 3 |
| \* | CENPL\_stlcld\_tube2\_122314\_01.07415.07415.3 | 3.1615 | 0.2598 | 98.7% | 2441.8442 | 2442.3916 | 16 | 5.018 | 26.1% | 1 | K.GAPHSS\*PARGT#PAGT#TPGASQAPK.A | 3 |
| \* | CENPL\_stlcld\_tube2\_122314\_01.18078.18078.2 | 3.1828 | 0.2377 | 99.4% | 2695.7722 | 2695.986 | 1 | 5.603 | 36.5% | 1 | R.AVPTWASVQVVDDPGSLASVESPGTPK.E | 2 |
| \* | CENPL\_stlcld\_tube2\_122314\_01.12632.12632.2 | 2.6855 | 0.3695 | 99.9% | 871.21216 | 871.0678 | 2 | 7.165 | 78.6% | 1 | K.LSLITAPR.R | 2 |
| \* | CENPL\_stlcld\_tube2\_122314\_01.13440.13440.2 | 2.9548 | 0.2358 | 98.8% | 1698.6122 | 1697.8448 | 12 | 5.435 | 43.3% | 2 | R.ASTPDWVSEGPQPGLR.R | 2 |
| \* | CENPL\_stlcld\_122314\_01.13623.13623.2 | 2.4535 | 0.2735 | 98.3% | 1580.1122 | 1580.6488 | 3 | 5.816 | 50.0% | 1 | R.ALSSDSILS\*PAPDAR.A | 2 |
| \* | CENPL\_stlcld\_122314\_01.17079.17079.3 | 5.627 | 0.4478 | 100.0% | 3013.1343 | 3014.3757 | 1 | 8.076 | 31.7% | 3 | R.FRAPDEPQQAQVPHVWGWEVAGAPALR.L | 3 |
| \* | CENPL\_stlcld\_122314\_01.18196.18196.3 | 4.0439 | 0.2788 | 99.9% | 2710.1943 | 2711.0115 | 1 | 6.323 | 33.3% | 3 | R.APDEPQQAQVPHVWGWEVAGAPALR.L | 3 |
| \* | CENPL\_stlcld\_122314\_01.17381.17381.3 | 4.5392 | 0.2681 | 99.9% | 2718.5344 | 2719.0264 | 1 | 5.391 | 37.0% | 2 | R.KKEQWYAGINPSDGINSEVLEAIR.V | 3 |

---

|  |  |  |  |  |  |  |  |  |
| --- | --- | --- | --- | --- | --- | --- | --- | --- |
| U | *gi|14165469|ref|NP\_00* | 5 | 9 | 32.3% | 130 | 14839 | 10.1 | ribosomal protein S15a [Homo sapiens] |
| U | *gi|71772415|ref|NP\_00* | 5 | 9 | 32.3% | 130 | 14839 | 10.1 | ribosomal protein S15a [Homo sapiens] |

| Filename XCorr DeltCN Conf% ObsM+H+ CalcM+H+ SpR ZScore Ion% # Sequence  | | | | | | | | | | | | |
| --- | --- | --- | --- | --- | --- | --- | --- | --- | --- | --- | --- | --- |
|  | CENPL\_stlcld\_122314\_01.15046.15046.2 | 2.4756 | 0.2331 | 98.7% | 975.27216 | 975.19135 | 13 | 5.222 | 75.0% | 2 | R.MNVLADALK.S | 2 |
|  | CENPL\_stlcld\_122314\_01.13824.13824.2 | 3.4672 | 0.4317 | 100.0% | 1701.9321 | 1701.8357 | 1 | 7.473 | 53.8% | 1 | K.HGYIGEFEIIDDHR.A | 2 |
|  | CENPL\_stlcld\_122314\_01.13829.13829.3 | 4.2683 | 0.3538 | 100.0% | 1702.1044 | 1701.8357 | 1 | 7.276 | 53.8% | 3 | K.HGYIGEFEIIDDHR.A | 3 |
|  | CENPL\_stlcld\_122314\_01.13386.13386.2 | 2.5854 | 0.1898 | 98.3% | 1235.5721 | 1235.4227 | 1 | 5.245 | 83.3% | 1 | R.FDVQLKDLEK.W | 2 |
|  | CENPL\_stlcld\_tube2\_122314\_01.12917.12917.2 | 2.5901 | 0.2543 | 99.3% | 1128.4122 | 1128.276 | 1 | 5.332 | 87.5% | 2 | K.WQNNLLPSR.Q | 2 |

---

|  |  |  |  |  |  |  |  |  |
| --- | --- | --- | --- | --- | --- | --- | --- | --- |
| U | *gi|11968182|ref|NP\_07* | 5 | 7 | 32.2% | 152 | 17719 | 11.0 | ribosomal protein S18 [Homo sapiens] |
| U | *gi|169168597|ref|XP\_0* | 5 | 7 | 32.2% | 152 | 17719 | 11.0 | PREDICTED: hypothetical protein [Homo sapiens] |

| Filename XCorr DeltCN Conf% ObsM+H+ CalcM+H+ SpR ZScore Ion% # Sequence  | | | | | | | | | | | | |
| --- | --- | --- | --- | --- | --- | --- | --- | --- | --- | --- | --- | --- |
|  | CENPL\_stlcld\_122314\_01.09023.09023.2 | 2.7152 | 0.3934 | 99.9% | 1002.59216 | 1002.1154 | 2 | 7.2 | 81.2% | 2 | R.VLNTNIDGR.R | 2 |
|  | CENPL\_stlcld\_122314\_01.09147.09147.2 | 3.2267 | 0.2569 | 99.9% | 1248.2722 | 1248.2891 | 1 | 6.268 | 70.0% | 1 | R.AGELTEDEVER.V | 2 |
|  | CENPL\_stlcld\_tube2\_122314\_01.11487.11487.2 | 2.3281 | 0.462 | 99.9% | 1071.8522 | 1072.311 | 1 | 7.388 | 81.2% | 1 | R.VITIMQNPR.Q | 2 |
|  | CENPL\_stlcld\_122314\_01.18848.18848.2 | 2.8004 | 0.326 | 99.9% | 1061.4521 | 1061.2285 | 2 | 5.812 | 78.6% | 2 | K.IPDWFLNR.Q | 2 |
|  | CENPL\_stlcld\_tube2\_122314\_01.10944.10944.2 | 3.2738 | 0.3917 | 100.0% | 1322.5122 | 1322.4606 | 1 | 7.012 | 77.3% | 1 | K.YSQVLANGLDNK.L | 2 |

---

|  |  |  |  |  |  |  |  |  |
| --- | --- | --- | --- | --- | --- | --- | --- | --- |
| U | *gi|4506691|ref|NP\_001* | 5 | 7 | 32.2% | 146 | 16445 | 10.2 | ribosomal protein S16 [Homo sapiens] |

| Filename XCorr DeltCN Conf% ObsM+H+ CalcM+H+ SpR ZScore Ion% # Sequence  | | | | | | | | | | | | |
| --- | --- | --- | --- | --- | --- | --- | --- | --- | --- | --- | --- | --- |
|  | CENPL\_stlcld\_122314\_01.14774.14774.2 | 3.0905 | 0.5222 | 100.0% | 1188.3121 | 1188.372 | 2 | 9.0 | 70.0% | 3 | K.GPLQSVQVFGR.K | 2 |
| \* | CENPL\_stlcld\_tube2\_122314\_01.11723.11723.2 | 2.5197 | 0.2151 | 98.0% | 1411.8522 | 1411.6622 | 6 | 4.402 | 59.1% | 1 | K.VNGRPLEMIEPR.T | 2 |
| \* | CENPL\_stlcld\_tube2\_122314\_01.11687.11687.3 | 3.0581 | 0.2508 | 99.9% | 1412.7544 | 1411.6622 | 4 | 5.025 | 47.7% | 1 | K.VNGRPLEMIEPR.T | 3 |
|  | CENPL\_stlcld\_tube2\_122314\_01.19640.19640.2 | 2.3735 | 0.1589 | 96.0% | 1095.1122 | 1095.4111 | 64 | 4.909 | 61.1% | 1 | K.LLEPVLLLGK.E | 2 |
| \* | CENPL\_stlcld\_122314\_01.09369.09369.3 | 3.0539 | 0.1825 | 96.3% | 1470.1743 | 1469.7299 | 6 | 4.391 | 44.2% | 1 | R.VKGGGHVAQIYAIR.Q | 3 |

---

|  |  |  |  |  |  |  |  |  |
| --- | --- | --- | --- | --- | --- | --- | --- | --- |
| U | *gi|14141152|ref|NP\_00* | 18 | 61 | 31.5% | 730 | 77516 | 8.7 | heterogeneous nuclear ribonucleoprotein M isoform a [Homo sapiens] |
| U | *gi|157412270|ref|NP\_1* | 18 | 61 | 33.3% | 691 | 73621 | 8.8 | heterogeneous nuclear ribonucleoprotein M isoform b [Homo sapiens] |

| Filename XCorr DeltCN Conf% ObsM+H+ CalcM+H+ SpR ZScore Ion% # Sequence  | | | | | | | | | | | | |
| --- | --- | --- | --- | --- | --- | --- | --- | --- | --- | --- | --- | --- |
|  | CENPL\_stlcld\_122314\_01.08547.08547.3 | 3.3909 | 0.1491 | 96.7% | 1607.7244 | 1607.769 | 14 | 4.439 | 42.3% | 1 | R.GGNRFEPYANPTKR.Y | 3 |
|  | CENPL\_stlcld\_122314\_01.19038.19038.2 | 3.3524 | 0.3472 | 100.0% | 1265.6721 | 1265.4949 | 1 | 7.095 | 80.0% | 3 | R.AFITNIPFDVK.W | 2 |
|  | CENPL\_stlcld\_122314\_02.13660.13660.2 | 3.1907 | 0.4306 | 100.0% | 1427.3722 | 1427.6403 | 1 | 7.902 | 66.7% | 3 | R.LGSTVFVANLDYK.V | 2 |
|  | CENPL\_stlcld\_122314\_01.15104.15104.2 | 2.8762 | 0.3726 | 99.9% | 1435.3722 | 1435.768 | 1 | 6.0 | 79.2% | 2 | K.LKEVFSMAGVVVR.A | 2 |
|  | CENPL\_stlcld\_122314\_01.20318.20318.2 | 5.1341 | 0.5278 | 100.0% | 2178.8323 | 2179.5752 | 1 | 10.192 | 52.3% | 3 | K.GIGMGNIGPAGMGMEGIGFGINK.M | 2 |
|  | CENPL\_stlcld\_122314\_01.15828.15828.2 | 4.8126 | 0.5544 | 100.0% | 1715.5721 | 1715.9724 | 1 | 9.787 | 65.6% | 1 | K.MGGMEGPFGGGMENMGR.F | 2 |
|  | CENPL\_stlcld\_122314\_02.08726.08726.2 | 1.809 | 0.2994 | 96.0% | 957.2522 | 957.11017 | 37 | 5.286 | 62.5% | 1 | R.FGSGMNMGR.I | 2 |
|  | CENPL\_stlcld\_122314\_01.14076.14076.2 | 3.0776 | 0.2341 | 99.7% | 1114.9922 | 1115.3152 | 36 | 4.666 | 61.1% | 1 | R.INEILSNALK.R | 2 |
|  | CENPL\_stlcld\_122314\_01.08757.08757.2 | 2.4784 | 0.3369 | 99.1% | 1286.3722 | 1285.3591 | 75 | 5.446 | 46.4% | 1 | K.QGGGGGGGSVPGIER.M | 2 |
|  | CENPL\_stlcld\_122314\_01.11614.11614.2 | 2.3703 | 0.2963 | 99.7% | 821.9922 | 822.0283 | 1 | 6.349 | 83.3% | 3 | R.MGLVMDR.M | 2 |
|  | CENPL\_stlcld\_tube2\_122314\_01.14400.14400.2 | 4.6274 | 0.491 | 100.0% | 1614.4122 | 1614.875 | 1 | 9.239 | 75.0% | 4 | R.MGPLGLDHMASSIER.M | 2 |
|  | CENPL\_stlcld\_tube2\_122314\_01.14427.14427.3 | 3.6683 | 0.4527 | 100.0% | 1615.6444 | 1614.875 | 1 | 8.495 | 53.6% | 4 | R.MGPLGLDHMASSIER.M | 3 |
|  | CENPL\_stlcld\_tube2\_122314\_01.14216.14216.2 | 3.3044 | 0.5525 | 100.0% | 1126.3722 | 1126.3337 | 1 | 9.732 | 90.0% | 8 | R.MGAGMGFGLER.M | 2 |
|  | CENPL\_stlcld\_122314\_01.10176.10176.2 | 1.8807 | 0.3721 | 98.9% | 824.39215 | 824.00085 | 1 | 7.425 | 83.3% | 1 | R.MGLSMER.M | 2 |
|  | CENPL\_stlcld\_tube2\_122314\_01.11643.11643.2 | 3.2184 | 0.3104 | 99.9% | 1190.1721 | 1189.4333 | 3 | 5.441 | 72.7% | 3 | R.MVPAGMGAGLER.M | 2 |
|  | CENPL\_stlcld\_tube2\_122314\_01.13664.13664.2 | 3.6332 | 0.4076 | 100.0% | 1428.6721 | 1428.7076 | 1 | 6.481 | 67.9% | 3 | R.MGPAMGPALGAGIER.M | 2 |
|  | CENPL\_stlcld\_122314\_02.11966.11966.2 | 4.3084 | 0.4818 | 100.0% | 1385.2922 | 1384.5677 | 1 | 9.221 | 75.0% | 9 | R.MGLAMGGGGGASFDR.A | 2 |
|  | CENPL\_stlcld\_122314\_01.13173.13173.3 | 3.6939 | 0.307 | 99.9% | 2037.2043 | 2036.1735 | 1 | 5.085 | 29.5% | 10 | R.GNFGGSFAGSFGGAGGHAPGVAR.K | 3 |

---

|  |  |  |  |  |  |  |  |  |
| --- | --- | --- | --- | --- | --- | --- | --- | --- |
| U | *gi|27436946|ref|NP\_73* | 20 | 50 | 31.5% | 664 | 74140 | 7.0 | lamin A/C isoform 1 precursor [Homo sapiens] |

| Filename XCorr DeltCN Conf% ObsM+H+ CalcM+H+ SpR ZScore Ion% # Sequence  | | | | | | | | | | | | |
| --- | --- | --- | --- | --- | --- | --- | --- | --- | --- | --- | --- | --- |
|  | CENPL\_stlcld\_122314\_02.08766.08766.3 | 3.591 | 0.1313 | 97.7% | 1630.6743 | 1630.7521 | 15 | 4.295 | 47.9% | 2 | R.LQEKEDLQELNDR.L | 3 |
|  | CENPL\_stlcld\_tube2\_122314\_01.09226.09226.2 | 4.3626 | 0.1808 | 99.9% | 1631.7722 | 1630.7521 | 1 | 6.203 | 75.0% | 1 | R.LQEKEDLQELNDR.L | 2 |
|  | CENPL\_stlcld\_tube2\_122314\_01.08780.08780.2 | 3.0746 | 0.1588 | 99.1% | 1090.2922 | 1090.1783 | 3 | 5.095 | 77.8% | 3 | R.SLETENAGLR.L | 2 |
|  | CENPL\_stlcld\_tube2\_122314\_01.07983.07983.2 | 3.2005 | 0.482 | 100.0% | 1149.1322 | 1149.2432 | 1 | 8.248 | 83.3% | 5 | R.ITESEEVVSR.E | 2 |
|  | CENPL\_stlcld\_tube2\_122314\_01.09658.09658.2 | 3.2484 | 0.4119 | 100.0% | 1166.2922 | 1166.2328 | 1 | 7.092 | 80.0% | 1 | K.AAYEAELGDAR.K | 2 |
|  | CENPL\_stlcld\_tube2\_122314\_01.09222.09222.2 | 2.5699 | 0.2626 | 99.1% | 1044.2722 | 1044.1527 | 4 | 5.787 | 72.2% | 1 | K.EGDLIAAQAR.L | 2 |
|  | CENPL\_stlcld\_122314\_01.14874.14874.2 | 3.4243 | 0.3397 | 100.0% | 1244.6522 | 1244.474 | 2 | 7.256 | 75.0% | 2 | R.LKDLEALLNSK.E | 2 |
|  | CENPL\_stlcld\_122314\_01.09707.09707.2 | 2.6273 | 0.3402 | 99.7% | 1276.1322 | 1276.4325 | 1 | 5.925 | 68.2% | 1 | K.EAALSTALSEKR.T | 2 |
|  | CENPL\_stlcld\_122314\_01.12117.12117.2 | 3.0346 | 0.2888 | 99.9% | 1183.5922 | 1183.3066 | 4 | 5.275 | 77.8% | 2 | R.TLEGELHDLR.G | 2 |
|  | CENPL\_stlcld\_tube2\_122314\_01.11650.11650.2 | 3.0679 | 0.2301 | 99.5% | 1511.4321 | 1510.7455 | 1 | 4.631 | 68.2% | 1 | R.LQTMKEELDFQK.N | 2 |
|  | CENPL\_stlcld\_tube2\_122314\_01.13305.13305.2 | 3.1863 | 0.3213 | 100.0% | 1028.9521 | 1029.1814 | 1 | 6.051 | 87.5% | 4 | R.LADALQELR.A | 2 |
|  | CENPL\_stlcld\_122314\_01.11222.11222.2 | 4.901 | 0.4825 | 100.0% | 1753.2122 | 1753.8693 | 1 | 8.401 | 70.0% | 3 | R.NSNLVGAAHEELQQSR.I | 2 |
|  | CENPL\_stlcld\_tube2\_122314\_01.10515.10515.3 | 2.6979 | 0.3547 | 99.9% | 1753.8243 | 1753.8693 | 2 | 5.309 | 36.7% | 1 | R.NSNLVGAAHEELQQSR.I | 3 |
|  | CENPL\_stlcld\_122314\_01.15551.15551.2 | 3.4966 | 0.2308 | 99.7% | 1702.2122 | 1700.9762 | 1 | 6.234 | 53.6% | 2 | R.IRIDSLSAQLSQLQK.Q | 2 |
|  | CENPL\_stlcld\_122314\_01.11302.11302.2 | 2.9033 | 0.3073 | 99.9% | 1188.3522 | 1188.3262 | 6 | 5.775 | 77.8% | 5 | K.LRDLEDSLAR.E | 2 |
|  | CENPL\_stlcld\_122314\_01.19108.19108.2 | 2.5815 | 0.2923 | 98.7% | 1893.5922 | 1895.1346 | 64 | 5.236 | 42.9% | 1 | R.MQQQLDEYQELLDIK.L | 2 |
|  | CENPL\_stlcld\_122314\_02.10649.10649.2 | 3.7163 | 0.5522 | 100.0% | 1606.1921 | 1606.7728 | 1 | 8.952 | 65.4% | 2 | R.VAVEEVDEEGKFVR.L | 2 |
|  | CENPL\_stlcld\_tube2\_122314\_01.10857.10857.3 | 2.6253 | 0.2719 | 98.5% | 1606.4944 | 1606.7728 | 1 | 5.542 | 42.3% | 5 | R.VAVEEVDEEGKFVR.L | 3 |
|  | CENPL\_stlcld\_122314\_02.11301.11301.2 | 4.1963 | 0.5473 | 100.0% | 1492.6322 | 1492.6874 | 1 | 9.134 | 69.2% | 6 | R.TALINSTGEEVAMR.K | 2 |
|  | CENPL\_stlcld\_122314\_02.11280.11280.2 | 3.9872 | 0.5606 | 100.0% | 1567.0322 | 1567.6555 | 1 | 9.007 | 50.0% | 2 | R.SVGGSGGGSFGDNLVTR.S | 2 |

---

|  |  |  |  |  |  |  |  |  |
| --- | --- | --- | --- | --- | --- | --- | --- | --- |
| U | *gi|148470397|ref|NP\_0* | 8 | 24 | 31.1% | 415 | 45672 | 5.6 | heterogeneous nuclear ribonucleoprotein F [Homo sapiens] |
| U | *gi|4826760|ref|NP\_004* | 8 | 24 | 31.1% | 415 | 45672 | 5.6 | heterogeneous nuclear ribonucleoprotein F [Homo sapiens] |
| U | *gi|148470406|ref|NP\_0* | 8 | 24 | 31.1% | 415 | 45672 | 5.6 | heterogeneous nuclear ribonucleoprotein F [Homo sapiens] |
| U | *gi|148470404|ref|NP\_0* | 8 | 24 | 31.1% | 415 | 45672 | 5.6 | heterogeneous nuclear ribonucleoprotein F [Homo sapiens] |
| U | *gi|148470402|ref|NP\_0* | 8 | 24 | 31.1% | 415 | 45672 | 5.6 | heterogeneous nuclear ribonucleoprotein F [Homo sapiens] |
| U | *gi|148470400|ref|NP\_0* | 8 | 24 | 31.1% | 415 | 45672 | 5.6 | heterogeneous nuclear ribonucleoprotein F [Homo sapiens] |

| Filename XCorr DeltCN Conf% ObsM+H+ CalcM+H+ SpR ZScore Ion% # Sequence  | | | | | | | | | | | | |
| --- | --- | --- | --- | --- | --- | --- | --- | --- | --- | --- | --- | --- |
|  | CENPL\_stlcld\_122314\_02.12152.12152.2 | 3.1387 | 0.4504 | 100.0% | 1710.4122 | 1710.7919 | 1 | 7.735 | 46.7% | 1 | R.QSGEAFVELGSEDDVK.M | 2 |
|  | CENPL\_stlcld\_122314\_01.08385.08385.3 | 3.2252 | 0.2611 | 99.9% | 1631.3043 | 1631.6584 | 68 | 5.574 | 31.7% | 1 | K.HSGPNSADSANDGFVR.L | 3 |
|  | CENPL\_stlcld\_122314\_01.08393.08393.2 | 3.7676 | 0.4939 | 100.0% | 1631.3121 | 1631.6584 | 1 | 9.577 | 63.3% | 1 | K.HSGPNSADSANDGFVR.L | 2 |
|  | CENPL\_stlcld\_tube2\_122314\_02.17267.17267.2 | 5.1811 | 0.378 | 100.0% | 1869.7722 | 1869.0813 | 1 | 7.731 | 62.5% | 4 | K.ITGEAFVQFASQELAEK.A | 2 |
|  | CENPL\_stlcld\_122314\_02.16148.16148.3 | 6.6359 | 0.4765 | 100.0% | 3477.3843 | 3476.7114 | 1 | 9.329 | 32.3% | 5 | R.MRPGAYSTGYGGYEEYSGLSDGYGFTTDLFGR.D | 3 |
|  | CENPL\_stlcld\_tube2\_122314\_01.20397.20397.2 | 4.5599 | 0.463 | 100.0% | 1997.8121 | 1998.2023 | 1 | 7.917 | 59.4% | 4 | K.ATENDIYNFFSPLNPVR.V | 22 |
|  | CENPL\_stlcld\_tube2\_122314\_01.09572.09572.2 | 3.1384 | 0.502 | 100.0% | 1093.4922 | 1093.2278 | 1 | 8.474 | 83.3% | 7 | R.VHIEIGPDGR.V | 22 |
|  | CENPL\_stlcld\_122314\_02.12220.12220.3 | 2.7563 | 0.3438 | 99.9% | 2192.4543 | 2193.39 | 9 | 4.871 | 28.8% | 1 | R.VTGEADVEFATHEEAVAAMSK.D | 3 |

Similarities:
gi|5031753|ref|NP\_005(2:6)  

---

|  |  |  |  |  |  |  |  |  |
| --- | --- | --- | --- | --- | --- | --- | --- | --- |
| U | *gi|38016911|ref|NP\_00* | 7 | 14 | 30.9% | 288 | 31731 | 7.9 | stomatin isoform a [Homo sapiens] |

| Filename XCorr DeltCN Conf% ObsM+H+ CalcM+H+ SpR ZScore Ion% # Sequence  | | | | | | | | | | | | |
| --- | --- | --- | --- | --- | --- | --- | --- | --- | --- | --- | --- | --- |
| \* | CENPL\_stlcld\_122314\_02.11925.11925.2 | 5.7872 | 0.3687 | 100.0% | 1930.5721 | 1931.113 | 1 | 10.404 | 66.7% | 1 | R.VQNATLAVANITNADSATR.L | 2 |
| \* | CENPL\_stlcld\_122314\_01.10106.10106.2 | 2.454 | 0.2197 | 98.7% | 916.3722 | 916.1087 | 9 | 4.59 | 71.4% | 2 | R.LLAQTTLR.N | 2 |
| \* | CENPL\_stlcld\_122314\_01.08500.08500.3 | 3.3167 | 0.2368 | 99.9% | 1448.7544 | 1447.6494 | 3 | 5.072 | 44.2% | 2 | R.AKVIAAEGEMNASR.A | 3 |
|  | CENPL\_stlcld\_tube2\_122314\_01.08670.08670.2 | 3.9837 | 0.3785 | 100.0% | 1249.1522 | 1248.3966 | 1 | 8.843 | 77.3% | 4 | K.VIAAEGEMNASR.A | 2 |
|  | CENPL\_stlcld\_tube2\_122314\_01.16103.16103.2 | 2.8992 | 0.249 | 98.9% | 1716.7922 | 1716.9904 | 12 | 4.771 | 43.3% | 1 | K.EASMVITESPAALQLR.Y | 2 |
|  | CENPL\_stlcld\_122314\_01.13959.13959.2 | 3.7308 | 0.3822 | 100.0% | 1352.5721 | 1352.5707 | 1 | 7.81 | 72.7% | 2 | R.YLQTLTTIAAEK.N | 2 |
|  | CENPL\_stlcld\_122314\_02.01574.01574.2 | 3.4593 | 0.2597 | 99.9% | 2128.9321 | 2128.5781 | 1 | 4.926 | 42.1% | 2 | K.NSTIVFPLPIDMLQGIIGAK.H | 2 |

---

|  |  |  |  |  |  |  |  |  |
| --- | --- | --- | --- | --- | --- | --- | --- | --- |
| U | *gi|4503571|ref|NP\_001* | 9 | 20 | 30.6% | 434 | 47169 | 7.4 | enolase 1 [Homo sapiens] |

| Filename XCorr DeltCN Conf% ObsM+H+ CalcM+H+ SpR ZScore Ion% # Sequence  | | | | | | | | | | | | |
| --- | --- | --- | --- | --- | --- | --- | --- | --- | --- | --- | --- | --- |
|  | CENPL\_stlcld\_122314\_01.18662.18662.2 | 4.4341 | 0.4869 | 100.0% | 1806.0322 | 1806.0258 | 1 | 9.231 | 61.8% | 3 | R.AAVPSGASTGIYEALELR.D | 2 |
| \* | CENPL\_stlcld\_tube2\_122314\_01.08108.08108.2 | 2.7304 | 0.2897 | 99.5% | 1317.4321 | 1317.482 | 333 | 5.014 | 55.0% | 1 | K.LNVTEQEKIDK.L | 2 |
| \* | CENPL\_stlcld\_122314\_01.18738.18738.3 | 5.9364 | 0.4767 | 100.0% | 3011.9644 | 3013.383 | 1 | 8.225 | 31.0% | 2 | R.HIADLAGNSEVILPVPAFNVINGGSHAGNK.L | 3 |
| \* | CENPL\_stlcld\_tube2\_122314\_01.21009.21009.2 | 4.9039 | 0.4607 | 100.0% | 1909.7722 | 1909.3148 | 1 | 7.197 | 68.8% | 2 | K.LAMQEFMILPVGAANFR.E | 2 |
| \* | CENPL\_stlcld\_tube2\_122314\_01.08853.08853.2 | 3.3794 | 0.2435 | 99.9% | 1144.3322 | 1144.3158 | 1 | 5.841 | 83.3% | 2 | R.IGAEVYHNLK.N | 2 |
| \* | CENPL\_stlcld\_122314\_01.17325.17325.2 | 3.1641 | 0.3834 | 99.9% | 1427.5922 | 1426.6091 | 2 | 6.261 | 77.3% | 4 | R.YISPDQLADLYK.S | 2 |
| \* | CENPL\_stlcld\_tube2\_122314\_02.14063.14063.3 | 3.8916 | 0.2758 | 99.9% | 2190.9243 | 2190.4612 | 1 | 6.082 | 36.2% | 2 | K.FTASAGIQVVGDDLTVTNPKR.I | 3 |
| \* | CENPL\_stlcld\_122314\_01.12300.12300.2 | 3.0598 | 0.3004 | 99.7% | 1526.5922 | 1526.7563 | 1 | 6.575 | 69.2% | 3 | K.LAQANGWGVMVSHR.S | 2 |
| \* | CENPL\_stlcld\_122314\_02.11631.11631.3 | 2.7335 | 0.3421 | 99.9% | 1527.1444 | 1526.7563 | 1 | 5.544 | 50.0% | 1 | K.LAQANGWGVMVSHR.S | 3 |

---

|  |  |  |  |  |  |  |  |  |
| --- | --- | --- | --- | --- | --- | --- | --- | --- |
| U | *gi|4826998|ref|NP\_005* | 20 | 64 | 30.4% | 707 | 76150 | 9.4 | splicing factor proline/glutamine rich (polypyrimidine tract binding protein associated) [Homo sapiens] |

| Filename XCorr DeltCN Conf% ObsM+H+ CalcM+H+ SpR ZScore Ion% # Sequence  | | | | | | | | | | | | |
| --- | --- | --- | --- | --- | --- | --- | --- | --- | --- | --- | --- | --- |
| \* | CENPL\_stlcld\_122314\_01.09195.09195.2 | 1.9113 | 0.2814 | 95.1% | 1267.8722 | 1268.4332 | 2 | 6.044 | 59.1% | 1 | R.SPPPGMGLNQNR.G | 2 |
| \* | CENPL\_stlcld\_122314\_01.08169.08169.3 | 4.9725 | 0.4485 | 100.0% | 2371.9143 | 2371.725 | 1 | 7.755 | 33.3% | 1 | K.MPGGPKPGGGPGLSTPGGHPKPPHR.G | 3 |
| \* | CENPL\_stlcld\_122314\_02.13000.13000.3 | 3.3751 | 0.3703 | 100.0% | 1652.3344 | 1650.8723 | 2 | 6.749 | 37.5% | 1 | K.ISDSEGFKANLSLLR.R | 3 |
| \* | CENPL\_stlcld\_122314\_01.19964.19964.2 | 2.9594 | 0.4794 | 100.0% | 1808.4722 | 1809.0258 | 2 | 7.665 | 50.0% | 1 | R.LFVGNLPADITEDEFK.R | 2 |
| \* | CENPL\_stlcld\_tube2\_122314\_01.17538.17538.2 | 4.5689 | 0.4778 | 100.0% | 1964.5122 | 1965.2133 | 1 | 8.009 | 56.2% | 4 | R.LFVGNLPADITEDEFKR.L | 2 |
| \* | CENPL\_stlcld\_tube2\_122314\_01.17494.17494.3 | 2.6538 | 0.2823 | 98.3% | 1964.9343 | 1965.2133 | 1 | 5.682 | 39.1% | 1 | R.LFVGNLPADITEDEFKR.L | 3 |
| \* | CENPL\_stlcld\_122314\_01.12650.12650.2 | 3.2176 | 0.397 | 100.0% | 1253.3121 | 1253.3971 | 1 | 7.878 | 65.0% | 4 | K.YGEPGEVFINK.G | 2 |
| \* | CENPL\_stlcld\_122314\_01.14886.14886.2 | 3.3656 | 0.4901 | 100.0% | 1744.2922 | 1745.0007 | 1 | 7.091 | 63.3% | 1 | R.ALAEIAKAELDDTPMR.G | 2 |
| \* | CENPL\_stlcld\_tube2\_122314\_01.09326.09326.2 | 2.6707 | 0.3554 | 99.9% | 1048.0521 | 1048.1559 | 1 | 7.073 | 81.2% | 5 | K.AELDDTPMR.G | 2 |
| \* | CENPL\_stlcld\_122314\_01.09394.09394.2 | 3.3112 | 0.4611 | 100.0% | 1143.7322 | 1144.3188 | 1 | 7.853 | 90.0% | 3 | R.FATHAAALSVR.N | 2 |
| \* | CENPL\_stlcld\_tube2\_122314\_01.20733.20733.3 | 5.2173 | 0.4873 | 100.0% | 3765.3245 | 3766.2046 | 1 | 8.504 | 22.7% | 1 | R.FATHAAALSVRNLSPYVSNELLEEAFSQFGPIER.A | 3 |
| \* | CENPL\_stlcld\_122314\_01.22671.22671.2 | 5.0402 | 0.5142 | 100.0% | 2641.172 | 2640.9092 | 1 | 8.691 | 47.7% | 3 | R.NLSPYVSNELLEEAFSQFGPIER.A | 2 |
|  | CENPL\_stlcld\_tube2\_122314\_01.09248.09248.2 | 2.399 | 0.2054 | 98.4% | 887.3722 | 887.0238 | 1 | 6.834 | 85.7% | 2 | R.AVVIVDDR.G | 22 |
| \* | CENPL\_stlcld\_122314\_01.09326.09326.3 | 2.9694 | 0.342 | 99.9% | 1620.0844 | 1619.8613 | 1 | 5.415 | 38.3% | 2 | R.STGKGIVEFASKPAAR.K | 3 |
| \* | CENPL\_stlcld\_122314\_01.10454.10454.2 | 2.9293 | 0.3832 | 99.9% | 1246.5922 | 1246.452 | 8 | 6.57 | 68.2% | 5 | K.GIVEFASKPAAR.K | 2 |
| \* | CENPL\_stlcld\_tube2\_122314\_02.11327.11327.2 | 4.4503 | 0.4741 | 100.0% | 1763.3922 | 1763.8632 | 1 | 8.912 | 73.1% | 4 | R.FAQHGTFEYEYSQR.W | 2 |
| \* | CENPL\_stlcld\_tube2\_122314\_02.11342.11342.3 | 4.4596 | 0.3043 | 100.0% | 1765.3143 | 1763.8632 | 1 | 5.755 | 44.2% | 12 | R.FAQHGTFEYEYSQR.W | 3 |
| \* | CENPL\_stlcld\_122314\_01.13731.13731.3 | 5.7922 | 0.4208 | 100.0% | 2429.7244 | 2429.6233 | 1 | 7.419 | 36.8% | 3 | K.DKLESEMEDAYHEHQANLLR.Q | 3 |
| \* | CENPL\_stlcld\_122314\_01.08847.08847.2 | 3.9389 | 0.5031 | 100.0% | 1342.4722 | 1342.4569 | 1 | 9.259 | 82.1% | 7 | R.FGQGGAGPVGGQGPR.G | 2 |
| \* | CENPL\_stlcld\_122314\_01.08997.08997.2 | 3.2544 | 0.4484 | 100.0% | 1121.2922 | 1121.2561 | 24 | 7.191 | 63.6% | 3 | R.GMGPGTPAGYGR.G | 2 |

Similarities:
gi|224028244|ref|NP\_0(1:19)  

---

|  |  |  |  |  |  |  |  |  |
| --- | --- | --- | --- | --- | --- | --- | --- | --- |
| U | *gi|5729877|ref|NP\_006* | 21 | 55 | 30.3% | 646 | 70898 | 5.5 | heat shock 70kDa protein 8 isoform 1 [Homo sapiens] |

| Filename XCorr DeltCN Conf% ObsM+H+ CalcM+H+ SpR ZScore Ion% # Sequence  | | | | | | | | | | | | |
| --- | --- | --- | --- | --- | --- | --- | --- | --- | --- | --- | --- | --- |
|  | CENPL\_stlcld\_tube2\_122314\_01.12731.12731.2 | 3.5901 | 0.5406 | 100.0% | 1488.3322 | 1488.5939 | 1 | 9.387 | 75.0% | 8 | R.TTPSYVAFTDTER.L | 222 |
|  | CENPL\_stlcld\_tube2\_122314\_01.12807.12807.2 | 4.2929 | 0.4337 | 100.0% | 1650.4722 | 1650.8468 | 1 | 8.613 | 78.6% | 3 | K.NQVAMNPTNTVFDAK.R | 2 |
|  | CENPL\_stlcld\_tube2\_122314\_01.09869.09869.2 | 2.9901 | 0.4009 | 99.9% | 1411.0922 | 1411.5725 | 1 | 7.497 | 72.7% | 3 | R.RFDDAVVQSDMK.H | 2 |
|  | CENPL\_stlcld\_122314\_01.13788.13788.2 | 2.1664 | 0.2467 | 95.8% | 1654.3722 | 1654.9298 | 12 | 4.762 | 46.2% | 1 | K.HWPFMVVNDAGRPK.V | 2 |
|  | CENPL\_stlcld\_tube2\_122314\_01.13670.13670.3 | 3.6775 | 0.4009 | 100.0% | 1655.1244 | 1654.9298 | 37 | 6.235 | 32.7% | 3 | K.HWPFMVVNDAGRPK.V | 3 |
|  | CENPL\_stlcld\_tube2\_122314\_01.17840.17840.2 | 2.7149 | 0.3328 | 99.6% | 1617.2722 | 1617.8542 | 2 | 5.676 | 50.0% | 2 | K.SFYPEEVSSMVLTK.M | 2 |
|  | CENPL\_stlcld\_122314\_01.11878.11878.2 | 3.1375 | 0.355 | 99.9% | 1253.3722 | 1253.4993 | 1 | 6.473 | 85.0% | 3 | K.MKEIAEAYLGK.T | 2 |
|  | CENPL\_stlcld\_122314\_01.11854.11854.3 | 2.94 | 0.2203 | 98.7% | 1253.6044 | 1253.4993 | 131 | 5.365 | 40.0% | 1 | K.MKEIAEAYLGK.T | 3 |
|  | CENPL\_stlcld\_122314\_01.15642.15642.2 | 3.0517 | 0.2955 | 99.7% | 1982.4722 | 1983.1882 | 4 | 5.393 | 41.2% | 3 | K.TVTNAVVTVPAYFNDSQR.Q | 2 |
|  | CENPL\_stlcld\_122314\_01.16466.16466.2 | 5.1137 | 0.5254 | 100.0% | 1660.8722 | 1660.9078 | 1 | 10.001 | 76.7% | 3 | R.IINEPTAAAIAYGLDK.K | 222 |
|  | CENPL\_stlcld\_122314\_01.14506.14506.2 | 4.4997 | 0.4966 | 100.0% | 1787.9521 | 1789.0819 | 1 | 8.975 | 68.8% | 1 | R.IINEPTAAAIAYGLDKK.V | 2 |
|  | CENPL\_stlcld\_122314\_01.09344.09344.2 | 3.9107 | 0.47 | 100.0% | 1692.8322 | 1692.6958 | 1 | 8.222 | 60.0% | 3 | K.STAGDTHLGGEDFDNR.M | 2 |
|  | CENPL\_stlcld\_122314\_02.08522.08522.3 | 2.5722 | 0.3155 | 99.2% | 1693.0443 | 1692.6958 | 171 | 5.858 | 31.7% | 1 | K.STAGDTHLGGEDFDNR.M | 3 |
|  | CENPL\_stlcld\_122314\_01.13317.13317.2 | 2.9834 | 0.4381 | 100.0% | 1235.7322 | 1236.4741 | 1 | 7.955 | 88.9% | 2 | R.MVNHFIAEFK.R | 2 |
|  | CENPL\_stlcld\_tube2\_122314\_01.13114.13114.3 | 3.2004 | 0.3661 | 100.0% | 1236.5044 | 1236.4741 | 1 | 6.002 | 50.0% | 2 | R.MVNHFIAEFK.R | 3 |
|  | CENPL\_stlcld\_tube2\_122314\_01.15429.15429.2 | 3.5715 | 0.3814 | 100.0% | 1481.2922 | 1481.6511 | 1 | 7.08 | 72.7% | 4 | R.ARFEELNADLFR.G | 2 |
|  | CENPL\_stlcld\_tube2\_122314\_01.15447.15447.3 | 3.962 | 0.3494 | 100.0% | 1481.9944 | 1481.6511 | 1 | 6.231 | 54.5% | 2 | R.ARFEELNADLFR.G | 3 |
|  | CENPL\_stlcld\_122314\_02.11390.11390.3 | 4.6653 | 0.4133 | 100.0% | 1839.4143 | 1839.1019 | 1 | 8.017 | 42.2% | 3 | K.LDKSQIHDIVLVGGSTR.I | 3 |
|  | CENPL\_stlcld\_122314\_02.11211.11211.2 | 4.4299 | 0.5745 | 100.0% | 1482.7522 | 1482.6798 | 1 | 10.282 | 80.8% | 2 | K.SQIHDIVLVGGSTR.I | 2 |
| \* | CENPL\_stlcld\_122314\_01.07757.07757.3 | 3.6006 | 0.2272 | 99.9% | 1985.1843 | 1983.2036 | 1 | 5.137 | 43.3% | 1 | R.MVQEAEKYKAEDEKQR.D | 3 |
| \* | CENPL\_stlcld\_122314\_01.14966.14966.2 | 3.5597 | 0.365 | 100.0% | 1305.3522 | 1304.4602 | 1 | 6.78 | 80.0% | 4 | K.NSLESYAFNMK.A | 2 |

Similarities:
gi|167466173|ref|NP\_0(1:20)  
gi|124256496|ref|NP\_0(2:19)  
contaminant\_GR78\_HUMA(1:20)  

---

|  |  |  |  |  |  |  |  |  |
| --- | --- | --- | --- | --- | --- | --- | --- | --- |
| U | *gi|62414289|ref|NP\_00* | 13 | 20 | 30.3% | 466 | 53652 | 5.1 | vimentin [Homo sapiens] |

| Filename XCorr DeltCN Conf% ObsM+H+ CalcM+H+ SpR ZScore Ion% # Sequence  | | | | | | | | | | | | |
| --- | --- | --- | --- | --- | --- | --- | --- | --- | --- | --- | --- | --- |
| \* | CENPL\_stlcld\_tube2\_122314\_01.07978.07978.2 | 2.7156 | 0.2533 | 98.5% | 1495.2722 | 1495.6531 | 39 | 5.116 | 46.4% | 2 | R.MFGGPGTASRPSSSR.S | 2 |
| \* | CENPL\_stlcld\_122314\_01.10961.10961.2 | 2.4762 | 0.141 | 97.1% | 1128.1322 | 1126.3005 | 147 | 4.31 | 62.5% | 1 | R.FANYIDKVR.F | 2 |
| \* | CENPL\_stlcld\_122314\_01.19197.19197.2 | 3.6953 | 0.2759 | 100.0% | 1170.3922 | 1170.4349 | 1 | 6.737 | 77.8% | 1 | K.ILLAELEQLK.G | 2 |
| \* | CENPL\_stlcld\_122314\_01.15287.15287.2 | 4.1147 | 0.3296 | 100.0% | 1541.8922 | 1540.8436 | 1 | 6.86 | 69.2% | 2 | K.ILLAELEQLKGQGK.S | 2 |
| \* | CENPL\_stlcld\_tube2\_122314\_01.12620.12620.2 | 2.9786 | 0.5159 | 100.0% | 1255.0122 | 1255.385 | 1 | 8.577 | 77.8% | 1 | R.LGDLYEEEMR.E | 2 |
| \* | CENPL\_stlcld\_tube2\_122314\_01.13614.13614.2 | 3.0808 | 0.1903 | 98.6% | 1690.4321 | 1689.881 | 1 | 4.684 | 61.5% | 1 | R.VEVERDNLAEDIMR.L | 2 |
| \* | CENPL\_stlcld\_tube2\_122314\_01.16614.16614.2 | 4.4233 | 0.4424 | 100.0% | 1535.1721 | 1534.793 | 1 | 7.652 | 75.0% | 3 | R.KVESLQEEIAFLK.K | 2 |
| \* | CENPL\_stlcld\_122314\_01.14236.14236.3 | 2.9786 | 0.2759 | 99.9% | 1663.4644 | 1662.967 | 172 | 5.037 | 32.7% | 1 | R.KVESLQEEIAFLKK.L | 3 |
|  | CENPL\_stlcld\_tube2\_122314\_01.13282.13282.2 | 2.5856 | 0.2956 | 99.5% | 1309.4122 | 1310.4056 | 3 | 5.076 | 66.7% | 1 | K.NLQEAEEWYK.S | 2 |
| \* | CENPL\_stlcld\_tube2\_122314\_01.10175.10175.2 | 2.4575 | 0.1937 | 97.8% | 1093.8522 | 1094.1692 | 1 | 4.596 | 77.8% | 3 | K.FADLSEAANR.N | 2 |
| \* | CENPL\_stlcld\_122314\_02.13542.13542.2 | 5.3839 | 0.5472 | 100.0% | 2187.8123 | 2188.33 | 1 | 10.857 | 72.2% | 2 | R.EMEENFAVEAANYQDTIGR.L | 2 |
| \* | CENPL\_stlcld\_tube2\_122314\_01.12053.12053.2 | 4.5855 | 0.4484 | 100.0% | 1735.5521 | 1735.9679 | 1 | 8.032 | 69.2% | 1 | R.LQDEIQNMKEEMAR.H | 2 |
|  | CENPL\_stlcld\_tube2\_122314\_01.11760.11760.3 | 2.9775 | 0.2191 | 98.7% | 1529.2444 | 1528.7513 | 1 | 4.81 | 50.0% | 1 | R.HLREYQDLLNVK.M | 3 |

---

|  |  |  |  |  |  |  |  |  |
| --- | --- | --- | --- | --- | --- | --- | --- | --- |
| U | *gi|67782365|ref|NP\_00* | 14 | 26 | 30.1% | 469 | 51386 | 5.5 | keratin 7 [Homo sapiens] |

| Filename XCorr DeltCN Conf% ObsM+H+ CalcM+H+ SpR ZScore Ion% # Sequence  | | | | | | | | | | | | |
| --- | --- | --- | --- | --- | --- | --- | --- | --- | --- | --- | --- | --- |
|  | CENPL\_stlcld\_122314\_01.11938.11938.3 | 4.7261 | 0.3617 | 100.0% | 2248.0144 | 2247.519 | 5 | 6.715 | 29.5% | 2 | R.LSSARPGGLGSSSLYGLGASRPR.V | 33 |
|  | CENPL\_stlcld\_122314\_02.08966.08966.2 | 2.7529 | 0.3773 | 99.9% | 1105.8922 | 1105.2388 | 8 | 6.395 | 59.1% | 3 | R.SAYGGPVGAGIR.E | 22 |
|  | CENPL\_stlcld\_tube2\_122314\_01.12512.12512.2 | 2.417 | 0.2122 | 98.9% | 828.09216 | 827.95544 | 5 | 4.999 | 91.7% | 4 | K.FASFIDK.V | 222222222 |
|  | CENPL\_stlcld\_122314\_01.12293.12293.2 | 2.815 | 0.2809 | 99.9% | 1082.9922 | 1083.2755 | 3 | 6.695 | 75.0% | 2 | K.FASFIDKVR.F | 222322223 |
|  | CENPL\_stlcld\_122314\_01.20612.20612.2 | 3.9207 | 0.3876 | 100.0% | 1443.7322 | 1443.686 | 4 | 8.265 | 58.3% | 3 | R.LPDIFEAQIAGLR.G | 22 |
|  | CENPL\_stlcld\_tube2\_122314\_02.10514.10514.2 | 2.0307 | 0.282 | 96.8% | 1243.9122 | 1243.3622 | 11 | 4.573 | 54.5% | 1 | R.GQLEALQVDGGR.L | 22 |
| \* | CENPL\_stlcld\_tube2\_122314\_01.16440.16440.2 | 3.1765 | 0.2082 | 99.0% | 1954.8922 | 1955.1783 | 11 | 5.076 | 41.2% | 1 | R.GQLEALQVDGGRLEAELR.S | 2 |
| \* | CENPL\_stlcld\_122314\_02.13816.13816.3 | 3.4199 | 0.3099 | 99.9% | 1955.4844 | 1955.1783 | 1 | 6.166 | 42.6% | 2 | R.GQLEALQVDGGRLEAELR.S | 3 |
|  | CENPL\_stlcld\_tube2\_122314\_01.10942.10942.2 | 2.6462 | 0.2259 | 98.8% | 1198.0122 | 1197.2897 | 1 | 5.375 | 77.8% | 1 | R.AEAEAWYQTK.F | 222 |
|  | CENPL\_stlcld\_tube2\_122314\_01.09035.09035.2 | 2.4033 | 0.2708 | 98.7% | 1093.3121 | 1093.2249 | 1 | 5.779 | 66.7% | 1 | K.FETLQAQAGK.H | 22 |
| \* | CENPL\_stlcld\_122314\_02.13656.13656.3 | 3.4079 | 0.354 | 99.9% | 2014.5844 | 2013.2987 | 1 | 4.947 | 43.1% | 1 | R.AKLEAAIAEAEERGELALK.D | 3 |
| \* | CENPL\_stlcld\_tube2\_122314\_01.16905.16905.3 | 3.9228 | 0.4463 | 100.0% | 2355.7744 | 2355.6536 | 1 | 6.346 | 33.3% | 1 | R.AKLEAAIAEAEERGELALKDAR.A | 3 |
|  | CENPL\_stlcld\_tube2\_122314\_01.09796.09796.2 | 3.62 | 0.3166 | 100.0% | 1386.4122 | 1386.548 | 3 | 6.125 | 68.2% | 2 | R.AKQEELEAALQR.G | 22 |
|  | CENPL\_stlcld\_122314\_02.13122.13122.2 | 3.3979 | 0.3814 | 100.0% | 1407.4122 | 1406.6653 | 2 | 6.236 | 68.2% | 2 | K.LALDIEIATYRK.L | 22222 |

Similarities:
gi|4504919|ref|NP\_002(3:11)  
gi|47132620|ref|NP\_00(2:12)  
contaminant\_KERATIN19(8:6)  
gi|119703753|ref|NP\_0(2:12)  
gi|32567786|ref|NP\_78(3:11)  
gi|119395754|ref|NP\_0(2:12)  
contaminant\_KERATIN16(1:13)  
gi|153791158|ref|NP\_0(2:12)  
gi|109255249|ref|NP\_0(2:12)  
gi|15618995|ref|NP\_25(2:12)  

---

|  |  |  |  |  |  |  |  |  |
| --- | --- | --- | --- | --- | --- | --- | --- | --- |
| U | *gi|38045913|ref|NP\_93* | 5 | 12 | 29.4% | 177 | 19654 | 5.6 | non-metastatic cells 1, protein (NM23A) expressed in isoform a [Homo sapiens] |
| U | *gi|4557797|ref|NP\_000* | 5 | 12 | 34.2% | 152 | 17149 | 6.2 | non-metastatic cells 1, protein (NM23A) expressed in isoform b [Homo sapiens] |

| Filename XCorr DeltCN Conf% ObsM+H+ CalcM+H+ SpR ZScore Ion% # Sequence  | | | | | | | | | | | | |
| --- | --- | --- | --- | --- | --- | --- | --- | --- | --- | --- | --- | --- |
|  | CENPL\_stlcld\_tube2\_122314\_01.11264.11264.2 | 2.9804 | 0.315 | 99.9% | 1345.5521 | 1345.5846 | 2 | 6.332 | 63.6% | 3 | R.TFIAIKPDGVQR.G | 22 |
|  | CENPL\_stlcld\_122314\_01.12003.12003.2 | 2.3734 | 0.2936 | 99.2% | 985.0522 | 985.21497 | 4 | 6.27 | 75.0% | 2 | R.GLVGEIIKR.F | 22 |
|  | CENPL\_stlcld\_tube2\_122314\_01.09785.09785.2 | 4.5302 | 0.4022 | 100.0% | 1787.5122 | 1787.041 | 1 | 7.109 | 71.9% | 3 | R.VMLGETNPADSKPGTIR.G | 22 |
|  | CENPL\_stlcld\_tube2\_122314\_01.09743.09743.3 | 2.3591 | 0.3203 | 98.3% | 1787.7544 | 1787.041 | 1 | 5.302 | 39.1% | 2 | R.VMLGETNPADSKPGTIR.G | 33 |
|  | CENPL\_stlcld\_122314\_01.08476.08476.2 | 3.4532 | 0.339 | 99.9% | 1486.1921 | 1486.5785 | 1 | 6.744 | 76.9% | 2 | R.NIIHGSDSVESAEK.E | 2 |

Similarities:
gi|4505409|ref|NP\_002(4:1)  

---

|  |  |  |  |  |  |  |  |  |
| --- | --- | --- | --- | --- | --- | --- | --- | --- |
| U | *contaminant\_KERATIN03* | 26 | 88 | 29.3% | 593 | 59519 | 5.2 | no description |
| U | *gi|195972866|ref|NP\_0* | 26 | 89 | 29.8% | 584 | 58801 | 5.2 | keratin 10 [Homo sapiens] |

| Filename XCorr DeltCN Conf% ObsM+H+ CalcM+H+ SpR ZScore Ion% # Sequence  | | | | | | | | | | | | |
| --- | --- | --- | --- | --- | --- | --- | --- | --- | --- | --- | --- | --- |
|  | CENPL\_stlcld\_tube2\_122314\_02.13767.13767.2 | 5.7817 | 0.5009 | 100.0% | 1708.4722 | 1708.7844 | 1 | 9.481 | 63.9% | 15 | K.GSLGGGFSSGGFSGGSFSR.G | 2 |
|  | CENPL\_stlcld\_tube2\_122314\_01.08714.08714.2 | 3.0686 | 0.3444 | 100.0% | 1091.2522 | 1091.2273 | 32 | 5.996 | 68.8% | 4 | K.VTMQNLNDR.L | 222 |
|  | CENPL\_stlcld\_tube2\_122314\_01.09831.09831.2 | 3.0041 | 0.1699 | 99.3% | 1065.3322 | 1065.2578 | 54 | 5.893 | 62.5% | 1 | R.LASYLDKVR.A | 2222 |
|  | CENPL\_stlcld\_tube2\_122314\_01.10260.10260.1 | 3.1611 | 0.3581 | 100.0% | 1382.63 | 1382.4668 | 3 | 7.008 | 59.1% | 1 | R.ALEESNYELEGK.I | 1 |
|  | CENPL\_stlcld\_tube2\_122314\_01.10286.10286.2 | 4.0478 | 0.4446 | 100.0% | 1383.2522 | 1382.4668 | 1 | 8.664 | 72.7% | 7 | R.ALEESNYELEGK.I | 2 |
|  | CENPL\_stlcld\_122314\_01.22322.22322.3 | 5.3002 | 0.4652 | 100.0% | 3054.4443 | 3054.4277 | 1 | 7.725 | 27.9% | 2 | K.TIDDLKNQILNLTTDNANILLQIDNAR.L | 3 |
|  | CENPL\_stlcld\_tube2\_122314\_01.21542.21542.2 | 5.2555 | 0.4659 | 100.0% | 3054.4722 | 3054.4277 | 1 | 10.234 | 48.1% | 1 | K.TIDDLKNQILNLTTDNANILLQIDNAR.L | 2 |
|  | CENPL\_stlcld\_tube2\_122314\_01.19924.19924.2 | 5.0269 | 0.3459 | 100.0% | 2369.0723 | 2368.6523 | 1 | 7.389 | 52.5% | 2 | K.NQILNLTTDNANILLQIDNAR.L | 2 |
|  | CENPL\_stlcld\_122314\_02.16607.16607.3 | 5.0614 | 0.2917 | 100.0% | 2369.2744 | 2368.6523 | 1 | 6.406 | 36.2% | 1 | K.NQILNLTTDNANILLQIDNAR.L | 3 |
|  | CENPL\_stlcld\_122314\_01.09788.09788.2 | 2.5662 | 0.2183 | 99.5% | 808.39215 | 807.8815 | 68 | 6.201 | 66.7% | 6 | R.LAADDFR.L | 22222 |
|  | CENPL\_stlcld\_tube2\_122314\_01.09578.09578.3 | 3.7295 | 0.3163 | 100.0% | 1236.0543 | 1235.4258 | 5 | 5.669 | 52.8% | 6 | R.LKYENEVALR.Q | 3 |
|  | CENPL\_stlcld\_tube2\_122314\_01.09522.09522.2 | 3.3363 | 0.359 | 100.0% | 1236.3322 | 1235.4258 | 1 | 7.077 | 83.3% | 4 | R.LKYENEVALR.Q | 2 |
|  | CENPL\_stlcld\_tube2\_122314\_01.09141.09141.2 | 2.7865 | 0.3172 | 99.9% | 994.15216 | 994.0923 | 3 | 6.631 | 78.6% | 1 | K.YENEVALR.Q | 2 |
|  | CENPL\_stlcld\_tube2\_122314\_01.13430.13430.2 | 3.3862 | 0.409 | 100.0% | 1032.2522 | 1032.2224 | 1 | 6.767 | 87.5% | 5 | R.VLDELTLTK.A | 2 |
|  | CENPL\_stlcld\_tube2\_122314\_01.08802.08802.2 | 3.7119 | 0.3024 | 100.0% | 1366.5322 | 1366.43 | 1 | 6.401 | 70.0% | 4 | R.SQYEQLAEQNR.K | 2 |
|  | CENPL\_stlcld\_tube2\_122314\_01.07820.07820.2 | 3.2576 | 0.3279 | 99.9% | 1494.3322 | 1494.6041 | 2 | 5.978 | 68.2% | 3 | R.SQYEQLAEQNRK.D | 2 |
|  | CENPL\_stlcld\_tube2\_122314\_01.14187.14187.1 | 2.0158 | 0.2794 | 96.4% | 1109.54 | 1110.1681 | 1 | 5.703 | 68.8% | 1 | K.DAEAWFNEK.S | 11 |
|  | CENPL\_stlcld\_tube2\_122314\_01.14228.14228.2 | 2.7953 | 0.1474 | 98.5% | 1109.6921 | 1110.1681 | 2 | 6.72 | 81.2% | 3 | K.DAEAWFNEK.S | 22 |
|  | CENPL\_stlcld\_tube2\_122314\_01.15884.15884.3 | 4.9353 | 0.3709 | 100.0% | 2212.8542 | 2213.4033 | 1 | 6.506 | 45.8% | 3 | K.SKELTTEIDNNIEQISSYK.S | 3 |
|  | CENPL\_stlcld\_tube2\_122314\_01.15834.15834.2 | 6.4326 | 0.575 | 100.0% | 2213.632 | 2213.4033 | 1 | 10.914 | 77.8% | 2 | K.SKELTTEIDNNIEQISSYK.S | 2 |
|  | CENPL\_stlcld\_tube2\_122314\_01.18091.18091.3 | 4.6433 | 0.4487 | 100.0% | 3041.6943 | 3042.324 | 1 | 7.006 | 35.0% | 1 | K.SKELTTEIDNNIEQISSYKSEITELR.R | 3 |
|  | CENPL\_stlcld\_tube2\_122314\_01.17039.17039.3 | 6.332 | 0.541 | 100.0% | 3197.8442 | 3198.5115 | 1 | 8.929 | 36.5% | 1 | K.SKELTTEIDNNIEQISSYKSEITELRR.N | 3 |
|  | CENPL\_stlcld\_tube2\_122314\_01.17796.17796.3 | 3.4376 | 0.3466 | 99.9% | 2983.1343 | 2983.259 | 5 | 5.636 | 24.0% | 1 | K.ELTTEIDNNIEQISSYKSEITELRR.N | 3 |
|  | CENPL\_stlcld\_tube2\_122314\_01.12418.12418.2 | 3.9049 | 0.4975 | 100.0% | 1391.4922 | 1391.4778 | 1 | 8.35 | 75.0% | 4 | K.QSLEASLAETEGR.Y | 2 |
|  | CENPL\_stlcld\_tube2\_122314\_01.11216.11216.2 | 3.9002 | 0.3433 | 100.0% | 1435.5322 | 1435.623 | 1 | 6.658 | 85.0% | 5 | K.IRLENEIQTYR.S | 2 |
|  | CENPL\_stlcld\_tube2\_122314\_01.09500.09500.2 | 3.4264 | 0.1181 | 99.5% | 1165.8922 | 1166.2761 | 1 | 5.437 | 87.5% | 4 | R.LENEIQTYR.S | 2 |

Similarities:
gi|40354195|ref|NP\_95(1:25)  
gi|4557701|ref|NP\_000(2:24)  
contaminant\_KERATIN05(3:23)  
gi|24234699|ref|NP\_00(2:24)  
gi|153945736|ref|NP\_8(3:23)  

---

|  |  |  |  |  |  |  |  |  |
| --- | --- | --- | --- | --- | --- | --- | --- | --- |
| U | *gi|10835063|ref|NP\_00* | 4 | 8 | 29.3% | 294 | 32575 | 4.8 | nucleophosmin 1 isoform 1 [Homo sapiens] |
| U | *gi|83641870|ref|NP\_00* | 4 | 8 | 33.2% | 259 | 28400 | 4.7 | nucleophosmin 1 isoform 3 [Homo sapiens] |
| U | *gi|40353734|ref|NP\_95* | 4 | 8 | 32.5% | 265 | 29465 | 4.6 | nucleophosmin 1 isoform 2 [Homo sapiens] |

| Filename XCorr DeltCN Conf% ObsM+H+ CalcM+H+ SpR ZScore Ion% # Sequence  | | | | | | | | | | | | |
| --- | --- | --- | --- | --- | --- | --- | --- | --- | --- | --- | --- | --- |
|  | CENPL\_stlcld\_tube2\_122314\_01.18017.18017.2 | 5.5824 | 0.4956 | 100.0% | 2931.2522 | 2931.2874 | 1 | 9.202 | 40.7% | 1 | R.TVSLGAGAKDELHIVEAEAMNYEGSPIK.V | 2 |
|  | CENPL\_stlcld\_tube2\_122314\_01.18033.18033.3 | 5.8349 | 0.3985 | 100.0% | 2932.0444 | 2931.2874 | 1 | 7.106 | 30.6% | 5 | R.TVSLGAGAKDELHIVEAEAMNYEGSPIK.V | 3 |
|  | CENPL\_stlcld\_tube2\_122314\_01.20216.20216.2 | 3.6723 | 0.4153 | 100.0% | 2228.8123 | 2228.655 | 1 | 6.302 | 45.0% | 1 | K.MSVQPTVSLGGFEITPPVVLR.L | 2 |
|  | CENPL\_stlcld\_122314\_02.09717.09717.3 | 5.553 | 0.5194 | 100.0% | 4248.0244 | 4248.9644 | 1 | 9.362 | 22.9% | 1 | K.LAADEDDDDDDEEDDDEDDDDDDFDDEEAEEKAPVKK.S | 3 |

---

|  |  |  |  |  |  |  |  |  |
| --- | --- | --- | --- | --- | --- | --- | --- | --- |
| U | *gi|4502891|ref|NP\_001* | 3 | 7 | 29.1% | 237 | 26215 | 4.1 | chloride channel, nucleotide-sensitive, 1A [Homo sapiens] |

| Filename XCorr DeltCN Conf% ObsM+H+ CalcM+H+ SpR ZScore Ion% # Sequence  | | | | | | | | | | | | |
| --- | --- | --- | --- | --- | --- | --- | --- | --- | --- | --- | --- | --- |
| \* | CENPL\_stlcld\_tube2\_122314\_01.13718.13718.2 | 3.7718 | 0.4632 | 100.0% | 1338.6122 | 1338.5034 | 1 | 8.057 | 70.8% | 5 | K.GLGTGTLYIAESR.L | 2 |
| \* | CENPL\_stlcld\_122314\_02.17549.17549.3 | 3.3597 | 0.2212 | 97.9% | 2719.5244 | 2721.085 | 63 | 5.395 | 24.0% | 1 | R.LSWLDGSGLGFSLEYPTISLHALSR.D | 3 |
| \* | CENPL\_stlcld\_122314\_02.13073.13073.3 | 7.0215 | 0.5508 | 100.0% | 3459.6543 | 3459.5435 | 1 | 8.67 | 31.7% | 1 | R.TEDSIRDYEDGMEVDTTPTVAGQFEDADVDH.- | 3 |

---

|  |  |  |  |  |  |  |  |  |
| --- | --- | --- | --- | --- | --- | --- | --- | --- |
| U | *gi|4506901|ref|NP\_003* | 5 | 11 | 28.7% | 164 | 19330 | 11.6 | splicing factor, arginine/serine-rich 3 [Homo sapiens] |

| Filename XCorr DeltCN Conf% ObsM+H+ CalcM+H+ SpR ZScore Ion% # Sequence  | | | | | | | | | | | | |
| --- | --- | --- | --- | --- | --- | --- | --- | --- | --- | --- | --- | --- |
| \* | CENPL\_stlcld\_tube2\_122314\_01.09621.09621.2 | 4.2803 | 0.2774 | 100.0% | 1877.5721 | 1878.0519 | 1 | 6.368 | 62.5% | 2 | K.VYVGNLGNNGNKTELER.A | 2 |
| \* | CENPL\_stlcld\_122314\_01.14294.14294.2 | 2.8136 | 0.432 | 100.0% | 1044.3522 | 1044.198 | 1 | 6.885 | 87.5% | 6 | R.AFGYYGPLR.S | 2 |
|  | CENPL\_stlcld\_122314\_01.19713.19713.2 | 2.6902 | 0.2983 | 99.2% | 1622.4321 | 1622.7771 | 3 | 5.074 | 50.0% | 1 | R.NPPGFAFVEFEDPR.D | 22 |
| \* | CENPL\_stlcld\_tube2\_122314\_01.18392.18392.3 | 2.633 | 0.2686 | 96.8% | 2321.2744 | 2321.5107 | 24 | 4.67 | 30.0% | 1 | R.NPPGFAFVEFEDPRDAADAVR.E | 3 |
| \* | CENPL\_stlcld\_122314\_01.18934.18934.2 | 3.2665 | 0.2538 | 99.6% | 2322.112 | 2321.5107 | 48 | 5.021 | 37.5% | 1 | R.NPPGFAFVEFEDPRDAADAVR.E | 2 |

Similarities:
gi|72534660|ref|NP\_00(1:4)  

---

|  |  |  |  |  |  |  |  |  |
| --- | --- | --- | --- | --- | --- | --- | --- | --- |
| U | *gi|4507651|ref|NP\_003* | 7 | 10 | 28.6% | 248 | 28522 | 4.7 | tropomyosin 4 isoform 2 [Homo sapiens] |

| Filename XCorr DeltCN Conf% ObsM+H+ CalcM+H+ SpR ZScore Ion% # Sequence  | | | | | | | | | | | | |
| --- | --- | --- | --- | --- | --- | --- | --- | --- | --- | --- | --- | --- |
| \* | CENPL\_stlcld\_tube2\_122314\_01.08523.08523.3 | 3.165 | 0.333 | 99.9% | 1744.3744 | 1743.8711 | 1 | 5.709 | 39.3% | 1 | R.KIQALQQQADEAEDR.A | 3 |
| \* | CENPL\_stlcld\_122314\_01.09682.09682.2 | 3.6063 | 0.4782 | 100.0% | 1615.9521 | 1615.697 | 1 | 7.429 | 69.2% | 1 | K.IQALQQQADEAEDR.A | 2 |
|  | CENPL\_stlcld\_tube2\_122314\_01.13634.13634.2 | 3.3821 | 0.2919 | 99.9% | 1244.1921 | 1244.3873 | 2 | 5.902 | 77.8% | 3 | R.IQLVEEELDR.A | 22 |
|  | CENPL\_stlcld\_122314\_01.12767.12767.2 | 3.0824 | 0.2575 | 99.6% | 1445.2922 | 1444.6683 | 1 | 5.012 | 70.8% | 1 | R.LATALQKLEEAEK.A | 22 |
|  | CENPL\_stlcld\_tube2\_122314\_01.13997.13997.3 | 3.2887 | 0.3151 | 99.9% | 2203.6443 | 2203.4111 | 1 | 5.586 | 31.6% | 2 | R.LATALQKLEEAEKAADESER.G | 33 |
|  | CENPL\_stlcld\_tube2\_122314\_01.07946.07946.3 | 2.9327 | 0.2576 | 99.4% | 1691.2144 | 1690.801 | 1 | 4.39 | 45.8% | 1 | K.YSEKEDKYEEEIK.L | 3 |
|  | CENPL\_stlcld\_tube2\_122314\_02.10545.10545.3 | 2.6313 | 0.2226 | 95.4% | 1493.7544 | 1492.7153 | 22 | 4.211 | 35.4% | 1 | R.AEFAERTVAKLEK.T | 3 |

Similarities:
gi|114155144|ref|NP\_0(3:4)  

---

|  |  |  |  |  |  |  |  |  |
| --- | --- | --- | --- | --- | --- | --- | --- | --- |
| U | *gi|4505773|ref|NP\_002* | 7 | 12 | 28.3% | 272 | 29804 | 5.8 | prohibitin [Homo sapiens] |

| Filename XCorr DeltCN Conf% ObsM+H+ CalcM+H+ SpR ZScore Ion% # Sequence  | | | | | | | | | | | | |
| --- | --- | --- | --- | --- | --- | --- | --- | --- | --- | --- | --- | --- |
| \* | CENPL\_stlcld\_122314\_01.13322.13322.2 | 2.8237 | 0.1798 | 98.4% | 1397.7322 | 1397.7067 | 2 | 4.5 | 72.7% | 2 | R.ILFRPVASQLPR.I | 2 |
| \* | CENPL\_stlcld\_122314\_01.13298.13298.3 | 3.0099 | 0.2808 | 99.9% | 1398.3544 | 1397.7067 | 1 | 5.451 | 45.5% | 1 | R.ILFRPVASQLPR.I | 3 |
| \* | CENPL\_stlcld\_tube2\_122314\_01.13125.13125.2 | 3.2053 | 0.3633 | 99.9% | 1445.2122 | 1445.5255 | 2 | 7.871 | 72.7% | 2 | R.IFTSIGEDYDER.V | 2 |
| \* | CENPL\_stlcld\_122314\_01.14099.14099.2 | 3.1235 | 0.4646 | 100.0% | 1150.4521 | 1150.2767 | 2 | 7.709 | 72.2% | 2 | R.FDAGELITQR.E | 2 |
| \* | CENPL\_stlcld\_122314\_02.17332.17332.2 | 2.2265 | 0.2441 | 95.9% | 2119.412 | 2120.4534 | 246 | 4.643 | 26.3% | 1 | R.AATFGLILDDVSLTHLTFGK.E | 2 |
| \* | CENPL\_stlcld\_122314\_02.17472.17472.3 | 3.3351 | 0.2233 | 97.9% | 3125.3943 | 3125.5457 | 4 | 5.492 | 21.4% | 1 | R.AATFGLILDDVSLTHLTFGKEFTEAVEAK.Q | 3 |
| \* | CENPL\_stlcld\_122314\_01.14777.14777.2 | 4.7048 | 0.4166 | 100.0% | 1607.5322 | 1607.804 | 1 | 8.163 | 84.6% | 3 | R.KLEAAEDIAYQLSR.S | 2 |

---

|  |  |  |  |  |  |  |  |  |
| --- | --- | --- | --- | --- | --- | --- | --- | --- |
| U | *gi|4504517|ref|NP\_001* | 4 | 11 | 28.3% | 205 | 22783 | 6.4 | heat shock protein beta-1 [Homo sapiens] |

| Filename XCorr DeltCN Conf% ObsM+H+ CalcM+H+ SpR ZScore Ion% # Sequence  | | | | | | | | | | | | |
| --- | --- | --- | --- | --- | --- | --- | --- | --- | --- | --- | --- | --- |
| \* | CENPL\_stlcld\_tube2\_122314\_01.17669.17669.3 | 2.8533 | 0.2909 | 99.8% | 1904.0944 | 1904.0537 | 1 | 4.821 | 41.1% | 1 | R.GPSWDPFRDWYPHSR.L | 3 |
| \* | CENPL\_stlcld\_tube2\_122314\_01.17529.17529.2 | 3.7673 | 0.4254 | 100.0% | 1164.6322 | 1164.3494 | 1 | 7.264 | 83.3% | 5 | R.LFDQAFGLPR.L | 2 |
| \* | CENPL\_stlcld\_122314\_01.15190.15190.2 | 4.4758 | 0.4433 | 100.0% | 1784.5122 | 1785.0068 | 1 | 7.96 | 56.7% | 2 | R.VSLDVNHFAPDELTVK.T | 2 |
| \* | CENPL\_stlcld\_122314\_01.16630.16630.2 | 3.2027 | 0.5167 | 100.0% | 1907.0122 | 1907.1307 | 50 | 8.393 | 37.5% | 3 | K.LATQSNEITIPVTFESR.A | 2 |

---

|  |  |  |  |  |  |  |  |  |
| --- | --- | --- | --- | --- | --- | --- | --- | --- |
| U | *gi|4504345|ref|NP\_000* | 4 | 7 | 28.2% | 142 | 15258 | 8.7 | alpha 2 globin [Homo sapiens] |
| U | *gi|4504347|ref|NP\_000* | 4 | 7 | 28.2% | 142 | 15258 | 8.7 | alpha 1 globin [Homo sapiens] |

| Filename XCorr DeltCN Conf% ObsM+H+ CalcM+H+ SpR ZScore Ion% # Sequence  | | | | | | | | | | | | |
| --- | --- | --- | --- | --- | --- | --- | --- | --- | --- | --- | --- | --- |
|  | CENPL\_stlcld\_tube2\_122314\_01.09362.09362.2 | 3.0552 | 0.2868 | 99.6% | 1530.6522 | 1530.6371 | 1 | 6.805 | 60.7% | 2 | K.VGAHAGEYGAEALER.M | 2 |
|  | CENPL\_stlcld\_tube2\_122314\_02.10563.10563.3 | 3.9096 | 0.1148 | 97.5% | 1531.1344 | 1530.6371 | 3 | 4.196 | 48.2% | 2 | K.VGAHAGEYGAEALER.M | 3 |
|  | CENPL\_stlcld\_122314\_01.17517.17517.2 | 2.5407 | 0.438 | 99.9% | 1072.7322 | 1072.3075 | 1 | 7.637 | 81.2% | 2 | R.MFLSFPTTK.T | 2 |
|  | CENPL\_stlcld\_tube2\_122314\_01.13366.13366.3 | 2.6092 | 0.32 | 99.5% | 1834.7043 | 1835.0287 | 1 | 6.642 | 36.7% | 1 | K.TYFPHFDLSHGSAQVK.G | 3 |

---

|  |  |  |  |  |  |  |  |  |
| --- | --- | --- | --- | --- | --- | --- | --- | --- |
| U | *gi|12667788|ref|NP\_00* | 43 | 87 | 27.9% | 1960 | 226530 | 5.6 | myosin, heavy polypeptide 9, non-muscle [Homo sapiens] |

| Filename XCorr DeltCN Conf% ObsM+H+ CalcM+H+ SpR ZScore Ion% # Sequence  | | | | | | | | | | | | |
| --- | --- | --- | --- | --- | --- | --- | --- | --- | --- | --- | --- | --- |
| \* | CENPL\_stlcld\_122314\_01.18442.18442.2 | 3.2012 | 0.2259 | 99.2% | 1673.7322 | 1673.8687 | 2 | 5.395 | 57.1% | 1 | K.NFINNPLAQADWAAK.K | 2 |
| \* | CENPL\_stlcld\_tube2\_122314\_01.11574.11574.3 | 3.0898 | 0.2324 | 98.7% | 1918.7943 | 1916.1614 | 1 | 4.415 | 36.7% | 1 | R.HEMPPHIYAITDTAYR.S | 3 |
| \* | CENPL\_stlcld\_122314\_01.14033.14033.2 | 3.8226 | 0.3569 | 100.0% | 1480.8922 | 1479.719 | 1 | 6.756 | 66.7% | 2 | K.VIQYLAYVASSHK.S | 2 |
|  | CENPL\_stlcld\_tube2\_122314\_01.20001.20001.2 | 3.992 | 0.4233 | 100.0% | 1727.7522 | 1728.0012 | 1 | 7.621 | 60.0% | 2 | R.QLLQANPILEAFGNAK.T | 2 |
| \* | CENPL\_stlcld\_122314\_02.16590.16590.3 | 4.9306 | 0.3734 | 100.0% | 1997.5743 | 1997.3037 | 2 | 8.22 | 42.2% | 3 | R.TFHIFYYLLSGAGEHLK.T | 3 |
| \* | CENPL\_stlcld\_122314\_01.17145.17145.3 | 4.8251 | 0.2522 | 99.9% | 3013.0144 | 3012.4 | 1 | 5.736 | 35.0% | 1 | R.FLSNGHVTIPGQQDKDMFQETMEAMR.I | 3 |
| \* | CENPL\_stlcld\_tube2\_122314\_01.17973.17973.2 | 3.4094 | 0.3705 | 99.9% | 1616.4922 | 1616.9313 | 1 | 6.865 | 80.8% | 4 | R.IMGIPEEEQMGLLR.V | 2 |
| \* | CENPL\_stlcld\_122314\_01.19590.19590.2 | 3.634 | 0.384 | 100.0% | 1615.4521 | 1616.0 | 2 | 6.455 | 53.6% | 2 | R.VISGVLQLGNIVFKK.E | 2 |
| \* | CENPL\_stlcld\_122314\_01.16703.16703.2 | 3.9535 | 0.3943 | 100.0% | 1572.6122 | 1572.8044 | 1 | 8.362 | 76.9% | 7 | K.VSHLLGINVTDFTR.G | 2 |
| \* | CENPL\_stlcld\_tube2\_122314\_01.16562.16562.3 | 3.8991 | 0.3339 | 100.0% | 1573.0443 | 1572.8044 | 1 | 5.765 | 53.8% | 3 | K.VSHLLGINVTDFTR.G | 3 |
|  | CENPL\_stlcld\_tube2\_122314\_01.13938.13938.2 | 2.3512 | 0.3614 | 99.5% | 1398.3922 | 1398.6166 | 1 | 5.677 | 65.0% | 1 | K.VDYKADEWLMK.N | 2 |
| \* | CENPL\_stlcld\_122314\_01.20782.20782.2 | 2.8144 | 0.1323 | 95.9% | 2021.1122 | 2019.3636 | 1 | 4.316 | 39.5% | 1 | R.IIGLDQVAGMSETALPGAFK.T | 2 |
|  | CENPL\_stlcld\_tube2\_122314\_01.16276.16276.2 | 2.8244 | 0.3362 | 99.9% | 1318.9122 | 1319.5468 | 3 | 6.072 | 70.0% | 4 | K.LDPHLVLDQLR.C | 2 |
| \* | CENPL\_stlcld\_122314\_01.14210.14210.2 | 2.3563 | 0.3068 | 98.9% | 1275.6721 | 1275.4875 | 7 | 5.45 | 60.0% | 1 | R.YEILTPNSIPK.G | 2 |
| \* | CENPL\_stlcld\_122314\_01.13925.13925.2 | 2.5506 | 0.3983 | 99.9% | 1194.6721 | 1194.33 | 389 | 6.671 | 50.0% | 1 | K.ALELDSNLYR.I | 2 |
|  | CENPL\_stlcld\_tube2\_122314\_01.11285.11285.2 | 3.4125 | 0.4856 | 100.0% | 1224.5521 | 1224.3591 | 1 | 8.046 | 75.0% | 2 | R.AGVLAHLEEER.D | 2 |
| \* | CENPL\_stlcld\_122314\_02.14301.14301.2 | 4.8252 | 0.4469 | 100.0% | 1752.6522 | 1753.0358 | 1 | 8.712 | 71.4% | 5 | R.LTEMETLQSQLMAEK.L | 2 |
| \* | CENPL\_stlcld\_tube2\_122314\_01.17327.17327.2 | 6.3685 | 0.4131 | 100.0% | 2334.8523 | 2334.4736 | 1 | 8.447 | 66.7% | 2 | K.MQQNIQELEEQLEEEESAR.Q | 2 |
| \* | CENPL\_stlcld\_122314\_02.11832.11832.2 | 4.7244 | 0.4671 | 100.0% | 1654.5122 | 1654.7681 | 1 | 8.265 | 73.1% | 6 | R.IAEFTTNLTEEEEK.S | 2 |
| \* | CENPL\_stlcld\_122314\_01.09989.09989.2 | 3.4565 | 0.4527 | 100.0% | 1586.4722 | 1586.76 | 1 | 7.697 | 70.8% | 1 | K.NKHEAMITDLEER.L | 2 |
| \* | CENPL\_stlcld\_122314\_01.09969.09969.3 | 3.0073 | 0.3152 | 99.9% | 1586.9644 | 1586.76 | 1 | 6.271 | 47.9% | 1 | K.NKHEAMITDLEER.L | 3 |
| \* | CENPL\_stlcld\_tube2\_122314\_01.11135.11135.2 | 2.9892 | 0.5223 | 100.0% | 1344.1522 | 1344.4822 | 1 | 8.301 | 85.0% | 1 | K.HEAMITDLEER.L | 2 |
| \* | CENPL\_stlcld\_tube2\_122314\_01.19815.19815.3 | 5.6937 | 0.3802 | 100.0% | 2773.5842 | 2773.0696 | 1 | 7.102 | 33.3% | 2 | R.RKLEGDSTDLSDQIAELQAQIAELK.M | 3 |
|  | CENPL\_stlcld\_122314\_01.09935.09935.2 | 3.24 | 0.2689 | 99.9% | 1258.5922 | 1258.4172 | 2 | 5.576 | 75.0% | 2 | K.KEEELQAALAR.V | 2 |
| \* | CENPL\_stlcld\_tube2\_122314\_01.17987.17987.2 | 5.5186 | 0.4852 | 100.0% | 2303.8523 | 2304.473 | 1 | 9.412 | 69.4% | 1 | K.IRELESQISELQEDLESER.A | 2 |
| \* | CENPL\_stlcld\_tube2\_122314\_01.17981.17981.3 | 3.6523 | 0.2784 | 99.9% | 2305.1643 | 2304.473 | 1 | 4.758 | 43.1% | 2 | K.IRELESQISELQEDLESER.A | 3 |
| \* | CENPL\_stlcld\_tube2\_122314\_01.11148.11148.3 | 3.5054 | 0.3083 | 99.9% | 2043.9543 | 2044.2439 | 1 | 6.2 | 37.5% | 1 | K.TLEEEAKTHEAQIQEMR.Q | 3 |
| \* | CENPL\_stlcld\_122314\_01.13611.13611.3 | 6.0507 | 0.3886 | 100.0% | 1997.4243 | 1997.1722 | 1 | 7.013 | 40.6% | 4 | K.HSQAVEELAEQLEQTKR.V | 3 |
| \* | CENPL\_stlcld\_122314\_01.09333.09333.3 | 3.4903 | 0.2378 | 99.9% | 1929.8043 | 1930.1252 | 2 | 4.604 | 40.6% | 1 | K.AKQTLENERGELANEVK.V | 3 |
| \* | CENPL\_stlcld\_tube2\_122314\_01.12087.12087.2 | 2.4073 | 0.2069 | 97.6% | 1286.1921 | 1285.4833 | 34 | 5.141 | 65.0% | 1 | K.VEAQLQELQVK.F | 2 |
| \* | CENPL\_stlcld\_122314\_01.08092.08092.3 | 3.0139 | 0.319 | 99.9% | 1494.2644 | 1493.6598 | 2 | 5.168 | 45.5% | 1 | K.LKQVEDEKNSFR.E | 3 |
| \* | CENPL\_stlcld\_tube2\_122314\_01.19354.19354.2 | 5.0739 | 0.3966 | 100.0% | 1950.9321 | 1951.1436 | 1 | 8.738 | 70.0% | 2 | R.LQQELDDLLVDLDHQR.Q | 2 |
| \* | CENPL\_stlcld\_tube2\_122314\_01.19328.19328.3 | 3.4244 | 0.2429 | 99.9% | 1951.3744 | 1951.1436 | 9 | 4.999 | 40.0% | 2 | R.LQQELDDLLVDLDHQR.Q | 3 |
| \* | CENPL\_stlcld\_122314\_02.10646.10646.2 | 3.3125 | 0.3469 | 99.9% | 1647.5721 | 1647.8407 | 1 | 5.812 | 57.7% | 1 | R.ALEEAMEQKAELER.L | 2 |
| \* | CENPL\_stlcld\_122314\_02.10625.10625.3 | 3.2662 | 0.2143 | 98.9% | 1648.5243 | 1647.8407 | 1 | 5.798 | 50.0% | 2 | R.ALEEAMEQKAELER.L | 3 |
| \* | CENPL\_stlcld\_122314\_02.12537.12537.2 | 2.4794 | 0.1986 | 97.7% | 1315.6322 | 1315.6171 | 1 | 5.749 | 60.0% | 1 | K.LRLEVNLQAMK.A | 2 |
| \* | CENPL\_stlcld\_122314\_01.08745.08745.2 | 3.4992 | 0.2725 | 99.9% | 1379.6122 | 1379.4816 | 10 | 5.19 | 65.0% | 1 | R.EMEAELEDERK.Q | 2 |
| \* | CENPL\_stlcld\_122314\_01.08736.08736.2 | 2.6474 | 0.1633 | 97.7% | 1213.4122 | 1213.2896 | 1 | 4.456 | 80.0% | 1 | K.DLEAHIDSANK.N | 2 |
| \* | CENPL\_stlcld\_122314\_02.13650.13650.3 | 4.3403 | 0.4188 | 100.0% | 2474.2744 | 2473.6099 | 2 | 6.733 | 32.5% | 1 | R.IAQLEEELEEEQGNTELINDR.L | 3 |
| \* | CENPL\_stlcld\_tube2\_122314\_01.16487.16487.2 | 3.7789 | 0.3465 | 100.0% | 1871.7522 | 1871.0574 | 2 | 6.492 | 50.0% | 1 | K.ANLQIDQINTDLNLER.S | 2 |
| \* | CENPL\_stlcld\_tube2\_122314\_01.11903.11903.2 | 2.9221 | 0.348 | 99.9% | 1531.2922 | 1531.6598 | 1 | 5.698 | 70.8% | 2 | K.IAQLEEQLDNETK.E | 2 |
| \* | CENPL\_stlcld\_tube2\_122314\_01.10155.10155.2 | 3.9781 | 0.5264 | 100.0% | 1566.3922 | 1566.6367 | 1 | 8.773 | 61.5% | 2 | R.ELEDATETADAMNR.E | 2 |
| \* | CENPL\_stlcld\_tube2\_122314\_01.13354.13354.2 | 3.3879 | 0.2388 | 99.9% | 1156.4722 | 1156.3732 | 1 | 6.649 | 88.9% | 4 | R.RGDLPFVVPR.R | 2 |

---

|  |  |  |  |  |  |  |  |  |
| --- | --- | --- | --- | --- | --- | --- | --- | --- |
| U | *gi|4503471|ref|NP\_001* | 14 | 86 | 27.7% | 462 | 50141 | 9.0 | eukaryotic translation elongation factor 1 alpha 1 [Homo sapiens] |

| Filename XCorr DeltCN Conf% ObsM+H+ CalcM+H+ SpR ZScore Ion% # Sequence  | | | | | | | | | | | | |
| --- | --- | --- | --- | --- | --- | --- | --- | --- | --- | --- | --- | --- |
|  | CENPL\_stlcld\_122314\_02.11104.11104.2 | 4.9065 | 0.4927 | 100.0% | 1589.7322 | 1589.835 | 1 | 9.297 | 75.0% | 7 | K.THINIVVIGHVDSGK.S | 2 |
|  | CENPL\_stlcld\_122314\_02.11138.11138.3 | 5.1523 | 0.4819 | 100.0% | 1590.5944 | 1589.835 | 1 | 8.434 | 57.1% | 32 | K.THINIVVIGHVDSGK.S | 3 |
| \* | CENPL\_stlcld\_tube2\_122314\_01.12580.12580.2 | 3.3336 | 0.4687 | 100.0% | 1405.4521 | 1405.5962 | 1 | 7.229 | 77.3% | 4 | K.YYVTIIDAPGHR.D | 2 |
| \* | CENPL\_stlcld\_122314\_01.12795.12795.3 | 3.4374 | 0.3168 | 100.0% | 1406.9944 | 1405.5962 | 8 | 5.991 | 43.2% | 5 | K.YYVTIIDAPGHR.D | 3 |
|  | CENPL\_stlcld\_122314\_01.14408.14408.2 | 3.5033 | 0.4636 | 100.0% | 1315.7522 | 1315.5553 | 1 | 8.217 | 72.7% | 9 | R.EHALLAYTLGVK.Q | 2 |
|  | CENPL\_stlcld\_122314\_02.12758.12758.3 | 3.808 | 0.3458 | 100.0% | 1315.9744 | 1315.5553 | 1 | 6.803 | 56.8% | 2 | R.EHALLAYTLGVK.Q | 3 |
|  | CENPL\_stlcld\_tube2\_122314\_01.15892.15892.2 | 2.669 | 0.2376 | 98.5% | 1602.0521 | 1601.837 | 3 | 4.869 | 54.2% | 1 | R.YEEIVKEVSTYIK.K | 2 |
| \* | CENPL\_stlcld\_122314\_01.21682.21682.3 | 4.3823 | 0.3957 | 100.0% | 3697.8245 | 3698.205 | 1 | 5.171 | 23.4% | 1 | K.KIGYNPDTVAFVPISGWNGDNMLEPSANMPWFK.G | 3 |
|  | CENPL\_stlcld\_122314\_01.12362.12362.2 | 2.4878 | 0.2506 | 99.2% | 976.3122 | 976.1607 | 16 | 5.897 | 71.4% | 5 | R.LPLQDVYK.I | 2 |
|  | CENPL\_stlcld\_122314\_01.11916.11916.1 | 1.4712 | 0.3088 | 95.9% | 1025.57 | 1026.2241 | 1 | 5.388 | 55.0% | 1 | K.IGGIGTVPVGR.V | 1 |
|  | CENPL\_stlcld\_122314\_01.11954.11954.2 | 3.5551 | 0.4155 | 100.0% | 1026.6122 | 1026.2241 | 2 | 8.081 | 75.0% | 8 | K.IGGIGTVPVGR.V | 2 |
| \* | CENPL\_stlcld\_tube2\_122314\_01.18169.18169.3 | 5.4263 | 0.4163 | 100.0% | 3524.0044 | 3524.2 | 1 | 7.075 | 27.2% | 1 | K.IGGIGTVPVGRVETGVLKPGMVVTFAPVNVTTEVK.S | 3 |
| \* | CENPL\_stlcld\_122314\_01.17986.17986.2 | 5.4923 | 0.4937 | 100.0% | 2517.2922 | 2516.999 | 1 | 9.244 | 47.8% | 5 | R.VETGVLKPGMVVTFAPVNVTTEVK.S | 2 |
| \* | CENPL\_stlcld\_122314\_01.17972.17972.3 | 4.9986 | 0.4172 | 100.0% | 2517.8342 | 2516.999 | 1 | 7.266 | 38.0% | 5 | R.VETGVLKPGMVVTFAPVNVTTEVK.S | 3 |

---

|  |  |  |  |  |  |  |  |  |
| --- | --- | --- | --- | --- | --- | --- | --- | --- |
| U | *gi|4507357|ref|NP\_003* | 4 | 10 | 27.6% | 199 | 22391 | 8.2 | transgelin 2 [Homo sapiens] |

| Filename XCorr DeltCN Conf% ObsM+H+ CalcM+H+ SpR ZScore Ion% # Sequence  | | | | | | | | | | | | |
| --- | --- | --- | --- | --- | --- | --- | --- | --- | --- | --- | --- | --- |
| \* | CENPL\_stlcld\_tube2\_122314\_01.21179.21179.2 | 3.8356 | 0.3533 | 100.0% | 2101.0522 | 2101.3203 | 20 | 6.747 | 38.2% | 2 | R.YGINTTDIFQTVDLWEGK.N | 2 |
| \* | CENPL\_stlcld\_tube2\_122314\_01.16949.16949.2 | 3.0006 | 0.3523 | 99.9% | 1216.6721 | 1216.4845 | 1 | 5.894 | 68.2% | 3 | R.TLMNLGGLAVAR.D | 2 |
| \* | CENPL\_stlcld\_122314\_02.08422.08422.2 | 2.4235 | 0.2666 | 98.5% | 1279.1322 | 1280.3367 | 3 | 4.684 | 65.0% | 2 | R.NFSDNQLQEGK.N | 2 |
|  | CENPL\_stlcld\_122314\_01.10958.10958.2 | 2.6283 | 0.2982 | 99.1% | 1384.4722 | 1384.5677 | 7 | 5.299 | 50.0% | 3 | R.GASQAGMTGYGMPR.Q | 2 |

---

|  |  |  |  |  |  |  |  |  |
| --- | --- | --- | --- | --- | --- | --- | --- | --- |
| U | *gi|46367787|ref|NP\_00* | 16 | 42 | 27.4% | 636 | 70671 | 9.5 | poly(A) binding protein, cytoplasmic 1 [Homo sapiens] |

| Filename XCorr DeltCN Conf% ObsM+H+ CalcM+H+ SpR ZScore Ion% # Sequence  | | | | | | | | | | | | |
| --- | --- | --- | --- | --- | --- | --- | --- | --- | --- | --- | --- | --- |
|  | CENPL\_stlcld\_122314\_01.16239.16239.2 | 2.5434 | 0.3347 | 99.7% | 1158.5721 | 1158.3861 | 12 | 5.887 | 65.0% | 2 | K.FSPAGPILSIR.V | 2 |
|  | CENPL\_stlcld\_tube2\_122314\_02.12795.12795.3 | 3.4609 | 0.2338 | 99.4% | 2086.3145 | 2086.2712 | 21 | 4.82 | 33.8% | 1 | R.RSLGYAYVNFQQPADAER.A | 33 |
|  | CENPL\_stlcld\_122314\_01.14967.14967.2 | 5.0053 | 0.4787 | 100.0% | 1930.5322 | 1930.0837 | 1 | 8.555 | 53.1% | 7 | R.SLGYAYVNFQQPADAER.A | 22 |
|  | CENPL\_stlcld\_tube2\_122314\_02.14073.14073.2 | 2.8218 | 0.525 | 100.0% | 1267.4521 | 1267.4828 | 1 | 8.158 | 75.0% | 2 | R.ALDTMNFDVIK.G | 22 |
| \* | CENPL\_stlcld\_tube2\_122314\_02.12986.12986.3 | 2.7748 | 0.324 | 99.9% | 1742.0944 | 1741.857 | 2 | 5.61 | 37.5% | 1 | K.GYGFVHFETQEAAER.A | 3 |
|  | CENPL\_stlcld\_122314\_01.12149.12149.2 | 2.0554 | 0.246 | 97.1% | 1064.5122 | 1064.263 | 6 | 4.937 | 68.8% | 1 | K.MNGMLLNDR.K | 22 |
|  | CENPL\_stlcld\_tube2\_122314\_01.10307.10307.2 | 2.8627 | 0.3205 | 99.9% | 1213.6921 | 1213.4191 | 37 | 6.612 | 61.1% | 3 | R.AKEFTNVYIK.N | 22 |
|  | CENPL\_stlcld\_122314\_01.17970.17970.2 | 2.2696 | 0.2532 | 98.4% | 1046.2322 | 1046.1704 | 2 | 5.508 | 68.8% | 2 | K.GFGFVSFER.H | 2 |
|  | CENPL\_stlcld\_tube2\_122314\_01.12173.12173.2 | 2.6441 | 0.2098 | 98.9% | 1085.3522 | 1084.2603 | 2 | 6.468 | 81.2% | 2 | R.YQGVNLYVK.N | 2 |
| \* | CENPL\_stlcld\_tube2\_122314\_01.13162.13162.2 | 3.3041 | 0.3245 | 99.9% | 1413.2522 | 1413.6134 | 1 | 6.386 | 70.8% | 2 | R.KEFSPFGTITSAK.V | 2 |
|  | CENPL\_stlcld\_122314\_01.13622.13622.3 | 3.4547 | 0.2709 | 99.9% | 1544.1543 | 1543.8931 | 135 | 5.597 | 36.5% | 5 | R.IVATKPLYVALAQR.K | 3 |
|  | CENPL\_stlcld\_tube2\_122314\_01.13454.13454.2 | 3.69 | 0.3312 | 99.9% | 1545.7322 | 1543.8931 | 1 | 5.975 | 61.5% | 4 | R.IVATKPLYVALAQR.K | 2 |
|  | CENPL\_stlcld\_122314\_01.18924.18924.2 | 4.0878 | 0.5031 | 100.0% | 1637.8922 | 1638.0239 | 1 | 8.322 | 71.4% | 1 | R.LFPLIQAMHPTLAGK.I | 2 |
|  | CENPL\_stlcld\_tube2\_122314\_01.21639.21639.2 | 5.0786 | 0.5494 | 100.0% | 2741.7522 | 2742.175 | 1 | 8.398 | 45.7% | 3 | K.ITGMLLEIDNSELLHMLESPESLR.S | 22 |
|  | CENPL\_stlcld\_122314\_01.22454.22454.3 | 5.3189 | 0.3378 | 100.0% | 2741.8145 | 2742.175 | 13 | 6.113 | 30.4% | 3 | K.ITGMLLEIDNSELLHMLESPESLR.S | 33 |
|  | CENPL\_stlcld\_tube2\_122314\_01.11156.11156.3 | 5.4407 | 0.4676 | 100.0% | 1695.5944 | 1694.9285 | 1 | 9.065 | 55.0% | 3 | R.SKVDEAVAVLQAHQAK.E | 3 |

Similarities:
gi|208431833|ref|NP\_0(7:9)  

---

|  |  |  |  |  |  |  |  |  |
| --- | --- | --- | --- | --- | --- | --- | --- | --- |
| U | *gi|16753227|ref|NP\_00* | 6 | 8 | 27.4% | 288 | 32728 | 10.6 | ribosomal protein L6 [Homo sapiens] |
| U | *gi|67189747|ref|NP\_00* | 6 | 8 | 27.4% | 288 | 32728 | 10.6 | ribosomal protein L6 [Homo sapiens] |

| Filename XCorr DeltCN Conf% ObsM+H+ CalcM+H+ SpR ZScore Ion% # Sequence  | | | | | | | | | | | | |
| --- | --- | --- | --- | --- | --- | --- | --- | --- | --- | --- | --- | --- |
|  | CENPL\_stlcld\_122314\_01.08409.08409.2 | 2.9422 | 0.4525 | 100.0% | 1284.7722 | 1285.5266 | 1 | 6.964 | 58.3% | 1 | K.VLATVTKPVGGDK.N | 2 |
|  | CENPL\_stlcld\_122314\_02.00158.00158.2 | 3.0762 | 0.3867 | 99.9% | 1526.9521 | 1526.8601 | 1 | 7.366 | 50.0% | 2 | R.ASITPGTILIILTGR.H | 2 |
|  | CENPL\_stlcld\_tube2\_122314\_01.09824.09824.2 | 2.0452 | 0.2678 | 97.9% | 995.0722 | 995.1228 | 4 | 5.752 | 71.4% | 1 | K.HLTDAYFK.K | 2 |
|  | CENPL\_stlcld\_122314\_02.10551.10551.3 | 3.6154 | 0.311 | 99.9% | 2510.0645 | 2510.6763 | 1 | 5.51 | 36.8% | 1 | R.HQEGEIFDTEKEKYEITEQR.K | 3 |
|  | CENPL\_stlcld\_tube2\_122314\_01.15206.15206.2 | 2.0325 | 0.3136 | 98.3% | 1159.4922 | 1159.3738 | 94 | 6.236 | 61.1% | 1 | K.AIPQLQGYLR.S | 2 |
|  | CENPL\_stlcld\_122314\_01.15022.15022.2 | 3.5403 | 0.3923 | 100.0% | 1448.5122 | 1447.6769 | 1 | 7.36 | 58.3% | 2 | R.SVFALTNGIYPHK.L | 2 |

---

|  |  |  |  |  |  |  |  |  |
| --- | --- | --- | --- | --- | --- | --- | --- | --- |
| U | *gi|156071459|ref|NP\_0* | 7 | 13 | 27.2% | 298 | 32852 | 9.7 | solute carrier family 25, member 5 [Homo sapiens] |

| Filename XCorr DeltCN Conf% ObsM+H+ CalcM+H+ SpR ZScore Ion% # Sequence  | | | | | | | | | | | | |
| --- | --- | --- | --- | --- | --- | --- | --- | --- | --- | --- | --- | --- |
| \* | CENPL\_stlcld\_122314\_02.14181.14181.2 | 3.8895 | 0.45 | 100.0% | 1220.0721 | 1220.4111 | 1 | 8.886 | 83.3% | 6 | K.DFLAGGVAAAISK.T | 2 |
|  | CENPL\_stlcld\_122314\_01.09808.09808.2 | 2.5277 | 0.2161 | 98.4% | 1138.4722 | 1137.3677 | 4 | 4.515 | 66.7% | 2 | K.LLLQVQHASK.Q | 22 |
|  | CENPL\_stlcld\_tube2\_122314\_01.16517.16517.2 | 2.1212 | 0.2444 | 95.9% | 1460.0521 | 1460.719 | 51 | 5.186 | 40.9% | 1 | R.IPKEQGVLSFWR.G | 22 |
|  | CENPL\_stlcld\_tube2\_122314\_01.18507.18507.2 | 2.2937 | 0.1945 | 97.5% | 1122.0922 | 1122.2688 | 60 | 5.048 | 56.2% | 1 | K.EQGVLSFWR.G | 22 |
|  | CENPL\_stlcld\_122314\_01.10493.10493.2 | 2.2972 | 0.1833 | 97.7% | 857.47217 | 857.00055 | 3 | 4.089 | 78.6% | 1 | R.GNLANVIR.Y | 22 |
|  | CENPL\_stlcld\_122314\_01.20482.20482.2 | 3.2415 | 0.386 | 100.0% | 1447.4122 | 1447.6763 | 1 | 6.952 | 77.3% | 1 | R.YFPTQALNFAFK.D | 22 |
| \* | CENPL\_stlcld\_tube2\_122314\_02.01950.01950.3 | 4.8647 | 0.2698 | 99.9% | 2890.1643 | 2889.385 | 1 | 4.724 | 29.0% | 1 | K.GAWSNVLRGMGGAFVLVLYDEIKKYT.- | 3 |

Similarities:
gi|156071462|ref|NP\_0(5:2)  

---

|  |  |  |  |  |  |  |  |  |
| --- | --- | --- | --- | --- | --- | --- | --- | --- |
| U | *gi|15431303|ref|NP\_00* | 3 | 3 | 27.1% | 192 | 21863 | 10.0 | ribosomal protein L9 [Homo sapiens] |
| U | *gi|67944630|ref|NP\_00* | 3 | 3 | 27.1% | 192 | 21863 | 10.0 | ribosomal protein L9 [Homo sapiens] |

| Filename XCorr DeltCN Conf% ObsM+H+ CalcM+H+ SpR ZScore Ion% # Sequence  | | | | | | | | | | | | |
| --- | --- | --- | --- | --- | --- | --- | --- | --- | --- | --- | --- | --- |
|  | CENPL\_stlcld\_tube2\_122314\_01.17697.17697.2 | 3.0884 | 0.317 | 99.9% | 2114.5322 | 2114.401 | 1 | 5.144 | 44.4% | 1 | K.TILSNQTVDIPENVDITLK.G | 2 |
|  | CENPL\_stlcld\_tube2\_122314\_01.19278.19278.2 | 4.4408 | 0.3918 | 100.0% | 2486.0923 | 2485.8455 | 1 | 8.074 | 54.8% | 1 | R.SVYAHFPINVVIQENGSLVEIR.N | 2 |
|  | CENPL\_stlcld\_tube2\_122314\_01.12780.12780.2 | 2.8622 | 0.1969 | 98.8% | 1299.1721 | 1299.5095 | 1 | 4.976 | 75.0% | 1 | R.KFLDGIYVSEK.G | 2 |

---

|  |  |  |  |  |  |  |  |  |
| --- | --- | --- | --- | --- | --- | --- | --- | --- |
| U | *gi|12056465|ref|NP\_00* | 6 | 7 | 25.9% | 321 | 33784 | 10.2 | fibrillarin [Homo sapiens] |

| Filename XCorr DeltCN Conf% ObsM+H+ CalcM+H+ SpR ZScore Ion% # Sequence  | | | | | | | | | | | | |
| --- | --- | --- | --- | --- | --- | --- | --- | --- | --- | --- | --- | --- |
| \* | CENPL\_stlcld\_122314\_01.09402.09402.3 | 3.0357 | 0.2812 | 99.9% | 1667.3644 | 1667.8162 | 119 | 4.97 | 32.7% | 1 | K.RVSISEGDDKIEYR.A | 3 |
| \* | CENPL\_stlcld\_122314\_01.20487.20487.3 | 7.0009 | 0.4469 | 100.0% | 3419.3943 | 3418.8308 | 1 | 8.362 | 34.4% | 1 | K.VLYLGAASGTTVSHVSDIVGPDGLVYAVEFSHR.S | 3 |
| \* | CENPL\_stlcld\_122314\_01.15383.15383.2 | 2.108 | 0.2844 | 97.7% | 1241.2322 | 1241.43 | 19 | 5.229 | 50.0% | 1 | R.TNIIPVIEDAR.H | 2 |
| \* | CENPL\_stlcld\_tube2\_122314\_01.13907.13907.2 | 2.5335 | 0.3384 | 99.7% | 1255.8322 | 1255.5057 | 1 | 6.9 | 65.0% | 2 | R.IVALNAHTFLR.N | 2 |
| \* | CENPL\_stlcld\_122314\_01.09342.09342.2 | 2.8575 | 0.4317 | 99.9% | 1534.0122 | 1534.8014 | 2 | 7.076 | 57.7% | 1 | R.DHAVVVGVYRPPPK.V | 2 |
| \* | CENPL\_stlcld\_122314\_01.09371.09371.3 | 3.0051 | 0.2273 | 98.6% | 1535.6344 | 1534.8014 | 164 | 6.862 | 34.6% | 1 | R.DHAVVVGVYRPPPK.V | 3 |

---

|  |  |  |  |  |  |  |  |  |
| --- | --- | --- | --- | --- | --- | --- | --- | --- |
| U | *gi|8923930|ref|NP\_060* | 2 | 2 | 25.9% | 108 | 12199 | 9.1 | CDGSH iron sulfur domain 1 [Homo sapiens] |

| Filename XCorr DeltCN Conf% ObsM+H+ CalcM+H+ SpR ZScore Ion% # Sequence  | | | | | | | | | | | | |
| --- | --- | --- | --- | --- | --- | --- | --- | --- | --- | --- | --- | --- |
| \* | CENPL\_stlcld\_tube2\_122314\_01.13620.13620.2 | 2.4693 | 0.2767 | 98.5% | 1490.3522 | 1490.6709 | 40 | 5.042 | 50.0% | 1 | K.IVHAFDMEDLGDK.A | 2 |
| \* | CENPL\_stlcld\_tube2\_122314\_01.11904.11904.2 | 2.9358 | 0.3707 | 99.9% | 1635.6322 | 1636.8021 | 5 | 5.602 | 53.6% | 1 | K.HNEETGDNVGPLIIK.K | 2 |

---

|  |  |  |  |  |  |  |  |  |
| --- | --- | --- | --- | --- | --- | --- | --- | --- |
| U | *gi|221307584|ref|NP\_0* | 7 | 24 | 25.8% | 299 | 33296 | 9.8 | prohibitin 2 isoform 1 [Homo sapiens] |
| U | *gi|6005854|ref|NP\_009* | 7 | 24 | 25.8% | 299 | 33296 | 9.8 | prohibitin 2 isoform 2 [Homo sapiens] |

| Filename XCorr DeltCN Conf% ObsM+H+ CalcM+H+ SpR ZScore Ion% # Sequence  | | | | | | | | | | | | |
| --- | --- | --- | --- | --- | --- | --- | --- | --- | --- | --- | --- | --- |
|  | CENPL\_stlcld\_tube2\_122314\_02.15035.15035.2 | 3.2769 | 0.5301 | 100.0% | 1260.4321 | 1260.5222 | 1 | 9.353 | 66.7% | 3 | K.LLLGAGAVAYGVR.E | 2 |
|  | CENPL\_stlcld\_tube2\_122314\_01.16835.16835.2 | 4.0732 | 0.5599 | 100.0% | 1854.7122 | 1855.1038 | 1 | 9.634 | 50.0% | 2 | R.IGGVQQDTILAEGLHFR.I | 2 |
|  | CENPL\_stlcld\_122314\_01.17007.17007.3 | 5.0879 | 0.5 | 100.0% | 1856.1244 | 1855.1038 | 1 | 9.044 | 45.3% | 10 | R.IGGVQQDTILAEGLHFR.I | 3 |
|  | CENPL\_stlcld\_tube2\_122314\_01.21501.21501.2 | 3.9091 | 0.4059 | 100.0% | 1725.0122 | 1725.0428 | 1 | 7.411 | 75.0% | 5 | R.IPWFQYPIIYDIR.A | 2 |
|  | CENPL\_stlcld\_tube2\_122314\_01.11372.11372.3 | 3.9337 | 0.1784 | 99.4% | 1891.2544 | 1890.1675 | 9 | 4.23 | 46.7% | 2 | R.VLSRPNAQELPSMYQR.L | 3 |
|  | CENPL\_stlcld\_tube2\_122314\_01.11414.11414.2 | 2.1877 | 0.1658 | 95.9% | 995.1122 | 995.077 | 33 | 4.297 | 71.4% | 1 | R.LGLDYEER.V | 2 |
|  | CENPL\_stlcld\_tube2\_122314\_01.13397.13397.2 | 2.9724 | 0.2775 | 99.9% | 1178.1322 | 1178.3335 | 4 | 5.292 | 77.8% | 1 | K.FNASQLITQR.A | 2 |

---

|  |  |  |  |  |  |  |  |  |
| --- | --- | --- | --- | --- | --- | --- | --- | --- |
| U | *gi|32455264|ref|NP\_85* | 8 | 23 | 25.6% | 199 | 22110 | 8.1 | peroxiredoxin 1 [Homo sapiens] |
| U | *gi|4505591|ref|NP\_002* | 8 | 23 | 25.6% | 199 | 22110 | 8.1 | peroxiredoxin 1 [Homo sapiens] |
| U | *gi|32455266|ref|NP\_85* | 8 | 23 | 25.6% | 199 | 22110 | 8.1 | peroxiredoxin 1 [Homo sapiens] |

| Filename XCorr DeltCN Conf% ObsM+H+ CalcM+H+ SpR ZScore Ion% # Sequence  | | | | | | | | | | | | |
| --- | --- | --- | --- | --- | --- | --- | --- | --- | --- | --- | --- | --- |
|  | CENPL\_stlcld\_tube2\_122314\_01.12274.12274.2 | 2.9955 | 0.2972 | 99.9% | 1108.2922 | 1108.2798 | 1 | 5.938 | 83.3% | 4 | R.TIAQDYGVLK.A | 2 |
|  | CENPL\_stlcld\_tube2\_122314\_02.14555.14555.3 | 2.5416 | 0.3248 | 99.1% | 1983.5044 | 1984.2163 | 1 | 5.554 | 38.2% | 1 | R.TIAQDYGVLKADEGISFR.G | 3 |
|  | CENPL\_stlcld\_122314\_01.10497.10497.2 | 2.2251 | 0.3281 | 99.4% | 895.2322 | 894.9597 | 1 | 5.737 | 78.6% | 3 | K.ADEGISFR.G | 2 |
|  | CENPL\_stlcld\_122314\_01.16572.16572.1 | 2.255 | 0.2753 | 95.6% | 920.61 | 921.0813 | 24 | 5.852 | 64.3% | 1 | R.GLFIIDDK.G | 1 |
|  | CENPL\_stlcld\_122314\_01.16638.16638.2 | 2.3263 | 0.3566 | 99.9% | 921.1922 | 921.0813 | 93 | 6.487 | 57.1% | 1 | R.GLFIIDDK.G | 2 |
|  | CENPL\_stlcld\_tube2\_122314\_01.17913.17913.2 | 3.2581 | 0.3134 | 99.9% | 1360.4521 | 1360.6395 | 1 | 6.625 | 77.3% | 3 | R.GLFIIDDKGILR.Q | 2 |
|  | CENPL\_stlcld\_tube2\_122314\_01.12825.12825.2 | 2.8075 | 0.3442 | 99.9% | 1212.2722 | 1212.3915 | 3 | 6.703 | 80.0% | 3 | R.QITVNDLPVGR.S | 22 |
|  | CENPL\_stlcld\_tube2\_122314\_01.14312.14312.2 | 3.5071 | 0.3796 | 100.0% | 1198.3322 | 1197.3763 | 1 | 7.748 | 88.9% | 7 | R.LVQAFQFTDK.H | 2 |

Similarities:
gi|32189392|ref|NP\_00(1:7)  

---

|  |  |  |  |  |  |  |  |  |
| --- | --- | --- | --- | --- | --- | --- | --- | --- |
| U | *contaminant\_KERATIN19* | 9 | 17 | 25.2% | 468 | 51203 | 5.5 | no description |

| Filename XCorr DeltCN Conf% ObsM+H+ CalcM+H+ SpR ZScore Ion% # Sequence  | | | | | | | | | | | | |
| --- | --- | --- | --- | --- | --- | --- | --- | --- | --- | --- | --- | --- |
|  | CENPL\_stlcld\_122314\_01.11938.11938.3 | 4.7261 | 0.3617 | 100.0% | 2248.0144 | 2247.519 | 5 | 6.715 | 29.5% | 2 | R.LSSARPGGLGSSSLYGLGASRPR.V | 33 |
|  | CENPL\_stlcld\_122314\_02.08966.08966.2 | 2.7529 | 0.3773 | 99.9% | 1105.8922 | 1105.2388 | 8 | 6.395 | 59.1% | 3 | R.SAYGGPVGAGIR.E | 22 |
|  | CENPL\_stlcld\_122314\_01.12293.12293.3 | 2.566 | 0.3055 | 99.3% | 1623.9844 | 1623.8955 | 4 | 5.197 | 40.4% | 2 | K.ALNNKFASFIDKVR.F | 222322223 |
|  | CENPL\_stlcld\_122314\_01.20612.20612.2 | 3.9207 | 0.3876 | 100.0% | 1443.7322 | 1443.686 | 4 | 8.265 | 58.3% | 3 | R.LPDIFEAQIAGLR.G | 22 |
|  | CENPL\_stlcld\_tube2\_122314\_02.10514.10514.2 | 2.0307 | 0.282 | 96.8% | 1243.9122 | 1243.3622 | 11 | 4.573 | 54.5% | 1 | R.GQLEALQVDGGR.L | 22 |
|  | CENPL\_stlcld\_tube2\_122314\_01.10942.10942.2 | 2.6462 | 0.2259 | 98.8% | 1198.0122 | 1197.2897 | 1 | 5.375 | 77.8% | 1 | R.AEAEAWYQTK.F | 222 |
|  | CENPL\_stlcld\_tube2\_122314\_01.09035.09035.2 | 2.4033 | 0.2708 | 98.7% | 1093.3121 | 1093.2249 | 1 | 5.779 | 66.7% | 1 | K.FETLQAQAGK.H | 22 |
|  | CENPL\_stlcld\_tube2\_122314\_01.09796.09796.2 | 3.62 | 0.3166 | 100.0% | 1386.4122 | 1386.548 | 3 | 6.125 | 68.2% | 2 | R.AKQEELEAALQR.A | 22 |
|  | CENPL\_stlcld\_122314\_02.13122.13122.2 | 3.3979 | 0.3814 | 100.0% | 1407.4122 | 1406.6653 | 2 | 6.236 | 68.2% | 2 | K.LALDIEIATYRK.L | 22222 |

Similarities:
gi|4504919|ref|NP\_002(1:8)  
gi|67782365|ref|NP\_00(8:1)  
gi|32567786|ref|NP\_78(1:8)  
contaminant\_KERATIN16(1:8)  
gi|109255249|ref|NP\_0(1:8)  
gi|15618995|ref|NP\_25(1:8)  

---

|  |  |  |  |  |  |  |  |  |
| --- | --- | --- | --- | --- | --- | --- | --- | --- |
| U | *gi|4506725|ref|NP\_000* | 8 | 11 | 25.1% | 263 | 29598 | 10.2 | ribosomal protein S4, X-linked X isoform [Homo sapiens] |

| Filename XCorr DeltCN Conf% ObsM+H+ CalcM+H+ SpR ZScore Ion% # Sequence  | | | | | | | | | | | | |
| --- | --- | --- | --- | --- | --- | --- | --- | --- | --- | --- | --- | --- |
|  | CENPL\_stlcld\_122314\_01.08483.08483.2 | 2.2841 | 0.2609 | 98.3% | 1124.6122 | 1124.2792 | 1 | 5.359 | 72.2% | 1 | K.YALTGDEVKK.I | 2 |
|  | CENPL\_stlcld\_122314\_02.08273.08273.3 | 1.8217 | 0.3428 | 95.4% | 1216.3744 | 1216.3854 | 429 | 4.904 | 32.5% | 1 | K.GIPHLVTHDAR.T | 3 |
|  | CENPL\_stlcld\_122314\_01.08602.08602.2 | 2.3163 | 0.3879 | 99.7% | 1216.4122 | 1216.3854 | 36 | 6.253 | 55.0% | 1 | K.GIPHLVTHDAR.T | 2 |
|  | CENPL\_stlcld\_tube2\_122314\_01.12300.12300.3 | 2.7212 | 0.2584 | 99.4% | 1217.1843 | 1216.4661 | 1 | 5.223 | 52.8% | 1 | R.TIRYPDPLIK.V | 3 |
| \* | CENPL\_stlcld\_tube2\_122314\_02.13349.13349.2 | 2.8583 | 0.3426 | 99.9% | 1447.3121 | 1446.5975 | 2 | 5.985 | 58.3% | 1 | K.VNDTIQIDLETGK.I | 2 |
|  | CENPL\_stlcld\_122314\_01.08826.08826.3 | 2.8237 | 0.2858 | 99.9% | 1508.4243 | 1507.692 | 5 | 5.585 | 45.8% | 1 | R.ERHPGSFDVVHVK.D | 3 |
|  | CENPL\_stlcld\_122314\_01.09424.09424.3 | 3.5705 | 0.332 | 100.0% | 1221.8344 | 1222.3892 | 1 | 5.62 | 52.5% | 2 | R.HPGSFDVVHVK.D | 3 |
| \* | CENPL\_stlcld\_122314\_01.16347.16347.2 | 3.201 | 0.3929 | 100.0% | 991.71216 | 991.2187 | 1 | 6.591 | 81.2% | 3 | R.LSNIFVIGK.G | 2 |

---

|  |  |  |  |  |  |  |  |  |
| --- | --- | --- | --- | --- | --- | --- | --- | --- |
| U | *gi|58743363|ref|NP\_20* | 6 | 20 | 24.7% | 344 | 38998 | 6.5 | centromere protein L isoform 2 [Homo sapiens] |

| Filename XCorr DeltCN Conf% ObsM+H+ CalcM+H+ SpR ZScore Ion% # Sequence  | | | | | | | | | | | | |
| --- | --- | --- | --- | --- | --- | --- | --- | --- | --- | --- | --- | --- |
|  | CENPL\_stlcld\_122314\_01.11669.11669.2 | 3.1868 | 0.3972 | 100.0% | 1395.5721 | 1394.5266 | 1 | 6.411 | 60.0% | 3 | K.FSYSNLKEYSR.L | 2 |
|  | CENPL\_stlcld\_tube2\_122314\_01.16520.16520.2 | 3.1025 | 0.4613 | 100.0% | 1118.4922 | 1118.3617 | 1 | 7.008 | 72.2% | 8 | R.LLNAFIVAEK.Q | 2 |
|  | CENPL\_stlcld\_122314\_02.13904.13904.2 | 3.5849 | 0.4398 | 100.0% | 1391.2722 | 1391.5638 | 1 | 7.215 | 75.0% | 3 | K.GLAVEVGEDFNIK.V | 2 |
|  | CENPL\_stlcld\_122314\_01.20163.20163.2 | 2.6852 | 0.4134 | 99.9% | 1109.1122 | 1109.4125 | 1 | 6.72 | 83.3% | 3 | K.VIFSTLLGMK.G | 2 |
| \* | CENPL\_stlcld\_122314\_01.18758.18758.2 | 4.2732 | 0.2532 | 99.9% | 1789.5721 | 1789.0415 | 1 | 6.351 | 60.0% | 2 | K.GTQRDPEAFLVQIVSK.S | 2 |
|  | CENPL\_stlcld\_tube2\_122314\_01.15384.15384.2 | 2.8693 | 0.1258 | 96.0% | 2750.8523 | 2753.0688 | 1 | 3.703 | 33.3% | 1 | K.IHLSATRLVRVSTS\*VASAHTDGK@IK@.I | 2 |

---

|  |  |  |  |  |  |  |  |  |
| --- | --- | --- | --- | --- | --- | --- | --- | --- |
| U | *gi|226530908|ref|NP\_0* | 5 | 9 | 24.6% | 285 | 30315 | 7.5 | protein-L-isoaspartate (D-aspartate) O-methyltransferase [Homo sapiens] |

| Filename XCorr DeltCN Conf% ObsM+H+ CalcM+H+ SpR ZScore Ion% # Sequence  | | | | | | | | | | | | |
| --- | --- | --- | --- | --- | --- | --- | --- | --- | --- | --- | --- | --- |
| \* | CENPL\_stlcld\_tube2\_122314\_01.08133.08133.3 | 3.6551 | 0.2624 | 99.9% | 1477.7344 | 1478.6078 | 1 | 6.372 | 46.2% | 3 | K.SGGASHSELIHNLR.K | 3 |
| \* | CENPL\_stlcld\_tube2\_122314\_01.15305.15305.2 | 2.8386 | 0.2957 | 99.5% | 1524.7522 | 1525.7601 | 1 | 6.141 | 62.5% | 1 | K.TDKVFEVMLATDR.S | 2 |
| \* | CENPL\_stlcld\_122314\_01.15730.15730.2 | 1.9215 | 0.3748 | 98.5% | 1180.5922 | 1181.3923 | 1 | 6.053 | 77.8% | 1 | K.VFEVMLATDR.S | 2 |
| \* | CENPL\_stlcld\_122314\_01.10173.10173.2 | 2.7069 | 0.2236 | 99.2% | 943.65216 | 943.091 | 1 | 5.452 | 87.5% | 2 | R.VQLVVGDGR.M | 2 |
| \* | CENPL\_stlcld\_tube2\_122314\_01.19076.19076.3 | 6.1203 | 0.4513 | 100.0% | 3506.6042 | 3507.0015 | 1 | 8.304 | 29.5% | 2 | R.MGYAEEAPYDAIHVGAAAPVVPQALIDQLKPGGR.L | 3 |

---

|  |  |  |  |  |  |  |  |  |
| --- | --- | --- | --- | --- | --- | --- | --- | --- |
| U | *gi|15431295|ref|NP\_15* | 5 | 14 | 24.6% | 211 | 24261 | 11.7 | ribosomal protein L13 [Homo sapiens] |
| U | *gi|15431297|ref|NP\_00* | 5 | 14 | 24.6% | 211 | 24261 | 11.7 | ribosomal protein L13 [Homo sapiens] |

| Filename XCorr DeltCN Conf% ObsM+H+ CalcM+H+ SpR ZScore Ion% # Sequence  | | | | | | | | | | | | |
| --- | --- | --- | --- | --- | --- | --- | --- | --- | --- | --- | --- | --- |
|  | CENPL\_stlcld\_tube2\_122314\_01.13558.13558.2 | 3.2072 | 0.3665 | 100.0% | 1190.0122 | 1190.3469 | 2 | 6.47 | 77.8% | 4 | R.VATWFNQPAR.K | 2 |
|  | CENPL\_stlcld\_122314\_01.15496.15496.2 | 2.1346 | 0.2428 | 97.9% | 951.2322 | 951.0672 | 4 | 5.635 | 78.6% | 2 | R.GFSLEELR.V | 2 |
|  | CENPL\_stlcld\_122314\_02.08112.08112.2 | 3.6296 | 0.3905 | 100.0% | 1232.9321 | 1233.3237 | 1 | 7.291 | 75.0% | 2 | K.STESLQANVQR.L | 2 |
|  | CENPL\_stlcld\_122314\_01.13874.13874.2 | 3.0144 | 0.3719 | 99.9% | 1383.4122 | 1383.6923 | 1 | 6.727 | 66.7% | 4 | K.LATQLTGPVMPVR.N | 2 |
|  | CENPL\_stlcld\_tube2\_122314\_01.08004.08004.2 | 3.1998 | 0.2813 | 99.9% | 1238.5721 | 1237.3953 | 2 | 5.168 | 72.2% | 2 | R.VITEEEKNFK.A | 2 |

---

|  |  |  |  |  |  |  |  |  |
| --- | --- | --- | --- | --- | --- | --- | --- | --- |
| U | *gi|10863927|ref|NP\_06* | 5 | 15 | 24.2% | 165 | 18012 | 7.8 | peptidylprolyl isomerase A [Homo sapiens] |
| U | *gi|169215435|ref|XP\_0* | 5 | 15 | 17.9% | 223 | 24376 | 6.9 | PREDICTED: similar to peptidylprolyl isomerase A-like [Homo sapiens] |

| Filename XCorr DeltCN Conf% ObsM+H+ CalcM+H+ SpR ZScore Ion% # Sequence  | | | | | | | | | | | | |
| --- | --- | --- | --- | --- | --- | --- | --- | --- | --- | --- | --- | --- |
|  | CENPL\_stlcld\_122314\_01.16134.16134.2 | 3.4578 | 0.3011 | 99.9% | 1381.1522 | 1380.6268 | 1 | 6.657 | 63.6% | 5 | R.VSFELFADKVPK.T | 2 |
|  | CENPL\_stlcld\_122314\_01.16220.16220.3 | 2.566 | 0.3619 | 99.9% | 1381.3444 | 1380.6268 | 158 | 5.535 | 40.9% | 2 | R.VSFELFADKVPK.T | 3 |
|  | CENPL\_stlcld\_tube2\_122314\_01.15633.15633.2 | 4.4878 | 0.3449 | 100.0% | 1833.5521 | 1833.0477 | 1 | 5.544 | 67.9% | 4 | K.SIYGEKFEDENFILK.H | 2 |
|  | CENPL\_stlcld\_122314\_01.13637.13637.2 | 4.0463 | 0.0532 | 98.9% | 1506.6322 | 1506.7755 | 1 | 8.333 | 79.2% | 2 | K.VKEGMNIVEAMER.F | 2 |
|  | CENPL\_stlcld\_122314\_01.16234.16234.2 | 2.5428 | 0.1956 | 97.9% | 1279.4321 | 1279.4689 | 1 | 6.666 | 75.0% | 2 | K.EGMNIVEAMER.F | 2 |

---

|  |  |  |  |  |  |  |  |  |
| --- | --- | --- | --- | --- | --- | --- | --- | --- |
| U | *contaminant\_KERATIN02* | 10 | 20 | 24.1% | 622 | 61987 | 5.2 | no description |
| U | *gi|55956899|ref|NP\_00* | 10 | 20 | 24.1% | 623 | 62064 | 5.2 | keratin 9 [Homo sapiens] |

| Filename XCorr DeltCN Conf% ObsM+H+ CalcM+H+ SpR ZScore Ion% # Sequence  | | | | | | | | | | | | |
| --- | --- | --- | --- | --- | --- | --- | --- | --- | --- | --- | --- | --- |
|  | CENPL\_stlcld\_122314\_01.15987.15987.3 | 3.8793 | 0.1676 | 98.2% | 2379.1143 | 2378.5981 | 1 | 4.971 | 37.5% | 1 | R.LASYLDKVQALEEANNDLENK.I | 3 |
|  | CENPL\_stlcld\_122314\_01.13184.13184.2 | 2.4608 | 0.3351 | 99.7% | 1061.3121 | 1061.1802 | 1 | 5.574 | 75.0% | 1 | K.TLLDIDNTR.M | 2 |
|  | CENPL\_stlcld\_122314\_01.11818.11818.2 | 2.1954 | 0.2624 | 97.7% | 1158.1322 | 1158.2566 | 3 | 5.167 | 60.0% | 1 | R.QGVDADINGLR.Q | 2 |
|  | CENPL\_stlcld\_122314\_01.19673.19673.2 | 4.433 | 0.5729 | 100.0% | 3265.612 | 3266.413 | 1 | 10.304 | 35.7% | 1 | K.DIENQYETQITQIEHEVSSSGQEVQSSAK.E | 2 |
|  | CENPL\_stlcld\_122314\_01.19661.19661.3 | 6.006 | 0.5169 | 100.0% | 3265.7043 | 3266.413 | 1 | 8.159 | 36.6% | 1 | K.DIENQYETQITQIEHEVSSSGQEVQSSAK.E | 3 |
|  | CENPL\_stlcld\_tube2\_122314\_01.16997.16997.2 | 5.9089 | 0.5689 | 100.0% | 1838.7722 | 1839.0557 | 1 | 9.566 | 76.7% | 2 | R.HGVQELEIELQSQLSK.K | 2 |
|  | CENPL\_stlcld\_122314\_02.14211.14211.3 | 3.6988 | 0.1495 | 97.9% | 1840.8243 | 1839.0557 | 1 | 3.504 | 41.7% | 1 | R.HGVQELEIELQSQLSK.K | 3 |
|  | CENPL\_stlcld\_122314\_01.15454.15454.3 | 5.9315 | 0.3915 | 100.0% | 1967.1543 | 1967.2297 | 1 | 8.08 | 46.9% | 4 | R.HGVQELEIELQSQLSKK.A | 3 |
|  | CENPL\_stlcld\_122314\_02.12531.12531.3 | 5.2394 | 0.2927 | 100.0% | 2512.6743 | 2511.6177 | 1 | 7.057 | 36.4% | 6 | K.EIETYHNLLEGGQEDFESSGAGK.I | 3 |
|  | CENPL\_stlcld\_122314\_02.08518.08518.3 | 7.4961 | 0.5996 | 100.0% | 3224.5745 | 3225.1118 | 1 | 9.935 | 25.6% | 2 | R.GGSGGSHGGGSGFGGESGGSYGGGEEASGSGGGYGGGSGK.S | 3 |

---

|  |  |  |  |  |  |  |  |  |
| --- | --- | --- | --- | --- | --- | --- | --- | --- |
| U | *gi|15809016|ref|NP\_29* | 4 | 8 | 23.8% | 172 | 19779 | 4.8 | myosin regulatory light chain MRCL2 isoform A [Homo sapiens] |
| U | *gi|5453740|ref|NP\_006* | 4 | 8 | 24.0% | 171 | 19794 | 4.8 | myosin, light chain 12A, regulatory, non-sarcomeric [Homo sapiens] |
| U | *gi|222144328|ref|NP\_0* | 4 | 8 | 26.6% | 154 | 17757 | 4.4 | myosin regulatory light chain MRCL2 isoform B [Homo sapiens] |
| U | *gi|222144326|ref|NP\_0* | 4 | 8 | 23.8% | 172 | 19779 | 4.8 | myosin regulatory light chain MRCL2 isoform A [Homo sapiens] |
| U | *gi|222144324|ref|NP\_0* | 4 | 8 | 23.8% | 172 | 19779 | 4.8 | myosin regulatory light chain MRCL2 isoform A [Homo sapiens] |

| Filename XCorr DeltCN Conf% ObsM+H+ CalcM+H+ SpR ZScore Ion% # Sequence  | | | | | | | | | | | | |
| --- | --- | --- | --- | --- | --- | --- | --- | --- | --- | --- | --- | --- |
|  | CENPL\_stlcld\_tube2\_122314\_01.11278.11278.2 | 2.4087 | 0.3275 | 99.3% | 1230.2922 | 1229.3324 | 19 | 5.463 | 60.0% | 1 | K.LNGTDPEDVIR.N | 2 |
|  | CENPL\_stlcld\_122314\_02.14879.14879.3 | 4.4505 | 0.4749 | 100.0% | 2432.9944 | 2433.649 | 1 | 7.545 | 36.8% | 3 | R.ELLTTMGDRFTDEEVDELYR.E | 3 |
|  | CENPL\_stlcld\_tube2\_122314\_01.13768.13768.2 | 2.795 | 0.2954 | 99.7% | 1416.9521 | 1416.4839 | 1 | 5.932 | 65.0% | 1 | R.FTDEEVDELYR.E | 2 |
|  | CENPL\_stlcld\_tube2\_122314\_01.15472.15472.2 | 2.7777 | 0.4525 | 100.0% | 1261.4321 | 1261.3794 | 1 | 7.283 | 66.7% | 3 | K.GNFNYIEFTR.I | 2 |

---

|  |  |  |  |  |  |  |  |  |
| --- | --- | --- | --- | --- | --- | --- | --- | --- |
| U | *gi|5032051|ref|NP\_005* | 2 | 3 | 23.8% | 151 | 16273 | 10.1 | ribosomal protein S14 [Homo sapiens] |
| U | *gi|68160922|ref|NP\_00* | 2 | 3 | 23.8% | 151 | 16273 | 10.1 | ribosomal protein S14 [Homo sapiens] |
| U | *gi|68160915|ref|NP\_00* | 2 | 3 | 23.8% | 151 | 16273 | 10.1 | ribosomal protein S14 [Homo sapiens] |

| Filename XCorr DeltCN Conf% ObsM+H+ CalcM+H+ SpR ZScore Ion% # Sequence  | | | | | | | | | | | | |
| --- | --- | --- | --- | --- | --- | --- | --- | --- | --- | --- | --- | --- |
|  | CENPL\_stlcld\_tube2\_122314\_01.13398.13398.3 | 4.0106 | 0.3862 | 100.0% | 2493.6843 | 2493.754 | 1 | 6.247 | 33.0% | 1 | K.VKADRDESSPYAAMLAAQDVAQR.C | 3 |
|  | CENPL\_stlcld\_tube2\_122314\_01.10824.10824.2 | 3.1361 | 0.2099 | 99.2% | 1430.5521 | 1430.5547 | 3 | 4.668 | 62.5% | 2 | R.IEDVTPIPSDSTR.R | 2 |

---

|  |  |  |  |  |  |  |  |  |
| --- | --- | --- | --- | --- | --- | --- | --- | --- |
| U | *gi|50592996|ref|NP\_00* | 14 | 74 | 23.6% | 450 | 50433 | 4.9 | tubulin, beta, 4 [Homo sapiens] |

| Filename XCorr DeltCN Conf% ObsM+H+ CalcM+H+ SpR ZScore Ion% # Sequence  | | | | | | | | | | | | |
| --- | --- | --- | --- | --- | --- | --- | --- | --- | --- | --- | --- | --- |
|  | CENPL\_stlcld\_122314\_02.13131.13131.2 | 4.2785 | 0.369 | 100.0% | 1617.9722 | 1616.8701 | 1 | 6.59 | 67.9% | 11 | R.AILVDLEPGTMDSVR.S | 22 |
|  | CENPL\_stlcld\_122314\_01.20825.20825.2 | 5.647 | 0.559 | 100.0% | 1959.0322 | 1960.151 | 1 | 11.798 | 79.4% | 2 | K.GHYTEGAELVDSVLDVVR.K | 222 |
|  | CENPL\_stlcld\_122314\_01.20822.20822.3 | 2.9108 | 0.2585 | 98.5% | 1959.7743 | 1960.151 | 7 | 4.629 | 32.4% | 1 | K.GHYTEGAELVDSVLDVVR.K | 333 |
|  | CENPL\_stlcld\_tube2\_122314\_01.19052.19052.3 | 5.1569 | 0.4766 | 100.0% | 2087.9644 | 2088.325 | 1 | 8.639 | 44.4% | 6 | K.GHYTEGAELVDSVLDVVRK.E | 333 |
|  | CENPL\_stlcld\_tube2\_122314\_01.19009.19009.2 | 6.3337 | 0.4925 | 100.0% | 2088.612 | 2088.325 | 1 | 9.687 | 66.7% | 3 | K.GHYTEGAELVDSVLDVVRK.E | 222 |
|  | CENPL\_stlcld\_tube2\_122314\_01.14282.14282.2 | 4.3911 | 0.4008 | 100.0% | 1320.4922 | 1320.5896 | 1 | 7.658 | 81.8% | 10 | R.IMNTFSVVPSPK.V | 222 |
|  | CENPL\_stlcld\_122314\_01.13025.13025.2 | 3.0807 | 0.3262 | 99.9% | 1130.8322 | 1131.2767 | 1 | 5.245 | 83.3% | 8 | R.FPGQLNADLR.K | 2222 |
|  | CENPL\_stlcld\_122314\_01.11442.11442.3 | 2.9929 | 0.3054 | 99.9% | 1259.5443 | 1259.4508 | 9 | 5.658 | 42.5% | 1 | R.FPGQLNADLRK.L | 3333 |
|  | CENPL\_stlcld\_tube2\_122314\_01.10454.10454.2 | 2.6368 | 0.2303 | 98.6% | 1259.7322 | 1259.4508 | 17 | 4.582 | 60.0% | 5 | R.FPGQLNADLRK.L | 2222 |
|  | CENPL\_stlcld\_tube2\_122314\_01.14181.14181.2 | 3.8965 | 0.404 | 100.0% | 1271.9922 | 1272.5945 | 1 | 7.764 | 75.0% | 4 | R.KLAVNMVPFPR.L | 2222 |
|  | CENPL\_stlcld\_122314\_01.16748.16748.2 | 3.646 | 0.4855 | 100.0% | 1143.9922 | 1144.4204 | 1 | 8.724 | 94.4% | 8 | K.LAVNMVPFPR.L | 2222 |
|  | CENPL\_stlcld\_122314\_01.20271.20271.2 | 3.407 | 0.4681 | 100.0% | 1692.8322 | 1692.9678 | 1 | 7.635 | 75.0% | 2 | R.ALTVPELTQQMFDAK.N | 22 |
|  | CENPL\_stlcld\_122314\_01.19247.19247.2 | 3.5195 | 0.346 | 99.9% | 1698.4321 | 1697.8877 | 3 | 5.888 | 53.8% | 4 | K.NSSYFVEWIPNNVK.V | 2222 |
|  | CENPL\_stlcld\_122314\_02.14308.14308.2 | 3.8472 | 0.492 | 100.0% | 1230.5122 | 1230.4241 | 1 | 7.151 | 94.4% | 9 | R.ISEQFTAMFR.R | 222 |

Similarities:
gi|29788785|ref|NP\_82(13:1)  
gi|5174735|ref|NP\_006(13:1)  
gi|14210536|ref|NP\_11(6:8)  

---

|  |  |  |  |  |  |  |  |  |
| --- | --- | --- | --- | --- | --- | --- | --- | --- |
| U | *gi|4506679|ref|NP\_001* | 4 | 7 | 23.6% | 165 | 18898 | 10.2 | ribosomal protein S10 [Homo sapiens] |

| Filename XCorr DeltCN Conf% ObsM+H+ CalcM+H+ SpR ZScore Ion% # Sequence  | | | | | | | | | | | | |
| --- | --- | --- | --- | --- | --- | --- | --- | --- | --- | --- | --- | --- |
|  | CENPL\_stlcld\_122314\_01.21677.21677.2 | 2.7788 | 0.5045 | 100.0% | 1110.5922 | 1110.3818 | 1 | 8.3 | 81.2% | 2 | R.IAIYELLFK.E | 2 |
| \* | CENPL\_stlcld\_122314\_02.15725.15725.2 | 4.4461 | 0.4968 | 100.0% | 2003.3121 | 2004.2548 | 1 | 8.397 | 64.3% | 2 | R.HFYWYLTNEGIQYLR.D | 2 |
| \* | CENPL\_stlcld\_122314\_02.15676.15676.3 | 4.5777 | 0.3367 | 100.0% | 2004.5643 | 2004.2548 | 4 | 6.89 | 48.2% | 2 | R.HFYWYLTNEGIQYLR.D | 3 |
|  | CENPL\_stlcld\_122314\_02.10794.10794.3 | 3.0874 | 0.228 | 98.7% | 1571.2444 | 1570.7019 | 51 | 4.81 | 39.3% | 1 | K.KAEAGAGSATEFQFR.G | 3 |

---

|  |  |  |  |  |  |  |  |  |
| --- | --- | --- | --- | --- | --- | --- | --- | --- |
| U | *gi|5031635|ref|NP\_005* | 2 | 3 | 23.5% | 166 | 18502 | 8.1 | cofilin 1 (non-muscle) [Homo sapiens] |

| Filename XCorr DeltCN Conf% ObsM+H+ CalcM+H+ SpR ZScore Ion% # Sequence  | | | | | | | | | | | | |
| --- | --- | --- | --- | --- | --- | --- | --- | --- | --- | --- | --- | --- |
| \* | CENPL\_stlcld\_tube2\_122314\_01.19840.19840.3 | 4.5672 | 0.3596 | 100.0% | 3063.8943 | 3064.4595 | 1 | 6.102 | 27.8% | 1 | K.NIILEEGKEILVGDVGQTVDDPYATFVK.M | 3 |
|  | CENPL\_stlcld\_122314\_01.12354.12354.2 | 2.4262 | 0.3314 | 99.4% | 1338.3522 | 1338.4564 | 5 | 5.885 | 60.0% | 2 | R.YALYDATYETK.E | 2 |

---

|  |  |  |  |  |  |  |  |  |
| --- | --- | --- | --- | --- | --- | --- | --- | --- |
| U | *gi|14043070|ref|NP\_11* | 10 | 56 | 23.4% | 372 | 38747 | 9.1 | heterogeneous nuclear ribonucleoprotein A1 isoform b [Homo sapiens] |
| U | *gi|4504445|ref|NP\_002* | 10 | 56 | 27.2% | 320 | 34196 | 9.2 | heterogeneous nuclear ribonucleoprotein A1 isoform a [Homo sapiens] |

| Filename XCorr DeltCN Conf% ObsM+H+ CalcM+H+ SpR ZScore Ion% # Sequence  | | | | | | | | | | | | |
| --- | --- | --- | --- | --- | --- | --- | --- | --- | --- | --- | --- | --- |
|  | CENPL\_stlcld\_122314\_02.14848.14848.3 | 4.0414 | 0.3999 | 100.0% | 1914.3243 | 1914.1656 | 1 | 6.467 | 45.3% | 3 | R.KLFIGGLSFETTDESLR.S | 3 |
|  | CENPL\_stlcld\_122314\_01.18153.18153.2 | 4.726 | 0.4372 | 100.0% | 1914.8121 | 1914.1656 | 1 | 7.189 | 71.9% | 6 | R.KLFIGGLSFETTDESLR.S | 2 |
|  | CENPL\_stlcld\_122314\_02.16388.16388.2 | 5.4519 | 0.5372 | 100.0% | 1785.1921 | 1785.9916 | 1 | 9.063 | 76.7% | 16 | K.LFIGGLSFETTDESLR.S | 2 |
|  | CENPL\_stlcld\_122314\_01.20044.20044.2 | 5.4324 | 0.5307 | 100.0% | 2511.112 | 2511.8145 | 1 | 8.335 | 43.2% | 1 | R.GFGFVTYATVEEVDAAMNARPHK.V | 2 |
|  | CENPL\_stlcld\_122314\_01.17714.17714.2 | 3.7122 | 0.3833 | 100.0% | 1219.4722 | 1219.4387 | 1 | 7.01 | 88.9% | 9 | K.IEVIEIMTDR.G | 2 |
|  | CENPL\_stlcld\_122314\_02.12688.12688.3 | 4.7594 | 0.3856 | 100.0% | 1857.7444 | 1856.989 | 1 | 7.373 | 43.3% | 1 | K.RGFAFVTFDDHDSVDK.I | 3 |
|  | CENPL\_stlcld\_122314\_02.13893.13893.2 | 4.3148 | 0.5374 | 100.0% | 1700.4722 | 1700.8016 | 1 | 9.029 | 67.9% | 7 | R.GFAFVTFDDHDSVDK.I | 2 |
|  | CENPL\_stlcld\_tube2\_122314\_01.17124.17124.2 | 4.7784 | 0.4727 | 100.0% | 2282.2722 | 2282.5579 | 1 | 8.53 | 60.5% | 1 | R.GFAFVTFDDHDSVDKIVIQK.Y | 2 |
|  | CENPL\_stlcld\_122314\_01.17330.17330.3 | 4.3778 | 0.2384 | 99.9% | 2283.0544 | 2282.5579 | 1 | 6.399 | 39.5% | 7 | R.GFAFVTFDDHDSVDKIVIQK.Y | 3 |
|  | CENPL\_stlcld\_122314\_01.09861.09861.2 | 3.9767 | 0.3817 | 100.0% | 1629.4722 | 1629.7721 | 1 | 7.452 | 63.3% | 5 | R.SSGPYGGGGQYFAKPR.N | 2 |

---

|  |  |  |  |  |  |  |  |  |
| --- | --- | --- | --- | --- | --- | --- | --- | --- |
| U | *gi|4503483|ref|NP\_001* | 15 | 46 | 23.2% | 858 | 95338 | 6.8 | eukaryotic translation elongation factor 2 [Homo sapiens] |

| Filename XCorr DeltCN Conf% ObsM+H+ CalcM+H+ SpR ZScore Ion% # Sequence  | | | | | | | | | | | | |
| --- | --- | --- | --- | --- | --- | --- | --- | --- | --- | --- | --- | --- |
| \* | CENPL\_stlcld\_tube2\_122314\_02.00506.00506.2 | 4.0018 | 0.3424 | 100.0% | 2205.5522 | 2205.4692 | 1 | 6.458 | 61.1% | 2 | K.STAISLFYELSENDLNFIK.Q | 2 |
| \* | CENPL\_stlcld\_122314\_01.21621.21621.2 | 3.8154 | 0.3832 | 100.0% | 2220.8123 | 2221.5151 | 1 | 6.686 | 52.9% | 1 | R.ALLELQLEPEELYQTFQR.I | 2 |
| \* | CENPL\_stlcld\_tube2\_122314\_01.21255.21255.3 | 6.1416 | 0.4926 | 100.0% | 2758.7944 | 2758.2976 | 1 | 8.539 | 36.5% | 2 | R.RWLPAGDALLQMITIHLPSPVTAQK.Y | 3 |
| \* | CENPL\_stlcld\_tube2\_122314\_02.00599.00599.2 | 5.0394 | 0.518 | 100.0% | 2601.672 | 2602.11 | 1 | 8.089 | 45.7% | 3 | R.WLPAGDALLQMITIHLPSPVTAQK.Y | 2 |
| \* | CENPL\_stlcld\_tube2\_122314\_02.00686.00686.3 | 3.1017 | 0.3105 | 99.9% | 2602.1643 | 2602.11 | 3 | 5.352 | 30.4% | 1 | R.WLPAGDALLQMITIHLPSPVTAQK.Y | 3 |
| \* | CENPL\_stlcld\_tube2\_122314\_01.15070.15070.2 | 2.5834 | 0.4021 | 99.9% | 1040.2522 | 1040.3241 | 21 | 6.997 | 62.5% | 3 | K.GPLMMYISK.M | 2 |
| \* | CENPL\_stlcld\_122314\_01.14301.14301.2 | 3.0277 | 0.3308 | 99.9% | 1108.4521 | 1108.3231 | 1 | 6.821 | 75.0% | 2 | R.VFSGLVSTGLK.V | 2 |
| \* | CENPL\_stlcld\_122314\_01.09345.09345.2 | 3.9838 | 0.3963 | 100.0% | 1616.4922 | 1616.7917 | 1 | 8.199 | 50.0% | 1 | K.TGTITTFEHAHNMR.V | 2 |
| \* | CENPL\_stlcld\_122314\_01.13671.13671.3 | 6.2341 | 0.4287 | 100.0% | 2144.6643 | 2144.3489 | 1 | 8.155 | 48.7% | 17 | K.ARPFPDGLAEDIDKGEVSAR.Q | 3 |
| \* | CENPL\_stlcld\_tube2\_122314\_02.13095.13095.3 | 3.3149 | 0.3532 | 100.0% | 1744.9143 | 1743.9133 | 1 | 6.067 | 48.1% | 3 | R.YLAEKYEWDVAEAR.K | 3 |
| \* | CENPL\_stlcld\_122314\_02.11224.11224.3 | 2.855 | 0.2528 | 98.7% | 1872.1444 | 1872.0874 | 1 | 5.191 | 41.1% | 1 | R.YLAEKYEWDVAEARK.I | 3 |
| \* | CENPL\_stlcld\_122314\_02.17259.17259.2 | 4.9326 | 0.5625 | 100.0% | 2353.3323 | 2354.6677 | 1 | 8.456 | 55.0% | 3 | K.GVQYLNEIKDSVVAGFQWATK.E | 2 |
| \* | CENPL\_stlcld\_122314\_01.14787.14787.2 | 4.0851 | 0.087 | 99.0% | 1962.8121 | 1963.2585 | 1 | 6.162 | 52.9% | 1 | R.GHVFEESQVAGTPMFVVK.A | 2 |
| \* | CENPL\_stlcld\_122314\_02.15944.15944.2 | 4.1632 | 0.3859 | 100.0% | 1801.8722 | 1801.0087 | 1 | 7.546 | 63.3% | 3 | K.AYLPVNESFGFTADLR.S | 2 |
| \* | CENPL\_stlcld\_tube2\_122314\_02.00018.00018.2 | 3.3435 | 0.4979 | 100.0% | 1445.2922 | 1445.6555 | 1 | 8.019 | 75.0% | 3 | K.EGIPALDNFLDKL.- | 2 |

---

|  |  |  |  |  |  |  |  |  |
| --- | --- | --- | --- | --- | --- | --- | --- | --- |
| U | *gi|32189392|ref|NP\_00* | 3 | 6 | 23.2% | 198 | 21892 | 6.0 | peroxiredoxin 2 isoform a [Homo sapiens] |

| Filename XCorr DeltCN Conf% ObsM+H+ CalcM+H+ SpR ZScore Ion% # Sequence  | | | | | | | | | | | | |
| --- | --- | --- | --- | --- | --- | --- | --- | --- | --- | --- | --- | --- |
| \* | CENPL\_stlcld\_tube2\_122314\_01.19931.19931.2 | 3.4943 | 0.3779 | 100.0% | 1863.5521 | 1864.1954 | 2 | 6.542 | 47.1% | 1 | R.KEGGLGPLNIPLLADVTR.R | 2 |
| \* | CENPL\_stlcld\_122314\_02.11820.11820.3 | 3.4838 | 0.3674 | 100.0% | 1930.1643 | 1930.1217 | 1 | 6.808 | 37.5% | 2 | R.LSEDYGVLKTDEGIAYR.G | 3 |
|  | CENPL\_stlcld\_tube2\_122314\_01.12825.12825.2 | 2.8075 | 0.3442 | 99.9% | 1212.2722 | 1212.3915 | 3 | 6.703 | 80.0% | 3 | R.QITVNDLPVGR.S | 22 |

Similarities:
gi|32455264|ref|NP\_85(1:2)  

---

|  |  |  |  |  |  |  |  |  |
| --- | --- | --- | --- | --- | --- | --- | --- | --- |
| U | *gi|34098946|ref|NP\_00* | 4 | 6 | 23.1% | 324 | 35924 | 9.9 | nuclease sensitive element binding protein 1 [Homo sapiens] |

| Filename XCorr DeltCN Conf% ObsM+H+ CalcM+H+ SpR ZScore Ion% # Sequence  | | | | | | | | | | | | |
| --- | --- | --- | --- | --- | --- | --- | --- | --- | --- | --- | --- | --- |
|  | CENPL\_stlcld\_tube2\_122314\_01.09208.09208.3 | 3.0767 | 0.2594 | 99.5% | 1746.7444 | 1745.9298 | 1 | 4.908 | 44.6% | 1 | R.NDTKEDVFVHQTAIK.K | 3 |
|  | CENPL\_stlcld\_122314\_01.08609.08609.3 | 4.326 | 0.3715 | 100.0% | 1874.1843 | 1874.1039 | 1 | 6.765 | 50.0% | 2 | R.NDTKEDVFVHQTAIKK.N | 3 |
| \* | CENPL\_stlcld\_tube2\_122314\_01.07586.07586.3 | 6.1058 | 0.5097 | 100.0% | 3259.3442 | 3259.2566 | 1 | 8.441 | 36.6% | 1 | R.NYQQNYQNSESGEKNEGSESAPEGQAQQR.R | 3 |
| \* | CENPL\_stlcld\_tube2\_122314\_01.09276.09276.3 | 5.0137 | 0.3482 | 100.0% | 3224.7244 | 3225.4795 | 1 | 6.997 | 26.7% | 2 | R.RPQYSNPPVQGEVMEGADNQGAGEQGRPVR.Q | 3 |

---

|  |  |  |  |  |  |  |  |  |
| --- | --- | --- | --- | --- | --- | --- | --- | --- |
| U | *gi|32189394|ref|NP\_00* | 8 | 13 | 22.9% | 529 | 56560 | 5.4 | mitochondrial ATP synthase beta subunit precursor [Homo sapiens] |

| Filename XCorr DeltCN Conf% ObsM+H+ CalcM+H+ SpR ZScore Ion% # Sequence  | | | | | | | | | | | | |
| --- | --- | --- | --- | --- | --- | --- | --- | --- | --- | --- | --- | --- |
| \* | CENPL\_stlcld\_122314\_02.12567.12567.3 | 2.8495 | 0.3975 | 100.0% | 1652.4844 | 1651.9034 | 1 | 6.471 | 50.0% | 1 | R.LVLEVAQHLGESTVR.T | 3 |
| \* | CENPL\_stlcld\_122314\_02.12591.12591.2 | 4.0686 | 0.4599 | 100.0% | 1652.9321 | 1651.9034 | 1 | 8.633 | 71.4% | 3 | R.LVLEVAQHLGESTVR.T | 2 |
| \* | CENPL\_stlcld\_122314\_01.14216.14216.2 | 3.6293 | 0.331 | 100.0% | 1386.4321 | 1386.6061 | 2 | 6.519 | 72.7% | 2 | R.IMNVIGEPIDER.G | 2 |
| \* | CENPL\_stlcld\_tube2\_122314\_01.21669.21669.2 | 3.6059 | 0.3576 | 100.0% | 1458.7522 | 1458.7998 | 1 | 6.847 | 62.5% | 1 | K.TVLIMELINNVAK.A | 2 |
| \* | CENPL\_stlcld\_122314\_01.17320.17320.3 | 4.0685 | 0.1936 | 99.4% | 2319.1743 | 2319.5945 | 1 | 5.02 | 38.2% | 1 | R.TREGNDLYHEMIESGVINLK.D | 3 |
| \* | CENPL\_stlcld\_tube2\_122314\_02.01996.01996.3 | 3.9573 | 0.3393 | 100.0% | 3344.6643 | 3344.7917 | 2 | 5.891 | 23.2% | 1 | R.VALTGLTVAEYFRDQEGQDVLLFIDNIFR.F | 3 |
| \* | CENPL\_stlcld\_122314\_01.16206.16206.2 | 3.7099 | 0.4376 | 100.0% | 1436.6721 | 1436.6078 | 1 | 7.543 | 65.4% | 3 | R.FTQAGSEVSALLGR.I | 2 |
| \* | CENPL\_stlcld\_122314\_01.20640.20640.2 | 3.9757 | 0.5437 | 100.0% | 2024.2522 | 2024.3447 | 1 | 9.128 | 44.1% | 1 | R.FLSQPFQVAEVFTGHMGK.L | 2 |

---

|  |  |  |  |  |  |  |  |  |
| --- | --- | --- | --- | --- | --- | --- | --- | --- |
| U | *gi|117190174|ref|NP\_0* | 6 | 28 | 22.9% | 293 | 32338 | 5.1 | heterogeneous nuclear ribonucleoprotein C isoform b [Homo sapiens] |
| U | *gi|117190254|ref|NP\_0* | 6 | 28 | 22.9% | 293 | 32338 | 5.1 | heterogeneous nuclear ribonucleoprotein C isoform b [Homo sapiens] |

| Filename XCorr DeltCN Conf% ObsM+H+ CalcM+H+ SpR ZScore Ion% # Sequence  | | | | | | | | | | | | |
| --- | --- | --- | --- | --- | --- | --- | --- | --- | --- | --- | --- | --- |
|  | CENPL\_stlcld\_122314\_01.18183.18183.2 | 3.8167 | 0.2692 | 99.9% | 1318.5922 | 1317.6145 | 1 | 6.173 | 77.3% | 7 | R.VFIGNLNTLVVK.K | 2 |
|  | CENPL\_stlcld\_tube2\_122314\_01.11516.11516.2 | 2.5137 | 0.3004 | 99.5% | 1124.2922 | 1124.2792 | 10 | 5.561 | 61.1% | 2 | K.KSDVEAIFSK.Y | 2 |
|  | CENPL\_stlcld\_tube2\_122314\_01.13388.13388.2 | 1.9762 | 0.3346 | 98.5% | 996.3722 | 996.10516 | 46 | 6.548 | 62.5% | 1 | K.SDVEAIFSK.Y | 2 |
|  | CENPL\_stlcld\_122314\_01.16809.16809.2 | 3.7952 | 0.4848 | 100.0% | 1330.6522 | 1330.4857 | 1 | 9.234 | 80.0% | 4 | K.GFAFVQYVNER.N | 2 |
|  | CENPL\_stlcld\_122314\_02.14744.14744.2 | 4.9356 | 0.403 | 100.0% | 1684.6322 | 1684.0038 | 1 | 7.952 | 80.0% | 12 | R.MIAGQVLDINLAAEPK.V | 2 |
|  | CENPL\_stlcld\_122314\_02.14967.14967.2 | 3.7251 | 0.1758 | 99.5% | 2105.2122 | 2103.2239 | 1 | 4.765 | 50.0% | 2 | R.SAAEMYGSSFDLDYDFQR.D | 2 |

---

|  |  |  |  |  |  |  |  |  |
| --- | --- | --- | --- | --- | --- | --- | --- | --- |
| U | *contaminant\_KERATIN05* | 11 | 30 | 22.3% | 471 | 51531 | 5.2 | no description |
| U | *gi|15431310|ref|NP\_00* | 11 | 32 | 22.2% | 472 | 51622 | 5.2 | keratin 14 [Homo sapiens] |

| Filename XCorr DeltCN Conf% ObsM+H+ CalcM+H+ SpR ZScore Ion% # Sequence  | | | | | | | | | | | | |
| --- | --- | --- | --- | --- | --- | --- | --- | --- | --- | --- | --- | --- |
|  | CENPL\_stlcld\_tube2\_122314\_01.08714.08714.2 | 3.0686 | 0.3444 | 100.0% | 1091.2522 | 1091.2273 | 32 | 5.996 | 68.8% | 4 | K.VTMQNLNDR.L | 222 |
|  | CENPL\_stlcld\_tube2\_122314\_01.09831.09831.2 | 3.0041 | 0.1699 | 99.3% | 1065.3322 | 1065.2578 | 54 | 5.893 | 62.5% | 1 | R.LASYLDKVR.A | 2222 |
|  | CENPL\_stlcld\_tube2\_122314\_02.11382.11382.2 | 3.8041 | 0.3441 | 100.0% | 1302.4922 | 1302.4241 | 1 | 7.466 | 77.3% | 2 | R.ALEEANADLEVK.I | 2 |
|  | CENPL\_stlcld\_122314\_01.09504.09504.2 | 2.335 | 0.0893 | 95.2% | 1037.4922 | 1037.1661 | 21 | 4.386 | 75.0% | 1 | K.IRDWYQR.Q | 22 |
|  | CENPL\_stlcld\_122314\_01.09788.09788.2 | 2.5662 | 0.2183 | 99.5% | 808.39215 | 807.8815 | 68 | 6.201 | 66.7% | 6 | R.LAADDFR.T | 22222 |
|  | CENPL\_stlcld\_122314\_01.14091.14091.2 | 3.0726 | 0.4118 | 100.0% | 1030.3522 | 1030.2096 | 3 | 6.871 | 81.2% | 5 | R.VLDELTLAR.A | 222 |
|  | CENPL\_stlcld\_122314\_02.10301.10301.3 | 3.6063 | 0.3568 | 100.0% | 2106.0244 | 2105.2664 | 8 | 5.981 | 34.7% | 1 | K.TEELNREVATNSELVQSGK.S | 33 |
|  | CENPL\_stlcld\_122314\_01.08586.08586.2 | 3.5852 | 0.459 | 100.0% | 1362.2322 | 1362.4796 | 1 | 8.37 | 66.7% | 4 | R.EVATNSELVQSGK.S | 22 |
|  | CENPL\_stlcld\_tube2\_122314\_01.11194.11194.2 | 3.5553 | 0.293 | 99.9% | 1380.4722 | 1380.5437 | 5 | 5.961 | 65.0% | 4 | K.TRLEQEIATYR.R | 22 |
|  | CENPL\_stlcld\_tube2\_122314\_01.09641.09641.2 | 2.9939 | 0.3424 | 99.9% | 1123.2722 | 1123.2511 | 2 | 6.024 | 81.2% | 1 | R.LEQEIATYR.R | 222 |
|  | CENPL\_stlcld\_122314\_02.10529.10529.3 | 4.0257 | 0.4484 | 100.0% | 2310.1143 | 2310.396 | 1 | 7.318 | 36.9% | 1 | R.LLEGEDAHLSSSQFSSGSQSSR.D | 3 |

Similarities:
gi|40354195|ref|NP\_95(1:10)  
gi|4557701|ref|NP\_000(8:3)  
contaminant\_KERATIN03(3:8)  
gi|24234699|ref|NP\_00(4:7)  
gi|153945736|ref|NP\_8(1:10)  

---

|  |  |  |  |  |  |  |  |  |
| --- | --- | --- | --- | --- | --- | --- | --- | --- |
| U | *gi|42734430|ref|NP\_03* | 6 | 11 | 22.1% | 390 | 43476 | 5.6 | polymerase I and transcript release factor [Homo sapiens] |

| Filename XCorr DeltCN Conf% ObsM+H+ CalcM+H+ SpR ZScore Ion% # Sequence  | | | | | | | | | | | | |
| --- | --- | --- | --- | --- | --- | --- | --- | --- | --- | --- | --- | --- |
| \* | CENPL\_stlcld\_122314\_02.16936.16936.2 | 2.818 | 0.3566 | 99.9% | 1600.5322 | 1600.8528 | 1 | 6.923 | 60.7% | 1 | K.SDQVNGVLVLSLLDK.I | 2 |
| \* | CENPL\_stlcld\_122314\_02.13582.13582.2 | 6.2293 | 0.4633 | 100.0% | 2025.6721 | 2026.2975 | 1 | 9.325 | 70.6% | 3 | K.IIGAVDQIQLTQAQLEER.Q | 2 |
| \* | CENPL\_stlcld\_tube2\_122314\_02.14907.14907.3 | 4.1885 | 0.3204 | 100.0% | 2026.6144 | 2026.2975 | 1 | 6.791 | 41.2% | 3 | K.IIGAVDQIQLTQAQLEER.Q | 3 |
| \* | CENPL\_stlcld\_122314\_01.15725.15725.2 | 3.0144 | 0.4132 | 99.9% | 1804.9922 | 1805.9977 | 11 | 6.556 | 40.6% | 1 | R.QAEMEGAVQSIQGELSK.L | 2 |
| \* | CENPL\_stlcld\_tube2\_122314\_01.12299.12299.2 | 3.3923 | 0.395 | 100.0% | 1314.3722 | 1314.5248 | 2 | 6.388 | 75.0% | 2 | K.KLEVNEAELLR.R | 2 |
| \* | CENPL\_stlcld\_tube2\_122314\_02.11361.11361.3 | 2.9174 | 0.2386 | 95.2% | 2551.1042 | 2550.627 | 1 | 4.726 | 27.1% | 1 | K.ATEMVEVGADDDEGGAERGEAGDLR.R | 3 |

---

|  |  |  |  |  |  |  |  |  |
| --- | --- | --- | --- | --- | --- | --- | --- | --- |
| U | *contaminant\_INT-STD1* | 15 | 75 | 21.4% | 607 | 69271 | 6.1 | BSA |

| Filename XCorr DeltCN Conf% ObsM+H+ CalcM+H+ SpR ZScore Ion% # Sequence  | | | | | | | | | | | | |
| --- | --- | --- | --- | --- | --- | --- | --- | --- | --- | --- | --- | --- |
| \* | CENPL\_stlcld\_tube2\_122314\_01.14720.14720.2 | 3.5758 | 0.3139 | 100.0% | 1164.3121 | 1164.344 | 1 | 6.776 | 83.3% | 7 | K.LVNELTEFAK.T | 2 |
|  | CENPL\_stlcld\_tube2\_122314\_02.11064.11064.2 | 2.3529 | 0.3243 | 99.9% | 928.3122 | 928.0758 | 2 | 5.345 | 75.0% | 2 | K.YLYEIAR.R | 2 |
| \* | CENPL\_stlcld\_tube2\_122314\_01.16304.16304.2 | 4.4709 | 0.4421 | 100.0% | 2046.5122 | 2046.3354 | 1 | 7.462 | 73.3% | 2 | R.RHPYFYAPELLYYANK.Y | 2 |
| \* | CENPL\_stlcld\_tube2\_122314\_01.16316.16316.3 | 3.9194 | 0.3213 | 100.0% | 2047.6144 | 2046.3354 | 1 | 5.679 | 43.3% | 3 | R.RHPYFYAPELLYYANK.Y | 3 |
| \* | CENPL\_stlcld\_tube2\_122314\_01.09794.09794.2 | 2.5353 | 0.3334 | 99.9% | 923.3522 | 923.05383 | 1 | 6.04 | 85.7% | 2 | K.AEFVEVTK.L | 2 |
| \* | CENPL\_stlcld\_tube2\_122314\_02.17898.17898.2 | 4.5196 | 0.4377 | 100.0% | 1569.0322 | 1568.7258 | 1 | 8.426 | 75.0% | 8 | K.DAFLGSFLYEYSR.R | 2 |
| \* | CENPL\_stlcld\_122314\_01.12212.12212.2 | 3.011 | 0.3211 | 99.9% | 1440.6721 | 1440.6884 | 3 | 5.812 | 63.6% | 3 | R.RHPEYAVSVLLR.L | 2 |
| \* | CENPL\_stlcld\_tube2\_122314\_01.12248.12248.3 | 4.3066 | 0.293 | 100.0% | 1441.2244 | 1440.6884 | 1 | 5.372 | 61.4% | 11 | R.RHPEYAVSVLLR.L | 3 |
| \* | CENPL\_stlcld\_tube2\_122314\_01.11240.11240.2 | 3.2717 | 0.4249 | 100.0% | 1306.5322 | 1306.5046 | 1 | 8.28 | 70.0% | 4 | K.HLVDEPQNLIK.Q | 2 |
| \* | CENPL\_stlcld\_122314\_01.17606.17606.2 | 4.3978 | 0.3976 | 100.0% | 1480.5322 | 1480.7068 | 1 | 8.095 | 75.0% | 17 | K.LGEYGFQNALIVR.Y | 2 |
| \* | CENPL\_stlcld\_122314\_02.14153.14153.3 | 3.1034 | 0.385 | 100.0% | 1901.5743 | 1901.1753 | 1 | 6.258 | 31.7% | 1 | K.LGEYGFQNALIVRYTR.K | 3 |
|  | CENPL\_stlcld\_122314\_01.12080.12080.2 | 3.7883 | 0.5449 | 100.0% | 1640.6522 | 1640.9205 | 1 | 9.288 | 71.4% | 3 | R.KVPQVSTPTLVEVSR.S | 2 |
|  | CENPL\_stlcld\_tube2\_122314\_01.11888.11888.3 | 4.4938 | 0.4089 | 100.0% | 1641.5944 | 1640.9205 | 6 | 7.326 | 41.1% | 4 | R.KVPQVSTPTLVEVSR.S | 3 |
| \* | CENPL\_stlcld\_122314\_01.13664.13664.2 | 2.84 | 0.2689 | 99.7% | 1143.4722 | 1143.4124 | 1 | 5.571 | 83.3% | 4 | K.KQTALVELLK.H | 2 |
| \* | CENPL\_stlcld\_122314\_02.16536.16536.2 | 3.7489 | 0.536 | 100.0% | 1400.4321 | 1400.6324 | 1 | 10.116 | 77.3% | 4 | K.TVMENFVAFVDK.C | 2 |

---

|  |  |  |  |  |  |  |  |  |
| --- | --- | --- | --- | --- | --- | --- | --- | --- |
| U | *gi|169161114|ref|XP\_0* | 5 | 6 | 21.2% | 184 | 21456 | 10.1 | PREDICTED: hypothetical protein isoform 1 [Homo sapiens] |
| U | *gi|78000186|ref|NP\_00* | 5 | 6 | 21.2% | 184 | 21397 | 10.2 | ribosomal protein L17 [Homo sapiens] |
| U | *gi|4506617|ref|NP\_000* | 5 | 6 | 21.2% | 184 | 21397 | 10.2 | ribosomal protein L17 [Homo sapiens] |
| U | *gi|169212979|ref|XP\_0* | 5 | 6 | 21.2% | 184 | 21397 | 10.2 | PREDICTED: hypothetical protein [Homo sapiens] |
| U | *gi|169161116|ref|XP\_0* | 5 | 6 | 21.2% | 184 | 21456 | 10.1 | PREDICTED: hypothetical protein isoform 2 [Homo sapiens] |

| Filename XCorr DeltCN Conf% ObsM+H+ CalcM+H+ SpR ZScore Ion% # Sequence  | | | | | | | | | | | | |
| --- | --- | --- | --- | --- | --- | --- | --- | --- | --- | --- | --- | --- |
|  | CENPL\_stlcld\_122314\_01.09282.09282.2 | 1.9549 | 0.2342 | 95.1% | 1107.5521 | 1108.3231 | 26 | 4.156 | 62.5% | 1 | K.YLKDVTLQK.Q | 2 |
|  | CENPL\_stlcld\_122314\_01.14331.14331.3 | 3.3899 | 0.3111 | 100.0% | 1318.2843 | 1317.6323 | 6 | 5.871 | 52.5% | 2 | K.KSAEFLLHMLK.N | 3 |
|  | CENPL\_stlcld\_122314\_01.17777.17777.2 | 3.1037 | 0.2704 | 99.9% | 1189.5922 | 1189.4583 | 2 | 6.609 | 66.7% | 1 | K.SAEFLLHMLK.N | 2 |
|  | CENPL\_stlcld\_122314\_02.14415.14415.3 | 2.7435 | 0.2901 | 99.1% | 1780.1943 | 1780.0311 | 92 | 4.827 | 31.7% | 1 | K.GLDVDSLVIEHIQVNK.A | 3 |
|  | CENPL\_stlcld\_122314\_02.13473.13473.3 | 2.781 | 0.3258 | 99.9% | 2077.3442 | 2076.4006 | 40 | 5.176 | 30.6% | 1 | K.GLDVDSLVIEHIQVNKAPK.M | 3 |

---

|  |  |  |  |  |  |  |  |  |
| --- | --- | --- | --- | --- | --- | --- | --- | --- |
| U | *gi|4502201|ref|NP\_001* | 2 | 3 | 21.0% | 181 | 20697 | 6.8 | ADP-ribosylation factor 1 [Homo sapiens] |
| U | *gi|66879664|ref|NP\_00* | 2 | 3 | 21.0% | 181 | 20697 | 6.8 | ADP-ribosylation factor 1 [Homo sapiens] |
| U | *gi|66879662|ref|NP\_00* | 2 | 3 | 21.0% | 181 | 20697 | 6.8 | ADP-ribosylation factor 1 [Homo sapiens] |
| U | *gi|66879660|ref|NP\_00* | 2 | 3 | 21.0% | 181 | 20697 | 6.8 | ADP-ribosylation factor 1 [Homo sapiens] |
| U | *gi|4502203|ref|NP\_001* | 2 | 3 | 21.0% | 181 | 20601 | 7.4 | ADP-ribosylation factor 3 [Homo sapiens] |

| Filename XCorr DeltCN Conf% ObsM+H+ CalcM+H+ SpR ZScore Ion% # Sequence  | | | | | | | | | | | | |
| --- | --- | --- | --- | --- | --- | --- | --- | --- | --- | --- | --- | --- |
|  | CENPL\_stlcld\_122314\_02.12905.12905.3 | 2.9114 | 0.2462 | 97.8% | 2439.8342 | 2439.6494 | 1 | 4.741 | 38.2% | 2 | R.HYFQNTQGLIFVVDSNDRER.V | 3 |
|  | CENPL\_stlcld\_122314\_01.23004.23004.2 | 2.8645 | 0.2361 | 98.6% | 2047.7722 | 2048.4053 | 1 | 5.015 | 44.1% | 1 | R.MLAEDELRDAVLLVFANK.Q | 2 |

---

|  |  |  |  |  |  |  |  |  |
| --- | --- | --- | --- | --- | --- | --- | --- | --- |
| U | *gi|4507879|ref|NP\_003* | 3 | 3 | 20.8% | 283 | 30773 | 8.5 | voltage-dependent anion channel 1 [Homo sapiens] |

| Filename XCorr DeltCN Conf% ObsM+H+ CalcM+H+ SpR ZScore Ion% # Sequence  | | | | | | | | | | | | |
| --- | --- | --- | --- | --- | --- | --- | --- | --- | --- | --- | --- | --- |
| \* | CENPL\_stlcld\_122314\_02.09262.09262.3 | 3.6744 | 0.2022 | 98.7% | 2190.6543 | 2190.2822 | 222 | 4.607 | 27.5% | 1 | K.TKSENGLEFTSSGSANTETTK.V | 3 |
| \* | CENPL\_stlcld\_tube2\_122314\_02.13143.13143.3 | 3.2182 | 0.2187 | 98.3% | 1947.6244 | 1947.1582 | 4 | 4.366 | 33.8% | 1 | K.KLETAVNLAWTAGNSNTR.F | 3 |
| \* | CENPL\_stlcld\_tube2\_122314\_02.12956.12956.3 | 3.1496 | 0.3039 | 99.9% | 2104.1643 | 2104.4546 | 38 | 4.946 | 27.6% | 1 | K.VNNSSLIGLGYTQTLKPGIK.L | 3 |

---

|  |  |  |  |  |  |  |  |  |
| --- | --- | --- | --- | --- | --- | --- | --- | --- |
| U | *gi|14916501|ref|NP\_14* | 2 | 6 | 20.8% | 130 | 15069 | 10.9 | ribosomal protein S24 isoform a [Homo sapiens] |
| U | *gi|4506703|ref|NP\_001* | 2 | 6 | 20.3% | 133 | 15423 | 10.8 | ribosomal protein S24 isoform c [Homo sapiens] |
| U | *gi|214829241|ref|NP\_0* | 2 | 6 | 20.6% | 131 | 15197 | 10.9 | ribosomal protein S24 isoform b [Homo sapiens] |
| U | *gi|214010226|ref|NP\_0* | 2 | 5 | 9.3% | 289 | 32431 | 10.2 | ribosomal protein S24 isoform d [Homo sapiens] |
| U | *gi|214010224|ref|NP\_0* | 2 | 6 | 20.6% | 131 | 15197 | 10.9 | ribosomal protein S24 isoform f [Homo sapiens] |
| U | *gi|214010222|ref|NP\_0* | 2 | 5 | 20.5% | 132 | 15325 | 10.9 | ribosomal protein S24 isoform e [Homo sapiens] |

| Filename XCorr DeltCN Conf% ObsM+H+ CalcM+H+ SpR ZScore Ion% # Sequence  | | | | | | | | | | | | |
| --- | --- | --- | --- | --- | --- | --- | --- | --- | --- | --- | --- | --- |
|  | CENPL\_stlcld\_tube2\_122314\_01.20930.20930.2 | 2.5013 | 0.3998 | 99.9% | 1399.8722 | 1399.6323 | 1 | 8.065 | 59.1% | 2 | K.TTPDVIFVFGFR.T | 2 |
|  | CENPL\_stlcld\_122314\_02.15951.15951.2 | 4.6271 | 0.5177 | 100.0% | 1682.4922 | 1682.8854 | 1 | 9.672 | 64.3% | 4 | K.TTGFGMIYDSLDYAK.K | 2 |

---

|  |  |  |  |  |  |  |  |  |
| --- | --- | --- | --- | --- | --- | --- | --- | --- |
| U | *gi|14141166|ref|NP\_11* | 4 | 8 | 20.7% | 362 | 38222 | 6.8 | poly(rC) binding protein 2 isoform b [Homo sapiens] |
| U | *gi|193083114|ref|NP\_0* | 4 | 8 | 23.6% | 318 | 33497 | 8.2 | poly(rC) binding protein 2 isoform g [Homo sapiens] |
| U | *gi|193083112|ref|NP\_0* | 4 | 8 | 22.4% | 335 | 35347 | 8.0 | poly(rC) binding protein 2 isoform f [Homo sapiens] |
| U | *gi|193083110|ref|NP\_0* | 4 | 8 | 20.8% | 361 | 38151 | 6.8 | poly(rC) binding protein 2 isoform e [Homo sapiens] |
| U | *gi|193083108|ref|NP\_0* | 4 | 8 | 20.5% | 365 | 38580 | 6.8 | poly(rC) binding protein 2 isoform d [Homo sapiens] |
| U | *gi|148833484|ref|NP\_0* | 4 | 8 | 22.7% | 331 | 34917 | 8.0 | poly(rC) binding protein 2 isoform c [Homo sapiens] |
| U | *gi|14141168|ref|NP\_00* | 4 | 8 | 20.5% | 366 | 38651 | 6.8 | poly(rC) binding protein 2 isoform a [Homo sapiens] |

| Filename XCorr DeltCN Conf% ObsM+H+ CalcM+H+ SpR ZScore Ion% # Sequence  | | | | | | | | | | | | |
| --- | --- | --- | --- | --- | --- | --- | --- | --- | --- | --- | --- | --- |
|  | CENPL\_stlcld\_122314\_01.18461.18461.2 | 3.4635 | 0.3868 | 100.0% | 1360.0322 | 1359.6519 | 1 | 7.007 | 62.5% | 2 | R.IITLAGPTNAIFK.A | 2 |
|  | CENPL\_stlcld\_tube2\_122314\_01.21159.21159.3 | 3.4129 | 0.2435 | 98.7% | 3354.5044 | 3353.8306 | 1 | 4.5 | 25.0% | 1 | K.AFAMIIDKLEEDISSSMTNSTAASRPPVTLR.L | 3 |
|  | CENPL\_stlcld\_tube2\_122314\_02.12410.12410.2 | 5.6809 | 0.5742 | 100.0% | 2090.4521 | 2091.2573 | 1 | 10.311 | 60.5% | 4 | R.ESTGAQVQVAGDMLPNSTER.A | 22 |
|  | CENPL\_stlcld\_tube2\_122314\_01.08374.08374.2 | 2.1895 | 0.215 | 95.9% | 1159.1721 | 1159.2413 | 5 | 5.789 | 70.0% | 1 | K.IANPVEGSTDR.Q | 2 |

Similarities:
gi|222352151|ref|NP\_0(1:3)  

---

|  |  |  |  |  |  |  |  |  |
| --- | --- | --- | --- | --- | --- | --- | --- | --- |
| U | *gi|38201714|ref|NP\_00* | 4 | 6 | 20.6% | 326 | 36092 | 9.2 | ELAV-like 1 [Homo sapiens] |

| Filename XCorr DeltCN Conf% ObsM+H+ CalcM+H+ SpR ZScore Ion% # Sequence  | | | | | | | | | | | | |
| --- | --- | --- | --- | --- | --- | --- | --- | --- | --- | --- | --- | --- |
| \* | CENPL\_stlcld\_122314\_02.12606.12606.3 | 4.4475 | 0.4693 | 100.0% | 2027.5144 | 2027.2871 | 1 | 8.795 | 43.1% | 2 | R.DKVAGHSLGYGFVNYVTAK.D | 3 |
| \* | CENPL\_stlcld\_tube2\_122314\_01.16306.16306.3 | 3.6524 | 0.2413 | 99.4% | 2536.4644 | 2536.8906 | 10 | 4.346 | 28.4% | 1 | K.VSYARPSSEVIKDANLYISGLPR.T | 3 |
| \* | CENPL\_stlcld\_tube2\_122314\_01.10152.10152.2 | 3.0817 | 0.3838 | 100.0% | 1189.4521 | 1189.3542 | 1 | 7.821 | 75.0% | 2 | R.VLVDQTTGLSR.G | 2 |
| \* | CENPL\_stlcld\_tube2\_122314\_01.14879.14879.2 | 2.9625 | 0.3781 | 99.9% | 1569.6921 | 1569.8038 | 1 | 6.464 | 50.0% | 1 | K.NVALLSQLYHSPAR.R | 2 |

---

|  |  |  |  |  |  |  |  |  |
| --- | --- | --- | --- | --- | --- | --- | --- | --- |
| U | *gi|4506623|ref|NP\_000* | 4 | 7 | 20.6% | 136 | 15798 | 10.6 | ribosomal protein L27 [Homo sapiens] |

| Filename XCorr DeltCN Conf% ObsM+H+ CalcM+H+ SpR ZScore Ion% # Sequence  | | | | | | | | | | | | |
| --- | --- | --- | --- | --- | --- | --- | --- | --- | --- | --- | --- | --- |
| \* | CENPL\_stlcld\_tube2\_122314\_01.12953.12953.2 | 2.3493 | 0.2951 | 99.4% | 827.4122 | 827.058 | 1 | 6.124 | 78.6% | 1 | K.VVLVLAGR.Y | 2 |
|  | CENPL\_stlcld\_tube2\_122314\_01.11022.11022.2 | 3.3982 | 0.3494 | 100.0% | 1408.4521 | 1408.6177 | 2 | 6.575 | 80.0% | 1 | K.VYNYNHLMPTR.Y | 2 |
|  | CENPL\_stlcld\_122314\_01.11752.11752.3 | 2.3097 | 0.37 | 99.9% | 1410.2043 | 1408.6177 | 4 | 5.478 | 50.0% | 2 | K.VYNYNHLMPTR.Y | 3 |
| \* | CENPL\_stlcld\_tube2\_122314\_01.13522.13522.2 | 2.4667 | 0.3522 | 99.9% | 1050.3522 | 1050.1968 | 9 | 7.098 | 62.5% | 3 | R.YSVDIPLDK.T | 2 |

---

|  |  |  |  |  |  |  |  |  |
| --- | --- | --- | --- | --- | --- | --- | --- | --- |
| U | *gi|73623035|ref|NP\_00* | 20 | 64 | 20.5% | 1193 | 134422 | 5.0 | sperm associated antigen 5 [Homo sapiens] |

| Filename XCorr DeltCN Conf% ObsM+H+ CalcM+H+ SpR ZScore Ion% # Sequence  | | | | | | | | | | | | |
| --- | --- | --- | --- | --- | --- | --- | --- | --- | --- | --- | --- | --- |
| \* | CENPL\_stlcld\_122314\_02.11074.11074.3 | 4.1013 | 0.2925 | 99.9% | 2221.4944 | 2220.3752 | 1 | 5.555 | 36.1% | 1 | R.VNHVDPEEEIVEHGAMEER.E | 3 |
| \* | CENPL\_stlcld\_122314\_02.01426.01426.2 | 7.1805 | 0.6365 | 100.0% | 2165.7322 | 2166.4795 | 1 | 11.472 | 66.7% | 4 | R.HDLEDNLLSSLVILEVLSR.Q | 2 |
| \* | CENPL\_stlcld\_122314\_01.20070.20070.3 | 3.7823 | 0.2662 | 99.9% | 2891.9043 | 2892.2793 | 1 | 5.372 | 29.2% | 2 | K.ELISLLHLSLLHLEEDKTTVSQESR.R | 3 |
| \* | CENPL\_stlcld\_122314\_01.16372.16372.2 | 3.4471 | 0.4664 | 100.0% | 1391.3322 | 1391.5823 | 1 | 8.034 | 68.2% | 4 | R.ISQLEQDLASMR.E | 2 |
| \* | CENPL\_stlcld\_tube2\_122314\_01.08021.08021.2 | 3.5427 | 0.3671 | 100.0% | 1402.9722 | 1403.5321 | 5 | 7.085 | 68.2% | 2 | R.DVAIEEKQEVSR.V | 2 |
| \* | CENPL\_stlcld\_122314\_02.14812.14812.2 | 5.4176 | 0.388 | 100.0% | 1574.5721 | 1573.848 | 1 | 7.224 | 76.9% | 6 | R.AQLQILANMDSQLK.E | 2 |
| \* | CENPL\_stlcld\_122314\_01.09785.09785.2 | 4.9889 | 0.48 | 100.0% | 1724.6522 | 1723.9879 | 1 | 8.463 | 75.0% | 5 | K.HMQAELQQQQAVLAK.E | 2 |
| \* | CENPL\_stlcld\_122314\_02.09650.09650.3 | 5.0215 | 0.2865 | 100.0% | 1725.2644 | 1723.9879 | 1 | 5.825 | 55.4% | 7 | K.HMQAELQQQQAVLAK.E | 3 |
| \* | CENPL\_stlcld\_tube2\_122314\_01.09856.09856.2 | 4.8294 | 0.4734 | 100.0% | 1675.5521 | 1675.7899 | 1 | 7.893 | 67.9% | 8 | K.LASTIADNQEQDLEK.T | 2 |
| \* | CENPL\_stlcld\_122314\_02.00782.00782.2 | 5.802 | 0.4576 | 100.0% | 2046.9321 | 2047.443 | 1 | 9.432 | 61.8% | 5 | K.LGLLTEQLQSLTLFLQTK.L | 2 |
| \* | CENPL\_stlcld\_tube2\_122314\_01.00110.00110.3 | 4.6272 | 0.4777 | 100.0% | 2047.5543 | 2047.443 | 1 | 7.803 | 47.1% | 1 | K.LGLLTEQLQSLTLFLQTK.L | 3 |
| \* | CENPL\_stlcld\_122314\_02.00434.00434.3 | 3.5049 | 0.3511 | 99.9% | 2788.3442 | 2788.121 | 1 | 5.658 | 24.0% | 1 | R.TFLGSILTAVADEEPESTPVPLLGSDK.S | 3 |
| \* | CENPL\_stlcld\_tube2\_122314\_02.00771.00771.2 | 5.1729 | 0.517 | 100.0% | 2788.412 | 2788.121 | 1 | 9.147 | 40.4% | 3 | R.TFLGSILTAVADEEPESTPVPLLGSDK.S | 2 |
| \* | CENPL\_stlcld\_tube2\_122314\_01.10826.10826.2 | 2.2252 | 0.1386 | 95.0% | 987.8122 | 988.1412 | 80 | 3.998 | 71.4% | 1 | K.LNQALCLR.Y | 2 |
| \* | CENPL\_stlcld\_tube2\_122314\_01.09510.09510.3 | 5.4116 | 0.2863 | 100.0% | 2149.5244 | 2149.3652 | 2 | 5.725 | 48.4% | 1 | R.YKNEKELQEVIQQQNEK.I | 3 |
| \* | CENPL\_stlcld\_tube2\_122314\_01.09526.09526.2 | 4.4042 | 0.2385 | 100.0% | 1486.2122 | 1486.622 | 1 | 6.09 | 86.4% | 3 | K.ELQEVIQQQNEK.I | 2 |
| \* | CENPL\_stlcld\_tube2\_122314\_01.17324.17324.3 | 4.1883 | 0.2733 | 99.9% | 2681.7244 | 2681.0618 | 1 | 5.765 | 34.1% | 1 | K.ILEQIDKSGELISLREEVTHLTR.S | 3 |
| \* | CENPL\_stlcld\_122314\_01.15254.15254.3 | 4.5678 | 0.3896 | 100.0% | 1841.2144 | 1841.0745 | 1 | 7.013 | 50.0% | 5 | K.SGELISLREEVTHLTR.S | 3 |
| \* | CENPL\_stlcld\_122314\_01.14462.14462.2 | 2.8501 | 0.2299 | 99.2% | 1373.5922 | 1373.595 | 1 | 5.462 | 80.0% | 3 | K.VWLSQEVDKLR.V | 2 |
| \* | CENPL\_stlcld\_122314\_01.17564.17564.2 | 2.4855 | 0.3221 | 99.5% | 1127.7922 | 1127.3696 | 4 | 5.509 | 72.2% | 1 | K.TLLSIPEVVR.G | 2 |

---

|  |  |  |  |  |  |  |  |  |
| --- | --- | --- | --- | --- | --- | --- | --- | --- |
| U | *gi|11024714|ref|NP\_06* | 4 | 12 | 20.5% | 229 | 25762 | 7.4 | ubiquitin B precursor [Homo sapiens] |
| U | *gi|77539055|ref|NP\_00* | 4 | 12 | 36.7% | 128 | 14728 | 9.8 | ubiquitin and ribosomal protein L40 precursor [Homo sapiens] |
| U | *gi|67191208|ref|NP\_06* | 4 | 12 | 6.9% | 685 | 77029 | 7.7 | ubiquitin C [Homo sapiens] |
| U | *gi|4507761|ref|NP\_003* | 4 | 12 | 36.7% | 128 | 14728 | 9.8 | ubiquitin and ribosomal protein L40 precursor [Homo sapiens] |
| U | *gi|4506713|ref|NP\_002* | 4 | 12 | 30.1% | 156 | 17965 | 9.6 | ubiquitin and ribosomal protein S27a precursor [Homo sapiens] |
| U | *gi|208022622|ref|NP\_0* | 4 | 12 | 30.1% | 156 | 17965 | 9.6 | ubiquitin and ribosomal protein S27a precursor [Homo sapiens] |

| Filename XCorr DeltCN Conf% ObsM+H+ CalcM+H+ SpR ZScore Ion% # Sequence  | | | | | | | | | | | | |
| --- | --- | --- | --- | --- | --- | --- | --- | --- | --- | --- | --- | --- |
|  | CENPL\_stlcld\_122314\_01.14877.14877.2 | 4.6778 | 0.4131 | 100.0% | 1789.5721 | 1788.9897 | 1 | 7.156 | 63.3% | 5 | K.TITLEVEPSDTIENVK.A | 22 |
|  | CENPL\_stlcld\_tube2\_122314\_01.07529.07529.2 | 3.7953 | 0.2849 | 99.9% | 1525.5521 | 1524.6738 | 1 | 6.556 | 79.2% | 2 | K.IQDKEGIPPDQQR.L | 22 |
|  | CENPL\_stlcld\_122314\_01.09752.09752.2 | 2.5433 | 0.3686 | 99.9% | 1082.4122 | 1082.1986 | 1 | 6.513 | 75.0% | 3 | R.TLSDYNIQK.E | 22 |
|  | CENPL\_stlcld\_tube2\_122314\_01.12200.12200.2 | 2.764 | 0.3448 | 99.9% | 1068.3121 | 1068.2615 | 2 | 5.517 | 87.5% | 2 | K.ESTLHLVLR.L | 2 |

Similarities:
contaminant\_UBIQUITIN(3:1)  

---

|  |  |  |  |  |  |  |  |  |
| --- | --- | --- | --- | --- | --- | --- | --- | --- |
| U | *gi|52632383|ref|NP\_00* | 9 | 16 | 20.0% | 589 | 64133 | 8.2 | heterogeneous nuclear ribonucleoprotein L isoform a [Homo sapiens] |

| Filename XCorr DeltCN Conf% ObsM+H+ CalcM+H+ SpR ZScore Ion% # Sequence  | | | | | | | | | | | | |
| --- | --- | --- | --- | --- | --- | --- | --- | --- | --- | --- | --- | --- |
| \* | CENPL\_stlcld\_122314\_02.02054.02054.3 | 4.0692 | 0.3805 | 100.0% | 3088.4043 | 3089.6143 | 1 | 5.83 | 25.0% | 1 | R.GLIDGVVEADLVEALQEFGPISYVVVMPK.K | 3 |
| \* | CENPL\_stlcld\_122314\_02.02056.02056.2 | 5.5594 | 0.4762 | 100.0% | 3090.672 | 3089.6143 | 1 | 7.508 | 39.3% | 2 | R.GLIDGVVEADLVEALQEFGPISYVVVMPK.K | 2 |
|  | CENPL\_stlcld\_tube2\_122314\_02.13892.13892.2 | 2.4807 | 0.2051 | 96.6% | 1866.7322 | 1867.0435 | 66 | 4.216 | 34.4% | 1 | K.NGVQAMVEFDSVQSAQR.A | 2 |
|  | CENPL\_stlcld\_122314\_01.20798.20798.2 | 2.2886 | 0.2174 | 96.0% | 1590.0521 | 1589.7974 | 315 | 4.337 | 45.8% | 1 | R.VFNVFCLYGNVEK.V | 2 |
|  | CENPL\_stlcld\_tube2\_122314\_02.12944.12944.3 | 3.6474 | 0.4837 | 100.0% | 1870.4043 | 1869.1176 | 1 | 7.778 | 36.8% | 4 | K.SKPGAAMVEMADGYAVDR.A | 3 |
|  | CENPL\_stlcld\_tube2\_122314\_01.13270.13270.2 | 4.3438 | 0.3832 | 100.0% | 1635.2922 | 1635.881 | 1 | 6.82 | 65.4% | 3 | R.AITHLNNNFMFGQK.L | 2 |
|  | CENPL\_stlcld\_122314\_01.13467.13467.3 | 2.6848 | 0.224 | 95.9% | 1635.8644 | 1635.881 | 38 | 4.637 | 34.6% | 1 | R.AITHLNNNFMFGQK.L | 3 |
|  | CENPL\_stlcld\_122314\_01.14500.14500.2 | 2.5033 | 0.2087 | 98.2% | 1223.3522 | 1223.3251 | 200 | 5.176 | 50.0% | 2 | R.SSSGLLEWESK.S | 2 |
|  | CENPL\_stlcld\_122314\_01.18786.18786.2 | 3.9865 | 0.3987 | 100.0% | 1868.6721 | 1868.1144 | 1 | 6.514 | 70.0% | 1 | K.SDALETLGFLNHYQMK.N | 2 |

---

|  |  |  |  |  |  |  |  |  |
| --- | --- | --- | --- | --- | --- | --- | --- | --- |
| U | *gi|4757810|ref|NP\_004* | 8 | 20 | 19.9% | 553 | 59751 | 9.1 | ATP synthase, H+ transporting, mitochondrial F1 complex, alpha subunit precursor [Homo sapiens] |
| U | *gi|50345984|ref|NP\_00* | 8 | 20 | 19.9% | 553 | 59751 | 9.1 | ATP synthase, H+ transporting, mitochondrial F1 complex, alpha subunit precursor [Homo sapiens] |

| Filename XCorr DeltCN Conf% ObsM+H+ CalcM+H+ SpR ZScore Ion% # Sequence  | | | | | | | | | | | | |
| --- | --- | --- | --- | --- | --- | --- | --- | --- | --- | --- | --- | --- |
|  | CENPL\_stlcld\_122314\_01.14010.14010.2 | 3.002 | 0.4279 | 100.0% | 1424.6921 | 1424.5659 | 1 | 6.906 | 62.5% | 1 | K.TGTAEMSSILEER.I | 2 |
|  | CENPL\_stlcld\_122314\_02.11795.11795.2 | 5.1118 | 0.5284 | 100.0% | 1577.6522 | 1576.7007 | 1 | 9.573 | 82.1% | 3 | R.ILGADTSVDLEETGR.V | 2 |
|  | CENPL\_stlcld\_tube2\_122314\_01.12556.12556.2 | 1.9585 | 0.2564 | 95.5% | 1001.21216 | 1001.171 | 7 | 4.432 | 66.7% | 1 | R.VLSIGDGIAR.V | 2 |
|  | CENPL\_stlcld\_122314\_02.13686.13686.2 | 3.3077 | 0.2093 | 99.2% | 1669.4722 | 1668.8591 | 1 | 6.2 | 53.6% | 3 | R.NVQAEEMVEFSSGLK.G | 2 |
|  | CENPL\_stlcld\_122314\_02.10216.10216.2 | 3.1082 | 0.461 | 100.0% | 1173.3922 | 1172.3237 | 5 | 7.19 | 59.1% | 2 | R.VVDALGNAIDGK.G | 2 |
|  | CENPL\_stlcld\_tube2\_122314\_01.13190.13190.2 | 2.9252 | 0.3886 | 99.9% | 1288.4321 | 1288.4863 | 1 | 8.37 | 80.0% | 4 | K.HALIIYDDLSK.Q | 2 |
|  | CENPL\_stlcld\_122314\_01.14208.14208.2 | 2.656 | 0.409 | 99.9% | 1555.6122 | 1554.7019 | 1 | 6.021 | 70.8% | 3 | R.EAYPGDVFYLHSR.L | 2 |
|  | CENPL\_stlcld\_122314\_01.18171.18171.3 | 5.5051 | 0.4532 | 100.0% | 2368.3743 | 2368.7007 | 1 | 8.841 | 43.8% | 3 | K.FENAFLSHVVSQHQALLGTIR.A | 3 |

---

|  |  |  |  |  |  |  |  |  |
| --- | --- | --- | --- | --- | --- | --- | --- | --- |
| U | *gi|169212778|ref|XP\_0* | 8 | 18 | 19.9% | 266 | 30042 | 10.6 | PREDICTED: similar to ribosomal protein L7a [Homo sapiens] |
| U | *gi|4506661|ref|NP\_000* | 8 | 18 | 19.9% | 266 | 29996 | 10.6 | ribosomal protein L7a [Homo sapiens] |
| U | *gi|169213130|ref|XP\_0* | 8 | 18 | 19.9% | 266 | 30042 | 10.6 | PREDICTED: similar to ribosomal protein L7a [Homo sapiens] |
| U | *gi|169212940|ref|XP\_0* | 8 | 18 | 19.9% | 266 | 30028 | 10.6 | PREDICTED: similar to ribosomal protein L7a [Homo sapiens] |

| Filename XCorr DeltCN Conf% ObsM+H+ CalcM+H+ SpR ZScore Ion% # Sequence  | | | | | | | | | | | | |
| --- | --- | --- | --- | --- | --- | --- | --- | --- | --- | --- | --- | --- |
|  | CENPL\_stlcld\_122314\_01.12358.12358.2 | 3.2967 | 0.3051 | 99.9% | 1217.4722 | 1217.3672 | 10 | 5.864 | 65.0% | 4 | K.NFGIGQDIQPK.R | 2 |
|  | CENPL\_stlcld\_122314\_01.18482.18482.2 | 3.0382 | 0.3632 | 99.9% | 1811.9521 | 1812.1222 | 23 | 6.239 | 43.3% | 2 | R.LKVPPAINQFTQALDR.Q | 2 |
|  | CENPL\_stlcld\_122314\_01.18473.18473.3 | 4.6611 | 0.3668 | 100.0% | 1813.3143 | 1812.1222 | 1 | 6.383 | 45.0% | 3 | R.LKVPPAINQFTQALDR.Q | 3 |
|  | CENPL\_stlcld\_122314\_01.18027.18027.2 | 3.4453 | 0.3939 | 100.0% | 1572.6322 | 1570.7886 | 1 | 6.013 | 65.4% | 3 | K.VPPAINQFTQALDR.Q | 2 |
|  | CENPL\_stlcld\_122314\_01.12764.12764.2 | 3.8565 | 0.4084 | 100.0% | 1346.5122 | 1346.5236 | 1 | 6.402 | 66.7% | 3 | R.AGVNTVTTLVENK.K | 2 |
|  | CENPL\_stlcld\_122314\_01.11604.11604.2 | 2.8877 | 0.1872 | 98.4% | 1474.3922 | 1474.6976 | 5 | 4.725 | 53.8% | 1 | R.AGVNTVTTLVENKK.A | 2 |
|  | CENPL\_stlcld\_tube2\_122314\_01.10918.10918.3 | 2.6012 | 0.338 | 99.9% | 1474.9143 | 1474.6976 | 1 | 6.231 | 36.5% | 1 | R.AGVNTVTTLVENKK.A | 3 |
|  | CENPL\_stlcld\_122314\_01.08378.08378.3 | 3.0974 | 0.1959 | 98.4% | 1616.7544 | 1615.7025 | 3 | 4.887 | 45.5% | 1 | R.TNYNDRYDEIRR.H | 3 |

---

|  |  |  |  |  |  |  |  |  |
| --- | --- | --- | --- | --- | --- | --- | --- | --- |
| U | *gi|39995082|ref|NP\_06* | 12 | 25 | 19.8% | 767 | 86471 | 6.8 | NOL1/NOP2/Sun domain family, member 2 [Homo sapiens] |

| Filename XCorr DeltCN Conf% ObsM+H+ CalcM+H+ SpR ZScore Ion% # Sequence  | | | | | | | | | | | | |
| --- | --- | --- | --- | --- | --- | --- | --- | --- | --- | --- | --- | --- |
| \* | CENPL\_stlcld\_122314\_01.13200.13200.2 | 2.7423 | 0.1191 | 97.6% | 1371.4122 | 1370.5474 | 1 | 3.816 | 72.2% | 2 | K.LFEHYYQELK.I | 2 |
| \* | CENPL\_stlcld\_122314\_02.11576.11576.3 | 4.5223 | 0.1979 | 99.9% | 1852.0443 | 1852.0134 | 1 | 6.473 | 45.0% | 2 | K.FHQFLVSETESGNISR.Q | 3 |
| \* | CENPL\_stlcld\_122314\_01.17375.17375.2 | 4.067 | 0.3566 | 100.0% | 1780.0922 | 1781.0677 | 1 | 6.572 | 71.4% | 3 | K.WTTLNSLQLHGLQLR.I | 2 |
| \* | CENPL\_stlcld\_tube2\_122314\_01.17150.17150.3 | 2.5729 | 0.2461 | 96.2% | 1782.0844 | 1781.0677 | 154 | 4.441 | 32.1% | 1 | K.WTTLNSLQLHGLQLR.I | 3 |
| \* | CENPL\_stlcld\_122314\_01.19168.19168.2 | 4.6599 | 0.4424 | 100.0% | 1843.9321 | 1843.041 | 1 | 7.386 | 55.9% | 2 | K.SEGALELADVSNELPGLK.W | 2 |
| \* | CENPL\_stlcld\_122314\_01.09046.09046.2 | 2.381 | 0.2861 | 99.4% | 998.4522 | 998.18823 | 3 | 5.314 | 78.6% | 1 | K.LQAMHLER.C | 2 |
| \* | CENPL\_stlcld\_122314\_02.13756.13756.3 | 3.703 | 0.3768 | 100.0% | 1977.8043 | 1978.3475 | 1 | 6.936 | 45.6% | 3 | R.ILPHHQNTGGFFVAVLVK.K | 3 |
| \* | CENPL\_stlcld\_122314\_01.16971.16971.2 | 2.7168 | 0.3429 | 99.9% | 1213.5322 | 1213.3782 | 2 | 6.585 | 72.2% | 2 | K.FYALDPSFPR.M | 2 |
| \* | CENPL\_stlcld\_122314\_01.13205.13205.2 | 2.3121 | 0.2678 | 98.5% | 1135.5122 | 1135.3612 | 92 | 5.155 | 61.1% | 3 | R.IITVSMEDVK.I | 2 |
| \* | CENPL\_stlcld\_122314\_01.19779.19779.2 | 3.7275 | 0.3585 | 100.0% | 1378.5122 | 1378.6139 | 1 | 6.864 | 65.0% | 2 | K.ILLTQENPFFR.K | 2 |
| \* | CENPL\_stlcld\_122314\_01.16733.16733.2 | 2.3941 | 0.3888 | 99.9% | 1107.0922 | 1107.3687 | 19 | 6.632 | 61.1% | 3 | R.MMGLEVLGEK.K | 2 |
| \* | CENPL\_stlcld\_122314\_02.10047.10047.3 | 4.6778 | 0.2349 | 99.9% | 2889.7444 | 2889.8633 | 1 | 5.908 | 32.0% | 1 | K.K@EGVILT#NES\*AASTGQPDNDVTEGQR.A | 3 |

---

|  |  |  |  |  |  |  |  |  |
| --- | --- | --- | --- | --- | --- | --- | --- | --- |
| U | *gi|15431301|ref|NP\_00* | 4 | 8 | 19.8% | 248 | 29226 | 10.7 | ribosomal protein L7 [Homo sapiens] |
| U | *gi|88988289|ref|XP\_94* | 4 | 8 | 18.9% | 259 | 30508 | 10.8 | PREDICTED: hypothetical protein LOC648000 isoform 3 [Homo sapiens] |
| U | *gi|169170622|ref|XP\_0* | 4 | 8 | 19.8% | 247 | 29009 | 10.7 | PREDICTED: hypothetical protein [Homo sapiens] |
| U | *gi|169168181|ref|XP\_0* | 4 | 8 | 18.9% | 259 | 30508 | 10.8 | PREDICTED: hypothetical protein LOC648000 [Homo sapiens] |
| U | *gi|169167651|ref|XP\_0* | 4 | 8 | 18.9% | 259 | 30508 | 10.8 | PREDICTED: hypothetical protein LOC648000 [Homo sapiens] |

| Filename XCorr DeltCN Conf% ObsM+H+ CalcM+H+ SpR ZScore Ion% # Sequence  | | | | | | | | | | | | |
| --- | --- | --- | --- | --- | --- | --- | --- | --- | --- | --- | --- | --- |
|  | CENPL\_stlcld\_122314\_01.10086.10086.2 | 3.261 | 0.0862 | 98.2% | 1321.2322 | 1321.5187 | 1 | 5.366 | 81.8% | 1 | R.KAGNFYVPAEPK.L | 2 |
|  | CENPL\_stlcld\_122314\_01.19781.19781.2 | 3.5401 | 0.4046 | 100.0% | 1663.9122 | 1663.9567 | 1 | 7.027 | 65.4% | 3 | R.IVEPYIAWGYPNLK.S | 2 |
|  | CENPL\_stlcld\_122314\_01.14264.14264.2 | 3.5467 | 0.4044 | 100.0% | 1171.5922 | 1171.3823 | 1 | 8.086 | 95.0% | 3 | R.IALTDNALIAR.S | 2 |
|  | CENPL\_stlcld\_122314\_01.19614.19614.2 | 2.8308 | 0.2495 | 99.2% | 1541.4521 | 1541.7924 | 1 | 5.115 | 68.2% | 1 | R.FKEANNFLWPFK.L | 2 |

---

|  |  |  |  |  |  |  |  |  |
| --- | --- | --- | --- | --- | --- | --- | --- | --- |
| U | *gi|87196351|ref|NP\_00* | 10 | 29 | 19.5% | 662 | 73244 | 7.2 | DEAD/H (Asp-Glu-Ala-Asp/His) box polypeptide 3 [Homo sapiens] |

| Filename XCorr DeltCN Conf% ObsM+H+ CalcM+H+ SpR ZScore Ion% # Sequence  | | | | | | | | | | | | |
| --- | --- | --- | --- | --- | --- | --- | --- | --- | --- | --- | --- | --- |
| \* | CENPL\_stlcld\_tube2\_122314\_02.01083.01083.2 | 4.0383 | 0.4755 | 100.0% | 2333.892 | 2333.6897 | 1 | 8.749 | 50.0% | 3 | K.TAAFLLPILSQIYSDGPGEALR.A | 2 |
|  | CENPL\_stlcld\_122314\_02.12611.12611.2 | 3.1429 | 0.4915 | 100.0% | 1321.6721 | 1321.4729 | 1 | 7.672 | 80.0% | 3 | R.ELAVQIYEEAR.K | 2 |
|  | CENPL\_stlcld\_122314\_01.12846.12846.2 | 2.5214 | 0.2106 | 98.5% | 1094.3522 | 1094.2096 | 1 | 6.122 | 87.5% | 1 | K.YLVLDEADR.M | 2 |
|  | CENPL\_stlcld\_122314\_01.17030.17030.2 | 4.1012 | 0.4447 | 100.0% | 1338.4521 | 1337.5946 | 1 | 7.711 | 85.0% | 8 | R.MLDMGFEPQIR.R | 222 |
|  | CENPL\_stlcld\_122314\_01.13572.13572.2 | 2.6563 | 0.4732 | 100.0% | 1298.3121 | 1298.5604 | 155 | 7.248 | 55.0% | 1 | R.HTMMFSATFPK.E | 2 |
| \* | CENPL\_stlcld\_tube2\_122314\_01.21148.21148.2 | 3.1378 | 0.4207 | 100.0% | 1292.5322 | 1292.5181 | 1 | 8.048 | 63.6% | 1 | R.SFLLDLLNATGK.D | 2 |
|  | CENPL\_stlcld\_122314\_01.08590.08590.2 | 2.915 | 0.121 | 98.4% | 1300.6721 | 1301.4043 | 34 | 4.906 | 66.7% | 1 | R.DREEALHQFR.S | 2 |
|  | CENPL\_stlcld\_122314\_01.13647.13647.2 | 3.607 | 0.4824 | 100.0% | 1169.4321 | 1169.4099 | 1 | 8.89 | 77.3% | 3 | K.SPILVATAVAAR.G | 2 |
|  | CENPL\_stlcld\_122314\_01.18752.18752.3 | 4.7733 | 0.3601 | 100.0% | 2084.6042 | 2084.2957 | 1 | 6.847 | 50.0% | 2 | K.HVINFDLPSDIEEYVHR.I | 3 |
| \* | CENPL\_stlcld\_122314\_01.19402.19402.2 | 4.137 | 0.4619 | 100.0% | 1525.3121 | 1525.7043 | 1 | 8.438 | 73.1% | 6 | R.VGNLGLATSFFNER.N | 2 |

Similarities:
gi|4758138|ref|NP\_004(1:9)  
gi|148613856|ref|NP\_0(1:9)  

---

|  |  |  |  |  |  |  |  |  |
| --- | --- | --- | --- | --- | --- | --- | --- | --- |
| U | *gi|163965362|ref|NP\_0* | 3 | 6 | 19.5% | 215 | 23384 | 4.6 | nascent polypeptide-associated complex alpha subunit isoform b [Homo sapiens] |
| U | *gi|5031931|ref|NP\_005* | 3 | 6 | 19.5% | 215 | 23384 | 4.6 | nascent polypeptide-associated complex alpha subunit isoform b [Homo sapiens] |
| U | *gi|163965366|ref|NP\_0* | 3 | 6 | 2.0% | 2078 | 205419 | 9.6 | nascent polypeptide-associated complex alpha subunit isoform a [Homo sapiens] |
| U | *gi|163965364|ref|NP\_0* | 3 | 6 | 19.5% | 215 | 23384 | 4.6 | nascent polypeptide-associated complex alpha subunit isoform b [Homo sapiens] |

| Filename XCorr DeltCN Conf% ObsM+H+ CalcM+H+ SpR ZScore Ion% # Sequence  | | | | | | | | | | | | |
| --- | --- | --- | --- | --- | --- | --- | --- | --- | --- | --- | --- | --- |
|  | CENPL\_stlcld\_tube2\_122314\_01.16950.16950.2 | 2.9674 | 0.4106 | 99.9% | 1550.7522 | 1550.8816 | 1 | 6.706 | 58.3% | 2 | K.NILFVITKPDVYK.S | 2 |
|  | CENPL\_stlcld\_tube2\_122314\_01.14920.14920.2 | 3.4178 | 0.4608 | 100.0% | 1486.3322 | 1485.6335 | 7 | 6.64 | 53.8% | 3 | K.SPASDTYIVFGEAK.I | 2 |
|  | CENPL\_stlcld\_122314\_02.10994.10994.2 | 4.7448 | 0.2915 | 100.0% | 1615.4922 | 1615.7808 | 2 | 8.643 | 75.0% | 1 | K.IEDLSQQAQLAAAEK.F | 2 |

---

|  |  |  |  |  |  |  |  |  |
| --- | --- | --- | --- | --- | --- | --- | --- | --- |
| U | *gi|34740329|ref|NP\_91* | 7 | 16 | 19.3% | 378 | 39595 | 9.0 | heterogeneous nuclear ribonucleoprotein A3 [Homo sapiens] |

| Filename XCorr DeltCN Conf% ObsM+H+ CalcM+H+ SpR ZScore Ion% # Sequence  | | | | | | | | | | | | |
| --- | --- | --- | --- | --- | --- | --- | --- | --- | --- | --- | --- | --- |
| \* | CENPL\_stlcld\_122314\_01.18372.18372.2 | 3.4055 | 0.2455 | 99.7% | 1900.0521 | 1900.1388 | 173 | 5.733 | 31.2% | 1 | R.KLFIGGLSFETTDDSLR.E | 2 |
| \* | CENPL\_stlcld\_tube2\_122314\_01.18040.18040.3 | 3.8026 | 0.2634 | 99.9% | 2443.7944 | 2442.6873 | 1 | 4.265 | 36.2% | 1 | K.LFIGGLSFETTDDSLREHFEK.W | 3 |
| \* | CENPL\_stlcld\_122314\_01.13461.13461.2 | 4.2039 | 0.346 | 100.0% | 1884.3322 | 1884.096 | 1 | 7.13 | 73.3% | 1 | K.IFVGGIKEDTEEYNLR.D | 2 |
| \* | CENPL\_stlcld\_122314\_02.11985.11985.3 | 4.1654 | 0.3977 | 100.0% | 1884.4443 | 1884.096 | 1 | 6.521 | 46.7% | 4 | K.IFVGGIKEDTEEYNLR.D | 3 |
| \* | CENPL\_stlcld\_122314\_01.16300.16300.3 | 3.7495 | 0.2594 | 99.9% | 2565.9543 | 2566.8267 | 1 | 4.96 | 33.8% | 2 | K.IFVGGIKEDTEEYNLRDYFEK.Y | 3 |
| \* | CENPL\_stlcld\_122314\_01.13119.13119.2 | 2.9913 | 0.4635 | 100.0% | 1235.3922 | 1235.3948 | 1 | 7.544 | 77.8% | 2 | K.IETIEVMEDR.Q | 2 |
|  | CENPL\_stlcld\_122314\_02.14069.14069.3 | 4.3519 | 0.2254 | 99.9% | 2283.2944 | 2282.5579 | 2 | 7.551 | 34.2% | 5 | R.GFAFVTFDDHDTVDKIVVQK.Y | 3 |

---

|  |  |  |  |  |  |  |  |  |
| --- | --- | --- | --- | --- | --- | --- | --- | --- |
| U | *gi|16905073|ref|NP\_07* | 3 | 3 | 19.3% | 269 | 31655 | 4.9 | SoxLZ/Sox6 leucine zipper binding protein [Homo sapiens] |

| Filename XCorr DeltCN Conf% ObsM+H+ CalcM+H+ SpR ZScore Ion% # Sequence  | | | | | | | | | | | | |
| --- | --- | --- | --- | --- | --- | --- | --- | --- | --- | --- | --- | --- |
| \* | CENPL\_stlcld\_122314\_02.12105.12105.2 | 3.2915 | 0.3098 | 99.9% | 1434.6721 | 1433.7063 | 1 | 6.217 | 63.6% | 1 | K.LRQDLEMVLSTK.E | 2 |
| \* | CENPL\_stlcld\_tube2\_122314\_01.21245.21245.2 | 3.991 | 0.2898 | 99.9% | 2100.5122 | 2100.379 | 1 | 6.197 | 44.1% | 1 | K.LLSTLGEFLEDHFPLPDR.S | 2 |
| \* | CENPL\_stlcld\_122314\_02.18576.18576.3 | 3.4125 | 0.3348 | 99.9% | 2581.0444 | 2581.0027 | 1 | 5.916 | 31.0% | 1 | K.NIQESSVNLITLHEMLEILINR.L | 3 |

---

|  |  |  |  |  |  |  |  |  |
| --- | --- | --- | --- | --- | --- | --- | --- | --- |
| U | *gi|4758086|ref|NP\_004* | 2 | 3 | 19.2% | 193 | 20567 | 8.6 | cysteine and glycine-rich protein 1 isoform 1 [Homo sapiens] |

| Filename XCorr DeltCN Conf% ObsM+H+ CalcM+H+ SpR ZScore Ion% # Sequence  | | | | | | | | | | | | |
| --- | --- | --- | --- | --- | --- | --- | --- | --- | --- | --- | --- | --- |
|  | CENPL\_stlcld\_122314\_02.11088.11088.3 | 3.32 | 0.2968 | 99.9% | 2160.7144 | 2160.3452 | 1 | 5.768 | 33.3% | 1 | K.GYGYGQGAGTLSTDKGESLGIK.H | 3 |
| \* | CENPL\_stlcld\_122314\_01.15276.15276.2 | 3.0454 | 0.3515 | 99.9% | 1434.4922 | 1434.551 | 4 | 5.848 | 57.1% | 2 | K.GFGFGQGAGALVHSE.- | 2 |

---

|  |  |  |  |  |  |  |  |  |
| --- | --- | --- | --- | --- | --- | --- | --- | --- |
| U | *gi|17986258|ref|NP\_06* | 3 | 11 | 19.2% | 151 | 16930 | 4.7 | myosin, light chain 6, alkali, smooth muscle and non-muscle isoform 1 [Homo sapiens] |
| U | *gi|88999583|ref|NP\_52* | 3 | 11 | 19.2% | 151 | 16961 | 4.6 | myosin, light chain 6, alkali, smooth muscle and non-muscle isoform 2 [Homo sapiens] |

| Filename XCorr DeltCN Conf% ObsM+H+ CalcM+H+ SpR ZScore Ion% # Sequence  | | | | | | | | | | | | |
| --- | --- | --- | --- | --- | --- | --- | --- | --- | --- | --- | --- | --- |
|  | CENPL\_stlcld\_tube2\_122314\_01.10203.10203.2 | 3.8666 | 0.3647 | 100.0% | 1355.3922 | 1355.5339 | 1 | 6.161 | 66.7% | 6 | R.ALGQNPTNAEVLK.V | 2 |
|  | CENPL\_stlcld\_122314\_01.21812.21812.3 | 4.3408 | 0.3221 | 100.0% | 1889.6044 | 1889.2628 | 2 | 6.023 | 43.3% | 3 | K.VLDFEHFLPMLQTVAK.N | 3 |
|  | CENPL\_stlcld\_tube2\_122314\_01.21065.21065.2 | 4.8547 | 0.4124 | 100.0% | 1889.8322 | 1889.2628 | 1 | 7.265 | 60.0% | 2 | K.VLDFEHFLPMLQTVAK.N | 2 |

---

|  |  |  |  |  |  |  |  |  |
| --- | --- | --- | --- | --- | --- | --- | --- | --- |
| U | *gi|4506607|ref|NP\_000* | 3 | 13 | 19.1% | 188 | 21634 | 11.7 | ribosomal protein L18 [Homo sapiens] |

| Filename XCorr DeltCN Conf% ObsM+H+ CalcM+H+ SpR ZScore Ion% # Sequence  | | | | | | | | | | | | |
| --- | --- | --- | --- | --- | --- | --- | --- | --- | --- | --- | --- | --- |
| \* | CENPL\_stlcld\_122314\_01.09254.09254.2 | 2.2328 | 0.2531 | 98.0% | 1141.2522 | 1141.3158 | 75 | 4.636 | 50.0% | 2 | R.TNRPPLSLSR.M | 2 |
| \* | CENPL\_stlcld\_tube2\_122314\_02.12807.12807.2 | 4.1538 | 0.3798 | 100.0% | 1347.6322 | 1346.5236 | 1 | 8.724 | 83.3% | 8 | K.TAVVVGTITDDVR.V | 2 |
| \* | CENPL\_stlcld\_tube2\_122314\_01.18450.18450.2 | 3.4962 | 0.4931 | 100.0% | 1461.8322 | 1461.6982 | 1 | 8.766 | 70.8% | 3 | K.ILTFDQLALDSPK.G | 2 |

---

|  |  |  |  |  |  |  |  |  |
| --- | --- | --- | --- | --- | --- | --- | --- | --- |
| U | *gi|226246671|ref|NP\_0* | 2 | 4 | 19.0% | 142 | 16006 | 9.3 | ribosomal protein S20 isoform 1 [Homo sapiens] |
| U | *gi|4506697|ref|NP\_001* | 2 | 4 | 22.7% | 119 | 13373 | 9.9 | ribosomal protein S20 isoform 2 [Homo sapiens] |

| Filename XCorr DeltCN Conf% ObsM+H+ CalcM+H+ SpR ZScore Ion% # Sequence  | | | | | | | | | | | | |
| --- | --- | --- | --- | --- | --- | --- | --- | --- | --- | --- | --- | --- |
|  | CENPL\_stlcld\_tube2\_122314\_01.08835.08835.3 | 3.7242 | 0.395 | 100.0% | 1650.1144 | 1649.8442 | 21 | 6.342 | 37.5% | 2 | K.DTGKTPVEPEVAIHR.I | 3 |
|  | CENPL\_stlcld\_122314\_01.12797.12797.2 | 3.2907 | 0.3994 | 100.0% | 1351.4722 | 1351.5858 | 3 | 5.543 | 63.6% | 2 | R.LIDLHSPSEIVK.Q | 2 |

---

|  |  |  |  |  |  |  |  |  |
| --- | --- | --- | --- | --- | --- | --- | --- | --- |
| U | *gi|4506743|ref|NP\_001* | 4 | 19 | 18.8% | 208 | 24205 | 10.3 | ribosomal protein S8 [Homo sapiens] |

| Filename XCorr DeltCN Conf% ObsM+H+ CalcM+H+ SpR ZScore Ion% # Sequence  | | | | | | | | | | | | |
| --- | --- | --- | --- | --- | --- | --- | --- | --- | --- | --- | --- | --- |
| \* | CENPL\_stlcld\_tube2\_122314\_02.14237.14237.3 | 3.7654 | 0.2878 | 99.9% | 1719.4443 | 1719.9353 | 59 | 5.904 | 35.7% | 2 | R.IIDVVYNASNNELVR.T | 3 |
| \* | CENPL\_stlcld\_122314\_02.12926.12926.2 | 4.8737 | 0.4557 | 100.0% | 1719.6721 | 1719.9353 | 1 | 8.467 | 71.4% | 8 | R.IIDVVYNASNNELVR.T | 2 |
|  | CENPL\_stlcld\_122314\_01.12170.12170.2 | 2.8768 | 0.2473 | 99.5% | 1315.4321 | 1315.4631 | 4 | 5.281 | 65.0% | 1 | K.LTPEEEEILNK.K | 2 |
| \* | CENPL\_stlcld\_tube2\_122314\_01.14200.14200.2 | 4.1945 | 0.3652 | 100.0% | 1508.4521 | 1507.6836 | 1 | 7.319 | 75.0% | 8 | K.ISSLLEEQFQQGK.L | 2 |

---

|  |  |  |  |  |  |  |  |  |
| --- | --- | --- | --- | --- | --- | --- | --- | --- |
| U | *gi|4506741|ref|NP\_001* | 3 | 4 | 18.6% | 194 | 22127 | 10.1 | ribosomal protein S7 [Homo sapiens] |

| Filename XCorr DeltCN Conf% ObsM+H+ CalcM+H+ SpR ZScore Ion% # Sequence  | | | | | | | | | | | | |
| --- | --- | --- | --- | --- | --- | --- | --- | --- | --- | --- | --- | --- |
| \* | CENPL\_stlcld\_122314\_01.18824.18824.2 | 3.9184 | 0.3502 | 100.0% | 1466.6322 | 1466.8937 | 1 | 7.221 | 66.7% | 1 | R.KAIIIFVPVPQLK.S | 2 |
| \* | CENPL\_stlcld\_tube2\_122314\_01.20669.20669.2 | 2.9419 | 0.3896 | 99.9% | 2367.8323 | 2368.7324 | 1 | 6.649 | 35.7% | 2 | R.TLTAVHDAILEDLVFPSEIVGK.R | 2 |
| \* | CENPL\_stlcld\_122314\_01.20849.20849.3 | 4.5666 | 0.4652 | 100.0% | 2522.8145 | 2524.92 | 1 | 7.015 | 35.2% | 1 | R.TLTAVHDAILEDLVFPSEIVGKR.I | 3 |

---

|  |  |  |  |  |  |  |  |  |
| --- | --- | --- | --- | --- | --- | --- | --- | --- |
| U | *gi|4502101|ref|NP\_000* | 4 | 5 | 18.5% | 346 | 38714 | 7.0 | annexin I [Homo sapiens] |

| Filename XCorr DeltCN Conf% ObsM+H+ CalcM+H+ SpR ZScore Ion% # Sequence  | | | | | | | | | | | | |
| --- | --- | --- | --- | --- | --- | --- | --- | --- | --- | --- | --- | --- |
| \* | CENPL\_stlcld\_tube2\_122314\_01.14201.14201.3 | 2.8768 | 0.2458 | 96.3% | 2358.0544 | 2357.5847 | 28 | 4.662 | 26.1% | 1 | K.GGPGSAVSPYPTFNPSSDVAALHK.A | 3 |
| \* | CENPL\_stlcld\_tube2\_122314\_01.19557.19557.2 | 2.3699 | 0.3569 | 99.2% | 1545.3322 | 1544.7887 | 16 | 5.552 | 42.3% | 1 | K.GVDEATIIDILTKR.N | 2 |
| \* | CENPL\_stlcld\_122314\_01.22232.22232.2 | 4.0653 | 0.2579 | 99.9% | 1607.6721 | 1606.9463 | 1 | 6.537 | 71.4% | 2 | K.ALTGHLEEVVLALLK.T | 2 |
| \* | CENPL\_stlcld\_tube2\_122314\_01.11793.11793.2 | 3.241 | 0.3869 | 100.0% | 1263.7322 | 1263.3495 | 1 | 6.254 | 80.0% | 1 | K.TPAQFDADELR.A | 2 |

---

|  |  |  |  |  |  |  |  |  |
| --- | --- | --- | --- | --- | --- | --- | --- | --- |
| U | *gi|156071462|ref|NP\_0* | 6 | 7 | 18.5% | 298 | 32866 | 9.7 | solute carrier family 25, member A6 [Homo sapiens] |

| Filename XCorr DeltCN Conf% ObsM+H+ CalcM+H+ SpR ZScore Ion% # Sequence  | | | | | | | | | | | | |
| --- | --- | --- | --- | --- | --- | --- | --- | --- | --- | --- | --- | --- |
| \* | CENPL\_stlcld\_122314\_02.15510.15510.2 | 4.3413 | 0.4691 | 100.0% | 1235.0122 | 1234.438 | 1 | 9.185 | 79.2% | 1 | K.DFLAGGIAAAISK.T | 2 |
|  | CENPL\_stlcld\_122314\_01.09808.09808.2 | 2.5277 | 0.2161 | 98.4% | 1138.4722 | 1137.3677 | 4 | 4.515 | 66.7% | 2 | K.LLLQVQHASK.Q | 22 |
|  | CENPL\_stlcld\_tube2\_122314\_01.16517.16517.2 | 2.1212 | 0.2444 | 95.9% | 1460.0521 | 1460.719 | 51 | 5.186 | 40.9% | 1 | R.IPKEQGVLSFWR.G | 22 |
|  | CENPL\_stlcld\_tube2\_122314\_01.18507.18507.2 | 2.2937 | 0.1945 | 97.5% | 1122.0922 | 1122.2688 | 60 | 5.048 | 56.2% | 1 | K.EQGVLSFWR.G | 22 |
|  | CENPL\_stlcld\_122314\_01.10493.10493.2 | 2.2972 | 0.1833 | 97.7% | 857.47217 | 857.00055 | 3 | 4.089 | 78.6% | 1 | R.GNLANVIR.Y | 22 |
|  | CENPL\_stlcld\_122314\_01.20482.20482.2 | 3.2415 | 0.386 | 100.0% | 1447.4122 | 1447.6763 | 1 | 6.952 | 77.3% | 1 | R.YFPTQALNFAFK.D | 22 |

Similarities:
gi|156071459|ref|NP\_0(5:1)  

---

|  |  |  |  |  |  |  |  |  |
| --- | --- | --- | --- | --- | --- | --- | --- | --- |
| U | *gi|34147630|ref|NP\_00* | 6 | 9 | 18.0% | 455 | 49875 | 7.6 | Tu translation elongation factor, mitochondrial precursor [Homo sapiens] |

| Filename XCorr DeltCN Conf% ObsM+H+ CalcM+H+ SpR ZScore Ion% # Sequence  | | | | | | | | | | | | |
| --- | --- | --- | --- | --- | --- | --- | --- | --- | --- | --- | --- | --- |
| \* | CENPL\_stlcld\_122314\_02.10850.10850.3 | 3.5649 | 0.4578 | 100.0% | 1676.4243 | 1674.854 | 1 | 6.895 | 43.3% | 3 | R.GITINAAHVEYSTAAR.H | 3 |
| \* | CENPL\_stlcld\_tube2\_122314\_01.17703.17703.2 | 2.2637 | 0.3782 | 99.1% | 1543.1721 | 1543.8033 | 9 | 5.817 | 53.8% | 1 | K.LLDAVDTYIPVPAR.D | 2 |
| \* | CENPL\_stlcld\_122314\_01.20823.20823.2 | 3.5249 | 0.3886 | 100.0% | 2130.4321 | 2130.4917 | 1 | 6.584 | 44.4% | 1 | R.DLEKPFLLPVEAVYSVPGR.G | 2 |
| \* | CENPL\_stlcld\_tube2\_122314\_01.09203.09203.2 | 2.5682 | 0.29 | 99.5% | 1033.5322 | 1033.1698 | 1 | 6.414 | 83.3% | 2 | R.GTVVTGTLER.G | 2 |
| \* | CENPL\_stlcld\_tube2\_122314\_01.13180.13180.2 | 2.1597 | 0.2414 | 96.9% | 1264.4922 | 1262.5096 | 1 | 4.687 | 70.0% | 1 | R.TVVTGIEMFHK.S | 2 |
| \* | CENPL\_stlcld\_tube2\_122314\_01.12621.12621.2 | 2.8086 | 0.1235 | 97.2% | 1186.3322 | 1186.3103 | 55 | 4.253 | 63.6% | 1 | R.AEAGDNLGALVR.G | 2 |

---

|  |  |  |  |  |  |  |  |  |
| --- | --- | --- | --- | --- | --- | --- | --- | --- |
| U | *gi|222352151|ref|NP\_0* | 3 | 11 | 18.0% | 356 | 37498 | 7.1 | poly(rC) binding protein 1 [Homo sapiens] |

| Filename XCorr DeltCN Conf% ObsM+H+ CalcM+H+ SpR ZScore Ion% # Sequence  | | | | | | | | | | | | |
| --- | --- | --- | --- | --- | --- | --- | --- | --- | --- | --- | --- | --- |
| \* | CENPL\_stlcld\_122314\_02.13575.13575.2 | 2.7734 | 0.4023 | 99.9% | 1389.5322 | 1389.6781 | 4 | 6.775 | 58.3% | 4 | R.IITLTGPTNAIFK.A | 2 |
| \* | CENPL\_stlcld\_tube2\_122314\_01.20638.20638.3 | 4.931 | 0.3326 | 100.0% | 3381.4443 | 3380.8562 | 1 | 5.854 | 35.8% | 3 | K.AFAMIIDKLEEDINSSMTNSTAASRPPVTLR.L | 3 |
|  | CENPL\_stlcld\_tube2\_122314\_02.12410.12410.2 | 5.6809 | 0.5742 | 100.0% | 2090.4521 | 2091.2573 | 1 | 10.311 | 60.5% | 4 | R.ESTGAQVQVAGDMLPNSTER.A | 22 |

Similarities:
gi|14141166|ref|NP\_11(1:2)  

---

|  |  |  |  |  |  |  |  |  |
| --- | --- | --- | --- | --- | --- | --- | --- | --- |
| U | *gi|4758138|ref|NP\_004* | 10 | 32 | 17.8% | 614 | 69148 | 8.9 | DEAD (Asp-Glu-Ala-Asp) box polypeptide 5 [Homo sapiens] |

| Filename XCorr DeltCN Conf% ObsM+H+ CalcM+H+ SpR ZScore Ion% # Sequence  | | | | | | | | | | | | |
| --- | --- | --- | --- | --- | --- | --- | --- | --- | --- | --- | --- | --- |
| \* | CENPL\_stlcld\_122314\_01.16715.16715.2 | 2.755 | 0.18 | 98.4% | 1419.7722 | 1419.6201 | 1 | 4.82 | 70.0% | 1 | K.WNLDELPKFEK.N | 2 |
| \* | CENPL\_stlcld\_tube2\_122314\_01.09753.09753.3 | 2.658 | 0.3516 | 99.9% | 1389.9543 | 1390.4978 | 22 | 6.109 | 42.5% | 1 | K.NFYQEHPDLAR.R | 3 |
| \* | CENPL\_stlcld\_122314\_01.14126.14126.2 | 3.1736 | 0.4867 | 100.0% | 1296.4521 | 1296.4198 | 1 | 8.189 | 75.0% | 5 | R.TTYLVLDEADR.M | 2 |
|  | CENPL\_stlcld\_122314\_01.17030.17030.2 | 4.1012 | 0.4447 | 100.0% | 1338.4521 | 1337.5946 | 1 | 7.711 | 85.0% | 8 | R.MLDMGFEPQIR.K | 222 |
|  | CENPL\_stlcld\_tube2\_122314\_01.17921.17921.2 | 3.0835 | 0.3183 | 99.9% | 1349.7122 | 1349.5902 | 7 | 6.614 | 60.0% | 2 | R.QTLMWSATWPK.E | 22 |
| \* | CENPL\_stlcld\_tube2\_122314\_01.10487.10487.2 | 3.4442 | 0.1904 | 99.7% | 1481.8121 | 1481.7194 | 3 | 4.904 | 72.7% | 1 | R.LMEEIMSEKENK.T | 2 |
|  | CENPL\_stlcld\_122314\_01.13792.13792.2 | 4.2232 | 0.289 | 100.0% | 1228.4521 | 1227.4465 | 2 | 7.323 | 81.8% | 8 | K.APILIATDVASR.G | 22 |
|  | CENPL\_stlcld\_tube2\_122314\_01.09857.09857.2 | 2.4344 | 0.3394 | 99.9% | 875.0122 | 874.96643 | 1 | 6.575 | 78.6% | 1 | R.GLDVEDVK.F | 22 |
| \* | CENPL\_stlcld\_122314\_01.16223.16223.2 | 3.398 | 0.2648 | 99.9% | 1575.6921 | 1575.7612 | 1 | 5.85 | 61.5% | 3 | K.TGTAYTFFTPNNIK.Q | 2 |
| \* | CENPL\_stlcld\_122314\_01.13334.13334.2 | 2.7419 | 0.2276 | 99.6% | 985.9322 | 986.1564 | 1 | 6.295 | 85.7% | 2 | K.LLQLVEDR.G | 2 |

Similarities:
gi|87196351|ref|NP\_00(1:9)  
gi|148613856|ref|NP\_0(4:6)  

---

|  |  |  |  |  |  |  |  |  |
| --- | --- | --- | --- | --- | --- | --- | --- | --- |
| U | *gi|4885375|ref|NP\_005* | 6 | 22 | 17.8% | 213 | 21365 | 10.9 | histone cluster 1, H1c [Homo sapiens] |
| U | *gi|4885379|ref|NP\_005* | 6 | 22 | 17.4% | 219 | 21865 | 11.0 | histone cluster 1, H1e [Homo sapiens] |
| U | *gi|4885377|ref|NP\_005* | 6 | 22 | 17.2% | 221 | 22350 | 11.0 | histone cluster 1, H1d [Homo sapiens] |

| Filename XCorr DeltCN Conf% ObsM+H+ CalcM+H+ SpR ZScore Ion% # Sequence  | | | | | | | | | | | | |
| --- | --- | --- | --- | --- | --- | --- | --- | --- | --- | --- | --- | --- |
|  | CENPL\_stlcld\_122314\_01.11258.11258.3 | 3.5635 | 0.3591 | 100.0% | 1327.7043 | 1327.5638 | 1 | 6.192 | 50.0% | 2 | R.KASGPPVSELITK.A | 3 |
|  | CENPL\_stlcld\_tube2\_122314\_01.10779.10779.2 | 4.2988 | 0.4393 | 100.0% | 1328.0721 | 1327.5638 | 1 | 7.325 | 70.8% | 7 | R.KASGPPVSELITK.A | 2 |
|  | CENPL\_stlcld\_tube2\_122314\_01.12681.12681.2 | 2.3266 | 0.3381 | 99.1% | 1199.5322 | 1199.3898 | 6 | 6.115 | 63.6% | 2 | K.ASGPPVSELITK.A | 2 |
|  | CENPL\_stlcld\_tube2\_122314\_01.09538.09538.2 | 3.2451 | 0.2072 | 99.8% | 973.9122 | 974.1887 | 3 | 6.231 | 77.8% | 4 | R.SGVSLAALKK.A | 2 |
|  | CENPL\_stlcld\_122314\_01.09930.09930.2 | 3.2607 | 0.4804 | 100.0% | 1108.4722 | 1108.2365 | 1 | 8.256 | 70.0% | 5 | K.ALAAAGYDVEK.N | 2 |
|  | CENPL\_stlcld\_122314\_01.08903.08903.2 | 4.2837 | 0.5256 | 100.0% | 1579.4321 | 1579.7098 | 1 | 9.562 | 78.6% | 2 | K.ALAAAGYDVEKNNSR.I | 2 |

---

|  |  |  |  |  |  |  |  |  |
| --- | --- | --- | --- | --- | --- | --- | --- | --- |
| U | *gi|17158044|ref|NP\_00* | 4 | 10 | 17.7% | 249 | 28681 | 10.8 | ribosomal protein S6 [Homo sapiens] |

| Filename XCorr DeltCN Conf% ObsM+H+ CalcM+H+ SpR ZScore Ion% # Sequence  | | | | | | | | | | | | |
| --- | --- | --- | --- | --- | --- | --- | --- | --- | --- | --- | --- | --- |
| \* | CENPL\_stlcld\_122314\_01.09508.09508.2 | 2.371 | 0.2569 | 99.0% | 988.7322 | 989.0703 | 4 | 4.699 | 78.6% | 2 | K.LIEVDDER.K | 2 |
| \* | CENPL\_stlcld\_122314\_02.13548.13548.2 | 4.1061 | 0.498 | 100.0% | 1621.4521 | 1621.8022 | 1 | 8.929 | 67.9% | 5 | R.MATEVAADALGEEWK.G | 2 |
| \* | CENPL\_stlcld\_122314\_02.14519.14519.3 | 3.2914 | 0.3547 | 99.9% | 2196.9243 | 2196.4827 | 1 | 6.043 | 34.2% | 2 | R.MATEVAADALGEEWKGYVVR.I | 3 |
| \* | CENPL\_stlcld\_tube2\_122314\_01.09862.09862.3 | 3.6008 | 0.397 | 100.0% | 1728.0543 | 1727.9554 | 1 | 7.41 | 41.7% | 1 | K.KGEKDIPGLTDTTVPR.R | 3 |

---

|  |  |  |  |  |  |  |  |  |
| --- | --- | --- | --- | --- | --- | --- | --- | --- |
| U | *gi|208431833|ref|NP\_0* | 10 | 24 | 17.6% | 660 | 72391 | 9.3 | poly A binding protein, cytoplasmic 4 isoform 1 [Homo sapiens] |
| U | *gi|4504715|ref|NP\_003* | 10 | 24 | 18.0% | 644 | 70783 | 9.3 | poly A binding protein, cytoplasmic 4 isoform 2 [Homo sapiens] |
| U | *gi|208431836|ref|NP\_0* | 10 | 24 | 18.4% | 631 | 69579 | 9.5 | poly A binding protein, cytoplasmic 4 isoform 3 [Homo sapiens] |

| Filename XCorr DeltCN Conf% ObsM+H+ CalcM+H+ SpR ZScore Ion% # Sequence  | | | | | | | | | | | | |
| --- | --- | --- | --- | --- | --- | --- | --- | --- | --- | --- | --- | --- |
|  | CENPL\_stlcld\_tube2\_122314\_01.14711.14711.2 | 2.6363 | 0.4766 | 100.0% | 1144.3922 | 1144.3591 | 3 | 7.018 | 70.0% | 1 | K.FSPAGPVLSIR.V | 2 |
|  | CENPL\_stlcld\_tube2\_122314\_02.12795.12795.3 | 3.4609 | 0.2338 | 99.4% | 2086.3145 | 2086.2712 | 21 | 4.82 | 33.8% | 1 | R.RSLGYAYVNFQQPADAER.A | 33 |
|  | CENPL\_stlcld\_122314\_01.14967.14967.2 | 5.0053 | 0.4787 | 100.0% | 1930.5322 | 1930.0837 | 1 | 8.555 | 53.1% | 7 | R.SLGYAYVNFQQPADAER.A | 22 |
|  | CENPL\_stlcld\_tube2\_122314\_02.14073.14073.2 | 2.8218 | 0.525 | 100.0% | 1267.4521 | 1267.4828 | 1 | 8.158 | 75.0% | 2 | R.ALDTMNFDVIK.G | 22 |
|  | CENPL\_stlcld\_122314\_02.11836.11836.3 | 3.8824 | 0.2385 | 99.9% | 2155.7043 | 2155.3716 | 1 | 6.952 | 44.4% | 1 | K.GYAFVHFETQEAADKAIEK.M | 3 |
|  | CENPL\_stlcld\_122314\_01.12149.12149.2 | 2.0554 | 0.246 | 97.1% | 1064.5122 | 1064.263 | 6 | 4.937 | 68.8% | 1 | K.MNGMLLNDR.K | 22 |
|  | CENPL\_stlcld\_tube2\_122314\_01.10307.10307.2 | 2.8627 | 0.3205 | 99.9% | 1213.6921 | 1213.4191 | 37 | 6.612 | 61.1% | 3 | K.AKEFTNVYIK.N | 22 |
|  | CENPL\_stlcld\_122314\_01.13145.13145.2 | 3.1376 | 0.3131 | 99.9% | 1516.4321 | 1515.8394 | 1 | 5.075 | 69.2% | 2 | R.IVGSKPLYVALAQR.K | 2 |
|  | CENPL\_stlcld\_tube2\_122314\_01.21639.21639.2 | 5.0786 | 0.5494 | 100.0% | 2741.7522 | 2742.175 | 1 | 8.398 | 45.7% | 3 | K.ITGMLLEIDNSELLHMLESPESLR.S | 22 |
|  | CENPL\_stlcld\_122314\_01.22454.22454.3 | 5.3189 | 0.3378 | 100.0% | 2741.8145 | 2742.175 | 13 | 6.113 | 30.4% | 3 | K.ITGMLLEIDNSELLHMLESPESLR.S | 33 |

Similarities:
gi|46367787|ref|NP\_00(7:3)  

---

|  |  |  |  |  |  |  |  |  |
| --- | --- | --- | --- | --- | --- | --- | --- | --- |
| U | *gi|193794814|ref|NP\_0* | 4 | 8 | 17.6% | 364 | 39420 | 8.1 | fructose-bisphosphate aldolase A [Homo sapiens] |
| U | *gi|4557305|ref|NP\_000* | 4 | 8 | 17.6% | 364 | 39420 | 8.1 | fructose-bisphosphate aldolase A [Homo sapiens] |
| U | *gi|34577112|ref|NP\_90* | 4 | 8 | 17.6% | 364 | 39420 | 8.1 | fructose-bisphosphate aldolase A [Homo sapiens] |

| Filename XCorr DeltCN Conf% ObsM+H+ CalcM+H+ SpR ZScore Ion% # Sequence  | | | | | | | | | | | | |
| --- | --- | --- | --- | --- | --- | --- | --- | --- | --- | --- | --- | --- |
|  | CENPL\_stlcld\_122314\_02.09610.09610.2 | 2.8584 | 0.3853 | 99.9% | 1333.6122 | 1333.4814 | 1 | 6.925 | 76.9% | 4 | K.GILAADESTGSIAK.R | 2 |
|  | CENPL\_stlcld\_122314\_01.08114.08114.2 | 2.5425 | 0.1535 | 95.1% | 1647.4922 | 1647.7422 | 7 | 4.324 | 61.5% | 1 | R.LQSIGTENTEENRR.F | 2 |
|  | CENPL\_stlcld\_122314\_01.18616.18616.3 | 3.8191 | 0.2154 | 99.5% | 2108.1843 | 2108.4204 | 4 | 5.957 | 32.9% | 2 | K.IGEHTPSALAIMENANVLAR.Y | 3 |
|  | CENPL\_stlcld\_122314\_02.12266.12266.3 | 2.4505 | 0.318 | 98.7% | 1833.4744 | 1833.1156 | 9 | 4.738 | 36.7% | 1 | K.FSHEEIAMATVTALRR.T | 3 |

---

|  |  |  |  |  |  |  |  |  |
| --- | --- | --- | --- | --- | --- | --- | --- | --- |
| U | *gi|14141161|ref|NP\_00* | 8 | 31 | 17.5% | 806 | 88980 | 5.8 | heterogeneous nuclear ribonucleoprotein U isoform b [Homo sapiens] |
| U | *gi|74136883|ref|NP\_11* | 8 | 31 | 17.1% | 825 | 90585 | 6.0 | heterogeneous nuclear ribonucleoprotein U isoform a [Homo sapiens] |

| Filename XCorr DeltCN Conf% ObsM+H+ CalcM+H+ SpR ZScore Ion% # Sequence  | | | | | | | | | | | | |
| --- | --- | --- | --- | --- | --- | --- | --- | --- | --- | --- | --- | --- |
|  | CENPL\_stlcld\_122314\_01.13278.13278.3 | 5.0041 | 0.5318 | 100.0% | 3127.0444 | 3128.311 | 1 | 9.101 | 31.5% | 1 | R.LQAALDDEEAGGRPAMEPGNGSLDLGGDSAGR.S | 3 |
|  | CENPL\_stlcld\_122314\_01.17612.17612.2 | 2.9288 | 0.2875 | 99.5% | 1716.4722 | 1715.9469 | 34 | 5.532 | 35.3% | 1 | K.SSGPTSLFAVTVAPPGAR.Q | 2 |
|  | CENPL\_stlcld\_tube2\_122314\_01.13506.13506.2 | 3.9191 | 0.4128 | 100.0% | 1698.4722 | 1698.8291 | 1 | 7.293 | 75.0% | 3 | R.GYFEYIEENKYSR.A | 2 |
|  | CENPL\_stlcld\_tube2\_122314\_01.21628.21628.2 | 2.3934 | 0.2078 | 95.9% | 2304.8123 | 2303.5977 | 1 | 4.233 | 37.5% | 1 | R.DRLSASSLTMESFAFLWAGGR.A | 2 |
|  | CENPL\_stlcld\_122314\_01.20384.20384.3 | 4.1125 | 0.313 | 99.9% | 2726.6042 | 2726.0576 | 1 | 5.772 | 28.6% | 1 | K.EKPYFPIPEEYTFIQNVPLEDR.V | 3 |
|  | CENPL\_stlcld\_122314\_01.12197.12197.3 | 4.2874 | 0.4375 | 100.0% | 2188.2244 | 2188.4631 | 1 | 7.428 | 39.5% | 2 | K.HAAENPGKYNILGTNTIMDK.M | 3 |
|  | CENPL\_stlcld\_tube2\_122314\_01.15388.15388.2 | 2.2637 | 0.228 | 96.9% | 1382.8922 | 1383.6025 | 2 | 4.405 | 59.1% | 1 | K.YNILGTNTIMDK.M | 2 |
|  | CENPL\_stlcld\_122314\_02.12392.12392.2 | 5.0606 | 0.389 | 100.0% | 1648.4521 | 1648.816 | 1 | 8.262 | 82.1% | 21 | R.NFILDQTNVSAAAQR.R | 2 |

---

|  |  |  |  |  |  |  |  |  |
| --- | --- | --- | --- | --- | --- | --- | --- | --- |
| U | *gi|72534660|ref|NP\_00* | 4 | 6 | 17.2% | 238 | 27367 | 11.8 | splicing factor, arginine/serine-rich 7 [Homo sapiens] |

| Filename XCorr DeltCN Conf% ObsM+H+ CalcM+H+ SpR ZScore Ion% # Sequence  | | | | | | | | | | | | |
| --- | --- | --- | --- | --- | --- | --- | --- | --- | --- | --- | --- | --- |
| \* | CENPL\_stlcld\_tube2\_122314\_01.14108.14108.2 | 2.129 | 0.2914 | 98.4% | 1074.5922 | 1074.2242 | 122 | 5.331 | 62.5% | 2 | R.AFSYYGPLR.T | 2 |
|  | CENPL\_stlcld\_122314\_01.19713.19713.2 | 2.6902 | 0.2983 | 99.2% | 1622.4321 | 1622.7771 | 3 | 5.074 | 50.0% | 1 | R.NPPGFAFVEFEDPR.D | 22 |
| \* | CENPL\_stlcld\_tube2\_122314\_01.18254.18254.2 | 2.8371 | 0.1592 | 97.4% | 2379.912 | 2379.5474 | 167 | 4.232 | 30.0% | 1 | R.NPPGFAFVEFEDPRDAEDAVR.G | 2 |
| \* | CENPL\_stlcld\_122314\_01.10997.10997.2 | 3.2057 | 0.3721 | 100.0% | 1245.2522 | 1245.4827 | 1 | 6.186 | 80.0% | 2 | R.VRVELSTGMPR.R | 2 |

Similarities:
gi|4506901|ref|NP\_003(1:3)  

---

|  |  |  |  |  |  |  |  |  |
| --- | --- | --- | --- | --- | --- | --- | --- | --- |
| U | *gi|153792590|ref|NP\_0* | 13 | 37 | 17.1% | 854 | 98161 | 5.2 | heat shock 90kDa protein 1, alpha isoform 1 [Homo sapiens] |
| U | *gi|154146191|ref|NP\_0* | 13 | 37 | 19.9% | 732 | 84660 | 5.0 | heat shock 90kDa protein 1, alpha isoform 2 [Homo sapiens] |

| Filename XCorr DeltCN Conf% ObsM+H+ CalcM+H+ SpR ZScore Ion% # Sequence  | | | | | | | | | | | | |
| --- | --- | --- | --- | --- | --- | --- | --- | --- | --- | --- | --- | --- |
|  | CENPL\_stlcld\_tube2\_122314\_01.15220.15220.2 | 3.8553 | 0.3359 | 100.0% | 1244.5521 | 1243.4459 | 1 | 7.087 | 72.7% | 5 | K.ADLINNLGTIAK.S | 22 |
|  | CENPL\_stlcld\_tube2\_122314\_02.13241.13241.3 | 3.0099 | 0.3033 | 99.9% | 2256.3843 | 2257.294 | 1 | 4.591 | 32.9% | 1 | K.HNDDEQYAWESSAGGSFTVR.T | 33 |
|  | CENPL\_stlcld\_tube2\_122314\_02.12039.12039.3 | 4.507 | 0.4049 | 100.0% | 2017.6743 | 2016.2584 | 1 | 7.479 | 48.3% | 6 | K.VILHLKEDQTEYLEER.R | 33 |
|  | CENPL\_stlcld\_122314\_02.10739.10739.3 | 3.6436 | 0.2957 | 99.9% | 2172.2644 | 2172.446 | 1 | 6.397 | 34.4% | 1 | K.VILHLKEDQTEYLEERR.I | 33 |
|  | CENPL\_stlcld\_122314\_01.20092.20092.2 | 3.1988 | 0.3616 | 99.9% | 1779.8322 | 1780.0764 | 1 | 6.174 | 50.0% | 1 | K.HSQFIGYPITLFVEK.E | 2 |
|  | CENPL\_stlcld\_122314\_01.09152.09152.2 | 3.4786 | 0.3417 | 100.0% | 1152.4521 | 1152.2462 | 1 | 5.806 | 87.5% | 5 | K.YIDQEELNK.T | 22 |
|  | CENPL\_stlcld\_tube2\_122314\_01.13653.13653.2 | 4.5186 | 0.4666 | 100.0% | 1528.1322 | 1528.6616 | 1 | 8.221 | 70.8% | 3 | K.SLTNDWEDHLAVK.H | 22 |
|  | CENPL\_stlcld\_122314\_01.13418.13418.2 | 3.3434 | 0.4267 | 100.0% | 1349.7322 | 1349.4886 | 3 | 7.174 | 65.0% | 10 | K.HFSVEGQLEFR.A | 22 |
|  | CENPL\_stlcld\_tube2\_122314\_02.13062.13062.3 | 2.6349 | 0.2058 | 95.2% | 1349.9043 | 1349.4886 | 279 | 4.978 | 40.0% | 1 | K.HFSVEGQLEFR.A | 33 |
|  | CENPL\_stlcld\_122314\_01.15690.15690.2 | 2.4646 | 0.1849 | 97.7% | 1265.5122 | 1265.4142 | 5 | 5.147 | 61.1% | 1 | R.RAPFDLFENR.K | 2 |
|  | CENPL\_stlcld\_122314\_02.11505.11505.2 | 2.3882 | 0.4463 | 99.9% | 1550.3121 | 1551.7083 | 2 | 7.164 | 53.8% | 1 | R.YYTSASGDEMVSLK.D | 2 |
|  | CENPL\_stlcld\_122314\_01.09792.09792.2 | 1.9032 | 0.3089 | 97.3% | 1225.1322 | 1225.3867 | 79 | 5.568 | 50.0% | 1 | K.HIYYITGETK.D | 2 |
|  | CENPL\_stlcld\_122314\_01.13692.13692.3 | 3.0639 | 0.3204 | 99.9% | 1789.4644 | 1788.0134 | 1 | 6.017 | 41.1% | 1 | K.HLEINPDHSIIETLR.Q | 3 |

Similarities:
gi|20149594|ref|NP\_03(8:5)  

---

|  |  |  |  |  |  |  |  |  |
| --- | --- | --- | --- | --- | --- | --- | --- | --- |
| U | *gi|59709758|ref|NP\_00* | 3 | 3 | 17.0% | 288 | 33165 | 6.3 | centromere protein P [Homo sapiens] |

| Filename XCorr DeltCN Conf% ObsM+H+ CalcM+H+ SpR ZScore Ion% # Sequence  | | | | | | | | | | | | |
| --- | --- | --- | --- | --- | --- | --- | --- | --- | --- | --- | --- | --- |
| \* | CENPL\_stlcld\_122314\_01.22830.22830.2 | 2.6124 | 0.3574 | 99.5% | 2442.3123 | 2443.7625 | 1 | 5.741 | 35.7% | 1 | K.NQLGHLESELSFLSTLTGINIR.N | 2 |
| \* | CENPL\_stlcld\_122314\_01.13936.13936.2 | 2.3922 | 0.2565 | 98.5% | 1106.3322 | 1105.2792 | 3 | 5.062 | 61.1% | 1 | R.AIETAPLSFR.T | 2 |
| \* | CENPL\_stlcld\_122314\_02.01995.01995.2 | 4.7907 | 0.4442 | 100.0% | 1741.7922 | 1741.1216 | 1 | 9.221 | 65.6% | 1 | R.TLVGLLGIEAALESLIK.S | 2 |

---

|  |  |  |  |  |  |  |  |  |
| --- | --- | --- | --- | --- | --- | --- | --- | --- |
| U | *gi|5454064|ref|NP\_006* | 8 | 22 | 16.7% | 669 | 69492 | 9.7 | RNA binding motif protein 14 [Homo sapiens] |

| Filename XCorr DeltCN Conf% ObsM+H+ CalcM+H+ SpR ZScore Ion% # Sequence  | | | | | | | | | | | | |
| --- | --- | --- | --- | --- | --- | --- | --- | --- | --- | --- | --- | --- |
| \* | CENPL\_stlcld\_tube2\_122314\_01.08018.08018.3 | 3.433 | 0.3657 | 100.0% | 1557.3243 | 1556.7677 | 1 | 6.742 | 46.2% | 2 | R.AIEALHGHELRPGR.A | 3 |
| \* | CENPL\_stlcld\_tube2\_122314\_01.12542.12542.3 | 3.5248 | 0.4135 | 100.0% | 1898.5743 | 1898.2767 | 5 | 6.715 | 33.3% | 2 | R.ALVVEMSRPRPLNTWK.I | 3 |
| \* | CENPL\_stlcld\_tube2\_122314\_01.12268.12268.2 | 3.2539 | 0.3297 | 99.9% | 1609.4122 | 1609.8223 | 1 | 7.478 | 67.9% | 2 | R.ASYVAPLTAQPATYR.A | 2 |
| \* | CENPL\_stlcld\_tube2\_122314\_01.08627.08627.2 | 2.9174 | 0.4746 | 100.0% | 1324.6721 | 1325.482 | 1 | 8.706 | 81.8% | 3 | R.TQPMTAQAASYR.A | 2 |
| \* | CENPL\_stlcld\_122314\_01.11829.11829.2 | 2.3212 | 0.2407 | 97.7% | 1246.3722 | 1246.4087 | 1 | 5.718 | 68.2% | 3 | R.AQPSVSLGAPYR.G | 2 |
| \* | CENPL\_stlcld\_tube2\_122314\_01.10272.10272.3 | 4.3733 | 0.3266 | 100.0% | 2467.1943 | 2466.6292 | 1 | 6.096 | 29.3% | 7 | R.TQSSASLAASYAAQQHPQAAASYR.G | 23 |
| \* | CENPL\_stlcld\_tube2\_122314\_01.12134.12134.2 | 2.2628 | 0.2194 | 97.8% | 1068.0521 | 1067.1869 | 2 | 5.152 | 75.0% | 1 | R.LSESQLSFR.R | 2 |
| \* | CENPL\_stlcld\_tube2\_122314\_01.11590.11590.2 | 2.854 | 0.372 | 99.9% | 1239.3522 | 1238.2988 | 1 | 6.846 | 72.2% | 2 | R.YSGSYNDYLR.A | 2 |

---

|  |  |  |  |  |  |  |  |  |
| --- | --- | --- | --- | --- | --- | --- | --- | --- |
| U | *gi|38201621|ref|NP\_88* | 18 | 29 | 16.4% | 1599 | 175460 | 5.3 | eukaryotic translation initiation factor 4 gamma, 1 isoform 1 [Homo sapiens] |
| U | *gi|38201627|ref|NP\_93* | 18 | 29 | 17.3% | 1512 | 166588 | 5.2 | eukaryotic translation initiation factor 4 gamma, 1 isoform 2 [Homo sapiens] |
| U | *gi|38201625|ref|NP\_93* | 18 | 29 | 18.3% | 1435 | 158516 | 5.2 | eukaryotic translation initiation factor 4 gamma, 1 isoform 3 [Homo sapiens] |
| U | *gi|38201623|ref|NP\_93* | 18 | 29 | 16.4% | 1599 | 175460 | 5.3 | eukaryotic translation initiation factor 4 gamma, 1 isoform 1 [Homo sapiens] |

| Filename XCorr DeltCN Conf% ObsM+H+ CalcM+H+ SpR ZScore Ion% # Sequence  | | | | | | | | | | | | |
| --- | --- | --- | --- | --- | --- | --- | --- | --- | --- | --- | --- | --- |
|  | CENPL\_stlcld\_122314\_01.12705.12705.3 | 4.6243 | 0.4615 | 100.0% | 2188.3743 | 2188.4204 | 2 | 7.657 | 36.8% | 4 | R.IRDPNQGGKDITEEIMSGAR.T | 3 |
|  | CENPL\_stlcld\_122314\_01.15743.15743.2 | 2.6757 | 0.3458 | 99.9% | 1222.0922 | 1222.3557 | 1 | 6.22 | 80.0% | 3 | K.DITEEIMSGAR.T | 2 |
|  | CENPL\_stlcld\_tube2\_122314\_01.13013.13013.3 | 4.5836 | 0.4455 | 100.0% | 3459.3542 | 3458.7666 | 1 | 7.45 | 26.5% | 2 | R.TASTPTPPQTGGGLEPQANGETPQVAVIVRPDDR.S | 3 |
|  | CENPL\_stlcld\_122314\_01.20510.20510.2 | 2.5895 | 0.4413 | 99.9% | 1178.2322 | 1178.3275 | 52 | 7.166 | 50.0% | 2 | K.EAVGDLLDAFK.E | 2 |
|  | CENPL\_stlcld\_122314\_01.08554.08554.3 | 3.825 | 0.2565 | 99.9% | 2062.5544 | 2062.2463 | 1 | 5.545 | 40.6% | 1 | K.IHNAENIQPGEQKYEYK.S | 3 |
|  | CENPL\_stlcld\_tube2\_122314\_01.12394.12394.2 | 2.7085 | 0.0967 | 95.0% | 1487.4321 | 1487.6525 | 11 | 4.08 | 54.5% | 1 | K.SDQWKPLNLEEK.K | 2 |
|  | CENPL\_stlcld\_122314\_01.14723.14723.2 | 3.5803 | 0.3162 | 99.9% | 1476.1921 | 1475.8273 | 1 | 6.811 | 70.8% | 1 | R.KIIATVLMTEDIK.L | 2 |
|  | CENPL\_stlcld\_122314\_01.09183.09183.3 | 3.7617 | 0.2384 | 99.9% | 2426.9644 | 2426.5156 | 2 | 4.735 | 32.1% | 1 | R.TAADKDRGEEDADGSKTQDLFR.R | 3 |
|  | CENPL\_stlcld\_122314\_01.18507.18507.2 | 2.5089 | 0.3464 | 99.7% | 1365.4122 | 1365.6918 | 6 | 6.588 | 70.0% | 2 | K.LTPQMFQQLMK.Q | 2 |
|  | CENPL\_stlcld\_tube2\_122314\_01.18980.18980.2 | 2.3473 | 0.2333 | 97.8% | 1276.4722 | 1275.5742 | 3 | 4.841 | 60.0% | 1 | R.LKGVIDLIFEK.A | 2 |
|  | CENPL\_stlcld\_122314\_01.11489.11489.3 | 3.1801 | 0.3642 | 100.0% | 1591.5543 | 1589.832 | 1 | 5.954 | 46.2% | 3 | K.VPTTEKPTVTVNFR.K | 3 |
|  | CENPL\_stlcld\_tube2\_122314\_01.11890.11890.2 | 3.2603 | 0.4304 | 100.0% | 1334.2522 | 1334.5043 | 1 | 7.449 | 83.3% | 2 | R.MDQYFNQMEK.I | 2 |
|  | CENPL\_stlcld\_122314\_01.08620.08620.2 | 2.0288 | 0.2475 | 96.0% | 934.8522 | 935.0708 | 8 | 7.17 | 61.1% | 1 | R.GGPPGPPISR.G | 2 |
|  | CENPL\_stlcld\_122314\_01.18784.18784.2 | 2.5532 | 0.3751 | 99.6% | 1768.5922 | 1769.0073 | 6 | 5.937 | 43.8% | 1 | R.GLPLVDDGGWNTVPISK.G | 2 |
|  | CENPL\_stlcld\_122314\_01.11978.11978.3 | 3.3408 | 0.2909 | 99.9% | 1973.6643 | 1973.196 | 130 | 5.24 | 27.8% | 1 | K.ITKPGSIDSNNQLFAPGGR.L | 3 |
|  | CENPL\_stlcld\_122314\_01.08764.08764.2 | 2.393 | 0.2199 | 97.8% | 1247.1122 | 1247.388 | 3 | 4.631 | 65.0% | 1 | K.AALSEEELEKK.S | 2 |
|  | CENPL\_stlcld\_tube2\_122314\_01.17063.17063.2 | 2.4415 | 0.164 | 95.1% | 1588.8322 | 1589.8468 | 78 | 4.184 | 45.8% | 1 | K.AIIEEYLHLNDMK.E | 2 |
|  | CENPL\_stlcld\_122314\_01.18390.18390.2 | 2.617 | 0.1979 | 97.4% | 1879.1921 | 1879.0764 | 134 | 5.286 | 31.2% | 1 | K.EFLPEGQDIGAFVAEQK.V | 2 |

---

|  |  |  |  |  |  |  |  |  |
| --- | --- | --- | --- | --- | --- | --- | --- | --- |
| U | *gi|167466173|ref|NP\_0* | 8 | 19 | 16.2% | 641 | 70052 | 5.6 | heat shock 70kDa protein 1B [Homo sapiens] |
| U | *gi|194248072|ref|NP\_0* | 8 | 19 | 16.2% | 641 | 70052 | 5.6 | heat shock 70kDa protein 1A [Homo sapiens] |

| Filename XCorr DeltCN Conf% ObsM+H+ CalcM+H+ SpR ZScore Ion% # Sequence  | | | | | | | | | | | | |
| --- | --- | --- | --- | --- | --- | --- | --- | --- | --- | --- | --- | --- |
|  | CENPL\_stlcld\_tube2\_122314\_01.12731.12731.2 | 3.5901 | 0.5406 | 100.0% | 1488.3322 | 1488.5939 | 1 | 9.387 | 75.0% | 8 | R.TTPSYVAFTDTER.L | 222 |
|  | CENPL\_stlcld\_tube2\_122314\_01.13192.13192.2 | 4.1484 | 0.1454 | 99.7% | 1659.7322 | 1659.8394 | 1 | 6.896 | 67.9% | 1 | K.NQVALNPQNTVFDAK.R | 2 |
|  | CENPL\_stlcld\_tube2\_122314\_01.19245.19245.2 | 2.7077 | 0.2462 | 98.5% | 1616.4321 | 1615.8817 | 1 | 5.626 | 65.4% | 2 | K.AFYPEEISSMVLTK.M | 22 |
|  | CENPL\_stlcld\_122314\_02.14764.14764.2 | 3.2162 | 0.3474 | 99.9% | 1198.7922 | 1198.408 | 1 | 6.851 | 81.8% | 1 | K.DAGVIAGLNVLR.I | 22 |
|  | CENPL\_stlcld\_122314\_01.17068.17068.2 | 5.0128 | 0.4714 | 100.0% | 1689.6721 | 1688.9213 | 1 | 9.062 | 76.7% | 3 | R.IINEPTAAAIAYGLDR.T | 2 |
|  | CENPL\_stlcld\_122314\_01.11831.11831.3 | 2.1964 | 0.2732 | 95.2% | 1419.4143 | 1418.6383 | 1 | 4.824 | 47.5% | 1 | R.LVNHFVEEFKR.K | 3 |
|  | CENPL\_stlcld\_tube2\_122314\_01.15980.15980.2 | 2.3167 | 0.1975 | 95.9% | 1543.9922 | 1543.6855 | 3 | 5.397 | 59.1% | 1 | R.ARFEELCSDLFR.S | 2 |
|  | CENPL\_stlcld\_122314\_01.15185.15185.2 | 3.0827 | 0.3993 | 100.0% | 1288.5721 | 1288.4608 | 1 | 8.185 | 75.0% | 2 | K.NALESYAFNMK.S | 22 |

Similarities:
gi|5729877|ref|NP\_006(1:7)  
gi|124256496|ref|NP\_0(4:4)  

---

|  |  |  |  |  |  |  |  |  |
| --- | --- | --- | --- | --- | --- | --- | --- | --- |
| U | *gi|13904870|ref|NP\_00* | 2 | 2 | 16.2% | 204 | 22876 | 9.7 | ribosomal protein S5 [Homo sapiens] |

| Filename XCorr DeltCN Conf% ObsM+H+ CalcM+H+ SpR ZScore Ion% # Sequence  | | | | | | | | | | | | |
| --- | --- | --- | --- | --- | --- | --- | --- | --- | --- | --- | --- | --- |
| \* | CENPL\_stlcld\_tube2\_122314\_02.01994.01994.3 | 3.7561 | 0.3778 | 100.0% | 3066.4744 | 3067.5613 | 1 | 6.109 | 25.9% | 1 | K.HAFEIIHLLTGENPLQVLVNAIINSGPR.E | 3 |
| \* | CENPL\_stlcld\_122314\_02.01392.01392.3 | 3.8789 | 0.3725 | 100.0% | 3656.0344 | 3656.1362 | 6 | 5.763 | 21.1% | 1 | K.HAFEIIHLLTGENPLQVLVNAIINSGPREDSTR.I | 3 |

---

|  |  |  |  |  |  |  |  |  |
| --- | --- | --- | --- | --- | --- | --- | --- | --- |
| U | *gi|169201338|ref|XP\_0* | 3 | 14 | 16.2% | 160 | 18565 | 10.5 | PREDICTED: hypothetical protein [Homo sapiens] |
| U | *gi|89040203|ref|XP\_93* | 3 | 14 | 16.2% | 160 | 18593 | 10.5 | PREDICTED: hypothetical protein [Homo sapiens] |
| U | *gi|18104948|ref|NP\_00* | 3 | 14 | 16.2% | 160 | 18565 | 10.5 | ribosomal protein L21 [Homo sapiens] |
| U | *gi|169213854|ref|XP\_0* | 3 | 14 | 16.2% | 160 | 18790 | 10.3 | PREDICTED: hypothetical protein [Homo sapiens] |
| U | *gi|169210381|ref|XP\_0* | 3 | 14 | 16.2% | 160 | 18535 | 10.6 | PREDICTED: hypothetical protein isoform 2 [Homo sapiens] |
| U | *gi|169210379|ref|XP\_0* | 3 | 14 | 16.2% | 160 | 18535 | 10.6 | PREDICTED: hypothetical protein isoform 3 [Homo sapiens] |
| U | *gi|169210377|ref|XP\_0* | 3 | 14 | 16.2% | 160 | 18535 | 10.6 | PREDICTED: hypothetical protein isoform 1 [Homo sapiens] |
| U | *gi|169202779|ref|XP\_0* | 3 | 14 | 16.2% | 160 | 18521 | 10.5 | PREDICTED: similar to ribosomal protein L21 isoform 1 [Homo sapiens] |
| U | *gi|169202777|ref|XP\_0* | 3 | 14 | 16.2% | 160 | 18521 | 10.5 | PREDICTED: similar to ribosomal protein L21 isoform 2 [Homo sapiens] |
| U | *gi|169201750|ref|XP\_0* | 3 | 14 | 16.2% | 160 | 18550 | 10.5 | PREDICTED: hypothetical protein [Homo sapiens] |

| Filename XCorr DeltCN Conf% ObsM+H+ CalcM+H+ SpR ZScore Ion% # Sequence  | | | | | | | | | | | | |
| --- | --- | --- | --- | --- | --- | --- | --- | --- | --- | --- | --- | --- |
|  | CENPL\_stlcld\_tube2\_122314\_01.12455.12455.2 | 2.8231 | 0.3834 | 99.9% | 1244.4922 | 1244.4973 | 1 | 7.346 | 65.0% | 4 | K.HGVVPLATYMR.I | 2 |
|  | CENPL\_stlcld\_122314\_02.11148.11148.2 | 4.6708 | 0.3995 | 100.0% | 1641.5322 | 1641.9108 | 1 | 8.362 | 85.7% | 3 | R.VYNVTQHAVGIVVNK.Q | 2 |
|  | CENPL\_stlcld\_122314\_02.11132.11132.3 | 4.2029 | 0.4711 | 100.0% | 1643.1244 | 1641.9108 | 1 | 8.042 | 50.0% | 7 | R.VYNVTQHAVGIVVNK.Q | 3 |

---

|  |  |  |  |  |  |  |  |  |
| --- | --- | --- | --- | --- | --- | --- | --- | --- |
| U | *gi|4506625|ref|NP\_000* | 2 | 4 | 16.2% | 148 | 16561 | 11.0 | ribosomal protein L27a [Homo sapiens] |

| Filename XCorr DeltCN Conf% ObsM+H+ CalcM+H+ SpR ZScore Ion% # Sequence  | | | | | | | | | | | | |
| --- | --- | --- | --- | --- | --- | --- | --- | --- | --- | --- | --- | --- |
| \* | CENPL\_stlcld\_tube2\_122314\_01.11799.11799.3 | 2.9486 | 0.3159 | 99.9% | 1587.0243 | 1586.7899 | 16 | 6.092 | 37.5% | 1 | R.INFDKYHPGYFGK.V | 3 |
| \* | CENPL\_stlcld\_tube2\_122314\_02.12191.12191.2 | 2.7147 | 0.2348 | 99.0% | 1112.7722 | 1112.3146 | 2 | 5.836 | 75.0% | 3 | K.TGAAPIIDVVR.S | 2 |

---

|  |  |  |  |  |  |  |  |  |
| --- | --- | --- | --- | --- | --- | --- | --- | --- |
| U | *gi|4502491|ref|NP\_001* | 2 | 5 | 16.0% | 282 | 31362 | 4.8 | complement component 1, q subcomponent binding protein precursor [Homo sapiens] |

| Filename XCorr DeltCN Conf% ObsM+H+ CalcM+H+ SpR ZScore Ion% # Sequence  | | | | | | | | | | | | |
| --- | --- | --- | --- | --- | --- | --- | --- | --- | --- | --- | --- | --- |
| \* | CENPL\_stlcld\_122314\_02.13384.13384.2 | 3.0253 | 0.3245 | 99.9% | 1622.1122 | 1622.79 | 1 | 5.832 | 53.6% | 3 | K.MSGGWELELNGTEAK.L | 2 |
| \* | CENPL\_stlcld\_122314\_02.01792.01792.3 | 3.8606 | 0.2895 | 99.9% | 3443.0645 | 3441.77 | 1 | 4.465 | 25.0% | 2 | R.GVDNTFADELVELSTALEHQEYITFLEDLK.S | 3 |

---

|  |  |  |  |  |  |  |  |  |
| --- | --- | --- | --- | --- | --- | --- | --- | --- |
| U | *gi|4506707|ref|NP\_001* | 2 | 3 | 16.0% | 125 | 13742 | 10.1 | ribosomal protein S25 [Homo sapiens] |

| Filename XCorr DeltCN Conf% ObsM+H+ CalcM+H+ SpR ZScore Ion% # Sequence  | | | | | | | | | | | | |
| --- | --- | --- | --- | --- | --- | --- | --- | --- | --- | --- | --- | --- |
| \* | CENPL\_stlcld\_122314\_01.15095.15095.2 | 3.092 | 0.2813 | 99.9% | 1319.3522 | 1319.5437 | 1 | 5.241 | 75.0% | 2 | R.DKLNNLVLFDK.A | 2 |
| \* | CENPL\_stlcld\_tube2\_122314\_01.14680.14680.2 | 2.2013 | 0.2697 | 98.4% | 972.1722 | 973.1576 | 22 | 4.775 | 68.8% | 1 | R.AALQELLSK.G | 2 |

---

|  |  |  |  |  |  |  |  |  |
| --- | --- | --- | --- | --- | --- | --- | --- | --- |
| U | *gi|56699409|ref|NP\_00* | 4 | 11 | 15.9% | 391 | 42332 | 10.1 | RNA binding motif protein, X-linked [Homo sapiens] |

| Filename XCorr DeltCN Conf% ObsM+H+ CalcM+H+ SpR ZScore Ion% # Sequence  | | | | | | | | | | | | |
| --- | --- | --- | --- | --- | --- | --- | --- | --- | --- | --- | --- | --- |
|  | CENPL\_stlcld\_tube2\_122314\_01.14229.14229.2 | 3.6345 | 0.3314 | 100.0% | 1436.3522 | 1436.6049 | 2 | 6.499 | 66.7% | 4 | K.LFIGGLNTETNEK.A | 2 |
|  | CENPL\_stlcld\_tube2\_122314\_02.15180.15180.2 | 2.6504 | 0.3224 | 99.4% | 1488.3322 | 1487.6519 | 2 | 5.122 | 57.7% | 2 | R.GFAFVTFESPADAK.D | 2 |
| \* | CENPL\_stlcld\_122314\_01.08919.08919.3 | 3.8775 | 0.3738 | 100.0% | 1749.2344 | 1748.9768 | 1 | 7.055 | 40.0% | 2 | K.AIKVEQATKPSFESGR.R | 3 |
|  | CENPL\_stlcld\_122314\_02.11524.11524.3 | 3.9289 | 0.4373 | 100.0% | 2051.0044 | 2051.1873 | 1 | 7.281 | 38.9% | 3 | R.GGHMDDGGYSMNFNMSSSR.G | 3 |

---

|  |  |  |  |  |  |  |  |  |
| --- | --- | --- | --- | --- | --- | --- | --- | --- |
| U | *gi|66933016|ref|NP\_00* | 7 | 16 | 15.8% | 514 | 55805 | 6.9 | inosine monophosphate dehydrogenase 2 [Homo sapiens] |

| Filename XCorr DeltCN Conf% ObsM+H+ CalcM+H+ SpR ZScore Ion% # Sequence  | | | | | | | | | | | | |
| --- | --- | --- | --- | --- | --- | --- | --- | --- | --- | --- | --- | --- |
| \* | CENPL\_stlcld\_122314\_01.17062.17062.2 | 2.9303 | 0.3495 | 99.9% | 1694.0521 | 1693.9371 | 3 | 6.454 | 50.0% | 1 | K.YEQGFITDPVVLSPK.D | 2 |
| \* | CENPL\_stlcld\_tube2\_122314\_01.13775.13775.2 | 4.7554 | 0.4994 | 100.0% | 1482.6921 | 1482.7635 | 1 | 8.763 | 76.9% | 2 | K.REDLVVAPAGITLK.E | 2 |
| \* | CENPL\_stlcld\_tube2\_122314\_01.16666.16666.2 | 2.9154 | 0.3812 | 99.9% | 1156.9521 | 1157.3121 | 1 | 7.514 | 80.0% | 3 | K.NLIDAGVDALR.V | 2 |
| \* | CENPL\_stlcld\_122314\_01.14120.14120.3 | 5.9549 | 0.4348 | 100.0% | 2049.7444 | 2049.3835 | 1 | 8.543 | 46.1% | 7 | R.RFGVPVIADGGIQNVGHIAK.A | 3 |
| \* | CENPL\_stlcld\_tube2\_122314\_01.15833.15833.2 | 3.72 | 0.4 | 100.0% | 1893.8121 | 1893.196 | 1 | 7.11 | 55.6% | 1 | R.FGVPVIADGGIQNVGHIAK.A | 2 |
| \* | CENPL\_stlcld\_tube2\_122314\_01.08687.08687.2 | 2.1013 | 0.3694 | 99.2% | 1102.1122 | 1101.2444 | 23 | 5.465 | 56.2% | 1 | R.YFSEADKIK.V | 2 |
| \* | CENPL\_stlcld\_122314\_02.11718.11718.2 | 2.7303 | 0.4075 | 99.9% | 1435.0322 | 1434.7084 | 5 | 6.74 | 54.5% | 1 | R.AMMYSGELKFEK.R | 2 |

---

|  |  |  |  |  |  |  |  |  |
| --- | --- | --- | --- | --- | --- | --- | --- | --- |
| U | *gi|28872732|ref|NP\_78* | 3 | 9 | 15.8% | 215 | 23431 | 8.6 | mitochondrial ribosomal protein L43 isoform b [Homo sapiens] |
| U | *gi|28872738|ref|NP\_78* | 3 | 9 | 14.5% | 235 | 25862 | 9.0 | mitochondrial ribosomal protein L43 isoform d [Homo sapiens] |
| U | *gi|28872736|ref|NP\_78* | 3 | 9 | 16.8% | 202 | 22580 | 9.3 | mitochondrial ribosomal protein L43 isoform c [Homo sapiens] |
| U | *gi|28872734|ref|NP\_11* | 3 | 9 | 21.4% | 159 | 17853 | 9.9 | mitochondrial ribosomal protein L43 isoform a [Homo sapiens] |

| Filename XCorr DeltCN Conf% ObsM+H+ CalcM+H+ SpR ZScore Ion% # Sequence  | | | | | | | | | | | | |
| --- | --- | --- | --- | --- | --- | --- | --- | --- | --- | --- | --- | --- |
|  | CENPL\_stlcld\_tube2\_122314\_01.13283.13283.2 | 2.9106 | 0.4786 | 100.0% | 1284.6322 | 1284.5039 | 1 | 8.296 | 68.2% | 3 | R.FLASVLHNGLGR.Y | 2 |
|  | CENPL\_stlcld\_tube2\_122314\_01.12778.12778.2 | 2.9998 | 0.4604 | 100.0% | 1232.7522 | 1233.4044 | 3 | 7.081 | 65.0% | 1 | K.SVEEISTLVQK.L | 2 |
|  | CENPL\_stlcld\_122314\_01.12455.12455.2 | 3.6284 | 0.4072 | 100.0% | 1188.1322 | 1187.3384 | 1 | 7.312 | 75.0% | 5 | K.LADQSGLDVIR.I | 2 |

---

|  |  |  |  |  |  |  |  |  |
| --- | --- | --- | --- | --- | --- | --- | --- | --- |
| U | *gi|219555707|ref|NP\_0* | 2 | 7 | 15.8% | 184 | 20170 | 7.0 | eukaryotic translation initiation factor 5A isoform A [Homo sapiens] |
| U | *gi|4503545|ref|NP\_001* | 2 | 7 | 18.8% | 154 | 16832 | 5.2 | eukaryotic translation initiation factor 5A isoform B [Homo sapiens] |
| U | *gi|219555712|ref|NP\_0* | 2 | 7 | 18.8% | 154 | 16832 | 5.2 | eukaryotic translation initiation factor 5A isoform B [Homo sapiens] |
| U | *gi|219555710|ref|NP\_0* | 2 | 7 | 18.8% | 154 | 16832 | 5.2 | eukaryotic translation initiation factor 5A isoform B [Homo sapiens] |

| Filename XCorr DeltCN Conf% ObsM+H+ CalcM+H+ SpR ZScore Ion% # Sequence  | | | | | | | | | | | | |
| --- | --- | --- | --- | --- | --- | --- | --- | --- | --- | --- | --- | --- |
|  | CENPL\_stlcld\_122314\_01.17835.17835.2 | 3.7981 | 0.5644 | 100.0% | 1299.5521 | 1299.5559 | 1 | 9.651 | 77.3% | 6 | K.VHLVGIDIFTGK.K | 2 |
|  | CENPL\_stlcld\_tube2\_122314\_01.12716.12716.3 | 3.9376 | 0.3859 | 100.0% | 1969.7344 | 1970.187 | 1 | 6.448 | 40.6% | 1 | R.EDLRLPEGDLGKEIEQK.Y | 3 |

---

|  |  |  |  |  |  |  |  |  |
| --- | --- | --- | --- | --- | --- | --- | --- | --- |
| U | *gi|23308577|ref|NP\_00* | 4 | 5 | 15.4% | 533 | 56651 | 6.7 | phosphoglycerate dehydrogenase [Homo sapiens] |

| Filename XCorr DeltCN Conf% ObsM+H+ CalcM+H+ SpR ZScore Ion% # Sequence  | | | | | | | | | | | | |
| --- | --- | --- | --- | --- | --- | --- | --- | --- | --- | --- | --- | --- |
| \* | CENPL\_stlcld\_122314\_01.11957.11957.2 | 2.5604 | 0.2871 | 98.6% | 1489.8722 | 1489.5822 | 54 | 4.632 | 46.4% | 1 | R.AGTGVDNVDLEAATR.K | 2 |
| \* | CENPL\_stlcld\_122314\_01.14092.14092.2 | 2.7582 | 0.2335 | 99.1% | 1101.4321 | 1100.2603 | 26 | 5.087 | 60.0% | 2 | R.GGIVDEGALLR.A | 2 |
| \* | CENPL\_stlcld\_tube2\_122314\_02.00459.00459.3 | 3.4337 | 0.3375 | 99.9% | 3539.1843 | 3540.118 | 1 | 4.949 | 24.2% | 1 | K.SLTGVVNAQALTSAFSPHTKPWIGLAEALGTLMR.A | 3 |
| \* | CENPL\_stlcld\_tube2\_122314\_02.00582.00582.2 | 4.0219 | 0.4243 | 100.0% | 2273.172 | 2273.668 | 1 | 8.031 | 38.1% | 1 | R.TQTSDPAMLPTMIGLLAEAGVR.L | 2 |

---

|  |  |  |  |  |  |  |  |  |
| --- | --- | --- | --- | --- | --- | --- | --- | --- |
| U | *gi|113412878|ref|XP\_0* | 3 | 6 | 15.4% | 293 | 31479 | 7.6 | PREDICTED: similar to voltage-dependent anion channel [Homo sapiens] |
| U | *gi|42476281|ref|NP\_00* | 3 | 6 | 15.3% | 294 | 31566 | 7.6 | voltage-dependent anion channel 2 [Homo sapiens] |
| U | *gi|169164151|ref|XP\_0* | 3 | 6 | 15.4% | 293 | 31445 | 7.6 | PREDICTED: similar to voltage-dependent anion channel [Homo sapiens] |

| Filename XCorr DeltCN Conf% ObsM+H+ CalcM+H+ SpR ZScore Ion% # Sequence  | | | | | | | | | | | | |
| --- | --- | --- | --- | --- | --- | --- | --- | --- | --- | --- | --- | --- |
|  | CENPL\_stlcld\_122314\_02.12110.12110.3 | 6.2584 | 0.4571 | 100.0% | 2530.0745 | 2529.682 | 1 | 8.114 | 47.7% | 3 | R.TGDFQLHTNVNDGTEFGGSIYQK.V | 3 |
|  | CENPL\_stlcld\_122314\_01.12161.12161.2 | 3.0847 | 0.4916 | 100.0% | 1294.4722 | 1294.4473 | 1 | 7.201 | 68.2% | 2 | K.YQLDPTASISAK.V | 2 |
|  | CENPL\_stlcld\_122314\_01.16222.16222.2 | 1.8163 | 0.3378 | 97.4% | 1017.2922 | 1017.21075 | 91 | 6.33 | 55.6% | 1 | K.LTLSALVDGK.S | 2 |

---

|  |  |  |  |  |  |  |  |  |
| --- | --- | --- | --- | --- | --- | --- | --- | --- |
| U | *gi|154355000|ref|NP\_0* | 7 | 11 | 15.3% | 711 | 73115 | 7.3 | KH-type splicing regulatory protein (FUSE binding protein 2) [Homo sapiens] |

| Filename XCorr DeltCN Conf% ObsM+H+ CalcM+H+ SpR ZScore Ion% # Sequence  | | | | | | | | | | | | |
| --- | --- | --- | --- | --- | --- | --- | --- | --- | --- | --- | --- | --- |
| \* | CENPL\_stlcld\_tube2\_122314\_01.07724.07724.3 | 3.6756 | 0.3781 | 100.0% | 2399.5745 | 2399.5447 | 3 | 5.831 | 28.3% | 1 | R.GGGGPGGGGPGGGSAGGPSQPPGGGGPGIRK.D | 3 |
| \* | CENPL\_stlcld\_tube2\_122314\_01.10961.10961.3 | 3.1667 | 0.2653 | 99.1% | 2187.8044 | 2186.4758 | 1 | 5.54 | 32.5% | 1 | K.KLASQGDSISSQLGPIHPPPR.T | 3 |
| \* | CENPL\_stlcld\_tube2\_122314\_01.10468.10468.2 | 2.9089 | 0.4028 | 99.9% | 1355.2922 | 1355.4906 | 1 | 6.977 | 62.5% | 1 | K.VQISPDSGGLPER.S | 2 |
| \* | CENPL\_stlcld\_tube2\_122314\_01.15428.15428.2 | 2.2214 | 0.2925 | 98.6% | 1079.1921 | 1080.3026 | 1 | 5.06 | 75.0% | 1 | K.MMLDDIVSR.G | 2 |
| \* | CENPL\_stlcld\_tube2\_122314\_01.12873.12873.2 | 4.1649 | 0.4411 | 100.0% | 1080.1122 | 1080.2725 | 1 | 8.002 | 90.0% | 3 | R.IGGGIDVPVPR.H | 2 |
| \* | CENPL\_stlcld\_122314\_01.18575.18575.2 | 2.9857 | 0.2906 | 99.9% | 1185.5922 | 1185.4093 | 1 | 6.419 | 83.3% | 2 | R.IINDLLQSLR.S | 2 |
| \* | CENPL\_stlcld\_tube2\_122314\_02.12719.12719.2 | 3.7999 | 0.3381 | 100.0% | 1534.4321 | 1534.7123 | 1 | 6.506 | 69.2% | 2 | K.AINQQTGAFVEISR.Q | 2 |

---

|  |  |  |  |  |  |  |  |  |
| --- | --- | --- | --- | --- | --- | --- | --- | --- |
| U | *gi|4505701|ref|NP\_003* | 3 | 4 | 15.1% | 312 | 35102 | 6.1 | pyridoxal kinase [Homo sapiens] |

| Filename XCorr DeltCN Conf% ObsM+H+ CalcM+H+ SpR ZScore Ion% # Sequence  | | | | | | | | | | | | |
| --- | --- | --- | --- | --- | --- | --- | --- | --- | --- | --- | --- | --- |
| \* | CENPL\_stlcld\_122314\_01.10888.10888.2 | 2.423 | 0.3724 | 99.9% | 1152.0322 | 1152.3823 | 35 | 6.298 | 61.1% | 1 | R.VLSIQSHVIR.G | 2 |
| \* | CENPL\_stlcld\_tube2\_122314\_02.02046.02046.2 | 3.7107 | 0.3213 | 99.9% | 1837.3522 | 1836.1969 | 1 | 6.453 | 53.3% | 1 | R.DKSFLAMVVDIVQELK.Q | 2 |
| \* | CENPL\_stlcld\_tube2\_122314\_01.21644.21644.2 | 3.9551 | 0.4164 | 100.0% | 2283.112 | 2283.63 | 1 | 7.743 | 42.5% | 2 | K.VVPLADIITPNQFEAELLSGR.K | 2 |

---

|  |  |  |  |  |  |  |  |  |
| --- | --- | --- | --- | --- | --- | --- | --- | --- |
| U | *gi|56118234|ref|NP\_85* | 3 | 7 | 15.1% | 272 | 29731 | 9.7 | proline rich 6 [Homo sapiens] |

| Filename XCorr DeltCN Conf% ObsM+H+ CalcM+H+ SpR ZScore Ion% # Sequence  | | | | | | | | | | | | |
| --- | --- | --- | --- | --- | --- | --- | --- | --- | --- | --- | --- | --- |
| \* | CENPL\_stlcld\_122314\_02.16244.16244.2 | 4.5959 | 0.4902 | 100.0% | 1539.7922 | 1539.8119 | 1 | 8.586 | 79.2% | 3 | K.LLLDTFEYQGLVK.H | 2 |
| \* | CENPL\_stlcld\_tube2\_122314\_01.08964.08964.3 | 2.906 | 0.2964 | 99.9% | 1621.6743 | 1620.7618 | 3 | 5.582 | 38.5% | 2 | K.GAEHITTYTFNTHK.A | 3 |
| \* | CENPL\_stlcld\_122314\_02.12695.12695.2 | 3.6 | 0.5102 | 100.0% | 1660.3322 | 1659.7644 | 1 | 8.126 | 61.5% | 2 | R.SMVTEEFNGSDWEK.A | 2 |

---

|  |  |  |  |  |  |  |  |  |
| --- | --- | --- | --- | --- | --- | --- | --- | --- |
| U | *gi|15055539|ref|NP\_00* | 3 | 7 | 15.0% | 293 | 31324 | 10.2 | ribosomal protein S2 [Homo sapiens] |
| U | *gi|169205508|ref|XP\_0* | 3 | 7 | 18.9% | 233 | 25545 | 9.9 | PREDICTED: hypothetical protein isoform 2 [Homo sapiens] |
| U | *gi|169205506|ref|XP\_0* | 3 | 7 | 15.0% | 293 | 31364 | 10.2 | PREDICTED: hypothetical protein isoform 1 [Homo sapiens] |
| U | *gi|169204986|ref|XP\_0* | 3 | 7 | 18.9% | 233 | 25545 | 9.9 | PREDICTED: hypothetical protein isoform 2 [Homo sapiens] |
| U | *gi|169204984|ref|XP\_0* | 3 | 7 | 15.0% | 293 | 31364 | 10.2 | PREDICTED: hypothetical protein isoform 1 [Homo sapiens] |
| U | *gi|169204456|ref|XP\_0* | 3 | 7 | 18.9% | 233 | 25619 | 9.8 | PREDICTED: hypothetical protein isoform 2 [Homo sapiens] |
| U | *gi|169204454|ref|XP\_0* | 3 | 7 | 15.0% | 293 | 31438 | 10.2 | PREDICTED: hypothetical protein isoform 1 [Homo sapiens] |

| Filename XCorr DeltCN Conf% ObsM+H+ CalcM+H+ SpR ZScore Ion% # Sequence  | | | | | | | | | | | | |
| --- | --- | --- | --- | --- | --- | --- | --- | --- | --- | --- | --- | --- |
|  | CENPL\_stlcld\_122314\_02.01850.01850.3 | 5.7463 | 0.3845 | 100.0% | 3689.3643 | 3688.293 | 1 | 7.643 | 23.4% | 1 | K.SLEEIYLFSLPIKESEIIDFFLGASLKDEVLK.I | 3 |
|  | CENPL\_stlcld\_tube2\_122314\_01.13880.13880.3 | 3.5205 | 0.414 | 100.0% | 1464.6244 | 1464.6177 | 1 | 8.316 | 52.3% | 3 | K.SPYQEFTDHLVK.T | 3 |
|  | CENPL\_stlcld\_122314\_01.13997.13997.2 | 3.2561 | 0.3276 | 99.9% | 1464.8322 | 1464.6177 | 1 | 5.591 | 72.7% | 3 | K.SPYQEFTDHLVK.T | 2 |

---

|  |  |  |  |  |  |  |  |  |
| --- | --- | --- | --- | --- | --- | --- | --- | --- |
| U | *gi|14141193|ref|NP\_00* | 4 | 7 | 14.9% | 194 | 22591 | 10.7 | ribosomal protein S9 [Homo sapiens] |

| Filename XCorr DeltCN Conf% ObsM+H+ CalcM+H+ SpR ZScore Ion% # Sequence  | | | | | | | | | | | | |
| --- | --- | --- | --- | --- | --- | --- | --- | --- | --- | --- | --- | --- |
| \* | CENPL\_stlcld\_tube2\_122314\_02.11866.11866.2 | 2.6199 | 0.176 | 98.3% | 1188.6122 | 1189.4031 | 1 | 4.29 | 77.8% | 2 | R.RLFEGNALLR.R | 2 |
| \* | CENPL\_stlcld\_tube2\_122314\_01.14704.14704.2 | 2.775 | 0.3259 | 99.9% | 1033.3922 | 1033.2156 | 5 | 6.304 | 75.0% | 2 | R.LFEGNALLR.R | 2 |
| \* | CENPL\_stlcld\_tube2\_122314\_01.15282.15282.2 | 3.4946 | 0.3142 | 99.9% | 1400.6522 | 1400.7074 | 1 | 6.078 | 72.7% | 2 | R.KQVVNIPSFIVR.L | 2 |
| \* | CENPL\_stlcld\_tube2\_122314\_01.11816.11816.2 | 2.4172 | 0.1779 | 98.5% | 887.7922 | 888.01404 | 3 | 5.106 | 83.3% | 1 | K.HIDFSLR.S | 2 |

---

|  |  |  |  |  |  |  |  |  |
| --- | --- | --- | --- | --- | --- | --- | --- | --- |
| U | *gi|55770864|ref|NP\_00* | 2 | 5 | 14.8% | 257 | 26888 | 11.2 | THO complex 4 [Homo sapiens] |

| Filename XCorr DeltCN Conf% ObsM+H+ CalcM+H+ SpR ZScore Ion% # Sequence  | | | | | | | | | | | | |
| --- | --- | --- | --- | --- | --- | --- | --- | --- | --- | --- | --- | --- |
| \* | CENPL\_stlcld\_tube2\_122314\_02.00653.00653.3 | 3.402 | 0.3654 | 99.9% | 2971.4043 | 2971.377 | 1 | 5.58 | 26.0% | 1 | K.LLVSNLDFGVSDADIQELFAEFGTLKK.A | 3 |
| \* | CENPL\_stlcld\_122314\_01.10852.10852.2 | 3.2234 | 0.4132 | 100.0% | 1232.2522 | 1232.3384 | 1 | 7.646 | 75.0% | 4 | R.SLGTADVHFER.K | 2 |

---

|  |  |  |  |  |  |  |  |  |
| --- | --- | --- | --- | --- | --- | --- | --- | --- |
| U | *gi|14210536|ref|NP\_11* | 9 | 37 | 14.3% | 446 | 49857 | 4.9 | tubulin, beta 6 [Homo sapiens] |

| Filename XCorr DeltCN Conf% ObsM+H+ CalcM+H+ SpR ZScore Ion% # Sequence  | | | | | | | | | | | | |
| --- | --- | --- | --- | --- | --- | --- | --- | --- | --- | --- | --- | --- |
| \* | CENPL\_stlcld\_tube2\_122314\_01.15359.15359.2 | 3.0894 | 0.1395 | 97.8% | 1575.4321 | 1574.7894 | 6 | 4.047 | 50.0% | 1 | R.AALVDLEPGTMDSVR.S | 2 |
|  | CENPL\_stlcld\_122314\_01.13025.13025.2 | 3.0807 | 0.3262 | 99.9% | 1130.8322 | 1131.2767 | 1 | 5.245 | 83.3% | 8 | R.FPGQLNADLR.K | 2222 |
|  | CENPL\_stlcld\_122314\_01.11442.11442.3 | 2.9929 | 0.3054 | 99.9% | 1259.5443 | 1259.4508 | 9 | 5.658 | 42.5% | 1 | R.FPGQLNADLRK.L | 3333 |
|  | CENPL\_stlcld\_tube2\_122314\_01.10454.10454.2 | 2.6368 | 0.2303 | 98.6% | 1259.7322 | 1259.4508 | 17 | 4.582 | 60.0% | 5 | R.FPGQLNADLRK.L | 2222 |
|  | CENPL\_stlcld\_tube2\_122314\_01.14181.14181.2 | 3.8965 | 0.404 | 100.0% | 1271.9922 | 1272.5945 | 1 | 7.764 | 75.0% | 4 | R.KLAVNMVPFPR.L | 2222 |
|  | CENPL\_stlcld\_122314\_01.16748.16748.2 | 3.646 | 0.4855 | 100.0% | 1143.9922 | 1144.4204 | 1 | 8.724 | 94.4% | 8 | K.LAVNMVPFPR.L | 2222 |
|  | CENPL\_stlcld\_122314\_01.20176.20176.3 | 4.2322 | 0.3658 | 100.0% | 1622.3644 | 1621.9403 | 1 | 6.191 | 55.8% | 3 | R.LHFFMPGFAPLTSR.G | 333 |
|  | CENPL\_stlcld\_122314\_01.20175.20175.2 | 3.8813 | 0.426 | 100.0% | 1622.5721 | 1621.9403 | 1 | 8.381 | 73.1% | 3 | R.LHFFMPGFAPLTSR.G | 222 |
|  | CENPL\_stlcld\_122314\_01.19247.19247.2 | 3.5195 | 0.346 | 99.9% | 1698.4321 | 1697.8877 | 3 | 5.888 | 53.8% | 4 | K.NSSYFVEWIPNNVK.V | 2222 |

Similarities:
gi|29788785|ref|NP\_82(8:1)  
gi|5174735|ref|NP\_006(8:1)  
gi|50592996|ref|NP\_00(6:3)  

---

|  |  |  |  |  |  |  |  |  |
| --- | --- | --- | --- | --- | --- | --- | --- | --- |
| U | *gi|5803036|ref|NP\_006* | 3 | 9 | 14.1% | 305 | 30841 | 9.3 | heterogeneous nuclear ribonucleoprotein A0 [Homo sapiens] |

| Filename XCorr DeltCN Conf% ObsM+H+ CalcM+H+ SpR ZScore Ion% # Sequence  | | | | | | | | | | | | |
| --- | --- | --- | --- | --- | --- | --- | --- | --- | --- | --- | --- | --- |
| \* | CENPL\_stlcld\_122314\_01.17656.17656.2 | 4.6254 | 0.5518 | 100.0% | 1691.3322 | 1691.9248 | 1 | 9.739 | 80.0% | 6 | K.LFIGGLNVQTSESGLR.G | 2 |
| \* | CENPL\_stlcld\_tube2\_122314\_01.19115.19115.3 | 4.2656 | 0.4302 | 100.0% | 2892.8044 | 2894.2537 | 1 | 6.904 | 26.0% | 2 | K.LFVGGLKGDVAEGDLIEHFSQFGTVEK.A | 3 |
| \* | CENPL\_stlcld\_122314\_02.14826.14826.3 | 5.0954 | 0.4538 | 100.0% | 2180.1543 | 2179.3477 | 1 | 7.788 | 36.8% | 1 | K.GDVAEGDLIEHFSQFGTVEK.A | 3 |

---

|  |  |  |  |  |  |  |  |  |
| --- | --- | --- | --- | --- | --- | --- | --- | --- |
| U | *gi|24307939|ref|NP\_03* | 4 | 4 | 13.5% | 541 | 59671 | 5.6 | chaperonin containing TCP1, subunit 5 (epsilon) [Homo sapiens] |

| Filename XCorr DeltCN Conf% ObsM+H+ CalcM+H+ SpR ZScore Ion% # Sequence  | | | | | | | | | | | | |
| --- | --- | --- | --- | --- | --- | --- | --- | --- | --- | --- | --- | --- |
| \* | CENPL\_stlcld\_tube2\_122314\_01.18902.18902.3 | 4.881 | 0.4485 | 100.0% | 3092.5444 | 3092.5571 | 1 | 7.349 | 26.9% | 1 | R.VAIEHLDKISDSVLVDIKDTEPLIQTAK.T | 3 |
| \* | CENPL\_stlcld\_122314\_01.15437.15437.2 | 2.5768 | 0.3043 | 99.3% | 1392.0922 | 1392.592 | 10 | 5.344 | 50.0% | 1 | R.DVDFELIKVEGK.V | 2 |
| \* | CENPL\_stlcld\_tube2\_122314\_01.21579.21579.2 | 4.0775 | 0.5139 | 100.0% | 1739.7322 | 1740.0122 | 1 | 8.83 | 56.2% | 1 | R.WVGGPEIELIAIATGGR.I | 2 |
| \* | CENPL\_stlcld\_122314\_02.17250.17250.2 | 4.2436 | 0.3942 | 100.0% | 1669.7322 | 1668.9304 | 1 | 8.516 | 73.3% | 1 | K.LGFAGLVQEISFGTTK.D | 2 |

---

|  |  |  |  |  |  |  |  |  |
| --- | --- | --- | --- | --- | --- | --- | --- | --- |
| U | *gi|16445419|ref|NP\_00* | 3 | 5 | 13.5% | 347 | 38287 | 7.6 | secretory carrier membrane protein 3 isoform 1 [Homo sapiens] |
| U | *gi|16445421|ref|NP\_44* | 3 | 5 | 14.6% | 321 | 35202 | 7.9 | secretory carrier membrane protein 3 isoform 2 [Homo sapiens] |

| Filename XCorr DeltCN Conf% ObsM+H+ CalcM+H+ SpR ZScore Ion% # Sequence  | | | | | | | | | | | | |
| --- | --- | --- | --- | --- | --- | --- | --- | --- | --- | --- | --- | --- |
|  | CENPL\_stlcld\_122314\_01.08098.08098.3 | 3.3333 | 0.2736 | 99.9% | 1611.1743 | 1610.7703 | 1 | 5.663 | 44.6% | 1 | R.ERELQHAALGGTATR.Q | 3 |
|  | CENPL\_stlcld\_tube2\_122314\_01.15030.15030.2 | 2.8977 | 0.3451 | 99.9% | 1692.4521 | 1692.8717 | 1 | 6.78 | 53.3% | 1 | K.AQQEFAAGVFSNPAVR.T | 2 |
|  | CENPL\_stlcld\_122314\_01.12114.12114.2 | 4.4924 | 0.5268 | 100.0% | 1477.3322 | 1477.5767 | 1 | 9.398 | 80.0% | 3 | R.TAAANAAAGAAENAFR.A | 2 |

---

|  |  |  |  |  |  |  |  |  |
| --- | --- | --- | --- | --- | --- | --- | --- | --- |
| U | *gi|48762932|ref|NP\_00* | 6 | 9 | 13.3% | 548 | 59621 | 5.6 | chaperonin containing TCP1, subunit 8 (theta) [Homo sapiens] |

| Filename XCorr DeltCN Conf% ObsM+H+ CalcM+H+ SpR ZScore Ion% # Sequence  | | | | | | | | | | | | |
| --- | --- | --- | --- | --- | --- | --- | --- | --- | --- | --- | --- | --- |
| \* | CENPL\_stlcld\_tube2\_122314\_01.11624.11624.2 | 2.0202 | 0.2498 | 95.5% | 1308.2722 | 1308.4362 | 245 | 4.36 | 55.0% | 1 | K.HFSGLEEAVYR.N | 2 |
| \* | CENPL\_stlcld\_122314\_01.16584.16584.2 | 2.5585 | 0.3232 | 99.5% | 1334.5721 | 1334.5583 | 9 | 5.62 | 54.5% | 2 | K.LFVTNDAATILR.E | 2 |
| \* | CENPL\_stlcld\_tube2\_122314\_01.16736.16736.2 | 3.529 | 0.2541 | 99.9% | 1531.0721 | 1530.7214 | 1 | 5.558 | 75.0% | 1 | K.NLRDIDEVSSLLR.T | 2 |
| \* | CENPL\_stlcld\_tube2\_122314\_02.10956.10956.2 | 4.2048 | 0.5671 | 100.0% | 1373.5922 | 1373.5492 | 1 | 9.42 | 64.3% | 2 | K.AIADTGANVVVTGGK.V | 2 |
| \* | CENPL\_stlcld\_122314\_01.14253.14253.2 | 2.6948 | 0.3517 | 99.9% | 1366.9722 | 1366.4425 | 1 | 5.949 | 72.7% | 1 | R.GSTDNLMDDIER.A | 2 |
| \* | CENPL\_stlcld\_122314\_01.14843.14843.2 | 2.5296 | 0.2798 | 99.2% | 1151.5322 | 1151.3073 | 2 | 5.25 | 66.7% | 2 | K.FAEAFEAIPR.A | 2 |

---

|  |  |  |  |  |  |  |  |  |
| --- | --- | --- | --- | --- | --- | --- | --- | --- |
| U | *gi|24234699|ref|NP\_00* | 6 | 22 | 13.2% | 400 | 44106 | 5.1 | keratin 19 [Homo sapiens] |

| Filename XCorr DeltCN Conf% ObsM+H+ CalcM+H+ SpR ZScore Ion% # Sequence  | | | | | | | | | | | | |
| --- | --- | --- | --- | --- | --- | --- | --- | --- | --- | --- | --- | --- |
|  | CENPL\_stlcld\_tube2\_122314\_01.09831.09831.2 | 3.0041 | 0.1699 | 99.3% | 1065.3322 | 1065.2578 | 54 | 5.893 | 62.5% | 1 | R.LASYLDKVR.A | 2222 |
|  | CENPL\_stlcld\_tube2\_122314\_01.11966.11966.2 | 3.0615 | 0.1615 | 99.4% | 1042.3722 | 1042.2235 | 3 | 6.157 | 87.5% | 6 | R.IVLQIDNAR.L | 22 |
|  | CENPL\_stlcld\_122314\_01.09788.09788.2 | 2.5662 | 0.2183 | 99.5% | 808.39215 | 807.8815 | 68 | 6.201 | 66.7% | 8 | R.LAADDFR.T | 22222 |
|  | CENPL\_stlcld\_122314\_01.08733.08733.2 | 2.3452 | 0.299 | 99.0% | 1223.3522 | 1223.3715 | 7 | 5.286 | 77.8% | 1 | R.TKFETEQALR.M | 22 |
|  | CENPL\_stlcld\_122314\_01.14091.14091.2 | 3.0726 | 0.4118 | 100.0% | 1030.3522 | 1030.2096 | 3 | 6.871 | 81.2% | 5 | R.VLDELTLAR.T | 222 |
|  | CENPL\_stlcld\_tube2\_122314\_01.09641.09641.2 | 2.9939 | 0.3424 | 99.9% | 1123.2722 | 1123.2511 | 2 | 6.024 | 81.2% | 1 | R.LEQEIATYR.S | 222 |

Similarities:
gi|40354195|ref|NP\_95(2:4)  
gi|4557701|ref|NP\_000(5:1)  
contaminant\_KERATIN03(2:4)  
contaminant\_KERATIN05(4:2)  

---

|  |  |  |  |  |  |  |  |  |
| --- | --- | --- | --- | --- | --- | --- | --- | --- |
| U | *gi|4826734|ref|NP\_004* | 5 | 10 | 13.1% | 526 | 53426 | 9.4 | fusion (involved in t(12;16) in malignant liposarcoma) [Homo sapiens] |

| Filename XCorr DeltCN Conf% ObsM+H+ CalcM+H+ SpR ZScore Ion% # Sequence  | | | | | | | | | | | | |
| --- | --- | --- | --- | --- | --- | --- | --- | --- | --- | --- | --- | --- |
| \* | CENPL\_stlcld\_122314\_01.13871.13871.2 | 2.5769 | 0.2383 | 98.5% | 1409.9521 | 1409.6 | 30 | 4.812 | 54.5% | 1 | K.TGQPMINLYTDR.E | 2 |
| \* | CENPL\_stlcld\_122314\_02.10106.10106.3 | 4.0662 | 0.3578 | 100.0% | 1663.1643 | 1662.837 | 1 | 6.171 | 48.3% | 4 | K.LKGEATVSFDDPPSAK.A | 3 |
|  | CENPL\_stlcld\_tube2\_122314\_01.10652.10652.2 | 2.3484 | 0.2194 | 96.3% | 1420.4722 | 1421.5034 | 89 | 5.7 | 46.2% | 1 | K.GEATVSFDDPPSAK.A | 2 |
| \* | CENPL\_stlcld\_122314\_01.18327.18327.2 | 4.4996 | 0.4582 | 100.0% | 1895.9722 | 1896.1094 | 1 | 7.554 | 68.8% | 2 | K.AAIDWFDGKEFSGNPIK.V | 2 |
| \* | CENPL\_stlcld\_tube2\_122314\_01.07626.07626.3 | 3.9178 | 0.4494 | 100.0% | 2254.4043 | 2254.355 | 1 | 7.201 | 43.5% | 2 | K.APKPDGPGGGPGGSHMGGNYGDDR.R | 3 |

---

|  |  |  |  |  |  |  |  |  |
| --- | --- | --- | --- | --- | --- | --- | --- | --- |
| U | *gi|15431290|ref|NP\_00* | 2 | 7 | 12.9% | 178 | 20252 | 9.6 | ribosomal protein L11 [Homo sapiens] |

| Filename XCorr DeltCN Conf% ObsM+H+ CalcM+H+ SpR ZScore Ion% # Sequence  | | | | | | | | | | | | |
| --- | --- | --- | --- | --- | --- | --- | --- | --- | --- | --- | --- | --- |
| \* | CENPL\_stlcld\_tube2\_122314\_01.14324.14324.2 | 4.2068 | 0.4376 | 100.0% | 1548.8922 | 1547.7917 | 1 | 7.496 | 61.5% | 5 | K.VLEQLTGQTPVFSK.A | 2 |
| \* | CENPL\_stlcld\_tube2\_122314\_01.13301.13301.2 | 2.1848 | 0.302 | 98.6% | 976.3522 | 976.1607 | 3 | 6.127 | 81.2% | 2 | K.YDGIILPGK.- | 2 |

---

|  |  |  |  |  |  |  |  |  |
| --- | --- | --- | --- | --- | --- | --- | --- | --- |
| U | *gi|4506605|ref|NP\_000* | 2 | 7 | 12.9% | 140 | 14865 | 10.5 | ribosomal protein L23 [Homo sapiens] |

| Filename XCorr DeltCN Conf% ObsM+H+ CalcM+H+ SpR ZScore Ion% # Sequence  | | | | | | | | | | | | |
| --- | --- | --- | --- | --- | --- | --- | --- | --- | --- | --- | --- | --- |
| \* | CENPL\_stlcld\_tube2\_122314\_01.16259.16259.3 | 3.2438 | 0.2981 | 99.9% | 1844.3344 | 1844.2408 | 79 | 6.298 | 30.9% | 1 | R.LNRLPAAGVGDMVMATVK.K | 3 |
| \* | CENPL\_stlcld\_122314\_01.17118.17118.2 | 4.251 | 0.5362 | 100.0% | 1460.6322 | 1460.7902 | 1 | 9.553 | 75.0% | 6 | R.LPAAGVGDMVMATVK.K | 2 |

---

|  |  |  |  |  |  |  |  |  |
| --- | --- | --- | --- | --- | --- | --- | --- | --- |
| U | *gi|55956919|ref|NP\_11* | 3 | 7 | 12.7% | 332 | 35968 | 6.9 | heterogeneous nuclear ribonucleoprotein A/B isoform a [Homo sapiens] |
| U | *gi|55956921|ref|NP\_00* | 3 | 7 | 14.7% | 285 | 30588 | 7.9 | heterogeneous nuclear ribonucleoprotein A/B isoform b [Homo sapiens] |

| Filename XCorr DeltCN Conf% ObsM+H+ CalcM+H+ SpR ZScore Ion% # Sequence  | | | | | | | | | | | | |
| --- | --- | --- | --- | --- | --- | --- | --- | --- | --- | --- | --- | --- |
|  | CENPL\_stlcld\_tube2\_122314\_01.14658.14658.2 | 2.735 | 0.3065 | 99.5% | 1457.1322 | 1456.6996 | 13 | 6.314 | 50.0% | 2 | K.MFVGGLSWDTSKK.D | 2 |
|  | CENPL\_stlcld\_122314\_01.13786.13786.2 | 3.9902 | 0.4033 | 100.0% | 1504.4722 | 1504.6799 | 1 | 7.594 | 73.1% | 3 | K.IFVGGLNPEATEEK.I | 2 |
|  | CENPL\_stlcld\_122314\_01.15086.15086.3 | 3.1699 | 0.3845 | 100.0% | 1799.4844 | 1799.1199 | 1 | 6.001 | 39.3% | 2 | R.GFVFITFKEEEPVKK.V | 3 |

---

|  |  |  |  |  |  |  |  |  |
| --- | --- | --- | --- | --- | --- | --- | --- | --- |
| U | *gi|189458817|ref|NP\_0* | 7 | 11 | 12.5% | 760 | 84871 | 6.6 | transferrin receptor [Homo sapiens] |
| U | *gi|189458819|ref|NP\_0* | 7 | 11 | 12.5% | 760 | 84871 | 6.6 | transferrin receptor [Homo sapiens] |

| Filename XCorr DeltCN Conf% ObsM+H+ CalcM+H+ SpR ZScore Ion% # Sequence  | | | | | | | | | | | | |
| --- | --- | --- | --- | --- | --- | --- | --- | --- | --- | --- | --- | --- |
|  | CENPL\_stlcld\_122314\_01.20328.20328.2 | 2.8084 | 0.3558 | 99.8% | 1746.2322 | 1746.917 | 122 | 7.18 | 36.7% | 1 | R.SAFSNLFGGEPLSYTR.F | 2 |
|  | CENPL\_stlcld\_tube2\_122314\_01.07911.07911.2 | 4.2415 | 0.4676 | 100.0% | 1562.7522 | 1562.5872 | 1 | 7.854 | 69.2% | 1 | K.LAVDEEENADNNTK.A | 2 |
|  | CENPL\_stlcld\_tube2\_122314\_01.11332.11332.3 | 2.6834 | 0.202 | 97.8% | 1265.7244 | 1265.4575 | 1 | 4.343 | 59.4% | 1 | R.RLYWDDLKR.K | 3 |
|  | CENPL\_stlcld\_122314\_01.11886.11886.2 | 2.4311 | 0.2952 | 99.2% | 1204.4722 | 1205.3561 | 45 | 5.099 | 55.6% | 3 | K.LLNENSYVPR.E | 2 |
|  | CENPL\_stlcld\_122314\_01.14225.14225.2 | 3.0574 | 0.2106 | 98.9% | 1470.7722 | 1469.6812 | 2 | 4.837 | 61.5% | 2 | R.SSGLPNIPVQTISR.A | 2 |
|  | CENPL\_stlcld\_122314\_01.20400.20400.2 | 3.3914 | 0.3726 | 100.0% | 1434.7922 | 1434.7161 | 1 | 7.446 | 70.8% | 1 | K.VSASPLLYTLIEK.T | 2 |
|  | CENPL\_stlcld\_122314\_01.18726.18726.3 | 4.2715 | 0.4855 | 100.0% | 2108.7244 | 2107.4197 | 1 | 7.955 | 40.3% | 2 | R.HVFWGSGSHTLPALLENLK.L | 3 |

---

|  |  |  |  |  |  |  |  |  |
| --- | --- | --- | --- | --- | --- | --- | --- | --- |
| U | *gi|57863257|ref|NP\_11* | 6 | 10 | 12.4% | 556 | 60344 | 6.1 | T-complex protein 1 isoform a [Homo sapiens] |

| Filename XCorr DeltCN Conf% ObsM+H+ CalcM+H+ SpR ZScore Ion% # Sequence  | | | | | | | | | | | | |
| --- | --- | --- | --- | --- | --- | --- | --- | --- | --- | --- | --- | --- |
| \* | CENPL\_stlcld\_122314\_01.16460.16460.2 | 3.7104 | 0.3683 | 100.0% | 1517.5721 | 1517.7838 | 1 | 6.464 | 53.6% | 4 | R.SQNVMAAASIANIVK.S | 2 |
| \* | CENPL\_stlcld\_122314\_01.08625.08625.2 | 2.6743 | 0.1722 | 98.3% | 1107.4321 | 1107.295 | 24 | 4.664 | 66.7% | 1 | K.LLEVEHPAAK.V | 2 |
|  | CENPL\_stlcld\_122314\_01.13427.13427.2 | 2.5957 | 0.2818 | 99.5% | 1148.0521 | 1147.3599 | 1 | 5.056 | 72.2% | 1 | R.YPVNSVNILK.A | 2 |
|  | CENPL\_stlcld\_tube2\_122314\_02.13234.13234.2 | 2.5228 | 0.2886 | 99.1% | 1214.5322 | 1214.4247 | 1 | 5.387 | 75.0% | 1 | K.YFVEAGAMAVR.R | 2 |
|  | CENPL\_stlcld\_122314\_01.14260.14260.2 | 2.4997 | 0.3613 | 99.7% | 1356.8922 | 1356.5675 | 1 | 5.956 | 68.2% | 1 | K.WIGLDLSNGKPR.D | 2 |
|  | CENPL\_stlcld\_122314\_01.16469.16469.2 | 2.692 | 0.2552 | 99.2% | 1206.4922 | 1206.4276 | 1 | 6.071 | 70.0% | 2 | K.FATEAAITILR.I | 2 |

---

|  |  |  |  |  |  |  |  |  |
| --- | --- | --- | --- | --- | --- | --- | --- | --- |
| U | *gi|154800483|ref|NP\_0* | 4 | 6 | 12.4% | 339 | 39541 | 9.1 | centromere protein N isoform 2 [Homo sapiens] |
| U | *gi|154800485|ref|NP\_0* | 4 | 6 | 11.9% | 353 | 41180 | 8.9 | centromere protein N isoform 1 [Homo sapiens] |

| Filename XCorr DeltCN Conf% ObsM+H+ CalcM+H+ SpR ZScore Ion% # Sequence  | | | | | | | | | | | | |
| --- | --- | --- | --- | --- | --- | --- | --- | --- | --- | --- | --- | --- |
|  | CENPL\_stlcld\_122314\_02.16419.16419.2 | 2.6668 | 0.26 | 98.5% | 1968.5721 | 1969.1637 | 40 | 5.763 | 36.7% | 1 | K.AWDFLSENQLQTVNFR.Q | 2 |
|  | CENPL\_stlcld\_tube2\_122314\_01.14538.14538.2 | 3.3003 | 0.3587 | 99.9% | 1584.7522 | 1583.8717 | 1 | 5.673 | 53.6% | 1 | R.RNTPLLGQALTIASK.H | 2 |
|  | CENPL\_stlcld\_122314\_01.17673.17673.2 | 4.1358 | 0.463 | 100.0% | 1427.8121 | 1427.6842 | 1 | 8.468 | 80.8% | 3 | R.NTPLLGQALTIASK.H | 2 |
|  | CENPL\_stlcld\_122314\_01.08223.08223.3 | 3.0807 | 0.3905 | 100.0% | 1381.3744 | 1380.587 | 1 | 6.602 | 55.0% | 1 | R.IIHENIVEKER.V | 3 |

---

|  |  |  |  |  |  |  |  |  |
| --- | --- | --- | --- | --- | --- | --- | --- | --- |
| U | *gi|11136628|ref|NP\_06* | 2 | 8 | 12.4% | 225 | 24764 | 4.7 | eukaryotic translation elongation factor 1 beta 2 [Homo sapiens] |
| U | *gi|83376130|ref|NP\_00* | 2 | 8 | 12.4% | 225 | 24764 | 4.7 | eukaryotic translation elongation factor 1 beta 2 [Homo sapiens] |
| U | *gi|4503477|ref|NP\_001* | 2 | 8 | 12.4% | 225 | 24764 | 4.7 | eukaryotic translation elongation factor 1 beta 2 [Homo sapiens] |

| Filename XCorr DeltCN Conf% ObsM+H+ CalcM+H+ SpR ZScore Ion% # Sequence  | | | | | | | | | | | | |
| --- | --- | --- | --- | --- | --- | --- | --- | --- | --- | --- | --- | --- |
|  | CENPL\_stlcld\_122314\_01.19316.19316.2 | 3.439 | 0.3382 | 99.9% | 1605.6721 | 1604.8003 | 24 | 5.958 | 46.4% | 1 | K.SPAGLQVLNDYLADK.S | 2 |
|  | CENPL\_stlcld\_tube2\_122314\_02.13904.13904.2 | 3.1878 | 0.4147 | 100.0% | 1347.7722 | 1348.4985 | 3 | 7.419 | 62.5% | 7 | R.SIQADGLVWGSSK.L | 2 |

---

|  |  |  |  |  |  |  |  |  |
| --- | --- | --- | --- | --- | --- | --- | --- | --- |
| U | *gi|17402900|ref|NP\_00* | 7 | 14 | 12.3% | 644 | 67560 | 7.6 | far upstream element-binding protein [Homo sapiens] |

| Filename XCorr DeltCN Conf% ObsM+H+ CalcM+H+ SpR ZScore Ion% # Sequence  | | | | | | | | | | | | |
| --- | --- | --- | --- | --- | --- | --- | --- | --- | --- | --- | --- | --- |
| \* | CENPL\_stlcld\_tube2\_122314\_02.11057.11057.2 | 2.5675 | 0.2105 | 97.5% | 1976.2922 | 1975.0355 | 1 | 4.342 | 36.8% | 1 | K.IGGDAGTSLNSNDYGYGGQK.R | 2 |
| \* | CENPL\_stlcld\_122314\_01.12018.12018.2 | 2.6385 | 0.2792 | 99.0% | 1353.0922 | 1353.5181 | 5 | 5.603 | 58.3% | 4 | K.IQIAPDSGGLPER.S | 2 |
| \* | CENPL\_stlcld\_tube2\_122314\_01.14038.14038.2 | 3.0187 | 0.3345 | 99.9% | 1338.6921 | 1337.5187 | 1 | 5.356 | 75.0% | 3 | R.IGGNEGIDVPIPR.F | 2 |
| \* | CENPL\_stlcld\_tube2\_122314\_01.08872.08872.2 | 3.2778 | 0.3896 | 100.0% | 1504.4321 | 1504.6396 | 1 | 6.153 | 75.0% | 2 | R.IQFKPDDGTTPER.I | 2 |
| \* | CENPL\_stlcld\_tube2\_122314\_01.08888.08888.3 | 2.4295 | 0.2629 | 97.2% | 1504.7043 | 1504.6396 | 208 | 4.612 | 33.3% | 1 | R.IQFKPDDGTTPER.I | 3 |
| \* | CENPL\_stlcld\_122314\_01.09308.09308.2 | 2.6498 | 0.1587 | 98.0% | 1068.2122 | 1068.218 | 32 | 5.102 | 61.1% | 2 | R.IAQITGPPDR.C | 2 |
| \* | CENPL\_stlcld\_tube2\_122314\_01.08540.08540.2 | 2.0577 | 0.2251 | 95.3% | 1149.1522 | 1149.2487 | 40 | 4.589 | 66.7% | 1 | R.GTPQQIDYAR.Q | 2 |

---

|  |  |  |  |  |  |  |  |  |
| --- | --- | --- | --- | --- | --- | --- | --- | --- |
| U | *gi|5174447|ref|NP\_006* | 3 | 4 | 12.3% | 317 | 35077 | 7.7 | guanine nucleotide binding protein (G protein), beta polypeptide 2-like 1 [Homo sapiens] |

| Filename XCorr DeltCN Conf% ObsM+H+ CalcM+H+ SpR ZScore Ion% # Sequence  | | | | | | | | | | | | |
| --- | --- | --- | --- | --- | --- | --- | --- | --- | --- | --- | --- | --- |
| \* | CENPL\_stlcld\_tube2\_122314\_01.14778.14778.2 | 2.4691 | 0.2661 | 98.7% | 1265.8722 | 1265.4087 | 1 | 5.32 | 75.0% | 1 | R.LWDLTTGTTTR.R | 2 |
| \* | CENPL\_stlcld\_122314\_02.13437.13437.2 | 2.8445 | 0.4049 | 99.9% | 1310.4122 | 1310.4062 | 1 | 7.143 | 77.3% | 1 | K.DVLSVAFSSDNR.Q | 2 |
| \* | CENPL\_stlcld\_122314\_01.14591.14591.2 | 4.7635 | 0.502 | 100.0% | 1789.1122 | 1790.0642 | 1 | 9.026 | 63.3% | 2 | K.IIVDELKQEVISTSSK.A | 2 |

---

|  |  |  |  |  |  |  |  |  |
| --- | --- | --- | --- | --- | --- | --- | --- | --- |
| U | *gi|31711992|ref|NP\_00* | 5 | 12 | 12.2% | 647 | 68997 | 7.8 | dihydrolipoamide S-acetyltransferase [Homo sapiens] |

| Filename XCorr DeltCN Conf% ObsM+H+ CalcM+H+ SpR ZScore Ion% # Sequence  | | | | | | | | | | | | |
| --- | --- | --- | --- | --- | --- | --- | --- | --- | --- | --- | --- | --- |
| \* | CENPL\_stlcld\_122314\_01.15814.15814.2 | 4.2706 | 0.5299 | 100.0% | 1740.6721 | 1740.0709 | 1 | 9.031 | 62.5% | 5 | K.VPLPSLSPTMQAGTIAR.W | 2 |
| \* | CENPL\_stlcld\_tube2\_122314\_01.14261.14261.2 | 2.5474 | 0.158 | 97.1% | 1266.4321 | 1264.5077 | 4 | 4.105 | 65.0% | 1 | R.SKISVNDFIIK.A | 2 |
| \* | CENPL\_stlcld\_122314\_01.17038.17038.2 | 3.6191 | 0.5332 | 100.0% | 1605.1322 | 1605.8059 | 1 | 8.851 | 61.5% | 1 | K.VPEANSSWMDTVIR.Q | 2 |
| \* | CENPL\_stlcld\_122314\_01.20348.20348.2 | 4.2157 | 0.5413 | 100.0% | 1517.4521 | 1517.7196 | 1 | 8.717 | 67.9% | 3 | K.GVETIANDVVSLATK.A | 2 |
| \* | CENPL\_stlcld\_122314\_02.16401.16401.3 | 3.8671 | 0.3431 | 100.0% | 2424.0842 | 2423.7917 | 12 | 6.091 | 27.4% | 2 | K.LQPHEFQGGTFTISNLGMFGIK.N | 3 |

---

|  |  |  |  |  |  |  |  |  |
| --- | --- | --- | --- | --- | --- | --- | --- | --- |
| U | *gi|21626466|ref|NP\_06* | 6 | 7 | 11.9% | 847 | 94623 | 6.3 | matrin 3 [Homo sapiens] |
| U | *gi|62750354|ref|NP\_95* | 6 | 7 | 11.9% | 847 | 94623 | 6.3 | matrin 3 [Homo sapiens] |

| Filename XCorr DeltCN Conf% ObsM+H+ CalcM+H+ SpR ZScore Ion% # Sequence  | | | | | | | | | | | | |
| --- | --- | --- | --- | --- | --- | --- | --- | --- | --- | --- | --- | --- |
|  | CENPL\_stlcld\_tube2\_122314\_01.00017.00017.2 | 2.5877 | 0.2405 | 98.2% | 2373.0122 | 2372.7424 | 1 | 5.838 | 37.5% | 1 | R.DLSAAGIGLLAAATQSLSMPASLGR.M | 2 |
|  | CENPL\_stlcld\_122314\_01.08327.08327.3 | 3.0818 | 0.2488 | 99.5% | 1523.2444 | 1522.6609 | 12 | 5.458 | 43.8% | 1 | R.RRTEEGPTLSYGR.D | 3 |
|  | CENPL\_stlcld\_122314\_01.08741.08741.2 | 2.6025 | 0.3166 | 99.2% | 1326.6322 | 1325.4269 | 2 | 5.404 | 57.7% | 1 | R.GNLGAGNGNLQGPR.H | 2 |
|  | CENPL\_stlcld\_122314\_01.12210.12210.2 | 2.5611 | 0.2954 | 99.7% | 1145.3121 | 1145.3647 | 1 | 5.941 | 81.2% | 2 | R.VVHIMDFQR.G | 2 |
|  | CENPL\_stlcld\_tube2\_122314\_01.21526.21526.3 | 2.7736 | 0.2482 | 96.3% | 2439.9243 | 2439.9036 | 1 | 4.24 | 30.0% | 1 | R.YQLLQLVEPFGVISNHLILNK.I | 3 |
|  | CENPL\_stlcld\_tube2\_122314\_01.11906.11906.3 | 3.2722 | 0.4142 | 100.0% | 2038.8844 | 2038.3109 | 1 | 7.161 | 36.1% | 1 | R.VIHLSNLPHSGYSDSAVLK.L | 3 |

---

|  |  |  |  |  |  |  |  |  |
| --- | --- | --- | --- | --- | --- | --- | --- | --- |
| U | *gi|4506649|ref|NP\_000* | 4 | 7 | 11.9% | 403 | 46109 | 10.2 | ribosomal protein L3 isoform a [Homo sapiens] |
| U | *gi|76496472|ref|NP\_00* | 4 | 7 | 13.6% | 354 | 40152 | 10.2 | ribosomal protein L3 isoform b [Homo sapiens] |

| Filename XCorr DeltCN Conf% ObsM+H+ CalcM+H+ SpR ZScore Ion% # Sequence  | | | | | | | | | | | | |
| --- | --- | --- | --- | --- | --- | --- | --- | --- | --- | --- | --- | --- |
|  | CENPL\_stlcld\_tube2\_122314\_01.11858.11858.2 | 3.1307 | 0.4863 | 100.0% | 983.9522 | 984.14594 | 1 | 8.259 | 75.0% | 3 | R.HGSLGFLPR.K | 2 |
|  | CENPL\_stlcld\_122314\_02.10288.10288.3 | 3.1398 | 0.1991 | 97.3% | 1826.4243 | 1826.1211 | 8 | 4.622 | 35.9% | 1 | K.KAHLMEIQVNGGTVAEK.L | 3 |
|  | CENPL\_stlcld\_tube2\_122314\_02.11902.11902.3 | 3.1675 | 0.2314 | 98.7% | 1698.4744 | 1697.947 | 28 | 5.513 | 33.3% | 1 | K.AHLMEIQVNGGTVAEK.L | 3 |
|  | CENPL\_stlcld\_122314\_02.16677.16677.3 | 4.2066 | 0.466 | 100.0% | 2438.6343 | 2438.8035 | 1 | 7.578 | 42.9% | 2 | K.SINPLGGFVHYGEVTNDFVMLK.G | 3 |

---

|  |  |  |  |  |  |  |  |  |
| --- | --- | --- | --- | --- | --- | --- | --- | --- |
| U | *gi|4503481|ref|NP\_001* | 4 | 9 | 11.7% | 437 | 50119 | 6.7 | eukaryotic translation elongation factor 1 gamma [Homo sapiens] |

| Filename XCorr DeltCN Conf% ObsM+H+ CalcM+H+ SpR ZScore Ion% # Sequence  | | | | | | | | | | | | |
| --- | --- | --- | --- | --- | --- | --- | --- | --- | --- | --- | --- | --- |
| \* | CENPL\_stlcld\_122314\_02.11000.11000.2 | 4.1398 | 0.269 | 100.0% | 1349.6322 | 1348.5448 | 2 | 7.323 | 66.7% | 4 | K.ALIAAQYSGAQVR.V | 2 |
| \* | CENPL\_stlcld\_122314\_01.14109.14109.2 | 2.8951 | 0.4152 | 100.0% | 1242.5721 | 1242.4172 | 2 | 8.083 | 66.7% | 3 | K.STFVLDEFKR.K | 2 |
| \* | CENPL\_stlcld\_122314\_01.09887.09887.2 | 3.5882 | 0.2579 | 99.9% | 1573.7522 | 1573.7434 | 1 | 6.186 | 76.9% | 1 | R.KLDPGSEETQTLVR.E | 2 |
| \* | CENPL\_stlcld\_122314\_01.17579.17579.2 | 2.5812 | 0.2493 | 98.4% | 1685.0322 | 1685.8357 | 7 | 5.661 | 46.2% | 1 | R.EYFSWEGAFQHVGK.A | 2 |

---

|  |  |  |  |  |  |  |  |  |
| --- | --- | --- | --- | --- | --- | --- | --- | --- |
| U | *gi|9951915|ref|NP\_000* | 4 | 5 | 11.6% | 432 | 47716 | 6.3 | S-adenosylhomocysteine hydrolase [Homo sapiens] |

| Filename XCorr DeltCN Conf% ObsM+H+ CalcM+H+ SpR ZScore Ion% # Sequence  | | | | | | | | | | | | |
| --- | --- | --- | --- | --- | --- | --- | --- | --- | --- | --- | --- | --- |
| \* | CENPL\_stlcld\_122314\_01.16200.16200.2 | 3.3448 | 0.1981 | 99.7% | 1129.9122 | 1129.3042 | 1 | 6.593 | 85.0% | 1 | K.VADIGLAAWGR.K | 2 |
| \* | CENPL\_stlcld\_122314\_01.17151.17151.2 | 2.8925 | 0.3507 | 99.9% | 1561.2322 | 1560.8237 | 30 | 6.145 | 46.2% | 1 | K.ALDIAENEMPGLMR.M | 2 |
| \* | CENPL\_stlcld\_tube2\_122314\_01.10600.10600.2 | 3.0327 | 0.4566 | 100.0% | 1257.3322 | 1257.4294 | 1 | 8.505 | 63.6% | 1 | K.VPAINVNDSVTK.S | 2 |
| \* | CENPL\_stlcld\_tube2\_122314\_01.08883.08883.3 | 4.0737 | 0.2643 | 100.0% | 1381.4043 | 1381.5718 | 1 | 5.58 | 50.0% | 2 | K.KLDEAVAEAHLGK.L | 3 |

---

|  |  |  |  |  |  |  |  |  |
| --- | --- | --- | --- | --- | --- | --- | --- | --- |
| U | *gi|4505763|ref|NP\_000* | 3 | 3 | 11.5% | 417 | 44615 | 8.1 | phosphoglycerate kinase 1 [Homo sapiens] |

| Filename XCorr DeltCN Conf% ObsM+H+ CalcM+H+ SpR ZScore Ion% # Sequence  | | | | | | | | | | | | |
| --- | --- | --- | --- | --- | --- | --- | --- | --- | --- | --- | --- | --- |
| \* | CENPL\_stlcld\_122314\_01.09957.09957.2 | 3.2452 | 0.4885 | 100.0% | 1368.7322 | 1368.5939 | 1 | 8.177 | 75.0% | 1 | R.AHSSMVGVNLPQK.A | 2 |
|  | CENPL\_stlcld\_122314\_01.19283.19283.2 | 3.2375 | 0.3022 | 99.9% | 1770.9521 | 1770.0819 | 9 | 5.469 | 43.8% | 1 | K.ALESPERPFLAILGGAK.V | 2 |
| \* | CENPL\_stlcld\_tube2\_122314\_01.18023.18023.2 | 2.501 | 0.3059 | 98.6% | 1967.9722 | 1968.1859 | 10 | 6.083 | 38.2% | 1 | K.VLNNMEIGTSLFDEEGAK.I | 2 |

---

|  |  |  |  |  |  |  |  |  |
| --- | --- | --- | --- | --- | --- | --- | --- | --- |
| U | *gi|5803137|ref|NP\_006* | 2 | 2 | 11.5% | 157 | 17170 | 8.9 | RNA binding motif protein 3 [Homo sapiens] |

| Filename XCorr DeltCN Conf% ObsM+H+ CalcM+H+ SpR ZScore Ion% # Sequence  | | | | | | | | | | | | |
| --- | --- | --- | --- | --- | --- | --- | --- | --- | --- | --- | --- | --- |
| \* | CENPL\_stlcld\_122314\_01.18424.18424.2 | 4.5873 | 0.527 | 100.0% | 1982.8121 | 1983.2518 | 1 | 9.907 | 55.9% | 1 | R.GFGFITFTNPEHASVAMR.A | 2 |
| \* | CENPL\_stlcld\_tube2\_122314\_01.17914.17914.3 | 3.1877 | 0.2657 | 99.5% | 1983.1444 | 1983.2518 | 1 | 4.941 | 38.2% | 1 | R.GFGFITFTNPEHASVAMR.A | 3 |

---

|  |  |  |  |  |  |  |  |  |
| --- | --- | --- | --- | --- | --- | --- | --- | --- |
| U | *gi|4506681|ref|NP\_001* | 2 | 4 | 11.4% | 158 | 18431 | 10.3 | ribosomal protein S11 [Homo sapiens] |

| Filename XCorr DeltCN Conf% ObsM+H+ CalcM+H+ SpR ZScore Ion% # Sequence  | | | | | | | | | | | | |
| --- | --- | --- | --- | --- | --- | --- | --- | --- | --- | --- | --- | --- |
| \* | CENPL\_stlcld\_tube2\_122314\_01.08572.08572.2 | 3.354 | 0.2975 | 99.9% | 1267.4521 | 1267.4216 | 1 | 5.95 | 80.0% | 3 | K.EAIEGTYIDKK.C | 2 |
|  | CENPL\_stlcld\_122314\_01.12231.12231.2 | 2.1286 | 0.2411 | 98.3% | 980.15216 | 980.1112 | 1 | 6.059 | 83.3% | 1 | R.DYLHYIR.K | 2 |

---

|  |  |  |  |  |  |  |  |  |
| --- | --- | --- | --- | --- | --- | --- | --- | --- |
| U | *gi|47271443|ref|NP\_00* | 2 | 6 | 11.3% | 221 | 25476 | 11.9 | splicing factor, arginine/serine-rich 2 [Homo sapiens] |

| Filename XCorr DeltCN Conf% ObsM+H+ CalcM+H+ SpR ZScore Ion% # Sequence  | | | | | | | | | | | | |
| --- | --- | --- | --- | --- | --- | --- | --- | --- | --- | --- | --- | --- |
|  | CENPL\_stlcld\_tube2\_122314\_01.11228.11228.2 | 2.7435 | 0.1971 | 99.3% | 918.7322 | 919.0684 | 1 | 4.858 | 85.7% | 3 | R.VGDVYIPR.D | 2 |
| \* | CENPL\_stlcld\_122314\_01.17643.17643.2 | 3.4417 | 0.3678 | 99.9% | 1752.2122 | 1752.8654 | 1 | 7.713 | 59.4% | 3 | R.DAEDAMDAMDGAVLDGR.E | 2 |

---

|  |  |  |  |  |  |  |  |  |
| --- | --- | --- | --- | --- | --- | --- | --- | --- |
| U | *gi|15431293|ref|NP\_00* | 2 | 2 | 11.3% | 204 | 24146 | 11.6 | ribosomal protein L15 [Homo sapiens] |
| U | *gi|88998868|ref|XP\_94* | 2 | 2 | 11.3% | 204 | 24174 | 11.6 | PREDICTED: hypothetical protein isoform 4 [Homo sapiens] |
| U | *gi|88992455|ref|XP\_93* | 2 | 2 | 11.3% | 204 | 24174 | 11.6 | PREDICTED: hypothetical protein isoform 1 [Homo sapiens] |
| U | *gi|169169711|ref|XP\_0* | 2 | 2 | 11.3% | 204 | 24174 | 11.6 | PREDICTED: hypothetical protein [Homo sapiens] |

| Filename XCorr DeltCN Conf% ObsM+H+ CalcM+H+ SpR ZScore Ion% # Sequence  | | | | | | | | | | | | |
| --- | --- | --- | --- | --- | --- | --- | --- | --- | --- | --- | --- | --- |
|  | CENPL\_stlcld\_tube2\_122314\_01.08746.08746.2 | 2.8484 | 0.0986 | 98.1% | 1019.3122 | 1019.0996 | 1 | 5.453 | 75.0% | 1 | R.SLQSVAEER.A | 2 |
|  | CENPL\_stlcld\_tube2\_122314\_01.14412.14412.2 | 3.5884 | 0.4665 | 100.0% | 1661.7122 | 1661.8083 | 1 | 7.655 | 65.4% | 1 | R.VLNSYWVGEDSTYK.F | 2 |

---

|  |  |  |  |  |  |  |  |  |
| --- | --- | --- | --- | --- | --- | --- | --- | --- |
| U | *gi|41352697|ref|NP\_00* | 6 | 7 | 11.2% | 756 | 86720 | 8.8 | centromere protein I [Homo sapiens] |

| Filename XCorr DeltCN Conf% ObsM+H+ CalcM+H+ SpR ZScore Ion% # Sequence  | | | | | | | | | | | | |
| --- | --- | --- | --- | --- | --- | --- | --- | --- | --- | --- | --- | --- |
| \* | CENPL\_stlcld\_tube2\_122314\_02.14628.14628.2 | 2.7815 | 0.4213 | 99.9% | 1717.5521 | 1716.8448 | 163 | 6.674 | 33.3% | 1 | R.TSQGSSSFQTTLSAWK.V | 2 |
| \* | CENPL\_stlcld\_122314\_01.20619.20619.3 | 5.6651 | 0.4682 | 100.0% | 3478.7944 | 3479.629 | 1 | 8.279 | 28.3% | 2 | K.HGQNNPVGDYEHADDQAEEDALQMAVGYFEK.G | 3 |
| \* | CENPL\_stlcld\_122314\_01.19068.19068.2 | 3.8022 | 0.4788 | 100.0% | 1731.0322 | 1731.1243 | 1 | 8.954 | 71.4% | 1 | K.MGMQPHLQALLSLYK.F | 2 |
| \* | CENPL\_stlcld\_122314\_01.19110.19110.3 | 2.818 | 0.5016 | 100.0% | 1731.6244 | 1731.1243 | 2 | 7.412 | 35.7% | 1 | K.MGMQPHLQALLSLYK.F | 3 |
| \* | CENPL\_stlcld\_122314\_01.12806.12806.2 | 2.3818 | 0.1994 | 96.8% | 1309.3522 | 1309.6561 | 1 | 3.972 | 63.6% | 1 | K.LMLGPANVRPLK.R | 2 |
| \* | CENPL\_stlcld\_122314\_01.17680.17680.2 | 2.7175 | 0.1617 | 98.0% | 1291.6322 | 1290.4991 | 2 | 4.348 | 65.0% | 1 | K.GIYIDPEILEK.T | 2 |

---

|  |  |  |  |  |  |  |  |  |
| --- | --- | --- | --- | --- | --- | --- | --- | --- |
| U | *gi|78000181|ref|NP\_00* | 2 | 8 | 11.2% | 215 | 23432 | 10.9 | ribosomal protein L14 [Homo sapiens] |
| U | *gi|78000183|ref|NP\_00* | 2 | 8 | 11.2% | 215 | 23432 | 10.9 | ribosomal protein L14 [Homo sapiens] |

| Filename XCorr DeltCN Conf% ObsM+H+ CalcM+H+ SpR ZScore Ion% # Sequence  | | | | | | | | | | | | |
| --- | --- | --- | --- | --- | --- | --- | --- | --- | --- | --- | --- | --- |
|  | CENPL\_stlcld\_122314\_01.11164.11164.2 | 3.0128 | 0.3978 | 99.9% | 1234.0322 | 1233.4124 | 1 | 6.328 | 63.6% | 1 | R.VAYVSFGPHAGK.L | 2 |
|  | CENPL\_stlcld\_122314\_01.18188.18188.2 | 4.427 | 0.4868 | 100.0% | 1355.7122 | 1355.5773 | 1 | 9.506 | 77.3% | 7 | K.LVAIVDVIDQNR.A | 2 |

---

|  |  |  |  |  |  |  |  |  |
| --- | --- | --- | --- | --- | --- | --- | --- | --- |
| U | *gi|5453555|ref|NP\_006* | 3 | 5 | 11.1% | 216 | 24423 | 7.5 | ras-related nuclear protein [Homo sapiens] |

| Filename XCorr DeltCN Conf% ObsM+H+ CalcM+H+ SpR ZScore Ion% # Sequence  | | | | | | | | | | | | |
| --- | --- | --- | --- | --- | --- | --- | --- | --- | --- | --- | --- | --- |
| \* | CENPL\_stlcld\_tube2\_122314\_01.12065.12065.2 | 2.5321 | 0.375 | 99.9% | 1215.5721 | 1215.3483 | 1 | 6.293 | 77.8% | 1 | K.NLQYYDISAK.S | 2 |
| \* | CENPL\_stlcld\_tube2\_122314\_01.19284.19284.2 | 4.0104 | 0.4042 | 100.0% | 1786.3722 | 1786.0427 | 1 | 7.102 | 65.4% | 2 | K.SNYNFEKPFLWLAR.K | 2 |
| \* | CENPL\_stlcld\_122314\_01.19996.19996.3 | 3.779 | 0.3229 | 100.0% | 1786.8544 | 1786.0427 | 1 | 7.349 | 48.1% | 2 | K.SNYNFEKPFLWLAR.K | 3 |

---

|  |  |  |  |  |  |  |  |  |
| --- | --- | --- | --- | --- | --- | --- | --- | --- |
| U | *gi|41872631|ref|NP\_00* | 18 | 28 | 10.8% | 2511 | 273424 | 6.4 | fatty acid synthase [Homo sapiens] |

| Filename XCorr DeltCN Conf% ObsM+H+ CalcM+H+ SpR ZScore Ion% # Sequence  | | | | | | | | | | | | |
| --- | --- | --- | --- | --- | --- | --- | --- | --- | --- | --- | --- | --- |
| \* | CENPL\_stlcld\_122314\_02.00887.00887.3 | 4.6211 | 0.4778 | 100.0% | 3164.4243 | 3165.415 | 1 | 7.213 | 32.7% | 1 | K.LPESENLQEFWDNLIGGVDMVTDDDRR.W | 3 |
| \* | CENPL\_stlcld\_tube2\_122314\_01.15143.15143.2 | 2.8493 | 0.4631 | 100.0% | 1252.4722 | 1252.4148 | 2 | 7.76 | 60.0% | 1 | R.FDASFFGVHPK.Q | 2 |
| \* | CENPL\_stlcld\_tube2\_122314\_01.10590.10590.2 | 2.5126 | 0.3247 | 99.4% | 1298.9722 | 1299.4264 | 2 | 5.822 | 68.2% | 1 | K.VGDPQELNGITR.A | 2 |
| \* | CENPL\_stlcld\_122314\_01.13110.13110.2 | 3.166 | 0.3227 | 99.9% | 1265.1322 | 1264.5106 | 61 | 5.762 | 60.0% | 3 | R.LQVVDQPLPVR.G | 2 |
| \* | CENPL\_stlcld\_tube2\_122314\_01.19340.19340.3 | 3.7647 | 0.2163 | 99.0% | 2473.9143 | 2473.837 | 16 | 4.752 | 29.5% | 2 | R.LHLSGIDANPNALFPPVEFPAPR.G | 3 |
| \* | CENPL\_stlcld\_tube2\_122314\_01.21428.21428.2 | 2.2131 | 0.3301 | 98.5% | 1595.0122 | 1594.9371 | 1 | 5.638 | 57.7% | 1 | R.VLFPATGYLSIVWK.T | 2 |
| \* | CENPL\_stlcld\_tube2\_122314\_02.00932.00932.3 | 3.4159 | 0.287 | 99.9% | 2787.7144 | 2787.3152 | 1 | 4.728 | 29.0% | 1 | R.ALGLGVEQLPVVFEDVVLHQATILPK.T | 3 |
| \* | CENPL\_stlcld\_122314\_02.11765.11765.2 | 2.7672 | 0.3396 | 99.7% | 1550.9922 | 1550.7086 | 2 | 6.411 | 57.1% | 1 | R.AFEVSENGNLVVSGK.V | 2 |
| \* | CENPL\_stlcld\_tube2\_122314\_01.09563.09563.2 | 2.1348 | 0.3424 | 99.2% | 957.5522 | 957.1203 | 1 | 6.205 | 71.4% | 2 | K.HGLYLPTR.V | 2 |
| \* | CENPL\_stlcld\_tube2\_122314\_02.11205.11205.3 | 3.8983 | 0.3709 | 100.0% | 1889.0643 | 1889.1215 | 3 | 6.548 | 36.1% | 2 | R.VTVAGGVHISGLHTESAPR.R | 3 |
| \* | CENPL\_stlcld\_122314\_02.10107.10107.3 | 2.6946 | 0.2965 | 99.4% | 1538.3043 | 1537.7617 | 1 | 4.922 | 55.8% | 1 | K.VVEVLAGHGHLYSR.I | 3 |
| \* | CENPL\_stlcld\_tube2\_122314\_01.14730.14730.2 | 2.4991 | 0.3199 | 98.9% | 1686.2122 | 1686.9054 | 1 | 5.959 | 50.0% | 1 | K.GILADEDSSRPVWLK.A | 2 |
| \* | CENPL\_stlcld\_122314\_01.15479.15479.2 | 2.6864 | 0.332 | 99.7% | 1407.4122 | 1407.6709 | 1 | 5.771 | 72.7% | 2 | K.VLQGDLVMNVYR.D | 2 |
| \* | CENPL\_stlcld\_tube2\_122314\_01.14148.14148.2 | 2.8547 | 0.0923 | 95.5% | 1472.3722 | 1470.5815 | 1 | 3.472 | 66.7% | 1 | R.FPQLDSTSFANSR.D | 2 |
| \* | CENPL\_stlcld\_122314\_01.17568.17568.2 | 3.7106 | 0.3805 | 100.0% | 1624.0721 | 1623.9304 | 3 | 6.946 | 57.1% | 1 | K.VVVQVLAEEPEAVLK.G | 2 |
| \* | CENPL\_stlcld\_tube2\_122314\_01.15779.15779.2 | 2.6391 | 0.3384 | 99.6% | 1428.2122 | 1427.702 | 9 | 5.76 | 58.3% | 3 | R.SLLVNPEGPTLMR.L | 2 |
| \* | CENPL\_stlcld\_122314\_02.00561.00561.3 | 3.3374 | 0.3926 | 100.0% | 2424.4143 | 2423.769 | 1 | 6.8 | 31.8% | 1 | R.TLLEGSGLESIISIIHSSLAEPR.V | 3 |
| \* | CENPL\_stlcld\_tube2\_122314\_02.00839.00839.2 | 4.4935 | 0.4063 | 100.0% | 2424.5322 | 2423.769 | 1 | 7.611 | 43.2% | 3 | R.TLLEGSGLESIISIIHSSLAEPR.V | 2 |

---

|  |  |  |  |  |  |  |  |  |
| --- | --- | --- | --- | --- | --- | --- | --- | --- |
| U | *gi|218505827|ref|NP\_1* | 3 | 5 | 10.8% | 316 | 35438 | 6.3 | TRAF4 associated factor 1 isoform a [Homo sapiens] |
| U | *gi|218505831|ref|NP\_0* | 3 | 5 | 11.9% | 286 | 31880 | 7.1 | TRAF4 associated factor 1 isoform b [Homo sapiens] |

| Filename XCorr DeltCN Conf% ObsM+H+ CalcM+H+ SpR ZScore Ion% # Sequence  | | | | | | | | | | | | |
| --- | --- | --- | --- | --- | --- | --- | --- | --- | --- | --- | --- | --- |
|  | CENPL\_stlcld\_tube2\_122314\_01.16955.16955.3 | 4.7857 | 0.3218 | 100.0% | 2316.2344 | 2316.6543 | 1 | 5.948 | 36.8% | 2 | K.LTETQGELKDLTQKVELLEK.F | 3 |
|  | CENPL\_stlcld\_tube2\_122314\_01.16980.16980.2 | 4.9922 | 0.3506 | 100.0% | 2317.132 | 2316.6543 | 1 | 6.179 | 55.3% | 1 | K.LTETQGELKDLTQKVELLEK.F | 2 |
|  | CENPL\_stlcld\_tube2\_122314\_01.12749.12749.2 | 3.7596 | 0.4402 | 100.0% | 1388.4521 | 1387.5327 | 1 | 7.964 | 65.4% | 2 | K.GLDPALGSETLASR.Q | 2 |

---

|  |  |  |  |  |  |  |  |  |
| --- | --- | --- | --- | --- | --- | --- | --- | --- |
| U | *gi|14110414|ref|NP\_00* | 2 | 6 | 10.8% | 306 | 32835 | 8.2 | heterogeneous nuclear ribonucleoprotein D isoform c [Homo sapiens] |
| U | *gi|51477708|ref|NP\_00* | 2 | 6 | 11.5% | 287 | 30672 | 8.4 | heterogeneous nuclear ribonucleoprotein D isoform d [Homo sapiens] |
| U | *gi|14110420|ref|NP\_11* | 2 | 6 | 9.3% | 355 | 38434 | 7.8 | heterogeneous nuclear ribonucleoprotein D isoform a [Homo sapiens] |
| U | *gi|14110417|ref|NP\_11* | 2 | 6 | 9.8% | 336 | 36272 | 8.1 | heterogeneous nuclear ribonucleoprotein D isoform b [Homo sapiens] |

| Filename XCorr DeltCN Conf% ObsM+H+ CalcM+H+ SpR ZScore Ion% # Sequence  | | | | | | | | | | | | |
| --- | --- | --- | --- | --- | --- | --- | --- | --- | --- | --- | --- | --- |
|  | CENPL\_stlcld\_122314\_01.13514.13514.2 | 3.9433 | 0.393 | 100.0% | 1489.3922 | 1489.6653 | 1 | 7.292 | 69.2% | 5 | K.IFVGGLSPDTPEEK.I | 2 |
|  | CENPL\_stlcld\_122314\_02.16426.16426.2 | 3.2977 | 0.2967 | 99.9% | 2161.892 | 2162.3755 | 5 | 5.202 | 38.9% | 1 | R.EYFGGFGEVESIELPMDNK.T | 2 |

---

|  |  |  |  |  |  |  |  |  |
| --- | --- | --- | --- | --- | --- | --- | --- | --- |
| U | *gi|5031857|ref|NP\_005* | 3 | 3 | 10.5% | 332 | 36689 | 8.3 | L-lactate dehydrogenase A isoform 1 [Homo sapiens] |

| Filename XCorr DeltCN Conf% ObsM+H+ CalcM+H+ SpR ZScore Ion% # Sequence  | | | | | | | | | | | | |
| --- | --- | --- | --- | --- | --- | --- | --- | --- | --- | --- | --- | --- |
|  | CENPL\_stlcld\_122314\_02.13154.13154.3 | 3.1882 | 0.4184 | 100.0% | 1876.5243 | 1876.2422 | 1 | 6.468 | 41.7% | 1 | K.LKGEMMDLQHGSLFLR.T | 3 |
| \* | CENPL\_stlcld\_122314\_02.10014.10014.2 | 2.3454 | 0.1628 | 96.8% | 914.47217 | 914.13617 | 1 | 5.133 | 81.2% | 1 | K.LVIITAGAR.Q | 2 |
|  | CENPL\_stlcld\_tube2\_122314\_01.14099.14099.2 | 2.3453 | 0.1974 | 97.5% | 1119.1122 | 1119.2627 | 5 | 4.278 | 66.7% | 1 | K.SADTLWGIQK.E | 2 |

---

|  |  |  |  |  |  |  |  |  |
| --- | --- | --- | --- | --- | --- | --- | --- | --- |
| U | *gi|28875797|ref|NP\_05* | 2 | 2 | 10.5% | 248 | 26397 | 12.2 | hypothetical protein LOC26097 [Homo sapiens] |

| Filename XCorr DeltCN Conf% ObsM+H+ CalcM+H+ SpR ZScore Ion% # Sequence  | | | | | | | | | | | | |
| --- | --- | --- | --- | --- | --- | --- | --- | --- | --- | --- | --- | --- |
| \* | CENPL\_stlcld\_122314\_01.08294.08294.2 | 4.1432 | 0.3883 | 100.0% | 1447.6921 | 1447.6091 | 1 | 7.403 | 70.8% | 1 | R.ASMQQQQQLASAR.N | 2 |
| \* | CENPL\_stlcld\_122314\_02.12370.12370.2 | 2.798 | 0.3415 | 99.9% | 1556.2722 | 1555.6997 | 6 | 6.159 | 50.0% | 1 | K.EQLDNQLDAYMSK.T | 2 |

---

|  |  |  |  |  |  |  |  |  |
| --- | --- | --- | --- | --- | --- | --- | --- | --- |
| U | *gi|14141157|ref|NP\_03* | 3 | 11 | 10.4% | 346 | 36926 | 6.9 | heterogeneous nuclear ribonucleoprotein H3 isoform a [Homo sapiens] |
| U | *gi|14141159|ref|NP\_06* | 3 | 11 | 10.9% | 331 | 35239 | 6.9 | heterogeneous nuclear ribonucleoprotein H3 isoform b [Homo sapiens] |

| Filename XCorr DeltCN Conf% ObsM+H+ CalcM+H+ SpR ZScore Ion% # Sequence  | | | | | | | | | | | | |
| --- | --- | --- | --- | --- | --- | --- | --- | --- | --- | --- | --- | --- |
|  | CENPL\_stlcld\_tube2\_122314\_01.13517.13517.2 | 3.4197 | 0.4278 | 100.0% | 1273.3722 | 1272.4001 | 1 | 8.707 | 72.7% | 8 | R.STGEAFVQFASK.E | 2 |
|  | CENPL\_stlcld\_122314\_01.09700.09700.2 | 2.2659 | 0.3339 | 99.2% | 1053.4521 | 1053.1631 | 1 | 6.049 | 77.8% | 1 | R.VHIDIGADGR.A | 2 |
|  | CENPL\_stlcld\_122314\_01.09095.09095.2 | 3.5379 | 0.391 | 100.0% | 1413.1921 | 1413.4613 | 8 | 7.262 | 53.8% | 2 | R.DGMDNQGGYGSVGR.M | 2 |

---

|  |  |  |  |  |  |  |  |  |
| --- | --- | --- | --- | --- | --- | --- | --- | --- |
| U | *gi|113408763|ref|XP\_0* | 2 | 2 | 10.4% | 249 | 26943 | 8.1 | PREDICTED: hypothetical protein isoform 5 [Homo sapiens] |
| U | *gi|169160691|ref|XP\_0* | 2 | 2 | 10.4% | 249 | 26999 | 8.1 | PREDICTED: similar to rcTPI1 isoform 2 [Homo sapiens] |

| Filename XCorr DeltCN Conf% ObsM+H+ CalcM+H+ SpR ZScore Ion% # Sequence  | | | | | | | | | | | | |
| --- | --- | --- | --- | --- | --- | --- | --- | --- | --- | --- | --- | --- |
|  | CENPL\_stlcld\_tube2\_122314\_01.10206.10206.2 | 2.0893 | 0.2482 | 96.3% | 1276.4722 | 1275.4471 | 294 | 5.527 | 40.0% | 1 | K.VIADNVKDWSK.V | 2 |
|  | CENPL\_stlcld\_122314\_01.19889.19889.2 | 3.1061 | 0.2682 | 99.5% | 1605.8322 | 1603.9017 | 6 | 5.201 | 50.0% | 1 | K.VVLAYEPVWAIGTGK.T | 2 |

---

|  |  |  |  |  |  |  |  |  |
| --- | --- | --- | --- | --- | --- | --- | --- | --- |
| U | *gi|124256496|ref|NP\_0* | 5 | 16 | 10.3% | 641 | 70375 | 6.0 | heat shock 70kDa protein 1-like [Homo sapiens] |

| Filename XCorr DeltCN Conf% ObsM+H+ CalcM+H+ SpR ZScore Ion% # Sequence  | | | | | | | | | | | | |
| --- | --- | --- | --- | --- | --- | --- | --- | --- | --- | --- | --- | --- |
|  | CENPL\_stlcld\_tube2\_122314\_01.12731.12731.2 | 3.5901 | 0.5406 | 100.0% | 1488.3322 | 1488.5939 | 1 | 9.387 | 75.0% | 8 | R.TTPSYVAFTDTER.L | 222 |
|  | CENPL\_stlcld\_tube2\_122314\_01.19245.19245.2 | 2.7077 | 0.2462 | 98.5% | 1616.4321 | 1615.8817 | 1 | 5.626 | 65.4% | 2 | K.AFYPEEISSMVLTK.L | 22 |
|  | CENPL\_stlcld\_122314\_02.14764.14764.2 | 3.2162 | 0.3474 | 99.9% | 1198.7922 | 1198.408 | 1 | 6.851 | 81.8% | 1 | K.DAGVIAGLNVLR.I | 22 |
|  | CENPL\_stlcld\_122314\_01.16466.16466.2 | 5.1137 | 0.5254 | 100.0% | 1660.8722 | 1660.9078 | 1 | 10.001 | 76.7% | 3 | R.IINEPTAAAIAYGLDK.G | 222 |
|  | CENPL\_stlcld\_122314\_01.15185.15185.2 | 3.0827 | 0.3993 | 100.0% | 1288.5721 | 1288.4608 | 1 | 8.185 | 75.0% | 2 | K.NALESYAFNMK.S | 22 |

Similarities:
gi|5729877|ref|NP\_006(2:3)  
gi|167466173|ref|NP\_0(4:1)  
contaminant\_GR78\_HUMA(1:4)  

---

|  |  |  |  |  |  |  |  |  |
| --- | --- | --- | --- | --- | --- | --- | --- | --- |
| U | *gi|21327708|ref|NP\_63* | 3 | 6 | 10.2% | 391 | 45374 | 4.5 | nucleosome assembly protein 1-like 1 [Homo sapiens] |
| U | *gi|4758756|ref|NP\_004* | 3 | 6 | 10.2% | 391 | 45374 | 4.5 | nucleosome assembly protein 1-like 1 [Homo sapiens] |

| Filename XCorr DeltCN Conf% ObsM+H+ CalcM+H+ SpR ZScore Ion% # Sequence  | | | | | | | | | | | | |
| --- | --- | --- | --- | --- | --- | --- | --- | --- | --- | --- | --- | --- |
|  | CENPL\_stlcld\_122314\_01.11463.11463.2 | 2.5835 | 0.3825 | 99.9% | 1337.4922 | 1337.4314 | 1 | 5.938 | 77.8% | 4 | K.FYEEVHDLER.K | 22 |
|  | CENPL\_stlcld\_122314\_01.15446.15446.2 | 1.9997 | 0.2632 | 95.3% | 1512.4521 | 1513.7795 | 1 | 4.364 | 68.2% | 1 | K.YAVLYQPLFDKR.F | 2 |
|  | CENPL\_stlcld\_tube2\_122314\_01.18043.18043.2 | 4.8676 | 0.3492 | 100.0% | 2095.7322 | 2096.36 | 1 | 7.89 | 64.7% | 1 | K.NVDLLSDMVQEHDEPILK.H | 2 |

Similarities:
gi|5174613|ref|NP\_005(1:2)  

---

|  |  |  |  |  |  |  |  |  |
| --- | --- | --- | --- | --- | --- | --- | --- | --- |
| U | *gi|4758256|ref|NP\_004* | 2 | 2 | 10.2% | 315 | 36112 | 5.1 | eukaryotic translation initiation factor 2, subunit 1 alpha, 35kDa [Homo sapiens] |

| Filename XCorr DeltCN Conf% ObsM+H+ CalcM+H+ SpR ZScore Ion% # Sequence  | | | | | | | | | | | | |
| --- | --- | --- | --- | --- | --- | --- | --- | --- | --- | --- | --- | --- |
| \* | CENPL\_stlcld\_tube2\_122314\_01.16791.16791.3 | 4.278 | 0.4733 | 100.0% | 2435.8145 | 2435.6958 | 1 | 7.325 | 43.4% | 1 | R.HVAEVLEYTKDEQLESLFQR.T | 3 |
| \* | CENPL\_stlcld\_tube2\_122314\_01.09156.09156.2 | 3.0739 | 0.4652 | 100.0% | 1349.6322 | 1349.4375 | 1 | 7.242 | 72.7% | 1 | K.VVTDTDETELAR.Q | 2 |

---

|  |  |  |  |  |  |  |  |  |
| --- | --- | --- | --- | --- | --- | --- | --- | --- |
| U | *gi|4503519|ref|NP\_003* | 2 | 2 | 10.1% | 357 | 37564 | 5.4 | eukaryotic translation initiation factor 3, subunit 5 epsilon, 47kDa [Homo sapiens] |

| Filename XCorr DeltCN Conf% ObsM+H+ CalcM+H+ SpR ZScore Ion% # Sequence  | | | | | | | | | | | | |
| --- | --- | --- | --- | --- | --- | --- | --- | --- | --- | --- | --- | --- |
| \* | CENPL\_stlcld\_122314\_02.12227.12227.2 | 4.4851 | 0.5563 | 100.0% | 1658.5122 | 1658.8522 | 2 | 9.77 | 53.1% | 1 | R.VIGLSSDLQQVGGASAR.I | 2 |
| \* | CENPL\_stlcld\_122314\_02.02013.02013.2 | 2.8259 | 0.4198 | 99.9% | 2051.0122 | 2051.3013 | 117 | 6.511 | 30.6% | 1 | R.IQDALSTVLQYAEDVLSGK.V | 2 |

---

|  |  |  |  |  |  |  |  |  |
| --- | --- | --- | --- | --- | --- | --- | --- | --- |
| U | *gi|20336746|ref|NP\_61* | 2 | 2 | 10.0% | 369 | 39183 | 9.8 | H2A histone family, member Y isoform 1 [Homo sapiens] |
| U | *gi|93141020|ref|NP\_00* | 2 | 2 | 10.0% | 371 | 39489 | 9.8 | H2A histone family, member Y isoform 2 [Homo sapiens] |
| U | *gi|93141018|ref|NP\_61* | 2 | 2 | 9.9% | 372 | 39617 | 9.8 | H2A histone family, member Y isoform 3 [Homo sapiens] |
| U | *gi|4758496|ref|NP\_004* | 2 | 2 | 10.0% | 371 | 39489 | 9.8 | H2A histone family, member Y isoform 2 [Homo sapiens] |

| Filename XCorr DeltCN Conf% ObsM+H+ CalcM+H+ SpR ZScore Ion% # Sequence  | | | | | | | | | | | | |
| --- | --- | --- | --- | --- | --- | --- | --- | --- | --- | --- | --- | --- |
|  | CENPL\_stlcld\_tube2\_122314\_01.18436.18436.2 | 3.7252 | 0.5198 | 100.0% | 1933.8121 | 1934.2432 | 1 | 8.334 | 40.6% | 1 | R.HILLAVANDEELNQLLK.G | 2 |
|  | CENPL\_stlcld\_tube2\_122314\_01.16983.16983.2 | 4.3286 | 0.4317 | 100.0% | 1988.1122 | 1987.35 | 1 | 7.603 | 57.9% | 1 | K.GVTIASGGVLPNIHPELLAK.K | 2 |

---

|  |  |  |  |  |  |  |  |  |
| --- | --- | --- | --- | --- | --- | --- | --- | --- |
| U | *gi|4758012|ref|NP\_004* | 11 | 22 | 9.9% | 1675 | 191613 | 5.7 | clathrin heavy chain 1 [Homo sapiens] |

| Filename XCorr DeltCN Conf% ObsM+H+ CalcM+H+ SpR ZScore Ion% # Sequence  | | | | | | | | | | | | |
| --- | --- | --- | --- | --- | --- | --- | --- | --- | --- | --- | --- | --- |
| \* | CENPL\_stlcld\_122314\_02.15983.15983.2 | 2.2062 | 0.2327 | 95.9% | 1499.8522 | 1500.741 | 10 | 4.854 | 54.2% | 1 | K.WLLLTGISAQQNR.V | 2 |
|  | CENPL\_stlcld\_tube2\_122314\_01.13784.13784.2 | 2.6151 | 0.1684 | 97.5% | 1338.4722 | 1338.5646 | 22 | 4.374 | 59.1% | 2 | R.VVGAMQLYSVDR.K | 2 |
| \* | CENPL\_stlcld\_122314\_02.10641.10641.3 | 3.2516 | 0.2102 | 98.3% | 1846.4944 | 1846.096 | 1 | 5.118 | 39.1% | 1 | R.KVSQPIEGHAASFAQFK.M | 3 |
| \* | CENPL\_stlcld\_tube2\_122314\_01.15741.15741.2 | 3.7085 | 0.4225 | 100.0% | 1305.8322 | 1305.4331 | 1 | 6.874 | 77.3% | 4 | R.NNLAGAEELFAR.K | 2 |
| \* | CENPL\_stlcld\_122314\_01.13314.13314.3 | 3.8578 | 0.4712 | 100.0% | 1622.5443 | 1621.8333 | 1 | 7.944 | 50.0% | 2 | R.ALEHFTDLYDIKR.A | 3 |
| \* | CENPL\_stlcld\_tube2\_122314\_01.08884.08884.2 | 3.1313 | 0.3525 | 99.9% | 1335.3522 | 1335.416 | 1 | 6.586 | 75.0% | 2 | K.IYIDSNNNPER.F | 2 |
| \* | CENPL\_stlcld\_122314\_02.14355.14355.3 | 4.0941 | 0.3303 | 100.0% | 2160.7144 | 2160.4375 | 3 | 6.021 | 35.3% | 1 | R.RKDPELWGSVLLESNPYR.R | 3 |
|  | CENPL\_stlcld\_122314\_02.00867.00867.2 | 2.586 | 0.2851 | 98.6% | 1948.5922 | 1948.2819 | 4 | 4.856 | 40.6% | 1 | K.AFMTADLPNELIELLEK.I | 2 |
| \* | CENPL\_stlcld\_122314\_01.16137.16137.3 | 4.0048 | 0.3942 | 100.0% | 2466.5645 | 2465.6848 | 1 | 6.918 | 34.5% | 1 | R.LAELEEFINGPNNAHIQQVGDR.C | 3 |
| \* | CENPL\_stlcld\_tube2\_122314\_01.13504.13504.2 | 3.1705 | 0.4502 | 100.0% | 1296.9122 | 1297.4563 | 1 | 8.243 | 75.0% | 4 | K.LLYNNVSNFGR.L | 2 |
| \* | CENPL\_stlcld\_tube2\_122314\_02.13659.13659.3 | 3.7377 | 0.3768 | 100.0% | 1972.4043 | 1972.2083 | 1 | 6.512 | 38.9% | 3 | R.LASTLVHLGEYQAAVDGAR.K | 3 |

---

|  |  |  |  |  |  |  |  |  |
| --- | --- | --- | --- | --- | --- | --- | --- | --- |
| U | *gi|24638454|ref|NP\_73* | 7 | 25 | 9.8% | 1042 | 114757 | 5.3 | ATPase, Ca++ transporting, slow twitch 2 isoform 1 [Homo sapiens] |
| U | *gi|4502285|ref|NP\_001* | 7 | 25 | 10.2% | 997 | 109691 | 5.4 | ATPase, Ca++ transporting, slow twitch 2 isoform 2 [Homo sapiens] |

| Filename XCorr DeltCN Conf% ObsM+H+ CalcM+H+ SpR ZScore Ion% # Sequence  | | | | | | | | | | | | |
| --- | --- | --- | --- | --- | --- | --- | --- | --- | --- | --- | --- | --- |
|  | CENPL\_stlcld\_tube2\_122314\_01.16793.16793.3 | 4.7888 | 0.4866 | 100.0% | 2601.8044 | 2601.9163 | 2 | 7.923 | 30.4% | 6 | K.TVEEVLGHFGVNESTGLSLEQVKK.L | 3 |
|  | CENPL\_stlcld\_122314\_02.12814.12814.2 | 4.5355 | 0.3587 | 100.0% | 1576.8722 | 1575.7997 | 1 | 7.297 | 67.9% | 9 | R.VDQSILTGESVSVIK.H | 22 |
|  | CENPL\_stlcld\_122314\_01.14544.14544.2 | 2.4158 | 0.231 | 97.7% | 1324.2322 | 1324.5377 | 11 | 5.098 | 54.2% | 1 | K.NMLFSGTNIAAGK.A | 22 |
|  | CENPL\_stlcld\_122314\_02.11996.11996.2 | 3.7109 | 0.4528 | 100.0% | 1547.2122 | 1546.8224 | 1 | 7.7 | 56.7% | 3 | K.AMGVVVATGVNTEIGK.I | 2 |
|  | CENPL\_stlcld\_tube2\_122314\_01.08091.08091.2 | 3.8322 | 0.3072 | 100.0% | 1477.6322 | 1477.6323 | 2 | 6.901 | 68.2% | 2 | K.IRDEMVATEQER.T | 2 |
|  | CENPL\_stlcld\_tube2\_122314\_01.14878.14878.2 | 1.9391 | 0.2693 | 96.6% | 1097.8922 | 1097.2714 | 10 | 4.597 | 68.8% | 1 | K.MNVFDTELK.G | 2 |
|  | CENPL\_stlcld\_tube2\_122314\_01.14507.14507.2 | 3.3198 | 0.3898 | 100.0% | 1409.2122 | 1409.5358 | 1 | 7.592 | 66.7% | 3 | R.IGIFGQDEDVTSK.A | 2 |

Similarities:
gi|10835220|ref|NP\_00(2:5)  

---

|  |  |  |  |  |  |  |  |  |
| --- | --- | --- | --- | --- | --- | --- | --- | --- |
| U | *gi|18375623|ref|NP\_54* | 4 | 9 | 9.8% | 428 | 48991 | 5.7 | HLA-B associated transcript 1 [Homo sapiens] |
| U | *gi|4758112|ref|NP\_004* | 4 | 8 | 9.8% | 428 | 48991 | 5.7 | HLA-B associated transcript 1 [Homo sapiens] |
| U | *gi|21040371|ref|NP\_00* | 4 | 9 | 9.8% | 427 | 49130 | 5.7 | DEAD (Asp-Glu-Ala-Asp) box polypeptide 39 [Homo sapiens] |

| Filename XCorr DeltCN Conf% ObsM+H+ CalcM+H+ SpR ZScore Ion% # Sequence  | | | | | | | | | | | | |
| --- | --- | --- | --- | --- | --- | --- | --- | --- | --- | --- | --- | --- |
|  | CENPL\_stlcld\_122314\_01.17884.17884.2 | 2.3783 | 0.252 | 98.5% | 1244.5521 | 1244.5199 | 2 | 5.226 | 77.8% | 2 | R.DFLLKPELLR.A | 2 |
|  | CENPL\_stlcld\_122314\_01.10241.10241.2 | 2.4212 | 0.089 | 95.1% | 1064.2522 | 1063.2015 | 6 | 4.051 | 85.7% | 1 | R.RDVQEIFR.M | 2 |
|  | CENPL\_stlcld\_tube2\_122314\_01.17440.17440.2 | 2.2548 | 0.1717 | 95.2% | 1103.3522 | 1104.3378 | 2 | 4.722 | 77.8% | 1 | R.ILVATNLFGR.G | 2 |
|  | CENPL\_stlcld\_122314\_01.15183.15183.2 | 3.6236 | 0.4948 | 100.0% | 1480.6322 | 1480.6146 | 1 | 7.758 | 73.1% | 5 | K.GLAITFVSDENDAK.I | 2 |

---

|  |  |  |  |  |  |  |  |  |
| --- | --- | --- | --- | --- | --- | --- | --- | --- |
| U | *gi|169213536|ref|XP\_0* | 2 | 3 | 9.8% | 214 | 24627 | 10.1 | PREDICTED: similar to QM protein isoform 1 [Homo sapiens] |
| U | *gi|41151097|ref|XP\_20* | 2 | 3 | 9.8% | 214 | 24627 | 10.1 | PREDICTED: similar to QM protein isoform 1 [Homo sapiens] |
| U | *gi|223890243|ref|NP\_0* | 2 | 3 | 9.8% | 214 | 24604 | 10.1 | ribosomal protein L10 [Homo sapiens] |
| U | *gi|18152783|ref|NP\_54* | 2 | 3 | 9.8% | 214 | 24519 | 10.0 | ribosomal protein L10-like protein [Homo sapiens] |
| U | *gi|169213732|ref|XP\_0* | 2 | 3 | 9.8% | 214 | 24600 | 10.1 | PREDICTED: similar to Q1Z 7F5 isoform 1 [Homo sapiens] |

| Filename XCorr DeltCN Conf% ObsM+H+ CalcM+H+ SpR ZScore Ion% # Sequence  | | | | | | | | | | | | |
| --- | --- | --- | --- | --- | --- | --- | --- | --- | --- | --- | --- | --- |
|  | CENPL\_stlcld\_tube2\_122314\_01.09010.09010.3 | 2.514 | 0.2838 | 99.3% | 1273.2544 | 1274.5576 | 332 | 5.915 | 41.7% | 1 | R.VRLHPFHVIR.I | 3 |
|  | CENPL\_stlcld\_122314\_01.14319.14319.2 | 2.9878 | 0.479 | 100.0% | 1253.3522 | 1253.5486 | 8 | 8.483 | 60.0% | 2 | R.VHIGQVIMSIR.T | 2 |

---

|  |  |  |  |  |  |  |  |  |
| --- | --- | --- | --- | --- | --- | --- | --- | --- |
| U | *gi|5453603|ref|NP\_006* | 4 | 7 | 9.7% | 535 | 57488 | 6.4 | chaperonin containing TCP1, subunit 2 [Homo sapiens] |

| Filename XCorr DeltCN Conf% ObsM+H+ CalcM+H+ SpR ZScore Ion% # Sequence  | | | | | | | | | | | | |
| --- | --- | --- | --- | --- | --- | --- | --- | --- | --- | --- | --- | --- |
| \* | CENPL\_stlcld\_tube2\_122314\_02.14320.14320.2 | 3.1828 | 0.1608 | 98.5% | 1550.5322 | 1549.7797 | 8 | 4.7 | 50.0% | 4 | R.DASLMVTNDGATILK.N | 2 |
| \* | CENPL\_stlcld\_tube2\_122314\_01.19003.19003.2 | 2.9873 | 0.0866 | 95.1% | 2530.9922 | 2529.8682 | 86 | 3.86 | 27.1% | 1 | R.DASLMVTNDGATILKNIGVDNPAAK.V | 2 |
| \* | CENPL\_stlcld\_tube2\_122314\_01.11700.11700.2 | 3.3548 | 0.4314 | 100.0% | 1331.2522 | 1331.4252 | 1 | 7.221 | 81.8% | 1 | R.GATQQILDEAER.S | 2 |
| \* | CENPL\_stlcld\_122314\_01.08495.08495.3 | 2.7781 | 0.2801 | 99.1% | 1532.1543 | 1531.6403 | 382 | 4.615 | 26.8% | 1 | R.AAHSEGNTTAGLDMR.E | 3 |

---

|  |  |  |  |  |  |  |  |  |
| --- | --- | --- | --- | --- | --- | --- | --- | --- |
| U | *gi|169204113|ref|XP\_0* | 2 | 3 | 9.5% | 295 | 32744 | 4.5 | PREDICTED: similar to 40S ribosomal protein SA (p40) (34/67 kDa laminin receptor) (Colon carcinoma laminin-binding protein) (NEM/1CHD4) (Multidrug resistance-associated protein MGr1-Ag) isoform 2 [Homo sapiens] |
| U | *gi|9845502|ref|NP\_002* | 2 | 3 | 9.5% | 295 | 32854 | 4.9 | ribosomal protein SA [Homo sapiens] |
| U | *gi|59859885|ref|NP\_00* | 2 | 3 | 9.5% | 295 | 32854 | 4.9 | ribosomal protein SA [Homo sapiens] |
| U | *gi|41201737|ref|XP\_37* | 2 | 3 | 9.5% | 295 | 32744 | 4.5 | PREDICTED: similar to 40S ribosomal protein SA (p40) (34/67 kDa laminin receptor) (Colon carcinoma laminin-binding protein) (NEM/1CHD4) (Multidrug resistance-associated protein MGr1-Ag) isoform 3 [Homo sapiens] |
| U | *gi|169205444|ref|XP\_0* | 2 | 3 | 9.5% | 295 | 32744 | 4.5 | PREDICTED: similar to 40S ribosomal protein SA (p40) (34/67 kDa laminin receptor) (Colon carcinoma laminin-binding protein) (NEM/1CHD4) (Multidrug resistance-associated protein MGr1-Ag) isoform 1 [Homo sapiens] |
| U | *gi|169205442|ref|XP\_0* | 2 | 3 | 9.5% | 295 | 32744 | 4.5 | PREDICTED: similar to 40S ribosomal protein SA (p40) (34/67 kDa laminin receptor) (Colon carcinoma laminin-binding protein) (NEM/1CHD4) (Multidrug resistance-associated protein MGr1-Ag) isoform 3 [Homo sapiens] |
| U | *gi|169205440|ref|XP\_0* | 2 | 3 | 9.5% | 295 | 32744 | 4.5 | PREDICTED: similar to 40S ribosomal protein SA (p40) (34/67 kDa laminin receptor) (Colon carcinoma laminin-binding protein) (NEM/1CHD4) (Multidrug resistance-associated protein MGr1-Ag) isoform 2 [Homo sapiens] |
| U | *gi|169204115|ref|XP\_0* | 2 | 3 | 9.5% | 295 | 32744 | 4.5 | PREDICTED: similar to 40S ribosomal protein SA (p40) (34/67 kDa laminin receptor) (Colon carcinoma laminin-binding protein) (NEM/1CHD4) (Multidrug resistance-associated protein MGr1-Ag) isoform 1 [Homo sapiens] |

| Filename XCorr DeltCN Conf% ObsM+H+ CalcM+H+ SpR ZScore Ion% # Sequence  | | | | | | | | | | | | |
| --- | --- | --- | --- | --- | --- | --- | --- | --- | --- | --- | --- | --- |
|  | CENPL\_stlcld\_122314\_01.09992.09992.2 | 3.7754 | 0.4597 | 100.0% | 1204.2922 | 1204.3713 | 1 | 8.911 | 87.5% | 2 | K.FAAATGATPIAGR.F | 2 |
|  | CENPL\_stlcld\_122314\_01.19542.19542.2 | 3.9069 | 0.5752 | 100.0% | 1700.3522 | 1699.9065 | 1 | 9.173 | 67.9% | 1 | R.FTPGTFTNQIQAAFR.E | 2 |

---

|  |  |  |  |  |  |  |  |  |
| --- | --- | --- | --- | --- | --- | --- | --- | --- |
| U | *gi|4506723|ref|NP\_000* | 2 | 2 | 9.5% | 264 | 29945 | 9.7 | ribosomal protein S3a [Homo sapiens] |

| Filename XCorr DeltCN Conf% ObsM+H+ CalcM+H+ SpR ZScore Ion% # Sequence  | | | | | | | | | | | | |
| --- | --- | --- | --- | --- | --- | --- | --- | --- | --- | --- | --- | --- |
|  | CENPL\_stlcld\_122314\_01.09766.09766.2 | 3.8507 | 0.4283 | 100.0% | 1516.1522 | 1516.6915 | 1 | 6.407 | 66.7% | 1 | R.EVQTNDLKEVVNK.L | 2 |
| \* | CENPL\_stlcld\_tube2\_122314\_01.11627.11627.2 | 2.3799 | 0.3217 | 99.0% | 1328.4321 | 1328.5486 | 149 | 5.303 | 45.5% | 1 | K.LIPDSIGKDIEK.A | 2 |

---

|  |  |  |  |  |  |  |  |  |
| --- | --- | --- | --- | --- | --- | --- | --- | --- |
| U | *gi|116875765|ref|NP\_7* | 9 | 13 | 9.4% | 1668 | 186965 | 6.8 | tight junction protein 1 isoform b [Homo sapiens] |
| U | *gi|116875767|ref|NP\_0* | 9 | 13 | 9.0% | 1748 | 195457 | 6.7 | tight junction protein 1 isoform a [Homo sapiens] |

| Filename XCorr DeltCN Conf% ObsM+H+ CalcM+H+ SpR ZScore Ion% # Sequence  | | | | | | | | | | | | |
| --- | --- | --- | --- | --- | --- | --- | --- | --- | --- | --- | --- | --- |
|  | CENPL\_stlcld\_tube2\_122314\_02.13460.13460.3 | 3.2245 | 0.3468 | 99.9% | 2242.3442 | 2241.4834 | 4 | 5.602 | 34.7% | 1 | K.STAMEETAIWEQHTVTLHR.A | 3 |
|  | CENPL\_stlcld\_122314\_02.11649.11649.3 | 3.6153 | 0.2272 | 99.5% | 1986.1743 | 1986.2786 | 9 | 5.518 | 33.8% | 1 | R.LASHIFVKEISQDSLAAR.D | 3 |
|  | CENPL\_stlcld\_122314\_01.07778.07778.3 | 3.7654 | 0.4678 | 100.0% | 2166.7744 | 2166.3555 | 2 | 7.036 | 39.5% | 1 | R.ISKPGAVS\*TPVKHADDHTPK.T | 3 |
|  | CENPL\_stlcld\_122314\_02.01121.01121.3 | 5.3509 | 0.4031 | 100.0% | 3742.8245 | 3743.3354 | 1 | 6.65 | 28.1% | 1 | R.VNNVDFTNIIREEAVLFLLDLPKGEEVTILAQK.K | 3 |
|  | CENPL\_stlcld\_tube2\_122314\_02.14411.14411.2 | 3.5586 | 0.4235 | 100.0% | 1501.7322 | 1500.6482 | 1 | 6.88 | 66.7% | 3 | R.IVESDVGDSFYIR.T | 2 |
|  | CENPL\_stlcld\_tube2\_122314\_01.19844.19844.3 | 4.2839 | 0.34 | 100.0% | 2030.6643 | 2030.3774 | 5 | 6.994 | 33.3% | 2 | R.EAGFLRPVTIFGPIADVAR.E | 3 |
|  | CENPL\_stlcld\_tube2\_122314\_01.19836.19836.2 | 3.9031 | 0.1268 | 99.2% | 2030.9321 | 2030.3774 | 3 | 5.418 | 47.2% | 1 | R.EAGFLRPVTIFGPIADVAR.E | 2 |
|  | CENPL\_stlcld\_tube2\_122314\_02.00516.00516.2 | 5.0393 | 0.3894 | 100.0% | 2169.4321 | 2168.4998 | 1 | 8.044 | 64.7% | 2 | R.LNYAQWYPIVVFLNPDSK.Q | 2 |
|  | CENPL\_stlcld\_122314\_02.12384.12384.2 | 4.4949 | 0.5003 | 100.0% | 1985.6721 | 1986.1918 | 1 | 9.156 | 56.2% | 1 | K.EAIQQQQNQLVWVSEGK.A | 2 |

---

|  |  |  |  |  |  |  |  |  |
| --- | --- | --- | --- | --- | --- | --- | --- | --- |
| U | *gi|4502643|ref|NP\_001* | 3 | 4 | 9.4% | 531 | 58024 | 6.7 | chaperonin containing TCP1, subunit 6A isoform a [Homo sapiens] |

| Filename XCorr DeltCN Conf% ObsM+H+ CalcM+H+ SpR ZScore Ion% # Sequence  | | | | | | | | | | | | |
| --- | --- | --- | --- | --- | --- | --- | --- | --- | --- | --- | --- | --- |
|  | CENPL\_stlcld\_122314\_02.11331.11331.2 | 2.4734 | 0.3465 | 99.4% | 1255.9521 | 1256.4478 | 3 | 6.21 | 58.3% | 2 | R.AQAALAVNISAAR.G | 2 |
| \* | CENPL\_stlcld\_122314\_01.15372.15372.3 | 4.5149 | 0.2541 | 99.9% | 2318.7244 | 2316.6807 | 2 | 5.189 | 32.5% | 1 | K.DGNVLLHEMQIQHPTASLIAK.V | 3 |
|  | CENPL\_stlcld\_122314\_01.16367.16367.2 | 3.7971 | 0.4993 | 100.0% | 1762.4521 | 1763.0006 | 1 | 8.346 | 56.7% | 1 | K.VLAQNSGFDLQETLVK.I | 2 |

---

|  |  |  |  |  |  |  |  |  |
| --- | --- | --- | --- | --- | --- | --- | --- | --- |
| U | *gi|148613856|ref|NP\_0* | 6 | 22 | 9.3% | 731 | 80458 | 8.4 | DEAD box polypeptide 17 isoform 3 [Homo sapiens] |
| U | *gi|38201710|ref|NP\_00* | 6 | 22 | 9.3% | 729 | 80273 | 8.3 | DEAD box polypeptide 17 isoform 1 [Homo sapiens] |

| Filename XCorr DeltCN Conf% ObsM+H+ CalcM+H+ SpR ZScore Ion% # Sequence  | | | | | | | | | | | | |
| --- | --- | --- | --- | --- | --- | --- | --- | --- | --- | --- | --- | --- |
|  | CENPL\_stlcld\_tube2\_122314\_01.09852.09852.2 | 3.1714 | 0.3713 | 100.0% | 1361.2122 | 1361.4998 | 1 | 6.075 | 65.0% | 1 | K.NFYVEHPEVAR.L | 2 |
|  | CENPL\_stlcld\_122314\_01.14229.14229.2 | 4.8126 | 0.4426 | 100.0% | 1693.5922 | 1692.8229 | 1 | 8.028 | 57.1% | 2 | R.ELAQQVQQVADDYGK.C | 2 |
|  | CENPL\_stlcld\_122314\_01.17030.17030.2 | 4.1012 | 0.4447 | 100.0% | 1338.4521 | 1337.5946 | 1 | 7.711 | 85.0% | 8 | R.MLDMGFEPQIR.K | 222 |
|  | CENPL\_stlcld\_tube2\_122314\_01.17921.17921.2 | 3.0835 | 0.3183 | 99.9% | 1349.7122 | 1349.5902 | 7 | 6.614 | 60.0% | 2 | R.QTLMWSATWPK.E | 22 |
|  | CENPL\_stlcld\_122314\_01.13792.13792.2 | 4.2232 | 0.289 | 100.0% | 1228.4521 | 1227.4465 | 2 | 7.323 | 81.8% | 8 | K.APILIATDVASR.G | 22 |
|  | CENPL\_stlcld\_tube2\_122314\_01.09857.09857.2 | 2.4344 | 0.3394 | 99.9% | 875.0122 | 874.96643 | 1 | 6.575 | 78.6% | 1 | R.GLDVEDVK.F | 22 |

Similarities:
gi|87196351|ref|NP\_00(1:5)  
gi|4758138|ref|NP\_004(4:2)  

---

|  |  |  |  |  |  |  |  |  |
| --- | --- | --- | --- | --- | --- | --- | --- | --- |
| U | *gi|58761484|ref|NP\_00* | 4 | 7 | 9.3% | 507 | 56431 | 6.5 | chaperonin containing TCP1, subunit 3 isoform c [Homo sapiens] |
| U | *gi|63162572|ref|NP\_00* | 4 | 7 | 8.6% | 545 | 60534 | 6.5 | chaperonin containing TCP1, subunit 3 isoform a [Homo sapiens] |
| U | *gi|58761486|ref|NP\_00* | 4 | 7 | 8.6% | 544 | 60463 | 6.5 | chaperonin containing TCP1, subunit 3 isoform b [Homo sapiens] |

| Filename XCorr DeltCN Conf% ObsM+H+ CalcM+H+ SpR ZScore Ion% # Sequence  | | | | | | | | | | | | |
| --- | --- | --- | --- | --- | --- | --- | --- | --- | --- | --- | --- | --- |
|  | CENPL\_stlcld\_tube2\_122314\_01.14933.14933.2 | 2.5783 | 0.3062 | 99.2% | 1429.9722 | 1429.6227 | 1 | 5.653 | 62.5% | 1 | K.IPGGIIEDSCVLR.G | 2 |
|  | CENPL\_stlcld\_122314\_01.16421.16421.2 | 2.213 | 0.2973 | 98.4% | 1280.5322 | 1280.504 | 1 | 5.315 | 60.0% | 1 | R.IVLLDSSLEYK.K | 2 |
|  | CENPL\_stlcld\_122314\_01.16156.16156.2 | 3.2643 | 0.5085 | 100.0% | 1405.5922 | 1404.6268 | 1 | 8.078 | 68.2% | 4 | K.GISDLAQHYLMR.A | 2 |
|  | CENPL\_stlcld\_tube2\_122314\_01.14217.14217.2 | 3.1365 | 0.2285 | 99.7% | 1167.5521 | 1167.3939 | 1 | 5.291 | 85.0% | 1 | R.AVAQALEVIPR.T | 2 |

---

|  |  |  |  |  |  |  |  |  |
| --- | --- | --- | --- | --- | --- | --- | --- | --- |
| U | *gi|55956788|ref|NP\_00* | 5 | 15 | 9.2% | 710 | 76615 | 4.7 | nucleolin [Homo sapiens] |

| Filename XCorr DeltCN Conf% ObsM+H+ CalcM+H+ SpR ZScore Ion% # Sequence  | | | | | | | | | | | | |
| --- | --- | --- | --- | --- | --- | --- | --- | --- | --- | --- | --- | --- |
| \* | CENPL\_stlcld\_122314\_02.14424.14424.2 | 4.1093 | 0.4767 | 100.0% | 1649.9122 | 1649.751 | 1 | 8.283 | 65.4% | 6 | K.FGYVDFESAEDLEK.A | 2 |
| \* | CENPL\_stlcld\_tube2\_122314\_01.19041.19041.3 | 2.9636 | 0.2077 | 95.9% | 1992.7743 | 1993.1779 | 1 | 5.038 | 37.5% | 2 | K.VTQDELKEVFEDAAEIR.L | 3 |
| \* | CENPL\_stlcld\_122314\_01.19667.19667.2 | 3.7268 | 0.1532 | 99.2% | 1994.2922 | 1993.1779 | 1 | 6.034 | 53.1% | 1 | K.VTQDELKEVFEDAAEIR.L | 2 |
| \* | CENPL\_stlcld\_tube2\_122314\_02.13646.13646.3 | 3.04 | 0.4224 | 100.0% | 2200.5244 | 2201.3057 | 1 | 6.902 | 39.5% | 2 | K.GLSEDTTEETLKESFDGSVR.A | 3 |
| \* | CENPL\_stlcld\_122314\_02.14564.14564.2 | 4.0747 | 0.492 | 100.0% | 1562.7922 | 1562.6323 | 1 | 8.332 | 73.1% | 4 | K.GFGFVDFNSEEDAK.A | 2 |

---

|  |  |  |  |  |  |  |  |  |
| --- | --- | --- | --- | --- | --- | --- | --- | --- |
| U | *gi|5453607|ref|NP\_006* | 3 | 5 | 8.8% | 543 | 59367 | 7.6 | chaperonin containing TCP1, subunit 7 isoform a [Homo sapiens] |

| Filename XCorr DeltCN Conf% ObsM+H+ CalcM+H+ SpR ZScore Ion% # Sequence  | | | | | | | | | | | | |
| --- | --- | --- | --- | --- | --- | --- | --- | --- | --- | --- | --- | --- |
| \* | CENPL\_stlcld\_tube2\_122314\_01.21173.21173.2 | 3.6388 | 0.4692 | 100.0% | 2254.0322 | 2253.468 | 1 | 8.258 | 45.2% | 2 | K.SQDAEVGDGTTSVTLLAAEFLK.Q | 2 |
|  | CENPL\_stlcld\_122314\_01.18177.18177.2 | 3.3022 | 0.0646 | 97.1% | 1567.8522 | 1566.7539 | 1 | 6.557 | 69.2% | 2 | K.LPIGDVATQYFADR.D | 2 |
|  | CENPL\_stlcld\_122314\_01.12104.12104.2 | 2.6589 | 0.161 | 97.5% | 1384.4122 | 1384.4602 | 13 | 4.684 | 59.1% | 1 | R.GGAEQFMEETER.S | 2 |

---

|  |  |  |  |  |  |  |  |  |
| --- | --- | --- | --- | --- | --- | --- | --- | --- |
| U | *gi|24233517|ref|NP\_71* | 2 | 2 | 8.8% | 377 | 43084 | 4.9 | IKK interacting protein isoform 1 [Homo sapiens] |

| Filename XCorr DeltCN Conf% ObsM+H+ CalcM+H+ SpR ZScore Ion% # Sequence  | | | | | | | | | | | | |
| --- | --- | --- | --- | --- | --- | --- | --- | --- | --- | --- | --- | --- |
| \* | CENPL\_stlcld\_122314\_02.10506.10506.3 | 4.4631 | 0.2786 | 100.0% | 2062.6743 | 2062.29 | 1 | 5.679 | 42.6% | 1 | K.HIHSQVTVQINSAEQEIK.L | 3 |
| \* | CENPL\_stlcld\_tube2\_122314\_01.17480.17480.3 | 2.697 | 0.2547 | 98.2% | 1676.5443 | 1674.9407 | 2 | 4.897 | 35.7% | 1 | K.THLPTIESAIHSVLR.V | 3 |

---

|  |  |  |  |  |  |  |  |  |
| --- | --- | --- | --- | --- | --- | --- | --- | --- |
| U | *gi|86991438|ref|NP\_00* | 2 | 3 | 8.8% | 272 | 31264 | 11.6 | splicing factor, arginine/serine-rich 5 [Homo sapiens] |
| U | *gi|86991440|ref|NP\_00* | 2 | 3 | 8.8% | 272 | 31264 | 11.6 | splicing factor, arginine/serine-rich 5 [Homo sapiens] |

| Filename XCorr DeltCN Conf% ObsM+H+ CalcM+H+ SpR ZScore Ion% # Sequence  | | | | | | | | | | | | |
| --- | --- | --- | --- | --- | --- | --- | --- | --- | --- | --- | --- | --- |
|  | CENPL\_stlcld\_tube2\_122314\_01.11540.11540.2 | 2.9904 | 0.2887 | 99.9% | 1031.5922 | 1031.1973 | 1 | 6.615 | 87.5% | 2 | R.LIVENLSSR.V | 22 |
|  | CENPL\_stlcld\_122314\_02.14148.14148.2 | 3.9222 | 0.3252 | 100.0% | 1641.9521 | 1641.8181 | 1 | 7.886 | 57.1% | 1 | K.LNEGVVEFASYGDLK.N | 2 |

Similarities:
gi|20127499|ref|NP\_00(1:1)  

---

|  |  |  |  |  |  |  |  |  |
| --- | --- | --- | --- | --- | --- | --- | --- | --- |
| U | *gi|10863945|ref|NP\_06* | 3 | 3 | 8.7% | 732 | 82705 | 5.8 | ATP-dependent DNA helicase II [Homo sapiens] |

| Filename XCorr DeltCN Conf% ObsM+H+ CalcM+H+ SpR ZScore Ion% # Sequence  | | | | | | | | | | | | |
| --- | --- | --- | --- | --- | --- | --- | --- | --- | --- | --- | --- | --- |
| \* | CENPL\_stlcld\_tube2\_122314\_01.20726.20726.2 | 3.9075 | 0.3393 | 100.0% | 1916.4521 | 1916.1963 | 1 | 7.123 | 70.0% | 1 | R.HLMLPDFDLLEDIESK.I | 2 |
| \* | CENPL\_stlcld\_122314\_02.02015.02015.2 | 4.8239 | 0.4766 | 100.0% | 2724.152 | 2725.0864 | 1 | 10.003 | 50.0% | 1 | R.DDEAAAVALSSLIHALDDLDMVAIVR.Y | 2 |
| \* | CENPL\_stlcld\_122314\_01.22707.22707.2 | 4.9079 | 0.5136 | 100.0% | 2323.2922 | 2322.6355 | 1 | 8.901 | 50.0% | 1 | K.YAPTEAQLNAVDALIDSMSLAK.K | 2 |

---

|  |  |  |  |  |  |  |  |  |
| --- | --- | --- | --- | --- | --- | --- | --- | --- |
| U | *gi|119703753|ref|NP\_0* | 8 | 22 | 8.7% | 564 | 60067 | 8.0 | keratin 6B [Homo sapiens] |

| Filename XCorr DeltCN Conf% ObsM+H+ CalcM+H+ SpR ZScore Ion% # Sequence  | | | | | | | | | | | | |
| --- | --- | --- | --- | --- | --- | --- | --- | --- | --- | --- | --- | --- |
|  | CENPL\_stlcld\_tube2\_122314\_01.12512.12512.2 | 2.417 | 0.2122 | 98.9% | 828.09216 | 827.95544 | 5 | 4.999 | 91.7% | 4 | K.FASFIDK.V | 222222222 |
|  | CENPL\_stlcld\_122314\_01.12293.12293.2 | 2.815 | 0.2809 | 99.9% | 1082.9922 | 1083.2755 | 3 | 6.695 | 75.0% | 2 | K.FASFIDKVR.F | 222322223 |
|  | CENPL\_stlcld\_tube2\_122314\_01.19944.19944.1 | 2.8562 | 0.3917 | 100.0% | 1329.95 | 1330.5211 | 1 | 6.619 | 72.7% | 1 | R.NLDLDSIIAEVK.A | 11111 |
|  | CENPL\_stlcld\_122314\_01.20872.20872.2 | 3.7762 | 0.4242 | 100.0% | 1330.7522 | 1330.5211 | 1 | 7.499 | 81.8% | 2 | R.NLDLDSIIAEVK.A | 22222 |
|  | CENPL\_stlcld\_122314\_01.08782.08782.2 | 2.6273 | 0.1146 | 97.5% | 1108.5721 | 1108.196 | 60 | 6.069 | 62.5% | 2 | K.AQYEEIAQR.S | 2222 |
|  | CENPL\_stlcld\_tube2\_122314\_02.10658.10658.2 | 4.0519 | 0.3315 | 100.0% | 1181.4122 | 1180.303 | 2 | 6.856 | 83.3% | 7 | K.YEELQITAGR.H | 22 |
|  | CENPL\_stlcld\_122314\_01.12065.12065.1 | 2.418 | 0.2607 | 95.8% | 1153.63 | 1154.3234 | 450 | 5.874 | 50.0% | 1 | K.EYQELMNVK.L | 11 |
|  | CENPL\_stlcld\_122314\_01.12050.12050.2 | 2.7001 | 0.2224 | 99.2% | 1154.3121 | 1154.3234 | 9 | 6.13 | 68.8% | 3 | K.EYQELMNVK.L | 22 |

Similarities:
gi|4504919|ref|NP\_002(4:4)  
gi|119395750|ref|NP\_0(1:7)  
gi|47132620|ref|NP\_00(5:3)  
gi|67782365|ref|NP\_00(2:6)  
gi|32567786|ref|NP\_78(4:4)  
gi|119395754|ref|NP\_0(4:4)  
contaminant\_KERATIN16(1:7)  
gi|153791158|ref|NP\_0(4:4)  
gi|109255249|ref|NP\_0(2:6)  
gi|15618995|ref|NP\_25(2:6)  

---

|  |  |  |  |  |  |  |  |  |
| --- | --- | --- | --- | --- | --- | --- | --- | --- |
| U | *gi|23397427|ref|NP\_00* | 4 | 5 | 8.5% | 623 | 69633 | 8.6 | synaptotagmin binding, cytoplasmic RNA interacting protein [Homo sapiens] |

| Filename XCorr DeltCN Conf% ObsM+H+ CalcM+H+ SpR ZScore Ion% # Sequence  | | | | | | | | | | | | |
| --- | --- | --- | --- | --- | --- | --- | --- | --- | --- | --- | --- | --- |
| \* | CENPL\_stlcld\_122314\_02.14224.14224.3 | 3.8527 | 0.2018 | 98.7% | 2442.8943 | 2443.716 | 1 | 5.641 | 35.7% | 2 | K.VAEKLDEIYVAGLVAHSDLDER.A | 3 |
|  | CENPL\_stlcld\_tube2\_122314\_01.11300.11300.2 | 2.4841 | 0.3913 | 99.9% | 1311.8121 | 1312.4221 | 1 | 6.754 | 68.2% | 1 | R.TGYTLDVTTGQR.K | 22 |
|  | CENPL\_stlcld\_tube2\_122314\_01.15983.15983.2 | 2.5986 | 0.2048 | 99.0% | 927.9322 | 928.0788 | 5 | 5.104 | 78.6% | 1 | K.AGPIWDLR.L | 22 |
| \* | CENPL\_stlcld\_122314\_01.12244.12244.2 | 3.035 | 0.3182 | 99.9% | 1352.4122 | 1352.5272 | 3 | 6.218 | 65.0% | 1 | K.TKEQILEEFSK.V | 2 |

Similarities:
gi|156151392|ref|NP\_0(2:2)  

---

|  |  |  |  |  |  |  |  |  |
| --- | --- | --- | --- | --- | --- | --- | --- | --- |
| U | *gi|205277463|ref|NP\_0* | 4 | 5 | 8.5% | 623 | 67878 | 7.7 | transketolase isoform 1 [Homo sapiens] |
| U | *gi|4507521|ref|NP\_001* | 4 | 5 | 8.5% | 623 | 67878 | 7.7 | transketolase isoform 1 [Homo sapiens] |
| U | *gi|205277465|ref|NP\_0* | 4 | 5 | 9.8% | 540 | 58982 | 7.7 | transketolase isoform 2 [Homo sapiens] |

| Filename XCorr DeltCN Conf% ObsM+H+ CalcM+H+ SpR ZScore Ion% # Sequence  | | | | | | | | | | | | |
| --- | --- | --- | --- | --- | --- | --- | --- | --- | --- | --- | --- | --- |
|  | CENPL\_stlcld\_tube2\_122314\_02.00888.00888.2 | 3.3529 | 0.4189 | 100.0% | 2023.6721 | 2024.2965 | 1 | 7.039 | 53.1% | 1 | K.NMAEQIIQEIYSQIQSK.K | 2 |
|  | CENPL\_stlcld\_122314\_02.00557.00557.3 | 3.5485 | 0.2528 | 99.9% | 2024.7843 | 2024.2965 | 1 | 4.696 | 40.6% | 1 | K.NMAEQIIQEIYSQIQSK.K | 3 |
|  | CENPL\_stlcld\_122314\_02.11234.11234.3 | 4.9541 | 0.4046 | 100.0% | 2509.6143 | 2509.6946 | 1 | 6.93 | 42.9% | 2 | R.TSRPENAIIYNNNEDFQVGQAK.V | 3 |
|  | CENPL\_stlcld\_122314\_01.15228.15228.2 | 2.3717 | 0.2901 | 98.4% | 1564.9122 | 1563.815 | 1 | 4.547 | 57.7% | 1 | K.MFGIDRDAIAQAVR.G | 2 |

---

|  |  |  |  |  |  |  |  |  |
| --- | --- | --- | --- | --- | --- | --- | --- | --- |
| U | *gi|156151392|ref|NP\_0* | 4 | 4 | 8.5% | 532 | 59682 | 9.2 | heterogeneous nuclear ribonucleoprotein R isoform 4 [Homo sapiens] |
| U | *gi|5031755|ref|NP\_005* | 4 | 4 | 7.1% | 633 | 70943 | 8.1 | heterogeneous nuclear ribonucleoprotein R isoform 2 [Homo sapiens] |
| U | *gi|156151396|ref|NP\_0* | 4 | 4 | 8.4% | 535 | 59953 | 9.2 | heterogeneous nuclear ribonucleoprotein R isoform 3 [Homo sapiens] |
| U | *gi|156151394|ref|NP\_0* | 4 | 4 | 7.1% | 636 | 71214 | 8.1 | heterogeneous nuclear ribonucleoprotein R isoform 1 [Homo sapiens] |

| Filename XCorr DeltCN Conf% ObsM+H+ CalcM+H+ SpR ZScore Ion% # Sequence  | | | | | | | | | | | | |
| --- | --- | --- | --- | --- | --- | --- | --- | --- | --- | --- | --- | --- |
|  | CENPL\_stlcld\_tube2\_122314\_01.11300.11300.2 | 2.4841 | 0.3913 | 99.9% | 1311.8121 | 1312.4221 | 1 | 6.754 | 68.2% | 1 | R.TGYTLDVTTGQR.K | 22 |
|  | CENPL\_stlcld\_tube2\_122314\_01.15983.15983.2 | 2.5986 | 0.2048 | 99.0% | 927.9322 | 928.0788 | 5 | 5.104 | 78.6% | 1 | K.AGPIWDLR.L | 22 |
|  | CENPL\_stlcld\_122314\_01.20484.20484.2 | 4.4895 | 0.4442 | 100.0% | 1461.6322 | 1461.6525 | 1 | 8.342 | 70.8% | 1 | R.NLATTVTEEILEK.S | 2 |
|  | CENPL\_stlcld\_122314\_01.12738.12738.2 | 2.9962 | 0.0867 | 97.1% | 1540.7722 | 1540.7184 | 35 | 4.34 | 50.0% | 1 | K.LKDYAFVHFEDR.G | 2 |

Similarities:
gi|23397427|ref|NP\_00(2:2)  

---

|  |  |  |  |  |  |  |  |  |
| --- | --- | --- | --- | --- | --- | --- | --- | --- |
| U | *gi|38455427|ref|NP\_00* | 3 | 8 | 8.5% | 539 | 57924 | 7.8 | chaperonin containing TCP1, subunit 4 (delta) [Homo sapiens] |

| Filename XCorr DeltCN Conf% ObsM+H+ CalcM+H+ SpR ZScore Ion% # Sequence  | | | | | | | | | | | | |
| --- | --- | --- | --- | --- | --- | --- | --- | --- | --- | --- | --- | --- |
| \* | CENPL\_stlcld\_122314\_02.11552.11552.3 | 4.9513 | 0.3472 | 100.0% | 2091.2944 | 2091.3845 | 1 | 7.087 | 43.4% | 3 | K.MIQDGKGDVTITNDGATILK.Q | 3 |
| \* | CENPL\_stlcld\_tube2\_122314\_01.12081.12081.2 | 2.4427 | 0.2172 | 97.5% | 1457.1721 | 1457.6696 | 3 | 5.154 | 54.2% | 1 | K.GIHPTIISESFQK.A | 2 |
| \* | CENPL\_stlcld\_tube2\_122314\_01.12711.12711.2 | 3.0985 | 0.4441 | 100.0% | 1358.4922 | 1358.5345 | 1 | 8.104 | 70.8% | 4 | K.VIDPATATSVDLR.D | 2 |

---

|  |  |  |  |  |  |  |  |  |
| --- | --- | --- | --- | --- | --- | --- | --- | --- |
| U | *gi|19923142|ref|NP\_00* | 5 | 7 | 8.4% | 876 | 97170 | 4.8 | karyopherin beta 1 [Homo sapiens] |

| Filename XCorr DeltCN Conf% ObsM+H+ CalcM+H+ SpR ZScore Ion% # Sequence  | | | | | | | | | | | | |
| --- | --- | --- | --- | --- | --- | --- | --- | --- | --- | --- | --- | --- |
| \* | CENPL\_stlcld\_122314\_01.21807.21807.2 | 3.9487 | 0.5012 | 100.0% | 1660.1122 | 1659.9231 | 1 | 7.824 | 64.3% | 2 | R.AAVENLPTFLVELSR.V | 2 |
| \* | CENPL\_stlcld\_122314\_01.08303.08303.2 | 2.3518 | 0.1914 | 96.0% | 1227.6522 | 1226.378 | 215 | 4.359 | 54.5% | 1 | R.VLANPGNSQVAR.V | 2 |
| \* | CENPL\_stlcld\_122314\_01.20079.20079.2 | 3.5711 | 0.4839 | 100.0% | 1607.0322 | 1606.8595 | 1 | 7.5 | 64.3% | 2 | K.LAATNALLNSLEFTK.A | 2 |
| \* | CENPL\_stlcld\_tube2\_122314\_01.07956.07956.3 | 3.1128 | 0.3598 | 99.9% | 1880.6044 | 1880.0275 | 1 | 6.052 | 35.0% | 1 | K.LLETTDRPDGHQNNLR.S | 3 |
| \* | CENPL\_stlcld\_122314\_01.14469.14469.3 | 2.8573 | 0.3262 | 99.9% | 1866.5343 | 1865.201 | 3 | 5.306 | 38.3% | 1 | K.LVEARPMIHELLTEGR.R | 3 |

---

|  |  |  |  |  |  |  |  |  |
| --- | --- | --- | --- | --- | --- | --- | --- | --- |
| U | *gi|24234756|ref|NP\_70* | 2 | 2 | 8.4% | 690 | 74607 | 8.2 | interleukin enhancer binding factor 3 isoform c [Homo sapiens] |

| Filename XCorr DeltCN Conf% ObsM+H+ CalcM+H+ SpR ZScore Ion% # Sequence  | | | | | | | | | | | | |
| --- | --- | --- | --- | --- | --- | --- | --- | --- | --- | --- | --- | --- |
|  | CENPL\_stlcld\_122314\_01.17304.17304.3 | 3.1721 | 0.2801 | 99.3% | 2672.5444 | 2672.886 | 1 | 5.463 | 28.4% | 1 | K.HSSVYPTQEELEAVQNMVSHTER.A | 3 |
| \* | CENPL\_stlcld\_122314\_02.00710.00710.3 | 2.7886 | 0.2547 | 95.6% | 3314.5144 | 3312.4695 | 8 | 4.309 | 19.1% | 1 | R.GFGGANHGGYMNAGAGYGSYGYGGNSATAGYTGFV.- | 3 |

---

|  |  |  |  |  |  |  |  |  |
| --- | --- | --- | --- | --- | --- | --- | --- | --- |
| U | *gi|16579885|ref|NP\_00* | 4 | 10 | 8.4% | 427 | 47697 | 11.1 | ribosomal protein L4 [Homo sapiens] |

| Filename XCorr DeltCN Conf% ObsM+H+ CalcM+H+ SpR ZScore Ion% # Sequence  | | | | | | | | | | | | |
| --- | --- | --- | --- | --- | --- | --- | --- | --- | --- | --- | --- | --- |
| \* | CENPL\_stlcld\_tube2\_122314\_01.15018.15018.3 | 4.7862 | 0.405 | 100.0% | 1863.0543 | 1863.1727 | 1 | 8.23 | 43.3% | 4 | K.APIRPDIVNFVHTNLR.K | 3 |
| \* | CENPL\_stlcld\_122314\_01.13552.13552.3 | 3.4338 | 0.2622 | 99.9% | 1992.2344 | 1991.3468 | 1 | 5.177 | 40.6% | 1 | K.APIRPDIVNFVHTNLRK.N | 3 |
| \* | CENPL\_stlcld\_122314\_01.13254.13254.2 | 3.4436 | 0.396 | 100.0% | 1281.8922 | 1281.4539 | 1 | 6.41 | 88.9% | 4 | R.KLDELYGTWR.K | 2 |
| \* | CENPL\_stlcld\_tube2\_122314\_01.08750.08750.2 | 2.3869 | 0.2666 | 98.9% | 1104.2722 | 1104.2689 | 15 | 5.058 | 62.5% | 1 | K.SNYNLPMHK.M | 2 |

---

|  |  |  |  |  |  |  |  |  |
| --- | --- | --- | --- | --- | --- | --- | --- | --- |
| U | *gi|12025678|ref|NP\_00* | 5 | 8 | 8.2% | 911 | 104854 | 5.4 | actinin, alpha 4 [Homo sapiens] |

| Filename XCorr DeltCN Conf% ObsM+H+ CalcM+H+ SpR ZScore Ion% # Sequence  | | | | | | | | | | | | |
| --- | --- | --- | --- | --- | --- | --- | --- | --- | --- | --- | --- | --- |
| \* | CENPL\_stlcld\_tube2\_122314\_01.14714.14714.2 | 2.5949 | 0.3105 | 99.4% | 1487.3722 | 1486.6731 | 1 | 5.446 | 72.7% | 1 | K.NVNVQNFHISWK.D | 2 |
| \* | CENPL\_stlcld\_122314\_01.20868.20868.3 | 3.8868 | 0.2843 | 99.9% | 3326.2144 | 3326.7742 | 1 | 5.255 | 27.8% | 2 | R.SIVDYKPNLDLLEQQHQLIQEALIFDNK.H | 3 |
|  | CENPL\_stlcld\_122314\_01.21695.21695.2 | 3.7554 | 0.4108 | 100.0% | 1387.4722 | 1387.6218 | 1 | 8.265 | 68.2% | 3 | R.VGWEQLLTTIAR.T | 22 |
|  | CENPL\_stlcld\_122314\_01.13869.13869.2 | 3.0085 | 0.1928 | 98.9% | 1430.6322 | 1430.6011 | 127 | 4.839 | 54.5% | 1 | R.TINEVENQILTR.D | 22 |
| \* | CENPL\_stlcld\_122314\_01.11898.11898.2 | 2.4967 | 0.3057 | 99.2% | 1353.6122 | 1353.4926 | 3 | 5.851 | 60.0% | 1 | K.GISQEQMQEFR.A | 2 |

Similarities:
gi|194097350|ref|NP\_0(2:3)  

---

|  |  |  |  |  |  |  |  |  |
| --- | --- | --- | --- | --- | --- | --- | --- | --- |
| U | *gi|4885225|ref|NP\_005* | 3 | 4 | 8.1% | 656 | 68478 | 9.3 | Ewing sarcoma breakpoint region 1 isoform EWS [Homo sapiens] |

| Filename XCorr DeltCN Conf% ObsM+H+ CalcM+H+ SpR ZScore Ion% # Sequence  | | | | | | | | | | | | |
| --- | --- | --- | --- | --- | --- | --- | --- | --- | --- | --- | --- | --- |
| \* | CENPL\_stlcld\_122314\_02.10474.10474.3 | 5.6406 | 0.5332 | 100.0% | 2482.0144 | 2481.572 | 1 | 8.495 | 33.7% | 1 | R.QDHPSSMGVYGQESGGFSGPGENR.S | 3 |
|  | CENPL\_stlcld\_122314\_02.08417.08417.2 | 2.704 | 0.1222 | 95.0% | 1452.6721 | 1451.5298 | 7 | 4.93 | 46.2% | 1 | K.GDATVSYEDPPTAK.A | 2 |
|  | CENPL\_stlcld\_122314\_01.14243.14243.2 | 3.8903 | 0.3961 | 100.0% | 1685.3322 | 1685.8333 | 1 | 6.975 | 64.3% | 2 | K.AAVEWFDGKDFQGSK.L | 2 |

---

|  |  |  |  |  |  |  |  |  |
| --- | --- | --- | --- | --- | --- | --- | --- | --- |
| U | *gi|24430149|ref|NP\_70* | 10 | 19 | 8.0% | 1391 | 155199 | 6.2 | nucleoporin 155kDa isoform 1 [Homo sapiens] |
| U | *gi|4758844|ref|NP\_004* | 10 | 19 | 8.3% | 1332 | 149016 | 6.3 | nucleoporin 155kDa isoform 2 [Homo sapiens] |

| Filename XCorr DeltCN Conf% ObsM+H+ CalcM+H+ SpR ZScore Ion% # Sequence  | | | | | | | | | | | | |
| --- | --- | --- | --- | --- | --- | --- | --- | --- | --- | --- | --- | --- |
|  | CENPL\_stlcld\_122314\_01.14771.14771.2 | 4.9536 | 0.5719 | 100.0% | 1823.9122 | 1824.0184 | 1 | 10.255 | 62.5% | 2 | K.GVIQVYDLGQDGQGMSR.V | 2 |
|  | CENPL\_stlcld\_tube2\_122314\_01.14410.14410.2 | 5.1299 | 0.4475 | 100.0% | 1728.8522 | 1728.9462 | 1 | 7.415 | 64.7% | 8 | R.VASVSQNAIVSAAGNIAR.T | 2 |
|  | CENPL\_stlcld\_tube2\_122314\_02.13740.13740.3 | 3.3816 | 0.1995 | 98.2% | 1729.6743 | 1728.9462 | 1 | 4.994 | 38.2% | 2 | R.VASVSQNAIVSAAGNIAR.T | 3 |
|  | CENPL\_stlcld\_tube2\_122314\_01.10883.10883.2 | 4.5936 | 0.6254 | 100.0% | 1724.3922 | 1724.868 | 1 | 11.076 | 83.3% | 1 | R.HLLVSNVGGDGEEIER.F | 2 |
|  | CENPL\_stlcld\_tube2\_122314\_01.10906.10906.3 | 3.0845 | 0.2273 | 98.3% | 1725.2644 | 1724.868 | 134 | 4.614 | 33.3% | 1 | R.HLLVSNVGGDGEEIER.F | 3 |
|  | CENPL\_stlcld\_122314\_02.16211.16211.2 | 4.1516 | 0.4054 | 100.0% | 1603.7122 | 1603.8767 | 1 | 7.929 | 69.2% | 1 | R.IMGNIWDASLVVER.I | 2 |
|  | CENPL\_stlcld\_122314\_01.19522.19522.2 | 2.4197 | 0.208 | 97.7% | 1269.6322 | 1269.5302 | 12 | 5.089 | 55.0% | 1 | K.ISLQAIQQLVR.K | 2 |
|  | CENPL\_stlcld\_122314\_02.10673.10673.3 | 2.74 | 0.2193 | 96.2% | 1630.0743 | 1629.8285 | 13 | 4.447 | 42.3% | 1 | R.LADMHSTEISLQQR.L | 3 |
|  | CENPL\_stlcld\_122314\_02.12339.12339.2 | 2.6063 | 0.1541 | 97.5% | 1370.0721 | 1370.5919 | 3 | 6.675 | 60.0% | 1 | R.IQLQIQETLQR.Q | 2 |
|  | CENPL\_stlcld\_122314\_01.20081.20081.2 | 2.6615 | 0.3848 | 99.9% | 1268.4722 | 1268.4955 | 1 | 6.313 | 66.7% | 1 | R.LLEVYDQLFK.S | 2 |

---

|  |  |  |  |  |  |  |  |  |
| --- | --- | --- | --- | --- | --- | --- | --- | --- |
| U | *gi|109240550|ref|NP\_0* | 3 | 4 | 8.0% | 523 | 58744 | 6.7 | paraspeckle protein 1 [Homo sapiens] |

| Filename XCorr DeltCN Conf% ObsM+H+ CalcM+H+ SpR ZScore Ion% # Sequence  | | | | | | | | | | | | |
| --- | --- | --- | --- | --- | --- | --- | --- | --- | --- | --- | --- | --- |
| \* | CENPL\_stlcld\_tube2\_122314\_01.17074.17074.2 | 4.0186 | 0.4547 | 100.0% | 1994.5721 | 1995.2395 | 1 | 8.126 | 50.0% | 1 | R.LFVGNLPTDITEEDFKR.L | 2 |
| \* | CENPL\_stlcld\_tube2\_122314\_01.12789.12789.2 | 2.9051 | 0.3929 | 99.9% | 1311.1322 | 1311.4368 | 1 | 6.721 | 65.0% | 1 | R.YGEPSEVFINR.D | 2 |
| \* | CENPL\_stlcld\_122314\_01.15784.15784.2 | 3.4068 | 0.475 | 100.0% | 1650.4722 | 1650.7875 | 1 | 7.255 | 76.9% | 2 | R.FAQPGTFEFEYASR.W | 2 |

---

|  |  |  |  |  |  |  |  |  |
| --- | --- | --- | --- | --- | --- | --- | --- | --- |
| U | *gi|32567786|ref|NP\_78* | 6 | 11 | 8.0% | 535 | 57836 | 7.2 | keratin 6L [Homo sapiens] |

| Filename XCorr DeltCN Conf% ObsM+H+ CalcM+H+ SpR ZScore Ion% # Sequence  | | | | | | | | | | | | |
| --- | --- | --- | --- | --- | --- | --- | --- | --- | --- | --- | --- | --- |
|  | CENPL\_stlcld\_tube2\_122314\_01.12512.12512.2 | 2.417 | 0.2122 | 98.9% | 828.09216 | 827.95544 | 5 | 4.999 | 91.7% | 4 | K.FASFIDK.V | 222222222 |
|  | CENPL\_stlcld\_122314\_01.12293.12293.2 | 2.815 | 0.2809 | 99.9% | 1082.9922 | 1083.2755 | 3 | 6.695 | 75.0% | 2 | K.FASFIDKVR.F | 222322223 |
|  | CENPL\_stlcld\_tube2\_122314\_01.19944.19944.1 | 2.8562 | 0.3917 | 100.0% | 1329.95 | 1330.5211 | 1 | 6.619 | 72.7% | 1 | R.NLDLDSIIAEVK.A | 11111 |
|  | CENPL\_stlcld\_122314\_01.20872.20872.2 | 3.7762 | 0.4242 | 100.0% | 1330.7522 | 1330.5211 | 1 | 7.499 | 81.8% | 2 | R.NLDLDSIIAEVK.A | 22222 |
|  | CENPL\_stlcld\_tube2\_122314\_01.10942.10942.2 | 2.6462 | 0.2259 | 98.8% | 1198.0122 | 1197.2897 | 1 | 5.375 | 77.8% | 1 | R.AEAEAWYQTK.Y | 222 |
|  | CENPL\_stlcld\_tube2\_122314\_01.13841.13841.2 | 2.9177 | 0.2074 | 98.9% | 1522.5322 | 1522.8029 | 2 | 5.216 | 63.6% | 1 | R.LLRDYQELMNVK.L | 22 |

Similarities:
gi|4504919|ref|NP\_002(2:4)  
gi|47132620|ref|NP\_00(5:1)  
gi|67782365|ref|NP\_00(3:3)  
contaminant\_KERATIN19(1:5)  
gi|119703753|ref|NP\_0(4:2)  
gi|119395754|ref|NP\_0(4:2)  
gi|153791158|ref|NP\_0(4:2)  
gi|109255249|ref|NP\_0(1:5)  
gi|15618995|ref|NP\_25(2:4)  

---

|  |  |  |  |  |  |  |  |  |
| --- | --- | --- | --- | --- | --- | --- | --- | --- |
| U | *gi|116063573|ref|NP\_0* | 15 | 25 | 7.8% | 2639 | 280016 | 6.0 | filamin A, alpha isoform 1 [Homo sapiens] |
| U | *gi|160420317|ref|NP\_0* | 15 | 25 | 7.8% | 2647 | 280737 | 6.1 | filamin A, alpha isoform 2 [Homo sapiens] |

| Filename XCorr DeltCN Conf% ObsM+H+ CalcM+H+ SpR ZScore Ion% # Sequence  | | | | | | | | | | | | |
| --- | --- | --- | --- | --- | --- | --- | --- | --- | --- | --- | --- | --- |
|  | CENPL\_stlcld\_tube2\_122314\_01.17358.17358.2 | 2.3577 | 0.3388 | 99.3% | 1286.7322 | 1286.5167 | 2 | 6.354 | 70.0% | 3 | K.LPQLPITNFSR.D | 2 |
|  | CENPL\_stlcld\_tube2\_122314\_01.11908.11908.2 | 2.5347 | 0.1943 | 97.7% | 1284.3922 | 1284.5443 | 57 | 5.622 | 45.5% | 1 | K.VTVLFAGQHIAK.S | 2 |
|  | CENPL\_stlcld\_tube2\_122314\_01.08904.08904.2 | 2.6791 | 0.2712 | 99.5% | 1100.3922 | 1100.2169 | 28 | 5.183 | 66.7% | 1 | K.GTVEPQLEAR.G | 2 |
|  | CENPL\_stlcld\_122314\_01.13650.13650.2 | 3.2321 | 0.4864 | 100.0% | 1227.2722 | 1227.4056 | 1 | 7.94 | 70.8% | 2 | R.AWGPGLEGGVVGK.S | 2 |
|  | CENPL\_stlcld\_tube2\_122314\_01.10450.10450.2 | 2.6149 | 0.3252 | 99.2% | 1648.2322 | 1647.8687 | 2 | 5.748 | 50.0% | 1 | K.TGVAVNKPAEFTVDAK.H | 2 |
|  | CENPL\_stlcld\_122314\_01.14368.14368.2 | 2.7296 | 0.2157 | 99.0% | 1107.6721 | 1108.2798 | 3 | 7.074 | 66.7% | 1 | K.LDVQFSGLTK.G | 2 |
|  | CENPL\_stlcld\_122314\_01.16974.16974.2 | 3.3971 | 0.3354 | 99.9% | 1535.4521 | 1534.7496 | 1 | 5.791 | 53.6% | 3 | K.SPFSVAVSPSLDLSK.I | 2 |
|  | CENPL\_stlcld\_tube2\_122314\_01.10043.10043.2 | 4.2904 | 0.4227 | 100.0% | 1430.4922 | 1430.5632 | 1 | 8.398 | 66.7% | 1 | K.AFGPGLQGGSAGSPAR.F | 2 |
|  | CENPL\_stlcld\_122314\_01.12274.12274.2 | 3.8537 | 0.5142 | 100.0% | 1572.3322 | 1571.7275 | 2 | 8.207 | 44.1% | 1 | R.GAGTGGLGLAVEGPSEAK.M | 2 |
|  | CENPL\_stlcld\_tube2\_122314\_01.11950.11950.2 | 2.8222 | 0.3867 | 99.9% | 1435.7522 | 1435.5767 | 1 | 6.876 | 66.7% | 1 | R.ANLPQSFQVDTSK.A | 2 |
|  | CENPL\_stlcld\_tube2\_122314\_01.15406.15406.3 | 4.3902 | 0.3112 | 100.0% | 2442.3542 | 2441.662 | 4 | 5.279 | 31.0% | 1 | R.VSGQGLHEGHTFEPAEFIIDTR.D | 3 |
|  | CENPL\_stlcld\_122314\_02.11600.11600.2 | 3.1237 | 0.4497 | 100.0% | 1427.6921 | 1427.5974 | 1 | 6.436 | 60.0% | 1 | R.EAGAGGLAIAVEGPSK.A | 2 |
|  | CENPL\_stlcld\_122314\_02.11984.11984.3 | 4.6261 | 0.4898 | 100.0% | 2201.2444 | 2201.4412 | 1 | 8.167 | 44.7% | 5 | R.LVSNHSLHETSSVFVDSLTK.A | 3 |
|  | CENPL\_stlcld\_122314\_01.12148.12148.3 | 2.6619 | 0.3025 | 99.5% | 1436.4543 | 1435.6873 | 2 | 6.021 | 46.2% | 1 | K.AGNNMLLVGVHGPR.T | 3 |
|  | CENPL\_stlcld\_tube2\_122314\_01.12071.12071.2 | 2.9157 | 0.3291 | 99.9% | 1436.6322 | 1435.6873 | 1 | 5.709 | 57.7% | 2 | K.AGNNMLLVGVHGPR.T | 2 |

---

|  |  |  |  |  |  |  |  |  |
| --- | --- | --- | --- | --- | --- | --- | --- | --- |
| U | *gi|154354962|ref|NP\_0* | 5 | 7 | 7.8% | 757 | 83549 | 6.6 | inner membrane protein, mitochondrial isoform 2 [Homo sapiens] |
| U | *gi|154354966|ref|NP\_0* | 5 | 7 | 7.9% | 747 | 82625 | 6.6 | inner membrane protein, mitochondrial isoform 3 [Homo sapiens] |
| U | *gi|154354964|ref|NP\_0* | 5 | 7 | 7.8% | 758 | 83678 | 6.5 | inner membrane protein, mitochondrial isoform 1 [Homo sapiens] |

| Filename XCorr DeltCN Conf% ObsM+H+ CalcM+H+ SpR ZScore Ion% # Sequence  | | | | | | | | | | | | |
| --- | --- | --- | --- | --- | --- | --- | --- | --- | --- | --- | --- | --- |
|  | CENPL\_stlcld\_122314\_02.08222.08222.3 | 2.831 | 0.2365 | 97.2% | 2010.4443 | 2011.2872 | 79 | 4.064 | 29.4% | 1 | K.KSIQSGPLK@ISS\*VSEVMK.E | 3 |
|  | CENPL\_stlcld\_122314\_02.10284.10284.3 | 3.0166 | 0.3317 | 99.9% | 1529.4543 | 1528.7513 | 2 | 5.579 | 41.7% | 1 | K.VVSQYHELVVQAR.D | 3 |
|  | CENPL\_stlcld\_122314\_01.12365.12365.2 | 2.288 | 0.2688 | 98.6% | 1150.3121 | 1150.2767 | 1 | 5.291 | 75.0% | 1 | K.LSEQELQFR.R | 2 |
|  | CENPL\_stlcld\_122314\_02.10385.10385.3 | 4.8869 | 0.4759 | 100.0% | 2094.3843 | 2094.292 | 1 | 8.313 | 34.7% | 3 | R.LRGIEQAVQSHAVAEEEAR.K | 3 |
|  | CENPL\_stlcld\_122314\_02.10557.10557.3 | 3.4483 | 0.272 | 99.9% | 1824.3844 | 1824.9451 | 1 | 5.102 | 40.6% | 1 | R.GIEQAVQSHAVAEEEAR.K | 3 |

---

|  |  |  |  |  |  |  |  |  |
| --- | --- | --- | --- | --- | --- | --- | --- | --- |
| U | *gi|25470886|ref|NP\_06* | 2 | 5 | 7.6% | 407 | 43383 | 8.6 | DAZ associated protein 1 isoform b [Homo sapiens] |
| U | *gi|25470890|ref|NP\_73* | 2 | 5 | 8.2% | 378 | 40530 | 8.3 | DAZ associated protein 1 isoform a [Homo sapiens] |

| Filename XCorr DeltCN Conf% ObsM+H+ CalcM+H+ SpR ZScore Ion% # Sequence  | | | | | | | | | | | | |
| --- | --- | --- | --- | --- | --- | --- | --- | --- | --- | --- | --- | --- |
|  | CENPL\_stlcld\_122314\_02.15875.15875.2 | 4.039 | 0.4656 | 100.0% | 1824.7522 | 1824.0435 | 1 | 7.855 | 66.7% | 4 | K.LFVGGLDWSTTQETLR.S | 2 |
|  | CENPL\_stlcld\_tube2\_122314\_01.08682.08682.2 | 3.8367 | 0.5286 | 100.0% | 1514.0721 | 1514.5974 | 1 | 8.901 | 82.1% | 1 | K.SQAPGQPGASQWGSR.V | 2 |

---

|  |  |  |  |  |  |  |  |  |
| --- | --- | --- | --- | --- | --- | --- | --- | --- |
| U | *gi|148227764|ref|NP\_0* | 2 | 2 | 7.6% | 184 | 20925 | 5.5 | RAP1B, member of RAS oncogene family-like [Homo sapiens] |
| U | *gi|7661678|ref|NP\_056* | 2 | 2 | 7.6% | 184 | 20825 | 5.8 | RAP1B, member of RAS oncogene family [Homo sapiens] |
| U | *gi|58331202|ref|NP\_00* | 2 | 2 | 7.6% | 184 | 20987 | 6.6 | RAP1A, member of RAS oncogene family [Homo sapiens] |
| U | *gi|58219792|ref|NP\_00* | 2 | 2 | 7.6% | 184 | 20825 | 5.8 | RAP1B, member of RAS oncogene family [Homo sapiens] |
| U | *gi|4506413|ref|NP\_002* | 2 | 2 | 7.6% | 184 | 20987 | 6.6 | RAP1A, member of RAS oncogene family [Homo sapiens] |

| Filename XCorr DeltCN Conf% ObsM+H+ CalcM+H+ SpR ZScore Ion% # Sequence  | | | | | | | | | | | | |
| --- | --- | --- | --- | --- | --- | --- | --- | --- | --- | --- | --- | --- |
|  | CENPL\_stlcld\_tube2\_122314\_01.19832.19832.2 | 3.2952 | 0.4064 | 100.0% | 1710.3522 | 1710.9707 | 8 | 8.085 | 53.8% | 1 | K.SKINVNEIFYDLVR.Q | 2 |
|  | CENPL\_stlcld\_122314\_01.22290.22290.2 | 3.1923 | 0.2428 | 99.7% | 1496.2322 | 1495.7184 | 1 | 5.569 | 63.6% | 1 | K.INVNEIFYDLVR.Q | 2 |

---

|  |  |  |  |  |  |  |  |  |
| --- | --- | --- | --- | --- | --- | --- | --- | --- |
| U | *gi|100913206|ref|NP\_0* | 8 | 10 | 7.5% | 1270 | 140958 | 6.8 | DEAH (Asp-Glu-Ala-His) box polypeptide 9 [Homo sapiens] |

| Filename XCorr DeltCN Conf% ObsM+H+ CalcM+H+ SpR ZScore Ion% # Sequence  | | | | | | | | | | | | |
| --- | --- | --- | --- | --- | --- | --- | --- | --- | --- | --- | --- | --- |
| \* | CENPL\_stlcld\_tube2\_122314\_01.08566.08566.2 | 2.2349 | 0.2341 | 97.9% | 1124.9122 | 1125.3281 | 1 | 5.074 | 75.0% | 1 | R.KMTPSYEIR.A | 2 |
| \* | CENPL\_stlcld\_tube2\_122314\_01.18109.18109.3 | 4.2172 | 0.323 | 100.0% | 3481.8542 | 3483.727 | 13 | 5.502 | 22.5% | 1 | R.KEEQEVQATLESEEVDLNAGLHGNWTLENAK.A | 3 |
| \* | CENPL\_stlcld\_tube2\_122314\_01.09086.09086.2 | 2.0661 | 0.2199 | 96.0% | 1076.3322 | 1076.1973 | 2 | 4.701 | 75.0% | 1 | K.LAQFEPSQR.Q | 2 |
| \* | CENPL\_stlcld\_tube2\_122314\_01.11078.11078.3 | 2.6814 | 0.3048 | 99.9% | 1504.7344 | 1504.686 | 7 | 5.26 | 41.7% | 1 | R.GISHVIVDEIHER.D | 3 |
| \* | CENPL\_stlcld\_122314\_01.08540.08540.3 | 3.7904 | 0.3799 | 100.0% | 1463.5144 | 1462.6287 | 1 | 6.146 | 50.0% | 1 | K.HLEMNPHFGSHR.Y | 3 |
| \* | CENPL\_stlcld\_122314\_01.13389.13389.3 | 3.9078 | 0.3759 | 100.0% | 1222.2544 | 1222.4703 | 3 | 6.645 | 55.0% | 1 | R.TPLHEIALSIK.L | 3 |
| \* | CENPL\_stlcld\_tube2\_122314\_01.13154.13154.2 | 2.1202 | 0.321 | 98.4% | 1222.5122 | 1222.4703 | 3 | 6.291 | 60.0% | 3 | R.TPLHEIALSIK.L | 2 |
| \* | CENPL\_stlcld\_tube2\_122314\_01.16043.16043.2 | 2.4161 | 0.2931 | 99.1% | 1004.2522 | 1004.21747 | 1 | 6.235 | 72.2% | 1 | R.LGGIGQFLAK.A | 2 |

---

|  |  |  |  |  |  |  |  |  |
| --- | --- | --- | --- | --- | --- | --- | --- | --- |
| U | *gi|50659095|ref|NP\_00* | 4 | 4 | 7.5% | 783 | 87344 | 9.3 | DEAD (Asp-Glu-Ala-Asp) box polypeptide 21 [Homo sapiens] |

| Filename XCorr DeltCN Conf% ObsM+H+ CalcM+H+ SpR ZScore Ion% # Sequence  | | | | | | | | | | | | |
| --- | --- | --- | --- | --- | --- | --- | --- | --- | --- | --- | --- | --- |
|  | CENPL\_stlcld\_122314\_01.16838.16838.2 | 2.7523 | 0.2384 | 98.5% | 1669.8121 | 1669.8285 | 1 | 5.478 | 46.4% | 1 | K.EGAFSNFPISEETIK.L | 2 |
| \* | CENPL\_stlcld\_122314\_01.13665.13665.2 | 2.7088 | 0.3989 | 99.9% | 1165.6322 | 1165.4215 | 1 | 6.385 | 75.0% | 1 | R.APQVLVLAPTR.E | 2 |
| \* | CENPL\_stlcld\_tube2\_122314\_01.11541.11541.2 | 2.151 | 0.2421 | 96.8% | 1196.9922 | 1196.4325 | 8 | 5.086 | 65.0% | 1 | K.TAITVEHLAIK.C | 2 |
| \* | CENPL\_stlcld\_tube2\_122314\_01.20818.20818.2 | 5.7313 | 0.4774 | 100.0% | 2110.112 | 2109.347 | 1 | 9.281 | 50.0% | 1 | K.GAVEALAAALAHISGATSVDQR.S | 2 |

---

|  |  |  |  |  |  |  |  |  |
| --- | --- | --- | --- | --- | --- | --- | --- | --- |
| U | *gi|32698730|ref|NP\_06* | 4 | 5 | 7.5% | 695 | 76121 | 8.7 | nuclear fragile X mental retardation protein interacting protein 2 [Homo sapiens] |

| Filename XCorr DeltCN Conf% ObsM+H+ CalcM+H+ SpR ZScore Ion% # Sequence  | | | | | | | | | | | | |
| --- | --- | --- | --- | --- | --- | --- | --- | --- | --- | --- | --- | --- |
| \* | CENPL\_stlcld\_tube2\_122314\_01.08554.08554.2 | 2.4315 | 0.2651 | 98.3% | 1337.4922 | 1338.3763 | 10 | 6.059 | 50.0% | 1 | K.TGYGELNGNAGER.E | 2 |
| \* | CENPL\_stlcld\_122314\_01.09777.09777.2 | 3.4183 | 0.385 | 100.0% | 1404.4122 | 1404.4764 | 11 | 6.523 | 58.3% | 2 | K.NLSSDEATNPISR.V | 2 |
| \* | CENPL\_stlcld\_tube2\_122314\_01.10560.10560.2 | 3.0028 | 0.3718 | 99.9% | 1515.3722 | 1515.7068 | 1 | 6.203 | 57.7% | 1 | R.VLNGNQQVVDTSLK.Q | 2 |
| \* | CENPL\_stlcld\_tube2\_122314\_01.11076.11076.2 | 3.2095 | 0.4038 | 100.0% | 1375.2122 | 1375.6233 | 27 | 5.73 | 54.5% | 1 | K.IMQQETSVPTLK.Q | 2 |

---

|  |  |  |  |  |  |  |  |  |
| --- | --- | --- | --- | --- | --- | --- | --- | --- |
| U | *gi|126722969|ref|NP\_0* | 3 | 5 | 7.5% | 561 | 60423 | 6.6 | centromere protein T [Homo sapiens] |

| Filename XCorr DeltCN Conf% ObsM+H+ CalcM+H+ SpR ZScore Ion% # Sequence  | | | | | | | | | | | | |
| --- | --- | --- | --- | --- | --- | --- | --- | --- | --- | --- | --- | --- |
| \* | CENPL\_stlcld\_tube2\_122314\_02.08642.08642.3 | 3.2621 | 0.3386 | 99.9% | 1763.2444 | 1761.8918 | 1 | 5.875 | 45.0% | 3 | R.SAHIQASGHLEEQTPR.T | 3 |
| \* | CENPL\_stlcld\_tube2\_122314\_01.16467.16467.2 | 1.8886 | 0.3317 | 97.9% | 948.6722 | 948.1099 | 8 | 5.833 | 75.0% | 1 | R.AVDVGAFLR.D | 2 |
| \* | CENPL\_stlcld\_122314\_02.12202.12202.3 | 4.5818 | 0.4387 | 100.0% | 1950.4443 | 1950.2485 | 2 | 7.378 | 37.5% | 1 | R.RQGLVTDQVSLHVLVER.H | 3 |

---

|  |  |  |  |  |  |  |  |  |
| --- | --- | --- | --- | --- | --- | --- | --- | --- |
| U | *gi|5031699|ref|NP\_005* | 2 | 3 | 7.0% | 427 | 47355 | 7.5 | flotillin 1 [Homo sapiens] |

| Filename XCorr DeltCN Conf% ObsM+H+ CalcM+H+ SpR ZScore Ion% # Sequence  | | | | | | | | | | | | |
| --- | --- | --- | --- | --- | --- | --- | --- | --- | --- | --- | --- | --- |
| \* | CENPL\_stlcld\_122314\_02.13458.13458.2 | 4.657 | 0.5119 | 100.0% | 1604.6921 | 1604.8187 | 1 | 9.344 | 75.0% | 1 | K.SQLIMQAEAEAASVR.M | 2 |
| \* | CENPL\_stlcld\_122314\_02.10662.10662.2 | 3.8215 | 0.4805 | 100.0% | 1381.4922 | 1380.5994 | 1 | 7.324 | 67.9% | 2 | K.ITLVSSGSGTMGAAK.V | 2 |

---

|  |  |  |  |  |  |  |  |  |
| --- | --- | --- | --- | --- | --- | --- | --- | --- |
| U | *gi|119395754|ref|NP\_0* | 6 | 11 | 6.9% | 590 | 62378 | 7.8 | keratin 5 [Homo sapiens] |

| Filename XCorr DeltCN Conf% ObsM+H+ CalcM+H+ SpR ZScore Ion% # Sequence  | | | | | | | | | | | | |
| --- | --- | --- | --- | --- | --- | --- | --- | --- | --- | --- | --- | --- |
| \* | CENPL\_stlcld\_tube2\_122314\_01.11331.11331.2 | 2.1654 | 0.3069 | 98.4% | 1111.9521 | 1112.2279 | 5 | 5.705 | 55.0% | 1 | R.ISISTSGGSFR.N | 2 |
|  | CENPL\_stlcld\_tube2\_122314\_01.12512.12512.2 | 2.417 | 0.2122 | 98.9% | 828.09216 | 827.95544 | 5 | 4.999 | 91.7% | 4 | K.FASFIDK.V | 222222222 |
|  | CENPL\_stlcld\_122314\_01.12293.12293.2 | 2.815 | 0.2809 | 99.9% | 1082.9922 | 1083.2755 | 3 | 6.695 | 75.0% | 2 | K.FASFIDKVR.F | 222322223 |
|  | CENPL\_stlcld\_tube2\_122314\_01.19944.19944.1 | 2.8562 | 0.3917 | 100.0% | 1329.95 | 1330.5211 | 1 | 6.619 | 72.7% | 1 | R.NLDLDSIIAEVK.A | 11111 |
|  | CENPL\_stlcld\_122314\_01.20872.20872.2 | 3.7762 | 0.4242 | 100.0% | 1330.7522 | 1330.5211 | 1 | 7.499 | 81.8% | 2 | R.NLDLDSIIAEVK.A | 22222 |
|  | CENPL\_stlcld\_tube2\_122314\_02.08830.08830.2 | 2.0541 | 0.213 | 95.5% | 1094.1122 | 1094.1692 | 329 | 5.323 | 62.5% | 1 | K.AQYEEIANR.S | 2 |

Similarities:
gi|4504919|ref|NP\_002(2:4)  
gi|47132620|ref|NP\_00(4:2)  
gi|67782365|ref|NP\_00(2:4)  
gi|119703753|ref|NP\_0(4:2)  
gi|32567786|ref|NP\_78(4:2)  
gi|153791158|ref|NP\_0(4:2)  
gi|109255249|ref|NP\_0(1:5)  
gi|15618995|ref|NP\_25(2:4)  

---

|  |  |  |  |  |  |  |  |  |
| --- | --- | --- | --- | --- | --- | --- | --- | --- |
| U | *gi|11415026|ref|NP\_00* | 3 | 3 | 6.8% | 176 | 20762 | 10.7 | ribosomal protein L18a [Homo sapiens] |
| U | *gi|88954764|ref|XP\_95* | 3 | 3 | 7.2% | 166 | 19523 | 10.7 | PREDICTED: similar to ribosomal protein L18a isoform 6 [Homo sapiens] |
| U | *gi|88954757|ref|XP\_94* | 3 | 3 | 6.8% | 176 | 20767 | 10.7 | PREDICTED: similar to ribosomal protein L18a isoform 4 [Homo sapiens] |
| U | *gi|88953058|ref|XP\_93* | 3 | 3 | 7.2% | 166 | 19523 | 10.7 | PREDICTED: similar to ribosomal protein L18a isoform 3 [Homo sapiens] |
| U | *gi|27480190|ref|XP\_20* | 3 | 3 | 6.8% | 176 | 20767 | 10.7 | PREDICTED: similar to ribosomal protein L18a isoform 1 [Homo sapiens] |
| U | *gi|169163933|ref|XP\_0* | 3 | 3 | 7.2% | 166 | 19523 | 10.7 | PREDICTED: similar to ribosomal protein L18a isoform 2 [Homo sapiens] |
| U | *gi|169163931|ref|XP\_0* | 3 | 3 | 6.8% | 176 | 20767 | 10.7 | PREDICTED: similar to ribosomal protein L18a isoform 1 [Homo sapiens] |

| Filename XCorr DeltCN Conf% ObsM+H+ CalcM+H+ SpR ZScore Ion% # Sequence  | | | | | | | | | | | | |
| --- | --- | --- | --- | --- | --- | --- | --- | --- | --- | --- | --- | --- |
|  | CENPL\_stlcld\_122314\_01.18918.18918.2 | 2.7984 | 0.1293 | 97.7% | 1461.7322 | 1461.7062 | 2 | 5.42 | 60.0% | 1 | K.SRFWYFVSQLK.K | 2 |
|  | CENPL\_stlcld\_tube2\_122314\_01.15837.15837.3 | 3.2077 | 0.2429 | 99.9% | 1589.4543 | 1589.8802 | 12 | 5.142 | 43.2% | 1 | K.SRFWYFVSQLKK.M | 3 |
|  | CENPL\_stlcld\_122314\_01.17182.17182.2 | 2.7981 | 0.3115 | 99.9% | 1347.1122 | 1346.6146 | 1 | 5.814 | 77.8% | 1 | R.FWYFVSQLKK.M | 2 |

---

|  |  |  |  |  |  |  |  |  |
| --- | --- | --- | --- | --- | --- | --- | --- | --- |
| U | *gi|30795212|ref|NP\_00* | 3 | 3 | 6.7% | 579 | 63705 | 8.9 | insulin-like growth factor 2 mRNA binding protein 3 [Homo sapiens] |

| Filename XCorr DeltCN Conf% ObsM+H+ CalcM+H+ SpR ZScore Ion% # Sequence  | | | | | | | | | | | | |
| --- | --- | --- | --- | --- | --- | --- | --- | --- | --- | --- | --- | --- |
|  | CENPL\_stlcld\_122314\_01.09190.09190.2 | 2.115 | 0.225 | 96.0% | 1141.3522 | 1141.3182 | 11 | 4.816 | 61.1% | 1 | K.ILAHNNFVGR.L | 2 |
| \* | CENPL\_stlcld\_tube2\_122314\_01.19932.19932.2 | 3.4399 | 0.3514 | 99.9% | 1888.2522 | 1888.1711 | 5 | 6.876 | 43.3% | 1 | K.ITISPLQELTLYNPER.T | 2 |
|  | CENPL\_stlcld\_tube2\_122314\_01.15222.15222.2 | 2.2819 | 0.2332 | 96.8% | 1431.6322 | 1431.7333 | 4 | 4.895 | 54.2% | 1 | R.MVIITGPPEAQFK.A | 22 |

Similarities:
gi|56118219|ref|NP\_00(1:2)  

---

|  |  |  |  |  |  |  |  |  |
| --- | --- | --- | --- | --- | --- | --- | --- | --- |
| U | *gi|4506787|ref|NP\_003* | 8 | 11 | 6.2% | 1657 | 189251 | 6.5 | IQ motif containing GTPase activating protein 1 [Homo sapiens] |

| Filename XCorr DeltCN Conf% ObsM+H+ CalcM+H+ SpR ZScore Ion% # Sequence  | | | | | | | | | | | | |
| --- | --- | --- | --- | --- | --- | --- | --- | --- | --- | --- | --- | --- |
| \* | CENPL\_stlcld\_122314\_01.18641.18641.2 | 3.0394 | 0.3989 | 99.9% | 1656.1122 | 1655.9371 | 1 | 6.682 | 50.0% | 1 | R.NGVYLAKLGNFFSPK.V | 2 |
| \* | CENPL\_stlcld\_122314\_01.15520.15520.2 | 2.4677 | 0.3009 | 99.3% | 1142.2922 | 1142.3582 | 1 | 5.894 | 72.2% | 1 | K.YGIQMPAFSK.I | 2 |
| \* | CENPL\_stlcld\_122314\_02.01893.01893.3 | 5.851 | 0.4527 | 100.0% | 2961.6543 | 2960.3574 | 1 | 7.671 | 33.9% | 2 | K.IGGILANELSVDEAALHAAVIAINEAIDR.R | 3 |
| \* | CENPL\_stlcld\_tube2\_122314\_01.16860.16860.2 | 3.099 | 0.1953 | 98.8% | 1453.5521 | 1452.7837 | 1 | 4.566 | 61.5% | 1 | R.RLAAVALINAAIQK.G | 2 |
| \* | CENPL\_stlcld\_122314\_01.19163.19163.2 | 3.5542 | 0.4164 | 100.0% | 1296.2122 | 1296.5962 | 1 | 7.299 | 70.8% | 2 | R.LAAVALINAAIQK.G | 2 |
| \* | CENPL\_stlcld\_122314\_01.20346.20346.2 | 5.0457 | 0.4503 | 100.0% | 2056.152 | 2056.3262 | 1 | 8.228 | 50.0% | 1 | K.LEGVLAEVAQHYQDTLIR.A | 2 |
| \* | CENPL\_stlcld\_122314\_01.20343.20343.3 | 4.519 | 0.3983 | 100.0% | 2057.0344 | 2056.3262 | 1 | 7.11 | 44.1% | 1 | K.LEGVLAEVAQHYQDTLIR.A | 3 |
| \* | CENPL\_stlcld\_122314\_02.11686.11686.2 | 2.7282 | 0.3944 | 99.9% | 1725.0721 | 1725.8528 | 1 | 7.29 | 46.7% | 2 | R.EEIQSSISGVTAAYNR.E | 2 |

---

|  |  |  |  |  |  |  |  |  |
| --- | --- | --- | --- | --- | --- | --- | --- | --- |
| U | *gi|35493811|ref|NP\_90* | 2 | 2 | 6.2% | 530 | 59380 | 10.1 | RNA binding motif protein 39 isoform a [Homo sapiens] |
| U | *gi|4757926|ref|NP\_004* | 2 | 2 | 6.3% | 524 | 58657 | 10.1 | RNA binding motif protein 39 isoform b [Homo sapiens] |

| Filename XCorr DeltCN Conf% ObsM+H+ CalcM+H+ SpR ZScore Ion% # Sequence  | | | | | | | | | | | | |
| --- | --- | --- | --- | --- | --- | --- | --- | --- | --- | --- | --- | --- |
|  | CENPL\_stlcld\_122314\_01.19803.19803.2 | 2.6965 | 0.3248 | 99.4% | 1909.9321 | 1909.2103 | 2 | 5.301 | 40.0% | 1 | R.LYVGSLHFNITEDMLR.G | 2 |
|  | CENPL\_stlcld\_122314\_01.14975.14975.2 | 2.7295 | 0.2621 | 98.6% | 1830.0721 | 1830.8566 | 1 | 5.447 | 43.8% | 1 | R.TDASSASSFLDSDELER.T | 2 |

---

|  |  |  |  |  |  |  |  |  |
| --- | --- | --- | --- | --- | --- | --- | --- | --- |
| U | *gi|24234688|ref|NP\_00* | 3 | 3 | 5.9% | 679 | 73681 | 6.2 | heat shock 70kDa protein 9 precursor [Homo sapiens] |

| Filename XCorr DeltCN Conf% ObsM+H+ CalcM+H+ SpR ZScore Ion% # Sequence  | | | | | | | | | | | | |
| --- | --- | --- | --- | --- | --- | --- | --- | --- | --- | --- | --- | --- |
| \* | CENPL\_stlcld\_122314\_01.20140.20140.2 | 4.3164 | 0.398 | 100.0% | 1363.2122 | 1362.5687 | 1 | 8.832 | 77.3% | 1 | R.AQFEGIVTDLIR.R | 2 |
| \* | CENPL\_stlcld\_122314\_01.14571.14571.2 | 2.3079 | 0.2324 | 97.7% | 1291.5721 | 1291.4496 | 3 | 4.973 | 60.0% | 1 | K.VQQTVQDLFGR.A | 2 |
| \* | CENPL\_stlcld\_tube2\_122314\_01.16356.16356.2 | 2.4932 | 0.2258 | 97.5% | 1856.9321 | 1858.0735 | 1 | 4.632 | 46.9% | 1 | R.VEAVNMAEGIIHDTETK.M | 2 |

---

|  |  |  |  |  |  |  |  |  |
| --- | --- | --- | --- | --- | --- | --- | --- | --- |
| U | *gi|153945736|ref|NP\_8* | 4 | 9 | 5.9% | 459 | 49822 | 5.0 | keratin 27 [Homo sapiens] |

| Filename XCorr DeltCN Conf% ObsM+H+ CalcM+H+ SpR ZScore Ion% # Sequence  | | | | | | | | | | | | |
| --- | --- | --- | --- | --- | --- | --- | --- | --- | --- | --- | --- | --- |
|  | CENPL\_stlcld\_tube2\_122314\_01.08714.08714.2 | 3.0686 | 0.3444 | 100.0% | 1091.2522 | 1091.2273 | 32 | 5.996 | 68.8% | 4 | K.VTMQNLNDR.L | 222 |
| \* | CENPL\_stlcld\_tube2\_122314\_02.09828.09828.2 | 2.5315 | 0.1122 | 96.5% | 1065.0322 | 1065.2145 | 75 | 4.869 | 62.5% | 1 | R.LASYLENVR.A | 2 |
|  | CENPL\_stlcld\_tube2\_122314\_01.14187.14187.1 | 2.0158 | 0.2794 | 96.4% | 1109.54 | 1110.1681 | 1 | 5.703 | 68.8% | 1 | R.DAEAWFNEK.S | 11 |
|  | CENPL\_stlcld\_tube2\_122314\_01.14228.14228.2 | 2.7953 | 0.1474 | 98.5% | 1109.6921 | 1110.1681 | 2 | 6.72 | 81.2% | 3 | R.DAEAWFNEK.S | 22 |

Similarities:
contaminant\_KERATIN03(3:1)  
contaminant\_KERATIN05(1:3)  

---

|  |  |  |  |  |  |  |  |  |
| --- | --- | --- | --- | --- | --- | --- | --- | --- |
| U | *contaminant\_KERATIN16* | 3 | 5 | 5.8% | 534 | 57265 | 6.6 | no description |

| Filename XCorr DeltCN Conf% ObsM+H+ CalcM+H+ SpR ZScore Ion% # Sequence  | | | | | | | | | | | | |
| --- | --- | --- | --- | --- | --- | --- | --- | --- | --- | --- | --- | --- |
|  | CENPL\_stlcld\_122314\_01.08782.08782.2 | 2.6273 | 0.1146 | 97.5% | 1108.5721 | 1108.196 | 60 | 6.069 | 62.5% | 2 | R.AQYEEIAQR.S | 2222 |
|  | CENPL\_stlcld\_122314\_02.13122.13122.2 | 3.3979 | 0.3814 | 100.0% | 1407.4122 | 1406.6653 | 2 | 6.236 | 68.2% | 2 | K.LALDIEIATYRK.L | 22222 |
| \* | CENPL\_stlcld\_122314\_01.08411.08411.2 | 2.7256 | 0.1229 | 97.6% | 1197.3121 | 1197.3341 | 7 | 5.034 | 66.7% | 1 | K.IIGTTT#LNKR.R | 2 |

Similarities:
gi|4504919|ref|NP\_002(1:2)  
gi|47132620|ref|NP\_00(1:2)  
gi|67782365|ref|NP\_00(1:2)  
contaminant\_KERATIN19(1:2)  
gi|119703753|ref|NP\_0(1:2)  
gi|109255249|ref|NP\_0(2:1)  

---

|  |  |  |  |  |  |  |  |  |
| --- | --- | --- | --- | --- | --- | --- | --- | --- |
[truncated: 57,101 more chars]
