# Supplementary material for: Astrin-SKAP complex reconstitution reveals its kinetochore interaction with microtubule-bound Ndc80
Source: eLife. 2017 Aug 25;6:e26866. doi: 10.7554/eLife.26866 (PMC5602300; doi:10.7554/eLife.26866)
Supplement: Source data 1. — Complete mass spectrometry searches using methods described in (Washburn et al., 2001) for affinity purification/mass spectrometry data sets described in this paper (data from this study; [Kern et al., 2016] [Gascoigne et al., 2011]). Individual Astrin cross-linking immunoprecipitations are listed based on the order in Figure 4—figure supplement 1. These samples have not been pruned for common or antibody-specific contaminants. [file elife-26866-data1.zip › CENPL_NocodazoleIP.html]

D CENPL\_Noc300
DTASelect v2.0.21  
/nfs/cheeseman\_massspec/David/CENPL\_Noc300  
/nfs/cheeseman\_massspec/Databases/NCBI-RefSeq\_human\_na\_04-13-2009\_con\_reversed.fasta  
SEQUEST 3.0 in SQT format.  
-p 1  
 Jump  to the summary table.  
  
sequest.params modifications:

|  |  |  |
| --- | --- | --- |
| \* | S | 80.0 |
| # | T | 80.0 |
| @ | Y | 80.0 |
| Static | C | 57.0 |

|  |  |
| --- | --- |
| true | Use criteria |
| 0.0 | Minimum peptide confidence |
| 0.05 | Peptide false positive rate |
| 0.0 | Minimum protein confidence |
| 1.0 | Protein false positive rate |
| 1 | Minimum charge state |
| 16 | Maximum charge state |
| 0.0 | Minimum ion proportion |
| 1000 | Maximum Sp rank |
| -1.0 | Minimum Sp score |
| Include | Modified peptide inclusion |
| Any | Tryptic status requirement |
| false | Multiple, ambiguous IDs allowed |
| Ignore | Peptide validation handling |
| XCorr | Purge duplicate peptides by protein |
| false | Include only loci with unique peptide |
| true | Remove subset proteins |
| Ignore | Locus validation handling |
| 0 | Minimum modified peptides per locus |
| 1000 | Minimum redundancy for low coverage loci |
| 1 | Minimum peptides per locus |

#### Locus Key:

|  |  |  |  |  |  |  |  |  |
| --- | --- | --- | --- | --- | --- | --- | --- | --- |
| Validation Status | Locus | Sequence Count | Spectrum Count | Sequence Coverage | Length | MolWt | pI | Descriptive Name |

#### Similarity Key:

|  |  |  |
| --- | --- | --- |
| Locus | # of identical peptides | # of differing peptides |

---

|  |  |  |  |  |  |  |  |  |
| --- | --- | --- | --- | --- | --- | --- | --- | --- |
| U | *gi|31542947|ref|NP\_00* | 92 | 588 | 84.6% | 573 | 61055 | 5.9 | chaperonin [Homo sapiens] |
| U | *gi|41399285|ref|NP\_95* | 92 | 588 | 84.6% | 573 | 61055 | 5.9 | chaperonin [Homo sapiens] |

| Filename XCorr DeltCN Conf% ObsM+H+ CalcM+H+ SpR ZScore Ion% # Sequence  | | | | | | | | | | | | |
| --- | --- | --- | --- | --- | --- | --- | --- | --- | --- | --- | --- | --- |
|  | CENPL\_Noc300\_122214\_01.20044.20044.2 | 5.7165 | 0.5792 | 100.0% | 2113.7122 | 2114.5667 | 1 | 11.224 | 62.5% | 13 | R.ALMLQGVDLLADAVAVTMGPK.G | 2 |
|  | CENPL\_Noc300\_122214\_01.19948.19948.3 | 5.857 | 0.4948 | 100.0% | 2114.9343 | 2114.5667 | 1 | 9.488 | 42.5% | 14 | R.ALMLQGVDLLADAVAVTMGPK.G | 3 |
|  | CENPL\_Noc300\_122214\_01.19012.19012.2 | 4.1321 | 0.4857 | 100.0% | 2328.112 | 2327.806 | 1 | 7.505 | 47.7% | 1 | R.ALMLQGVDLLADAVAVTMGPKGR.T | 2 |
|  | CENPL\_Noc300\_122214\_02.15952.15952.3 | 3.4066 | 0.2916 | 99.9% | 2328.5344 | 2327.806 | 10 | 5.271 | 30.7% | 3 | R.ALMLQGVDLLADAVAVTMGPKGR.T | 3 |
|  | CENPL\_Noc300\_122214\_01.10592.10592.2 | 3.9209 | 0.4848 | 100.0% | 1345.3121 | 1345.5382 | 1 | 7.778 | 81.8% | 26 | R.TVIIEQSWGSPK.V | 2 |
|  | CENPL\_Noc300\_tube2\_122214\_01.14310.14310.1 | 2.7908 | 0.1297 | 95.0% | 1346.72 | 1345.5382 | 1 | 4.914 | 50.0% | 1 | R.TVIIEQSWGSPK.V | 1 |
|  | CENPL\_Noc300\_122214\_01.11388.11388.2 | 2.3188 | 0.1904 | 97.2% | 1425.4521 | 1425.5382 | 180 | 4.088 | 45.5% | 2 | R.TVIIEQSWGS\*PK.V | 2 |
|  | CENPL\_Noc300\_122214\_01.06242.06242.2 | 2.7729 | 0.2418 | 99.7% | 1110.1522 | 1110.2957 | 30 | 5.865 | 62.5% | 2 | K.SIDLKDKYK.N | 2 |
|  | CENPL\_Noc300\_tube2\_122214\_01.09341.09341.3 | 3.7098 | 0.2203 | 99.9% | 1594.3143 | 1593.8638 | 6 | 6.07 | 36.5% | 2 | K.SIDLKDKYKNIGAK.L | 3 |
|  | CENPL\_Noc300\_tube2\_122214\_01.09352.09352.2 | 3.6227 | 0.2053 | 99.8% | 1594.4521 | 1593.8638 | 1 | 5.818 | 69.2% | 1 | K.SIDLKDKYKNIGAK.L | 2 |
|  | CENPL\_Noc300\_122214\_01.09342.09342.2 | 6.8552 | 0.6589 | 100.0% | 2560.9521 | 2561.7222 | 1 | 12.674 | 58.3% | 14 | K.LVQDVANNTNEEAGDGTTTATVLAR.S | 2 |
|  | CENPL\_Noc300\_tube2\_122214\_01.12440.12440.3 | 5.483 | 0.5242 | 100.0% | 2562.0544 | 2561.7222 | 1 | 9.28 | 33.3% | 7 | K.LVQDVANNTNEEAGDGTTTATVLAR.S | 3 |
|  | CENPL\_Noc300\_122214\_01.06363.06363.2 | 2.3106 | 0.1443 | 97.8% | 855.7522 | 855.9694 | 23 | 3.698 | 71.4% | 5 | K.GANPVEIR.R | 2 |
|  | CENPL\_Noc300\_122214\_01.15978.15978.2 | 4.0823 | 0.3241 | 100.0% | 1585.3722 | 1585.9458 | 1 | 6.575 | 78.6% | 3 | R.RGVMLAVDAVIAELK.K | 2 |
|  | CENPL\_Noc300\_122214\_02.13535.13535.3 | 4.124 | 0.3304 | 100.0% | 1586.4844 | 1585.9458 | 3 | 7.331 | 50.0% | 1 | R.RGVMLAVDAVIAELK.K | 3 |
|  | CENPL\_Noc300\_122214\_01.15012.15012.2 | 3.6218 | 0.2968 | 100.0% | 1713.7122 | 1714.1199 | 1 | 6.978 | 63.3% | 1 | R.RGVMLAVDAVIAELKK.Q | 2 |
|  | CENPL\_Noc300\_122214\_01.15010.15010.3 | 6.1849 | 0.3986 | 100.0% | 1714.3744 | 1714.1199 | 1 | 8.478 | 51.7% | 9 | R.RGVMLAVDAVIAELKK.Q | 3 |
|  | CENPL\_Noc300\_122214\_02.15296.15296.3 | 3.9593 | 0.3488 | 100.0% | 1430.3043 | 1429.7583 | 1 | 7.094 | 53.8% | 2 | R.GVMLAVDAVIAELK.K | 3 |
|  | CENPL\_Noc300\_122214\_02.15398.15398.2 | 5.2014 | 0.4705 | 100.0% | 1430.5521 | 1429.7583 | 1 | 7.772 | 80.8% | 8 | R.GVMLAVDAVIAELK.K | 2 |
|  | CENPL\_Noc300\_122214\_02.14102.14102.2 | 5.4491 | 0.4706 | 100.0% | 1557.7722 | 1557.9324 | 1 | 8.743 | 75.0% | 12 | R.GVMLAVDAVIAELKK.Q | 2 |
|  | CENPL\_Noc300\_122214\_01.16676.16676.1 | 3.3224 | 0.1569 | 95.9% | 1558.96 | 1557.9324 | 1 | 4.697 | 57.1% | 2 | R.GVMLAVDAVIAELKK.Q | 1 |
|  | CENPL\_Noc300\_tube2\_122214\_01.15542.15542.3 | 3.7381 | 0.5095 | 100.0% | 2385.8044 | 2385.6335 | 1 | 7.24 | 30.7% | 7 | K.QSKPVTTPEEIAQVATISANGDK.E | 3 |
|  | CENPL\_Noc300\_tube2\_122214\_01.15605.15605.2 | 3.9531 | 0.4697 | 100.0% | 2385.8523 | 2385.6335 | 1 | 8.064 | 36.4% | 2 | K.QSKPVTTPEEIAQVATISANGDK.E | 2 |
|  | CENPL\_Noc300\_tube2\_122214\_01.20782.20782.3 | 4.0474 | 0.2484 | 99.9% | 3559.5244 | 3557.9954 | 1 | 4.772 | 22.0% | 1 | K.QSKPVTTPEEIAQVATISANGDKEIGNIISDAMK.K | 3 |
|  | CENPL\_Noc300\_tube2\_122214\_01.20614.20614.3 | 3.6653 | 0.5215 | 100.0% | 3685.4343 | 3686.1694 | 35 | 8.609 | 18.4% | 4 | K.QSKPVTTPEEIAQVATISANGDKEIGNIISDAMKK.V | 3 |
|  | CENPL\_Noc300\_122214\_01.12653.12653.2 | 2.8203 | 0.2541 | 99.5% | 1192.4521 | 1191.385 | 1 | 6.222 | 75.0% | 4 | K.EIGNIISDAMK.K | 2 |
|  | CENPL\_Noc300\_tube2\_122214\_01.15819.15819.2 | 2.5616 | 0.1262 | 97.2% | 1320.3722 | 1319.5591 | 40 | 4.563 | 59.1% | 2 | K.EIGNIISDAMKK.V | 2 |
|  | CENPL\_Noc300\_122214\_01.14871.14871.2 | 5.0727 | 0.3556 | 100.0% | 1505.5122 | 1505.7235 | 1 | 8.944 | 79.2% | 9 | K.TLNDELEIIEGMK.F | 2 |
|  | CENPL\_Noc300\_tube2\_122214\_01.19976.19976.2 | 4.7628 | 0.488 | 100.0% | 1924.1122 | 1924.1761 | 1 | 8.132 | 63.3% | 11 | K.TLNDELEIIEGMKFDR.G | 2 |
|  | CENPL\_Noc300\_122214\_02.12044.12044.3 | 2.728 | 0.2915 | 99.6% | 1924.7043 | 1924.1761 | 14 | 5.064 | 36.7% | 2 | K.TLNDELEIIEGMKFDR.G | 3 |
|  | CENPL\_Noc300\_tube2\_122214\_01.16530.16530.3 | 3.5151 | 0.1718 | 99.5% | 1809.0844 | 1809.0312 | 15 | 5.078 | 41.1% | 2 | K.FDRGYISPYFINTSK.G | 3 |
|  | CENPL\_Noc300\_122214\_01.12106.12106.2 | 3.6649 | 0.4847 | 100.0% | 1391.5122 | 1390.5786 | 1 | 8.273 | 86.4% | 7 | R.GYISPYFINTSK.G | 2 |
|  | CENPL\_Noc300\_tube2\_122214\_01.17048.17048.1 | 2.5324 | 0.232 | 95.5% | 1391.64 | 1390.5786 | 1 | 4.942 | 50.0% | 1 | R.GYISPYFINTSK.G | 1 |
|  | CENPL\_Noc300\_tube2\_122214\_01.19310.19310.2 | 4.6655 | 0.5867 | 100.0% | 1602.7122 | 1602.7473 | 1 | 10.665 | 87.5% | 4 | K.CEFQDAYVLLSEK.K | 2 |
|  | CENPL\_Noc300\_tube2\_122214\_01.17310.17310.3 | 3.7438 | 0.2644 | 99.9% | 1730.5743 | 1730.9214 | 1 | 6.126 | 50.0% | 2 | K.CEFQDAYVLLSEKK.I | 3 |
|  | CENPL\_Noc300\_tube2\_122214\_01.17907.17907.2 | 5.8403 | 0.4973 | 100.0% | 2048.632 | 2048.3933 | 1 | 9.241 | 63.9% | 5 | K.KISSIQSIVPALEIANAHR.K | 2 |
|  | CENPL\_Noc300\_122214\_01.12791.12791.3 | 6.1297 | 0.4644 | 100.0% | 2049.4443 | 2048.3933 | 1 | 8.744 | 51.4% | 7 | K.KISSIQSIVPALEIANAHR.K | 3 |
|  | CENPL\_Noc300\_122214\_01.14142.14142.2 | 3.9134 | 0.3615 | 100.0% | 1920.1122 | 1920.2192 | 8 | 6.956 | 41.2% | 5 | K.ISSIQSIVPALEIANAHR.K | 2 |
|  | CENPL\_Noc300\_tube2\_122214\_01.19322.19322.3 | 3.6605 | 0.2805 | 99.9% | 1920.8043 | 1920.2192 | 2 | 6.548 | 39.7% | 7 | K.ISSIQSIVPALEIANAHR.K | 3 |
|  | CENPL\_Noc300\_122214\_01.17376.17376.3 | 5.6468 | 0.5285 | 100.0% | 4266.6846 | 4267.9565 | 1 | 8.926 | 25.6% | 1 | K.ISSIQSIVPALEIANAHRKPLVIIAEDVDGEALSTLVLNR.L | 3 |
|  | CENPL\_Noc300\_122214\_01.15851.15851.2 | 7.7688 | 0.6456 | 100.0% | 2367.0322 | 2366.7605 | 1 | 12.519 | 71.4% | 10 | R.KPLVIIAEDVDGEALSTLVLNR.L | 2 |
|  | CENPL\_Noc300\_tube2\_122214\_01.20726.20726.3 | 3.643 | 0.3975 | 100.0% | 2367.0544 | 2366.7605 | 2 | 6.367 | 33.3% | 21 | R.KPLVIIAEDVDGEALSTLVLNR.L | 3 |
|  | CENPL\_Noc300\_122214\_02.08870.08870.2 | 3.3923 | 0.3781 | 100.0% | 1153.9321 | 1154.4819 | 1 | 6.697 | 90.0% | 9 | R.LKVGLQVVAVK.A | 2 |
|  | CENPL\_Noc300\_122214\_02.09550.09550.3 | 3.6783 | 0.4231 | 100.0% | 1970.0044 | 1969.3378 | 1 | 6.336 | 33.3% | 1 | R.LKVGLQVVAVKAPGFGDNR.K | 3 |
|  | CENPL\_Noc300\_122214\_02.08931.08931.3 | 3.1231 | 0.382 | 99.9% | 2097.0544 | 2097.5117 | 12 | 5.542 | 28.9% | 2 | R.LKVGLQVVAVKAPGFGDNRK.N | 3 |
|  | CENPL\_Noc300\_tube2\_122214\_01.12677.12677.1 | 1.9489 | 0.2728 | 95.3% | 912.59 | 913.14844 | 1 | 5.711 | 68.8% | 2 | K.VGLQVVAVK.A | 1 |
|  | CENPL\_Noc300\_tube2\_122214\_01.12770.12770.2 | 3.5239 | 0.2869 | 100.0% | 913.1922 | 913.14844 | 1 | 7.017 | 87.5% | 8 | K.VGLQVVAVK.A | 2 |
|  | CENPL\_Noc300\_122214\_01.10038.10038.3 | 4.6472 | 0.5044 | 100.0% | 1856.4243 | 1856.1783 | 1 | 8.099 | 50.0% | 3 | K.VGLQVVAVKAPGFGDNRK.N | 3 |
|  | CENPL\_Noc300\_122214\_02.12286.12286.3 | 5.9039 | 0.5094 | 100.0% | 3583.2544 | 3583.0076 | 1 | 8.998 | 28.0% | 3 | K.NQLKDMAIATGGAVFGEEGLTLNLEDVQPHDLGK.V | 3 |
|  | CENPL\_Noc300\_122214\_02.12408.12408.3 | 5.6585 | 0.5429 | 100.0% | 3098.2744 | 3099.4395 | 1 | 8.252 | 24.1% | 13 | K.DMAIATGGAVFGEEGLTLNLEDVQPHDLGK.V | 3 |
|  | CENPL\_Noc300\_122214\_01.15464.15464.2 | 4.612 | 0.4781 | 100.0% | 3099.0522 | 3099.4395 | 1 | 8.488 | 32.8% | 2 | K.DMAIATGGAVFGEEGLTLNLEDVQPHDLGK.V | 2 |
|  | CENPL\_Noc300\_122214\_01.06472.06472.1 | 2.0787 | 0.2596 | 95.2% | 844.46 | 845.0269 | 1 | 5.486 | 78.6% | 4 | K.VGEVIVTK.D | 1 |
|  | CENPL\_Noc300\_122214\_01.06507.06507.2 | 2.9446 | 0.3101 | 100.0% | 844.9122 | 845.0269 | 1 | 6.18 | 92.9% | 9 | K.VGEVIVTK.D | 2 |
|  | CENPL\_Noc300\_122214\_02.09524.09524.2 | 4.6038 | 0.5033 | 100.0% | 1631.6122 | 1631.9684 | 1 | 9.623 | 75.0% | 36 | K.VGEVIVTKDDAMLLK.G | 2 |
|  | CENPL\_Noc300\_122214\_02.09512.09512.3 | 4.1771 | 0.5078 | 100.0% | 1633.0144 | 1631.9684 | 1 | 7.988 | 48.2% | 17 | K.VGEVIVTKDDAMLLK.G | 3 |
|  | CENPL\_Noc300\_tube2\_122214\_01.11236.11236.1 | 2.0233 | 0.2703 | 95.0% | 805.38 | 805.9647 | 1 | 6.101 | 83.3% | 4 | K.DDAMLLK.G | 1 |
|  | CENPL\_Noc300\_122214\_01.09112.09112.2 | 2.655 | 0.1444 | 99.5% | 806.0522 | 805.9647 | 4 | 5.622 | 83.3% | 8 | K.DDAMLLK.G | 2 |
|  | CENPL\_Noc300\_122214\_01.14033.14033.2 | 5.7323 | 0.5628 | 100.0% | 2194.652 | 2195.4314 | 1 | 11.254 | 73.5% | 2 | K.RIQEIIEQLDVTTSEYEK.E | 2 |
|  | CENPL\_Noc300\_122214\_02.11224.11224.3 | 6.446 | 0.4583 | 100.0% | 2452.0745 | 2452.721 | 1 | 8.889 | 42.1% | 5 | K.RIQEIIEQLDVTTSEYEKEK.L | 3 |
|  | CENPL\_Noc300\_122214\_02.11240.11240.2 | 6.6068 | 0.4854 | 100.0% | 2038.5922 | 2039.2439 | 1 | 9.114 | 75.0% | 12 | R.IQEIIEQLDVTTSEYEK.E | 2 |
|  | CENPL\_Noc300\_122214\_02.11247.11247.3 | 4.3336 | 0.3715 | 100.0% | 2039.3944 | 2039.2439 | 1 | 6.886 | 51.6% | 1 | R.IQEIIEQLDVTTSEYEK.E | 3 |
|  | CENPL\_Noc300\_122214\_02.10842.10842.2 | 5.9493 | 0.5229 | 100.0% | 2295.672 | 2296.5334 | 1 | 9.488 | 63.9% | 9 | R.IQEIIEQLDVTTSEYEKEK.L | 2 |
|  | CENPL\_Noc300\_tube2\_122214\_01.19101.19101.3 | 3.5618 | 0.301 | 99.9% | 2296.3145 | 2296.5334 | 2 | 4.755 | 34.7% | 11 | R.IQEIIEQLDVTTSEYEKEK.L | 3 |
|  | CENPL\_Noc300\_122214\_02.10850.10850.3 | 4.5774 | 0.2598 | 99.9% | 2808.5044 | 2809.0996 | 1 | 5.448 | 31.8% | 2 | R.IQEIIEQLDVTTSEYEKEKLNER.L | 3 |
|  | CENPL\_Noc300\_tube2\_122214\_01.11404.11404.1 | 1.8331 | 0.2719 | 95.9% | 901.7 | 902.0788 | 1 | 5.784 | 62.5% | 3 | K.LSDGVAVLK.V | 1 |
|  | CENPL\_Noc300\_tube2\_122214\_01.11450.11450.2 | 3.2736 | 0.4098 | 100.0% | 901.97217 | 902.0788 | 2 | 7.16 | 93.8% | 12 | K.LSDGVAVLK.V | 2 |
|  | CENPL\_Noc300\_122214\_01.05630.05630.2 | 4.0953 | 0.5749 | 100.0% | 1234.2722 | 1234.3055 | 1 | 8.651 | 72.7% | 6 | K.VGGTSDVEVNEK.K | 2 |
|  | CENPL\_Noc300\_tube2\_122214\_01.07899.07899.3 | 2.3464 | 0.29 | 98.4% | 1362.3844 | 1362.4796 | 110 | 5.129 | 35.4% | 1 | K.VGGTSDVEVNEKK.D | 3 |
|  | CENPL\_Noc300\_122214\_01.05235.05235.2 | 3.3466 | 0.2772 | 99.8% | 1632.8522 | 1633.7556 | 5 | 5.997 | 50.0% | 1 | K.VGGTSDVEVNEKKDR.V | 2 |
|  | CENPL\_Noc300\_122214\_01.08272.08272.2 | 3.5765 | 0.4101 | 100.0% | 1232.2922 | 1232.339 | 1 | 7.421 | 85.0% | 6 | K.DRVTDALNATR.A | 2 |
|  | CENPL\_Noc300\_tube2\_122214\_01.08745.08745.2 | 3.0067 | 0.4228 | 100.0% | 961.3122 | 961.0629 | 1 | 7.365 | 87.5% | 2 | R.VTDALNATR.A | 2 |
|  | CENPL\_Noc300\_122214\_02.10073.10073.2 | 4.8273 | 0.555 | 100.0% | 1685.7522 | 1685.9274 | 1 | 9.766 | 71.9% | 9 | R.AAVEEGIVLGGGCALLR.C | 2 |
|  | CENPL\_Noc300\_122214\_02.11061.11061.3 | 3.3271 | 0.2736 | 99.9% | 1686.1444 | 1685.9274 | 1 | 5.736 | 46.9% | 2 | R.AAVEEGIVLGGGCALLR.C | 3 |
|  | CENPL\_Noc300\_122214\_01.11786.11786.2 | 3.7092 | 0.3251 | 100.0% | 1773.8722 | 1772.9159 | 3 | 5.578 | 50.0% | 1 | R.CIPALDSLTPANEDQK.I | 2 |
|  | CENPL\_Noc300\_122214\_02.07534.07534.2 | 2.8454 | 0.0454 | 98.7% | 942.2922 | 942.18994 | 11 | 4.267 | 85.7% | 6 | K.IGIEIIKR.T | 2 |
|  | CENPL\_Noc300\_tube2\_122214\_01.14444.14444.2 | 2.6829 | 0.1641 | 98.7% | 1187.4122 | 1187.5267 | 1 | 4.63 | 65.0% | 7 | R.TLKIPAMTIAK.N | 2 |
|  | CENPL\_Noc300\_tube2\_122214\_01.14307.14307.3 | 3.535 | 0.3905 | 100.0% | 1188.0844 | 1187.5267 | 1 | 7.082 | 52.5% | 11 | R.TLKIPAMTIAK.N | 3 |
|  | CENPL\_Noc300\_122214\_01.08704.08704.1 | 1.6674 | 0.293 | 95.4% | 844.43 | 845.0881 | 1 | 5.673 | 71.4% | 6 | K.IPAMTIAK.N | 1 |
|  | CENPL\_Noc300\_tube2\_122214\_01.11432.11432.2 | 2.4086 | 0.4449 | 100.0% | 845.2522 | 845.0881 | 1 | 7.195 | 85.7% | 8 | K.IPAMTIAK.N | 2 |
|  | CENPL\_Noc300\_tube2\_122214\_01.12296.12296.1 | 2.2727 | 0.2491 | 95.6% | 1215.61 | 1216.377 | 16 | 5.056 | 54.5% | 4 | K.NAGVEGSLIVEK.I | 1 |
|  | CENPL\_Noc300\_tube2\_122214\_01.12200.12200.2 | 4.0869 | 0.3686 | 100.0% | 1216.1322 | 1216.377 | 1 | 7.499 | 86.4% | 66 | K.NAGVEGSLIVEK.I | 2 |
|  | CENPL\_Noc300\_122214\_02.15578.15578.3 | 3.4518 | 0.3376 | 99.9% | 3706.3145 | 3707.177 | 1 | 6.406 | 24.3% | 1 | K.NAGVEGSLIVEKIMQSSSEVGYDAMAGDFVNMVEK.G | 3 |
|  | CENPL\_Noc300\_122214\_01.15322.15322.3 | 3.728 | 0.3553 | 100.0% | 3788.1243 | 3787.177 | 1 | 5.728 | 18.4% | 3 | K.NAGVEGS\*LIVEKIMQSSSEVGYDAMAGDFVNMVEK.G | 3 |
|  | CENPL\_Noc300\_tube2\_122214\_01.20588.20588.2 | 6.3745 | 0.6728 | 100.0% | 2508.612 | 2509.8235 | 1 | 12.097 | 59.1% | 8 | K.IMQSSSEVGYDAMAGDFVNMVEK.G | 2 |
|  | CENPL\_Noc300\_122214\_02.12705.12705.3 | 6.2426 | 0.4509 | 100.0% | 2509.5244 | 2509.8235 | 1 | 9.324 | 48.9% | 4 | K.IMQSSSEVGYDAMAGDFVNMVEK.G | 3 |
|  | CENPL\_Noc300\_tube2\_122214\_01.12563.12563.2 | 2.4232 | 0.2911 | 99.5% | 1097.8121 | 1098.331 | 2 | 5.915 | 77.8% | 4 | K.GIIDPTKVVR.T | 2 |
|  | CENPL\_Noc300\_122214\_02.17031.17031.3 | 3.0981 | 0.2061 | 95.5% | 3723.8943 | 3723.2134 | 1 | 5.583 | 18.4% | 2 | K.GIIDPT#KVVRT#ALLDAAGVASLLTTAEVVVTEIPK.E | 3 |
|  | CENPL\_Noc300\_122214\_02.17031.17031.2 | 5.2669 | 0.6005 | 100.0% | 2482.9321 | 2483.9055 | 1 | 10.668 | 58.3% | 2 | R.TALLDAAGVASLLTTAEVVVTEIPK.E | 3 |
|  | CENPL\_Noc300\_122214\_02.17024.17024.3 | 6.915 | 0.5025 | 100.0% | 2484.5044 | 2483.9055 | 1 | 8.834 | 44.8% | 1 | R.TALLDAAGVASLLTTAEVVVTEIPK.E | 3 |
|  | CENPL\_Noc300\_122214\_01.19798.19798.2 | 5.0506 | 0.4162 | 100.0% | 2869.9321 | 2870.3105 | 1 | 9.498 | 44.4% | 1 | R.TALLDAAGVASLLTTAEVVVTEIPKEEK.D | 23 |
|  | CENPL\_Noc300\_122214\_02.16545.16545.3 | 5.2037 | 0.5214 | 100.0% | 2870.3943 | 2870.3105 | 1 | 7.87 | 29.6% | 3 | R.TALLDAAGVASLLTTAEVVVTEIPKEEK.D | 3 |
|  | CENPL\_Noc300\_122214\_02.16850.16850.3 | 6.3658 | 0.611 | 100.0% | 4526.3643 | 4527.253 | 1 | 13.16 | 24.5% | 2 | R.TALLDAAGVASLLTTAEVVVTEIPKEEKDPGMGAMGGMGGGMGGGMF.- | 3 |

---

|  |  |  |  |  |  |  |  |  |
| --- | --- | --- | --- | --- | --- | --- | --- | --- |
| U | *gi|24432106|ref|NP\_06* | 58 | 253 | 63.8% | 923 | 102902 | 5.2 | p30 DBC protein [Homo sapiens] |
| U | *gi|40548408|ref|NP\_95* | 58 | 253 | 63.8% | 923 | 102902 | 5.2 | p30 DBC protein [Homo sapiens] |

| Filename XCorr DeltCN Conf% ObsM+H+ CalcM+H+ SpR ZScore Ion% # Sequence  | | | | | | | | | | | | |
| --- | --- | --- | --- | --- | --- | --- | --- | --- | --- | --- | --- | --- |
|  | CENPL\_Noc300\_tube2\_122214\_01.20582.20582.2 | 4.5733 | 0.5543 | 100.0% | 3094.8523 | 3095.4795 | 1 | 8.902 | 33.3% | 5 | R.NFSGTASTSLLGPPPGLLTPPVATELSQNAR.H | 2 |
|  | CENPL\_Noc300\_122214\_02.12608.12608.3 | 4.9661 | 0.3261 | 100.0% | 3096.7144 | 3095.4795 | 1 | 5.123 | 27.5% | 3 | R.NFSGTASTSLLGPPPGLLTPPVATELSQNAR.H | 3 |
|  | CENPL\_Noc300\_122214\_01.19833.19833.3 | 4.0808 | 0.4036 | 100.0% | 3176.3643 | 3176.636 | 1 | 6.653 | 22.2% | 4 | R.VFTGIVTSLHDYFGVVDEEVFFQLSVVK.G | 3 |
|  | CENPL\_Noc300\_122214\_01.19777.19777.2 | 5.0629 | 0.4969 | 100.0% | 3176.7922 | 3176.636 | 1 | 8.691 | 40.7% | 1 | R.VFTGIVTSLHDYFGVVDEEVFFQLSVVK.G | 2 |
|  | CENPL\_Noc300\_122214\_01.07017.07017.2 | 2.96 | 0.3248 | 100.0% | 998.09216 | 998.1704 | 2 | 5.931 | 75.0% | 4 | K.GRLPQLGEK.V | 2 |
|  | CENPL\_Noc300\_tube2\_122214\_01.15830.15830.2 | 4.4459 | 0.4982 | 100.0% | 1586.4922 | 1586.7904 | 1 | 9.832 | 67.9% | 9 | K.AAYNPGQAVPWNAVK.V | 2 |
|  | CENPL\_Noc300\_tube2\_122214\_01.12500.12500.2 | 3.258 | 0.3357 | 100.0% | 1241.4922 | 1241.4734 | 1 | 5.834 | 80.0% | 8 | K.VQTLSNQPLLK.S | 2 |
|  | CENPL\_Noc300\_tube2\_122214\_01.18608.18608.3 | 4.5073 | 0.3444 | 100.0% | 2802.2043 | 2802.2468 | 1 | 6.197 | 34.0% | 5 | K.VQTLSNQPLLKS\*PAPPLLHVAALGQK.Q | 3 |
|  | CENPL\_Noc300\_tube2\_122214\_01.15434.15434.2 | 4.3879 | 0.5867 | 100.0% | 1499.5521 | 1499.7965 | 1 | 10.116 | 75.0% | 9 | K.SPAPPLLHVAALGQK.Q | 2 |
|  | CENPL\_Noc300\_122214\_01.11146.11146.3 | 3.2422 | 0.4858 | 100.0% | 1500.1444 | 1499.7965 | 1 | 7.945 | 46.4% | 9 | K.SPAPPLLHVAALGQK.Q | 3 |
|  | CENPL\_Noc300\_tube2\_122214\_01.18056.18056.2 | 3.6772 | 0.3941 | 100.0% | 1803.5322 | 1804.105 | 1 | 6.888 | 60.0% | 3 | K.QGILGAQPQLIFQPHR.I | 2 |
|  | CENPL\_Noc300\_122214\_01.12906.12906.3 | 4.3073 | 0.4561 | 100.0% | 1804.2843 | 1804.105 | 1 | 8.032 | 46.7% | 7 | K.QGILGAQPQLIFQPHR.I | 3 |
|  | CENPL\_Noc300\_tube2\_122214\_01.18442.18442.3 | 3.1225 | 0.2007 | 95.3% | 3023.0344 | 3023.554 | 1 | 4.389 | 32.0% | 2 | R.IPPLFPQKPLSLFQTSHTLHLSHLNR.F | 3 |
|  | CENPL\_Noc300\_122214\_01.06026.06026.2 | 2.4391 | 0.2759 | 99.7% | 898.0522 | 898.00916 | 14 | 5.124 | 75.0% | 8 | R.HDLPPYR.V | 2 |
|  | CENPL\_Noc300\_tube2\_122214\_01.20789.20789.3 | 4.3608 | 0.3795 | 100.0% | 2404.2844 | 2404.6912 | 1 | 6.387 | 40.8% | 1 | R.VHLTPYTVDSPICDFLELQR.R | 3 |
|  | CENPL\_Noc300\_tube2\_122214\_01.20798.20798.2 | 5.7262 | 0.5563 | 100.0% | 2404.7922 | 2404.6912 | 1 | 10.495 | 63.2% | 2 | R.VHLTPYTVDSPICDFLELQR.R | 2 |
|  | CENPL\_Noc300\_122214\_01.18145.18145.3 | 5.1606 | 0.4527 | 100.0% | 3603.5942 | 3604.1484 | 1 | 9.266 | 29.8% | 2 | R.SLLVPSDFLSVHLSWLSAFPLSQPFSLHHPSR.I | 3 |
|  | CENPL\_Noc300\_tube2\_122214\_01.11074.11074.3 | 6.0163 | 0.4173 | 100.0% | 2935.3442 | 2936.1125 | 1 | 7.813 | 41.1% | 6 | R.IQVSSEKEAAPDAGAEPITADSDPAYSSK.V | 3 |
|  | CENPL\_Noc300\_tube2\_122214\_01.11002.11002.2 | 4.4977 | 0.5225 | 100.0% | 2935.4521 | 2936.1125 | 1 | 8.099 | 50.0% | 1 | R.IQVSSEKEAAPDAGAEPITADSDPAYSSK.V | 2 |
|  | CENPL\_Noc300\_tube2\_122214\_01.11060.11060.2 | 5.2348 | 0.616 | 100.0% | 2163.5723 | 2164.244 | 1 | 10.486 | 71.4% | 8 | K.EAAPDAGAEPITADSDPAYSSK.V | 2 |
|  | CENPL\_Noc300\_tube2\_122214\_01.11042.11042.3 | 4.8844 | 0.4517 | 100.0% | 2164.3442 | 2164.244 | 1 | 7.921 | 39.3% | 3 | K.EAAPDAGAEPITADSDPAYSSK.V | 3 |
|  | CENPL\_Noc300\_tube2\_122214\_01.20348.20348.2 | 3.7468 | 0.5031 | 100.0% | 1589.3722 | 1589.8724 | 1 | 7.574 | 84.6% | 9 | K.VLLLSSPGLEELYR.C | 2 |
|  | CENPL\_Noc300\_tube2\_122214\_01.19608.19608.2 | 3.8524 | 0.5322 | 100.0% | 3234.3323 | 3235.575 | 1 | 9.489 | 32.8% | 2 | R.KEEEAVLVGGEWSPSLDGLDPQADPQVLVR.T | 2 |
|  | CENPL\_Noc300\_122214\_02.11042.11042.3 | 6.6945 | 0.5412 | 100.0% | 3235.0745 | 3235.575 | 1 | 8.921 | 35.3% | 8 | R.KEEEAVLVGGEWSPSLDGLDPQADPQVLVR.T | 3 |
|  | CENPL\_Noc300\_tube2\_122214\_01.17630.17630.2 | 3.4451 | 0.4668 | 100.0% | 1551.3121 | 1550.757 | 1 | 7.551 | 83.3% | 9 | R.FAEFQYLQPGPPR.R | 2 |
|  | CENPL\_Noc300\_tube2\_122214\_01.15543.15543.2 | 2.6596 | 0.0935 | 96.0% | 1706.1721 | 1706.9443 | 18 | 3.959 | 50.0% | 1 | R.FAEFQYLQPGPPRR.L | 2 |
|  | CENPL\_Noc300\_tube2\_122214\_01.15506.15506.3 | 3.8022 | 0.3821 | 100.0% | 1706.9343 | 1706.9443 | 2 | 7.648 | 46.2% | 6 | R.FAEFQYLQPGPPRR.L | 3 |
|  | CENPL\_Noc300\_122214\_01.19402.19402.3 | 4.2121 | 0.1825 | 99.8% | 3548.0344 | 3548.1006 | 3 | 5.389 | 25.0% | 1 | R.RLQTVVVYLPDVWTIMPTLEEWEALCQQK.A | 3 |
|  | CENPL\_Noc300\_tube2\_122214\_01.13040.13040.3 | 4.8171 | 0.4797 | 100.0% | 3381.8643 | 3382.4895 | 1 | 7.835 | 28.9% | 3 | K.AAEAAPPTQEAQGETEPTEQAPDALEQAADTSR.R | 3 |
|  | CENPL\_Noc300\_tube2\_122214\_01.12994.12994.2 | 3.798 | 0.5636 | 100.0% | 3382.0923 | 3382.4895 | 1 | 7.403 | 34.4% | 3 | K.AAEAAPPTQEAQGETEPTEQAPDALEQAADTSR.R | 2 |
|  | CENPL\_Noc300\_122214\_02.07824.07824.3 | 4.4805 | 0.5543 | 100.0% | 3537.9844 | 3538.677 | 1 | 8.895 | 33.3% | 7 | K.AAEAAPPTQEAQGETEPTEQAPDALEQAADTSRR.N | 3 |
|  | CENPL\_Noc300\_tube2\_122214\_01.16426.16426.2 | 2.3539 | 0.1412 | 98.1% | 930.6322 | 931.1785 | 126 | 4.525 | 71.4% | 4 | K.MLLSLPEK.V | 2 |
|  | CENPL\_Noc300\_122214\_01.06041.06041.2 | 3.2732 | 0.4166 | 100.0% | 1510.0322 | 1510.6842 | 1 | 7.488 | 69.2% | 1 | K.VVSPPEPEKEEAAK.E | 2 |
|  | CENPL\_Noc300\_tube2\_122214\_01.11218.11218.3 | 3.7852 | 0.3292 | 100.0% | 1861.8544 | 1862.041 | 3 | 7.378 | 40.0% | 3 | K.EEATKEEEAIKEEVVK.E | 3 |
|  | CENPL\_Noc300\_122214\_01.17944.17944.2 | 5.2521 | 0.5248 | 100.0% | 2274.5522 | 2275.5422 | 1 | 9.301 | 52.6% | 2 | R.GEASEDLCEMALDPELLLLR.D | 2 |
|  | CENPL\_Noc300\_122214\_02.06503.06503.2 | 3.1538 | 0.3481 | 100.0% | 1167.5922 | 1168.1589 | 1 | 6.943 | 85.0% | 4 | R.DDGEEEFAGAK.L | 2 |
|  | CENPL\_Noc300\_tube2\_122214\_01.13042.13042.3 | 3.271 | 0.2734 | 99.9% | 1997.0643 | 1997.0361 | 1 | 5.166 | 33.8% | 1 | R.DDGEEEFAGAKLEDSEVR.S | 3 |
|  | CENPL\_Noc300\_122214\_02.10089.10089.3 | 3.9123 | 0.3213 | 100.0% | 2115.5942 | 2116.3223 | 1 | 6.067 | 45.8% | 2 | R.SVASNQSEMEFSSLQDMPK.E | 3 |
|  | CENPL\_Noc300\_tube2\_122214\_01.16940.16940.2 | 5.1737 | 0.4862 | 100.0% | 2116.5122 | 2116.3223 | 1 | 9.525 | 61.1% | 7 | R.SVASNQSEMEFSSLQDMPK.E | 2 |
|  | CENPL\_Noc300\_122214\_02.09891.09891.2 | 4.9273 | 0.456 | 100.0% | 2195.5322 | 2196.3223 | 1 | 8.072 | 55.6% | 1 | R.SVASNQSEMEFSS\*LQDMPK.E | 2 |
|  | CENPL\_Noc300\_122214\_01.13634.13634.2 | 2.7339 | 0.2433 | 99.8% | 899.3122 | 899.1649 | 2 | 5.82 | 85.7% | 6 | R.ILLTLGIR.L | 2 |
|  | CENPL\_Noc300\_122214\_01.07654.07654.2 | 3.4042 | 0.4307 | 100.0% | 1281.2522 | 1281.4204 | 1 | 8.43 | 77.8% | 4 | R.VVTQNICQYR.S | 2 |
|  | CENPL\_Noc300\_122214\_01.17894.17894.2 | 4.783 | 0.5292 | 100.0% | 2605.0322 | 2605.9475 | 1 | 10.01 | 43.8% | 5 | R.QEGLDGGLPEEVLFGNLDLLPPPGK.S | 2 |
|  | CENPL\_Noc300\_122214\_01.17906.17906.3 | 3.8807 | 0.4809 | 100.0% | 2605.6443 | 2605.9475 | 3 | 7.046 | 28.1% | 1 | R.QEGLDGGLPEEVLFGNLDLLPPPGK.S | 3 |
|  | CENPL\_Noc300\_tube2\_122214\_01.18387.18387.2 | 6.319 | 0.5534 | 100.0% | 1878.2922 | 1879.1698 | 1 | 9.648 | 79.4% | 9 | K.ALVSHNGSLINVGSLLQR.A | 2 |
|  | CENPL\_Noc300\_tube2\_122214\_01.18422.18422.3 | 4.2598 | 0.2859 | 100.0% | 1879.4944 | 1879.1698 | 1 | 6.478 | 44.1% | 6 | K.ALVSHNGSLINVGSLLQR.A | 3 |
|  | CENPL\_Noc300\_122214\_02.07617.07617.3 | 2.8155 | 0.3996 | 100.0% | 1719.8043 | 1719.9382 | 1 | 6.187 | 44.2% | 2 | K.IHTLELKLEESHNR.F | 3 |
|  | CENPL\_Noc300\_122214\_01.06813.06813.2 | 2.8598 | 0.3897 | 100.0% | 997.15216 | 997.0929 | 3 | 7.061 | 81.2% | 3 | R.FSATEVTNK.T | 2 |
|  | CENPL\_Noc300\_tube2\_122214\_01.15755.15755.1 | 2.1286 | 0.273 | 95.2% | 1161.47 | 1162.3466 | 9 | 4.776 | 50.0% | 4 | K.TLAAEMQELR.V | 1 |
|  | CENPL\_Noc300\_122214\_01.11266.11266.2 | 3.8146 | 0.3501 | 100.0% | 1162.2122 | 1162.3466 | 1 | 7.75 | 88.9% | 10 | K.TLAAEMQELR.V | 2 |
|  | CENPL\_Noc300\_122214\_01.06254.06254.2 | 3.4071 | 0.4117 | 100.0% | 1245.1921 | 1245.378 | 1 | 7.359 | 85.0% | 1 | R.VRLAEAEETAR.T | 2 |
|  | CENPL\_Noc300\_tube2\_122214\_01.07988.07988.2 | 2.5972 | 0.2236 | 99.5% | 991.33215 | 990.05804 | 7 | 5.617 | 68.8% | 2 | R.LAEAEETAR.T | 2 |
|  | CENPL\_Noc300\_tube2\_122214\_01.12338.12338.3 | 3.4432 | 0.2925 | 100.0% | 1524.5044 | 1523.8221 | 1 | 5.246 | 59.1% | 3 | R.RRLTPLQLEIQR.V | 3 |
|  | CENPL\_Noc300\_tube2\_122214\_01.14288.14288.2 | 3.7941 | 0.3751 | 100.0% | 1367.3922 | 1367.6346 | 1 | 5.783 | 80.0% | 5 | R.RLTPLQLEIQR.V | 2 |
|  | CENPL\_Noc300\_122214\_01.10594.10594.3 | 3.7689 | 0.2552 | 100.0% | 1367.6943 | 1367.6346 | 1 | 5.513 | 50.0% | 3 | R.RLTPLQLEIQR.V | 3 |
|  | CENPL\_Noc300\_122214\_02.08990.08990.2 | 3.1519 | 0.2619 | 99.9% | 1211.3322 | 1211.4471 | 1 | 5.457 | 77.8% | 6 | R.LTPLQLEIQR.V | 2 |
|  | CENPL\_Noc300\_tube2\_122214\_01.10680.10680.2 | 4.7137 | 0.4915 | 100.0% | 2014.3922 | 2015.184 | 1 | 9.108 | 61.8% | 3 | R.VVEKADSWVEKEEPAPSN.- | 2 |
|  | CENPL\_Noc300\_tube2\_122214\_01.10712.10712.2 | 3.6198 | 0.5728 | 100.0% | 1559.0521 | 1559.6293 | 1 | 9.207 | 65.4% | 6 | K.ADSWVEKEEPAPSN.- | 2 |

---

|  |  |  |  |  |  |  |  |  |
| --- | --- | --- | --- | --- | --- | --- | --- | --- |
| U | *gi|10800130|ref|NP\_06* | 6 | 12 | 57.7% | 130 | 14107 | 10.9 | histone cluster 1, H2ad [Homo sapiens] |
| U | *gi|4504243|ref|NP\_003* | 6 | 12 | 57.7% | 130 | 14091 | 10.9 | histone cluster 1, H2al [Homo sapiens] |
| U | *gi|4504239|ref|NP\_003* | 6 | 12 | 57.7% | 130 | 14091 | 10.9 | histone cluster 1, H2ai [Homo sapiens] |
| U | *gi|18105045|ref|NP\_54* | 6 | 12 | 58.6% | 128 | 13906 | 10.9 | histone cluster 1, H2ah [Homo sapiens] |
| U | *gi|10800144|ref|NP\_06* | 6 | 12 | 58.6% | 128 | 13936 | 10.9 | histone cluster 1, H2aj [Homo sapiens] |
| U | *gi|10800132|ref|NP\_06* | 6 | 12 | 57.7% | 130 | 14091 | 10.9 | histone cluster 1, H2ag [Homo sapiens] |

| Filename XCorr DeltCN Conf% ObsM+H+ CalcM+H+ SpR ZScore Ion% # Sequence  | | | | | | | | | | | | |
| --- | --- | --- | --- | --- | --- | --- | --- | --- | --- | --- | --- | --- |
|  | CENPL\_Noc300\_tube2\_122214\_01.14990.14990.2 | 2.9694 | 0.2377 | 99.8% | 945.27216 | 945.1093 | 3 | 5.186 | 81.2% | 6 | R.AGLQFPVGR.V | 222 |
|  | CENPL\_Noc300\_122214\_01.20898.20898.2 | 4.6385 | 0.6003 | 100.0% | 2916.5723 | 2917.3752 | 1 | 11.021 | 44.6% | 1 | R.VGAGAPVYLAAVLEYLTAEILELAGNAAR.D | 2 |
|  | CENPL\_Noc300\_122214\_01.20861.20861.3 | 3.6938 | 0.389 | 100.0% | 2917.4944 | 2917.3752 | 1 | 5.55 | 27.7% | 1 | R.VGAGAPVYLAAVLEYLTAEILELAGNAAR.D | 3 |
|  | CENPL\_Noc300\_122214\_01.07954.07954.2 | 2.374 | 0.2222 | 99.5% | 851.2322 | 851.0396 | 1 | 5.231 | 91.7% | 2 | R.HLQLAIR.N | 22 |
|  | CENPL\_Noc300\_tube2\_122214\_01.14238.14238.2 | 3.4142 | 0.3899 | 100.0% | 1273.1122 | 1273.4288 | 1 | 6.528 | 70.0% | 1 | R.NDEELNKLLGK.V | 2 |
|  | CENPL\_Noc300\_tube2\_122214\_01.20801.20801.2 | 3.0263 | 0.2922 | 99.7% | 1932.2122 | 1932.3573 | 4 | 4.929 | 38.9% | 1 | K.VTIAQGGVLPNIQAVLLPK.K | 2 |

Similarities:
gi|20357599|ref|NP\_61(2:4)  
gi|113425815|ref|XP\_9(1:5)  

---

|  |  |  |  |  |  |  |  |  |
| --- | --- | --- | --- | --- | --- | --- | --- | --- |
| U | *contaminant\_gi|746301* | 17 | 81 | 56.9% | 269 | 27961 | 6.7 | lysyl endopeptidase (EC 3.4.21.50) - Lysobacter enzymogenes |

| Filename XCorr DeltCN Conf% ObsM+H+ CalcM+H+ SpR ZScore Ion% # Sequence  | | | | | | | | | | | | |
| --- | --- | --- | --- | --- | --- | --- | --- | --- | --- | --- | --- | --- |
| \* | CENPL\_Noc300\_tube2\_122214\_01.12782.12782.3 | 2.9749 | 0.2369 | 98.5% | 2112.7444 | 2112.288 | 16 | 4.006 | 27.8% | 1 | K.QGTMWCTGSLVNNSANDKK.M | 3 |
| \* | CENPL\_Noc300\_122214\_01.07287.07287.2 | 6.4855 | 0.6306 | 100.0% | 2261.652 | 2262.355 | 1 | 11.109 | 56.2% | 13 | R.APGSSSSGANGDGSLAQSQTGAVVR.A | 2 |
| \* | CENPL\_Noc300\_tube2\_122214\_01.09519.09519.3 | 4.633 | 0.4213 | 100.0% | 2262.3245 | 2262.355 | 1 | 8.0 | 39.6% | 7 | R.APGSSSSGANGDGSLAQSQTGAVVR.A | 3 |
| \* | CENPL\_Noc300\_122214\_02.15998.15998.3 | 6.5967 | 0.5196 | 100.0% | 3315.7744 | 3315.6257 | 1 | 9.246 | 26.7% | 2 | R.ATNAASDFTLLELNTAANPAYNLFWAGWDR.R | 3 |
| \* | CENPL\_Noc300\_122214\_01.19191.19191.2 | 5.3727 | 0.4952 | 100.0% | 3316.5122 | 3315.6257 | 1 | 9.581 | 39.7% | 4 | R.ATNAASDFTLLELNTAANPAYNLFWAGWDR.R | 2 |
| \* | CENPL\_Noc300\_122214\_02.15134.15134.3 | 6.6992 | 0.5501 | 100.0% | 3472.8843 | 3471.813 | 1 | 10.63 | 30.0% | 5 | R.ATNAASDFTLLELNTAANPAYNLFWAGWDRR.D | 3 |
| \* | CENPL\_Noc300\_122214\_01.18181.18181.3 | 4.4978 | 0.2886 | 100.0% | 3474.7744 | 3475.6257 | 1 | 4.681 | 24.1% | 1 | R.ATNAASDFTLLELNT#AANPAY@NLFWAGWDR.R | 3 |
| \* | CENPL\_Noc300\_122214\_01.06569.06569.3 | 4.7213 | 0.4011 | 100.0% | 2077.0745 | 2077.2668 | 1 | 6.892 | 41.7% | 4 | R.RDQNFAGATAIHHPNVAEK.R | 3 |
| \* | CENPL\_Noc300\_122214\_01.06236.06236.3 | 4.6132 | 0.3338 | 100.0% | 2233.7344 | 2233.4543 | 1 | 6.445 | 39.5% | 1 | R.RDQNFAGATAIHHPNVAEKR.I | 3 |
| \* | CENPL\_Noc300\_tube2\_122214\_01.09620.09620.2 | 4.5236 | 0.5538 | 100.0% | 1920.4321 | 1921.0793 | 1 | 9.45 | 58.8% | 2 | R.DQNFAGATAIHHPNVAEK.R | 2 |
| \* | CENPL\_Noc300\_122214\_01.07270.07270.3 | 2.564 | 0.3219 | 99.6% | 1920.9243 | 1921.0793 | 5 | 5.672 | 30.9% | 3 | R.DQNFAGATAIHHPNVAEK.R | 3 |
| \* | CENPL\_Noc300\_tube2\_122214\_01.08962.08962.2 | 5.4338 | 0.433 | 100.0% | 2076.5723 | 2077.2668 | 1 | 7.627 | 55.6% | 4 | R.DQNFAGATAIHHPNVAEKR.I | 2 |
| \* | CENPL\_Noc300\_tube2\_122214\_01.08990.08990.3 | 4.369 | 0.4768 | 100.0% | 2077.5544 | 2077.2668 | 1 | 7.583 | 40.3% | 7 | R.DQNFAGATAIHHPNVAEKR.I | 3 |
| \* | CENPL\_Noc300\_122214\_01.06772.06772.3 | 2.7203 | 0.2708 | 98.4% | 1871.2144 | 1870.983 | 1 | 5.445 | 37.5% | 1 | R.VLGQLHGGPSSCSATGADR.S | 3 |
| \* | CENPL\_Noc300\_tube2\_122214\_01.12917.12917.1 | 2.0619 | 0.2475 | 95.6% | 1427.64 | 1428.5443 | 13 | 5.216 | 38.5% | 4 | R.VFTSWTGGGTSATR.L | 1 |
| \* | CENPL\_Noc300\_tube2\_122214\_01.12986.12986.2 | 4.8673 | 0.519 | 100.0% | 1428.3322 | 1428.5443 | 1 | 8.941 | 76.9% | 20 | R.VFTSWTGGGTSATR.L | 2 |
| \* | CENPL\_Noc300\_122214\_02.14538.14538.2 | 2.8127 | 0.3006 | 99.6% | 2605.652 | 2605.8174 | 14 | 6.003 | 26.0% | 2 | R.LSDWLDAAGTGAQFIDGLDSTGTPPV.- | 2 |

---

|  |  |  |  |  |  |  |  |  |
| --- | --- | --- | --- | --- | --- | --- | --- | --- |
| U | *gi|47132620|ref|NP\_00* | 30 | 75 | 55.1% | 639 | 65433 | 8.0 | keratin 2 [Homo sapiens] |

| Filename XCorr DeltCN Conf% ObsM+H+ CalcM+H+ SpR ZScore Ion% # Sequence  | | | | | | | | | | | | |
| --- | --- | --- | --- | --- | --- | --- | --- | --- | --- | --- | --- | --- |
|  | CENPL\_Noc300\_122214\_01.07005.07005.2 | 4.3327 | 0.4594 | 100.0% | 1255.0922 | 1255.3298 | 1 | 9.789 | 76.9% | 2 | R.GFSSGSAVVSGGSR.R | 2 |
|  | CENPL\_Noc300\_122214\_01.07161.07161.2 | 4.9356 | 0.6477 | 100.0% | 1321.2122 | 1321.3542 | 1 | 11.634 | 86.7% | 2 | R.HGGGGGGFGGGGFGSR.S | 2 |
|  | CENPL\_Noc300\_122214\_02.06549.06549.3 | 3.2845 | 0.2472 | 99.8% | 1322.2743 | 1321.3542 | 1 | 5.142 | 46.7% | 1 | R.HGGGGGGFGGGGFGSR.S | 3 |
|  | CENPL\_Noc300\_122214\_01.12488.12488.2 | 5.904 | 0.571 | 100.0% | 1839.5721 | 1840.0055 | 1 | 10.418 | 64.3% | 3 | K.SISISVAGGGGGFGAAGGFGGR.G | 2 |
| \* | CENPL\_Noc300\_122214\_02.10619.10619.2 | 6.3258 | 0.6473 | 100.0% | 2399.612 | 2400.4446 | 1 | 11.648 | 50.0% | 1 | R.GGGFGGGSSFGGGSGFSGGGFGGGGFGGGR.F | 2 |
| \* | CENPL\_Noc300\_122214\_02.10587.10587.3 | 5.8957 | 0.4643 | 100.0% | 2400.0842 | 2400.4446 | 1 | 10.695 | 48.3% | 1 | R.GGGFGGGSSFGGGSGFSGGGFGGGGFGGGR.F | 3 |
|  | CENPL\_Noc300\_122214\_01.15382.15382.3 | 5.1897 | 0.4963 | 100.0% | 4093.5244 | 4094.5786 | 1 | 9.365 | 22.1% | 2 | R.FGGFGGPGGVGGLGGPGGFGPGGYPGGIHEVSVNQSLLQPLNVK.V | 3 |
|  | CENPL\_Noc300\_122214\_02.06465.06465.2 | 2.0273 | 0.1958 | 96.1% | 1043.2122 | 1042.1771 | 130 | 4.785 | 75.0% | 1 | K.VDPEIQNVK.A | 2 |
|  | CENPL\_Noc300\_tube2\_122214\_01.13599.13599.2 | 2.3642 | 0.1974 | 99.3% | 827.9922 | 827.95544 | 1 | 5.382 | 100.0% | 5 | K.FASFIDK.V | 222222 |
|  | CENPL\_Noc300\_tube2\_122214\_01.13469.13469.2 | 2.5321 | 0.0875 | 97.5% | 1083.2122 | 1083.2755 | 2 | 6.11 | 81.2% | 2 | K.FASFIDKVR.F | 222222 |
|  | CENPL\_Noc300\_tube2\_122214\_01.11840.11840.2 | 4.361 | 0.1003 | 100.0% | 1475.8121 | 1476.6726 | 1 | 7.365 | 90.9% | 5 | R.FLEQQNQVLQTK.W | 22 |
|  | CENPL\_Noc300\_122214\_01.17520.17520.3 | 4.1006 | 0.3766 | 100.0% | 3462.5044 | 3461.019 | 1 | 5.251 | 25.0% | 1 | K.WELLQQMNVGTRPINLEPIFQGYIDSLKR.Y | 3 |
|  | CENPL\_Noc300\_122214\_01.09445.09445.2 | 3.4147 | 0.4103 | 100.0% | 1038.1122 | 1038.1454 | 1 | 6.606 | 93.8% | 4 | R.YLDGLTAER.T | 2 |
|  | CENPL\_Noc300\_tube2\_122214\_01.16734.16734.3 | 3.5943 | 0.2989 | 99.9% | 2257.0444 | 2257.4338 | 17 | 5.191 | 31.9% | 2 | R.TSQNSELNNMQDLVEDYKK.K | 3 |
|  | CENPL\_Noc300\_122214\_01.10083.10083.2 | 3.0485 | 0.489 | 100.0% | 1209.4722 | 1209.3416 | 1 | 7.39 | 75.0% | 2 | R.TAAENDFVTLK.K | 2 |
|  | CENPL\_Noc300\_122214\_01.08104.08104.2 | 3.3534 | 0.412 | 100.0% | 1337.1921 | 1337.5156 | 1 | 6.781 | 81.8% | 2 | R.TAAENDFVTLKK.D | 2 |
|  | CENPL\_Noc300\_122214\_01.15485.15485.2 | 4.1348 | 0.2707 | 100.0% | 1461.5521 | 1461.6982 | 1 | 7.015 | 77.3% | 3 | K.VDLLNQEIEFLK.V | 2 |
|  | CENPL\_Noc300\_tube2\_122214\_01.20704.20704.2 | 4.3469 | 0.4382 | 100.0% | 1330.3522 | 1330.5211 | 1 | 8.484 | 86.4% | 3 | R.NLDLDSIIAEVK.A | 2222 |
|  | CENPL\_Noc300\_122214\_01.06802.06802.2 | 2.7697 | 0.0862 | 98.7% | 1107.5922 | 1108.196 | 1 | 6.289 | 81.2% | 2 | K.AQYEEIAQR.S | 22 |
|  | CENPL\_Noc300\_122214\_01.09832.09832.3 | 2.9931 | 0.2154 | 96.3% | 2565.3542 | 2567.815 | 1 | 4.338 | 33.3% | 1 | R.SKEEAEALYHSKYEELQVTVGR.H | 3 |
|  | CENPL\_Noc300\_tube2\_122214\_01.12652.12652.2 | 3.6915 | 0.3139 | 100.0% | 1195.1721 | 1194.33 | 1 | 8.288 | 88.9% | 6 | K.YEELQVTVGR.H | 2 |
|  | CENPL\_Noc300\_122214\_01.09166.09166.2 | 3.0491 | 0.1856 | 99.8% | 973.9922 | 974.102 | 1 | 5.374 | 92.9% | 6 | K.IEISELNR.V | 22 |
|  | CENPL\_Noc300\_122214\_01.09391.09391.2 | 4.1134 | 0.5192 | 100.0% | 1330.2522 | 1330.3971 | 1 | 8.62 | 81.8% | 3 | K.NVQDAIADAEQR.G | 2 |
|  | CENPL\_Noc300\_122214\_01.12072.12072.3 | 3.1127 | 0.338 | 99.9% | 2309.0645 | 2308.4727 | 1 | 5.329 | 35.0% | 1 | K.NVQDAIADAEQRGEHALKDAR.N | 3 |
|  | CENPL\_Noc300\_122214\_01.12181.12181.2 | 4.6169 | 0.4191 | 100.0% | 1614.1921 | 1614.796 | 1 | 8.388 | 80.8% | 4 | R.NKLNDLEEALQQAK.E | 2 |
|  | CENPL\_Noc300\_tube2\_122214\_01.19889.19889.3 | 4.519 | 0.3799 | 100.0% | 2199.5942 | 2199.4258 | 1 | 6.57 | 40.3% | 3 | R.NKLNDLEEALQQAKEDLAR.L | 3 |
|  | CENPL\_Noc300\_122214\_01.12047.12047.2 | 4.1636 | 0.4782 | 100.0% | 1372.1522 | 1372.5181 | 1 | 8.007 | 81.8% | 1 | K.LNDLEEALQQAK.E | 2 |
|  | CENPL\_Noc300\_tube2\_122214\_01.15606.15606.2 | 2.5513 | 0.254 | 99.2% | 1521.7522 | 1522.8029 | 5 | 5.161 | 54.5% | 3 | R.LLRDYQELMNVK.L | 2 |
|  | CENPL\_Noc300\_122214\_01.12967.12967.2 | 2.6979 | 0.4056 | 100.0% | 1264.4722 | 1264.4644 | 5 | 7.073 | 60.0% | 2 | K.LALDVEIATYR.K | 2222 |
|  | CENPL\_Noc300\_122214\_02.09616.09616.2 | 2.8991 | 0.271 | 99.7% | 1392.8121 | 1392.6384 | 12 | 5.484 | 59.1% | 1 | K.LALDVEIATYRK.L | 2222 |

Similarities:
gi|4504919|ref|NP\_002(2:28)  
gi|119395750|ref|NP\_0(2:28)  
gi|119703753|ref|NP\_0(6:24)  
gi|119395754|ref|NP\_0(5:25)  
gi|153791158|ref|NP\_0(5:25)  
gi|67782365|ref|NP\_00(2:28)  

---

|  |  |  |  |  |  |  |  |  |
| --- | --- | --- | --- | --- | --- | --- | --- | --- |
| U | *gi|5902102|ref|NP\_008* | 5 | 6 | 54.6% | 119 | 13282 | 11.6 | small nuclear ribonucleoprotein D1 polypeptide 16kDa [Homo sapiens] |

| Filename XCorr DeltCN Conf% ObsM+H+ CalcM+H+ SpR ZScore Ion% # Sequence  | | | | | | | | | | | | |
| --- | --- | --- | --- | --- | --- | --- | --- | --- | --- | --- | --- | --- |
| \* | CENPL\_Noc300\_122214\_02.08174.08174.3 | 3.0871 | 0.2418 | 99.9% | 1270.5844 | 1270.4686 | 103 | 4.669 | 40.0% | 1 | K.LSHETVTIELK.N | 3 |
| \* | CENPL\_Noc300\_122214\_02.08160.08160.2 | 2.463 | 0.3198 | 99.6% | 1270.6522 | 1270.4686 | 13 | 4.84 | 60.0% | 1 | K.LSHETVTIELK.N | 2 |
| \* | CENPL\_Noc300\_122214\_02.08190.08190.3 | 5.2099 | 0.5339 | 100.0% | 2209.9143 | 2210.47 | 1 | 7.867 | 42.5% | 1 | K.NGTQVHGTITGVDVSMNTHLK.A | 3 |
|  | CENPL\_Noc300\_tube2\_122214\_01.14109.14109.2 | 4.1081 | 0.391 | 100.0% | 1556.5721 | 1555.7745 | 1 | 6.694 | 70.8% | 2 | K.NREPVQLETLSIR.G | 2 |
| \* | CENPL\_Noc300\_122214\_01.19500.19500.2 | 4.9568 | 0.5085 | 100.0% | 2288.5322 | 2288.6863 | 1 | 9.574 | 65.8% | 1 | R.YFILPDSLPLDTLLVDVEPK.V | 2 |

---

|  |  |  |  |  |  |  |  |  |
| --- | --- | --- | --- | --- | --- | --- | --- | --- |
| U | *gi|73623035|ref|NP\_00* | 55 | 138 | 54.4% | 1193 | 134422 | 5.0 | sperm associated antigen 5 [Homo sapiens] |

| Filename XCorr DeltCN Conf% ObsM+H+ CalcM+H+ SpR ZScore Ion% # Sequence  | | | | | | | | | | | | |
| --- | --- | --- | --- | --- | --- | --- | --- | --- | --- | --- | --- | --- |
| \* | CENPL\_Noc300\_tube2\_122214\_01.13925.13925.2 | 2.9363 | 0.1791 | 99.0% | 1431.6122 | 1429.6133 | 2 | 4.132 | 69.2% | 1 | R.ELTLQPGALTNSGK.R | 2 |
| \* | CENPL\_Noc300\_tube2\_122214\_01.13623.13623.3 | 3.5513 | 0.3143 | 99.9% | 2176.2844 | 2176.3506 | 1 | 5.596 | 38.2% | 1 | K.LGLQEGSNNSSPVDFVNNKR.T | 3 |
| \* | CENPL\_Noc300\_122214\_01.12257.12257.2 | 4.102 | 0.2657 | 100.0% | 1654.3121 | 1653.8445 | 1 | 7.643 | 64.3% | 2 | K.TSEEAVDPLGNYMVK.T | 2 |
| \* | CENPL\_Noc300\_122214\_01.15679.15679.2 | 3.1292 | 0.4904 | 100.0% | 2322.7722 | 2323.6262 | 1 | 8.095 | 52.6% | 2 | K.TIVLVPS\*PLGQQQDMIFEAR.L | 2 |
| \* | CENPL\_Noc300\_tube2\_122214\_01.16811.16811.2 | 4.6807 | 0.3577 | 100.0% | 1833.3722 | 1833.0668 | 1 | 6.321 | 62.5% | 4 | R.LDTMAETNSISLNGPLR.T | 2 |
| \* | CENPL\_Noc300\_122214\_02.10438.10438.3 | 2.9925 | 0.2524 | 98.4% | 2532.8943 | 2532.8286 | 23 | 4.123 | 22.7% | 1 | R.LDTMAETNSISLNGPLRTDDLVR.E | 32 |
| \* | CENPL\_Noc300\_122214\_01.17876.17876.3 | 5.7069 | 0.2162 | 100.0% | 3858.5645 | 3858.2102 | 1 | 5.831 | 25.0% | 4 | R.TEAVREDLVPSESNAFLPSSVLWLS\*PSTALAADFR.V | 3 |
| \* | CENPL\_Noc300\_tube2\_122214\_01.12417.12417.3 | 4.8213 | 0.3876 | 100.0% | 2220.7144 | 2220.3752 | 1 | 6.289 | 40.3% | 5 | R.VNHVDPEEEIVEHGAMEER.E | 3 |
| \* | CENPL\_Noc300\_122214\_01.18604.18604.2 | 4.0611 | 0.2486 | 100.0% | 2065.9722 | 2064.3606 | 3 | 5.734 | 47.1% | 1 | R.ILGSDTESWMSPLAWLEK.G | 2 |
| \* | CENPL\_Noc300\_122214\_01.19162.19162.2 | 5.0755 | 0.5386 | 100.0% | 2144.132 | 2144.3606 | 1 | 8.048 | 70.6% | 1 | R.ILGSDTESWMS\*PLAWLEK.G | 2 |
| \* | CENPL\_Noc300\_tube2\_122214\_01.16890.16890.2 | 2.1755 | 0.2145 | 96.8% | 1332.5922 | 1333.5457 | 2 | 4.457 | 59.1% | 2 | K.GVNTSVMLENLR.Q | 2 |
| \* | CENPL\_Noc300\_122214\_01.12895.12895.2 | 2.1223 | 0.3039 | 98.7% | 1132.3121 | 1132.3635 | 13 | 5.51 | 66.7% | 2 | R.QSLSLPSMLR.D | 2 |
| \* | CENPL\_Noc300\_122214\_01.11472.11472.3 | 3.1128 | 0.3527 | 99.9% | 2469.1143 | 2469.68 | 1 | 5.522 | 35.7% | 1 | K.HSTSETEQLLCGRPPDLTALSR.H | 3 |
| \* | CENPL\_Noc300\_122214\_01.19810.19810.2 | 6.1874 | 0.586 | 100.0% | 2166.152 | 2166.4795 | 1 | 10.245 | 75.0% | 3 | R.HDLEDNLLSSLVILEVLSR.Q | 2 |
| \* | CENPL\_Noc300\_122214\_01.07187.07187.3 | 5.4104 | 0.4999 | 100.0% | 2867.5444 | 2868.0 | 1 | 8.12 | 34.6% | 5 | K.SQLAVPHPETQDSSTQTDTSHSGITNK.L | 3 |
| \* | CENPL\_Noc300\_tube2\_122214\_01.08969.08969.3 | 5.0927 | 0.3915 | 100.0% | 2105.1843 | 2105.3794 | 2 | 7.35 | 45.6% | 1 | K.LQHLKESHEMGQALQQAR.N | 3 |
| \* | CENPL\_Noc300\_122214\_01.06489.06489.2 | 3.2836 | 0.3131 | 100.0% | 1485.1721 | 1485.6146 | 1 | 6.842 | 75.0% | 1 | K.ESHEMGQALQQAR.N | 2 |
| \* | CENPL\_Noc300\_tube2\_122214\_01.19456.19456.2 | 3.9979 | 0.4537 | 100.0% | 1305.7322 | 1305.578 | 1 | 8.269 | 75.0% | 2 | R.NVMQSWVLISK.E | 2 |
| \* | CENPL\_Noc300\_122214\_01.14971.14971.3 | 6.1716 | 0.5091 | 100.0% | 2891.6643 | 2892.2793 | 1 | 8.157 | 32.3% | 2 | K.ELISLLHLSLLHLEEDKTTVSQESR.R | 3 |
| \* | CENPL\_Noc300\_tube2\_122214\_01.18147.18147.2 | 3.9693 | 0.3607 | 100.0% | 1390.8522 | 1391.5823 | 1 | 8.216 | 68.2% | 4 | R.ISQLEQDLASMR.E | 2 |
| \* | CENPL\_Noc300\_tube2\_122214\_01.14199.14199.3 | 3.9409 | 0.2577 | 99.9% | 1694.2444 | 1692.9994 | 6 | 5.686 | 41.7% | 2 | R.GLLKDAQTQLVGLHAK.Q | 3 |
| \* | CENPL\_Noc300\_tube2\_122214\_01.10110.10110.2 | 3.7406 | 0.4424 | 100.0% | 1280.8922 | 1281.4545 | 1 | 7.131 | 81.8% | 2 | K.DAQTQLVGLHAK.Q | 2 |
| \* | CENPL\_Noc300\_tube2\_122214\_01.10168.10168.3 | 2.8915 | 0.3095 | 99.9% | 1281.9844 | 1281.4545 | 6 | 5.817 | 40.9% | 2 | K.DAQTQLVGLHAK.Q | 3 |
| \* | CENPL\_Noc300\_122214\_01.15639.15639.2 | 4.4069 | 0.4806 | 100.0% | 2390.8323 | 2390.612 | 1 | 9.334 | 44.7% | 3 | K.QEELVQQTVSLTSTLQQDWR.S | 2 |
| \* | CENPL\_Noc300\_122214\_02.12680.12680.3 | 3.0438 | 0.2703 | 99.6% | 2391.0842 | 2390.612 | 13 | 4.956 | 27.6% | 1 | K.QEELVQQTVSLTSTLQQDWR.S | 3 |
| \* | CENPL\_Noc300\_122214\_02.13546.13546.2 | 4.992 | 0.4353 | 100.0% | 1786.5922 | 1787.0405 | 1 | 9.21 | 71.4% | 2 | R.SMQLDYTTWTALLSR.S | 2 |
| \* | CENPL\_Noc300\_122214\_01.06349.06349.2 | 3.4831 | 0.3628 | 100.0% | 1402.7322 | 1403.5321 | 1 | 7.274 | 81.8% | 1 | R.DVAIEEKQEVSR.V | 2 |
| \* | CENPL\_Noc300\_122214\_02.06282.06282.3 | 2.4327 | 0.3009 | 99.6% | 1405.0443 | 1403.5321 | 1 | 5.311 | 40.9% | 1 | R.DVAIEEKQEVSR.V | 3 |
| \* | CENPL\_Noc300\_122214\_02.08588.08588.2 | 3.6134 | 0.4085 | 100.0% | 1533.4122 | 1533.6849 | 1 | 8.114 | 62.5% | 1 | R.VLEQVSAQLEECK.G | 2 |
| \* | CENPL\_Noc300\_122214\_01.11238.11238.3 | 4.8537 | 0.3776 | 100.0% | 2918.7244 | 2919.1382 | 1 | 8.081 | 32.3% | 2 | R.VLEQVSAQLEECKGQTEQLELENSR.L | 3 |
| \* | CENPL\_Noc300\_122214\_01.07681.07681.2 | 3.6481 | 0.2967 | 100.0% | 1404.2722 | 1404.4764 | 1 | 7.104 | 68.2% | 3 | K.GQTEQLELENSR.L | 2 |
| \* | CENPL\_Noc300\_tube2\_122214\_01.19172.19172.2 | 5.2461 | 0.429 | 100.0% | 1573.8322 | 1573.848 | 1 | 8.03 | 80.8% | 7 | R.AQLQILANMDSQLK.E | 2 |
| \* | CENPL\_Noc300\_tube2\_122214\_01.09962.09962.2 | 5.4957 | 0.6233 | 100.0% | 1723.2922 | 1723.9879 | 1 | 11.455 | 85.7% | 4 | K.HMQAELQQQQAVLAK.E | 2 |
| \* | CENPL\_Noc300\_tube2\_122214\_01.09998.09998.3 | 4.6322 | 0.3385 | 100.0% | 1724.5443 | 1723.9879 | 1 | 6.078 | 57.1% | 4 | K.HMQAELQQQQAVLAK.E | 3 |
| \* | CENPL\_Noc300\_122214\_02.12534.12534.3 | 6.1097 | 0.4701 | 100.0% | 3286.8843 | 3286.5862 | 1 | 8.184 | 32.4% | 1 | R.DLKETLEFADQENQVAHLELGQVECQLK.T | 3 |
| \* | CENPL\_Noc300\_122214\_01.09260.09260.2 | 2.1247 | 0.1943 | 98.2% | 833.2922 | 831.9878 | 6 | 5.129 | 83.3% | 3 | K.TTLEVLR.E | 2 |
| \* | CENPL\_Noc300\_tube2\_122214\_01.15842.15842.3 | 3.2599 | 0.1612 | 96.6% | 1964.7544 | 1964.1462 | 1 | 4.479 | 40.6% | 1 | R.SLQCENLKDTVENLTAK.L | 3 |
| \* | CENPL\_Noc300\_tube2\_122214\_01.10667.10667.2 | 4.7456 | 0.4463 | 100.0% | 1676.4321 | 1675.7899 | 1 | 7.47 | 75.0% | 7 | K.LASTIADNQEQDLEK.T | 2 |
| \* | CENPL\_Noc300\_122214\_02.16048.16048.3 | 5.0592 | 0.4022 | 100.0% | 2047.2244 | 2047.443 | 1 | 8.775 | 50.0% | 3 | K.LGLLTEQLQSLTLFLQTK.L | 3 |
| \* | CENPL\_Noc300\_122214\_01.19248.19248.2 | 6.1125 | 0.5067 | 100.0% | 2047.2722 | 2047.443 | 1 | 9.755 | 64.7% | 4 | K.LGLLTEQLQSLTLFLQTK.L | 2 |
| \* | CENPL\_Noc300\_122214\_01.19034.19034.2 | 5.4353 | 0.3981 | 100.0% | 2789.632 | 2788.121 | 1 | 7.919 | 46.2% | 2 | R.TFLGSILTAVADEEPESTPVPLLGSDK.S | 2 |
| \* | CENPL\_Noc300\_122214\_01.18848.18848.3 | 4.6634 | 0.2791 | 100.0% | 3432.1743 | 3430.7473 | 2 | 6.128 | 25.8% | 1 | R.TFLGSILTAVADEEPESTPVPLLGS\*DKSAFTR.V | 3 |
| \* | CENPL\_Noc300\_tube2\_122214\_01.11373.11373.2 | 2.2772 | 0.3344 | 99.5% | 987.4522 | 988.1412 | 1 | 4.775 | 78.6% | 3 | K.LNQALCLR.Y | 2 |
| \* | CENPL\_Noc300\_tube2\_122214\_01.10248.10248.3 | 5.7444 | 0.3629 | 100.0% | 2148.5344 | 2149.3652 | 1 | 6.854 | 54.7% | 2 | R.YKNEKELQEVIQQQNEK.I | 3 |
| \* | CENPL\_Noc300\_tube2\_122214\_01.10076.10076.2 | 4.5667 | 0.2271 | 100.0% | 1486.2522 | 1486.622 | 1 | 6.604 | 86.4% | 5 | K.ELQEVIQQQNEK.I | 2 |
| \* | CENPL\_Noc300\_tube2\_122214\_01.17121.17121.2 | 3.6691 | 0.4207 | 100.0% | 1840.5322 | 1841.0745 | 1 | 6.165 | 56.7% | 1 | K.SGELISLREEVTHLTR.S | 2 |
| \* | CENPL\_Noc300\_122214\_01.12256.12256.3 | 4.9358 | 0.433 | 100.0% | 1841.4844 | 1841.0745 | 1 | 7.36 | 50.0% | 4 | K.SGELISLREEVTHLTR.S | 3 |
| \* | CENPL\_Noc300\_tube2\_122214\_01.13414.13414.2 | 2.5823 | 0.2782 | 99.6% | 1103.7522 | 1104.248 | 3 | 5.765 | 81.2% | 2 | K.VWLSQEVDK.L | 2 |
| \* | CENPL\_Noc300\_122214\_01.11704.11704.2 | 3.4779 | 0.3574 | 100.0% | 1373.6122 | 1373.595 | 1 | 6.408 | 85.0% | 4 | K.VWLSQEVDKLR.V | 2 |
| \* | CENPL\_Noc300\_tube2\_122214\_01.18040.18040.2 | 2.2687 | 0.4136 | 99.9% | 899.0122 | 898.16644 | 1 | 8.231 | 91.7% | 2 | R.VMFLEMK.N | 2 |
| \* | CENPL\_Noc300\_tube2\_122214\_01.13446.13446.1 | 2.2003 | 0.2342 | 95.8% | 1000.39 | 1001.1277 | 1 | 4.357 | 78.6% | 3 | R.NILEENLR.R | 1 |
| \* | CENPL\_Noc300\_tube2\_122214\_01.13394.13394.2 | 2.7093 | 0.0492 | 98.3% | 1000.9522 | 1001.1277 | 7 | 4.54 | 85.7% | 3 | R.NILEENLR.R | 2 |
| \* | CENPL\_Noc300\_tube2\_122214\_01.17094.17094.3 | 5.5941 | 0.4581 | 100.0% | 2231.7244 | 2230.526 | 1 | 8.849 | 47.1% | 2 | R.RSDKELEKLDDIVQHIYK.T | 3 |
| \* | CENPL\_Noc300\_tube2\_122214\_01.13227.13227.2 | 2.9147 | 0.2494 | 99.8% | 1244.4722 | 1244.4331 | 13 | 5.437 | 72.2% | 2 | K.LDDIVQHIYK.T | 2 |
| \* | CENPL\_Noc300\_122214\_01.13381.13381.2 | 2.8093 | 0.1735 | 99.4% | 1128.5721 | 1127.3696 | 4 | 4.243 | 72.2% | 3 | K.TLLSIPEVVR.G | 2 |

---

|  |  |  |  |  |  |  |  |  |
| --- | --- | --- | --- | --- | --- | --- | --- | --- |
| U | *gi|226530908|ref|NP\_0* | 24 | 101 | 54.4% | 285 | 30315 | 7.5 | protein-L-isoaspartate (D-aspartate) O-methyltransferase [Homo sapiens] |

| Filename XCorr DeltCN Conf% ObsM+H+ CalcM+H+ SpR ZScore Ion% # Sequence  | | | | | | | | | | | | |
| --- | --- | --- | --- | --- | --- | --- | --- | --- | --- | --- | --- | --- |
| \* | CENPL\_Noc300\_122214\_01.06759.06759.2 | 4.1685 | 0.3941 | 100.0% | 1478.3722 | 1478.6078 | 1 | 7.97 | 61.5% | 2 | K.SGGASHSELIHNLR.K | 2 |
| \* | CENPL\_Noc300\_122214\_02.06446.06446.3 | 4.2633 | 0.2933 | 100.0% | 1479.9543 | 1478.6078 | 1 | 6.477 | 44.2% | 3 | K.SGGASHSELIHNLR.K | 3 |
| \* | CENPL\_Noc300\_122214\_02.06194.06194.3 | 3.7963 | 0.3823 | 100.0% | 1607.0643 | 1606.7819 | 1 | 6.817 | 39.3% | 2 | K.SGGASHSELIHNLRK.N | 3 |
| \* | CENPL\_Noc300\_122214\_01.06058.06058.2 | 3.9654 | 0.4197 | 100.0% | 1607.4122 | 1606.7819 | 1 | 7.562 | 60.7% | 2 | K.SGGASHSELIHNLRK.N | 2 |
| \* | CENPL\_Noc300\_122214\_02.10810.10810.3 | 4.0289 | 0.5238 | 100.0% | 2050.2844 | 2051.409 | 1 | 8.489 | 35.3% | 1 | K.NGIIKTDKVFEVMLATDR.S | 3 |
| \* | CENPL\_Noc300\_122214\_02.10260.10260.3 | 4.2509 | 0.3993 | 100.0% | 1525.5243 | 1525.7601 | 1 | 6.708 | 54.2% | 6 | K.TDKVFEVMLATDR.S | 3 |
| \* | CENPL\_Noc300\_122214\_02.10262.10262.2 | 4.4086 | 0.5175 | 100.0% | 1526.8322 | 1525.7601 | 1 | 8.256 | 79.2% | 14 | K.TDKVFEVMLATDR.S | 2 |
| \* | CENPL\_Noc300\_tube2\_122214\_01.17486.17486.2 | 4.1983 | 0.4956 | 100.0% | 1181.1322 | 1181.3923 | 1 | 9.311 | 88.9% | 15 | K.VFEVMLATDR.S | 2 |
| \* | CENPL\_Noc300\_122214\_02.10994.10994.2 | 5.45 | 0.6714 | 100.0% | 1695.5322 | 1695.8792 | 1 | 12.129 | 81.2% | 5 | K.ALDVGSGSGILTACFAR.M | 2 |
| \* | CENPL\_Noc300\_122214\_01.07782.07782.1 | 1.2919 | 0.253 | 95.0% | 894.61 | 895.0898 | 16 | 4.324 | 64.3% | 1 | K.VIGIDHIK.E | 1 |
| \* | CENPL\_Noc300\_122214\_01.07810.07810.2 | 3.0513 | 0.2965 | 100.0% | 895.1322 | 895.0898 | 1 | 5.897 | 92.9% | 3 | K.VIGIDHIK.E | 2 |
| \* | CENPL\_Noc300\_122214\_01.06856.06856.2 | 2.7826 | 0.3804 | 100.0% | 1188.8121 | 1189.3109 | 1 | 6.698 | 75.0% | 4 | R.KDDPTLLSSGR.V | 2 |
| \* | CENPL\_Noc300\_122214\_01.06867.06867.3 | 3.0991 | 0.2921 | 99.9% | 1189.6743 | 1189.3109 | 4 | 5.542 | 47.5% | 2 | R.KDDPTLLSSGR.V | 3 |
| \* | CENPL\_Noc300\_tube2\_122214\_01.10607.10607.2 | 2.4098 | 0.2025 | 98.5% | 1060.9922 | 1061.1368 | 1 | 5.46 | 88.9% | 3 | K.DDPTLLSSGR.V | 2 |
| \* | CENPL\_Noc300\_tube2\_122214\_01.10298.10298.2 | 2.874 | 0.3636 | 100.0% | 943.0722 | 943.091 | 1 | 7.36 | 81.2% | 5 | R.VQLVVGDGR.M | 2 |
| \* | CENPL\_Noc300\_122214\_01.14651.14651.3 | 6.7845 | 0.5968 | 100.0% | 3505.1643 | 3507.0015 | 1 | 12.154 | 34.8% | 6 | R.MGYAEEAPYDAIHVGAAAPVVPQALIDQLKPGGR.L | 3 |
| \* | CENPL\_Noc300\_122214\_01.14653.14653.2 | 3.622 | 0.4603 | 100.0% | 3506.2922 | 3507.0015 | 1 | 9.309 | 25.8% | 1 | R.MGYAEEAPYDAIHVGAAAPVVPQALIDQLKPGGR.L | 2 |
| \* | CENPL\_Noc300\_tube2\_122214\_01.19202.19202.2 | 5.0955 | 0.4938 | 100.0% | 2044.6322 | 2044.3734 | 1 | 8.555 | 75.0% | 6 | R.LILPVGPAGGNQMLEQYDK.L | 2 |
| \* | CENPL\_Noc300\_122214\_01.14229.14229.3 | 2.1971 | 0.3273 | 95.5% | 2785.0444 | 2786.2158 | 1 | 4.961 | 30.0% | 1 | R.LILPVGPAGGNQMLEQYDKLQDGSIK.M | 3 |
| \* | CENPL\_Noc300\_122214\_01.14154.14154.2 | 4.5056 | 0.4694 | 100.0% | 2785.2122 | 2786.2158 | 1 | 8.928 | 44.0% | 4 | R.LILPVGPAGGNQMLEQYDKLQDGSIK.M | 2 |
| \* | CENPL\_Noc300\_tube2\_122214\_01.19328.19328.2 | 3.9423 | 0.4813 | 100.0% | 1705.4922 | 1706.1549 | 1 | 8.1 | 75.0% | 3 | K.MKPLMGVIYVPLTDK.E | 2 |
| \* | CENPL\_Noc300\_122214\_01.13966.13966.3 | 3.9173 | 0.273 | 100.0% | 1706.3944 | 1706.1549 | 1 | 6.028 | 57.1% | 2 | K.MKPLMGVIYVPLTDK.E | 3 |
| \* | CENPL\_Noc300\_122214\_01.12624.12624.2 | 5.0346 | 0.5864 | 100.0% | 1962.4922 | 1963.4445 | 1 | 9.645 | 75.0% | 4 | K.MKPLMGVIYVPLTDKEK.Q | 2 |
| \* | CENPL\_Noc300\_tube2\_122214\_01.17757.17757.3 | 3.9241 | 0.3847 | 100.0% | 1964.9043 | 1963.4445 | 1 | 6.876 | 45.3% | 6 | K.MKPLMGVIYVPLTDKEK.Q | 3 |

---

|  |  |  |  |  |  |  |  |  |
| --- | --- | --- | --- | --- | --- | --- | --- | --- |
| U | *gi|4504919|ref|NP\_002* | 27 | 62 | 54.0% | 483 | 53704 | 5.6 | keratin 8 [Homo sapiens] |

| Filename XCorr DeltCN Conf% ObsM+H+ CalcM+H+ SpR ZScore Ion% # Sequence  | | | | | | | | | | | | |
| --- | --- | --- | --- | --- | --- | --- | --- | --- | --- | --- | --- | --- |
| \* | CENPL\_Noc300\_122214\_02.14523.14523.3 | 5.2812 | 0.4339 | 100.0% | 3928.4644 | 3927.465 | 1 | 8.896 | 18.8% | 1 | R.GGLGGGYGGASGMGGITAVTVNQSLLSPLVLEVDPNIQAVR.T | 3 |
|  | CENPL\_Noc300\_tube2\_122214\_01.13599.13599.2 | 2.3642 | 0.1974 | 99.3% | 827.9922 | 827.95544 | 1 | 5.382 | 100.0% | 5 | K.FASFIDK.V | 222222 |
|  | CENPL\_Noc300\_tube2\_122214\_01.13469.13469.2 | 2.5321 | 0.0875 | 97.5% | 1083.2122 | 1083.2755 | 2 | 6.11 | 81.2% | 2 | K.FASFIDKVR.F | 222222 |
|  | CENPL\_Noc300\_tube2\_122214\_01.15422.15422.2 | 2.7314 | 0.1417 | 99.4% | 1030.5122 | 1031.1997 | 1 | 3.903 | 92.9% | 2 | K.WSLLQQQK.T | 2 |
|  | CENPL\_Noc300\_122214\_02.12446.12446.3 | 5.2224 | 0.2438 | 100.0% | 2035.8243 | 2035.363 | 1 | 8.247 | 50.0% | 2 | K.LKLEAELGNMQGLVEDFK.N | 3 |
|  | CENPL\_Noc300\_122214\_01.05936.05936.2 | 2.8336 | 0.2811 | 99.8% | 1309.1322 | 1309.4215 | 177 | 5.12 | 61.1% | 2 | K.NKYEDEINKR.T | 2222 |
|  | CENPL\_Noc300\_tube2\_122214\_01.18492.18492.2 | 3.0627 | 0.3628 | 100.0% | 1353.2322 | 1353.5732 | 1 | 6.527 | 75.0% | 2 | R.TEMENEFVLIK.K | 2 |
|  | CENPL\_Noc300\_122214\_01.10991.10991.2 | 3.341 | 0.2405 | 99.9% | 1481.6322 | 1481.7473 | 4 | 5.666 | 59.1% | 2 | R.TEMENEFVLIKK.D | 2 |
|  | CENPL\_Noc300\_tube2\_122214\_01.15087.15087.3 | 2.1102 | 0.3049 | 96.9% | 1481.6344 | 1481.7473 | 249 | 5.516 | 29.5% | 1 | R.TEMENEFVLIKK.D | 3 |
|  | CENPL\_Noc300\_122214\_02.08702.08702.3 | 3.8782 | 0.3722 | 100.0% | 1799.2144 | 1798.9623 | 1 | 6.007 | 44.6% | 1 | K.DVDEAYMNKVELESR.L | 3 |
|  | CENPL\_Noc300\_tube2\_122214\_01.13181.13181.2 | 3.7371 | 0.4268 | 100.0% | 1799.4922 | 1798.9623 | 1 | 7.241 | 53.6% | 1 | K.DVDEAYMNKVELESR.L | 2 |
|  | CENPL\_Noc300\_122214\_01.14761.14761.2 | 4.0467 | 0.4861 | 100.0% | 1420.7322 | 1420.6055 | 1 | 8.511 | 86.4% | 3 | R.LEGLTDEINFLR.Q | 2 |
|  | CENPL\_Noc300\_122214\_02.10056.10056.2 | 5.1331 | 0.5923 | 100.0% | 2109.412 | 2110.3008 | 1 | 9.795 | 63.9% | 1 | R.ELQSQISDTSVVLSMDNSR.S | 2 |
|  | CENPL\_Noc300\_122214\_01.14477.14477.2 | 3.8843 | 0.4903 | 100.0% | 1321.6921 | 1321.5286 | 1 | 7.752 | 77.3% | 2 | R.SLDMDSIIAEVK.A | 2 |
|  | CENPL\_Noc300\_122214\_01.06760.06760.2 | 2.6334 | 0.3364 | 99.9% | 1080.0521 | 1080.1423 | 1 | 6.231 | 87.5% | 3 | K.AQYEDIANR.S | 22 |
|  | CENPL\_Noc300\_tube2\_122214\_01.09689.09689.2 | 3.4253 | 0.2981 | 100.0% | 1413.0122 | 1413.5884 | 1 | 6.635 | 86.4% | 1 | R.SRAEAESMYQIK.Y | 2 |
|  | CENPL\_Noc300\_tube2\_122214\_01.11016.11016.2 | 2.7727 | 0.2872 | 99.8% | 1170.0721 | 1170.3228 | 12 | 5.805 | 72.2% | 3 | R.AEAESMYQIK.Y | 2 |
|  | CENPL\_Noc300\_tube2\_122214\_01.12279.12279.2 | 3.782 | 0.0629 | 99.8% | 1137.9321 | 1138.2627 | 1 | 7.317 | 83.3% | 5 | K.YEELQSLAGK.H | 2 |
|  | CENPL\_Noc300\_tube2\_122214\_01.12320.12320.2 | 2.8987 | 0.2027 | 99.7% | 1001.2322 | 1001.168 | 49 | 4.92 | 75.0% | 2 | R.LQAEIEGLK.G | 2 |
|  | CENPL\_Noc300\_tube2\_122214\_01.10058.10058.2 | 3.3096 | 0.2516 | 99.9% | 1342.4521 | 1342.5381 | 1 | 6.915 | 68.2% | 2 | R.LQAEIEGLKGQR.A | 2 |
|  | CENPL\_Noc300\_122214\_01.11926.11926.2 | 4.2358 | 0.4307 | 100.0% | 1345.2922 | 1345.452 | 1 | 7.196 | 66.7% | 4 | R.ASLEAAIADAEQR.G | 2 |
|  | CENPL\_Noc300\_tube2\_122214\_01.19380.19380.3 | 3.4026 | 0.3289 | 99.9% | 1957.6144 | 1957.1912 | 1 | 6.496 | 43.1% | 1 | R.ASLEAAIADAEQRGELAIK.D | 3 |
|  | CENPL\_Noc300\_tube2\_122214\_01.15159.15159.2 | 4.1238 | 0.2435 | 100.0% | 1130.0922 | 1130.2865 | 1 | 6.194 | 83.3% | 3 | K.LSELEAALQR.A | 2 |
|  | CENPL\_Noc300\_tube2\_122214\_01.12657.12657.2 | 2.3715 | 0.2391 | 99.1% | 1153.8322 | 1154.3234 | 6 | 6.628 | 62.5% | 2 | R.EYQELMNVK.L | 22 |
|  | CENPL\_Noc300\_tube2\_122214\_01.09790.09790.3 | 2.6442 | 0.3642 | 99.9% | 1476.2344 | 1476.7058 | 3 | 5.98 | 41.7% | 2 | R.LESGMQNMSIHTK.T | 3 |
|  | CENPL\_Noc300\_tube2\_122214\_01.09834.09834.2 | 4.1956 | 0.5017 | 100.0% | 1476.3922 | 1476.7058 | 1 | 8.273 | 87.5% | 1 | R.LESGMQNMSIHTK.T | 2 |
|  | CENPL\_Noc300\_122214\_01.08511.08511.2 | 3.0371 | 0.4809 | 100.0% | 1174.2722 | 1174.3367 | 1 | 7.28 | 75.0% | 6 | K.LVSESSDVLPK.- | 2 |

Similarities:
gi|47132620|ref|NP\_00(2:25)  
gi|119395750|ref|NP\_0(1:26)  
gi|119703753|ref|NP\_0(4:23)  
gi|119395754|ref|NP\_0(3:24)  
gi|153791158|ref|NP\_0(3:24)  
gi|67782365|ref|NP\_00(2:25)  

---

|  |  |  |  |  |  |  |  |  |
| --- | --- | --- | --- | --- | --- | --- | --- | --- |
| U | *gi|40354195|ref|NP\_95* | 18 | 45 | 54.0% | 430 | 48058 | 5.5 | keratin 18 [Homo sapiens] |
| U | *gi|4557888|ref|NP\_000* | 18 | 45 | 54.0% | 430 | 48058 | 5.5 | keratin 18 [Homo sapiens] |

| Filename XCorr DeltCN Conf% ObsM+H+ CalcM+H+ SpR ZScore Ion% # Sequence  | | | | | | | | | | | | |
| --- | --- | --- | --- | --- | --- | --- | --- | --- | --- | --- | --- | --- |
|  | CENPL\_Noc300\_tube2\_122214\_01.12783.12783.3 | 4.2339 | 0.3432 | 100.0% | 2856.2344 | 2856.0813 | 1 | 6.707 | 24.2% | 4 | R.SLGSVQAPSYGARPVSSAASVYAGAGGSGSR.I | 3 |
|  | CENPL\_Noc300\_tube2\_122214\_01.19104.19104.2 | 5.0051 | 0.5297 | 100.0% | 2261.3123 | 2262.561 | 1 | 8.757 | 40.0% | 1 | R.GGMGSGGLATGIAGGLAGMGGIQNEK.E | 2 |
|  | CENPL\_Noc300\_tube2\_122214\_01.19094.19094.3 | 4.3964 | 0.3037 | 100.0% | 3337.1042 | 3337.7224 | 1 | 5.288 | 27.2% | 1 | R.GGMGSGGLATGIAGGLAGMGGIQNEKETMQSLNDR.L | 32 |
|  | CENPL\_Noc300\_tube2\_122214\_01.10034.10034.2 | 1.904 | 0.2863 | 98.3% | 838.2322 | 837.9511 | 2 | 5.808 | 91.7% | 1 | R.LASYLDR.V | 2 |
|  | CENPL\_Noc300\_tube2\_122214\_01.15789.15789.2 | 2.0922 | 0.1125 | 95.4% | 983.2522 | 983.0709 | 3 | 4.251 | 83.3% | 1 | R.DWSHYFK.I | 2 |
|  | CENPL\_Noc300\_tube2\_122214\_01.11990.11990.2 | 3.917 | 0.5004 | 100.0% | 1320.2322 | 1320.4478 | 1 | 8.542 | 81.8% | 5 | R.AQIFANTVDNAR.I | 2 |
|  | CENPL\_Noc300\_tube2\_122214\_01.12938.12938.2 | 3.3745 | 0.1748 | 99.9% | 1042.0521 | 1042.2235 | 1 | 6.53 | 87.5% | 5 | R.IVLQIDNAR.L | 22 |
|  | CENPL\_Noc300\_122214\_01.07888.07888.2 | 2.5135 | 0.3649 | 100.0% | 808.2522 | 807.8815 | 1 | 6.681 | 83.3% | 6 | R.LAADDFR.V | 2222222 |
|  | CENPL\_Noc300\_tube2\_122214\_01.10468.10468.2 | 2.657 | 0.24 | 99.5% | 1240.0922 | 1240.4601 | 223 | 5.135 | 61.1% | 3 | R.VKYETELAMR.Q | 2 |
|  | CENPL\_Noc300\_122214\_01.06330.06330.2 | 3.215 | 0.2718 | 100.0% | 1175.2922 | 1175.3274 | 1 | 6.447 | 83.3% | 1 | R.KVIDDTNITR.L | 2 |
|  | CENPL\_Noc300\_122214\_01.17985.17985.2 | 6.2046 | 0.5873 | 100.0% | 2178.2322 | 2178.589 | 1 | 10.325 | 55.9% | 2 | R.LQLETEIEALKEELLFMK.K | 2 |
|  | CENPL\_Noc300\_122214\_01.12143.12143.2 | 4.2479 | 0.5134 | 100.0% | 1884.6921 | 1885.1246 | 1 | 9.022 | 58.3% | 1 | K.GLQAQIASSGLTVEVDAPK.S | 2 |
|  | CENPL\_Noc300\_122214\_01.13640.13640.2 | 3.9916 | 0.5284 | 100.0% | 1507.5521 | 1507.699 | 1 | 8.575 | 75.0% | 3 | R.TVQSLEIDLDSMR.N | 2 |
|  | CENPL\_Noc300\_122214\_01.07481.07481.2 | 2.1479 | 0.2166 | 98.2% | 890.27216 | 889.9841 | 1 | 5.977 | 85.7% | 1 | K.ASLENSLR.E | 2 |
|  | CENPL\_Noc300\_122214\_01.18890.18890.3 | 5.8909 | 0.4265 | 100.0% | 2672.9043 | 2672.0715 | 1 | 7.226 | 43.2% | 3 | R.YALQMEQLNGILLHLESELAQTR.A | 3 |
|  | CENPL\_Noc300\_122214\_01.18900.18900.2 | 5.3896 | 0.3652 | 100.0% | 2673.5923 | 2672.0715 | 1 | 7.632 | 54.5% | 1 | R.YALQMEQLNGILLHLESELAQTR.A | 2 |
|  | CENPL\_Noc300\_tube2\_122214\_01.17901.17901.2 | 3.3342 | 0.3924 | 100.0% | 1420.6522 | 1420.6055 | 1 | 5.887 | 72.7% | 4 | R.QAQEYEALLNIK.V | 2 |
|  | CENPL\_Noc300\_tube2\_122214\_01.12389.12389.2 | 2.9288 | 0.3312 | 99.9% | 1292.8121 | 1293.5059 | 1 | 6.383 | 80.0% | 2 | K.VKLEAEIATYR.R | 2 |

Similarities:
contaminant\_KERATIN03(1:17)  
gi|15431310|ref|NP\_00(1:17)  
gi|4557701|ref|NP\_000(1:17)  
gi|24430192|ref|NP\_00(1:17)  
gi|131412225|ref|NP\_7(1:17)  
gi|24234699|ref|NP\_00(2:16)  

---

|  |  |  |  |  |  |  |  |  |
| --- | --- | --- | --- | --- | --- | --- | --- | --- |
| U | *gi|154800483|ref|NP\_0* | 19 | 44 | 53.7% | 339 | 39541 | 9.1 | centromere protein N isoform 2 [Homo sapiens] |
| U | *gi|154800485|ref|NP\_0* | 19 | 44 | 51.6% | 353 | 41180 | 8.9 | centromere protein N isoform 1 [Homo sapiens] |

| Filename XCorr DeltCN Conf% ObsM+H+ CalcM+H+ SpR ZScore Ion% # Sequence  | | | | | | | | | | | | |
| --- | --- | --- | --- | --- | --- | --- | --- | --- | --- | --- | --- | --- |
|  | CENPL\_Noc300\_122214\_01.15296.15296.2 | 2.5531 | 0.3296 | 99.5% | 1728.7522 | 1729.1719 | 40 | 5.619 | 42.9% | 1 | R.TILKIPMNELTTILK.A | 2 |
|  | CENPL\_Noc300\_122214\_01.14121.14121.2 | 3.6308 | 0.4007 | 100.0% | 1273.4521 | 1273.5737 | 1 | 7.522 | 90.0% | 3 | K.IPMNELTTILK.A | 2 |
|  | CENPL\_Noc300\_tube2\_122214\_01.20457.20457.2 | 4.0319 | 0.479 | 100.0% | 1968.4521 | 1969.1637 | 1 | 9.434 | 63.3% | 4 | K.AWDFLSENQLQTVNFR.Q | 2 |
|  | CENPL\_Noc300\_122214\_01.15927.15927.2 | 4.2436 | 0.5096 | 100.0% | 2429.6921 | 2430.784 | 1 | 9.017 | 37.5% | 1 | R.ASISDAALLDIIYMQFHQHQK.V | 2 |
|  | CENPL\_Noc300\_122214\_01.13090.13090.2 | 4.2228 | 0.561 | 100.0% | 1324.2722 | 1323.4602 | 1 | 10.076 | 72.7% | 5 | K.GPGEDVDLFDMK.Q | 2 |
|  | CENPL\_Noc300\_122214\_01.10352.10352.2 | 2.2582 | 0.2597 | 98.7% | 1247.5721 | 1247.3501 | 2 | 5.689 | 72.2% | 1 | R.ETEENAVWIR.I | 2 |
|  | CENPL\_Noc300\_tube2\_122214\_01.16548.16548.2 | 3.2687 | 0.296 | 99.8% | 1585.3322 | 1583.8717 | 1 | 6.053 | 64.3% | 1 | R.RNTPLLGQALTIASK.H | 2 |
|  | CENPL\_Noc300\_122214\_01.13465.13465.2 | 4.2407 | 0.5237 | 100.0% | 1427.5122 | 1427.6842 | 1 | 9.388 | 80.8% | 5 | R.NTPLLGQALTIASK.H | 2 |
|  | CENPL\_Noc300\_tube2\_122214\_01.10174.10174.2 | 3.2727 | 0.5392 | 100.0% | 2194.4722 | 2195.3098 | 1 | 8.028 | 52.9% | 2 | K.QYNQTFETHNSTTPLQER.S | 2 |
|  | CENPL\_Noc300\_tube2\_122214\_01.10214.10214.3 | 2.9947 | 0.2892 | 99.8% | 2195.2444 | 2195.3098 | 2 | 5.172 | 32.4% | 3 | K.QYNQTFETHNSTTPLQER.S | 3 |
|  | CENPL\_Noc300\_122214\_02.09894.09894.2 | 3.4719 | 0.4832 | 100.0% | 1222.2522 | 1221.371 | 1 | 8.272 | 80.0% | 4 | R.SLGLDINMDSR.I | 2 |
|  | CENPL\_Noc300\_122214\_01.06508.06508.2 | 2.3552 | 0.1662 | 98.1% | 1096.1921 | 1095.284 | 1 | 4.321 | 87.5% | 1 | R.IIHENIVEK.E | 2 |
|  | CENPL\_Noc300\_122214\_01.06197.06197.2 | 2.986 | 0.2639 | 99.8% | 1380.4122 | 1380.587 | 1 | 6.759 | 80.0% | 1 | R.IIHENIVEKER.V | 2 |
|  | CENPL\_Noc300\_122214\_01.06153.06153.3 | 2.9236 | 0.3757 | 100.0% | 1380.5643 | 1380.587 | 1 | 6.695 | 55.0% | 1 | R.IIHENIVEKER.V | 3 |
|  | CENPL\_Noc300\_tube2\_122214\_01.18898.18898.3 | 3.8972 | 0.4225 | 100.0% | 2403.7444 | 2404.638 | 1 | 7.219 | 39.5% | 1 | R.ITQETFGDYPQPQLEFAQYK.L | 3 |
|  | CENPL\_Noc300\_tube2\_122214\_01.18939.18939.2 | 5.1886 | 0.5625 | 100.0% | 2404.6921 | 2404.638 | 1 | 10.14 | 68.4% | 3 | R.ITQETFGDYPQPQLEFAQYK.L | 2 |
|  | CENPL\_Noc300\_tube2\_122214\_01.18695.18695.3 | 3.2618 | 0.23 | 98.6% | 2875.0745 | 2876.192 | 4 | 4.919 | 25.0% | 1 | R.ITQETFGDYPQPQLEFAQYKLETK.F | 3 |
|  | CENPL\_Noc300\_tube2\_122214\_01.14002.14002.3 | 3.215 | 0.3069 | 99.9% | 2098.0144 | 2097.2925 | 202 | 4.959 | 30.9% | 2 | K.FKSGLNGS\*ILAEREEPLR.C | 3 |
|  | CENPL\_Noc300\_tube2\_122214\_01.15950.15950.2 | 3.5414 | 0.3384 | 100.0% | 1242.3722 | 1242.4606 | 1 | 6.724 | 85.0% | 4 | K.FSSPHLLEALK.S | 2 |

---

|  |  |  |  |  |  |  |  |  |
| --- | --- | --- | --- | --- | --- | --- | --- | --- |
| U | *gi|30795231|ref|NP\_00* | 5 | 5 | 53.7% | 227 | 22693 | 4.6 | brain abundant, membrane attached signal protein 1 [Homo sapiens] |

| Filename XCorr DeltCN Conf% ObsM+H+ CalcM+H+ SpR ZScore Ion% # Sequence  | | | | | | | | | | | | |
| --- | --- | --- | --- | --- | --- | --- | --- | --- | --- | --- | --- | --- |
| \* | CENPL\_Noc300\_tube2\_122214\_01.08404.08404.3 | 4.89 | 0.4584 | 100.0% | 2699.8145 | 2699.7986 | 1 | 7.813 | 30.8% | 1 | K.AEGAATEEEGTPKESEPQAAAEPAEAK.E | 3 |
| \* | CENPL\_Noc300\_122214\_01.06149.06149.3 | 4.079 | 0.3923 | 100.0% | 2299.8245 | 2299.5022 | 1 | 6.182 | 45.7% | 1 | K.AEPPKAPEQEQAAPGPAAGGEAPK.A | 3 |
| \* | CENPL\_Noc300\_tube2\_122214\_01.08612.08612.3 | 2.4203 | 0.3213 | 97.8% | 2636.9043 | 2637.7734 | 2 | 5.214 | 24.1% | 1 | K.AAEAAAAPAESAAPAAGEEPSKEEGEPK.K | 3 |
| \* | CENPL\_Noc300\_122214\_01.05542.05542.2 | 2.5716 | 0.3586 | 99.5% | 1387.2922 | 1387.4863 | 4 | 6.876 | 53.8% | 1 | K.ETPAATEAPSSTPK.A | 2 |
| \* | CENPL\_Noc300\_122214\_02.07250.07250.3 | 4.6135 | 0.4022 | 100.0% | 2893.5544 | 2894.1216 | 1 | 6.621 | 31.2% | 1 | K.AQGPAASAEEPKPVEAPAANSDQTVTVKE.- | 3 |

---

|  |  |  |  |  |  |  |  |  |
| --- | --- | --- | --- | --- | --- | --- | --- | --- |
| U | *gi|7657315|ref|NP\_055* | 3 | 7 | 52.9% | 102 | 11845 | 4.7 | Lsm3 protein [Homo sapiens] |

| Filename XCorr DeltCN Conf% ObsM+H+ CalcM+H+ SpR ZScore Ion% # Sequence  | | | | | | | | | | | | |
| --- | --- | --- | --- | --- | --- | --- | --- | --- | --- | --- | --- | --- |
| \* | CENPL\_Noc300\_122214\_02.16336.16336.3 | 6.1163 | 0.4726 | 100.0% | 4056.0544 | 4056.441 | 1 | 7.249 | 25.8% | 2 | R.LHAYDQHLNMILGDVEETVTTIEIDEETYEEIYK.S | 3 |
| \* | CENPL\_Noc300\_122214\_01.14181.14181.2 | 1.8835 | 0.28 | 97.5% | 990.0722 | 990.2518 | 8 | 6.141 | 64.3% | 1 | R.NIPMLFVR.G | 2 |
| \* | CENPL\_Noc300\_tube2\_122214\_01.16562.16562.2 | 2.8893 | 0.4156 | 100.0% | 1193.2522 | 1193.4319 | 94 | 6.878 | 59.1% | 4 | R.GDGVVLVAPPLR.V | 2 |

---

|  |  |  |  |  |  |  |  |  |
| --- | --- | --- | --- | --- | --- | --- | --- | --- |
| U | *gi|10863977|ref|NP\_06* | 3 | 9 | 52.6% | 95 | 10835 | 6.5 | LSM2 homolog, U6 small nuclear RNA associated [Homo sapiens] |

| Filename XCorr DeltCN Conf% ObsM+H+ CalcM+H+ SpR ZScore Ion% # Sequence  | | | | | | | | | | | | |
| --- | --- | --- | --- | --- | --- | --- | --- | --- | --- | --- | --- | --- |
| \* | CENPL\_Noc300\_tube2\_122214\_01.13356.13356.2 | 2.3954 | 0.1975 | 97.7% | 1285.5922 | 1286.5547 | 21 | 4.182 | 63.6% | 1 | K.SLVGKDVVVELK.N | 2 |
| \* | CENPL\_Noc300\_tube2\_122214\_01.15266.15266.3 | 4.3978 | 0.4606 | 100.0% | 2173.7344 | 2174.517 | 1 | 7.412 | 37.5% | 5 | K.LTDISVTDPEKYPHMLSVK.N | 3 |
| \* | CENPL\_Noc300\_tube2\_122214\_01.19218.19218.2 | 6.0775 | 0.4959 | 100.0% | 2145.4722 | 2146.3618 | 1 | 9.239 | 75.0% | 3 | R.YVQLPADEVDTQLLQDAAR.K | 2 |

---

|  |  |  |  |  |  |  |  |  |
| --- | --- | --- | --- | --- | --- | --- | --- | --- |
| U | *gi|119395750|ref|NP\_0* | 44 | 147 | 52.3% | 644 | 66039 | 8.1 | keratin 1 [Homo sapiens] |

| Filename XCorr DeltCN Conf% ObsM+H+ CalcM+H+ SpR ZScore Ion% # Sequence  | | | | | | | | | | | | |
| --- | --- | --- | --- | --- | --- | --- | --- | --- | --- | --- | --- | --- |
| \* | CENPL\_Noc300\_tube2\_122214\_01.13245.13245.2 | 4.357 | 0.4313 | 100.0% | 1659.5721 | 1658.7678 | 1 | 7.408 | 68.8% | 4 | R.SGGGFSSGSAGIINYQR.R | 2 |
| \* | CENPL\_Noc300\_tube2\_122214\_01.10635.10635.1 | 1.5653 | 0.3056 | 95.7% | 874.49 | 875.0128 | 22 | 5.545 | 56.2% | 3 | R.SLVNLGGSK.S | 1 |
| \* | CENPL\_Noc300\_122214\_02.07104.07104.2 | 2.5133 | 0.1707 | 98.8% | 875.1322 | 875.0128 | 5 | 4.287 | 75.0% | 2 | R.SLVNLGGSK.S | 2 |
|  | CENPL\_Noc300\_tube2\_122214\_01.18566.18566.2 | 4.0615 | 0.4172 | 100.0% | 1384.1921 | 1384.5315 | 1 | 7.371 | 72.7% | 8 | K.SLNNQFASFIDK.V | 2 |
|  | CENPL\_Noc300\_tube2\_122214\_01.19176.19176.2 | 4.1232 | 0.4089 | 100.0% | 1639.2122 | 1639.8516 | 1 | 6.945 | 73.1% | 2 | K.SLNNQFASFIDKVR.F | 2 |
|  | CENPL\_Noc300\_tube2\_122214\_01.11840.11840.2 | 4.361 | 0.1003 | 100.0% | 1475.8121 | 1476.6726 | 1 | 7.365 | 90.9% | 5 | R.FLEQQNQVLQTK.W | 22 |
|  | CENPL\_Noc300\_122214\_01.12924.12924.2 | 4.6285 | 0.4751 | 100.0% | 1477.4122 | 1476.6293 | 1 | 8.551 | 77.3% | 11 | K.WELLQQVDTSTR.T | 2 |
|  | CENPL\_Noc300\_tube2\_122214\_01.20786.20786.2 | 4.4815 | 0.4353 | 100.0% | 1994.6921 | 1995.2017 | 1 | 7.817 | 70.0% | 3 | R.THNLEPYFESFINNLR.R | 2 |
|  | CENPL\_Noc300\_tube2\_122214\_01.20768.20768.3 | 4.2228 | 0.3771 | 100.0% | 1994.7843 | 1995.2017 | 1 | 7.092 | 50.0% | 2 | R.THNLEPYFESFINNLR.R | 3 |
|  | CENPL\_Noc300\_tube2\_122214\_01.20091.20091.3 | 3.1152 | 0.4388 | 100.0% | 2152.0144 | 2151.3892 | 100 | 5.663 | 28.1% | 1 | R.THNLEPYFESFINNLRR.R | 3 |
|  | CENPL\_Noc300\_tube2\_122214\_01.14033.14033.2 | 3.434 | 0.4151 | 100.0% | 1301.1522 | 1301.4316 | 5 | 7.339 | 66.7% | 4 | K.NMQDMVEDYR.N | 2 |
|  | CENPL\_Noc300\_tube2\_122214\_01.16314.16314.3 | 4.0786 | 0.3752 | 100.0% | 2591.4243 | 2591.8298 | 1 | 5.837 | 34.2% | 1 | K.NMQDMVEDYRNKYEDEINKR.T | 3 |
|  | CENPL\_Noc300\_122214\_01.05936.05936.2 | 2.8336 | 0.2811 | 99.8% | 1309.1322 | 1309.4215 | 177 | 5.12 | 61.1% | 2 | R.NKYEDEINKR.T | 2222 |
|  | CENPL\_Noc300\_tube2\_122214\_01.13149.13149.2 | 3.496 | 0.3709 | 100.0% | 1266.2722 | 1266.3934 | 1 | 7.447 | 75.0% | 5 | R.TNAENEFVTIK.K | 2 |
|  | CENPL\_Noc300\_tube2\_122214\_01.10282.10282.2 | 3.625 | 0.3466 | 100.0% | 1393.7322 | 1394.5675 | 1 | 7.438 | 68.2% | 6 | R.TNAENEFVTIKK.D | 2 |
|  | CENPL\_Noc300\_tube2\_122214\_01.10346.10346.3 | 2.7321 | 0.3155 | 99.9% | 1395.2043 | 1394.5675 | 1 | 5.818 | 40.9% | 2 | R.TNAENEFVTIKK.D | 3 |
|  | CENPL\_Noc300\_122214\_02.06468.06468.2 | 2.1666 | 0.3077 | 99.2% | 999.8722 | 1000.1114 | 1 | 5.015 | 81.2% | 1 | K.DVDGAYMTK.V | 2 |
| \* | CENPL\_Noc300\_tube2\_122214\_01.20745.20745.1 | 2.2065 | 0.2699 | 95.1% | 1302.79 | 1303.4955 | 7 | 5.038 | 54.5% | 1 | R.SLDLDSIIAEVK.A | 1 |
| \* | CENPL\_Noc300\_tube2\_122214\_01.20709.20709.2 | 4.3618 | 0.454 | 100.0% | 1303.3121 | 1303.4955 | 1 | 9.141 | 86.4% | 3 | R.SLDLDSIIAEVK.A | 2 |
| \* | CENPL\_Noc300\_122214\_01.06389.06389.1 | 2.558 | 0.2124 | 96.0% | 1066.16 | 1066.1558 | 56 | 4.667 | 56.2% | 1 | K.AQYEDIAQK.S | 1 |
| \* | CENPL\_Noc300\_122214\_01.06370.06370.2 | 2.7193 | 0.2719 | 99.8% | 1067.1721 | 1066.1558 | 1 | 5.063 | 81.2% | 2 | K.AQYEDIAQK.S | 2 |
|  | CENPL\_Noc300\_122214\_01.06032.06032.3 | 3.0958 | 0.2443 | 99.8% | 1340.9343 | 1341.4607 | 5 | 4.872 | 40.9% | 1 | K.SKAEAESLYQSK.Y | 3 |
|  | CENPL\_Noc300\_122214\_01.05955.05955.2 | 4.4132 | 0.4529 | 100.0% | 1342.1122 | 1341.4607 | 1 | 7.952 | 77.3% | 6 | K.SKAEAESLYQSK.Y | 2 |
|  | CENPL\_Noc300\_122214\_02.08907.08907.3 | 5.5693 | 0.4402 | 100.0% | 2503.9143 | 2502.7405 | 1 | 8.201 | 40.5% | 2 | K.SKAEAESLYQSKYEELQITAGR.H | 3 |
|  | CENPL\_Noc300\_122214\_02.06393.06393.2 | 2.8846 | 0.3793 | 100.0% | 1126.2522 | 1126.2084 | 1 | 7.176 | 83.3% | 2 | K.AEAESLYQSK.Y | 2 |
|  | CENPL\_Noc300\_122214\_01.09420.09420.2 | 3.9725 | 0.3349 | 100.0% | 1180.1522 | 1180.303 | 1 | 7.434 | 88.9% | 10 | K.YEELQITAGR.H | 22 |
|  | CENPL\_Noc300\_tube2\_122214\_01.10385.10385.3 | 3.2863 | 0.2597 | 99.9% | 1303.1943 | 1303.4581 | 4 | 4.694 | 50.0% | 2 | R.NSKIEISELNR.V | 3 |
|  | CENPL\_Noc300\_tube2\_122214\_01.10361.10361.2 | 3.3687 | 0.3422 | 100.0% | 1303.2522 | 1303.4581 | 1 | 6.083 | 80.0% | 3 | R.NSKIEISELNR.V | 2 |
|  | CENPL\_Noc300\_122214\_01.09166.09166.2 | 3.0491 | 0.1856 | 99.8% | 973.9922 | 974.102 | 1 | 5.374 | 92.9% | 6 | K.IEISELNR.V | 22 |
|  | CENPL\_Noc300\_tube2\_122214\_01.13826.13826.2 | 4.5158 | 0.4752 | 100.0% | 1717.3922 | 1717.8333 | 1 | 7.906 | 71.4% | 5 | K.QISNLQQSISDAEQR.G | 2 |
|  | CENPL\_Noc300\_tube2\_122214\_01.16418.16418.2 | 4.954 | 0.461 | 100.0% | 1600.3522 | 1600.769 | 1 | 8.65 | 80.8% | 4 | K.NKLNDLEDALQQAK.E | 2 |
|  | CENPL\_Noc300\_122214\_01.11848.11848.3 | 4.2313 | 0.3942 | 100.0% | 1600.5844 | 1600.769 | 1 | 6.561 | 55.8% | 1 | K.NKLNDLEDALQQAK.E | 3 |
| \* | CENPL\_Noc300\_122214\_01.14421.14421.2 | 5.1571 | 0.4825 | 100.0% | 2184.872 | 2185.399 | 1 | 9.359 | 75.0% | 2 | K.NKLNDLEDALQQAKEDLAR.L | 2 |
| \* | CENPL\_Noc300\_tube2\_122214\_01.19739.19739.3 | 5.7426 | 0.4925 | 100.0% | 2185.9744 | 2185.399 | 1 | 8.013 | 50.0% | 5 | K.NKLNDLEDALQQAKEDLAR.L | 3 |
|  | CENPL\_Noc300\_tube2\_122214\_01.15872.15872.2 | 4.8615 | 0.3312 | 100.0% | 1359.0922 | 1358.4912 | 1 | 7.818 | 86.4% | 5 | K.LNDLEDALQQAK.E | 2 |
|  | CENPL\_Noc300\_tube2\_122214\_01.13854.13854.2 | 3.9917 | 0.3631 | 100.0% | 1523.9521 | 1524.7754 | 1 | 6.749 | 77.3% | 4 | R.LLRDYQELMNTK.L | 2 |
|  | CENPL\_Noc300\_tube2\_122214\_01.13928.13928.3 | 3.3799 | 0.2945 | 99.9% | 1524.3844 | 1524.7754 | 1 | 5.42 | 56.8% | 5 | R.LLRDYQELMNTK.L | 3 |
|  | CENPL\_Noc300\_tube2\_122214\_01.12327.12327.2 | 2.4946 | 0.2727 | 99.6% | 1141.6721 | 1142.2689 | 36 | 6.519 | 62.5% | 5 | R.DYQELMNTK.L | 2 |
|  | CENPL\_Noc300\_122214\_01.07234.07234.2 | 2.6122 | 0.364 | 99.9% | 1033.7922 | 1034.1112 | 5 | 5.795 | 75.0% | 3 | R.TLLEGEESR.M | 2 |
|  | CENPL\_Noc300\_122214\_01.06267.06267.2 | 6.0777 | 0.5988 | 100.0% | 2384.5522 | 2385.298 | 1 | 11.235 | 36.7% | 1 | R.GGGGGGYGSGGSSYGSGGGSYGSGGGGGGGR.G | 2 |
|  | CENPL\_Noc300\_122214\_01.06276.06276.3 | 6.2477 | 0.4715 | 100.0% | 2384.6943 | 2385.298 | 1 | 10.046 | 33.3% | 1 | R.GGGGGGYGSGGSSYGSGGGSYGSGGGGGGGR.G | 3 |
| \* | CENPL\_Noc300\_tube2\_122214\_01.08876.08876.3 | 6.334 | 0.1893 | 100.0% | 3313.7043 | 3314.2085 | 1 | 9.311 | 27.6% | 2 | R.GSYGSGGSSYGSGGGSYGSGGGGGGHGSYGSGSSSGGYR.G | 3 |
| \* | CENPL\_Noc300\_tube2\_122214\_01.16826.16826.3 | 3.783 | 0.1606 | 97.4% | 2240.3943 | 2241.0396 | 2 | 4.352 | 30.6% | 2 | R.GGSGGGGGGS\*S\*GGRGSGGGSSGGSIGGR.G | 3 |
| \* | CENPL\_Noc300\_122214\_02.05907.05907.3 | 4.369 | 0.342 | 100.0% | 2740.3743 | 2740.7366 | 1 | 6.823 | 25.7% | 1 | R.GGSGGGGGGSSGGRGSGGGSSGGSIGGRGSSSGGVK.S | 3 |

Similarities:
gi|47132620|ref|NP\_00(2:42)  
gi|4504919|ref|NP\_002(1:43)  
gi|119703753|ref|NP\_0(2:42)  
gi|119395754|ref|NP\_0(1:43)  

---

|  |  |  |  |  |  |  |  |  |
| --- | --- | --- | --- | --- | --- | --- | --- | --- |
| U | *gi|7657385|ref|NP\_055* | 18 | 49 | 50.7% | 540 | 59738 | 7.7 | CCR4-NOT transcription complex, subunit 2 [Homo sapiens] |

| Filename XCorr DeltCN Conf% ObsM+H+ CalcM+H+ SpR ZScore Ion% # Sequence  | | | | | | | | | | | | |
| --- | --- | --- | --- | --- | --- | --- | --- | --- | --- | --- | --- | --- |
| \* | CENPL\_Noc300\_tube2\_122214\_01.13872.13872.2 | 4.1878 | 0.4119 | 100.0% | 1475.6721 | 1475.6189 | 1 | 8.004 | 70.8% | 4 | R.NYQVTNSMFGASR.K | 2 |
| \* | CENPL\_Noc300\_tube2\_122214\_01.15395.15395.3 | 5.9561 | 0.5446 | 100.0% | 3041.6643 | 3042.2368 | 1 | 10.299 | 36.5% | 3 | K.FVEGVDSDYHDENMYYSQSSMFPHR.S | 3 |
| \* | CENPL\_Noc300\_122214\_01.06448.06448.2 | 3.2932 | 0.3184 | 100.0% | 1232.3121 | 1232.3568 | 1 | 5.701 | 75.0% | 2 | R.GMSNNTPQLNR.S | 2 |
| \* | CENPL\_Noc300\_tube2\_122214\_01.13293.13293.2 | 1.9389 | 0.3781 | 99.3% | 898.09216 | 898.11127 | 85 | 6.005 | 64.3% | 1 | R.GILPMNPR.N | 2 |
| \* | CENPL\_Noc300\_tube2\_122214\_01.11919.11919.2 | 5.4621 | 0.5416 | 100.0% | 1826.3522 | 1827.0863 | 1 | 9.505 | 75.0% | 3 | R.NMMNHSQVGQGIGIPSR.T | 2 |
| \* | CENPL\_Noc300\_tube2\_122214\_01.11901.11901.3 | 4.6939 | 0.3431 | 100.0% | 1827.9243 | 1827.0863 | 1 | 7.191 | 53.1% | 6 | R.NMMNHSQVGQGIGIPSR.T | 3 |
| \* | CENPL\_Noc300\_122214\_01.06600.06600.2 | 3.7437 | 0.3805 | 100.0% | 1475.0922 | 1475.4869 | 1 | 7.736 | 69.2% | 1 | R.TNSMSSSGLGS\*PNR.S | 2 |
| \* | CENPL\_Noc300\_tube2\_122214\_01.17834.17834.2 | 3.31 | 0.524 | 100.0% | 1673.4521 | 1673.9019 | 1 | 7.974 | 57.1% | 3 | R.QPFTVNSMSGFGMNR.N | 2 |
| \* | CENPL\_Noc300\_122214\_02.13506.13506.3 | 5.744 | 0.3565 | 100.0% | 3992.7844 | 3991.2483 | 1 | 7.741 | 23.0% | 1 | R.NQAFGMNNSLSSNIFNGTDGSENVTGLDLSDFPALADR.N | 3 |
| \* | CENPL\_Noc300\_122214\_01.11938.11938.2 | 4.1034 | 0.5123 | 100.0% | 1594.3522 | 1593.78 | 1 | 8.029 | 63.3% | 7 | R.EGSGNPTPLINPLAGR.A | 2 |
| \* | CENPL\_Noc300\_122214\_01.06466.06466.2 | 3.3063 | 0.4231 | 100.0% | 1438.5122 | 1438.5339 | 1 | 7.758 | 65.4% | 1 | K.TTSSTDGPKFPGDK.S | 2 |
| \* | CENPL\_Noc300\_tube2\_122214\_01.09969.09969.2 | 2.8138 | 0.2468 | 99.7% | 1082.8121 | 1083.276 | 1 | 6.062 | 83.3% | 2 | K.KGIQVLPDGR.V | 2 |
| \* | CENPL\_Noc300\_122214\_01.09071.09071.2 | 2.2519 | 0.289 | 99.3% | 955.2522 | 955.102 | 13 | 6.435 | 62.5% | 1 | K.GIQVLPDGR.V | 2 |
| \* | CENPL\_Noc300\_122214\_02.16211.16211.2 | 5.1595 | 0.5932 | 100.0% | 2551.912 | 2553.0527 | 1 | 11.068 | 59.1% | 3 | R.VTNIPQGMVTDQFGMIGLLTFIR.A | 2 |
| \* | CENPL\_Noc300\_122214\_01.19439.19439.3 | 4.5176 | 0.494 | 100.0% | 2553.9243 | 2553.0527 | 1 | 7.332 | 34.1% | 2 | R.VTNIPQGMVTDQFGMIGLLTFIR.A | 3 |
| \* | CENPL\_Noc300\_122214\_02.13065.13065.3 | 4.0971 | 0.221 | 99.8% | 3452.8442 | 3453.8892 | 1 | 5.688 | 23.4% | 2 | R.AAETDPGMVHLALGSDLTTLGLNLNSPENLYPK.F | 3 |
| \* | CENPL\_Noc300\_122214\_02.06404.06404.2 | 2.3632 | 0.401 | 99.8% | 961.89215 | 962.16705 | 68 | 6.486 | 68.8% | 4 | R.APGMEPTMK.T | 2 |
| \* | CENPL\_Noc300\_122214\_01.13207.13207.3 | 5.1358 | 0.5073 | 100.0% | 3422.1243 | 3422.7373 | 1 | 7.37 | 28.7% | 3 | K.EFHLEYDKLEERPHLPSTFNYNPAQQAF.- | 3 |

---

|  |  |  |  |  |  |  |  |  |
| --- | --- | --- | --- | --- | --- | --- | --- | --- |
| U | *gi|4501885|ref|NP\_001* | 16 | 57 | 50.1% | 375 | 41737 | 5.5 | beta actin [Homo sapiens] |
| U | *gi|4501887|ref|NP\_001* | 16 | 57 | 50.1% | 375 | 41793 | 5.5 | actin, gamma 1 propeptide [Homo sapiens] |

| Filename XCorr DeltCN Conf% ObsM+H+ CalcM+H+ SpR ZScore Ion% # Sequence  | | | | | | | | | | | | |
| --- | --- | --- | --- | --- | --- | --- | --- | --- | --- | --- | --- | --- |
|  | CENPL\_Noc300\_122214\_01.06198.06198.2 | 3.1915 | 0.4707 | 100.0% | 976.71216 | 977.02136 | 2 | 7.675 | 77.8% | 6 | K.AGFAGDDAPR.A | 22 |
|  | CENPL\_Noc300\_tube2\_122214\_01.13340.13340.2 | 2.9245 | 0.3281 | 99.9% | 1198.9122 | 1199.4415 | 1 | 5.967 | 70.0% | 5 | R.AVFPSIVGRPR.H | 22 |
|  | CENPL\_Noc300\_122214\_01.09106.09106.3 | 3.1819 | 0.2237 | 99.9% | 1517.3043 | 1516.7019 | 1 | 5.905 | 52.5% | 4 | K.IWHHTFYNELR.V | 33 |
|  | CENPL\_Noc300\_tube2\_122214\_01.11913.11913.2 | 3.4213 | 0.3999 | 100.0% | 1517.4521 | 1516.7019 | 1 | 6.479 | 80.0% | 3 | K.IWHHTFYNELR.V | 22 |
|  | CENPL\_Noc300\_tube2\_122214\_01.14588.14588.3 | 4.4103 | 0.0629 | 97.1% | 1954.4343 | 1955.2615 | 2 | 6.555 | 38.2% | 6 | R.VAPEEHPVLLTEAPLNPK.A | 3 |
|  | CENPL\_Noc300\_122214\_01.10705.10705.2 | 4.6602 | 0.3495 | 100.0% | 1954.6322 | 1955.2615 | 1 | 8.931 | 61.8% | 6 | R.VAPEEHPVLLTEAPLNPK.A | 2 |
|  | CENPL\_Noc300\_122214\_01.13570.13570.3 | 6.2592 | 0.5575 | 100.0% | 3185.7844 | 3185.622 | 1 | 8.76 | 35.3% | 3 | R.TTGIVMDSGDGVTHTVPIYEGYALPHAILR.L | 3 |
|  | CENPL\_Noc300\_tube2\_122214\_01.18828.18828.1 | 1.9308 | 0.2636 | 95.6% | 998.55 | 999.167 | 1 | 4.895 | 71.4% | 1 | R.DLTDYLMK.I | 11 |
|  | CENPL\_Noc300\_tube2\_122214\_01.18800.18800.2 | 2.085 | 0.3819 | 99.6% | 999.3122 | 999.167 | 8 | 6.884 | 71.4% | 1 | R.DLTDYLMK.I | 22 |
|  | CENPL\_Noc300\_tube2\_122214\_01.10971.10971.2 | 3.326 | 0.4903 | 100.0% | 1133.4122 | 1133.2029 | 1 | 9.886 | 83.3% | 6 | R.GYSFTTTAER.E | 2 |
|  | CENPL\_Noc300\_tube2\_122214\_01.18374.18374.2 | 3.9887 | 0.3196 | 100.0% | 1791.3722 | 1791.9554 | 1 | 8.636 | 76.7% | 6 | K.SYELPDGQVITIGNER.F | 22 |
|  | CENPL\_Noc300\_122214\_02.12784.12784.3 | 3.7112 | 0.2405 | 99.8% | 3233.9043 | 3233.6104 | 1 | 4.977 | 25.0% | 1 | R.CPEALFQPSFLGMESCGIHETTFNSIMK.C | 3 |
|  | CENPL\_Noc300\_122214\_02.10058.10058.3 | 5.6407 | 0.5326 | 100.0% | 2344.5842 | 2344.6448 | 1 | 8.74 | 42.9% | 1 | R.KDLYANTVLSGGTTMYPGIADR.M | 3 |
|  | CENPL\_Noc300\_122214\_02.11178.11178.2 | 3.4365 | 0.5123 | 100.0% | 2215.5923 | 2216.4705 | 1 | 9.091 | 32.5% | 2 | K.DLYANTVLSGGTTMYPGIADR.M | 2 |
|  | CENPL\_Noc300\_122214\_01.09378.09378.2 | 2.7457 | 0.3903 | 100.0% | 1162.3722 | 1162.3868 | 1 | 6.981 | 90.0% | 5 | K.EITALAPSTMK.I | 22 |
|  | CENPL\_Noc300\_122214\_01.07133.07133.3 | 2.2539 | 0.3133 | 98.6% | 1517.0643 | 1517.595 | 1 | 5.068 | 37.5% | 1 | K.QEYDESGPSIVHR.K | 3 |

Similarities:
gi|4501881|ref|NP\_001(8:8)  

---

|  |  |  |  |  |  |  |  |  |
| --- | --- | --- | --- | --- | --- | --- | --- | --- |
| U | *gi|14165270|ref|NP\_05* | 10 | 38 | 49.4% | 178 | 20692 | 9.1 | mitochondrial ribosomal protein L13 [Homo sapiens] |

| Filename XCorr DeltCN Conf% ObsM+H+ CalcM+H+ SpR ZScore Ion% # Sequence  | | | | | | | | | | | | |
| --- | --- | --- | --- | --- | --- | --- | --- | --- | --- | --- | --- | --- |
| \* | CENPL\_Noc300\_tube2\_122214\_01.14655.14655.2 | 3.4047 | 0.4231 | 100.0% | 1175.8121 | 1176.3201 | 1 | 7.698 | 94.4% | 7 | R.APQQWATFAR.I | 2 |
| \* | CENPL\_Noc300\_tube2\_122214\_01.19638.19638.2 | 2.3836 | 0.2158 | 99.2% | 1008.09216 | 1008.2053 | 6 | 5.364 | 71.4% | 2 | R.IWYLLDGK.M | 2 |
| \* | CENPL\_Noc300\_tube2\_122214\_01.10391.10391.2 | 2.1006 | 0.2992 | 99.1% | 832.5122 | 833.03674 | 1 | 5.7 | 78.6% | 1 | K.LAAMASIR.L | 2 |
| \* | CENPL\_Noc300\_122214\_01.07567.07567.2 | 4.1111 | 0.4951 | 100.0% | 1428.2522 | 1428.5468 | 1 | 9.177 | 75.0% | 2 | K.VYSSHTGYPGGFR.Q | 2 |
| \* | CENPL\_Noc300\_tube2\_122214\_01.09849.09849.3 | 3.3022 | 0.4075 | 100.0% | 1428.7444 | 1428.5468 | 1 | 6.735 | 43.8% | 4 | K.VYSSHTGYPGGFR.Q | 3 |
| \* | CENPL\_Noc300\_tube2\_122214\_01.18582.18582.2 | 2.262 | 0.3119 | 99.4% | 1005.9922 | 1006.29156 | 1 | 5.551 | 75.0% | 2 | K.LAIYGMLPK.N | 2 |
| \* | CENPL\_Noc300\_122214\_01.15103.15103.2 | 2.7964 | 0.4385 | 100.0% | 1842.8922 | 1843.1296 | 1 | 6.732 | 57.1% | 2 | R.LHLFPDEYIPEDILK.N | 2 |
| \* | CENPL\_Noc300\_122214\_01.10533.10533.2 | 3.0036 | 0.3251 | 100.0% | 1195.2922 | 1195.361 | 1 | 5.613 | 83.3% | 6 | K.NLVEELPQPR.K | 2 |
| \* | CENPL\_Noc300\_tube2\_122214\_01.15549.15549.2 | 4.7929 | 0.5379 | 100.0% | 1882.3322 | 1883.0245 | 1 | 8.984 | 89.3% | 6 | K.RLDEYTQEEIDAFPR.L | 2 |
| \* | CENPL\_Noc300\_tube2\_122214\_01.15609.15609.3 | 3.2826 | 0.4254 | 100.0% | 1882.8243 | 1883.0245 | 1 | 5.729 | 50.0% | 6 | K.RLDEYTQEEIDAFPR.L | 3 |

---

|  |  |  |  |  |  |  |  |  |
| --- | --- | --- | --- | --- | --- | --- | --- | --- |
| U | *gi|39995082|ref|NP\_06* | 34 | 98 | 48.8% | 767 | 86471 | 6.8 | NOL1/NOP2/Sun domain family, member 2 [Homo sapiens] |

| Filename XCorr DeltCN Conf% ObsM+H+ CalcM+H+ SpR ZScore Ion% # Sequence  | | | | | | | | | | | | |
| --- | --- | --- | --- | --- | --- | --- | --- | --- | --- | --- | --- | --- |
| \* | CENPL\_Noc300\_122214\_01.05259.05259.2 | 4.3309 | 0.491 | 100.0% | 2013.0521 | 2014.0728 | 1 | 8.347 | 55.6% | 1 | R.LQQQQRPEDAEDGAEGGGK.R | 2 |
| \* | CENPL\_Noc300\_122214\_01.05294.05294.3 | 4.9759 | 0.4991 | 100.0% | 2015.0044 | 2014.0728 | 1 | 8.302 | 45.8% | 4 | R.LQQQQRPEDAEDGAEGGGK.R | 3 |
| \* | CENPL\_Noc300\_tube2\_122214\_01.13847.13847.2 | 3.9488 | 0.4223 | 100.0% | 1648.5721 | 1648.8156 | 1 | 6.934 | 71.4% | 4 | K.RGEAGWEGGYPEIVK.E | 2 |
| \* | CENPL\_Noc300\_tube2\_122214\_01.13740.13740.3 | 3.7156 | 0.2326 | 99.9% | 1648.8243 | 1648.8156 | 41 | 5.054 | 37.5% | 1 | K.RGEAGWEGGYPEIVK.E | 3 |
| \* | CENPL\_Noc300\_122214\_01.09302.09302.3 | 4.6285 | 0.3555 | 100.0% | 2020.4043 | 2020.209 | 1 | 6.6 | 45.6% | 3 | K.RGEAGWEGGYPEIVKENK.L | 3 |
| \* | CENPL\_Noc300\_tube2\_122214\_01.15983.15983.2 | 3.758 | 0.4121 | 100.0% | 1492.1921 | 1492.628 | 1 | 7.531 | 65.4% | 2 | R.GEAGWEGGYPEIVK.E | 2 |
| \* | CENPL\_Noc300\_tube2\_122214\_01.14216.14216.2 | 2.9159 | 0.2124 | 99.5% | 1370.2722 | 1370.5474 | 1 | 4.28 | 72.2% | 6 | K.LFEHYYQELK.I | 2 |
| \* | CENPL\_Noc300\_122214\_01.10665.10665.3 | 2.7836 | 0.3079 | 99.9% | 1370.9343 | 1370.5474 | 9 | 5.373 | 50.0% | 1 | K.LFEHYYQELK.I | 3 |
| \* | CENPL\_Noc300\_tube2\_122214\_02.00029.00029.2 | 3.1084 | 0.2193 | 99.5% | 2627.2722 | 2627.0332 | 98 | 4.726 | 27.3% | 1 | K.IVPEGEWGQFMDALREPLPATLR.I | 2 |
| \* | CENPL\_Noc300\_tube2\_122214\_01.12011.12011.2 | 3.3091 | 0.4086 | 100.0% | 1275.4321 | 1275.355 | 1 | 6.378 | 70.0% | 1 | K.ELEDLEVDGQK.V | 2 |
| \* | CENPL\_Noc300\_tube2\_122214\_01.20680.20680.3 | 5.5322 | 0.4501 | 100.0% | 3908.0942 | 3909.3008 | 1 | 7.957 | 30.5% | 1 | K.ELEDLEVDGQKVEVPQPLSWYPEELAWHTNLSR.K | 3 |
| \* | CENPL\_Noc300\_tube2\_122214\_01.20592.20592.2 | 2.7795 | 0.3625 | 99.8% | 2652.652 | 2652.969 | 4 | 6.36 | 33.3% | 1 | K.VEVPQPLSWYPEELAWHTNLSR.K | 2 |
| \* | CENPL\_Noc300\_122214\_02.08860.08860.2 | 5.4791 | 0.547 | 100.0% | 1851.2722 | 1852.0134 | 1 | 9.44 | 86.7% | 3 | K.FHQFLVSETESGNISR.Q | 2 |
| \* | CENPL\_Noc300\_122214\_02.08888.08888.3 | 4.9309 | 0.2601 | 100.0% | 1852.5844 | 1852.0134 | 1 | 6.069 | 48.3% | 5 | K.FHQFLVSETESGNISR.Q | 3 |
| \* | CENPL\_Noc300\_122214\_02.13563.13563.3 | 6.174 | 0.3848 | 100.0% | 3415.1042 | 3416.8916 | 1 | 7.633 | 31.0% | 4 | K.TTQLIEMLHADMNVPFPEGFVIANDVDNKR.C | 3 |
| \* | CENPL\_Noc300\_122214\_01.13418.13418.2 | 4.0966 | 0.3911 | 100.0% | 1781.3922 | 1781.0677 | 1 | 6.189 | 60.7% | 3 | K.WTTLNSLQLHGLQLR.I | 2 |
| \* | CENPL\_Noc300\_122214\_01.18871.18871.3 | 3.5325 | 0.2442 | 99.8% | 3791.5144 | 3792.9417 | 1 | 4.62 | 18.8% | 1 | R.GAEQLAEGGRMVYS\*T#CS\*LNPIEDEAVIASLLEK.S | 3 |
| \* | CENPL\_Noc300\_122214\_01.18228.18228.2 | 4.8679 | 0.4966 | 100.0% | 2583.7322 | 2583.9197 | 1 | 9.587 | 47.7% | 1 | R.MVYSTCSLNPIEDEAVIASLLEK.S | 2 |
| \* | CENPL\_Noc300\_tube2\_122214\_01.19565.19565.2 | 3.5896 | 0.0637 | 98.9% | 1845.6721 | 1843.041 | 1 | 4.235 | 55.9% | 3 | K.SEGALELADVSNELPGLK.W | 2 |
| \* | CENPL\_Noc300\_tube2\_122214\_01.18796.18796.2 | 2.5351 | 0.4009 | 100.0% | 1146.8121 | 1147.3801 | 2 | 6.734 | 81.2% | 3 | K.WMPGITQWK.V | 2 |
| \* | CENPL\_Noc300\_122214\_01.13867.13867.3 | 3.1147 | 0.4699 | 100.0% | 1818.1444 | 1817.9146 | 1 | 7.545 | 53.6% | 3 | K.DGQWFTDWDAVPHSR.H | 3 |
| \* | CENPL\_Noc300\_122214\_01.13817.13817.2 | 3.2187 | 0.342 | 99.9% | 1818.6721 | 1817.9146 | 1 | 5.638 | 60.7% | 2 | K.DGQWFTDWDAVPHSR.H | 2 |
| \* | CENPL\_Noc300\_122214\_01.07176.07176.2 | 2.6922 | 0.1641 | 99.5% | 999.2322 | 998.18823 | 1 | 4.78 | 85.7% | 1 | K.LQAMHLER.C | 2 |
| \* | CENPL\_Noc300\_122214\_02.10630.10630.3 | 5.6412 | 0.5513 | 100.0% | 1979.0343 | 1978.3475 | 1 | 9.104 | 48.5% | 5 | R.ILPHHQNTGGFFVAVLVK.K | 3 |
| \* | CENPL\_Noc300\_122214\_02.09687.09687.3 | 3.514 | 0.368 | 100.0% | 2106.7144 | 2106.5217 | 1 | 6.143 | 37.5% | 1 | R.ILPHHQNTGGFFVAVLVKK.S | 3 |
| \* | CENPL\_Noc300\_122214\_01.18296.18296.3 | 3.6857 | 0.3564 | 100.0% | 2837.6343 | 2838.272 | 1 | 5.59 | 32.6% | 1 | K.LFGFKEDPFVFIPEDDPLFPPIEK.F | 3 |
| \* | CENPL\_Noc300\_122214\_01.18399.18399.2 | 4.2232 | 0.467 | 100.0% | 2838.2522 | 2838.272 | 1 | 7.899 | 47.8% | 3 | K.LFGFKEDPFVFIPEDDPLFPPIEK.F | 2 |
| \* | CENPL\_Noc300\_122214\_01.17742.17742.2 | 3.3423 | 0.2413 | 99.7% | 2245.5923 | 2245.5334 | 1 | 6.931 | 50.0% | 1 | K.EDPFVFIPEDDPLFPPIEK.F | 2 |
| \* | CENPL\_Noc300\_tube2\_122214\_01.18573.18573.2 | 3.2648 | 0.4059 | 100.0% | 1214.2122 | 1213.3782 | 1 | 6.883 | 77.8% | 12 | K.FYALDPSFPR.M | 2 |
| \* | CENPL\_Noc300\_tube2\_122214\_01.20517.20517.2 | 3.524 | 0.5266 | 100.0% | 1884.6322 | 1886.1577 | 1 | 8.944 | 56.7% | 2 | R.LAQEGIYTLYPFINSR.I | 2 |
| \* | CENPL\_Noc300\_122214\_01.10550.10550.2 | 3.4236 | 0.4167 | 100.0% | 1136.4321 | 1135.3612 | 1 | 6.346 | 83.3% | 5 | R.IITVSMEDVK.I | 2 |
| \* | CENPL\_Noc300\_tube2\_122214\_01.20032.20032.2 | 4.2376 | 0.3074 | 100.0% | 1379.6721 | 1378.6139 | 10 | 6.985 | 60.0% | 4 | K.ILLTQENPFFR.K | 2 |
| \* | CENPL\_Noc300\_122214\_01.13010.13010.2 | 3.2076 | 0.4042 | 100.0% | 1107.8121 | 1107.3687 | 2 | 6.988 | 72.2% | 6 | R.MMGLEVLGEK.K | 2 |
| \* | CENPL\_Noc300\_tube2\_122214\_01.09350.09350.3 | 6.9884 | 0.4043 | 100.0% | 2846.5745 | 2846.0374 | 1 | 8.87 | 47.1% | 3 | K.KKEGVILTNESAASTGQPDNDVTEGQR.A | 3 |

---

|  |  |  |  |  |  |  |  |  |
| --- | --- | --- | --- | --- | --- | --- | --- | --- |
| U | *contaminant\_KERATIN03* | 34 | 123 | 47.9% | 593 | 59519 | 5.2 | no description |
| U | *gi|195972866|ref|NP\_0* | 34 | 123 | 48.6% | 584 | 58801 | 5.2 | keratin 10 [Homo sapiens] |

| Filename XCorr DeltCN Conf% ObsM+H+ CalcM+H+ SpR ZScore Ion% # Sequence  | | | | | | | | | | | | |
| --- | --- | --- | --- | --- | --- | --- | --- | --- | --- | --- | --- | --- |
|  | CENPL\_Noc300\_122214\_02.09692.09692.2 | 5.5415 | 0.5777 | 100.0% | 1708.2122 | 1708.7844 | 1 | 10.44 | 63.9% | 14 | K.GSLGGGFSSGGFSGGSFSR.G | 2 |
|  | CENPL\_Noc300\_122214\_01.07045.07045.1 | 1.7771 | 0.24 | 96.1% | 1090.58 | 1091.2273 | 4 | 4.459 | 62.5% | 1 | K.VTMQNLNDR.L | 111 |
|  | CENPL\_Noc300\_122214\_02.06500.06500.2 | 2.7854 | 0.4106 | 100.0% | 1091.0922 | 1091.2273 | 1 | 6.291 | 87.5% | 4 | K.VTMQNLNDR.L | 222 |
|  | CENPL\_Noc300\_122214\_01.07294.07294.1 | 1.6519 | 0.2663 | 95.7% | 809.37 | 809.93774 | 1 | 5.658 | 75.0% | 1 | R.LASYLDK.V | 111111 |
|  | CENPL\_Noc300\_tube2\_122214\_01.10668.10668.2 | 2.9757 | 0.1828 | 99.7% | 1065.1921 | 1065.2578 | 7 | 6.164 | 75.0% | 5 | R.LASYLDKVR.A | 22222 |
|  | CENPL\_Noc300\_122214\_01.08374.08374.2 | 4.1958 | 0.484 | 100.0% | 1382.4122 | 1382.4668 | 1 | 9.312 | 77.3% | 8 | R.ALEESNYELEGK.I | 2 |
|  | CENPL\_Noc300\_122214\_01.07684.07684.2 | 2.4061 | 0.1447 | 98.8% | 996.1722 | 996.15094 | 63 | 3.868 | 83.3% | 1 | K.IKEWYEK.H | 2 |
|  | CENPL\_Noc300\_122214\_01.17754.17754.3 | 4.8934 | 0.4301 | 100.0% | 3053.5444 | 3054.4277 | 1 | 7.031 | 30.8% | 4 | K.TIDDLKNQILNLTTDNANILLQIDNAR.L | 3 |
|  | CENPL\_Noc300\_122214\_01.17760.17760.2 | 4.6163 | 0.3796 | 100.0% | 3056.5522 | 3054.4277 | 1 | 6.982 | 40.4% | 1 | K.TIDDLKNQILNLTTDNANILLQIDNAR.L | 2 |
|  | CENPL\_Noc300\_122214\_02.12725.12725.2 | 5.6643 | 0.4824 | 100.0% | 2368.912 | 2368.6523 | 1 | 9.155 | 57.5% | 2 | K.NQILNLTTDNANILLQIDNAR.L | 2 |
|  | CENPL\_Noc300\_122214\_02.12706.12706.3 | 6.2605 | 0.4156 | 100.0% | 2369.0044 | 2368.6523 | 1 | 6.953 | 45.0% | 2 | K.NQILNLTTDNANILLQIDNAR.L | 3 |
|  | CENPL\_Noc300\_122214\_01.07888.07888.2 | 2.5135 | 0.3649 | 100.0% | 808.2522 | 807.8815 | 1 | 6.681 | 83.3% | 6 | R.LAADDFR.L | 2222222 |
|  | CENPL\_Noc300\_122214\_01.07854.07854.3 | 3.3999 | 0.2112 | 99.9% | 1235.2144 | 1235.4258 | 6 | 5.195 | 52.8% | 3 | R.LKYENEVALR.Q | 3 |
|  | CENPL\_Noc300\_tube2\_122214\_01.10281.10281.2 | 3.4098 | 0.3893 | 100.0% | 1235.3522 | 1235.4258 | 3 | 7.538 | 83.3% | 5 | R.LKYENEVALR.Q | 2 |
|  | CENPL\_Noc300\_122214\_01.07372.07372.2 | 2.7511 | 0.3595 | 100.0% | 993.7922 | 994.0923 | 2 | 6.748 | 85.7% | 2 | K.YENEVALR.Q | 2 |
|  | CENPL\_Noc300\_tube2\_122214\_01.13263.13263.2 | 3.0428 | 0.3738 | 100.0% | 1202.1122 | 1202.3097 | 1 | 6.656 | 80.0% | 3 | R.QSVEADINGLR.R | 22 |
|  | CENPL\_Noc300\_122214\_01.09923.09923.2 | 2.9097 | 0.3044 | 99.9% | 1189.3522 | 1188.4099 | 1 | 5.321 | 77.8% | 3 | R.RVLDELTLTK.A | 2 |
|  | CENPL\_Noc300\_tube2\_122214\_01.14638.14638.2 | 3.4285 | 0.4387 | 100.0% | 1031.7322 | 1032.2224 | 1 | 7.481 | 87.5% | 10 | R.VLDELTLTK.A | 2 |
|  | CENPL\_Noc300\_122214\_02.14087.14087.3 | 2.6373 | 0.3223 | 99.7% | 2225.2144 | 2225.5593 | 25 | 4.809 | 26.4% | 1 | K.ADLEMQIESLTEELAYLKK.N | 3 |
|  | CENPL\_Noc300\_122214\_02.13779.13779.3 | 3.3679 | 0.2599 | 99.8% | 2873.9644 | 2874.2134 | 1 | 6.295 | 25.0% | 1 | R.NVSTGDVNVEMNAAPGVDLTQLLNNMR.S | 3 |
|  | CENPL\_Noc300\_tube2\_122214\_01.09321.09321.2 | 4.2067 | 0.3257 | 100.0% | 1366.2122 | 1366.43 | 25 | 6.779 | 60.0% | 5 | R.SQYEQLAEQNR.K | 2 |
|  | CENPL\_Noc300\_122214\_01.06345.06345.2 | 3.273 | 0.2941 | 100.0% | 1494.1122 | 1494.6041 | 2 | 6.211 | 68.2% | 3 | R.SQYEQLAEQNRK.D | 2 |
|  | CENPL\_Noc300\_122214\_01.11424.11424.1 | 2.1279 | 0.2451 | 96.0% | 1109.58 | 1110.1681 | 1 | 5.845 | 68.8% | 2 | K.DAEAWFNEK.S | 1 |
|  | CENPL\_Noc300\_122214\_01.11482.11482.2 | 2.9864 | 0.2709 | 99.9% | 1110.0521 | 1110.1681 | 2 | 7.411 | 75.0% | 6 | K.DAEAWFNEK.S | 2 |
|  | CENPL\_Noc300\_tube2\_122214\_01.17728.17728.2 | 6.2398 | 0.5865 | 100.0% | 2212.612 | 2213.4033 | 1 | 11.141 | 77.8% | 1 | K.SKELTTEIDNNIEQISSYK.S | 2 |
|  | CENPL\_Noc300\_122214\_01.12667.12667.3 | 5.3487 | 0.4212 | 100.0% | 2212.6143 | 2213.4033 | 1 | 7.068 | 45.8% | 3 | K.SKELTTEIDNNIEQISSYK.S | 3 |
|  | CENPL\_Noc300\_122214\_02.10962.10962.2 | 5.2103 | 0.5656 | 100.0% | 1997.4122 | 1998.151 | 1 | 9.873 | 68.8% | 3 | K.ELTTEIDNNIEQISSYK.S | 2 |
|  | CENPL\_Noc300\_122214\_01.15461.15461.2 | 5.0828 | 0.1458 | 100.0% | 1798.7522 | 1798.0898 | 1 | 6.756 | 63.3% | 1 | R.NVQALEIELQSQLALK.Q | 2 |
|  | CENPL\_Noc300\_122214\_01.10185.10185.2 | 4.1246 | 0.5439 | 100.0% | 1391.3322 | 1391.4778 | 1 | 9.059 | 79.2% | 7 | K.QSLEASLAETEGR.Y | 2 |
|  | CENPL\_Noc300\_122214\_01.18713.18713.2 | 6.1569 | 0.388 | 100.0% | 2748.7922 | 2748.0747 | 1 | 7.468 | 56.8% | 2 | R.YCVQLSQIQAQISALEEQLQQIR.A | 2 |
|  | CENPL\_Noc300\_tube2\_122214\_01.12105.12105.2 | 3.9912 | 0.3192 | 100.0% | 1435.5122 | 1435.623 | 1 | 6.799 | 85.0% | 4 | K.IRLENEIQTYR.S | 2 |
|  | CENPL\_Noc300\_122214\_02.08298.08298.3 | 3.0723 | 0.2244 | 99.8% | 1436.0343 | 1435.623 | 4 | 4.725 | 50.0% | 1 | K.IRLENEIQTYR.S | 3 |
|  | CENPL\_Noc300\_tube2\_122214\_01.10082.10082.2 | 3.7026 | 0.0962 | 99.9% | 1167.1322 | 1166.2761 | 5 | 5.676 | 81.2% | 5 | R.LENEIQTYR.S | 2 |
|  | CENPL\_Noc300\_122214\_01.07051.07051.2 | 3.3753 | 0.3898 | 100.0% | 1263.0721 | 1263.3066 | 1 | 7.968 | 76.9% | 3 | R.SLLEGEGSSGGGGR.G | 2 |

Similarities:
gi|40354195|ref|NP\_95(1:33)  
contaminant\_KERATIN02(1:33)  
gi|15431310|ref|NP\_00(5:29)  
gi|4557701|ref|NP\_000(3:31)  
gi|24430192|ref|NP\_00(5:29)  
gi|131412225|ref|NP\_7(2:32)  
gi|24234699|ref|NP\_00(3:31)  

---

|  |  |  |  |  |  |  |  |  |
| --- | --- | --- | --- | --- | --- | --- | --- | --- |
| U | *gi|57242777|ref|NP\_03* | 3 | 4 | 46.6% | 103 | 11967 | 5.9 | c-myc binding protein [Homo sapiens] |

| Filename XCorr DeltCN Conf% ObsM+H+ CalcM+H+ SpR ZScore Ion% # Sequence  | | | | | | | | | | | | |
| --- | --- | --- | --- | --- | --- | --- | --- | --- | --- | --- | --- | --- |
| \* | CENPL\_Noc300\_tube2\_122214\_01.19612.19612.3 | 4.9273 | 0.4732 | 100.0% | 2277.1443 | 2276.6348 | 1 | 7.234 | 39.5% | 1 | K.VLVALYEEPEKPNSALDFLK.H | 3 |
| \* | CENPL\_Noc300\_tube2\_122214\_01.13348.13348.3 | 4.0061 | 0.1665 | 99.8% | 1897.4944 | 1898.1289 | 1 | 5.362 | 46.9% | 2 | K.HHLGAATPENPEIELLR.L | 3 |
| \* | CENPL\_Noc300\_122214\_01.06865.06865.2 | 2.6578 | 0.211 | 99.2% | 1331.6522 | 1332.4528 | 15 | 5.065 | 55.0% | 1 | K.LAQYEPPQEEK.R | 2 |

---

|  |  |  |  |  |  |  |  |  |
| --- | --- | --- | --- | --- | --- | --- | --- | --- |
| U | *gi|224028244|ref|NP\_0* | 35 | 141 | 46.3% | 471 | 54232 | 8.9 | non-POU domain containing, octamer-binding isoform 1 [Homo sapiens] |
| U | *gi|34932414|ref|NP\_03* | 35 | 141 | 46.3% | 471 | 54232 | 8.9 | non-POU domain containing, octamer-binding isoform 1 [Homo sapiens] |
| U | *gi|224028246|ref|NP\_0* | 35 | 141 | 46.3% | 471 | 54232 | 8.9 | non-POU domain containing, octamer-binding isoform 1 [Homo sapiens] |

| Filename XCorr DeltCN Conf% ObsM+H+ CalcM+H+ SpR ZScore Ion% # Sequence  | | | | | | | | | | | | |
| --- | --- | --- | --- | --- | --- | --- | --- | --- | --- | --- | --- | --- |
|  | CENPL\_Noc300\_122214\_01.13567.13567.2 | 4.0677 | 0.4841 | 100.0% | 1860.6522 | 1861.12 | 1 | 8.338 | 73.3% | 12 | R.LFVGNLPPDITEEEMR.K | 2 |
|  | CENPL\_Noc300\_tube2\_122214\_01.16916.16916.3 | 3.3548 | 0.2281 | 99.8% | 1989.1743 | 1989.2941 | 146 | 5.077 | 28.1% | 1 | R.LFVGNLPPDITEEEMRK.L | 3 |
|  | CENPL\_Noc300\_122214\_01.14604.14604.2 | 3.3043 | 0.4085 | 100.0% | 1814.0521 | 1814.1504 | 1 | 7.966 | 53.3% | 4 | R.TLAEIAKVELDNMPLR.G | 2 |
|  | CENPL\_Noc300\_tube2\_122214\_01.19828.19828.3 | 5.1248 | 0.472 | 100.0% | 1815.5044 | 1814.1504 | 1 | 7.83 | 51.7% | 2 | R.TLAEIAKVELDNMPLR.G | 3 |
|  | CENPL\_Noc300\_tube2\_122214\_01.18894.18894.2 | 3.0027 | 0.4172 | 100.0% | 1998.5721 | 1999.3765 | 1 | 6.967 | 41.2% | 1 | R.TLAEIAKVELDNMPLRGK.Q | 2 |
|  | CENPL\_Noc300\_tube2\_122214\_01.18875.18875.3 | 5.1157 | 0.4192 | 100.0% | 2000.6943 | 1999.3765 | 1 | 7.424 | 45.6% | 2 | R.TLAEIAKVELDNMPLRGK.Q | 3 |
|  | CENPL\_Noc300\_tube2\_122214\_01.14190.14190.1 | 2.1677 | 0.2724 | 95.0% | 1086.42 | 1087.2793 | 1 | 5.161 | 75.0% | 1 | K.VELDNMPLR.G | 1 |
|  | CENPL\_Noc300\_tube2\_122214\_01.14228.14228.2 | 3.4001 | 0.2645 | 100.0% | 1087.1721 | 1087.2793 | 14 | 5.048 | 75.0% | 6 | K.VELDNMPLR.G | 2 |
|  | CENPL\_Noc300\_tube2\_122214\_01.11664.11664.2 | 3.3969 | 0.2939 | 100.0% | 1272.4122 | 1272.5052 | 1 | 5.586 | 90.0% | 3 | K.VELDNMPLRGK.Q | 2 |
|  | CENPL\_Noc300\_122214\_01.06931.06931.2 | 2.7109 | 0.3395 | 99.8% | 1249.5922 | 1249.3782 | 1 | 6.207 | 80.0% | 1 | R.FACHSASLTVR.N | 2 |
|  | CENPL\_Noc300\_122214\_01.06934.06934.3 | 3.7654 | 0.4298 | 100.0% | 1250.0044 | 1249.3782 | 1 | 6.585 | 57.5% | 2 | R.FACHSASLTVR.N | 3 |
|  | CENPL\_Noc300\_122214\_02.16724.16724.3 | 3.8388 | 0.2958 | 99.9% | 2668.7043 | 2669.9507 | 1 | 7.346 | 34.1% | 1 | R.NLPQYVSNELLEEAFSVFGQVER.A | 3 |
|  | CENPL\_Noc300\_122214\_01.20045.20045.2 | 5.5482 | 0.518 | 100.0% | 2669.8123 | 2669.9507 | 1 | 10.476 | 50.0% | 8 | R.NLPQYVSNELLEEAFSVFGQVER.A | 2 |
|  | CENPL\_Noc300\_122214\_01.07408.07408.1 | 2.1379 | 0.2466 | 95.5% | 886.56 | 887.0238 | 27 | 6.392 | 64.3% | 1 | R.AVVIVDDR.G | 11 |
|  | CENPL\_Noc300\_122214\_01.07359.07359.2 | 2.5797 | 0.3261 | 99.9% | 887.1722 | 887.0238 | 2 | 7.415 | 92.9% | 3 | R.AVVIVDDR.G | 22 |
|  | CENPL\_Noc300\_tube2\_122214\_01.09944.09944.3 | 2.2607 | 0.2855 | 97.3% | 1233.2943 | 1232.4252 | 3 | 6.024 | 43.2% | 3 | K.GIVEFSGKPAAR.K | 3 |
|  | CENPL\_Noc300\_tube2\_122214\_01.09958.09958.2 | 3.3211 | 0.2851 | 100.0% | 1234.3322 | 1232.4252 | 1 | 5.892 | 77.3% | 5 | K.GIVEFSGKPAAR.K | 2 |
|  | CENPL\_Noc300\_tube2\_122214\_01.16344.16344.3 | 3.3352 | 0.3054 | 99.9% | 2304.2344 | 2304.5415 | 1 | 5.27 | 36.1% | 1 | R.EQPPRFAQPGSFEYEYAMR.W | 3 |
|  | CENPL\_Noc300\_122214\_02.10160.10160.2 | 4.2742 | 0.5185 | 100.0% | 1696.0922 | 1696.8744 | 1 | 10.081 | 73.1% | 10 | R.FAQPGSFEYEYAMR.W | 2 |
|  | CENPL\_Noc300\_tube2\_122214\_01.11056.11056.2 | 2.4462 | 0.1539 | 99.1% | 833.9922 | 834.0185 | 4 | 5.714 | 83.3% | 5 | K.ALIEMEK.Q | 2 |
|  | CENPL\_Noc300\_tube2\_122214\_01.10490.10490.3 | 2.4327 | 0.2889 | 98.4% | 1833.0844 | 1832.0386 | 29 | 5.144 | 37.5% | 1 | K.ALIEMEKQQQDQVDR.N | 3 |
|  | CENPL\_Noc300\_tube2\_122214\_01.10930.10930.3 | 3.0199 | 0.1541 | 97.2% | 1337.7244 | 1337.5488 | 2 | 5.107 | 57.5% | 4 | R.EKLEMEMEAAR.H | 3 |
|  | CENPL\_Noc300\_tube2\_122214\_01.11140.11140.2 | 3.4649 | 0.424 | 100.0% | 1338.0721 | 1337.5488 | 2 | 6.914 | 70.0% | 12 | R.EKLEMEMEAAR.H | 2 |
|  | CENPL\_Noc300\_tube2\_122214\_01.12724.12724.3 | 3.2038 | 0.1831 | 96.6% | 2498.3643 | 2499.942 | 7 | 3.877 | 28.9% | 2 | R.EKLEMEMEAARHEHQVMLMR.Q | 32 |
|  | CENPL\_Noc300\_122214\_01.08936.08936.2 | 2.1223 | 0.1948 | 97.0% | 1079.9722 | 1080.2593 | 1 | 4.97 | 81.2% | 3 | K.LEMEMEAAR.H | 2 |
|  | CENPL\_Noc300\_tube2\_122214\_01.07917.07917.3 | 3.4818 | 0.2236 | 99.9% | 1541.6344 | 1541.7222 | 2 | 4.773 | 56.8% | 1 | R.RMEELHNQEVQK.R | 3 |
|  | CENPL\_Noc300\_tube2\_122214\_01.09470.09470.3 | 3.0042 | 0.3331 | 99.9% | 1637.9644 | 1637.7953 | 1 | 5.651 | 44.2% | 4 | R.RQQEGFKGTFPDAR.E | 3 |
|  | CENPL\_Noc300\_122214\_01.08132.08132.2 | 2.6997 | 0.4724 | 100.0% | 1481.3522 | 1481.6078 | 80 | 7.64 | 50.0% | 4 | R.QQEGFKGTFPDAR.E | 2 |
|  | CENPL\_Noc300\_tube2\_122214\_01.13280.13280.2 | 4.6948 | 0.5304 | 100.0% | 1539.3121 | 1539.8441 | 1 | 8.449 | 64.3% | 18 | R.MGQMAMGGAMGINNR.G | 2 |
|  | CENPL\_Noc300\_tube2\_122214\_01.18464.18464.2 | 5.9787 | 0.6152 | 100.0% | 2163.632 | 2164.4436 | 1 | 11.167 | 57.1% | 9 | R.FGQAATMEGIGAIGGTPPAFNR.A | 2 |
|  | CENPL\_Noc300\_122214\_02.10773.10773.3 | 4.553 | 0.5106 | 100.0% | 2165.3342 | 2164.4436 | 1 | 7.81 | 39.3% | 2 | R.FGQAATMEGIGAIGGTPPAFNR.A | 3 |
|  | CENPL\_Noc300\_122214\_01.13588.13588.2 | 5.2353 | 0.4973 | 100.0% | 2244.6921 | 2244.4436 | 1 | 9.851 | 59.5% | 4 | R.FGQAATMEGIGAIGGT#PPAFNR.A | 2 |
|  | CENPL\_Noc300\_122214\_02.10916.10916.3 | 4.1003 | 0.2053 | 99.8% | 2245.4944 | 2244.4436 | 1 | 5.209 | 44.0% | 2 | R.FGQAATMEGIGAIGGT#PPAFNR.A | 3 |
|  | CENPL\_Noc300\_122214\_01.07096.07096.2 | 2.0619 | 0.4573 | 99.5% | 1072.2122 | 1073.1936 | 24 | 6.812 | 60.0% | 1 | R.AAPGAEFAPNK.R | 2 |
|  | CENPL\_Noc300\_122214\_02.06297.06297.2 | 1.9326 | 0.3598 | 98.2% | 1229.4922 | 1229.3811 | 2 | 6.263 | 63.6% | 2 | R.AAPGAEFAPNKR.R | 2 |

Similarities:
gi|4826998|ref|NP\_005(2:33)  

---

|  |  |  |  |  |  |  |  |  |
| --- | --- | --- | --- | --- | --- | --- | --- | --- |
| U | *gi|16507237|ref|NP\_00* | 33 | 92 | 46.2% | 654 | 72333 | 5.2 | heat shock 70kDa protein 5 [Homo sapiens] |

| Filename XCorr DeltCN Conf% ObsM+H+ CalcM+H+ SpR ZScore Ion% # Sequence  | | | | | | | | | | | | |
| --- | --- | --- | --- | --- | --- | --- | --- | --- | --- | --- | --- | --- |
|  | CENPL\_Noc300\_tube2\_122214\_01.15634.15634.2 | 3.598 | 0.4607 | 100.0% | 1567.2922 | 1567.7386 | 1 | 7.449 | 61.5% | 6 | R.ITPSYVAFTPEGER.L | 2 |
|  | CENPL\_Noc300\_122214\_01.09970.09970.2 | 4.8539 | 0.493 | 100.0% | 1678.1122 | 1678.796 | 1 | 9.43 | 75.0% | 5 | K.NQLTSNPENTVFDAK.R | 2 |
|  | CENPL\_Noc300\_tube2\_122214\_01.12056.12056.2 | 3.9571 | 0.3599 | 100.0% | 1431.1322 | 1431.5449 | 1 | 7.265 | 81.8% | 6 | R.TWNDPSVQQDIK.F | 2 |
|  | CENPL\_Noc300\_tube2\_122214\_01.11968.11968.2 | 4.1956 | 0.4778 | 100.0% | 1605.4122 | 1605.8314 | 1 | 7.409 | 85.7% | 3 | K.TKPYIQVDIGGGQTK.T | 2 |
|  | CENPL\_Noc300\_tube2\_122214\_01.11954.11954.3 | 4.059 | 0.221 | 99.9% | 1607.3944 | 1605.8314 | 1 | 5.818 | 44.6% | 4 | K.TKPYIQVDIGGGQTK.T | 3 |
|  | CENPL\_Noc300\_122214\_01.14820.14820.2 | 4.2691 | 0.4833 | 100.0% | 1537.2722 | 1537.8114 | 1 | 8.692 | 69.2% | 2 | K.TFAPEEISAMVLTK.M | 2 |
|  | CENPL\_Noc300\_tube2\_122214\_01.09658.09658.2 | 3.4122 | 0.3629 | 100.0% | 1241.2522 | 1241.445 | 1 | 7.029 | 85.0% | 1 | K.MKETAEAYLGK.K | 2 |
|  | CENPL\_Noc300\_122214\_01.06551.06551.2 | 3.3236 | 0.3196 | 100.0% | 1368.7322 | 1369.619 | 2 | 6.053 | 72.7% | 1 | K.MKETAEAYLGKK.V | 2 |
|  | CENPL\_Noc300\_122214\_01.06533.06533.3 | 3.9451 | 0.4204 | 100.0% | 1369.8844 | 1369.619 | 1 | 7.147 | 45.5% | 2 | K.MKETAEAYLGKK.V | 3 |
|  | CENPL\_Noc300\_tube2\_122214\_01.14668.14668.2 | 3.8076 | 0.5395 | 100.0% | 1888.3522 | 1889.121 | 20 | 9.327 | 37.5% | 2 | K.VTHAVVTVPAYFNDAQR.Q | 23 |
|  | CENPL\_Noc300\_tube2\_122214\_01.14650.14650.3 | 5.3442 | 0.3179 | 100.0% | 1890.5944 | 1889.121 | 4 | 6.942 | 40.6% | 4 | K.VTHAVVTVPAYFNDAQR.Q | 3 |
|  | CENPL\_Noc300\_122214\_01.11722.11722.2 | 3.714 | 0.4369 | 100.0% | 1218.3922 | 1218.4137 | 1 | 8.111 | 90.9% | 2 | K.DAGTIAGLNVMR.I | 2 |
|  | CENPL\_Noc300\_tube2\_122214\_01.18059.18059.3 | 3.4522 | 0.1839 | 99.4% | 1660.7943 | 1660.9078 | 1 | 4.919 | 50.0% | 1 | R.IINEPTAAAIAYGLDK.R | 333 |
|  | CENPL\_Noc300\_tube2\_122214\_01.18074.18074.2 | 5.3155 | 0.5593 | 100.0% | 1660.8322 | 1660.9078 | 1 | 10.712 | 86.7% | 4 | R.IINEPTAAAIAYGLDK.R | 222 |
|  | CENPL\_Noc300\_tube2\_122214\_01.16577.16577.2 | 4.2238 | 0.4865 | 100.0% | 1815.9321 | 1817.0953 | 1 | 7.519 | 71.9% | 2 | R.IINEPTAAAIAYGLDKR.E | 2 |
|  | CENPL\_Noc300\_122214\_01.07571.07571.2 | 2.0251 | 0.168 | 96.8% | 904.1322 | 904.115 | 3 | 4.659 | 83.3% | 1 | R.VMEHFIK.L | 2 |
|  | CENPL\_Noc300\_122214\_02.11922.11922.2 | 5.5445 | 0.5986 | 100.0% | 2165.6921 | 2166.3025 | 1 | 10.854 | 61.8% | 1 | R.IEIESFYEGEDFSETLTR.A | 2 |
|  | CENPL\_Noc300\_122214\_01.13649.13649.2 | 3.9948 | 0.4241 | 100.0% | 1513.4722 | 1513.7516 | 1 | 7.589 | 72.7% | 2 | R.AKFEELNMDLFR.S | 2 |
|  | CENPL\_Noc300\_122214\_01.13659.13659.3 | 4.1563 | 0.3677 | 100.0% | 1514.2743 | 1513.7516 | 1 | 6.722 | 56.8% | 2 | R.AKFEELNMDLFR.S | 3 |
|  | CENPL\_Noc300\_122214\_02.09950.09950.3 | 4.4828 | 0.3937 | 100.0% | 2489.7244 | 2489.7827 | 1 | 7.021 | 35.2% | 3 | K.VLEDSDLKKSDIDEIVLVGGSTR.I | 3 |
|  | CENPL\_Noc300\_tube2\_122214\_01.15050.15050.2 | 4.8318 | 0.2829 | 100.0% | 1589.7722 | 1589.7863 | 1 | 8.35 | 82.1% | 4 | K.KSDIDEIVLVGGSTR.I | 2 |
|  | CENPL\_Noc300\_122214\_02.10296.10296.2 | 3.2546 | 0.0774 | 98.5% | 1461.3722 | 1461.6122 | 1 | 6.877 | 65.4% | 1 | K.SDIDEIVLVGGSTR.I | 2 |
|  | CENPL\_Noc300\_tube2\_122214\_01.13834.13834.2 | 4.9976 | 0.5554 | 100.0% | 1837.4722 | 1838.0245 | 1 | 10.066 | 65.6% | 5 | K.SQIFSTASDNQPTVTIK.V | 2 |
|  | CENPL\_Noc300\_122214\_01.05699.05699.2 | 2.3072 | 0.3471 | 99.6% | 1191.4321 | 1192.3574 | 2 | 6.264 | 61.1% | 2 | K.VYEGERPLTK.D | 2 |
|  | CENPL\_Noc300\_122214\_01.14045.14045.2 | 2.6084 | 0.2094 | 98.3% | 1935.8522 | 1935.19 | 7 | 4.792 | 38.2% | 1 | K.DNHLLGTFDLTGIPPAPR.G | 2 |
|  | CENPL\_Noc300\_tube2\_122214\_01.16151.16151.2 | 3.3102 | 0.2369 | 99.9% | 1319.1322 | 1317.4381 | 1 | 5.301 | 85.0% | 2 | R.NELESYAYSLK.N | 2 |
|  | CENPL\_Noc300\_tube2\_122214\_01.16948.16948.3 | 4.4178 | 0.3189 | 100.0% | 2530.9143 | 2532.723 | 1 | 6.435 | 33.8% | 1 | K.AVEEKIEWLESHQDADIEDFK.A | 3 |
|  | CENPL\_Noc300\_122214\_02.10299.10299.3 | 3.9444 | 0.343 | 100.0% | 1975.8243 | 1976.1064 | 1 | 6.362 | 46.7% | 3 | K.IEWLESHQDADIEDFK.A | 3 |
|  | CENPL\_Noc300\_tube2\_122214\_01.15426.15426.3 | 4.8854 | 0.2958 | 100.0% | 2176.2244 | 2175.3594 | 1 | 6.53 | 44.1% | 4 | K.IEWLESHQDADIEDFKAK.K | 3 |
|  | CENPL\_Noc300\_tube2\_122214\_01.13302.13302.2 | 5.0152 | 0.4249 | 100.0% | 1654.5521 | 1654.9878 | 1 | 7.68 | 88.5% | 3 | K.KKELEEIVQPIISK.L | 2 |
|  | CENPL\_Noc300\_tube2\_122214\_01.13347.13347.3 | 3.8681 | 0.0966 | 98.3% | 1656.4744 | 1654.9878 | 12 | 4.342 | 44.2% | 2 | K.KKELEEIVQPIISK.L | 3 |
|  | CENPL\_Noc300\_tube2\_122214\_01.17261.17261.2 | 3.8394 | 0.4449 | 100.0% | 1398.3121 | 1398.6396 | 1 | 6.873 | 81.8% | 3 | K.ELEEIVQPIISK.L | 2 |
|  | CENPL\_Noc300\_122214\_01.09655.09655.2 | 5.6317 | 0.6169 | 100.0% | 2176.4521 | 2177.283 | 1 | 10.334 | 60.0% | 7 | K.LYGSAGPPPTGEEDTAEKDEL.- | 2 |

Similarities:
gi|5729877|ref|NP\_006(2:31)  
gi|124256496|ref|NP\_0(2:31)  

---

|  |  |  |  |  |  |  |  |  |
| --- | --- | --- | --- | --- | --- | --- | --- | --- |
| U | *gi|189217919|ref|NP\_6* | 41 | 121 | 45.5% | 770 | 86850 | 6.7 | protein associated with topoisomerase II homolog 1 [Homo sapiens] |

| Filename XCorr DeltCN Conf% ObsM+H+ CalcM+H+ SpR ZScore Ion% # Sequence  | | | | | | | | | | | | |
| --- | --- | --- | --- | --- | --- | --- | --- | --- | --- | --- | --- | --- |
| \* | CENPL\_Noc300\_tube2\_122214\_01.09558.09558.2 | 3.9103 | 0.5658 | 100.0% | 1484.3121 | 1484.609 | 1 | 10.056 | 80.8% | 1 | K.LPVAVNEQTGNGER.D | 2 |
| \* | CENPL\_Noc300\_tube2\_122214\_01.15652.15652.3 | 6.5504 | 0.4877 | 100.0% | 3351.8943 | 3352.5708 | 1 | 6.964 | 28.4% | 3 | K.LPVAVNEQTGNGERDEMDLLGDHEENLAER.L | 3 |
| \* | CENPL\_Noc300\_tube2\_122214\_01.15666.15666.2 | 4.0953 | 0.3574 | 100.0% | 1885.6522 | 1886.985 | 1 | 6.75 | 73.3% | 1 | R.DEMDLLGDHEENLAER.L | 2 |
| \* | CENPL\_Noc300\_tube2\_122214\_01.18352.18352.2 | 4.2721 | 0.2765 | 100.0% | 1662.3522 | 1660.941 | 1 | 5.992 | 69.2% | 8 | K.MVIENELEDPAIMR.A | 2 |
| \* | CENPL\_Noc300\_tube2\_122214\_01.18494.18494.3 | 7.3975 | 0.5409 | 100.0% | 2935.6743 | 2936.2974 | 1 | 10.204 | 38.5% | 6 | R.AVQTRPVLQPQPGSLNSSIWDGSEVLR.R | 3 |
| \* | CENPL\_Noc300\_tube2\_122214\_01.18468.18468.2 | 5.0849 | 0.5134 | 100.0% | 2935.9922 | 2936.2974 | 1 | 7.897 | 44.2% | 3 | R.AVQTRPVLQPQPGSLNSSIWDGSEVLR.R | 2 |
| \* | CENPL\_Noc300\_122214\_01.12233.12233.3 | 4.2265 | 0.39 | 100.0% | 3091.3442 | 3092.4849 | 1 | 5.477 | 26.9% | 2 | R.AVQTRPVLQPQPGSLNSSIWDGSEVLRR.I | 3 |
| \* | CENPL\_Noc300\_tube2\_122214\_01.12545.12545.2 | 2.3072 | 0.2452 | 98.0% | 1607.5922 | 1607.5913 | 1 | 4.63 | 66.7% | 1 | R.RS\*TS\*PIIGS\*PPVR.A | 2 |
| \* | CENPL\_Noc300\_tube2\_122214\_01.12837.12837.2 | 3.7878 | 0.3508 | 100.0% | 1309.2922 | 1309.5112 | 1 | 7.647 | 79.2% | 6 | R.AQLLGGAQLQPGR.M | 2 |
| \* | CENPL\_Noc300\_122214\_01.13159.13159.2 | 3.9034 | 0.4693 | 100.0% | 1485.2722 | 1485.7845 | 1 | 8.605 | 71.4% | 7 | R.VPGFVGSPLAAMNPK.L | 2 |
| \* | CENPL\_Noc300\_tube2\_122214\_01.15292.15292.2 | 2.4759 | 0.1546 | 97.4% | 1271.1522 | 1270.5353 | 28 | 4.825 | 59.1% | 4 | R.VGQMLPPAPGFR.A | 2 |
| \* | CENPL\_Noc300\_tube2\_122214\_01.09191.09191.3 | 4.077 | 0.3771 | 100.0% | 2156.8145 | 2157.4172 | 1 | 6.988 | 41.2% | 2 | R.SQAPMFRPDTTHLHPQHR.R | 3 |
| \* | CENPL\_Noc300\_122214\_01.10026.10026.3 | 3.4041 | 0.3087 | 100.0% | 1349.1543 | 1349.5908 | 26 | 5.425 | 47.5% | 2 | R.KDPYANLMLQR.E | 3 |
| \* | CENPL\_Noc300\_122214\_01.10005.10005.2 | 3.4357 | 0.2778 | 100.0% | 1350.1522 | 1349.5908 | 1 | 6.527 | 75.0% | 5 | R.KDPYANLMLQR.E | 2 |
| \* | CENPL\_Noc300\_tube2\_122214\_01.16353.16353.2 | 3.5674 | 0.3509 | 100.0% | 1221.2122 | 1221.4167 | 1 | 6.749 | 83.3% | 4 | K.DPYANLMLQR.E | 2 |
| \* | CENPL\_Noc300\_tube2\_122214\_01.21052.21052.2 | 4.2395 | 0.5141 | 100.0% | 3081.132 | 3082.4258 | 1 | 9.834 | 47.8% | 1 | K.IQMMQLQSTDPYLDDFYYQNYFEK.L | 2 |
| \* | CENPL\_Noc300\_tube2\_122214\_01.20746.20746.3 | 4.9614 | 0.473 | 100.0% | 3451.7043 | 3452.8748 | 1 | 7.08 | 33.7% | 2 | K.IQMMQLQSTDPYLDDFYYQNYFEKLEK.L | 3 |
| \* | CENPL\_Noc300\_tube2\_122214\_01.10239.10239.2 | 4.0546 | 0.3972 | 100.0% | 1315.4922 | 1315.4227 | 1 | 7.332 | 79.2% | 5 | K.LSAAEEIQGDGPK.K | 2 |
| \* | CENPL\_Noc300\_122214\_01.06829.06829.2 | 3.626 | 0.3621 | 100.0% | 1443.5521 | 1443.5968 | 1 | 6.428 | 69.2% | 2 | K.LSAAEEIQGDGPKK.E | 2 |
| \* | CENPL\_Noc300\_122214\_01.06433.06433.2 | 3.6883 | 0.2811 | 100.0% | 1728.4122 | 1728.8998 | 1 | 5.216 | 63.3% | 1 | K.LSAAEEIQGDGPKKER.T | 2 |
| \* | CENPL\_Noc300\_122214\_01.06465.06465.3 | 2.961 | 0.2583 | 99.6% | 1729.1943 | 1728.8998 | 2 | 4.966 | 36.7% | 1 | K.LSAAEEIQGDGPKKER.T | 3 |
| \* | CENPL\_Noc300\_122214\_01.06875.06875.2 | 2.4785 | 0.1418 | 97.7% | 1100.2522 | 1099.3591 | 320 | 4.517 | 44.4% | 1 | R.TKLITPQVAK.L | 2 |
| \* | CENPL\_Noc300\_tube2\_122214\_01.11258.11258.2 | 4.2839 | 0.5942 | 100.0% | 1803.4321 | 1804.0557 | 1 | 9.507 | 76.7% | 3 | K.LEHAYKPVQFEGSLGK.L | 2 |
| \* | CENPL\_Noc300\_122214\_01.08536.08536.3 | 3.581 | 0.4326 | 100.0% | 1804.6743 | 1804.0557 | 1 | 7.513 | 38.3% | 6 | K.LEHAYKPVQFEGSLGK.L | 3 |
| \* | CENPL\_Noc300\_122214\_01.06627.06627.2 | 3.2522 | 0.4927 | 100.0% | 1087.2322 | 1087.2211 | 1 | 8.696 | 94.4% | 3 | K.LTVSSVNNPR.K | 2 |
| \* | CENPL\_Noc300\_122214\_01.05794.05794.2 | 2.5008 | 0.2498 | 99.2% | 1215.4122 | 1215.3951 | 1 | 5.546 | 65.0% | 1 | K.LTVSSVNNPRK.M | 2 |
| \* | CENPL\_Noc300\_122214\_01.08038.08038.2 | 3.1181 | 0.4822 | 100.0% | 992.1722 | 992.1785 | 1 | 8.251 | 87.5% | 4 | K.MIDAVVTSR.S | 2 |
| \* | CENPL\_Noc300\_122214\_01.09390.09390.2 | 2.2091 | 0.0817 | 96.1% | 816.3722 | 816.02875 | 47 | 4.506 | 83.3% | 2 | K.TLVIIEK.T | 2 |
| \* | CENPL\_Noc300\_122214\_02.12292.12292.2 | 4.1831 | 0.5357 | 100.0% | 1616.4722 | 1616.7649 | 1 | 9.46 | 70.8% | 4 | K.TYSLLLDVEDYER.R | 2 |
| \* | CENPL\_Noc300\_122214\_01.14054.14054.2 | 3.2929 | 0.199 | 99.5% | 1773.5521 | 1772.9524 | 1 | 4.778 | 57.7% | 1 | K.TYSLLLDVEDYERR.Y | 2 |
| \* | CENPL\_Noc300\_tube2\_122214\_01.17864.17864.3 | 3.508 | 0.3769 | 100.0% | 1950.8644 | 1951.2018 | 1 | 6.017 | 43.3% | 3 | R.YLLSLEEERPALMDDR.K | 3 |
| \* | CENPL\_Noc300\_tube2\_122214\_01.17823.17823.2 | 3.3203 | 0.1256 | 99.1% | 1951.1721 | 1951.2018 | 3 | 5.415 | 43.3% | 3 | R.YLLSLEEERPALMDDR.K | 2 |
| \* | CENPL\_Noc300\_tube2\_122214\_01.15956.15956.3 | 2.9977 | 0.277 | 99.8% | 2078.9644 | 2079.376 | 1 | 5.376 | 39.1% | 1 | R.YLLSLEEERPALMDDRK.H | 3 |
| \* | CENPL\_Noc300\_122214\_01.09366.09366.2 | 2.6908 | 0.1882 | 99.4% | 1172.2522 | 1172.3075 | 12 | 4.952 | 81.2% | 1 | K.ICSMYDNLR.G | 2 |
| \* | CENPL\_Noc300\_122214\_01.17740.17740.2 | 5.768 | 0.6262 | 100.0% | 2092.4521 | 2092.4583 | 1 | 10.999 | 72.2% | 4 | R.ILPFLSTEQAADILMTTAR.N | 2 |
| \* | CENPL\_Noc300\_122214\_02.14660.14660.3 | 4.2002 | 0.4534 | 100.0% | 2092.6743 | 2092.4583 | 1 | 8.473 | 43.1% | 2 | R.ILPFLSTEQAADILMTTAR.N | 3 |
| \* | CENPL\_Noc300\_122214\_01.16548.16548.3 | 5.3931 | 0.2822 | 100.0% | 2849.7544 | 2849.433 | 1 | 5.835 | 35.0% | 2 | R.ELLRIPQAALAKPISIPTNLVSLFSR.Y | 3 |
| \* | CENPL\_Noc300\_tube2\_122214\_01.21128.21128.3 | 6.515 | 0.5079 | 100.0% | 2337.4143 | 2337.8113 | 1 | 8.588 | 44.0% | 5 | R.IPQAALAKPISIPTNLVSLFSR.Y | 3 |
| \* | CENPL\_Noc300\_tube2\_122214\_01.21159.21159.2 | 5.3853 | 0.3913 | 100.0% | 2337.632 | 2337.8113 | 1 | 7.427 | 71.4% | 2 | R.IPQAALAKPISIPTNLVSLFSR.Y | 2 |
| \* | CENPL\_Noc300\_tube2\_122214\_01.13364.13364.2 | 2.6147 | 0.0141 | 97.7% | 831.15216 | 831.0 | 4 | 3.851 | 91.7% | 1 | K.LNLLETK.L | 2 |
| \* | CENPL\_Noc300\_tube2\_122214\_01.13718.13718.2 | 3.1822 | 0.2552 | 100.0% | 927.1322 | 927.13495 | 2 | 5.091 | 85.7% | 5 | K.LQLVQGIR.- | 2 |

---

|  |  |  |  |  |  |  |  |  |
| --- | --- | --- | --- | --- | --- | --- | --- | --- |
| U | *Reverse\_gi|169217412|* | 1 | 1 | 45.0% | 40 | 4656 | 5.9 | PREDICTED: hypothetical protein [Homo sapiens] |
| U | *Reverse\_gi|28212220|r* | 1 | 1 | 10.0% | 180 | 19723 | 7.5 | XG glycoprotein isoform 1 precursor [Homo sapiens] |
| U | *Reverse\_gi|213688348|* | 1 | 1 | 9.9% | 181 | 19810 | 7.5 | XG glycoprotein isoform 3 precursor [Homo sapiens] |
| U | *Reverse\_gi|213688346|* | 1 | 1 | 9.2% | 195 | 21439 | 8.5 | XG glycoprotein isoform 2 precursor [Homo sapiens] |

| Filename XCorr DeltCN Conf% ObsM+H+ CalcM+H+ SpR ZScore Ion% # Sequence  | | | | | | | | | | | | |
| --- | --- | --- | --- | --- | --- | --- | --- | --- | --- | --- | --- | --- |
|  | CENPL\_Noc300\_122214\_01.18809.18809.2 | 1.984 | 0.2989 | 96.6% | 2031.1721 | 2032.1307 | 33 | 4.701 | 29.4% | 1 | K.TPEPDDLADALDFDRQGR.A | 2 |

---

|  |  |  |  |  |  |  |  |  |
| --- | --- | --- | --- | --- | --- | --- | --- | --- |
| U | *gi|62414289|ref|NP\_00* | 22 | 39 | 44.2% | 466 | 53652 | 5.1 | vimentin [Homo sapiens] |

| Filename XCorr DeltCN Conf% ObsM+H+ CalcM+H+ SpR ZScore Ion% # Sequence  | | | | | | | | | | | | |
| --- | --- | --- | --- | --- | --- | --- | --- | --- | --- | --- | --- | --- |
| \* | CENPL\_Noc300\_122214\_01.08980.08980.2 | 2.1553 | 0.2949 | 97.9% | 1496.3922 | 1496.6633 | 3 | 4.77 | 53.8% | 1 | R.TYSLGSALRPSTSR.S | 2 |
| \* | CENPL\_Noc300\_tube2\_122214\_01.11745.11745.2 | 4.0349 | 0.4756 | 100.0% | 1429.0521 | 1429.5724 | 1 | 7.968 | 76.9% | 2 | R.SLYASSPGGVYATR.S | 2 |
| \* | CENPL\_Noc300\_tube2\_122214\_01.12750.12750.2 | 3.014 | 0.3957 | 100.0% | 1509.2722 | 1509.5724 | 1 | 6.393 | 61.5% | 1 | R.SLYASS\*PGGVYATR.S | 2 |
| \* | CENPL\_Noc300\_122214\_02.13460.13460.2 | 3.5367 | 0.4821 | 100.0% | 2126.9521 | 2127.3557 | 1 | 8.306 | 44.4% | 1 | R.LLQDSVDFSLADAINTEFK.N | 2 |
|  | CENPL\_Noc300\_tube2\_122214\_01.10443.10443.2 | 3.8185 | 0.4388 | 100.0% | 1588.2922 | 1588.7147 | 1 | 7.743 | 79.2% | 2 | R.TNEKVELQELNDR.F | 2 |
|  | CENPL\_Noc300\_tube2\_122214\_01.10409.10409.3 | 3.4168 | 0.2922 | 99.9% | 1589.5443 | 1588.7147 | 1 | 5.842 | 54.2% | 1 | R.TNEKVELQELNDR.F | 3 |
| \* | CENPL\_Noc300\_tube2\_122214\_01.11208.11208.2 | 2.2829 | 0.1388 | 97.0% | 1127.4922 | 1126.3005 | 8 | 4.369 | 68.8% | 1 | R.FANYIDKVR.F | 2 |
| \* | CENPL\_Noc300\_tube2\_122214\_01.17062.17062.2 | 3.5509 | 0.4678 | 100.0% | 1540.0721 | 1540.8436 | 1 | 8.291 | 65.4% | 3 | K.ILLAELEQLKGQGK.S | 2 |
| \* | CENPL\_Noc300\_tube2\_122214\_01.17074.17074.3 | 2.2418 | 0.3009 | 97.3% | 1541.0343 | 1540.8436 | 11 | 4.872 | 28.8% | 1 | K.ILLAELEQLKGQGK.S | 3 |
| \* | CENPL\_Noc300\_tube2\_122214\_01.13841.13841.2 | 3.2075 | 0.4979 | 100.0% | 1255.3922 | 1255.385 | 1 | 7.947 | 77.8% | 3 | R.LGDLYEEEMR.E | 2 |
| \* | CENPL\_Noc300\_122214\_01.11087.11087.2 | 2.8574 | 0.1893 | 98.9% | 1689.4521 | 1689.881 | 1 | 5.464 | 57.7% | 1 | R.VEVERDNLAEDIMR.L | 2 |
| \* | CENPL\_Noc300\_tube2\_122214\_01.10085.10085.2 | 2.4157 | 0.2089 | 99.3% | 1046.9722 | 1047.2146 | 108 | 4.317 | 71.4% | 1 | K.LQEEMLQR.E | 2 |
| \* | CENPL\_Noc300\_tube2\_122214\_01.12520.12520.2 | 2.7687 | 0.4101 | 100.0% | 1324.3322 | 1324.3898 | 4 | 6.392 | 65.0% | 1 | R.EEAENTLQSFR.Q | 2 |
| \* | CENPL\_Noc300\_tube2\_122214\_01.17469.17469.3 | 3.2081 | 0.3664 | 99.9% | 2395.3145 | 2394.5168 | 1 | 5.523 | 28.8% | 1 | R.EEAENTLQSFRQDVDNASLAR.L | 3 |
| \* | CENPL\_Noc300\_122214\_01.13088.13088.2 | 4.2336 | 0.4482 | 100.0% | 1535.4521 | 1534.793 | 1 | 7.557 | 87.5% | 4 | R.KVESLQEEIAFLK.K | 2 |
| \* | CENPL\_Noc300\_122214\_01.11551.11551.3 | 4.5703 | 0.3139 | 100.0% | 1663.0144 | 1662.967 | 3 | 6.425 | 42.3% | 2 | R.KVESLQEEIAFLKK.L | 3 |
|  | CENPL\_Noc300\_tube2\_122214\_01.14841.14841.2 | 3.0619 | 0.3344 | 100.0% | 1309.9521 | 1310.4056 | 1 | 6.338 | 77.8% | 4 | K.NLQEAEEWYK.S | 2 |
| \* | CENPL\_Noc300\_tube2\_122214\_01.10772.10772.2 | 3.618 | 0.4429 | 100.0% | 1094.0322 | 1094.1692 | 1 | 8.583 | 94.4% | 3 | K.FADLSEAANR.N | 2 |
| \* | CENPL\_Noc300\_tube2\_122214\_01.11619.11619.3 | 2.708 | 0.288 | 99.6% | 1779.6543 | 1777.8912 | 1 | 4.838 | 35.0% | 1 | K.FADLSEAANRNNDALR.Q | 3 |
| \* | CENPL\_Noc300\_tube2\_122214\_01.13120.13120.2 | 4.2984 | 0.4054 | 100.0% | 1736.3722 | 1735.9679 | 1 | 7.279 | 73.1% | 2 | R.LQDEIQNMKEEMAR.H | 2 |
| \* | CENPL\_Noc300\_122214\_01.09933.09933.3 | 2.6914 | 0.2793 | 99.6% | 1737.4744 | 1735.9679 | 16 | 4.086 | 36.5% | 1 | R.LQDEIQNMKEEMAR.H | 3 |
| \* | CENPL\_Noc300\_122214\_01.07073.07073.2 | 3.4833 | 0.3371 | 100.0% | 1838.1322 | 1837.854 | 1 | 6.656 | 63.3% | 2 | R.DGQVINETSQHHDDLE.- | 2 |

---

|  |  |  |  |  |  |  |  |  |
| --- | --- | --- | --- | --- | --- | --- | --- | --- |
| U | *gi|5729877|ref|NP\_006* | 34 | 130 | 44.1% | 646 | 70898 | 5.5 | heat shock 70kDa protein 8 isoform 1 [Homo sapiens] |

| Filename XCorr DeltCN Conf% ObsM+H+ CalcM+H+ SpR ZScore Ion% # Sequence  | | | | | | | | | | | | |
| --- | --- | --- | --- | --- | --- | --- | --- | --- | --- | --- | --- | --- |
|  | CENPL\_Noc300\_122214\_01.10323.10323.2 | 3.3577 | 0.4739 | 100.0% | 1488.4722 | 1488.5939 | 1 | 8.67 | 79.2% | 6 | R.TTPSYVAFTDTER.L | 2222 |
|  | CENPL\_Noc300\_tube2\_122214\_01.14043.14043.2 | 5.2323 | 0.5512 | 100.0% | 1651.2522 | 1650.8468 | 1 | 9.746 | 78.6% | 6 | K.NQVAMNPTNTVFDAK.R | 2 |
|  | CENPL\_Noc300\_122214\_01.09321.09321.2 | 4.0485 | 0.4466 | 100.0% | 1806.7922 | 1807.0343 | 1 | 8.045 | 63.3% | 2 | K.NQVAMNPTNTVFDAKR.L | 2 |
|  | CENPL\_Noc300\_tube2\_122214\_01.10646.10646.2 | 3.4753 | 0.4728 | 100.0% | 1411.1721 | 1411.5725 | 1 | 8.398 | 77.3% | 6 | R.RFDDAVVQSDMK.H | 2 |
|  | CENPL\_Noc300\_tube2\_122214\_01.11830.11830.2 | 4.1301 | 0.5392 | 100.0% | 1255.2122 | 1255.385 | 1 | 9.328 | 90.0% | 2 | R.FDDAVVQSDMK.H | 2 |
|  | CENPL\_Noc300\_122214\_01.11239.11239.2 | 2.7935 | 0.4136 | 99.9% | 1654.1721 | 1654.9298 | 1 | 7.059 | 69.2% | 1 | K.HWPFMVVNDAGRPK.V | 2 |
|  | CENPL\_Noc300\_tube2\_122214\_01.15573.15573.3 | 4.4553 | 0.4506 | 100.0% | 1655.2444 | 1654.9298 | 1 | 7.687 | 46.2% | 8 | K.HWPFMVVNDAGRPK.V | 3 |
|  | CENPL\_Noc300\_tube2\_122214\_01.19206.19206.2 | 4.5463 | 0.4948 | 100.0% | 1618.2122 | 1617.8542 | 1 | 8.997 | 84.6% | 4 | K.SFYPEEVSSMVLTK.M | 2 |
|  | CENPL\_Noc300\_tube2\_122214\_01.12086.12086.2 | 3.8824 | 0.3518 | 100.0% | 1253.4922 | 1253.4993 | 1 | 6.85 | 90.0% | 4 | K.MKEIAEAYLGK.T | 2 |
|  | CENPL\_Noc300\_122214\_01.09190.09190.3 | 3.115 | 0.1248 | 96.2% | 1253.9944 | 1253.4993 | 8 | 5.02 | 45.0% | 5 | K.MKEIAEAYLGK.T | 3 |
|  | CENPL\_Noc300\_tube2\_122214\_01.17187.17187.2 | 4.0335 | 0.4025 | 100.0% | 1982.6122 | 1983.1882 | 1 | 6.814 | 64.7% | 6 | K.TVTNAVVTVPAYFNDSQR.Q | 2 |
|  | CENPL\_Noc300\_tube2\_122214\_01.17184.17184.3 | 4.1712 | 0.495 | 100.0% | 1982.6344 | 1983.1882 | 1 | 7.924 | 39.7% | 3 | K.TVTNAVVTVPAYFNDSQR.Q | 3 |
|  | CENPL\_Noc300\_tube2\_122214\_01.18059.18059.3 | 3.4522 | 0.1839 | 99.4% | 1660.7943 | 1660.9078 | 1 | 4.919 | 50.0% | 1 | R.IINEPTAAAIAYGLDK.K | 333 |
|  | CENPL\_Noc300\_tube2\_122214\_01.18074.18074.2 | 5.3155 | 0.5593 | 100.0% | 1660.8322 | 1660.9078 | 1 | 10.712 | 86.7% | 4 | R.IINEPTAAAIAYGLDK.K | 222 |
|  | CENPL\_Noc300\_122214\_01.11627.11627.2 | 4.5595 | 0.4242 | 100.0% | 1789.7522 | 1789.0819 | 1 | 7.105 | 65.6% | 4 | R.IINEPTAAAIAYGLDKK.V | 2 |
|  | CENPL\_Noc300\_tube2\_122214\_01.09509.09509.2 | 4.431 | 0.5125 | 100.0% | 1691.9722 | 1692.6958 | 1 | 8.361 | 66.7% | 5 | K.STAGDTHLGGEDFDNR.M | 2 |
|  | CENPL\_Noc300\_122214\_02.06626.06626.3 | 3.1587 | 0.5316 | 100.0% | 1692.1444 | 1692.6958 | 3 | 7.545 | 35.0% | 5 | K.STAGDTHLGGEDFDNR.M | 3 |
|  | CENPL\_Noc300\_122214\_01.10853.10853.2 | 3.5286 | 0.5567 | 100.0% | 1235.6921 | 1236.4741 | 1 | 9.863 | 88.9% | 7 | R.MVNHFIAEFK.R | 2 |
|  | CENPL\_Noc300\_tube2\_122214\_01.14762.14762.3 | 3.3039 | 0.4844 | 100.0% | 1237.3444 | 1236.4741 | 1 | 7.573 | 52.8% | 9 | R.MVNHFIAEFK.R | 3 |
|  | CENPL\_Noc300\_tube2\_122214\_01.12480.12480.3 | 2.6979 | 0.2325 | 99.0% | 1392.9844 | 1392.6616 | 1 | 5.045 | 50.0% | 2 | R.MVNHFIAEFKR.K | 3 |
|  | CENPL\_Noc300\_122214\_01.18524.18524.2 | 2.928 | 0.3108 | 99.7% | 2999.2522 | 2999.255 | 1 | 6.045 | 28.8% | 2 | R.TLSSSTQASIEIDSLYEGIDFYTSITR.A | 2 |
|  | CENPL\_Noc300\_tube2\_122214\_01.17318.17318.2 | 3.8981 | 0.3888 | 100.0% | 1481.3522 | 1481.6511 | 1 | 7.6 | 77.3% | 4 | R.ARFEELNADLFR.G | 2 |
|  | CENPL\_Noc300\_tube2\_122214\_01.17319.17319.3 | 4.004 | 0.3681 | 100.0% | 1482.1444 | 1481.6511 | 1 | 6.522 | 56.8% | 3 | R.ARFEELNADLFR.G | 3 |
|  | CENPL\_Noc300\_122214\_01.13526.13526.2 | 3.4499 | 0.432 | 100.0% | 1254.3322 | 1254.3849 | 1 | 7.773 | 72.2% | 4 | R.FEELNADLFR.G | 2 |
|  | CENPL\_Noc300\_tube2\_122214\_01.12948.12948.3 | 5.1313 | 0.3967 | 100.0% | 1838.9043 | 1839.1019 | 1 | 7.818 | 50.0% | 4 | K.LDKSQIHDIVLVGGSTR.I | 3 |
|  | CENPL\_Noc300\_tube2\_122214\_01.12554.12554.3 | 3.243 | 0.2933 | 99.9% | 1483.0443 | 1482.6798 | 2 | 6.051 | 40.4% | 1 | K.SQIHDIVLVGGSTR.I | 3 |
|  | CENPL\_Noc300\_tube2\_122214\_01.12518.12518.2 | 4.8422 | 0.6011 | 100.0% | 1483.5721 | 1482.6798 | 1 | 11.32 | 80.8% | 3 | K.SQIHDIVLVGGSTR.I | 2 |
|  | CENPL\_Noc300\_tube2\_122214\_01.18572.18572.2 | 3.0614 | 0.3609 | 100.0% | 1082.0922 | 1082.2444 | 1 | 6.351 | 81.2% | 6 | K.LLQDFFNGK.E | 22 |
|  | CENPL\_Noc300\_tube2\_122214\_01.17592.17592.2 | 3.4331 | 0.2156 | 99.8% | 1566.1322 | 1566.7972 | 1 | 5.244 | 66.7% | 2 | K.LLQDFFNGKELNK.S | 22 |
|  | CENPL\_Noc300\_122214\_02.13146.13146.2 | 3.1705 | 0.5478 | 100.0% | 2260.0522 | 2261.4937 | 1 | 8.299 | 40.9% | 1 | K.SINPDEAVAYGAAVQAAILSGDK.S | 2 |
|  | CENPL\_Noc300\_122214\_02.13140.13140.3 | 5.0746 | 0.4763 | 100.0% | 2261.6343 | 2261.4937 | 1 | 8.623 | 34.1% | 1 | K.SINPDEAVAYGAAVQAAILSGDK.S | 3 |
|  | CENPL\_Noc300\_tube2\_122214\_01.19184.19184.2 | 4.2351 | 0.5492 | 100.0% | 2774.912 | 2775.9885 | 1 | 10.299 | 37.0% | 2 | K.QTQTFTTYSDNQPGVLIQVYEGER.A | 2 |
| \* | CENPL\_Noc300\_122214\_01.05392.05392.3 | 4.2717 | 0.3787 | 100.0% | 1983.1144 | 1983.2036 | 1 | 6.898 | 51.7% | 1 | R.MVQEAEKYKAEDEKQR.D | 3 |
| \* | CENPL\_Noc300\_122214\_01.12028.12028.2 | 3.6867 | 0.5212 | 100.0% | 1304.3722 | 1304.4602 | 1 | 8.503 | 85.0% | 6 | K.NSLESYAFNMK.A | 2 |

Similarities:
gi|16507237|ref|NP\_00(2:32)  
gi|167466173|ref|NP\_0(1:33)  
gi|124256496|ref|NP\_0(3:31)  
gi|34419635|ref|NP\_00(3:31)  

---

|  |  |  |  |  |  |  |  |  |
| --- | --- | --- | --- | --- | --- | --- | --- | --- |
| U | *gi|34098946|ref|NP\_00* | 11 | 18 | 44.1% | 324 | 35924 | 9.9 | nuclease sensitive element binding protein 1 [Homo sapiens] |

| Filename XCorr DeltCN Conf% ObsM+H+ CalcM+H+ SpR ZScore Ion% # Sequence  | | | | | | | | | | | | |
| --- | --- | --- | --- | --- | --- | --- | --- | --- | --- | --- | --- | --- |
|  | CENPL\_Noc300\_122214\_01.09186.09186.2 | 2.0055 | 0.4436 | 99.7% | 941.4122 | 941.0342 | 3 | 6.871 | 71.4% | 1 | R.NGYGFINR.N | 2 |
|  | CENPL\_Noc300\_122214\_01.07664.07664.2 | 4.0075 | 0.4245 | 100.0% | 1745.4922 | 1745.9298 | 1 | 7.905 | 71.4% | 1 | R.NDTKEDVFVHQTAIK.K | 2 |
|  | CENPL\_Noc300\_tube2\_122214\_01.09916.09916.3 | 3.7061 | 0.3925 | 100.0% | 1746.2644 | 1745.9298 | 1 | 6.379 | 41.1% | 2 | R.NDTKEDVFVHQTAIK.K | 3 |
|  | CENPL\_Noc300\_122214\_01.06678.06678.2 | 4.5157 | 0.4567 | 100.0% | 1873.4722 | 1874.1039 | 1 | 7.422 | 70.0% | 1 | R.NDTKEDVFVHQTAIKK.N | 2 |
|  | CENPL\_Noc300\_122214\_01.06663.06663.3 | 3.5541 | 0.2785 | 99.9% | 1873.9143 | 1874.1039 | 1 | 6.752 | 46.7% | 1 | R.NDTKEDVFVHQTAIKK.N | 3 |
|  | CENPL\_Noc300\_122214\_02.09680.09680.2 | 4.8131 | 0.3773 | 100.0% | 1797.3922 | 1796.8822 | 1 | 8.455 | 71.9% | 2 | R.SVGDGETVEFDVVEGEK.G | 2 |
| \* | CENPL\_Noc300\_tube2\_122214\_01.09459.09459.2 | 4.4224 | 0.5816 | 100.0% | 1696.3522 | 1696.8577 | 1 | 9.166 | 66.7% | 3 | K.GAEAANVTGPGGVPVQGSK.Y | 2 |
| \* | CENPL\_Noc300\_122214\_01.05902.05902.3 | 6.1765 | 0.4842 | 100.0% | 3258.6243 | 3259.2566 | 1 | 8.184 | 34.8% | 2 | R.NYQQNYQNSESGEKNEGSESAPEGQAQQR.R | 3 |
| \* | CENPL\_Noc300\_122214\_01.07651.07651.3 | 5.6713 | 0.3714 | 100.0% | 3226.6143 | 3225.4795 | 1 | 8.56 | 31.0% | 3 | R.RPQYSNPPVQGEVMEGADNQGAGEQGRPVR.Q | 3 |
| \* | CENPL\_Noc300\_122214\_01.05159.05159.3 | 3.9743 | 0.3428 | 100.0% | 2628.8943 | 2629.5835 | 5 | 6.645 | 29.5% | 1 | R.EDGNEEDKENQGDETQGQQPPQR.R | 3 |
| \* | CENPL\_Noc300\_122214\_01.05085.05085.3 | 3.4243 | 0.2535 | 99.8% | 2786.1543 | 2785.771 | 1 | 6.177 | 27.2% | 1 | R.EDGNEEDKENQGDETQGQQPPQRR.Y | 3 |

---

|  |  |  |  |  |  |  |  |  |
| --- | --- | --- | --- | --- | --- | --- | --- | --- |
| U | *gi|5031753|ref|NP\_005* | 12 | 31 | 43.7% | 449 | 49229 | 6.3 | heterogeneous nuclear ribonucleoprotein H1 [Homo sapiens] |

| Filename XCorr DeltCN Conf% ObsM+H+ CalcM+H+ SpR ZScore Ion% # Sequence  | | | | | | | | | | | | |
| --- | --- | --- | --- | --- | --- | --- | --- | --- | --- | --- | --- | --- |
| \* | CENPL\_Noc300\_tube2\_122214\_01.18807.18807.2 | 3.884 | 0.3315 | 100.0% | 1335.3722 | 1335.5176 | 2 | 6.895 | 65.0% | 6 | K.SNNVEMDWVLK.H | 2 |
|  | CENPL\_Noc300\_122214\_01.06873.06873.3 | 4.5277 | 0.2838 | 100.0% | 1686.0543 | 1685.7501 | 1 | 5.686 | 41.7% | 2 | K.HTGPNSPDTANDGFVR.L | 3 |
|  | CENPL\_Noc300\_122214\_01.06863.06863.2 | 3.6511 | 0.4393 | 100.0% | 1686.4722 | 1685.7501 | 1 | 8.285 | 73.3% | 1 | K.HTGPNSPDTANDGFVR.L | 2 |
| \* | CENPL\_Noc300\_122214\_01.18962.18962.2 | 3.6913 | 0.4255 | 100.0% | 2907.8123 | 2906.3079 | 1 | 5.786 | 36.0% | 1 | K.EEIVQFFSGLEIVPNGITLPVDFQGR.S | 2 |
|  | CENPL\_Noc300\_122214\_01.14122.14122.2 | 4.984 | 0.5875 | 100.0% | 1842.8722 | 1843.0001 | 1 | 10.863 | 68.8% | 5 | R.STGEAFVQFASQEIAEK.A | 2 |
| \* | CENPL\_Noc300\_tube2\_122214\_01.16247.16247.2 | 3.3825 | 0.1367 | 99.3% | 2719.872 | 2718.6814 | 1 | 6.619 | 36.0% | 2 | R.GAYGGGYGGYDDYNGYNDGYGFGSDR.F | 2 |
|  | CENPL\_Noc300\_122214\_01.10341.10341.2 | 2.542 | 0.1678 | 97.6% | 1602.0721 | 1602.6844 | 8 | 5.497 | 50.0% | 1 | R.DLNYCFSGMSDHR.Y | 2 |
| \* | CENPL\_Noc300\_tube2\_122214\_01.09312.09312.3 | 3.7227 | 0.4494 | 100.0% | 2098.4944 | 2099.208 | 1 | 6.953 | 43.1% | 2 | R.YGDGGSTFQSTTGHCVHMR.G | 3 |
|  | CENPL\_Noc300\_tube2\_122214\_01.21291.21291.2 | 4.1446 | 0.5164 | 100.0% | 1997.8722 | 1998.2023 | 1 | 8.504 | 59.4% | 3 | R.ATENDIYNFFSPLNPVR.V | 22 |
|  | CENPL\_Noc300\_tube2\_122214\_01.10130.10130.2 | 3.3992 | 0.4785 | 100.0% | 1093.2922 | 1093.2278 | 1 | 7.675 | 66.7% | 5 | R.VHIEIGPDGR.V | 22 |
| \* | CENPL\_Noc300\_122214\_02.09255.09255.3 | 3.0362 | 0.5466 | 100.0% | 2178.3843 | 2179.363 | 1 | 8.258 | 35.0% | 1 | R.VTGEADVEFATHEDAVAAMSK.D | 3 |
| \* | CENPL\_Noc300\_tube2\_122214\_01.16875.16875.3 | 2.7016 | 0.2941 | 99.2% | 2142.7444 | 2143.32 | 3 | 6.447 | 35.5% | 2 | R.YVELFLNSTAGASGGAYEHR.Y | 3 |

Similarities:
gi|148470397|ref|NP\_0(2:10)  

---

|  |  |  |  |  |  |  |  |  |
| --- | --- | --- | --- | --- | --- | --- | --- | --- |
| U | *gi|7669492|ref|NP\_002* | 11 | 24 | 43.0% | 335 | 36053 | 8.5 | glyceraldehyde-3-phosphate dehydrogenase [Homo sapiens] |

| Filename XCorr DeltCN Conf% ObsM+H+ CalcM+H+ SpR ZScore Ion% # Sequence  | | | | | | | | | | | | |
| --- | --- | --- | --- | --- | --- | --- | --- | --- | --- | --- | --- | --- |
| \* | CENPL\_Noc300\_122214\_02.06731.06731.2 | 2.1628 | 0.1337 | 96.5% | 806.33215 | 805.912 | 14 | 5.572 | 64.3% | 2 | K.VGVNGFGR.I | 2 |
| \* | CENPL\_Noc300\_tube2\_122214\_01.19968.19968.2 | 4.0668 | 0.367 | 100.0% | 1615.3922 | 1614.8851 | 1 | 8.22 | 73.1% | 3 | K.LVINGNPITIFQER.D | 2 |
| \* | CENPL\_Noc300\_tube2\_122214\_01.20463.20463.2 | 2.3555 | 0.2559 | 98.0% | 2276.6921 | 2278.495 | 1 | 5.337 | 37.5% | 1 | K.WGDAGAEYVVESTGVFTTMEK.A | 2 |
| \* | CENPL\_Noc300\_tube2\_122214\_01.16624.16624.3 | 5.717 | 0.3499 | 100.0% | 2372.0645 | 2370.79 | 1 | 7.262 | 40.5% | 1 | K.RVIISAPSADAPMFVMGVNHEK.Y | 3 |
| \* | CENPL\_Noc300\_tube2\_122214\_01.18330.18330.2 | 2.5094 | 0.2133 | 97.9% | 2214.0122 | 2214.6025 | 1 | 4.608 | 42.5% | 1 | R.VIISAPSADAPMFVMGVNHEK.Y | 2 |
| \* | CENPL\_Noc300\_122214\_01.17242.17242.2 | 5.7057 | 0.5821 | 100.0% | 2596.6921 | 2597.0044 | 1 | 10.64 | 50.0% | 1 | K.VIHDNFGIVEGLMTTVHAITATQK.T | 2 |
| \* | CENPL\_Noc300\_122214\_01.17276.17276.3 | 7.0177 | 0.6431 | 100.0% | 2596.7043 | 2597.0044 | 1 | 12.387 | 42.4% | 2 | K.VIHDNFGIVEGLMTTVHAITATQK.T | 3 |
| \* | CENPL\_Noc300\_tube2\_122214\_01.14846.14846.2 | 3.6266 | 0.3534 | 100.0% | 1412.6322 | 1412.6292 | 1 | 5.877 | 71.4% | 6 | R.GALQNIIPASTGAAK.A | 2 |
| \* | CENPL\_Noc300\_122214\_01.11066.11066.2 | 2.4746 | 0.3229 | 99.4% | 1532.3522 | 1531.7155 | 1 | 5.512 | 57.7% | 1 | R.VPTANVSVVDLTCR.L | 2 |
|  | CENPL\_Noc300\_122214\_01.13921.13921.2 | 3.7457 | 0.4024 | 100.0% | 1764.5721 | 1764.8914 | 1 | 7.94 | 61.5% | 2 | K.LISWYDNEFGYSNR.V | 2 |
| \* | CENPL\_Noc300\_tube2\_122214\_01.13114.13114.2 | 3.2019 | 0.4955 | 100.0% | 1331.2522 | 1331.5879 | 1 | 7.862 | 72.7% | 4 | R.VVDLMAHMASKE.- | 2 |

---

|  |  |  |  |  |  |  |  |  |
| --- | --- | --- | --- | --- | --- | --- | --- | --- |
| U | *gi|123173757|ref|NP\_5* | 21 | 40 | 42.9% | 756 | 79579 | 8.9 | RAVER1 [Homo sapiens] |

| Filename XCorr DeltCN Conf% ObsM+H+ CalcM+H+ SpR ZScore Ion% # Sequence  | | | | | | | | | | | | |
| --- | --- | --- | --- | --- | --- | --- | --- | --- | --- | --- | --- | --- |
| \* | CENPL\_Noc300\_tube2\_122214\_01.13184.13184.3 | 4.1082 | 0.3713 | 100.0% | 2019.9543 | 2019.2621 | 19 | 6.997 | 37.5% | 3 | R.RAPEEELPPLDPEEIRK.R | 3 |
| \* | CENPL\_Noc300\_122214\_01.14936.14936.2 | 3.7777 | 0.4982 | 100.0% | 2343.0322 | 2343.5522 | 1 | 7.882 | 52.5% | 2 | R.GLPGDVTNQEVHDLLSDYELK.Y | 2 |
| \* | CENPL\_Noc300\_122214\_01.07504.07504.3 | 3.0332 | 0.353 | 100.0% | 1353.2043 | 1352.62 | 1 | 6.422 | 41.7% | 2 | R.AKSDLLGKPLGPR.T | 3 |
| \* | CENPL\_Noc300\_122214\_01.08854.08854.2 | 2.7551 | 0.2238 | 99.5% | 1153.2122 | 1153.3671 | 1 | 5.499 | 75.0% | 2 | K.SDLLGKPLGPR.T | 2 |
| \* | CENPL\_Noc300\_122214\_02.10742.10742.3 | 5.345 | 0.3301 | 100.0% | 2280.2043 | 2280.5913 | 1 | 7.128 | 40.8% | 1 | R.TLYVHWTDAGQLTPALLHSR.C | 3 |
| \* | CENPL\_Noc300\_122214\_01.16567.16567.3 | 4.9648 | 0.5274 | 100.0% | 3309.1443 | 3309.5884 | 1 | 9.191 | 29.2% | 1 | K.GFAVLEYETAEMAEEAQQQADGLSLGGSHLR.V | 3 |
| \* | CENPL\_Noc300\_122214\_02.13679.13679.2 | 3.96 | 0.4356 | 100.0% | 1616.0521 | 1615.9316 | 1 | 7.18 | 70.0% | 1 | R.SMLAALIAAQATALNR.G | 2 |
| \* | CENPL\_Noc300\_122214\_01.20272.20272.3 | 3.7197 | 0.4279 | 100.0% | 3673.4944 | 3674.322 | 1 | 6.535 | 26.4% | 1 | K.GLLPEPNILQLLNNLGPSASLQLLLNPLLHGSAGGK.Q | 3 |
| \* | CENPL\_Noc300\_122214\_01.19584.19584.3 | 3.5145 | 0.2516 | 99.8% | 3313.3442 | 3312.939 | 1 | 5.542 | 19.5% | 1 | K.QGLLGAPPAMPLLNGPALSTALLQLALQTQGQK.K | 3 |
| \* | CENPL\_Noc300\_tube2\_122214\_01.16534.16534.2 | 2.351 | 0.3991 | 99.6% | 1728.3522 | 1729.0323 | 7 | 6.136 | 44.1% | 1 | R.GKPPPLLPSVLGPAGGDR.E | 2 |
| \* | CENPL\_Noc300\_tube2\_122214\_01.16499.16499.3 | 3.7962 | 0.3333 | 100.0% | 1729.4043 | 1729.0323 | 6 | 6.487 | 38.2% | 1 | R.GKPPPLLPSVLGPAGGDR.E | 3 |
| \* | CENPL\_Noc300\_tube2\_122214\_01.20369.20369.2 | 4.637 | 0.5547 | 100.0% | 2246.0522 | 2246.658 | 1 | 9.95 | 57.5% | 2 | R.IPLNPYLNLHSLLPASNLAGK.E | 2 |
| \* | CENPL\_Noc300\_122214\_01.15358.15358.3 | 5.2028 | 0.4731 | 100.0% | 2246.2144 | 2246.658 | 1 | 8.234 | 43.8% | 4 | R.IPLNPYLNLHSLLPASNLAGK.E | 3 |
| \* | CENPL\_Noc300\_122214\_01.05301.05301.3 | 4.7571 | 0.4709 | 100.0% | 2191.9744 | 2192.353 | 1 | 8.221 | 35.2% | 3 | R.SRRPAEGPPTNPPAPGGGSSSSK.A | 3 |
| \* | CENPL\_Noc300\_122214\_01.05608.05608.2 | 5.8408 | 0.66 | 100.0% | 1948.4922 | 1949.0873 | 1 | 10.472 | 75.0% | 1 | R.RPAEGPPTNPPAPGGGSSSSK.A | 2 |
| \* | CENPL\_Noc300\_122214\_01.05566.05566.3 | 3.6642 | 0.2307 | 99.8% | 1948.7344 | 1949.0873 | 1 | 5.184 | 37.5% | 2 | R.RPAEGPPTNPPAPGGGSSSSK.A | 3 |
| \* | CENPL\_Noc300\_tube2\_122214\_01.15878.15878.2 | 2.1923 | 0.2065 | 97.7% | 1023.8722 | 1024.1191 | 1 | 4.799 | 68.8% | 1 | R.LLS\*PLSSAR.L | 2 |
| \* | CENPL\_Noc300\_tube2\_122214\_01.18694.18694.3 | 3.1365 | 0.2605 | 99.6% | 2428.4644 | 2428.6318 | 85 | 3.927 | 23.8% | 1 | R.LPPEPGLSDSYSFDYPSDMGPR.R | 3 |
| \* | CENPL\_Noc300\_122214\_01.13283.13283.2 | 4.4694 | 0.3425 | 100.0% | 2428.4922 | 2428.6318 | 1 | 7.739 | 50.0% | 5 | R.LPPEPGLSDSYSFDYPSDMGPR.R | 2 |
| \* | CENPL\_Noc300\_122214\_01.06699.06699.3 | 3.1353 | 0.3414 | 100.0% | 1507.7043 | 1507.6207 | 9 | 5.624 | 45.8% | 2 | R.HKMS\*PPPSGFGER.S | 3 |
| \* | CENPL\_Noc300\_122214\_02.12370.12370.3 | 4.1947 | 0.2492 | 99.9% | 3072.7144 | 3073.2158 | 1 | 5.99 | 26.7% | 3 | R.SSGGSGGGPLSHFYSGS\*PTSYFTSGLQAGLK.Q | 3 |

---

|  |  |  |  |  |  |  |  |  |
| --- | --- | --- | --- | --- | --- | --- | --- | --- |
| U | *gi|4502891|ref|NP\_001* | 5 | 10 | 42.2% | 237 | 26215 | 4.1 | chloride channel, nucleotide-sensitive, 1A [Homo sapiens] |

| Filename XCorr DeltCN Conf% ObsM+H+ CalcM+H+ SpR ZScore Ion% # Sequence  | | | | | | | | | | | | |
| --- | --- | --- | --- | --- | --- | --- | --- | --- | --- | --- | --- | --- |
| \* | CENPL\_Noc300\_tube2\_122214\_01.09670.09670.2 | 1.8784 | 0.2892 | 96.0% | 1299.9521 | 1300.4111 | 128 | 5.728 | 50.0% | 2 | R.QQPDTEAVLNGK.G | 2 |
| \* | CENPL\_Noc300\_122214\_01.11050.11050.2 | 4.0953 | 0.5526 | 100.0% | 1338.2122 | 1338.5034 | 1 | 9.775 | 66.7% | 5 | K.GLGTGTLYIAESR.L | 2 |
| \* | CENPL\_Noc300\_122214\_02.14366.14366.3 | 3.2832 | 0.4402 | 100.0% | 2720.1843 | 2721.085 | 1 | 6.071 | 26.0% | 1 | R.LSWLDGSGLGFSLEYPTISLHALSR.D | 3 |
| \* | CENPL\_Noc300\_122214\_02.09905.09905.2 | 4.854 | 0.5503 | 100.0% | 2057.2922 | 2058.332 | 1 | 10.167 | 61.1% | 1 | R.LEGMLSQSVSSQYNMAGVR.T | 2 |
| \* | CENPL\_Noc300\_122214\_02.10136.10136.3 | 6.7372 | 0.5554 | 100.0% | 3459.5044 | 3459.5435 | 1 | 9.268 | 33.3% | 1 | R.TEDSIRDYEDGMEVDTTPTVAGQFEDADVDH.- | 3 |

---

|  |  |  |  |  |  |  |  |  |
| --- | --- | --- | --- | --- | --- | --- | --- | --- |
| U | *gi|5032161|ref|NP\_005* | 5 | 17 | 42.0% | 112 | 12473 | 4.8 | elongin C [Homo sapiens] |

| Filename XCorr DeltCN Conf% ObsM+H+ CalcM+H+ SpR ZScore Ion% # Sequence  | | | | | | | | | | | | |
| --- | --- | --- | --- | --- | --- | --- | --- | --- | --- | --- | --- | --- |
| \* | CENPL\_Noc300\_122214\_01.08917.08917.2 | 4.1517 | 0.4819 | 100.0% | 1548.4321 | 1548.6736 | 1 | 8.362 | 73.1% | 2 | K.TYGGCEGPDAMYVK.L | 2 |
|  | CENPL\_Noc300\_tube2\_122214\_01.12366.12366.2 | 3.7702 | 0.3538 | 100.0% | 1345.3922 | 1345.5382 | 1 | 7.082 | 77.3% | 5 | K.LISSDGHEFIVK.R | 2 |
|  | CENPL\_Noc300\_tube2\_122214\_01.10398.10398.2 | 3.8507 | 0.437 | 100.0% | 1501.2922 | 1501.7257 | 1 | 7.456 | 83.3% | 2 | K.LISSDGHEFIVKR.E | 2 |
| \* | CENPL\_Noc300\_tube2\_122214\_01.17493.17493.2 | 5.9625 | 0.5826 | 100.0% | 2212.5923 | 2212.3984 | 1 | 10.638 | 63.2% | 6 | K.AMLSGPGQFAENETNEVNFR.E | 2 |
| \* | CENPL\_Noc300\_122214\_02.10280.10280.3 | 4.1094 | 0.3749 | 100.0% | 2213.4543 | 2212.3984 | 1 | 6.002 | 42.1% | 2 | K.AMLSGPGQFAENETNEVNFR.E | 3 |

---

|  |  |  |  |  |  |  |  |  |
| --- | --- | --- | --- | --- | --- | --- | --- | --- |
| U | *gi|148470397|ref|NP\_0* | 9 | 22 | 41.4% | 415 | 45672 | 5.6 | heterogeneous nuclear ribonucleoprotein F [Homo sapiens] |
| U | *gi|4826760|ref|NP\_004* | 9 | 22 | 41.4% | 415 | 45672 | 5.6 | heterogeneous nuclear ribonucleoprotein F [Homo sapiens] |
| U | *gi|148470406|ref|NP\_0* | 9 | 22 | 41.4% | 415 | 45672 | 5.6 | heterogeneous nuclear ribonucleoprotein F [Homo sapiens] |
| U | *gi|148470404|ref|NP\_0* | 9 | 22 | 41.4% | 415 | 45672 | 5.6 | heterogeneous nuclear ribonucleoprotein F [Homo sapiens] |
| U | *gi|148470402|ref|NP\_0* | 9 | 22 | 41.4% | 415 | 45672 | 5.6 | heterogeneous nuclear ribonucleoprotein F [Homo sapiens] |
| U | *gi|148470400|ref|NP\_0* | 9 | 22 | 41.4% | 415 | 45672 | 5.6 | heterogeneous nuclear ribonucleoprotein F [Homo sapiens] |

| Filename XCorr DeltCN Conf% ObsM+H+ CalcM+H+ SpR ZScore Ion% # Sequence  | | | | | | | | | | | | |
| --- | --- | --- | --- | --- | --- | --- | --- | --- | --- | --- | --- | --- |
|  | CENPL\_Noc300\_122214\_02.09368.09368.2 | 3.2553 | 0.3511 | 100.0% | 1710.0721 | 1710.7919 | 1 | 6.366 | 50.0% | 2 | R.QSGEAFVELGSEDDVK.M | 2 |
|  | CENPL\_Noc300\_122214\_01.06425.06425.2 | 3.9961 | 0.5799 | 100.0% | 1631.2722 | 1631.6584 | 1 | 10.248 | 63.3% | 1 | K.HSGPNSADSANDGFVR.L | 2 |
|  | CENPL\_Noc300\_122214\_01.18507.18507.2 | 4.5799 | 0.5328 | 100.0% | 2829.2122 | 2829.2195 | 1 | 8.665 | 44.0% | 1 | K.EEIVQFFSGLEIVPNGITLPVDPEGK.I | 2 |
|  | CENPL\_Noc300\_122214\_02.12777.12777.2 | 5.902 | 0.4156 | 100.0% | 1869.5521 | 1869.0813 | 1 | 9.189 | 78.1% | 5 | K.ITGEAFVQFASQELAEK.A | 2 |
|  | CENPL\_Noc300\_122214\_01.07463.07463.3 | 2.6485 | 0.2768 | 98.7% | 1935.5044 | 1936.1986 | 1 | 4.339 | 34.4% | 1 | K.FMSVQRPGPYDRPGTAR.R | 3 |
|  | CENPL\_Noc300\_122214\_02.12357.12357.3 | 7.5574 | 0.5735 | 100.0% | 3475.7644 | 3476.7114 | 1 | 11.162 | 30.6% | 2 | R.MRPGAYSTGYGGYEEYSGLSDGYGFTTDLFGR.D | 32 |
|  | CENPL\_Noc300\_tube2\_122214\_01.21291.21291.2 | 4.1446 | 0.5164 | 100.0% | 1997.8722 | 1998.2023 | 1 | 8.504 | 59.4% | 3 | K.ATENDIYNFFSPLNPVR.V | 22 |
|  | CENPL\_Noc300\_tube2\_122214\_01.10130.10130.2 | 3.3992 | 0.4785 | 100.0% | 1093.2922 | 1093.2278 | 1 | 7.675 | 66.7% | 5 | R.VHIEIGPDGR.V | 22 |
|  | CENPL\_Noc300\_122214\_02.09496.09496.3 | 3.3134 | 0.3851 | 100.0% | 2193.5645 | 2193.39 | 1 | 5.769 | 30.0% | 2 | R.VTGEADVEFATHEEAVAAMSK.D | 3 |

Similarities:
gi|5031753|ref|NP\_005(2:7)  

---

|  |  |  |  |  |  |  |  |  |
| --- | --- | --- | --- | --- | --- | --- | --- | --- |
| U | *gi|29788785|ref|NP\_82* | 19 | 49 | 40.8% | 444 | 49671 | 4.9 | tubulin, beta [Homo sapiens] |

| Filename XCorr DeltCN Conf% ObsM+H+ CalcM+H+ SpR ZScore Ion% # Sequence  | | | | | | | | | | | | |
| --- | --- | --- | --- | --- | --- | --- | --- | --- | --- | --- | --- | --- |
| \* | CENPL\_Noc300\_122214\_01.08524.08524.2 | 3.1945 | 0.5483 | 100.0% | 1302.0521 | 1302.4265 | 1 | 8.721 | 77.3% | 4 | R.ISVYYNEATGGK.Y | 2 |
|  | CENPL\_Noc300\_122214\_01.13395.13395.2 | 4.2809 | 0.4699 | 100.0% | 1617.6322 | 1616.8701 | 1 | 8.713 | 82.1% | 4 | R.AILVDLEPGTMDSVR.S | 2 |
|  | CENPL\_Noc300\_tube2\_122214\_01.20349.20349.3 | 6.9426 | 0.4442 | 100.0% | 2799.5044 | 2800.0647 | 1 | 7.521 | 38.0% | 5 | R.SGPFGQIFRPDNFVFGQSGAGNNWAK.G | 33 |
|  | CENPL\_Noc300\_tube2\_122214\_01.20703.20703.3 | 3.7681 | 0.2527 | 99.9% | 1959.3844 | 1960.151 | 1 | 5.742 | 42.6% | 1 | K.GHYTEGAELVDSVLDVVR.K | 33 |
|  | CENPL\_Noc300\_122214\_01.15753.15753.2 | 6.9684 | 0.5741 | 100.0% | 1959.7522 | 1960.151 | 1 | 10.753 | 79.4% | 2 | K.GHYTEGAELVDSVLDVVR.K | 22 |
|  | CENPL\_Noc300\_tube2\_122214\_01.19962.19962.3 | 4.848 | 0.4608 | 100.0% | 2088.1743 | 2088.325 | 1 | 7.606 | 37.5% | 3 | K.GHYTEGAELVDSVLDVVRK.E | 33 |
|  | CENPL\_Noc300\_tube2\_122214\_01.08062.08062.2 | 2.3719 | 0.2213 | 99.3% | 1077.5922 | 1078.1698 | 58 | 4.748 | 64.3% | 1 | K.IREEYPDR.I | 22 |
|  | CENPL\_Noc300\_tube2\_122214\_01.15951.15951.2 | 4.2126 | 0.4177 | 100.0% | 1320.5721 | 1320.5896 | 1 | 8.013 | 72.7% | 7 | R.IMNTFSVVPSPK.V | 22 |
|  | CENPL\_Noc300\_tube2\_122214\_01.15953.15953.2 | 2.9791 | 0.283 | 99.8% | 1273.8522 | 1272.5945 | 2 | 6.138 | 65.0% | 2 | R.KLAVNMVPFPR.L | 22 |
|  | CENPL\_Noc300\_tube2\_122214\_01.18262.18262.2 | 2.9656 | 0.3872 | 100.0% | 1144.1921 | 1144.4204 | 1 | 8.43 | 77.8% | 3 | K.LAVNMVPFPR.L | 22 |
|  | CENPL\_Noc300\_122214\_01.15001.15001.2 | 3.8413 | 0.4088 | 100.0% | 1621.3722 | 1621.9403 | 1 | 8.844 | 69.2% | 2 | R.LHFFMPGFAPLTSR.G | 22 |
|  | CENPL\_Noc300\_tube2\_122214\_01.20181.20181.3 | 3.532 | 0.2572 | 99.9% | 1621.9143 | 1621.9403 | 2 | 5.041 | 46.2% | 2 | R.LHFFMPGFAPLTSR.G | 33 |
| \* | CENPL\_Noc300\_tube2\_122214\_01.19982.19982.2 | 4.044 | 0.5077 | 100.0% | 1660.4922 | 1660.9078 | 1 | 8.077 | 67.9% | 2 | R.ALTVPELTQQVFDAK.N | 2 |
|  | CENPL\_Noc300\_122214\_01.13623.13623.2 | 2.3091 | 0.3204 | 99.6% | 1041.6522 | 1040.2505 | 2 | 5.88 | 75.0% | 2 | R.YLTVAAVFR.G | 22 |
|  | CENPL\_Noc300\_tube2\_122214\_01.11680.11680.2 | 4.1504 | 0.2215 | 100.0% | 1448.3121 | 1447.6031 | 1 | 5.86 | 77.3% | 4 | K.EVDEQMLNVQNK.N | 22 |
| \* | CENPL\_Noc300\_122214\_02.13763.13763.2 | 3.7004 | 0.46 | 100.0% | 1869.7722 | 1871.2018 | 1 | 8.912 | 56.2% | 1 | K.MAVTFIGNSTAIQELFK.R | 2 |
| \* | CENPL\_Noc300\_122214\_02.12707.12707.3 | 3.8564 | 0.4248 | 100.0% | 2027.6044 | 2027.3893 | 1 | 7.297 | 39.7% | 1 | K.MAVTFIGNSTAIQELFKR.I | 3 |
|  | CENPL\_Noc300\_122214\_01.11970.11970.2 | 2.8968 | 0.3507 | 100.0% | 1386.4922 | 1386.6116 | 2 | 5.864 | 65.0% | 1 | K.RISEQFTAMFR.R | 22 |
|  | CENPL\_Noc300\_tube2\_122214\_01.18759.18759.2 | 3.3107 | 0.4636 | 100.0% | 1230.1921 | 1230.4241 | 1 | 7.602 | 94.4% | 2 | R.ISEQFTAMFR.R | 22 |

Similarities:
gi|5174735|ref|NP\_006(14:5)  

---

|  |  |  |  |  |  |  |  |  |
| --- | --- | --- | --- | --- | --- | --- | --- | --- |
| U | *contaminant\_KERATIN02* | 21 | 48 | 40.7% | 622 | 61987 | 5.2 | no description |
| U | *gi|55956899|ref|NP\_00* | 21 | 48 | 40.6% | 623 | 62064 | 5.2 | keratin 9 [Homo sapiens] |

| Filename XCorr DeltCN Conf% ObsM+H+ CalcM+H+ SpR ZScore Ion% # Sequence  | | | | | | | | | | | | |
| --- | --- | --- | --- | --- | --- | --- | --- | --- | --- | --- | --- | --- |
|  | CENPL\_Noc300\_tube2\_122214\_01.08150.08150.2 | 3.3228 | 0.4631 | 100.0% | 1236.1921 | 1236.2401 | 1 | 7.176 | 66.7% | 1 | R.FSSSSGYGGGSSR.V | 2 |
|  | CENPL\_Noc300\_122214\_01.07294.07294.1 | 1.6519 | 0.2663 | 95.7% | 809.37 | 809.93774 | 1 | 5.658 | 75.0% | 1 | R.LASYLDK.V | 111111 |
|  | CENPL\_Noc300\_122214\_01.08642.08642.2 | 5.4932 | 0.4759 | 100.0% | 1587.2922 | 1587.6836 | 1 | 9.683 | 84.6% | 3 | K.VQALEEANNDLENK.I | 2 |
|  | CENPL\_Noc300\_122214\_01.07867.07867.3 | 3.6863 | 0.3988 | 100.0% | 1763.1843 | 1762.0183 | 14 | 6.045 | 37.5% | 1 | K.IQDWYDKKGPAAIQK.N | 3 |
|  | CENPL\_Noc300\_122214\_01.16598.16598.2 | 3.8814 | 0.5394 | 100.0% | 2902.2722 | 2904.1597 | 1 | 10.514 | 41.7% | 2 | K.NYSPYYNTIDDLKDQIVDLTVGNNK.T | 2 |
|  | CENPL\_Noc300\_tube2\_122214\_01.14188.14188.2 | 2.8011 | 0.381 | 100.0% | 1061.0322 | 1061.1802 | 1 | 6.079 | 87.5% | 4 | K.TLLDIDNTR.M | 2 |
|  | CENPL\_Noc300\_122214\_01.11108.11108.2 | 1.7078 | 0.4404 | 99.1% | 898.0122 | 898.02155 | 92 | 6.479 | 50.0% | 1 | R.MTLDDFR.I | 2 |
|  | CENPL\_Noc300\_122214\_02.08712.08712.2 | 2.2796 | 0.2449 | 98.6% | 1308.2322 | 1308.5383 | 5 | 4.823 | 61.1% | 1 | R.IKFEMEQNLR.Q | 2 |
|  | CENPL\_Noc300\_122214\_02.08741.08741.3 | 2.7268 | 0.1794 | 96.9% | 1309.0743 | 1308.5383 | 35 | 4.42 | 44.4% | 1 | R.IKFEMEQNLR.Q | 3 |
|  | CENPL\_Noc300\_tube2\_122214\_01.11898.11898.2 | 2.8226 | 0.3147 | 99.8% | 1158.3522 | 1158.2566 | 5 | 5.679 | 70.0% | 6 | R.QGVDADINGLR.Q | 2 |
|  | CENPL\_Noc300\_122214\_01.16912.16912.2 | 3.4841 | 0.3816 | 100.0% | 2172.632 | 2172.4702 | 1 | 6.941 | 52.9% | 2 | K.SDLEMQYETLQEELMALK.K | 2 |
|  | CENPL\_Noc300\_122214\_01.12857.12857.3 | 3.4554 | 0.3379 | 100.0% | 1852.6743 | 1853.1003 | 1 | 6.145 | 42.9% | 2 | K.TLNDMRQEYEQLIAK.N | 3 |
|  | CENPL\_Noc300\_122214\_01.12859.12859.2 | 3.2305 | 0.1833 | 99.4% | 1853.7522 | 1853.1003 | 1 | 4.278 | 60.7% | 2 | K.TLNDMRQEYEQLIAK.N | 2 |
|  | CENPL\_Noc300\_122214\_01.14718.14718.2 | 2.682 | 0.2319 | 98.8% | 3266.112 | 3266.413 | 1 | 6.155 | 25.0% | 1 | K.DIENQYETQITQIEHEVSSSGQEVQSSAK.E | 2 |
|  | CENPL\_Noc300\_122214\_01.14754.14754.3 | 6.9147 | 0.5647 | 100.0% | 3266.8145 | 3266.413 | 1 | 9.079 | 33.0% | 2 | K.DIENQYETQITQIEHEVSSSGQEVQSSAK.E | 3 |
|  | CENPL\_Noc300\_122214\_01.13364.13364.2 | 5.9381 | 0.5793 | 100.0% | 1838.5922 | 1839.0557 | 1 | 10.288 | 80.0% | 4 | R.HGVQELEIELQSQLSK.K | 2 |
|  | CENPL\_Noc300\_122214\_02.10970.10970.3 | 4.2556 | 0.2604 | 100.0% | 1840.3143 | 1839.0557 | 1 | 5.054 | 45.0% | 1 | R.HGVQELEIELQSQLSK.K | 3 |
|  | CENPL\_Noc300\_122214\_02.10334.10334.3 | 6.1198 | 0.3714 | 100.0% | 1967.0643 | 1967.2297 | 1 | 7.977 | 51.6% | 5 | R.HGVQELEIELQSQLSKK.A | 3 |
|  | CENPL\_Noc300\_122214\_02.09806.09806.3 | 5.8509 | 0.1756 | 99.9% | 2511.5344 | 2511.6177 | 1 | 7.168 | 39.8% | 6 | K.EIETYHNLLEGGQEDFESSGAGK.I | 3 |
|  | CENPL\_Noc300\_122214\_01.11666.11666.2 | 4.9791 | 0.427 | 100.0% | 2511.5923 | 2511.6177 | 1 | 8.78 | 52.3% | 1 | K.EIETYHNLLEGGQEDFESSGAGK.I | 2 |
|  | CENPL\_Noc300\_122214\_02.06681.06681.3 | 6.9502 | 0.5301 | 100.0% | 3225.2944 | 3225.1118 | 1 | 9.038 | 27.6% | 1 | R.GGSGGSHGGGSGFGGESGGSYGGGEEASGSGGGYGGGSGK.S | 3 |

Similarities:
contaminant\_KERATIN03(1:20)  
gi|15431310|ref|NP\_00(1:20)  
gi|4557701|ref|NP\_000(1:20)  
gi|24430192|ref|NP\_00(1:20)  
gi|24234699|ref|NP\_00(1:20)  

---

|  |  |  |  |  |  |  |  |  |
| --- | --- | --- | --- | --- | --- | --- | --- | --- |
| U | *gi|23097250|ref|NP\_69* | 9 | 13 | 40.4% | 272 | 31444 | 5.9 | TIP41, TOR signalling pathway regulator-like isoform 1 [Homo sapiens] |

| Filename XCorr DeltCN Conf% ObsM+H+ CalcM+H+ SpR ZScore Ion% # Sequence  | | | | | | | | | | | | |
| --- | --- | --- | --- | --- | --- | --- | --- | --- | --- | --- | --- | --- |
|  | CENPL\_Noc300\_tube2\_122214\_02.00122.00122.3 | 4.4896 | 0.4286 | 100.0% | 2416.1943 | 2416.8306 | 1 | 7.797 | 41.2% | 1 | K.LADELHMPSLPEMMFGDNVLR.I | 3 |
|  | CENPL\_Noc300\_122214\_01.16810.16810.2 | 3.2632 | 0.4302 | 100.0% | 2417.3123 | 2416.8306 | 1 | 6.362 | 45.0% | 1 | K.LADELHMPSLPEMMFGDNVLR.I | 2 |
|  | CENPL\_Noc300\_tube2\_122214\_01.17505.17505.2 | 5.487 | 0.6216 | 100.0% | 1933.9521 | 1934.1185 | 1 | 10.119 | 67.6% | 4 | R.IQHGSGFGIEFNATDALR.C | 2 |
|  | CENPL\_Noc300\_122214\_02.10388.10388.3 | 3.9672 | 0.308 | 100.0% | 1934.1843 | 1934.1185 | 2 | 6.237 | 38.2% | 2 | R.IQHGSGFGIEFNATDALR.C | 3 |
|  | CENPL\_Noc300\_tube2\_122214\_01.18960.18960.3 | 4.4582 | 0.3643 | 100.0% | 2822.1243 | 2822.184 | 1 | 6.858 | 29.3% | 1 | K.EVIKPYDWTYTTDYKGTLLGESLK.L | 3 |
|  | CENPL\_Noc300\_122214\_01.10103.10103.2 | 2.1149 | 0.3295 | 99.3% | 918.1322 | 918.07825 | 2 | 5.625 | 68.8% | 1 | K.GTLLGESLK.L | 2 |
|  | CENPL\_Noc300\_122214\_01.06152.06152.2 | 2.7359 | 0.5497 | 100.0% | 1355.2322 | 1355.4875 | 1 | 8.243 | 68.2% | 1 | K.VVPTTDHIDTEK.L | 2 |
|  | CENPL\_Noc300\_122214\_02.13349.13349.3 | 4.8775 | 0.4558 | 100.0% | 2677.6143 | 2677.9702 | 1 | 7.365 | 36.4% | 1 | K.FFEEVLLFEDELHDHGVSSLSVK.I | 3 |
| \* | CENPL\_Noc300\_122214\_01.07585.07585.2 | 3.7232 | 0.3059 | 100.0% | 1540.8322 | 1540.7772 | 1 | 6.631 | 77.3% | 1 | R.LYHEADKTYMLR.E | 2 |

---

|  |  |  |  |  |  |  |  |  |
| --- | --- | --- | --- | --- | --- | --- | --- | --- |
| U | *gi|20357599|ref|NP\_61* | 3 | 9 | 39.5% | 114 | 12146 | 10.5 | H2A histone family, member V isoform 2 [Homo sapiens] |
| U | *gi|6912616|ref|NP\_036* | 3 | 9 | 35.2% | 128 | 13509 | 10.6 | H2A histone family, member V isoform 1 [Homo sapiens] |
| U | *gi|4504255|ref|NP\_002* | 3 | 9 | 35.2% | 128 | 13553 | 10.6 | H2A histone family, member Z [Homo sapiens] |

| Filename XCorr DeltCN Conf% ObsM+H+ CalcM+H+ SpR ZScore Ion% # Sequence  | | | | | | | | | | | | |
| --- | --- | --- | --- | --- | --- | --- | --- | --- | --- | --- | --- | --- |
|  | CENPL\_Noc300\_tube2\_122214\_01.14990.14990.2 | 2.9694 | 0.2377 | 99.8% | 945.27216 | 945.1093 | 3 | 5.186 | 81.2% | 6 | R.AGLQFPVGR.I | 222 |
|  | CENPL\_Noc300\_122214\_01.20519.20519.3 | 5.6495 | 0.4217 | 100.0% | 2898.1143 | 2897.2952 | 1 | 8.878 | 29.5% | 1 | R.VGATAAVYSAAILEYLTAEVLELAGNASK.D | 3 |
|  | CENPL\_Noc300\_122214\_01.07954.07954.2 | 2.374 | 0.2222 | 99.5% | 851.2322 | 851.0396 | 1 | 5.231 | 91.7% | 2 | R.HLQLAIR.G | 22 |

Similarities:
gi|10800130|ref|NP\_06(2:1)  
gi|113425815|ref|XP\_9(1:2)  

---

|  |  |  |  |  |  |  |  |  |
| --- | --- | --- | --- | --- | --- | --- | --- | --- |
| U | *gi|4758302|ref|NP\_004* | 5 | 20 | 39.4% | 104 | 12259 | 5.9 | enhancer of rudimentary homolog [Homo sapiens] |

| Filename XCorr DeltCN Conf% ObsM+H+ CalcM+H+ SpR ZScore Ion% # Sequence  | | | | | | | | | | | | |
| --- | --- | --- | --- | --- | --- | --- | --- | --- | --- | --- | --- | --- |
| \* | CENPL\_Noc300\_122214\_02.08864.08864.2 | 5.3593 | 0.5373 | 100.0% | 2055.112 | 2056.1375 | 1 | 10.874 | 68.8% | 1 | R.TYADYESVNECMEGVCK.M | 2 |
| \* | CENPL\_Noc300\_122214\_01.06076.06076.2 | 3.0716 | 0.4175 | 100.0% | 1329.1721 | 1329.4087 | 1 | 7.064 | 70.0% | 5 | R.ADTQTYQPYNK.D | 2 |
| \* | CENPL\_Noc300\_tube2\_122214\_01.12794.12794.2 | 3.8682 | 0.3689 | 100.0% | 1871.4722 | 1872.0441 | 1 | 6.819 | 64.3% | 7 | R.ADTQTYQPYNKDWIK.E | 2 |
| \* | CENPL\_Noc300\_tube2\_122214\_01.11320.11320.3 | 3.5363 | 0.4649 | 100.0% | 2128.7644 | 2129.3337 | 1 | 6.906 | 45.3% | 2 | R.ADTQTYQPYNKDWIKEK.I | 3 |
| \* | CENPL\_Noc300\_122214\_01.09361.09361.2 | 2.4357 | 0.0868 | 98.2% | 934.21216 | 933.185 | 1 | 4.768 | 91.7% | 5 | K.IYVLLRR.Q | 2 |

---

|  |  |  |  |  |  |  |  |  |
| --- | --- | --- | --- | --- | --- | --- | --- | --- |
| U | *gi|4502923|ref|NP\_001* | 6 | 9 | 38.6% | 329 | 36414 | 6.1 | calponin 3 [Homo sapiens] |

| Filename XCorr DeltCN Conf% ObsM+H+ CalcM+H+ SpR ZScore Ion% # Sequence  | | | | | | | | | | | | |
| --- | --- | --- | --- | --- | --- | --- | --- | --- | --- | --- | --- | --- |
|  | CENPL\_Noc300\_tube2\_122214\_01.11229.11229.2 | 2.4468 | 0.303 | 99.5% | 1108.3722 | 1108.2365 | 1 | 5.955 | 70.0% | 2 | K.GPSYGLSAEVK.N | 2 |
| \* | CENPL\_Noc300\_122214\_01.18812.18812.3 | 3.8101 | 0.2606 | 99.9% | 3491.8442 | 3491.8567 | 1 | 4.777 | 26.7% | 1 | K.YDHQAEEDLRNWIEEVTGMSIGPNFQLGLK.D | 3 |
| \* | CENPL\_Noc300\_122214\_01.17934.17934.3 | 5.6718 | 0.3598 | 100.0% | 4224.1445 | 4222.821 | 1 | 6.247 | 23.7% | 1 | K.AIQAYGMKPHDIFEANDLFENGNMTQVQTTLVALAGLAK.T | 3 |
| \* | CENPL\_Noc300\_122214\_02.07986.07986.3 | 2.7126 | 0.4196 | 100.0% | 1417.9143 | 1417.6482 | 57 | 6.122 | 33.3% | 1 | K.TKGFHTTIDIGVK.Y | 3 |
| \* | CENPL\_Noc300\_tube2\_122214\_01.13275.13275.2 | 4.4164 | 0.5074 | 100.0% | 1404.3121 | 1404.6244 | 1 | 9.782 | 80.8% | 3 | K.AGQSVIGLQMGTNK.C | 2 |
| \* | CENPL\_Noc300\_122214\_02.09430.09430.3 | 4.0094 | 0.4386 | 100.0% | 2284.4343 | 2285.5923 | 1 | 7.268 | 38.2% | 1 | K.MQTDKPFDQTTISLQMGTNK.G | 3 |

---

|  |  |  |  |  |  |  |  |  |
| --- | --- | --- | --- | --- | --- | --- | --- | --- |
| U | *gi|167234419|ref|NP\_0* | 46 | 146 | 38.4% | 955 | 108666 | 10.2 | thyroid hormone receptor associated protein 3 [Homo sapiens] |

| Filename XCorr DeltCN Conf% ObsM+H+ CalcM+H+ SpR ZScore Ion% # Sequence  | | | | | | | | | | | | |
| --- | --- | --- | --- | --- | --- | --- | --- | --- | --- | --- | --- | --- |
| \* | CENPL\_Noc300\_122214\_01.05585.05585.3 | 5.1355 | 0.4464 | 100.0% | 3503.3342 | 3503.545 | 1 | 6.714 | 28.0% | 1 | K.SSSKDSRPSQAAGDNQGDEAKEQTFSGGTSQDTK.A | 3 |
| \* | CENPL\_Noc300\_122214\_01.05950.05950.3 | 4.8958 | 0.5377 | 100.0% | 3112.8843 | 3114.1362 | 1 | 8.599 | 32.8% | 1 | K.DSRPSQAAGDNQGDEAKEQTFSGGTSQDTK.A | 3 |
| \* | CENPL\_Noc300\_tube2\_122214\_01.10502.10502.3 | 4.5607 | 0.4466 | 100.0% | 2056.2244 | 2056.1528 | 1 | 7.579 | 39.5% | 6 | K.ASESSKPWPDATYGTGSASR.A | 3 |
| \* | CENPL\_Noc300\_tube2\_122214\_01.10552.10552.2 | 5.3564 | 0.4613 | 100.0% | 2056.3123 | 2056.1528 | 1 | 7.667 | 57.9% | 5 | K.ASESSKPWPDATYGTGSASR.A | 2 |
| \* | CENPL\_Noc300\_tube2\_122214\_01.11646.11646.3 | 3.9448 | 0.3288 | 100.0% | 2136.0544 | 2136.1528 | 3 | 5.056 | 34.2% | 1 | K.ASESSKPWPDATYGTGS\*ASR.A | 3 |
| \* | CENPL\_Noc300\_tube2\_122214\_01.09702.09702.2 | 3.1103 | 0.3464 | 100.0% | 1017.2522 | 1017.1271 | 1 | 7.102 | 77.8% | 6 | R.ASAVSELSPR.E | 2 |
| \* | CENPL\_Noc300\_tube2\_122214\_01.09566.09566.2 | 2.6691 | 0.3139 | 99.8% | 1097.2122 | 1097.1271 | 2 | 6.577 | 83.3% | 6 | R.ASAVSELS\*PR.E | 2 |
| \* | CENPL\_Noc300\_tube2\_122214\_01.12285.12285.3 | 3.1481 | 0.1654 | 96.2% | 1847.3043 | 1847.0818 | 1 | 4.82 | 41.7% | 1 | R.ERSPALKS\*PLQSVVVR.R | 3 |
| \* | CENPL\_Noc300\_tube2\_122214\_01.13803.13803.3 | 2.702 | 0.2896 | 99.6% | 1847.3344 | 1847.0818 | 3 | 5.147 | 35.0% | 1 | R.ERS\*PALKSPLQSVVVR.R | 3 |
| \* | CENPL\_Noc300\_tube2\_122214\_01.13550.13550.3 | 2.7962 | 0.3312 | 99.9% | 1926.9243 | 1927.0818 | 11 | 5.178 | 33.3% | 3 | R.ERS\*PALKS\*PLQSVVVR.R | 3 |
| \* | CENPL\_Noc300\_122214\_01.10229.10229.2 | 4.1064 | 0.4343 | 100.0% | 1562.5322 | 1561.7788 | 1 | 6.593 | 61.5% | 2 | R.SPALKS\*PLQSVVVR.R | 2 |
| \* | CENPL\_Noc300\_tube2\_122214\_01.12015.12015.2 | 3.1027 | 0.2557 | 100.0% | 984.65216 | 985.17163 | 1 | 6.304 | 87.5% | 3 | K.SPLQSVVVR.R | 2 |
| \* | CENPL\_Noc300\_tube2\_122214\_01.08558.08558.3 | 4.7674 | 0.5336 | 100.0% | 2608.3145 | 2607.7039 | 1 | 8.489 | 36.5% | 1 | K.SPPSTGSTYGSSQKEESAASGGAAYTK.R | 3 |
| \* | CENPL\_Noc300\_122214\_01.07780.07780.2 | 3.3788 | 0.5678 | 100.0% | 1140.7122 | 1141.1797 | 1 | 9.124 | 72.7% | 4 | K.GSFSDTGLGDGK.M | 2 |
| \* | CENPL\_Noc300\_122214\_01.05712.05712.3 | 4.0685 | 0.1921 | 99.9% | 2112.3245 | 2111.3057 | 6 | 5.141 | 40.6% | 1 | K.KMADFHKEEMDDQDKDK.A | 3 |
| \* | CENPL\_Noc300\_122214\_01.05466.05466.3 | 4.7705 | 0.2792 | 100.0% | 2312.1843 | 2310.5586 | 1 | 5.384 | 38.9% | 1 | K.KMADFHKEEMDDQDKDKAK.G | 3 |
| \* | CENPL\_Noc300\_122214\_01.06078.06078.2 | 4.1692 | 0.3426 | 100.0% | 1982.2722 | 1983.1317 | 2 | 5.846 | 60.0% | 1 | K.MADFHKEEMDDQDKDK.A | 2 |
| \* | CENPL\_Noc300\_122214\_01.06093.06093.3 | 4.9529 | 0.2815 | 100.0% | 1984.0743 | 1983.1317 | 3 | 5.707 | 46.7% | 4 | K.MADFHKEEMDDQDKDK.A | 3 |
| \* | CENPL\_Noc300\_122214\_01.05720.05720.3 | 3.8313 | 0.3303 | 100.0% | 2181.4744 | 2182.3845 | 1 | 6.42 | 38.2% | 1 | K.MADFHKEEMDDQDKDKAK.G | 3 |
| \* | CENPL\_Noc300\_122214\_01.06445.06445.2 | 1.9074 | 0.2204 | 95.0% | 1095.5122 | 1096.0924 | 35 | 5.758 | 62.5% | 2 | K.ESEFDDEPK.F | 2 |
| \* | CENPL\_Noc300\_122214\_01.10268.10268.3 | 3.1037 | 0.2851 | 99.9% | 1490.2444 | 1489.7141 | 15 | 4.815 | 40.4% | 3 | K.SGKWEGLVYAPPGK.E | 3 |
| \* | CENPL\_Noc300\_122214\_01.10256.10256.2 | 4.3326 | 0.4655 | 100.0% | 1490.4521 | 1489.7141 | 1 | 8.097 | 73.1% | 4 | K.SGKWEGLVYAPPGK.E | 2 |
| \* | CENPL\_Noc300\_tube2\_122214\_01.12026.12026.3 | 4.144 | 0.3489 | 100.0% | 1747.1943 | 1747.0037 | 1 | 6.132 | 51.7% | 4 | K.SGKWEGLVYAPPGKEK.Q | 3 |
| \* | CENPL\_Noc300\_122214\_01.11650.11650.2 | 3.6667 | 0.4149 | 100.0% | 1218.1721 | 1217.4099 | 1 | 7.66 | 80.0% | 4 | K.WEGLVYAPPGK.E | 2 |
| \* | CENPL\_Noc300\_122214\_01.10178.10178.3 | 2.7483 | 0.2776 | 99.8% | 1474.5543 | 1474.6995 | 2 | 5.402 | 43.8% | 1 | K.WEGLVYAPPGKEK.Q | 3 |
| \* | CENPL\_Noc300\_tube2\_122214\_01.10533.10533.3 | 3.7402 | 0.204 | 99.9% | 1623.8944 | 1623.7136 | 1 | 5.449 | 43.8% | 5 | R.KTEELEEESFPER.S | 3 |
| \* | CENPL\_Noc300\_tube2\_122214\_01.10631.10631.2 | 4.6331 | 0.3857 | 100.0% | 1624.0721 | 1623.7136 | 1 | 8.237 | 66.7% | 8 | R.KTEELEEESFPER.S | 2 |
| \* | CENPL\_Noc300\_122214\_01.09324.09324.2 | 4.3092 | 0.491 | 100.0% | 1496.2322 | 1495.5396 | 1 | 8.226 | 81.8% | 7 | K.TEELEEESFPER.S | 2 |
| \* | CENPL\_Noc300\_122214\_01.07811.07811.2 | 2.0816 | 0.1586 | 97.0% | 798.1122 | 797.88635 | 3 | 3.787 | 83.3% | 1 | K.SSFSITR.E | 23 |
| \* | CENPL\_Noc300\_tube2\_122214\_01.17432.17432.3 | 3.9073 | 0.4618 | 100.0% | 2151.5645 | 2151.3557 | 1 | 7.417 | 38.9% | 6 | R.MDSFDEDLARPSGLLAQER.K | 3 |
| \* | CENPL\_Noc300\_122214\_01.05920.05920.3 | 2.9965 | 0.3675 | 100.0% | 1631.2444 | 1630.8008 | 2 | 6.301 | 39.6% | 1 | R.DLVHSNKKEQEFR.S | 3 |
| \* | CENPL\_Noc300\_122214\_01.08758.08758.2 | 4.341 | 0.4692 | 100.0% | 1531.0721 | 1530.6835 | 1 | 7.937 | 70.8% | 7 | R.SIFQHIQSAQSQR.S | 2 |
| \* | CENPL\_Noc300\_tube2\_122214\_01.11486.11486.3 | 3.665 | 0.2988 | 100.0% | 1531.1044 | 1530.6835 | 1 | 7.194 | 52.1% | 7 | R.SIFQHIQSAQSQR.S | 3 |
| \* | CENPL\_Noc300\_tube2\_122214\_01.17168.17168.3 | 5.3968 | 0.4677 | 100.0% | 2043.9543 | 2043.3762 | 1 | 8.402 | 48.5% | 7 | R.SPSELFAQHIVTIVHHVK.E | 3 |
| \* | CENPL\_Noc300\_122214\_01.06214.06214.2 | 3.2096 | 0.4614 | 100.0% | 1624.7722 | 1625.7588 | 1 | 7.503 | 69.2% | 1 | K.EHHFGSSGMTLHER.F | 2 |
| \* | CENPL\_Noc300\_122214\_01.06266.06266.3 | 3.228 | 0.4242 | 100.0% | 1625.9043 | 1625.7588 | 2 | 6.89 | 42.3% | 3 | K.EHHFGSSGMTLHER.F | 3 |
| \* | CENPL\_Noc300\_122214\_01.11916.11916.2 | 2.4656 | 0.2657 | 99.5% | 1115.6122 | 1116.1729 | 1 | 5.268 | 81.2% | 4 | R.IDIS\*PSTFR.K | 2 |
| \* | CENPL\_Noc300\_122214\_01.05376.05376.3 | 2.8087 | 0.2425 | 97.8% | 2160.4143 | 2161.357 | 1 | 4.843 | 42.6% | 1 | R.KHGLAHDEMKS\*PREPGYK.A | 3 |
| \* | CENPL\_Noc300\_122214\_01.05732.05732.3 | 2.8825 | 0.2463 | 98.7% | 2032.6144 | 2033.1827 | 2 | 5.393 | 37.5% | 2 | K.HGLAHDEMKS\*PREPGYK.A | 3 |
| \* | CENPL\_Noc300\_122214\_01.05535.05535.3 | 2.9849 | 0.2321 | 97.6% | 2419.2544 | 2418.603 | 1 | 4.866 | 36.2% | 1 | K.HGLAHDEMKS\*PREPGYKAEGK.Y | 3 |
| \* | CENPL\_Noc300\_122214\_01.07361.07361.2 | 2.9346 | 0.2595 | 99.9% | 1122.4922 | 1121.2352 | 2 | 4.915 | 68.8% | 3 | K.YKDDPVDLR.L | 2 |
| \* | CENPL\_Noc300\_122214\_02.07492.07492.3 | 4.069 | 0.5244 | 100.0% | 2380.7344 | 2381.379 | 1 | 7.636 | 35.5% | 1 | K.AEEYTEETEEREESTTGFDK.S | 3 |
| \* | CENPL\_Noc300\_122214\_01.08796.08796.2 | 3.9688 | 0.4424 | 100.0% | 1693.1122 | 1693.7667 | 1 | 7.944 | 79.2% | 4 | R.NREEEWDPEYTPK.S | 2 |
| \* | CENPL\_Noc300\_tube2\_122214\_01.11463.11463.3 | 3.5263 | 0.2669 | 99.9% | 1694.2444 | 1693.7667 | 7 | 6.302 | 47.9% | 4 | R.NREEEWDPEYTPK.S | 3 |
|  | CENPL\_Noc300\_122214\_01.09801.09801.2 | 2.746 | 0.4413 | 100.0% | 1423.2922 | 1423.4755 | 1 | 6.708 | 65.0% | 3 | R.EEEWDPEYTPK.S | 22 |
| \* | CENPL\_Noc300\_122214\_01.09173.09173.3 | 4.4123 | 0.3914 | 100.0% | 4083.6243 | 4083.9636 | 1 | 6.426 | 25.0% | 3 | K.WAHDKFS\*GEEGEIEDDES\*GTENREEKDNIQPTTE.- | 3 |

Similarities:
gi|7661958|ref|NP\_055(1:45)  

---

|  |  |  |  |  |  |  |  |  |
| --- | --- | --- | --- | --- | --- | --- | --- | --- |
| U | *gi|14141152|ref|NP\_00* | 20 | 47 | 38.1% | 730 | 77516 | 8.7 | heterogeneous nuclear ribonucleoprotein M isoform a [Homo sapiens] |
| U | *gi|157412270|ref|NP\_1* | 20 | 47 | 40.2% | 691 | 73621 | 8.8 | heterogeneous nuclear ribonucleoprotein M isoform b [Homo sapiens] |

| Filename XCorr DeltCN Conf% ObsM+H+ CalcM+H+ SpR ZScore Ion% # Sequence  | | | | | | | | | | | | |
| --- | --- | --- | --- | --- | --- | --- | --- | --- | --- | --- | --- | --- |
|  | CENPL\_Noc300\_tube2\_122214\_01.19548.19548.2 | 3.0233 | 0.4583 | 100.0% | 1265.2522 | 1265.4949 | 1 | 8.27 | 75.0% | 1 | R.AFITNIPFDVK.W | 2 |
|  | CENPL\_Noc300\_tube2\_122214\_01.14958.14958.2 | 2.3254 | 0.2641 | 99.3% | 1117.4521 | 1117.3335 | 6 | 4.939 | 68.8% | 1 | K.WQSLKDLVK.E | 2 |
|  | CENPL\_Noc300\_122214\_02.13084.13084.2 | 4.3774 | 0.5524 | 100.0% | 1753.5122 | 1754.0051 | 1 | 9.872 | 60.0% | 1 | K.VGEVTYVELLMDAEGK.S | 2 |
|  | CENPL\_Noc300\_122214\_02.10449.10449.2 | 3.6257 | 0.5428 | 100.0% | 1426.6721 | 1427.6403 | 1 | 8.649 | 70.8% | 2 | R.LGSTVFVANLDYK.V | 2 |
|  | CENPL\_Noc300\_tube2\_122214\_01.16884.16884.2 | 3.5122 | 0.3931 | 100.0% | 1435.4122 | 1435.768 | 1 | 7.211 | 75.0% | 2 | K.LKEVFSMAGVVVR.A | 2 |
|  | CENPL\_Noc300\_122214\_01.18726.18726.3 | 5.332 | 0.4583 | 100.0% | 3665.0942 | 3666.2478 | 1 | 8.187 | 22.7% | 1 | R.GIGTVTFEQSIEAVQAISMFNGQLLFDRPMHVK.M | 3 |
|  | CENPL\_Noc300\_tube2\_122214\_01.20196.20196.2 | 4.2337 | 0.3632 | 100.0% | 2180.2122 | 2179.5752 | 1 | 7.966 | 50.0% | 2 | K.GIGMGNIGPAGMGMEGIGFGINK.M | 2 |
|  | CENPL\_Noc300\_tube2\_122214\_01.17574.17574.2 | 4.1336 | 0.5411 | 100.0% | 1714.9722 | 1715.9724 | 1 | 9.021 | 59.4% | 3 | K.MGGMEGPFGGGMENMGR.F | 2 |
|  | CENPL\_Noc300\_122214\_01.07727.07727.2 | 2.4356 | 0.4137 | 99.9% | 957.3522 | 957.11017 | 2 | 6.916 | 75.0% | 1 | R.FGSGMNMGR.I | 2 |
|  | CENPL\_Noc300\_tube2\_122214\_01.15680.15680.2 | 3.3213 | 0.3288 | 100.0% | 1115.1921 | 1115.3152 | 1 | 5.319 | 88.9% | 4 | R.INEILSNALK.R | 2 |
|  | CENPL\_Noc300\_122214\_01.06847.06847.2 | 2.8155 | 0.3879 | 99.8% | 1285.2122 | 1285.3591 | 3 | 5.849 | 50.0% | 1 | K.QGGGGGGGSVPGIER.M | 2 |
|  | CENPL\_Noc300\_122214\_01.06406.06406.2 | 3.2189 | 0.2925 | 100.0% | 1103.2722 | 1102.2714 | 1 | 6.205 | 80.0% | 1 | R.MGAGLGHGMDR.V | 2 |
|  | CENPL\_Noc300\_122214\_01.09199.09199.2 | 2.1343 | 0.3828 | 99.8% | 822.1922 | 822.0283 | 1 | 7.151 | 91.7% | 1 | R.MGLVMDR.M | 2 |
|  | CENPL\_Noc300\_tube2\_122214\_01.16115.16115.2 | 4.6017 | 0.4995 | 100.0% | 1613.8722 | 1614.875 | 1 | 9.52 | 71.4% | 4 | R.MGPLGLDHMASSIER.M | 2 |
|  | CENPL\_Noc300\_tube2\_122214\_01.16166.16166.3 | 3.5298 | 0.4777 | 100.0% | 1615.1943 | 1614.875 | 2 | 8.131 | 46.4% | 4 | R.MGPLGLDHMASSIER.M | 3 |
|  | CENPL\_Noc300\_tube2\_122214\_01.15999.15999.2 | 3.068 | 0.5026 | 100.0% | 1125.4321 | 1126.3337 | 1 | 8.727 | 80.0% | 4 | R.MGAGMGFGLER.M | 2 |
|  | CENPL\_Noc300\_tube2\_122214\_01.12548.12548.2 | 2.5379 | 0.2467 | 99.1% | 1189.1122 | 1189.4333 | 3 | 5.081 | 68.2% | 2 | R.MVPAGMGAGLER.M | 2 |
|  | CENPL\_Noc300\_122214\_01.10943.10943.2 | 3.3243 | 0.3926 | 100.0% | 1428.1921 | 1428.7076 | 1 | 6.516 | 75.0% | 5 | R.MGPAMGPALGAGIER.M | 2 |
|  | CENPL\_Noc300\_122214\_02.09138.09138.2 | 3.3662 | 0.4975 | 100.0% | 1384.4922 | 1384.5677 | 1 | 8.498 | 67.9% | 3 | R.MGLAMGGGGGASFDR.A | 2 |
|  | CENPL\_Noc300\_tube2\_122214\_01.14272.14272.3 | 3.4383 | 0.4713 | 100.0% | 2036.0944 | 2036.1735 | 1 | 7.231 | 29.5% | 4 | R.GNFGGSFAGSFGGAGGHAPGVAR.K | 3 |

---

|  |  |  |  |  |  |  |  |  |
| --- | --- | --- | --- | --- | --- | --- | --- | --- |
| U | *gi|21361320|ref|NP\_00* | 15 | 46 | 38.0% | 400 | 43448 | 5.1 | TRK-fused [Homo sapiens] |
| U | *gi|56090139|ref|NP\_00* | 15 | 46 | 38.0% | 400 | 43448 | 5.1 | TRK-fused [Homo sapiens] |

| Filename XCorr DeltCN Conf% ObsM+H+ CalcM+H+ SpR ZScore Ion% # Sequence  | | | | | | | | | | | | |
| --- | --- | --- | --- | --- | --- | --- | --- | --- | --- | --- | --- | --- |
|  | CENPL\_Noc300\_122214\_01.07047.07047.2 | 2.7385 | 0.1378 | 99.4% | 902.1122 | 901.9951 | 1 | 5.153 | 85.7% | 2 | K.AQLGEDIR.R | 2 |
|  | CENPL\_Noc300\_tube2\_122214\_01.19319.19319.3 | 4.6571 | 0.4559 | 100.0% | 2487.4443 | 2487.8945 | 1 | 7.775 | 38.2% | 1 | R.RIPIHNEDITYDELVLMMQR.V | 3 |
|  | CENPL\_Noc300\_tube2\_122214\_01.20070.20070.2 | 5.8002 | 0.4816 | 100.0% | 2330.8523 | 2331.707 | 1 | 10.112 | 69.4% | 2 | R.IPIHNEDITYDELVLMMQR.V | 2 |
|  | CENPL\_Noc300\_122214\_01.14938.14938.3 | 3.8671 | 0.3419 | 100.0% | 2331.6843 | 2331.707 | 2 | 6.378 | 34.7% | 3 | R.IPIHNEDITYDELVLMMQR.V | 3 |
|  | CENPL\_Noc300\_tube2\_122214\_01.10833.10833.2 | 2.6383 | 0.1654 | 98.3% | 1317.2122 | 1317.5254 | 2 | 5.055 | 72.7% | 2 | R.GKLLSNDEVTIK.Y | 2 |
|  | CENPL\_Noc300\_tube2\_122214\_01.11918.11918.2 | 3.0169 | 0.3482 | 100.0% | 1132.3322 | 1132.2994 | 1 | 5.559 | 88.9% | 4 | K.LLSNDEVTIK.Y | 2 |
|  | CENPL\_Noc300\_122214\_01.11162.11162.2 | 4.5055 | 0.4482 | 100.0% | 2013.8522 | 2014.3323 | 1 | 7.279 | 44.1% | 5 | K.LTLFVNGQPRPLESSQVK.Y | 2 |
|  | CENPL\_Noc300\_tube2\_122214\_01.15435.15435.3 | 3.4504 | 0.3897 | 100.0% | 2015.3043 | 2014.3323 | 1 | 6.372 | 33.8% | 3 | K.LTLFVNGQPRPLESSQVK.Y | 3 |
|  | CENPL\_Noc300\_tube2\_122214\_01.16310.16310.2 | 3.9508 | 0.5377 | 100.0% | 2720.7322 | 2720.9067 | 1 | 8.671 | 44.0% | 3 | R.LLDSLEPPGEPGPSTNIPENDTVDGR.E | 2 |
|  | CENPL\_Noc300\_tube2\_122214\_01.14555.14555.3 | 3.8788 | 0.3549 | 100.0% | 3107.4243 | 3107.3118 | 1 | 5.579 | 28.6% | 6 | R.LLDSLEPPGEPGPSTNIPENDTVDGREEK.S | 3 |
|  | CENPL\_Noc300\_tube2\_122214\_01.19547.19547.2 | 3.6627 | 0.5286 | 100.0% | 1811.6522 | 1813.0938 | 1 | 9.994 | 59.4% | 1 | K.QSTQVMAASMSAFDPLK.N | 2 |
|  | CENPL\_Noc300\_tube2\_122214\_01.18827.18827.3 | 5.4493 | 0.5242 | 100.0% | 3303.5344 | 3304.5261 | 1 | 7.552 | 28.3% | 1 | K.NQDEINKNVMSAFGLTDDQVSGPPSAPAEDR.S | 3 |
|  | CENPL\_Noc300\_tube2\_122214\_01.18938.18938.2 | 4.8965 | 0.603 | 100.0% | 2461.5723 | 2462.6501 | 1 | 10.444 | 54.3% | 2 | K.NVMSAFGLTDDQVSGPPSAPAEDR.S | 2 |
|  | CENPL\_Noc300\_122214\_01.08318.08318.3 | 4.4563 | 0.3823 | 100.0% | 1892.6643 | 1893.0714 | 1 | 6.309 | 51.6% | 7 | R.NRPPFGQGYTQPGPGYR.- | 3 |
|  | CENPL\_Noc300\_tube2\_122214\_01.10824.10824.2 | 4.2193 | 0.4588 | 100.0% | 1893.2122 | 1893.0714 | 1 | 8.105 | 65.6% | 4 | R.NRPPFGQGYTQPGPGYR.- | 2 |

---

|  |  |  |  |  |  |  |  |  |
| --- | --- | --- | --- | --- | --- | --- | --- | --- |
| U | *gi|7706423|ref|NP\_057* | 6 | 11 | 37.9% | 103 | 11602 | 5.3 | U6 snRNA-associated Sm-like protein LSm7 [Homo sapiens] |

| Filename XCorr DeltCN Conf% ObsM+H+ CalcM+H+ SpR ZScore Ion% # Sequence  | | | | | | | | | | | | |
| --- | --- | --- | --- | --- | --- | --- | --- | --- | --- | --- | --- | --- |
| \* | CENPL\_Noc300\_tube2\_122214\_01.15400.15400.1 | 1.8776 | 0.2582 | 95.3% | 904.57 | 905.0361 | 10 | 5.244 | 64.3% | 3 | K.ESILDLSK.Y | 1 |
| \* | CENPL\_Noc300\_tube2\_122214\_01.15380.15380.2 | 2.1157 | 0.2269 | 98.2% | 905.1322 | 905.0361 | 1 | 4.741 | 85.7% | 2 | K.ESILDLSK.Y | 2 |
| \* | CENPL\_Noc300\_122214\_02.16516.16516.2 | 3.8274 | 0.4433 | 100.0% | 2066.4321 | 2067.4077 | 1 | 7.924 | 50.0% | 1 | K.GFDPLLNLVLDGTIEYMR.D | 2 |
| \* | CENPL\_Noc300\_122214\_01.19158.19158.2 | 3.1638 | 0.329 | 99.9% | 2929.1921 | 2929.271 | 15 | 4.973 | 27.1% | 1 | K.GFDPLLNLVLDGTIEYMRDPDDQYK.L | 2 |
| \* | CENPL\_Noc300\_122214\_02.16010.16010.3 | 3.2981 | 0.2222 | 98.4% | 2930.3943 | 2929.271 | 1 | 5.071 | 30.2% | 2 | K.GFDPLLNLVLDGTIEYMRDPDDQYK.L | 3 |
| \* | CENPL\_Noc300\_122214\_01.18637.18637.3 | 4.4649 | 0.4183 | 100.0% | 3643.9143 | 3645.032 | 1 | 7.963 | 31.7% | 2 | K.GFDPLLNLVLDGTIEYMRDPDDQYKLTEDTR.Q | 3 |

---

|  |  |  |  |  |  |  |  |  |
| --- | --- | --- | --- | --- | --- | --- | --- | --- |
| U | *Reverse\_gi|169166523|* | 1 | 1 | 36.7% | 79 | 8984 | 9.0 | PREDICTED: hypothetical protein [Homo sapiens] |
| U | *Reverse\_gi|169166699|* | 1 | 1 | 36.7% | 79 | 8984 | 9.0 | PREDICTED: hypothetical protein [Homo sapiens] |
| U | *Reverse\_gi|169166641|* | 1 | 1 | 36.7% | 79 | 8984 | 9.0 | PREDICTED: hypothetical protein [Homo sapiens] |

| Filename XCorr DeltCN Conf% ObsM+H+ CalcM+H+ SpR ZScore Ion% # Sequence  | | | | | | | | | | | | |
| --- | --- | --- | --- | --- | --- | --- | --- | --- | --- | --- | --- | --- |
|  | CENPL\_Noc300\_122214\_01.14976.14976.3 | 2.9694 | 0.2733 | 99.0% | 3813.9543 | 3813.063 | 77 | 4.188 | 17.9% | 1 | K.IISRT#PKQWY@QT#SDSETFVGYRFIFDVRM.- | 3 |

---

|  |  |  |  |  |  |  |  |  |
| --- | --- | --- | --- | --- | --- | --- | --- | --- |
| U | *gi|85067507|ref|NP\_03* | 9 | 17 | 36.5% | 285 | 32745 | 4.8 | CCR4-NOT transcription complex, subunit 7 isoform 1 [Homo sapiens] |

| Filename XCorr DeltCN Conf% ObsM+H+ CalcM+H+ SpR ZScore Ion% # Sequence  | | | | | | | | | | | | |
| --- | --- | --- | --- | --- | --- | --- | --- | --- | --- | --- | --- | --- |
|  | CENPL\_Noc300\_tube2\_122214\_01.18489.18489.3 | 2.8735 | 0.3501 | 99.9% | 2660.7244 | 2661.0503 | 10 | 5.651 | 23.9% | 1 | R.KYNYVAMDTEFPGVVARPIGEFR.S | 3 |
|  | CENPL\_Noc300\_tube2\_122214\_01.14678.14678.2 | 3.1732 | 0.2998 | 100.0% | 1370.8922 | 1371.4924 | 1 | 6.184 | 65.0% | 6 | R.SNADYQYQLLR.C | 2 |
|  | CENPL\_Noc300\_122214\_01.18388.18388.2 | 4.4338 | 0.4017 | 100.0% | 2293.632 | 2293.5786 | 1 | 7.271 | 55.6% | 1 | K.ILTNSNLPEEELDFFEILR.L | 2 |
|  | CENPL\_Noc300\_tube2\_122214\_01.19325.19325.2 | 3.7044 | 0.5314 | 100.0% | 1926.8522 | 1927.1649 | 1 | 8.168 | 62.5% | 2 | K.NLKGGLQEVAEQLELER.I | 2 |
|  | CENPL\_Noc300\_122214\_01.14026.14026.2 | 3.9684 | 0.3249 | 100.0% | 1571.9722 | 1571.7275 | 1 | 7.081 | 61.5% | 2 | K.GGLQEVAEQLELER.I | 2 |
|  | CENPL\_Noc300\_122214\_01.14441.14441.2 | 3.8164 | 0.5838 | 100.0% | 2104.892 | 2106.404 | 1 | 10.231 | 55.3% | 1 | R.IGPQHQAGSDSLLTGMAFFK.M | 2 |
|  | CENPL\_Noc300\_tube2\_122214\_01.19762.19762.3 | 3.1763 | 0.22 | 98.5% | 2107.7344 | 2106.404 | 36 | 4.762 | 30.3% | 2 | R.IGPQHQAGSDSLLTGMAFFK.M | 3 |
| \* | CENPL\_Noc300\_122214\_02.09198.09198.3 | 2.4341 | 0.2979 | 99.0% | 1784.3043 | 1784.9991 | 39 | 4.654 | 36.5% | 1 | K.MREMFFEDHIDDAK.Y | 3 |
| \* | CENPL\_Noc300\_122214\_01.11292.11292.2 | 3.8402 | 0.4948 | 100.0% | 1497.1921 | 1497.619 | 1 | 7.956 | 86.4% | 1 | R.EMFFEDHIDDAK.Y | 2 |

---

|  |  |  |  |  |  |  |  |  |
| --- | --- | --- | --- | --- | --- | --- | --- | --- |
| U | *gi|9910596|ref|NP\_064* | 14 | 30 | 36.4% | 390 | 44264 | 6.0 | tuftelin 1 isoform 1 [Homo sapiens] |

| Filename XCorr DeltCN Conf% ObsM+H+ CalcM+H+ SpR ZScore Ion% # Sequence  | | | | | | | | | | | | |
| --- | --- | --- | --- | --- | --- | --- | --- | --- | --- | --- | --- | --- |
| \* | CENPL\_Noc300\_122214\_02.10407.10407.3 | 2.7894 | 0.2392 | 97.4% | 1996.0743 | 1996.2249 | 3 | 6.311 | 32.4% | 2 | R.LTLQGELTGDELEHIAQK.A | 3 |
|  | CENPL\_Noc300\_tube2\_122214\_01.10424.10424.2 | 3.4295 | 0.3057 | 100.0% | 1223.9521 | 1223.328 | 2 | 6.849 | 72.2% | 6 | K.SEVQYIQEAR.N | 2 |
|  | CENPL\_Noc300\_122214\_01.06736.06736.2 | 2.7635 | 0.2392 | 99.6% | 1332.2722 | 1332.4996 | 2 | 4.898 | 65.0% | 1 | K.LREDISSKLDR.N | 2 |
|  | CENPL\_Noc300\_122214\_01.06707.06707.3 | 2.7026 | 0.2223 | 98.6% | 1332.4143 | 1332.4996 | 54 | 4.673 | 47.5% | 1 | K.LREDISSKLDR.N | 3 |
|  | CENPL\_Noc300\_tube2\_122214\_01.10307.10307.2 | 2.7971 | 0.0873 | 98.7% | 1016.03217 | 1016.226 | 148 | 5.218 | 68.8% | 1 | R.KTVQDLLAK.L | 2 |
|  | CENPL\_Noc300\_122214\_01.06586.06586.2 | 2.2816 | 0.1606 | 97.5% | 1031.3121 | 1031.1106 | 12 | 4.791 | 68.8% | 1 | K.EAEVGELQR.R | 2 |
|  | CENPL\_Noc300\_tube2\_122214\_01.13982.13982.2 | 4.2984 | 0.4631 | 100.0% | 1554.2522 | 1554.845 | 1 | 8.148 | 65.4% | 3 | R.LLGMETEHQALLAK.V | 2 |
|  | CENPL\_Noc300\_tube2\_122214\_01.12656.12656.2 | 3.8993 | 0.3235 | 100.0% | 1399.6721 | 1400.5748 | 1 | 6.947 | 77.3% | 3 | K.VREGEVALEELR.S | 2 |
|  | CENPL\_Noc300\_tube2\_122214\_01.14198.14198.2 | 2.6969 | 0.2941 | 99.8% | 1144.6721 | 1145.2548 | 1 | 5.562 | 94.4% | 1 | R.EGEVALEELR.S | 2 |
|  | CENPL\_Noc300\_122214\_01.09438.09438.2 | 2.8032 | 0.23 | 99.5% | 1257.9122 | 1258.4606 | 1 | 5.27 | 63.6% | 1 | K.AATLEKEVAGLR.E | 2 |
|  | CENPL\_Noc300\_tube2\_122214\_01.10245.10245.2 | 2.0638 | 0.3556 | 99.3% | 1122.9521 | 1122.3275 | 2 | 5.77 | 75.0% | 1 | K.IHHLDDMLK.S | 2 |
|  | CENPL\_Noc300\_122214\_01.07721.07721.2 | 2.9286 | 0.3293 | 100.0% | 1218.7522 | 1219.3984 | 6 | 5.215 | 77.8% | 5 | R.QMIEQLQNSK.A | 2 |
|  | CENPL\_Noc300\_122214\_01.10691.10691.2 | 3.8306 | 0.3659 | 100.0% | 1705.3922 | 1704.8951 | 1 | 7.416 | 65.4% | 3 | K.IAYLEAENLEMHDR.M | 2 |
|  | CENPL\_Noc300\_tube2\_122214\_01.10240.10240.3 | 2.3847 | 0.3674 | 99.8% | 1626.4744 | 1627.9426 | 1 | 6.022 | 38.5% | 1 | R.ISKPPS\*PKPMPVIR.V | 3 |

---

|  |  |  |  |  |  |  |  |  |
| --- | --- | --- | --- | --- | --- | --- | --- | --- |
| U | *gi|14211889|ref|NP\_11* | 2 | 2 | 36.4% | 99 | 11250 | 4.9 | dpy-30-like protein [Homo sapiens] |

| Filename XCorr DeltCN Conf% ObsM+H+ CalcM+H+ SpR ZScore Ion% # Sequence  | | | | | | | | | | | | |
| --- | --- | --- | --- | --- | --- | --- | --- | --- | --- | --- | --- | --- |
| \* | CENPL\_Noc300\_122214\_01.20376.20376.3 | 4.8619 | 0.4833 | 100.0% | 2126.9944 | 2126.5876 | 1 | 8.742 | 36.8% | 1 | R.AYLDQTVVPILLQGLAVLAK.E | 3 |
| \* | CENPL\_Noc300\_122214\_01.19367.19367.3 | 2.7494 | 0.2747 | 99.5% | 1887.7444 | 1888.2169 | 1 | 4.588 | 36.7% | 1 | K.ERPPNPIEFLASYLLK.N | 3 |

---

|  |  |  |  |  |  |  |  |  |
| --- | --- | --- | --- | --- | --- | --- | --- | --- |
| U | *gi|4826998|ref|NP\_005* | 23 | 68 | 36.2% | 707 | 76150 | 9.4 | splicing factor proline/glutamine rich (polypyrimidine tract binding protein associated) [Homo sapiens] |

| Filename XCorr DeltCN Conf% ObsM+H+ CalcM+H+ SpR ZScore Ion% # Sequence  | | | | | | | | | | | | |
| --- | --- | --- | --- | --- | --- | --- | --- | --- | --- | --- | --- | --- |
| \* | CENPL\_Noc300\_122214\_01.07103.07103.2 | 2.2323 | 0.4312 | 99.6% | 1267.6921 | 1268.4332 | 16 | 7.463 | 59.1% | 2 | R.SPPPGMGLNQNR.G | 2 |
| \* | CENPL\_Noc300\_122214\_01.06111.06111.3 | 4.8494 | 0.4526 | 100.0% | 2372.3943 | 2371.725 | 1 | 7.402 | 30.2% | 2 | K.MPGGPKPGGGPGLSTPGGHPKPPHR.G | 3 |
| \* | CENPL\_Noc300\_tube2\_122214\_01.16691.16691.2 | 2.9605 | 0.1532 | 98.6% | 1649.6122 | 1650.8723 | 65 | 4.125 | 42.9% | 2 | K.ISDSEGFKANLSLLR.R | 2 |
| \* | CENPL\_Noc300\_122214\_02.10100.10100.3 | 4.4367 | 0.4313 | 100.0% | 1651.6444 | 1650.8723 | 1 | 6.944 | 48.2% | 1 | K.ISDSEGFKANLSLLR.R | 3 |
| \* | CENPL\_Noc300\_122214\_01.14830.14830.2 | 4.018 | 0.5389 | 100.0% | 1808.8322 | 1809.0258 | 1 | 8.022 | 66.7% | 2 | R.LFVGNLPADITEDEFK.R | 2 |
| \* | CENPL\_Noc300\_122214\_01.13672.13672.2 | 4.2042 | 0.4283 | 100.0% | 1965.5122 | 1965.2133 | 1 | 7.508 | 56.2% | 3 | R.LFVGNLPADITEDEFKR.L | 2 |
| \* | CENPL\_Noc300\_tube2\_122214\_01.13538.13538.2 | 3.4629 | 0.4688 | 100.0% | 1253.3121 | 1253.3971 | 1 | 7.965 | 85.0% | 9 | K.YGEPGEVFINK.G | 2 |
| \* | CENPL\_Noc300\_tube2\_122214\_01.16618.16618.2 | 4.4976 | 0.4997 | 100.0% | 1744.9722 | 1745.0007 | 1 | 9.311 | 70.0% | 1 | R.ALAEIAKAELDDTPMR.G | 2 |
| \* | CENPL\_Noc300\_122214\_01.07553.07553.2 | 2.8586 | 0.3631 | 100.0% | 1048.2322 | 1048.1559 | 1 | 6.515 | 87.5% | 7 | K.AELDDTPMR.G | 2 |
| \* | CENPL\_Noc300\_tube2\_122214\_01.09596.09596.2 | 3.5891 | 0.5189 | 100.0% | 1144.1921 | 1144.3188 | 1 | 8.759 | 85.0% | 5 | R.FATHAAALSVR.N | 2 |
| \* | CENPL\_Noc300\_122214\_01.18125.18125.2 | 4.9084 | 0.5472 | 100.0% | 2640.372 | 2640.9092 | 1 | 9.221 | 52.3% | 2 | R.NLSPYVSNELLEEAFSQFGPIER.A | 2 |
|  | CENPL\_Noc300\_122214\_01.07408.07408.1 | 2.1379 | 0.2466 | 95.5% | 886.56 | 887.0238 | 27 | 6.392 | 64.3% | 1 | R.AVVIVDDR.G | 11 |
|  | CENPL\_Noc300\_122214\_01.07359.07359.2 | 2.5797 | 0.3261 | 99.9% | 887.1722 | 887.0238 | 2 | 7.415 | 92.9% | 3 | R.AVVIVDDR.G | 22 |
| \* | CENPL\_Noc300\_tube2\_122214\_01.10737.10737.2 | 3.0069 | 0.4539 | 100.0% | 1246.3121 | 1246.452 | 1 | 6.951 | 72.7% | 6 | K.GIVEFASKPAAR.K | 2 |
| \* | CENPL\_Noc300\_tube2\_122214\_01.11336.11336.3 | 4.4337 | 0.3067 | 100.0% | 1763.8444 | 1763.8632 | 18 | 5.93 | 40.4% | 6 | R.FAQHGTFEYEYSQR.W | 3 |
| \* | CENPL\_Noc300\_122214\_02.07979.07979.2 | 4.716 | 0.4504 | 100.0% | 1764.3922 | 1763.8632 | 1 | 8.137 | 69.2% | 1 | R.FAQHGTFEYEYSQR.W | 2 |
| \* | CENPL\_Noc300\_122214\_01.11140.11140.3 | 5.8423 | 0.4909 | 100.0% | 2428.9744 | 2429.6233 | 1 | 8.359 | 51.3% | 4 | K.DKLESEMEDAYHEHQANLLR.Q | 3 |
| \* | CENPL\_Noc300\_122214\_01.05930.05930.2 | 4.117 | 0.3616 | 100.0% | 1417.1721 | 1417.5946 | 1 | 7.053 | 85.0% | 1 | R.MEELHNQEMQK.R | 2 |
| \* | CENPL\_Noc300\_122214\_01.06948.06948.2 | 2.7175 | 0.1969 | 99.5% | 1094.0122 | 1094.2892 | 3 | 4.541 | 78.6% | 1 | R.REEEMMIR.Q | 2 |
| \* | CENPL\_Noc300\_122214\_01.07690.07690.2 | 2.3519 | 0.2347 | 99.5% | 870.0122 | 870.029 | 11 | 6.481 | 75.0% | 1 | R.MGYMDPR.E | 2 |
| \* | CENPL\_Noc300\_tube2\_122214\_01.11597.11597.2 | 4.0001 | 0.5579 | 100.0% | 1772.2322 | 1772.9631 | 1 | 9.984 | 58.3% | 2 | R.MGGGGAMNMGDPYGSGGQK.F | 2 |
| \* | CENPL\_Noc300\_122214\_01.06814.06814.2 | 4.0962 | 0.5961 | 100.0% | 1341.6322 | 1342.4569 | 1 | 9.581 | 82.1% | 4 | R.FGQGGAGPVGGQGPR.G | 2 |
| \* | CENPL\_Noc300\_122214\_01.06969.06969.2 | 2.6229 | 0.5491 | 100.0% | 1120.7522 | 1121.2561 | 94 | 8.351 | 59.1% | 2 | R.GMGPGTPAGYGR.G | 2 |

Similarities:
gi|224028244|ref|NP\_0(2:21)  

---

|  |  |  |  |  |  |  |  |  |
| --- | --- | --- | --- | --- | --- | --- | --- | --- |
| U | *gi|5901998|ref|NP\_009* | 4 | 12 | 36.2% | 80 | 9128 | 9.6 | Sm protein F [Homo sapiens] |

| Filename XCorr DeltCN Conf% ObsM+H+ CalcM+H+ SpR ZScore Ion% # Sequence  | | | | | | | | | | | | |
| --- | --- | --- | --- | --- | --- | --- | --- | --- | --- | --- | --- | --- |
| \* | CENPL\_Noc300\_122214\_01.07420.07420.2 | 2.2549 | 0.1728 | 97.0% | 1109.3922 | 1109.4006 | 3 | 4.576 | 66.7% | 3 | K.QIIGRPVVVK.L | 2 |
| \* | CENPL\_Noc300\_tube2\_122214\_01.12218.12218.2 | 2.4821 | 0.3416 | 99.9% | 841.77216 | 841.94196 | 1 | 7.967 | 83.3% | 3 | K.YGDAFIR.G | 2 |
| \* | CENPL\_Noc300\_tube2\_122214\_01.12236.12236.2 | 3.3761 | 0.409 | 100.0% | 1237.2122 | 1237.3983 | 1 | 7.456 | 75.0% | 5 | R.GNNVLYISTQK.R | 2 |
| \* | CENPL\_Noc300\_tube2\_122214\_01.10216.10216.2 | 2.9188 | 0.3311 | 99.9% | 1393.2722 | 1393.5858 | 1 | 6.101 | 63.6% | 1 | R.GNNVLYISTQKR.R | 2 |

---

|  |  |  |  |  |  |  |  |  |
| --- | --- | --- | --- | --- | --- | --- | --- | --- |
| U | *gi|218505827|ref|NP\_1* | 14 | 37 | 36.1% | 316 | 35438 | 6.3 | TRAF4 associated factor 1 isoform a [Homo sapiens] |

| Filename XCorr DeltCN Conf% ObsM+H+ CalcM+H+ SpR ZScore Ion% # Sequence  | | | | | | | | | | | | |
| --- | --- | --- | --- | --- | --- | --- | --- | --- | --- | --- | --- | --- |
|  | CENPL\_Noc300\_tube2\_122214\_01.14817.14817.2 | 5.862 | 0.5549 | 100.0% | 2274.5723 | 2275.4802 | 1 | 9.789 | 66.7% | 6 | K.TVYSLQPPSALSGGQPADTQTR.A | 2 |
|  | CENPL\_Noc300\_tube2\_122214\_01.14757.14757.3 | 4.1441 | 0.4463 | 100.0% | 2275.2244 | 2275.4802 | 1 | 6.632 | 39.3% | 1 | K.TVYSLQPPSALSGGQPADTQTR.A | 3 |
|  | CENPL\_Noc300\_122214\_01.07894.07894.3 | 3.3168 | 0.4057 | 100.0% | 2244.3245 | 2244.507 | 11 | 6.203 | 31.9% | 3 | K.QKSEEELKDKNQLLEAVNK.Q | 3 |
|  | CENPL\_Noc300\_tube2\_122214\_01.11681.11681.3 | 4.8016 | 0.5351 | 100.0% | 1988.0343 | 1988.2023 | 1 | 7.964 | 42.2% | 2 | K.SEEELKDKNQLLEAVNK.Q | 3 |
|  | CENPL\_Noc300\_tube2\_122214\_01.11298.11298.2 | 2.5379 | 0.2187 | 99.0% | 1273.0322 | 1272.4441 | 1 | 4.81 | 75.0% | 1 | K.DKNQLLEAVNK.Q | 2 |
|  | CENPL\_Noc300\_122214\_01.08762.08762.2 | 2.5475 | 0.1641 | 98.8% | 1029.4722 | 1029.1814 | 1 | 4.305 | 87.5% | 4 | K.NQLLEAVNK.Q | 2 |
|  | CENPL\_Noc300\_122214\_01.08531.08531.2 | 3.9176 | 0.3266 | 100.0% | 1604.5322 | 1604.7979 | 1 | 6.766 | 61.5% | 2 | K.LTETQGELKDLTQK.V | 2 |
|  | CENPL\_Noc300\_122214\_01.13370.13370.3 | 5.0957 | 0.3323 | 100.0% | 2317.1943 | 2316.6543 | 1 | 6.575 | 47.4% | 2 | K.LTETQGELKDLTQKVELLEK.F | 3 |
|  | CENPL\_Noc300\_tube2\_122214\_01.14464.14464.2 | 2.6055 | 0.2538 | 99.4% | 1314.9321 | 1316.5376 | 1 | 5.426 | 75.0% | 2 | K.DLTQKVELLEK.F | 2 |
|  | CENPL\_Noc300\_tube2\_122214\_01.13910.13910.2 | 4.3986 | 0.6457 | 100.0% | 1387.2322 | 1387.5327 | 1 | 10.844 | 84.6% | 9 | K.GLDPALGSETLASR.Q | 2 |
|  | CENPL\_Noc300\_tube2\_122214\_01.21412.21412.2 | 3.7465 | 0.481 | 100.0% | 2592.132 | 2592.8928 | 8 | 7.744 | 31.0% | 2 | R.QESTTDHMDSMLLLETLQEELK.L | 2 |
|  | CENPL\_Noc300\_122214\_01.18133.18133.3 | 4.0857 | 0.4898 | 100.0% | 3394.3442 | 3396.8062 | 2 | 7.77 | 21.4% | 1 | R.QESTTDHMDSMLLLETLQEELKLFNETAK.K | 3 |
| \* | CENPL\_Noc300\_tube2\_122214\_01.10108.10108.2 | 3.3077 | 0.1831 | 99.8% | 1217.6122 | 1218.454 | 39 | 4.771 | 72.2% | 1 | K.KQMEELQALK.V | 2 |
| \* | CENPL\_Noc300\_tube2\_122214\_01.10128.10128.3 | 3.7295 | 0.0625 | 98.2% | 1218.4143 | 1218.454 | 85 | 3.964 | 50.0% | 1 | K.KQMEELQALK.V | 3 |

---

|  |  |  |  |  |  |  |  |  |
| --- | --- | --- | --- | --- | --- | --- | --- | --- |
| U | *gi|4503571|ref|NP\_001* | 10 | 12 | 35.7% | 434 | 47169 | 7.4 | enolase 1 [Homo sapiens] |

| Filename XCorr DeltCN Conf% ObsM+H+ CalcM+H+ SpR ZScore Ion% # Sequence  | | | | | | | | | | | | |
| --- | --- | --- | --- | --- | --- | --- | --- | --- | --- | --- | --- | --- |
| \* | CENPL\_Noc300\_122214\_02.10288.10288.2 | 2.8092 | 0.2509 | 99.5% | 1408.6122 | 1407.5634 | 1 | 5.347 | 62.5% | 1 | R.GNPTVEVDLFTSK.G | 2 |
|  | CENPL\_Noc300\_122214\_02.11435.11435.2 | 4.8659 | 0.4289 | 100.0% | 1805.9321 | 1806.0258 | 1 | 8.404 | 58.8% | 1 | R.AAVPSGASTGIYEALELR.D | 2 |
| \* | CENPL\_Noc300\_tube2\_122214\_01.10296.10296.1 | 1.3473 | 0.2711 | 95.7% | 899.51 | 900.1063 | 3 | 4.779 | 56.2% | 1 | K.TIAPALVSK.K | 1 |
| \* | CENPL\_Noc300\_122214\_01.05886.05886.3 | 3.4393 | 0.3125 | 100.0% | 1445.3644 | 1445.6561 | 22 | 5.631 | 38.6% | 1 | K.KLNVTEQEKIDK.L | 3 |
| \* | CENPL\_Noc300\_122214\_01.14056.14056.3 | 4.5511 | 0.3554 | 100.0% | 3014.3643 | 3013.383 | 1 | 5.933 | 23.3% | 1 | R.HIADLAGNSEVILPVPAFNVINGGSHAGNK.L | 3 |
| \* | CENPL\_Noc300\_122214\_01.07195.07195.2 | 2.9108 | 0.2886 | 99.9% | 1144.0122 | 1144.3158 | 1 | 5.693 | 77.8% | 2 | R.IGAEVYHNLK.N | 2 |
| \* | CENPL\_Noc300\_122214\_02.12954.12954.2 | 2.7631 | 0.3163 | 99.5% | 1542.8522 | 1541.8053 | 1 | 6.122 | 50.0% | 1 | K.VVIGMDVAASEFFR.S | 2 |
| \* | CENPL\_Noc300\_122214\_01.08556.08556.3 | 2.9377 | 0.3563 | 99.9% | 1828.4043 | 1827.9451 | 1 | 5.479 | 40.0% | 1 | R.SGKYDLDFKSPDDPSR.Y | 3 |
| \* | CENPL\_Noc300\_tube2\_122214\_01.18754.18754.2 | 2.7335 | 0.4853 | 100.0% | 1426.2722 | 1426.6091 | 1 | 8.065 | 72.7% | 2 | R.YISPDQLADLYK.S | 2 |
| \* | CENPL\_Noc300\_122214\_02.10010.10010.3 | 3.5053 | 0.4052 | 100.0% | 2192.1243 | 2190.4612 | 1 | 6.059 | 31.2% | 1 | K.FTASAGIQVVGDDLTVTNPKR.I | 3 |

---

|  |  |  |  |  |  |  |  |  |
| --- | --- | --- | --- | --- | --- | --- | --- | --- |
| U | *gi|197333695|ref|NP\_0* | 31 | 92 | 35.5% | 578 | 66574 | 6.4 | poly(A)-specific ribonuclease (deadenylation nuclease) isoform 2 [Homo sapiens] |
| U | *gi|4505611|ref|NP\_002* | 31 | 92 | 32.1% | 639 | 73451 | 6.2 | poly(A)-specific ribonuclease (deadenylation nuclease) isoform 1 [Homo sapiens] |

| Filename XCorr DeltCN Conf% ObsM+H+ CalcM+H+ SpR ZScore Ion% # Sequence  | | | | | | | | | | | | |
| --- | --- | --- | --- | --- | --- | --- | --- | --- | --- | --- | --- | --- |
|  | CENPL\_Noc300\_tube2\_122214\_01.14192.14192.2 | 3.1591 | 0.2932 | 99.9% | 1462.3121 | 1462.559 | 1 | 6.445 | 77.3% | 4 | R.NGIPYLNQEEER.Q | 2 |
|  | CENPL\_Noc300\_122214\_01.08568.08568.2 | 5.1186 | 0.5404 | 100.0% | 1832.2122 | 1832.8784 | 1 | 7.907 | 64.7% | 4 | R.SQANGAGALSYVS\*PNTSK.C | 2 |
|  | CENPL\_Noc300\_122214\_01.08367.08367.2 | 2.9877 | 0.1712 | 99.8% | 978.1722 | 978.13324 | 10 | 3.985 | 85.7% | 4 | K.FIDQVVEK.I | 2 |
|  | CENPL\_Noc300\_122214\_01.18234.18234.3 | 4.0414 | 0.176 | 99.8% | 2278.2844 | 2277.5334 | 1 | 5.104 | 40.3% | 1 | K.FIDQVVEKIEDLLQSEENK.N | 3 |
|  | CENPL\_Noc300\_tube2\_122214\_01.13121.13121.2 | 3.6666 | 0.3022 | 100.0% | 1318.0122 | 1318.4235 | 1 | 6.86 | 80.0% | 3 | K.IEDLLQSEENK.N | 2 |
|  | CENPL\_Noc300\_tube2\_122214\_01.16143.16143.2 | 3.3942 | 0.3631 | 100.0% | 1279.7722 | 1280.5529 | 1 | 7.238 | 72.2% | 5 | R.KLIYQTLSWK.Y | 2 |
|  | CENPL\_Noc300\_tube2\_122214\_01.17931.17931.2 | 2.7924 | 0.4095 | 100.0% | 1151.9521 | 1152.3788 | 1 | 7.35 | 81.2% | 5 | K.LIYQTLSWK.Y | 2 |
|  | CENPL\_Noc300\_tube2\_122214\_01.16802.16802.2 | 2.4758 | 0.2008 | 98.3% | 1540.8322 | 1540.8455 | 385 | 4.639 | 45.5% | 1 | K.LIYQTLSWKYPK.G | 2 |
|  | CENPL\_Noc300\_tube2\_122214\_01.09922.09922.2 | 3.4906 | 0.4955 | 100.0% | 1256.3922 | 1256.3983 | 1 | 8.066 | 65.0% | 3 | K.GIHVETLETEK.K | 2 |
|  | CENPL\_Noc300\_122214\_01.06785.06785.3 | 3.7411 | 0.3888 | 100.0% | 1385.2144 | 1384.5724 | 2 | 7.328 | 52.3% | 2 | K.GIHVETLETEKK.E | 3 |
|  | CENPL\_Noc300\_122214\_01.06731.06731.2 | 3.2184 | 0.3741 | 100.0% | 1385.2322 | 1384.5724 | 2 | 6.411 | 63.6% | 1 | K.GIHVETLETEKK.E | 2 |
|  | CENPL\_Noc300\_122214\_01.06298.06298.3 | 2.6484 | 0.2525 | 98.5% | 1669.8243 | 1669.8754 | 1 | 5.88 | 32.7% | 1 | K.GIHVETLETEKKER.Y | 3 |
|  | CENPL\_Noc300\_122214\_01.09776.09776.2 | 3.7299 | 0.4075 | 100.0% | 1494.1721 | 1494.5577 | 1 | 8.101 | 70.8% | 8 | K.EQEELNDAVGFSR.V | 2 |
|  | CENPL\_Noc300\_tube2\_122214\_01.11288.11288.2 | 2.396 | 0.3253 | 99.5% | 1023.1122 | 1023.23535 | 10 | 5.951 | 75.0% | 2 | K.LMASTQPFK.D | 2 |
|  | CENPL\_Noc300\_122214\_01.14634.14634.2 | 4.4827 | 0.419 | 100.0% | 2464.8123 | 2464.8364 | 1 | 8.36 | 54.8% | 1 | K.LMASTQPFKDIINNTSLAELEK.R | 2 |
|  | CENPL\_Noc300\_122214\_01.14572.14572.3 | 5.6289 | 0.3085 | 100.0% | 2465.0645 | 2464.8364 | 1 | 6.016 | 40.5% | 2 | K.LMASTQPFKDIINNTSLAELEK.R | 3 |
|  | CENPL\_Noc300\_tube2\_122214\_01.19252.19252.3 | 6.1908 | 0.2856 | 100.0% | 2620.6743 | 2621.024 | 1 | 6.077 | 38.6% | 3 | K.LMASTQPFKDIINNTSLAELEKR.L | 3 |
|  | CENPL\_Noc300\_tube2\_122214\_01.17418.17418.2 | 3.669 | 0.3766 | 100.0% | 1462.0521 | 1460.6244 | 1 | 6.511 | 75.0% | 3 | K.DIINNTSLAELEK.R | 2 |
|  | CENPL\_Noc300\_tube2\_122214\_01.09617.09617.2 | 2.8606 | 0.2653 | 99.8% | 1171.3322 | 1171.3818 | 1 | 5.909 | 83.3% | 2 | R.LKETPFNPPK.V | 2 |
|  | CENPL\_Noc300\_122214\_01.11176.11176.2 | 3.0982 | 0.0907 | 99.3% | 1223.2722 | 1223.4575 | 9 | 5.148 | 66.7% | 4 | R.SKLIEPFFNK.L | 2 |
|  | CENPL\_Noc300\_tube2\_122214\_01.17954.17954.2 | 2.8001 | 0.1945 | 99.7% | 1008.2522 | 1008.2053 | 3 | 4.674 | 85.7% | 3 | K.LIEPFFNK.L | 2 |
|  | CENPL\_Noc300\_122214\_01.15430.15430.2 | 5.2407 | 0.5605 | 100.0% | 1942.8322 | 1943.2654 | 1 | 9.78 | 68.8% | 4 | R.VMDIPYLNLEGPDLQPK.R | 2 |
|  | CENPL\_Noc300\_tube2\_122214\_01.10119.10119.3 | 4.0476 | 0.3736 | 100.0% | 1349.9644 | 1349.5784 | 1 | 6.167 | 55.0% | 4 | K.RDHVLHVTFPK.E | 3 |
|  | CENPL\_Noc300\_122214\_01.07778.07778.2 | 3.2751 | 0.3656 | 100.0% | 1350.1122 | 1349.5784 | 2 | 6.198 | 70.0% | 2 | K.RDHVLHVTFPK.E | 2 |
|  | CENPL\_Noc300\_tube2\_122214\_01.12059.12059.2 | 2.693 | 0.3819 | 100.0% | 1193.4922 | 1193.391 | 1 | 6.375 | 77.8% | 1 | R.DHVLHVTFPK.E | 2 |
|  | CENPL\_Noc300\_tube2\_122214\_01.12062.12062.3 | 2.604 | 0.3098 | 99.9% | 1193.6643 | 1193.391 | 1 | 6.333 | 55.6% | 4 | R.DHVLHVTFPK.E | 3 |
|  | CENPL\_Noc300\_122214\_02.07616.07616.2 | 2.8873 | 0.4473 | 100.0% | 1232.4122 | 1232.3966 | 2 | 6.891 | 72.2% | 5 | R.IQTYAEYMGR.K | 2 |
|  | CENPL\_Noc300\_tube2\_122214\_01.10779.10779.2 | 2.1609 | 0.3212 | 98.7% | 1482.2122 | 1482.5463 | 11 | 5.172 | 63.6% | 1 | K.WTEDSWKEADSK.R | 2 |
|  | CENPL\_Noc300\_122214\_01.07293.07293.2 | 3.1749 | 0.3496 | 100.0% | 1223.0322 | 1223.328 | 1 | 6.891 | 72.7% | 6 | R.NNSFTAPSTVGK.R | 2 |
|  | CENPL\_Noc300\_122214\_01.06509.06509.2 | 2.8526 | 0.3224 | 99.8% | 1380.3722 | 1379.5155 | 1 | 6.186 | 62.5% | 2 | R.NNSFTAPSTVGKR.N | 2 |
|  | CENPL\_Noc300\_122214\_02.06564.06564.2 | 1.828 | 0.3722 | 98.2% | 1069.3121 | 1069.1136 | 5 | 6.456 | 50.0% | 1 | K.ELS\*PAGSISK.N | 2 |

---

|  |  |  |  |  |  |  |  |  |
| --- | --- | --- | --- | --- | --- | --- | --- | --- |
| U | *gi|169210992|ref|XP\_0* | 11 | 31 | 35.5% | 217 | 25126 | 6.4 | PREDICTED: similar to hCG1777996 [Homo sapiens] |
| U | *gi|4503535|ref|NP\_001* | 11 | 31 | 35.5% | 217 | 25097 | 6.1 | eukaryotic translation initiation factor 4E isoform 1 [Homo sapiens] |
| U | *gi|194578909|ref|NP\_0* | 11 | 31 | 31.0% | 248 | 28778 | 6.4 | eukaryotic translation initiation factor 4E isoform 2 [Homo sapiens] |
| U | *gi|194578907|ref|NP\_0* | 11 | 31 | 32.5% | 237 | 27260 | 6.8 | eukaryotic translation initiation factor 4E isoform 3 [Homo sapiens] |
| U | *gi|169211874|ref|XP\_0* | 11 | 31 | 35.5% | 217 | 25126 | 6.4 | PREDICTED: similar to hCG1777996 [Homo sapiens] |
| U | *gi|169211405|ref|XP\_0* | 11 | 31 | 35.5% | 217 | 25126 | 6.4 | PREDICTED: similar to hCG1777996 [Homo sapiens] |

| Filename XCorr DeltCN Conf% ObsM+H+ CalcM+H+ SpR ZScore Ion% # Sequence  | | | | | | | | | | | | |
| --- | --- | --- | --- | --- | --- | --- | --- | --- | --- | --- | --- | --- |
|  | CENPL\_Noc300\_tube2\_122214\_01.11004.11004.3 | 2.8594 | 0.2418 | 99.0% | 1759.7644 | 1759.87 | 1 | 4.719 | 48.2% | 2 | K.TESNQEVANPEHYIK.H | 3 |
|  | CENPL\_Noc300\_122214\_01.17834.17834.2 | 2.6807 | 0.0967 | 99.3% | 997.53217 | 998.2151 | 38 | 4.906 | 83.3% | 2 | R.WALWFFK.N | 2 |
|  | CENPL\_Noc300\_122214\_01.15370.15370.2 | 2.6244 | 0.3443 | 99.8% | 1355.9122 | 1355.5815 | 1 | 5.769 | 77.8% | 1 | R.WALWFFKNDK.S | 2 |
|  | CENPL\_Noc300\_tube2\_122214\_01.16352.16352.2 | 3.1447 | 0.2796 | 99.9% | 1349.2322 | 1349.4547 | 1 | 6.464 | 70.0% | 5 | K.DGIEPMWEDEK.N | 2 |
|  | CENPL\_Noc300\_tube2\_122214\_01.13336.13336.2 | 3.4863 | 0.299 | 100.0% | 1591.4122 | 1591.7327 | 1 | 6.31 | 70.8% | 3 | K.DGIEPMWEDEKNK.R | 2 |
|  | CENPL\_Noc300\_tube2\_122214\_01.11808.11808.2 | 3.8081 | 0.2387 | 100.0% | 1748.2722 | 1747.9202 | 1 | 5.414 | 69.2% | 1 | K.DGIEPMWEDEKNKR.G | 2 |
|  | CENPL\_Noc300\_tube2\_122214\_01.18626.18626.1 | 2.1191 | 0.185 | 96.2% | 887.62 | 888.0977 | 7 | 5.237 | 75.0% | 1 | R.WLITLNK.Q | 1 |
|  | CENPL\_Noc300\_tube2\_122214\_01.18513.18513.2 | 2.4091 | 0.0701 | 97.6% | 888.03217 | 888.0977 | 10 | 3.416 | 91.7% | 2 | R.WLITLNK.Q | 2 |
|  | CENPL\_Noc300\_tube2\_122214\_01.14888.14888.2 | 3.265 | 0.3603 | 100.0% | 1394.0322 | 1393.5054 | 1 | 6.819 | 75.0% | 5 | K.IAIWTTECENR.E | 2 |
|  | CENPL\_Noc300\_122214\_02.08979.08979.3 | 4.7425 | 0.4696 | 100.0% | 2110.8245 | 2110.4612 | 1 | 7.527 | 43.4% | 2 | R.LGLPPKIVIGYQSHADTATK.S | 3 |
|  | CENPL\_Noc300\_122214\_01.07299.07299.2 | 4.5161 | 0.6057 | 100.0% | 1504.4122 | 1504.683 | 1 | 10.775 | 73.1% | 7 | K.IVIGYQSHADTATK.S | 2 |

---

|  |  |  |  |  |  |  |  |  |
| --- | --- | --- | --- | --- | --- | --- | --- | --- |
| U | *gi|117938251|ref|NP\_0* | 32 | 78 | 35.3% | 869 | 100232 | 10.0 | BCL2-associated transcription factor 1 isoform 2 [Homo sapiens] |

| Filename XCorr DeltCN Conf% ObsM+H+ CalcM+H+ SpR ZScore Ion% # Sequence  | | | | | | | | | | | | |
| --- | --- | --- | --- | --- | --- | --- | --- | --- | --- | --- | --- | --- |
|  | CENPL\_Noc300\_122214\_01.05812.05812.2 | 3.4053 | 0.3024 | 100.0% | 1523.6322 | 1522.5658 | 1 | 6.044 | 75.0% | 3 | K.KAEGEPQEES\*PLK.S | 22 |
|  | CENPL\_Noc300\_122214\_01.09348.09348.3 | 4.7172 | 0.4594 | 100.0% | 2609.7244 | 2609.6758 | 2 | 7.058 | 33.3% | 4 | K.SQEEPKDTFEHDPSESIDEFNK.S | 33 |
|  | CENPL\_Noc300\_tube2\_122214\_01.18689.18689.2 | 5.3878 | 0.5413 | 100.0% | 2082.3323 | 2082.1907 | 1 | 8.996 | 55.3% | 1 | K.SSATSGDIWPGLSAYDNSPR.S | 22 |
|  | CENPL\_Noc300\_122214\_01.06257.06257.3 | 2.6762 | 0.3986 | 99.9% | 1958.7244 | 1957.0726 | 3 | 5.578 | 33.3% | 1 | R.YSPSQNS\*PIHHIPSRR.S | 33 |
|  | CENPL\_Noc300\_tube2\_122214\_01.10023.10023.2 | 3.4266 | 0.3631 | 100.0% | 1502.1721 | 1502.534 | 1 | 7.652 | 65.4% | 5 | R.SSFYPDGGDQETAK.T | 22 |
|  | CENPL\_Noc300\_122214\_02.09845.09845.3 | 4.4465 | 0.2878 | 100.0% | 2647.3743 | 2647.7278 | 1 | 5.164 | 30.7% | 1 | K.GRAEGEWEDQEALDYFSDKESGK.Q | 33 |
|  | CENPL\_Noc300\_122214\_02.09698.09698.3 | 4.3791 | 0.3835 | 100.0% | 2727.9844 | 2727.7278 | 1 | 6.362 | 38.6% | 2 | K.GRAEGEWEDQEALDYFS\*DKESGK.Q | 33 |
|  | CENPL\_Noc300\_122214\_02.10220.10220.3 | 3.5122 | 0.2813 | 99.9% | 2515.4944 | 2514.4883 | 2 | 5.38 | 32.5% | 1 | R.AEGEWEDQEALDYFS\*DKESGK.Q | 33 |
|  | CENPL\_Noc300\_tube2\_122214\_01.08834.08834.3 | 4.5354 | 0.4542 | 100.0% | 2179.5544 | 2179.1284 | 1 | 7.928 | 44.1% | 1 | K.QKFNDSEGDDTEETEDYR.Q | 33 |
|  | CENPL\_Noc300\_tube2\_122214\_01.09310.09310.3 | 2.9947 | 0.2377 | 98.7% | 2259.0244 | 2259.1284 | 7 | 4.747 | 30.9% | 1 | K.QKFNDS\*EGDDTEETEDYR.Q | 33 |
|  | CENPL\_Noc300\_122214\_01.07284.07284.2 | 4.3914 | 0.3062 | 100.0% | 1922.4722 | 1922.8236 | 1 | 7.412 | 56.7% | 2 | K.FNDSEGDDTEETEDYR.Q | 22 |
|  | CENPL\_Noc300\_122214\_01.06471.06471.2 | 4.3581 | 0.4647 | 100.0% | 1708.4122 | 1708.9524 | 1 | 8.621 | 60.7% | 2 | K.LKETGYVVERPSTTK.D | 22 |
|  | CENPL\_Noc300\_122214\_02.06321.06321.3 | 2.829 | 0.3118 | 99.9% | 1709.7244 | 1708.9524 | 415 | 5.139 | 28.6% | 2 | K.LKETGYVVERPSTTK.D | 33 |
|  | CENPL\_Noc300\_122214\_01.06309.06309.2 | 3.3411 | 0.4355 | 100.0% | 1467.3922 | 1467.6189 | 1 | 6.538 | 75.0% | 2 | K.ETGYVVERPSTTK.D | 22 |
|  | CENPL\_Noc300\_122214\_01.05256.05256.3 | 2.9876 | 0.2492 | 99.5% | 2041.3143 | 2040.2352 | 13 | 4.372 | 35.9% | 1 | R.ITVKKETQS\*PEQVKSEK.L | 33 |
|  | CENPL\_Noc300\_tube2\_122214\_01.15420.15420.2 | 3.0942 | 0.2568 | 99.7% | 1574.2722 | 1573.832 | 2 | 5.904 | 50.0% | 1 | K.LKDLFDYSPPLHK.N | 22 |
|  | CENPL\_Noc300\_tube2\_122214\_01.17123.17123.2 | 3.8173 | 0.2685 | 100.0% | 1654.3522 | 1653.832 | 1 | 6.576 | 70.8% | 4 | K.LKDLFDYS\*PPLHK.N | 22 |
|  | CENPL\_Noc300\_122214\_01.08844.08844.2 | 3.9493 | 0.4758 | 100.0% | 1813.4922 | 1813.079 | 1 | 8.118 | 59.4% | 5 | K.MAPVPLDDSNRPASLTK.D | 22 |
|  | CENPL\_Noc300\_tube2\_122214\_01.12902.12902.3 | 3.4006 | 0.2718 | 99.9% | 1440.0243 | 1439.6981 | 1 | 4.96 | 54.2% | 3 | K.DRLLASTLVHSVK.K | 33 |
|  | CENPL\_Noc300\_tube2\_122214\_01.12680.12680.2 | 3.3827 | 0.3642 | 100.0% | 1168.2922 | 1168.4221 | 1 | 7.747 | 80.0% | 5 | R.LLASTLVHSVK.K | 22 |
|  | CENPL\_Noc300\_tube2\_122214\_01.13157.13157.2 | 2.2235 | 0.2546 | 98.2% | 1248.6122 | 1248.4221 | 3 | 4.766 | 65.0% | 1 | R.LLASTLVHS\*VK.K | 22 |
|  | CENPL\_Noc300\_tube2\_122214\_01.10546.10546.2 | 2.638 | 0.1583 | 98.2% | 1298.3522 | 1296.5962 | 1 | 4.289 | 68.2% | 2 | R.LLASTLVHSVKK.E | 22 |
|  | CENPL\_Noc300\_tube2\_122214\_01.11924.11924.3 | 3.2191 | 0.322 | 99.9% | 2066.5745 | 2066.322 | 1 | 5.368 | 43.8% | 1 | R.LLASTLVHS\*VKKEQEFR.S | 33 |
|  | CENPL\_Noc300\_tube2\_122214\_01.16137.16137.2 | 3.6086 | 0.4891 | 100.0% | 1484.4321 | 1484.7385 | 1 | 7.81 | 70.8% | 2 | R.SIFDHIKLPQASK.S | 22 |
|  | CENPL\_Noc300\_tube2\_122214\_01.16144.16144.3 | 3.1478 | 0.2367 | 99.8% | 1485.4744 | 1484.7385 | 305 | 5.354 | 39.6% | 1 | R.SIFDHIKLPQASK.S | 33 |
|  | CENPL\_Noc300\_tube2\_122214\_01.19985.19985.2 | 4.4756 | 0.357 | 100.0% | 2049.132 | 2049.3372 | 1 | 8.403 | 55.9% | 3 | K.STSESFIQHIVSLVHHVK.E | 22 |
|  | CENPL\_Noc300\_122214\_01.14783.14783.3 | 4.3596 | 0.4643 | 100.0% | 2049.5344 | 2049.3372 | 1 | 7.926 | 44.1% | 4 | K.STSESFIQHIVSLVHHVK.E | 33 |
|  | CENPL\_Noc300\_122214\_01.06526.06526.2 | 2.9165 | 0.3903 | 100.0% | 993.3522 | 993.1229 | 5 | 7.686 | 81.2% | 6 | K.SAAMTLNER.F | 22 |
|  | CENPL\_Noc300\_tube2\_122214\_01.14942.14942.2 | 2.2465 | 0.2566 | 98.9% | 1082.4521 | 1082.1558 | 1 | 4.246 | 81.2% | 3 | R.IDIS\*PSTLR.K | 22 |
|  | CENPL\_Noc300\_122214\_01.07764.07764.2 | 2.4692 | 0.214 | 98.5% | 1326.2522 | 1326.4545 | 44 | 4.293 | 50.0% | 1 | K.EYSGFAGVSRPR.G | 22 |
|  | CENPL\_Noc300\_tube2\_122214\_01.11980.11980.3 | 3.4245 | 0.2126 | 99.8% | 1898.2144 | 1897.9548 | 1 | 5.744 | 36.7% | 5 | R.GTFHDDRDDGVDYWAK.R | 3 |
|  | CENPL\_Noc300\_122214\_01.09584.09584.3 | 3.3662 | 0.398 | 100.0% | 2758.7344 | 2758.8408 | 1 | 6.311 | 34.1% | 2 | K.YQGDGIVEDEEETMENNEEKKDR.R | 33 |

Similarities:
gi|7661958|ref|NP\_055(31:1)  

---

|  |  |  |  |  |  |  |  |  |
| --- | --- | --- | --- | --- | --- | --- | --- | --- |
| U | *gi|27735067|ref|NP\_77* | 18 | 46 | 35.1% | 679 | 75357 | 6.8 | hypothetical protein LOC126353 [Homo sapiens] |

| Filename XCorr DeltCN Conf% ObsM+H+ CalcM+H+ SpR ZScore Ion% # Sequence  | | | | | | | | | | | | |
| --- | --- | --- | --- | --- | --- | --- | --- | --- | --- | --- | --- | --- |
| \* | CENPL\_Noc300\_tube2\_122214\_01.14354.14354.2 | 2.791 | 0.2515 | 99.5% | 1265.1921 | 1265.5009 | 3 | 5.526 | 70.0% | 3 | R.YPILGIPQAHR.G | 2 |
| \* | CENPL\_Noc300\_122214\_01.10635.10635.3 | 2.9011 | 0.3884 | 100.0% | 1266.2344 | 1265.5009 | 1 | 6.554 | 47.5% | 5 | R.YPILGIPQAHR.G | 3 |
| \* | CENPL\_Noc300\_122214\_02.07836.07836.2 | 2.4352 | 0.1835 | 96.9% | 1914.2722 | 1914.986 | 1 | 4.774 | 46.9% | 1 | R.QGVSYSVHAYTGQPS\*PR.G | 2 |
| \* | CENPL\_Noc300\_tube2\_122214\_01.10656.10656.2 | 4.6303 | 0.406 | 100.0% | 1975.4122 | 1976.072 | 1 | 7.009 | 63.3% | 3 | R.GLHSENREDEGWQVYR.L | 2 |
| \* | CENPL\_Noc300\_tube2\_122214\_01.10632.10632.3 | 3.1218 | 0.3648 | 100.0% | 1975.7644 | 1976.072 | 1 | 6.871 | 41.7% | 2 | R.GLHSENREDEGWQVYR.L | 3 |
| \* | CENPL\_Noc300\_tube2\_122214\_01.09868.09868.3 | 3.5504 | 0.2895 | 99.9% | 2567.0044 | 2567.7954 | 1 | 5.347 | 32.1% | 1 | R.DAHQGRPTWALRPEDGEDKEMK.T | 3 |
| \* | CENPL\_Noc300\_tube2\_122214\_01.13124.13124.2 | 3.215 | 0.3185 | 100.0% | 1128.4321 | 1128.3195 | 1 | 7.163 | 94.4% | 7 | R.WAVIQGQAVR.K | 2 |
| \* | CENPL\_Noc300\_122214\_01.06670.06670.2 | 3.8089 | 0.483 | 100.0% | 1739.4321 | 1739.8394 | 1 | 7.494 | 56.2% | 1 | K.SSTVATLQGTPDHGDPR.T | 2 |
| \* | CENPL\_Noc300\_122214\_02.10761.10761.3 | 3.2939 | 0.3461 | 99.9% | 2303.3643 | 2303.534 | 1 | 6.279 | 31.6% | 3 | R.STPLEENVVDREQIDFLAAR.Q | 3 |
| \* | CENPL\_Noc300\_tube2\_122214\_01.18369.18369.2 | 2.6837 | 0.3318 | 99.6% | 2303.8523 | 2303.534 | 1 | 5.183 | 39.5% | 1 | R.STPLEENVVDREQIDFLAAR.Q | 2 |
| \* | CENPL\_Noc300\_122214\_01.10371.10371.2 | 2.8145 | 0.391 | 100.0% | 1306.3722 | 1306.4612 | 18 | 6.298 | 65.0% | 5 | R.QQFLSLEQANK.G | 2 |
| \* | CENPL\_Noc300\_tube2\_122214\_01.09377.09377.3 | 5.0306 | 0.3955 | 100.0% | 2195.1843 | 2195.6187 | 1 | 6.704 | 43.4% | 2 | K.AFNKPHLANGHVVPIKPQVK.G | 3 |
| \* | CENPL\_Noc300\_tube2\_122214\_01.14540.14540.3 | 2.9671 | 0.385 | 99.9% | 2190.1143 | 2190.5046 | 101 | 5.775 | 33.3% | 1 | R.QATDHQELVEIPTRPLLTK.L | 3 |
| \* | CENPL\_Noc300\_tube2\_122214\_01.13732.13732.2 | 2.7554 | 0.2876 | 99.9% | 871.0522 | 871.0678 | 2 | 6.212 | 85.7% | 3 | K.LSLITAPR.R | 2 |
| \* | CENPL\_Noc300\_122214\_01.06568.06568.2 | 2.7625 | 0.3281 | 99.9% | 1076.4122 | 1076.2437 | 1 | 5.377 | 75.0% | 1 | R.GRPSLYVQR.D | 2 |
| \* | CENPL\_Noc300\_tube2\_122214\_01.14837.14837.2 | 3.2513 | 0.4677 | 100.0% | 1698.2522 | 1697.8448 | 16 | 7.348 | 40.0% | 3 | R.ASTPDWVSEGPQPGLR.R | 2 |
| \* | CENPL\_Noc300\_tube2\_122214\_01.13647.13647.2 | 4.4283 | 0.5013 | 100.0% | 1500.5122 | 1500.6488 | 1 | 8.093 | 78.6% | 3 | R.ALSSDSILSPAPDAR.A | 2 |
| \* | CENPL\_Noc300\_tube2\_122214\_01.18699.18699.3 | 5.3825 | 0.4169 | 100.0% | 3014.0044 | 3014.3757 | 1 | 7.283 | 34.6% | 1 | R.FRAPDEPQQAQVPHVWGWEVAGAPALR.L | 3 |

---

|  |  |  |  |  |  |  |  |  |
| --- | --- | --- | --- | --- | --- | --- | --- | --- |
| U | *gi|153791372|ref|NP\_0* | 16 | 34 | 35.1% | 490 | 58588 | 9.5 | CDC-like kinase 3 isoform b [Homo sapiens] |
| U | *gi|194097436|ref|NP\_0* | 16 | 34 | 27.0% | 638 | 73515 | 9.9 | CDC-like kinase 3 isoform a [Homo sapiens] |

| Filename XCorr DeltCN Conf% ObsM+H+ CalcM+H+ SpR ZScore Ion% # Sequence  | | | | | | | | | | | | |
| --- | --- | --- | --- | --- | --- | --- | --- | --- | --- | --- | --- | --- |
|  | CENPL\_Noc300\_tube2\_122214\_01.13041.13041.2 | 2.9581 | 0.3265 | 99.8% | 1723.1322 | 1723.796 | 1 | 5.942 | 70.8% | 3 | R.YRS\*PEPDPYLSYR.W | 2 |
|  | CENPL\_Noc300\_122214\_01.05843.05843.3 | 3.0855 | 0.2275 | 99.8% | 1544.7843 | 1544.6274 | 1 | 5.465 | 56.2% | 1 | R.SVEDDKEGHLVCR.I | 3 |
|  | CENPL\_Noc300\_122214\_01.11254.11254.2 | 2.8302 | 0.3278 | 100.0% | 1017.15216 | 1017.1295 | 1 | 6.609 | 85.7% | 6 | R.IGDWLQER.Y | 2 |
|  | CENPL\_Noc300\_tube2\_122214\_01.17073.17073.2 | 4.3679 | 0.5329 | 100.0% | 1484.4722 | 1484.6488 | 1 | 8.842 | 84.6% | 6 | R.YEIVGNLGEGTFGK.V | 2 |
|  | CENPL\_Noc300\_tube2\_122214\_01.11831.11831.2 | 2.427 | 0.1596 | 98.7% | 957.53217 | 957.2016 | 5 | 5.03 | 85.7% | 1 | R.LEINVLKK.I | 2 |
|  | CENPL\_Noc300\_tube2\_122214\_01.10070.10070.3 | 3.9155 | 0.3641 | 100.0% | 1400.8744 | 1400.6074 | 1 | 6.902 | 50.0% | 1 | R.HMAYQLCHALR.F | 3 |
|  | CENPL\_Noc300\_122214\_02.07844.07844.3 | 4.4883 | 0.5259 | 100.0% | 2313.3843 | 2313.4912 | 1 | 7.919 | 38.8% | 2 | R.VADFGSATFDHEHHTTIVATR.H | 3 |
|  | CENPL\_Noc300\_122214\_01.10951.10951.2 | 2.4979 | 0.0879 | 96.0% | 1350.2922 | 1350.4764 | 2 | 4.518 | 65.0% | 1 | R.GFTLFQTHENR.E | 2 |
|  | CENPL\_Noc300\_122214\_01.09037.09037.2 | 2.561 | 0.2909 | 99.5% | 1371.2922 | 1371.6866 | 1 | 5.394 | 72.7% | 1 | K.ILGPIPSHMIHR.T | 2 |
|  | CENPL\_Noc300\_tube2\_122214\_01.13796.13796.2 | 3.849 | 0.4087 | 100.0% | 1392.0721 | 1392.4246 | 1 | 8.587 | 66.7% | 3 | K.GGLVWDENSSDGR.Y | 2 |
|  | CENPL\_Noc300\_tube2\_122214\_01.21233.21233.3 | 2.6487 | 0.3154 | 99.7% | 2226.6243 | 2226.5703 | 9 | 5.012 | 32.4% | 1 | K.SYMLQDSLEHVQLFDLMR.R | 3 |
|  | CENPL\_Noc300\_122214\_01.14959.14959.2 | 2.272 | 0.2391 | 97.2% | 2383.3523 | 2382.7578 | 61 | 4.069 | 30.6% | 1 | K.SYMLQDSLEHVQLFDLMRR.M | 2 |
|  | CENPL\_Noc300\_tube2\_122214\_01.12593.12593.2 | 2.2394 | 0.1495 | 96.3% | 1263.3522 | 1263.457 | 10 | 4.666 | 66.7% | 1 | R.RMLEFDPAQR.I | 2 |
|  | CENPL\_Noc300\_tube2\_122214\_01.14883.14883.2 | 2.8675 | 0.015 | 97.7% | 1108.2122 | 1107.2695 | 1 | 6.189 | 93.8% | 3 | R.MLEFDPAQR.I | 2 |
|  | CENPL\_Noc300\_122214\_01.18887.18887.3 | 5.6905 | 0.5047 | 100.0% | 2227.2544 | 2226.5803 | 1 | 7.502 | 48.7% | 1 | R.ITLAEALLHPFFAGLTPEER.S | 3 |
|  | CENPL\_Noc300\_122214\_01.18844.18844.2 | 3.223 | 0.2174 | 99.6% | 2228.672 | 2226.5803 | 1 | 4.588 | 47.4% | 2 | R.ITLAEALLHPFFAGLTPEER.S | 2 |

---

|  |  |  |  |  |  |  |  |  |
| --- | --- | --- | --- | --- | --- | --- | --- | --- |
| U | *gi|7661958|ref|NP\_055* | 35 | 81 | 34.9% | 920 | 106122 | 10.0 | BCL2-associated transcription factor 1 isoform 1 [Homo sapiens] |

| Filename XCorr DeltCN Conf% ObsM+H+ CalcM+H+ SpR ZScore Ion% # Sequence  | | | | | | | | | | | | |
| --- | --- | --- | --- | --- | --- | --- | --- | --- | --- | --- | --- | --- |
|  | CENPL\_Noc300\_122214\_01.05812.05812.2 | 3.4053 | 0.3024 | 100.0% | 1523.6322 | 1522.5658 | 1 | 6.044 | 75.0% | 3 | K.KAEGEPQEES\*PLK.S | 22 |
|  | CENPL\_Noc300\_122214\_01.09348.09348.3 | 4.7172 | 0.4594 | 100.0% | 2609.7244 | 2609.6758 | 2 | 7.058 | 33.3% | 4 | K.SQEEPKDTFEHDPSESIDEFNK.S | 33 |
|  | CENPL\_Noc300\_tube2\_122214\_01.18689.18689.2 | 5.3878 | 0.5413 | 100.0% | 2082.3323 | 2082.1907 | 1 | 8.996 | 55.3% | 1 | K.SSATSGDIWPGLSAYDNSPR.S | 22 |
|  | CENPL\_Noc300\_122214\_01.06257.06257.3 | 2.6762 | 0.3986 | 99.9% | 1958.7244 | 1957.0726 | 3 | 5.578 | 33.3% | 1 | R.YSPSQNS\*PIHHIPSRR.S | 33 |
|  | CENPL\_Noc300\_tube2\_122214\_01.10023.10023.2 | 3.4266 | 0.3631 | 100.0% | 1502.1721 | 1502.534 | 1 | 7.652 | 65.4% | 5 | R.SSFYPDGGDQETAK.T | 22 |
|  | CENPL\_Noc300\_122214\_02.09845.09845.3 | 4.4465 | 0.2878 | 100.0% | 2647.3743 | 2647.7278 | 1 | 5.164 | 30.7% | 1 | K.GRAEGEWEDQEALDYFSDKESGK.Q | 33 |
|  | CENPL\_Noc300\_122214\_02.09698.09698.3 | 4.3791 | 0.3835 | 100.0% | 2727.9844 | 2727.7278 | 1 | 6.362 | 38.6% | 2 | K.GRAEGEWEDQEALDYFS\*DKESGK.Q | 33 |
|  | CENPL\_Noc300\_122214\_02.10220.10220.3 | 3.5122 | 0.2813 | 99.9% | 2515.4944 | 2514.4883 | 2 | 5.38 | 32.5% | 1 | R.AEGEWEDQEALDYFS\*DKESGK.Q | 33 |
|  | CENPL\_Noc300\_tube2\_122214\_01.08834.08834.3 | 4.5354 | 0.4542 | 100.0% | 2179.5544 | 2179.1284 | 1 | 7.928 | 44.1% | 1 | K.QKFNDSEGDDTEETEDYR.Q | 33 |
|  | CENPL\_Noc300\_tube2\_122214\_01.09310.09310.3 | 2.9947 | 0.2377 | 98.7% | 2259.0244 | 2259.1284 | 7 | 4.747 | 30.9% | 1 | K.QKFNDS\*EGDDTEETEDYR.Q | 33 |
|  | CENPL\_Noc300\_122214\_01.07284.07284.2 | 4.3914 | 0.3062 | 100.0% | 1922.4722 | 1922.8236 | 1 | 7.412 | 56.7% | 2 | K.FNDSEGDDTEETEDYR.Q | 22 |
|  | CENPL\_Noc300\_122214\_01.06471.06471.2 | 4.3581 | 0.4647 | 100.0% | 1708.4122 | 1708.9524 | 1 | 8.621 | 60.7% | 2 | K.LKETGYVVERPSTTK.D | 22 |
|  | CENPL\_Noc300\_122214\_02.06321.06321.3 | 2.829 | 0.3118 | 99.9% | 1709.7244 | 1708.9524 | 415 | 5.139 | 28.6% | 2 | K.LKETGYVVERPSTTK.D | 33 |
|  | CENPL\_Noc300\_122214\_01.06309.06309.2 | 3.3411 | 0.4355 | 100.0% | 1467.3922 | 1467.6189 | 1 | 6.538 | 75.0% | 2 | K.ETGYVVERPSTTK.D | 22 |
|  | CENPL\_Noc300\_122214\_01.05256.05256.3 | 2.9876 | 0.2492 | 99.5% | 2041.3143 | 2040.2352 | 13 | 4.372 | 35.9% | 1 | R.ITVKKETQS\*PEQVKSEK.L | 33 |
|  | CENPL\_Noc300\_tube2\_122214\_01.15420.15420.2 | 3.0942 | 0.2568 | 99.7% | 1574.2722 | 1573.832 | 2 | 5.904 | 50.0% | 1 | K.LKDLFDYSPPLHK.N | 22 |
|  | CENPL\_Noc300\_tube2\_122214\_01.17123.17123.2 | 3.8173 | 0.2685 | 100.0% | 1654.3522 | 1653.832 | 1 | 6.576 | 70.8% | 4 | K.LKDLFDYS\*PPLHK.N | 22 |
|  | CENPL\_Noc300\_122214\_01.08844.08844.2 | 3.9493 | 0.4758 | 100.0% | 1813.4922 | 1813.079 | 1 | 8.118 | 59.4% | 5 | K.MAPVPLDDSNRPASLTK.D | 22 |
|  | CENPL\_Noc300\_tube2\_122214\_01.12902.12902.3 | 3.4006 | 0.2718 | 99.9% | 1440.0243 | 1439.6981 | 1 | 4.96 | 54.2% | 3 | K.DRLLASTLVHSVK.K | 33 |
|  | CENPL\_Noc300\_tube2\_122214\_01.12680.12680.2 | 3.3827 | 0.3642 | 100.0% | 1168.2922 | 1168.4221 | 1 | 7.747 | 80.0% | 5 | R.LLASTLVHSVK.K | 22 |
|  | CENPL\_Noc300\_tube2\_122214\_01.13157.13157.2 | 2.2235 | 0.2546 | 98.2% | 1248.6122 | 1248.4221 | 3 | 4.766 | 65.0% | 1 | R.LLASTLVHS\*VK.K | 22 |
|  | CENPL\_Noc300\_tube2\_122214\_01.10546.10546.2 | 2.638 | 0.1583 | 98.2% | 1298.3522 | 1296.5962 | 1 | 4.289 | 68.2% | 2 | R.LLASTLVHSVKK.E | 22 |
|  | CENPL\_Noc300\_tube2\_122214\_01.11924.11924.3 | 3.2191 | 0.322 | 99.9% | 2066.5745 | 2066.322 | 1 | 5.368 | 43.8% | 1 | R.LLASTLVHS\*VKKEQEFR.S | 33 |
|  | CENPL\_Noc300\_tube2\_122214\_01.16137.16137.2 | 3.6086 | 0.4891 | 100.0% | 1484.4321 | 1484.7385 | 1 | 7.81 | 70.8% | 2 | R.SIFDHIKLPQASK.S | 22 |
|  | CENPL\_Noc300\_tube2\_122214\_01.16144.16144.3 | 3.1478 | 0.2367 | 99.8% | 1485.4744 | 1484.7385 | 305 | 5.354 | 39.6% | 1 | R.SIFDHIKLPQASK.S | 33 |
|  | CENPL\_Noc300\_tube2\_122214\_01.19985.19985.2 | 4.4756 | 0.357 | 100.0% | 2049.132 | 2049.3372 | 1 | 8.403 | 55.9% | 3 | K.STSESFIQHIVSLVHHVK.E | 22 |
|  | CENPL\_Noc300\_122214\_01.14783.14783.3 | 4.3596 | 0.4643 | 100.0% | 2049.5344 | 2049.3372 | 1 | 7.926 | 44.1% | 4 | K.STSESFIQHIVSLVHHVK.E | 33 |
|  | CENPL\_Noc300\_122214\_01.06526.06526.2 | 2.9165 | 0.3903 | 100.0% | 993.3522 | 993.1229 | 5 | 7.686 | 81.2% | 6 | K.SAAMTLNER.F | 22 |
|  | CENPL\_Noc300\_tube2\_122214\_01.14942.14942.2 | 2.2465 | 0.2566 | 98.9% | 1082.4521 | 1082.1558 | 1 | 4.246 | 81.2% | 3 | R.IDIS\*PSTLR.K | 22 |
|  | CENPL\_Noc300\_122214\_01.07764.07764.2 | 2.4692 | 0.214 | 98.5% | 1326.2522 | 1326.4545 | 44 | 4.293 | 50.0% | 1 | K.EYSGFAGVSRPR.G | 22 |
|  | CENPL\_Noc300\_122214\_01.07763.07763.3 | 2.709 | 0.2611 | 99.2% | 1803.2943 | 1804.9536 | 1 | 4.742 | 42.3% | 1 | K.RPKEEEWDPEYTPK.S | 3 |
|  | CENPL\_Noc300\_tube2\_122214\_01.10091.10091.2 | 4.0868 | 0.4721 | 100.0% | 1804.7122 | 1804.9536 | 1 | 7.163 | 73.1% | 1 | K.RPKEEEWDPEYTPK.S | 2 |
|  | CENPL\_Noc300\_122214\_01.09801.09801.2 | 2.746 | 0.4413 | 100.0% | 1423.2922 | 1423.4755 | 1 | 6.708 | 65.0% | 3 | K.EEEWDPEYTPK.S | 22 |
|  | CENPL\_Noc300\_tube2\_122214\_01.15237.15237.3 | 3.3144 | 0.4216 | 100.0% | 2015.8143 | 2016.1333 | 1 | 7.02 | 41.7% | 3 | K.YFLHDDRDDGVDYWAK.R | 3 |
|  | CENPL\_Noc300\_122214\_01.09584.09584.3 | 3.3662 | 0.398 | 100.0% | 2758.7344 | 2758.8408 | 1 | 6.311 | 34.1% | 2 | K.YQGDGIVEDEEETMENNEEKKDR.R | 33 |

Similarities:
gi|167234419|ref|NP\_0(1:34)  
gi|117938251|ref|NP\_0(31:4)  

---

|  |  |  |  |  |  |  |  |  |
| --- | --- | --- | --- | --- | --- | --- | --- | --- |
| U | *gi|167466173|ref|NP\_0* | 21 | 71 | 34.8% | 641 | 70052 | 5.6 | heat shock 70kDa protein 1B [Homo sapiens] |
| U | *gi|194248072|ref|NP\_0* | 21 | 71 | 34.8% | 641 | 70052 | 5.6 | heat shock 70kDa protein 1A [Homo sapiens] |

| Filename XCorr DeltCN Conf% ObsM+H+ CalcM+H+ SpR ZScore Ion% # Sequence  | | | | | | | | | | | | |
| --- | --- | --- | --- | --- | --- | --- | --- | --- | --- | --- | --- | --- |
|  | CENPL\_Noc300\_122214\_01.10323.10323.2 | 3.3577 | 0.4739 | 100.0% | 1488.4722 | 1488.5939 | 1 | 8.67 | 79.2% | 6 | R.TTPSYVAFTDTER.L | 2222 |
|  | CENPL\_Noc300\_tube2\_122214\_01.14321.14321.2 | 5.8073 | 0.4828 | 100.0% | 1660.2122 | 1659.8394 | 1 | 9.744 | 82.1% | 7 | K.NQVALNPQNTVFDAK.R | 2 |
|  | CENPL\_Noc300\_tube2\_122214\_01.12586.12586.2 | 4.0359 | 0.3064 | 100.0% | 1816.3322 | 1816.0269 | 1 | 6.099 | 66.7% | 2 | K.NQVALNPQNTVFDAKR.L | 2 |
|  | CENPL\_Noc300\_tube2\_122214\_01.10229.10229.2 | 3.3914 | 0.3621 | 100.0% | 1351.3121 | 1351.5603 | 1 | 7.517 | 72.7% | 2 | R.KFGDPVVQSDMK.H | 2 |
|  | CENPL\_Noc300\_122214\_01.08945.08945.2 | 2.5033 | 0.5506 | 100.0% | 1223.3322 | 1223.3862 | 1 | 8.621 | 70.0% | 4 | K.FGDPVVQSDMK.H | 2 |
|  | CENPL\_Noc300\_122214\_01.10801.10801.3 | 4.7638 | 0.3705 | 100.0% | 1682.5443 | 1681.8912 | 1 | 6.706 | 55.8% | 10 | K.HWPFQVINDGDKPK.V | 3 |
|  | CENPL\_Noc300\_tube2\_122214\_01.20031.20031.2 | 3.4205 | 0.5316 | 100.0% | 1615.3121 | 1615.8817 | 1 | 8.039 | 69.2% | 2 | K.AFYPEEISSMVLTK.M | 22 |
|  | CENPL\_Noc300\_122214\_02.12480.12480.3 | 3.6344 | 0.3857 | 100.0% | 3262.6443 | 3262.7046 | 1 | 5.493 | 25.9% | 1 | K.MKEIAEAYLGYPVTNAVITVPAYFNDSQR.Q | 3 |
|  | CENPL\_Noc300\_122214\_01.13712.13712.2 | 3.7132 | 0.3227 | 100.0% | 1199.2922 | 1198.408 | 1 | 7.244 | 86.4% | 4 | K.DAGVIAGLNVLR.I | 22 |
|  | CENPL\_Noc300\_tube2\_122214\_01.18592.18592.2 | 5.2641 | 0.5029 | 100.0% | 1688.5721 | 1688.9213 | 1 | 10.235 | 83.3% | 5 | R.IINEPTAAAIAYGLDR.T | 22 |
|  | CENPL\_Noc300\_tube2\_122214\_01.18629.18629.3 | 3.6013 | 0.2861 | 99.9% | 1688.8744 | 1688.9213 | 2 | 6.1 | 48.3% | 1 | R.IINEPTAAAIAYGLDR.T | 33 |
|  | CENPL\_Noc300\_122214\_01.07337.07337.2 | 4.5821 | 0.5001 | 100.0% | 1676.2322 | 1676.6964 | 1 | 8.059 | 70.0% | 3 | K.ATAGDTHLGGEDFDNR.L | 222 |
|  | CENPL\_Noc300\_122214\_01.07385.07385.3 | 3.5425 | 0.4619 | 100.0% | 1676.5144 | 1676.6964 | 1 | 7.623 | 51.7% | 5 | K.ATAGDTHLGGEDFDNR.L | 333 |
|  | CENPL\_Noc300\_122214\_01.10239.10239.2 | 3.0535 | 0.4123 | 100.0% | 1262.3121 | 1262.4508 | 1 | 7.518 | 83.3% | 3 | R.LVNHFVEEFK.R | 2 |
|  | CENPL\_Noc300\_tube2\_122214\_01.13730.13730.3 | 2.7265 | 0.4016 | 100.0% | 1263.0243 | 1262.4508 | 1 | 6.335 | 50.0% | 2 | R.LVNHFVEEFK.R | 3 |
|  | CENPL\_Noc300\_tube2\_122214\_01.11973.11973.3 | 2.8541 | 0.329 | 99.9% | 1418.8444 | 1418.6383 | 2 | 6.384 | 45.0% | 2 | R.LVNHFVEEFKR.K | 3 |
|  | CENPL\_Noc300\_122214\_01.13409.13409.2 | 2.2018 | 0.2641 | 98.5% | 1316.6322 | 1316.4193 | 4 | 4.839 | 66.7% | 1 | R.FEELCSDLFR.S | 22 |
|  | CENPL\_Noc300\_122214\_02.08961.08961.3 | 3.3302 | 0.392 | 100.0% | 1823.3944 | 1823.1025 | 1 | 6.725 | 45.3% | 1 | K.LDKAQIHDLVLVGGSTR.I | 3 |
|  | CENPL\_Noc300\_tube2\_122214\_01.18867.18867.2 | 2.4494 | 0.2986 | 99.5% | 1110.1921 | 1110.2578 | 1 | 6.082 | 87.5% | 3 | K.LLQDFFNGR.D | 2 |
|  | CENPL\_Noc300\_122214\_02.13851.13851.3 | 5.284 | 0.5335 | 100.0% | 2305.4343 | 2305.608 | 1 | 9.436 | 38.6% | 1 | K.SINPDEAVAYGAAVQAAILMGDK.S | 33 |
|  | CENPL\_Noc300\_tube2\_122214\_01.16994.16994.2 | 3.7772 | 0.4871 | 100.0% | 1288.1122 | 1288.4608 | 1 | 8.312 | 80.0% | 6 | K.NALESYAFNMK.S | 22 |

Similarities:
gi|5729877|ref|NP\_006(1:20)  
gi|124256496|ref|NP\_0(7:14)  
gi|34419635|ref|NP\_00(6:15)  

---

|  |  |  |  |  |  |  |  |  |
| --- | --- | --- | --- | --- | --- | --- | --- | --- |
| U | *gi|117189975|ref|NP\_1* | 10 | 23 | 34.6% | 306 | 33670 | 5.1 | heterogeneous nuclear ribonucleoprotein C isoform a [Homo sapiens] |
| U | *gi|117190254|ref|NP\_0* | 10 | 23 | 36.2% | 293 | 32338 | 5.1 | heterogeneous nuclear ribonucleoprotein C isoform b [Homo sapiens] |
| U | *gi|117190192|ref|NP\_0* | 10 | 23 | 34.6% | 306 | 33670 | 5.1 | heterogeneous nuclear ribonucleoprotein C isoform a [Homo sapiens] |
| U | *gi|117190174|ref|NP\_0* | 10 | 23 | 36.2% | 293 | 32338 | 5.1 | heterogeneous nuclear ribonucleoprotein C isoform b [Homo sapiens] |

| Filename XCorr DeltCN Conf% ObsM+H+ CalcM+H+ SpR ZScore Ion% # Sequence  | | | | | | | | | | | | |
| --- | --- | --- | --- | --- | --- | --- | --- | --- | --- | --- | --- | --- |
|  | CENPL\_Noc300\_122214\_01.13695.13695.3 | 2.7422 | 0.3332 | 99.9% | 1975.7043 | 1973.2548 | 1 | 5.249 | 32.8% | 1 | R.SMNS\*RVFIGNLNTLVVK.K | 3 |
|  | CENPL\_Noc300\_122214\_02.11231.11231.2 | 3.6936 | 0.2459 | 100.0% | 1318.5122 | 1317.6145 | 1 | 6.561 | 81.8% | 4 | R.VFIGNLNTLVVK.K | 2 |
|  | CENPL\_Noc300\_tube2\_122214\_01.12429.12429.2 | 2.6958 | 0.3285 | 99.8% | 1124.6122 | 1124.2792 | 1 | 5.899 | 72.2% | 4 | K.KSDVEAIFSK.Y | 2 |
|  | CENPL\_Noc300\_tube2\_122214\_01.18570.18570.2 | 3.738 | 0.5257 | 100.0% | 1330.0922 | 1330.4857 | 1 | 9.587 | 80.0% | 4 | K.GFAFVQYVNER.N | 2 |
|  | CENPL\_Noc300\_122214\_02.11174.11174.2 | 4.5875 | 0.4569 | 100.0% | 1683.6522 | 1684.0038 | 1 | 7.978 | 80.0% | 4 | R.MIAGQVLDINLAAEPK.V | 2 |
|  | CENPL\_Noc300\_122214\_01.07281.07281.2 | 2.0054 | 0.2935 | 98.3% | 944.39215 | 944.1649 | 37 | 4.971 | 56.2% | 1 | R.VPPPPPIAR.A | 2 |
|  | CENPL\_Noc300\_122214\_01.06136.06136.2 | 3.1989 | 0.2952 | 100.0% | 1229.4722 | 1229.4624 | 2 | 6.765 | 75.0% | 1 | K.LKGDDLQAIKK.E | 2 |
|  | CENPL\_Noc300\_122214\_01.06188.06188.3 | 3.3493 | 0.2143 | 99.9% | 1229.7843 | 1229.4624 | 133 | 5.04 | 37.5% | 1 | K.LKGDDLQAIKK.E | 3 |
|  | CENPL\_Noc300\_tube2\_122214\_01.15944.15944.2 | 2.1916 | 0.3585 | 99.3% | 1416.4521 | 1416.6146 | 47 | 5.826 | 59.1% | 2 | K.QKVDSLLENLEK.I | 2 |
|  | CENPL\_Noc300\_122214\_01.05286.05286.3 | 4.0166 | 0.4139 | 100.0% | 2243.1543 | 2241.3887 | 2 | 6.665 | 30.3% | 1 | K.QAVEMKNDKSEEEQSSSSVK.K | 3 |

---

|  |  |  |  |  |  |  |  |  |
| --- | --- | --- | --- | --- | --- | --- | --- | --- |
| U | *gi|46276893|ref|NP\_99* | 5 | 14 | 34.2% | 161 | 17911 | 6.1 | elongin B isoform b [Homo sapiens] |
| U | *gi|6005890|ref|NP\_009* | 5 | 14 | 46.6% | 118 | 13133 | 4.9 | elongin B isoform a [Homo sapiens] |

| Filename XCorr DeltCN Conf% ObsM+H+ CalcM+H+ SpR ZScore Ion% # Sequence  | | | | | | | | | | | | |
| --- | --- | --- | --- | --- | --- | --- | --- | --- | --- | --- | --- | --- |
|  | CENPL\_Noc300\_122214\_02.09725.09725.3 | 4.0529 | 0.334 | 100.0% | 2073.9844 | 2074.3384 | 1 | 5.352 | 36.8% | 1 | K.TTIFTDAKESSTVFELKR.I | 3 |
|  | CENPL\_Noc300\_tube2\_122214\_01.11264.11264.2 | 2.6304 | 0.2456 | 99.5% | 1197.3922 | 1196.3458 | 29 | 4.221 | 55.6% | 4 | K.ESSTVFELKR.I | 2 |
|  | CENPL\_Noc300\_tube2\_122214\_01.10864.10864.2 | 4.247 | 0.4129 | 100.0% | 1423.3121 | 1423.5627 | 1 | 8.568 | 81.8% | 5 | R.LYKDDQLLDDGK.T | 2 |
|  | CENPL\_Noc300\_tube2\_122214\_01.10850.10850.3 | 3.6263 | 0.2918 | 100.0% | 1424.0343 | 1423.5627 | 1 | 5.475 | 59.1% | 3 | R.LYKDDQLLDDGK.T | 3 |
|  | CENPL\_Noc300\_tube2\_122214\_01.16479.16479.3 | 3.6312 | 0.2133 | 99.4% | 2638.9443 | 2637.9214 | 1 | 4.662 | 33.3% | 1 | K.TLGECGFTSQTARPQAPATVGLAFR.A | 3 |

---

|  |  |  |  |  |  |  |  |  |
| --- | --- | --- | --- | --- | --- | --- | --- | --- |
| U | *gi|5174735|ref|NP\_006* | 16 | 39 | 33.5% | 445 | 49831 | 4.9 | tubulin, beta, 2 [Homo sapiens] |

| Filename XCorr DeltCN Conf% ObsM+H+ CalcM+H+ SpR ZScore Ion% # Sequence  | | | | | | | | | | | | |
| --- | --- | --- | --- | --- | --- | --- | --- | --- | --- | --- | --- | --- |
| \* | CENPL\_Noc300\_tube2\_122214\_01.11364.11364.2 | 2.4205 | 0.1573 | 97.1% | 1330.0521 | 1329.4521 | 10 | 4.211 | 63.6% | 1 | R.INVYYNEATGGK.Y | 2 |
|  | CENPL\_Noc300\_tube2\_122214\_01.17874.17874.2 | 3.2383 | 0.4252 | 100.0% | 1602.5322 | 1602.8431 | 2 | 7.651 | 57.1% | 1 | R.AVLVDLEPGTMDSVR.S | 2 |
|  | CENPL\_Noc300\_tube2\_122214\_01.20349.20349.3 | 6.9426 | 0.4442 | 100.0% | 2799.5044 | 2800.0647 | 1 | 7.521 | 38.0% | 5 | R.SGPFGQIFRPDNFVFGQSGAGNNWAK.G | 33 |
|  | CENPL\_Noc300\_tube2\_122214\_01.20703.20703.3 | 3.7681 | 0.2527 | 99.9% | 1959.3844 | 1960.151 | 1 | 5.742 | 42.6% | 1 | K.GHYTEGAELVDSVLDVVR.K | 33 |
|  | CENPL\_Noc300\_122214\_01.15753.15753.2 | 6.9684 | 0.5741 | 100.0% | 1959.7522 | 1960.151 | 1 | 10.753 | 79.4% | 2 | K.GHYTEGAELVDSVLDVVR.K | 22 |
|  | CENPL\_Noc300\_tube2\_122214\_01.19962.19962.3 | 4.848 | 0.4608 | 100.0% | 2088.1743 | 2088.325 | 1 | 7.606 | 37.5% | 3 | K.GHYTEGAELVDSVLDVVRK.E | 33 |
|  | CENPL\_Noc300\_tube2\_122214\_01.08062.08062.2 | 2.3719 | 0.2213 | 99.3% | 1077.5922 | 1078.1698 | 58 | 4.748 | 64.3% | 1 | K.IREEYPDR.I | 22 |
|  | CENPL\_Noc300\_tube2\_122214\_01.15951.15951.2 | 4.2126 | 0.4177 | 100.0% | 1320.5721 | 1320.5896 | 1 | 8.013 | 72.7% | 7 | R.IMNTFSVVPSPK.V | 22 |
|  | CENPL\_Noc300\_tube2\_122214\_01.15953.15953.2 | 2.9791 | 0.283 | 99.8% | 1273.8522 | 1272.5945 | 2 | 6.138 | 65.0% | 2 | R.KLAVNMVPFPR.L | 22 |
|  | CENPL\_Noc300\_tube2\_122214\_01.18262.18262.2 | 2.9656 | 0.3872 | 100.0% | 1144.1921 | 1144.4204 | 1 | 8.43 | 77.8% | 3 | K.LAVNMVPFPR.L | 22 |
|  | CENPL\_Noc300\_122214\_01.15001.15001.2 | 3.8413 | 0.4088 | 100.0% | 1621.3722 | 1621.9403 | 1 | 8.844 | 69.2% | 2 | R.LHFFMPGFAPLTSR.G | 22 |
|  | CENPL\_Noc300\_tube2\_122214\_01.20181.20181.3 | 3.532 | 0.2572 | 99.9% | 1621.9143 | 1621.9403 | 2 | 5.041 | 46.2% | 2 | R.LHFFMPGFAPLTSR.G | 33 |
|  | CENPL\_Noc300\_122214\_01.13623.13623.2 | 2.3091 | 0.3204 | 99.6% | 1041.6522 | 1040.2505 | 2 | 5.88 | 75.0% | 2 | R.YLTVAAVFR.G | 22 |
|  | CENPL\_Noc300\_tube2\_122214\_01.11680.11680.2 | 4.1504 | 0.2215 | 100.0% | 1448.3121 | 1447.6031 | 1 | 5.86 | 77.3% | 4 | K.EVDEQMLNVQNK.N | 22 |
|  | CENPL\_Noc300\_122214\_01.11970.11970.2 | 2.8968 | 0.3507 | 100.0% | 1386.4922 | 1386.6116 | 2 | 5.864 | 65.0% | 1 | K.RISEQFTAMFR.R | 22 |
|  | CENPL\_Noc300\_tube2\_122214\_01.18759.18759.2 | 3.3107 | 0.4636 | 100.0% | 1230.1921 | 1230.4241 | 1 | 7.602 | 94.4% | 2 | R.ISEQFTAMFR.R | 22 |

Similarities:
gi|29788785|ref|NP\_82(14:2)  

---

|  |  |  |  |  |  |  |  |  |
| --- | --- | --- | --- | --- | --- | --- | --- | --- |
| U | *gi|38201621|ref|NP\_88* | 50 | 136 | 33.0% | 1599 | 175460 | 5.3 | eukaryotic translation initiation factor 4 gamma, 1 isoform 1 [Homo sapiens] |
| U | *gi|38201627|ref|NP\_93* | 50 | 136 | 34.9% | 1512 | 166588 | 5.2 | eukaryotic translation initiation factor 4 gamma, 1 isoform 2 [Homo sapiens] |
| U | *gi|38201625|ref|NP\_93* | 50 | 136 | 36.8% | 1435 | 158516 | 5.2 | eukaryotic translation initiation factor 4 gamma, 1 isoform 3 [Homo sapiens] |
| U | *gi|38201623|ref|NP\_93* | 50 | 136 | 33.0% | 1599 | 175460 | 5.3 | eukaryotic translation initiation factor 4 gamma, 1 isoform 1 [Homo sapiens] |

| Filename XCorr DeltCN Conf% ObsM+H+ CalcM+H+ SpR ZScore Ion% # Sequence  | | | | | | | | | | | | |
| --- | --- | --- | --- | --- | --- | --- | --- | --- | --- | --- | --- | --- |
|  | CENPL\_Noc300\_tube2\_122214\_01.13677.13677.2 | 5.0766 | 0.5574 | 100.0% | 2187.5723 | 2188.4204 | 1 | 8.623 | 73.7% | 1 | R.IRDPNQGGKDITEEIMSGAR.T | 2 |
|  | CENPL\_Noc300\_tube2\_122214\_01.13688.13688.3 | 5.275 | 0.5039 | 100.0% | 2188.7043 | 2188.4204 | 1 | 8.125 | 44.7% | 7 | R.IRDPNQGGKDITEEIMSGAR.T | 3 |
|  | CENPL\_Noc300\_tube2\_122214\_01.17271.17271.2 | 3.126 | 0.3839 | 100.0% | 1221.6721 | 1222.3557 | 1 | 7.785 | 75.0% | 3 | K.DITEEIMSGAR.T | 2 |
|  | CENPL\_Noc300\_tube2\_122214\_01.14025.14025.3 | 5.0769 | 0.5626 | 100.0% | 3459.7444 | 3458.7666 | 1 | 10.103 | 32.6% | 9 | R.TASTPTPPQTGGGLEPQANGETPQVAVIVRPDDR.S | 3 |
|  | CENPL\_Noc300\_tube2\_122214\_01.20372.20372.2 | 3.1267 | 0.264 | 99.9% | 1178.3522 | 1178.3275 | 1 | 8.245 | 75.0% | 3 | K.EAVGDLLDAFK.E | 2 |
|  | CENPL\_Noc300\_tube2\_122214\_01.08726.08726.3 | 4.2266 | 0.3732 | 100.0% | 2062.3145 | 2062.2463 | 1 | 6.367 | 45.3% | 4 | K.IHNAENIQPGEQKYEYK.S | 3 |
|  | CENPL\_Noc300\_122214\_01.10226.10226.2 | 2.8838 | 0.0124 | 96.1% | 1487.4122 | 1487.6525 | 1 | 3.618 | 63.6% | 5 | K.SDQWKPLNLEEK.K | 2 |
|  | CENPL\_Noc300\_tube2\_122214\_01.13557.13557.3 | 3.7957 | 0.1912 | 99.9% | 1488.2344 | 1487.6525 | 1 | 5.612 | 56.8% | 5 | K.SDQWKPLNLEEK.K | 3 |
|  | CENPL\_Noc300\_122214\_01.17958.17958.3 | 6.3411 | 0.4025 | 100.0% | 3741.8943 | 3742.3271 | 1 | 7.903 | 31.5% | 1 | R.YDREFLLGFQFIFASMQKPEGLPHISDVVLDK.A | 3 |
|  | CENPL\_Noc300\_122214\_01.18607.18607.3 | 5.9346 | 0.4541 | 100.0% | 3309.1143 | 3307.875 | 1 | 8.855 | 26.8% | 3 | R.EFLLGFQFIFASMQKPEGLPHISDVVLDK.A | 3 |
|  | CENPL\_Noc300\_122214\_01.06691.06691.3 | 2.2712 | 0.2733 | 96.2% | 1478.8143 | 1479.7225 | 7 | 5.037 | 39.6% | 1 | K.ANKTPLRPLDPTR.L | 3 |
|  | CENPL\_Noc300\_122214\_01.07527.07527.2 | 2.0849 | 0.2214 | 96.9% | 1167.3121 | 1166.3658 | 13 | 4.157 | 61.1% | 1 | K.TPLRPLDPTR.L | 2 |
|  | CENPL\_Noc300\_122214\_02.10077.10077.2 | 4.2433 | 0.4368 | 100.0% | 1475.6522 | 1475.8273 | 1 | 8.275 | 75.0% | 5 | R.KIIATVLMTEDIK.L | 2 |
|  | CENPL\_Noc300\_122214\_02.10022.10022.3 | 3.1972 | 0.3331 | 100.0% | 1476.2043 | 1475.8273 | 1 | 6.055 | 50.0% | 1 | R.KIIATVLMTEDIK.L | 3 |
|  | CENPL\_Noc300\_122214\_02.10053.10053.3 | 4.9275 | 0.4225 | 100.0% | 1832.2144 | 1831.2646 | 1 | 7.256 | 45.0% | 2 | R.KIIATVLMTEDIKLNK.A | 3 |
|  | CENPL\_Noc300\_122214\_02.11056.11056.2 | 4.0566 | 0.4727 | 100.0% | 1347.6322 | 1347.6532 | 1 | 10.014 | 86.4% | 2 | K.IIATVLMTEDIK.L | 2 |
|  | CENPL\_Noc300\_tube2\_122214\_01.09402.09402.3 | 4.3691 | 0.406 | 100.0% | 2425.7644 | 2426.5156 | 1 | 7.877 | 36.9% | 1 | R.TAADKDRGEEDADGSKTQDLFR.R | 3 |
|  | CENPL\_Noc300\_122214\_01.13933.13933.2 | 2.6351 | 0.4427 | 100.0% | 1365.7722 | 1365.6918 | 1 | 7.628 | 80.0% | 2 | K.LTPQMFQQLMK.Q | 2 |
|  | CENPL\_Noc300\_tube2\_122214\_01.11996.11996.2 | 3.5226 | 0.5056 | 100.0% | 1403.2722 | 1403.5321 | 1 | 8.727 | 77.3% | 5 | K.QVTQLAIDTEER.L | 2 |
|  | CENPL\_Noc300\_tube2\_122214\_01.17441.17441.2 | 2.8001 | 0.2538 | 99.3% | 1830.5521 | 1831.0192 | 29 | 6.477 | 40.0% | 2 | K.AISEPNFSVAYANMCR.C | 2 |
|  | CENPL\_Noc300\_tube2\_122214\_01.11585.11585.2 | 3.7145 | 0.4522 | 100.0% | 1589.3922 | 1589.832 | 1 | 7.786 | 73.1% | 2 | K.VPTTEKPTVTVNFR.K | 2 |
|  | CENPL\_Noc300\_tube2\_122214\_01.11631.11631.3 | 3.5809 | 0.4301 | 100.0% | 1591.2544 | 1589.832 | 1 | 6.515 | 50.0% | 7 | K.VPTTEKPTVTVNFR.K | 3 |
|  | CENPL\_Noc300\_122214\_01.07469.07469.3 | 3.2134 | 0.2897 | 99.9% | 1717.9443 | 1718.0061 | 48 | 5.391 | 33.9% | 1 | K.VPTTEKPTVTVNFRK.L | 3 |
|  | CENPL\_Noc300\_122214\_01.05706.05706.2 | 2.8234 | 0.5117 | 100.0% | 1508.2522 | 1508.6001 | 1 | 8.111 | 66.7% | 1 | K.QKEMDEAATAEER.G | 2 |
|  | CENPL\_Noc300\_122214\_01.05708.05708.3 | 2.7418 | 0.2196 | 97.8% | 1508.2743 | 1508.6001 | 73 | 4.247 | 35.4% | 1 | K.QKEMDEAATAEER.G | 3 |
|  | CENPL\_Noc300\_122214\_02.06173.06173.2 | 3.3874 | 0.3803 | 100.0% | 1252.2922 | 1252.2953 | 1 | 6.658 | 75.0% | 5 | K.EMDEAATAEER.G | 2 |
|  | CENPL\_Noc300\_122214\_01.06070.06070.2 | 3.194 | 0.2534 | 100.0% | 1117.4321 | 1117.2444 | 1 | 5.591 | 81.2% | 2 | R.LKEELEEAR.D | 2 |
|  | CENPL\_Noc300\_122214\_01.11960.11960.2 | 3.4494 | 0.2689 | 99.9% | 1494.5521 | 1493.7405 | 1 | 6.769 | 66.7% | 1 | R.LLTTIGKDLDFEK.A | 2 |
|  | CENPL\_Noc300\_tube2\_122214\_01.12842.12842.2 | 3.8062 | 0.4782 | 100.0% | 1334.0721 | 1334.5043 | 1 | 8.215 | 83.3% | 7 | R.MDQYFNQMEK.I | 2 |
|  | CENPL\_Noc300\_122214\_01.05975.05975.2 | 2.7705 | 0.1199 | 98.5% | 1091.2722 | 1091.2583 | 4 | 4.328 | 65.0% | 1 | R.RGGPPGPPISR.G | 2 |
|  | CENPL\_Noc300\_122214\_01.06622.06622.2 | 2.1366 | 0.2899 | 98.6% | 935.03217 | 935.0708 | 3 | 5.996 | 66.7% | 2 | R.GGPPGPPISR.G | 2 |
|  | CENPL\_Noc300\_122214\_01.14032.14032.2 | 3.8446 | 0.3356 | 100.0% | 1769.3722 | 1769.0073 | 1 | 8.08 | 62.5% | 2 | R.GLPLVDDGGWNTVPISK.G | 2 |
|  | CENPL\_Noc300\_tube2\_122214\_01.12345.12345.2 | 4.4222 | 0.4886 | 100.0% | 1972.5521 | 1973.196 | 1 | 8.556 | 61.1% | 2 | K.ITKPGSIDSNNQLFAPGGR.L | 2 |
|  | CENPL\_Noc300\_tube2\_122214\_01.12381.12381.3 | 3.6969 | 0.384 | 100.0% | 1973.4543 | 1973.196 | 3 | 6.103 | 34.7% | 6 | K.ITKPGSIDSNNQLFAPGGR.L | 3 |
|  | CENPL\_Noc300\_122214\_01.11988.11988.3 | 3.4229 | 0.2046 | 98.4% | 2624.1543 | 2624.8728 | 4 | 5.216 | 29.3% | 3 | K.ITKPGSIDSNNQLFAPGGRLS\*WGK.G | 3 |
|  | CENPL\_Noc300\_tube2\_122214\_01.08730.08730.2 | 2.7584 | 0.238 | 99.1% | 2504.0122 | 2504.6328 | 50 | 5.218 | 23.1% | 1 | K.GSSGGSGAKPSDAASEAARPATSTLNR.F | 2 |
|  | CENPL\_Noc300\_tube2\_122214\_01.08714.08714.3 | 3.6178 | 0.3867 | 100.0% | 2504.6643 | 2504.6328 | 1 | 6.154 | 29.8% | 4 | K.GSSGGSGAKPSDAASEAARPATSTLNR.F | 3 |
|  | CENPL\_Noc300\_122214\_01.08562.08562.2 | 3.1593 | 0.1678 | 99.3% | 1920.2522 | 1921.0769 | 2 | 5.475 | 43.8% | 1 | R.FSALQQAVPTESTDNRR.V | 2 |
|  | CENPL\_Noc300\_122214\_01.07742.07742.2 | 2.3552 | 0.3037 | 99.4% | 1118.9722 | 1119.2139 | 5 | 5.75 | 66.7% | 2 | K.AALSEEELEK.K | 2 |
|  | CENPL\_Noc300\_122214\_01.06837.06837.2 | 2.2121 | 0.2234 | 97.6% | 1247.1322 | 1247.388 | 9 | 5.132 | 65.0% | 1 | K.AALSEEELEKK.S | 2 |
|  | CENPL\_Noc300\_122214\_01.13281.13281.2 | 4.6021 | 0.4031 | 100.0% | 1590.3522 | 1589.8468 | 1 | 7.891 | 75.0% | 5 | K.AIIEEYLHLNDMK.E | 2 |
|  | CENPL\_Noc300\_122214\_01.18944.18944.2 | 3.2853 | 0.51 | 100.0% | 2306.5723 | 2307.6611 | 1 | 8.221 | 44.7% | 1 | K.EAVQCVQELASPSLLFIFVR.H | 2 |
|  | CENPL\_Noc300\_122214\_01.06649.06649.2 | 2.5128 | 0.2714 | 99.4% | 1252.7122 | 1252.543 | 5 | 4.949 | 65.0% | 1 | R.EITKPLRPLGK.A | 2 |
|  | CENPL\_Noc300\_122214\_01.07914.07914.2 | 2.138 | 0.1469 | 97.3% | 860.59216 | 860.047 | 4 | 4.111 | 83.3% | 1 | K.KVGTLWR.E | 2 |
|  | CENPL\_Noc300\_tube2\_122214\_01.19217.19217.2 | 4.2441 | 0.4119 | 100.0% | 1879.2322 | 1879.0764 | 1 | 7.691 | 62.5% | 1 | K.EFLPEGQDIGAFVAEQK.V | 2 |
|  | CENPL\_Noc300\_122214\_02.12105.12105.3 | 4.1329 | 0.4559 | 100.0% | 3525.6543 | 3525.8071 | 1 | 7.027 | 22.6% | 1 | K.EFLPEGQDIGAFVAEQKVEYTLGEESEAPGQR.A | 3 |
|  | CENPL\_Noc300\_tube2\_122214\_01.11444.11444.2 | 4.0266 | 0.5188 | 100.0% | 1665.2722 | 1665.7539 | 1 | 8.924 | 71.4% | 5 | K.VEYTLGEESEAPGQR.A | 2 |
|  | CENPL\_Noc300\_tube2\_122214\_01.09684.09684.2 | 2.3073 | 0.2903 | 99.4% | 1028.5922 | 1029.1381 | 26 | 5.158 | 75.0% | 2 | R.ALPSEELNR.Q | 2 |
|  | CENPL\_Noc300\_122214\_02.13647.13647.3 | 5.2657 | 0.5186 | 100.0% | 2462.7544 | 2462.765 | 1 | 8.692 | 43.8% | 2 | R.VFDWIEANLSEQQIVSNTLVR.A | 3 |
|  | CENPL\_Noc300\_tube2\_122214\_01.20513.20513.3 | 6.6608 | 0.4956 | 100.0% | 3777.2344 | 3777.0632 | 1 | 8.538 | 28.2% | 2 | R.MFFDALYDEDVVKEDAFYSWESSKDPAEQQGK.G | 3 |

---

|  |  |  |  |  |  |  |  |  |
| --- | --- | --- | --- | --- | --- | --- | --- | --- |
| U | *gi|42716275|ref|NP\_05* | 62 | 152 | 32.7% | 2376 | 266937 | 7.1 | CCR4-NOT transcription complex, subunit 1 isoform a [Homo sapiens] |

| Filename XCorr DeltCN Conf% ObsM+H+ CalcM+H+ SpR ZScore Ion% # Sequence  | | | | | | | | | | | | |
| --- | --- | --- | --- | --- | --- | --- | --- | --- | --- | --- | --- | --- |
|  | CENPL\_Noc300\_tube2\_122214\_01.09506.09506.3 | 3.442 | 0.3532 | 100.0% | 1423.6743 | 1423.5717 | 1 | 6.474 | 56.8% | 1 | R.ASQQEIQHIVNR.H | 3 |
|  | CENPL\_Noc300\_tube2\_122214\_01.17307.17307.2 | 2.9489 | 0.2376 | 99.8% | 1046.2522 | 1046.2577 | 2 | 5.514 | 93.8% | 1 | R.NIIVQFGVR.E | 2 |
|  | CENPL\_Noc300\_tube2\_122214\_01.17688.17688.3 | 3.4726 | 0.3539 | 99.9% | 2825.2444 | 2826.0923 | 1 | 5.603 | 26.9% | 1 | R.THSGLTDGIPLQSISAPGSGIWSDGKDK.S | 3 |
|  | CENPL\_Noc300\_122214\_02.13607.13607.3 | 4.3059 | 0.3014 | 100.0% | 2096.4243 | 2096.3477 | 1 | 5.734 | 38.9% | 2 | K.SDGAQAHTWNVEVLIDVLK.E | 3 |
|  | CENPL\_Noc300\_tube2\_122214\_01.13989.13989.2 | 2.0165 | 0.3029 | 98.6% | 1062.1122 | 1062.2108 | 44 | 5.018 | 56.2% | 1 | K.ELNPSLNFK.E | 2 |
|  | CENPL\_Noc300\_tube2\_122214\_01.15478.15478.2 | 4.3299 | 0.4517 | 100.0% | 1705.3522 | 1704.8798 | 2 | 7.315 | 57.7% | 2 | K.EVTYELDHPGFQIR.D | 2 |
|  | CENPL\_Noc300\_122214\_01.08299.08299.2 | 3.4292 | 0.4675 | 100.0% | 1256.2522 | 1256.4502 | 1 | 8.946 | 80.0% | 5 | K.GLHNVVYGIQR.G | 2 |
|  | CENPL\_Noc300\_122214\_01.17093.17093.2 | 3.7182 | 0.0829 | 99.8% | 1160.6522 | 1159.3684 | 10 | 4.992 | 72.2% | 2 | K.SLDLIESLLR.L | 2 |
|  | CENPL\_Noc300\_tube2\_122214\_01.10596.10596.2 | 3.8561 | 0.3577 | 100.0% | 1265.4122 | 1264.4209 | 1 | 6.394 | 85.0% | 3 | R.LAEVGQYEQVK.Q | 2 |
|  | CENPL\_Noc300\_tube2\_122214\_01.14711.14711.2 | 2.8487 | 0.2503 | 99.8% | 1014.7522 | 1015.1949 | 3 | 6.335 | 75.0% | 3 | R.ILDVAQDLK.A | 2 |
|  | CENPL\_Noc300\_122214\_01.20560.20560.2 | 4.8529 | 0.4913 | 100.0% | 2392.0322 | 2392.862 | 1 | 8.222 | 47.7% | 1 | K.ALSMLLNGTPFAFVIDLAALASR.R | 2 |
|  | CENPL\_Noc300\_tube2\_122214\_01.09970.09970.2 | 4.8947 | 0.5497 | 100.0% | 1734.5322 | 1734.95 | 1 | 9.395 | 70.0% | 1 | R.KLGTSGLNQPTFQQSK.M | 2 |
|  | CENPL\_Noc300\_122214\_01.08595.08595.2 | 4.7386 | 0.4559 | 100.0% | 1606.6721 | 1606.7759 | 1 | 7.92 | 64.3% | 3 | K.LGTSGLNQPTFQQSK.M | 2 |
|  | CENPL\_Noc300\_tube2\_122214\_01.15753.15753.3 | 3.9779 | 0.2929 | 100.0% | 2230.4343 | 2230.503 | 1 | 5.599 | 41.7% | 5 | K.MKPSDLSQVWPEANQHFSK.E | 3 |
|  | CENPL\_Noc300\_tube2\_122214\_01.13190.13190.2 | 4.069 | 0.4574 | 100.0% | 1487.3322 | 1487.5223 | 1 | 7.769 | 77.3% | 4 | K.EIDDEANSYFQR.I | 2 |
|  | CENPL\_Noc300\_tube2\_122214\_01.20028.20028.3 | 5.6364 | 0.4067 | 100.0% | 2508.5044 | 2507.8848 | 1 | 7.292 | 35.0% | 2 | R.IYNHPPHPTMSVDEVLEMLQR.F | 3 |
|  | CENPL\_Noc300\_tube2\_122214\_01.16401.16401.2 | 5.3214 | 0.5758 | 100.0% | 2467.9321 | 2468.8308 | 1 | 10.924 | 52.1% | 1 | K.MQGSITTPGSIALAQAQAQAQVPAK.A | 2 |
|  | CENPL\_Noc300\_tube2\_122214\_01.16463.16463.3 | 3.7107 | 0.4858 | 100.0% | 2468.3342 | 2468.8308 | 1 | 7.532 | 33.3% | 2 | K.MQGSITTPGSIALAQAQAQAQVPAK.A | 3 |
|  | CENPL\_Noc300\_tube2\_122214\_01.10839.10839.2 | 3.3728 | 0.4329 | 100.0% | 1393.4722 | 1393.6261 | 1 | 7.505 | 66.7% | 5 | K.TVTVTRPTGVSFK.K | 2 |
|  | CENPL\_Noc300\_122214\_01.07570.07570.2 | 2.6906 | 0.205 | 99.3% | 1296.3922 | 1296.4631 | 1 | 5.575 | 80.0% | 4 | R.IVEPPENIQEK.I | 2 |
|  | CENPL\_Noc300\_122214\_01.14100.14100.2 | 4.3411 | 0.3469 | 100.0% | 1856.6322 | 1857.1345 | 1 | 8.403 | 63.3% | 2 | K.IAFIFNNLSQSNMTQK.V | 2 |
|  | CENPL\_Noc300\_122214\_02.13077.13077.3 | 3.4282 | 0.2308 | 99.6% | 2843.0645 | 2844.3535 | 17 | 4.92 | 28.4% | 3 | K.VEELKETVKEEFMPWVSQYLVMK.R | 3 |
|  | CENPL\_Noc300\_tube2\_122214\_01.20415.20415.3 | 3.4029 | 0.2373 | 99.8% | 2381.8442 | 2381.6934 | 17 | 4.616 | 27.6% | 1 | K.RVSIEPNFHSLYSNFLDTLK.N | 3 |
|  | CENPL\_Noc300\_122214\_01.15925.15925.3 | 4.5737 | 0.3966 | 100.0% | 2954.3044 | 2955.2964 | 1 | 5.808 | 28.1% | 2 | R.VSIEPNFHSLYSNFLDTLKNPEFNK.M | 3 |
|  | CENPL\_Noc300\_122214\_01.08390.08390.2 | 2.8869 | 0.3254 | 100.0% | 1026.5521 | 1026.1957 | 8 | 6.128 | 85.7% | 5 | K.MVLNETYR.N | 2 |
|  | CENPL\_Noc300\_122214\_02.08009.08009.3 | 2.5955 | 0.3339 | 99.8% | 1609.0144 | 1608.7917 | 1 | 5.511 | 41.1% | 1 | K.VLLTSDKAAANFSDR.S | 3 |
|  | CENPL\_Noc300\_tube2\_122214\_01.19992.19992.2 | 4.052 | 0.4999 | 100.0% | 1454.5922 | 1454.7734 | 1 | 8.171 | 79.2% | 3 | K.NLGHWLGMITLAK.N | 2 |
|  | CENPL\_Noc300\_tube2\_122214\_01.09699.09699.2 | 3.5482 | 0.393 | 100.0% | 1393.5721 | 1393.6261 | 1 | 6.664 | 77.3% | 2 | K.NKPILHTDLDVK.S | 2 |
|  | CENPL\_Noc300\_tube2\_122214\_01.09710.09710.3 | 2.3828 | 0.2541 | 96.8% | 1393.8544 | 1393.6261 | 1 | 4.535 | 52.3% | 1 | K.NKPILHTDLDVK.S | 3 |
|  | CENPL\_Noc300\_tube2\_122214\_01.20207.20207.2 | 3.576 | 0.3994 | 100.0% | 1592.3722 | 1591.8907 | 1 | 6.685 | 69.2% | 1 | K.GQQELLYVVPFVAK.V | 2 |
|  | CENPL\_Noc300\_122214\_01.14322.14322.2 | 3.7371 | 0.3701 | 100.0% | 1766.2122 | 1766.0911 | 2 | 6.457 | 50.0% | 3 | K.NLALDINELKPGNLLK.D | 2 |
|  | CENPL\_Noc300\_tube2\_122214\_01.10139.10139.2 | 4.0014 | 0.4369 | 100.0% | 1356.1921 | 1356.562 | 1 | 7.055 | 77.3% | 1 | R.LKNLDEQLSAPK.K | 2 |
|  | CENPL\_Noc300\_tube2\_122214\_01.10132.10132.3 | 4.5193 | 0.2669 | 100.0% | 1357.5243 | 1356.562 | 2 | 6.123 | 54.5% | 2 | R.LKNLDEQLSAPK.K | 3 |
|  | CENPL\_Noc300\_tube2\_122214\_01.10221.10221.2 | 3.3943 | 0.3525 | 100.0% | 1114.8121 | 1115.2285 | 1 | 6.664 | 88.9% | 4 | K.NLDEQLSAPK.K | 2 |
|  | CENPL\_Noc300\_122214\_01.08407.08407.3 | 2.4448 | 0.3241 | 99.8% | 1363.1344 | 1362.5718 | 1 | 5.872 | 47.7% | 3 | R.AVQELVHPVVDR.S | 3 |
|  | CENPL\_Noc300\_tube2\_122214\_01.10992.10992.2 | 3.8836 | 0.361 | 100.0% | 1363.3722 | 1362.5718 | 1 | 6.963 | 72.7% | 6 | R.AVQELVHPVVDR.S | 2 |
|  | CENPL\_Noc300\_122214\_01.07297.07297.2 | 3.312 | 0.4424 | 100.0% | 1297.2522 | 1297.3641 | 1 | 6.649 | 85.0% | 2 | R.KDFALDSEESR.M | 2 |
|  | CENPL\_Noc300\_tube2\_122214\_01.16605.16605.2 | 2.2524 | 0.2981 | 98.8% | 1338.9722 | 1339.577 | 1 | 4.956 | 63.6% | 1 | R.NLTAGMAMITCR.E | 2 |
|  | CENPL\_Noc300\_tube2\_122214\_01.19636.19636.2 | 2.2616 | 0.3238 | 99.1% | 1346.4122 | 1346.6251 | 6 | 5.551 | 50.0% | 2 | R.EPLLMSISTNLK.N | 2 |
|  | CENPL\_Noc300\_tube2\_122214\_01.11087.11087.2 | 1.8901 | 0.253 | 96.9% | 865.9522 | 865.96454 | 2 | 4.901 | 78.6% | 1 | K.NSFASALR.T | 2 |
| \* | CENPL\_Noc300\_tube2\_122214\_01.11331.11331.2 | 2.8369 | 0.2305 | 99.8% | 1106.5322 | 1107.295 | 1 | 4.799 | 75.0% | 3 | R.LATEFELRK.H | 2 |
| \* | CENPL\_Noc300\_tube2\_122214\_01.16952.16952.2 | 2.4501 | 0.3747 | 99.8% | 1225.7322 | 1226.3745 | 1 | 7.205 | 83.3% | 5 | K.QLAVYEEFAR.N | 2 |
| \* | CENPL\_Noc300\_122214\_01.15030.15030.2 | 4.024 | 0.4985 | 100.0% | 2473.412 | 2473.8494 | 1 | 7.106 | 50.0% | 2 | R.NVPGFLPTNDLSQPTGFLAQPMK.Q | 2 |
| \* | CENPL\_Noc300\_122214\_02.09838.09838.2 | 4.4966 | 0.5199 | 100.0% | 1624.3922 | 1624.7472 | 1 | 9.561 | 80.8% | 6 | K.QAWATDDVAQIYDK.C | 2 |
| \* | CENPL\_Noc300\_tube2\_122214\_01.19888.19888.2 | 2.6085 | 0.1877 | 99.0% | 1115.4521 | 1115.3585 | 1 | 5.403 | 83.3% | 1 | R.SLLEVVVLSR.N | 2 |
| \* | CENPL\_Noc300\_122214\_01.16981.16981.2 | 6.422 | 0.5496 | 100.0% | 2170.8123 | 2171.476 | 1 | 11.675 | 69.4% | 1 | R.SVAHVTEADLFHTIETLMR.I | 2 |
| \* | CENPL\_Noc300\_122214\_01.17030.17030.3 | 4.4813 | 0.5421 | 100.0% | 2171.3643 | 2171.476 | 1 | 9.526 | 37.5% | 2 | R.SVAHVTEADLFHTIETLMR.I | 3 |
| \* | CENPL\_Noc300\_tube2\_122214\_01.19629.19629.2 | 4.0272 | 0.503 | 100.0% | 1610.2922 | 1610.8688 | 1 | 8.31 | 67.9% | 4 | R.GNAPEGLPQLMEVVR.S | 2 |
| \* | CENPL\_Noc300\_tube2\_122214\_01.11816.11816.2 | 3.1709 | 0.4883 | 100.0% | 1099.1522 | 1099.2036 | 1 | 7.998 | 81.2% | 4 | R.SNYEAMIDR.A | 2 |
| \* | CENPL\_Noc300\_tube2\_122214\_01.14176.14176.3 | 3.3506 | 0.34 | 99.9% | 2772.8643 | 2773.0266 | 1 | 5.537 | 34.0% | 1 | R.AHGGPNFMMHSGISQASEYDDPPGLR.E | 3 |
| \* | CENPL\_Noc300\_122214\_01.10080.10080.2 | 4.0149 | 0.4942 | 100.0% | 1474.2322 | 1474.6188 | 1 | 8.073 | 75.0% | 5 | R.EWVNLYHSAAAGR.D | 2 |
| \* | CENPL\_Noc300\_tube2\_122214\_01.16787.16787.2 | 4.6068 | 0.4769 | 100.0% | 1762.4122 | 1763.0673 | 1 | 9.201 | 63.3% | 4 | K.AFSAFVGQMHQQGILK.T | 2 |
| \* | CENPL\_Noc300\_122214\_01.06787.06787.3 | 2.9193 | 0.1909 | 95.3% | 1878.1144 | 1878.0728 | 1 | 4.591 | 37.5% | 2 | R.AQAEQQHNPAANPTMIR.A | 3 |
| \* | CENPL\_Noc300\_122214\_01.18747.18747.3 | 2.8145 | 0.3453 | 99.9% | 1759.9744 | 1760.0482 | 1 | 5.977 | 44.6% | 1 | K.APGFVYAWLELISHR.I | 3 |
| \* | CENPL\_Noc300\_tube2\_122214\_01.19121.19121.2 | 2.838 | 0.0211 | 97.7% | 1031.0922 | 1031.243 | 1 | 5.359 | 87.5% | 3 | R.NLILSAFPR.N | 23 |
| \* | CENPL\_Noc300\_tube2\_122214\_01.17570.17570.2 | 2.5009 | 0.2691 | 99.5% | 1142.2122 | 1142.3403 | 7 | 5.234 | 61.1% | 4 | R.LPDPFTPNLK.V | 2 |
| \* | CENPL\_Noc300\_tube2\_122214\_01.20666.20666.3 | 4.7277 | 0.4249 | 100.0% | 2481.7144 | 2481.9128 | 2 | 7.429 | 36.9% | 1 | R.LPDPFTPNLKVDMLSEINIAPR.I | 3 |
| \* | CENPL\_Noc300\_122214\_01.13519.13519.2 | 3.2803 | 0.3782 | 100.0% | 1358.3121 | 1358.5957 | 1 | 5.902 | 72.7% | 2 | K.VDMLSEINIAPR.I | 2 |
| \* | CENPL\_Noc300\_tube2\_122214\_01.19413.19413.2 | 3.1233 | 0.402 | 100.0% | 1593.4122 | 1593.9244 | 1 | 7.91 | 76.9% | 1 | R.ILTNFTGVMPPQFK.K | 2 |
| \* | CENPL\_Noc300\_tube2\_122214\_01.15106.15106.1 | 1.7493 | 0.2075 | 95.2% | 853.36 | 853.9475 | 2 | 4.725 | 66.7% | 1 | K.DLDSYLK.T | 1 |
| \* | CENPL\_Noc300\_122214\_01.14123.14123.2 | 4.2785 | 0.4161 | 100.0% | 1135.3922 | 1135.3054 | 1 | 8.201 | 88.9% | 3 | R.SPVTFLSDLR.S | 2 |
| \* | CENPL\_Noc300\_122214\_01.18237.18237.3 | 5.0653 | 0.3559 | 100.0% | 4005.1443 | 4004.5413 | 1 | 5.557 | 22.1% | 1 | R.SNLQVSNEPGNRYNLQLINALVLYVGTQAIAHIHNK.G | 3 |

---

|  |  |  |  |  |  |  |  |  |
| --- | --- | --- | --- | --- | --- | --- | --- | --- |
| U | *gi|146134388|ref|NP\_0* | 60 | 142 | 32.7% | 2146 | 241643 | 6.6 | YLP motif containing 1 [Homo sapiens] |

| Filename XCorr DeltCN Conf% ObsM+H+ CalcM+H+ SpR ZScore Ion% # Sequence  | | | | | | | | | | | | |
| --- | --- | --- | --- | --- | --- | --- | --- | --- | --- | --- | --- | --- |
| \* | CENPL\_Noc300\_tube2\_122214\_01.11399.11399.2 | 5.4263 | 0.3982 | 100.0% | 1989.0721 | 1989.2596 | 1 | 8.162 | 66.7% | 1 | R.EQHLAQLQQLQQMHQK.Q | 2 |
| \* | CENPL\_Noc300\_122214\_01.08714.08714.3 | 3.5048 | 0.2426 | 99.9% | 1989.1444 | 1989.2596 | 12 | 5.023 | 35.0% | 5 | R.EQHLAQLQQLQQMHQK.Q | 3 |
| \* | CENPL\_Noc300\_tube2\_122214\_01.11326.11326.2 | 3.3109 | 0.3116 | 99.9% | 1572.3121 | 1571.8168 | 1 | 6.169 | 63.3% | 5 | K.SQLLAPPPPSAPPGNK.T | 2 |
| \* | CENPL\_Noc300\_122214\_01.06435.06435.2 | 3.6054 | 0.3051 | 100.0% | 1388.3722 | 1388.5175 | 1 | 6.523 | 70.8% | 3 | K.TTVQQEPLESGAK.N | 2 |
| \* | CENPL\_Noc300\_122214\_01.05984.05984.3 | 2.9476 | 0.3465 | 99.9% | 1939.3744 | 1939.1061 | 1 | 5.41 | 35.0% | 1 | K.QLQAAAAHWQQHQQHR.V | 3 |
| \* | CENPL\_Noc300\_122214\_01.11014.11014.2 | 3.8452 | 0.4167 | 100.0% | 1299.3922 | 1299.5304 | 1 | 6.592 | 75.0% | 5 | R.VGFQYQGIMQK.H | 2 |
| \* | CENPL\_Noc300\_tube2\_122214\_01.20519.20519.3 | 6.6079 | 0.5407 | 100.0% | 3742.6443 | 3743.3367 | 1 | 8.868 | 35.8% | 1 | K.HTQLQQILQQYQQIIQPPPHIQTMSVDMQLR.H | 3 |
| \* | CENPL\_Noc300\_122214\_01.12253.12253.3 | 5.2956 | 0.3959 | 100.0% | 2510.0942 | 2509.7173 | 1 | 6.501 | 52.9% | 4 | R.HYEMQQQQFQHLYQEWER.E | 3 |
| \* | CENPL\_Noc300\_tube2\_122214\_01.15869.15869.3 | 4.0143 | 0.3228 | 100.0% | 3006.1743 | 3006.2573 | 1 | 5.645 | 35.2% | 1 | R.EFQLWEEQLHSYPHKDQLQEYEK.Q | 3 |
| \* | CENPL\_Noc300\_tube2\_122214\_01.16773.16773.2 | 3.8601 | 0.4391 | 100.0% | 2882.912 | 2884.126 | 1 | 9.059 | 37.5% | 1 | K.SALPYSSFSSDQGLGESSAAPSQPITAVK.D | 2 |
| \* | CENPL\_Noc300\_tube2\_122214\_01.12682.12682.2 | 2.7124 | 0.2986 | 99.8% | 1009.5122 | 1009.15027 | 9 | 6.492 | 66.7% | 3 | R.SGGLLPDPPR.S | 2 |
| \* | CENPL\_Noc300\_122214\_02.06479.06479.2 | 2.3504 | 0.2055 | 99.0% | 939.3522 | 939.0129 | 1 | 5.497 | 92.9% | 3 | R.SSYLESPR.G | 2 |
| \* | CENPL\_Noc300\_122214\_02.06375.06375.2 | 2.5878 | 0.172 | 99.3% | 980.21216 | 980.06836 | 1 | 4.458 | 92.9% | 3 | R.RFEDLGSR.C | 2 |
| \* | CENPL\_Noc300\_122214\_01.11872.11872.2 | 4.7341 | 0.3567 | 100.0% | 1809.3922 | 1810.0165 | 1 | 8.375 | 59.4% | 6 | R.GPASQFYITPSTSLSPR.Q | 2 |
| \* | CENPL\_Noc300\_tube2\_122214\_01.09160.09160.2 | 5.3954 | 0.4919 | 100.0% | 2329.5723 | 2330.4705 | 1 | 9.834 | 59.5% | 2 | K.SQAEPLSGNKEPLADTSSNQQK.N | 2 |
| \* | CENPL\_Noc300\_122214\_01.06935.06935.3 | 4.9494 | 0.4343 | 100.0% | 2330.5444 | 2330.4705 | 1 | 6.516 | 45.2% | 5 | K.SQAEPLSGNKEPLADTSSNQQK.N | 3 |
| \* | CENPL\_Noc300\_122214\_02.09790.09790.2 | 4.4506 | 0.4974 | 100.0% | 1340.2322 | 1339.5493 | 1 | 9.145 | 83.3% | 8 | K.MQSAAFSIAADVK.D | 2 |
| \* | CENPL\_Noc300\_122214\_02.09693.09693.3 | 2.9386 | 0.2932 | 99.8% | 1681.8844 | 1681.9446 | 1 | 5.654 | 50.0% | 1 | K.MQSAAFSIAADVKDVK.A | 3 |
| \* | CENPL\_Noc300\_tube2\_122214\_01.12114.12114.2 | 4.249 | 0.4573 | 100.0% | 2476.9321 | 2477.8662 | 1 | 7.657 | 39.1% | 1 | K.AQAVTQPVPLANKPVPAQSTFPSK.T | 2 |
| \* | CENPL\_Noc300\_122214\_01.09232.09232.3 | 3.7434 | 0.4318 | 100.0% | 2477.9944 | 2477.8662 | 3 | 6.695 | 27.2% | 6 | K.AQAVTQPVPLANKPVPAQSTFPSK.T | 3 |
| \* | CENPL\_Noc300\_122214\_01.05884.05884.2 | 2.59 | 0.2624 | 99.3% | 1372.2122 | 1371.4929 | 104 | 5.369 | 50.0% | 2 | R.EKVPGGLQGSQDR.G | 2 |
| \* | CENPL\_Noc300\_122214\_01.05994.05994.2 | 2.1331 | 0.2893 | 97.5% | 1614.0721 | 1614.7147 | 1 | 5.754 | 53.6% | 1 | R.DRGPFRPEPGDGGEK.M | 2 |
| \* | CENPL\_Noc300\_122214\_01.07975.07975.2 | 2.8137 | 0.5142 | 100.0% | 1157.1921 | 1157.2279 | 1 | 8.008 | 72.2% | 3 | R.GHEEFPLDGR.N | 2 |
| \* | CENPL\_Noc300\_tube2\_122214\_01.10222.10222.2 | 2.0514 | 0.1553 | 95.5% | 1104.9321 | 1105.1521 | 7 | 3.82 | 71.4% | 1 | R.ERLDDWDR.E | 2 |
| \* | CENPL\_Noc300\_122214\_01.07555.07555.2 | 2.6233 | 0.2919 | 99.6% | 1390.3522 | 1390.4551 | 118 | 4.985 | 55.6% | 1 | R.ERLDDWDRER.Y | 2 |
| \* | CENPL\_Noc300\_tube2\_122214\_01.13624.13624.3 | 2.9798 | 0.3703 | 99.9% | 1946.1543 | 1945.994 | 6 | 5.054 | 37.5% | 1 | R.DYQDDTLELYNREDR.F | 3 |
| \* | CENPL\_Noc300\_tube2\_122214\_01.19192.19192.2 | 2.9876 | 0.3053 | 100.0% | 1403.4722 | 1403.499 | 1 | 5.313 | 77.8% | 2 | R.RGPWWDDWER.D | 2 |
| \* | CENPL\_Noc300\_tube2\_122214\_01.20386.20386.2 | 2.7215 | 0.289 | 99.8% | 1247.1122 | 1247.3115 | 1 | 5.978 | 75.0% | 1 | R.GPWWDDWER.D | 2 |
| \* | CENPL\_Noc300\_122214\_01.07799.07799.2 | 2.4179 | 0.1555 | 97.7% | 1284.3522 | 1284.4907 | 1 | 3.939 | 77.8% | 1 | R.ISRPMDMYDR.S | 2 |
| \* | CENPL\_Noc300\_tube2\_122214\_01.15010.15010.3 | 3.1855 | 0.4511 | 100.0% | 2755.7944 | 2756.8613 | 1 | 6.737 | 29.8% | 2 | R.SLDNEWDRDYGRPLDEQESQFR.E | 3 |
| \* | CENPL\_Noc300\_tube2\_122214\_01.11601.11601.2 | 2.3335 | 0.1897 | 96.6% | 1740.2122 | 1740.8265 | 25 | 6.002 | 46.2% | 1 | R.DYGRPLDEQESQFR.E | 2 |
| \* | CENPL\_Noc300\_tube2\_122214\_01.11609.11609.3 | 3.023 | 0.2335 | 99.6% | 1741.9443 | 1740.8265 | 1 | 4.801 | 48.1% | 1 | R.DYGRPLDEQESQFR.E | 3 |
| \* | CENPL\_Noc300\_122214\_01.17255.17255.2 | 2.4099 | 0.1685 | 96.2% | 2044.8121 | 2045.4729 | 3 | 4.951 | 38.9% | 2 | R.DIPSLPPLPPLPPLPPLDR.Y | 2 |
| \* | CENPL\_Noc300\_122214\_01.08518.08518.3 | 4.4518 | 0.3254 | 100.0% | 2301.0544 | 2301.5537 | 1 | 6.534 | 43.4% | 2 | K.MKDFGSEPQMADHLPPQESR.L | 3 |
| \* | CENPL\_Noc300\_122214\_01.16379.16379.2 | 3.762 | 0.5533 | 100.0% | 2796.3123 | 2796.8762 | 1 | 9.808 | 43.2% | 2 | R.WDEDSFYGLWDTNDEQGLNSEFK.S | 2 |
| \* | CENPL\_Noc300\_tube2\_122214\_01.11615.11615.2 | 4.0063 | 0.381 | 100.0% | 1749.4922 | 1749.9182 | 1 | 6.887 | 53.6% | 3 | R.DISTNKVEQIPYGER.I | 2 |
| \* | CENPL\_Noc300\_tube2\_122214\_01.11625.11625.3 | 3.1839 | 0.1594 | 96.7% | 1750.4944 | 1749.9182 | 83 | 3.799 | 33.9% | 2 | R.DISTNKVEQIPYGER.I | 3 |
| \* | CENPL\_Noc300\_tube2\_122214\_01.09888.09888.2 | 2.3047 | 0.2778 | 99.3% | 1091.1522 | 1091.209 | 3 | 5.701 | 81.2% | 2 | K.VEQIPYGER.I | 2 |
| \* | CENPL\_Noc300\_122214\_01.09124.09124.3 | 3.0526 | 0.2619 | 99.9% | 1307.3043 | 1307.5356 | 10 | 5.657 | 47.5% | 3 | R.ITLRPDPLPER.S | 3 |
| \* | CENPL\_Noc300\_122214\_01.09281.09281.3 | 5.0037 | 0.4294 | 100.0% | 1913.3944 | 1913.115 | 1 | 6.692 | 50.0% | 4 | R.KSDRPVYEGPSMFGGER.R | 3 |
| \* | CENPL\_Noc300\_tube2\_122214\_01.12254.12254.3 | 3.6741 | 0.4177 | 100.0% | 1794.6244 | 1794.1248 | 1 | 6.376 | 43.8% | 6 | R.MPLPAPSLSHQPPPAPR.V | 3 |
| \* | CENPL\_Noc300\_tube2\_122214\_01.12749.12749.2 | 2.9602 | 0.3308 | 100.0% | 1224.3322 | 1224.4026 | 1 | 5.945 | 80.0% | 5 | K.NVDDILKPPGR.E | 2 |
| \* | CENPL\_Noc300\_tube2\_122214\_01.09870.09870.3 | 2.6971 | 0.3188 | 99.8% | 1684.4043 | 1684.9358 | 4 | 5.254 | 33.9% | 1 | K.LIRDKEVEFGGPAPR.V | 3 |
| \* | CENPL\_Noc300\_122214\_01.07582.07582.2 | 3.3748 | 0.2605 | 99.8% | 1684.5721 | 1684.9358 | 1 | 5.769 | 60.7% | 1 | K.LIRDKEVEFGGPAPR.V | 2 |
| \* | CENPL\_Noc300\_122214\_01.07269.07269.2 | 3.0938 | 0.4507 | 100.0% | 1302.0322 | 1302.4294 | 4 | 6.636 | 72.7% | 1 | R.DKEVEFGGPAPR.V | 2 |
| \* | CENPL\_Noc300\_122214\_01.07263.07263.3 | 2.6179 | 0.2814 | 99.6% | 1302.8043 | 1302.4294 | 51 | 5.062 | 34.1% | 1 | R.DKEVEFGGPAPR.V | 3 |
| \* | CENPL\_Noc300\_122214\_02.12864.12864.2 | 4.4035 | 0.5276 | 100.0% | 1672.2922 | 1671.8846 | 1 | 9.403 | 69.2% | 1 | R.VLSLDDYFITEVEK.E | 2 |
| \* | CENPL\_Noc300\_tube2\_122214\_01.19137.19137.3 | 2.5509 | 0.2928 | 97.2% | 2785.8843 | 2786.062 | 13 | 4.539 | 22.8% | 1 | R.VLSLDDYFITEVEKEEKDPDSGKK.V | 3 |
| \* | CENPL\_Noc300\_122214\_02.09976.09976.2 | 2.8119 | 0.4273 | 100.0% | 1972.5322 | 1973.133 | 1 | 6.807 | 42.9% | 1 | K.VMEYEYEAEMEETYR.T | 2 |
| \* | CENPL\_Noc300\_122214\_01.19950.19950.2 | 5.0962 | 0.5031 | 100.0% | 2083.112 | 2083.3484 | 1 | 9.391 | 64.7% | 2 | K.TLDDGFFPFIILDAINDR.V | 2 |
| \* | CENPL\_Noc300\_122214\_01.10651.10651.2 | 3.1596 | 0.4305 | 100.0% | 1238.2322 | 1237.3599 | 1 | 6.797 | 83.3% | 5 | R.HFDQFWSAAK.T | 2 |
| \* | CENPL\_Noc300\_122214\_01.07043.07043.2 | 3.3466 | 0.435 | 100.0% | 1213.2322 | 1214.341 | 1 | 7.23 | 83.3% | 1 | K.MADHWETAPR.H | 2 |
| \* | CENPL\_Noc300\_122214\_01.07077.07077.3 | 2.7917 | 0.3023 | 99.9% | 1213.7943 | 1214.341 | 7 | 6.113 | 44.4% | 1 | K.MADHWETAPR.H | 3 |
| \* | CENPL\_Noc300\_122214\_02.12690.12690.3 | 3.1567 | 0.2871 | 99.8% | 3154.9443 | 3154.3804 | 1 | 5.261 | 28.8% | 1 | R.SLLQDAAIEEVEMEDFDANIEEQKEEK.K | 3 |
| \* | CENPL\_Noc300\_122214\_01.07132.07132.2 | 2.8222 | 0.3112 | 99.8% | 1312.0721 | 1312.437 | 1 | 6.189 | 75.0% | 1 | K.SKWEMDTSEAK.L | 2 |
| \* | CENPL\_Noc300\_tube2\_122214\_01.13860.13860.3 | 3.2506 | 0.4768 | 100.0% | 2223.4143 | 2223.5059 | 2 | 6.646 | 33.3% | 2 | K.SKWEMDTSEAKLDKLDGLR.T | 3 |
| \* | CENPL\_Noc300\_122214\_01.07119.07119.3 | 3.6347 | 0.3141 | 100.0% | 1232.7244 | 1232.3848 | 4 | 5.983 | 52.8% | 1 | R.KRDWEAIASR.M | 3 |
| \* | CENPL\_Noc300\_122214\_01.13118.13118.2 | 2.6669 | 0.2395 | 99.0% | 1774.4722 | 1774.8964 | 1 | 5.561 | 61.5% | 1 | R.MEDYLQLPDDYDTR.A | 2 |
| \* | CENPL\_Noc300\_tube2\_122214\_01.18304.18304.2 | 4.0255 | 0.3751 | 100.0% | 1450.8522 | 1450.6342 | 1 | 7.267 | 79.2% | 6 | R.AIGFVVGQTDWEK.I | 2 |
| \* | CENPL\_Noc300\_122214\_02.10973.10973.3 | 2.9927 | 0.2163 | 95.5% | 2631.8943 | 2631.9019 | 1 | 5.029 | 30.4% | 1 | R.AIGFVVGQTDWEKITDESGHLAEK.A | 3 |

---

|  |  |  |  |  |  |  |  |  |
| --- | --- | --- | --- | --- | --- | --- | --- | --- |
| U | *gi|4506691|ref|NP\_001* | 4 | 8 | 32.2% | 146 | 16445 | 10.2 | ribosomal protein S16 [Homo sapiens] |

| Filename XCorr DeltCN Conf% ObsM+H+ CalcM+H+ SpR ZScore Ion% # Sequence  | | | | | | | | | | | | |
| --- | --- | --- | --- | --- | --- | --- | --- | --- | --- | --- | --- | --- |
|  | CENPL\_Noc300\_tube2\_122214\_01.16545.16545.2 | 3.3319 | 0.4173 | 100.0% | 1188.2922 | 1188.372 | 1 | 7.69 | 75.0% | 4 | K.GPLQSVQVFGR.K | 2 |
| \* | CENPL\_Noc300\_tube2\_122214\_01.12660.12660.2 | 2.2734 | 0.3024 | 98.9% | 1411.3722 | 1411.6622 | 8 | 4.29 | 50.0% | 1 | K.VNGRPLEMIEPR.T | 2 |
|  | CENPL\_Noc300\_tube2\_122214\_01.20404.20404.2 | 2.2426 | 0.2671 | 98.7% | 1095.1721 | 1095.4111 | 5 | 5.243 | 66.7% | 2 | K.LLEPVLLLGK.E | 2 |
| \* | CENPL\_Noc300\_tube2\_122214\_01.09533.09533.3 | 2.4054 | 0.2492 | 95.3% | 1468.7943 | 1469.7299 | 1 | 5.534 | 44.2% | 1 | R.VKGGGHVAQIYAIR.Q | 3 |

---

|  |  |  |  |  |  |  |  |  |
| --- | --- | --- | --- | --- | --- | --- | --- | --- |
| U | *gi|14389309|ref|NP\_11* | 12 | 43 | 31.2% | 449 | 49895 | 5.1 | tubulin alpha 6 [Homo sapiens] |
| U | *gi|57013276|ref|NP\_00* | 12 | 43 | 31.0% | 451 | 50152 | 5.1 | tubulin, alpha, ubiquitous [Homo sapiens] |

| Filename XCorr DeltCN Conf% ObsM+H+ CalcM+H+ SpR ZScore Ion% # Sequence  | | | | | | | | | | | | |
| --- | --- | --- | --- | --- | --- | --- | --- | --- | --- | --- | --- | --- |
|  | CENPL\_Noc300\_122214\_02.11319.11319.2 | 5.2417 | 0.6618 | 100.0% | 2008.7322 | 2009.093 | 1 | 11.194 | 60.5% | 4 | K.TIGGGDDSFNTFFSETGAGK.H | 2 |
|  | CENPL\_Noc300\_122214\_01.14725.14725.2 | 4.0142 | 0.5298 | 100.0% | 1702.3522 | 1702.9451 | 1 | 9.19 | 67.9% | 5 | R.AVFVDLEPTVIDEVR.T | 2 |
|  | CENPL\_Noc300\_tube2\_122214\_01.14741.14741.3 | 4.0854 | 0.4334 | 100.0% | 2415.6843 | 2416.6555 | 1 | 6.159 | 33.8% | 5 | R.QLFHPEQLITGKEDAANNYAR.G | 3 |
|  | CENPL\_Noc300\_122214\_01.08153.08153.3 | 3.18 | 0.3273 | 99.9% | 1876.9744 | 1876.0824 | 1 | 5.505 | 41.1% | 1 | R.RNLDIERPTYTNLNR.L | 3 |
|  | CENPL\_Noc300\_tube2\_122214\_01.12418.12418.2 | 3.2742 | 0.2886 | 99.9% | 1718.7322 | 1719.8949 | 1 | 5.599 | 69.2% | 4 | R.NLDIERPTYTNLNR.L | 2 |
|  | CENPL\_Noc300\_tube2\_122214\_01.12368.12368.3 | 2.7321 | 0.3195 | 99.9% | 1719.8344 | 1719.8949 | 2 | 5.599 | 46.2% | 2 | R.NLDIERPTYTNLNR.L | 3 |
|  | CENPL\_Noc300\_122214\_02.14318.14318.2 | 4.4742 | 0.5198 | 100.0% | 1488.6522 | 1488.7678 | 1 | 8.744 | 69.2% | 4 | R.LISQIVSSITASLR.F | 2 |
|  | CENPL\_Noc300\_tube2\_122214\_01.18767.18767.2 | 3.6704 | 0.4723 | 100.0% | 1757.5721 | 1758.0703 | 1 | 7.742 | 56.7% | 5 | R.IHFPLATYAPVISAEK.A | 2 |
|  | CENPL\_Noc300\_tube2\_122214\_01.18878.18878.3 | 2.811 | 0.2666 | 99.5% | 1758.6543 | 1758.0703 | 1 | 5.025 | 43.3% | 1 | R.IHFPLATYAPVISAEK.A | 3 |
|  | CENPL\_Noc300\_tube2\_122214\_01.12993.12993.2 | 2.8942 | 0.3823 | 100.0% | 1015.7322 | 1016.1827 | 1 | 6.894 | 94.4% | 5 | K.DVNAAIATIK.T | 2 |
|  | CENPL\_Noc300\_122214\_01.11572.11572.2 | 4.3546 | 0.4889 | 100.0% | 1825.5721 | 1826.1027 | 1 | 7.423 | 67.6% | 5 | K.VGINYQPPTVVPGGDLAK.V | 2 |
|  | CENPL\_Noc300\_tube2\_122214\_01.12620.12620.3 | 3.8053 | 0.3509 | 100.0% | 1381.8544 | 1381.6324 | 1 | 6.263 | 52.5% | 2 | R.LDHKFDLMYAK.R | 3 |

---

|  |  |  |  |  |  |  |  |  |
| --- | --- | --- | --- | --- | --- | --- | --- | --- |
| U | *gi|38016911|ref|NP\_00* | 5 | 9 | 31.2% | 288 | 31731 | 7.9 | stomatin isoform a [Homo sapiens] |

| Filename XCorr DeltCN Conf% ObsM+H+ CalcM+H+ SpR ZScore Ion% # Sequence  | | | | | | | | | | | | |
| --- | --- | --- | --- | --- | --- | --- | --- | --- | --- | --- | --- | --- |
| \* | CENPL\_Noc300\_122214\_01.15968.15968.3 | 4.836 | 0.4447 | 100.0% | 3373.2844 | 3373.6758 | 1 | 6.837 | 25.0% | 1 | K.NLSQILSDREEIAHNMQSTLDDATDAWGIK.V | 3 |
|  | CENPL\_Noc300\_122214\_01.06993.06993.2 | 3.0175 | 0.5183 | 100.0% | 1248.3322 | 1248.3966 | 1 | 8.52 | 81.8% | 1 | K.VIAAEGEMNASR.A | 2 |
|  | CENPL\_Noc300\_tube2\_122214\_01.17724.17724.2 | 2.787 | 0.4484 | 100.0% | 1717.0122 | 1716.9904 | 1 | 6.964 | 46.7% | 1 | K.EASMVITESPAALQLR.Y | 2 |
|  | CENPL\_Noc300\_tube2\_122214\_01.15340.15340.2 | 3.2762 | 0.3397 | 100.0% | 1352.3922 | 1352.5707 | 1 | 7.983 | 86.4% | 5 | R.YLQTLTTIAAEK.N | 2 |
|  | CENPL\_Noc300\_122214\_01.19758.19758.2 | 3.5825 | 0.3897 | 100.0% | 2128.6921 | 2128.5781 | 1 | 5.737 | 55.3% | 1 | K.NSTIVFPLPIDMLQGIIGAK.H | 2 |

---

|  |  |  |  |  |  |  |  |  |
| --- | --- | --- | --- | --- | --- | --- | --- | --- |
| U | *gi|32455264|ref|NP\_85* | 5 | 7 | 31.2% | 199 | 22110 | 8.1 | peroxiredoxin 1 [Homo sapiens] |
| U | *gi|4505591|ref|NP\_002* | 5 | 7 | 31.2% | 199 | 22110 | 8.1 | peroxiredoxin 1 [Homo sapiens] |
| U | *gi|32455266|ref|NP\_85* | 5 | 7 | 31.2% | 199 | 22110 | 8.1 | peroxiredoxin 1 [Homo sapiens] |

| Filename XCorr DeltCN Conf% ObsM+H+ CalcM+H+ SpR ZScore Ion% # Sequence  | | | | | | | | | | | | |
| --- | --- | --- | --- | --- | --- | --- | --- | --- | --- | --- | --- | --- |
|  | CENPL\_Noc300\_tube2\_122214\_01.11495.11495.2 | 2.1894 | 0.3029 | 98.8% | 1165.1921 | 1165.3496 | 1 | 6.05 | 60.0% | 1 | K.ATAVMPDGQFK.D | 2 |
|  | CENPL\_Noc300\_tube2\_122214\_01.14786.14786.3 | 3.1843 | 0.2011 | 98.3% | 1907.8143 | 1908.2694 | 1 | 4.653 | 39.7% | 1 | K.KQGGLGPMNIPLVSDPKR.T | 3 |
|  | CENPL\_Noc300\_tube2\_122214\_01.19254.19254.2 | 2.3193 | 0.2296 | 98.0% | 1360.0122 | 1360.6395 | 1 | 6.301 | 63.6% | 1 | R.GLFIIDDKGILR.Q | 2 |
|  | CENPL\_Noc300\_tube2\_122214\_01.13941.13941.2 | 2.5037 | 0.3355 | 99.5% | 1212.3322 | 1212.3915 | 13 | 6.956 | 75.0% | 1 | R.QITVNDLPVGR.S | 22 |
|  | CENPL\_Noc300\_122214\_01.11462.11462.2 | 3.0352 | 0.4297 | 100.0% | 1197.3522 | 1197.3763 | 1 | 7.821 | 88.9% | 3 | R.LVQAFQFTDK.H | 2 |

Similarities:
gi|32189392|ref|NP\_00(1:4)  

---

|  |  |  |  |  |  |  |  |  |
| --- | --- | --- | --- | --- | --- | --- | --- | --- |
| U | *gi|14150155|ref|NP\_11* | 2 | 3 | 30.7% | 176 | 20748 | 6.3 | vacuolar protein sorting 25 [Homo sapiens] |

| Filename XCorr DeltCN Conf% ObsM+H+ CalcM+H+ SpR ZScore Ion% # Sequence  | | | | | | | | | | | | |
| --- | --- | --- | --- | --- | --- | --- | --- | --- | --- | --- | --- | --- |
| \* | CENPL\_Noc300\_122214\_02.12899.12899.3 | 5.2357 | 0.4735 | 100.0% | 3845.2144 | 3846.0227 | 1 | 8.196 | 25.8% | 1 | R.SGQNNSVFTLYELTNGEDTEDEEFHGLDEATLLR.A | 3 |
| \* | CENPL\_Noc300\_122214\_02.08123.08123.3 | 5.1942 | 0.3595 | 100.0% | 2208.0842 | 2208.4795 | 1 | 8.07 | 38.2% | 2 | R.ALQALQQEHKAEIITVSDGR.G | 3 |

---

|  |  |  |  |  |  |  |  |  |
| --- | --- | --- | --- | --- | --- | --- | --- | --- |
| U | *gi|14916501|ref|NP\_14* | 3 | 3 | 30.0% | 130 | 15069 | 10.9 | ribosomal protein S24 isoform a [Homo sapiens] |
| U | *gi|4506703|ref|NP\_001* | 3 | 3 | 29.3% | 133 | 15423 | 10.8 | ribosomal protein S24 isoform c [Homo sapiens] |
| U | *gi|214829241|ref|NP\_0* | 3 | 3 | 29.8% | 131 | 15197 | 10.9 | ribosomal protein S24 isoform b [Homo sapiens] |
| U | *gi|214010226|ref|NP\_0* | 3 | 3 | 13.5% | 289 | 32431 | 10.2 | ribosomal protein S24 isoform d [Homo sapiens] |
| U | *gi|214010224|ref|NP\_0* | 3 | 3 | 29.8% | 131 | 15197 | 10.9 | ribosomal protein S24 isoform f [Homo sapiens] |
| U | *gi|214010222|ref|NP\_0* | 3 | 3 | 29.5% | 132 | 15325 | 10.9 | ribosomal protein S24 isoform e [Homo sapiens] |

| Filename XCorr DeltCN Conf% ObsM+H+ CalcM+H+ SpR ZScore Ion% # Sequence  | | | | | | | | | | | | |
| --- | --- | --- | --- | --- | --- | --- | --- | --- | --- | --- | --- | --- |
|  | CENPL\_Noc300\_tube2\_122214\_01.12095.12095.3 | 3.6593 | 0.3028 | 100.0% | 1364.8744 | 1365.6769 | 11 | 5.6 | 43.2% | 1 | R.KQMVIDVLHPGK.A | 3 |
|  | CENPL\_Noc300\_122214\_01.16900.16900.2 | 1.9252 | 0.3494 | 98.0% | 1399.8522 | 1399.6323 | 2 | 6.305 | 59.1% | 1 | K.TTPDVIFVFGFR.T | 2 |
|  | CENPL\_Noc300\_122214\_02.12155.12155.2 | 2.088 | 0.3566 | 98.6% | 1682.6522 | 1682.8854 | 4 | 5.952 | 46.4% | 1 | K.TTGFGMIYDSLDYAK.K | 2 |

---

|  |  |  |  |  |  |  |  |  |
| --- | --- | --- | --- | --- | --- | --- | --- | --- |
| U | *gi|46367787|ref|NP\_00* | 23 | 74 | 29.9% | 636 | 70671 | 9.5 | poly(A) binding protein, cytoplasmic 1 [Homo sapiens] |

| Filename XCorr DeltCN Conf% ObsM+H+ CalcM+H+ SpR ZScore Ion% # Sequence  | | | | | | | | | | | | |
| --- | --- | --- | --- | --- | --- | --- | --- | --- | --- | --- | --- | --- |
|  | CENPL\_Noc300\_122214\_01.12689.12689.2 | 2.2787 | 0.3401 | 99.4% | 1158.0322 | 1158.3861 | 2 | 6.243 | 80.0% | 4 | K.FSPAGPILSIR.V | 22 |
|  | CENPL\_Noc300\_tube2\_122214\_01.14126.14126.2 | 2.514 | 0.1648 | 96.9% | 2085.5122 | 2086.2712 | 245 | 4.3 | 29.4% | 1 | R.RSLGYAYVNFQQPADAER.A | 22 |
|  | CENPL\_Noc300\_tube2\_122214\_01.16640.16640.2 | 5.1516 | 0.5274 | 100.0% | 1930.4321 | 1930.0837 | 1 | 9.166 | 62.5% | 4 | R.SLGYAYVNFQQPADAER.A | 22 |
|  | CENPL\_Noc300\_tube2\_122214\_01.18668.18668.2 | 3.6712 | 0.5155 | 100.0% | 1267.1921 | 1267.4828 | 1 | 8.883 | 85.0% | 6 | R.ALDTMNFDVIK.G | 22 |
|  | CENPL\_Noc300\_tube2\_122214\_01.15716.15716.2 | 2.954 | 0.2678 | 99.9% | 935.1322 | 935.1112 | 1 | 5.547 | 81.2% | 4 | K.SGVGNIFIK.N | 2 |
| \* | CENPL\_Noc300\_tube2\_122214\_01.14274.14274.2 | 4.2256 | 0.4063 | 100.0% | 1741.5721 | 1741.857 | 1 | 8.61 | 67.9% | 2 | K.GYGFVHFETQEAAER.A | 2 |
|  | CENPL\_Noc300\_tube2\_122214\_01.12850.12850.2 | 2.2965 | 0.2754 | 99.3% | 1064.1122 | 1064.263 | 1 | 5.757 | 75.0% | 3 | K.MNGMLLNDR.K | 22 |
|  | CENPL\_Noc300\_tube2\_122214\_01.11039.11039.2 | 2.8856 | 0.3055 | 99.9% | 1213.5322 | 1213.4191 | 1 | 5.708 | 77.8% | 5 | R.AKEFTNVYIK.N | 22 |
|  | CENPL\_Noc300\_122214\_02.07188.07188.3 | 3.4716 | 0.3385 | 100.0% | 1214.0044 | 1213.4191 | 10 | 6.461 | 50.0% | 3 | R.AKEFTNVYIK.N | 33 |
|  | CENPL\_Noc300\_tube2\_122214\_01.10122.10122.2 | 3.0571 | 0.4048 | 100.0% | 1228.1122 | 1228.2323 | 1 | 5.89 | 72.2% | 3 | K.NFGEDMDDER.L | 2 |
|  | CENPL\_Noc300\_122214\_01.08584.08584.2 | 2.5925 | 0.2938 | 99.5% | 1470.6122 | 1469.5659 | 1 | 5.257 | 68.2% | 1 | K.NFGEDMDDERLK.D | 2 |
|  | CENPL\_Noc300\_122214\_01.13718.13718.2 | 3.0326 | 0.4288 | 100.0% | 1046.2522 | 1046.1704 | 1 | 8.538 | 75.0% | 3 | K.GFGFVSFER.H | 2 |
|  | CENPL\_Noc300\_tube2\_122214\_01.13160.13160.2 | 2.6136 | 0.2183 | 99.5% | 1083.8121 | 1084.2603 | 8 | 6.074 | 75.0% | 5 | R.YQGVNLYVK.N | 2 |
|  | CENPL\_Noc300\_tube2\_122214\_01.09632.09632.2 | 2.9966 | 0.4428 | 100.0% | 1162.0721 | 1162.1553 | 1 | 7.342 | 83.3% | 3 | K.NLDDGIDDER.L | 2 |
| \* | CENPL\_Noc300\_tube2\_122214\_01.14651.14651.2 | 3.8824 | 0.4453 | 100.0% | 1413.6522 | 1413.6134 | 1 | 8.111 | 79.2% | 4 | R.KEFSPFGTITSAK.V | 2 |
| \* | CENPL\_Noc300\_122214\_01.12663.12663.2 | 2.6375 | 0.5172 | 100.0% | 1285.4722 | 1285.4393 | 1 | 8.338 | 68.2% | 3 | K.EFSPFGTITSAK.V | 2 |
|  | CENPL\_Noc300\_tube2\_122214\_01.15086.15086.3 | 3.6043 | 0.2841 | 99.9% | 1544.2743 | 1543.8931 | 36 | 6.253 | 40.4% | 4 | R.IVATKPLYVALAQR.K | 3 |
|  | CENPL\_Noc300\_tube2\_122214\_01.15032.15032.2 | 4.0268 | 0.4567 | 100.0% | 1544.4922 | 1543.8931 | 1 | 7.42 | 61.5% | 4 | R.IVATKPLYVALAQR.K | 2 |
|  | CENPL\_Noc300\_122214\_01.17849.17849.2 | 5.6116 | 0.5639 | 100.0% | 2741.632 | 2742.175 | 1 | 10.706 | 47.8% | 1 | K.ITGMLLEIDNSELLHMLESPESLR.S | 22 |
|  | CENPL\_Noc300\_122214\_01.17916.17916.3 | 4.9443 | 0.3608 | 100.0% | 2743.0745 | 2742.175 | 1 | 6.65 | 38.0% | 3 | K.ITGMLLEIDNSELLHMLESPESLR.S | 33 |
|  | CENPL\_Noc300\_122214\_01.09176.09176.2 | 5.192 | 0.5611 | 100.0% | 1694.4922 | 1694.9285 | 1 | 10.465 | 80.0% | 2 | R.SKVDEAVAVLQAHQAK.E | 2 |
|  | CENPL\_Noc300\_122214\_01.09208.09208.3 | 5.6269 | 0.4639 | 100.0% | 1695.0543 | 1694.9285 | 1 | 8.576 | 56.7% | 5 | R.SKVDEAVAVLQAHQAK.E | 3 |
|  | CENPL\_Noc300\_122214\_01.08709.08709.3 | 2.6834 | 0.2697 | 99.5% | 1481.6344 | 1479.6763 | 1 | 4.779 | 48.1% | 1 | K.VDEAVAVLQAHQAK.E | 3 |

Similarities:
gi|208431833|ref|NP\_0(8:15)  
gi|109948285|ref|NP\_0(1:22)  

---

|  |  |  |  |  |  |  |  |  |
| --- | --- | --- | --- | --- | --- | --- | --- | --- |
| U | *gi|5032051|ref|NP\_005* | 3 | 4 | 29.8% | 151 | 16273 | 10.1 | ribosomal protein S14 [Homo sapiens] |
| U | *gi|68160922|ref|NP\_00* | 3 | 4 | 29.8% | 151 | 16273 | 10.1 | ribosomal protein S14 [Homo sapiens] |
| U | *gi|68160915|ref|NP\_00* | 3 | 4 | 29.8% | 151 | 16273 | 10.1 | ribosomal protein S14 [Homo sapiens] |

| Filename XCorr DeltCN Conf% ObsM+H+ CalcM+H+ SpR ZScore Ion% # Sequence  | | | | | | | | | | | | |
| --- | --- | --- | --- | --- | --- | --- | --- | --- | --- | --- | --- | --- |
|  | CENPL\_Noc300\_tube2\_122214\_01.16445.16445.3 | 3.5633 | 0.1722 | 97.9% | 2266.4944 | 2266.4473 | 1 | 4.584 | 36.2% | 1 | K.ADRDESSPYAAMLAAQDVAQR.C | 3 |
|  | CENPL\_Noc300\_122214\_01.06252.06252.2 | 2.4995 | 0.4341 | 99.9% | 1054.4722 | 1055.179 | 1 | 7.022 | 75.0% | 2 | K.TPGPGAQSALR.A | 2 |
|  | CENPL\_Noc300\_tube2\_122214\_01.11375.11375.2 | 2.6659 | 0.0837 | 96.3% | 1431.3922 | 1430.5547 | 4 | 4.603 | 62.5% | 1 | R.IEDVTPIPSDSTR.R | 2 |

---

|  |  |  |  |  |  |  |  |  |
| --- | --- | --- | --- | --- | --- | --- | --- | --- |
| U | *gi|190360566|ref|NP\_4* | 13 | 20 | 29.7% | 357 | 40675 | 5.7 | protein-L-isoaspartate (D-aspartate) O-methyltransferase domain containing 1 [Homo sapiens] |

| Filename XCorr DeltCN Conf% ObsM+H+ CalcM+H+ SpR ZScore Ion% # Sequence  | | | | | | | | | | | | |
| --- | --- | --- | --- | --- | --- | --- | --- | --- | --- | --- | --- | --- |
| \* | CENPL\_Noc300\_122214\_01.20766.20766.2 | 3.9836 | 0.4364 | 100.0% | 2014.5122 | 2015.0698 | 1 | 6.613 | 58.3% | 1 | -.MGGAVS\*AGEDNDDLIDNLK.E | 2 |
| \* | CENPL\_Noc300\_122214\_01.20518.20518.2 | 3.9315 | 0.508 | 100.0% | 2774.132 | 2775.9177 | 1 | 7.525 | 37.5% | 1 | -.MGGAVS\*AGEDNDDLIDNLKEAQYIR.T | 2 |
| \* | CENPL\_Noc300\_tube2\_122214\_01.12876.12876.2 | 2.8723 | 0.4111 | 100.0% | 1590.6122 | 1591.7202 | 1 | 6.084 | 58.3% | 2 | R.AIDRGDYYLEGYR.D | 2 |
| \* | CENPL\_Noc300\_tube2\_122214\_01.12284.12284.3 | 3.9964 | 0.3307 | 100.0% | 2183.9043 | 2183.3416 | 1 | 5.712 | 45.6% | 2 | R.AIDRGDYYLEGYRDNAYK.D | 3 |
| \* | CENPL\_Noc300\_tube2\_122214\_01.13431.13431.2 | 2.4577 | 0.2656 | 99.5% | 1136.4521 | 1136.2059 | 1 | 5.951 | 75.0% | 1 | R.GDYYLEGYR.D | 2 |
| \* | CENPL\_Noc300\_122214\_01.12082.12082.3 | 5.2492 | 0.4671 | 100.0% | 2341.9443 | 2341.5413 | 1 | 8.165 | 40.3% | 2 | R.GDYYLEGYRDNAYKDLAWK.H | 3 |
| \* | CENPL\_Noc300\_tube2\_122214\_01.12812.12812.2 | 2.7506 | 0.1871 | 99.4% | 1223.7322 | 1224.3586 | 17 | 5.686 | 72.2% | 2 | R.DNAYKDLAWK.H | 2 |
| \* | CENPL\_Noc300\_122214\_01.17550.17550.3 | 3.3771 | 0.4571 | 100.0% | 2013.4744 | 2014.4492 | 1 | 7.595 | 39.7% | 1 | K.VGGILVMPIEDQLTQIMR.T | 3 |
| \* | CENPL\_Noc300\_122214\_01.17483.17483.2 | 4.0004 | 0.4357 | 100.0% | 2014.7922 | 2014.4492 | 1 | 7.176 | 55.9% | 1 | K.VGGILVMPIEDQLTQIMR.T | 2 |
| \* | CENPL\_Noc300\_tube2\_122214\_01.20248.20248.2 | 3.8568 | 0.4994 | 100.0% | 1584.8121 | 1584.8992 | 1 | 8.434 | 60.7% | 2 | K.NILAVSFAPLVQPSK.N | 2 |
| \* | CENPL\_Noc300\_tube2\_122214\_01.13352.13352.2 | 2.5644 | 0.1661 | 98.6% | 1210.4722 | 1210.3472 | 9 | 4.293 | 72.2% | 3 | R.NFINDEMQAK.G | 2 |
| \* | CENPL\_Noc300\_122214\_02.08069.08069.2 | 2.2353 | 0.2515 | 99.1% | 896.6922 | 897.10266 | 3 | 5.153 | 78.6% | 1 | K.LPLPESLK.A | 2 |
| \* | CENPL\_Noc300\_tube2\_122214\_01.16370.16370.2 | 1.9595 | 0.2776 | 98.6% | 933.6922 | 934.0825 | 3 | 6.36 | 83.3% | 1 | K.AYLTYFR.D | 2 |

---

|  |  |  |  |  |  |  |  |  |
| --- | --- | --- | --- | --- | --- | --- | --- | --- |
| U | *gi|4506687|ref|NP\_001* | 2 | 2 | 28.3% | 145 | 17040 | 10.4 | ribosomal protein S15 [Homo sapiens] |

| Filename XCorr DeltCN Conf% ObsM+H+ CalcM+H+ SpR ZScore Ion% # Sequence  | | | | | | | | | | | | |
| --- | --- | --- | --- | --- | --- | --- | --- | --- | --- | --- | --- | --- |
|  | CENPL\_Noc300\_122214\_01.19840.19840.2 | 3.5362 | 0.0536 | 98.7% | 2592.892 | 2589.938 | 1 | 3.98 | 47.6% | 1 | R.GVDLDQLLDMSYEQLMQLYSAR.Q | 23 |
| \* | CENPL\_Noc300\_122214\_02.14652.14652.2 | 2.9328 | 0.2288 | 99.4% | 2055.0923 | 2054.4856 | 1 | 6.395 | 36.1% | 1 | R.DMIILPEMVGSMVGVYNGK.T | 2 |

---

|  |  |  |  |  |  |  |  |  |
| --- | --- | --- | --- | --- | --- | --- | --- | --- |
| U | *gi|222352151|ref|NP\_0* | 5 | 6 | 27.0% | 356 | 37498 | 7.1 | poly(rC) binding protein 1 [Homo sapiens] |

| Filename XCorr DeltCN Conf% ObsM+H+ CalcM+H+ SpR ZScore Ion% # Sequence  | | | | | | | | | | | | |
| --- | --- | --- | --- | --- | --- | --- | --- | --- | --- | --- | --- | --- |
| \* | CENPL\_Noc300\_122214\_01.13779.13779.2 | 2.9981 | 0.4576 | 100.0% | 1389.5122 | 1389.6781 | 1 | 7.262 | 58.3% | 1 | R.IITLTGPTNAIFK.A | 2 |
| \* | CENPL\_Noc300\_tube2\_122214\_02.00136.00136.3 | 3.2544 | 0.2264 | 98.4% | 3380.0942 | 3380.8562 | 1 | 4.852 | 26.7% | 2 | K.AFAMIIDKLEEDINSSMTNSTAASRPPVTLR.L | 3 |
|  | CENPL\_Noc300\_122214\_02.08782.08782.2 | 4.4038 | 0.5004 | 100.0% | 2090.3323 | 2091.2573 | 1 | 9.449 | 50.0% | 1 | R.ESTGAQVQVAGDMLPNSTER.A | 22 |
| \* | CENPL\_Noc300\_122214\_01.06341.06341.2 | 2.279 | 0.1572 | 96.4% | 1087.0521 | 1087.1777 | 21 | 5.636 | 60.0% | 1 | K.IANPVEGSSGR.Q | 2 |
| \* | CENPL\_Noc300\_122214\_02.13103.13103.3 | 3.9072 | 0.4025 | 100.0% | 2178.4443 | 2178.4937 | 1 | 6.728 | 32.5% | 1 | R.QVTITGSAASISLAQYLINAR.L | 3 |

Similarities:
gi|14141166|ref|NP\_11(1:4)  

---

|  |  |  |  |  |  |  |  |  |
| --- | --- | --- | --- | --- | --- | --- | --- | --- |
| U | *gi|11968182|ref|NP\_07* | 4 | 6 | 27.0% | 152 | 17719 | 11.0 | ribosomal protein S18 [Homo sapiens] |
| U | *gi|169168597|ref|XP\_0* | 4 | 6 | 27.0% | 152 | 17719 | 11.0 | PREDICTED: hypothetical protein [Homo sapiens] |

| Filename XCorr DeltCN Conf% ObsM+H+ CalcM+H+ SpR ZScore Ion% # Sequence  | | | | | | | | | | | | |
| --- | --- | --- | --- | --- | --- | --- | --- | --- | --- | --- | --- | --- |
|  | CENPL\_Noc300\_122214\_01.07068.07068.2 | 2.4281 | 0.3261 | 99.7% | 1002.0722 | 1002.1154 | 8 | 5.897 | 68.8% | 1 | R.VLNTNIDGR.R | 2 |
|  | CENPL\_Noc300\_122214\_01.07074.07074.2 | 2.169 | 0.2724 | 98.3% | 1247.7522 | 1248.2891 | 1 | 4.877 | 75.0% | 1 | R.AGELTEDEVER.V | 2 |
|  | CENPL\_Noc300\_tube2\_122214\_01.12179.12179.2 | 2.1898 | 0.3844 | 99.5% | 1072.1721 | 1072.311 | 1 | 5.999 | 81.2% | 2 | R.VITIMQNPR.Q | 2 |
|  | CENPL\_Noc300\_tube2\_122214\_01.11727.11727.2 | 3.5905 | 0.368 | 100.0% | 1322.3922 | 1322.4606 | 1 | 7.778 | 77.3% | 2 | K.YSQVLANGLDNK.L | 2 |

---

|  |  |  |  |  |  |  |  |  |
| --- | --- | --- | --- | --- | --- | --- | --- | --- |
| U | *gi|4505813|ref|NP\_003* | 2 | 3 | 27.0% | 89 | 10366 | 7.4 | dynein light chain 1 [Homo sapiens] |
| U | *gi|83267868|ref|NP\_00* | 2 | 3 | 27.0% | 89 | 10366 | 7.4 | dynein light chain 1 [Homo sapiens] |
| U | *gi|83267866|ref|NP\_00* | 2 | 3 | 27.0% | 89 | 10366 | 7.4 | dynein light chain 1 [Homo sapiens] |

| Filename XCorr DeltCN Conf% ObsM+H+ CalcM+H+ SpR ZScore Ion% # Sequence  | | | | | | | | | | | | |
| --- | --- | --- | --- | --- | --- | --- | --- | --- | --- | --- | --- | --- |
|  | CENPL\_Noc300\_tube2\_122214\_01.12131.12131.3 | 3.5149 | 0.3043 | 100.0% | 1544.3344 | 1543.8064 | 1 | 5.825 | 45.8% | 2 | K.YNIEKDIAAHIKK.E | 3 |
|  | CENPL\_Noc300\_tube2\_122214\_01.09776.09776.2 | 2.9972 | 0.4861 | 100.0% | 1283.1322 | 1283.383 | 1 | 8.397 | 70.0% | 1 | R.NFGSYVTHETK.H | 2 |

---

|  |  |  |  |  |  |  |  |  |
| --- | --- | --- | --- | --- | --- | --- | --- | --- |
| U | *gi|4501881|ref|NP\_001* | 11 | 38 | 26.8% | 377 | 42051 | 5.4 | actin, alpha 1, skeletal muscle [Homo sapiens] |
| U | *gi|4885049|ref|NP\_005* | 11 | 38 | 26.8% | 377 | 42019 | 5.4 | cardiac muscle alpha actin 1 proprotein [Homo sapiens] |

| Filename XCorr DeltCN Conf% ObsM+H+ CalcM+H+ SpR ZScore Ion% # Sequence  | | | | | | | | | | | | |
| --- | --- | --- | --- | --- | --- | --- | --- | --- | --- | --- | --- | --- |
|  | CENPL\_Noc300\_122214\_01.06198.06198.2 | 3.1915 | 0.4707 | 100.0% | 976.71216 | 977.02136 | 2 | 7.675 | 77.8% | 6 | K.AGFAGDDAPR.A | 22 |
|  | CENPL\_Noc300\_tube2\_122214\_01.13340.13340.2 | 2.9245 | 0.3281 | 99.9% | 1198.9122 | 1199.4415 | 1 | 5.967 | 70.0% | 5 | R.AVFPSIVGRPR.H | 22 |
|  | CENPL\_Noc300\_122214\_01.11932.11932.2 | 4.5532 | 0.4374 | 100.0% | 1961.5122 | 1962.1841 | 1 | 7.612 | 70.0% | 3 | K.YPIEHGIITNWDDMEK.I | 2 |
|  | CENPL\_Noc300\_tube2\_122214\_01.16700.16700.3 | 4.4283 | 0.1561 | 99.9% | 1963.2544 | 1962.1841 | 7 | 5.486 | 41.7% | 3 | K.YPIEHGIITNWDDMEK.I | 3 |
|  | CENPL\_Noc300\_122214\_01.09106.09106.3 | 3.1819 | 0.2237 | 99.9% | 1517.3043 | 1516.7019 | 1 | 5.905 | 52.5% | 4 | K.IWHHTFYNELR.V | 33 |
|  | CENPL\_Noc300\_tube2\_122214\_01.11913.11913.2 | 3.4213 | 0.3999 | 100.0% | 1517.4521 | 1516.7019 | 1 | 6.479 | 80.0% | 3 | K.IWHHTFYNELR.V | 22 |
|  | CENPL\_Noc300\_tube2\_122214\_01.14639.14639.3 | 4.0682 | 0.2309 | 99.9% | 1958.9944 | 1957.234 | 467 | 4.702 | 26.5% | 1 | R.VAPEEHPTLLTEAPLNPK.A | 3 |
|  | CENPL\_Noc300\_tube2\_122214\_01.18828.18828.1 | 1.9308 | 0.2636 | 95.6% | 998.55 | 999.167 | 1 | 4.895 | 71.4% | 1 | R.DLTDYLMK.I | 11 |
|  | CENPL\_Noc300\_tube2\_122214\_01.18800.18800.2 | 2.085 | 0.3819 | 99.6% | 999.3122 | 999.167 | 8 | 6.884 | 71.4% | 1 | R.DLTDYLMK.I | 22 |
|  | CENPL\_Noc300\_tube2\_122214\_01.18374.18374.2 | 3.9887 | 0.3196 | 100.0% | 1791.3722 | 1791.9554 | 1 | 8.636 | 76.7% | 6 | K.SYELPDGQVITIGNER.F | 22 |
|  | CENPL\_Noc300\_122214\_01.09378.09378.2 | 2.7457 | 0.3903 | 100.0% | 1162.3722 | 1162.3868 | 1 | 6.981 | 90.0% | 5 | K.EITALAPSTMK.I | 22 |

Similarities:
gi|4501885|ref|NP\_001(8:3)  

---

|  |  |  |  |  |  |  |  |  |
| --- | --- | --- | --- | --- | --- | --- | --- | --- |
| U | *Reverse\_gi|55956777|r* | 1 | 1 | 26.6% | 143 | 15401 | 8.9 | sterol carrier protein 2 isoform 1 precursor [Homo sapiens] |
| U | *Reverse\_gi|55956781|r* | 1 | 1 | 64.4% | 59 | 6699 | 7.3 | sterol carrier protein 2 isoform 3 precursor [Homo sapiens] |

| Filename XCorr DeltCN Conf% ObsM+H+ CalcM+H+ SpR ZScore Ion% # Sequence  | | | | | | | | | | | | |
| --- | --- | --- | --- | --- | --- | --- | --- | --- | --- | --- | --- | --- |
|  | CENPL\_Noc300\_122214\_02.17080.17080.3 | 2.6736 | 0.2561 | 95.3% | 4449.744 | 4449.634 | 11 | 4.534 | 14.9% | 1 | K.KEIEKFVLNAKFGDSASST#PVAEIQHT#RFS\*S\*AAEPFGM.- | 3 |

---

|  |  |  |  |  |  |  |  |  |
| --- | --- | --- | --- | --- | --- | --- | --- | --- |
| U | *gi|40254446|ref|NP\_00* | 20 | 28 | 26.5% | 780 | 90955 | 8.0 | Vasopressin-activated calcium-mobilizing receptor-1 [Homo sapiens] |

| Filename XCorr DeltCN Conf% ObsM+H+ CalcM+H+ SpR ZScore Ion% # Sequence  | | | | | | | | | | | | |
| --- | --- | --- | --- | --- | --- | --- | --- | --- | --- | --- | --- | --- |
| \* | CENPL\_Noc300\_tube2\_122214\_01.18551.18551.3 | 3.7715 | 0.4765 | 100.0% | 1698.7144 | 1698.0153 | 1 | 7.695 | 42.3% | 1 | K.IHQALKEDILEFIK.Q | 3 |
| \* | CENPL\_Noc300\_122214\_01.13162.13162.2 | 4.6326 | 0.431 | 100.0% | 1698.8522 | 1698.0153 | 1 | 8.561 | 65.4% | 1 | K.IHQALKEDILEFIK.Q | 2 |
| \* | CENPL\_Noc300\_122214\_01.14884.14884.2 | 2.4025 | 0.2098 | 97.4% | 1940.5521 | 1940.2646 | 1 | 4.76 | 46.7% | 1 | R.KLMLDTWNESIFSNIK.N | 2 |
| \* | CENPL\_Noc300\_122214\_02.13379.13379.2 | 2.4347 | 0.4429 | 99.8% | 1811.7722 | 1812.0906 | 3 | 7.111 | 39.3% | 1 | K.LMLDTWNESIFSNIK.N | 2 |
| \* | CENPL\_Noc300\_122214\_02.11118.11118.2 | 3.4255 | 0.3791 | 100.0% | 1504.3922 | 1504.7263 | 1 | 7.875 | 61.5% | 1 | R.LGEAFDSQLVIGVR.E | 2 |
| \* | CENPL\_Noc300\_122214\_01.10645.10645.2 | 4.7136 | 0.6411 | 100.0% | 1970.2522 | 1971.1951 | 1 | 10.532 | 78.1% | 2 | R.TQAPSYLQQNGVQNYMK.Y | 2 |
| \* | CENPL\_Noc300\_tube2\_122214\_01.14069.14069.2 | 2.4051 | 0.4191 | 99.9% | 1078.3522 | 1078.2535 | 1 | 8.638 | 83.3% | 1 | K.AVVNDATIFK.L | 2 |
| \* | CENPL\_Noc300\_122214\_01.14617.14617.2 | 3.0141 | 0.2808 | 99.6% | 1772.3922 | 1772.1381 | 1 | 5.696 | 50.0% | 1 | K.AVVNDATIFKLELPLK.Q | 2 |
| \* | CENPL\_Noc300\_122214\_01.11401.11401.2 | 2.586 | 0.2395 | 99.6% | 1055.7722 | 1055.3898 | 2 | 4.674 | 81.2% | 1 | K.LKEVLLVLK.Y | 2 |
| \* | CENPL\_Noc300\_122214\_01.08845.08845.2 | 2.1534 | 0.2971 | 98.6% | 1223.3121 | 1223.3862 | 111 | 5.355 | 50.0% | 2 | R.EVGMPADYVNK.L | 2 |
| \* | CENPL\_Noc300\_tube2\_122214\_01.11974.11974.2 | 2.7824 | 0.3622 | 100.0% | 1150.9122 | 1151.2615 | 1 | 8.583 | 77.8% | 2 | K.VSEDLNQAFK.E | 2 |
| \* | CENPL\_Noc300\_tube2\_122214\_01.14361.14361.2 | 2.2281 | 0.3529 | 99.4% | 1141.4122 | 1141.3531 | 46 | 5.929 | 55.0% | 1 | K.LALPADSVNIK.I | 2 |
| \* | CENPL\_Noc300\_122214\_01.09694.09694.2 | 2.7734 | 0.2795 | 99.8% | 988.2922 | 988.13434 | 1 | 6.197 | 75.0% | 3 | K.ILNAGAWSR.S | 2 |
| \* | CENPL\_Noc300\_tube2\_122214\_01.14136.14136.2 | 2.3276 | 0.1365 | 96.1% | 1228.3522 | 1228.388 | 1 | 4.276 | 75.0% | 1 | K.LATELPDAELR.R | 2 |
| \* | CENPL\_Noc300\_tube2\_122214\_01.14399.14399.2 | 3.4137 | 0.3226 | 100.0% | 1515.4722 | 1515.7496 | 1 | 6.335 | 75.0% | 3 | R.QVLLYEPQVNSPK.D | 2 |
| \* | CENPL\_Noc300\_122214\_02.13542.13542.3 | 3.8259 | 0.26 | 99.9% | 3572.4543 | 3573.0369 | 13 | 4.685 | 20.0% | 1 | R.QVLLYEPQVNSPKDFTEGTLFSVNQEFSLIK.N | 3 |
| \* | CENPL\_Noc300\_122214\_02.13901.13901.2 | 2.3059 | 0.3774 | 99.4% | 2075.9922 | 2076.3105 | 11 | 5.946 | 32.4% | 1 | K.DFTEGTLFSVNQEFSLIK.N | 2 |
| \* | CENPL\_Noc300\_tube2\_122214\_01.11271.11271.2 | 3.7804 | 0.1839 | 99.9% | 1604.2122 | 1603.7905 | 1 | 4.985 | 75.0% | 1 | R.MREEENEGIVQLR.I | 2 |
| \* | CENPL\_Noc300\_tube2\_122214\_01.16377.16377.2 | 3.2426 | 0.3481 | 100.0% | 1175.4521 | 1175.429 | 1 | 6.568 | 77.8% | 2 | R.TQEAIIQIMK.M | 2 |
| \* | CENPL\_Noc300\_tube2\_122214\_01.20813.20813.2 | 4.5095 | 0.4885 | 100.0% | 1699.6522 | 1699.9854 | 1 | 8.33 | 60.7% | 1 | K.ISNAQLQTELVEILK.N | 2 |

---

|  |  |  |  |  |  |  |  |  |
| --- | --- | --- | --- | --- | --- | --- | --- | --- |
| U | *gi|28875797|ref|NP\_05* | 6 | 16 | 26.2% | 248 | 26397 | 12.2 | hypothetical protein LOC26097 [Homo sapiens] |

| Filename XCorr DeltCN Conf% ObsM+H+ CalcM+H+ SpR ZScore Ion% # Sequence  | | | | | | | | | | | | |
| --- | --- | --- | --- | --- | --- | --- | --- | --- | --- | --- | --- | --- |
| \* | CENPL\_Noc300\_122214\_01.06208.06208.2 | 4.2365 | 0.3768 | 100.0% | 1447.3722 | 1447.6091 | 4 | 6.966 | 62.5% | 3 | R.ASMQQQQQLASAR.N | 2 |
| \* | CENPL\_Noc300\_tube2\_122214\_01.09720.09720.3 | 3.9032 | 0.4061 | 100.0% | 1942.3143 | 1941.259 | 1 | 6.2 | 37.5% | 1 | R.RLAQQMENRPSVQAALK.L | 3 |
| \* | CENPL\_Noc300\_tube2\_122214\_01.10145.10145.2 | 4.6073 | 0.2789 | 100.0% | 1784.5721 | 1785.0715 | 1 | 6.09 | 63.3% | 2 | R.LAQQMENRPSVQAALK.L | 2 |
| \* | CENPL\_Noc300\_tube2\_122214\_01.10193.10193.3 | 3.4291 | 0.353 | 100.0% | 1785.0543 | 1785.0715 | 1 | 6.281 | 45.0% | 2 | R.LAQQMENRPSVQAALK.L | 3 |
| \* | CENPL\_Noc300\_tube2\_122214\_01.15758.15758.2 | 3.9017 | 0.4279 | 100.0% | 1556.5322 | 1555.6997 | 1 | 7.542 | 70.8% | 7 | K.EQLDNQLDAYMSK.T | 2 |
| \* | CENPL\_Noc300\_122214\_02.09676.09676.3 | 3.5208 | 0.3307 | 99.9% | 2435.9043 | 2436.566 | 1 | 6.493 | 38.1% | 1 | K.TKGHLDAELDAYMAQTDPETND.- | 3 |

---

|  |  |  |  |  |  |  |  |  |
| --- | --- | --- | --- | --- | --- | --- | --- | --- |
| U | *gi|4506749|ref|NP\_001* | 14 | 18 | 26.0% | 792 | 90070 | 7.1 | ribonucleoside-diphosphate reductase M1 chain [Homo sapiens] |

| Filename XCorr DeltCN Conf% ObsM+H+ CalcM+H+ SpR ZScore Ion% # Sequence  | | | | | | | | | | | | |
| --- | --- | --- | --- | --- | --- | --- | --- | --- | --- | --- | --- | --- |
| \* | CENPL\_Noc300\_122214\_01.18411.18411.2 | 3.4939 | 0.3931 | 100.0% | 2868.0122 | 2868.2512 | 33 | 6.611 | 22.2% | 1 | K.VIQGLYSGVTTVELDTLAAETAATLTTK.H | 2 |
| \* | CENPL\_Noc300\_122214\_01.17548.17548.3 | 5.6484 | 0.4115 | 100.0% | 3976.4944 | 3976.5164 | 1 | 7.82 | 18.9% | 1 | K.VIQGLYSGVTTVELDTLAAETAATLTTKHPDYAILAAR.I | 3 |
| \* | CENPL\_Noc300\_tube2\_122214\_01.20747.20747.3 | 4.7285 | 0.5373 | 100.0% | 2384.9644 | 2384.6685 | 1 | 7.826 | 39.5% | 1 | K.KVFSDVMEDLYNYINPHNGK.H | 3 |
| \* | CENPL\_Noc300\_122214\_01.16822.16822.2 | 3.0279 | 0.2753 | 99.5% | 2256.7722 | 2256.4944 | 3 | 4.906 | 36.1% | 2 | K.VFSDVMEDLYNYINPHNGK.H | 2 |
| \* | CENPL\_Noc300\_tube2\_122214\_01.13385.13385.2 | 2.5565 | 0.1128 | 96.8% | 1345.1921 | 1345.5388 | 1 | 4.674 | 68.2% | 1 | K.STLDIVLANKDR.L | 2 |
| \* | CENPL\_Noc300\_122214\_01.06430.06430.3 | 3.287 | 0.45 | 100.0% | 1368.5643 | 1368.6581 | 1 | 7.266 | 47.5% | 1 | K.VAERPQHMLMR.V | 3 |
| \* | CENPL\_Noc300\_122214\_01.20488.20488.2 | 2.8598 | 0.1599 | 98.6% | 1756.8322 | 1757.1432 | 1 | 5.961 | 53.8% | 1 | R.DLFFALWIPDLFMK.R | 2 |
| \* | CENPL\_Noc300\_122214\_02.13002.13002.3 | 5.4551 | 0.4808 | 100.0% | 2065.3442 | 2065.4875 | 1 | 10.424 | 44.4% | 1 | R.HRPIGIGVQGLADAFILMR.Y | 3 |
| \* | CENPL\_Noc300\_tube2\_122214\_01.17964.17964.2 | 3.0663 | 0.2735 | 99.8% | 1511.7722 | 1510.6866 | 1 | 5.054 | 70.8% | 1 | R.YPFESAEAQLLNK.Q | 2 |
| \* | CENPL\_Noc300\_tube2\_122214\_01.10673.10673.2 | 3.3757 | 0.4236 | 100.0% | 1670.8322 | 1671.7576 | 1 | 6.741 | 64.3% | 1 | K.EQGPYETYEGSPVSK.G | 2 |
| \* | CENPL\_Noc300\_122214\_01.18669.18669.2 | 5.3901 | 0.4799 | 100.0% | 2382.8323 | 2382.6953 | 1 | 9.303 | 66.7% | 2 | K.GILQYDMWNVTPTDLWDWK.V | 2 |
| \* | CENPL\_Noc300\_122214\_02.09968.09968.3 | 4.0538 | 0.3125 | 100.0% | 2304.6243 | 2303.5364 | 2 | 6.018 | 35.0% | 1 | R.GAFIDQSQSLNIHIAEPNYGK.L | 3 |
| \* | CENPL\_Noc300\_tube2\_122214\_01.15933.15933.2 | 2.3236 | 0.1572 | 97.2% | 1270.5122 | 1270.4913 | 3 | 4.953 | 66.7% | 2 | K.LTSMHFYGWK.Q | 2 |
| \* | CENPL\_Noc300\_122214\_01.10283.10283.2 | 4.4589 | 0.4427 | 100.0% | 1571.5521 | 1571.8197 | 1 | 7.677 | 69.2% | 2 | R.TRPAANPIQFTLNK.E | 2 |

---

|  |  |  |  |  |  |  |  |  |
| --- | --- | --- | --- | --- | --- | --- | --- | --- |
| U | *gi|208431833|ref|NP\_0* | 16 | 37 | 25.9% | 660 | 72391 | 9.3 | poly A binding protein, cytoplasmic 4 isoform 1 [Homo sapiens] |
| U | *gi|4504715|ref|NP\_003* | 16 | 37 | 26.6% | 644 | 70783 | 9.3 | poly A binding protein, cytoplasmic 4 isoform 2 [Homo sapiens] |
| U | *gi|208431836|ref|NP\_0* | 16 | 37 | 27.1% | 631 | 69579 | 9.5 | poly A binding protein, cytoplasmic 4 isoform 3 [Homo sapiens] |

| Filename XCorr DeltCN Conf% ObsM+H+ CalcM+H+ SpR ZScore Ion% # Sequence  | | | | | | | | | | | | |
| --- | --- | --- | --- | --- | --- | --- | --- | --- | --- | --- | --- | --- |
|  | CENPL\_Noc300\_tube2\_122214\_01.16456.16456.2 | 2.3474 | 0.1326 | 96.2% | 1145.4521 | 1144.3591 | 1 | 3.999 | 80.0% | 1 | K.FSPAGPVLSIR.V | 2 |
|  | CENPL\_Noc300\_tube2\_122214\_01.14126.14126.2 | 2.514 | 0.1648 | 96.9% | 2085.5122 | 2086.2712 | 245 | 4.3 | 29.4% | 1 | R.RSLGYAYVNFQQPADAER.A | 22 |
|  | CENPL\_Noc300\_tube2\_122214\_01.16640.16640.2 | 5.1516 | 0.5274 | 100.0% | 1930.4321 | 1930.0837 | 1 | 9.166 | 62.5% | 4 | R.SLGYAYVNFQQPADAER.A | 22 |
|  | CENPL\_Noc300\_tube2\_122214\_01.18668.18668.2 | 3.6712 | 0.5155 | 100.0% | 1267.1921 | 1267.4828 | 1 | 8.883 | 85.0% | 6 | R.ALDTMNFDVIK.G | 22 |
|  | CENPL\_Noc300\_tube2\_122214\_01.13396.13396.2 | 2.3206 | 0.1853 | 98.2% | 921.1722 | 921.0843 | 9 | 5.486 | 81.2% | 1 | K.SGVGNVFIK.N | 22 |
|  | CENPL\_Noc300\_122214\_02.09173.09173.3 | 4.6899 | 0.4196 | 100.0% | 2155.1042 | 2155.3716 | 1 | 7.997 | 43.1% | 2 | K.GYAFVHFETQEAADKAIEK.M | 3 |
|  | CENPL\_Noc300\_tube2\_122214\_01.12850.12850.2 | 2.2965 | 0.2754 | 99.3% | 1064.1122 | 1064.263 | 1 | 5.757 | 75.0% | 3 | K.MNGMLLNDR.K | 22 |
|  | CENPL\_Noc300\_tube2\_122214\_01.11039.11039.2 | 2.8856 | 0.3055 | 99.9% | 1213.5322 | 1213.4191 | 1 | 5.708 | 77.8% | 5 | K.AKEFTNVYIK.N | 22 |
|  | CENPL\_Noc300\_122214\_02.07188.07188.3 | 3.4716 | 0.3385 | 100.0% | 1214.0044 | 1213.4191 | 10 | 6.461 | 50.0% | 3 | K.AKEFTNVYIK.N | 33 |
|  | CENPL\_Noc300\_tube2\_122214\_01.14894.14894.2 | 2.4589 | 0.2643 | 99.5% | 1098.2122 | 1098.2871 | 1 | 5.666 | 81.2% | 1 | R.YQGVNLYIK.N | 2 |
|  | CENPL\_Noc300\_122214\_01.07694.07694.2 | 2.465 | 0.3136 | 99.5% | 1178.4521 | 1178.195 | 1 | 5.588 | 72.2% | 1 | K.NLDDTIDDEK.L | 2 |
|  | CENPL\_Noc300\_tube2\_122214\_01.17835.17835.2 | 2.4895 | 0.412 | 99.8% | 1271.1921 | 1271.4125 | 5 | 6.765 | 54.5% | 1 | K.EFSPFGSITSAK.V | 2 |
|  | CENPL\_Noc300\_tube2\_122214\_01.14243.14243.2 | 3.6448 | 0.4444 | 100.0% | 1515.7922 | 1515.8394 | 1 | 7.134 | 76.9% | 3 | R.IVGSKPLYVALAQR.K | 2 |
|  | CENPL\_Noc300\_tube2\_122214\_01.19008.19008.2 | 3.0526 | 0.3455 | 99.9% | 1670.5922 | 1671.0105 | 1 | 5.999 | 50.0% | 1 | R.LFPLIQTMHSNLAGK.I | 2 |
|  | CENPL\_Noc300\_122214\_01.17849.17849.2 | 5.6116 | 0.5639 | 100.0% | 2741.632 | 2742.175 | 1 | 10.706 | 47.8% | 1 | K.ITGMLLEIDNSELLHMLESPESLR.S | 22 |
|  | CENPL\_Noc300\_122214\_01.17916.17916.3 | 4.9443 | 0.3608 | 100.0% | 2743.0745 | 2742.175 | 1 | 6.65 | 38.0% | 3 | K.ITGMLLEIDNSELLHMLESPESLR.S | 33 |

Similarities:
gi|46367787|ref|NP\_00(8:8)  
gi|109948285|ref|NP\_0(1:15)  

---

|  |  |  |  |  |  |  |  |  |
| --- | --- | --- | --- | --- | --- | --- | --- | --- |
| U | *gi|4505409|ref|NP\_002* | 3 | 4 | 25.7% | 152 | 17298 | 8.4 | non-metastatic cells 2, protein (NM23B) expressed in [Homo sapiens] |
| U | *gi|66392227|ref|NP\_00* | 3 | 4 | 25.7% | 152 | 17298 | 8.4 | non-metastatic cells 2, protein (NM23B) expressed in [Homo sapiens] |
| U | *gi|66392205|ref|NP\_00* | 3 | 4 | 25.7% | 152 | 17298 | 8.4 | non-metastatic cells 2, protein (NM23B) expressed in [Homo sapiens] |
| U | *gi|66392203|ref|NP\_00* | 3 | 4 | 14.6% | 267 | 30137 | 8.9 | NME1-NME2 protein [Homo sapiens] |
| U | *gi|66392192|ref|NP\_00* | 3 | 4 | 25.7% | 152 | 17298 | 8.4 | non-metastatic cells 2, protein (NM23B) expressed in [Homo sapiens] |

| Filename XCorr DeltCN Conf% ObsM+H+ CalcM+H+ SpR ZScore Ion% # Sequence  | | | | | | | | | | | | |
| --- | --- | --- | --- | --- | --- | --- | --- | --- | --- | --- | --- | --- |
|  | CENPL\_Noc300\_tube2\_122214\_01.12042.12042.2 | 2.8887 | 0.316 | 99.8% | 1345.2722 | 1345.5846 | 1 | 6.051 | 59.1% | 1 | R.TFIAIKPDGVQR.G | 2 |
|  | CENPL\_Noc300\_tube2\_122214\_01.16932.16932.2 | 2.649 | 0.2951 | 99.7% | 1175.7722 | 1176.4038 | 67 | 5.656 | 55.6% | 2 | K.DRPFFPGLVK.Y | 2 |
|  | CENPL\_Noc300\_tube2\_122214\_01.10558.10558.3 | 2.3758 | 0.2784 | 96.2% | 1787.5443 | 1787.041 | 2 | 5.22 | 31.2% | 1 | R.VMLGETNPADSKPGTIR.G | 3 |

---

|  |  |  |  |  |  |  |  |  |
| --- | --- | --- | --- | --- | --- | --- | --- | --- |
| U | *gi|14141161|ref|NP\_00* | 17 | 39 | 25.3% | 806 | 88980 | 5.8 | heterogeneous nuclear ribonucleoprotein U isoform b [Homo sapiens] |
| U | *gi|74136883|ref|NP\_11* | 17 | 39 | 24.7% | 825 | 90585 | 6.0 | heterogeneous nuclear ribonucleoprotein U isoform a [Homo sapiens] |

| Filename XCorr DeltCN Conf% ObsM+H+ CalcM+H+ SpR ZScore Ion% # Sequence  | | | | | | | | | | | | |
| --- | --- | --- | --- | --- | --- | --- | --- | --- | --- | --- | --- | --- |
|  | CENPL\_Noc300\_122214\_01.06637.06637.2 | 2.3019 | 0.1185 | 96.6% | 1076.2122 | 1075.2474 | 9 | 4.523 | 75.0% | 1 | K.VSELKEELK.K | 2 |
|  | CENPL\_Noc300\_122214\_02.09076.09076.3 | 5.2217 | 0.4459 | 100.0% | 3128.3342 | 3128.311 | 1 | 6.802 | 28.2% | 2 | R.LQAALDDEEAGGRPAMEPGNGSLDLGGDSAGR.S | 3 |
|  | CENPL\_Noc300\_tube2\_122214\_01.18934.18934.2 | 2.3186 | 0.2377 | 97.4% | 1716.3522 | 1715.9469 | 1 | 4.716 | 44.1% | 1 | K.SSGPTSLFAVTVAPPGAR.Q | 2 |
|  | CENPL\_Noc300\_tube2\_122214\_01.15179.15179.2 | 4.0273 | 0.4217 | 100.0% | 1698.3322 | 1698.8291 | 1 | 7.719 | 79.2% | 3 | R.GYFEYIEENKYSR.A | 2 |
|  | CENPL\_Noc300\_tube2\_122214\_01.15184.15184.3 | 2.3085 | 0.2623 | 95.6% | 1700.2444 | 1698.8291 | 7 | 4.466 | 37.5% | 1 | R.GYFEYIEENKYSR.A | 3 |
|  | CENPL\_Noc300\_tube2\_122214\_01.09813.09813.2 | 2.374 | 0.2839 | 99.6% | 997.6122 | 997.0959 | 2 | 5.939 | 78.6% | 2 | K.DIDIHEVR.I | 2 |
|  | CENPL\_Noc300\_tube2\_122214\_01.12400.12400.2 | 2.4066 | 0.2916 | 99.5% | 1049.1122 | 1049.1716 | 17 | 6.137 | 61.1% | 2 | K.NGQDLGVAFK.I | 2 |
|  | CENPL\_Noc300\_tube2\_122214\_01.20273.20273.2 | 2.9238 | 0.3011 | 99.6% | 2725.1921 | 2726.0576 | 4 | 5.155 | 31.0% | 1 | K.EKPYFPIPEEYTFIQNVPLEDR.V | 2 |
|  | CENPL\_Noc300\_122214\_01.15204.15204.3 | 4.773 | 0.3742 | 100.0% | 2726.0645 | 2726.0576 | 1 | 7.107 | 39.3% | 3 | K.EKPYFPIPEEYTFIQNVPLEDR.V | 3 |
|  | CENPL\_Noc300\_122214\_01.10008.10008.3 | 4.8289 | 0.4334 | 100.0% | 2188.9443 | 2188.4631 | 1 | 6.968 | 40.8% | 2 | K.HAAENPGKYNILGTNTIMDK.M | 3 |
|  | CENPL\_Noc300\_122214\_01.12182.12182.2 | 3.4512 | 0.3935 | 100.0% | 1384.0521 | 1383.6025 | 1 | 6.64 | 63.6% | 3 | K.YNILGTNTIMDK.M | 2 |
|  | CENPL\_Noc300\_122214\_01.07404.07404.2 | 2.5307 | 0.2685 | 99.7% | 912.2922 | 912.19635 | 1 | 5.12 | 85.7% | 1 | K.MMVAGFKK.Q | 2 |
|  | CENPL\_Noc300\_tube2\_122214\_01.15514.15514.2 | 4.9337 | 0.4069 | 100.0% | 1649.3722 | 1648.816 | 1 | 8.022 | 78.6% | 10 | R.NFILDQTNVSAAAQR.R | 2 |
|  | CENPL\_Noc300\_tube2\_122214\_01.09748.09748.2 | 2.0775 | 0.2732 | 98.3% | 1022.39215 | 1022.1894 | 11 | 5.121 | 68.8% | 2 | K.DLPEHAVLK.M | 2 |
|  | CENPL\_Noc300\_122214\_01.06493.06493.2 | 2.9786 | 0.27 | 99.9% | 1267.4321 | 1267.4216 | 1 | 5.957 | 72.2% | 1 | K.LLEQYKEESK.K | 2 |
|  | CENPL\_Noc300\_122214\_01.05727.05727.2 | 2.4533 | 0.3446 | 99.5% | 1394.4521 | 1395.5957 | 1 | 5.082 | 80.0% | 1 | K.LLEQYKEESKK.A | 2 |
|  | CENPL\_Noc300\_tube2\_122214\_01.16965.16965.3 | 4.5115 | 0.4964 | 100.0% | 3659.5444 | 3660.8645 | 1 | 7.457 | 27.7% | 3 | K.NQSQGYNQWQQGQFWGQKPWSQHYHQGYY.- | 3 |

---

|  |  |  |  |  |  |  |  |  |
| --- | --- | --- | --- | --- | --- | --- | --- | --- |
| U | *gi|4506003|ref|NP\_002* | 8 | 13 | 24.8% | 330 | 37512 | 6.3 | protein phosphatase 1, catalytic subunit, alpha isoform 1 [Homo sapiens] |

| Filename XCorr DeltCN Conf% ObsM+H+ CalcM+H+ SpR ZScore Ion% # Sequence  | | | | | | | | | | | | |
| --- | --- | --- | --- | --- | --- | --- | --- | --- | --- | --- | --- | --- |
| \* | CENPL\_Noc300\_122214\_01.06382.06382.2 | 2.9515 | 0.204 | 99.6% | 1184.3522 | 1184.3811 | 1 | 5.784 | 75.0% | 1 | R.LLEVQGSRPGK.N | 2 |
|  | CENPL\_Noc300\_122214\_01.08295.08295.2 | 2.6774 | 0.2055 | 99.4% | 1216.0922 | 1216.3365 | 34 | 5.604 | 66.7% | 1 | K.NVQLTENEIR.G | 2 |
|  | CENPL\_Noc300\_122214\_01.18116.18116.2 | 4.9446 | 0.4967 | 100.0% | 1954.1721 | 1954.3574 | 1 | 9.269 | 68.8% | 1 | R.EIFLSQPILLELEAPLK.I | 22 |
|  | CENPL\_Noc300\_122214\_01.14013.14013.3 | 3.7838 | 0.3656 | 100.0% | 1440.8344 | 1440.7281 | 1 | 5.994 | 47.5% | 1 | K.IKYPENFFLLR.G | 33 |
|  | CENPL\_Noc300\_tube2\_122214\_01.15603.15603.2 | 3.4319 | 0.182 | 99.5% | 1640.4922 | 1640.7924 | 1 | 6.457 | 73.1% | 5 | R.AHQVVEDGYEFFAK.R | 22 |
|  | CENPL\_Noc300\_122214\_02.09682.09682.3 | 4.2877 | 0.4404 | 100.0% | 1641.8344 | 1640.7924 | 1 | 7.732 | 53.8% | 1 | R.AHQVVEDGYEFFAK.R | 33 |
|  | CENPL\_Noc300\_tube2\_122214\_01.14273.14273.3 | 3.4586 | 0.4634 | 100.0% | 1797.1743 | 1796.9799 | 1 | 7.337 | 46.4% | 1 | R.AHQVVEDGYEFFAKR.Q | 33 |
|  | CENPL\_Noc300\_tube2\_122214\_01.13036.13036.3 | 2.7297 | 0.3068 | 99.8% | 1914.5343 | 1915.1619 | 1 | 5.636 | 36.8% | 2 | K.YGQFSGLNPGGRPITPPR.N | 3 |

Similarities:
gi|4506007|ref|NP\_002(5:3)  

---

|  |  |  |  |  |  |  |  |  |
| --- | --- | --- | --- | --- | --- | --- | --- | --- |
| U | *gi|7657387|ref|NP\_055* | 14 | 31 | 24.4% | 753 | 81872 | 6.2 | CCR4-NOT transcription complex, subunit 3 [Homo sapiens] |

| Filename XCorr DeltCN Conf% ObsM+H+ CalcM+H+ SpR ZScore Ion% # Sequence  | | | | | | | | | | | | |
| --- | --- | --- | --- | --- | --- | --- | --- | --- | --- | --- | --- | --- |
| \* | CENPL\_Noc300\_122214\_01.12867.12867.2 | 4.5305 | 0.4076 | 100.0% | 1822.4922 | 1823.0123 | 1 | 7.642 | 67.9% | 2 | K.KVSEGVEQFEDIWQK.L | 2 |
| \* | CENPL\_Noc300\_122214\_01.06655.06655.2 | 2.7376 | 0.1466 | 99.3% | 1148.4722 | 1148.363 | 51 | 4.268 | 62.5% | 1 | R.KLIETQMER.F | 2 |
| \* | CENPL\_Noc300\_122214\_01.07217.07217.2 | 2.2717 | 0.2602 | 99.3% | 1019.6722 | 1020.1889 | 2 | 5.473 | 78.6% | 1 | K.LIETQMER.F | 2 |
| \* | CENPL\_Noc300\_122214\_01.20320.20320.3 | 4.9642 | 0.3479 | 100.0% | 4087.2844 | 4085.4426 | 1 | 6.191 | 22.1% | 1 | K.EKEEVGQWLTNTIDTLNMQVDQFESEVESLSVQTR.K | 3 |
| \* | CENPL\_Noc300\_tube2\_122214\_01.15388.15388.2 | 1.9574 | 0.3052 | 98.8% | 875.6322 | 876.1021 | 2 | 5.582 | 91.7% | 1 | R.MLETILR.M | 2 |
| \* | CENPL\_Noc300\_122214\_01.14043.14043.2 | 2.5031 | 0.2313 | 98.6% | 1475.8722 | 1475.7002 | 1 | 5.134 | 62.5% | 1 | R.MLDNDSILVDAIR.K | 2 |
| \* | CENPL\_Noc300\_tube2\_122214\_01.17162.17162.2 | 4.5268 | 0.4819 | 100.0% | 1882.7922 | 1883.1545 | 1 | 9.146 | 58.3% | 5 | K.AAGALLNGPPQFSTAPEIK.A | 2 |
| \* | CENPL\_Noc300\_122214\_01.08896.08896.2 | 2.4624 | 0.205 | 99.0% | 942.3722 | 942.1002 | 7 | 6.715 | 68.8% | 8 | K.APEPLSSLK.S | 2 |
| \* | CENPL\_Noc300\_tube2\_122214\_01.16679.16679.2 | 4.942 | 0.5058 | 100.0% | 2006.6721 | 2007.2511 | 1 | 9.194 | 61.1% | 3 | R.AAISSGIEDPVPTLHLTER.D | 2 |
| \* | CENPL\_Noc300\_tube2\_122214\_01.16736.16736.3 | 3.3398 | 0.3398 | 99.9% | 2007.3844 | 2007.2511 | 4 | 6.493 | 29.2% | 2 | R.AAISSGIEDPVPTLHLTER.D | 3 |
| \* | CENPL\_Noc300\_tube2\_122214\_01.15870.15870.3 | 4.1145 | 0.4313 | 100.0% | 2908.1643 | 2909.1365 | 1 | 7.734 | 34.8% | 2 | K.EQLYQQAMEEAAWHHMPHPSDSER.I | 3 |
| \* | CENPL\_Noc300\_122214\_01.19722.19722.2 | 2.9872 | 0.3651 | 99.9% | 2072.632 | 2073.393 | 1 | 7.203 | 40.6% | 2 | R.LSTETLFFIFYYLEGTK.A | 2 |
| \* | CENPL\_Noc300\_tube2\_122214\_01.19431.19431.2 | 1.8918 | 0.1875 | 95.3% | 1061.7922 | 1062.2922 | 5 | 4.48 | 66.7% | 1 | K.YMMWFQR.H | 2 |
| \* | CENPL\_Noc300\_122214\_01.08441.08441.2 | 2.3596 | 0.2812 | 99.3% | 1305.4922 | 1305.476 | 7 | 4.711 | 55.6% | 1 | R.KKEGFTFEYR.Y | 2 |

---

|  |  |  |  |  |  |  |  |  |
| --- | --- | --- | --- | --- | --- | --- | --- | --- |
| U | *gi|42734430|ref|NP\_03* | 8 | 14 | 24.4% | 390 | 43476 | 5.6 | polymerase I and transcript release factor [Homo sapiens] |

| Filename XCorr DeltCN Conf% ObsM+H+ CalcM+H+ SpR ZScore Ion% # Sequence  | | | | | | | | | | | | |
| --- | --- | --- | --- | --- | --- | --- | --- | --- | --- | --- | --- | --- |
| \* | CENPL\_Noc300\_122214\_02.13174.13174.2 | 3.5963 | 0.4063 | 100.0% | 1600.7322 | 1600.8528 | 1 | 7.086 | 57.1% | 1 | K.SDQVNGVLVLSLLDK.I | 2 |
| \* | CENPL\_Noc300\_122214\_02.10425.10425.2 | 6.3695 | 0.5433 | 100.0% | 2026.0322 | 2026.2975 | 1 | 9.537 | 70.6% | 2 | K.IIGAVDQIQLTQAQLEER.Q | 2 |
| \* | CENPL\_Noc300\_122214\_02.10404.10404.3 | 3.5596 | 0.4195 | 100.0% | 2027.1244 | 2026.2975 | 1 | 6.418 | 44.1% | 1 | K.IIGAVDQIQLTQAQLEER.Q | 32 |
| \* | CENPL\_Noc300\_122214\_01.12415.12415.2 | 5.2428 | 0.463 | 100.0% | 1805.4122 | 1805.9977 | 1 | 8.076 | 71.9% | 2 | R.QAEMEGAVQSIQGELSK.L | 2 |
| \* | CENPL\_Noc300\_122214\_01.10134.10134.2 | 3.678 | 0.3768 | 100.0% | 1314.3322 | 1314.5248 | 1 | 6.305 | 80.0% | 4 | K.KLEVNEAELLR.R | 2 |
| \* | CENPL\_Noc300\_tube2\_122214\_01.13450.13450.2 | 2.9614 | 0.3719 | 100.0% | 1534.4122 | 1534.8541 | 1 | 6.151 | 66.7% | 2 | K.VMIYQDEVKLPAK.L | 2 |
| \* | CENPL\_Noc300\_tube2\_122214\_01.10781.10781.2 | 2.5438 | 0.3958 | 99.8% | 1292.3522 | 1292.4368 | 1 | 7.793 | 70.0% | 1 | K.SFTPDHVVYAR.S | 2 |
| \* | CENPL\_Noc300\_122214\_01.07181.07181.2 | 2.4679 | 0.1975 | 98.7% | 1171.5322 | 1171.3823 | 4 | 4.68 | 72.2% | 1 | K.IREGQVEVLK.A | 2 |

---

|  |  |  |  |  |  |  |  |  |
| --- | --- | --- | --- | --- | --- | --- | --- | --- |
| U | *gi|66933016|ref|NP\_00* | 12 | 28 | 24.3% | 514 | 55805 | 6.9 | inosine monophosphate dehydrogenase 2 [Homo sapiens] |

| Filename XCorr DeltCN Conf% ObsM+H+ CalcM+H+ SpR ZScore Ion% # Sequence  | | | | | | | | | | | | |
| --- | --- | --- | --- | --- | --- | --- | --- | --- | --- | --- | --- | --- |
| \* | CENPL\_Noc300\_tube2\_122214\_01.16270.16270.2 | 5.3671 | 0.5719 | 100.0% | 1821.4521 | 1822.1112 | 1 | 9.629 | 76.7% | 3 | K.KYEQGFITDPVVLSPK.D | 2 |
| \* | CENPL\_Noc300\_tube2\_122214\_01.18436.18436.2 | 3.5604 | 0.3893 | 100.0% | 1692.7922 | 1693.9371 | 1 | 6.948 | 60.7% | 1 | K.YEQGFITDPVVLSPK.D | 2 |
| \* | CENPL\_Noc300\_tube2\_122214\_01.12107.12107.2 | 2.4262 | 0.1471 | 98.6% | 844.9122 | 845.0299 | 1 | 5.244 | 78.6% | 1 | R.LVGIISSR.D | 2 |
| \* | CENPL\_Noc300\_122214\_01.11078.11078.2 | 4.5084 | 0.4701 | 100.0% | 1481.8922 | 1482.7635 | 1 | 8.397 | 80.8% | 6 | K.REDLVVAPAGITLK.E | 2 |
| \* | CENPL\_Noc300\_tube2\_122214\_01.19978.19978.2 | 5.1449 | 0.6075 | 100.0% | 1965.8722 | 1966.2853 | 1 | 11.51 | 76.5% | 1 | K.GKLPIVNEDDELVAIIAR.T | 2 |
| \* | CENPL\_Noc300\_122214\_02.13101.13101.2 | 4.578 | 0.5639 | 100.0% | 1780.6122 | 1781.0593 | 1 | 10.681 | 73.3% | 1 | K.LPIVNEDDELVAIIAR.T | 2 |
| \* | CENPL\_Noc300\_122214\_02.09982.09982.3 | 5.2483 | 0.458 | 100.0% | 2087.9944 | 2087.3835 | 1 | 8.261 | 42.1% | 3 | K.DKYPNLQVIGGNVVTAAQAK.N | 3 |
| \* | CENPL\_Noc300\_tube2\_122214\_01.18359.18359.2 | 3.363 | 0.2716 | 100.0% | 1158.3121 | 1157.3121 | 3 | 5.772 | 70.0% | 2 | K.NLIDAGVDALR.V | 2 |
| \* | CENPL\_Noc300\_122214\_01.11291.11291.3 | 6.6289 | 0.5049 | 100.0% | 2049.6843 | 2049.3835 | 1 | 8.77 | 47.4% | 7 | R.RFGVPVIADGGIQNVGHIAK.A | 3 |
| \* | CENPL\_Noc300\_tube2\_122214\_01.17532.17532.2 | 4.2198 | 0.6002 | 100.0% | 1892.8722 | 1893.196 | 1 | 9.375 | 61.1% | 1 | R.FGVPVIADGGIQNVGHIAK.A | 2 |
| \* | CENPL\_Noc300\_tube2\_122214\_01.09137.09137.2 | 5.0757 | 0.5638 | 100.0% | 1916.4521 | 1917.0422 | 1 | 9.1 | 55.9% | 1 | R.TSSAQVEGGVHSLHSYEK.R | 2 |
| \* | CENPL\_Noc300\_tube2\_122214\_01.09116.09116.3 | 3.5331 | 0.3444 | 100.0% | 1916.7244 | 1917.0422 | 1 | 6.139 | 33.8% | 1 | R.TSSAQVEGGVHSLHSYEK.R | 3 |

---

|  |  |  |  |  |  |  |  |  |
| --- | --- | --- | --- | --- | --- | --- | --- | --- |
| U | *gi|4506671|ref|NP\_000* | 2 | 4 | 24.3% | 115 | 11665 | 4.5 | ribosomal protein P2 [Homo sapiens] |

| Filename XCorr DeltCN Conf% ObsM+H+ CalcM+H+ SpR ZScore Ion% # Sequence  | | | | | | | | | | | | |
| --- | --- | --- | --- | --- | --- | --- | --- | --- | --- | --- | --- | --- |
| \* | CENPL\_Noc300\_tube2\_122214\_01.12784.12784.2 | 3.9021 | 0.4741 | 100.0% | 1773.5721 | 1773.9377 | 1 | 7.693 | 56.7% | 1 | K.ILDSVGIEADDDRLNK.V | 2 |
| \* | CENPL\_Noc300\_tube2\_122214\_01.18852.18852.2 | 3.8811 | 0.5403 | 100.0% | 1257.2722 | 1257.4294 | 1 | 9.201 | 81.8% | 3 | K.NIEDVIAQGIGK.L | 2 |

---

|  |  |  |  |  |  |  |  |  |
| --- | --- | --- | --- | --- | --- | --- | --- | --- |
| U | *gi|20149594|ref|NP\_03* | 18 | 41 | 24.0% | 724 | 83264 | 5.0 | heat shock 90kDa protein 1, beta [Homo sapiens] |

| Filename XCorr DeltCN Conf% ObsM+H+ CalcM+H+ SpR ZScore Ion% # Sequence  | | | | | | | | | | | | |
| --- | --- | --- | --- | --- | --- | --- | --- | --- | --- | --- | --- | --- |
|  | CENPL\_Noc300\_tube2\_122214\_01.12330.12330.2 | 3.1294 | 0.4258 | 100.0% | 1276.4122 | 1276.3861 | 4 | 7.487 | 63.6% | 2 | R.ELISNASDALDK.I | 2 |
|  | CENPL\_Noc300\_tube2\_122214\_01.15346.15346.2 | 3.3548 | 0.2677 | 99.8% | 1545.3922 | 1545.733 | 1 | 5.796 | 65.4% | 1 | R.ELISNASDALDKIR.Y | 2 |
|  | CENPL\_Noc300\_tube2\_122214\_01.16956.16956.2 | 3.2517 | 0.4773 | 100.0% | 1243.5721 | 1243.4459 | 1 | 7.572 | 72.7% | 3 | K.ADLINNLGTIAK.S | 22 |
|  | CENPL\_Noc300\_122214\_02.08541.08541.3 | 4.9851 | 0.4233 | 100.0% | 2016.5944 | 2016.2584 | 1 | 8.357 | 45.0% | 4 | K.VILHLKEDQTEYLEER.R | 33 |
|  | CENPL\_Noc300\_122214\_01.07110.07110.2 | 2.7256 | 0.359 | 100.0% | 1151.4521 | 1152.2462 | 1 | 5.671 | 81.2% | 1 | K.YIDQEELNK.T | 22 |
| \* | CENPL\_Noc300\_tube2\_122214\_01.15492.15492.2 | 4.7324 | 0.4631 | 100.0% | 1848.4722 | 1848.9171 | 1 | 8.728 | 71.4% | 3 | R.NPDDITQEEYGEFYK.S | 2 |
|  | CENPL\_Noc300\_tube2\_122214\_01.15382.15382.2 | 3.503 | 0.309 | 100.0% | 1527.9122 | 1528.6616 | 2 | 6.055 | 62.5% | 1 | K.SLTNDWEDHLAVK.H | 22 |
|  | CENPL\_Noc300\_tube2\_122214\_01.14814.14814.2 | 2.8075 | 0.3826 | 100.0% | 1349.8922 | 1349.4886 | 4 | 6.552 | 65.0% | 4 | K.HFSVEGQLEFR.A | 22 |
|  | CENPL\_Noc300\_122214\_02.09340.09340.3 | 2.7079 | 0.194 | 97.0% | 1350.0543 | 1349.4886 | 32 | 5.299 | 40.0% | 1 | K.HFSVEGQLEFR.A | 33 |
| \* | CENPL\_Noc300\_tube2\_122214\_01.17321.17321.2 | 2.9133 | 0.1376 | 99.3% | 1237.4122 | 1237.4008 | 1 | 4.765 | 83.3% | 4 | R.RAPFDLFENK.K | 2 |
| \* | CENPL\_Noc300\_tube2\_122214\_01.12182.12182.2 | 1.6978 | 0.3143 | 97.0% | 892.09216 | 891.99884 | 1 | 5.085 | 83.3% | 1 | K.FYEAFSK.N | 2 |
| \* | CENPL\_Noc300\_tube2\_122214\_01.14868.14868.2 | 3.4824 | 0.433 | 100.0% | 2179.0122 | 2178.2915 | 1 | 7.209 | 47.2% | 1 | R.YHTSQSGDEMTSLSEYVSR.M | 2 |
| \* | CENPL\_Noc300\_tube2\_122214\_01.14877.14877.3 | 3.9083 | 0.3828 | 100.0% | 2179.1943 | 2178.2915 | 2 | 6.622 | 36.1% | 3 | R.YHTSQSGDEMTSLSEYVSR.M | 3 |
| \* | CENPL\_Noc300\_tube2\_122214\_01.12638.12638.2 | 2.5861 | 0.358 | 99.8% | 1161.1522 | 1161.297 | 1 | 6.833 | 72.2% | 2 | K.SIYYITGESK.E | 2 |
| \* | CENPL\_Noc300\_tube2\_122214\_01.10047.10047.2 | 2.5322 | 0.3719 | 99.8% | 1250.3322 | 1250.3538 | 1 | 7.681 | 65.0% | 2 | K.EQVANSAFVER.V | 2 |
| \* | CENPL\_Noc300\_122214\_01.09740.09740.2 | 2.2475 | 0.328 | 99.3% | 1248.3322 | 1249.4574 | 1 | 6.295 | 75.0% | 3 | R.DNSTMGYMMAK.K | 2 |
| \* | CENPL\_Noc300\_tube2\_122214\_01.11566.11566.3 | 3.3224 | 0.1987 | 99.4% | 1913.9944 | 1912.1991 | 15 | 4.083 | 35.0% | 1 | K.KHLEINPDHPIVETLR.Q | 3 |
| \* | CENPL\_Noc300\_tube2\_122214\_01.13209.13209.3 | 3.9903 | 0.4088 | 100.0% | 1786.5844 | 1784.025 | 1 | 6.389 | 46.4% | 4 | K.HLEINPDHPIVETLR.Q | 3 |

Similarities:
gi|153792590|ref|NP\_0(6:12)  

---

|  |  |  |  |  |  |  |  |  |
| --- | --- | --- | --- | --- | --- | --- | --- | --- |
| U | *gi|24234688|ref|NP\_00* | 11 | 25 | 23.4% | 679 | 73681 | 6.2 | heat shock 70kDa protein 9 precursor [Homo sapiens] |

| Filename XCorr DeltCN Conf% ObsM+H+ CalcM+H+ SpR ZScore Ion% # Sequence  | | | | | | | | | | | | |
| --- | --- | --- | --- | --- | --- | --- | --- | --- | --- | --- | --- | --- |
| \* | CENPL\_Noc300\_122214\_01.10427.10427.2 | 3.3019 | 0.5186 | 100.0% | 1451.0521 | 1451.576 | 1 | 8.82 | 73.1% | 3 | R.TTPSVVAFTADGER.L | 2 |
| \* | CENPL\_Noc300\_122214\_01.07927.07927.2 | 3.6194 | 0.5156 | 100.0% | 1569.6522 | 1569.7141 | 1 | 7.996 | 73.1% | 2 | R.QAVTNPNNTFYATK.R | 2 |
| \* | CENPL\_Noc300\_tube2\_122214\_01.20657.20657.2 | 3.2751 | 0.3305 | 100.0% | 1555.4722 | 1554.8878 | 1 | 6.561 | 73.1% | 2 | K.LYSPSQIGAFVLMK.M | 2 |
| \* | CENPL\_Noc300\_122214\_01.06635.06635.3 | 3.5077 | 0.1748 | 99.6% | 1595.6044 | 1593.7949 | 9 | 5.231 | 32.7% | 1 | K.MKETAENYLGHTAK.N | 3 |
| \* | CENPL\_Noc300\_tube2\_122214\_01.17500.17500.2 | 3.2714 | 0.3317 | 99.9% | 1695.2122 | 1695.8723 | 1 | 6.814 | 64.3% | 1 | K.NAVITVPAYFNDSQR.Q | 2 |
| \* | CENPL\_Noc300\_tube2\_122214\_01.16527.16527.2 | 3.7012 | 0.3182 | 100.0% | 1244.6721 | 1243.4056 | 2 | 5.977 | 77.3% | 4 | K.DAGQISGLNVLR.V | 2 |
| \* | CENPL\_Noc300\_tube2\_122214\_01.19950.19950.2 | 5.0613 | 0.4844 | 100.0% | 2056.5122 | 2057.181 | 1 | 9.552 | 58.3% | 2 | K.STNGDTFLGGEDFDQALLR.H | 2 |
| \* | CENPL\_Noc300\_tube2\_122214\_01.20136.20136.2 | 4.2924 | 0.3972 | 100.0% | 1362.5322 | 1362.5687 | 1 | 8.275 | 68.2% | 2 | R.AQFEGIVTDLIR.R | 2 |
| \* | CENPL\_Noc300\_tube2\_122214\_01.16257.16257.2 | 3.3797 | 0.3485 | 100.0% | 1291.4521 | 1291.4496 | 1 | 8.568 | 80.0% | 4 | K.VQQTVQDLFGR.A | 2 |
| \* | CENPL\_Noc300\_122214\_02.09730.09730.3 | 2.647 | 0.2607 | 96.6% | 2421.0244 | 2419.7095 | 1 | 4.743 | 34.5% | 1 | R.EQQIVIQSSGGLSKDDIENMVK.N | 3 |
| \* | CENPL\_Noc300\_122214\_01.06870.06870.2 | 3.0336 | 0.3833 | 100.0% | 1232.7722 | 1232.3794 | 1 | 5.704 | 72.7% | 3 | R.QAASSLQQASLK.L | 2 |

---

|  |  |  |  |  |  |  |  |  |
| --- | --- | --- | --- | --- | --- | --- | --- | --- |
| U | *gi|4506695|ref|NP\_001* | 3 | 4 | 23.4% | 145 | 16060 | 10.3 | ribosomal protein S19 [Homo sapiens] |

| Filename XCorr DeltCN Conf% ObsM+H+ CalcM+H+ SpR ZScore Ion% # Sequence  | | | | | | | | | | | | |
| --- | --- | --- | --- | --- | --- | --- | --- | --- | --- | --- | --- | --- |
| \* | CENPL\_Noc300\_122214\_02.06772.06772.2 | 2.978 | 0.2265 | 99.8% | 1134.9922 | 1135.2217 | 8 | 6.852 | 68.8% | 2 | K.DVNQQEFVR.A | 2 |
| \* | CENPL\_Noc300\_tube2\_122214\_01.15957.15957.3 | 4.3467 | 0.3479 | 100.0% | 1970.6943 | 1970.151 | 1 | 6.159 | 50.0% | 1 | K.HKELAPYDENWFYTR.A | 3 |
| \* | CENPL\_Noc300\_tube2\_122214\_01.13256.13256.2 | 2.3289 | 0.0993 | 95.0% | 1127.5521 | 1127.3726 | 9 | 3.844 | 66.7% | 1 | R.RVLQALEGLK.M | 2 |

---

|  |  |  |  |  |  |  |  |  |
| --- | --- | --- | --- | --- | --- | --- | --- | --- |
| U | *gi|157388995|ref|NP\_0* | 8 | 14 | 23.3% | 361 | 41072 | 6.1 | protein-L-isoaspartate (D-aspartate) O-methyltransferase domain containing 2 isoform 1 [Homo sapiens] |

| Filename XCorr DeltCN Conf% ObsM+H+ CalcM+H+ SpR ZScore Ion% # Sequence  | | | | | | | | | | | | |
| --- | --- | --- | --- | --- | --- | --- | --- | --- | --- | --- | --- | --- |
|  | CENPL\_Noc300\_tube2\_122214\_01.13888.13888.2 | 3.0573 | 0.2841 | 100.0% | 1093.5521 | 1093.2249 | 1 | 6.296 | 87.5% | 2 | R.TELVEQAFR.A | 2 |
|  | CENPL\_Noc300\_tube2\_122214\_01.19532.19532.3 | 4.27 | 0.3656 | 100.0% | 2397.6243 | 2397.6458 | 1 | 5.478 | 40.3% | 2 | R.ADYYLEEFKENAYKDLAWK.H | 3 |
|  | CENPL\_Noc300\_122214\_01.14304.14304.2 | 3.5721 | 0.3296 | 100.0% | 2398.652 | 2397.6458 | 1 | 5.716 | 50.0% | 2 | R.ADYYLEEFKENAYKDLAWK.H | 2 |
| \* | CENPL\_Noc300\_122214\_01.13621.13621.2 | 3.6365 | 0.2888 | 100.0% | 1286.4722 | 1285.5848 | 1 | 5.921 | 72.7% | 3 | K.VGGILVMPLEEK.L | 2 |
|  | CENPL\_Noc300\_tube2\_122214\_01.15579.15579.2 | 2.5182 | 0.1376 | 97.9% | 1093.3922 | 1092.3701 | 15 | 4.941 | 66.7% | 2 | R.LVQLPPVAVR.S | 2 |
|  | CENPL\_Noc300\_122214\_02.11121.11121.3 | 4.9706 | 0.4075 | 100.0% | 1943.4243 | 1942.2836 | 1 | 7.041 | 48.3% | 1 | R.RMETIVFLDKEVFASR.I | 3 |
|  | CENPL\_Noc300\_122214\_01.14797.14797.2 | 2.7269 | 0.2366 | 99.5% | 1192.2322 | 1192.4845 | 1 | 6.571 | 70.0% | 1 | K.VLSLPLPDPLK.Y | 2 |
|  | CENPL\_Noc300\_122214\_01.18147.18147.2 | 2.775 | 0.4185 | 99.9% | 2226.7522 | 2227.6946 | 1 | 6.056 | 38.2% | 1 | K.VLSLPLPDPLKYYLLYYR.E | 2 |

---

|  |  |  |  |  |  |  |  |  |
| --- | --- | --- | --- | --- | --- | --- | --- | --- |
| U | *gi|56118234|ref|NP\_85* | 5 | 17 | 22.8% | 272 | 29731 | 9.7 | proline rich 6 [Homo sapiens] |

| Filename XCorr DeltCN Conf% ObsM+H+ CalcM+H+ SpR ZScore Ion% # Sequence  | | | | | | | | | | | | |
| --- | --- | --- | --- | --- | --- | --- | --- | --- | --- | --- | --- | --- |
| \* | CENPL\_Noc300\_tube2\_122214\_01.12755.12755.2 | 4.6303 | 0.5267 | 100.0% | 1770.2322 | 1770.9401 | 1 | 9.447 | 50.0% | 3 | R.SGASAAPAASAAAALAPSATR.T | 2 |
| \* | CENPL\_Noc300\_122214\_02.12294.12294.2 | 4.8749 | 0.5565 | 100.0% | 1539.3322 | 1539.8119 | 1 | 9.864 | 91.7% | 5 | K.LLLDTFEYQGLVK.H | 2 |
| \* | CENPL\_Noc300\_tube2\_122214\_01.09698.09698.3 | 3.9264 | 0.3624 | 100.0% | 1621.1344 | 1620.7618 | 2 | 6.303 | 38.5% | 2 | K.GAEHITTYTFNTHK.A | 3 |
| \* | CENPL\_Noc300\_tube2\_122214\_01.09718.09718.2 | 4.0523 | 0.4113 | 100.0% | 1621.5122 | 1620.7618 | 1 | 6.824 | 76.9% | 2 | K.GAEHITTYTFNTHK.A | 2 |
| \* | CENPL\_Noc300\_tube2\_122214\_01.16438.16438.2 | 4.704 | 0.5184 | 100.0% | 1660.3121 | 1659.7644 | 1 | 9.172 | 73.1% | 5 | R.SMVTEEFNGSDWEK.A | 2 |

---

|  |  |  |  |  |  |  |  |  |
| --- | --- | --- | --- | --- | --- | --- | --- | --- |
| U | *gi|4757834|ref|NP\_004* | 2 | 2 | 22.7% | 211 | 23772 | 6.7 | BCL2-associated athanogene 2 [Homo sapiens] |

| Filename XCorr DeltCN Conf% ObsM+H+ CalcM+H+ SpR ZScore Ion% # Sequence  | | | | | | | | | | | | |
| --- | --- | --- | --- | --- | --- | --- | --- | --- | --- | --- | --- | --- |
| \* | CENPL\_Noc300\_122214\_01.16803.16803.3 | 4.0365 | 0.358 | 100.0% | 3115.8542 | 3115.5007 | 1 | 6.724 | 25.0% | 1 | R.EAATAVEQEKEILLEMIHSIQNSQDMR.Q | 3 |
| \* | CENPL\_Noc300\_122214\_02.09321.09321.3 | 3.4848 | 0.3758 | 100.0% | 2400.1743 | 2400.6917 | 1 | 5.689 | 33.8% | 1 | R.TLTVEVSVETIRNPQQQESLK.H | 3 |

---

|  |  |  |  |  |  |  |  |  |
| --- | --- | --- | --- | --- | --- | --- | --- | --- |
| U | *gi|15208660|ref|NP\_00* | 12 | 26 | 21.9% | 475 | 54170 | 6.4 | tripartite motif protein 21 [Homo sapiens] |

| Filename XCorr DeltCN Conf% ObsM+H+ CalcM+H+ SpR ZScore Ion% # Sequence  | | | | | | | | | | | | |
| --- | --- | --- | --- | --- | --- | --- | --- | --- | --- | --- | --- | --- |
| \* | CENPL\_Noc300\_tube2\_122214\_01.13168.13168.2 | 2.9403 | 0.2802 | 99.9% | 1146.3322 | 1145.3623 | 2 | 5.326 | 72.2% | 3 | R.QLANMVNNLK.E | 2 |
| \* | CENPL\_Noc300\_tube2\_122214\_01.15806.15806.3 | 4.0165 | 0.2554 | 99.9% | 1989.2344 | 1989.164 | 1 | 5.629 | 43.8% | 1 | R.DHAMVPLEEAAQEYQEK.L | 3 |
| \* | CENPL\_Noc300\_tube2\_122214\_01.15839.15839.2 | 5.7291 | 0.5611 | 100.0% | 1989.3722 | 1989.164 | 1 | 10.517 | 71.9% | 2 | R.DHAMVPLEEAAQEYQEK.L | 2 |
| \* | CENPL\_Noc300\_tube2\_122214\_01.15783.15783.2 | 2.9524 | 0.2649 | 99.9% | 999.5122 | 999.1985 | 1 | 6.242 | 87.5% | 4 | K.LQVALGELR.R | 2 |
| \* | CENPL\_Noc300\_tube2\_122214\_01.12798.12798.2 | 2.4019 | 0.1396 | 97.4% | 1156.1122 | 1155.386 | 144 | 3.539 | 61.1% | 1 | K.LQVALGELRR.K | 2 |
| \* | CENPL\_Noc300\_122214\_01.06453.06453.2 | 2.0574 | 0.3581 | 99.3% | 1100.2322 | 1100.2627 | 6 | 5.454 | 68.8% | 2 | R.IHAEFVQQK.N | 2 |
| \* | CENPL\_Noc300\_122214\_01.09738.09738.2 | 2.8326 | 0.1265 | 99.3% | 1164.3322 | 1164.2603 | 17 | 3.84 | 75.0% | 4 | K.NFLVEEEQR.Q | 2 |
| \* | CENPL\_Noc300\_tube2\_122214\_01.21484.21484.2 | 3.8631 | 0.4965 | 100.0% | 1943.5322 | 1943.1643 | 1 | 8.251 | 50.0% | 2 | K.LAQQSQALQELISELDR.R | 2 |
| \* | CENPL\_Noc300\_122214\_01.15443.15443.2 | 3.7401 | 0.3126 | 100.0% | 2098.8123 | 2099.3518 | 18 | 6.119 | 38.2% | 1 | K.LAQQSQALQELISELDRR.C | 2 |
| \* | CENPL\_Noc300\_tube2\_122214\_01.20445.20445.3 | 3.0106 | 0.2735 | 99.8% | 2099.2444 | 2099.3518 | 1 | 5.014 | 35.3% | 1 | K.LAQQSQALQELISELDRR.C | 3 |
| \* | CENPL\_Noc300\_tube2\_122214\_01.19086.19086.2 | 3.4793 | 0.0944 | 99.1% | 2006.1322 | 2004.2041 | 1 | 4.625 | 50.0% | 1 | R.SESWNLKDLDITSPELR.S | 2 |
| \* | CENPL\_Noc300\_tube2\_122214\_01.09010.09010.2 | 3.5997 | 0.4458 | 100.0% | 1544.0521 | 1544.6182 | 1 | 6.605 | 69.2% | 4 | R.LGDTQQSIPGNEER.F | 2 |

---

|  |  |  |  |  |  |  |  |  |
| --- | --- | --- | --- | --- | --- | --- | --- | --- |
| U | *gi|33286418|ref|NP\_00* | 7 | 10 | 21.8% | 531 | 57937 | 7.8 | pyruvate kinase, muscle isoform M2 [Homo sapiens] |
| U | *gi|33286422|ref|NP\_87* | 7 | 10 | 21.8% | 531 | 58062 | 7.7 | pyruvate kinase, muscle isoform M1 [Homo sapiens] |
| U | *gi|33286420|ref|NP\_87* | 7 | 10 | 21.8% | 531 | 58062 | 7.7 | pyruvate kinase, muscle isoform M1 [Homo sapiens] |

| Filename XCorr DeltCN Conf% ObsM+H+ CalcM+H+ SpR ZScore Ion% # Sequence  | | | | | | | | | | | | |
| --- | --- | --- | --- | --- | --- | --- | --- | --- | --- | --- | --- | --- |
|  | CENPL\_Noc300\_tube2\_122214\_01.13540.13540.2 | 2.7351 | 0.2959 | 99.7% | 1199.0521 | 1198.3617 | 46 | 4.919 | 60.0% | 2 | R.LDIDSPPITAR.N | 2 |
|  | CENPL\_Noc300\_tube2\_122214\_01.08967.08967.3 | 3.8624 | 0.3965 | 100.0% | 1884.5944 | 1885.0458 | 1 | 7.058 | 48.3% | 2 | R.LNFSHGTHEYHAETIK.N | 3 |
|  | CENPL\_Noc300\_tube2\_122214\_01.19481.19481.2 | 2.7611 | 0.3462 | 99.7% | 2466.632 | 2466.7937 | 1 | 5.406 | 31.8% | 1 | R.TATESFASDPILYRPVAVALDTK.G | 2 |
|  | CENPL\_Noc300\_122214\_01.09894.09894.2 | 2.6548 | 0.3087 | 99.8% | 1198.3322 | 1198.3765 | 3 | 5.855 | 66.7% | 2 | K.ITLDNAYMEK.C | 2 |
|  | CENPL\_Noc300\_122214\_01.12650.12650.3 | 5.0214 | 0.438 | 100.0% | 2479.4043 | 2478.8486 | 1 | 7.583 | 41.3% | 1 | K.KGVNLPGAAVDLPAVSEKDIQDLK.F | 3 |
|  | CENPL\_Noc300\_122214\_01.19335.19335.2 | 4.3447 | 0.5525 | 100.0% | 1860.6522 | 1861.1224 | 1 | 9.076 | 66.7% | 1 | K.FGVEQDVDMVFASFIR.K | 2 |
|  | CENPL\_Noc300\_tube2\_122214\_01.18887.18887.2 | 2.5022 | 0.2668 | 98.7% | 1822.4521 | 1823.0741 | 15 | 5.074 | 36.7% | 1 | R.RFDEILEASDGIMVAR.G | 2 |

---

|  |  |  |  |  |  |  |  |  |
| --- | --- | --- | --- | --- | --- | --- | --- | --- |
| U | *gi|4506699|ref|NP\_001* | 1 | 1 | 21.7% | 83 | 9111 | 8.5 | ribosomal protein S21 [Homo sapiens] |

| Filename XCorr DeltCN Conf% ObsM+H+ CalcM+H+ SpR ZScore Ion% # Sequence  | | | | | | | | | | | | |
| --- | --- | --- | --- | --- | --- | --- | --- | --- | --- | --- | --- | --- |
| \* | CENPL\_Noc300\_122214\_02.08806.08806.3 | 4.7806 | 0.5394 | 100.0% | 1971.8644 | 1971.1956 | 1 | 8.346 | 41.2% | 1 | K.DHASIQMNVAEVDKVTGR.F | 3 |

---

|  |  |  |  |  |  |  |  |  |
| --- | --- | --- | --- | --- | --- | --- | --- | --- |
| U | *gi|4506439|ref|NP\_002* | 4 | 5 | 21.4% | 425 | 47820 | 5.0 | retinoblastoma binding protein 7 [Homo sapiens] |

| Filename XCorr DeltCN Conf% ObsM+H+ CalcM+H+ SpR ZScore Ion% # Sequence  | | | | | | | | | | | | |
| --- | --- | --- | --- | --- | --- | --- | --- | --- | --- | --- | --- | --- |
| \* | CENPL\_Noc300\_122214\_02.11075.11075.3 | 3.3451 | 0.4372 | 100.0% | 2776.6743 | 2776.0837 | 3 | 6.305 | 27.2% | 1 | K.DYALHWLVLGTHTSDEQNHLVVAR.V | 3 |
| \* | CENPL\_Noc300\_122214\_01.17362.17362.3 | 4.6256 | 0.4845 | 100.0% | 3380.8442 | 3380.741 | 1 | 6.955 | 23.3% | 1 | K.AIFTGHSAVVEDVAWHLLHESLFGSVADDQK.L | 3 |
|  | CENPL\_Noc300\_tube2\_122214\_01.14998.14998.2 | 2.4534 | 0.0627 | 96.2% | 1131.4722 | 1131.3201 | 11 | 3.53 | 62.5% | 2 | R.RLNVWDLSK.I | 2 |
| \* | CENPL\_Noc300\_tube2\_122214\_01.16590.16590.3 | 4.1776 | 0.3254 | 100.0% | 2849.7544 | 2849.0398 | 2 | 5.559 | 26.9% | 1 | K.IGEEQSAEDAEDGPPELLFIHGGHTAK.I | 3 |

---

|  |  |  |  |  |  |  |  |  |
| --- | --- | --- | --- | --- | --- | --- | --- | --- |
| U | *gi|119703753|ref|NP\_0* | 13 | 35 | 21.3% | 564 | 60067 | 8.0 | keratin 6B [Homo sapiens] |

| Filename XCorr DeltCN Conf% ObsM+H+ CalcM+H+ SpR ZScore Ion% # Sequence  | | | | | | | | | | | | |
| --- | --- | --- | --- | --- | --- | --- | --- | --- | --- | --- | --- | --- |
|  | CENPL\_Noc300\_122214\_01.08909.08909.2 | 1.9098 | 0.3419 | 98.2% | 1027.1522 | 1027.1222 | 1 | 6.175 | 72.2% | 1 | R.SGFSSISVSR.S | 2 |
|  | CENPL\_Noc300\_tube2\_122214\_01.13599.13599.2 | 2.3642 | 0.1974 | 99.3% | 827.9922 | 827.95544 | 1 | 5.382 | 100.0% | 5 | K.FASFIDK.V | 222222 |
|  | CENPL\_Noc300\_tube2\_122214\_01.13469.13469.2 | 2.5321 | 0.0875 | 97.5% | 1083.2122 | 1083.2755 | 2 | 6.11 | 81.2% | 2 | K.FASFIDKVR.F | 222222 |
|  | CENPL\_Noc300\_122214\_01.11215.11215.2 | 2.7607 | 0.3764 | 100.0% | 1204.1522 | 1204.3684 | 1 | 7.489 | 77.8% | 3 | K.WTLLQEQGTK.T | 22 |
|  | CENPL\_Noc300\_122214\_01.05936.05936.2 | 2.8336 | 0.2811 | 99.8% | 1309.1322 | 1309.4215 | 177 | 5.12 | 61.1% | 2 | K.NKYEDEINKR.T | 2222 |
|  | CENPL\_Noc300\_tube2\_122214\_01.20704.20704.2 | 4.3469 | 0.4382 | 100.0% | 1330.3522 | 1330.5211 | 1 | 8.484 | 86.4% | 3 | R.NLDLDSIIAEVK.A | 2222 |
|  | CENPL\_Noc300\_122214\_01.06802.06802.2 | 2.7697 | 0.0862 | 98.7% | 1107.5922 | 1108.196 | 1 | 6.289 | 81.2% | 2 | K.AQYEEIAQR.S | 22 |
|  | CENPL\_Noc300\_122214\_01.09420.09420.2 | 3.9725 | 0.3349 | 100.0% | 1180.1522 | 1180.303 | 1 | 7.434 | 88.9% | 10 | K.YEELQITAGR.H | 22 |
|  | CENPL\_Noc300\_122214\_01.10187.10187.2 | 3.335 | 0.3042 | 100.0% | 1358.4521 | 1358.5345 | 1 | 6.156 | 77.3% | 1 | K.NKLEGLEDALQK.A | 2 |
|  | CENPL\_Noc300\_tube2\_122214\_01.12657.12657.2 | 2.3715 | 0.2391 | 99.1% | 1153.8322 | 1154.3234 | 6 | 6.628 | 62.5% | 2 | K.EYQELMNVK.L | 22 |
|  | CENPL\_Noc300\_122214\_01.12967.12967.2 | 2.6979 | 0.4056 | 100.0% | 1264.4722 | 1264.4644 | 5 | 7.073 | 60.0% | 2 | K.LALDVEIATYR.K | 2222 |
|  | CENPL\_Noc300\_122214\_02.09616.09616.2 | 2.8991 | 0.271 | 99.7% | 1392.8121 | 1392.6384 | 12 | 5.484 | 59.1% | 1 | K.LALDVEIATYRK.L | 2222 |
|  | CENPL\_Noc300\_122214\_01.07417.07417.2 | 2.4739 | 0.2866 | 98.9% | 1436.1322 | 1436.562 | 1 | 6.046 | 53.1% | 1 | R.ATGGGLSSVGGGSSTIK.Y | 2 |

Similarities:
gi|47132620|ref|NP\_00(6:7)  
gi|4504919|ref|NP\_002(4:9)  
gi|119395750|ref|NP\_0(2:11)  
gi|119395754|ref|NP\_0(7:6)  
gi|153791158|ref|NP\_0(5:8)  
gi|67782365|ref|NP\_00(2:11)  

---

|  |  |  |  |  |  |  |  |  |
| --- | --- | --- | --- | --- | --- | --- | --- | --- |
| U | *Reverse\_gi|11345462|r* | 1 | 1 | 21.1% | 180 | 20313 | 8.6 | signal peptidase complex subunit 3 [Homo sapiens] |

| Filename XCorr DeltCN Conf% ObsM+H+ CalcM+H+ SpR ZScore Ion% # Sequence  | | | | | | | | | | | | |
| --- | --- | --- | --- | --- | --- | --- | --- | --- | --- | --- | --- | --- |
| \* | CENPL\_Noc300\_122214\_01.19798.19798.3 | 3.0256 | 0.2661 | 98.9% | 4304.3945 | 4306.6465 | 3 | 4.668 | 16.9% | 1 | K.TIEYTDPFPVSVHGS\*GT#VLPLIGANPVVNWSLTLT#VNR.N | 23 |

---

|  |  |  |  |  |  |  |  |  |
| --- | --- | --- | --- | --- | --- | --- | --- | --- |
| U | *gi|4504517|ref|NP\_001* | 4 | 6 | 21.0% | 205 | 22783 | 6.4 | heat shock protein beta-1 [Homo sapiens] |

| Filename XCorr DeltCN Conf% ObsM+H+ CalcM+H+ SpR ZScore Ion% # Sequence  | | | | | | | | | | | | |
| --- | --- | --- | --- | --- | --- | --- | --- | --- | --- | --- | --- | --- |
| \* | CENPL\_Noc300\_tube2\_122214\_01.19079.19079.2 | 2.8841 | 0.3519 | 100.0% | 1163.7522 | 1164.3494 | 1 | 6.976 | 88.9% | 1 | R.LFDQAFGLPR.L | 2 |
| \* | CENPL\_Noc300\_122214\_01.12078.12078.3 | 2.7888 | 0.2931 | 99.8% | 1784.6943 | 1785.0068 | 33 | 4.716 | 33.3% | 2 | R.VSLDVNHFAPDELTVK.T | 3 |
| \* | CENPL\_Noc300\_tube2\_122214\_01.16854.16854.2 | 4.5704 | 0.4431 | 100.0% | 1784.7322 | 1785.0068 | 1 | 8.662 | 53.3% | 2 | R.VSLDVNHFAPDELTVK.T | 2 |
| \* | CENPL\_Noc300\_tube2\_122214\_01.18029.18029.2 | 3.6827 | 0.4951 | 100.0% | 1906.5322 | 1907.1307 | 1 | 7.897 | 53.1% | 1 | K.LATQSNEITIPVTFESR.A | 2 |

---

|  |  |  |  |  |  |  |  |  |
| --- | --- | --- | --- | --- | --- | --- | --- | --- |
| U | *contaminant\_INT-STD1* | 15 | 54 | 20.9% | 607 | 69271 | 6.1 | BSA |

| Filename XCorr DeltCN Conf% ObsM+H+ CalcM+H+ SpR ZScore Ion% # Sequence  | | | | | | | | | | | | |
| --- | --- | --- | --- | --- | --- | --- | --- | --- | --- | --- | --- | --- |
| \* | CENPL\_Noc300\_tube2\_122214\_01.16532.16532.2 | 3.4493 | 0.3435 | 100.0% | 1164.2122 | 1164.344 | 1 | 6.299 | 77.8% | 5 | K.LVNELTEFAK.T | 2 |
|  | CENPL\_Noc300\_122214\_01.09974.09974.2 | 2.2803 | 0.1107 | 97.6% | 928.1122 | 928.0758 | 2 | 5.29 | 91.7% | 1 | K.YLYEIAR.R | 2 |
| \* | CENPL\_Noc300\_tube2\_122214\_01.18136.18136.2 | 4.2794 | 0.4777 | 100.0% | 2045.6522 | 2046.3354 | 1 | 8.52 | 53.3% | 1 | R.RHPYFYAPELLYYANK.Y | 2 |
| \* | CENPL\_Noc300\_tube2\_122214\_01.18176.18176.3 | 3.8064 | 0.2804 | 99.9% | 2046.9543 | 2046.3354 | 1 | 5.423 | 41.7% | 4 | R.RHPYFYAPELLYYANK.Y | 3 |
| \* | CENPL\_Noc300\_tube2\_122214\_01.10144.10144.2 | 2.1999 | 0.2711 | 99.1% | 923.1122 | 923.05383 | 1 | 5.897 | 85.7% | 2 | K.AEFVEVTK.L | 2 |
| \* | CENPL\_Noc300\_122214\_02.13454.13454.2 | 4.6473 | 0.4727 | 100.0% | 1568.3522 | 1568.7258 | 1 | 8.642 | 79.2% | 3 | K.DAFLGSFLYEYSR.R | 2 |
| \* | CENPL\_Noc300\_122214\_01.10130.10130.3 | 4.5585 | 0.3409 | 100.0% | 1440.2943 | 1440.6884 | 1 | 7.107 | 63.6% | 8 | R.RHPEYAVSVLLR.L | 3 |
| \* | CENPL\_Noc300\_tube2\_122214\_01.13468.13468.2 | 2.8553 | 0.4021 | 100.0% | 1440.3922 | 1440.6884 | 1 | 6.835 | 72.7% | 1 | R.RHPEYAVSVLLR.L | 2 |
| \* | CENPL\_Noc300\_tube2\_122214\_01.11978.11978.2 | 3.2236 | 0.3971 | 100.0% | 1306.1921 | 1306.5046 | 1 | 7.776 | 70.0% | 5 | K.HLVDEPQNLIK.Q | 2 |
| \* | CENPL\_Noc300\_122214\_02.11079.11079.2 | 4.4114 | 0.3787 | 100.0% | 1480.4922 | 1480.7068 | 1 | 8.242 | 75.0% | 7 | K.LGEYGFQNALIVR.Y | 2 |
|  | CENPL\_Noc300\_tube2\_122214\_01.12746.12746.2 | 4.0588 | 0.4832 | 100.0% | 1640.3121 | 1640.9205 | 1 | 9.294 | 78.6% | 3 | R.KVPQVSTPTLVEVSR.S | 2 |
|  | CENPL\_Noc300\_tube2\_122214\_01.12686.12686.3 | 5.1954 | 0.4599 | 100.0% | 1641.5643 | 1640.9205 | 1 | 8.443 | 57.1% | 6 | R.KVPQVSTPTLVEVSR.S | 3 |
| \* | CENPL\_Noc300\_tube2\_122214\_01.15172.15172.2 | 2.9283 | 0.2307 | 99.7% | 1143.6721 | 1143.4124 | 2 | 5.664 | 77.8% | 3 | K.KQTALVELLK.H | 2 |
| \* | CENPL\_Noc300\_tube2\_122214\_01.18414.18414.2 | 1.6897 | 0.3694 | 97.4% | 1014.9522 | 1015.2383 | 9 | 6.439 | 75.0% | 2 | K.QTALVELLK.H | 2 |
| \* | CENPL\_Noc300\_tube2\_122214\_01.20583.20583.2 | 3.9593 | 0.4942 | 100.0% | 1400.5322 | 1400.6324 | 1 | 9.17 | 81.8% | 3 | K.TVMENFVAFVDK.C | 2 |

---

|  |  |  |  |  |  |  |  |  |
| --- | --- | --- | --- | --- | --- | --- | --- | --- |
| U | *Reverse\_gi|113415819|* | 1 | 1 | 20.8% | 120 | 13234 | 11.0 | PREDICTED: hypothetical protein [Homo sapiens] |
| U | *Reverse\_gi|169167227|* | 1 | 1 | 20.8% | 120 | 13234 | 11.0 | PREDICTED: hypothetical protein [Homo sapiens] |
| U | *Reverse\_gi|113416320|* | 1 | 1 | 20.8% | 120 | 13234 | 11.0 | PREDICTED: hypothetical protein [Homo sapiens] |

| Filename XCorr DeltCN Conf% ObsM+H+ CalcM+H+ SpR ZScore Ion% # Sequence  | | | | | | | | | | | | |
| --- | --- | --- | --- | --- | --- | --- | --- | --- | --- | --- | --- | --- |
|  | CENPL\_Noc300\_tube2\_122214\_01.15131.15131.3 | 2.9889 | 0.244 | 97.5% | 2872.4944 | 2869.8516 | 1 | 4.517 | 24.0% | 1 | R.T#PYGRREGADDGY@GEAPARSRDPSK.S | 3 |

---

|  |  |  |  |  |  |  |  |  |
| --- | --- | --- | --- | --- | --- | --- | --- | --- |
| U | *gi|4885579|ref|NP\_005* | 6 | 12 | 20.7% | 299 | 33631 | 8.0 | RCD1 required for cell differentiation1 homolog [Homo sapiens] |

| Filename XCorr DeltCN Conf% ObsM+H+ CalcM+H+ SpR ZScore Ion% # Sequence  | | | | | | | | | | | | |
| --- | --- | --- | --- | --- | --- | --- | --- | --- | --- | --- | --- | --- |
| \* | CENPL\_Noc300\_122214\_01.13792.13792.2 | 3.3548 | 0.398 | 100.0% | 1736.2322 | 1736.9219 | 1 | 6.999 | 69.2% | 2 | K.IYQWINELSSPETR.E | 2 |
| \* | CENPL\_Noc300\_tube2\_122214\_01.15464.15464.2 | 2.3676 | 0.14 | 97.6% | 1017.4322 | 1017.1674 | 1 | 4.966 | 87.5% | 2 | R.ENALLELSK.K | 2 |
| \* | CENPL\_Noc300\_122214\_01.17705.17705.2 | 4.9362 | 0.5131 | 100.0% | 2374.2722 | 2373.8462 | 1 | 7.91 | 47.5% | 2 | R.SAFLAAHIPLFLYPFLHTVSK.T | 2 |
| \* | CENPL\_Noc300\_122214\_01.17681.17681.3 | 5.3029 | 0.3454 | 100.0% | 2375.7544 | 2373.8462 | 1 | 6.025 | 42.5% | 2 | R.SAFLAAHIPLFLYPFLHTVSK.T | 3 |
| \* | CENPL\_Noc300\_tube2\_122214\_01.16976.16976.2 | 2.1836 | 0.1897 | 98.5% | 858.0522 | 858.0715 | 1 | 4.028 | 100.0% | 2 | R.WLAQLVK.N | 2 |
| \* | CENPL\_Noc300\_122214\_01.06537.06537.2 | 3.6445 | 0.3898 | 100.0% | 1257.0922 | 1257.3457 | 1 | 7.623 | 80.0% | 2 | K.NLQEGQVTDPR.G | 2 |

---

|  |  |  |  |  |  |  |  |  |
| --- | --- | --- | --- | --- | --- | --- | --- | --- |
| U | *gi|58743363|ref|NP\_20* | 7 | 22 | 20.6% | 344 | 38998 | 6.5 | centromere protein L isoform 2 [Homo sapiens] |

| Filename XCorr DeltCN Conf% ObsM+H+ CalcM+H+ SpR ZScore Ion% # Sequence  | | | | | | | | | | | | |
| --- | --- | --- | --- | --- | --- | --- | --- | --- | --- | --- | --- | --- |
|  | CENPL\_Noc300\_tube2\_122214\_01.12648.12648.2 | 2.9919 | 0.3417 | 100.0% | 1274.4922 | 1274.5057 | 3 | 6.347 | 60.0% | 2 | R.KQSSFILTPPR.R | 2 |
|  | CENPL\_Noc300\_tube2\_122214\_01.15626.15626.2 | 2.8044 | 0.2409 | 99.6% | 1146.5721 | 1146.3317 | 1 | 4.75 | 77.8% | 9 | K.QSSFILTPPR.R | 2 |
|  | CENPL\_Noc300\_tube2\_122214\_01.11849.11849.2 | 3.4485 | 0.46 | 100.0% | 1393.9521 | 1394.5266 | 1 | 7.579 | 75.0% | 1 | K.FSYSNLKEYSR.L | 2 |
|  | CENPL\_Noc300\_tube2\_122214\_01.18272.18272.2 | 3.1118 | 0.4338 | 100.0% | 1118.6721 | 1118.3617 | 1 | 7.125 | 72.2% | 7 | R.LLNAFIVAEK.Q | 2 |
|  | CENPL\_Noc300\_122214\_02.10628.10628.2 | 2.9216 | 0.3951 | 100.0% | 1391.5122 | 1391.5638 | 1 | 7.048 | 62.5% | 1 | K.GLAVEVGEDFNIK.V | 2 |
|  | CENPL\_Noc300\_122214\_01.14948.14948.2 | 3.1431 | 0.4077 | 100.0% | 1109.8722 | 1109.4125 | 1 | 6.474 | 77.8% | 1 | K.VIFSTLLGMK.G | 2 |
| \* | CENPL\_Noc300\_122214\_01.14129.14129.2 | 3.0134 | 0.1178 | 98.2% | 1787.6921 | 1789.0415 | 1 | 4.966 | 53.3% | 1 | K.GTQRDPEAFLVQIVSK.S | 2 |

---

|  |  |  |  |  |  |  |  |  |
| --- | --- | --- | --- | --- | --- | --- | --- | --- |
| U | *gi|11024714|ref|NP\_06* | 4 | 13 | 20.5% | 229 | 25762 | 7.4 | ubiquitin B precursor [Homo sapiens] |
| U | *gi|77539055|ref|NP\_00* | 4 | 13 | 36.7% | 128 | 14728 | 9.8 | ubiquitin and ribosomal protein L40 precursor [Homo sapiens] |
| U | *gi|67191208|ref|NP\_06* | 4 | 13 | 6.9% | 685 | 77029 | 7.7 | ubiquitin C [Homo sapiens] |
| U | *gi|4507761|ref|NP\_003* | 4 | 13 | 36.7% | 128 | 14728 | 9.8 | ubiquitin and ribosomal protein L40 precursor [Homo sapiens] |
| U | *gi|4506713|ref|NP\_002* | 4 | 13 | 30.1% | 156 | 17965 | 9.6 | ubiquitin and ribosomal protein S27a precursor [Homo sapiens] |
| U | *gi|208022622|ref|NP\_0* | 4 | 13 | 30.1% | 156 | 17965 | 9.6 | ubiquitin and ribosomal protein S27a precursor [Homo sapiens] |

| Filename XCorr DeltCN Conf% ObsM+H+ CalcM+H+ SpR ZScore Ion% # Sequence  | | | | | | | | | | | | |
| --- | --- | --- | --- | --- | --- | --- | --- | --- | --- | --- | --- | --- |
|  | CENPL\_Noc300\_tube2\_122214\_01.16385.16385.2 | 3.9933 | 0.4467 | 100.0% | 1788.5922 | 1788.9897 | 1 | 7.835 | 70.0% | 6 | K.TITLEVEPSDTIENVK.A | 22 |
|  | CENPL\_Noc300\_122214\_01.05559.05559.2 | 3.3893 | 0.3644 | 100.0% | 1524.1322 | 1524.6738 | 1 | 6.425 | 79.2% | 1 | K.IQDKEGIPPDQQR.L | 22 |
|  | CENPL\_Noc300\_tube2\_122214\_01.09839.09839.2 | 2.7621 | 0.3921 | 100.0% | 1082.2522 | 1082.1986 | 1 | 6.45 | 75.0% | 3 | R.TLSDYNIQK.E | 22 |
|  | CENPL\_Noc300\_tube2\_122214\_01.13268.13268.2 | 2.8645 | 0.3242 | 100.0% | 1068.3722 | 1068.2615 | 7 | 5.33 | 75.0% | 3 | K.ESTLHLVLR.L | 2 |

Similarities:
contaminant\_UBIQUITIN(3:1)  

---

|  |  |  |  |  |  |  |  |  |
| --- | --- | --- | --- | --- | --- | --- | --- | --- |
| U | *gi|113422487|ref|XP\_0* | 1 | 1 | 20.5% | 78 | 8632 | 9.5 | PREDICTED: similar to beta-defensin 130 [Homo sapiens] |
| U | *gi|83699414|ref|NP\_00* | 1 | 1 | 20.3% | 79 | 8736 | 9.3 | beta-defensin 130 [Homo sapiens] |
| U | *gi|169203490|ref|XP\_0* | 1 | 1 | 20.5% | 78 | 8632 | 9.5 | PREDICTED: hypothetical protein [Homo sapiens] |
| U | *gi|169202991|ref|XP\_0* | 1 | 1 | 20.5% | 78 | 8632 | 9.5 | PREDICTED: similar to beta-defensin 130 [Homo sapiens] |
| U | *gi|169172908|ref|XP\_0* | 1 | 1 | 20.3% | 79 | 8736 | 9.3 | PREDICTED: similar to beta-defensin 130 [Homo sapiens] |

| Filename XCorr DeltCN Conf% ObsM+H+ CalcM+H+ SpR ZScore Ion% # Sequence  | | | | | | | | | | | | |
| --- | --- | --- | --- | --- | --- | --- | --- | --- | --- | --- | --- | --- |
|  | CENPL\_Noc300\_122214\_01.17991.17991.2 | 2.282 | 0.1938 | 95.8% | 1797.6921 | 1796.9014 | 6 | 4.623 | 40.0% | 1 | K.LCSTLDDTIGICNEGK.K | 2 |

---

|  |  |  |  |  |  |  |  |  |
| --- | --- | --- | --- | --- | --- | --- | --- | --- |
| U | *gi|15431310|ref|NP\_00* | 11 | 26 | 20.1% | 472 | 51622 | 5.2 | keratin 14 [Homo sapiens] |

| Filename XCorr DeltCN Conf% ObsM+H+ CalcM+H+ SpR ZScore Ion% # Sequence  | | | | | | | | | | | | |
| --- | --- | --- | --- | --- | --- | --- | --- | --- | --- | --- | --- | --- |
| \* | CENPL\_Noc300\_122214\_01.07758.07758.2 | 3.5159 | 0.4829 | 100.0% | 1426.3522 | 1426.526 | 1 | 7.866 | 60.7% | 1 | R.APSTYGGGLSVSSSR.F | 2 |
|  | CENPL\_Noc300\_122214\_01.07045.07045.1 | 1.7771 | 0.24 | 96.1% | 1090.58 | 1091.2273 | 4 | 4.459 | 62.5% | 1 | K.VTMQNLNDR.L | 111 |
|  | CENPL\_Noc300\_122214\_02.06500.06500.2 | 2.7854 | 0.4106 | 100.0% | 1091.0922 | 1091.2273 | 1 | 6.291 | 87.5% | 4 | K.VTMQNLNDR.L | 222 |
|  | CENPL\_Noc300\_122214\_01.07294.07294.1 | 1.6519 | 0.2663 | 95.7% | 809.37 | 809.93774 | 1 | 5.658 | 75.0% | 1 | R.LASYLDK.V | 111111 |
|  | CENPL\_Noc300\_tube2\_122214\_01.10668.10668.2 | 2.9757 | 0.1828 | 99.7% | 1065.1921 | 1065.2578 | 7 | 6.164 | 75.0% | 5 | R.LASYLDKVR.A | 22222 |
|  | CENPL\_Noc300\_122214\_02.07913.07913.2 | 3.6976 | 0.3822 | 100.0% | 1302.2922 | 1302.4241 | 1 | 7.464 | 77.3% | 2 | R.ALEEANADLEVK.I | 222 |
|  | CENPL\_Noc300\_122214\_01.07888.07888.2 | 2.5135 | 0.3649 | 100.0% | 808.2522 | 807.8815 | 1 | 6.681 | 83.3% | 6 | R.LAADDFR.T | 2222222 |
|  | CENPL\_Noc300\_tube2\_122214\_01.15556.15556.2 | 3.2708 | 0.3771 | 100.0% | 1030.4321 | 1030.2096 | 1 | 7.026 | 87.5% | 3 | R.VLDELTLAR.A | 2222 |
|  | CENPL\_Noc300\_122214\_01.06383.06383.2 | 2.6033 | 0.3459 | 99.8% | 1243.1322 | 1243.3367 | 1 | 6.613 | 83.3% | 1 | K.NHEEEMNALR.G | 22 |
|  | CENPL\_Noc300\_122214\_01.06579.06579.2 | 3.1107 | 0.4002 | 100.0% | 1362.1921 | 1362.4796 | 15 | 8.218 | 58.3% | 1 | R.EVATNSELVQSGK.S | 22 |
|  | CENPL\_Noc300\_122214\_01.09282.09282.2 | 3.3604 | 0.2669 | 100.0% | 1380.1322 | 1380.5437 | 1 | 5.797 | 80.0% | 1 | K.TRLEQEIATYR.R | 2222 |

Similarities:
gi|40354195|ref|NP\_95(1:10)  
contaminant\_KERATIN03(5:6)  
contaminant\_KERATIN02(1:10)  
gi|4557701|ref|NP\_000(7:4)  
gi|24430192|ref|NP\_00(8:3)  
gi|131412225|ref|NP\_7(3:8)  
gi|24234699|ref|NP\_00(4:7)  

---

|  |  |  |  |  |  |  |  |  |
| --- | --- | --- | --- | --- | --- | --- | --- | --- |
| U | *gi|6912486|ref|NP\_036* | 5 | 21 | 20.1% | 139 | 15350 | 10.0 | U6 snRNA-associated Sm-like protein 4 [Homo sapiens] |

| Filename XCorr DeltCN Conf% ObsM+H+ CalcM+H+ SpR ZScore Ion% # Sequence  | | | | | | | | | | | | |
| --- | --- | --- | --- | --- | --- | --- | --- | --- | --- | --- | --- | --- |
| \* | CENPL\_Noc300\_tube2\_122214\_01.12092.12092.2 | 3.3438 | 0.3755 | 100.0% | 1381.2922 | 1381.6329 | 1 | 5.683 | 77.3% | 5 | K.TAQNHPMLVELK.N | 2 |
| \* | CENPL\_Noc300\_tube2\_122214\_01.12074.12074.3 | 2.9233 | 0.4671 | 100.0% | 1382.2444 | 1381.6329 | 3 | 6.931 | 43.2% | 4 | K.TAQNHPMLVELK.N | 3 |
| \* | CENPL\_Noc300\_tube2\_122214\_01.18813.18813.2 | 3.4572 | 0.4123 | 100.0% | 1172.9722 | 1173.4102 | 1 | 7.083 | 83.3% | 4 | R.IPDEIIDMVK.E | 2 |
| \* | CENPL\_Noc300\_122214\_02.12076.12076.3 | 4.0156 | 0.471 | 100.0% | 1829.0343 | 1829.159 | 1 | 8.558 | 46.7% | 3 | R.IPDEIIDMVKEEVVAK.G | 3 |
| \* | CENPL\_Noc300\_122214\_01.14829.14829.2 | 5.43 | 0.5531 | 100.0% | 1829.0721 | 1829.159 | 1 | 9.8 | 76.7% | 5 | R.IPDEIIDMVKEEVVAK.G | 2 |

---

|  |  |  |  |  |  |  |  |  |
| --- | --- | --- | --- | --- | --- | --- | --- | --- |
| U | *gi|113421553|ref|XP\_0* | 1 | 1 | 20.0% | 125 | 14287 | 10.6 | PREDICTED: hypothetical protein [Homo sapiens] |
| U | *gi|169194846|ref|XP\_0* | 1 | 1 | 20.0% | 125 | 14287 | 10.6 | PREDICTED: hypothetical protein [Homo sapiens] |
| U | *gi|113422012|ref|XP\_0* | 1 | 1 | 20.0% | 125 | 14287 | 10.6 | PREDICTED: hypothetical protein [Homo sapiens] |

| Filename XCorr DeltCN Conf% ObsM+H+ CalcM+H+ SpR ZScore Ion% # Sequence  | | | | | | | | | | | | |
| --- | --- | --- | --- | --- | --- | --- | --- | --- | --- | --- | --- | --- |
|  | CENPL\_Noc300\_122214\_01.15719.15719.2 | 2.2316 | 0.2084 | 95.8% | 2903.132 | 2901.166 | 38 | 3.281 | 22.9% | 1 | R.ENRGKAT#DLARWTLSALVPATLIS\*R.V | 2 |

---

|  |  |  |  |  |  |  |  |  |
| --- | --- | --- | --- | --- | --- | --- | --- | --- |
| U | *gi|21536320|ref|NP\_65* | 9 | 14 | 19.7% | 756 | 84794 | 8.8 | heterogeneous nuclear ribonucleoprotein U-like 1 isoform d [Homo sapiens] |
| U | *gi|21536326|ref|NP\_00* | 9 | 14 | 17.4% | 856 | 95739 | 6.9 | heterogeneous nuclear ribonucleoprotein U-like 1 isoform a [Homo sapiens] |

| Filename XCorr DeltCN Conf% ObsM+H+ CalcM+H+ SpR ZScore Ion% # Sequence  | | | | | | | | | | | | |
| --- | --- | --- | --- | --- | --- | --- | --- | --- | --- | --- | --- | --- |
|  | CENPL\_Noc300\_122214\_01.07500.07500.3 | 4.0999 | 0.2998 | 100.0% | 2034.8644 | 2035.3435 | 1 | 5.782 | 41.7% | 3 | R.RPLEMEQQQAYRPEMK.T | 3 |
|  | CENPL\_Noc300\_tube2\_122214\_01.15447.15447.3 | 3.1206 | 0.3341 | 99.9% | 2040.1144 | 2040.2834 | 2 | 5.258 | 33.3% | 1 | K.QGAPTSFLPPEASQLKPDR.Q | 3 |
|  | CENPL\_Noc300\_122214\_02.15028.15028.2 | 2.5314 | 0.4511 | 99.8% | 2074.5923 | 2076.316 | 1 | 7.271 | 44.4% | 1 | R.SSGYPLTIEGFAYLWSGAR.A | 2 |
|  | CENPL\_Noc300\_122214\_01.06457.06457.3 | 2.7028 | 0.2202 | 97.4% | 1486.4343 | 1484.6548 | 57 | 5.487 | 29.2% | 1 | K.HLPSTEPDPHVVR.I | 3 |
|  | CENPL\_Noc300\_122214\_01.11018.11018.2 | 3.6711 | 0.39 | 100.0% | 1596.6322 | 1595.8821 | 1 | 6.884 | 64.3% | 2 | K.EALGGQALYPHVLVK.N | 2 |
|  | CENPL\_Noc300\_tube2\_122214\_01.14195.14195.2 | 2.8486 | 0.3748 | 99.9% | 1481.6122 | 1481.7502 | 3 | 5.944 | 62.5% | 1 | K.KYNILGTNAIMDK.M | 2 |
|  | CENPL\_Noc300\_122214\_02.09135.09135.2 | 4.7596 | 0.5348 | 100.0% | 1743.2122 | 1742.8857 | 1 | 9.074 | 78.6% | 3 | R.NYILDQTNVYGSAQR.R | 2 |
|  | CENPL\_Noc300\_tube2\_122214\_01.10709.10709.3 | 2.3211 | 0.2874 | 96.2% | 1914.6543 | 1914.0944 | 3 | 4.779 | 35.9% | 1 | R.TDEEGKDVPDHAVLEMK.A | 3 |
|  | CENPL\_Noc300\_122214\_01.20364.20364.2 | 4.6764 | 0.4513 | 100.0% | 2552.7722 | 2552.8865 | 1 | 9.354 | 54.8% | 1 | K.ANFTLPDVGDFLDEVLFIELQR.E | 2 |

---

|  |  |  |  |  |  |  |  |  |
| --- | --- | --- | --- | --- | --- | --- | --- | --- |
| U | *gi|169204265|ref|XP\_0* | 1 | 1 | 19.6% | 51 | 6352 | 12.5 | PREDICTED: similar to mCG146274 [Homo sapiens] |
| U | *gi|4506647|ref|NP\_000* | 1 | 1 | 19.6% | 51 | 6407 | 12.6 | ribosomal protein L39 [Homo sapiens] |
| U | *gi|169205206|ref|XP\_0* | 1 | 1 | 19.6% | 51 | 6352 | 12.5 | PREDICTED: similar to mCG146274 [Homo sapiens] |
| U | *gi|169204607|ref|XP\_0* | 1 | 1 | 19.6% | 51 | 6352 | 12.5 | PREDICTED: similar to mCG146274 [Homo sapiens] |

| Filename XCorr DeltCN Conf% ObsM+H+ CalcM+H+ SpR ZScore Ion% # Sequence  | | | | | | | | | | | | |
| --- | --- | --- | --- | --- | --- | --- | --- | --- | --- | --- | --- | --- |
|  | CENPL\_Noc300\_122214\_01.09842.09842.3 | 2.0931 | 0.3122 | 97.9% | 1309.1344 | 1308.5289 | 74 | 4.827 | 38.9% | 1 | K.QNRPIPQWIR.M | 3 |

---

|  |  |  |  |  |  |  |  |  |
| --- | --- | --- | --- | --- | --- | --- | --- | --- |
| U | *gi|62955833|ref|NP\_11* | 5 | 8 | 19.3% | 399 | 44523 | 5.0 | DNA-damage inducible protein 2 [Homo sapiens] |

| Filename XCorr DeltCN Conf% ObsM+H+ CalcM+H+ SpR ZScore Ion% # Sequence  | | | | | | | | | | | | |
| --- | --- | --- | --- | --- | --- | --- | --- | --- | --- | --- | --- | --- |
| \* | CENPL\_Noc300\_tube2\_122214\_01.17598.17598.2 | 3.3262 | 0.4193 | 100.0% | 1535.4521 | 1534.7092 | 1 | 6.188 | 64.3% | 3 | R.IDFSSIAVPGTSSPR.Q | 2 |
| \* | CENPL\_Noc300\_tube2\_122214\_01.19152.19152.2 | 4.1319 | 0.4899 | 100.0% | 1595.6522 | 1594.9098 | 1 | 8.581 | 76.9% | 2 | R.DMLLANPHELSLLK.E | 2 |
| \* | CENPL\_Noc300\_122214\_02.12173.12173.2 | 2.8702 | 0.4464 | 100.0% | 1567.2722 | 1567.7795 | 1 | 7.462 | 46.4% | 1 | R.NPPLAEALLSGDLEK.F | 2 |
| \* | CENPL\_Noc300\_tube2\_122214\_01.19630.19630.2 | 3.3324 | 0.4175 | 100.0% | 1552.5322 | 1552.724 | 1 | 7.904 | 57.7% | 1 | R.LFSADPFDLEAQAK.I | 2 |
| \* | CENPL\_Noc300\_tube2\_122214\_01.19142.19142.3 | 3.9024 | 0.1425 | 98.6% | 2184.6243 | 2184.3647 | 1 | 4.338 | 38.9% | 1 | R.EDVRPEEIADQELAEALQK.S | 3 |

---

|  |  |  |  |  |  |  |  |  |
| --- | --- | --- | --- | --- | --- | --- | --- | --- |
| U | *gi|16905073|ref|NP\_07* | 4 | 4 | 19.3% | 269 | 31655 | 4.9 | SoxLZ/Sox6 leucine zipper binding protein [Homo sapiens] |

| Filename XCorr DeltCN Conf% ObsM+H+ CalcM+H+ SpR ZScore Ion% # Sequence  | | | | | | | | | | | | |
| --- | --- | --- | --- | --- | --- | --- | --- | --- | --- | --- | --- | --- |
| \* | CENPL\_Noc300\_122214\_01.10862.10862.2 | 3.2321 | 0.3861 | 100.0% | 1433.7522 | 1433.7063 | 1 | 6.69 | 72.7% | 1 | K.LRQDLEMVLSTK.E | 2 |
| \* | CENPL\_Noc300\_122214\_01.17382.17382.2 | 4.6329 | 0.3994 | 100.0% | 2100.5322 | 2100.379 | 1 | 8.269 | 52.9% | 1 | K.LLSTLGEFLEDHFPLPDR.S | 2 |
| \* | CENPL\_Noc300\_122214\_01.17383.17383.3 | 3.0762 | 0.2656 | 99.8% | 2101.3442 | 2100.379 | 5 | 5.466 | 33.8% | 1 | K.LLSTLGEFLEDHFPLPDR.S | 3 |
| \* | CENPL\_Noc300\_122214\_02.15801.15801.3 | 3.4977 | 0.2521 | 99.8% | 2581.7944 | 2581.0027 | 3 | 5.545 | 28.6% | 1 | K.NIQESSVNLITLHEMLEILINR.L | 3 |

---

|  |  |  |  |  |  |  |  |  |
| --- | --- | --- | --- | --- | --- | --- | --- | --- |
| U | *gi|10800138|ref|NP\_06* | 2 | 3 | 19.0% | 126 | 13936 | 10.3 | histone cluster 1, H2bd [Homo sapiens] |
| U | *gi|66912162|ref|NP\_00* | 2 | 3 | 19.0% | 126 | 13920 | 10.3 | histone cluster 2, H2bf [Homo sapiens] |
| U | *gi|4504277|ref|NP\_003* | 2 | 3 | 19.0% | 126 | 13920 | 10.3 | histone cluster 2, H2be [Homo sapiens] |
| U | *gi|4504271|ref|NP\_003* | 2 | 3 | 19.0% | 126 | 13906 | 10.3 | histone cluster 1, H2bi [Homo sapiens] |
| U | *gi|4504269|ref|NP\_003* | 2 | 3 | 19.0% | 126 | 13892 | 10.3 | histone cluster 1, H2bh [Homo sapiens] |
| U | *gi|4504265|ref|NP\_003* | 2 | 3 | 19.0% | 126 | 13906 | 10.3 | histone cluster 1, H2bf [Homo sapiens] |
| U | *gi|4504263|ref|NP\_003* | 2 | 3 | 19.0% | 126 | 13989 | 10.3 | histone cluster 1, H2bm [Homo sapiens] |
| U | *gi|4504261|ref|NP\_003* | 2 | 3 | 19.0% | 126 | 13922 | 10.3 | histone cluster 1, H2bn [Homo sapiens] |
| U | *gi|4504259|ref|NP\_003* | 2 | 3 | 19.0% | 126 | 13952 | 10.3 | histone cluster 1, H2bl [Homo sapiens] |
| U | *gi|4504257|ref|NP\_003* | 2 | 3 | 19.0% | 126 | 13906 | 10.3 | histone cluster 1, H2bg [Homo sapiens] |
| U | *gi|28173554|ref|NP\_77* | 2 | 3 | 19.0% | 126 | 13908 | 10.3 | histone cluster 3, H2bb [Homo sapiens] |
| U | *gi|21396484|ref|NP\_00* | 2 | 3 | 19.0% | 126 | 13906 | 10.3 | histone cluster 1, H2be [Homo sapiens] |
| U | *gi|21166389|ref|NP\_00* | 2 | 3 | 19.0% | 126 | 13906 | 10.3 | histone cluster 1, H2bc [Homo sapiens] |
| U | *gi|20336754|ref|NP\_06* | 2 | 3 | 19.0% | 126 | 13904 | 10.3 | histone cluster 1, H2bj [Homo sapiens] |
| U | *gi|20336752|ref|NP\_61* | 2 | 3 | 19.0% | 126 | 13936 | 10.3 | histone cluster 1, H2bd [Homo sapiens] |
| U | *gi|18105048|ref|NP\_54* | 2 | 3 | 19.0% | 126 | 13890 | 10.3 | histone cluster 1, H2bk [Homo sapiens] |
| U | *gi|16306566|ref|NP\_00* | 2 | 3 | 19.0% | 126 | 13906 | 10.3 | histone cluster 1, H2bo [Homo sapiens] |
| U | *gi|10800140|ref|NP\_06* | 2 | 3 | 19.0% | 126 | 13950 | 10.3 | histone cluster 1, H2bb [Homo sapiens] |

| Filename XCorr DeltCN Conf% ObsM+H+ CalcM+H+ SpR ZScore Ion% # Sequence  | | | | | | | | | | | | |
| --- | --- | --- | --- | --- | --- | --- | --- | --- | --- | --- | --- | --- |
|  | CENPL\_Noc300\_122214\_01.17403.17403.2 | 5.0501 | 0.5164 | 100.0% | 1745.5122 | 1745.0211 | 1 | 9.446 | 75.0% | 2 | K.AMGIMNSFVNDIFER.I | 2 |
|  | CENPL\_Noc300\_tube2\_122214\_01.16718.16718.2 | 2.1303 | 0.1626 | 96.2% | 954.27216 | 954.19794 | 2 | 4.146 | 81.2% | 1 | R.LLLPGELAK.H | 2 |

---

|  |  |  |  |  |  |  |  |  |
| --- | --- | --- | --- | --- | --- | --- | --- | --- |
| U | *gi|31711992|ref|NP\_00* | 11 | 30 | 18.9% | 647 | 68997 | 7.8 | dihydrolipoamide S-acetyltransferase [Homo sapiens] |

| Filename XCorr DeltCN Conf% ObsM+H+ CalcM+H+ SpR ZScore Ion% # Sequence  | | | | | | | | | | | | |
| --- | --- | --- | --- | --- | --- | --- | --- | --- | --- | --- | --- | --- |
| \* | CENPL\_Noc300\_tube2\_122214\_01.17229.17229.2 | 4.4274 | 0.6265 | 100.0% | 1739.5922 | 1740.0709 | 1 | 9.673 | 71.9% | 6 | K.VPLPSLSPTMQAGTIAR.W | 2 |
| \* | CENPL\_Noc300\_tube2\_122214\_01.11907.11907.2 | 2.8114 | 0.2322 | 99.8% | 874.33215 | 874.0251 | 7 | 5.924 | 78.6% | 3 | K.GIDLTQVK.G | 2 |
| \* | CENPL\_Noc300\_tube2\_122214\_01.14020.14020.2 | 2.2902 | 0.2704 | 99.2% | 1008.1122 | 1008.11615 | 1 | 6.275 | 81.2% | 2 | K.DIDSFVPSK.V | 2 |
| \* | CENPL\_Noc300\_tube2\_122214\_01.16125.16125.2 | 3.3078 | 0.2452 | 99.9% | 1264.8922 | 1264.5077 | 2 | 6.917 | 65.0% | 3 | R.SKISVNDFIIK.A | 2 |
| \* | CENPL\_Noc300\_tube2\_122214\_01.19170.19170.2 | 2.3474 | 0.3239 | 99.5% | 1049.3922 | 1049.2554 | 3 | 6.582 | 68.8% | 1 | K.ISVNDFIIK.A | 2 |
| \* | CENPL\_Noc300\_tube2\_122214\_01.18646.18646.2 | 4.4443 | 0.5589 | 100.0% | 1605.6322 | 1605.8059 | 1 | 9.075 | 61.5% | 3 | K.VPEANSSWMDTVIR.Q | 2 |
| \* | CENPL\_Noc300\_122214\_01.15104.15104.2 | 4.5732 | 0.5318 | 100.0% | 1517.5922 | 1517.7196 | 1 | 9.515 | 78.6% | 4 | K.GVETIANDVVSLATK.A | 2 |
| \* | CENPL\_Noc300\_tube2\_122214\_01.20459.20459.2 | 4.5791 | 0.4096 | 100.0% | 2424.2922 | 2423.7917 | 1 | 7.328 | 50.0% | 1 | K.LQPHEFQGGTFTISNLGMFGIK.N | 2 |
| \* | CENPL\_Noc300\_tube2\_122214\_01.20451.20451.3 | 4.5978 | 0.3173 | 100.0% | 2425.2244 | 2423.7917 | 1 | 6.258 | 34.5% | 5 | K.LQPHEFQGGTFTISNLGMFGIK.N | 3 |
| \* | CENPL\_Noc300\_122214\_02.12540.12540.2 | 4.3412 | 0.4864 | 100.0% | 1619.7522 | 1618.8326 | 1 | 8.671 | 67.9% | 1 | R.VVDGAVGAQWLAEFR.K | 2 |
| \* | CENPL\_Noc300\_122214\_01.13190.13190.2 | 2.2015 | 0.1622 | 95.4% | 1349.2122 | 1349.7148 | 34 | 3.872 | 55.0% | 1 | R.KYLEKPITMLL.- | 2 |

---

|  |  |  |  |  |  |  |  |  |
| --- | --- | --- | --- | --- | --- | --- | --- | --- |
| U | *gi|109240550|ref|NP\_0* | 7 | 17 | 18.9% | 523 | 58744 | 6.7 | paraspeckle protein 1 [Homo sapiens] |

| Filename XCorr DeltCN Conf% ObsM+H+ CalcM+H+ SpR ZScore Ion% # Sequence  | | | | | | | | | | | | |
| --- | --- | --- | --- | --- | --- | --- | --- | --- | --- | --- | --- | --- |
| \* | CENPL\_Noc300\_tube2\_122214\_01.18718.18718.2 | 3.6373 | 0.4683 | 100.0% | 1993.6721 | 1995.2395 | 1 | 7.165 | 62.5% | 1 | R.LFVGNLPTDITEEDFKR.L | 2 |
| \* | CENPL\_Noc300\_122214\_01.10386.10386.2 | 3.5024 | 0.5214 | 100.0% | 1311.6322 | 1311.4368 | 1 | 8.609 | 75.0% | 4 | R.YGEPSEVFINR.D | 2 |
| \* | CENPL\_Noc300\_122214\_01.07009.07009.2 | 2.355 | 0.2899 | 99.2% | 1116.2122 | 1116.3054 | 1 | 6.113 | 75.0% | 1 | R.FATHGAALTVK.N | 2 |
| \* | CENPL\_Noc300\_122214\_01.17128.17128.2 | 5.7376 | 0.5681 | 100.0% | 2533.6921 | 2533.8406 | 1 | 10.237 | 52.3% | 1 | K.NLSPVVSNELLEQAFSQFGPVEK.A | 2 |
| \* | CENPL\_Noc300\_122214\_01.09477.09477.2 | 2.8819 | 0.3644 | 99.9% | 1290.2922 | 1290.5076 | 23 | 6.176 | 50.0% | 2 | K.GFVEFAAKPPAR.K | 2 |
| \* | CENPL\_Noc300\_122214\_01.12474.12474.2 | 3.4967 | 0.5096 | 100.0% | 1649.9521 | 1650.7875 | 1 | 8.159 | 69.2% | 6 | R.FAQPGTFEFEYASR.W | 2 |
| \* | CENPL\_Noc300\_tube2\_122214\_01.09756.09756.2 | 2.2595 | 0.3637 | 99.5% | 1276.3722 | 1277.435 | 2 | 5.895 | 70.0% | 2 | K.EKLEAEMEAAR.H | 2 |

---

|  |  |  |  |  |  |  |  |  |
| --- | --- | --- | --- | --- | --- | --- | --- | --- |
| U | *gi|169191957|ref|XP\_0* | 1 | 3 | 18.9% | 53 | 5987 | 7.5 | PREDICTED: hypothetical protein [Homo sapiens] |
| U | *gi|169201919|ref|XP\_0* | 1 | 3 | 18.9% | 53 | 5987 | 7.5 | PREDICTED: hypothetical protein [Homo sapiens] |
| U | *gi|169194699|ref|XP\_0* | 1 | 3 | 18.9% | 53 | 5987 | 7.5 | PREDICTED: hypothetical protein [Homo sapiens] |

| Filename XCorr DeltCN Conf% ObsM+H+ CalcM+H+ SpR ZScore Ion% # Sequence  | | | | | | | | | | | | |
| --- | --- | --- | --- | --- | --- | --- | --- | --- | --- | --- | --- | --- |
|  | CENPL\_Noc300\_122214\_01.10681.10681.2 | 2.9871 | 0.0521 | 98.6% | 1190.5521 | 1189.3597 | 89 | 3.551 | 55.6% | 3 | K.ITQILHQHAQ.- | 2 |

---

|  |  |  |  |  |  |  |  |  |
| --- | --- | --- | --- | --- | --- | --- | --- | --- |
| U | *gi|209862831|ref|NP\_0* | 4 | 9 | 18.6% | 339 | 38604 | 7.8 | annexin A2 isoform 2 [Homo sapiens] |
| U | *gi|50845388|ref|NP\_00* | 4 | 9 | 17.6% | 357 | 40411 | 8.4 | annexin A2 isoform 1 [Homo sapiens] |
| U | *gi|50845386|ref|NP\_00* | 4 | 9 | 18.6% | 339 | 38604 | 7.8 | annexin A2 isoform 2 [Homo sapiens] |
| U | *gi|4757756|ref|NP\_004* | 4 | 9 | 18.6% | 339 | 38604 | 7.8 | annexin A2 isoform 2 [Homo sapiens] |

| Filename XCorr DeltCN Conf% ObsM+H+ CalcM+H+ SpR ZScore Ion% # Sequence  | | | | | | | | | | | | |
| --- | --- | --- | --- | --- | --- | --- | --- | --- | --- | --- | --- | --- |
|  | CENPL\_Noc300\_tube2\_122214\_01.18491.18491.2 | 4.3147 | 0.4765 | 100.0% | 2155.0322 | 2156.357 | 1 | 8.901 | 52.8% | 2 | K.AYTNFDAERDALNIETAIK.T | 2 |
|  | CENPL\_Noc300\_122214\_01.17179.17179.2 | 4.1726 | 0.339 | 100.0% | 1653.2922 | 1651.9872 | 1 | 7.412 | 70.0% | 1 | K.SALSGHLETVILGLLK.T | 2 |
|  | CENPL\_Noc300\_tube2\_122214\_01.09668.09668.2 | 3.8509 | 0.5177 | 100.0% | 1223.2322 | 1223.3251 | 1 | 8.724 | 85.0% | 3 | K.TPAQYDASELK.A | 2 |
|  | CENPL\_Noc300\_tube2\_122214\_01.15344.15344.3 | 5.0626 | 0.465 | 100.0% | 1941.1144 | 1941.102 | 1 | 7.851 | 45.3% | 3 | K.TDLEKDIISDTSGDFRK.L | 3 |

---

|  |  |  |  |  |  |  |  |  |
| --- | --- | --- | --- | --- | --- | --- | --- | --- |
| U | *gi|4506901|ref|NP\_003* | 2 | 3 | 18.3% | 164 | 19330 | 11.6 | splicing factor, arginine/serine-rich 3 [Homo sapiens] |

| Filename XCorr DeltCN Conf% ObsM+H+ CalcM+H+ SpR ZScore Ion% # Sequence  | | | | | | | | | | | | |
| --- | --- | --- | --- | --- | --- | --- | --- | --- | --- | --- | --- | --- |
| \* | CENPL\_Noc300\_tube2\_122214\_01.16103.16103.2 | 2.4518 | 0.404 | 99.9% | 1043.7122 | 1044.198 | 1 | 6.517 | 81.2% | 2 | R.AFGYYGPLR.S | 2 |
| \* | CENPL\_Noc300\_tube2\_122214\_01.19475.19475.3 | 2.4426 | 0.3486 | 99.6% | 2321.7844 | 2321.5107 | 9 | 5.409 | 31.2% | 1 | R.NPPGFAFVEFEDPRDAADAVR.E | 3 |

---

|  |  |  |  |  |  |  |  |  |
| --- | --- | --- | --- | --- | --- | --- | --- | --- |
| U | *gi|4506679|ref|NP\_001* | 3 | 3 | 18.2% | 165 | 18898 | 10.2 | ribosomal protein S10 [Homo sapiens] |

| Filename XCorr DeltCN Conf% ObsM+H+ CalcM+H+ SpR ZScore Ion% # Sequence  | | | | | | | | | | | | |
| --- | --- | --- | --- | --- | --- | --- | --- | --- | --- | --- | --- | --- |
| \* | CENPL\_Noc300\_tube2\_122214\_01.19845.19845.2 | 2.5833 | 0.2353 | 98.6% | 2004.8121 | 2004.2548 | 1 | 5.058 | 46.4% | 1 | R.HFYWYLTNEGIQYLR.D | 2 |
|  | CENPL\_Noc300\_122214\_02.08232.08232.3 | 3.8212 | 0.2166 | 99.9% | 1570.9744 | 1570.7019 | 1 | 5.514 | 42.9% | 1 | K.KAEAGAGSATEFQFR.G | 3 |
|  | CENPL\_Noc300\_122214\_02.08934.08934.2 | 3.0225 | 0.1395 | 98.7% | 1443.3322 | 1442.5278 | 1 | 5.109 | 61.5% | 1 | K.AEAGAGSATEFQFR.G | 2 |

---

|  |  |  |  |  |  |  |  |  |
| --- | --- | --- | --- | --- | --- | --- | --- | --- |
| U | *gi|4503471|ref|NP\_001* | 8 | 17 | 17.7% | 462 | 50141 | 9.0 | eukaryotic translation elongation factor 1 alpha 1 [Homo sapiens] |

| Filename XCorr DeltCN Conf% ObsM+H+ CalcM+H+ SpR ZScore Ion% # Sequence  | | | | | | | | | | | | |
| --- | --- | --- | --- | --- | --- | --- | --- | --- | --- | --- | --- | --- |
|  | CENPL\_Noc300\_122214\_02.08474.08474.3 | 5.0149 | 0.454 | 100.0% | 1590.2344 | 1589.835 | 1 | 8.532 | 53.6% | 3 | K.THINIVVIGHVDSGK.S | 3 |
| \* | CENPL\_Noc300\_tube2\_122214\_01.13750.13750.3 | 3.0197 | 0.313 | 99.9% | 1406.0643 | 1405.5962 | 2 | 5.862 | 50.0% | 1 | K.YYVTIIDAPGHR.D | 3 |
| \* | CENPL\_Noc300\_tube2\_122214\_01.13770.13770.2 | 3.3868 | 0.3781 | 100.0% | 1406.3121 | 1405.5962 | 2 | 7.276 | 68.2% | 1 | K.YYVTIIDAPGHR.D | 2 |
|  | CENPL\_Noc300\_122214\_02.09921.09921.2 | 3.2541 | 0.4217 | 100.0% | 1315.2322 | 1315.5553 | 1 | 8.258 | 81.8% | 2 | R.EHALLAYTLGVK.Q | 2 |
|  | CENPL\_Noc300\_tube2\_122214\_01.13354.13354.2 | 2.2249 | 0.2326 | 98.8% | 975.83215 | 976.1607 | 1 | 5.851 | 92.9% | 3 | R.LPLQDVYK.I | 2 |
|  | CENPL\_Noc300\_tube2\_122214\_01.12057.12057.2 | 3.7327 | 0.3974 | 100.0% | 1026.2322 | 1026.2241 | 1 | 8.1 | 85.0% | 4 | K.IGGIGTVPVGR.V | 2 |
| \* | CENPL\_Noc300\_tube2\_122214\_01.19035.19035.2 | 4.8826 | 0.5099 | 100.0% | 2516.5122 | 2516.999 | 1 | 9.549 | 47.8% | 1 | R.VETGVLKPGMVVTFAPVNVTTEVK.S | 2 |
| \* | CENPL\_Noc300\_tube2\_122214\_01.19052.19052.3 | 4.3425 | 0.3361 | 100.0% | 2517.9844 | 2516.999 | 1 | 6.406 | 33.7% | 2 | R.VETGVLKPGMVVTFAPVNVTTEVK.S | 3 |

---

|  |  |  |  |  |  |  |  |  |
| --- | --- | --- | --- | --- | --- | --- | --- | --- |
| U | *gi|14141166|ref|NP\_11* | 3 | 3 | 17.7% | 362 | 38222 | 6.8 | poly(rC) binding protein 2 isoform b [Homo sapiens] |
| U | *gi|193083114|ref|NP\_0* | 3 | 3 | 20.1% | 318 | 33497 | 8.2 | poly(rC) binding protein 2 isoform g [Homo sapiens] |
| U | *gi|193083112|ref|NP\_0* | 3 | 3 | 19.1% | 335 | 35347 | 8.0 | poly(rC) binding protein 2 isoform f [Homo sapiens] |
| U | *gi|193083110|ref|NP\_0* | 3 | 3 | 17.7% | 361 | 38151 | 6.8 | poly(rC) binding protein 2 isoform e [Homo sapiens] |
| U | *gi|193083108|ref|NP\_0* | 3 | 3 | 17.5% | 365 | 38580 | 6.8 | poly(rC) binding protein 2 isoform d [Homo sapiens] |
| U | *gi|148833484|ref|NP\_0* | 3 | 3 | 19.3% | 331 | 34917 | 8.0 | poly(rC) binding protein 2 isoform c [Homo sapiens] |
| U | *gi|14141168|ref|NP\_00* | 3 | 3 | 17.5% | 366 | 38651 | 6.8 | poly(rC) binding protein 2 isoform a [Homo sapiens] |

| Filename XCorr DeltCN Conf% ObsM+H+ CalcM+H+ SpR ZScore Ion% # Sequence  | | | | | | | | | | | | |
| --- | --- | --- | --- | --- | --- | --- | --- | --- | --- | --- | --- | --- |
|  | CENPL\_Noc300\_122214\_01.13778.13778.2 | 2.241 | 0.2755 | 98.2% | 1359.9321 | 1359.6519 | 1 | 5.331 | 66.7% | 1 | R.IITLAGPTNAIFK.A | 2 |
|  | CENPL\_Noc300\_122214\_01.17409.17409.3 | 3.2506 | 0.3581 | 99.9% | 3352.2244 | 3353.8306 | 6 | 4.445 | 21.7% | 1 | K.AFAMIIDKLEEDISSSMTNSTAASRPPVTLR.L | 3 |
|  | CENPL\_Noc300\_122214\_02.08782.08782.2 | 4.4038 | 0.5004 | 100.0% | 2090.3323 | 2091.2573 | 1 | 9.449 | 50.0% | 1 | R.ESTGAQVQVAGDMLPNSTER.A | 22 |

Similarities:
gi|222352151|ref|NP\_0(1:2)  

---

|  |  |  |  |  |  |  |  |  |
| --- | --- | --- | --- | --- | --- | --- | --- | --- |
| U | *gi|208973238|ref|NP\_0* | 2 | 2 | 17.6% | 245 | 27745 | 4.8 | tyrosine 3/tryptophan 5 -monooxygenase activation protein, zeta polypeptide [Homo sapiens] |
| U | *gi|4507953|ref|NP\_003* | 2 | 2 | 17.6% | 245 | 27745 | 4.8 | tyrosine 3/tryptophan 5 -monooxygenase activation protein, zeta polypeptide [Homo sapiens] |
| U | *gi|21735625|ref|NP\_66* | 2 | 2 | 17.6% | 245 | 27745 | 4.8 | tyrosine 3/tryptophan 5 -monooxygenase activation protein, zeta polypeptide [Homo sapiens] |
| U | *gi|208973244|ref|NP\_0* | 2 | 2 | 17.6% | 245 | 27745 | 4.8 | tyrosine 3/tryptophan 5 -monooxygenase activation protein, zeta polypeptide [Homo sapiens] |
| U | *gi|208973242|ref|NP\_0* | 2 | 2 | 17.6% | 245 | 27745 | 4.8 | tyrosine 3/tryptophan 5 -monooxygenase activation protein, zeta polypeptide [Homo sapiens] |
| U | *gi|208973240|ref|NP\_0* | 2 | 2 | 17.6% | 245 | 27745 | 4.8 | tyrosine 3/tryptophan 5 -monooxygenase activation protein, zeta polypeptide [Homo sapiens] |

| Filename XCorr DeltCN Conf% ObsM+H+ CalcM+H+ SpR ZScore Ion% # Sequence  | | | | | | | | | | | | |
| --- | --- | --- | --- | --- | --- | --- | --- | --- | --- | --- | --- | --- |
|  | CENPL\_Noc300\_tube2\_122214\_01.08981.08981.2 | 3.2402 | 0.3193 | 99.9% | 1548.1522 | 1549.5914 | 1 | 6.343 | 69.2% | 1 | K.SVTEQGAELSNEER.N | 2 |
|  | CENPL\_Noc300\_122214\_01.20399.20399.3 | 4.6942 | 0.4981 | 100.0% | 3303.8342 | 3304.6907 | 1 | 8.287 | 28.6% | 1 | K.TAFDEAIAELDTLSEESYKDSTLIMQLLR.D | 3 |

---

|  |  |  |  |  |  |  |  |  |
| --- | --- | --- | --- | --- | --- | --- | --- | --- |
| U | *gi|11415030|ref|NP\_06* | 2 | 3 | 17.5% | 103 | 11367 | 11.4 | histone cluster 1, H4j [Homo sapiens] |
| U | *gi|77539758|ref|NP\_00* | 2 | 3 | 17.5% | 103 | 11367 | 11.4 | histone cluster 2, H4b [Homo sapiens] |
| U | *gi|4504323|ref|NP\_003* | 2 | 3 | 17.5% | 103 | 11367 | 11.4 | histone cluster 2, H4a [Homo sapiens] |
| U | *gi|4504321|ref|NP\_003* | 2 | 3 | 17.5% | 103 | 11367 | 11.4 | histone cluster 1, H4i [Homo sapiens] |
| U | *gi|4504317|ref|NP\_003* | 2 | 3 | 17.5% | 103 | 11367 | 11.4 | histone cluster 1, H4l [Homo sapiens] |
| U | *gi|4504315|ref|NP\_003* | 2 | 3 | 17.5% | 103 | 11367 | 11.4 | histone cluster 1, H4e [Homo sapiens] |
| U | *gi|4504313|ref|NP\_003* | 2 | 3 | 17.5% | 103 | 11367 | 11.4 | histone cluster 1, H4b [Homo sapiens] |
| U | *gi|4504311|ref|NP\_003* | 2 | 3 | 17.5% | 103 | 11367 | 11.4 | histone cluster 1, H4h [Homo sapiens] |
| U | *gi|4504309|ref|NP\_003* | 2 | 3 | 17.5% | 103 | 11367 | 11.4 | histone cluster 1, H4c [Homo sapiens] |
| U | *gi|4504307|ref|NP\_003* | 2 | 3 | 17.5% | 103 | 11367 | 11.4 | histone cluster 1, H4k [Homo sapiens] |
| U | *gi|4504305|ref|NP\_003* | 2 | 3 | 17.5% | 103 | 11367 | 11.4 | histone cluster 1, H4f [Homo sapiens] |
| U | *gi|4504303|ref|NP\_003* | 2 | 3 | 17.5% | 103 | 11367 | 11.4 | histone cluster 1, H4d [Homo sapiens] |
| U | *gi|4504301|ref|NP\_003* | 2 | 3 | 17.5% | 103 | 11367 | 11.4 | histone cluster 1, H4a [Homo sapiens] |
| U | *gi|28173560|ref|NP\_77* | 2 | 3 | 17.5% | 103 | 11367 | 11.4 | histone cluster 4, H4 [Homo sapiens] |

| Filename XCorr DeltCN Conf% ObsM+H+ CalcM+H+ SpR ZScore Ion% # Sequence  | | | | | | | | | | | | |
| --- | --- | --- | --- | --- | --- | --- | --- | --- | --- | --- | --- | --- |
|  | CENPL\_Noc300\_tube2\_122214\_01.13728.13728.2 | 3.1716 | 0.3797 | 100.0% | 1181.4922 | 1181.3312 | 1 | 6.802 | 72.2% | 2 | R.ISGLIYEETR.G | 2 |
|  | CENPL\_Noc300\_tube2\_122214\_01.17548.17548.2 | 2.3606 | 0.3082 | 99.5% | 990.21216 | 990.19055 | 1 | 5.363 | 92.9% | 1 | K.VFLENVIR.D | 2 |

---

|  |  |  |  |  |  |  |  |  |
| --- | --- | --- | --- | --- | --- | --- | --- | --- |
| U | *gi|5031699|ref|NP\_005* | 5 | 5 | 17.3% | 427 | 47355 | 7.5 | flotillin 1 [Homo sapiens] |

| Filename XCorr DeltCN Conf% ObsM+H+ CalcM+H+ SpR ZScore Ion% # Sequence  | | | | | | | | | | | | |
| --- | --- | --- | --- | --- | --- | --- | --- | --- | --- | --- | --- | --- |
| \* | CENPL\_Noc300\_122214\_02.09084.09084.3 | 2.9859 | 0.3576 | 99.9% | 1808.3043 | 1808.1206 | 1 | 5.612 | 42.9% | 1 | R.AIMAHMTVEEIYKDR.Q | 3 |
| \* | CENPL\_Noc300\_122214\_01.17620.17620.2 | 3.733 | 0.252 | 99.9% | 1984.9122 | 1984.3154 | 1 | 6.979 | 52.8% | 1 | K.VASSDLVNMGISVVSYTLK.D | 2 |
| \* | CENPL\_Noc300\_tube2\_122214\_01.12462.12462.3 | 2.4247 | 0.2434 | 95.2% | 1657.7043 | 1656.749 | 21 | 4.687 | 38.5% | 1 | K.DIHDDQDYLHSLGK.A | 3 |
| \* | CENPL\_Noc300\_tube2\_122214\_01.14183.14183.2 | 2.0866 | 0.3133 | 98.1% | 1419.5721 | 1419.6206 | 11 | 5.14 | 54.2% | 1 | R.AQADLAYQLQVAK.T | 2 |
| \* | CENPL\_Noc300\_122214\_02.06636.06636.2 | 3.4283 | 0.4458 | 100.0% | 1470.5122 | 1470.6255 | 1 | 7.167 | 62.5% | 1 | R.AQQVAVQEQEIAR.R | 2 |

---

|  |  |  |  |  |  |  |  |  |
| --- | --- | --- | --- | --- | --- | --- | --- | --- |
| U | *gi|24762225|ref|NP\_00* | 3 | 3 | 17.1% | 381 | 41542 | 6.3 | Meis homeobox 2 isoform f [Homo sapiens] |
| U | *gi|27502381|ref|NP\_75* | 3 | 3 | 16.8% | 388 | 42272 | 6.2 | Meis homeobox 2 isoform g [Homo sapiens] |
| U | *gi|24762250|ref|NP\_73* | 3 | 3 | 13.8% | 470 | 51060 | 6.5 | Meis homeobox 2 isoform d [Homo sapiens] |
| U | *gi|24762246|ref|NP\_73* | 3 | 3 | 16.5% | 394 | 43061 | 6.2 | Meis homeobox 2 isoform b [Homo sapiens] |
| U | *gi|24762244|ref|NP\_73* | 3 | 3 | 16.2% | 401 | 43791 | 6.2 | Meis homeobox 2 isoform a [Homo sapiens] |
| U | *gi|24762241|ref|NP\_73* | 3 | 3 | 13.6% | 477 | 51790 | 6.4 | Meis homeobox 2 isoform c [Homo sapiens] |

| Filename XCorr DeltCN Conf% ObsM+H+ CalcM+H+ SpR ZScore Ion% # Sequence  | | | | | | | | | | | | |
| --- | --- | --- | --- | --- | --- | --- | --- | --- | --- | --- | --- | --- |
|  | CENPL\_Noc300\_122214\_01.19082.19082.3 | 4.9558 | 0.3697 | 100.0% | 2287.5842 | 2287.7068 | 1 | 7.243 | 42.1% | 1 | R.DKDAIYGHPLFPLLALVFEK.C | 3 |
|  | CENPL\_Noc300\_122214\_01.18702.18702.2 | 5.5045 | 0.5523 | 100.0% | 2726.912 | 2728.1792 | 1 | 11.605 | 50.0% | 1 | R.AEKPLFSSNPELDNLMIQAIQVLR.F | 2 |
|  | CENPL\_Noc300\_122214\_02.14382.14382.3 | 4.6364 | 0.4616 | 100.0% | 2416.4944 | 2416.7422 | 2 | 7.947 | 31.2% | 1 | K.QLAQDTGLTILQVNNWFINAR.R | 3 |

---

|  |  |  |  |  |  |  |  |  |
| --- | --- | --- | --- | --- | --- | --- | --- | --- |
| U | *gi|4506645|ref|NP\_000* | 1 | 1 | 17.1% | 70 | 8218 | 10.1 | ribosomal protein L38 [Homo sapiens] |
| U | *gi|78214522|ref|NP\_00* | 1 | 1 | 17.1% | 70 | 8218 | 10.1 | ribosomal protein L38 [Homo sapiens] |

| Filename XCorr DeltCN Conf% ObsM+H+ CalcM+H+ SpR ZScore Ion% # Sequence  | | | | | | | | | | | | |
| --- | --- | --- | --- | --- | --- | --- | --- | --- | --- | --- | --- | --- |
|  | CENPL\_Noc300\_tube2\_122214\_01.16013.16013.2 | 3.0519 | 0.2867 | 99.8% | 1486.7122 | 1486.7484 | 1 | 6.307 | 68.2% | 1 | R.YLYTLVITDKEK.A | 2 |

---

|  |  |  |  |  |  |  |  |  |
| --- | --- | --- | --- | --- | --- | --- | --- | --- |
| U | *Reverse\_gi|169207738|* | 1 | 1 | 17.0% | 182 | 18973 | 8.5 | PREDICTED: hypothetical protein [Homo sapiens] |

| Filename XCorr DeltCN Conf% ObsM+H+ CalcM+H+ SpR ZScore Ion% # Sequence  | | | | | | | | | | | | |
| --- | --- | --- | --- | --- | --- | --- | --- | --- | --- | --- | --- | --- |
| \* | CENPL\_Noc300\_122214\_01.16749.16749.3 | 2.9195 | 0.2819 | 99.0% | 3376.3442 | 3374.5251 | 212 | 4.47 | 20.8% | 1 | R.S\*GAPSS\*LARCS\*LGAGVRGSCLETPLAPMAQM.- | 3 |

---

|  |  |  |  |  |  |  |  |  |
| --- | --- | --- | --- | --- | --- | --- | --- | --- |
| U | *gi|14043070|ref|NP\_11* | 5 | 6 | 16.7% | 372 | 38747 | 9.1 | heterogeneous nuclear ribonucleoprotein A1 isoform b [Homo sapiens] |
| U | *gi|88958985|ref|XP\_94* | 5 | 6 | 20.6% | 301 | 32399 | 9.0 | PREDICTED: similar to heterogeneous nuclear ribonucleoprotein A1 [Homo sapiens] |
| U | *gi|88953883|ref|XP\_93* | 5 | 6 | 20.6% | 301 | 32380 | 8.9 | PREDICTED: similar to heterogeneous nuclear ribonucleoprotein A1 [Homo sapiens] |
| U | *gi|4504445|ref|NP\_002* | 5 | 6 | 19.4% | 320 | 34196 | 9.2 | heterogeneous nuclear ribonucleoprotein A1 isoform a [Homo sapiens] |
| U | *gi|169164476|ref|XP\_0* | 5 | 6 | 20.6% | 301 | 32399 | 9.0 | PREDICTED: similar to heterogeneous nuclear ribonucleoprotein A1 [Homo sapiens] |

| Filename XCorr DeltCN Conf% ObsM+H+ CalcM+H+ SpR ZScore Ion% # Sequence  | | | | | | | | | | | | |
| --- | --- | --- | --- | --- | --- | --- | --- | --- | --- | --- | --- | --- |
|  | CENPL\_Noc300\_122214\_02.12447.12447.2 | 5.0436 | 0.5377 | 100.0% | 1785.4122 | 1785.9916 | 1 | 8.982 | 73.3% | 1 | K.LFIGGLSFETTDESLR.S | 2 |
|  | CENPL\_Noc300\_tube2\_122214\_01.19029.19029.2 | 2.965 | 0.3349 | 100.0% | 1219.5122 | 1219.4387 | 1 | 6.352 | 88.9% | 2 | K.IEVIEIMTDR.G | 2 |
|  | CENPL\_Noc300\_tube2\_122214\_01.18030.18030.2 | 2.7758 | 0.3801 | 99.8% | 1700.1322 | 1700.8016 | 59 | 6.081 | 42.9% | 1 | R.GFAFVTFDDHDSVDK.I | 2 |
|  | CENPL\_Noc300\_122214\_02.10978.10978.3 | 3.5991 | 0.2502 | 99.9% | 2282.4543 | 2282.5579 | 1 | 5.941 | 34.2% | 1 | R.GFAFVTFDDHDSVDKIVIQK.Y | 3 |
|  | CENPL\_Noc300\_122214\_01.07823.07823.2 | 3.8169 | 0.3468 | 100.0% | 1629.6522 | 1629.7721 | 1 | 7.012 | 63.3% | 1 | R.SSGPYGGGGQYFAKPR.N | 2 |

---

|  |  |  |  |  |  |  |  |  |
| --- | --- | --- | --- | --- | --- | --- | --- | --- |
| U | *gi|124256496|ref|NP\_0* | 9 | 32 | 16.4% | 641 | 70375 | 6.0 | heat shock 70kDa protein 1-like [Homo sapiens] |

| Filename XCorr DeltCN Conf% ObsM+H+ CalcM+H+ SpR ZScore Ion% # Sequence  | | | | | | | | | | | | |
| --- | --- | --- | --- | --- | --- | --- | --- | --- | --- | --- | --- | --- |
|  | CENPL\_Noc300\_122214\_01.10323.10323.2 | 3.3577 | 0.4739 | 100.0% | 1488.4722 | 1488.5939 | 1 | 8.67 | 79.2% | 6 | R.TTPSYVAFTDTER.L | 2222 |
|  | CENPL\_Noc300\_tube2\_122214\_01.20031.20031.2 | 3.4205 | 0.5316 | 100.0% | 1615.3121 | 1615.8817 | 1 | 8.039 | 69.2% | 2 | K.AFYPEEISSMVLTK.L | 22 |
|  | CENPL\_Noc300\_122214\_01.13712.13712.2 | 3.7132 | 0.3227 | 100.0% | 1199.2922 | 1198.408 | 1 | 7.244 | 86.4% | 4 | K.DAGVIAGLNVLR.I | 22 |
|  | CENPL\_Noc300\_tube2\_122214\_01.18059.18059.3 | 3.4522 | 0.1839 | 99.4% | 1660.7943 | 1660.9078 | 1 | 4.919 | 50.0% | 1 | R.IINEPTAAAIAYGLDK.G | 333 |
|  | CENPL\_Noc300\_tube2\_122214\_01.18074.18074.2 | 5.3155 | 0.5593 | 100.0% | 1660.8322 | 1660.9078 | 1 | 10.712 | 86.7% | 4 | R.IINEPTAAAIAYGLDK.G | 222 |
|  | CENPL\_Noc300\_122214\_01.07337.07337.2 | 4.5821 | 0.5001 | 100.0% | 1676.2322 | 1676.6964 | 1 | 8.059 | 70.0% | 3 | K.ATAGDTHLGGEDFDNR.L | 222 |
|  | CENPL\_Noc300\_122214\_01.07385.07385.3 | 3.5425 | 0.4619 | 100.0% | 1676.5144 | 1676.6964 | 1 | 7.623 | 51.7% | 5 | K.ATAGDTHLGGEDFDNR.L | 333 |
|  | CENPL\_Noc300\_122214\_02.13851.13851.3 | 5.284 | 0.5335 | 100.0% | 2305.4343 | 2305.608 | 1 | 9.436 | 38.6% | 1 | K.SINPDEAVAYGAAVQAAILMGDK.S | 33 |
|  | CENPL\_Noc300\_tube2\_122214\_01.16994.16994.2 | 3.7772 | 0.4871 | 100.0% | 1288.1122 | 1288.4608 | 1 | 8.312 | 80.0% | 6 | K.NALESYAFNMK.S | 22 |

Similarities:
gi|16507237|ref|NP\_00(2:7)  
gi|5729877|ref|NP\_006(3:6)  
gi|167466173|ref|NP\_0(7:2)  
gi|34419635|ref|NP\_00(3:6)  

---

|  |  |  |  |  |  |  |  |  |
| --- | --- | --- | --- | --- | --- | --- | --- | --- |
| U | *gi|4557701|ref|NP\_000* | 8 | 19 | 16.4% | 432 | 48106 | 5.0 | keratin 17 [Homo sapiens] |

| Filename XCorr DeltCN Conf% ObsM+H+ CalcM+H+ SpR ZScore Ion% # Sequence  | | | | | | | | | | | | |
| --- | --- | --- | --- | --- | --- | --- | --- | --- | --- | --- | --- | --- |
|  | CENPL\_Noc300\_122214\_01.07294.07294.1 | 1.6519 | 0.2663 | 95.7% | 809.37 | 809.93774 | 1 | 5.658 | 75.0% | 1 | R.LASYLDK.V | 111111 |
|  | CENPL\_Noc300\_tube2\_122214\_01.10668.10668.2 | 2.9757 | 0.1828 | 99.7% | 1065.1921 | 1065.2578 | 7 | 6.164 | 75.0% | 5 | R.LASYLDKVR.A | 22222 |
|  | CENPL\_Noc300\_122214\_01.08804.08804.2 | 2.8835 | 0.3315 | 99.8% | 1345.8121 | 1346.4772 | 1 | 5.726 | 68.2% | 1 | R.ALEEANTELEVK.I | 2 |
|  | CENPL\_Noc300\_122214\_01.07888.07888.2 | 2.5135 | 0.3649 | 100.0% | 808.2522 | 807.8815 | 1 | 6.681 | 83.3% | 6 | R.LAADDFR.T | 2222222 |
|  | CENPL\_Noc300\_tube2\_122214\_01.15556.15556.2 | 3.2708 | 0.3771 | 100.0% | 1030.4321 | 1030.2096 | 1 | 7.026 | 87.5% | 3 | R.VLDELTLAR.A | 2222 |
|  | CENPL\_Noc300\_122214\_01.06383.06383.2 | 2.6033 | 0.3459 | 99.8% | 1243.1322 | 1243.3367 | 1 | 6.613 | 83.3% | 1 | K.NHEEEMNALR.G | 22 |
|  | CENPL\_Noc300\_122214\_01.06579.06579.2 | 3.1107 | 0.4002 | 100.0% | 1362.1921 | 1362.4796 | 15 | 8.218 | 58.3% | 1 | R.EVATNSELVQSGK.S | 22 |
|  | CENPL\_Noc300\_122214\_01.09282.09282.2 | 3.3604 | 0.2669 | 100.0% | 1380.1322 | 1380.5437 | 1 | 5.797 | 80.0% | 1 | K.TRLEQEIATYR.R | 2222 |

Similarities:
gi|40354195|ref|NP\_95(1:7)  
contaminant\_KERATIN03(3:5)  
contaminant\_KERATIN02(1:7)  
gi|15431310|ref|NP\_00(7:1)  
gi|24430192|ref|NP\_00(5:3)  
gi|131412225|ref|NP\_7(2:6)  
gi|24234699|ref|NP\_00(4:4)  

---

|  |  |  |  |  |  |  |  |  |
| --- | --- | --- | --- | --- | --- | --- | --- | --- |
| U | *gi|4506007|ref|NP\_002* | 6 | 11 | 16.4% | 323 | 36984 | 6.5 | protein phosphatase 1, catalytic subunit, gamma isoform [Homo sapiens] |

| Filename XCorr DeltCN Conf% ObsM+H+ CalcM+H+ SpR ZScore Ion% # Sequence  | | | | | | | | | | | | |
| --- | --- | --- | --- | --- | --- | --- | --- | --- | --- | --- | --- | --- |
| \* | CENPL\_Noc300\_tube2\_122214\_01.10192.10192.2 | 3.1843 | 0.2357 | 99.9% | 1243.2722 | 1243.3622 | 57 | 5.399 | 61.1% | 2 | K.NVQLQENEIR.G | 2 |
|  | CENPL\_Noc300\_122214\_01.18116.18116.2 | 4.9446 | 0.4967 | 100.0% | 1954.1721 | 1954.3574 | 1 | 9.269 | 68.8% | 1 | R.EIFLSQPILLELEAPLK.I | 22 |
|  | CENPL\_Noc300\_122214\_01.14013.14013.3 | 3.7838 | 0.3656 | 100.0% | 1440.8344 | 1440.7281 | 1 | 5.994 | 47.5% | 1 | K.IKYPENFFLLR.G | 33 |
|  | CENPL\_Noc300\_tube2\_122214\_01.15603.15603.2 | 3.4319 | 0.182 | 99.5% | 1640.4922 | 1640.7924 | 1 | 6.457 | 73.1% | 5 | R.AHQVVEDGYEFFAK.R | 22 |
|  | CENPL\_Noc300\_122214\_02.09682.09682.3 | 4.2877 | 0.4404 | 100.0% | 1641.8344 | 1640.7924 | 1 | 7.732 | 53.8% | 1 | R.AHQVVEDGYEFFAK.R | 33 |
|  | CENPL\_Noc300\_tube2\_122214\_01.14273.14273.3 | 3.4586 | 0.4634 | 100.0% | 1797.1743 | 1796.9799 | 1 | 7.337 | 46.4% | 1 | R.AHQVVEDGYEFFAKR.Q | 33 |

Similarities:
gi|4506003|ref|NP\_002(5:1)  

---

|  |  |  |  |  |  |  |  |  |
| --- | --- | --- | --- | --- | --- | --- | --- | --- |
| U | *gi|10863927|ref|NP\_06* | 2 | 4 | 16.4% | 165 | 18012 | 7.8 | peptidylprolyl isomerase A [Homo sapiens] |
| U | *gi|169215435|ref|XP\_0* | 2 | 4 | 12.1% | 223 | 24376 | 6.9 | PREDICTED: similar to peptidylprolyl isomerase A-like [Homo sapiens] |

| Filename XCorr DeltCN Conf% ObsM+H+ CalcM+H+ SpR ZScore Ion% # Sequence  | | | | | | | | | | | | |
| --- | --- | --- | --- | --- | --- | --- | --- | --- | --- | --- | --- | --- |
|  | CENPL\_Noc300\_tube2\_122214\_01.17958.17958.2 | 3.4725 | 0.3227 | 100.0% | 1380.7722 | 1380.6268 | 4 | 6.439 | 68.2% | 2 | R.VSFELFADKVPK.T | 2 |
|  | CENPL\_Noc300\_tube2\_122214\_01.17476.17476.2 | 3.7356 | 0.4219 | 100.0% | 1833.5521 | 1833.0477 | 1 | 5.897 | 53.6% | 2 | K.SIYGEKFEDENFILK.H | 2 |

---

|  |  |  |  |  |  |  |  |  |
| --- | --- | --- | --- | --- | --- | --- | --- | --- |
| U | *gi|83776600|ref|NP\_00* | 4 | 8 | 16.3% | 344 | 39311 | 9.3 | aurora kinase B [Homo sapiens] |

| Filename XCorr DeltCN Conf% ObsM+H+ CalcM+H+ SpR ZScore Ion% # Sequence  | | | | | | | | | | | | |
| --- | --- | --- | --- | --- | --- | --- | --- | --- | --- | --- | --- | --- |
| \* | CENPL\_Noc300\_tube2\_122214\_01.12725.12725.2 | 4.2097 | 0.4169 | 100.0% | 1521.4722 | 1520.6978 | 1 | 7.499 | 69.2% | 1 | K.VMENSSGTPDILTR.H | 2 |
| \* | CENPL\_Noc300\_122214\_01.12587.12587.3 | 4.1695 | 0.4676 | 100.0% | 1745.7544 | 1745.9756 | 1 | 7.844 | 42.9% | 3 | R.HFTIDDFEIGRPLGK.G | 3 |
| \* | CENPL\_Noc300\_tube2\_122214\_01.17771.17771.2 | 2.7024 | 0.2235 | 98.9% | 1556.4922 | 1556.7642 | 1 | 4.533 | 50.0% | 1 | K.IADFGWSVHAPSLR.R | 2 |
| \* | CENPL\_Noc300\_122214\_01.11149.11149.3 | 2.9116 | 0.3209 | 99.9% | 1474.5243 | 1474.7489 | 1 | 5.774 | 45.8% | 3 | R.LPLAQVSAHPWVR.A | 3 |

---

|  |  |  |  |  |  |  |  |  |
| --- | --- | --- | --- | --- | --- | --- | --- | --- |
| U | *gi|15431295|ref|NP\_15* | 3 | 6 | 16.1% | 211 | 24261 | 11.7 | ribosomal protein L13 [Homo sapiens] |
| U | *gi|15431297|ref|NP\_00* | 3 | 6 | 16.1% | 211 | 24261 | 11.7 | ribosomal protein L13 [Homo sapiens] |

| Filename XCorr DeltCN Conf% ObsM+H+ CalcM+H+ SpR ZScore Ion% # Sequence  | | | | | | | | | | | | |
| --- | --- | --- | --- | --- | --- | --- | --- | --- | --- | --- | --- | --- |
|  | CENPL\_Noc300\_tube2\_122214\_01.15246.15246.2 | 2.2239 | 0.2763 | 98.8% | 1190.1921 | 1190.3469 | 1 | 5.916 | 77.8% | 1 | R.VATWFNQPAR.K | 2 |
|  | CENPL\_Noc300\_122214\_01.06364.06364.2 | 3.1309 | 0.3367 | 100.0% | 1233.2322 | 1233.3237 | 1 | 6.228 | 90.0% | 2 | K.STESLQANVQR.L | 2 |
|  | CENPL\_Noc300\_tube2\_122214\_01.15148.15148.2 | 3.4222 | 0.4949 | 100.0% | 1383.5122 | 1383.6923 | 1 | 7.912 | 66.7% | 3 | K.LATQLTGPVMPVR.N | 2 |

---

|  |  |  |  |  |  |  |  |  |
| --- | --- | --- | --- | --- | --- | --- | --- | --- |
| U | *gi|4502491|ref|NP\_001* | 2 | 2 | 16.0% | 282 | 31362 | 4.8 | complement component 1, q subcomponent binding protein precursor [Homo sapiens] |

| Filename XCorr DeltCN Conf% ObsM+H+ CalcM+H+ SpR ZScore Ion% # Sequence  | | | | | | | | | | | | |
| --- | --- | --- | --- | --- | --- | --- | --- | --- | --- | --- | --- | --- |
| \* | CENPL\_Noc300\_122214\_02.10271.10271.2 | 3.7107 | 0.3967 | 100.0% | 1621.9722 | 1622.79 | 1 | 7.016 | 57.1% | 1 | K.MSGGWELELNGTEAK.L | 2 |
| \* | CENPL\_Noc300\_122214\_01.20104.20104.3 | 5.0156 | 0.4684 | 100.0% | 3440.8442 | 3441.77 | 2 | 7.483 | 23.3% | 1 | R.GVDNTFADELVELSTALEHQEYITFLEDLK.S | 3 |

---

|  |  |  |  |  |  |  |  |  |
| --- | --- | --- | --- | --- | --- | --- | --- | --- |
| U | *gi|4506707|ref|NP\_001* | 2 | 2 | 16.0% | 125 | 13742 | 10.1 | ribosomal protein S25 [Homo sapiens] |

| Filename XCorr DeltCN Conf% ObsM+H+ CalcM+H+ SpR ZScore Ion% # Sequence  | | | | | | | | | | | | |
| --- | --- | --- | --- | --- | --- | --- | --- | --- | --- | --- | --- | --- |
| \* | CENPL\_Noc300\_122214\_01.12107.12107.2 | 2.6479 | 0.193 | 99.0% | 1320.3522 | 1319.5437 | 3 | 4.362 | 70.0% | 1 | R.DKLNNLVLFDK.A | 2 |
| \* | CENPL\_Noc300\_tube2\_122214\_01.16500.16500.2 | 2.995 | 0.2561 | 99.9% | 973.21216 | 973.1576 | 1 | 6.488 | 87.5% | 1 | R.AALQELLSK.G | 2 |

---

|  |  |  |  |  |  |  |  |  |
| --- | --- | --- | --- | --- | --- | --- | --- | --- |
| U | *gi|113418864|ref|XP\_0* | 1 | 1 | 15.7% | 197 | 20691 | 11.2 | PREDICTED: hypothetical protein [Homo sapiens] |
| U | *gi|169171580|ref|XP\_0* | 1 | 1 | 15.7% | 197 | 20691 | 11.2 | PREDICTED: hypothetical protein [Homo sapiens] |
| U | *gi|113419843|ref|XP\_0* | 1 | 1 | 15.7% | 197 | 20691 | 11.2 | PREDICTED: hypothetical protein [Homo sapiens] |
| U | *gi|113419324|ref|XP\_0* | 1 | 1 | 15.7% | 197 | 20691 | 11.2 | PREDICTED: hypothetical protein [Homo sapiens] |

| Filename XCorr DeltCN Conf% ObsM+H+ CalcM+H+ SpR ZScore Ion% # Sequence  | | | | | | | | | | | | |
| --- | --- | --- | --- | --- | --- | --- | --- | --- | --- | --- | --- | --- |
|  | CENPL\_Noc300\_122214\_02.13185.13185.3 | 3.3733 | 0.3036 | 99.9% | 3346.6743 | 3348.6265 | 32 | 5.581 | 20.8% | 1 | R.VPGSLPNPLTT#VAGGWCAAEILLHGPALCS\*K.V | 3 |

---

|  |  |  |  |  |  |  |  |  |
| --- | --- | --- | --- | --- | --- | --- | --- | --- |
| U | *gi|4885375|ref|NP\_005* | 3 | 6 | 15.5% | 213 | 21365 | 10.9 | histone cluster 1, H1c [Homo sapiens] |
| U | *gi|4885379|ref|NP\_005* | 3 | 6 | 15.1% | 219 | 21865 | 11.0 | histone cluster 1, H1e [Homo sapiens] |
| U | *gi|4885377|ref|NP\_005* | 3 | 6 | 14.9% | 221 | 22350 | 11.0 | histone cluster 1, H1d [Homo sapiens] |

| Filename XCorr DeltCN Conf% ObsM+H+ CalcM+H+ SpR ZScore Ion% # Sequence  | | | | | | | | | | | | |
| --- | --- | --- | --- | --- | --- | --- | --- | --- | --- | --- | --- | --- |
|  | CENPL\_Noc300\_tube2\_122214\_01.13694.13694.2 | 3.1037 | 0.4422 | 100.0% | 1199.2922 | 1199.3898 | 1 | 7.478 | 63.6% | 3 | K.ASGPPVSELITK.A | 2 |
|  | CENPL\_Noc300\_122214\_01.07859.07859.2 | 2.9247 | 0.2293 | 99.7% | 973.65216 | 974.1887 | 1 | 5.707 | 77.8% | 2 | R.SGVSLAALKK.A | 2 |
|  | CENPL\_Noc300\_tube2\_122214\_01.10149.10149.2 | 2.8686 | 0.4189 | 100.0% | 1108.2122 | 1108.2365 | 3 | 8.666 | 70.0% | 1 | K.ALAAAGYDVEK.N | 2 |

---

|  |  |  |  |  |  |  |  |  |
| --- | --- | --- | --- | --- | --- | --- | --- | --- |
| U | *gi|169160598|ref|XP\_0* | 1 | 2 | 15.5% | 84 | 9461 | 9.5 | PREDICTED: similar to hCG1783679 [Homo sapiens] |
| U | *gi|7705706|ref|NP\_057* | 1 | 2 | 15.5% | 84 | 9477 | 9.5 | ribosomal protein S27-like [Homo sapiens] |
| U | *gi|4506711|ref|NP\_001* | 1 | 2 | 15.5% | 84 | 9461 | 9.5 | ribosomal protein S27 [Homo sapiens] |
| U | *gi|169214231|ref|XP\_0* | 1 | 2 | 15.7% | 83 | 9378 | 9.6 | PREDICTED: similar to hCG2027326 [Homo sapiens] |
| U | *gi|169213802|ref|XP\_0* | 1 | 2 | 15.7% | 83 | 9378 | 9.6 | PREDICTED: similar to hCG2027326 [Homo sapiens] |
| U | *gi|169213575|ref|XP\_0* | 1 | 2 | 15.7% | 83 | 9378 | 9.6 | PREDICTED: similar to hCG2027326 [Homo sapiens] |
| U | *gi|169166679|ref|XP\_0* | 1 | 2 | 15.5% | 84 | 9450 | 9.4 | PREDICTED: similar to metallopanstimulin [Homo sapiens] |
| U | *gi|169166621|ref|XP\_0* | 1 | 2 | 15.5% | 84 | 9465 | 9.5 | PREDICTED: similar to metallopanstimulin [Homo sapiens] |
| U | *gi|169166508|ref|XP\_0* | 1 | 2 | 15.5% | 84 | 9450 | 9.4 | PREDICTED: similar to metallopanstimulin [Homo sapiens] |
| U | *gi|169161552|ref|XP\_0* | 1 | 2 | 15.5% | 84 | 9461 | 9.5 | PREDICTED: similar to hCG1783679 [Homo sapiens] |
| U | *gi|169161255|ref|XP\_0* | 1 | 2 | 15.5% | 84 | 9461 | 9.5 | PREDICTED: hypothetical protein [Homo sapiens] |

| Filename XCorr DeltCN Conf% ObsM+H+ CalcM+H+ SpR ZScore Ion% # Sequence  | | | | | | | | | | | | |
| --- | --- | --- | --- | --- | --- | --- | --- | --- | --- | --- | --- | --- |
|  | CENPL\_Noc300\_tube2\_122214\_01.16839.16839.2 | 3.3935 | 0.4489 | 100.0% | 1528.7122 | 1528.7632 | 1 | 7.505 | 70.8% | 2 | R.LVQSPNSYFMDVK.C | 2 |

---

|  |  |  |  |  |  |  |  |  |
| --- | --- | --- | --- | --- | --- | --- | --- | --- |
| U | *gi|170763498|ref|NP\_0* | 2 | 2 | 15.2% | 277 | 32103 | 4.2 | SET translocation (myeloid leukemia-associated) isoform 2 [Homo sapiens] |
| U | *gi|170763500|ref|NP\_0* | 2 | 2 | 14.5% | 290 | 33489 | 4.3 | SET translocation (myeloid leukemia-associated) isoform 1 [Homo sapiens] |

| Filename XCorr DeltCN Conf% ObsM+H+ CalcM+H+ SpR ZScore Ion% # Sequence  | | | | | | | | | | | | |
| --- | --- | --- | --- | --- | --- | --- | --- | --- | --- | --- | --- | --- |
|  | CENPL\_Noc300\_122214\_01.17610.17610.3 | 6.1542 | 0.5644 | 100.0% | 3727.6743 | 3728.1553 | 1 | 9.133 | 31.5% | 1 | K.IPNFWVTTFVNHPQVSALLGEEDEEALHYLTR.V | 3 |
|  | CENPL\_Noc300\_tube2\_122214\_01.15401.15401.2 | 2.9715 | 0.4048 | 100.0% | 1209.5922 | 1209.3385 | 1 | 7.232 | 77.8% | 1 | R.VEVTEFEDIK.S | 2 |

---

|  |  |  |  |  |  |  |  |  |
| --- | --- | --- | --- | --- | --- | --- | --- | --- |
| U | *gi|15055539|ref|NP\_00* | 3 | 3 | 15.0% | 293 | 31324 | 10.2 | ribosomal protein S2 [Homo sapiens] |
| U | *gi|169205508|ref|XP\_0* | 3 | 3 | 18.9% | 233 | 25545 | 9.9 | PREDICTED: hypothetical protein isoform 2 [Homo sapiens] |
| U | *gi|169205506|ref|XP\_0* | 3 | 3 | 15.0% | 293 | 31364 | 10.2 | PREDICTED: hypothetical protein isoform 1 [Homo sapiens] |
| U | *gi|169204986|ref|XP\_0* | 3 | 3 | 18.9% | 233 | 25545 | 9.9 | PREDICTED: hypothetical protein isoform 2 [Homo sapiens] |
| U | *gi|169204984|ref|XP\_0* | 3 | 3 | 15.0% | 293 | 31364 | 10.2 | PREDICTED: hypothetical protein isoform 1 [Homo sapiens] |
| U | *gi|169204456|ref|XP\_0* | 3 | 3 | 18.9% | 233 | 25619 | 9.8 | PREDICTED: hypothetical protein isoform 2 [Homo sapiens] |
| U | *gi|169204454|ref|XP\_0* | 3 | 3 | 15.0% | 293 | 31438 | 10.2 | PREDICTED: hypothetical protein isoform 1 [Homo sapiens] |

| Filename XCorr DeltCN Conf% ObsM+H+ CalcM+H+ SpR ZScore Ion% # Sequence  | | | | | | | | | | | | |
| --- | --- | --- | --- | --- | --- | --- | --- | --- | --- | --- | --- | --- |
|  | CENPL\_Noc300\_122214\_01.20263.20263.3 | 5.5446 | 0.3528 | 100.0% | 3687.2944 | 3688.293 | 1 | 7.335 | 27.4% | 1 | K.SLEEIYLFSLPIKESEIIDFFLGASLKDEVLK.I | 3 |
|  | CENPL\_Noc300\_tube2\_122214\_01.15621.15621.2 | 2.8118 | 0.3288 | 99.8% | 1464.4722 | 1464.6177 | 1 | 6.249 | 68.2% | 1 | K.SPYQEFTDHLVK.T | 2 |
|  | CENPL\_Noc300\_tube2\_122214\_01.15730.15730.3 | 2.7524 | 0.3516 | 99.9% | 1464.8944 | 1464.6177 | 1 | 6.551 | 52.3% | 1 | K.SPYQEFTDHLVK.T | 3 |

---

|  |  |  |  |  |  |  |  |  |
| --- | --- | --- | --- | --- | --- | --- | --- | --- |
| U | *gi|59859885|ref|NP\_00* | 3 | 6 | 14.9% | 295 | 32854 | 4.9 | ribosomal protein SA [Homo sapiens] |
| U | *gi|9845502|ref|NP\_002* | 3 | 6 | 14.9% | 295 | 32854 | 4.9 | ribosomal protein SA [Homo sapiens] |

| Filename XCorr DeltCN Conf% ObsM+H+ CalcM+H+ SpR ZScore Ion% # Sequence  | | | | | | | | | | | | |
| --- | --- | --- | --- | --- | --- | --- | --- | --- | --- | --- | --- | --- |
|  | CENPL\_Noc300\_122214\_02.11604.11604.2 | 3.0912 | 0.3807 | 100.0% | 2619.172 | 2618.9666 | 1 | 6.435 | 36.4% | 1 | K.FLAAGTHLGGTNLDFQMEQYIYK.R | 2 |
|  | CENPL\_Noc300\_122214\_02.06824.06824.2 | 3.6735 | 0.4 | 100.0% | 1204.5122 | 1204.3713 | 1 | 8.403 | 79.2% | 3 | K.FAAATGATPIAGR.F | 2 |
|  | CENPL\_Noc300\_122214\_01.09160.09160.2 | 2.2387 | 0.3125 | 99.5% | 913.89215 | 913.10504 | 1 | 5.759 | 78.6% | 2 | R.LLVVTDPR.A | 2 |

---

|  |  |  |  |  |  |  |  |  |
| --- | --- | --- | --- | --- | --- | --- | --- | --- |
| U | *gi|148229144|ref|NP\_0* | 1 | 1 | 14.8% | 169 | 18154 | 8.6 | small proline-rich protein 3 [Homo sapiens] |
| U | *gi|4885607|ref|NP\_005* | 1 | 1 | 14.8% | 169 | 18154 | 8.6 | small proline-rich protein 3 [Homo sapiens] |

| Filename XCorr DeltCN Conf% ObsM+H+ CalcM+H+ SpR ZScore Ion% # Sequence  | | | | | | | | | | | | |
| --- | --- | --- | --- | --- | --- | --- | --- | --- | --- | --- | --- | --- |
|  | CENPL\_Noc300\_122214\_01.18968.18968.2 | 2.9551 | 0.07 | 96.9% | 2810.7322 | 2810.013 | 3 | 2.941 | 29.2% | 1 | K.VPVPGY@T#KLPEPCPSTVTPGPAQQK.T | 2 |

---

|  |  |  |  |  |  |  |  |  |
| --- | --- | --- | --- | --- | --- | --- | --- | --- |
| U | *gi|27436946|ref|NP\_73* | 8 | 9 | 14.6% | 664 | 74140 | 7.0 | lamin A/C isoform 1 precursor [Homo sapiens] |
| U | *gi|5031875|ref|NP\_005* | 8 | 9 | 17.0% | 572 | 65135 | 6.8 | lamin A/C isoform 2 [Homo sapiens] |

| Filename XCorr DeltCN Conf% ObsM+H+ CalcM+H+ SpR ZScore Ion% # Sequence  | | | | | | | | | | | | |
| --- | --- | --- | --- | --- | --- | --- | --- | --- | --- | --- | --- | --- |
|  | CENPL\_Noc300\_tube2\_122214\_01.09952.09952.3 | 3.1527 | 0.1296 | 95.1% | 1630.9143 | 1630.7521 | 23 | 4.698 | 41.7% | 1 | R.LQEKEDLQELNDR.L | 3 |
|  | CENPL\_Noc300\_122214\_01.06427.06427.2 | 3.08 | 0.4075 | 100.0% | 1149.4122 | 1149.2432 | 2 | 7.562 | 83.3% | 1 | R.ITESEEVVSR.E | 2 |
|  | CENPL\_Noc300\_tube2\_122214\_01.16727.16727.2 | 2.8587 | 0.2524 | 99.6% | 1244.2122 | 1244.474 | 1 | 5.862 | 75.0% | 1 | R.LKDLEALLNSK.E | 2 |
|  | CENPL\_Noc300\_tube2\_122214\_01.13182.13182.2 | 2.589 | 0.1965 | 99.1% | 1183.9521 | 1183.3066 | 10 | 5.445 | 66.7% | 2 | R.TLEGELHDLR.G | 2 |
|  | CENPL\_Noc300\_tube2\_122214\_01.11513.11513.2 | 2.5627 | 0.0963 | 97.4% | 1189.9521 | 1188.3262 | 12 | 3.828 | 66.7% | 1 | K.LRDLEDSLAR.E | 2 |
|  | CENPL\_Noc300\_tube2\_122214\_01.19563.19563.2 | 2.5159 | 0.3206 | 99.3% | 1893.3121 | 1895.1346 | 1 | 4.888 | 53.6% | 1 | R.MQQQLDEYQELLDIK.L | 2 |
|  | CENPL\_Noc300\_tube2\_122214\_01.11633.11633.3 | 2.9523 | 0.3684 | 99.9% | 1606.8243 | 1606.7728 | 1 | 6.882 | 42.3% | 1 | R.VAVEEVDEEGKFVR.L | 3 |
|  | CENPL\_Noc300\_tube2\_122214\_01.12881.12881.2 | 3.6649 | 0.496 | 100.0% | 1492.4922 | 1492.6874 | 1 | 8.062 | 73.1% | 1 | R.TALINSTGEEVAMR.K | 2 |

---

|  |  |  |  |  |  |  |  |  |
| --- | --- | --- | --- | --- | --- | --- | --- | --- |
| U | *gi|32189392|ref|NP\_00* | 2 | 2 | 14.6% | 198 | 21892 | 6.0 | peroxiredoxin 2 isoform a [Homo sapiens] |

| Filename XCorr DeltCN Conf% ObsM+H+ CalcM+H+ SpR ZScore Ion% # Sequence  | | | | | | | | | | | | |
| --- | --- | --- | --- | --- | --- | --- | --- | --- | --- | --- | --- | --- |
| \* | CENPL\_Noc300\_tube2\_122214\_01.20688.20688.2 | 3.205 | 0.4337 | 100.0% | 1862.9122 | 1864.1954 | 33 | 6.487 | 32.4% | 1 | R.KEGGLGPLNIPLLADVTR.R | 2 |
|  | CENPL\_Noc300\_tube2\_122214\_01.13941.13941.2 | 2.5037 | 0.3355 | 99.5% | 1212.3322 | 1212.3915 | 13 | 6.956 | 75.0% | 1 | R.QITVNDLPVGR.S | 22 |

Similarities:
gi|32455264|ref|NP\_85(1:1)  

---

|  |  |  |  |  |  |  |  |  |
| --- | --- | --- | --- | --- | --- | --- | --- | --- |
| U | *gi|164664518|ref|NP\_0* | 3 | 3 | 14.5% | 483 | 54417 | 8.7 | DEAD (Asp-Glu-Ala-Asp) box polypeptide 6 [Homo sapiens] |

| Filename XCorr DeltCN Conf% ObsM+H+ CalcM+H+ SpR ZScore Ion% # Sequence  | | | | | | | | | | | | |
| --- | --- | --- | --- | --- | --- | --- | --- | --- | --- | --- | --- | --- |
| \* | CENPL\_Noc300\_tube2\_122214\_01.08922.08922.3 | 5.3799 | 0.3782 | 100.0% | 2366.7844 | 2367.642 | 1 | 6.716 | 41.3% | 1 | R.GPVKPTGGPGGGGTQTQQQMNQLK.N | 3 |
| \* | CENPL\_Noc300\_122214\_02.14811.14811.3 | 4.0999 | 0.4261 | 100.0% | 3259.8245 | 3259.803 | 1 | 7.116 | 25.0% | 1 | R.ELLMGIFEMGWEKPSPIQEESIPIALSGR.D | 3 |
| \* | CENPL\_Noc300\_122214\_01.09193.09193.2 | 2.1073 | 0.2821 | 97.1% | 1991.5721 | 1993.1339 | 73 | 5.215 | 34.4% | 1 | K.SLYVAEYHSEPVEDEKP.- | 2 |

---

|  |  |  |  |  |  |  |  |  |
| --- | --- | --- | --- | --- | --- | --- | --- | --- |
| U | *gi|113425815|ref|XP\_9* | 2 | 9 | 14.5% | 172 | 18080 | 10.2 | PREDICTED: similar to Histone H2AV (H2A.F/Z) [Homo sapiens] |
| U | *gi|169209156|ref|XP\_0* | 2 | 9 | 14.5% | 172 | 18066 | 10.1 | PREDICTED: similar to Histone H2AV (H2A.F/Z) [Homo sapiens] |

| Filename XCorr DeltCN Conf% ObsM+H+ CalcM+H+ SpR ZScore Ion% # Sequence  | | | | | | | | | | | | |
| --- | --- | --- | --- | --- | --- | --- | --- | --- | --- | --- | --- | --- |
|  | CENPL\_Noc300\_122214\_01.20326.20326.2 | 3.4081 | 0.1171 | 99.2% | 1602.2922 | 1601.6821 | 10 | 4.593 | 53.3% | 3 | K.ESGGGGAGRQREGARR.R | 2 |
|  | CENPL\_Noc300\_tube2\_122214\_01.14990.14990.2 | 2.9694 | 0.2377 | 99.8% | 945.27216 | 945.1093 | 3 | 5.186 | 81.2% | 6 | R.AGLQFPVGR.I | 222 |

Similarities:
gi|10800130|ref|NP\_06(1:1)  
gi|20357599|ref|NP\_61(1:1)  

---

|  |  |  |  |  |  |  |  |  |
| --- | --- | --- | --- | --- | --- | --- | --- | --- |
| U | *Reverse\_gi|169214576|* | 1 | 1 | 14.5% | 131 | 14506 | 8.7 | PREDICTED: hypothetical protein [Homo sapiens] |
| U | *Reverse\_gi|169215052|* | 1 | 1 | 14.5% | 131 | 14506 | 8.7 | PREDICTED: hypothetical protein [Homo sapiens] |
| U | *Reverse\_gi|169214881|* | 1 | 1 | 14.5% | 131 | 14506 | 8.7 | PREDICTED: hypothetical protein [Homo sapiens] |

| Filename XCorr DeltCN Conf% ObsM+H+ CalcM+H+ SpR ZScore Ion% # Sequence  | | | | | | | | | | | | |
| --- | --- | --- | --- | --- | --- | --- | --- | --- | --- | --- | --- | --- |
|  | CENPL\_Noc300\_tube2\_122214\_01.19094.19094.2 | 2.4239 | 0.1797 | 96.8% | 2225.0723 | 2227.4392 | 18 | 5.155 | 33.3% | 1 | R.RPTLGQEKYALIEQLGSS\*R.W | 32 |

---

|  |  |  |  |  |  |  |  |  |
| --- | --- | --- | --- | --- | --- | --- | --- | --- |
| U | *gi|24430192|ref|NP\_00* | 9 | 24 | 14.4% | 473 | 51268 | 5.0 | keratin 16 [Homo sapiens] |

| Filename XCorr DeltCN Conf% ObsM+H+ CalcM+H+ SpR ZScore Ion% # Sequence  | | | | | | | | | | | | |
| --- | --- | --- | --- | --- | --- | --- | --- | --- | --- | --- | --- | --- |
|  | CENPL\_Noc300\_122214\_01.07045.07045.1 | 1.7771 | 0.24 | 96.1% | 1090.58 | 1091.2273 | 4 | 4.459 | 62.5% | 1 | K.VTMQNLNDR.L | 111 |
|  | CENPL\_Noc300\_122214\_02.06500.06500.2 | 2.7854 | 0.4106 | 100.0% | 1091.0922 | 1091.2273 | 1 | 6.291 | 87.5% | 4 | K.VTMQNLNDR.L | 222 |
|  | CENPL\_Noc300\_122214\_01.07294.07294.1 | 1.6519 | 0.2663 | 95.7% | 809.37 | 809.93774 | 1 | 5.658 | 75.0% | 1 | R.LASYLDK.V | 111111 |
|  | CENPL\_Noc300\_tube2\_122214\_01.10668.10668.2 | 2.9757 | 0.1828 | 99.7% | 1065.1921 | 1065.2578 | 7 | 6.164 | 75.0% | 5 | R.LASYLDKVR.A | 22222 |
|  | CENPL\_Noc300\_122214\_02.07913.07913.2 | 3.6976 | 0.3822 | 100.0% | 1302.2922 | 1302.4241 | 1 | 7.464 | 77.3% | 2 | R.ALEEANADLEVK.I | 222 |
|  | CENPL\_Noc300\_122214\_01.07888.07888.2 | 2.5135 | 0.3649 | 100.0% | 808.2522 | 807.8815 | 1 | 6.681 | 83.3% | 6 | R.LAADDFR.T | 2222222 |
|  | CENPL\_Noc300\_122214\_01.12711.12711.2 | 2.5195 | 0.0972 | 96.6% | 1202.9922 | 1202.3097 | 1 | 3.859 | 70.0% | 1 | R.QTVEADVNGLR.R | 2 |
|  | CENPL\_Noc300\_tube2\_122214\_01.15556.15556.2 | 3.2708 | 0.3771 | 100.0% | 1030.4321 | 1030.2096 | 1 | 7.026 | 87.5% | 3 | R.VLDELTLAR.T | 2222 |
|  | CENPL\_Noc300\_122214\_01.09282.09282.2 | 3.3604 | 0.2669 | 100.0% | 1380.1322 | 1380.5437 | 1 | 5.797 | 80.0% | 1 | K.TRLEQEIATYR.R | 2222 |

Similarities:
gi|40354195|ref|NP\_95(1:8)  
contaminant\_KERATIN03(5:4)  
contaminant\_KERATIN02(1:8)  
gi|15431310|ref|NP\_00(8:1)  
gi|4557701|ref|NP\_000(5:4)  
gi|131412225|ref|NP\_7(3:6)  
gi|24234699|ref|NP\_00(4:5)  

---

|  |  |  |  |  |  |  |  |  |
| --- | --- | --- | --- | --- | --- | --- | --- | --- |
| U | *gi|14043072|ref|NP\_11* | 3 | 4 | 14.4% | 353 | 37430 | 8.9 | heterogeneous nuclear ribonucleoprotein A2/B1 isoform B1 [Homo sapiens] |
| U | *gi|4504447|ref|NP\_002* | 3 | 4 | 15.0% | 341 | 36006 | 8.6 | heterogeneous nuclear ribonucleoprotein A2/B1 isoform A2 [Homo sapiens] |

| Filename XCorr DeltCN Conf% ObsM+H+ CalcM+H+ SpR ZScore Ion% # Sequence  | | | | | | | | | | | | |
| --- | --- | --- | --- | --- | --- | --- | --- | --- | --- | --- | --- | --- |
|  | CENPL\_Noc300\_122214\_02.12585.12585.2 | 3.7114 | 0.3443 | 100.0% | 1799.2522 | 1800.0184 | 1 | 7.034 | 63.3% | 1 | K.LFIGGLSFETTEESLR.N | 2 |
|  | CENPL\_Noc300\_tube2\_122214\_01.19264.19264.3 | 3.7129 | 0.3842 | 100.0% | 2279.4243 | 2278.5693 | 1 | 6.337 | 38.2% | 1 | R.GFGFVTFDDHDPVDKIVLQK.Y | 3 |
|  | CENPL\_Noc300\_tube2\_122214\_01.11314.11314.2 | 2.3324 | 0.3504 | 99.3% | 1377.9722 | 1378.4465 | 6 | 5.571 | 50.0% | 2 | R.GGGGNFGPGPGSNFR.G | 2 |

---

|  |  |  |  |  |  |  |  |  |
| --- | --- | --- | --- | --- | --- | --- | --- | --- |
| U | *gi|148612809|ref|NP\_0* | 27 | 63 | 14.1% | 2382 | 250775 | 6.3 | WNK lysine deficient protein kinase 1 [Homo sapiens] |

| Filename XCorr DeltCN Conf% ObsM+H+ CalcM+H+ SpR ZScore Ion% # Sequence  | | | | | | | | | | | | |
| --- | --- | --- | --- | --- | --- | --- | --- | --- | --- | --- | --- | --- |
| \* | CENPL\_Noc300\_tube2\_122214\_01.09401.09401.2 | 4.215 | 0.5138 | 100.0% | 1072.8522 | 1073.1942 | 1 | 9.765 | 90.9% | 5 | K.LGAAAADAVTGR.T | 2 |
| \* | CENPL\_Noc300\_tube2\_122214\_01.09003.09003.3 | 4.5341 | 0.4284 | 100.0% | 2147.7544 | 2147.3958 | 1 | 7.634 | 36.8% | 1 | K.DRPVSQPSLVGSKEEPPPAR.S | 3 |
| \* | CENPL\_Noc300\_tube2\_122214\_01.12088.12088.2 | 4.4353 | 0.4068 | 100.0% | 1563.1921 | 1563.6152 | 1 | 8.439 | 83.3% | 1 | R.SQQQDDIEELETK.A | 2 |
|  | CENPL\_Noc300\_tube2\_122214\_01.09801.09801.2 | 2.2495 | 0.1811 | 97.7% | 1125.2922 | 1125.3252 | 29 | 4.708 | 62.5% | 1 | R.FKEEAEMLK.G | 2 |
|  | CENPL\_Noc300\_122214\_01.11780.11780.2 | 2.6033 | 0.2404 | 99.5% | 1001.03217 | 1001.21136 | 2 | 5.888 | 77.8% | 3 | K.IGDLGLATLK.R | 2 |
|  | CENPL\_Noc300\_122214\_01.10055.10055.2 | 2.9188 | 0.2214 | 99.5% | 1157.8922 | 1157.3988 | 5 | 5.363 | 70.0% | 1 | K.IGDLGLATLKR.A | 2 |
| \* | CENPL\_Noc300\_122214\_01.06262.06262.2 | 3.1422 | 0.3851 | 100.0% | 1236.2122 | 1236.4105 | 1 | 6.285 | 72.7% | 1 | R.VTSGVKPASFDK.V | 2 |
| \* | CENPL\_Noc300\_122214\_01.15559.15559.3 | 4.5251 | 0.4603 | 100.0% | 2268.2344 | 2268.534 | 1 | 7.888 | 37.5% | 1 | R.YSIKDLLNHAFFQEETGVR.V | 3 |
| \* | CENPL\_Noc300\_tube2\_122214\_01.18351.18351.3 | 4.0672 | 0.4622 | 100.0% | 2888.5144 | 2889.2993 | 1 | 7.42 | 28.7% | 1 | K.QPIPASSMPQQIGIPTSSLTQVVHSAGR.R | 3 |
| \* | CENPL\_Noc300\_122214\_01.10086.10086.2 | 2.7353 | 0.2906 | 99.8% | 1131.3922 | 1131.317 | 1 | 5.58 | 83.3% | 4 | R.FIVSPVPESR.L | 2 |
| \* | CENPL\_Noc300\_tube2\_122214\_01.15042.15042.2 | 2.6771 | 0.07 | 97.4% | 1211.2722 | 1211.317 | 2 | 4.18 | 77.8% | 1 | R.FIVS\*PVPESR.L | 2 |
| \* | CENPL\_Noc300\_tube2\_122214\_01.09844.09844.2 | 2.1072 | 0.199 | 97.6% | 829.8722 | 828.9408 | 5 | 4.08 | 78.6% | 3 | K.APGIDDIK.T | 2 |
| \* | CENPL\_Noc300\_tube2\_122214\_01.13211.13211.2 | 3.1685 | 0.2711 | 99.8% | 1430.4922 | 1429.6104 | 5 | 6.355 | 58.3% | 1 | K.APGIDDIKTLEEK.L | 2 |
| \* | CENPL\_Noc300\_122214\_01.15822.15822.3 | 3.8349 | 0.4299 | 100.0% | 4663.3145 | 4663.2847 | 1 | 7.77 | 19.2% | 1 | K.APVLPVGTELPAGTLPSEQLPPFPGPSLTQSQQPLEDLDAQLRR.T | 3 |
| \* | CENPL\_Noc300\_122214\_01.10574.10574.2 | 4.0141 | 0.4639 | 100.0% | 1421.4521 | 1420.6055 | 1 | 8.755 | 71.4% | 8 | K.EGPVLATSSGAGVFK.M | 2 |
| \* | CENPL\_Noc300\_tube2\_122214\_01.10744.10744.2 | 3.4731 | 0.411 | 100.0% | 1220.9722 | 1221.3556 | 1 | 7.434 | 77.3% | 7 | R.FQVSVAADGAQK.E | 2 |
| \* | CENPL\_Noc300\_122214\_02.07258.07258.2 | 3.9495 | 0.357 | 100.0% | 1535.7522 | 1535.697 | 1 | 6.099 | 64.3% | 2 | R.FQVSVAADGAQKEGK.N | 2 |
| \* | CENPL\_Noc300\_122214\_01.06348.06348.2 | 2.3409 | 0.1757 | 98.2% | 1010.27216 | 1010.135 | 120 | 4.078 | 56.2% | 2 | R.FQVTTTANK.V | 2 |
| \* | CENPL\_Noc300\_122214\_01.17706.17706.2 | 4.3736 | 0.5014 | 100.0% | 2406.5322 | 2406.8425 | 1 | 9.16 | 50.0% | 2 | K.EGPVASPPFMDLEQAVLPAVIPK.K | 2 |
| \* | CENPL\_Noc300\_122214\_01.18483.18483.2 | 3.5341 | 0.2656 | 99.9% | 2487.7522 | 2486.8425 | 1 | 5.34 | 40.9% | 2 | K.EGPVAS\*PPFMDLEQAVLPAVIPK.K | 2 |
| \* | CENPL\_Noc300\_tube2\_122214\_01.13521.13521.3 | 3.27 | 0.3803 | 99.9% | 2903.8442 | 2904.0764 | 1 | 5.815 | 37.0% | 1 | K.KEKPELSEPSHLNGPSS\*DPEAAFLSR.D | 3 |
| \* | CENPL\_Noc300\_tube2\_122214\_01.14194.14194.3 | 3.4512 | 0.2421 | 99.6% | 2696.7244 | 2695.9023 | 76 | 4.554 | 22.9% | 1 | K.EKPELSEPSHLNGPSSDPEAAFLSR.D | 3 |
| \* | CENPL\_Noc300\_122214\_01.06636.06636.2 | 2.6026 | 0.3741 | 99.8% | 1375.9922 | 1376.5522 | 19 | 5.447 | 60.0% | 1 | R.QKHEIESLYTK.L | 2 |
| \* | CENPL\_Noc300\_122214\_01.12127.12127.2 | 3.2688 | 0.3718 | 100.0% | 1556.5721 | 1555.904 | 1 | 6.307 | 53.3% | 9 | K.VPPAVIIPPAAPLSGR.R | 2 |
| \* | CENPL\_Noc300\_tube2\_122214\_01.10750.10750.2 | 2.126 | 0.1493 | 97.2% | 873.83215 | 873.9871 | 3 | 4.323 | 83.3% | 1 | K.LVDNWAR.D | 2 |
| \* | CENPL\_Noc300\_122214\_01.06768.06768.2 | 3.3598 | 0.5723 | 100.0% | 1319.9722 | 1320.4834 | 1 | 9.647 | 77.3% | 1 | K.GHMNYEGPGMAR.K | 2 |
| \* | CENPL\_Noc300\_122214\_01.06783.06783.2 | 2.3138 | 0.2038 | 97.7% | 1141.5521 | 1142.2571 | 3 | 4.841 | 70.0% | 1 | K.SISNPPGSNLR.T | 2 |

---

|  |  |  |  |  |  |  |  |  |
| --- | --- | --- | --- | --- | --- | --- | --- | --- |
| U | *gi|32698730|ref|NP\_06* | 7 | 18 | 14.1% | 695 | 76121 | 8.7 | nuclear fragile X mental retardation protein interacting protein 2 [Homo sapiens] |

| Filename XCorr DeltCN Conf% ObsM+H+ CalcM+H+ SpR ZScore Ion% # Sequence  | | | | | | | | | | | | |
| --- | --- | --- | --- | --- | --- | --- | --- | --- | --- | --- | --- | --- |
| \* | CENPL\_Noc300\_122214\_01.06910.06910.2 | 2.7551 | 0.1126 | 97.6% | 1337.9722 | 1338.3763 | 1 | 4.557 | 66.7% | 1 | K.TGYGELNGNAGER.E | 2 |
| \* | CENPL\_Noc300\_tube2\_122214\_01.09999.09999.2 | 3.9088 | 0.1608 | 99.9% | 1404.0721 | 1404.4764 | 1 | 7.409 | 70.8% | 3 | K.NLSSDEATNPISR.V | 2 |
| \* | CENPL\_Noc300\_tube2\_122214\_01.11157.11157.2 | 3.8976 | 0.5221 | 100.0% | 1515.5122 | 1515.7068 | 1 | 8.467 | 73.1% | 6 | R.VLNGNQQVVDTSLK.Q | 2 |
| \* | CENPL\_Noc300\_tube2\_122214\_01.11786.11786.2 | 3.7662 | 0.351 | 100.0% | 1376.3121 | 1375.6233 | 1 | 6.843 | 81.8% | 4 | K.IMQQETSVPTLK.Q | 2 |
| \* | CENPL\_Noc300\_tube2\_122214\_01.12718.12718.2 | 2.4924 | 0.2037 | 98.8% | 1074.6122 | 1074.3239 | 1 | 5.276 | 77.8% | 1 | R.LSQVPMSALK.S | 2 |
| \* | CENPL\_Noc300\_tube2\_122214\_01.13994.13994.3 | 3.0776 | 0.2442 | 98.7% | 2215.8245 | 2215.379 | 2 | 4.9 | 31.0% | 2 | K.SGTTSESGALSLEPSHIGDLQK.A | 3 |
| \* | CENPL\_Noc300\_122214\_01.12434.12434.2 | 3.1096 | 0.2001 | 99.5% | 1424.4521 | 1424.5939 | 1 | 5.764 | 53.8% | 1 | K.ADTSSQGALVFLSK.D | 2 |

---

|  |  |  |  |  |  |  |  |  |
| --- | --- | --- | --- | --- | --- | --- | --- | --- |
| U | *gi|119395754|ref|NP\_0* | 10 | 21 | 14.1% | 590 | 62378 | 7.8 | keratin 5 [Homo sapiens] |

| Filename XCorr DeltCN Conf% ObsM+H+ CalcM+H+ SpR ZScore Ion% # Sequence  | | | | | | | | | | | | |
| --- | --- | --- | --- | --- | --- | --- | --- | --- | --- | --- | --- | --- |
| \* | CENPL\_Noc300\_122214\_01.09154.09154.2 | 2.4972 | 0.4085 | 99.8% | 1112.0521 | 1112.2279 | 1 | 6.903 | 70.0% | 1 | R.ISISTSGGSFR.N | 2 |
|  | CENPL\_Noc300\_tube2\_122214\_01.13599.13599.2 | 2.3642 | 0.1974 | 99.3% | 827.9922 | 827.95544 | 1 | 5.382 | 100.0% | 5 | K.FASFIDK.V | 222222 |
|  | CENPL\_Noc300\_tube2\_122214\_01.13469.13469.2 | 2.5321 | 0.0875 | 97.5% | 1083.2122 | 1083.2755 | 2 | 6.11 | 81.2% | 2 | K.FASFIDKVR.F | 222222 |
|  | CENPL\_Noc300\_122214\_01.11215.11215.2 | 2.7607 | 0.3764 | 100.0% | 1204.1522 | 1204.3684 | 1 | 7.489 | 77.8% | 3 | K.WTLLQEQGTK.T | 22 |
|  | CENPL\_Noc300\_122214\_01.05936.05936.2 | 2.8336 | 0.2811 | 99.8% | 1309.1322 | 1309.4215 | 177 | 5.12 | 61.1% | 2 | K.NKYEDEINKR.T | 2222 |
|  | CENPL\_Noc300\_tube2\_122214\_01.20704.20704.2 | 4.3469 | 0.4382 | 100.0% | 1330.3522 | 1330.5211 | 1 | 8.484 | 86.4% | 3 | R.NLDLDSIIAEVK.A | 2222 |
|  | CENPL\_Noc300\_122214\_01.06727.06727.2 | 1.7568 | 0.3257 | 97.0% | 1093.5322 | 1094.1692 | 7 | 5.73 | 75.0% | 1 | K.AQYEEIANR.S | 2 |
|  | CENPL\_Noc300\_122214\_01.06414.06414.2 | 3.3225 | 0.298 | 100.0% | 1195.2322 | 1195.2743 | 1 | 7.405 | 83.3% | 1 | K.YEELQQTAGR.H | 2 |
|  | CENPL\_Noc300\_122214\_01.12967.12967.2 | 2.6979 | 0.4056 | 100.0% | 1264.4722 | 1264.4644 | 5 | 7.073 | 60.0% | 2 | K.LALDVEIATYR.K | 2222 |
|  | CENPL\_Noc300\_122214\_02.09616.09616.2 | 2.8991 | 0.271 | 99.7% | 1392.8121 | 1392.6384 | 12 | 5.484 | 59.1% | 1 | K.LALDVEIATYRK.L | 2222 |

Similarities:
gi|47132620|ref|NP\_00(5:5)  
gi|4504919|ref|NP\_002(3:7)  
gi|119395750|ref|NP\_0(1:9)  
gi|119703753|ref|NP\_0(7:3)  
gi|153791158|ref|NP\_0(5:5)  
gi|67782365|ref|NP\_00(2:8)  

---

|  |  |  |  |  |  |  |  |  |
| --- | --- | --- | --- | --- | --- | --- | --- | --- |
| U | *gi|9506543|ref|NP\_062* | 1 | 1 | 14.1% | 213 | 24086 | 7.8 | motile sperm domain containing 1 [Homo sapiens] |

| Filename XCorr DeltCN Conf% ObsM+H+ CalcM+H+ SpR ZScore Ion% # Sequence  | | | | | | | | | | | | |
| --- | --- | --- | --- | --- | --- | --- | --- | --- | --- | --- | --- | --- |
| \* | CENPL\_Noc300\_122214\_01.18063.18063.3 | 6.8069 | 0.4665 | 100.0% | 3491.6042 | 3491.925 | 1 | 6.643 | 29.3% | 1 | K.RQPELVEGNLPVFVFPTELIFYADDQSTHK.Q | 3 |

---

|  |  |  |  |  |  |  |  |  |
| --- | --- | --- | --- | --- | --- | --- | --- | --- |
| U | *gi|17986258|ref|NP\_06* | 2 | 4 | 13.9% | 151 | 16930 | 4.7 | myosin, light chain 6, alkali, smooth muscle and non-muscle isoform 1 [Homo sapiens] |
| U | *gi|88999583|ref|NP\_52* | 2 | 4 | 13.9% | 151 | 16961 | 4.6 | myosin, light chain 6, alkali, smooth muscle and non-muscle isoform 2 [Homo sapiens] |

| Filename XCorr DeltCN Conf% ObsM+H+ CalcM+H+ SpR ZScore Ion% # Sequence  | | | | | | | | | | | | |
| --- | --- | --- | --- | --- | --- | --- | --- | --- | --- | --- | --- | --- |
|  | CENPL\_Noc300\_tube2\_122214\_01.18095.18095.2 | 2.1288 | 0.1713 | 97.1% | 1025.6122 | 1026.1368 | 15 | 5.564 | 71.4% | 1 | K.EAFQLFDR.T | 2 |
|  | CENPL\_Noc300\_122214\_01.08183.08183.2 | 3.094 | 0.3532 | 100.0% | 1355.5721 | 1355.5339 | 1 | 5.367 | 66.7% | 3 | R.ALGQNPTNAEVLK.V | 2 |

---

|  |  |  |  |  |  |  |  |  |
| --- | --- | --- | --- | --- | --- | --- | --- | --- |
| U | *gi|87196351|ref|NP\_00* | 7 | 13 | 13.6% | 662 | 73244 | 7.2 | DEAD/H (Asp-Glu-Ala-Asp/His) box polypeptide 3 [Homo sapiens] |

| Filename XCorr DeltCN Conf% ObsM+H+ CalcM+H+ SpR ZScore Ion% # Sequence  | | | | | | | | | | | | |
| --- | --- | --- | --- | --- | --- | --- | --- | --- | --- | --- | --- | --- |
|  | CENPL\_Noc300\_122214\_01.07179.07179.2 | 2.4476 | 0.3197 | 99.8% | 791.97217 | 792.0122 | 3 | 6.008 | 83.3% | 1 | K.HAIPIIK.E | 2 |
| \* | CENPL\_Noc300\_122214\_01.19244.19244.2 | 5.2958 | 0.4948 | 100.0% | 2334.4722 | 2333.6897 | 1 | 8.367 | 54.8% | 1 | K.TAAFLLPILSQIYSDGPGEALR.A | 2 |
|  | CENPL\_Noc300\_122214\_02.09726.09726.2 | 2.2722 | 0.2759 | 98.7% | 1322.6721 | 1321.4729 | 5 | 5.515 | 65.0% | 1 | R.ELAVQIYEEAR.K | 2 |
|  | CENPL\_Noc300\_tube2\_122214\_01.18660.18660.2 | 3.7233 | 0.4411 | 100.0% | 1338.2322 | 1337.5946 | 1 | 7.031 | 85.0% | 3 | R.MLDMGFEPQIR.R | 22 |
|  | CENPL\_Noc300\_122214\_02.15692.15692.2 | 2.7521 | 0.2767 | 99.6% | 1558.1721 | 1558.774 | 6 | 5.673 | 50.0% | 2 | R.DFLDEYIFLAVGR.V | 2 |
|  | CENPL\_Noc300\_tube2\_122214\_01.14873.14873.2 | 3.0863 | 0.4211 | 100.0% | 1169.3722 | 1169.4099 | 1 | 8.124 | 72.7% | 4 | K.SPILVATAVAAR.G | 2 |
| \* | CENPL\_Noc300\_tube2\_122214\_01.19823.19823.2 | 2.5016 | 0.377 | 99.5% | 1524.9122 | 1525.7043 | 25 | 6.65 | 42.3% | 1 | R.VGNLGLATSFFNER.N | 2 |

Similarities:
gi|4758138|ref|NP\_004(1:6)  

---

|  |  |  |  |  |  |  |  |  |
| --- | --- | --- | --- | --- | --- | --- | --- | --- |
| U | *gi|21361380|ref|NP\_00* | 3 | 5 | 13.6% | 258 | 28864 | 6.7 | EAP30 subunit of ELL complex [Homo sapiens] |

| Filename XCorr DeltCN Conf% ObsM+H+ CalcM+H+ SpR ZScore Ion% # Sequence  | | | | | | | | | | | | |
| --- | --- | --- | --- | --- | --- | --- | --- | --- | --- | --- | --- | --- |
| \* | CENPL\_Noc300\_tube2\_122214\_01.14333.14333.2 | 3.628 | 0.4724 | 100.0% | 1491.2522 | 1491.6996 | 1 | 8.266 | 69.2% | 2 | R.GTVLAEDQLAQMSK.Q | 2 |
| \* | CENPL\_Noc300\_122214\_01.09019.09019.2 | 1.9504 | 0.3216 | 98.5% | 1039.2922 | 1039.1301 | 1 | 6.009 | 87.5% | 1 | K.TNLEEFASK.H | 2 |
| \* | CENPL\_Noc300\_tube2\_122214\_01.13278.13278.2 | 3.5692 | 0.4391 | 100.0% | 1408.1921 | 1407.523 | 1 | 6.492 | 72.7% | 2 | K.FAQDVSQDDLIR.A | 2 |

---

|  |  |  |  |  |  |  |  |  |
| --- | --- | --- | --- | --- | --- | --- | --- | --- |
| U | *gi|169161114|ref|XP\_0* | 2 | 2 | 13.6% | 184 | 21456 | 10.1 | PREDICTED: hypothetical protein isoform 1 [Homo sapiens] |
| U | *gi|78000186|ref|NP\_00* | 2 | 2 | 13.6% | 184 | 21397 | 10.2 | ribosomal protein L17 [Homo sapiens] |
| U | *gi|4506617|ref|NP\_000* | 2 | 2 | 13.6% | 184 | 21397 | 10.2 | ribosomal protein L17 [Homo sapiens] |
| U | *gi|169212979|ref|XP\_0* | 2 | 2 | 13.6% | 184 | 21397 | 10.2 | PREDICTED: hypothetical protein [Homo sapiens] |
| U | *gi|169161116|ref|XP\_0* | 2 | 2 | 13.6% | 184 | 21456 | 10.1 | PREDICTED: hypothetical protein isoform 2 [Homo sapiens] |

| Filename XCorr DeltCN Conf% ObsM+H+ CalcM+H+ SpR ZScore Ion% # Sequence  | | | | | | | | | | | | |
| --- | --- | --- | --- | --- | --- | --- | --- | --- | --- | --- | --- | --- |
|  | CENPL\_Noc300\_122214\_01.11665.11665.2 | 2.6898 | 0.2372 | 99.5% | 1316.8121 | 1317.6323 | 4 | 4.623 | 65.0% | 1 | K.KSAEFLLHMLK.N | 2 |
|  | CENPL\_Noc300\_tube2\_122214\_01.09449.09449.2 | 3.3282 | 0.3461 | 100.0% | 1624.5122 | 1624.8314 | 1 | 6.298 | 69.2% | 1 | K.EQIVPKPEEEVAQK.K | 2 |

---

|  |  |  |  |  |  |  |  |  |
| --- | --- | --- | --- | --- | --- | --- | --- | --- |
| U | *gi|20070130|ref|NP\_00* | 1 | 1 | 13.6% | 162 | 17699 | 7.5 | basic transcription factor 3 isoform B [Homo sapiens] |
| U | *gi|83641885|ref|NP\_00* | 1 | 1 | 10.7% | 206 | 22168 | 9.4 | basic transcription factor 3 isoform A [Homo sapiens] |

| Filename XCorr DeltCN Conf% ObsM+H+ CalcM+H+ SpR ZScore Ion% # Sequence  | | | | | | | | | | | | |
| --- | --- | --- | --- | --- | --- | --- | --- | --- | --- | --- | --- | --- |
|  | CENPL\_Noc300\_122214\_01.18641.18641.2 | 2.8185 | 0.2342 | 99.2% | 2403.5723 | 2401.7808 | 28 | 4.155 | 28.6% | 1 | K.QLTEMLPSILNQLGADSLTSLR.R | 2 |

---

|  |  |  |  |  |  |  |  |  |
| --- | --- | --- | --- | --- | --- | --- | --- | --- |
| U | *Reverse\_gi|169168848|* | 1 | 1 | 13.6% | 118 | 12914 | 8.5 | PREDICTED: hypothetical protein [Homo sapiens] |
| U | *Reverse\_gi|169169431|* | 1 | 1 | 13.6% | 118 | 12914 | 8.5 | PREDICTED: hypothetical protein [Homo sapiens] |
| U | *Reverse\_gi|169169107|* | 1 | 1 | 13.6% | 118 | 12914 | 8.5 | PREDICTED: hypothetical protein [Homo sapiens] |

| Filename XCorr DeltCN Conf% ObsM+H+ CalcM+H+ SpR ZScore Ion% # Sequence  | | | | | | | | | | | | |
| --- | --- | --- | --- | --- | --- | --- | --- | --- | --- | --- | --- | --- |
|  | CENPL\_Noc300\_tube2\_122214\_01.17189.17189.2 | 2.6326 | 0.2292 | 98.7% | 1779.2522 | 1780.9849 | 7 | 4.301 | 36.7% | 1 | R.ANVSCQLFATEGLLTR.S | 2 |

---

|  |  |  |  |  |  |  |  |  |
| --- | --- | --- | --- | --- | --- | --- | --- | --- |
| U | *gi|169211725|ref|XP\_9* | 1 | 3 | 13.6% | 88 | 9949 | 11.3 | PREDICTED: similar to 40S ribosomal protein S28 [Homo sapiens] |
| U | *gi|4506715|ref|NP\_001* | 1 | 3 | 17.4% | 69 | 7841 | 10.7 | ribosomal protein S28 [Homo sapiens] |
| U | *gi|169212081|ref|XP\_0* | 1 | 3 | 13.2% | 91 | 10272 | 11.2 | PREDICTED: similar to 40S ribosomal protein S28 [Homo sapiens] |

| Filename XCorr DeltCN Conf% ObsM+H+ CalcM+H+ SpR ZScore Ion% # Sequence  | | | | | | | | | | | | |
| --- | --- | --- | --- | --- | --- | --- | --- | --- | --- | --- | --- | --- |
|  | CENPL\_Noc300\_tube2\_122214\_01.19821.19821.2 | 2.8199 | 0.4538 | 100.0% | 1361.9122 | 1361.4918 | 1 | 7.21 | 63.6% | 3 | R.EGDVLTLLESER.E | 2 |

---

|  |  |  |  |  |  |  |  |  |
| --- | --- | --- | --- | --- | --- | --- | --- | --- |
| U | *gi|4759098|ref|NP\_004* | 3 | 4 | 13.5% | 288 | 33666 | 11.2 | splicing factor, arginine/serine-rich 10 [Homo sapiens] |

| Filename XCorr DeltCN Conf% ObsM+H+ CalcM+H+ SpR ZScore Ion% # Sequence  | | | | | | | | | | | | |
| --- | --- | --- | --- | --- | --- | --- | --- | --- | --- | --- | --- | --- |
| \* | CENPL\_Noc300\_122214\_01.12570.12570.2 | 4.38 | 0.5731 | 100.0% | 1812.0922 | 1811.989 | 1 | 9.661 | 63.3% | 1 | K.YGPIADVSIVYDQQSR.R | 2 |
| \* | CENPL\_Noc300\_122214\_02.12424.12424.2 | 4.1901 | 0.5518 | 100.0% | 1622.4922 | 1622.774 | 1 | 9.153 | 69.2% | 2 | R.GFAFVYFENVDDAK.E | 2 |
| \* | CENPL\_Noc300\_122214\_01.10222.10222.2 | 2.0872 | 0.2134 | 97.2% | 1079.8322 | 1079.2847 | 1 | 5.08 | 75.0% | 1 | R.IRVDFSITK.R | 2 |

---

|  |  |  |  |  |  |  |  |  |
| --- | --- | --- | --- | --- | --- | --- | --- | --- |
| U | *gi|34419635|ref|NP\_00* | 9 | 30 | 13.4% | 643 | 71028 | 6.1 | heat shock 70kDa protein 6 (HSP70B') [Homo sapiens] |

| Filename XCorr DeltCN Conf% ObsM+H+ CalcM+H+ SpR ZScore Ion% # Sequence  | | | | | | | | | | | | |
| --- | --- | --- | --- | --- | --- | --- | --- | --- | --- | --- | --- | --- |
|  | CENPL\_Noc300\_122214\_01.10323.10323.2 | 3.3577 | 0.4739 | 100.0% | 1488.4722 | 1488.5939 | 1 | 8.67 | 79.2% | 6 | R.TTPSYVAFTDTER.L | 2222 |
|  | CENPL\_Noc300\_tube2\_122214\_01.18592.18592.2 | 5.2641 | 0.5029 | 100.0% | 1688.5721 | 1688.9213 | 1 | 10.235 | 83.3% | 5 | R.IINEPTAAAIAYGLDR.R | 22 |
|  | CENPL\_Noc300\_tube2\_122214\_01.18629.18629.3 | 3.6013 | 0.2861 | 99.9% | 1688.8744 | 1688.9213 | 2 | 6.1 | 48.3% | 1 | R.IINEPTAAAIAYGLDR.R | 33 |
|  | CENPL\_Noc300\_122214\_01.07337.07337.2 | 4.5821 | 0.5001 | 100.0% | 1676.2322 | 1676.6964 | 1 | 8.059 | 70.0% | 3 | K.ATAGDTHLGGEDFDNR.L | 222 |
|  | CENPL\_Noc300\_122214\_01.07385.07385.3 | 3.5425 | 0.4619 | 100.0% | 1676.5144 | 1676.6964 | 1 | 7.623 | 51.7% | 5 | K.ATAGDTHLGGEDFDNR.L | 333 |
|  | CENPL\_Noc300\_122214\_01.13409.13409.2 | 2.2018 | 0.2641 | 98.5% | 1316.6322 | 1316.4193 | 4 | 4.839 | 66.7% | 1 | R.FEELCSDLFR.S | 22 |
|  | CENPL\_Noc300\_tube2\_122214\_01.18572.18572.2 | 3.0614 | 0.3609 | 100.0% | 1082.0922 | 1082.2444 | 1 | 6.351 | 81.2% | 6 | K.LLQDFFNGK.E | 22 |
|  | CENPL\_Noc300\_tube2\_122214\_01.17592.17592.2 | 3.4331 | 0.2156 | 99.8% | 1566.1322 | 1566.7972 | 1 | 5.244 | 66.7% | 2 | K.LLQDFFNGKELNK.S | 22 |
| \* | CENPL\_Noc300\_tube2\_122214\_01.18924.18924.2 | 3.0335 | 0.0117 | 95.1% | 1801.9521 | 1801.8334 | 19 | 3.397 | 41.2% | 1 | R.LYGGPGVPGGS\*SCGTQAR.Q | 2 |

Similarities:
gi|5729877|ref|NP\_006(3:6)  
gi|167466173|ref|NP\_0(6:3)  
gi|124256496|ref|NP\_0(3:6)  

---

|  |  |  |  |  |  |  |  |  |
| --- | --- | --- | --- | --- | --- | --- | --- | --- |
| U | *gi|4506619|ref|NP\_000* | 2 | 4 | 13.4% | 157 | 17779 | 11.3 | ribosomal protein L24 [Homo sapiens] |

| Filename XCorr DeltCN Conf% ObsM+H+ CalcM+H+ SpR ZScore Ion% # Sequence  | | | | | | | | | | | | |
| --- | --- | --- | --- | --- | --- | --- | --- | --- | --- | --- | --- | --- |
| \* | CENPL\_Noc300\_tube2\_122214\_01.17160.17160.2 | 2.436 | 0.3466 | 99.8% | 967.1322 | 967.15576 | 1 | 6.05 | 78.6% | 1 | K.VFQFLNAK.C | 2 |
| \* | CENPL\_Noc300\_tube2\_122214\_01.18113.18113.2 | 4.3039 | 0.478 | 100.0% | 1262.4321 | 1262.5072 | 1 | 9.758 | 79.2% | 3 | R.AITGASLADIMAK.R | 2 |

---

|  |  |  |  |  |  |  |  |  |
| --- | --- | --- | --- | --- | --- | --- | --- | --- |
| U | *gi|169188544|ref|XP\_0* | 1 | 1 | 13.3% | 173 | 17576 | 11.8 | PREDICTED: hypothetical protein [Homo sapiens] |
| U | *gi|169201741|ref|XP\_0* | 1 | 1 | 13.3% | 173 | 17576 | 11.8 | PREDICTED: hypothetical protein [Homo sapiens] |
| U | *gi|169201345|ref|XP\_0* | 1 | 1 | 13.3% | 173 | 17576 | 11.8 | PREDICTED: hypothetical protein [Homo sapiens] |

| Filename XCorr DeltCN Conf% ObsM+H+ CalcM+H+ SpR ZScore Ion% # Sequence  | | | | | | | | | | | | |
| --- | --- | --- | --- | --- | --- | --- | --- | --- | --- | --- | --- | --- |
|  | CENPL\_Noc300\_122214\_02.14333.14333.2 | 2.0682 | 0.2613 | 96.2% | 2341.5723 | 2340.6633 | 38 | 4.489 | 27.3% | 1 | R.RVCVRASVAGARPSVAGVRGSAR.G | 2 |

---

|  |  |  |  |  |  |  |  |  |
| --- | --- | --- | --- | --- | --- | --- | --- | --- |
| U | *gi|169212674|ref|XP\_0* | 1 | 2 | 13.1% | 130 | 14502 | 11.4 | PREDICTED: hypothetical protein [Homo sapiens] |
| U | *gi|169213082|ref|XP\_0* | 1 | 2 | 13.1% | 130 | 14502 | 11.4 | PREDICTED: hypothetical protein [Homo sapiens] |
| U | *gi|169212885|ref|XP\_0* | 1 | 2 | 13.1% | 130 | 14502 | 11.4 | PREDICTED: hypothetical protein [Homo sapiens] |

| Filename XCorr DeltCN Conf% ObsM+H+ CalcM+H+ SpR ZScore Ion% # Sequence  | | | | | | | | | | | | |
| --- | --- | --- | --- | --- | --- | --- | --- | --- | --- | --- | --- | --- |
|  | CENPL\_Noc300\_tube2\_122214\_01.17942.17942.2 | 2.9175 | 0.058 | 96.5% | 1944.3922 | 1945.3218 | 4 | 3.275 | 40.6% | 2 | R.LHRQPLSVARQAVLVTR.A | 2 |

---

|  |  |  |  |  |  |  |  |  |
| --- | --- | --- | --- | --- | --- | --- | --- | --- |
| U | *gi|4758138|ref|NP\_004* | 7 | 16 | 12.9% | 614 | 69148 | 8.9 | DEAD (Asp-Glu-Ala-Asp) box polypeptide 5 [Homo sapiens] |

| Filename XCorr DeltCN Conf% ObsM+H+ CalcM+H+ SpR ZScore Ion% # Sequence  | | | | | | | | | | | | |
| --- | --- | --- | --- | --- | --- | --- | --- | --- | --- | --- | --- | --- |
| \* | CENPL\_Noc300\_tube2\_122214\_01.15575.15575.2 | 3.8626 | 0.4987 | 100.0% | 1296.4521 | 1296.4198 | 1 | 8.575 | 85.0% | 2 | R.TTYLVLDEADR.M | 2 |
|  | CENPL\_Noc300\_tube2\_122214\_01.18660.18660.2 | 3.7233 | 0.4411 | 100.0% | 1338.2322 | 1337.5946 | 1 | 7.031 | 85.0% | 3 | R.MLDMGFEPQIR.K | 22 |
|  | CENPL\_Noc300\_tube2\_122214\_01.19313.19313.2 | 2.7353 | 0.4502 | 100.0% | 1349.4521 | 1349.5902 | 1 | 6.155 | 75.0% | 1 | R.QTLMWSATWPK.E | 2 |
| \* | CENPL\_Noc300\_122214\_01.08615.08615.2 | 3.114 | 0.0903 | 98.8% | 1481.9122 | 1481.7194 | 4 | 4.368 | 63.6% | 1 | R.LMEEIMSEKENK.T | 2 |
|  | CENPL\_Noc300\_122214\_01.10961.10961.2 | 3.9611 | 0.2494 | 100.0% | 1227.3522 | 1227.4465 | 1 | 7.564 | 81.8% | 5 | K.APILIATDVASR.G | 2 |
| \* | CENPL\_Noc300\_tube2\_122214\_01.17976.17976.2 | 3.5468 | 0.4129 | 100.0% | 1575.2522 | 1575.7612 | 1 | 7.176 | 65.4% | 2 | K.TGTAYTFFTPNNIK.Q | 2 |
| \* | CENPL\_Noc300\_122214\_01.10571.10571.2 | 2.5644 | 0.2372 | 99.5% | 986.21216 | 986.1564 | 1 | 4.205 | 78.6% | 2 | K.LLQLVEDR.G | 2 |

Similarities:
gi|87196351|ref|NP\_00(1:6)  

---

|  |  |  |  |  |  |  |  |  |
| --- | --- | --- | --- | --- | --- | --- | --- | --- |
| U | *gi|13443018|ref|NP\_07* | 1 | 1 | 12.9% | 132 | 15270 | 8.0 | U11/U12 snRNP 25K protein [Homo sapiens] |

| Filename XCorr DeltCN Conf% ObsM+H+ CalcM+H+ SpR ZScore Ion% # Sequence  | | | | | | | | | | | | |
| --- | --- | --- | --- | --- | --- | --- | --- | --- | --- | --- | --- | --- |
| \* | CENPL\_Noc300\_tube2\_122214\_01.14997.14997.2 | 2.8336 | 0.0886 | 96.8% | 2217.4321 | 2218.2126 | 5 | 3.724 | 46.9% | 1 | R.T#Y@HLT#SAGEKLTEDRKK.L | 2 |

---

|  |  |  |  |  |  |  |  |  |
| --- | --- | --- | --- | --- | --- | --- | --- | --- |
| U | *gi|5031887|ref|NP\_005* | 5 | 8 | 12.7% | 612 | 65746 | 7.4 | LIM domain containing preferred translocation partner in lipoma [Homo sapiens] |

| Filename XCorr DeltCN Conf% ObsM+H+ CalcM+H+ SpR ZScore Ion% # Sequence  | | | | | | | | | | | | |
| --- | --- | --- | --- | --- | --- | --- | --- | --- | --- | --- | --- | --- |
| \* | CENPL\_Noc300\_122214\_01.06421.06421.2 | 2.42 | 0.2644 | 98.9% | 1221.3722 | 1221.3585 | 3 | 5.007 | 63.6% | 1 | K.STGEPLGHVPAR.M | 2 |
| \* | CENPL\_Noc300\_tube2\_122214\_01.10114.10114.3 | 6.4577 | 0.4824 | 100.0% | 2402.8743 | 2402.6843 | 1 | 8.01 | 44.0% | 2 | R.METTHSFGNPSISVSTQQPPKK.F | 3 |
| \* | CENPL\_Noc300\_tube2\_122214\_01.13499.13499.2 | 2.5485 | 0.3054 | 99.5% | 1410.1921 | 1410.7148 | 1 | 5.208 | 58.3% | 2 | R.MVIPNQPPLTATK.K | 2 |
| \* | CENPL\_Noc300\_122214\_01.09537.09537.2 | 4.7791 | 0.6186 | 100.0% | 1644.1721 | 1644.7399 | 1 | 10.608 | 82.1% | 2 | R.YYEGYYAAGPGYGGR.N | 2 |
| \* | CENPL\_Noc300\_tube2\_122214\_01.18869.18869.2 | 4.2529 | 0.462 | 100.0% | 1948.5322 | 1949.1598 | 1 | 7.913 | 63.3% | 1 | K.MLYDMENPPADEYFGR.C | 2 |

---

|  |  |  |  |  |  |  |  |  |
| --- | --- | --- | --- | --- | --- | --- | --- | --- |
| U | *Reverse\_gi|116642878|* | 1 | 1 | 12.7% | 166 | 17239 | 11.3 | hypothetical protein LOC400223 [Homo sapiens] |

| Filename XCorr DeltCN Conf% ObsM+H+ CalcM+H+ SpR ZScore Ion% # Sequence  | | | | | | | | | | | | |
| --- | --- | --- | --- | --- | --- | --- | --- | --- | --- | --- | --- | --- |
| \* | CENPL\_Noc300\_122214\_02.09748.09748.3 | 3.1156 | 0.2506 | 99.4% | 2087.1543 | 2088.1558 | 34 | 4.5 | 26.2% | 1 | R.VRAT#GLS\*AAAEGASGSGVPLR.H | 3 |

---

|  |  |  |  |  |  |  |  |  |
| --- | --- | --- | --- | --- | --- | --- | --- | --- |
| U | *gi|13128970|ref|NP\_07* | 1 | 1 | 12.7% | 102 | 12266 | 11.5 | mitochondrial ribosomal protein 63 [Homo sapiens] |

| Filename XCorr DeltCN Conf% ObsM+H+ CalcM+H+ SpR ZScore Ion% # Sequence  | | | | | | | | | | | | |
| --- | --- | --- | --- | --- | --- | --- | --- | --- | --- | --- | --- | --- |
| \* | CENPL\_Noc300\_tube2\_122214\_01.15285.15285.2 | 2.4935 | 0.2228 | 98.5% | 1514.4521 | 1514.7214 | 34 | 4.902 | 50.0% | 1 | R.FIADQLDHLNVTK.K | 2 |

---

|  |  |  |  |  |  |  |  |  |
| --- | --- | --- | --- | --- | --- | --- | --- | --- |
| U | *gi|163965362|ref|NP\_0* | 2 | 4 | 12.6% | 215 | 23384 | 4.6 | nascent polypeptide-associated complex alpha subunit isoform b [Homo sapiens] |
| U | *gi|5031931|ref|NP\_005* | 2 | 4 | 12.6% | 215 | 23384 | 4.6 | nascent polypeptide-associated complex alpha subunit isoform b [Homo sapiens] |
| U | *gi|163965366|ref|NP\_0* | 2 | 4 | 1.3% | 2078 | 205419 | 9.6 | nascent polypeptide-associated complex alpha subunit isoform a [Homo sapiens] |
| U | *gi|163965364|ref|NP\_0* | 2 | 4 | 12.6% | 215 | 23384 | 4.6 | nascent polypeptide-associated complex alpha subunit isoform b [Homo sapiens] |

| Filename XCorr DeltCN Conf% ObsM+H+ CalcM+H+ SpR ZScore Ion% # Sequence  | | | | | | | | | | | | |
| --- | --- | --- | --- | --- | --- | --- | --- | --- | --- | --- | --- | --- |
|  | CENPL\_Noc300\_tube2\_122214\_01.18741.18741.2 | 2.7809 | 0.3969 | 99.9% | 1550.3722 | 1550.8816 | 1 | 6.456 | 70.8% | 2 | K.NILFVITKPDVYK.S | 2 |
|  | CENPL\_Noc300\_122214\_01.11903.11903.2 | 3.2589 | 0.5472 | 100.0% | 1485.1921 | 1485.6335 | 1 | 8.422 | 65.4% | 2 | K.SPASDTYIVFGEAK.I | 2 |

---

|  |  |  |  |  |  |  |  |  |
| --- | --- | --- | --- | --- | --- | --- | --- | --- |
| U | *Reverse\_gi|59859880|r* | 1 | 1 | 12.4% | 137 | 15622 | 7.7 | baculoviral IAP repeat-containing protein 5 isoform 2 [Homo sapiens] |

| Filename XCorr DeltCN Conf% ObsM+H+ CalcM+H+ SpR ZScore Ion% # Sequence  | | | | | | | | | | | | |
| --- | --- | --- | --- | --- | --- | --- | --- | --- | --- | --- | --- | --- |
| \* | CENPL\_Noc300\_122214\_02.00380.00380.3 | 2.554 | 0.2447 | 95.5% | 2034.3844 | 2036.0851 | 10 | 4.182 | 34.4% | 1 | R.GSAEIWPLWS\*S\*SPVACK.R | 3 |

---

|  |  |  |  |  |  |  |  |  |
| --- | --- | --- | --- | --- | --- | --- | --- | --- |
| U | *gi|224586804|ref|NP\_0* | 1 | 1 | 12.2% | 147 | 16860 | 4.7 | polymerase (DNA directed), epsilon 3 (p17 subunit) [Homo sapiens] |

| Filename XCorr DeltCN Conf% ObsM+H+ CalcM+H+ SpR ZScore Ion% # Sequence  | | | | | | | | | | | | |
| --- | --- | --- | --- | --- | --- | --- | --- | --- | --- | --- | --- | --- |
| \* | CENPL\_Noc300\_122214\_02.14055.14055.2 | 4.6823 | 0.5521 | 100.0% | 2071.392 | 2072.3125 | 1 | 10.897 | 61.8% | 1 | K.TLNASDVLSAMEEMEFQR.F | 2 |

---

|  |  |  |  |  |  |  |  |  |
| --- | --- | --- | --- | --- | --- | --- | --- | --- |
| U | *gi|225690491|ref|NP\_0* | 6 | 7 | 12.1% | 547 | 60721 | 5.9 | tight junction associated protein 1 isoform b [Homo sapiens] |
| U | *gi|225690497|ref|NP\_0* | 6 | 7 | 12.1% | 547 | 60721 | 5.9 | tight junction associated protein 1 isoform b [Homo sapiens] |
| U | *gi|225690495|ref|NP\_0* | 6 | 7 | 12.1% | 547 | 60721 | 5.9 | tight junction associated protein 1 isoform b [Homo sapiens] |
| U | *gi|225690493|ref|NP\_5* | 6 | 7 | 12.1% | 547 | 60721 | 5.9 | tight junction associated protein 1 isoform b [Homo sapiens] |

| Filename XCorr DeltCN Conf% ObsM+H+ CalcM+H+ SpR ZScore Ion% # Sequence  | | | | | | | | | | | | |
| --- | --- | --- | --- | --- | --- | --- | --- | --- | --- | --- | --- | --- |
|  | CENPL\_Noc300\_tube2\_122214\_01.14866.14866.3 | 3.3684 | 0.2682 | 99.9% | 1682.8444 | 1682.9146 | 1 | 5.67 | 42.3% | 1 | R.TNQELEDKLHTLIK.K | 3 |
|  | CENPL\_Noc300\_122214\_01.08822.08822.2 | 2.6051 | 0.2104 | 98.9% | 1472.2722 | 1473.6261 | 1 | 5.463 | 59.1% | 1 | K.NTINKLEELNER.Y | 2 |
|  | CENPL\_Noc300\_tube2\_122214\_01.09508.09508.3 | 3.4329 | 0.2991 | 100.0% | 1379.7544 | 1378.5712 | 5 | 6.13 | 50.0% | 1 | R.RVIEFSEDKVR.I | 3 |
|  | CENPL\_Noc300\_tube2\_122214\_01.10626.10626.2 | 2.7845 | 0.1916 | 99.5% | 1222.4922 | 1222.3837 | 1 | 6.554 | 72.2% | 1 | R.VIEFSEDKVR.I | 2 |
|  | CENPL\_Noc300\_122214\_02.09992.09992.2 | 3.5958 | 0.5639 | 100.0% | 1518.3922 | 1518.6641 | 1 | 9.56 | 61.5% | 1 | R.QAISLSLVEEGSER.A | 2 |
|  | CENPL\_Noc300\_tube2\_122214\_01.10121.10121.3 | 2.7864 | 0.2624 | 99.5% | 1678.3143 | 1676.8295 | 1 | 5.77 | 51.8% | 2 | R.AFVDRT#PPPAAVAQR.T | 3 |

---

|  |  |  |  |  |  |  |  |  |
| --- | --- | --- | --- | --- | --- | --- | --- | --- |
| U | *Reverse\_gi|169170762|* | 1 | 1 | 12.0% | 316 | 35163 | 9.4 | PREDICTED: hypothetical protein [Homo sapiens] |
| U | *Reverse\_gi|169171962|* | 1 | 1 | 12.0% | 316 | 35163 | 9.4 | PREDICTED: hypothetical protein [Homo sapiens] |
| U | *Reverse\_gi|169171538|* | 1 | 1 | 12.0% | 316 | 35163 | 9.4 | PREDICTED: hypothetical protein [Homo sapiens] |
[truncated: 483,397 more chars]
