# Supplementary material for: Astrin-SKAP complex reconstitution reveals its kinetochore interaction with microtubule-bound Ndc80
Source: eLife. 2017 Aug 25;6:e26866. doi: 10.7554/eLife.26866 (PMC5602300; doi:10.7554/eLife.26866)
Supplement: Source data 1. — Complete mass spectrometry searches using methods described in (Washburn et al., 2001) for affinity purification/mass spectrometry data sets described in this paper (data from this study; [Kern et al., 2016] [Gascoigne et al., 2011]). Individual Astrin cross-linking immunoprecipitations are listed based on the order in Figure 4—figure supplement 1. These samples have not been pruned for common or antibody-specific contaminants. [file elife-26866-data1.zip › Mis12_CrosslinkingIP.html]

D LAPMis12DANCE
DTASelect v2.0.21  
/nfs/cheeseman\_massspec/David/LAPMis12DANCE  
/nfs/cheeseman\_massspec/Databases/NCBI-RefSeq\_human\_na\_04-13-2009\_con\_reversed.fasta  
SEQUEST 3.0 in SQT format.  
-p 1  
 Jump  to the summary table.  
  
sequest.params modifications:

|  |  |  |
| --- | --- | --- |
| \* | S | 80.0 |
| # | T | 80.0 |
| @ | K | 12.0 |
| Static | C | 57.0 |

|  |  |
| --- | --- |
| true | Use criteria |
| 0.0 | Minimum peptide confidence |
| 0.05 | Peptide false positive rate |
| 0.0 | Minimum protein confidence |
| 1.0 | Protein false positive rate |
| 1 | Minimum charge state |
| 16 | Maximum charge state |
| 0.0 | Minimum ion proportion |
| 1000 | Maximum Sp rank |
| -1.0 | Minimum Sp score |
| Include | Modified peptide inclusion |
| Any | Tryptic status requirement |
| false | Multiple, ambiguous IDs allowed |
| Ignore | Peptide validation handling |
| XCorr | Purge duplicate peptides by protein |
| false | Include only loci with unique peptide |
| true | Remove subset proteins |
| Ignore | Locus validation handling |
| 0 | Minimum modified peptides per locus |
| 1000 | Minimum redundancy for low coverage loci |
| 1 | Minimum peptides per locus |

#### Locus Key:

|  |  |  |  |  |  |  |  |  |
| --- | --- | --- | --- | --- | --- | --- | --- | --- |
| Validation Status | Locus | Sequence Count | Spectrum Count | Sequence Coverage | Length | MolWt | pI | Descriptive Name |

#### Similarity Key:

|  |  |  |
| --- | --- | --- |
| Locus | # of identical peptides | # of differing peptides |

---

|  |  |  |  |  |  |  |  |  |
| --- | --- | --- | --- | --- | --- | --- | --- | --- |
| U | *gi|88900509|ref|NP\_00* | 26 | 93 | 77.1% | 205 | 23339 | 5.5 | polyamine-modulated factor 1 [Homo sapiens] |

| Filename XCorr DeltCN Conf% ObsM+H+ CalcM+H+ SpR ZScore Ion% # Sequence  | | | | | | | | | | | | |
| --- | --- | --- | --- | --- | --- | --- | --- | --- | --- | --- | --- | --- |
| \* | LAPMis12DANCE\_011813\_01.03921.03921.2 | 5.5565 | 0.5206 | 100.0% | 1884.2722 | 1885.0006 | 1 | 8.814 | 64.7% | 21 | K.RHEGSSSESVPPGTTISR.V | 2 |
| \* | LAPMis12DANCE2\_011813\_01.04018.04018.2 | 4.9843 | 0.5078 | 100.0% | 1728.2522 | 1728.8131 | 1 | 9.679 | 65.6% | 3 | R.HEGSSSESVPPGTTISR.V | 2 |
| \* | LAPMis12DANCE\_011813\_01.12647.12647.2 | 4.0775 | 0.2368 | 100.0% | 1653.3522 | 1652.0021 | 1 | 5.632 | 76.9% | 3 | R.VKLLDTMVDTFLQK.L | 2 |
| \* | LAPMis12DANCE\_011813\_01.15522.15522.3 | 5.1723 | 0.3972 | 100.0% | 2597.0942 | 2598.076 | 1 | 6.985 | 35.2% | 1 | R.VKLLDTMVDTFLQKLVAAGSYQR.F | 3 |
| \* | LAPMis12DANCE2\_011813\_01.10503.10503.2 | 4.254 | 0.4608 | 100.0% | 1424.2922 | 1424.6954 | 1 | 8.282 | 86.4% | 7 | K.LLDTMVDTFLQK.L | 2 |
| \* | LAPMis12DANCE2\_011813\_01.14140.14140.2 | 3.4699 | 0.4028 | 100.0% | 2370.632 | 2370.7693 | 4 | 6.02 | 37.5% | 1 | K.LLDTMVDTFLQKLVAAGSYQR.F | 2 |
| \* | LAPMis12DANCE\_011813\_01.04079.04079.2 | 2.3025 | 0.3456 | 100.0% | 964.4122 | 965.0971 | 1 | 6.34 | 81.2% | 1 | K.LVAAGSYQR.F | 2 |
| \* | LAPMis12DANCE\_011813\_01.09149.09149.2 | 3.0701 | 0.2586 | 100.0% | 1177.5521 | 1177.3892 | 1 | 5.408 | 94.4% | 4 | K.FIAQLQTSIR.E | 2 |
| \* | LAPMis12DANCE\_011813\_02.10132.10132.3 | 5.7766 | 0.3826 | 100.0% | 3487.1943 | 3488.917 | 1 | 7.139 | 22.5% | 2 | K.FIAQLQTSIREEISDIKEEGNLEAVLNALDK.I | 3 |
| \* | LAPMis12DANCE2\_011813\_01.15039.15039.3 | 4.6257 | 0.5287 | 100.0% | 4144.344 | 4144.666 | 1 | 8.313 | 27.8% | 1 | K.FIAQLQTSIREEISDIKEEGNLEAVLNALDKIVEEGK.V | 3 |
| \* | LAPMis12DANCE\_011813\_02.08649.08649.3 | 3.6802 | 0.329 | 99.7% | 2331.5942 | 2330.551 | 105 | 5.911 | 26.2% | 2 | R.EEISDIKEEGNLEAVLNALDK.I | 3 |
| \* | LAPMis12DANCE2\_011813\_01.13362.13362.3 | 6.6821 | 0.5912 | 100.0% | 2984.2144 | 2986.3 | 1 | 9.588 | 33.7% | 4 | R.EEISDIKEEGNLEAVLNALDKIVEEGK.V | 3 |
| \* | LAPMis12DANCE\_011813\_01.10923.10923.3 | 5.9285 | 0.3838 | 100.0% | 3182.8442 | 3182.6702 | 1 | 6.994 | 37.5% | 2 | R.KEPAWRPSGIPEKDLHSVMAPYFLQQR.D | 3 |
| \* | LAPMis12DANCE2\_011813\_01.09564.09564.3 | 3.7041 | 0.2304 | 96.9% | 3054.1143 | 3054.496 | 15 | 5.283 | 26.0% | 1 | K.EPAWRPSGIPEKDLHSVMAPYFLQQR.D | 3 |
| \* | LAPMis12DANCE\_011813\_01.11289.11289.2 | 3.7407 | 0.2268 | 100.0% | 1705.9722 | 1705.972 | 15 | 5.611 | 50.0% | 1 | K.DLHSVMAPYFLQQR.D | 2 |
| \* | LAPMis12DANCE\_011813\_01.07232.07232.3 | 4.8189 | 0.4251 | 100.0% | 2362.3743 | 2362.6108 | 1 | 7.625 | 37.5% | 3 | R.RHVQKQEAENQQLADAVLAGR.R | 3 |
| \* | LAPMis12DANCE\_011813\_01.07870.07870.2 | 5.768 | 0.644 | 100.0% | 2205.392 | 2206.4233 | 1 | 13.035 | 68.4% | 1 | R.HVQKQEAENQQLADAVLAGR.R | 2 |
| \* | LAPMis12DANCE\_011813\_01.07928.07928.3 | 6.251 | 0.5183 | 100.0% | 2206.6743 | 2206.4233 | 1 | 9.0 | 36.8% | 8 | R.HVQKQEAENQQLADAVLAGR.R | 3 |
| \* | LAPMis12DANCE\_011813\_01.07166.07166.3 | 4.1167 | 0.4406 | 100.0% | 2362.5842 | 2362.6108 | 6 | 7.06 | 26.2% | 1 | R.HVQKQEAENQQLADAVLAGRR.Q | 3 |
| \* | LAPMis12DANCE\_011813\_01.09806.09806.2 | 4.7855 | 0.4402 | 100.0% | 1712.7522 | 1713.8448 | 1 | 7.986 | 70.0% | 6 | K.QEAENQQLADAVLAGR.R | 2 |
| \* | LAPMis12DANCE\_011813\_02.06765.06765.3 | 7.6042 | 0.4257 | 100.0% | 2688.5044 | 2689.009 | 1 | 8.986 | 45.2% | 5 | R.RQVEELQLQVQAQQQAWQALHR.E | 3 |
| \* | LAPMis12DANCE\_011813\_02.07316.07316.3 | 4.4072 | 0.3717 | 100.0% | 2531.8442 | 2532.8215 | 1 | 6.124 | 40.0% | 4 | R.QVEELQLQVQAQQQAWQALHR.E | 3 |
| \* | LAPMis12DANCE2\_011813\_01.09177.09177.2 | 4.2126 | 0.4732 | 100.0% | 2532.9722 | 2532.8215 | 1 | 7.894 | 55.0% | 2 | R.QVEELQLQVQAQQQAWQALHR.E | 2 |
| \* | LAPMis12DANCE2\_011813\_01.06063.06063.1 | 1.636 | 0.3425 | 100.0% | 799.52 | 799.98895 | 64 | 4.677 | 66.7% | 1 | R.ELVAVLR.E | 1 |
| \* | LAPMis12DANCE\_011813\_01.09278.09278.1 | 2.3763 | 0.1983 | 100.0% | 1154.58 | 1155.3367 | 3 | 6.667 | 61.1% | 2 | R.ELVAVLREPE.- | 1 |
| \* | LAPMis12DANCE\_011813\_01.09224.09224.2 | 3.6157 | 0.3755 | 100.0% | 1156.0521 | 1155.3367 | 2 | 7.476 | 77.8% | 6 | R.ELVAVLREPE.- | 2 |

---

|  |  |  |  |  |  |  |  |  |
| --- | --- | --- | --- | --- | --- | --- | --- | --- |
| U | *TEV-Speptide* | 4 | 39 | 70.6% | 51 | 5423 | 9.4 | no description |

| Filename XCorr DeltCN Conf% ObsM+H+ CalcM+H+ SpR ZScore Ion% # Sequence  | | | | | | | | | | | | |
| --- | --- | --- | --- | --- | --- | --- | --- | --- | --- | --- | --- | --- |
| \* | LAPMis12DANCE\_011813\_01.03484.03484.2 | 2.0936 | 0.2693 | 96.8% | 1279.4122 | 1280.2993 | 8 | 5.015 | 54.2% | 1 | -.SGGDRWSSTGGGR.S | 2 |
| \* | LAPMis12DANCE\_011813\_01.05450.05450.2 | 4.1101 | 0.41 | 100.0% | 1383.6322 | 1384.5345 | 1 | 7.96 | 72.7% | 34 | R.SRENLYFQGAAK.F | 2 |
| \* | LAPMis12DANCE2\_011813\_01.05507.05507.1 | 2.679 | 0.2417 | 100.0% | 1140.5 | 1141.2688 | 1 | 5.273 | 72.2% | 2 | R.ENLYFQGAAK.F | 1 |
| \* | LAPMis12DANCE\_011813\_01.04103.04103.2 | 3.0211 | 0.3235 | 100.0% | 1297.7722 | 1298.4844 | 1 | 5.68 | 70.0% | 2 | K.FKETAAAKFER.Q | 2 |

---

|  |  |  |  |  |  |  |  |  |
| --- | --- | --- | --- | --- | --- | --- | --- | --- |
| U | *gi|13128994|ref|NP\_07* | 17 | 51 | 66.3% | 205 | 24140 | 5.7 | MIS12 homolog [Homo sapiens] |

| Filename XCorr DeltCN Conf% ObsM+H+ CalcM+H+ SpR ZScore Ion% # Sequence  | | | | | | | | | | | | |
| --- | --- | --- | --- | --- | --- | --- | --- | --- | --- | --- | --- | --- |
| \* | LAPMis12DANCE\_011813\_01.18726.18726.2 | 5.5674 | 0.4253 | 100.0% | 2662.892 | 2662.1592 | 1 | 8.744 | 59.5% | 3 | R.IYIAFQDYLFEVMQAVEQVILK.K | 2 |
| \* | LAPMis12DANCE2\_011813\_01.16922.16922.3 | 3.5759 | 0.3045 | 99.5% | 2789.3643 | 2790.3333 | 1 | 5.264 | 31.8% | 2 | R.IYIAFQDYLFEVMQAVEQVILKK.L | 3 |
| \* | LAPMis12DANCE\_011813\_01.18417.18417.3 | 4.1516 | 0.2907 | 99.5% | 3672.8943 | 3671.3354 | 1 | 5.607 | 26.7% | 1 | K.GHFDNLFSKMEQLFLQLILRIPSNILLPEDK.C | 3 |
| \* | LAPMis12DANCE\_011813\_01.09687.09687.2 | 3.5129 | 0.2539 | 100.0% | 1241.2922 | 1239.4546 | 1 | 5.129 | 80.0% | 5 | R.IPSNILLPEDK.C | 2 |
| \* | LAPMis12DANCE\_011813\_01.07413.07413.2 | 3.6289 | 0.366 | 100.0% | 1752.3121 | 1751.8468 | 1 | 5.323 | 61.5% | 3 | K.ETPYSEEDFQHLQK.E | 2 |
| \* | LAPMis12DANCE\_011813\_01.10863.10863.3 | 3.083 | 0.2875 | 98.1% | 2716.5842 | 2716.9146 | 10 | 4.901 | 26.2% | 1 | K.T#ELCT#K@QALLAELEEQKIVQAK.L | 3 |
| \* | LAPMis12DANCE\_011813\_01.10508.10508.2 | 3.6734 | 0.3406 | 100.0% | 1272.3322 | 1272.441 | 1 | 6.718 | 80.0% | 5 | K.QALLAELEEQK.I | 2 |
| \* | LAPMis12DANCE\_011813\_01.10928.10928.2 | 4.8505 | 0.4652 | 100.0% | 1811.5521 | 1812.1167 | 1 | 7.589 | 66.7% | 3 | K.QALLAELEEQKIVQAK.L | 2 |
| \* | LAPMis12DANCE\_011813\_01.10922.10922.3 | 2.9298 | 0.2716 | 97.6% | 1812.0543 | 1812.1167 | 4 | 5.654 | 41.7% | 1 | K.QALLAELEEQKIVQAK.L | 3 |
| \* | LAPMis12DANCE\_011813\_01.10970.10970.3 | 5.2874 | 0.4708 | 100.0% | 1918.6144 | 1919.1906 | 1 | 8.266 | 55.0% | 6 | K.LKQTLTFFDELHNVGR.D | 3 |
| \* | LAPMis12DANCE\_011813\_01.10971.10971.2 | 5.6792 | 0.5125 | 100.0% | 1919.2122 | 1919.1906 | 1 | 9.91 | 76.7% | 5 | K.LKQTLTFFDELHNVGR.D | 2 |
| \* | LAPMis12DANCE\_011813\_01.11258.11258.2 | 2.5447 | 0.246 | 99.0% | 1676.7922 | 1677.857 | 1 | 6.005 | 57.7% | 2 | K.QTLTFFDELHNVGR.D | 2 |
| \* | LAPMis12DANCE\_011813\_01.11115.11115.3 | 4.1202 | 0.3735 | 100.0% | 2147.8442 | 2148.2969 | 1 | 6.821 | 38.9% | 1 | R.DHGTSDFRESLVSLVQNSR.K | 3 |
| \* | LAPMis12DANCE\_011813\_01.10232.10232.1 | 1.6162 | 0.3933 | 100.0% | 1231.58 | 1232.3794 | 38 | 5.869 | 50.0% | 1 | R.ESLVSLVQNSR.K | 1 |
| \* | LAPMis12DANCE\_011813\_01.10208.10208.2 | 4.0898 | 0.3795 | 100.0% | 1232.0521 | 1232.3794 | 1 | 7.266 | 85.0% | 7 | R.ESLVSLVQNSR.K | 2 |
| \* | LAPMis12DANCE2\_011813\_01.03788.03788.2 | 3.5336 | 0.1444 | 99.6% | 1358.0721 | 1357.5527 | 1 | 4.148 | 80.0% | 2 | R.KLQNIRDNVEK.E | 2 |
| \* | LAPMis12DANCE\_011813\_01.03651.03651.2 | 3.0248 | 0.1767 | 99.5% | 1229.5521 | 1229.3787 | 40 | 4.336 | 66.7% | 3 | K.LQNIRDNVEK.E | 2 |

---

|  |  |  |  |  |  |  |  |  |
| --- | --- | --- | --- | --- | --- | --- | --- | --- |
| U | *gi|11415030|ref|NP\_06* | 14 | 71 | 65.0% | 103 | 11367 | 11.4 | histone cluster 1, H4j [Homo sapiens] |
| U | *gi|77539758|ref|NP\_00* | 14 | 71 | 65.0% | 103 | 11367 | 11.4 | histone cluster 2, H4b [Homo sapiens] |
| U | *gi|4504323|ref|NP\_003* | 14 | 71 | 65.0% | 103 | 11367 | 11.4 | histone cluster 2, H4a [Homo sapiens] |
| U | *gi|4504321|ref|NP\_003* | 14 | 71 | 65.0% | 103 | 11367 | 11.4 | histone cluster 1, H4i [Homo sapiens] |
| U | *gi|4504317|ref|NP\_003* | 14 | 71 | 65.0% | 103 | 11367 | 11.4 | histone cluster 1, H4l [Homo sapiens] |
| U | *gi|4504315|ref|NP\_003* | 14 | 71 | 65.0% | 103 | 11367 | 11.4 | histone cluster 1, H4e [Homo sapiens] |
| U | *gi|4504313|ref|NP\_003* | 14 | 71 | 65.0% | 103 | 11367 | 11.4 | histone cluster 1, H4b [Homo sapiens] |
| U | *gi|4504311|ref|NP\_003* | 14 | 71 | 65.0% | 103 | 11367 | 11.4 | histone cluster 1, H4h [Homo sapiens] |
| U | *gi|4504309|ref|NP\_003* | 14 | 71 | 65.0% | 103 | 11367 | 11.4 | histone cluster 1, H4c [Homo sapiens] |
| U | *gi|4504307|ref|NP\_003* | 14 | 71 | 65.0% | 103 | 11367 | 11.4 | histone cluster 1, H4k [Homo sapiens] |
| U | *gi|4504305|ref|NP\_003* | 14 | 71 | 65.0% | 103 | 11367 | 11.4 | histone cluster 1, H4f [Homo sapiens] |
| U | *gi|4504303|ref|NP\_003* | 14 | 71 | 65.0% | 103 | 11367 | 11.4 | histone cluster 1, H4d [Homo sapiens] |
| U | *gi|4504301|ref|NP\_003* | 14 | 71 | 65.0% | 103 | 11367 | 11.4 | histone cluster 1, H4a [Homo sapiens] |
| U | *gi|28173560|ref|NP\_77* | 14 | 71 | 65.0% | 103 | 11367 | 11.4 | histone cluster 4, H4 [Homo sapiens] |

| Filename XCorr DeltCN Conf% ObsM+H+ CalcM+H+ SpR ZScore Ion% # Sequence  | | | | | | | | | | | | |
| --- | --- | --- | --- | --- | --- | --- | --- | --- | --- | --- | --- | --- |
|  | LAPMis12DANCE\_011813\_01.06284.06284.2 | 3.4255 | 0.1758 | 99.6% | 1325.6322 | 1326.5387 | 1 | 7.046 | 81.8% | 44 | R.DNIQGITKPAIR.R | 2 |
|  | LAPMis12DANCE\_011813\_01.07385.07385.2 | 2.629 | 0.2677 | 99.4% | 1337.1322 | 1337.5187 | 147 | 4.987 | 55.0% | 2 | K.RISGLIYEETR.G | 2 |
|  | LAPMis12DANCE\_011813\_01.08309.08309.1 | 2.4406 | 0.1728 | 97.4% | 1180.62 | 1181.3312 | 1 | 4.411 | 66.7% | 1 | R.ISGLIYEETR.G | 1 |
|  | LAPMis12DANCE\_011813\_01.08312.08312.2 | 3.808 | 0.4067 | 100.0% | 1181.1721 | 1181.3312 | 1 | 7.379 | 88.9% | 8 | R.ISGLIYEETR.G | 2 |
|  | LAPMis12DANCE\_011813\_02.06584.06584.2 | 2.5742 | 0.214 | 98.0% | 1578.0521 | 1578.8491 | 5 | 4.669 | 57.7% | 1 | R.ISGLIYEETRGVLK.V | 2 |
|  | LAPMis12DANCE2\_011813\_01.16574.16574.3 | 3.5565 | 0.3337 | 99.7% | 3666.0244 | 3666.212 | 2 | 5.897 | 21.8% | 1 | R.ISGLIYEETRGVLKVFLENVIRDAVTYTEHAK.R | 3 |
|  | LAPMis12DANCE\_011813\_01.10396.10396.2 | 2.9548 | 0.2797 | 100.0% | 990.0122 | 990.19055 | 2 | 5.507 | 85.7% | 3 | K.VFLENVIR.D | 2 |
|  | LAPMis12DANCE2\_011813\_01.11489.11489.3 | 4.0019 | 0.3785 | 100.0% | 2105.9944 | 2106.386 | 3 | 6.294 | 38.2% | 1 | K.VFLENVIRDAVTYTEHAK.R | 3 |
|  | LAPMis12DANCE2\_011813\_01.03730.03730.2 | 2.5168 | 0.1898 | 98.4% | 1134.9722 | 1135.2188 | 2 | 5.841 | 72.2% | 3 | R.DAVTYTEHAK.R | 2 |
|  | LAPMis12DANCE\_011813\_01.03473.03473.2 | 2.3466 | 0.206 | 97.2% | 1292.1921 | 1291.4062 | 1 | 3.905 | 75.0% | 1 | R.DAVTYTEHAKR.K | 2 |
|  | LAPMis12DANCE\_011813\_01.03258.03258.2 | 2.9939 | 0.2778 | 100.0% | 1418.1721 | 1419.5803 | 13 | 4.786 | 63.6% | 1 | R.DAVTYTEHAKRK.T | 2 |
|  | LAPMis12DANCE\_011813\_01.10164.10164.3 | 3.0274 | 0.3015 | 99.0% | 1596.8644 | 1595.9409 | 187 | 4.724 | 36.5% | 1 | R.KTVTAMDVVYALKR.Q | 3 |
|  | LAPMis12DANCE\_011813\_01.11108.11108.2 | 3.3802 | 0.4147 | 100.0% | 1468.3322 | 1467.7667 | 1 | 7.015 | 70.8% | 2 | K.TVTAMDVVYALKR.Q | 2 |
|  | LAPMis12DANCE\_011813\_01.10184.10184.1 | 1.7257 | 0.4186 | 100.0% | 714.31 | 714.796 | 2 | 7.177 | 66.7% | 2 | R.TLYGFGG.- | 1 |

---

|  |  |  |  |  |  |  |  |  |
| --- | --- | --- | --- | --- | --- | --- | --- | --- |
| U | *gi|10645195|ref|NP\_06* | 13 | 34 | 60.8% | 130 | 14135 | 11.1 | histone cluster 1, H2ae [Homo sapiens] |
| U | *gi|19557656|ref|NP\_00* | 13 | 34 | 60.8% | 130 | 14135 | 11.1 | histone cluster 1, H2ab [Homo sapiens] |
| U | *gi|15617199|ref|NP\_25* | 13 | 34 | 60.8% | 130 | 14121 | 11.1 | histone cluster 3, H2a [Homo sapiens] |

| Filename XCorr DeltCN Conf% ObsM+H+ CalcM+H+ SpR ZScore Ion% # Sequence  | | | | | | | | | | | | |
| --- | --- | --- | --- | --- | --- | --- | --- | --- | --- | --- | --- | --- |
|  | LAPMis12DANCE\_011813\_01.09038.09038.1 | 2.051 | 0.2284 | 100.0% | 944.57 | 945.1093 | 6 | 5.27 | 56.2% | 1 | R.AGLQFPVGR.V | 11111 |
|  | LAPMis12DANCE\_011813\_01.09014.09014.2 | 2.9676 | 0.2668 | 100.0% | 944.9522 | 945.1093 | 1 | 5.313 | 87.5% | 3 | R.AGLQFPVGR.V | 22222 |
|  | LAPMis12DANCE\_011813\_01.18950.18950.3 | 3.5679 | 0.3886 | 100.0% | 2916.3843 | 2917.3752 | 1 | 6.071 | 31.2% | 2 | R.VGAGAPVYLAAVLEYLTAEILELAGNAAR.D | 333 |
|  | LAPMis12DANCE2\_011813\_01.17427.17427.2 | 4.5588 | 0.3271 | 100.0% | 2919.892 | 2917.3752 | 1 | 5.555 | 42.9% | 4 | R.VGAGAPVYLAAVLEYLTAEILELAGNAAR.D | 222 |
|  | LAPMis12DANCE\_011813\_01.18603.18603.3 | 4.5431 | 0.4716 | 100.0% | 3274.2844 | 3274.7417 | 1 | 7.498 | 28.2% | 1 | R.VGAGAPVYLAAVLEYLTAEILELAGNAARDNK.K | 333 |
|  | LAPMis12DANCE\_011813\_01.18419.18419.3 | 4.2155 | 0.3305 | 99.7% | 3401.7544 | 3402.9158 | 1 | 6.529 | 25.0% | 2 | R.VGAGAPVYLAAVLEYLTAEILELAGNAARDNKK.T | 333 |
|  | LAPMis12DANCE\_011813\_01.05489.05489.1 | 1.4835 | 0.2754 | 100.0% | 850.5 | 851.0396 | 2 | 5.039 | 66.7% | 1 | R.HLQLAIR.N | 11111 |
|  | LAPMis12DANCE\_011813\_02.04797.04797.3 | 2.889 | 0.317 | 99.5% | 1693.3744 | 1693.9004 | 1 | 5.647 | 42.3% | 3 | R.HLQLAIRNDEELNK.L | 3333 |
|  | LAPMis12DANCE2\_011813\_01.05418.05418.2 | 3.6747 | 0.2629 | 100.0% | 1693.5721 | 1693.9004 | 1 | 5.791 | 69.2% | 3 | R.HLQLAIRNDEELNK.L | 2222 |
|  | LAPMis12DANCE2\_011813\_01.08337.08337.3 | 3.7998 | 0.399 | 100.0% | 2132.6042 | 2133.4587 | 1 | 5.582 | 35.3% | 1 | R.HLQLAIRNDEELNKLLGR.V | 3 |
|  | LAPMis12DANCE2\_011813\_01.06957.06957.2 | 3.1438 | 0.1794 | 99.5% | 1301.3922 | 1301.4423 | 1 | 5.597 | 75.0% | 2 | R.NDEELNKLLGR.V | 2 |
|  | LAPMis12DANCE\_011813\_01.13010.13010.2 | 4.9595 | 0.4768 | 100.0% | 1931.5122 | 1932.3573 | 1 | 7.387 | 66.7% | 8 | R.VTIAQGGVLPNIQAVLLPK.K | 222 |
|  | LAPMis12DANCE2\_011813\_01.11367.11367.3 | 4.6427 | 0.4198 | 100.0% | 1932.1743 | 1932.3573 | 1 | 6.601 | 41.7% | 3 | R.VTIAQGGVLPNIQAVLLPK.K | 333 |

Similarities:
gi|10800130|ref|NP\_06(11:2)  
gi|106775678|ref|NP\_0(7:6)  
gi|4504253|ref|NP\_002(9:4)  
gi|20357599|ref|NP\_61(3:10)  

---

|  |  |  |  |  |  |  |  |  |
| --- | --- | --- | --- | --- | --- | --- | --- | --- |
| U | *gi|10800130|ref|NP\_06* | 13 | 40 | 60.8% | 130 | 14107 | 10.9 | histone cluster 1, H2ad [Homo sapiens] |
| U | *gi|4504243|ref|NP\_003* | 13 | 40 | 60.8% | 130 | 14091 | 10.9 | histone cluster 1, H2al [Homo sapiens] |
| U | *gi|4504239|ref|NP\_003* | 13 | 40 | 60.8% | 130 | 14091 | 10.9 | histone cluster 1, H2ai [Homo sapiens] |
| U | *gi|18105045|ref|NP\_54* | 13 | 39 | 61.7% | 128 | 13906 | 10.9 | histone cluster 1, H2ah [Homo sapiens] |
| U | *gi|10800144|ref|NP\_06* | 13 | 40 | 61.7% | 128 | 13936 | 10.9 | histone cluster 1, H2aj [Homo sapiens] |
| U | *gi|10800132|ref|NP\_06* | 13 | 40 | 60.8% | 130 | 14091 | 10.9 | histone cluster 1, H2ag [Homo sapiens] |

| Filename XCorr DeltCN Conf% ObsM+H+ CalcM+H+ SpR ZScore Ion% # Sequence  | | | | | | | | | | | | |
| --- | --- | --- | --- | --- | --- | --- | --- | --- | --- | --- | --- | --- |
|  | LAPMis12DANCE\_011813\_01.09038.09038.1 | 2.051 | 0.2284 | 100.0% | 944.57 | 945.1093 | 6 | 5.27 | 56.2% | 1 | R.AGLQFPVGR.V | 11111 |
|  | LAPMis12DANCE\_011813\_01.09014.09014.2 | 2.9676 | 0.2668 | 100.0% | 944.9522 | 945.1093 | 1 | 5.313 | 87.5% | 3 | R.AGLQFPVGR.V | 22222 |
|  | LAPMis12DANCE\_011813\_01.18950.18950.3 | 3.5679 | 0.3886 | 100.0% | 2916.3843 | 2917.3752 | 1 | 6.071 | 31.2% | 2 | R.VGAGAPVYLAAVLEYLTAEILELAGNAAR.D | 333 |
|  | LAPMis12DANCE2\_011813\_01.17427.17427.2 | 4.5588 | 0.3271 | 100.0% | 2919.892 | 2917.3752 | 1 | 5.555 | 42.9% | 4 | R.VGAGAPVYLAAVLEYLTAEILELAGNAAR.D | 222 |
|  | LAPMis12DANCE\_011813\_01.18603.18603.3 | 4.5431 | 0.4716 | 100.0% | 3274.2844 | 3274.7417 | 1 | 7.498 | 28.2% | 1 | R.VGAGAPVYLAAVLEYLTAEILELAGNAARDNK.K | 333 |
|  | LAPMis12DANCE\_011813\_01.18419.18419.3 | 4.2155 | 0.3305 | 99.7% | 3401.7544 | 3402.9158 | 1 | 6.529 | 25.0% | 2 | R.VGAGAPVYLAAVLEYLTAEILELAGNAARDNKK.T | 333 |
|  | LAPMis12DANCE\_011813\_01.05489.05489.1 | 1.4835 | 0.2754 | 100.0% | 850.5 | 851.0396 | 2 | 5.039 | 66.7% | 1 | R.HLQLAIR.N | 11111 |
|  | LAPMis12DANCE\_011813\_02.04797.04797.3 | 2.889 | 0.317 | 99.5% | 1693.3744 | 1693.9004 | 1 | 5.647 | 42.3% | 3 | R.HLQLAIRNDEELNK.L | 3333 |
|  | LAPMis12DANCE2\_011813\_01.05418.05418.2 | 3.6747 | 0.2629 | 100.0% | 1693.5721 | 1693.9004 | 1 | 5.791 | 69.2% | 3 | R.HLQLAIRNDEELNK.L | 2222 |
|  | LAPMis12DANCE2\_011813\_01.08104.08104.3 | 4.9186 | 0.5102 | 100.0% | 2105.1243 | 2105.4453 | 1 | 7.487 | 39.7% | 2 | R.HLQLAIRNDEELNKLLGK.V | 33 |
|  | LAPMis12DANCE\_011813\_01.08824.08824.2 | 3.5443 | 0.0354 | 97.8% | 1275.3922 | 1273.4288 | 1 | 4.522 | 75.0% | 7 | R.NDEELNKLLGK.V | 223 |
|  | LAPMis12DANCE\_011813\_01.13010.13010.2 | 4.9595 | 0.4768 | 100.0% | 1931.5122 | 1932.3573 | 1 | 7.387 | 66.7% | 8 | K.VTIAQGGVLPNIQAVLLPK.K | 222 |
|  | LAPMis12DANCE2\_011813\_01.11367.11367.3 | 4.6427 | 0.4198 | 100.0% | 1932.1743 | 1932.3573 | 1 | 6.601 | 41.7% | 3 | K.VTIAQGGVLPNIQAVLLPK.K | 333 |

Similarities:
gi|10645195|ref|NP\_06(11:2)  
gi|106775678|ref|NP\_0(9:4)  
gi|4504253|ref|NP\_002(9:4)  
gi|20357599|ref|NP\_61(3:10)  

---

|  |  |  |  |  |  |  |  |  |
| --- | --- | --- | --- | --- | --- | --- | --- | --- |
| U | *gi|106775678|ref|NP\_0* | 11 | 36 | 60.8% | 130 | 14095 | 10.9 | histone cluster 2, H2aa4 [Homo sapiens] |
| U | *gi|4504251|ref|NP\_003* | 11 | 36 | 60.8% | 130 | 14095 | 10.9 | histone cluster 2, H2aa3 [Homo sapiens] |
| U | *gi|24638446|ref|NP\_00* | 11 | 36 | 61.2% | 129 | 13988 | 10.9 | histone cluster 2, H2ac [Homo sapiens] |

| Filename XCorr DeltCN Conf% ObsM+H+ CalcM+H+ SpR ZScore Ion% # Sequence  | | | | | | | | | | | | |
| --- | --- | --- | --- | --- | --- | --- | --- | --- | --- | --- | --- | --- |
|  | LAPMis12DANCE\_011813\_01.09038.09038.1 | 2.051 | 0.2284 | 100.0% | 944.57 | 945.1093 | 6 | 5.27 | 56.2% | 1 | R.AGLQFPVGR.V | 11111 |
|  | LAPMis12DANCE\_011813\_01.09014.09014.2 | 2.9676 | 0.2668 | 100.0% | 944.9522 | 945.1093 | 1 | 5.313 | 87.5% | 3 | R.AGLQFPVGR.V | 22222 |
|  | LAPMis12DANCE2\_011813\_01.17259.17259.2 | 5.557 | 0.5238 | 100.0% | 2935.8123 | 2935.4082 | 1 | 9.621 | 42.9% | 3 | R.VGAGAPVYMAAVLEYLTAEILELAGNAAR.D | 2 |
|  | LAPMis12DANCE\_011813\_01.18279.18279.3 | 4.3586 | 0.4408 | 100.0% | 3420.9243 | 3420.949 | 44 | 7.069 | 17.2% | 2 | R.VGAGAPVYMAAVLEYLTAEILELAGNAARDNKK.T | 3 |
|  | LAPMis12DANCE\_011813\_01.05489.05489.1 | 1.4835 | 0.2754 | 100.0% | 850.5 | 851.0396 | 2 | 5.039 | 66.7% | 1 | R.HLQLAIR.N | 11111 |
|  | LAPMis12DANCE\_011813\_02.04797.04797.3 | 2.889 | 0.317 | 99.5% | 1693.3744 | 1693.9004 | 1 | 5.647 | 42.3% | 3 | R.HLQLAIRNDEELNK.L | 3333 |
|  | LAPMis12DANCE2\_011813\_01.05418.05418.2 | 3.6747 | 0.2629 | 100.0% | 1693.5721 | 1693.9004 | 1 | 5.791 | 69.2% | 3 | R.HLQLAIRNDEELNK.L | 2222 |
|  | LAPMis12DANCE2\_011813\_01.08104.08104.3 | 4.9186 | 0.5102 | 100.0% | 2105.1243 | 2105.4453 | 1 | 7.487 | 39.7% | 2 | R.HLQLAIRNDEELNKLLGK.V | 33 |
|  | LAPMis12DANCE\_011813\_01.08824.08824.2 | 3.5443 | 0.0354 | 97.8% | 1275.3922 | 1273.4288 | 1 | 4.522 | 75.0% | 7 | R.NDEELNKLLGK.V | 223 |
|  | LAPMis12DANCE\_011813\_01.13010.13010.2 | 4.9595 | 0.4768 | 100.0% | 1931.5122 | 1932.3573 | 1 | 7.387 | 66.7% | 8 | K.VTIAQGGVLPNIQAVLLPK.K | 222 |
|  | LAPMis12DANCE2\_011813\_01.11367.11367.3 | 4.6427 | 0.4198 | 100.0% | 1932.1743 | 1932.3573 | 1 | 6.601 | 41.7% | 3 | K.VTIAQGGVLPNIQAVLLPK.K | 333 |

Similarities:
gi|10645195|ref|NP\_06(7:4)  
gi|10800130|ref|NP\_06(9:2)  
gi|4504253|ref|NP\_002(5:6)  
gi|20357599|ref|NP\_61(3:8)  

---

|  |  |  |  |  |  |  |  |  |
| --- | --- | --- | --- | --- | --- | --- | --- | --- |
| U | *gi|32698866|ref|NP\_87* | 9 | 21 | 58.9% | 197 | 22443 | 4.7 | spindle pole body component 24 homolog [Homo sapiens] |

| Filename XCorr DeltCN Conf% ObsM+H+ CalcM+H+ SpR ZScore Ion% # Sequence  | | | | | | | | | | | | |
| --- | --- | --- | --- | --- | --- | --- | --- | --- | --- | --- | --- | --- |
| \* | LAPMis12DANCE\_011813\_01.15260.15260.2 | 3.9162 | 0.3405 | 100.0% | 1815.0721 | 1815.0336 | 1 | 7.059 | 62.5% | 3 | R.DIEEVSQGLLSLLGANR.A | 2 |
| \* | LAPMis12DANCE\_011813\_01.03957.03957.2 | 2.2053 | 0.2061 | 96.2% | 1104.1522 | 1104.2023 | 2 | 5.428 | 77.8% | 1 | R.LLETQDGAEK.Q | 2 |
| \* | LAPMis12DANCE\_011813\_01.11144.11144.3 | 5.451 | 0.4307 | 100.0% | 2850.9543 | 2852.0007 | 1 | 7.704 | 33.0% | 2 | K.EQVHQGGVELQQLEAGLQEAGEEDTR.L | 3 |
| \* | LAPMis12DANCE2\_011813\_01.09621.09621.3 | 4.7939 | 0.4622 | 100.0% | 3092.6643 | 3093.3342 | 53 | 7.472 | 24.1% | 1 | K.EQVHQGGVELQQLEAGLQEAGEEDTRLK.A | 3 |
| \* | LAPMis12DANCE\_011813\_01.09222.09222.2 | 2.4073 | 0.1741 | 98.2% | 902.1122 | 902.08185 | 6 | 5.576 | 85.7% | 1 | K.ASLLQLTR.E | 2 |
| \* | LAPMis12DANCE\_011813\_01.11817.11817.2 | 3.7214 | 0.3593 | 100.0% | 1716.6921 | 1716.8828 | 1 | 7.136 | 73.1% | 3 | R.ELEELKEIEADLER.Q | 2 |
| \* | LAPMis12DANCE2\_011813\_01.09939.09939.3 | 3.022 | 0.2717 | 97.6% | 2102.2444 | 2102.303 | 1 | 5.431 | 37.5% | 1 | R.ELEELKEIEADLERQEK.E | 3 |
| \* | LAPMis12DANCE\_011813\_01.06915.06915.3 | 5.0389 | 0.4752 | 100.0% | 2250.3542 | 2251.51 | 1 | 8.074 | 43.8% | 6 | K.GIHHGPSVAQPIHLDSTQLSR.K | 3 |
| \* | LAPMis12DANCE\_011813\_01.15992.15992.2 | 3.8605 | 0.5443 | 100.0% | 1902.1122 | 1903.1412 | 1 | 9.512 | 64.3% | 3 | R.KFISDYLWSLVDTEW.- | 2 |

---

|  |  |  |  |  |  |  |  |  |
| --- | --- | --- | --- | --- | --- | --- | --- | --- |
| U | *gi|4504919|ref|NP\_002* | 37 | 81 | 58.8% | 483 | 53704 | 5.6 | keratin 8 [Homo sapiens] |

| Filename XCorr DeltCN Conf% ObsM+H+ CalcM+H+ SpR ZScore Ion% # Sequence  | | | | | | | | | | | | |
| --- | --- | --- | --- | --- | --- | --- | --- | --- | --- | --- | --- | --- |
| \* | LAPMis12DANCE\_011813\_01.14486.14486.3 | 3.8823 | 0.2453 | 97.6% | 3927.0244 | 3927.465 | 2 | 5.56 | 20.0% | 1 | R.GGLGGGYGGASGMGGITAVTVNQSLLSPLVLEVDPNIQAVR.T | 3 |
|  | LAPMis12DANCE\_011813\_01.08360.08360.2 | 2.5656 | 0.1541 | 98.0% | 1082.3722 | 1083.2755 | 7 | 6.493 | 68.8% | 1 | K.FASFIDKVR.F | 222222 |
|  | LAPMis12DANCE\_011813\_01.09134.09134.2 | 3.1437 | 0.1237 | 99.5% | 1031.0322 | 1031.1997 | 2 | 3.587 | 92.9% | 2 | K.WSLLQQQK.T | 2 |
|  | LAPMis12DANCE\_011813\_01.12870.12870.2 | 4.478 | 0.3881 | 100.0% | 1849.0322 | 1849.0431 | 1 | 7.829 | 64.3% | 2 | R.SNMDNMFESYINNLR.R | 2 |
|  | LAPMis12DANCE\_011813\_01.11918.11918.3 | 2.6402 | 0.2898 | 97.6% | 2004.9243 | 2005.2306 | 21 | 4.525 | 30.0% | 1 | R.SNMDNMFESYINNLRR.Q | 3 |
|  | LAPMis12DANCE2\_011813\_01.03915.03915.2 | 2.4217 | 0.157 | 95.9% | 1201.7522 | 1202.353 | 18 | 4.195 | 66.7% | 1 | R.RQLETLGQEK.L | 2 |
|  | LAPMis12DANCE\_011813\_01.12413.12413.2 | 5.8805 | 0.3173 | 100.0% | 2034.4321 | 2035.363 | 2 | 10.663 | 67.6% | 1 | K.LKLEAELGNMQGLVEDFK.N | 2 |
|  | LAPMis12DANCE\_011813\_02.08676.08676.3 | 4.8388 | 0.207 | 98.5% | 2034.9243 | 2035.363 | 1 | 8.487 | 44.1% | 1 | K.LKLEAELGNMQGLVEDFK.N | 3 |
|  | LAPMis12DANCE\_011813\_01.12185.12185.2 | 2.5985 | 0.2251 | 98.3% | 1792.4122 | 1794.0295 | 1 | 6.019 | 50.0% | 1 | K.LEAELGNMQGLVEDFK.N | 2 |
|  | LAPMis12DANCE\_011813\_01.03897.03897.2 | 2.7653 | 0.3015 | 100.0% | 1308.5322 | 1309.4215 | 1 | 5.673 | 77.8% | 2 | K.NKYEDEINKR.T | 2222 |
|  | LAPMis12DANCE\_011813\_01.11020.11020.2 | 2.8974 | 0.4492 | 100.0% | 1353.3121 | 1353.5732 | 1 | 7.52 | 70.0% | 4 | R.TEMENEFVLIK.K | 2 |
|  | LAPMis12DANCE\_011813\_01.09369.09369.2 | 3.2799 | 0.3531 | 100.0% | 1481.2522 | 1481.7473 | 1 | 5.871 | 77.3% | 4 | R.TEMENEFVLIKK.D | 2 |
|  | LAPMis12DANCE\_011813\_01.08472.08472.2 | 4.1531 | 0.5132 | 100.0% | 1797.9321 | 1798.9623 | 1 | 8.868 | 71.4% | 3 | K.DVDEAYMNKVELESR.L | 2 |
|  | LAPMis12DANCE\_011813\_02.05615.05615.3 | 3.6494 | 0.2854 | 99.5% | 1799.5743 | 1798.9623 | 7 | 5.55 | 41.1% | 1 | K.DVDEAYMNKVELESR.L | 3 |
|  | LAPMis12DANCE\_011813\_01.11894.11894.2 | 3.9485 | 0.4252 | 100.0% | 1420.2722 | 1420.6055 | 1 | 8.308 | 86.4% | 8 | R.LEGLTDEINFLR.Q | 2 |
|  | LAPMis12DANCE\_011813\_01.06720.06720.2 | 2.4396 | 0.228 | 99.4% | 1080.1921 | 1080.1827 | 23 | 4.619 | 64.3% | 1 | R.QLYEEEIR.E | 2 |
|  | LAPMis12DANCE\_011813\_01.11578.11578.2 | 4.4652 | 0.453 | 100.0% | 1322.1122 | 1321.5286 | 1 | 8.379 | 72.7% | 4 | R.SLDMDSIIAEVK.A | 2 |
|  | LAPMis12DANCE2\_011813\_01.04202.04202.1 | 1.9412 | 0.3297 | 100.0% | 1079.46 | 1080.1423 | 4 | 5.717 | 62.5% | 2 | K.AQYEDIANR.S | 11 |
|  | LAPMis12DANCE\_011813\_01.04676.04676.2 | 2.3902 | 0.3451 | 100.0% | 1079.9321 | 1080.1423 | 21 | 6.799 | 62.5% | 1 | K.AQYEDIANR.S | 22 |
|  | LAPMis12DANCE2\_011813\_01.04673.04673.2 | 3.6069 | 0.1906 | 100.0% | 1414.1122 | 1413.5884 | 2 | 5.515 | 72.7% | 2 | R.SRAEAESMYQIK.Y | 2 |
|  | LAPMis12DANCE\_011813\_02.06554.06554.3 | 5.3795 | 0.3504 | 100.0% | 2531.9644 | 2532.828 | 1 | 6.378 | 35.7% | 1 | R.SRAEAESMYQIKYEELQSLAGK.H | 3 |
|  | LAPMis12DANCE\_011813\_01.06653.06653.2 | 3.1749 | 0.3619 | 100.0% | 1169.6721 | 1170.3228 | 3 | 7.024 | 77.8% | 1 | R.AEAESMYQIK.Y | 2 |
|  | LAPMis12DANCE\_011813\_02.06916.06916.3 | 3.8071 | 0.4096 | 100.0% | 2289.3542 | 2289.5623 | 1 | 6.643 | 31.6% | 1 | R.AEAESMYQIKYEELQSLAGK.H | 3 |
|  | LAPMis12DANCE\_011813\_01.07526.07526.2 | 3.3994 | 0.066 | 98.8% | 1137.6322 | 1138.2627 | 1 | 6.593 | 77.8% | 2 | K.YEELQSLAGK.H | 2 |
|  | LAPMis12DANCE2\_011813\_01.03767.03767.2 | 3.1404 | 0.412 | 100.0% | 1210.1322 | 1209.36 | 1 | 6.387 | 77.8% | 4 | R.TKTEISEMNR.N | 2 |
|  | LAPMis12DANCE\_011813\_01.07480.07480.1 | 2.0899 | 0.2026 | 97.3% | 1000.46 | 1001.168 | 4 | 5.121 | 68.8% | 1 | R.LQAEIEGLK.G | 1 |
|  | LAPMis12DANCE\_011813\_01.05549.05549.2 | 3.2537 | 0.3074 | 100.0% | 1341.8522 | 1342.5381 | 1 | 7.072 | 77.3% | 3 | R.LQAEIEGLKGQR.A | 2 |
|  | LAPMis12DANCE\_011813\_01.10143.10143.2 | 3.6528 | 0.4525 | 100.0% | 1345.0521 | 1345.452 | 1 | 7.019 | 66.7% | 5 | R.ASLEAAIADAEQR.G | 2 |
|  | LAPMis12DANCE\_011813\_02.07914.07914.3 | 3.2584 | 0.3049 | 99.0% | 1956.8944 | 1957.1912 | 44 | 5.636 | 33.3% | 1 | R.ASLEAAIADAEQRGELAIK.D | 3 |
|  | LAPMis12DANCE2\_011813\_01.09977.09977.2 | 5.1369 | 0.3959 | 100.0% | 1957.4122 | 1957.1912 | 1 | 7.458 | 52.8% | 2 | R.ASLEAAIADAEQRGELAIK.D | 2 |
|  | LAPMis12DANCE\_011813\_01.11476.11476.3 | 4.7789 | 0.3866 | 100.0% | 2456.1843 | 2456.7153 | 1 | 6.791 | 30.4% | 3 | R.ASLEAAIADAEQRGELAIKDANAK.L | 3 |
|  | LAPMis12DANCE\_011813\_01.03916.03916.2 | 3.1796 | 0.2258 | 99.9% | 1130.0922 | 1130.2865 | 1 | 6.386 | 75.0% | 2 | R.GELAIKDANAK.L | 2 |
|  | LAPMis12DANCE\_011813\_01.09214.09214.1 | 2.122 | 0.2633 | 100.0% | 1129.5 | 1130.2865 | 11 | 5.546 | 55.6% | 1 | K.LSELEAALQR.A | 1 |
|  | LAPMis12DANCE\_011813\_01.09200.09200.2 | 4.3029 | 0.2649 | 100.0% | 1130.4922 | 1130.2865 | 1 | 5.977 | 88.9% | 3 | K.LSELEAALQR.A | 2 |
|  | LAPMis12DANCE\_011813\_01.05404.05404.2 | 4.0492 | 0.4532 | 100.0% | 1475.7522 | 1476.7058 | 1 | 7.636 | 83.3% | 2 | R.LESGMQNMSIHTK.T | 2 |
|  | LAPMis12DANCE\_011813\_01.07307.07307.2 | 2.8807 | 0.2806 | 99.9% | 1474.2522 | 1474.6512 | 30 | 5.545 | 46.2% | 1 | R.DGKLVSESSDVLPK.- | 2 |
|  | LAPMis12DANCE2\_011813\_01.05242.05242.2 | 3.0379 | 0.3369 | 100.0% | 1174.2122 | 1174.3367 | 102 | 6.582 | 60.0% | 5 | K.LVSESSDVLPK.- | 2 |

Similarities:
gi|47132620|ref|NP\_00(1:36)  
gi|119395750|ref|NP\_0(1:36)  
gi|119395754|ref|NP\_0(2:35)  
gi|119703753|ref|NP\_0(2:35)  
gi|67782365|ref|NP\_00(1:36)  
gi|153791158|ref|NP\_0(3:34)  

---

|  |  |  |  |  |  |  |  |  |
| --- | --- | --- | --- | --- | --- | --- | --- | --- |
| U | *gi|4504253|ref|NP\_002* | 10 | 21 | 55.2% | 143 | 15145 | 10.7 | H2A histone family, member X [Homo sapiens] |

| Filename XCorr DeltCN Conf% ObsM+H+ CalcM+H+ SpR ZScore Ion% # Sequence  | | | | | | | | | | | | |
| --- | --- | --- | --- | --- | --- | --- | --- | --- | --- | --- | --- | --- |
|  | LAPMis12DANCE\_011813\_01.09038.09038.1 | 2.051 | 0.2284 | 100.0% | 944.57 | 945.1093 | 6 | 5.27 | 56.2% | 1 | R.AGLQFPVGR.V | 11111 |
|  | LAPMis12DANCE\_011813\_01.09014.09014.2 | 2.9676 | 0.2668 | 100.0% | 944.9522 | 945.1093 | 1 | 5.313 | 87.5% | 3 | R.AGLQFPVGR.V | 22222 |
|  | LAPMis12DANCE\_011813\_01.18950.18950.3 | 3.5679 | 0.3886 | 100.0% | 2916.3843 | 2917.3752 | 1 | 6.071 | 31.2% | 2 | R.VGAGAPVYLAAVLEYLTAEILELAGNAAR.D | 333 |
|  | LAPMis12DANCE2\_011813\_01.17427.17427.2 | 4.5588 | 0.3271 | 100.0% | 2919.892 | 2917.3752 | 1 | 5.555 | 42.9% | 4 | R.VGAGAPVYLAAVLEYLTAEILELAGNAAR.D | 222 |
|  | LAPMis12DANCE\_011813\_01.18603.18603.3 | 4.5431 | 0.4716 | 100.0% | 3274.2844 | 3274.7417 | 1 | 7.498 | 28.2% | 1 | R.VGAGAPVYLAAVLEYLTAEILELAGNAARDNK.K | 333 |
|  | LAPMis12DANCE\_011813\_01.18419.18419.3 | 4.2155 | 0.3305 | 99.7% | 3401.7544 | 3402.9158 | 1 | 6.529 | 25.0% | 2 | R.VGAGAPVYLAAVLEYLTAEILELAGNAARDNKK.T | 333 |
|  | LAPMis12DANCE\_011813\_01.05489.05489.1 | 1.4835 | 0.2754 | 100.0% | 850.5 | 851.0396 | 2 | 5.039 | 66.7% | 1 | R.HLQLAIR.N | 11111 |
|  | LAPMis12DANCE\_011813\_02.04797.04797.3 | 2.889 | 0.317 | 99.5% | 1693.3744 | 1693.9004 | 1 | 5.647 | 42.3% | 3 | R.HLQLAIRNDEELNK.L | 3333 |
|  | LAPMis12DANCE2\_011813\_01.05418.05418.2 | 3.6747 | 0.2629 | 100.0% | 1693.5721 | 1693.9004 | 1 | 5.791 | 69.2% | 3 | R.HLQLAIRNDEELNK.L | 2222 |
|  | LAPMis12DANCE\_011813\_01.13883.13883.2 | 3.2427 | 0.2651 | 99.9% | 2273.912 | 2272.78 | 6 | 4.984 | 31.8% | 1 | K.LLGGVTIAQGGVLPNIQAVLLPK.K | 2 |

Similarities:
gi|10645195|ref|NP\_06(9:1)  
gi|10800130|ref|NP\_06(9:1)  
gi|106775678|ref|NP\_0(5:5)  
gi|20357599|ref|NP\_61(3:7)  

---

|  |  |  |  |  |  |  |  |  |
| --- | --- | --- | --- | --- | --- | --- | --- | --- |
| U | *gi|47132620|ref|NP\_00* | 26 | 35 | 52.1% | 639 | 65433 | 8.0 | keratin 2 [Homo sapiens] |

| Filename XCorr DeltCN Conf% ObsM+H+ CalcM+H+ SpR ZScore Ion% # Sequence  | | | | | | | | | | | | |
| --- | --- | --- | --- | --- | --- | --- | --- | --- | --- | --- | --- | --- |
|  | LAPMis12DANCE\_011813\_01.04797.04797.2 | 4.019 | 0.4698 | 100.0% | 1255.0922 | 1255.3298 | 1 | 8.739 | 73.1% | 1 | R.GFSSGSAVVSGGSR.R | 2 |
|  | LAPMis12DANCE2\_011813\_01.04442.04442.2 | 3.014 | 0.4649 | 100.0% | 1321.3722 | 1321.3542 | 2 | 8.437 | 43.3% | 1 | R.HGGGGGGFGGGGFGSR.S | 2 |
|  | LAPMis12DANCE\_011813\_01.05974.05974.1 | 1.7539 | 0.4329 | 100.0% | 831.47 | 831.9878 | 216 | 6.638 | 37.5% | 2 | R.SLVGLGGTK.S | 1 |
|  | LAPMis12DANCE\_011813\_01.10794.10794.2 | 3.3142 | 0.4209 | 100.0% | 1839.9922 | 1840.0055 | 4 | 6.598 | 35.7% | 2 | K.SISISVAGGGGGFGAAGGFGGR.G | 2 |
| \* | LAPMis12DANCE\_011813\_01.11055.11055.2 | 4.2463 | 0.4843 | 100.0% | 2399.3323 | 2400.4446 | 1 | 8.164 | 46.6% | 1 | R.GGGFGGGSSFGGGSGFSGGGFGGGGFGGGR.F | 2 |
| \* | LAPMis12DANCE\_011813\_02.07108.07108.3 | 4.1887 | 0.3821 | 100.0% | 2400.0842 | 2400.4446 | 1 | 6.11 | 30.2% | 1 | R.GGGFGGGSSFGGGSGFSGGGFGGGGFGGGR.F | 3 |
|  | LAPMis12DANCE\_011813\_01.12540.12540.3 | 1.7124 | 0.3655 | 97.4% | 4094.6643 | 4094.5786 | 1 | 6.09 | 26.2% | 1 | R.FGGFGGPGGVGGLGGPGGFGPGGYPGGIHEVSVNQSLLQPLNVK.V | 3 |
|  | LAPMis12DANCE\_011813\_01.08360.08360.2 | 2.5656 | 0.1541 | 98.0% | 1082.3722 | 1083.2755 | 7 | 6.493 | 68.8% | 1 | K.FASFIDKVR.F | 222222 |
|  | LAPMis12DANCE\_011813\_01.07401.07401.2 | 4.4166 | 0.1231 | 100.0% | 1475.7122 | 1476.6726 | 1 | 7.41 | 90.9% | 4 | R.FLEQQNQVLQTK.W | 22 |
|  | LAPMis12DANCE\_011813\_01.07581.07581.1 | 1.4058 | 0.3078 | 100.0% | 1037.46 | 1038.1454 | 10 | 5.015 | 56.2% | 1 | R.YLDGLTAER.T | 1 |
|  | LAPMis12DANCE\_011813\_01.07575.07575.2 | 2.5329 | 0.1797 | 98.7% | 1038.2322 | 1038.1454 | 7 | 4.737 | 75.0% | 1 | R.YLDGLTAER.T | 2 |
|  | LAPMis12DANCE\_011813\_01.10324.10324.2 | 3.7128 | 0.4667 | 100.0% | 2256.0723 | 2257.4338 | 26 | 6.942 | 38.9% | 1 | R.TSQNSELNNMQDLVEDYKK.K | 2 |
|  | LAPMis12DANCE\_011813\_01.03411.03411.2 | 2.8767 | 0.2688 | 100.0% | 1194.3322 | 1195.3176 | 6 | 5.463 | 75.0% | 1 | K.KYEDEINKR.T | 2 |
|  | LAPMis12DANCE\_011813\_01.06034.06034.2 | 3.426 | 0.4199 | 100.0% | 1337.2322 | 1337.5156 | 1 | 5.878 | 81.8% | 1 | R.TAAENDFVTLKK.D | 2 |
|  | LAPMis12DANCE\_011813\_01.12453.12453.2 | 3.8393 | 0.1232 | 99.6% | 1462.1921 | 1461.6982 | 4 | 5.59 | 72.7% | 2 | K.VDLLNQEIEFLK.V | 2 |
|  | LAPMis12DANCE\_011813\_01.12718.12718.2 | 3.0679 | 0.3399 | 100.0% | 1329.7522 | 1330.5211 | 1 | 6.91 | 77.3% | 2 | R.NLDLDSIIAEVK.A | 2222 |
|  | LAPMis12DANCE\_011813\_01.03725.03725.2 | 4.4244 | 0.4503 | 100.0% | 1391.7522 | 1392.5083 | 1 | 7.633 | 77.3% | 1 | R.SKEEAEALYHSK.Y | 2 |
|  | LAPMis12DANCE\_011813\_01.07756.07756.2 | 3.0557 | 0.4035 | 100.0% | 1194.2522 | 1194.33 | 1 | 6.625 | 77.8% | 2 | K.YEELQVTVGR.H | 2 |
|  | LAPMis12DANCE\_011813\_01.07370.07370.1 | 1.9174 | 0.2765 | 100.0% | 973.43 | 974.102 | 7 | 4.277 | 64.3% | 1 | K.IEISELNR.V | 11 |
|  | LAPMis12DANCE\_011813\_01.07371.07371.2 | 2.2684 | 0.1613 | 96.2% | 974.0122 | 974.102 | 23 | 4.01 | 71.4% | 1 | K.IEISELNR.V | 22 |
|  | LAPMis12DANCE\_011813\_01.03815.03815.2 | 2.2238 | 0.2572 | 99.1% | 994.97217 | 995.16675 | 1 | 4.623 | 75.0% | 1 | R.LQGEIAHVK.K | 2 |
|  | LAPMis12DANCE\_011813\_01.07703.07703.2 | 3.0657 | 0.3632 | 100.0% | 1329.9321 | 1330.3971 | 1 | 6.591 | 81.8% | 1 | K.NVQDAIADAEQR.G | 2 |
|  | LAPMis12DANCE\_011813\_01.10538.10538.2 | 4.2223 | 0.3516 | 100.0% | 1613.7522 | 1614.796 | 1 | 7.137 | 76.9% | 1 | R.NKLNDLEEALQQAK.E | 2 |
|  | LAPMis12DANCE\_011813\_01.11712.11712.3 | 4.2427 | 0.3877 | 100.0% | 2199.5942 | 2199.4258 | 1 | 6.341 | 34.7% | 2 | R.NKLNDLEEALQQAKEDLAR.L | 3 |
|  | LAPMis12DANCE\_011813\_02.07132.07132.2 | 2.9898 | 0.376 | 100.0% | 1264.1522 | 1264.4644 | 1 | 7.517 | 75.0% | 1 | K.LALDVEIATYR.K | 2222 |
|  | LAPMis12DANCE\_011813\_01.03777.03777.2 | 5.0133 | 0.3129 | 100.0% | 1742.1122 | 1742.716 | 1 | 10.173 | 40.9% | 1 | R.GGSGGGGSISGGGYGSGGGSGGR.Y | 2 |

Similarities:
gi|4504919|ref|NP\_002(1:25)  
gi|119395750|ref|NP\_0(3:23)  
gi|119395754|ref|NP\_0(3:23)  
gi|119703753|ref|NP\_0(3:23)  
gi|67782365|ref|NP\_00(1:25)  
gi|153791158|ref|NP\_0(3:23)  

---

|  |  |  |  |  |  |  |  |  |
| --- | --- | --- | --- | --- | --- | --- | --- | --- |
| U | *gi|29788785|ref|NP\_82* | 19 | 53 | 52.0% | 444 | 49671 | 4.9 | tubulin, beta [Homo sapiens] |

| Filename XCorr DeltCN Conf% ObsM+H+ CalcM+H+ SpR ZScore Ion% # Sequence  | | | | | | | | | | | | |
| --- | --- | --- | --- | --- | --- | --- | --- | --- | --- | --- | --- | --- |
| \* | LAPMis12DANCE\_011813\_02.07214.07214.3 | 5.6398 | 0.4026 | 100.0% | 3103.4343 | 3104.2725 | 1 | 8.584 | 36.5% | 2 | K.FWEVISDEHGIDPTGTYHGDSDLQLDR.I | 3 |
| \* | LAPMis12DANCE\_011813\_01.06962.06962.2 | 3.5011 | 0.5507 | 100.0% | 1302.2722 | 1302.4265 | 1 | 9.207 | 81.8% | 4 | R.ISVYYNEATGGK.Y | 2 |
|  | LAPMis12DANCE2\_011813\_01.08850.08850.2 | 4.685 | 0.4758 | 100.0% | 1617.3922 | 1616.8701 | 1 | 8.537 | 67.9% | 4 | R.AILVDLEPGTMDSVR.S | 2 |
|  | LAPMis12DANCE\_011813\_01.12368.12368.3 | 6.8452 | 0.5048 | 100.0% | 2799.8044 | 2800.0647 | 1 | 9.192 | 36.0% | 7 | R.SGPFGQIFRPDNFVFGQSGAGNNWAK.G | 33 |
|  | LAPMis12DANCE\_011813\_01.12752.12752.2 | 6.7078 | 0.4112 | 100.0% | 1960.4321 | 1960.151 | 1 | 8.744 | 76.5% | 3 | K.GHYTEGAELVDSVLDVVR.K | 22 |
|  | LAPMis12DANCE\_011813\_01.11830.11830.2 | 5.614 | 0.5661 | 100.0% | 2087.5923 | 2088.325 | 1 | 10.318 | 69.4% | 2 | K.GHYTEGAELVDSVLDVVRK.E | 22 |
|  | LAPMis12DANCE\_011813\_01.11865.11865.3 | 4.2402 | 0.4278 | 100.0% | 2088.5344 | 2088.325 | 2 | 7.312 | 34.7% | 4 | K.GHYTEGAELVDSVLDVVRK.E | 33 |
|  | LAPMis12DANCE2\_011813\_01.03633.03633.2 | 2.72 | 0.1715 | 99.4% | 1078.0922 | 1078.1698 | 3 | 4.599 | 85.7% | 3 | K.IREEYPDR.I | 22 |
|  | LAPMis12DANCE\_011813\_01.09722.09722.2 | 4.1598 | 0.429 | 100.0% | 1320.2722 | 1320.5896 | 1 | 8.109 | 77.3% | 6 | R.IMNTFSVVPSPK.V | 22 |
|  | LAPMis12DANCE\_011813\_01.08648.08648.2 | 2.5957 | 0.2336 | 99.4% | 1131.6921 | 1131.2767 | 107 | 5.098 | 61.1% | 3 | R.FPGQLNADLR.K | 22 |
|  | LAPMis12DANCE\_011813\_01.09826.09826.2 | 3.3678 | 0.349 | 100.0% | 1272.4521 | 1272.5945 | 5 | 7.066 | 60.0% | 2 | R.KLAVNMVPFPR.L | 22 |
|  | LAPMis12DANCE\_011813\_01.11000.11000.2 | 3.7645 | 0.4603 | 100.0% | 1144.0122 | 1144.4204 | 1 | 8.466 | 88.9% | 1 | K.LAVNMVPFPR.L | 22 |
|  | LAPMis12DANCE\_011813\_01.12036.12036.2 | 3.4224 | 0.464 | 100.0% | 1620.7922 | 1621.9403 | 1 | 9.29 | 76.9% | 2 | R.LHFFMPGFAPLTSR.G | 22 |
| \* | LAPMis12DANCE\_011813\_01.11811.11811.2 | 3.2482 | 0.4718 | 100.0% | 1660.4321 | 1660.9078 | 1 | 7.307 | 60.7% | 4 | R.ALTVPELTQQVFDAK.N | 2 |
|  | LAPMis12DANCE\_011813\_01.11200.11200.1 | 1.4887 | 0.2888 | 100.0% | 1039.53 | 1040.2505 | 98 | 4.805 | 50.0% | 1 | R.YLTVAAVFR.G | 11 |
|  | LAPMis12DANCE\_011813\_01.07258.07258.2 | 4.0244 | 0.2359 | 100.0% | 1446.8922 | 1447.6031 | 2 | 6.543 | 68.2% | 2 | K.EVDEQMLNVQNK.N | 22 |
|  | LAPMis12DANCE2\_011813\_01.09810.09810.2 | 3.4367 | 0.3512 | 100.0% | 1696.9722 | 1697.8877 | 2 | 6.103 | 53.8% | 1 | K.NSSYFVEWIPNNVK.T | 22 |
| \* | LAPMis12DANCE\_011813\_02.09472.09472.2 | 3.7482 | 0.4463 | 100.0% | 1871.3522 | 1871.2018 | 1 | 6.486 | 59.4% | 1 | K.MAVTFIGNSTAIQELFK.R | 2 |
|  | LAPMis12DANCE2\_011813\_01.08520.08520.2 | 2.8584 | 0.3778 | 100.0% | 1229.9722 | 1230.4241 | 2 | 6.75 | 66.7% | 1 | R.ISEQFTAMFR.R | 22 |

Similarities:
gi|5174735|ref|NP\_006(14:5)  

---

|  |  |  |  |  |  |  |  |  |
| --- | --- | --- | --- | --- | --- | --- | --- | --- |
| U | *gi|10190716|ref|NP\_06* | 10 | 16 | 50.9% | 224 | 26153 | 8.0 | spindle pole body component 25 [Homo sapiens] |

| Filename XCorr DeltCN Conf% ObsM+H+ CalcM+H+ SpR ZScore Ion% # Sequence  | | | | | | | | | | | | |
| --- | --- | --- | --- | --- | --- | --- | --- | --- | --- | --- | --- | --- |
| \* | LAPMis12DANCE\_011813\_01.09443.09443.1 | 2.0981 | 0.201 | 100.0% | 1037.29 | 1038.1478 | 3 | 4.537 | 64.3% | 1 | K.SINEFWNK.F | 1 |
| \* | LAPMis12DANCE\_011813\_01.11394.11394.2 | 2.3212 | 0.2074 | 95.3% | 1788.4922 | 1789.0742 | 60 | 4.739 | 42.3% | 1 | R.MVEMFLEYQNQISR.Q | 2 |
| \* | LAPMis12DANCE\_011813\_01.11019.11019.3 | 6.271 | 0.4458 | 100.0% | 2592.7444 | 2592.909 | 1 | 8.327 | 42.9% | 1 | K.GKKQELEVLTANIQDLKEEYSR.K | 3 |
| \* | LAPMis12DANCE2\_011813\_01.09543.09543.3 | 5.593 | 0.3377 | 100.0% | 2408.3044 | 2407.683 | 1 | 6.717 | 44.7% | 1 | K.KQELEVLTANIQDLKEEYSR.K | 3 |
| \* | LAPMis12DANCE\_011813\_01.03468.03468.2 | 2.3841 | 0.1324 | 95.9% | 968.0122 | 968.0544 | 6 | 5.071 | 78.6% | 1 | K.SADLYKDR.L | 2 |
| \* | LAPMis12DANCE\_011813\_01.11606.11606.2 | 3.476 | 0.3336 | 100.0% | 1336.4122 | 1336.5736 | 1 | 6.226 | 75.0% | 4 | K.LQFIFTNIDPK.N | 2 |
| \* | LAPMis12DANCE2\_011813\_01.09419.09419.2 | 4.0069 | 0.443 | 100.0% | 1889.9122 | 1890.1235 | 1 | 7.928 | 70.0% | 1 | K.NPESPFMFSLHLNEAR.D | 2 |
| \* | LAPMis12DANCE\_011813\_02.07262.07262.3 | 5.5717 | 0.5048 | 100.0% | 2505.6843 | 2506.6445 | 1 | 9.168 | 47.6% | 3 | R.DYEVSDSAPHLEGLAEFQENVR.K | 3 |
| \* | LAPMis12DANCE\_011813\_01.10421.10421.2 | 3.8341 | 0.4364 | 100.0% | 1482.5322 | 1482.6823 | 1 | 7.414 | 83.3% | 2 | R.KTNNFSAFLANVR.K | 2 |
| \* | LAPMis12DANCE\_011813\_01.11352.11352.2 | 2.6431 | 0.241 | 99.4% | 1353.8922 | 1354.5082 | 2 | 5.437 | 59.1% | 1 | K.TNNFSAFLANVR.K | 2 |

---

|  |  |  |  |  |  |  |  |  |
| --- | --- | --- | --- | --- | --- | --- | --- | --- |
| U | *gi|223890147|ref|NP\_0* | 22 | 69 | 50.3% | 356 | 40067 | 7.0 | DSN1, MIND kinetochore complex component, homolog isoform 1 [Homo sapiens] |
| U | *gi|223972618|ref|NP\_0* | 22 | 69 | 50.3% | 356 | 40067 | 7.0 | DSN1, MIND kinetochore complex component, homolog isoform 1 [Homo sapiens] |
| U | *gi|223890149|ref|NP\_0* | 22 | 69 | 50.3% | 356 | 40067 | 7.0 | DSN1, MIND kinetochore complex component, homolog isoform 1 [Homo sapiens] |

| Filename XCorr DeltCN Conf% ObsM+H+ CalcM+H+ SpR ZScore Ion% # Sequence  | | | | | | | | | | | | |
| --- | --- | --- | --- | --- | --- | --- | --- | --- | --- | --- | --- | --- |
|  | LAPMis12DANCE\_011813\_01.06000.06000.2 | 3.7046 | 0.3294 | 100.0% | 1433.9321 | 1433.6599 | 1 | 6.184 | 75.0% | 10 | R.SEIIDEKGPVMSK.T | 2 |
|  | LAPMis12DANCE\_011813\_01.10010.10010.3 | 5.5278 | 0.479 | 100.0% | 2105.3943 | 2105.2253 | 1 | 7.353 | 45.6% | 2 | K.THDHQLESSLS\*PVEVFAK.T | 3 |
|  | LAPMis12DANCE\_011813\_01.10131.10131.2 | 4.1291 | 0.4536 | 100.0% | 2184.5522 | 2185.2253 | 1 | 6.747 | 58.8% | 1 | K.THDHQLESS\*LS\*PVEVFAK.T | 2 |
|  | LAPMis12DANCE\_011813\_02.04425.04425.2 | 4.4845 | 0.3923 | 100.0% | 1639.1522 | 1638.7467 | 5 | 7.939 | 53.6% | 16 | K.TSASLEMNQGVSEER.I | 2 |
|  | LAPMis12DANCE\_011813\_02.04630.04630.2 | 2.7457 | 0.2348 | 99.1% | 1718.0521 | 1718.7467 | 1 | 6.01 | 60.7% | 1 | K.TSAS\*LEMNQGVSEER.I | 2 |
|  | LAPMis12DANCE\_011813\_01.06873.06873.2 | 4.1283 | 0.4107 | 100.0% | 1926.1322 | 1926.9512 | 1 | 6.968 | 70.0% | 3 | K.SLHLS\*PQEQSASYQDR.R | 2 |
|  | LAPMis12DANCE\_011813\_01.04962.04962.3 | 3.7744 | 0.2324 | 97.9% | 1953.1444 | 1953.1685 | 5 | 4.9 | 38.3% | 3 | R.RKS\*LHPIHQGITELSR.S | 3 |
|  | LAPMis12DANCE\_011813\_01.06632.06632.2 | 3.6295 | 0.4557 | 100.0% | 1588.1522 | 1588.8069 | 1 | 7.085 | 73.1% | 3 | K.SLHPIHQGITELSR.S | 2 |
|  | LAPMis12DANCE\_011813\_01.06644.06644.3 | 4.2445 | 0.478 | 100.0% | 1588.8243 | 1588.8069 | 1 | 6.815 | 51.9% | 2 | K.SLHPIHQGITELSR.S | 3 |
|  | LAPMis12DANCE\_011813\_01.05571.05571.2 | 2.8788 | 0.2643 | 99.9% | 1204.4722 | 1205.3538 | 5 | 5.978 | 65.0% | 3 | R.SISVDLAESKR.L | 2 |
|  | LAPMis12DANCE2\_011813\_01.10402.10402.2 | 4.1115 | 0.5679 | 100.0% | 2476.4321 | 2477.6873 | 1 | 9.322 | 45.5% | 1 | K.ASSLSEELKHFADGLETDGTLQK.C | 2 |
|  | LAPMis12DANCE\_011813\_01.11720.11720.3 | 5.5295 | 0.3601 | 100.0% | 2478.9243 | 2477.6873 | 1 | 6.408 | 35.2% | 3 | K.ASSLSEELKHFADGLETDGTLQK.C | 3 |
|  | LAPMis12DANCE\_011813\_01.07055.07055.2 | 3.8188 | 0.4356 | 100.0% | 1532.3722 | 1532.65 | 1 | 8.241 | 73.1% | 7 | K.HFADGLETDGTLQK.C | 2 |
|  | LAPMis12DANCE\_011813\_02.07070.07070.2 | 3.7373 | 0.4365 | 100.0% | 1485.6322 | 1485.6489 | 1 | 7.829 | 57.7% | 2 | K.ASDFSLEASVAEMK.E | 2 |
|  | LAPMis12DANCE\_011813\_01.12612.12612.2 | 4.7278 | 0.5125 | 100.0% | 2118.672 | 2120.379 | 1 | 8.923 | 61.1% | 1 | K.ASDFSLEASVAEMKEYITK.F | 2 |
|  | LAPMis12DANCE\_011813\_02.08806.08806.3 | 3.2038 | 0.2874 | 98.7% | 2120.0044 | 2120.379 | 1 | 5.675 | 36.1% | 2 | K.ASDFSLEASVAEMKEYITK.F | 3 |
|  | LAPMis12DANCE\_011813\_01.10071.10071.3 | 5.7634 | 0.4478 | 100.0% | 3213.3542 | 3213.627 | 1 | 7.183 | 36.1% | 2 | K.ITEVKVEPMTYLGSSQNEVLNTKPDYQK.I | 3 |
|  | LAPMis12DANCE\_011813\_01.09789.09789.3 | 3.5178 | 0.4033 | 100.0% | 2641.8542 | 2642.9404 | 3 | 5.862 | 30.7% | 1 | K.VEPMTYLGSSQNEVLNTKPDYQK.I | 3 |
|  | LAPMis12DANCE\_011813\_01.09760.09760.2 | 4.5182 | 0.4347 | 100.0% | 2642.2722 | 2642.9404 | 3 | 7.155 | 45.5% | 1 | K.VEPMTYLGSSQNEVLNTKPDYQK.I | 2 |
|  | LAPMis12DANCE\_011813\_01.03909.03909.2 | 2.1249 | 0.2342 | 98.2% | 887.03217 | 887.0702 | 48 | 4.89 | 71.4% | 1 | K.VSVQLGKR.S | 2 |
|  | LAPMis12DANCE\_011813\_01.05688.05688.2 | 2.809 | 0.2156 | 99.5% | 1309.5322 | 1310.3813 | 1 | 5.491 | 65.0% | 3 | R.SMQQLDPS\*PAR.K | 2 |
|  | LAPMis12DANCE\_011813\_01.04472.04472.2 | 2.3226 | 0.2608 | 98.7% | 1438.0122 | 1438.5554 | 1 | 5.352 | 63.6% | 1 | R.SMQQLDPS\*PARK.L | 2 |

---

|  |  |  |  |  |  |  |  |  |
| --- | --- | --- | --- | --- | --- | --- | --- | --- |
| U | *contaminant\_KERATIN09* | 19 | 61 | 49.7% | 429 | 47927 | 5.5 | no description |
| U | *gi|4557888|ref|NP\_000* | 19 | 60 | 49.5% | 430 | 48058 | 5.5 | keratin 18 [Homo sapiens] |
| U | *gi|40354195|ref|NP\_95* | 19 | 60 | 49.5% | 430 | 48058 | 5.5 | keratin 18 [Homo sapiens] |

| Filename XCorr DeltCN Conf% ObsM+H+ CalcM+H+ SpR ZScore Ion% # Sequence  | | | | | | | | | | | | |
| --- | --- | --- | --- | --- | --- | --- | --- | --- | --- | --- | --- | --- |
|  | LAPMis12DANCE\_011813\_01.08468.08468.3 | 4.4568 | 0.5655 | 100.0% | 2855.0044 | 2856.0813 | 1 | 9.164 | 30.8% | 5 | R.SLGSVQAPSYGARPVSSAASVYAGAGGSGSR.I | 3 |
|  | LAPMis12DANCE\_011813\_01.11256.11256.2 | 5.0844 | 0.5399 | 100.0% | 2262.612 | 2262.561 | 1 | 8.341 | 52.0% | 2 | R.GGMGSGGLATGIAGGLAGMGGIQNEK.E | 2 |
|  | LAPMis12DANCE\_011813\_01.11223.11223.3 | 6.0827 | 0.4228 | 100.0% | 3336.7744 | 3337.7224 | 1 | 8.564 | 25.0% | 3 | R.GGMGSGGLATGIAGGLAGMGGIQNEKETMQSLNDR.L | 3 |
|  | LAPMis12DANCE\_011813\_01.04262.04262.2 | 2.339 | 0.2357 | 99.0% | 1093.9122 | 1094.1846 | 228 | 4.557 | 56.2% | 1 | K.ETMQSLNDR.L | 2 |
|  | LAPMis12DANCE\_011813\_02.07155.07155.3 | 3.9221 | 0.387 | 100.0% | 2059.8843 | 2060.3176 | 1 | 6.113 | 39.7% | 1 | K.IIEDLRAQIFANTVDNAR.I | 3 |
|  | LAPMis12DANCE\_011813\_01.07341.07341.2 | 3.5336 | 0.4432 | 100.0% | 1321.3121 | 1320.4478 | 1 | 7.48 | 77.3% | 3 | R.AQIFANTVDNAR.I | 2 |
|  | LAPMis12DANCE\_011813\_01.07811.07811.1 | 2.1522 | 0.2588 | 100.0% | 1041.41 | 1042.2235 | 10 | 5.465 | 62.5% | 1 | R.IVLQIDNAR.L | 11 |
|  | LAPMis12DANCE\_011813\_01.07768.07768.2 | 2.6317 | 0.2885 | 100.0% | 1042.0521 | 1042.2235 | 1 | 6.3 | 87.5% | 2 | R.IVLQIDNAR.L | 22 |
|  | LAPMis12DANCE\_011813\_01.06508.06508.2 | 2.9629 | 0.4049 | 100.0% | 1241.1122 | 1240.4601 | 25 | 6.793 | 72.2% | 4 | R.VKYETELAMR.Q | 2 |
|  | LAPMis12DANCE\_011813\_01.04256.04256.2 | 3.2727 | 0.2508 | 100.0% | 1175.2322 | 1175.3274 | 1 | 6.155 | 77.8% | 2 | R.KVIDDTNITR.L | 2 |
|  | LAPMis12DANCE\_011813\_01.14678.14678.2 | 6.0437 | 0.5266 | 100.0% | 2177.5322 | 2178.589 | 1 | 10.557 | 64.7% | 4 | R.LQLETEIEALKEELLFMK.K | 2 |
|  | LAPMis12DANCE2\_011813\_01.13226.13226.3 | 3.2289 | 0.2298 | 96.1% | 2179.2244 | 2178.589 | 10 | 5.582 | 36.8% | 2 | R.LQLETEIEALKEELLFMK.K | 3 |
|  | LAPMis12DANCE\_011813\_01.10119.10119.3 | 4.8729 | 0.4425 | 100.0% | 2750.7844 | 2751.0227 | 1 | 7.737 | 38.0% | 3 | K.NHEEEVKGLQAQIASSGLTVEVDAPK.S | 3 |
|  | LAPMis12DANCE\_011813\_01.10598.10598.2 | 3.9325 | 0.4012 | 100.0% | 1885.3722 | 1885.1246 | 1 | 7.055 | 55.6% | 2 | K.GLQAQIASSGLTVEVDAPK.S | 2 |
|  | LAPMis12DANCE\_011813\_01.05081.05081.1 | 1.8167 | 0.2135 | 96.2% | 965.56 | 966.0385 | 37 | 4.477 | 50.0% | 2 | R.AQYDELAR.K | 1 |
|  | LAPMis12DANCE\_011813\_02.07446.07446.2 | 3.6717 | 0.4863 | 100.0% | 1508.3322 | 1507.699 | 1 | 7.989 | 75.0% | 7 | R.TVQSLEIDLDSMR.N | 2 |
|  | LAPMis12DANCE2\_011813\_01.14108.14108.3 | 6.2626 | 0.54 | 100.0% | 2670.9543 | 2672.0715 | 1 | 9.543 | 45.5% | 14 | R.YALQMEQLNGILLHLESELAQTR.A | 3 |
|  | LAPMis12DANCE2\_011813\_01.14078.14078.2 | 3.4327 | 0.4836 | 100.0% | 2671.612 | 2672.0715 | 1 | 8.437 | 54.5% | 2 | R.YALQMEQLNGILLHLESELAQTR.A | 2 |
|  | LAPMis12DANCE\_011813\_01.10869.10869.2 | 3.5037 | 0.3746 | 100.0% | 1420.4521 | 1420.6055 | 3 | 6.203 | 68.2% | 1 | R.QAQEYEALLNIK.V | 2 |

Similarities:
contaminant\_KERATIN10(2:17)  

---

|  |  |  |  |  |  |  |  |  |
| --- | --- | --- | --- | --- | --- | --- | --- | --- |
| U | *gi|55956899|ref|NP\_00* | 25 | 60 | 49.3% | 623 | 62064 | 5.2 | keratin 9 [Homo sapiens] |

| Filename XCorr DeltCN Conf% ObsM+H+ CalcM+H+ SpR ZScore Ion% # Sequence  | | | | | | | | | | | | |
| --- | --- | --- | --- | --- | --- | --- | --- | --- | --- | --- | --- | --- |
| \* | LAPMis12DANCE\_011813\_01.04096.04096.1 | 2.5213 | 0.2731 | 100.0% | 1232.53 | 1233.2833 | 61 | 5.775 | 33.3% | 1 | R.SGGGGGGGLGSGGSIR.S | 1 |
| \* | LAPMis12DANCE\_011813\_01.04130.04130.2 | 4.3168 | 0.5289 | 100.0% | 1232.6122 | 1233.2833 | 1 | 8.033 | 80.0% | 4 | R.SGGGGGGGLGSGGSIR.S | 2 |
|  | LAPMis12DANCE\_011813\_01.03693.03693.2 | 3.4852 | 0.487 | 100.0% | 1237.2522 | 1236.2401 | 1 | 8.684 | 79.2% | 2 | R.FSSSSGYGGGSSR.V | 2 |
|  | LAPMis12DANCE\_011813\_02.07034.07034.3 | 5.2138 | 0.4401 | 100.0% | 2706.6843 | 2706.7605 | 1 | 8.555 | 31.5% | 1 | R.GGGGSFGYSYGGGSGGGFSASSLGGGFGGGSR.G | 3 |
|  | LAPMis12DANCE\_011813\_01.04240.04240.2 | 2.6743 | 0.281 | 100.0% | 1065.9922 | 1066.1742 | 1 | 5.684 | 87.5% | 3 | K.STMQELNSR.L | 2 |
|  | LAPMis12DANCE\_011813\_01.05114.05114.1 | 1.7551 | 0.2197 | 97.3% | 809.35 | 809.93774 | 6 | 5.101 | 66.7% | 2 | R.LASYLDK.V | 11111 |
|  | LAPMis12DANCE\_011813\_01.10941.10941.2 | 5.8718 | 0.5403 | 100.0% | 2378.4722 | 2378.5981 | 1 | 9.937 | 65.0% | 1 | R.LASYLDKVQALEEANNDLENK.I | 2 |
|  | LAPMis12DANCE\_011813\_01.12630.12630.3 | 3.9734 | 0.3311 | 99.7% | 3327.3542 | 3327.6287 | 1 | 4.706 | 25.9% | 1 | R.LASYLDKVQALEEANNDLENKIQDWYDK.K | 3 |
|  | LAPMis12DANCE\_011813\_01.07202.07202.2 | 4.2793 | 0.3654 | 100.0% | 1589.5322 | 1587.6836 | 1 | 6.449 | 69.2% | 3 | K.VQALEEANNDLENK.I | 2 |
|  | LAPMis12DANCE\_011813\_01.13466.13466.3 | 3.6837 | 0.4315 | 100.0% | 2903.1543 | 2904.1597 | 1 | 7.203 | 27.1% | 2 | K.NYSPYYNTIDDLKDQIVDLTVGNNK.T | 3 |
|  | LAPMis12DANCE\_011813\_01.08546.08546.1 | 2.4288 | 0.3996 | 100.0% | 1060.44 | 1061.1802 | 2 | 6.644 | 62.5% | 2 | K.TLLDIDNTR.M | 1 |
|  | LAPMis12DANCE\_011813\_01.08528.08528.2 | 2.8898 | 0.3857 | 100.0% | 1060.9922 | 1061.1802 | 1 | 6.323 | 87.5% | 3 | K.TLLDIDNTR.M | 2 |
|  | LAPMis12DANCE\_011813\_01.09237.09237.2 | 2.1744 | 0.2562 | 99.4% | 897.53217 | 898.02155 | 2 | 5.448 | 75.0% | 2 | R.MTLDDFR.I | 2 |
|  | LAPMis12DANCE\_011813\_02.05548.05548.2 | 2.6168 | 0.2603 | 99.5% | 1308.1921 | 1308.5383 | 3 | 5.112 | 66.7% | 3 | R.IKFEMEQNLR.Q | 2 |
|  | LAPMis12DANCE\_011813\_01.07348.07348.2 | 2.9649 | 0.3224 | 100.0% | 1158.3922 | 1158.2566 | 1 | 6.295 | 70.0% | 3 | R.QGVDADINGLR.Q | 2 |
|  | LAPMis12DANCE\_011813\_01.11006.11006.2 | 3.0452 | 0.1214 | 96.3% | 1852.3522 | 1853.1003 | 1 | 4.577 | 57.1% | 1 | K.TLNDMRQEYEQLIAK.N | 2 |
|  | LAPMis12DANCE\_011813\_01.11015.11015.3 | 3.249 | 0.382 | 100.0% | 1853.8744 | 1853.1003 | 1 | 6.015 | 48.2% | 1 | K.TLNDMRQEYEQLIAK.N | 3 |
|  | LAPMis12DANCE\_011813\_01.11883.11883.3 | 7.0309 | 0.4814 | 100.0% | 3267.0244 | 3266.413 | 1 | 8.881 | 33.9% | 4 | K.DIENQYETQITQIEHEVSSSGQEVQSSAK.E | 3 |
|  | LAPMis12DANCE\_011813\_02.07391.07391.2 | 6.1549 | 0.5615 | 100.0% | 1838.3522 | 1839.0557 | 1 | 10.068 | 80.0% | 3 | R.HGVQELEIELQSQLSK.K | 2 |
|  | LAPMis12DANCE\_011813\_02.07396.07396.3 | 4.1022 | 0.2669 | 99.5% | 1838.8143 | 1839.0557 | 1 | 5.756 | 40.0% | 1 | R.HGVQELEIELQSQLSK.K | 3 |
|  | LAPMis12DANCE\_011813\_02.06944.06944.3 | 5.6147 | 0.3893 | 100.0% | 1967.2144 | 1967.2297 | 1 | 6.998 | 48.4% | 5 | R.HGVQELEIELQSQLSKK.A | 3 |
|  | LAPMis12DANCE\_011813\_01.10091.10091.2 | 4.6686 | 0.5375 | 100.0% | 2510.5122 | 2511.6177 | 1 | 8.61 | 52.3% | 2 | K.EIETYHNLLEGGQEDFESSGAGK.I | 2 |
|  | LAPMis12DANCE\_011813\_02.06512.06512.3 | 6.0784 | 0.2628 | 99.7% | 2511.0544 | 2511.6177 | 1 | 8.168 | 45.5% | 3 | K.EIETYHNLLEGGQEDFESSGAGK.I | 3 |
|  | LAPMis12DANCE\_011813\_01.03626.03626.2 | 5.36 | 0.4236 | 100.0% | 1792.1921 | 1792.7324 | 1 | 11.624 | 50.0% | 3 | R.GGSGGSYGGGGSGGGYGGGSGSR.G | 2 |
|  | LAPMis12DANCE\_011813\_02.03968.03968.3 | 8.049 | 0.5546 | 100.0% | 3223.9443 | 3225.1118 | 1 | 10.572 | 28.8% | 4 | R.GGSGGSHGGGSGFGGESGGSYGGGEEASGSGGGYGGGSGK.S | 3 |

Similarities:
contaminant\_KERATIN03(1:24)  
gi|15431310|ref|NP\_00(1:24)  
contaminant\_KERATIN12(1:24)  
contaminant\_KERATIN10(1:24)  

---

|  |  |  |  |  |  |  |  |  |
| --- | --- | --- | --- | --- | --- | --- | --- | --- |
| U | *gi|4501885|ref|NP\_001* | 16 | 39 | 48.5% | 375 | 41737 | 5.5 | beta actin [Homo sapiens] |
| U | *gi|4501887|ref|NP\_001* | 16 | 39 | 48.5% | 375 | 41793 | 5.5 | actin, gamma 1 propeptide [Homo sapiens] |

| Filename XCorr DeltCN Conf% ObsM+H+ CalcM+H+ SpR ZScore Ion% # Sequence  | | | | | | | | | | | | |
| --- | --- | --- | --- | --- | --- | --- | --- | --- | --- | --- | --- | --- |
|  | LAPMis12DANCE\_011813\_01.04340.04340.2 | 3.195 | 0.3595 | 100.0% | 977.0122 | 977.02136 | 1 | 6.573 | 77.8% | 1 | K.AGFAGDDAPR.A | 22 |
|  | LAPMis12DANCE\_011813\_01.08306.08306.2 | 2.5563 | 0.1578 | 96.6% | 1199.2522 | 1199.4415 | 6 | 5.24 | 65.0% | 4 | R.AVFPSIVGRPR.H | 22 |
|  | LAPMis12DANCE\_011813\_01.04196.04196.1 | 2.8041 | 0.4625 | 100.0% | 1171.37 | 1172.4058 | 1 | 7.949 | 70.0% | 3 | R.HQGVMVGMGQK.D | 11 |
|  | LAPMis12DANCE\_011813\_01.04214.04214.2 | 3.2151 | 0.3352 | 100.0% | 1172.0721 | 1172.4058 | 1 | 6.264 | 85.0% | 3 | R.HQGVMVGMGQK.D | 22 |
|  | LAPMis12DANCE2\_011813\_01.03634.03634.2 | 2.9425 | 0.1034 | 96.2% | 1355.2522 | 1355.4038 | 1 | 6.437 | 77.3% | 2 | K.DSYVGDEAQSKR.G | 22 |
|  | LAPMis12DANCE\_011813\_01.07454.07454.3 | 2.9923 | 0.2437 | 97.5% | 1516.6144 | 1516.7019 | 3 | 5.55 | 52.5% | 1 | K.IWHHTFYNELR.V | 33 |
|  | LAPMis12DANCE\_011813\_01.07502.07502.2 | 3.1529 | 0.3794 | 100.0% | 1517.1921 | 1516.7019 | 1 | 5.859 | 85.0% | 8 | K.IWHHTFYNELR.V | 22 |
|  | LAPMis12DANCE\_011813\_01.09182.09182.2 | 4.3576 | 0.2974 | 100.0% | 1954.5122 | 1955.2615 | 1 | 9.187 | 50.0% | 4 | R.VAPEEHPVLLTEAPLNPK.A | 2 |
|  | LAPMis12DANCE2\_011813\_01.09723.09723.3 | 6.9366 | 0.6006 | 100.0% | 3184.2244 | 3185.622 | 1 | 10.036 | 31.0% | 1 | R.TTGIVMDSGDGVTHTVPIYEGYALPHAILR.L | 3 |
|  | LAPMis12DANCE\_011813\_01.11112.11112.1 | 1.8036 | 0.2511 | 100.0% | 998.37 | 999.167 | 13 | 5.216 | 64.3% | 1 | R.DLTDYLMK.I | 11 |
|  | LAPMis12DANCE\_011813\_01.06573.06573.2 | 2.6996 | 0.4341 | 100.0% | 1132.9122 | 1133.2029 | 1 | 7.619 | 77.8% | 2 | R.GYSFTTTAER.E | 2 |
|  | LAPMis12DANCE2\_011813\_01.08888.08888.2 | 3.961 | 0.2645 | 100.0% | 1791.4122 | 1791.9554 | 2 | 8.179 | 66.7% | 4 | K.SYELPDGQVITIGNER.F | 22 |
|  | LAPMis12DANCE\_011813\_02.07584.07584.2 | 3.9887 | 0.4676 | 100.0% | 2216.2722 | 2216.4705 | 1 | 7.042 | 47.5% | 2 | K.DLYANTVLSGGTTMYPGIADR.M | 2 |
|  | LAPMis12DANCE\_011813\_01.07542.07542.1 | 2.3831 | 0.4599 | 100.0% | 1161.44 | 1162.3868 | 1 | 7.36 | 65.0% | 1 | K.EITALAPSTMK.I | 11 |
|  | LAPMis12DANCE\_011813\_01.07574.07574.2 | 2.2003 | 0.3331 | 99.5% | 1161.4922 | 1162.3868 | 7 | 5.913 | 60.0% | 1 | K.EITALAPSTMK.I | 22 |
|  | LAPMis12DANCE\_011813\_01.04997.04997.2 | 2.165 | 0.3932 | 99.9% | 1517.3522 | 1517.595 | 11 | 5.866 | 45.8% | 1 | K.QEYDESGPSIVHR.K | 2 |

Similarities:
gi|4501881|ref|NP\_001(11:5)  

---

|  |  |  |  |  |  |  |  |  |
| --- | --- | --- | --- | --- | --- | --- | --- | --- |
| U | *gi|20357599|ref|NP\_61* | 5 | 8 | 48.2% | 114 | 12146 | 10.5 | H2A histone family, member V isoform 2 [Homo sapiens] |
| U | *gi|6912616|ref|NP\_036* | 5 | 8 | 43.0% | 128 | 13509 | 10.6 | H2A histone family, member V isoform 1 [Homo sapiens] |
| U | *gi|4504255|ref|NP\_002* | 5 | 8 | 43.0% | 128 | 13553 | 10.6 | H2A histone family, member Z [Homo sapiens] |

| Filename XCorr DeltCN Conf% ObsM+H+ CalcM+H+ SpR ZScore Ion% # Sequence  | | | | | | | | | | | | |
| --- | --- | --- | --- | --- | --- | --- | --- | --- | --- | --- | --- | --- |
|  | LAPMis12DANCE\_011813\_01.09038.09038.1 | 2.051 | 0.2284 | 100.0% | 944.57 | 945.1093 | 6 | 5.27 | 56.2% | 1 | R.AGLQFPVGR.I | 11111 |
|  | LAPMis12DANCE\_011813\_01.09014.09014.2 | 2.9676 | 0.2668 | 100.0% | 944.9522 | 945.1093 | 1 | 5.313 | 87.5% | 3 | R.AGLQFPVGR.I | 22222 |
|  | LAPMis12DANCE2\_011813\_01.16917.16917.3 | 4.0168 | 0.4589 | 100.0% | 2896.0444 | 2897.2952 | 1 | 6.823 | 28.6% | 2 | R.VGATAAVYSAAILEYLTAEVLELAGNASK.D | 3 |
|  | LAPMis12DANCE\_011813\_01.05489.05489.1 | 1.4835 | 0.2754 | 100.0% | 850.5 | 851.0396 | 2 | 5.039 | 66.7% | 1 | R.HLQLAIR.G | 11111 |
|  | LAPMis12DANCE2\_011813\_01.08812.08812.3 | 2.6615 | 0.3637 | 99.7% | 1951.4644 | 1951.2303 | 8 | 5.367 | 34.4% | 1 | R.HLQLAIRGDEELDSLIK.A | 32 |

Similarities:
gi|10645195|ref|NP\_06(3:2)  
gi|10800130|ref|NP\_06(3:2)  
gi|106775678|ref|NP\_0(3:2)  
gi|4504253|ref|NP\_002(3:2)  

---

|  |  |  |  |  |  |  |  |  |
| --- | --- | --- | --- | --- | --- | --- | --- | --- |
| U | *gi|119395750|ref|NP\_0* | 39 | 99 | 48.0% | 644 | 66039 | 8.1 | keratin 1 [Homo sapiens] |

| Filename XCorr DeltCN Conf% ObsM+H+ CalcM+H+ SpR ZScore Ion% # Sequence  | | | | | | | | | | | | |
| --- | --- | --- | --- | --- | --- | --- | --- | --- | --- | --- | --- | --- |
| \* | LAPMis12DANCE\_011813\_01.08416.08416.2 | 4.2639 | 0.4347 | 100.0% | 1658.0721 | 1658.7678 | 1 | 7.009 | 59.4% | 3 | R.SGGGFSSGSAGIINYQR.R | 2 |
| \* | LAPMis12DANCE\_011813\_01.06357.06357.1 | 1.5736 | 0.3247 | 100.0% | 874.4 | 875.0128 | 18 | 5.209 | 56.2% | 3 | R.SLVNLGGSK.S | 1 |
|  | LAPMis12DANCE\_011813\_01.11078.11078.2 | 3.5906 | 0.4728 | 100.0% | 1384.1522 | 1384.5315 | 4 | 7.157 | 54.5% | 1 | K.SLNNQFASFIDK.V | 2 |
|  | LAPMis12DANCE\_011813\_01.07401.07401.2 | 4.4166 | 0.1231 | 100.0% | 1475.7122 | 1476.6726 | 1 | 7.41 | 90.9% | 4 | R.FLEQQNQVLQTK.W | 22 |
|  | LAPMis12DANCE\_011813\_02.07764.07764.3 | 5.7079 | 0.4671 | 100.0% | 2934.4143 | 2934.2786 | 1 | 8.47 | 35.9% | 1 | R.FLEQQNQVLQTKWELLQQVDTSTR.T | 3 |
|  | LAPMis12DANCE\_011813\_02.07041.07041.2 | 4.2815 | 0.4495 | 100.0% | 1477.2122 | 1476.6293 | 1 | 8.102 | 86.4% | 5 | K.WELLQQVDTSTR.T | 2 |
|  | LAPMis12DANCE\_011813\_01.12890.12890.2 | 4.7872 | 0.4567 | 100.0% | 1994.4321 | 1995.2017 | 1 | 8.525 | 70.0% | 4 | R.THNLEPYFESFINNLR.R | 2 |
|  | LAPMis12DANCE\_011813\_01.12938.12938.3 | 4.9642 | 0.387 | 100.0% | 1995.0543 | 1995.2017 | 1 | 7.075 | 51.7% | 3 | R.THNLEPYFESFINNLR.R | 3 |
|  | LAPMis12DANCE\_011813\_01.12220.12220.2 | 3.1549 | 0.3073 | 100.0% | 2150.7322 | 2151.3892 | 3 | 5.396 | 43.8% | 2 | R.THNLEPYFESFINNLRR.R | 2 |
| \* | LAPMis12DANCE\_011813\_01.03165.03165.2 | 2.6879 | 0.2929 | 99.9% | 1487.8322 | 1488.6467 | 1 | 5.497 | 63.6% | 1 | R.RRVDQLKSDQSR.L | 2 |
|  | LAPMis12DANCE\_011813\_01.03897.03897.2 | 2.7653 | 0.3015 | 100.0% | 1308.5322 | 1309.4215 | 1 | 5.673 | 77.8% | 2 | R.NKYEDEINKR.T | 2222 |
|  | LAPMis12DANCE\_011813\_01.08084.08084.2 | 3.2847 | 0.4156 | 100.0% | 1266.1322 | 1266.3934 | 1 | 7.409 | 75.0% | 3 | R.TNAENEFVTIK.K | 2 |
|  | LAPMis12DANCE\_011813\_01.05870.05870.2 | 3.6927 | 0.2899 | 100.0% | 1394.3322 | 1394.5675 | 4 | 6.257 | 63.6% | 7 | R.TNAENEFVTIKK.D | 2 |
|  | LAPMis12DANCE\_011813\_01.04781.04781.1 | 1.8829 | 0.2801 | 100.0% | 999.32 | 1000.1114 | 6 | 6.657 | 56.2% | 1 | K.DVDGAYMTK.V | 1 |
| \* | LAPMis12DANCE\_011813\_01.12716.12716.2 | 4.4737 | 0.4667 | 100.0% | 1302.8922 | 1303.4955 | 1 | 8.849 | 81.8% | 5 | R.SLDLDSIIAEVK.A | 2 |
| \* | LAPMis12DANCE\_011813\_01.14910.14910.2 | 2.6653 | 0.2992 | 99.4% | 2350.5923 | 2350.6282 | 10 | 4.939 | 30.0% | 1 | R.SLDLDSIIAEVKAQYEDIAQK.S | 2 |
| \* | LAPMis12DANCE\_011813\_01.04436.04436.2 | 2.6158 | 0.269 | 99.9% | 1065.9521 | 1066.1558 | 1 | 5.398 | 75.0% | 2 | K.AQYEDIAQK.S | 2 |
|  | LAPMis12DANCE\_011813\_01.03993.03993.2 | 3.9107 | 0.464 | 100.0% | 1341.0922 | 1341.4607 | 1 | 7.806 | 77.3% | 3 | K.SKAEAESLYQSK.Y | 2 |
|  | LAPMis12DANCE\_011813\_01.08859.08859.3 | 5.7167 | 0.5645 | 100.0% | 2501.7244 | 2502.7405 | 1 | 9.097 | 45.2% | 2 | K.SKAEAESLYQSKYEELQITAGR.H | 3 |
|  | LAPMis12DANCE\_011813\_01.04538.04538.1 | 2.2954 | 0.3818 | 100.0% | 1125.48 | 1126.2084 | 2 | 6.671 | 61.1% | 1 | K.AEAESLYQSK.Y | 1 |
|  | LAPMis12DANCE\_011813\_01.04516.04516.2 | 2.8029 | 0.375 | 100.0% | 1126.0922 | 1126.2084 | 1 | 6.651 | 83.3% | 1 | K.AEAESLYQSK.Y | 2 |
|  | LAPMis12DANCE\_011813\_02.05306.05306.2 | 3.7619 | 0.3376 | 100.0% | 1179.5922 | 1180.303 | 2 | 7.4 | 83.3% | 8 | K.YEELQITAGR.H | 22 |
|  | LAPMis12DANCE\_011813\_01.06143.06143.2 | 2.829 | 0.246 | 99.7% | 1304.0322 | 1303.4581 | 1 | 5.446 | 70.0% | 3 | R.NSKIEISELNR.V | 2 |
|  | LAPMis12DANCE\_011813\_01.07370.07370.1 | 1.9174 | 0.2765 | 100.0% | 973.43 | 974.102 | 7 | 4.277 | 64.3% | 1 | K.IEISELNR.V | 11 |
|  | LAPMis12DANCE\_011813\_01.07371.07371.2 | 2.2684 | 0.1613 | 96.2% | 974.0122 | 974.102 | 23 | 4.01 | 71.4% | 1 | K.IEISELNR.V | 22 |
|  | LAPMis12DANCE\_011813\_01.03896.03896.2 | 2.4877 | 0.1283 | 95.7% | 1075.3522 | 1074.2223 | 50 | 4.154 | 56.2% | 1 | R.LRSEIDNVK.K | 2 |
|  | LAPMis12DANCE\_011813\_01.08816.08816.2 | 4.8296 | 0.4564 | 100.0% | 1718.3322 | 1717.8333 | 1 | 8.109 | 64.3% | 3 | K.QISNLQQSISDAEQR.G | 2 |
|  | LAPMis12DANCE\_011813\_01.10190.10190.2 | 4.9733 | 0.4078 | 100.0% | 1601.5122 | 1600.769 | 1 | 7.2 | 80.8% | 2 | K.NKLNDLEDALQQAK.E | 2 |
| \* | LAPMis12DANCE\_011813\_01.11529.11529.3 | 5.5004 | 0.3793 | 100.0% | 2185.7644 | 2185.399 | 1 | 7.434 | 41.7% | 4 | K.NKLNDLEDALQQAKEDLAR.L | 3 |
|  | LAPMis12DANCE\_011813\_01.09690.09690.2 | 3.1613 | 0.242 | 99.9% | 1357.6322 | 1358.4912 | 1 | 5.876 | 81.8% | 3 | K.LNDLEDALQQAK.E | 2 |
| \* | LAPMis12DANCE\_011813\_01.11823.11823.2 | 4.3303 | 0.4636 | 100.0% | 1941.6921 | 1943.121 | 1 | 7.825 | 62.5% | 2 | K.LNDLEDALQQAKEDLAR.L | 2 |
|  | LAPMis12DANCE\_011813\_01.08876.08876.3 | 3.3919 | 0.244 | 98.3% | 1524.9243 | 1524.7754 | 116 | 5.251 | 38.6% | 1 | R.LLRDYQELMNTK.L | 3 |
|  | LAPMis12DANCE\_011813\_01.08846.08846.2 | 3.8245 | 0.2667 | 100.0% | 1525.1522 | 1524.7754 | 1 | 5.874 | 77.3% | 2 | R.LLRDYQELMNTK.L | 2 |
|  | LAPMis12DANCE\_011813\_01.07596.07596.2 | 2.2111 | 0.3001 | 99.5% | 1142.4521 | 1142.2689 | 4 | 5.86 | 68.8% | 1 | R.DYQELMNTK.L | 2 |
|  | LAPMis12DANCE\_011813\_01.04988.04988.1 | 2.2147 | 0.3831 | 100.0% | 1033.51 | 1034.1112 | 1 | 6.997 | 62.5% | 2 | R.TLLEGEESR.M | 1 |
|  | LAPMis12DANCE\_011813\_01.04970.04970.2 | 2.416 | 0.2923 | 99.8% | 1033.9922 | 1034.1112 | 4 | 5.063 | 75.0% | 2 | R.TLLEGEESR.M | 2 |
|  | LAPMis12DANCE\_011813\_01.04312.04312.2 | 5.6745 | 0.5644 | 100.0% | 2384.5723 | 2385.298 | 1 | 10.135 | 43.3% | 2 | R.GGGGGGYGSGGSSYGSGGGSYGSGGGGGGGR.G | 2 |
|  | LAPMis12DANCE\_011813\_02.03663.03663.3 | 6.9592 | 0.4451 | 100.0% | 2386.2844 | 2385.298 | 1 | 10.852 | 37.5% | 3 | R.GGGGGGYGSGGSSYGSGGGSYGSGGGGGGGR.G | 3 |
| \* | LAPMis12DANCE\_011813\_02.03986.03986.3 | 5.1841 | 0.2206 | 98.7% | 3314.1543 | 3314.2085 | 1 | 7.772 | 22.4% | 3 | R.GSYGSGGSSYGSGGGSYGSGGGGGGHGSYGSGSSSGGYR.G | 3 |

Similarities:
gi|4504919|ref|NP\_002(1:38)  
gi|47132620|ref|NP\_00(3:36)  
gi|119395754|ref|NP\_0(1:38)  
gi|119703753|ref|NP\_0(2:37)  

---

|  |  |  |  |  |  |  |  |  |
| --- | --- | --- | --- | --- | --- | --- | --- | --- |
| U | *GFP* | 19 | 127 | 46.6% | 238 | 26813 | 5.8 | no description |

| Filename XCorr DeltCN Conf% ObsM+H+ CalcM+H+ SpR ZScore Ion% # Sequence  | | | | | | | | | | | | |
| --- | --- | --- | --- | --- | --- | --- | --- | --- | --- | --- | --- | --- |
| \* | LAPMis12DANCE2\_011813\_01.13250.13250.3 | 3.7439 | 0.3132 | 99.7% | 2438.4243 | 2438.7397 | 1 | 5.98 | 33.0% | 9 | K.GEELFTGVVPILVELDGDVNGHK.F | 3 |
| \* | LAPMis12DANCE2\_011813\_01.13098.13098.2 | 5.0988 | 0.5638 | 100.0% | 2438.632 | 2438.7397 | 1 | 9.475 | 50.0% | 5 | K.GEELFTGVVPILVELDGDVNGHK.F | 2 |
| \* | LAPMis12DANCE\_011813\_02.04790.04790.2 | 4.2752 | 0.5385 | 100.0% | 1505.0721 | 1504.5499 | 1 | 8.981 | 67.9% | 26 | K.FSVSGEGEGDATYGK.L | 2 |
| \* | LAPMis12DANCE\_011813\_01.05984.05984.2 | 3.0915 | 0.4772 | 100.0% | 1266.8322 | 1267.399 | 1 | 7.871 | 75.0% | 10 | K.SAMPEGYVQER.T | 2 |
| \* | LAPMis12DANCE\_011813\_01.07694.07694.2 | 3.5698 | 0.435 | 100.0% | 1348.9521 | 1348.4979 | 1 | 6.976 | 80.0% | 22 | R.TIFFKDDGNYK.T | 2 |
| \* | LAPMis12DANCE\_011813\_02.04804.04804.3 | 3.8658 | 0.3873 | 100.0% | 1735.7644 | 1735.9376 | 1 | 7.019 | 44.6% | 1 | K.TRAEVKFEGDTLVNR.I | 3 |
| \* | LAPMis12DANCE\_011813\_02.05204.05204.2 | 4.0264 | 0.4096 | 100.0% | 1478.3322 | 1478.6451 | 1 | 7.918 | 79.2% | 23 | R.AEVKFEGDTLVNR.I | 2 |
| \* | LAPMis12DANCE\_011813\_02.05198.05198.3 | 3.1996 | 0.3155 | 99.7% | 1478.5443 | 1478.6451 | 1 | 6.029 | 50.0% | 1 | R.AEVKFEGDTLVNR.I | 3 |
| \* | LAPMis12DANCE\_011813\_01.09984.09984.2 | 4.4627 | 0.4448 | 100.0% | 1961.4722 | 1962.2535 | 2 | 7.386 | 43.8% | 1 | R.AEVKFEGDTLVNRIELK.G | 2 |
| \* | LAPMis12DANCE\_011813\_01.09998.09998.3 | 2.975 | 0.3177 | 99.5% | 1961.5144 | 1962.2535 | 1 | 4.954 | 39.1% | 1 | R.AEVKFEGDTLVNRIELK.G | 3 |
| \* | LAPMis12DANCE\_011813\_01.06614.06614.1 | 2.1451 | 0.3862 | 100.0% | 1050.44 | 1051.1442 | 1 | 6.507 | 62.5% | 4 | K.FEGDTLVNR.I | 1 |
| \* | LAPMis12DANCE\_011813\_01.06608.06608.2 | 2.7406 | 0.3948 | 100.0% | 1050.9521 | 1051.1442 | 1 | 6.222 | 87.5% | 2 | K.FEGDTLVNR.I | 2 |
| \* | LAPMis12DANCE\_011813\_01.08085.08085.2 | 4.255 | 0.4427 | 100.0% | 1543.4722 | 1543.7196 | 1 | 8.238 | 69.2% | 8 | K.GIDFKEDGNILGHK.L | 2 |
| \* | LAPMis12DANCE\_011813\_02.05555.05555.3 | 3.9742 | 0.3369 | 100.0% | 1543.6444 | 1543.7196 | 3 | 6.361 | 46.2% | 3 | K.GIDFKEDGNILGHK.L | 3 |
| \* | LAPMis12DANCE2\_011813\_01.08866.08866.3 | 5.8995 | 0.3807 | 100.0% | 3499.7944 | 3499.8792 | 1 | 7.938 | 31.9% | 2 | K.GIDFKEDGNILGHKLEYNYNSHNVYIMADK.Q | 3 |
| \* | LAPMis12DANCE\_011813\_01.09820.09820.3 | 3.9855 | 0.3842 | 100.0% | 2938.1943 | 2939.2288 | 1 | 6.383 | 34.4% | 1 | K.EDGNILGHKLEYNYNSHNVYIMADK.Q | 3 |
| \* | LAPMis12DANCE\_011813\_01.08756.08756.3 | 2.7091 | 0.3398 | 99.5% | 1973.7843 | 1975.1829 | 2 | 5.993 | 38.3% | 2 | K.LEYNYNSHNVYIMADK.Q | 3 |
| \* | LAPMis12DANCE\_011813\_01.08768.08768.2 | 4.6451 | 0.46 | 100.0% | 1974.1322 | 1975.1829 | 1 | 8.239 | 66.7% | 5 | K.LEYNYNSHNVYIMADK.Q | 2 |
| \* | LAPMis12DANCE\_011813\_01.07578.07578.3 | 3.7805 | 0.3248 | 99.7% | 2231.5444 | 2231.4875 | 21 | 5.682 | 33.8% | 1 | K.LEYNYNSHNVYIMADKQK.N | 3 |

---

|  |  |  |  |  |  |  |  |  |
| --- | --- | --- | --- | --- | --- | --- | --- | --- |
| U | *gi|4504517|ref|NP\_001* | 6 | 8 | 45.9% | 205 | 22783 | 6.4 | heat shock protein beta-1 [Homo sapiens] |

| Filename XCorr DeltCN Conf% ObsM+H+ CalcM+H+ SpR ZScore Ion% # Sequence  | | | | | | | | | | | | |
| --- | --- | --- | --- | --- | --- | --- | --- | --- | --- | --- | --- | --- |
| \* | LAPMis12DANCE\_011813\_01.11207.11207.2 | 2.8801 | 0.4421 | 100.0% | 1164.2722 | 1164.3494 | 1 | 7.235 | 72.2% | 1 | R.LFDQAFGLPR.L | 2 |
| \* | LAPMis12DANCE\_011813\_01.13212.13212.3 | 2.5091 | 0.5678 | 100.0% | 4094.4243 | 4095.606 | 1 | 8.647 | 29.7% | 2 | R.LPEEWSQWLGGSSWPGYVRPLPPAAIESPAVAAPAYSR.A | 3 |
| \* | LAPMis12DANCE\_011813\_01.10496.10496.2 | 4.4104 | 0.4995 | 100.0% | 1783.6921 | 1785.0068 | 1 | 8.086 | 60.0% | 1 | R.VSLDVNHFAPDELTVK.T | 2 |
| \* | LAPMis12DANCE\_011813\_01.10527.10527.3 | 2.736 | 0.3148 | 99.1% | 1784.7544 | 1785.0068 | 23 | 5.595 | 31.7% | 1 | R.VSLDVNHFAPDELTVK.T | 3 |
| \* | LAPMis12DANCE\_011813\_01.03298.03298.3 | 3.0781 | 0.2466 | 97.5% | 1655.5443 | 1656.7117 | 8 | 5.4 | 39.6% | 1 | K.HEERQDEHGYISR.C | 3 |
| \* | LAPMis12DANCE\_011813\_01.11008.11008.2 | 3.2288 | 0.2305 | 99.6% | 1907.3522 | 1907.1307 | 1 | 5.384 | 46.9% | 2 | K.LATQSNEITIPVTFESR.A | 2 |

---

|  |  |  |  |  |  |  |  |  |
| --- | --- | --- | --- | --- | --- | --- | --- | --- |
| U | *gi|4503571|ref|NP\_001* | 12 | 20 | 45.2% | 434 | 47169 | 7.4 | enolase 1 [Homo sapiens] |

| Filename XCorr DeltCN Conf% ObsM+H+ CalcM+H+ SpR ZScore Ion% # Sequence  | | | | | | | | | | | | |
| --- | --- | --- | --- | --- | --- | --- | --- | --- | --- | --- | --- | --- |
| \* | LAPMis12DANCE\_011813\_01.10511.10511.2 | 3.4266 | 0.3711 | 100.0% | 1406.9722 | 1407.5634 | 1 | 7.918 | 62.5% | 1 | R.GNPTVEVDLFTSK.G | 2 |
|  | LAPMis12DANCE2\_011813\_01.09437.09437.2 | 3.9749 | 0.3145 | 100.0% | 1806.2722 | 1806.0258 | 1 | 7.574 | 58.8% | 3 | R.AAVPSGASTGIYEALELR.D | 2 |
| \* | LAPMis12DANCE\_011813\_01.03831.03831.2 | 4.023 | 0.2991 | 100.0% | 1445.1921 | 1445.6561 | 1 | 5.989 | 72.7% | 1 | K.KLNVTEQEKIDK.L | 2 |
| \* | LAPMis12DANCE\_011813\_02.05583.05583.2 | 2.7977 | 0.2927 | 100.0% | 1281.2322 | 1281.4817 | 17 | 6.816 | 65.0% | 1 | K.LMIEMDGTENK.S | 2 |
| \* | LAPMis12DANCE2\_011813\_01.09963.09963.3 | 4.5659 | 0.3961 | 100.0% | 3012.4143 | 3013.383 | 1 | 6.161 | 30.2% | 2 | R.HIADLAGNSEVILPVPAFNVINGGSHAGNK.L | 3 |
| \* | LAPMis12DANCE\_011813\_01.13638.13638.2 | 4.7472 | 0.4753 | 100.0% | 1909.0922 | 1909.3148 | 1 | 8.135 | 75.0% | 2 | K.LAMQEFMILPVGAANFR.E | 2 |
| \* | LAPMis12DANCE\_011813\_01.04996.04996.2 | 3.2447 | 0.2693 | 100.0% | 1143.9722 | 1144.3158 | 1 | 6.065 | 83.3% | 2 | R.IGAEVYHNLK.N | 2 |
| \* | LAPMis12DANCE\_011813\_01.12657.12657.2 | 3.4156 | 0.0759 | 96.9% | 1542.5521 | 1541.8053 | 1 | 6.008 | 69.2% | 2 | K.VVIGMDVAASEFFR.S | 2 |
| \* | LAPMis12DANCE\_011813\_01.11139.11139.2 | 3.378 | 0.4445 | 100.0% | 1427.1122 | 1426.6091 | 1 | 6.71 | 77.3% | 3 | R.YISPDQLADLYK.S | 2 |
| \* | LAPMis12DANCE2\_011813\_01.11589.11589.3 | 3.6799 | 0.233 | 97.1% | 2985.7744 | 2987.2512 | 1 | 4.465 | 34.4% | 1 | K.SFIKDYPVVSIEDPFDQDDWGAWQK.F | 3 |
| \* | LAPMis12DANCE2\_011813\_01.08766.08766.2 | 2.5887 | 0.2266 | 98.3% | 2034.4722 | 2034.2737 | 22 | 4.149 | 31.6% | 1 | K.FTASAGIQVVGDDLTVTNPK.R | 2 |
| \* | LAPMis12DANCE\_011813\_01.08399.08399.2 | 2.9045 | 0.1416 | 96.8% | 1527.8121 | 1526.7563 | 4 | 3.71 | 57.7% | 1 | K.LAQANGWGVMVSHR.S | 2 |

---

|  |  |  |  |  |  |  |  |  |
| --- | --- | --- | --- | --- | --- | --- | --- | --- |
| U | *gi|226530908|ref|NP\_0* | 16 | 72 | 43.9% | 285 | 30315 | 7.5 | protein-L-isoaspartate (D-aspartate) O-methyltransferase [Homo sapiens] |

| Filename XCorr DeltCN Conf% ObsM+H+ CalcM+H+ SpR ZScore Ion% # Sequence  | | | | | | | | | | | | |
| --- | --- | --- | --- | --- | --- | --- | --- | --- | --- | --- | --- | --- |
| \* | LAPMis12DANCE\_011813\_01.04437.04437.3 | 3.7424 | 0.339 | 99.6% | 1478.1543 | 1478.6078 | 1 | 6.493 | 48.1% | 6 | K.SGGASHSELIHNLR.K | 3 |
| \* | LAPMis12DANCE2\_011813\_01.04755.04755.2 | 4.5157 | 0.4014 | 100.0% | 1478.2522 | 1478.6078 | 1 | 8.051 | 69.2% | 8 | K.SGGASHSELIHNLR.K | 2 |
| \* | LAPMis12DANCE2\_011813\_01.04048.04048.2 | 3.8717 | 0.3472 | 100.0% | 1606.4122 | 1606.7819 | 1 | 6.309 | 57.1% | 11 | K.SGGASHSELIHNLRK.N | 2 |
| \* | LAPMis12DANCE\_011813\_01.03836.03836.3 | 2.9763 | 0.2428 | 96.8% | 1606.5543 | 1606.7819 | 30 | 4.962 | 33.9% | 1 | K.SGGASHSELIHNLRK.N | 3 |
| \* | LAPMis12DANCE\_011813\_01.10356.10356.3 | 3.1911 | 0.2826 | 99.0% | 1525.5844 | 1525.7601 | 6 | 5.502 | 47.9% | 1 | K.TDKVFEVMLATDR.S | 3 |
| \* | LAPMis12DANCE\_011813\_01.10376.10376.2 | 4.276 | 0.5147 | 100.0% | 1526.4321 | 1525.7601 | 1 | 8.92 | 75.0% | 8 | K.TDKVFEVMLATDR.S | 2 |
| \* | LAPMis12DANCE\_011813\_01.10350.10350.2 | 3.4536 | 0.4996 | 100.0% | 1181.3121 | 1181.3923 | 1 | 9.259 | 88.9% | 2 | K.VFEVMLATDR.S | 2 |
| \* | LAPMis12DANCE2\_011813\_01.04250.04250.2 | 2.8228 | 0.3304 | 100.0% | 1189.1522 | 1189.3109 | 2 | 5.881 | 65.0% | 15 | R.KDDPTLLSSGR.V | 2 |
| \* | LAPMis12DANCE\_011813\_01.05888.05888.2 | 2.6333 | 0.3383 | 100.0% | 942.3722 | 943.091 | 1 | 6.866 | 87.5% | 1 | R.VQLVVGDGR.M | 2 |
| \* | LAPMis12DANCE\_011813\_01.05876.05876.1 | 2.2634 | 0.3404 | 100.0% | 942.53 | 943.091 | 1 | 7.143 | 68.8% | 3 | R.VQLVVGDGR.M | 1 |
| \* | LAPMis12DANCE\_011813\_01.11644.11644.3 | 7.4406 | 0.4958 | 100.0% | 3506.4543 | 3507.0015 | 1 | 9.182 | 32.6% | 9 | R.MGYAEEAPYDAIHVGAAAPVVPQALIDQLKPGGR.L | 3 |
| \* | LAPMis12DANCE2\_011813\_01.09521.09521.2 | 4.8752 | 0.4429 | 100.0% | 2044.3722 | 2044.3734 | 1 | 8.373 | 69.4% | 3 | R.LILPVGPAGGNQMLEQYDK.L | 2 |
| \* | LAPMis12DANCE2\_011813\_01.10238.10238.3 | 2.4972 | 0.2796 | 95.0% | 2784.8643 | 2786.2158 | 1 | 4.535 | 28.0% | 1 | R.LILPVGPAGGNQMLEQYDKLQDGSIK.M | 3 |
| \* | LAPMis12DANCE2\_011813\_01.10146.10146.2 | 3.2543 | 0.3223 | 100.0% | 2785.8323 | 2786.2158 | 60 | 6.115 | 34.0% | 1 | R.LILPVGPAGGNQMLEQYDKLQDGSIK.M | 2 |
| \* | LAPMis12DANCE\_011813\_01.10840.10840.2 | 3.9846 | 0.5624 | 100.0% | 1961.9922 | 1963.4445 | 1 | 10.026 | 68.8% | 1 | K.MKPLMGVIYVPLTDKEK.Q | 2 |
| \* | LAPMis12DANCE\_011813\_01.10892.10892.3 | 3.247 | 0.2481 | 97.5% | 1962.5044 | 1963.4445 | 256 | 5.341 | 29.7% | 1 | K.MKPLMGVIYVPLTDKEK.Q | 3 |

---

|  |  |  |  |  |  |  |  |  |
| --- | --- | --- | --- | --- | --- | --- | --- | --- |
| U | *gi|34098946|ref|NP\_00* | 6 | 10 | 41.7% | 324 | 35924 | 9.9 | nuclease sensitive element binding protein 1 [Homo sapiens] |

| Filename XCorr DeltCN Conf% ObsM+H+ CalcM+H+ SpR ZScore Ion% # Sequence  | | | | | | | | | | | | |
| --- | --- | --- | --- | --- | --- | --- | --- | --- | --- | --- | --- | --- |
|  | LAPMis12DANCE\_011813\_01.04413.04413.3 | 3.4492 | 0.3378 | 99.7% | 1874.0343 | 1874.1039 | 1 | 5.617 | 35.0% | 1 | R.NDTKEDVFVHQTAIKK.N | 33 |
|  | LAPMis12DANCE\_011813\_02.06380.06380.2 | 4.6756 | 0.5344 | 100.0% | 1796.0721 | 1796.8822 | 1 | 9.537 | 59.4% | 4 | R.SVGDGETVEFDVVEGEK.G | 22 |
| \* | LAPMis12DANCE\_011813\_01.05241.05241.2 | 3.921 | 0.4685 | 100.0% | 1696.3722 | 1696.8577 | 1 | 7.045 | 63.9% | 1 | K.GAEAANVTGPGGVPVQGSK.Y | 2 |
| \* | LAPMis12DANCE\_011813\_01.04002.04002.3 | 6.0566 | 0.4645 | 100.0% | 3257.8743 | 3259.2566 | 1 | 8.764 | 34.8% | 1 | R.NYQQNYQNSESGEKNEGSESAPEGQAQQR.R | 3 |
| \* | LAPMis12DANCE\_011813\_01.06556.06556.3 | 4.7661 | 0.2213 | 98.1% | 3223.5244 | 3225.4795 | 1 | 5.74 | 25.0% | 1 | R.RPQYSNPPVQGEVMEGADNQGAGEQGRPVR.Q | 3 |
| \* | LAPMis12DANCE2\_011813\_01.03347.03347.3 | 3.2538 | 0.3008 | 98.8% | 2784.3542 | 2785.771 | 1 | 5.863 | 31.5% | 2 | R.EDGNEEDKENQGDETQGQQPPQRR.Y | 3 |

Similarities:
gi|224586882|ref|NP\_0(2:4)  

---

|  |  |  |  |  |  |  |  |  |
| --- | --- | --- | --- | --- | --- | --- | --- | --- |
| U | *gi|4758638|ref|NP\_004* | 9 | 14 | 39.3% | 224 | 25035 | 6.4 | peroxiredoxin 6 [Homo sapiens] |

| Filename XCorr DeltCN Conf% ObsM+H+ CalcM+H+ SpR ZScore Ion% # Sequence  | | | | | | | | | | | | |
| --- | --- | --- | --- | --- | --- | --- | --- | --- | --- | --- | --- | --- |
| \* | LAPMis12DANCE2\_011813\_01.11346.11346.3 | 4.5986 | 0.4492 | 100.0% | 2300.9944 | 2301.615 | 1 | 6.833 | 44.4% | 1 | R.IRFHDFLGDSWGILFSHPR.D | 3 |
| \* | LAPMis12DANCE\_011813\_01.12867.12867.2 | 5.5675 | 0.5921 | 100.0% | 2031.2722 | 2032.2682 | 1 | 10.402 | 81.2% | 1 | R.FHDFLGDSWGILFSHPR.D | 2 |
| \* | LAPMis12DANCE2\_011813\_01.11303.11303.3 | 4.1809 | 0.4777 | 100.0% | 2032.3444 | 2032.2682 | 1 | 6.879 | 48.4% | 5 | R.FHDFLGDSWGILFSHPR.D | 3 |
| \* | LAPMis12DANCE2\_011813\_01.10383.10383.2 | 2.9969 | 0.444 | 100.0% | 1896.3322 | 1898.1663 | 2 | 7.981 | 40.6% | 1 | K.LIALSIDSVEDHLAWSK.D | 2 |
| \* | LAPMis12DANCE\_011813\_01.12804.12804.3 | 4.191 | 0.4074 | 100.0% | 2154.8044 | 2156.5022 | 15 | 6.414 | 31.9% | 2 | R.NRELAILLGMLDPAEKDEK.G | 3 |
| \* | LAPMis12DANCE\_011813\_01.12945.12945.2 | 4.631 | 0.3773 | 100.0% | 1884.8322 | 1886.2109 | 1 | 7.842 | 65.6% | 1 | R.ELAILLGMLDPAEKDEK.G | 2 |
| \* | LAPMis12DANCE\_011813\_01.08798.08798.2 | 2.7631 | 0.3553 | 100.0% | 1135.9122 | 1136.3794 | 1 | 6.911 | 77.8% | 1 | R.VVFVFGPDKK.L | 2 |
| \* | LAPMis12DANCE\_011813\_01.09549.09549.2 | 1.9232 | 0.2732 | 96.0% | 1192.1921 | 1192.4008 | 181 | 5.207 | 45.0% | 1 | K.LSILYPATTGR.N | 23 |
| \* | LAPMis12DANCE\_011813\_02.05990.05990.2 | 2.2751 | 0.3855 | 100.0% | 1357.3722 | 1357.6365 | 22 | 5.88 | 54.5% | 1 | R.VVISLQLTAEKR.V | 2 |

---

|  |  |  |  |  |  |  |  |  |
| --- | --- | --- | --- | --- | --- | --- | --- | --- |
| U | *gi|110349759|ref|NP\_0* | 9 | 33 | 39.1% | 281 | 32162 | 6.8 | NSL1, MIND kinetochore complex component isoform 1 [Homo sapiens] |

| Filename XCorr DeltCN Conf% ObsM+H+ CalcM+H+ SpR ZScore Ion% # Sequence  | | | | | | | | | | | | |
| --- | --- | --- | --- | --- | --- | --- | --- | --- | --- | --- | --- | --- |
|  | LAPMis12DANCE\_011813\_02.05450.05450.2 | 3.1205 | 0.4192 | 100.0% | 1701.4321 | 1701.8741 | 3 | 7.452 | 46.9% | 1 | K.ELAAGTESQALVSATPR.E | 2 |
|  | LAPMis12DANCE\_011813\_01.09434.09434.3 | 2.4662 | 0.2873 | 97.4% | 1679.2743 | 1679.9138 | 1 | 5.086 | 41.1% | 1 | K.LGDALPEEIREPALR.D | 3 |
|  | LAPMis12DANCE\_011813\_01.09440.09440.2 | 3.275 | 0.2592 | 99.9% | 1679.2922 | 1679.9138 | 26 | 4.814 | 53.6% | 1 | K.LGDALPEEIREPALR.D | 2 |
|  | LAPMis12DANCE\_011813\_02.09990.09990.2 | 5.2095 | 0.5087 | 100.0% | 1848.4521 | 1849.0881 | 1 | 9.853 | 73.3% | 10 | K.VLEDQFDEIIVDIATK.R | 2 |
| \* | LAPMis12DANCE\_011813\_01.06760.06760.2 | 2.807 | 0.3632 | 100.0% | 1528.1522 | 1527.6917 | 1 | 6.035 | 75.0% | 3 | K.YDPDPAPHMENLK.C | 2 |
| \* | LAPMis12DANCE\_011813\_01.12512.12512.2 | 5.3854 | 0.5164 | 100.0% | 1844.3522 | 1845.1057 | 1 | 9.033 | 71.9% | 9 | K.SLPALIEQGEGFSQVLR.M | 2 |
| \* | LAPMis12DANCE2\_011813\_01.05278.05278.2 | 2.6801 | 0.2789 | 100.0% | 1121.8922 | 1122.3739 | 1 | 6.2 | 87.5% | 3 | R.MQPVIHLQR.I | 2 |
| \* | LAPMis12DANCE\_011813\_01.09686.09686.3 | 5.0743 | 0.3957 | 100.0% | 2575.6143 | 2575.8352 | 1 | 6.601 | 30.7% | 3 | R.KPDAKPENFITQIETTPTETASR.K | 3 |
| \* | LAPMis12DANCE\_011813\_01.09363.09363.3 | 5.4988 | 0.1995 | 98.5% | 2655.1743 | 2655.8352 | 1 | 6.559 | 35.2% | 2 | R.KPDAKPENFITQIETT#PTETASR.K | 3 |

---

|  |  |  |  |  |  |  |  |  |
| --- | --- | --- | --- | --- | --- | --- | --- | --- |
| U | *gi|5174735|ref|NP\_006* | 16 | 41 | 38.7% | 445 | 49831 | 4.9 | tubulin, beta, 2 [Homo sapiens] |

| Filename XCorr DeltCN Conf% ObsM+H+ CalcM+H+ SpR ZScore Ion% # Sequence  | | | | | | | | | | | | |
| --- | --- | --- | --- | --- | --- | --- | --- | --- | --- | --- | --- | --- |
| \* | LAPMis12DANCE\_011813\_01.06976.06976.2 | 2.9005 | 0.2789 | 99.9% | 1329.2922 | 1329.4521 | 1 | 5.716 | 72.7% | 1 | R.INVYYNEATGGK.Y | 2 |
|  | LAPMis12DANCE\_011813\_01.10880.10880.2 | 4.5435 | 0.3722 | 100.0% | 1602.4122 | 1602.8431 | 1 | 9.502 | 75.0% | 2 | R.AVLVDLEPGTMDSVR.S | 2 |
|  | LAPMis12DANCE\_011813\_01.12368.12368.3 | 6.8452 | 0.5048 | 100.0% | 2799.8044 | 2800.0647 | 1 | 9.192 | 36.0% | 7 | R.SGPFGQIFRPDNFVFGQSGAGNNWAK.G | 33 |
|  | LAPMis12DANCE\_011813\_01.12752.12752.2 | 6.7078 | 0.4112 | 100.0% | 1960.4321 | 1960.151 | 1 | 8.744 | 76.5% | 3 | K.GHYTEGAELVDSVLDVVR.K | 22 |
|  | LAPMis12DANCE\_011813\_01.11830.11830.2 | 5.614 | 0.5661 | 100.0% | 2087.5923 | 2088.325 | 1 | 10.318 | 69.4% | 2 | K.GHYTEGAELVDSVLDVVRK.E | 22 |
|  | LAPMis12DANCE\_011813\_01.11865.11865.3 | 4.2402 | 0.4278 | 100.0% | 2088.5344 | 2088.325 | 2 | 7.312 | 34.7% | 4 | K.GHYTEGAELVDSVLDVVRK.E | 33 |
|  | LAPMis12DANCE2\_011813\_01.03633.03633.2 | 2.72 | 0.1715 | 99.4% | 1078.0922 | 1078.1698 | 3 | 4.599 | 85.7% | 3 | K.IREEYPDR.I | 22 |
|  | LAPMis12DANCE\_011813\_01.09722.09722.2 | 4.1598 | 0.429 | 100.0% | 1320.2722 | 1320.5896 | 1 | 8.109 | 77.3% | 6 | R.IMNTFSVVPSPK.V | 22 |
|  | LAPMis12DANCE\_011813\_01.08648.08648.2 | 2.5957 | 0.2336 | 99.4% | 1131.6921 | 1131.2767 | 107 | 5.098 | 61.1% | 3 | R.FPGQLNADLR.K | 22 |
|  | LAPMis12DANCE\_011813\_01.09826.09826.2 | 3.3678 | 0.349 | 100.0% | 1272.4521 | 1272.5945 | 5 | 7.066 | 60.0% | 2 | R.KLAVNMVPFPR.L | 22 |
|  | LAPMis12DANCE\_011813\_01.11000.11000.2 | 3.7645 | 0.4603 | 100.0% | 1144.0122 | 1144.4204 | 1 | 8.466 | 88.9% | 1 | K.LAVNMVPFPR.L | 22 |
|  | LAPMis12DANCE\_011813\_01.12036.12036.2 | 3.4224 | 0.464 | 100.0% | 1620.7922 | 1621.9403 | 1 | 9.29 | 76.9% | 2 | R.LHFFMPGFAPLTSR.G | 22 |
|  | LAPMis12DANCE\_011813\_01.11200.11200.1 | 1.4887 | 0.2888 | 100.0% | 1039.53 | 1040.2505 | 98 | 4.805 | 50.0% | 1 | R.YLTVAAVFR.G | 11 |
|  | LAPMis12DANCE\_011813\_01.07258.07258.2 | 4.0244 | 0.2359 | 100.0% | 1446.8922 | 1447.6031 | 2 | 6.543 | 68.2% | 2 | K.EVDEQMLNVQNK.N | 22 |
|  | LAPMis12DANCE2\_011813\_01.09810.09810.2 | 3.4367 | 0.3512 | 100.0% | 1696.9722 | 1697.8877 | 2 | 6.103 | 53.8% | 1 | K.NSSYFVEWIPNNVK.T | 22 |
|  | LAPMis12DANCE2\_011813\_01.08520.08520.2 | 2.8584 | 0.3778 | 100.0% | 1229.9722 | 1230.4241 | 2 | 6.75 | 66.7% | 1 | R.ISEQFTAMFR.R | 22 |

Similarities:
gi|29788785|ref|NP\_82(14:2)  

---

|  |  |  |  |  |  |  |  |  |
| --- | --- | --- | --- | --- | --- | --- | --- | --- |
| U | *gi|4759302|ref|NP\_004* | 9 | 20 | 37.9% | 243 | 27228 | 7.3 | VAMP-associated protein B/C [Homo sapiens] |

| Filename XCorr DeltCN Conf% ObsM+H+ CalcM+H+ SpR ZScore Ion% # Sequence  | | | | | | | | | | | | |
| --- | --- | --- | --- | --- | --- | --- | --- | --- | --- | --- | --- | --- |
| \* | LAPMis12DANCE\_011813\_01.08410.08410.2 | 3.942 | 0.3929 | 100.0% | 1649.0322 | 1649.8845 | 1 | 6.36 | 76.9% | 3 | K.VEQVLSLEPQHELK.F | 2 |
| \* | LAPMis12DANCE\_011813\_01.08420.08420.3 | 3.4814 | 0.3282 | 99.7% | 1651.4343 | 1649.8845 | 5 | 5.813 | 42.3% | 1 | K.VEQVLSLEPQHELK.F | 3 |
| \* | LAPMis12DANCE\_011813\_01.10266.10266.2 | 4.4637 | 0.4215 | 100.0% | 1595.3722 | 1595.8387 | 1 | 8.343 | 84.6% | 3 | K.FRGPFTDVVTTNLK.L | 2 |
|  | LAPMis12DANCE\_011813\_01.10106.10106.2 | 4.1522 | 0.4164 | 100.0% | 1292.0521 | 1292.4747 | 1 | 8.461 | 72.7% | 6 | R.GPFTDVVTTNLK.L | 22 |
| \* | LAPMis12DANCE\_011813\_01.13017.13017.2 | 3.6192 | 0.4728 | 100.0% | 2321.372 | 2322.6707 | 1 | 8.382 | 57.9% | 1 | K.FMVQSMFAPTDTSDMEAVWK.E | 2 |
| \* | LAPMis12DANCE2\_011813\_01.04580.04580.2 | 3.1589 | 0.415 | 100.0% | 1677.6322 | 1676.9481 | 1 | 6.396 | 56.7% | 1 | K.IISTTASKTETPIVSK.S | 2 |
| \* | LAPMis12DANCE\_011813\_01.07079.07079.2 | 2.9501 | 0.307 | 100.0% | 1282.1721 | 1281.3593 | 1 | 5.906 | 68.2% | 1 | K.SLSSSLDDTEVK.K | 2 |
| \* | LAPMis12DANCE2\_011813\_01.04634.04634.2 | 3.0039 | 0.2845 | 100.0% | 1410.5322 | 1409.5333 | 1 | 5.472 | 58.3% | 3 | K.SLSSSLDDTEVKK.V | 2 |
| \* | LAPMis12DANCE\_011813\_01.03992.03992.3 | 3.5 | 0.2845 | 99.5% | 1892.1244 | 1892.0787 | 1 | 5.585 | 46.4% | 1 | R.LREENKQFKEEDGLR.M | 3 |

Similarities:
gi|94721250|ref|NP\_00(1:8)  

---

|  |  |  |  |  |  |  |  |  |
| --- | --- | --- | --- | --- | --- | --- | --- | --- |
| U | *gi|14389309|ref|NP\_11* | 10 | 30 | 37.6% | 449 | 49895 | 5.1 | tubulin alpha 6 [Homo sapiens] |
| U | *gi|57013276|ref|NP\_00* | 10 | 30 | 37.5% | 451 | 50152 | 5.1 | tubulin, alpha, ubiquitous [Homo sapiens] |

| Filename XCorr DeltCN Conf% ObsM+H+ CalcM+H+ SpR ZScore Ion% # Sequence  | | | | | | | | | | | | |
| --- | --- | --- | --- | --- | --- | --- | --- | --- | --- | --- | --- | --- |
|  | LAPMis12DANCE2\_011813\_01.09314.09314.2 | 5.0039 | 0.6664 | 100.0% | 2008.4922 | 2009.093 | 1 | 12.009 | 60.5% | 4 | K.TIGGGDDSFNTFFSETGAGK.H | 2 |
|  | LAPMis12DANCE\_011813\_01.11768.11768.2 | 5.0105 | 0.4889 | 100.0% | 1702.3922 | 1702.9451 | 1 | 7.789 | 71.4% | 7 | R.AVFVDLEPTVIDEVR.T | 2 |
|  | LAPMis12DANCE\_011813\_01.09476.09476.3 | 3.6457 | 0.3154 | 99.7% | 2416.8542 | 2416.6555 | 1 | 6.039 | 33.8% | 2 | R.QLFHPEQLITGKEDAANNYAR.G | 3 |
|  | LAPMis12DANCE\_011813\_01.07872.07872.2 | 3.1354 | 0.0899 | 95.7% | 1720.2922 | 1719.8949 | 17 | 4.766 | 50.0% | 2 | R.NLDIERPTYTNLNR.L | 2 |
|  | LAPMis12DANCE\_011813\_02.09818.09818.2 | 4.5201 | 0.4909 | 100.0% | 1489.5521 | 1488.7678 | 1 | 9.136 | 76.9% | 4 | R.LISQIVSSITASLR.F | 2 |
|  | LAPMis12DANCE2\_011813\_01.11643.11643.2 | 4.2685 | 0.4186 | 100.0% | 2410.5923 | 2410.6885 | 1 | 7.983 | 47.5% | 3 | R.FDGALNVDLTEFQTNLVPYPR.I | 2 |
|  | LAPMis12DANCE\_011813\_01.11141.11141.2 | 3.2611 | 0.4485 | 100.0% | 1756.8121 | 1758.0703 | 1 | 7.429 | 53.3% | 1 | R.IHFPLATYAPVISAEK.A | 2 |
|  | LAPMis12DANCE\_011813\_01.08006.08006.1 | 1.7496 | 0.4105 | 100.0% | 1015.44 | 1016.1827 | 2 | 6.265 | 61.1% | 1 | K.DVNAAIATIK.T | 1 |
|  | LAPMis12DANCE\_011813\_01.10014.10014.2 | 4.1515 | 0.4215 | 100.0% | 1826.1921 | 1826.1027 | 2 | 6.458 | 52.9% | 4 | K.VGINYQPPTVVPGGDLAK.V | 2 |
|  | LAPMis12DANCE\_011813\_02.07412.07412.3 | 4.4585 | 0.4175 | 100.0% | 2331.0244 | 2331.5208 | 1 | 6.633 | 36.8% | 2 | R.AFVHWYVGEGMEEGEFSEAR.E | 3 |

---

|  |  |  |  |  |  |  |  |  |
| --- | --- | --- | --- | --- | --- | --- | --- | --- |
| U | *gi|33286420|ref|NP\_87* | 13 | 18 | 37.1% | 531 | 58062 | 7.7 | pyruvate kinase, muscle isoform M1 [Homo sapiens] |
| U | *gi|33286422|ref|NP\_87* | 13 | 18 | 37.1% | 531 | 58062 | 7.7 | pyruvate kinase, muscle isoform M1 [Homo sapiens] |

| Filename XCorr DeltCN Conf% ObsM+H+ CalcM+H+ SpR ZScore Ion% # Sequence  | | | | | | | | | | | | |
| --- | --- | --- | --- | --- | --- | --- | --- | --- | --- | --- | --- | --- |
|  | LAPMis12DANCE\_011813\_01.08380.08380.2 | 2.1384 | 0.2465 | 97.1% | 1198.0322 | 1198.3617 | 234 | 5.074 | 50.0% | 1 | R.LDIDSPPITAR.N | 2 |
|  | LAPMis12DANCE\_011813\_01.04577.04577.2 | 3.9576 | 0.5101 | 100.0% | 1884.6921 | 1885.0458 | 1 | 8.531 | 63.3% | 2 | R.LNFSHGTHEYHAETIK.N | 2 |
|  | LAPMis12DANCE\_011813\_01.11402.11402.3 | 3.2792 | 0.3476 | 99.7% | 2466.4443 | 2466.7937 | 1 | 5.625 | 33.0% | 1 | R.TATESFASDPILYRPVAVALDTK.G | 3 |
|  | LAPMis12DANCE2\_011813\_01.09900.09900.3 | 4.335 | 0.4244 | 100.0% | 3018.4744 | 3019.4246 | 103 | 7.298 | 22.2% | 1 | R.TATESFASDPILYRPVAVALDTKGPEIR.T | 3 |
|  | LAPMis12DANCE\_011813\_01.03609.03609.2 | 2.5267 | 0.2426 | 99.2% | 1119.1721 | 1119.2603 | 40 | 5.278 | 55.0% | 1 | K.GSGTAEVELKK.G | 2 |
|  | LAPMis12DANCE2\_011813\_01.09250.09250.2 | 3.1407 | 0.3921 | 100.0% | 1463.2122 | 1463.7142 | 6 | 7.251 | 54.2% | 3 | K.IYVDDGLISLQVK.Q | 2 |
|  | LAPMis12DANCE2\_011813\_01.09023.09023.2 | 3.594 | 0.4253 | 100.0% | 1780.3722 | 1780.9292 | 1 | 6.376 | 55.9% | 1 | K.GADFLVTEVENGGSLGSK.K | 2 |
|  | LAPMis12DANCE2\_011813\_01.08998.08998.3 | 4.0965 | 0.3456 | 99.7% | 2479.7644 | 2478.8486 | 1 | 5.359 | 33.7% | 1 | K.KGVNLPGAAVDLPAVSEKDIQDLK.F | 3 |
|  | LAPMis12DANCE\_011813\_01.15753.15753.2 | 3.9928 | 0.4199 | 100.0% | 1860.1921 | 1861.1224 | 1 | 8.251 | 63.3% | 1 | K.FGVEQDVDMVFASFIR.K | 2 |
|  | LAPMis12DANCE\_011813\_01.11157.11157.2 | 4.2276 | 0.4493 | 100.0% | 1822.4722 | 1823.0741 | 1 | 7.521 | 66.7% | 3 | R.RFDEILEASDGIMVAR.G | 2 |
|  | LAPMis12DANCE\_011813\_01.06020.06020.1 | 1.8065 | 0.3728 | 100.0% | 840.49 | 841.0415 | 42 | 5.362 | 50.0% | 1 | R.APIIAVTR.N | 1 |
|  | LAPMis12DANCE2\_011813\_01.08844.08844.2 | 2.4982 | 0.3071 | 99.5% | 1643.9521 | 1643.7502 | 3 | 5.413 | 53.8% | 1 | K.DPVQEAWAEDVDLR.V | 2 |
|  | LAPMis12DANCE\_011813\_01.11140.11140.3 | 3.8678 | 0.2789 | 99.0% | 2392.4043 | 2392.7815 | 1 | 5.488 | 33.3% | 1 | K.KGDVVIVLTGWRPGSGFTNTMR.V | 3 |

---

|  |  |  |  |  |  |  |  |  |
| --- | --- | --- | --- | --- | --- | --- | --- | --- |
| U | *gi|62414289|ref|NP\_00* | 15 | 20 | 36.9% | 466 | 53652 | 5.1 | vimentin [Homo sapiens] |

| Filename XCorr DeltCN Conf% ObsM+H+ CalcM+H+ SpR ZScore Ion% # Sequence  | | | | | | | | | | | | |
| --- | --- | --- | --- | --- | --- | --- | --- | --- | --- | --- | --- | --- |
| \* | LAPMis12DANCE\_011813\_01.07958.07958.2 | 3.4892 | 0.3906 | 100.0% | 1509.1122 | 1509.5724 | 1 | 6.317 | 65.4% | 2 | R.SLYASS\*PGGVYATR.S | 2 |
|  | LAPMis12DANCE\_011813\_01.06496.06496.2 | 3.3319 | 0.341 | 100.0% | 1588.0922 | 1588.7147 | 1 | 5.871 | 79.2% | 1 | R.TNEKVELQELNDR.F | 2 |
| \* | LAPMis12DANCE\_011813\_01.06975.06975.2 | 2.2361 | 0.3171 | 99.5% | 1126.1721 | 1126.3005 | 113 | 5.184 | 62.5% | 1 | R.FANYIDKVR.F | 2 |
| \* | LAPMis12DANCE\_011813\_01.08506.08506.2 | 3.2524 | 0.4675 | 100.0% | 1254.4521 | 1255.385 | 1 | 8.432 | 77.8% | 2 | R.LGDLYEEEMR.E | 2 |
| \* | LAPMis12DANCE\_011813\_01.03538.03538.2 | 2.5157 | 0.2351 | 99.2% | 1216.9922 | 1217.3243 | 1 | 5.22 | 83.3% | 1 | R.RQVDQLTNDK.A | 2 |
| \* | LAPMis12DANCE\_011813\_01.09442.09442.2 | 3.1901 | 0.2111 | 99.5% | 1689.0122 | 1689.881 | 1 | 5.156 | 61.5% | 1 | R.VEVERDNLAEDIMR.L | 2 |
| \* | LAPMis12DANCE\_011813\_02.06482.06482.3 | 3.3299 | 0.3303 | 99.7% | 2351.5144 | 2352.581 | 59 | 6.428 | 29.2% | 1 | K.LQEEMLQREEAENTLQSFR.Q | 3 |
| \* | LAPMis12DANCE\_011813\_01.07589.07589.2 | 2.5071 | 0.3246 | 99.9% | 1324.0922 | 1324.3898 | 60 | 6.082 | 50.0% | 2 | R.EEAENTLQSFR.Q | 2 |
| \* | LAPMis12DANCE\_011813\_01.09945.09945.2 | 3.4603 | 0.3097 | 100.0% | 1662.8522 | 1662.967 | 1 | 4.916 | 76.9% | 1 | R.KVESLQEEIAFLKK.L | 2 |
| \* | LAPMis12DANCE\_011813\_01.09958.09958.3 | 3.7543 | 0.2805 | 99.5% | 1662.9243 | 1662.967 | 4 | 5.377 | 44.2% | 1 | R.KVESLQEEIAFLKK.L | 3 |
|  | LAPMis12DANCE\_011813\_01.09105.09105.2 | 2.7363 | 0.2564 | 99.9% | 1309.9321 | 1310.4056 | 1 | 4.811 | 77.8% | 2 | K.NLQEAEEWYK.S | 2 |
| \* | LAPMis12DANCE\_011813\_01.08394.08394.2 | 4.4187 | 0.3695 | 100.0% | 1736.0721 | 1735.9679 | 1 | 7.858 | 73.1% | 1 | R.LQDEIQNMKEEMAR.H | 2 |
| \* | LAPMis12DANCE\_011813\_01.13048.13048.2 | 2.9269 | 0.2998 | 100.0% | 1572.3922 | 1571.8601 | 1 | 5.739 | 69.2% | 2 | R.ISLPLPNFSSLNLR.E | 2 |
| \* | LAPMis12DANCE\_011813\_01.09276.09276.2 | 2.7932 | 0.3078 | 99.9% | 1670.4122 | 1669.829 | 3 | 6.428 | 50.0% | 1 | R.ETNLDSLPLVDTHSK.R | 2 |
| \* | LAPMis12DANCE\_011813\_01.05016.05016.2 | 3.1879 | 0.3205 | 100.0% | 1837.0922 | 1837.854 | 1 | 5.655 | 53.3% | 1 | R.DGQVINETSQHHDDLE.- | 2 |

---

|  |  |  |  |  |  |  |  |  |
| --- | --- | --- | --- | --- | --- | --- | --- | --- |
| U | *gi|10800140|ref|NP\_06* | 4 | 17 | 36.5% | 126 | 13950 | 10.3 | histone cluster 1, H2bb [Homo sapiens] |
| U | *gi|4504277|ref|NP\_003* | 4 | 17 | 36.5% | 126 | 13920 | 10.3 | histone cluster 2, H2be [Homo sapiens] |
| U | *gi|20336754|ref|NP\_06* | 4 | 17 | 36.5% | 126 | 13904 | 10.3 | histone cluster 1, H2bj [Homo sapiens] |
| U | *gi|16306566|ref|NP\_00* | 4 | 17 | 36.5% | 126 | 13906 | 10.3 | histone cluster 1, H2bo [Homo sapiens] |

| Filename XCorr DeltCN Conf% ObsM+H+ CalcM+H+ SpR ZScore Ion% # Sequence  | | | | | | | | | | | | |
| --- | --- | --- | --- | --- | --- | --- | --- | --- | --- | --- | --- | --- |
|  | LAPMis12DANCE\_011813\_01.07317.07317.2 | 2.2275 | 0.2711 | 99.0% | 1280.3322 | 1280.4631 | 74 | 5.31 | 55.6% | 1 | R.KESYSIYVYK.V | 2 |
|  | LAPMis12DANCE\_011813\_01.13952.13952.2 | 5.447 | 0.4635 | 100.0% | 1744.2922 | 1745.0211 | 1 | 8.945 | 75.0% | 12 | K.AMGIMNSFVNDIFER.I | 22 |
|  | LAPMis12DANCE2\_011813\_01.04980.04980.2 | 2.9332 | 0.1598 | 98.4% | 1462.3722 | 1462.6462 | 9 | 4.63 | 54.2% | 3 | R.STITSREIQTAVR.L | 22 |
|  | LAPMis12DANCE2\_011813\_01.02100.02100.2 | 1.8306 | 0.3716 | 99.4% | 829.2322 | 828.9004 | 5 | 7.216 | 71.4% | 1 | K.HAVSEGTK.A | 22 |

Similarities:
gi|10800138|ref|NP\_06(3:1)  

---

|  |  |  |  |  |  |  |  |  |
| --- | --- | --- | --- | --- | --- | --- | --- | --- |
| U | *gi|10800138|ref|NP\_06* | 6 | 24 | 36.5% | 126 | 13936 | 10.3 | histone cluster 1, H2bd [Homo sapiens] |
| U | *gi|66912162|ref|NP\_00* | 6 | 24 | 36.5% | 126 | 13920 | 10.3 | histone cluster 2, H2bf [Homo sapiens] |
| U | *gi|4504271|ref|NP\_003* | 6 | 24 | 36.5% | 126 | 13906 | 10.3 | histone cluster 1, H2bi [Homo sapiens] |
| U | *gi|4504269|ref|NP\_003* | 6 | 24 | 36.5% | 126 | 13892 | 10.3 | histone cluster 1, H2bh [Homo sapiens] |
| U | *gi|4504265|ref|NP\_003* | 6 | 24 | 36.5% | 126 | 13906 | 10.3 | histone cluster 1, H2bf [Homo sapiens] |
| U | *gi|4504263|ref|NP\_003* | 6 | 24 | 36.5% | 126 | 13989 | 10.3 | histone cluster 1, H2bm [Homo sapiens] |
| U | *gi|4504261|ref|NP\_003* | 6 | 24 | 36.5% | 126 | 13922 | 10.3 | histone cluster 1, H2bn [Homo sapiens] |
| U | *gi|4504259|ref|NP\_003* | 6 | 24 | 36.5% | 126 | 13952 | 10.3 | histone cluster 1, H2bl [Homo sapiens] |
| U | *gi|4504257|ref|NP\_003* | 6 | 24 | 36.5% | 126 | 13906 | 10.3 | histone cluster 1, H2bg [Homo sapiens] |
| U | *gi|21396484|ref|NP\_00* | 6 | 24 | 36.5% | 126 | 13906 | 10.3 | histone cluster 1, H2be [Homo sapiens] |
| U | *gi|21166389|ref|NP\_00* | 6 | 24 | 36.5% | 126 | 13906 | 10.3 | histone cluster 1, H2bc [Homo sapiens] |
| U | *gi|20336752|ref|NP\_61* | 6 | 24 | 36.5% | 126 | 13936 | 10.3 | histone cluster 1, H2bd [Homo sapiens] |
| U | *gi|18105048|ref|NP\_54* | 6 | 24 | 36.5% | 126 | 13890 | 10.3 | histone cluster 1, H2bk [Homo sapiens] |

| Filename XCorr DeltCN Conf% ObsM+H+ CalcM+H+ SpR ZScore Ion% # Sequence  | | | | | | | | | | | | |
| --- | --- | --- | --- | --- | --- | --- | --- | --- | --- | --- | --- | --- |
|  | LAPMis12DANCE\_011813\_01.06405.06405.2 | 2.9292 | 0.347 | 100.0% | 1266.0322 | 1266.4363 | 1 | 5.825 | 72.2% | 4 | R.KESYSVYVYK.V | 2 |
|  | LAPMis12DANCE\_011813\_01.07728.07728.1 | 1.895 | 0.3271 | 100.0% | 1137.39 | 1138.2622 | 1 | 5.868 | 68.8% | 3 | K.ESYSVYVYK.V | 1 |
|  | LAPMis12DANCE\_011813\_01.07772.07772.2 | 2.259 | 0.3802 | 100.0% | 1137.6721 | 1138.2622 | 2 | 6.092 | 62.5% | 1 | K.ESYSVYVYK.V | 2 |
|  | LAPMis12DANCE\_011813\_01.13952.13952.2 | 5.447 | 0.4635 | 100.0% | 1744.2922 | 1745.0211 | 1 | 8.945 | 75.0% | 12 | K.AMGIMNSFVNDIFER.I | 22 |
|  | LAPMis12DANCE2\_011813\_01.04980.04980.2 | 2.9332 | 0.1598 | 98.4% | 1462.3722 | 1462.6462 | 9 | 4.63 | 54.2% | 3 | R.STITSREIQTAVR.L | 22 |
|  | LAPMis12DANCE2\_011813\_01.02100.02100.2 | 1.8306 | 0.3716 | 99.4% | 829.2322 | 828.9004 | 5 | 7.216 | 71.4% | 1 | K.HAVSEGTK.A | 22 |

Similarities:
gi|10800140|ref|NP\_06(3:3)  

---

|  |  |  |  |  |  |  |  |  |
| --- | --- | --- | --- | --- | --- | --- | --- | --- |
| U | *gi|10863927|ref|NP\_06* | 6 | 8 | 35.8% | 165 | 18012 | 7.8 | peptidylprolyl isomerase A [Homo sapiens] |

| Filename XCorr DeltCN Conf% ObsM+H+ CalcM+H+ SpR ZScore Ion% # Sequence  | | | | | | | | | | | | |
| --- | --- | --- | --- | --- | --- | --- | --- | --- | --- | --- | --- | --- |
| \* | LAPMis12DANCE\_011813\_01.16841.16841.2 | 2.3744 | 0.2084 | 95.6% | 2157.9722 | 2158.3906 | 8 | 4.228 | 36.1% | 1 | -.MVNPT#VFFDIAVDGEPLGR.V | 2 |
|  | LAPMis12DANCE\_011813\_01.10946.10946.2 | 3.2853 | 0.3341 | 100.0% | 1380.1921 | 1380.6268 | 1 | 6.286 | 72.7% | 2 | R.VSFELFADKVPK.T | 2 |
|  | LAPMis12DANCE2\_011813\_01.08435.08435.2 | 3.702 | 0.2716 | 100.0% | 1833.9122 | 1833.0477 | 1 | 5.013 | 60.7% | 1 | K.SIYGEKFEDENFILK.H | 2 |
|  | LAPMis12DANCE\_011813\_01.10252.10252.2 | 2.6033 | 0.1395 | 97.7% | 1155.2922 | 1155.2927 | 33 | 5.312 | 68.8% | 1 | K.FEDENFILK.H | 2 |
|  | LAPMis12DANCE\_011813\_01.09398.09398.2 | 4.0358 | 0.0716 | 99.3% | 1506.1522 | 1506.7755 | 2 | 8.248 | 75.0% | 2 | K.VKEGMNIVEAMER.F | 2 |
|  | LAPMis12DANCE\_011813\_01.10767.10767.2 | 2.4586 | 0.2136 | 98.4% | 1278.9122 | 1279.4689 | 8 | 6.513 | 60.0% | 1 | K.EGMNIVEAMER.F | 2 |

---

|  |  |  |  |  |  |  |  |  |
| --- | --- | --- | --- | --- | --- | --- | --- | --- |
| U | *contaminant\_gi|746301* | 15 | 301 | 35.7% | 269 | 27961 | 6.7 | lysyl endopeptidase (EC 3.4.21.50) - Lysobacter enzymogenes |

| Filename XCorr DeltCN Conf% ObsM+H+ CalcM+H+ SpR ZScore Ion% # Sequence  | | | | | | | | | | | | |
| --- | --- | --- | --- | --- | --- | --- | --- | --- | --- | --- | --- | --- |
| \* | LAPMis12DANCE2\_011813\_01.03526.03526.1 | 1.6809 | 0.2928 | 100.0% | 725.33 | 725.8198 | 1 | 5.261 | 75.0% | 2 | R.SVAAYSK.Q | 1 |
| \* | LAPMis12DANCE\_011813\_01.05348.05348.2 | 6.5353 | 0.6073 | 100.0% | 2261.412 | 2262.355 | 1 | 10.973 | 62.5% | 75 | R.APGSSSSGANGDGSLAQSQTGAVVR.A | 2 |
| \* | LAPMis12DANCE\_011813\_01.05288.05288.3 | 5.0589 | 0.4074 | 100.0% | 2263.1042 | 2262.355 | 1 | 7.399 | 42.7% | 19 | R.APGSSSSGANGDGSLAQSQTGAVVR.A | 3 |
| \* | LAPMis12DANCE\_011813\_02.11078.11078.3 | 7.3383 | 0.5222 | 100.0% | 3315.2043 | 3315.6257 | 1 | 10.819 | 34.5% | 4 | R.ATNAASDFTLLELNTAANPAYNLFWAGWDR.R | 3 |
| \* | LAPMis12DANCE\_011813\_01.14906.14906.3 | 6.7842 | 0.5835 | 100.0% | 3470.1243 | 3471.813 | 1 | 10.562 | 27.5% | 16 | R.ATNAASDFTLLELNTAANPAYNLFWAGWDRR.D | 3 |
| \* | LAPMis12DANCE2\_011813\_01.04524.04524.2 | 3.6694 | 0.4392 | 100.0% | 2076.372 | 2077.2668 | 1 | 6.608 | 50.0% | 2 | R.RDQNFAGATAIHHPNVAEK.R | 2 |
| \* | LAPMis12DANCE2\_011813\_01.04550.04550.3 | 4.5723 | 0.2489 | 99.5% | 2077.2844 | 2077.2668 | 1 | 6.054 | 43.1% | 4 | R.RDQNFAGATAIHHPNVAEK.R | 3 |
| \* | LAPMis12DANCE2\_011813\_01.04316.04316.2 | 5.1359 | 0.4386 | 100.0% | 2232.5322 | 2233.4543 | 1 | 9.234 | 52.6% | 2 | R.RDQNFAGATAIHHPNVAEKR.I | 2 |
| \* | LAPMis12DANCE\_011813\_01.04166.04166.3 | 5.1671 | 0.3961 | 100.0% | 2234.0942 | 2233.4543 | 1 | 6.559 | 42.1% | 14 | R.RDQNFAGATAIHHPNVAEKR.I | 3 |
| \* | LAPMis12DANCE\_011813\_01.05480.05480.2 | 4.816 | 0.558 | 100.0% | 1920.2522 | 1921.0793 | 1 | 8.64 | 55.9% | 20 | R.DQNFAGATAIHHPNVAEK.R | 2 |
| \* | LAPMis12DANCE2\_011813\_01.05025.05025.3 | 2.493 | 0.2927 | 97.5% | 1920.5343 | 1921.0793 | 1 | 5.116 | 38.2% | 1 | R.DQNFAGATAIHHPNVAEK.R | 3 |
| \* | LAPMis12DANCE\_011813\_01.04706.04706.2 | 5.443 | 0.417 | 100.0% | 2076.372 | 2077.2668 | 1 | 7.357 | 58.3% | 23 | R.DQNFAGATAIHHPNVAEKR.I | 2 |
| \* | LAPMis12DANCE\_011813\_01.04754.04754.3 | 4.2284 | 0.4269 | 100.0% | 2077.3145 | 2077.2668 | 1 | 7.454 | 41.7% | 13 | R.DQNFAGATAIHHPNVAEKR.I | 3 |
| \* | LAPMis12DANCE\_011813\_01.07970.07970.2 | 4.9971 | 0.4856 | 100.0% | 1429.0721 | 1428.5443 | 1 | 8.684 | 73.1% | 105 | R.VFTSWTGGGTSATR.L | 2 |
| \* | LAPMis12DANCE\_011813\_01.07833.07833.1 | 1.5091 | 0.3899 | 100.0% | 1429.57 | 1428.5443 | 45 | 5.756 | 38.5% | 1 | R.VFTSWTGGGTSATR.L | 1 |

---

|  |  |  |  |  |  |  |  |  |
| --- | --- | --- | --- | --- | --- | --- | --- | --- |
| U | *gi|4506645|ref|NP\_000* | 2 | 3 | 35.7% | 70 | 8218 | 10.1 | ribosomal protein L38 [Homo sapiens] |
| U | *gi|78214522|ref|NP\_00* | 2 | 3 | 35.7% | 70 | 8218 | 10.1 | ribosomal protein L38 [Homo sapiens] |

| Filename XCorr DeltCN Conf% ObsM+H+ CalcM+H+ SpR ZScore Ion% # Sequence  | | | | | | | | | | | | |
| --- | --- | --- | --- | --- | --- | --- | --- | --- | --- | --- | --- | --- |
|  | LAPMis12DANCE\_011813\_01.10600.10600.2 | 3.2539 | 0.0572 | 95.3% | 1578.1522 | 1576.8766 | 2 | 3.78 | 66.7% | 2 | R.KIEEIKDFLLTAR.R | 2 |
|  | LAPMis12DANCE\_011813\_01.09736.09736.2 | 2.3583 | 0.2828 | 99.2% | 1486.1522 | 1486.7484 | 1 | 5.255 | 59.1% | 1 | R.YLYTLVITDKEK.A | 2 |

---

|  |  |  |  |  |  |  |  |  |
| --- | --- | --- | --- | --- | --- | --- | --- | --- |
| U | *contaminant\_KERATIN03* | 22 | 50 | 35.2% | 593 | 59519 | 5.2 | no description |
| U | *gi|195972866|ref|NP\_0* | 22 | 50 | 35.8% | 584 | 58801 | 5.2 | keratin 10 [Homo sapiens] |

| Filename XCorr DeltCN Conf% ObsM+H+ CalcM+H+ SpR ZScore Ion% # Sequence  | | | | | | | | | | | | |
| --- | --- | --- | --- | --- | --- | --- | --- | --- | --- | --- | --- | --- |
|  | LAPMis12DANCE\_011813\_02.06356.06356.2 | 5.3025 | 0.4894 | 100.0% | 1708.3522 | 1708.7844 | 1 | 8.757 | 61.1% | 5 | K.GSLGGGFSSGGFSGGSFSR.G | 2 |
|  | LAPMis12DANCE\_011813\_01.04844.04844.2 | 2.9602 | 0.3484 | 100.0% | 1090.6322 | 1091.2273 | 24 | 6.022 | 68.8% | 3 | K.VTMQNLNDR.L | 222 |
|  | LAPMis12DANCE\_011813\_01.05114.05114.1 | 1.7551 | 0.2197 | 97.3% | 809.35 | 809.93774 | 6 | 5.101 | 66.7% | 2 | R.LASYLDK.V | 11111 |
|  | LAPMis12DANCE\_011813\_01.06436.06436.2 | 3.0274 | 0.1822 | 99.7% | 1065.3322 | 1065.2578 | 68 | 5.735 | 62.5% | 2 | R.LASYLDKVR.A | 2222 |
|  | LAPMis12DANCE\_011813\_01.06759.06759.2 | 4.3567 | 0.4314 | 100.0% | 1382.0122 | 1382.4668 | 1 | 8.091 | 72.7% | 5 | R.ALEESNYELEGK.I | 2 |
|  | LAPMis12DANCE\_011813\_01.14558.14558.3 | 5.5519 | 0.4113 | 100.0% | 3054.2344 | 3054.4277 | 1 | 7.464 | 32.7% | 5 | K.TIDDLKNQILNLTTDNANILLQIDNAR.L | 3 |
|  | LAPMis12DANCE\_011813\_01.15232.15232.2 | 3.3027 | 0.3758 | 100.0% | 2367.8323 | 2368.6523 | 279 | 5.73 | 30.0% | 1 | K.NQILNLTTDNANILLQIDNAR.L | 2 |
|  | LAPMis12DANCE\_011813\_02.08826.08826.3 | 5.108 | 0.3482 | 100.0% | 2368.1943 | 2368.6523 | 3 | 7.066 | 43.8% | 1 | K.NQILNLTTDNANILLQIDNAR.L | 3 |
|  | LAPMis12DANCE\_011813\_01.06388.06388.2 | 3.4156 | 0.3985 | 100.0% | 1236.0922 | 1235.4258 | 2 | 7.349 | 83.3% | 4 | R.LKYENEVALR.Q | 2 |
|  | LAPMis12DANCE\_011813\_01.08164.08164.2 | 2.6594 | 0.2612 | 99.7% | 1188.5322 | 1188.4099 | 2 | 5.418 | 66.7% | 1 | R.RVLDELTLTK.A | 2 |
|  | LAPMis12DANCE\_011813\_01.08918.08918.2 | 3.3296 | 0.4305 | 100.0% | 1032.1522 | 1032.2224 | 1 | 7.489 | 87.5% | 3 | R.VLDELTLTK.A | 2 |
|  | LAPMis12DANCE\_011813\_01.03640.03640.2 | 2.4611 | 0.3127 | 99.9% | 1301.1322 | 1301.4167 | 10 | 5.223 | 66.7% | 1 | K.NHEEEMKDLR.N | 2 |
|  | LAPMis12DANCE\_011813\_02.09549.09549.3 | 5.5688 | 0.4959 | 100.0% | 2872.8843 | 2874.2134 | 1 | 9.018 | 31.7% | 1 | R.NVSTGDVNVEMNAAPGVDLTQLLNNMR.S | 3 |
|  | LAPMis12DANCE\_011813\_01.05004.05004.2 | 4.0163 | 0.326 | 100.0% | 1366.0721 | 1366.43 | 3 | 6.736 | 70.0% | 1 | R.SQYEQLAEQNR.K | 2 |
|  | LAPMis12DANCE\_011813\_01.04220.04220.2 | 3.2907 | 0.3178 | 100.0% | 1494.2522 | 1494.6041 | 2 | 6.082 | 63.6% | 3 | R.SQYEQLAEQNRK.D | 2 |
|  | LAPMis12DANCE\_011813\_01.09472.09472.1 | 1.7805 | 0.2419 | 98.6% | 1109.37 | 1110.1681 | 1 | 5.322 | 62.5% | 1 | K.DAEAWFNEK.S | 11 |
|  | LAPMis12DANCE\_011813\_01.09488.09488.2 | 3.0337 | 0.288 | 100.0% | 1110.0721 | 1110.1681 | 1 | 6.896 | 75.0% | 1 | K.DAEAWFNEK.S | 22 |
|  | LAPMis12DANCE\_011813\_02.07451.07451.2 | 2.4458 | 0.2305 | 97.5% | 1996.8121 | 1998.151 | 1 | 4.81 | 37.5% | 1 | K.ELTTEIDNNIEQISSYK.S | 2 |
|  | LAPMis12DANCE\_011813\_01.08514.08514.2 | 3.9463 | 0.4442 | 100.0% | 1392.3522 | 1391.4778 | 1 | 6.911 | 70.8% | 3 | K.QSLEASLAETEGR.Y | 2 |
|  | LAPMis12DANCE\_011813\_01.07539.07539.2 | 3.5412 | 0.3525 | 100.0% | 1434.9321 | 1435.623 | 1 | 6.877 | 80.0% | 2 | K.IRLENEIQTYR.S | 2 |
|  | LAPMis12DANCE\_011813\_01.05607.05607.2 | 2.8326 | 0.1411 | 99.1% | 1165.9122 | 1166.2761 | 1 | 5.189 | 87.5% | 1 | R.LENEIQTYR.S | 2 |
|  | LAPMis12DANCE\_011813\_01.04845.04845.2 | 3.6286 | 0.4582 | 100.0% | 1263.3121 | 1263.3066 | 2 | 8.33 | 65.4% | 3 | R.SLLEGEGSSGGGGR.G | 2 |

Similarities:
gi|55956899|ref|NP\_00(1:21)  
gi|15431310|ref|NP\_00(3:19)  
contaminant\_KERATIN12(2:20)  
gi|114431246|ref|NP\_8(3:19)  
contaminant\_KERATIN10(2:20)  

---

|  |  |  |  |  |  |  |  |  |
| --- | --- | --- | --- | --- | --- | --- | --- | --- |
| U | *gi|94721250|ref|NP\_00* | 13 | 42 | 35.0% | 294 | 32614 | 8.9 | vesicle-associated membrane protein-associated protein A isoform 1 [Homo sapiens] |
| U | *gi|94721252|ref|NP\_91* | 13 | 42 | 41.4% | 249 | 27893 | 8.6 | vesicle-associated membrane protein-associated protein A isoform 2 [Homo sapiens] |

| Filename XCorr DeltCN Conf% ObsM+H+ CalcM+H+ SpR ZScore Ion% # Sequence  | | | | | | | | | | | | |
| --- | --- | --- | --- | --- | --- | --- | --- | --- | --- | --- | --- | --- |
|  | LAPMis12DANCE2\_011813\_01.07844.07844.2 | 4.5119 | 0.4818 | 100.0% | 1618.3322 | 1618.8705 | 1 | 8.012 | 80.8% | 9 | K.HEQILVLDPPTDLK.F | 2 |
|  | LAPMis12DANCE\_011813\_01.10125.10125.3 | 3.3893 | 0.2732 | 99.1% | 1618.3744 | 1618.8705 | 1 | 5.498 | 53.8% | 1 | K.HEQILVLDPPTDLK.F | 3 |
|  | LAPMis12DANCE\_011813\_01.10142.10142.2 | 4.6212 | 0.4373 | 100.0% | 1567.2522 | 1567.8253 | 1 | 7.826 | 88.5% | 7 | K.FKGPFTDVVTTNLK.L | 2 |
|  | LAPMis12DANCE\_011813\_01.10228.10228.3 | 3.2975 | 0.221 | 96.9% | 1567.8544 | 1567.8253 | 1 | 5.5 | 51.9% | 1 | K.FKGPFTDVVTTNLK.L | 3 |
|  | LAPMis12DANCE\_011813\_01.10106.10106.2 | 4.1522 | 0.4164 | 100.0% | 1292.0521 | 1292.4747 | 1 | 8.461 | 72.7% | 6 | K.GPFTDVVTTNLK.L | 22 |
|  | LAPMis12DANCE\_011813\_01.13224.13224.2 | 2.9088 | 0.4145 | 100.0% | 2312.3323 | 2313.6912 | 1 | 7.45 | 47.4% | 3 | K.FMVQTIFAPPNTSDMEAVWK.E | 2 |
|  | LAPMis12DANCE\_011813\_01.04430.04430.2 | 1.7301 | 0.415 | 99.3% | 1263.0922 | 1263.4053 | 140 | 6.372 | 55.0% | 1 | K.EAKPDELMDSK.L | 2 |
|  | LAPMis12DANCE\_011813\_01.05199.05199.3 | 3.5031 | 0.4252 | 100.0% | 2109.8643 | 2110.3062 | 76 | 6.722 | 29.2% | 2 | K.QDGPMPKPHSVSLNDTETR.K | 3 |
|  | LAPMis12DANCE\_011813\_01.03278.03278.2 | 2.7433 | 0.3537 | 100.0% | 996.0522 | 996.11414 | 45 | 5.364 | 71.4% | 4 | R.HLRDEGLR.L | 2 |
|  | LAPMis12DANCE2\_011813\_01.03491.03491.2 | 3.7286 | 0.305 | 100.0% | 1775.5721 | 1776.9468 | 27 | 6.174 | 53.1% | 1 | R.KVAHSDKPGSTSTASFR.D | 2 |
|  | LAPMis12DANCE\_011813\_01.03314.03314.3 | 3.8582 | 0.4004 | 100.0% | 1776.2344 | 1776.9468 | 1 | 7.087 | 32.8% | 1 | R.KVAHSDKPGSTSTASFR.D | 3 |
|  | LAPMis12DANCE\_011813\_01.03465.03465.2 | 3.7998 | 0.439 | 100.0% | 1647.9521 | 1648.7727 | 1 | 6.741 | 70.0% | 4 | K.VAHSDKPGSTSTASFR.D | 2 |
|  | LAPMis12DANCE\_011813\_01.03459.03459.3 | 3.8454 | 0.5284 | 100.0% | 1648.4644 | 1648.7727 | 4 | 8.19 | 38.3% | 2 | K.VAHSDKPGSTSTASFR.D | 3 |

Similarities:
gi|4759302|ref|NP\_004(1:12)  

---

|  |  |  |  |  |  |  |  |  |
| --- | --- | --- | --- | --- | --- | --- | --- | --- |
| U | *gi|32455264|ref|NP\_85* | 6 | 10 | 34.7% | 199 | 22110 | 8.1 | peroxiredoxin 1 [Homo sapiens] |
| U | *gi|4505591|ref|NP\_002* | 6 | 10 | 34.7% | 199 | 22110 | 8.1 | peroxiredoxin 1 [Homo sapiens] |
| U | *gi|32455266|ref|NP\_85* | 6 | 10 | 34.7% | 199 | 22110 | 8.1 | peroxiredoxin 1 [Homo sapiens] |

| Filename XCorr DeltCN Conf% ObsM+H+ CalcM+H+ SpR ZScore Ion% # Sequence  | | | | | | | | | | | | |
| --- | --- | --- | --- | --- | --- | --- | --- | --- | --- | --- | --- | --- |
|  | LAPMis12DANCE\_011813\_01.09287.09287.3 | 3.3305 | 0.2426 | 97.5% | 1907.9644 | 1908.2694 | 1 | 5.104 | 38.2% | 1 | K.KQGGLGPMNIPLVSDPKR.T | 3 |
|  | LAPMis12DANCE\_011813\_01.08223.08223.2 | 2.4796 | 0.3495 | 100.0% | 1108.1122 | 1108.2798 | 1 | 6.145 | 77.8% | 2 | R.TIAQDYGVLK.A | 2 |
|  | LAPMis12DANCE\_011813\_02.06910.06910.3 | 2.6887 | 0.322 | 98.8% | 1983.5944 | 1984.2163 | 1 | 5.854 | 33.8% | 1 | R.TIAQDYGVLKADEGISFR.G | 3 |
|  | LAPMis12DANCE2\_011813\_01.09287.09287.2 | 2.8792 | 0.3116 | 100.0% | 1361.4122 | 1360.6395 | 1 | 5.558 | 68.2% | 1 | R.GLFIIDDKGILR.Q | 2 |
|  | LAPMis12DANCE\_011813\_01.08806.08806.2 | 2.9103 | 0.3007 | 100.0% | 1212.2522 | 1212.3915 | 5 | 5.506 | 70.0% | 3 | R.QITVNDLPVGR.S | 2 |
|  | LAPMis12DANCE\_011813\_01.09705.09705.2 | 3.0814 | 0.4757 | 100.0% | 1197.2122 | 1197.3763 | 1 | 7.417 | 83.3% | 2 | R.LVQAFQFTDK.H | 2 |

---

|  |  |  |  |  |  |  |  |  |
| --- | --- | --- | --- | --- | --- | --- | --- | --- |
| U | *gi|14043072|ref|NP\_11* | 10 | 17 | 34.6% | 353 | 37430 | 8.9 | heterogeneous nuclear ribonucleoprotein A2/B1 isoform B1 [Homo sapiens] |
| U | *gi|4504447|ref|NP\_002* | 10 | 17 | 35.8% | 341 | 36006 | 8.6 | heterogeneous nuclear ribonucleoprotein A2/B1 isoform A2 [Homo sapiens] |

| Filename XCorr DeltCN Conf% ObsM+H+ CalcM+H+ SpR ZScore Ion% # Sequence  | | | | | | | | | | | | |
| --- | --- | --- | --- | --- | --- | --- | --- | --- | --- | --- | --- | --- |
|  | LAPMis12DANCE\_011813\_01.12332.12332.2 | 4.4966 | 0.5118 | 100.0% | 1798.9922 | 1800.0184 | 1 | 8.07 | 73.3% | 2 | K.LFIGGLSFETTEESLR.N | 2 |
|  | LAPMis12DANCE2\_011813\_01.03318.03318.2 | 3.0094 | 0.266 | 99.9% | 1339.0521 | 1339.4911 | 2 | 5.428 | 66.7% | 2 | R.EESGKPGAHVTVK.K | 2 |
|  | LAPMis12DANCE\_011813\_01.10336.10336.2 | 3.2326 | 0.3753 | 100.0% | 1188.8922 | 1189.3513 | 1 | 7.048 | 94.4% | 1 | K.IDTIEIITDR.Q | 2 |
|  | LAPMis12DANCE\_011813\_01.10980.10980.2 | 4.0002 | 0.4661 | 100.0% | 1696.1721 | 1696.8132 | 1 | 8.429 | 71.4% | 1 | R.GFGFVTFDDHDPVDK.I | 2 |
|  | LAPMis12DANCE2\_011813\_01.09837.09837.3 | 4.2012 | 0.4834 | 100.0% | 2277.7744 | 2278.5693 | 1 | 7.959 | 34.2% | 1 | R.GFGFVTFDDHDPVDKIVLQK.Y | 3 |
|  | LAPMis12DANCE2\_011813\_01.03598.03598.2 | 3.6428 | 0.3557 | 100.0% | 1412.0721 | 1411.5198 | 1 | 5.875 | 72.7% | 2 | K.YHTINGHNAEVR.K | 2 |
|  | LAPMis12DANCE\_011813\_01.03250.03250.2 | 3.3795 | 0.243 | 100.0% | 1539.2922 | 1539.6938 | 1 | 5.672 | 75.0% | 1 | K.YHTINGHNAEVRK.A | 2 |
|  | LAPMis12DANCE\_011813\_01.03620.03620.2 | 2.4244 | 0.3127 | 99.9% | 1222.0721 | 1222.3153 | 12 | 5.783 | 72.2% | 1 | R.QEMQEVQSSR.S | 2 |
|  | LAPMis12DANCE\_011813\_01.06974.06974.2 | 3.2026 | 0.4774 | 100.0% | 1378.0521 | 1378.4465 | 1 | 6.953 | 57.1% | 4 | R.GGGGNFGPGPGSNFR.G | 2 |
|  | LAPMis12DANCE\_011813\_01.07034.07034.2 | 5.316 | 0.6139 | 100.0% | 2190.3323 | 2191.2554 | 1 | 10.647 | 50.0% | 2 | R.NMGGPYGGGNYGPGGSGGSGGYGGR.S | 2 |

---

|  |  |  |  |  |  |  |  |  |
| --- | --- | --- | --- | --- | --- | --- | --- | --- |
| U | *gi|5729877|ref|NP\_006* | 20 | 36 | 34.5% | 646 | 70898 | 5.5 | heat shock 70kDa protein 8 isoform 1 [Homo sapiens] |

| Filename XCorr DeltCN Conf% ObsM+H+ CalcM+H+ SpR ZScore Ion% # Sequence  | | | | | | | | | | | | |
| --- | --- | --- | --- | --- | --- | --- | --- | --- | --- | --- | --- | --- |
|  | LAPMis12DANCE\_011813\_01.08684.08684.2 | 3.1796 | 0.5386 | 100.0% | 1487.6721 | 1488.5939 | 1 | 9.759 | 70.8% | 5 | R.TTPSYVAFTDTER.L | 222 |
|  | LAPMis12DANCE\_011813\_01.08728.08728.2 | 4.7469 | 0.5129 | 100.0% | 1650.0521 | 1650.8468 | 1 | 9.932 | 75.0% | 2 | K.NQVAMNPTNTVFDAK.R | 2 |
|  | LAPMis12DANCE\_011813\_01.06602.06602.2 | 3.1692 | 0.4652 | 100.0% | 1411.1322 | 1411.5725 | 1 | 7.126 | 68.2% | 3 | R.RFDDAVVQSDMK.H | 2 |
|  | LAPMis12DANCE\_011813\_01.07343.07343.2 | 2.5405 | 0.4452 | 100.0% | 1255.2722 | 1255.385 | 1 | 7.149 | 80.0% | 1 | R.FDDAVVQSDMK.H | 2 |
|  | LAPMis12DANCE\_011813\_01.09473.09473.3 | 3.5131 | 0.3179 | 99.7% | 1654.9443 | 1654.9298 | 19 | 5.798 | 36.5% | 2 | K.HWPFMVVNDAGRPK.V | 3 |
|  | LAPMis12DANCE\_011813\_01.03632.03632.2 | 2.4568 | 0.332 | 100.0% | 1180.1721 | 1181.3312 | 6 | 5.892 | 61.1% | 1 | K.VQVEYKGETK.S | 2 |
|  | LAPMis12DANCE\_011813\_01.11265.11265.2 | 2.913 | 0.3263 | 100.0% | 1618.7122 | 1617.8542 | 1 | 5.973 | 69.2% | 1 | K.SFYPEEVSSMVLTK.M | 2 |
|  | LAPMis12DANCE\_011813\_02.05259.05259.2 | 3.1599 | 0.2641 | 100.0% | 1253.2322 | 1253.4993 | 1 | 5.265 | 90.0% | 1 | K.MKEIAEAYLGK.T | 2 |
|  | LAPMis12DANCE2\_011813\_01.08200.08200.2 | 3.1613 | 0.3462 | 100.0% | 1983.6721 | 1983.1882 | 267 | 5.699 | 26.5% | 1 | K.TVTNAVVTVPAYFNDSQR.Q | 2 |
|  | LAPMis12DANCE\_011813\_02.06268.06268.2 | 2.5299 | 0.3136 | 99.5% | 1660.5521 | 1660.9078 | 1 | 5.605 | 60.0% | 1 | R.IINEPTAAAIAYGLDK.K | 222 |
|  | LAPMis12DANCE\_011813\_01.10062.10062.2 | 3.6639 | 0.3845 | 100.0% | 1787.4521 | 1789.0819 | 1 | 8.325 | 75.0% | 1 | R.IINEPTAAAIAYGLDKK.V | 2 |
|  | LAPMis12DANCE\_011813\_01.05189.05189.2 | 4.3808 | 0.4391 | 100.0% | 1693.3121 | 1692.6958 | 1 | 7.989 | 63.3% | 2 | K.STAGDTHLGGEDFDNR.M | 2 |
|  | LAPMis12DANCE\_011813\_01.09062.09062.2 | 3.0519 | 0.461 | 100.0% | 1237.0521 | 1236.4741 | 1 | 7.199 | 83.3% | 3 | R.MVNHFIAEFK.R | 2 |
|  | LAPMis12DANCE\_011813\_01.10552.10552.2 | 3.3215 | 0.3223 | 100.0% | 1481.2522 | 1481.6511 | 1 | 5.962 | 77.3% | 2 | R.ARFEELNADLFR.G | 2 |
|  | LAPMis12DANCE\_011813\_02.05624.05624.3 | 4.2422 | 0.3535 | 100.0% | 1838.5144 | 1839.1019 | 3 | 6.357 | 35.9% | 2 | K.LDKSQIHDIVLVGGSTR.I | 3 |
|  | LAPMis12DANCE\_011813\_02.05385.05385.2 | 4.2076 | 0.5333 | 100.0% | 1482.5322 | 1482.6798 | 1 | 9.284 | 73.1% | 3 | K.SQIHDIVLVGGSTR.I | 2 |
|  | LAPMis12DANCE\_011813\_01.03429.03429.2 | 2.4704 | 0.4034 | 100.0% | 1017.5522 | 1018.1582 | 8 | 6.412 | 62.5% | 1 | K.ITITNDKGR.L | 222 |
| \* | LAPMis12DANCE\_011813\_01.03728.03728.2 | 2.1252 | 0.2351 | 98.2% | 990.1122 | 990.10144 | 360 | 4.518 | 57.1% | 1 | R.LSKEDIER.M | 2 |
| \* | LAPMis12DANCE\_011813\_01.03510.03510.3 | 3.6443 | 0.2187 | 97.4% | 1982.1843 | 1983.2036 | 3 | 5.796 | 40.0% | 1 | R.MVQEAEKYKAEDEKQR.D | 3 |
| \* | LAPMis12DANCE\_011813\_02.05711.05711.2 | 3.019 | 0.3036 | 100.0% | 1304.4321 | 1304.4602 | 1 | 5.736 | 70.0% | 2 | K.NSLESYAFNMK.A | 2 |

Similarities:
gi|167466173|ref|NP\_0(2:18)  
contaminant\_GR78\_MESA(1:19)  
gi|124256496|ref|NP\_0(3:17)  

---

|  |  |  |  |  |  |  |  |  |
| --- | --- | --- | --- | --- | --- | --- | --- | --- |
| U | *gi|55770864|ref|NP\_00* | 4 | 8 | 31.5% | 257 | 26888 | 11.2 | THO complex 4 [Homo sapiens] |

| Filename XCorr DeltCN Conf% ObsM+H+ CalcM+H+ SpR ZScore Ion% # Sequence  | | | | | | | | | | | | |
| --- | --- | --- | --- | --- | --- | --- | --- | --- | --- | --- | --- | --- |
| \* | LAPMis12DANCE\_011813\_02.10916.10916.3 | 3.9608 | 0.4058 | 100.0% | 2970.8943 | 2971.377 | 1 | 6.162 | 24.0% | 3 | K.LLVSNLDFGVSDADIQELFAEFGTLKK.A | 3 |
| \* | LAPMis12DANCE\_011813\_01.06831.06831.2 | 2.8606 | 0.4089 | 100.0% | 1231.5922 | 1232.3384 | 1 | 7.039 | 75.0% | 3 | R.SLGTADVHFER.K | 2 |
| \* | LAPMis12DANCE\_011813\_01.11146.11146.3 | 3.3144 | 0.2322 | 95.0% | 2814.2043 | 2815.1765 | 1 | 4.916 | 27.1% | 1 | K.QYNGVPLDGRPMNIQLVTSQIDAQR.R | 3 |
| \* | LAPMis12DANCE\_011813\_01.10608.10608.2 | 4.4573 | 0.53 | 100.0% | 2036.0922 | 2036.1626 | 1 | 8.977 | 64.7% | 1 | K.QQLSAEELDAQLDAYNAR.M | 2 |

---

|  |  |  |  |  |  |  |  |  |
| --- | --- | --- | --- | --- | --- | --- | --- | --- |
| U | *gi|32483377|ref|NP\_05* | 9 | 40 | 31.5% | 238 | 25839 | 7.5 | peroxiredoxin 3 isoform b [Homo sapiens] |
| U | *gi|5802974|ref|NP\_006* | 9 | 40 | 29.3% | 256 | 27693 | 7.8 | peroxiredoxin 3 isoform a precursor [Homo sapiens] |

| Filename XCorr DeltCN Conf% ObsM+H+ CalcM+H+ SpR ZScore Ion% # Sequence  | | | | | | | | | | | | |
| --- | --- | --- | --- | --- | --- | --- | --- | --- | --- | --- | --- | --- |
|  | LAPMis12DANCE2\_011813\_01.04515.04515.1 | 1.6968 | 0.2778 | 100.0% | 1021.46 | 1022.146 | 34 | 5.727 | 44.4% | 1 | K.GTAVVNGEFK.D | 1 |
|  | LAPMis12DANCE\_011813\_02.06566.06566.3 | 3.8515 | 0.3275 | 99.7% | 2141.0044 | 2141.3855 | 1 | 5.136 | 36.8% | 1 | K.GTAVVNGEFKDLSLDDFKGK.Y | 3 |
|  | LAPMis12DANCE\_011813\_01.08660.08660.2 | 2.6047 | 0.2939 | 99.9% | 1138.1122 | 1138.2627 | 2 | 4.856 | 66.7% | 1 | K.DLSLDDFKGK.Y | 2 |
|  | LAPMis12DANCE\_011813\_01.11085.11085.2 | 4.8025 | 0.5489 | 100.0% | 1882.3722 | 1883.2163 | 1 | 8.849 | 64.7% | 2 | R.KNGGLGHMNIALLSDLTK.Q | 2 |
|  | LAPMis12DANCE\_011813\_01.11082.11082.3 | 5.0918 | 0.4869 | 100.0% | 1883.0643 | 1883.2163 | 1 | 8.236 | 51.5% | 4 | R.KNGGLGHMNIALLSDLTK.Q | 3 |
|  | LAPMis12DANCE\_011813\_01.11408.11408.2 | 5.5518 | 0.5702 | 100.0% | 1754.3522 | 1755.0422 | 1 | 10.057 | 62.5% | 5 | K.NGGLGHMNIALLSDLTK.Q | 2 |
|  | LAPMis12DANCE\_011813\_02.08072.08072.2 | 5.1531 | 0.4866 | 100.0% | 1464.0922 | 1463.6738 | 1 | 8.796 | 80.8% | 12 | R.DYGVLLEGSGLALR.G | 2 |
|  | LAPMis12DANCE\_011813\_01.11943.11943.2 | 3.5968 | 0.2219 | 100.0% | 1287.3121 | 1286.5571 | 4 | 5.852 | 72.7% | 3 | R.GLFIIDPNGVIK.H | 2 |
|  | LAPMis12DANCE\_011813\_01.07442.07442.2 | 3.1761 | 0.3599 | 100.0% | 1207.1122 | 1207.375 | 1 | 6.982 | 65.0% | 11 | K.HLSVNDLPVGR.S | 2 |

---

|  |  |  |  |  |  |  |  |  |
| --- | --- | --- | --- | --- | --- | --- | --- | --- |
| U | *gi|169160598|ref|XP\_0* | 2 | 3 | 31.0% | 84 | 9461 | 9.5 | PREDICTED: similar to hCG1783679 [Homo sapiens] |
| U | *gi|4506711|ref|NP\_001* | 2 | 3 | 31.0% | 84 | 9461 | 9.5 | ribosomal protein S27 [Homo sapiens] |
| U | *gi|169161552|ref|XP\_0* | 2 | 3 | 31.0% | 84 | 9461 | 9.5 | PREDICTED: similar to hCG1783679 [Homo sapiens] |
| U | *gi|169161255|ref|XP\_0* | 2 | 3 | 31.0% | 84 | 9461 | 9.5 | PREDICTED: hypothetical protein [Homo sapiens] |

| Filename XCorr DeltCN Conf% ObsM+H+ CalcM+H+ SpR ZScore Ion% # Sequence  | | | | | | | | | | | | |
| --- | --- | --- | --- | --- | --- | --- | --- | --- | --- | --- | --- | --- |
|  | LAPMis12DANCE\_011813\_01.04110.04110.2 | 2.802 | 0.1677 | 98.0% | 1578.2522 | 1578.7654 | 3 | 5.017 | 58.3% | 1 | K.DLLHPSPEEEKRK.H | 2 |
|  | LAPMis12DANCE\_011813\_01.10258.10258.2 | 3.6838 | 0.3951 | 100.0% | 1528.1122 | 1528.7632 | 1 | 7.399 | 70.8% | 2 | R.LVQSPNSYFMDVK.C | 2 |

---

|  |  |  |  |  |  |  |  |  |
| --- | --- | --- | --- | --- | --- | --- | --- | --- |
| U | *gi|20149594|ref|NP\_03* | 19 | 31 | 30.4% | 724 | 83264 | 5.0 | heat shock 90kDa protein 1, beta [Homo sapiens] |

| Filename XCorr DeltCN Conf% ObsM+H+ CalcM+H+ SpR ZScore Ion% # Sequence  | | | | | | | | | | | | |
| --- | --- | --- | --- | --- | --- | --- | --- | --- | --- | --- | --- | --- |
|  | LAPMis12DANCE\_011813\_01.09648.09648.2 | 2.8001 | 0.1863 | 98.3% | 1545.0322 | 1545.733 | 1 | 5.147 | 65.4% | 2 | R.ELISNASDALDKIR.Y | 22 |
|  | LAPMis12DANCE\_011813\_01.06597.06597.2 | 3.8101 | 0.4687 | 100.0% | 1540.2322 | 1540.6672 | 1 | 6.953 | 65.4% | 1 | R.YESLTDPSKLDSGK.E | 22 |
|  | LAPMis12DANCE\_011813\_01.10278.10278.2 | 3.4458 | 0.4477 | 100.0% | 1243.3922 | 1243.4459 | 1 | 7.532 | 77.3% | 2 | K.ADLINNLGTIAK.S | 22 |
|  | LAPMis12DANCE\_011813\_02.05552.05552.3 | 4.3415 | 0.49 | 100.0% | 2015.5144 | 2016.2584 | 1 | 8.175 | 48.3% | 1 | K.VILHLKEDQTEYLEER.R | 33 |
|  | LAPMis12DANCE2\_011813\_01.04391.04391.1 | 2.7038 | 0.1407 | 96.1% | 1151.46 | 1152.2462 | 5 | 4.475 | 75.0% | 2 | K.YIDQEELNK.T | 11 |
|  | LAPMis12DANCE\_011813\_01.04958.04958.2 | 2.7262 | 0.0852 | 95.8% | 1153.3922 | 1152.2462 | 3 | 3.717 | 81.2% | 1 | K.YIDQEELNK.T | 22 |
| \* | LAPMis12DANCE\_011813\_02.06207.06207.2 | 4.9094 | 0.5294 | 100.0% | 1848.2522 | 1848.9171 | 1 | 9.312 | 78.6% | 3 | R.NPDDITQEEYGEFYK.S | 2 |
|  | LAPMis12DANCE\_011813\_01.09534.09534.2 | 4.0346 | 0.426 | 100.0% | 1528.0521 | 1528.6616 | 2 | 7.458 | 62.5% | 2 | K.SLTNDWEDHLAVK.H | 22 |
|  | LAPMis12DANCE\_011813\_01.09167.09167.2 | 2.9063 | 0.4308 | 100.0% | 1349.1921 | 1349.4886 | 6 | 6.774 | 60.0% | 2 | K.HFSVEGQLEFR.A | 22 |
| \* | LAPMis12DANCE\_011813\_01.10316.10316.2 | 2.8096 | 0.1981 | 99.4% | 1237.1921 | 1237.4008 | 1 | 5.069 | 72.2% | 2 | R.RAPFDLFENK.K | 2 |
| \* | LAPMis12DANCE\_011813\_01.03700.03700.2 | 2.1499 | 0.2331 | 97.1% | 1141.6522 | 1142.2137 | 16 | 4.327 | 55.6% | 1 | K.LGIHEDSTNR.R | 2 |
| \* | LAPMis12DANCE\_011813\_02.06123.06123.2 | 5.2707 | 0.6073 | 100.0% | 2176.6921 | 2178.2915 | 1 | 11.927 | 66.7% | 1 | R.YHTSQSGDEMTSLSEYVSR.M | 2 |
| \* | LAPMis12DANCE\_011813\_02.06146.06146.3 | 3.6785 | 0.4682 | 100.0% | 2177.7544 | 2178.2915 | 2 | 7.121 | 33.3% | 3 | R.YHTSQSGDEMTSLSEYVSR.M | 3 |
| \* | LAPMis12DANCE\_011813\_01.07642.07642.2 | 2.1018 | 0.3264 | 99.4% | 1161.3322 | 1161.297 | 2 | 6.143 | 66.7% | 1 | K.SIYYITGESK.E | 2 |
| \* | LAPMis12DANCE\_011813\_01.05588.05588.2 | 2.2441 | 0.3727 | 99.9% | 1250.1122 | 1250.3538 | 290 | 6.77 | 45.0% | 1 | K.EQVANSAFVER.V | 2 |
| \* | LAPMis12DANCE\_011813\_01.07878.07878.1 | 2.2975 | 0.3766 | 100.0% | 1248.83 | 1249.4574 | 1 | 6.664 | 55.0% | 1 | R.DNSTMGYMMAK.K | 1 |
| \* | LAPMis12DANCE\_011813\_01.08534.08534.2 | 4.3558 | 0.3731 | 100.0% | 1783.5122 | 1784.025 | 1 | 7.488 | 64.3% | 1 | K.HLEINPDHPIVETLR.Q | 2 |
| \* | LAPMis12DANCE\_011813\_01.08501.08501.3 | 4.4448 | 0.4884 | 100.0% | 1783.9143 | 1784.025 | 1 | 8.135 | 50.0% | 2 | K.HLEINPDHPIVETLR.Q | 3 |
| \* | LAPMis12DANCE2\_011813\_01.16420.16420.3 | 4.9865 | 0.4008 | 100.0% | 3287.8442 | 3288.725 | 1 | 7.377 | 31.9% | 2 | K.AVKDLVVLLFETALLSSGFSLEDPQTHSNR.I | 3 |

Similarities:
gi|153792590|ref|NP\_0(7:12)  
gi|4507677|ref|NP\_003(1:18)  

---

|  |  |  |  |  |  |  |  |  |
| --- | --- | --- | --- | --- | --- | --- | --- | --- |
| U | *gi|4506671|ref|NP\_000* | 2 | 3 | 30.4% | 115 | 11665 | 4.5 | ribosomal protein P2 [Homo sapiens] |

| Filename XCorr DeltCN Conf% ObsM+H+ CalcM+H+ SpR ZScore Ion% # Sequence  | | | | | | | | | | | | |
| --- | --- | --- | --- | --- | --- | --- | --- | --- | --- | --- | --- | --- |
| \* | LAPMis12DANCE\_011813\_01.12281.12281.2 | 2.8854 | 0.3115 | 99.9% | 1869.6322 | 1870.1124 | 1 | 5.662 | 47.2% | 1 | R.YVASYLLAALGGNSSPSAK.D | 2 |
| \* | LAPMis12DANCE\_011813\_01.08127.08127.2 | 4.2022 | 0.4076 | 100.0% | 1773.3722 | 1773.9377 | 1 | 7.054 | 63.3% | 2 | K.ILDSVGIEADDDRLNK.V | 2 |

---

|  |  |  |  |  |  |  |  |  |
| --- | --- | --- | --- | --- | --- | --- | --- | --- |
| U | *gi|24234688|ref|NP\_00* | 17 | 24 | 29.2% | 679 | 73681 | 6.2 | heat shock 70kDa protein 9 precursor [Homo sapiens] |

| Filename XCorr DeltCN Conf% ObsM+H+ CalcM+H+ SpR ZScore Ion% # Sequence  | | | | | | | | | | | | |
| --- | --- | --- | --- | --- | --- | --- | --- | --- | --- | --- | --- | --- |
| \* | LAPMis12DANCE\_011813\_01.08757.08757.2 | 3.2371 | 0.4664 | 100.0% | 1451.1122 | 1451.576 | 1 | 8.622 | 65.4% | 3 | R.TTPSVVAFTADGER.L | 2 |
| \* | LAPMis12DANCE\_011813\_01.06382.06382.2 | 3.4515 | 0.4114 | 100.0% | 1569.2922 | 1569.7141 | 1 | 7.569 | 73.1% | 2 | R.QAVTNPNNTFYATK.R | 2 |
| \* | LAPMis12DANCE\_011813\_01.04388.04388.2 | 4.0372 | 0.508 | 100.0% | 1342.1322 | 1342.4105 | 1 | 8.825 | 66.7% | 1 | R.ASNGDAWVEAHGK.L | 2 |
| \* | LAPMis12DANCE\_011813\_01.12543.12543.2 | 3.5597 | 0.4116 | 100.0% | 1554.2322 | 1554.8878 | 1 | 6.997 | 76.9% | 2 | K.LYSPSQIGAFVLMK.M | 2 |
| \* | LAPMis12DANCE\_011813\_01.04371.04371.3 | 3.0911 | 0.2256 | 96.4% | 1592.8444 | 1593.7949 | 13 | 5.026 | 38.5% | 1 | K.MKETAENYLGHTAK.N | 3 |
| \* | LAPMis12DANCE\_011813\_01.04358.04358.2 | 4.6885 | 0.4127 | 100.0% | 1593.4321 | 1593.7949 | 1 | 7.998 | 73.1% | 1 | K.MKETAENYLGHTAK.N | 2 |
| \* | LAPMis12DANCE\_011813\_01.04574.04574.2 | 3.017 | 0.3619 | 100.0% | 1333.4321 | 1334.4282 | 88 | 6.892 | 50.0% | 1 | K.ETAENYLGHTAK.N | 2 |
| \* | LAPMis12DANCE\_011813\_01.10012.10012.2 | 2.7662 | 0.307 | 100.0% | 1241.6721 | 1243.4056 | 1 | 6.501 | 81.8% | 2 | K.DAGQISGLNVLR.V | 2 |
| \* | LAPMis12DANCE\_011813\_01.11711.11711.2 | 4.2644 | 0.4636 | 100.0% | 2056.412 | 2057.181 | 1 | 8.292 | 52.8% | 1 | K.STNGDTFLGGEDFDQALLR.H | 2 |
| \* | LAPMis12DANCE\_011813\_01.07548.07548.2 | 2.4641 | 0.2254 | 97.2% | 1692.4722 | 1691.8969 | 1 | 5.015 | 57.1% | 1 | R.ETGVDLTKDNMALQR.V | 2 |
| \* | LAPMis12DANCE\_011813\_01.12077.12077.2 | 3.7002 | 0.367 | 100.0% | 1362.0922 | 1362.5687 | 1 | 7.619 | 68.2% | 3 | R.AQFEGIVTDLIR.R | 2 |
| \* | LAPMis12DANCE\_011813\_02.07516.07516.2 | 2.6068 | 0.2216 | 98.6% | 1447.3522 | 1447.6898 | 1 | 6.22 | 61.5% | 1 | K.SDIGEVILVGGMTR.M | 2 |
| \* | LAPMis12DANCE\_011813\_01.09776.09776.2 | 3.6418 | 0.22 | 100.0% | 1291.2722 | 1291.4496 | 1 | 7.531 | 75.0% | 1 | K.VQQTVQDLFGR.A | 2 |
| \* | LAPMis12DANCE\_011813\_02.04779.04779.2 | 2.7662 | 0.3062 | 99.9% | 1809.9521 | 1809.9707 | 16 | 5.454 | 40.6% | 1 | K.SQVFSTAADGQTQVEIK.V | 2 |
| \* | LAPMis12DANCE\_011813\_01.11058.11058.2 | 3.5823 | 0.4818 | 100.0% | 1857.7722 | 1858.0735 | 1 | 7.923 | 62.5% | 1 | R.VEAVNMAEGIIHDTETK.M | 2 |
| \* | LAPMis12DANCE\_011813\_01.11066.11066.3 | 2.8658 | 0.2745 | 97.4% | 1858.4043 | 1858.0735 | 1 | 5.359 | 39.1% | 1 | R.VEAVNMAEGIIHDTETK.M | 3 |
| \* | LAPMis12DANCE\_011813\_01.04678.04678.2 | 2.6671 | 0.2163 | 99.1% | 1231.9521 | 1232.3794 | 1 | 4.57 | 72.7% | 1 | R.QAASSLQQASLK.L | 2 |

---

|  |  |  |  |  |  |  |  |  |
| --- | --- | --- | --- | --- | --- | --- | --- | --- |
| U | *gi|17986258|ref|NP\_06* | 3 | 3 | 29.1% | 151 | 16930 | 4.7 | myosin, light chain 6, alkali, smooth muscle and non-muscle isoform 1 [Homo sapiens] |
| U | *gi|88999583|ref|NP\_52* | 3 | 3 | 29.1% | 151 | 16961 | 4.6 | myosin, light chain 6, alkali, smooth muscle and non-muscle isoform 2 [Homo sapiens] |

| Filename XCorr DeltCN Conf% ObsM+H+ CalcM+H+ SpR ZScore Ion% # Sequence  | | | | | | | | | | | | |
| --- | --- | --- | --- | --- | --- | --- | --- | --- | --- | --- | --- | --- |
|  | LAPMis12DANCE\_011813\_01.06695.06695.2 | 2.9389 | 0.2139 | 99.4% | 1356.1721 | 1355.5339 | 1 | 4.438 | 62.5% | 1 | R.ALGQNPTNAEVLK.V | 2 |
|  | LAPMis12DANCE\_011813\_01.13791.13791.2 | 3.4016 | 0.4262 | 100.0% | 1888.1322 | 1889.2628 | 1 | 6.384 | 40.0% | 1 | K.VLDFEHFLPMLQTVAK.N | 2 |
|  | LAPMis12DANCE\_011813\_01.08913.08913.2 | 3.1191 | 0.4619 | 100.0% | 1786.3722 | 1787.8804 | 2 | 7.363 | 50.0% | 1 | K.NKDQGTYEDYVEGLR.V | 2 |

---

|  |  |  |  |  |  |  |  |  |
| --- | --- | --- | --- | --- | --- | --- | --- | --- |
| U | *gi|17105394|ref|NP\_00* | 3 | 4 | 28.8% | 156 | 17695 | 10.4 | ribosomal protein L23a [Homo sapiens] |

| Filename XCorr DeltCN Conf% ObsM+H+ CalcM+H+ SpR ZScore Ion% # Sequence  | | | | | | | | | | | | |
| --- | --- | --- | --- | --- | --- | --- | --- | --- | --- | --- | --- | --- |
|  | LAPMis12DANCE\_011813\_01.10241.10241.3 | 3.8074 | 0.2636 | 98.9% | 2323.1343 | 2321.74 | 14 | 5.414 | 31.6% | 1 | R.NKLDHYAIIKFPLTTESAMK.K | 3 |
|  | LAPMis12DANCE\_011813\_01.03802.03802.2 | 2.4917 | 0.2399 | 99.0% | 1370.2122 | 1370.5919 | 22 | 5.076 | 54.5% | 1 | K.VNTLIRPDGEKK.A | 2 |
| \* | LAPMis12DANCE\_011813\_01.08984.08984.2 | 3.1235 | 0.4882 | 100.0% | 1405.6921 | 1405.5474 | 18 | 6.72 | 62.5% | 2 | R.LAPDYDALDVANK.I | 2 |

---

|  |  |  |  |  |  |  |  |  |
| --- | --- | --- | --- | --- | --- | --- | --- | --- |
| U | *gi|4506687|ref|NP\_001* | 2 | 2 | 28.3% | 145 | 17040 | 10.4 | ribosomal protein S15 [Homo sapiens] |

| Filename XCorr DeltCN Conf% ObsM+H+ CalcM+H+ SpR ZScore Ion% # Sequence  | | | | | | | | | | | | |
| --- | --- | --- | --- | --- | --- | --- | --- | --- | --- | --- | --- | --- |
|  | LAPMis12DANCE2\_011813\_01.15210.15210.2 | 5.1591 | 0.5845 | 100.0% | 2588.8523 | 2589.938 | 1 | 11.297 | 54.8% | 1 | R.GVDLDQLLDMSYEQLMQLYSAR.Q | 2 |
| \* | LAPMis12DANCE\_011813\_01.14442.14442.2 | 4.0893 | 0.5887 | 100.0% | 2053.8123 | 2054.4856 | 1 | 9.987 | 61.1% | 1 | R.DMIILPEMVGSMVGVYNGK.T | 2 |

---

|  |  |  |  |  |  |  |  |  |
| --- | --- | --- | --- | --- | --- | --- | --- | --- |
| U | *gi|4501881|ref|NP\_001* | 13 | 31 | 28.1% | 377 | 42051 | 5.4 | actin, alpha 1, skeletal muscle [Homo sapiens] |
| U | *gi|4885049|ref|NP\_005* | 13 | 31 | 28.1% | 377 | 42019 | 5.4 | cardiac muscle alpha actin 1 proprotein [Homo sapiens] |

| Filename XCorr DeltCN Conf% ObsM+H+ CalcM+H+ SpR ZScore Ion% # Sequence  | | | | | | | | | | | | |
| --- | --- | --- | --- | --- | --- | --- | --- | --- | --- | --- | --- | --- |
|  | LAPMis12DANCE\_011813\_01.04340.04340.2 | 3.195 | 0.3595 | 100.0% | 977.0122 | 977.02136 | 1 | 6.573 | 77.8% | 1 | K.AGFAGDDAPR.A | 22 |
|  | LAPMis12DANCE\_011813\_01.08306.08306.2 | 2.5563 | 0.1578 | 96.6% | 1199.2522 | 1199.4415 | 6 | 5.24 | 65.0% | 4 | R.AVFPSIVGRPR.H | 22 |
|  | LAPMis12DANCE\_011813\_01.04196.04196.1 | 2.8041 | 0.4625 | 100.0% | 1171.37 | 1172.4058 | 1 | 7.949 | 70.0% | 3 | R.HQGVMVGMGQK.D | 11 |
|  | LAPMis12DANCE\_011813\_01.04214.04214.2 | 3.2151 | 0.3352 | 100.0% | 1172.0721 | 1172.4058 | 1 | 6.264 | 85.0% | 3 | R.HQGVMVGMGQK.D | 22 |
|  | LAPMis12DANCE2\_011813\_01.03634.03634.2 | 2.9425 | 0.1034 | 96.2% | 1355.2522 | 1355.4038 | 1 | 6.437 | 77.3% | 2 | K.DSYVGDEAQSKR.G | 22 |
|  | LAPMis12DANCE\_011813\_01.10263.10263.3 | 4.2383 | 0.2255 | 98.7% | 1960.7344 | 1962.1841 | 1 | 6.213 | 50.0% | 1 | K.YPIEHGIITNWDDMEK.I | 3 |
|  | LAPMis12DANCE2\_011813\_01.08060.08060.2 | 3.8522 | 0.36 | 100.0% | 1961.2522 | 1962.1841 | 1 | 7.267 | 56.7% | 1 | K.YPIEHGIITNWDDMEK.I | 2 |
|  | LAPMis12DANCE\_011813\_01.07454.07454.3 | 2.9923 | 0.2437 | 97.5% | 1516.6144 | 1516.7019 | 3 | 5.55 | 52.5% | 1 | K.IWHHTFYNELR.V | 33 |
|  | LAPMis12DANCE\_011813\_01.07502.07502.2 | 3.1529 | 0.3794 | 100.0% | 1517.1921 | 1516.7019 | 1 | 5.859 | 85.0% | 8 | K.IWHHTFYNELR.V | 22 |
|  | LAPMis12DANCE\_011813\_01.11112.11112.1 | 1.8036 | 0.2511 | 100.0% | 998.37 | 999.167 | 13 | 5.216 | 64.3% | 1 | R.DLTDYLMK.I | 11 |
|  | LAPMis12DANCE2\_011813\_01.08888.08888.2 | 3.961 | 0.2645 | 100.0% | 1791.4122 | 1791.9554 | 2 | 8.179 | 66.7% | 4 | K.SYELPDGQVITIGNER.F | 22 |
|  | LAPMis12DANCE\_011813\_01.07542.07542.1 | 2.3831 | 0.4599 | 100.0% | 1161.44 | 1162.3868 | 1 | 7.36 | 65.0% | 1 | K.EITALAPSTMK.I | 11 |
|  | LAPMis12DANCE\_011813\_01.07574.07574.2 | 2.2003 | 0.3331 | 99.5% | 1161.4922 | 1162.3868 | 7 | 5.913 | 60.0% | 1 | K.EITALAPSTMK.I | 22 |

Similarities:
gi|4501885|ref|NP\_001(11:2)  

---

|  |  |  |  |  |  |  |  |  |
| --- | --- | --- | --- | --- | --- | --- | --- | --- |
| U | *gi|4758086|ref|NP\_004* | 3 | 5 | 28.0% | 193 | 20567 | 8.6 | cysteine and glycine-rich protein 1 isoform 1 [Homo sapiens] |

| Filename XCorr DeltCN Conf% ObsM+H+ CalcM+H+ SpR ZScore Ion% # Sequence  | | | | | | | | | | | | |
| --- | --- | --- | --- | --- | --- | --- | --- | --- | --- | --- | --- | --- |
|  | LAPMis12DANCE\_011813\_02.05426.05426.3 | 3.0907 | 0.2558 | 97.1% | 2159.8145 | 2160.3452 | 27 | 4.655 | 25.0% | 1 | K.GYGYGQGAGTLSTDKGESLGIK.H | 3 |
|  | LAPMis12DANCE2\_011813\_01.03201.03201.2 | 2.74 | 0.2751 | 99.5% | 1842.6721 | 1843.9535 | 1 | 5.974 | 40.6% | 1 | K.HEEAPGHRPTTNPNASK.F | 2 |
| \* | LAPMis12DANCE\_011813\_02.06681.06681.2 | 3.3657 | 0.3798 | 100.0% | 1435.2722 | 1434.551 | 1 | 5.99 | 60.7% | 3 | K.GFGFGQGAGALVHSE.- | 2 |

---

|  |  |  |  |  |  |  |  |  |
| --- | --- | --- | --- | --- | --- | --- | --- | --- |
| U | *gi|5902102|ref|NP\_008* | 2 | 3 | 27.7% | 119 | 13282 | 11.6 | small nuclear ribonucleoprotein D1 polypeptide 16kDa [Homo sapiens] |

| Filename XCorr DeltCN Conf% ObsM+H+ CalcM+H+ SpR ZScore Ion% # Sequence  | | | | | | | | | | | | |
| --- | --- | --- | --- | --- | --- | --- | --- | --- | --- | --- | --- | --- |
|  | LAPMis12DANCE\_011813\_01.08866.08866.2 | 3.5289 | 0.3155 | 100.0% | 1556.5922 | 1555.7745 | 10 | 5.746 | 54.2% | 1 | K.NREPVQLETLSIR.G | 2 |
| \* | LAPMis12DANCE2\_011813\_01.14453.14453.2 | 4.4182 | 0.4485 | 100.0% | 2287.5122 | 2288.6863 | 1 | 8.033 | 50.0% | 2 | R.YFILPDSLPLDTLLVDVEPK.V | 2 |

---

|  |  |  |  |  |  |  |  |  |
| --- | --- | --- | --- | --- | --- | --- | --- | --- |
| U | *gi|167466173|ref|NP\_0* | 15 | 22 | 27.3% | 641 | 70052 | 5.6 | heat shock 70kDa protein 1B [Homo sapiens] |
| U | *gi|194248072|ref|NP\_0* | 15 | 22 | 27.3% | 641 | 70052 | 5.6 | heat shock 70kDa protein 1A [Homo sapiens] |

| Filename XCorr DeltCN Conf% ObsM+H+ CalcM+H+ SpR ZScore Ion% # Sequence  | | | | | | | | | | | | |
| --- | --- | --- | --- | --- | --- | --- | --- | --- | --- | --- | --- | --- |
|  | LAPMis12DANCE\_011813\_01.08684.08684.2 | 3.1796 | 0.5386 | 100.0% | 1487.6721 | 1488.5939 | 1 | 9.759 | 70.8% | 5 | R.TTPSYVAFTDTER.L | 222 |
|  | LAPMis12DANCE\_011813\_01.09113.09113.2 | 4.271 | 0.3651 | 100.0% | 1659.5322 | 1659.8394 | 1 | 7.587 | 75.0% | 2 | K.NQVALNPQNTVFDAK.R | 2 |
|  | LAPMis12DANCE\_011813\_01.05907.05907.2 | 2.3359 | 0.208 | 96.6% | 1352.1122 | 1351.5603 | 4 | 5.162 | 63.6% | 1 | R.KFGDPVVQSDMK.H | 2 |
|  | LAPMis12DANCE\_011813\_01.07294.07294.2 | 2.2178 | 0.3305 | 99.5% | 1222.7922 | 1223.3862 | 1 | 5.668 | 70.0% | 1 | K.FGDPVVQSDMK.H | 2 |
|  | LAPMis12DANCE\_011813\_01.09084.09084.2 | 3.0657 | 0.2057 | 99.4% | 1681.5322 | 1681.8912 | 7 | 5.285 | 50.0% | 1 | K.HWPFQVINDGDKPK.V | 2 |
|  | LAPMis12DANCE\_011813\_01.09116.09116.3 | 3.3462 | 0.2849 | 99.0% | 1681.7344 | 1681.8912 | 2 | 5.703 | 42.3% | 1 | K.HWPFQVINDGDKPK.V | 3 |
|  | LAPMis12DANCE\_011813\_01.11934.11934.2 | 2.539 | 0.3173 | 99.6% | 1615.1721 | 1615.8817 | 2 | 5.08 | 50.0% | 2 | K.AFYPEEISSMVLTK.M | 22 |
|  | LAPMis12DANCE\_011813\_01.12560.12560.3 | 3.2747 | 0.3188 | 99.5% | 3263.1543 | 3262.7046 | 19 | 4.872 | 20.5% | 1 | K.MKEIAEAYLGYPVTNAVITVPAYFNDSQR.Q | 3 |
|  | LAPMis12DANCE2\_011813\_01.08877.08877.2 | 2.8805 | 0.1668 | 97.7% | 1688.2122 | 1688.9213 | 1 | 5.433 | 56.7% | 1 | R.IINEPTAAAIAYGLDR.T | 2 |
|  | LAPMis12DANCE\_011813\_01.05212.05212.2 | 3.7838 | 0.5043 | 100.0% | 1675.8322 | 1676.6964 | 1 | 8.957 | 66.7% | 2 | K.ATAGDTHLGGEDFDNR.L | 22 |
|  | LAPMis12DANCE\_011813\_01.08550.08550.2 | 2.7632 | 0.3809 | 100.0% | 1261.6522 | 1262.4508 | 1 | 6.853 | 83.3% | 1 | R.LVNHFVEEFK.R | 2 |
|  | LAPMis12DANCE\_011813\_01.07463.07463.2 | 2.2394 | 0.2004 | 95.6% | 1419.1522 | 1418.6383 | 5 | 4.504 | 60.0% | 1 | R.LVNHFVEEFKR.K | 2 |
|  | LAPMis12DANCE\_011813\_02.05840.05840.2 | 2.0963 | 0.3433 | 99.1% | 1823.1122 | 1823.1025 | 2 | 5.797 | 34.4% | 1 | K.LDKAQIHDLVLVGGSTR.I | 2 |
|  | LAPMis12DANCE\_011813\_01.03429.03429.2 | 2.4704 | 0.4034 | 100.0% | 1017.5522 | 1018.1582 | 8 | 6.412 | 62.5% | 1 | K.ITITNDKGR.L | 222 |
|  | LAPMis12DANCE\_011813\_01.03428.03428.2 | 2.776 | 0.3658 | 100.0% | 1137.3922 | 1138.2224 | 1 | 6.496 | 75.0% | 1 | K.YKAEDEVQR.E | 22 |

Similarities:
gi|5729877|ref|NP\_006(2:13)  
gi|124256496|ref|NP\_0(5:10)  

---

|  |  |  |  |  |  |  |  |  |
| --- | --- | --- | --- | --- | --- | --- | --- | --- |
| U | *gi|14165435|ref|NP\_11* | 7 | 10 | 26.3% | 463 | 50976 | 5.5 | heterogeneous nuclear ribonucleoprotein K isoform b [Homo sapiens] |
| U | *gi|14165439|ref|NP\_00* | 7 | 10 | 26.3% | 464 | 51028 | 5.3 | heterogeneous nuclear ribonucleoprotein K isoform a [Homo sapiens] |
| U | *gi|14165437|ref|NP\_11* | 7 | 10 | 26.3% | 464 | 51028 | 5.3 | heterogeneous nuclear ribonucleoprotein K isoform a [Homo sapiens] |

| Filename XCorr DeltCN Conf% ObsM+H+ CalcM+H+ SpR ZScore Ion% # Sequence  | | | | | | | | | | | | |
| --- | --- | --- | --- | --- | --- | --- | --- | --- | --- | --- | --- | --- |
|  | LAPMis12DANCE\_011813\_01.06730.06730.2 | 3.4496 | 0.4604 | 100.0% | 1781.1921 | 1781.8302 | 4 | 7.935 | 53.1% | 2 | R.TDYNASVSVPDSSGPER.I | 2 |
|  | LAPMis12DANCE\_011813\_02.10042.10042.2 | 2.2032 | 0.2526 | 96.3% | 1842.3121 | 1844.1992 | 14 | 4.998 | 37.5% | 1 | R.ILSISADIETIGEILKK.I | 2 |
|  | LAPMis12DANCE2\_011813\_01.07977.07977.2 | 3.2038 | 0.3301 | 100.0% | 1520.3522 | 1519.8711 | 1 | 6.437 | 64.3% | 1 | R.LLIHQSLAGGIIGVK.G | 2 |
|  | LAPMis12DANCE\_011813\_01.12099.12099.2 | 3.2334 | 0.4041 | 100.0% | 1341.5322 | 1341.6311 | 1 | 6.727 | 68.2% | 1 | K.IILDLISESPIK.G | 2 |
|  | LAPMis12DANCE2\_011813\_01.08949.08949.2 | 3.6969 | 0.5623 | 100.0% | 1917.4321 | 1918.1974 | 1 | 9.104 | 44.4% | 3 | R.GSYGDLGGPIITTQVTIPK.D | 2 |
|  | LAPMis12DANCE\_011813\_02.04912.04912.3 | 3.562 | 0.2377 | 97.5% | 2069.9944 | 2070.1772 | 6 | 5.225 | 30.6% | 1 | R.HESGASIKIDEPLEGSEDR.I | 3 |
|  | LAPMis12DANCE\_011813\_01.12075.12075.3 | 4.2163 | 0.4295 | 100.0% | 2590.1943 | 2590.9365 | 1 | 7.176 | 34.1% | 1 | R.IITITGTQDQIQNAQYLLQNSVK.Q | 3 |

---

|  |  |  |  |  |  |  |  |  |
| --- | --- | --- | --- | --- | --- | --- | --- | --- |
| U | *gi|27436946|ref|NP\_73* | 13 | 19 | 25.8% | 664 | 74140 | 7.0 | lamin A/C isoform 1 precursor [Homo sapiens] |

| Filename XCorr DeltCN Conf% ObsM+H+ CalcM+H+ SpR ZScore Ion% # Sequence  | | | | | | | | | | | | |
| --- | --- | --- | --- | --- | --- | --- | --- | --- | --- | --- | --- | --- |
|  | LAPMis12DANCE\_011813\_01.05712.05712.2 | 4.1847 | 0.3722 | 100.0% | 1630.0122 | 1630.7521 | 1 | 7.172 | 75.0% | 1 | R.LQEKEDLQELNDR.L | 2 |
|  | LAPMis12DANCE\_011813\_01.10169.10169.2 | 3.0405 | 0.3081 | 100.0% | 1243.9122 | 1244.474 | 2 | 6.38 | 75.0% | 1 | R.LKDLEALLNSK.E | 2 |
|  | LAPMis12DANCE\_011813\_01.08934.08934.2 | 3.1313 | 0.3405 | 100.0% | 1029.1322 | 1029.1814 | 1 | 6.503 | 87.5% | 1 | R.LADALQELR.A | 2 |
|  | LAPMis12DANCE\_011813\_01.03286.03286.2 | 3.8424 | 0.3678 | 100.0% | 1502.8722 | 1503.6115 | 1 | 6.877 | 68.2% | 1 | R.AQHEDQVEQYKK.E | 2 |
|  | LAPMis12DANCE\_011813\_01.07233.07233.2 | 4.791 | 0.5435 | 100.0% | 1753.2922 | 1753.8693 | 1 | 8.546 | 66.7% | 3 | R.NSNLVGAAHEELQQSR.I | 2 |
|  | LAPMis12DANCE\_011813\_01.10787.10787.2 | 3.8664 | 0.3745 | 100.0% | 1700.5521 | 1700.9762 | 1 | 6.028 | 64.3% | 1 | R.IRIDSLSAQLSQLQK.Q | 2 |
|  | LAPMis12DANCE\_011813\_01.07059.07059.2 | 2.3494 | 0.2412 | 99.0% | 1187.6322 | 1188.3262 | 1 | 4.688 | 77.8% | 1 | K.LRDLEDSLAR.E | 2 |
|  | LAPMis12DANCE\_011813\_01.11464.11464.2 | 3.9229 | 0.427 | 100.0% | 1894.6122 | 1895.1346 | 1 | 7.029 | 64.3% | 1 | R.MQQQLDEYQELLDIK.L | 2 |
|  | LAPMis12DANCE\_011813\_02.05096.05096.2 | 2.9014 | 0.4976 | 100.0% | 1604.8322 | 1606.7728 | 1 | 8.519 | 57.7% | 2 | R.VAVEEVDEEGKFVR.L | 2 |
|  | LAPMis12DANCE\_011813\_02.05105.05105.3 | 2.3072 | 0.3479 | 99.0% | 1606.7043 | 1606.7728 | 8 | 5.256 | 40.4% | 1 | R.VAVEEVDEEGKFVR.L | 3 |
|  | LAPMis12DANCE\_011813\_02.07822.07822.3 | 3.0234 | 0.3137 | 98.9% | 2534.3643 | 2534.8772 | 11 | 5.133 | 26.0% | 1 | K.AGQVVTIWAAGAGATHSPPTDLVWK.A | 3 |
|  | LAPMis12DANCE\_011813\_02.05481.05481.2 | 3.8349 | 0.5295 | 100.0% | 1492.2722 | 1492.6874 | 1 | 8.207 | 69.2% | 2 | R.TALINSTGEEVAMR.K | 2 |
|  | LAPMis12DANCE\_011813\_02.05445.05445.2 | 3.3896 | 0.4441 | 100.0% | 1566.0122 | 1567.6555 | 1 | 7.835 | 46.9% | 3 | R.SVGGSGGGSFGDNLVTR.S | 23 |

---

|  |  |  |  |  |  |  |  |  |
| --- | --- | --- | --- | --- | --- | --- | --- | --- |
| U | *gi|93588500|ref|NP\_06* | 4 | 14 | 25.8% | 159 | 17419 | 6.1 | angiotensin II receptor-associated protein isoform a [Homo sapiens] |

| Filename XCorr DeltCN Conf% ObsM+H+ CalcM+H+ SpR ZScore Ion% # Sequence  | | | | | | | | | | | | |
| --- | --- | --- | --- | --- | --- | --- | --- | --- | --- | --- | --- | --- |
| \* | LAPMis12DANCE\_011813\_01.08932.08932.3 | 4.2302 | 0.3519 | 100.0% | 2060.6943 | 2059.246 | 138 | 5.616 | 30.6% | 2 | R.ERGGELLVHTGFLGSSQDR.S | 3 |
| \* | LAPMis12DANCE\_011813\_01.09674.09674.2 | 4.6321 | 0.4828 | 100.0% | 1774.0922 | 1773.9432 | 1 | 8.696 | 59.4% | 6 | R.GGELLVHTGFLGSSQDR.S | 2 |
|  | LAPMis12DANCE\_011813\_01.10796.10796.2 | 4.4579 | 0.5546 | 100.0% | 2292.3123 | 2293.4517 | 1 | 9.459 | 52.4% | 5 | R.SAYQTIDSAEAPADPFAVPEGR.S | 2 |
|  | LAPMis12DANCE\_011813\_02.06134.06134.3 | 2.631 | 0.3391 | 99.0% | 2292.5044 | 2293.4517 | 17 | 5.045 | 28.6% | 1 | R.SAYQTIDSAEAPADPFAVPEGR.S | 3 |

---

|  |  |  |  |  |  |  |  |  |
| --- | --- | --- | --- | --- | --- | --- | --- | --- |
| U | *gi|4505409|ref|NP\_002* | 3 | 3 | 25.7% | 152 | 17298 | 8.4 | non-metastatic cells 2, protein (NM23B) expressed in [Homo sapiens] |
| U | *gi|66392227|ref|NP\_00* | 3 | 3 | 25.7% | 152 | 17298 | 8.4 | non-metastatic cells 2, protein (NM23B) expressed in [Homo sapiens] |
| U | *gi|66392205|ref|NP\_00* | 3 | 3 | 25.7% | 152 | 17298 | 8.4 | non-metastatic cells 2, protein (NM23B) expressed in [Homo sapiens] |
| U | *gi|66392203|ref|NP\_00* | 3 | 3 | 14.6% | 267 | 30137 | 8.9 | NME1-NME2 protein [Homo sapiens] |
| U | *gi|66392192|ref|NP\_00* | 3 | 3 | 25.7% | 152 | 17298 | 8.4 | non-metastatic cells 2, protein (NM23B) expressed in [Homo sapiens] |

| Filename XCorr DeltCN Conf% ObsM+H+ CalcM+H+ SpR ZScore Ion% # Sequence  | | | | | | | | | | | | |
| --- | --- | --- | --- | --- | --- | --- | --- | --- | --- | --- | --- | --- |
|  | LAPMis12DANCE\_011813\_01.07425.07425.2 | 3.0612 | 0.2698 | 100.0% | 1346.1122 | 1345.5846 | 7 | 5.497 | 59.1% | 1 | R.TFIAIKPDGVQR.G | 2 |
|  | LAPMis12DANCE\_011813\_01.10176.10176.2 | 2.1817 | 0.2706 | 98.9% | 1176.0922 | 1176.4038 | 79 | 5.263 | 61.1% | 1 | K.DRPFFPGLVK.Y | 2 |
|  | LAPMis12DANCE\_011813\_01.06684.06684.2 | 3.6804 | 0.4412 | 100.0% | 1786.0322 | 1787.041 | 1 | 6.925 | 50.0% | 1 | R.VMLGETNPADSKPGTIR.G | 2 |

---

|  |  |  |  |  |  |  |  |  |
| --- | --- | --- | --- | --- | --- | --- | --- | --- |
| U | *gi|15431310|ref|NP\_00* | 11 | 16 | 25.6% | 472 | 51622 | 5.2 | keratin 14 [Homo sapiens] |

| Filename XCorr DeltCN Conf% ObsM+H+ CalcM+H+ SpR ZScore Ion% # Sequence  | | | | | | | | | | | | |
| --- | --- | --- | --- | --- | --- | --- | --- | --- | --- | --- | --- | --- |
| \* | LAPMis12DANCE\_011813\_01.05886.05886.2 | 3.4664 | 0.4939 | 100.0% | 1426.0521 | 1426.526 | 1 | 9.011 | 64.3% | 2 | R.APSTYGGGLSVSSSR.F | 2 |
|  | LAPMis12DANCE\_011813\_01.04844.04844.2 | 2.9602 | 0.3484 | 100.0% | 1090.6322 | 1091.2273 | 24 | 6.022 | 68.8% | 3 | K.VTMQNLNDR.L | 222 |
|  | LAPMis12DANCE\_011813\_01.05114.05114.1 | 1.7551 | 0.2197 | 97.3% | 809.35 | 809.93774 | 6 | 5.101 | 66.7% | 2 | R.LASYLDK.V | 11111 |
|  | LAPMis12DANCE\_011813\_01.06436.06436.2 | 3.0274 | 0.1822 | 99.7% | 1065.3322 | 1065.2578 | 68 | 5.735 | 62.5% | 2 | R.LASYLDKVR.A | 2222 |
|  | LAPMis12DANCE\_011813\_02.04922.04922.2 | 3.7704 | 0.2987 | 100.0% | 1303.2322 | 1302.4241 | 1 | 7.162 | 77.3% | 1 | R.ALEEANADLEVK.I | 2 |
|  | LAPMis12DANCE\_011813\_01.06546.06546.2 | 2.3623 | 0.1715 | 96.2% | 1267.9321 | 1267.4246 | 5 | 4.611 | 61.1% | 1 | R.TKYETELNLR.M | 2 |
|  | LAPMis12DANCE\_011813\_01.09338.09338.2 | 2.6607 | 0.2826 | 100.0% | 1030.0122 | 1030.2096 | 2 | 6.157 | 81.2% | 1 | R.VLDELTLAR.A | 222 |
|  | LAPMis12DANCE\_011813\_01.05410.05410.2 | 2.8753 | 0.1865 | 99.2% | 1438.9321 | 1439.6263 | 2 | 4.216 | 75.0% | 1 | R.ILNEMRDQYEK.M | 22 |
|  | LAPMis12DANCE\_011813\_01.04502.04502.2 | 4.0545 | 0.4775 | 100.0% | 1361.8722 | 1362.4796 | 1 | 9.708 | 66.7% | 1 | R.EVATNSELVQSGK.S | 22 |
|  | LAPMis12DANCE\_011813\_01.07610.07610.2 | 2.4647 | 0.1921 | 97.6% | 1381.2522 | 1380.5437 | 7 | 4.454 | 60.0% | 1 | K.TRLEQEIATYR.R | 22 |
|  | LAPMis12DANCE\_011813\_02.05024.05024.3 | 4.2557 | 0.4414 | 100.0% | 2308.9143 | 2310.396 | 1 | 6.934 | 36.9% | 1 | R.LLEGEDAHLSSSQFSSGSQSSR.D | 3 |

Similarities:
gi|55956899|ref|NP\_00(1:10)  
contaminant\_KERATIN03(3:8)  
contaminant\_KERATIN12(6:5)  
gi|114431246|ref|NP\_8(1:10)  
contaminant\_KERATIN10(3:8)  

---

|  |  |  |  |  |  |  |  |  |
| --- | --- | --- | --- | --- | --- | --- | --- | --- |
| U | *gi|15431295|ref|NP\_15* | 4 | 5 | 25.6% | 211 | 24261 | 11.7 | ribosomal protein L13 [Homo sapiens] |
| U | *gi|15431297|ref|NP\_00* | 4 | 5 | 25.6% | 211 | 24261 | 11.7 | ribosomal protein L13 [Homo sapiens] |

| Filename XCorr DeltCN Conf% ObsM+H+ CalcM+H+ SpR ZScore Ion% # Sequence  | | | | | | | | | | | | |
| --- | --- | --- | --- | --- | --- | --- | --- | --- | --- | --- | --- | --- |
|  | LAPMis12DANCE\_011813\_01.08974.08974.2 | 2.6574 | 0.3016 | 100.0% | 1190.2122 | 1190.3469 | 1 | 5.696 | 77.8% | 1 | R.VATWFNQPAR.K | 2 |
|  | LAPMis12DANCE\_011813\_01.04322.04322.2 | 2.6516 | 0.2777 | 99.8% | 1232.3322 | 1233.3237 | 1 | 5.379 | 75.0% | 2 | K.STESLQANVQR.L | 2 |
|  | LAPMis12DANCE\_011813\_02.06958.06958.3 | 4.1748 | 0.397 | 100.0% | 2428.5842 | 2428.8064 | 4 | 6.071 | 27.3% | 1 | K.KGDSSAEELKLATQLTGPVMPVR.N | 3 |
|  | LAPMis12DANCE2\_011813\_01.04175.04175.2 | 2.8226 | 0.3296 | 100.0% | 1237.5122 | 1237.3953 | 18 | 5.31 | 66.7% | 1 | R.VITEEEKNFK.A | 2 |

---

|  |  |  |  |  |  |  |  |  |
| --- | --- | --- | --- | --- | --- | --- | --- | --- |
| U | *gi|209862831|ref|NP\_0* | 7 | 13 | 25.1% | 339 | 38604 | 7.8 | annexin A2 isoform 2 [Homo sapiens] |
| U | *gi|50845388|ref|NP\_00* | 7 | 13 | 23.8% | 357 | 40411 | 8.4 | annexin A2 isoform 1 [Homo sapiens] |
| U | *gi|50845386|ref|NP\_00* | 7 | 13 | 25.1% | 339 | 38604 | 7.8 | annexin A2 isoform 2 [Homo sapiens] |
| U | *gi|4757756|ref|NP\_004* | 7 | 13 | 25.1% | 339 | 38604 | 7.8 | annexin A2 isoform 2 [Homo sapiens] |

| Filename XCorr DeltCN Conf% ObsM+H+ CalcM+H+ SpR ZScore Ion% # Sequence  | | | | | | | | | | | | |
| --- | --- | --- | --- | --- | --- | --- | --- | --- | --- | --- | --- | --- |
|  | LAPMis12DANCE\_011813\_01.13800.13800.2 | 4.8112 | 0.4142 | 100.0% | 1652.3722 | 1651.9872 | 1 | 7.898 | 63.3% | 3 | K.SALSGHLETVILGLLK.T | 2 |
|  | LAPMis12DANCE2\_011813\_01.04571.04571.2 | 3.6201 | 0.4823 | 100.0% | 1223.3322 | 1223.3251 | 1 | 8.796 | 80.0% | 3 | K.TPAQYDASELK.A | 2 |
|  | LAPMis12DANCE\_011813\_01.04904.04904.2 | 2.922 | 0.1768 | 99.4% | 1244.8722 | 1245.3347 | 2 | 5.329 | 77.8% | 2 | R.TNQELQEINR.V | 2 |
|  | LAPMis12DANCE\_011813\_01.10734.10734.2 | 3.1735 | 0.2944 | 100.0% | 1813.1322 | 1812.928 | 1 | 5.137 | 53.3% | 1 | K.TDLEKDIISDTSGDFR.K | 2 |
|  | LAPMis12DANCE\_011813\_01.09752.09752.3 | 4.3725 | 0.4495 | 100.0% | 1941.0543 | 1941.102 | 1 | 7.351 | 48.4% | 2 | K.TDLEKDIISDTSGDFRK.L | 3 |
|  | LAPMis12DANCE\_011813\_01.09936.09936.2 | 4.5366 | 0.3426 | 100.0% | 2066.3323 | 2066.1887 | 1 | 5.897 | 58.8% | 1 | R.RAEDGSVIDYELIDQDAR.D | 2 |
|  | LAPMis12DANCE\_011813\_01.11100.11100.2 | 3.0052 | 0.3638 | 100.0% | 1589.6122 | 1589.8035 | 1 | 5.422 | 58.3% | 1 | K.SYSPYDMLESIRK.E | 2 |

---

|  |  |  |  |  |  |  |  |  |
| --- | --- | --- | --- | --- | --- | --- | --- | --- |
| U | *gi|14043070|ref|NP\_11* | 9 | 26 | 24.7% | 372 | 38747 | 9.1 | heterogeneous nuclear ribonucleoprotein A1 isoform b [Homo sapiens] |
| U | *gi|4504445|ref|NP\_002* | 9 | 26 | 28.8% | 320 | 34196 | 9.2 | heterogeneous nuclear ribonucleoprotein A1 isoform a [Homo sapiens] |

| Filename XCorr DeltCN Conf% ObsM+H+ CalcM+H+ SpR ZScore Ion% # Sequence  | | | | | | | | | | | | |
| --- | --- | --- | --- | --- | --- | --- | --- | --- | --- | --- | --- | --- |
|  | LAPMis12DANCE2\_011813\_01.03773.03773.2 | 2.7266 | 0.3386 | 100.0% | 1299.7522 | 1300.4111 | 1 | 5.037 | 65.0% | 3 | K.SESPKEPEQLR.K | 2 |
|  | LAPMis12DANCE2\_011813\_01.03534.03534.2 | 2.7294 | 0.1792 | 98.4% | 1428.4122 | 1428.5852 | 3 | 4.539 | 68.2% | 2 | K.SESPKEPEQLRK.L | 2 |
|  | LAPMis12DANCE2\_011813\_01.09371.09371.2 | 5.3089 | 0.4779 | 100.0% | 1913.6322 | 1914.1656 | 1 | 7.887 | 68.8% | 3 | R.KLFIGGLSFETTDESLR.S | 2 |
|  | LAPMis12DANCE\_011813\_02.08476.08476.2 | 4.9659 | 0.382 | 100.0% | 1786.3922 | 1785.9916 | 1 | 7.042 | 66.7% | 4 | K.LFIGGLSFETTDESLR.S | 2 |
|  | LAPMis12DANCE2\_011813\_01.08698.08698.2 | 2.9139 | 0.3224 | 100.0% | 1219.2122 | 1219.4387 | 1 | 6.662 | 77.8% | 1 | K.IEVIEIMTDR.G | 2 |
|  | LAPMis12DANCE2\_011813\_01.08355.08355.2 | 3.3942 | 0.455 | 100.0% | 1699.5322 | 1700.8016 | 1 | 7.495 | 60.7% | 3 | R.GFAFVTFDDHDSVDK.I | 2 |
|  | LAPMis12DANCE\_011813\_02.07430.07430.3 | 3.8538 | 0.2369 | 97.6% | 2281.8245 | 2282.5579 | 1 | 6.889 | 31.6% | 1 | R.GFAFVTFDDHDSVDKIVIQK.Y | 3 |
|  | LAPMis12DANCE\_011813\_01.06201.06201.2 | 3.7668 | 0.3572 | 100.0% | 1629.1322 | 1629.7721 | 1 | 7.54 | 63.3% | 7 | R.SSGPYGGGGQYFAKPR.N | 2 |
|  | LAPMis12DANCE\_011813\_01.03662.03662.2 | 5.0441 | 0.5286 | 100.0% | 1695.1322 | 1695.6561 | 1 | 10.96 | 70.6% | 2 | R.NQGGYGGSSSSSSYGSGR.R | 2 |

---

|  |  |  |  |  |  |  |  |  |
| --- | --- | --- | --- | --- | --- | --- | --- | --- |
| U | *gi|4506743|ref|NP\_001* | 5 | 8 | 24.5% | 208 | 24205 | 10.3 | ribosomal protein S8 [Homo sapiens] |

| Filename XCorr DeltCN Conf% ObsM+H+ CalcM+H+ SpR ZScore Ion% # Sequence  | | | | | | | | | | | | |
| --- | --- | --- | --- | --- | --- | --- | --- | --- | --- | --- | --- | --- |
| \* | LAPMis12DANCE2\_011813\_01.03738.03738.2 | 2.8247 | 0.2861 | 99.9% | 1348.0922 | 1348.5448 | 4 | 5.65 | 63.6% | 2 | R.KYELGRPAANTK.I | 2 |
| \* | LAPMis12DANCE\_011813\_01.04048.04048.2 | 2.6433 | 0.292 | 99.9% | 1220.0521 | 1220.3707 | 1 | 5.073 | 60.0% | 1 | K.YELGRPAANTK.I | 2 |
| \* | LAPMis12DANCE\_011813\_02.06572.06572.2 | 3.0266 | 0.2614 | 99.7% | 1719.4521 | 1719.9353 | 2 | 6.138 | 50.0% | 1 | R.IIDVVYNASNNELVR.T | 2 |
|  | LAPMis12DANCE\_011813\_01.08147.08147.2 | 2.9194 | 0.296 | 100.0% | 1315.1921 | 1315.4631 | 129 | 5.834 | 60.0% | 1 | K.LTPEEEEILNK.K | 2 |
| \* | LAPMis12DANCE\_011813\_02.06297.06297.2 | 4.113 | 0.3989 | 100.0% | 1508.0122 | 1507.6836 | 1 | 7.331 | 75.0% | 3 | K.ISSLLEEQFQQGK.L | 2 |

---

|  |  |  |  |  |  |  |  |  |
| --- | --- | --- | --- | --- | --- | --- | --- | --- |
| U | *gi|194018550|ref|NP\_9* | 17 | 44 | 24.1% | 773 | 86331 | 6.7 | c-Maf-inducing protein isoform C-mip [Homo sapiens] |

| Filename XCorr DeltCN Conf% ObsM+H+ CalcM+H+ SpR ZScore Ion% # Sequence  | | | | | | | | | | | | |
| --- | --- | --- | --- | --- | --- | --- | --- | --- | --- | --- | --- | --- |
| \* | LAPMis12DANCE\_011813\_01.07562.07562.2 | 3.778 | 0.4737 | 100.0% | 2070.4722 | 2070.3042 | 1 | 7.271 | 57.9% | 3 | R.QIEETKPLLGGDVSAPEGTK.M | 2 |
| \* | LAPMis12DANCE\_011813\_01.07544.07544.3 | 3.8792 | 0.3353 | 99.7% | 2071.2844 | 2070.3042 | 122 | 5.492 | 31.6% | 2 | R.QIEETKPLLGGDVSAPEGTK.M | 3 |
|  | LAPMis12DANCE\_011813\_01.09884.09884.2 | 2.7502 | 0.2084 | 99.0% | 1527.4122 | 1527.8069 | 10 | 4.646 | 54.2% | 1 | K.VLSNPSRWEVVLK.E | 2 |
|  | LAPMis12DANCE\_011813\_01.12862.12862.2 | 4.2563 | 0.5047 | 100.0% | 2799.912 | 2800.1938 | 1 | 8.833 | 48.0% | 2 | R.TLVDMALTSPLQDDSINQAPLEIVSK.L | 2 |
|  | LAPMis12DANCE\_011813\_02.08492.08492.2 | 3.5963 | 0.3916 | 100.0% | 1604.7922 | 1604.9482 | 1 | 7.043 | 65.4% | 5 | R.SMVVIEVFTPVVQR.I | 2 |
|  | LAPMis12DANCE\_011813\_01.03527.03527.2 | 2.0354 | 0.3306 | 99.5% | 849.2522 | 848.95184 | 4 | 5.181 | 75.0% | 1 | K.HNMDFGK.C | 2 |
|  | LAPMis12DANCE\_011813\_02.09675.09675.3 | 4.7069 | 0.2533 | 99.0% | 2792.5745 | 2794.3252 | 41 | 5.224 | 26.1% | 1 | R.LRLFTQEYILALNELNAGMEVVKK.F | 3 |
|  | LAPMis12DANCE\_011813\_02.10211.10211.3 | 3.5382 | 0.2478 | 97.4% | 2397.2644 | 2396.8042 | 1 | 4.81 | 32.5% | 1 | R.LFTQEYILALNELNAGMEVVK.K | 3 |
|  | LAPMis12DANCE\_011813\_01.13665.13665.3 | 4.1053 | 0.4955 | 100.0% | 2525.2744 | 2524.9783 | 1 | 7.348 | 38.1% | 3 | R.LFTQEYILALNELNAGMEVVKK.F | 3 |
|  | LAPMis12DANCE\_011813\_01.09428.09428.2 | 4.7935 | 0.6027 | 100.0% | 2063.5723 | 2064.303 | 1 | 9.542 | 66.7% | 3 | R.LLHPSPDLVSQEATLSEAR.L | 2 |
|  | LAPMis12DANCE\_011813\_01.09464.09464.3 | 4.1863 | 0.3914 | 100.0% | 2064.2644 | 2064.303 | 1 | 7.301 | 38.9% | 3 | R.LLHPSPDLVSQEATLSEAR.L | 3 |
|  | LAPMis12DANCE2\_011813\_01.07984.07984.2 | 4.4587 | 0.5203 | 100.0% | 2143.7922 | 2144.303 | 1 | 7.823 | 63.9% | 3 | R.LLHPS\*PDLVSQEATLSEAR.L | 2 |
|  | LAPMis12DANCE\_011813\_02.05374.05374.2 | 3.9832 | 0.3493 | 100.0% | 1641.4321 | 1640.8339 | 1 | 6.49 | 57.1% | 7 | K.SVVVASSEIHVEVER.T | 2 |
|  | LAPMis12DANCE\_011813\_01.14445.14445.3 | 4.9529 | 0.3778 | 100.0% | 2671.7944 | 2673.0837 | 1 | 6.248 | 40.9% | 3 | K.LLSDYDDWRPSLASLLQPIPFPK.E | 3 |
|  | LAPMis12DANCE\_011813\_01.14374.14374.2 | 2.5208 | 0.2722 | 99.1% | 2672.7122 | 2673.0837 | 1 | 6.154 | 47.7% | 1 | K.LLSDYDDWRPSLASLLQPIPFPK.E | 2 |
|  | LAPMis12DANCE\_011813\_02.08069.08069.2 | 4.2373 | 0.4909 | 100.0% | 1706.2122 | 1706.8986 | 1 | 8.82 | 67.9% | 4 | K.QLNLWSTQFGDAGLR.L | 2 |
|  | LAPMis12DANCE\_011813\_01.07304.07304.2 | 3.099 | 0.3684 | 100.0% | 1155.3121 | 1155.247 | 2 | 6.917 | 77.8% | 1 | K.LSADTYEDLK.A | 2 |

---

|  |  |  |  |  |  |  |  |  |
| --- | --- | --- | --- | --- | --- | --- | --- | --- |
| U | *gi|117189975|ref|NP\_1* | 5 | 6 | 23.5% | 306 | 33670 | 5.1 | heterogeneous nuclear ribonucleoprotein C isoform a [Homo sapiens] |
| U | *gi|117190254|ref|NP\_0* | 5 | 6 | 24.6% | 293 | 32338 | 5.1 | heterogeneous nuclear ribonucleoprotein C isoform b [Homo sapiens] |
| U | *gi|117190192|ref|NP\_0* | 5 | 6 | 23.5% | 306 | 33670 | 5.1 | heterogeneous nuclear ribonucleoprotein C isoform a [Homo sapiens] |
| U | *gi|117190174|ref|NP\_0* | 5 | 6 | 24.6% | 293 | 32338 | 5.1 | heterogeneous nuclear ribonucleoprotein C isoform b [Homo sapiens] |

| Filename XCorr DeltCN Conf% ObsM+H+ CalcM+H+ SpR ZScore Ion% # Sequence  | | | | | | | | | | | | |
| --- | --- | --- | --- | --- | --- | --- | --- | --- | --- | --- | --- | --- |
|  | LAPMis12DANCE\_011813\_02.07533.07533.2 | 2.9262 | 0.2417 | 99.6% | 1316.8722 | 1317.6145 | 1 | 6.605 | 72.7% | 1 | R.VFIGNLNTLVVK.K | 2 |
|  | LAPMis12DANCE\_011813\_02.07550.07550.2 | 4.6608 | 0.4043 | 100.0% | 1683.4922 | 1684.0038 | 1 | 7.738 | 80.0% | 2 | R.MIAGQVLDINLAAEPK.V | 2 |
|  | LAPMis12DANCE\_011813\_01.04019.04019.2 | 2.3555 | 0.2861 | 99.4% | 1228.5122 | 1229.4624 | 10 | 4.81 | 65.0% | 1 | K.LKGDDLQAIKK.E | 2 |
|  | LAPMis12DANCE\_011813\_01.09804.09804.2 | 2.0285 | 0.3179 | 98.7% | 1416.4922 | 1416.6146 | 307 | 5.766 | 50.0% | 1 | K.QKVDSLLENLEK.I | 2 |
|  | LAPMis12DANCE\_011813\_01.03308.03308.3 | 4.044 | 0.3122 | 99.7% | 2368.6143 | 2369.4583 | 1 | 5.585 | 42.5% | 1 | K.NDKSEEEQSSSSVKKDETNVK.M | 3 |

---

|  |  |  |  |  |  |  |  |  |
| --- | --- | --- | --- | --- | --- | --- | --- | --- |
| U | *gi|15082258|ref|NP\_00* | 4 | 4 | 23.5% | 183 | 20811 | 5.3 | chromobox homolog 3 [Homo sapiens] |
| U | *gi|89038889|ref|XP\_94* | 4 | 4 | 23.5% | 183 | 20811 | 5.3 | PREDICTED: similar to Chromobox homolog 3 (HP1 gamma homolog, Drosophila) [Homo sapiens] |
| U | *gi|20544151|ref|NP\_05* | 4 | 4 | 23.5% | 183 | 20811 | 5.3 | chromobox homolog 3 [Homo sapiens] |

| Filename XCorr DeltCN Conf% ObsM+H+ CalcM+H+ SpR ZScore Ion% # Sequence  | | | | | | | | | | | | |
| --- | --- | --- | --- | --- | --- | --- | --- | --- | --- | --- | --- | --- |
|  | LAPMis12DANCE\_011813\_01.07445.07445.2 | 4.6408 | 0.4355 | 100.0% | 1662.3322 | 1662.834 | 1 | 7.881 | 76.9% | 1 | K.KVEEAEPEEFVVEK.V | 2 |
|  | LAPMis12DANCE\_011813\_01.08612.08612.2 | 3.8307 | 0.3631 | 100.0% | 1534.1122 | 1534.6599 | 2 | 5.717 | 62.5% | 1 | K.VEEAEPEEFVVEK.V | 2 |
|  | LAPMis12DANCE\_011813\_02.08554.08554.2 | 4.5812 | 0.56 | 100.0% | 1714.1921 | 1714.0449 | 1 | 9.36 | 70.0% | 1 | R.IIGATDSSGELMFLMK.W | 2 |
|  | LAPMis12DANCE\_011813\_02.05676.05676.2 | 2.8902 | 0.2154 | 99.4% | 1491.3722 | 1490.6531 | 1 | 5.246 | 75.0% | 1 | K.WKDSDEADLVLAK.E | 2 |

---

|  |  |  |  |  |  |  |  |  |
| --- | --- | --- | --- | --- | --- | --- | --- | --- |
| U | *gi|4503471|ref|NP\_001* | 11 | 16 | 23.4% | 462 | 50141 | 9.0 | eukaryotic translation elongation factor 1 alpha 1 [Homo sapiens] |

| Filename XCorr DeltCN Conf% ObsM+H+ CalcM+H+ SpR ZScore Ion% # Sequence  | | | | | | | | | | | | |
| --- | --- | --- | --- | --- | --- | --- | --- | --- | --- | --- | --- | --- |
|  | LAPMis12DANCE\_011813\_02.05417.05417.2 | 3.207 | 0.436 | 100.0% | 1589.6721 | 1589.835 | 1 | 6.852 | 64.3% | 1 | K.THINIVVIGHVDSGK.S | 2 |
|  | LAPMis12DANCE\_011813\_02.05369.05369.3 | 4.6114 | 0.4313 | 100.0% | 1590.0243 | 1589.835 | 1 | 6.82 | 46.4% | 2 | K.THINIVVIGHVDSGK.S | 3 |
|  | LAPMis12DANCE2\_011813\_01.04121.04121.2 | 2.4772 | 0.3698 | 100.0% | 1120.8722 | 1121.2786 | 1 | 5.775 | 61.1% | 2 | K.STTTGHLIYK.C | 2 |
| \* | LAPMis12DANCE\_011813\_01.08548.08548.2 | 2.53 | 0.2227 | 98.7% | 1406.1522 | 1405.5962 | 1 | 4.984 | 68.2% | 1 | K.YYVTIIDAPGHR.D | 2 |
|  | LAPMis12DANCE\_011813\_02.06474.06474.2 | 2.3583 | 0.3298 | 99.5% | 1315.1522 | 1315.5553 | 1 | 5.474 | 59.1% | 1 | R.EHALLAYTLGVK.Q | 23 |
|  | LAPMis12DANCE\_011813\_01.04721.04721.1 | 1.8044 | 0.2817 | 100.0% | 839.32 | 839.96405 | 5 | 4.483 | 66.7% | 1 | K.EVSTYIK.K | 1 |
|  | LAPMis12DANCE\_011813\_01.08176.08176.2 | 2.4355 | 0.2224 | 99.4% | 976.21216 | 976.1607 | 126 | 6.312 | 64.3% | 1 | R.LPLQDVYK.I | 2 |
|  | LAPMis12DANCE\_011813\_01.07364.07364.2 | 3.3843 | 0.3319 | 100.0% | 1026.0521 | 1026.2241 | 1 | 5.748 | 80.0% | 2 | K.IGGIGTVPVGR.V | 2 |
| \* | LAPMis12DANCE\_011813\_01.11208.11208.2 | 4.6245 | 0.4212 | 100.0% | 2516.6921 | 2516.999 | 1 | 8.583 | 54.3% | 1 | R.VETGVLKPGMVVTFAPVNVTTEVK.S | 2 |
| \* | LAPMis12DANCE2\_011813\_01.09590.09590.3 | 4.5743 | 0.3474 | 100.0% | 2517.4143 | 2516.999 | 2 | 6.063 | 32.6% | 1 | R.VETGVLKPGMVVTFAPVNVTTEVK.S | 3 |
|  | LAPMis12DANCE2\_011813\_01.04869.04869.1 | 1.744 | 0.3609 | 100.0% | 914.55 | 915.1209 | 177 | 5.168 | 50.0% | 3 | R.QTVAVGVIK.A | 1 |

---

|  |  |  |  |  |  |  |  |  |
| --- | --- | --- | --- | --- | --- | --- | --- | --- |
| U | *gi|7669492|ref|NP\_002* | 6 | 16 | 23.0% | 335 | 36053 | 8.5 | glyceraldehyde-3-phosphate dehydrogenase [Homo sapiens] |

| Filename XCorr DeltCN Conf% ObsM+H+ CalcM+H+ SpR ZScore Ion% # Sequence  | | | | | | | | | | | | |
| --- | --- | --- | --- | --- | --- | --- | --- | --- | --- | --- | --- | --- |
| \* | LAPMis12DANCE\_011813\_01.13894.13894.3 | 7.3123 | 0.5751 | 100.0% | 2596.6743 | 2597.0044 | 1 | 9.881 | 41.3% | 4 | K.VIHDNFGIVEGLMTTVHAITATQK.T | 3 |
| \* | LAPMis12DANCE\_011813\_01.09183.09183.2 | 3.8673 | 0.4074 | 100.0% | 1412.1522 | 1412.6292 | 1 | 6.308 | 67.9% | 4 | R.GALQNIIPASTGAAK.A | 2 |
| \* | LAPMis12DANCE\_011813\_01.03520.03520.2 | 3.2789 | 0.2306 | 100.0% | 1448.4521 | 1448.7025 | 15 | 5.533 | 59.1% | 1 | R.LEKPAKYDDIKK.V | 2 |
|  | LAPMis12DANCE\_011813\_02.07616.07616.2 | 4.3282 | 0.4845 | 100.0% | 1764.2922 | 1764.8914 | 1 | 8.329 | 61.5% | 3 | K.LISWYDNEFGYSNR.V | 2 |
| \* | LAPMis12DANCE\_011813\_01.07731.07731.2 | 2.289 | 0.2436 | 98.3% | 1201.4521 | 1202.4724 | 9 | 5.23 | 65.0% | 1 | R.VVDLMAHMASK.E | 2 |
| \* | LAPMis12DANCE\_011813\_02.05578.05578.2 | 3.0857 | 0.3478 | 100.0% | 1331.2922 | 1331.5879 | 1 | 7.349 | 72.7% | 3 | R.VVDLMAHMASKE.- | 2 |

---

|  |  |  |  |  |  |  |  |  |
| --- | --- | --- | --- | --- | --- | --- | --- | --- |
| U | *gi|15718687|ref|NP\_00* | 4 | 4 | 23.0% | 243 | 26688 | 9.7 | ribosomal protein S3 [Homo sapiens] |

| Filename XCorr DeltCN Conf% ObsM+H+ CalcM+H+ SpR ZScore Ion% # Sequence  | | | | | | | | | | | | |
| --- | --- | --- | --- | --- | --- | --- | --- | --- | --- | --- | --- | --- |
| \* | LAPMis12DANCE\_011813\_02.05073.05073.2 | 3.4583 | 0.4789 | 100.0% | 1423.6122 | 1424.5071 | 1 | 8.615 | 75.0% | 1 | R.ELAEDGYSGVEVR.V | 2 |
| \* | LAPMis12DANCE\_011813\_01.09706.09706.2 | 2.9355 | 0.1425 | 97.2% | 1584.9521 | 1584.8998 | 5 | 4.399 | 50.0% | 1 | R.VTPTRTEIIILATR.T | 2 |
| \* | LAPMis12DANCE\_011813\_01.08825.08825.2 | 3.3516 | 0.3119 | 100.0% | 1472.4722 | 1471.6476 | 10 | 4.918 | 62.5% | 1 | K.DEILPTTPISEQK.G | 2 |
| \* | LAPMis12DANCE\_011813\_01.07550.07550.2 | 3.1453 | 0.3793 | 100.0% | 1574.4922 | 1574.8352 | 12 | 6.07 | 46.7% | 1 | K.GGKPEPPAMPQPVPTA.- | 2 |

---

|  |  |  |  |  |  |  |  |  |
| --- | --- | --- | --- | --- | --- | --- | --- | --- |
| U | *gi|225690529|ref|NP\_6* | 8 | 10 | 22.8% | 394 | 44993 | 7.1 | golgin, RAB6-interacting isoform a [Homo sapiens] |

| Filename XCorr DeltCN Conf% ObsM+H+ CalcM+H+ SpR ZScore Ion% # Sequence  | | | | | | | | | | | | |
| --- | --- | --- | --- | --- | --- | --- | --- | --- | --- | --- | --- | --- |
|  | LAPMis12DANCE\_011813\_01.12249.12249.2 | 4.6564 | 0.5335 | 100.0% | 2067.632 | 2068.3752 | 1 | 9.013 | 52.6% | 1 | K.LGLQDGSTSLLPEQLLSAPK.Q | 2 |
|  | LAPMis12DANCE\_011813\_01.07103.07103.2 | 3.1556 | 0.1391 | 99.1% | 1460.3522 | 1459.6047 | 4 | 4.437 | 70.0% | 3 | K.SRWEVLQQEQR.L | 2 |
|  | LAPMis12DANCE2\_011813\_01.11849.11849.2 | 2.2447 | 0.2573 | 97.1% | 1858.9122 | 1860.1327 | 2 | 5.586 | 43.8% | 1 | K.ELQALDDMVSADIGILR.N | 2 |
|  | LAPMis12DANCE\_011813\_01.07266.07266.2 | 3.1776 | 0.3438 | 100.0% | 1672.1122 | 1672.7948 | 1 | 6.023 | 65.4% | 1 | R.NRIDQASLDYSYAR.K | 2 |
|  | LAPMis12DANCE\_011813\_01.07685.07685.2 | 2.4669 | 0.1817 | 96.3% | 1403.8322 | 1402.5034 | 1 | 3.64 | 63.6% | 1 | R.IDQASLDYSYAR.K | 2 |
| \* | LAPMis12DANCE\_011813\_01.03905.03905.2 | 2.2027 | 0.2041 | 96.2% | 1239.9122 | 1240.3585 | 20 | 4.433 | 66.7% | 1 | R.LLHEQEVESR.R | 2 |
| \* | LAPMis12DANCE\_011813\_01.10799.10799.2 | 4.9218 | 0.4631 | 100.0% | 2091.3323 | 2092.3562 | 1 | 8.617 | 76.5% | 1 | R.LERPFQPAEESVTLEFAK.E | 2 |
| \* | LAPMis12DANCE2\_011813\_01.08468.08468.3 | 4.2663 | 0.4646 | 100.0% | 2091.9844 | 2092.3562 | 1 | 8.022 | 42.6% | 1 | R.LERPFQPAEESVTLEFAK.E | 3 |

---

|  |  |  |  |  |  |  |  |  |
| --- | --- | --- | --- | --- | --- | --- | --- | --- |
| U | *gi|4885375|ref|NP\_005* | 7 | 12 | 22.5% | 213 | 21365 | 10.9 | histone cluster 1, H1c [Homo sapiens] |

| Filename XCorr DeltCN Conf% ObsM+H+ CalcM+H+ SpR ZScore Ion% # Sequence  | | | | | | | | | | | | |
| --- | --- | --- | --- | --- | --- | --- | --- | --- | --- | --- | --- | --- |
|  | LAPMis12DANCE\_011813\_01.07040.07040.2 | 3.6183 | 0.4257 | 100.0% | 1327.2122 | 1327.5638 | 1 | 7.012 | 75.0% | 4 | R.KASGPPVSELITK.A | 2 |
|  | LAPMis12DANCE\_011813\_01.05505.05505.2 | 2.8484 | 0.1904 | 99.4% | 974.3522 | 974.1887 | 7 | 5.026 | 66.7% | 2 | R.SGVSLAALKK.A | 2 |
|  | LAPMis12DANCE\_011813\_01.04511.04511.2 | 3.411 | 0.4543 | 100.0% | 1236.0721 | 1236.4105 | 1 | 7.285 | 77.3% | 1 | K.KALAAAGYDVEK.N | 2 |
|  | LAPMis12DANCE2\_011813\_01.04800.04800.1 | 2.8034 | 0.4152 | 100.0% | 1107.54 | 1108.2365 | 4 | 8.306 | 60.0% | 1 | K.ALAAAGYDVEK.N | 1 |
|  | LAPMis12DANCE\_011813\_01.05788.05788.2 | 2.6333 | 0.3154 | 100.0% | 1109.0521 | 1108.2365 | 20 | 6.239 | 65.0% | 1 | K.ALAAAGYDVEK.N | 2 |
|  | LAPMis12DANCE\_011813\_01.04680.04680.2 | 4.184 | 0.515 | 100.0% | 1579.0521 | 1579.7098 | 1 | 8.906 | 67.9% | 2 | K.ALAAAGYDVEKNNSR.I | 2 |
| \* | LAPMis12DANCE2\_011813\_01.02848.02848.2 | 1.8069 | 0.2868 | 95.6% | 1015.1722 | 1015.2413 | 36 | 4.631 | 61.1% | 1 | K.KPAAATVTKK.V | 2 |

---

|  |  |  |  |  |  |  |  |  |
| --- | --- | --- | --- | --- | --- | --- | --- | --- |
| U | *contaminant\_GR78\_MESA* | 10 | 12 | 21.7% | 654 | 72379 | 5.2 | owl|P07823| 78 KD GLUCOSE REGULATED PROTEIN PRECURSOR (GRP 78) (IMMUNOGLOBULIN... |
| U | *gi|16507237|ref|NP\_00* | 10 | 12 | 21.7% | 654 | 72333 | 5.2 | heat shock 70kDa protein 5 [Homo sapiens] |
| U | *contaminant\_GR78\_RAT* | 10 | 12 | 21.7% | 654 | 72347 | 5.2 | owl|P06761| 78 KD GLUCOSE REGULATED PROTEIN PRECURSOR (GRP 78) (IMMUNOGLOBULIN... |
| U | *contaminant\_GR78\_MOUS* | 10 | 12 | 21.7% | 655 | 72421 | 5.2 | owl|P20029| 78 KD GLUCOSE REGULATED PROTEIN PRECURSOR (GRP 78) (IMMUNOGLOBULIN... |

| Filename XCorr DeltCN Conf% ObsM+H+ CalcM+H+ SpR ZScore Ion% # Sequence  | | | | | | | | | | | | |
| --- | --- | --- | --- | --- | --- | --- | --- | --- | --- | --- | --- | --- |
|  | LAPMis12DANCE\_011813\_01.09555.09555.2 | 3.842 | 0.2996 | 100.0% | 1567.2522 | 1567.7386 | 1 | 7.488 | 65.4% | 1 | R.ITPSYVAFTPEGER.L | 2 |
|  | LAPMis12DANCE\_011813\_01.08421.08421.2 | 3.4789 | 0.3955 | 100.0% | 1679.5322 | 1678.796 | 1 | 6.445 | 60.7% | 1 | K.NQLTSNPENTVFDAK.R | 2 |
|  | LAPMis12DANCE\_011813\_01.07487.07487.2 | 3.0266 | 0.2504 | 99.9% | 1430.7922 | 1431.5449 | 1 | 5.379 | 77.3% | 1 | R.TWNDPSVQQDIK.F | 2 |
|  | LAPMis12DANCE\_011813\_02.06268.06268.2 | 2.5299 | 0.3136 | 99.5% | 1660.5521 | 1660.9078 | 1 | 5.605 | 60.0% | 1 | R.IINEPTAAAIAYGLDK.R | 222 |
|  | LAPMis12DANCE\_011813\_02.06138.06138.2 | 3.299 | 0.2801 | 100.0% | 1590.2722 | 1589.7863 | 1 | 6.577 | 67.9% | 2 | K.KSDIDEIVLVGGSTR.I | 2 |
|  | LAPMis12DANCE\_011813\_01.08861.08861.2 | 2.8896 | 0.3076 | 99.9% | 1837.2322 | 1838.0245 | 25 | 4.869 | 37.5% | 1 | K.SQIFSTASDNQPTVTIK.V | 2 |
|  | LAPMis12DANCE\_011813\_01.03884.03884.2 | 2.2123 | 0.1974 | 95.8% | 1193.1721 | 1192.3574 | 1 | 4.418 | 72.2% | 2 | K.VYEGERPLTK.D | 2 |
|  | LAPMis12DANCE\_011813\_01.09910.09910.2 | 2.6975 | 0.2242 | 99.4% | 1317.2922 | 1317.4381 | 2 | 4.234 | 80.0% | 1 | R.NELESYAYSLK.N | 2 |
|  | LAPMis12DANCE\_011813\_02.06340.06340.3 | 2.9741 | 0.2368 | 95.0% | 2174.0645 | 2175.3594 | 17 | 4.614 | 30.9% | 1 | K.IEWLESHQDADIEDFKAK.K | 3 |
|  | LAPMis12DANCE\_011813\_01.08616.08616.2 | 3.3521 | 0.2404 | 99.9% | 1654.1921 | 1654.9878 | 1 | 6.046 | 73.1% | 1 | K.KKELEEIVQPIISK.L | 2 |

Similarities:
gi|5729877|ref|NP\_006(1:9)  
gi|124256496|ref|NP\_0(1:9)  

---

|  |  |  |  |  |  |  |  |  |
| --- | --- | --- | --- | --- | --- | --- | --- | --- |
| U | *gi|4506699|ref|NP\_001* | 1 | 1 | 21.7% | 83 | 9111 | 8.5 | ribosomal protein S21 [Homo sapiens] |

| Filename XCorr DeltCN Conf% ObsM+H+ CalcM+H+ SpR ZScore Ion% # Sequence  | | | | | | | | | | | | |
| --- | --- | --- | --- | --- | --- | --- | --- | --- | --- | --- | --- | --- |
| \* | LAPMis12DANCE\_011813\_02.05750.05750.3 | 5.6226 | 0.5511 | 100.0% | 1970.9644 | 1971.1956 | 1 | 9.74 | 45.6% | 1 | K.DHASIQMNVAEVDKVTGR.F | 3 |

---

|  |  |  |  |  |  |  |  |  |
| --- | --- | --- | --- | --- | --- | --- | --- | --- |
| U | *gi|4502107|ref|NP\_001* | 4 | 5 | 21.6% | 320 | 35937 | 5.0 | annexin 5 [Homo sapiens] |

| Filename XCorr DeltCN Conf% ObsM+H+ CalcM+H+ SpR ZScore Ion% # Sequence  | | | | | | | | | | | | |
| --- | --- | --- | --- | --- | --- | --- | --- | --- | --- | --- | --- | --- |
| \* | LAPMis12DANCE\_011813\_01.09957.09957.2 | 3.1386 | 0.4569 | 100.0% | 1341.5521 | 1341.4198 | 1 | 8.011 | 63.6% | 1 | R.GTVTDFPGFDER.A | 2 |
| \* | LAPMis12DANCE2\_011813\_01.12026.12026.2 | 4.188 | 0.4546 | 100.0% | 1705.2722 | 1705.903 | 1 | 8.874 | 56.7% | 2 | K.GLGTDEESILTLLTSR.S | 2 |
| \* | LAPMis12DANCE\_011813\_02.11806.11806.3 | 3.1588 | 0.2841 | 97.9% | 3400.5842 | 3399.881 | 142 | 4.455 | 19.0% | 1 | K.YMTISGFQIEETIDRETSGNLEQLLLAVVK.S | 3 |
| \* | LAPMis12DANCE\_011813\_01.04121.04121.2 | 2.9608 | 0.1749 | 99.2% | 1155.9722 | 1156.2406 | 3 | 4.119 | 70.0% | 1 | K.GAGTDDHTLIR.V | 2 |

---

|  |  |  |  |  |  |  |  |  |
| --- | --- | --- | --- | --- | --- | --- | --- | --- |
| U | *gi|10835063|ref|NP\_00* | 3 | 6 | 21.4% | 294 | 32575 | 4.8 | nucleophosmin 1 isoform 1 [Homo sapiens] |
| U | *gi|40353734|ref|NP\_95* | 3 | 6 | 23.8% | 265 | 29465 | 4.6 | nucleophosmin 1 isoform 2 [Homo sapiens] |

| Filename XCorr DeltCN Conf% ObsM+H+ CalcM+H+ SpR ZScore Ion% # Sequence  | | | | | | | | | | | | |
| --- | --- | --- | --- | --- | --- | --- | --- | --- | --- | --- | --- | --- |
|  | LAPMis12DANCE\_011813\_02.07956.07956.3 | 4.7113 | 0.4734 | 100.0% | 2930.0645 | 2931.2874 | 1 | 8.98 | 28.7% | 2 | R.TVSLGAGAKDELHIVEAEAMNYEGSPIK.V | 3 |
|  | LAPMis12DANCE2\_011813\_01.11583.11583.2 | 3.6634 | 0.3168 | 100.0% | 2230.5723 | 2228.655 | 2 | 5.584 | 45.0% | 2 | K.MSVQPTVSLGGFEITPPVVLR.L | 2 |
|  | LAPMis12DANCE\_011813\_01.13026.13026.2 | 3.9043 | 0.2205 | 100.0% | 1822.1122 | 1821.0172 | 1 | 5.146 | 65.4% | 2 | R.MTDQEAIQDLWQWR.K | 2 |

---

|  |  |  |  |  |  |  |  |  |
| --- | --- | --- | --- | --- | --- | --- | --- | --- |
| U | *gi|4826898|ref|NP\_005* | 2 | 2 | 21.4% | 140 | 15054 | 8.3 | profilin 1 [Homo sapiens] |

| Filename XCorr DeltCN Conf% ObsM+H+ CalcM+H+ SpR ZScore Ion% # Sequence  | | | | | | | | | | | | |
| --- | --- | --- | --- | --- | --- | --- | --- | --- | --- | --- | --- | --- |
| \* | LAPMis12DANCE\_011813\_01.12017.12017.2 | 4.4038 | 0.4418 | 100.0% | 1645.3922 | 1644.9518 | 1 | 7.241 | 73.3% | 1 | K.TFVNITPAEVGVLVGK.D | 2 |
| \* | LAPMis12DANCE\_011813\_01.07766.07766.2 | 3.0244 | 0.4147 | 100.0% | 1380.2922 | 1380.5406 | 2 | 5.654 | 57.7% | 1 | K.STGGAPTFNVTVTK.T | 2 |

---

|  |  |  |  |  |  |  |  |  |
| --- | --- | --- | --- | --- | --- | --- | --- | --- |
| U | *gi|4506607|ref|NP\_000* | 3 | 5 | 21.3% | 188 | 21634 | 11.7 | ribosomal protein L18 [Homo sapiens] |

| Filename XCorr DeltCN Conf% ObsM+H+ CalcM+H+ SpR ZScore Ion% # Sequence  | | | | | | | | | | | | |
| --- | --- | --- | --- | --- | --- | --- | --- | --- | --- | --- | --- | --- |
| \* | LAPMis12DANCE\_011813\_02.05806.05806.2 | 3.5662 | 0.4138 | 100.0% | 1347.0721 | 1346.5236 | 1 | 6.531 | 70.8% | 3 | K.TAVVVGTITDDVR.V | 2 |
| \* | LAPMis12DANCE\_011813\_01.11423.11423.2 | 4.051 | 0.5079 | 100.0% | 1461.5521 | 1461.6982 | 1 | 8.574 | 79.2% | 1 | K.ILTFDQLALDSPK.G | 2 |
| \* | LAPMis12DANCE\_011813\_01.03202.03202.3 | 3.3212 | 0.4359 | 100.0% | 1548.2344 | 1548.7446 | 1 | 7.048 | 42.3% | 1 | K.APGTPHSHTKPYVR.S | 3 |

---

|  |  |  |  |  |  |  |  |  |
| --- | --- | --- | --- | --- | --- | --- | --- | --- |
| U | *Reverse\_gi|169210817|* | 1 | 1 | 20.3% | 79 | 8556 | 7.4 | PREDICTED: hypothetical protein [Homo sapiens] |
| U | *Reverse\_gi|169212068|* | 1 | 1 | 20.3% | 79 | 8556 | 7.4 | PREDICTED: hypothetical protein [Homo sapiens] |

| Filename XCorr DeltCN Conf% ObsM+H+ CalcM+H+ SpR ZScore Ion% # Sequence  | | | | | | | | | | | | |
| --- | --- | --- | --- | --- | --- | --- | --- | --- | --- | --- | --- | --- |
|  | LAPMis12DANCE2\_011813\_01.09966.09966.2 | 2.6374 | 0.2577 | 99.1% | 1662.0122 | 1661.9045 | 250 | 4.904 | 36.7% | 1 | K.EQGRGLSHGLAGLPIR.T | 2 |

---

|  |  |  |  |  |  |  |  |  |
| --- | --- | --- | --- | --- | --- | --- | --- | --- |
| U | *Reverse\_gi|20149322|r* | 1 | 1 | 20.2% | 99 | 11140 | 9.5 | acylphosphatase 2 [Homo sapiens] |

| Filename XCorr DeltCN Conf% ObsM+H+ CalcM+H+ SpR ZScore Ion% # Sequence  | | | | | | | | | | | | |
| --- | --- | --- | --- | --- | --- | --- | --- | --- | --- | --- | --- | --- |
| \* | LAPMis12DANCE2\_011813\_01.15638.15638.2 | 2.2 | 0.2548 | 96.5% | 2308.612 | 2311.553 | 232 | 4.467 | 26.3% | 1 | R.DIRSS\*PSGVK@SLWSKMSNVK@.D | 2 |

---

|  |  |  |  |  |  |  |  |  |
| --- | --- | --- | --- | --- | --- | --- | --- | --- |
| U | *gi|4503529|ref|NP\_001* | 4 | 6 | 20.0% | 406 | 46154 | 5.5 | eukaryotic translation initiation factor 4A isoform 1 [Homo sapiens] |

| Filename XCorr DeltCN Conf% ObsM+H+ CalcM+H+ SpR ZScore Ion% # Sequence  | | | | | | | | | | | | |
| --- | --- | --- | --- | --- | --- | --- | --- | --- | --- | --- | --- | --- |
| \* | LAPMis12DANCE\_011813\_01.16076.16076.3 | 3.8937 | 0.4532 | 100.0% | 4168.0444 | 4169.451 | 1 | 8.757 | 25.0% | 2 | R.SRDNGPDGMEPEGVIESNWNEIVDSFDDMNLSESLLR.G | 3 |
|  | LAPMis12DANCE\_011813\_01.08714.08714.2 | 4.8885 | 0.541 | 100.0% | 1828.4321 | 1829.0654 | 1 | 9.009 | 73.3% | 2 | R.GIYAYGFEKPSAIQQR.A | 2 |
| \* | LAPMis12DANCE\_011813\_01.09244.09244.3 | 2.6602 | 0.2625 | 96.6% | 1619.6643 | 1619.9225 | 70 | 5.12 | 37.5% | 1 | K.LQMEAPHIIVGTPGR.V | 3 |
|  | LAPMis12DANCE\_011813\_01.12480.12480.2 | 4.1536 | 0.4048 | 100.0% | 1556.3121 | 1556.789 | 1 | 8.183 | 79.2% | 1 | K.MFVLDEADEMLSR.G | 2 |

---

|  |  |  |  |  |  |  |  |  |
| --- | --- | --- | --- | --- | --- | --- | --- | --- |
| U | *gi|4506643|ref|NP\_000* | 1 | 1 | 19.6% | 92 | 10275 | 10.4 | ribosomal protein L37a [Homo sapiens] |

| Filename XCorr DeltCN Conf% ObsM+H+ CalcM+H+ SpR ZScore Ion% # Sequence  | | | | | | | | | | | | |
| --- | --- | --- | --- | --- | --- | --- | --- | --- | --- | --- | --- | --- |
| \* | LAPMis12DANCE\_011813\_02.05988.05988.2 | 2.956 | 0.2381 | 99.5% | 1828.4122 | 1828.0319 | 2 | 5.03 | 44.1% | 1 | K.TVAGGAWTYNTTSAVTVK.S | 2 |

---

|  |  |  |  |  |  |  |  |  |
| --- | --- | --- | --- | --- | --- | --- | --- | --- |
| U | *gi|226246671|ref|NP\_0* | 2 | 3 | 19.0% | 142 | 16006 | 9.3 | ribosomal protein S20 isoform 1 [Homo sapiens] |
| U | *gi|4506697|ref|NP\_001* | 2 | 3 | 22.7% | 119 | 13373 | 9.9 | ribosomal protein S20 isoform 2 [Homo sapiens] |

| Filename XCorr DeltCN Conf% ObsM+H+ CalcM+H+ SpR ZScore Ion% # Sequence  | | | | | | | | | | | | |
| --- | --- | --- | --- | --- | --- | --- | --- | --- | --- | --- | --- | --- |
|  | LAPMis12DANCE\_011813\_01.05040.05040.2 | 2.8855 | 0.302 | 99.9% | 1649.9922 | 1649.8442 | 88 | 5.641 | 46.4% | 1 | K.DTGKTPVEPEVAIHR.I | 2 |
|  | LAPMis12DANCE\_011813\_01.08710.08710.2 | 3.2483 | 0.3497 | 100.0% | 1351.5122 | 1351.5858 | 2 | 5.739 | 63.6% | 2 | R.LIDLHSPSEIVK.Q | 2 |

---

|  |  |  |  |  |  |  |  |  |
| --- | --- | --- | --- | --- | --- | --- | --- | --- |
| U | *gi|15055539|ref|NP\_00* | 3 | 3 | 18.8% | 293 | 31324 | 10.2 | ribosomal protein S2 [Homo sapiens] |
| U | *gi|169205506|ref|XP\_0* | 3 | 3 | 18.8% | 293 | 31364 | 10.2 | PREDICTED: hypothetical protein isoform 1 [Homo sapiens] |
| U | *gi|169204984|ref|XP\_0* | 3 | 3 | 18.8% | 293 | 31364 | 10.2 | PREDICTED: hypothetical protein isoform 1 [Homo sapiens] |
| U | *gi|169204454|ref|XP\_0* | 3 | 3 | 18.8% | 293 | 31438 | 10.2 | PREDICTED: hypothetical protein isoform 1 [Homo sapiens] |

| Filename XCorr DeltCN Conf% ObsM+H+ CalcM+H+ SpR ZScore Ion% # Sequence  | | | | | | | | | | | | |
| --- | --- | --- | --- | --- | --- | --- | --- | --- | --- | --- | --- | --- |
|  | LAPMis12DANCE2\_011813\_01.05200.05200.2 | 2.4233 | 0.1589 | 95.1% | 1334.6921 | 1334.5298 | 1 | 4.512 | 70.0% | 1 | K.AEDKEWMPVTK.L | 2 |
|  | LAPMis12DANCE2\_011813\_01.16479.16479.3 | 4.0208 | 0.2558 | 98.5% | 3687.8342 | 3688.293 | 22 | 4.906 | 18.5% | 1 | K.SLEEIYLFSLPIKESEIIDFFLGASLKDEVLK.I | 3 |
|  | LAPMis12DANCE\_011813\_01.09716.09716.2 | 3.3483 | 0.313 | 100.0% | 1464.2922 | 1464.6177 | 1 | 6.464 | 72.7% | 1 | K.SPYQEFTDHLVK.T | 2 |

---

|  |  |  |  |  |  |  |  |  |
| --- | --- | --- | --- | --- | --- | --- | --- | --- |
| U | *gi|4506597|ref|NP\_000* | 2 | 4 | 18.8% | 165 | 17819 | 9.4 | ribosomal protein L12 [Homo sapiens] |

| Filename XCorr DeltCN Conf% ObsM+H+ CalcM+H+ SpR ZScore Ion% # Sequence  | | | | | | | | | | | | |
| --- | --- | --- | --- | --- | --- | --- | --- | --- | --- | --- | --- | --- |
|  | LAPMis12DANCE\_011813\_02.07389.07389.2 | 3.7134 | 0.3705 | 100.0% | 1667.2122 | 1667.9866 | 2 | 6.311 | 56.7% | 2 | R.QAQIEVVPSASALIIK.A | 2 |
| \* | LAPMis12DANCE\_011813\_01.11189.11189.2 | 4.6953 | 0.5871 | 100.0% | 1686.3722 | 1686.865 | 1 | 9.906 | 64.3% | 2 | K.HSGNITFDEIVNIAR.Q | 2 |

---

|  |  |  |  |  |  |  |  |  |
| --- | --- | --- | --- | --- | --- | --- | --- | --- |
| U | *gi|207028494|ref|NP\_0* | 3 | 3 | 18.6% | 274 | 30205 | 7.1 | L-lactate dehydrogenase A isoform 2 [Homo sapiens] |
| U | *gi|5031857|ref|NP\_005* | 3 | 3 | 15.4% | 332 | 36689 | 8.3 | L-lactate dehydrogenase A isoform 1 [Homo sapiens] |

| Filename XCorr DeltCN Conf% ObsM+H+ CalcM+H+ SpR ZScore Ion% # Sequence  | | | | | | | | | | | | |
| --- | --- | --- | --- | --- | --- | --- | --- | --- | --- | --- | --- | --- |
|  | LAPMis12DANCE2\_011813\_01.15530.15530.3 | 3.9349 | 0.292 | 99.5% | 3516.8044 | 3516.062 | 91 | 5.266 | 20.0% | 1 | K.DLADELALVDVIEDKLKGEMMDLQHGSLFLR.T | 3 |
|  | LAPMis12DANCE\_011813\_01.04533.04533.2 | 2.8571 | 0.4508 | 100.0% | 1135.2722 | 1135.2163 | 1 | 7.365 | 88.9% | 1 | K.VTLTSEEEAR.L | 2 |
|  | LAPMis12DANCE\_011813\_01.09370.09370.2 | 2.5562 | 0.336 | 100.0% | 1120.1921 | 1119.2627 | 1 | 5.615 | 83.3% | 1 | K.SADTLWGIQK.E | 2 |

---

|  |  |  |  |  |  |  |  |  |
| --- | --- | --- | --- | --- | --- | --- | --- | --- |
| U | *gi|13904870|ref|NP\_00* | 2 | 2 | 18.6% | 204 | 22876 | 9.7 | ribosomal protein S5 [Homo sapiens] |

| Filename XCorr DeltCN Conf% ObsM+H+ CalcM+H+ SpR ZScore Ion% # Sequence  | | | | | | | | | | | | |
| --- | --- | --- | --- | --- | --- | --- | --- | --- | --- | --- | --- | --- |
| \* | LAPMis12DANCE\_011813\_01.04396.04396.2 | 3.0439 | 0.3541 | 100.0% | 1177.7122 | 1178.428 | 1 | 6.105 | 77.8% | 1 | R.LTNSMMMHGR.N | 2 |
| \* | LAPMis12DANCE\_011813\_01.17927.17927.3 | 3.6078 | 0.398 | 100.0% | 3066.4143 | 3067.5613 | 3 | 6.398 | 25.0% | 1 | K.HAFEIIHLLTGENPLQVLVNAIINSGPR.E | 3 |

---

|  |  |  |  |  |  |  |  |  |
| --- | --- | --- | --- | --- | --- | --- | --- | --- |
| U | *gi|14602427|ref|NP\_12* | 5 | 7 | 18.4% | 277 | 31293 | 5.2 | ZW10 interactor isoform a [Homo sapiens] |
| U | *gi|14602429|ref|NP\_00* | 5 | 7 | 18.4% | 277 | 31293 | 5.2 | ZW10 interactor isoform a [Homo sapiens] |

| Filename XCorr DeltCN Conf% ObsM+H+ CalcM+H+ SpR ZScore Ion% # Sequence  | | | | | | | | | | | | |
| --- | --- | --- | --- | --- | --- | --- | --- | --- | --- | --- | --- | --- |
|  | LAPMis12DANCE\_011813\_01.07570.07570.2 | 2.0523 | 0.2485 | 95.7% | 1262.1122 | 1261.3312 | 1 | 4.468 | 68.2% | 1 | K.GLDPLASEDTSR.Q | 2 |
|  | LAPMis12DANCE\_011813\_01.06779.06779.2 | 3.8889 | 0.3295 | 100.0% | 1500.1322 | 1500.6542 | 3 | 5.7 | 68.2% | 1 | R.AVQNQWQLQQEK.H | 2 |
|  | LAPMis12DANCE\_011813\_02.04377.04377.3 | 4.3378 | 0.3795 | 100.0% | 1489.8844 | 1489.6743 | 1 | 7.458 | 54.2% | 2 | K.HLQHLAEVSAEVR.E | 3 |
|  | LAPMis12DANCE\_011813\_01.05076.05076.2 | 3.3195 | 0.4494 | 100.0% | 1490.0122 | 1489.6743 | 1 | 7.964 | 79.2% | 2 | K.HLQHLAEVSAEVR.E | 2 |
|  | LAPMis12DANCE\_011813\_01.15081.15081.2 | 3.9827 | 0.5408 | 100.0% | 1716.8922 | 1717.0178 | 1 | 9.556 | 69.2% | 1 | R.YQTFLQLLYTLQGK.L | 2 |

---

|  |  |  |  |  |  |  |  |  |
| --- | --- | --- | --- | --- | --- | --- | --- | --- |
| U | *gi|208973238|ref|NP\_0* | 2 | 4 | 17.6% | 245 | 27745 | 4.8 | tyrosine 3/tryptophan 5 -monooxygenase activation protein, zeta polypeptide [Homo sapiens] |
| U | *gi|4507953|ref|NP\_003* | 2 | 4 | 17.6% | 245 | 27745 | 4.8 | tyrosine 3/tryptophan 5 -monooxygenase activation protein, zeta polypeptide [Homo sapiens] |
| U | *gi|21735625|ref|NP\_66* | 2 | 4 | 17.6% | 245 | 27745 | 4.8 | tyrosine 3/tryptophan 5 -monooxygenase activation protein, zeta polypeptide [Homo sapiens] |
| U | *gi|208973244|ref|NP\_0* | 2 | 4 | 17.6% | 245 | 27745 | 4.8 | tyrosine 3/tryptophan 5 -monooxygenase activation protein, zeta polypeptide [Homo sapiens] |
| U | *gi|208973242|ref|NP\_0* | 2 | 4 | 17.6% | 245 | 27745 | 4.8 | tyrosine 3/tryptophan 5 -monooxygenase activation protein, zeta polypeptide [Homo sapiens] |
| U | *gi|208973240|ref|NP\_0* | 2 | 4 | 17.6% | 245 | 27745 | 4.8 | tyrosine 3/tryptophan 5 -monooxygenase activation protein, zeta polypeptide [Homo sapiens] |

| Filename XCorr DeltCN Conf% ObsM+H+ CalcM+H+ SpR ZScore Ion% # Sequence  | | | | | | | | | | | | |
| --- | --- | --- | --- | --- | --- | --- | --- | --- | --- | --- | --- | --- |
|  | LAPMis12DANCE2\_011813\_01.04337.04337.2 | 3.3486 | 0.3182 | 100.0% | 1549.5521 | 1549.5914 | 1 | 6.014 | 69.2% | 2 | K.SVTEQGAELSNEER.N | 2 |
|  | LAPMis12DANCE2\_011813\_01.16712.16712.3 | 4.9239 | 0.4879 | 100.0% | 3303.3843 | 3304.6907 | 1 | 8.051 | 30.4% | 2 | K.TAFDEAIAELDTLSEESYKDSTLIMQLLR.D | 3 |

---

|  |  |  |  |  |  |  |  |  |
| --- | --- | --- | --- | --- | --- | --- | --- | --- |
| U | *Reverse\_gi|169213308|* | 1 | 1 | 17.5% | 120 | 13147 | 11.8 | PREDICTED: hypothetical protein [Homo sapiens] |
| U | *Reverse\_gi|89057547|r* | 1 | 1 | 17.5% | 120 | 13147 | 11.8 | PREDICTED: hypothetical protein [Homo sapiens] |

| Filename XCorr DeltCN Conf% ObsM+H+ CalcM+H+ SpR ZScore Ion% # Sequence  | | | | | | | | | | | | |
| --- | --- | --- | --- | --- | --- | --- | --- | --- | --- | --- | --- | --- |
|  | LAPMis12DANCE2\_011813\_01.07697.07697.3 | 2.7319 | 0.2827 | 97.3% | 2261.2744 | 2258.6902 | 34 | 4.214 | 26.2% | 1 | R.TAPQPIMALPLSSRLGHSKPR.M | 3 |

---

|  |  |  |  |  |  |  |  |  |
| --- | --- | --- | --- | --- | --- | --- | --- | --- |
| U | *contaminant\_KERATIN12* | 8 | 12 | 17.4% | 431 | 47974 | 5.0 | no description |
| U | *gi|4557701|ref|NP\_000* | 8 | 12 | 17.4% | 432 | 48106 | 5.0 | keratin 17 [Homo sapiens] |

| Filename XCorr DeltCN Conf% ObsM+H+ CalcM+H+ SpR ZScore Ion% # Sequence  | | | | | | | | | | | | |
| --- | --- | --- | --- | --- | --- | --- | --- | --- | --- | --- | --- | --- |
|  | LAPMis12DANCE\_011813\_01.05114.05114.1 | 1.7551 | 0.2197 | 97.3% | 809.35 | 809.93774 | 6 | 5.101 | 66.7% | 2 | R.LASYLDK.V | 11111 |
|  | LAPMis12DANCE\_011813\_01.06436.06436.2 | 3.0274 | 0.1822 | 99.7% | 1065.3322 | 1065.2578 | 68 | 5.735 | 62.5% | 2 | R.LASYLDKVR.A | 2222 |
|  | LAPMis12DANCE\_011813\_02.04994.04994.2 | 3.3212 | 0.4698 | 100.0% | 1345.4122 | 1346.4772 | 2 | 7.234 | 68.2% | 3 | R.ALEEANTELEVK.I | 2 |
|  | LAPMis12DANCE\_011813\_01.09338.09338.2 | 2.6607 | 0.2826 | 100.0% | 1030.0122 | 1030.2096 | 2 | 6.157 | 81.2% | 1 | R.VLDELTLAR.A | 222 |
|  | LAPMis12DANCE\_011813\_01.05410.05410.2 | 2.8753 | 0.1865 | 99.2% | 1438.9321 | 1439.6263 | 2 | 4.216 | 75.0% | 1 | R.ILNEMRDQYEK.M | 22 |
|  | LAPMis12DANCE\_011813\_01.04502.04502.2 | 4.0545 | 0.4775 | 100.0% | 1361.8722 | 1362.4796 | 1 | 9.708 | 66.7% | 1 | R.EVATNSELVQSGK.S | 22 |
|  | LAPMis12DANCE\_011813\_01.07610.07610.2 | 2.4647 | 0.1921 | 97.6% | 1381.2522 | 1380.5437 | 7 | 4.454 | 60.0% | 1 | K.TRLEQEIATYR.R | 22 |
|  | LAPMis12DANCE\_011813\_01.05813.05813.2 | 2.3313 | 0.2474 | 99.1% | 1119.3722 | 1118.2291 | 1 | 5.176 | 77.8% | 1 | R.TIVEEVQDGK.V | 2 |

Similarities:
gi|55956899|ref|NP\_00(1:7)  
contaminant\_KERATIN03(2:6)  
gi|15431310|ref|NP\_00(6:2)  
contaminant\_KERATIN10(3:5)  

---

|  |  |  |  |  |  |  |  |  |
| --- | --- | --- | --- | --- | --- | --- | --- | --- |
| U | *gi|68508961|ref|NP\_00* | 11 | 16 | 17.1% | 943 | 106834 | 9.4 | centromere protein C 1 [Homo sapiens] |

| Filename XCorr DeltCN Conf% ObsM+H+ CalcM+H+ SpR ZScore Ion% # Sequence  | | | | | | | | | | | | |
| --- | --- | --- | --- | --- | --- | --- | --- | --- | --- | --- | --- | --- |
| \* | LAPMis12DANCE\_011813\_01.06615.06615.2 | 3.3323 | 0.4342 | 100.0% | 1285.2722 | 1285.3531 | 1 | 7.803 | 77.3% | 2 | K.SLANDFSTNSTK.S | 2 |
| \* | LAPMis12DANCE2\_011813\_01.07601.07601.2 | 3.4864 | 0.1848 | 99.5% | 1778.5122 | 1777.9286 | 1 | 4.935 | 63.3% | 2 | K.EASLQFVVEPSEATNR.S | 2 |
| \* | LAPMis12DANCE2\_011813\_01.02964.02964.2 | 2.8677 | 0.4256 | 100.0% | 1162.9722 | 1163.2784 | 1 | 6.949 | 77.8% | 1 | R.SVQAHEVHQK.I | 2 |
| \* | LAPMis12DANCE2\_011813\_01.03980.03980.2 | 3.317 | 0.4057 | 100.0% | 1656.0721 | 1656.7441 | 1 | 6.741 | 64.3% | 1 | K.ILATDVSSKNT#PDSK.K | 2 |
| \* | LAPMis12DANCE\_011813\_01.05270.05270.2 | 3.683 | 0.3684 | 100.0% | 1446.1522 | 1446.6005 | 1 | 6.558 | 76.9% | 3 | K.TSVSQNVIPSSAQK.R | 2 |
| \* | LAPMis12DANCE\_011813\_01.03198.03198.2 | 2.9838 | 0.2083 | 99.3% | 1608.9521 | 1609.6006 | 1 | 4.532 | 61.5% | 1 | K.VSDEEDKTSEGQER.K | 2 |
| \* | LAPMis12DANCE\_011813\_01.04404.04404.2 | 3.1492 | 0.2615 | 100.0% | 1309.2522 | 1309.4215 | 1 | 5.768 | 77.8% | 2 | R.IRDSEYEIQR.Q | 2 |
| \* | LAPMis12DANCE\_011813\_01.03176.03176.3 | 4.6214 | 0.4056 | 100.0% | 2454.2043 | 2454.6616 | 1 | 7.607 | 39.3% | 1 | K.TLANDKHSHKPHPVETSQPSDK.T | 3 |
| \* | LAPMis12DANCE2\_011813\_01.10990.10990.2 | 4.6136 | 0.4591 | 100.0% | 2029.5922 | 2030.242 | 1 | 8.346 | 61.8% | 1 | K.TVLDTSYALIGETVNNYR.S | 2 |
| \* | LAPMis12DANCE\_011813\_01.03257.03257.2 | 2.8399 | 0.335 | 100.0% | 1322.2322 | 1322.441 | 182 | 5.699 | 60.0% | 1 | R.NELPMHHNSSR.K | 2 |
| \* | LAPMis12DANCE\_011813\_01.13716.13716.2 | 3.7878 | 0.441 | 100.0% | 2356.3523 | 2356.7852 | 1 | 6.996 | 52.8% | 1 | R.EIILMDLVRPQDTYQFFVK.H | 2 |

---

|  |  |  |  |  |  |  |  |  |
| --- | --- | --- | --- | --- | --- | --- | --- | --- |
| U | *gi|153792590|ref|NP\_0* | 13 | 21 | 16.9% | 854 | 98161 | 5.2 | heat shock 90kDa protein 1, alpha isoform 1 [Homo sapiens] |
| U | *gi|154146191|ref|NP\_0* | 13 | 21 | 19.7% | 732 | 84660 | 5.0 | heat shock 90kDa protein 1, alpha isoform 2 [Homo sapiens] |

| Filename XCorr DeltCN Conf% ObsM+H+ CalcM+H+ SpR ZScore Ion% # Sequence  | | | | | | | | | | | | |
| --- | --- | --- | --- | --- | --- | --- | --- | --- | --- | --- | --- | --- |
|  | LAPMis12DANCE\_011813\_01.08333.08333.2 | 3.1825 | 0.3522 | 100.0% | 1561.9722 | 1561.7324 | 1 | 5.834 | 69.2% | 1 | R.ELISNSSDALDKIR.Y | 2 |
|  | LAPMis12DANCE\_011813\_01.06597.06597.2 | 3.8101 | 0.4687 | 100.0% | 1540.2322 | 1540.6672 | 1 | 6.953 | 65.4% | 1 | R.YESLTDPSKLDSGK.E | 22 |
|  | LAPMis12DANCE\_011813\_01.10278.10278.2 | 3.4458 | 0.4477 | 100.0% | 1243.3922 | 1243.4459 | 1 | 7.532 | 77.3% | 2 | K.ADLINNLGTIAK.S | 22 |
|  | LAPMis12DANCE\_011813\_02.05552.05552.3 | 4.3415 | 0.49 | 100.0% | 2015.5144 | 2016.2584 | 1 | 8.175 | 48.3% | 1 | K.VILHLKEDQTEYLEER.R | 33 |
|  | LAPMis12DANCE2\_011813\_01.04391.04391.1 | 2.7038 | 0.1407 | 96.1% | 1151.46 | 1152.2462 | 5 | 4.475 | 75.0% | 2 | K.YIDQEELNK.T | 11 |
|  | LAPMis12DANCE\_011813\_01.04958.04958.2 | 2.7262 | 0.0852 | 95.8% | 1153.3922 | 1152.2462 | 3 | 3.717 | 81.2% | 1 | K.YIDQEELNK.T | 22 |
|  | LAPMis12DANCE\_011813\_02.06142.06142.2 | 4.6469 | 0.3562 | 100.0% | 1835.0922 | 1834.8903 | 1 | 7.472 | 82.1% | 3 | R.NPDDITNEEYGEFYK.S | 2 |
|  | LAPMis12DANCE\_011813\_01.09534.09534.2 | 4.0346 | 0.426 | 100.0% | 1528.0521 | 1528.6616 | 2 | 7.458 | 62.5% | 2 | K.SLTNDWEDHLAVK.H | 22 |
|  | LAPMis12DANCE\_011813\_01.09167.09167.2 | 2.9063 | 0.4308 | 100.0% | 1349.1921 | 1349.4886 | 6 | 6.774 | 60.0% | 2 | K.HFSVEGQLEFR.A | 22 |
|  | LAPMis12DANCE\_011813\_02.05625.05625.2 | 3.2118 | 0.398 | 100.0% | 1551.1122 | 1551.7083 | 1 | 7.492 | 57.7% | 1 | R.YYTSASGDEMVSLK.D | 2 |
|  | LAPMis12DANCE\_011813\_01.05754.05754.2 | 1.9295 | 0.3011 | 97.7% | 1188.2322 | 1189.3436 | 39 | 5.109 | 55.0% | 1 | R.DNSTMGYMAAK.K | 2 |
|  | LAPMis12DANCE\_011813\_01.09533.09533.2 | 4.109 | 0.465 | 100.0% | 1787.5322 | 1788.0134 | 1 | 8.421 | 71.4% | 1 | K.HLEINPDHSIIETLR.Q | 2 |
|  | LAPMis12DANCE\_011813\_01.09549.09549.3 | 3.4087 | 0.4376 | 100.0% | 1787.7843 | 1788.0134 | 1 | 7.952 | 48.2% | 3 | K.HLEINPDHSIIETLR.Q | 23 |

Similarities:
gi|20149594|ref|NP\_03(7:6)  

---

|  |  |  |  |  |  |  |  |  |
| --- | --- | --- | --- | --- | --- | --- | --- | --- |
| U | *gi|115270970|ref|NP\_0* | 12 | 20 | 16.9% | 551 | 62023 | 5.6 | Mid-1-related chloride channel 1 isoform 1 [Homo sapiens] |
| U | *gi|13194195|ref|NP\_05* | 12 | 20 | 18.6% | 501 | 56267 | 6.0 | Mid-1-related chloride channel 1 isoform 2 [Homo sapiens] |

| Filename XCorr DeltCN Conf% ObsM+H+ CalcM+H+ SpR ZScore Ion% # Sequence  | | | | | | | | | | | | |
| --- | --- | --- | --- | --- | --- | --- | --- | --- | --- | --- | --- | --- |
|  | LAPMis12DANCE\_011813\_01.04794.04794.3 | 3.5376 | 0.2429 | 98.3% | 1755.0543 | 1755.8845 | 1 | 5.255 | 42.3% | 1 | K.KREDYESQSNPVFR.R | 3 |
|  | LAPMis12DANCE\_011813\_01.04827.04827.2 | 3.6429 | 0.2628 | 100.0% | 1755.2522 | 1755.8845 | 1 | 6.197 | 69.2% | 1 | K.KREDYESQSNPVFR.R | 2 |
|  | LAPMis12DANCE\_011813\_01.06419.06419.2 | 2.5102 | 0.2139 | 98.0% | 1626.7922 | 1627.7104 | 49 | 4.569 | 50.0% | 1 | K.REDYESQSNPVFR.R | 2 |
|  | LAPMis12DANCE\_011813\_01.07433.07433.2 | 2.9387 | 0.3306 | 100.0% | 1470.8722 | 1471.523 | 1 | 6.886 | 63.6% | 1 | R.EDYESQSNPVFR.R | 2 |
|  | LAPMis12DANCE\_011813\_01.04602.04602.2 | 2.5269 | 0.2578 | 99.3% | 1313.4722 | 1313.499 | 5 | 5.407 | 59.1% | 1 | K.LAFAQHQAEVAK.M | 2 |
|  | LAPMis12DANCE\_011813\_02.09827.09827.2 | 2.3552 | 0.2367 | 97.8% | 1742.4922 | 1742.9922 | 1 | 5.264 | 58.3% | 1 | K.KMDWTGSIWEWFR.S | 2 |
|  | LAPMis12DANCE2\_011813\_01.13498.13498.2 | 3.8102 | 0.4424 | 100.0% | 1615.2922 | 1614.8181 | 1 | 7.322 | 72.7% | 3 | K.MDWTGSIWEWFR.S | 2 |
|  | LAPMis12DANCE2\_011813\_01.04180.04180.2 | 4.0781 | 0.3301 | 100.0% | 1365.1122 | 1364.5157 | 1 | 6.764 | 66.7% | 5 | R.GQMGPTEQGPYAK.T | 2 |
|  | LAPMis12DANCE\_011813\_01.05138.05138.2 | 3.3094 | 0.2483 | 100.0% | 1456.1522 | 1456.5553 | 2 | 5.596 | 72.7% | 2 | R.FQTGNKS\*PEVLR.A | 2 |
|  | LAPMis12DANCE2\_011813\_01.05454.05454.1 | 1.7167 | 0.4177 | 100.0% | 1090.59 | 1091.1655 | 8 | 6.239 | 50.0% | 2 | R.AFDVPDAEAR.E | 1 |
|  | LAPMis12DANCE\_011813\_01.07316.07316.2 | 1.9878 | 0.2968 | 98.4% | 1090.7522 | 1091.1655 | 1 | 6.607 | 77.8% | 1 | R.AFDVPDAEAR.E | 2 |
|  | LAPMis12DANCE\_011813\_01.04000.04000.3 | 2.9943 | 0.351 | 99.7% | 2177.0344 | 2177.422 | 5 | 5.981 | 31.9% | 1 | R.EHPTVVPSHKS\*PVLDTKPK.E | 3 |

---

|  |  |  |  |  |  |  |  |  |
| --- | --- | --- | --- | --- | --- | --- | --- | --- |
| U | *gi|5901922|ref|NP\_008* | 5 | 5 | 16.9% | 378 | 44468 | 5.2 | cell division cycle 37 protein [Homo sapiens] |

| Filename XCorr DeltCN Conf% ObsM+H+ CalcM+H+ SpR ZScore Ion% # Sequence  | | | | | | | | | | | | |
| --- | --- | --- | --- | --- | --- | --- | --- | --- | --- | --- | --- | --- |
| \* | LAPMis12DANCE\_011813\_02.04865.04865.3 | 3.629 | 0.3159 | 99.7% | 1772.0643 | 1772.0085 | 4 | 6.171 | 41.7% | 1 | K.LKELEVAEGGKAELER.L | 3 |
| \* | LAPMis12DANCE\_011813\_01.09567.09567.2 | 2.4767 | 0.1472 | 96.0% | 1337.1522 | 1336.5054 | 1 | 4.601 | 83.3% | 1 | R.SWEQKLEEMR.K | 2 |
| \* | LAPMis12DANCE\_011813\_01.03674.03674.2 | 2.3925 | 0.2226 | 98.7% | 1314.3322 | 1314.4095 | 8 | 4.297 | 61.1% | 1 | K.AMKEYEEEER.K | 2 |
| \* | LAPMis12DANCE\_011813\_01.09491.09491.2 | 2.6908 | 0.212 | 99.2% | 1248.3722 | 1248.4369 | 1 | 6.143 | 70.0% | 1 | K.DVQMLQDAISK.M | 2 |
| \* | LAPMis12DANCE\_011813\_01.10602.10602.2 | 2.2628 | 0.2219 | 95.1% | 1707.9722 | 1708.8627 | 317 | 4.973 | 31.2% | 1 | K.EGEEAGPGDPLLEAVPK.T | 2 |

---

|  |  |  |  |  |  |  |  |  |
| --- | --- | --- | --- | --- | --- | --- | --- | --- |
| U | *gi|11024714|ref|NP\_06* | 3 | 6 | 16.6% | 229 | 25762 | 7.4 | ubiquitin B precursor [Homo sapiens] |
| U | *gi|77539055|ref|NP\_00* | 3 | 6 | 29.7% | 128 | 14728 | 9.8 | ubiquitin and ribosomal protein L40 precursor [Homo sapiens] |
| U | *gi|67191208|ref|NP\_06* | 3 | 6 | 5.5% | 685 | 77029 | 7.7 | ubiquitin C [Homo sapiens] |
| U | *gi|4507761|ref|NP\_003* | 3 | 6 | 29.7% | 128 | 14728 | 9.8 | ubiquitin and ribosomal protein L40 precursor [Homo sapiens] |
| U | *gi|4506713|ref|NP\_002* | 3 | 6 | 24.4% | 156 | 17965 | 9.6 | ubiquitin and ribosomal protein S27a precursor [Homo sapiens] |
| U | *gi|208022622|ref|NP\_0* | 3 | 6 | 24.4% | 156 | 17965 | 9.6 | ubiquitin and ribosomal protein S27a precursor [Homo sapiens] |

| Filename XCorr DeltCN Conf% ObsM+H+ CalcM+H+ SpR ZScore Ion% # Sequence  | | | | | | | | | | | | |
| --- | --- | --- | --- | --- | --- | --- | --- | --- | --- | --- | --- | --- |
|  | LAPMis12DANCE\_011813\_01.10191.10191.2 | 4.1939 | 0.4602 | 100.0% | 1789.2722 | 1788.9897 | 1 | 7.872 | 73.3% | 2 | K.TITLEVEPSDTIENVK.A | 2 |
|  | LAPMis12DANCE\_011813\_01.03666.03666.2 | 3.7132 | 0.3343 | 100.0% | 1524.3322 | 1524.6738 | 1 | 6.911 | 83.3% | 2 | K.IQDKEGIPPDQQR.L | 2 |
|  | LAPMis12DANCE\_011813\_01.08072.08072.2 | 2.5486 | 0.2984 | 100.0% | 1067.4922 | 1068.2615 | 3 | 4.982 | 81.2% | 2 | K.ESTLHLVLR.L | 2 |

---

|  |  |  |  |  |  |  |  |  |
| --- | --- | --- | --- | --- | --- | --- | --- | --- |
| U | *gi|4506693|ref|NP\_001* | 1 | 1 | 16.3% | 135 | 15550 | 9.8 | ribosomal protein S17 [Homo sapiens] |

| Filename XCorr DeltCN Conf% ObsM+H+ CalcM+H+ SpR ZScore Ion% # Sequence  | | | | | | | | | | | | |
| --- | --- | --- | --- | --- | --- | --- | --- | --- | --- | --- | --- | --- |
| \* | LAPMis12DANCE2\_011813\_01.11442.11442.2 | 3.6825 | 0.4646 | 100.0% | 2410.112 | 2410.7908 | 1 | 7.789 | 38.1% | 1 | K.LLDFGSLSNLQVTQPTVGMNFK.T | 2 |

---

|  |  |  |  |  |  |  |  |  |
| --- | --- | --- | --- | --- | --- | --- | --- | --- |
| U | *gi|38016907|ref|NP\_93* | 1 | 1 | 16.3% | 123 | 13475 | 8.0 | stomatin isoform b [Homo sapiens] |
| U | *gi|38016911|ref|NP\_00* | 1 | 1 | 6.9% | 288 | 31731 | 7.9 | stomatin isoform a [Homo sapiens] |

| Filename XCorr DeltCN Conf% ObsM+H+ CalcM+H+ SpR ZScore Ion% # Sequence  | | | | | | | | | | | | |
| --- | --- | --- | --- | --- | --- | --- | --- | --- | --- | --- | --- | --- |
|  | LAPMis12DANCE\_011813\_01.16607.16607.2 | 2.7933 | 0.3052 | 99.9% | 2128.112 | 2128.5781 | 1 | 5.442 | 36.8% | 1 | K.NSTIVFPLPIDMLQGIIGAK.H | 2 |

---

|  |  |  |  |  |  |  |  |  |
| --- | --- | --- | --- | --- | --- | --- | --- | --- |
| U | *gi|4503483|ref|NP\_001* | 9 | 19 | 16.2% | 858 | 95338 | 6.8 | eukaryotic translation elongation factor 2 [Homo sapiens] |

| Filename XCorr DeltCN Conf% ObsM+H+ CalcM+H+ SpR ZScore Ion% # Sequence  | | | | | | | | | | | | |
| --- | --- | --- | --- | --- | --- | --- | --- | --- | --- | --- | --- | --- |
| \* | LAPMis12DANCE\_011813\_01.04276.04276.2 | 3.4777 | 0.3299 | 100.0% | 1307.9722 | 1308.4979 | 1 | 6.189 | 63.6% | 2 | R.NMSVIAHVDHGK.S | 2 |
| \* | LAPMis12DANCE\_011813\_01.09638.09638.2 | 2.0635 | 0.2433 | 96.1% | 1108.0322 | 1108.3231 | 1 | 4.986 | 70.0% | 1 | R.VFSGLVSTGLK.V | 2 |
| \* | LAPMis12DANCE\_011813\_01.05050.05050.2 | 3.0765 | 0.3087 | 100.0% | 1617.8522 | 1616.7917 | 62 | 5.985 | 42.3% | 1 | K.TGTITTFEHAHNMR.V | 2 |
| \* | LAPMis12DANCE\_011813\_01.09471.09471.3 | 5.773 | 0.4583 | 100.0% | 2144.4543 | 2144.3489 | 1 | 8.056 | 44.7% | 5 | K.ARPFPDGLAEDIDKGEVSAR.Q | 3 |
| \* | LAPMis12DANCE\_011813\_01.09305.09305.2 | 4.3528 | 0.5078 | 100.0% | 1743.8522 | 1743.9133 | 1 | 8.194 | 69.2% | 1 | R.YLAEKYEWDVAEAR.K | 2 |
| \* | LAPMis12DANCE2\_011813\_01.12153.12153.2 | 3.8451 | 0.3912 | 100.0% | 2355.2322 | 2354.6677 | 3 | 6.425 | 37.5% | 1 | K.GVQYLNEIKDSVVAGFQWATK.E | 2 |
| \* | LAPMis12DANCE\_011813\_01.10218.10218.2 | 3.7496 | 0.1593 | 99.5% | 1962.4321 | 1963.2585 | 1 | 6.64 | 58.8% | 1 | R.GHVFEESQVAGTPMFVVK.A | 2 |
| \* | LAPMis12DANCE\_011813\_01.11808.11808.2 | 3.3017 | 0.3948 | 100.0% | 1800.1122 | 1801.0087 | 1 | 6.54 | 56.7% | 4 | K.AYLPVNESFGFTADLR.S | 2 |
| \* | LAPMis12DANCE\_011813\_01.14630.14630.2 | 3.1915 | 0.4886 | 100.0% | 1445.1921 | 1445.6555 | 1 | 7.939 | 66.7% | 3 | K.EGIPALDNFLDKL.- | 2 |

---

|  |  |  |  |  |  |  |  |  |
| --- | --- | --- | --- | --- | --- | --- | --- | --- |
| U | *gi|4757880|ref|NP\_004* | 3 | 4 | 16.2% | 328 | 37155 | 6.8 | budding uninhibited by benzimidazoles 3 isoform a [Homo sapiens] |
| U | *gi|56550081|ref|NP\_00* | 3 | 4 | 16.3% | 326 | 36955 | 6.8 | budding uninhibited by benzimidazoles 3 isoform b [Homo sapiens] |

| Filename XCorr DeltCN Conf% ObsM+H+ CalcM+H+ SpR ZScore Ion% # Sequence  | | | | | | | | | | | | |
| --- | --- | --- | --- | --- | --- | --- | --- | --- | --- | --- | --- | --- |
|  | LAPMis12DANCE\_011813\_02.08825.08825.2 | 3.9603 | 0.5639 | 100.0% | 2171.7122 | 2172.402 | 1 | 9.046 | 47.2% | 1 | K.FSPNTSQFLLVSSWDTSVR.L | 2 |
|  | LAPMis12DANCE\_011813\_01.07298.07298.3 | 4.7808 | 0.4879 | 100.0% | 2276.5144 | 2277.4736 | 1 | 8.261 | 46.1% | 1 | K.MHDLNTDQENLVGTHDAPIR.C | 3 |
|  | LAPMis12DANCE\_011813\_01.08703.08703.2 | 3.5955 | 0.3763 | 100.0% | 1575.2522 | 1574.771 | 1 | 6.234 | 69.2% | 2 | R.VAVEYLDPSPEVQK.K | 2 |

---

|  |  |  |  |  |  |  |  |  |
| --- | --- | --- | --- | --- | --- | --- | --- | --- |
| U | *gi|4506625|ref|NP\_000* | 2 | 2 | 16.2% | 148 | 16561 | 11.0 | ribosomal protein L27a [Homo sapiens] |

| Filename XCorr DeltCN Conf% ObsM+H+ CalcM+H+ SpR ZScore Ion% # Sequence  | | | | | | | | | | | | |
| --- | --- | --- | --- | --- | --- | --- | --- | --- | --- | --- | --- | --- |
| \* | LAPMis12DANCE\_011813\_01.08243.08243.2 | 3.9736 | 0.3816 | 100.0% | 1586.0322 | 1586.7899 | 1 | 7.118 | 75.0% | 1 | R.INFDKYHPGYFGK.V | 2 |
| \* | LAPMis12DANCE\_011813\_01.09206.09206.2 | 2.4906 | 0.1719 | 96.7% | 1113.2722 | 1112.3146 | 2 | 5.393 | 75.0% | 1 | K.TGAAPIIDVVR.S | 2 |

---

|  |  |  |  |  |  |  |  |  |
| --- | --- | --- | --- | --- | --- | --- | --- | --- |
| U | *gi|5032051|ref|NP\_005* | 2 | 4 | 15.9% | 151 | 16273 | 10.1 | ribosomal protein S14 [Homo sapiens] |
| U | *gi|68160922|ref|NP\_00* | 2 | 4 | 15.9% | 151 | 16273 | 10.1 | ribosomal protein S14 [Homo sapiens] |
| U | *gi|68160915|ref|NP\_00* | 2 | 4 | 15.9% | 151 | 16273 | 10.1 | ribosomal protein S14 [Homo sapiens] |

| Filename XCorr DeltCN Conf% ObsM+H+ CalcM+H+ SpR ZScore Ion% # Sequence  | | | | | | | | | | | | |
| --- | --- | --- | --- | --- | --- | --- | --- | --- | --- | --- | --- | --- |
|  | LAPMis12DANCE\_011813\_01.04161.04161.2 | 2.9461 | 0.4385 | 100.0% | 1054.4321 | 1055.179 | 7 | 8.564 | 65.0% | 1 | K.TPGPGAQSALR.A | 2 |
|  | LAPMis12DANCE\_011813\_01.07038.07038.2 | 3.2539 | 0.2729 | 100.0% | 1431.1522 | 1430.5547 | 2 | 5.293 | 66.7% | 3 | R.IEDVTPIPSDSTR.R | 2 |

---

|  |  |  |  |  |  |  |  |  |
| --- | --- | --- | --- | --- | --- | --- | --- | --- |
| U | *gi|14591909|ref|NP\_00* | 4 | 5 | 15.8% | 297 | 34363 | 9.7 | ribosomal protein L5 [Homo sapiens] |

| Filename XCorr DeltCN Conf% ObsM+H+ CalcM+H+ SpR ZScore Ion% # Sequence  | | | | | | | | | | | | |
| --- | --- | --- | --- | --- | --- | --- | --- | --- | --- | --- | --- | --- |
|  | LAPMis12DANCE\_011813\_01.06372.06372.2 | 3.3211 | 0.2858 | 100.0% | 1339.2522 | 1339.4911 | 1 | 5.945 | 61.5% | 2 | K.GAVDGGLSIPHSTK.R | 2 |
|  | LAPMis12DANCE\_011813\_01.03321.03321.2 | 2.7389 | 0.2301 | 99.7% | 1129.8722 | 1130.2485 | 29 | 5.12 | 68.8% | 1 | K.EFNAEVHRK.H | 2 |
| \* | LAPMis12DANCE\_011813\_01.07977.07977.2 | 3.2671 | 0.3417 | 100.0% | 1436.2522 | 1435.6587 | 1 | 6.752 | 77.3% | 1 | K.HIMGQNVADYMR.Y | 2 |
| \* | LAPMis12DANCE\_011813\_01.06406.06406.2 | 3.6447 | 0.4136 | 100.0% | 1533.9122 | 1534.6777 | 1 | 6.812 | 81.8% | 1 | R.YLMEEDEDAYKK.Q | 2 |

---

|  |  |  |  |  |  |  |  |  |
| --- | --- | --- | --- | --- | --- | --- | --- | --- |
| U | *gi|219555707|ref|NP\_0* | 3 | 4 | 15.8% | 184 | 20170 | 7.0 | eukaryotic translation initiation factor 5A isoform A [Homo sapiens] |
| U | *gi|4503545|ref|NP\_001* | 3 | 4 | 18.8% | 154 | 16832 | 5.2 | eukaryotic translation initiation factor 5A isoform B [Homo sapiens] |
| U | *gi|219555712|ref|NP\_0* | 3 | 4 | 18.8% | 154 | 16832 | 5.2 | eukaryotic translation initiation factor 5A isoform B [Homo sapiens] |
| U | *gi|219555710|ref|NP\_0* | 3 | 4 | 18.8% | 154 | 16832 | 5.2 | eukaryotic translation initiation factor 5A isoform B [Homo sapiens] |

| Filename XCorr DeltCN Conf% ObsM+H+ CalcM+H+ SpR ZScore Ion% # Sequence  | | | | | | | | | | | | |
| --- | --- | --- | --- | --- | --- | --- | --- | --- | --- | --- | --- | --- |
|  | LAPMis12DANCE\_011813\_01.11212.11212.2 | 2.4019 | 0.35 | 99.9% | 1298.3322 | 1299.5559 | 1 | 6.856 | 68.2% | 1 | K.VHLVGIDIFTGK.K | 2 |
|  | LAPMis12DANCE\_011813\_01.08045.08045.2 | 2.4463 | 0.2473 | 99.0% | 1342.2922 | 1342.4918 | 36 | 5.132 | 54.5% | 1 | R.EDLRLPEGDLGK.E | 23 |
|  | LAPMis12DANCE\_011813\_01.08993.08993.3 | 4.0126 | 0.3087 | 99.7% | 1969.6444 | 1970.187 | 2 | 5.56 | 34.4% | 2 | R.EDLRLPEGDLGKEIEQK.Y | 3 |

---

|  |  |  |  |  |  |  |  |  |
| --- | --- | --- | --- | --- | --- | --- | --- | --- |
| U | *gi|4506691|ref|NP\_001* | 2 | 2 | 15.8% | 146 | 16445 | 10.2 | ribosomal protein S16 [Homo sapiens] |

| Filename XCorr DeltCN Conf% ObsM+H+ CalcM+H+ SpR ZScore Ion% # Sequence  | | | | | | | | | | | | |
| --- | --- | --- | --- | --- | --- | --- | --- | --- | --- | --- | --- | --- |
|  | LAPMis12DANCE\_011813\_01.10002.10002.2 | 3.1834 | 0.4122 | 100.0% | 1188.2922 | 1188.372 | 1 | 7.675 | 75.0% | 1 | K.GPLQSVQVFGR.K | 2 |
| \* | LAPMis12DANCE\_011813\_01.07809.07809.2 | 2.5025 | 0.3062 | 99.6% | 1411.8522 | 1411.6622 | 2 | 4.699 | 59.1% | 1 | K.VNGRPLEMIEPR.T | 2 |

---

|  |  |  |  |  |  |  |  |  |
| --- | --- | --- | --- | --- | --- | --- | --- | --- |
| U | *gi|224586882|ref|NP\_0* | 3 | 6 | 15.6% | 372 | 40090 | 9.8 | cold shock domain protein A isoform a [Homo sapiens] |

| Filename XCorr DeltCN Conf% ObsM+H+ CalcM+H+ SpR ZScore Ion% # Sequence  | | | | | | | | | | | | |
| --- | --- | --- | --- | --- | --- | --- | --- | --- | --- | --- | --- | --- |
|  | LAPMis12DANCE\_011813\_01.04413.04413.3 | 3.4492 | 0.3378 | 99.7% | 1874.0343 | 1874.1039 | 1 | 5.617 | 35.0% | 1 | R.NDTKEDVFVHQTAIKK.N | 33 |
|  | LAPMis12DANCE\_011813\_02.06380.06380.2 | 4.6756 | 0.5344 | 100.0% | 1796.0721 | 1796.8822 | 1 | 9.537 | 59.4% | 4 | R.SVGDGETVEFDVVEGEK.G | 22 |
| \* | LAPMis12DANCE\_011813\_01.08442.08442.3 | 4.6421 | 0.3843 | 100.0% | 2617.4644 | 2617.9397 | 1 | 6.575 | 36.5% | 1 | R.IQAGEIGEMKDGVPEGAQLQGPVHR.N | 3 |

Similarities:
gi|34098946|ref|NP\_00(2:1)  

---

|  |  |  |  |  |  |  |  |  |
| --- | --- | --- | --- | --- | --- | --- | --- | --- |
| U | *gi|59859885|ref|NP\_00* | 3 | 6 | 15.3% | 295 | 32854 | 4.9 | ribosomal protein SA [Homo sapiens] |
| U | *gi|9845502|ref|NP\_002* | 3 | 6 | 15.3% | 295 | 32854 | 4.9 | ribosomal protein SA [Homo sapiens] |

| Filename XCorr DeltCN Conf% ObsM+H+ CalcM+H+ SpR ZScore Ion% # Sequence  | | | | | | | | | | | | |
| --- | --- | --- | --- | --- | --- | --- | --- | --- | --- | --- | --- | --- |
|  | LAPMis12DANCE\_011813\_01.10680.10680.2 | 3.2228 | 0.4672 | 100.0% | 1741.3322 | 1741.9823 | 1 | 8.467 | 53.1% | 1 | R.AIVAIENPADVSVISSR.N | 2 |
|  | LAPMis12DANCE\_011813\_01.05799.05799.2 | 3.2361 | 0.3976 | 100.0% | 1203.7322 | 1204.3713 | 1 | 7.411 | 75.0% | 4 | K.FAAATGATPIAGR.F | 2 |
|  | LAPMis12DANCE2\_011813\_01.10077.10077.2 | 3.106 | 0.4268 | 100.0% | 1700.0322 | 1699.9065 | 1 | 6.654 | 57.1% | 1 | R.FTPGTFTNQIQAAFR.E | 2 |

---

|  |  |  |  |  |  |  |  |  |
| --- | --- | --- | --- | --- | --- | --- | --- | --- |
| U | *gi|169171281|ref|XP\_0* | 1 | 1 | 15.2% | 99 | 11432 | 5.5 | PREDICTED: similar to Chromosome 7 open reading frame 28B [Homo sapiens] |
| U | *gi|56699480|ref|NP\_05* | 1 | 1 | 3.1% | 482 | 55866 | 6.5 | hypothetical protein LOC51622 [Homo sapiens] |
| U | *gi|37674289|ref|NP\_93* | 1 | 1 | 3.1% | 482 | 55866 | 6.5 | hypothetical protein LOC221960 [Homo sapiens] |
| U | *gi|169217893|ref|XP\_0* | 1 | 1 | 15.2% | 99 | 11432 | 5.5 | PREDICTED: similar to Chromosome 7 open reading frame 28B [Homo sapiens] |

| Filename XCorr DeltCN Conf% ObsM+H+ CalcM+H+ SpR ZScore Ion% # Sequence  | | | | | | | | | | | | |
| --- | --- | --- | --- | --- | --- | --- | --- | --- | --- | --- | --- | --- |
|  | LAPMis12DANCE2\_011813\_01.06604.06604.2 | 2.8541 | 0.2364 | 99.4% | 1901.2122 | 1903.0575 | 17 | 4.285 | 42.9% | 1 | K.AMS\*DYWVVGK@K@SDRR.E | 2 |

---

|  |  |  |  |  |  |  |  |  |
| --- | --- | --- | --- | --- | --- | --- | --- | --- |
| U | *gi|5031635|ref|NP\_005* | 2 | 5 | 15.1% | 166 | 18502 | 8.1 | cofilin 1 (non-muscle) [Homo sapiens] |

| Filename XCorr DeltCN Conf% ObsM+H+ CalcM+H+ SpR ZScore Ion% # Sequence  | | | | | | | | | | | | |
| --- | --- | --- | --- | --- | --- | --- | --- | --- | --- | --- | --- | --- |
|  | LAPMis12DANCE\_011813\_01.08190.08190.2 | 2.9265 | 0.3475 | 100.0% | 1337.9521 | 1338.4564 | 1 | 6.239 | 75.0% | 4 | R.YALYDATYETK.E | 2 |
| \* | LAPMis12DANCE\_011813\_02.06899.06899.2 | 2.3876 | 0.3163 | 99.5% | 1340.7322 | 1341.5907 | 1 | 5.509 | 53.8% | 1 | K.LGGSAVISLEGKPL.- | 2 |

---

|  |  |  |  |  |  |  |  |  |
| --- | --- | --- | --- | --- | --- | --- | --- | --- |
| U | *gi|11968182|ref|NP\_07* | 2 | 2 | 15.1% | 152 | 17719 | 11.0 | ribosomal protein S18 [Homo sapiens] |
| U | *gi|169168597|ref|XP\_0* | 2 | 2 | 15.1% | 152 | 17719 | 11.0 | PREDICTED: hypothetical protein [Homo sapiens] |

| Filename XCorr DeltCN Conf% ObsM+H+ CalcM+H+ SpR ZScore Ion% # Sequence  | | | | | | | | | | | | |
| --- | --- | --- | --- | --- | --- | --- | --- | --- | --- | --- | --- | --- |
|  | LAPMis12DANCE\_011813\_01.04949.04949.2 | 3.1995 | 0.3605 | 100.0% | 1247.9922 | 1248.2891 | 9 | 6.188 | 65.0% | 1 | R.AGELTEDEVER.V | 2 |
|  | LAPMis12DANCE\_011813\_01.07181.07181.2 | 3.2502 | 0.3675 | 100.0% | 1322.4321 | 1322.4606 | 1 | 6.677 | 77.3% | 1 | K.YSQVLANGLDNK.L | 2 |

---

|  |  |  |  |  |  |  |  |  |
| --- | --- | --- | --- | --- | --- | --- | --- | --- |
| U | *gi|157779135|ref|NP\_0* | 5 | 7 | 15.0% | 432 | 46511 | 6.4 | succinate-CoA ligase, GDP-forming, beta subunit precursor [Homo sapiens] |

| Filename XCorr DeltCN Conf% ObsM+H+ CalcM+H+ SpR ZScore Ion% # Sequence  | | | | | | | | | | | | |
| --- | --- | --- | --- | --- | --- | --- | --- | --- | --- | --- | --- | --- |
|  | LAPMis12DANCE\_011813\_02.06970.06970.2 | 4.4775 | 0.481 | 100.0% | 1597.1721 | 1597.7649 | 1 | 8.978 | 75.0% | 2 | R.FFVADTANEALEAAK.R | 2 |
| \* | LAPMis12DANCE\_011813\_01.07089.07089.3 | 3.7624 | 0.4449 | 100.0% | 1733.8444 | 1734.0085 | 1 | 6.335 | 40.6% | 1 | K.GGVHLTKDPNVVGQLAK.Q | 3 |
|  | LAPMis12DANCE\_011813\_01.10630.10630.2 | 3.2163 | 0.5257 | 100.0% | 1276.2922 | 1276.5365 | 1 | 7.684 | 81.8% | 1 | R.MAENLGFVGPLK.S | 2 |
| \* | LAPMis12DANCE\_011813\_01.08936.08936.2 | 2.9544 | 0.3281 | 100.0% | 1241.2322 | 1241.3024 | 1 | 6.393 | 77.8% | 2 | K.INFDDNAEFR.Q | 2 |
| \* | LAPMis12DANCE\_011813\_01.03664.03664.2 | 2.7104 | 0.1674 | 98.3% | 1218.0922 | 1217.3213 | 1 | 4.411 | 70.0% | 1 | R.LEGTNVQEAQK.I | 2 |

---

|  |  |  |  |  |  |  |  |  |
| --- | --- | --- | --- | --- | --- | --- | --- | --- |
| U | *gi|16418357|ref|NP\_44* | 3 | 3 | 14.7% | 368 | 41089 | 5.0 | ankyrin repeat domain 40 [Homo sapiens] |

| Filename XCorr DeltCN Conf% ObsM+H+ CalcM+H+ SpR ZScore Ion% # Sequence  | | | | | | | | | | | | |
| --- | --- | --- | --- | --- | --- | --- | --- | --- | --- | --- | --- | --- |
| \* | LAPMis12DANCE\_011813\_01.03963.03963.2 | 2.8658 | 0.347 | 100.0% | 1162.5721 | 1163.3135 | 2 | 6.607 | 60.0% | 1 | K.SGADKEILTTK.G | 2 |
| \* | LAPMis12DANCE\_011813\_01.08716.08716.3 | 4.1865 | 0.3774 | 100.0% | 2549.0942 | 2549.6333 | 4 | 6.078 | 29.8% | 1 | K.IMGVEEEDDDDDDDDNLPQLKK.E | 3 |
| \* | LAPMis12DANCE2\_011813\_01.15588.15588.2 | 2.5186 | 0.3521 | 99.9% | 2598.892 | 2600.0044 | 6 | 4.649 | 32.5% | 1 | R.LQDFQELELVLMISENNFLFR.N | 2 |

---

|  |  |  |  |  |  |  |  |  |
| --- | --- | --- | --- | --- | --- | --- | --- | --- |
| U | *gi|15431293|ref|NP\_00* | 2 | 3 | 14.7% | 204 | 24146 | 11.6 | ribosomal protein L15 [Homo sapiens] |
| U | *gi|88998868|ref|XP\_94* | 2 | 3 | 14.7% | 204 | 24174 | 11.6 | PREDICTED: hypothetical protein isoform 4 [Homo sapiens] |
| U | *gi|88992455|ref|XP\_93* | 2 | 3 | 14.7% | 204 | 24174 | 11.6 | PREDICTED: hypothetical protein isoform 1 [Homo sapiens] |
| U | *gi|169169711|ref|XP\_0* | 2 | 3 | 14.7% | 204 | 24174 | 11.6 | PREDICTED: hypothetical protein [Homo sapiens] |

| Filename XCorr DeltCN Conf% ObsM+H+ CalcM+H+ SpR ZScore Ion% # Sequence  | | | | | | | | | | | | |
| --- | --- | --- | --- | --- | --- | --- | --- | --- | --- | --- | --- | --- |
|  | LAPMis12DANCE2\_011813\_01.03623.03623.2 | 3.1821 | 0.4351 | 100.0% | 1705.5521 | 1706.945 | 2 | 7.204 | 43.3% | 1 | K.GATYGKPVHHGVNQLK.F | 2 |
|  | LAPMis12DANCE\_011813\_02.06374.06374.2 | 3.742 | 0.4387 | 100.0% | 1661.6522 | 1661.8083 | 1 | 8.12 | 57.7% | 2 | R.VLNSYWVGEDSTYK.F | 2 |

---

|  |  |  |  |  |  |  |  |  |
| --- | --- | --- | --- | --- | --- | --- | --- | --- |
| U | *gi|4506623|ref|NP\_000* | 2 | 2 | 14.7% | 136 | 15798 | 10.6 | ribosomal protein L27 [Homo sapiens] |

| Filename XCorr DeltCN Conf% ObsM+H+ CalcM+H+ SpR ZScore Ion% # Sequence  | | | | | | | | | | | | |
| --- | --- | --- | --- | --- | --- | --- | --- | --- | --- | --- | --- | --- |
|  | LAPMis12DANCE\_011813\_01.07415.07415.2 | 3.3098 | 0.3599 | 100.0% | 1408.0521 | 1408.6177 | 3 | 7.095 | 75.0% | 1 | K.VYNYNHLMPTR.Y | 2 |
| \* | LAPMis12DANCE\_011813\_01.09148.09148.2 | 1.9387 | 0.3704 | 99.5% | 1050.1522 | 1050.1968 | 5 | 6.298 | 56.2% | 1 | R.YSVDIPLDK.T | 2 |

---

|  |  |  |  |  |  |  |  |  |
| --- | --- | --- | --- | --- | --- | --- | --- | --- |
| U | *gi|10440560|ref|NP\_06* | 3 | 5 | 14.7% | 136 | 15404 | 11.1 | histone cluster 1, H3f [Homo sapiens] |
| U | *gi|88976633|ref|XP\_93* | 3 | 5 | 14.7% | 136 | 15226 | 11.0 | PREDICTED: hypothetical protein [Homo sapiens] |
| U | *gi|53793688|ref|NP\_00* | 3 | 5 | 14.7% | 136 | 15388 | 11.3 | histone cluster 2, H3a [Homo sapiens] |
| U | *gi|4885385|ref|NP\_005* | 3 | 5 | 14.7% | 136 | 15328 | 11.3 | H3 histone, family 3B [Homo sapiens] |
| U | *gi|4504299|ref|NP\_003* | 3 | 5 | 14.7% | 136 | 15508 | 11.1 | histone cluster 3, H3 [Homo sapiens] |
| U | *gi|4504297|ref|NP\_003* | 3 | 5 | 14.7% | 136 | 15404 | 11.1 | histone cluster 1, H3b [Homo sapiens] |
| U | *gi|4504295|ref|NP\_003* | 3 | 5 | 14.7% | 136 | 15404 | 11.1 | histone cluster 1, H3h [Homo sapiens] |
| U | *gi|4504293|ref|NP\_003* | 3 | 5 | 14.7% | 136 | 15404 | 11.1 | histone cluster 1, H3j [Homo sapiens] |
| U | *gi|4504291|ref|NP\_003* | 3 | 5 | 14.7% | 136 | 15404 | 11.1 | H3 histone family, member H [Homo sapiens] |
| U | *gi|4504289|ref|NP\_003* | 3 | 5 | 14.7% | 136 | 15404 | 11.1 | histone cluster 1, H3i [Homo sapiens] |
| U | *gi|4504287|ref|NP\_003* | 3 | 5 | 14.7% | 136 | 15404 | 11.1 | histone cluster 1, H3e [Homo sapiens] |
| U | *gi|4504285|ref|NP\_003* | 3 | 5 | 14.7% | 136 | 15404 | 11.1 | histone cluster 1, H3c [Homo sapiens] |
| U | *gi|4504281|ref|NP\_003* | 3 | 5 | 14.7% | 136 | 15404 | 11.1 | histone cluster 1, H3a [Homo sapiens] |
| U | *gi|4504279|ref|NP\_002* | 3 | 5 | 14.7% | 136 | 15328 | 11.3 | H3 histone, family 3A [Homo sapiens] |
| U | *gi|31742503|ref|NP\_06* | 3 | 5 | 14.7% | 136 | 15388 | 11.3 | histone cluster 2, H3c [Homo sapiens] |
| U | *gi|21071021|ref|NP\_00* | 3 | 5 | 14.7% | 136 | 15404 | 11.1 | histone cluster 1, H3d [Homo sapiens] |
| U | *gi|183076548|ref|NP\_0* | 3 | 5 | 14.7% | 136 | 15388 | 11.3 | histone cluster 2, H3d [Homo sapiens] |
| U | *gi|169167131|ref|XP\_0* | 3 | 5 | 14.7% | 136 | 15226 | 11.0 | PREDICTED: hypothetical protein [Homo sapiens] |
| U | *gi|113416274|ref|XP\_0* | 3 | 5 | 14.7% | 136 | 15226 | 11.0 | PREDICTED: hypothetical protein [Homo sapiens] |

| Filename XCorr DeltCN Conf% ObsM+H+ CalcM+H+ SpR ZScore Ion% # Sequence  | | | | | | | | | | | | |
| --- | --- | --- | --- | --- | --- | --- | --- | --- | --- | --- | --- | --- |
|  | LAPMis12DANCE2\_011813\_01.06720.06720.2 | 2.2814 | 0.2422 | 99.0% | 1034.0322 | 1033.2186 | 4 | 5.163 | 62.5% | 2 | R.YRPGTVALR.E | 2 |
|  | LAPMis12DANCE2\_011813\_01.04354.04354.1 | 1.8163 | 0.206 | 96.2% | 850.55 | 850.9469 | 75 | 4.519 | 58.3% | 1 | R.EIAQDFK.T | 1 |
|  | LAPMis12DANCE2\_011813\_01.05759.05759.2 | 2.8366 | 0.3309 | 100.0% | 1336.4521 | 1336.4875 | 10 | 5.55 | 65.0% | 2 | R.EIAQDFKTDLR.F | 2 |

---

|  |  |  |  |  |  |  |  |  |
| --- | --- | --- | --- | --- | --- | --- | --- | --- |
| U | *gi|164519146|ref|NP\_0* | 7 | 8 | 14.5% | 633 | 68063 | 9.9 | GATA zinc finger domain containing 2A [Homo sapiens] |

| Filename XCorr DeltCN Conf% ObsM+H+ CalcM+H+ SpR ZScore Ion% # Sequence  | | | | | | | | | | | | |
| --- | --- | --- | --- | --- | --- | --- | --- | --- | --- | --- | --- | --- |
| \* | LAPMis12DANCE\_011813\_01.04114.04114.2 | 2.8839 | 0.1952 | 99.0% | 1605.6721 | 1604.6677 | 1 | 3.949 | 61.5% | 1 | R.ALERDPTEDDVESK.K | 2 |
| \* | LAPMis12DANCE\_011813\_01.09246.09246.2 | 3.7492 | 0.2869 | 100.0% | 1479.0521 | 1478.6172 | 1 | 7.164 | 69.2% | 1 | R.GLLASDLNTDGDMR.V | 2 |
| \* | LAPMis12DANCE\_011813\_01.08015.08015.2 | 2.4285 | 0.3624 | 99.9% | 1494.2122 | 1493.7031 | 5 | 5.829 | 50.0% | 1 | R.VTPEPGAGPTQGLLR.A | 2 |
| \* | LAPMis12DANCE\_011813\_01.08205.08205.2 | 3.3 | 0.2398 | 100.0% | 1302.0922 | 1302.4448 | 1 | 5.366 | 70.8% | 2 | R.GEGLVGDGPVDMR.T | 2 |
|  | LAPMis12DANCE\_011813\_01.04625.04625.2 | 2.6555 | 0.139 | 96.5% | 1372.0322 | 1372.4777 | 9 | 4.636 | 60.0% | 1 | K.ALQQEQEIEQR.L | 2 |
| \* | LAPMis12DANCE\_011813\_01.03952.03952.2 | 2.7751 | 0.3078 | 100.0% | 1225.7122 | 1226.4185 | 1 | 6.003 | 72.7% | 1 | R.LLQQGTAPAQAK.A | 2 |
| \* | LAPMis12DANCE\_011813\_02.04534.04534.2 | 3.7709 | 0.5131 | 100.0% | 1318.1322 | 1318.4728 | 1 | 8.564 | 66.7% | 1 | K.LQNSASATALVSR.T | 2 |

---

|  |  |  |  |  |  |  |  |  |
| --- | --- | --- | --- | --- | --- | --- | --- | --- |
| U | *gi|148470397|ref|NP\_0* | 4 | 7 | 14.5% | 415 | 45672 | 5.6 | heterogeneous nuclear ribonucleoprotein F [Homo sapiens] |
| U | *gi|4826760|ref|NP\_004* | 4 | 7 | 14.5% | 415 | 45672 | 5.6 | heterogeneous nuclear ribonucleoprotein F [Homo sapiens] |
| U | *gi|148470406|ref|NP\_0* | 4 | 7 | 14.5% | 415 | 45672 | 5.6 | heterogeneous nuclear ribonucleoprotein F [Homo sapiens] |
| U | *gi|148470404|ref|NP\_0* | 4 | 7 | 14.5% | 415 | 45672 | 5.6 | heterogeneous nuclear ribonucleoprotein F [Homo sapiens] |
| U | *gi|148470402|ref|NP\_0* | 4 | 7 | 14.5% | 415 | 45672 | 5.6 | heterogeneous nuclear ribonucleoprotein F [Homo sapiens] |
| U | *gi|148470400|ref|NP\_0* | 4 | 7 | 14.5% | 415 | 45672 | 5.6 | heterogeneous nuclear ribonucleoprotein F [Homo sapiens] |

| Filename XCorr DeltCN Conf% ObsM+H+ CalcM+H+ SpR ZScore Ion% # Sequence  | | | | | | | | | | | | |
| --- | --- | --- | --- | --- | --- | --- | --- | --- | --- | --- | --- | --- |
|  | LAPMis12DANCE\_011813\_02.06084.06084.2 | 2.4944 | 0.2447 | 98.4% | 1710.0922 | 1710.7919 | 45 | 5.083 | 40.0% | 1 | R.QSGEAFVELGSEDDVK.M | 2 |
|  | LAPMis12DANCE\_011813\_02.08832.08832.2 | 4.0105 | 0.4085 | 100.0% | 1868.7122 | 1869.0813 | 1 | 7.727 | 59.4% | 2 | K.ITGEAFVQFASQELAEK.A | 2 |
|  | LAPMis12DANCE2\_011813\_01.11433.11433.2 | 4.4772 | 0.4136 | 100.0% | 1998.2722 | 1998.2023 | 1 | 6.912 | 56.2% | 2 | K.ATENDIYNFFSPLNPVR.V | 22 |
|  | LAPMis12DANCE\_011813\_01.05564.05564.2 | 2.774 | 0.4876 | 100.0% | 1093.1721 | 1093.2278 | 1 | 8.153 | 83.3% | 2 | R.VHIEIGPDGR.V | 22 |

Similarities:
gi|5031753|ref|NP\_005(2:2)  

---

|  |  |  |  |  |  |  |  |  |
| --- | --- | --- | --- | --- | --- | --- | --- | --- |
| U | *gi|4758304|ref|NP\_004* | 7 | 7 | 14.4% | 645 | 72933 | 5.1 | protein disulfide isomerase-associated 4 [Homo sapiens] |

| Filename XCorr DeltCN Conf% ObsM+H+ CalcM+H+ SpR ZScore Ion% # Sequence  | | | | | | | | | | | | |
| --- | --- | --- | --- | --- | --- | --- | --- | --- | --- | --- | --- | --- |
| \* | LAPMis12DANCE\_011813\_01.07251.07251.2 | 3.5145 | 0.3728 | 100.0% | 1192.1921 | 1191.3268 | 1 | 6.562 | 72.7% | 1 | K.IDATSASVLASR.F | 2 |
| \* | LAPMis12DANCE\_011813\_01.04858.04858.2 | 2.254 | 0.2227 | 97.1% | 1133.8722 | 1134.2285 | 6 | 5.704 | 60.0% | 1 | K.VDATAETDLAK.R | 2 |
| \* | LAPMis12DANCE\_011813\_01.04474.04474.2 | 2.318 | 0.2351 | 97.8% | 1289.6322 | 1290.416 | 8 | 4.579 | 59.1% | 1 | K.VDATAETDLAKR.F | 2 |
| \* | LAPMis12DANCE\_011813\_01.12585.12585.3 | 3.7065 | 0.3119 | 99.7% | 2484.1143 | 2482.8945 | 3 | 5.809 | 27.4% | 1 | K.YGIVDYMIEQSGPPSKEILTLK.Q | 3 |
| \* | LAPMis12DANCE\_011813\_01.10022.10022.3 | 4.4333 | 0.4912 | 100.0% | 2338.1343 | 2338.6558 | 1 | 8.588 | 31.2% | 1 | R.SHMMDVQGSTQDSAIKDFVLK.Y | 3 |
| \* | LAPMis12DANCE\_011813\_01.10887.10887.2 | 3.1905 | 0.3916 | 100.0% | 1687.5521 | 1687.8182 | 1 | 6.333 | 73.1% | 1 | K.FAMEPEEFDSDTLR.E | 2 |
| \* | LAPMis12DANCE\_011813\_01.04301.04301.2 | 2.722 | 0.3988 | 100.0% | 1291.8121 | 1292.363 | 2 | 5.887 | 72.7% | 1 | K.MDATANDVPSDR.Y | 2 |

---

|  |  |  |  |  |  |  |  |  |
| --- | --- | --- | --- | --- | --- | --- | --- | --- |
| U | *gi|116235460|ref|NP\_6* | 6 | 7 | 14.4% | 585 | 63861 | 9.0 | YTH domain family, member 3 [Homo sapiens] |

| Filename XCorr DeltCN Conf% ObsM+H+ CalcM+H+ SpR ZScore Ion% # Sequence  | | | | | | | | | | | | |
| --- | --- | --- | --- | --- | --- | --- | --- | --- | --- | --- | --- | --- |
| \* | LAPMis12DANCE2\_011813\_01.05758.05758.2 | 4.2317 | 0.3843 | 100.0% | 1595.0721 | 1595.7062 | 1 | 7.225 | 66.7% | 2 | R.AITDGQAGFGNDTLSK.V | 2 |
| \* | LAPMis12DANCE\_011813\_01.11130.11130.2 | 3.5768 | 0.4647 | 100.0% | 1518.5322 | 1517.7809 | 1 | 7.78 | 60.7% | 1 | K.VPGISSIEQGMTGLK.I | 2 |
| \* | LAPMis12DANCE\_011813\_01.08200.08200.2 | 3.545 | 0.441 | 100.0% | 1460.3722 | 1460.7166 | 1 | 7.195 | 53.3% | 1 | K.GNVGIGGSAVPPPPIK.H | 2 |
|  | LAPMis12DANCE\_011813\_01.03188.03188.2 | 4.4057 | 0.4603 | 100.0% | 1501.0322 | 1501.5962 | 1 | 7.076 | 79.2% | 1 | R.LENNDNKPVTNSR.D | 2 |
|  | LAPMis12DANCE\_011813\_01.10354.10354.3 | 2.7456 | 0.2506 | 96.5% | 1712.4243 | 1711.8279 | 90 | 5.392 | 38.5% | 1 | K.HTTSIFDDFAHYEK.R | 3 |
| \* | LAPMis12DANCE\_011813\_01.03203.03203.2 | 2.9322 | 0.2014 | 99.4% | 1334.2122 | 1334.4497 | 29 | 5.075 | 61.1% | 1 | K.RQEEEEAMRR.E | 2 |

---

|  |  |  |  |  |  |  |  |  |
| --- | --- | --- | --- | --- | --- | --- | --- | --- |
| U | *contaminant\_INT-STD1* | 9 | 15 | 14.2% | 607 | 69271 | 6.1 | BSA |

| Filename XCorr DeltCN Conf% ObsM+H+ CalcM+H+ SpR ZScore Ion% # Sequence  | | | | | | | | | | | | |
| --- | --- | --- | --- | --- | --- | --- | --- | --- | --- | --- | --- | --- |
| \* | LAPMis12DANCE\_011813\_01.09999.09999.2 | 2.7567 | 0.3515 | 100.0% | 1164.1522 | 1164.344 | 1 | 6.324 | 83.3% | 1 | K.LVNELTEFAK.T | 2 |
| \* | LAPMis12DANCE\_011813\_02.09208.09208.2 | 3.4959 | 0.4499 | 100.0% | 1568.2722 | 1568.7258 | 1 | 7.052 | 70.8% | 1 | K.DAFLGSFLYEYSR.R | 2 |
| \* | LAPMis12DANCE\_011813\_01.08466.08466.3 | 3.9726 | 0.2595 | 99.5% | 1440.4143 | 1440.6884 | 1 | 4.681 | 56.8% | 1 | R.RHPEYAVSVLLR.L | 3 |
| \* | LAPMis12DANCE\_011813\_01.08434.08434.2 | 2.7309 | 0.3207 | 100.0% | 1441.2122 | 1440.6884 | 9 | 5.344 | 54.5% | 2 | R.RHPEYAVSVLLR.L | 2 |
| \* | LAPMis12DANCE\_011813\_01.07437.07437.2 | 2.6848 | 0.3924 | 100.0% | 1306.1122 | 1306.5046 | 1 | 6.793 | 65.0% | 2 | K.HLVDEPQNLIK.Q | 2 |
| \* | LAPMis12DANCE\_011813\_02.07384.07384.2 | 3.8628 | 0.3715 | 100.0% | 1479.6522 | 1480.7068 | 1 | 8.169 | 83.3% | 3 | K.LGEYGFQNALIVR.Y | 2 |
|  | LAPMis12DANCE\_011813\_01.08204.08204.2 | 3.6144 | 0.4822 | 100.0% | 1641.2722 | 1640.9205 | 1 | 7.516 | 50.0% | 3 | R.KVPQVSTPTLVEVSR.S | 2 |
|  | LAPMis12DANCE\_011813\_01.09208.09208.2 | 2.4917 | 0.18 | 95.4% | 1513.7122 | 1512.7465 | 1 | 4.752 | 57.7% | 1 | K.VPQVSTPTLVEVSR.S | 2 |
| \* | LAPMis12DANCE\_011813\_01.12429.12429.2 | 3.779 | 0.5107 | 100.0% | 1400.5922 | 1400.6324 | 1 | 8.444 | 68.2% | 1 | K.TVMENFVAFVDK.C | 2 |

---

|  |  |  |  |  |  |  |  |  |
| --- | --- | --- | --- | --- | --- | --- | --- | --- |
| U | *gi|169215551|ref|XP\_0* | 2 | 2 | 14.2% | 211 | 24218 | 7.4 | PREDICTED: similar to high-mobility group (nonhistone chromosomal) protein 1-like 10 [Homo sapiens] |
| U | *gi|169215956|ref|XP\_0* | 2 | 2 | 14.2% | 211 | 24188 | 7.4 | PREDICTED: similar to high-mobility group (nonhistone chromosomal) protein 1-like 10 isoform 1 [Homo sapiens] |
| U | *gi|169215954|ref|XP\_0* | 2 | 2 | 14.2% | 211 | 24188 | 7.4 | PREDICTED: similar to high-mobility group (nonhistone chromosomal) protein 1-like 10 isoform 2 [Homo sapiens] |
| U | *gi|169215763|ref|XP\_0* | 2 | 2 | 14.2% | 211 | 24188 | 7.4 | PREDICTED: similar to high-mobility group (nonhistone chromosomal) protein 1-like 10 isoform 1 [Homo sapiens] |
| U | *gi|169215761|ref|XP\_0* | 2 | 2 | 14.2% | 211 | 24188 | 7.4 | PREDICTED: similar to high-mobility group (nonhistone chromosomal) protein 1-like 10 isoform 2 [Homo sapiens] |

| Filename XCorr DeltCN Conf% ObsM+H+ CalcM+H+ SpR ZScore Ion% # Sequence  | | | | | | | | | | | | |
| --- | --- | --- | --- | --- | --- | --- | --- | --- | --- | --- | --- | --- |
|  | LAPMis12DANCE2\_011813\_01.04313.04313.2 | 2.0937 | 0.2462 | 96.6% | 1329.3322 | 1329.5881 | 3 | 4.622 | 65.0% | 1 | K.KKFKDPNAPKR.T | 2 |
|  | LAPMis12DANCE\_011813\_01.07132.07132.3 | 3.6019 | 0.2483 | 98.0% | 2239.5842 | 2239.4644 | 2 | 5.142 | 36.1% | 1 | K.KLGEMWNNTAADDKQPYEK.K | 3 |

---

|  |  |  |  |  |  |  |  |  |
| --- | --- | --- | --- | --- | --- | --- | --- | --- |
| U | *gi|169163422|ref|XP\_0* | 1 | 3 | 14.0% | 114 | 11577 | 4.7 | PREDICTED: similar to 60S acidic ribosomal protein P1 [Homo sapiens] |
| U | *gi|4506669|ref|NP\_000* | 1 | 3 | 14.0% | 114 | 11514 | 4.3 | ribosomal protein P1 isoform 1 [Homo sapiens] |
| U | *gi|169164724|ref|XP\_0* | 1 | 3 | 14.0% | 114 | 11577 | 4.7 | PREDICTED: similar to 60S acidic ribosomal protein P1 [Homo sapiens] |
| U | *gi|169164389|ref|XP\_0* | 1 | 3 | 14.0% | 114 | 11577 | 4.7 | PREDICTED: similar to 60S acidic ribosomal protein P1 [Homo sapiens] |

| Filename XCorr DeltCN Conf% ObsM+H+ CalcM+H+ SpR ZScore Ion% # Sequence  | | | | | | | | | | | | |
| --- | --- | --- | --- | --- | --- | --- | --- | --- | --- | --- | --- | --- |
|  | LAPMis12DANCE\_011813\_01.13137.13137.2 | 3.0693 | 0.3545 | 100.0% | 1704.5922 | 1703.9811 | 1 | 6.274 | 53.3% | 3 | K.AAGVNVEPFWPGLFAK.A | 2 |

---

|  |  |  |  |  |  |  |  |  |
| --- | --- | --- | --- | --- | --- | --- | --- | --- |
| U | *gi|126722969|ref|NP\_0* | 5 | 6 | 13.9% | 561 | 60423 | 6.6 | centromere protein T [Homo sapiens] |

| Filename XCorr DeltCN Conf% ObsM+H+ CalcM+H+ SpR ZScore Ion% # Sequence  | | | | | | | | | | | | |
| --- | --- | --- | --- | --- | --- | --- | --- | --- | --- | --- | --- | --- |
| \* | LAPMis12DANCE\_011813\_01.04074.04074.2 | 2.2195 | 0.2448 | 97.9% | 1321.0922 | 1321.3896 | 14 | 4.434 | 60.0% | 1 | R.VLDTADPRT#PR.R | 2 |
| \* | LAPMis12DANCE\_011813\_02.03488.03488.3 | 3.5079 | 0.3892 | 100.0% | 1762.0743 | 1761.8918 | 1 | 5.988 | 48.3% | 2 | R.SAHIQASGHLEEQTPR.T | 3 |
| \* | LAPMis12DANCE\_011813\_01.11050.11050.3 | 2.8164 | 0.3042 | 98.1% | 2394.6543 | 2395.767 | 3 | 4.852 | 27.4% | 1 | R.SLNLTFATPLQPQSVQRPGLAR.R | 3 |
| \* | LAPMis12DANCE\_011813\_01.10907.10907.2 | 3.0157 | 0.3851 | 100.0% | 1445.4521 | 1444.7295 | 1 | 6.202 | 68.2% | 1 | K.TVKPEDLELLMR.R | 2 |
| \* | LAPMis12DANCE\_011813\_02.06258.06258.3 | 3.9253 | 0.3132 | 99.7% | 1949.7843 | 1950.2485 | 1 | 5.664 | 43.8% | 1 | R.RQGLVTDQVSLHVLVER.H | 3 |

---

|  |  |  |  |  |  |  |  |  |
| --- | --- | --- | --- | --- | --- | --- | --- | --- |
| U | *gi|12025678|ref|NP\_00* | 8 | 8 | 13.7% | 911 | 104854 | 5.4 | actinin, alpha 4 [Homo sapiens] |

| Filename XCorr DeltCN Conf% ObsM+H+ CalcM+H+ SpR ZScore Ion% # Sequence  | | | | | | | | | | | | |
| --- | --- | --- | --- | --- | --- | --- | --- | --- | --- | --- | --- | --- |
| \* | LAPMis12DANCE\_011813\_01.09366.09366.2 | 3.9803 | 0.4236 | 100.0% | 1920.1122 | 1921.1577 | 2 | 7.936 | 44.1% | 1 | K.LSGSNPYTTVTPQIINSK.W | 2 |
| \* | LAPMis12DANCE\_011813\_01.03522.03522.2 | 2.8422 | 0.2792 | 100.0% | 1325.9521 | 1326.452 | 12 | 5.972 | 60.0% | 1 | K.RDHALLEEQSK.Q | 2 |
| \* | LAPMis12DANCE\_011813\_01.10805.10805.2 | 2.599 | 0.2703 | 99.2% | 1774.1522 | 1775.0171 | 48 | 4.442 | 36.7% | 1 | R.QFASQANVVGPWIQTK.M | 2 |
| \* | LAPMis12DANCE2\_011813\_01.11661.11661.3 | 5.0379 | 0.518 | 100.0% | 3325.5842 | 3326.7742 | 1 | 7.552 | 28.7% | 1 | R.SIVDYKPNLDLLEQQHQLIQEALIFDNK.H | 3 |
|  | LAPMis12DANCE\_011813\_01.13491.13491.2 | 3.9996 | 0.3793 | 100.0% | 1387.1322 | 1387.6218 | 1 | 7.917 | 72.7% | 1 | R.VGWEQLLTTIAR.T | 2 |
|  | LAPMis12DANCE\_011813\_01.09537.09537.2 | 2.9722 | 0.2517 | 99.9% | 1430.4321 | 1430.6011 | 7 | 4.954 | 63.6% | 1 | R.TINEVENQILTR.D | 2 |
| \* | LAPMis12DANCE\_011813\_01.07475.07475.2 | 2.3295 | 0.2685 | 99.1% | 1353.3722 | 1353.4926 | 1 | 5.506 | 60.0% | 1 | K.GISQEQMQEFR.A | 2 |
| \* | LAPMis12DANCE\_011813\_01.09710.09710.2 | 3.3729 | 0.3392 | 100.0% | 1794.1122 | 1794.032 | 4 | 6.195 | 50.0% | 1 | R.MAPYQGPDAVPGALDYK.S | 2 |

---

|  |  |  |  |  |  |  |  |  |
| --- | --- | --- | --- | --- | --- | --- | --- | --- |
| U | *gi|4504523|ref|NP\_002* | 1 | 2 | 13.7% | 102 | 10932 | 8.9 | heat shock 10kDa protein 1 [Homo sapiens] |

| Filename XCorr DeltCN Conf% ObsM+H+ CalcM+H+ SpR ZScore Ion% # Sequence  | | | | | | | | | | | | |
| --- | --- | --- | --- | --- | --- | --- | --- | --- | --- | --- | --- | --- |
| \* | LAPMis12DANCE\_011813\_02.04942.04942.2 | 3.9418 | 0.4845 | 100.0% | 1316.4521 | 1316.5406 | 1 | 9.74 | 76.9% | 2 | K.VLQATVVAVGSGSK.G | 2 |

---

|  |  |  |  |  |  |  |  |  |
| --- | --- | --- | --- | --- | --- | --- | --- | --- |
| U | *gi|4506723|ref|NP\_000* | 3 | 3 | 13.6% | 264 | 29945 | 9.7 | ribosomal protein S3a [Homo sapiens] |

| Filename XCorr DeltCN Conf% ObsM+H+ CalcM+H+ SpR ZScore Ion% # Sequence  | | | | | | | | | | | | |
| --- | --- | --- | --- | --- | --- | --- | --- | --- | --- | --- | --- | --- |
|  | LAPMis12DANCE\_011813\_01.03230.03230.2 | 3.0567 | 0.4302 | 100.0% | 1218.0521 | 1218.3146 | 1 | 6.731 | 77.8% | 1 | K.TSYAQHQQVR.Q | 2 |
|  | LAPMis12DANCE\_011813\_01.05652.05652.2 | 3.4119 | 0.3497 | 100.0% | 1516.5322 | 1516.6915 | 1 | 5.042 | 62.5% | 1 | R.EVQTNDLKEVVNK.L | 2 |
| \* | LAPMis12DANCE\_011813\_01.04205.04205.2 | 3.3968 | 0.4331 | 100.0% | 1332.1322 | 1332.4712 | 1 | 6.24 | 70.8% | 1 | K.LMELHGEGSSSGK.A | 2 |

---

|  |  |  |  |  |  |  |  |  |
| --- | --- | --- | --- | --- | --- | --- | --- | --- |
| U | *gi|60302883|ref|NP\_00* | 1 | 1 | 13.6% | 88 | 10061 | 11.3 | hypothetical protein LOC387103 [Homo sapiens] |

| Filename XCorr DeltCN Conf% ObsM+H+ CalcM+H+ SpR ZScore Ion% # Sequence  | | | | | | | | | | | | |
| --- | --- | --- | --- | --- | --- | --- | --- | --- | --- | --- | --- | --- |
| \* | LAPMis12DANCE\_011813\_01.03822.03822.2 | 2.4106 | 0.2711 | 99.2% | 1293.4521 | 1293.5521 | 7 | 5.022 | 59.1% | 1 | R.VINKEHVLAAAK.V | 2 |

---

|  |  |  |  |  |  |  |  |  |
| --- | --- | --- | --- | --- | --- | --- | --- | --- |
| U | *gi|169211725|ref|XP\_9* | 1 | 3 | 13.6% | 88 | 9949 | 11.3 | PREDICTED: similar to 40S ribosomal protein S28 [Homo sapiens] |
| U | *gi|4506715|ref|NP\_001* | 1 | 3 | 17.4% | 69 | 7841 | 10.7 | ribosomal protein S28 [Homo sapiens] |
| U | *gi|169212081|ref|XP\_0* | 1 | 3 | 13.2% | 91 | 10272 | 11.2 | PREDICTED: similar to 40S ribosomal protein S28 [Homo sapiens] |

| Filename XCorr DeltCN Conf% ObsM+H+ CalcM+H+ SpR ZScore Ion% # Sequence  | | | | | | | | | | | | |
| --- | --- | --- | --- | --- | --- | --- | --- | --- | --- | --- | --- | --- |
|  | LAPMis12DANCE2\_011813\_01.09671.09671.2 | 3.0086 | 0.3959 | 100.0% | 1361.3522 | 1361.4918 | 5 | 6.79 | 63.6% | 3 | R.EGDVLTLLESER.E | 2 |

---

|  |  |  |  |  |  |  |  |  |
| --- | --- | --- | --- | --- | --- | --- | --- | --- |
| U | *gi|4557032|ref|NP\_002* | 2 | 2 | 13.5% | 334 | 36639 | 6.1 | L-lactate dehydrogenase B [Homo sapiens] |

| Filename XCorr DeltCN Conf% ObsM+H+ CalcM+H+ SpR ZScore Ion% # Sequence  | | | | | | | | | | | | |
| --- | --- | --- | --- | --- | --- | --- | --- | --- | --- | --- | --- | --- |
| \* | LAPMis12DANCE2\_011813\_01.14968.14968.3 | 6.5148 | 0.5559 | 100.0% | 3785.6343 | 3786.3906 | 1 | 8.797 | 30.3% | 1 | K.SLADELALVDVLEDKLKGEMMDLQHGSLFLQTPK.I | 3 |
| \* | LAPMis12DANCE\_011813\_01.03936.03936.2 | 2.4486 | 0.2128 | 98.3% | 1287.9722 | 1287.4991 | 6 | 3.98 | 65.0% | 1 | K.LKDDEVAQLKK.S | 2 |

---

|  |  |  |  |  |  |  |  |  |
| --- | --- | --- | --- | --- | --- | --- | --- | --- |
| U | *gi|163965362|ref|NP\_0* | 2 | 2 | 13.5% | 215 | 23384 | 4.6 | nascent polypeptide-associated complex alpha subunit isoform b [Homo sapiens] |
| U | *gi|5031931|ref|NP\_005* | 2 | 2 | 13.5% | 215 | 23384 | 4.6 | nascent polypeptide-associated complex alpha subunit isoform b [Homo sapiens] |
| U | *gi|163965366|ref|NP\_0* | 2 | 2 | 1.4% | 2078 | 205419 | 9.6 | nascent polypeptide-associated complex alpha subunit isoform a [Homo sapiens] |
| U | *gi|163965364|ref|NP\_0* | 2 | 2 | 13.5% | 215 | 23384 | 4.6 | nascent polypeptide-associated complex alpha subunit isoform b [Homo sapiens] |

| Filename XCorr DeltCN Conf% ObsM+H+ CalcM+H+ SpR ZScore Ion% # Sequence  | | | | | | | | | | | | |
| --- | --- | --- | --- | --- | --- | --- | --- | --- | --- | --- | --- | --- |
|  | LAPMis12DANCE\_011813\_01.10068.10068.2 | 3.3072 | 0.4702 | 100.0% | 1485.1921 | 1485.6335 | 1 | 7.981 | 69.2% | 1 | K.SPASDTYIVFGEAK.I | 23 |
|  | LAPMis12DANCE\_011813\_02.05318.05318.2 | 3.9064 | 0.2162 | 100.0% | 1614.2322 | 1615.7808 | 1 | 8.704 | 82.1% | 1 | K.IEDLSQQAQLAAAEK.F | 2 |

---

|  |  |  |  |  |  |  |  |  |
| --- | --- | --- | --- | --- | --- | --- | --- | --- |
| U | *gi|5031753|ref|NP\_005* | 4 | 7 | 13.4% | 449 | 49229 | 6.3 | heterogeneous nuclear ribonucleoprotein H1 [Homo sapiens] |

| Filename XCorr DeltCN Conf% ObsM+H+ CalcM+H+ SpR ZScore Ion% # Sequence  | | | | | | | | | | | | |
| --- | --- | --- | --- | --- | --- | --- | --- | --- | --- | --- | --- | --- |
|  | LAPMis12DANCE\_011813\_01.04540.04540.2 | 2.6301 | 0.1883 | 96.7% | 1686.4321 | 1685.7501 | 1 | 4.037 | 53.3% | 1 | K.HTGPNSPDTANDGFVR.L | 2 |
|  | LAPMis12DANCE\_011813\_02.07836.07836.2 | 4.0943 | 0.3857 | 100.0% | 1843.3522 | 1843.0001 | 1 | 7.32 | 65.6% | 2 | R.STGEAFVQFASQEIAEK.A | 2 |
|  | LAPMis12DANCE2\_011813\_01.11433.11433.2 | 4.4772 | 0.4136 | 100.0% | 1998.2722 | 1998.2023 | 1 | 6.912 | 56.2% | 2 | R.ATENDIYNFFSPLNPVR.V | 22 |
|  | LAPMis12DANCE\_011813\_01.05564.05564.2 | 2.774 | 0.4876 | 100.0% | 1093.1721 | 1093.2278 | 1 | 8.153 | 83.3% | 2 | R.VHIEIGPDGR.V | 22 |

Similarities:
gi|148470397|ref|NP\_0(2:2)  

---

|  |  |  |  |  |  |  |  |  |
| --- | --- | --- | --- | --- | --- | --- | --- | --- |
| U | *gi|4758158|ref|NP\_004* | 3 | 3 | 13.3% | 361 | 41487 | 6.6 | septin 2 [Homo sapiens] |
| U | *gi|56549640|ref|NP\_00* | 3 | 3 | 13.3% | 361 | 41487 | 6.6 | septin 2 [Homo sapiens] |
| U | *gi|56549638|ref|NP\_00* | 3 | 3 | 13.3% | 361 | 41487 | 6.6 | septin 2 [Homo sapiens] |
| U | *gi|56549636|ref|NP\_00* | 3 | 3 | 13.3% | 361 | 41487 | 6.6 | septin 2 [Homo sapiens] |

| Filename XCorr DeltCN Conf% ObsM+H+ CalcM+H+ SpR ZScore Ion% # Sequence  | | | | | | | | | | | | |
| --- | --- | --- | --- | --- | --- | --- | --- | --- | --- | --- | --- | --- |
|  | LAPMis12DANCE\_011813\_02.05690.05690.2 | 3.8566 | 0.414 | 100.0% | 1604.1522 | 1604.7545 | 1 | 6.61 | 73.1% | 1 | R.TVQIEASTVEIEER.G | 2 |
|  | LAPMis12DANCE\_011813\_01.07302.07302.2 | 3.3871 | 0.2638 | 100.0% | 1354.0922 | 1353.515 | 1 | 5.027 | 75.0% | 1 | R.ILDEIEEHNIK.I | 2 |
|  | LAPMis12DANCE\_011813\_02.05349.05349.3 | 4.0916 | 0.3798 | 100.0% | 2384.9043 | 2385.6675 | 1 | 5.898 | 37.5% | 1 | R.MQAQMQMQMQGGDGDGGALGHHV.- | 3 |

---

|  |  |  |  |  |  |  |  |  |
| --- | --- | --- | --- | --- | --- | --- | --- | --- |
| U | *gi|4506609|ref|NP\_000* | 2 | 2 | 13.3% | 196 | 23466 | 11.5 | ribosomal protein L19 [Homo sapiens] |

| Filename XCorr DeltCN Conf% ObsM+H+ CalcM+H+ SpR ZScore Ion% # Sequence  | | | | | | | | | | | | |
| --- | --- | --- | --- | --- | --- | --- | --- | --- | --- | --- | --- | --- |
| \* | LAPMis12DANCE\_011813\_01.09796.09796.2 | 4.3165 | 0.5854 | 100.0% | 1943.3322 | 1944.0679 | 1 | 10.062 | 56.2% | 1 | K.VWLDPNETNEIANANSR.Q | 2 |
| \* | LAPMis12DANCE\_011813\_01.04937.04937.1 | 1.9636 | 0.3048 | 100.0% | 1191.56 | 1192.421 | 8 | 5.475 | 56.2% | 1 | R.HMYHSLYLK.V | 1 |

---

|  |  |  |  |  |  |  |  |  |
| --- | --- | --- | --- | --- | --- | --- | --- | --- |
| U | *gi|5174457|ref|NP\_006* | 6 | 8 | 13.2% | 642 | 73913 | 5.6 | kinetochore associated 2 [Homo sapiens] |

| Filename XCorr DeltCN Conf% ObsM+H+ CalcM+H+ SpR ZScore Ion% # Sequence  | | | | | | | | | | | | |
| --- | --- | --- | --- | --- | --- | --- | --- | --- | --- | --- | --- | --- |
| \* | LAPMis12DANCE2\_011813\_01.09951.09951.3 | 3.3997 | 0.272 | 98.7% | 2080.6443 | 2081.4167 | 1 | 5.262 | 44.1% | 1 | K.LKDLFNVDAFKLESLEAK.N | 3 |
| \* | LAPMis12DANCE\_011813\_02.06153.06153.3 | 3.1464 | 0.3844 | 99.6% | 2099.0645 | 2099.3228 | 1 | 6.917 | 36.8% | 1 | K.YQAYMSNLESHSAILDQK.L | 3 |
| \* | LAPMis12DANCE\_011813\_01.06346.06346.2 | 2.8109 | 0.3125 | 99.9% | 1619.2722 | 1617.7991 | 1 | 5.259 | 65.4% | 1 | R.GKEAIETQLAEYHK.L | 2 |
| \* | LAPMis12DANCE\_011813\_02.05448.05448.2 | 2.6129 | 0.2111 | 98.6% | 1597.2122 | 1596.7344 | 1 | 5.024 | 58.3% | 1 | R.EYQLVVQTTTEER.R | 2 |
| \* | LAPMis12DANCE\_011813\_02.05823.05823.2 | 3.7333 | 0.398 | 100.0% | 1513.2122 | 1513.7925 | 1 | 6.601 | 76.9% | 3 | R.LLEMVATHVGSVEK.H | 2 |
| \* | LAPMis12DANCE\_011813\_01.03530.03530.2 | 2.2708 | 0.2074 | 98.4% | 967.97217 | 968.0977 | 10 | 5.488 | 57.1% | 1 | K.HLEEQIAK.V | 2 |

---

|  |  |  |  |  |  |  |  |  |
| --- | --- | --- | --- | --- | --- | --- | --- | --- |
| U | *gi|224028244|ref|NP\_0* | 5 | 7 | 13.2% | 471 | 54232 | 8.9 | non-POU domain containing, octamer-binding isoform 1 [Homo sapiens] |
| U | *gi|34932414|ref|NP\_03* | 5 | 7 | 13.2% | 471 | 54232 | 8.9 | non-POU domain containing, octamer-binding isoform 1 [Homo sapiens] |
| U | *gi|224028248|ref|NP\_0* | 5 | 7 | 16.2% | 382 | 43866 | 8.6 | non-POU domain containing, octamer-binding isoform 2 [Homo sapiens] |
| U | *gi|224028246|ref|NP\_0* | 5 | 7 | 13.2% | 471 | 54232 | 8.9 | non-POU domain containing, octamer-binding isoform 1 [Homo sapiens] |

| Filename XCorr DeltCN Conf% ObsM+H+ CalcM+H+ SpR ZScore Ion% # Sequence  | | | | | | | | | | | | |
| --- | --- | --- | --- | --- | --- | --- | --- | --- | --- | --- | --- | --- |
|  | LAPMis12DANCE\_011813\_01.05452.05452.2 | 2.5114 | 0.1694 | 96.2% | 1232.0922 | 1232.4252 | 3 | 5.14 | 63.6% | 1 | K.GIVEFSGKPAAR.K | 2 |
|  | LAPMis12DANCE\_011813\_01.10287.10287.2 | 2.9373 | 0.2659 | 99.7% | 1697.8322 | 1696.8744 | 117 | 4.935 | 38.5% | 2 | R.FAQPGSFEYEYAMR.W | 2 |
|  | LAPMis12DANCE\_011813\_01.04118.04118.2 | 2.4728 | 0.2917 | 99.9% | 1182.2322 | 1181.4161 | 1 | 5.325 | 68.8% | 1 | R.HEHQVMLMR.Q | 2 |
|  | LAPMis12DANCE\_011813\_02.05630.05630.2 | 3.9882 | 0.5262 | 100.0% | 1538.5122 | 1539.8441 | 1 | 8.602 | 67.9% | 2 | R.MGQMAMGGAMGINNR.G | 2 |
|  | LAPMis12DANCE\_011813\_01.04182.04182.2 | 2.188 | 0.309 | 99.1% | 1229.2322 | 1229.3811 | 8 | 5.317 | 59.1% | 1 | R.AAPGAEFAPNKR.R | 2 |

---

|  |  |  |  |  |  |  |  |  |
| --- | --- | --- | --- | --- | --- | --- | --- | --- |
| U | *gi|4504511|ref|NP\_001* | 3 | 3 | 13.1% | 397 | 44868 | 7.1 | DnaJ (Hsp40) homolog, subfamily A, member 1 [Homo sapiens] |

| Filename XCorr DeltCN Conf% ObsM+H+ CalcM+H+ SpR ZScore Ion% # Sequence  | | | | | | | | | | | | |
| --- | --- | --- | --- | --- | --- | --- | --- | --- | --- | --- | --- | --- |
| \* | LAPMis12DANCE\_011813\_01.14864.14864.2 | 2.0391 | 0.353 | 99.1% | 1736.1921 | 1736.9658 | 122 | 5.16 | 32.1% | 1 | K.QISQAYEVLSDAKKR.E | 2 |
| \* | LAPMis12DANCE2\_011813\_01.15089.15089.2 | 2.9162 | 0.3688 | 100.0% | 2324.9321 | 2324.547 | 4 | 5.289 | 28.3% | 1 | K.EGGAGGGFGSPMDIFDMFFGGGGR.M | 2 |
| \* | LAPMis12DANCE\_011813\_01.06862.06862.2 | 2.3607 | 0.2593 | 98.7% | 1392.4122 | 1393.6696 | 2 | 4.805 | 58.3% | 1 | R.TIVITSHPGQIVK.H | 2 |

---

|  |  |  |  |  |  |  |  |  |
| --- | --- | --- | --- | --- | --- | --- | --- | --- |
| U | *gi|15011936|ref|NP\_00* | 1 | 1 | 13.0% | 115 | 13015 | 11.0 | ribosomal protein S26 [Homo sapiens] |
| U | *gi|89039015|ref|XP\_94* | 1 | 1 | 13.0% | 115 | 13063 | 11.0 | PREDICTED: hypothetical protein isoform 1 [Homo sapiens] |
| U | *gi|89038315|ref|XP\_93* | 1 | 1 | 13.0% | 115 | 13063 | 11.0 | PREDICTED: hypothetical protein isoform 1 [Homo sapiens] |
| U | *gi|89027474|ref|XP\_94* | 1 | 1 | 13.0% | 115 | 13029 | 11.0 | PREDICTED: similar to ribosomal protein S26 isoform 2 [Homo sapiens] |
| U | *gi|89026311|ref|XP\_94* | 1 | 1 | 13.0% | 115 | 13029 | 11.0 | PREDICTED: similar to ribosomal protein S26 isoform 2 [Homo sapiens] |
| U | *gi|89025350|ref|XP\_94* | 1 | 1 | 13.0% | 115 | 13029 | 11.0 | PREDICTED: similar to ribosomal protein S26 isoform 2 [Homo sapiens] |
| U | *gi|169208906|ref|XP\_0* | 1 | 1 | 13.0% | 115 | 13063 | 11.0 | PREDICTED: similar to ribosomal protein S26 [Homo sapiens] |
| U | *gi|169171442|ref|XP\_0* | 1 | 1 | 13.0% | 115 | 13029 | 11.0 | PREDICTED: similar to ribosomal protein S26 [Homo sapiens] |
| U | *gi|169167044|ref|XP\_0* | 1 | 1 | 13.0% | 115 | 13015 | 11.0 | PREDICTED: similar to ribosomal protein S26 [Homo sapiens] |
| U | *gi|169166765|ref|XP\_0* | 1 | 1 | 13.0% | 115 | 13015 | 11.0 | PREDICTED: similar to ribosomal protein S26 [Homo sapiens] |
| U | *gi|169166210|ref|XP\_0* | 1 | 1 | 13.0% | 115 | 13015 | 11.0 | PREDICTED: hypothetical protein LOC728937 [Homo sapiens] |

| Filename XCorr DeltCN Conf% ObsM+H+ CalcM+H+ SpR ZScore Ion% # Sequence  | | | | | | | | | | | | |
| --- | --- | --- | --- | --- | --- | --- | --- | --- | --- | --- | --- | --- |
|  | LAPMis12DANCE\_011813\_01.04530.04530.2 | 2.7853 | 0.32 | 99.9% | 1590.1921 | 1590.9298 | 2 | 5.166 | 60.7% | 1 | R.FRPAGAAPRPPPKPM.- | 2 |

---

|  |  |  |  |  |  |  |  |  |
| --- | --- | --- | --- | --- | --- | --- | --- | --- |
| U | *gi|16753227|ref|NP\_00* | 3 | 4 | 12.8% | 288 | 32728 | 10.6 | ribosomal protein L6 [Homo sapiens] |
| U | *gi|67189747|ref|NP\_00* | 3 | 4 | 12.8% | 288 | 32728 | 10.6 | ribosomal protein L6 [Homo sapiens] |

| Filename XCorr DeltCN Conf% ObsM+H+ CalcM+H+ SpR ZScore Ion% # Sequence  | | | | | | | | | | | | |
| --- | --- | --- | --- | --- | --- | --- | --- | --- | --- | --- | --- | --- |
|  | LAPMis12DANCE\_011813\_01.04180.04180.2 | 2.557 | 0.3616 | 100.0% | 1285.4122 | 1285.5266 | 36 | 5.932 | 50.0% | 2 | K.VLATVTKPVGGDK.N | 2 |
|  | LAPMis12DANCE\_011813\_01.05044.05044.1 | 1.5727 | 0.3113 | 100.0% | 1332.75 | 1333.3971 | 11 | 5.959 | 50.0% | 1 | R.HQEGEIFDTEK.E | 1 |
|  | LAPMis12DANCE\_011813\_01.10245.10245.2 | 2.934 | 0.3276 | 100.0% | 1447.2922 | 1447.6769 | 4 | 6.695 | 50.0% | 1 | R.SVFALTNGIYPHK.L | 2 |

---

|  |  |  |  |  |  |  |  |  |
| --- | --- | --- | --- | --- | --- | --- | --- | --- |
| U | *Reverse\_gi|5729997|re* | 1 | 1 | 12.8% | 218 | 24608 | 5.5 | RAB27B, member RAS oncogene family [Homo sapiens] |

| Filename XCorr DeltCN Conf% ObsM+H+ CalcM+H+ SpR ZScore Ion% # Sequence  | | | | | | | | | | | | |
| --- | --- | --- | --- | --- | --- | --- | --- | --- | --- | --- | --- | --- |
| \* | LAPMis12DANCE2\_011813\_01.16626.16626.3 | 3.1253 | 0.3255 | 99.2% | 3616.8542 | 3616.958 | 126 | 4.578 | 20.4% | 1 | R.K@ERFDIGVTT#IFKPNFKNDTYRYLFTT#K.G | 3 |

---

|  |  |  |  |  |  |  |  |  |
| --- | --- | --- | --- | --- | --- | --- | --- | --- |
| U | *gi|30795231|ref|NP\_00* | 1 | 1 | 12.8% | 227 | 22693 | 4.6 | brain abundant, membrane attached signal protein 1 [Homo sapiens] |

| Filename XCorr DeltCN Conf% ObsM+H+ CalcM+H+ SpR ZScore Ion% # Sequence  | | | | | | | | | | | | |
| --- | --- | --- | --- | --- | --- | --- | --- | --- | --- | --- | --- | --- |
| \* | LAPMis12DANCE\_011813\_02.04526.04526.3 | 5.1547 | 0.4601 | 100.0% | 2893.6443 | 2894.1216 | 1 | 7.45 | 26.8% | 1 | K.AQGPAASAEEPKPVEAPAANSDQTVTVKE.- | 3 |

---

|  |  |  |  |  |  |  |  |  |
| --- | --- | --- | --- | --- | --- | --- | --- | --- |
| U | *gi|14141161|ref|NP\_00* | 5 | 7 | 12.7% | 806 | 88980 | 5.8 | heterogeneous nuclear ribonucleoprotein U isoform b [Homo sapiens] |
| U | *gi|74136883|ref|NP\_11* | 5 | 7 | 12.4% | 825 | 90585 | 6.0 | heterogeneous nuclear ribonucleoprotein U isoform a [Homo sapiens] |

| Filename XCorr DeltCN Conf% ObsM+H+ CalcM+H+ SpR ZScore Ion% # Sequence  | | | | | | | | | | | | |
| --- | --- | --- | --- | --- | --- | --- | --- | --- | --- | --- | --- | --- |
|  | LAPMis12DANCE\_011813\_02.05984.05984.3 | 5.5487 | 0.4925 | 100.0% | 3127.6743 | 3128.311 | 1 | 8.226 | 28.2% | 2 | R.LQAALDDEEAGGRPAMEPGNGSLDLGGDSAGR.S | 3 |
|  | LAPMis12DANCE\_011813\_01.09483.09483.2 | 3.6318 | 0.4139 | 100.0% | 1697.8922 | 1698.8291 | 1 | 8.063 | 75.0% | 1 | R.GYFEYIEENKYSR.A | 2 |
|  | LAPMis12DANCE2\_011813\_01.10878.10878.3 | 4.464 | 0.2585 | 99.0% | 2726.4543 | 2726.0576 | 1 | 4.995 | 34.5% | 1 | K.EKPYFPIPEEYTFIQNVPLEDR.V | 3 |
|  | LAPMis12DANCE\_011813\_01.08530.08530.3 | 3.4061 | 0.2523 | 97.4% | 2187.0544 | 2188.4631 | 1 | 5.895 | 38.2% | 1 | K.HAAENPGKYNILGTNTIMDK.M | 3 |
|  | LAPMis12DANCE\_011813\_01.09566.09566.2 | 4.1046 | 0.4352 | 100.0% | 1647.9122 | 1648.816 | 1 | 7.12 | 78.6% | 2 | R.NFILDQTNVSAAAQR.R | 2 |

---

|  |  |  |  |  |  |  |  |  |
| --- | --- | --- | --- | --- | --- | --- | --- | --- |
| U | *gi|5174449|ref|NP\_006* | 2 | 2 | 12.7% | 213 | 22487 | 10.8 | H1 histone family, member X [Homo sapiens] |

| Filename XCorr DeltCN Conf% ObsM+H+ CalcM+H+ SpR ZScore Ion% # Sequence  | | | | | | | | | | | | |
| --- | --- | --- | --- | --- | --- | --- | --- | --- | --- | --- | --- | --- |
| \* | LAPMis12DANCE\_011813\_02.06173.06173.2 | 3.0087 | 0.2721 | 100.0% | 1342.2522 | 1342.5785 | 1 | 5.622 | 72.7% | 1 | K.ALVQNDTLLQVK.G | 2 |
| \* | LAPMis12DANCE2\_011813\_01.03477.03477.2 | 3.8258 | 0.4937 | 100.0% | 1331.6122 | 1332.5021 | 1 | 8.471 | 67.9% | 1 | R.GAPAAATAPAPTAHK.A | 2 |

---

|  |  |  |  |  |  |  |  |  |
| --- | --- | --- | --- | --- | --- | --- | --- | --- |
| U | *gi|169218202|ref|XP\_0* | 1 | 1 | 12.6% | 95 | 10814 | 7.4 | PREDICTED: similar to SMT3B protein [Homo sapiens] |
| U | *gi|54792071|ref|NP\_00* | 1 | 1 | 16.9% | 71 | 8111 | 5.4 | SMT3 suppressor of mif two 3 homolog 2 isoform b precursor [Homo sapiens] |
| U | *gi|54792069|ref|NP\_00* | 1 | 1 | 12.6% | 95 | 10871 | 5.5 | SMT3 suppressor of mif two 3 homolog 2 isoform a precursor [Homo sapiens] |
| U | *gi|50400081|ref|NP\_00* | 1 | 1 | 12.6% | 95 | 10653 | 7.2 | SMT3 suppressor of mif two 3 homolog 4 [Homo sapiens] |
| U | *gi|48928058|ref|NP\_00* | 1 | 1 | 11.7% | 103 | 11637 | 5.5 | small ubiquitin-like modifier protein 3 [Homo sapiens] |

| Filename XCorr DeltCN Conf% ObsM+H+ CalcM+H+ SpR ZScore Ion% # Sequence  | | | | | | | | | | | | |
| --- | --- | --- | --- | --- | --- | --- | --- | --- | --- | --- | --- | --- |
|  | LAPMis12DANCE\_011813\_01.06796.06796.2 | 3.1674 | 0.4128 | 100.0% | 1236.0521 | 1235.3824 | 8 | 7.586 | 63.6% | 1 | K.VAGQDGSVVQFK.I | 2 |

---

|  |  |  |  |  |  |  |  |  |
| --- | --- | --- | --- | --- | --- | --- | --- | --- |
| U | *gi|119395754|ref|NP\_0* | 7 | 9 | 12.5% | 590 | 62378 | 7.8 | keratin 5 [Homo sapiens] |

| Filename XCorr DeltCN Conf% ObsM+H+ CalcM+H+ SpR ZScore Ion% # Sequence  | | | | | | | | | | | | |
| --- | --- | --- | --- | --- | --- | --- | --- | --- | --- | --- | --- | --- |
|  | LAPMis12DANCE\_011813\_01.08360.08360.2 | 2.5656 | 0.1541 | 98.0% | 1082.3722 | 1083.2755 | 7 | 6.493 | 68.8% | 1 | K.FASFIDKVR.F | 222222 |
|  | LAPMis12DANCE\_011813\_01.09275.09275.2 | 2.6952 | 0.3655 | 100.0% | 1203.8922 | 1204.3684 | 3 | 6.309 | 77.8% | 1 | K.WTLLQEQGTK.T | 22 |
|  | LAPMis12DANCE\_011813\_01.03897.03897.2 | 2.7653 | 0.3015 | 100.0% | 1308.5322 | 1309.4215 | 1 | 5.673 | 77.8% | 2 | K.NKYEDEINKR.T | 2222 |
|  | LAPMis12DANCE\_011813\_01.12718.12718.2 | 3.0679 | 0.3399 | 100.0% | 1329.7522 | 1330.5211 | 1 | 6.91 | 77.3% | 2 | R.NLDLDSIIAEVK.A | 2222 |
|  | LAPMis12DANCE\_011813\_01.04349.04349.2 | 3.0849 | 0.4309 | 100.0% | 1195.0721 | 1195.2743 | 1 | 7.308 | 77.8% | 1 | K.YEELQQTAGR.H | 2 |
|  | LAPMis12DANCE\_011813\_01.10198.10198.2 | 2.9647 | 0.2575 | 99.9% | 1386.7122 | 1386.5883 | 19 | 4.367 | 59.1% | 1 | R.NKLAELEEALQK.A | 2 |
|  | LAPMis12DANCE\_011813\_02.07132.07132.2 | 2.9898 | 0.376 | 100.0% | 1264.1522 | 1264.4644 | 1 | 7.517 | 75.0% | 1 | K.LALDVEIATYR.K | 2222 |

Similarities:
gi|4504919|ref|NP\_002(2:5)  
gi|47132620|ref|NP\_00(3:4)  
gi|119395750|ref|NP\_0(1:6)  
gi|119703753|ref|NP\_0(5:2)  
gi|67782365|ref|NP\_00(1:6)  
gi|153791158|ref|NP\_0(3:4)  

---

|  |  |  |  |  |  |  |  |  |
| --- | --- | --- | --- | --- | --- | --- | --- | --- |
| U | *gi|169167049|ref|XP\_0* | 1 | 1 | 12.5% | 104 | 11872 | 10.4 | PREDICTED: hypothetical protein [Homo sapiens] |
| U | *gi|88976416|ref|XP\_93* | 1 | 1 | 12.5% | 104 | 11872 | 10.4 | PREDICTED: hypothetical protein [Homo sapiens] |

| Filename XCorr DeltCN Conf% ObsM+H+ CalcM+H+ SpR ZScore Ion% # Sequence  | | | | | | | | | | | | |
| --- | --- | --- | --- | --- | --- | --- | --- | --- | --- | --- | --- | --- |
|  | LAPMis12DANCE\_011813\_01.09071.09071.2 | 4.1985 | 0.0918 | 99.4% | 1518.4722 | 1517.7435 | 1 | 5.648 | 70.8% | 1 | R.RQEELSNVLAAMR.K | 2 |

---

|  |  |  |  |  |  |  |  |  |
| --- | --- | --- | --- | --- | --- | --- | --- | --- |
| U | *gi|162417971|ref|NP\_0* | 1 | 1 | 12.4% | 226 | 25003 | 8.5 | signal peptidase complex subunit 2 homolog [Homo sapiens] |

| Filename XCorr DeltCN Conf% ObsM+H+ CalcM+H+ SpR ZScore Ion% # Sequence  | | | | | | | | | | | | |
| --- | --- | --- | --- | --- | --- | --- | --- | --- | --- | --- | --- | --- |
| \* | LAPMis12DANCE\_011813\_01.16233.16233.3 | 3.3269 | 0.321 | 99.5% | 2710.7944 | 2712.4768 | 8 | 5.076 | 25.0% | 1 | R.SGGSGGCSGAGGASNCGT#GS\*GRS\*GLLDK.W | 3 |

---

|  |  |  |  |  |  |  |  |  |
| --- | --- | --- | --- | --- | --- | --- | --- | --- |
| U | *gi|11136628|ref|NP\_06* | 2 | 2 | 12.4% | 225 | 24764 | 4.7 | eukaryotic translation elongation factor 1 beta 2 [Homo sapiens] |
| U | *gi|83376130|ref|NP\_00* | 2 | 2 | 12.4% | 225 | 24764 | 4.7 | eukaryotic translation elongation factor 1 beta 2 [Homo sapiens] |
| U | *gi|4503477|ref|NP\_001* | 2 | 2 | 12.4% | 225 | 24764 | 4.7 | eukaryotic translation elongation factor 1 beta 2 [Homo sapiens] |

| Filename XCorr DeltCN Conf% ObsM+H+ CalcM+H+ SpR ZScore Ion% # Sequence  | | | | | | | | | | | | |
| --- | --- | --- | --- | --- | --- | --- | --- | --- | --- | --- | --- | --- |
|  | LAPMis12DANCE\_011813\_01.11525.11525.2 | 4.4245 | 0.5324 | 100.0% | 1603.5322 | 1604.8003 | 1 | 9.148 | 64.3% | 1 | K.SPAGLQVLNDYLADK.S | 2 |
|  | LAPMis12DANCE\_011813\_02.06310.06310.2 | 2.9203 | 0.3648 | 100.0% | 1348.6522 | 1348.4985 | 1 | 6.701 | 70.8% | 1 | R.SIQADGLVWGSSK.L | 2 |

---

|  |  |  |  |  |  |  |  |  |
| --- | --- | --- | --- | --- | --- | --- | --- | --- |
| U | *gi|5901926|ref|NP\_008* | 2 | 2 | 12.3% | 227 | 26227 | 8.8 | cleavage and polyadenylation specific factor 5 [Homo sapiens] |

| Filename XCorr DeltCN Conf% ObsM+H+ CalcM+H+ SpR ZScore Ion% # Sequence  | | | | | | | | | | | | |
| --- | --- | --- | --- | --- | --- | --- | --- | --- | --- | --- | --- | --- |
| \* | LAPMis12DANCE\_011813\_01.08291.08291.3 | 3.6818 | 0.2625 | 98.6% | 1909.7943 | 1910.0911 | 1 | 5.74 | 36.8% | 1 | K.LPGGELNPGEDEVEGLKR.L | 3 |
| \* | LAPMis12DANCE\_011813\_01.09650.09650.2 | 2.3085 | 0.2364 | 98.7% | 1246.5721 | 1246.5358 | 12 | 4.51 | 66.7% | 1 | K.KLFLVQLQEK.A | 2 |

---

|  |  |  |  |  |  |  |  |  |
| --- | --- | --- | --- | --- | --- | --- | --- | --- |
| U | *gi|4826998|ref|NP\_005* | 6 | 7 | 12.0% | 707 | 76150 | 9.4 | splicing factor proline/glutamine rich (polypyrimidine tract binding protein associated) [Homo sapiens] |

| Filename XCorr DeltCN Conf% ObsM+H+ CalcM+H+ SpR ZScore Ion% # Sequence  | | | | | | | | | | | | |
| --- | --- | --- | --- | --- | --- | --- | --- | --- | --- | --- | --- | --- |
| \* | LAPMis12DANCE\_011813\_01.05068.05068.2 | 2.3838 | 0.2859 | 99.4% | 1143.3322 | 1144.3188 | 1 | 5.722 | 70.0% | 1 | R.FATHAAALSVR.N | 2 |
| \* | LAPMis12DANCE\_011813\_02.04986.04986.2 | 2.6142 | 0.3619 | 100.0% | 1762.6522 | 1763.8632 | 23 | 5.976 | 46.2% | 1 | R.FAQHGTFEYEYSQR.W | 2 |
| \* | LAPMis12DANCE\_011813\_01.09756.09756.3 | 3.5987 | 0.2887 | 99.0% | 2429.0645 | 2429.6233 | 53 | 4.769 | 26.3% | 1 | K.DKLESEMEDAYHEHQANLLR.Q | 3 |
| \* | LAPMis12DANCE\_011813\_01.03603.03603.3 | 3.3622 | 0.2918 | 99.5% | 1573.5243 | 1573.7821 | 11 | 5.313 | 40.9% | 1 | R.RMEELHNQEMQK.R | 3 |
| \* | LAPMis12DANCE\_011813\_01.04596.04596.2 | 2.7405 | 0.3588 | 100.0% | 1341.9521 | 1342.4569 | 1 | 6.147 | 71.4% | 2 | R.FGQGGAGPVGGQGPR.G | 2 |
| \* | LAPMis12DANCE\_011813\_01.03149.03149.2 | 3.7491 | 0.412 | 100.0% | 1559.1721 | 1560.7098 | 1 | 6.783 | 66.7% | 1 | R.GREEYEGPNKKPR.F | 2 |

---

|  |  |  |  |  |  |  |  |  |
| --- | --- | --- | --- | --- | --- | --- | --- | --- |
| U | *gi|124256496|ref|NP\_0* | 6 | 12 | 12.0% | 641 | 70375 | 6.0 | heat shock 70kDa protein 1-like [Homo sapiens] |

| Filename XCorr DeltCN Conf% ObsM+H+ CalcM+H+ SpR ZScore Ion% # Sequence  | | | | | | | | | | | | |
| --- | --- | --- | --- | --- | --- | --- | --- | --- | --- | --- | --- | --- |
|  | LAPMis12DANCE\_011813\_01.08684.08684.2 | 3.1796 | 0.5386 | 100.0% | 1487.6721 | 1488.5939 | 1 | 9.759 | 70.8% | 5 | R.TTPSYVAFTDTER.L | 222 |
|  | LAPMis12DANCE\_011813\_01.11934.11934.2 | 2.539 | 0.3173 | 99.6% | 1615.1721 | 1615.8817 | 2 | 5.08 | 50.0% | 2 | K.AFYPEEISSMVLTK.L | 22 |
|  | LAPMis12DANCE\_011813\_02.06268.06268.2 | 2.5299 | 0.3136 | 99.5% | 1660.5521 | 1660.9078 | 1 | 5.605 | 60.0% | 1 | R.IINEPTAAAIAYGLDK.G | 222 |
|  | LAPMis12DANCE\_011813\_01.05212.05212.2 | 3.7838 | 0.5043 | 100.0% | 1675.8322 | 1676.6964 | 1 | 8.957 | 66.7% | 2 | K.ATAGDTHLGGEDFDNR.L | 22 |
|  | LAPMis12DANCE\_011813\_01.03429.03429.2 | 2.4704 | 0.4034 | 100.0% | 1017.5522 | 1018.1582 | 8 | 6.412 | 62.5% | 1 | K.ITITNDKGR.L | 222 |
|  | LAPMis12DANCE\_011813\_01.03428.03428.2 | 2.776 | 0.3658 | 100.0% | 1137.3922 | 1138.2224 | 1 | 6.496 | 75.0% | 1 | K.YKAEDEVQR.E | 22 |

Similarities:
gi|5729877|ref|NP\_006(3:3)  
gi|167466173|ref|NP\_0(5:1)  
contaminant\_GR78\_MESA(1:5)  

---

|  |  |  |  |  |  |  |  |  |
| --- | --- | --- | --- | --- | --- | --- | --- | --- |
| U | *gi|4506741|ref|NP\_001* | 1 | 1 | 11.9% | 194 | 22127 | 10.1 | ribosomal protein S7 [Homo sapiens] |

| Filename XCorr DeltCN Conf% ObsM+H+ CalcM+H+ SpR ZScore Ion% # Sequence  | | | | | | | | | | | | |
| --- | --- | --- | --- | --- | --- | --- | --- | --- | --- | --- | --- | --- |
| \* | LAPMis12DANCE\_011813\_01.12800.12800.3 | 3.4679 | 0.2244 | 95.3% | 2525.5144 | 2524.92 | 1 | 4.451 | 31.8% | 1 | R.TLTAVHDAILEDLVFPSEIVGKR.I | 3 |

---

|  |  |  |  |  |  |  |  |  |
| --- | --- | --- | --- | --- | --- | --- | --- | --- |
| U | *gi|4506649|ref|NP\_000* | 3 | 4 | 11.7% | 403 | 46109 | 10.2 | ribosomal protein L3 isoform a [Homo sapiens] |
| U | *gi|76496472|ref|NP\_00* | 3 | 4 | 13.3% | 354 | 40152 | 10.2 | ribosomal protein L3 isoform b [Homo sapiens] |

| Filename XCorr DeltCN Conf% ObsM+H+ CalcM+H+ SpR ZScore Ion% # Sequence  | | | | | | | | | | | | |
| --- | --- | --- | --- | --- | --- | --- | --- | --- | --- | --- | --- | --- |
|  | LAPMis12DANCE\_011813\_01.07666.07666.2 | 2.232 | 0.4421 | 100.0% | 984.4322 | 984.14594 | 5 | 7.729 | 56.2% | 2 | R.HGSLGFLPR.K | 2 |
|  | LAPMis12DANCE\_011813\_02.08062.08062.3 | 3.4901 | 0.36 | 99.7% | 2973.5942 | 2973.371 | 2 | 5.677 | 27.0% | 1 | R.ERLEQQVPVNQVFGQDEMIDVIGVTK.G | 3 |
|  | LAPMis12DANCE\_011813\_01.04798.04798.2 | 2.5523 | 0.2076 | 98.4% | 1343.9321 | 1343.3464 | 16 | 4.951 | 50.0% | 1 | K.NNASTDYDLSDK.S | 2 |

---

|  |  |  |  |  |  |  |  |  |
| --- | --- | --- | --- | --- | --- | --- | --- | --- |
| U | *gi|4505119|ref|NP\_003* | 2 | 3 | 11.7% | 291 | 32844 | 5.3 | methyl-CpG binding domain protein 3 [Homo sapiens] |

| Filename XCorr DeltCN Conf% ObsM+H+ CalcM+H+ SpR ZScore Ion% # Sequence  | | | | | | | | | | | | |
| --- | --- | --- | --- | --- | --- | --- | --- | --- | --- | --- | --- | --- |
| \* | LAPMis12DANCE\_011813\_01.11958.11958.2 | 3.6847 | 0.4332 | 100.0% | 1610.2522 | 1609.7937 | 1 | 8.793 | 73.1% | 2 | R.YLGGSMDLSTFDFR.T | 2 |
| \* | LAPMis12DANCE2\_011813\_01.11795.11795.3 | 5.8499 | 0.5386 | 100.0% | 2326.1042 | 2326.7346 | 1 | 9.043 | 46.1% | 1 | R.KRLEEALMADMLAHVEELAR.D | 3 |

---

|  |  |  |  |  |  |  |  |  |
| --- | --- | --- | --- | --- | --- | --- | --- | --- |
| U | *gi|169213536|ref|XP\_0* | 3 | 3 | 11.7% | 214 | 24627 | 10.1 | PREDICTED: similar to QM protein isoform 1 [Homo sapiens] |
| U | *gi|41151097|ref|XP\_20* | 3 | 3 | 11.7% | 214 | 24627 | 10.1 | PREDICTED: similar to QM protein isoform 1 [Homo sapiens] |
| U | *gi|223890243|ref|NP\_0* | 3 | 3 | 11.7% | 214 | 24604 | 10.1 | ribosomal protein L10 [Homo sapiens] |
| U | *gi|169213734|ref|XP\_0* | 3 | 3 | 15.0% | 167 | 19409 | 9.9 | PREDICTED: similar to Q1Z 7F5 isoform 2 [Homo sapiens] |
| U | *gi|169213732|ref|XP\_0* | 3 | 3 | 11.7% | 214 | 24600 | 10.1 | PREDICTED: similar to Q1Z 7F5 isoform 1 [Homo sapiens] |
| U | *gi|169213538|ref|XP\_0* | 3 | 3 | 15.0% | 167 | 19436 | 9.9 | PREDICTED: similar to QM protein isoform 2 [Homo sapiens] |

| Filename XCorr DeltCN Conf% ObsM+H+ CalcM+H+ SpR ZScore Ion% # Sequence  | | | | | | | | | | | | |
| --- | --- | --- | --- | --- | --- | --- | --- | --- | --- | --- | --- | --- |
|  | LAPMis12DANCE\_011813\_01.09923.09923.2 | 2.3918 | 0.3834 | 100.0% | 1252.6721 | 1253.5486 | 1 | 7.555 | 60.0% | 1 | R.VHIGQVIMSIR.T | 2 |
|  | LAPMis12DANCE2\_011813\_01.08987.08987.2 | 2.1316 | 0.2428 | 95.9% | 1545.7522 | 1545.6606 | 4 | 4.495 | 54.2% | 1 | K.FNADEFEDMVAEK.R | 2 |
|  | LAPMis12DANCE\_011813\_01.10715.10715.2 | 3.5313 | 0.3134 | 100.0% | 1701.5922 | 1701.8481 | 1 | 6.27 | 53.8% | 1 | K.FNADEFEDMVAEKR.L | 2 |

---

|  |  |  |  |  |  |  |  |  |
| --- | --- | --- | --- | --- | --- | --- | --- | --- |
| U | *gi|9951915|ref|NP\_000* | 4 | 9 | 11.3% | 432 | 47716 | 6.3 | S-adenosylhomocysteine hydrolase [Homo sapiens] |

| Filename XCorr DeltCN Conf% ObsM+H+ CalcM+H+ SpR ZScore Ion% # Sequence  | | | | | | | | | | | | |
| --- | --- | --- | --- | --- | --- | --- | --- | --- | --- | --- | --- | --- |
| \* | LAPMis12DANCE\_011813\_01.07052.07052.2 | 3.8096 | 0.3473 | 100.0% | 1649.1522 | 1649.7979 | 26 | 6.398 | 42.9% | 3 | R.GISEETTTGVHNLYK.M | 2 |
| \* | LAPMis12DANCE\_011813\_01.06977.06977.2 | 3.5563 | 0.5493 | 100.0% | 1256.5721 | 1257.4294 | 1 | 8.94 | 77.3% | 4 | K.VPAINVNDSVTK.S | 2 |
| \* | LAPMis12DANCE\_011813\_01.03968.03968.2 | 2.3509 | 0.2917 | 99.4% | 1069.3121 | 1069.2491 | 42 | 4.769 | 62.5% | 1 | K.VNIKPQVDR.Y | 2 |
| \* | LAPMis12DANCE\_011813\_01.05154.05154.2 | 4.1425 | 0.4665 | 100.0% | 1381.7722 | 1381.5718 | 1 | 8.269 | 79.2% | 1 | K.KLDEAVAEAHLGK.L | 2 |

---

|  |  |  |  |  |  |  |  |  |
| --- | --- | --- | --- | --- | --- | --- | --- | --- |
| U | *gi|63025212|ref|NP\_98* | 2 | 2 | 11.3% | 266 | 27202 | 4.9 | hypothetical protein LOC255374 [Homo sapiens] |

| Filename XCorr DeltCN Conf% ObsM+H+ CalcM+H+ SpR ZScore Ion% # Sequence  | | | | | | | | | | | | |
| --- | --- | --- | --- | --- | --- | --- | --- | --- | --- | --- | --- | --- |
| \* | LAPMis12DANCE\_011813\_01.10558.10558.2 | 2.7704 | 0.4262 | 100.0% | 1339.5322 | 1339.537 | 1 | 7.592 | 58.3% | 1 | R.GPILVDTGGPWAR.E | 2 |
| \* | LAPMis12DANCE\_011813\_01.09546.09546.2 | 2.9588 | 0.3479 | 100.0% | 1733.0521 | 1732.9395 | 1 | 6.199 | 46.9% | 1 | R.LGPGLEVWATPGHGGQR.D | 2 |

---

|  |  |  |  |  |  |  |  |  |
| --- | --- | --- | --- | --- | --- | --- | --- | --- |
| U | *gi|4757810|ref|NP\_004* | 4 | 5 | 11.2% | 553 | 59751 | 9.1 | ATP synthase, H+ transporting, mitochondrial F1 complex, alpha subunit precursor [Homo sapiens] |
| U | *gi|50345984|ref|NP\_00* | 4 | 5 | 11.2% | 553 | 59751 | 9.1 | ATP synthase, H+ transporting, mitochondrial F1 complex, alpha subunit precursor [Homo sapiens] |

| Filename XCorr DeltCN Conf% ObsM+H+ CalcM+H+ SpR ZScore Ion% # Sequence  | | | | | | | | | | | | |
| --- | --- | --- | --- | --- | --- | --- | --- | --- | --- | --- | --- | --- |
|  | LAPMis12DANCE\_011813\_01.09556.09556.2 | 2.5677 | 0.3709 | 100.0% | 1424.3722 | 1424.5659 | 2 | 5.492 | 58.3% | 1 | K.TGTAEMSSILEER.I | 2 |
|  | LAPMis12DANCE\_011813\_02.05901.05901.2 | 3.4765 | 0.4073 | 100.0% | 1576.0922 | 1576.7007 | 1 | 6.722 | 53.6% | 2 | R.ILGADTSVDLEETGR.V | 2 |
|  | LAPMis12DANCE\_011813\_01.09749.09749.2 | 2.0071 | 0.3057 | 97.9% | 1554.1721 | 1554.7019 | 79 | 4.884 | 45.8% | 1 | R.EAYPGDVFYLHSR.L | 2 |
|  | LAPMis12DANCE2\_011813\_01.09868.09868.3 | 3.093 | 0.2584 | 97.4% | 2367.2944 | 2368.7007 | 1 | 5.417 | 30.0% | 1 | K.FENAFLSHVVSQHQALLGTIR.A | 3 |

---

|  |  |  |  |  |  |  |  |  |
| --- | --- | --- | --- | --- | --- | --- | --- | --- |
| U | *gi|119703753|ref|NP\_0* | 6 | 15 | 11.0% | 564 | 60067 | 8.0 | keratin 6B [Homo sapiens] |

| Filename XCorr DeltCN Conf% ObsM+H+ CalcM+H+ SpR ZScore Ion% # Sequence  | | | | | | | | | | | | |
| --- | --- | --- | --- | --- | --- | --- | --- | --- | --- | --- | --- | --- |
|  | LAPMis12DANCE\_011813\_01.08360.08360.2 | 2.5656 | 0.1541 | 98.0% | 1082.3722 | 1083.2755 | 7 | 6.493 | 68.8% | 1 | K.FASFIDKVR.F | 222222 |
|  | LAPMis12DANCE\_011813\_01.09275.09275.2 | 2.6952 | 0.3655 | 100.0% | 1203.8922 | 1204.3684 | 3 | 6.309 | 77.8% | 1 | K.WTLLQEQGTK.T | 22 |
|  | LAPMis12DANCE\_011813\_01.03897.03897.2 | 2.7653 | 0.3015 | 100.0% | 1308.5322 | 1309.4215 | 1 | 5.673 | 77.8% | 2 | K.NKYEDEINKR.T | 2222 |
|  | LAPMis12DANCE\_011813\_01.12718.12718.2 | 3.0679 | 0.3399 | 100.0% | 1329.7522 | 1330.5211 | 1 | 6.91 | 77.3% | 2 | R.NLDLDSIIAEVK.A | 2222 |
|  | LAPMis12DANCE\_011813\_02.05306.05306.2 | 3.7619 | 0.3376 | 100.0% | 1179.5922 | 1180.303 | 2 | 7.4 | 83.3% | 8 | K.YEELQITAGR.H | 22 |
|  | LAPMis12DANCE\_011813\_02.07132.07132.2 | 2.9898 | 0.376 | 100.0% | 1264.1522 | 1264.4644 | 1 | 7.517 | 75.0% | 1 | K.LALDVEIATYR.K | 2222 |

Similarities:
gi|4504919|ref|NP\_002(2:4)  
gi|47132620|ref|NP\_00(3:3)  
gi|119395750|ref|NP\_0(2:4)  
gi|119395754|ref|NP\_0(5:1)  
gi|67782365|ref|NP\_00(1:5)  
gi|153791158|ref|NP\_0(3:3)  

---

|  |  |  |  |  |  |  |  |  |
| --- | --- | --- | --- | --- | --- | --- | --- | --- |
| U | *gi|4506457|ref|NP\_002* | 2 | 2 | 11.0% | 317 | 36876 | 4.4 | reticulocalbin 2 precursor [Homo sapiens] |

| Filename XCorr DeltCN Conf% ObsM+H+ CalcM+H+ SpR ZScore Ion% # Sequence  | | | | | | | | | | | | |
| --- | --- | --- | --- | --- | --- | --- | --- | --- | --- | --- | --- | --- |
| \* | LAPMis12DANCE\_011813\_02.07334.07334.2 | 3.8682 | 0.3577 | 100.0% | 1706.9722 | 1707.8749 | 1 | 7.462 | 64.3% | 1 | R.EALLGVQEDVDEYVK.L | 2 |
| \* | LAPMis12DANCE\_011813\_02.06927.06927.3 | 2.2996 | 0.3595 | 99.0% | 2344.1042 | 2344.4504 | 1 | 5.499 | 31.6% | 1 | R.VIDFDENTALDDAEEESFRK.L | 3 |

---

|  |  |  |  |  |  |  |  |  |
| --- | --- | --- | --- | --- | --- | --- | --- | --- |
| U | *gi|19526475|ref|NP\_44* | 1 | 1 | 11.0% | 254 | 27710 | 9.9 | outer dense fiber of sperm tails 3 [Homo sapiens] |

| Filename XCorr DeltCN Conf% ObsM+H+ CalcM+H+ SpR ZScore Ion% # Sequence  | | | | | | | | | | | | |
| --- | --- | --- | --- | --- | --- | --- | --- | --- | --- | --- | --- | --- |
| \* | LAPMis12DANCE2\_011813\_01.14889.14889.3 | 3.0278 | 0.2615 | 96.6% | 3147.8943 | 3150.4128 | 158 | 4.301 | 20.4% | 1 | R.SK@LGGFSDDLHKTPGPAAYRQTDVRVT#K@.F | 3 |

---

|  |  |  |  |  |  |  |  |  |
| --- | --- | --- | --- | --- | --- | --- | --- | --- |
| U | *Reverse\_gi|20070252|r* | 1 | 1 | 11.0% | 164 | 18522 | 9.5 | interleukin 1 family, member 8 isoform 1 [Homo sapiens] |

| Filename XCorr DeltCN Conf% ObsM+H+ CalcM+H+ SpR ZScore Ion% # Sequence  | | | | | | | | | | | | |
| --- | --- | --- | --- | --- | --- | --- | --- | --- | --- | --- | --- | --- |
| \* | LAPMis12DANCE2\_011813\_01.12812.12812.2 | 2.7251 | 0.1708 | 96.6% | 2265.4321 | 2265.4045 | 1 | 3.501 | 38.2% | 1 | R.GPMGINTRMS\*S\*FDK@DK@K@R.L | 2 |

---

|  |  |  |  |  |  |  |  |  |
| --- | --- | --- | --- | --- | --- | --- | --- | --- |
| U | *gi|56243533|ref|NP\_07* | 1 | 1 | 10.9% | 221 | 23598 | 7.0 | stromal cell-derived factor 2-like 1 precursor [Homo sapiens] |

| Filename XCorr DeltCN Conf% ObsM+H+ CalcM+H+ SpR ZScore Ion% # Sequence  | | | | | | | | | | | | |
| --- | --- | --- | --- | --- | --- | --- | --- | --- | --- | --- | --- | --- |
| \* | LAPMis12DANCE\_011813\_02.05909.05909.3 | 3.3083 | 0.3679 | 99.7% | 2521.8542 | 2522.5603 | 1 | 5.936 | 31.5% | 1 | K.YGSGSGQQSVTGVEASDDANSYWR.I | 3 |

---

|  |  |  |  |  |  |  |  |  |
| --- | --- | --- | --- | --- | --- | --- | --- | --- |
| U | *gi|16306492|ref|NP\_20* | 2 | 2 | 10.8% | 240 | 27503 | 7.1 | cell division cycle 2 isoform 2 [Homo sapiens] |
| U | *gi|4502709|ref|NP\_001* | 2 | 2 | 8.8% | 297 | 34095 | 8.4 | cell division cycle 2 isoform 1 [Homo sapiens] |
| U | *gi|195927041|ref|NP\_0* | 2 | 2 | 8.8% | 297 | 34081 | 8.4 | cell division cycle 2 isoform 3 [Homo sapiens] |

| Filename XCorr DeltCN Conf% ObsM+H+ CalcM+H+ SpR ZScore Ion% # Sequence  | | | | | | | | | | | | |
| --- | --- | --- | --- | --- | --- | --- | --- | --- | --- | --- | --- | --- |
|  | LAPMis12DANCE\_011813\_01.07432.07432.2 | 2.349 | 0.2972 | 99.5% | 1186.3121 | 1186.3501 | 1 | 6.063 | 70.0% | 1 | K.IGEGTYGVVYK.G | 2 |
|  | LAPMis12DANCE\_011813\_01.10696.10696.3 | 3.0194 | 0.2917 | 98.9% | 1803.9243 | 1803.0275 | 1 | 4.649 | 42.9% | 1 | K.KPLFHGDSEIDQLFR.I | 3 |

---

|  |  |  |  |  |  |  |  |  |
| --- | --- | --- | --- | --- | --- | --- | --- | --- |
| U | *gi|166235186|ref|NP\_0* | 2 | 2 | 10.7% | 242 | 27125 | 7.1 | APAF1 interacting protein [Homo sapiens] |

| Filename XCorr DeltCN Conf% ObsM+H+ CalcM+H+ SpR ZScore Ion% # Sequence  | | | | | | | | | | | | |
| --- | --- | --- | --- | --- | --- | --- | --- | --- | --- | --- | --- | --- |
| \* | LAPMis12DANCE\_011813\_01.06353.06353.2 | 3.712 | 0.4807 | 100.0% | 1514.9321 | 1514.6781 | 1 | 9.585 | 69.2% | 1 | K.HGDEIYIAPSGVQK.E | 2 |
| \* | LAPMis12DANCE\_011813\_01.09890.09890.2 | 2.7326 | 0.2607 | 99.4% | 1491.8121 | 1491.6458 | 30 | 6.077 | 54.5% | 1 | R.HGVYVWGETWEK.A | 2 |

---

|  |  |  |  |  |  |  |  |  |
| --- | --- | --- | --- | --- | --- | --- | --- | --- |
| U | *gi|4506605|ref|NP\_000* | 1 | 3 | 10.7% | 140 | 14865 | 10.5 | ribosomal protein L23 [Homo sapiens] |

| Filename XCorr DeltCN Conf% ObsM+H+ CalcM+H+ SpR ZScore Ion% # Sequence  | | | | | | | | | | | | |
| --- | --- | --- | --- | --- | --- | --- | --- | --- | --- | --- | --- | --- |
| \* | LAPMis12DANCE\_011813\_02.07263.07263.2 | 3.8292 | 0.5532 | 100.0% | 1459.8322 | 1460.7902 | 1 | 9.34 | 75.0% | 3 | R.LPAAGVGDMVMATVK.K | 2 |

---

|  |  |  |  |  |  |  |  |  |
| --- | --- | --- | --- | --- | --- | --- | --- | --- |
| U | *gi|19913406|ref|NP\_00* | 11 | 15 | 10.6% | 1531 | 174384 | 8.7 | DNA topoisomerase II, alpha isozyme [Homo sapiens] |

| Filename XCorr DeltCN Conf% ObsM+H+ CalcM+H+ SpR ZScore Ion% # Sequence  | | | | | | | | | | | | |
| --- | --- | --- | --- | --- | --- | --- | --- | --- | --- | --- | --- | --- |
|  | LAPMis12DANCE\_011813\_01.11062.11062.2 | 3.2267 | 0.2846 | 100.0% | 1462.2722 | 1462.6427 | 1 | 5.54 | 75.0% | 1 | K.IFDEILVNAADNK.Q | 2 |
| \* | LAPMis12DANCE\_011813\_01.12233.12233.2 | 3.6351 | 0.2577 | 100.0% | 1928.1921 | 1928.1521 | 1 | 5.921 | 46.9% | 2 | R.VTIDPENNLISIWNNGK.G | 2 |
| \* | LAPMis12DANCE\_011813\_01.06663.06663.2 | 3.4231 | 0.4765 | 100.0% | 1387.8722 | 1388.5632 | 1 | 7.419 | 63.6% | 2 | R.HVDYVADQIVTK.L | 2 |
| \* | LAPMis12DANCE\_011813\_01.08942.08942.2 | 1.7888 | 0.2944 | 96.4% | 1094.3322 | 1095.2865 | 1 | 5.212 | 81.2% | 1 | R.DKYGVFPLR.G | 2 |
| \* | LAPMis12DANCE\_011813\_02.07043.07043.2 | 4.1956 | 0.567 | 100.0% | 1671.1322 | 1671.801 | 1 | 9.87 | 63.3% | 1 | K.YSGPEDDAAISLAFSK.K | 2 |
|  | LAPMis12DANCE\_011813\_01.10480.10480.2 | 3.663 | 0.3672 | 100.0% | 1438.1721 | 1437.5492 | 1 | 7.128 | 77.3% | 1 | K.ELILFSNSDNER.S | 2 |
| \* | LAPMis12DANCE\_011813\_01.08364.08364.2 | 2.7245 | 0.2845 | 99.4% | 1485.6122 | 1485.7417 | 2 | 5.358 | 53.8% | 1 | R.SIPSMVDGLKPGQR.K | 2 |
| \* | LAPMis12DANCE\_011813\_01.08878.08878.2 | 2.5269 | 0.1685 | 95.7% | 1488.1921 | 1488.6959 | 425 | 4.119 | 41.7% | 1 | K.EQVLEPMLNGTEK.T | 2 |
| \* | LAPMis12DANCE\_011813\_01.03975.03975.3 | 4.5097 | 0.3851 | 100.0% | 1772.1843 | 1772.9994 | 1 | 6.92 | 57.7% | 1 | R.LRNEKEQELDTLKR.K | 3 |
| \* | LAPMis12DANCE2\_011813\_01.14427.14427.3 | 5.4169 | 0.4011 | 100.0% | 2750.3044 | 2750.0747 | 1 | 7.151 | 33.7% | 2 | R.KSPSDLWKEDLATFIEELEAVEAK.E | 3 |
| \* | LAPMis12DANCE\_011813\_02.05957.05957.3 | 4.0497 | 0.4332 | 100.0% | 2111.9944 | 2112.2349 | 1 | 8.476 | 41.7% | 2 | K.SKGESDDFHMDFDSAVAPR.A | 3 |

---

|  |  |  |  |  |  |  |  |  |
| --- | --- | --- | --- | --- | --- | --- | --- | --- |
| U | *gi|4502491|ref|NP\_001* | 1 | 1 | 10.6% | 282 | 31362 | 4.8 | complement component 1, q subcomponent binding protein precursor [Homo sapiens] |

| Filename XCorr DeltCN Conf% ObsM+H+ CalcM+H+ SpR ZScore Ion% # Sequence  | | | | | | | | | | | | |
| --- | --- | --- | --- | --- | --- | --- | --- | --- | --- | --- | --- | --- |
| \* | LAPMis12DANCE\_011813\_01.17286.17286.3 | 4.7449 | 0.4368 | 100.0% | 3440.1243 | 3441.77 | 2 | 6.699 | 24.1% | 1 | R.GVDNTFADELVELSTALEHQEYITFLEDLK.S | 3 |

---

|  |  |  |  |  |  |  |  |  |
| --- | --- | --- | --- | --- | --- | --- | --- | --- |
| U | *Reverse\_gi|17986258|r* | 1 | 5 | 10.6% | 151 | 16930 | 4.7 | myosin, light chain 6, alkali, smooth muscle and non-muscle isoform 1 [Homo sapiens] |
| U | *Reverse\_gi|88999583|r* | 1 | 5 | 10.6% | 151 | 16961 | 4.6 | myosin, light chain 6, alkali, smooth muscle and non-muscle isoform 2 [Homo sapiens] |

| Filename XCorr DeltCN Conf% ObsM+H+ CalcM+H+ SpR ZScore Ion% # Sequence  | | | | | | | | | | | | |
| --- | --- | --- | --- | --- | --- | --- | --- | --- | --- | --- | --- | --- |
|  | LAPMis12DANCE\_011813\_02.07085.07085.2 | 4.2675 | 0.1295 | 99.9% | 1801.1921 | 1800.9905 | 227 | 4.72 | 40.0% | 5 | R.MVDGCQSYLIKGDGTR.D | 2 |

---

|  |  |  |  |  |  |  |  |  |
| --- | --- | --- | --- | --- | --- | --- | --- | --- |
| U | *gi|14110414|ref|NP\_00* | 2 | 2 | 10.5% | 306 | 32835 | 8.2 | heterogeneous nuclear ribonucleoprotein D isoform c [Homo sapiens] |
| U | *gi|14110420|ref|NP\_11* | 2 | 2 | 9.0% | 355 | 38434 | 7.8 | heterogeneous nuclear ribonucleoprotein D isoform a [Homo sapiens] |

| Filename XCorr DeltCN Conf% ObsM+H+ CalcM+H+ SpR ZScore Ion% # Sequence  | | | | | | | | | | | | |
| --- | --- | --- | --- | --- | --- | --- | --- | --- | --- | --- | --- | --- |
|  | LAPMis12DANCE\_011813\_01.03246.03246.3 | 3.1903 | 0.2819 | 98.7% | 2053.1042 | 2052.9768 | 1 | 5.175 | 36.8% | 1 | K.IDASKNEEDEGHSNSS\*PR.H | 3 |
|  | LAPMis12DANCE\_011813\_01.09075.09075.2 | 3.9683 | 0.4257 | 100.0% | 1489.2322 | 1489.6653 | 1 | 7.397 | 69.2% | 1 | K.IFVGGLSPDTPEEK.I | 2 |

---

|  |  |  |  |  |  |  |  |  |
| --- | --- | --- | --- | --- | --- | --- | --- | --- |
| U | *gi|28875797|ref|NP\_05* | 2 | 3 | 10.5% | 248 | 26397 | 12.2 | hypothetical protein LOC26097 [Homo sapiens] |

| Filename XCorr DeltCN Conf% ObsM+H+ CalcM+H+ SpR ZScore Ion% # Sequence  | | | | | | | | | | | | |
| --- | --- | --- | --- | --- | --- | --- | --- | --- | --- | --- | --- | --- |
| \* | LAPMis12DANCE\_011813\_01.04191.04191.2 | 4.2038 | 0.4077 | 100.0% | 1447.8922 | 1447.6091 | 1 | 7.35 | 70.8% | 2 | R.ASMQQQQQLASAR.N | 2 |
| \* | LAPMis12DANCE\_011813\_02.06288.06288.2 | 3.5943 | 0.4053 | 100.0% | 1556.1122 | 1555.6997 | 1 | 6.604 | 66.7% | 1 | K.EQLDNQLDAYMSK.T | 2 |

---

|  |  |  |  |  |  |  |  |  |
| --- | --- | --- | --- | --- | --- | --- | --- | --- |
| U | *gi|169163644|ref|XP\_0* | 1 | 1 | 10.4% | 135 | 15572 | 5.6 | PREDICTED: similar to adrenal gland protein AD-001 [Homo sapiens] |
| U | *gi|88955003|ref|XP\_94* | 1 | 1 | 10.4% | 135 | 15572 | 5.6 | PREDICTED: similar to adrenal gland protein AD-001 [Homo sapiens] |
| U | *gi|7705477|ref|NP\_057* | 1 | 1 | 11.2% | 125 | 14199 | 5.3 | hypothetical protein LOC51504 [Homo sapiens] |
| U | *gi|41190515|ref|XP\_37* | 1 | 1 | 10.4% | 135 | 15572 | 5.6 | PREDICTED: similar to adrenal gland protein AD-001 [Homo sapiens] |

| Filename XCorr DeltCN Conf% ObsM+H+ CalcM+H+ SpR ZScore Ion% # Sequence  | | | | | | | | | | | | |
| --- | --- | --- | --- | --- | --- | --- | --- | --- | --- | --- | --- | --- |
|  | LAPMis12DANCE\_011813\_02.06111.06111.2 | 3.1153 | 0.352 | 100.0% | 1667.9521 | 1668.757 | 1 | 6.571 | 61.5% | 1 | K.GPVEGYEENEEFLR.T | 2 |

---

|  |  |  |  |  |  |  |  |  |
| --- | --- | --- | --- | --- | --- | --- | --- | --- |
| U | *gi|18375623|ref|NP\_54* | 4 | 6 | 10.3% | 428 | 48991 | 5.7 | HLA-B associated transcript 1 [Homo sapiens] |
| U | *gi|4758112|ref|NP\_004* | 4 | 6 | 10.3% | 428 | 48991 | 5.7 | HLA-B associated transcript 1 [Homo sapiens] |

| Filename XCorr DeltCN Conf% ObsM+H+ CalcM+H+ SpR ZScore Ion% # Sequence  | | | | | | | | | | | | |
| --- | --- | --- | --- | --- | --- | --- | --- | --- | --- | --- | --- | --- |
|  | LAPMis12DANCE\_011813\_01.10122.10122.2 | 1.9108 | 0.2961 | 97.1% | 1259.6921 | 1260.5253 | 2 | 4.905 | 65.0% | 1 | R.RILVATNLFGR.G | 2 |
|  | LAPMis12DANCE\_011813\_01.10919.10919.2 | 3.3699 | 0.494 | 100.0% | 2299.8523 | 2301.492 | 10 | 8.261 | 30.6% | 1 | R.VNIAFNYDMPEDSDTYLHR.V | 2 |
|  | LAPMis12DANCE2\_011813\_01.08649.08649.3 | 3.2034 | 0.2704 | 97.9% | 2302.1343 | 2301.492 | 1 | 4.991 | 29.2% | 1 | R.VNIAFNYDMPEDSDTYLHR.V | 3 |
|  | LAPMis12DANCE\_011813\_02.06585.06585.2 | 4.4619 | 0.5486 | 100.0% | 1480.2322 | 1480.6146 | 1 | 9.845 | 76.9% | 3 | K.GLAITFVSDENDAK.I | 2 |

---

|  |  |  |  |  |  |  |  |  |
| --- | --- | --- | --- | --- | --- | --- | --- | --- |
| U | *gi|6912634|ref|NP\_036* | 2 | 2 | 10.3% | 203 | 23577 | 10.9 | ribosomal protein L13a [Homo sapiens] |

| Filename XCorr DeltCN Conf% ObsM+H+ CalcM+H+ SpR ZScore Ion% # Sequence  | | | | | | | | | | | | |
| --- | --- | --- | --- | --- | --- | --- | --- | --- | --- | --- | --- | --- |
| \* | LAPMis12DANCE\_011813\_02.04889.04889.2 | 1.993 | 0.393 | 99.5% | 1252.8322 | 1253.3947 | 3 | 6.759 | 50.0% | 1 | K.YQAVTATLEEK.R | 2 |
| \* | LAPMis12DANCE\_011813\_01.04792.04792.2 | 2.6075 | 0.162 | 98.0% | 1238.3722 | 1237.482 | 2 | 3.78 | 77.8% | 1 | K.KIDKYTEVLK.T | 2 |

---

|  |  |  |  |  |  |  |  |  |
| --- | --- | --- | --- | --- | --- | --- | --- | --- |
| U | *gi|14141193|ref|NP\_00* | 2 | 2 | 10.3% | 194 | 22591 | 10.7 | ribosomal protein S9 [Homo sapiens] |

| Filename XCorr DeltCN Conf% ObsM+H+ CalcM+H+ SpR ZScore Ion% # Sequence  | | | | | | | | | | | | |
| --- | --- | --- | --- | --- | --- | --- | --- | --- | --- | --- | --- | --- |
| \* | LAPMis12DANCE\_011813\_01.08808.08808.2 | 3.131 | 0.2324 | 100.0% | 1189.7122 | 1189.4031 | 1 | 4.96 | 72.2% | 1 | R.RLFEGNALLR.R | 2 |
| \* | LAPMis12DANCE\_011813\_01.03243.03243.2 | 2.5258 | 0.1772 | 98.0% | 1003.9922 | 1004.0934 | 5 | 4.632 | 61.1% | 1 | R.SPYGGGRPGR.V | 2 |

---

|  |  |  |  |  |  |  |  |  |
| --- | --- | --- | --- | --- | --- | --- | --- | --- |
| U | *gi|4506695|ref|NP\_001* | 1 | 1 | 10.3% | 145 | 16060 | 10.3 | ribosomal protein S19 [Homo sapiens] |

| Filename XCorr DeltCN Conf% ObsM+H+ CalcM+H+ SpR ZScore Ion% # Sequence  | | | | | | | | | | | | |
| --- | --- | --- | --- | --- | --- | --- | --- | --- | --- | --- | --- | --- |
| \* | LAPMis12DANCE\_011813\_01.09908.09908.3 | 2.8387 | 0.3146 | 99.0% | 1970.1843 | 1970.151 | 1 | 5.876 | 42.9% | 1 | K.HKELAPYDENWFYTR.A | 3 |

---

|  |  |  |  |  |  |  |  |  |
| --- | --- | --- | --- | --- | --- | --- | --- | --- |
| U | *gi|4506761|ref|NP\_002* | 1 | 1 | 10.3% | 97 | 11203 | 7.4 | S100 calcium binding protein A10 [Homo sapiens] |

| Filename XCorr DeltCN Conf% ObsM+H+ CalcM+H+ SpR ZScore Ion% # Sequence  | | | | | | | | | | | | |
| --- | --- | --- | --- | --- | --- | --- | --- | --- | --- | --- | --- | --- |
| \* | LAPMis12DANCE\_011813\_01.04566.04566.2 | 2.353 | 0.3475 | 99.9% | 1100.1322 | 1100.2596 | 1 | 5.879 | 72.2% | 1 | K.FAGDKGYLTK.E | 2 |

---

|  |  |  |  |  |  |  |  |  |
| --- | --- | --- | --- | --- | --- | --- | --- | --- |
| U | *gi|4506613|ref|NP\_000* | 1 | 1 | 10.2% | 128 | 14787 | 9.2 | ribosomal protein L22 proprotein [Homo sapiens] |

| Filename XCorr DeltCN Conf% ObsM+H+ CalcM+H+ SpR ZScore Ion% # Sequence  | | | | | | | | | | | | |
| --- | --- | --- | --- | --- | --- | --- | --- | --- | --- | --- | --- | --- |
| \* | LAPMis12DANCE\_011813\_01.07558.07558.2 | 2.994 | 0.3857 | 100.0% | 1243.2122 | 1243.4056 | 3 | 6.219 | 66.7% | 1 | K.AGNLGGGVVTIER.S | 2 |

---

|  |  |  |  |  |  |  |  |  |
| --- | --- | --- | --- | --- | --- | --- | --- | --- |
| U | *gi|4507231|ref|NP\_003* | 1 | 1 | 10.1% | 148 | 17260 | 9.6 | single-stranded DNA binding protein 1 [Homo sapiens] |

| Filename XCorr DeltCN Conf% ObsM+H+ CalcM+H+ SpR ZScore Ion% # Sequence  | | | | | | | | | | | | |
| --- | --- | --- | --- | --- | --- | --- | --- | --- | --- | --- | --- | --- |
| \* | LAPMis12DANCE\_011813\_01.07826.07826.2 | 3.6782 | 0.2864 | 100.0% | 1613.5721 | 1612.6904 | 1 | 5.33 | 57.1% | 1 | R.SGDSEVYQLGDVSQK.T | 2 |

---

|  |  |  |  |  |  |  |  |  |
| --- | --- | --- | --- | --- | --- | --- | --- | --- |
| U | *gi|33239451|ref|NP\_87* | 2 | 2 | 10.0% | 261 | 28769 | 4.7 | proliferating cell nuclear antigen [Homo sapiens] |
| U | *gi|4505641|ref|NP\_002* | 2 | 2 | 10.0% | 261 | 28769 | 4.7 | proliferating cell nuclear antigen [Homo sapiens] |

| Filename XCorr DeltCN Conf% ObsM+H+ CalcM+H+ SpR ZScore Ion% # Sequence  | | | | | | | | | | | | |
| --- | --- | --- | --- | --- | --- | --- | --- | --- | --- | --- | --- | --- |
|  | LAPMis12DANCE\_011813\_02.06548.06548.2 | 2.6509 | 0.2947 | 99.6% | 1366.1122 | 1366.6338 | 96 | 5.488 | 50.0% | 1 | R.NLAMGVNLTSMSK.I | 2 |
|  | LAPMis12DANCE\_011813\_01.07037.07037.2 | 2.2924 | 0.2627 | 98.4% | 1295.3322 | 1294.4069 | 24 | 4.569 | 58.3% | 1 | K.FSASGELGNGNIK.L | 2 |

---

|  |  |  |  |  |  |  |  |  |
| --- | --- | --- | --- | --- | --- | --- | --- | --- |
| U | *gi|31542947|ref|NP\_00* | 4 | 6 | 9.9% | 573 | 61055 | 5.9 | chaperonin [Homo sapiens] |
| U | *gi|41399285|ref|NP\_95* | 4 | 6 | 9.9% | 573 | 61055 | 5.9 | chaperonin [Homo sapiens] |

| Filename XCorr DeltCN Conf% ObsM+H+ CalcM+H+ SpR ZScore Ion% # Sequence  | | | | | | | | | | | | |
| --- | --- | --- | --- | --- | --- | --- | --- | --- | --- | --- | --- | --- |
|  | LAPMis12DANCE\_011813\_01.16780.16780.2 | 2.9209 | 0.3313 | 100.0% | 2113.5522 | 2114.5667 | 1 | 6.324 | 40.0% | 2 | R.ALMLQGVDLLADAVAVTMGPK.G | 2 |
|  | LAPMis12DANCE\_011813\_01.08967.08967.2 | 2.9976 | 0.2808 | 100.0% | 1345.6122 | 1345.5382 | 1 | 5.919 | 68.2% | 2 | R.TVIIEQSWGSPK.V | 2 |
|  | LAPMis12DANCE\_011813\_01.03945.03945.2 | 3.1337 | 0.4409 | 100.0% | 1233.9122 | 1234.3055 | 9 | 7.406 | 59.1% | 1 | K.VGGTSDVEVNEK.K | 2 |
|  | LAPMis12DANCE\_011813\_02.05218.05218.2 | 3.3382 | 0.3767 | 100.0% | 1216.2522 | 1216.377 | 1 | 7.044 | 68.2% | 1 | K.NAGVEGSLIVEK.I | 2 |

---

|  |  |  |  |  |  |  |  |  |
| --- | --- | --- | --- | --- | --- | --- | --- | --- |
| U | *gi|50053795|ref|NP\_00* | 4 | 4 | 9.7% | 611 | 69151 | 5.7 | eukaryotic translation initiation factor 4B [Homo sapiens] |

| Filename XCorr DeltCN Conf% ObsM+H+ CalcM+H+ SpR ZScore Ion% # Sequence  | | | | | | | | | | | | |
| --- | --- | --- | --- | --- | --- | --- | --- | --- | --- | --- | --- | --- |
| \* | LAPMis12DANCE2\_011813\_01.14200.14200.3 | 3.69 | 0.3338 | 99.7% | 2921.1843 | 2922.2634 | 5 | 5.357 | 26.0% | 1 | K.SPPYTAFLGNLPYDVTEESIKEFFR.G | 3 |
| \* | LAPMis12DANCE\_011813\_01.03863.03863.2 | 2.4793 | 0.1787 | 96.9% | 1316.0322 | 1316.3727 | 14 | 4.453 | 60.0% | 1 | R.RGDDSFGDKYR.D | 2 |
| \* | LAPMis12DANCE\_011813\_01.03146.03146.3 | 2.5642 | 0.3193 | 98.1% | 2675.6943 | 2678.689 | 2 | 5.346 | 23.9% | 1 | K.LNLK@PRS\*T#PKEDDSSASTSQSTR.A | 3 |
| \* | LAPMis12DANCE\_011813\_01.03146.03146.2 | 3.6694 | 0.4996 | 100.0% | 1784.1322 | 1784.7881 | 1 | 8.936 | 68.8% | 1 | R.STPKEDDSSASTSQSTR.A | 3 |

---

|  |  |  |  |  |  |  |  |  |
| --- | --- | --- | --- | --- | --- | --- | --- | --- |
| U | *gi|169167803|ref|XP\_0* | 1 | 2 | 9.7% | 155 | 17138 | 10.8 | PREDICTED: similar to ribosomal protein L29 [Homo sapiens] |
| U | *gi|89042710|ref|XP\_94* | 1 | 2 | 9.7% | 155 | 17219 | 11.1 | PREDICTED: similar to ribosomal protein L29 [Homo sapiens] |
| U | *gi|88987619|ref|XP\_94* | 1 | 2 | 9.7% | 155 | 17138 | 10.8 | PREDICTED: similar to ribosomal protein L29 [Homo sapiens] |
| U | *gi|88982429|ref|XP\_49* | 1 | 2 | 9.7% | 155 | 17138 | 10.8 | PREDICTED: hypothetical protein [Homo sapiens] |
| U | *gi|4506629|ref|NP\_000* | 1 | 2 | 9.4% | 159 | 17752 | 11.7 | ribosomal protein L29 [Homo sapiens] |
| U | *gi|27482992|ref|XP\_21* | 1 | 2 | 9.7% | 155 | 17219 | 11.1 | PREDICTED: similar to ribosomal protein L29 [Homo sapiens] |
| U | *gi|169211322|ref|XP\_0* | 1 | 2 | 9.7% | 155 | 17219 | 11.1 | PREDICTED: similar to ribosomal protein L29 [Homo sapiens] |
| U | *gi|169208890|ref|XP\_0* | 1 | 2 | 7.4% | 204 | 22304 | 10.6 | PREDICTED: similar to hCG1641491 isoform 2 [Homo sapiens] |
| U | *gi|169208888|ref|XP\_0* | 1 | 2 | 6.5% | 232 | 25745 | 11.0 | PREDICTED: similar to hCG1641491 isoform 1 [Homo sapiens] |
| U | *gi|169208686|ref|XP\_0* | 1 | 2 | 6.5% | 232 | 25745 | 11.0 | PREDICTED: hypothetical protein [Homo sapiens] |
| U | *gi|169208101|ref|XP\_0* | 1 | 2 | 6.5% | 232 | 25745 | 11.0 | PREDICTED: hypothetical protein [Homo sapiens] |

| Filename XCorr DeltCN Conf% ObsM+H+ CalcM+H+ SpR ZScore Ion% # Sequence  | | | | | | | | | | | | |
| --- | --- | --- | --- | --- | --- | --- | --- | --- | --- | --- | --- | --- |
|  | LAPMis12DANCE\_011813\_01.04299.04299.2 | 3.2363 | 0.2181 | 99.5% | 1378.0122 | 1378.5712 | 2 | 6.888 | 64.3% | 2 | K.AQAAAPASVPAQAPK.G | 2 |

---

|  |  |  |  |  |  |  |  |  |
| --- | --- | --- | --- | --- | --- | --- | --- | --- |
| U | *Reverse\_gi|61744445|r* | 1 | 1 | 9.6% | 313 | 34001 | 6.8 | ficolin 2 isoform a precursor [Homo sapiens] |
| U | *Reverse\_gi|8051586|re* | 1 | 1 | 10.9% | 275 | 30228 | 6.3 | ficolin 2 isoform b precursor [Homo sapiens] |

| Filename XCorr DeltCN Conf% ObsM+H+ CalcM+H+ SpR ZScore Ion% # Sequence  | | | | | | | | | | | | |
| --- | --- | --- | --- | --- | --- | --- | --- | --- | --- | --- | --- | --- |
|  | LAPMis12DANCE2\_011813\_01.10372.10372.3 | 3.391 | 0.2645 | 97.4% | 3542.4543 | 3539.766 | 25 | 4.412 | 19.0% | 1 | R.VK@MESVKYSYNYGKGS\*KWNIGNAFSGHT#GR.L | 3 |

---

|  |  |  |  |  |  |  |  |  |
| --- | --- | --- | --- | --- | --- | --- | --- | --- |
| U | *gi|4505773|ref|NP\_002* | 2 | 3 | 9.6% | 272 | 29804 | 5.8 | prohibitin [Homo sapiens] |

| Filename XCorr DeltCN Conf% ObsM+H+ CalcM+H+ SpR ZScore Ion% # Sequence  | | | | | | | | | | | | |
| --- | --- | --- | --- | --- | --- | --- | --- | --- | --- | --- | --- | --- |
| \* | LAPMis12DANCE\_011813\_02.05892.05892.2 | 2.8474 | 0.4061 | 100.0% | 1445.2122 | 1445.5255 | 1 | 6.155 | 72.7% | 1 | R.IFTSIGEDYDER.V | 2 |
| \* | LAPMis12DANCE\_011813\_01.10238.10238.2 | 3.6503 | 0.2408 | 100.0% | 1608.4722 | 1607.804 | 1 | 5.102 | 69.2% | 2 | R.KLEAAEDIAYQLSR.S | 2 |

---

|  |  |  |  |  |  |  |  |  |
| --- | --- | --- | --- | --- | --- | --- | --- | --- |
| U | *gi|67782365|ref|NP\_00* | 4 | 4 | 9.4% | 469 | 51386 | 5.5 | keratin 7 [Homo sapiens] |

| Filename XCorr DeltCN Conf% ObsM+H+ CalcM+H+ SpR ZScore Ion% # Sequence  | | | | | | | | | | | | |
| --- | --- | --- | --- | --- | --- | --- | --- | --- | --- | --- | --- | --- |
|  | LAPMis12DANCE\_011813\_01.03248.03248.2 | 3.092 | 0.2437 | 100.0% | 1245.4722 | 1246.3629 | 1 | 5.079 | 77.8% | 1 | R.VRQEESEQIK.T | 2 |
|  | LAPMis12DANCE\_011813\_01.08360.08360.2 | 2.5656 | 0.1541 | 98.0% | 1082.3722 | 1083.2755 | 7 | 6.493 | 68.8% | 1 | K.FASFIDKVR.F | 222222 |
|  | LAPMis12DANCE\_011813\_01.12341.12341.2 | 3.7647 | 0.4656 | 100.0% | 1443.2722 | 1443.686 | 1 | 8.666 | 70.8% | 1 | R.LPDIFEAQIAGLR.G | 2 |
|  | LAPMis12DANCE\_011813\_01.06580.06580.2 | 2.9281 | 0.2269 | 99.5% | 1385.3121 | 1386.548 | 1 | 5.81 | 72.7% | 1 | R.AKQEELEAALQR.G | 2 |

Similarities:
gi|4504919|ref|NP\_002(1:3)  
gi|47132620|ref|NP\_00(1:3)  
gi|119395754|ref|NP\_0(1:3)  
gi|119703753|ref|NP\_0(1:3)  
gi|153791158|ref|NP\_0(1:3)  

---

|  |  |  |  |  |  |  |  |  |
| --- | --- | --- | --- | --- | --- | --- | --- | --- |
| U | *gi|222352151|ref|NP\_0* | 2 | 3 | 9.3% | 356 | 37498 | 7.1 | poly(rC) binding protein 1 [Homo sapiens] |

| Filename XCorr DeltCN Conf% ObsM+H+ CalcM+H+ SpR ZScore Ion% # Sequence  | | | | | | | | | | | | |
| --- | --- | --- | --- | --- | --- | --- | --- | --- | --- | --- | --- | --- |
| \* | LAPMis12DANCE\_011813\_01.11252.11252.2 | 2.7209 | 0.3726 | 100.0% | 1388.7922 | 1389.6781 | 101 | 6.414 | 45.8% | 2 | R.IITLTGPTNAIFK.A | 2 |
|  | LAPMis12DANCE\_011813\_02.05693.05693.2 | 5.0761 | 0.528 | 100.0% | 2091.412 | 2091.2573 | 1 | 10.023 | 60.5% | 1 | R.ESTGAQVQVAGDMLPNSTER.A | 2 |

---

|  |  |  |  |  |  |  |  |  |
| --- | --- | --- | --- | --- | --- | --- | --- | --- |
| U | *Reverse\_gi|61966781|r* | 1 | 1 | 9.3% | 140 | 15805 | 9.1 | hypothetical protein LOC391356 [Homo sapiens] |

| Filename XCorr DeltCN Conf% ObsM+H+ CalcM+H+ SpR ZScore Ion% # Sequence  | | | | | | | | | | | | |
| --- | --- | --- | --- | --- | --- | --- | --- | --- | --- | --- | --- | --- |
| \* | LAPMis12DANCE\_011813\_02.08255.08255.2 | 2.5065 | 0.2284 | 98.5% | 1563.0721 | 1563.694 | 1 | 4.303 | 58.3% | 1 | K.LTT#EDPAELVVK@R.M | 2 |

---

|  |  |  |  |  |  |  |  |  |
| --- | --- | --- | --- | --- | --- | --- | --- | --- |
| U | *gi|113412837|ref|XP\_2* | 1 | 1 | 9.2% | 348 | 38821 | 5.6 | PREDICTED: similar to unr-interacting protein [Homo sapiens] |

| Filename XCorr DeltCN Conf% ObsM+H+ CalcM+H+ SpR ZScore Ion% # Sequence  | | | | | | | | | | | | |
| --- | --- | --- | --- | --- | --- | --- | --- | --- | --- | --- | --- | --- |
| \* | LAPMis12DANCE\_011813\_02.08315.08315.3 | 3.5672 | 0.2323 | 96.5% | 3583.5244 | 3584.8699 | 73 | 3.818 | 18.5% | 1 | R.FSRDGELYASGS\*EDGTLRLGQTVVGKTYGLWK@.C | 3 |

---

|  |  |  |  |  |  |  |  |  |
| --- | --- | --- | --- | --- | --- | --- | --- | --- |
| U | *gi|10864047|ref|NP\_06* | 6 | 7 | 9.1% | 864 | 94255 | 5.1 | epidermal growth factor receptor pathway substrate 15-like 1 [Homo sapiens] |

| Filename XCorr DeltCN Conf% ObsM+H+ CalcM+H+ SpR ZScore Ion% # Sequence  | | | | | | | | | | | | |
| --- | --- | --- | --- | --- | --- | --- | --- | --- | --- | --- | --- | --- |
| \* | LAPMis12DANCE\_011813\_01.14451.14451.2 | 2.8259 | 0.3662 | 100.0% | 2234.172 | 2235.5852 | 1 | 5.917 | 40.0% | 1 | K.AKFDGIFESLLPINGLLSGDK.V | 2 |
| \* | LAPMis12DANCE\_011813\_01.04257.04257.2 | 2.4528 | 0.1649 | 96.0% | 1277.7122 | 1277.3739 | 166 | 4.696 | 50.0% | 1 | K.TQIQSQESDLK.S | 2 |
| \* | LAPMis12DANCE\_011813\_02.05656.05656.2 | 5.4026 | 0.4001 | 100.0% | 1858.3722 | 1859.003 | 1 | 8.184 | 80.0% | 2 | R.LQQEETQLEQSIQAGR.V | 2 |
| \* | LAPMis12DANCE\_011813\_01.03749.03749.2 | 2.6307 | 0.2235 | 99.4% | 1162.0521 | 1162.2015 | 1 | 5.085 | 77.8% | 1 | K.STQDEINQAR.S | 2 |
| \* | LAPMis12DANCE\_011813\_01.03287.03287.2 | 2.1916 | 0.1711 | 95.7% | 969.83215 | 970.07324 | 7 | 5.193 | 78.6% | 1 | K.LSQLHESR.Q | 2 |
| \* | LAPMis12DANCE\_011813\_01.08853.08853.2 | 2.7423 | 0.1496 | 96.6% | 1413.8722 | 1414.5756 | 2 | 4.403 | 58.3% | 1 | R.GSFGAMDDPFKNK.A | 2 |

---

|  |  |  |  |  |  |  |  |  |
| --- | --- | --- | --- | --- | --- | --- | --- | --- |
| U | *gi|32129199|ref|NP\_14* | 1 | 1 | 9.0% | 210 | 23671 | 6.4 | cytokine induced protein 29 kDa [Homo sapiens] |

| Filename XCorr DeltCN Conf% ObsM+H+ CalcM+H+ SpR ZScore Ion% # Sequence  | | | | | | | | | | | | |
| --- | --- | --- | --- | --- | --- | --- | --- | --- | --- | --- | --- | --- |
| \* | LAPMis12DANCE\_011813\_02.05145.05145.2 | 4.8062 | 0.5232 | 100.0% | 1872.2122 | 1872.9806 | 1 | 9.563 | 63.9% | 1 | R.FGIVTSSAGTGTTEDTEAK.K | 2 |

---

|  |  |  |  |  |  |  |  |  |
| --- | --- | --- | --- | --- | --- | --- | --- | --- |
| U | *gi|16933546|ref|NP\_44* | 2 | 4 | 8.8% | 317 | 34274 | 6.0 | ribosomal protein P0 [Homo sapiens] |
| U | *gi|4506667|ref|NP\_000* | 2 | 4 | 8.8% | 317 | 34274 | 6.0 | ribosomal protein P0 [Homo sapiens] |

| Filename XCorr DeltCN Conf% ObsM+H+ CalcM+H+ SpR ZScore Ion% # Sequence  | | | | | | | | | | | | |
| --- | --- | --- | --- | --- | --- | --- | --- | --- | --- | --- | --- | --- |
|  | LAPMis12DANCE\_011813\_01.03807.03807.2 | 2.6697 | 0.2538 | 99.5% | 1222.2522 | 1222.3433 | 1 | 5.915 | 65.0% | 3 | R.GHLENNPALEK.L | 2 |
|  | LAPMis12DANCE\_011813\_02.08373.08373.2 | 3.3935 | 0.272 | 100.0% | 1897.3322 | 1897.1754 | 6 | 5.614 | 37.5% | 1 | R.VLALSVETDYTFPLAEK.V | 2 |

---

|  |  |  |  |  |  |  |  |  |
| --- | --- | --- | --- | --- | --- | --- | --- | --- |
| U | *gi|13699824|ref|NP\_00* | 7 | 7 | 8.7% | 1056 | 119159 | 5.6 | kinesin family member 11 [Homo sapiens] |

| Filename XCorr DeltCN Conf% ObsM+H+ CalcM+H+ SpR ZScore Ion% # Sequence  | | | | | | | | | | | | |
| --- | --- | --- | --- | --- | --- | --- | --- | --- | --- | --- | --- | --- |
| \* | LAPMis12DANCE\_011813\_01.13101.13101.2 | 3.8995 | 0.3522 | 100.0% | 2216.3123 | 2217.3965 | 1 | 6.876 | 44.4% | 1 | R.SPNEEYTWEEDPLAGIIPR.T | 2 |
| \* | LAPMis12DANCE\_011813\_01.10408.10408.2 | 2.5557 | 0.2203 | 98.1% | 1485.9521 | 1486.6682 | 2 | 4.077 | 50.0% | 1 | R.EAGNINQSLLTLGR.V | 2 |
| \* | LAPMis12DANCE\_011813\_01.04278.04278.2 | 3.0176 | 0.1965 | 99.5% | 1296.4922 | 1297.4972 | 1 | 5.092 | 75.0% | 1 | K.NILNKPEVNQK.L | 2 |
| \* | LAPMis12DANCE\_011813\_01.12497.12497.2 | 3.1532 | 0.3009 | 100.0% | 1671.5721 | 1671.9286 | 1 | 5.672 | 61.5% | 1 | K.LTVQEEQIVELIEK.I | 2 |
| \* | LAPMis12DANCE\_011813\_01.06718.06718.2 | 1.9791 | 0.2758 | 97.2% | 1129.5521 | 1130.2432 | 45 | 4.396 | 66.7% | 1 | K.IGAVEEELNR.V | 2 |
| \* | LAPMis12DANCE\_011813\_01.03299.03299.2 | 3.6339 | 0.3463 | 100.0% | 1256.0721 | 1255.3732 | 1 | 6.285 | 75.0% | 1 | K.HSDKLNGNLEK.I | 2 |
| \* | LAPMis12DANCE\_011813\_01.04347.04347.2 | 4.0336 | 0.3752 | 100.0% | 1501.3722 | 1501.6769 | 1 | 7.626 | 70.8% | 1 | R.SKVEETTEHLVTK.S | 2 |

---

|  |  |  |  |  |  |  |  |  |
| --- | --- | --- | --- | --- | --- | --- | --- | --- |
| U | *gi|56699409|ref|NP\_00* | 3 | 4 | 8.7% | 391 | 42332 | 10.1 | RNA binding motif protein, X-linked [Homo sapiens] |

| Filename XCorr DeltCN Conf% ObsM+H+ CalcM+H+ SpR ZScore Ion% # Sequence  | | | | | | | | | | | | |
| --- | --- | --- | --- | --- | --- | --- | --- | --- | --- | --- | --- | --- |
|  | LAPMis12DANCE\_011813\_02.06279.06279.2 | 3.1229 | 0.2329 | 99.8% | 1436.7722 | 1436.6049 | 3 | 4.915 | 66.7% | 2 | K.LFIGGLNTETNEK.A | 2 |
| \* | LAPMis12DANCE\_011813\_01.03971.03971.2 | 2.7311 | 0.2486 | 99.4% | 1435.9922 | 1436.5645 | 11 | 5.028 | 58.3% | 1 | K.VEQATKPSFESGR.R | 2 |
| \* | LAPMis12DANCE\_011813\_01.03201.03201.2 | 2.6098 | 0.2705 | 100.0% | 874.09216 | 874.0335 | 2 | 5.711 | 85.7% | 1 | R.RGPPPPPR.S | 2 |

---

|  |  |  |  |  |  |  |  |  |
| --- | --- | --- | --- | --- | --- | --- | --- | --- |
| U | *gi|8923173|ref|NP\_060* | 1 | 1 | 8.7% | 219 | 25507 | 6.0 | hypothetical protein LOC54848 [Homo sapiens] |

| Filename XCorr DeltCN Conf% ObsM+H+ CalcM+H+ SpR ZScore Ion% # Sequence  | | | | | | | | | | | | |
| --- | --- | --- | --- | --- | --- | --- | --- | --- | --- | --- | --- | --- |
| \* | LAPMis12DANCE\_011813\_01.15975.15975.2 | 2.1219 | 0.2884 | 97.6% | 2055.0923 | 2056.1074 | 1 | 4.206 | 38.9% | 1 | R.KT#DTVVESSVSGDHSGTLR.R | 2 |

---

|  |  |  |  |  |  |  |  |  |
| --- | --- | --- | --- | --- | --- | --- | --- | --- |
| U | *gi|217330646|ref|NP\_0* | 1 | 1 | 8.7% | 127 | 14395 | 9.6 | activated RNA polymerase II transcription cofactor 4 [Homo sapiens] |

| Filename XCorr DeltCN Conf% ObsM+H+ CalcM+H+ SpR ZScore Ion% # Sequence  | | | | | | | | | | | | |
| --- | --- | --- | --- | --- | --- | --- | --- | --- | --- | --- | --- | --- |
| \* | LAPMis12DANCE\_011813\_01.07494.07494.2 | 2.1165 | 0.2517 | 97.2% | 1261.4922 | 1261.3312 | 1 | 5.261 | 70.0% | 1 | K.EQISDIDDAVR.K | 2 |

---

|  |  |  |  |  |  |  |  |  |
| --- | --- | --- | --- | --- | --- | --- | --- | --- |
| U | *gi|4506685|ref|NP\_001* | 1 | 1 | 8.6% | 151 | 17222 | 10.5 | ribosomal protein S13 [Homo sapiens] |

| Filename XCorr DeltCN Conf% ObsM+H+ CalcM+H+ SpR ZScore Ion% # Sequence  | | | | | | | | | | | | |
| --- | --- | --- | --- | --- | --- | --- | --- | --- | --- | --- | --- | --- |
| \* | LAPMis12DANCE\_011813\_01.09801.09801.2 | 2.8459 | 0.3401 | 100.0% | 1382.0922 | 1382.6896 | 1 | 6.416 | 70.8% | 1 | K.KGLTPSQIGVILR.D | 2 |

---

|  |  |  |  |  |  |  |  |  |
| --- | --- | --- | --- | --- | --- | --- | --- | --- |
| U | *gi|23618867|ref|NP\_07* | 2 | 2 | 8.4% | 322 | 35619 | 9.1 | sideroflexin 1 [Homo sapiens] |

| Filename XCorr DeltCN Conf% ObsM+H+ CalcM+H+ SpR ZScore Ion% # Sequence  | | | | | | | | | | | | |
| --- | --- | --- | --- | --- | --- | --- | --- | --- | --- | --- | --- | --- |
| \* | LAPMis12DANCE\_011813\_01.09231.09231.2 | 3.2019 | 0.3978 | 100.0% | 1500.6721 | 1501.6799 | 1 | 6.698 | 70.8% | 1 | R.NILLTNEQLESAR.K | 2 |
| \* | LAPMis12DANCE\_011813\_01.07919.07919.2 | 2.8246 | 0.2341 | 99.4% | 1644.4722 | 1643.7496 | 1 | 4.171 | 61.5% | 1 | K.YIYDSAFHPDTGEK.M | 2 |

---

|  |  |  |  |  |  |  |  |  |
| --- | --- | --- | --- | --- | --- | --- | --- | --- |
| U | *gi|16905073|ref|NP\_07* | 1 | 1 | 8.2% | 269 | 31655 | 4.9 | SoxLZ/Sox6 leucine zipper binding protein [Homo sapiens] |

| Filename XCorr DeltCN Conf% ObsM+H+ CalcM+H+ SpR ZScore Ion% # Sequence  | | | | | | | | | | | | |
| --- | --- | --- | --- | --- | --- | --- | --- | --- | --- | --- | --- | --- |
| \* | LAPMis12DANCE\_011813\_02.10937.10937.3 | 3.0264 | 0.3472 | 99.7% | 2581.0745 | 2581.0027 | 1 | 6.167 | 27.4% | 1 | K.NIQESSVNLITLHEMLEILINR.L | 3 |

---

|  |  |  |  |  |  |  |  |  |
| --- | --- | --- | --- | --- | --- | --- | --- | --- |
| U | *gi|55956919|ref|NP\_11* | 2 | 2 | 8.1% | 332 | 35968 | 6.9 | heterogeneous nuclear ribonucleoprotein A/B isoform a [Homo sapiens] |
| U | *gi|55956921|ref|NP\_00* | 2 | 2 | 9.5% | 285 | 30588 | 7.9 | heterogeneous nuclear ribonucleoprotein A/B isoform b [Homo sapiens] |

| Filename XCorr DeltCN Conf% ObsM+H+ CalcM+H+ SpR ZScore Ion% # Sequence  | | | | | | | | | | | | |
| --- | --- | --- | --- | --- | --- | --- | --- | --- | --- | --- | --- | --- |
|  | LAPMis12DANCE\_011813\_01.09184.09184.2 | 3.7857 | 0.4742 | 100.0% | 1504.4722 | 1504.6799 | 1 | 7.636 | 69.2% | 1 | K.IFVGGLNPEATEEK.I | 2 |
|  | LAPMis12DANCE\_011813\_01.04098.04098.2 | 2.5781 | 0.3674 | 100.0% | 1500.1322 | 1500.5675 | 4 | 6.207 | 54.2% | 1 | K.EVYQQQQYGSGGR.G | 2 |

---

|  |  |  |  |  |  |  |  |  |
| --- | --- | --- | --- | --- | --- | --- | --- | --- |
| U | *gi|56847616|ref|NP\_00* | 1 | 1 | 8.1% | 333 | 34713 | 8.1 | collagen triple helix repeat-containing [Homo sapiens] |
| U | *gi|65301115|ref|NP\_84* | 1 | 1 | 8.1% | 333 | 34681 | 8.5 | C1q and tumor necrosis factor related protein 9 [Homo sapiens] |

| Filename XCorr DeltCN Conf% ObsM+H+ CalcM+H+ SpR ZScore Ion% # Sequence  | | | | | | | | | | | | |
| --- | --- | --- | --- | --- | --- | --- | --- | --- | --- | --- | --- | --- |
|  | LAPMis12DANCE\_011813\_01.11770.11770.3 | 3.2566 | 0.2443 | 96.4% | 2752.9443 | 2751.8674 | 43 | 4.613 | 24.0% | 1 | K.GLRGETGPQGQK@GNK@GDVGPT#GPEGPR.G | 3 |

---

|  |  |  |  |  |  |  |  |  |
| --- | --- | --- | --- | --- | --- | --- | --- | --- |
| U | *gi|72534660|ref|NP\_00* | 2 | 2 | 8.0% | 238 | 27367 | 11.8 | splicing factor, arginine/serine-rich 7 [Homo sapiens] |

| Filename XCorr DeltCN Conf% ObsM+H+ CalcM+H+ SpR ZScore Ion% # Sequence  | | | | | | | | | | | | |
| --- | --- | --- | --- | --- | --- | --- | --- | --- | --- | --- | --- | --- |
| \* | LAPMis12DANCE2\_011813\_01.05423.05423.2 | 2.1645 | 0.2386 | 96.9% | 1245.2722 | 1245.4827 | 89 | 5.437 | 50.0% | 1 | R.VRVELSTGMPR.R | 2 |
| \* | LAPMis12DANCE\_011813\_01.03736.03736.2 | 2.4116 | 0.2097 | 99.1% | 1017.0522 | 1017.0891 | 4 | 4.273 | 85.7% | 1 | R.RPFDPNDR.C | 2 |

---

|  |  |  |  |  |  |  |  |  |
| --- | --- | --- | --- | --- | --- | --- | --- | --- |
| U | *Reverse\_gi|169188953|* | 1 | 1 | 8.0% | 137 | 15565 | 8.4 | PREDICTED: similar to hCG2023449 [Homo sapiens] |
| U | *Reverse\_gi|169201783|* | 1 | 1 | 8.0% | 137 | 15550 | 8.4 | PREDICTED: similar to hCG2023449 [Homo sapiens] |
| U | *Reverse\_gi|169201463|* | 1 | 1 | 8.0% | 137 | 15550 | 8.4 | PREDICTED: similar to hCG2023449 [Homo sapiens] |

| Filename XCorr DeltCN Conf% ObsM+H+ CalcM+H+ SpR ZScore Ion% # Sequence  | | | | | | | | | | | | |
| --- | --- | --- | --- | --- | --- | --- | --- | --- | --- | --- | --- | --- |
|  | LAPMis12DANCE2\_011813\_01.07289.07289.2 | 2.4546 | 0.2024 | 98.0% | 1250.4321 | 1251.3818 | 1 | 4.411 | 65.0% | 1 | -.SSPKAYNDQIK.T | 2 |

---

|  |  |  |  |  |  |  |  |  |
| --- | --- | --- | --- | --- | --- | --- | --- | --- |
| U | *gi|5174447|ref|NP\_006* | 2 | 2 | 7.9% | 317 | 35077 | 7.7 | guanine nucleotide binding protein (G protein), beta polypeptide 2-like 1 [Homo sapiens] |

| Filename XCorr DeltCN Conf% ObsM+H+ CalcM+H+ SpR ZScore Ion% # Sequence  | | | | | | | | | | | | |
| --- | --- | --- | --- | --- | --- | --- | --- | --- | --- | --- | --- | --- |
| \* | LAPMis12DANCE\_011813\_01.10126.10126.2 | 4.5659 | 0.4037 | 100.0% | 1789.2922 | 1790.0642 | 1 | 8.039 | 60.0% | 1 | K.IIVDELKQEVISTSSK.A | 2 |
| \* | LAPMis12DANCE\_011813\_01.09132.09132.2 | 2.5716 | 0.2362 | 99.5% | 1059.7722 | 1060.2412 | 1 | 4.726 | 81.2% | 1 | R.VWQVTIGTR.- | 2 |

---

|  |  |  |  |  |  |  |  |  |
| --- | --- | --- | --- | --- | --- | --- | --- | --- |
| U | *gi|15431290|ref|NP\_00* | 1 | 2 | 7.9% | 178 | 20252 | 9.6 | ribosomal protein L11 [Homo sapiens] |

| Filename XCorr DeltCN Conf% ObsM+H+ CalcM+H+ SpR ZScore Ion% # Sequence  | | | | | | | | | | | | |
| --- | --- | --- | --- | --- | --- | --- | --- | --- | --- | --- | --- | --- |
| \* | LAPMis12DANCE\_011813\_01.09879.09879.2 | 3.8579 | 0.5089 | 100.0% | 1547.3522 | 1547.7917 | 1 | 8.933 | 69.2% | 2 | K.VLEQLTGQTPVFSK.A | 2 |

---

|  |  |  |  |  |  |  |  |  |
| --- | --- | --- | --- | --- | --- | --- | --- | --- |
| U | *gi|169165045|ref|XP\_0* | 1 | 1 | 7.9% | 164 | 18111 | 6.9 | PREDICTED: similar to peptidyl-Pro cis trans isomerase [Homo sapiens] |
| U | *gi|88969090|ref|XP\_94* | 1 | 1 | 7.9% | 164 | 18111 | 6.9 | PREDICTED: similar to peptidyl-Pro cis trans isomerase [Homo sapiens] |
| U | *gi|169165350|ref|XP\_0* | 1 | 1 | 7.9% | 164 | 18111 | 6.9 | PREDICTED: similar to peptidyl-Pro cis trans isomerase [Homo sapiens] |

| Filename XCorr DeltCN Conf% ObsM+H+ CalcM+H+ SpR ZScore Ion% # Sequence  | | | | | | | | | | | | |
| --- | --- | --- | --- | --- | --- | --- | --- | --- | --- | --- | --- | --- |
|  | LAPMis12DANCE\_011813\_01.09374.09374.2 | 3.0154 | 0.2716 | 99.9% | 1509.1522 | 1507.7603 | 1 | 5.691 | 75.0% | 1 | R.VKEGMDIVEAMER.F | 2 |

---

|  |  |  |  |  |  |  |  |  |
| --- | --- | --- | --- | --- | --- | --- | --- | --- |
| U | *gi|4759160|ref|NP\_004* | 1 | 1 | 7.9% | 126 | 13916 | 10.3 | small nuclear ribonucleoprotein polypeptide D3 [Homo sapiens] |

| Filename XCorr DeltCN Conf% ObsM+H+ CalcM+H+ SpR ZScore Ion% # Sequence  | | | | | | | | | | | | |
| --- | --- | --- | --- | --- | --- | --- | --- | --- | --- | --- | --- | --- |
| \* | LAPMis12DANCE\_011813\_01.08646.08646.2 | 2.7834 | 0.3174 | 100.0% | 1219.3322 | 1219.4264 | 1 | 6.052 | 77.8% | 1 | R.VAQLEQVYIR.G | 2 |

---

|  |  |  |  |  |  |  |  |  |
| --- | --- | --- | --- | --- | --- | --- | --- | --- |
| U | *gi|14141152|ref|NP\_00* | 4 | 5 | 7.8% | 730 | 77516 | 8.7 | heterogeneous nuclear ribonucleoprotein M isoform a [Homo sapiens] |
| U | *gi|157412270|ref|NP\_1* | 4 | 5 | 8.2% | 691 | 73621 | 8.8 | heterogeneous nuclear ribonucleoprotein M isoform b [Homo sapiens] |

| Filename XCorr DeltCN Conf% ObsM+H+ CalcM+H+ SpR ZScore Ion% # Sequence  | | | | | | | | | | | | |
| --- | --- | --- | --- | --- | --- | --- | --- | --- | --- | --- | --- | --- |
|  | LAPMis12DANCE\_011813\_01.12923.12923.2 | 2.4082 | 0.2541 | 98.3% | 1753.6921 | 1754.0051 | 1 | 4.88 | 50.0% | 1 | K.VGEVTYVELLMDAEGK.S | 2 |
|  | LAPMis12DANCE2\_011813\_01.04118.04118.2 | 2.7988 | 0.1618 | 98.6% | 1102.1322 | 1102.2714 | 2 | 3.682 | 75.0% | 2 | R.MGAGLGHGMDR.V | 2 |
|  | LAPMis12DANCE2\_011813\_01.07799.07799.2 | 2.7505 | 0.3341 | 100.0% | 1613.6322 | 1614.875 | 2 | 5.799 | 46.4% | 1 | R.MGPLGLDHMASSIER.M | 2 |
|  | LAPMis12DANCE\_011813\_01.09322.09322.2 | 3.2112 | 0.2015 | 99.4% | 1429.5122 | 1428.7076 | 1 | 5.424 | 67.9% | 1 | R.MGPAMGPALGAGIER.M | 2 |

---

|  |  |  |  |  |  |  |  |  |
| --- | --- | --- | --- | --- | --- | --- | --- | --- |
| U | *gi|66346679|ref|NP\_00* | 2 | 2 | 7.8% | 408 | 44965 | 8.6 | SERPINE1 mRNA binding protein 1 isoform 1 [Homo sapiens] |
| U | *gi|66346685|ref|NP\_05* | 2 | 2 | 8.3% | 387 | 42427 | 8.4 | SERPINE1 mRNA binding protein 1 isoform 4 [Homo sapiens] |
| U | *gi|66346683|ref|NP\_00* | 2 | 2 | 8.1% | 393 | 43135 | 8.4 | SERPINE1 mRNA binding protein 1 isoform 3 [Homo sapiens] |
| U | *gi|66346681|ref|NP\_00* | 2 | 2 | 8.0% | 402 | 44257 | 8.7 | SERPINE1 mRNA binding protein 1 isoform 2 [Homo sapiens] |

| Filename XCorr DeltCN Conf% ObsM+H+ CalcM+H+ SpR ZScore Ion% # Sequence  | | | | | | | | | | | | |
| --- | --- | --- | --- | --- | --- | --- | --- | --- | --- | --- | --- | --- |
|  | LAPMis12DANCE2\_011813\_01.11242.11242.2 | 3.3767 | 0.3943 | 100.0% | 1945.1921 | 1945.0894 | 1 | 6.288 | 63.3% | 1 | R.FDQLFDDESDPFEVLK.A | 2 |
|  | LAPMis12DANCE\_011813\_01.03244.03244.2 | 5.2383 | 0.5667 | 100.0% | 1461.1122 | 1461.5314 | 1 | 9.92 | 70.0% | 1 | K.SAAQAAAQTNSNAAGK.Q | 2 |

---

|  |  |  |  |  |  |  |  |  |
| --- | --- | --- | --- | --- | --- | --- | --- | --- |
| U | *gi|113412878|ref|XP\_0* | 1 | 1 | 7.8% | 293 | 31479 | 7.6 | PREDICTED: similar to voltage-dependent anion channel [Homo sapiens] |
| U | *gi|42476281|ref|NP\_00* | 1 | 1 | 7.8% | 294 | 31566 | 7.6 | voltage-dependent anion channel 2 [Homo sapiens] |
| U | *gi|169164151|ref|XP\_0* | 1 | 1 | 7.8% | 293 | 31445 | 7.6 | PREDICTED: similar to voltage-dependent anion channel [Homo sapiens] |
| U | *gi|169163967|ref|XP\_0* | 1 | 1 | 7.8% | 293 | 31419 | 7.6 | PREDICTED: similar to voltage-dependent anion channel [Homo sapiens] |

| Filename XCorr DeltCN Conf% ObsM+H+ CalcM+H+ SpR ZScore Ion% # Sequence  | | | | | | | | | | | | |
| --- | --- | --- | --- | --- | --- | --- | --- | --- | --- | --- | --- | --- |
|  | LAPMis12DANCE\_011813\_02.06251.06251.3 | 3.5917 | 0.2645 | 98.1% | 2528.8145 | 2529.682 | 36 | 4.852 | 26.1% | 1 | R.TGDFQLHTNVNDGTEFGGSIYQK.V | 3 |

---

|  |  |  |  |  |  |  |  |  |
| --- | --- | --- | --- | --- | --- | --- | --- | --- |
| U | *gi|12408675|ref|NP\_03* | 1 | 1 | 7.8% | 154 | 16648 | 6.6 | prefoldin subunit 2 [Homo sapiens] |

| Filename XCorr DeltCN Conf% ObsM+H+ CalcM+H+ SpR ZScore Ion% # Sequence  | | | | | | | | | | | | |
| --- | --- | --- | --- | --- | --- | --- | --- | --- | --- | --- | --- | --- |
| \* | LAPMis12DANCE\_011813\_02.06045.06045.2 | 2.6945 | 0.1687 | 97.8% | 1387.3121 | 1386.6317 | 2 | 4.233 | 68.2% | 1 | K.IIETLTQQLQAK.G | 2 |

---

|  |  |  |  |  |  |  |  |  |
| --- | --- | --- | --- | --- | --- | --- | --- | --- |
| U | *gi|83776600|ref|NP\_00* | 2 | 2 | 7.6% | 344 | 39311 | 9.3 | aurora kinase B [Homo sapiens] |

| Filename XCorr DeltCN Conf% ObsM+H+ CalcM+H+ SpR ZScore Ion% # Sequence  | | | | | | | | | | | | |
| --- | --- | --- | --- | --- | --- | --- | --- | --- | --- | --- | --- | --- |
| \* | LAPMis12DANCE\_011813\_01.03394.03394.2 | 2.1659 | 0.2878 | 98.7% | 1197.3922 | 1198.3213 | 14 | 4.915 | 54.5% | 1 | R.SNVQPTAAPGQK.V | 2 |
| \* | LAPMis12DANCE\_011813\_01.07803.07803.2 | 3.2903 | 0.2616 | 100.0% | 1521.5521 | 1520.6978 | 1 | 4.586 | 61.5% | 1 | K.VMENSSGTPDILTR.H | 2 |

---

|  |  |  |  |  |  |  |  |  |
| --- | --- | --- | --- | --- | --- | --- | --- | --- |
| U | *gi|14277700|ref|NP\_00* | 1 | 1 | 7.6% | 132 | 14515 | 7.2 | ribosomal protein S12 [Homo sapiens] |

| Filename XCorr DeltCN Conf% ObsM+H+ CalcM+H+ SpR ZScore Ion% # Sequence  | | | | | | | | | | | | |
| --- | --- | --- | --- | --- | --- | --- | --- | --- | --- | --- | --- | --- |
| \* | LAPMis12DANCE\_011813\_01.05183.05183.2 | 2.1983 | 0.223 | 97.1% | 1066.4521 | 1067.2333 | 22 | 4.495 | 72.2% | 1 | K.TALIHDGLAR.G | 2 |

---

|  |  |  |  |  |  |  |  |  |
| --- | --- | --- | --- | --- | --- | --- | --- | --- |
| U | *gi|114431246|ref|NP\_8* | 4 | 6 | 7.5% | 464 | 50567 | 5.5 | keratin 25D [Homo sapiens] |

| Filename XCorr DeltCN Conf% ObsM+H+ CalcM+H+ SpR ZScore Ion% # Sequence  | | | | | | | | | | | | |
| --- | --- | --- | --- | --- | --- | --- | --- | --- | --- | --- | --- | --- |
|  | LAPMis12DANCE\_011813\_01.04844.04844.2 | 2.9602 | 0.3484 | 100.0% | 1090.6322 | 1091.2273 | 24 | 6.022 | 68.8% | 3 | K.VTMQNLNDR.L | 222 |
|  | LAPMis12DANCE\_011813\_01.09472.09472.1 | 1.7805 | 0.2419 | 98.6% | 1109.37 | 1110.1681 | 1 | 5.322 | 62.5% | 1 | K.DAEAWFNEK.S | 11 |
|  | LAPMis12DANCE\_011813\_01.09488.09488.2 | 3.0337 | 0.288 | 100.0% | 1110.0721 | 1110.1681 | 1 | 6.896 | 75.0% | 1 | K.DAEAWFNEK.S | 22 |
| \* | LAPMis12DANCE\_011813\_01.16748.16748.2 | 2.4192 | 0.294 | 99.2% | 1991.1322 | 1992.1857 | 193 | 4.216 | 31.2% | 1 | R.GKVLSSRIHSIEEK@TS\*K.M | 2 |

Similarities:
contaminant\_KERATIN03(3:1)  
gi|15431310|ref|NP\_00(1:3)  

---

|  |  |  |  |  |  |  |  |  |
| --- | --- | --- | --- | --- | --- | --- | --- | --- |
| U | *gi|169212778|ref|XP\_0* | 2 | 5 | 7.5% | 266 | 30042 | 10.6 | PREDICTED: similar to ribosomal protein L7a [Homo sapiens] |
| U | *gi|4506661|ref|NP\_000* | 2 | 5 | 7.5% | 266 | 29996 | 10.6 | ribosomal protein L7a [Homo sapiens] |
| U | *gi|169213130|ref|XP\_0* | 2 | 5 | 7.5% | 266 | 30042 | 10.6 | PREDICTED: similar to ribosomal protein L7a [Homo sapiens] |
| U | *gi|169212940|ref|XP\_0* | 2 | 5 | 7.5% | 266 | 30028 | 10.6 | PREDICTED: similar to ribosomal protein L7a [Homo sapiens] |

| Filename XCorr DeltCN Conf% ObsM+H+ CalcM+H+ SpR ZScore Ion% # Sequence  | | | | | | | | | | | | |
| --- | --- | --- | --- | --- | --- | --- | --- | --- | --- | --- | --- | --- |
|  | LAPMis12DANCE2\_011813\_01.03935.03935.1 | 2.0507 | 0.4149 | 100.0% | 851.23 | 852.06476 | 1 | 6.98 | 75.0% | 3 | K.VAPAPAVVK.K | 1 |
|  | LAPMis12DANCE\_011813\_01.08278.08278.2 | 2.916 | 0.4016 | 100.0% | 1217.2122 | 1217.3672 | 2 | 6.744 | 75.0% | 2 | K.NFGIGQDIQPK.R | 2 |

---

|  |  |  |  |  |  |  |  |  |
| --- | --- | --- | --- | --- | --- | --- | --- | --- |
| U | *gi|153791158|ref|NP\_0* | 5 | 7 | 7.4% | 551 | 59560 | 7.7 | keratin 75 [Homo sapiens] |

| Filename XCorr DeltCN Conf% ObsM+H+ CalcM+H+ SpR ZScore Ion% # Sequence  | | | | | | | | | | | | |
| --- | --- | --- | --- | --- | --- | --- | --- | --- | --- | --- | --- | --- |
|  | LAPMis12DANCE\_011813\_01.08360.08360.2 | 2.5656 | 0.1541 | 98.0% | 1082.3722 | 1083.2755 | 7 | 6.493 | 68.8% | 1 | K.FASFIDKVR.F | 222222 |
|  | LAPMis12DANCE\_011813\_01.12718.12718.2 | 3.0679 | 0.3399 | 100.0% | 1329.7522 | 1330.5211 | 1 | 6.91 | 77.3% | 2 | R.NLDLDSIIAEVK.A | 2222 |
|  | LAPMis12DANCE2\_011813\_01.04202.04202.1 | 1.9412 | 0.3297 | 100.0% | 1079.46 | 1080.1423 | 4 | 5.717 | 62.5% | 2 | K.AQYEDIANR.S | 11 |
|  | LAPMis12DANCE\_011813\_01.04676.04676.2 | 2.3902 | 0.3451 | 100.0% | 1079.9321 | 1080.1423 | 21 | 6.799 | 62.5% | 1 | K.AQYEDIANR.S | 22 |
|  | LAPMis12DANCE\_011813\_02.07132.07132.2 | 2.9898 | 0.376 | 100.0% | 1264.1522 | 1264.4644 | 1 | 7.517 | 75.0% | 1 | K.LALDVEIATYR.K | 2222 |

Similarities:
gi|4504919|ref|NP\_002(3:2)  
gi|47132620|ref|NP\_00(3:2)  
gi|119395754|ref|NP\_0(3:2)  
gi|119703753|ref|NP\_0(3:2)  
gi|67782365|ref|NP\_00(1:4)  

---

|  |  |  |  |  |  |  |  |  |
| --- | --- | --- | --- | --- | --- | --- | --- | --- |
| U | *gi|61676091|ref|NP\_00* | 1 | 2 | 7.4% | 283 | 33243 | 7.3 | cyclin C isoform a [Homo sapiens] |
| U | *gi|61676093|ref|NP\_00* | 1 | 2 | 10.6% | 198 | 22945 | 5.9 | cyclin C isoform b [Homo sapiens] |

| Filename XCorr DeltCN Conf% ObsM+H+ CalcM+H+ SpR ZScore Ion% # Sequence  | | | | | | | | | | | | |
| --- | --- | --- | --- | --- | --- | --- | --- | --- | --- | --- | --- | --- |
|  | LAPMis12DANCE2\_011813\_01.14435.14435.2 | 2.6046 | 0.1885 | 96.6% | 2284.0122 | 2285.5596 | 263 | 4.427 | 25.0% | 2 | K.VEEFGVVSNT#RLIAAATSVLK.T | 2 |

---

|  |  |  |  |  |  |  |  |  |
| --- | --- | --- | --- | --- | --- | --- | --- | --- |
| U | *gi|52632383|ref|NP\_00* | 2 | 2 | 7.3% | 589 | 64133 | 8.2 | heterogeneous nuclear ribonucleoprotein L isoform a [Homo sapiens] |

| Filename XCorr DeltCN Conf% ObsM+H+ CalcM+H+ SpR ZScore Ion% # Sequence  | | | | | | | | | | | | |
| --- | --- | --- | --- | --- | --- | --- | --- | --- | --- | --- | --- | --- |
| \* | LAPMis12DANCE\_011813\_01.18326.18326.3 | 3.9056 | 0.2185 | 96.7% | 3088.5842 | 3089.6143 | 1 | 4.11 | 27.7% | 1 | R.GLIDGVVEADLVEALQEFGPISYVVVMPK.K | 3 |
|  | LAPMis12DANCE\_011813\_01.09286.09286.2 | 3.7498 | 0.2359 | 100.0% | 1636.1522 | 1635.881 | 1 | 6.467 | 61.5% | 1 | R.AITHLNNNFMFGQK.L | 2 |

---

|  |  |  |  |  |  |  |  |  |
| --- | --- | --- | --- | --- | --- | --- | --- | --- |
| U | *gi|10835143|ref|NP\_00* | 1 | 1 | 7.3% | 381 | 41400 | 7.6 | decay accelerating factor for complement isoform 1 preproprotein [Homo sapiens] |
| U | *gi|168693643|ref|NP\_0* | 1 | 1 | 6.4% | 440 | 48717 | 8.4 | decay accelerating factor for complement isoform 2 precursor [Homo sapiens] |

| Filename XCorr DeltCN Conf% ObsM+H+ CalcM+H+ SpR ZScore Ion% # Sequence  | | | | | | | | | | | | |
| --- | --- | --- | --- | --- | --- | --- | --- | --- | --- | --- | --- | --- |
|  | LAPMis12DANCE2\_011813\_01.16313.16313.3 | 3.3703 | 0.3139 | 99.5% | 3550.0144 | 3550.7834 | 19 | 4.229 | 21.3% | 1 | K.LTCLQNLK@WS\*T#AVEFCKK@KSCPNPGEIR.N | 3 |

---

|  |  |  |  |  |  |  |  |  |
| --- | --- | --- | --- | --- | --- | --- | --- | --- |
| U | *Reverse\_gi|169167696|* | 1 | 1 | 7.3% | 313 | 35310 | 8.8 | PREDICTED: hypothetical protein [Homo sapiens] |
| U | *Reverse\_gi|169168219|* | 1 | 1 | 6.6% | 349 | 39472 | 9.5 | PREDICTED: hypothetical protein [Homo sapiens] |
| U | *Reverse\_gi|169167981|* | 1 | 1 | 8.7% | 263 | 29852 | 8.2 | PREDICTED: hypothetical protein [Homo sapiens] |

| Filename XCorr DeltCN Conf% ObsM+H+ CalcM+H+ SpR ZScore Ion% # Sequence  | | | | | | | | | | | | |
| --- | --- | --- | --- | --- | --- | --- | --- | --- | --- | --- | --- | --- |
|  | LAPMis12DANCE\_011813\_01.15006.15006.3 | 2.8933 | 0.2712 | 97.3% | 2803.4944 | 2804.9124 | 91 | 4.71 | 21.6% | 1 | R.EGMMKAK@EAQYPT#PNS\*S\*TIRTAK@.L | 3 |

---

|  |  |  |  |  |  |  |  |  |
| --- | --- | --- | --- | --- | --- | --- | --- | --- |
| U | *gi|15431303|ref|NP\_00* | 1 | 2 | 7.3% | 192 | 21863 | 10.0 | ribosomal protein L9 [Homo sapiens] |
| U | *gi|67944630|ref|NP\_00* | 1 | 2 | 7.3% | 192 | 21863 | 10.0 | ribosomal protein L9 [Homo sapiens] |
| U | *gi|169208958|ref|XP\_0* | 1 | 2 | 6.0% | 234 | 26188 | 10.5 | PREDICTED: similar to rat ribosomal protein L9 homologue [Homo sapiens] |
| U | *gi|169208716|ref|XP\_9* | 1 | 2 | 6.0% | 234 | 26205 | 10.4 | PREDICTED: hypothetical protein [Homo sapiens] |
| U | *gi|169208204|ref|XP\_9* | 1 | 2 | 6.0% | 234 | 26205 | 10.4 | PREDICTED: similar to rat ribosomal protein L9 homologue [Homo sapiens] |

| Filename XCorr DeltCN Conf% ObsM+H+ CalcM+H+ SpR ZScore Ion% # Sequence  | | | | | | | | | | | | |
| --- | --- | --- | --- | --- | --- | --- | --- | --- | --- | --- | --- | --- |
|  | LAPMis12DANCE2\_011813\_01.09681.09681.2 | 3.5372 | 0.4217 | 100.0% | 1600.1721 | 1599.8271 | 1 | 7.616 | 61.5% | 2 | R.DFNHINVELSLLGK.K | 2 |

---

|  |  |  |  |  |  |  |  |  |
| --- | --- | --- | --- | --- | --- | --- | --- | --- |
| U | *gi|4826734|ref|NP\_004* | 2 | 2 | 7.2% | 526 | 53426 | 9.4 | fusion (involved in t(12;16) in malignant liposarcoma) [Homo sapiens] |

| Filename XCorr DeltCN Conf% ObsM+H+ CalcM+H+ SpR ZScore Ion% # Sequence  | | | | | | | | | | | | |
| --- | --- | --- | --- | --- | --- | --- | --- | --- | --- | --- | --- | --- |
|  | LAPMis12DANCE\_011813\_01.06945.06945.2 | 3.1332 | 0.4062 | 100.0% | 1420.9321 | 1421.5034 | 1 | 6.377 | 57.7% | 1 | K.GEATVSFDDPPSAK.A | 2 |
| \* | LAPMis12DANCE\_011813\_01.03950.03950.3 | 3.0397 | 0.318 | 99.1% | 2253.9243 | 2254.355 | 1 | 5.624 | 35.9% | 1 | K.APKPDGPGGGPGGSHMGGNYGDDR.R | 3 |

---

|  |  |  |  |  |  |  |  |  |
| --- | --- | --- | --- | --- | --- | --- | --- | --- |
| U | *gi|13569879|ref|NP\_11* | 1 | 1 | 7.1% | 268 | 30692 | 3.8 | acidic (leucine-rich) nuclear phosphoprotein 32 family, member E isoform 1 [Homo sapiens] |
| U | *gi|210147571|ref|NP\_0* | 1 | 1 | 8.6% | 220 | 25125 | 3.7 | acidic (leucine-rich) nuclear phosphoprotein 32 family, member E isoform 3 [Homo sapiens] |

| Filename XCorr DeltCN Conf% ObsM+H+ CalcM+H+ SpR ZScore Ion% # Sequence  | | | | | | | | | | | | |
| --- | --- | --- | --- | --- | --- | --- | --- | --- | --- | --- | --- | --- |
|  | LAPMis12DANCE\_011813\_01.12593.12593.2 | 4.4793 | 0.3659 | 100.0% | 2028.3922 | 2029.3385 | 1 | 7.785 | 69.4% | 1 | R.KLELSDNIISGGLEVLAEK.C | 2 |

---

|  |  |  |  |  |  |  |  |  |
| --- | --- | --- | --- | --- | --- | --- | --- | --- |
| U | *Reverse\_gi|38570158|r* | 1 | 1 | 7.1% | 267 | 30259 | 8.7 | OVO-like 1 binding protein [Homo sapiens] |

| Filename XCorr DeltCN Conf% ObsM+H+ CalcM+H+ SpR ZScore Ion% # Sequence  | | | | | | | | | | | | |
| --- | --- | --- | --- | --- | --- | --- | --- | --- | --- | --- | --- | --- |
| \* | LAPMis12DANCE\_011813\_01.09452.09452.3 | 2.4459 | 0.3233 | 98.1% | 2375.3943 | 2378.3877 | 13 | 4.524 | 31.9% | 1 | R.CT#FLDGS\*PSDGLTVK@MK@T#R.L | 3 |

---

|  |  |  |  |  |  |  |  |  |
| --- | --- | --- | --- | --- | --- | --- | --- | --- |
| U | *gi|169161796|ref|XP\_0* | 7 | 7 | 7.0% | 1910 | 190392 | 9.8 | PREDICTED: similar to Hornerin [Homo sapiens] |
| U | *gi|57864582|ref|NP\_00* | 7 | 7 | 4.7% | 2850 | 282389 | 10.0 | hornerin [Homo sapiens] |

| Filename XCorr DeltCN Conf% ObsM+H+ CalcM+H+ SpR ZScore Ion% # Sequence  | | | | | | | | | | | | |
| --- | --- | --- | --- | --- | --- | --- | --- | --- | --- | --- | --- | --- |
|  | LAPMis12DANCE\_011813\_01.03122.03122.3 | 4.5725 | 0.5167 | 100.0% | 2390.3943 | 2391.351 | 1 | 8.094 | 35.9% | 1 | R.HGSGSGHSSSHGQHGSGSSYSYSR.G | 3 |
|  | LAPMis12DANCE\_011813\_01.03386.03386.3 | 4.1919 | 0.5101 | 100.0% | 1930.8243 | 1929.9573 | 1 | 7.422 | 36.1% | 1 | R.GPYESGSGHSSGLGHQESR.S | 3 |
|  | LAPMis12DANCE\_011813\_01.03210.03210.3 | 5.4994 | 0.518 | 100.0% | 2870.6943 | 2870.8418 | 1 | 8.493 | 27.7% | 1 | R.SEQHGSSSGLSSSYGQHGSGSHQSSGHGR.Q | 3 |
|  | LAPMis12DANCE\_011813\_01.03155.03155.3 | 2.5692 | 0.3494 | 99.0% | 1948.7644 | 1949.0066 | 19 | 5.221 | 32.9% | 1 | R.QSLGHGQHGSGSGQSPSPSR.G | 3 |
|  | LAPMis12DANCE\_011813\_01.03350.03350.2 | 5.0293 | 0.6008 | 100.0% | 1585.2322 | 1585.6328 | 1 | 10.724 | 63.3% | 1 | R.GPYESGSGHSSGLGHR.E | 2 |
|  | LAPMis12DANCE\_011813\_01.03356.03356.3 | 2.5662 | 0.3209 | 98.7% | 1586.4543 | 1585.6328 | 8 | 5.386 | 38.3% | 1 | R.GPYESGSGHSSGLGHR.E | 3 |
|  | LAPMis12DANCE\_011813\_01.03322.03322.3 | 5.1476 | 0.5338 | 100.0% | 2478.8044 | 2478.4294 | 2 | 8.572 | 34.0% | 1 | R.HGSGSGHSSSYGQHGSGSGWSSSSGR.H | 3 |

---

|  |  |  |  |  |  |  |  |  |
| --- | --- | --- | --- | --- | --- | --- | --- | --- |
| U | *gi|149193323|ref|NP\_0* | 1 | 1 | 7.0% | 484 | 54116 | 6.5 | threonine synthase-like 2 [Homo sapiens] |

| Filename XCorr DeltCN Conf% ObsM+H+ CalcM+H+ SpR ZScore Ion% # Sequence  | | | | | | | | | | | | |
| --- | --- | --- | --- | --- | --- | --- | --- | --- | --- | --- | --- | --- |
| \* | LAPMis12DANCE2\_011813\_01.16014.16014.3 | 2.8677 | 0.3283 | 99.1% | 3595.8245 | 3592.9353 | 29 | 4.962 | 19.7% | 1 | K.HVTVVVGTSGDT#GSAAIES\*VQGAK@NMDIIVLLPK@.G | 3 |

---

|  |  |  |  |  |  |  |  |  |
| --- | --- | --- | --- | --- | --- | --- | --- | --- |
| U | *Reverse\_gi|4504403|re* | 1 | 1 | 7.0% | 273 | 30822 | 6.8 | major histocompatibility complex, class II, DO beta precursor [Homo sapiens] |

| Filename XCorr DeltCN Conf% ObsM+H+ CalcM+H+ SpR ZScore Ion% # Sequence  | | | | | | | | | | | | |
| --- | --- | --- | --- | --- | --- | --- | --- | --- | --- | --- | --- | --- |
| \* | LAPMis12DANCE2\_011813\_01.09008.09008.2 | 3.5243 | 0.0543 | 95.9% | 2212.5322 | 2214.3813 | 2 | 3.503 | 47.2% | 1 | -.CS\*QPLLVARSVENGSMQTR.V | 2 |

---

|  |  |  |  |  |  |  |  |  |
| --- | --- | --- | --- | --- | --- | --- | --- | --- |
| U | *gi|7657326|ref|NP\_055* | 1 | 1 | 7.0% | 185 | 19905 | 4.2 | male-enhanced antigen [Homo sapiens] |

| Filename XCorr DeltCN Conf% ObsM+H+ CalcM+H+ SpR ZScore Ion% # Sequence  | | | | | | | | | | | | |
| --- | --- | --- | --- | --- | --- | --- | --- | --- | --- | --- | --- | --- |
| \* | LAPMis12DANCE\_011813\_01.09651.09651.2 | 3.6895 | 0.2989 | 100.0% | 1548.2722 | 1547.6616 | 1 | 5.675 | 66.7% | 1 | R.EISDAQWEDVVQK.A | 2 |

---

|  |  |  |  |  |  |  |  |  |
| --- | --- | --- | --- | --- | --- | --- | --- | --- |
| U | *gi|4506681|ref|NP\_001* | 1 | 1 | 7.0% | 158 | 18431 | 10.3 | ribosomal protein S11 [Homo sapiens] |

| Filename XCorr DeltCN Conf% ObsM+H+ CalcM+H+ SpR ZScore Ion% # Sequence  | | | | | | | | | | | | |
| --- | --- | --- | --- | --- | --- | --- | --- | --- | --- | --- | --- | --- |
| \* | LAPMis12DANCE\_011813\_01.04776.04776.2 | 2.7389 | 0.1819 | 99.0% | 1267.0922 | 1267.4216 | 11 | 4.615 | 65.0% | 1 | K.EAIEGTYIDKK.C | 2 |

---

|  |  |  |  |  |  |  |  |  |
| --- | --- | --- | --- | --- | --- | --- | --- | --- |
| U | *gi|23308577|ref|NP\_00* | 2 | 2 | 6.9% | 533 | 56651 | 6.7 | phosphoglycerate dehydrogenase [Homo sapiens] |

| Filename XCorr DeltCN Conf% ObsM+H+ CalcM+H+ SpR ZScore Ion% # Sequence  | | | | | | | | | | | | |
| --- | --- | --- | --- | --- | --- | --- | --- | --- | --- | --- | --- | --- |
| \* | LAPMis12DANCE\_011813\_01.07504.07504.2 | 2.8411 | 0.3136 | 99.9% | 1488.1921 | 1489.5822 | 1 | 5.23 | 60.7% | 1 | R.AGTGVDNVDLEAATR.K | 2 |
| \* | LAPMis12DANCE\_011813\_01.15177.15177.2 | 4.1054 | 0.4 | 100.0% | 2273.132 | 2273.668 | 15 | 6.962 | 28.6% | 1 | R.TQTSDPAMLPTMIGLLAEAGVR.L | 2 |

---

|  |  |  |  |  |  |  |  |  |
| --- | --- | --- | --- | --- | --- | --- | --- | --- |
| U | *gi|12597655|ref|NP\_07* | 1 | 1 | 6.9% | 247 | 28481 | 5.3 | centromere protein H [Homo sapiens] |

| Filename XCorr DeltCN Conf% ObsM+H+ CalcM+H+ SpR ZScore Ion% # Sequence  | | | | | | | | | | | | |
| --- | --- | --- | --- | --- | --- | --- | --- | --- | --- | --- | --- | --- |
| \* | LAPMis12DANCE\_011813\_02.07756.07756.3 | 2.8175 | 0.3421 | 99.5% | 2168.3342 | 2166.332 | 305 | 4.594 | 29.7% | 1 | K.NLEK@IS\*RQSS\*VLMDNMK.H | 3 |

---

|  |  |  |  |  |  |  |  |  |
| --- | --- | --- | --- | --- | --- | --- | --- | --- |
| U | *gi|189095269|ref|NP\_0* | 4 | 4 | 6.8% | 702 | 77048 | 7.3 | propionyl-Coenzyme A carboxylase, alpha polypeptide isoform b [Homo sapiens] |
| U | *gi|65506442|ref|NP\_00* | 4 | 4 | 6.6% | 728 | 80059 | 7.5 | propionyl-Coenzyme A carboxylase, alpha polypeptide isoform a precursor [Homo sapiens] |

| Filename XCorr DeltCN Conf% ObsM+H+ CalcM+H+ SpR ZScore Ion% # Sequence  | | | | | | | | | | | | |
| --- | --- | --- | --- | --- | --- | --- | --- | --- | --- | --- | --- | --- |
|  | LAPMis12DANCE\_011813\_01.07715.07715.2 | 2.3385 | 0.2663 | 98.3% | 1617.5922 | 1618.7893 | 71 | 4.166 | 42.9% | 1 | R.AQAVHPGYGFLSENK.E | 2 |
|  | LAPMis12DANCE\_011813\_01.08654.08654.3 | 2.7958 | 0.2543 | 95.6% | 2122.2844 | 2122.3477 | 20 | 4.975 | 30.6% | 1 | R.AQAVHPGYGFLSENKEFAR.C | 3 |
|  | LAPMis12DANCE\_011813\_01.09023.09023.2 | 2.896 | 0.2135 | 99.1% | 1574.4521 | 1574.8174 | 34 | 4.789 | 46.4% | 1 | K.KAEVNTIPGFDGVVK.D | 2 |
|  | LAPMis12DANCE\_011813\_01.06136.06136.2 | 2.6491 | 0.2381 | 99.0% | 1472.0922 | 1470.4924 | 1 | 5.016 | 57.7% | 1 | R.LSSQEAASSFGDDR.L | 2 |

---

|  |  |  |  |  |  |  |  |  |
| --- | --- | --- | --- | --- | --- | --- | --- | --- |
| U | *contaminant\_KERATIN10* | 5 | 8 | 6.8% | 400 | 44106 | 5.1 | no description |
| U | *gi|24234699|ref|NP\_00* | 5 | 8 | 6.8% | 400 | 44106 | 5.1 | keratin 19 [Homo sapiens] |

| Filename XCorr DeltCN Conf% ObsM+H+ CalcM+H+ SpR ZScore Ion% # Sequence  | | | | | | | | | | | | |
| --- | --- | --- | --- | --- | --- | --- | --- | --- | --- | --- | --- | --- |
|  | LAPMis12DANCE\_011813\_01.05114.05114.1 | 1.7551 | 0.2197 | 97.3% | 809.35 | 809.93774 | 6 | 5.101 | 66.7% | 2 | R.LASYLDK.V | 11111 |
|  | LAPMis12DANCE\_011813\_01.06436.06436.2 | 3.0274 | 0.1822 | 99.7% | 1065.3322 | 1065.2578 | 68 | 5.735 | 62.5% | 2 | R.LASYLDKVR.A | 2222 |
|  | LAPMis12DANCE\_011813\_01.07811.07811.1 | 2.1522 | 0.2588 | 100.0% | 1041.41 | 1042.2235 | 10 | 5.465 | 62.5% | 1 | R.IVLQIDNAR.L | 11 |
|  | LAPMis12DANCE\_011813\_01.07768.07768.2 | 2.6317 | 0.2885 | 100.0% | 1042.0521 | 1042.2235 | 1 | 6.3 | 87.5% | 2 | R.IVLQIDNAR.L | 22 |
|  | LAPMis12DANCE\_011813\_01.09338.09338.2 | 2.6607 | 0.2826 | 100.0% | 1030.0122 | 1030.2096 | 2 | 6.157 | 81.2% | 1 | R.VLDELTLAR.T | 222 |

Similarities:
contaminant\_KERATIN09(2:3)  
gi|55956899|ref|NP\_00(1:4)  
contaminant\_KERATIN03(2:3)  
gi|15431310|ref|NP\_00(3:2)  
contaminant\_KERATIN12(3:2)  

---

|  |  |  |  |  |  |  |  |  |
| --- | --- | --- | --- | --- | --- | --- | --- | --- |
| U | *gi|4505073|ref|NP\_002* | 1 | 1 | 6.8% | 162 | 17850 | 10.0 | v-maf musculoaponeurotic fibrosarcoma oncogene homolog G [Homo sapiens] |
| U | *gi|49619228|ref|NP\_11* | 1 | 1 | 6.8% | 162 | 17850 | 10.0 | v-maf musculoaponeurotic fibrosarcoma oncogene homolog G [Homo sapiens] |
| U | *gi|4505075|ref|NP\_002* | 1 | 1 | 7.1% | 156 | 17523 | 10.1 | v-maf musculoaponeurotic fibrosarcoma oncogene homolog K [Homo sapiens] |

| Filename XCorr DeltCN Conf% ObsM+H+ CalcM+H+ SpR ZScore Ion% # Sequence  | | | | | | | | | | | | |
| --- | --- | --- | --- | --- | --- | --- | --- | --- | --- | --- | --- | --- |
|  | LAPMis12DANCE\_011813\_01.07349.07349.2 | 2.3916 | 0.187 | 96.6% | 1315.1921 | 1314.4838 | 1 | 4.437 | 80.0% | 1 | R.SKYEALQTFAR.T | 2 |

---

|  |  |  |  |  |  |  |  |  |
| --- | --- | --- | --- | --- | --- | --- | --- | --- |
| U | *Reverse\_gi|13376844|r* | 1 | 1 | 6.7% | 420 | 45524 | 8.4 | ring finger protein 39 isoform 1 [Homo sapiens] |
| U | *Reverse\_gi|25777716|r* | 1 | 1 | 7.9% | 354 | 38179 | 8.3 | ring finger protein 39 isoform 2 [Homo sapiens] |

| Filename XCorr DeltCN Conf% ObsM+H+ CalcM+H+ SpR ZScore Ion% # Sequence  | | | | | | | | | | | | |
| --- | --- | --- | --- | --- | --- | --- | --- | --- | --- | --- | --- | --- |
|  | LAPMis12DANCE2\_011813\_01.11244.11244.3 | 3.4957 | 0.2275 | 95.3% | 3339.0544 | 3341.6196 | 291 | 4.218 | 17.6% | 1 | R.HLMKKVVPYDEPLDDES\*NSSK@STPVEFR.R | 3 |

---

|  |  |  |  |  |  |  |  |  |
| --- | --- | --- | --- | --- | --- | --- | --- | --- |
| U | *Reverse\_gi|19718751|r* | 1 | 1 | 6.7% | 313 | 34645 | 9.3 | uracil-DNA glycosylase isoform UNG2 [Homo sapiens] |
| U | *Reverse\_gi|6224979|re* | 1 | 1 | 6.9% | 304 | 33924 | 9.5 | uracil-DNA glycosylase isoform UNG1 precursor [Homo sapiens] |

| Filename XCorr DeltCN Conf% ObsM+H+ CalcM+H+ SpR ZScore Ion% # Sequence  | | | | | | | | | | | | |
| --- | --- | --- | --- | --- | --- | --- | --- | --- | --- | --- | --- | --- |
|  | LAPMis12DANCE\_011813\_01.10068.10068.3 | 2.6143 | 0.3294 | 98.6% | 2227.2844 | 2227.6174 | 77 | 4.695 | 26.2% | 1 | K.WSEGFGVPVNRAALRLLAAAK.N | 23 |

---

|  |  |  |  |  |  |  |  |  |
| --- | --- | --- | --- | --- | --- | --- | --- | --- |
| U | *gi|169162122|ref|XP\_0* | 1 | 3 | 6.7% | 165 | 19047 | 10.4 | PREDICTED: hypothetical LOC440575 [Homo sapiens] |
| U | *gi|89040203|ref|XP\_93* | 1 | 3 | 6.9% | 160 | 18593 | 10.5 | PREDICTED: hypothetical protein [Homo sapiens] |
| U | *gi|18104948|ref|NP\_00* | 1 | 3 | 6.9% | 160 | 18565 | 10.5 | ribosomal protein L21 [Homo sapiens] |
| U | *gi|169213854|ref|XP\_0* | 1 | 3 | 6.9% | 160 | 18790 | 10.3 | PREDICTED: hypothetical protein [Homo sapiens] |
| U | *gi|169210381|ref|XP\_0* | 1 | 3 | 6.9% | 160 | 18535 | 10.6 | PREDICTED: hypothetical protein isoform 2 [Homo sapiens] |
| U | *gi|169210379|ref|XP\_0* | 1 | 3 | 6.9% | 160 | 18535 | 10.6 | PREDICTED: hypothetical protein isoform 3 [Homo sapiens] |
| U | *gi|169210377|ref|XP\_0* | 1 | 3 | 6.9% | 160 | 18535 | 10.6 | PREDICTED: hypothetical protein isoform 1 [Homo sapiens] |
| U | *gi|169202779|ref|XP\_0* | 1 | 3 | 6.9% | 160 | 18521 | 10.5 | PREDICTED: similar to ribosomal protein L21 isoform 1 [Homo sapiens] |
| U | *gi|169202777|ref|XP\_0* | 1 | 3 | 6.9% | 160 | 18521 | 10.5 | PREDICTED: similar to ribosomal protein L21 isoform 2 [Homo sapiens] |
| U | *gi|169201912|ref|XP\_0* | 1 | 3 | 7.5% | 147 | 17070 | 10.2 | PREDICTED: similar to mCG4465 [Homo sapiens] |
| U | *gi|169201750|ref|XP\_0* | 1 | 3 | 6.9% | 160 | 18550 | 10.5 | PREDICTED: hypothetical protein [Homo sapiens] |
| U | *gi|169201338|ref|XP\_0* | 1 | 3 | 6.9% | 160 | 18565 | 10.5 | PREDICTED: hypothetical protein [Homo sapiens] |
| U | *gi|169194577|ref|XP\_0* | 1 | 3 | 6.7% | 163 | 18991 | 10.4 | PREDICTED: similar to mCG4465, partial [Homo sapiens] |
| U | *gi|169192131|ref|XP\_0* | 1 | 3 | 7.5% | 147 | 17070 | 10.2 | PREDICTED: similar to mCG4465 [Homo sapiens] |
| U | *gi|169162801|ref|XP\_0* | 1 | 3 | 7.5% | 147 | 16969 | 10.2 | PREDICTED: similar to ribosomal protein L21 [Homo sapiens] |

| Filename XCorr DeltCN Conf% ObsM+H+ CalcM+H+ SpR ZScore Ion% # Sequence  | | | | | | | | | | | | |
| --- | --- | --- | --- | --- | --- | --- | --- | --- | --- | --- | --- | --- |
|  | LAPMis12DANCE\_011813\_01.08573.08573.2 | 2.6223 | 0.417 | 100.0% | 1244.0922 | 1244.4973 | 1 | 6.66 | 65.0% | 3 | K.HGVVPLATYMR.I | 2 |

---

|  |  |  |  |  |  |  |  |  |
| --- | --- | --- | --- | --- | --- | --- | --- | --- |
| U | *gi|5031699|ref|NP\_005* | 2 | 2 | 6.6% | 427 | 47355 | 7.5 | flotillin 1 [Homo sapiens] |

| Filename XCorr DeltCN Conf% ObsM+H+ CalcM+H+ SpR ZScore Ion% # Sequence  | | | | | | | | | | | | |
| --- | --- | --- | --- | --- | --- | --- | --- | --- | --- | --- | --- | --- |
| \* | LAPMis12DANCE\_011813\_02.06462.06462.2 | 2.8484 | 0.2985 | 99.9% | 1469.9722 | 1469.693 | 5 | 6.13 | 54.2% | 1 | K.VSAQYLSEIEMAK.A | 2 |
| \* | LAPMis12DANCE\_011813\_02.04982.04982.2 | 3.9242 | 0.4285 | 100.0% | 1379.9321 | 1380.5994 | 1 | 8.144 | 67.9% | 1 | K.ITLVSSGSGTMGAAK.V | 2 |

---

|  |  |  |  |  |  |  |  |  |
| --- | --- | --- | --- | --- | --- | --- | --- | --- |
| U | *Reverse\_gi|5901938|re* | 1 | 1 | 6.6% | 212 | 23406 | 10.7 | fasting-induced protein [Homo sapiens] |

| Filename XCorr DeltCN Conf% ObsM+H+ CalcM+H+ SpR ZScore Ion% # Sequence  | | | | | | | | | | | | |
| --- | --- | --- | --- | --- | --- | --- | --- | --- | --- | --- | --- | --- |
| \* | LAPMis12DANCE\_011813\_02.06098.06098.2 | 2.0828 | 0.2579 | 95.7% | 1608.3322 | 1608.6825 | 22 | 4.31 | 38.5% | 1 | R.SAMAQASQGFT#WSR.L | 2 |

---

|  |  |  |  |  |  |  |  |  |
| --- | --- | --- | --- | --- | --- | --- | --- | --- |
| U | *gi|87196351|ref|NP\_00* | 3 | 3 | 6.5% | 662 | 73244 | 7.2 | DEAD/H (Asp-Glu-Ala-Asp/His) box polypeptide 3 [Homo sapiens] |

| Filename XCorr DeltCN Conf% ObsM+H+ CalcM+H+ SpR ZScore Ion% # Sequence  | | | | | | | | | | | | |
| --- | --- | --- | --- | --- | --- | --- | --- | --- | --- | --- | --- | --- |
|  | LAPMis12DANCE\_011813\_01.09056.09056.2 | 2.8143 | 0.3999 | 100.0% | 1169.5721 | 1169.4099 | 13 | 8.0 | 59.1% | 1 | K.SPILVATAVAAR.G | 22 |
|  | LAPMis12DANCE2\_011813\_01.10036.10036.3 | 4.2399 | 0.4156 | 100.0% | 2083.4644 | 2084.2957 | 1 | 8.041 | 48.4% | 1 | K.HVINFDLPSDIEEYVHR.I | 33 |
| \* | LAPMis12DANCE\_011813\_01.11550.11550.2 | 3.6673 | 0.4791 | 100.0% | 1525.5922 | 1525.7043 | 1 | 9.366 | 69.2% | 1 | R.VGNLGLATSFFNER.N | 2 |

Similarities:
gi|13514809|ref|NP\_00(2:1)  

---

|  |  |  |  |  |  |  |  |  |
| --- | --- | --- | --- | --- | --- | --- | --- | --- |
| U | *Reverse\_gi|32171233|r* | 1 | 1 | 6.5% | 292 | 33200 | 6.7 | BCDIN3 domain containing [Homo sapiens] |

| Filename XCorr DeltCN Conf% ObsM+H+ CalcM+H+ SpR ZScore Ion% # Sequence  | | | | | | | | | | | | |
| --- | --- | --- | --- | --- | --- | --- | --- | --- | --- | --- | --- | --- |
| \* | LAPMis12DANCE2\_011813\_01.09386.09386.3 | 3.9385 | 0.2363 | 97.9% | 2288.9644 | 2288.5862 | 3 | 5.012 | 38.9% | 1 | R.QNMFDLTIFTLADPFPCEK.E | 3 |

---

|  |  |  |  |  |  |  |  |  |
| --- | --- | --- | --- | --- | --- | --- | --- | --- |
| U | *gi|94429050|ref|NP\_00* | 1 | 1 | 6.5% | 215 | 24593 | 6.9 | SEC22 vesicle trafficking protein homolog B [Homo sapiens] |

| Filename XCorr DeltCN Conf% ObsM+H+ CalcM+H+ SpR ZScore Ion% # Sequence  | | | | | | | | | | | | |
| --- | --- | --- | --- | --- | --- | --- | --- | --- | --- | --- | --- | --- |
| \* | LAPMis12DANCE\_011813\_01.09450.09450.2 | 3.6159 | 0.2369 | 100.0% | 1587.7722 | 1587.73 | 16 | 5.149 | 61.5% | 1 | R.NLGSINTELQDVQR.I | 2 |

---

|  |  |  |  |  |  |  |  |  |
| --- | --- | --- | --- | --- | --- | --- | --- | --- |
| U | *gi|5453555|ref|NP\_006* | 1 | 1 | 6.5% | 216 | 24423 | 7.5 | ras-related nuclear protein [Homo sapiens] |

| Filename XCorr DeltCN Conf% ObsM+H+ CalcM+H+ SpR ZScore Ion% # Sequence  | | | | | | | | | | | | |
| --- | --- | --- | --- | --- | --- | --- | --- | --- | --- | --- | --- | --- |
| \* | LAPMis12DANCE\_011813\_01.11999.11999.2 | 3.3106 | 0.4518 | 100.0% | 1785.4722 | 1786.0427 | 1 | 7.444 | 65.4% | 1 | K.SNYNFEKPFLWLAR.K | 2 |

---

|  |  |  |  |  |  |  |  |  |
| --- | --- | --- | --- | --- | --- | --- | --- | --- |
| U | *Reverse\_gi|169163248|* | 1 | 1 | 6.5% | 184 | 21104 | 9.5 | PREDICTED: similar to hCG1644578 [Homo sapiens] |
| U | *Reverse\_gi|169164229|* | 1 | 1 | 6.5% | 184 | 21104 | 9.5 | PREDICTED: similar to hCG1644578 [Homo sapiens] |
| U | *Reverse\_gi|169164029|* | 1 | 1 | 6.5% | 184 | 21105 | 9.5 | PREDICTED: similar to hCG1644578 [Homo sapiens] |

| Filename XCorr DeltCN Conf% ObsM+H+ CalcM+H+ SpR ZScore Ion% # Sequence  | | | | | | | | | | | | |
| --- | --- | --- | --- | --- | --- | --- | --- | --- | --- | --- | --- | --- |
|  | LAPMis12DANCE\_011813\_01.08915.08915.2 | 3.5685 | 0.1642 | 99.8% | 1275.3722 | 1275.506 | 4 | 4.718 | 63.6% | 1 | R.NNMELVSIGIGK.H | 2 |

---

|  |  |  |  |  |  |  |  |  |
| --- | --- | --- | --- | --- | --- | --- | --- | --- |
| U | *gi|21626466|ref|NP\_06* | 3 | 3 | 6.4% | 847 | 94623 | 6.3 | matrin 3 [Homo sapiens] |
| U | *gi|62750354|ref|NP\_95* | 3 | 3 | 6.4% | 847 | 94623 | 6.3 | matrin 3 [Homo sapiens] |

| Filename XCorr DeltCN Conf% ObsM+H+ CalcM+H+ SpR ZScore Ion% # Sequence  | | | | | | | | | | | | |
| --- | --- | --- | --- | --- | --- | --- | --- | --- | --- | --- | --- | --- |
|  | LAPMis12DANCE\_011813\_01.04564.04564.2 | 2.8501 | 0.3003 | 99.9% | 1324.6322 | 1325.4269 | 2 | 5.685 | 65.4% | 1 | R.GNLGAGNGNLQGPR.H | 2 |
|  | LAPMis12DANCE\_011813\_01.14404.14404.3 | 3.6424 | 0.2973 | 99.5% | 2440.3743 | 2439.9036 | 8 | 5.191 | 28.8% | 1 | R.YQLLQLVEPFGVISNHLILNK.I | 3 |
|  | LAPMis12DANCE\_011813\_01.08435.08435.3 | 2.8882 | 0.2938 | 98.1% | 2037.1144 | 2038.3109 | 1 | 4.946 | 33.3% | 1 | R.VIHLSNLPHSGYSDSAVLK.L | 3 |

---

|  |  |  |  |  |  |  |  |  |
| --- | --- | --- | --- | --- | --- | --- | --- | --- |
| U | *gi|12707580|ref|NP\_00* | 1 | 1 | 6.4% | 314 | 31621 | 8.8 | paired-like homeobox 2b [Homo sapiens] |

| Filename XCorr DeltCN Conf% ObsM+H+ CalcM+H+ SpR ZScore Ion% # Sequence  | | | | | | | | | | | | |
| --- | --- | --- | --- | --- | --- | --- | --- | --- | --- | --- | --- | --- |
| \* | LAPMis12DANCE\_011813\_01.13187.13187.2 | 3.1208 | 0.2411 | 99.5% | 2228.5522 | 2229.1594 | 6 | 4.507 | 36.8% | 1 | K.NGSSGKK@SDSS\*RDDESK@EAK@.S | 2 |

---

|  |  |  |  |  |  |  |  |  |
| --- | --- | --- | --- | --- | --- | --- | --- | --- |
| U | *gi|193794814|ref|NP\_0* | 2 | 4 | 6.3% | 364 | 39420 | 8.1 | fructose-bisphosphate aldolase A [Homo sapiens] |
| U | *gi|4557305|ref|NP\_000* | 2 | 4 | 6.3% | 364 | 39420 | 8.1 | fructose-bisphosphate aldolase A [Homo sapiens] |
| U | *gi|34577112|ref|NP\_90* | 2 | 4 | 6.3% | 364 | 39420 | 8.1 | fructose-bisphosphate aldolase A [Homo sapiens] |
| U | *gi|34577110|ref|NP\_90* | 2 | 4 | 6.3% | 364 | 39420 | 8.1 | fructose-bisphosphate aldolase A [Homo sapiens] |

| Filename XCorr DeltCN Conf% ObsM+H+ CalcM+H+ SpR ZScore Ion% # Sequence  | | | | | | | | | | | | |
| --- | --- | --- | --- | --- | --- | --- | --- | --- | --- | --- | --- | --- |
|  | LAPMis12DANCE\_011813\_01.07274.07274.2 | 3.4263 | 0.4333 | 100.0% | 1333.3922 | 1333.4814 | 1 | 7.196 | 73.1% | 3 | K.GILAADESTGSIAK.R | 2 |
|  | LAPMis12DANCE\_011813\_01.03348.03348.2 | 2.8437 | 0.287 | 100.0% | 1093.7922 | 1094.2125 | 4 | 5.518 | 75.0% | 1 | K.AAQEEYVKR.A | 2 |

---

|  |  |  |  |  |  |  |  |  |
| --- | --- | --- | --- | --- | --- | --- | --- | --- |
| U | *gi|15431306|ref|NP\_15* | 1 | 1 | 6.2% | 257 | 28025 | 11.0 | ribosomal protein L8 [Homo sapiens] |
| U | *gi|4506663|ref|NP\_000* | 1 | 1 | 6.2% | 257 | 28025 | 11.0 | ribosomal protein L8 [Homo sapiens] |

| Filename XCorr DeltCN Conf% ObsM+H+ CalcM+H+ SpR ZScore Ion% # Sequence  | | | | | | | | | | | | |
| --- | --- | --- | --- | --- | --- | --- | --- | --- | --- | --- | --- | --- |
|  | LAPMis12DANCE\_011813\_01.05286.05286.2 | 4.2048 | 0.5018 | 100.0% | 1690.0122 | 1689.8223 | 1 | 8.614 | 60.0% | 1 | R.ASGNYATVISHNPETK.K | 2 |

---

|  |  |  |  |  |  |  |  |  |
| --- | --- | --- | --- | --- | --- | --- | --- | --- |
| U | *gi|23308579|ref|NP\_00* | 1 | 1 | 6.2% | 160 | 18697 | 4.5 | unactive progesterone receptor, 23 kD [Homo sapiens] |

| Filename XCorr DeltCN Conf% ObsM+H+ CalcM+H+ SpR ZScore Ion% # Sequence  | | | | | | | | | | | | |
| --- | --- | --- | --- | --- | --- | --- | --- | --- | --- | --- | --- | --- |
| \* | LAPMis12DANCE\_011813\_01.03755.03755.2 | 2.761 | 0.1664 | 99.0% | 1131.2922 | 1132.2211 | 1 | 4.787 | 88.9% | 1 | R.KGESGQSWPR.L | 2 |

---

|  |  |  |  |  |  |  |  |  |
| --- | --- | --- | --- | --- | --- | --- | --- | --- |
| U | *gi|13514809|ref|NP\_00* | 3 | 3 | 6.1% | 660 | 73154 | 7.6 | DEAD (Asp-Glu-Ala-Asp) box polypeptide 3, Y-linked [Homo sapiens] |
| U | *gi|169881239|ref|NP\_0* | 3 | 3 | 6.1% | 660 | 73154 | 7.6 | DEAD (Asp-Glu-Ala-Asp) box polypeptide 3, Y-linked [Homo sapiens] |
| U | *gi|169216131|ref|XP\_0* | 3 | 3 | 6.1% | 660 | 73154 | 7.6 | PREDICTED: hypothetical protein [Homo sapiens] |

| Filename XCorr DeltCN Conf% ObsM+H+ CalcM+H+ SpR ZScore Ion% # Sequence  | | | | | | | | | | | | |
| --- | --- | --- | --- | --- | --- | --- | --- | --- | --- | --- | --- | --- |
|  | LAPMis12DANCE\_011813\_01.03567.03567.2 | 2.6083 | 0.2309 | 99.2% | 1164.1122 | 1164.2578 | 303 | 5.845 | 50.0% | 1 | R.VGSTSENITQK.V | 2 |
|  | LAPMis12DANCE\_011813\_01.09056.09056.2 | 2.8143 | 0.3999 | 100.0% | 1169.5721 | 1169.4099 | 13 | 8.0 | 59.1% | 1 | K.SPILVATAVAAR.G | 22 |
|  | LAPMis12DANCE2\_011813\_01.10036.10036.3 | 4.2399 | 0.4156 | 100.0% | 2083.4644 | 2084.2957 | 1 | 8.041 | 48.4% | 1 | R.HVINFDLPSDIEEYVHR.I | 33 |

Similarities:
gi|87196351|ref|NP\_00(2:1)  

---

|  |  |  |  |  |  |  |  |  |
| --- | --- | --- | --- | --- | --- | --- | --- | --- |
| U | *gi|19923403|ref|NP\_05* | 1 | 1 | 6.1% | 293 | 32789 | 7.5 | exosome component 2 [Homo sapiens] |

| Filename XCorr DeltCN Conf% ObsM+H+ CalcM+H+ SpR ZScore Ion% # Sequence  | | | | | | | | | | | | |
| --- | --- | --- | --- | --- | --- | --- | --- | --- | --- | --- | --- | --- |
| \* | LAPMis12DANCE\_011813\_01.18701.18701.2 | 2.2345 | 0.2295 | 95.3% | 2056.912 | 2057.246 | 62 | 4.522 | 32.4% | 1 | K.YGK@LGQGVLVQVS\*PS\*LVK@.R | 2 |

---

|  |  |  |  |  |  |  |  |  |
| --- | --- | --- | --- | --- | --- | --- | --- | --- |
| U | *gi|4758012|ref|NP\_004* | 6 | 6 | 6.0% | 1675 | 191613 | 5.7 | clathrin heavy chain 1 [Homo sapiens] |

| Filename XCorr DeltCN Conf% ObsM+H+ CalcM+H+ SpR ZScore Ion% # Sequence  | | | | | | | | | | | | |
| --- | --- | --- | --- | --- | --- | --- | --- | --- | --- | --- | --- | --- |
| \* | LAPMis12DANCE\_011813\_01.08603.08603.2 | 3.3153 | 0.0924 | 96.7% | 1759.7322 | 1759.9591 | 111 | 5.22 | 40.0% | 1 | R.KFNALFAQGNYSEAAK.V | 2 |
| \* | LAPMis12DANCE\_011813\_02.06360.06360.2 | 3.7979 | 0.4555 | 100.0% | 1631.9122 | 1631.785 | 1 | 6.915 | 57.1% | 1 | K.FNALFAQGNYSEAAK.V | 2 |
| \* | LAPMis12DANCE2\_011813\_01.11820.11820.3 | 6.3228 | 0.459 | 100.0% | 3129.7444 | 3130.6248 | 1 | 8.918 | 34.6% | 1 | R.LLEMNLMHAPQVADAILGNQMFTHYDR.A | 3 |
|  | LAPMis12DANCE2\_011813\_01.14134.14134.2 | 3.6581 | 0.3458 | 100.0% | 1948.3722 | 1948.2819 | 1 | 6.471 | 46.9% | 1 | K.AFMTADLPNELIELLEK.I | 2 |
| \* | LAPMis12DANCE2\_011813\_01.11790.11790.3 | 6.4554 | 0.5349 | 100.0% | 2369.9343 | 2369.6829 | 1 | 8.487 | 41.2% | 1 | R.KFDVNTSAVQVLIEHIGNLDR.A | 3 |
| \* | LAPMis12DANCE\_011813\_02.06474.06474.3 | 3.0284 | 0.3618 | 99.7% | 1972.2244 | 1972.2083 | 2 | 5.947 | 34.7% | 1 | R.LASTLVHLGEYQAAVDGAR.K | 23 |

---

|  |  |  |  |  |  |  |  |  |
| --- | --- | --- | --- | --- | --- | --- | --- | --- |
| U | *gi|195539395|ref|NP\_0* | 2 | 2 | 6.0% | 403 | 45797 | 7.8 | proteasome 26S ATPase subunit 6 [Homo sapiens] |

| Filename XCorr DeltCN Conf% ObsM+H+ CalcM+H+ SpR ZScore Ion% # Sequence  | | | | | | | | | | | | |
| --- | --- | --- | --- | --- | --- | --- | --- | --- | --- | --- | --- | --- |
| \* | LAPMis12DANCE\_011813\_01.07967.07967.2 | 2.1787 | 0.2076 | 95.2% | 1273.9122 | 1274.416 | 24 | 4.862 | 55.0% | 1 | K.HGEIDYEAIVK.L | 2 |
| \* | LAPMis12DANCE\_011813\_01.09732.09732.2 | 2.8082 | 0.3386 | 100.0% | 1581.6122 | 1581.74 | 1 | 5.794 | 70.8% | 1 | R.ADHDFVVQEDFMK.A | 2 |

---

|  |  |  |  |  |  |  |  |  |
| --- | --- | --- | --- | --- | --- | --- | --- | --- |
| U | *gi|17158044|ref|NP\_00* | 1 | 2 | 6.0% | 249 | 28681 | 10.8 | ribosomal protein S6 [Homo sapiens] |

| Filename XCorr DeltCN Conf% ObsM+H+ CalcM+H+ SpR ZScore Ion% # Sequence  | | | | | | | | | | | | |
| --- | --- | --- | --- | --- | --- | --- | --- | --- | --- | --- | --- | --- |
| \* | LAPMis12DANCE\_011813\_01.10809.10809.2 | 3.0379 | 0.3635 | 100.0% | 1621.1522 | 1621.8022 | 1 | 6.206 | 60.7% | 2 | R.MATEVAADALGEEWK.G | 2 |

---

|  |  |  |  |  |  |  |  |  |
| --- | --- | --- | --- | --- | --- | --- | --- | --- |
| U | *gi|15431288|ref|NP\_00* | 1 | 2 | 6.0% | 217 | 24831 | 9.9 | ribosomal protein L10a [Homo sapiens] |
| U | *gi|169165866|ref|XP\_0* | 1 | 2 | 9.8% | 133 | 15021 | 10.1 | PREDICTED: similar to ribosomal protein L10a [Homo sapiens] |
| U | *gi|169165348|ref|XP\_0* | 1 | 2 | 9.8% | 133 | 15065 | 10.0 | PREDICTED: similar to ribosomal protein L10a [Homo sapiens] |
| U | *gi|169165048|ref|XP\_0* | 1 | 2 | 9.8% | 133 | 15021 | 10.1 | PREDICTED: similar to ribosomal protein L10a [Homo sapiens] |

| Filename XCorr DeltCN Conf% ObsM+H+ CalcM+H+ SpR ZScore Ion% # Sequence  | | | | | | | | | | | | |
| --- | --- | --- | --- | --- | --- | --- | --- | --- | --- | --- | --- | --- |
|  | LAPMis12DANCE\_011813\_01.10342.10342.2 | 3.0577 | 0.4217 | 100.0% | 1485.0922 | 1485.7203 | 1 | 7.407 | 66.7% | 2 | K.KYDAFLASESLIK.Q | 2 |

---

|  |  |  |  |  |  |  |  |  |
| --- | --- | --- | --- | --- | --- | --- | --- | --- |
| U | *gi|60218913|ref|NP\_00* | 1 | 1 | 5.9% | 185 | 21228 | 7.9 | calcyclin binding protein isoform 2 [Homo sapiens] |
| U | *gi|7656952|ref|NP\_055* | 1 | 1 | 4.8% | 228 | 26210 | 8.2 | calcyclin binding protein isoform 1 [Homo sapiens] |

| Filename XCorr DeltCN Conf% ObsM+H+ CalcM+H+ SpR ZScore Ion% # Sequence  | | | | | | | | | | | | |
| --- | --- | --- | --- | --- | --- | --- | --- | --- | --- | --- | --- | --- |
|  | LAPMis12DANCE\_011813\_01.03890.03890.2 | 2.1426 | 0.2571 | 97.8% | 1355.7522 | 1357.4346 | 1 | 4.589 | 75.0% | 1 | K.IYEDGDDDMKR.T | 2 |

---

|  |  |  |  |  |  |  |  |  |
| --- | --- | --- | --- | --- | --- | --- | --- | --- |
| U | *gi|8923900|ref|NP\_061* | 1 | 1 | 5.8% | 434 | 48379 | 7.9 | cytidine 5'-monophosphate N-acetylneuraminic acid synthetase [Homo sapiens] |

| Filename XCorr DeltCN Conf% ObsM+H+ CalcM+H+ SpR ZScore Ion% # Sequence  | | | | | | | | | | | | |
| --- | --- | --- | --- | --- | --- | --- | --- | --- | --- | --- | --- | --- |
| \* | LAPMis12DANCE\_011813\_02.10952.10952.3 | 2.7725 | 0.2607 | 95.0% | 2822.6042 | 2825.2017 | 149 | 4.141 | 21.9% | 1 | K.DAIGISLLKKSGIEVRLIS\*ERACSK.Q | 3 |

---

|  |  |  |  |  |  |  |  |  |
| --- | --- | --- | --- | --- | --- | --- | --- | --- |
| U | *gi|7019485|ref|NP\_037* | 1 | 1 | 5.8% | 191 | 21868 | 5.4 | programmed cell death 6 [Homo sapiens] |

| Filename XCorr DeltCN Conf% ObsM+H+ CalcM+H+ SpR ZScore Ion% # Sequence  | | | | | | | | | | | | |
| --- | --- | --- | --- | --- | --- | --- | --- | --- | --- | --- | --- | --- |
| \* | LAPMis12DANCE\_011813\_01.10224.10224.2 | 2.4134 | 0.2773 | 99.4% | 1340.8121 | 1340.5371 | 56 | 4.66 | 50.0% | 1 | R.SIISMFDRENK.A | 2 |

---

|  |  |  |  |  |  |  |  |  |
| --- | --- | --- | --- | --- | --- | --- | --- | --- |
| U | *gi|41872631|ref|NP\_00* | 8 | 11 | 5.7% | 2511 | 273424 | 6.4 | fatty acid synthase [Homo sapiens] |

| Filename XCorr DeltCN Conf% ObsM+H+ CalcM+H+ SpR ZScore Ion% # Sequence  | | | | | | | | | | | | |
| --- | --- | --- | --- | --- | --- | --- | --- | --- | --- | --- | --- | --- |
| \* | LAPMis12DANCE\_011813\_01.16049.16049.3 | 4.7675 | 0.371 | 100.0% | 3165.4744 | 3165.415 | 1 | 6.688 | 27.9% | 2 | K.LPESENLQEFWDNLIGGVDMVTDDDRR.W | 3 |
| \* | LAPMis12DANCE\_011813\_01.07005.07005.2 | 2.4957 | 0.3447 | 99.9% | 1299.2122 | 1299.4264 | 8 | 5.758 | 59.1% | 1 | K.VGDPQELNGITR.A | 2 |
| \* | LAPMis12DANCE2\_011813\_01.11848.11848.2 | 3.0221 | 0.3585 | 100.0% | 1778.8922 | 1779.0837 | 4 | 7.025 | 43.8% | 1 | K.LPEDPLLSGLLDSPALK.A | 2 |
| \* | LAPMis12DANCE\_011813\_01.10206.10206.3 | 4.0089 | 0.3059 | 99.7% | 2669.2444 | 2668.9658 | 1 | 5.282 | 29.5% | 1 | R.HFLLEEDKPEEPTAHAFVSTLTR.G | 3 |
| \* | LAPMis12DANCE\_011813\_01.09714.09714.2 | 3.1659 | 0.2733 | 100.0% | 1469.9521 | 1470.5815 | 11 | 5.26 | 62.5% | 1 | R.FPQLDSTSFANSR.D | 2 |
| \* | LAPMis12DANCE\_011813\_01.07680.07680.2 | 3.3078 | 0.3844 | 100.0% | 1613.8922 | 1614.7556 | 1 | 6.44 | 53.8% | 2 | K.EDGLAQQQTQLNLR.S | 2 |
| \* | LAPMis12DANCE\_011813\_01.10545.10545.2 | 2.4373 | 0.2296 | 98.1% | 1427.4722 | 1427.702 | 44 | 5.501 | 50.0% | 1 | R.SLLVNPEGPTLMR.L | 2 |
| \* | LAPMis12DANCE2\_011813\_01.14109.14109.3 | 3.6654 | 0.3295 | 99.7% | 2424.3843 | 2423.769 | 13 | 6.209 | 26.1% | 2 | R.TLLEGSGLESIISIIHSSLAEPR.V | 3 |

---

|  |  |  |  |  |  |  |  |  |
| --- | --- | --- | --- | --- | --- | --- | --- | --- |
| U | *contaminant\_SPA1\_STAA* | 1 | 1 | 5.7% | 524 | 57320 | 5.7 | owl|P02976| IMMUNOGLOBULIN G BINDING PROTEIN A PRECURSOR (PROTEIN A). - STAPHYLOCOCCUS... |

| Filename XCorr DeltCN Conf% ObsM+H+ CalcM+H+ SpR ZScore Ion% # Sequence  | | | | | | | | | | | | |
| --- | --- | --- | --- | --- | --- | --- | --- | --- | --- | --- | --- | --- |
| \* | LAPMis12DANCE2\_011813\_01.10606.10606.3 | 4.0835 | 0.3666 | 100.0% | 3513.3843 | 3513.7795 | 41 | 5.918 | 20.7% | 1 | K.ADAQQNNFNKDQQSAFYEILNMPNLNEAQR.N | 3 |

---

|  |  |  |  |  |  |  |  |  |
| --- | --- | --- | --- | --- | --- | --- | --- | --- |
| U | *Reverse\_gi|205360930|* | 1 | 1 | 5.7% | 456 | 50994 | 6.2 | hypothetical protein LOC57707 [Homo sapiens] |

| Filename XCorr DeltCN Conf% ObsM+H+ CalcM+H+ SpR ZScore Ion% # Sequence  | | | | | | | | | | | | |
| --- | --- | --- | --- | --- | --- | --- | --- | --- | --- | --- | --- | --- |
| \* | LAPMis12DANCE\_011813\_02.07791.07791.3 | 2.7346 | 0.2682 | 95.4% | 2872.7944 | 2873.2163 | 330 | 3.902 | 23.0% | 1 | K.MDS\*LLQAALVQVRPNPGPAEKGTWGR.L | 3 |

---

|  |  |  |  |  |  |  |  |  |
| --- | --- | --- | --- | --- | --- | --- | --- | --- |
| U | *gi|47575849|ref|NP\_68* | 1 | 1 | 5.7% | 331 | 38318 | 8.5 | docking protein 6 [Homo sapiens] |

| Filename XCorr DeltCN Conf% ObsM+H+ CalcM+H+ SpR ZScore Ion% # Sequence  | | | | | | | | | | | | |
| --- | --- | --- | --- | --- | --- | --- | --- | --- | --- | --- | --- | --- |
| \* | LAPMis12DANCE2\_011813\_01.08375.08375.2 | 2.9016 | 0.15 | 96.8% | 2225.2722 | 2227.5896 | 289 | 4.062 | 33.3% | 1 | -.MASNFNDIVKQGYVKIRSR.K | 2 |

---

|  |  |  |  |  |  |  |  |  |
| --- | --- | --- | --- | --- | --- | --- | --- | --- |
| U | *gi|5454102|ref|NP\_006* | 3 | 4 | 5.6% | 838 | 90360 | 5.0 | transforming, acidic coiled-coil containing protein 3 [Homo sapiens] |

| Filename XCorr DeltCN Conf% ObsM+H+ CalcM+H+ SpR ZScore Ion% # Sequence  | | | | | | | | | | | | |
| --- | --- | --- | --- | --- | --- | --- | --- | --- | --- | --- | --- | --- |
| \* | LAPMis12DANCE\_011813\_02.05824.05824.2 | 2.9741 | 0.3964 | 100.0% | 1737.6322 | 1737.9042 | 1 | 6.988 | 50.0% | 1 | R.SGPVKLEFDVSDGATSK.R | 2 |
| \* | LAPMis12DANCE2\_011813\_01.12030.12030.2 | 3.0474 | 0.2757 | 99.9% | 2245.3123 | 2245.5703 | 1 | 5.404 | 41.2% | 1 | K.IMDRFEEVVYQAMEEVQK.Q | 2 |
| \* | LAPMis12DANCE\_011813\_01.08880.08880.2 | 2.7319 | 0.1238 | 95.6% | 1384.0922 | 1384.5754 | 2 | 5.348 | 68.2% | 2 | K.LQLANEEIAQVR.S | 2 |

---

|  |  |  |  |  |  |  |  |  |
| --- | --- | --- | --- | --- | --- | --- | --- | --- |
| U | *gi|55956788|ref|NP\_00* | 3 | 4 | 5.6% | 710 | 76615 | 4.7 | nucleolin [Homo sapiens] |

| Filename XCorr DeltCN Conf% ObsM+H+ CalcM+H+ SpR ZScore Ion% # Sequence  | | | | | | | | | | | | |
| --- | --- | --- | --- | --- | --- | --- | --- | --- | --- | --- | --- | --- |
| \* | LAPMis12DANCE\_011813\_02.07424.07424.2 | 2.8939 | 0.1574 | 97.8% | 1649.5322 | 1649.751 | 1 | 5.581 | 65.4% | 2 | K.FGYVDFESAEDLEK.A | 2 |
| \* | LAPMis12DANCE\_011813\_01.04398.04398.2 | 3.0262 | 0.24 | 99.9% | 1397.8722 | 1398.4662 | 1 | 5.792 | 63.6% | 1 | K.TEADAEKTFEEK.Q | 2 |
| \* | LAPMis12DANCE\_011813\_02.07353.07353.2 | 3.0767 | 0.2262 | 99.5% | 1563.0322 | 1562.6323 | 1 | 5.228 | 61.5% | 1 | K.GFGFVDFNSEEDAK.A | 2 |

---

|  |  |  |  |  |  |  |  |  |
| --- | --- | --- | --- | --- | --- | --- | --- | --- |
| U | *gi|194239723|ref|NP\_0* | 2 | 3 | 5.6% | 647 | 71408 | 6.4 | eukaryotic translation elongation factor 1 delta isoform 1 [Homo sapiens] |
| U | *gi|25453474|ref|NP\_11* | 2 | 3 | 5.6% | 647 | 71408 | 6.4 | eukaryotic translation elongation factor 1 delta isoform 1 [Homo sapiens] |
| U | *gi|25453472|ref|NP\_00* | 2 | 3 | 12.8% | 281 | 31122 | 5.0 | eukaryotic translation elongation factor 1 delta isoform 2 [Homo sapiens] |
| U | *gi|194239731|ref|NP\_0* | 2 | 3 | 12.8% | 281 | 31122 | 5.0 | eukaryotic translation elongation factor 1 delta isoform 2 [Homo sapiens] |
| U | *gi|194239727|ref|NP\_0* | 2 | 3 | 12.8% | 281 | 31122 | 5.0 | eukaryotic translation elongation factor 1 delta isoform 2 [Homo sapiens] |
| U | *gi|194239725|ref|NP\_0* | 2 | 3 | 5.6% | 646 | 71266 | 6.4 | eukaryotic translation elongation factor 1 delta isoform 3 [Homo sapiens] |

| Filename XCorr DeltCN Conf% ObsM+H+ CalcM+H+ SpR ZScore Ion% # Sequence  | | | | | | | | | | | | |
| --- | --- | --- | --- | --- | --- | --- | --- | --- | --- | --- | --- | --- |
|  | LAPMis12DANCE2\_011813\_01.05010.05010.2 | 3.9568 | 0.474 | 100.0% | 2185.9922 | 2186.3 | 1 | 8.437 | 41.3% | 1 | K.SLAGSSGPGASSGTSGDHGELVVR.I | 2 |
|  | LAPMis12DANCE\_011813\_01.07888.07888.2 | 3.1343 | 0.2288 | 99.9% | 1360.2922 | 1359.5223 | 2 | 4.555 | 68.2% | 2 | R.IASLEVENQSLR.G | 2 |

---

|  |  |  |  |  |  |  |  |  |
| --- | --- | --- | --- | --- | --- | --- | --- | --- |
| U | *gi|14165464|ref|NP\_11* | 2 | 2 | 5.6% | 550 | 59037 | 9.2 | polypyrimidine tract-binding protein 1 isoform b [Homo sapiens] |
| U | *gi|4506243|ref|NP\_002* | 2 | 2 | 5.6% | 557 | 59633 | 9.2 | polypyrimidine tract-binding protein 1 isoform a [Homo sapiens] |
| U | *gi|14165466|ref|NP\_11* | 2 | 2 | 5.8% | 531 | 57221 | 9.2 | polypyrimidine tract-binding protein 1 isoform c [Homo sapiens] |

| Filename XCorr DeltCN Conf% ObsM+H+ CalcM+H+ SpR ZScore Ion% # Sequence  | | | | | | | | | | | | |
| --- | --- | --- | --- | --- | --- | --- | --- | --- | --- | --- | --- | --- |
|  | LAPMis12DANCE2\_011813\_01.11315.11315.2 | 3.2225 | 0.3171 | 100.0% | 2275.9922 | 2276.6414 | 35 | 6.82 | 29.5% | 1 | R.IAIPGLAGAGNSVLLVSNLNPER.V | 2 |
|  | LAPMis12DANCE\_011813\_01.03969.03969.2 | 2.6186 | 0.357 | 100.0% | 991.8122 | 992.12573 | 3 | 5.965 | 64.3% | 1 | K.HQNVQLPR.E | 2 |

---

|  |  |  |  |  |  |  |  |  |
| --- | --- | --- | --- | --- | --- | --- | --- | --- |
| U | *Reverse\_gi|16945972|r* | 1 | 1 | 5.5% | 382 | 43088 | 8.1 | kelch domain containing 3 [Homo sapiens] |

| Filename XCorr DeltCN Conf% ObsM+H+ CalcM+H+ SpR ZScore Ion% # Sequence  | | | | | | | | | | | | |
| --- | --- | --- | --- | --- | --- | --- | --- | --- | --- | --- | --- | --- |
| \* | LAPMis12DANCE\_011813\_01.07767.07767.3 | 3.1742 | 0.3003 | 98.8% | 2615.7844 | 2614.8127 | 48 | 4.905 | 25.0% | 1 | R.WRAPS\*GKT#CILTWTMTSTDLK.H | 3 |

---

|  |  |  |  |  |  |  |  |  |
| --- | --- | --- | --- | --- | --- | --- | --- | --- |
| U | *gi|4506901|ref|NP\_003* | 1 | 1 | 5.5% | 164 | 19330 | 11.6 | splicing factor, arginine/serine-rich 3 [Homo sapiens] |

| Filename XCorr DeltCN Conf% ObsM+H+ CalcM+H+ SpR ZScore Ion% # Sequence  | | | | | | | | | | | | |
| --- | --- | --- | --- | --- | --- | --- | --- | --- | --- | --- | --- | --- |
| \* | LAPMis12DANCE\_011813\_01.09660.09660.2 | 2.2968 | 0.1755 | 96.3% | 1043.8322 | 1044.198 | 3 | 5.16 | 81.2% | 1 | R.AFGYYGPLR.S | 2 |

---

|  |  |  |  |  |  |  |  |  |
| --- | --- | --- | --- | --- | --- | --- | --- | --- |
| U | *gi|12667788|ref|NP\_00* | 7 | 7 | 5.4% | 1960 | 226530 | 5.6 | myosin, heavy polypeptide 9, non-muscle [Homo sapiens] |

| Filename XCorr DeltCN Conf% ObsM+H+ CalcM+H+ SpR ZScore Ion% # Sequence  | | | | | | | | | | | | |
| --- | --- | --- | --- | --- | --- | --- | --- | --- | --- | --- | --- | --- |
|  | LAPMis12DANCE\_011813\_01.12707.12707.2 | 2.4255 | 0.3529 | 99.8% | 1727.1122 | 1728.0012 | 204 | 5.521 | 30.0% | 1 | R.QLLQANPILEAFGNAK.T | 2 |
| \* | LAPMis12DANCE\_011813\_02.05866.05866.2 | 3.2502 | 0.3853 | 100.0% | 1653.1721 | 1654.7681 | 1 | 6.73 | 65.4% | 1 | R.IAEFTTNLTEEEEK.S | 2 |
| \* | LAPMis12DANCE\_011813\_01.09575.09575.3 | 3.624 | 0.2086 | 96.5% | 1997.8444 | 1997.1722 | 1 | 5.525 | 37.5% | 1 | K.HSQAVEELAEQLEQTKR.V | 3 |
| \* | LAPMis12DANCE\_011813\_02.07094.07094.3 | 3.9471 | 0.3656 | 100.0% | 2473.6143 | 2473.6099 | 1 | 6.214 | 32.5% | 1 | R.IAQLEEELEEEQGNTELINDR.L | 3 |
| \* | LAPMis12DANCE\_011813\_01.08046.08046.2 | 3.2883 | 0.3617 | 100.0% | 1531.0922 | 1531.6598 | 1 | 6.235 | 62.5% | 1 | K.IAQLEEQLDNETK.E | 2 |
| \* | LAPMis12DANCE\_011813\_01.06762.06762.2 | 2.3677 | 0.2876 | 99.1% | 1566.1721 | 1566.6367 | 37 | 4.722 | 50.0% | 1 | R.ELEDATETADAMNR.E | 2 |
| \* | LAPMis12DANCE\_011813\_01.09140.09140.2 | 3.1995 | 0.2976 | 100.0% | 1156.1921 | 1156.3732 | 2 | 5.69 | 83.3% | 1 | R.RGDLPFVVPR.R | 2 |

---

|  |  |  |  |  |  |  |  |  |
| --- | --- | --- | --- | --- | --- | --- | --- | --- |
| U | *gi|4505257|ref|NP\_002* | 1 | 1 | 5.4% | 577 | 67820 | 6.4 | moesin [Homo sapiens] |

| Filename XCorr DeltCN Conf% ObsM+H+ CalcM+H+ SpR ZScore Ion% # Sequence  | | | | | | | | | | | | |
| --- | --- | --- | --- | --- | --- | --- | --- | --- | --- | --- | --- | --- |
| \* | LAPMis12DANCE\_011813\_02.04970.04970.3 | 6.4111 | 0.4196 | 100.0% | 3313.7644 | 3314.3887 | 1 | 8.998 | 37.5% | 1 | K.TAMSTPHVAEPAENEQDEQDENGAEASADLR.A | 3 |

---

|  |  |  |  |  |  |  |  |  |
| --- | --- | --- | --- | --- | --- | --- | --- | --- |
| U | *gi|5453710|ref|NP\_006* | 1 | 1 | 5.4% | 261 | 29717 | 7.0 | LIM and SH3 protein 1 [Homo sapiens] |

| Filename XCorr DeltCN Conf% ObsM+H+ CalcM+H+ SpR ZScore Ion% # Sequence  | | | | | | | | | | | | |
| --- | --- | --- | --- | --- | --- | --- | --- | --- | --- | --- | --- | --- |
| \* | LAPMis12DANCE\_011813\_01.09574.09574.2 | 3.1294 | 0.2581 | 99.9% | 1610.1522 | 1609.7943 | 1 | 5.267 | 57.7% | 1 | K.QSFTMVADTPENLR.L | 2 |

---

|  |  |  |  |  |  |  |  |  |
| --- | --- | --- | --- | --- | --- | --- | --- | --- |
| U | *gi|73760405|ref|NP\_00* | 1 | 1 | 5.3% | 454 | 50670 | 9.4 | thymopoietin isoform beta [Homo sapiens] |

| Filename XCorr DeltCN Conf% ObsM+H+ CalcM+H+ SpR ZScore Ion% # Sequence  | | | | | | | | | | | | |
| --- | --- | --- | --- | --- | --- | --- | --- | --- | --- | --- | --- | --- |
| \* | LAPMis12DANCE\_011813\_02.04823.04823.3 | 4.0892 | 0.4476 | 100.0% | 2683.8843 | 2683.8076 | 1 | 6.798 | 31.5% | 1 | R.RVEHNQSYSQAGITETEWTSGSSK.G | 3 |

---

|  |  |  |  |  |  |  |  |  |
| --- | --- | --- | --- | --- | --- | --- | --- | --- |
| U | *gi|4503481|ref|NP\_001* | 2 | 2 | 5.3% | 437 | 50119 | 6.7 | eukaryotic translation elongation factor 1 gamma [Homo sapiens] |

| Filename XCorr DeltCN Conf% ObsM+H+ CalcM+H+ SpR ZScore Ion% # Sequence  | | | | | | | | | | | | |
| --- | --- | --- | --- | --- | --- | --- | --- | --- | --- | --- | --- | --- |
| \* | LAPMis12DANCE\_011813\_02.05244.05244.2 | 3.7859 | 0.361 | 100.0% | 1349.2322 | 1348.5448 | 1 | 7.134 | 66.7% | 1 | K.ALIAAQYSGAQVR.V | 2 |
| \* | LAPMis12DANCE\_011813\_01.09620.09620.2 | 2.4909 | 0.3758 | 100.0% | 1241.3922 | 1242.4172 | 3 | 6.474 | 72.2% | 1 | K.STFVLDEFKR.K | 2 |

---

|  |  |  |  |  |  |  |  |  |
| --- | --- | --- | --- | --- | --- | --- | --- | --- |
| U | *gi|74048514|ref|NP\_73* | 7 | 8 | 5.2% | 2342 | 265290 | 5.4 | cancer susceptibility candidate 5 isoform 1 [Homo sapiens] |
| U | *gi|74048554|ref|NP\_65* | 7 | 8 | 5.2% | 2316 | 262530 | 5.4 | cancer susceptibility candidate 5 isoform 2 [Homo sapiens] |

| Filename XCorr DeltCN Conf% ObsM+H+ CalcM+H+ SpR ZScore Ion% # Sequence  | | | | | | | | | | | | |
| --- | --- | --- | --- | --- | --- | --- | --- | --- | --- | --- | --- | --- |
|  | LAPMis12DANCE\_011813\_02.05190.05190.2 | 3.1732 | 0.4268 | 100.0% | 1500.4122 | 1501.6484 | 1 | 7.384 | 75.0% | 1 | K.TIYSGEENMDITK.S | 2 |
|  | LAPMis12DANCE\_011813\_01.03254.03254.2 | 3.2592 | 0.3783 | 100.0% | 1309.8522 | 1310.4093 | 1 | 7.85 | 68.2% | 1 | K.NHDTAISSHTVK.S | 2 |
|  | LAPMis12DANCE\_011813\_01.09983.09983.2 | 2.7154 | 0.3313 | 100.0% | 1611.3522 | 1611.9403 | 11 | 6.006 | 50.0% | 1 | K.TLLPNEIAIRPMDK.T | 2 |
|  | LAPMis12DANCE\_011813\_01.04559.04559.2 | 4.3075 | 0.455 | 100.0% | 1660.0521 | 1660.9083 | 2 | 7.585 | 64.3% | 1 | K.KGQSSINKEEVILSK.A | 2 |
|  | LAPMis12DANCE2\_011813\_01.12832.12832.3 | 4.1551 | 0.2563 | 98.7% | 2795.1543 | 2793.2354 | 14 | 5.182 | 28.1% | 1 | K.TGEFLAFQTVHLPPLPEQLLELGNK.A | 3 |
|  | LAPMis12DANCE\_011813\_02.05974.05974.3 | 4.0788 | 0.4165 | 100.0% | 2671.1943 | 2671.991 | 1 | 6.526 | 33.7% | 1 | K.AHNDMHIVQATEIHNINIISSNAK.D | 3 |
|  | LAPMis12DANCE\_011813\_01.09252.09252.3 | 3.9858 | 0.3242 | 99.7% | 2175.2043 | 2174.3696 | 1 | 6.164 | 39.7% | 2 | R.AAEKELEQLKTEEEELQR.N | 3 |

---

|  |  |  |  |  |  |  |  |  |
| --- | --- | --- | --- | --- | --- | --- | --- | --- |
| U | *gi|5803036|ref|NP\_006* | 1 | 1 | 5.2% | 305 | 30841 | 9.3 | heterogeneous nuclear ribonucleoprotein A0 [Homo sapiens] |

| Filename XCorr DeltCN Conf% ObsM+H+ CalcM+H+ SpR ZScore Ion% # Sequence  | | | | | | | | | | | | |
| --- | --- | --- | --- | --- | --- | --- | --- | --- | --- | --- | --- | --- |
| \* | LAPMis12DANCE\_011813\_02.07400.07400.2 | 3.8247 | 0.3841 | 100.0% | 1691.2122 | 1691.9248 | 1 | 7.153 | 70.0% | 1 | K.LFIGGLNVQTSESGLR.G | 2 |

---

|  |  |  |  |  |  |  |  |  |
| --- | --- | --- | --- | --- | --- | --- | --- | --- |
| U | *gi|40068061|ref|NP\_06* | 1 | 1 | 5.2% | 268 | 30595 | 9.4 | centromere protein Q [Homo sapiens] |

| Filename XCorr DeltCN Conf% ObsM+H+ CalcM+H+ SpR ZScore Ion% # Sequence  | | | | | | | | | | | | |
| --- | --- | --- | --- | --- | --- | --- | --- | --- | --- | --- | --- | --- |
| \* | LAPMis12DANCE\_011813\_02.06245.06245.2 | 2.672 | 0.2506 | 99.2% | 1676.5521 | 1674.8021 | 27 | 4.774 | 46.2% | 1 | K.IQILASEVEEEEER.V | 2 |

---

|  |  |  |  |  |  |  |  |  |
| --- | --- | --- | --- | --- | --- | --- | --- | --- |
| U | *gi|122891870|ref|NP\_9* | 7 | 8 | 5.1% | 1907 | 213700 | 4.8 | melanoma inhibitory activity family, member 3 [Homo sapiens] |

| Filename XCorr DeltCN Conf% ObsM+H+ CalcM+H+ SpR ZScore Ion% # Sequence  | | | | | | | | | | | | |
| --- | --- | --- | --- | --- | --- | --- | --- | --- | --- | --- | --- | --- |
| \* | LAPMis12DANCE\_011813\_02.05338.05338.2 | 3.8999 | 0.5413 | 100.0% | 1685.5922 | 1685.7417 | 1 | 7.98 | 53.3% | 1 | K.FGSTADALVSDDETTR.L | 2 |
| \* | LAPMis12DANCE\_011813\_01.03194.03194.3 | 4.1009 | 0.4873 | 100.0% | 1649.1543 | 1649.7605 | 1 | 7.61 | 44.2% | 1 | K.TNNDKEVNAEHHIK.G | 3 |
| \* | LAPMis12DANCE\_011813\_02.04844.04844.3 | 4.9612 | 0.3083 | 99.7% | 2572.5544 | 2571.7754 | 1 | 6.622 | 40.2% | 2 | K.GMLHEEKPGEQILEGGSESESAQK.A | 3 |
| \* | LAPMis12DANCE\_011813\_01.07716.07716.2 | 2.8653 | 0.1567 | 99.0% | 1229.8522 | 1230.4442 | 77 | 3.886 | 66.7% | 1 | R.EELKEELVLK.T | 2 |
| \* | LAPMis12DANCE\_011813\_01.09324.09324.2 | 2.7492 | 0.2661 | 99.9% | 1276.0922 | 1276.3885 | 1 | 6.882 | 80.0% | 1 | R.FSSPDEIDLPR.E | 2 |
| \* | LAPMis12DANCE2\_011813\_01.11522.11522.3 | 3.083 | 0.3384 | 99.5% | 2527.1042 | 2527.7905 | 2 | 5.252 | 32.1% | 1 | R.FSSPDEIDLPRELEDEVPILGR.N | 3 |
| \* | LAPMis12DANCE\_011813\_02.05964.05964.2 | 2.4639 | 0.2637 | 99.2% | 1276.1322 | 1276.4294 | 1 | 5.391 | 63.6% | 1 | R.ASESQILSIAEK.M | 2 |

---

|  |  |  |  |  |  |  |  |  |
| --- | --- | --- | --- | --- | --- | --- | --- | --- |
| U | *gi|4757878|ref|NP\_004* | 4 | 5 | 5.1% | 1085 | 122375 | 6.5 | budding uninhibited by benzimidazoles 1 [Homo sapiens] |

| Filename XCorr DeltCN Conf% ObsM+H+ CalcM+H+ SpR ZScore Ion% # Sequence  | | | | | | | | | | | | |
| --- | --- | --- | --- | --- | --- | --- | --- | --- | --- | --- | --- | --- |
| \* | LAPMis12DANCE\_011813\_01.04163.04163.2 | 2.7195 | 0.1961 | 99.1% | 1237.2922 | 1237.4012 | 12 | 5.099 | 70.0% | 1 | R.LTETHLPAQAR.T | 2 |
| \* | LAPMis12DANCE\_011813\_01.05331.05331.2 | 2.6516 | 0.3186 | 99.9% | 1289.4722 | 1289.4491 | 1 | 5.757 | 63.6% | 1 | R.MGPSVGSQQELR.A | 2 |
| \* | LAPMis12DANCE\_011813\_02.06210.06210.2 | 2.6206 | 0.5129 | 100.0% | 1456.1721 | 1456.6538 | 2 | 7.826 | 50.0% | 2 | K.AQTVTDSMFAVASK.D | 2 |
| \* | LAPMis12DANCE\_011813\_01.08670.08670.3 | 3.8917 | 0.3909 | 100.0% | 2020.6444 | 2020.3336 | 1 | 6.42 | 38.2% | 1 | K.LPVESVHILEDKENVVAK.Q | 3 |

---

|  |  |  |  |  |  |  |  |  |
| --- | --- | --- | --- | --- | --- | --- | --- | --- |
| U | *gi|156523968|ref|NP\_0* | 3 | 3 | 5.1% | 1014 | 113084 | 8.9 | poly (ADP-ribose) polymerase family, member 1 [Homo sapiens] |

| Filename XCorr DeltCN Conf% ObsM+H+ CalcM+H+ SpR ZScore Ion% # Sequence  | | | | | | | | | | | | |
| --- | --- | --- | --- | --- | --- | --- | --- | --- | --- | --- | --- | --- |
| \* | LAPMis12DANCE\_011813\_02.06986.06986.2 | 3.6277 | 0.5741 | 100.0% | 1624.6721 | 1625.7728 | 1 | 9.761 | 60.7% | 1 | R.VVSEDFLQDVSASTK.S | 2 |
| \* | LAPMis12DANCE\_011813\_01.14538.14538.2 | 5.1513 | 0.5248 | 100.0% | 2138.632 | 2139.5022 | 1 | 9.503 | 58.3% | 1 | K.SLQELFLAHILSPWGAEVK.A | 2 |
| \* | LAPMis12DANCE\_011813\_02.11286.11286.2 | 2.4587 | 0.2083 | 96.3% | 2106.7122 | 2107.47 | 336 | 4.56 | 26.5% | 1 | K.VEMLDNLLDIEVAYSLLR.G | 2 |

---

|  |  |  |  |  |  |  |  |  |
| --- | --- | --- | --- | --- | --- | --- | --- | --- |
| U | *gi|17402900|ref|NP\_00* | 2 | 2 | 5.1% | 644 | 67560 | 7.6 | far upstream element-binding protein [Homo sapiens] |

| Filename XCorr DeltCN Conf% ObsM+H+ CalcM+H+ SpR ZScore Ion% # Sequence  | | | | | | | | | | | | |
| --- | --- | --- | --- | --- | --- | --- | --- | --- | --- | --- | --- | --- |
| \* | LAPMis12DANCE\_011813\_02.04877.04877.2 | 4.0402 | 0.4156 | 100.0% | 1974.2122 | 1975.0355 | 1 | 7.953 | 55.3% | 1 | K.IGGDAGTSLNSNDYGYGGQK.R | 2 |
| \* | LAPMis12DANCE\_011813\_01.07698.07698.2 | 2.4059 | 0.2282 | 97.8% | 1353.1522 | 1353.5181 | 9 | 5.015 | 58.3% | 1 | K.IQIAPDSGGLPER.S | 2 |

---

|  |  |  |  |  |  |  |  |  |
| --- | --- | --- | --- | --- | --- | --- | --- | --- |
| U | *gi|21361114|ref|NP\_00* | 1 | 1 | 5.1% | 314 | 34062 | 9.9 | solute carrier family 25 (mitochondrial carrier; oxoglutarate carrier), member 11 [Homo sapiens] |

| Filename XCorr DeltCN Conf% ObsM+H+ CalcM+H+ SpR ZScore Ion% # Sequence  | | | | | | | | | | | | |
| --- | --- | --- | --- | --- | --- | --- | --- | --- | --- | --- | --- | --- |
| \* | LAPMis12DANCE\_011813\_02.05932.05932.2 | 2.5217 | 0.181 | 95.3% | 1638.2322 | 1636.8455 | 89 | 4.149 | 36.7% | 1 | R.AVVVNAAQLASYSQSK.Q | 2 |

---

|  |  |  |  |  |  |  |  |  |
| --- | --- | --- | --- | --- | --- | --- | --- | --- |
| U | *gi|90669931|ref|NP\_06* | 1 | 1 | 5.0% | 705 | 78756 | 5.7 | tetratricopeptide repeat domain 12 [Homo sapiens] |

| Filename XCorr DeltCN Conf% ObsM+H+ CalcM+H+ SpR ZScore Ion% # Sequence  | | | | | | | | | | | | |
| --- | --- | --- | --- | --- | --- | --- | --- | --- | --- | --- | --- | --- |
| \* | LAPMis12DANCE\_011813\_01.18424.18424.3 | 2.9064 | 0.2595 | 95.8% | 4126.9146 | 4127.301 | 70 | 4.664 | 19.1% | 1 | R.T#T#LNK@T#MISPPQTAMKS\*AEEINSEAFLASVEKDAK@.E | 3 |

---

|  |  |  |  |  |  |  |  |  |
| --- | --- | --- | --- | --- | --- | --- | --- | --- |
| U | *Reverse\_gi|109240538|* | 1 | 1 | 5.0% | 259 | 30346 | 8.9 | polycomb group ring finger 1 [Homo sapiens] |

| Filename XCorr DeltCN Conf% ObsM+H+ CalcM+H+ SpR ZScore Ion% # Sequence  | | | | | | | | | | | | |
| --- | --- | --- | --- | --- | --- | --- | --- | --- | --- | --- | --- | --- |
| \* | LAPMis12DANCE\_011813\_02.07176.07176.2 | 2.3981 | 0.1873 | 95.4% | 1660.7922 | 1661.8217 | 29 | 4.004 | 41.7% | 1 | R.VS\*CRVYKNQLVSK.N | 2 |

---

|  |  |  |  |  |  |  |  |  |
| --- | --- | --- | --- | --- | --- | --- | --- | --- |
| U | *gi|118582269|ref|NP\_0* | 1 | 1 | 5.0% | 201 | 22460 | 8.0 | splicing factor, arginine/serine-rich 1 isoform 2 [Homo sapiens] |
| U | *gi|5902076|ref|NP\_008* | 1 | 1 | 4.0% | 248 | 27745 | 10.4 | splicing factor, arginine/serine-rich 1 isoform 1 [Homo sapiens] |

| Filename XCorr DeltCN Conf% ObsM+H+ CalcM+H+ SpR ZScore Ion% # Sequence  | | | | | | | | | | | | |
| --- | --- | --- | --- | --- | --- | --- | --- | --- | --- | --- | --- | --- |
|  | LAPMis12DANCE\_011813\_01.08452.08452.2 | 2.5228 | 0.1493 | 96.5% | 1258.3722 | 1258.4137 | 2 | 5.052 | 72.2% | 1 | R.TKDIEDVFYK.Y | 2 |

---

|  |  |  |  |  |  |  |  |  |
| --- | --- | --- | --- | --- | --- | --- | --- | --- |
| U | *Reverse\_gi|30425446|r* | 1 | 1 | 4.9% | 142 | 14668 | 10.7 | hypothetical protein LOC255783 [Homo sapiens] |

| Filename XCorr DeltCN Conf% ObsM+H+ CalcM+H+ SpR ZScore Ion% # Sequence  | | | | | | | | | | | | |
| --- | --- | --- | --- | --- | --- | --- | --- | --- | --- | --- | --- | --- |
| \* | LAPMis12DANCE\_011813\_01.09773.09773.2 | 2.2602 | 0.1533 | 96.7% | 908.03217 | 908.9462 | 2 | 4.097 | 91.7% | 1 | R.RDPDSYR.R | 2 |

---

|  |  |  |  |  |  |  |  |  |
| --- | --- | --- | --- | --- | --- | --- | --- | --- |
| U | *gi|62241042|ref|NP\_00* | 4 | 4 | 4.8% | 1512 | 170590 | 7.3 | glutamyl-prolyl tRNA synthetase [Homo sapiens] |

| Filename XCorr DeltCN Conf% ObsM+H+ CalcM+H+ SpR ZScore Ion% # Sequence  | | | | | | | | | | | | |
| --- | --- | --- | --- | --- | --- | --- | --- | --- | --- | --- | --- | --- |
| \* | LAPMis12DANCE\_011813\_01.09350.09350.2 | 2.622 | 0.1818 | 96.7% | 1613.8722 | 1614.8845 | 132 | 4.117 | 46.2% | 1 | K.NPEVGLKPVWYSPK.V | 2 |
| \* | LAPMis12DANCE\_011813\_02.05981.05981.3 | 4.9542 | 0.4483 | 100.0% | 3105.2344 | 3105.3423 | 1 | 7.774 | 26.7% | 1 | K.TGQEYKPGNPPAEIGQNISSNSSASILESK.S | 3 |
| \* | LAPMis12DANCE\_011813\_02.06339.06339.2 | 3.0019 | 0.2727 | 99.9% | 1537.4122 | 1536.6818 | 1 | 5.645 | 57.7% | 1 | K.SLYDEVAAQGEVVR.K | 2 |
| \* | LAPMis12DANCE\_011813\_01.14453.14453.2 | 2.4854 | 0.2532 | 98.7% | 1761.1122 | 1761.0715 | 113 | 5.011 | 39.3% | 1 | K.LQAILEDIQVTLFTR.A | 2 |

---

|  |  |  |  |  |  |  |  |  |
| --- | --- | --- | --- | --- | --- | --- | --- | --- |
| U | *gi|169646441|ref|NP\_0* | 1 | 1 | 4.8% | 400 | 45619 | 6.2 | GDP dissociation inhibitor 2 isoform 2 [Homo sapiens] |
| U | *gi|6598323|ref|NP\_001* | 1 | 1 | 4.3% | 445 | 50663 | 6.5 | GDP dissociation inhibitor 2 isoform 1 [Homo sapiens] |
| U | *gi|4503971|ref|NP\_001* | 1 | 1 | 4.3% | 447 | 50583 | 5.1 | GDP dissociation inhibitor 1 [Homo sapiens] |

| Filename XCorr DeltCN Conf% ObsM+H+ CalcM+H+ SpR ZScore Ion% # Sequence  | | | | | | | | | | | | |
| --- | --- | --- | --- | --- | --- | --- | --- | --- | --- | --- | --- | --- |
|  | LAPMis12DANCE2\_011813\_01.11753.11753.2 | 3.0492 | 0.4156 | 100.0% | 2141.3123 | 2142.462 | 1 | 6.327 | 41.7% | 1 | K.SPYLYPLYGLGELPQGFAR.L | 2 |

---

|  |  |  |  |  |  |  |  |  |
| --- | --- | --- | --- | --- | --- | --- | --- | --- |
| U | *gi|4885259|ref|NP\_005* | 1 | 1 | 4.8% | 375 | 42750 | 6.8 | myostatin [Homo sapiens] |

| Filename XCorr DeltCN Conf% ObsM+H+ CalcM+H+ SpR ZScore Ion% # Sequence  | | | | | | | | | | | | |
| --- | --- | --- | --- | --- | --- | --- | --- | --- | --- | --- | --- | --- |
| \* | LAPMis12DANCE\_011813\_01.15780.15780.2 | 3.1539 | 0.1772 | 99.1% | 2114.5122 | 2116.4226 | 1 | 4.061 | 47.1% | 1 | R.LET#APNISKDVIRQLLPK.A | 2 |

---

|  |  |  |  |  |  |  |  |  |
| --- | --- | --- | --- | --- | --- | --- | --- | --- |
| U | *gi|51896031|ref|NP\_78* | 1 | 1 | 4.8% | 294 | 33620 | 5.3 | hypothetical protein LOC285636 [Homo sapiens] |

| Filename XCorr DeltCN Conf% ObsM+H+ CalcM+H+ SpR ZScore Ion% # Sequence  | | | | | | | | | | | | |
| --- | --- | --- | --- | --- | --- | --- | --- | --- | --- | --- | --- | --- |
| \* | LAPMis12DANCE2\_011813\_01.11502.11502.2 | 3.9896 | 0.3703 | 100.0% | 1617.8922 | 1617.9231 | 1 | 7.111 | 61.5% | 1 | K.ELISFLSEPEILVK.E | 2 |

---

|  |  |  |  |  |  |  |  |  |
| --- | --- | --- | --- | --- | --- | --- | --- | --- |
| U | *gi|153281202|ref|NP\_0* | 1 | 1 | 4.8% | 165 | 18048 | 11.6 | neuropeptide W preproprotein [Homo sapiens] |

| Filename XCorr DeltCN Conf% ObsM+H+ CalcM+H+ SpR ZScore Ion% # Sequence  | | | | | | | | | | | | |
| --- | --- | --- | --- | --- | --- | --- | --- | --- | --- | --- | --- | --- |
| \* | LAPMis12DANCE2\_011813\_01.18654.18654.1 | 1.0753 | 0.3586 | 100.0% | 727.5 | 726.85394 | 344 | 4.907 | 42.9% | 1 | R.AAAGPLAR.D | 1 |

---

|  |  |  |  |  |  |  |  |  |
| --- | --- | --- | --- | --- | --- | --- | --- | --- |
| U | *gi|18640750|ref|NP\_57* | 1 | 1 | 4.7% | 235 | 27582 | 7.9 | regulator of G-protein signalling 18 [Homo sapiens] |

| Filename XCorr DeltCN Conf% ObsM+H+ CalcM+H+ SpR ZScore Ion% # Sequence  | | | | | | | | | | | | |
| --- | --- | --- | --- | --- | --- | --- | --- | --- | --- | --- | --- | --- |
| \* | LAPMis12DANCE\_011813\_01.12683.12683.2 | 2.4722 | 0.1836 | 97.2% | 1325.3322 | 1325.3751 | 1 | 4.029 | 65.0% | 1 | K.ET#RVSPEEAVK.W | 2 |

---

|  |  |  |  |  |  |  |  |  |
| --- | --- | --- | --- | --- | --- | --- | --- | --- |
| U | *gi|5729982|ref|NP\_006* | 1 | 1 | 4.6% | 713 | 78413 | 8.4 | polymerase (DNA directed), eta [Homo sapiens] |

| Filename XCorr DeltCN Conf% ObsM+H+ CalcM+H+ SpR ZScore Ion% # Sequence  | | | | | | | | | | | | |
| --- | --- | --- | --- | --- | --- | --- | --- | --- | --- | --- | --- | --- |
| \* | LAPMis12DANCE2\_011813\_01.15971.15971.3 | 2.4527 | 0.377 | 99.5% | 3637.5544 | 3637.7961 | 21 | 2.766 | 20.3% | 1 | K.VPVTS\*S\*EAKTQGSGPAVTAT#KK@ATTSLESFFQK.A | 3 |

---

|  |  |  |  |  |  |  |  |  |
| --- | --- | --- | --- | --- | --- | --- | --- | --- |
| U | *gi|58219064|ref|NP\_00* | 1 | 1 | 4.6% | 495 | 53950 | 9.2 | Src homology 2 domain containing E [Homo sapiens] |

| Filename XCorr DeltCN Conf% ObsM+H+ CalcM+H+ SpR ZScore Ion% # Sequence  | | | | | | | | | | | | |
| --- | --- | --- | --- | --- | --- | --- | --- | --- | --- | --- | --- | --- |
| \* | LAPMis12DANCE\_011813\_01.10499.10499.3 | 2.8984 | 0.2753 | 97.4% | 2484.5344 | 2486.5667 | 11 | 4.588 | 28.4% | 1 | R.KNS\*AAELGS\*GRAGVGPK@DSRLSR.D | 3 |

---

|  |  |  |  |  |  |  |  |  |
| --- | --- | --- | --- | --- | --- | --- | --- | --- |
| U | *gi|157168360|ref|NP\_0* | 1 | 1 | 4.6% | 373 | 41661 | 5.7 | acetylserotonin O-methyltransferase [Homo sapiens] |

| Filename XCorr DeltCN Conf% ObsM+H+ CalcM+H+ SpR ZScore Ion% # Sequence  | | | | | | | | | | | | |
| --- | --- | --- | --- | --- | --- | --- | --- | --- | --- | --- | --- | --- |
| \* | LAPMis12DANCE2\_011813\_01.04114.04114.3 | 2.7191 | 0.2621 | 96.5% | 2001.2043 | 2000.082 | 13 | 4.444 | 32.8% | 1 | R.AS\*AHGTELLLDICVS\*LK@.L | 3 |

---

|  |  |  |  |  |  |  |  |  |
| --- | --- | --- | --- | --- | --- | --- | --- | --- |
| U | *gi|147903292|ref|NP\_9* | 1 | 1 | 4.6% | 369 | 39744 | 11.3 | nuclear localized factor 1 [Homo sapiens] |

| Filename XCorr DeltCN Conf% ObsM+H+ CalcM+H+ SpR ZScore Ion% # Sequence  | | | | | | | | | | | | |
| --- | --- | --- | --- | --- | --- | --- | --- | --- | --- | --- | --- | --- |
| \* | LAPMis12DANCE\_011813\_02.15303.15303.3 | 2.3302 | 0.298 | 97.4% | 2099.4844 | 2102.3738 | 127 | 4.224 | 29.7% | 1 | R.RPRGCRLLRVPDGLLS\*R.A | 3 |

---

|  |  |  |  |  |  |  |  |  |
| --- | --- | --- | --- | --- | --- | --- | --- | --- |
| U | *gi|114796640|ref|NP\_0* | 2 | 2 | 4.5% | 421 | 44969 | 7.5 | regulator of chromosome condensation 1 [Homo sapiens] |
| U | *gi|4502801|ref|NP\_001* | 2 | 2 | 4.5% | 421 | 44969 | 7.5 | regulator of chromosome condensation 1 isoform c [Homo sapiens] |
| U | *gi|114796648|ref|NP\_0* | 2 | 2 | 4.5% | 421 | 44969 | 7.5 | regulator of chromosome condensation 1 [Homo sapiens] |
| U | *gi|114796646|ref|NP\_0* | 2 | 2 | 4.3% | 438 | 46753 | 8.1 | regulator of chromosome condensation 1 isoform b [Homo sapiens] |
| U | *gi|114796644|ref|NP\_0* | 2 | 2 | 4.2% | 452 | 48146 | 8.2 | regulator of chromosome condensation 1 isoform a [Homo sapiens] |
| U | *gi|114796642|ref|NP\_0* | 2 | 2 | 4.5% | 421 | 44969 | 7.5 | regulator of chromosome condensation 1 [Homo sapiens] |

| Filename XCorr DeltCN Conf% ObsM+H+ CalcM+H+ SpR ZScore Ion% # Sequence  | | | | | | | | | | | | |
| --- | --- | --- | --- | --- | --- | --- | --- | --- | --- | --- | --- | --- |
|  | LAPMis12DANCE2\_011813\_01.04739.04739.2 | 2.3531 | 0.3572 | 99.6% | 1899.3322 | 1900.0122 | 149 | 5.339 | 36.1% | 1 | K.VVQVSAGDSHTAALTDDGR.V | 2 |
|  | LAPMis12DANCE\_011813\_02.04282.04282.3 | 4.5155 | 0.3604 | 100.0% | 1900.6743 | 1900.0122 | 1 | 7.156 | 41.7% | 1 | K.VVQVSAGDSHTAALTDDGR.V | 3 |

---

|  |  |  |  |  |  |  |  |  |
| --- | --- | --- | --- | --- | --- | --- | --- | --- |
| U | *Reverse\_gi|66346733|r* | 1 | 1 | 4.5% | 247 | 28356 | 5.6 | chloride intracellular channel 2 [Homo sapiens] |

| Filename XCorr DeltCN Conf% ObsM+H+ CalcM+H+ SpR ZScore Ion% # Sequence  | | | | | | | | | | | | |
| --- | --- | --- | --- | --- | --- | --- | --- | --- | --- | --- | --- | --- |
| \* | LAPMis12DANCE\_011813\_01.09755.09755.2 | 2.1549 | 0.2519 | 97.6% | 1333.2522 | 1332.3666 | 1 | 4.18 | 65.0% | 1 | K.AVNAYT#NEIEK.D | 2 |

---

|  |  |  |  |  |  |  |  |  |
| --- | --- | --- | --- | --- | --- | --- | --- | --- |
| U | *gi|46367787|ref|NP\_00* | 2 | 2 | 4.4% | 636 | 70671 | 9.5 | poly(A) binding protein, cytoplasmic 1 [Homo sapiens] |

| Filename XCorr DeltCN Conf% ObsM+H+ CalcM+H+ SpR ZScore Ion% # Sequence  | | | | | | | | | | | | |
| --- | --- | --- | --- | --- | --- | --- | --- | --- | --- | --- | --- | --- |
| \* | LAPMis12DANCE\_011813\_02.05992.05992.2 | 3.1746 | 0.4137 | 100.0% | 1741.9321 | 1741.857 | 1 | 6.864 | 53.6% | 1 | K.GYGFVHFETQEAAER.A | 2 |
| \* | LAPMis12DANCE\_011813\_01.09053.09053.2 | 2.9375 | 0.2863 | 99.9% | 1412.8722 | 1413.6134 | 1 | 5.766 | 62.5% | 1 | R.KEFSPFGTITSAK.V | 2 |

---

|  |  |  |  |  |  |  |  |  |
| --- | --- | --- | --- | --- | --- | --- | --- | --- |
| U | *gi|169169195|ref|XP\_0* | 1 | 1 | 4.4% | 365 | 41062 | 6.5 | PREDICTED: hypothetical protein [Homo sapiens] |

| Filename XCorr DeltCN Conf% ObsM+H+ CalcM+H+ SpR ZScore Ion% # Sequence  | | | | | | | | | | | | |
| --- | --- | --- | --- | --- | --- | --- | --- | --- | --- | --- | --- | --- |
| \* | LAPMis12DANCE\_011813\_01.03305.03305.3 | 3.4872 | 0.4036 | 100.0% | 1843.3444 | 1844.0146 | 100 | 5.986 | 31.7% | 1 | K.THMTHHAVSDHEATLR.C | 3 |

---

|  |  |  |  |  |  |  |  |  |
| --- | --- | --- | --- | --- | --- | --- | --- | --- |
| U | *Reverse\_gi|7710154|re* | 1 | 1 | 4.4% | 360 | 40147 | 9.3 | mitochondrial tryptophanyl tRNA synthetase 2 isoform 1 precursor [Homo sapiens] |

| Filename XCorr DeltCN Conf% ObsM+H+ CalcM+H+ SpR ZScore Ion% # Sequence  | | | | | | | | | | | | |
| --- | --- | --- | --- | --- | --- | --- | --- | --- | --- | --- | --- | --- |
| \* | LAPMis12DANCE\_011813\_02.09036.09036.2 | 2.5951 | 0.2281 | 98.4% | 1932.9321 | 1934.9757 | 27 | 4.004 | 40.0% | 1 | K.DPDS\*KSMKAS\*PDRLSK@.V | 2 |

---

|  |  |  |  |  |  |  |  |  |
| --- | --- | --- | --- | --- | --- | --- | --- | --- |
| U | *Reverse\_gi|7657345|re* | 1 | 1 | 4.3% | 372 | 39920 | 9.5 | mitochondrial carrier homolog 1 [Homo sapiens] |

| Filename XCorr DeltCN Conf% ObsM+H+ CalcM+H+ SpR ZScore Ion% # Sequence  | | | | | | | | | | | | |
| --- | --- | --- | --- | --- | --- | --- | --- | --- | --- | --- | --- | --- |
| \* | LAPMis12DANCE2\_011813\_01.05920.05920.2 | 2.3176 | 0.2299 | 96.2% | 1204.1921 | 1203.3184 | 7 | 4.692 | 56.7% | 1 | R.AGAGAGAGAMGAAGGR.A | 2 |

---

|  |  |  |  |  |  |  |  |  |
| --- | --- | --- | --- | --- | --- | --- | --- | --- |
| U | *gi|19743838|ref|NP\_00* | 1 | 1 | 4.3% | 345 | 36916 | 7.4 | TAR RNA binding protein 2 isoform b [Homo sapiens] |
| U | *gi|85068512|ref|NP\_59* | 1 | 1 | 4.3% | 345 | 36916 | 7.4 | TAR RNA binding protein 2 isoform b [Homo sapiens] |
| U | *gi|19743840|ref|NP\_59* | 1 | 1 | 4.1% | 366 | 39039 | 6.5 | TAR RNA binding protein 2 isoform a [Homo sapiens] |

| Filename XCorr DeltCN Conf% ObsM+H+ CalcM+H+ SpR ZScore Ion% # Sequence  | | | | | | | | | | | | |
| --- | --- | --- | --- | --- | --- | --- | --- | --- | --- | --- | --- | --- |
|  | LAPMis12DANCE2\_011813\_01.15960.15960.2 | 2.3984 | 0.2226 | 96.7% | 1873.7722 | 1875.9474 | 80 | 4.434 | 39.3% | 1 | R.FIEIGS\*GT#S\*KKLAKR.N | 2 |

---

|  |  |  |  |  |  |  |  |  |
| --- | --- | --- | --- | --- | --- | --- | --- | --- |
| U | *gi|221307584|ref|NP\_0* | 1 | 1 | 4.3% | 299 | 33296 | 9.8 | prohibitin 2 isoform 1 [Homo sapiens] |
| U | *gi|6005854|ref|NP\_009* | 1 | 1 | 4.3% | 299 | 33296 | 9.8 | prohibitin 2 isoform 2 [Homo sapiens] |

| Filename XCorr DeltCN Conf% ObsM+H+ CalcM+H+ SpR ZScore Ion% # Sequence  | | | | | | | | | | | | |
| --- | --- | --- | --- | --- | --- | --- | --- | --- | --- | --- | --- | --- |
|  | LAPMis12DANCE\_011813\_01.14124.14124.2 | 3.5922 | 0.3328 | 100.0% | 1724.3722 | 1725.0428 | 2 | 7.406 | 50.0% | 1 | R.IPWFQYPIIYDIR.A | 2 |

---

|  |  |  |  |  |  |  |  |  |
| --- | --- | --- | --- | --- | --- | --- | --- | --- |
| U | *gi|21361122|ref|NP\_00* | 1 | 1 | 4.3% | 280 | 31895 | 8.3 | four and a half LIM domains 1 [Homo sapiens] |

| Filename XCorr DeltCN Conf% ObsM+H+ CalcM+H+ SpR ZScore Ion% # Sequence  | | | | | | | | | | | | |
| --- | --- | --- | --- | --- | --- | --- | --- | --- | --- | --- | --- | --- |
| \* | LAPMis12DANCE\_011813\_01.05355.05355.2 | 2.6953 | 0.2656 | 99.4% | 1307.1721 | 1307.4459 | 1 | 6.628 | 63.6% | 1 | K.AIVAGDQNVEYK.G | 2 |

---

|  |  |  |  |  |  |  |  |  |
| --- | --- | --- | --- | --- | --- | --- | --- | --- |
| U | *gi|113429434|ref|XP\_9* | 1 | 1 | 4.2% | 601 | 69363 | 9.1 | PREDICTED: similar to OTTHUMP00000028720 [Homo sapiens] |
| U | *gi|169216050|ref|XP\_0* | 1 | 1 | 4.2% | 601 | 69363 | 9.1 | PREDICTED: similar to OTTHUMP00000028720 [Homo sapiens] |
| U | *gi|169215842|ref|XP\_0* | 1 | 1 | 4.2% | 601 | 69363 | 9.1 | PREDICTED: similar to OTTHUMP00000028720 [Homo sapiens] |

| Filename XCorr DeltCN Conf% ObsM+H+ CalcM+H+ SpR ZScore Ion% # Sequence  | | | | | | | | | | | | |
| --- | --- | --- | --- | --- | --- | --- | --- | --- | --- | --- | --- | --- |
|  | LAPMis12DANCE\_011813\_01.10372.10372.3 | 2.6308 | 0.3453 | 99.0% | 2807.7544 | 2807.8608 | 4 | 3.874 | 27.1% | 1 | K.FYHPQSSSANSPSEKT#SSAKQNSEK.S | 3 |

---

|  |  |  |  |  |  |  |  |  |
| --- | --- | --- | --- | --- | --- | --- | --- | --- |
| U | *gi|24307933|ref|NP\_00* | 1 | 1 | 4.2% | 476 | 52865 | 6.3 | transcription factor EB [Homo sapiens] |

| Filename XCorr DeltCN Conf% ObsM+H+ CalcM+H+ SpR ZScore Ion% # Sequence  | | | | | | | | | | | | |
| --- | --- | --- | --- | --- | --- | --- | --- | --- | --- | --- | --- | --- |
| \* | LAPMis12DANCE\_011813\_01.11548.11548.3 | 2.8793 | 0.2603 | 96.6% | 2523.2043 | 2524.812 | 32 | 4.925 | 28.9% | 1 | K.GT#ILKAS\*VDYIRRMQKDLQK.S | 3 |

---

|  |  |  |  |  |  |  |  |  |
| --- | --- | --- | --- | --- | --- | --- | --- | --- |
| U | *gi|169213873|ref|XP\_0* | 1 | 1 | 4.2% | 355 | 39862 | 7.2 | PREDICTED: similar to Glucose phosphate isomerase [Homo sapiens] |
| U | *gi|18201905|ref|NP\_00* | 1 | 1 | 2.7% | 558 | 63147 | 8.3 | glucose phosphate isomerase [Homo sapiens] |

| Filename XCorr DeltCN Conf% ObsM+H+ CalcM+H+ SpR ZScore Ion% # Sequence  | | | | | | | | | | | | |
| --- | --- | --- | --- | --- | --- | --- | --- | --- | --- | --- | --- | --- |
|  | LAPMis12DANCE\_011813\_01.15438.15438.2 | 2.4732 | 0.2724 | 99.1% | 1704.7722 | 1705.0685 | 11 | 4.558 | 50.0% | 1 | K.ILLANFLAQTEALMR.G | 2 |

---

|  |  |  |  |  |  |  |  |  |
| --- | --- | --- | --- | --- | --- | --- | --- | --- |
| U | *gi|7305503|ref|NP\_038* | 1 | 1 | 4.2% | 356 | 38534 | 7.4 | stomatin (EPB72)-like 2 [Homo sapiens] |

| Filename XCorr DeltCN Conf% ObsM+H+ CalcM+H+ SpR ZScore Ion% # Sequence  | | | | | | | | | | | | |
| --- | --- | --- | --- | --- | --- | --- | --- | --- | --- | --- | --- | --- |
| \* | LAPMis12DANCE\_011813\_02.05610.05610.2 | 3.1428 | 0.403 | 100.0% | 1663.2122 | 1663.6923 | 1 | 7.018 | 57.1% | 1 | R.DVQGTDASLDEELDR.V | 2 |

---

|  |  |  |  |  |  |  |  |  |
| --- | --- | --- | --- | --- | --- | --- | --- | --- |
| U | *gi|4506003|ref|NP\_002* | 1 | 1 | 4.2% | 330 | 37512 | 6.3 | protein phosphatase 1, catalytic subunit, alpha isoform 1 [Homo sapiens] |
| U | *gi|56790945|ref|NP\_00* | 1 | 1 | 4.1% | 341 | 38631 | 6.6 | protein phosphatase 1, catalytic subunit, alpha isoform 3 [Homo sapiens] |
| U | *gi|46249376|ref|NP\_99* | 1 | 1 | 4.3% | 327 | 37187 | 6.2 | protein phosphatase 1, catalytic subunit, beta isoform 1 [Homo sapiens] |
| U | *gi|45827798|ref|NP\_99* | 1 | 1 | 4.9% | 286 | 32595 | 6.1 | protein phosphatase 1, catalytic subunit, alpha isoform 2 [Homo sapiens] |
| U | *gi|4506007|ref|NP\_002* | 1 | 1 | 4.3% | 323 | 36984 | 6.5 | protein phosphatase 1, catalytic subunit, gamma isoform [Homo sapiens] |
| U | *gi|4506005|ref|NP\_002* | 1 | 1 | 4.3% | 327 | 37187 | 6.2 | protein phosphatase 1, catalytic subunit, beta isoform 1 [Homo sapiens] |

| Filename XCorr DeltCN Conf% ObsM+H+ CalcM+H+ SpR ZScore Ion% # Sequence  | | | | | | | | | | | | |
| --- | --- | --- | --- | --- | --- | --- | --- | --- | --- | --- | --- | --- |
|  | LAPMis12DANCE\_011813\_01.09711.09711.2 | 2.5729 | 0.3135 | 99.6% | 1640.3722 | 1640.7924 | 1 | 5.746 | 65.4% | 1 | R.AHQVVEDGYEFFAK.R | 2 |

---

|  |  |  |  |  |  |  |  |  |
| --- | --- | --- | --- | --- | --- | --- | --- | --- |
| U | *gi|38788380|ref|NP\_06* | 2 | 2 | 4.1% | 919 | 103043 | 6.9 | dehydrogenase E1 and transketolase domain containing protein 1 [Homo sapiens] |

| Filename XCorr DeltCN Conf% ObsM+H+ CalcM+H+ SpR ZScore Ion% # Sequence  | | | | | | | | | | | | |
| --- | --- | --- | --- | --- | --- | --- | --- | --- | --- | --- | --- | --- |
| \* | LAPMis12DANCE2\_011813\_01.14949.14949.2 | 2.9721 | 0.2862 | 99.9% | 2002.1522 | 2003.4534 | 1 | 6.47 | 46.9% | 1 | R.LNLLTGLLQFPPELMFR.K | 2 |
| \* | LAPMis12DANCE\_011813\_02.06182.06182.3 | 3.156 | 0.2374 | 95.6% | 2240.5444 | 2240.5242 | 2 | 5.067 | 32.5% | 1 | R.IGGSVHLIVNNQLGYTTPAER.G | 3 |

---

|  |  |  |  |  |  |  |  |  |
| --- | --- | --- | --- | --- | --- | --- | --- | --- |
| U | *gi|4507677|ref|NP\_003* | 2 | 3 | 4.1% | 803 | 92469 | 4.8 | heat shock protein 90kDa beta, member 1 [Homo sapiens] |

| Filename XCorr DeltCN Conf% ObsM+H+ CalcM+H+ SpR ZScore Ion% # Sequence  | | | | | | | | | | | | |
| --- | --- | --- | --- | --- | --- | --- | --- | --- | --- | --- | --- | --- |
|  | LAPMis12DANCE\_011813\_01.09648.09648.2 | 2.8001 | 0.1863 | 98.3% | 1545.0322 | 1545.733 | 1 | 5.147 | 65.4% | 2 | R.ELISNASDALDKIR.L | 22 |
| \* | LAPMis12DANCE\_011813\_01.08933.08933.3 | 3.7155 | 0.4392 | 100.0% | 2260.8542 | 2261.4124 | 2 | 6.511 | 31.9% | 1 | R.FQSSHHPTDITSLDQYVER.M | 3 |

Similarities:
gi|20149594|ref|NP\_03(1:1)  

---

|  |  |  |  |  |  |  |  |  |
| --- | --- | --- | --- | --- | --- | --- | --- | --- |
| U | *gi|21536320|ref|NP\_65* | 2 | 3 | 4.1% | 756 | 84794 | 8.8 | heterogeneous nuclear ribonucleoprotein U-like 1 isoform d [Homo sapiens] |
| U | *gi|21536326|ref|NP\_00* | 2 | 3 | 3.6% | 856 | 95739 | 6.9 | heterogeneous nuclear ribonucleoprotein U-like 1 isoform a [Homo sapiens] |

| Filename XCorr DeltCN Conf% ObsM+H+ CalcM+H+ SpR ZScore Ion% # Sequence  | | | | | | | | | | | | |
| --- | --- | --- | --- | --- | --- | --- | --- | --- | --- | --- | --- | --- |
|  | LAPMis12DANCE\_011813\_01.05620.05620.3 | 4.0637 | 0.3272 | 99.7% | 2034.3844 | 2035.3435 | 1 | 5.907 | 43.3% | 2 | R.RPLEMEQQQAYRPEMK.T | 3 |
|  | LAPMis12DANCE\_011813\_02.05924.05924.2 | 4.5753 | 0.5181 | 100.0% | 1742.2122 | 1742.8857 | 1 | 8.614 | 67.9% | 1 | R.NYILDQTNVYGSAQR.R | 2 |

---

|  |  |  |  |  |  |  |  |  |
| --- | --- | --- | --- | --- | --- | --- | --- | --- |
| U | *gi|194097436|ref|NP\_0* | 1 | 1 | 4.1% | 638 | 73515 | 9.9 | CDC-like kinase 3 isoform a [Homo sapiens] |

| Filename XCorr DeltCN Conf% ObsM+H+ CalcM+H+ SpR ZScore Ion% # Sequence  | | | | | | | | | | | | |
| --- | --- | --- | --- | --- | --- | --- | --- | --- | --- | --- | --- | --- |
| \* | LAPMis12DANCE\_011813\_01.18700.18700.3 | 3.0693 | 0.2748 | 97.5% | 2535.9543 | 2533.7236 | 1 | 4.75 | 29.0% | 1 | R.LAT#AASREGAGLPRAEVAAGSGRGAR.S | 3 |

---

|  |  |  |  |  |  |  |  |  |
| --- | --- | --- | --- | --- | --- | --- | --- | --- |
| U | *gi|4503729|ref|NP\_002* | 1 | 1 | 4.1% | 459 | 51805 | 5.4 | FK506 binding protein 52 [Homo sapiens] |

| Filename XCorr DeltCN Conf% ObsM+H+ CalcM+H+ SpR ZScore Ion% # Sequence  | | | | | | | | | | | | |
| --- | --- | --- | --- | --- | --- | --- | --- | --- | --- | --- | --- | --- |
| \* | LAPMis12DANCE\_011813\_01.03345.03345.3 | 3.0764 | 0.2759 | 97.9% | 2090.5144 | 2091.1677 | 6 | 5.358 | 33.3% | 1 | K.AEASSGDHPTDTEMKEEQK.S | 3 |

---

|  |  |  |  |  |  |  |  |  |
| --- | --- | --- | --- | --- | --- | --- | --- | --- |
| U | *gi|109134336|ref|NP\_0* | 1 | 1 | 4.0% | 455 | 50425 | 5.0 | SH3-domain binding protein 5 (BTK-associated) isoform a [Homo sapiens] |

| Filename XCorr DeltCN Conf% ObsM+H+ CalcM+H+ SpR ZScore Ion% # Sequence  | | | | | | | | | | | | |
| --- | --- | --- | --- | --- | --- | --- | --- | --- | --- | --- | --- | --- |
| \* | LAPMis12DANCE\_011813\_01.04726.04726.2 | 3.7831 | 0.1233 | 99.4% | 2079.672 | 2079.264 | 2 | 3.589 | 52.9% | 1 | R.ATEVLRAAK@ET#ISLAEQR.L | 2 |

---

|  |  |  |  |  |  |  |  |  |
| --- | --- | --- | --- | --- | --- | --- | --- | --- |
| U | *gi|34147630|ref|NP\_00* | 1 | 1 | 4.0% | 455 | 49875 | 7.6 | Tu translation elongation factor, mitochondrial precursor [Homo sapiens] |

| Filename XCorr DeltCN Conf% ObsM+H+ CalcM+H+ SpR ZScore Ion% # Sequence  | | | | | | | | | | | | |
| --- | --- | --- | --- | --- | --- | --- | --- | --- | --- | --- | --- | --- |
| \* | LAPMis12DANCE\_011813\_02.07482.07482.2 | 2.8316 | 0.3228 | 100.0% | 1908.0521 | 1909.159 | 1 | 6.206 | 38.2% | 1 | R.TIGTGLVTNTLAMTEEEK.N | 2 |

---

|  |  |  |  |  |  |  |  |  |
| --- | --- | --- | --- | --- | --- | --- | --- | --- |
| U | *Reverse\_gi|224591430|* | 1 | 1 | 4.0% | 379 | 43634 | 9.6 | Ly1 antibody reactive homolog [Homo sapiens] |
| U | *Reverse\_gi|8923398|re* | 1 | 1 | 4.0% | 379 | 43634 | 9.6 | Ly1 antibody reactive homolog [Homo sapiens] |

| Filename XCorr DeltCN Conf% ObsM+H+ CalcM+H+ SpR ZScore Ion% # Sequence  | | | | | | | | | | | | |
| --- | --- | --- | --- | --- | --- | --- | --- | --- | --- | --- | --- | --- |
|  | LAPMis12DANCE2\_011813\_01.13062.13062.2 | 2.691 | 0.1908 | 97.3% | 1863.2322 | 1864.2278 | 30 | 3.99 | 39.3% | 1 | K.VLKVKDKLLK@FT#PNK.S | 2 |

---

|  |  |  |  |  |  |  |  |  |
| --- | --- | --- | --- | --- | --- | --- | --- | --- |
| U | *gi|12056465|ref|NP\_00* | 1 | 1 | 4.0% | 321 | 33784 | 10.2 | fibrillarin [Homo sapiens] |

| Filename XCorr DeltCN Conf% ObsM+H+ CalcM+H+ SpR ZScore Ion% # Sequence  | | | | | | | | | | | | |
| --- | --- | --- | --- | --- | --- | --- | --- | --- | --- | --- | --- | --- |
| \* | LAPMis12DANCE\_011813\_01.06755.06755.2 | 3.1313 | 0.3597 | 100.0% | 1512.5122 | 1511.6287 | 2 | 6.436 | 58.3% | 1 | R.VSISEGDDKIEYR.A | 2 |

---

|  |  |  |  |  |  |  |  |  |
| --- | --- | --- | --- | --- | --- | --- | --- | --- |
| U | *gi|11559923|ref|NP\_07* | 1 | 1 | 4.0% | 248 | 27385 | 7.2 | eukaryotic translation initiation factor 4H isoform 1 [Homo sapiens] |
| U | *gi|14702180|ref|NP\_11* | 1 | 1 | 4.4% | 228 | 25200 | 8.1 | eukaryotic translation initiation factor 4H isoform 2 [Homo sapiens] |

| Filename XCorr DeltCN Conf% ObsM+H+ CalcM+H+ SpR ZScore Ion% # Sequence  | | | | | | | | | | | | |
| --- | --- | --- | --- | --- | --- | --- | --- | --- | --- | --- | --- | --- |
|  | LAPMis12DANCE\_011813\_01.05741.05741.2 | 2.5886 | 0.2588 | 99.4% | 1116.1122 | 1116.2626 | 5 | 5.428 | 66.7% | 1 | R.SLRVDIAEGR.K | 2 |

---

|  |  |  |  |  |  |  |  |  |
| --- | --- | --- | --- | --- | --- | --- | --- | --- |
| U | *gi|206597505|ref|NP\_0* | 1 | 1 | 3.9% | 584 | 60767 | 8.6 | HIV-1 Rev binding protein isoform 1 [Homo sapiens] |
| U | *gi|38570132|ref|NP\_00* | 1 | 1 | 4.1% | 562 | 58260 | 8.6 | HIV-1 Rev binding protein isoform 2 [Homo sapiens] |
| U | *gi|206597509|ref|NP\_0* | 1 | 1 | 4.4% | 522 | 54182 | 8.9 | HIV-1 Rev binding protein isoform 4 [Homo sapiens] |
| U | *gi|206597507|ref|NP\_0* | 1 | 1 | 4.1% | 560 | 58132 | 8.6 | HIV-1 Rev binding protein isoform 3 [Homo sapiens] |

| Filename XCorr DeltCN Conf% ObsM+H+ CalcM+H+ SpR ZScore Ion% # Sequence  | | | | | | | | | | | | |
| --- | --- | --- | --- | --- | --- | --- | --- | --- | --- | --- | --- | --- |
|  | LAPMis12DANCE\_011813\_02.04786.04786.3 | 3.0974 | 0.2519 | 96.5% | 2348.0044 | 2346.429 | 1 | 4.923 | 30.7% | 1 | K.SSSADFGTFNTSQSHQTASAVSK.V | 3 |

---

|  |  |  |  |  |  |  |  |  |
| --- | --- | --- | --- | --- | --- | --- | --- | --- |
| U | *contaminant\_SPA2\_STAA* | 1 | 1 | 3.9% | 508 | 55439 | 5.7 | owl|P38507| IMMUNOGLOBULIN G BINDING PROTEIN A PRECURSOR (PROTEIN A). - STAPHYLOCOCCUS... |

| Filename XCorr DeltCN Conf% ObsM+H+ CalcM+H+ SpR ZScore Ion% # Sequence  | | | | | | | | | | | | |
| --- | --- | --- | --- | --- | --- | --- | --- | --- | --- | --- | --- | --- |
| \* | LAPMis12DANCE2\_011813\_01.11222.11222.3 | 3.5667 | 0.2568 | 98.1% | 2473.3145 | 2473.7043 | 3 | 4.895 | 31.6% | 1 | K.EQQNAFYEILHLPNLTEEQR.N | 3 |

---

|  |  |  |  |  |  |  |  |  |
| --- | --- | --- | --- | --- | --- | --- | --- | --- |
| U | *Reverse\_gi|4758092|re* | 1 | 1 | 3.9% | 385 | 43760 | 6.6 | chitobiase, di-N-acetyl- [Homo sapiens] |

| Filename XCorr DeltCN Conf% ObsM+H+ CalcM+H+ SpR ZScore Ion% # Sequence  | | | | | | | | | | | | |
| --- | --- | --- | --- | --- | --- | --- | --- | --- | --- | --- | --- | --- |
| \* | LAPMis12DANCE2\_011813\_01.08849.08849.2 | 2.7767 | 0.293 | 99.6% | 1863.9521 | 1864.8767 | 9 | 4.609 | 42.9% | 1 | K.GFTAVT#T#IQSWDYSK.W | 2 |

---

|  |  |  |  |  |  |  |  |  |
| --- | --- | --- | --- | --- | --- | --- | --- | --- |
| U | *Reverse\_gi|68303555|r* | 1 | 1 | 3.8% | 717 | 78691 | 6.7 | aryl hydrocarbon receptor nuclear translocator 2 [Homo sapiens] |

| Filename XCorr DeltCN Conf% ObsM+H+ CalcM+H+ SpR ZScore Ion% # Sequence  | | | | | | | | | | | | |
| --- | --- | --- | --- | --- | --- | --- | --- | --- | --- | --- | --- | --- |
| \* | LAPMis12DANCE2\_011813\_01.10156.10156.3 | 2.8391 | 0.2656 | 96.1% | 2943.5044 | 2940.9575 | 293 | 4.379 | 21.2% | 1 | K.GS\*HGSPFPS\*GQSYIQQTGAASASS\*MMK.K | 3 |

---

|  |  |  |  |  |  |  |  |  |
| --- | --- | --- | --- | --- | --- | --- | --- | --- |
| U | *Reverse\_gi|4506567|re* | 1 | 1 | 3.8% | 476 | 54844 | 6.6 | RNA (guanine-7-) methyltransferase [Homo sapiens] |

| Filename XCorr DeltCN Conf% ObsM+H+ CalcM+H+ SpR ZScore Ion% # Sequence  | | | | | | | | | | | | |
| --- | --- | --- | --- | --- | --- | --- | --- | --- | --- | --- | --- | --- |
| \* | LAPMis12DANCE2\_011813\_01.10617.10617.2 | 3.3961 | 0.0844 | 96.7% | 2211.612 | 2213.3428 | 35 | 3.88 | 38.2% | 1 | K.VS\*VDAIDT#CVLK@NIRGK@K@.W | 2 |

---

|  |  |  |  |  |  |  |  |  |
| --- | --- | --- | --- | --- | --- | --- | --- | --- |
| U | *gi|58761500|ref|NP\_03* | 1 | 5 | 3.8% | 396 | 44744 | 7.8 | GTP-binding protein PTD004 isoform 1 [Homo sapiens] |

| Filename XCorr DeltCN Conf% ObsM+H+ CalcM+H+ SpR ZScore Ion% # Sequence  | | | | | | | | | | | | |
| --- | --- | --- | --- | --- | --- | --- | --- | --- | --- | --- | --- | --- |
| \* | LAPMis12DANCE\_011813\_01.14498.14498.2 | 4.7801 | 0.5067 | 100.0% | 1569.9122 | 1569.928 | 1 | 9.101 | 82.1% | 5 | K.IPAFLNVVDIAGLVK.G | 2 |

---

|  |  |  |  |  |  |  |  |  |
| --- | --- | --- | --- | --- | --- | --- | --- | --- |
| U | *gi|61743952|ref|NP\_06* | 1 | 1 | 3.8% | 364 | 42072 | 9.1 | 1-acylglycerol-3-phosphate O-acyltransferase 5 [Homo sapiens] |

| Filename XCorr DeltCN Conf% ObsM+H+ CalcM+H+ SpR ZScore Ion% # Sequence  | | | | | | | | | | | | |
| --- | --- | --- | --- | --- | --- | --- | --- | --- | --- | --- | --- | --- |
| \* | LAPMis12DANCE2\_011813\_01.09674.09674.2 | 2.6421 | 0.2473 | 99.1% | 1692.6921 | 1693.8546 | 16 | 5.175 | 42.3% | 1 | R.FPGKS\*VNS\*KLSIKK.T | 2 |

---

|  |  |  |  |  |  |  |  |  |
| --- | --- | --- | --- | --- | --- | --- | --- | --- |
| U | *gi|4506725|ref|NP\_000* | 1 | 1 | 3.8% | 263 | 29598 | 10.2 | ribosomal protein S4, X-linked X isoform [Homo sapiens] |

| Filename XCorr DeltCN Conf% ObsM+H+ CalcM+H+ SpR ZScore Ion% # Sequence  | | | | | | | | | | | | |
| --- | --- | --- | --- | --- | --- | --- | --- | --- | --- | --- | --- | --- |
| \* | LAPMis12DANCE\_011813\_01.08844.08844.2 | 2.5971 | 0.3735 | 100.0% | 1168.1921 | 1168.3842 | 13 | 5.876 | 61.1% | 1 | K.GNKPWISLPR.G | 2 |

---

|  |  |  |  |  |  |  |  |  |
| --- | --- | --- | --- | --- | --- | --- | --- | --- |
| U | *gi|4758138|ref|NP\_004* | 2 | 4 | 3.7% | 614 | 69148 | 8.9 | DEAD (Asp-Glu-Ala-Asp) box polypeptide 5 [Homo sapiens] |

| Filename XCorr DeltCN Conf% ObsM+H+ CalcM+H+ SpR ZScore Ion% # Sequence  | | | | | | | | | | | | |
| --- | --- | --- | --- | --- | --- | --- | --- | --- | --- | --- | --- | --- |
| \* | LAPMis12DANCE\_011813\_01.06581.06581.2 | 3.344 | 0.3867 | 100.0% | 1390.2522 | 1390.4978 | 1 | 6.567 | 75.0% | 1 | K.NFYQEHPDLAR.R | 2 |
|  | LAPMis12DANCE2\_011813\_01.06765.06765.2 | 2.8785 | 0.272 | 99.9% | 1227.6721 | 1227.4465 | 1 | 7.282 | 81.8% | 3 | K.APILIATDVASR.G | 22 |

Similarities:
gi|148613856|ref|NP\_0(1:1)  

---

|  |  |  |  |  |  |  |  |  |
| --- | --- | --- | --- | --- | --- | --- | --- | --- |
| U | *gi|4885225|ref|NP\_005* | 1 | 1 | 3.7% | 656 | 68478 | 9.3 | Ewing sarcoma breakpoint region 1 isoform EWS [Homo sapiens] |

| Filename XCorr DeltCN Conf% ObsM+H+ CalcM+H+ SpR ZScore Ion% # Sequence  | | | | | | | | | | | | |
| --- | --- | --- | --- | --- | --- | --- | --- | --- | --- | --- | --- | --- |
| \* | LAPMis12DANCE\_011813\_02.05031.05031.3 | 4.8583 | 0.4874 | 100.0% | 2480.0645 | 2481.572 | 1 | 8.833 | 31.5% | 1 | R.QDHPSSMGVYGQESGGFSGPGENR.S | 3 |

---

|  |  |  |  |  |  |  |  |  |
| --- | --- | --- | --- | --- | --- | --- | --- | --- |
| U | *gi|58761486|ref|NP\_00* | 1 | 1 | 3.7% | 544 | 60463 | 6.5 | chaperonin containing TCP1, subunit 3 isoform b [Homo sapiens] |
| U | *gi|63162572|ref|NP\_00* | 1 | 1 | 3.7% | 545 | 60534 | 6.5 | chaperonin containing TCP1, subunit 3 isoform a [Homo sapiens] |

| Filename XCorr DeltCN Conf% ObsM+H+ CalcM+H+ SpR ZScore Ion% # Sequence  | | | | | | | | | | | | |
| --- | --- | --- | --- | --- | --- | --- | --- | --- | --- | --- | --- | --- |
|  | LAPMis12DANCE\_011813\_01.12905.12905.2 | 3.6755 | 0.363 | 100.0% | 2132.2122 | 2132.5593 | 1 | 6.674 | 44.7% | 1 | K.MLLDPMGGIVMTNDGNAILR.E | 2 |

---

|  |  |  |  |  |  |  |  |  |
| --- | --- | --- | --- | --- | --- | --- | --- | --- |
| U | *gi|13375618|ref|NP\_05* | 1 | 1 | 3.7% | 516 | 60101 | 8.2 | 24-dehydrocholesterol reductase precursor [Homo sapiens] |

| Filename XCorr DeltCN Conf% ObsM+H+ CalcM+H+ SpR ZScore Ion% # Sequence  | | | | | | | | | | | | |
| --- | --- | --- | --- | --- | --- | --- | --- | --- | --- | --- | --- | --- |
| \* | LAPMis12DANCE2\_011813\_01.14836.14836.2 | 2.8516 | 0.3877 | 100.0% | 2307.3323 | 2307.6567 | 2 | 5.774 | 36.1% | 1 | R.SIFWELQDIIPFGNNPIFR.Y | 2 |

---

|  |  |  |  |  |  |  |  |  |
| --- | --- | --- | --- | --- | --- | --- | --- | --- |
| U | *gi|54792131|ref|NP\_05* | 1 | 1 | 3.7% | 482 | 53250 | 9.5 | hypothetical protein LOC25854 [Homo sapiens] |
| U | *gi|54792133|ref|NP\_00* | 1 | 1 | 3.7% | 482 | 53250 | 9.5 | hypothetical protein LOC25854 [Homo sapiens] |

| Filename XCorr DeltCN Conf% ObsM+H+ CalcM+H+ SpR ZScore Ion% # Sequence  | | | | | | | | | | | | |
| --- | --- | --- | --- | --- | --- | --- | --- | --- | --- | --- | --- | --- |
|  | LAPMis12DANCE\_011813\_01.04014.04014.3 | 3.3278 | 0.2872 | 99.1% | 1760.3043 | 1760.7635 | 1 | 4.816 | 38.2% | 1 | K.EDK@ASGGGAGALSS\*APHR.L | 3 |

---

|  |  |  |  |  |  |  |  |  |
| --- | --- | --- | --- | --- | --- | --- | --- | --- |
| U | *gi|50659095|ref|NP\_00* | 2 | 2 | 3.6% | 783 | 87344 | 9.3 | DEAD (Asp-Glu-Ala-Asp) box polypeptide 21 [Homo sapiens] |

| Filename XCorr DeltCN Conf% ObsM+H+ CalcM+H+ SpR ZScore Ion% # Sequence  | | | | | | | | | | | | |
| --- | --- | --- | --- | --- | --- | --- | --- | --- | --- | --- | --- | --- |
|  | LAPMis12DANCE\_011813\_01.11081.11081.2 | 2.2101 | 0.2706 | 97.4% | 1669.3722 | 1669.8285 | 1 | 5.132 | 46.4% | 1 | K.EGAFSNFPISEETIK.L | 2 |
| \* | LAPMis12DANCE\_011813\_01.07673.07673.2 | 3.0721 | 0.1909 | 99.4% | 1377.5721 | 1378.6609 | 19 | 6.066 | 50.0% | 1 | R.GRAPQVLVLAPTR.E | 2 |

---

|  |  |  |  |  |  |  |  |  |
| --- | --- | --- | --- | --- | --- | --- | --- | --- |
| U | *gi|5454064|ref|NP\_006* | 1 | 2 | 3.6% | 669 | 69492 | 9.7 | RNA binding motif protein 14 [Homo sapiens] |

| Filename XCorr DeltCN Conf% ObsM+H+ CalcM+H+ SpR ZScore Ion% # Sequence  | | | | | | | | | | | | |
| --- | --- | --- | --- | --- | --- | --- | --- | --- | --- | --- | --- | --- |
| \* | LAPMis12DANCE\_011813\_01.07278.07278.3 | 3.0973 | 0.275 | 97.5% | 2466.0544 | 2466.6292 | 1 | 5.081 | 26.1% | 2 | R.TQSSASLAASYAAQQHPQAAASYR.G | 3 |

---

|  |  |  |  |  |  |  |  |  |
| --- | --- | --- | --- | --- | --- | --- | --- | --- |
| U | *gi|20357552|ref|NP\_00* | 1 | 1 | 3.6% | 550 | 61586 | 5.4 | cortactin isoform a [Homo sapiens] |
| U | *gi|20357556|ref|NP\_61* | 1 | 1 | 3.9% | 513 | 57467 | 5.3 | cortactin isoform b [Homo sapiens] |

| Filename XCorr DeltCN Conf% ObsM+H+ CalcM+H+ SpR ZScore Ion% # Sequence  | | | | | | | | | | | | |
| --- | --- | --- | --- | --- | --- | --- | --- | --- | --- | --- | --- | --- |
|  | LAPMis12DANCE\_011813\_02.05782.05782.3 | 3.1293 | 0.2613 | 97.5% | 2249.9644 | 2250.442 | 25 | 4.848 | 30.3% | 1 | R.MDKNASTFEDVTQVSSAYQK.T | 3 |

---

|  |  |  |  |  |  |  |  |  |
| --- | --- | --- | --- | --- | --- | --- | --- | --- |
| U | *gi|113205085|ref|NP\_0* | 1 | 1 | 3.6% | 552 | 61489 | 6.0 | leucine rich repeat containing 31 [Homo sapiens] |

| Filename XCorr DeltCN Conf% ObsM+H+ CalcM+H+ SpR ZScore Ion% # Sequence  | | | | | | | | | | | | |
| --- | --- | --- | --- | --- | --- | --- | --- | --- | --- | --- | --- | --- |
| \* | LAPMis12DANCE2\_011813\_01.13793.13793.2 | 2.3084 | 0.2995 | 99.1% | 2246.0122 | 2248.6921 | 55 | 4.48 | 26.3% | 1 | K.KMGSSSENLLSRLRFLPALK.S | 2 |

---

|  |  |  |  |  |  |  |  |  |
| --- | --- | --- | --- | --- | --- | --- | --- | --- |
| U | *gi|148596968|ref|NP\_1* | 1 | 1 | 3.6% | 551 | 61489 | 7.4 | tripartite motif-containing 8 [Homo sapiens] |

| Filename XCorr DeltCN Conf% ObsM+H+ CalcM+H+ SpR ZScore Ion% # Sequence  | | | | | | | | | | | | |
| --- | --- | --- | --- | --- | --- | --- | --- | --- | --- | --- | --- | --- |
| \* | LAPMis12DANCE\_011813\_02.09341.09341.2 | 2.3005 | 0.2572 | 97.7% | 2240.6921 | 2240.344 | 1 | 4.426 | 42.1% | 1 | R.T#QTCTSSSLSPTKIGHLNSK@.L | 2 |

---

|  |  |  |  |  |  |  |  |  |
| --- | --- | --- | --- | --- | --- | --- | --- | --- |
| U | *gi|8923870|ref|NP\_060* | 1 | 1 | 3.6% | 550 | 59972 | 8.6 | solute carrier family 22 member 11 [Homo sapiens] |

| Filename XCorr DeltCN Conf% ObsM+H+ CalcM+H+ SpR ZScore Ion% # Sequence  | | | | | | | | | | | | |
| --- | --- | --- | --- | --- | --- | --- | --- | --- | --- | --- | --- | --- |
| \* | LAPMis12DANCE2\_011813\_01.16570.16570.2 | 2.9363 | 0.1175 | 95.1% | 2211.652 | 2212.6128 | 3 | 3.966 | 36.8% | 1 | R.INGHKEAKNLTIEVLMSSVK.E | 2 |

---

|  |  |  |  |  |  |  |  |  |
| --- | --- | --- | --- | --- | --- | --- | --- | --- |
| U | *gi|160707950|ref|NP\_0* | 1 | 1 | 3.6% | 498 | 53323 | 9.9 | methyl CpG binding protein 2 isoform 2 [Homo sapiens] |
| U | *gi|4826830|ref|NP\_004* | 1 | 1 | 3.7% | 486 | 52441 | 9.9 | methyl CpG binding protein 2 isoform 1 [Homo sapiens] |

| Filename XCorr DeltCN Conf% ObsM+H+ CalcM+H+ SpR ZScore Ion% # Sequence  | | | | | | | | | | | | |
| --- | --- | --- | --- | --- | --- | --- | --- | --- | --- | --- | --- | --- |
|  | LAPMis12DANCE\_011813\_01.15711.15711.2 | 2.2684 | 0.2462 | 96.7% | 1979.2122 | 1976.9548 | 211 | 4.849 | 29.4% | 1 | K.S\*KESS\*PK@GRSSSASSPPK.K | 2 |

---

|  |  |  |  |  |  |  |  |  |
| --- | --- | --- | --- | --- | --- | --- | --- | --- |
| U | *gi|31543397|ref|NP\_62* | 1 | 1 | 3.6% | 417 | 44796 | 8.5 | phosphoglycerate kinase 2 [Homo sapiens] |
| U | *gi|4505763|ref|NP\_000* | 1 | 1 | 3.6% | 417 | 44615 | 8.1 | phosphoglycerate kinase 1 [Homo sapiens] |

| Filename XCorr DeltCN Conf% ObsM+H+ CalcM+H+ SpR ZScore Ion% # Sequence  | | | | | | | | | | | | |
| --- | --- | --- | --- | --- | --- | --- | --- | --- | --- | --- | --- | --- |
|  | LAPMis12DANCE\_011813\_01.08964.08964.2 | 2.5708 | 0.2472 | 98.9% | 1637.3522 | 1635.7764 | 1 | 5.565 | 60.7% | 1 | K.LGDVYVNDAFGTAHR.A | 2 |

---

|  |  |  |  |  |  |  |  |  |
| --- | --- | --- | --- | --- | --- | --- | --- | --- |
| U | *gi|4507447|ref|NP\_003* | 1 | 1 | 3.6% | 338 | 38726 | 5.9 | transcription factor AP-4 (activating enhancer binding protein 4) [Homo sapiens] |

| Filename XCorr DeltCN Conf% ObsM+H+ CalcM+H+ SpR ZScore Ion% # Sequence  | | | | | | | | | | | | |
| --- | --- | --- | --- | --- | --- | --- | --- | --- | --- | --- | --- | --- |
| \* | LAPMis12DANCE\_011813\_01.09094.09094.2 | 2.3329 | 0.2023 | 96.3% | 1323.9321 | 1324.5377 | 1 | 4.297 | 63.6% | 1 | R.MQSINAGFQSLK.T | 2 |

---

|  |  |  |  |  |  |  |  |  |
| --- | --- | --- | --- | --- | --- | --- | --- | --- |
| U | *gi|118442839|ref|NP\_0* | 1 | 1 | 3.6% | 330 | 37651 | 7.4 | complement factor H-related 1 [Homo sapiens] |
| U | *gi|62739186|ref|NP\_00* | 1 | 1 | 1.0% | 1231 | 139070 | 6.6 | complement factor H isoform a precursor [Homo sapiens] |

| Filename XCorr DeltCN Conf% ObsM+H+ CalcM+H+ SpR ZScore Ion% # Sequence  | | | | | | | | | | | | |
| --- | --- | --- | --- | --- | --- | --- | --- | --- | --- | --- | --- | --- |
|  | LAPMis12DANCE2\_011813\_01.04314.04314.2 | 2.4412 | 0.1731 | 95.6% | 1438.2322 | 1438.6445 | 166 | 3.84 | 50.0% | 1 | R.QMSKYPSGERVR.Y | 2 |

---

|  |  |  |  |  |  |  |  |  |
| --- | --- | --- | --- | --- | --- | --- | --- | --- |
| U | *gi|116063573|ref|NP\_0* | 5 | 7 | 3.5% | 2639 | 280016 | 6.0 | filamin A, alpha isoform 1 [Homo sapiens] |
| U | *gi|160420317|ref|NP\_0* | 5 | 7 | 3.5% | 2647 | 280737 | 6.1 | filamin A, alpha isoform 2 [Homo sapiens] |

| Filename XCorr DeltCN Conf% ObsM+H+ CalcM+H+ SpR ZScore Ion% # Sequence  | | | | | | | | | | | | |
| --- | --- | --- | --- | --- | --- | --- | --- | --- | --- | --- | --- | --- |
|  | LAPMis12DANCE\_011813\_02.05948.05948.3 | 3.7072 | 0.3238 | 99.7% | 2313.9844 | 2314.4705 | 1 | 6.166 | 35.7% | 1 | R.SAGQGEVLVYVEDPAGHQEEAK.V | 3 |
|  | LAPMis12DANCE\_011813\_02.05543.05543.2 | 3.9284 | 0.5404 | 100.0% | 1571.1921 | 1571.7275 | 1 | 9.331 | 55.9% | 2 | R.GAGTGGLGLAVEGPSEAK.M | 2 |
|  | LAPMis12DANCE\_011813\_02.06257.06257.2 | 2.8342 | 0.3158 | 99.9% | 1501.9922 | 1501.6335 | 1 | 5.539 | 60.0% | 1 | K.DAGEGGLSLAIEGPSK.A | 2 |
|  | LAPMis12DANCE\_011813\_02.05636.05636.2 | 3.576 | 0.3505 | 100.0% | 1428.0922 | 1427.5974 | 1 | 6.014 | 56.7% | 1 | R.EAGAGGLAIAVEGPSK.A | 2 |
|  | LAPMis12DANCE\_011813\_01.09327.09327.3 | 3.5417 | 0.3474 | 99.7% | 2201.3044 | 2201.4412 | 2 | 6.067 | 32.9% | 2 | R.LVSNHSLHETSSVFVDSLTK.A | 3 |

---

|  |  |  |  |  |  |  |  |  |
| --- | --- | --- | --- | --- | --- | --- | --- | --- |
| U | *gi|21450665|ref|NP\_65* | 1 | 1 | 3.5% | 665 | 76219 | 8.5 | coiled-coil domain containing 138 [Homo sapiens] |

| Filename XCorr DeltCN Conf% ObsM+H+ CalcM+H+ SpR ZScore Ion% # Sequence  | | | | | | | | | | | | |
| --- | --- | --- | --- | --- | --- | --- | --- | --- | --- | --- | --- | --- |
| \* | LAPMis12DANCE\_011813\_02.07186.07186.3 | 3.2234 | 0.2697 | 97.6% | 2545.0745 | 2546.754 | 108 | 4.774 | 22.7% | 1 | K.RRTLTS\*PGDLDIYSGDKVGSSLK.Y | 3 |

---

|  |  |  |  |  |  |  |  |  |
| --- | --- | --- | --- | --- | --- | --- | --- | --- |
| U | *gi|23397427|ref|NP\_00* | 1 | 1 | 3.5% | 623 | 69633 | 8.6 | synaptotagmin binding, cytoplasmic RNA interacting protein [Homo sapiens] |

| Filename XCorr DeltCN Conf% ObsM+H+ CalcM+H+ SpR ZScore Ion% # Sequence  | | | | | | | | | | | | |
| --- | --- | --- | --- | --- | --- | --- | --- | --- | --- | --- | --- | --- |
| \* | LAPMis12DANCE\_011813\_02.07437.07437.3 | 4.5286 | 0.4165 | 100.0% | 2442.5044 | 2443.716 | 1 | 6.437 | 35.7% | 1 | K.VAEKLDEIYVAGLVAHSDLDER.A | 3 |

---

|  |  |  |  |  |  |  |  |  |
| --- | --- | --- | --- | --- | --- | --- | --- | --- |
| U | *gi|205277463|ref|NP\_0* | 1 | 1 | 3.5% | 623 | 67878 | 7.7 | transketolase isoform 1 [Homo sapiens] |
| U | *gi|4507521|ref|NP\_001* | 1 | 1 | 3.5% | 623 | 67878 | 7.7 | transketolase isoform 1 [Homo sapiens] |
| U | *gi|205277465|ref|NP\_0* | 1 | 1 | 4.1% | 540 | 58982 | 7.7 | transketolase isoform 2 [Homo sapiens] |

| Filename XCorr DeltCN Conf% ObsM+H+ CalcM+H+ SpR ZScore Ion% # Sequence  | | | | | | | | | | | | |
| --- | --- | --- | --- | --- | --- | --- | --- | --- | --- | --- | --- | --- |
|  | LAPMis12DANCE\_011813\_02.05537.05537.3 | 4.1362 | 0.2691 | 99.0% | 2509.7043 | 2509.6946 | 13 | 5.056 | 29.8% | 1 | R.TSRPENAIIYNNNEDFQVGQAK.V | 3 |

---

|  |  |  |  |  |  |  |  |  |
| --- | --- | --- | --- | --- | --- | --- | --- | --- |
| U | *gi|40068518|ref|NP\_00* | 1 | 1 | 3.5% | 483 | 53140 | 7.2 | phosphogluconate dehydrogenase [Homo sapiens] |

| Filename XCorr DeltCN Conf% ObsM+H+ CalcM+H+ SpR ZScore Ion% # Sequence  | | | | | | | | | | | | |
| --- | --- | --- | --- | --- | --- | --- | --- | --- | --- | --- | --- | --- |
| \* | LAPMis12DANCE\_011813\_02.06618.06618.2 | 3.2427 | 0.2557 | 99.9% | 1594.0721 | 1592.7489 | 1 | 4.654 | 59.4% | 1 | K.GILFVGSGVSGGEEGAR.Y | 2 |

---

|  |  |  |  |  |  |  |  |  |
| --- | --- | --- | --- | --- | --- | --- | --- | --- |
| U | *Reverse\_gi|169205096|* | 1 | 1 | 3.5% | 372 | 40017 | 11.2 | PREDICTED: hypothetical protein [Homo sapiens] |
| U | *Reverse\_gi|169217688|* | 1 | 1 | 3.5% | 372 | 39958 | 11.1 | PREDICTED: hypothetical protein [Homo sapiens] |

| Filename XCorr DeltCN Conf% ObsM+H+ CalcM+H+ SpR ZScore Ion% # Sequence  | | | | | | | | | | | | |
| --- | --- | --- | --- | --- | --- | --- | --- | --- | --- | --- | --- | --- |
|  | LAPMis12DANCE2\_011813\_01.09508.09508.2 | 2.6988 | 0.2892 | 99.7% | 1470.1122 | 1470.5442 | 1 | 4.857 | 50.0% | 1 | R.FPRPAT#AGWSSGR.D | 2 |

---

|  |  |  |  |  |  |  |  |  |
| --- | --- | --- | --- | --- | --- | --- | --- | --- |
| U | *gi|194440660|ref|NP\_0* | 4 | 4 | 3.4% | 1522 | 170678 | 7.0 | topoisomerase (DNA) II binding protein 1 [Homo sapiens] |

| Filename XCorr DeltCN Conf% ObsM+H+ CalcM+H+ SpR ZScore Ion% # Sequence  | | | | | | | | | | | | |
| --- | --- | --- | --- | --- | --- | --- | --- | --- | --- | --- | --- | --- |
| \* | LAPMis12DANCE\_011813\_01.09466.09466.2 | 2.568 | 0.3625 | 100.0% | 1275.9722 | 1276.4034 | 1 | 6.331 | 72.2% | 1 | R.YTDINMEDFK.C | 2 |
| \* | LAPMis12DANCE\_011813\_01.04094.04094.2 | 2.7937 | 0.3487 | 100.0% | 1119.1921 | 1119.2836 | 1 | 6.026 | 77.8% | 1 | K.HGGQYMGQLK.M | 2 |
| \* | LAPMis12DANCE\_011813\_01.16922.16922.2 | 2.8333 | 0.4427 | 100.0% | 1854.3322 | 1855.19 | 5 | 7.685 | 40.0% | 1 | K.WNLPAVTIAWLLETAR.T | 2 |
| \* | LAPMis12DANCE\_011813\_01.03252.03252.2 | 2.6881 | 0.2563 | 99.2% | 1721.1322 | 1721.9078 | 4 | 6.91 | 50.0% | 1 | K.EAQSEKEEAPKPLHK.V | 2 |

---

|  |  |  |  |  |  |  |  |  |
| --- | --- | --- | --- | --- | --- | --- | --- | --- |
| U | *gi|148613856|ref|NP\_0* | 2 | 4 | 3.4% | 731 | 80458 | 8.4 | DEAD box polypeptide 17 isoform 3 [Homo sapiens] |
| U | *gi|38201710|ref|NP\_00* | 2 | 4 | 3.4% | 729 | 80273 | 8.3 | DEAD box polypeptide 17 isoform 1 [Homo sapiens] |

| Filename XCorr DeltCN Conf% ObsM+H+ CalcM+H+ SpR ZScore Ion% # Sequence  | | | | | | | | | | | | |
| --- | --- | --- | --- | --- | --- | --- | --- | --- | --- | --- | --- | --- |
|  | LAPMis12DANCE\_011813\_01.10553.10553.2 | 2.7851 | 0.2797 | 99.8% | 1676.7322 | 1676.829 | 27 | 4.839 | 45.8% | 1 | K.SQPERDWVLNEFR.S | 2 |
|  | LAPMis12DANCE2\_011813\_01.06765.06765.2 | 2.8785 | 0.272 | 99.9% | 1227.6721 | 1227.4465 | 1 | 7.282 | 81.8% | 3 | K.APILIATDVASR.G | 22 |

Similarities:
gi|4758138|ref|NP\_004(1:1)  

---

|  |  |  |  |  |  |  |  |  |
| --- | --- | --- | --- | --- | --- | --- | --- | --- |
| U | *gi|193083178|ref|NP\_0* | 1 | 1 | 3.4% | 524 | 60146 | 6.7 | cytochrome P450 family 4 subfamily F polypeptide 11 [Homo sapiens] |
| U | *gi|193083180|ref|NP\_0* | 1 | 1 | 3.4% | 524 | 60146 | 6.7 | cytochrome P450 family 4 subfamily F polypeptide 11 [Homo sapiens] |

| Filename XCorr DeltCN Conf% ObsM+H+ CalcM+H+ SpR ZScore Ion% # Sequence  | | | | | | | | | | | | |
| --- | --- | --- | --- | --- | --- | --- | --- | --- | --- | --- | --- | --- |
|  | LAPMis12DANCE\_011813\_01.10556.10556.2 | 3.9329 | 0.0205 | 96.5% | 2229.632 | 2230.4473 | 13 | 2.933 | 47.1% | 1 | K.NK@AK@SKT#LDFIDVLLLS\*K@.D | 2 |

---

|  |  |  |  |  |  |  |  |  |
| --- | --- | --- | --- | --- | --- | --- | --- | --- |
| U | *gi|217416366|ref|NP\_1* | 1 | 1 | 3.4% | 474 | 50619 | 9.2 | major facilitator superfamily domain containing 9 [Homo sapiens] |

| Filename XCorr DeltCN Conf% ObsM+H+ CalcM+H+ SpR ZScore Ion% # Sequence  | | | | | | | | | | | | |
| --- | --- | --- | --- | --- | --- | --- | --- | --- | --- | --- | --- | --- |
| \* | LAPMis12DANCE2\_011813\_01.11415.11415.2 | 2.7377 | 0.2218 | 98.9% | 1463.6522 | 1463.501 | 1 | 4.48 | 53.3% | 1 | K.TGTEAEAADSGAVGAR.R | 2 |

---

|  |  |  |  |  |  |  |  |  |
| --- | --- | --- | --- | --- | --- | --- | --- | --- |
| U | *gi|5453629|ref|NP\_006* | 1 | 1 | 3.4% | 406 | 44820 | 5.2 | dynactin 2 [Homo sapiens] |

| Filename XCorr DeltCN Conf% ObsM+H+ CalcM+H+ SpR ZScore Ion% # Sequence  | | | | | | | | | | | | |
| --- | --- | --- | --- | --- | --- | --- | --- | --- | --- | --- | --- | --- |
| \* | LAPMis12DANCE\_011813\_01.10000.10000.2 | 3.0483 | 0.4056 | 100.0% | 1668.4321 | 1668.8846 | 1 | 6.665 | 65.4% | 1 | R.LLHEVQELTTEVEK.I | 2 |

---

|  |  |  |  |  |  |  |  |  |
| --- | --- | --- | --- | --- | --- | --- | --- | --- |
| U | *gi|31542331|ref|NP\_00* | 1 | 1 | 3.4% | 381 | 42027 | 8.2 | cysteine-rich, angiogenic inducer, 61 [Homo sapiens] |

| Filename XCorr DeltCN Conf% ObsM+H+ CalcM+H+ SpR ZScore Ion% # Sequence  | | | | | | | | | | | | |
| --- | --- | --- | --- | --- | --- | --- | --- | --- | --- | --- | --- | --- |
| \* | LAPMis12DANCE\_011813\_01.10667.10667.2 | 2.5368 | 0.3011 | 99.4% | 1467.0721 | 1466.5878 | 12 | 4.876 | 50.0% | 1 | K.ELGFDASEVELTR.N | 2 |

---

|  |  |  |  |  |  |  |  |  |
| --- | --- | --- | --- | --- | --- | --- | --- | --- |
| U | *gi|34740329|ref|NP\_91* | 1 | 1 | 3.4% | 378 | 39595 | 9.0 | heterogeneous nuclear ribonucleoprotein A3 [Homo sapiens] |

| Filename XCorr DeltCN Conf% ObsM+H+ CalcM+H+ SpR ZScore Ion% # Sequence  | | | | | | | | | | | | |
| --- | --- | --- | --- | --- | --- | --- | --- | --- | --- | --- | --- | --- |
| \* | LAPMis12DANCE\_011813\_01.08549.08549.2 | 3.3536 | 0.3511 | 100.0% | 1583.7122 | 1583.7968 | 1 | 6.43 | 70.8% | 1 | K.YGKIETIEVMEDR.Q | 2 |

---

|  |  |  |  |  |  |  |  |  |
| --- | --- | --- | --- | --- | --- | --- | --- | --- |
| U | *Reverse\_gi|154800487|* | 1 | 1 | 3.4% | 348 | 39171 | 7.9 | ER lipid raft associated 1 [Homo sapiens] |
| U | *Reverse\_gi|6005721|re* | 1 | 1 | 3.5% | 339 | 37840 | 5.6 | ER lipid raft associated 2 isoform 1 [Homo sapiens] |
| U | *Reverse\_gi|154800489|* | 1 | 1 | 3.4% | 348 | 39171 | 7.9 | ER lipid raft associated 1 [Homo sapiens] |

| Filename XCorr DeltCN Conf% ObsM+H+ CalcM+H+ SpR ZScore Ion% # Sequence  | | | | | | | | | | | | |
| --- | --- | --- | --- | --- | --- | --- | --- | --- | --- | --- | --- | --- |
|  | LAPMis12DANCE\_011813\_01.06593.06593.2 | 2.2556 | 0.2071 | 95.6% | 1526.0322 | 1526.6439 | 128 | 4.138 | 50.0% | 1 | K.KRET#EAEKEVVK.Q | 2 |

---

|  |  |  |  |  |  |  |  |  |
| --- | --- | --- | --- | --- | --- | --- | --- | --- |
| U | *gi|38201714|ref|NP\_00* | 1 | 1 | 3.4% | 326 | 36092 | 9.2 | ELAV-like 1 [Homo sapiens] |

| Filename XCorr DeltCN Conf% ObsM+H+ CalcM+H+ SpR ZScore Ion% # Sequence  | | | | | | | | | | | | |
| --- | --- | --- | --- | --- | --- | --- | --- | --- | --- | --- | --- | --- |
| \* | LAPMis12DANCE\_011813\_01.06490.06490.2 | 2.0712 | 0.3389 | 99.4% | 1190.7722 | 1189.3542 | 1 | 5.399 | 65.0% | 1 | R.VLVDQTTGLSR.G | 2 |

---

|  |  |  |  |  |  |  |  |  |
| --- | --- | --- | --- | --- | --- | --- | --- | --- |
| U | *Reverse\_gi|223555968|* | 1 | 1 | 3.3% | 658 | 73815 | 6.3 | integrator complex subunit 9 isoform 1 [Homo sapiens] |
| U | *Reverse\_gi|223555970|* | 1 | 1 | 3.5% | 637 | 71468 | 6.6 | integrator complex subunit 9 isoform 2 [Homo sapiens] |

| Filename XCorr DeltCN Conf% ObsM+H+ CalcM+H+ SpR ZScore Ion% # Sequence  | | | | | | | | | | | | |
| --- | --- | --- | --- | --- | --- | --- | --- | --- | --- | --- | --- | --- |
|  | LAPMis12DANCE\_011813\_01.16763.16763.2 | 1.8004 | 0.3535 | 97.4% | 2575.0723 | 2574.9058 | 33 | 4.615 | 23.8% | 1 | K.RKKGST#PQAPRPPPQLLHKNDK.T | 2 |

---

|  |  |  |  |  |  |  |  |  |
| --- | --- | --- | --- | --- | --- | --- | --- | --- |
[truncated: 151,458 more chars]
