# Supplementary material for: Astrin-SKAP complex reconstitution reveals its kinetochore interaction with microtubule-bound Ndc80
Source: eLife. 2017 Aug 25;6:e26866. doi: 10.7554/eLife.26866 (PMC5602300; doi:10.7554/eLife.26866)
Supplement: Source data 1. — Complete mass spectrometry searches using methods described in (Washburn et al., 2001) for affinity purification/mass spectrometry data sets described in this paper (data from this study; [Kern et al., 2016] [Gascoigne et al., 2011]). Individual Astrin cross-linking immunoprecipitations are listed based on the order in Figure 4—figure supplement 1. These samples have not been pruned for common or antibody-specific contaminants. [file elife-26866-data1.zip › Mis12_CrosslinkingSupernatant.html]

D Mis12XLAPLSS
DTASelect v2.0.21  
/nfs/cheeseman\_massspec/David/Mis12XLAPLSS  
/nfs/cheeseman\_massspec/Databases/NCBI-RefSeq\_human\_na\_04-13-2009\_con\_reversed.fasta  
SEQUEST 3.0 in SQT format.  
  
 Jump  to the summary table.  
  
sequest.params modifications:

|  |  |  |
| --- | --- | --- |
| \* | S | 80.0 |
| # | T | 80.0 |
| @ | K | 12.0 |
| Static | C | 57.0 |

|  |  |
| --- | --- |
| true | Use criteria |
| 0.0 | Minimum peptide confidence |
| 0.05 | Peptide false positive rate |
| 0.0 | Minimum protein confidence |
| 1.0 | Protein false positive rate |
| 1 | Minimum charge state |
| 16 | Maximum charge state |
| 0.0 | Minimum ion proportion |
| 1000 | Maximum Sp rank |
| -1.0 | Minimum Sp score |
| Include | Modified peptide inclusion |
| Any | Tryptic status requirement |
| false | Multiple, ambiguous IDs allowed |
| Ignore | Peptide validation handling |
| XCorr | Purge duplicate peptides by protein |
| false | Include only loci with unique peptide |
| true | Remove subset proteins |
| Ignore | Locus validation handling |
| 0 | Minimum modified peptides per locus |
| 1000 | Minimum redundancy for low coverage loci |
| 2 | Minimum peptides per locus |

#### Locus Key:

|  |  |  |  |  |  |  |  |  |
| --- | --- | --- | --- | --- | --- | --- | --- | --- |
| Validation Status | Locus | Sequence Count | Spectrum Count | Sequence Coverage | Length | MolWt | pI | Descriptive Name |

#### Similarity Key:

|  |  |  |
| --- | --- | --- |
| Locus | # of identical peptides | # of differing peptides |

---

|  |  |  |  |  |  |  |  |  |
| --- | --- | --- | --- | --- | --- | --- | --- | --- |
| U | *gi|4506671|ref|NP\_000* | 14 | 35 | 95.7% | 115 | 11665 | 4.5 | ribosomal protein P2 [Homo sapiens] |

| Filename XCorr DeltCN Conf% ObsM+H+ CalcM+H+ SpR ZScore Ion% # Sequence  | | | | | | | | | | | | |
| --- | --- | --- | --- | --- | --- | --- | --- | --- | --- | --- | --- | --- |
| \* | Mis12XLAPLSS\_011913\_01.12794.12794.2 | 5.3948 | 0.4228 | 100.0% | 1870.0922 | 1870.1124 | 1 | 9.335 | 61.1% | 4 | R.YVASYLLAALGGNSSPSAK.D | 2 |
| \* | Mis12XLAPLSS\_011913\_01.12782.12782.3 | 4.9772 | 0.4397 | 100.0% | 1870.2244 | 1870.1124 | 1 | 8.328 | 38.9% | 2 | R.YVASYLLAALGGNSSPSAK.D | 3 |
| \* | Mis12XLAPLSS\_011913\_01.07886.07886.3 | 3.536 | 0.3426 | 100.0% | 1900.4944 | 1902.1118 | 1 | 6.273 | 39.1% | 1 | K.KILDSVGIEADDDRLNK.V | 3 |
| \* | Mis12XLAPLSS\_011913\_02.05913.05913.2 | 4.5817 | 0.493 | 100.0% | 1417.9122 | 1418.5004 | 1 | 8.857 | 83.3% | 1 | K.ILDSVGIEADDDR.L | 2 |
| \* | Mis12XLAPLSS\_011913\_01.08330.08330.2 | 4.5151 | 0.4444 | 100.0% | 1773.1522 | 1773.9377 | 1 | 7.392 | 63.3% | 4 | K.ILDSVGIEADDDRLNK.V | 2 |
| \* | Mis12XLAPLSS\_011913\_01.08306.08306.3 | 2.4104 | 0.291 | 97.1% | 1774.1344 | 1773.9377 | 31 | 4.273 | 35.0% | 1 | K.ILDSVGIEADDDRLNK.V | 3 |
| \* | Mis12XLAPLSS\_011913\_01.12312.12312.3 | 5.4678 | 0.4973 | 100.0% | 2614.4343 | 2614.9126 | 1 | 7.706 | 35.9% | 1 | K.ILDSVGIEADDDRLNKVISELNGK.N | 3 |
| \* | Mis12XLAPLSS\_011913\_01.12353.12353.3 | 3.0237 | 0.2507 | 97.6% | 2098.7644 | 2098.4045 | 1 | 4.457 | 31.6% | 1 | K.VISELNGKNIEDVIAQGIGK.L | 3 |
| \* | Mis12XLAPLSS\_011913\_01.11330.11330.1 | 2.2046 | 0.4165 | 100.0% | 1256.55 | 1257.4294 | 1 | 6.908 | 59.1% | 1 | K.NIEDVIAQGIGK.L | 1 |
| \* | Mis12XLAPLSS\_011913\_01.11384.11384.2 | 4.7297 | 0.4796 | 100.0% | 1257.1122 | 1257.4294 | 1 | 8.511 | 81.8% | 5 | K.NIEDVIAQGIGK.L | 2 |
| \* | Mis12XLAPLSS\_011913\_01.08124.08124.2 | 1.8685 | 0.5539 | 99.8% | 2775.0522 | 2776.0757 | 1 | 7.161 | 37.5% | 1 | K.LASVPAGGAVAVSAAPGSAAPAAGSAPAAAEEK.K | 2 |
| \* | Mis12XLAPLSS\_011913\_02.05786.05786.3 | 5.8866 | 0.531 | 100.0% | 2775.3542 | 2776.0757 | 1 | 9.019 | 32.8% | 2 | K.LASVPAGGAVAVSAAPGSAAPAAGSAPAAAEEK.K | 3 |
| \* | Mis12XLAPLSS\_011913\_01.07065.07065.3 | 4.1583 | 0.2791 | 99.7% | 3277.5842 | 3276.628 | 1 | 6.424 | 20.8% | 2 | K.LASVPAGGAVAVSAAPGSAAPAAGSAPAAAEEKKDEK.K | 3 |
|  | Mis12XLAPLSS\_011913\_01.22512.22512.2 | 2.7357 | 0.4455 | 99.8% | 2110.0923 | 2110.979 | 1 | 8.226 | 59.4% | 9 | K.KEES\*EES\*DDDMGFGLFD.- | 22 |

Similarities:
gi|4506669|ref|NP\_000(1:13)  

---

|  |  |  |  |  |  |  |  |  |
| --- | --- | --- | --- | --- | --- | --- | --- | --- |
| U | *gi|4504517|ref|NP\_001* | 12 | 24 | 81.5% | 205 | 22783 | 6.4 | heat shock protein beta-1 [Homo sapiens] |

| Filename XCorr DeltCN Conf% ObsM+H+ CalcM+H+ SpR ZScore Ion% # Sequence  | | | | | | | | | | | | |
| --- | --- | --- | --- | --- | --- | --- | --- | --- | --- | --- | --- | --- |
| \* | Mis12XLAPLSS\_011913\_01.11781.11781.2 | 2.6332 | 0.3533 | 99.8% | 1903.0521 | 1904.0537 | 3 | 5.601 | 46.4% | 1 | R.GPSWDPFRDWYPHSR.L | 2 |
| \* | Mis12XLAPLSS\_011913\_01.11738.11738.3 | 3.1766 | 0.3341 | 99.7% | 1903.6444 | 1904.0537 | 1 | 6.062 | 41.1% | 2 | R.GPSWDPFRDWYPHSR.L | 3 |
| \* | Mis12XLAPLSS\_011913\_01.11343.11343.1 | 2.0338 | 0.2977 | 95.5% | 1163.61 | 1164.3494 | 31 | 5.672 | 50.0% | 1 | R.LFDQAFGLPR.L | 1 |
| \* | Mis12XLAPLSS\_011913\_01.11324.11324.2 | 4.4682 | 0.4621 | 100.0% | 1164.1322 | 1164.3494 | 1 | 8.02 | 83.3% | 4 | R.LFDQAFGLPR.L | 2 |
| \* | Mis12XLAPLSS\_011913\_01.13516.13516.3 | 3.5229 | 0.5828 | 100.0% | 4094.7544 | 4095.606 | 1 | 8.895 | 29.1% | 2 | R.LPEEWSQWLGGSSWPGYVRPLPPAAIESPAVAAPAYSR.A | 3 |
| \* | Mis12XLAPLSS\_011913\_01.05009.05009.2 | 2.5493 | 0.1731 | 98.4% | 1077.2322 | 1076.1948 | 54 | 4.199 | 66.7% | 1 | R.QLSSGVSEIR.H | 2 |
| \* | Mis12XLAPLSS\_011913\_01.10118.10118.2 | 4.6943 | 0.4706 | 100.0% | 1784.2322 | 1785.0068 | 1 | 7.781 | 63.3% | 2 | R.VSLDVNHFAPDELTVK.T | 2 |
| \* | Mis12XLAPLSS\_011913\_01.10136.10136.3 | 2.7666 | 0.3509 | 99.7% | 1785.1743 | 1785.0068 | 169 | 5.76 | 28.3% | 2 | R.VSLDVNHFAPDELTVK.T | 3 |
| \* | Mis12XLAPLSS\_011913\_01.03341.03341.3 | 3.6233 | 0.4313 | 100.0% | 1656.4443 | 1656.7117 | 1 | 6.785 | 41.7% | 1 | K.HEERQDEHGYISR.C | 3 |
| \* | Mis12XLAPLSS\_011913\_02.07715.07715.3 | 4.9341 | 0.3507 | 100.0% | 3227.2744 | 3228.6821 | 1 | 6.22 | 30.0% | 3 | R.KYTLPPGVDPTQVSSSLSPEGTLTVEAPMPK.L | 3 |
| \* | Mis12XLAPLSS\_011913\_01.10850.10850.2 | 3.4719 | 0.4682 | 100.0% | 1906.3522 | 1907.1307 | 1 | 7.825 | 50.0% | 4 | K.LATQSNEITIPVTFESR.A | 2 |
| \* | Mis12XLAPLSS\_011913\_01.03700.03700.2 | 4.6881 | 0.5127 | 100.0% | 1644.2922 | 1644.7789 | 1 | 9.722 | 68.8% | 1 | R.AQLGGPEAAKSDETAAK.- | 2 |

---

|  |  |  |  |  |  |  |  |  |
| --- | --- | --- | --- | --- | --- | --- | --- | --- |
| U | *TEV-Speptide* | 5 | 10 | 70.6% | 51 | 5423 | 9.4 | no description |

| Filename XCorr DeltCN Conf% ObsM+H+ CalcM+H+ SpR ZScore Ion% # Sequence  | | | | | | | | | | | | |
| --- | --- | --- | --- | --- | --- | --- | --- | --- | --- | --- | --- | --- |
| \* | Mis12XLAPLSS\_011913\_01.03449.03449.2 | 2.6769 | 0.3193 | 99.8% | 1280.1921 | 1280.2993 | 1 | 5.455 | 62.5% | 1 | -.SGGDRWSSTGGGR.S | 2 |
| \* | Mis12XLAPLSS\_011913\_01.04996.04996.2 | 4.0343 | 0.4028 | 100.0% | 1384.2122 | 1384.5345 | 1 | 7.461 | 72.7% | 5 | R.SRENLYFQGAAK.F | 2 |
| \* | Mis12XLAPLSS\_011913\_01.07310.07310.2 | 2.0897 | 0.2205 | 96.2% | 1140.4521 | 1141.2688 | 265 | 6.302 | 50.0% | 2 | R.ENLYFQGAAK.F | 2 |
| \* | Mis12XLAPLSS\_011913\_01.04054.04054.2 | 2.3281 | 0.1703 | 96.2% | 1297.9922 | 1298.4844 | 1 | 4.407 | 75.0% | 1 | K.FKETAAAKFER.Q | 2 |
| \* | Mis12XLAPLSS\_011913\_01.04040.04040.3 | 3.6173 | 0.2907 | 100.0% | 1298.8143 | 1298.4844 | 1 | 5.624 | 52.5% | 1 | K.FKETAAAKFER.Q | 3 |

---

|  |  |  |  |  |  |  |  |  |
| --- | --- | --- | --- | --- | --- | --- | --- | --- |
| U | *gi|10645195|ref|NP\_06* | 12 | 25 | 63.1% | 130 | 14135 | 11.1 | histone cluster 1, H2ae [Homo sapiens] |
| U | *gi|4504245|ref|NP\_003* | 11 | 21 | 63.1% | 130 | 14105 | 11.1 | histone cluster 1, H2ac [Homo sapiens] |
| U | *gi|19557656|ref|NP\_00* | 12 | 25 | 63.1% | 130 | 14135 | 11.1 | histone cluster 1, H2ab [Homo sapiens] |
| U | *gi|15617199|ref|NP\_25* | 12 | 25 | 63.1% | 130 | 14121 | 11.1 | histone cluster 3, H2a [Homo sapiens] |

| Filename XCorr DeltCN Conf% ObsM+H+ CalcM+H+ SpR ZScore Ion% # Sequence  | | | | | | | | | | | | |
| --- | --- | --- | --- | --- | --- | --- | --- | --- | --- | --- | --- | --- |
|  | Mis12XLAPLSS\_011913\_01.07319.07319.2 | 2.77 | 0.0989 | 96.9% | 1275.2722 | 1275.4531 | 1 | 4.75 | 68.2% | 1 | R.SSRAGLQFPVGR.V | 222 |
|  | Mis12XLAPLSS\_011913\_01.09008.09008.2 | 3.1059 | 0.329 | 99.9% | 944.59216 | 945.1093 | 2 | 5.574 | 81.2% | 4 | R.AGLQFPVGR.V | 2222 |
|  | Mis12XLAPLSS\_011913\_01.18992.18992.3 | 3.6724 | 0.2894 | 99.8% | 2918.4243 | 2917.3752 | 1 | 5.976 | 29.5% | 1 | R.VGAGAPVYLAAVLEYLTAEILELAGNAAR.D | 33 |
|  | Mis12XLAPLSS\_011913\_01.18956.18956.2 | 4.2915 | 0.2816 | 99.9% | 2919.7922 | 2917.3752 | 1 | 5.25 | 41.1% | 2 | R.VGAGAPVYLAAVLEYLTAEILELAGNAAR.D | 22 |
|  | Mis12XLAPLSS\_011913\_01.18684.18684.3 | 3.7222 | 0.2081 | 97.5% | 3274.8245 | 3274.7417 | 28 | 4.71 | 20.2% | 1 | R.VGAGAPVYLAAVLEYLTAEILELAGNAARDNK.K | 33 |
|  | Mis12XLAPLSS\_011913\_01.18458.18458.3 | 4.6811 | 0.3898 | 100.0% | 3402.3843 | 3402.9158 | 1 | 6.183 | 25.0% | 1 | R.VGAGAPVYLAAVLEYLTAEILELAGNAARDNKK.T | 33 |
|  | Mis12XLAPLSS\_011913\_01.05156.05156.2 | 2.4241 | 0.2579 | 99.8% | 851.09216 | 851.0396 | 3 | 5.349 | 83.3% | 5 | R.HLQLAIR.N | 2222 |
|  | Mis12XLAPLSS\_011913\_02.05366.05366.3 | 3.1821 | 0.3259 | 99.7% | 1694.9343 | 1693.9004 | 1 | 5.774 | 44.2% | 3 | R.HLQLAIRNDEELNK.L | 333 |
|  | Mis12XLAPLSS\_011913\_01.09910.09910.3 | 5.0033 | 0.4852 | 100.0% | 2133.2344 | 2133.4587 | 1 | 8.144 | 33.8% | 1 | R.HLQLAIRNDEELNKLLGR.V | 3 |
|  | Mis12XLAPLSS\_011913\_01.09198.09198.2 | 3.5604 | 0.3463 | 100.0% | 1301.1322 | 1301.4423 | 1 | 5.728 | 70.0% | 1 | R.NDEELNKLLGR.V | 2 |
|  | Mis12XLAPLSS\_011913\_01.13262.13262.2 | 5.2273 | 0.5136 | 100.0% | 1931.7122 | 1932.3573 | 1 | 8.573 | 63.9% | 3 | R.VTIAQGGVLPNIQAVLLPK.K | 222 |
|  | Mis12XLAPLSS\_011913\_01.13299.13299.3 | 4.5036 | 0.3796 | 100.0% | 1931.9343 | 1932.3573 | 1 | 7.342 | 45.8% | 2 | R.VTIAQGGVLPNIQAVLLPK.K | 333 |

Similarities:
gi|10800130|ref|NP\_06(10:2)  
gi|106775678|ref|NP\_0(6:6)  
gi|20357599|ref|NP\_61(2:10)  

---

|  |  |  |  |  |  |  |  |  |
| --- | --- | --- | --- | --- | --- | --- | --- | --- |
| U | *gi|10800130|ref|NP\_06* | 12 | 28 | 63.1% | 130 | 14107 | 10.9 | histone cluster 1, H2ad [Homo sapiens] |
| U | *gi|4504249|ref|NP\_003* | 11 | 24 | 63.1% | 130 | 14091 | 10.9 | histone cluster 1, H2am [Homo sapiens] |
| U | *gi|4504243|ref|NP\_003* | 12 | 28 | 63.1% | 130 | 14091 | 10.9 | histone cluster 1, H2al [Homo sapiens] |
| U | *gi|4504241|ref|NP\_003* | 10 | 19 | 63.1% | 130 | 14091 | 10.9 | histone cluster 1, H2ak [Homo sapiens] |
| U | *gi|4504239|ref|NP\_003* | 12 | 28 | 63.1% | 130 | 14091 | 10.9 | histone cluster 1, H2ai [Homo sapiens] |
| U | *gi|29553970|ref|NP\_80* | 10 | 19 | 63.6% | 129 | 14019 | 10.9 | H2A histone family, member J [Homo sapiens] |
| U | *gi|18105045|ref|NP\_54* | 12 | 28 | 64.1% | 128 | 13906 | 10.9 | histone cluster 1, H2ah [Homo sapiens] |
| U | *gi|10800144|ref|NP\_06* | 12 | 28 | 64.1% | 128 | 13936 | 10.9 | histone cluster 1, H2aj [Homo sapiens] |
| U | *gi|10800132|ref|NP\_06* | 12 | 28 | 63.1% | 130 | 14091 | 10.9 | histone cluster 1, H2ag [Homo sapiens] |

| Filename XCorr DeltCN Conf% ObsM+H+ CalcM+H+ SpR ZScore Ion% # Sequence  | | | | | | | | | | | | |
| --- | --- | --- | --- | --- | --- | --- | --- | --- | --- | --- | --- | --- |
|  | Mis12XLAPLSS\_011913\_01.07319.07319.2 | 2.77 | 0.0989 | 96.9% | 1275.2722 | 1275.4531 | 1 | 4.75 | 68.2% | 1 | R.SSRAGLQFPVGR.V | 222 |
|  | Mis12XLAPLSS\_011913\_01.09008.09008.2 | 3.1059 | 0.329 | 99.9% | 944.59216 | 945.1093 | 2 | 5.574 | 81.2% | 4 | R.AGLQFPVGR.V | 2222 |
|  | Mis12XLAPLSS\_011913\_01.18992.18992.3 | 3.6724 | 0.2894 | 99.8% | 2918.4243 | 2917.3752 | 1 | 5.976 | 29.5% | 1 | R.VGAGAPVYLAAVLEYLTAEILELAGNAAR.D | 33 |
|  | Mis12XLAPLSS\_011913\_01.18956.18956.2 | 4.2915 | 0.2816 | 99.9% | 2919.7922 | 2917.3752 | 1 | 5.25 | 41.1% | 2 | R.VGAGAPVYLAAVLEYLTAEILELAGNAAR.D | 22 |
|  | Mis12XLAPLSS\_011913\_01.18684.18684.3 | 3.7222 | 0.2081 | 97.5% | 3274.8245 | 3274.7417 | 28 | 4.71 | 20.2% | 1 | R.VGAGAPVYLAAVLEYLTAEILELAGNAARDNK.K | 33 |
|  | Mis12XLAPLSS\_011913\_01.18458.18458.3 | 4.6811 | 0.3898 | 100.0% | 3402.3843 | 3402.9158 | 1 | 6.183 | 25.0% | 1 | R.VGAGAPVYLAAVLEYLTAEILELAGNAARDNKK.T | 33 |
|  | Mis12XLAPLSS\_011913\_01.05156.05156.2 | 2.4241 | 0.2579 | 99.8% | 851.09216 | 851.0396 | 3 | 5.349 | 83.3% | 5 | R.HLQLAIR.N | 2222 |
|  | Mis12XLAPLSS\_011913\_02.05366.05366.3 | 3.1821 | 0.3259 | 99.7% | 1694.9343 | 1693.9004 | 1 | 5.774 | 44.2% | 3 | R.HLQLAIRNDEELNK.L | 333 |
|  | Mis12XLAPLSS\_011913\_01.09788.09788.3 | 4.491 | 0.3984 | 100.0% | 2104.7644 | 2105.4453 | 1 | 6.511 | 39.7% | 3 | R.HLQLAIRNDEELNKLLGK.V | 33 |
|  | Mis12XLAPLSS\_011913\_01.08937.08937.2 | 3.4431 | 0.3727 | 100.0% | 1272.7322 | 1273.4288 | 19 | 6.452 | 60.0% | 2 | R.NDEELNKLLGK.V | 22 |
|  | Mis12XLAPLSS\_011913\_01.13262.13262.2 | 5.2273 | 0.5136 | 100.0% | 1931.7122 | 1932.3573 | 1 | 8.573 | 63.9% | 3 | K.VTIAQGGVLPNIQAVLLPK.K | 222 |
|  | Mis12XLAPLSS\_011913\_01.13299.13299.3 | 4.5036 | 0.3796 | 100.0% | 1931.9343 | 1932.3573 | 1 | 7.342 | 45.8% | 2 | K.VTIAQGGVLPNIQAVLLPK.K | 333 |

Similarities:
gi|10645195|ref|NP\_06(10:2)  
gi|106775678|ref|NP\_0(8:4)  
gi|20357599|ref|NP\_61(2:10)  

---

|  |  |  |  |  |  |  |  |  |
| --- | --- | --- | --- | --- | --- | --- | --- | --- |
| U | *gi|22748615|ref|NP\_68* | 5 | 9 | 62.9% | 116 | 11748 | 9.3 | hypothetical protein LOC90488 [Homo sapiens] |

| Filename XCorr DeltCN Conf% ObsM+H+ CalcM+H+ SpR ZScore Ion% # Sequence  | | | | | | | | | | | | |
| --- | --- | --- | --- | --- | --- | --- | --- | --- | --- | --- | --- | --- |
| \* | Mis12XLAPLSS\_011913\_01.04065.04065.2 | 2.2349 | 0.3843 | 99.6% | 1393.8922 | 1394.548 | 7 | 5.822 | 54.5% | 1 | K.DHPQQQPGMLSR.V | 2 |
| \* | Mis12XLAPLSS\_011913\_01.08622.08622.2 | 2.8387 | 0.3446 | 99.8% | 1009.5522 | 1009.1906 | 1 | 6.755 | 77.8% | 2 | R.VTGGIFSVTK.G | 2 |
| \* | Mis12XLAPLSS\_011913\_01.10833.10833.2 | 3.7353 | 0.3329 | 99.9% | 1414.1921 | 1414.6475 | 1 | 6.349 | 73.3% | 3 | K.GAVGATIGGVAWIGGK.S | 2 |
| \* | Mis12XLAPLSS\_011913\_01.10340.10340.2 | 3.02 | 0.429 | 99.9% | 1475.7122 | 1474.7992 | 1 | 6.958 | 57.1% | 2 | K.TAVTTVPSMGIGLVK.G | 2 |
| \* | Mis12XLAPLSS\_011913\_01.09615.09615.2 | 2.4181 | 0.2684 | 98.4% | 1701.2922 | 1700.9327 | 275 | 5.034 | 28.9% | 1 | K.GGVSAVAGGVTAVGSAVVNK.V | 2 |

---

|  |  |  |  |  |  |  |  |  |
| --- | --- | --- | --- | --- | --- | --- | --- | --- |
| U | *gi|11415030|ref|NP\_06* | 13 | 26 | 61.2% | 103 | 11367 | 11.4 | histone cluster 1, H4j [Homo sapiens] |
| U | *gi|77539758|ref|NP\_00* | 13 | 26 | 61.2% | 103 | 11367 | 11.4 | histone cluster 2, H4b [Homo sapiens] |
| U | *gi|4504323|ref|NP\_003* | 13 | 26 | 61.2% | 103 | 11367 | 11.4 | histone cluster 2, H4a [Homo sapiens] |
| U | *gi|4504321|ref|NP\_003* | 13 | 26 | 61.2% | 103 | 11367 | 11.4 | histone cluster 1, H4i [Homo sapiens] |
| U | *gi|4504317|ref|NP\_003* | 13 | 26 | 61.2% | 103 | 11367 | 11.4 | histone cluster 1, H4l [Homo sapiens] |
| U | *gi|4504315|ref|NP\_003* | 13 | 26 | 61.2% | 103 | 11367 | 11.4 | histone cluster 1, H4e [Homo sapiens] |
| U | *gi|4504313|ref|NP\_003* | 13 | 26 | 61.2% | 103 | 11367 | 11.4 | histone cluster 1, H4b [Homo sapiens] |
| U | *gi|4504311|ref|NP\_003* | 13 | 26 | 61.2% | 103 | 11367 | 11.4 | histone cluster 1, H4h [Homo sapiens] |
| U | *gi|4504309|ref|NP\_003* | 13 | 26 | 61.2% | 103 | 11367 | 11.4 | histone cluster 1, H4c [Homo sapiens] |
| U | *gi|4504307|ref|NP\_003* | 13 | 26 | 61.2% | 103 | 11367 | 11.4 | histone cluster 1, H4k [Homo sapiens] |
| U | *gi|4504305|ref|NP\_003* | 13 | 26 | 61.2% | 103 | 11367 | 11.4 | histone cluster 1, H4f [Homo sapiens] |
| U | *gi|4504303|ref|NP\_003* | 13 | 26 | 61.2% | 103 | 11367 | 11.4 | histone cluster 1, H4d [Homo sapiens] |
| U | *gi|4504301|ref|NP\_003* | 13 | 26 | 61.2% | 103 | 11367 | 11.4 | histone cluster 1, H4a [Homo sapiens] |
| U | *gi|28173560|ref|NP\_77* | 13 | 26 | 61.2% | 103 | 11367 | 11.4 | histone cluster 4, H4 [Homo sapiens] |

| Filename XCorr DeltCN Conf% ObsM+H+ CalcM+H+ SpR ZScore Ion% # Sequence  | | | | | | | | | | | | |
| --- | --- | --- | --- | --- | --- | --- | --- | --- | --- | --- | --- | --- |
|  | Mis12XLAPLSS\_011913\_01.05558.05558.2 | 3.3382 | 0.2124 | 99.8% | 1325.5122 | 1326.5387 | 1 | 6.482 | 81.8% | 5 | R.DNIQGITKPAIR.R | 2 |
|  | Mis12XLAPLSS\_011913\_01.07469.07469.2 | 2.8373 | 0.2244 | 99.6% | 1337.1921 | 1337.5187 | 4 | 4.932 | 70.0% | 1 | K.RISGLIYEETR.G | 2 |
|  | Mis12XLAPLSS\_011913\_01.08469.08469.1 | 2.3552 | 0.176 | 95.5% | 1180.53 | 1181.3312 | 69 | 4.443 | 55.6% | 1 | R.ISGLIYEETR.G | 1 |
|  | Mis12XLAPLSS\_011913\_02.06176.06176.2 | 3.5748 | 0.3938 | 100.0% | 1181.1122 | 1181.3312 | 1 | 7.555 | 88.9% | 4 | R.ISGLIYEETR.G | 2 |
|  | Mis12XLAPLSS\_011913\_02.06340.06340.2 | 2.9017 | 0.2998 | 99.8% | 989.9922 | 990.19055 | 3 | 5.358 | 85.7% | 4 | K.VFLENVIR.D | 2 |
|  | Mis12XLAPLSS\_011913\_01.13269.13269.3 | 4.5208 | 0.3517 | 100.0% | 2106.3245 | 2106.386 | 1 | 6.419 | 44.1% | 1 | K.VFLENVIRDAVTYTEHAK.R | 3 |
|  | Mis12XLAPLSS\_011913\_01.03681.03681.1 | 2.0825 | 0.4157 | 100.0% | 1134.33 | 1135.2188 | 2 | 7.126 | 66.7% | 1 | R.DAVTYTEHAK.R | 1 |
|  | Mis12XLAPLSS\_011913\_01.03657.03657.2 | 2.8743 | 0.3146 | 99.8% | 1134.3922 | 1135.2188 | 5 | 6.904 | 66.7% | 2 | R.DAVTYTEHAK.R | 2 |
|  | Mis12XLAPLSS\_011913\_01.03503.03503.2 | 2.677 | 0.2525 | 99.5% | 1291.8922 | 1291.4062 | 1 | 5.099 | 65.0% | 1 | R.DAVTYTEHAKR.K | 2 |
|  | Mis12XLAPLSS\_011913\_01.09820.09820.3 | 3.6733 | 0.3971 | 100.0% | 1595.9343 | 1595.9409 | 3 | 6.091 | 42.3% | 1 | R.KTVTAMDVVYALKR.Q | 3 |
|  | Mis12XLAPLSS\_011913\_01.12239.12239.2 | 2.9605 | 0.4207 | 99.9% | 1312.3121 | 1311.5793 | 1 | 6.736 | 77.3% | 1 | K.TVTAMDVVYALK.R | 2 |
|  | Mis12XLAPLSS\_011913\_01.11158.11158.2 | 3.2258 | 0.5107 | 100.0% | 1466.6921 | 1467.7667 | 1 | 7.959 | 70.8% | 3 | K.TVTAMDVVYALKR.Q | 2 |
|  | Mis12XLAPLSS\_011913\_01.10016.10016.1 | 1.816 | 0.4793 | 100.0% | 714.33 | 714.796 | 1 | 8.573 | 75.0% | 1 | R.TLYGFGG.- | 1 |

---

|  |  |  |  |  |  |  |  |  |
| --- | --- | --- | --- | --- | --- | --- | --- | --- |
| U | *gi|106775678|ref|NP\_0* | 10 | 26 | 60.0% | 130 | 14095 | 10.9 | histone cluster 2, H2aa4 [Homo sapiens] |
| U | *gi|4504251|ref|NP\_003* | 10 | 26 | 60.0% | 130 | 14095 | 10.9 | histone cluster 2, H2aa3 [Homo sapiens] |
| U | *gi|24638446|ref|NP\_00* | 10 | 26 | 60.5% | 129 | 13988 | 10.9 | histone cluster 2, H2ac [Homo sapiens] |

| Filename XCorr DeltCN Conf% ObsM+H+ CalcM+H+ SpR ZScore Ion% # Sequence  | | | | | | | | | | | | |
| --- | --- | --- | --- | --- | --- | --- | --- | --- | --- | --- | --- | --- |
|  | Mis12XLAPLSS\_011913\_01.07319.07319.2 | 2.77 | 0.0989 | 96.9% | 1275.2722 | 1275.4531 | 1 | 4.75 | 68.2% | 1 | R.SSRAGLQFPVGR.V | 222 |
|  | Mis12XLAPLSS\_011913\_01.09008.09008.2 | 3.1059 | 0.329 | 99.9% | 944.59216 | 945.1093 | 2 | 5.574 | 81.2% | 4 | R.AGLQFPVGR.V | 2222 |
|  | Mis12XLAPLSS\_011913\_01.18866.18866.3 | 4.5155 | 0.4163 | 100.0% | 2934.7144 | 2935.4082 | 1 | 7.57 | 32.1% | 1 | R.VGAGAPVYMAAVLEYLTAEILELAGNAAR.D | 3 |
|  | Mis12XLAPLSS\_011913\_01.18814.18814.2 | 4.783 | 0.2942 | 100.0% | 2937.912 | 2935.4082 | 1 | 5.343 | 39.3% | 2 | R.VGAGAPVYMAAVLEYLTAEILELAGNAAR.D | 2 |
|  | Mis12XLAPLSS\_011913\_01.05156.05156.2 | 2.4241 | 0.2579 | 99.8% | 851.09216 | 851.0396 | 3 | 5.349 | 83.3% | 5 | R.HLQLAIR.N | 2222 |
|  | Mis12XLAPLSS\_011913\_02.05366.05366.3 | 3.1821 | 0.3259 | 99.7% | 1694.9343 | 1693.9004 | 1 | 5.774 | 44.2% | 3 | R.HLQLAIRNDEELNK.L | 333 |
|  | Mis12XLAPLSS\_011913\_01.09788.09788.3 | 4.491 | 0.3984 | 100.0% | 2104.7644 | 2105.4453 | 1 | 6.511 | 39.7% | 3 | R.HLQLAIRNDEELNKLLGK.V | 33 |
|  | Mis12XLAPLSS\_011913\_01.08937.08937.2 | 3.4431 | 0.3727 | 100.0% | 1272.7322 | 1273.4288 | 19 | 6.452 | 60.0% | 2 | R.NDEELNKLLGK.V | 22 |
|  | Mis12XLAPLSS\_011913\_01.13262.13262.2 | 5.2273 | 0.5136 | 100.0% | 1931.7122 | 1932.3573 | 1 | 8.573 | 63.9% | 3 | K.VTIAQGGVLPNIQAVLLPK.K | 222 |
|  | Mis12XLAPLSS\_011913\_01.13299.13299.3 | 4.5036 | 0.3796 | 100.0% | 1931.9343 | 1932.3573 | 1 | 7.342 | 45.8% | 2 | K.VTIAQGGVLPNIQAVLLPK.K | 333 |

Similarities:
gi|10645195|ref|NP\_06(6:4)  
gi|10800130|ref|NP\_06(8:2)  
gi|20357599|ref|NP\_61(2:8)  

---

|  |  |  |  |  |  |  |  |  |
| --- | --- | --- | --- | --- | --- | --- | --- | --- |
| U | *gi|88900509|ref|NP\_00* | 9 | 10 | 58.0% | 205 | 23339 | 5.5 | polyamine-modulated factor 1 [Homo sapiens] |

| Filename XCorr DeltCN Conf% ObsM+H+ CalcM+H+ SpR ZScore Ion% # Sequence  | | | | | | | | | | | | |
| --- | --- | --- | --- | --- | --- | --- | --- | --- | --- | --- | --- | --- |
| \* | Mis12XLAPLSS\_011913\_01.03663.03663.2 | 5.2805 | 0.499 | 100.0% | 1884.2322 | 1885.0006 | 1 | 8.58 | 64.7% | 1 | K.RHEGSSSESVPPGTTISR.V | 2 |
| \* | Mis12XLAPLSS\_011913\_01.13040.13040.2 | 3.4314 | 0.4185 | 100.0% | 1651.3522 | 1652.0021 | 1 | 7.386 | 69.2% | 1 | R.VKLLDTMVDTFLQK.L | 2 |
| \* | Mis12XLAPLSS\_011913\_01.12923.12923.2 | 4.0288 | 0.4727 | 100.0% | 1424.5521 | 1424.6954 | 1 | 8.469 | 81.8% | 2 | K.LLDTMVDTFLQK.L | 2 |
| \* | Mis12XLAPLSS\_011913\_02.03683.03683.2 | 2.0417 | 0.3025 | 98.7% | 964.9522 | 965.0971 | 28 | 5.456 | 62.5% | 1 | K.LVAAGSYQR.F | 2 |
| \* | Mis12XLAPLSS\_011913\_02.09132.09132.3 | 3.3124 | 0.3303 | 99.7% | 2330.5745 | 2330.551 | 52 | 5.282 | 27.5% | 1 | R.EEISDIKEEGNLEAVLNALDK.I | 3 |
| \* | Mis12XLAPLSS\_011913\_01.14724.14724.3 | 3.6611 | 0.3159 | 99.7% | 2986.4343 | 2986.3 | 4 | 4.908 | 21.2% | 1 | R.EEISDIKEEGNLEAVLNALDKIVEEGK.V | 3 |
| \* | Mis12XLAPLSS\_011913\_01.08069.08069.3 | 5.5552 | 0.4992 | 100.0% | 2205.2644 | 2206.4233 | 1 | 8.528 | 39.5% | 1 | R.HVQKQEAENQQLADAVLAGR.R | 3 |
| \* | Mis12XLAPLSS\_011913\_01.11290.11290.3 | 3.0704 | 0.2577 | 97.6% | 2531.6943 | 2532.8215 | 12 | 4.906 | 30.0% | 1 | R.QVEELQLQVQAQQQAWQALHR.E | 3 |
| \* | Mis12XLAPLSS\_011913\_01.09219.09219.2 | 3.1326 | 0.2278 | 99.8% | 1155.1322 | 1155.3367 | 1 | 6.481 | 77.8% | 1 | R.ELVAVLREPE.- | 2 |

---

|  |  |  |  |  |  |  |  |  |
| --- | --- | --- | --- | --- | --- | --- | --- | --- |
| U | *gi|7669492|ref|NP\_002* | 17 | 30 | 57.0% | 335 | 36053 | 8.5 | glyceraldehyde-3-phosphate dehydrogenase [Homo sapiens] |

| Filename XCorr DeltCN Conf% ObsM+H+ CalcM+H+ SpR ZScore Ion% # Sequence  | | | | | | | | | | | | |
| --- | --- | --- | --- | --- | --- | --- | --- | --- | --- | --- | --- | --- |
| \* | Mis12XLAPLSS\_011913\_01.05093.05093.2 | 2.0377 | 0.2175 | 97.1% | 805.8522 | 805.912 | 133 | 5.61 | 64.3% | 1 | K.VGVNGFGR.I | 2 |
| \* | Mis12XLAPLSS\_011913\_01.16718.16718.3 | 3.9524 | 0.3971 | 100.0% | 3309.1743 | 3310.7634 | 1 | 6.166 | 28.7% | 1 | K.VDIVAINDPFIDLNYMVYMFQYDSTHGK.F | 3 |
| \* | Mis12XLAPLSS\_011913\_01.12419.12419.2 | 4.0243 | 0.362 | 100.0% | 1614.5922 | 1614.8851 | 1 | 8.174 | 73.1% | 1 | K.LVINGNPITIFQER.D | 2 |
| \* | Mis12XLAPLSS\_011913\_01.11528.11528.2 | 3.7222 | 0.3673 | 99.9% | 2041.4922 | 2042.3427 | 1 | 7.166 | 47.1% | 1 | K.LVINGNPITIFQERDPSK.I | 2 |
| \* | Mis12XLAPLSS\_011913\_01.12881.12881.2 | 5.4516 | 0.6249 | 100.0% | 2277.1921 | 2278.495 | 1 | 10.539 | 57.5% | 2 | K.WGDAGAEYVVESTGVFTTMEK.A | 2 |
| \* | Mis12XLAPLSS\_011913\_01.03125.03125.2 | 2.8079 | 0.2807 | 99.8% | 1065.5122 | 1066.2081 | 4 | 5.458 | 70.0% | 2 | K.AGAHLQGGAKR.V | 2 |
| \* | Mis12XLAPLSS\_011913\_01.09885.09885.3 | 5.6561 | 0.4229 | 100.0% | 2370.0244 | 2370.79 | 1 | 7.86 | 47.6% | 1 | K.RVIISAPSADAPMFVMGVNHEK.Y | 3 |
| \* | Mis12XLAPLSS\_011913\_01.11004.11004.2 | 3.2336 | 0.1388 | 99.0% | 2215.7722 | 2214.6025 | 1 | 4.789 | 40.0% | 1 | R.VIISAPSADAPMFVMGVNHEK.Y | 2 |
| \* | Mis12XLAPLSS\_011913\_01.14116.14116.3 | 5.9114 | 0.6118 | 100.0% | 2594.4844 | 2597.0044 | 1 | 11.326 | 43.5% | 4 | K.VIHDNFGIVEGLMTTVHAITATQK.T | 3 |
| \* | Mis12XLAPLSS\_011913\_01.14106.14106.2 | 5.7907 | 0.5934 | 100.0% | 2595.9321 | 2597.0044 | 1 | 10.742 | 52.2% | 1 | K.VIHDNFGIVEGLMTTVHAITATQK.T | 2 |
| \* | Mis12XLAPLSS\_011913\_01.09092.09092.2 | 3.9037 | 0.3901 | 100.0% | 1412.1921 | 1412.6292 | 1 | 6.239 | 82.1% | 3 | R.GALQNIIPASTGAAK.A | 2 |
|  | Mis12XLAPLSS\_011913\_01.07094.07094.2 | 1.8446 | 0.3527 | 99.1% | 795.83215 | 795.97504 | 3 | 5.538 | 75.0% | 1 | K.LTGMAFR.V | 2 |
| \* | Mis12XLAPLSS\_011913\_01.03534.03534.2 | 3.4814 | 0.2625 | 99.8% | 1448.1522 | 1448.7025 | 5 | 5.733 | 68.2% | 1 | R.LEKPAKYDDIKK.V | 2 |
| \* | Mis12XLAPLSS\_011913\_01.03530.03530.3 | 3.0752 | 0.1818 | 95.5% | 1448.8143 | 1448.7025 | 1 | 4.452 | 50.0% | 1 | R.LEKPAKYDDIKK.V | 3 |
|  | Mis12XLAPLSS\_011913\_02.08042.08042.2 | 4.5219 | 0.5116 | 100.0% | 1765.1721 | 1764.8914 | 1 | 9.179 | 65.4% | 5 | K.LISWYDNEFGYSNR.V | 2 |
| \* | Mis12XLAPLSS\_011913\_01.07851.07851.2 | 3.5219 | 0.4854 | 100.0% | 1201.8322 | 1202.4724 | 1 | 7.79 | 85.0% | 1 | R.VVDLMAHMASK.E | 2 |
| \* | Mis12XLAPLSS\_011913\_01.08216.08216.2 | 3.7133 | 0.4853 | 100.0% | 1331.0521 | 1331.5879 | 1 | 8.557 | 72.7% | 3 | R.VVDLMAHMASKE.- | 2 |

---

|  |  |  |  |  |  |  |  |  |
| --- | --- | --- | --- | --- | --- | --- | --- | --- |
| U | *gi|4503571|ref|NP\_001* | 19 | 34 | 55.5% | 434 | 47169 | 7.4 | enolase 1 [Homo sapiens] |

| Filename XCorr DeltCN Conf% ObsM+H+ CalcM+H+ SpR ZScore Ion% # Sequence  | | | | | | | | | | | | |
| --- | --- | --- | --- | --- | --- | --- | --- | --- | --- | --- | --- | --- |
| \* | Mis12XLAPLSS\_011913\_01.10234.10234.2 | 3.7752 | 0.3038 | 99.9% | 1408.1122 | 1407.5634 | 1 | 7.634 | 66.7% | 3 | R.GNPTVEVDLFTSK.G | 2 |
|  | Mis12XLAPLSS\_011913\_01.11900.11900.2 | 4.6149 | 0.4171 | 100.0% | 1805.3121 | 1806.0258 | 1 | 8.349 | 55.9% | 5 | R.AAVPSGASTGIYEALELR.D | 2 |
| \* | Mis12XLAPLSS\_011913\_01.05400.05400.1 | 1.4725 | 0.2674 | 95.2% | 899.58 | 900.1063 | 1 | 5.218 | 62.5% | 1 | K.TIAPALVSK.K | 1 |
| \* | Mis12XLAPLSS\_011913\_01.03788.03788.3 | 3.5187 | 0.2371 | 99.8% | 1445.5743 | 1445.6561 | 13 | 5.815 | 43.2% | 1 | K.KLNVTEQEKIDK.L | 3 |
| \* | Mis12XLAPLSS\_011913\_01.03804.03804.2 | 4.1269 | 0.296 | 100.0% | 1445.9922 | 1445.6561 | 2 | 5.923 | 72.7% | 1 | K.KLNVTEQEKIDK.L | 2 |
| \* | Mis12XLAPLSS\_011913\_01.04233.04233.2 | 3.0973 | 0.3324 | 99.8% | 1317.4122 | 1317.482 | 5 | 5.941 | 70.0% | 1 | K.LNVTEQEKIDK.L | 2 |
| \* | Mis12XLAPLSS\_011913\_01.08333.08333.2 | 3.5663 | 0.4099 | 100.0% | 1281.1122 | 1281.4817 | 1 | 7.54 | 80.0% | 2 | K.LMIEMDGTENK.S | 2 |
| \* | Mis12XLAPLSS\_011913\_01.12070.12070.3 | 5.671 | 0.4374 | 100.0% | 3013.0444 | 3013.383 | 1 | 6.39 | 34.5% | 1 | R.HIADLAGNSEVILPVPAFNVINGGSHAGNK.L | 3 |
| \* | Mis12XLAPLSS\_011913\_01.13911.13911.2 | 4.9276 | 0.4094 | 100.0% | 1909.0521 | 1909.3148 | 1 | 7.862 | 65.6% | 2 | K.LAMQEFMILPVGAANFR.E | 2 |
| \* | Mis12XLAPLSS\_011913\_01.04760.04760.2 | 3.3573 | 0.2734 | 99.9% | 1144.1322 | 1144.3158 | 1 | 5.729 | 77.8% | 2 | R.IGAEVYHNLK.N | 2 |
| \* | Mis12XLAPLSS\_011913\_01.10218.10218.2 | 3.746 | 0.3624 | 99.9% | 1961.2722 | 1962.0801 | 1 | 7.321 | 44.4% | 1 | K.DATNVGDEGGFAPNILENK.E | 2 |
| \* | Mis12XLAPLSS\_011913\_02.09285.09285.2 | 3.7832 | 0.5051 | 100.0% | 1541.5521 | 1541.8053 | 1 | 9.141 | 69.2% | 2 | K.VVIGMDVAASEFFR.S | 2 |
| \* | Mis12XLAPLSS\_011913\_01.06956.06956.3 | 3.1983 | 0.2178 | 97.6% | 1828.1344 | 1827.9451 | 1 | 4.862 | 46.7% | 3 | R.SGKYDLDFKSPDDPSR.Y | 3 |
| \* | Mis12XLAPLSS\_011913\_01.11180.11180.2 | 2.9377 | 0.4404 | 99.9% | 1425.4722 | 1426.6091 | 1 | 7.112 | 77.3% | 3 | R.YISPDQLADLYK.S | 2 |
| \* | Mis12XLAPLSS\_011913\_01.13272.13272.3 | 4.8431 | 0.4094 | 100.0% | 2986.2844 | 2987.2512 | 1 | 7.462 | 35.4% | 1 | K.SFIKDYPVVSIEDPFDQDDWGAWQK.F | 3 |
| \* | Mis12XLAPLSS\_011913\_02.07497.07497.2 | 5.806 | 0.5858 | 100.0% | 2033.3922 | 2034.2737 | 1 | 11.293 | 55.3% | 1 | K.FTASAGIQVVGDDLTVTNPK.R | 2 |
| \* | Mis12XLAPLSS\_011913\_01.09897.09897.3 | 3.1798 | 0.2911 | 99.4% | 2189.9343 | 2190.4612 | 1 | 5.262 | 33.8% | 1 | K.FTASAGIQVVGDDLTVTNPKR.I | 3 |
| \* | Mis12XLAPLSS\_011913\_02.06221.06221.2 | 2.431 | 0.2128 | 97.4% | 1526.2922 | 1526.7563 | 1 | 4.056 | 61.5% | 1 | K.LAQANGWGVMVSHR.S | 2 |
| \* | Mis12XLAPLSS\_011913\_02.06213.06213.3 | 3.2025 | 0.3342 | 99.7% | 1526.3644 | 1526.7563 | 1 | 5.97 | 55.8% | 2 | K.LAQANGWGVMVSHR.S | 3 |

---

|  |  |  |  |  |  |  |  |  |
| --- | --- | --- | --- | --- | --- | --- | --- | --- |
| U | *gi|29788785|ref|NP\_82* | 25 | 64 | 53.8% | 444 | 49671 | 4.9 | tubulin, beta [Homo sapiens] |

| Filename XCorr DeltCN Conf% ObsM+H+ CalcM+H+ SpR ZScore Ion% # Sequence  | | | | | | | | | | | | |
| --- | --- | --- | --- | --- | --- | --- | --- | --- | --- | --- | --- | --- |
| \* | Mis12XLAPLSS\_011913\_01.11076.11076.3 | 5.3357 | 0.3797 | 100.0% | 3103.7644 | 3104.2725 | 1 | 8.394 | 29.8% | 2 | K.FWEVISDEHGIDPTGTYHGDSDLQLDR.I | 3 |
| \* | Mis12XLAPLSS\_011913\_01.06542.06542.2 | 3.9881 | 0.5231 | 100.0% | 1303.1522 | 1302.4265 | 1 | 9.116 | 86.4% | 9 | R.ISVYYNEATGGK.Y | 2 |
| \* | Mis12XLAPLSS\_011913\_02.05864.05864.3 | 2.7125 | 0.3494 | 99.8% | 1817.6643 | 1818.0392 | 173 | 4.98 | 28.3% | 1 | R.ISVYYNEATGGKYVPR.A | 3 |
|  | Mis12XLAPLSS\_011913\_01.11252.11252.2 | 4.408 | 0.5175 | 100.0% | 1616.3722 | 1616.8701 | 1 | 8.763 | 71.4% | 5 | R.AILVDLEPGTMDSVR.S | 22 |
|  | Mis12XLAPLSS\_011913\_01.12800.12800.3 | 6.5294 | 0.5256 | 100.0% | 2799.4443 | 2800.0647 | 1 | 9.134 | 40.0% | 3 | R.SGPFGQIFRPDNFVFGQSGAGNNWAK.G | 33 |
|  | Mis12XLAPLSS\_011913\_01.13076.13076.2 | 6.5905 | 0.5843 | 100.0% | 1959.6522 | 1960.151 | 1 | 11.152 | 79.4% | 2 | K.GHYTEGAELVDSVLDVVR.K | 222 |
|  | Mis12XLAPLSS\_011913\_01.13106.13106.3 | 4.5785 | 0.393 | 100.0% | 1961.5144 | 1960.151 | 1 | 7.056 | 45.6% | 1 | K.GHYTEGAELVDSVLDVVR.K | 333 |
|  | Mis12XLAPLSS\_011913\_01.12470.12470.2 | 5.7772 | 0.4953 | 100.0% | 2087.9521 | 2088.325 | 1 | 9.485 | 66.7% | 1 | K.GHYTEGAELVDSVLDVVRK.E | 222 |
|  | Mis12XLAPLSS\_011913\_01.12422.12422.3 | 4.0642 | 0.4487 | 100.0% | 2088.2644 | 2088.325 | 2 | 7.577 | 38.9% | 2 | K.GHYTEGAELVDSVLDVVRK.E | 333 |
|  | Mis12XLAPLSS\_011913\_01.03590.03590.2 | 2.5923 | 0.2503 | 99.8% | 1077.7522 | 1078.1698 | 20 | 4.976 | 71.4% | 1 | K.IREEYPDR.I | 22 |
|  | Mis12XLAPLSS\_011913\_01.09478.09478.2 | 4.27 | 0.3449 | 100.0% | 1321.9122 | 1320.5896 | 1 | 6.366 | 81.8% | 4 | R.IMNTFSVVPSPK.V | 222 |
|  | Mis12XLAPLSS\_011913\_01.08709.08709.2 | 2.7937 | 0.1227 | 98.4% | 1131.1721 | 1131.2767 | 10 | 4.38 | 72.2% | 3 | R.FPGQLNADLR.K | 222 |
|  | Mis12XLAPLSS\_011913\_01.06874.06874.2 | 2.5666 | 0.1608 | 98.1% | 1259.6322 | 1259.4508 | 74 | 4.363 | 55.0% | 1 | R.FPGQLNADLRK.L | 222 |
|  | Mis12XLAPLSS\_011913\_01.09525.09525.2 | 3.5594 | 0.3946 | 100.0% | 1271.7322 | 1272.5945 | 1 | 7.783 | 75.0% | 2 | R.KLAVNMVPFPR.L | 222 |
|  | Mis12XLAPLSS\_011913\_01.10730.10730.2 | 3.5516 | 0.5021 | 100.0% | 1144.1721 | 1144.4204 | 1 | 8.538 | 94.4% | 3 | K.LAVNMVPFPR.L | 222 |
|  | Mis12XLAPLSS\_011913\_01.12581.12581.3 | 3.8947 | 0.3599 | 100.0% | 1622.0643 | 1621.9403 | 2 | 6.47 | 50.0% | 1 | R.LHFFMPGFAPLTSR.G | 33 |
|  | Mis12XLAPLSS\_011913\_01.12591.12591.2 | 3.5785 | 0.326 | 99.9% | 1623.4722 | 1621.9403 | 1 | 6.785 | 73.1% | 4 | R.LHFFMPGFAPLTSR.G | 22 |
| \* | Mis12XLAPLSS\_011913\_01.12462.12462.2 | 3.4344 | 0.5142 | 100.0% | 1660.1322 | 1660.9078 | 1 | 8.645 | 64.3% | 2 | R.ALTVPELTQQVFDAK.N | 2 |
|  | Mis12XLAPLSS\_011913\_01.11268.11268.1 | 1.6377 | 0.3909 | 100.0% | 1039.55 | 1040.2505 | 4 | 6.253 | 56.2% | 2 | R.YLTVAAVFR.G | 11 |
|  | Mis12XLAPLSS\_011913\_01.11306.11306.2 | 3.0968 | 0.4003 | 100.0% | 1040.2122 | 1040.2505 | 1 | 6.856 | 87.5% | 3 | R.YLTVAAVFR.G | 22 |
|  | Mis12XLAPLSS\_011913\_02.06605.06605.3 | 3.4205 | 0.2917 | 99.7% | 1923.4443 | 1925.2405 | 1 | 5.078 | 40.0% | 1 | R.MSMKEVDEQMLNVQNK.N | 33 |
|  | Mis12XLAPLSS\_011913\_01.07232.07232.2 | 4.2221 | 0.2425 | 99.9% | 1447.1522 | 1447.6031 | 1 | 5.681 | 72.7% | 3 | K.EVDEQMLNVQNK.N | 22 |
|  | Mis12XLAPLSS\_011913\_01.12202.12202.2 | 2.8146 | 0.3212 | 99.8% | 1697.7522 | 1697.8877 | 1 | 6.074 | 57.7% | 2 | K.NSSYFVEWIPNNVK.T | 222 |
| \* | Mis12XLAPLSS\_011913\_01.13703.13703.2 | 3.8528 | 0.3173 | 99.9% | 1871.7122 | 1871.2018 | 1 | 6.953 | 62.5% | 1 | K.MAVTFIGNSTAIQELFK.R | 2 |
|  | Mis12XLAPLSS\_011913\_01.11018.11018.2 | 3.7611 | 0.4743 | 100.0% | 1230.2122 | 1230.4241 | 1 | 7.691 | 94.4% | 5 | R.ISEQFTAMFR.R | 222 |

Similarities:
gi|5174735|ref|NP\_006(19:6)  
gi|50592996|ref|NP\_00(12:13)  

---

|  |  |  |  |  |  |  |  |  |
| --- | --- | --- | --- | --- | --- | --- | --- | --- |
| U | *gi|40354195|ref|NP\_95* | 18 | 40 | 52.3% | 430 | 48058 | 5.5 | keratin 18 [Homo sapiens] |
| U | *gi|4557888|ref|NP\_000* | 18 | 40 | 52.3% | 430 | 48058 | 5.5 | keratin 18 [Homo sapiens] |

| Filename XCorr DeltCN Conf% ObsM+H+ CalcM+H+ SpR ZScore Ion% # Sequence  | | | | | | | | | | | | |
| --- | --- | --- | --- | --- | --- | --- | --- | --- | --- | --- | --- | --- |
|  | Mis12XLAPLSS\_011913\_02.06074.06074.3 | 5.2121 | 0.5544 | 100.0% | 2855.5144 | 2856.0813 | 1 | 9.733 | 28.3% | 5 | R.SLGSVQAPSYGARPVSSAASVYAGAGGSGSR.I | 3 |
|  | Mis12XLAPLSS\_011913\_02.06166.06166.3 | 3.2867 | 0.3045 | 99.8% | 2936.0942 | 2936.0813 | 1 | 5.431 | 24.2% | 1 | R.SLGSVQAPSYGARPVSSAAS\*VYAGAGGSGSR.I | 3 |
|  | Mis12XLAPLSS\_011913\_02.07976.07976.3 | 4.507 | 0.3763 | 100.0% | 2261.9043 | 2262.561 | 1 | 6.25 | 34.0% | 1 | R.GGMGSGGLATGIAGGLAGMGGIQNEK.E | 3 |
|  | Mis12XLAPLSS\_011913\_01.11886.11886.3 | 5.1938 | 0.393 | 100.0% | 3337.1042 | 3337.7224 | 1 | 7.0 | 27.2% | 1 | R.GGMGSGGLATGIAGGLAGMGGIQNEKETMQSLNDR.L | 3 |
|  | Mis12XLAPLSS\_011913\_01.07226.07226.2 | 3.801 | 0.4878 | 100.0% | 1320.1921 | 1320.4478 | 1 | 9.086 | 77.3% | 3 | R.AQIFANTVDNAR.I | 2 |
|  | Mis12XLAPLSS\_011913\_01.07946.07946.2 | 3.3034 | 0.1194 | 99.8% | 1041.6921 | 1042.2235 | 3 | 5.916 | 87.5% | 3 | R.IVLQIDNAR.L | 2 |
|  | Mis12XLAPLSS\_011913\_01.05369.05369.2 | 2.0125 | 0.208 | 97.3% | 807.9122 | 807.8815 | 90 | 5.564 | 66.7% | 1 | R.LAADDFR.V | 222 |
|  | Mis12XLAPLSS\_011913\_01.05724.05724.2 | 2.6223 | 0.3622 | 99.8% | 1240.2122 | 1240.4601 | 43 | 6.296 | 61.1% | 3 | R.VKYETELAMR.Q | 2 |
|  | Mis12XLAPLSS\_011913\_01.04137.04137.2 | 2.6688 | 0.1036 | 97.4% | 1176.1921 | 1175.3274 | 3 | 4.211 | 77.8% | 1 | R.KVIDDTNITR.L | 2 |
|  | Mis12XLAPLSS\_011913\_01.14717.14717.2 | 5.5914 | 0.4942 | 100.0% | 2178.4521 | 2178.589 | 1 | 8.067 | 64.7% | 2 | R.LQLETEIEALKEELLFMK.K | 2 |
|  | Mis12XLAPLSS\_011913\_01.09726.09726.3 | 4.207 | 0.3883 | 100.0% | 2749.8843 | 2751.0227 | 1 | 6.916 | 32.0% | 2 | K.NHEEEVKGLQAQIASSGLTVEVDAPK.S | 3 |
|  | Mis12XLAPLSS\_011913\_01.11558.11558.2 | 3.9174 | 0.4873 | 100.0% | 1508.2122 | 1507.699 | 1 | 8.915 | 75.0% | 3 | R.TVQSLEIDLDSMR.N | 2 |
|  | Mis12XLAPLSS\_011913\_01.04922.04922.2 | 2.6554 | 0.2114 | 99.9% | 889.9322 | 889.9841 | 33 | 5.078 | 78.6% | 1 | K.ASLENSLR.E | 2 |
|  | Mis12XLAPLSS\_011913\_01.15500.15500.2 | 2.9046 | 0.4965 | 99.9% | 2670.9521 | 2672.0715 | 1 | 8.977 | 50.0% | 1 | R.YALQMEQLNGILLHLESELAQTR.A | 2 |
|  | Mis12XLAPLSS\_011913\_01.15458.15458.3 | 5.2524 | 0.5172 | 100.0% | 2672.1843 | 2672.0715 | 1 | 8.202 | 43.2% | 7 | R.YALQMEQLNGILLHLESELAQTR.A | 3 |
|  | Mis12XLAPLSS\_011913\_01.10589.10589.2 | 3.0363 | 0.3548 | 99.8% | 1420.3522 | 1420.6055 | 49 | 5.466 | 54.5% | 2 | R.QAQEYEALLNIK.V | 2 |
|  | Mis12XLAPLSS\_011913\_01.07979.07979.2 | 2.6863 | 0.2832 | 99.8% | 1293.6721 | 1293.5059 | 1 | 5.217 | 75.0% | 2 | K.VKLEAEIATYR.R | 2 |
|  | Mis12XLAPLSS\_011913\_01.06951.06951.2 | 2.7564 | 0.2607 | 99.8% | 1066.1721 | 1066.1992 | 2 | 5.52 | 81.2% | 1 | K.LEAEIATYR.R | 2 |

Similarities:
contaminant\_KERATIN03(1:17)  
gi|4557701|ref|NP\_000(1:17)  

---

|  |  |  |  |  |  |  |  |  |
| --- | --- | --- | --- | --- | --- | --- | --- | --- |
| U | *gi|4506695|ref|NP\_001* | 9 | 11 | 50.3% | 145 | 16060 | 10.3 | ribosomal protein S19 [Homo sapiens] |

| Filename XCorr DeltCN Conf% ObsM+H+ CalcM+H+ SpR ZScore Ion% # Sequence  | | | | | | | | | | | | |
| --- | --- | --- | --- | --- | --- | --- | --- | --- | --- | --- | --- | --- |
| \* | Mis12XLAPLSS\_011913\_02.04220.04220.2 | 2.9854 | 0.2122 | 99.8% | 1135.1122 | 1135.2217 | 2 | 6.059 | 81.2% | 3 | K.DVNQQEFVR.A | 2 |
| \* | Mis12XLAPLSS\_011913\_01.06768.06768.2 | 2.0525 | 0.3244 | 99.4% | 862.0122 | 862.1032 | 13 | 6.277 | 71.4% | 1 | R.ALAAFLKK.S | 2 |
|  | Mis12XLAPLSS\_011913\_01.08972.08972.2 | 2.5571 | 0.3255 | 99.8% | 1073.2522 | 1073.234 | 3 | 5.505 | 68.8% | 1 | K.VPEWVDTVK.L | 2 |
| \* | Mis12XLAPLSS\_011913\_01.09548.09548.2 | 4.2274 | 0.437 | 100.0% | 1969.2122 | 1970.151 | 1 | 8.129 | 71.4% | 1 | K.HKELAPYDENWFYTR.A | 2 |
| \* | Mis12XLAPLSS\_011913\_01.09554.09554.3 | 4.2125 | 0.3691 | 100.0% | 1970.4844 | 1970.151 | 1 | 6.271 | 50.0% | 1 | K.HKELAPYDENWFYTR.A | 3 |
| \* | Mis12XLAPLSS\_011913\_01.12045.12045.2 | 3.0742 | 0.2911 | 99.8% | 1704.1921 | 1704.8358 | 1 | 6.822 | 62.5% | 1 | K.ELAPYDENWFYTR.A | 2 |
| \* | Mis12XLAPLSS\_011913\_01.04499.04499.2 | 2.2881 | 0.3488 | 99.9% | 1131.8922 | 1132.2823 | 19 | 5.802 | 66.7% | 1 | R.NGVMPSHFSR.G | 2 |
| \* | Mis12XLAPLSS\_011913\_01.08462.08462.2 | 2.3635 | 0.2104 | 98.4% | 1126.5721 | 1127.3726 | 1 | 4.816 | 72.2% | 1 | R.RVLQALEGLK.M | 2 |
| \* | Mis12XLAPLSS\_011913\_01.03158.03158.2 | 3.2856 | 0.4347 | 100.0% | 1208.2722 | 1208.4061 | 1 | 6.936 | 68.2% | 1 | R.IAGQVAAANKKH.- | 2 |

---

|  |  |  |  |  |  |  |  |  |
| --- | --- | --- | --- | --- | --- | --- | --- | --- |
| U | *contaminant\_KERATIN20* | 28 | 55 | 49.7% | 483 | 53748 | 5.4 | no description |
| U | *gi|4504919|ref|NP\_002* | 28 | 55 | 49.7% | 483 | 53704 | 5.6 | keratin 8 [Homo sapiens] |

| Filename XCorr DeltCN Conf% ObsM+H+ CalcM+H+ SpR ZScore Ion% # Sequence  | | | | | | | | | | | | |
| --- | --- | --- | --- | --- | --- | --- | --- | --- | --- | --- | --- | --- |
|  | Mis12XLAPLSS\_011913\_01.03556.03556.2 | 2.1995 | 0.3279 | 99.5% | 1081.7522 | 1082.2015 | 2 | 5.134 | 61.1% | 1 | K.SYKVSTSGPR.A | 2 |
|  | Mis12XLAPLSS\_011913\_01.09146.09146.2 | 2.6257 | 0.1264 | 98.9% | 1030.4122 | 1031.1997 | 3 | 4.272 | 92.9% | 2 | K.WSLLQQQK.T | 2 |
|  | Mis12XLAPLSS\_011913\_01.13205.13205.2 | 2.9129 | 0.2447 | 99.5% | 1849.9521 | 1849.0431 | 4 | 4.667 | 42.9% | 1 | R.SNMDNMFESYINNLR.R | 2 |
|  | Mis12XLAPLSS\_011913\_01.12810.12810.3 | 4.9364 | 0.2012 | 99.7% | 2036.0044 | 2035.363 | 1 | 7.745 | 47.1% | 2 | K.LKLEAELGNMQGLVEDFK.N | 3 |
|  | Mis12XLAPLSS\_011913\_01.03807.03807.2 | 2.6964 | 0.2533 | 99.9% | 1309.1721 | 1309.4215 | 1 | 5.17 | 72.2% | 1 | K.NKYEDEINKR.T | 222 |
|  | Mis12XLAPLSS\_011913\_01.10845.10845.2 | 2.4391 | 0.1571 | 96.9% | 1354.5322 | 1353.5732 | 1 | 4.653 | 65.0% | 1 | R.TEMENEFVLIK.K | 2 |
|  | Mis12XLAPLSS\_011913\_01.08674.08674.2 | 3.8263 | 0.5048 | 100.0% | 1797.9922 | 1798.9623 | 1 | 7.951 | 67.9% | 2 | K.DVDEAYMNKVELESR.L | 2 |
|  | Mis12XLAPLSS\_011913\_02.06122.06122.3 | 4.1135 | 0.3571 | 100.0% | 1798.7344 | 1798.9623 | 1 | 6.772 | 50.0% | 2 | K.DVDEAYMNKVELESR.L | 3 |
|  | Mis12XLAPLSS\_011913\_01.12435.12435.2 | 3.7753 | 0.4597 | 100.0% | 1421.1721 | 1420.6055 | 1 | 7.863 | 86.4% | 3 | R.LEGLTDEINFLR.Q | 2 |
|  | Mis12XLAPLSS\_011913\_01.12298.12298.2 | 3.8624 | 0.4607 | 100.0% | 1321.3522 | 1321.5286 | 1 | 8.56 | 72.7% | 2 | R.SLDMDSIIAEVK.A | 2 |
|  | Mis12XLAPLSS\_011913\_01.04542.04542.2 | 2.7527 | 0.27 | 99.8% | 1080.1721 | 1080.1423 | 19 | 5.987 | 68.8% | 3 | K.AQYEDIANR.S | 2 |
|  | Mis12XLAPLSS\_011913\_01.04812.04812.2 | 3.0193 | 0.283 | 99.8% | 1413.0322 | 1413.5884 | 1 | 5.352 | 72.7% | 1 | R.SRAEAESMYQIK.Y | 2 |
|  | Mis12XLAPLSS\_011913\_02.07043.07043.3 | 4.8425 | 0.3286 | 100.0% | 2531.7844 | 2532.828 | 1 | 6.915 | 34.5% | 2 | R.SRAEAESMYQIKYEELQSLAGK.H | 3 |
|  | Mis12XLAPLSS\_011913\_01.06053.06053.2 | 2.7127 | 0.3363 | 99.8% | 1170.0521 | 1170.3228 | 1 | 6.486 | 77.8% | 2 | R.AEAESMYQIK.Y | 2 |
|  | Mis12XLAPLSS\_011913\_01.07617.07617.2 | 3.4963 | 0.0908 | 99.7% | 1138.6322 | 1138.2627 | 2 | 7.434 | 77.8% | 3 | K.YEELQSLAGK.H | 2 |
|  | Mis12XLAPLSS\_011913\_01.03723.03723.2 | 2.9126 | 0.3584 | 99.9% | 1208.2722 | 1209.36 | 1 | 6.694 | 77.8% | 2 | R.TKTEISEMNR.N | 2 |
|  | Mis12XLAPLSS\_011913\_01.07568.07568.2 | 2.5497 | 0.214 | 99.5% | 1000.39215 | 1001.168 | 37 | 5.325 | 75.0% | 2 | R.LQAEIEGLK.G | 2 |
|  | Mis12XLAPLSS\_011913\_01.05246.05246.2 | 3.1322 | 0.3009 | 99.8% | 1342.5322 | 1342.5381 | 1 | 6.183 | 77.3% | 2 | R.LQAEIEGLKGQR.A | 2 |
|  | Mis12XLAPLSS\_011913\_01.09839.09839.2 | 3.773 | 0.3343 | 99.8% | 1346.1122 | 1345.452 | 1 | 5.89 | 66.7% | 2 | R.ASLEAAIADAEQR.G | 2 |
|  | Mis12XLAPLSS\_011913\_01.12150.12150.2 | 4.6284 | 0.4333 | 100.0% | 1956.4722 | 1957.1912 | 2 | 8.539 | 55.6% | 1 | R.ASLEAAIADAEQRGELAIK.D | 2 |
|  | Mis12XLAPLSS\_011913\_01.12335.12335.3 | 5.1326 | 0.4142 | 100.0% | 2457.2344 | 2456.7153 | 3 | 7.354 | 32.6% | 1 | R.ASLEAAIADAEQRGELAIKDANAK.L | 3 |
|  | Mis12XLAPLSS\_011913\_01.03872.03872.2 | 2.8171 | 0.2453 | 99.9% | 1129.9922 | 1130.2865 | 1 | 5.026 | 75.0% | 1 | R.GELAIKDANAK.L | 2 |
|  | Mis12XLAPLSS\_011913\_01.09195.09195.2 | 4.2538 | 0.1756 | 99.9% | 1131.0521 | 1130.2865 | 1 | 5.62 | 77.8% | 2 | K.LSELEAALQR.A | 2 |
|  | Mis12XLAPLSS\_011913\_01.07844.07844.2 | 2.3187 | 0.2251 | 98.7% | 1153.9922 | 1154.3234 | 166 | 5.891 | 50.0% | 2 | R.EYQELMNVK.L | 22 |
|  | Mis12XLAPLSS\_011913\_01.08964.08964.3 | 3.6457 | 0.2164 | 97.8% | 2520.4143 | 2518.8628 | 1 | 4.339 | 31.0% | 1 | R.KLLEGEESRLESGMQNMSIHTK.T | 3 |
|  | Mis12XLAPLSS\_011913\_01.05050.05050.3 | 2.5277 | 0.2463 | 95.3% | 1476.3544 | 1476.7058 | 98 | 4.653 | 35.4% | 1 | R.LESGMQNMSIHTK.T | 3 |
|  | Mis12XLAPLSS\_011913\_01.05069.05069.2 | 4.0405 | 0.4073 | 100.0% | 1476.4122 | 1476.7058 | 1 | 8.387 | 79.2% | 1 | R.LESGMQNMSIHTK.T | 2 |
|  | Mis12XLAPLSS\_011913\_01.06326.06326.2 | 3.319 | 0.4558 | 100.0% | 1174.2122 | 1174.3367 | 1 | 7.721 | 75.0% | 9 | K.LVSESSDVLPK.- | 2 |

Similarities:
gi|119395750|ref|NP\_0(1:27)  
gi|119703753|ref|NP\_0(2:26)  

---

|  |  |  |  |  |  |  |  |  |
| --- | --- | --- | --- | --- | --- | --- | --- | --- |
| U | *gi|5174735|ref|NP\_006* | 23 | 57 | 49.0% | 445 | 49831 | 4.9 | tubulin, beta, 2 [Homo sapiens] |

| Filename XCorr DeltCN Conf% ObsM+H+ CalcM+H+ SpR ZScore Ion% # Sequence  | | | | | | | | | | | | |
| --- | --- | --- | --- | --- | --- | --- | --- | --- | --- | --- | --- | --- |
|  | Mis12XLAPLSS\_011913\_02.07672.07672.3 | 4.7258 | 0.2814 | 99.7% | 3119.2144 | 3118.2996 | 1 | 4.385 | 29.8% | 1 | K.FWEVISDEHGIDPTGTYHGDSDLQLER.I | 3 |
| \* | Mis12XLAPLSS\_011913\_01.06752.06752.2 | 3.6773 | 0.2539 | 99.8% | 1328.9321 | 1329.4521 | 1 | 6.924 | 81.8% | 6 | R.INVYYNEATGGK.Y | 2 |
|  | Mis12XLAPLSS\_011913\_01.10575.10575.2 | 4.3594 | 0.4623 | 100.0% | 1602.3722 | 1602.8431 | 1 | 9.058 | 75.0% | 4 | R.AVLVDLEPGTMDSVR.S | 2 |
|  | Mis12XLAPLSS\_011913\_01.12800.12800.3 | 6.5294 | 0.5256 | 100.0% | 2799.4443 | 2800.0647 | 1 | 9.134 | 40.0% | 3 | R.SGPFGQIFRPDNFVFGQSGAGNNWAK.G | 33 |
|  | Mis12XLAPLSS\_011913\_01.13076.13076.2 | 6.5905 | 0.5843 | 100.0% | 1959.6522 | 1960.151 | 1 | 11.152 | 79.4% | 2 | K.GHYTEGAELVDSVLDVVR.K | 222 |
|  | Mis12XLAPLSS\_011913\_01.13106.13106.3 | 4.5785 | 0.393 | 100.0% | 1961.5144 | 1960.151 | 1 | 7.056 | 45.6% | 1 | K.GHYTEGAELVDSVLDVVR.K | 333 |
|  | Mis12XLAPLSS\_011913\_01.12470.12470.2 | 5.7772 | 0.4953 | 100.0% | 2087.9521 | 2088.325 | 1 | 9.485 | 66.7% | 1 | K.GHYTEGAELVDSVLDVVRK.E | 222 |
|  | Mis12XLAPLSS\_011913\_01.12422.12422.3 | 4.0642 | 0.4487 | 100.0% | 2088.2644 | 2088.325 | 2 | 7.577 | 38.9% | 2 | K.GHYTEGAELVDSVLDVVRK.E | 333 |
|  | Mis12XLAPLSS\_011913\_01.03590.03590.2 | 2.5923 | 0.2503 | 99.8% | 1077.7522 | 1078.1698 | 20 | 4.976 | 71.4% | 1 | K.IREEYPDR.I | 22 |
|  | Mis12XLAPLSS\_011913\_01.09478.09478.2 | 4.27 | 0.3449 | 100.0% | 1321.9122 | 1320.5896 | 1 | 6.366 | 81.8% | 4 | R.IMNTFSVVPSPK.V | 222 |
|  | Mis12XLAPLSS\_011913\_01.08709.08709.2 | 2.7937 | 0.1227 | 98.4% | 1131.1721 | 1131.2767 | 10 | 4.38 | 72.2% | 3 | R.FPGQLNADLR.K | 222 |
|  | Mis12XLAPLSS\_011913\_01.06874.06874.2 | 2.5666 | 0.1608 | 98.1% | 1259.6322 | 1259.4508 | 74 | 4.363 | 55.0% | 1 | R.FPGQLNADLRK.L | 222 |
|  | Mis12XLAPLSS\_011913\_01.09525.09525.2 | 3.5594 | 0.3946 | 100.0% | 1271.7322 | 1272.5945 | 1 | 7.783 | 75.0% | 2 | R.KLAVNMVPFPR.L | 222 |
|  | Mis12XLAPLSS\_011913\_01.10730.10730.2 | 3.5516 | 0.5021 | 100.0% | 1144.1721 | 1144.4204 | 1 | 8.538 | 94.4% | 3 | K.LAVNMVPFPR.L | 222 |
|  | Mis12XLAPLSS\_011913\_01.12581.12581.3 | 3.8947 | 0.3599 | 100.0% | 1622.0643 | 1621.9403 | 2 | 6.47 | 50.0% | 1 | R.LHFFMPGFAPLTSR.G | 33 |
|  | Mis12XLAPLSS\_011913\_01.12591.12591.2 | 3.5785 | 0.326 | 99.9% | 1623.4722 | 1621.9403 | 1 | 6.785 | 73.1% | 4 | R.LHFFMPGFAPLTSR.G | 22 |
|  | Mis12XLAPLSS\_011913\_01.12669.12669.2 | 3.4785 | 0.3826 | 99.9% | 1692.4922 | 1692.9678 | 1 | 7.084 | 71.4% | 2 | R.ALTVPELTQQMFDAK.N | 22 |
|  | Mis12XLAPLSS\_011913\_01.11268.11268.1 | 1.6377 | 0.3909 | 100.0% | 1039.55 | 1040.2505 | 4 | 6.253 | 56.2% | 2 | R.YLTVAAVFR.G | 11 |
|  | Mis12XLAPLSS\_011913\_01.11306.11306.2 | 3.0968 | 0.4003 | 100.0% | 1040.2122 | 1040.2505 | 1 | 6.856 | 87.5% | 3 | R.YLTVAAVFR.G | 22 |
|  | Mis12XLAPLSS\_011913\_02.06605.06605.3 | 3.4205 | 0.2917 | 99.7% | 1923.4443 | 1925.2405 | 1 | 5.078 | 40.0% | 1 | R.MSMKEVDEQMLNVQNK.N | 33 |
|  | Mis12XLAPLSS\_011913\_01.07232.07232.2 | 4.2221 | 0.2425 | 99.9% | 1447.1522 | 1447.6031 | 1 | 5.681 | 72.7% | 3 | K.EVDEQMLNVQNK.N | 22 |
|  | Mis12XLAPLSS\_011913\_01.12202.12202.2 | 2.8146 | 0.3212 | 99.8% | 1697.7522 | 1697.8877 | 1 | 6.074 | 57.7% | 2 | K.NSSYFVEWIPNNVK.T | 222 |
|  | Mis12XLAPLSS\_011913\_01.11018.11018.2 | 3.7611 | 0.4743 | 100.0% | 1230.2122 | 1230.4241 | 1 | 7.691 | 94.4% | 5 | R.ISEQFTAMFR.R | 222 |

Similarities:
gi|29788785|ref|NP\_82(19:4)  
gi|50592996|ref|NP\_00(12:11)  

---

|  |  |  |  |  |  |  |  |  |
| --- | --- | --- | --- | --- | --- | --- | --- | --- |
| U | *gi|10835063|ref|NP\_00* | 7 | 8 | 48.3% | 294 | 32575 | 4.8 | nucleophosmin 1 isoform 1 [Homo sapiens] |
| U | *gi|40353734|ref|NP\_95* | 7 | 8 | 53.6% | 265 | 29465 | 4.6 | nucleophosmin 1 isoform 2 [Homo sapiens] |

| Filename XCorr DeltCN Conf% ObsM+H+ CalcM+H+ SpR ZScore Ion% # Sequence  | | | | | | | | | | | | |
| --- | --- | --- | --- | --- | --- | --- | --- | --- | --- | --- | --- | --- |
|  | Mis12XLAPLSS\_011913\_01.06200.06200.3 | 4.7226 | 0.2319 | 99.7% | 2573.7544 | 2574.7258 | 1 | 7.46 | 36.2% | 1 | K.ADKDYHFKVDNDENEHQLSLR.T | 3 |
|  | Mis12XLAPLSS\_011913\_02.08420.08420.3 | 5.5583 | 0.5329 | 100.0% | 2929.9443 | 2931.2874 | 1 | 7.813 | 33.3% | 2 | R.TVSLGAGAKDELHIVEAEAMNYEGSPIK.V | 3 |
|  | Mis12XLAPLSS\_011913\_01.13412.13412.2 | 2.2665 | 0.3335 | 99.1% | 2226.8123 | 2228.655 | 1 | 5.696 | 35.0% | 1 | K.MSVQPTVSLGGFEITPPVVLR.L | 2 |
|  | Mis12XLAPLSS\_011913\_01.03132.03132.2 | 2.3561 | 0.2824 | 98.9% | 1241.1721 | 1241.4331 | 23 | 5.05 | 58.3% | 1 | R.SAPGGGSKVPQKK.V | 2 |
|  | Mis12XLAPLSS\_011913\_02.05030.05030.3 | 5.055 | 0.4943 | 100.0% | 4120.074 | 4120.7905 | 1 | 8.171 | 27.9% | 1 | K.LAADEDDDDDDEEDDDEDDDDDDFDDEEAEEKAPVK.K | 3 |
|  | Mis12XLAPLSS\_011913\_01.04154.04154.2 | 2.228 | 0.2037 | 97.8% | 932.15216 | 932.0184 | 316 | 4.832 | 56.2% | 1 | K.GPSSVEDIK.A | 2 |
|  | Mis12XLAPLSS\_011913\_01.13352.13352.2 | 4.3044 | 0.2863 | 99.8% | 1821.1322 | 1821.0172 | 1 | 5.746 | 69.2% | 1 | R.MTDQEAIQDLWQWR.K | 2 |

---

|  |  |  |  |  |  |  |  |  |
| --- | --- | --- | --- | --- | --- | --- | --- | --- |
| U | *GFP* | 15 | 40 | 46.6% | 238 | 26813 | 5.8 | no description |

| Filename XCorr DeltCN Conf% ObsM+H+ CalcM+H+ SpR ZScore Ion% # Sequence  | | | | | | | | | | | | |
| --- | --- | --- | --- | --- | --- | --- | --- | --- | --- | --- | --- | --- |
| \* | Mis12XLAPLSS\_011913\_01.14808.14808.2 | 3.4698 | 0.5924 | 100.0% | 2436.4521 | 2438.7397 | 1 | 9.583 | 40.9% | 1 | K.GEELFTGVVPILVELDGDVNGHK.F | 2 |
| \* | Mis12XLAPLSS\_011913\_01.14810.14810.3 | 3.9403 | 0.4017 | 100.0% | 2439.0244 | 2438.7397 | 1 | 6.442 | 31.8% | 3 | K.GEELFTGVVPILVELDGDVNGHK.F | 3 |
| \* | Mis12XLAPLSS\_011913\_02.05138.05138.2 | 3.9736 | 0.5697 | 100.0% | 1503.7522 | 1504.5499 | 1 | 10.945 | 71.4% | 8 | K.FSVSGEGEGDATYGK.L | 2 |
| \* | Mis12XLAPLSS\_011913\_01.05348.05348.2 | 2.983 | 0.42 | 99.9% | 1268.1522 | 1267.399 | 1 | 7.101 | 75.0% | 4 | K.SAMPEGYVQER.T | 2 |
| \* | Mis12XLAPLSS\_011913\_01.07826.07826.2 | 3.1595 | 0.4545 | 100.0% | 1348.1122 | 1348.4979 | 1 | 7.442 | 80.0% | 4 | R.TIFFKDDGNYK.T | 2 |
| \* | Mis12XLAPLSS\_011913\_01.06048.06048.3 | 2.6899 | 0.2468 | 97.4% | 1607.0944 | 1605.7905 | 1 | 5.521 | 41.7% | 1 | R.TIFFKDDGNYKTR.A | 3 |
| \* | Mis12XLAPLSS\_011913\_02.05696.05696.3 | 3.0466 | 0.3142 | 99.7% | 1478.4543 | 1478.6451 | 3 | 5.623 | 45.8% | 2 | R.AEVKFEGDTLVNR.I | 3 |
| \* | Mis12XLAPLSS\_011913\_01.07611.07611.2 | 3.9505 | 0.3699 | 100.0% | 1479.4521 | 1478.6451 | 1 | 8.103 | 75.0% | 4 | R.AEVKFEGDTLVNR.I | 2 |
| \* | Mis12XLAPLSS\_011913\_01.09608.09608.3 | 2.9807 | 0.35 | 99.7% | 1961.2743 | 1962.2535 | 14 | 6.206 | 35.9% | 2 | R.AEVKFEGDTLVNRIELK.G | 3 |
| \* | Mis12XLAPLSS\_011913\_01.06009.06009.2 | 3.0147 | 0.4237 | 100.0% | 1050.4321 | 1051.1442 | 1 | 6.933 | 87.5% | 3 | K.FEGDTLVNR.I | 2 |
| \* | Mis12XLAPLSS\_011913\_02.06051.06051.3 | 3.67 | 0.3648 | 100.0% | 1542.6543 | 1543.7196 | 1 | 6.183 | 46.2% | 2 | K.GIDFKEDGNILGHK.L | 3 |
| \* | Mis12XLAPLSS\_011913\_01.08301.08301.2 | 4.2484 | 0.4936 | 100.0% | 1542.8922 | 1543.7196 | 1 | 8.466 | 65.4% | 1 | K.GIDFKEDGNILGHK.L | 2 |
| \* | Mis12XLAPLSS\_011913\_02.06257.06257.2 | 4.6976 | 0.4886 | 100.0% | 1974.4321 | 1975.1829 | 1 | 7.944 | 60.0% | 2 | K.LEYNYNSHNVYIMADK.Q | 2 |
| \* | Mis12XLAPLSS\_011913\_02.06302.06302.3 | 3.2774 | 0.35 | 99.7% | 1975.2544 | 1975.1829 | 1 | 6.452 | 46.7% | 2 | K.LEYNYNSHNVYIMADK.Q | 3 |
| \* | Mis12XLAPLSS\_011913\_02.05742.05742.3 | 3.508 | 0.335 | 99.7% | 2231.6943 | 2231.4875 | 121 | 5.485 | 30.9% | 1 | K.LEYNYNSHNVYIMADKQK.N | 3 |

---

|  |  |  |  |  |  |  |  |  |
| --- | --- | --- | --- | --- | --- | --- | --- | --- |
| U | *gi|4501885|ref|NP\_001* | 20 | 48 | 46.1% | 375 | 41737 | 5.5 | beta actin [Homo sapiens] |
| U | *gi|4501887|ref|NP\_001* | 20 | 48 | 46.1% | 375 | 41793 | 5.5 | actin, gamma 1 propeptide [Homo sapiens] |

| Filename XCorr DeltCN Conf% ObsM+H+ CalcM+H+ SpR ZScore Ion% # Sequence  | | | | | | | | | | | | |
| --- | --- | --- | --- | --- | --- | --- | --- | --- | --- | --- | --- | --- |
|  | Mis12XLAPLSS\_011913\_01.04238.04238.1 | 1.6701 | 0.2372 | 95.6% | 976.27 | 977.02136 | 36 | 4.581 | 50.0% | 1 | K.AGFAGDDAPR.A | 11 |
|  | Mis12XLAPLSS\_011913\_01.04286.04286.2 | 3.0985 | 0.3788 | 99.9% | 976.4322 | 977.02136 | 4 | 6.932 | 72.2% | 3 | K.AGFAGDDAPR.A | 22 |
|  | Mis12XLAPLSS\_011913\_01.08354.08354.2 | 2.8671 | 0.242 | 99.8% | 1199.1721 | 1199.4415 | 15 | 5.579 | 65.0% | 3 | R.AVFPSIVGRPR.H | 22 |
|  | Mis12XLAPLSS\_011913\_01.04090.04090.1 | 2.6388 | 0.4538 | 100.0% | 1171.44 | 1172.4058 | 1 | 7.705 | 70.0% | 1 | R.HQGVMVGMGQK.D | 11 |
|  | Mis12XLAPLSS\_011913\_01.04067.04067.2 | 2.9524 | 0.213 | 99.7% | 1173.2122 | 1172.4058 | 1 | 5.212 | 75.0% | 2 | R.HQGVMVGMGQK.D | 22 |
|  | Mis12XLAPLSS\_011913\_02.03558.03558.2 | 2.8314 | 0.0791 | 97.3% | 1198.4122 | 1199.2163 | 2 | 6.833 | 60.0% | 1 | K.DSYVGDEAQSK.R | 22 |
|  | Mis12XLAPLSS\_011913\_01.03634.03634.2 | 3.1046 | 0.0939 | 98.4% | 1354.5922 | 1355.4038 | 1 | 7.877 | 77.3% | 1 | K.DSYVGDEAQSKR.G | 22 |
|  | Mis12XLAPLSS\_011913\_01.07736.07736.2 | 3.2339 | 0.4221 | 100.0% | 1516.3922 | 1516.7019 | 1 | 6.318 | 80.0% | 3 | K.IWHHTFYNELR.V | 22 |
|  | Mis12XLAPLSS\_011913\_01.07730.07730.3 | 3.3097 | 0.2091 | 99.4% | 1516.9143 | 1516.7019 | 1 | 5.643 | 55.0% | 3 | K.IWHHTFYNELR.V | 33 |
|  | Mis12XLAPLSS\_011913\_01.09033.09033.2 | 4.4149 | 0.1877 | 99.8% | 1955.5322 | 1955.2615 | 1 | 7.039 | 52.9% | 2 | R.VAPEEHPVLLTEAPLNPK.A | 2 |
|  | Mis12XLAPLSS\_011913\_01.11810.11810.3 | 6.5211 | 0.524 | 100.0% | 3184.5244 | 3185.622 | 1 | 9.234 | 37.1% | 2 | R.TTGIVMDSGDGVTHTVPIYEGYALPHAILR.L | 3 |
|  | Mis12XLAPLSS\_011913\_01.10998.10998.2 | 2.236 | 0.2609 | 99.4% | 999.0122 | 999.167 | 44 | 5.601 | 71.4% | 2 | R.DLTDYLMK.I | 22 |
|  | Mis12XLAPLSS\_011913\_01.05907.05907.1 | 2.0778 | 0.3885 | 100.0% | 1132.33 | 1133.2029 | 98 | 6.032 | 44.4% | 2 | R.GYSFTTTAER.E | 1 |
|  | Mis12XLAPLSS\_011913\_01.05906.05906.2 | 3.3714 | 0.4836 | 100.0% | 1132.9922 | 1133.2029 | 1 | 8.233 | 77.8% | 5 | R.GYSFTTTAER.E | 2 |
|  | Mis12XLAPLSS\_011913\_01.11108.11108.2 | 4.8425 | 0.3335 | 100.0% | 1791.4321 | 1791.9554 | 1 | 8.207 | 83.3% | 6 | K.SYELPDGQVITIGNER.F | 22 |
|  | Mis12XLAPLSS\_011913\_02.07245.07245.3 | 5.0272 | 0.3827 | 100.0% | 2343.7144 | 2344.6448 | 1 | 6.365 | 40.5% | 2 | R.KDLYANTVLSGGTTMYPGIADR.M | 3 |
|  | Mis12XLAPLSS\_011913\_02.08002.08002.2 | 4.0403 | 0.4056 | 100.0% | 2215.5723 | 2216.4705 | 1 | 8.547 | 50.0% | 3 | K.DLYANTVLSGGTTMYPGIADR.M | 2 |
|  | Mis12XLAPLSS\_011913\_01.07324.07324.2 | 3.9946 | 0.3349 | 100.0% | 1549.1721 | 1549.8843 | 1 | 6.577 | 76.9% | 1 | R.MQKEITALAPSTMK.I | 22 |
|  | Mis12XLAPLSS\_011913\_01.07623.07623.1 | 2.3118 | 0.4052 | 100.0% | 1161.46 | 1162.3868 | 2 | 7.007 | 60.0% | 2 | K.EITALAPSTMK.I | 11 |
|  | Mis12XLAPLSS\_011913\_01.07652.07652.2 | 2.9102 | 0.4108 | 99.9% | 1162.0922 | 1162.3868 | 1 | 6.495 | 65.0% | 3 | K.EITALAPSTMK.I | 22 |

Similarities:
gi|4501881|ref|NP\_001(14:6)  

---

|  |  |  |  |  |  |  |  |  |
| --- | --- | --- | --- | --- | --- | --- | --- | --- |
| U | *gi|226530908|ref|NP\_0* | 17 | 44 | 46.0% | 285 | 30315 | 7.5 | protein-L-isoaspartate (D-aspartate) O-methyltransferase [Homo sapiens] |

| Filename XCorr DeltCN Conf% ObsM+H+ CalcM+H+ SpR ZScore Ion% # Sequence  | | | | | | | | | | | | |
| --- | --- | --- | --- | --- | --- | --- | --- | --- | --- | --- | --- | --- |
| \* | Mis12XLAPLSS\_011913\_01.04310.04310.2 | 4.3385 | 0.4025 | 100.0% | 1478.1721 | 1478.6078 | 1 | 7.749 | 73.1% | 2 | K.SGGASHSELIHNLR.K | 2 |
| \* | Mis12XLAPLSS\_011913\_02.03969.03969.3 | 4.2877 | 0.3185 | 100.0% | 1478.4543 | 1478.6078 | 1 | 7.341 | 50.0% | 4 | K.SGGASHSELIHNLR.K | 3 |
| \* | Mis12XLAPLSS\_011913\_01.03869.03869.2 | 3.5979 | 0.312 | 99.8% | 1606.3922 | 1606.7819 | 1 | 5.102 | 57.1% | 2 | K.SGGASHSELIHNLRK.N | 2 |
| \* | Mis12XLAPLSS\_011913\_01.03800.03800.3 | 3.6523 | 0.2874 | 99.7% | 1606.5243 | 1606.7819 | 1 | 5.975 | 41.1% | 2 | K.SGGASHSELIHNLRK.N | 3 |
| \* | Mis12XLAPLSS\_011913\_02.07827.07827.3 | 3.794 | 0.48 | 100.0% | 2051.1543 | 2051.409 | 1 | 7.531 | 39.7% | 2 | K.NGIIKTDKVFEVMLATDR.S | 3 |
| \* | Mis12XLAPLSS\_011913\_01.10036.10036.2 | 4.5884 | 0.4688 | 100.0% | 1526.3722 | 1525.7601 | 1 | 9.026 | 75.0% | 6 | K.TDKVFEVMLATDR.S | 2 |
| \* | Mis12XLAPLSS\_011913\_01.10088.10088.2 | 3.7766 | 0.5336 | 100.0% | 1181.1322 | 1181.3923 | 1 | 9.879 | 88.9% | 6 | K.VFEVMLATDR.S | 2 |
| \* | Mis12XLAPLSS\_011913\_01.05138.05138.2 | 2.2177 | 0.3029 | 99.5% | 894.9122 | 895.0898 | 37 | 4.854 | 78.6% | 1 | K.VIGIDHIK.E | 2 |
| \* | Mis12XLAPLSS\_011913\_01.04407.04407.2 | 2.8214 | 0.3538 | 99.8% | 1188.5521 | 1189.3109 | 2 | 6.751 | 75.0% | 3 | R.KDDPTLLSSGR.V | 2 |
| \* | Mis12XLAPLSS\_011913\_01.06052.06052.2 | 2.274 | 0.1749 | 96.4% | 1060.9321 | 1061.1368 | 3 | 4.817 | 66.7% | 1 | K.DDPTLLSSGR.V | 2 |
| \* | Mis12XLAPLSS\_011913\_01.05486.05486.1 | 2.0768 | 0.2957 | 100.0% | 942.59 | 943.091 | 1 | 6.574 | 68.8% | 2 | R.VQLVVGDGR.M | 1 |
| \* | Mis12XLAPLSS\_011913\_01.05468.05468.2 | 3.2746 | 0.3496 | 100.0% | 943.2522 | 943.091 | 1 | 7.718 | 87.5% | 4 | R.VQLVVGDGR.M | 2 |
| \* | Mis12XLAPLSS\_011913\_01.12494.12494.3 | 6.8294 | 0.4874 | 100.0% | 3507.0842 | 3507.0015 | 1 | 8.488 | 30.3% | 2 | R.MGYAEEAPYDAIHVGAAAPVVPQALIDQLKPGGR.L | 3 |
| \* | Mis12XLAPLSS\_011913\_01.11870.11870.2 | 5.088 | 0.494 | 100.0% | 2043.3922 | 2044.3734 | 1 | 9.039 | 72.2% | 2 | R.LILPVGPAGGNQMLEQYDK.L | 2 |
| \* | Mis12XLAPLSS\_011913\_01.11889.11889.2 | 3.3781 | 0.3569 | 99.9% | 1705.6322 | 1706.1549 | 1 | 6.822 | 57.1% | 1 | K.MKPLMGVIYVPLTDK.E | 2 |
| \* | Mis12XLAPLSS\_011913\_01.10432.10432.2 | 4.8589 | 0.6056 | 100.0% | 1962.5322 | 1963.4445 | 1 | 10.532 | 65.6% | 2 | K.MKPLMGVIYVPLTDKEK.Q | 2 |
| \* | Mis12XLAPLSS\_011913\_01.10436.10436.3 | 3.5783 | 0.3256 | 99.7% | 1963.4043 | 1963.4445 | 98 | 6.279 | 37.5% | 2 | K.MKPLMGVIYVPLTDKEK.Q | 3 |

---

|  |  |  |  |  |  |  |  |  |
| --- | --- | --- | --- | --- | --- | --- | --- | --- |
| U | *gi|14389309|ref|NP\_11* | 19 | 44 | 45.7% | 449 | 49895 | 5.1 | tubulin alpha 6 [Homo sapiens] |
| U | *gi|57013276|ref|NP\_00* | 19 | 44 | 45.5% | 451 | 50152 | 5.1 | tubulin, alpha, ubiquitous [Homo sapiens] |

| Filename XCorr DeltCN Conf% ObsM+H+ CalcM+H+ SpR ZScore Ion% # Sequence  | | | | | | | | | | | | |
| --- | --- | --- | --- | --- | --- | --- | --- | --- | --- | --- | --- | --- |
|  | Mis12XLAPLSS\_011913\_02.08066.08066.2 | 5.7284 | 0.5715 | 100.0% | 2008.2522 | 2009.093 | 1 | 10.946 | 57.9% | 3 | K.TIGGGDDSFNTFFSETGAGK.H | 2 |
|  | Mis12XLAPLSS\_011913\_01.12460.12460.2 | 3.8744 | 0.1783 | 99.8% | 1705.4922 | 1702.9451 | 1 | 4.528 | 64.3% | 6 | R.AVFVDLEPTVIDEVR.T | 2 |
|  | Mis12XLAPLSS\_011913\_01.09231.09231.3 | 3.5966 | 0.338 | 99.7% | 2415.7444 | 2416.6555 | 1 | 5.256 | 36.2% | 2 | R.QLFHPEQLITGKEDAANNYAR.G | 3 |
|  | Mis12XLAPLSS\_011913\_01.13079.13079.2 | 2.8649 | 0.2477 | 99.5% | 1843.8522 | 1843.1332 | 3 | 5.435 | 46.7% | 1 | R.GHYTIGKEIIDLVLDR.I | 2 |
|  | Mis12XLAPLSS\_011913\_01.12095.12095.2 | 2.0455 | 0.2894 | 98.4% | 1086.1721 | 1086.2737 | 20 | 5.035 | 68.8% | 1 | K.EIIDLVLDR.I | 2 |
|  | Mis12XLAPLSS\_011913\_01.03885.03885.2 | 2.1509 | 0.2997 | 99.5% | 909.6322 | 910.05804 | 2 | 5.29 | 71.4% | 1 | R.LSVDYGKK.S | 2 |
|  | Mis12XLAPLSS\_011913\_01.06659.06659.3 | 3.2632 | 0.2209 | 97.9% | 1876.3143 | 1876.0824 | 2 | 4.696 | 42.9% | 3 | R.RNLDIERPTYTNLNR.L | 3 |
|  | Mis12XLAPLSS\_011913\_01.08139.08139.2 | 3.0673 | 0.0845 | 97.4% | 1719.0721 | 1719.8949 | 2 | 4.373 | 57.7% | 2 | R.NLDIERPTYTNLNR.L | 2 |
|  | Mis12XLAPLSS\_011913\_01.08132.08132.3 | 2.8376 | 0.3286 | 99.8% | 1720.1643 | 1719.8949 | 62 | 5.458 | 42.3% | 1 | R.NLDIERPTYTNLNR.L | 3 |
|  | Mis12XLAPLSS\_011913\_01.14102.14102.2 | 4.5903 | 0.4927 | 100.0% | 1488.3322 | 1488.7678 | 1 | 9.662 | 76.9% | 4 | R.LISQIVSSITASLR.F | 2 |
|  | Mis12XLAPLSS\_011913\_02.09594.09594.2 | 4.9399 | 0.4722 | 100.0% | 2410.412 | 2410.6885 | 1 | 9.166 | 50.0% | 2 | R.FDGALNVDLTEFQTNLVPYPR.I | 2 |
|  | Mis12XLAPLSS\_011913\_02.09573.09573.3 | 3.1579 | 0.3066 | 99.8% | 2411.5144 | 2410.6885 | 2 | 5.807 | 28.8% | 1 | R.FDGALNVDLTEFQTNLVPYPR.I | 3 |
|  | Mis12XLAPLSS\_011913\_01.11378.11378.2 | 3.9896 | 0.4116 | 100.0% | 1757.6322 | 1758.0703 | 1 | 7.308 | 73.3% | 6 | R.IHFPLATYAPVISAEK.A | 2 |
|  | Mis12XLAPLSS\_011913\_01.11372.11372.3 | 3.4683 | 0.3314 | 99.7% | 1757.7544 | 1758.0703 | 47 | 5.796 | 36.7% | 2 | R.IHFPLATYAPVISAEK.A | 3 |
|  | Mis12XLAPLSS\_011913\_01.08154.08154.1 | 1.6328 | 0.3908 | 100.0% | 1015.51 | 1016.1827 | 24 | 5.967 | 55.6% | 1 | K.DVNAAIATIK.T | 1 |
|  | Mis12XLAPLSS\_011913\_01.08159.08159.2 | 2.9896 | 0.4043 | 99.9% | 1016.1722 | 1016.1827 | 1 | 7.655 | 88.9% | 1 | K.DVNAAIATIK.T | 2 |
|  | Mis12XLAPLSS\_011913\_01.09716.09716.2 | 4.2795 | 0.4557 | 100.0% | 1825.3322 | 1826.1027 | 1 | 7.092 | 61.8% | 3 | K.VGINYQPPTVVPGGDLAK.V | 2 |
|  | Mis12XLAPLSS\_011913\_01.07934.07934.3 | 3.3735 | 0.226 | 99.8% | 1382.0944 | 1381.6324 | 3 | 4.642 | 55.0% | 2 | R.LDHKFDLMYAK.R | 3 |
|  | Mis12XLAPLSS\_011913\_02.07940.07940.3 | 4.3761 | 0.4014 | 100.0% | 2331.0244 | 2331.5208 | 1 | 7.738 | 40.8% | 2 | R.AFVHWYVGEGMEEGEFSEAR.E | 3 |

---

|  |  |  |  |  |  |  |  |  |
| --- | --- | --- | --- | --- | --- | --- | --- | --- |
| U | *gi|5803225|ref|NP\_006* | 7 | 9 | 45.5% | 255 | 29174 | 4.7 | tyrosine 3/tryptophan 5 -monooxygenase activation protein, epsilon polypeptide [Homo sapiens] |

| Filename XCorr DeltCN Conf% ObsM+H+ CalcM+H+ SpR ZScore Ion% # Sequence  | | | | | | | | | | | | |
| --- | --- | --- | --- | --- | --- | --- | --- | --- | --- | --- | --- | --- |
| \* | Mis12XLAPLSS\_011913\_02.06754.06754.2 | 3.5386 | 0.3975 | 100.0% | 1448.2722 | 1448.6312 | 1 | 7.992 | 79.2% | 2 | K.VAGMDVELTVEER.N | 2 |
| \* | Mis12XLAPLSS\_011913\_01.04056.04056.3 | 4.218 | 0.2844 | 99.7% | 2147.1843 | 2146.4026 | 1 | 5.803 | 37.5% | 1 | R.IISSIEQKEENKGGEDKLK.M | 3 |
| \* | Mis12XLAPLSS\_011913\_01.03929.03929.2 | 2.7799 | 0.2841 | 99.8% | 1237.5122 | 1238.3861 | 1 | 5.505 | 68.2% | 1 | K.HLIPAANTGESK.V | 2 |
| \* | Mis12XLAPLSS\_011913\_01.08880.08880.3 | 3.7572 | 0.2987 | 99.7% | 2561.1843 | 2561.8108 | 2 | 5.135 | 28.4% | 1 | R.YLAEFATGNDRKEAAENSLVAYK.A | 3 |
| \* | Mis12XLAPLSS\_011913\_01.05807.05807.2 | 2.0756 | 0.2982 | 98.4% | 1194.9722 | 1195.3147 | 2 | 5.557 | 65.0% | 1 | K.EAAENSLVAYK.A | 2 |
| \* | Mis12XLAPLSS\_011913\_02.11760.11760.2 | 3.178 | 0.3026 | 99.8% | 2332.2122 | 2332.6611 | 35 | 5.584 | 31.6% | 1 | R.LGLALNFSVFYYEILNSPDR.A | 2 |
| \* | Mis12XLAPLSS\_011913\_01.18200.18200.3 | 5.8245 | 0.4813 | 100.0% | 3259.4944 | 3260.6375 | 1 | 8.185 | 30.4% | 2 | K.AAFDDAIAELDTLSEESYKDSTLIMQLLR.D | 3 |

---

|  |  |  |  |  |  |  |  |  |
| --- | --- | --- | --- | --- | --- | --- | --- | --- |
| U | *gi|13128994|ref|NP\_07* | 7 | 9 | 45.4% | 205 | 24140 | 5.7 | MIS12 homolog [Homo sapiens] |

| Filename XCorr DeltCN Conf% ObsM+H+ CalcM+H+ SpR ZScore Ion% # Sequence  | | | | | | | | | | | | |
| --- | --- | --- | --- | --- | --- | --- | --- | --- | --- | --- | --- | --- |
| \* | Mis12XLAPLSS\_011913\_01.18801.18801.2 | 3.7683 | 0.4966 | 100.0% | 2660.9321 | 2662.1592 | 1 | 8.25 | 40.5% | 1 | R.IYIAFQDYLFEVMQAVEQVILK.K | 2 |
| \* | Mis12XLAPLSS\_011913\_01.14622.14622.2 | 3.2665 | 0.3332 | 99.9% | 1405.5122 | 1404.7539 | 1 | 6.162 | 80.0% | 1 | K.MEQLFLQLILR.I | 2 |
| \* | Mis12XLAPLSS\_011913\_01.09489.09489.2 | 2.8039 | 0.14 | 98.5% | 1240.8922 | 1239.4546 | 1 | 4.55 | 80.0% | 1 | R.IPSNILLPEDK.C | 2 |
| \* | Mis12XLAPLSS\_011913\_01.07637.07637.2 | 2.9422 | 0.1869 | 99.1% | 1750.4321 | 1751.8468 | 4 | 3.832 | 50.0% | 1 | K.ETPYSEEDFQHLQK.E | 2 |
| \* | Mis12XLAPLSS\_011913\_01.10682.10682.3 | 4.6712 | 0.4637 | 100.0% | 1919.1244 | 1919.1906 | 1 | 7.804 | 46.7% | 2 | K.LKQTLTFFDELHNVGR.D | 3 |
| \* | Mis12XLAPLSS\_011913\_01.11129.11129.3 | 4.1889 | 0.5004 | 100.0% | 2148.5344 | 2148.2969 | 1 | 7.42 | 43.1% | 1 | R.DHGTSDFRESLVSLVQNSR.K | 3 |
| \* | Mis12XLAPLSS\_011913\_01.09888.09888.2 | 3.787 | 0.2833 | 99.9% | 1232.3522 | 1232.3794 | 1 | 6.67 | 85.0% | 2 | R.ESLVSLVQNSR.K | 2 |

---

|  |  |  |  |  |  |  |  |  |
| --- | --- | --- | --- | --- | --- | --- | --- | --- |
| U | *gi|32698866|ref|NP\_87* | 5 | 7 | 45.2% | 197 | 22443 | 4.7 | spindle pole body component 24 homolog [Homo sapiens] |

| Filename XCorr DeltCN Conf% ObsM+H+ CalcM+H+ SpR ZScore Ion% # Sequence  | | | | | | | | | | | | |
| --- | --- | --- | --- | --- | --- | --- | --- | --- | --- | --- | --- | --- |
| \* | Mis12XLAPLSS\_011913\_01.15417.15417.2 | 4.7596 | 0.3421 | 100.0% | 1815.3922 | 1815.0336 | 1 | 7.913 | 68.8% | 1 | R.DIEEVSQGLLSLLGANR.A | 2 |
| \* | Mis12XLAPLSS\_011913\_01.07004.07004.2 | 2.2109 | 0.263 | 98.4% | 1072.5721 | 1073.2346 | 1 | 5.134 | 88.9% | 1 | K.EVAQSLLNAK.E | 2 |
| \* | Mis12XLAPLSS\_011913\_01.11502.11502.3 | 5.5012 | 0.4133 | 100.0% | 2852.1543 | 2852.0007 | 1 | 7.383 | 33.0% | 2 | K.EQVHQGGVELQQLEAGLQEAGEEDTR.L | 3 |
| \* | Mis12XLAPLSS\_011913\_01.06387.06387.3 | 4.8029 | 0.442 | 100.0% | 2251.5544 | 2251.51 | 1 | 7.588 | 42.5% | 2 | K.GIHHGPSVAQPIHLDSTQLSR.K | 3 |
| \* | Mis12XLAPLSS\_011913\_01.16161.16161.2 | 4.2232 | 0.5665 | 100.0% | 1902.1322 | 1903.1412 | 1 | 9.668 | 64.3% | 1 | R.KFISDYLWSLVDTEW.- | 2 |

---

|  |  |  |  |  |  |  |  |  |
| --- | --- | --- | --- | --- | --- | --- | --- | --- |
| U | *gi|209862831|ref|NP\_0* | 12 | 18 | 42.8% | 339 | 38604 | 7.8 | annexin A2 isoform 2 [Homo sapiens] |
| U | *gi|50845386|ref|NP\_00* | 12 | 18 | 42.8% | 339 | 38604 | 7.8 | annexin A2 isoform 2 [Homo sapiens] |
| U | *gi|4757756|ref|NP\_004* | 12 | 18 | 42.8% | 339 | 38604 | 7.8 | annexin A2 isoform 2 [Homo sapiens] |

| Filename XCorr DeltCN Conf% ObsM+H+ CalcM+H+ SpR ZScore Ion% # Sequence  | | | | | | | | | | | | |
| --- | --- | --- | --- | --- | --- | --- | --- | --- | --- | --- | --- | --- |
|  | Mis12XLAPLSS\_011913\_01.07067.07067.2 | 5.0611 | 0.5017 | 100.0% | 1845.3922 | 1846.0038 | 1 | 8.418 | 52.9% | 1 | K.LSLEGDHSTPPSAYGSVK.A | 2 |
|  | Mis12XLAPLSS\_011913\_01.13095.13095.2 | 4.1278 | 0.3775 | 100.0% | 1544.5122 | 1543.7605 | 1 | 7.173 | 73.1% | 1 | K.GVDEVTIVNILTNR.S | 2 |
|  | Mis12XLAPLSS\_011913\_01.07954.07954.2 | 2.3089 | 0.2473 | 99.1% | 1112.3121 | 1112.2303 | 1 | 5.475 | 75.0% | 1 | R.QDIAFAYQR.R | 2 |
|  | Mis12XLAPLSS\_011913\_01.14042.14042.3 | 4.3366 | 0.2594 | 99.7% | 1652.2444 | 1651.9872 | 12 | 6.203 | 45.0% | 1 | K.SALSGHLETVILGLLK.T | 3 |
|  | Mis12XLAPLSS\_011913\_01.14067.14067.2 | 5.0046 | 0.3803 | 100.0% | 1652.8121 | 1651.9872 | 1 | 8.091 | 63.3% | 4 | K.SALSGHLETVILGLLK.T | 2 |
|  | Mis12XLAPLSS\_011913\_01.05001.05001.2 | 2.3599 | 0.2819 | 99.4% | 1222.0922 | 1223.3251 | 2 | 4.857 | 65.0% | 2 | K.TPAQYDASELK.A | 2 |
|  | Mis12XLAPLSS\_011913\_01.04755.04755.2 | 3.2404 | 0.301 | 99.9% | 1245.3121 | 1245.3347 | 3 | 6.115 | 77.8% | 2 | R.TNQELQEINR.V | 2 |
|  | Mis12XLAPLSS\_011913\_01.09362.09362.3 | 4.2191 | 0.4605 | 100.0% | 1940.2743 | 1941.102 | 1 | 7.751 | 48.4% | 1 | K.TDLEKDIISDTSGDFRK.L | 3 |
|  | Mis12XLAPLSS\_011913\_01.09610.09610.3 | 4.2533 | 0.1647 | 98.3% | 2066.8743 | 2066.1887 | 1 | 5.1 | 47.1% | 1 | R.RAEDGSVIDYELIDQDAR.D | 3 |
|  | Mis12XLAPLSS\_011913\_01.10029.10029.2 | 2.0413 | 0.1952 | 95.9% | 1035.6921 | 1036.2341 | 11 | 5.394 | 64.3% | 1 | K.WISIMTER.S | 2 |
|  | Mis12XLAPLSS\_011913\_01.11128.11128.2 | 2.5276 | 0.2869 | 99.5% | 1589.4521 | 1589.8035 | 2 | 6.024 | 54.2% | 1 | K.SYSPYDMLESIRK.E | 2 |
|  | Mis12XLAPLSS\_011913\_01.09641.09641.2 | 2.8741 | 0.3852 | 99.9% | 1422.2722 | 1422.5774 | 1 | 6.024 | 70.0% | 2 | K.SLYYYIQQDTK.G | 2 |

---

|  |  |  |  |  |  |  |  |  |
| --- | --- | --- | --- | --- | --- | --- | --- | --- |
| U | *contaminant\_gi|746301* | 13 | 85 | 42.8% | 269 | 27961 | 6.7 | lysyl endopeptidase (EC 3.4.21.50) - Lysobacter enzymogenes |

| Filename XCorr DeltCN Conf% ObsM+H+ CalcM+H+ SpR ZScore Ion% # Sequence  | | | | | | | | | | | | |
| --- | --- | --- | --- | --- | --- | --- | --- | --- | --- | --- | --- | --- |
| \* | Mis12XLAPLSS\_011913\_01.04941.04941.2 | 6.3239 | 0.5593 | 100.0% | 2262.5723 | 2262.355 | 1 | 10.313 | 52.1% | 14 | R.APGSSSSGANGDGSLAQSQTGAVVR.A | 2 |
| \* | Mis12XLAPLSS\_011913\_01.05066.05066.3 | 4.8023 | 0.4216 | 100.0% | 2262.7144 | 2262.355 | 1 | 7.274 | 40.6% | 12 | R.APGSSSSGANGDGSLAQSQTGAVVR.A | 3 |
| \* | Mis12XLAPLSS\_011913\_01.15854.15854.3 | 6.4876 | 0.5319 | 100.0% | 3315.6543 | 3315.6257 | 1 | 8.595 | 31.0% | 2 | R.ATNAASDFTLLELNTAANPAYNLFWAGWDR.R | 3 |
| \* | Mis12XLAPLSS\_011913\_01.14979.14979.3 | 6.5934 | 0.4914 | 100.0% | 3471.6543 | 3471.813 | 1 | 9.749 | 30.0% | 10 | R.ATNAASDFTLLELNTAANPAYNLFWAGWDRR.D | 3 |
| \* | Mis12XLAPLSS\_011913\_01.04329.04329.2 | 3.2226 | 0.4164 | 99.9% | 2076.4722 | 2077.2668 | 1 | 6.72 | 50.0% | 1 | R.RDQNFAGATAIHHPNVAEK.R | 2 |
| \* | Mis12XLAPLSS\_011913\_01.04364.04364.3 | 5.0832 | 0.3449 | 100.0% | 2077.9443 | 2077.2668 | 1 | 6.591 | 45.8% | 3 | R.RDQNFAGATAIHHPNVAEK.R | 3 |
| \* | Mis12XLAPLSS\_011913\_01.04167.04167.3 | 4.2643 | 0.2809 | 99.7% | 2233.6143 | 2233.4543 | 1 | 5.903 | 36.8% | 3 | R.RDQNFAGATAIHHPNVAEKR.I | 3 |
| \* | Mis12XLAPLSS\_011913\_01.05042.05042.2 | 4.7199 | 0.5454 | 100.0% | 1920.3722 | 1921.0793 | 1 | 8.775 | 64.7% | 3 | R.DQNFAGATAIHHPNVAEK.R | 2 |
| \* | Mis12XLAPLSS\_011913\_01.05048.05048.3 | 2.7427 | 0.2471 | 95.2% | 1921.9143 | 1921.0793 | 1 | 4.825 | 39.7% | 1 | R.DQNFAGATAIHHPNVAEK.R | 3 |
| \* | Mis12XLAPLSS\_011913\_01.04463.04463.2 | 5.3658 | 0.4189 | 100.0% | 2076.5723 | 2077.2668 | 1 | 7.578 | 61.1% | 3 | R.DQNFAGATAIHHPNVAEKR.I | 2 |
| \* | Mis12XLAPLSS\_011913\_01.04514.04514.3 | 4.2881 | 0.471 | 100.0% | 2077.2544 | 2077.2668 | 1 | 8.404 | 41.7% | 5 | R.DQNFAGATAIHHPNVAEKR.I | 3 |
| \* | Mis12XLAPLSS\_011913\_01.07943.07943.2 | 5.0176 | 0.3706 | 100.0% | 1430.1721 | 1428.5443 | 1 | 8.142 | 73.1% | 27 | R.VFTSWTGGGTSATR.L | 2 |
| \* | Mis12XLAPLSS\_011913\_01.14762.14762.2 | 2.6045 | 0.2257 | 98.4% | 2604.4722 | 2605.8174 | 59 | 5.183 | 26.0% | 1 | R.LSDWLDAAGTGAQFIDGLDSTGTPPV.- | 2 |

---

|  |  |  |  |  |  |  |  |  |
| --- | --- | --- | --- | --- | --- | --- | --- | --- |
| U | *gi|32455264|ref|NP\_85* | 11 | 20 | 42.7% | 199 | 22110 | 8.1 | peroxiredoxin 1 [Homo sapiens] |
| U | *gi|4505591|ref|NP\_002* | 11 | 20 | 42.7% | 199 | 22110 | 8.1 | peroxiredoxin 1 [Homo sapiens] |
| U | *gi|32455266|ref|NP\_85* | 11 | 20 | 42.7% | 199 | 22110 | 8.1 | peroxiredoxin 1 [Homo sapiens] |

| Filename XCorr DeltCN Conf% ObsM+H+ CalcM+H+ SpR ZScore Ion% # Sequence  | | | | | | | | | | | | |
| --- | --- | --- | --- | --- | --- | --- | --- | --- | --- | --- | --- | --- |
|  | Mis12XLAPLSS\_011913\_01.03965.03965.1 | 1.8122 | 0.4193 | 100.0% | 980.49 | 981.1423 | 4 | 6.818 | 62.5% | 1 | K.IGHPAPNFK.A | 1 |
|  | Mis12XLAPLSS\_011913\_01.03947.03947.2 | 2.2984 | 0.2934 | 99.5% | 981.2522 | 981.1423 | 1 | 4.995 | 68.8% | 1 | K.IGHPAPNFK.A | 2 |
|  | Mis12XLAPLSS\_011913\_01.06723.06723.2 | 2.8969 | 0.3251 | 99.8% | 1166.2522 | 1165.3496 | 3 | 5.709 | 65.0% | 5 | K.ATAVMPDGQFK.D | 2 |
|  | Mis12XLAPLSS\_011913\_01.09093.09093.3 | 3.397 | 0.2544 | 99.4% | 1907.9644 | 1908.2694 | 1 | 5.217 | 38.2% | 1 | K.KQGGLGPMNIPLVSDPKR.T | 3 |
|  | Mis12XLAPLSS\_011913\_01.08355.08355.2 | 3.0131 | 0.4531 | 100.0% | 1107.6921 | 1108.2798 | 1 | 6.278 | 88.9% | 2 | R.TIAQDYGVLK.A | 2 |
|  | Mis12XLAPLSS\_011913\_02.07400.07400.3 | 2.9437 | 0.4138 | 100.0% | 1983.9844 | 1984.2163 | 1 | 7.059 | 44.1% | 1 | R.TIAQDYGVLKADEGISFR.G | 3 |
|  | Mis12XLAPLSS\_011913\_01.06153.06153.2 | 2.0024 | 0.2054 | 95.6% | 894.3122 | 894.9597 | 3 | 5.409 | 85.7% | 2 | K.ADEGISFR.G | 2 |
|  | Mis12XLAPLSS\_011913\_01.10468.10468.1 | 2.4939 | 0.2988 | 100.0% | 920.57 | 921.0813 | 19 | 5.7 | 64.3% | 1 | R.GLFIIDDK.G | 1 |
|  | Mis12XLAPLSS\_011913\_01.10544.10544.2 | 1.9282 | 0.2633 | 97.3% | 920.9122 | 921.0813 | 32 | 5.634 | 78.6% | 1 | R.GLFIIDDK.G | 2 |
|  | Mis12XLAPLSS\_011913\_01.08840.08840.2 | 2.8089 | 0.326 | 99.8% | 1212.1721 | 1212.3915 | 8 | 7.022 | 75.0% | 2 | R.QITVNDLPVGR.S | 22 |
|  | Mis12XLAPLSS\_011913\_01.09482.09482.2 | 3.4556 | 0.4659 | 100.0% | 1197.1721 | 1197.3763 | 1 | 7.607 | 83.3% | 3 | R.LVQAFQFTDK.H | 2 |

Similarities:
gi|32189392|ref|NP\_00(1:10)  

---

|  |  |  |  |  |  |  |  |  |
| --- | --- | --- | --- | --- | --- | --- | --- | --- |
| U | *gi|209693439|ref|NP\_0* | 4 | 5 | 41.7% | 127 | 13331 | 4.9 | synuclein, gamma (breast cancer-specific protein 1) [Homo sapiens] |

| Filename XCorr DeltCN Conf% ObsM+H+ CalcM+H+ SpR ZScore Ion% # Sequence  | | | | | | | | | | | | |
| --- | --- | --- | --- | --- | --- | --- | --- | --- | --- | --- | --- | --- |
| \* | Mis12XLAPLSS\_011913\_01.07739.07739.2 | 4.7542 | 0.4749 | 100.0% | 1619.3322 | 1619.8125 | 1 | 9.039 | 78.6% | 1 | K.TKENVVQSVTSVAEK.T | 2 |
| \* | Mis12XLAPLSS\_011913\_02.06911.06911.3 | 3.2805 | 0.2292 | 97.4% | 2233.3145 | 2234.4685 | 2 | 4.329 | 31.0% | 1 | K.TKEQANAVSEAVVSSVNTVATK.T | 3 |
| \* | Mis12XLAPLSS\_011913\_02.06440.06440.2 | 4.1029 | 0.4339 | 100.0% | 1674.1122 | 1674.8485 | 1 | 9.171 | 76.7% | 2 | K.TVEEAENIAVTSGVVR.K | 2 |
| \* | Mis12XLAPLSS\_011913\_02.06418.06418.3 | 4.2614 | 0.2992 | 100.0% | 1674.7743 | 1674.8485 | 2 | 6.068 | 43.3% | 1 | K.TVEEAENIAVTSGVVR.K | 3 |

---

|  |  |  |  |  |  |  |  |  |
| --- | --- | --- | --- | --- | --- | --- | --- | --- |
| U | *gi|10800140|ref|NP\_06* | 5 | 8 | 41.3% | 126 | 13950 | 10.3 | histone cluster 1, H2bb [Homo sapiens] |
| U | *gi|4504277|ref|NP\_003* | 5 | 8 | 41.3% | 126 | 13920 | 10.3 | histone cluster 2, H2be [Homo sapiens] |
| U | *gi|28173554|ref|NP\_77* | 5 | 8 | 41.3% | 126 | 13908 | 10.3 | histone cluster 3, H2bb [Homo sapiens] |
| U | *gi|20336754|ref|NP\_06* | 5 | 8 | 41.3% | 126 | 13904 | 10.3 | histone cluster 1, H2bj [Homo sapiens] |
| U | *gi|16306566|ref|NP\_00* | 5 | 8 | 41.3% | 126 | 13906 | 10.3 | histone cluster 1, H2bo [Homo sapiens] |

| Filename XCorr DeltCN Conf% ObsM+H+ CalcM+H+ SpR ZScore Ion% # Sequence  | | | | | | | | | | | | |
| --- | --- | --- | --- | --- | --- | --- | --- | --- | --- | --- | --- | --- |
|  | Mis12XLAPLSS\_011913\_01.07287.07287.2 | 1.8586 | 0.3382 | 98.1% | 1278.5521 | 1280.4631 | 121 | 5.085 | 61.1% | 1 | R.KESYSIYVYK.V | 2 |
|  | Mis12XLAPLSS\_011913\_01.03431.03431.1 | 1.4382 | 0.3852 | 100.0% | 1168.49 | 1169.2798 | 2 | 5.418 | 65.0% | 1 | K.QVHPDTGISSK.A | 11 |
|  | Mis12XLAPLSS\_011913\_01.14162.14162.2 | 4.9389 | 0.4991 | 100.0% | 1744.3522 | 1745.0211 | 1 | 9.933 | 82.1% | 4 | K.AMGIMNSFVNDIFER.I | 22 |
|  | Mis12XLAPLSS\_011913\_01.03130.03130.2 | 2.4313 | 0.2281 | 99.6% | 901.7322 | 902.0439 | 8 | 4.645 | 83.3% | 1 | R.LAHYNKR.S | 22 |
|  | Mis12XLAPLSS\_011913\_01.09692.09692.2 | 2.3993 | 0.14 | 97.3% | 954.0122 | 954.19794 | 17 | 4.076 | 81.2% | 1 | R.LLLPGELAK.H | 22 |

Similarities:
gi|10800138|ref|NP\_06(4:1)  

---

|  |  |  |  |  |  |  |  |  |
| --- | --- | --- | --- | --- | --- | --- | --- | --- |
| U | *gi|10800138|ref|NP\_06* | 6 | 11 | 41.3% | 126 | 13936 | 10.3 | histone cluster 1, H2bd [Homo sapiens] |
| U | *gi|66912162|ref|NP\_00* | 6 | 11 | 41.3% | 126 | 13920 | 10.3 | histone cluster 2, H2bf [Homo sapiens] |
| U | *gi|4504271|ref|NP\_003* | 6 | 11 | 41.3% | 126 | 13906 | 10.3 | histone cluster 1, H2bi [Homo sapiens] |
| U | *gi|4504269|ref|NP\_003* | 6 | 11 | 41.3% | 126 | 13892 | 10.3 | histone cluster 1, H2bh [Homo sapiens] |
| U | *gi|4504265|ref|NP\_003* | 6 | 11 | 41.3% | 126 | 13906 | 10.3 | histone cluster 1, H2bf [Homo sapiens] |
| U | *gi|4504263|ref|NP\_003* | 6 | 11 | 41.3% | 126 | 13989 | 10.3 | histone cluster 1, H2bm [Homo sapiens] |
| U | *gi|4504261|ref|NP\_003* | 6 | 11 | 41.3% | 126 | 13922 | 10.3 | histone cluster 1, H2bn [Homo sapiens] |
| U | *gi|4504259|ref|NP\_003* | 6 | 11 | 41.3% | 126 | 13952 | 10.3 | histone cluster 1, H2bl [Homo sapiens] |
| U | *gi|4504257|ref|NP\_003* | 6 | 11 | 41.3% | 126 | 13906 | 10.3 | histone cluster 1, H2bg [Homo sapiens] |
| U | *gi|21396484|ref|NP\_00* | 6 | 11 | 41.3% | 126 | 13906 | 10.3 | histone cluster 1, H2be [Homo sapiens] |
| U | *gi|21166389|ref|NP\_00* | 6 | 11 | 41.3% | 126 | 13906 | 10.3 | histone cluster 1, H2bc [Homo sapiens] |
| U | *gi|20336752|ref|NP\_61* | 6 | 11 | 41.3% | 126 | 13936 | 10.3 | histone cluster 1, H2bd [Homo sapiens] |
| U | *gi|18105048|ref|NP\_54* | 6 | 11 | 41.3% | 126 | 13890 | 10.3 | histone cluster 1, H2bk [Homo sapiens] |

| Filename XCorr DeltCN Conf% ObsM+H+ CalcM+H+ SpR ZScore Ion% # Sequence  | | | | | | | | | | | | |
| --- | --- | --- | --- | --- | --- | --- | --- | --- | --- | --- | --- | --- |
|  | Mis12XLAPLSS\_011913\_01.05553.05553.2 | 2.569 | 0.2959 | 99.8% | 1266.1522 | 1266.4363 | 2 | 5.571 | 77.8% | 2 | R.KESYSVYVYK.V | 2 |
|  | Mis12XLAPLSS\_011913\_02.05021.05021.2 | 1.992 | 0.3295 | 99.0% | 1137.2722 | 1138.2622 | 7 | 6.035 | 62.5% | 2 | K.ESYSVYVYK.V | 2 |
|  | Mis12XLAPLSS\_011913\_01.03431.03431.1 | 1.4382 | 0.3852 | 100.0% | 1168.49 | 1169.2798 | 2 | 5.418 | 65.0% | 1 | K.QVHPDTGISSK.A | 11 |
|  | Mis12XLAPLSS\_011913\_01.14162.14162.2 | 4.9389 | 0.4991 | 100.0% | 1744.3522 | 1745.0211 | 1 | 9.933 | 82.1% | 4 | K.AMGIMNSFVNDIFER.I | 22 |
|  | Mis12XLAPLSS\_011913\_01.03130.03130.2 | 2.4313 | 0.2281 | 99.6% | 901.7322 | 902.0439 | 8 | 4.645 | 83.3% | 1 | R.LAHYNKR.S | 22 |
|  | Mis12XLAPLSS\_011913\_01.09692.09692.2 | 2.3993 | 0.14 | 97.3% | 954.0122 | 954.19794 | 17 | 4.076 | 81.2% | 1 | R.LLLPGELAK.H | 22 |

Similarities:
gi|10800140|ref|NP\_06(4:2)  

---

|  |  |  |  |  |  |  |  |  |
| --- | --- | --- | --- | --- | --- | --- | --- | --- |
| U | *gi|16933546|ref|NP\_44* | 10 | 13 | 40.1% | 317 | 34274 | 6.0 | ribosomal protein P0 [Homo sapiens] |
| U | *gi|4506667|ref|NP\_000* | 10 | 13 | 40.1% | 317 | 34274 | 6.0 | ribosomal protein P0 [Homo sapiens] |

| Filename XCorr DeltCN Conf% ObsM+H+ CalcM+H+ SpR ZScore Ion% # Sequence  | | | | | | | | | | | | |
| --- | --- | --- | --- | --- | --- | --- | --- | --- | --- | --- | --- | --- |
|  | Mis12XLAPLSS\_011913\_01.10582.10582.2 | 3.1631 | 0.3083 | 99.9% | 1217.7122 | 1218.4357 | 1 | 7.374 | 83.3% | 1 | K.IIQLLDDYPK.C | 2 |
|  | Mis12XLAPLSS\_011913\_01.03722.03722.2 | 3.0687 | 0.2787 | 99.8% | 1222.2722 | 1222.3433 | 2 | 7.481 | 65.0% | 1 | R.GHLENNPALEK.L | 2 |
|  | Mis12XLAPLSS\_011913\_02.07647.07647.3 | 3.7881 | 0.4306 | 100.0% | 1826.1543 | 1826.0593 | 9 | 6.719 | 38.3% | 1 | R.GNVGFVFTKEDLTEIR.D | 3 |
|  | Mis12XLAPLSS\_011913\_01.13175.13175.3 | 3.8772 | 0.1934 | 97.4% | 3179.3044 | 3177.6892 | 2 | 4.321 | 23.2% | 1 | R.GNVGFVFTKEDLTEIRDMLLANKVPAAAR.A | 3 |
|  | Mis12XLAPLSS\_011913\_01.11855.11855.3 | 3.5269 | 0.2911 | 99.8% | 2226.3245 | 2227.584 | 59 | 4.808 | 26.3% | 1 | K.EDLTEIRDMLLANKVPAAAR.A | 3 |
|  | Mis12XLAPLSS\_011913\_01.07906.07906.2 | 3.5032 | 0.4423 | 100.0% | 1370.3322 | 1370.6531 | 1 | 7.233 | 79.2% | 2 | R.DMLLANKVPAAAR.A | 2 |
|  | Mis12XLAPLSS\_011913\_01.12825.12825.2 | 3.1238 | 0.4561 | 100.0% | 1314.3121 | 1314.5242 | 1 | 8.571 | 77.3% | 1 | K.TSFFQALGITTK.I | 2 |
|  | Mis12XLAPLSS\_011913\_02.08800.08800.2 | 3.4415 | 0.2761 | 99.8% | 1429.5122 | 1429.697 | 1 | 6.578 | 62.5% | 2 | R.GTIEILSDVQLIK.T | 2 |
|  | Mis12XLAPLSS\_011913\_01.12599.12599.2 | 4.2409 | 0.4955 | 100.0% | 1896.3722 | 1897.1754 | 1 | 8.56 | 50.0% | 2 | R.VLALSVETDYTFPLAEK.V | 2 |
|  | Mis12XLAPLSS\_011913\_01.12075.12075.3 | 4.124 | 0.36 | 100.0% | 3181.3442 | 3181.6592 | 1 | 6.673 | 25.7% | 1 | K.AFLADPSAFVAAAPVAAATTAAPAAAAAPAKVEAK.E | 3 |

---

|  |  |  |  |  |  |  |  |  |
| --- | --- | --- | --- | --- | --- | --- | --- | --- |
| U | *gi|4826898|ref|NP\_005* | 4 | 7 | 39.3% | 140 | 15054 | 8.3 | profilin 1 [Homo sapiens] |

| Filename XCorr DeltCN Conf% ObsM+H+ CalcM+H+ SpR ZScore Ion% # Sequence  | | | | | | | | | | | | |
| --- | --- | --- | --- | --- | --- | --- | --- | --- | --- | --- | --- | --- |
| \* | Mis12XLAPLSS\_011913\_01.12551.12551.2 | 2.4673 | 0.2692 | 98.7% | 1644.9122 | 1644.9518 | 2 | 5.184 | 43.3% | 1 | K.TFVNITPAEVGVLVGK.D | 2 |
| \* | Mis12XLAPLSS\_011913\_02.07218.07218.2 | 3.6167 | 0.4609 | 100.0% | 1471.3121 | 1471.6531 | 1 | 7.932 | 73.1% | 3 | R.SSFYVNGLTLGGQK.C | 2 |
| \* | Mis12XLAPLSS\_011913\_01.07944.07944.2 | 3.1479 | 0.4227 | 99.9% | 1381.2322 | 1380.5406 | 1 | 6.327 | 61.5% | 2 | K.STGGAPTFNVTVTK.T | 2 |
| \* | Mis12XLAPLSS\_011913\_01.09501.09501.2 | 2.5519 | 0.4037 | 99.8% | 1220.0721 | 1219.5255 | 122 | 5.98 | 55.0% | 1 | K.TDKTLVLLMGK.E | 2 |

---

|  |  |  |  |  |  |  |  |  |
| --- | --- | --- | --- | --- | --- | --- | --- | --- |
| U | *gi|31542947|ref|NP\_00* | 22 | 36 | 38.7% | 573 | 61055 | 5.9 | chaperonin [Homo sapiens] |
| U | *gi|41399285|ref|NP\_95* | 22 | 36 | 38.7% | 573 | 61055 | 5.9 | chaperonin [Homo sapiens] |

| Filename XCorr DeltCN Conf% ObsM+H+ CalcM+H+ SpR ZScore Ion% # Sequence  | | | | | | | | | | | | |
| --- | --- | --- | --- | --- | --- | --- | --- | --- | --- | --- | --- | --- |
|  | Mis12XLAPLSS\_011913\_02.12470.12470.3 | 5.3705 | 0.4819 | 100.0% | 2114.8145 | 2114.5667 | 1 | 8.751 | 43.8% | 3 | R.ALMLQGVDLLADAVAVTMGPK.G | 3 |
|  | Mis12XLAPLSS\_011913\_01.16797.16797.2 | 5.4099 | 0.5278 | 100.0% | 2114.8323 | 2114.5667 | 1 | 9.435 | 60.0% | 3 | R.ALMLQGVDLLADAVAVTMGPK.G | 2 |
|  | Mis12XLAPLSS\_011913\_02.06410.06410.2 | 3.3472 | 0.3952 | 99.8% | 1345.1921 | 1345.5382 | 1 | 6.803 | 72.7% | 2 | R.TVIIEQSWGSPK.V | 2 |
|  | Mis12XLAPLSS\_011913\_02.05818.05818.2 | 4.2049 | 0.6719 | 100.0% | 2560.612 | 2561.7222 | 1 | 12.262 | 54.2% | 1 | K.LVQDVANNTNEEAGDGTTTATVLAR.S | 2 |
|  | Mis12XLAPLSS\_011913\_02.05846.05846.3 | 4.7779 | 0.4981 | 100.0% | 2561.3643 | 2561.7222 | 1 | 8.179 | 35.4% | 1 | K.LVQDVANNTNEEAGDGTTTATVLAR.S | 3 |
|  | Mis12XLAPLSS\_011913\_01.09964.09964.2 | 2.4386 | 0.2859 | 99.4% | 1390.0721 | 1390.5786 | 1 | 5.719 | 72.7% | 1 | R.GYISPYFINTSK.G | 2 |
|  | Mis12XLAPLSS\_011913\_01.10744.10744.3 | 4.1874 | 0.3756 | 100.0% | 2048.3643 | 2048.3933 | 1 | 6.972 | 44.4% | 2 | K.KISSIQSIVPALEIANAHR.K | 3 |
|  | Mis12XLAPLSS\_011913\_01.11954.11954.2 | 4.3478 | 0.5472 | 100.0% | 1919.2322 | 1920.2192 | 2 | 8.708 | 41.2% | 1 | K.ISSIQSIVPALEIANAHR.K | 2 |
|  | Mis12XLAPLSS\_011913\_01.11966.11966.3 | 3.0828 | 0.3158 | 99.8% | 1919.8444 | 1920.2192 | 28 | 4.873 | 30.9% | 1 | K.ISSIQSIVPALEIANAHR.K | 3 |
|  | Mis12XLAPLSS\_011913\_01.13220.13220.2 | 4.1079 | 0.462 | 100.0% | 2365.5923 | 2366.7605 | 1 | 8.517 | 47.6% | 1 | R.KPLVIIAEDVDGEALSTLVLNR.L | 2 |
|  | Mis12XLAPLSS\_011913\_01.13206.13206.3 | 3.0761 | 0.2853 | 98.8% | 2366.5444 | 2366.7605 | 23 | 4.839 | 27.4% | 1 | R.KPLVIIAEDVDGEALSTLVLNR.L | 3 |
|  | Mis12XLAPLSS\_011913\_01.07804.07804.2 | 2.8844 | 0.2103 | 99.8% | 913.4522 | 913.14844 | 1 | 6.717 | 87.5% | 1 | K.VGLQVVAVK.A | 2 |
|  | Mis12XLAPLSS\_011913\_02.06723.06723.2 | 4.1778 | 0.4098 | 100.0% | 1631.4321 | 1631.9684 | 1 | 8.566 | 67.9% | 2 | K.VGEVIVTKDDAMLLK.G | 2 |
|  | Mis12XLAPLSS\_011913\_02.06694.06694.3 | 4.085 | 0.5232 | 100.0% | 1631.6643 | 1631.9684 | 2 | 8.864 | 44.6% | 2 | K.VGEVIVTKDDAMLLK.G | 3 |
|  | Mis12XLAPLSS\_011913\_02.08171.08171.3 | 3.3235 | 0.2212 | 97.5% | 2451.0244 | 2452.721 | 55 | 4.279 | 28.9% | 1 | K.RIQEIIEQLDVTTSEYEKEK.L | 3 |
|  | Mis12XLAPLSS\_011913\_01.03861.03861.2 | 3.1426 | 0.3986 | 99.9% | 1234.4122 | 1234.3055 | 2 | 6.9 | 68.2% | 1 | K.VGGTSDVEVNEK.K | 2 |
|  | Mis12XLAPLSS\_011913\_01.03490.03490.2 | 3.2568 | 0.2978 | 99.8% | 1362.2322 | 1362.4796 | 1 | 5.683 | 66.7% | 1 | K.VGGTSDVEVNEKK.D | 2 |
|  | Mis12XLAPLSS\_011913\_01.03404.03404.2 | 4.2809 | 0.398 | 100.0% | 1633.2722 | 1633.7556 | 1 | 7.976 | 64.3% | 1 | K.VGGTSDVEVNEKKDR.V | 2 |
|  | Mis12XLAPLSS\_011913\_01.04118.04118.2 | 3.2614 | 0.3987 | 100.0% | 961.1322 | 961.0629 | 1 | 7.54 | 81.2% | 3 | R.VTDALNATR.A | 2 |
|  | Mis12XLAPLSS\_011913\_01.07498.07498.2 | 2.325 | 0.1492 | 97.6% | 942.0522 | 942.18994 | 9 | 4.613 | 85.7% | 1 | K.IGIEIIKR.T | 2 |
|  | Mis12XLAPLSS\_011913\_02.05660.05660.2 | 3.8661 | 0.3671 | 100.0% | 1215.9321 | 1216.377 | 1 | 7.404 | 77.3% | 5 | K.NAGVEGSLIVEK.I | 2 |
|  | Mis12XLAPLSS\_011913\_02.09195.09195.3 | 4.8433 | 0.4081 | 100.0% | 2509.3743 | 2509.8235 | 1 | 7.652 | 39.8% | 1 | K.IMQSSSEVGYDAMAGDFVNMVEK.G | 3 |

---

|  |  |  |  |  |  |  |  |  |
| --- | --- | --- | --- | --- | --- | --- | --- | --- |
| U | *gi|225690602|ref|NP\_0* | 6 | 7 | 38.6% | 140 | 14460 | 4.7 | alpha-synuclein isoform NACP140 [Homo sapiens] |
| U | *gi|6806898|ref|NP\_009* | 6 | 7 | 48.2% | 112 | 11372 | 8.3 | alpha-synuclein isoform NACP112 [Homo sapiens] |
| U | *gi|4507109|ref|NP\_000* | 6 | 7 | 38.6% | 140 | 14460 | 4.7 | alpha-synuclein isoform NACP140 [Homo sapiens] |
| U | *gi|225690604|ref|NP\_0* | 6 | 7 | 38.6% | 140 | 14460 | 4.7 | alpha-synuclein isoform NACP140 [Homo sapiens] |

| Filename XCorr DeltCN Conf% ObsM+H+ CalcM+H+ SpR ZScore Ion% # Sequence  | | | | | | | | | | | | |
| --- | --- | --- | --- | --- | --- | --- | --- | --- | --- | --- | --- | --- |
|  | Mis12XLAPLSS\_011913\_01.03972.03972.2 | 4.4803 | 0.4614 | 100.0% | 1524.6522 | 1525.7452 | 1 | 8.388 | 75.0% | 1 | K.TKEGVVHGVATVAEK.T | 2 |
|  | Mis12XLAPLSS\_011913\_01.04376.04376.2 | 2.9941 | 0.4068 | 99.9% | 1296.0322 | 1296.4661 | 21 | 7.308 | 58.3% | 1 | K.EGVVHGVATVAEK.T | 2 |
|  | Mis12XLAPLSS\_011913\_02.06704.06704.3 | 5.0121 | 0.391 | 100.0% | 2158.7944 | 2158.46 | 1 | 7.599 | 39.3% | 2 | K.TKEQVTNVGGAVVTGVTAVAQK.T | 3 |
|  | Mis12XLAPLSS\_011913\_02.07113.07113.3 | 4.6913 | 0.4563 | 100.0% | 1929.5044 | 1929.1808 | 1 | 8.527 | 42.1% | 1 | K.EQVTNVGGAVVTGVTAVAQK.T | 3 |
|  | Mis12XLAPLSS\_011913\_02.06902.06902.2 | 4.1121 | 0.5175 | 100.0% | 1479.2922 | 1479.6732 | 1 | 9.92 | 73.3% | 1 | K.TVEGAGSIAAATGFVK.K | 2 |
|  | Mis12XLAPLSS\_011913\_02.06250.06250.2 | 2.4666 | 0.3845 | 99.8% | 1606.0521 | 1607.8473 | 1 | 6.182 | 56.2% | 1 | K.TVEGAGSIAAATGFVKK.D | 2 |

---

|  |  |  |  |  |  |  |  |  |
| --- | --- | --- | --- | --- | --- | --- | --- | --- |
| U | *gi|11968182|ref|NP\_07* | 7 | 14 | 38.2% | 152 | 17719 | 11.0 | ribosomal protein S18 [Homo sapiens] |
| U | *gi|169168597|ref|XP\_0* | 7 | 14 | 38.2% | 152 | 17719 | 11.0 | PREDICTED: hypothetical protein [Homo sapiens] |

| Filename XCorr DeltCN Conf% ObsM+H+ CalcM+H+ SpR ZScore Ion% # Sequence  | | | | | | | | | | | | |
| --- | --- | --- | --- | --- | --- | --- | --- | --- | --- | --- | --- | --- |
|  | Mis12XLAPLSS\_011913\_01.04710.04710.2 | 2.4711 | 0.3122 | 99.8% | 1003.2322 | 1002.1154 | 16 | 5.371 | 75.0% | 1 | R.VLNTNIDGR.R | 2 |
|  | Mis12XLAPLSS\_011913\_01.03666.03666.2 | 2.293 | 0.134 | 95.4% | 1060.1921 | 1060.2388 | 14 | 4.394 | 68.8% | 1 | R.KADIDLTKR.A | 2 |
|  | Mis12XLAPLSS\_011913\_01.04169.04169.2 | 3.4827 | 0.3477 | 99.9% | 1404.2522 | 1404.4764 | 1 | 5.847 | 68.2% | 1 | K.RAGELTEDEVER.V | 2 |
|  | Mis12XLAPLSS\_011913\_01.04788.04788.2 | 3.1756 | 0.1813 | 99.8% | 1249.2722 | 1248.2891 | 1 | 5.041 | 70.0% | 2 | R.AGELTEDEVER.V | 2 |
|  | Mis12XLAPLSS\_011913\_01.07281.07281.2 | 2.9267 | 0.3571 | 99.9% | 1072.1721 | 1072.311 | 1 | 6.748 | 87.5% | 4 | R.VITIMQNPR.Q | 2 |
|  | Mis12XLAPLSS\_011913\_01.11861.11861.2 | 2.9663 | 0.3135 | 99.9% | 1061.2122 | 1061.2285 | 1 | 6.357 | 85.7% | 2 | K.IPDWFLNR.Q | 2 |
|  | Mis12XLAPLSS\_011913\_01.07023.07023.2 | 4.0869 | 0.4164 | 100.0% | 1322.2522 | 1322.4606 | 1 | 7.21 | 72.7% | 3 | K.YSQVLANGLDNK.L | 2 |

---

|  |  |  |  |  |  |  |  |  |
| --- | --- | --- | --- | --- | --- | --- | --- | --- |
| U | *gi|14043072|ref|NP\_11* | 14 | 32 | 37.4% | 353 | 37430 | 8.9 | heterogeneous nuclear ribonucleoprotein A2/B1 isoform B1 [Homo sapiens] |
| U | *gi|4504447|ref|NP\_002* | 14 | 32 | 38.7% | 341 | 36006 | 8.6 | heterogeneous nuclear ribonucleoprotein A2/B1 isoform A2 [Homo sapiens] |

| Filename XCorr DeltCN Conf% ObsM+H+ CalcM+H+ SpR ZScore Ion% # Sequence  | | | | | | | | | | | | |
| --- | --- | --- | --- | --- | --- | --- | --- | --- | --- | --- | --- | --- |
|  | Mis12XLAPLSS\_011913\_01.11921.11921.2 | 4.275 | 0.1951 | 99.8% | 1928.9122 | 1928.1925 | 4 | 5.211 | 43.8% | 1 | R.KLFIGGLSFETTEESLR.N | 2 |
|  | Mis12XLAPLSS\_011913\_01.12885.12885.2 | 4.5865 | 0.4058 | 100.0% | 1800.4122 | 1800.0184 | 1 | 7.091 | 66.7% | 3 | K.LFIGGLSFETTEESLR.N | 2 |
|  | Mis12XLAPLSS\_011913\_01.13961.13961.3 | 3.7975 | 0.2201 | 98.1% | 2869.5244 | 2869.1597 | 4 | 4.207 | 29.3% | 1 | K.LFIGGLSFETTEESLRNYYEQWGK.L | 3 |
|  | Mis12XLAPLSS\_011913\_01.03257.03257.2 | 2.6548 | 0.2479 | 99.4% | 1339.2322 | 1339.4911 | 1 | 5.923 | 75.0% | 1 | R.EESGKPGAHVTVK.K | 2 |
|  | Mis12XLAPLSS\_011913\_01.03189.03189.2 | 2.5089 | 0.1636 | 96.0% | 1467.2922 | 1467.6652 | 15 | 4.296 | 57.7% | 1 | R.EESGKPGAHVTVKK.L | 2 |
|  | Mis12XLAPLSS\_011913\_01.10691.10691.2 | 3.4817 | 0.3805 | 99.9% | 1697.4321 | 1696.8132 | 1 | 6.729 | 71.4% | 2 | R.GFGFVTFDDHDPVDK.I | 2 |
|  | Mis12XLAPLSS\_011913\_01.12002.12002.3 | 3.9754 | 0.476 | 100.0% | 2278.2244 | 2278.5693 | 1 | 7.85 | 36.8% | 1 | R.GFGFVTFDDHDPVDKIVLQK.Y | 3 |
|  | Mis12XLAPLSS\_011913\_01.03472.03472.2 | 3.7303 | 0.3505 | 100.0% | 1410.5521 | 1411.5198 | 1 | 6.688 | 72.7% | 1 | K.YHTINGHNAEVR.K | 2 |
|  | Mis12XLAPLSS\_011913\_01.03300.03300.2 | 3.2251 | 0.2825 | 99.8% | 1538.5122 | 1539.6938 | 1 | 6.318 | 83.3% | 1 | K.YHTINGHNAEVRK.A | 2 |
|  | Mis12XLAPLSS\_011913\_01.03628.03628.2 | 2.5335 | 0.1728 | 98.4% | 1222.1522 | 1222.3153 | 3 | 4.715 | 77.8% | 2 | R.QEMQEVQSSR.S | 2 |
|  | Mis12XLAPLSS\_011913\_01.06903.06903.2 | 3.2009 | 0.1687 | 99.8% | 1014.0522 | 1014.0421 | 2 | 5.116 | 77.8% | 4 | R.GGNFGFGDSR.G | 2 |
|  | Mis12XLAPLSS\_011913\_01.06500.06500.2 | 3.161 | 0.4206 | 99.9% | 1379.1921 | 1378.4465 | 1 | 6.64 | 60.7% | 9 | R.GGGGNFGPGPGSNFR.G | 2 |
|  | Mis12XLAPLSS\_011913\_01.06831.06831.2 | 6.1218 | 0.6718 | 100.0% | 2190.4321 | 2191.2554 | 1 | 12.116 | 56.2% | 3 | R.NMGGPYGGGNYGPGGSGGSGGYGGR.S | 2 |
|  | Mis12XLAPLSS\_011913\_01.06950.06950.3 | 4.4738 | 0.5625 | 100.0% | 2190.5645 | 2191.2554 | 1 | 8.86 | 36.5% | 2 | R.NMGGPYGGGNYGPGGSGGSGGYGGR.S | 3 |

---

|  |  |  |  |  |  |  |  |  |
| --- | --- | --- | --- | --- | --- | --- | --- | --- |
| U | *gi|4506687|ref|NP\_001* | 4 | 5 | 37.2% | 145 | 17040 | 10.4 | ribosomal protein S15 [Homo sapiens] |

| Filename XCorr DeltCN Conf% ObsM+H+ CalcM+H+ SpR ZScore Ion% # Sequence  | | | | | | | | | | | | |
| --- | --- | --- | --- | --- | --- | --- | --- | --- | --- | --- | --- | --- |
|  | Mis12XLAPLSS\_011913\_01.16958.16958.2 | 5.6382 | 0.4325 | 100.0% | 2590.5923 | 2589.938 | 1 | 8.603 | 54.8% | 2 | R.GVDLDQLLDMSYEQLMQLYSAR.Q | 2 |
| \* | Mis12XLAPLSS\_011913\_01.14590.14590.2 | 4.821 | 0.4673 | 100.0% | 2054.652 | 2054.4856 | 1 | 7.714 | 66.7% | 1 | R.DMIILPEMVGSMVGVYNGK.T | 2 |
| \* | Mis12XLAPLSS\_011913\_01.03166.03166.2 | 2.7091 | 0.2339 | 99.4% | 1333.3922 | 1333.4525 | 2 | 5.051 | 54.2% | 1 | K.HGRPGIGATHSSR.F | 2 |
| \* | Mis12XLAPLSS\_011913\_01.03167.03167.3 | 4.1705 | 0.3096 | 100.0% | 1333.5844 | 1333.4525 | 1 | 7.528 | 54.2% | 1 | K.HGRPGIGATHSSR.F | 3 |

---

|  |  |  |  |  |  |  |  |  |
| --- | --- | --- | --- | --- | --- | --- | --- | --- |
| U | *gi|21464101|ref|NP\_03* | 6 | 9 | 36.4% | 247 | 28303 | 4.9 | tyrosine 3-monooxygenase/tryptophan 5-monooxygenase activation protein, gamma polypeptide [Homo sapiens] |

| Filename XCorr DeltCN Conf% ObsM+H+ CalcM+H+ SpR ZScore Ion% # Sequence  | | | | | | | | | | | | |
| --- | --- | --- | --- | --- | --- | --- | --- | --- | --- | --- | --- | --- |
|  | Mis12XLAPLSS\_011913\_01.08100.08100.2 | 3.8296 | 0.4533 | 100.0% | 1813.9321 | 1814.0382 | 1 | 7.965 | 56.7% | 1 | R.LAEQAERYDDMAAAMK.N | 22 |
| \* | Mis12XLAPLSS\_011913\_01.07550.07550.2 | 4.9301 | 0.5206 | 100.0% | 1644.2522 | 1644.7356 | 1 | 8.649 | 76.9% | 3 | K.NVTELNEPLSNEER.N | 2 |
| \* | Mis12XLAPLSS\_011913\_01.04320.04320.2 | 2.6855 | 0.3688 | 99.8% | 1237.4521 | 1237.3983 | 1 | 5.497 | 75.0% | 1 | R.YLAEVATGEKR.A | 2 |
| \* | Mis12XLAPLSS\_011913\_01.03491.03491.2 | 3.0613 | 0.335 | 99.9% | 1135.3121 | 1135.2188 | 1 | 5.32 | 66.7% | 1 | K.AYSEAHEISK.E | 2 |
| \* | Mis12XLAPLSS\_011913\_01.03401.03401.2 | 2.5019 | 0.37 | 99.8% | 1245.5922 | 1246.4294 | 80 | 6.283 | 55.6% | 1 | K.EHMQPTHPIR.L | 2 |
| \* | Mis12XLAPLSS\_011913\_01.17234.17234.3 | 5.1272 | 0.4316 | 100.0% | 3302.7244 | 3303.6626 | 1 | 8.099 | 30.4% | 2 | K.TAFDDAIAELDTLNEDSYKDSTLIMQLLR.D | 3 |

Similarities:
gi|21328448|ref|NP\_64(1:5)  

---

|  |  |  |  |  |  |  |  |  |
| --- | --- | --- | --- | --- | --- | --- | --- | --- |
| U | *gi|193794814|ref|NP\_0* | 12 | 21 | 36.3% | 364 | 39420 | 8.1 | fructose-bisphosphate aldolase A [Homo sapiens] |
| U | *gi|4557305|ref|NP\_000* | 12 | 21 | 36.3% | 364 | 39420 | 8.1 | fructose-bisphosphate aldolase A [Homo sapiens] |
| U | *gi|34577112|ref|NP\_90* | 12 | 21 | 36.3% | 364 | 39420 | 8.1 | fructose-bisphosphate aldolase A [Homo sapiens] |
| U | *gi|34577110|ref|NP\_90* | 12 | 21 | 36.3% | 364 | 39420 | 8.1 | fructose-bisphosphate aldolase A [Homo sapiens] |

| Filename XCorr DeltCN Conf% ObsM+H+ CalcM+H+ SpR ZScore Ion% # Sequence  | | | | | | | | | | | | |
| --- | --- | --- | --- | --- | --- | --- | --- | --- | --- | --- | --- | --- |
|  | Mis12XLAPLSS\_011913\_01.04042.04042.2 | 2.0405 | 0.1834 | 95.2% | 941.1922 | 941.03174 | 81 | 5.095 | 64.3% | 2 | K.ELSDIAHR.I | 2 |
|  | Mis12XLAPLSS\_011913\_02.04774.04774.2 | 4.3258 | 0.5279 | 100.0% | 1333.4122 | 1333.4814 | 1 | 9.63 | 76.9% | 5 | K.GILAADESTGSIAK.R | 2 |
|  | Mis12XLAPLSS\_011913\_01.05472.05472.2 | 3.1114 | 0.3557 | 99.8% | 1489.7922 | 1489.669 | 1 | 5.812 | 64.3% | 1 | K.GILAADESTGSIAKR.L | 2 |
|  | Mis12XLAPLSS\_011913\_01.03906.03906.2 | 2.9668 | 0.192 | 99.3% | 1646.7322 | 1647.7422 | 144 | 4.614 | 46.2% | 1 | K.RLQSIGTENTEENR.R | 2 |
|  | Mis12XLAPLSS\_011913\_01.07646.07646.3 | 2.5733 | 0.3276 | 99.8% | 1343.7843 | 1343.5254 | 28 | 5.972 | 40.9% | 1 | K.ADDGRPFPQVIK.S | 3 |
|  | Mis12XLAPLSS\_011913\_01.11972.11972.3 | 4.1393 | 0.3896 | 100.0% | 2107.6143 | 2108.4204 | 1 | 6.887 | 35.5% | 3 | K.IGEHTPSALAIMENANVLAR.Y | 3 |
|  | Mis12XLAPLSS\_011913\_01.11981.11981.2 | 5.2091 | 0.4452 | 100.0% | 2108.412 | 2108.4204 | 1 | 8.11 | 65.8% | 1 | K.IGEHTPSALAIMENANVLAR.Y | 2 |
|  | Mis12XLAPLSS\_011913\_01.04799.04799.1 | 1.7363 | 0.2297 | 95.1% | 763.53 | 763.95544 | 2 | 5.268 | 66.7% | 1 | K.VLAAVYK.A | 11 |
|  | Mis12XLAPLSS\_011913\_01.09915.09915.2 | 4.9098 | 0.5201 | 100.0% | 1676.2722 | 1676.9281 | 1 | 9.249 | 78.6% | 1 | K.FSHEEIAMATVTALR.R | 2 |
|  | Mis12XLAPLSS\_011913\_01.03380.03380.2 | 3.1693 | 0.3298 | 99.9% | 1094.0922 | 1094.2125 | 7 | 6.007 | 75.0% | 1 | K.AAQEEYVKR.A | 2 |
|  | Mis12XLAPLSS\_011913\_02.07090.07090.3 | 4.4555 | 0.4204 | 100.0% | 3341.9043 | 3343.6 | 1 | 8.287 | 21.9% | 2 | R.ALANSLACQGKYTPSGQAGAAASESLFVSNHAY.- | 3 |
|  | Mis12XLAPLSS\_011913\_01.09911.09911.2 | 5.6556 | 0.5399 | 100.0% | 2228.8323 | 2229.3672 | 1 | 10.075 | 54.8% | 2 | K.YTPSGQAGAAASESLFVSNHAY.- | 2 |

Similarities:
gi|4885063|ref|NP\_005(1:11)  

---

|  |  |  |  |  |  |  |  |  |
| --- | --- | --- | --- | --- | --- | --- | --- | --- |
| U | *gi|66392203|ref|NP\_00* | 9 | 14 | 36.3% | 267 | 30137 | 8.9 | NME1-NME2 protein [Homo sapiens] |

| Filename XCorr DeltCN Conf% ObsM+H+ CalcM+H+ SpR ZScore Ion% # Sequence  | | | | | | | | | | | | |
| --- | --- | --- | --- | --- | --- | --- | --- | --- | --- | --- | --- | --- |
|  | Mis12XLAPLSS\_011913\_01.07478.07478.2 | 3.3745 | 0.2477 | 99.8% | 1346.1721 | 1345.5846 | 1 | 5.657 | 63.6% | 3 | R.TFIAIKPDGVQR.G | 22 |
|  | Mis12XLAPLSS\_011913\_01.10235.10235.2 | 2.3937 | 0.282 | 99.5% | 1150.1921 | 1150.3658 | 9 | 4.611 | 61.1% | 1 | K.DRPFFAGLVK.Y | 22 |
|  | Mis12XLAPLSS\_011913\_01.06041.06041.3 | 2.537 | 0.3747 | 99.8% | 1786.3143 | 1787.041 | 1 | 5.724 | 39.1% | 1 | R.VMLGETNPADSKPGTIR.G | 33 |
|  | Mis12XLAPLSS\_011913\_01.05949.05949.2 | 4.3476 | 0.3567 | 100.0% | 1787.3121 | 1787.041 | 1 | 6.921 | 62.5% | 3 | R.VMLGETNPADSKPGTIR.G | 22 |
|  | Mis12XLAPLSS\_011913\_01.09879.09879.2 | 2.5721 | 0.2692 | 99.6% | 1176.4122 | 1176.4038 | 12 | 5.085 | 66.7% | 1 | K.DRPFFPGLVK.Y | 2 |
|  | Mis12XLAPLSS\_011913\_01.14908.14908.2 | 5.0213 | 0.5447 | 100.0% | 2093.672 | 2094.4944 | 1 | 9.309 | 52.8% | 2 | K.YMNSGPVVAMVWEGLNVVK.T | 2 |
|  | Mis12XLAPLSS\_011913\_01.03682.03682.1 | 2.1285 | 0.3909 | 100.0% | 1069.51 | 1070.1906 | 1 | 6.828 | 72.2% | 1 | R.NIIHGSDSVK.S | 1 |
|  | Mis12XLAPLSS\_011913\_01.12240.12240.3 | 3.5187 | 0.2392 | 99.2% | 2313.2644 | 2312.6245 | 1 | 4.789 | 30.6% | 1 | K.SAEKEISLWFKPEELVDYK.S | 3 |
|  | Mis12XLAPLSS\_011913\_01.12837.12837.2 | 3.37 | 0.3387 | 99.8% | 1896.2922 | 1897.1779 | 1 | 6.919 | 46.4% | 1 | K.EISLWFKPEELVDYK.S | 2 |

Similarities:
gi|38045913|ref|NP\_93(4:5)  

---

|  |  |  |  |  |  |  |  |  |
| --- | --- | --- | --- | --- | --- | --- | --- | --- |
| U | *gi|5729877|ref|NP\_006* | 26 | 49 | 36.2% | 646 | 70898 | 5.5 | heat shock 70kDa protein 8 isoform 1 [Homo sapiens] |

| Filename XCorr DeltCN Conf% ObsM+H+ CalcM+H+ SpR ZScore Ion% # Sequence  | | | | | | | | | | | | |
| --- | --- | --- | --- | --- | --- | --- | --- | --- | --- | --- | --- | --- |
|  | Mis12XLAPLSS\_011913\_02.05422.05422.2 | 3.1076 | 0.4678 | 100.0% | 1488.2722 | 1488.5939 | 1 | 8.504 | 70.8% | 3 | R.TTPSYVAFTDTER.L | 2222 |
|  | Mis12XLAPLSS\_011913\_01.08882.08882.2 | 4.5799 | 0.4867 | 100.0% | 1650.2122 | 1650.8468 | 1 | 9.977 | 78.6% | 2 | K.NQVAMNPTNTVFDAK.R | 2 |
|  | Mis12XLAPLSS\_011913\_01.07799.07799.2 | 4.1307 | 0.3801 | 100.0% | 1806.2522 | 1807.0343 | 1 | 6.838 | 60.0% | 1 | K.NQVAMNPTNTVFDAKR.L | 2 |
|  | Mis12XLAPLSS\_011913\_01.05943.05943.2 | 3.4896 | 0.4819 | 100.0% | 1410.9122 | 1411.5725 | 1 | 7.804 | 77.3% | 3 | R.RFDDAVVQSDMK.H | 2 |
|  | Mis12XLAPLSS\_011913\_01.07341.07341.2 | 3.6557 | 0.5024 | 100.0% | 1254.7322 | 1255.385 | 1 | 8.018 | 85.0% | 2 | R.FDDAVVQSDMK.H | 2 |
|  | Mis12XLAPLSS\_011913\_01.09314.09314.3 | 3.8546 | 0.3819 | 100.0% | 1654.8544 | 1654.9298 | 11 | 6.153 | 38.5% | 2 | K.HWPFMVVNDAGRPK.V | 3 |
|  | Mis12XLAPLSS\_011913\_01.03633.03633.2 | 2.7539 | 0.3196 | 99.8% | 1180.4521 | 1181.3312 | 1 | 6.119 | 77.8% | 1 | K.VQVEYKGETK.S | 2 |
|  | Mis12XLAPLSS\_011913\_01.11811.11811.2 | 3.4194 | 0.5536 | 100.0% | 1616.6921 | 1617.8542 | 1 | 8.489 | 73.1% | 3 | K.SFYPEEVSSMVLTK.M | 2 |
|  | Mis12XLAPLSS\_011913\_01.07551.07551.2 | 3.6448 | 0.2653 | 99.9% | 1253.1721 | 1253.4993 | 1 | 5.902 | 85.0% | 2 | K.MKEIAEAYLGK.T | 2 |
|  | Mis12XLAPLSS\_011913\_01.10229.10229.2 | 3.9012 | 0.3806 | 100.0% | 1983.1921 | 1983.1882 | 1 | 7.803 | 55.9% | 2 | K.TVTNAVVTVPAYFNDSQR.Q | 2 |
|  | Mis12XLAPLSS\_011913\_01.10821.10821.2 | 4.3886 | 0.3529 | 100.0% | 1662.2522 | 1660.9078 | 1 | 8.101 | 80.0% | 3 | R.IINEPTAAAIAYGLDK.K | 222 |
|  | Mis12XLAPLSS\_011913\_01.09681.09681.2 | 3.9126 | 0.3563 | 99.8% | 1787.9922 | 1789.0819 | 1 | 6.306 | 62.5% | 2 | R.IINEPTAAAIAYGLDKK.V | 2 |
|  | Mis12XLAPLSS\_011913\_01.04947.04947.2 | 3.8655 | 0.5115 | 100.0% | 1692.2322 | 1692.6958 | 1 | 8.677 | 56.7% | 1 | K.STAGDTHLGGEDFDNR.M | 2 |
|  | Mis12XLAPLSS\_011913\_01.09053.09053.3 | 3.3885 | 0.3484 | 100.0% | 1236.5643 | 1236.4741 | 1 | 5.885 | 52.8% | 1 | R.MVNHFIAEFK.R | 3 |
|  | Mis12XLAPLSS\_011913\_01.09039.09039.2 | 3.3129 | 0.4935 | 100.0% | 1236.8922 | 1236.4741 | 1 | 8.279 | 88.9% | 3 | R.MVNHFIAEFK.R | 2 |
|  | Mis12XLAPLSS\_011913\_01.07887.07887.2 | 2.8681 | 0.0772 | 97.4% | 1392.2522 | 1392.6616 | 1 | 5.069 | 75.0% | 1 | R.MVNHFIAEFKR.K | 2 |
|  | Mis12XLAPLSS\_011913\_01.07911.07911.3 | 2.7169 | 0.2419 | 97.6% | 1392.7144 | 1392.6616 | 1 | 5.192 | 52.5% | 1 | R.MVNHFIAEFKR.K | 3 |
|  | Mis12XLAPLSS\_011913\_01.10232.10232.2 | 3.3834 | 0.3567 | 99.9% | 1481.2722 | 1481.6511 | 1 | 6.077 | 77.3% | 1 | R.ARFEELNADLFR.G | 2 |
|  | Mis12XLAPLSS\_011913\_01.11350.11350.2 | 2.8253 | 0.3868 | 99.9% | 1253.7722 | 1254.3849 | 4 | 6.874 | 61.1% | 2 | R.FEELNADLFR.G | 2 |
|  | Mis12XLAPLSS\_011913\_01.08498.08498.3 | 4.4374 | 0.4427 | 100.0% | 1838.7544 | 1839.1019 | 6 | 6.956 | 35.9% | 2 | K.LDKSQIHDIVLVGGSTR.I | 3 |
|  | Mis12XLAPLSS\_011913\_02.05903.05903.2 | 4.1988 | 0.574 | 100.0% | 1482.0122 | 1482.6798 | 1 | 9.423 | 73.1% | 4 | K.SQIHDIVLVGGSTR.I | 2 |
|  | Mis12XLAPLSS\_011913\_01.10815.10815.2 | 2.5878 | 0.3949 | 99.8% | 1082.2322 | 1082.2444 | 1 | 5.822 | 81.2% | 2 | K.LLQDFFNGK.E | 22 |
|  | Mis12XLAPLSS\_011913\_01.03447.03447.2 | 2.4481 | 0.4255 | 99.8% | 1017.4922 | 1018.1582 | 6 | 6.628 | 62.5% | 1 | K.ITITNDKGR.L | 2222 |
| \* | Mis12XLAPLSS\_011913\_01.03717.03717.2 | 2.7628 | 0.0826 | 98.6% | 990.0522 | 990.10144 | 50 | 4.865 | 71.4% | 1 | R.LSKEDIER.M | 2 |
| \* | Mis12XLAPLSS\_011913\_01.03538.03538.3 | 4.29 | 0.2846 | 100.0% | 1982.8744 | 1983.2036 | 1 | 7.025 | 41.7% | 1 | R.MVQEAEKYKAEDEKQR.D | 3 |
| \* | Mis12XLAPLSS\_011913\_01.09872.09872.2 | 2.7173 | 0.4653 | 99.9% | 1303.8722 | 1304.4602 | 2 | 6.881 | 75.0% | 2 | K.NSLESYAFNMK.A | 2 |

Similarities:
gi|167466173|ref|NP\_0(2:24)  
contaminant\_GR78\_HUMA(1:25)  
gi|124256496|ref|NP\_0(3:23)  
gi|34419635|ref|NP\_00(3:23)  

---

|  |  |  |  |  |  |  |  |  |
| --- | --- | --- | --- | --- | --- | --- | --- | --- |
| U | *gi|4506645|ref|NP\_000* | 3 | 5 | 35.7% | 70 | 8218 | 10.1 | ribosomal protein L38 [Homo sapiens] |
| U | *gi|78214522|ref|NP\_00* | 3 | 5 | 35.7% | 70 | 8218 | 10.1 | ribosomal protein L38 [Homo sapiens] |

| Filename XCorr DeltCN Conf% ObsM+H+ CalcM+H+ SpR ZScore Ion% # Sequence  | | | | | | | | | | | | |
| --- | --- | --- | --- | --- | --- | --- | --- | --- | --- | --- | --- | --- |
|  | Mis12XLAPLSS\_011913\_01.10269.10269.3 | 3.9685 | 0.338 | 100.0% | 1576.1643 | 1576.8766 | 26 | 6.342 | 43.8% | 1 | R.KIEEIKDFLLTAR.R | 3 |
|  | Mis12XLAPLSS\_011913\_01.10281.10281.2 | 4.8896 | 0.3812 | 100.0% | 1576.4922 | 1576.8766 | 1 | 7.283 | 70.8% | 3 | R.KIEEIKDFLLTAR.R | 2 |
|  | Mis12XLAPLSS\_011913\_01.09483.09483.2 | 3.2057 | 0.3367 | 99.8% | 1486.1122 | 1486.7484 | 1 | 6.631 | 77.3% | 1 | R.YLYTLVITDKEK.A | 2 |

---

|  |  |  |  |  |  |  |  |  |
| --- | --- | --- | --- | --- | --- | --- | --- | --- |
| U | *gi|4505763|ref|NP\_000* | 11 | 12 | 35.3% | 417 | 44615 | 8.1 | phosphoglycerate kinase 1 [Homo sapiens] |

| Filename XCorr DeltCN Conf% ObsM+H+ CalcM+H+ SpR ZScore Ion% # Sequence  | | | | | | | | | | | | |
| --- | --- | --- | --- | --- | --- | --- | --- | --- | --- | --- | --- | --- |
| \* | Mis12XLAPLSS\_011913\_01.11375.11375.3 | 5.9649 | 0.4308 | 100.0% | 3264.5645 | 3265.8538 | 1 | 6.923 | 30.2% | 1 | K.SVVLMSHLGRPDGVPMPDKYSLEPVAVELK.S | 3 |
| \* | Mis12XLAPLSS\_011913\_01.03320.03320.3 | 2.7101 | 0.284 | 97.9% | 1732.5543 | 1732.8473 | 3 | 5.151 | 38.3% | 1 | R.FHVEEEGKGKDASGNK.V | 3 |
| \* | Mis12XLAPLSS\_011913\_01.03339.03339.3 | 3.4218 | 0.2754 | 99.8% | 1959.8043 | 1960.1539 | 1 | 5.861 | 35.3% | 1 | R.FHVEEEGKGKDASGNKVK.A | 3 |
|  | Mis12XLAPLSS\_011913\_02.06386.06386.3 | 3.3485 | 0.4473 | 100.0% | 1634.9644 | 1635.7764 | 2 | 7.042 | 42.9% | 1 | K.LGDVYVNDAFGTAHR.A | 3 |
|  | Mis12XLAPLSS\_011913\_01.08957.08957.2 | 3.9773 | 0.4904 | 100.0% | 1635.0721 | 1635.7764 | 1 | 9.367 | 78.6% | 1 | K.LGDVYVNDAFGTAHR.A | 2 |
| \* | Mis12XLAPLSS\_011913\_01.05228.05228.2 | 4.0649 | 0.5216 | 100.0% | 1368.2122 | 1368.5939 | 1 | 9.412 | 79.2% | 1 | R.AHSSMVGVNLPQK.A | 2 |
|  | Mis12XLAPLSS\_011913\_01.12256.12256.2 | 4.035 | 0.3745 | 100.0% | 1769.3522 | 1770.0819 | 1 | 6.396 | 50.0% | 1 | K.ALESPERPFLAILGGAK.V | 2 |
|  | Mis12XLAPLSS\_011913\_01.12261.12261.3 | 3.1167 | 0.4085 | 100.0% | 1770.3844 | 1770.0819 | 74 | 6.16 | 32.8% | 1 | K.ALESPERPFLAILGGAK.V | 3 |
| \* | Mis12XLAPLSS\_011913\_01.11927.11927.2 | 4.4635 | 0.5502 | 100.0% | 2023.2322 | 2024.2781 | 1 | 9.565 | 52.9% | 1 | K.ITLPVDFVTADKFDENAK.T | 2 |
|  | Mis12XLAPLSS\_011913\_01.14568.14568.2 | 4.0229 | 0.4609 | 100.0% | 2105.872 | 2106.3909 | 1 | 7.886 | 38.2% | 2 | K.QIVWNGPVGVFEWEAFAR.G | 2 |
|  | Mis12XLAPLSS\_011913\_02.06408.06408.3 | 2.7643 | 0.2945 | 98.4% | 1741.8544 | 1741.939 | 15 | 5.14 | 30.9% | 1 | K.VSHVSTGGGASLELLEGK.V | 3 |

---

|  |  |  |  |  |  |  |  |  |
| --- | --- | --- | --- | --- | --- | --- | --- | --- |
| U | *gi|17986258|ref|NP\_06* | 5 | 12 | 35.1% | 151 | 16930 | 4.7 | myosin, light chain 6, alkali, smooth muscle and non-muscle isoform 1 [Homo sapiens] |
| U | *gi|88999583|ref|NP\_52* | 5 | 12 | 35.1% | 151 | 16961 | 4.6 | myosin, light chain 6, alkali, smooth muscle and non-muscle isoform 2 [Homo sapiens] |

| Filename XCorr DeltCN Conf% ObsM+H+ CalcM+H+ SpR ZScore Ion% # Sequence  | | | | | | | | | | | | |
| --- | --- | --- | --- | --- | --- | --- | --- | --- | --- | --- | --- | --- |
|  | Mis12XLAPLSS\_011913\_01.05954.05954.2 | 3.9612 | 0.3413 | 100.0% | 1356.1921 | 1355.5339 | 1 | 6.285 | 70.8% | 4 | R.ALGQNPTNAEVLK.V | 2 |
|  | Mis12XLAPLSS\_011913\_01.14009.14009.2 | 4.3473 | 0.5102 | 100.0% | 1887.9722 | 1889.2628 | 1 | 8.517 | 60.0% | 2 | K.VLDFEHFLPMLQTVAK.N | 2 |
|  | Mis12XLAPLSS\_011913\_01.14032.14032.3 | 4.7614 | 0.3985 | 100.0% | 1889.2444 | 1889.2628 | 3 | 6.589 | 45.0% | 2 | K.VLDFEHFLPMLQTVAK.N | 3 |
|  | Mis12XLAPLSS\_011913\_01.08919.08919.2 | 4.0067 | 0.4955 | 100.0% | 1787.2322 | 1787.8804 | 1 | 7.356 | 60.7% | 1 | K.NKDQGTYEDYVEGLR.V | 2 |
|  | Mis12XLAPLSS\_011913\_01.04934.04934.2 | 2.4457 | 0.2113 | 99.1% | 996.2522 | 996.1949 | 1 | 6.661 | 81.2% | 3 | R.HVLVTLGEK.M | 2 |

---

|  |  |  |  |  |  |  |  |  |
| --- | --- | --- | --- | --- | --- | --- | --- | --- |
| U | *gi|4506699|ref|NP\_001* | 4 | 13 | 34.9% | 83 | 9111 | 8.5 | ribosomal protein S21 [Homo sapiens] |

| Filename XCorr DeltCN Conf% ObsM+H+ CalcM+H+ SpR ZScore Ion% # Sequence  | | | | | | | | | | | | |
| --- | --- | --- | --- | --- | --- | --- | --- | --- | --- | --- | --- | --- |
| \* | Mis12XLAPLSS\_011913\_01.08781.08781.2 | 5.2326 | 0.5731 | 100.0% | 1970.0122 | 1971.1956 | 1 | 10.072 | 64.7% | 1 | K.DHASIQMNVAEVDKVTGR.F | 2 |
| \* | Mis12XLAPLSS\_011913\_02.06173.06173.3 | 5.2003 | 0.4351 | 100.0% | 1970.2743 | 1971.1956 | 1 | 8.564 | 38.2% | 4 | K.DHASIQMNVAEVDKVTGR.F | 3 |
| \* | Mis12XLAPLSS\_011913\_01.04913.04913.2 | 2.925 | 0.261 | 99.8% | 1279.5322 | 1279.4106 | 1 | 5.311 | 80.0% | 1 | R.RMGESDDSILR.L | 2 |
| \* | Mis12XLAPLSS\_011913\_01.06614.06614.2 | 3.7266 | 0.4815 | 100.0% | 1122.9122 | 1123.2231 | 1 | 8.864 | 88.9% | 7 | R.MGESDDSILR.L | 2 |

---

|  |  |  |  |  |  |  |  |  |
| --- | --- | --- | --- | --- | --- | --- | --- | --- |
| U | *gi|20149594|ref|NP\_03* | 23 | 43 | 34.3% | 724 | 83264 | 5.0 | heat shock 90kDa protein 1, beta [Homo sapiens] |

| Filename XCorr DeltCN Conf% ObsM+H+ CalcM+H+ SpR ZScore Ion% # Sequence  | | | | | | | | | | | | |
| --- | --- | --- | --- | --- | --- | --- | --- | --- | --- | --- | --- | --- |
|  | Mis12XLAPLSS\_011913\_01.07862.07862.2 | 2.8237 | 0.407 | 99.8% | 1276.0322 | 1276.3861 | 15 | 6.409 | 54.5% | 2 | R.ELISNASDALDK.I | 2 |
|  | Mis12XLAPLSS\_011913\_01.09339.09339.2 | 3.2069 | 0.3213 | 99.8% | 1545.3322 | 1545.733 | 1 | 5.662 | 61.5% | 1 | R.ELISNASDALDKIR.Y | 2 |
|  | Mis12XLAPLSS\_011913\_01.09962.09962.2 | 3.6701 | 0.488 | 100.0% | 1242.6122 | 1243.4459 | 1 | 8.954 | 72.7% | 1 | K.ADLINNLGTIAK.S | 22 |
|  | Mis12XLAPLSS\_011913\_02.06044.06044.3 | 4.4058 | 0.4311 | 100.0% | 2015.8744 | 2016.2584 | 1 | 7.628 | 48.3% | 4 | K.VILHLKEDQTEYLEER.R | 33 |
| \* | Mis12XLAPLSS\_011913\_01.12311.12311.2 | 3.6412 | 0.4276 | 100.0% | 1810.5122 | 1810.1027 | 1 | 7.713 | 67.9% | 1 | K.HSQFIGYPITLYLEK.E | 2 |
| \* | Mis12XLAPLSS\_011913\_01.11546.11546.3 | 4.7023 | 0.4202 | 100.0% | 2095.4944 | 2095.4058 | 2 | 7.515 | 40.6% | 2 | K.HSQFIGYPITLYLEKER.E | 3 |
| \* | Mis12XLAPLSS\_011913\_01.03555.03555.3 | 3.2342 | 0.2407 | 98.3% | 1945.9143 | 1946.8871 | 1 | 4.453 | 42.2% | 1 | K.IEDVGS\*DEEDDSGKDKK.K | 3 |
|  | Mis12XLAPLSS\_011913\_01.04832.04832.2 | 3.4034 | 0.2713 | 99.9% | 1151.9521 | 1152.2462 | 1 | 5.333 | 81.2% | 3 | K.YIDQEELNK.T | 22 |
| \* | Mis12XLAPLSS\_011913\_02.06674.06674.2 | 5.127 | 0.4896 | 100.0% | 1848.1921 | 1848.9171 | 1 | 8.694 | 82.1% | 3 | R.NPDDITQEEYGEFYK.S | 2 |
|  | Mis12XLAPLSS\_011913\_01.09360.09360.2 | 4.3397 | 0.4125 | 100.0% | 1528.4922 | 1528.6616 | 1 | 7.008 | 66.7% | 4 | K.SLTNDWEDHLAVK.H | 22 |
|  | Mis12XLAPLSS\_011913\_01.09105.09105.2 | 3.5028 | 0.4222 | 100.0% | 1349.9122 | 1349.4886 | 2 | 7.281 | 70.0% | 3 | K.HFSVEGQLEFR.A | 22 |
| \* | Mis12XLAPLSS\_011913\_01.10072.10072.2 | 3.1995 | 0.1838 | 99.8% | 1237.8121 | 1237.4008 | 1 | 5.167 | 72.2% | 2 | R.RAPFDLFENK.K | 2 |
| \* | Mis12XLAPLSS\_011913\_01.07280.07280.2 | 1.819 | 0.2542 | 96.2% | 891.9122 | 891.99884 | 36 | 4.129 | 75.0% | 1 | K.FYEAFSK.N | 2 |
| \* | Mis12XLAPLSS\_011913\_01.03650.03650.2 | 3.0049 | 0.4311 | 100.0% | 1142.1921 | 1142.2137 | 4 | 7.424 | 66.7% | 1 | K.LGIHEDSTNR.R | 2 |
| \* | Mis12XLAPLSS\_011913\_02.06590.06590.3 | 4.6187 | 0.4371 | 100.0% | 2177.4243 | 2178.2915 | 1 | 7.702 | 37.5% | 3 | R.YHTSQSGDEMTSLSEYVSR.M | 3 |
| \* | Mis12XLAPLSS\_011913\_01.07784.07784.2 | 2.5868 | 0.3803 | 99.8% | 1161.0122 | 1161.297 | 1 | 7.67 | 72.2% | 1 | K.SIYYITGESK.E | 2 |
| \* | Mis12XLAPLSS\_011913\_01.05236.05236.2 | 3.0161 | 0.4322 | 99.8% | 1250.0122 | 1250.3538 | 4 | 6.978 | 65.0% | 2 | K.EQVANSAFVER.V | 2 |
| \* | Mis12XLAPLSS\_011913\_02.04948.04948.2 | 2.9988 | 0.4072 | 99.9% | 1249.4722 | 1249.4574 | 1 | 7.572 | 70.0% | 1 | R.DNSTMGYMMAK.K | 2 |
| \* | Mis12XLAPLSS\_011913\_01.07580.07580.3 | 3.4125 | 0.3732 | 100.0% | 1911.9543 | 1912.1991 | 18 | 6.101 | 33.3% | 1 | K.KHLEINPDHPIVETLR.Q | 3 |
| \* | Mis12XLAPLSS\_011913\_01.08667.08667.2 | 4.2904 | 0.4198 | 100.0% | 1783.4321 | 1784.025 | 1 | 7.953 | 60.7% | 1 | K.HLEINPDHPIVETLR.Q | 2 |
| \* | Mis12XLAPLSS\_011913\_01.08638.08638.3 | 4.0535 | 0.5003 | 100.0% | 1784.6943 | 1784.025 | 1 | 7.595 | 44.6% | 3 | K.HLEINPDHPIVETLR.Q | 3 |
| \* | Mis12XLAPLSS\_011913\_01.17506.17506.3 | 5.6574 | 0.3985 | 100.0% | 3289.1343 | 3288.725 | 1 | 7.107 | 31.9% | 1 | K.AVKDLVVLLFETALLSSGFSLEDPQTHSNR.I | 3 |
| \* | Mis12XLAPLSS\_011913\_01.17858.17858.3 | 3.6879 | 0.4208 | 100.0% | 2990.0942 | 2990.3398 | 2 | 6.261 | 25.0% | 1 | K.DLVVLLFETALLSSGFSLEDPQTHSNR.I | 3 |

Similarities:
gi|153792590|ref|NP\_0(5:18)  

---

|  |  |  |  |  |  |  |  |  |
| --- | --- | --- | --- | --- | --- | --- | --- | --- |
| U | *gi|119395750|ref|NP\_0* | 14 | 20 | 34.3% | 644 | 66039 | 8.1 | keratin 1 [Homo sapiens] |

| Filename XCorr DeltCN Conf% ObsM+H+ CalcM+H+ SpR ZScore Ion% # Sequence  | | | | | | | | | | | | |
| --- | --- | --- | --- | --- | --- | --- | --- | --- | --- | --- | --- | --- |
|  | Mis12XLAPLSS\_011913\_01.10996.10996.2 | 3.38 | 0.4381 | 100.0% | 1384.1921 | 1384.5315 | 1 | 7.621 | 63.6% | 2 | K.SLNNQFASFIDK.V | 2 |
|  | Mis12XLAPLSS\_011913\_01.07400.07400.2 | 4.339 | 0.0625 | 99.8% | 1476.1522 | 1476.6726 | 1 | 6.654 | 90.9% | 3 | R.FLEQQNQVLQTK.W | 22 |
|  | Mis12XLAPLSS\_011913\_02.07540.07540.2 | 3.5701 | 0.3554 | 99.8% | 1476.2122 | 1476.6293 | 1 | 7.327 | 81.8% | 1 | K.WELLQQVDTSTR.T | 2 |
|  | Mis12XLAPLSS\_011913\_01.13251.13251.2 | 2.9127 | 0.2091 | 99.3% | 1995.4321 | 1995.2017 | 1 | 4.813 | 53.3% | 1 | R.THNLEPYFESFINNLR.R | 2 |
|  | Mis12XLAPLSS\_011913\_01.03807.03807.2 | 2.6964 | 0.2533 | 99.9% | 1309.1721 | 1309.4215 | 1 | 5.17 | 72.2% | 1 | R.NKYEDEINKR.T | 222 |
| \* | Mis12XLAPLSS\_011913\_01.13080.13080.2 | 3.8508 | 0.4989 | 100.0% | 1303.3922 | 1303.4955 | 1 | 9.504 | 86.4% | 1 | R.SLDLDSIIAEVK.A | 2 |
|  | Mis12XLAPLSS\_011913\_01.03946.03946.2 | 3.7516 | 0.4427 | 100.0% | 1340.4122 | 1341.4607 | 1 | 7.665 | 68.2% | 1 | K.SKAEAESLYQSK.Y | 2 |
|  | Mis12XLAPLSS\_011913\_02.05747.05747.2 | 3.574 | 0.2762 | 99.9% | 1180.1322 | 1180.303 | 2 | 6.851 | 83.3% | 4 | K.YEELQITAGR.H | 22 |
|  | Mis12XLAPLSS\_011913\_01.07412.07412.2 | 2.2297 | 0.157 | 97.1% | 974.0122 | 974.102 | 39 | 3.753 | 71.4% | 1 | K.IEISELNR.V | 22 |
|  | Mis12XLAPLSS\_011913\_01.09827.09827.2 | 3.7253 | 0.3776 | 100.0% | 1601.3522 | 1600.769 | 1 | 6.552 | 76.9% | 1 | K.NKLNDLEDALQQAK.E | 2 |
| \* | Mis12XLAPLSS\_011913\_01.12341.12341.3 | 4.9349 | 0.3584 | 100.0% | 2184.8943 | 2185.399 | 1 | 6.38 | 47.2% | 1 | K.NKLNDLEDALQQAKEDLAR.L | 3 |
|  | Mis12XLAPLSS\_011913\_02.04127.04127.3 | 3.5483 | 0.3662 | 100.0% | 2384.7244 | 2385.298 | 1 | 6.639 | 28.3% | 1 | R.GGGGGGYGSGGSSYGSGGGSYGSGGGGGGGR.G | 3 |
| \* | Mis12XLAPLSS\_011913\_02.04434.04434.3 | 4.2596 | 0.4395 | 100.0% | 3313.2544 | 3314.2085 | 1 | 6.236 | 18.4% | 1 | R.GSYGSGGSSYGSGGGSYGSGGGGGGHGSYGSGSSSGGYR.G | 3 |
| \* | Mis12XLAPLSS\_011913\_01.10148.10148.3 | 3.6241 | 0.1947 | 95.5% | 2240.2744 | 2241.0396 | 2 | 3.953 | 32.4% | 1 | R.GGSGGGGGGS\*S\*GGRGSGGGSSGGSIGGR.G | 3 |

Similarities:
contaminant\_KERATIN20(1:13)  
contaminant\_KERATIN22(2:12)  
gi|119703753|ref|NP\_0(2:12)  

---

|  |  |  |  |  |  |  |  |  |
| --- | --- | --- | --- | --- | --- | --- | --- | --- |
| U | *gi|163965362|ref|NP\_0* | 5 | 8 | 34.0% | 215 | 23384 | 4.6 | nascent polypeptide-associated complex alpha subunit isoform b [Homo sapiens] |
| U | *gi|5031931|ref|NP\_005* | 5 | 8 | 34.0% | 215 | 23384 | 4.6 | nascent polypeptide-associated complex alpha subunit isoform b [Homo sapiens] |
| U | *gi|163965366|ref|NP\_0* | 5 | 8 | 3.5% | 2078 | 205419 | 9.6 | nascent polypeptide-associated complex alpha subunit isoform a [Homo sapiens] |
| U | *gi|163965364|ref|NP\_0* | 5 | 8 | 34.0% | 215 | 23384 | 4.6 | nascent polypeptide-associated complex alpha subunit isoform b [Homo sapiens] |

| Filename XCorr DeltCN Conf% ObsM+H+ CalcM+H+ SpR ZScore Ion% # Sequence  | | | | | | | | | | | | |
| --- | --- | --- | --- | --- | --- | --- | --- | --- | --- | --- | --- | --- |
|  | Mis12XLAPLSS\_011913\_01.11061.11061.2 | 3.4171 | 0.3576 | 99.9% | 1550.2322 | 1550.8816 | 1 | 6.6 | 75.0% | 1 | K.NILFVITKPDVYK.S | 2 |
|  | Mis12XLAPLSS\_011913\_02.06989.06989.2 | 3.0374 | 0.3969 | 99.9% | 1484.2322 | 1485.6335 | 2 | 6.915 | 57.7% | 2 | K.SPASDTYIVFGEAK.I | 2 |
|  | Mis12XLAPLSS\_011913\_01.08058.08058.2 | 4.8963 | 0.237 | 99.8% | 1615.5322 | 1615.7808 | 2 | 8.356 | 78.6% | 3 | K.IEDLSQQAQLAAAEK.F | 2 |
|  | Mis12XLAPLSS\_011913\_02.06869.06869.2 | 2.946 | 0.4005 | 99.9% | 1463.0521 | 1462.6611 | 1 | 7.09 | 58.3% | 1 | K.DIELVMSQANVSR.A | 2 |
|  | Mis12XLAPLSS\_011913\_01.15225.15225.2 | 3.3394 | 0.429 | 99.8% | 1991.8722 | 1992.3131 | 1 | 6.972 | 44.1% | 1 | R.ALKNNSNDIVNAIMELTM.- | 2 |

---

|  |  |  |  |  |  |  |  |  |
| --- | --- | --- | --- | --- | --- | --- | --- | --- |
| U | *gi|14165435|ref|NP\_11* | 13 | 20 | 33.9% | 463 | 50976 | 5.5 | heterogeneous nuclear ribonucleoprotein K isoform b [Homo sapiens] |
| U | *gi|14165439|ref|NP\_00* | 13 | 20 | 33.8% | 464 | 51028 | 5.3 | heterogeneous nuclear ribonucleoprotein K isoform a [Homo sapiens] |
| U | *gi|14165437|ref|NP\_11* | 13 | 20 | 33.8% | 464 | 51028 | 5.3 | heterogeneous nuclear ribonucleoprotein K isoform a [Homo sapiens] |

| Filename XCorr DeltCN Conf% ObsM+H+ CalcM+H+ SpR ZScore Ion% # Sequence  | | | | | | | | | | | | |
| --- | --- | --- | --- | --- | --- | --- | --- | --- | --- | --- | --- | --- |
|  | Mis12XLAPLSS\_011913\_01.04400.04400.3 | 4.1394 | 0.299 | 100.0% | 1736.6344 | 1736.8969 | 1 | 5.851 | 48.1% | 1 | K.RPAEDMEEEQAFKR.S | 3 |
|  | Mis12XLAPLSS\_011913\_01.05062.05062.2 | 2.8943 | 0.2191 | 99.6% | 1352.1522 | 1350.4894 | 1 | 4.174 | 75.0% | 1 | R.SRNTDEMVELR.I | 2 |
|  | Mis12XLAPLSS\_011913\_01.07059.07059.2 | 2.7225 | 0.2662 | 99.8% | 1106.9521 | 1107.2238 | 2 | 5.566 | 75.0% | 2 | R.NTDEMVELR.I | 2 |
|  | Mis12XLAPLSS\_011913\_01.06164.06164.2 | 3.8063 | 0.4814 | 100.0% | 1781.1921 | 1781.8302 | 20 | 9.027 | 46.9% | 3 | R.TDYNASVSVPDSSGPER.I | 2 |
|  | Mis12XLAPLSS\_011913\_01.15381.15381.2 | 3.4631 | 0.2962 | 99.8% | 1717.4922 | 1716.0251 | 1 | 6.204 | 56.7% | 1 | R.ILSISADIETIGEILK.K | 2 |
|  | Mis12XLAPLSS\_011913\_01.14289.14289.2 | 3.1851 | 0.3166 | 99.8% | 1843.2922 | 1844.1992 | 96 | 6.965 | 34.4% | 1 | R.ILSISADIETIGEILKK.I | 2 |
|  | Mis12XLAPLSS\_011913\_01.09831.09831.2 | 3.9325 | 0.4485 | 100.0% | 1519.5922 | 1519.8711 | 1 | 7.783 | 64.3% | 2 | R.LLIHQSLAGGIIGVK.G | 2 |
|  | Mis12XLAPLSS\_011913\_02.07151.07151.3 | 3.1415 | 0.2862 | 99.8% | 1519.8243 | 1519.8711 | 37 | 5.111 | 37.5% | 1 | R.LLIHQSLAGGIIGVK.G | 3 |
|  | Mis12XLAPLSS\_011913\_01.04415.04415.2 | 2.5811 | 0.3505 | 99.8% | 1053.9922 | 1054.278 | 19 | 6.185 | 61.1% | 1 | R.VVLIGGKPDR.V | 2 |
|  | Mis12XLAPLSS\_011913\_01.12614.12614.2 | 2.7998 | 0.3932 | 99.8% | 1341.1921 | 1341.6311 | 1 | 7.22 | 77.3% | 1 | K.IILDLISESPIK.G | 2 |
|  | Mis12XLAPLSS\_011913\_01.11156.11156.2 | 5.4024 | 0.4511 | 100.0% | 1918.4922 | 1918.1974 | 1 | 9.227 | 58.3% | 3 | R.GSYGDLGGPIITTQVTIPK.D | 2 |
|  | Mis12XLAPLSS\_011913\_02.05426.05426.3 | 3.4635 | 0.3865 | 100.0% | 2069.5444 | 2070.1772 | 1 | 5.687 | 37.5% | 2 | R.HESGASIKIDEPLEGSEDR.I | 3 |
|  | Mis12XLAPLSS\_011913\_01.12645.12645.2 | 4.8528 | 0.5403 | 100.0% | 2589.7322 | 2590.9365 | 1 | 9.746 | 50.0% | 1 | R.IITITGTQDQIQNAQYLLQNSVK.Q | 2 |

---

|  |  |  |  |  |  |  |  |  |
| --- | --- | --- | --- | --- | --- | --- | --- | --- |
| U | *gi|34098946|ref|NP\_00* | 6 | 7 | 32.7% | 324 | 35924 | 9.9 | nuclease sensitive element binding protein 1 [Homo sapiens] |

| Filename XCorr DeltCN Conf% ObsM+H+ CalcM+H+ SpR ZScore Ion% # Sequence  | | | | | | | | | | | | |
| --- | --- | --- | --- | --- | --- | --- | --- | --- | --- | --- | --- | --- |
|  | Mis12XLAPLSS\_011913\_01.05103.05103.3 | 3.4595 | 0.3746 | 100.0% | 1745.8143 | 1745.9298 | 1 | 6.711 | 46.4% | 1 | R.NDTKEDVFVHQTAIK.K | 33 |
|  | Mis12XLAPLSS\_011913\_01.04263.04263.3 | 3.1658 | 0.3703 | 100.0% | 1874.3344 | 1874.1039 | 6 | 5.588 | 35.0% | 1 | R.NDTKEDVFVHQTAIKK.N | 33 |
|  | Mis12XLAPLSS\_011913\_02.06794.06794.2 | 4.3245 | 0.3461 | 100.0% | 1796.9321 | 1796.8822 | 1 | 8.079 | 65.6% | 2 | R.SVGDGETVEFDVVEGEK.G | 22 |
| \* | Mis12XLAPLSS\_011913\_02.07082.07082.3 | 5.2786 | 0.4826 | 100.0% | 3473.6643 | 3474.7168 | 1 | 8.33 | 27.1% | 1 | R.SVGDGETVEFDVVEGEKGAEAANVTGPGGVPVQGSK.Y | 3 |
| \* | Mis12XLAPLSS\_011913\_01.05760.05760.3 | 5.0366 | 0.3454 | 100.0% | 3224.6042 | 3225.4795 | 1 | 6.881 | 26.7% | 1 | R.RPQYSNPPVQGEVMEGADNQGAGEQGRPVR.Q | 3 |
| \* | Mis12XLAPLSS\_011913\_01.03314.03314.3 | 3.7405 | 0.4102 | 100.0% | 2785.1343 | 2785.771 | 1 | 5.962 | 29.3% | 1 | R.EDGNEEDKENQGDETQGQQPPQRR.Y | 3 |

Similarities:
gi|224586884|ref|NP\_0(3:3)  

---

|  |  |  |  |  |  |  |  |  |
| --- | --- | --- | --- | --- | --- | --- | --- | --- |
| U | *gi|33286418|ref|NP\_00* | 11 | 18 | 32.4% | 531 | 57937 | 7.8 | pyruvate kinase, muscle isoform M2 [Homo sapiens] |
| U | *gi|33286422|ref|NP\_87* | 11 | 17 | 32.4% | 531 | 58062 | 7.7 | pyruvate kinase, muscle isoform M1 [Homo sapiens] |
| U | *gi|33286420|ref|NP\_87* | 11 | 18 | 32.4% | 531 | 58062 | 7.7 | pyruvate kinase, muscle isoform M1 [Homo sapiens] |

| Filename XCorr DeltCN Conf% ObsM+H+ CalcM+H+ SpR ZScore Ion% # Sequence  | | | | | | | | | | | | |
| --- | --- | --- | --- | --- | --- | --- | --- | --- | --- | --- | --- | --- |
|  | Mis12XLAPLSS\_011913\_01.08552.08552.2 | 3.1488 | 0.2991 | 99.8% | 1198.3522 | 1198.3617 | 3 | 6.207 | 75.0% | 2 | R.LDIDSPPITAR.N | 2 |
|  | Mis12XLAPLSS\_011913\_01.08260.08260.2 | 2.6702 | 0.2873 | 99.8% | 1178.1721 | 1178.4296 | 4 | 6.577 | 66.7% | 1 | R.SVETLKEMIK.S | 2 |
|  | Mis12XLAPLSS\_011913\_01.11961.11961.3 | 5.1859 | 0.4997 | 100.0% | 3019.1643 | 3019.4246 | 1 | 8.639 | 32.4% | 1 | R.TATESFASDPILYRPVAVALDTKGPEIR.T | 3 |
|  | Mis12XLAPLSS\_011913\_01.03597.03597.2 | 2.5768 | 0.274 | 99.5% | 1118.5122 | 1119.2603 | 6 | 5.06 | 60.0% | 1 | K.GSGTAEVELKK.G | 2 |
|  | Mis12XLAPLSS\_011913\_02.08093.08093.2 | 3.8086 | 0.348 | 100.0% | 1464.5322 | 1463.7142 | 1 | 7.301 | 75.0% | 4 | K.IYVDDGLISLQVK.Q | 2 |
|  | Mis12XLAPLSS\_011913\_01.11460.11460.2 | 3.1018 | 0.2774 | 99.8% | 1779.8722 | 1780.9292 | 3 | 6.558 | 47.1% | 3 | K.GADFLVTEVENGGSLGSK.K | 2 |
|  | Mis12XLAPLSS\_011913\_01.10830.10830.3 | 5.2156 | 0.3639 | 100.0% | 2478.4744 | 2478.8486 | 1 | 6.474 | 39.1% | 1 | K.KGVNLPGAAVDLPAVSEKDIQDLK.F | 3 |
|  | Mis12XLAPLSS\_011913\_01.15790.15790.2 | 4.3123 | 0.4251 | 100.0% | 1861.6522 | 1861.1224 | 1 | 8.468 | 73.3% | 2 | K.FGVEQDVDMVFASFIR.K | 2 |
|  | Mis12XLAPLSS\_011913\_01.09160.09160.2 | 2.8859 | 0.2432 | 99.8% | 1141.9922 | 1142.2946 | 6 | 4.803 | 65.0% | 1 | R.GDLGIEIPAEK.V | 2 |
|  | Mis12XLAPLSS\_011913\_01.05514.05514.1 | 1.8869 | 0.4051 | 100.0% | 840.55 | 841.0415 | 56 | 5.862 | 50.0% | 1 | R.APIIAVTR.N | 1 |
|  | Mis12XLAPLSS\_011913\_01.11463.11463.3 | 4.0217 | 0.2725 | 99.7% | 2392.8843 | 2392.7815 | 1 | 5.02 | 36.9% | 1 | K.KGDVVIVLTGWRPGSGFTNTMR.V | 3 |

---

|  |  |  |  |  |  |  |  |  |
| --- | --- | --- | --- | --- | --- | --- | --- | --- |
| U | *gi|5031635|ref|NP\_005* | 3 | 6 | 31.9% | 166 | 18502 | 8.1 | cofilin 1 (non-muscle) [Homo sapiens] |

| Filename XCorr DeltCN Conf% ObsM+H+ CalcM+H+ SpR ZScore Ion% # Sequence  | | | | | | | | | | | | |
| --- | --- | --- | --- | --- | --- | --- | --- | --- | --- | --- | --- | --- |
| \* | Mis12XLAPLSS\_011913\_01.13088.13088.3 | 4.4552 | 0.3925 | 100.0% | 3064.2844 | 3064.4595 | 1 | 6.513 | 28.7% | 1 | K.NIILEEGKEILVGDVGQTVDDPYATFVK.M | 3 |
|  | Mis12XLAPLSS\_011913\_01.08369.08369.2 | 3.4232 | 0.4146 | 100.0% | 1339.1322 | 1338.4564 | 1 | 6.696 | 80.0% | 2 | R.YALYDATYETK.E | 2 |
| \* | Mis12XLAPLSS\_011913\_02.07430.07430.2 | 3.1009 | 0.3575 | 99.8% | 1341.5122 | 1341.5907 | 1 | 5.901 | 61.5% | 3 | K.LGGSAVISLEGKPL.- | 2 |

---

|  |  |  |  |  |  |  |  |  |
| --- | --- | --- | --- | --- | --- | --- | --- | --- |
| U | *gi|169211725|ref|XP\_9* | 6 | 14 | 31.8% | 88 | 9949 | 11.3 | PREDICTED: similar to 40S ribosomal protein S28 [Homo sapiens] |
| U | *gi|4506715|ref|NP\_001* | 6 | 14 | 40.6% | 69 | 7841 | 10.7 | ribosomal protein S28 [Homo sapiens] |
| U | *gi|169212081|ref|XP\_0* | 6 | 14 | 30.8% | 91 | 10272 | 11.2 | PREDICTED: similar to 40S ribosomal protein S28 [Homo sapiens] |

| Filename XCorr DeltCN Conf% ObsM+H+ CalcM+H+ SpR ZScore Ion% # Sequence  | | | | | | | | | | | | |
| --- | --- | --- | --- | --- | --- | --- | --- | --- | --- | --- | --- | --- |
|  | Mis12XLAPLSS\_011913\_01.06600.06600.1 | 1.8975 | 0.3705 | 100.0% | 1099.4 | 1100.1884 | 1 | 6.226 | 68.8% | 1 | R.VEFMDDTSR.S | 1 |
|  | Mis12XLAPLSS\_011913\_01.06590.06590.2 | 2.9085 | 0.3394 | 99.9% | 1099.9922 | 1100.1884 | 1 | 7.263 | 81.2% | 7 | R.VEFMDDTSR.S | 2 |
|  | Mis12XLAPLSS\_011913\_01.10425.10425.3 | 3.8519 | 0.4559 | 100.0% | 2112.3542 | 2112.3909 | 1 | 7.499 | 37.5% | 2 | R.NVKGPVREGDVLTLLESER.E | 3 |
|  | Mis12XLAPLSS\_011913\_01.11518.11518.2 | 3.5817 | 0.3524 | 99.9% | 1771.1721 | 1770.9805 | 1 | 5.658 | 66.7% | 1 | K.GPVREGDVLTLLESER.E | 2 |
|  | Mis12XLAPLSS\_011913\_01.11523.11523.3 | 3.9206 | 0.241 | 99.7% | 1771.2244 | 1770.9805 | 31 | 6.37 | 36.7% | 1 | K.GPVREGDVLTLLESER.E | 3 |
|  | Mis12XLAPLSS\_011913\_01.12284.12284.2 | 3.7141 | 0.3848 | 100.0% | 1362.1122 | 1361.4918 | 1 | 6.892 | 72.7% | 2 | R.EGDVLTLLESER.E | 2 |

---

|  |  |  |  |  |  |  |  |  |
| --- | --- | --- | --- | --- | --- | --- | --- | --- |
| U | *gi|4557032|ref|NP\_002* | 6 | 7 | 31.7% | 334 | 36639 | 6.1 | L-lactate dehydrogenase B [Homo sapiens] |

| Filename XCorr DeltCN Conf% ObsM+H+ CalcM+H+ SpR ZScore Ion% # Sequence  | | | | | | | | | | | | |
| --- | --- | --- | --- | --- | --- | --- | --- | --- | --- | --- | --- | --- |
| \* | Mis12XLAPLSS\_011913\_01.08136.08136.2 | 3.345 | 0.3534 | 99.8% | 1695.3522 | 1695.9103 | 3 | 5.708 | 56.7% | 2 | K.LIAPVAEEEATVPNNK.I | 2 |
| \* | Mis12XLAPLSS\_011913\_01.16043.16043.3 | 3.7226 | 0.2517 | 99.4% | 3787.5244 | 3786.3906 | 1 | 4.009 | 22.7% | 1 | K.SLADELALVDVLEDKLKGEMMDLQHGSLFLQTPK.I | 3 |
| \* | Mis12XLAPLSS\_011913\_01.04174.04174.2 | 3.9688 | 0.4553 | 100.0% | 1511.3522 | 1511.672 | 1 | 8.453 | 80.8% | 1 | K.IVADKDYSVTANSK.I | 2 |
| \* | Mis12XLAPLSS\_011913\_02.06699.06699.2 | 2.8737 | 0.4728 | 100.0% | 1267.5721 | 1268.5109 | 1 | 7.786 | 75.0% | 1 | K.MVVESAYEVIK.L | 2 |
| \* | Mis12XLAPLSS\_011913\_02.13608.13608.2 | 3.9381 | 0.4825 | 100.0% | 2181.392 | 2182.5396 | 1 | 8.37 | 44.7% | 1 | K.GYTNWAIGLSVADLIESMLK.N | 2 |
| \* | Mis12XLAPLSS\_011913\_01.03875.03875.2 | 3.1333 | 0.2225 | 99.8% | 1288.0521 | 1287.4991 | 2 | 5.228 | 70.0% | 1 | K.LKDDEVAQLKK.S | 2 |

---

|  |  |  |  |  |  |  |  |  |
| --- | --- | --- | --- | --- | --- | --- | --- | --- |
| U | *gi|7706337|ref|NP\_057* | 2 | 3 | 31.6% | 177 | 20198 | 4.8 | coatomer protein complex, subunit zeta 1 [Homo sapiens] |

| Filename XCorr DeltCN Conf% ObsM+H+ CalcM+H+ SpR ZScore Ion% # Sequence  | | | | | | | | | | | | |
| --- | --- | --- | --- | --- | --- | --- | --- | --- | --- | --- | --- | --- |
| \* | Mis12XLAPLSS\_011913\_02.13592.13592.3 | 6.1061 | 0.4943 | 100.0% | 3607.8542 | 3608.101 | 1 | 10.082 | 26.6% | 2 | R.ALLENMEGLFLAVDEIVDGGVILESDPQQVVHR.V | 3 |
| \* | Mis12XLAPLSS\_011913\_01.11878.11878.3 | 4.2316 | 0.3803 | 100.0% | 2469.8643 | 2469.7979 | 1 | 6.642 | 27.3% | 1 | R.VALRGEDVPLTEQTVSQVLQSAK.E | 3 |

---

|  |  |  |  |  |  |  |  |  |
| --- | --- | --- | --- | --- | --- | --- | --- | --- |
| U | *gi|4506613|ref|NP\_000* | 4 | 5 | 31.2% | 128 | 14787 | 9.2 | ribosomal protein L22 proprotein [Homo sapiens] |

| Filename XCorr DeltCN Conf% ObsM+H+ CalcM+H+ SpR ZScore Ion% # Sequence  | | | | | | | | | | | | |
| --- | --- | --- | --- | --- | --- | --- | --- | --- | --- | --- | --- | --- |
| \* | Mis12XLAPLSS\_011913\_01.07719.07719.2 | 3.7695 | 0.4341 | 100.0% | 1243.2122 | 1243.4056 | 1 | 7.088 | 66.7% | 2 | K.AGNLGGGVVTIER.S | 2 |
| \* | Mis12XLAPLSS\_011913\_01.08914.08914.2 | 2.687 | 0.2882 | 99.8% | 1207.9521 | 1208.3971 | 4 | 5.613 | 70.0% | 1 | K.ITVTSEVPFSK.R | 2 |
| \* | Mis12XLAPLSS\_011913\_01.07726.07726.2 | 2.221 | 0.2041 | 95.6% | 1364.0521 | 1364.5846 | 2 | 4.528 | 63.6% | 1 | K.ITVTSEVPFSKR.Y | 2 |
| \* | Mis12XLAPLSS\_011913\_02.06258.06258.2 | 2.7341 | 0.3101 | 99.7% | 1932.0922 | 1932.8591 | 1 | 7.721 | 57.1% | 1 | R.YFQINQDEEEEEDED.- | 2 |

---

|  |  |  |  |  |  |  |  |  |
| --- | --- | --- | --- | --- | --- | --- | --- | --- |
| U | *gi|4506901|ref|NP\_003* | 4 | 5 | 31.1% | 164 | 19330 | 11.6 | splicing factor, arginine/serine-rich 3 [Homo sapiens] |

| Filename XCorr DeltCN Conf% ObsM+H+ CalcM+H+ SpR ZScore Ion% # Sequence  | | | | | | | | | | | | |
| --- | --- | --- | --- | --- | --- | --- | --- | --- | --- | --- | --- | --- |
| \* | Mis12XLAPLSS\_011913\_01.09428.09428.2 | 2.2964 | 0.1325 | 95.4% | 1043.7322 | 1044.198 | 3 | 4.95 | 81.2% | 2 | R.AFGYYGPLR.S | 2 |
| \* | Mis12XLAPLSS\_011913\_01.12134.12134.3 | 2.9507 | 0.4264 | 100.0% | 2321.2744 | 2321.5107 | 2 | 5.89 | 30.0% | 1 | R.NPPGFAFVEFEDPRDAADAVR.E | 3 |
| \* | Mis12XLAPLSS\_011913\_01.03575.03575.3 | 3.8815 | 0.24 | 99.7% | 1288.8844 | 1287.4618 | 2 | 5.483 | 52.5% | 1 | R.VRVELSNGEKR.S | 3 |
| \* | Mis12XLAPLSS\_011913\_01.03953.03953.2 | 2.5932 | 0.2944 | 99.8% | 1123.9122 | 1124.2473 | 12 | 5.531 | 72.2% | 1 | R.NRGPPPSWGR.R | 2 |

---

|  |  |  |  |  |  |  |  |  |
| --- | --- | --- | --- | --- | --- | --- | --- | --- |
| U | *gi|5453559|ref|NP\_006* | 3 | 3 | 31.1% | 161 | 18491 | 5.3 | ATP synthase, H+ transporting, mitochondrial F0 complex, subunit d isoform a [Homo sapiens] |

| Filename XCorr DeltCN Conf% ObsM+H+ CalcM+H+ SpR ZScore Ion% # Sequence  | | | | | | | | | | | | |
| --- | --- | --- | --- | --- | --- | --- | --- | --- | --- | --- | --- | --- |
|  | Mis12XLAPLSS\_011913\_01.12184.12184.2 | 3.3594 | 0.4669 | 100.0% | 1932.6721 | 1933.2139 | 1 | 7.632 | 50.0% | 1 | R.LAALPENPPAIDWAYYK.A | 2 |
| \* | Mis12XLAPLSS\_011913\_02.05352.05352.3 | 2.9968 | 0.3372 | 99.8% | 2390.3044 | 2390.6062 | 1 | 5.601 | 32.5% | 1 | K.VPVPEDKYTAQVDAEEKEDVK.S | 3 |
|  | Mis12XLAPLSS\_011913\_01.11543.11543.2 | 2.2654 | 0.1858 | 95.3% | 1557.5122 | 1557.7484 | 40 | 3.932 | 50.0% | 1 | K.YPYWPHQPIENL.- | 2 |

---

|  |  |  |  |  |  |  |  |  |
| --- | --- | --- | --- | --- | --- | --- | --- | --- |
| U | *gi|169160598|ref|XP\_0* | 2 | 2 | 31.0% | 84 | 9461 | 9.5 | PREDICTED: similar to hCG1783679 [Homo sapiens] |
| U | *gi|4506711|ref|NP\_001* | 2 | 2 | 31.0% | 84 | 9461 | 9.5 | ribosomal protein S27 [Homo sapiens] |
| U | *gi|169161552|ref|XP\_0* | 2 | 2 | 31.0% | 84 | 9461 | 9.5 | PREDICTED: similar to hCG1783679 [Homo sapiens] |
| U | *gi|169161255|ref|XP\_0* | 2 | 2 | 31.0% | 84 | 9461 | 9.5 | PREDICTED: hypothetical protein [Homo sapiens] |

| Filename XCorr DeltCN Conf% ObsM+H+ CalcM+H+ SpR ZScore Ion% # Sequence  | | | | | | | | | | | | |
| --- | --- | --- | --- | --- | --- | --- | --- | --- | --- | --- | --- | --- |
|  | Mis12XLAPLSS\_011913\_01.04037.04037.2 | 2.9601 | 0.3084 | 99.8% | 1578.3121 | 1578.7654 | 1 | 5.898 | 54.2% | 1 | K.DLLHPSPEEEKRK.H | 2 |
|  | Mis12XLAPLSS\_011913\_01.09875.09875.2 | 3.3681 | 0.3834 | 99.9% | 1529.4722 | 1528.7632 | 1 | 6.252 | 75.0% | 1 | R.LVQSPNSYFMDVK.C | 2 |

---

|  |  |  |  |  |  |  |  |  |
| --- | --- | --- | --- | --- | --- | --- | --- | --- |
| U | *gi|15431295|ref|NP\_15* | 6 | 7 | 30.8% | 211 | 24261 | 11.7 | ribosomal protein L13 [Homo sapiens] |
| U | *gi|15431297|ref|NP\_00* | 6 | 7 | 30.8% | 211 | 24261 | 11.7 | ribosomal protein L13 [Homo sapiens] |

| Filename XCorr DeltCN Conf% ObsM+H+ CalcM+H+ SpR ZScore Ion% # Sequence  | | | | | | | | | | | | |
| --- | --- | --- | --- | --- | --- | --- | --- | --- | --- | --- | --- | --- |
|  | Mis12XLAPLSS\_011913\_01.04048.04048.2 | 2.7442 | 0.1175 | 98.0% | 1307.8121 | 1308.5872 | 2 | 4.224 | 70.0% | 1 | R.NGMVLKPHFHK.D | 2 |
|  | Mis12XLAPLSS\_011913\_01.08984.08984.2 | 3.0896 | 0.3268 | 99.9% | 1190.2722 | 1190.3469 | 75 | 5.667 | 66.7% | 1 | R.VATWFNQPAR.K | 2 |
|  | Mis12XLAPLSS\_011913\_01.04191.04191.2 | 3.0402 | 0.2411 | 99.8% | 1234.2522 | 1233.3237 | 1 | 5.115 | 75.0% | 1 | K.STESLQANVQR.L | 2 |
|  | Mis12XLAPLSS\_011913\_01.10329.10329.3 | 3.9387 | 0.2508 | 99.8% | 2427.4744 | 2428.8064 | 3 | 5.063 | 28.4% | 2 | K.KGDSSAEELKLATQLTGPVMPVR.N | 3 |
|  | Mis12XLAPLSS\_011913\_01.09218.09218.2 | 3.1386 | 0.4401 | 99.8% | 1383.1522 | 1383.6923 | 2 | 7.45 | 62.5% | 1 | K.LATQLTGPVMPVR.N | 2 |
|  | Mis12XLAPLSS\_011913\_01.04216.04216.2 | 3.3527 | 0.3355 | 99.9% | 1237.9922 | 1237.3953 | 3 | 6.607 | 66.7% | 1 | R.VITEEEKNFK.A | 2 |

---

|  |  |  |  |  |  |  |  |  |
| --- | --- | --- | --- | --- | --- | --- | --- | --- |
| U | *gi|59859885|ref|NP\_00* | 6 | 10 | 30.5% | 295 | 32854 | 4.9 | ribosomal protein SA [Homo sapiens] |
| U | *gi|9845502|ref|NP\_002* | 6 | 10 | 30.5% | 295 | 32854 | 4.9 | ribosomal protein SA [Homo sapiens] |

| Filename XCorr DeltCN Conf% ObsM+H+ CalcM+H+ SpR ZScore Ion% # Sequence  | | | | | | | | | | | | |
| --- | --- | --- | --- | --- | --- | --- | --- | --- | --- | --- | --- | --- |
|  | Mis12XLAPLSS\_011913\_02.08409.08409.3 | 4.7348 | 0.2509 | 99.7% | 2618.9343 | 2618.9666 | 2 | 5.617 | 35.2% | 1 | K.FLAAGTHLGGTNLDFQMEQYIYK.R | 3 |
|  | Mis12XLAPLSS\_011913\_01.10211.10211.2 | 3.2252 | 0.4073 | 99.9% | 1741.3922 | 1741.9823 | 1 | 7.121 | 53.1% | 1 | R.AIVAIENPADVSVISSR.N | 2 |
|  | Mis12XLAPLSS\_011913\_01.05298.05298.2 | 4.3826 | 0.4725 | 100.0% | 1204.1921 | 1204.3713 | 1 | 8.601 | 79.2% | 4 | K.FAAATGATPIAGR.F | 2 |
|  | Mis12XLAPLSS\_011913\_01.12346.12346.2 | 3.1627 | 0.3993 | 99.9% | 1699.1522 | 1699.9065 | 3 | 6.556 | 50.0% | 1 | R.FTPGTFTNQIQAAFR.E | 2 |
|  | Mis12XLAPLSS\_011913\_01.07198.07198.2 | 2.895 | 0.2656 | 99.8% | 914.0522 | 913.10504 | 1 | 6.888 | 85.7% | 2 | R.LLVVTDPR.A | 2 |
|  | Mis12XLAPLSS\_011913\_01.13168.13168.2 | 3.7898 | 0.57 | 100.0% | 1615.0521 | 1615.9543 | 1 | 8.511 | 57.7% | 1 | K.GAHSVGLMWWMLAR.E | 2 |

---

|  |  |  |  |  |  |  |  |  |
| --- | --- | --- | --- | --- | --- | --- | --- | --- |
| U | *gi|32189392|ref|NP\_00* | 5 | 6 | 30.3% | 198 | 21892 | 6.0 | peroxiredoxin 2 isoform a [Homo sapiens] |

| Filename XCorr DeltCN Conf% ObsM+H+ CalcM+H+ SpR ZScore Ion% # Sequence  | | | | | | | | | | | | |
| --- | --- | --- | --- | --- | --- | --- | --- | --- | --- | --- | --- | --- |
|  | Mis12XLAPLSS\_011913\_02.05411.05411.2 | 2.9328 | 0.2816 | 99.8% | 1335.4122 | 1335.5431 | 3 | 4.937 | 62.5% | 1 | K.ATAVVDGAFKEVK.L | 2 |
| \* | Mis12XLAPLSS\_011913\_01.13038.13038.2 | 4.4619 | 0.4946 | 100.0% | 1863.5122 | 1864.1954 | 1 | 8.77 | 61.8% | 1 | R.KEGGLGPLNIPLLADVTR.R | 2 |
| \* | Mis12XLAPLSS\_011913\_02.06114.06114.3 | 3.4094 | 0.2728 | 99.8% | 2087.3643 | 2086.309 | 12 | 4.634 | 32.4% | 1 | R.RLSEDYGVLKTDEGIAYR.G | 3 |
| \* | Mis12XLAPLSS\_011913\_02.06416.06416.3 | 3.3381 | 0.2892 | 99.8% | 1929.2644 | 1930.1217 | 1 | 5.086 | 39.1% | 1 | R.LSEDYGVLKTDEGIAYR.G | 3 |
|  | Mis12XLAPLSS\_011913\_01.08840.08840.2 | 2.8089 | 0.326 | 99.8% | 1212.1721 | 1212.3915 | 8 | 7.022 | 75.0% | 2 | R.QITVNDLPVGR.S | 22 |

Similarities:
gi|32455264|ref|NP\_85(1:4)  

---

|  |  |  |  |  |  |  |  |  |
| --- | --- | --- | --- | --- | --- | --- | --- | --- |
| U | *gi|4506691|ref|NP\_001* | 4 | 6 | 30.1% | 146 | 16445 | 10.2 | ribosomal protein S16 [Homo sapiens] |

| Filename XCorr DeltCN Conf% ObsM+H+ CalcM+H+ SpR ZScore Ion% # Sequence  | | | | | | | | | | | | |
| --- | --- | --- | --- | --- | --- | --- | --- | --- | --- | --- | --- | --- |
|  | Mis12XLAPLSS\_011913\_01.09734.09734.2 | 3.3677 | 0.5532 | 100.0% | 1187.8922 | 1188.372 | 1 | 8.71 | 75.0% | 3 | K.GPLQSVQVFGR.K | 2 |
| \* | Mis12XLAPLSS\_011913\_01.07958.07958.2 | 2.7773 | 0.2586 | 99.6% | 1412.3121 | 1411.6622 | 3 | 4.466 | 59.1% | 1 | K.VNGRPLEMIEPR.T | 2 |
|  | Mis12XLAPLSS\_011913\_01.12598.12598.2 | 2.4009 | 0.1975 | 98.4% | 1095.2522 | 1095.4111 | 111 | 5.516 | 55.6% | 1 | K.LLEPVLLLGK.E | 2 |
| \* | Mis12XLAPLSS\_011913\_01.09711.09711.2 | 1.9274 | 0.2857 | 96.4% | 1406.2322 | 1406.622 | 16 | 5.565 | 50.0% | 1 | K.EIKDILIQYDR.T | 2 |

---

|  |  |  |  |  |  |  |  |  |
| --- | --- | --- | --- | --- | --- | --- | --- | --- |
| U | *gi|38045913|ref|NP\_93* | 5 | 9 | 29.9% | 177 | 19654 | 5.6 | non-metastatic cells 1, protein (NM23A) expressed in isoform a [Homo sapiens] |
| U | *gi|4557797|ref|NP\_000* | 5 | 9 | 34.9% | 152 | 17149 | 6.2 | non-metastatic cells 1, protein (NM23A) expressed in isoform b [Homo sapiens] |

| Filename XCorr DeltCN Conf% ObsM+H+ CalcM+H+ SpR ZScore Ion% # Sequence  | | | | | | | | | | | | |
| --- | --- | --- | --- | --- | --- | --- | --- | --- | --- | --- | --- | --- |
|  | Mis12XLAPLSS\_011913\_01.07478.07478.2 | 3.3745 | 0.2477 | 99.8% | 1346.1721 | 1345.5846 | 1 | 5.657 | 63.6% | 3 | R.TFIAIKPDGVQR.G | 22 |
|  | Mis12XLAPLSS\_011913\_01.10235.10235.2 | 2.3937 | 0.282 | 99.5% | 1150.1921 | 1150.3658 | 9 | 4.611 | 61.1% | 1 | K.DRPFFAGLVK.Y | 22 |
|  | Mis12XLAPLSS\_011913\_01.06041.06041.3 | 2.537 | 0.3747 | 99.8% | 1786.3143 | 1787.041 | 1 | 5.724 | 39.1% | 1 | R.VMLGETNPADSKPGTIR.G | 33 |
|  | Mis12XLAPLSS\_011913\_01.05949.05949.2 | 4.3476 | 0.3567 | 100.0% | 1787.3121 | 1787.041 | 1 | 6.921 | 62.5% | 3 | R.VMLGETNPADSKPGTIR.G | 22 |
|  | Mis12XLAPLSS\_011913\_01.04198.04198.2 | 3.7229 | 0.3767 | 100.0% | 1485.9722 | 1486.5785 | 1 | 7.22 | 84.6% | 1 | R.NIIHGSDSVESAEK.E | 2 |

Similarities:
gi|66392203|ref|NP\_00(4:1)  

---

|  |  |  |  |  |  |  |  |  |
| --- | --- | --- | --- | --- | --- | --- | --- | --- |
| U | *gi|4503529|ref|NP\_001* | 7 | 10 | 29.8% | 406 | 46154 | 5.5 | eukaryotic translation initiation factor 4A isoform 1 [Homo sapiens] |

| Filename XCorr DeltCN Conf% ObsM+H+ CalcM+H+ SpR ZScore Ion% # Sequence  | | | | | | | | | | | | |
| --- | --- | --- | --- | --- | --- | --- | --- | --- | --- | --- | --- | --- |
| \* | Mis12XLAPLSS\_011913\_01.16130.16130.3 | 4.6485 | 0.5125 | 100.0% | 4170.2046 | 4169.451 | 1 | 7.639 | 27.8% | 2 | R.SRDNGPDGMEPEGVIESNWNEIVDSFDDMNLSESLLR.G | 3 |
|  | Mis12XLAPLSS\_011913\_01.08770.08770.2 | 4.8327 | 0.5288 | 100.0% | 1828.3322 | 1829.0654 | 1 | 8.936 | 76.7% | 1 | R.GIYAYGFEKPSAIQQR.A | 22 |
|  | Mis12XLAPLSS\_011913\_01.06926.06926.2 | 3.2058 | 0.4372 | 99.9% | 1395.3722 | 1395.512 | 1 | 7.935 | 69.2% | 2 | K.GYDVIAQAQSGTGK.T | 2 |
| \* | Mis12XLAPLSS\_011913\_01.09068.09068.3 | 3.4922 | 0.3606 | 100.0% | 1620.0243 | 1619.9225 | 1 | 6.579 | 48.2% | 1 | K.LQMEAPHIIVGTPGR.V | 3 |
|  | Mis12XLAPLSS\_011913\_01.12939.12939.2 | 4.0477 | 0.4531 | 100.0% | 1556.3522 | 1556.789 | 1 | 7.967 | 75.0% | 1 | K.MFVLDEADEMLSR.G | 2 |
| \* | Mis12XLAPLSS\_011913\_01.11534.11534.2 | 3.578 | 0.244 | 99.8% | 1502.3322 | 1502.71 | 5 | 5.998 | 68.2% | 2 | R.GFKDQIYDIFQK.L | 2 |
| \* | Mis12XLAPLSS\_011913\_01.04802.04802.3 | 3.8681 | 0.433 | 100.0% | 1591.9744 | 1590.8352 | 1 | 6.621 | 51.9% | 1 | R.KGVAINMVTEEDKR.T | 3 |

Similarities:
gi|7661920|ref|NP\_055(1:6)  

---

|  |  |  |  |  |  |  |  |  |
| --- | --- | --- | --- | --- | --- | --- | --- | --- |
| U | *gi|208973238|ref|NP\_0* | 4 | 7 | 29.8% | 245 | 27745 | 4.8 | tyrosine 3/tryptophan 5 -monooxygenase activation protein, zeta polypeptide [Homo sapiens] |
| U | *gi|4507953|ref|NP\_003* | 4 | 7 | 29.8% | 245 | 27745 | 4.8 | tyrosine 3/tryptophan 5 -monooxygenase activation protein, zeta polypeptide [Homo sapiens] |
| U | *gi|21735625|ref|NP\_66* | 4 | 7 | 29.8% | 245 | 27745 | 4.8 | tyrosine 3/tryptophan 5 -monooxygenase activation protein, zeta polypeptide [Homo sapiens] |
| U | *gi|208973244|ref|NP\_0* | 4 | 7 | 29.8% | 245 | 27745 | 4.8 | tyrosine 3/tryptophan 5 -monooxygenase activation protein, zeta polypeptide [Homo sapiens] |
| U | *gi|208973242|ref|NP\_0* | 4 | 7 | 29.8% | 245 | 27745 | 4.8 | tyrosine 3/tryptophan 5 -monooxygenase activation protein, zeta polypeptide [Homo sapiens] |
| U | *gi|208973240|ref|NP\_0* | 4 | 7 | 29.8% | 245 | 27745 | 4.8 | tyrosine 3/tryptophan 5 -monooxygenase activation protein, zeta polypeptide [Homo sapiens] |

| Filename XCorr DeltCN Conf% ObsM+H+ CalcM+H+ SpR ZScore Ion% # Sequence  | | | | | | | | | | | | |
| --- | --- | --- | --- | --- | --- | --- | --- | --- | --- | --- | --- | --- |
|  | Mis12XLAPLSS\_011913\_02.03976.03976.2 | 4.2556 | 0.4044 | 100.0% | 1550.0922 | 1549.5914 | 1 | 6.829 | 69.2% | 2 | K.SVTEQGAELSNEER.N | 2 |
|  | Mis12XLAPLSS\_011913\_01.04252.04252.2 | 2.9955 | 0.2713 | 99.8% | 1280.1122 | 1280.4203 | 1 | 5.666 | 68.2% | 1 | R.YLAEVAAGDDKK.G | 2 |
|  | Mis12XLAPLSS\_011913\_01.11208.11208.2 | 4.4172 | 0.4388 | 100.0% | 2041.7522 | 2042.2096 | 1 | 7.741 | 50.0% | 3 | K.GIVDQSQQAYQEAFEISK.K | 2 |
|  | Mis12XLAPLSS\_011913\_01.18320.18320.3 | 3.8991 | 0.385 | 100.0% | 3303.6543 | 3304.6907 | 3 | 7.741 | 24.1% | 1 | K.TAFDEAIAELDTLSEESYKDSTLIMQLLR.D | 3 |

---

|  |  |  |  |  |  |  |  |  |
| --- | --- | --- | --- | --- | --- | --- | --- | --- |
| U | *gi|5032051|ref|NP\_005* | 3 | 8 | 29.8% | 151 | 16273 | 10.1 | ribosomal protein S14 [Homo sapiens] |
| U | *gi|68160922|ref|NP\_00* | 3 | 8 | 29.8% | 151 | 16273 | 10.1 | ribosomal protein S14 [Homo sapiens] |
| U | *gi|68160915|ref|NP\_00* | 3 | 8 | 29.8% | 151 | 16273 | 10.1 | ribosomal protein S14 [Homo sapiens] |

| Filename XCorr DeltCN Conf% ObsM+H+ CalcM+H+ SpR ZScore Ion% # Sequence  | | | | | | | | | | | | |
| --- | --- | --- | --- | --- | --- | --- | --- | --- | --- | --- | --- | --- |
|  | Mis12XLAPLSS\_011913\_01.09842.09842.3 | 3.4333 | 0.3471 | 99.7% | 2265.9844 | 2266.4473 | 1 | 6.84 | 32.5% | 1 | K.ADRDESSPYAAMLAAQDVAQR.C | 3 |
|  | Mis12XLAPLSS\_011913\_01.04066.04066.2 | 3.0738 | 0.3402 | 99.8% | 1056.2722 | 1055.179 | 18 | 6.826 | 60.0% | 2 | K.TPGPGAQSALR.A | 2 |
|  | Mis12XLAPLSS\_011913\_01.06872.06872.2 | 3.5678 | 0.4115 | 100.0% | 1430.2922 | 1430.5547 | 1 | 7.66 | 83.3% | 5 | R.IEDVTPIPSDSTR.R | 2 |

---

|  |  |  |  |  |  |  |  |  |
| --- | --- | --- | --- | --- | --- | --- | --- | --- |
| U | *gi|24234688|ref|NP\_00* | 15 | 18 | 29.3% | 679 | 73681 | 6.2 | heat shock 70kDa protein 9 precursor [Homo sapiens] |

| Filename XCorr DeltCN Conf% ObsM+H+ CalcM+H+ SpR ZScore Ion% # Sequence  | | | | | | | | | | | | |
| --- | --- | --- | --- | --- | --- | --- | --- | --- | --- | --- | --- | --- |
| \* | Mis12XLAPLSS\_011913\_02.05462.05462.2 | 3.1488 | 0.4208 | 99.9% | 1451.6322 | 1451.576 | 1 | 7.271 | 57.7% | 2 | R.TTPSVVAFTADGER.L | 2 |
| \* | Mis12XLAPLSS\_011913\_01.04288.04288.2 | 3.5378 | 0.3433 | 99.9% | 1341.9521 | 1342.4105 | 1 | 7.422 | 66.7% | 1 | R.ASNGDAWVEAHGK.L | 2 |
| \* | Mis12XLAPLSS\_011913\_01.04221.04221.3 | 3.4912 | 0.2036 | 98.6% | 1593.0844 | 1593.7949 | 6 | 4.534 | 38.5% | 1 | K.MKETAENYLGHTAK.N | 3 |
| \* | Mis12XLAPLSS\_011913\_01.10334.10334.2 | 2.72 | 0.2498 | 99.3% | 1695.3722 | 1695.8723 | 1 | 5.292 | 60.7% | 1 | K.NAVITVPAYFNDSQR.Q | 2 |
| \* | Mis12XLAPLSS\_011913\_01.09764.09764.2 | 4.1345 | 0.3327 | 100.0% | 1243.4122 | 1243.4056 | 1 | 6.59 | 81.8% | 3 | K.DAGQISGLNVLR.V | 2 |
| \* | Mis12XLAPLSS\_011913\_01.07742.07742.2 | 3.328 | 0.2248 | 99.8% | 1692.1921 | 1691.8969 | 1 | 5.462 | 67.9% | 1 | R.ETGVDLTKDNMALQR.V | 2 |
| \* | Mis12XLAPLSS\_011913\_01.12573.12573.2 | 3.9433 | 0.4244 | 100.0% | 1362.2522 | 1362.5687 | 2 | 8.644 | 63.6% | 1 | R.AQFEGIVTDLIR.R | 2 |
| \* | Mis12XLAPLSS\_011913\_02.07841.07841.3 | 3.0851 | 0.274 | 97.9% | 2407.3743 | 2407.7595 | 1 | 5.215 | 30.7% | 1 | K.AMQDAEVSKSDIGEVILVGGMTR.M | 3 |
| \* | Mis12XLAPLSS\_011913\_02.08015.08015.2 | 2.8619 | 0.1261 | 97.4% | 1448.6122 | 1447.6898 | 19 | 4.288 | 46.2% | 1 | K.SDIGEVILVGGMTR.M | 2 |
| \* | Mis12XLAPLSS\_011913\_01.09567.09567.2 | 2.7599 | 0.1389 | 98.4% | 1293.1921 | 1291.4496 | 1 | 4.77 | 75.0% | 1 | K.VQQTVQDLFGR.A | 2 |
| \* | Mis12XLAPLSS\_011913\_02.05936.05936.2 | 4.7262 | 0.5252 | 100.0% | 1809.6721 | 1809.9707 | 1 | 10.291 | 59.4% | 1 | K.SQVFSTAADGQTQVEIK.V | 2 |
| \* | Mis12XLAPLSS\_011913\_02.06986.06986.3 | 2.9012 | 0.3057 | 99.2% | 2419.4644 | 2419.7095 | 1 | 5.808 | 35.7% | 1 | R.EQQIVIQSSGGLSKDDIENMVK.N | 3 |
| \* | Mis12XLAPLSS\_011913\_01.10346.10346.3 | 4.2079 | 0.3865 | 100.0% | 2142.1143 | 2143.3765 | 1 | 5.906 | 38.9% | 1 | K.ERVEAVNMAEGIIHDTETK.M | 3 |
| \* | Mis12XLAPLSS\_011913\_01.11058.11058.2 | 2.6896 | 0.3679 | 99.8% | 1855.7922 | 1858.0735 | 1 | 5.951 | 43.8% | 1 | R.VEAVNMAEGIIHDTETK.M | 2 |
| \* | Mis12XLAPLSS\_011913\_01.04506.04506.2 | 2.5571 | 0.1975 | 98.4% | 1232.2322 | 1232.3794 | 22 | 4.193 | 59.1% | 1 | R.QAASSLQQASLK.L | 2 |

---

|  |  |  |  |  |  |  |  |  |
| --- | --- | --- | --- | --- | --- | --- | --- | --- |
| U | *gi|167466173|ref|NP\_0* | 18 | 26 | 29.0% | 641 | 70052 | 5.6 | heat shock 70kDa protein 1B [Homo sapiens] |
| U | *gi|194248072|ref|NP\_0* | 18 | 26 | 29.0% | 641 | 70052 | 5.6 | heat shock 70kDa protein 1A [Homo sapiens] |

| Filename XCorr DeltCN Conf% ObsM+H+ CalcM+H+ SpR ZScore Ion% # Sequence  | | | | | | | | | | | | |
| --- | --- | --- | --- | --- | --- | --- | --- | --- | --- | --- | --- | --- |
|  | Mis12XLAPLSS\_011913\_02.05422.05422.2 | 3.1076 | 0.4678 | 100.0% | 1488.2722 | 1488.5939 | 1 | 8.504 | 70.8% | 3 | R.TTPSYVAFTDTER.L | 2222 |
|  | Mis12XLAPLSS\_011913\_01.09000.09000.2 | 4.1724 | 0.3131 | 99.8% | 1659.2122 | 1659.8394 | 1 | 6.525 | 75.0% | 2 | K.NQVALNPQNTVFDAK.R | 2 |
|  | Mis12XLAPLSS\_011913\_01.07305.07305.2 | 2.9504 | 0.4083 | 99.9% | 1223.3522 | 1223.3862 | 9 | 7.496 | 60.0% | 1 | K.FGDPVVQSDMK.H | 2 |
|  | Mis12XLAPLSS\_011913\_01.09016.09016.3 | 3.9576 | 0.3464 | 100.0% | 1682.0343 | 1681.8912 | 1 | 6.1 | 50.0% | 1 | K.HWPFQVINDGDKPK.V | 3 |
|  | Mis12XLAPLSS\_011913\_01.12483.12483.2 | 3.292 | 0.3526 | 99.8% | 1616.3322 | 1615.8817 | 1 | 7.069 | 65.4% | 1 | K.AFYPEEISSMVLTK.M | 22 |
|  | Mis12XLAPLSS\_011913\_02.07888.07888.2 | 3.0144 | 0.3046 | 99.8% | 1198.3522 | 1198.408 | 1 | 6.584 | 72.7% | 3 | K.DAGVIAGLNVLR.I | 22 |
|  | Mis12XLAPLSS\_011913\_01.11126.11126.2 | 5.0548 | 0.4514 | 100.0% | 1689.2922 | 1688.9213 | 1 | 9.079 | 76.7% | 4 | R.IINEPTAAAIAYGLDR.T | 22 |
|  | Mis12XLAPLSS\_011913\_01.04986.04986.2 | 3.8448 | 0.4962 | 100.0% | 1676.1522 | 1676.6964 | 1 | 8.128 | 63.3% | 1 | K.ATAGDTHLGGEDFDNR.L | 222 |
|  | Mis12XLAPLSS\_011913\_01.04985.04985.3 | 2.7193 | 0.3103 | 99.4% | 1679.1543 | 1676.6964 | 23 | 5.282 | 36.7% | 1 | K.ATAGDTHLGGEDFDNR.L | 333 |
|  | Mis12XLAPLSS\_011913\_01.08699.08699.2 | 3.04 | 0.4005 | 99.8% | 1263.1522 | 1262.4508 | 1 | 7.151 | 72.2% | 1 | R.LVNHFVEEFK.R | 2 |
|  | Mis12XLAPLSS\_011913\_01.07625.07625.2 | 3.0756 | 0.3975 | 99.9% | 1418.1522 | 1418.6383 | 1 | 7.271 | 75.0% | 1 | R.LVNHFVEEFKR.K | 2 |
|  | Mis12XLAPLSS\_011913\_01.07601.07601.3 | 2.7613 | 0.291 | 99.8% | 1418.3344 | 1418.6383 | 1 | 5.727 | 47.5% | 1 | R.LVNHFVEEFKR.K | 3 |
|  | Mis12XLAPLSS\_011913\_02.06324.06324.3 | 4.0064 | 0.253 | 99.7% | 1823.4243 | 1823.1025 | 4 | 5.567 | 39.1% | 1 | K.LDKAQIHDLVLVGGSTR.I | 3 |
|  | Mis12XLAPLSS\_011913\_01.10977.10977.2 | 2.5053 | 0.3679 | 99.8% | 1110.2122 | 1110.2578 | 1 | 6.904 | 87.5% | 1 | K.LLQDFFNGR.D | 2 |
|  | Mis12XLAPLSS\_011913\_01.03447.03447.2 | 2.4481 | 0.4255 | 99.8% | 1017.4922 | 1018.1582 | 6 | 6.628 | 62.5% | 1 | K.ITITNDKGR.L | 2222 |
|  | Mis12XLAPLSS\_011913\_02.04217.04217.3 | 3.2562 | 0.279 | 99.8% | 1953.1144 | 1954.1621 | 4 | 4.889 | 38.3% | 1 | R.MVQEAEKYKAEDEVQR.E | 3 |
|  | Mis12XLAPLSS\_011913\_01.03494.03494.3 | 2.7556 | 0.3395 | 99.7% | 1423.5844 | 1423.5254 | 44 | 4.968 | 37.5% | 1 | K.YKAEDEVQRER.V | 3 |
|  | Mis12XLAPLSS\_011913\_01.09957.09957.2 | 3.6473 | 0.4529 | 100.0% | 1289.1522 | 1288.4608 | 1 | 7.703 | 85.0% | 1 | K.NALESYAFNMK.S | 22 |

Similarities:
gi|5729877|ref|NP\_006(2:16)  
gi|124256496|ref|NP\_0(7:11)  
gi|34419635|ref|NP\_00(5:13)  

---

|  |  |  |  |  |  |  |  |  |
| --- | --- | --- | --- | --- | --- | --- | --- | --- |
| U | *gi|4501881|ref|NP\_001* | 17 | 35 | 28.9% | 377 | 42051 | 5.4 | actin, alpha 1, skeletal muscle [Homo sapiens] |
| U | *gi|4885049|ref|NP\_005* | 17 | 35 | 28.9% | 377 | 42019 | 5.4 | cardiac muscle alpha actin 1 proprotein [Homo sapiens] |

| Filename XCorr DeltCN Conf% ObsM+H+ CalcM+H+ SpR ZScore Ion% # Sequence  | | | | | | | | | | | | |
| --- | --- | --- | --- | --- | --- | --- | --- | --- | --- | --- | --- | --- |
|  | Mis12XLAPLSS\_011913\_01.04238.04238.1 | 1.6701 | 0.2372 | 95.6% | 976.27 | 977.02136 | 36 | 4.581 | 50.0% | 1 | K.AGFAGDDAPR.A | 11 |
|  | Mis12XLAPLSS\_011913\_01.04286.04286.2 | 3.0985 | 0.3788 | 99.9% | 976.4322 | 977.02136 | 4 | 6.932 | 72.2% | 3 | K.AGFAGDDAPR.A | 22 |
|  | Mis12XLAPLSS\_011913\_01.08354.08354.2 | 2.8671 | 0.242 | 99.8% | 1199.1721 | 1199.4415 | 15 | 5.579 | 65.0% | 3 | R.AVFPSIVGRPR.H | 22 |
|  | Mis12XLAPLSS\_011913\_01.04090.04090.1 | 2.6388 | 0.4538 | 100.0% | 1171.44 | 1172.4058 | 1 | 7.705 | 70.0% | 1 | R.HQGVMVGMGQK.D | 11 |
|  | Mis12XLAPLSS\_011913\_01.04067.04067.2 | 2.9524 | 0.213 | 99.7% | 1173.2122 | 1172.4058 | 1 | 5.212 | 75.0% | 2 | R.HQGVMVGMGQK.D | 22 |
|  | Mis12XLAPLSS\_011913\_02.03558.03558.2 | 2.8314 | 0.0791 | 97.3% | 1198.4122 | 1199.2163 | 2 | 6.833 | 60.0% | 1 | K.DSYVGDEAQSK.R | 22 |
|  | Mis12XLAPLSS\_011913\_01.03634.03634.2 | 3.1046 | 0.0939 | 98.4% | 1354.5922 | 1355.4038 | 1 | 7.877 | 77.3% | 1 | K.DSYVGDEAQSKR.G | 22 |
|  | Mis12XLAPLSS\_011913\_01.09856.09856.2 | 3.3871 | 0.0224 | 96.4% | 1961.7722 | 1962.1841 | 1 | 5.02 | 56.7% | 1 | K.YPIEHGIITNWDDMEK.I | 2 |
|  | Mis12XLAPLSS\_011913\_01.09890.09890.3 | 4.3177 | 0.1131 | 97.1% | 1963.5243 | 1962.1841 | 10 | 4.972 | 41.7% | 1 | K.YPIEHGIITNWDDMEK.I | 3 |
|  | Mis12XLAPLSS\_011913\_01.12935.12935.3 | 5.208 | 0.2881 | 100.0% | 3460.0144 | 3459.8628 | 1 | 5.619 | 29.8% | 1 | K.YPIEHGIITNWDDMEKIWHHTFYNELR.V | 3 |
|  | Mis12XLAPLSS\_011913\_01.07736.07736.2 | 3.2339 | 0.4221 | 100.0% | 1516.3922 | 1516.7019 | 1 | 6.318 | 80.0% | 3 | K.IWHHTFYNELR.V | 22 |
|  | Mis12XLAPLSS\_011913\_01.07730.07730.3 | 3.3097 | 0.2091 | 99.4% | 1516.9143 | 1516.7019 | 1 | 5.643 | 55.0% | 3 | K.IWHHTFYNELR.V | 33 |
|  | Mis12XLAPLSS\_011913\_01.10998.10998.2 | 2.236 | 0.2609 | 99.4% | 999.0122 | 999.167 | 44 | 5.601 | 71.4% | 2 | R.DLTDYLMK.I | 22 |
|  | Mis12XLAPLSS\_011913\_01.11108.11108.2 | 4.8425 | 0.3335 | 100.0% | 1791.4321 | 1791.9554 | 1 | 8.207 | 83.3% | 6 | K.SYELPDGQVITIGNER.F | 22 |
|  | Mis12XLAPLSS\_011913\_01.07324.07324.2 | 3.9946 | 0.3349 | 100.0% | 1549.1721 | 1549.8843 | 1 | 6.577 | 76.9% | 1 | R.MQKEITALAPSTMK.I | 22 |
|  | Mis12XLAPLSS\_011913\_01.07623.07623.1 | 2.3118 | 0.4052 | 100.0% | 1161.46 | 1162.3868 | 2 | 7.007 | 60.0% | 2 | K.EITALAPSTMK.I | 11 |
|  | Mis12XLAPLSS\_011913\_01.07652.07652.2 | 2.9102 | 0.4108 | 99.9% | 1162.0922 | 1162.3868 | 1 | 6.495 | 65.0% | 3 | K.EITALAPSTMK.I | 22 |

Similarities:
gi|4501885|ref|NP\_001(14:3)  

---

|  |  |  |  |  |  |  |  |  |
| --- | --- | --- | --- | --- | --- | --- | --- | --- |
| U | *gi|4506669|ref|NP\_000* | 2 | 11 | 28.9% | 114 | 11514 | 4.3 | ribosomal protein P1 isoform 1 [Homo sapiens] |

| Filename XCorr DeltCN Conf% ObsM+H+ CalcM+H+ SpR ZScore Ion% # Sequence  | | | | | | | | | | | | |
| --- | --- | --- | --- | --- | --- | --- | --- | --- | --- | --- | --- | --- |
|  | Mis12XLAPLSS\_011913\_01.13511.13511.2 | 2.7798 | 0.3574 | 99.8% | 1702.8522 | 1703.9811 | 1 | 7.131 | 40.0% | 2 | K.AAGVNVEPFWPGLFAK.A | 2 |
|  | Mis12XLAPLSS\_011913\_01.22512.22512.2 | 2.7357 | 0.4455 | 99.8% | 2110.0923 | 2110.979 | 1 | 8.226 | 59.4% | 9 | K.KEES\*EES\*DDDMGFGLFD.- | 22 |

Similarities:
gi|4506671|ref|NP\_000(1:1)  

---

|  |  |  |  |  |  |  |  |  |
| --- | --- | --- | --- | --- | --- | --- | --- | --- |
| U | *gi|15718687|ref|NP\_00* | 5 | 9 | 28.8% | 243 | 26688 | 9.7 | ribosomal protein S3 [Homo sapiens] |

| Filename XCorr DeltCN Conf% ObsM+H+ CalcM+H+ SpR ZScore Ion% # Sequence  | | | | | | | | | | | | |
| --- | --- | --- | --- | --- | --- | --- | --- | --- | --- | --- | --- | --- |
| \* | Mis12XLAPLSS\_011913\_02.05576.05576.2 | 3.9649 | 0.4049 | 100.0% | 1424.1921 | 1424.5071 | 1 | 8.43 | 79.2% | 3 | R.ELAEDGYSGVEVR.V | 2 |
| \* | Mis12XLAPLSS\_011913\_01.09436.09436.2 | 3.5575 | 0.1911 | 99.8% | 1584.4922 | 1584.8998 | 3 | 4.744 | 53.8% | 1 | R.VTPTRTEIIILATR.T | 2 |
| \* | Mis12XLAPLSS\_011913\_01.11314.11314.2 | 3.1422 | 0.3895 | 99.9% | 1573.2922 | 1573.7423 | 1 | 7.628 | 73.1% | 2 | R.FGFPEGSVELYAEK.V | 2 |
| \* | Mis12XLAPLSS\_011913\_01.08885.08885.2 | 2.5638 | 0.2115 | 98.4% | 1471.0122 | 1471.6476 | 51 | 4.648 | 54.2% | 1 | K.DEILPTTPISEQK.G | 2 |
| \* | Mis12XLAPLSS\_011913\_01.07702.07702.2 | 3.1554 | 0.3843 | 99.8% | 1574.0721 | 1574.8352 | 4 | 5.91 | 50.0% | 2 | K.GGKPEPPAMPQPVPTA.- | 2 |

---

|  |  |  |  |  |  |  |  |  |
| --- | --- | --- | --- | --- | --- | --- | --- | --- |
| U | *gi|10863927|ref|NP\_06* | 7 | 13 | 28.5% | 165 | 18012 | 7.8 | peptidylprolyl isomerase A [Homo sapiens] |
| U | *gi|169215435|ref|XP\_0* | 7 | 13 | 21.1% | 223 | 24376 | 6.9 | PREDICTED: similar to peptidylprolyl isomerase A-like [Homo sapiens] |

| Filename XCorr DeltCN Conf% ObsM+H+ CalcM+H+ SpR ZScore Ion% # Sequence  | | | | | | | | | | | | |
| --- | --- | --- | --- | --- | --- | --- | --- | --- | --- | --- | --- | --- |
|  | Mis12XLAPLSS\_011913\_01.10640.10640.2 | 3.6099 | 0.3392 | 99.9% | 1380.3322 | 1380.6268 | 1 | 7.218 | 68.2% | 3 | R.VSFELFADKVPK.T | 2 |
|  | Mis12XLAPLSS\_011913\_01.10610.10610.3 | 2.9645 | 0.4276 | 100.0% | 1380.8644 | 1380.6268 | 456 | 6.599 | 38.6% | 1 | R.VSFELFADKVPK.T | 3 |
|  | Mis12XLAPLSS\_011913\_01.10318.10318.2 | 4.8551 | 0.4637 | 100.0% | 1832.4521 | 1833.0477 | 1 | 7.583 | 64.3% | 2 | K.SIYGEKFEDENFILK.H | 2 |
|  | Mis12XLAPLSS\_011913\_01.09928.09928.2 | 3.063 | 0.0835 | 99.3% | 1156.1721 | 1155.2927 | 16 | 4.881 | 75.0% | 1 | K.FEDENFILK.H | 2 |
|  | Mis12XLAPLSS\_011913\_01.06471.06471.2 | 2.2478 | 0.1401 | 97.4% | 849.9522 | 848.931 | 1 | 4.129 | 75.0% | 2 | K.TEWLDGK.H | 2 |
|  | Mis12XLAPLSS\_011913\_01.09197.09197.2 | 3.9123 | 0.0438 | 99.5% | 1505.9922 | 1506.7755 | 1 | 8.084 | 79.2% | 2 | K.VKEGMNIVEAMER.F | 2 |
|  | Mis12XLAPLSS\_011913\_01.10484.10484.2 | 2.9833 | 0.0693 | 98.0% | 1279.9321 | 1279.4689 | 2 | 6.448 | 70.0% | 2 | K.EGMNIVEAMER.F | 2 |

---

|  |  |  |  |  |  |  |  |  |
| --- | --- | --- | --- | --- | --- | --- | --- | --- |
| U | *gi|16306492|ref|NP\_20* | 5 | 6 | 28.3% | 240 | 27503 | 7.1 | cell division cycle 2 isoform 2 [Homo sapiens] |
| U | *gi|4502709|ref|NP\_001* | 5 | 6 | 22.9% | 297 | 34095 | 8.4 | cell division cycle 2 isoform 1 [Homo sapiens] |
| U | *gi|195927041|ref|NP\_0* | 5 | 6 | 22.9% | 297 | 34081 | 8.4 | cell division cycle 2 isoform 3 [Homo sapiens] |

| Filename XCorr DeltCN Conf% ObsM+H+ CalcM+H+ SpR ZScore Ion% # Sequence  | | | | | | | | | | | | |
| --- | --- | --- | --- | --- | --- | --- | --- | --- | --- | --- | --- | --- |
|  | Mis12XLAPLSS\_011913\_01.07502.07502.2 | 3.1678 | 0.2797 | 99.8% | 1186.2122 | 1186.3501 | 1 | 8.066 | 75.0% | 2 | K.IGEGTYGVVYK.G | 2 |
|  | Mis12XLAPLSS\_011913\_01.07251.07251.2 | 2.1984 | 0.2731 | 98.7% | 1027.9922 | 1029.1814 | 1 | 5.564 | 83.3% | 1 | R.SPEVLLGSAR.Y | 2 |
|  | Mis12XLAPLSS\_011913\_01.17270.17270.2 | 3.2509 | 0.2872 | 99.8% | 2212.392 | 2213.5352 | 4 | 6.498 | 42.1% | 1 | R.YSTPVDIWSIGTIFAELATK.K | 2 |
|  | Mis12XLAPLSS\_011913\_01.10312.10312.2 | 2.9581 | 0.3116 | 99.8% | 1802.7122 | 1803.0275 | 1 | 6.215 | 50.0% | 1 | K.KPLFHGDSEIDQLFR.I | 2 |
|  | Mis12XLAPLSS\_011913\_01.10259.10259.2 | 3.1775 | 0.3022 | 99.8% | 1331.5922 | 1331.4656 | 1 | 6.913 | 77.3% | 1 | K.NLDENGLDLLSK.M | 2 |

---

|  |  |  |  |  |  |  |  |  |
| --- | --- | --- | --- | --- | --- | --- | --- | --- |
| U | *gi|23308577|ref|NP\_00* | 8 | 10 | 28.0% | 533 | 56651 | 6.7 | phosphoglycerate dehydrogenase [Homo sapiens] |

| Filename XCorr DeltCN Conf% ObsM+H+ CalcM+H+ SpR ZScore Ion% # Sequence  | | | | | | | | | | | | |
| --- | --- | --- | --- | --- | --- | --- | --- | --- | --- | --- | --- | --- |
| \* | Mis12XLAPLSS\_011913\_01.08166.08166.2 | 3.3996 | 0.3773 | 99.8% | 1299.2122 | 1299.5101 | 1 | 6.91 | 86.4% | 1 | K.ILQDGGLQVVEK.Q | 2 |
| \* | Mis12XLAPLSS\_011913\_01.05274.05274.2 | 2.9482 | 0.3227 | 99.8% | 1131.1122 | 1131.2712 | 1 | 7.031 | 75.0% | 1 | K.VTADVINAAEK.L | 2 |
| \* | Mis12XLAPLSS\_011913\_01.07724.07724.2 | 4.3681 | 0.4965 | 100.0% | 1489.3322 | 1489.5822 | 1 | 9.235 | 60.7% | 3 | R.AGTGVDNVDLEAATR.K | 2 |
| \* | Mis12XLAPLSS\_011913\_01.09315.09315.2 | 2.0517 | 0.2453 | 96.1% | 1100.1322 | 1100.2603 | 47 | 4.911 | 60.0% | 1 | R.GGIVDEGALLR.A | 2 |
| \* | Mis12XLAPLSS\_011913\_01.15477.15477.3 | 4.1614 | 0.2741 | 99.7% | 3541.7043 | 3540.118 | 1 | 4.653 | 25.0% | 1 | K.SLTGVVNAQALTSAFSPHTKPWIGLAEALGTLMR.A | 3 |
| \* | Mis12XLAPLSS\_011913\_01.08727.08727.2 | 3.6325 | 0.1623 | 99.8% | 1346.1122 | 1346.5669 | 2 | 6.839 | 66.7% | 1 | K.GTIQVITQGTSLK.N | 2 |
| \* | Mis12XLAPLSS\_011913\_01.15303.15303.2 | 4.2253 | 0.475 | 100.0% | 2272.0322 | 2273.668 | 1 | 7.474 | 47.6% | 1 | R.TQTSDPAMLPTMIGLLAEAGVR.L | 2 |
| \* | Mis12XLAPLSS\_011913\_02.10940.10940.3 | 3.5481 | 0.2749 | 99.4% | 3448.9443 | 3449.9434 | 53 | 5.376 | 19.2% | 1 | R.LLSYQTSLVSDGETWHVMGISSLLPSLEAWK.Q | 3 |

---

|  |  |  |  |  |  |  |  |  |
| --- | --- | --- | --- | --- | --- | --- | --- | --- |
| U | *gi|5902102|ref|NP\_008* | 2 | 3 | 27.7% | 119 | 13282 | 11.6 | small nuclear ribonucleoprotein D1 polypeptide 16kDa [Homo sapiens] |

| Filename XCorr DeltCN Conf% ObsM+H+ CalcM+H+ SpR ZScore Ion% # Sequence  | | | | | | | | | | | | |
| --- | --- | --- | --- | --- | --- | --- | --- | --- | --- | --- | --- | --- |
|  | Mis12XLAPLSS\_011913\_01.08918.08918.2 | 3.7586 | 0.3415 | 99.8% | 1555.7922 | 1555.7745 | 5 | 5.948 | 62.5% | 1 | K.NREPVQLETLSIR.G | 2 |
| \* | Mis12XLAPLSS\_011913\_01.16184.16184.2 | 4.7111 | 0.4945 | 100.0% | 2288.4321 | 2288.6863 | 1 | 8.591 | 68.4% | 2 | R.YFILPDSLPLDTLLVDVEPK.V | 2 |

---

|  |  |  |  |  |  |  |  |  |
| --- | --- | --- | --- | --- | --- | --- | --- | --- |
| U | *gi|4507357|ref|NP\_003* | 4 | 7 | 27.6% | 199 | 22391 | 8.2 | transgelin 2 [Homo sapiens] |

| Filename XCorr DeltCN Conf% ObsM+H+ CalcM+H+ SpR ZScore Ion% # Sequence  | | | | | | | | | | | | |
| --- | --- | --- | --- | --- | --- | --- | --- | --- | --- | --- | --- | --- |
| \* | Mis12XLAPLSS\_011913\_01.14166.14166.2 | 5.259 | 0.5498 | 100.0% | 2100.392 | 2101.3203 | 1 | 10.446 | 61.8% | 1 | R.YGINTTDIFQTVDLWEGK.N | 2 |
| \* | Mis12XLAPLSS\_011913\_01.10978.10978.2 | 3.6734 | 0.4004 | 100.0% | 1216.5521 | 1216.4845 | 1 | 7.603 | 72.7% | 2 | R.TLMNLGGLAVAR.D | 2 |
| \* | Mis12XLAPLSS\_011913\_01.08438.08438.2 | 2.8338 | 0.4524 | 99.9% | 1203.3522 | 1203.402 | 1 | 7.697 | 85.0% | 1 | K.NVIGLQMGTNR.G | 2 |
|  | Mis12XLAPLSS\_011913\_01.06297.06297.2 | 3.5618 | 0.4718 | 100.0% | 1383.9321 | 1384.5677 | 1 | 7.998 | 73.1% | 3 | R.GASQAGMTGYGMPR.Q | 2 |

---

|  |  |  |  |  |  |  |  |  |
| --- | --- | --- | --- | --- | --- | --- | --- | --- |
| U | *gi|169201338|ref|XP\_0* | 4 | 7 | 27.5% | 160 | 18565 | 10.5 | PREDICTED: hypothetical protein [Homo sapiens] |
| U | *gi|18104948|ref|NP\_00* | 4 | 7 | 27.5% | 160 | 18565 | 10.5 | ribosomal protein L21 [Homo sapiens] |
| U | *gi|169210381|ref|XP\_0* | 4 | 7 | 27.5% | 160 | 18535 | 10.6 | PREDICTED: hypothetical protein isoform 2 [Homo sapiens] |
| U | *gi|169210379|ref|XP\_0* | 4 | 7 | 27.5% | 160 | 18535 | 10.6 | PREDICTED: hypothetical protein isoform 3 [Homo sapiens] |
| U | *gi|169210377|ref|XP\_0* | 4 | 7 | 27.5% | 160 | 18535 | 10.6 | PREDICTED: hypothetical protein isoform 1 [Homo sapiens] |
| U | *gi|169201750|ref|XP\_0* | 4 | 7 | 27.5% | 160 | 18550 | 10.5 | PREDICTED: hypothetical protein [Homo sapiens] |

| Filename XCorr DeltCN Conf% ObsM+H+ CalcM+H+ SpR ZScore Ion% # Sequence  | | | | | | | | | | | | |
| --- | --- | --- | --- | --- | --- | --- | --- | --- | --- | --- | --- | --- |
|  | Mis12XLAPLSS\_011913\_01.08690.08690.2 | 2.8526 | 0.415 | 99.9% | 1244.1721 | 1244.4973 | 1 | 7.249 | 75.0% | 2 | K.HGVVPLATYMR.I | 2 |
|  | Mis12XLAPLSS\_011913\_01.08157.08157.3 | 4.2413 | 0.2859 | 100.0% | 1642.1643 | 1641.9108 | 1 | 7.242 | 46.4% | 2 | R.VYNVTQHAVGIVVNK.Q | 3 |
|  | Mis12XLAPLSS\_011913\_02.05932.05932.2 | 4.8216 | 0.4007 | 100.0% | 1642.3522 | 1641.9108 | 1 | 7.233 | 71.4% | 2 | R.VYNVTQHAVGIVVNK.Q | 2 |
|  | Mis12XLAPLSS\_011913\_01.13265.13265.2 | 4.3408 | 0.2631 | 99.9% | 2079.4521 | 2079.3723 | 1 | 6.594 | 52.9% | 1 | R.TNGKEPELLEPIPYEFMA.- | 2 |

---

|  |  |  |  |  |  |  |  |  |
| --- | --- | --- | --- | --- | --- | --- | --- | --- |
| U | *gi|27436946|ref|NP\_73* | 14 | 22 | 27.3% | 664 | 74140 | 7.0 | lamin A/C isoform 1 precursor [Homo sapiens] |

| Filename XCorr DeltCN Conf% ObsM+H+ CalcM+H+ SpR ZScore Ion% # Sequence  | | | | | | | | | | | | |
| --- | --- | --- | --- | --- | --- | --- | --- | --- | --- | --- | --- | --- |
|  | Mis12XLAPLSS\_011913\_01.04737.04737.2 | 2.5342 | 0.2588 | 99.5% | 1089.8522 | 1090.1783 | 8 | 5.156 | 72.2% | 1 | R.SLETENAGLR.L | 2 |
|  | Mis12XLAPLSS\_011913\_01.04311.04311.2 | 2.9455 | 0.3498 | 99.9% | 1148.4321 | 1149.2432 | 2 | 7.227 | 72.2% | 1 | R.ITESEEVVSR.E | 2 |
|  | Mis12XLAPLSS\_011913\_01.08087.08087.2 | 3.5958 | 0.3666 | 100.0% | 1510.6122 | 1510.7455 | 1 | 7.195 | 72.7% | 1 | R.LQTMKEELDFQK.N | 2 |
|  | Mis12XLAPLSS\_011913\_01.08952.08952.2 | 3.2798 | 0.3058 | 99.9% | 1029.4321 | 1029.1814 | 2 | 5.97 | 87.5% | 2 | R.LADALQELR.A | 2 |
|  | Mis12XLAPLSS\_011913\_01.03316.03316.2 | 4.1614 | 0.3468 | 100.0% | 1503.0922 | 1503.6115 | 1 | 7.317 | 77.3% | 1 | R.AQHEDQVEQYKK.E | 2 |
|  | Mis12XLAPLSS\_011913\_01.07210.07210.2 | 4.6441 | 0.4539 | 100.0% | 1754.2922 | 1753.8693 | 1 | 8.329 | 63.3% | 2 | R.NSNLVGAAHEELQQSR.I | 2 |
|  | Mis12XLAPLSS\_011913\_01.10335.10335.2 | 4.0437 | 0.3317 | 99.8% | 1701.0721 | 1700.9762 | 1 | 7.142 | 71.4% | 1 | R.IRIDSLSAQLSQLQK.Q | 2 |
|  | Mis12XLAPLSS\_011913\_01.06801.06801.2 | 2.7681 | 0.2302 | 99.7% | 1188.2922 | 1188.3262 | 6 | 4.859 | 72.2% | 3 | K.LRDLEDSLAR.E | 2 |
|  | Mis12XLAPLSS\_011913\_01.12225.12225.2 | 3.1286 | 0.3708 | 99.8% | 1894.0721 | 1895.1346 | 1 | 6.512 | 50.0% | 1 | R.MQQQLDEYQELLDIK.L | 2 |
|  | Mis12XLAPLSS\_011913\_02.05564.05564.3 | 2.5784 | 0.4062 | 99.7% | 1606.4343 | 1606.7728 | 4 | 6.187 | 42.3% | 1 | R.VAVEEVDEEGKFVR.L | 3 |
|  | Mis12XLAPLSS\_011913\_02.05577.05577.2 | 3.9704 | 0.4389 | 100.0% | 1607.5122 | 1606.7728 | 1 | 7.981 | 65.4% | 2 | R.VAVEEVDEEGKFVR.L | 2 |
|  | Mis12XLAPLSS\_011913\_02.05938.05938.2 | 4.2961 | 0.5704 | 100.0% | 1492.0122 | 1492.6874 | 1 | 8.605 | 73.1% | 3 | R.TALINSTGEEVAMR.K | 2 |
|  | Mis12XLAPLSS\_011913\_02.05109.05109.3 | 3.0364 | 0.2996 | 99.0% | 2366.9944 | 2366.504 | 1 | 5.604 | 28.8% | 1 | K.ASASGSGAQVGGPISSGSSASSVTVTR.S | 3 |
|  | Mis12XLAPLSS\_011913\_02.05880.05880.2 | 3.1127 | 0.465 | 99.9% | 1567.0721 | 1567.6555 | 1 | 6.547 | 43.8% | 2 | R.SVGGSGGGSFGDNLVTR.S | 2 |

---

|  |  |  |  |  |  |  |  |  |
| --- | --- | --- | --- | --- | --- | --- | --- | --- |
| U | *gi|4506679|ref|NP\_001* | 3 | 5 | 27.3% | 165 | 18898 | 10.2 | ribosomal protein S10 [Homo sapiens] |

| Filename XCorr DeltCN Conf% ObsM+H+ CalcM+H+ SpR ZScore Ion% # Sequence  | | | | | | | | | | | | |
| --- | --- | --- | --- | --- | --- | --- | --- | --- | --- | --- | --- | --- |
| \* | Mis12XLAPLSS\_011913\_01.12359.12359.2 | 3.3927 | 0.3877 | 99.9% | 2002.9122 | 2004.2548 | 1 | 7.233 | 64.3% | 1 | R.HFYWYLTNEGIQYLR.D | 2 |
| \* | Mis12XLAPLSS\_011913\_01.11169.11169.3 | 3.2978 | 0.293 | 99.8% | 1891.6144 | 1891.2235 | 2 | 5.043 | 40.0% | 2 | R.DYLHLPPEIVPATLRR.S | 3 |
|  | Mis12XLAPLSS\_011913\_02.06146.06146.2 | 3.192 | 0.4595 | 100.0% | 1441.8121 | 1442.5278 | 1 | 7.555 | 69.2% | 2 | K.AEAGAGSATEFQFR.G | 2 |

---

|  |  |  |  |  |  |  |  |  |
| --- | --- | --- | --- | --- | --- | --- | --- | --- |
| U | *gi|4503471|ref|NP\_001* | 10 | 33 | 27.1% | 462 | 50141 | 9.0 | eukaryotic translation elongation factor 1 alpha 1 [Homo sapiens] |

| Filename XCorr DeltCN Conf% ObsM+H+ CalcM+H+ SpR ZScore Ion% # Sequence  | | | | | | | | | | | | |
| --- | --- | --- | --- | --- | --- | --- | --- | --- | --- | --- | --- | --- |
|  | Mis12XLAPLSS\_011913\_01.08044.08044.2 | 2.7137 | 0.1885 | 98.2% | 1588.3522 | 1589.835 | 1 | 5.089 | 71.4% | 4 | K.THINIVVIGHVDSGK.S | 2 |
|  | Mis12XLAPLSS\_011913\_02.05962.05962.3 | 4.7345 | 0.4773 | 100.0% | 1589.3344 | 1589.835 | 1 | 7.814 | 50.0% | 7 | K.THINIVVIGHVDSGK.S | 3 |
|  | Mis12XLAPLSS\_011913\_01.04060.04060.2 | 3.1097 | 0.4576 | 100.0% | 1121.9521 | 1121.2786 | 1 | 6.641 | 66.7% | 2 | K.STTTGHLIYK.C | 2 |
| \* | Mis12XLAPLSS\_011913\_01.08741.08741.2 | 2.8866 | 0.3781 | 99.8% | 1405.3522 | 1405.5962 | 1 | 6.496 | 68.2% | 2 | K.YYVTIIDAPGHR.D | 2 |
|  | Mis12XLAPLSS\_011913\_02.07016.07016.2 | 3.1639 | 0.4972 | 100.0% | 1315.5922 | 1315.5553 | 1 | 8.519 | 72.7% | 1 | R.EHALLAYTLGVK.Q | 23 |
| \* | Mis12XLAPLSS\_011913\_01.13944.13944.3 | 4.3861 | 0.4164 | 100.0% | 3698.9043 | 3698.205 | 1 | 5.442 | 26.6% | 1 | K.KIGYNPDTVAFVPISGWNGDNMLEPSANMPWFK.G | 3 |
|  | Mis12XLAPLSS\_011913\_01.08307.08307.2 | 2.7197 | 0.2423 | 99.8% | 976.0522 | 976.1607 | 1 | 5.906 | 85.7% | 2 | R.LPLQDVYK.I | 2 |
|  | Mis12XLAPLSS\_011913\_01.07448.07448.2 | 3.4755 | 0.4212 | 100.0% | 1026.0122 | 1026.2241 | 1 | 8.161 | 85.0% | 5 | K.IGGIGTVPVGR.V | 2 |
| \* | Mis12XLAPLSS\_011913\_01.11750.11750.2 | 4.4043 | 0.4665 | 100.0% | 2515.912 | 2516.999 | 1 | 8.51 | 52.2% | 2 | R.VETGVLKPGMVVTFAPVNVTTEVK.S | 2 |
| \* | Mis12XLAPLSS\_011913\_02.08006.08006.3 | 4.5592 | 0.395 | 100.0% | 2518.0444 | 2516.999 | 1 | 6.401 | 35.9% | 7 | R.VETGVLKPGMVVTFAPVNVTTEVK.S | 3 |

---

|  |  |  |  |  |  |  |  |  |
| --- | --- | --- | --- | --- | --- | --- | --- | --- |
| U | *gi|13654278|ref|NP\_11* | 2 | 2 | 25.7% | 109 | 12349 | 10.2 | SRA stem-loop-interacting RNA-binding protein [Homo sapiens] |

| Filename XCorr DeltCN Conf% ObsM+H+ CalcM+H+ SpR ZScore Ion% # Sequence  | | | | | | | | | | | | |
| --- | --- | --- | --- | --- | --- | --- | --- | --- | --- | --- | --- | --- |
| \* | Mis12XLAPLSS\_011913\_02.08205.08205.2 | 3.267 | 0.2827 | 99.8% | 1566.5922 | 1565.7257 | 1 | 5.42 | 53.8% | 1 | R.GLGWVQFSSEEGLR.N | 2 |
| \* | Mis12XLAPLSS\_011913\_01.07517.07517.2 | 3.9181 | 0.1501 | 99.8% | 1580.2322 | 1579.7532 | 1 | 7.027 | 61.5% | 1 | R.NALQQENHIIDGVK.V | 2 |

---

|  |  |  |  |  |  |  |  |  |
| --- | --- | --- | --- | --- | --- | --- | --- | --- |
| U | *gi|4504523|ref|NP\_002* | 2 | 8 | 25.5% | 102 | 10932 | 8.9 | heat shock 10kDa protein 1 [Homo sapiens] |

| Filename XCorr DeltCN Conf% ObsM+H+ CalcM+H+ SpR ZScore Ion% # Sequence  | | | | | | | | | | | | |
| --- | --- | --- | --- | --- | --- | --- | --- | --- | --- | --- | --- | --- |
| \* | Mis12XLAPLSS\_011913\_02.05463.05463.2 | 4.3352 | 0.5784 | 100.0% | 1316.0721 | 1316.5406 | 1 | 9.629 | 84.6% | 6 | K.VLQATVVAVGSGSK.G | 2 |
|  | Mis12XLAPLSS\_011913\_02.08051.08051.2 | 2.7272 | 0.3409 | 99.8% | 1530.3121 | 1530.7637 | 1 | 5.953 | 63.6% | 2 | K.VVLDDKDYFLFR.D | 2 |

---

|  |  |  |  |  |  |  |  |  |
| --- | --- | --- | --- | --- | --- | --- | --- | --- |
| U | *gi|223890147|ref|NP\_0* | 7 | 12 | 25.3% | 356 | 40067 | 7.0 | DSN1, MIND kinetochore complex component, homolog isoform 1 [Homo sapiens] |
| U | *gi|223972618|ref|NP\_0* | 7 | 12 | 25.3% | 356 | 40067 | 7.0 | DSN1, MIND kinetochore complex component, homolog isoform 1 [Homo sapiens] |
| U | *gi|223890149|ref|NP\_0* | 7 | 12 | 25.3% | 356 | 40067 | 7.0 | DSN1, MIND kinetochore complex component, homolog isoform 1 [Homo sapiens] |

| Filename XCorr DeltCN Conf% ObsM+H+ CalcM+H+ SpR ZScore Ion% # Sequence  | | | | | | | | | | | | |
| --- | --- | --- | --- | --- | --- | --- | --- | --- | --- | --- | --- | --- |
|  | Mis12XLAPLSS\_011913\_01.05358.05358.2 | 3.1346 | 0.3665 | 99.9% | 1433.5122 | 1433.6599 | 1 | 6.702 | 70.8% | 1 | R.SEIIDEKGPVMSK.T | 2 |
|  | Mis12XLAPLSS\_011913\_02.04994.04994.2 | 4.3685 | 0.4424 | 100.0% | 1638.3922 | 1638.7467 | 1 | 7.676 | 64.3% | 2 | K.TSASLEMNQGVSEER.I | 2 |
|  | Mis12XLAPLSS\_011913\_01.06184.06184.3 | 3.8327 | 0.475 | 100.0% | 1588.7644 | 1588.8069 | 1 | 7.523 | 46.2% | 3 | K.SLHPIHQGITELSR.S | 3 |
|  | Mis12XLAPLSS\_011913\_01.05139.05139.2 | 2.4542 | 0.1815 | 97.8% | 1205.1921 | 1205.3538 | 3 | 4.188 | 65.0% | 1 | R.SISVDLAESKR.L | 2 |
|  | Mis12XLAPLSS\_011913\_01.12449.12449.3 | 4.2344 | 0.4238 | 100.0% | 2477.6643 | 2477.6873 | 2 | 6.68 | 31.8% | 1 | K.ASSLSEELKHFADGLETDGTLQK.C | 3 |
|  | Mis12XLAPLSS\_011913\_01.06917.06917.2 | 3.6659 | 0.3237 | 99.9% | 1533.1122 | 1532.65 | 1 | 6.385 | 73.1% | 1 | K.HFADGLETDGTLQK.C | 2 |
|  | Mis12XLAPLSS\_011913\_02.07509.07509.2 | 2.8385 | 0.3752 | 99.8% | 1485.2722 | 1485.6489 | 4 | 6.482 | 57.7% | 3 | K.ASDFSLEASVAEMK.E | 2 |

---

|  |  |  |  |  |  |  |  |  |
| --- | --- | --- | --- | --- | --- | --- | --- | --- |
| U | *gi|5803227|ref|NP\_006* | 3 | 3 | 25.3% | 245 | 27764 | 4.8 | tyrosine 3/tryptophan 5 -monooxygenase activation protein, theta polypeptide [Homo sapiens] |

| Filename XCorr DeltCN Conf% ObsM+H+ CalcM+H+ SpR ZScore Ion% # Sequence  | | | | | | | | | | | | |
| --- | --- | --- | --- | --- | --- | --- | --- | --- | --- | --- | --- | --- |
| \* | Mis12XLAPLSS\_011913\_01.04452.04452.2 | 4.774 | 0.4951 | 100.0% | 1533.4321 | 1533.592 | 1 | 8.605 | 76.9% | 1 | K.AVTEQGAELSNEER.N | 2 |
| \* | Mis12XLAPLSS\_011913\_02.06208.06208.3 | 3.4097 | 0.2387 | 98.6% | 2143.7043 | 2144.3025 | 29 | 4.57 | 29.2% | 1 | K.QTIDNSQGAYQEAFDISKK.E | 3 |
| \* | Mis12XLAPLSS\_011913\_01.17336.17336.3 | 4.2944 | 0.3735 | 100.0% | 3317.5144 | 3317.6895 | 1 | 6.262 | 25.9% | 1 | K.TAFDEAIAELDTLNEDSYKDSTLIMQLLR.D | 3 |

---

|  |  |  |  |  |  |  |  |  |
| --- | --- | --- | --- | --- | --- | --- | --- | --- |
| U | *gi|14043070|ref|NP\_11* | 10 | 21 | 25.0% | 372 | 38747 | 9.1 | heterogeneous nuclear ribonucleoprotein A1 isoform b [Homo sapiens] |
| U | *gi|4504445|ref|NP\_002* | 10 | 21 | 29.1% | 320 | 34196 | 9.2 | heterogeneous nuclear ribonucleoprotein A1 isoform a [Homo sapiens] |

| Filename XCorr DeltCN Conf% ObsM+H+ CalcM+H+ SpR ZScore Ion% # Sequence  | | | | | | | | | | | | |
| --- | --- | --- | --- | --- | --- | --- | --- | --- | --- | --- | --- | --- |
|  | Mis12XLAPLSS\_011913\_01.03741.03741.2 | 2.4547 | 0.2035 | 98.4% | 1300.2722 | 1300.4111 | 4 | 4.416 | 60.0% | 1 | K.SESPKEPEQLR.K | 2 |
|  | Mis12XLAPLSS\_011913\_01.03437.03437.2 | 2.6896 | 0.1537 | 98.1% | 1427.4521 | 1428.5852 | 13 | 4.37 | 63.6% | 1 | K.SESPKEPEQLRK.L | 2 |
|  | Mis12XLAPLSS\_011913\_02.08140.08140.3 | 4.6849 | 0.3166 | 100.0% | 1913.6943 | 1914.1656 | 3 | 5.925 | 39.1% | 1 | R.KLFIGGLSFETTDESLR.S | 3 |
|  | Mis12XLAPLSS\_011913\_01.12758.12758.2 | 4.7673 | 0.5627 | 100.0% | 1785.3922 | 1785.9916 | 1 | 10.264 | 70.0% | 5 | K.LFIGGLSFETTDESLR.S | 2 |
|  | Mis12XLAPLSS\_011913\_01.11282.11282.2 | 3.8131 | 0.3985 | 100.0% | 1220.0122 | 1219.4387 | 1 | 7.224 | 88.9% | 4 | K.IEVIEIMTDR.G | 2 |
|  | Mis12XLAPLSS\_011913\_02.07018.07018.3 | 3.2413 | 0.3067 | 99.7% | 1856.3944 | 1856.989 | 7 | 5.481 | 35.0% | 1 | K.RGFAFVTFDDHDSVDK.I | 3 |
|  | Mis12XLAPLSS\_011913\_01.10556.10556.2 | 3.2025 | 0.4322 | 99.9% | 1701.8722 | 1700.8016 | 1 | 7.109 | 57.1% | 2 | R.GFAFVTFDDHDSVDK.I | 2 |
|  | Mis12XLAPLSS\_011913\_01.11444.11444.3 | 4.3166 | 0.21 | 99.8% | 2282.3943 | 2282.5579 | 1 | 6.713 | 34.2% | 2 | R.GFAFVTFDDHDSVDKIVIQK.Y | 3 |
|  | Mis12XLAPLSS\_011913\_01.05374.05374.2 | 3.8712 | 0.445 | 100.0% | 1629.3722 | 1629.7721 | 1 | 7.064 | 63.3% | 2 | R.SSGPYGGGGQYFAKPR.N | 2 |
|  | Mis12XLAPLSS\_011913\_01.03658.03658.2 | 5.1288 | 0.5366 | 100.0% | 1696.0721 | 1695.6561 | 1 | 11.709 | 70.6% | 2 | R.NQGGYGGSSSSSSYGSGR.R | 2 |

---

|  |  |  |  |  |  |  |  |  |
| --- | --- | --- | --- | --- | --- | --- | --- | --- |
| U | *gi|5453603|ref|NP\_006* | 8 | 8 | 24.7% | 535 | 57488 | 6.4 | chaperonin containing TCP1, subunit 2 [Homo sapiens] |

| Filename XCorr DeltCN Conf% ObsM+H+ CalcM+H+ SpR ZScore Ion% # Sequence  | | | | | | | | | | | | |
| --- | --- | --- | --- | --- | --- | --- | --- | --- | --- | --- | --- | --- |
| \* | Mis12XLAPLSS\_011913\_01.13782.13782.2 | 2.6024 | 0.3295 | 99.6% | 1518.7322 | 1518.8369 | 14 | 5.503 | 42.9% | 1 | R.LTSFIGAIAIGDLVK.S | 2 |
| \* | Mis12XLAPLSS\_011913\_02.07041.07041.2 | 2.8195 | 0.1952 | 98.6% | 1549.0322 | 1549.7797 | 2 | 5.368 | 57.1% | 1 | R.DASLMVTNDGATILK.N | 2 |
| \* | Mis12XLAPLSS\_011913\_01.14086.14086.2 | 3.1865 | 0.3776 | 99.8% | 2289.9521 | 2289.5017 | 1 | 6.482 | 38.1% | 1 | R.VQDDEVGDGTTSVTVLAAELLR.E | 2 |
| \* | Mis12XLAPLSS\_011913\_01.07554.07554.2 | 4.4334 | 0.4689 | 100.0% | 1657.1322 | 1657.7747 | 1 | 7.595 | 66.7% | 1 | R.EALLSSAVDHGSDEVK.F | 2 |
| \* | Mis12XLAPLSS\_011913\_01.12141.12141.3 | 3.4689 | 0.3787 | 100.0% | 2042.4844 | 2042.2933 | 1 | 5.924 | 31.9% | 1 | K.LGGSLADSYLDEGFLLDKK.I | 3 |
| \* | Mis12XLAPLSS\_011913\_01.03326.03326.2 | 3.1606 | 0.3451 | 99.9% | 1283.1522 | 1283.424 | 1 | 5.937 | 80.0% | 1 | K.VAEIEHAEKEK.M | 2 |
| \* | Mis12XLAPLSS\_011913\_01.08086.08086.2 | 3.7541 | 0.4322 | 100.0% | 1330.8322 | 1331.4252 | 1 | 7.369 | 77.3% | 1 | R.GATQQILDEAER.S | 2 |
| \* | Mis12XLAPLSS\_011913\_02.09314.09314.3 | 3.2412 | 0.2147 | 95.5% | 2348.1843 | 2348.6765 | 6 | 4.229 | 31.0% | 1 | R.MLPTIIADNAGYDSADLVAQLR.A | 3 |

---

|  |  |  |  |  |  |  |  |  |
| --- | --- | --- | --- | --- | --- | --- | --- | --- |
| U | *gi|5901926|ref|NP\_008* | 3 | 4 | 24.7% | 227 | 26227 | 8.8 | cleavage and polyadenylation specific factor 5 [Homo sapiens] |

| Filename XCorr DeltCN Conf% ObsM+H+ CalcM+H+ SpR ZScore Ion% # Sequence  | | | | | | | | | | | | |
| --- | --- | --- | --- | --- | --- | --- | --- | --- | --- | --- | --- | --- |
| \* | Mis12XLAPLSS\_011913\_01.08512.08512.3 | 4.006 | 0.2939 | 99.7% | 1909.9744 | 1910.0911 | 2 | 6.567 | 39.7% | 2 | K.LPGGELNPGEDEVEGLKR.L | 3 |
| \* | Mis12XLAPLSS\_011913\_01.10734.10734.2 | 2.1924 | 0.1701 | 95.9% | 1118.0521 | 1118.3617 | 9 | 4.063 | 75.0% | 1 | K.LFLVQLQEK.A | 2 |
| \* | Mis12XLAPLSS\_011913\_01.16293.16293.3 | 5.0647 | 0.4556 | 100.0% | 3117.2043 | 3116.6274 | 1 | 7.601 | 27.7% | 1 | K.LVAAPLFELYDNAPGYGPIISSLPQLLSR.F | 3 |

---

|  |  |  |  |  |  |  |  |  |
| --- | --- | --- | --- | --- | --- | --- | --- | --- |
| U | *gi|4506743|ref|NP\_001* | 6 | 9 | 24.5% | 208 | 24205 | 10.3 | ribosomal protein S8 [Homo sapiens] |

| Filename XCorr DeltCN Conf% ObsM+H+ CalcM+H+ SpR ZScore Ion% # Sequence  | | | | | | | | | | | | |
| --- | --- | --- | --- | --- | --- | --- | --- | --- | --- | --- | --- | --- |
| \* | Mis12XLAPLSS\_011913\_01.03606.03606.2 | 3.4544 | 0.3652 | 99.8% | 1347.6322 | 1348.5448 | 2 | 6.828 | 72.7% | 1 | R.KYELGRPAANTK.I | 2 |
| \* | Mis12XLAPLSS\_011913\_01.03982.03982.2 | 2.9945 | 0.3439 | 99.8% | 1220.0322 | 1220.3707 | 1 | 6.476 | 65.0% | 1 | K.YELGRPAANTK.I | 2 |
| \* | Mis12XLAPLSS\_011913\_02.07052.07052.3 | 3.1596 | 0.2755 | 99.8% | 1719.2344 | 1719.9353 | 36 | 5.609 | 41.1% | 1 | R.IIDVVYNASNNELVR.T | 3 |
| \* | Mis12XLAPLSS\_011913\_01.09894.09894.2 | 2.7948 | 0.3037 | 99.9% | 1720.0721 | 1719.9353 | 1 | 5.261 | 50.0% | 2 | R.IIDVVYNASNNELVR.T | 2 |
|  | Mis12XLAPLSS\_011913\_01.08294.08294.2 | 2.8248 | 0.1783 | 99.4% | 1315.1322 | 1315.4631 | 3 | 5.427 | 70.0% | 2 | K.LTPEEEEILNK.K | 2 |
| \* | Mis12XLAPLSS\_011913\_01.09460.09460.2 | 4.0158 | 0.4127 | 100.0% | 1507.2122 | 1507.6836 | 1 | 7.754 | 79.2% | 2 | K.ISSLLEEQFQQGK.L | 2 |

---

|  |  |  |  |  |  |  |  |  |
| --- | --- | --- | --- | --- | --- | --- | --- | --- |
| U | *gi|93588500|ref|NP\_06* | 3 | 6 | 24.5% | 159 | 17419 | 6.1 | angiotensin II receptor-associated protein isoform a [Homo sapiens] |

| Filename XCorr DeltCN Conf% ObsM+H+ CalcM+H+ SpR ZScore Ion% # Sequence  | | | | | | | | | | | | |
| --- | --- | --- | --- | --- | --- | --- | --- | --- | --- | --- | --- | --- |
| \* | Mis12XLAPLSS\_011913\_01.09381.09381.2 | 4.0617 | 0.4851 | 100.0% | 1773.4321 | 1773.9432 | 1 | 7.875 | 56.2% | 1 | R.GGELLVHTGFLGSSQDR.S | 2 |
| \* | Mis12XLAPLSS\_011913\_02.06836.06836.3 | 3.9213 | 0.3474 | 100.0% | 1774.2843 | 1773.9432 | 1 | 5.55 | 43.8% | 1 | R.GGELLVHTGFLGSSQDR.S | 3 |
|  | Mis12XLAPLSS\_011913\_01.10539.10539.2 | 4.6238 | 0.5361 | 100.0% | 2292.4321 | 2293.4517 | 1 | 10.092 | 47.6% | 4 | R.SAYQTIDSAEAPADPFAVPEGR.S | 2 |

---

|  |  |  |  |  |  |  |  |  |
| --- | --- | --- | --- | --- | --- | --- | --- | --- |
| U | *gi|4506597|ref|NP\_000* | 3 | 6 | 24.2% | 165 | 17819 | 9.4 | ribosomal protein L12 [Homo sapiens] |

| Filename XCorr DeltCN Conf% ObsM+H+ CalcM+H+ SpR ZScore Ion% # Sequence  | | | | | | | | | | | | |
| --- | --- | --- | --- | --- | --- | --- | --- | --- | --- | --- | --- | --- |
| \* | Mis12XLAPLSS\_011913\_01.08308.08308.1 | 1.823 | 0.3232 | 100.0% | 881.53 | 882.091 | 51 | 5.588 | 56.2% | 1 | K.IGPLGLSPK.K | 1 |
|  | Mis12XLAPLSS\_011913\_01.11349.11349.2 | 3.4859 | 0.2966 | 99.8% | 1667.3322 | 1667.9866 | 1 | 5.561 | 63.3% | 2 | R.QAQIEVVPSASALIIK.A | 2 |
| \* | Mis12XLAPLSS\_011913\_01.11582.11582.2 | 4.9124 | 0.5665 | 100.0% | 1686.2522 | 1686.865 | 1 | 11.242 | 71.4% | 3 | K.HSGNITFDEIVNIAR.Q | 2 |

---

|  |  |  |  |  |  |  |  |  |
| --- | --- | --- | --- | --- | --- | --- | --- | --- |
| U | *gi|10190716|ref|NP\_06* | 3 | 4 | 24.1% | 224 | 26153 | 8.0 | spindle pole body component 25 [Homo sapiens] |

| Filename XCorr DeltCN Conf% ObsM+H+ CalcM+H+ SpR ZScore Ion% # Sequence  | | | | | | | | | | | | |
| --- | --- | --- | --- | --- | --- | --- | --- | --- | --- | --- | --- | --- |
| \* | Mis12XLAPLSS\_011913\_01.11754.11754.3 | 5.5466 | 0.4289 | 100.0% | 2406.9543 | 2407.683 | 1 | 7.743 | 42.1% | 1 | K.KQELEVLTANIQDLKEEYSR.K | 3 |
| \* | Mis12XLAPLSS\_011913\_02.07683.07683.3 | 4.7744 | 0.4767 | 100.0% | 2506.0444 | 2506.6445 | 1 | 7.633 | 35.7% | 2 | R.DYEVSDSAPHLEGLAEFQENVR.K | 3 |
| \* | Mis12XLAPLSS\_011913\_01.11884.11884.2 | 2.6859 | 0.1703 | 98.4% | 1354.2722 | 1354.5082 | 2 | 5.409 | 68.2% | 1 | K.TNNFSAFLANVR.K | 2 |

---

|  |  |  |  |  |  |  |  |  |
| --- | --- | --- | --- | --- | --- | --- | --- | --- |
| U | *gi|21328448|ref|NP\_64* | 4 | 4 | 24.0% | 246 | 28082 | 4.8 | tyrosine 3-monooxygenase/tryptophan 5-monooxygenase activation protein, beta polypeptide [Homo sapiens] |
| U | *gi|4507949|ref|NP\_003* | 4 | 4 | 24.0% | 246 | 28082 | 4.8 | tyrosine 3-monooxygenase/tryptophan 5-monooxygenase activation protein, beta polypeptide [Homo sapiens] |

| Filename XCorr DeltCN Conf% ObsM+H+ CalcM+H+ SpR ZScore Ion% # Sequence  | | | | | | | | | | | | |
| --- | --- | --- | --- | --- | --- | --- | --- | --- | --- | --- | --- | --- |
|  | Mis12XLAPLSS\_011913\_01.08100.08100.2 | 3.8296 | 0.4533 | 100.0% | 1813.9321 | 1814.0382 | 1 | 7.965 | 56.7% | 1 | K.LAEQAERYDDMAAAMK.A | 22 |
|  | Mis12XLAPLSS\_011913\_01.03704.03704.2 | 4.3614 | 0.4838 | 100.0% | 1598.6122 | 1599.6543 | 1 | 8.379 | 76.9% | 1 | K.AVTEQGHELSNEER.N | 2 |
|  | Mis12XLAPLSS\_011913\_01.03707.03707.3 | 2.9406 | 0.2435 | 97.8% | 1601.3644 | 1599.6543 | 194 | 4.529 | 30.8% | 1 | K.AVTEQGHELSNEER.N | 3 |
|  | Mis12XLAPLSS\_011913\_01.17570.17570.3 | 4.9568 | 0.5025 | 100.0% | 3330.6243 | 3331.7163 | 1 | 7.99 | 25.0% | 1 | K.TAFDEAIAELDTLNEESYKDSTLIMQLLR.D | 3 |

Similarities:
gi|21464101|ref|NP\_03(1:3)  

---

|  |  |  |  |  |  |  |  |  |
| --- | --- | --- | --- | --- | --- | --- | --- | --- |
| U | *gi|50592996|ref|NP\_00* | 13 | 33 | 23.6% | 450 | 50433 | 4.9 | tubulin, beta, 4 [Homo sapiens] |

| Filename XCorr DeltCN Conf% ObsM+H+ CalcM+H+ SpR ZScore Ion% # Sequence  | | | | | | | | | | | | |
| --- | --- | --- | --- | --- | --- | --- | --- | --- | --- | --- | --- | --- |
|  | Mis12XLAPLSS\_011913\_01.11252.11252.2 | 4.408 | 0.5175 | 100.0% | 1616.3722 | 1616.8701 | 1 | 8.763 | 71.4% | 5 | R.AILVDLEPGTMDSVR.S | 22 |
|  | Mis12XLAPLSS\_011913\_01.13076.13076.2 | 6.5905 | 0.5843 | 100.0% | 1959.6522 | 1960.151 | 1 | 11.152 | 79.4% | 2 | K.GHYTEGAELVDSVLDVVR.K | 222 |
|  | Mis12XLAPLSS\_011913\_01.13106.13106.3 | 4.5785 | 0.393 | 100.0% | 1961.5144 | 1960.151 | 1 | 7.056 | 45.6% | 1 | K.GHYTEGAELVDSVLDVVR.K | 333 |
|  | Mis12XLAPLSS\_011913\_01.12470.12470.2 | 5.7772 | 0.4953 | 100.0% | 2087.9521 | 2088.325 | 1 | 9.485 | 66.7% | 1 | K.GHYTEGAELVDSVLDVVRK.E | 222 |
|  | Mis12XLAPLSS\_011913\_01.12422.12422.3 | 4.0642 | 0.4487 | 100.0% | 2088.2644 | 2088.325 | 2 | 7.577 | 38.9% | 2 | K.GHYTEGAELVDSVLDVVRK.E | 333 |
|  | Mis12XLAPLSS\_011913\_01.09478.09478.2 | 4.27 | 0.3449 | 100.0% | 1321.9122 | 1320.5896 | 1 | 6.366 | 81.8% | 4 | R.IMNTFSVVPSPK.V | 222 |
|  | Mis12XLAPLSS\_011913\_01.08709.08709.2 | 2.7937 | 0.1227 | 98.4% | 1131.1721 | 1131.2767 | 10 | 4.38 | 72.2% | 3 | R.FPGQLNADLR.K | 222 |
|  | Mis12XLAPLSS\_011913\_01.06874.06874.2 | 2.5666 | 0.1608 | 98.1% | 1259.6322 | 1259.4508 | 74 | 4.363 | 55.0% | 1 | R.FPGQLNADLRK.L | 222 |
|  | Mis12XLAPLSS\_011913\_01.09525.09525.2 | 3.5594 | 0.3946 | 100.0% | 1271.7322 | 1272.5945 | 1 | 7.783 | 75.0% | 2 | R.KLAVNMVPFPR.L | 222 |
|  | Mis12XLAPLSS\_011913\_01.10730.10730.2 | 3.5516 | 0.5021 | 100.0% | 1144.1721 | 1144.4204 | 1 | 8.538 | 94.4% | 3 | K.LAVNMVPFPR.L | 222 |
|  | Mis12XLAPLSS\_011913\_01.12669.12669.2 | 3.4785 | 0.3826 | 99.9% | 1692.4922 | 1692.9678 | 1 | 7.084 | 71.4% | 2 | R.ALTVPELTQQMFDAK.N | 22 |
|  | Mis12XLAPLSS\_011913\_01.12202.12202.2 | 2.8146 | 0.3212 | 99.8% | 1697.7522 | 1697.8877 | 1 | 6.074 | 57.7% | 2 | K.NSSYFVEWIPNNVK.V | 222 |
|  | Mis12XLAPLSS\_011913\_01.11018.11018.2 | 3.7611 | 0.4743 | 100.0% | 1230.2122 | 1230.4241 | 1 | 7.691 | 94.4% | 5 | R.ISEQFTAMFR.R | 222 |

Similarities:
gi|29788785|ref|NP\_82(12:1)  
gi|5174735|ref|NP\_006(12:1)  

---

|  |  |  |  |  |  |  |  |  |
| --- | --- | --- | --- | --- | --- | --- | --- | --- |
| U | *gi|15082258|ref|NP\_00* | 4 | 5 | 23.5% | 183 | 20811 | 5.3 | chromobox homolog 3 [Homo sapiens] |
| U | *gi|89038889|ref|XP\_94* | 4 | 5 | 23.5% | 183 | 20811 | 5.3 | PREDICTED: similar to Chromobox homolog 3 (HP1 gamma homolog, Drosophila) [Homo sapiens] |
| U | *gi|20544151|ref|NP\_05* | 4 | 5 | 23.5% | 183 | 20811 | 5.3 | chromobox homolog 3 [Homo sapiens] |

| Filename XCorr DeltCN Conf% ObsM+H+ CalcM+H+ SpR ZScore Ion% # Sequence  | | | | | | | | | | | | |
| --- | --- | --- | --- | --- | --- | --- | --- | --- | --- | --- | --- | --- |
|  | Mis12XLAPLSS\_011913\_01.07565.07565.2 | 4.8679 | 0.4427 | 100.0% | 1662.3322 | 1662.834 | 1 | 7.705 | 76.9% | 1 | K.KVEEAEPEEFVVEK.V | 2 |
|  | Mis12XLAPLSS\_011913\_01.08735.08735.2 | 3.4742 | 0.3719 | 99.9% | 1533.8722 | 1534.6599 | 2 | 6.216 | 62.5% | 1 | K.VEEAEPEEFVVEK.V | 2 |
|  | Mis12XLAPLSS\_011913\_01.12749.12749.2 | 4.3321 | 0.5565 | 100.0% | 1713.6721 | 1714.0449 | 1 | 10.502 | 73.3% | 2 | R.IIGATDSSGELMFLMK.W | 2 |
|  | Mis12XLAPLSS\_011913\_02.06155.06155.2 | 2.8789 | 0.3114 | 99.8% | 1490.3722 | 1490.6531 | 1 | 5.078 | 62.5% | 1 | K.WKDSDEADLVLAK.E | 2 |

---

|  |  |  |  |  |  |  |  |  |
| --- | --- | --- | --- | --- | --- | --- | --- | --- |
| U | *gi|13904866|ref|NP\_00* | 3 | 3 | 23.4% | 137 | 15747 | 12.0 | ribosomal protein L28 isoform 2 [Homo sapiens] |
| U | *gi|209915588|ref|NP\_0* | 3 | 3 | 46.4% | 69 | 7888 | 10.7 | ribosomal protein L28 isoform 5 [Homo sapiens] |
| U | *gi|209915586|ref|NP\_0* | 3 | 3 | 36.8% | 87 | 10088 | 10.7 | ribosomal protein L28 isoform 4 [Homo sapiens] |
| U | *gi|209915584|ref|NP\_0* | 3 | 3 | 18.9% | 169 | 19148 | 10.8 | ribosomal protein L28 isoform 3 [Homo sapiens] |
| U | *gi|209915581|ref|NP\_0* | 3 | 3 | 19.6% | 163 | 18430 | 10.8 | ribosomal protein L28 isoform 1 [Homo sapiens] |

| Filename XCorr DeltCN Conf% ObsM+H+ CalcM+H+ SpR ZScore Ion% # Sequence  | | | | | | | | | | | | |
| --- | --- | --- | --- | --- | --- | --- | --- | --- | --- | --- | --- | --- |
|  | Mis12XLAPLSS\_011913\_01.03702.03702.2 | 4.2754 | 0.3358 | 100.0% | 1537.1721 | 1537.6696 | 1 | 6.728 | 70.8% | 1 | R.NKQTYSTEPNNLK.A | 2 |
|  | Mis12XLAPLSS\_011913\_01.04046.04046.2 | 1.9542 | 0.2214 | 97.1% | 872.83215 | 873.0024 | 34 | 4.367 | 75.0% | 1 | R.YNGLIHR.K | 2 |
|  | Mis12XLAPLSS\_011913\_01.03395.03395.2 | 3.5049 | 0.324 | 99.9% | 1173.1921 | 1172.3237 | 1 | 6.76 | 77.3% | 1 | R.KTVGVEPAADGK.G | 2 |

---

|  |  |  |  |  |  |  |  |  |
| --- | --- | --- | --- | --- | --- | --- | --- | --- |
| U | *gi|30795231|ref|NP\_00* | 2 | 2 | 23.3% | 227 | 22693 | 4.6 | brain abundant, membrane attached signal protein 1 [Homo sapiens] |

| Filename XCorr DeltCN Conf% ObsM+H+ CalcM+H+ SpR ZScore Ion% # Sequence  | | | | | | | | | | | | |
| --- | --- | --- | --- | --- | --- | --- | --- | --- | --- | --- | --- | --- |
| \* | Mis12XLAPLSS\_011913\_01.04138.04138.3 | 3.8742 | 0.4037 | 100.0% | 2298.0544 | 2299.5022 | 1 | 6.792 | 37.0% | 1 | K.AEPPKAPEQEQAAPGPAAGGEAPK.A | 3 |
| \* | Mis12XLAPLSS\_011913\_02.04956.04956.3 | 5.2494 | 0.4738 | 100.0% | 2892.5044 | 2894.1216 | 1 | 8.278 | 31.2% | 1 | K.AQGPAASAEEPKPVEAPAANSDQTVTVKE.- | 3 |

---

|  |  |  |  |  |  |  |  |  |
| --- | --- | --- | --- | --- | --- | --- | --- | --- |
| U | *gi|117189975|ref|NP\_1* | 5 | 13 | 23.2% | 306 | 33670 | 5.1 | heterogeneous nuclear ribonucleoprotein C isoform a [Homo sapiens] |
| U | *gi|117190254|ref|NP\_0* | 5 | 13 | 24.2% | 293 | 32338 | 5.1 | heterogeneous nuclear ribonucleoprotein C isoform b [Homo sapiens] |
| U | *gi|117190192|ref|NP\_0* | 5 | 13 | 23.2% | 306 | 33670 | 5.1 | heterogeneous nuclear ribonucleoprotein C isoform a [Homo sapiens] |
| U | *gi|117190174|ref|NP\_0* | 5 | 13 | 24.2% | 293 | 32338 | 5.1 | heterogeneous nuclear ribonucleoprotein C isoform b [Homo sapiens] |

| Filename XCorr DeltCN Conf% ObsM+H+ CalcM+H+ SpR ZScore Ion% # Sequence  | | | | | | | | | | | | |
| --- | --- | --- | --- | --- | --- | --- | --- | --- | --- | --- | --- | --- |
|  | Mis12XLAPLSS\_011913\_02.07971.07971.2 | 3.5672 | 0.2391 | 99.8% | 1317.4521 | 1317.6145 | 1 | 6.874 | 72.7% | 3 | R.VFIGNLNTLVVK.K | 2 |
|  | Mis12XLAPLSS\_011913\_01.10946.10946.2 | 3.6172 | 0.4595 | 100.0% | 1330.3121 | 1330.4857 | 1 | 7.498 | 80.0% | 4 | K.GFAFVQYVNER.N | 2 |
|  | Mis12XLAPLSS\_011913\_02.08018.08018.2 | 5.1048 | 0.4212 | 100.0% | 1683.3322 | 1684.0038 | 1 | 8.066 | 83.3% | 4 | R.MIAGQVLDINLAAEPK.V | 2 |
|  | Mis12XLAPLSS\_011913\_01.03944.03944.2 | 3.1832 | 0.3065 | 99.8% | 1230.1122 | 1229.4624 | 1 | 6.01 | 80.0% | 1 | K.LKGDDLQAIKK.E | 2 |
|  | Mis12XLAPLSS\_011913\_01.03359.03359.3 | 4.3406 | 0.2549 | 99.7% | 2369.1843 | 2369.4583 | 1 | 5.279 | 41.2% | 1 | K.NDKSEEEQSSSSVKKDETNVK.M | 3 |

---

|  |  |  |  |  |  |  |  |  |
| --- | --- | --- | --- | --- | --- | --- | --- | --- |
| U | *gi|32483377|ref|NP\_05* | 5 | 10 | 23.1% | 238 | 25839 | 7.5 | peroxiredoxin 3 isoform b [Homo sapiens] |
| U | *gi|5802974|ref|NP\_006* | 5 | 10 | 21.5% | 256 | 27693 | 7.8 | peroxiredoxin 3 isoform a precursor [Homo sapiens] |

| Filename XCorr DeltCN Conf% ObsM+H+ CalcM+H+ SpR ZScore Ion% # Sequence  | | | | | | | | | | | | |
| --- | --- | --- | --- | --- | --- | --- | --- | --- | --- | --- | --- | --- |
|  | Mis12XLAPLSS\_011913\_01.11186.11186.3 | 4.827 | 0.5092 | 100.0% | 1883.4844 | 1883.2163 | 1 | 8.477 | 45.6% | 2 | R.KNGGLGHMNIALLSDLTK.Q | 3 |
|  | Mis12XLAPLSS\_011913\_01.12929.12929.2 | 2.5265 | 0.2939 | 99.4% | 1755.2322 | 1755.0422 | 2 | 5.126 | 40.6% | 1 | K.NGGLGHMNIALLSDLTK.Q | 2 |
|  | Mis12XLAPLSS\_011913\_02.08504.08504.2 | 4.4314 | 0.5501 | 100.0% | 1463.3121 | 1463.6738 | 1 | 9.497 | 73.1% | 2 | R.DYGVLLEGSGLALR.G | 2 |
|  | Mis12XLAPLSS\_011913\_01.12490.12490.2 | 3.092 | 0.3439 | 99.8% | 1286.3922 | 1286.5571 | 14 | 6.232 | 63.6% | 1 | R.GLFIIDPNGVIK.H | 2 |
|  | Mis12XLAPLSS\_011913\_01.07538.07538.2 | 3.0272 | 0.3833 | 99.9% | 1207.1122 | 1207.375 | 2 | 6.915 | 65.0% | 4 | K.HLSVNDLPVGR.S | 2 |

---

|  |  |  |  |  |  |  |  |  |
| --- | --- | --- | --- | --- | --- | --- | --- | --- |
| U | *gi|15431303|ref|NP\_00* | 3 | 4 | 22.9% | 192 | 21863 | 10.0 | ribosomal protein L9 [Homo sapiens] |
| U | *gi|67944630|ref|NP\_00* | 3 | 4 | 22.9% | 192 | 21863 | 10.0 | ribosomal protein L9 [Homo sapiens] |

| Filename XCorr DeltCN Conf% ObsM+H+ CalcM+H+ SpR ZScore Ion% # Sequence  | | | | | | | | | | | | |
| --- | --- | --- | --- | --- | --- | --- | --- | --- | --- | --- | --- | --- |
|  | Mis12XLAPLSS\_011913\_01.11790.11790.2 | 3.1609 | 0.307 | 99.8% | 2113.872 | 2114.401 | 1 | 6.147 | 38.9% | 1 | K.TILSNQTVDIPENVDITLK.G | 2 |
|  | Mis12XLAPLSS\_011913\_01.12076.12076.2 | 2.7957 | 0.3648 | 99.8% | 1598.8522 | 1599.8271 | 1 | 6.083 | 53.8% | 2 | R.DFNHINVELSLLGK.K | 2 |
|  | Mis12XLAPLSS\_011913\_01.08828.08828.2 | 2.8835 | 0.1723 | 99.4% | 1299.1921 | 1299.5095 | 1 | 4.728 | 70.0% | 1 | R.KFLDGIYVSEK.G | 2 |

---

|  |  |  |  |  |  |  |  |  |
| --- | --- | --- | --- | --- | --- | --- | --- | --- |
| U | *gi|20357599|ref|NP\_61* | 3 | 13 | 22.8% | 114 | 12146 | 10.5 | H2A histone family, member V isoform 2 [Homo sapiens] |
| U | *gi|6912616|ref|NP\_036* | 3 | 13 | 20.3% | 128 | 13509 | 10.6 | H2A histone family, member V isoform 1 [Homo sapiens] |
| U | *gi|4504255|ref|NP\_002* | 3 | 13 | 20.3% | 128 | 13553 | 10.6 | H2A histone family, member Z [Homo sapiens] |

| Filename XCorr DeltCN Conf% ObsM+H+ CalcM+H+ SpR ZScore Ion% # Sequence  | | | | | | | | | | | | |
| --- | --- | --- | --- | --- | --- | --- | --- | --- | --- | --- | --- | --- |
|  | Mis12XLAPLSS\_011913\_01.09008.09008.2 | 3.1059 | 0.329 | 99.9% | 944.59216 | 945.1093 | 2 | 5.574 | 81.2% | 4 | R.AGLQFPVGR.I | 2222 |
|  | Mis12XLAPLSS\_011913\_01.05156.05156.2 | 2.4241 | 0.2579 | 99.8% | 851.09216 | 851.0396 | 3 | 5.349 | 83.3% | 5 | R.HLQLAIR.G | 2222 |
|  | Mis12XLAPLSS\_011913\_02.07642.07642.3 | 3.2934 | 0.4931 | 100.0% | 1950.5944 | 1951.2303 | 1 | 7.314 | 39.1% | 4 | R.HLQLAIRGDEELDSLIK.A | 3 |

Similarities:
gi|10645195|ref|NP\_06(2:1)  
gi|10800130|ref|NP\_06(2:1)  
gi|106775678|ref|NP\_0(2:1)  

---

|  |  |  |  |  |  |  |  |  |
| --- | --- | --- | --- | --- | --- | --- | --- | --- |
| U | *gi|4757834|ref|NP\_004* | 2 | 2 | 22.7% | 211 | 23772 | 6.7 | BCL2-associated athanogene 2 [Homo sapiens] |

| Filename XCorr DeltCN Conf% ObsM+H+ CalcM+H+ SpR ZScore Ion% # Sequence  | | | | | | | | | | | | |
| --- | --- | --- | --- | --- | --- | --- | --- | --- | --- | --- | --- | --- |
| \* | Mis12XLAPLSS\_011913\_01.13875.13875.3 | 5.0874 | 0.4007 | 100.0% | 3114.1743 | 3115.5007 | 1 | 7.6 | 29.8% | 1 | R.EAATAVEQEKEILLEMIHSIQNSQDMR.Q | 3 |
| \* | Mis12XLAPLSS\_011913\_02.06656.06656.3 | 4.3219 | 0.3301 | 100.0% | 2401.1343 | 2400.6917 | 1 | 6.966 | 37.5% | 1 | R.TLTVEVSVETIRNPQQQESLK.H | 3 |

---

|  |  |  |  |  |  |  |  |  |
| --- | --- | --- | --- | --- | --- | --- | --- | --- |
| U | *gi|153792590|ref|NP\_0* | 14 | 26 | 22.6% | 854 | 98161 | 5.2 | heat shock 90kDa protein 1, alpha isoform 1 [Homo sapiens] |
| U | *gi|154146191|ref|NP\_0* | 14 | 26 | 26.4% | 732 | 84660 | 5.0 | heat shock 90kDa protein 1, alpha isoform 2 [Homo sapiens] |

| Filename XCorr DeltCN Conf% ObsM+H+ CalcM+H+ SpR ZScore Ion% # Sequence  | | | | | | | | | | | | |
| --- | --- | --- | --- | --- | --- | --- | --- | --- | --- | --- | --- | --- |
|  | Mis12XLAPLSS\_011913\_01.08505.08505.2 | 3.3872 | 0.3398 | 99.9% | 1561.3922 | 1561.7324 | 1 | 6.231 | 69.2% | 1 | R.ELISNSSDALDKIR.Y | 2 |
|  | Mis12XLAPLSS\_011913\_01.08105.08105.2 | 2.8602 | 0.2779 | 99.8% | 1591.3722 | 1590.8228 | 1 | 4.851 | 70.8% | 1 | K.ELHINLIPNKQDR.T | 2 |
|  | Mis12XLAPLSS\_011913\_01.09962.09962.2 | 3.6701 | 0.488 | 100.0% | 1242.6122 | 1243.4459 | 1 | 8.954 | 72.7% | 1 | K.ADLINNLGTIAK.S | 22 |
|  | Mis12XLAPLSS\_011913\_02.06044.06044.3 | 4.4058 | 0.4311 | 100.0% | 2015.8744 | 2016.2584 | 1 | 7.628 | 48.3% | 4 | K.VILHLKEDQTEYLEER.R | 33 |
|  | Mis12XLAPLSS\_011913\_01.04832.04832.2 | 3.4034 | 0.2713 | 99.9% | 1151.9521 | 1152.2462 | 1 | 5.333 | 81.2% | 3 | K.YIDQEELNK.T | 22 |
|  | Mis12XLAPLSS\_011913\_02.06594.06594.2 | 4.9792 | 0.4492 | 100.0% | 1834.1122 | 1834.8903 | 1 | 9.092 | 85.7% | 2 | R.NPDDITNEEYGEFYK.S | 2 |
|  | Mis12XLAPLSS\_011913\_01.09360.09360.2 | 4.3397 | 0.4125 | 100.0% | 1528.4922 | 1528.6616 | 1 | 7.008 | 66.7% | 4 | K.SLTNDWEDHLAVK.H | 22 |
|  | Mis12XLAPLSS\_011913\_01.09105.09105.2 | 3.5028 | 0.4222 | 100.0% | 1349.9122 | 1349.4886 | 2 | 7.281 | 70.0% | 3 | K.HFSVEGQLEFR.A | 22 |
|  | Mis12XLAPLSS\_011913\_01.10193.10193.2 | 2.6622 | 0.2968 | 99.8% | 1264.8322 | 1265.4142 | 6 | 6.463 | 61.1% | 1 | R.RAPFDLFENR.K | 2 |
|  | Mis12XLAPLSS\_011913\_01.08474.08474.2 | 2.5523 | 0.2602 | 99.1% | 1551.6522 | 1551.7083 | 5 | 4.843 | 57.7% | 1 | R.YYTSASGDEMVSLK.D | 2 |
|  | Mis12XLAPLSS\_011913\_02.06177.06177.3 | 4.2088 | 0.5218 | 100.0% | 2441.8145 | 2442.6904 | 1 | 8.178 | 35.0% | 1 | K.HIYYITGETKDQVANSAFVER.L | 3 |
|  | Mis12XLAPLSS\_011913\_01.09242.09242.2 | 3.7064 | 0.3733 | 99.8% | 1788.4521 | 1788.0134 | 1 | 6.843 | 57.1% | 1 | K.HLEINPDHSIIETLR.Q | 2 |
|  | Mis12XLAPLSS\_011913\_01.09297.09297.3 | 3.6777 | 0.3162 | 100.0% | 1788.8043 | 1788.0134 | 1 | 7.433 | 50.0% | 2 | K.HLEINPDHSIIETLR.Q | 3 |
|  | Mis12XLAPLSS\_011913\_01.17091.17091.3 | 3.6347 | 0.2745 | 99.8% | 3317.9644 | 3318.7515 | 1 | 4.996 | 25.0% | 1 | K.SVKDLVILLYETALLSSGFSLEDPQTHANR.I | 3 |

Similarities:
gi|20149594|ref|NP\_03(5:9)  

---

|  |  |  |  |  |  |  |  |  |
| --- | --- | --- | --- | --- | --- | --- | --- | --- |
| U | *gi|4758302|ref|NP\_004* | 2 | 2 | 22.1% | 104 | 12259 | 5.9 | enhancer of rudimentary homolog [Homo sapiens] |

| Filename XCorr DeltCN Conf% ObsM+H+ CalcM+H+ SpR ZScore Ion% # Sequence  | | | | | | | | | | | | |
| --- | --- | --- | --- | --- | --- | --- | --- | --- | --- | --- | --- | --- |
| \* | Mis12XLAPLSS\_011913\_01.03526.03526.2 | 2.4432 | 0.1478 | 98.4% | 1105.2322 | 1106.2848 | 57 | 4.044 | 78.6% | 1 | K.MYEEHLKR.M | 2 |
| \* | Mis12XLAPLSS\_011913\_01.08274.08274.2 | 3.4575 | 0.2866 | 99.8% | 1871.0122 | 1872.0441 | 1 | 6.053 | 50.0% | 1 | R.ADTQTYQPYNKDWIK.E | 2 |

---

|  |  |  |  |  |  |  |  |  |
| --- | --- | --- | --- | --- | --- | --- | --- | --- |
| U | *gi|4506407|ref|NP\_002* | 4 | 5 | 21.9% | 201 | 23310 | 5.3 | RAN binding protein 1 [Homo sapiens] |

| Filename XCorr DeltCN Conf% ObsM+H+ CalcM+H+ SpR ZScore Ion% # Sequence  | | | | | | | | | | | | |
| --- | --- | --- | --- | --- | --- | --- | --- | --- | --- | --- | --- | --- |
|  | Mis12XLAPLSS\_011913\_01.10773.10773.2 | 3.0913 | 0.2822 | 99.8% | 1381.9321 | 1382.4639 | 1 | 6.013 | 80.0% | 2 | K.TLEEDEEELFK.M | 2 |
|  | Mis12XLAPLSS\_011913\_01.09434.09434.2 | 2.6865 | 0.2134 | 99.4% | 1336.1921 | 1336.4436 | 73 | 4.928 | 55.0% | 1 | R.FASENDLPEWK.E | 2 |
| \* | Mis12XLAPLSS\_011913\_02.04978.04978.3 | 5.206 | 0.4871 | 100.0% | 2503.4944 | 2504.751 | 1 | 7.816 | 42.9% | 1 | K.VAEKLEALSVKEETKEDAEEKQ.- | 3 |
| \* | Mis12XLAPLSS\_011913\_02.04565.04565.3 | 3.4248 | 0.3065 | 99.7% | 2076.3245 | 2077.25 | 32 | 5.498 | 35.3% | 1 | K.LEALSVKEETKEDAEEKQ.- | 3 |

---

|  |  |  |  |  |  |  |  |  |
| --- | --- | --- | --- | --- | --- | --- | --- | --- |
| U | *gi|222352151|ref|NP\_0* | 4 | 5 | 21.3% | 356 | 37498 | 7.1 | poly(rC) binding protein 1 [Homo sapiens] |

| Filename XCorr DeltCN Conf% ObsM+H+ CalcM+H+ SpR ZScore Ion% # Sequence  | | | | | | | | | | | | |
| --- | --- | --- | --- | --- | --- | --- | --- | --- | --- | --- | --- | --- |
| \* | Mis12XLAPLSS\_011913\_01.13883.13883.3 | 3.6819 | 0.2061 | 97.4% | 3380.4243 | 3380.8562 | 1 | 3.955 | 27.5% | 1 | K.AFAMIIDKLEEDINSSMTNSTAASRPPVTLR.L | 3 |
|  | Mis12XLAPLSS\_011913\_02.06128.06128.2 | 5.1662 | 0.6278 | 100.0% | 2090.4321 | 2091.2573 | 1 | 11.202 | 57.9% | 2 | R.ESTGAQVQVAGDMLPNSTER.A | 2 |
|  | Mis12XLAPLSS\_011913\_02.06117.06117.3 | 4.0093 | 0.2252 | 99.8% | 2091.8943 | 2091.2573 | 1 | 4.984 | 39.5% | 1 | R.ESTGAQVQVAGDMLPNSTER.A | 3 |
| \* | Mis12XLAPLSS\_011913\_02.06515.06515.3 | 3.8713 | 0.3284 | 99.7% | 2686.2844 | 2687.875 | 3 | 6.082 | 32.3% | 1 | R.QQSHFAMMHGGTGFAGIDS\*SSPEVK.G | 3 |

---

|  |  |  |  |  |  |  |  |  |
| --- | --- | --- | --- | --- | --- | --- | --- | --- |
| U | *gi|4506607|ref|NP\_000* | 4 | 7 | 21.3% | 188 | 21634 | 11.7 | ribosomal protein L18 [Homo sapiens] |

| Filename XCorr DeltCN Conf% ObsM+H+ CalcM+H+ SpR ZScore Ion% # Sequence  | | | | | | | | | | | | |
| --- | --- | --- | --- | --- | --- | --- | --- | --- | --- | --- | --- | --- |
| \* | Mis12XLAPLSS\_011913\_02.06334.06334.2 | 3.832 | 0.3922 | 100.0% | 1346.2322 | 1346.5236 | 1 | 7.945 | 75.0% | 4 | K.TAVVVGTITDDVR.V | 2 |
| \* | Mis12XLAPLSS\_011913\_01.12048.12048.2 | 3.6436 | 0.4504 | 100.0% | 1461.0322 | 1461.6982 | 1 | 8.665 | 66.7% | 1 | K.ILTFDQLALDSPK.G | 2 |
| \* | Mis12XLAPLSS\_011913\_01.03261.03261.2 | 2.5704 | 0.367 | 99.8% | 1548.3121 | 1548.7446 | 1 | 6.302 | 53.8% | 1 | K.APGTPHSHTKPYVR.S | 2 |
| \* | Mis12XLAPLSS\_011913\_01.03262.03262.3 | 2.9388 | 0.4472 | 100.0% | 1548.6843 | 1548.7446 | 1 | 6.988 | 44.2% | 1 | K.APGTPHSHTKPYVR.S | 3 |

---

|  |  |  |  |  |  |  |  |  |
| --- | --- | --- | --- | --- | --- | --- | --- | --- |
| U | *gi|13904870|ref|NP\_00* | 3 | 5 | 21.1% | 204 | 22876 | 9.7 | ribosomal protein S5 [Homo sapiens] |

| Filename XCorr DeltCN Conf% ObsM+H+ CalcM+H+ SpR ZScore Ion% # Sequence  | | | | | | | | | | | | |
| --- | --- | --- | --- | --- | --- | --- | --- | --- | --- | --- | --- | --- |
| \* | Mis12XLAPLSS\_011913\_01.04259.04259.2 | 3.048 | 0.4532 | 100.0% | 1177.9722 | 1178.428 | 1 | 6.827 | 83.3% | 1 | R.LTNSMMMHGR.N | 2 |
| \* | Mis12XLAPLSS\_011913\_01.18033.18033.3 | 4.8985 | 0.3656 | 100.0% | 3066.7744 | 3067.5613 | 1 | 7.308 | 29.6% | 2 | K.HAFEIIHLLTGENPLQVLVNAIINSGPR.E | 3 |
| \* | Mis12XLAPLSS\_011913\_01.16995.16995.3 | 6.6792 | 0.5863 | 100.0% | 3655.3442 | 3656.1362 | 1 | 11.053 | 28.9% | 2 | K.HAFEIIHLLTGENPLQVLVNAIINSGPREDSTR.I | 3 |

---

|  |  |  |  |  |  |  |  |  |
| --- | --- | --- | --- | --- | --- | --- | --- | --- |
| U | *gi|14916501|ref|NP\_14* | 2 | 2 | 20.8% | 130 | 15069 | 10.9 | ribosomal protein S24 isoform a [Homo sapiens] |
| U | *gi|4506703|ref|NP\_001* | 2 | 2 | 20.3% | 133 | 15423 | 10.8 | ribosomal protein S24 isoform c [Homo sapiens] |
| U | *gi|214829241|ref|NP\_0* | 2 | 2 | 20.6% | 131 | 15197 | 10.9 | ribosomal protein S24 isoform b [Homo sapiens] |
| U | *gi|214010226|ref|NP\_0* | 2 | 2 | 9.3% | 289 | 32431 | 10.2 | ribosomal protein S24 isoform d [Homo sapiens] |
| U | *gi|214010224|ref|NP\_0* | 2 | 2 | 20.6% | 131 | 15197 | 10.9 | ribosomal protein S24 isoform f [Homo sapiens] |
| U | *gi|214010222|ref|NP\_0* | 2 | 2 | 20.5% | 132 | 15325 | 10.9 | ribosomal protein S24 isoform e [Homo sapiens] |

| Filename XCorr DeltCN Conf% ObsM+H+ CalcM+H+ SpR ZScore Ion% # Sequence  | | | | | | | | | | | | |
| --- | --- | --- | --- | --- | --- | --- | --- | --- | --- | --- | --- | --- |
|  | Mis12XLAPLSS\_011913\_01.13742.13742.2 | 2.6778 | 0.2816 | 99.6% | 1400.6322 | 1399.6323 | 3 | 5.795 | 59.1% | 1 | K.TTPDVIFVFGFR.T | 2 |
|  | Mis12XLAPLSS\_011913\_02.08717.08717.2 | 3.487 | 0.3953 | 99.9% | 1682.1322 | 1682.8854 | 1 | 6.862 | 64.3% | 1 | K.TTGFGMIYDSLDYAK.K | 2 |

---

|  |  |  |  |  |  |  |  |  |
| --- | --- | --- | --- | --- | --- | --- | --- | --- |
| U | *gi|4506605|ref|NP\_000* | 6 | 11 | 20.7% | 140 | 14865 | 10.5 | ribosomal protein L23 [Homo sapiens] |

| Filename XCorr DeltCN Conf% ObsM+H+ CalcM+H+ SpR ZScore Ion% # Sequence  | | | | | | | | | | | | |
| --- | --- | --- | --- | --- | --- | --- | --- | --- | --- | --- | --- | --- |
| \* | Mis12XLAPLSS\_011913\_01.10800.10800.2 | 4.3253 | 0.4613 | 100.0% | 1843.0721 | 1844.2408 | 1 | 7.723 | 61.8% | 2 | R.LNRLPAAGVGDMVMATVK.K | 2 |
| \* | Mis12XLAPLSS\_011913\_01.10887.10887.3 | 4.2757 | 0.4802 | 100.0% | 1845.0243 | 1844.2408 | 1 | 7.545 | 42.6% | 2 | R.LNRLPAAGVGDMVMATVK.K | 3 |
| \* | Mis12XLAPLSS\_011913\_01.09836.09836.3 | 4.8241 | 0.4299 | 100.0% | 1970.1543 | 1972.415 | 3 | 6.895 | 37.5% | 1 | R.LNRLPAAGVGDMVMATVKK.G | 3 |
| \* | Mis12XLAPLSS\_011913\_01.11123.11123.2 | 4.1688 | 0.5453 | 100.0% | 1459.6322 | 1460.7902 | 1 | 9.48 | 78.6% | 4 | R.LPAAGVGDMVMATVK.K | 2 |
| \* | Mis12XLAPLSS\_011913\_01.09808.09808.3 | 2.9906 | 0.4701 | 100.0% | 1589.7843 | 1588.9642 | 3 | 7.096 | 31.7% | 1 | R.LPAAGVGDMVMATVKK.G | 3 |
| \* | Mis12XLAPLSS\_011913\_01.03368.03368.2 | 2.8991 | 0.1671 | 99.5% | 1147.4521 | 1147.4525 | 1 | 5.644 | 77.8% | 1 | R.KKVHPAVVIR.Q | 2 |

---

|  |  |  |  |  |  |  |  |  |
| --- | --- | --- | --- | --- | --- | --- | --- | --- |
| U | *gi|170763498|ref|NP\_0* | 3 | 4 | 20.2% | 277 | 32103 | 4.2 | SET translocation (myeloid leukemia-associated) isoform 2 [Homo sapiens] |
| U | *gi|170763500|ref|NP\_0* | 3 | 4 | 19.3% | 290 | 33489 | 4.3 | SET translocation (myeloid leukemia-associated) isoform 1 [Homo sapiens] |

| Filename XCorr DeltCN Conf% ObsM+H+ CalcM+H+ SpR ZScore Ion% # Sequence  | | | | | | | | | | | | |
| --- | --- | --- | --- | --- | --- | --- | --- | --- | --- | --- | --- | --- |
|  | Mis12XLAPLSS\_011913\_01.14513.14513.3 | 5.6315 | 0.6001 | 100.0% | 3727.8245 | 3728.1553 | 1 | 9.673 | 28.2% | 1 | K.IPNFWVTTFVNHPQVSALLGEEDEEALHYLTR.V | 3 |
|  | Mis12XLAPLSS\_011913\_02.06645.06645.2 | 3.0777 | 0.3747 | 99.9% | 1209.1522 | 1209.3385 | 1 | 6.933 | 77.8% | 2 | R.VEVTEFEDIK.S | 2 |
|  | Mis12XLAPLSS\_011913\_01.12576.12576.2 | 2.4405 | 0.1974 | 97.1% | 1840.7122 | 1841.9708 | 3 | 4.504 | 50.0% | 1 | R.IDFYFDENPYFENK.V | 2 |

---

|  |  |  |  |  |  |  |  |  |
| --- | --- | --- | --- | --- | --- | --- | --- | --- |
| U | *gi|10440560|ref|NP\_06* | 3 | 4 | 19.9% | 136 | 15404 | 11.1 | histone cluster 1, H3f [Homo sapiens] |
| U | *gi|53793688|ref|NP\_00* | 3 | 4 | 19.9% | 136 | 15388 | 11.3 | histone cluster 2, H3a [Homo sapiens] |
| U | *gi|4885385|ref|NP\_005* | 3 | 4 | 19.9% | 136 | 15328 | 11.3 | H3 histone, family 3B [Homo sapiens] |
| U | *gi|4504299|ref|NP\_003* | 3 | 4 | 19.9% | 136 | 15508 | 11.1 | histone cluster 3, H3 [Homo sapiens] |
| U | *gi|4504297|ref|NP\_003* | 3 | 4 | 19.9% | 136 | 15404 | 11.1 | histone cluster 1, H3b [Homo sapiens] |
| U | *gi|4504295|ref|NP\_003* | 3 | 4 | 19.9% | 136 | 15404 | 11.1 | histone cluster 1, H3h [Homo sapiens] |
| U | *gi|4504293|ref|NP\_003* | 3 | 4 | 19.9% | 136 | 15404 | 11.1 | histone cluster 1, H3j [Homo sapiens] |
| U | *gi|4504291|ref|NP\_003* | 3 | 4 | 19.9% | 136 | 15404 | 11.1 | H3 histone family, member H [Homo sapiens] |
| U | *gi|4504289|ref|NP\_003* | 3 | 4 | 19.9% | 136 | 15404 | 11.1 | histone cluster 1, H3i [Homo sapiens] |
| U | *gi|4504287|ref|NP\_003* | 3 | 4 | 19.9% | 136 | 15404 | 11.1 | histone cluster 1, H3e [Homo sapiens] |
| U | *gi|4504285|ref|NP\_003* | 3 | 4 | 19.9% | 136 | 15404 | 11.1 | histone cluster 1, H3c [Homo sapiens] |
| U | *gi|4504281|ref|NP\_003* | 3 | 4 | 19.9% | 136 | 15404 | 11.1 | histone cluster 1, H3a [Homo sapiens] |
| U | *gi|4504279|ref|NP\_002* | 3 | 4 | 19.9% | 136 | 15328 | 11.3 | H3 histone, family 3A [Homo sapiens] |
| U | *gi|31742503|ref|NP\_06* | 3 | 4 | 19.9% | 136 | 15388 | 11.3 | histone cluster 2, H3c [Homo sapiens] |
| U | *gi|183076548|ref|NP\_0* | 3 | 4 | 19.9% | 136 | 15388 | 11.3 | histone cluster 2, H3d [Homo sapiens] |

| Filename XCorr DeltCN Conf% ObsM+H+ CalcM+H+ SpR ZScore Ion% # Sequence  | | | | | | | | | | | | |
| --- | --- | --- | --- | --- | --- | --- | --- | --- | --- | --- | --- | --- |
|  | Mis12XLAPLSS\_011913\_02.03932.03932.2 | 2.1054 | 0.2299 | 97.4% | 1033.5721 | 1033.2186 | 20 | 4.365 | 62.5% | 1 | R.YRPGTVALR.E | 2 |
|  | Mis12XLAPLSS\_011913\_01.07322.07322.2 | 2.3267 | 0.1079 | 97.3% | 833.15216 | 831.9878 | 14 | 4.322 | 83.3% | 1 | K.STELLIR.K | 2 |
|  | Mis12XLAPLSS\_011913\_01.07598.07598.2 | 2.6239 | 0.338 | 99.8% | 1335.9722 | 1336.4875 | 1 | 6.474 | 70.0% | 2 | R.EIAQDFKTDLR.F | 2 |

---

|  |  |  |  |  |  |  |  |  |
| --- | --- | --- | --- | --- | --- | --- | --- | --- |
| U | *gi|4503483|ref|NP\_001* | 12 | 18 | 19.7% | 858 | 95338 | 6.8 | eukaryotic translation elongation factor 2 [Homo sapiens] |

| Filename XCorr DeltCN Conf% ObsM+H+ CalcM+H+ SpR ZScore Ion% # Sequence  | | | | | | | | | | | | |
| --- | --- | --- | --- | --- | --- | --- | --- | --- | --- | --- | --- | --- |
| \* | Mis12XLAPLSS\_011913\_01.04170.04170.2 | 3.4085 | 0.3912 | 100.0% | 1308.1122 | 1308.4979 | 1 | 6.348 | 77.3% | 1 | R.NMSVIAHVDHGK.S | 2 |
| \* | Mis12XLAPLSS\_011913\_01.15314.15314.2 | 3.5867 | 0.3416 | 99.9% | 2206.392 | 2205.4692 | 1 | 5.621 | 50.0% | 1 | K.STAISLFYELSENDLNFIK.Q | 2 |
| \* | Mis12XLAPLSS\_011913\_01.15444.15444.2 | 3.7639 | 0.4805 | 100.0% | 2600.8123 | 2602.11 | 1 | 8.228 | 43.5% | 1 | R.WLPAGDALLQMITIHLPSPVTAQK.Y | 2 |
| \* | Mis12XLAPLSS\_011913\_01.09862.09862.2 | 2.5715 | 0.4465 | 99.9% | 1039.7522 | 1040.3241 | 18 | 6.968 | 62.5% | 1 | K.GPLMMYISK.M | 2 |
| \* | Mis12XLAPLSS\_011913\_02.06807.06807.2 | 3.0365 | 0.4092 | 99.9% | 1108.0521 | 1108.3231 | 1 | 7.064 | 75.0% | 2 | R.VFSGLVSTGLK.V | 2 |
| \* | Mis12XLAPLSS\_011913\_01.09386.09386.3 | 5.2424 | 0.4492 | 100.0% | 2144.2144 | 2144.3489 | 1 | 8.229 | 42.1% | 5 | K.ARPFPDGLAEDIDKGEVSAR.Q | 3 |
| \* | Mis12XLAPLSS\_011913\_02.06604.06604.3 | 3.3832 | 0.3946 | 100.0% | 1742.8143 | 1743.9133 | 1 | 6.307 | 46.2% | 1 | R.YLAEKYEWDVAEAR.K | 3 |
| \* | Mis12XLAPLSS\_011913\_02.06629.06629.2 | 3.444 | 0.4302 | 100.0% | 1743.5721 | 1743.9133 | 1 | 7.152 | 65.4% | 1 | R.YLAEKYEWDVAEAR.K | 2 |
| \* | Mis12XLAPLSS\_011913\_01.13962.13962.2 | 5.1833 | 0.5519 | 100.0% | 2353.9922 | 2354.6677 | 1 | 8.553 | 57.5% | 1 | K.GVQYLNEIKDSVVAGFQWATK.E | 2 |
|  | Mis12XLAPLSS\_011913\_01.04956.04956.2 | 2.3765 | 0.2641 | 99.4% | 970.15216 | 970.11664 | 5 | 5.093 | 72.2% | 1 | R.GGGQIIPTAR.R | 2 |
| \* | Mis12XLAPLSS\_011913\_01.12466.12466.2 | 3.9693 | 0.5631 | 100.0% | 1800.1522 | 1801.0087 | 1 | 9.175 | 63.3% | 1 | K.AYLPVNESFGFTADLR.S | 2 |
| \* | Mis12XLAPLSS\_011913\_01.14792.14792.2 | 3.7339 | 0.5536 | 100.0% | 1445.0521 | 1445.6555 | 1 | 8.67 | 70.8% | 2 | K.EGIPALDNFLDKL.- | 2 |

---

|  |  |  |  |  |  |  |  |  |
| --- | --- | --- | --- | --- | --- | --- | --- | --- |
| U | *gi|58761486|ref|NP\_00* | 7 | 10 | 19.3% | 544 | 60463 | 6.5 | chaperonin containing TCP1, subunit 3 isoform b [Homo sapiens] |
| U | *gi|63162572|ref|NP\_00* | 7 | 10 | 19.3% | 545 | 60534 | 6.5 | chaperonin containing TCP1, subunit 3 isoform a [Homo sapiens] |

| Filename XCorr DeltCN Conf% ObsM+H+ CalcM+H+ SpR ZScore Ion% # Sequence  | | | | | | | | | | | | |
| --- | --- | --- | --- | --- | --- | --- | --- | --- | --- | --- | --- | --- |
|  | Mis12XLAPLSS\_011913\_01.13222.13222.2 | 3.3257 | 0.3225 | 99.8% | 2132.5723 | 2132.5593 | 1 | 6.339 | 42.1% | 1 | K.MLLDPMGGIVMTNDGNAILR.E | 2 |
|  | Mis12XLAPLSS\_011913\_01.03525.03525.2 | 3.1008 | 0.2095 | 99.8% | 1120.4122 | 1121.2816 | 3 | 5.496 | 77.8% | 1 | R.EIQVQHPAAK.S | 2 |
|  | Mis12XLAPLSS\_011913\_02.07479.07479.2 | 2.508 | 0.1444 | 97.1% | 1279.9321 | 1280.504 | 1 | 5.529 | 75.0% | 2 | R.IVLLDSSLEYK.K | 2 |
|  | Mis12XLAPLSS\_011913\_02.05529.05529.3 | 2.6469 | 0.2631 | 95.8% | 2154.8342 | 2155.283 | 1 | 5.103 | 33.8% | 1 | K.KGESQTDIEITREEDFTR.I | 3 |
|  | Mis12XLAPLSS\_011913\_01.10570.10570.2 | 3.4843 | 0.5107 | 100.0% | 1404.9521 | 1404.6268 | 1 | 8.815 | 63.6% | 2 | K.GISDLAQHYLMR.A | 2 |
|  | Mis12XLAPLSS\_011913\_02.06876.06876.3 | 4.1885 | 0.3076 | 99.7% | 2498.6343 | 2497.808 | 4 | 5.308 | 31.8% | 1 | R.IVSRPEELREDDVGTGAGLLEIK.K | 3 |
|  | Mis12XLAPLSS\_011913\_01.09414.09414.2 | 3.0674 | 0.2955 | 99.8% | 1167.3522 | 1167.3939 | 1 | 5.528 | 90.0% | 2 | R.AVAQALEVIPR.T | 2 |

---

|  |  |  |  |  |  |  |  |  |
| --- | --- | --- | --- | --- | --- | --- | --- | --- |
| U | *gi|4506723|ref|NP\_000* | 5 | 5 | 19.3% | 264 | 29945 | 9.7 | ribosomal protein S3a [Homo sapiens] |

| Filename XCorr DeltCN Conf% ObsM+H+ CalcM+H+ SpR ZScore Ion% # Sequence  | | | | | | | | | | | | |
| --- | --- | --- | --- | --- | --- | --- | --- | --- | --- | --- | --- | --- |
| \* | Mis12XLAPLSS\_011913\_01.09076.09076.2 | 1.9348 | 0.3671 | 99.4% | 919.89215 | 920.1174 | 2 | 6.279 | 71.4% | 1 | K.APAMFNIR.N | 2 |
|  | Mis12XLAPLSS\_011913\_02.09112.09112.2 | 3.2854 | 0.3731 | 99.9% | 1952.3322 | 1953.1589 | 18 | 7.629 | 37.5% | 1 | R.VFEVSLADLQNDEVAFR.K | 2 |
|  | Mis12XLAPLSS\_011913\_02.08340.08340.3 | 2.9245 | 0.4163 | 100.0% | 2082.0244 | 2081.333 | 2 | 6.214 | 35.3% | 1 | R.VFEVSLADLQNDEVAFRK.F | 3 |
| \* | Mis12XLAPLSS\_011913\_01.07988.07988.2 | 2.3818 | 0.2143 | 98.0% | 1328.2722 | 1328.5486 | 43 | 4.196 | 54.5% | 1 | K.LIPDSIGKDIEK.A | 2 |
| \* | Mis12XLAPLSS\_011913\_01.04119.04119.2 | 3.733 | 0.3354 | 99.9% | 1333.3522 | 1332.4712 | 1 | 5.948 | 66.7% | 1 | K.LMELHGEGSSSGK.A | 2 |

---

|  |  |  |  |  |  |  |  |  |
| --- | --- | --- | --- | --- | --- | --- | --- | --- |
| U | *gi|5902076|ref|NP\_008* | 5 | 5 | 19.0% | 248 | 27745 | 10.4 | splicing factor, arginine/serine-rich 1 isoform 1 [Homo sapiens] |

| Filename XCorr DeltCN Conf% ObsM+H+ CalcM+H+ SpR ZScore Ion% # Sequence  | | | | | | | | | | | | |
| --- | --- | --- | --- | --- | --- | --- | --- | --- | --- | --- | --- | --- |
|  | Mis12XLAPLSS\_011913\_01.09338.09338.2 | 2.5513 | 0.2799 | 99.5% | 1257.3121 | 1257.4752 | 3 | 5.008 | 65.0% | 1 | R.IYVGNLPPDIR.T | 2 |
|  | Mis12XLAPLSS\_011913\_01.08586.08586.2 | 2.5753 | 0.3497 | 99.8% | 1257.3922 | 1258.4137 | 1 | 6.067 | 77.8% | 1 | R.TKDIEDVFYK.Y | 2 |
|  | Mis12XLAPLSS\_011913\_01.07402.07402.2 | 2.2127 | 0.1077 | 95.3% | 917.03217 | 917.0989 | 2 | 3.713 | 83.3% | 1 | R.LRVEFPR.S | 2 |
|  | Mis12XLAPLSS\_011913\_01.04143.04143.2 | 2.2015 | 0.1592 | 95.4% | 1114.1522 | 1113.2738 | 196 | 3.804 | 62.5% | 1 | R.KEDMTYAVR.K | 2 |
| \* | Mis12XLAPLSS\_011913\_01.03842.03842.2 | 2.8166 | 0.2725 | 99.8% | 1163.0721 | 1163.2322 | 2 | 6.094 | 77.8% | 1 | R.SHEGETAYIR.V | 2 |

---

|  |  |  |  |  |  |  |  |  |
| --- | --- | --- | --- | --- | --- | --- | --- | --- |
| U | *gi|226246671|ref|NP\_0* | 3 | 4 | 19.0% | 142 | 16006 | 9.3 | ribosomal protein S20 isoform 1 [Homo sapiens] |
| U | *gi|4506697|ref|NP\_001* | 3 | 4 | 22.7% | 119 | 13373 | 9.9 | ribosomal protein S20 isoform 2 [Homo sapiens] |

| Filename XCorr DeltCN Conf% ObsM+H+ CalcM+H+ SpR ZScore Ion% # Sequence  | | | | | | | | | | | | |
| --- | --- | --- | --- | --- | --- | --- | --- | --- | --- | --- | --- | --- |
|  | Mis12XLAPLSS\_011913\_01.04818.04818.3 | 3.721 | 0.3728 | 100.0% | 1650.7144 | 1649.8442 | 1 | 6.268 | 37.5% | 1 | K.DTGKTPVEPEVAIHR.I | 3 |
|  | Mis12XLAPLSS\_011913\_01.04992.04992.2 | 2.5967 | 0.2646 | 99.5% | 1247.7922 | 1248.4246 | 1 | 5.503 | 65.0% | 1 | K.TPVEPEVAIHR.I | 2 |
|  | Mis12XLAPLSS\_011913\_01.08780.08780.2 | 3.3625 | 0.4356 | 100.0% | 1350.6721 | 1351.5858 | 2 | 6.359 | 63.6% | 2 | R.LIDLHSPSEIVK.Q | 2 |

---

|  |  |  |  |  |  |  |  |  |
| --- | --- | --- | --- | --- | --- | --- | --- | --- |
| U | *gi|224586884|ref|NP\_0* | 4 | 5 | 18.8% | 303 | 31947 | 9.7 | cold shock domain protein A isoform b [Homo sapiens] |

| Filename XCorr DeltCN Conf% ObsM+H+ CalcM+H+ SpR ZScore Ion% # Sequence  | | | | | | | | | | | | |
| --- | --- | --- | --- | --- | --- | --- | --- | --- | --- | --- | --- | --- |
|  | Mis12XLAPLSS\_011913\_01.05103.05103.3 | 3.4595 | 0.3746 | 100.0% | 1745.8143 | 1745.9298 | 1 | 6.711 | 46.4% | 1 | R.NDTKEDVFVHQTAIK.K | 33 |
|  | Mis12XLAPLSS\_011913\_01.04263.04263.3 | 3.1658 | 0.3703 | 100.0% | 1874.3344 | 1874.1039 | 6 | 5.588 | 35.0% | 1 | R.NDTKEDVFVHQTAIKK.N | 33 |
|  | Mis12XLAPLSS\_011913\_02.06794.06794.2 | 4.3245 | 0.3461 | 100.0% | 1796.9321 | 1796.8822 | 1 | 8.079 | 65.6% | 2 | R.SVGDGETVEFDVVEGEK.G | 22 |
| \* | Mis12XLAPLSS\_011913\_01.07938.07938.3 | 3.921 | 0.3322 | 99.7% | 2490.1143 | 2490.7534 | 228 | 5.803 | 25.0% | 1 | R.NAGEIGEMKDGVPEGAQLQGPVHR.N | 3 |

Similarities:
gi|34098946|ref|NP\_00(3:1)  

---

|  |  |  |  |  |  |  |  |  |
| --- | --- | --- | --- | --- | --- | --- | --- | --- |
| U | *gi|4826860|ref|NP\_004* | 2 | 2 | 18.8% | 128 | 14174 | 8.5 | NHP2 non-histone chromosome protein 2-like 1 [Homo sapiens] |
| U | *gi|51317376|ref|NP\_00* | 2 | 2 | 18.8% | 128 | 14174 | 8.5 | NHP2 non-histone chromosome protein 2-like 1 [Homo sapiens] |

| Filename XCorr DeltCN Conf% ObsM+H+ CalcM+H+ SpR ZScore Ion% # Sequence  | | | | | | | | | | | | |
| --- | --- | --- | --- | --- | --- | --- | --- | --- | --- | --- | --- | --- |
|  | Mis12XLAPLSS\_011913\_01.04574.04574.2 | 2.7006 | 0.2628 | 99.5% | 1328.0122 | 1328.5541 | 1 | 5.097 | 72.7% | 1 | K.AYPLADAHLTKK.L | 2 |
|  | Mis12XLAPLSS\_011913\_01.07547.07547.2 | 2.8166 | 0.2682 | 99.8% | 1458.1322 | 1458.6145 | 31 | 5.685 | 50.0% | 1 | K.QQIQSIQQSIER.L | 2 |

---

|  |  |  |  |  |  |  |  |  |
| --- | --- | --- | --- | --- | --- | --- | --- | --- |
| U | *gi|27436901|ref|NP\_00* | 2 | 2 | 18.7% | 198 | 21348 | 8.9 | mitochondrial ribosomal protein L12 [Homo sapiens] |

| Filename XCorr DeltCN Conf% ObsM+H+ CalcM+H+ SpR ZScore Ion% # Sequence  | | | | | | | | | | | | |
| --- | --- | --- | --- | --- | --- | --- | --- | --- | --- | --- | --- | --- |
| \* | Mis12XLAPLSS\_011913\_01.18246.18246.2 | 2.6611 | 0.3717 | 99.8% | 2840.8523 | 2839.3428 | 49 | 6.529 | 25.0% | 1 | K.IQQLVQDIASLTLLEISDLNELLKK.T | 2 |
| \* | Mis12XLAPLSS\_011913\_01.09570.09570.2 | 2.2846 | 0.1957 | 96.2% | 1362.9122 | 1361.584 | 3 | 4.709 | 54.5% | 1 | K.NYIQGINLVQAK.K | 2 |

---

|  |  |  |  |  |  |  |  |  |
| --- | --- | --- | --- | --- | --- | --- | --- | --- |
| U | *gi|48762932|ref|NP\_00* | 9 | 13 | 18.6% | 548 | 59621 | 5.6 | chaperonin containing TCP1, subunit 8 (theta) [Homo sapiens] |

| Filename XCorr DeltCN Conf% ObsM+H+ CalcM+H+ SpR ZScore Ion% # Sequence  | | | | | | | | | | | | |
| --- | --- | --- | --- | --- | --- | --- | --- | --- | --- | --- | --- | --- |
| \* | Mis12XLAPLSS\_011913\_01.09447.09447.2 | 2.5455 | 0.3812 | 99.8% | 963.3522 | 963.1828 | 3 | 6.595 | 75.0% | 1 | K.APGFAQMLK.E | 2 |
| \* | Mis12XLAPLSS\_011913\_01.07971.07971.2 | 3.0311 | 0.5212 | 100.0% | 1308.6322 | 1308.4362 | 3 | 7.501 | 70.0% | 2 | K.HFSGLEEAVYR.N | 2 |
| \* | Mis12XLAPLSS\_011913\_01.10731.10731.2 | 2.6869 | 0.4175 | 99.8% | 1333.7722 | 1334.5583 | 1 | 6.543 | 77.3% | 1 | K.LFVTNDAATILR.E | 2 |
| \* | Mis12XLAPLSS\_011913\_01.03659.03659.2 | 2.6045 | 0.2382 | 99.5% | 1123.2322 | 1122.2664 | 66 | 4.745 | 66.7% | 1 | R.ELEVQHPAAK.M | 2 |
| \* | Mis12XLAPLSS\_011913\_01.11106.11106.2 | 3.4958 | 0.3873 | 99.8% | 1531.1721 | 1530.7214 | 1 | 6.136 | 62.5% | 2 | K.NLRDIDEVSSLLR.T | 2 |
| \* | Mis12XLAPLSS\_011913\_02.05122.05122.2 | 4.1722 | 0.6004 | 100.0% | 1372.4922 | 1373.5492 | 1 | 10.777 | 60.7% | 3 | K.AIADTGANVVVTGGK.V | 2 |
| \* | Mis12XLAPLSS\_011913\_01.09468.09468.2 | 2.7527 | 0.3703 | 99.8% | 1366.6522 | 1366.4425 | 2 | 5.797 | 72.7% | 1 | R.GSTDNLMDDIER.A | 2 |
| \* | Mis12XLAPLSS\_011913\_01.09771.09771.2 | 3.1245 | 0.445 | 100.0% | 1151.2322 | 1151.3073 | 1 | 7.175 | 88.9% | 1 | K.FAEAFEAIPR.A | 2 |
| \* | Mis12XLAPLSS\_011913\_01.03533.03533.2 | 2.227 | 0.2209 | 97.8% | 1158.8522 | 1159.2871 | 1 | 4.796 | 66.7% | 1 | K.LYAVHQEGNK.N | 2 |

---

|  |  |  |  |  |  |  |  |  |
| --- | --- | --- | --- | --- | --- | --- | --- | --- |
| U | *gi|224028244|ref|NP\_0* | 7 | 9 | 18.5% | 471 | 54232 | 8.9 | non-POU domain containing, octamer-binding isoform 1 [Homo sapiens] |
| U | *gi|34932414|ref|NP\_03* | 7 | 9 | 18.5% | 471 | 54232 | 8.9 | non-POU domain containing, octamer-binding isoform 1 [Homo sapiens] |
| U | *gi|224028248|ref|NP\_0* | 7 | 9 | 22.8% | 382 | 43866 | 8.6 | non-POU domain containing, octamer-binding isoform 2 [Homo sapiens] |
| U | *gi|224028246|ref|NP\_0* | 7 | 9 | 18.5% | 471 | 54232 | 8.9 | non-POU domain containing, octamer-binding isoform 1 [Homo sapiens] |

| Filename XCorr DeltCN Conf% ObsM+H+ CalcM+H+ SpR ZScore Ion% # Sequence  | | | | | | | | | | | | |
| --- | --- | --- | --- | --- | --- | --- | --- | --- | --- | --- | --- | --- |
|  | Mis12XLAPLSS\_011913\_01.17114.17114.3 | 3.9983 | 0.4344 | 100.0% | 2669.3943 | 2669.9507 | 1 | 6.866 | 30.7% | 1 | R.NLPQYVSNELLEEAFSVFGQVER.A | 3 |
|  | Mis12XLAPLSS\_011913\_01.17099.17099.2 | 5.4728 | 0.497 | 100.0% | 2669.9722 | 2669.9507 | 1 | 9.525 | 54.5% | 1 | R.NLPQYVSNELLEEAFSVFGQVER.A | 2 |
|  | Mis12XLAPLSS\_011913\_01.09998.09998.2 | 3.4078 | 0.42 | 100.0% | 1695.9321 | 1696.8744 | 1 | 7.59 | 76.9% | 1 | R.FAQPGSFEYEYAMR.W | 2 |
|  | Mis12XLAPLSS\_011913\_01.03510.03510.2 | 3.5918 | 0.1486 | 99.8% | 1541.1721 | 1541.7222 | 17 | 4.373 | 63.6% | 1 | R.RMEELHNQEVQK.R | 2 |
|  | Mis12XLAPLSS\_011913\_01.03398.03398.3 | 3.7006 | 0.1317 | 95.8% | 1697.2444 | 1697.9097 | 1 | 4.134 | 47.9% | 1 | R.RMEELHNQEVQKR.K | 3 |
|  | Mis12XLAPLSS\_011913\_02.06098.06098.2 | 3.9295 | 0.4672 | 100.0% | 1538.7322 | 1539.8441 | 1 | 7.557 | 64.3% | 3 | R.MGQMAMGGAMGINNR.G | 2 |
|  | Mis12XLAPLSS\_011913\_02.07821.07821.3 | 3.1639 | 0.2688 | 98.3% | 2243.8145 | 2244.4436 | 29 | 5.499 | 31.0% | 1 | R.FGQAATMEGIGAIGGT#PPAFNR.A | 3 |

---

|  |  |  |  |  |  |  |  |  |
| --- | --- | --- | --- | --- | --- | --- | --- | --- |
| U | *gi|62414289|ref|NP\_00* | 7 | 8 | 18.5% | 466 | 53652 | 5.1 | vimentin [Homo sapiens] |

| Filename XCorr DeltCN Conf% ObsM+H+ CalcM+H+ SpR ZScore Ion% # Sequence  | | | | | | | | | | | | |
| --- | --- | --- | --- | --- | --- | --- | --- | --- | --- | --- | --- | --- |
| \* | Mis12XLAPLSS\_011913\_01.08992.08992.2 | 2.3686 | 0.265 | 98.9% | 1500.0521 | 1498.6508 | 7 | 4.419 | 59.1% | 1 | K.SRLGDLYEEEMR.E | 2 |
| \* | Mis12XLAPLSS\_011913\_01.07738.07738.2 | 2.5685 | 0.3046 | 99.8% | 1323.8922 | 1324.3898 | 6 | 5.753 | 60.0% | 1 | R.EEAENTLQSFR.Q | 2 |
| \* | Mis12XLAPLSS\_011913\_01.04025.04025.2 | 2.3171 | 0.1479 | 95.5% | 1089.1921 | 1089.1503 | 3 | 3.761 | 77.8% | 1 | R.QDVDNASLAR.L | 2 |
|  | Mis12XLAPLSS\_011913\_01.09104.09104.2 | 2.7412 | 0.3021 | 99.8% | 1310.1522 | 1310.4056 | 3 | 4.871 | 66.7% | 1 | K.NLQEAEEWYK.S | 2 |
| \* | Mis12XLAPLSS\_011913\_01.05988.05988.2 | 3.2397 | 0.2979 | 99.9% | 1093.6921 | 1094.1692 | 2 | 6.001 | 72.2% | 2 | K.FADLSEAANR.N | 2 |
| \* | Mis12XLAPLSS\_011913\_02.07466.07466.3 | 3.3359 | 0.3288 | 99.7% | 2188.8843 | 2188.33 | 3 | 5.686 | 36.1% | 1 | R.EMEENFAVEAANYQDTIGR.L | 3 |
| \* | Mis12XLAPLSS\_011913\_01.08603.08603.2 | 3.8495 | 0.4996 | 100.0% | 1734.9722 | 1735.9679 | 1 | 9.055 | 73.1% | 1 | R.LQDEIQNMKEEMAR.H | 2 |

---

|  |  |  |  |  |  |  |  |  |
| --- | --- | --- | --- | --- | --- | --- | --- | --- |
| U | *gi|5453555|ref|NP\_006* | 4 | 4 | 18.5% | 216 | 24423 | 7.5 | ras-related nuclear protein [Homo sapiens] |

| Filename XCorr DeltCN Conf% ObsM+H+ CalcM+H+ SpR ZScore Ion% # Sequence  | | | | | | | | | | | | |
| --- | --- | --- | --- | --- | --- | --- | --- | --- | --- | --- | --- | --- |
| \* | Mis12XLAPLSS\_011913\_01.03881.03881.2 | 2.0234 | 0.2045 | 96.2% | 960.1922 | 961.0623 | 11 | 5.45 | 78.6% | 1 | R.HLTGEFEK.K | 2 |
| \* | Mis12XLAPLSS\_011913\_01.09768.09768.3 | 2.8376 | 0.2677 | 97.6% | 2054.8743 | 2053.3713 | 1 | 4.859 | 30.9% | 1 | K.YVATLGVEVHPLVFHTNR.G | 3 |
| \* | Mis12XLAPLSS\_011913\_01.12549.12549.2 | 4.0342 | 0.4437 | 100.0% | 1784.8121 | 1786.0427 | 1 | 7.657 | 73.1% | 1 | K.SNYNFEKPFLWLAR.K | 2 |
| \* | Mis12XLAPLSS\_011913\_01.12546.12546.3 | 3.2934 | 0.4059 | 100.0% | 1786.1943 | 1786.0427 | 1 | 7.044 | 46.2% | 1 | K.SNYNFEKPFLWLAR.K | 3 |

---

|  |  |  |  |  |  |  |  |  |
| --- | --- | --- | --- | --- | --- | --- | --- | --- |
| U | *gi|21327708|ref|NP\_63* | 4 | 5 | 18.4% | 391 | 45374 | 4.5 | nucleosome assembly protein 1-like 1 [Homo sapiens] |
| U | *gi|4758756|ref|NP\_004* | 4 | 5 | 18.4% | 391 | 45374 | 4.5 | nucleosome assembly protein 1-like 1 [Homo sapiens] |

| Filename XCorr DeltCN Conf% ObsM+H+ CalcM+H+ SpR ZScore Ion% # Sequence  | | | | | | | | | | | | |
| --- | --- | --- | --- | --- | --- | --- | --- | --- | --- | --- | --- | --- |
|  | Mis12XLAPLSS\_011913\_01.12100.12100.2 | 3.5101 | 0.2857 | 99.8% | 1861.1322 | 1861.102 | 1 | 5.412 | 56.2% | 1 | R.LDGLVETPTGYIESLPR.V | 2 |
|  | Mis12XLAPLSS\_011913\_01.07263.07263.2 | 2.6409 | 0.3706 | 99.8% | 1336.9521 | 1337.4314 | 1 | 6.325 | 77.8% | 2 | K.FYEEVHDLER.K | 22 |
|  | Mis12XLAPLSS\_011913\_01.03210.03210.2 | 3.3211 | 0.3052 | 99.8% | 1931.3722 | 1932.0918 | 1 | 6.463 | 63.3% | 1 | K.AKIEDEKKDEEKEDPK.G | 2 |
|  | Mis12XLAPLSS\_011913\_02.10473.10473.3 | 3.1495 | 0.2282 | 95.3% | 3423.5942 | 3425.7932 | 2 | 4.628 | 21.4% | 1 | K.FSDAGQPMSFVLEFHFEPNEYFTNEVLTK.T | 3 |

Similarities:
gi|5174613|ref|NP\_005(1:3)  

---

|  |  |  |  |  |  |  |  |  |
| --- | --- | --- | --- | --- | --- | --- | --- | --- |
| U | *gi|23110942|ref|NP\_00* | 3 | 4 | 18.3% | 241 | 26411 | 4.8 | proteasome alpha 5 subunit [Homo sapiens] |

| Filename XCorr DeltCN Conf% ObsM+H+ CalcM+H+ SpR ZScore Ion% # Sequence  | | | | | | | | | | | | |
| --- | --- | --- | --- | --- | --- | --- | --- | --- | --- | --- | --- | --- |
| \* | Mis12XLAPLSS\_011913\_01.12629.12629.2 | 2.4525 | 0.2387 | 98.7% | 1423.7322 | 1424.6799 | 12 | 4.835 | 54.5% | 1 | R.LFQVEYAIEAIK.L | 2 |
| \* | Mis12XLAPLSS\_011913\_01.09106.09106.2 | 2.887 | 0.2925 | 99.8% | 1432.9521 | 1432.6721 | 1 | 5.617 | 66.7% | 2 | R.ITSPLMEPSSIEK.I | 2 |
| \* | Mis12XLAPLSS\_011913\_01.07476.07476.2 | 5.3451 | 0.5249 | 100.0% | 1962.3121 | 1963.1112 | 1 | 9.529 | 66.7% | 1 | R.AIGSASEGAQSSLQEVYHK.S | 2 |

---

|  |  |  |  |  |  |  |  |  |
| --- | --- | --- | --- | --- | --- | --- | --- | --- |
| U | *gi|74136883|ref|NP\_11* | 11 | 14 | 18.1% | 825 | 90585 | 6.0 | heterogeneous nuclear ribonucleoprotein U isoform a [Homo sapiens] |

| Filename XCorr DeltCN Conf% ObsM+H+ CalcM+H+ SpR ZScore Ion% # Sequence  | | | | | | | | | | | | |
| --- | --- | --- | --- | --- | --- | --- | --- | --- | --- | --- | --- | --- |
|  | Mis12XLAPLSS\_011913\_01.04257.04257.2 | 2.4048 | 0.1332 | 97.1% | 1075.1921 | 1075.2474 | 2 | 5.225 | 75.0% | 1 | K.VSELKEELK.K | 2 |
|  | Mis12XLAPLSS\_011913\_02.06431.06431.3 | 5.3592 | 0.553 | 100.0% | 3127.2244 | 3128.311 | 1 | 7.974 | 32.3% | 2 | R.LQAALDDEEAGGRPAMEPGNGSLDLGGDSAGR.S | 3 |
|  | Mis12XLAPLSS\_011913\_01.11354.11354.2 | 2.705 | 0.4159 | 99.8% | 1715.4521 | 1715.9469 | 88 | 6.372 | 29.4% | 1 | K.SSGPTSLFAVTVAPPGAR.Q | 2 |
| \* | Mis12XLAPLSS\_011913\_01.03218.03218.3 | 5.4105 | 0.4905 | 100.0% | 2025.9844 | 2026.13 | 1 | 8.352 | 40.9% | 1 | K.AEGGGGGGRPGAPAAGDGKTEQK.G | 3 |
|  | Mis12XLAPLSS\_011913\_01.09261.09261.2 | 4.0306 | 0.473 | 100.0% | 1698.4521 | 1698.8291 | 1 | 8.087 | 75.0% | 1 | R.GYFEYIEENKYSR.A | 2 |
|  | Mis12XLAPLSS\_011913\_02.06710.06710.3 | 2.3645 | 0.2965 | 97.6% | 1698.5643 | 1698.8291 | 1 | 5.276 | 41.7% | 1 | R.GYFEYIEENKYSR.A | 3 |
|  | Mis12XLAPLSS\_011913\_01.05117.05117.2 | 2.3201 | 0.2365 | 99.4% | 997.1922 | 997.0959 | 15 | 5.429 | 78.6% | 1 | K.DIDIHEVR.I | 2 |
|  | Mis12XLAPLSS\_011913\_01.08661.08661.3 | 4.4107 | 0.4194 | 100.0% | 2188.6443 | 2188.4631 | 1 | 6.644 | 34.2% | 1 | K.HAAENPGKYNILGTNTIMDK.M | 3 |
|  | Mis12XLAPLSS\_011913\_01.10059.10059.2 | 3.0226 | 0.2065 | 99.6% | 1383.6122 | 1383.6025 | 1 | 5.273 | 72.7% | 1 | K.YNILGTNTIMDK.M | 2 |
|  | Mis12XLAPLSS\_011913\_01.09369.09369.2 | 4.6011 | 0.4523 | 100.0% | 1648.4122 | 1648.816 | 1 | 7.676 | 82.1% | 3 | R.NFILDQTNVSAAAQR.R | 2 |
|  | Mis12XLAPLSS\_011913\_01.03792.03792.2 | 3.5364 | 0.3756 | 100.0% | 1395.6322 | 1395.5957 | 1 | 5.873 | 80.0% | 1 | K.LLEQYKEESKK.A | 2 |

---

|  |  |  |  |  |  |  |  |  |
| --- | --- | --- | --- | --- | --- | --- | --- | --- |
| U | *gi|5031753|ref|NP\_005* | 6 | 7 | 17.8% | 449 | 49229 | 6.3 | heterogeneous nuclear ribonucleoprotein H1 [Homo sapiens] |

| Filename XCorr DeltCN Conf% ObsM+H+ CalcM+H+ SpR ZScore Ion% # Sequence  | | | | | | | | | | | | |
| --- | --- | --- | --- | --- | --- | --- | --- | --- | --- | --- | --- | --- |
| \* | Mis12XLAPLSS\_011913\_01.03497.03497.2 | 2.5655 | 0.2352 | 99.5% | 958.33215 | 957.0775 | 166 | 4.083 | 62.5% | 1 | K.IQNGAQGIR.F | 2 |
| \* | Mis12XLAPLSS\_011913\_02.07725.07725.2 | 3.4819 | 0.3122 | 99.9% | 1335.7322 | 1335.5176 | 1 | 6.571 | 80.0% | 1 | K.SNNVEMDWVLK.H | 2 |
|  | Mis12XLAPLSS\_011913\_01.04414.04414.2 | 3.3849 | 0.5071 | 100.0% | 1685.2722 | 1685.7501 | 1 | 7.938 | 63.3% | 1 | K.HTGPNSPDTANDGFVR.L | 2 |
|  | Mis12XLAPLSS\_011913\_02.08278.08278.2 | 4.9553 | 0.4982 | 100.0% | 1842.5922 | 1843.0001 | 1 | 8.65 | 68.8% | 2 | R.STGEAFVQFASQEIAEK.A | 2 |
|  | Mis12XLAPLSS\_011913\_01.13463.13463.2 | 2.3486 | 0.2144 | 96.3% | 1996.7722 | 1998.2023 | 77 | 5.884 | 34.4% | 1 | R.ATENDIYNFFSPLNPVR.V | 22 |
|  | Mis12XLAPLSS\_011913\_01.05208.05208.2 | 2.8214 | 0.4817 | 100.0% | 1093.4321 | 1093.2278 | 1 | 7.479 | 72.2% | 1 | R.VHIEIGPDGR.V | 22 |

Similarities:
gi|148470397|ref|NP\_0(2:4)  

---

|  |  |  |  |  |  |  |  |  |
| --- | --- | --- | --- | --- | --- | --- | --- | --- |
| U | *gi|4885375|ref|NP\_005* | 4 | 9 | 17.8% | 213 | 21365 | 10.9 | histone cluster 1, H1c [Homo sapiens] |
| U | *gi|4885379|ref|NP\_005* | 4 | 9 | 17.4% | 219 | 21865 | 11.0 | histone cluster 1, H1e [Homo sapiens] |
| U | *gi|4885377|ref|NP\_005* | 4 | 9 | 17.2% | 221 | 22350 | 11.0 | histone cluster 1, H1d [Homo sapiens] |

| Filename XCorr DeltCN Conf% ObsM+H+ CalcM+H+ SpR ZScore Ion% # Sequence  | | | | | | | | | | | | |
| --- | --- | --- | --- | --- | --- | --- | --- | --- | --- | --- | --- | --- |
|  | Mis12XLAPLSS\_011913\_01.06413.06413.2 | 4.0696 | 0.3465 | 100.0% | 1328.0322 | 1327.5638 | 1 | 6.584 | 66.7% | 4 | R.KASGPPVSELITK.A | 2 |
|  | Mis12XLAPLSS\_011913\_01.05120.05120.2 | 3.1482 | 0.2553 | 99.8% | 973.6922 | 974.1887 | 7 | 5.487 | 72.2% | 2 | R.SGVSLAALKK.A | 2 |
|  | Mis12XLAPLSS\_011913\_01.05408.05408.2 | 2.8725 | 0.4037 | 99.9% | 1108.0721 | 1108.2365 | 6 | 7.372 | 65.0% | 2 | K.ALAAAGYDVEK.N | 2 |
|  | Mis12XLAPLSS\_011913\_01.04546.04546.2 | 4.4303 | 0.4638 | 100.0% | 1579.1921 | 1579.7098 | 1 | 8.98 | 78.6% | 1 | K.ALAAAGYDVEKNNSR.I | 2 |

---

|  |  |  |  |  |  |  |  |  |
| --- | --- | --- | --- | --- | --- | --- | --- | --- |
| U | *gi|94721250|ref|NP\_00* | 6 | 7 | 17.7% | 294 | 32614 | 8.9 | vesicle-associated membrane protein-associated protein A isoform 1 [Homo sapiens] |
| U | *gi|94721252|ref|NP\_91* | 6 | 7 | 20.9% | 249 | 27893 | 8.6 | vesicle-associated membrane protein-associated protein A isoform 2 [Homo sapiens] |

| Filename XCorr DeltCN Conf% ObsM+H+ CalcM+H+ SpR ZScore Ion% # Sequence  | | | | | | | | | | | | |
| --- | --- | --- | --- | --- | --- | --- | --- | --- | --- | --- | --- | --- |
|  | Mis12XLAPLSS\_011913\_01.09810.09810.2 | 3.5444 | 0.0668 | 98.8% | 1618.5922 | 1618.8705 | 1 | 5.451 | 65.4% | 1 | K.HEQILVLDPPTDLK.F | 2 |
|  | Mis12XLAPLSS\_011913\_01.09818.09818.2 | 3.3187 | 0.3656 | 99.9% | 1568.0322 | 1567.8253 | 1 | 6.435 | 76.9% | 1 | K.FKGPFTDVVTTNLK.L | 2 |
|  | Mis12XLAPLSS\_011913\_01.09789.09789.2 | 3.1348 | 0.3155 | 99.8% | 1292.1522 | 1292.4747 | 29 | 5.556 | 63.6% | 2 | K.GPFTDVVTTNLK.L | 2 |
|  | Mis12XLAPLSS\_011913\_01.03348.03348.2 | 2.2251 | 0.2231 | 98.6% | 995.39215 | 996.11414 | 225 | 4.12 | 64.3% | 1 | R.HLRDEGLR.L | 2 |
|  | Mis12XLAPLSS\_011913\_01.03508.03508.3 | 3.4758 | 0.3883 | 100.0% | 1648.1044 | 1648.7727 | 2 | 5.87 | 38.3% | 1 | K.VAHSDKPGSTSTASFR.D | 3 |
|  | Mis12XLAPLSS\_011913\_01.03512.03512.2 | 3.4549 | 0.3558 | 99.9% | 1648.3121 | 1648.7727 | 1 | 6.416 | 70.0% | 1 | K.VAHSDKPGSTSTASFR.D | 2 |

---

|  |  |  |  |  |  |  |  |  |
| --- | --- | --- | --- | --- | --- | --- | --- | --- |
| U | *gi|15431288|ref|NP\_00* | 3 | 4 | 17.5% | 217 | 24831 | 9.9 | ribosomal protein L10a [Homo sapiens] |

| Filename XCorr DeltCN Conf% ObsM+H+ CalcM+H+ SpR ZScore Ion% # Sequence  | | | | | | | | | | | | |
| --- | --- | --- | --- | --- | --- | --- | --- | --- | --- | --- | --- | --- |
| \* | Mis12XLAPLSS\_011913\_01.06850.06850.2 | 2.0615 | 0.2718 | 98.6% | 967.1922 | 967.06665 | 27 | 4.799 | 71.4% | 1 | R.DTLYEAVR.E | 2 |
|  | Mis12XLAPLSS\_011913\_01.09965.09965.2 | 3.4613 | 0.3491 | 99.9% | 1485.8121 | 1485.7203 | 3 | 6.925 | 58.3% | 2 | K.KYDAFLASESLIK.Q | 2 |
|  | Mis12XLAPLSS\_011913\_01.08217.08217.3 | 3.2715 | 0.3395 | 99.7% | 1858.1044 | 1858.1656 | 4 | 5.65 | 32.8% | 1 | K.AGKFPSLLTHNENMVAK.V | 3 |

---

|  |  |  |  |  |  |  |  |  |
| --- | --- | --- | --- | --- | --- | --- | --- | --- |
| U | *gi|15809016|ref|NP\_29* | 3 | 3 | 17.4% | 172 | 19779 | 4.8 | myosin regulatory light chain MRCL2 isoform A [Homo sapiens] |
| U | *gi|5453740|ref|NP\_006* | 3 | 3 | 17.5% | 171 | 19794 | 4.8 | myosin, light chain 12A, regulatory, non-sarcomeric [Homo sapiens] |
| U | *gi|222144328|ref|NP\_0* | 3 | 3 | 19.5% | 154 | 17757 | 4.4 | myosin regulatory light chain MRCL2 isoform B [Homo sapiens] |
| U | *gi|222144326|ref|NP\_0* | 3 | 3 | 17.4% | 172 | 19779 | 4.8 | myosin regulatory light chain MRCL2 isoform A [Homo sapiens] |
| U | *gi|222144324|ref|NP\_0* | 3 | 3 | 17.4% | 172 | 19779 | 4.8 | myosin regulatory light chain MRCL2 isoform A [Homo sapiens] |

| Filename XCorr DeltCN Conf% ObsM+H+ CalcM+H+ SpR ZScore Ion% # Sequence  | | | | | | | | | | | | |
| --- | --- | --- | --- | --- | --- | --- | --- | --- | --- | --- | --- | --- |
|  | Mis12XLAPLSS\_011913\_01.12059.12059.3 | 3.7541 | 0.2823 | 99.7% | 2434.4043 | 2433.649 | 1 | 5.134 | 35.5% | 1 | R.ELLTTMGDRFTDEEVDELYR.E | 3 |
|  | Mis12XLAPLSS\_011913\_01.09300.09300.2 | 3.5565 | 0.4305 | 100.0% | 1415.9321 | 1416.4839 | 1 | 7.304 | 75.0% | 1 | R.FTDEEVDELYR.E | 2 |
|  | Mis12XLAPLSS\_011913\_01.10214.10214.2 | 2.6501 | 0.2574 | 99.7% | 1261.3922 | 1261.3794 | 3 | 6.917 | 66.7% | 1 | K.GNFNYIEFTR.I | 2 |

---

|  |  |  |  |  |  |  |  |  |
| --- | --- | --- | --- | --- | --- | --- | --- | --- |
| U | *gi|14277700|ref|NP\_00* | 2 | 2 | 17.4% | 132 | 14515 | 7.2 | ribosomal protein S12 [Homo sapiens] |

| Filename XCorr DeltCN Conf% ObsM+H+ CalcM+H+ SpR ZScore Ion% # Sequence  | | | | | | | | | | | | |
| --- | --- | --- | --- | --- | --- | --- | --- | --- | --- | --- | --- | --- |
| \* | Mis12XLAPLSS\_011913\_01.04946.04946.2 | 2.422 | 0.2335 | 99.1% | 1066.5521 | 1067.2333 | 3 | 4.915 | 72.2% | 1 | K.TALIHDGLAR.G | 2 |
|  | Mis12XLAPLSS\_011913\_01.10480.10480.2 | 3.8523 | 0.4729 | 100.0% | 1586.2122 | 1586.7386 | 2 | 7.261 | 62.5% | 1 | K.ESQAKDVIEEYFK.C | 2 |

---

|  |  |  |  |  |  |  |  |  |
| --- | --- | --- | --- | --- | --- | --- | --- | --- |
| U | *gi|14141152|ref|NP\_00* | 10 | 15 | 17.3% | 730 | 77516 | 8.7 | heterogeneous nuclear ribonucleoprotein M isoform a [Homo sapiens] |
| U | *gi|157412270|ref|NP\_1* | 10 | 15 | 18.2% | 691 | 73621 | 8.8 | heterogeneous nuclear ribonucleoprotein M isoform b [Homo sapiens] |

| Filename XCorr DeltCN Conf% ObsM+H+ CalcM+H+ SpR ZScore Ion% # Sequence  | | | | | | | | | | | | |
| --- | --- | --- | --- | --- | --- | --- | --- | --- | --- | --- | --- | --- |
|  | Mis12XLAPLSS\_011913\_01.13275.13275.2 | 2.3699 | 0.2867 | 98.6% | 1753.6522 | 1754.0051 | 1 | 5.327 | 53.3% | 1 | K.VGEVTYVELLMDAEGK.S | 2 |
|  | Mis12XLAPLSS\_011913\_01.04132.04132.2 | 2.7833 | 0.3385 | 99.8% | 1102.2722 | 1102.2714 | 1 | 6.132 | 80.0% | 1 | R.MGAGLGHGMDR.V | 2 |
|  | Mis12XLAPLSS\_011913\_01.09687.09687.2 | 4.2686 | 0.4778 | 100.0% | 1614.5922 | 1614.875 | 1 | 8.847 | 75.0% | 1 | R.MGPLGLDHMASSIER.M | 2 |
|  | Mis12XLAPLSS\_011913\_01.09668.09668.3 | 2.7486 | 0.3023 | 99.4% | 1614.6843 | 1614.875 | 1 | 5.587 | 39.3% | 2 | R.MGPLGLDHMASSIER.M | 3 |
|  | Mis12XLAPLSS\_011913\_02.06779.06779.2 | 3.2781 | 0.5154 | 100.0% | 1125.8922 | 1126.3337 | 1 | 9.395 | 75.0% | 2 | R.MGAGMGFGLER.M | 2 |
|  | Mis12XLAPLSS\_011913\_01.07745.07745.2 | 2.901 | 0.3706 | 99.8% | 1190.1522 | 1189.4333 | 1 | 5.409 | 63.6% | 2 | R.MVPAGMGAGLER.M | 2 |
|  | Mis12XLAPLSS\_011913\_01.04005.04005.2 | 2.2752 | 0.259 | 99.4% | 877.9322 | 877.99097 | 26 | 5.679 | 71.4% | 1 | R.MGANSLER.M | 2 |
|  | Mis12XLAPLSS\_011913\_01.09212.09212.2 | 2.7573 | 0.2374 | 99.2% | 1428.1522 | 1428.7076 | 1 | 4.873 | 60.7% | 1 | R.MGPAMGPALGAGIER.M | 2 |
|  | Mis12XLAPLSS\_011913\_02.06364.06364.2 | 3.5709 | 0.2129 | 99.8% | 1384.3722 | 1384.5677 | 1 | 7.54 | 71.4% | 2 | R.MGLAMGGGGGASFDR.A | 2 |
|  | Mis12XLAPLSS\_011913\_02.06465.06465.3 | 3.2388 | 0.3821 | 99.7% | 2035.6144 | 2036.1735 | 5 | 5.748 | 27.3% | 2 | R.GNFGGSFAGSFGGAGGHAPGVAR.K | 3 |

---

|  |  |  |  |  |  |  |  |  |
| --- | --- | --- | --- | --- | --- | --- | --- | --- |
| U | *gi|9951915|ref|NP\_000* | 7 | 13 | 17.1% | 432 | 47716 | 6.3 | S-adenosylhomocysteine hydrolase [Homo sapiens] |

| Filename XCorr DeltCN Conf% ObsM+H+ CalcM+H+ SpR ZScore Ion% # Sequence  | | | | | | | | | | | | |
| --- | --- | --- | --- | --- | --- | --- | --- | --- | --- | --- | --- | --- |
| \* | Mis12XLAPLSS\_011913\_02.07434.07434.2 | 3.6717 | 0.4209 | 100.0% | 1129.1921 | 1129.3042 | 1 | 8.323 | 80.0% | 2 | K.VADIGLAAWGR.K | 2 |
| \* | Mis12XLAPLSS\_011913\_01.09752.09752.2 | 2.8045 | 0.2757 | 99.5% | 1688.8722 | 1688.9978 | 20 | 4.798 | 42.9% | 1 | R.KALDIAENEMPGLMR.M | 2 |
| \* | Mis12XLAPLSS\_011913\_01.11114.11114.2 | 2.5803 | 0.3184 | 99.6% | 1560.2122 | 1560.8237 | 8 | 5.742 | 53.8% | 1 | K.ALDIAENEMPGLMR.M | 2 |
| \* | Mis12XLAPLSS\_011913\_01.10420.10420.2 | 2.6093 | 0.1479 | 98.6% | 1057.4521 | 1057.281 | 27 | 5.068 | 68.8% | 2 | K.YPQLLPGIR.G | 2 |
| \* | Mis12XLAPLSS\_011913\_01.06965.06965.2 | 3.6707 | 0.3648 | 99.9% | 1650.5521 | 1649.7979 | 2 | 6.576 | 53.6% | 2 | R.GISEETTTGVHNLYK.M | 2 |
| \* | Mis12XLAPLSS\_011913\_01.06687.06687.2 | 3.6979 | 0.4499 | 100.0% | 1258.2722 | 1257.4294 | 1 | 7.717 | 77.3% | 4 | K.VPAINVNDSVTK.S | 2 |
| \* | Mis12XLAPLSS\_011913\_02.05346.05346.2 | 2.8241 | 0.3984 | 99.8% | 1135.8322 | 1135.3054 | 2 | 8.132 | 63.6% | 1 | K.VAVVAGYGDVGK.G | 2 |

---

|  |  |  |  |  |  |  |  |  |
| --- | --- | --- | --- | --- | --- | --- | --- | --- |
| U | *gi|12408675|ref|NP\_03* | 2 | 2 | 16.9% | 154 | 16648 | 6.6 | prefoldin subunit 2 [Homo sapiens] |

| Filename XCorr DeltCN Conf% ObsM+H+ CalcM+H+ SpR ZScore Ion% # Sequence  | | | | | | | | | | | | |
| --- | --- | --- | --- | --- | --- | --- | --- | --- | --- | --- | --- | --- |
| \* | Mis12XLAPLSS\_011913\_01.10504.10504.2 | 2.4664 | 0.166 | 95.5% | 1419.8722 | 1419.5803 | 1 | 5.356 | 61.5% | 1 | K.GAVSAEQVIAGFNR.L | 2 |
| \* | Mis12XLAPLSS\_011913\_02.06537.06537.2 | 3.1705 | 0.3076 | 99.8% | 1386.6122 | 1386.6317 | 1 | 5.211 | 72.7% | 1 | K.IIETLTQQLQAK.G | 2 |

---

|  |  |  |  |  |  |  |  |  |
| --- | --- | --- | --- | --- | --- | --- | --- | --- |
| U | *gi|4758086|ref|NP\_004* | 3 | 3 | 16.6% | 193 | 20567 | 8.6 | cysteine and glycine-rich protein 1 isoform 1 [Homo sapiens] |

| Filename XCorr DeltCN Conf% ObsM+H+ CalcM+H+ SpR ZScore Ion% # Sequence  | | | | | | | | | | | | |
| --- | --- | --- | --- | --- | --- | --- | --- | --- | --- | --- | --- | --- |
|  | Mis12XLAPLSS\_011913\_01.03192.03192.2 | 4.1149 | 0.46 | 100.0% | 1842.7722 | 1843.9535 | 1 | 8.608 | 56.2% | 1 | K.HEEAPGHRPTTNPNASK.F | 2 |
|  | Mis12XLAPLSS\_011913\_01.03182.03182.3 | 2.819 | 0.2463 | 96.9% | 1843.9443 | 1843.9535 | 15 | 5.578 | 31.2% | 1 | K.HEEAPGHRPTTNPNASK.F | 3 |
| \* | Mis12XLAPLSS\_011913\_02.07176.07176.2 | 4.0062 | 0.4768 | 100.0% | 1435.0721 | 1434.551 | 1 | 8.376 | 60.7% | 1 | K.GFGFGQGAGALVHSE.- | 2 |

---

|  |  |  |  |  |  |  |  |  |
| --- | --- | --- | --- | --- | --- | --- | --- | --- |
| U | *gi|4502227|ref|NP\_001* | 2 | 3 | 16.6% | 181 | 20418 | 5.7 | ADP-ribosylation factor-like 1 [Homo sapiens] |

| Filename XCorr DeltCN Conf% ObsM+H+ CalcM+H+ SpR ZScore Ion% # Sequence  | | | | | | | | | | | | |
| --- | --- | --- | --- | --- | --- | --- | --- | --- | --- | --- | --- | --- |
| \* | Mis12XLAPLSS\_011913\_01.11097.11097.2 | 3.2207 | 0.2847 | 99.8% | 1677.0721 | 1676.9226 | 6 | 5.47 | 53.8% | 1 | K.SELVAMLEEEELRK.A | 2 |
| \* | Mis12XLAPLSS\_011913\_01.16571.16571.2 | 3.6476 | 0.5183 | 100.0% | 1792.4722 | 1793.0417 | 14 | 8.953 | 36.7% | 2 | K.GTGLDEAMEWLVETLK.S | 2 |

---

|  |  |  |  |  |  |  |  |  |
| --- | --- | --- | --- | --- | --- | --- | --- | --- |
| U | *gi|4506685|ref|NP\_001* | 2 | 3 | 16.6% | 151 | 17222 | 10.5 | ribosomal protein S13 [Homo sapiens] |

| Filename XCorr DeltCN Conf% ObsM+H+ CalcM+H+ SpR ZScore Ion% # Sequence  | | | | | | | | | | | | |
| --- | --- | --- | --- | --- | --- | --- | --- | --- | --- | --- | --- | --- |
| \* | Mis12XLAPLSS\_011913\_01.05956.05956.2 | 3.0103 | 0.4137 | 99.9% | 1437.9722 | 1439.6055 | 1 | 7.551 | 68.2% | 2 | K.LTSDDVKEQIYK.L | 2 |
| \* | Mis12XLAPLSS\_011913\_01.09454.09454.2 | 2.7701 | 0.2629 | 99.5% | 1383.5521 | 1382.6896 | 1 | 4.975 | 66.7% | 1 | K.KGLTPSQIGVILR.D | 2 |

---

|  |  |  |  |  |  |  |  |  |
| --- | --- | --- | --- | --- | --- | --- | --- | --- |
| U | *gi|63025212|ref|NP\_98* | 3 | 4 | 16.5% | 266 | 27202 | 4.9 | hypothetical protein LOC255374 [Homo sapiens] |

| Filename XCorr DeltCN Conf% ObsM+H+ CalcM+H+ SpR ZScore Ion% # Sequence  | | | | | | | | | | | | |
| --- | --- | --- | --- | --- | --- | --- | --- | --- | --- | --- | --- | --- |
| \* | Mis12XLAPLSS\_011913\_01.07527.07527.2 | 3.3336 | 0.3292 | 99.8% | 1290.4521 | 1289.3445 | 2 | 5.878 | 65.4% | 1 | R.GSGGAEAALEEAAR.G | 2 |
| \* | Mis12XLAPLSS\_011913\_01.10186.10186.2 | 3.7592 | 0.5018 | 100.0% | 1339.1921 | 1339.537 | 1 | 9.149 | 66.7% | 2 | R.GPILVDTGGPWAR.E | 2 |
| \* | Mis12XLAPLSS\_011913\_01.09284.09284.2 | 3.8549 | 0.4863 | 100.0% | 1732.4722 | 1732.9395 | 1 | 8.723 | 53.1% | 1 | R.LGPGLEVWATPGHGGQR.D | 2 |

---

|  |  |  |  |  |  |  |  |  |
| --- | --- | --- | --- | --- | --- | --- | --- | --- |
| U | *contaminant\_GR78\_HUMA* | 8 | 10 | 16.4% | 653 | 72116 | 5.1 | owl|P11021| 78 KD GLUCOSE REGULATED PROTEIN PRECURSOR (GRP 78) (IMMUNOGLOBULIN... |
| U | *gi|16507237|ref|NP\_00* | 8 | 10 | 16.4% | 654 | 72333 | 5.2 | heat shock 70kDa protein 5 [Homo sapiens] |
| U | *contaminant\_GR78\_RAT* | 8 | 10 | 16.4% | 654 | 72347 | 5.2 | owl|P06761| 78 KD GLUCOSE REGULATED PROTEIN PRECURSOR (GRP 78) (IMMUNOGLOBULIN... |
| U | *contaminant\_GR78\_MOUS* | 8 | 10 | 16.3% | 655 | 72421 | 5.2 | owl|P20029| 78 KD GLUCOSE REGULATED PROTEIN PRECURSOR (GRP 78) (IMMUNOGLOBULIN... |
| U | *contaminant\_GR78\_MESA* | 8 | 10 | 16.4% | 654 | 72379 | 5.2 | owl|P07823| 78 KD GLUCOSE REGULATED PROTEIN PRECURSOR (GRP 78) (IMMUNOGLOBULIN... |

| Filename XCorr DeltCN Conf% ObsM+H+ CalcM+H+ SpR ZScore Ion% # Sequence  | | | | | | | | | | | | |
| --- | --- | --- | --- | --- | --- | --- | --- | --- | --- | --- | --- | --- |
|  | Mis12XLAPLSS\_011913\_01.09352.09352.2 | 3.563 | 0.2677 | 99.8% | 1568.1322 | 1567.7386 | 1 | 7.343 | 69.2% | 1 | R.ITPSYVAFTPEGER.L | 2 |
|  | Mis12XLAPLSS\_011913\_01.08609.08609.2 | 3.8243 | 0.4568 | 100.0% | 1678.2122 | 1678.796 | 1 | 7.151 | 78.6% | 1 | K.NQLTSNPENTVFDAK.R | 2 |
|  | Mis12XLAPLSS\_011913\_01.07635.07635.2 | 3.2562 | 0.3384 | 99.9% | 1431.2922 | 1431.5449 | 1 | 6.768 | 81.8% | 1 | R.TWNDPSVQQDIK.F | 2 |
|  | Mis12XLAPLSS\_011913\_01.09107.09107.3 | 3.5294 | 0.1924 | 97.6% | 1889.6344 | 1889.121 | 24 | 4.499 | 35.9% | 1 | K.VTHAVVTVPAYFNDAQR.Q | 3 |
|  | Mis12XLAPLSS\_011913\_01.09699.09699.2 | 2.5624 | 0.2674 | 99.5% | 1218.1522 | 1218.4137 | 1 | 5.309 | 81.8% | 1 | K.DAGTIAGLNVMR.I | 2 |
|  | Mis12XLAPLSS\_011913\_01.10821.10821.2 | 4.3886 | 0.3529 | 100.0% | 1662.2522 | 1660.9078 | 1 | 8.101 | 80.0% | 3 | R.IINEPTAAAIAYGLDK.R | 222 |
|  | Mis12XLAPLSS\_011913\_01.11540.11540.2 | 3.0357 | 0.3512 | 99.8% | 1513.2522 | 1513.7516 | 1 | 6.979 | 68.2% | 1 | R.AKFEELNMDLFR.S | 2 |
|  | Mis12XLAPLSS\_011913\_01.03983.03983.2 | 2.254 | 0.1775 | 97.3% | 1074.9122 | 1075.1667 | 1 | 4.6 | 81.2% | 1 | K.ITITNDQNR.L | 2 |

Similarities:
gi|5729877|ref|NP\_006(1:7)  
gi|124256496|ref|NP\_0(1:7)  

---

|  |  |  |  |  |  |  |  |  |
| --- | --- | --- | --- | --- | --- | --- | --- | --- |
| U | *gi|18375623|ref|NP\_54* | 7 | 9 | 16.4% | 428 | 48991 | 5.7 | HLA-B associated transcript 1 [Homo sapiens] |
| U | *gi|4758112|ref|NP\_004* | 7 | 9 | 16.4% | 428 | 48991 | 5.7 | HLA-B associated transcript 1 [Homo sapiens] |

| Filename XCorr DeltCN Conf% ObsM+H+ CalcM+H+ SpR ZScore Ion% # Sequence  | | | | | | | | | | | | |
| --- | --- | --- | --- | --- | --- | --- | --- | --- | --- | --- | --- | --- |
|  | Mis12XLAPLSS\_011913\_01.11451.11451.2 | 2.3081 | 0.2471 | 98.8% | 1243.9122 | 1244.5199 | 3 | 4.199 | 61.1% | 1 | R.DFLLKPELLR.A | 2 |
|  | Mis12XLAPLSS\_011913\_01.08607.08607.2 | 2.37 | 0.3199 | 99.8% | 1036.1122 | 1036.2495 | 1 | 5.958 | 92.9% | 1 | K.MLEQLDMR.R | 2 |
|  | Mis12XLAPLSS\_011913\_01.05562.05562.2 | 2.4438 | 0.0706 | 95.4% | 1063.7922 | 1063.2015 | 61 | 4.573 | 78.6% | 1 | R.RDVQEIFR.M | 2 |
|  | Mis12XLAPLSS\_011913\_01.09815.09815.2 | 3.3253 | 0.4618 | 100.0% | 1260.3922 | 1260.5253 | 1 | 7.815 | 65.0% | 1 | R.RILVATNLFGR.G | 2 |
|  | Mis12XLAPLSS\_011913\_01.11178.11178.2 | 2.9464 | 0.465 | 100.0% | 1104.0922 | 1104.3378 | 1 | 7.086 | 72.2% | 2 | R.ILVATNLFGR.G | 2 |
|  | Mis12XLAPLSS\_011913\_01.10562.10562.3 | 3.9592 | 0.2738 | 99.7% | 2301.2644 | 2301.492 | 1 | 5.514 | 36.1% | 2 | R.VNIAFNYDMPEDSDTYLHR.V | 3 |
|  | Mis12XLAPLSS\_011913\_02.07067.07067.2 | 4.7555 | 0.5616 | 100.0% | 1480.3722 | 1480.6146 | 1 | 9.582 | 76.9% | 1 | K.GLAITFVSDENDAK.I | 2 |

---

|  |  |  |  |  |  |  |  |  |
| --- | --- | --- | --- | --- | --- | --- | --- | --- |
| U | *gi|34740329|ref|NP\_91* | 4 | 5 | 16.4% | 378 | 39595 | 9.0 | heterogeneous nuclear ribonucleoprotein A3 [Homo sapiens] |

| Filename XCorr DeltCN Conf% ObsM+H+ CalcM+H+ SpR ZScore Ion% # Sequence  | | | | | | | | | | | | |
| --- | --- | --- | --- | --- | --- | --- | --- | --- | --- | --- | --- | --- |
| \* | Mis12XLAPLSS\_011913\_01.03825.03825.2 | 3.0893 | 0.258 | 99.8% | 1381.5721 | 1381.5718 | 2 | 5.948 | 62.5% | 1 | R.EDSVKPGAHLTVK.K | 2 |
| \* | Mis12XLAPLSS\_011913\_02.06567.06567.3 | 3.8608 | 0.4009 | 100.0% | 1883.1543 | 1884.096 | 1 | 6.212 | 41.7% | 1 | K.IFVGGIKEDTEEYNLR.D | 3 |
| \* | Mis12XLAPLSS\_011913\_01.08692.08692.2 | 3.4286 | 0.4201 | 100.0% | 1582.8722 | 1583.7968 | 1 | 7.56 | 70.8% | 1 | K.YGKIETIEVMEDR.Q | 2 |
|  | Mis12XLAPLSS\_011913\_01.11265.11265.3 | 4.1657 | 0.3722 | 100.0% | 2283.2644 | 2282.5579 | 1 | 6.761 | 32.9% | 2 | R.GFAFVTFDDHDTVDKIVVQK.Y | 3 |

---

|  |  |  |  |  |  |  |  |  |
| --- | --- | --- | --- | --- | --- | --- | --- | --- |
| U | *gi|4506725|ref|NP\_000* | 4 | 5 | 16.3% | 263 | 29598 | 10.2 | ribosomal protein S4, X-linked X isoform [Homo sapiens] |

| Filename XCorr DeltCN Conf% ObsM+H+ CalcM+H+ SpR ZScore Ion% # Sequence  | | | | | | | | | | | | |
| --- | --- | --- | --- | --- | --- | --- | --- | --- | --- | --- | --- | --- |
|  | Mis12XLAPLSS\_011913\_01.04247.04247.2 | 2.2534 | 0.2942 | 99.1% | 1215.8121 | 1216.3854 | 24 | 5.928 | 60.0% | 1 | K.GIPHLVTHDAR.T | 2 |
| \* | Mis12XLAPLSS\_011913\_01.09280.09280.2 | 3.4059 | 0.3987 | 99.8% | 1446.2722 | 1446.5975 | 1 | 7.606 | 66.7% | 2 | K.VNDTIQIDLETGK.I | 2 |
| \* | Mis12XLAPLSS\_011913\_01.10414.10414.2 | 2.2544 | 0.3303 | 99.6% | 991.0722 | 991.2187 | 1 | 5.975 | 75.0% | 1 | R.LSNIFVIGK.G | 2 |
| \* | Mis12XLAPLSS\_011913\_01.08886.08886.2 | 2.1932 | 0.2149 | 97.4% | 1169.1921 | 1168.3842 | 4 | 4.891 | 66.7% | 1 | K.GNKPWISLPR.G | 2 |

---

|  |  |  |  |  |  |  |  |  |
| --- | --- | --- | --- | --- | --- | --- | --- | --- |
| U | *gi|4506693|ref|NP\_001* | 2 | 3 | 16.3% | 135 | 15550 | 9.8 | ribosomal protein S17 [Homo sapiens] |

| Filename XCorr DeltCN Conf% ObsM+H+ CalcM+H+ SpR ZScore Ion% # Sequence  | | | | | | | | | | | | |
| --- | --- | --- | --- | --- | --- | --- | --- | --- | --- | --- | --- | --- |
| \* | Mis12XLAPLSS\_011913\_01.13229.13229.2 | 3.0837 | 0.3814 | 99.8% | 2409.5923 | 2410.7908 | 4 | 6.598 | 33.3% | 1 | K.LLDFGSLSNLQVTQPTVGMNFK.T | 2 |
| \* | Mis12XLAPLSS\_011913\_01.13238.13238.3 | 4.2223 | 0.3546 | 100.0% | 2410.4644 | 2410.7908 | 1 | 6.262 | 34.5% | 2 | K.LLDFGSLSNLQVTQPTVGMNFK.T | 3 |

---

|  |  |  |  |  |  |  |  |  |
| --- | --- | --- | --- | --- | --- | --- | --- | --- |
| U | *gi|116235460|ref|NP\_6* | 7 | 9 | 16.2% | 585 | 63861 | 9.0 | YTH domain family, member 3 [Homo sapiens] |

| Filename XCorr DeltCN Conf% ObsM+H+ CalcM+H+ SpR ZScore Ion% # Sequence  | | | | | | | | | | | | |
| --- | --- | --- | --- | --- | --- | --- | --- | --- | --- | --- | --- | --- |
| \* | Mis12XLAPLSS\_011913\_01.03442.03442.2 | 2.537 | 0.1346 | 96.9% | 1197.3322 | 1197.3365 | 23 | 4.589 | 60.0% | 1 | K.VSVQNGSIHQK.D | 2 |
| \* | Mis12XLAPLSS\_011913\_01.07710.07710.2 | 3.0867 | 0.2022 | 99.5% | 1597.8322 | 1595.7062 | 1 | 4.541 | 60.0% | 1 | R.AITDGQAGFGNDTLSK.V | 2 |
| \* | Mis12XLAPLSS\_011913\_01.11214.11214.2 | 3.8672 | 0.5011 | 100.0% | 1518.2922 | 1517.7809 | 1 | 8.162 | 64.3% | 1 | K.VPGISSIEQGMTGLK.I | 2 |
| \* | Mis12XLAPLSS\_011913\_01.06971.06971.2 | 2.9283 | 0.4388 | 99.9% | 1047.2122 | 1046.209 | 1 | 7.838 | 85.0% | 3 | K.IGGDLTAAVTK.T | 2 |
| \* | Mis12XLAPLSS\_011913\_01.08370.08370.2 | 3.4126 | 0.4079 | 99.9% | 1460.3322 | 1460.7166 | 1 | 6.944 | 60.0% | 1 | K.GNVGIGGSAVPPPPIK.H | 2 |
| \* | Mis12XLAPLSS\_011913\_01.07149.07149.3 | 3.4032 | 0.3893 | 100.0% | 1815.7444 | 1816.0416 | 1 | 6.105 | 38.3% | 1 | K.HNMNIGTWDEKGSVVK.A | 3 |
| \* | Mis12XLAPLSS\_011913\_01.03252.03252.3 | 3.0861 | 0.1823 | 97.5% | 1334.6044 | 1334.4497 | 13 | 6.041 | 44.4% | 1 | K.RQEEEEAMRR.E | 3 |

---

|  |  |  |  |  |  |  |  |  |
| --- | --- | --- | --- | --- | --- | --- | --- | --- |
| U | *gi|7657326|ref|NP\_055* | 2 | 3 | 16.2% | 185 | 19905 | 4.2 | male-enhanced antigen [Homo sapiens] |

| Filename XCorr DeltCN Conf% ObsM+H+ CalcM+H+ SpR ZScore Ion% # Sequence  | | | | | | | | | | | | |
| --- | --- | --- | --- | --- | --- | --- | --- | --- | --- | --- | --- | --- |
| \* | Mis12XLAPLSS\_011913\_01.11727.11727.2 | 2.934 | 0.1268 | 97.6% | 1681.0721 | 1681.9934 | 1 | 4.427 | 53.1% | 2 | R.TMAGVSLPAPGVPAWAR.E | 2 |
| \* | Mis12XLAPLSS\_011913\_01.09404.09404.2 | 3.9137 | 0.3657 | 100.0% | 1547.1122 | 1547.6616 | 2 | 5.982 | 66.7% | 1 | R.EISDAQWEDVVQK.A | 2 |

---

|  |  |  |  |  |  |  |  |  |
| --- | --- | --- | --- | --- | --- | --- | --- | --- |
| U | *gi|4506625|ref|NP\_000* | 2 | 2 | 16.2% | 148 | 16561 | 11.0 | ribosomal protein L27a [Homo sapiens] |

| Filename XCorr DeltCN Conf% ObsM+H+ CalcM+H+ SpR ZScore Ion% # Sequence  | | | | | | | | | | | | |
| --- | --- | --- | --- | --- | --- | --- | --- | --- | --- | --- | --- | --- |
| \* | Mis12XLAPLSS\_011913\_01.08384.08384.3 | 2.9041 | 0.2879 | 99.8% | 1586.8143 | 1586.7899 | 32 | 5.102 | 35.4% | 1 | R.INFDKYHPGYFGK.V | 3 |
| \* | Mis12XLAPLSS\_011913\_01.09141.09141.2 | 2.1207 | 0.2226 | 96.0% | 1112.1721 | 1112.3146 | 4 | 5.554 | 70.0% | 1 | K.TGAAPIIDVVR.S | 2 |

---

|  |  |  |  |  |  |  |  |  |
| --- | --- | --- | --- | --- | --- | --- | --- | --- |
| U | *gi|4502891|ref|NP\_001* | 2 | 2 | 16.0% | 237 | 26215 | 4.1 | chloride channel, nucleotide-sensitive, 1A [Homo sapiens] |

| Filename XCorr DeltCN Conf% ObsM+H+ CalcM+H+ SpR ZScore Ion% # Sequence  | | | | | | | | | | | | |
| --- | --- | --- | --- | --- | --- | --- | --- | --- | --- | --- | --- | --- |
| \* | Mis12XLAPLSS\_011913\_02.06615.06615.2 | 2.7991 | 0.4201 | 99.8% | 1338.1322 | 1338.5034 | 1 | 6.951 | 58.3% | 1 | K.GLGTGTLYIAESR.L | 2 |
| \* | Mis12XLAPLSS\_011913\_02.10421.10421.3 | 3.277 | 0.3252 | 99.8% | 2720.5144 | 2721.085 | 261 | 5.075 | 20.8% | 1 | R.LSWLDGSGLGFSLEYPTISLHALSR.D | 3 |

---

|  |  |  |  |  |  |  |  |  |
| --- | --- | --- | --- | --- | --- | --- | --- | --- |
| U | *gi|17105394|ref|NP\_00* | 2 | 3 | 16.0% | 156 | 17695 | 10.4 | ribosomal protein L23a [Homo sapiens] |

| Filename XCorr DeltCN Conf% ObsM+H+ CalcM+H+ SpR ZScore Ion% # Sequence  | | | | | | | | | | | | |
| --- | --- | --- | --- | --- | --- | --- | --- | --- | --- | --- | --- | --- |
|  | Mis12XLAPLSS\_011913\_01.03756.03756.2 | 2.5716 | 0.2165 | 98.8% | 1369.9321 | 1370.5919 | 9 | 5.203 | 59.1% | 1 | K.VNTLIRPDGEKK.A | 2 |
| \* | Mis12XLAPLSS\_011913\_01.08973.08973.2 | 3.224 | 0.5273 | 100.0% | 1405.0122 | 1405.5474 | 13 | 8.815 | 58.3% | 2 | R.LAPDYDALDVANK.I | 2 |

---

|  |  |  |  |  |  |  |  |  |
| --- | --- | --- | --- | --- | --- | --- | --- | --- |
| U | *gi|4506707|ref|NP\_001* | 3 | 4 | 16.0% | 125 | 13742 | 10.1 | ribosomal protein S25 [Homo sapiens] |

| Filename XCorr DeltCN Conf% ObsM+H+ CalcM+H+ SpR ZScore Ion% # Sequence  | | | | | | | | | | | | |
| --- | --- | --- | --- | --- | --- | --- | --- | --- | --- | --- | --- | --- |
| \* | Mis12XLAPLSS\_011913\_01.09923.09923.2 | 3.2167 | 0.3785 | 99.9% | 1318.4722 | 1319.5437 | 1 | 5.755 | 80.0% | 1 | R.DKLNNLVLFDK.A | 2 |
| \* | Mis12XLAPLSS\_011913\_01.10326.10326.2 | 2.8781 | 0.2964 | 99.8% | 1077.2722 | 1076.281 | 26 | 5.418 | 68.8% | 1 | K.LNNLVLFDK.A | 2 |
| \* | Mis12XLAPLSS\_011913\_01.09633.09633.2 | 2.4 | 0.2574 | 99.5% | 973.0722 | 973.1576 | 24 | 5.47 | 68.8% | 2 | R.AALQELLSK.G | 2 |

---

|  |  |  |  |  |  |  |  |  |
| --- | --- | --- | --- | --- | --- | --- | --- | --- |
| U | *gi|219555707|ref|NP\_0* | 2 | 5 | 15.8% | 184 | 20170 | 7.0 | eukaryotic translation initiation factor 5A isoform A [Homo sapiens] |
| U | *gi|4503545|ref|NP\_001* | 2 | 5 | 18.8% | 154 | 16832 | 5.2 | eukaryotic translation initiation factor 5A isoform B [Homo sapiens] |
| U | *gi|219555712|ref|NP\_0* | 2 | 5 | 18.8% | 154 | 16832 | 5.2 | eukaryotic translation initiation factor 5A isoform B [Homo sapiens] |
| U | *gi|219555710|ref|NP\_0* | 2 | 5 | 18.8% | 154 | 16832 | 5.2 | eukaryotic translation initiation factor 5A isoform B [Homo sapiens] |

| Filename XCorr DeltCN Conf% ObsM+H+ CalcM+H+ SpR ZScore Ion% # Sequence  | | | | | | | | | | | | |
| --- | --- | --- | --- | --- | --- | --- | --- | --- | --- | --- | --- | --- |
|  | Mis12XLAPLSS\_011913\_01.11493.11493.2 | 3.2236 | 0.5163 | 100.0% | 1298.7522 | 1299.5559 | 1 | 8.91 | 77.3% | 4 | K.VHLVGIDIFTGK.K | 2 |
|  | Mis12XLAPLSS\_011913\_01.08943.08943.3 | 3.5664 | 0.3151 | 99.7% | 1970.2444 | 1970.187 | 1 | 5.521 | 40.6% | 1 | R.EDLRLPEGDLGKEIEQK.Y | 3 |

---

|  |  |  |  |  |  |  |  |  |
| --- | --- | --- | --- | --- | --- | --- | --- | --- |
| U | *gi|169212778|ref|XP\_0* | 4 | 6 | 15.0% | 266 | 30042 | 10.6 | PREDICTED: similar to ribosomal protein L7a [Homo sapiens] |
| U | *gi|4506661|ref|NP\_000* | 4 | 6 | 15.0% | 266 | 29996 | 10.6 | ribosomal protein L7a [Homo sapiens] |
| U | *gi|169213130|ref|XP\_0* | 4 | 6 | 15.0% | 266 | 30042 | 10.6 | PREDICTED: similar to ribosomal protein L7a [Homo sapiens] |
| U | *gi|169212940|ref|XP\_0* | 4 | 6 | 15.0% | 266 | 30028 | 10.6 | PREDICTED: similar to ribosomal protein L7a [Homo sapiens] |

| Filename XCorr DeltCN Conf% ObsM+H+ CalcM+H+ SpR ZScore Ion% # Sequence  | | | | | | | | | | | | |
| --- | --- | --- | --- | --- | --- | --- | --- | --- | --- | --- | --- | --- |
|  | Mis12XLAPLSS\_011913\_01.08399.08399.2 | 3.2731 | 0.4447 | 100.0% | 1217.0521 | 1217.3672 | 3 | 6.705 | 70.0% | 3 | K.NFGIGQDIQPK.R | 2 |
|  | Mis12XLAPLSS\_011913\_01.11986.11986.2 | 3.5058 | 0.4667 | 100.0% | 1811.6322 | 1812.1222 | 1 | 7.475 | 70.0% | 1 | R.LKVPPAINQFTQALDR.Q | 2 |
|  | Mis12XLAPLSS\_011913\_01.11957.11957.3 | 4.291 | 0.3668 | 100.0% | 1811.9343 | 1812.1222 | 1 | 6.649 | 48.3% | 1 | R.LKVPPAINQFTQALDR.Q | 3 |
|  | Mis12XLAPLSS\_011913\_02.06201.06201.2 | 3.1385 | 0.154 | 99.4% | 1346.1122 | 1346.5236 | 144 | 5.413 | 45.8% | 1 | R.AGVNTVTTLVENK.K | 2 |

---

|  |  |  |  |  |  |  |  |  |
| --- | --- | --- | --- | --- | --- | --- | --- | --- |
| U | *gi|55770864|ref|NP\_00* | 2 | 4 | 14.8% | 257 | 26888 | 11.2 | THO complex 4 [Homo sapiens] |

| Filename XCorr DeltCN Conf% ObsM+H+ CalcM+H+ SpR ZScore Ion% # Sequence  | | | | | | | | | | | | |
| --- | --- | --- | --- | --- | --- | --- | --- | --- | --- | --- | --- | --- |
| \* | Mis12XLAPLSS\_011913\_01.15584.15584.3 | 3.5206 | 0.3366 | 99.7% | 2970.0544 | 2971.377 | 4 | 5.09 | 23.1% | 1 | K.LLVSNLDFGVSDADIQELFAEFGTLKK.A | 3 |
| \* | Mis12XLAPLSS\_011913\_01.06189.06189.2 | 3.0467 | 0.3298 | 99.8% | 1231.2922 | 1232.3384 | 1 | 7.289 | 75.0% | 3 | R.SLGTADVHFER.K | 2 |

---

|  |  |  |  |  |  |  |  |  |
| --- | --- | --- | --- | --- | --- | --- | --- | --- |
| U | *gi|14110414|ref|NP\_00* | 4 | 5 | 14.7% | 306 | 32835 | 8.2 | heterogeneous nuclear ribonucleoprotein D isoform c [Homo sapiens] |
| U | *gi|14110420|ref|NP\_11* | 4 | 5 | 12.7% | 355 | 38434 | 7.8 | heterogeneous nuclear ribonucleoprotein D isoform a [Homo sapiens] |

| Filename XCorr DeltCN Conf% ObsM+H+ CalcM+H+ SpR ZScore Ion% # Sequence  | | | | | | | | | | | | |
| --- | --- | --- | --- | --- | --- | --- | --- | --- | --- | --- | --- | --- |
|  | Mis12XLAPLSS\_011913\_01.03281.03281.3 | 2.9208 | 0.2734 | 98.1% | 2053.7344 | 2052.9768 | 14 | 4.545 | 33.8% | 1 | K.IDASKNEEDEGHSNSS\*PR.H | 3 |
|  | Mis12XLAPLSS\_011913\_01.12057.12057.2 | 1.9587 | 0.2687 | 95.3% | 1356.5922 | 1356.5793 | 4 | 5.03 | 54.5% | 1 | K.MFIGGLSWDTTK.K | 2 |
|  | Mis12XLAPLSS\_011913\_01.10534.10534.2 | 2.6652 | 0.3056 | 99.9% | 1484.1522 | 1484.7534 | 9 | 4.82 | 50.0% | 1 | K.MFIGGLSWDTTKK.D | 2 |
|  | Mis12XLAPLSS\_011913\_01.09018.09018.2 | 3.7899 | 0.3606 | 99.8% | 1490.3121 | 1489.6653 | 2 | 5.753 | 69.2% | 2 | K.IFVGGLSPDTPEEK.I | 2 |

---

|  |  |  |  |  |  |  |  |  |
| --- | --- | --- | --- | --- | --- | --- | --- | --- |
| U | *gi|23397427|ref|NP\_00* | 6 | 11 | 14.4% | 623 | 69633 | 8.6 | synaptotagmin binding, cytoplasmic RNA interacting protein [Homo sapiens] |

| Filename XCorr DeltCN Conf% ObsM+H+ CalcM+H+ SpR ZScore Ion% # Sequence  | | | | | | | | | | | | |
| --- | --- | --- | --- | --- | --- | --- | --- | --- | --- | --- | --- | --- |
| \* | Mis12XLAPLSS\_011913\_02.07899.07899.3 | 5.387 | 0.5522 | 100.0% | 2442.8342 | 2443.716 | 1 | 8.772 | 40.5% | 2 | K.VAEKLDEIYVAGLVAHSDLDER.A | 3 |
| \* | Mis12XLAPLSS\_011913\_01.13353.13353.2 | 3.4514 | 0.4229 | 99.8% | 2335.3323 | 2335.6624 | 2 | 7.593 | 37.5% | 1 | R.AIEALKEFNEDGALAVLQQFK.D | 2 |
|  | Mis12XLAPLSS\_011913\_01.07436.07436.2 | 2.9127 | 0.345 | 99.8% | 1312.1122 | 1312.4221 | 1 | 7.154 | 72.7% | 5 | R.TGYTLDVTTGQR.K | 22 |
| \* | Mis12XLAPLSS\_011913\_01.11271.11271.2 | 2.5018 | 0.3704 | 99.8% | 1261.2122 | 1261.5403 | 1 | 6.587 | 75.0% | 1 | R.LMMDPLTGLNR.G | 2 |
| \* | Mis12XLAPLSS\_011913\_01.08470.08470.2 | 2.6583 | 0.0983 | 96.4% | 1353.3522 | 1352.5272 | 26 | 4.066 | 60.0% | 1 | K.TKEQILEEFSK.V | 2 |
| \* | Mis12XLAPLSS\_011913\_01.12053.12053.2 | 2.8085 | 0.2492 | 99.5% | 1475.3922 | 1474.6512 | 1 | 5.764 | 66.7% | 1 | R.NLANTVTEEILEK.A | 2 |

Similarities:
gi|156151392|ref|NP\_0(1:5)  

---

|  |  |  |  |  |  |  |  |  |
| --- | --- | --- | --- | --- | --- | --- | --- | --- |
| U | *gi|124256496|ref|NP\_0* | 8 | 14 | 14.2% | 641 | 70375 | 6.0 | heat shock 70kDa protein 1-like [Homo sapiens] |

| Filename XCorr DeltCN Conf% ObsM+H+ CalcM+H+ SpR ZScore Ion% # Sequence  | | | | | | | | | | | | |
| --- | --- | --- | --- | --- | --- | --- | --- | --- | --- | --- | --- | --- |
|  | Mis12XLAPLSS\_011913\_02.05422.05422.2 | 3.1076 | 0.4678 | 100.0% | 1488.2722 | 1488.5939 | 1 | 8.504 | 70.8% | 3 | R.TTPSYVAFTDTER.L | 2222 |
|  | Mis12XLAPLSS\_011913\_01.12483.12483.2 | 3.292 | 0.3526 | 99.8% | 1616.3322 | 1615.8817 | 1 | 7.069 | 65.4% | 1 | K.AFYPEEISSMVLTK.L | 22 |
|  | Mis12XLAPLSS\_011913\_02.07888.07888.2 | 3.0144 | 0.3046 | 99.8% | 1198.3522 | 1198.408 | 1 | 6.584 | 72.7% | 3 | K.DAGVIAGLNVLR.I | 22 |
|  | Mis12XLAPLSS\_011913\_01.10821.10821.2 | 4.3886 | 0.3529 | 100.0% | 1662.2522 | 1660.9078 | 1 | 8.101 | 80.0% | 3 | R.IINEPTAAAIAYGLDK.G | 222 |
|  | Mis12XLAPLSS\_011913\_01.04986.04986.2 | 3.8448 | 0.4962 | 100.0% | 1676.1522 | 1676.6964 | 1 | 8.128 | 63.3% | 1 | K.ATAGDTHLGGEDFDNR.L | 222 |
|  | Mis12XLAPLSS\_011913\_01.04985.04985.3 | 2.7193 | 0.3103 | 99.4% | 1679.1543 | 1676.6964 | 23 | 5.282 | 36.7% | 1 | K.ATAGDTHLGGEDFDNR.L | 333 |
|  | Mis12XLAPLSS\_011913\_01.03447.03447.2 | 2.4481 | 0.4255 | 99.8% | 1017.4922 | 1018.1582 | 6 | 6.628 | 62.5% | 1 | K.ITITNDKGR.L | 2222 |
|  | Mis12XLAPLSS\_011913\_01.09957.09957.2 | 3.6473 | 0.4529 | 100.0% | 1289.1522 | 1288.4608 | 1 | 7.703 | 85.0% | 1 | K.NALESYAFNMK.S | 22 |

Similarities:
gi|5729877|ref|NP\_006(3:5)  
gi|167466173|ref|NP\_0(7:1)  
contaminant\_GR78\_HUMA(1:7)  
gi|34419635|ref|NP\_00(4:4)  

---

|  |  |  |  |  |  |  |  |  |
| --- | --- | --- | --- | --- | --- | --- | --- | --- |
| U | *contaminant\_INT-STD1* | 9 | 17 | 14.2% | 607 | 69271 | 6.1 | BSA |

| Filename XCorr DeltCN Conf% ObsM+H+ CalcM+H+ SpR ZScore Ion% # Sequence  | | | | | | | | | | | | |
| --- | --- | --- | --- | --- | --- | --- | --- | --- | --- | --- | --- | --- |
| \* | Mis12XLAPLSS\_011913\_01.09747.09747.2 | 3.1428 | 0.2724 | 99.8% | 1163.9521 | 1164.344 | 3 | 6.415 | 77.8% | 1 | K.LVNELTEFAK.T | 2 |
| \* | Mis12XLAPLSS\_011913\_01.13547.13547.2 | 3.8289 | 0.4212 | 100.0% | 1569.3722 | 1568.7258 | 1 | 7.161 | 62.5% | 2 | K.DAFLGSFLYEYSR.R | 2 |
| \* | Mis12XLAPLSS\_011913\_01.08584.08584.3 | 4.1683 | 0.3335 | 100.0% | 1440.5343 | 1440.6884 | 1 | 6.492 | 61.4% | 2 | R.RHPEYAVSVLLR.L | 3 |
| \* | Mis12XLAPLSS\_011913\_01.08646.08646.2 | 2.6732 | 0.262 | 99.5% | 1441.7722 | 1440.6884 | 9 | 4.855 | 54.5% | 1 | R.RHPEYAVSVLLR.L | 2 |
| \* | Mis12XLAPLSS\_011913\_01.07575.07575.2 | 3.1605 | 0.3587 | 99.9% | 1305.9922 | 1306.5046 | 1 | 7.257 | 70.0% | 2 | K.HLVDEPQNLIK.Q | 2 |
| \* | Mis12XLAPLSS\_011913\_01.11319.11319.2 | 4.0415 | 0.425 | 100.0% | 1480.1522 | 1480.7068 | 1 | 8.504 | 70.8% | 5 | K.LGEYGFQNALIVR.Y | 2 |
|  | Mis12XLAPLSS\_011913\_01.08336.08336.2 | 3.8751 | 0.4755 | 100.0% | 1640.4922 | 1640.9205 | 1 | 9.43 | 57.1% | 2 | R.KVPQVSTPTLVEVSR.S | 2 |
|  | Mis12XLAPLSS\_011913\_01.08349.08349.3 | 3.2577 | 0.3199 | 99.7% | 1641.0543 | 1640.9205 | 52 | 5.995 | 35.7% | 1 | R.KVPQVSTPTLVEVSR.S | 3 |
| \* | Mis12XLAPLSS\_011913\_01.12857.12857.2 | 3.7241 | 0.447 | 100.0% | 1401.0721 | 1400.6324 | 1 | 8.14 | 72.7% | 1 | K.TVMENFVAFVDK.C | 2 |

---

|  |  |  |  |  |  |  |  |  |
| --- | --- | --- | --- | --- | --- | --- | --- | --- |
| U | *gi|14602427|ref|NP\_12* | 3 | 5 | 14.1% | 277 | 31293 | 5.2 | ZW10 interactor isoform a [Homo sapiens] |
| U | *gi|14602429|ref|NP\_00* | 3 | 5 | 14.1% | 277 | 31293 | 5.2 | ZW10 interactor isoform a [Homo sapiens] |

| Filename XCorr DeltCN Conf% ObsM+H+ CalcM+H+ SpR ZScore Ion% # Sequence  | | | | | | | | | | | | |
| --- | --- | --- | --- | --- | --- | --- | --- | --- | --- | --- | --- | --- |
|  | Mis12XLAPLSS\_011913\_01.06233.06233.2 | 4.0658 | 0.326 | 100.0% | 1500.0922 | 1500.6542 | 3 | 5.963 | 68.2% | 2 | R.AVQNQWQLQQEK.H | 2 |
|  | Mis12XLAPLSS\_011913\_02.05012.05012.3 | 4.0061 | 0.3471 | 100.0% | 1489.8243 | 1489.6743 | 1 | 7.919 | 45.8% | 2 | K.HLQHLAEVSAEVR.E | 3 |
|  | Mis12XLAPLSS\_011913\_01.15216.15216.2 | 3.4436 | 0.3836 | 99.9% | 1718.2922 | 1717.0178 | 1 | 6.349 | 57.7% | 1 | R.YQTFLQLLYTLQGK.L | 2 |

---

|  |  |  |  |  |  |  |  |  |
| --- | --- | --- | --- | --- | --- | --- | --- | --- |
| U | *gi|14141193|ref|NP\_00* | 3 | 4 | 13.9% | 194 | 22591 | 10.7 | ribosomal protein S9 [Homo sapiens] |

| Filename XCorr DeltCN Conf% ObsM+H+ CalcM+H+ SpR ZScore Ion% # Sequence  | | | | | | | | | | | | |
| --- | --- | --- | --- | --- | --- | --- | --- | --- | --- | --- | --- | --- |
| \* | Mis12XLAPLSS\_011913\_01.08236.08236.2 | 2.2616 | 0.1617 | 97.4% | 921.0722 | 921.0843 | 24 | 4.674 | 85.7% | 1 | K.LIGEYGLR.N | 2 |
| \* | Mis12XLAPLSS\_011913\_01.10061.10061.2 | 2.8611 | 0.1917 | 99.4% | 1400.4922 | 1400.7074 | 12 | 5.335 | 54.5% | 1 | R.KQVVNIPSFIVR.L | 2 |
| \* | Mis12XLAPLSS\_011913\_01.07820.07820.2 | 2.2821 | 0.189 | 98.8% | 887.71216 | 888.01404 | 1 | 4.969 | 91.7% | 2 | K.HIDFSLR.S | 2 |

---

|  |  |  |  |  |  |  |  |  |
| --- | --- | --- | --- | --- | --- | --- | --- | --- |
| U | *gi|205277463|ref|NP\_0* | 5 | 5 | 13.8% | 623 | 67878 | 7.7 | transketolase isoform 1 [Homo sapiens] |
| U | *gi|4507521|ref|NP\_001* | 5 | 5 | 13.8% | 623 | 67878 | 7.7 | transketolase isoform 1 [Homo sapiens] |
| U | *gi|205277465|ref|NP\_0* | 5 | 5 | 15.9% | 540 | 58982 | 7.7 | transketolase isoform 2 [Homo sapiens] |

| Filename XCorr DeltCN Conf% ObsM+H+ CalcM+H+ SpR ZScore Ion% # Sequence  | | | | | | | | | | | | |
| --- | --- | --- | --- | --- | --- | --- | --- | --- | --- | --- | --- | --- |
|  | Mis12XLAPLSS\_011913\_01.03680.03680.2 | 2.7619 | 0.1605 | 99.2% | 1130.1122 | 1130.2896 | 18 | 5.423 | 66.7% | 1 | K.LQALKDTANR.L | 2 |
|  | Mis12XLAPLSS\_011913\_01.15748.15748.2 | 3.4087 | 0.4851 | 100.0% | 2022.7722 | 2024.2965 | 1 | 8.863 | 53.1% | 1 | K.NMAEQIIQEIYSQIQSK.K | 2 |
|  | Mis12XLAPLSS\_011913\_01.10066.10066.2 | 3.8196 | 0.4244 | 100.0% | 2020.3522 | 2021.278 | 1 | 8.027 | 47.2% | 1 | K.ILATPPQEDAPSVDIANIR.M | 2 |
|  | Mis12XLAPLSS\_011913\_01.09120.09120.2 | 3.15 | 0.4246 | 99.9% | 1885.1522 | 1886.0656 | 1 | 7.759 | 44.1% | 1 | R.SVPTSTVFYPSDGVATEK.A | 2 |
|  | Mis12XLAPLSS\_011913\_02.06021.06021.3 | 4.8955 | 0.3807 | 100.0% | 2508.6843 | 2509.6946 | 1 | 5.98 | 41.7% | 1 | R.TSRPENAIIYNNNEDFQVGQAK.V | 3 |

---

|  |  |  |  |  |  |  |  |  |
| --- | --- | --- | --- | --- | --- | --- | --- | --- |
| U | *gi|32129199|ref|NP\_14* | 2 | 2 | 13.8% | 210 | 23671 | 6.4 | cytokine induced protein 29 kDa [Homo sapiens] |

| Filename XCorr DeltCN Conf% ObsM+H+ CalcM+H+ SpR ZScore Ion% # Sequence  | | | | | | | | | | | | |
| --- | --- | --- | --- | --- | --- | --- | --- | --- | --- | --- | --- | --- |
| \* | Mis12XLAPLSS\_011913\_01.09321.09321.2 | 3.0105 | 0.309 | 99.8% | 1079.9321 | 1080.229 | 1 | 7.664 | 88.9% | 1 | R.FGLNVSSISR.K | 2 |
| \* | Mis12XLAPLSS\_011913\_02.05606.05606.2 | 4.8406 | 0.602 | 100.0% | 1872.1721 | 1872.9806 | 1 | 9.89 | 61.1% | 1 | R.FGIVTSSAGTGTTEDTEAK.K | 2 |

---

|  |  |  |  |  |  |  |  |  |
| --- | --- | --- | --- | --- | --- | --- | --- | --- |
| U | *gi|4503481|ref|NP\_001* | 5 | 9 | 13.7% | 437 | 50119 | 6.7 | eukaryotic translation elongation factor 1 gamma [Homo sapiens] |

| Filename XCorr DeltCN Conf% ObsM+H+ CalcM+H+ SpR ZScore Ion% # Sequence  | | | | | | | | | | | | |
| --- | --- | --- | --- | --- | --- | --- | --- | --- | --- | --- | --- | --- |
| \* | Mis12XLAPLSS\_011913\_01.07694.07694.2 | 4.1138 | 0.432 | 100.0% | 1348.0922 | 1348.5448 | 2 | 8.427 | 70.8% | 4 | K.ALIAAQYSGAQVR.V | 2 |
| \* | Mis12XLAPLSS\_011913\_01.04955.04955.2 | 2.2898 | 0.3136 | 99.5% | 1124.0122 | 1124.328 | 44 | 4.996 | 61.1% | 1 | K.AKDPFAHLPK.S | 2 |
| \* | Mis12XLAPLSS\_011913\_01.09422.09422.2 | 2.95 | 0.36 | 99.9% | 1242.0922 | 1242.4172 | 1 | 6.618 | 77.8% | 2 | K.STFVLDEFKR.K | 2 |
| \* | Mis12XLAPLSS\_011913\_01.07100.07100.2 | 2.6277 | 0.146 | 97.1% | 1446.3722 | 1445.5693 | 44 | 4.03 | 58.3% | 1 | K.LDPGSEETQTLVR.E | 2 |
| \* | Mis12XLAPLSS\_011913\_01.11452.11452.2 | 3.4512 | 0.3457 | 99.9% | 1686.4521 | 1685.8357 | 1 | 6.242 | 61.5% | 1 | R.EYFSWEGAFQHVGK.A | 2 |

---

|  |  |  |  |  |  |  |  |  |
| --- | --- | --- | --- | --- | --- | --- | --- | --- |
| U | *gi|4506439|ref|NP\_002* | 2 | 3 | 13.6% | 425 | 47820 | 5.0 | retinoblastoma binding protein 7 [Homo sapiens] |

| Filename XCorr DeltCN Conf% ObsM+H+ CalcM+H+ SpR ZScore Ion% # Sequence  | | | | | | | | | | | | |
| --- | --- | --- | --- | --- | --- | --- | --- | --- | --- | --- | --- | --- |
| \* | Mis12XLAPLSS\_011913\_01.14342.14342.3 | 3.8771 | 0.364 | 100.0% | 3380.5144 | 3380.741 | 1 | 6.0 | 22.5% | 2 | K.AIFTGHSAVVEDVAWHLLHESLFGSVADDQK.L | 3 |
| \* | Mis12XLAPLSS\_011913\_01.09849.09849.3 | 4.0258 | 0.2848 | 99.7% | 2848.3743 | 2849.0398 | 1 | 4.404 | 32.7% | 1 | K.IGEEQSAEDAEDGPPELLFIHGGHTAK.I | 3 |

---

|  |  |  |  |  |  |  |  |  |
| --- | --- | --- | --- | --- | --- | --- | --- | --- |
| U | *gi|14141157|ref|NP\_03* | 3 | 3 | 13.6% | 346 | 36926 | 6.9 | heterogeneous nuclear ribonucleoprotein H3 isoform a [Homo sapiens] |
| U | *gi|14141159|ref|NP\_06* | 3 | 3 | 14.2% | 331 | 35239 | 6.9 | heterogeneous nuclear ribonucleoprotein H3 isoform b [Homo sapiens] |

| Filename XCorr DeltCN Conf% ObsM+H+ CalcM+H+ SpR ZScore Ion% # Sequence  | | | | | | | | | | | | |
| --- | --- | --- | --- | --- | --- | --- | --- | --- | --- | --- | --- | --- |
|  | Mis12XLAPLSS\_011913\_02.06524.06524.2 | 2.4322 | 0.3752 | 99.8% | 1271.0322 | 1272.4001 | 17 | 6.466 | 54.5% | 1 | R.STGEAFVQFASK.E | 2 |
|  | Mis12XLAPLSS\_011913\_02.06874.06874.3 | 3.2617 | 0.3155 | 99.8% | 2178.9543 | 2179.363 | 1 | 5.28 | 35.0% | 1 | R.ATGEADVEFVTHEDAVAAMSK.D | 3 |
|  | Mis12XLAPLSS\_011913\_02.03952.03952.2 | 2.4748 | 0.3194 | 99.5% | 1413.7922 | 1413.4613 | 1 | 6.062 | 53.8% | 1 | R.DGMDNQGGYGSVGR.M | 2 |

---

|  |  |  |  |  |  |  |  |  |
| --- | --- | --- | --- | --- | --- | --- | --- | --- |
| U | *gi|4826998|ref|NP\_005* | 6 | 8 | 13.4% | 707 | 76150 | 9.4 | splicing factor proline/glutamine rich (polypyrimidine tract binding protein associated) [Homo sapiens] |

| Filename XCorr DeltCN Conf% ObsM+H+ CalcM+H+ SpR ZScore Ion% # Sequence  | | | | | | | | | | | | |
| --- | --- | --- | --- | --- | --- | --- | --- | --- | --- | --- | --- | --- |
| \* | Mis12XLAPLSS\_011913\_01.08532.08532.2 | 2.5915 | 0.2594 | 99.5% | 1253.8722 | 1253.3971 | 8 | 6.22 | 55.0% | 2 | K.YGEPGEVFINK.G | 2 |
| \* | Mis12XLAPLSS\_011913\_01.14859.14859.2 | 3.9877 | 0.5789 | 100.0% | 2639.6921 | 2640.9092 | 1 | 10.663 | 45.5% | 1 | R.NLSPYVSNELLEEAFSQFGPIER.A | 2 |
| \* | Mis12XLAPLSS\_011913\_02.05470.05470.3 | 3.5281 | 0.2377 | 99.8% | 1762.4043 | 1763.8632 | 3 | 4.901 | 38.5% | 1 | R.FAQHGTFEYEYSQR.W | 3 |
| \* | Mis12XLAPLSS\_011913\_01.09382.09382.3 | 3.9058 | 0.3444 | 100.0% | 2429.7244 | 2429.6233 | 6 | 6.086 | 31.6% | 1 | K.DKLESEMEDAYHEHQANLLR.Q | 3 |
| \* | Mis12XLAPLSS\_011913\_01.03609.03609.3 | 3.9201 | 0.3239 | 100.0% | 1573.8243 | 1573.7821 | 3 | 6.455 | 45.5% | 1 | R.RMEELHNQEMQK.R | 3 |
| \* | Mis12XLAPLSS\_011913\_01.04464.04464.2 | 3.702 | 0.4844 | 100.0% | 1342.1522 | 1342.4569 | 1 | 8.184 | 75.0% | 2 | R.FGQGGAGPVGGQGPR.G | 2 |

---

|  |  |  |  |  |  |  |  |  |
| --- | --- | --- | --- | --- | --- | --- | --- | --- |
| U | *gi|4757880|ref|NP\_004* | 3 | 4 | 13.4% | 328 | 37155 | 6.8 | budding uninhibited by benzimidazoles 3 isoform a [Homo sapiens] |
| U | *gi|56550081|ref|NP\_00* | 3 | 4 | 13.5% | 326 | 36955 | 6.8 | budding uninhibited by benzimidazoles 3 isoform b [Homo sapiens] |

| Filename XCorr DeltCN Conf% ObsM+H+ CalcM+H+ SpR ZScore Ion% # Sequence  | | | | | | | | | | | | |
| --- | --- | --- | --- | --- | --- | --- | --- | --- | --- | --- | --- | --- |
|  | Mis12XLAPLSS\_011913\_01.07107.07107.2 | 2.376 | 0.1483 | 96.4% | 1166.2122 | 1166.3373 | 22 | 4.762 | 61.1% | 1 | R.LYDVPANSMR.L | 2 |
|  | Mis12XLAPLSS\_011913\_01.07420.07420.3 | 4.1374 | 0.3895 | 100.0% | 2276.9343 | 2277.4736 | 1 | 6.354 | 42.1% | 2 | K.MHDLNTDQENLVGTHDAPIR.C | 3 |
|  | Mis12XLAPLSS\_011913\_01.08782.08782.2 | 2.6331 | 0.1406 | 96.2% | 1573.9722 | 1574.771 | 92 | 4.736 | 46.2% | 1 | R.VAVEYLDPSPEVQK.K | 2 |

---

|  |  |  |  |  |  |  |  |  |
| --- | --- | --- | --- | --- | --- | --- | --- | --- |
| U | *gi|4506609|ref|NP\_000* | 2 | 2 | 13.3% | 196 | 23466 | 11.5 | ribosomal protein L19 [Homo sapiens] |

| Filename XCorr DeltCN Conf% ObsM+H+ CalcM+H+ SpR ZScore Ion% # Sequence  | | | | | | | | | | | | |
| --- | --- | --- | --- | --- | --- | --- | --- | --- | --- | --- | --- | --- |
| \* | Mis12XLAPLSS\_011913\_01.09514.09514.2 | 4.8347 | 0.5888 | 100.0% | 1943.3722 | 1944.0679 | 1 | 10.141 | 68.8% | 1 | K.VWLDPNETNEIANANSR.Q | 2 |
| \* | Mis12XLAPLSS\_011913\_01.04196.04196.2 | 2.7672 | 0.1748 | 99.5% | 987.1922 | 987.10077 | 1 | 4.473 | 81.2% | 1 | K.LLADQAEAR.R | 2 |

---

|  |  |  |  |  |  |  |  |  |
| --- | --- | --- | --- | --- | --- | --- | --- | --- |
| U | *gi|4503519|ref|NP\_003* | 3 | 3 | 13.2% | 357 | 37564 | 5.4 | eukaryotic translation initiation factor 3, subunit 5 epsilon, 47kDa [Homo sapiens] |

| Filename XCorr DeltCN Conf% ObsM+H+ CalcM+H+ SpR ZScore Ion% # Sequence  | | | | | | | | | | | | |
| --- | --- | --- | --- | --- | --- | --- | --- | --- | --- | --- | --- | --- |
| \* | Mis12XLAPLSS\_011913\_02.06591.06591.2 | 4.3991 | 0.4501 | 100.0% | 1657.8522 | 1658.8522 | 2 | 8.786 | 59.4% | 1 | R.VIGLSSDLQQVGGASAR.I | 2 |
| \* | Mis12XLAPLSS\_011913\_01.18189.18189.2 | 4.5474 | 0.5972 | 100.0% | 2049.9922 | 2051.3013 | 1 | 11.189 | 50.0% | 1 | R.IQDALSTVLQYAEDVLSGK.V | 2 |
| \* | Mis12XLAPLSS\_011913\_01.11334.11334.2 | 2.9615 | 0.3798 | 99.9% | 1276.1122 | 1276.5798 | 1 | 7.948 | 80.0% | 1 | R.FLMSLVNQVPK.I | 2 |

---

|  |  |  |  |  |  |  |  |  |
| --- | --- | --- | --- | --- | --- | --- | --- | --- |
| U | *gi|16753227|ref|NP\_00* | 4 | 4 | 13.2% | 288 | 32728 | 10.6 | ribosomal protein L6 [Homo sapiens] |
| U | *gi|67189747|ref|NP\_00* | 4 | 4 | 13.2% | 288 | 32728 | 10.6 | ribosomal protein L6 [Homo sapiens] |

| Filename XCorr DeltCN Conf% ObsM+H+ CalcM+H+ SpR ZScore Ion% # Sequence  | | | | | | | | | | | | |
| --- | --- | --- | --- | --- | --- | --- | --- | --- | --- | --- | --- | --- |
|  | Mis12XLAPLSS\_011913\_01.04076.04076.2 | 2.7929 | 0.3009 | 99.8% | 1285.3722 | 1285.5266 | 18 | 6.443 | 50.0% | 1 | K.VLATVTKPVGGDK.N | 2 |
|  | Mis12XLAPLSS\_011913\_01.03728.03728.3 | 2.9657 | 0.3949 | 100.0% | 1770.8944 | 1771.0269 | 1 | 5.721 | 30.9% | 1 | K.VLATVTKPVGGDKNGGTR.V | 3 |
|  | Mis12XLAPLSS\_011913\_01.04816.04816.1 | 1.8517 | 0.3183 | 100.0% | 1332.89 | 1333.3971 | 1 | 5.248 | 60.0% | 1 | R.HQEGEIFDTEK.E | 1 |
|  | Mis12XLAPLSS\_011913\_01.07626.07626.3 | 3.0815 | 0.2802 | 99.0% | 2510.1843 | 2510.6763 | 5 | 4.232 | 30.3% | 1 | R.HQEGEIFDTEKEKYEITEQR.K | 3 |

---

|  |  |  |  |  |  |  |  |  |
| --- | --- | --- | --- | --- | --- | --- | --- | --- |
| U | *gi|46367787|ref|NP\_00* | 6 | 7 | 13.1% | 636 | 70671 | 9.5 | poly(A) binding protein, cytoplasmic 1 [Homo sapiens] |

| Filename XCorr DeltCN Conf% ObsM+H+ CalcM+H+ SpR ZScore Ion% # Sequence  | | | | | | | | | | | | |
| --- | --- | --- | --- | --- | --- | --- | --- | --- | --- | --- | --- | --- |
|  | Mis12XLAPLSS\_011913\_01.11008.11008.2 | 2.2963 | 0.2398 | 98.4% | 1266.2722 | 1267.4828 | 1 | 6.164 | 75.0% | 2 | R.ALDTMNFDVIK.G | 2 |
|  | Mis12XLAPLSS\_011913\_01.06065.06065.2 | 2.4705 | 0.3838 | 99.8% | 1213.1322 | 1213.4191 | 89 | 6.125 | 55.6% | 1 | R.AKEFTNVYIK.N | 2 |
|  | Mis12XLAPLSS\_011913\_01.08198.08198.2 | 2.5754 | 0.1695 | 99.0% | 1083.9521 | 1084.2603 | 3 | 5.953 | 75.0% | 1 | R.YQGVNLYVK.N | 2 |
| \* | Mis12XLAPLSS\_011913\_01.09030.09030.2 | 3.2233 | 0.2114 | 99.8% | 1413.1721 | 1413.6134 | 1 | 5.623 | 75.0% | 1 | R.KEFSPFGTITSAK.V | 2 |
|  | Mis12XLAPLSS\_011913\_01.14680.14680.3 | 3.9667 | 0.3904 | 100.0% | 2740.6443 | 2742.175 | 1 | 6.076 | 33.7% | 1 | K.ITGMLLEIDNSELLHMLESPESLR.S | 3 |
|  | Mis12XLAPLSS\_011913\_01.07845.07845.3 | 4.6163 | 0.4255 | 100.0% | 1694.5443 | 1694.9285 | 1 | 7.679 | 53.3% | 1 | R.SKVDEAVAVLQAHQAK.E | 3 |

---

|  |  |  |  |  |  |  |  |  |
| --- | --- | --- | --- | --- | --- | --- | --- | --- |
| U | *gi|30410792|ref|NP\_00* | 2 | 2 | 13.0% | 239 | 27402 | 5.7 | proteasome activator subunit 2 [Homo sapiens] |

| Filename XCorr DeltCN Conf% ObsM+H+ CalcM+H+ SpR ZScore Ion% # Sequence  | | | | | | | | | | | | |
| --- | --- | --- | --- | --- | --- | --- | --- | --- | --- | --- | --- | --- |
| \* | Mis12XLAPLSS\_011913\_01.04965.04965.3 | 3.0403 | 0.2686 | 99.4% | 1729.6444 | 1729.89 | 20 | 4.981 | 39.3% | 1 | R.ALVHERDEAAYGELR.A | 3 |
| \* | Mis12XLAPLSS\_011913\_01.13670.13670.2 | 3.8744 | 0.3068 | 99.9% | 1899.4521 | 1899.1534 | 1 | 6.36 | 50.0% | 1 | R.AFYAELYHIISSNLEK.I | 2 |

---

|  |  |  |  |  |  |  |  |  |
| --- | --- | --- | --- | --- | --- | --- | --- | --- |
| U | *gi|4827038|ref|NP\_005* | 2 | 3 | 13.0% | 184 | 19863 | 5.0 | tumor protein D52 isoform 3 [Homo sapiens] |
| U | *gi|70608174|ref|NP\_00* | 2 | 3 | 11.6% | 207 | 22477 | 5.4 | tumor protein D52 isoform 2 [Homo sapiens] |
| U | *gi|70608172|ref|NP\_00* | 2 | 3 | 10.7% | 224 | 24327 | 4.8 | tumor protein D52 isoform 1 [Homo sapiens] |

| Filename XCorr DeltCN Conf% ObsM+H+ CalcM+H+ SpR ZScore Ion% # Sequence  | | | | | | | | | | | | |
| --- | --- | --- | --- | --- | --- | --- | --- | --- | --- | --- | --- | --- |
|  | Mis12XLAPLSS\_011913\_02.06434.06434.2 | 3.6444 | 0.4332 | 100.0% | 1325.3322 | 1325.5046 | 1 | 7.868 | 73.1% | 1 | K.ASAAFSSVGSVITK.K | 2 |
|  | Mis12XLAPLSS\_011913\_01.06285.06285.2 | 2.7261 | 0.2869 | 99.8% | 1223.3121 | 1223.3684 | 1 | 4.946 | 72.2% | 2 | K.SFEEKVENLK.S | 2 |

---

|  |  |  |  |  |  |  |  |  |
| --- | --- | --- | --- | --- | --- | --- | --- | --- |
| U | *gi|5174449|ref|NP\_006* | 2 | 2 | 12.7% | 213 | 22487 | 10.8 | H1 histone family, member X [Homo sapiens] |

| Filename XCorr DeltCN Conf% ObsM+H+ CalcM+H+ SpR ZScore Ion% # Sequence  | | | | | | | | | | | | |
| --- | --- | --- | --- | --- | --- | --- | --- | --- | --- | --- | --- | --- |
| \* | Mis12XLAPLSS\_011913\_02.06636.06636.2 | 3.0064 | 0.1289 | 98.9% | 1342.5122 | 1342.5785 | 3 | 4.811 | 68.2% | 1 | K.ALVQNDTLLQVK.G | 2 |
| \* | Mis12XLAPLSS\_011913\_01.03452.03452.2 | 3.8084 | 0.5427 | 100.0% | 1331.5721 | 1332.5021 | 1 | 8.809 | 75.0% | 1 | R.GAPAAATAPAPTAHK.A | 2 |

---

|  |  |  |  |  |  |  |  |  |
| --- | --- | --- | --- | --- | --- | --- | --- | --- |
| U | *gi|55956788|ref|NP\_00* | 6 | 9 | 12.4% | 710 | 76615 | 4.7 | nucleolin [Homo sapiens] |

| Filename XCorr DeltCN Conf% ObsM+H+ CalcM+H+ SpR ZScore Ion% # Sequence  | | | | | | | | | | | | |
| --- | --- | --- | --- | --- | --- | --- | --- | --- | --- | --- | --- | --- |
| \* | Mis12XLAPLSS\_011913\_01.07755.07755.2 | 2.5011 | 0.2568 | 99.5% | 1000.4522 | 1001.1277 | 3 | 5.159 | 75.0% | 1 | K.NDLAVVDVR.I | 2 |
| \* | Mis12XLAPLSS\_011913\_01.11420.11420.2 | 3.61 | 0.4091 | 100.0% | 1649.4321 | 1649.751 | 1 | 7.437 | 76.9% | 2 | K.FGYVDFESAEDLEK.A | 2 |
| \* | Mis12XLAPLSS\_011913\_01.08548.08548.2 | 2.568 | 0.3599 | 99.8% | 1161.2722 | 1161.297 | 2 | 6.046 | 72.2% | 1 | R.SISLYYTGEK.G | 2 |
| \* | Mis12XLAPLSS\_011913\_02.06922.06922.3 | 3.0514 | 0.365 | 99.7% | 2200.2844 | 2201.3057 | 2 | 5.532 | 35.5% | 1 | K.GLSEDTTEETLKESFDGSVR.A | 3 |
| \* | Mis12XLAPLSS\_011913\_02.07835.07835.2 | 4.1668 | 0.514 | 100.0% | 1562.1322 | 1562.6323 | 1 | 8.512 | 73.1% | 3 | K.GFGFVDFNSEEDAK.A | 2 |
| \* | Mis12XLAPLSS\_011913\_01.09168.09168.3 | 3.5575 | 0.2667 | 99.8% | 2347.1343 | 2347.602 | 1 | 4.934 | 40.0% | 1 | K.EAMEDGEIDGNKVTLDWAKPK.G | 3 |

---

|  |  |  |  |  |  |  |  |  |
| --- | --- | --- | --- | --- | --- | --- | --- | --- |
| U | *gi|17158044|ref|NP\_00* | 3 | 5 | 12.4% | 249 | 28681 | 10.8 | ribosomal protein S6 [Homo sapiens] |

| Filename XCorr DeltCN Conf% ObsM+H+ CalcM+H+ SpR ZScore Ion% # Sequence  | | | | | | | | | | | | |
| --- | --- | --- | --- | --- | --- | --- | --- | --- | --- | --- | --- | --- |
| \* | Mis12XLAPLSS\_011913\_02.07419.07419.2 | 2.9806 | 0.3615 | 99.8% | 1621.6921 | 1621.8022 | 1 | 5.92 | 53.6% | 3 | R.MATEVAADALGEEWK.G | 2 |
| \* | Mis12XLAPLSS\_011913\_02.08022.08022.3 | 3.1468 | 0.3456 | 99.7% | 2197.7043 | 2196.4827 | 2 | 5.208 | 32.9% | 1 | R.MATEVAADALGEEWKGYVVR.I | 3 |
| \* | Mis12XLAPLSS\_011913\_01.07854.07854.2 | 2.4042 | 0.1446 | 95.6% | 1335.5322 | 1336.4875 | 107 | 4.786 | 55.0% | 1 | K.LFNLSKEDDVR.Q | 2 |

---

|  |  |  |  |  |  |  |  |  |
| --- | --- | --- | --- | --- | --- | --- | --- | --- |
| U | *gi|11136628|ref|NP\_06* | 2 | 6 | 12.4% | 225 | 24764 | 4.7 | eukaryotic translation elongation factor 1 beta 2 [Homo sapiens] |
| U | *gi|83376130|ref|NP\_00* | 2 | 6 | 12.4% | 225 | 24764 | 4.7 | eukaryotic translation elongation factor 1 beta 2 [Homo sapiens] |
| U | *gi|4503477|ref|NP\_001* | 2 | 6 | 12.4% | 225 | 24764 | 4.7 | eukaryotic translation elongation factor 1 beta 2 [Homo sapiens] |

| Filename XCorr DeltCN Conf% ObsM+H+ CalcM+H+ SpR ZScore Ion% # Sequence  | | | | | | | | | | | | |
| --- | --- | --- | --- | --- | --- | --- | --- | --- | --- | --- | --- | --- |
|  | Mis12XLAPLSS\_011913\_01.12236.12236.2 | 4.8699 | 0.4945 | 100.0% | 1604.6921 | 1604.8003 | 1 | 9.58 | 71.4% | 1 | K.SPAGLQVLNDYLADK.S | 2 |
|  | Mis12XLAPLSS\_011913\_02.06830.06830.2 | 4.7613 | 0.5723 | 100.0% | 1348.1522 | 1348.4985 | 1 | 9.106 | 79.2% | 5 | R.SIQADGLVWGSSK.L | 2 |

---

|  |  |  |  |  |  |  |  |  |
| --- | --- | --- | --- | --- | --- | --- | --- | --- |
| U | *gi|24307939|ref|NP\_03* | 5 | 6 | 12.2% | 541 | 59671 | 5.6 | chaperonin containing TCP1, subunit 5 (epsilon) [Homo sapiens] |

| Filename XCorr DeltCN Conf% ObsM+H+ CalcM+H+ SpR ZScore Ion% # Sequence  | | | | | | | | | | | | |
| --- | --- | --- | --- | --- | --- | --- | --- | --- | --- | --- | --- | --- |
| \* | Mis12XLAPLSS\_011913\_01.04140.04140.2 | 3.3399 | 0.4595 | 100.0% | 1093.9521 | 1094.1692 | 1 | 8.425 | 94.4% | 1 | R.IADGYEQAAR.V | 2 |
| \* | Mis12XLAPLSS\_011913\_01.10116.10116.2 | 2.7181 | 0.3779 | 99.8% | 1391.7922 | 1392.592 | 1 | 6.634 | 68.2% | 1 | R.DVDFELIKVEGK.V | 2 |
| \* | Mis12XLAPLSS\_011913\_01.09557.09557.2 | 3.2117 | 0.2372 | 99.8% | 1423.2722 | 1423.6672 | 3 | 5.044 | 70.0% | 1 | K.EKFEEMIQQIK.E | 2 |
| \* | Mis12XLAPLSS\_011913\_01.14361.14361.2 | 4.8232 | 0.5285 | 100.0% | 1739.2922 | 1740.0122 | 1 | 8.825 | 62.5% | 2 | R.WVGGPEIELIAIATGGR.I | 2 |
| \* | Mis12XLAPLSS\_011913\_01.13671.13671.2 | 4.1806 | 0.5184 | 100.0% | 1669.3322 | 1668.9304 | 1 | 9.095 | 66.7% | 1 | K.LGFAGLVQEISFGTTK.D | 23 |

---

|  |  |  |  |  |  |  |  |  |
| --- | --- | --- | --- | --- | --- | --- | --- | --- |
| U | *gi|119220557|ref|NP\_0* | 3 | 3 | 12.2% | 425 | 47079 | 7.2 | phosphoribosylaminoimidazole carboxylase, phosphoribosylaminoimidazole succinocarboxamide synthetase isoform 2 [Homo sapiens] |
| U | *gi|5453539|ref|NP\_006* | 3 | 3 | 12.2% | 425 | 47079 | 7.2 | phosphoribosylaminoimidazole carboxylase, phosphoribosylaminoimidazole succinocarboxamide synthetase isoform 2 [Homo sapiens] |
| U | *gi|119220559|ref|NP\_0* | 3 | 3 | 12.0% | 432 | 47958 | 7.7 | phosphoribosylaminoimidazole carboxylase, phosphoribosylaminoimidazole succinocarboxamide synthetase isoform 1 [Homo sapiens] |

| Filename XCorr DeltCN Conf% ObsM+H+ CalcM+H+ SpR ZScore Ion% # Sequence  | | | | | | | | | | | | |
| --- | --- | --- | --- | --- | --- | --- | --- | --- | --- | --- | --- | --- |
|  | Mis12XLAPLSS\_011913\_01.11559.11559.3 | 3.616 | 0.2707 | 99.8% | 2228.0344 | 2228.5046 | 20 | 4.759 | 30.6% | 1 | K.TKEVYELLDS\*PGKVLLQSK.D | 3 |
|  | Mis12XLAPLSS\_011913\_01.03381.03381.2 | 3.538 | 0.3815 | 99.8% | 1410.5721 | 1411.5577 | 1 | 7.068 | 66.7% | 1 | R.VTSAHKGPDETLR.I | 2 |
|  | Mis12XLAPLSS\_011913\_02.07980.07980.3 | 2.728 | 0.254 | 95.3% | 2093.1243 | 2093.3875 | 4 | 4.302 | 30.3% | 1 | R.IKAEYEGDGIPTVFVAVAGR.S | 3 |

---

|  |  |  |  |  |  |  |  |  |
| --- | --- | --- | --- | --- | --- | --- | --- | --- |
| U | *gi|4758638|ref|NP\_004* | 2 | 2 | 12.1% | 224 | 25035 | 6.4 | peroxiredoxin 6 [Homo sapiens] |

| Filename XCorr DeltCN Conf% ObsM+H+ CalcM+H+ SpR ZScore Ion% # Sequence  | | | | | | | | | | | | |
| --- | --- | --- | --- | --- | --- | --- | --- | --- | --- | --- | --- | --- |
| \* | Mis12XLAPLSS\_011913\_01.13239.13239.3 | 3.814 | 0.405 | 100.0% | 2032.2244 | 2032.2682 | 1 | 6.65 | 50.0% | 1 | R.FHDFLGDSWGILFSHPR.D | 3 |
| \* | Mis12XLAPLSS\_011913\_01.08876.08876.2 | 1.9855 | 0.3276 | 98.4% | 1136.2122 | 1136.3794 | 38 | 5.212 | 61.1% | 1 | R.VVFVFGPDKK.L | 2 |

---

|  |  |  |  |  |  |  |  |  |
| --- | --- | --- | --- | --- | --- | --- | --- | --- |
| U | *gi|110349759|ref|NP\_0* | 2 | 3 | 11.7% | 281 | 32162 | 6.8 | NSL1, MIND kinetochore complex component isoform 1 [Homo sapiens] |

| Filename XCorr DeltCN Conf% ObsM+H+ CalcM+H+ SpR ZScore Ion% # Sequence  | | | | | | | | | | | | |
| --- | --- | --- | --- | --- | --- | --- | --- | --- | --- | --- | --- | --- |
|  | Mis12XLAPLSS\_011913\_01.14504.14504.2 | 4.8749 | 0.4678 | 100.0% | 1849.4922 | 1849.0881 | 1 | 7.491 | 63.3% | 1 | K.VLEDQFDEIIVDIATK.R | 2 |
| \* | Mis12XLAPLSS\_011913\_01.12928.12928.2 | 4.7183 | 0.3377 | 100.0% | 1845.7122 | 1845.1057 | 1 | 6.487 | 71.9% | 2 | K.SLPALIEQGEGFSQVLR.M | 2 |

---

|  |  |  |  |  |  |  |  |  |
| --- | --- | --- | --- | --- | --- | --- | --- | --- |
| U | *gi|169213536|ref|XP\_0* | 3 | 3 | 11.7% | 214 | 24627 | 10.1 | PREDICTED: similar to QM protein isoform 1 [Homo sapiens] |
| U | *gi|41151097|ref|XP\_20* | 3 | 3 | 11.7% | 214 | 24627 | 10.1 | PREDICTED: similar to QM protein isoform 1 [Homo sapiens] |
| U | *gi|223890243|ref|NP\_0* | 3 | 3 | 11.7% | 214 | 24604 | 10.1 | ribosomal protein L10 [Homo sapiens] |
| U | *gi|169213734|ref|XP\_0* | 3 | 3 | 15.0% | 167 | 19409 | 9.9 | PREDICTED: similar to Q1Z 7F5 isoform 2 [Homo sapiens] |
| U | *gi|169213732|ref|XP\_0* | 3 | 3 | 11.7% | 214 | 24600 | 10.1 | PREDICTED: similar to Q1Z 7F5 isoform 1 [Homo sapiens] |
| U | *gi|169213538|ref|XP\_0* | 3 | 3 | 15.0% | 167 | 19436 | 9.9 | PREDICTED: similar to QM protein isoform 2 [Homo sapiens] |

| Filename XCorr DeltCN Conf% ObsM+H+ CalcM+H+ SpR ZScore Ion% # Sequence  | | | | | | | | | | | | |
| --- | --- | --- | --- | --- | --- | --- | --- | --- | --- | --- | --- | --- |
|  | Mis12XLAPLSS\_011913\_01.09662.09662.2 | 3.2452 | 0.523 | 100.0% | 1253.3322 | 1253.5486 | 1 | 9.621 | 65.0% | 1 | R.VHIGQVIMSIR.T | 2 |
|  | Mis12XLAPLSS\_011913\_01.11415.11415.2 | 3.8542 | 0.3221 | 99.8% | 1546.4722 | 1545.6606 | 1 | 6.138 | 70.8% | 1 | K.FNADEFEDMVAEK.R | 2 |
|  | Mis12XLAPLSS\_011913\_01.10270.10270.2 | 3.6855 | 0.3158 | 99.9% | 1701.7922 | 1701.8481 | 2 | 6.829 | 57.7% | 1 | K.FNADEFEDMVAEKR.L | 2 |

---

|  |  |  |  |  |  |  |  |  |
| --- | --- | --- | --- | --- | --- | --- | --- | --- |
| U | *gi|194239723|ref|NP\_0* | 5 | 13 | 11.6% | 647 | 71408 | 6.4 | eukaryotic translation elongation factor 1 delta isoform 1 [Homo sapiens] |
| U | *gi|25453474|ref|NP\_11* | 5 | 13 | 11.6% | 647 | 71408 | 6.4 | eukaryotic translation elongation factor 1 delta isoform 1 [Homo sapiens] |
| U | *gi|25453472|ref|NP\_00* | 5 | 13 | 26.7% | 281 | 31122 | 5.0 | eukaryotic translation elongation factor 1 delta isoform 2 [Homo sapiens] |
| U | *gi|194239731|ref|NP\_0* | 5 | 13 | 26.7% | 281 | 31122 | 5.0 | eukaryotic translation elongation factor 1 delta isoform 2 [Homo sapiens] |
| U | *gi|194239729|ref|NP\_0* | 5 | 13 | 29.2% | 257 | 28558 | 4.9 | eukaryotic translation elongation factor 1 delta isoform 4 [Homo sapiens] |
| U | *gi|194239727|ref|NP\_0* | 5 | 13 | 26.7% | 281 | 31122 | 5.0 | eukaryotic translation elongation factor 1 delta isoform 2 [Homo sapiens] |
| U | *gi|194239725|ref|NP\_0* | 5 | 13 | 11.6% | 646 | 71266 | 6.4 | eukaryotic translation elongation factor 1 delta isoform 3 [Homo sapiens] |

| Filename XCorr DeltCN Conf% ObsM+H+ CalcM+H+ SpR ZScore Ion% # Sequence  | | | | | | | | | | | | |
| --- | --- | --- | --- | --- | --- | --- | --- | --- | --- | --- | --- | --- |
|  | Mis12XLAPLSS\_011913\_01.07227.07227.2 | 4.4941 | 0.4154 | 100.0% | 1527.1322 | 1527.6947 | 1 | 7.772 | 73.1% | 4 | R.FYEQMNGPVAGASR.Q | 2 |
|  | Mis12XLAPLSS\_011913\_01.08042.08042.2 | 4.2195 | 0.4338 | 100.0% | 1359.0922 | 1359.5223 | 1 | 8.199 | 81.8% | 5 | R.IASLEVENQSLR.G | 2 |
|  | Mis12XLAPLSS\_011913\_01.11829.11829.2 | 3.4698 | 0.3554 | 99.9% | 1300.0322 | 1300.4978 | 1 | 6.719 | 81.8% | 1 | R.GVVQELQQAISK.L | 2 |
|  | Mis12XLAPLSS\_011913\_01.12700.12700.3 | 4.3386 | 0.3666 | 100.0% | 2792.6042 | 2793.084 | 1 | 6.19 | 33.7% | 1 | K.SSILLDVKPWDDETDMAQLEACVR.S | 3 |
|  | Mis12XLAPLSS\_011913\_01.12020.12020.2 | 3.4524 | 0.3742 | 99.9% | 1374.2522 | 1374.5797 | 2 | 6.68 | 66.7% | 2 | R.SIQLDGLVWGASK.L | 2 |

---

|  |  |  |  |  |  |  |  |  |
| --- | --- | --- | --- | --- | --- | --- | --- | --- |
| U | *contaminant\_KERATIN03* | 6 | 8 | 11.6% | 593 | 59519 | 5.2 | no description |
| U | *gi|195972866|ref|NP\_0* | 6 | 8 | 11.8% | 584 | 58801 | 5.2 | keratin 10 [Homo sapiens] |

| Filename XCorr DeltCN Conf% ObsM+H+ CalcM+H+ SpR ZScore Ion% # Sequence  | | | | | | | | | | | | |
| --- | --- | --- | --- | --- | --- | --- | --- | --- | --- | --- | --- | --- |
|  | Mis12XLAPLSS\_011913\_02.06826.06826.2 | 2.482 | 0.2969 | 99.3% | 1708.2122 | 1708.7844 | 2 | 6.588 | 36.1% | 1 | K.GSLGGGFSSGGFSGGSFSR.G | 2 |
|  | Mis12XLAPLSS\_011913\_01.04665.04665.2 | 2.589 | 0.2496 | 99.7% | 1092.2522 | 1091.2273 | 2 | 5.382 | 81.2% | 1 | K.VTMQNLNDR.L | 2 |
|  | Mis12XLAPLSS\_011913\_01.06232.06232.2 | 3.561 | 0.3985 | 100.0% | 1381.9722 | 1382.4668 | 1 | 8.362 | 68.2% | 3 | R.ALEESNYELEGK.I | 2 |
|  | Mis12XLAPLSS\_011913\_01.05369.05369.2 | 2.0125 | 0.208 | 97.3% | 807.9122 | 807.8815 | 90 | 5.564 | 66.7% | 1 | R.LAADDFR.L | 222 |
|  | Mis12XLAPLSS\_011913\_01.05550.05550.2 | 2.4577 | 0.3513 | 99.8% | 1235.7522 | 1235.4258 | 1 | 5.406 | 83.3% | 1 | R.LKYENEVALR.Q | 2 |
|  | Mis12XLAPLSS\_011913\_01.04094.04094.2 | 2.678 | 0.2762 | 99.6% | 1494.1921 | 1494.6041 | 14 | 5.389 | 54.5% | 1 | R.SQYEQLAEQNRK.D | 2 |

Similarities:
gi|40354195|ref|NP\_95(1:5)  
gi|4557701|ref|NP\_000(1:5)  

---

|  |  |  |  |  |  |  |  |  |
| --- | --- | --- | --- | --- | --- | --- | --- | --- |
| U | *gi|217416369|ref|NP\_0* | 2 | 2 | 11.6% | 198 | 22367 | 6.7 | SAR1a gene homolog 1 [Homo sapiens] |
| U | *gi|9910542|ref|NP\_064* | 2 | 2 | 11.6% | 198 | 22367 | 6.7 | SAR1a gene homolog 1 [Homo sapiens] |

| Filename XCorr DeltCN Conf% ObsM+H+ CalcM+H+ SpR ZScore Ion% # Sequence  | | | | | | | | | | | | |
| --- | --- | --- | --- | --- | --- | --- | --- | --- | --- | --- | --- | --- |
|  | Mis12XLAPLSS\_011913\_01.08189.08189.2 | 2.7557 | 0.3479 | 99.8% | 1343.6522 | 1343.5841 | 1 | 6.073 | 75.0% | 1 | K.TTLLHMLKDDR.L | 2 |
|  | Mis12XLAPLSS\_011913\_01.09773.09773.2 | 2.1538 | 0.3199 | 98.9% | 1314.5122 | 1314.4808 | 1 | 5.239 | 68.2% | 1 | R.EIFGLYGQTTGK.G | 2 |

---

|  |  |  |  |  |  |  |  |  |
| --- | --- | --- | --- | --- | --- | --- | --- | --- |
| U | *gi|38455427|ref|NP\_00* | 4 | 6 | 11.5% | 539 | 57924 | 7.8 | chaperonin containing TCP1, subunit 4 (delta) [Homo sapiens] |

| Filename XCorr DeltCN Conf% ObsM+H+ CalcM+H+ SpR ZScore Ion% # Sequence  | | | | | | | | | | | | |
| --- | --- | --- | --- | --- | --- | --- | --- | --- | --- | --- | --- | --- |
| \* | Mis12XLAPLSS\_011913\_02.06251.06251.3 | 4.1739 | 0.3305 | 100.0% | 2092.5244 | 2091.3845 | 1 | 5.638 | 39.5% | 1 | K.MIQDGKGDVTITNDGATILK.Q | 3 |
| \* | Mis12XLAPLSS\_011913\_01.08402.08402.2 | 2.4081 | 0.2988 | 99.4% | 1457.0521 | 1457.6696 | 2 | 5.574 | 50.0% | 1 | K.GIHPTIISESFQK.A | 2 |
| \* | Mis12XLAPLSS\_011913\_01.08826.08826.2 | 3.1532 | 0.4571 | 100.0% | 1357.6721 | 1358.5345 | 1 | 8.38 | 70.8% | 2 | K.VIDPATATSVDLR.D | 2 |
| \* | Mis12XLAPLSS\_011913\_01.12022.12022.2 | 3.4657 | 0.3741 | 99.9% | 1552.1322 | 1551.8265 | 2 | 5.91 | 60.0% | 2 | R.ALIAGGGAPEIELALR.L | 2 |

---

|  |  |  |  |  |  |  |  |  |
| --- | --- | --- | --- | --- | --- | --- | --- | --- |
| U | *gi|14110407|ref|NP\_11* | 2 | 2 | 11.4% | 420 | 46437 | 9.6 | heterogeneous nuclear ribonucleoprotein D-like [Homo sapiens] |

| Filename XCorr DeltCN Conf% ObsM+H+ CalcM+H+ SpR ZScore Ion% # Sequence  | | | | | | | | | | | | |
| --- | --- | --- | --- | --- | --- | --- | --- | --- | --- | --- | --- | --- |
| \* | Mis12XLAPLSS\_011913\_01.10422.10422.2 | 2.7396 | 0.3103 | 99.8% | 1471.3322 | 1470.7264 | 8 | 4.865 | 54.2% | 1 | K.MFIGGLSWDTSKK.D | 2 |
| \* | Mis12XLAPLSS\_011913\_02.10618.10618.3 | 4.9457 | 0.4141 | 100.0% | 3890.6643 | 3892.323 | 1 | 6.345 | 27.2% | 1 | K.VFVGGLSPDTSEEQIKEYFGAFGEIENIELPMDTK.T | 3 |

---

|  |  |  |  |  |  |  |  |  |
| --- | --- | --- | --- | --- | --- | --- | --- | --- |
| U | *gi|12025678|ref|NP\_00* | 9 | 11 | 11.2% | 911 | 104854 | 5.4 | actinin, alpha 4 [Homo sapiens] |

| Filename XCorr DeltCN Conf% ObsM+H+ CalcM+H+ SpR ZScore Ion% # Sequence  | | | | | | | | | | | | |
| --- | --- | --- | --- | --- | --- | --- | --- | --- | --- | --- | --- | --- |
|  | Mis12XLAPLSS\_011913\_01.10236.10236.2 | 2.4078 | 0.2718 | 99.4% | 1227.2722 | 1227.4086 | 20 | 5.065 | 65.0% | 1 | K.DGLAFNALIHR.H | 2 |
| \* | Mis12XLAPLSS\_011913\_01.09183.09183.2 | 4.1873 | 0.447 | 100.0% | 1920.2722 | 1921.1577 | 1 | 9.235 | 61.8% | 1 | K.LSGSNPYTTVTPQIINSK.W | 2 |
| \* | Mis12XLAPLSS\_011913\_01.03544.03544.2 | 3.2487 | 0.2607 | 99.8% | 1326.2322 | 1326.452 | 2 | 5.281 | 70.0% | 1 | K.RDHALLEEQSK.Q | 2 |
|  | Mis12XLAPLSS\_011913\_01.03888.03888.2 | 2.7807 | 0.464 | 99.8% | 1302.1122 | 1302.4503 | 4 | 7.292 | 72.2% | 1 | K.HTNYTMEHIR.V | 22 |
|  | Mis12XLAPLSS\_011913\_01.03874.03874.3 | 3.7737 | 0.4195 | 100.0% | 1302.9543 | 1302.4503 | 25 | 6.885 | 47.2% | 1 | K.HTNYTMEHIR.V | 33 |
|  | Mis12XLAPLSS\_011913\_01.13774.13774.2 | 4.1026 | 0.3868 | 100.0% | 1387.4321 | 1387.6218 | 1 | 8.228 | 72.7% | 2 | R.VGWEQLLTTIAR.T | 22 |
|  | Mis12XLAPLSS\_011913\_01.09320.09320.2 | 3.0688 | 0.2526 | 99.8% | 1430.1122 | 1430.6011 | 9 | 5.42 | 63.6% | 1 | R.TINEVENQILTR.D | 22 |
| \* | Mis12XLAPLSS\_011913\_01.07613.07613.2 | 2.8709 | 0.4314 | 99.9% | 1353.3121 | 1353.4926 | 1 | 6.686 | 70.0% | 2 | K.GISQEQMQEFR.A | 2 |
| \* | Mis12XLAPLSS\_011913\_01.09418.09418.2 | 3.092 | 0.2777 | 99.8% | 1794.4122 | 1794.032 | 1 | 5.23 | 50.0% | 1 | R.MAPYQGPDAVPGALDYK.S | 2 |

Similarities:
gi|194097350|ref|NP\_0(4:5)  

---

|  |  |  |  |  |  |  |  |  |
| --- | --- | --- | --- | --- | --- | --- | --- | --- |
| U | *gi|21735621|ref|NP\_00* | 2 | 2 | 11.2% | 338 | 35503 | 8.7 | mitochondrial malate dehydrogenase precursor [Homo sapiens] |

| Filename XCorr DeltCN Conf% ObsM+H+ CalcM+H+ SpR ZScore Ion% # Sequence  | | | | | | | | | | | | |
| --- | --- | --- | --- | --- | --- | --- | --- | --- | --- | --- | --- | --- |
| \* | Mis12XLAPLSS\_011913\_01.12004.12004.3 | 5.4336 | 0.4594 | 100.0% | 2366.5444 | 2366.6763 | 1 | 8.637 | 47.6% | 1 | R.LTLYDIAHTPGVAADLSHIETK.A | 3 |
| \* | Mis12XLAPLSS\_011913\_02.05904.05904.2 | 3.1801 | 0.3694 | 99.8% | 1455.5122 | 1455.6287 | 1 | 6.579 | 56.7% | 1 | K.AGAGSATLSMAYAGAR.F | 2 |

---

|  |  |  |  |  |  |  |  |  |
| --- | --- | --- | --- | --- | --- | --- | --- | --- |
| U | *gi|78000181|ref|NP\_00* | 2 | 4 | 11.2% | 215 | 23432 | 10.9 | ribosomal protein L14 [Homo sapiens] |
| U | *gi|78000183|ref|NP\_00* | 2 | 4 | 11.2% | 215 | 23432 | 10.9 | ribosomal protein L14 [Homo sapiens] |

| Filename XCorr DeltCN Conf% ObsM+H+ CalcM+H+ SpR ZScore Ion% # Sequence  | | | | | | | | | | | | |
| --- | --- | --- | --- | --- | --- | --- | --- | --- | --- | --- | --- | --- |
|  | Mis12XLAPLSS\_011913\_01.06414.06414.2 | 2.0808 | 0.321 | 98.4% | 1232.2322 | 1233.4124 | 7 | 5.803 | 59.1% | 2 | R.VAYVSFGPHAGK.L | 2 |
|  | Mis12XLAPLSS\_011913\_02.07936.07936.2 | 3.1432 | 0.4929 | 100.0% | 1355.2122 | 1355.5773 | 1 | 8.491 | 77.3% | 2 | K.LVAIVDVIDQNR.A | 2 |

---

|  |  |  |  |  |  |  |  |  |
| --- | --- | --- | --- | --- | --- | --- | --- | --- |
| U | *gi|148727341|ref|NP\_0* | 3 | 3 | 10.9% | 350 | 38438 | 5.1 | serine/threonine kinase receptor associated protein [Homo sapiens] |

| Filename XCorr DeltCN Conf% ObsM+H+ CalcM+H+ SpR ZScore Ion% # Sequence  | | | | | | | | | | | | |
| --- | --- | --- | --- | --- | --- | --- | --- | --- | --- | --- | --- | --- |
|  | Mis12XLAPLSS\_011913\_01.07071.07071.2 | 2.2337 | 0.2335 | 97.8% | 1332.9321 | 1331.5292 | 14 | 4.835 | 60.0% | 1 | R.LWDHATMTEVK.S | 2 |
| \* | Mis12XLAPLSS\_011913\_01.09575.09575.2 | 2.5734 | 0.2969 | 99.5% | 1485.9922 | 1485.7233 | 1 | 4.746 | 57.7% | 1 | R.SIAFHSAVSLDPIK.S | 2 |
| \* | Mis12XLAPLSS\_011913\_01.08050.08050.2 | 3.8143 | 0.4563 | 100.0% | 1596.9722 | 1597.6318 | 1 | 8.584 | 70.8% | 1 | K.YDYNSGEELESYK.G | 2 |

---

|  |  |  |  |  |  |  |  |  |
| --- | --- | --- | --- | --- | --- | --- | --- | --- |
| U | *gi|148470397|ref|NP\_0* | 3 | 3 | 10.6% | 415 | 45672 | 5.6 | heterogeneous nuclear ribonucleoprotein F [Homo sapiens] |
| U | *gi|4826760|ref|NP\_004* | 3 | 3 | 10.6% | 415 | 45672 | 5.6 | heterogeneous nuclear ribonucleoprotein F [Homo sapiens] |
| U | *gi|148470406|ref|NP\_0* | 3 | 3 | 10.6% | 415 | 45672 | 5.6 | heterogeneous nuclear ribonucleoprotein F [Homo sapiens] |
| U | *gi|148470404|ref|NP\_0* | 3 | 3 | 10.6% | 415 | 45672 | 5.6 | heterogeneous nuclear ribonucleoprotein F [Homo sapiens] |
| U | *gi|148470402|ref|NP\_0* | 3 | 3 | 10.6% | 415 | 45672 | 5.6 | heterogeneous nuclear ribonucleoprotein F [Homo sapiens] |
| U | *gi|148470400|ref|NP\_0* | 3 | 3 | 10.6% | 415 | 45672 | 5.6 | heterogeneous nuclear ribonucleoprotein F [Homo sapiens] |

| Filename XCorr DeltCN Conf% ObsM+H+ CalcM+H+ SpR ZScore Ion% # Sequence  | | | | | | | | | | | | |
| --- | --- | --- | --- | --- | --- | --- | --- | --- | --- | --- | --- | --- |
|  | Mis12XLAPLSS\_011913\_02.09261.09261.2 | 3.7915 | 0.3169 | 99.9% | 1868.3522 | 1869.0813 | 1 | 7.677 | 53.1% | 1 | K.ITGEAFVQFASQELAEK.A | 2 |
|  | Mis12XLAPLSS\_011913\_01.13463.13463.2 | 2.3486 | 0.2144 | 96.3% | 1996.7722 | 1998.2023 | 77 | 5.884 | 34.4% | 1 | K.ATENDIYNFFSPLNPVR.V | 22 |
|  | Mis12XLAPLSS\_011913\_01.05208.05208.2 | 2.8214 | 0.4817 | 100.0% | 1093.4321 | 1093.2278 | 1 | 7.479 | 72.2% | 1 | R.VHIEIGPDGR.V | 22 |

Similarities:
gi|5031753|ref|NP\_005(2:1)  

---

|  |  |  |  |  |  |  |  |  |
| --- | --- | --- | --- | --- | --- | --- | --- | --- |
| U | *gi|4503515|ref|NP\_003* | 2 | 2 | 10.5% | 352 | 39930 | 6.5 | eukaryotic translation initiation factor 3, subunit 3 gamma, 40kDa [Homo sapiens] |

| Filename XCorr DeltCN Conf% ObsM+H+ CalcM+H+ SpR ZScore Ion% # Sequence  | | | | | | | | | | | | |
| --- | --- | --- | --- | --- | --- | --- | --- | --- | --- | --- | --- | --- |
| \* | Mis12XLAPLSS\_011913\_01.03245.03245.2 | 4.487 | 0.4577 | 100.0% | 1629.2722 | 1628.647 | 1 | 8.356 | 52.8% | 1 | K.EGTGSTATSSSSTAGAAGK.G | 2 |
| \* | Mis12XLAPLSS\_011913\_01.13416.13416.2 | 3.4858 | 0.3047 | 99.8% | 2148.8123 | 2148.4976 | 1 | 4.97 | 50.0% | 1 | K.NLQLLMDRVDEMSQDIVK.Y | 2 |

---

|  |  |  |  |  |  |  |  |  |
| --- | --- | --- | --- | --- | --- | --- | --- | --- |
| U | *gi|28875797|ref|NP\_05* | 2 | 3 | 10.5% | 248 | 26397 | 12.2 | hypothetical protein LOC26097 [Homo sapiens] |

| Filename XCorr DeltCN Conf% ObsM+H+ CalcM+H+ SpR ZScore Ion% # Sequence  | | | | | | | | | | | | |
| --- | --- | --- | --- | --- | --- | --- | --- | --- | --- | --- | --- | --- |
| \* | Mis12XLAPLSS\_011913\_01.04102.04102.2 | 4.1311 | 0.4017 | 100.0% | 1448.2522 | 1447.6091 | 1 | 7.127 | 70.8% | 1 | R.ASMQQQQQLASAR.N | 2 |
| \* | Mis12XLAPLSS\_011913\_02.06772.06772.2 | 3.3792 | 0.4402 | 100.0% | 1554.9122 | 1555.6997 | 1 | 7.81 | 62.5% | 2 | K.EQLDNQLDAYMSK.T | 2 |

---

|  |  |  |  |  |  |  |  |  |
| --- | --- | --- | --- | --- | --- | --- | --- | --- |
| U | *gi|5730023|ref|NP\_006* | 4 | 5 | 10.4% | 463 | 51157 | 5.6 | RuvB-like 2 [Homo sapiens] |

| Filename XCorr DeltCN Conf% ObsM+H+ CalcM+H+ SpR ZScore Ion% # Sequence  | | | | | | | | | | | | |
| --- | --- | --- | --- | --- | --- | --- | --- | --- | --- | --- | --- | --- |
| \* | Mis12XLAPLSS\_011913\_01.09536.09536.2 | 2.5221 | 0.3166 | 99.8% | 1156.1721 | 1156.281 | 8 | 6.678 | 60.0% | 1 | R.GLGLDDALEPR.Q | 2 |
| \* | Mis12XLAPLSS\_011913\_01.06197.06197.2 | 2.8804 | 0.332 | 99.8% | 1317.4122 | 1317.5059 | 3 | 5.606 | 66.7% | 2 | R.QASQGMVGQLAAR.R | 2 |
| \* | Mis12XLAPLSS\_011913\_01.09216.09216.2 | 2.3595 | 0.1878 | 97.4% | 1213.6522 | 1215.4998 | 2 | 4.056 | 65.0% | 1 | R.RAAGVVLEMIR.E | 2 |
| \* | Mis12XLAPLSS\_011913\_01.10223.10223.2 | 2.4027 | 0.1909 | 96.8% | 1502.9122 | 1502.6765 | 1 | 4.985 | 66.7% | 1 | K.TTEMETIYDLGTK.M | 2 |

---

|  |  |  |  |  |  |  |  |  |
| --- | --- | --- | --- | --- | --- | --- | --- | --- |
| U | *gi|6912634|ref|NP\_036* | 2 | 4 | 10.3% | 203 | 23577 | 10.9 | ribosomal protein L13a [Homo sapiens] |

| Filename XCorr DeltCN Conf% ObsM+H+ CalcM+H+ SpR ZScore Ion% # Sequence  | | | | | | | | | | | | |
| --- | --- | --- | --- | --- | --- | --- | --- | --- | --- | --- | --- | --- |
| \* | Mis12XLAPLSS\_011913\_01.06393.06393.2 | 2.8353 | 0.2714 | 99.8% | 1253.2922 | 1253.3947 | 1 | 6.503 | 65.0% | 3 | K.YQAVTATLEEK.R | 2 |
| \* | Mis12XLAPLSS\_011913\_01.04601.04601.2 | 3.029 | 0.2033 | 99.8% | 1238.1322 | 1237.482 | 4 | 4.223 | 72.2% | 1 | K.KIDKYTEVLK.T | 2 |

---

|  |  |  |  |  |  |  |  |  |
| --- | --- | --- | --- | --- | --- | --- | --- | --- |
| U | *gi|42558250|ref|NP\_00* | 3 | 3 | 10.2% | 709 | 78366 | 5.2 | membrane component chromosome 11 surface marker 1 isoform 1 [Homo sapiens] |
| U | *gi|42734503|ref|NP\_97* | 3 | 3 | 10.4% | 694 | 76862 | 5.1 | membrane component chromosome 11 surface marker 1 isoform 2 [Homo sapiens] |

| Filename XCorr DeltCN Conf% ObsM+H+ CalcM+H+ SpR ZScore Ion% # Sequence  | | | | | | | | | | | | |
| --- | --- | --- | --- | --- | --- | --- | --- | --- | --- | --- | --- | --- |
|  | Mis12XLAPLSS\_011913\_02.04739.04739.3 | 3.4283 | 0.3594 | 99.7% | 3703.7644 | 3705.949 | 1 | 6.125 | 20.7% | 1 | K.SSGPPPPSGSSGSEAAAGAGAAAPASQHPATGTGAVQTEAMK.Q | 3 |
|  | Mis12XLAPLSS\_011913\_01.09530.09530.2 | 2.3477 | 0.2811 | 99.3% | 1268.1721 | 1268.4706 | 9 | 5.368 | 60.0% | 1 | R.SFMALSQDIQK.T | 2 |
|  | Mis12XLAPLSS\_011913\_02.08910.08910.3 | 3.5292 | 0.2077 | 97.6% | 2295.7744 | 2296.5444 | 6 | 4.469 | 30.6% | 1 | R.LNEQYEHASIHLWDLLEGK.E | 3 |

---

|  |  |  |  |  |  |  |  |  |
| --- | --- | --- | --- | --- | --- | --- | --- | --- |
| U | *gi|33239451|ref|NP\_87* | 2 | 3 | 10.0% | 261 | 28769 | 4.7 | proliferating cell nuclear antigen [Homo sapiens] |
| U | *gi|4505641|ref|NP\_002* | 2 | 3 | 10.0% | 261 | 28769 | 4.7 | proliferating cell nuclear antigen [Homo sapiens] |

| Filename XCorr DeltCN Conf% ObsM+H+ CalcM+H+ SpR ZScore Ion% # Sequence  | | | | | | | | | | | | |
| --- | --- | --- | --- | --- | --- | --- | --- | --- | --- | --- | --- | --- |
|  | Mis12XLAPLSS\_011913\_02.07026.07026.2 | 2.2202 | 0.2636 | 97.8% | 1366.4321 | 1366.6338 | 12 | 5.115 | 50.0% | 1 | R.NLAMGVNLTSMSK.I | 2 |
|  | Mis12XLAPLSS\_011913\_01.06798.06798.2 | 3.2719 | 0.3224 | 99.8% | 1294.2522 | 1294.4069 | 1 | 5.896 | 83.3% | 2 | K.FSASGELGNGNIK.L | 2 |

---

|  |  |  |  |  |  |  |  |  |
| --- | --- | --- | --- | --- | --- | --- | --- | --- |
| U | *gi|87196351|ref|NP\_00* | 5 | 7 | 9.8% | 662 | 73244 | 7.2 | DEAD/H (Asp-Glu-Ala-Asp/His) box polypeptide 3 [Homo sapiens] |

| Filename XCorr DeltCN Conf% ObsM+H+ CalcM+H+ SpR ZScore Ion% # Sequence  | | | | | | | | | | | | |
| --- | --- | --- | --- | --- | --- | --- | --- | --- | --- | --- | --- | --- |
|  | Mis12XLAPLSS\_011913\_02.06867.06867.2 | 2.8378 | 0.2681 | 99.8% | 1321.4521 | 1321.4729 | 1 | 5.282 | 75.0% | 1 | R.ELAVQIYEEAR.K | 2 |
|  | Mis12XLAPLSS\_011913\_01.11007.11007.2 | 3.7277 | 0.4571 | 100.0% | 1337.3922 | 1337.5946 | 1 | 8.328 | 85.0% | 2 | R.MLDMGFEPQIR.R | 22 |
|  | Mis12XLAPLSS\_011913\_02.06513.06513.2 | 3.2738 | 0.4542 | 100.0% | 1169.6122 | 1169.4099 | 1 | 7.702 | 68.2% | 2 | K.SPILVATAVAAR.G | 2 |
|  | Mis12XLAPLSS\_011913\_01.12173.12173.3 | 4.2992 | 0.4023 | 100.0% | 2083.9443 | 2084.2957 | 1 | 7.583 | 40.6% | 1 | K.HVINFDLPSDIEEYVHR.I | 3 |
| \* | Mis12XLAPLSS\_011913\_01.12258.12258.2 | 3.6063 | 0.3883 | 99.8% | 1525.3722 | 1525.7043 | 1 | 7.249 | 65.4% | 1 | R.VGNLGLATSFFNER.N | 2 |

Similarities:
gi|4758138|ref|NP\_004(1:4)  

---

|  |  |  |  |  |  |  |  |  |
| --- | --- | --- | --- | --- | --- | --- | --- | --- |
| U | *gi|34419635|ref|NP\_00* | 6 | 12 | 9.8% | 643 | 71028 | 6.1 | heat shock 70kDa protein 6 (HSP70B') [Homo sapiens] |

| Filename XCorr DeltCN Conf% ObsM+H+ CalcM+H+ SpR ZScore Ion% # Sequence  | | | | | | | | | | | | |
| --- | --- | --- | --- | --- | --- | --- | --- | --- | --- | --- | --- | --- |
|  | Mis12XLAPLSS\_011913\_02.05422.05422.2 | 3.1076 | 0.4678 | 100.0% | 1488.2722 | 1488.5939 | 1 | 8.504 | 70.8% | 3 | R.TTPSYVAFTDTER.L | 2222 |
|  | Mis12XLAPLSS\_011913\_01.11126.11126.2 | 5.0548 | 0.4514 | 100.0% | 1689.2922 | 1688.9213 | 1 | 9.079 | 76.7% | 4 | R.IINEPTAAAIAYGLDR.R | 22 |
|  | Mis12XLAPLSS\_011913\_01.04986.04986.2 | 3.8448 | 0.4962 | 100.0% | 1676.1522 | 1676.6964 | 1 | 8.128 | 63.3% | 1 | K.ATAGDTHLGGEDFDNR.L | 222 |
|  | Mis12XLAPLSS\_011913\_01.04985.04985.3 | 2.7193 | 0.3103 | 99.4% | 1679.1543 | 1676.6964 | 23 | 5.282 | 36.7% | 1 | K.ATAGDTHLGGEDFDNR.L | 333 |
|  | Mis12XLAPLSS\_011913\_01.10815.10815.2 | 2.5878 | 0.3949 | 99.8% | 1082.2322 | 1082.2444 | 1 | 5.822 | 81.2% | 2 | K.LLQDFFNGK.E | 22 |
|  | Mis12XLAPLSS\_011913\_01.03447.03447.2 | 2.4481 | 0.4255 | 99.8% | 1017.4922 | 1018.1582 | 6 | 6.628 | 62.5% | 1 | K.ITITNDKGR.L | 2222 |

Similarities:
gi|5729877|ref|NP\_006(3:3)  
gi|167466173|ref|NP\_0(5:1)  
gi|124256496|ref|NP\_0(4:2)  

---

|  |  |  |  |  |  |  |  |  |
| --- | --- | --- | --- | --- | --- | --- | --- | --- |
| U | *gi|5453597|ref|NP\_006* | 2 | 2 | 9.8% | 286 | 32923 | 5.7 | F-actin capping protein alpha-1 subunit [Homo sapiens] |

| Filename XCorr DeltCN Conf% ObsM+H+ CalcM+H+ SpR ZScore Ion% # Sequence  | | | | | | | | | | | | |
| --- | --- | --- | --- | --- | --- | --- | --- | --- | --- | --- | --- | --- |
| \* | Mis12XLAPLSS\_011913\_01.10605.10605.3 | 3.0058 | 0.2195 | 95.2% | 2090.2144 | 2090.3025 | 49 | 5.395 | 29.4% | 1 | K.FITHAPPGEFNEVFNDVR.L | 3 |
|  | Mis12XLAPLSS\_011913\_01.10107.10107.2 | 2.6292 | 0.1935 | 99.2% | 1197.9122 | 1198.408 | 19 | 5.145 | 72.2% | 1 | R.LLLNNDNLLR.E | 2 |

---

|  |  |  |  |  |  |  |  |  |
| --- | --- | --- | --- | --- | --- | --- | --- | --- |
| U | *gi|4506649|ref|NP\_000* | 3 | 4 | 9.7% | 403 | 46109 | 10.2 | ribosomal protein L3 isoform a [Homo sapiens] |
| U | *gi|76496472|ref|NP\_00* | 3 | 4 | 11.0% | 354 | 40152 | 10.2 | ribosomal protein L3 isoform b [Homo sapiens] |

| Filename XCorr DeltCN Conf% ObsM+H+ CalcM+H+ SpR ZScore Ion% # Sequence  | | | | | | | | | | | | |
| --- | --- | --- | --- | --- | --- | --- | --- | --- | --- | --- | --- | --- |
|  | Mis12XLAPLSS\_011913\_01.07856.07856.2 | 2.3919 | 0.3465 | 99.8% | 984.9922 | 984.14594 | 22 | 7.373 | 62.5% | 2 | R.HGSLGFLPR.K | 2 |
|  | Mis12XLAPLSS\_011913\_01.03651.03651.2 | 2.6067 | 0.2888 | 99.8% | 884.83215 | 885.07214 | 3 | 5.385 | 78.6% | 1 | K.AGMTHIVR.E | 2 |
|  | Mis12XLAPLSS\_011913\_02.09429.09429.3 | 3.0683 | 0.266 | 97.8% | 2437.7944 | 2438.8035 | 1 | 4.995 | 32.1% | 1 | K.SINPLGGFVHYGEVTNDFVMLK.G | 3 |

---

|  |  |  |  |  |  |  |  |  |
| --- | --- | --- | --- | --- | --- | --- | --- | --- |
| U | *gi|221307584|ref|NP\_0* | 2 | 3 | 9.7% | 299 | 33296 | 9.8 | prohibitin 2 isoform 1 [Homo sapiens] |
| U | *gi|6005854|ref|NP\_009* | 2 | 3 | 9.7% | 299 | 33296 | 9.8 | prohibitin 2 isoform 2 [Homo sapiens] |

| Filename XCorr DeltCN Conf% ObsM+H+ CalcM+H+ SpR ZScore Ion% # Sequence  | | | | | | | | | | | | |
| --- | --- | --- | --- | --- | --- | --- | --- | --- | --- | --- | --- | --- |
|  | Mis12XLAPLSS\_011913\_02.07806.07806.3 | 3.3215 | 0.3757 | 100.0% | 1855.7043 | 1855.1038 | 1 | 6.776 | 39.1% | 2 | R.IGGVQQDTILAEGLHFR.I | 3 |
|  | Mis12XLAPLSS\_011913\_01.03820.03820.2 | 3.2775 | 0.4441 | 100.0% | 1215.4521 | 1216.3336 | 1 | 8.126 | 72.7% | 1 | K.IVQAEGEAEAAK.M | 2 |

---

|  |  |  |  |  |  |  |  |  |
| --- | --- | --- | --- | --- | --- | --- | --- | --- |
| U | *gi|4758138|ref|NP\_004* | 5 | 9 | 9.6% | 614 | 69148 | 8.9 | DEAD (Asp-Glu-Ala-Asp) box polypeptide 5 [Homo sapiens] |

| Filename XCorr DeltCN Conf% ObsM+H+ CalcM+H+ SpR ZScore Ion% # Sequence  | | | | | | | | | | | | |
| --- | --- | --- | --- | --- | --- | --- | --- | --- | --- | --- | --- | --- |
| \* | Mis12XLAPLSS\_011913\_01.03440.03440.3 | 2.7771 | 0.2802 | 99.4% | 1409.5743 | 1409.5448 | 10 | 5.157 | 40.0% | 1 | R.RTAQEVETYRR.S | 3 |
| \* | Mis12XLAPLSS\_011913\_02.06663.06663.2 | 2.5044 | 0.3278 | 99.8% | 1296.5122 | 1296.4198 | 1 | 5.413 | 70.0% | 1 | R.TTYLVLDEADR.M | 2 |
|  | Mis12XLAPLSS\_011913\_01.11007.11007.2 | 3.7277 | 0.4571 | 100.0% | 1337.3922 | 1337.5946 | 1 | 8.328 | 85.0% | 2 | R.MLDMGFEPQIR.K | 22 |
|  | Mis12XLAPLSS\_011913\_02.05638.05638.2 | 3.4016 | 0.3284 | 99.9% | 1227.4722 | 1227.4465 | 1 | 7.311 | 77.3% | 4 | K.APILIATDVASR.G | 2 |
| \* | Mis12XLAPLSS\_011913\_01.10497.10497.2 | 3.0001 | 0.241 | 99.6% | 1575.2122 | 1575.7612 | 1 | 5.927 | 57.7% | 1 | K.TGTAYTFFTPNNIK.Q | 2 |

Similarities:
gi|87196351|ref|NP\_00(1:4)  

---

|  |  |  |  |  |  |  |  |  |
| --- | --- | --- | --- | --- | --- | --- | --- | --- |
| U | *gi|4758256|ref|NP\_004* | 2 | 2 | 9.5% | 315 | 36112 | 5.1 | eukaryotic translation initiation factor 2, subunit 1 alpha, 35kDa [Homo sapiens] |

| Filename XCorr DeltCN Conf% ObsM+H+ CalcM+H+ SpR ZScore Ion% # Sequence  | | | | | | | | | | | | |
| --- | --- | --- | --- | --- | --- | --- | --- | --- | --- | --- | --- | --- |
| \* | Mis12XLAPLSS\_011913\_01.11344.11344.3 | 3.4495 | 0.2583 | 99.4% | 2435.9644 | 2435.6958 | 1 | 5.53 | 32.9% | 1 | R.HVAEVLEYTKDEQLESLFQR.T | 3 |
| \* | Mis12XLAPLSS\_011913\_01.07258.07258.2 | 1.9303 | 0.2617 | 95.8% | 1215.3922 | 1215.407 | 1 | 4.717 | 77.8% | 1 | R.YVMTTTTLER.T | 2 |

---

|  |  |  |  |  |  |  |  |  |
| --- | --- | --- | --- | --- | --- | --- | --- | --- |
| U | *gi|4758158|ref|NP\_004* | 2 | 2 | 9.4% | 361 | 41487 | 6.6 | septin 2 [Homo sapiens] |
| U | *gi|56549640|ref|NP\_00* | 2 | 2 | 9.4% | 361 | 41487 | 6.6 | septin 2 [Homo sapiens] |
| U | *gi|56549638|ref|NP\_00* | 2 | 2 | 9.4% | 361 | 41487 | 6.6 | septin 2 [Homo sapiens] |
| U | *gi|56549636|ref|NP\_00* | 2 | 2 | 9.4% | 361 | 41487 | 6.6 | septin 2 [Homo sapiens] |

| Filename XCorr DeltCN Conf% ObsM+H+ CalcM+H+ SpR ZScore Ion% # Sequence  | | | | | | | | | | | | |
| --- | --- | --- | --- | --- | --- | --- | --- | --- | --- | --- | --- | --- |
|  | Mis12XLAPLSS\_011913\_01.07335.07335.2 | 2.5059 | 0.2475 | 99.4% | 1351.6322 | 1353.515 | 134 | 4.812 | 60.0% | 1 | R.ILDEIEEHNIK.I | 2 |
|  | Mis12XLAPLSS\_011913\_02.05826.05826.3 | 4.1568 | 0.2359 | 99.8% | 2385.1143 | 2385.6675 | 1 | 6.037 | 33.0% | 1 | R.MQAQMQMQMQGGDGDGGALGHHV.- | 3 |

---

|  |  |  |  |  |  |  |  |  |
| --- | --- | --- | --- | --- | --- | --- | --- | --- |
| U | *gi|4504511|ref|NP\_001* | 2 | 2 | 9.3% | 397 | 44868 | 7.1 | DnaJ (Hsp40) homolog, subfamily A, member 1 [Homo sapiens] |

| Filename XCorr DeltCN Conf% ObsM+H+ CalcM+H+ SpR ZScore Ion% # Sequence  | | | | | | | | | | | | |
| --- | --- | --- | --- | --- | --- | --- | --- | --- | --- | --- | --- | --- |
| \* | Mis12XLAPLSS\_011913\_01.17082.17082.2 | 3.8771 | 0.5446 | 100.0% | 2323.1921 | 2324.547 | 1 | 8.7 | 34.8% | 1 | K.EGGAGGGFGSPMDIFDMFFGGGGR.M | 2 |
| \* | Mis12XLAPLSS\_011913\_01.06246.06246.2 | 3.1818 | 0.417 | 99.9% | 1392.4521 | 1393.6696 | 1 | 6.7 | 62.5% | 1 | R.TIVITSHPGQIVK.H | 2 |

---

|  |  |  |  |  |  |  |  |  |
| --- | --- | --- | --- | --- | --- | --- | --- | --- |
| U | *gi|23110944|ref|NP\_00* | 2 | 3 | 9.3% | 246 | 27399 | 6.7 | proteasome alpha 6 subunit [Homo sapiens] |

| Filename XCorr DeltCN Conf% ObsM+H+ CalcM+H+ SpR ZScore Ion% # Sequence  | | | | | | | | | | | | |
| --- | --- | --- | --- | --- | --- | --- | --- | --- | --- | --- | --- | --- |
| \* | Mis12XLAPLSS\_011913\_01.07593.07593.2 | 2.2191 | 0.2626 | 98.6% | 1156.6122 | 1157.3146 | 1 | 5.486 | 88.9% | 1 | R.HITIFSPEGR.L | 2 |
|  | Mis12XLAPLSS\_011913\_01.07334.07334.2 | 3.6149 | 0.404 | 100.0% | 1286.1921 | 1286.474 | 1 | 7.491 | 79.2% | 2 | K.AINQGGLTSVAVR.G | 2 |

---

|  |  |  |  |  |  |  |  |  |
| --- | --- | --- | --- | --- | --- | --- | --- | --- |
| U | *gi|23110925|ref|NP\_00* | 2 | 2 | 9.2% | 239 | 25358 | 4.9 | proteasome beta 6 subunit [Homo sapiens] |

| Filename XCorr DeltCN Conf% ObsM+H+ CalcM+H+ SpR ZScore Ion% # Sequence  | | | | | | | | | | | | |
| --- | --- | --- | --- | --- | --- | --- | --- | --- | --- | --- | --- | --- |
| \* | Mis12XLAPLSS\_011913\_01.03852.03852.2 | 3.1926 | 0.3468 | 99.9% | 1295.0922 | 1295.4813 | 1 | 6.436 | 75.0% | 1 | R.VTDKLTPIHDR.I | 2 |
| \* | Mis12XLAPLSS\_011913\_01.05268.05268.2 | 3.624 | 0.3489 | 100.0% | 1116.2922 | 1116.2596 | 1 | 7.751 | 85.0% | 1 | R.LAAIAESGVER.Q | 2 |

---

|  |  |  |  |  |  |  |  |  |
| --- | --- | --- | --- | --- | --- | --- | --- | --- |
| U | *gi|169211155|ref|XP\_0* | 3 | 3 | 9.1% | 529 | 57862 | 5.4 | PREDICTED: similar to Karyopherin alpha 2 (RAG cohort 1, importin alpha 1) isoform 1 [Homo sapiens] |
| U | *gi|4504897|ref|NP\_002* | 3 | 3 | 9.1% | 529 | 57862 | 5.4 | karyopherin alpha 2 [Homo sapiens] |
| U | *gi|169211157|ref|XP\_0* | 3 | 3 | 9.1% | 529 | 57862 | 5.4 | PREDICTED: similar to Karyopherin alpha 2 (RAG cohort 1, importin alpha 1) isoform 2 [Homo sapiens] |

| Filename XCorr DeltCN Conf% ObsM+H+ CalcM+H+ SpR ZScore Ion% # Sequence  | | | | | | | | | | | | |
| --- | --- | --- | --- | --- | --- | --- | --- | --- | --- | --- | --- | --- |
|  | Mis12XLAPLSS\_011913\_02.07391.07391.2 | 3.8775 | 0.4276 | 100.0% | 1689.4321 | 1689.8223 | 1 | 7.206 | 67.9% | 1 | R.NNQGTVNWSVDDIVK.G | 2 |
|  | Mis12XLAPLSS\_011913\_01.07834.07834.2 | 3.845 | 0.4188 | 100.0% | 1900.5521 | 1902.0311 | 1 | 8.73 | 58.8% | 1 | K.GINSSNVENQLQATQAAR.K | 2 |
|  | Mis12XLAPLSS\_011913\_01.12222.12222.2 | 2.8394 | 0.2481 | 99.5% | 1550.2522 | 1550.8821 | 13 | 5.718 | 50.0% | 1 | K.LLGASELPIVTPALR.A | 2 |

---

|  |  |  |  |  |  |  |  |  |
| --- | --- | --- | --- | --- | --- | --- | --- | --- |
| U | *gi|50053795|ref|NP\_00* | 3 | 3 | 9.0% | 611 | 69151 | 5.7 | eukaryotic translation initiation factor 4B [Homo sapiens] |

| Filename XCorr DeltCN Conf% ObsM+H+ CalcM+H+ SpR ZScore Ion% # Sequence  | | | | | | | | | | | | |
| --- | --- | --- | --- | --- | --- | --- | --- | --- | --- | --- | --- | --- |
| \* | Mis12XLAPLSS\_011913\_01.15725.15725.3 | 3.5228 | 0.219 | 97.4% | 3104.5444 | 3104.357 | 1 | 4.928 | 25.9% | 1 | K.GFGYAEFEDLDSLLSALSLNEESLGNRR.I | 3 |
| \* | Mis12XLAPLSS\_011913\_01.03813.03813.3 | 2.797 | 0.2716 | 99.4% | 1316.3644 | 1316.3727 | 1 | 5.118 | 50.0% | 1 | R.RGDDSFGDKYR.D | 3 |
| \* | Mis12XLAPLSS\_011913\_01.07002.07002.3 | 2.7159 | 0.3429 | 99.8% | 1533.1144 | 1532.7397 | 2 | 6.055 | 33.3% | 1 | R.AASIFGGAKPVDTAAR.E | 3 |

---

|  |  |  |  |  |  |  |  |  |
| --- | --- | --- | --- | --- | --- | --- | --- | --- |
| U | *gi|56699409|ref|NP\_00* | 3 | 4 | 9.0% | 391 | 42332 | 10.1 | RNA binding motif protein, X-linked [Homo sapiens] |

| Filename XCorr DeltCN Conf% ObsM+H+ CalcM+H+ SpR ZScore Ion% # Sequence  | | | | | | | | | | | | |
| --- | --- | --- | --- | --- | --- | --- | --- | --- | --- | --- | --- | --- |
|  | Mis12XLAPLSS\_011913\_01.09387.09387.2 | 3.7494 | 0.3514 | 100.0% | 1436.1322 | 1436.6049 | 1 | 6.576 | 75.0% | 2 | K.LFIGGLNTETNEK.A | 2 |
|  | Mis12XLAPLSS\_011913\_01.11943.11943.2 | 2.9042 | 0.3047 | 99.8% | 1487.4922 | 1487.6519 | 1 | 7.006 | 61.5% | 1 | R.GFAFVTFESPADAK.D | 2 |
| \* | Mis12XLAPLSS\_011913\_01.03250.03250.2 | 2.0909 | 0.1982 | 96.9% | 873.9922 | 874.0335 | 1 | 4.52 | 85.7% | 1 | R.RGPPPPPR.S | 2 |

---

|  |  |  |  |  |  |  |  |  |
| --- | --- | --- | --- | --- | --- | --- | --- | --- |
| U | *gi|32454741|ref|NP\_00* | 2 | 4 | 8.9% | 418 | 46441 | 8.7 | serine (or cysteine) proteinase inhibitor, clade H, member 1 precursor [Homo sapiens] |

| Filename XCorr DeltCN Conf% ObsM+H+ CalcM+H+ SpR ZScore Ion% # Sequence  | | | | | | | | | | | | |
| --- | --- | --- | --- | --- | --- | --- | --- | --- | --- | --- | --- | --- |
| \* | Mis12XLAPLSS\_011913\_01.11484.11484.3 | 4.9857 | 0.4533 | 100.0% | 2406.1143 | 2406.7012 | 1 | 8.557 | 45.2% | 2 | K.AVLSAEQLRDEEVHAGLGELLR.S | 3 |
| \* | Mis12XLAPLSS\_011913\_01.11988.11988.2 | 3.3974 | 0.4032 | 99.9% | 1660.3722 | 1660.8235 | 1 | 7.716 | 53.6% | 2 | R.LYGPSSVSFADDFVR.S | 2 |

---

|  |  |  |  |  |  |  |  |  |
| --- | --- | --- | --- | --- | --- | --- | --- | --- |
| U | *gi|5453607|ref|NP\_006* | 4 | 5 | 8.8% | 543 | 59367 | 7.6 | chaperonin containing TCP1, subunit 7 isoform a [Homo sapiens] |

| Filename XCorr DeltCN Conf% ObsM+H+ CalcM+H+ SpR ZScore Ion% # Sequence  | | | | | | | | | | | | |
| --- | --- | --- | --- | --- | --- | --- | --- | --- | --- | --- | --- | --- |
| \* | Mis12XLAPLSS\_011913\_01.06970.06970.2 | 2.7893 | 0.2598 | 99.6% | 1204.2122 | 1204.366 | 5 | 5.211 | 63.6% | 2 | K.ATISNDGATILK.L | 2 |
|  | Mis12XLAPLSS\_011913\_01.11585.11585.2 | 3.8861 | 0.321 | 99.9% | 1567.4521 | 1566.7539 | 1 | 7.835 | 80.8% | 1 | K.LPIGDVATQYFADR.D | 2 |
|  | Mis12XLAPLSS\_011913\_01.08002.08002.2 | 2.4101 | 0.2011 | 97.7% | 1384.3722 | 1384.4602 | 4 | 4.109 | 59.1% | 1 | R.GGAEQFMEETER.S | 2 |
|  | Mis12XLAPLSS\_011913\_01.08704.08704.2 | 2.7135 | 0.4239 | 99.9% | 1154.4722 | 1155.4009 | 12 | 6.885 | 61.1% | 1 | R.SLHDAIMIVR.R | 2 |

---

|  |  |  |  |  |  |  |  |  |
| --- | --- | --- | --- | --- | --- | --- | --- | --- |
| U | *gi|16579885|ref|NP\_00* | 3 | 3 | 8.7% | 427 | 47697 | 11.1 | ribosomal protein L4 [Homo sapiens] |

| Filename XCorr DeltCN Conf% ObsM+H+ CalcM+H+ SpR ZScore Ion% # Sequence  | | | | | | | | | | | | |
| --- | --- | --- | --- | --- | --- | --- | --- | --- | --- | --- | --- | --- |
| \* | Mis12XLAPLSS\_011913\_01.09232.09232.3 | 3.9202 | 0.2694 | 99.7% | 1991.3944 | 1991.3468 | 2 | 5.491 | 42.2% | 1 | K.APIRPDIVNFVHTNLRK.N | 3 |
| \* | Mis12XLAPLSS\_011913\_01.04643.04643.2 | 2.3279 | 0.2357 | 99.1% | 1104.0122 | 1104.2689 | 75 | 5.002 | 56.2% | 1 | K.SNYNLPMHK.M | 2 |
| \* | Mis12XLAPLSS\_011913\_01.04032.04032.2 | 3.6349 | 0.311 | 99.9% | 957.15216 | 957.11786 | 1 | 7.97 | 90.0% | 1 | K.AAAAAAALQAK.S | 2 |

---

|  |  |  |  |  |  |  |  |  |
| --- | --- | --- | --- | --- | --- | --- | --- | --- |
| U | *gi|156627571|ref|NP\_0* | 2 | 3 | 8.7% | 357 | 38325 | 8.0 | sorbitol dehydrogenase [Homo sapiens] |

| Filename XCorr DeltCN Conf% ObsM+H+ CalcM+H+ SpR ZScore Ion% # Sequence  | | | | | | | | | | | | |
| --- | --- | --- | --- | --- | --- | --- | --- | --- | --- | --- | --- | --- |
| \* | Mis12XLAPLSS\_011913\_01.03917.03917.3 | 2.7363 | 0.2383 | 95.5% | 1583.6943 | 1583.8431 | 19 | 4.832 | 33.9% | 1 | K.KPMVLGHEASGTVEK.V | 3 |
| \* | Mis12XLAPLSS\_011913\_01.10923.10923.2 | 2.3433 | 0.2362 | 97.4% | 1590.1522 | 1590.8352 | 1 | 5.516 | 53.3% | 2 | K.AMGAAQVVVTDLSATR.L | 2 |

---

|  |  |  |  |  |  |  |  |  |
| --- | --- | --- | --- | --- | --- | --- | --- | --- |
| U | *gi|169161114|ref|XP\_0* | 2 | 2 | 8.7% | 184 | 21456 | 10.1 | PREDICTED: hypothetical protein isoform 1 [Homo sapiens] |
| U | *gi|78000186|ref|NP\_00* | 2 | 2 | 8.7% | 184 | 21397 | 10.2 | ribosomal protein L17 [Homo sapiens] |
| U | *gi|4506617|ref|NP\_000* | 2 | 2 | 8.7% | 184 | 21397 | 10.2 | ribosomal protein L17 [Homo sapiens] |
| U | *gi|169212979|ref|XP\_0* | 2 | 2 | 8.7% | 184 | 21397 | 10.2 | PREDICTED: hypothetical protein [Homo sapiens] |
| U | *gi|169161116|ref|XP\_0* | 2 | 2 | 8.7% | 184 | 21456 | 10.1 | PREDICTED: hypothetical protein isoform 2 [Homo sapiens] |

| Filename XCorr DeltCN Conf% ObsM+H+ CalcM+H+ SpR ZScore Ion% # Sequence  | | | | | | | | | | | | |
| --- | --- | --- | --- | --- | --- | --- | --- | --- | --- | --- | --- | --- |
|  | Mis12XLAPLSS\_011913\_02.07913.07913.2 | 3.2768 | 0.3354 | 99.8% | 1778.7122 | 1780.0311 | 1 | 6.534 | 46.7% | 1 | K.GLDVDSLVIEHIQVNK.A | 2 |
|  | Mis12XLAPLSS\_011913\_02.07926.07926.3 | 2.4659 | 0.2726 | 95.5% | 1779.8043 | 1780.0311 | 5 | 4.811 | 36.7% | 1 | K.GLDVDSLVIEHIQVNK.A | 3 |

---

|  |  |  |  |  |  |  |  |  |
| --- | --- | --- | --- | --- | --- | --- | --- | --- |
| U | *gi|41872631|ref|NP\_00* | 14 | 21 | 8.6% | 2511 | 273424 | 6.4 | fatty acid synthase [Homo sapiens] |

| Filename XCorr DeltCN Conf% ObsM+H+ CalcM+H+ SpR ZScore Ion% # Sequence  | | | | | | | | | | | | |
| --- | --- | --- | --- | --- | --- | --- | --- | --- | --- | --- | --- | --- |
| \* | Mis12XLAPLSS\_011913\_01.16134.16134.3 | 4.3085 | 0.3454 | 100.0% | 3164.4844 | 3165.415 | 1 | 6.514 | 26.0% | 1 | K.LPESENLQEFWDNLIGGVDMVTDDDRR.W | 3 |
| \* | Mis12XLAPLSS\_011913\_01.09929.09929.2 | 2.9917 | 0.4322 | 99.8% | 1253.4122 | 1252.4148 | 1 | 7.863 | 65.0% | 1 | R.FDASFFGVHPK.Q | 2 |
| \* | Mis12XLAPLSS\_011913\_01.06497.06497.2 | 3.3082 | 0.3384 | 99.9% | 1299.5521 | 1299.4264 | 1 | 6.727 | 77.3% | 5 | K.VGDPQELNGITR.A | 2 |
| \* | Mis12XLAPLSS\_011913\_01.08861.08861.2 | 2.3949 | 0.2562 | 99.1% | 1263.5721 | 1264.5106 | 308 | 5.407 | 50.0% | 1 | R.LQVVDQPLPVR.G | 2 |
| \* | Mis12XLAPLSS\_011913\_01.15390.15390.2 | 2.8628 | 0.0949 | 95.4% | 2720.3123 | 2718.161 | 2 | 3.227 | 41.7% | 1 | R.HSQDLAFLSMLNDIAAVPATAMPFR.G | 2 |
| \* | Mis12XLAPLSS\_011913\_02.05291.05291.3 | 3.1269 | 0.2408 | 98.8% | 1537.7644 | 1537.7617 | 1 | 5.492 | 44.2% | 1 | K.VVEVLAGHGHLYSR.I | 3 |
| \* | Mis12XLAPLSS\_011913\_01.12260.12260.3 | 3.1999 | 0.2662 | 98.8% | 2193.1143 | 2193.551 | 4 | 4.452 | 32.9% | 1 | R.IPGLLSPHPLLQLSYTATDR.H | 3 |
| \* | Mis12XLAPLSS\_011913\_01.11408.11408.2 | 3.4711 | 0.2112 | 99.8% | 1623.7522 | 1623.9304 | 2 | 6.066 | 60.7% | 2 | K.VVVQVLAEEPEAVLK.G | 2 |
| \* | Mis12XLAPLSS\_011913\_01.07673.07673.2 | 4.4798 | 0.5154 | 100.0% | 1774.2522 | 1774.862 | 1 | 7.911 | 56.2% | 1 | R.GNAGQSNYGFANSAMER.I | 2 |
| \* | Mis12XLAPLSS\_011913\_01.16242.16242.2 | 3.4355 | 0.3488 | 99.9% | 1406.3121 | 1406.6682 | 1 | 7.152 | 66.7% | 1 | R.DLVEAVAHILGIR.D | 2 |
| \* | Mis12XLAPLSS\_011913\_01.07872.07872.2 | 3.7736 | 0.3719 | 100.0% | 1615.6122 | 1614.7556 | 1 | 6.009 | 61.5% | 1 | K.EDGLAQQQTQLNLR.S | 2 |
| \* | Mis12XLAPLSS\_011913\_01.10227.10227.2 | 3.5717 | 0.4091 | 100.0% | 1427.3121 | 1427.702 | 26 | 6.406 | 54.2% | 1 | R.SLLVNPEGPTLMR.L | 2 |
| \* | Mis12XLAPLSS\_011913\_01.15648.15648.2 | 4.8532 | 0.3489 | 100.0% | 2423.672 | 2423.769 | 1 | 8.865 | 36.4% | 2 | R.TLLEGSGLESIISIIHSSLAEPR.V | 2 |
| \* | Mis12XLAPLSS\_011913\_01.15692.15692.3 | 4.5583 | 0.4918 | 100.0% | 2424.3843 | 2423.769 | 2 | 7.7 | 30.7% | 2 | R.TLLEGSGLESIISIIHSSLAEPR.V | 3 |

---

|  |  |  |  |  |  |  |  |  |
| --- | --- | --- | --- | --- | --- | --- | --- | --- |
| U | *gi|4503513|ref|NP\_003* | 2 | 2 | 8.6% | 325 | 36502 | 5.6 | eukaryotic translation initiation factor 3, subunit 2 beta, 36kDa [Homo sapiens] |

| Filename XCorr DeltCN Conf% ObsM+H+ CalcM+H+ SpR ZScore Ion% # Sequence  | | | | | | | | | | | | |
| --- | --- | --- | --- | --- | --- | --- | --- | --- | --- | --- | --- | --- |
| \* | Mis12XLAPLSS\_011913\_01.08229.08229.3 | 3.4181 | 0.4295 | 100.0% | 1680.6244 | 1680.8632 | 1 | 7.653 | 38.3% | 1 | K.GHFGPINSVAFHPDGK.S | 3 |
| \* | Mis12XLAPLSS\_011913\_01.04353.04353.2 | 2.0917 | 0.2779 | 97.6% | 1276.2922 | 1277.2897 | 24 | 5.275 | 54.5% | 1 | K.SYSSGGEDGYVR.I | 2 |

---

|  |  |  |  |  |  |  |  |  |
| --- | --- | --- | --- | --- | --- | --- | --- | --- |
| U | *gi|4503659|ref|NP\_001* | 2 | 2 | 8.3% | 133 | 14390 | 10.2 | ubiquitin-like protein fubi and ribosomal protein S30 precursor [Homo sapiens] |

| Filename XCorr DeltCN Conf% ObsM+H+ CalcM+H+ SpR ZScore Ion% # Sequence  | | | | | | | | | | | | |
| --- | --- | --- | --- | --- | --- | --- | --- | --- | --- | --- | --- | --- |
| \* | Mis12XLAPLSS\_011913\_01.10047.10047.2 | 2.8567 | 0.3765 | 99.9% | 1108.2322 | 1108.3256 | 5 | 6.337 | 72.2% | 1 | R.FVNVVPTFGK.K | 2 |
| \* | Mis12XLAPLSS\_011913\_01.08907.08907.2 | 2.6135 | 0.1713 | 98.4% | 1235.7122 | 1236.4998 | 99 | 5.483 | 55.0% | 1 | R.FVNVVPTFGKK.K | 2 |

---

|  |  |  |  |  |  |  |  |  |
| --- | --- | --- | --- | --- | --- | --- | --- | --- |
| U | *gi|4885063|ref|NP\_005* | 2 | 2 | 8.2% | 364 | 39456 | 6.9 | fructose-bisphosphate aldolase C [Homo sapiens] |

| Filename XCorr DeltCN Conf% ObsM+H+ CalcM+H+ SpR ZScore Ion% # Sequence  | | | | | | | | | | | | |
| --- | --- | --- | --- | --- | --- | --- | --- | --- | --- | --- | --- | --- |
| \* | Mis12XLAPLSS\_011913\_01.09714.09714.2 | 6.22 | 0.0446 | 99.9% | 2273.5923 | 2274.4465 | 1 | 10.043 | 61.4% | 1 | K.GVVPLAGTDGETTTQGLDGLSER.C | 2 |
|  | Mis12XLAPLSS\_011913\_01.04799.04799.1 | 1.7363 | 0.2297 | 95.1% | 763.53 | 763.95544 | 2 | 5.268 | 66.7% | 1 | K.VLAAVYK.A | 11 |

Similarities:
gi|193794814|ref|NP\_0(1:1)  

---

|  |  |  |  |  |  |  |  |  |
| --- | --- | --- | --- | --- | --- | --- | --- | --- |
| U | *gi|4505773|ref|NP\_002* | 2 | 2 | 8.1% | 272 | 29804 | 5.8 | prohibitin [Homo sapiens] |

| Filename XCorr DeltCN Conf% ObsM+H+ CalcM+H+ SpR ZScore Ion% # Sequence  | | | | | | | | | | | | |
| --- | --- | --- | --- | --- | --- | --- | --- | --- | --- | --- | --- | --- |
| \* | Mis12XLAPLSS\_011913\_02.06387.06387.2 | 2.9649 | 0.3136 | 99.8% | 1446.1522 | 1445.5255 | 2 | 5.976 | 68.2% | 1 | R.IFTSIGEDYDER.V | 2 |
| \* | Mis12XLAPLSS\_011913\_01.09351.09351.2 | 2.9508 | 0.3899 | 99.9% | 1150.2922 | 1150.2767 | 3 | 6.668 | 72.2% | 1 | R.FDAGELITQR.E | 2 |

---

|  |  |  |  |  |  |  |  |  |
| --- | --- | --- | --- | --- | --- | --- | --- | --- |
| U | *gi|169202568|ref|XP\_9* | 2 | 2 | 8.0% | 349 | 39567 | 8.1 | PREDICTED: similar to high mobility group 1 protein [Homo sapiens] |
| U | *gi|4504425|ref|NP\_002* | 2 | 2 | 13.0% | 215 | 24894 | 5.7 | high-mobility group box 1 [Homo sapiens] |
| U | *gi|169215956|ref|XP\_0* | 2 | 2 | 13.3% | 211 | 24188 | 7.4 | PREDICTED: similar to high-mobility group (nonhistone chromosomal) protein 1-like 10 isoform 1 [Homo sapiens] |
| U | *gi|169215954|ref|XP\_0* | 2 | 2 | 13.3% | 211 | 24188 | 7.4 | PREDICTED: similar to high-mobility group (nonhistone chromosomal) protein 1-like 10 isoform 2 [Homo sapiens] |
| U | *gi|169215763|ref|XP\_0* | 2 | 2 | 13.3% | 211 | 24188 | 7.4 | PREDICTED: similar to high-mobility group (nonhistone chromosomal) protein 1-like 10 isoform 1 [Homo sapiens] |
| U | *gi|169215761|ref|XP\_0* | 2 | 2 | 13.3% | 211 | 24188 | 7.4 | PREDICTED: similar to high-mobility group (nonhistone chromosomal) protein 1-like 10 isoform 2 [Homo sapiens] |
| U | *gi|169215551|ref|XP\_0* | 2 | 2 | 13.3% | 211 | 24218 | 7.4 | PREDICTED: similar to high-mobility group (nonhistone chromosomal) protein 1-like 10 [Homo sapiens] |
| U | *gi|169203391|ref|XP\_0* | 2 | 2 | 8.0% | 350 | 39696 | 7.9 | PREDICTED: similar to high mobility group 1 protein [Homo sapiens] |
| U | *gi|169202818|ref|XP\_0* | 2 | 2 | 5.7% | 488 | 55106 | 6.7 | PREDICTED: similar to high mobility group 1 protein [Homo sapiens] |

| Filename XCorr DeltCN Conf% ObsM+H+ CalcM+H+ SpR ZScore Ion% # Sequence  | | | | | | | | | | | | |
| --- | --- | --- | --- | --- | --- | --- | --- | --- | --- | --- | --- | --- |
|  | Mis12XLAPLSS\_011913\_01.07036.07036.3 | 3.9933 | 0.3551 | 100.0% | 2238.9243 | 2239.4644 | 11 | 5.841 | 33.3% | 1 | K.KLGEMWNNTAADDKQPYEK.R | 3 |
|  | Mis12XLAPLSS\_011913\_01.04331.04331.2 | 2.3762 | 0.1838 | 98.4% | 1129.4521 | 1129.2578 | 1 | 4.982 | 81.2% | 1 | K.YEKDIAAYR.A | 2 |

---

|  |  |  |  |  |  |  |  |  |
| --- | --- | --- | --- | --- | --- | --- | --- | --- |
| U | *gi|4758012|ref|NP\_004* | 8 | 9 | 7.9% | 1675 | 191613 | 5.7 | clathrin heavy chain 1 [Homo sapiens] |

| Filename XCorr DeltCN Conf% ObsM+H+ CalcM+H+ SpR ZScore Ion% # Sequence  | | | | | | | | | | | | |
| --- | --- | --- | --- | --- | --- | --- | --- | --- | --- | --- | --- | --- |
| \* | Mis12XLAPLSS\_011913\_02.07662.07662.3 | 3.9208 | 0.3889 | 100.0% | 2355.8943 | 2354.6682 | 2 | 6.99 | 27.3% | 1 | R.ISGETIFVTAPHEATAGIIGVNR.K | 3 |
| \* | Mis12XLAPLSS\_011913\_01.10282.10282.2 | 3.0866 | 0.4756 | 100.0% | 1305.1721 | 1305.4331 | 1 | 7.002 | 72.7% | 2 | R.NNLAGAEELFAR.K | 2 |
| \* | Mis12XLAPLSS\_011913\_02.06320.06320.3 | 3.3298 | 0.2262 | 98.3% | 1759.8844 | 1759.9591 | 1 | 4.588 | 43.3% | 1 | R.KFNALFAQGNYSEAAK.V | 3 |
|  | Mis12XLAPLSS\_011913\_01.13176.13176.2 | 2.1305 | 0.2715 | 97.3% | 1608.5721 | 1608.9237 | 3 | 4.197 | 45.8% | 1 | K.KVGYTPDWIFLLR.N | 2 |
| \* | Mis12XLAPLSS\_011913\_01.04829.04829.2 | 2.5006 | 0.2971 | 99.6% | 1334.2122 | 1335.416 | 67 | 6.289 | 60.0% | 1 | K.IYIDSNNNPER.F | 2 |
|  | Mis12XLAPLSS\_011913\_01.15846.15846.2 | 2.9045 | 0.2733 | 99.6% | 1948.2922 | 1948.2819 | 1 | 5.648 | 43.8% | 1 | K.AFMTADLPNELIELLEK.I | 2 |
| \* | Mis12XLAPLSS\_011913\_01.13436.13436.3 | 4.8242 | 0.3708 | 100.0% | 2371.7344 | 2369.6829 | 1 | 7.068 | 38.8% | 1 | R.KFDVNTSAVQVLIEHIGNLDR.A | 3 |
| \* | Mis12XLAPLSS\_011913\_02.07016.07016.3 | 2.8438 | 0.2627 | 97.5% | 1972.8844 | 1972.2083 | 1 | 5.079 | 33.3% | 1 | R.LASTLVHLGEYQAAVDGAR.K | 23 |

---

|  |  |  |  |  |  |  |  |  |
| --- | --- | --- | --- | --- | --- | --- | --- | --- |
| U | *gi|7657015|ref|NP\_055* | 3 | 3 | 7.9% | 505 | 55210 | 7.2 | hypothetical protein LOC51493 [Homo sapiens] |

| Filename XCorr DeltCN Conf% ObsM+H+ CalcM+H+ SpR ZScore Ion% # Sequence  | | | | | | | | | | | | |
| --- | --- | --- | --- | --- | --- | --- | --- | --- | --- | --- | --- | --- |
| \* | Mis12XLAPLSS\_011913\_01.10487.10487.2 | 2.5071 | 0.2183 | 98.9% | 1386.3322 | 1386.501 | 3 | 5.547 | 75.0% | 1 | R.SYNDELQFLEK.I | 2 |
| \* | Mis12XLAPLSS\_011913\_01.09605.09605.2 | 3.4219 | 0.404 | 99.9% | 1639.7322 | 1640.895 | 1 | 7.46 | 60.0% | 1 | R.GLGHQVATDALVAMEK.A | 2 |
| \* | Mis12XLAPLSS\_011913\_02.06291.06291.2 | 2.8118 | 0.3828 | 99.8% | 1381.0521 | 1381.5518 | 1 | 5.928 | 66.7% | 1 | K.GMAAAGNYAWVNR.S | 2 |

---

|  |  |  |  |  |  |  |  |  |
| --- | --- | --- | --- | --- | --- | --- | --- | --- |
| U | *gi|15055539|ref|NP\_00* | 3 | 4 | 7.8% | 293 | 31324 | 10.2 | ribosomal protein S2 [Homo sapiens] |
| U | *gi|169205506|ref|XP\_0* | 3 | 4 | 7.8% | 293 | 31364 | 10.2 | PREDICTED: hypothetical protein isoform 1 [Homo sapiens] |
| U | *gi|169204984|ref|XP\_0* | 3 | 4 | 7.8% | 293 | 31364 | 10.2 | PREDICTED: hypothetical protein isoform 1 [Homo sapiens] |
| U | *gi|169204454|ref|XP\_0* | 3 | 4 | 7.8% | 293 | 31438 | 10.2 | PREDICTED: hypothetical protein isoform 1 [Homo sapiens] |

| Filename XCorr DeltCN Conf% ObsM+H+ CalcM+H+ SpR ZScore Ion% # Sequence  | | | | | | | | | | | | |
| --- | --- | --- | --- | --- | --- | --- | --- | --- | --- | --- | --- | --- |
|  | Mis12XLAPLSS\_011913\_01.06042.06042.2 | 2.5292 | 0.1869 | 98.4% | 1333.9722 | 1334.5298 | 1 | 5.506 | 70.0% | 1 | K.AEDKEWMPVTK.L | 2 |
|  | Mis12XLAPLSS\_011913\_01.09440.09440.3 | 2.8731 | 0.3529 | 99.7% | 1463.7244 | 1464.6177 | 1 | 5.887 | 52.3% | 1 | K.SPYQEFTDHLVK.T | 3 |
|  | Mis12XLAPLSS\_011913\_01.09407.09407.2 | 3.3143 | 0.2849 | 99.8% | 1464.3121 | 1464.6177 | 1 | 5.894 | 77.3% | 2 | K.SPYQEFTDHLVK.T | 2 |

---

|  |  |  |  |  |  |  |  |  |
| --- | --- | --- | --- | --- | --- | --- | --- | --- |
| U | *gi|116063573|ref|NP\_0* | 13 | 15 | 7.7% | 2639 | 280016 | 6.0 | filamin A, alpha isoform 1 [Homo sapiens] |
| U | *gi|160420317|ref|NP\_0* | 13 | 15 | 7.6% | 2647 | 280737 | 6.1 | filamin A, alpha isoform 2 [Homo sapiens] |

| Filename XCorr DeltCN Conf% ObsM+H+ CalcM+H+ SpR ZScore Ion% # Sequence  | | | | | | | | | | | | |
| --- | --- | --- | --- | --- | --- | --- | --- | --- | --- | --- | --- | --- |
|  | Mis12XLAPLSS\_011913\_01.11195.11195.2 | 2.3684 | 0.3109 | 99.5% | 1286.2922 | 1286.5167 | 1 | 5.264 | 70.0% | 1 | K.LPQLPITNFSR.D | 2 |
|  | Mis12XLAPLSS\_011913\_01.07797.07797.2 | 3.2754 | 0.3874 | 99.9% | 1434.0322 | 1434.6501 | 2 | 6.666 | 57.7% | 1 | R.AYGPGIEPTGNMVK.K | 2 |
|  | Mis12XLAPLSS\_011913\_02.06442.06442.3 | 3.1353 | 0.2645 | 97.9% | 2314.2244 | 2314.4705 | 1 | 4.998 | 28.6% | 1 | R.SAGQGEVLVYVEDPAGHQEEAK.V | 3 |
|  | Mis12XLAPLSS\_011913\_02.06234.06234.2 | 2.8266 | 0.2942 | 99.8% | 1294.0322 | 1293.5059 | 1 | 4.717 | 68.2% | 2 | K.GKLDVQFSGLTK.G | 2 |
|  | Mis12XLAPLSS\_011913\_02.07660.07660.2 | 2.8651 | 0.359 | 99.8% | 1534.0922 | 1534.7496 | 1 | 6.347 | 50.0% | 1 | K.SPFSVAVSPSLDLSK.I | 2 |
|  | Mis12XLAPLSS\_011913\_01.05825.05825.2 | 2.5469 | 0.2855 | 99.3% | 1430.4321 | 1430.5632 | 1 | 6.15 | 56.7% | 1 | K.AFGPGLQGGSAGSPAR.F | 2 |
|  | Mis12XLAPLSS\_011913\_01.07761.07761.2 | 4.2235 | 0.4978 | 100.0% | 1764.1122 | 1764.8894 | 1 | 8.334 | 60.0% | 1 | R.VANPSGNLTETYVQDR.G | 2 |
|  | Mis12XLAPLSS\_011913\_01.03321.03321.3 | 3.0406 | 0.4605 | 100.0% | 1635.4744 | 1635.8203 | 78 | 6.778 | 28.3% | 1 | R.VHGPGIQSGTTNKPNK.F | 3 |
|  | Mis12XLAPLSS\_011913\_02.06028.06028.2 | 4.2385 | 0.5145 | 100.0% | 1572.6122 | 1571.7275 | 1 | 9.008 | 52.9% | 1 | R.GAGTGGLGLAVEGPSEAK.M | 2 |
|  | Mis12XLAPLSS\_011913\_01.06245.06245.3 | 3.0352 | 0.2683 | 99.4% | 1603.4644 | 1603.7343 | 152 | 5.682 | 36.5% | 2 | K.YNEQHVPGSPFTAR.V | 3 |
|  | Mis12XLAPLSS\_011913\_02.06142.06142.2 | 3.3084 | 0.258 | 99.8% | 1428.1522 | 1427.5974 | 1 | 5.846 | 63.3% | 1 | R.EAGAGGLAIAVEGPSK.A | 2 |
|  | Mis12XLAPLSS\_011913\_02.06638.06638.3 | 4.4556 | 0.4851 | 100.0% | 2201.0645 | 2201.4412 | 1 | 7.755 | 38.2% | 1 | R.LVSNHSLHETSSVFVDSLTK.A | 3 |
|  | Mis12XLAPLSS\_011913\_01.07848.07848.2 | 2.8499 | 0.161 | 98.9% | 1414.0122 | 1414.5199 | 2 | 4.437 | 68.2% | 1 | K.WGDEHIPGSPYR.V | 2 |

---

|  |  |  |  |  |  |  |  |  |
| --- | --- | --- | --- | --- | --- | --- | --- | --- |
| U | *gi|14165464|ref|NP\_11* | 3 | 3 | 7.6% | 550 | 59037 | 9.2 | polypyrimidine tract-binding protein 1 isoform b [Homo sapiens] |
| U | *gi|4506243|ref|NP\_002* | 3 | 3 | 7.5% | 557 | 59633 | 9.2 | polypyrimidine tract-binding protein 1 isoform a [Homo sapiens] |
| U | *gi|14165466|ref|NP\_11* | 3 | 3 | 7.9% | 531 | 57221 | 9.2 | polypyrimidine tract-binding protein 1 isoform c [Homo sapiens] |

| Filename XCorr DeltCN Conf% ObsM+H+ CalcM+H+ SpR ZScore Ion% # Sequence  | | | | | | | | | | | | |
| --- | --- | --- | --- | --- | --- | --- | --- | --- | --- | --- | --- | --- |
|  | Mis12XLAPLSS\_011913\_01.13142.13142.2 | 4.8617 | 0.4276 | 100.0% | 2276.612 | 2276.6414 | 1 | 8.063 | 52.3% | 1 | R.IAIPGLAGAGNSVLLVSNLNPER.V | 2 |
|  | Mis12XLAPLSS\_011913\_01.03884.03884.2 | 2.7972 | 0.2776 | 99.8% | 992.0122 | 992.12573 | 2 | 6.572 | 64.3% | 1 | K.HQNVQLPR.E | 2 |
|  | Mis12XLAPLSS\_011913\_01.07768.07768.2 | 2.6795 | 0.3648 | 99.8% | 1107.0922 | 1107.295 | 1 | 6.367 | 65.0% | 1 | K.VLFSSNGGVVK.G | 2 |

---

|  |  |  |  |  |  |  |  |  |
| --- | --- | --- | --- | --- | --- | --- | --- | --- |
| U | *contaminant\_KERATIN19* | 2 | 2 | 7.5% | 468 | 51203 | 5.5 | no description |
| U | *gi|67782365|ref|NP\_00* | 2 | 2 | 7.5% | 469 | 51386 | 5.5 | keratin 7 [Homo sapiens] |

| Filename XCorr DeltCN Conf% ObsM+H+ CalcM+H+ SpR ZScore Ion% # Sequence  | | | | | | | | | | | | |
| --- | --- | --- | --- | --- | --- | --- | --- | --- | --- | --- | --- | --- |
|  | Mis12XLAPLSS\_011913\_01.08043.08043.3 | 3.4364 | 0.2727 | 99.4% | 2246.9944 | 2247.519 | 13 | 4.901 | 27.3% | 1 | R.LSSARPGGLGSSSLYGLGASRPR.V | 3 |
|  | Mis12XLAPLSS\_011913\_01.05520.05520.2 | 2.202 | 0.2496 | 97.6% | 1104.9722 | 1105.2388 | 7 | 4.934 | 63.6% | 1 | R.SAYGGPVGAGIR.E | 2 |

---

|  |  |  |  |  |  |  |  |  |
| --- | --- | --- | --- | --- | --- | --- | --- | --- |
| U | *gi|23397429|ref|NP\_00* | 2 | 4 | 7.5% | 374 | 42503 | 5.6 | eukaryotic translation initiation factor 3, subunit M [Homo sapiens] |

| Filename XCorr DeltCN Conf% ObsM+H+ CalcM+H+ SpR ZScore Ion% # Sequence  | | | | | | | | | | | | |
| --- | --- | --- | --- | --- | --- | --- | --- | --- | --- | --- | --- | --- |
| \* | Mis12XLAPLSS\_011913\_01.17894.17894.2 | 5.0873 | 0.4917 | 100.0% | 2045.8522 | 2046.4143 | 1 | 9.734 | 52.9% | 3 | K.FLEGELIHDLLTIFVSAK.L | 2 |
| \* | Mis12XLAPLSS\_011913\_01.03120.03120.2 | 3.3844 | 0.3644 | 100.0% | 1149.6122 | 1150.326 | 1 | 6.971 | 94.4% | 1 | R.KVVVSHSTHR.T | 2 |

---

|  |  |  |  |  |  |  |  |  |
| --- | --- | --- | --- | --- | --- | --- | --- | --- |
| U | *gi|55956899|ref|NP\_00* | 3 | 3 | 7.4% | 623 | 62064 | 5.2 | keratin 9 [Homo sapiens] |

| Filename XCorr DeltCN Conf% ObsM+H+ CalcM+H+ SpR ZScore Ion% # Sequence  | | | | | | | | | | | | |
| --- | --- | --- | --- | --- | --- | --- | --- | --- | --- | --- | --- | --- |
| \* | Mis12XLAPLSS\_011913\_01.04011.04011.2 | 3.8501 | 0.3949 | 100.0% | 1233.1322 | 1233.2833 | 1 | 7.172 | 66.7% | 1 | R.SGGGGGGGLGSGGSIR.S | 2 |
|  | Mis12XLAPLSS\_011913\_01.03693.03693.2 | 2.4727 | 0.18 | 97.1% | 1236.2122 | 1236.2401 | 18 | 3.92 | 50.0% | 1 | R.FSSSSGYGGGSSR.V | 2 |
|  | Mis12XLAPLSS\_011913\_02.07485.07485.3 | 4.6487 | 0.2963 | 100.0% | 1966.2244 | 1967.2297 | 1 | 6.067 | 42.2% | 1 | R.HGVQELEIELQSQLSKK.A | 3 |

---

|  |  |  |  |  |  |  |  |  |
| --- | --- | --- | --- | --- | --- | --- | --- | --- |
| U | *gi|4503729|ref|NP\_002* | 3 | 5 | 7.4% | 459 | 51805 | 5.4 | FK506 binding protein 52 [Homo sapiens] |

| Filename XCorr DeltCN Conf% ObsM+H+ CalcM+H+ SpR ZScore Ion% # Sequence  | | | | | | | | | | | | |
| --- | --- | --- | --- | --- | --- | --- | --- | --- | --- | --- | --- | --- |
| \* | Mis12XLAPLSS\_011913\_01.08516.08516.3 | 4.3753 | 0.3332 | 100.0% | 1698.6843 | 1698.879 | 1 | 6.484 | 50.0% | 3 | R.RGEAHLAVNDFELAR.A | 3 |
| \* | Mis12XLAPLSS\_011913\_01.03394.03394.3 | 3.6331 | 0.2748 | 99.8% | 2090.0344 | 2091.1677 | 2 | 5.894 | 33.3% | 1 | K.AEASSGDHPTDTEMKEEQK.S | 3 |
| \* | Mis12XLAPLSS\_011913\_01.03392.03392.2 | 4.3114 | 0.4352 | 100.0% | 2090.672 | 2091.1677 | 1 | 7.824 | 52.8% | 1 | K.AEASSGDHPTDTEMKEEQK.S | 2 |

---

|  |  |  |  |  |  |  |  |  |
| --- | --- | --- | --- | --- | --- | --- | --- | --- |
| U | *gi|38201714|ref|NP\_00* | 2 | 2 | 7.4% | 326 | 36092 | 9.2 | ELAV-like 1 [Homo sapiens] |

| Filename XCorr DeltCN Conf% ObsM+H+ CalcM+H+ SpR ZScore Ion% # Sequence  | | | | | | | | | | | | |
| --- | --- | --- | --- | --- | --- | --- | --- | --- | --- | --- | --- | --- |
| \* | Mis12XLAPLSS\_011913\_01.10916.10916.2 | 3.2186 | 0.3424 | 99.8% | 1353.9922 | 1354.4998 | 1 | 6.674 | 70.8% | 1 | R.SLFSSIGEVESAK.L | 2 |
| \* | Mis12XLAPLSS\_011913\_01.05770.05770.2 | 2.3245 | 0.2571 | 98.7% | 1189.4922 | 1189.3542 | 8 | 5.956 | 60.0% | 1 | R.VLVDQTTGLSR.G | 2 |

---

|  |  |  |  |  |  |  |  |  |
| --- | --- | --- | --- | --- | --- | --- | --- | --- |
| U | *gi|15431306|ref|NP\_15* | 2 | 2 | 7.4% | 257 | 28025 | 11.0 | ribosomal protein L8 [Homo sapiens] |
| U | *gi|4506663|ref|NP\_000* | 2 | 2 | 7.4% | 257 | 28025 | 11.0 | ribosomal protein L8 [Homo sapiens] |

| Filename XCorr DeltCN Conf% ObsM+H+ CalcM+H+ SpR ZScore Ion% # Sequence  | | | | | | | | | | | | |
| --- | --- | --- | --- | --- | --- | --- | --- | --- | --- | --- | --- | --- |
|  | Mis12XLAPLSS\_011913\_02.03917.03917.2 | 2.4056 | 0.3329 | 99.7% | 942.0522 | 942.1062 | 1 | 7.169 | 85.0% | 1 | R.AVVGVVAGGGR.I | 2 |
|  | Mis12XLAPLSS\_011913\_01.04024.04024.2 | 2.1315 | 0.2024 | 97.4% | 828.0122 | 828.0457 | 1 | 5.088 | 78.6% | 1 | R.KVGLIAAR.R | 2 |

---

|  |  |  |  |  |  |  |  |  |
| --- | --- | --- | --- | --- | --- | --- | --- | --- |
| U | *gi|5031973|ref|NP\_005* | 2 | 3 | 7.3% | 440 | 48121 | 5.1 | protein disulfide isomerase-associated 6 [Homo sapiens] |

| Filename XCorr DeltCN Conf% ObsM+H+ CalcM+H+ SpR ZScore Ion% # Sequence  | | | | | | | | | | | | |
| --- | --- | --- | --- | --- | --- | --- | --- | --- | --- | --- | --- | --- |
| \* | Mis12XLAPLSS\_011913\_01.10912.10912.2 | 2.7281 | 0.2421 | 99.2% | 1529.2522 | 1528.7489 | 26 | 4.844 | 42.9% | 2 | K.LAAVDATVNQVLASR.Y | 2 |
| \* | Mis12XLAPLSS\_011913\_01.09524.09524.2 | 2.6641 | 0.2958 | 99.5% | 1618.3121 | 1616.8143 | 24 | 5.261 | 43.8% | 1 | R.GSTAPVGGGAFPTIVER.E | 2 |

---

|  |  |  |  |  |  |  |  |  |
| --- | --- | --- | --- | --- | --- | --- | --- | --- |
| U | *gi|19923193|ref|NP\_00* | 2 | 2 | 7.3% | 369 | 41332 | 5.3 | heat shock 70kD protein binding protein [Homo sapiens] |

| Filename XCorr DeltCN Conf% ObsM+H+ CalcM+H+ SpR ZScore Ion% # Sequence  | | | | | | | | | | | | |
| --- | --- | --- | --- | --- | --- | --- | --- | --- | --- | --- | --- | --- |
| \* | Mis12XLAPLSS\_011913\_01.09011.09011.2 | 3.5847 | 0.2655 | 99.8% | 1472.7122 | 1471.6506 | 1 | 5.416 | 76.9% | 1 | K.VAAIEALNDGELQK.A | 2 |
| \* | Mis12XLAPLSS\_011913\_01.07721.07721.2 | 2.8553 | 0.2738 | 99.9% | 1446.3121 | 1446.6 | 13 | 5.488 | 54.2% | 1 | R.AIEINPDSAQPYK.W | 2 |

---

|  |  |  |  |  |  |  |  |  |
| --- | --- | --- | --- | --- | --- | --- | --- | --- |
| U | *gi|207028494|ref|NP\_0* | 2 | 3 | 7.3% | 274 | 30205 | 7.1 | L-lactate dehydrogenase A isoform 2 [Homo sapiens] |
| U | *gi|5031857|ref|NP\_005* | 2 | 3 | 6.0% | 332 | 36689 | 8.3 | L-lactate dehydrogenase A isoform 1 [Homo sapiens] |

| Filename XCorr DeltCN Conf% ObsM+H+ CalcM+H+ SpR ZScore Ion% # Sequence  | | | | | | | | | | | | |
| --- | --- | --- | --- | --- | --- | --- | --- | --- | --- | --- | --- | --- |
|  | Mis12XLAPLSS\_011913\_02.03867.03867.2 | 2.799 | 0.3135 | 99.8% | 1134.9922 | 1135.2163 | 2 | 6.609 | 66.7% | 2 | K.VTLTSEEEAR.L | 2 |
|  | Mis12XLAPLSS\_011913\_01.09316.09316.2 | 2.6452 | 0.3273 | 99.8% | 1119.3522 | 1119.2627 | 1 | 5.681 | 72.2% | 1 | K.SADTLWGIQK.E | 2 |

---

|  |  |  |  |  |  |  |  |  |
| --- | --- | --- | --- | --- | --- | --- | --- | --- |
| U | *gi|21626466|ref|NP\_06* | 4 | 4 | 7.2% | 847 | 94623 | 6.3 | matrin 3 [Homo sapiens] |
| U | *gi|62750354|ref|NP\_95* | 4 | 4 | 7.2% | 847 | 94623 | 6.3 | matrin 3 [Homo sapiens] |

| Filename XCorr DeltCN Conf% ObsM+H+ CalcM+H+ SpR ZScore Ion% # Sequence  | | | | | | | | | | | | |
| --- | --- | --- | --- | --- | --- | --- | --- | --- | --- | --- | --- | --- |
|  | Mis12XLAPLSS\_011913\_01.08313.08313.2 | 2.3552 | 0.2901 | 99.5% | 1145.3522 | 1145.3647 | 3 | 5.408 | 62.5% | 1 | R.VVHIMDFQR.G | 2 |
|  | Mis12XLAPLSS\_011913\_01.14511.14511.3 | 3.854 | 0.3101 | 99.7% | 2440.0444 | 2439.9036 | 2 | 5.503 | 33.8% | 1 | R.YQLLQLVEPFGVISNHLILNK.I | 3 |
|  | Mis12XLAPLSS\_011913\_01.08577.08577.3 | 3.3727 | 0.3363 | 99.7% | 2038.1344 | 2038.3109 | 18 | 5.563 | 30.6% | 1 | R.VIHLSNLPHSGYSDSAVLK.L | 3 |
|  | Mis12XLAPLSS\_011913\_02.04329.04329.2 | 2.2523 | 0.2476 | 98.0% | 1345.1122 | 1346.3317 | 1 | 4.59 | 63.6% | 1 | K.K@DGS\*AS\*AAAK@KK.L | 2 |

---

|  |  |  |  |  |  |  |  |  |
| --- | --- | --- | --- | --- | --- | --- | --- | --- |
| U | *contaminant\_KERATIN22* | 4 | 6 | 7.1% | 645 | 65865 | 8.0 | no description |
| U | *gi|47132620|ref|NP\_00* | 4 | 6 | 7.2% | 639 | 65433 | 8.0 | keratin 2 [Homo sapiens] |

| Filename XCorr DeltCN Conf% ObsM+H+ CalcM+H+ SpR ZScore Ion% # Sequence  | | | | | | | | | | | | |
| --- | --- | --- | --- | --- | --- | --- | --- | --- | --- | --- | --- | --- |
|  | Mis12XLAPLSS\_011913\_01.04488.04488.2 | 3.8478 | 0.6195 | 100.0% | 1321.7122 | 1321.3542 | 1 | 11.123 | 60.0% | 1 | R.HGGGGGGFGGGGFGSR.S | 2 |
|  | Mis12XLAPLSS\_011913\_01.07400.07400.2 | 4.339 | 0.0625 | 99.8% | 1476.1522 | 1476.6726 | 1 | 6.654 | 90.9% | 3 | R.FLEQQNQVLQTK.W | 22 |
|  | Mis12XLAPLSS\_011913\_02.05855.05855.2 | 2.4652 | 0.3961 | 99.8% | 1193.7322 | 1194.33 | 2 | 7.215 | 66.7% | 1 | K.YEELQVTVGR.H | 2 |
|  | Mis12XLAPLSS\_011913\_01.07412.07412.2 | 2.2297 | 0.157 | 97.1% | 974.0122 | 974.102 | 39 | 3.753 | 71.4% | 1 | K.IEISELNR.V | 22 |

Similarities:
gi|119395750|ref|NP\_0(2:2)  

---

|  |  |  |  |  |  |  |  |  |
| --- | --- | --- | --- | --- | --- | --- | --- | --- |
| U | *gi|7661920|ref|NP\_055* | 2 | 2 | 7.1% | 411 | 46871 | 6.7 | eukaryotic translation initiation factor 4A, isoform 3 [Homo sapiens] |

| Filename XCorr DeltCN Conf% ObsM+H+ CalcM+H+ SpR ZScore Ion% # Sequence  | | | | | | | | | | | | |
| --- | --- | --- | --- | --- | --- | --- | --- | --- | --- | --- | --- | --- |
|  | Mis12XLAPLSS\_011913\_01.08770.08770.2 | 4.8327 | 0.5288 | 100.0% | 1828.3322 | 1829.0654 | 1 | 8.936 | 76.7% | 1 | R.GIYAYGFEKPSAIQQR.A | 22 |
| \* | Mis12XLAPLSS\_011913\_01.12263.12263.2 | 3.7235 | 0.4648 | 100.0% | 1521.5322 | 1521.784 | 1 | 8.293 | 87.5% | 1 | K.MLVLDEADEMLNK.G | 2 |

Similarities:
gi|4503529|ref|NP\_001(1:1)  

---

|  |  |  |  |  |  |  |  |  |
| --- | --- | --- | --- | --- | --- | --- | --- | --- |
| U | *gi|4557237|ref|NP\_000* | 2 | 2 | 7.0% | 427 | 45200 | 8.8 | acetyl-Coenzyme A acetyltransferase 1 precursor [Homo sapiens] |

| Filename XCorr DeltCN Conf% ObsM+H+ CalcM+H+ SpR ZScore Ion% # Sequence  | | | | | | | | | | | | |
| --- | --- | --- | --- | --- | --- | --- | --- | --- | --- | --- | --- | --- |
| \* | Mis12XLAPLSS\_011913\_01.14406.14406.2 | 2.3209 | 0.3152 | 99.0% | 1701.8522 | 1703.0319 | 15 | 5.586 | 34.4% | 1 | R.TPIGSFLGSLSLLPATK.L | 2 |
| \* | Mis12XLAPLSS\_011913\_01.07408.07408.2 | 3.3331 | 0.3685 | 99.9% | 1545.4321 | 1545.6055 | 43 | 5.951 | 54.2% | 1 | R.NEQDAYAINSYTR.S | 2 |

---

|  |  |  |  |  |  |  |  |  |
| --- | --- | --- | --- | --- | --- | --- | --- | --- |
| U | *gi|194097350|ref|NP\_0* | 6 | 7 | 6.9% | 914 | 105568 | 5.4 | actinin, alpha 1 isoform a [Homo sapiens] |
| U | *gi|4501891|ref|NP\_001* | 6 | 7 | 7.1% | 892 | 103058 | 5.4 | actinin, alpha 1 isoform b [Homo sapiens] |
| U | *gi|194097352|ref|NP\_0* | 6 | 7 | 7.1% | 887 | 102709 | 5.5 | actinin, alpha 1 isoform c [Homo sapiens] |

| Filename XCorr DeltCN Conf% ObsM+H+ CalcM+H+ SpR ZScore Ion% # Sequence  | | | | | | | | | | | | |
| --- | --- | --- | --- | --- | --- | --- | --- | --- | --- | --- | --- | --- |
|  | Mis12XLAPLSS\_011913\_01.08675.08675.2 | 2.733 | 0.4007 | 99.8% | 1294.1122 | 1294.537 | 1 | 7.054 | 68.2% | 1 | R.LAILGIHNEVSK.I | 2 |
|  | Mis12XLAPLSS\_011913\_02.07468.07468.3 | 3.2426 | 0.3087 | 99.7% | 1979.8143 | 1980.2463 | 1 | 5.211 | 40.6% | 1 | R.ISIEMHGTLEDQLSHLR.Q | 3 |
|  | Mis12XLAPLSS\_011913\_01.03888.03888.2 | 2.7807 | 0.464 | 99.8% | 1302.1122 | 1302.4503 | 4 | 7.292 | 72.2% | 1 | K.HTNYTMEHIR.V | 22 |
|  | Mis12XLAPLSS\_011913\_01.03874.03874.3 | 3.7737 | 0.4195 | 100.0% | 1302.9543 | 1302.4503 | 25 | 6.885 | 47.2% | 1 | K.HTNYTMEHIR.V | 33 |
|  | Mis12XLAPLSS\_011913\_01.13774.13774.2 | 4.1026 | 0.3868 | 100.0% | 1387.4321 | 1387.6218 | 1 | 8.228 | 72.7% | 2 | R.VGWEQLLTTIAR.T | 22 |
|  | Mis12XLAPLSS\_011913\_01.09320.09320.2 | 3.0688 | 0.2526 | 99.8% | 1430.1122 | 1430.6011 | 9 | 5.42 | 63.6% | 1 | R.TINEVENQILTR.D | 22 |

Similarities:
gi|12025678|ref|NP\_00(4:2)  

---

|  |  |  |  |  |  |  |  |  |
| --- | --- | --- | --- | --- | --- | --- | --- | --- |
| U | *gi|194018550|ref|NP\_9* | 3 | 5 | 6.9% | 773 | 86331 | 6.7 | c-Maf-inducing protein isoform C-mip [Homo sapiens] |

| Filename XCorr DeltCN Conf% ObsM+H+ CalcM+H+ SpR ZScore Ion% # Sequence  | | | | | | | | | | | | |
| --- | --- | --- | --- | --- | --- | --- | --- | --- | --- | --- | --- | --- |
| \* | Mis12XLAPLSS\_011913\_01.07737.07737.3 | 3.2808 | 0.2663 | 99.4% | 2070.0842 | 2070.3042 | 52 | 4.712 | 30.3% | 1 | R.QIEETKPLLGGDVSAPEGTK.M | 3 |
|  | Mis12XLAPLSS\_011913\_01.12650.12650.2 | 2.8454 | 0.2639 | 99.5% | 1604.2122 | 1604.9482 | 1 | 6.363 | 69.2% | 1 | R.SMVVIEVFTPVVQR.I | 2 |
|  | Mis12XLAPLSS\_011913\_02.06598.06598.3 | 4.0857 | 0.2842 | 99.7% | 2065.3145 | 2064.303 | 1 | 6.765 | 38.9% | 3 | R.LLHPSPDLVSQEATLSEAR.L | 3 |

---

|  |  |  |  |  |  |  |  |  |
| --- | --- | --- | --- | --- | --- | --- | --- | --- |
| U | *gi|148277065|ref|NP\_0* | 2 | 2 | 6.9% | 551 | 60269 | 6.7 | thioredoxin reductase 1 isoform 1 [Homo sapiens] |
| U | *gi|33519430|ref|NP\_87* | 2 | 2 | 7.6% | 499 | 54604 | 6.5 | thioredoxin reductase 1 isoform 2 [Homo sapiens] |
| U | *gi|33519428|ref|NP\_87* | 2 | 2 | 7.6% | 499 | 54604 | 6.5 | thioredoxin reductase 1 isoform 2 [Homo sapiens] |
| U | *gi|33519426|ref|NP\_87* | 2 | 2 | 7.6% | 499 | 54604 | 6.5 | thioredoxin reductase 1 isoform 2 [Homo sapiens] |
| U | *gi|148277071|ref|NP\_0* | 2 | 2 | 5.9% | 649 | 70756 | 7.4 | thioredoxin reductase 1 isoform 3 [Homo sapiens] |

| Filename XCorr DeltCN Conf% ObsM+H+ CalcM+H+ SpR ZScore Ion% # Sequence  | | | | | | | | | | | | |
| --- | --- | --- | --- | --- | --- | --- | --- | --- | --- | --- | --- | --- |
|  | Mis12XLAPLSS\_011913\_01.12903.12903.2 | 2.9205 | 0.2482 | 99.5% | 1646.7522 | 1646.9855 | 9 | 5.881 | 46.4% | 1 | K.VMVLDFVTPTPLGTR.W | 2 |
|  | Mis12XLAPLSS\_011913\_01.14572.14572.3 | 4.3429 | 0.2938 | 99.7% | 2900.3943 | 2901.2065 | 2 | 5.305 | 28.4% | 1 | K.FGEENIEVYHSYFWPLEWTIPSR.D | 3 |

---

|  |  |  |  |  |  |  |  |  |
| --- | --- | --- | --- | --- | --- | --- | --- | --- |
| U | *gi|15011918|ref|NP\_00* | 2 | 2 | 6.9% | 350 | 39008 | 6.1 | ATPase, H+ transporting, lysosomal accessory protein 2 [Homo sapiens] |

| Filename XCorr DeltCN Conf% ObsM+H+ CalcM+H+ SpR ZScore Ion% # Sequence  | | | | | | | | | | | | |
| --- | --- | --- | --- | --- | --- | --- | --- | --- | --- | --- | --- | --- |
| \* | Mis12XLAPLSS\_011913\_01.18302.18302.3 | 3.9689 | 0.2907 | 99.7% | 2755.4644 | 2756.1284 | 1 | 6.069 | 32.6% | 1 | R.NNEVDLLFLSELQVLHDISSLLSR.H | 3 |
| \* | Mis12XLAPLSS\_011913\_01.18308.18308.2 | 4.2913 | 0.5261 | 100.0% | 2755.8323 | 2756.1284 | 1 | 8.061 | 45.7% | 1 | R.NNEVDLLFLSELQVLHDISSLLSR.H | 2 |

---

|  |  |  |  |  |  |  |  |  |
| --- | --- | --- | --- | --- | --- | --- | --- | --- |
| U | *gi|21536320|ref|NP\_65* | 3 | 3 | 6.7% | 756 | 84794 | 8.8 | heterogeneous nuclear ribonucleoprotein U-like 1 isoform d [Homo sapiens] |
| U | *gi|21536326|ref|NP\_00* | 3 | 3 | 6.0% | 856 | 95739 | 6.9 | heterogeneous nuclear ribonucleoprotein U-like 1 isoform a [Homo sapiens] |

| Filename XCorr DeltCN Conf% ObsM+H+ CalcM+H+ SpR ZScore Ion% # Sequence  | | | | | | | | | | | | |
| --- | --- | --- | --- | --- | --- | --- | --- | --- | --- | --- | --- | --- |
|  | Mis12XLAPLSS\_011913\_02.05806.05806.3 | 2.8121 | 0.2942 | 98.3% | 2477.1243 | 2477.606 | 48 | 4.534 | 27.6% | 1 | R.QNQFYDTQVIKQENESGYER.R | 3 |
|  | Mis12XLAPLSS\_011913\_01.05111.05111.3 | 3.5706 | 0.2215 | 99.4% | 2035.1643 | 2035.3435 | 6 | 4.925 | 38.3% | 1 | R.RPLEMEQQQAYRPEMK.T | 3 |
|  | Mis12XLAPLSS\_011913\_02.06393.06393.2 | 4.973 | 0.5748 | 100.0% | 1742.3121 | 1742.8857 | 1 | 9.504 | 82.1% | 1 | R.NYILDQTNVYGSAQR.R | 2 |

---

|  |  |  |  |  |  |  |  |  |
| --- | --- | --- | --- | --- | --- | --- | --- | --- |
| U | *gi|21361144|ref|NP\_00* | 2 | 2 | 6.6% | 439 | 49204 | 5.2 | proteasome 26S ATPase subunit 3 [Homo sapiens] |

| Filename XCorr DeltCN Conf% ObsM+H+ CalcM+H+ SpR ZScore Ion% # Sequence  | | | | | | | | | | | | |
| --- | --- | --- | --- | --- | --- | --- | --- | --- | --- | --- | --- | --- |
| \* | Mis12XLAPLSS\_011913\_01.06279.06279.2 | 2.4064 | 0.1027 | 95.3% | 1107.5521 | 1107.2671 | 77 | 4.562 | 62.5% | 1 | K.MSTEEIIQR.T | 2 |
| \* | Mis12XLAPLSS\_011913\_01.08645.08645.3 | 3.2704 | 0.2556 | 98.6% | 2255.3643 | 2254.4304 | 3 | 4.816 | 34.2% | 1 | K.AMEVDERPTEQYSDIGGLDK.Q | 3 |

---

|  |  |  |  |  |  |  |  |  |
| --- | --- | --- | --- | --- | --- | --- | --- | --- |
| U | *gi|66346679|ref|NP\_00* | 2 | 2 | 6.6% | 408 | 44965 | 8.6 | SERPINE1 mRNA binding protein 1 isoform 1 [Homo sapiens] |
| U | *gi|66346685|ref|NP\_05* | 2 | 2 | 7.0% | 387 | 42427 | 8.4 | SERPINE1 mRNA binding protein 1 isoform 4 [Homo sapiens] |
| U | *gi|66346683|ref|NP\_00* | 2 | 2 | 6.9% | 393 | 43135 | 8.4 | SERPINE1 mRNA binding protein 1 isoform 3 [Homo sapiens] |
| U | *gi|66346681|ref|NP\_00* | 2 | 2 | 6.7% | 402 | 44257 | 8.7 | SERPINE1 mRNA binding protein 1 isoform 2 [Homo sapiens] |

| Filename XCorr DeltCN Conf% ObsM+H+ CalcM+H+ SpR ZScore Ion% # Sequence  | | | | | | | | | | | | |
| --- | --- | --- | --- | --- | --- | --- | --- | --- | --- | --- | --- | --- |
|  | Mis12XLAPLSS\_011913\_01.03272.03272.2 | 5.41 | 0.4892 | 100.0% | 1462.9722 | 1461.5314 | 1 | 8.929 | 63.3% | 1 | K.SAAQAAAQTNSNAAGK.Q | 2 |
|  | Mis12XLAPLSS\_011913\_01.03346.03346.2 | 3.3015 | 0.4367 | 100.0% | 1255.2522 | 1256.361 | 1 | 6.886 | 75.0% | 1 | R.RPDQQLQGEGK.I | 2 |

---

|  |  |  |  |  |  |  |  |  |
| --- | --- | --- | --- | --- | --- | --- | --- | --- |
| U | *gi|19923142|ref|NP\_00* | 4 | 5 | 6.4% | 876 | 97170 | 4.8 | karyopherin beta 1 [Homo sapiens] |

| Filename XCorr DeltCN Conf% ObsM+H+ CalcM+H+ SpR ZScore Ion% # Sequence  | | | | | | | | | | | | |
| --- | --- | --- | --- | --- | --- | --- | --- | --- | --- | --- | --- | --- |
| \* | Mis12XLAPLSS\_011913\_01.06341.06341.2 | 2.9422 | 0.1978 | 99.3% | 1557.0122 | 1557.744 | 6 | 4.86 | 53.8% | 1 | K.TVSPDRLELEAAQK.F | 2 |
| \* | Mis12XLAPLSS\_011913\_01.13934.13934.2 | 4.152 | 0.5081 | 100.0% | 1658.7922 | 1659.9231 | 1 | 8.933 | 71.4% | 1 | R.AAVENLPTFLVELSR.V | 2 |
| \* | Mis12XLAPLSS\_011913\_01.04082.04082.2 | 3.042 | 0.4326 | 99.9% | 1226.0521 | 1226.378 | 1 | 7.148 | 81.8% | 2 | R.VLANPGNSQVAR.V | 2 |
| \* | Mis12XLAPLSS\_011913\_01.12586.12586.2 | 4.6152 | 0.4649 | 100.0% | 1606.4122 | 1606.8595 | 1 | 8.654 | 75.0% | 1 | K.LAATNALLNSLEFTK.A | 2 |

---

|  |  |  |  |  |  |  |  |  |
| --- | --- | --- | --- | --- | --- | --- | --- | --- |
| U | *gi|4502643|ref|NP\_001* | 2 | 2 | 6.4% | 531 | 58024 | 6.7 | chaperonin containing TCP1, subunit 6A isoform a [Homo sapiens] |

| Filename XCorr DeltCN Conf% ObsM+H+ CalcM+H+ SpR ZScore Ion% # Sequence  | | | | | | | | | | | | |
| --- | --- | --- | --- | --- | --- | --- | --- | --- | --- | --- | --- | --- |
|  | Mis12XLAPLSS\_011913\_02.05931.05931.2 | 3.0186 | 0.4181 | 99.9% | 1255.8722 | 1256.4478 | 1 | 6.781 | 70.8% | 1 | R.AQAALAVNISAAR.G | 2 |
| \* | Mis12XLAPLSS\_011913\_01.10286.10286.3 | 5.1327 | 0.4037 | 100.0% | 2316.4744 | 2316.6807 | 2 | 7.307 | 36.2% | 1 | K.DGNVLLHEMQIQHPTASLIAK.V | 3 |

---

|  |  |  |  |  |  |  |  |  |
| --- | --- | --- | --- | --- | --- | --- | --- | --- |
| U | *gi|4826734|ref|NP\_004* | 2 | 2 | 6.3% | 526 | 53426 | 9.4 | fusion (involved in t(12;16) in malignant liposarcoma) [Homo sapiens] |

| Filename XCorr DeltCN Conf% ObsM+H+ CalcM+H+ SpR ZScore Ion% # Sequence  | | | | | | | | | | | | |
| --- | --- | --- | --- | --- | --- | --- | --- | --- | --- | --- | --- | --- |
| \* | Mis12XLAPLSS\_011913\_02.05241.05241.3 | 3.6643 | 0.2543 | 99.8% | 1662.2644 | 1662.837 | 1 | 4.787 | 45.0% | 1 | K.LKGEATVSFDDPPSAK.A | 3 |
| \* | Mis12XLAPLSS\_011913\_01.11854.11854.2 | 4.1551 | 0.4157 | 100.0% | 1895.4122 | 1896.1094 | 1 | 7.276 | 65.6% | 1 | K.AAIDWFDGKEFSGNPIK.V | 2 |

---

|  |  |  |  |  |  |  |  |  |
| --- | --- | --- | --- | --- | --- | --- | --- | --- |
| U | *gi|124494254|ref|NP\_0* | 2 | 2 | 6.3% | 394 | 43787 | 6.6 | ErbB3-binding protein 1 [Homo sapiens] |

| Filename XCorr DeltCN Conf% ObsM+H+ CalcM+H+ SpR ZScore Ion% # Sequence  | | | | | | | | | | | | |
| --- | --- | --- | --- | --- | --- | --- | --- | --- | --- | --- | --- | --- |
| \* | Mis12XLAPLSS\_011913\_01.11416.11416.2 | 2.1865 | 0.294 | 98.4% | 1628.9521 | 1630.884 | 5 | 4.903 | 50.0% | 1 | K.HELLQPFNVLYEK.E | 2 |
| \* | Mis12XLAPLSS\_011913\_01.09612.09612.2 | 2.2182 | 0.3486 | 99.5% | 1366.9922 | 1367.5414 | 2 | 5.424 | 59.1% | 1 | R.ITSGPFEPDLYK.S | 2 |

---

|  |  |  |  |  |  |  |  |  |
| --- | --- | --- | --- | --- | --- | --- | --- | --- |
| U | *gi|4557469|ref|NP\_001* | 3 | 3 | 6.2% | 937 | 104553 | 5.4 | adaptor-related protein complex 2, beta 1 subunit isoform b [Homo sapiens] |
| U | *gi|71773106|ref|NP\_00* | 3 | 3 | 6.1% | 951 | 105692 | 5.3 | adaptor-related protein complex 2, beta 1 subunit isoform a [Homo sapiens] |

| Filename XCorr DeltCN Conf% ObsM+H+ CalcM+H+ SpR ZScore Ion% # Sequence  | | | | | | | | | | | | |
| --- | --- | --- | --- | --- | --- | --- | --- | --- | --- | --- | --- | --- |
|  | Mis12XLAPLSS\_011913\_01.16012.16012.3 | 3.5166 | 0.209 | 96.3% | 2993.6042 | 2994.3716 | 1 | 4.544 | 24.0% | 1 | R.LASQANIAQVLAELKEYATEVDVDFVR.K | 3 |
|  | Mis12XLAPLSS\_011913\_02.06796.06796.2 | 2.683 | 0.1238 | 96.5% | 1526.4722 | 1525.7168 | 3 | 4.975 | 54.2% | 1 | R.NVEGQDMLYQSLK.L | 2 |
|  | Mis12XLAPLSS\_011913\_01.14506.14506.2 | 3.2631 | 0.2425 | 99.8% | 2132.1921 | 2131.39 | 1 | 4.935 | 41.2% | 1 | R.APEVSQYIYQVYDSILKN.- | 2 |

---

|  |  |  |  |  |  |  |  |  |
| --- | --- | --- | --- | --- | --- | --- | --- | --- |
| U | *gi|35493811|ref|NP\_90* | 2 | 2 | 6.0% | 530 | 59380 | 10.1 | RNA binding motif protein 39 isoform a [Homo sapiens] |
| U | *gi|4757926|ref|NP\_004* | 2 | 2 | 6.1% | 524 | 58657 | 10.1 | RNA binding motif protein 39 isoform b [Homo sapiens] |

| Filename XCorr DeltCN Conf% ObsM+H+ CalcM+H+ SpR ZScore Ion% # Sequence  | | | | | | | | | | | | |
| --- | --- | --- | --- | --- | --- | --- | --- | --- | --- | --- | --- | --- |
|  | Mis12XLAPLSS\_011913\_01.11013.11013.2 | 2.5219 | 0.3192 | 99.5% | 1553.5521 | 1552.8546 | 4 | 5.0 | 53.6% | 1 | R.VLGVPIIVQASQAEK.N | 2 |
|  | Mis12XLAPLSS\_011913\_01.09867.09867.2 | 3.3714 | 0.2739 | 99.8% | 1830.1122 | 1830.8566 | 6 | 4.965 | 53.1% | 1 | R.TDASSASSFLDSDELER.T | 2 |

---

|  |  |  |  |  |  |  |  |  |
| --- | --- | --- | --- | --- | --- | --- | --- | --- |
| U | *gi|4885225|ref|NP\_005* | 2 | 2 | 5.9% | 656 | 68478 | 9.3 | Ewing sarcoma breakpoint region 1 isoform EWS [Homo sapiens] |

| Filename XCorr DeltCN Conf% ObsM+H+ CalcM+H+ SpR ZScore Ion% # Sequence  | | | | | | | | | | | | |
| --- | --- | --- | --- | --- | --- | --- | --- | --- | --- | --- | --- | --- |
| \* | Mis12XLAPLSS\_011913\_02.05465.05465.3 | 4.1198 | 0.4229 | 100.0% | 2481.0544 | 2481.572 | 1 | 7.183 | 28.3% | 1 | R.QDHPSSMGVYGQESGGFSGPGENR.S | 3 |
|  | Mis12XLAPLSS\_011913\_01.09513.09513.2 | 3.5622 | 0.4077 | 99.8% | 1684.9521 | 1685.8333 | 1 | 7.065 | 64.3% | 1 | K.AAVEWFDGKDFQGSK.L | 2 |

---

|  |  |  |  |  |  |  |  |  |
| --- | --- | --- | --- | --- | --- | --- | --- | --- |
| U | *gi|10864047|ref|NP\_06* | 2 | 2 | 5.6% | 864 | 94255 | 5.1 | epidermal growth factor receptor pathway substrate 15-like 1 [Homo sapiens] |

| Filename XCorr DeltCN Conf% ObsM+H+ CalcM+H+ SpR ZScore Ion% # Sequence  | | | | | | | | | | | | |
| --- | --- | --- | --- | --- | --- | --- | --- | --- | --- | --- | --- | --- |
| \* | Mis12XLAPLSS\_011913\_02.06119.06119.3 | 2.8678 | 0.2644 | 97.9% | 1858.9143 | 1859.003 | 9 | 4.566 | 33.3% | 1 | R.LQQEETQLEQSIQAGR.V | 3 |
| \* | Mis12XLAPLSS\_011913\_02.09194.09194.3 | 3.5449 | 0.2879 | 99.8% | 3359.0645 | 3360.616 | 1 | 5.679 | 22.6% | 1 | R.SLEQYDQVLDGAHGASLTDLANLSEGVSLAER.G | 3 |

---

|  |  |  |  |  |  |  |  |  |
| --- | --- | --- | --- | --- | --- | --- | --- | --- |
| U | *gi|5174613|ref|NP\_005* | 2 | 3 | 5.6% | 375 | 42823 | 4.7 | nucleosome assembly protein 1-like 4 [Homo sapiens] |

| Filename XCorr DeltCN Conf% ObsM+H+ CalcM+H+ SpR ZScore Ion% # Sequence  | | | | | | | | | | | | |
| --- | --- | --- | --- | --- | --- | --- | --- | --- | --- | --- | --- | --- |
|  | Mis12XLAPLSS\_011913\_01.07263.07263.2 | 2.6409 | 0.3706 | 99.8% | 1336.9521 | 1337.4314 | 1 | 6.325 | 77.8% | 2 | K.FYEEVHDLER.K | 22 |
| \* | Mis12XLAPLSS\_011913\_01.15599.15599.2 | 2.2523 | 0.2473 | 98.3% | 1413.2122 | 1413.6616 | 428 | 5.601 | 45.0% | 1 | K.GIPEFWFTIFR.N | 2 |

Similarities:
gi|21327708|ref|NP\_63(1:1)  

---

|  |  |  |  |  |  |  |  |  |
| --- | --- | --- | --- | --- | --- | --- | --- | --- |
| U | *gi|154355000|ref|NP\_0* | 3 | 5 | 5.3% | 711 | 73115 | 7.3 | KH-type splicing regulatory protein (FUSE binding protein 2) [Homo sapiens] |

| Filename XCorr DeltCN Conf% ObsM+H+ CalcM+H+ SpR ZScore Ion% # Sequence  | | | | | | | | | | | | |
| --- | --- | --- | --- | --- | --- | --- | --- | --- | --- | --- | --- | --- |
| \* | Mis12XLAPLSS\_011913\_01.06690.06690.2 | 3.1487 | 0.2926 | 99.8% | 1355.4922 | 1355.4906 | 3 | 5.593 | 62.5% | 3 | K.VQISPDSGGLPER.S | 2 |
| \* | Mis12XLAPLSS\_011913\_01.08820.08820.2 | 3.4211 | 0.4013 | 100.0% | 1080.2322 | 1080.2725 | 1 | 9.559 | 90.0% | 1 | R.IGGGIDVPVPR.H | 2 |
| \* | Mis12XLAPLSS\_011913\_02.06212.06212.2 | 3.8254 | 0.267 | 99.8% | 1534.1921 | 1534.7123 | 1 | 7.127 | 73.1% | 1 | K.AINQQTGAFVEISR.Q | 2 |

---

|  |  |  |  |  |  |  |  |  |
| --- | --- | --- | --- | --- | --- | --- | --- | --- |
| U | *gi|33239445|ref|NP\_00* | 2 | 2 | 5.2% | 814 | 92482 | 5.0 | eukaryotic translation initiation factor 3, subunit 9 eta, 116kDa [Homo sapiens] |
| U | *gi|83367072|ref|NP\_00* | 2 | 2 | 5.2% | 814 | 92482 | 5.0 | eukaryotic translation initiation factor 3, subunit 9 eta, 116kDa [Homo sapiens] |

| Filename XCorr DeltCN Conf% ObsM+H+ CalcM+H+ SpR ZScore Ion% # Sequence  | | | | | | | | | | | | |
| --- | --- | --- | --- | --- | --- | --- | --- | --- | --- | --- | --- | --- |
|  | Mis12XLAPLSS\_011913\_01.03389.03389.2 | 3.856 | 0.4422 | 100.0% | 1361.0521 | 1361.3678 | 1 | 9.476 | 76.9% | 1 | R.AQAVSEDAGGNEGR.A | 2 |
|  | Mis12XLAPLSS\_011913\_01.16172.16172.3 | 3.9448 | 0.3559 | 100.0% | 3230.7844 | 3232.7188 | 30 | 5.157 | 21.3% | 1 | R.YLVTFSPLMDTQDDPQAIIIWDILTGHK.K | 3 |

---

|  |  |  |  |  |  |  |  |  |
| --- | --- | --- | --- | --- | --- | --- | --- | --- |
| U | *gi|5454064|ref|NP\_006* | 2 | 2 | 5.1% | 669 | 69492 | 9.7 | RNA binding motif protein 14 [Homo sapiens] |

| Filename XCorr DeltCN Conf% ObsM+H+ CalcM+H+ SpR ZScore Ion% # Sequence  | | | | | | | | | | | | |
| --- | --- | --- | --- | --- | --- | --- | --- | --- | --- | --- | --- | --- |
| \* | Mis12XLAPLSS\_011913\_02.05392.05392.3 | 3.4558 | 0.4135 | 100.0% | 2465.8743 | 2466.6292 | 1 | 6.664 | 30.4% | 1 | R.TQSSASLAASYAAQQHPQAAASYR.G | 3 |
| \* | Mis12XLAPLSS\_011913\_01.07824.07824.2 | 2.2442 | 0.3477 | 99.6% | 1238.1322 | 1238.2988 | 15 | 5.91 | 61.1% | 1 | R.YSGSYNDYLR.A | 2 |

---

|  |  |  |  |  |  |  |  |  |
| --- | --- | --- | --- | --- | --- | --- | --- | --- |
| U | *gi|21361399|ref|NP\_05* | 2 | 2 | 5.1% | 589 | 65309 | 5.1 | alpha isoform of regulatory subunit A, protein phosphatase 2 [Homo sapiens] |

| Filename XCorr DeltCN Conf% ObsM+H+ CalcM+H+ SpR ZScore Ion% # Sequence  | | | | | | | | | | | | |
| --- | --- | --- | --- | --- | --- | --- | --- | --- | --- | --- | --- | --- |
| \* | Mis12XLAPLSS\_011913\_01.08979.08979.3 | 3.7129 | 0.3159 | 99.7% | 2214.2944 | 2214.4856 | 1 | 5.531 | 40.8% | 1 | R.AISHEHSPSDLEAHFVPLVK.R | 3 |
| \* | Mis12XLAPLSS\_011913\_01.03978.03978.2 | 2.959 | 0.2764 | 99.8% | 1161.1122 | 1161.2542 | 1 | 5.578 | 77.8% | 1 | K.LTQDQDVDVK.Y | 2 |

---

|  |  |  |  |  |  |  |  |  |
| --- | --- | --- | --- | --- | --- | --- | --- | --- |
| U | *gi|119703753|ref|NP\_0* | 3 | 7 | 5.1% | 564 | 60067 | 8.0 | keratin 6B [Homo sapiens] |

| Filename XCorr DeltCN Conf% ObsM+H+ CalcM+H+ SpR ZScore Ion% # Sequence  | | | | | | | | | | | | |
| --- | --- | --- | --- | --- | --- | --- | --- | --- | --- | --- | --- | --- |
|  | Mis12XLAPLSS\_011913\_01.03807.03807.2 | 2.6964 | 0.2533 | 99.9% | 1309.1721 | 1309.4215 | 1 | 5.17 | 72.2% | 1 | K.NKYEDEINKR.T | 222 |
|  | Mis12XLAPLSS\_011913\_02.05747.05747.2 | 3.574 | 0.2762 | 99.9% | 1180.1322 | 1180.303 | 2 | 6.851 | 83.3% | 4 | K.YEELQITAGR.H | 22 |
|  | Mis12XLAPLSS\_011913\_01.07844.07844.2 | 2.3187 | 0.2251 | 98.7% | 1153.9922 | 1154.3234 | 166 | 5.891 | 50.0% | 2 | K.EYQELMNVK.L | 22 |

Similarities:
contaminant\_KERATIN20(2:1)  
gi|119395750|ref|NP\_0(2:1)  

---

|  |  |  |  |  |  |  |  |  |
| --- | --- | --- | --- | --- | --- | --- | --- | --- |
| U | *gi|169216498|ref|XP\_0* | 2 | 2 | 4.9% | 729 | 82929 | 6.0 | PREDICTED: hypothetical LOC389901 [Homo sapiens] |
| U | *gi|4503841|ref|NP\_001* | 2 | 2 | 5.9% | 609 | 69843 | 6.6 | ATP-dependent DNA helicase II, 70 kDa subunit [Homo sapiens] |
| U | *gi|169217303|ref|XP\_0* | 2 | 2 | 4.2% | 859 | 96818 | 6.0 | PREDICTED: hypothetical LOC389901 [Homo sapiens] |
| U | *gi|169216723|ref|XP\_0* | 2 | 2 | 7.6% | 475 | 54430 | 8.5 | PREDICTED: hypothetical protein [Homo sapiens] |

| Filename XCorr DeltCN Conf% ObsM+H+ CalcM+H+ SpR ZScore Ion% # Sequence  | | | | | | | | | | | | |
| --- | --- | --- | --- | --- | --- | --- | --- | --- | --- | --- | --- | --- |
|  | Mis12XLAPLSS\_011913\_01.09108.09108.2 | 3.1114 | 0.3983 | 99.9% | 1704.9722 | 1704.8394 | 4 | 6.488 | 50.0% | 1 | R.SDSFENPVLQQHFR.N | 2 |
|  | Mis12XLAPLSS\_011913\_01.15424.15424.2 | 4.2935 | 0.3656 | 100.0% | 2424.3323 | 2424.812 | 1 | 6.839 | 42.9% | 1 | R.NLEALALDLMEPEQAVDLTLPK.V | 2 |

---

|  |  |  |  |  |  |  |  |  |
| --- | --- | --- | --- | --- | --- | --- | --- | --- |
| U | *gi|52632383|ref|NP\_00* | 2 | 2 | 4.9% | 589 | 64133 | 8.2 | heterogeneous nuclear ribonucleoprotein L isoform a [Homo sapiens] |
| U | *gi|52632385|ref|NP\_00* | 2 | 2 | 6.4% | 456 | 50561 | 7.5 | heterogeneous nuclear ribonucleoprotein L isoform b [Homo sapiens] |

| Filename XCorr DeltCN Conf% ObsM+H+ CalcM+H+ SpR ZScore Ion% # Sequence  | | | | | | | | | | | | |
| --- | --- | --- | --- | --- | --- | --- | --- | --- | --- | --- | --- | --- |
|  | Mis12XLAPLSS\_011913\_02.06568.06568.3 | 3.7561 | 0.4169 | 100.0% | 1869.7444 | 1869.1176 | 1 | 7.432 | 38.2% | 1 | K.SKPGAAMVEMADGYAVDR.A | 3 |
|  | Mis12XLAPLSS\_011913\_01.07618.07618.2 | 2.238 | 0.217 | 97.4% | 1264.2322 | 1264.4233 | 90 | 4.829 | 45.0% | 1 | K.NPNGPYPYTLK.L | 2 |

---

|  |  |  |  |  |  |  |  |  |
| --- | --- | --- | --- | --- | --- | --- | --- | --- |
| U | *gi|148529014|ref|NP\_0* | 2 | 2 | 4.7% | 1140 | 126968 | 5.3 | damage-specific DNA binding protein 1 [Homo sapiens] |

| Filename XCorr DeltCN Conf% ObsM+H+ CalcM+H+ SpR ZScore Ion% # Sequence  | | | | | | | | | | | | |
| --- | --- | --- | --- | --- | --- | --- | --- | --- | --- | --- | --- | --- |
| \* | Mis12XLAPLSS\_011913\_02.05766.05766.3 | 4.5708 | 0.4196 | 100.0% | 2953.0144 | 2954.1748 | 1 | 6.985 | 26.7% | 1 | R.IEVQDTSGGTTALRPSASTQALSSSVSSSK.L | 3 |
| \* | Mis12XLAPLSS\_011913\_01.13970.13970.3 | 5.0548 | 0.4158 | 100.0% | 2651.2444 | 2650.9885 | 1 | 7.555 | 37.0% | 1 | R.KTEPATGFIDGDLIESFLDISRPK.M | 3 |

---

|  |  |  |  |  |  |  |  |  |
| --- | --- | --- | --- | --- | --- | --- | --- | --- |
| U | *gi|33469922|ref|NP\_87* | 2 | 2 | 4.6% | 543 | 60643 | 6.3 | minichromosome maintenance complex component 7 isoform 2 [Homo sapiens] |
| U | *gi|33469968|ref|NP\_00* | 2 | 2 | 3.5% | 719 | 81308 | 6.5 | minichromosome maintenance complex component 7 isoform 1 [Homo sapiens] |

| Filename XCorr DeltCN Conf% ObsM+H+ CalcM+H+ SpR ZScore Ion% # Sequence  | | | | | | | | | | | | |
| --- | --- | --- | --- | --- | --- | --- | --- | --- | --- | --- | --- | --- |
|  | Mis12XLAPLSS\_011913\_02.06107.06107.2 | 3.0507 | 0.3586 | 99.8% | 1318.1522 | 1318.4697 | 11 | 6.522 | 54.5% | 1 | R.SITVLVEGENTR.I | 2 |
|  | Mis12XLAPLSS\_011913\_01.09370.09370.2 | 2.9028 | 0.3006 | 99.8% | 1474.2522 | 1474.703 | 1 | 5.611 | 62.5% | 1 | R.TQRPADVIFATVR.E | 2 |

---

|  |  |  |  |  |  |  |  |  |
| --- | --- | --- | --- | --- | --- | --- | --- | --- |
| U | *gi|57863257|ref|NP\_11* | 2 | 3 | 4.5% | 556 | 60344 | 6.1 | T-complex protein 1 isoform a [Homo sapiens] |

| Filename XCorr DeltCN Conf% ObsM+H+ CalcM+H+ SpR ZScore Ion% # Sequence  | | | | | | | | | | | | |
| --- | --- | --- | --- | --- | --- | --- | --- | --- | --- | --- | --- | --- |
| \* | Mis12XLAPLSS\_011913\_02.07570.07570.2 | 4.7107 | 0.4665 | 100.0% | 1517.8322 | 1517.7838 | 1 | 9.428 | 82.1% | 2 | R.SQNVMAAASIANIVK.S | 2 |
| \* | Mis12XLAPLSS\_011913\_01.04295.04295.2 | 2.2035 | 0.2386 | 98.1% | 1107.0521 | 1107.295 | 37 | 4.947 | 61.1% | 1 | K.LLEVEHPAAK.V | 2 |

---

|  |  |  |  |  |  |  |  |  |
| --- | --- | --- | --- | --- | --- | --- | --- | --- |
| U | *gi|19913424|ref|NP\_00* | 2 | 2 | 4.4% | 617 | 68304 | 5.5 | ATPase, H+ transporting, lysosomal V1 subunit A [Homo sapiens] |

| Filename XCorr DeltCN Conf% ObsM+H+ CalcM+H+ SpR ZScore Ion% # Sequence  | | | | | | | | | | | | |
| --- | --- | --- | --- | --- | --- | --- | --- | --- | --- | --- | --- | --- |
| \* | Mis12XLAPLSS\_011913\_01.08517.08517.2 | 2.1304 | 0.2648 | 97.4% | 1309.4122 | 1309.5082 | 14 | 4.489 | 59.1% | 1 | R.VGHSELVGEIIR.L | 2 |
| \* | Mis12XLAPLSS\_011913\_01.07486.07486.2 | 2.8734 | 0.2729 | 99.6% | 1518.1921 | 1516.7557 | 1 | 4.831 | 57.1% | 1 | R.TALVANTSNMPVAAR.E | 2 |

---

|  |  |  |  |  |  |  |  |  |
| --- | --- | --- | --- | --- | --- | --- | --- | --- |
| U | *gi|4557701|ref|NP\_000* | 2 | 2 | 4.4% | 432 | 48106 | 5.0 | keratin 17 [Homo sapiens] |

| Filename XCorr DeltCN Conf% ObsM+H+ CalcM+H+ SpR ZScore Ion% # Sequence  | | | | | | | | | | | | |
| --- | --- | --- | --- | --- | --- | --- | --- | --- | --- | --- | --- | --- |
|  | Mis12XLAPLSS\_011913\_02.05438.05438.2 | 3.0933 | 0.42 | 99.9% | 1345.3322 | 1346.4772 | 1 | 6.799 | 63.6% | 1 | R.ALEEANTELEVK.I | 2 |
|  | Mis12XLAPLSS\_011913\_01.05369.05369.2 | 2.0125 | 0.208 | 97.3% | 807.9122 | 807.8815 | 90 | 5.564 | 66.7% | 1 | R.LAADDFR.T | 222 |

Similarities:
gi|40354195|ref|NP\_95(1:1)  
contaminant\_KERATIN03(1:1)  

---

|  |  |  |  |  |  |  |  |  |
| --- | --- | --- | --- | --- | --- | --- | --- | --- |
| U | *gi|156151392|ref|NP\_0* | 2 | 6 | 4.3% | 532 | 59682 | 9.2 | heterogeneous nuclear ribonucleoprotein R isoform 4 [Homo sapiens] |
| U | *gi|5031755|ref|NP\_005* | 2 | 6 | 3.6% | 633 | 70943 | 8.1 | heterogeneous nuclear ribonucleoprotein R isoform 2 [Homo sapiens] |
| U | *gi|156151396|ref|NP\_0* | 2 | 6 | 4.3% | 535 | 59953 | 9.2 | heterogeneous nuclear ribonucleoprotein R isoform 3 [Homo sapiens] |
| U | *gi|156151394|ref|NP\_0* | 2 | 6 | 3.6% | 636 | 71214 | 8.1 | heterogeneous nuclear ribonucleoprotein R isoform 1 [Homo sapiens] |

| Filename XCorr DeltCN Conf% ObsM+H+ CalcM+H+ SpR ZScore Ion% # Sequence  | | | | | | | | | | | | |
| --- | --- | --- | --- | --- | --- | --- | --- | --- | --- | --- | --- | --- |
|  | Mis12XLAPLSS\_011913\_01.07436.07436.2 | 2.9127 | 0.345 | 99.8% | 1312.1122 | 1312.4221 | 1 | 7.154 | 72.7% | 5 | R.TGYTLDVTTGQR.K | 22 |
|  | Mis12XLAPLSS\_011913\_01.09179.09179.2 | 2.2143 | 0.3119 | 99.3% | 1262.2922 | 1262.4846 | 1 | 5.161 | 65.0% | 1 | R.LMMDPLSGQNR.G | 2 |

Similarities:
gi|23397427|ref|NP\_00(1:1)  

---

|  |  |  |  |  |  |  |  |  |
| --- | --- | --- | --- | --- | --- | --- | --- | --- |
| U | *gi|16753203|ref|NP\_03* | 2 | 2 | 4.2% | 589 | 62519 | 5.1 | ubiquilin 1 isoform 1 [Homo sapiens] |
| U | *gi|16753207|ref|NP\_03* | 2 | 2 | 4.0% | 624 | 65696 | 5.2 | ubiquilin 2 [Homo sapiens] |
| U | *gi|16753205|ref|NP\_44* | 2 | 2 | 4.5% | 561 | 59220 | 5.1 | ubiquilin 1 isoform 2 [Homo sapiens] |

| Filename XCorr DeltCN Conf% ObsM+H+ CalcM+H+ SpR ZScore Ion% # Sequence  | | | | | | | | | | | | |
| --- | --- | --- | --- | --- | --- | --- | --- | --- | --- | --- | --- | --- |
|  | Mis12XLAPLSS\_011913\_01.11033.11033.2 | 3.0759 | 0.2884 | 99.8% | 1813.2322 | 1813.1865 | 1 | 5.068 | 50.0% | 1 | R.QLIMANPQMQQLIQR.N | 2 |
|  | Mis12XLAPLSS\_011913\_01.08872.08872.2 | 2.2839 | 0.3868 | 99.8% | 1239.3522 | 1239.5265 | 117 | 5.439 | 55.6% | 1 | R.NPAMMQEMMR.N | 2 |

---

|  |  |  |  |  |  |  |  |  |
| --- | --- | --- | --- | --- | --- | --- | --- | --- |
| U | *gi|212549553|ref|NP\_0* | 2 | 2 | 4.1% | 898 | 95808 | 8.8 | interleukin enhancer binding factor 3 isoform d [Homo sapiens] |
| U | *gi|24234756|ref|NP\_70* | 2 | 2 | 5.4% | 690 | 74607 | 8.2 | interleukin enhancer binding factor 3 isoform c [Homo sapiens] |
| U | *gi|24234753|ref|NP\_00* | 2 | 2 | 5.3% | 702 | 76033 | 7.8 | interleukin enhancer binding factor 3 isoform b [Homo sapiens] |
| U | *gi|24234750|ref|NP\_03* | 2 | 2 | 4.1% | 894 | 95339 | 8.8 | interleukin enhancer binding factor 3 isoform a [Homo sapiens] |
| U | *gi|212549555|ref|NP\_0* | 2 | 2 | 5.2% | 706 | 76502 | 7.9 | interleukin enhancer binding factor 3 isoform e [Homo sapiens] |

| Filename XCorr DeltCN Conf% ObsM+H+ CalcM+H+ SpR ZScore Ion% # Sequence  | | | | | | | | | | | | |
| --- | --- | --- | --- | --- | --- | --- | --- | --- | --- | --- | --- | --- |
|  | Mis12XLAPLSS\_011913\_01.11550.11550.3 | 3.2617 | 0.2192 | 95.6% | 2671.6143 | 2672.886 | 46 | 5.044 | 25.0% | 1 | K.HSSVYPTQEELEAVQNMVSHTER.A | 3 |
|  | Mis12XLAPLSS\_011913\_01.08836.08836.2 | 2.7863 | 0.1273 | 97.1% | 1444.1921 | 1444.6459 | 4 | 4.26 | 57.7% | 1 | K.VLQDMGLPTGAEGR.D | 2 |

---

|  |  |  |  |  |  |  |  |  |
| --- | --- | --- | --- | --- | --- | --- | --- | --- |
| U | *gi|29029559|ref|NP\_00* | 2 | 3 | 4.0% | 971 | 110417 | 5.8 | CSE1 chromosome segregation 1-like protein [Homo sapiens] |

| Filename XCorr DeltCN Conf% ObsM+H+ CalcM+H+ SpR ZScore Ion% # Sequence  | | | | | | | | | | | | |
| --- | --- | --- | --- | --- | --- | --- | --- | --- | --- | --- | --- | --- |
| \* | Mis12XLAPLSS\_011913\_01.09798.09798.2 | 2.7533 | 0.12 | 96.4% | 1590.2322 | 1589.7941 | 4 | 3.472 | 61.5% | 1 | R.FQSGDFHVINGVLR.T | 2 |
| \* | Mis12XLAPLSS\_011913\_01.16989.16989.3 | 5.443 | 0.3781 | 100.0% | 2863.4944 | 2864.2737 | 1 | 7.052 | 32.3% | 2 | K.HGITQANELVNLTEFFVNHILPDLK.S | 3 |

---

|  |  |  |  |  |  |  |  |  |
| --- | --- | --- | --- | --- | --- | --- | --- | --- |
| U | *gi|62241042|ref|NP\_00* | 3 | 3 | 3.9% | 1512 | 170590 | 7.3 | glutamyl-prolyl tRNA synthetase [Homo sapiens] |

| Filename XCorr DeltCN Conf% ObsM+H+ CalcM+H+ SpR ZScore Ion% # Sequence  | | | | | | | | | | | | |
| --- | --- | --- | --- | --- | --- | --- | --- | --- | --- | --- | --- | --- |
| \* | Mis12XLAPLSS\_011913\_02.06435.06435.3 | 5.4903 | 0.4335 | 100.0% | 3105.2344 | 3105.3423 | 1 | 8.244 | 29.3% | 1 | K.TGQEYKPGNPPAEIGQNISSNSSASILESK.S | 3 |
| \* | Mis12XLAPLSS\_011913\_02.06842.06842.2 | 3.4646 | 0.4276 | 100.0% | 1535.9122 | 1536.6818 | 1 | 7.328 | 61.5% | 1 | K.SLYDEVAAQGEVVR.K | 2 |
| \* | Mis12XLAPLSS\_011913\_01.11165.11165.2 | 3.9953 | 0.4684 | 100.0% | 1699.6721 | 1699.9065 | 1 | 8.114 | 71.4% | 1 | K.THVADFAPEVAWVTR.S | 2 |

---

|  |  |  |  |  |  |  |  |  |
| --- | --- | --- | --- | --- | --- | --- | --- | --- |
| U | *gi|164519146|ref|NP\_0* | 2 | 2 | 3.8% | 633 | 68063 | 9.9 | GATA zinc finger domain containing 2A [Homo sapiens] |

| Filename XCorr DeltCN Conf% ObsM+H+ CalcM+H+ SpR ZScore Ion% # Sequence  | | | | | | | | | | | | |
| --- | --- | --- | --- | --- | --- | --- | --- | --- | --- | --- | --- | --- |
| \* | Mis12XLAPLSS\_011913\_01.04836.04836.2 | 2.2007 | 0.3992 | 99.8% | 1110.1322 | 1110.2886 | 27 | 6.567 | 55.0% | 1 | R.ATEATAMAMGR.G | 2 |
| \* | Mis12XLAPLSS\_011913\_02.05027.05027.2 | 3.3121 | 0.4923 | 100.0% | 1318.0122 | 1318.4728 | 1 | 8.018 | 66.7% | 1 | K.LQNSASATALVSR.T | 2 |

---

|  |  |  |  |  |  |  |  |  |
| --- | --- | --- | --- | --- | --- | --- | --- | --- |
| U | *gi|4506411|ref|NP\_002* | 2 | 2 | 3.7% | 587 | 63542 | 4.7 | Ran GTPase activating protein 1 [Homo sapiens] |

| Filename XCorr DeltCN Conf% ObsM+H+ CalcM+H+ SpR ZScore Ion% # Sequence  | | | | | | | | | | | | |
| --- | --- | --- | --- | --- | --- | --- | --- | --- | --- | --- | --- | --- |
| \* | Mis12XLAPLSS\_011913\_01.08849.08849.2 | 2.6367 | 0.3323 | 99.8% | 1408.5122 | 1408.551 | 1 | 6.492 | 72.7% | 1 | R.VINLNDNTFTEK.G | 2 |
| \* | Mis12XLAPLSS\_011913\_01.10060.10060.2 | 2.1907 | 0.3053 | 99.3% | 1201.7922 | 1202.4392 | 268 | 5.775 | 44.4% | 1 | R.HSLLQTLYKV.- | 2 |

---

|  |  |  |  |  |  |  |  |  |
| --- | --- | --- | --- | --- | --- | --- | --- | --- |
| U | *gi|20143967|ref|NP\_61* | 2 | 2 | 3.4% | 960 | 110059 | 8.5 | kinesin family member 23 isoform 1 [Homo sapiens] |
| U | *gi|6754472|ref|NP\_004* | 2 | 2 | 3.9% | 856 | 98105 | 8.5 | kinesin family member 23 isoform 2 [Homo sapiens] |

| Filename XCorr DeltCN Conf% ObsM+H+ CalcM+H+ SpR ZScore Ion% # Sequence  | | | | | | | | | | | | |
| --- | --- | --- | --- | --- | --- | --- | --- | --- | --- | --- | --- | --- |
|  | Mis12XLAPLSS\_011913\_02.05728.05728.3 | 2.934 | 0.3974 | 100.0% | 1817.9644 | 1818.0367 | 5 | 6.109 | 36.7% | 1 | R.FAEVTQEVEVARPVDK.A | 3 |
|  | Mis12XLAPLSS\_011913\_02.05764.05764.3 | 3.2853 | 0.2023 | 97.1% | 1964.5443 | 1966.17 | 3 | 4.511 | 35.9% | 1 | K.YMLTHQELASDGEIETK.L | 3 |

---

|  |  |  |  |  |  |  |  |  |
| --- | --- | --- | --- | --- | --- | --- | --- | --- |
| U | *gi|156523968|ref|NP\_0* | 2 | 2 | 3.3% | 1014 | 113084 | 8.9 | poly (ADP-ribose) polymerase family, member 1 [Homo sapiens] |

| Filename XCorr DeltCN Conf% ObsM+H+ CalcM+H+ SpR ZScore Ion% # Sequence  | | | | | | | | | | | | |
| --- | --- | --- | --- | --- | --- | --- | --- | --- | --- | --- | --- | --- |
| \* | Mis12XLAPLSS\_011913\_02.07493.07493.2 | 3.1291 | 0.5059 | 100.0% | 1625.6322 | 1625.7728 | 1 | 8.749 | 53.6% | 1 | R.VVSEDFLQDVSASTK.S | 2 |
| \* | Mis12XLAPLSS\_011913\_02.11685.11685.2 | 2.4803 | 0.1838 | 96.2% | 2107.392 | 2107.47 | 3 | 4.307 | 38.2% | 1 | K.VEMLDNLLDIEVAYSLLR.G | 2 |

---

|  |  |  |  |  |  |  |  |  |
| --- | --- | --- | --- | --- | --- | --- | --- | --- |
| U | *gi|5454102|ref|NP\_006* | 2 | 3 | 3.3% | 838 | 90360 | 5.0 | transforming, acidic coiled-coil containing protein 3 [Homo sapiens] |

| Filename XCorr DeltCN Conf% ObsM+H+ CalcM+H+ SpR ZScore Ion% # Sequence  | | | | | | | | | | | | |
| --- | --- | --- | --- | --- | --- | --- | --- | --- | --- | --- | --- | --- |
| \* | Mis12XLAPLSS\_011913\_02.06297.06297.2 | 3.7238 | 0.3674 | 100.0% | 1384.8722 | 1384.5754 | 1 | 7.524 | 68.2% | 2 | K.LQLANEEIAQVR.S | 2 |
| \* | Mis12XLAPLSS\_011913\_02.05807.05807.3 | 2.9517 | 0.2937 | 99.3% | 1685.5743 | 1685.9645 | 3 | 4.564 | 36.7% | 1 | R.SKAQAEALALQASLRK.E | 3 |

---

|  |  |  |  |  |  |  |  |  |
| --- | --- | --- | --- | --- | --- | --- | --- | --- |
| U | *gi|12667788|ref|NP\_00* | 4 | 5 | 3.2% | 1960 | 226530 | 5.6 | myosin, heavy polypeptide 9, non-muscle [Homo sapiens] |

| Filename XCorr DeltCN Conf% ObsM+H+ CalcM+H+ SpR ZScore Ion% # Sequence  | | | | | | | | | | | | |
| --- | --- | --- | --- | --- | --- | --- | --- | --- | --- | --- | --- | --- |
| \* | Mis12XLAPLSS\_011913\_02.06350.06350.2 | 4.5357 | 0.4673 | 100.0% | 1654.1721 | 1654.7681 | 1 | 7.085 | 73.1% | 1 | R.IAEFTTNLTEEEEK.S | 2 |
| \* | Mis12XLAPLSS\_011913\_01.09249.09249.3 | 3.3969 | 0.2855 | 99.8% | 1998.1144 | 1997.1722 | 13 | 5.548 | 32.8% | 1 | K.HSQAVEELAEQLEQTKR.V | 3 |
| \* | Mis12XLAPLSS\_011913\_02.08352.08352.2 | 3.934 | 0.4463 | 100.0% | 1946.3121 | 1947.1498 | 1 | 7.659 | 47.1% | 1 | K.LQVELDNVTGLLSQSDSK.S | 2 |
| \* | Mis12XLAPLSS\_011913\_01.06108.06108.2 | 3.5225 | 0.4565 | 100.0% | 1566.3722 | 1566.6367 | 1 | 7.284 | 65.4% | 2 | R.ELEDATETADAMNR.E | 2 |

---

|  |  |  |  |  |  |  |  |  |
| --- | --- | --- | --- | --- | --- | --- | --- | --- |
| U | *gi|6631095|ref|NP\_002* | 2 | 2 | 3.1% | 808 | 90981 | 5.8 | minichromosome maintenance complex component 3 [Homo sapiens] |

| Filename XCorr DeltCN Conf% ObsM+H+ CalcM+H+ SpR ZScore Ion% # Sequence  | | | | | | | | | | | | |
| --- | --- | --- | --- | --- | --- | --- | --- | --- | --- | --- | --- | --- |
| \* | Mis12XLAPLSS\_011913\_01.08856.08856.2 | 2.1224 | 0.2573 | 98.1% | 1165.5521 | 1165.3318 | 6 | 5.542 | 55.6% | 1 | R.SKDIFDQLAK.S | 2 |
| \* | Mis12XLAPLSS\_011913\_01.10992.10992.2 | 2.8725 | 0.2176 | 99.3% | 1674.2122 | 1674.9098 | 1 | 4.67 | 57.1% | 1 | K.TPMENIGLQDSLLSR.F | 2 |

---

|  |  |  |  |  |  |  |  |  |
| --- | --- | --- | --- | --- | --- | --- | --- | --- |
| U | *gi|122891870|ref|NP\_9* | 3 | 3 | 2.7% | 1907 | 213700 | 4.8 | melanoma inhibitory activity family, member 3 [Homo sapiens] |

| Filename XCorr DeltCN Conf% ObsM+H+ CalcM+H+ SpR ZScore Ion% # Sequence  | | | | | | | | | | | | |
| --- | --- | --- | --- | --- | --- | --- | --- | --- | --- | --- | --- | --- |
| \* | Mis12XLAPLSS\_011913\_02.05842.05842.2 | 4.6489 | 0.5822 | 100.0% | 1685.3722 | 1685.7417 | 1 | 9.868 | 63.3% | 1 | K.FGSTADALVSDDETTR.L | 2 |
| \* | Mis12XLAPLSS\_011913\_01.09214.09214.2 | 2.2247 | 0.2854 | 98.8% | 1276.2122 | 1276.3885 | 1 | 5.099 | 70.0% | 1 | R.FSSPDEIDLPR.E | 2 |
| \* | Mis12XLAPLSS\_011913\_02.06172.06172.3 | 3.6684 | 0.239 | 98.8% | 2428.5244 | 2427.602 | 3 | 4.861 | 29.3% | 1 | K.AMQGTEVGQTDQTDSTGGPAFLSK.V | 3 |

---

|  |  |  |  |  |  |  |  |  |
| --- | --- | --- | --- | --- | --- | --- | --- | --- |
| U | *gi|133925811|ref|NP\_0* | 2 | 2 | 2.6% | 898 | 102355 | 5.0 | transportin 1 isoform 1 [Homo sapiens] |
| U | *gi|23510381|ref|NP\_69* | 2 | 2 | 2.6% | 890 | 101310 | 5.0 | transportin 1 isoform 2 [Homo sapiens] |

| Filename XCorr DeltCN Conf% ObsM+H+ CalcM+H+ SpR ZScore Ion% # Sequence  | | | | | | | | | | | | |
| --- | --- | --- | --- | --- | --- | --- | --- | --- | --- | --- | --- | --- |
|  | Mis12XLAPLSS\_011913\_01.03778.03778.2 | 2.5204 | 0.2147 | 98.9% | 1262.0122 | 1262.3188 | 4 | 5.068 | 65.0% | 1 | K.ESQSPDTTIQR.T | 2 |
|  | Mis12XLAPLSS\_011913\_02.07025.07025.2 | 2.8642 | 0.2052 | 99.5% | 1302.0721 | 1302.5138 | 1 | 7.252 | 72.7% | 1 | K.TLLENTAITIGR.L | 2 |

---

|  |  |  |  |  |  |  |  |  |
| --- | --- | --- | --- | --- | --- | --- | --- | --- |
| U | *gi|222136639|ref|NP\_0* | 2 | 2 | 2.6% | 935 | 101531 | 7.3 | methylenetetrahydrofolate dehydrogenase 1 [Homo sapiens] |

| Filename XCorr DeltCN Conf% ObsM+H+ CalcM+H+ SpR ZScore Ion% # Sequence  | | | | | | | | | | | | |
| --- | --- | --- | --- | --- | --- | --- | --- | --- | --- | --- | --- | --- |
| \* | Mis12XLAPLSS\_011913\_01.11189.11189.2 | 2.4246 | 0.2133 | 98.2% | 1488.6322 | 1487.6525 | 91 | 4.538 | 50.0% | 1 | R.LDIDPETITWQR.V | 2 |
| \* | Mis12XLAPLSS\_011913\_01.09389.09389.2 | 2.3847 | 0.1624 | 95.6% | 1379.4122 | 1379.4637 | 1 | 4.053 | 72.7% | 1 | K.TDTESELDLISR.L | 2 |

---

|  |  |  |  |  |  |  |  |  |
| --- | --- | --- | --- | --- | --- | --- | --- | --- |
| U | *gi|194440660|ref|NP\_0* | 3 | 3 | 2.4% | 1522 | 170678 | 7.0 | topoisomerase (DNA) II binding protein 1 [Homo sapiens] |

| Filename XCorr DeltCN Conf% ObsM+H+ CalcM+H+ SpR ZScore Ion% # Sequence  | | | | | | | | | | | | |
| --- | --- | --- | --- | --- | --- | --- | --- | --- | --- | --- | --- | --- |
| \* | Mis12XLAPLSS\_011913\_01.10802.10802.2 | 2.3216 | 0.219 | 98.4% | 1194.8922 | 1195.5339 | 148 | 4.392 | 55.6% | 1 | K.KPILLPSWIK.T | 2 |
| \* | Mis12XLAPLSS\_011913\_01.03608.03608.2 | 2.155 | 0.2131 | 96.7% | 1029.0322 | 1029.1874 | 3 | 4.323 | 72.2% | 1 | R.RLINSGGGVR.F | 2 |
| \* | Mis12XLAPLSS\_011913\_01.16959.16959.2 | 2.928 | 0.3915 | 99.8% | 1854.8322 | 1855.19 | 1 | 6.288 | 40.0% | 1 | K.WNLPAVTIAWLLETAR.T | 2 |

---

|  |  |  |  |  |  |  |  |  |
| --- | --- | --- | --- | --- | --- | --- | --- | --- |
| U | *gi|4507877|ref|NP\_003* | 2 | 2 | 2.3% | 1066 | 116722 | 6.1 | vinculin isoform VCL [Homo sapiens] |
| U | *gi|7669550|ref|NP\_054* | 2 | 2 | 2.1% | 1134 | 123799 | 5.7 | vinculin isoform meta-VCL [Homo sapiens] |

| Filename XCorr DeltCN Conf% ObsM+H+ CalcM+H+ SpR ZScore Ion% # Sequence  | | | | | | | | | | | | |
| --- | --- | --- | --- | --- | --- | --- | --- | --- | --- | --- | --- | --- |
|  | Mis12XLAPLSS\_011913\_01.07439.07439.2 | 2.3644 | 0.1375 | 95.6% | 1231.2922 | 1231.3073 | 1 | 4.308 | 77.8% | 1 | R.WIDNPTVDDR.G | 2 |
|  | Mis12XLAPLSS\_011913\_01.08990.08990.2 | 2.515 | 0.1895 | 97.4% | 1520.3322 | 1519.8354 | 4 | 5.057 | 61.5% | 1 | K.AGEVINQPMMMAAR.Q | 2 |

---

|  |  |  |  |  |  |  |  |  |
| --- | --- | --- | --- | --- | --- | --- | --- | --- |
| U | *gi|207452735|ref|NP\_1* | 3 | 4 | 1.4% | 5090 | 555629 | 5.6 | epiplakin 1 [Homo sapiens] |

| Filename XCorr DeltCN Conf% ObsM+H+ CalcM+H+ SpR ZScore Ion% # Sequence  | | | | | | | | | | | | |
| --- | --- | --- | --- | --- | --- | --- | --- | --- | --- | --- | --- | --- |
| \* | Mis12XLAPLSS\_011913\_01.14856.14856.2 | 3.673 | 0.3429 | 99.9% | 2395.7522 | 2395.7202 | 1 | 6.012 | 45.2% | 1 | R.VTPGSGALQGQSVSVWELLFYR.E | 2 |
| \* | Mis12XLAPLSS\_011913\_01.16503.16503.3 | 3.9663 | 0.4167 | 100.0% | 2514.4744 | 2514.8386 | 1 | 7.036 | 30.2% | 2 | R.AGTLTVEELGATLTSLLAQAQAQAR.A | 3 |
| \* | Mis12XLAPLSS\_011913\_01.13671.13671.3 | 3.0331 | 0.3054 | 99.4% | 2503.4944 | 2500.904 | 8 | 5.017 | 27.2% | 1 | R.RDELLAQHAAGALGLPDLVAVLTR.V | 23 |

---

|  |  |  |  |  |  |  |  |  |
| --- | --- | --- | --- | --- | --- | --- | --- | --- |
| U | *gi|103472005|ref|NP\_0* | 3 | 3 | 1.4% | 3256 | 358695 | 9.4 | antigen identified by monoclonal antibody Ki-67 isoform 1 [Homo sapiens] |
| U | *gi|225543215|ref|NP\_0* | 3 | 3 | 1.5% | 2896 | 319444 | 9.5 | antigen identified by monoclonal antibody Ki-67 isoform 2 [Homo sapiens] |

| Filename XCorr DeltCN Conf% ObsM+H+ CalcM+H+ SpR ZScore Ion% # Sequence  | | | | | | | | | | | | |
| --- | --- | --- | --- | --- | --- | --- | --- | --- | --- | --- | --- | --- |
|  | Mis12XLAPLSS\_011913\_01.10421.10421.2 | 3.3479 | 0.4208 | 100.0% | 1420.4321 | 1420.6055 | 1 | 7.913 | 68.2% | 1 | R.KADVEEEFLALR.K | 2 |
|  | Mis12XLAPLSS\_011913\_01.11530.11530.2 | 2.8356 | 0.2744 | 99.8% | 1482.2522 | 1482.6763 | 1 | 5.67 | 72.7% | 1 | R.KVDVEEEFFALR.K | 2 |
|  | Mis12XLAPLSS\_011913\_01.03288.03288.3 | 3.4757 | 0.2674 | 99.8% | 2099.5444 | 2099.1753 | 41 | 5.244 | 28.9% | 1 | K.LTQTSGETTHTHTEPTGDGK.S | 3 |

---

|  |  |  |  |  |  |  |  |  |
| --- | --- | --- | --- | --- | --- | --- | --- | --- |
| U | *gi|74048514|ref|NP\_73* | 2 | 2 | 1.3% | 2342 | 265290 | 5.4 | cancer susceptibility candidate 5 isoform 1 [Homo sapiens] |
| U | *gi|74048554|ref|NP\_65* | 2 | 2 | 1.3% | 2316 | 262530 | 5.4 | cancer susceptibility candidate 5 isoform 2 [Homo sapiens] |

| Filename XCorr DeltCN Conf% ObsM+H+ CalcM+H+ SpR ZScore Ion% # Sequence  | | | | | | | | | | | | |
| --- | --- | --- | --- | --- | --- | --- | --- | --- | --- | --- | --- | --- |
|  | Mis12XLAPLSS\_011913\_02.05650.05650.2 | 3.4158 | 0.3623 | 99.9% | 1500.9922 | 1501.6484 | 1 | 7.023 | 70.8% | 1 | K.TIYSGEENMDITK.S | 2 |
|  | Mis12XLAPLSS\_011913\_01.09058.09058.3 | 3.9486 | 0.3145 | 100.0% | 2174.6343 | 2174.3696 | 15 | 6.704 | 30.9% | 1 | R.AAEKELEQLKTEEEELQR.N | 3 |

---

|  |  |  |  |  |  |  |  |  |
| --- | --- | --- | --- | --- | --- | --- | --- | --- |
| U | *gi|41322908|ref|NP\_95* | 3 | 3 | 1.0% | 4525 | 513712 | 5.8 | plectin 1 isoform 3 [Homo sapiens] |
| U | *gi|47607492|ref|NP\_00* | 3 | 3 | 1.0% | 4574 | 518478 | 5.7 | plectin 1 isoform 1 [Homo sapiens] |
| U | *gi|41322923|ref|NP\_95* | 3 | 3 | 1.0% | 4547 | 516204 | 5.8 | plectin 1 isoform 11 [Homo sapiens] |
| U | *gi|41322919|ref|NP\_95* | 3 | 3 | 1.0% | 4547 | 516282 | 5.8 | plectin 1 isoform 8 [Homo sapiens] |
| U | *gi|41322916|ref|NP\_95* | 3 | 3 | 0.9% | 4684 | 531796 | 6.0 | plectin 1 isoform 6 [Homo sapiens] |
| U | *gi|41322914|ref|NP\_95* | 3 | 3 | 1.0% | 4551 | 516484 | 5.8 | plectin 1 isoform 10 [Homo sapiens] |
| U | *gi|41322912|ref|NP\_95* | 3 | 3 | 1.0% | 4533 | 514780 | 5.7 | plectin 1 isoform 2 [Homo sapiens] |
| U | *gi|41322910|ref|NP\_95* | 3 | 3 | 1.0% | 4515 | 512609 | 5.8 | plectin 1 isoform 7 [Homo sapiens] |

| Filename XCorr DeltCN Conf% ObsM+H+ CalcM+H+ SpR ZScore Ion% # Sequence  | | | | | | | | | | | | |
| --- | --- | --- | --- | --- | --- | --- | --- | --- | --- | --- | --- | --- |
|  | Mis12XLAPLSS\_011913\_01.09646.09646.2 | 2.6236 | 0.2249 | 99.0% | 1520.7722 | 1519.738 | 1 | 4.371 | 66.7% | 1 | K.AKLEQLFQDEVAK.A | 2 |
|  | Mis12XLAPLSS\_011913\_02.04851.04851.3 | 3.0208 | 0.218 | 95.2% | 1784.8143 | 1784.964 | 2 | 4.777 | 36.8% | 1 | R.AALAHSEEVTASQVAATK.T | 3 |
|  | Mis12XLAPLSS\_011913\_02.06753.06753.2 | 2.5372 | 0.1758 | 97.4% | 1462.2522 | 1462.6611 | 21 | 4.229 | 54.2% | 1 | R.SQVMDEATALQLR.E | 2 |

---

|  |  |  |  |  |  |  |  |  |
| --- | --- | --- | --- | --- | --- | --- | --- | --- |
| U | *gi|47578105|ref|NP\_59* | 2 | 2 | 1.0% | 2804 | 316051 | 7.9 | delangin isoform A [Homo sapiens] |
| U | *gi|47578107|ref|NP\_05* | 2 | 2 | 1.0% | 2697 | 304343 | 7.8 | delangin isoform B [Homo sapiens] |

| Filename XCorr DeltCN Conf% ObsM+H+ CalcM+H+ SpR ZScore Ion% # Sequence  | | | | | | | | | | | | |
| --- | --- | --- | --- | --- | --- | --- | --- | --- | --- | --- | --- | --- |
|  | Mis12XLAPLSS\_011913\_01.19223.19223.2 | 1.9451 | 0.3178 | 96.6% | 1853.9922 | 1853.9841 | 90 | 4.967 | 36.7% | 1 | R.RDSGKPST#EKKPEVSK.H | 2 |
|  | Mis12XLAPLSS\_011913\_01.09485.09485.2 | 2.6209 | 0.1219 | 97.3% | 1447.2122 | 1447.4044 | 78 | 3.366 | 55.0% | 1 | K.QDT#K@S\*DSPRLK.S | 2 |

---

|  |  |  |  |  |  |  |  |  |
| --- | --- | --- | --- | --- | --- | --- | --- | --- |
| U | *gi|122937398|ref|NP\_0* | 2 | 2 | 0.8% | 4314 | 493425 | 6.6 | dynein, cytoplasmic 2, heavy chain 1 [Homo sapiens] |

| Filename XCorr DeltCN Conf% ObsM+H+ CalcM+H+ SpR ZScore Ion% # Sequence  | | | | | | | | | | | | |
| --- | --- | --- | --- | --- | --- | --- | --- | --- | --- | --- | --- | --- |
| \* | Mis12XLAPLSS\_011913\_02.08739.08739.2 | 2.1571 | 0.3137 | 98.3% | 1914.4922 | 1915.1489 | 160 | 4.901 | 30.0% | 1 | K.KELLK@RQS\*HLQAGVSK.L | 2 |
| \* | Mis12XLAPLSS\_011913\_01.16350.16350.2 | 2.4057 | 0.2016 | 96.3% | 2265.4722 | 2264.527 | 73 | 3.92 | 27.8% | 1 | R.KLEELLNSVGQKVS\*ELK@EK.F | 2 |

---

|  |  |  |  |  |  |  |  |  |
| --- | --- | --- | --- | --- | --- | --- | --- | --- |
| U | *gi|33350932|ref|NP\_00* | 2 | 2 | 0.6% | 4646 | 532412 | 6.4 | cytoplasmic dynein 1 heavy chain 1 [Homo sapiens] |

| Filename XCorr DeltCN Conf% ObsM+H+ CalcM+H+ SpR ZScore Ion% # Sequence  | | | | | | | | | | | | |
| --- | --- | --- | --- | --- | --- | --- | --- | --- | --- | --- | --- | --- |
| \* | Mis12XLAPLSS\_011913\_02.06952.06952.2 | 3.304 | 0.4689 | 100.0% | 1512.5322 | 1513.7343 | 1 | 7.831 | 73.1% | 1 | R.IQGLTVEQAEAVVR.L | 2 |
| \* | Mis12XLAPLSS\_011913\_02.07007.07007.2 | 3.336 | 0.375 | 99.9% | 1406.3722 | 1405.6929 | 1 | 6.256 | 75.0% | 1 | R.VLLTTQGVDMISK.M | 2 |

---

|  |  |  |  |  |  |  |  |  |
| --- | --- | --- | --- | --- | --- | --- | --- | --- |
| U | *gi|61743954|ref|NP\_00* | 2 | 2 | 0.4% | 5890 | 629114 | 6.1 | AHNAK nucleoprotein isoform 1 [Homo sapiens] |

| Filename XCorr DeltCN Conf% ObsM+H+ CalcM+H+ SpR ZScore Ion% # Sequence  | | | | | | | | | | | | |
| --- | --- | --- | --- | --- | --- | --- | --- | --- | --- | --- | --- | --- |
| \* | Mis12XLAPLSS\_011913\_01.08282.08282.2 | 2.572 | 0.3588 | 99.8% | 1268.0721 | 1268.4093 | 1 | 5.812 | 72.7% | 1 | K.AEGPEVDVNLPK.A | 2 |
| \* | Mis12XLAPLSS\_011913\_01.09819.09819.2 | 2.0042 | 0.3096 | 98.1% | 1268.4722 | 1268.5139 | 3 | 5.109 | 55.0% | 1 | K.ISMPDVDLHLK.G | 2 |

---

|  |  |  |  |  |  |  |  |  |
| --- | --- | --- | --- | --- | --- | --- | --- | --- |
| U | *contaminant\_UBIQUITIN* | 4 | 5 | 0.0% | 1118 | 127523 | 8.5 | no description |

| Filename XCorr DeltCN Conf% ObsM+H+ CalcM+H+ SpR ZScore Ion% # Sequence  | | | | | | | | | | | | |
| --- | --- | --- | --- | --- | --- | --- | --- | --- | --- | --- | --- | --- |
|  | Mis12XLAPLSS\_011913\_01.05248.05248.2 | 1.8851 | 0.4154 | 99.5% | 1082.0322 | 1082.1986 | 1 | 6.113 | 75.0% | 1 | R.TLSDYNIQK.E | 2 |
|  | Mis12XLAPLSS\_011913\_01.03671.03671.2 | 3.9211 | 0.3621 | 100.0% | 1524.0721 | 1524.6738 | 1 | 7.512 | 83.3% | 1 | K.IQDKEGIPPDQQR.L | 2 |
|  | Mis12XLAPLSS\_011913\_01.09882.09882.2 | 4.3989 | 0.4293 | 100.0% | 1789.4521 | 1788.9897 | 1 | 7.639 | 70.0% | 2 | K.TITLEVEPSDTIENVK.A | 2 |
|  | Mis12XLAPLSS\_011913\_01.13191.13191.2 | 2.346 | 0.3635 | 99.5% | 1909.4722 | 1910.1742 | 30 | 5.578 | 32.4% | 1 | K.SLVASLAEPDFVVTDFAK.F | 2 |

|  |  |  |  |
| --- | --- | --- | --- |
|  | Proteins | Peptide IDs | Spectra |
| Unfiltered | 32957 | 48465 | 84776 |
| Filtered | 295 | 1414 | 2293 |
| Forward matches | 295 | 1414 | 2293 |
| Decoy matches | 0 | 0 | 0 |
| Forward FP rate | 0.0% | 0.0% | 0.0% |

  
/nfs/cheeseman\_massspec/David/Mis12XLAPLSS
