# Supplementary material for: Astrin-SKAP complex reconstitution reveals its kinetochore interaction with microtubule-bound Ndc80
Source: eLife. 2017 Aug 25;6:e26866. doi: 10.7554/eLife.26866 (PMC5602300; doi:10.7554/eLife.26866)
Supplement: Source data 1. — Complete mass spectrometry searches using methods described in (Washburn et al., 2001) for affinity purification/mass spectrometry data sets described in this paper (data from this study; [Kern et al., 2016] [Gascoigne et al., 2011]). Individual Astrin cross-linking immunoprecipitations are listed based on the order in Figure 4—figure supplement 1. These samples have not been pruned for common or antibody-specific contaminants. [file elife-26866-data1.zip › Mis12_NocodazoleIP_Gascoigne2011.html]

D Nocodazole1
DTASelect v2.0.21  
/nfs/cheeseman\_massspec/Jenny/Nocodazole1  
/nfs/cheeseman\_massspec/Databases/NCBI-RefSeq\_human\_na\_05-04-2007\_con\_reversed.fasta  
SEQUEST 3.0 in SQT format.  
  
 Jump  to the summary table.  
  
sequest.params modifications:

|  |  |  |
| --- | --- | --- |
| \* | S | 80.0 |
| # | T | 80.0 |
| @ | Y | 80.0 |
| Static | C | 57.0 |

|  |  |
| --- | --- |
| true | Use criteria |
| 0.0 | Minimum peptide confidence |
| 0.05 | Peptide false positive rate |
| 0.0 | Minimum protein confidence |
| 1.0 | Protein false positive rate |
| 1 | Minimum charge state |
| 16 | Maximum charge state |
| 0.0 | Minimum ion proportion |
| 1000 | Maximum Sp rank |
| -1.0 | Minimum Sp score |
| Include | Modified peptide inclusion |
| Any | Tryptic status requirement |
| false | Multiple, ambiguous IDs allowed |
| Ignore | Peptide validation handling |
| XCorr | Purge duplicate peptides by protein |
| false | Include only loci with unique peptide |
| true | Remove subset proteins |
| Ignore | Locus validation handling |
| 0 | Minimum modified peptides per locus |
| 1000 | Minimum redundancy for low coverage loci |
| 2 | Minimum peptides per locus |

#### Locus Key:

|  |  |  |  |  |  |  |  |  |
| --- | --- | --- | --- | --- | --- | --- | --- | --- |
| Validation Status | Locus | Sequence Count | Spectrum Count | Sequence Coverage | Length | MolWt | pI | Descriptive Name |

#### Similarity Key:

|  |  |  |
| --- | --- | --- |
| Locus | # of identical peptides | # of differing peptides |

---

|  |  |  |  |  |  |  |  |  |
| --- | --- | --- | --- | --- | --- | --- | --- | --- |
| U | *gi|4506671|ref|NP\_000* | 4 | 4 | 62.6% | 115 | 11665 | 4.5 | ribosomal protein P2 [Homo sapiens] |

| Filename XCorr DeltCN Conf% ObsM+H+ CalcM+H+ SpR ZScore Ion% # Sequence  | | | | | | | | | | | | |
| --- | --- | --- | --- | --- | --- | --- | --- | --- | --- | --- | --- | --- |
| \* | Mis12IP\_Nocodazole\_MudPIT\_040709\_03.07675.07675.2 | 2.4493 | 0.2344 | 98.9% | 1949.2722 | 1950.1124 | 1 | 5.683 | 50.0% | 1 | R.YVASYLLAALGGNSS\*PSAK.D | 2 |
|  | Mis12IP\_Nocodazole\_MudPIT\_040709\_03.04364.04364.1 | 2.1354 | 0.2679 | 98.7% | 859.42 | 859.99817 | 16 | 5.501 | 64.3% | 1 | K.VISELNGK.N | 1 |
| \* | Mis12IP\_Nocodazole\_MudPIT\_040709\_06.06851.06851.2 | 4.4243 | 0.5092 | 100.0% | 1256.9722 | 1257.4294 | 1 | 8.128 | 81.8% | 1 | K.NIEDVIAQGIGK.L | 2 |
| \* | Mis12IP\_Nocodazole\_MudPIT\_040709\_06.05741.05741.3 | 2.6803 | 0.2807 | 98.1% | 2775.3843 | 2776.0757 | 7 | 5.857 | 21.9% | 1 | K.LASVPAGGAVAVSAAPGSAAPAAGSAPAAAEEK.K | 3 |

---

|  |  |  |  |  |  |  |  |  |
| --- | --- | --- | --- | --- | --- | --- | --- | --- |
| U | *gi|4504919|ref|NP\_002* | 24 | 66 | 58.0% | 483 | 53704 | 5.6 | keratin 8 [Homo sapiens] |

| Filename XCorr DeltCN Conf% ObsM+H+ CalcM+H+ SpR ZScore Ion% # Sequence  | | | | | | | | | | | | |
| --- | --- | --- | --- | --- | --- | --- | --- | --- | --- | --- | --- | --- |
|  | Mis12IP\_Nocodazole\_MudPIT\_040709\_06.05458.05458.2 | 2.8017 | 0.3925 | 100.0% | 1082.0322 | 1082.2015 | 1 | 8.128 | 77.8% | 3 | K.SYKVSTSGPR.A | 22 |
|  | Mis12IP\_Nocodazole\_MudPIT\_040709\_04.05217.05217.2 | 2.04 | 0.2398 | 98.9% | 871.1122 | 870.9377 | 1 | 4.924 | 78.6% | 1 | R.ISSSSFSR.V | 22 |
|  | Mis12IP\_Nocodazole\_MudPIT\_040709\_04.09547.09547.3 | 5.8647 | 0.4865 | 100.0% | 4007.7844 | 4007.465 | 1 | 9.4 | 24.4% | 4 | R.GGLGGGYGGASGMGGITAVTVNQSLLS\*PLVLEVDPNIQAVR.T | 33 |
|  | Mis12IP\_Nocodazole\_MudPIT\_040709\_06.02941.02941.2 | 3.2482 | 0.0717 | 97.2% | 1574.1522 | 1574.7745 | 2 | 6.37 | 62.5% | 1 | R.TQEKEQIKTLNNK.F | 22 |
|  | Mis12IP\_Nocodazole\_MudPIT\_040709\_05.05262.05262.1 | 2.1158 | 0.1126 | 95.8% | 827.46 | 827.95544 | 11 | 4.725 | 66.7% | 1 | K.FASFIDK.V | 1111111 |
|  | Mis12IP\_Nocodazole\_MudPIT\_040709\_06.07756.07756.2 | 3.7804 | 0.4759 | 100.0% | 1847.9922 | 1849.0431 | 1 | 7.332 | 60.7% | 1 | R.SNMDNMFESYINNLR.R | 22 |
|  | Mis12IP\_Nocodazole\_MudPIT\_040709\_06.05271.05271.2 | 2.1334 | 0.2349 | 98.9% | 1202.0721 | 1202.353 | 9 | 5.014 | 55.6% | 1 | R.RQLETLGQEK.L | 22 |
|  | Mis12IP\_Nocodazole\_MudPIT\_040709\_03.04345.04345.2 | 2.0307 | 0.2685 | 99.0% | 1047.0721 | 1046.1655 | 139 | 5.178 | 62.5% | 1 | R.QLETLGQEK.L | 22 |
|  | Mis12IP\_Nocodazole\_MudPIT\_040709\_06.06670.06670.2 | 4.0109 | 0.3775 | 100.0% | 1353.0322 | 1353.5732 | 2 | 7.479 | 75.0% | 2 | R.TEMENEFVLIK.K | 22 |
|  | Mis12IP\_Nocodazole\_MudPIT\_040709\_06.07360.07360.2 | 3.9924 | 0.4708 | 100.0% | 1420.1522 | 1420.6055 | 1 | 8.141 | 77.3% | 2 | R.LEGLTDEINFLR.Q | 22 |
|  | Mis12IP\_Nocodazole\_MudPIT\_040709\_05.05837.05837.2 | 5.6594 | 0.5529 | 100.0% | 2110.2922 | 2110.3008 | 1 | 10.168 | 75.0% | 2 | R.ELQSQISDTSVVLSMDNSR.S | 22 |
|  | Mis12IP\_Nocodazole\_MudPIT\_040709\_06.07186.07186.2 | 4.1385 | 0.4433 | 100.0% | 1321.1322 | 1321.5286 | 1 | 8.084 | 81.8% | 2 | R.SLDMDSIIAEVK.A | 22 |
|  | Mis12IP\_Nocodazole\_MudPIT\_040709\_03.04595.04595.2 | 3.3908 | 0.4701 | 100.0% | 1169.7922 | 1170.3228 | 2 | 7.3 | 77.8% | 1 | R.AEAESMYQIK.Y | 22 |
|  | Mis12IP\_Nocodazole\_MudPIT\_040709\_04.05588.05588.2 | 3.7095 | 0.079 | 99.3% | 1138.2122 | 1138.2627 | 1 | 6.668 | 83.3% | 2 | K.YEELQSLAGK.H | 22 |
|  | Mis12IP\_Nocodazole\_MudPIT\_040709\_03.04789.04789.2 | 2.6721 | 0.3396 | 99.9% | 1209.2722 | 1209.36 | 18 | 5.53 | 61.1% | 22 | R.TKTEISEMNR.N | 22 |
|  | Mis12IP\_Nocodazole\_MudPIT\_040709\_05.05002.05002.1 | 2.3045 | 0.1171 | 96.2% | 1000.66 | 1001.168 | 1 | 4.764 | 75.0% | 1 | R.LQAEIEGLK.G | 1 |
|  | Mis12IP\_Nocodazole\_MudPIT\_040709\_04.05632.05632.2 | 2.4709 | 0.1379 | 97.5% | 1000.83215 | 1001.168 | 2 | 4.475 | 75.0% | 2 | R.LQAEIEGLK.G | 2 |
|  | Mis12IP\_Nocodazole\_MudPIT\_040709\_06.06112.06112.2 | 4.5976 | 0.4236 | 100.0% | 1345.0922 | 1345.452 | 1 | 7.575 | 75.0% | 3 | R.ASLEAAIADAEQR.G | 22 |
|  | Mis12IP\_Nocodazole\_MudPIT\_040709\_06.06046.06046.2 | 4.3029 | 0.1016 | 99.9% | 1130.0521 | 1130.2865 | 1 | 5.68 | 83.3% | 2 | K.LSELEAALQR.A | 22 |
|  | Mis12IP\_Nocodazole\_MudPIT\_040709\_06.06055.06055.1 | 2.2588 | 0.218 | 99.1% | 1131.62 | 1130.2865 | 1 | 4.016 | 61.1% | 2 | K.LSELEAALQR.A | 11 |
|  | Mis12IP\_Nocodazole\_MudPIT\_040709\_05.05044.05044.1 | 2.1894 | 0.2198 | 99.1% | 1153.49 | 1154.3234 | 28 | 5.686 | 68.8% | 2 | R.EYQELMNVK.L | 1111111 |
|  | Mis12IP\_Nocodazole\_MudPIT\_040709\_04.05650.05650.2 | 2.737 | 0.2522 | 99.8% | 1154.1721 | 1154.3234 | 2 | 6.243 | 75.0% | 2 | R.EYQELMNVK.L | 2222222 |
|  | Mis12IP\_Nocodazole\_MudPIT\_040709\_02.06588.06588.3 | 4.4604 | 0.3011 | 100.0% | 3807.7444 | 3807.9336 | 1 | 7.074 | 23.7% | 3 | K.TTSGYAGGLSSAYGGLTS\*PGLSYSLGSSFGSGAGSSSFSR.T | 33 |
|  | Mis12IP\_Nocodazole\_MudPIT\_040709\_03.04626.04626.2 | 3.5508 | 0.4541 | 100.0% | 1174.0322 | 1174.3367 | 1 | 7.437 | 80.0% | 3 | K.LVSESSDVLPK.- | 22 |

Similarities:
gi|62897441|dbj|BAD96(19:5)  
gi|27465517|ref|NP\_77(2:22)  
gi|46812692|gb|AAH692(2:22)  
gi|21961227|gb|AAH345(2:22)  
gi|17505189|ref|NP\_49(3:21)  
gi|15559584|gb|AAH141(3:21)  
gi|5031841|ref|NP\_005(3:21)  
gi|1200072|emb|CAA316(1:23)  
gi|32567786|ref|NP\_78(1:23)  
gi|45597458|ref|NP\_77(1:23)  

---

|  |  |  |  |  |  |  |  |  |
| --- | --- | --- | --- | --- | --- | --- | --- | --- |
| U | *gi|62897441|dbj|BAD96* | 20 | 57 | 54.7% | 483 | 53738 | 5.6 | keratin 8 variant [Homo sapiens] |

| Filename XCorr DeltCN Conf% ObsM+H+ CalcM+H+ SpR ZScore Ion% # Sequence  | | | | | | | | | | | | |
| --- | --- | --- | --- | --- | --- | --- | --- | --- | --- | --- | --- | --- |
|  | Mis12IP\_Nocodazole\_MudPIT\_040709\_06.05458.05458.2 | 2.8017 | 0.3925 | 100.0% | 1082.0322 | 1082.2015 | 1 | 8.128 | 77.8% | 3 | K.SYKVSTSGPR.A | 22 |
|  | Mis12IP\_Nocodazole\_MudPIT\_040709\_04.05217.05217.2 | 2.04 | 0.2398 | 98.9% | 871.1122 | 870.9377 | 1 | 4.924 | 78.6% | 1 | R.ISSSSFSR.V | 22 |
|  | Mis12IP\_Nocodazole\_MudPIT\_040709\_04.09547.09547.3 | 5.8647 | 0.4865 | 100.0% | 4007.7844 | 4007.465 | 1 | 9.4 | 24.4% | 4 | R.GGLGGGYGGASGMGGITAVTVNQSLLS\*PLVLEVDPNIQAVR.T | 33 |
|  | Mis12IP\_Nocodazole\_MudPIT\_040709\_06.02941.02941.2 | 3.2482 | 0.0717 | 97.2% | 1574.1522 | 1574.7745 | 2 | 6.37 | 62.5% | 1 | R.TQEKEQIKTLNNK.F | 22 |
|  | Mis12IP\_Nocodazole\_MudPIT\_040709\_06.07756.07756.2 | 3.7804 | 0.4759 | 100.0% | 1847.9922 | 1849.0431 | 1 | 7.332 | 60.7% | 1 | R.SNMDNMFESYINNLR.R | 22 |
|  | Mis12IP\_Nocodazole\_MudPIT\_040709\_06.05271.05271.2 | 2.1334 | 0.2349 | 98.9% | 1202.0721 | 1202.353 | 9 | 5.014 | 55.6% | 1 | R.RQLETLGQEK.L | 22 |
|  | Mis12IP\_Nocodazole\_MudPIT\_040709\_03.04345.04345.2 | 2.0307 | 0.2685 | 99.0% | 1047.0721 | 1046.1655 | 139 | 5.178 | 62.5% | 1 | R.QLETLGQEK.L | 22 |
|  | Mis12IP\_Nocodazole\_MudPIT\_040709\_06.06670.06670.2 | 4.0109 | 0.3775 | 100.0% | 1353.0322 | 1353.5732 | 2 | 7.479 | 75.0% | 2 | R.TEMENEFVLIK.K | 22 |
|  | Mis12IP\_Nocodazole\_MudPIT\_040709\_06.07360.07360.2 | 3.9924 | 0.4708 | 100.0% | 1420.1522 | 1420.6055 | 1 | 8.141 | 77.3% | 2 | R.LEGLTDEINFLR.Q | 22 |
|  | Mis12IP\_Nocodazole\_MudPIT\_040709\_05.05837.05837.2 | 5.6594 | 0.5529 | 100.0% | 2110.2922 | 2110.3008 | 1 | 10.168 | 75.0% | 2 | R.ELQSQISDTSVVLSMDNSR.S | 22 |
|  | Mis12IP\_Nocodazole\_MudPIT\_040709\_06.07186.07186.2 | 4.1385 | 0.4433 | 100.0% | 1321.1322 | 1321.5286 | 1 | 8.084 | 81.8% | 2 | R.SLDMDSIIAEVK.A | 22 |
|  | Mis12IP\_Nocodazole\_MudPIT\_040709\_03.04595.04595.2 | 3.3908 | 0.4701 | 100.0% | 1169.7922 | 1170.3228 | 2 | 7.3 | 77.8% | 1 | R.AEAESMYQIK.Y | 22 |
|  | Mis12IP\_Nocodazole\_MudPIT\_040709\_04.05588.05588.2 | 3.7095 | 0.079 | 99.3% | 1138.2122 | 1138.2627 | 1 | 6.668 | 83.3% | 2 | K.YEELQSLAGK.H | 22 |
|  | Mis12IP\_Nocodazole\_MudPIT\_040709\_03.04789.04789.2 | 2.6721 | 0.3396 | 99.9% | 1209.2722 | 1209.36 | 18 | 5.53 | 61.1% | 20 | R.TKTEISEMNR.N | 22 |
|  | Mis12IP\_Nocodazole\_MudPIT\_040709\_06.06112.06112.2 | 4.5976 | 0.4236 | 100.0% | 1345.0922 | 1345.452 | 1 | 7.575 | 75.0% | 3 | R.ASLEAAIADAEQR.G | 22 |
|  | Mis12IP\_Nocodazole\_MudPIT\_040709\_06.06046.06046.2 | 4.3029 | 0.1016 | 99.9% | 1130.0521 | 1130.2865 | 1 | 5.68 | 83.3% | 2 | K.LSELEAALQR.A | 22 |
|  | Mis12IP\_Nocodazole\_MudPIT\_040709\_06.06055.06055.1 | 2.2588 | 0.218 | 99.1% | 1131.62 | 1130.2865 | 1 | 4.016 | 61.1% | 2 | K.LSELEAALQR.A | 11 |
|  | Mis12IP\_Nocodazole\_MudPIT\_040709\_04.05655.05655.1 | 1.9931 | 0.1882 | 98.2% | 1153.45 | 1154.3234 | 90 | 5.252 | 56.2% | 1 | R.EYQELMNVK.L | 1 |
|  | Mis12IP\_Nocodazole\_MudPIT\_040709\_02.06588.06588.3 | 4.4604 | 0.3011 | 100.0% | 3807.7444 | 3807.9336 | 1 | 7.074 | 23.7% | 3 | K.TTSGYAGGLSSAYGGLTS\*PGLSYSLGSSFGSGAGSSSFSR.T | 33 |
|  | Mis12IP\_Nocodazole\_MudPIT\_040709\_03.04626.04626.2 | 3.5508 | 0.4541 | 100.0% | 1174.0322 | 1174.3367 | 1 | 7.437 | 80.0% | 3 | K.LVSESSDVLPK.- | 22 |

Similarities:
gi|4504919|ref|NP\_002(19:1)  

---

|  |  |  |  |  |  |  |  |  |
| --- | --- | --- | --- | --- | --- | --- | --- | --- |
| U | *gi|4432756|dbj|BAA258* | 2 | 2 | 40.0% | 45 | 5146 | 11.4 | ribosomal protein L34 [Homo sapiens] |
| U | *gi|89057301|ref|XP\_94* | 2 | 2 | 15.4% | 117 | 13200 | 11.6 | PREDICTED: similar to ribosomal protein L34 [Homo sapiens] |

| Filename XCorr DeltCN Conf% ObsM+H+ CalcM+H+ SpR ZScore Ion% # Sequence  | | | | | | | | | | | | |
| --- | --- | --- | --- | --- | --- | --- | --- | --- | --- | --- | --- | --- |
|  | Mis12IP\_Nocodazole\_MudPIT\_040709\_06.05348.05348.2 | 1.5631 | 0.3035 | 95.8% | 1256.0322 | 1255.3732 | 311 | 5.683 | 35.0% | 1 | R.LSYNTASNKTR.L | 2 |
|  | Mis12IP\_Nocodazole\_MudPIT\_040709\_06.05843.05843.1 | 1.6874 | 0.2067 | 97.1% | 899.5 | 900.1057 | 5 | 5.136 | 66.7% | 1 | R.IVYLYTK.K | 11 |

Similarities:
gi|113427343|ref|XP\_0(1:1)  

---

|  |  |  |  |  |  |  |  |  |
| --- | --- | --- | --- | --- | --- | --- | --- | --- |
| U | *gi|30048193|gb|AAH507* | 11 | 20 | 39.2% | 199 | 22763 | 5.7 | PMF1 protein [Homo sapiens] |
| U | *gi|88900509|ref|NP\_00* | 11 | 20 | 38.0% | 205 | 23339 | 5.5 | polyamine-modulated factor 1 [Homo sapiens] |
| U | *gi|40787752|gb|AAH650* | 11 | 20 | 38.0% | 205 | 23349 | 5.7 | Polyamine-modulated factor 1 [Homo sapiens] |
| U | *gi|33990589|gb|AAH564* | 11 | 20 | 38.6% | 202 | 23008 | 5.7 | PMF1 protein [Homo sapiens] |

| Filename XCorr DeltCN Conf% ObsM+H+ CalcM+H+ SpR ZScore Ion% # Sequence  | | | | | | | | | | | | |
| --- | --- | --- | --- | --- | --- | --- | --- | --- | --- | --- | --- | --- |
|  | Mis12IP\_Nocodazole\_MudPIT\_040709\_04.02592.02592.2 | 2.3619 | 0.3459 | 99.7% | 1728.4521 | 1728.8131 | 53 | 6.856 | 28.1% | 1 | R.HEGSSSESVPPGTTISR.V | 2 |
|  | Mis12IP\_Nocodazole\_MudPIT\_040709\_06.07782.07782.1 | 2.1322 | 0.3589 | 97.4% | 1423.48 | 1424.6954 | 1 | 6.346 | 54.5% | 1 | K.LLDTMVDTFLQK.L | 1 |
|  | Mis12IP\_Nocodazole\_MudPIT\_040709\_06.07766.07766.2 | 4.3114 | 0.4969 | 100.0% | 1423.5122 | 1424.6954 | 1 | 8.704 | 86.4% | 2 | K.LLDTMVDTFLQK.L | 2 |
|  | Mis12IP\_Nocodazole\_MudPIT\_040709\_04.05206.05206.1 | 1.8995 | 0.3337 | 98.4% | 964.49 | 965.0971 | 5 | 4.851 | 62.5% | 1 | K.LVAAGSYQR.F | 1 |
|  | Mis12IP\_Nocodazole\_MudPIT\_040709\_04.05208.05208.2 | 2.8644 | 0.4073 | 100.0% | 965.0522 | 965.0971 | 1 | 7.565 | 81.2% | 1 | K.LVAAGSYQR.F | 2 |
|  | Mis12IP\_Nocodazole\_MudPIT\_040709\_06.07462.07462.2 | 2.9084 | 0.2616 | 99.7% | 1514.9722 | 1515.6604 | 1 | 6.591 | 65.4% | 2 | K.EEGNLEAVLNALDK.I | 2 |
|  | Mis12IP\_Nocodazole\_MudPIT\_040709\_06.06064.06064.2 | 4.8165 | 0.4057 | 100.0% | 1713.4321 | 1713.8448 | 1 | 8.029 | 73.3% | 2 | K.QEAENQQLADAVLAGR.R | 2 |
|  | Mis12IP\_Nocodazole\_MudPIT\_040709\_05.05488.05488.3 | 3.4731 | 0.3045 | 100.0% | 1713.5343 | 1713.8448 | 1 | 5.436 | 50.0% | 1 | K.QEAENQQLADAVLAGR.R | 3 |
|  | Mis12IP\_Nocodazole\_MudPIT\_040709\_06.05953.05953.1 | 2.1933 | 0.1742 | 99.2% | 800.48 | 799.98895 | 28 | 4.655 | 66.7% | 1 | R.ELVAVLR.E | 1 |
|  | Mis12IP\_Nocodazole\_MudPIT\_040709\_05.05536.05536.1 | 2.1987 | 0.1738 | 98.1% | 1154.07 | 1155.3367 | 1 | 6.209 | 61.1% | 2 | R.ELVAVLREPE.- | 1 |
|  | Mis12IP\_Nocodazole\_MudPIT\_040709\_06.06145.06145.2 | 3.6063 | 0.3613 | 100.0% | 1155.0721 | 1155.3367 | 1 | 7.69 | 66.7% | 6 | R.ELVAVLREPE.- | 2 |

---

|  |  |  |  |  |  |  |  |  |
| --- | --- | --- | --- | --- | --- | --- | --- | --- |
| U | *gi|5729877|ref|NP\_006* | 22 | 73 | 39.0% | 646 | 70898 | 5.5 | heat shock 70kDa protein 8 isoform 1 [Homo sapiens] |

| Filename XCorr DeltCN Conf% ObsM+H+ CalcM+H+ SpR ZScore Ion% # Sequence  | | | | | | | | | | | | |
| --- | --- | --- | --- | --- | --- | --- | --- | --- | --- | --- | --- | --- |
|  | Mis12IP\_Nocodazole\_MudPIT\_040709\_05.05229.05229.1 | 1.9614 | 0.3836 | 97.4% | 1487.44 | 1488.5939 | 1 | 7.022 | 45.8% | 1 | R.TTPSYVAFTDTER.L | 11111 |
|  | Mis12IP\_Nocodazole\_MudPIT\_040709\_06.05909.05909.2 | 3.3634 | 0.4566 | 100.0% | 1488.0721 | 1488.5939 | 1 | 8.603 | 75.0% | 3 | R.TTPSYVAFTDTER.L | 22222 |
|  | Mis12IP\_Nocodazole\_MudPIT\_040709\_06.05944.05944.2 | 4.8557 | 0.5068 | 100.0% | 1650.2122 | 1650.8468 | 1 | 9.113 | 82.1% | 1 | K.NQVAMNPTNTVFDAK.R | 2 |
|  | Mis12IP\_Nocodazole\_MudPIT\_040709\_03.03686.03686.2 | 2.9267 | 0.4214 | 100.0% | 1180.9521 | 1181.3312 | 1 | 6.976 | 77.8% | 7 | K.VQVEYKGETK.S | 22 |
|  | Mis12IP\_Nocodazole\_MudPIT\_040709\_06.06962.06962.1 | 1.5349 | 0.2707 | 98.2% | 1616.79 | 1617.8542 | 76 | 5.738 | 26.9% | 1 | K.SFYPEEVSSMVLTK.M | 1 |
|  | Mis12IP\_Nocodazole\_MudPIT\_040709\_05.06632.06632.2 | 4.1133 | 0.4911 | 100.0% | 1619.1921 | 1617.8542 | 1 | 7.657 | 76.9% | 2 | K.SFYPEEVSSMVLTK.M | 2 |
|  | Mis12IP\_Nocodazole\_MudPIT\_040709\_04.05574.05574.1 | 1.8859 | 0.2445 | 99.2% | 993.54 | 994.1326 | 62 | 6.703 | 56.2% | 1 | K.EIAEAYLGK.T | 11 |
|  | Mis12IP\_Nocodazole\_MudPIT\_040709\_06.06496.06496.2 | 3.246 | 0.475 | 100.0% | 1982.2522 | 1983.1882 | 1 | 7.314 | 61.8% | 2 | K.TVTNAVVTVPAYFNDSQR.Q | 2 |
|  | Mis12IP\_Nocodazole\_MudPIT\_040709\_06.06503.06503.3 | 4.2175 | 0.3549 | 100.0% | 1982.8143 | 1983.1882 | 1 | 6.706 | 47.1% | 2 | K.TVTNAVVTVPAYFNDSQR.Q | 3 |
|  | Mis12IP\_Nocodazole\_MudPIT\_040709\_06.06454.06454.2 | 4.0225 | 0.3278 | 100.0% | 1199.6122 | 1200.3805 | 1 | 6.967 | 86.4% | 3 | K.DAGTIAGLNVLR.I | 22 |
|  | Mis12IP\_Nocodazole\_MudPIT\_040709\_06.06455.06455.1 | 1.6319 | 0.2981 | 99.2% | 1199.65 | 1200.3805 | 214 | 5.188 | 45.5% | 1 | K.DAGTIAGLNVLR.I | 11 |
|  | Mis12IP\_Nocodazole\_MudPIT\_040709\_06.06545.06545.2 | 5.2019 | 0.5351 | 100.0% | 1660.3322 | 1660.9078 | 1 | 9.75 | 73.3% | 1 | R.IINEPTAAAIAYGLDK.K | 2222 |
|  | Mis12IP\_Nocodazole\_MudPIT\_040709\_04.05504.05504.2 | 3.6406 | 0.4415 | 100.0% | 1692.3922 | 1692.6958 | 1 | 8.02 | 63.3% | 13 | K.STAGDTHLGGEDFDNR.M | 22 |
|  | Mis12IP\_Nocodazole\_MudPIT\_040709\_06.06760.06760.2 | 3.5071 | 0.3609 | 100.0% | 1254.0122 | 1254.3849 | 1 | 7.204 | 77.8% | 1 | R.FEELNADLFR.G | 22 |
|  | Mis12IP\_Nocodazole\_MudPIT\_040709\_06.07914.07914.2 | 4.3902 | 0.619 | 100.0% | 2260.5322 | 2261.4937 | 1 | 10.723 | 54.5% | 1 | K.SINPDEAVAYGAAVQAAILSGDK.S | 2 |
|  | Mis12IP\_Nocodazole\_MudPIT\_040709\_03.03625.03625.1 | 1.4309 | 0.3276 | 99.1% | 774.34 | 774.8925 | 50 | 4.779 | 58.3% | 1 | R.NTTIPTK.Q | 111 |
|  | Mis12IP\_Nocodazole\_MudPIT\_040709\_06.06892.06892.3 | 5.7396 | 0.5431 | 100.0% | 2775.3245 | 2775.9885 | 1 | 10.563 | 32.6% | 2 | K.QTQTFTTYSDNQPGVLIQVYEGER.A | 3 |
|  | Mis12IP\_Nocodazole\_MudPIT\_040709\_06.06910.06910.2 | 3.8525 | 0.3105 | 100.0% | 2776.6921 | 2775.9885 | 1 | 6.281 | 41.3% | 2 | K.QTQTFTTYSDNQPGVLIQVYEGER.A | 2 |
|  | Mis12IP\_Nocodazole\_MudPIT\_040709\_04.05654.05654.2 | 2.3494 | 0.4197 | 100.0% | 1018.2922 | 1018.1582 | 2 | 6.376 | 68.8% | 14 | K.ITITNDKGR.L | 22222 |
|  | Mis12IP\_Nocodazole\_MudPIT\_040709\_03.03897.03897.2 | 2.7088 | 0.1491 | 99.2% | 989.97217 | 990.10144 | 1 | 5.416 | 85.7% | 11 | R.LSKEDIER.M | 2 |
|  | Mis12IP\_Nocodazole\_MudPIT\_040709\_06.06316.06316.2 | 3.8991 | 0.4403 | 100.0% | 1304.9321 | 1304.4602 | 2 | 7.398 | 80.0% | 2 | K.NSLESYAFNMK.A | 2 |
|  | Mis12IP\_Nocodazole\_MudPIT\_040709\_02.06552.06552.3 | 2.8657 | 0.3376 | 100.0% | 3348.3245 | 3348.64 | 1 | 5.285 | 19.4% | 1 | K.LYQSAGGMPGGMPGGFPGGGAPPSGGASSGPTIEEVD.- | 3 |

Similarities:
gi|16507237|ref|NP\_00(1:21)  
gi|12653415|gb|AAH004(1:21)  
gi|13676857|ref|NP\_06(8:14)  
gi|12803275|gb|AAH024(3:19)  
gi|51095055|gb|EAL242(3:19)  
gi|27436929|ref|NP\_00(4:18)  
gi|34419635|ref|NP\_00(3:19)  

---

|  |  |  |  |  |  |  |  |  |
| --- | --- | --- | --- | --- | --- | --- | --- | --- |
| U | *gi|181402|gb|AAC83410* | 25 | 41 | 37.5% | 645 | 65865 | 8.0 | epidermal cytokeratin 2 [Homo sapiens] |
| U | *gi|47132620|ref|NP\_00* | 25 | 41 | 37.9% | 639 | 65433 | 8.0 | keratin 2 [Homo sapiens] |

| Filename XCorr DeltCN Conf% ObsM+H+ CalcM+H+ SpR ZScore Ion% # Sequence  | | | | | | | | | | | | |
| --- | --- | --- | --- | --- | --- | --- | --- | --- | --- | --- | --- | --- |
|  | Mis12IP\_Nocodazole\_MudPIT\_040709\_05.04674.04674.1 | 2.0588 | 0.3359 | 98.0% | 1254.58 | 1255.3298 | 9 | 6.704 | 42.3% | 1 | R.GFSSGSAVVSGGSR.R | 1 |
|  | Mis12IP\_Nocodazole\_MudPIT\_040709\_05.04665.04665.2 | 4.7915 | 0.4112 | 100.0% | 1256.0721 | 1255.3298 | 1 | 8.971 | 76.9% | 2 | R.GFSSGSAVVSGGSR.R | 2 |
|  | Mis12IP\_Nocodazole\_MudPIT\_040709\_05.04956.04956.1 | 1.4381 | 0.4089 | 98.6% | 831.54 | 831.9878 | 17 | 6.661 | 43.8% | 1 | R.SLVGLGGTK.S | 1 |
|  | Mis12IP\_Nocodazole\_MudPIT\_040709\_06.05713.05713.2 | 2.2218 | 0.1921 | 98.0% | 831.77216 | 831.9878 | 1 | 6.24 | 75.0% | 1 | R.SLVGLGGTK.S | 2 |
|  | Mis12IP\_Nocodazole\_MudPIT\_040709\_06.05752.05752.2 | 4.2312 | 0.0778 | 99.6% | 1476.3522 | 1476.6726 | 3 | 7.319 | 86.4% | 1 | R.FLEQQNQVLQTK.W | 222 |
|  | Mis12IP\_Nocodazole\_MudPIT\_040709\_04.05710.05710.2 | 1.7806 | 0.2896 | 98.2% | 1037.3322 | 1038.1454 | 22 | 5.459 | 62.5% | 1 | R.YLDGLTAER.T | 2 |
|  | Mis12IP\_Nocodazole\_MudPIT\_040709\_04.05669.05669.1 | 2.001 | 0.3069 | 98.6% | 1037.5 | 1038.1454 | 87 | 6.388 | 50.0% | 2 | R.YLDGLTAER.T | 1 |
|  | Mis12IP\_Nocodazole\_MudPIT\_040709\_06.06582.06582.2 | 5.5264 | 0.426 | 100.0% | 2129.5122 | 2129.2598 | 1 | 10.1 | 61.8% | 2 | R.TSQNSELNNMQDLVEDYK.K | 2 |
|  | Mis12IP\_Nocodazole\_MudPIT\_040709\_04.05780.05780.1 | 1.606 | 0.2305 | 95.8% | 1208.67 | 1209.3416 | 2 | 4.693 | 55.0% | 1 | R.TAAENDFVTLK.K | 1 |
|  | Mis12IP\_Nocodazole\_MudPIT\_040709\_04.05764.05764.2 | 2.9612 | 0.3183 | 99.9% | 1208.9922 | 1209.3416 | 1 | 5.603 | 75.0% | 2 | R.TAAENDFVTLK.K | 2 |
|  | Mis12IP\_Nocodazole\_MudPIT\_040709\_06.07585.07585.2 | 3.8346 | 0.2466 | 100.0% | 1461.0322 | 1461.6982 | 1 | 6.061 | 77.3% | 2 | K.VDLLNQEIEFLK.V | 2 |
|  | Mis12IP\_Nocodazole\_MudPIT\_040709\_06.07734.07734.1 | 2.6237 | 0.3674 | 100.0% | 1329.6 | 1330.5211 | 1 | 7.145 | 68.2% | 3 | R.NLDLDSIIAEVK.A | 111111111 |
|  | Mis12IP\_Nocodazole\_MudPIT\_040709\_06.07727.07727.2 | 4.4229 | 0.3694 | 100.0% | 1331.0122 | 1330.5211 | 1 | 7.672 | 81.8% | 2 | R.NLDLDSIIAEVK.A | 222222222 |
|  | Mis12IP\_Nocodazole\_MudPIT\_040709\_03.04273.04273.2 | 3.0135 | 0.0704 | 98.3% | 1108.1721 | 1108.196 | 1 | 6.972 | 81.2% | 1 | K.AQYEEIAQR.S | 2222222 |
|  | Mis12IP\_Nocodazole\_MudPIT\_040709\_05.05032.05032.2 | 3.7256 | 0.3652 | 100.0% | 1193.4321 | 1194.33 | 1 | 8.55 | 88.9% | 3 | K.YEELQVTVGR.H | 2 |
|  | Mis12IP\_Nocodazole\_MudPIT\_040709\_03.04772.04772.2 | 2.3524 | 0.2026 | 99.2% | 973.7522 | 974.102 | 4 | 4.285 | 78.6% | 1 | K.IEISELNR.V | 22 |
|  | Mis12IP\_Nocodazole\_MudPIT\_040709\_03.04710.04710.2 | 3.7655 | 0.4746 | 100.0% | 1330.3922 | 1330.3971 | 1 | 7.556 | 77.3% | 1 | K.NVQDAIADAEQR.G | 2 |
|  | Mis12IP\_Nocodazole\_MudPIT\_040709\_04.05859.05859.2 | 2.3297 | 0.2954 | 99.7% | 1139.6322 | 1140.2965 | 2 | 6.109 | 75.0% | 1 | R.DYQELMNVK.L | 22 |
|  | Mis12IP\_Nocodazole\_MudPIT\_040709\_06.06269.06269.2 | 1.9951 | 0.3116 | 98.9% | 2458.392 | 2459.685 | 97 | 4.848 | 22.9% | 1 | R.MSGDLSSNVTVSVTSSTISSNVASK.A | 2 |
|  | Mis12IP\_Nocodazole\_MudPIT\_040709\_06.06240.06240.3 | 3.2238 | 0.4387 | 100.0% | 2459.7244 | 2459.685 | 1 | 6.853 | 28.1% | 1 | R.MSGDLSSNVTVSVTSSTISSNVASK.A | 3 |
|  | Mis12IP\_Nocodazole\_MudPIT\_040709\_06.05431.05431.2 | 3.0269 | 0.4337 | 100.0% | 2502.5122 | 2502.4893 | 1 | 7.415 | 33.9% | 1 | K.AAFGGSGGRGSSSGGGYSSGSSSYGSGGR.Q | 2 |
|  | Mis12IP\_Nocodazole\_MudPIT\_040709\_05.04650.04650.3 | 4.1094 | 0.4328 | 100.0% | 2502.8342 | 2502.4893 | 1 | 6.432 | 34.8% | 2 | K.AAFGGSGGRGSSSGGGYSSGSSSYGSGGR.Q | 3 |
|  | Mis12IP\_Nocodazole\_MudPIT\_040709\_04.05043.05043.3 | 3.797 | 0.2778 | 100.0% | 2257.9744 | 2258.2424 | 1 | 5.974 | 33.3% | 2 | R.QSGSRGGSGGGGSISGGGYGSGGGSGGR.Y | 3 |
|  | Mis12IP\_Nocodazole\_MudPIT\_040709\_04.04966.04966.2 | 4.8164 | 0.4227 | 100.0% | 1742.0322 | 1742.716 | 1 | 8.847 | 47.7% | 4 | R.GGSGGGGSISGGGYGSGGGSGGR.Y | 2 |
|  | Mis12IP\_Nocodazole\_MudPIT\_040709\_03.03912.03912.2 | 2.5899 | 0.4242 | 100.0% | 1197.6721 | 1198.2346 | 1 | 7.709 | 53.6% | 2 | K.GGSISGGGYGSGGGK.H | 2 |

Similarities:
gi|17318569|ref|NP\_00(2:23)  
gi|27465517|ref|NP\_77(3:22)  
gi|46812692|gb|AAH692(3:22)  
gi|21961227|gb|AAH345(3:22)  
gi|17505189|ref|NP\_49(3:22)  
gi|15559584|gb|AAH141(3:22)  
gi|5031841|ref|NP\_005(3:22)  
gi|18999435|gb|AAH242(2:23)  
gi|32567786|ref|NP\_78(3:22)  
gi|45597458|ref|NP\_77(1:24)  

---

|  |  |  |  |  |  |  |  |  |
| --- | --- | --- | --- | --- | --- | --- | --- | --- |
| U | *gi|17318569|ref|NP\_00* | 28 | 77 | 36.3% | 644 | 66067 | 8.1 | keratin 1 [Homo sapiens] |

| Filename XCorr DeltCN Conf% ObsM+H+ CalcM+H+ SpR ZScore Ion% # Sequence  | | | | | | | | | | | | |
| --- | --- | --- | --- | --- | --- | --- | --- | --- | --- | --- | --- | --- |
|  | Mis12IP\_Nocodazole\_MudPIT\_040709\_05.04987.04987.1 | 1.7116 | 0.3829 | 98.0% | 874.56 | 875.0128 | 1 | 6.577 | 62.5% | 2 | R.SLVNLGGSK.S | 1 |
|  | Mis12IP\_Nocodazole\_MudPIT\_040709\_05.04907.04907.2 | 2.2621 | 0.3554 | 99.9% | 833.0122 | 832.9755 | 1 | 6.231 | 78.6% | 2 | K.SISISVAR.G | 2 |
|  | Mis12IP\_Nocodazole\_MudPIT\_040709\_06.05752.05752.2 | 4.2312 | 0.0778 | 99.6% | 1476.3522 | 1476.6726 | 3 | 7.319 | 86.4% | 1 | R.FLEQQNQVLQTK.W | 222 |
|  | Mis12IP\_Nocodazole\_MudPIT\_040709\_06.06605.06605.2 | 4.3458 | 0.5433 | 100.0% | 1476.2522 | 1476.6293 | 1 | 8.753 | 86.4% | 2 | K.WELLQQVDTSTR.T | 2 |
|  | Mis12IP\_Nocodazole\_MudPIT\_040709\_04.05846.05846.2 | 2.9869 | 0.4781 | 100.0% | 1300.9722 | 1301.4316 | 1 | 7.765 | 72.2% | 1 | K.NMQDMVEDYR.N | 2 |
|  | Mis12IP\_Nocodazole\_MudPIT\_040709\_05.05082.05082.2 | 3.5036 | 0.3533 | 100.0% | 1266.0322 | 1266.3934 | 1 | 7.307 | 90.0% | 3 | R.TNAENEFVTIK.K | 2 |
|  | Mis12IP\_Nocodazole\_MudPIT\_040709\_02.03963.03963.1 | 1.8627 | 0.3537 | 98.1% | 999.51 | 1000.1114 | 2 | 5.995 | 62.5% | 1 | K.DVDGAYMTK.V | 1 |
|  | Mis12IP\_Nocodazole\_MudPIT\_040709\_02.03962.03962.2 | 2.6863 | 0.3944 | 100.0% | 999.9322 | 1000.1114 | 1 | 6.537 | 87.5% | 1 | K.DVDGAYMTK.V | 2 |
|  | Mis12IP\_Nocodazole\_MudPIT\_040709\_06.07726.07726.2 | 4.3776 | 0.4189 | 100.0% | 1303.1721 | 1303.4955 | 1 | 8.437 | 81.8% | 7 | R.SLDLDSIIAEVK.A | 2 |
|  | Mis12IP\_Nocodazole\_MudPIT\_040709\_06.07738.07738.1 | 2.6819 | 0.1543 | 99.2% | 1304.59 | 1303.4955 | 7 | 4.379 | 54.5% | 3 | R.SLDLDSIIAEVK.A | 1 |
| \* | Mis12IP\_Nocodazole\_MudPIT\_040709\_02.03938.03938.1 | 2.2063 | 0.2357 | 98.9% | 1065.47 | 1066.1558 | 4 | 4.953 | 68.8% | 1 | K.AQYEDIAQK.S | 1 |
| \* | Mis12IP\_Nocodazole\_MudPIT\_040709\_02.03932.03932.2 | 3.2465 | 0.2827 | 100.0% | 1065.6122 | 1066.1558 | 1 | 6.466 | 75.0% | 1 | K.AQYEDIAQK.S | 2 |
|  | Mis12IP\_Nocodazole\_MudPIT\_040709\_03.06933.06933.2 | 3.0163 | 0.2339 | 99.7% | 1340.9922 | 1341.4607 | 1 | 6.237 | 63.6% | 25 | K.SKAEAESLYQSK.Y | 2 |
|  | Mis12IP\_Nocodazole\_MudPIT\_040709\_02.03977.03977.1 | 2.1853 | 0.2803 | 98.8% | 1125.48 | 1126.2084 | 2 | 6.301 | 66.7% | 1 | K.AEAESLYQSK.Y | 1 |
|  | Mis12IP\_Nocodazole\_MudPIT\_040709\_02.03968.03968.2 | 3.6358 | 0.4384 | 100.0% | 1126.0922 | 1126.2084 | 1 | 7.702 | 88.9% | 1 | K.AEAESLYQSK.Y | 2 |
|  | Mis12IP\_Nocodazole\_MudPIT\_040709\_04.05612.05612.1 | 2.7425 | 0.1273 | 98.6% | 1179.53 | 1180.303 | 2 | 4.69 | 66.7% | 3 | K.YEELQITAGR.H | 11 |
|  | Mis12IP\_Nocodazole\_MudPIT\_040709\_05.04993.04993.2 | 3.9395 | 0.3218 | 100.0% | 1181.1122 | 1180.303 | 1 | 7.266 | 88.9% | 5 | K.YEELQITAGR.H | 22 |
|  | Mis12IP\_Nocodazole\_MudPIT\_040709\_03.04772.04772.2 | 2.3524 | 0.2026 | 99.2% | 973.7522 | 974.102 | 4 | 4.285 | 78.6% | 1 | K.IEISELNR.V | 22 |
|  | Mis12IP\_Nocodazole\_MudPIT\_040709\_06.05801.05801.2 | 4.2553 | 0.5914 | 100.0% | 1717.2122 | 1717.8333 | 1 | 9.369 | 60.7% | 2 | K.QISNLQQSISDAEQR.G | 2 |
|  | Mis12IP\_Nocodazole\_MudPIT\_040709\_05.05505.05505.2 | 4.5507 | 0.3942 | 100.0% | 1358.1921 | 1358.4912 | 1 | 7.849 | 81.8% | 1 | K.LNDLEDALQQAK.E | 2 |
|  | Mis12IP\_Nocodazole\_MudPIT\_040709\_03.04712.04712.2 | 2.9482 | 0.3465 | 100.0% | 1141.4722 | 1142.2689 | 9 | 6.201 | 68.8% | 1 | R.DYQELMNTK.L | 2 |
|  | Mis12IP\_Nocodazole\_MudPIT\_040709\_02.04143.04143.1 | 2.0251 | 0.2845 | 98.8% | 1033.49 | 1034.1112 | 1 | 5.991 | 62.5% | 2 | R.TLLEGEESR.M | 1 |
|  | Mis12IP\_Nocodazole\_MudPIT\_040709\_02.04077.04077.2 | 2.5873 | 0.2541 | 99.7% | 1034.2122 | 1034.1112 | 2 | 5.065 | 75.0% | 2 | R.TLLEGEESR.M | 2 |
|  | Mis12IP\_Nocodazole\_MudPIT\_040709\_04.05187.05187.3 | 7.5059 | 0.5046 | 100.0% | 2385.2944 | 2385.298 | 1 | 10.05 | 38.3% | 1 | R.GGGGGGYGSGGSSYGSGGGSYGSGGGGGGGR.G | 3 |
|  | Mis12IP\_Nocodazole\_MudPIT\_040709\_04.05192.05192.2 | 5.7919 | 0.58 | 100.0% | 2385.372 | 2385.298 | 1 | 10.709 | 40.0% | 1 | R.GGGGGGYGSGGSSYGSGGGSYGSGGGGGGGR.G | 2 |
|  | Mis12IP\_Nocodazole\_MudPIT\_040709\_06.04765.04765.3 | 3.9667 | 0.4848 | 100.0% | 2080.3442 | 2081.0396 | 1 | 8.557 | 32.4% | 2 | R.GGSGGGGGGSSGGRGSGGGSSGGSIGGR.G | 3 |
|  | Mis12IP\_Nocodazole\_MudPIT\_040709\_03.03573.03573.2 | 2.6149 | 0.4206 | 100.0% | 1093.0922 | 1093.0983 | 3 | 7.079 | 46.2% | 1 | R.GSGGGSSGGSIGGR.G | 2 |
|  | Mis12IP\_Nocodazole\_MudPIT\_040709\_06.04886.04886.2 | 4.7438 | 0.5028 | 100.0% | 1751.7722 | 1752.7953 | 1 | 9.242 | 57.1% | 3 | R.GSGGGSSGGSIGGRGSSSGGVK.S | 2 |

Similarities:
gi|181402|gb|AAC83410(2:26)  
gi|21961227|gb|AAH345(2:26)  
gi|45597458|ref|NP\_77(1:27)  

---

|  |  |  |  |  |  |  |  |  |
| --- | --- | --- | --- | --- | --- | --- | --- | --- |
| U | *gi|37852|emb|CAA79613* | 20 | 37 | 36.3% | 466 | 53686 | 5.1 | vimentin [Homo sapiens] |
| U | *gi|62414289|ref|NP\_00* | 20 | 37 | 36.3% | 466 | 53652 | 5.1 | vimentin [Homo sapiens] |

| Filename XCorr DeltCN Conf% ObsM+H+ CalcM+H+ SpR ZScore Ion% # Sequence  | | | | | | | | | | | | |
| --- | --- | --- | --- | --- | --- | --- | --- | --- | --- | --- | --- | --- |
|  | Mis12IP\_Nocodazole\_MudPIT\_040709\_03.03795.03795.2 | 2.1218 | 0.273 | 99.3% | 914.71216 | 914.99084 | 1 | 6.65 | 78.6% | 2 | R.SYVTTSTR.T | 2 |
|  | Mis12IP\_Nocodazole\_MudPIT\_040709\_06.05675.05675.2 | 4.3321 | 0.4903 | 100.0% | 1429.0922 | 1429.5724 | 1 | 7.887 | 76.9% | 1 | R.SLYASSPGGVYATR.S | 2 |
|  | Mis12IP\_Nocodazole\_MudPIT\_040709\_02.04251.04251.2 | 4.2197 | 0.4215 | 100.0% | 1509.3722 | 1509.5724 | 1 | 6.767 | 76.9% | 1 | R.SLYASS\*PGGVYATR.S | 2 |
|  | Mis12IP\_Nocodazole\_MudPIT\_040709\_06.08141.08141.3 | 4.0173 | 0.4277 | 100.0% | 2126.5745 | 2127.3557 | 1 | 7.324 | 43.1% | 1 | R.LLQDSVDFSLADAINTEFK.N | 3 |
|  | Mis12IP\_Nocodazole\_MudPIT\_040709\_06.08122.08122.2 | 5.5132 | 0.5623 | 100.0% | 2126.7322 | 2127.3557 | 1 | 10.543 | 63.9% | 7 | R.LLQDSVDFSLADAINTEFK.N | 2 |
|  | Mis12IP\_Nocodazole\_MudPIT\_040709\_03.04591.04591.2 | 2.7141 | 0.2481 | 99.7% | 1116.2322 | 1116.2163 | 16 | 5.363 | 75.0% | 1 | K.VELQELNDR.F | 2 |
|  | Mis12IP\_Nocodazole\_MudPIT\_040709\_03.04616.04616.1 | 1.8916 | 0.1719 | 97.6% | 870.43 | 870.9805 | 24 | 4.97 | 66.7% | 1 | R.FANYIDK.V | 11 |
|  | Mis12IP\_Nocodazole\_MudPIT\_040709\_03.04920.04920.2 | 3.4746 | 0.4453 | 100.0% | 1254.9521 | 1255.385 | 1 | 7.98 | 83.3% | 1 | R.LGDLYEEEMR.E | 22 |
|  | Mis12IP\_Nocodazole\_MudPIT\_040709\_02.05153.05153.2 | 2.8547 | 0.2459 | 99.9% | 1077.2722 | 1077.1975 | 1 | 6.521 | 75.0% | 2 | R.DNLAEDIMR.L | 22 |
|  | Mis12IP\_Nocodazole\_MudPIT\_040709\_03.04515.04515.2 | 2.6693 | 0.1923 | 99.4% | 1046.5122 | 1047.2146 | 11 | 4.629 | 78.6% | 1 | K.LQEEMLQR.E | 22 |
|  | Mis12IP\_Nocodazole\_MudPIT\_040709\_03.04723.04723.2 | 2.9584 | 0.4529 | 100.0% | 1324.0922 | 1324.3898 | 1 | 7.196 | 70.0% | 1 | R.EEAENTLQSFR.Q | 2 |
|  | Mis12IP\_Nocodazole\_MudPIT\_040709\_02.03408.03408.2 | 2.2736 | 0.3047 | 99.6% | 1088.9521 | 1089.1503 | 4 | 5.788 | 66.7% | 1 | R.QDVDNASLAR.L | 22 |
|  | Mis12IP\_Nocodazole\_MudPIT\_040709\_06.05981.05981.1 | 2.2422 | 0.2644 | 98.8% | 1309.61 | 1310.4056 | 8 | 5.566 | 61.1% | 2 | K.NLQEAEEWYK.S | 11 |
|  | Mis12IP\_Nocodazole\_MudPIT\_040709\_05.05333.05333.2 | 3.7986 | 0.3583 | 100.0% | 1309.6721 | 1310.4056 | 8 | 6.262 | 66.7% | 2 | K.NLQEAEEWYK.S | 22 |
|  | Mis12IP\_Nocodazole\_MudPIT\_040709\_04.05448.05448.2 | 3.854 | 0.443 | 100.0% | 1093.5521 | 1094.1692 | 1 | 8.376 | 83.3% | 2 | K.FADLSEAANR.N | 22 |
|  | Mis12IP\_Nocodazole\_MudPIT\_040709\_05.06029.06029.3 | 3.8596 | 0.3854 | 100.0% | 2187.3245 | 2188.33 | 1 | 7.314 | 45.8% | 1 | R.EMEENFAVEAANYQDTIGR.L | 3 |
|  | Mis12IP\_Nocodazole\_MudPIT\_040709\_06.06456.06456.2 | 5.4613 | 0.5755 | 100.0% | 2188.5322 | 2188.33 | 1 | 10.09 | 63.9% | 3 | R.EMEENFAVEAANYQDTIGR.L | 2 |
|  | Mis12IP\_Nocodazole\_MudPIT\_040709\_05.05409.05409.1 | 2.0294 | 0.2104 | 98.6% | 1121.51 | 1122.2633 | 30 | 4.922 | 62.5% | 1 | R.EYQDLLNVK.M | 11 |
|  | Mis12IP\_Nocodazole\_MudPIT\_040709\_06.06061.06061.2 | 2.3286 | 0.3869 | 99.9% | 1121.9922 | 1122.2633 | 1 | 6.184 | 87.5% | 2 | R.EYQDLLNVK.M | 22 |
|  | Mis12IP\_Nocodazole\_MudPIT\_040709\_02.04997.04997.2 | 2.7906 | 0.2874 | 99.7% | 1837.0521 | 1837.854 | 1 | 5.278 | 50.0% | 4 | R.DGQVINETSQHHDDLE.- | 22 |

Similarities:
gi|5030431|gb|AAA6128(11:9)  

---

|  |  |  |  |  |  |  |  |  |
| --- | --- | --- | --- | --- | --- | --- | --- | --- |
| U | *gi|4557888|ref|NP\_000* | 20 | 35 | 36.0% | 430 | 48058 | 5.5 | keratin 18 [Homo sapiens] |

| Filename XCorr DeltCN Conf% ObsM+H+ CalcM+H+ SpR ZScore Ion% # Sequence  | | | | | | | | | | | | |
| --- | --- | --- | --- | --- | --- | --- | --- | --- | --- | --- | --- | --- |
|  | Mis12IP\_Nocodazole\_MudPIT\_040709\_04.05292.05292.2 | 1.7969 | 0.3544 | 99.3% | 976.1922 | 976.0336 | 1 | 6.524 | 78.6% | 1 | R.STFSTNYR.S | 22 |
|  | Mis12IP\_Nocodazole\_MudPIT\_040709\_06.05686.05686.2 | 4.3034 | 0.4516 | 100.0% | 1320.0521 | 1320.4478 | 1 | 7.86 | 77.3% | 2 | R.AQIFANTVDNAR.I | 22 |
|  | Mis12IP\_Nocodazole\_MudPIT\_040709\_05.05122.05122.2 | 2.6251 | 0.2664 | 99.8% | 1041.4722 | 1042.2235 | 1 | 5.746 | 87.5% | 1 | R.IVLQIDNAR.L | 222 |
|  | Mis12IP\_Nocodazole\_MudPIT\_040709\_06.05825.05825.1 | 2.4765 | 0.3556 | 100.0% | 1041.51 | 1042.2235 | 2 | 6.107 | 68.8% | 1 | R.IVLQIDNAR.L | 111 |
|  | Mis12IP\_Nocodazole\_MudPIT\_040709\_02.04220.04220.1 | 1.5217 | 0.3222 | 99.0% | 807.44 | 807.8815 | 2 | 4.741 | 58.3% | 1 | R.LAADDFR.V | 11111111 |
|  | Mis12IP\_Nocodazole\_MudPIT\_040709\_03.04543.04543.2 | 2.2948 | 0.233 | 99.3% | 808.0522 | 807.8815 | 1 | 5.671 | 91.7% | 3 | R.LAADDFR.V | 22222222 |
|  | Mis12IP\_Nocodazole\_MudPIT\_040709\_03.04619.04619.2 | 2.2043 | 0.3927 | 99.9% | 1013.1122 | 1013.1535 | 1 | 5.883 | 85.7% | 1 | K.YETELAMR.Q | 22 |
|  | Mis12IP\_Nocodazole\_MudPIT\_040709\_06.05437.05437.2 | 3.2128 | 0.2343 | 99.9% | 1174.4321 | 1175.3274 | 2 | 6.191 | 83.3% | 1 | R.KVIDDTNITR.L | 22 |
|  | Mis12IP\_Nocodazole\_MudPIT\_040709\_02.03994.03994.2 | 2.8496 | 0.4555 | 100.0% | 1046.6122 | 1047.1533 | 1 | 7.974 | 87.5% | 1 | K.VIDDTNITR.L | 22 |
|  | Mis12IP\_Nocodazole\_MudPIT\_040709\_06.06536.06536.2 | 2.4077 | 0.41 | 99.9% | 1885.4521 | 1885.1246 | 1 | 6.115 | 50.0% | 1 | K.GLQAQIASSGLTVEVDAPK.S | 22 |
|  | Mis12IP\_Nocodazole\_MudPIT\_040709\_02.04068.04068.1 | 1.8018 | 0.3376 | 98.5% | 965.26 | 966.0385 | 22 | 5.82 | 57.1% | 1 | R.AQYDELAR.K | 11 |
|  | Mis12IP\_Nocodazole\_MudPIT\_040709\_05.06341.06341.2 | 3.9991 | 0.5389 | 100.0% | 1507.2722 | 1507.699 | 1 | 8.888 | 62.5% | 4 | R.TVQSLEIDLDSMR.N | 22 |
|  | Mis12IP\_Nocodazole\_MudPIT\_040709\_06.06544.06544.2 | 3.4647 | 0.422 | 100.0% | 1420.1122 | 1420.6055 | 1 | 6.905 | 81.8% | 1 | R.QAQEYEALLNIK.V | 22 |
|  | Mis12IP\_Nocodazole\_MudPIT\_040709\_03.04677.04677.2 | 2.8426 | 0.4463 | 100.0% | 1065.6522 | 1066.1992 | 1 | 7.162 | 81.2% | 1 | K.LEAEIATYR.R | 22 |
|  | Mis12IP\_Nocodazole\_MudPIT\_040709\_06.07085.07085.3 | 5.9061 | 0.5761 | 100.0% | 2741.2144 | 2741.9404 | 1 | 10.901 | 41.7% | 3 | R.LLEDGEDFNLGDALDSSNSMQTIQK.T | 33 |
|  | Mis12IP\_Nocodazole\_MudPIT\_040709\_05.06833.06833.2 | 5.1411 | 0.4192 | 100.0% | 2743.652 | 2741.9404 | 1 | 8.364 | 47.9% | 5 | R.LLEDGEDFNLGDALDSSNSMQTIQK.T | 22 |
|  | Mis12IP\_Nocodazole\_MudPIT\_040709\_02.06177.06177.3 | 5.7195 | 0.4857 | 100.0% | 2821.7644 | 2821.9404 | 2 | 7.146 | 41.7% | 1 | R.LLEDGEDFNLGDALDSSNS\*MQTIQK.T | 33 |
|  | Mis12IP\_Nocodazole\_MudPIT\_040709\_06.05346.05346.2 | 3.1981 | 0.52 | 100.0% | 1660.3522 | 1661.8528 | 1 | 8.175 | 60.7% | 1 | R.RIVDGKVVSETNDTK.V | 22 |
|  | Mis12IP\_Nocodazole\_MudPIT\_040709\_06.05344.05344.3 | 2.7637 | 0.3661 | 100.0% | 1660.8544 | 1661.8528 | 3 | 6.087 | 35.7% | 1 | R.RIVDGKVVSETNDTK.V | 33 |
|  | Mis12IP\_Nocodazole\_MudPIT\_040709\_03.03626.03626.2 | 3.3044 | 0.4687 | 100.0% | 1505.3922 | 1505.6653 | 1 | 8.05 | 73.1% | 4 | R.IVDGKVVSETNDTK.V | 22 |

Similarities:
gi|62897747|dbj|BAD96(18:2)  
gi|24430192|ref|NP\_00(2:18)  
gi|12803709|gb|AAH026(2:18)  
gi|4557701|ref|NP\_000(2:18)  
gi|14043271|gb|AAH076(4:16)  
gi|24430190|ref|NP\_00(2:18)  
gi|34526448|dbj|BAC85(2:18)  
gi|85566621|gb|AAI119(2:18)  

---

|  |  |  |  |  |  |  |  |  |
| --- | --- | --- | --- | --- | --- | --- | --- | --- |
| U | *gi|62897747|dbj|BAD96* | 19 | 32 | 34.4% | 430 | 47986 | 5.5 | keratin 18 variant [Homo sapiens] |

| Filename XCorr DeltCN Conf% ObsM+H+ CalcM+H+ SpR ZScore Ion% # Sequence  | | | | | | | | | | | | |
| --- | --- | --- | --- | --- | --- | --- | --- | --- | --- | --- | --- | --- |
|  | Mis12IP\_Nocodazole\_MudPIT\_040709\_04.05292.05292.2 | 1.7969 | 0.3544 | 99.3% | 976.1922 | 976.0336 | 1 | 6.524 | 78.6% | 1 | R.STFSTNYR.S | 22 |
|  | Mis12IP\_Nocodazole\_MudPIT\_040709\_06.05686.05686.2 | 4.3034 | 0.4516 | 100.0% | 1320.0521 | 1320.4478 | 1 | 7.86 | 77.3% | 2 | R.AQIFANTVDNAR.I | 22 |
|  | Mis12IP\_Nocodazole\_MudPIT\_040709\_05.05122.05122.2 | 2.6251 | 0.2664 | 99.8% | 1041.4722 | 1042.2235 | 1 | 5.746 | 87.5% | 1 | R.IVLQIDNAR.L | 222 |
|  | Mis12IP\_Nocodazole\_MudPIT\_040709\_06.05825.05825.1 | 2.4765 | 0.3556 | 100.0% | 1041.51 | 1042.2235 | 2 | 6.107 | 68.8% | 1 | R.IVLQIDNAR.L | 111 |
|  | Mis12IP\_Nocodazole\_MudPIT\_040709\_03.04619.04619.2 | 2.2043 | 0.3927 | 99.9% | 1013.1122 | 1013.1535 | 1 | 5.883 | 85.7% | 1 | K.YETELAMR.Q | 22 |
|  | Mis12IP\_Nocodazole\_MudPIT\_040709\_06.05437.05437.2 | 3.2128 | 0.2343 | 99.9% | 1174.4321 | 1175.3274 | 2 | 6.191 | 83.3% | 1 | R.KVIDDTNITR.L | 22 |
|  | Mis12IP\_Nocodazole\_MudPIT\_040709\_02.03994.03994.2 | 2.8496 | 0.4555 | 100.0% | 1046.6122 | 1047.1533 | 1 | 7.974 | 87.5% | 1 | K.VIDDTNITR.L | 22 |
|  | Mis12IP\_Nocodazole\_MudPIT\_040709\_06.06536.06536.2 | 2.4077 | 0.41 | 99.9% | 1885.4521 | 1885.1246 | 1 | 6.115 | 50.0% | 1 | K.GLQAQIASSGLTVEVDAPK.S | 22 |
|  | Mis12IP\_Nocodazole\_MudPIT\_040709\_02.04068.04068.1 | 1.8018 | 0.3376 | 98.5% | 965.26 | 966.0385 | 22 | 5.82 | 57.1% | 1 | R.AQYDELAR.K | 11 |
|  | Mis12IP\_Nocodazole\_MudPIT\_040709\_06.06703.06703.1 | 1.3905 | 0.3509 | 99.1% | 1506.63 | 1507.699 | 94 | 5.053 | 33.3% | 1 | R.TVQSLEIDLDSMR.N | 1 |
|  | Mis12IP\_Nocodazole\_MudPIT\_040709\_05.06341.06341.2 | 3.9991 | 0.5389 | 100.0% | 1507.2722 | 1507.699 | 1 | 8.888 | 62.5% | 4 | R.TVQSLEIDLDSMR.N | 22 |
|  | Mis12IP\_Nocodazole\_MudPIT\_040709\_06.06544.06544.2 | 3.4647 | 0.422 | 100.0% | 1420.1122 | 1420.6055 | 1 | 6.905 | 81.8% | 1 | R.QAQEYEALLNIK.V | 22 |
|  | Mis12IP\_Nocodazole\_MudPIT\_040709\_03.04677.04677.2 | 2.8426 | 0.4463 | 100.0% | 1065.6522 | 1066.1992 | 1 | 7.162 | 81.2% | 1 | K.LEAEIATYR.R | 22 |
|  | Mis12IP\_Nocodazole\_MudPIT\_040709\_06.07085.07085.3 | 5.9061 | 0.5761 | 100.0% | 2741.2144 | 2741.9404 | 1 | 10.901 | 41.7% | 3 | R.LLEDGEDFNLGDALDSSNSMQTIQK.T | 33 |
|  | Mis12IP\_Nocodazole\_MudPIT\_040709\_05.06833.06833.2 | 5.1411 | 0.4192 | 100.0% | 2743.652 | 2741.9404 | 1 | 8.364 | 47.9% | 5 | R.LLEDGEDFNLGDALDSSNSMQTIQK.T | 22 |
|  | Mis12IP\_Nocodazole\_MudPIT\_040709\_02.06177.06177.3 | 5.7195 | 0.4857 | 100.0% | 2821.7644 | 2821.9404 | 2 | 7.146 | 41.7% | 1 | R.LLEDGEDFNLGDALDSSNS\*MQTIQK.T | 33 |
|  | Mis12IP\_Nocodazole\_MudPIT\_040709\_06.05346.05346.2 | 3.1981 | 0.52 | 100.0% | 1660.3522 | 1661.8528 | 1 | 8.175 | 60.7% | 1 | R.RIVDGKVVSETNDTK.V | 22 |
|  | Mis12IP\_Nocodazole\_MudPIT\_040709\_06.05344.05344.3 | 2.7637 | 0.3661 | 100.0% | 1660.8544 | 1661.8528 | 3 | 6.087 | 35.7% | 1 | R.RIVDGKVVSETNDTK.V | 33 |
|  | Mis12IP\_Nocodazole\_MudPIT\_040709\_03.03626.03626.2 | 3.3044 | 0.4687 | 100.0% | 1505.3922 | 1505.6653 | 1 | 8.05 | 73.1% | 4 | R.IVDGKVVSETNDTK.V | 22 |

Similarities:
gi|4557888|ref|NP\_000(18:1)  
gi|14043271|gb|AAH076(2:17)  

---

|  |  |  |  |  |  |  |  |  |
| --- | --- | --- | --- | --- | --- | --- | --- | --- |
| U | *gi|16507237|ref|NP\_00* | 17 | 29 | 31.2% | 654 | 72333 | 5.2 | heat shock 70kDa protein 5 [Homo sapiens] |

| Filename XCorr DeltCN Conf% ObsM+H+ CalcM+H+ SpR ZScore Ion% # Sequence  | | | | | | | | | | | | |
| --- | --- | --- | --- | --- | --- | --- | --- | --- | --- | --- | --- | --- |
|  | Mis12IP\_Nocodazole\_MudPIT\_040709\_06.06179.06179.2 | 3.0249 | 0.4617 | 100.0% | 1566.5322 | 1567.7386 | 1 | 9.537 | 61.5% | 2 | R.ITPSYVAFTPEGER.L | 2 |
|  | Mis12IP\_Nocodazole\_MudPIT\_040709\_06.05812.05812.2 | 4.6177 | 0.5268 | 100.0% | 1678.2322 | 1678.796 | 1 | 9.238 | 78.6% | 2 | K.NQLTSNPENTVFDAK.R | 2 |
|  | Mis12IP\_Nocodazole\_MudPIT\_040709\_03.04687.04687.2 | 3.619 | 0.3574 | 100.0% | 1430.3922 | 1431.5449 | 1 | 6.987 | 86.4% | 1 | R.TWNDPSVQQDIK.F | 2 |
|  | Mis12IP\_Nocodazole\_MudPIT\_040709\_06.07332.07332.1 | 2.1051 | 0.3907 | 100.0% | 1536.85 | 1537.8114 | 1 | 7.41 | 42.3% | 1 | K.TFAPEEISAMVLTK.M | 1 |
|  | Mis12IP\_Nocodazole\_MudPIT\_040709\_06.07316.07316.2 | 3.2426 | 0.4733 | 100.0% | 1538.5122 | 1537.8114 | 1 | 7.289 | 65.4% | 2 | K.TFAPEEISAMVLTK.M | 2 |
|  | Mis12IP\_Nocodazole\_MudPIT\_040709\_06.06184.06184.2 | 3.5475 | 0.4311 | 100.0% | 1219.0122 | 1218.4137 | 1 | 7.725 | 77.3% | 2 | K.DAGTIAGLNVMR.I | 2 |
|  | Mis12IP\_Nocodazole\_MudPIT\_040709\_06.06545.06545.2 | 5.2019 | 0.5351 | 100.0% | 1660.3322 | 1660.9078 | 1 | 9.75 | 73.3% | 1 | R.IINEPTAAAIAYGLDK.R | 2222 |
|  | Mis12IP\_Nocodazole\_MudPIT\_040709\_06.04454.04454.2 | 2.5245 | 0.3298 | 99.9% | 997.8122 | 998.0867 | 27 | 6.626 | 62.5% | 2 | R.ALSSQHQAR.I | 2 |
|  | Mis12IP\_Nocodazole\_MudPIT\_040709\_04.07795.07795.2 | 5.0468 | 0.6466 | 100.0% | 2166.152 | 2166.3025 | 1 | 10.689 | 64.7% | 5 | R.IEIESFYEGEDFSETLTR.A | 2 |
|  | Mis12IP\_Nocodazole\_MudPIT\_040709\_06.06486.06486.2 | 3.0692 | 0.3421 | 99.9% | 1461.1522 | 1461.6122 | 2 | 7.086 | 61.5% | 2 | K.SDIDEIVLVGGSTR.I | 2 |
|  | Mis12IP\_Nocodazole\_MudPIT\_040709\_06.05934.05934.2 | 5.046 | 0.5081 | 100.0% | 1838.3722 | 1838.0245 | 1 | 9.452 | 75.0% | 2 | K.SQIFSTASDNQPTVTIK.V | 2 |
|  | Mis12IP\_Nocodazole\_MudPIT\_040709\_05.03006.03006.2 | 2.2215 | 0.168 | 96.6% | 1193.2322 | 1192.3574 | 1 | 4.615 | 61.1% | 1 | K.VYEGERPLTK.D | 2 |
|  | Mis12IP\_Nocodazole\_MudPIT\_040709\_03.03739.03739.2 | 1.7667 | 0.2521 | 96.5% | 1074.1921 | 1075.1667 | 2 | 5.505 | 68.8% | 1 | K.ITITNDQNR.L | 2 |
|  | Mis12IP\_Nocodazole\_MudPIT\_040709\_06.06140.06140.1 | 1.7973 | 0.2504 | 98.6% | 1316.37 | 1317.4381 | 13 | 4.522 | 50.0% | 1 | R.NELESYAYSLK.N | 1 |
|  | Mis12IP\_Nocodazole\_MudPIT\_040709\_05.05563.05563.2 | 3.4098 | 0.444 | 100.0% | 1316.7322 | 1317.4381 | 1 | 6.37 | 80.0% | 1 | R.NELESYAYSLK.N | 2 |
|  | Mis12IP\_Nocodazole\_MudPIT\_040709\_05.06058.06058.2 | 3.6183 | 0.3647 | 100.0% | 1398.2722 | 1398.6396 | 1 | 7.171 | 86.4% | 1 | K.ELEEIVQPIISK.L | 2 |
|  | Mis12IP\_Nocodazole\_MudPIT\_040709\_03.04735.04735.2 | 4.9683 | 0.6226 | 100.0% | 2176.0522 | 2177.283 | 1 | 11.462 | 55.0% | 2 | K.LYGSAGPPPTGEEDTAEKDEL.- | 2 |

Similarities:
gi|5729877|ref|NP\_006(1:16)  
gi|13676857|ref|NP\_06(1:16)  
gi|27436929|ref|NP\_00(1:16)  

---

|  |  |  |  |  |  |  |  |  |
| --- | --- | --- | --- | --- | --- | --- | --- | --- |
| U | *gi|28317|emb|CAA32649* | 19 | 76 | 31.2% | 593 | 59528 | 5.3 | unnamed protein product [Homo sapiens] |
| U | *gi|40354192|ref|NP\_00* | 19 | 77 | 31.7% | 584 | 58827 | 5.2 | keratin 10 [Homo sapiens] |

| Filename XCorr DeltCN Conf% ObsM+H+ CalcM+H+ SpR ZScore Ion% # Sequence  | | | | | | | | | | | | |
| --- | --- | --- | --- | --- | --- | --- | --- | --- | --- | --- | --- | --- |
|  | Mis12IP\_Nocodazole\_MudPIT\_040709\_04.05302.05302.2 | 2.5626 | 0.2281 | 99.5% | 1090.2922 | 1091.2273 | 1 | 5.497 | 81.2% | 2 | K.VTMQNLNDR.L | 2222 |
|  | Mis12IP\_Nocodazole\_MudPIT\_040709\_03.04508.04508.1 | 1.9048 | 0.2501 | 99.0% | 809.5 | 809.93774 | 160 | 5.178 | 66.7% | 1 | R.LASYLDK.V | 1111111 |
|  | Mis12IP\_Nocodazole\_MudPIT\_040709\_02.04180.04180.1 | 2.8956 | 0.3242 | 100.0% | 1381.59 | 1382.4668 | 1 | 6.94 | 63.6% | 1 | R.ALEESNYELEGK.I | 1 |
|  | Mis12IP\_Nocodazole\_MudPIT\_040709\_02.04190.04190.2 | 4.4926 | 0.4631 | 100.0% | 1382.3322 | 1382.4668 | 1 | 8.359 | 77.3% | 1 | R.ALEESNYELEGK.I | 2 |
|  | Mis12IP\_Nocodazole\_MudPIT\_040709\_06.05425.05425.1 | 1.7097 | 0.2439 | 99.3% | 966.32 | 967.06604 | 61 | 4.848 | 58.3% | 1 | R.DYSKYYK.T | 1 |
|  | Mis12IP\_Nocodazole\_MudPIT\_040709\_06.07754.07754.2 | 4.9413 | 0.4242 | 100.0% | 2368.7322 | 2368.6523 | 1 | 8.573 | 60.0% | 1 | K.NQILNLTTDNANILLQIDNAR.L | 2 |
|  | Mis12IP\_Nocodazole\_MudPIT\_040709\_02.04120.04120.2 | 2.7363 | 0.31 | 99.9% | 993.7522 | 994.0923 | 1 | 6.573 | 92.9% | 1 | K.YENEVALR.Q | 2 |
|  | Mis12IP\_Nocodazole\_MudPIT\_040709\_05.05008.05008.2 | 2.7343 | 0.3118 | 99.9% | 1201.9521 | 1202.3097 | 1 | 5.738 | 80.0% | 2 | R.QSVEADINGLR.R | 222 |
|  | Mis12IP\_Nocodazole\_MudPIT\_040709\_05.05448.05448.2 | 3.3374 | 0.4141 | 100.0% | 1031.9722 | 1032.2224 | 1 | 6.814 | 87.5% | 2 | R.VLDELTLTK.A | 2 |
|  | Mis12IP\_Nocodazole\_MudPIT\_040709\_06.08192.08192.3 | 4.6231 | 0.5084 | 100.0% | 2872.7644 | 2874.2134 | 1 | 8.689 | 34.6% | 6 | R.NVSTGDVNVEMNAAPGVDLTQLLNNMR.S | 3 |
|  | Mis12IP\_Nocodazole\_MudPIT\_040709\_06.08201.08201.2 | 4.9217 | 0.3498 | 100.0% | 2876.632 | 2874.2134 | 1 | 6.048 | 42.3% | 6 | R.NVSTGDVNVEMNAAPGVDLTQLLNNMR.S | 2 |
|  | Mis12IP\_Nocodazole\_MudPIT\_040709\_03.04399.04399.2 | 4.2496 | 0.3038 | 100.0% | 1366.2322 | 1366.43 | 6 | 7.006 | 65.0% | 1 | R.SQYEQLAEQNR.K | 2 |
|  | Mis12IP\_Nocodazole\_MudPIT\_040709\_05.05271.05271.2 | 3.6651 | 0.3559 | 100.0% | 1495.5721 | 1494.6041 | 1 | 6.746 | 72.7% | 34 | R.SQYEQLAEQNRK.D | 2 |
|  | Mis12IP\_Nocodazole\_MudPIT\_040709\_02.04797.04797.1 | 2.2793 | 0.4367 | 100.0% | 1109.51 | 1110.1681 | 2 | 7.211 | 62.5% | 4 | K.DAEAWFNEK.S | 11 |
|  | Mis12IP\_Nocodazole\_MudPIT\_040709\_03.05189.05189.2 | 3.1018 | 0.3908 | 100.0% | 1109.7522 | 1110.1681 | 1 | 6.675 | 75.0% | 4 | K.DAEAWFNEK.S | 22 |
|  | Mis12IP\_Nocodazole\_MudPIT\_040709\_06.06784.06784.2 | 5.026 | 0.5497 | 100.0% | 1998.3522 | 1998.151 | 1 | 9.71 | 62.5% | 2 | K.ELTTEIDNNIEQISSYK.S | 2 |
|  | Mis12IP\_Nocodazole\_MudPIT\_040709\_05.05085.05085.2 | 3.4986 | 0.5 | 100.0% | 1391.1322 | 1391.4778 | 1 | 8.505 | 79.2% | 3 | K.QSLEASLAETEGR.Y | 2 |
|  | Mis12IP\_Nocodazole\_MudPIT\_040709\_03.04472.04472.2 | 3.1417 | 0.1086 | 99.3% | 1167.3522 | 1166.2761 | 9 | 5.569 | 81.2% | 2 | R.LENEIQTYR.S | 2 |
|  | Mis12IP\_Nocodazole\_MudPIT\_040709\_03.04430.04430.2 | 3.7895 | 0.466 | 100.0% | 1264.0122 | 1263.3066 | 1 | 8.422 | 76.9% | 2 | R.SLLEGEGSSGGGGR.G | 2 |

Similarities:
gi|24430192|ref|NP\_00(2:17)  
gi|12803709|gb|AAH026(2:17)  
gi|4557701|ref|NP\_000(1:18)  
gi|435476|emb|CAA8231(1:18)  
gi|14043271|gb|AAH076(1:18)  
gi|24234696|ref|NP\_70(1:18)  
gi|24430190|ref|NP\_00(1:18)  
gi|34526448|dbj|BAC85(3:16)  
gi|85566621|gb|AAI119(1:18)  

---

|  |  |  |  |  |  |  |  |  |
| --- | --- | --- | --- | --- | --- | --- | --- | --- |
| U | *gi|10190716|ref|NP\_06* | 6 | 7 | 30.4% | 224 | 26153 | 8.0 | spindle pole body component 25 [Homo sapiens] |

| Filename XCorr DeltCN Conf% ObsM+H+ CalcM+H+ SpR ZScore Ion% # Sequence  | | | | | | | | | | | | |
| --- | --- | --- | --- | --- | --- | --- | --- | --- | --- | --- | --- | --- |
| \* | Mis12IP\_Nocodazole\_MudPIT\_040709\_06.07120.07120.2 | 4.2779 | 0.3719 | 100.0% | 1789.1721 | 1789.0742 | 2 | 7.195 | 69.2% | 2 | R.MVEMFLEYQNQISR.Q | 2 |
| \* | Mis12IP\_Nocodazole\_MudPIT\_040709\_05.06591.06591.2 | 4.0458 | 0.2812 | 100.0% | 1615.0122 | 1614.8363 | 2 | 7.076 | 65.4% | 1 | K.QELEVLTANIQDLK.E | 2 |
| \* | Mis12IP\_Nocodazole\_MudPIT\_040709\_02.02988.02988.1 | 1.5603 | 0.2386 | 97.2% | 863.39 | 863.94324 | 136 | 5.047 | 57.1% | 1 | K.ETISTANK.A | 1 |
| \* | Mis12IP\_Nocodazole\_MudPIT\_040709\_06.07369.07369.2 | 2.9755 | 0.3881 | 100.0% | 1336.2522 | 1336.5736 | 1 | 6.89 | 65.0% | 1 | K.LQFIFTNIDPK.N | 2 |
| \* | Mis12IP\_Nocodazole\_MudPIT\_040709\_06.07031.07031.2 | 2.5272 | 0.3059 | 99.7% | 1355.2922 | 1354.5082 | 1 | 5.204 | 59.1% | 1 | K.TNNFSAFLANVR.K | 2 |
| \* | Mis12IP\_Nocodazole\_MudPIT\_040709\_06.05594.05594.2 | 2.4627 | 0.3774 | 99.9% | 1015.1322 | 1015.154 | 1 | 6.536 | 68.8% | 1 | R.KAFTATVYN.- | 2 |

---

|  |  |  |  |  |  |  |  |  |
| --- | --- | --- | --- | --- | --- | --- | --- | --- |
| U | *gi|32698866|ref|NP\_87* | 7 | 27 | 29.4% | 197 | 22443 | 4.7 | spindle pole body component 24 homolog [Homo sapiens] |

| Filename XCorr DeltCN Conf% ObsM+H+ CalcM+H+ SpR ZScore Ion% # Sequence  | | | | | | | | | | | | |
| --- | --- | --- | --- | --- | --- | --- | --- | --- | --- | --- | --- | --- |
| \* | Mis12IP\_Nocodazole\_MudPIT\_040709\_05.09208.09208.3 | 3.6805 | 0.3749 | 100.0% | 1813.9443 | 1815.0336 | 1 | 6.833 | 51.6% | 1 | R.DIEEVSQGLLSLLGANR.A | 3 |
| \* | Mis12IP\_Nocodazole\_MudPIT\_040709\_06.09283.09283.2 | 4.7549 | 0.4572 | 100.0% | 1814.3922 | 1815.0336 | 1 | 8.152 | 81.2% | 12 | R.DIEEVSQGLLSLLGANR.A | 2 |
| \* | Mis12IP\_Nocodazole\_MudPIT\_040709\_02.03830.03830.2 | 3.149 | 0.2524 | 99.9% | 1104.0922 | 1104.2023 | 1 | 5.616 | 88.9% | 2 | R.LLETQDGAEK.Q | 2 |
| \* | Mis12IP\_Nocodazole\_MudPIT\_040709\_03.04627.04627.1 | 1.7353 | 0.2449 | 99.3% | 863.44 | 864.0448 | 18 | 4.061 | 75.0% | 2 | R.EILTMEK.E | 1 |
| \* | Mis12IP\_Nocodazole\_MudPIT\_040709\_05.04953.04953.1 | 2.3842 | 0.3076 | 97.8% | 1072.59 | 1073.2346 | 15 | 5.665 | 61.1% | 3 | K.EVAQSLLNAK.E | 1 |
| \* | Mis12IP\_Nocodazole\_MudPIT\_040709\_05.04959.04959.2 | 3.0975 | 0.2273 | 99.9% | 1074.9122 | 1073.2346 | 2 | 5.555 | 72.2% | 3 | K.EVAQSLLNAK.E | 2 |
| \* | Mis12IP\_Nocodazole\_MudPIT\_040709\_03.00592.00592.2 | 4.3067 | 0.542 | 100.0% | 1775.4122 | 1774.9672 | 1 | 9.674 | 80.8% | 4 | K.FISDYLWSLVDTEW.- | 2 |

---

|  |  |  |  |  |  |  |  |  |
| --- | --- | --- | --- | --- | --- | --- | --- | --- |
| U | *gi|15082258|ref|NP\_00* | 5 | 18 | 29.0% | 183 | 20811 | 5.3 | chromobox homolog 3 [Homo sapiens] |
| U | *gi|7416937|gb|AAF6237* | 5 | 18 | 29.0% | 183 | 20839 | 5.3 | heterochromatin-like protein 1 [Homo sapiens] |

| Filename XCorr DeltCN Conf% ObsM+H+ CalcM+H+ SpR ZScore Ion% # Sequence  | | | | | | | | | | | | |
| --- | --- | --- | --- | --- | --- | --- | --- | --- | --- | --- | --- | --- |
|  | Mis12IP\_Nocodazole\_MudPIT\_040709\_06.05764.05764.2 | 5.5741 | 0.4655 | 100.0% | 1662.3522 | 1662.834 | 1 | 8.319 | 88.5% | 9 | K.KVEEAEPEEFVVEK.V | 2 |
|  | Mis12IP\_Nocodazole\_MudPIT\_040709\_02.04552.04552.2 | 4.3762 | 0.46 | 100.0% | 1535.1721 | 1534.6599 | 1 | 7.496 | 79.2% | 1 | K.VEEAEPEEFVVEK.V | 2 |
|  | Mis12IP\_Nocodazole\_MudPIT\_040709\_06.07600.07600.2 | 4.7457 | 0.4801 | 100.0% | 1712.7522 | 1714.0449 | 1 | 10.712 | 70.0% | 6 | R.IIGATDSSGELMFLMK.W | 22 |
|  | Mis12IP\_Nocodazole\_MudPIT\_040709\_02.04388.04388.2 | 3.9249 | 0.1109 | 99.7% | 1176.0721 | 1176.2659 | 1 | 6.866 | 80.0% | 1 | K.DSDEADLVLAK.E | 2 |
|  | Mis12IP\_Nocodazole\_MudPIT\_040709\_02.07848.07848.2 | 2.9518 | 0.2115 | 99.5% | 1526.3322 | 1525.7106 | 1 | 4.874 | 72.7% | 1 | K.CPQIVIAFYEER.L | 22 |

Similarities:
gi|5803076|ref|NP\_006(1:4)  
gi|48146953|emb|CAG33(1:4)  

---

|  |  |  |  |  |  |  |  |  |
| --- | --- | --- | --- | --- | --- | --- | --- | --- |
| U | *gi|3901272|gb|AAC7862* | 13 | 24 | 28.9% | 277 | 31194 | 5.1 | ZW10 interactor Zwint [Homo sapiens] |

| Filename XCorr DeltCN Conf% ObsM+H+ CalcM+H+ SpR ZScore Ion% # Sequence  | | | | | | | | | | | | |
| --- | --- | --- | --- | --- | --- | --- | --- | --- | --- | --- | --- | --- |
|  | Mis12IP\_Nocodazole\_MudPIT\_040709\_06.06864.06864.2 | 3.1069 | 0.4266 | 100.0% | 1278.1921 | 1277.5034 | 1 | 6.316 | 75.0% | 1 | K.ILVEFVVDSQK.K | 2 |
|  | Mis12IP\_Nocodazole\_MudPIT\_040709\_02.04392.04392.1 | 1.5772 | 0.5578 | 100.0% | 1260.45 | 1261.3312 | 2 | 7.984 | 54.5% | 1 | K.GLDPLASEDTSR.Q | 1 |
|  | Mis12IP\_Nocodazole\_MudPIT\_040709\_02.04373.04373.2 | 3.0106 | 0.5315 | 100.0% | 1260.9521 | 1261.3312 | 1 | 8.25 | 81.8% | 2 | K.GLDPLASEDTSR.Q | 2 |
|  | Mis12IP\_Nocodazole\_MudPIT\_040709\_02.04020.04020.1 | 2.2054 | 0.2543 | 98.9% | 1176.53 | 1177.3177 | 1 | 4.883 | 66.7% | 1 | K.ALTQMEEAQR.K | 1 |
|  | Mis12IP\_Nocodazole\_MudPIT\_040709\_02.04005.04005.2 | 3.5991 | 0.2776 | 100.0% | 1177.3922 | 1177.3177 | 1 | 6.198 | 83.3% | 6 | K.ALTQMEEAQR.K | 2 |
|  | Mis12IP\_Nocodazole\_MudPIT\_040709\_04.05436.05436.1 | 2.1168 | 0.1545 | 97.1% | 1063.53 | 1064.1833 | 239 | 4.468 | 56.2% | 1 | R.EAFEQLQAK.K | 1 |
|  | Mis12IP\_Nocodazole\_MudPIT\_040709\_04.05426.05426.2 | 2.6857 | 0.2509 | 99.7% | 1064.0122 | 1064.1833 | 2 | 5.335 | 68.8% | 2 | R.EAFEQLQAK.K | 2 |
|  | Mis12IP\_Nocodazole\_MudPIT\_040709\_06.05628.05628.1 | 3.021 | 0.3544 | 100.0% | 1499.6 | 1500.6542 | 1 | 6.378 | 54.5% | 2 | R.AVQNQWQLQQEK.H | 1 |
|  | Mis12IP\_Nocodazole\_MudPIT\_040709\_05.04865.04865.2 | 4.069 | 0.294 | 100.0% | 1501.1721 | 1500.6542 | 1 | 5.727 | 68.2% | 1 | R.AVQNQWQLQQEK.H | 2 |
| \* | Mis12IP\_Nocodazole\_MudPIT\_040709\_05.05173.05173.1 | 1.7249 | 0.2995 | 99.1% | 1450.71 | 1451.576 | 46 | 5.53 | 41.7% | 1 | K.TGTQQELDGVFQK.L | 1 |
| \* | Mis12IP\_Nocodazole\_MudPIT\_040709\_06.05836.05836.2 | 4.7316 | 0.3728 | 100.0% | 1451.2722 | 1451.576 | 1 | 6.343 | 75.0% | 3 | K.TGTQQELDGVFQK.L | 2 |
|  | Mis12IP\_Nocodazole\_MudPIT\_040709\_02.05134.05134.1 | 1.7715 | 0.2042 | 95.7% | 1250.55 | 1251.4252 | 10 | 3.972 | 50.0% | 1 | K.AVGLQPAGDVNLP.- | 1 |
|  | Mis12IP\_Nocodazole\_MudPIT\_040709\_02.05123.05123.2 | 3.1385 | 0.2575 | 99.8% | 1252.3121 | 1251.4252 | 1 | 4.856 | 70.8% | 2 | K.AVGLQPAGDVNLP.- | 2 |

---

|  |  |  |  |  |  |  |  |  |
| --- | --- | --- | --- | --- | --- | --- | --- | --- |
| U | *gi|4506687|ref|NP\_001* | 2 | 2 | 28.3% | 145 | 17040 | 10.4 | ribosomal protein S15 [Homo sapiens] |

| Filename XCorr DeltCN Conf% ObsM+H+ CalcM+H+ SpR ZScore Ion% # Sequence  | | | | | | | | | | | | |
| --- | --- | --- | --- | --- | --- | --- | --- | --- | --- | --- | --- | --- |
|  | Mis12IP\_Nocodazole\_MudPIT\_040709\_06.10193.10193.2 | 4.8519 | 0.5385 | 100.0% | 2588.7922 | 2589.938 | 1 | 11.574 | 52.4% | 1 | R.GVDLDQLLDMSYEQLMQLYSAR.Q | 2 |
|  | Mis12IP\_Nocodazole\_MudPIT\_040709\_05.08775.08775.2 | 3.0921 | 0.3835 | 100.0% | 2055.1921 | 2054.4856 | 1 | 7.021 | 44.4% | 1 | R.DMIILPEMVGSMVGVYNGK.T | 2 |

---

|  |  |  |  |  |  |  |  |  |
| --- | --- | --- | --- | --- | --- | --- | --- | --- |
| U | *gi|5174457|ref|NP\_006* | 24 | 50 | 28.2% | 642 | 73913 | 5.6 | kinetochore associated 2 [Homo sapiens] |

| Filename XCorr DeltCN Conf% ObsM+H+ CalcM+H+ SpR ZScore Ion% # Sequence  | | | | | | | | | | | | |
| --- | --- | --- | --- | --- | --- | --- | --- | --- | --- | --- | --- | --- |
|  | Mis12IP\_Nocodazole\_MudPIT\_040709\_06.05693.05693.2 | 2.2965 | 0.1366 | 97.6% | 876.8722 | 877.0466 | 20 | 4.731 | 75.0% | 3 | R.LSMQELR.S | 2 |
|  | Mis12IP\_Nocodazole\_MudPIT\_040709\_04.05312.05312.2 | 1.7992 | 0.2167 | 95.3% | 1037.0922 | 1036.1729 | 5 | 5.489 | 75.0% | 2 | K.QGLYTPQTK.E | 2 |
|  | Mis12IP\_Nocodazole\_MudPIT\_040709\_06.05543.05543.2 | 3.1314 | 0.279 | 99.9% | 1144.8922 | 1145.3011 | 1 | 5.247 | 77.8% | 2 | K.LSINKPTSER.K | 2 |
|  | Mis12IP\_Nocodazole\_MudPIT\_040709\_06.06088.06088.1 | 1.9652 | 0.1896 | 97.1% | 1296.63 | 1297.4075 | 1 | 5.223 | 63.6% | 1 | R.NSQLGIFSSSEK.I | 1 |
|  | Mis12IP\_Nocodazole\_MudPIT\_040709\_06.06094.06094.2 | 3.8517 | 0.4104 | 100.0% | 1297.1721 | 1297.4075 | 1 | 8.269 | 86.4% | 3 | R.NSQLGIFSSSEK.I | 2 |
|  | Mis12IP\_Nocodazole\_MudPIT\_040709\_06.06710.06710.1 | 1.8313 | 0.3587 | 98.3% | 1110.53 | 1111.283 | 5 | 6.181 | 55.6% | 3 | K.DLGYPFALSK.S | 1 |
|  | Mis12IP\_Nocodazole\_MudPIT\_040709\_06.06702.06702.2 | 2.8212 | 0.476 | 100.0% | 1110.9521 | 1111.283 | 1 | 7.654 | 77.8% | 3 | K.DLGYPFALSK.S | 2 |
|  | Mis12IP\_Nocodazole\_MudPIT\_040709\_06.06904.06904.2 | 1.9419 | 0.1869 | 95.9% | 1012.4322 | 1013.2218 | 22 | 3.984 | 64.3% | 1 | K.LFLDYTIK.C | 2 |
|  | Mis12IP\_Nocodazole\_MudPIT\_040709\_06.06887.06887.1 | 1.8604 | 0.2119 | 98.7% | 1012.56 | 1013.2218 | 78 | 4.667 | 57.1% | 1 | K.LFLDYTIK.C | 1 |
|  | Mis12IP\_Nocodazole\_MudPIT\_040709\_05.06686.06686.1 | 2.2975 | 0.4915 | 100.0% | 1068.52 | 1069.2023 | 1 | 7.241 | 62.5% | 1 | K.DLFNVDAFK.L | 1 |
|  | Mis12IP\_Nocodazole\_MudPIT\_040709\_03.04361.04361.2 | 2.828 | 0.1068 | 99.0% | 914.97217 | 915.03723 | 2 | 5.317 | 92.9% | 2 | R.ALNEQIAR.L | 2 |
|  | Mis12IP\_Nocodazole\_MudPIT\_040709\_02.03086.03086.1 | 1.512 | 0.249 | 96.1% | 945.51 | 946.0483 | 40 | 4.224 | 56.2% | 1 | K.ASLQGDVQK.Y | 1 |
|  | Mis12IP\_Nocodazole\_MudPIT\_040709\_03.04655.04655.2 | 3.1031 | 0.3435 | 100.0% | 1128.6921 | 1129.2584 | 1 | 6.306 | 88.9% | 3 | K.LNGLNEEIAR.V | 2 |
|  | Mis12IP\_Nocodazole\_MudPIT\_040709\_03.04567.04567.1 | 2.5138 | 0.1845 | 99.0% | 1085.65 | 1086.2334 | 12 | 4.552 | 68.8% | 3 | R.LQNIIDNQK.Y | 1 |
|  | Mis12IP\_Nocodazole\_MudPIT\_040709\_03.04547.04547.2 | 3.1573 | 0.2185 | 99.9% | 1086.0122 | 1086.2334 | 1 | 4.552 | 87.5% | 3 | R.LQNIIDNQK.Y | 2 |
|  | Mis12IP\_Nocodazole\_MudPIT\_040709\_03.04734.04734.2 | 2.4464 | 0.3743 | 100.0% | 952.8522 | 953.03973 | 1 | 7.215 | 78.6% | 1 | K.YSVADIER.I | 2 |
|  | Mis12IP\_Nocodazole\_MudPIT\_040709\_04.05767.05767.2 | 2.6246 | 0.2334 | 99.8% | 932.27216 | 932.0642 | 46 | 4.697 | 83.3% | 2 | K.LWNEELK.Y | 2 |
|  | Mis12IP\_Nocodazole\_MudPIT\_040709\_05.05241.05241.1 | 1.8121 | 0.2175 | 98.6% | 871.45 | 871.9653 | 24 | 5.268 | 66.7% | 1 | K.GYDFEIK.F | 1 |
|  | Mis12IP\_Nocodazole\_MudPIT\_040709\_06.05728.05728.1 | 1.9805 | 0.1558 | 96.2% | 917.37 | 918.124 | 74 | 4.763 | 64.3% | 1 | R.AQVYVPLK.E | 1 |
|  | Mis12IP\_Nocodazole\_MudPIT\_040709\_03.04776.04776.2 | 2.9754 | 0.4267 | 100.0% | 1460.2922 | 1461.5658 | 1 | 6.246 | 68.2% | 1 | K.ELLNETEEEINK.A | 2 |
|  | Mis12IP\_Nocodazole\_MudPIT\_040709\_06.09315.09315.3 | 4.3669 | 0.3599 | 100.0% | 2023.5243 | 2024.3088 | 1 | 6.616 | 51.5% | 1 | K.MGLEDTLEQLNAMITESK.R | 3 |
|  | Mis12IP\_Nocodazole\_MudPIT\_040709\_06.09288.09288.2 | 5.1659 | 0.5394 | 100.0% | 2023.5721 | 2024.3088 | 1 | 9.956 | 61.8% | 2 | K.MGLEDTLEQLNAMITESK.R | 2 |
| \* | Mis12IP\_Nocodazole\_MudPIT\_040709\_04.05696.05696.2 | 4.5082 | 0.4005 | 100.0% | 1597.2122 | 1596.7344 | 1 | 8.062 | 83.3% | 7 | R.EYQLVVQTTTEER.R | 2 |
| \* | Mis12IP\_Nocodazole\_MudPIT\_040709\_04.04512.04512.2 | 2.5926 | 0.1493 | 99.2% | 801.1322 | 800.893 | 12 | 3.901 | 83.3% | 2 | K.VGNNLQR.L | 2 |

---

|  |  |  |  |  |  |  |  |  |
| --- | --- | --- | --- | --- | --- | --- | --- | --- |
| U | *gi|111309351|gb|AAI20* | 3 | 5 | 28.2% | 103 | 11314 | 11.0 | HIST1H4H protein [Homo sapiens] |
| U | *gi|49457374|emb|CAG46* | 3 | 5 | 28.2% | 103 | 11393 | 11.4 | HIST1H4F [Homo sapiens] |
| U | *gi|45767731|gb|AAH674* | 3 | 5 | 28.2% | 103 | 11339 | 11.4 | HIST1H4I protein [Homo sapiens] |
| U | *gi|4504301|ref|NP\_003* | 3 | 5 | 28.2% | 103 | 11367 | 11.4 | H4 histone family, member A [Homo sapiens] |

| Filename XCorr DeltCN Conf% ObsM+H+ CalcM+H+ SpR ZScore Ion% # Sequence  | | | | | | | | | | | | |
| --- | --- | --- | --- | --- | --- | --- | --- | --- | --- | --- | --- | --- |
|  | Mis12IP\_Nocodazole\_MudPIT\_040709\_06.05928.05928.2 | 3.6196 | 0.3628 | 100.0% | 1180.9922 | 1181.3312 | 1 | 6.889 | 88.9% | 2 | R.ISGLIYEETR.G | 2 |
|  | Mis12IP\_Nocodazole\_MudPIT\_040709\_06.07219.07219.2 | 2.7849 | 0.41 | 100.0% | 1311.1122 | 1311.5793 | 1 | 7.252 | 72.7% | 1 | K.TVTAMDVVYALK.R | 2 |
|  | Mis12IP\_Nocodazole\_MudPIT\_040709\_02.04871.04871.1 | 2.0319 | 0.4152 | 100.0% | 716.3 | 714.796 | 1 | 7.1 | 66.7% | 2 | R.TLYGFGG.- | 1 |

---

|  |  |  |  |  |  |  |  |  |
| --- | --- | --- | --- | --- | --- | --- | --- | --- |
| U | *gi|25777711|ref|NP\_00* | 2 | 3 | 28.1% | 160 | 18063 | 4.5 | S-phase kinase-associated protein 1A isoform a [Homo sapiens] |
| U | *gi|25777713|ref|NP\_73* | 2 | 2 | 27.6% | 163 | 18658 | 4.5 | S-phase kinase-associated protein 1A isoform b [Homo sapiens] |

| Filename XCorr DeltCN Conf% ObsM+H+ CalcM+H+ SpR ZScore Ion% # Sequence  | | | | | | | | | | | | |
| --- | --- | --- | --- | --- | --- | --- | --- | --- | --- | --- | --- | --- |
|  | Mis12IP\_Nocodazole\_MudPIT\_040709\_06.06833.06833.2 | 3.8522 | 0.4963 | 100.0% | 1879.1921 | 1880.0588 | 1 | 9.367 | 56.2% | 2 | K.LQSSDGEIFEVDVEIAK.Q | 2 |
|  | Mis12IP\_Nocodazole\_MudPIT\_040709\_05.07939.07939.3 | 3.4517 | 0.4153 | 100.0% | 2998.2244 | 2999.3315 | 48 | 6.433 | 23.1% | 1 | K.TMLEDLGMDDEGDDDPVPLPNVNAAILK.K | 3 |

---

|  |  |  |  |  |  |  |  |  |
| --- | --- | --- | --- | --- | --- | --- | --- | --- |
| U | *gi|15126735|gb|AAH122* | 3 | 4 | 27.8% | 205 | 22768 | 6.4 | Heat shock 27kDa protein 1 [Homo sapiens] |
| U | *gi|662841|gb|AAA62175* | 3 | 4 | 28.6% | 199 | 22327 | 8.0 | heat shock protein 27 [Homo sapiens] |
| U | *gi|4504517|ref|NP\_001* | 3 | 4 | 27.8% | 205 | 22783 | 6.4 | heat shock 27kDa protein 1 [Homo sapiens] |
| U | *gi|15928913|gb|AAH149* | 3 | 4 | 29.8% | 191 | 21142 | 5.8 | Unknown (protein for IMAGE:3906970) [Homo sapiens] |

| Filename XCorr DeltCN Conf% ObsM+H+ CalcM+H+ SpR ZScore Ion% # Sequence  | | | | | | | | | | | | |
| --- | --- | --- | --- | --- | --- | --- | --- | --- | --- | --- | --- | --- |
|  | Mis12IP\_Nocodazole\_MudPIT\_040709\_03.04506.04506.2 | 2.1905 | 0.1398 | 95.1% | 1075.6921 | 1076.1948 | 1 | 5.128 | 72.2% | 1 | R.QLSSGVSEIR.H | 2 |
|  | Mis12IP\_Nocodazole\_MudPIT\_040709\_06.07187.07187.3 | 3.7699 | 0.394 | 100.0% | 3100.0144 | 3100.508 | 1 | 6.534 | 25.0% | 1 | K.YTLPPGVDPTQVSSSLSPEGTLTVEAPMPK.L | 3 |
|  | Mis12IP\_Nocodazole\_MudPIT\_040709\_06.06676.06676.2 | 3.0192 | 0.4889 | 100.0% | 1906.3322 | 1907.1307 | 2 | 8.829 | 50.0% | 2 | K.LATQSNEITIPVTFESR.A | 2 |

---

|  |  |  |  |  |  |  |  |  |
| --- | --- | --- | --- | --- | --- | --- | --- | --- |
| U | *gi|12653415|gb|AAH004* | 17 | 25 | 26.2% | 679 | 73728 | 6.4 | Heat shock 70kDa protein 9 (mortalin) [Homo sapiens] |
| U | *gi|24234688|ref|NP\_00* | 17 | 25 | 26.2% | 679 | 73681 | 6.2 | heat shock 70kDa protein 9B precursor [Homo sapiens] |
| U | *gi|21040386|gb|AAH306* | 17 | 25 | 26.1% | 681 | 73854 | 6.4 | HSPA9 protein [Homo sapiens] |

| Filename XCorr DeltCN Conf% ObsM+H+ CalcM+H+ SpR ZScore Ion% # Sequence  | | | | | | | | | | | | |
| --- | --- | --- | --- | --- | --- | --- | --- | --- | --- | --- | --- | --- |
|  | Mis12IP\_Nocodazole\_MudPIT\_040709\_02.03365.03365.1 | 1.7526 | 0.3448 | 98.7% | 958.54 | 959.047 | 7 | 4.952 | 56.2% | 1 | K.VLENAEGAR.T | 1 |
|  | Mis12IP\_Nocodazole\_MudPIT\_040709\_02.03326.03326.2 | 2.7665 | 0.216 | 99.7% | 958.9922 | 959.047 | 3 | 5.105 | 81.2% | 1 | K.VLENAEGAR.T | 2 |
|  | Mis12IP\_Nocodazole\_MudPIT\_040709\_06.05956.05956.1 | 2.4186 | 0.5105 | 100.0% | 1450.61 | 1451.576 | 2 | 8.419 | 42.3% | 1 | R.TTPSVVAFTADGER.L | 1 |
|  | Mis12IP\_Nocodazole\_MudPIT\_040709\_06.05945.05945.2 | 3.648 | 0.5714 | 100.0% | 1451.1522 | 1451.576 | 1 | 8.82 | 73.1% | 2 | R.TTPSVVAFTADGER.L | 2 |
|  | Mis12IP\_Nocodazole\_MudPIT\_040709\_05.04792.04792.2 | 3.6491 | 0.4777 | 100.0% | 1568.9922 | 1569.7141 | 1 | 7.992 | 73.1% | 2 | R.QAVTNPNNTFYATK.R | 2 |
|  | Mis12IP\_Nocodazole\_MudPIT\_040709\_04.06061.06061.2 | 2.6607 | 0.4996 | 100.0% | 1342.0521 | 1342.4105 | 1 | 7.913 | 50.0% | 2 | R.ASNGDAWVEAHGK.L | 2 |
|  | Mis12IP\_Nocodazole\_MudPIT\_040709\_06.06533.06533.2 | 2.1624 | 0.2753 | 98.9% | 1695.1721 | 1695.8723 | 13 | 5.169 | 53.6% | 1 | K.NAVITVPAYFNDSQR.Q | 2 |
|  | Mis12IP\_Nocodazole\_MudPIT\_040709\_06.06198.06198.1 | 2.3681 | 0.4346 | 100.0% | 1242.55 | 1243.4056 | 12 | 6.552 | 59.1% | 1 | K.DAGQISGLNVLR.V | 1 |
|  | Mis12IP\_Nocodazole\_MudPIT\_040709\_06.06190.06190.2 | 4.0614 | 0.3469 | 100.0% | 1243.0922 | 1243.4056 | 1 | 6.298 | 81.8% | 2 | K.DAGQISGLNVLR.V | 2 |
|  | Mis12IP\_Nocodazole\_MudPIT\_040709\_06.06490.06490.2 | 4.6266 | 0.5576 | 100.0% | 1646.3322 | 1646.881 | 1 | 9.584 | 66.7% | 1 | R.VINEPTAAALAYGLDK.S | 2 |
|  | Mis12IP\_Nocodazole\_MudPIT\_040709\_06.07208.07208.2 | 5.2517 | 0.5541 | 100.0% | 2057.0723 | 2057.181 | 1 | 9.345 | 61.1% | 5 | K.STNGDTFLGGEDFDQALLR.H | 2 |
|  | Mis12IP\_Nocodazole\_MudPIT\_040709\_06.07381.07381.2 | 4.0101 | 0.4613 | 100.0% | 1362.0521 | 1362.5687 | 1 | 8.754 | 81.8% | 1 | R.AQFEGIVTDLIR.R | 2 |
|  | Mis12IP\_Nocodazole\_MudPIT\_040709\_06.06894.06894.2 | 4.0935 | 0.4381 | 100.0% | 1447.1322 | 1447.6898 | 1 | 8.334 | 76.9% | 1 | K.SDIGEVILVGGMTR.M | 2 |
|  | Mis12IP\_Nocodazole\_MudPIT\_040709\_06.06244.06244.2 | 3.8888 | 0.307 | 100.0% | 1291.0322 | 1291.4496 | 1 | 7.943 | 80.0% | 1 | K.VQQTVQDLFGR.A | 2 |
|  | Mis12IP\_Nocodazole\_MudPIT\_040709\_03.03625.03625.1 | 1.4309 | 0.3276 | 99.1% | 774.34 | 774.8925 | 50 | 4.779 | 58.3% | 1 | R.NTTIPTK.K | 111 |
|  | Mis12IP\_Nocodazole\_MudPIT\_040709\_02.02985.02985.2 | 2.3702 | 0.2855 | 99.6% | 1149.2322 | 1149.2029 | 13 | 5.435 | 66.7% | 1 | R.KDSETGENIR.Q | 2 |
|  | Mis12IP\_Nocodazole\_MudPIT\_040709\_03.04464.04464.2 | 2.7155 | 0.3067 | 99.9% | 1231.2122 | 1232.3794 | 1 | 4.977 | 77.3% | 1 | R.QAASSLQQASLK.L | 2 |

Similarities:
gi|5729877|ref|NP\_006(1:16)  
gi|13676857|ref|NP\_06(1:16)  

---

|  |  |  |  |  |  |  |  |  |
| --- | --- | --- | --- | --- | --- | --- | --- | --- |
| U | *gi|5030431|gb|AAA6128* | 12 | 20 | 25.1% | 354 | 41562 | 4.9 | vimentin [Homo sapiens] |

| Filename XCorr DeltCN Conf% ObsM+H+ CalcM+H+ SpR ZScore Ion% # Sequence  | | | | | | | | | | | | |
| --- | --- | --- | --- | --- | --- | --- | --- | --- | --- | --- | --- | --- |
|  | Mis12IP\_Nocodazole\_MudPIT\_040709\_03.04616.04616.1 | 1.8916 | 0.1719 | 97.6% | 870.43 | 870.9805 | 24 | 4.97 | 66.7% | 1 | R.FANYIDK.V | 11 |
|  | Mis12IP\_Nocodazole\_MudPIT\_040709\_03.04920.04920.2 | 3.4746 | 0.4453 | 100.0% | 1254.9521 | 1255.385 | 1 | 7.98 | 83.3% | 1 | R.LGDLYEEEMR.E | 22 |
| \* | Mis12IP\_Nocodazole\_MudPIT\_040709\_03.05546.05546.1 | 1.3793 | 0.3423 | 99.2% | 1076.42 | 1077.1975 | 153 | 4.98 | 31.2% | 1 | R.DNLAEDIMR.L | 1 |
|  | Mis12IP\_Nocodazole\_MudPIT\_040709\_02.05153.05153.2 | 2.8547 | 0.2459 | 99.9% | 1077.2722 | 1077.1975 | 1 | 6.521 | 75.0% | 2 | R.DNLAEDIMR.L | 22 |
|  | Mis12IP\_Nocodazole\_MudPIT\_040709\_03.04515.04515.2 | 2.6693 | 0.1923 | 99.4% | 1046.5122 | 1047.2146 | 11 | 4.629 | 78.6% | 1 | K.LQEEMLQR.E | 22 |
|  | Mis12IP\_Nocodazole\_MudPIT\_040709\_02.03408.03408.2 | 2.2736 | 0.3047 | 99.6% | 1088.9521 | 1089.1503 | 4 | 5.788 | 66.7% | 1 | R.QDVDNASLAR.L | 22 |
|  | Mis12IP\_Nocodazole\_MudPIT\_040709\_06.05981.05981.1 | 2.2422 | 0.2644 | 98.8% | 1309.61 | 1310.4056 | 8 | 5.566 | 61.1% | 2 | K.NLQEAEEWYK.S | 11 |
|  | Mis12IP\_Nocodazole\_MudPIT\_040709\_05.05333.05333.2 | 3.7986 | 0.3583 | 100.0% | 1309.6721 | 1310.4056 | 8 | 6.262 | 66.7% | 2 | K.NLQEAEEWYK.S | 22 |
|  | Mis12IP\_Nocodazole\_MudPIT\_040709\_04.05448.05448.2 | 3.854 | 0.443 | 100.0% | 1093.5521 | 1094.1692 | 1 | 8.376 | 83.3% | 2 | K.FADLSEAANR.N | 22 |
|  | Mis12IP\_Nocodazole\_MudPIT\_040709\_05.05409.05409.1 | 2.0294 | 0.2104 | 98.6% | 1121.51 | 1122.2633 | 30 | 4.922 | 62.5% | 1 | R.EYQDLLNVK.M | 11 |
|  | Mis12IP\_Nocodazole\_MudPIT\_040709\_06.06061.06061.2 | 2.3286 | 0.3869 | 99.9% | 1121.9922 | 1122.2633 | 1 | 6.184 | 87.5% | 2 | R.EYQDLLNVK.M | 22 |
|  | Mis12IP\_Nocodazole\_MudPIT\_040709\_02.04997.04997.2 | 2.7906 | 0.2874 | 99.7% | 1837.0521 | 1837.854 | 1 | 5.278 | 50.0% | 4 | R.DGQVINETSQHHDDLE.- | 22 |

Similarities:
gi|37852|emb|CAA79613(11:1)  

---

|  |  |  |  |  |  |  |  |  |
| --- | --- | --- | --- | --- | --- | --- | --- | --- |
| U | *gi|12005829|gb|AAG446* | 7 | 23 | 24.6% | 281 | 32130 | 6.8 | DC31 [Homo sapiens] |
| U | *gi|13937916|gb|AAH070* | 7 | 23 | 24.6% | 281 | 32190 | 6.8 | NSL1, MIND kinetochore complex component, homolog (S. cerevisiae) [Homo sapiens] |

| Filename XCorr DeltCN Conf% ObsM+H+ CalcM+H+ SpR ZScore Ion% # Sequence  | | | | | | | | | | | | |
| --- | --- | --- | --- | --- | --- | --- | --- | --- | --- | --- | --- | --- |
|  | Mis12IP\_Nocodazole\_MudPIT\_040709\_05.05040.05040.2 | 4.4241 | 0.4848 | 100.0% | 1701.4521 | 1701.8741 | 1 | 9.368 | 65.6% | 2 | K.ELAAGTESQALVSATPR.E | 22 |
|  | Mis12IP\_Nocodazole\_MudPIT\_040709\_03.04915.04915.2 | 2.7933 | 0.2352 | 99.7% | 1113.0721 | 1113.256 | 1 | 4.992 | 77.8% | 1 | K.LGDALPEEIR.E | 22 |
|  | Mis12IP\_Nocodazole\_MudPIT\_040709\_06.08649.08649.2 | 5.3761 | 0.4939 | 100.0% | 1848.1522 | 1849.0881 | 1 | 10.28 | 73.3% | 5 | K.VLEDQFDEIIVDIATK.R | 22 |
|  | Mis12IP\_Nocodazole\_MudPIT\_040709\_06.07564.07564.2 | 4.6069 | 0.4468 | 100.0% | 1844.2522 | 1845.1057 | 1 | 9.013 | 68.8% | 2 | K.SLPALIEQGEGFSQVLR.M | 22 |
|  | Mis12IP\_Nocodazole\_MudPIT\_040709\_04.05758.05758.1 | 1.3725 | 0.3008 | 98.1% | 889.49 | 890.0678 | 131 | 4.204 | 50.0% | 1 | R.KTSDVVLK.R | 1 |
|  | Mis12IP\_Nocodazole\_MudPIT\_040709\_02.03908.03908.1 | 2.0697 | 0.1312 | 97.2% | 763.44 | 761.89374 | 83 | 4.146 | 58.3% | 3 | K.TSDVVLK.R | 1 |
|  | Mis12IP\_Nocodazole\_MudPIT\_040709\_04.06399.06399.2 | 2.651 | 0.1756 | 99.3% | 917.71216 | 918.08124 | 1 | 5.091 | 78.6% | 9 | K.TSDVVLKR.K | 2 |

Similarities:
gi|110349759|ref|NP\_0(4:3)  

---

|  |  |  |  |  |  |  |  |  |
| --- | --- | --- | --- | --- | --- | --- | --- | --- |
| U | *gi|110349759|ref|NP\_0* | 5 | 11 | 23.8% | 281 | 32162 | 6.8 | NSL1, MIND kinetochore complex component isoform 1 [Homo sapiens] |

| Filename XCorr DeltCN Conf% ObsM+H+ CalcM+H+ SpR ZScore Ion% # Sequence  | | | | | | | | | | | | |
| --- | --- | --- | --- | --- | --- | --- | --- | --- | --- | --- | --- | --- |
|  | Mis12IP\_Nocodazole\_MudPIT\_040709\_05.05040.05040.2 | 4.4241 | 0.4848 | 100.0% | 1701.4521 | 1701.8741 | 1 | 9.368 | 65.6% | 2 | K.ELAAGTESQALVSATPR.E | 22 |
|  | Mis12IP\_Nocodazole\_MudPIT\_040709\_03.04915.04915.2 | 2.7933 | 0.2352 | 99.7% | 1113.0721 | 1113.256 | 1 | 4.992 | 77.8% | 1 | K.LGDALPEEIR.E | 22 |
|  | Mis12IP\_Nocodazole\_MudPIT\_040709\_06.08649.08649.2 | 5.3761 | 0.4939 | 100.0% | 1848.1522 | 1849.0881 | 1 | 10.28 | 73.3% | 5 | K.VLEDQFDEIIVDIATK.R | 22 |
|  | Mis12IP\_Nocodazole\_MudPIT\_040709\_06.07564.07564.2 | 4.6069 | 0.4468 | 100.0% | 1844.2522 | 1845.1057 | 1 | 9.013 | 68.8% | 2 | K.SLPALIEQGEGFSQVLR.M | 22 |
|  | Mis12IP\_Nocodazole\_MudPIT\_040709\_02.04018.04018.1 | 1.7209 | 0.3101 | 98.8% | 793.49 | 793.95374 | 1 | 5.623 | 75.0% | 1 | K.TSDMVLK.R | 1 |

Similarities:
gi|12005829|gb|AAG446(4:1)  

---

|  |  |  |  |  |  |  |  |  |
| --- | --- | --- | --- | --- | --- | --- | --- | --- |
| U | *gi|117968353|ref|NP\_1* | 13 | 44 | 23.3% | 464 | 54304 | 8.3 | NUF2, NDC80 kinetochore complex component [Homo sapiens] |
| U | *gi|34783025|gb|AAH211* | 13 | 44 | 33.8% | 320 | 37333 | 8.5 | NUF2 protein [Homo sapiens] |
| U | *gi|14317904|dbj|BAB59* | 13 | 44 | 35.6% | 303 | 35428 | 8.5 | kinetochore protein Nuf2 [Homo sapiens] |
| U | *gi|12667401|gb|AAK014* | 13 | 44 | 23.3% | 464 | 54330 | 8.3 | NUF2R [Homo sapiens] |

| Filename XCorr DeltCN Conf% ObsM+H+ CalcM+H+ SpR ZScore Ion% # Sequence  | | | | | | | | | | | | |
| --- | --- | --- | --- | --- | --- | --- | --- | --- | --- | --- | --- | --- |
|  | Mis12IP\_Nocodazole\_MudPIT\_040709\_02.04432.04432.2 | 3.1972 | 0.4616 | 100.0% | 1549.0521 | 1549.6746 | 1 | 7.6 | 75.0% | 1 | R.LDSVPVEEQEEFK.Q | 2 |
|  | Mis12IP\_Nocodazole\_MudPIT\_040709\_03.04446.04446.1 | 1.9571 | 0.3879 | 97.1% | 1216.63 | 1217.3647 | 6 | 6.095 | 55.0% | 1 | K.TIVLQEGNSQK.K | 1 |
|  | Mis12IP\_Nocodazole\_MudPIT\_040709\_03.04469.04469.2 | 3.5873 | 0.4135 | 100.0% | 1217.0721 | 1217.3647 | 1 | 6.383 | 80.0% | 1 | K.TIVLQEGNSQK.K | 2 |
|  | Mis12IP\_Nocodazole\_MudPIT\_040709\_05.05062.05062.2 | 3.1513 | 0.2177 | 99.7% | 1345.0521 | 1345.5388 | 1 | 4.97 | 77.3% | 27 | K.TIVLQEGNSQKK.S | 2 |
|  | Mis12IP\_Nocodazole\_MudPIT\_040709\_06.05448.05448.2 | 3.0749 | 0.4264 | 100.0% | 1338.1522 | 1338.501 | 18 | 6.599 | 70.0% | 1 | K.TKIVDS\*PEKLK.N | 2 |
|  | Mis12IP\_Nocodazole\_MudPIT\_040709\_02.03320.03320.2 | 2.5451 | 0.1713 | 98.9% | 1217.9722 | 1218.3091 | 11 | 3.817 | 66.7% | 3 | K.IQDLSDNREK.L | 2 |
|  | Mis12IP\_Nocodazole\_MudPIT\_040709\_04.06938.06938.2 | 4.6552 | 0.5234 | 100.0% | 1978.3322 | 1979.0588 | 1 | 9.18 | 75.0% | 3 | K.ESLNLEDQIESDESELK.K | 2 |
|  | Mis12IP\_Nocodazole\_MudPIT\_040709\_06.05372.05372.2 | 2.0153 | 0.2432 | 98.0% | 1252.5122 | 1252.413 | 11 | 4.885 | 61.1% | 1 | K.LKTEENSFKR.L | 2 |
|  | Mis12IP\_Nocodazole\_MudPIT\_040709\_06.05485.05485.2 | 2.299 | 0.3099 | 99.7% | 1037.0521 | 1036.2163 | 93 | 5.03 | 62.5% | 1 | K.EKLATAQFK.I | 2 |
|  | Mis12IP\_Nocodazole\_MudPIT\_040709\_06.05178.05178.2 | 2.4342 | 0.1448 | 98.9% | 851.0722 | 850.9529 | 4 | 4.614 | 91.7% | 2 | K.RGAVYER.V | 2 |
|  | Mis12IP\_Nocodazole\_MudPIT\_040709\_03.04397.04397.2 | 3.0646 | 0.2601 | 99.9% | 1174.3522 | 1174.3396 | 1 | 5.792 | 77.8% | 1 | R.VTTINQEIQK.I | 2 |
|  | Mis12IP\_Nocodazole\_MudPIT\_040709\_06.06487.06487.2 | 2.8419 | 0.1602 | 99.3% | 1091.5521 | 1092.2804 | 1 | 5.933 | 81.2% | 1 | K.SQEIFLNLK.T | 2 |
|  | Mis12IP\_Nocodazole\_MudPIT\_040709\_06.06491.06491.1 | 2.1049 | 0.175 | 98.2% | 1091.6 | 1092.2804 | 1 | 5.176 | 68.8% | 1 | K.SQEIFLNLK.T | 1 |

---

|  |  |  |  |  |  |  |  |  |
| --- | --- | --- | --- | --- | --- | --- | --- | --- |
| U | *gi|14717079|emb|CAC10* | 11 | 21 | 23.3% | 356 | 40067 | 7.0 | C20orf172 [Homo sapiens] |
| U | *gi|71051472|gb|AAH260* | 11 | 21 | 24.4% | 340 | 38295 | 7.3 | DSN1 protein [Homo sapiens] |
| U | *gi|56204749|emb|CAI21* | 11 | 21 | 24.4% | 340 | 38323 | 7.3 | C20orf172 [Homo sapiens] |
| U | *gi|31542257|ref|NP\_07* | 11 | 21 | 23.3% | 356 | 40007 | 7.0 | DSN1, MIND kinetochore complex component, homolog [Homo sapiens] |

| Filename XCorr DeltCN Conf% ObsM+H+ CalcM+H+ SpR ZScore Ion% # Sequence  | | | | | | | | | | | | |
| --- | --- | --- | --- | --- | --- | --- | --- | --- | --- | --- | --- | --- |
|  | Mis12IP\_Nocodazole\_MudPIT\_040709\_02.04162.04162.2 | 4.6688 | 0.4261 | 100.0% | 1638.3922 | 1638.7467 | 1 | 8.51 | 60.7% | 5 | K.TSASLEMNQGVSEER.I | 2 |
|  | Mis12IP\_Nocodazole\_MudPIT\_040709\_03.04484.04484.1 | 1.9448 | 0.1748 | 95.3% | 1639.7 | 1638.7467 | 1 | 4.043 | 46.4% | 2 | K.TSASLEMNQGVSEER.I | 1 |
|  | Mis12IP\_Nocodazole\_MudPIT\_040709\_06.05567.05567.3 | 4.1876 | 0.2338 | 100.0% | 1926.3544 | 1926.9512 | 2 | 6.177 | 38.3% | 1 | K.SLHLS\*PQEQSASYQDR.R | 3 |
|  | Mis12IP\_Nocodazole\_MudPIT\_040709\_05.04819.04819.2 | 4.1329 | 0.4179 | 100.0% | 1926.4321 | 1926.9512 | 1 | 7.474 | 73.3% | 2 | K.SLHLS\*PQEQSASYQDR.R | 2 |
|  | Mis12IP\_Nocodazole\_MudPIT\_040709\_02.04130.04130.2 | 3.1724 | 0.332 | 99.9% | 2006.4521 | 2006.9512 | 1 | 6.229 | 66.7% | 1 | K.SLHLS\*PQEQS\*ASYQDR.R | 2 |
|  | Mis12IP\_Nocodazole\_MudPIT\_040709\_03.04716.04716.1 | 1.7324 | 0.2055 | 95.3% | 1048.66 | 1049.1663 | 59 | 5.565 | 50.0% | 2 | R.SISVDLAESK.R | 1 |
|  | Mis12IP\_Nocodazole\_MudPIT\_040709\_04.05552.05552.2 | 2.8246 | 0.3519 | 100.0% | 1048.9122 | 1049.1663 | 1 | 6.829 | 77.8% | 2 | R.SISVDLAESK.R | 2 |
|  | Mis12IP\_Nocodazole\_MudPIT\_040709\_06.06478.06478.2 | 4.7136 | 0.3659 | 100.0% | 1486.9521 | 1485.6489 | 1 | 7.228 | 73.1% | 2 | K.ASDFSLEASVAEMK.E | 2 |
|  | Mis12IP\_Nocodazole\_MudPIT\_040709\_06.08585.08585.2 | 1.7156 | 0.2694 | 95.3% | 2000.5721 | 2001.2833 | 45 | 5.12 | 28.1% | 1 | K.VFDCMELVMDELQGSVK.Q | 2 |
|  | Mis12IP\_Nocodazole\_MudPIT\_040709\_04.05330.05330.2 | 3.7396 | 0.4619 | 100.0% | 1230.0122 | 1230.3813 | 1 | 7.442 | 75.0% | 2 | R.SMQQLDPSPAR.K | 2 |
|  | Mis12IP\_Nocodazole\_MudPIT\_040709\_02.04016.04016.2 | 2.8132 | 0.4054 | 100.0% | 1310.1721 | 1310.3813 | 1 | 6.424 | 80.0% | 1 | R.SMQQLDPS\*PAR.K | 2 |

---

|  |  |  |  |  |  |  |  |  |
| --- | --- | --- | --- | --- | --- | --- | --- | --- |
| U | *gi|4506457|ref|NP\_002* | 5 | 8 | 23.0% | 317 | 36876 | 4.4 | reticulocalbin 2, EF-hand calcium binding domain [Homo sapiens] |

| Filename XCorr DeltCN Conf% ObsM+H+ CalcM+H+ SpR ZScore Ion% # Sequence  | | | | | | | | | | | | |
| --- | --- | --- | --- | --- | --- | --- | --- | --- | --- | --- | --- | --- |
| \* | Mis12IP\_Nocodazole\_MudPIT\_040709\_05.06336.06336.2 | 3.8141 | 0.4181 | 100.0% | 1707.4321 | 1707.8749 | 1 | 7.622 | 64.3% | 1 | R.EALLGVQEDVDEYVK.L | 2 |
| \* | Mis12IP\_Nocodazole\_MudPIT\_040709\_05.06466.06466.2 | 3.8042 | 0.5939 | 100.0% | 2214.672 | 2216.2764 | 1 | 8.825 | 50.0% | 2 | R.VIDFDENTALDDAEEESFR.K | 2 |
| \* | Mis12IP\_Nocodazole\_MudPIT\_040709\_05.06946.06946.2 | 2.9592 | 0.4568 | 100.0% | 1942.4922 | 1943.1199 | 3 | 6.311 | 46.7% | 1 | R.WDPTANEDPEWILVEK.D | 2 |
| \* | Mis12IP\_Nocodazole\_MudPIT\_040709\_05.07838.07838.3 | 3.637 | 0.4147 | 100.0% | 2642.2444 | 2642.8328 | 1 | 7.433 | 30.7% | 1 | K.LSEEEILENPDLFLTSEATDYGR.Q | 3 |
| \* | Mis12IP\_Nocodazole\_MudPIT\_040709\_05.07829.07829.2 | 4.7004 | 0.3597 | 100.0% | 2643.7322 | 2642.8328 | 1 | 7.803 | 50.0% | 3 | K.LSEEEILENPDLFLTSEATDYGR.Q | 2 |

---

|  |  |  |  |  |  |  |  |  |
| --- | --- | --- | --- | --- | --- | --- | --- | --- |
| U | *gi|24430192|ref|NP\_00* | 14 | 21 | 22.6% | 473 | 51268 | 5.0 | keratin 16 [Homo sapiens] |

| Filename XCorr DeltCN Conf% ObsM+H+ CalcM+H+ SpR ZScore Ion% # Sequence  | | | | | | | | | | | | |
| --- | --- | --- | --- | --- | --- | --- | --- | --- | --- | --- | --- | --- |
|  | Mis12IP\_Nocodazole\_MudPIT\_040709\_06.05544.05544.1 | 2.2968 | 0.4769 | 100.0% | 1338.58 | 1339.4478 | 12 | 7.361 | 42.3% | 1 | R.APSTYGGGLSVSSR.F | 1 |
|  | Mis12IP\_Nocodazole\_MudPIT\_040709\_06.05542.05542.2 | 3.6859 | 0.3987 | 100.0% | 1338.7722 | 1339.4478 | 1 | 9.761 | 80.8% | 1 | R.APSTYGGGLSVSSR.F | 2 |
|  | Mis12IP\_Nocodazole\_MudPIT\_040709\_04.05302.05302.2 | 2.5626 | 0.2281 | 99.5% | 1090.2922 | 1091.2273 | 1 | 5.497 | 81.2% | 2 | K.VTMQNLNDR.L | 2222 |
|  | Mis12IP\_Nocodazole\_MudPIT\_040709\_03.04508.04508.1 | 1.9048 | 0.2501 | 99.0% | 809.5 | 809.93774 | 160 | 5.178 | 66.7% | 1 | R.LASYLDK.V | 1111111 |
|  | Mis12IP\_Nocodazole\_MudPIT\_040709\_06.06820.06820.2 | 5.235 | 0.4724 | 100.0% | 2064.5322 | 2065.3774 | 1 | 8.716 | 61.1% | 1 | K.IIAATIENAQPILQIDNAR.L | 2 |
|  | Mis12IP\_Nocodazole\_MudPIT\_040709\_06.06828.06828.3 | 6.0231 | 0.5144 | 100.0% | 2065.0745 | 2065.3774 | 1 | 9.25 | 55.6% | 1 | K.IIAATIENAQPILQIDNAR.L | 3 |
|  | Mis12IP\_Nocodazole\_MudPIT\_040709\_02.04220.04220.1 | 1.5217 | 0.3222 | 99.0% | 807.44 | 807.8815 | 2 | 4.741 | 58.3% | 1 | R.LAADDFR.T | 11111111 |
|  | Mis12IP\_Nocodazole\_MudPIT\_040709\_03.04543.04543.2 | 2.2948 | 0.233 | 99.3% | 808.0522 | 807.8815 | 1 | 5.671 | 91.7% | 3 | R.LAADDFR.T | 22222222 |
|  | Mis12IP\_Nocodazole\_MudPIT\_040709\_06.06210.06210.2 | 3.7407 | 0.4163 | 100.0% | 1030.0122 | 1030.2096 | 1 | 7.093 | 93.8% | 2 | R.VLDELTLAR.T | 22222 |
|  | Mis12IP\_Nocodazole\_MudPIT\_040709\_06.06209.06209.1 | 2.5279 | 0.3193 | 100.0% | 1030.56 | 1030.2096 | 6 | 5.521 | 62.5% | 1 | R.VLDELTLAR.T | 11111 |
|  | Mis12IP\_Nocodazole\_MudPIT\_040709\_05.05291.05291.2 | 5.1663 | 0.4738 | 100.0% | 2088.3523 | 2089.2415 | 1 | 10.504 | 62.5% | 1 | R.GQTGGDVNVEMDAAPGVDLSR.I | 2 |
|  | Mis12IP\_Nocodazole\_MudPIT\_040709\_05.06191.06191.1 | 1.624 | 0.3814 | 98.4% | 1096.53 | 1097.2126 | 9 | 6.17 | 50.0% | 2 | R.DAETWFLSK.T | 1 |
|  | Mis12IP\_Nocodazole\_MudPIT\_040709\_04.06865.06865.2 | 3.1615 | 0.4477 | 100.0% | 1096.9122 | 1097.2126 | 1 | 7.482 | 81.2% | 3 | R.DAETWFLSK.T | 2 |
|  | Mis12IP\_Nocodazole\_MudPIT\_040709\_02.03765.03765.2 | 2.3717 | 0.4658 | 100.0% | 1260.8522 | 1261.2877 | 1 | 7.433 | 68.2% | 1 | R.EVFTSSSSSSSR.Q | 2 |

Similarities:
gi|4557888|ref|NP\_000(2:12)  
gi|28317|emb|CAA32649(2:12)  
gi|12803709|gb|AAH026(6:8)  
gi|4557701|ref|NP\_000(5:9)  
gi|435476|emb|CAA8231(1:13)  
gi|14043271|gb|AAH076(5:9)  
gi|24430190|ref|NP\_00(5:9)  
gi|34526448|dbj|BAC85(3:11)  
gi|85566621|gb|AAI119(2:12)  

---

|  |  |  |  |  |  |  |  |  |
| --- | --- | --- | --- | --- | --- | --- | --- | --- |
| U | *gi|13436434|gb|AAH049* | 2 | 2 | 22.5% | 213 | 23540 | 4.6 | Unknown (protein for IMAGE:2905857) [Homo sapiens] |
| U | *gi|4506411|ref|NP\_002* | 2 | 2 | 8.2% | 587 | 63542 | 4.7 | Ran GTPase activating protein 1 [Homo sapiens] |
| U | *gi|14017887|dbj|BAB47* | 2 | 2 | 7.7% | 623 | 67334 | 4.7 | KIAA1835 protein [Homo sapiens] |

| Filename XCorr DeltCN Conf% ObsM+H+ CalcM+H+ SpR ZScore Ion% # Sequence  | | | | | | | | | | | | |
| --- | --- | --- | --- | --- | --- | --- | --- | --- | --- | --- | --- | --- |
|  | Mis12IP\_Nocodazole\_MudPIT\_040709\_02.08234.08234.3 | 3.53 | 0.2016 | 96.6% | 3833.6343 | 3835.2163 | 1 | 5.476 | 23.5% | 1 | K.ILDPNTGEPAPVLSS\*PPPADVSTFLAFPS\*PEKLLR.L | 3 |
|  | Mis12IP\_Nocodazole\_MudPIT\_040709\_06.07298.07298.2 | 2.0174 | 0.2469 | 97.1% | 1420.3922 | 1420.682 | 5 | 4.811 | 50.0% | 1 | R.MAVQDAVDALMQK.A | 2 |

---

|  |  |  |  |  |  |  |  |  |
| --- | --- | --- | --- | --- | --- | --- | --- | --- |
| U | *gi|13128994|ref|NP\_07* | 6 | 7 | 21.0% | 205 | 24140 | 5.7 | MIS12 homolog [Homo sapiens] |

| Filename XCorr DeltCN Conf% ObsM+H+ CalcM+H+ SpR ZScore Ion% # Sequence  | | | | | | | | | | | | |
| --- | --- | --- | --- | --- | --- | --- | --- | --- | --- | --- | --- | --- |
|  | Mis12IP\_Nocodazole\_MudPIT\_040709\_06.06307.06307.2 | 4.1551 | 0.4725 | 100.0% | 1238.6122 | 1239.4546 | 6 | 7.398 | 70.0% | 1 | R.IPSNILLPEDK.C | 2 |
|  | Mis12IP\_Nocodazole\_MudPIT\_040709\_06.06421.06421.1 | 2.5472 | 0.4046 | 100.0% | 1271.72 | 1272.441 | 6 | 5.859 | 60.0% | 1 | K.QALLAELEEQK.I | 1 |
|  | Mis12IP\_Nocodazole\_MudPIT\_040709\_06.06412.06412.2 | 3.6565 | 0.3595 | 100.0% | 1272.2122 | 1272.441 | 1 | 6.835 | 85.0% | 2 | K.QALLAELEEQK.I | 2 |
| \* | Mis12IP\_Nocodazole\_MudPIT\_040709\_06.06300.06300.1 | 1.786 | 0.2616 | 99.3% | 1231.61 | 1232.3794 | 31 | 4.513 | 55.0% | 1 | R.ESLVSLVQNSR.K | 1 |
| \* | Mis12IP\_Nocodazole\_MudPIT\_040709\_06.06292.06292.2 | 3.7128 | 0.3629 | 100.0% | 1231.6122 | 1232.3794 | 1 | 6.791 | 85.0% | 1 | R.ESLVSLVQNSR.K | 2 |
| \* | Mis12IP\_Nocodazole\_MudPIT\_040709\_05.03002.03002.2 | 3.2769 | 0.3398 | 100.0% | 1228.6522 | 1229.3787 | 1 | 5.766 | 83.3% | 1 | K.LQNIRDNVEK.E | 2 |

---

|  |  |  |  |  |  |  |  |  |
| --- | --- | --- | --- | --- | --- | --- | --- | --- |
| U | *gi|10047351|dbj|BAB13* | 4 | 4 | 20.7% | 479 | 53432 | 8.7 | KIAA1637 protein [Homo sapiens] |
| U | *gi|15147335|ref|NP\_06* | 4 | 4 | 17.1% | 579 | 65536 | 9.6 | nuclear receptor coactivator 5 [Homo sapiens] |
| U | *gi|11526821|gb|AAG367* | 4 | 4 | 15.9% | 621 | 69783 | 9.8 | nuclear receptor coactivator CIA [Homo sapiens] |

| Filename XCorr DeltCN Conf% ObsM+H+ CalcM+H+ SpR ZScore Ion% # Sequence  | | | | | | | | | | | | |
| --- | --- | --- | --- | --- | --- | --- | --- | --- | --- | --- | --- | --- |
|  | Mis12IP\_Nocodazole\_MudPIT\_040709\_06.06323.06323.2 | 3.2129 | 0.2693 | 99.9% | 1416.1921 | 1416.6967 | 1 | 6.187 | 79.2% | 1 | R.NMPQADAMVLVAR.N | 2 |
|  | Mis12IP\_Nocodazole\_MudPIT\_040709\_03.04675.04675.2 | 3.6768 | 0.0904 | 99.3% | 1177.8121 | 1176.3301 | 1 | 4.794 | 88.9% | 1 | K.MADEAILQER.E | 2 |
|  | Mis12IP\_Nocodazole\_MudPIT\_040709\_03.04866.04866.3 | 2.785 | 0.2966 | 98.9% | 3568.1343 | 3568.8333 | 80 | 5.004 | 15.9% | 1 | K.TQPSSQPLQSGQVLPSATPT#PSAPPTSQQELQAK.I | 3 |
|  | Mis12IP\_Nocodazole\_MudPIT\_040709\_03.06209.06209.3 | 2.0556 | 0.387 | 100.0% | 4303.7046 | 4304.504 | 83 | 6.035 | 12.2% | 1 | K.ILSLFNSGTVTANSSSASPSVAAGNT#PNQNFSTAANSQPQQR.S | 3 |

---

|  |  |  |  |  |  |  |  |  |
| --- | --- | --- | --- | --- | --- | --- | --- | --- |
| U | *gi|18308012|gb|AAL678* | 50 | 76 | 19.9% | 2316 | 262516 | 5.4 | AF15q14 isoform 2 [Homo sapiens] |
| U | *gi|74048554|ref|NP\_65* | 50 | 76 | 19.9% | 2316 | 262530 | 5.4 | cancer susceptibility candidate 5 isoform 2 [Homo sapiens] |

| Filename XCorr DeltCN Conf% ObsM+H+ CalcM+H+ SpR ZScore Ion% # Sequence  | | | | | | | | | | | | |
| --- | --- | --- | --- | --- | --- | --- | --- | --- | --- | --- | --- | --- |
|  | Mis12IP\_Nocodazole\_MudPIT\_040709\_03.04743.04743.2 | 2.7917 | 0.2914 | 99.7% | 2341.152 | 2342.3696 | 1 | 5.59 | 47.4% | 1 | -.MDGVSS\*EANEENDNIERPVR.R | 22 |
|  | Mis12IP\_Nocodazole\_MudPIT\_040709\_05.05109.05109.1 | 1.5489 | 0.2537 | 98.2% | 880.49 | 881.01654 | 2 | 6.001 | 71.4% | 2 | R.VSFADTIK.V | 11 |
|  | Mis12IP\_Nocodazole\_MudPIT\_040709\_06.05483.05483.2 | 2.4654 | 0.2526 | 99.6% | 1106.5521 | 1107.2695 | 1 | 5.949 | 87.5% | 2 | K.VFQTESHMK.I | 22 |
|  | Mis12IP\_Nocodazole\_MudPIT\_040709\_05.05891.05891.2 | 5.2025 | 0.4081 | 100.0% | 2079.3323 | 2079.2837 | 1 | 7.828 | 67.6% | 1 | K.SEMEETETGENLLLIQNK.K | 2 |
|  | Mis12IP\_Nocodazole\_MudPIT\_040709\_06.06208.06208.1 | 1.7858 | 0.2821 | 99.1% | 1085.58 | 1086.2303 | 10 | 4.96 | 44.4% | 1 | K.GLLDNPISEK.S | 11 |
|  | Mis12IP\_Nocodazole\_MudPIT\_040709\_05.05538.05538.2 | 2.9789 | 0.353 | 100.0% | 1086.1721 | 1086.2303 | 1 | 6.652 | 72.2% | 2 | K.GLLDNPISEK.S | 22 |
|  | Mis12IP\_Nocodazole\_MudPIT\_040709\_06.06994.06994.1 | 2.4847 | 0.2732 | 98.3% | 1222.61 | 1223.4117 | 2 | 5.2 | 65.0% | 1 | K.IDTTSFLANLK.L | 11 |
|  | Mis12IP\_Nocodazole\_MudPIT\_040709\_06.06988.06988.2 | 3.0828 | 0.3145 | 100.0% | 1223.0122 | 1223.4117 | 1 | 6.513 | 80.0% | 1 | K.IDTTSFLANLK.L | 22 |
|  | Mis12IP\_Nocodazole\_MudPIT\_040709\_06.05296.05296.2 | 2.6359 | 0.2628 | 99.8% | 951.8722 | 952.0983 | 1 | 5.975 | 87.5% | 2 | K.ASGNKTVFK.S | 22 |
|  | Mis12IP\_Nocodazole\_MudPIT\_040709\_05.05331.05331.2 | 3.1278 | 0.4594 | 100.0% | 1651.7322 | 1652.7582 | 1 | 8.144 | 50.0% | 1 | K.QNTAFQDLSINSADK.I | 22 |
|  | Mis12IP\_Nocodazole\_MudPIT\_040709\_04.05556.05556.1 | 2.5836 | 0.2808 | 97.9% | 1501.67 | 1501.6484 | 1 | 5.858 | 50.0% | 1 | K.TIYSGEENMDITK.S | 11 |
|  | Mis12IP\_Nocodazole\_MudPIT\_040709\_04.05546.05546.2 | 4.1509 | 0.5249 | 100.0% | 1502.1921 | 1501.6484 | 1 | 7.791 | 87.5% | 2 | K.TIYSGEENMDITK.S | 22 |
|  | Mis12IP\_Nocodazole\_MudPIT\_040709\_06.06940.06940.3 | 3.4885 | 0.3873 | 100.0% | 3486.7744 | 3487.8074 | 1 | 6.174 | 24.2% | 2 | K.QDQSNVQIAAAPT#PEKEMMLQNLMTTSEDGK.M | 33 |
|  | Mis12IP\_Nocodazole\_MudPIT\_040709\_06.06838.06838.2 | 3.2121 | 0.4934 | 100.0% | 1727.8722 | 1728.9883 | 3 | 8.097 | 46.4% | 2 | K.EMMLQNLMTTSEDGK.M | 22 |
|  | Mis12IP\_Nocodazole\_MudPIT\_040709\_06.06774.06774.2 | 4.776 | 0.4986 | 100.0% | 1772.5521 | 1772.9963 | 1 | 8.468 | 73.3% | 1 | R.IQQSLSNPLSISLTDR.K | 22 |
|  | Mis12IP\_Nocodazole\_MudPIT\_040709\_03.06065.06065.2 | 2.6083 | 0.4098 | 99.9% | 1852.2522 | 1852.9963 | 3 | 6.109 | 46.7% | 1 | R.IQQSLSNPLSIS\*LTDR.K | 22 |
|  | Mis12IP\_Nocodazole\_MudPIT\_040709\_06.06024.06024.2 | 3.1876 | 0.1925 | 99.9% | 1034.1921 | 1034.1196 | 1 | 4.517 | 85.7% | 3 | K.SRNEPFQR.S | 22 |
|  | Mis12IP\_Nocodazole\_MudPIT\_040709\_06.02972.02972.2 | 3.3662 | 0.4475 | 100.0% | 1309.7522 | 1310.4093 | 1 | 7.503 | 68.2% | 8 | K.NHDTAISSHTVK.S | 22 |
|  | Mis12IP\_Nocodazole\_MudPIT\_040709\_06.02979.02979.3 | 3.3128 | 0.2646 | 100.0% | 1310.5443 | 1310.4093 | 4 | 5.262 | 52.3% | 1 | K.NHDTAISSHTVK.S | 33 |
|  | Mis12IP\_Nocodazole\_MudPIT\_040709\_03.04097.04097.1 | 1.5184 | 0.2497 | 97.1% | 832.39 | 832.9322 | 4 | 4.95 | 57.1% | 1 | K.SVLGQNSK.L | 11 |
|  | Mis12IP\_Nocodazole\_MudPIT\_040709\_06.05488.05488.2 | 2.1592 | 0.1036 | 95.1% | 826.6122 | 827.01465 | 11 | 3.518 | 83.3% | 1 | K.LAEPLRK.S | 22 |
|  | Mis12IP\_Nocodazole\_MudPIT\_040709\_06.06743.06743.2 | 3.681 | 0.5597 | 100.0% | 2086.2722 | 2087.2534 | 1 | 9.106 | 52.9% | 1 | R.LVANDSQLTPLEEWSNNR.G | 22 |
|  | Mis12IP\_Nocodazole\_MudPIT\_040709\_06.07086.07086.3 | 3.2162 | 0.376 | 100.0% | 3535.4944 | 3537.7937 | 1 | 6.854 | 25.0% | 1 | R.LVANDSQLT#PLEEWSNNRGPVEVADNMELSK.S | 33 |
|  | Mis12IP\_Nocodazole\_MudPIT\_040709\_06.07402.07402.3 | 3.2588 | 0.3978 | 100.0% | 3536.6943 | 3537.7937 | 1 | 5.794 | 24.2% | 1 | R.LVANDS\*QLTPLEEWSNNRGPVEVADNMELSK.S | 33 |
|  | Mis12IP\_Nocodazole\_MudPIT\_040709\_03.06989.06989.3 | 3.5717 | 0.2741 | 100.0% | 3616.9143 | 3617.7937 | 1 | 6.218 | 30.0% | 2 | R.LVANDS\*QLT#PLEEWSNNRGPVEVADNMELSK.S | 33 |
|  | Mis12IP\_Nocodazole\_MudPIT\_040709\_02.04862.04862.2 | 3.6347 | 0.4387 | 100.0% | 1698.2922 | 1698.7838 | 1 | 7.314 | 67.9% | 1 | K.DVQS\*PGFLNEPLSSK.S | 22 |
|  | Mis12IP\_Nocodazole\_MudPIT\_040709\_02.03549.03549.1 | 2.082 | 0.1287 | 97.2% | 842.54 | 842.96765 | 28 | 3.443 | 75.0% | 1 | K.ILEENPK.F | 11 |
|  | Mis12IP\_Nocodazole\_MudPIT\_040709\_04.04848.04848.1 | 1.7729 | 0.3303 | 98.7% | 800.51 | 800.93335 | 58 | 6.56 | 57.1% | 2 | K.QALAVGNK.I | 11 |
|  | Mis12IP\_Nocodazole\_MudPIT\_040709\_06.05396.05396.2 | 2.0028 | 0.1668 | 95.7% | 967.9122 | 968.1411 | 4 | 4.852 | 64.3% | 1 | K.IVLHTEQK.Q | 22 |
|  | Mis12IP\_Nocodazole\_MudPIT\_040709\_06.05634.05634.1 | 1.5708 | 0.3048 | 99.2% | 1048.53 | 1049.1747 | 76 | 5.399 | 62.5% | 1 | K.QQLFAATNR.T | 11 |
|  | Mis12IP\_Nocodazole\_MudPIT\_040709\_06.05633.05633.2 | 1.8662 | 0.4045 | 99.7% | 1049.0322 | 1049.1747 | 1 | 7.151 | 75.0% | 1 | K.QQLFAATNR.T | 22 |
|  | Mis12IP\_Nocodazole\_MudPIT\_040709\_06.06970.06970.2 | 5.9643 | 0.6152 | 100.0% | 2138.5322 | 2139.4285 | 1 | 12.55 | 75.0% | 5 | R.NLLANQTLVYSQDLGEMTK.L | 22 |
|  | Mis12IP\_Nocodazole\_MudPIT\_040709\_06.06973.06973.3 | 5.8764 | 0.4978 | 100.0% | 2138.7544 | 2139.4285 | 1 | 9.534 | 54.2% | 1 | R.NLLANQTLVYSQDLGEMTK.L | 33 |
|  | Mis12IP\_Nocodazole\_MudPIT\_040709\_05.02979.02979.2 | 2.5169 | 0.3688 | 99.9% | 1339.1122 | 1339.4478 | 3 | 5.822 | 58.3% | 1 | K.SHNGAETTSLPPK.T | 22 |
|  | Mis12IP\_Nocodazole\_MudPIT\_040709\_02.03813.03813.1 | 1.9595 | 0.3975 | 96.9% | 890.47 | 890.9689 | 1 | 6.526 | 72.2% | 1 | K.DSGIGSVAGK.L | 11 |
|  | Mis12IP\_Nocodazole\_MudPIT\_040709\_02.03851.03851.2 | 2.7925 | 0.2494 | 99.7% | 893.1322 | 890.9689 | 1 | 5.019 | 77.8% | 1 | K.DSGIGSVAGK.L | 22 |
|  | Mis12IP\_Nocodazole\_MudPIT\_040709\_02.06784.06784.3 | 3.3881 | 0.3671 | 100.0% | 3975.6243 | 3976.164 | 1 | 7.439 | 20.5% | 1 | K.LNLS\*PSQYINEENLPVYPDEINSSDSINIETEEK.A | 3 |
|  | Mis12IP\_Nocodazole\_MudPIT\_040709\_03.04604.04604.2 | 2.0502 | 0.2002 | 97.4% | 965.4922 | 966.1222 | 3 | 4.891 | 85.7% | 1 | K.ALIETYQK.E | 2 |
|  | Mis12IP\_Nocodazole\_MudPIT\_040709\_05.04811.04811.1 | 1.6368 | 0.2132 | 96.2% | 965.51 | 966.1222 | 6 | 4.496 | 64.3% | 1 | K.ALIETYQK.E | 1 |
|  | Mis12IP\_Nocodazole\_MudPIT\_040709\_06.05612.05612.2 | 2.8408 | 0.1971 | 99.3% | 1415.7522 | 1414.5144 | 2 | 4.571 | 75.0% | 1 | R.TWVQEEEDIHK.E | 2 |
|  | Mis12IP\_Nocodazole\_MudPIT\_040709\_04.05892.05892.2 | 2.9551 | 0.463 | 100.0% | 1332.4922 | 1333.3971 | 5 | 7.5 | 68.2% | 2 | K.ADGTSLDFSTYR.S | 2 |
|  | Mis12IP\_Nocodazole\_MudPIT\_040709\_06.05772.05772.2 | 2.9677 | 0.2559 | 99.9% | 1212.4321 | 1213.3508 | 1 | 4.922 | 77.8% | 1 | R.SSQMESQFLR.D | 2 |
|  | Mis12IP\_Nocodazole\_MudPIT\_040709\_03.03494.03494.2 | 3.0182 | 0.3559 | 100.0% | 1302.0322 | 1302.43 | 1 | 7.209 | 70.0% | 3 | K.LVQSAQNEREK.L | 2 |
|  | Mis12IP\_Nocodazole\_MudPIT\_040709\_05.05738.05738.2 | 2.7576 | 0.4694 | 100.0% | 1506.0122 | 1506.5867 | 1 | 7.356 | 59.1% | 1 | K.NNPVEEWDSEMR.A | 2 |
|  | Mis12IP\_Nocodazole\_MudPIT\_040709\_06.06355.06355.1 | 2.5972 | 0.1845 | 98.9% | 1085.74 | 1086.2737 | 3 | 4.478 | 75.0% | 1 | R.NLLELEVQK.E | 1 |
|  | Mis12IP\_Nocodazole\_MudPIT\_040709\_06.06352.06352.2 | 3.192 | 0.1543 | 99.7% | 1085.8922 | 1086.2737 | 1 | 4.909 | 81.2% | 1 | R.NLLELEVQK.E | 2 |
|  | Mis12IP\_Nocodazole\_MudPIT\_040709\_06.06472.06472.2 | 4.3144 | 0.44 | 100.0% | 1452.2722 | 1452.6654 | 1 | 8.464 | 77.3% | 2 | K.EQTLAQIDFMQK.Q | 2 |
|  | Mis12IP\_Nocodazole\_MudPIT\_040709\_06.06480.06480.2 | 2.698 | 0.3344 | 99.9% | 1168.5122 | 1169.3629 | 1 | 5.702 | 81.2% | 1 | K.LIFQYVEEK.E | 2 |
|  | Mis12IP\_Nocodazole\_MudPIT\_040709\_06.06624.06624.2 | 2.6751 | 0.1087 | 97.1% | 1207.2322 | 1207.4094 | 13 | 5.702 | 55.6% | 1 | R.LLGEEIEYLK.R | 2 |
|  | Mis12IP\_Nocodazole\_MudPIT\_040709\_06.05765.05765.2 | 2.4939 | 0.3134 | 99.9% | 1089.4722 | 1090.2645 | 7 | 6.34 | 68.8% | 1 | K.VPLENNYLK.N | 2 |

Similarities:
gi|21912964|dbj|BAC05(35:15)  

---

|  |  |  |  |  |  |  |  |  |
| --- | --- | --- | --- | --- | --- | --- | --- | --- |
| U | *gi|13676857|ref|NP\_06* | 11 | 44 | 19.1% | 639 | 70021 | 5.7 | heat shock 70kDa protein 2 [Homo sapiens] |
| U | *gi|4204880|gb|AAD1146* | 11 | 44 | 19.1% | 639 | 69995 | 5.7 | heat shock protein [Homo sapiens] |
| U | *gi|23271312|gb|AAH361* | 11 | 44 | 19.1% | 639 | 69945 | 5.8 | HSPA2 protein [Homo sapiens] |

| Filename XCorr DeltCN Conf% ObsM+H+ CalcM+H+ SpR ZScore Ion% # Sequence  | | | | | | | | | | | | |
| --- | --- | --- | --- | --- | --- | --- | --- | --- | --- | --- | --- | --- |
|  | Mis12IP\_Nocodazole\_MudPIT\_040709\_05.05229.05229.1 | 1.9614 | 0.3836 | 97.4% | 1487.44 | 1488.5939 | 1 | 7.022 | 45.8% | 1 | R.TTPSYVAFTDTER.L | 11111 |
|  | Mis12IP\_Nocodazole\_MudPIT\_040709\_06.05909.05909.2 | 3.3634 | 0.4566 | 100.0% | 1488.0721 | 1488.5939 | 1 | 8.603 | 75.0% | 3 | R.TTPSYVAFTDTER.L | 22222 |
|  | Mis12IP\_Nocodazole\_MudPIT\_040709\_06.06247.06247.2 | 2.2492 | 0.1714 | 95.2% | 1664.2322 | 1664.8737 | 2 | 5.54 | 53.6% | 1 | K.NQVAMNPTNTIFDAK.R | 2 |
|  | Mis12IP\_Nocodazole\_MudPIT\_040709\_03.03686.03686.2 | 2.9267 | 0.4214 | 100.0% | 1180.9521 | 1181.3312 | 1 | 6.976 | 77.8% | 7 | K.VQVEYKGETK.T | 22 |
|  | Mis12IP\_Nocodazole\_MudPIT\_040709\_06.07883.07883.2 | 2.5784 | 0.4772 | 100.0% | 1629.9722 | 1629.9086 | 1 | 6.912 | 50.0% | 1 | K.TFFPEEISSMVLTK.M | 2 |
|  | Mis12IP\_Nocodazole\_MudPIT\_040709\_05.05844.05844.2 | 2.9936 | 0.2199 | 99.6% | 1230.0521 | 1230.4069 | 2 | 5.884 | 72.7% | 1 | K.DAGTITGLNVLR.I | 2 |
|  | Mis12IP\_Nocodazole\_MudPIT\_040709\_06.06545.06545.2 | 5.2019 | 0.5351 | 100.0% | 1660.3322 | 1660.9078 | 1 | 9.75 | 73.3% | 1 | R.IINEPTAAAIAYGLDK.K | 2222 |
|  | Mis12IP\_Nocodazole\_MudPIT\_040709\_04.05504.05504.2 | 3.6406 | 0.4415 | 100.0% | 1692.3922 | 1692.6958 | 1 | 8.02 | 63.3% | 13 | K.STAGDTHLGGEDFDNR.M | 22 |
|  | Mis12IP\_Nocodazole\_MudPIT\_040709\_06.06760.06760.2 | 3.5071 | 0.3609 | 100.0% | 1254.0122 | 1254.3849 | 1 | 7.204 | 77.8% | 1 | R.FEELNADLFR.G | 22 |
|  | Mis12IP\_Nocodazole\_MudPIT\_040709\_03.03625.03625.1 | 1.4309 | 0.3276 | 99.1% | 774.34 | 774.8925 | 50 | 4.779 | 58.3% | 1 | R.NTTIPTK.Q | 111 |
|  | Mis12IP\_Nocodazole\_MudPIT\_040709\_04.05654.05654.2 | 2.3494 | 0.4197 | 100.0% | 1018.2922 | 1018.1582 | 2 | 6.376 | 68.8% | 14 | K.ITITNDKGR.L | 22222 |

Similarities:
gi|5729877|ref|NP\_006(8:3)  
gi|16507237|ref|NP\_00(1:10)  
gi|12653415|gb|AAH004(1:10)  
gi|12803275|gb|AAH024(3:8)  
gi|27436929|ref|NP\_00(4:7)  
gi|34419635|ref|NP\_00(3:8)  

---

|  |  |  |  |  |  |  |  |  |
| --- | --- | --- | --- | --- | --- | --- | --- | --- |
| U | *gi|14250401|gb|AAH086* | 6 | 9 | 19.0% | 368 | 41005 | 5.8 | actin, beta [Homo sapiens] |
| U | *gi|4501887|ref|NP\_001* | 6 | 9 | 18.7% | 375 | 41793 | 5.5 | actin, gamma 1 propeptide [Homo sapiens] |
| U | *gi|4501885|ref|NP\_001* | 6 | 9 | 18.7% | 375 | 41737 | 5.5 | beta actin [Homo sapiens] |
| U | *gi|16924319|gb|AAH174* | 6 | 9 | 19.3% | 363 | 40503 | 6.1 | Unknown (protein for IMAGE:3538275) [Homo sapiens] |
| U | *gi|16359158|gb|AAH160* | 6 | 9 | 18.7% | 375 | 41763 | 5.5 | Actin, beta [Homo sapiens] |
| U | *gi|15277503|gb|AAH128* | 6 | 9 | 19.4% | 360 | 40220 | 5.8 | ACTB protein [Homo sapiens] |

| Filename XCorr DeltCN Conf% ObsM+H+ CalcM+H+ SpR ZScore Ion% # Sequence  | | | | | | | | | | | | |
| --- | --- | --- | --- | --- | --- | --- | --- | --- | --- | --- | --- | --- |
|  | Mis12IP\_Nocodazole\_MudPIT\_040709\_02.03898.03898.2 | 2.2051 | 0.303 | 99.5% | 977.0722 | 977.02136 | 1 | 6.033 | 66.7% | 1 | K.AGFAGDDAPR.A | 2 |
|  | Mis12IP\_Nocodazole\_MudPIT\_040709\_02.03082.03082.2 | 1.9581 | 0.3021 | 99.1% | 1355.2722 | 1355.4038 | 2 | 4.992 | 59.1% | 1 | K.DSYVGDEAQSKR.G | 2 |
|  | Mis12IP\_Nocodazole\_MudPIT\_040709\_06.06704.06704.2 | 4.2586 | 0.3635 | 100.0% | 1792.2322 | 1791.9554 | 1 | 7.741 | 73.3% | 1 | K.SYELPDGQVITIGNER.F | 2 |
|  | Mis12IP\_Nocodazole\_MudPIT\_040709\_05.06563.06563.2 | 2.8607 | 0.5296 | 100.0% | 2215.4922 | 2216.4705 | 4 | 7.63 | 35.0% | 2 | K.DLYANTVLSGGTTMYPGIADR.M | 2 |
|  | Mis12IP\_Nocodazole\_MudPIT\_040709\_06.05784.05784.1 | 2.0441 | 0.5159 | 100.0% | 1161.52 | 1162.3868 | 1 | 7.002 | 55.0% | 2 | K.EITALAPSTMK.I | 1 |
|  | Mis12IP\_Nocodazole\_MudPIT\_040709\_05.05061.05061.2 | 2.6462 | 0.4852 | 100.0% | 1161.9922 | 1162.3868 | 1 | 6.876 | 60.0% | 2 | K.EITALAPSTMK.I | 2 |

---

|  |  |  |  |  |  |  |  |  |
| --- | --- | --- | --- | --- | --- | --- | --- | --- |
| U | *gi|15431295|ref|NP\_15* | 5 | 11 | 19.0% | 211 | 24261 | 11.7 | ribosomal protein L13 [Homo sapiens] |
| U | *gi|42490910|gb|AAH663* | 5 | 11 | 19.0% | 211 | 24265 | 11.7 | Ribosomal protein L13 [Homo sapiens] |

| Filename XCorr DeltCN Conf% ObsM+H+ CalcM+H+ SpR ZScore Ion% # Sequence  | | | | | | | | | | | | |
| --- | --- | --- | --- | --- | --- | --- | --- | --- | --- | --- | --- | --- |
|  | Mis12IP\_Nocodazole\_MudPIT\_040709\_06.06407.06407.2 | 2.5638 | 0.4156 | 100.0% | 950.8722 | 951.0672 | 1 | 7.154 | 78.6% | 1 | R.GFSLEELR.V | 2 |
|  | Mis12IP\_Nocodazole\_MudPIT\_040709\_05.05033.05033.2 | 2.3963 | 0.2972 | 99.7% | 957.77216 | 958.1026 | 1 | 5.243 | 75.0% | 2 | R.TIGISVDPR.R | 2 |
|  | Mis12IP\_Nocodazole\_MudPIT\_040709\_05.03004.03004.2 | 3.5556 | 0.4006 | 100.0% | 1476.2122 | 1475.6017 | 1 | 7.389 | 83.3% | 3 | R.NKSTESLQANVQR.L | 2 |
|  | Mis12IP\_Nocodazole\_MudPIT\_040709\_03.03896.03896.2 | 3.4198 | 0.3983 | 100.0% | 1232.8522 | 1233.3237 | 1 | 7.025 | 75.0% | 4 | K.STESLQANVQR.L | 2 |
|  | Mis12IP\_Nocodazole\_MudPIT\_040709\_03.05529.05529.2 | 2.1274 | 0.2854 | 99.3% | 1237.1322 | 1237.3953 | 11 | 4.673 | 55.6% | 1 | R.VITEEEKNFK.A | 2 |

---

|  |  |  |  |  |  |  |  |  |
| --- | --- | --- | --- | --- | --- | --- | --- | --- |
| U | *gi|21912964|dbj|BAC05* | 37 | 60 | 18.8% | 1746 | 195450 | 5.5 | D40 [Homo sapiens] |

| Filename XCorr DeltCN Conf% ObsM+H+ CalcM+H+ SpR ZScore Ion% # Sequence  | | | | | | | | | | | | |
| --- | --- | --- | --- | --- | --- | --- | --- | --- | --- | --- | --- | --- |
|  | Mis12IP\_Nocodazole\_MudPIT\_040709\_03.04743.04743.2 | 2.7917 | 0.2914 | 99.7% | 2341.152 | 2342.3696 | 1 | 5.59 | 47.4% | 1 | -.MDGVSS\*EANEENDNIERPVR.R | 22 |
|  | Mis12IP\_Nocodazole\_MudPIT\_040709\_02.03910.03910.2 | 3.6185 | 0.3269 | 100.0% | 1375.3722 | 1375.438 | 1 | 6.256 | 70.8% | 1 | R.GGNETVQESNALR.N | 2 |
|  | Mis12IP\_Nocodazole\_MudPIT\_040709\_05.05109.05109.1 | 1.5489 | 0.2537 | 98.2% | 880.49 | 881.01654 | 2 | 6.001 | 71.4% | 2 | R.VSFADTIK.V | 11 |
|  | Mis12IP\_Nocodazole\_MudPIT\_040709\_06.05483.05483.2 | 2.4654 | 0.2526 | 99.6% | 1106.5521 | 1107.2695 | 1 | 5.949 | 87.5% | 2 | K.VFQTESHMK.I | 22 |
|  | Mis12IP\_Nocodazole\_MudPIT\_040709\_05.05986.05986.2 | 4.9394 | 0.5156 | 100.0% | 2048.412 | 2049.2573 | 1 | 8.215 | 67.6% | 2 | K.SEMEETEAGENLLLIQNK.K | 2 |
|  | Mis12IP\_Nocodazole\_MudPIT\_040709\_06.06208.06208.1 | 1.7858 | 0.2821 | 99.1% | 1085.58 | 1086.2303 | 10 | 4.96 | 44.4% | 1 | K.GLLDNPISEK.S | 11 |
|  | Mis12IP\_Nocodazole\_MudPIT\_040709\_05.05538.05538.2 | 2.9789 | 0.353 | 100.0% | 1086.1721 | 1086.2303 | 1 | 6.652 | 72.2% | 2 | K.GLLDNPISEK.S | 22 |
|  | Mis12IP\_Nocodazole\_MudPIT\_040709\_06.06994.06994.1 | 2.4847 | 0.2732 | 98.3% | 1222.61 | 1223.4117 | 2 | 5.2 | 65.0% | 1 | K.IDTTSFLANLK.L | 11 |
|  | Mis12IP\_Nocodazole\_MudPIT\_040709\_06.06988.06988.2 | 3.0828 | 0.3145 | 100.0% | 1223.0122 | 1223.4117 | 1 | 6.513 | 80.0% | 1 | K.IDTTSFLANLK.L | 22 |
|  | Mis12IP\_Nocodazole\_MudPIT\_040709\_06.05296.05296.2 | 2.6359 | 0.2628 | 99.8% | 951.8722 | 952.0983 | 1 | 5.975 | 87.5% | 2 | K.ASGNKTVFK.S | 22 |
|  | Mis12IP\_Nocodazole\_MudPIT\_040709\_05.05331.05331.2 | 3.1278 | 0.4594 | 100.0% | 1651.7322 | 1652.7582 | 1 | 8.144 | 50.0% | 1 | K.QNTAFQDLSINSADK.I | 22 |
|  | Mis12IP\_Nocodazole\_MudPIT\_040709\_04.05556.05556.1 | 2.5836 | 0.2808 | 97.9% | 1501.67 | 1501.6484 | 1 | 5.858 | 50.0% | 1 | K.TIYSGEENMDITK.S | 11 |
|  | Mis12IP\_Nocodazole\_MudPIT\_040709\_04.05546.05546.2 | 4.1509 | 0.5249 | 100.0% | 1502.1921 | 1501.6484 | 1 | 7.791 | 87.5% | 2 | K.TIYSGEENMDITK.S | 22 |
|  | Mis12IP\_Nocodazole\_MudPIT\_040709\_06.06940.06940.3 | 3.4885 | 0.3873 | 100.0% | 3486.7744 | 3487.8074 | 1 | 6.174 | 24.2% | 2 | K.QDQSNVQIAAAPT#PEKEMMLQNLMTTSEDGK.M | 33 |
|  | Mis12IP\_Nocodazole\_MudPIT\_040709\_06.06838.06838.2 | 3.2121 | 0.4934 | 100.0% | 1727.8722 | 1728.9883 | 3 | 8.097 | 46.4% | 2 | K.EMMLQNLMTTSEDGK.M | 22 |
|  | Mis12IP\_Nocodazole\_MudPIT\_040709\_06.06774.06774.2 | 4.776 | 0.4986 | 100.0% | 1772.5521 | 1772.9963 | 1 | 8.468 | 73.3% | 1 | R.IQQSLSNPLSISLTDR.K | 22 |
|  | Mis12IP\_Nocodazole\_MudPIT\_040709\_03.06065.06065.2 | 2.6083 | 0.4098 | 99.9% | 1852.2522 | 1852.9963 | 3 | 6.109 | 46.7% | 1 | R.IQQSLSNPLSIS\*LTDR.K | 22 |
|  | Mis12IP\_Nocodazole\_MudPIT\_040709\_06.06024.06024.2 | 3.1876 | 0.1925 | 99.9% | 1034.1921 | 1034.1196 | 1 | 4.517 | 85.7% | 3 | K.SRNEPFQR.S | 22 |
|  | Mis12IP\_Nocodazole\_MudPIT\_040709\_06.02972.02972.2 | 3.3662 | 0.4475 | 100.0% | 1309.7522 | 1310.4093 | 1 | 7.503 | 68.2% | 8 | K.NHDTAISSHTVK.S | 22 |
|  | Mis12IP\_Nocodazole\_MudPIT\_040709\_06.02979.02979.3 | 3.3128 | 0.2646 | 100.0% | 1310.5443 | 1310.4093 | 4 | 5.262 | 52.3% | 1 | K.NHDTAISSHTVK.S | 33 |
|  | Mis12IP\_Nocodazole\_MudPIT\_040709\_03.04097.04097.1 | 1.5184 | 0.2497 | 97.1% | 832.39 | 832.9322 | 4 | 4.95 | 57.1% | 1 | K.SVLGQNSK.L | 11 |
|  | Mis12IP\_Nocodazole\_MudPIT\_040709\_06.05488.05488.2 | 2.1592 | 0.1036 | 95.1% | 826.6122 | 827.01465 | 11 | 3.518 | 83.3% | 1 | K.LAEPLRK.S | 22 |
|  | Mis12IP\_Nocodazole\_MudPIT\_040709\_06.06743.06743.2 | 3.681 | 0.5597 | 100.0% | 2086.2722 | 2087.2534 | 1 | 9.106 | 52.9% | 1 | R.LVANDSQLTPLEEWSNNR.G | 22 |
|  | Mis12IP\_Nocodazole\_MudPIT\_040709\_06.07086.07086.3 | 3.2162 | 0.376 | 100.0% | 3535.4944 | 3537.7937 | 1 | 6.854 | 25.0% | 1 | R.LVANDSQLT#PLEEWSNNRGPVEVADNMELSK.S | 33 |
|  | Mis12IP\_Nocodazole\_MudPIT\_040709\_06.07402.07402.3 | 3.2588 | 0.3978 | 100.0% | 3536.6943 | 3537.7937 | 1 | 5.794 | 24.2% | 1 | R.LVANDS\*QLTPLEEWSNNRGPVEVADNMELSK.S | 33 |
|  | Mis12IP\_Nocodazole\_MudPIT\_040709\_03.06989.06989.3 | 3.5717 | 0.2741 | 100.0% | 3616.9143 | 3617.7937 | 1 | 6.218 | 30.0% | 2 | R.LVANDS\*QLT#PLEEWSNNRGPVEVADNMELSK.S | 33 |
|  | Mis12IP\_Nocodazole\_MudPIT\_040709\_02.04862.04862.2 | 3.6347 | 0.4387 | 100.0% | 1698.2922 | 1698.7838 | 1 | 7.314 | 67.9% | 1 | K.DVQS\*PGFLNEPLSSK.S | 22 |
|  | Mis12IP\_Nocodazole\_MudPIT\_040709\_02.03549.03549.1 | 2.082 | 0.1287 | 97.2% | 842.54 | 842.96765 | 28 | 3.443 | 75.0% | 1 | K.ILEENPK.F | 11 |
|  | Mis12IP\_Nocodazole\_MudPIT\_040709\_04.04848.04848.1 | 1.7729 | 0.3303 | 98.7% | 800.51 | 800.93335 | 58 | 6.56 | 57.1% | 2 | K.QALAVGNK.I | 11 |
|  | Mis12IP\_Nocodazole\_MudPIT\_040709\_06.05396.05396.2 | 2.0028 | 0.1668 | 95.7% | 967.9122 | 968.1411 | 4 | 4.852 | 64.3% | 1 | K.IVLHTEQK.Q | 22 |
|  | Mis12IP\_Nocodazole\_MudPIT\_040709\_06.05634.05634.1 | 1.5708 | 0.3048 | 99.2% | 1048.53 | 1049.1747 | 76 | 5.399 | 62.5% | 1 | K.QQLFAATNR.T | 11 |
|  | Mis12IP\_Nocodazole\_MudPIT\_040709\_06.05633.05633.2 | 1.8662 | 0.4045 | 99.7% | 1049.0322 | 1049.1747 | 1 | 7.151 | 75.0% | 1 | K.QQLFAATNR.T | 22 |
|  | Mis12IP\_Nocodazole\_MudPIT\_040709\_06.06970.06970.2 | 5.9643 | 0.6152 | 100.0% | 2138.5322 | 2139.4285 | 1 | 12.55 | 75.0% | 5 | R.NLLANQTLVYSQDLGEMTK.L | 22 |
|  | Mis12IP\_Nocodazole\_MudPIT\_040709\_06.06973.06973.3 | 5.8764 | 0.4978 | 100.0% | 2138.7544 | 2139.4285 | 1 | 9.534 | 54.2% | 1 | R.NLLANQTLVYSQDLGEMTK.L | 33 |
|  | Mis12IP\_Nocodazole\_MudPIT\_040709\_05.02979.02979.2 | 2.5169 | 0.3688 | 99.9% | 1339.1122 | 1339.4478 | 3 | 5.822 | 58.3% | 1 | K.SHNGAETTSLPPK.T | 22 |
|  | Mis12IP\_Nocodazole\_MudPIT\_040709\_02.03813.03813.1 | 1.9595 | 0.3975 | 96.9% | 890.47 | 890.9689 | 1 | 6.526 | 72.2% | 1 | K.DSGIGSVAGK.L | 11 |
|  | Mis12IP\_Nocodazole\_MudPIT\_040709\_02.03851.03851.2 | 2.7925 | 0.2494 | 99.7% | 893.1322 | 890.9689 | 1 | 5.019 | 77.8% | 1 | K.DSGIGSVAGK.L | 22 |

Similarities:
gi|18308012|gb|AAL678(35:2)  

---

|  |  |  |  |  |  |  |  |  |
| --- | --- | --- | --- | --- | --- | --- | --- | --- |
| U | *gi|27465517|ref|NP\_77* | 13 | 21 | 18.8% | 564 | 60223 | 8.0 | keratin 6 isoform K6e [Homo sapiens] |
| U | *gi|84040267|gb|AAI106* | 13 | 21 | 18.8% | 564 | 59997 | 7.7 | Keratin 6C [Homo sapiens] |

| Filename XCorr DeltCN Conf% ObsM+H+ CalcM+H+ SpR ZScore Ion% # Sequence  | | | | | | | | | | | | |
| --- | --- | --- | --- | --- | --- | --- | --- | --- | --- | --- | --- | --- |
|  | Mis12IP\_Nocodazole\_MudPIT\_040709\_06.05696.05696.1 | 1.9506 | 0.2944 | 98.9% | 1026.46 | 1027.1222 | 1 | 4.92 | 77.8% | 1 | R.SGFSSISVSR.S | 1111 |
|  | Mis12IP\_Nocodazole\_MudPIT\_040709\_06.05698.05698.2 | 3.316 | 0.4216 | 100.0% | 1027.0322 | 1027.1222 | 1 | 7.618 | 88.9% | 1 | R.SGFSSISVSR.S | 2222 |
|  | Mis12IP\_Nocodazole\_MudPIT\_040709\_06.05770.05770.1 | 1.6858 | 0.3688 | 98.4% | 881.6 | 882.0043 | 53 | 5.36 | 43.8% | 1 | R.SLYGLGGSK.R | 111 |
|  | Mis12IP\_Nocodazole\_MudPIT\_040709\_06.06226.06226.2 | 3.2189 | 0.3799 | 100.0% | 1204.2322 | 1204.3684 | 1 | 6.933 | 83.3% | 1 | K.WTLLQEQGTK.T | 2222222 |
|  | Mis12IP\_Nocodazole\_MudPIT\_040709\_05.04381.04381.2 | 2.3872 | 0.1809 | 99.0% | 945.6722 | 946.0513 | 10 | 5.236 | 78.6% | 2 | R.GRLDSELR.N | 22222222 |
|  | Mis12IP\_Nocodazole\_MudPIT\_040709\_06.07156.07156.2 | 3.4404 | 0.3725 | 100.0% | 1408.0521 | 1408.551 | 1 | 6.1 | 68.2% | 2 | K.ADTLTDEINFLR.A | 222222 |
|  | Mis12IP\_Nocodazole\_MudPIT\_040709\_06.07734.07734.1 | 2.6237 | 0.3674 | 100.0% | 1329.6 | 1330.5211 | 1 | 7.145 | 68.2% | 3 | R.NLDLDSIIAEVK.A | 111111111 |
|  | Mis12IP\_Nocodazole\_MudPIT\_040709\_06.07727.07727.2 | 4.4229 | 0.3694 | 100.0% | 1331.0122 | 1330.5211 | 1 | 7.672 | 81.8% | 2 | R.NLDLDSIIAEVK.A | 222222222 |
|  | Mis12IP\_Nocodazole\_MudPIT\_040709\_03.04273.04273.2 | 3.0135 | 0.0704 | 98.3% | 1108.1721 | 1108.196 | 1 | 6.972 | 81.2% | 1 | K.AQYEEIAQR.S | 2222222 |
|  | Mis12IP\_Nocodazole\_MudPIT\_040709\_03.04571.04571.2 | 3.6642 | 0.4559 | 100.0% | 1165.6721 | 1166.2761 | 1 | 8.196 | 88.9% | 1 | K.YEELQVTAGR.H | 22222 |
|  | Mis12IP\_Nocodazole\_MudPIT\_040709\_05.05044.05044.1 | 2.1894 | 0.2198 | 99.1% | 1153.49 | 1154.3234 | 28 | 5.686 | 68.8% | 2 | K.EYQELMNVK.L | 1111111 |
|  | Mis12IP\_Nocodazole\_MudPIT\_040709\_04.05650.05650.2 | 2.737 | 0.2522 | 99.8% | 1154.1721 | 1154.3234 | 2 | 6.243 | 75.0% | 2 | K.EYQELMNVK.L | 2222222 |
|  | Mis12IP\_Nocodazole\_MudPIT\_040709\_06.05682.05682.2 | 4.4575 | 0.5877 | 100.0% | 1447.5721 | 1448.6163 | 1 | 10.159 | 71.9% | 2 | R.AIGGGLSSVGGGSSTIK.Y | 2222 |

Similarities:
gi|4504919|ref|NP\_002(2:11)  
gi|181402|gb|AAC83410(3:10)  
gi|46812692|gb|AAH692(11:2)  
gi|21961227|gb|AAH345(11:2)  
gi|17505189|ref|NP\_49(12:1)  
gi|15559584|gb|AAH141(10:3)  
gi|5031841|ref|NP\_005(11:2)  
gi|18999435|gb|AAH242(4:9)  
gi|32567786|ref|NP\_78(3:10)  

---

|  |  |  |  |  |  |  |  |  |
| --- | --- | --- | --- | --- | --- | --- | --- | --- |
| U | *gi|46812692|gb|AAH692* | 12 | 20 | 18.8% | 564 | 60075 | 7.7 | Keratin 6A [Homo sapiens] |

| Filename XCorr DeltCN Conf% ObsM+H+ CalcM+H+ SpR ZScore Ion% # Sequence  | | | | | | | | | | | | |
| --- | --- | --- | --- | --- | --- | --- | --- | --- | --- | --- | --- | --- |
|  | Mis12IP\_Nocodazole\_MudPIT\_040709\_05.04755.04755.2 | 3.0035 | 0.4267 | 100.0% | 1012.3522 | 1013.0953 | 1 | 7.522 | 83.3% | 1 | R.SGFSSVSVSR.S | 22 |
|  | Mis12IP\_Nocodazole\_MudPIT\_040709\_06.05770.05770.1 | 1.6858 | 0.3688 | 98.4% | 881.6 | 882.0043 | 53 | 5.36 | 43.8% | 1 | R.SLYGLGGSK.R | 111 |
|  | Mis12IP\_Nocodazole\_MudPIT\_040709\_06.06226.06226.2 | 3.2189 | 0.3799 | 100.0% | 1204.2322 | 1204.3684 | 1 | 6.933 | 83.3% | 1 | K.WTLLQEQGTK.T | 2222222 |
|  | Mis12IP\_Nocodazole\_MudPIT\_040709\_05.04381.04381.2 | 2.3872 | 0.1809 | 99.0% | 945.6722 | 946.0513 | 10 | 5.236 | 78.6% | 2 | R.GRLDSELR.G | 22222222 |
|  | Mis12IP\_Nocodazole\_MudPIT\_040709\_06.07156.07156.2 | 3.4404 | 0.3725 | 100.0% | 1408.0521 | 1408.551 | 1 | 6.1 | 68.2% | 2 | K.ADTLTDEINFLR.A | 222222 |
|  | Mis12IP\_Nocodazole\_MudPIT\_040709\_06.07734.07734.1 | 2.6237 | 0.3674 | 100.0% | 1329.6 | 1330.5211 | 1 | 7.145 | 68.2% | 3 | R.NLDLDSIIAEVK.A | 111111111 |
|  | Mis12IP\_Nocodazole\_MudPIT\_040709\_06.07727.07727.2 | 4.4229 | 0.3694 | 100.0% | 1331.0122 | 1330.5211 | 1 | 7.672 | 81.8% | 2 | R.NLDLDSIIAEVK.A | 222222222 |
|  | Mis12IP\_Nocodazole\_MudPIT\_040709\_03.04273.04273.2 | 3.0135 | 0.0704 | 98.3% | 1108.1721 | 1108.196 | 1 | 6.972 | 81.2% | 1 | K.AQYEEIAQR.S | 2222222 |
|  | Mis12IP\_Nocodazole\_MudPIT\_040709\_03.04571.04571.2 | 3.6642 | 0.4559 | 100.0% | 1165.6721 | 1166.2761 | 1 | 8.196 | 88.9% | 1 | K.YEELQVTAGR.H | 22222 |
|  | Mis12IP\_Nocodazole\_MudPIT\_040709\_05.05044.05044.1 | 2.1894 | 0.2198 | 99.1% | 1153.49 | 1154.3234 | 28 | 5.686 | 68.8% | 2 | K.EYQELMNVK.L | 1111111 |
|  | Mis12IP\_Nocodazole\_MudPIT\_040709\_04.05650.05650.2 | 2.737 | 0.2522 | 99.8% | 1154.1721 | 1154.3234 | 2 | 6.243 | 75.0% | 2 | K.EYQELMNVK.L | 2222222 |
|  | Mis12IP\_Nocodazole\_MudPIT\_040709\_06.05682.05682.2 | 4.4575 | 0.5877 | 100.0% | 1447.5721 | 1448.6163 | 1 | 10.159 | 71.9% | 2 | R.AIGGGLSSVGGGSSTIK.Y | 2222 |

Similarities:
gi|4504919|ref|NP\_002(2:10)  
gi|181402|gb|AAC83410(3:9)  
gi|27465517|ref|NP\_77(11:1)  
gi|21961227|gb|AAH345(9:3)  
gi|17505189|ref|NP\_49(10:2)  
gi|15559584|gb|AAH141(11:1)  
gi|5031841|ref|NP\_005(9:3)  
gi|18999435|gb|AAH242(4:8)  
gi|32567786|ref|NP\_78(3:9)  

---

|  |  |  |  |  |  |  |  |  |
| --- | --- | --- | --- | --- | --- | --- | --- | --- |
| U | *gi|21961227|gb|AAH345* | 14 | 27 | 18.8% | 564 | 60067 | 8.0 | Keratin 6B [Homo sapiens] |

| Filename XCorr DeltCN Conf% ObsM+H+ CalcM+H+ SpR ZScore Ion% # Sequence  | | | | | | | | | | | | |
| --- | --- | --- | --- | --- | --- | --- | --- | --- | --- | --- | --- | --- |
|  | Mis12IP\_Nocodazole\_MudPIT\_040709\_06.05696.05696.1 | 1.9506 | 0.2944 | 98.9% | 1026.46 | 1027.1222 | 1 | 4.92 | 77.8% | 1 | R.SGFSSISVSR.S | 1111 |
|  | Mis12IP\_Nocodazole\_MudPIT\_040709\_06.05698.05698.2 | 3.316 | 0.4216 | 100.0% | 1027.0322 | 1027.1222 | 1 | 7.618 | 88.9% | 1 | R.SGFSSISVSR.S | 2222 |
|  | Mis12IP\_Nocodazole\_MudPIT\_040709\_06.05770.05770.1 | 1.6858 | 0.3688 | 98.4% | 881.6 | 882.0043 | 53 | 5.36 | 43.8% | 1 | R.SLYGLGGSK.R | 111 |
|  | Mis12IP\_Nocodazole\_MudPIT\_040709\_06.06226.06226.2 | 3.2189 | 0.3799 | 100.0% | 1204.2322 | 1204.3684 | 1 | 6.933 | 83.3% | 1 | K.WTLLQEQGTK.T | 2222222 |
|  | Mis12IP\_Nocodazole\_MudPIT\_040709\_05.04381.04381.2 | 2.3872 | 0.1809 | 99.0% | 945.6722 | 946.0513 | 10 | 5.236 | 78.6% | 2 | R.GRLDSELR.N | 22222222 |
|  | Mis12IP\_Nocodazole\_MudPIT\_040709\_06.07156.07156.2 | 3.4404 | 0.3725 | 100.0% | 1408.0521 | 1408.551 | 1 | 6.1 | 68.2% | 2 | K.ADTLTDEINFLR.A | 222222 |
|  | Mis12IP\_Nocodazole\_MudPIT\_040709\_06.07734.07734.1 | 2.6237 | 0.3674 | 100.0% | 1329.6 | 1330.5211 | 1 | 7.145 | 68.2% | 3 | R.NLDLDSIIAEVK.A | 111111111 |
|  | Mis12IP\_Nocodazole\_MudPIT\_040709\_06.07727.07727.2 | 4.4229 | 0.3694 | 100.0% | 1331.0122 | 1330.5211 | 1 | 7.672 | 81.8% | 2 | R.NLDLDSIIAEVK.A | 222222222 |
|  | Mis12IP\_Nocodazole\_MudPIT\_040709\_03.04273.04273.2 | 3.0135 | 0.0704 | 98.3% | 1108.1721 | 1108.196 | 1 | 6.972 | 81.2% | 1 | K.AQYEEIAQR.S | 2222222 |
|  | Mis12IP\_Nocodazole\_MudPIT\_040709\_04.05612.05612.1 | 2.7425 | 0.1273 | 98.6% | 1179.53 | 1180.303 | 2 | 4.69 | 66.7% | 3 | K.YEELQITAGR.H | 11 |
|  | Mis12IP\_Nocodazole\_MudPIT\_040709\_05.04993.04993.2 | 3.9395 | 0.3218 | 100.0% | 1181.1122 | 1180.303 | 1 | 7.266 | 88.9% | 5 | K.YEELQITAGR.H | 22 |
|  | Mis12IP\_Nocodazole\_MudPIT\_040709\_05.05044.05044.1 | 2.1894 | 0.2198 | 99.1% | 1153.49 | 1154.3234 | 28 | 5.686 | 68.8% | 2 | K.EYQELMNVK.L | 1111111 |
|  | Mis12IP\_Nocodazole\_MudPIT\_040709\_04.05650.05650.2 | 2.737 | 0.2522 | 99.8% | 1154.1721 | 1154.3234 | 2 | 6.243 | 75.0% | 2 | K.EYQELMNVK.L | 2222222 |
|  | Mis12IP\_Nocodazole\_MudPIT\_040709\_05.04702.04702.2 | 4.7933 | 0.5673 | 100.0% | 1436.4722 | 1436.562 | 1 | 9.688 | 78.1% | 1 | R.ATGGGLSSVGGGSSTIK.Y | 22 |

Similarities:
gi|4504919|ref|NP\_002(2:12)  
gi|181402|gb|AAC83410(3:11)  
gi|17318569|ref|NP\_00(2:12)  
gi|27465517|ref|NP\_77(11:3)  
gi|46812692|gb|AAH692(9:5)  
gi|17505189|ref|NP\_49(10:4)  
gi|15559584|gb|AAH141(8:6)  
gi|5031841|ref|NP\_005(11:3)  
gi|18999435|gb|AAH242(4:10)  
gi|32567786|ref|NP\_78(3:11)  

---

|  |  |  |  |  |  |  |  |  |
| --- | --- | --- | --- | --- | --- | --- | --- | --- |
| U | *gi|12803709|gb|AAH026* | 12 | 16 | 18.6% | 472 | 51652 | 5.2 | Keratin 14 (epidermolysis bullosa simplex, Dowling-Meara, Koebner) [Homo sapiens] |
| U | *gi|15431310|ref|NP\_00* | 12 | 16 | 18.6% | 472 | 51622 | 5.2 | keratin 14 [Homo sapiens] |

| Filename XCorr DeltCN Conf% ObsM+H+ CalcM+H+ SpR ZScore Ion% # Sequence  | | | | | | | | | | | | |
| --- | --- | --- | --- | --- | --- | --- | --- | --- | --- | --- | --- | --- |
|  | Mis12IP\_Nocodazole\_MudPIT\_040709\_06.05537.05537.1 | 1.8425 | 0.2626 | 99.2% | 1425.58 | 1426.526 | 20 | 6.076 | 32.1% | 1 | R.APSTYGGGLSVSSSR.F | 1 |
|  | Mis12IP\_Nocodazole\_MudPIT\_040709\_06.05531.05531.2 | 3.8902 | 0.6175 | 100.0% | 1426.1322 | 1426.526 | 1 | 11.323 | 67.9% | 1 | R.APSTYGGGLSVSSSR.F | 2 |
|  | Mis12IP\_Nocodazole\_MudPIT\_040709\_04.05302.05302.2 | 2.5626 | 0.2281 | 99.5% | 1090.2922 | 1091.2273 | 1 | 5.497 | 81.2% | 2 | K.VTMQNLNDR.L | 2222 |
|  | Mis12IP\_Nocodazole\_MudPIT\_040709\_03.04508.04508.1 | 1.9048 | 0.2501 | 99.0% | 809.5 | 809.93774 | 160 | 5.178 | 66.7% | 1 | R.LASYLDK.V | 1111111 |
|  | Mis12IP\_Nocodazole\_MudPIT\_040709\_06.07056.07056.2 | 3.6586 | 0.4471 | 100.0% | 2053.612 | 2055.339 | 1 | 9.196 | 61.1% | 1 | K.ILTATVDNANVLLQIDNAR.L | 2 |
|  | Mis12IP\_Nocodazole\_MudPIT\_040709\_06.07064.07064.3 | 3.9727 | 0.1822 | 97.3% | 2056.4043 | 2055.339 | 1 | 4.638 | 45.8% | 1 | K.ILTATVDNANVLLQIDNAR.L | 3 |
|  | Mis12IP\_Nocodazole\_MudPIT\_040709\_02.04220.04220.1 | 1.5217 | 0.3222 | 99.0% | 807.44 | 807.8815 | 2 | 4.741 | 58.3% | 1 | R.LAADDFR.T | 11111111 |
|  | Mis12IP\_Nocodazole\_MudPIT\_040709\_03.04543.04543.2 | 2.2948 | 0.233 | 99.3% | 808.0522 | 807.8815 | 1 | 5.671 | 91.7% | 3 | R.LAADDFR.T | 22222222 |
|  | Mis12IP\_Nocodazole\_MudPIT\_040709\_06.06210.06210.2 | 3.7407 | 0.4163 | 100.0% | 1030.0122 | 1030.2096 | 1 | 7.093 | 93.8% | 2 | R.VLDELTLAR.A | 22222 |
|  | Mis12IP\_Nocodazole\_MudPIT\_040709\_06.06209.06209.1 | 2.5279 | 0.3193 | 100.0% | 1030.56 | 1030.2096 | 6 | 5.521 | 62.5% | 1 | R.VLDELTLAR.A | 11111 |
|  | Mis12IP\_Nocodazole\_MudPIT\_040709\_05.06545.06545.1 | 1.5313 | 0.5226 | 100.0% | 1172.46 | 1173.2671 | 1 | 6.59 | 43.8% | 1 | K.DAEEWFFTK.T | 1 |
|  | Mis12IP\_Nocodazole\_MudPIT\_040709\_02.03972.03972.2 | 3.5993 | 0.5203 | 100.0% | 1361.5521 | 1362.4796 | 1 | 9.18 | 79.2% | 1 | R.EVATNSELVQSGK.S | 22 |

Similarities:
gi|4557888|ref|NP\_000(2:10)  
gi|28317|emb|CAA32649(2:10)  
gi|24430192|ref|NP\_00(6:6)  
gi|4557701|ref|NP\_000(6:6)  
gi|435476|emb|CAA8231(1:11)  
gi|14043271|gb|AAH076(5:7)  
gi|24430190|ref|NP\_00(5:7)  
gi|34526448|dbj|BAC85(3:9)  
gi|85566621|gb|AAI119(2:10)  

---

|  |  |  |  |  |  |  |  |  |
| --- | --- | --- | --- | --- | --- | --- | --- | --- |
| U | *gi|12803275|gb|AAH024* | 10 | 28 | 18.4% | 641 | 70052 | 5.6 | Heat shock 70kDa protein 1A [Homo sapiens] |
| U | *gi|62089222|dbj|BAD93* | 10 | 28 | 16.6% | 709 | 77496 | 6.3 | heat shock 70kDa protein 1A variant [Homo sapiens] |
| U | *gi|5123454|ref|NP\_005* | 10 | 28 | 18.4% | 641 | 70038 | 5.6 | heat shock 70kDa protein 1A [Homo sapiens] |
| U | *gi|4885431|ref|NP\_005* | 10 | 28 | 18.4% | 641 | 70025 | 5.6 | heat shock 70kDa protein 1B [Homo sapiens] |

| Filename XCorr DeltCN Conf% ObsM+H+ CalcM+H+ SpR ZScore Ion% # Sequence  | | | | | | | | | | | | |
| --- | --- | --- | --- | --- | --- | --- | --- | --- | --- | --- | --- | --- |
|  | Mis12IP\_Nocodazole\_MudPIT\_040709\_05.05229.05229.1 | 1.9614 | 0.3836 | 97.4% | 1487.44 | 1488.5939 | 1 | 7.022 | 45.8% | 1 | R.TTPSYVAFTDTER.L | 11111 |
|  | Mis12IP\_Nocodazole\_MudPIT\_040709\_06.05909.05909.2 | 3.3634 | 0.4566 | 100.0% | 1488.0721 | 1488.5939 | 1 | 8.603 | 75.0% | 3 | R.TTPSYVAFTDTER.L | 22222 |
|  | Mis12IP\_Nocodazole\_MudPIT\_040709\_03.04674.04674.2 | 2.4675 | 0.1627 | 97.4% | 1223.4521 | 1223.3862 | 1 | 5.331 | 70.0% | 1 | K.FGDPVVQSDMK.H | 2 |
|  | Mis12IP\_Nocodazole\_MudPIT\_040709\_06.07336.07336.2 | 3.8514 | 0.5353 | 100.0% | 1615.1921 | 1615.8817 | 1 | 8.222 | 80.8% | 2 | K.AFYPEEISSMVLTK.M | 22 |
|  | Mis12IP\_Nocodazole\_MudPIT\_040709\_05.08031.08031.3 | 2.2808 | 0.278 | 96.5% | 3003.9243 | 3003.338 | 13 | 4.987 | 24.0% | 1 | K.EIAEAYLGYPVTNAVITVPAYFNDSQR.Q | 3 |
|  | Mis12IP\_Nocodazole\_MudPIT\_040709\_06.06778.06778.1 | 2.2033 | 0.4122 | 100.0% | 1197.72 | 1198.408 | 11 | 6.354 | 50.0% | 1 | K.DAGVIAGLNVLR.I | 11 |
|  | Mis12IP\_Nocodazole\_MudPIT\_040709\_05.06407.06407.2 | 4.221 | 0.315 | 100.0% | 1199.0721 | 1198.408 | 1 | 6.871 | 90.9% | 2 | K.DAGVIAGLNVLR.I | 22 |
|  | Mis12IP\_Nocodazole\_MudPIT\_040709\_06.06619.06619.2 | 5.3251 | 0.5404 | 100.0% | 1688.7322 | 1688.9213 | 1 | 10.629 | 80.0% | 1 | R.IINEPTAAAIAYGLDR.T | 22 |
|  | Mis12IP\_Nocodazole\_MudPIT\_040709\_04.05631.05631.2 | 2.8556 | 0.3643 | 99.9% | 1676.4521 | 1676.6964 | 4 | 6.052 | 40.0% | 2 | K.ATAGDTHLGGEDFDNR.L | 222 |
|  | Mis12IP\_Nocodazole\_MudPIT\_040709\_04.05654.05654.2 | 2.3494 | 0.4197 | 100.0% | 1018.2922 | 1018.1582 | 2 | 6.376 | 68.8% | 14 | K.ITITNDKGR.L | 22222 |

Similarities:
gi|5729877|ref|NP\_006(3:7)  
gi|13676857|ref|NP\_06(3:7)  
gi|27436929|ref|NP\_00(7:3)  
gi|34419635|ref|NP\_00(5:5)  

---

|  |  |  |  |  |  |  |  |  |
| --- | --- | --- | --- | --- | --- | --- | --- | --- |
| U | *gi|17505189|ref|NP\_49* | 13 | 21 | 18.4% | 564 | 60200 | 8.0 | keratin 6C [Homo sapiens] |
| U | *gi|32964837|tpg|DAA01* | 13 | 21 | 18.4% | 564 | 60025 | 8.0 | TPA\_exp: type II keratin K6h [Homo sapiens] |

| Filename XCorr DeltCN Conf% ObsM+H+ CalcM+H+ SpR ZScore Ion% # Sequence  | | | | | | | | | | | | |
| --- | --- | --- | --- | --- | --- | --- | --- | --- | --- | --- | --- | --- |
|  | Mis12IP\_Nocodazole\_MudPIT\_040709\_06.05696.05696.1 | 1.9506 | 0.2944 | 98.9% | 1026.46 | 1027.1222 | 1 | 4.92 | 77.8% | 1 | R.SGFSSISVSR.S | 1111 |
|  | Mis12IP\_Nocodazole\_MudPIT\_040709\_06.05698.05698.2 | 3.316 | 0.4216 | 100.0% | 1027.0322 | 1027.1222 | 1 | 7.618 | 88.9% | 1 | R.SGFSSISVSR.S | 2222 |
|  | Mis12IP\_Nocodazole\_MudPIT\_040709\_05.05262.05262.1 | 2.1158 | 0.1126 | 95.8% | 827.46 | 827.95544 | 11 | 4.725 | 66.7% | 1 | K.FASFIDK.V | 1111111 |
|  | Mis12IP\_Nocodazole\_MudPIT\_040709\_06.06226.06226.2 | 3.2189 | 0.3799 | 100.0% | 1204.2322 | 1204.3684 | 1 | 6.933 | 83.3% | 1 | K.WTLLQEQGTK.T | 2222222 |
|  | Mis12IP\_Nocodazole\_MudPIT\_040709\_05.04381.04381.2 | 2.3872 | 0.1809 | 99.0% | 945.6722 | 946.0513 | 10 | 5.236 | 78.6% | 2 | R.GRLDSELR.G | 22222222 |
|  | Mis12IP\_Nocodazole\_MudPIT\_040709\_06.07156.07156.2 | 3.4404 | 0.3725 | 100.0% | 1408.0521 | 1408.551 | 1 | 6.1 | 68.2% | 2 | K.ADTLTDEINFLR.A | 222222 |
|  | Mis12IP\_Nocodazole\_MudPIT\_040709\_06.07734.07734.1 | 2.6237 | 0.3674 | 100.0% | 1329.6 | 1330.5211 | 1 | 7.145 | 68.2% | 3 | R.NLDLDSIIAEVK.A | 111111111 |
|  | Mis12IP\_Nocodazole\_MudPIT\_040709\_06.07727.07727.2 | 4.4229 | 0.3694 | 100.0% | 1331.0122 | 1330.5211 | 1 | 7.672 | 81.8% | 2 | R.NLDLDSIIAEVK.A | 222222222 |
|  | Mis12IP\_Nocodazole\_MudPIT\_040709\_03.04273.04273.2 | 3.0135 | 0.0704 | 98.3% | 1108.1721 | 1108.196 | 1 | 6.972 | 81.2% | 1 | K.AQYEEIAQR.S | 2222222 |
|  | Mis12IP\_Nocodazole\_MudPIT\_040709\_03.04571.04571.2 | 3.6642 | 0.4559 | 100.0% | 1165.6721 | 1166.2761 | 1 | 8.196 | 88.9% | 1 | K.YEELQVTAGR.H | 22222 |
|  | Mis12IP\_Nocodazole\_MudPIT\_040709\_05.05044.05044.1 | 2.1894 | 0.2198 | 99.1% | 1153.49 | 1154.3234 | 28 | 5.686 | 68.8% | 2 | K.EYQELMNVK.L | 1111111 |
|  | Mis12IP\_Nocodazole\_MudPIT\_040709\_04.05650.05650.2 | 2.737 | 0.2522 | 99.8% | 1154.1721 | 1154.3234 | 2 | 6.243 | 75.0% | 2 | K.EYQELMNVK.L | 2222222 |
|  | Mis12IP\_Nocodazole\_MudPIT\_040709\_06.05682.05682.2 | 4.4575 | 0.5877 | 100.0% | 1447.5721 | 1448.6163 | 1 | 10.159 | 71.9% | 2 | R.AIGGGLSSVGGGSSTIK.Y | 2222 |

Similarities:
gi|4504919|ref|NP\_002(3:10)  
gi|181402|gb|AAC83410(3:10)  
gi|27465517|ref|NP\_77(12:1)  
gi|46812692|gb|AAH692(10:3)  
gi|21961227|gb|AAH345(10:3)  
gi|15559584|gb|AAH141(11:2)  
gi|5031841|ref|NP\_005(12:1)  
gi|18999435|gb|AAH242(4:9)  
gi|1200072|emb|CAA316(1:12)  
gi|32567786|ref|NP\_78(4:9)  
gi|45597458|ref|NP\_77(1:12)  

---

|  |  |  |  |  |  |  |  |  |
| --- | --- | --- | --- | --- | --- | --- | --- | --- |
| U | *gi|15559584|gb|AAH141* | 12 | 19 | 18.4% | 564 | 60018 | 8.0 | Keratin 6A [Homo sapiens] |
| U | *gi|5031839|ref|NP\_005* | 12 | 20 | 18.4% | 564 | 60045 | 8.0 | keratin 6A [Homo sapiens] |

| Filename XCorr DeltCN Conf% ObsM+H+ CalcM+H+ SpR ZScore Ion% # Sequence  | | | | | | | | | | | | |
| --- | --- | --- | --- | --- | --- | --- | --- | --- | --- | --- | --- | --- |
|  | Mis12IP\_Nocodazole\_MudPIT\_040709\_05.04755.04755.2 | 3.0035 | 0.4267 | 100.0% | 1012.3522 | 1013.0953 | 1 | 7.522 | 83.3% | 1 | R.SGFSSVSVSR.S | 22 |
|  | Mis12IP\_Nocodazole\_MudPIT\_040709\_05.05262.05262.1 | 2.1158 | 0.1126 | 95.8% | 827.46 | 827.95544 | 11 | 4.725 | 66.7% | 1 | K.FASFIDK.V | 1111111 |
|  | Mis12IP\_Nocodazole\_MudPIT\_040709\_06.06226.06226.2 | 3.2189 | 0.3799 | 100.0% | 1204.2322 | 1204.3684 | 1 | 6.933 | 83.3% | 1 | K.WTLLQEQGTK.T | 2222222 |
|  | Mis12IP\_Nocodazole\_MudPIT\_040709\_05.04381.04381.2 | 2.3872 | 0.1809 | 99.0% | 945.6722 | 946.0513 | 10 | 5.236 | 78.6% | 2 | R.GRLDSELR.G | 22222222 |
|  | Mis12IP\_Nocodazole\_MudPIT\_040709\_06.07156.07156.2 | 3.4404 | 0.3725 | 100.0% | 1408.0521 | 1408.551 | 1 | 6.1 | 68.2% | 2 | K.ADTLTDEINFLR.A | 222222 |
|  | Mis12IP\_Nocodazole\_MudPIT\_040709\_06.07734.07734.1 | 2.6237 | 0.3674 | 100.0% | 1329.6 | 1330.5211 | 1 | 7.145 | 68.2% | 3 | R.NLDLDSIIAEVK.A | 111111111 |
|  | Mis12IP\_Nocodazole\_MudPIT\_040709\_06.07727.07727.2 | 4.4229 | 0.3694 | 100.0% | 1331.0122 | 1330.5211 | 1 | 7.672 | 81.8% | 2 | R.NLDLDSIIAEVK.A | 222222222 |
|  | Mis12IP\_Nocodazole\_MudPIT\_040709\_03.04273.04273.2 | 3.0135 | 0.0704 | 98.3% | 1108.1721 | 1108.196 | 1 | 6.972 | 81.2% | 1 | K.AQYEEIAQR.S | 2222222 |
|  | Mis12IP\_Nocodazole\_MudPIT\_040709\_03.04571.04571.2 | 3.6642 | 0.4559 | 100.0% | 1165.6721 | 1166.2761 | 1 | 8.196 | 88.9% | 1 | K.YEELQVTAGR.H | 22222 |
|  | Mis12IP\_Nocodazole\_MudPIT\_040709\_05.05044.05044.1 | 2.1894 | 0.2198 | 99.1% | 1153.49 | 1154.3234 | 28 | 5.686 | 68.8% | 1 | K.EYQELMNVK.L | 1111111 |
|  | Mis12IP\_Nocodazole\_MudPIT\_040709\_04.05650.05650.2 | 2.737 | 0.2522 | 99.8% | 1154.1721 | 1154.3234 | 2 | 6.243 | 75.0% | 2 | K.EYQELMNVK.L | 2222222 |
|  | Mis12IP\_Nocodazole\_MudPIT\_040709\_06.05682.05682.2 | 4.4575 | 0.5877 | 100.0% | 1447.5721 | 1448.6163 | 1 | 10.159 | 71.9% | 2 | R.AIGGGLSSVGGGSSTIK.Y | 2222 |

Similarities:
gi|4504919|ref|NP\_002(3:9)  
gi|181402|gb|AAC83410(3:9)  
gi|27465517|ref|NP\_77(10:2)  
gi|46812692|gb|AAH692(11:1)  
gi|21961227|gb|AAH345(8:4)  
gi|17505189|ref|NP\_49(11:1)  
gi|5031841|ref|NP\_005(10:2)  
gi|18999435|gb|AAH242(4:8)  
gi|1200072|emb|CAA316(1:11)  
gi|32567786|ref|NP\_78(4:8)  
gi|45597458|ref|NP\_77(1:11)  

---

|  |  |  |  |  |  |  |  |  |
| --- | --- | --- | --- | --- | --- | --- | --- | --- |
| U | *gi|5031841|ref|NP\_005* | 13 | 20 | 18.4% | 564 | 59999 | 8.0 | keratin 6B [Homo sapiens] |

| Filename XCorr DeltCN Conf% ObsM+H+ CalcM+H+ SpR ZScore Ion% # Sequence  | | | | | | | | | | | | |
| --- | --- | --- | --- | --- | --- | --- | --- | --- | --- | --- | --- | --- |
|  | Mis12IP\_Nocodazole\_MudPIT\_040709\_06.05696.05696.1 | 1.9506 | 0.2944 | 98.9% | 1026.46 | 1027.1222 | 1 | 4.92 | 77.8% | 1 | R.SGFSSISVSR.S | 1111 |
|  | Mis12IP\_Nocodazole\_MudPIT\_040709\_06.05698.05698.2 | 3.316 | 0.4216 | 100.0% | 1027.0322 | 1027.1222 | 1 | 7.618 | 88.9% | 1 | R.SGFSSISVSR.S | 2222 |
|  | Mis12IP\_Nocodazole\_MudPIT\_040709\_05.05262.05262.1 | 2.1158 | 0.1126 | 95.8% | 827.46 | 827.95544 | 11 | 4.725 | 66.7% | 1 | K.FASFIDK.V | 1111111 |
|  | Mis12IP\_Nocodazole\_MudPIT\_040709\_06.06226.06226.2 | 3.2189 | 0.3799 | 100.0% | 1204.2322 | 1204.3684 | 1 | 6.933 | 83.3% | 1 | K.WTLLQEQGTK.T | 2222222 |
|  | Mis12IP\_Nocodazole\_MudPIT\_040709\_05.04381.04381.2 | 2.3872 | 0.1809 | 99.0% | 945.6722 | 946.0513 | 10 | 5.236 | 78.6% | 2 | R.GRLDSELR.N | 22222222 |
|  | Mis12IP\_Nocodazole\_MudPIT\_040709\_06.07156.07156.2 | 3.4404 | 0.3725 | 100.0% | 1408.0521 | 1408.551 | 1 | 6.1 | 68.2% | 2 | K.ADTLTDEINFLR.A | 222222 |
|  | Mis12IP\_Nocodazole\_MudPIT\_040709\_06.07734.07734.1 | 2.6237 | 0.3674 | 100.0% | 1329.6 | 1330.5211 | 1 | 7.145 | 68.2% | 3 | R.NLDLDSIIAEVK.A | 111111111 |
|  | Mis12IP\_Nocodazole\_MudPIT\_040709\_06.07727.07727.2 | 4.4229 | 0.3694 | 100.0% | 1331.0122 | 1330.5211 | 1 | 7.672 | 81.8% | 2 | R.NLDLDSIIAEVK.A | 222222222 |
|  | Mis12IP\_Nocodazole\_MudPIT\_040709\_03.04273.04273.2 | 3.0135 | 0.0704 | 98.3% | 1108.1721 | 1108.196 | 1 | 6.972 | 81.2% | 1 | K.AQYEEIAQR.S | 2222222 |
|  | Mis12IP\_Nocodazole\_MudPIT\_040709\_03.04571.04571.2 | 3.6642 | 0.4559 | 100.0% | 1165.6721 | 1166.2761 | 1 | 8.196 | 88.9% | 1 | K.YEELQVTAGR.H | 22222 |
|  | Mis12IP\_Nocodazole\_MudPIT\_040709\_05.05044.05044.1 | 2.1894 | 0.2198 | 99.1% | 1153.49 | 1154.3234 | 28 | 5.686 | 68.8% | 2 | K.EYQELMNVK.L | 1111111 |
|  | Mis12IP\_Nocodazole\_MudPIT\_040709\_04.05650.05650.2 | 2.737 | 0.2522 | 99.8% | 1154.1721 | 1154.3234 | 2 | 6.243 | 75.0% | 2 | K.EYQELMNVK.L | 2222222 |
|  | Mis12IP\_Nocodazole\_MudPIT\_040709\_05.04702.04702.2 | 4.7933 | 0.5673 | 100.0% | 1436.4722 | 1436.562 | 1 | 9.688 | 78.1% | 1 | R.ATGGGLSSVGGGSSTIK.Y | 22 |

Similarities:
gi|4504919|ref|NP\_002(3:10)  
gi|181402|gb|AAC83410(3:10)  
gi|27465517|ref|NP\_77(11:2)  
gi|46812692|gb|AAH692(9:4)  
gi|21961227|gb|AAH345(11:2)  
gi|17505189|ref|NP\_49(12:1)  
gi|15559584|gb|AAH141(10:3)  
gi|18999435|gb|AAH242(4:9)  
gi|1200072|emb|CAA316(1:12)  
gi|32567786|ref|NP\_78(4:9)  
gi|45597458|ref|NP\_77(1:12)  

---

|  |  |  |  |  |  |  |  |  |
| --- | --- | --- | --- | --- | --- | --- | --- | --- |
| U | *gi|14042579|dbj|BAB55* | 10 | 15 | 18.1% | 707 | 78475 | 4.9 | unnamed protein product [Homo sapiens] |
| U | *gi|7018505|emb|CAB756* | 10 | 15 | 27.3% | 469 | 51130 | 4.9 | hypothetical protein [Homo sapiens] |
| U | *gi|38044290|ref|NP\_06* | 10 | 15 | 18.1% | 707 | 78577 | 4.9 | zinc finger, CCHC domain containing 8 [Homo sapiens] |
| U | *gi|34190917|gb|AAH177* | 10 | 15 | 26.0% | 492 | 53779 | 5.0 | ZCCHC8 protein [Homo sapiens] |

| Filename XCorr DeltCN Conf% ObsM+H+ CalcM+H+ SpR ZScore Ion% # Sequence  | | | | | | | | | | | | |
| --- | --- | --- | --- | --- | --- | --- | --- | --- | --- | --- | --- | --- |
|  | Mis12IP\_Nocodazole\_MudPIT\_040709\_04.06117.06117.2 | 2.2402 | 0.2548 | 98.9% | 1609.5122 | 1609.7301 | 32 | 5.144 | 39.3% | 1 | K.EAELENSGLALYDGK.D | 2 |
|  | Mis12IP\_Nocodazole\_MudPIT\_040709\_05.05479.05479.3 | 3.3065 | 0.3507 | 100.0% | 3391.8843 | 3391.451 | 1 | 5.504 | 20.8% | 1 | K.EAELENS\*GLALYDGKDGTDGETEVGEIQQNK.S | 3 |
|  | Mis12IP\_Nocodazole\_MudPIT\_040709\_03.05718.05718.2 | 2.3589 | 0.4591 | 100.0% | 1558.1322 | 1558.6909 | 1 | 6.361 | 54.2% | 1 | K.LVNYPGFNIST#PR.G | 2 |
|  | Mis12IP\_Nocodazole\_MudPIT\_040709\_06.07282.07282.2 | 3.6384 | 0.5305 | 100.0% | 1870.8922 | 1872.0874 | 1 | 8.454 | 56.2% | 2 | K.DVFANYLTSNFQAPGVK.S | 2 |
|  | Mis12IP\_Nocodazole\_MudPIT\_040709\_05.06002.06002.3 | 4.9135 | 0.3773 | 100.0% | 2304.4744 | 2305.4143 | 1 | 6.71 | 38.8% | 2 | R.TASGAVDEDALTLEELEEQQR.R | 3 |
|  | Mis12IP\_Nocodazole\_MudPIT\_040709\_05.05992.05992.2 | 5.5239 | 0.5178 | 100.0% | 2304.5923 | 2305.4143 | 1 | 8.789 | 62.5% | 2 | R.TASGAVDEDALTLEELEEQQR.R | 2 |
|  | Mis12IP\_Nocodazole\_MudPIT\_040709\_06.06698.06698.2 | 3.3196 | 0.4233 | 100.0% | 1646.2522 | 1646.8346 | 1 | 7.066 | 57.7% | 2 | K.QTLDEPEVPEIFTK.K | 2 |
|  | Mis12IP\_Nocodazole\_MudPIT\_040709\_06.05506.05506.2 | 2.8661 | 0.3419 | 100.0% | 1205.0521 | 1205.3281 | 5 | 5.696 | 66.7% | 1 | K.IHS\*PIPDMSK.F | 2 |
|  | Mis12IP\_Nocodazole\_MudPIT\_040709\_06.08512.08512.2 | 4.471 | 0.5367 | 100.0% | 2512.4521 | 2513.8423 | 1 | 9.574 | 54.8% | 2 | K.FATGITPFEFENMAESTGMYLR.I | 2 |
|  | Mis12IP\_Nocodazole\_MudPIT\_040709\_06.08516.08516.3 | 3.7858 | 0.5355 | 100.0% | 2512.7043 | 2513.8423 | 1 | 8.858 | 35.7% | 1 | K.FATGITPFEFENMAESTGMYLR.I | 3 |

---

|  |  |  |  |  |  |  |  |  |
| --- | --- | --- | --- | --- | --- | --- | --- | --- |
| U | *gi|14250587|gb|AAH087* | 7 | 23 | 18.1% | 227 | 24650 | 7.2 | Protein-L-isoaspartate (D-aspartate) O-methyltransferase [Homo sapiens] |

| Filename XCorr DeltCN Conf% ObsM+H+ CalcM+H+ SpR ZScore Ion% # Sequence  | | | | | | | | | | | | |
| --- | --- | --- | --- | --- | --- | --- | --- | --- | --- | --- | --- | --- |
|  | Mis12IP\_Nocodazole\_MudPIT\_040709\_06.06574.06574.2 | 4.0845 | 0.4691 | 100.0% | 1180.9321 | 1181.3923 | 3 | 8.673 | 77.8% | 2 | K.VFEVMLATDR.S | 2 |
|  | Mis12IP\_Nocodazole\_MudPIT\_040709\_06.06583.06583.1 | 2.3995 | 0.1852 | 99.2% | 1182.54 | 1181.3923 | 1 | 4.109 | 66.7% | 1 | K.VFEVMLATDR.S | 1 |
| \* | Mis12IP\_Nocodazole\_MudPIT\_040709\_02.04306.04306.2 | 3.3207 | 0.4094 | 100.0% | 1274.1122 | 1274.3733 | 2 | 7.038 | 70.0% | 3 | K.ELVDDSINNVR.K | 2 |
|  | Mis12IP\_Nocodazole\_MudPIT\_040709\_05.05050.05050.2 | 2.8071 | 0.3933 | 100.0% | 1189.0122 | 1189.3109 | 28 | 6.178 | 70.0% | 9 | R.KDDPTLLSSGR.V | 2 |
|  | Mis12IP\_Nocodazole\_MudPIT\_040709\_02.04148.04148.2 | 2.9706 | 0.2888 | 99.9% | 1061.3121 | 1061.1368 | 1 | 5.848 | 83.3% | 1 | K.DDPTLLSSGR.V | 2 |
|  | Mis12IP\_Nocodazole\_MudPIT\_040709\_03.04623.04623.1 | 2.1779 | 0.3123 | 97.9% | 942.56 | 943.091 | 12 | 6.822 | 56.2% | 2 | R.VQLVVGDGR.M | 1 |
|  | Mis12IP\_Nocodazole\_MudPIT\_040709\_05.04839.04839.2 | 3.0508 | 0.4073 | 100.0% | 942.97217 | 943.091 | 1 | 6.874 | 81.2% | 5 | R.VQLVVGDGR.M | 2 |

---

|  |  |  |  |  |  |  |  |  |
| --- | --- | --- | --- | --- | --- | --- | --- | --- |
| U | *gi|4557701|ref|NP\_000* | 9 | 13 | 17.8% | 432 | 48106 | 5.0 | keratin 17 [Homo sapiens] |

| Filename XCorr DeltCN Conf% ObsM+H+ CalcM+H+ SpR ZScore Ion% # Sequence  | | | | | | | | | | | | |
| --- | --- | --- | --- | --- | --- | --- | --- | --- | --- | --- | --- | --- |
|  | Mis12IP\_Nocodazole\_MudPIT\_040709\_03.04508.04508.1 | 1.9048 | 0.2501 | 99.0% | 809.5 | 809.93774 | 160 | 5.178 | 66.7% | 1 | R.LASYLDK.V | 1111111 |
|  | Mis12IP\_Nocodazole\_MudPIT\_040709\_02.04254.04254.2 | 3.8013 | 0.3554 | 100.0% | 1347.6921 | 1346.4772 | 1 | 6.512 | 77.3% | 2 | R.ALEEANTELEVK.I | 2 |
|  | Mis12IP\_Nocodazole\_MudPIT\_040709\_06.07300.07300.2 | 4.8194 | 0.4517 | 100.0% | 2068.5122 | 2069.366 | 1 | 8.416 | 63.9% | 1 | K.ILTATVDNANILLQIDNAR.L | 2 |
|  | Mis12IP\_Nocodazole\_MudPIT\_040709\_02.04220.04220.1 | 1.5217 | 0.3222 | 99.0% | 807.44 | 807.8815 | 2 | 4.741 | 58.3% | 1 | R.LAADDFR.T | 11111111 |
|  | Mis12IP\_Nocodazole\_MudPIT\_040709\_03.04543.04543.2 | 2.2948 | 0.233 | 99.3% | 808.0522 | 807.8815 | 1 | 5.671 | 91.7% | 3 | R.LAADDFR.T | 22222222 |
|  | Mis12IP\_Nocodazole\_MudPIT\_040709\_06.06210.06210.2 | 3.7407 | 0.4163 | 100.0% | 1030.0122 | 1030.2096 | 1 | 7.093 | 93.8% | 2 | R.VLDELTLAR.A | 22222 |
|  | Mis12IP\_Nocodazole\_MudPIT\_040709\_06.06209.06209.1 | 2.5279 | 0.3193 | 100.0% | 1030.56 | 1030.2096 | 6 | 5.521 | 62.5% | 1 | R.VLDELTLAR.A | 11111 |
|  | Mis12IP\_Nocodazole\_MudPIT\_040709\_02.03972.03972.2 | 3.5993 | 0.5203 | 100.0% | 1361.5521 | 1362.4796 | 1 | 9.18 | 79.2% | 1 | R.EVATNSELVQSGK.S | 22 |
|  | Mis12IP\_Nocodazole\_MudPIT\_040709\_02.04140.04140.2 | 2.2 | 0.3205 | 99.6% | 1118.1522 | 1118.2291 | 1 | 6.43 | 72.2% | 1 | R.TIVEEVQDGK.V | 2 |

Similarities:
gi|4557888|ref|NP\_000(2:7)  
gi|28317|emb|CAA32649(1:8)  
gi|24430192|ref|NP\_00(5:4)  
gi|12803709|gb|AAH026(6:3)  
gi|435476|emb|CAA8231(1:8)  
gi|14043271|gb|AAH076(5:4)  
gi|24430190|ref|NP\_00(5:4)  
gi|34526448|dbj|BAC85(2:7)  
gi|85566621|gb|AAI119(2:7)  

---

|  |  |  |  |  |  |  |  |  |
| --- | --- | --- | --- | --- | --- | --- | --- | --- |
| U | *gi|4503377|ref|NP\_001* | 8 | 15 | 17.7% | 572 | 62294 | 6.4 | dihydropyrimidinase-like 2 [Homo sapiens] |
| U | *gi|62898846|dbj|BAD97* | 8 | 15 | 17.7% | 572 | 62271 | 6.3 | dihydropyrimidinase-like 2 variant [Homo sapiens] |
| U | *gi|62087970|dbj|BAD92* | 8 | 15 | 16.1% | 628 | 68184 | 6.2 | dihydropyrimidinase-like 2 variant [Homo sapiens] |

| Filename XCorr DeltCN Conf% ObsM+H+ CalcM+H+ SpR ZScore Ion% # Sequence  | | | | | | | | | | | | |
| --- | --- | --- | --- | --- | --- | --- | --- | --- | --- | --- | --- | --- |
|  | Mis12IP\_Nocodazole\_MudPIT\_040709\_05.07367.07367.2 | 4.0573 | 0.3989 | 100.0% | 2349.4321 | 2350.602 | 1 | 7.299 | 47.4% | 2 | K.IVNDDQSFYADIYMEDGLIK.Q | 22 |
|  | Mis12IP\_Nocodazole\_MudPIT\_040709\_06.06942.06942.2 | 3.4951 | 0.6176 | 100.0% | 2150.2722 | 2152.3577 | 1 | 11.142 | 58.3% | 2 | R.FQMPDQGMTSADDFFQGTK.A | 2 |
|  | Mis12IP\_Nocodazole\_MudPIT\_040709\_03.04082.04082.2 | 2.8413 | 0.1737 | 99.3% | 1032.0721 | 1032.1417 | 1 | 4.465 | 88.9% | 2 | K.SSAEVIAQAR.K | 2 |
|  | Mis12IP\_Nocodazole\_MudPIT\_040709\_06.06136.06136.1 | 2.1294 | 0.1816 | 99.2% | 878.39 | 879.0619 | 9 | 5.554 | 66.7% | 1 | R.MSVIWDK.A | 11 |
|  | Mis12IP\_Nocodazole\_MudPIT\_040709\_05.05145.05145.2 | 5.4121 | 0.0873 | 99.9% | 1726.0521 | 1726.8988 | 2 | 9.357 | 60.0% | 2 | K.MDENQFVAVTSTNAAK.V | 222 |
|  | Mis12IP\_Nocodazole\_MudPIT\_040709\_05.06965.06965.2 | 3.897 | 0.5375 | 100.0% | 1900.5122 | 1901.1235 | 1 | 9.13 | 64.7% | 4 | R.IAVGSDADLVIWDPDSVK.T | 2 |
|  | Mis12IP\_Nocodazole\_MudPIT\_040709\_06.05940.05940.1 | 2.1182 | 0.3765 | 100.0% | 1084.61 | 1085.289 | 1 | 7.345 | 55.0% | 1 | R.GSPLVVISQGK.I | 1 |
|  | Mis12IP\_Nocodazole\_MudPIT\_040709\_06.05938.05938.2 | 3.5785 | 0.4034 | 100.0% | 1085.0521 | 1085.289 | 1 | 6.874 | 75.0% | 1 | R.GSPLVVISQGK.I | 2 |

Similarities:
gi|4503051|ref|NP\_001(1:7)  
gi|50417352|gb|AAH770(3:5)  

---

|  |  |  |  |  |  |  |  |  |
| --- | --- | --- | --- | --- | --- | --- | --- | --- |
| U | *gi|5901922|ref|NP\_008* | 6 | 10 | 17.7% | 378 | 44468 | 5.2 | CDC37 homolog [Homo sapiens] |

| Filename XCorr DeltCN Conf% ObsM+H+ CalcM+H+ SpR ZScore Ion% # Sequence  | | | | | | | | | | | | |
| --- | --- | --- | --- | --- | --- | --- | --- | --- | --- | --- | --- | --- |
|  | Mis12IP\_Nocodazole\_MudPIT\_040709\_03.04308.04308.2 | 3.126 | 0.1913 | 99.8% | 1057.1322 | 1057.1948 | 1 | 5.321 | 81.2% | 2 | R.LQAEAQQLR.K | 2 |
|  | Mis12IP\_Nocodazole\_MudPIT\_040709\_06.07895.07895.2 | 3.6705 | 0.4209 | 100.0% | 2269.632 | 2270.5413 | 1 | 9.013 | 50.0% | 2 | R.LGPGGLDPVEVYESLPEELQK.C | 2 |
|  | Mis12IP\_Nocodazole\_MudPIT\_040709\_06.07906.07906.3 | 3.5274 | 0.2394 | 99.0% | 2270.4243 | 2270.5413 | 1 | 6.476 | 35.0% | 2 | R.LGPGGLDPVEVYESLPEELQK.C | 3 |
| \* | Mis12IP\_Nocodazole\_MudPIT\_040709\_05.05362.05362.2 | 2.9555 | 0.1618 | 99.2% | 1248.2122 | 1248.4369 | 1 | 6.734 | 75.0% | 1 | K.DVQMLQDAISK.M | 2 |
|  | Mis12IP\_Nocodazole\_MudPIT\_040709\_05.05874.05874.2 | 3.7512 | 0.4897 | 100.0% | 1708.1522 | 1708.8627 | 1 | 7.931 | 56.2% | 2 | K.EGEEAGPGDPLLEAVPK.T | 2 |
|  | Mis12IP\_Nocodazole\_MudPIT\_040709\_02.03024.03024.1 | 1.4531 | 0.3449 | 99.1% | 949.38 | 949.9903 | 1 | 5.679 | 56.2% | 1 | K.TGDEKDVSV.- | 1 |

---

|  |  |  |  |  |  |  |  |  |
| --- | --- | --- | --- | --- | --- | --- | --- | --- |
| U | *gi|435476|emb|CAA8231* | 12 | 39 | 17.5% | 623 | 62129 | 5.3 | cytokeratin 9 [Homo sapiens] |
| U | *gi|55956899|ref|NP\_00* | 12 | 39 | 17.5% | 623 | 62064 | 5.2 | keratin 9 [Homo sapiens] |

| Filename XCorr DeltCN Conf% ObsM+H+ CalcM+H+ SpR ZScore Ion% # Sequence  | | | | | | | | | | | | |
| --- | --- | --- | --- | --- | --- | --- | --- | --- | --- | --- | --- | --- |
|  | Mis12IP\_Nocodazole\_MudPIT\_040709\_04.05198.05198.2 | 4.6443 | 0.5673 | 100.0% | 1233.0122 | 1233.2833 | 1 | 9.117 | 80.0% | 4 | R.SGGGGGGGLGSGGSIR.S | 2 |
|  | Mis12IP\_Nocodazole\_MudPIT\_040709\_03.04247.04247.2 | 3.6502 | 0.4919 | 100.0% | 1235.4521 | 1236.2401 | 1 | 9.329 | 75.0% | 7 | R.FSSSSGYGGGSSR.V | 2 |
|  | Mis12IP\_Nocodazole\_MudPIT\_040709\_03.03972.03972.2 | 2.9848 | 0.2459 | 99.9% | 1065.8522 | 1066.1742 | 1 | 6.701 | 87.5% | 6 | K.STMQELNSR.L | 2 |
|  | Mis12IP\_Nocodazole\_MudPIT\_040709\_03.04508.04508.1 | 1.9048 | 0.2501 | 99.0% | 809.5 | 809.93774 | 160 | 5.178 | 66.7% | 1 | R.LASYLDK.V | 1111111 |
|  | Mis12IP\_Nocodazole\_MudPIT\_040709\_03.04519.04519.3 | 2.5366 | 0.2606 | 97.3% | 2379.4143 | 2378.5981 | 30 | 4.689 | 22.5% | 1 | R.LASYLDKVQALEEANNDLENK.I | 3 |
|  | Mis12IP\_Nocodazole\_MudPIT\_040709\_03.04519.04519.2 | 5.3006 | 0.5098 | 100.0% | 1586.6122 | 1587.6836 | 1 | 10.522 | 80.8% | 1 | K.VQALEEANNDLENK.I | 3 |
|  | Mis12IP\_Nocodazole\_MudPIT\_040709\_05.05320.05320.1 | 1.857 | 0.4539 | 100.0% | 1060.63 | 1061.1802 | 16 | 6.236 | 50.0% | 1 | K.TLLDIDNTR.M | 1 |
|  | Mis12IP\_Nocodazole\_MudPIT\_040709\_05.05319.05319.2 | 2.9094 | 0.3866 | 100.0% | 1061.0922 | 1061.1802 | 15 | 6.044 | 68.8% | 3 | K.TLLDIDNTR.M | 2 |
|  | Mis12IP\_Nocodazole\_MudPIT\_040709\_06.06131.06131.2 | 1.9286 | 0.4283 | 99.9% | 897.33215 | 898.02155 | 3 | 6.609 | 66.7% | 2 | R.MTLDDFR.I | 2 |
|  | Mis12IP\_Nocodazole\_MudPIT\_040709\_06.06126.06126.1 | 1.5351 | 0.2681 | 98.7% | 897.39 | 898.02155 | 344 | 4.946 | 50.0% | 1 | R.MTLDDFR.I | 1 |
|  | Mis12IP\_Nocodazole\_MudPIT\_040709\_04.05522.05522.2 | 2.7778 | 0.3272 | 99.9% | 1157.9521 | 1158.2566 | 1 | 6.655 | 85.0% | 3 | R.QGVDADINGLR.Q | 2 |
|  | Mis12IP\_Nocodazole\_MudPIT\_040709\_03.03995.03995.2 | 4.8257 | 0.4637 | 100.0% | 1793.2722 | 1792.7324 | 1 | 9.842 | 43.2% | 9 | R.GGSGGSYGGGGSGGGYGGGSGSR.G | 2 |

Similarities:
gi|28317|emb|CAA32649(1:11)  
gi|24430192|ref|NP\_00(1:11)  
gi|12803709|gb|AAH026(1:11)  
gi|4557701|ref|NP\_000(1:11)  
gi|14043271|gb|AAH076(1:11)  
gi|24430190|ref|NP\_00(1:11)  

---

|  |  |  |  |  |  |  |  |  |
| --- | --- | --- | --- | --- | --- | --- | --- | --- |
| U | *gi|5803076|ref|NP\_006* | 2 | 7 | 17.3% | 185 | 21418 | 4.9 | chromobox homolog 1 (HP1 beta homolog Drosophila ) [Homo sapiens] |

| Filename XCorr DeltCN Conf% ObsM+H+ CalcM+H+ SpR ZScore Ion% # Sequence  | | | | | | | | | | | | |
| --- | --- | --- | --- | --- | --- | --- | --- | --- | --- | --- | --- | --- |
| \* | Mis12IP\_Nocodazole\_MudPIT\_040709\_03.05313.05313.2 | 4.4702 | 0.4238 | 100.0% | 1981.1322 | 1982.1023 | 1 | 9.185 | 70.0% | 1 | K.VEEVLEEEEEEYVVEK.V | 2 |
|  | Mis12IP\_Nocodazole\_MudPIT\_040709\_06.07600.07600.2 | 4.7457 | 0.4801 | 100.0% | 1712.7522 | 1714.0449 | 1 | 10.712 | 70.0% | 6 | R.IIGATDSSGELMFLMK.W | 22 |

Similarities:
gi|15082258|ref|NP\_00(1:1)  

---

|  |  |  |  |  |  |  |  |  |
| --- | --- | --- | --- | --- | --- | --- | --- | --- |
| U | *gi|16751921|ref|NP\_44* | 2 | 3 | 17.3% | 110 | 11284 | 6.5 | dermcidin preproprotein [Homo sapiens] |

| Filename XCorr DeltCN Conf% ObsM+H+ CalcM+H+ SpR ZScore Ion% # Sequence  | | | | | | | | | | | | |
| --- | --- | --- | --- | --- | --- | --- | --- | --- | --- | --- | --- | --- |
| \* | Mis12IP\_Nocodazole\_MudPIT\_040709\_06.05234.05234.1 | 1.6306 | 0.2599 | 98.6% | 729.55 | 729.89795 | 7 | 4.631 | 57.1% | 1 | K.KAVGGLGK.L | 1 |
| \* | Mis12IP\_Nocodazole\_MudPIT\_040709\_03.04981.04981.2 | 3.5044 | 0.4401 | 100.0% | 1161.7322 | 1162.2389 | 1 | 7.544 | 80.0% | 2 | K.DAVEDLESVGK.G | 2 |

---

|  |  |  |  |  |  |  |  |  |
| --- | --- | --- | --- | --- | --- | --- | --- | --- |
| U | *gi|4757880|ref|NP\_004* | 5 | 7 | 17.1% | 328 | 37155 | 6.8 | BUB3 budding uninhibited by benzimidazoles 3 isoform a [Homo sapiens] |
| U | *gi|56550081|ref|NP\_00* | 5 | 7 | 17.2% | 326 | 36955 | 6.8 | BUB3 budding uninhibited by benzimidazoles 3 isoform b [Homo sapiens] |

| Filename XCorr DeltCN Conf% ObsM+H+ CalcM+H+ SpR ZScore Ion% # Sequence  | | | | | | | | | | | | |
| --- | --- | --- | --- | --- | --- | --- | --- | --- | --- | --- | --- | --- |
|  | Mis12IP\_Nocodazole\_MudPIT\_040709\_03.04442.04442.2 | 2.1808 | 0.2283 | 97.5% | 1384.0521 | 1384.529 | 1 | 4.725 | 54.2% | 1 | K.LNQPPEDGISSVK.F | 2 |
|  | Mis12IP\_Nocodazole\_MudPIT\_040709\_05.04895.04895.2 | 2.6248 | 0.3238 | 99.9% | 1165.8322 | 1166.3373 | 1 | 6.021 | 77.8% | 2 | R.LYDVPANSMR.L | 2 |
|  | Mis12IP\_Nocodazole\_MudPIT\_040709\_04.05237.05237.2 | 2.1621 | 0.1621 | 96.8% | 995.2922 | 996.12897 | 1 | 4.145 | 85.7% | 1 | R.NMGYVQQR.R | 2 |
|  | Mis12IP\_Nocodazole\_MudPIT\_040709\_06.05995.05995.2 | 2.5568 | 0.4611 | 100.0% | 1208.8922 | 1209.3445 | 1 | 6.489 | 75.0% | 1 | K.QGYVLSSIEGR.V | 2 |
|  | Mis12IP\_Nocodazole\_MudPIT\_040709\_05.05236.05236.2 | 3.7691 | 0.4193 | 100.0% | 1574.3522 | 1574.771 | 2 | 7.294 | 61.5% | 2 | R.VAVEYLDPSPEVQK.K | 2 |

---

|  |  |  |  |  |  |  |  |  |
| --- | --- | --- | --- | --- | --- | --- | --- | --- |
| U | *gi|4503051|ref|NP\_001* | 8 | 12 | 16.8% | 572 | 62184 | 7.0 | collapsin response mediator protein 1 isoform 2 [Homo sapiens] |

| Filename XCorr DeltCN Conf% ObsM+H+ CalcM+H+ SpR ZScore Ion% # Sequence  | | | | | | | | | | | | |
| --- | --- | --- | --- | --- | --- | --- | --- | --- | --- | --- | --- | --- |
|  | Mis12IP\_Nocodazole\_MudPIT\_040709\_06.07433.07433.2 | 4.8903 | 0.4749 | 100.0% | 2298.6921 | 2298.5518 | 1 | 9.066 | 60.5% | 3 | R.IINDDQSLYADVYLEDGLIK.Q | 2 |
|  | Mis12IP\_Nocodazole\_MudPIT\_040709\_06.06413.06413.1 | 2.2111 | 0.3846 | 100.0% | 1000.52 | 1001.171 | 34 | 6.216 | 55.6% | 1 | K.SAADIIALAR.K | 1 |
|  | Mis12IP\_Nocodazole\_MudPIT\_040709\_06.06419.06419.2 | 3.4273 | 0.3494 | 100.0% | 1000.6322 | 1001.171 | 2 | 6.538 | 83.3% | 1 | K.SAADIIALAR.K | 2 |
|  | Mis12IP\_Nocodazole\_MudPIT\_040709\_05.06518.06518.2 | 2.1841 | 0.355 | 99.6% | 1803.1721 | 1803.9664 | 46 | 5.387 | 36.7% | 1 | K.DNFTLIPEGVNGIEER.M | 2 |
|  | Mis12IP\_Nocodazole\_MudPIT\_040709\_06.05911.05911.1 | 2.0766 | 0.3303 | 97.2% | 878.46 | 879.0619 | 16 | 5.915 | 66.7% | 1 | R.MTVVWDK.A | 1 |
|  | Mis12IP\_Nocodazole\_MudPIT\_040709\_05.05145.05145.2 | 5.4121 | 0.0873 | 99.9% | 1726.0521 | 1726.8988 | 2 | 9.357 | 60.0% | 2 | K.MDENQFVAVTSTNAAK.I | 222 |
|  | Mis12IP\_Nocodazole\_MudPIT\_040709\_06.06054.06054.2 | 3.8635 | 0.3843 | 100.0% | 1362.1522 | 1362.5254 | 1 | 6.546 | 86.4% | 1 | K.IVFEDGNINVNK.G | 2 |
|  | Mis12IP\_Nocodazole\_MudPIT\_040709\_06.06056.06056.2 | 4.4417 | 0.5335 | 100.0% | 1625.8322 | 1624.8488 | 1 | 8.947 | 67.9% | 2 | R.GMYDGPVYEVPATPK.Y | 2 |

Similarities:
gi|4503377|ref|NP\_001(1:7)  
gi|50417352|gb|AAH770(1:7)  

---

|  |  |  |  |  |  |  |  |  |
| --- | --- | --- | --- | --- | --- | --- | --- | --- |
| U | *gi|5032051|ref|NP\_005* | 2 | 2 | 15.9% | 151 | 16273 | 10.1 | ribosomal protein S14 [Homo sapiens] |

| Filename XCorr DeltCN Conf% ObsM+H+ CalcM+H+ SpR ZScore Ion% # Sequence  | | | | | | | | | | | | |
| --- | --- | --- | --- | --- | --- | --- | --- | --- | --- | --- | --- | --- |
| \* | Mis12IP\_Nocodazole\_MudPIT\_040709\_06.05368.05368.2 | 2.7192 | 0.4464 | 100.0% | 1054.6721 | 1055.179 | 1 | 8.16 | 80.0% | 1 | K.TPGPGAQSALR.A | 2 |
| \* | Mis12IP\_Nocodazole\_MudPIT\_040709\_02.04205.04205.2 | 3.0878 | 0.3604 | 100.0% | 1430.3322 | 1430.5547 | 1 | 6.242 | 70.8% | 1 | R.IEDVTPIPSDSTR.R | 2 |

---

|  |  |  |  |  |  |  |  |  |
| --- | --- | --- | --- | --- | --- | --- | --- | --- |
| U | *gi|51095055|gb|EAL242* | 4 | 6 | 15.6% | 231 | 25598 | 7.6 | similar to Chain , Heat-Shock Cognate 70kd Protein (44kd Atpase N-Terminal Fragment) (E.C.3.6.1.3) Mutant With Asp 206 Replaced By Ser (D206s) [Homo sapiens] |

| Filename XCorr DeltCN Conf% ObsM+H+ CalcM+H+ SpR ZScore Ion% # Sequence  | | | | | | | | | | | | |
| --- | --- | --- | --- | --- | --- | --- | --- | --- | --- | --- | --- | --- |
| \* | Mis12IP\_Nocodazole\_MudPIT\_040709\_06.05952.05952.2 | 2.8238 | 0.3425 | 99.9% | 1673.2522 | 1673.884 | 1 | 5.55 | 57.1% | 1 | K.HQVAMNPTNTVFDAK.R | 2 |
|  | Mis12IP\_Nocodazole\_MudPIT\_040709\_04.05574.05574.1 | 1.8859 | 0.2445 | 99.2% | 993.54 | 994.1326 | 62 | 6.703 | 56.2% | 1 | K.EIAEAYLGK.T | 11 |
|  | Mis12IP\_Nocodazole\_MudPIT\_040709\_06.06454.06454.2 | 4.0225 | 0.3278 | 100.0% | 1199.6122 | 1200.3805 | 1 | 6.967 | 86.4% | 3 | K.DAGTIAGLNVLR.I | 22 |
|  | Mis12IP\_Nocodazole\_MudPIT\_040709\_06.06455.06455.1 | 1.6319 | 0.2981 | 99.2% | 1199.65 | 1200.3805 | 214 | 5.188 | 45.5% | 1 | K.DAGTIAGLNVLR.I | 11 |

Similarities:
gi|5729877|ref|NP\_006(3:1)  

---

|  |  |  |  |  |  |  |  |  |
| --- | --- | --- | --- | --- | --- | --- | --- | --- |
| U | *gi|31542947|ref|NP\_00* | 7 | 8 | 15.4% | 573 | 61055 | 5.9 | chaperonin [Homo sapiens] |
| U | *gi|77702086|gb|ABB010* | 7 | 8 | 15.3% | 575 | 61213 | 5.9 | heat shock protein 60 [Homo sapiens] |

| Filename XCorr DeltCN Conf% ObsM+H+ CalcM+H+ SpR ZScore Ion% # Sequence  | | | | | | | | | | | | |
| --- | --- | --- | --- | --- | --- | --- | --- | --- | --- | --- | --- | --- |
|  | Mis12IP\_Nocodazole\_MudPIT\_040709\_06.10217.10217.3 | 5.005 | 0.524 | 100.0% | 2114.0645 | 2114.5667 | 1 | 10.324 | 45.0% | 1 | R.ALMLQGVDLLADAVAVTMGPK.G | 3 |
|  | Mis12IP\_Nocodazole\_MudPIT\_040709\_05.05001.05001.2 | 6.0423 | 0.631 | 100.0% | 2560.892 | 2561.7222 | 1 | 12.67 | 60.4% | 1 | K.LVQDVANNTNEEAGDGTTTATVLAR.S | 2 |
|  | Mis12IP\_Nocodazole\_MudPIT\_040709\_05.05007.05007.3 | 4.5614 | 0.4367 | 100.0% | 2561.8145 | 2561.7222 | 1 | 8.47 | 37.5% | 1 | K.LVQDVANNTNEEAGDGTTTATVLAR.S | 3 |
|  | Mis12IP\_Nocodazole\_MudPIT\_040709\_04.05608.05608.2 | 3.2534 | 0.344 | 100.0% | 1216.0521 | 1216.377 | 1 | 6.404 | 81.8% | 2 | K.NAGVEGSLIVEK.I | 2 |
|  | Mis12IP\_Nocodazole\_MudPIT\_040709\_06.07535.07535.3 | 4.8907 | 0.4575 | 100.0% | 2508.6843 | 2509.8235 | 1 | 7.892 | 37.5% | 1 | K.IMQSSSEVGYDAMAGDFVNMVEK.G | 3 |
|  | Mis12IP\_Nocodazole\_MudPIT\_040709\_06.07537.07537.2 | 3.6772 | 0.6076 | 100.0% | 2508.9321 | 2509.8235 | 1 | 10.478 | 50.0% | 1 | K.IMQSSSEVGYDAMAGDFVNMVEK.G | 2 |
|  | Mis12IP\_Nocodazole\_MudPIT\_040709\_03.04524.04524.1 | 1.6823 | 0.2736 | 99.1% | 743.64 | 743.8785 | 30 | 4.927 | 58.3% | 1 | K.GIIDPTK.V | 1 |

---

|  |  |  |  |  |  |  |  |  |
| --- | --- | --- | --- | --- | --- | --- | --- | --- |
| U | *gi|4506619|ref|NP\_000* | 3 | 4 | 15.3% | 157 | 17779 | 11.3 | ribosomal protein L24 [Homo sapiens] |

| Filename XCorr DeltCN Conf% ObsM+H+ CalcM+H+ SpR ZScore Ion% # Sequence  | | | | | | | | | | | | |
| --- | --- | --- | --- | --- | --- | --- | --- | --- | --- | --- | --- | --- |
| \* | Mis12IP\_Nocodazole\_MudPIT\_040709\_06.06666.06666.1 | 2.6532 | 0.3961 | 100.0% | 1261.46 | 1262.5072 | 1 | 8.44 | 66.7% | 1 | R.AITGASLADIMAK.R | 1 |
| \* | Mis12IP\_Nocodazole\_MudPIT\_040709\_06.06664.06664.2 | 4.3058 | 0.4758 | 100.0% | 1262.1122 | 1262.5072 | 1 | 9.392 | 83.3% | 1 | R.AITGASLADIMAK.R | 2 |
| \* | Mis12IP\_Nocodazole\_MudPIT\_040709\_06.05320.05320.2 | 2.7458 | 0.3609 | 100.0% | 1061.0122 | 1061.2848 | 3 | 6.65 | 65.0% | 2 | K.TAMAAAKAPTK.A | 2 |

---

|  |  |  |  |  |  |  |  |  |
| --- | --- | --- | --- | --- | --- | --- | --- | --- |
| U | *gi|5174419|ref|NP\_006* | 3 | 3 | 15.2% | 277 | 30180 | 8.1 | endopeptidase Clp precursor [Homo sapiens] |

| Filename XCorr DeltCN Conf% ObsM+H+ CalcM+H+ SpR ZScore Ion% # Sequence  | | | | | | | | | | | | |
| --- | --- | --- | --- | --- | --- | --- | --- | --- | --- | --- | --- | --- |
| \* | Mis12IP\_Nocodazole\_MudPIT\_040709\_06.06779.06779.2 | 5.412 | 0.6201 | 100.0% | 1618.1721 | 1618.8425 | 1 | 10.508 | 82.1% | 1 | R.GQATDIAIQAEEIMK.L | 2 |
| \* | Mis12IP\_Nocodazole\_MudPIT\_040709\_06.06270.06270.2 | 2.71 | 0.4776 | 100.0% | 1391.2522 | 1391.5823 | 1 | 6.949 | 59.1% | 1 | K.QSLQVIESAMER.D | 2 |
| \* | Mis12IP\_Nocodazole\_MudPIT\_040709\_06.06884.06884.2 | 3.4196 | 0.4732 | 100.0% | 1759.2522 | 1760.0297 | 1 | 8.57 | 78.6% | 1 | R.YMSPMEAQEFGILDK.V | 2 |

---

|  |  |  |  |  |  |  |  |  |
| --- | --- | --- | --- | --- | --- | --- | --- | --- |
| U | *gi|20070228|ref|NP\_00* | 6 | 10 | 15.0% | 461 | 53879 | 5.2 | nucleobindin 1 [Homo sapiens] |
| U | *gi|62897169|dbj|BAD96* | 6 | 10 | 15.0% | 461 | 53907 | 5.2 | nucleobindin 1 variant [Homo sapiens] |

| Filename XCorr DeltCN Conf% ObsM+H+ CalcM+H+ SpR ZScore Ion% # Sequence  | | | | | | | | | | | | |
| --- | --- | --- | --- | --- | --- | --- | --- | --- | --- | --- | --- | --- |
|  | Mis12IP\_Nocodazole\_MudPIT\_040709\_03.04311.04311.2 | 3.9497 | 0.4983 | 100.0% | 1345.2122 | 1345.4954 | 1 | 7.996 | 70.8% | 4 | K.LQAANAEDIKSGK.L | 2 |
|  | Mis12IP\_Nocodazole\_MudPIT\_040709\_06.07289.07289.2 | 3.2954 | 0.2657 | 99.9% | 1273.0521 | 1273.4722 | 1 | 7.347 | 80.0% | 2 | R.DLELLIQTATR.D | 2 |
|  | Mis12IP\_Nocodazole\_MudPIT\_040709\_06.05474.05474.2 | 2.4955 | 0.3767 | 99.9% | 1140.6322 | 1141.3127 | 1 | 6.508 | 70.0% | 1 | K.VNVPGSQAQLK.E | 2 |
|  | Mis12IP\_Nocodazole\_MudPIT\_040709\_05.06412.06412.2 | 2.6554 | 0.3338 | 99.9% | 1572.1921 | 1572.6714 | 4 | 5.806 | 54.2% | 1 | K.EVWEELDGLDPNR.F | 2 |
|  | Mis12IP\_Nocodazole\_MudPIT\_040709\_06.05276.05276.2 | 2.4452 | 0.244 | 99.3% | 1119.2322 | 1119.2657 | 7 | 5.33 | 50.0% | 1 | K.APAAHPEGQLK.F | 2 |
|  | Mis12IP\_Nocodazole\_MudPIT\_040709\_06.06232.06232.2 | 3.4706 | 0.5124 | 100.0% | 1160.4122 | 1161.3434 | 2 | 7.809 | 72.2% | 1 | R.LPEVEVPQHL.- | 2 |

---

|  |  |  |  |  |  |  |  |  |
| --- | --- | --- | --- | --- | --- | --- | --- | --- |
| U | *gi|24308386|ref|NP\_44* | 4 | 6 | 14.8% | 357 | 40674 | 5.7 | protein-L-isoaspartate (D-aspartate) O-methyltransferase domain containing 1 [Homo sapiens] |

| Filename XCorr DeltCN Conf% ObsM+H+ CalcM+H+ SpR ZScore Ion% # Sequence  | | | | | | | | | | | | |
| --- | --- | --- | --- | --- | --- | --- | --- | --- | --- | --- | --- | --- |
| \* | Mis12IP\_Nocodazole\_MudPIT\_040709\_04.01449.01449.2 | 2.591 | 0.3563 | 99.9% | 2013.4122 | 2015.0698 | 1 | 6.616 | 55.6% | 1 | -.MGGAVS\*AGEDNDDLIDNLK.E | 2 |
| \* | Mis12IP\_Nocodazole\_MudPIT\_040709\_06.10744.10744.2 | 3.7461 | 0.4357 | 100.0% | 2775.132 | 2775.9177 | 1 | 7.374 | 33.3% | 2 | -.MGGAVS\*AGEDNDDLIDNLKEAQYIR.T | 2 |
|  | Mis12IP\_Nocodazole\_MudPIT\_040709\_06.08698.08698.2 | 3.8621 | 0.475 | 100.0% | 2014.0322 | 2014.4492 | 1 | 8.455 | 58.8% | 1 | K.VGGILVMPIEDQLTQIMR.T | 2 |
|  | Mis12IP\_Nocodazole\_MudPIT\_040709\_05.05205.05205.2 | 3.2258 | 0.2528 | 99.9% | 1210.1122 | 1210.3472 | 10 | 5.349 | 66.7% | 2 | R.NFINDEMQAK.G | 2 |

---

|  |  |  |  |  |  |  |  |  |
| --- | --- | --- | --- | --- | --- | --- | --- | --- |
| U | *gi|14389309|ref|NP\_11* | 5 | 12 | 14.7% | 449 | 49895 | 5.1 | tubulin alpha 6 [Homo sapiens] |
| U | *gi|62897609|dbj|BAD96* | 5 | 12 | 14.7% | 449 | 49823 | 5.1 | tubulin alpha 6 variant [Homo sapiens] |
| U | *gi|57013276|ref|NP\_00* | 5 | 12 | 14.6% | 451 | 50152 | 5.1 | tubulin, alpha, ubiquitous [Homo sapiens] |
| U | *gi|37492|emb|CAA25855* | 5 | 12 | 14.6% | 451 | 50158 | 5.1 | alpha-tubulin [Homo sapiens] |
| U | *gi|18204869|gb|AAH215* | 5 | 12 | 19.7% | 335 | 37218 | 5.0 | K-ALPHA-1 protein [Homo sapiens] |
| U | *gi|17986283|ref|NP\_00* | 5 | 12 | 14.6% | 451 | 50136 | 5.1 | tubulin, alpha 3 [Homo sapiens] |

| Filename XCorr DeltCN Conf% ObsM+H+ CalcM+H+ SpR ZScore Ion% # Sequence  | | | | | | | | | | | | |
| --- | --- | --- | --- | --- | --- | --- | --- | --- | --- | --- | --- | --- |
|  | Mis12IP\_Nocodazole\_MudPIT\_040709\_06.06928.06928.2 | 4.6297 | 0.5538 | 100.0% | 2007.9521 | 2009.093 | 1 | 10.986 | 52.6% | 1 | K.TIGGGDDSFNTFFSETGAGK.H | 2 |
|  | Mis12IP\_Nocodazole\_MudPIT\_040709\_06.07313.07313.2 | 5.1783 | 0.4691 | 100.0% | 1703.3522 | 1702.9451 | 1 | 8.591 | 75.0% | 6 | R.AVFVDLEPTVIDEVR.T | 2 |
|  | Mis12IP\_Nocodazole\_MudPIT\_040709\_06.08024.08024.2 | 4.0805 | 0.5597 | 100.0% | 2410.8123 | 2410.6885 | 1 | 9.93 | 47.5% | 1 | R.FDGALNVDLTEFQTNLVPYPR.I | 22 |
|  | Mis12IP\_Nocodazole\_MudPIT\_040709\_04.05667.05667.1 | 1.9256 | 0.3741 | 97.7% | 1015.66 | 1016.1827 | 1 | 6.872 | 66.7% | 3 | K.DVNAAIATIK.T | 1 |
|  | Mis12IP\_Nocodazole\_MudPIT\_040709\_03.04849.04849.2 | 3.0463 | 0.3676 | 100.0% | 1015.9522 | 1016.1827 | 1 | 7.279 | 77.8% | 1 | K.DVNAAIATIK.T | 2 |

Similarities:
gi|17921989|ref|NP\_00(1:4)  

---

|  |  |  |  |  |  |  |  |  |
| --- | --- | --- | --- | --- | --- | --- | --- | --- |
| U | *gi|4506717|ref|NP\_001* | 2 | 6 | 14.3% | 56 | 6677 | 10.1 | ribosomal protein S29 isoform 1 [Homo sapiens] |

| Filename XCorr DeltCN Conf% ObsM+H+ CalcM+H+ SpR ZScore Ion% # Sequence  | | | | | | | | | | | | |
| --- | --- | --- | --- | --- | --- | --- | --- | --- | --- | --- | --- | --- |
| \* | Mis12IP\_Nocodazole\_MudPIT\_040709\_03.06600.06600.1 | 2.0571 | 0.3523 | 97.0% | 920.44 | 921.0813 | 1 | 7.074 | 64.3% | 5 | K.DIGFIKLD.- | 1 |
| \* | Mis12IP\_Nocodazole\_MudPIT\_040709\_02.06083.06083.2 | 1.655 | 0.2563 | 95.8% | 920.8722 | 921.0813 | 1 | 6.563 | 78.6% | 1 | K.DIGFIKLD.- | 2 |

---

|  |  |  |  |  |  |  |  |  |
| --- | --- | --- | --- | --- | --- | --- | --- | --- |
| U | *gi|11968182|ref|NP\_07* | 2 | 2 | 13.8% | 152 | 17719 | 11.0 | ribosomal protein S18 [Homo sapiens] |

| Filename XCorr DeltCN Conf% ObsM+H+ CalcM+H+ SpR ZScore Ion% # Sequence  | | | | | | | | | | | | |
| --- | --- | --- | --- | --- | --- | --- | --- | --- | --- | --- | --- | --- |
|  | Mis12IP\_Nocodazole\_MudPIT\_040709\_03.04465.04465.2 | 2.39 | 0.3192 | 99.9% | 1002.1722 | 1002.1154 | 1 | 6.224 | 87.5% | 1 | R.VLNTNIDGR.R | 2 |
|  | Mis12IP\_Nocodazole\_MudPIT\_040709\_05.04912.04912.2 | 3.8102 | 0.4255 | 100.0% | 1321.9122 | 1322.4606 | 1 | 7.453 | 77.3% | 1 | K.YSQVLANGLDNK.L | 2 |

---

|  |  |  |  |  |  |  |  |  |
| --- | --- | --- | --- | --- | --- | --- | --- | --- |
| U | *gi|4758648|ref|NP\_004* | 11 | 13 | 13.7% | 963 | 109685 | 6.5 | kinesin family member 5B [Homo sapiens] |

| Filename XCorr DeltCN Conf% ObsM+H+ CalcM+H+ SpR ZScore Ion% # Sequence  | | | | | | | | | | | | |
| --- | --- | --- | --- | --- | --- | --- | --- | --- | --- | --- | --- | --- |
|  | Mis12IP\_Nocodazole\_MudPIT\_040709\_05.06366.06366.2 | 3.6102 | 0.4843 | 100.0% | 2108.892 | 2108.2688 | 1 | 7.184 | 42.1% | 1 | K.DVLEGYNGTIFAYGQTSSGK.T | 2 |
|  | Mis12IP\_Nocodazole\_MudPIT\_040709\_06.06734.06734.1 | 1.8944 | 0.3514 | 98.2% | 1207.58 | 1208.3971 | 1 | 5.453 | 60.0% | 1 | K.LYLVDLAGSEK.V | 1 |
|  | Mis12IP\_Nocodazole\_MudPIT\_040709\_06.06730.06730.2 | 3.4707 | 0.4204 | 100.0% | 1208.0521 | 1208.3971 | 1 | 7.106 | 75.0% | 1 | K.LYLVDLAGSEK.V | 2 |
| \* | Mis12IP\_Nocodazole\_MudPIT\_040709\_05.05626.05626.2 | 3.7878 | 0.5035 | 100.0% | 1635.8322 | 1635.8297 | 1 | 8.655 | 73.1% | 1 | K.TQMLDQEELLASTR.R | 2 |
| \* | Mis12IP\_Nocodazole\_MudPIT\_040709\_05.05748.05748.2 | 2.9265 | 0.3186 | 99.9% | 1480.6522 | 1481.5994 | 1 | 6.226 | 72.7% | 1 | K.EYELLSDELNQK.S | 2 |
| \* | Mis12IP\_Nocodazole\_MudPIT\_040709\_06.06142.06142.2 | 4.4976 | 0.284 | 100.0% | 1348.4122 | 1347.5083 | 1 | 6.663 | 75.0% | 1 | K.SATLASIDAELQK.L | 2 |
| \* | Mis12IP\_Nocodazole\_MudPIT\_040709\_06.06390.06390.2 | 3.5206 | 0.3904 | 100.0% | 1452.0322 | 1452.6042 | 1 | 7.269 | 77.3% | 1 | K.SLTEYLQNVEQK.K | 2 |
| \* | Mis12IP\_Nocodazole\_MudPIT\_040709\_06.07816.07816.2 | 3.9657 | 0.4484 | 100.0% | 1959.1522 | 1959.1608 | 1 | 8.488 | 62.5% | 2 | R.QLEESVDALSEELVQLR.A | 2 |
| \* | Mis12IP\_Nocodazole\_MudPIT\_040709\_03.04602.04602.2 | 4.0245 | 0.3189 | 100.0% | 1315.9722 | 1316.4539 | 2 | 6.641 | 75.0% | 2 | K.LITDLQDQNQK.M | 2 |
| \* | Mis12IP\_Nocodazole\_MudPIT\_040709\_06.06238.06238.2 | 3.3082 | 0.3362 | 100.0% | 1063.1522 | 1063.242 | 3 | 6.133 | 81.2% | 1 | K.LFVQDLATR.V | 2 |
|  | Mis12IP\_Nocodazole\_MudPIT\_040709\_06.07222.07222.2 | 4.2235 | 0.3514 | 100.0% | 1549.1522 | 1549.7643 | 1 | 7.515 | 75.0% | 1 | K.ISFLENNLEQLTK.V | 2 |

---

|  |  |  |  |  |  |  |  |  |
| --- | --- | --- | --- | --- | --- | --- | --- | --- |
| U | *gi|14043732|gb|AAH078* | 3 | 4 | 13.6% | 294 | 32188 | 9.2 | FAM83A protein [Homo sapiens] |
| U | *gi|46255017|ref|NP\_99* | 3 | 4 | 10.9% | 367 | 40576 | 8.8 | hypothetical protein LOC84985 isoform b [Homo sapiens] |
| U | *gi|40254997|ref|NP\_11* | 3 | 4 | 9.2% | 434 | 47458 | 8.8 | hypothetical protein LOC84985 isoform a [Homo sapiens] |
| U | *gi|33323041|gb|AAQ072* | 3 | 4 | 10.9% | 367 | 40606 | 8.8 | BJ-TSA-9 [Homo sapiens] |

| Filename XCorr DeltCN Conf% ObsM+H+ CalcM+H+ SpR ZScore Ion% # Sequence  | | | | | | | | | | | | |
| --- | --- | --- | --- | --- | --- | --- | --- | --- | --- | --- | --- | --- |
|  | Mis12IP\_Nocodazole\_MudPIT\_040709\_05.06584.06584.2 | 4.7399 | 0.4669 | 100.0% | 1738.5322 | 1738.8944 | 1 | 8.972 | 60.0% | 2 | R.LATDALLDGGSEAYWR.V | 2 |
|  | Mis12IP\_Nocodazole\_MudPIT\_040709\_06.07900.07900.2 | 3.3991 | 0.3912 | 100.0% | 2668.5923 | 2668.9202 | 3 | 5.918 | 32.6% | 1 | R.VLSQEGEVDFLSSVEAQYIQAQAR.E | 2 |
|  | Mis12IP\_Nocodazole\_MudPIT\_040709\_06.07908.07908.3 | 4.5236 | 0.4074 | 100.0% | 2669.1843 | 2668.9202 | 1 | 7.871 | 34.8% | 1 | R.VLSQEGEVDFLSSVEAQYIQAQAR.E | 3 |

---

|  |  |  |  |  |  |  |  |  |
| --- | --- | --- | --- | --- | --- | --- | --- | --- |
| U | *gi|116283293|gb|AAH18* | 2 | 4 | 13.5% | 267 | 29505 | 10.1 | Unknown (protein for IMAGE:3882730) [Homo sapiens] |
| U | *gi|34098946|ref|NP\_00* | 2 | 4 | 11.1% | 324 | 35924 | 9.9 | nuclease sensitive element binding protein 1 [Homo sapiens] |
| U | *gi|33875177|gb|AAH000* | 2 | 4 | 13.5% | 266 | 29374 | 10.2 | YBX1 protein [Homo sapiens] |

| Filename XCorr DeltCN Conf% ObsM+H+ CalcM+H+ SpR ZScore Ion% # Sequence  | | | | | | | | | | | | |
| --- | --- | --- | --- | --- | --- | --- | --- | --- | --- | --- | --- | --- |
|  | Mis12IP\_Nocodazole\_MudPIT\_040709\_03.05379.05379.2 | 4.5521 | 0.5824 | 100.0% | 1796.1921 | 1796.8822 | 1 | 9.673 | 65.6% | 2 | R.SVGDGETVEFDVVEGEK.G | 22 |
|  | Mis12IP\_Nocodazole\_MudPIT\_040709\_05.04677.04677.2 | 4.587 | 0.2168 | 100.0% | 1696.2522 | 1696.8577 | 1 | 8.366 | 69.4% | 2 | K.GAEAANVTGPGGVPVQGSK.Y | 2 |

Similarities:
gi|1167838|emb|CAA646(1:1)  

---

|  |  |  |  |  |  |  |  |  |
| --- | --- | --- | --- | --- | --- | --- | --- | --- |
| U | *gi|10800138|ref|NP\_06* | 2 | 2 | 13.5% | 126 | 13936 | 10.3 | H2B histone family, member B [Homo sapiens] |
| U | *gi|78070466|gb|AAI069* | 2 | 2 | 13.6% | 125 | 13775 | 10.3 | HIST1H2BG protein [Homo sapiens] |
| U | *gi|73487310|gb|AAI008* | 2 | 2 | 12.8% | 133 | 14736 | 10.3 | Unknown (protein for IMAGE:40002416) [Homo sapiens] |
| U | *gi|68532405|gb|AAH981* | 2 | 2 | 13.5% | 126 | 13936 | 10.3 | HIST1H2BB protein [Homo sapiens] |
| U | *gi|66912162|ref|NP\_00* | 2 | 2 | 13.5% | 126 | 13920 | 10.3 | histone 2, H2bf [Homo sapiens] |
| U | *gi|49257898|gb|AAH739* | 2 | 2 | 25.4% | 67 | 7395 | 9.9 | HIST2H3PS2 protein [Homo sapiens] |
| U | *gi|4504277|ref|NP\_003* | 2 | 2 | 13.5% | 126 | 13920 | 10.3 | H2B histone family, member Q [Homo sapiens] |
| U | *gi|4504269|ref|NP\_003* | 2 | 2 | 13.5% | 126 | 13892 | 10.3 | H2B histone family, member J [Homo sapiens] |
| U | *gi|4504263|ref|NP\_003* | 2 | 2 | 13.5% | 126 | 13989 | 10.3 | H2B histone family, member E [Homo sapiens] |
| U | *gi|4504261|ref|NP\_003* | 2 | 2 | 13.5% | 126 | 13922 | 10.3 | H2B histone family, member D [Homo sapiens] |
| U | *gi|4504259|ref|NP\_003* | 2 | 2 | 13.5% | 126 | 13952 | 10.3 | H2B histone family, member C [Homo sapiens] |
| U | *gi|4504257|ref|NP\_003* | 2 | 2 | 13.5% | 126 | 13906 | 10.3 | H2B histone family, member A [Homo sapiens] |
| U | *gi|31979|emb|CAA40416* | 2 | 2 | 13.5% | 126 | 13907 | 10.2 | histone H2A.2 [Homo sapiens] |
| U | *gi|28173554|ref|NP\_77* | 2 | 2 | 13.5% | 126 | 13908 | 10.3 | histone H2B [Homo sapiens] |
| U | *gi|24586679|ref|NP\_73* | 2 | 2 | 13.4% | 127 | 14167 | 10.3 | testis-specific histone H2B [Homo sapiens] |
| U | *gi|16306566|ref|NP\_00* | 2 | 2 | 13.5% | 126 | 13906 | 10.3 | histone H2B [Homo sapiens] |
| U | *gi|1568557|emb|CAB025* | 2 | 2 | 13.5% | 126 | 13936 | 10.3 | histone H2B [Homo sapiens] |
| U | *gi|10800140|ref|NP\_06* | 2 | 2 | 13.5% | 126 | 13950 | 10.3 | H2B histone family, member F [Homo sapiens] |

| Filename XCorr DeltCN Conf% ObsM+H+ CalcM+H+ SpR ZScore Ion% # Sequence  | | | | | | | | | | | | |
| --- | --- | --- | --- | --- | --- | --- | --- | --- | --- | --- | --- | --- |
|  | Mis12IP\_Nocodazole\_MudPIT\_040709\_06.05215.05215.2 | 1.72 | 0.2941 | 96.8% | 1227.7522 | 1228.391 | 62 | 5.255 | 45.5% | 1 | K.HAVSEGTKAVTK.Y | 2 |
|  | Mis12IP\_Nocodazole\_MudPIT\_040709\_05.03941.03941.2 | 2.1716 | 0.3149 | 99.6% | 985.33215 | 985.12524 | 1 | 6.065 | 75.0% | 1 | K.AVTKYTSSK.- | 2 |

---

|  |  |  |  |  |  |  |  |  |
| --- | --- | --- | --- | --- | --- | --- | --- | --- |
| U | *gi|7657146|ref|NP\_055* | 2 | 4 | 13.3% | 196 | 21604 | 5.1 | heat shock 27kDa protein 8 [Homo sapiens] |

| Filename XCorr DeltCN Conf% ObsM+H+ CalcM+H+ SpR ZScore Ion% # Sequence  | | | | | | | | | | | | |
| --- | --- | --- | --- | --- | --- | --- | --- | --- | --- | --- | --- | --- |
| \* | Mis12IP\_Nocodazole\_MudPIT\_040709\_05.00057.00057.2 | 2.7443 | 0.5283 | 100.0% | 2948.5923 | 2949.2637 | 1 | 7.104 | 24.0% | 1 | R.LLDDGFGMDPFPDDLTASWPDWALPR.L | 2 |
| \* | Mis12IP\_Nocodazole\_MudPIT\_040709\_03.00070.00070.3 | 4.5432 | 0.4758 | 100.0% | 2949.5645 | 2949.2637 | 1 | 7.723 | 34.0% | 3 | R.LLDDGFGMDPFPDDLTASWPDWALPR.L | 3 |

---

|  |  |  |  |  |  |  |  |  |
| --- | --- | --- | --- | --- | --- | --- | --- | --- |
| U | *gi|27436929|ref|NP\_00* | 9 | 27 | 13.1% | 641 | 70405 | 6.0 | heat shock 70kDa protein 1-like [Homo sapiens] |
| U | *gi|62898333|dbj|BAD97* | 9 | 27 | 13.1% | 641 | 70404 | 6.3 | heat shock 70kDa protein 1-like variant [Homo sapiens] |
| U | *gi|55961919|emb|CAI18* | 9 | 27 | 13.1% | 641 | 70375 | 6.0 | heat shock 10kDa protein 1-like [Homo sapiens] |
| U | *gi|3461866|dbj|BAA325* | 9 | 27 | 13.1% | 641 | 70437 | 6.0 | Heat shock protein 70 testis variant [Homo sapiens] |

| Filename XCorr DeltCN Conf% ObsM+H+ CalcM+H+ SpR ZScore Ion% # Sequence  | | | | | | | | | | | | |
| --- | --- | --- | --- | --- | --- | --- | --- | --- | --- | --- | --- | --- |
|  | Mis12IP\_Nocodazole\_MudPIT\_040709\_05.05229.05229.1 | 1.9614 | 0.3836 | 97.4% | 1487.44 | 1488.5939 | 1 | 7.022 | 45.8% | 1 | R.TTPSYVAFTDTER.L | 11111 |
|  | Mis12IP\_Nocodazole\_MudPIT\_040709\_06.05909.05909.2 | 3.3634 | 0.4566 | 100.0% | 1488.0721 | 1488.5939 | 1 | 8.603 | 75.0% | 3 | R.TTPSYVAFTDTER.L | 22222 |
|  | Mis12IP\_Nocodazole\_MudPIT\_040709\_02.05319.05319.2 | 2.0012 | 0.2708 | 97.4% | 2043.4521 | 2044.327 | 75 | 4.859 | 35.3% | 1 | K.GENKAFYPEEISSMVLTK.L | 2 |
|  | Mis12IP\_Nocodazole\_MudPIT\_040709\_06.07336.07336.2 | 3.8514 | 0.5353 | 100.0% | 1615.1921 | 1615.8817 | 1 | 8.222 | 80.8% | 2 | K.AFYPEEISSMVLTK.L | 22 |
|  | Mis12IP\_Nocodazole\_MudPIT\_040709\_06.06778.06778.1 | 2.2033 | 0.4122 | 100.0% | 1197.72 | 1198.408 | 11 | 6.354 | 50.0% | 1 | K.DAGVIAGLNVLR.I | 11 |
|  | Mis12IP\_Nocodazole\_MudPIT\_040709\_05.06407.06407.2 | 4.221 | 0.315 | 100.0% | 1199.0721 | 1198.408 | 1 | 6.871 | 90.9% | 2 | K.DAGVIAGLNVLR.I | 22 |
|  | Mis12IP\_Nocodazole\_MudPIT\_040709\_06.06545.06545.2 | 5.2019 | 0.5351 | 100.0% | 1660.3322 | 1660.9078 | 1 | 9.75 | 73.3% | 1 | R.IINEPTAAAIAYGLDK.G | 2222 |
|  | Mis12IP\_Nocodazole\_MudPIT\_040709\_04.05631.05631.2 | 2.8556 | 0.3643 | 99.9% | 1676.4521 | 1676.6964 | 4 | 6.052 | 40.0% | 2 | K.ATAGDTHLGGEDFDNR.L | 222 |
|  | Mis12IP\_Nocodazole\_MudPIT\_040709\_04.05654.05654.2 | 2.3494 | 0.4197 | 100.0% | 1018.2922 | 1018.1582 | 2 | 6.376 | 68.8% | 14 | K.ITITNDKGR.L | 22222 |

Similarities:
gi|5729877|ref|NP\_006(4:5)  
gi|16507237|ref|NP\_00(1:8)  
gi|13676857|ref|NP\_06(4:5)  
gi|12803275|gb|AAH024(7:2)  
gi|34419635|ref|NP\_00(4:5)  

---

|  |  |  |  |  |  |  |  |  |
| --- | --- | --- | --- | --- | --- | --- | --- | --- |
| U | *gi|48146953|emb|CAG33* | 3 | 5 | 13.1% | 191 | 22167 | 6.2 | CBX5 [Homo sapiens] |
| U | *gi|6912292|ref|NP\_036* | 3 | 5 | 13.1% | 191 | 22225 | 5.9 | chromobox homolog 5 (HP1 alpha homolog, Drosophila) [Homo sapiens] |

| Filename XCorr DeltCN Conf% ObsM+H+ CalcM+H+ SpR ZScore Ion% # Sequence  | | | | | | | | | | | | |
| --- | --- | --- | --- | --- | --- | --- | --- | --- | --- | --- | --- | --- |
|  | Mis12IP\_Nocodazole\_MudPIT\_040709\_02.04002.04002.2 | 2.7299 | 0.3093 | 99.9% | 1198.2922 | 1198.2316 | 1 | 6.511 | 80.0% | 1 | K.SNFSNSADDIK.S | 2 |
|  | Mis12IP\_Nocodazole\_MudPIT\_040709\_03.04285.04285.2 | 3.3632 | 0.3621 | 100.0% | 1412.5122 | 1413.4839 | 1 | 7.259 | 66.7% | 3 | K.SNFSNSADDIKSK.K | 2 |
|  | Mis12IP\_Nocodazole\_MudPIT\_040709\_02.07848.07848.2 | 2.9518 | 0.2115 | 99.5% | 1526.3322 | 1525.7106 | 1 | 4.874 | 72.7% | 1 | K.CPQIVIAFYEER.L | 22 |

Similarities:
gi|15082258|ref|NP\_00(1:2)  

---

|  |  |  |  |  |  |  |  |  |
| --- | --- | --- | --- | --- | --- | --- | --- | --- |
| U | *gi|42542645|gb|AAH663* | 2 | 3 | 13.0% | 184 | 21416 | 10.2 | Ribosomal protein L17 [Homo sapiens] |
| U | *gi|89038350|ref|XP\_93* | 2 | 3 | 13.0% | 184 | 21313 | 10.1 | PREDICTED: similar to 60S ribosomal protein L17 (L23) isoform 1 [Homo sapiens] |
| U | *gi|88943956|ref|XP\_93* | 2 | 3 | 13.0% | 184 | 21455 | 10.2 | PREDICTED: similar to 60S ribosomal protein L17 (L23) isoform 5 [Homo sapiens] |
| U | *gi|88942751|ref|XP\_93* | 2 | 3 | 13.0% | 184 | 21399 | 10.1 | PREDICTED: similar to 60S ribosomal protein L17 (L23) isoform 1 [Homo sapiens] |
| U | *gi|4506617|ref|NP\_000* | 2 | 3 | 13.0% | 184 | 21397 | 10.2 | ribosomal protein L17 [Homo sapiens] |

| Filename XCorr DeltCN Conf% ObsM+H+ CalcM+H+ SpR ZScore Ion% # Sequence  | | | | | | | | | | | | |
| --- | --- | --- | --- | --- | --- | --- | --- | --- | --- | --- | --- | --- |
|  | Mis12IP\_Nocodazole\_MudPIT\_040709\_03.04638.04638.2 | 2.9756 | 0.238 | 99.8% | 1165.1122 | 1164.2572 | 1 | 5.425 | 66.7% | 2 | R.YSLDPENPTK.S | 2 |
|  | Mis12IP\_Nocodazole\_MudPIT\_040709\_06.05477.05477.2 | 3.2053 | 0.3158 | 99.9% | 1624.2722 | 1624.8314 | 1 | 6.024 | 69.2% | 1 | K.EQIVPKPEEEVAQK.K | 2 |

---

|  |  |  |  |  |  |  |  |  |
| --- | --- | --- | --- | --- | --- | --- | --- | --- |
| U | *gi|47682782|gb|AAH702* | 3 | 4 | 12.8% | 266 | 30532 | 9.6 | RNA binding motif protein 7 [Homo sapiens] |
| U | *gi|9994185|ref|NP\_057* | 3 | 4 | 12.8% | 266 | 30503 | 9.6 | RNA binding motif protein 7 [Homo sapiens] |
| U | *gi|7023641|dbj|BAA920* | 3 | 4 | 12.7% | 267 | 30617 | 9.5 | unnamed protein product [Homo sapiens] |

| Filename XCorr DeltCN Conf% ObsM+H+ CalcM+H+ SpR ZScore Ion% # Sequence  | | | | | | | | | | | | |
| --- | --- | --- | --- | --- | --- | --- | --- | --- | --- | --- | --- | --- |
|  | Mis12IP\_Nocodazole\_MudPIT\_040709\_06.05716.05716.2 | 4.8507 | 0.4526 | 100.0% | 1596.2322 | 1596.814 | 1 | 7.657 | 76.9% | 2 | R.TMDNMTSSAQIIQR.S | 2 |
|  | Mis12IP\_Nocodazole\_MudPIT\_040709\_02.04206.04206.2 | 2.0291 | 0.2041 | 96.5% | 1279.1721 | 1279.2651 | 1 | 4.589 | 66.7% | 1 | R.SFSS\*PENFQR.Q | 2 |
|  | Mis12IP\_Nocodazole\_MudPIT\_040709\_05.05105.05105.2 | 2.3921 | 0.3177 | 99.8% | 1229.7722 | 1230.3807 | 382 | 5.812 | 50.0% | 1 | R.MNSYPYLADR.H | 2 |

---

|  |  |  |  |  |  |  |  |  |
| --- | --- | --- | --- | --- | --- | --- | --- | --- |
| U | *gi|109389358|ref|NP\_0* | 7 | 11 | 12.7% | 433 | 47440 | 8.4 | transcription factor AP-2 alpha isoform c [Homo sapiens] |
| U | *gi|73760407|ref|NP\_00* | 7 | 11 | 12.8% | 431 | 47183 | 8.4 | transcription factor AP-2 alpha isoform b [Homo sapiens] |
| U | *gi|4507441|ref|NP\_003* | 7 | 11 | 12.6% | 437 | 48062 | 8.0 | transcription factor AP-2 alpha isoform a [Homo sapiens] |
| U | *gi|14031075|emb|CAC38* | 7 | 11 | 12.5% | 439 | 48321 | 8.2 | TFAP2A [Homo sapiens] |

| Filename XCorr DeltCN Conf% ObsM+H+ CalcM+H+ SpR ZScore Ion% # Sequence  | | | | | | | | | | | | |
| --- | --- | --- | --- | --- | --- | --- | --- | --- | --- | --- | --- | --- |
|  | Mis12IP\_Nocodazole\_MudPIT\_040709\_06.05603.05603.2 | 3.0663 | 0.3457 | 100.0% | 1316.4521 | 1315.4691 | 2 | 6.559 | 66.7% | 1 | K.SNSNAVSAIPINK.D | 2 |
|  | Mis12IP\_Nocodazole\_MudPIT\_040709\_06.05861.05861.1 | 1.9361 | 0.3268 | 98.5% | 935.43 | 936.0935 | 7 | 5.947 | 62.5% | 2 | R.LSLLSSTSK.Y | 1 |
|  | Mis12IP\_Nocodazole\_MudPIT\_040709\_06.05867.05867.2 | 2.2456 | 0.1507 | 96.5% | 935.77216 | 936.0935 | 1 | 5.081 | 75.0% | 1 | R.LSLLSSTSK.Y | 2 |
|  | Mis12IP\_Nocodazole\_MudPIT\_040709\_03.04078.04078.2 | 1.9956 | 0.2746 | 99.2% | 901.4922 | 902.03845 | 72 | 5.632 | 71.4% | 1 | K.VTVAEVQR.R | 2 |
|  | Mis12IP\_Nocodazole\_MudPIT\_040709\_06.05870.05870.2 | 2.3906 | 0.2936 | 99.8% | 919.2922 | 920.05615 | 2 | 6.883 | 78.6% | 1 | K.AVAEFLNR.Q | 2 |
|  | Mis12IP\_Nocodazole\_MudPIT\_040709\_05.04971.04971.1 | 2.4751 | 0.2217 | 98.4% | 790.49 | 790.99646 | 1 | 5.213 | 83.3% | 3 | K.NMLLATK.Q | 1 |
|  | Mis12IP\_Nocodazole\_MudPIT\_040709\_06.06416.06416.2 | 2.6752 | 0.3752 | 100.0% | 1208.2722 | 1208.3135 | 29 | 6.276 | 61.1% | 2 | K.EFTDLLAQDR.S | 2 |

---

|  |  |  |  |  |  |  |  |  |
| --- | --- | --- | --- | --- | --- | --- | --- | --- |
| U | *gi|13448009|gb|AAK268* | 2 | 2 | 12.5% | 88 | 9748 | 9.1 | immunoglobulin kappa light chain variable region [Homo sapiens] |
| U | *gi|77379536|gb|ABA714* | 2 | 2 | 10.7% | 103 | 11301 | 9.2 | immunoglobulin kappa chain variable region [Homo sapiens] |
| U | *gi|77378154|gb|ABA708* | 2 | 2 | 9.4% | 117 | 12634 | 8.9 | immunoglobulin kappa light chain variable region [Homo sapiens] |
| U | *gi|77378148|gb|ABA707* | 2 | 2 | 9.3% | 118 | 12718 | 8.3 | immunoglobulin kappa light chain variable region [Homo sapiens] |
| U | *gi|77378142|gb|ABA707* | 2 | 2 | 9.3% | 118 | 12898 | 8.7 | immunoglobulin kappa light chain variable region [Homo sapiens] |
| U | *gi|77378138|gb|ABA707* | 2 | 2 | 9.3% | 118 | 12726 | 9.2 | immunoglobulin kappa light chain variable region [Homo sapiens] |
| U | *gi|70798799|gb|AAZ091* | 2 | 2 | 10.6% | 104 | 11478 | 9.2 | immunoglobulin kappa light chain variable region [Homo sapiens] |
| U | *gi|58222682|gb|AAW689* | 2 | 2 | 10.3% | 107 | 11544 | 8.9 | anti-tetanus toxoid immunoglobulin light chain variable region [Homo sapiens] |
| U | *gi|51103403|gb|AAT964* | 2 | 2 | 10.3% | 107 | 11751 | 8.4 | immunoglobulin variable region VL kappa domain [Homo sapiens] |
| U | *gi|51103401|gb|AAT964* | 2 | 2 | 10.3% | 107 | 11790 | 8.5 | immunoglobulin variable region VL kappa domain [Homo sapiens] |
| U | *gi|51103399|gb|AAT964* | 2 | 2 | 10.3% | 107 | 11790 | 8.0 | immunoglobulin variable region VL kappa domain [Homo sapiens] |
| U | *gi|4378226|gb|AAD1944* | 2 | 2 | 10.4% | 106 | 11411 | 8.5 | immunoglobulin kappa light chain variable region [Homo sapiens] |
| U | *gi|415960|emb|CAA8169* | 2 | 2 | 9.4% | 117 | 12950 | 9.1 | IG light chain variable region (VJ) [Homo sapiens] |
| U | *gi|33570607|gb|AAQ221* | 2 | 2 | 12.5% | 88 | 9664 | 8.0 | immunoglobulin kappa chain variable region [Homo sapiens] |
| U | *gi|2654052|gb|AAB8797* | 2 | 2 | 11.0% | 100 | 10980 | 9.2 | variable immunoglobulin anti-HLA kappa light chain [Homo sapiens] |
| U | *gi|15722903|emb|CAC79* | 2 | 2 | 12.8% | 86 | 9453 | 6.3 | immunoglobulin kappa chain variable region [Homo sapiens] |

| Filename XCorr DeltCN Conf% ObsM+H+ CalcM+H+ SpR ZScore Ion% # Sequence  | | | | | | | | | | | | |
| --- | --- | --- | --- | --- | --- | --- | --- | --- | --- | --- | --- | --- |
|  | Mis12IP\_Nocodazole\_MudPIT\_040709\_03.04359.04359.2 | 2.5661 | 0.2409 | 99.3% | 1103.4122 | 1104.2052 | 2 | 6.546 | 80.0% | 1 | K.ASTLESGVPSR.F | 2 |
|  | Mis12IP\_Nocodazole\_MudPIT\_040709\_03.04369.04369.1 | 1.7755 | 0.3135 | 98.9% | 1103.68 | 1104.2052 | 325 | 5.443 | 40.0% | 1 | K.ASTLESGVPSR.F | 1 |

---

|  |  |  |  |  |  |  |  |  |
| --- | --- | --- | --- | --- | --- | --- | --- | --- |
| U | *gi|4502491|ref|NP\_001* | 2 | 2 | 12.4% | 282 | 31362 | 4.8 | complement component 1, q subcomponent binding protein precursor [Homo sapiens] |
| U | *gi|8699626|gb|AAF7876* | 2 | 2 | 17.8% | 197 | 22437 | 4.4 | hyaluronan-binding protein precursor [Homo sapiens] |

| Filename XCorr DeltCN Conf% ObsM+H+ CalcM+H+ SpR ZScore Ion% # Sequence  | | | | | | | | | | | | |
| --- | --- | --- | --- | --- | --- | --- | --- | --- | --- | --- | --- | --- |
|  | Mis12IP\_Nocodazole\_MudPIT\_040709\_06.06485.06485.2 | 4.1005 | 0.4367 | 100.0% | 1622.0521 | 1622.79 | 1 | 7.585 | 71.4% | 1 | K.MSGGWELELNGTEAK.L | 2 |
|  | Mis12IP\_Nocodazole\_MudPIT\_040709\_05.07151.07151.2 | 1.6666 | 0.2926 | 95.8% | 2287.3123 | 2288.5566 | 20 | 5.067 | 26.3% | 1 | K.VEEQEPELTSTPNFVVEVIK.N | 2 |

---

|  |  |  |  |  |  |  |  |  |
| --- | --- | --- | --- | --- | --- | --- | --- | --- |
| U | *gi|113427343|ref|XP\_0* | 2 | 2 | 12.3% | 138 | 15759 | 11.0 | PREDICTED: similar to ribosomal protein L34 [Homo sapiens] |
| U | *gi|16117787|ref|NP\_00* | 2 | 2 | 14.5% | 117 | 13293 | 11.5 | ribosomal protein L34 [Homo sapiens] |

| Filename XCorr DeltCN Conf% ObsM+H+ CalcM+H+ SpR ZScore Ion% # Sequence  | | | | | | | | | | | | |
| --- | --- | --- | --- | --- | --- | --- | --- | --- | --- | --- | --- | --- |
|  | Mis12IP\_Nocodazole\_MudPIT\_040709\_06.05843.05843.1 | 1.6874 | 0.2067 | 97.1% | 899.5 | 900.1057 | 5 | 5.136 | 66.7% | 1 | R.IVYLYTK.K | 11 |
|  | Mis12IP\_Nocodazole\_MudPIT\_040709\_06.04735.04735.2 | 2.299 | 0.3214 | 99.7% | 1100.9722 | 1101.2914 | 1 | 5.285 | 55.6% | 1 | K.VLKAQAQSQK.T | 2 |

Similarities:
gi|4432756|dbj|BAA258(1:1)  

---

|  |  |  |  |  |  |  |  |  |
| --- | --- | --- | --- | --- | --- | --- | --- | --- |
| U | *gi|30409766|gb|AAO625* | 7 | 9 | 12.1% | 556 | 63293 | 5.9 | kinesin light chain 1C [Homo sapiens] |
| U | *gi|8101107|gb|AAF7254* | 7 | 9 | 11.8% | 569 | 64786 | 6.3 | kinesin light-chain protein [Homo sapiens] |
| U | *gi|33620730|ref|NP\_00* | 7 | 9 | 12.0% | 560 | 63816 | 5.9 | kinesin light chain 1 isoform 1 [Homo sapiens] |
| U | *gi|33186846|tpg|DAA01* | 7 | 9 | 12.0% | 560 | 63796 | 6.4 | TPA: TPA\_exp: kinesin light chain 1G [Homo sapiens] |
| U | *gi|33186844|tpg|DAA01* | 7 | 9 | 11.6% | 580 | 65768 | 6.0 | TPA: TPA\_exp: kinesin light chain 1P [Homo sapiens] |
| U | *gi|33186838|tpg|DAA01* | 7 | 9 | 12.2% | 547 | 62302 | 6.0 | TPA: TPA\_exp: kinesin light chain 1S [Homo sapiens] |
| U | *gi|32452915|tpg|DAA01* | 7 | 9 | 10.7% | 626 | 70885 | 7.7 | TPA: TPA\_exp: kinesin light chain 1M [Homo sapiens] |
| U | *gi|32452911|tpg|DAA01* | 7 | 9 | 10.6% | 635 | 71874 | 7.7 | TPA: TPA\_exp: kinesin light chain 1I [Homo sapiens] |
| U | *gi|32452909|tpg|DAA01* | 7 | 9 | 12.2% | 547 | 62192 | 5.7 | TPA: TPA\_exp: kinesin light chain 1F [Homo sapiens] |
| U | *gi|32452907|tpg|DAA01* | 7 | 9 | 11.0% | 607 | 68964 | 7.3 | TPA: TPA\_exp: kinesin light chain 1O [Homo sapiens] |

| Filename XCorr DeltCN Conf% ObsM+H+ CalcM+H+ SpR ZScore Ion% # Sequence  | | | | | | | | | | | | |
| --- | --- | --- | --- | --- | --- | --- | --- | --- | --- | --- | --- | --- |
|  | Mis12IP\_Nocodazole\_MudPIT\_040709\_06.07355.07355.2 | 2.9558 | 0.2878 | 99.9% | 1370.0122 | 1370.5486 | 2 | 6.6 | 66.7% | 1 | K.DAANLLNDALAIR.E | 2 |
|  | Mis12IP\_Nocodazole\_MudPIT\_040709\_04.05520.05520.2 | 2.3217 | 0.1869 | 98.9% | 965.5122 | 966.1222 | 3 | 5.923 | 78.6% | 1 | R.ALEIYQTK.L | 2 |
|  | Mis12IP\_Nocodazole\_MudPIT\_040709\_04.05518.05518.1 | 1.9147 | 0.2322 | 99.2% | 965.61 | 966.1222 | 16 | 4.728 | 64.3% | 1 | R.ALEIYQTK.L | 1 |
|  | Mis12IP\_Nocodazole\_MudPIT\_040709\_05.05835.05835.2 | 3.5822 | 0.5813 | 100.0% | 1466.7122 | 1467.534 | 1 | 8.787 | 66.7% | 2 | K.DGTSFGEYGGWYK.A | 2 |
|  | Mis12IP\_Nocodazole\_MudPIT\_040709\_06.05717.05717.1 | 2.0378 | 0.1808 | 98.6% | 806.53 | 806.9401 | 100 | 4.406 | 58.3% | 1 | K.NLGALYR.R | 1 |
|  | Mis12IP\_Nocodazole\_MudPIT\_040709\_06.06376.06376.2 | 4.366 | 0.5823 | 100.0% | 1468.2122 | 1468.6215 | 1 | 9.948 | 83.3% | 1 | K.FEAAETLEEAAMR.S | 2 |
|  | Mis12IP\_Nocodazole\_MudPIT\_040709\_03.04649.04649.2 | 4.2872 | 0.4239 | 100.0% | 1488.1322 | 1488.6526 | 1 | 7.324 | 70.8% | 2 | R.VAEVLNDPENMEK.R | 2 |

---

|  |  |  |  |  |  |  |  |  |
| --- | --- | --- | --- | --- | --- | --- | --- | --- |
| U | *gi|18999435|gb|AAH242* | 8 | 12 | 12.0% | 590 | 62378 | 7.8 | Keratin 5 (epidermolysis bullosa simplex, Dowling-Meara/Kobner/Weber-Cockayne types) [Homo sapiens] |
| U | *gi|4557890|ref|NP\_000* | 8 | 12 | 12.0% | 590 | 62461 | 8.1 | keratin 5 [Homo sapiens] |

| Filename XCorr DeltCN Conf% ObsM+H+ CalcM+H+ SpR ZScore Ion% # Sequence  | | | | | | | | | | | | |
| --- | --- | --- | --- | --- | --- | --- | --- | --- | --- | --- | --- | --- |
|  | Mis12IP\_Nocodazole\_MudPIT\_040709\_06.05746.05746.2 | 3.633 | 0.3642 | 100.0% | 1411.3922 | 1411.5547 | 1 | 7.128 | 65.4% | 1 | R.SFSTASAITPSVSR.T | 2 |
|  | Mis12IP\_Nocodazole\_MudPIT\_040709\_03.04449.04449.2 | 1.6807 | 0.2972 | 97.7% | 885.09216 | 884.9646 | 3 | 5.505 | 71.4% | 1 | R.TSFTSVSR.S | 2 |
|  | Mis12IP\_Nocodazole\_MudPIT\_040709\_06.06226.06226.2 | 3.2189 | 0.3799 | 100.0% | 1204.2322 | 1204.3684 | 1 | 6.933 | 83.3% | 1 | K.WTLLQEQGTK.T | 2222222 |
|  | Mis12IP\_Nocodazole\_MudPIT\_040709\_05.04381.04381.2 | 2.3872 | 0.1809 | 99.0% | 945.6722 | 946.0513 | 10 | 5.236 | 78.6% | 2 | R.GRLDSELR.N | 22222222 |
|  | Mis12IP\_Nocodazole\_MudPIT\_040709\_06.07734.07734.1 | 2.6237 | 0.3674 | 100.0% | 1329.6 | 1330.5211 | 1 | 7.145 | 68.2% | 3 | R.NLDLDSIIAEVK.A | 111111111 |
|  | Mis12IP\_Nocodazole\_MudPIT\_040709\_06.07727.07727.2 | 4.4229 | 0.3694 | 100.0% | 1331.0122 | 1330.5211 | 1 | 7.672 | 81.8% | 2 | R.NLDLDSIIAEVK.A | 222222222 |
|  | Mis12IP\_Nocodazole\_MudPIT\_040709\_02.03990.03990.2 | 2.6853 | 0.3328 | 99.9% | 1094.0322 | 1094.1692 | 1 | 6.545 | 81.2% | 1 | K.AQYEEIANR.S | 2 |
|  | Mis12IP\_Nocodazole\_MudPIT\_040709\_02.03936.03936.2 | 3.2019 | 0.3678 | 100.0% | 1194.4922 | 1195.2743 | 1 | 7.616 | 88.9% | 1 | K.YEELQQTAGR.H | 2 |

Similarities:
gi|181402|gb|AAC83410(2:6)  
gi|27465517|ref|NP\_77(4:4)  
gi|46812692|gb|AAH692(4:4)  
gi|21961227|gb|AAH345(4:4)  
gi|17505189|ref|NP\_49(4:4)  
gi|15559584|gb|AAH141(4:4)  
gi|5031841|ref|NP\_005(4:4)  
gi|32567786|ref|NP\_78(3:5)  

---

|  |  |  |  |  |  |  |  |  |
| --- | --- | --- | --- | --- | --- | --- | --- | --- |
| U | *gi|16876910|gb|AAH167* | 3 | 4 | 12.0% | 415 | 45700 | 5.6 | HNRPF protein [Homo sapiens] |
| U | *gi|4826760|ref|NP\_004* | 3 | 4 | 12.0% | 415 | 45672 | 5.6 | heterogeneous nuclear ribonucleoprotein F [Homo sapiens] |

| Filename XCorr DeltCN Conf% ObsM+H+ CalcM+H+ SpR ZScore Ion% # Sequence  | | | | | | | | | | | | |
| --- | --- | --- | --- | --- | --- | --- | --- | --- | --- | --- | --- | --- |
|  | Mis12IP\_Nocodazole\_MudPIT\_040709\_03.05153.05153.2 | 2.8961 | 0.4923 | 100.0% | 1709.9521 | 1710.7919 | 1 | 7.96 | 56.7% | 2 | R.QSGEAFVELGSEDDVK.M | 2 |
|  | Mis12IP\_Nocodazole\_MudPIT\_040709\_06.07680.07680.2 | 4.4587 | 0.3644 | 100.0% | 1868.3121 | 1869.0813 | 1 | 7.124 | 68.8% | 1 | K.ITGEAFVQFASQELAEK.A | 2 |
|  | Mis12IP\_Nocodazole\_MudPIT\_040709\_06.08107.08107.2 | 4.5763 | 0.489 | 100.0% | 1997.3722 | 1998.2023 | 1 | 7.96 | 62.5% | 1 | K.ATENDIYNFFSPLNPVR.V | 22 |

Similarities:
gi|48145673|emb|CAG33(1:2)  

---

|  |  |  |  |  |  |  |  |  |
| --- | --- | --- | --- | --- | --- | --- | --- | --- |
| U | *gi|4929561|gb|AAD3404* | 5 | 5 | 11.8% | 442 | 48291 | 6.7 | CGI-46 protein [Homo sapiens] |
| U | *gi|5730023|ref|NP\_006* | 5 | 5 | 11.2% | 463 | 51157 | 5.6 | RuvB-like 2 [Homo sapiens] |

| Filename XCorr DeltCN Conf% ObsM+H+ CalcM+H+ SpR ZScore Ion% # Sequence  | | | | | | | | | | | | |
| --- | --- | --- | --- | --- | --- | --- | --- | --- | --- | --- | --- | --- |
|  | Mis12IP\_Nocodazole\_MudPIT\_040709\_05.05644.05644.2 | 3.0343 | 0.2023 | 99.6% | 1155.2922 | 1156.281 | 1 | 5.843 | 80.0% | 1 | R.GLGLDDALEPR.Q | 2 |
|  | Mis12IP\_Nocodazole\_MudPIT\_040709\_06.05582.05582.1 | 1.7092 | 0.4947 | 100.0% | 1111.58 | 1112.3146 | 2 | 6.65 | 40.9% | 1 | R.AVLIAGQPGTGK.T | 1 |
|  | Mis12IP\_Nocodazole\_MudPIT\_040709\_06.05574.05574.2 | 3.5552 | 0.4755 | 100.0% | 1112.2322 | 1112.3146 | 1 | 8.242 | 77.3% | 1 | R.AVLIAGQPGTGK.T | 2 |
|  | Mis12IP\_Nocodazole\_MudPIT\_040709\_05.05897.05897.2 | 4.1451 | 0.5394 | 100.0% | 1502.3121 | 1502.6765 | 1 | 9.219 | 70.8% | 1 | K.TTEMETIYDLGTK.M | 2 |
|  | Mis12IP\_Nocodazole\_MudPIT\_040709\_06.07240.07240.2 | 3.1965 | 0.4022 | 100.0% | 1731.9521 | 1733.051 | 1 | 8.257 | 60.0% | 1 | R.ALESDMAPVLIMATNR.G | 2 |

---

|  |  |  |  |  |  |  |  |  |
| --- | --- | --- | --- | --- | --- | --- | --- | --- |
| U | *gi|12054070|emb|CAC20* | 3 | 3 | 11.6% | 353 | 37647 | 6.5 | immunoglobulin heavy chain constant region alpha 1 [Homo sapiens] |
| U | *gi|34535864|dbj|BAC87* | 3 | 3 | 8.3% | 492 | 52666 | 6.8 | unnamed protein product [Homo sapiens] |
| U | *gi|34535045|dbj|BAC87* | 3 | 3 | 8.2% | 500 | 53992 | 7.5 | unnamed protein product [Homo sapiens] |
| U | *gi|34533060|dbj|BAC86* | 3 | 3 | 8.3% | 496 | 53158 | 6.1 | unnamed protein product [Homo sapiens] |
| U | *gi|34532724|dbj|BAC86* | 3 | 3 | 8.2% | 497 | 53905 | 7.0 | unnamed protein product [Homo sapiens] |
| U | *gi|34531264|dbj|BAC86* | 3 | 3 | 8.3% | 496 | 53752 | 5.8 | unnamed protein product [Homo sapiens] |
| U | *gi|34527351|dbj|BAC85* | 3 | 3 | 8.1% | 508 | 55186 | 6.9 | unnamed protein product [Homo sapiens] |
| U | *gi|34527290|dbj|BAC85* | 3 | 3 | 8.2% | 499 | 53265 | 7.0 | unnamed protein product [Homo sapiens] |
| U | *gi|34527280|dbj|BAC85* | 3 | 3 | 8.1% | 504 | 54743 | 6.6 | unnamed protein product [Homo sapiens] |
| U | *gi|34527275|dbj|BAC85* | 3 | 3 | 8.2% | 501 | 53787 | 7.7 | unnamed protein product [Homo sapiens] |
| U | *gi|34527259|dbj|BAC85* | 3 | 3 | 8.2% | 502 | 53818 | 6.0 | unnamed protein product [Homo sapiens] |
| U | *gi|34527233|dbj|BAC85* | 3 | 3 | 8.3% | 495 | 53134 | 6.5 | unnamed protein product [Homo sapiens] |
| U | *gi|34526199|dbj|BAC85* | 3 | 3 | 8.3% | 494 | 53389 | 7.6 | unnamed protein product [Homo sapiens] |
| U | *gi|34526163|dbj|BAC85* | 3 | 3 | 8.0% | 512 | 55537 | 7.3 | unnamed protein product [Homo sapiens] |
| U | *gi|22760231|dbj|BAC11* | 3 | 3 | 8.3% | 493 | 53224 | 6.5 | unnamed protein product [Homo sapiens] |
| U | *gi|16554028|dbj|BAB71* | 3 | 3 | 8.3% | 496 | 53533 | 6.7 | unnamed protein product [Homo sapiens] |
| U | *gi|14042015|dbj|BAB55* | 3 | 3 | 8.3% | 494 | 53088 | 6.9 | unnamed protein product [Homo sapiens] |

| Filename XCorr DeltCN Conf% ObsM+H+ CalcM+H+ SpR ZScore Ion% # Sequence  | | | | | | | | | | | | |
| --- | --- | --- | --- | --- | --- | --- | --- | --- | --- | --- | --- | --- |
|  | Mis12IP\_Nocodazole\_MudPIT\_040709\_05.05677.05677.2 | 2.9951 | 0.4467 | 100.0% | 1540.9521 | 1541.6573 | 1 | 7.681 | 50.0% | 1 | R.DASGVTFTWTPSSGK.S | 2 |
|  | Mis12IP\_Nocodazole\_MudPIT\_040709\_06.05629.05629.1 | 1.6709 | 0.2217 | 96.1% | 931.44 | 932.1051 | 18 | 5.05 | 50.0% | 1 | K.TPLTATLSK.S | 1 |
|  | Mis12IP\_Nocodazole\_MudPIT\_040709\_06.06817.06817.2 | 2.8573 | 0.5375 | 100.0% | 1836.4321 | 1837.0398 | 2 | 8.108 | 43.8% | 1 | R.QEPSQGTTTFAVTSILR.V | 2 |

---

|  |  |  |  |  |  |  |  |  |
| --- | --- | --- | --- | --- | --- | --- | --- | --- |
| U | *gi|21618338|ref|NP\_00* | 6 | 10 | 11.4% | 769 | 87981 | 6.3 | signal transducer and activator of transcription 3 isoform 2 [Homo sapiens] |
| U | *gi|47458820|ref|NP\_99* | 6 | 10 | 12.2% | 722 | 83126 | 7.1 | signal transducer and activator of transcription 3 isoform 3 [Homo sapiens] |
| U | *gi|21618340|ref|NP\_64* | 6 | 10 | 11.4% | 770 | 88068 | 6.3 | signal transducer and activator of transcription 3 isoform 1 [Homo sapiens] |

| Filename XCorr DeltCN Conf% ObsM+H+ CalcM+H+ SpR ZScore Ion% # Sequence  | | | | | | | | | | | | |
| --- | --- | --- | --- | --- | --- | --- | --- | --- | --- | --- | --- | --- |
|  | Mis12IP\_Nocodazole\_MudPIT\_040709\_06.08008.08008.2 | 3.3791 | 0.4561 | 100.0% | 2112.172 | 2112.3489 | 1 | 7.848 | 55.9% | 1 | R.QFLAPWIESQDWAYAASK.E | 2 |
|  | Mis12IP\_Nocodazole\_MudPIT\_040709\_06.06659.06659.2 | 5.1073 | 0.449 | 100.0% | 1746.3121 | 1746.8706 | 1 | 8.331 | 73.1% | 3 | K.VVENLQDDFDFNYK.T | 2 |
|  | Mis12IP\_Nocodazole\_MudPIT\_040709\_02.03989.03989.2 | 4.9815 | 0.4975 | 100.0% | 1864.3121 | 1864.9414 | 1 | 8.975 | 81.2% | 1 | K.SQGDMQDLNGNNQSVTR.Q | 2 |
|  | Mis12IP\_Nocodazole\_MudPIT\_040709\_04.06271.06271.2 | 3.2748 | 0.284 | 99.9% | 1321.9722 | 1321.4265 | 2 | 6.997 | 65.0% | 2 | K.TLTDEELADWK.R | 2 |
|  | Mis12IP\_Nocodazole\_MudPIT\_040709\_06.05968.05968.2 | 5.1644 | 0.5047 | 100.0% | 1887.2122 | 1888.0742 | 1 | 9.276 | 81.2% | 2 | K.VMNMEESNNGSLSAEFK.H | 2 |
|  | Mis12IP\_Nocodazole\_MudPIT\_040709\_04.05372.05372.2 | 3.3666 | 0.3467 | 100.0% | 1294.1122 | 1294.4473 | 1 | 6.126 | 70.0% | 1 | K.TQIQSVEPYTK.Q | 2 |

---

|  |  |  |  |  |  |  |  |  |
| --- | --- | --- | --- | --- | --- | --- | --- | --- |
| U | *gi|38016935|ref|NP\_07* | 4 | 5 | 11.2% | 418 | 47522 | 9.1 | MLF1 interacting protein [Homo sapiens] |
| U | *gi|78394921|gb|AAI077* | 4 | 5 | 18.4% | 256 | 29298 | 9.4 | MLF1IP protein [Homo sapiens] |

| Filename XCorr DeltCN Conf% ObsM+H+ CalcM+H+ SpR ZScore Ion% # Sequence  | | | | | | | | | | | | |
| --- | --- | --- | --- | --- | --- | --- | --- | --- | --- | --- | --- | --- |
|  | Mis12IP\_Nocodazole\_MudPIT\_040709\_04.05316.05316.2 | 2.0566 | 0.2705 | 98.8% | 1213.7922 | 1214.3611 | 2 | 4.944 | 54.5% | 1 | K.TGPLSAQPSVEK.E | 2 |
|  | Mis12IP\_Nocodazole\_MudPIT\_040709\_06.07205.07205.2 | 2.6937 | 0.3848 | 100.0% | 1331.3522 | 1331.5518 | 1 | 6.479 | 70.0% | 1 | K.ELNIVLPEFEK.T | 2 |
|  | Mis12IP\_Nocodazole\_MudPIT\_040709\_06.06388.06388.2 | 3.6938 | 0.3213 | 100.0% | 1246.6322 | 1246.4645 | 4 | 6.247 | 83.3% | 1 | R.MIEVQDELLR.L | 2 |
|  | Mis12IP\_Nocodazole\_MudPIT\_040709\_06.07475.07475.2 | 3.1949 | 0.4368 | 100.0% | 1571.1921 | 1571.7673 | 1 | 6.68 | 57.7% | 2 | K.ETYDSSSLPALLFK.A | 2 |

---

|  |  |  |  |  |  |  |  |  |
| --- | --- | --- | --- | --- | --- | --- | --- | --- |
| U | *gi|16306978|gb|AAH095* | 3 | 3 | 11.2% | 339 | 38618 | 7.8 | Annexin A2 [Homo sapiens] |
| U | *gi|73909156|gb|AAH669* | 3 | 3 | 10.6% | 357 | 40528 | 8.3 | ANXA2 protein [Homo sapiens] |
| U | *gi|50845388|ref|NP\_00* | 3 | 3 | 10.6% | 357 | 40411 | 8.4 | annexin A2 isoform 1 [Homo sapiens] |
| U | *gi|4757756|ref|NP\_004* | 3 | 3 | 11.2% | 339 | 38604 | 7.8 | annexin A2 isoform 2 [Homo sapiens] |
| U | *gi|34364597|emb|CAE45* | 3 | 3 | 10.6% | 357 | 40353 | 8.5 | hypothetical protein [Homo sapiens] |
| U | *gi|18645167|gb|AAH239* | 3 | 3 | 11.2% | 339 | 38576 | 7.8 | Annexin A2 [Homo sapiens] |

| Filename XCorr DeltCN Conf% ObsM+H+ CalcM+H+ SpR ZScore Ion% # Sequence  | | | | | | | | | | | | |
| --- | --- | --- | --- | --- | --- | --- | --- | --- | --- | --- | --- | --- |
|  | Mis12IP\_Nocodazole\_MudPIT\_040709\_06.07740.07740.2 | 3.8127 | 0.4144 | 100.0% | 1543.4922 | 1543.7605 | 1 | 7.137 | 61.5% | 1 | K.GVDEVTIVNILTNR.S | 2 |
|  | Mis12IP\_Nocodazole\_MudPIT\_040709\_06.05830.05830.1 | 1.967 | 0.1645 | 98.2% | 745.37 | 745.9989 | 11 | 4.79 | 75.0% | 1 | K.LMVALAK.G | 1 |
|  | Mis12IP\_Nocodazole\_MudPIT\_040709\_04.06575.06575.2 | 3.5605 | 0.4096 | 100.0% | 1908.9922 | 1910.0013 | 1 | 7.935 | 50.0% | 1 | R.AEDGSVIDYELIDQDAR.D | 2 |

---

|  |  |  |  |  |  |  |  |  |
| --- | --- | --- | --- | --- | --- | --- | --- | --- |
| U | *gi|48146259|emb|CAG33* | 4 | 4 | 11.0% | 535 | 57460 | 6.4 | CCT2 [Homo sapiens] |
| U | *gi|5453603|ref|NP\_006* | 4 | 4 | 11.0% | 535 | 57488 | 6.4 | chaperonin containing TCP1, subunit 2 [Homo sapiens] |

| Filename XCorr DeltCN Conf% ObsM+H+ CalcM+H+ SpR ZScore Ion% # Sequence  | | | | | | | | | | | | |
| --- | --- | --- | --- | --- | --- | --- | --- | --- | --- | --- | --- | --- |
|  | Mis12IP\_Nocodazole\_MudPIT\_040709\_04.06372.06372.2 | 2.8786 | 0.4891 | 100.0% | 1548.7922 | 1549.7797 | 1 | 7.668 | 53.6% | 1 | R.DASLMVTNDGATILK.N | 2 |
|  | Mis12IP\_Nocodazole\_MudPIT\_040709\_06.08335.08335.2 | 3.0099 | 0.4605 | 100.0% | 2289.5723 | 2289.5017 | 1 | 7.352 | 33.3% | 1 | R.VQDDEVGDGTTSVTVLAAELLR.E | 2 |
|  | Mis12IP\_Nocodazole\_MudPIT\_040709\_06.07702.07702.3 | 3.4837 | 0.4058 | 100.0% | 2347.7644 | 2348.6765 | 1 | 6.292 | 33.3% | 1 | R.MLPTIIADNAGYDSADLVAQLR.A | 3 |
|  | Mis12IP\_Nocodazole\_MudPIT\_040709\_06.07697.07697.2 | 3.2943 | 0.4823 | 100.0% | 2347.7922 | 2348.6765 | 1 | 8.355 | 47.6% | 1 | R.MLPTIIADNAGYDSADLVAQLR.A | 2 |

---

|  |  |  |  |  |  |  |  |  |
| --- | --- | --- | --- | --- | --- | --- | --- | --- |
| U | *gi|62899065|ref|NP\_06* | 6 | 10 | 10.8% | 647 | 71718 | 6.5 | centrosomal protein 72 kDa [Homo sapiens] |
| U | *gi|7959305|dbj|BAA960* | 6 | 10 | 10.8% | 648 | 71802 | 6.5 | KIAA1519 protein [Homo sapiens] |

| Filename XCorr DeltCN Conf% ObsM+H+ CalcM+H+ SpR ZScore Ion% # Sequence  | | | | | | | | | | | | |
| --- | --- | --- | --- | --- | --- | --- | --- | --- | --- | --- | --- | --- |
|  | Mis12IP\_Nocodazole\_MudPIT\_040709\_03.04751.04751.1 | 1.4792 | 0.2509 | 96.3% | 1015.42 | 1016.1827 | 25 | 5.034 | 56.2% | 1 | R.LVLSEEAVR.A | 1 |
|  | Mis12IP\_Nocodazole\_MudPIT\_040709\_03.04747.04747.2 | 2.8608 | 0.3087 | 99.9% | 1016.15216 | 1016.1827 | 1 | 5.622 | 87.5% | 2 | R.LVLSEEAVR.A | 2 |
|  | Mis12IP\_Nocodazole\_MudPIT\_040709\_05.06651.06651.2 | 2.2624 | 0.3631 | 99.7% | 1892.4521 | 1893.101 | 1 | 5.695 | 43.8% | 2 | R.DLAELQSLSIPGTYQEK.I | 2 |
|  | Mis12IP\_Nocodazole\_MudPIT\_040709\_06.05440.05440.2 | 2.7003 | 0.2414 | 99.7% | 1240.1522 | 1240.4049 | 1 | 4.788 | 72.2% | 1 | K.LQQLDDRPVR.A | 2 |
|  | Mis12IP\_Nocodazole\_MudPIT\_040709\_02.03812.03812.2 | 3.5726 | 0.3653 | 100.0% | 1606.2322 | 1605.6555 | 1 | 5.735 | 65.4% | 3 | R.TLSQPEASETEEQR.S | 2 |
|  | Mis12IP\_Nocodazole\_MudPIT\_040709\_06.06798.06798.2 | 4.0381 | 0.4105 | 100.0% | 2001.9521 | 2003.2603 | 1 | 8.271 | 50.0% | 1 | K.SADTAATLNLQIAGLQTSVK.R | 2 |

---

|  |  |  |  |  |  |  |  |  |
| --- | --- | --- | --- | --- | --- | --- | --- | --- |
| U | *gi|34419635|ref|NP\_00* | 6 | 22 | 10.7% | 643 | 71028 | 6.1 | heat shock 70kDa protein 6 (HSP70B') [Homo sapiens] |
| U | *gi|62898285|dbj|BAD97* | 6 | 22 | 10.7% | 643 | 71004 | 6.1 | heat shock 70kDa protein 6 (HSP70B') variant [Homo sapiens] |
| U | *gi|35222|emb|CAA36061* | 6 | 22 | 10.7% | 643 | 70854 | 5.9 | unnamed protein product [Homo sapiens] |

| Filename XCorr DeltCN Conf% ObsM+H+ CalcM+H+ SpR ZScore Ion% # Sequence  | | | | | | | | | | | | |
| --- | --- | --- | --- | --- | --- | --- | --- | --- | --- | --- | --- | --- |
|  | Mis12IP\_Nocodazole\_MudPIT\_040709\_05.05229.05229.1 | 1.9614 | 0.3836 | 97.4% | 1487.44 | 1488.5939 | 1 | 7.022 | 45.8% | 1 | R.TTPSYVAFTDTER.L | 11111 |
|  | Mis12IP\_Nocodazole\_MudPIT\_040709\_06.05909.05909.2 | 3.3634 | 0.4566 | 100.0% | 1488.0721 | 1488.5939 | 1 | 8.603 | 75.0% | 3 | R.TTPSYVAFTDTER.L | 22222 |
|  | Mis12IP\_Nocodazole\_MudPIT\_040709\_06.06546.06546.2 | 2.2271 | 0.175 | 95.2% | 1717.3322 | 1718.9095 | 2 | 3.989 | 50.0% | 1 | K.HAVITVPAYFNDSQR.Q | 2 |
|  | Mis12IP\_Nocodazole\_MudPIT\_040709\_06.06619.06619.2 | 5.3251 | 0.5404 | 100.0% | 1688.7322 | 1688.9213 | 1 | 10.629 | 80.0% | 1 | R.IINEPTAAAIAYGLDR.R | 22 |
|  | Mis12IP\_Nocodazole\_MudPIT\_040709\_04.05631.05631.2 | 2.8556 | 0.3643 | 99.9% | 1676.4521 | 1676.6964 | 4 | 6.052 | 40.0% | 2 | K.ATAGDTHLGGEDFDNR.L | 222 |
|  | Mis12IP\_Nocodazole\_MudPIT\_040709\_04.05654.05654.2 | 2.3494 | 0.4197 | 100.0% | 1018.2922 | 1018.1582 | 2 | 6.376 | 68.8% | 14 | K.ITITNDKGR.L | 22222 |

Similarities:
gi|5729877|ref|NP\_006(3:3)  
gi|13676857|ref|NP\_06(3:3)  
gi|12803275|gb|AAH024(5:1)  
gi|27436929|ref|NP\_00(4:2)  

---

|  |  |  |  |  |  |  |  |  |
| --- | --- | --- | --- | --- | --- | --- | --- | --- |
| U | *gi|17511777|gb|AAH187* | 8 | 16 | 10.5% | 1050 | 119507 | 5.3 | BUB1 budding uninhibited by benzimidazoles 1 homolog beta (yeast) [Homo sapiens] |
| U | *gi|62087144|dbj|BAD92* | 8 | 16 | 10.1% | 1091 | 124063 | 5.5 | Mitotic checkpoint serine/threonine-protein kinase BUB1 beta variant [Homo sapiens] |
| U | *gi|59814247|ref|NP\_00* | 8 | 16 | 10.5% | 1050 | 119517 | 5.3 | BUB1 budding uninhibited by benzimidazoles 1 homolog beta [Homo sapiens] |
| U | *gi|4050084|gb|AAD1194* | 8 | 16 | 10.5% | 1050 | 119545 | 5.3 | mitotic checkpoint protein kinase BUB1B [Homo sapiens] |
| U | *gi|3493533|gb|AAC3343* | 8 | 16 | 10.5% | 1050 | 119516 | 5.3 | mitotic checkpoint protein kinase Bub1A [Homo sapiens] |
| U | *gi|2992634|gb|AAC2373* | 8 | 16 | 10.5% | 1050 | 119579 | 5.3 | protein kinase [Homo sapiens] |
| U | *gi|2981235|gb|AAC0626* | 8 | 16 | 10.5% | 1050 | 119577 | 5.3 | mitotic checkpoint kinase Mad3L [Homo sapiens] |

| Filename XCorr DeltCN Conf% ObsM+H+ CalcM+H+ SpR ZScore Ion% # Sequence  | | | | | | | | | | | | |
| --- | --- | --- | --- | --- | --- | --- | --- | --- | --- | --- | --- | --- |
|  | Mis12IP\_Nocodazole\_MudPIT\_040709\_06.07193.07193.2 | 4.1266 | 0.591 | 100.0% | 2138.6921 | 2139.2954 | 1 | 10.928 | 55.3% | 1 | K.EGGALSEAMSLEGDEWELSK.E | 2 |
|  | Mis12IP\_Nocodazole\_MudPIT\_040709\_06.07102.07102.2 | 3.8638 | 0.5139 | 100.0% | 1598.0922 | 1598.7118 | 1 | 8.749 | 66.7% | 2 | R.FYTGNDPLDVWDR.Y | 2 |
|  | Mis12IP\_Nocodazole\_MudPIT\_040709\_04.05691.05691.2 | 3.5247 | 0.2337 | 99.9% | 1348.1721 | 1348.4985 | 1 | 5.366 | 77.3% | 2 | K.ADAIFQEGIQQK.A | 2 |
|  | Mis12IP\_Nocodazole\_MudPIT\_040709\_06.07183.07183.2 | 1.9035 | 0.2444 | 95.8% | 1617.6522 | 1617.7985 | 21 | 4.514 | 38.5% | 1 | K.IYAGVGEFSFEEIR.A | 2 |
|  | Mis12IP\_Nocodazole\_MudPIT\_040709\_06.05536.05536.2 | 3.011 | 0.3035 | 99.9% | 1247.1322 | 1247.391 | 3 | 6.097 | 75.0% | 1 | R.EAELLTSAEKR.A | 2 |
|  | Mis12IP\_Nocodazole\_MudPIT\_040709\_04.05103.05103.2 | 2.818 | 0.286 | 99.9% | 1373.9122 | 1374.537 | 2 | 5.671 | 65.0% | 5 | K.LKEIQTTQQER.T | 2 |
|  | Mis12IP\_Nocodazole\_MudPIT\_040709\_02.03060.03060.2 | 3.3133 | 0.4025 | 100.0% | 1824.0521 | 1824.9545 | 1 | 7.227 | 46.7% | 1 | R.TGDQQEETMPTKETTK.L | 2 |
|  | Mis12IP\_Nocodazole\_MudPIT\_040709\_04.07352.07352.2 | 3.0747 | 0.3169 | 99.9% | 1411.4722 | 1411.598 | 1 | 6.677 | 66.7% | 3 | K.VGKLTS\*PGALLFQ.- | 2 |

---

|  |  |  |  |  |  |  |  |  |
| --- | --- | --- | --- | --- | --- | --- | --- | --- |
| U | *gi|28875797|ref|NP\_05* | 2 | 2 | 10.5% | 248 | 26397 | 12.2 | hypothetical protein LOC26097 [Homo sapiens] |
| U | *gi|83318452|gb|AAI087* | 2 | 2 | 10.4% | 249 | 26525 | 12.2 | Chromosome 1 open reading frame 77 [Homo sapiens] |
| U | *gi|56205591|emb|CAI19* | 2 | 2 | 11.7% | 223 | 23661 | 12.3 | DKFZP547E1010 protein [Homo sapiens] |
| U | *gi|56205590|emb|CAI19* | 2 | 2 | 12.9% | 202 | 21918 | 12.0 | DKFZP547E1010 protein [Homo sapiens] |
| U | *gi|52545660|emb|CAC21* | 2 | 2 | 11.5% | 226 | 24024 | 12.3 | hypothetical protein [Homo sapiens] |

| Filename XCorr DeltCN Conf% ObsM+H+ CalcM+H+ SpR ZScore Ion% # Sequence  | | | | | | | | | | | | |
| --- | --- | --- | --- | --- | --- | --- | --- | --- | --- | --- | --- | --- |
|  | Mis12IP\_Nocodazole\_MudPIT\_040709\_04.05174.05174.2 | 4.1207 | 0.3959 | 100.0% | 1447.1122 | 1447.6091 | 4 | 6.781 | 62.5% | 1 | R.ASMQQQQQLASAR.N | 2 |
|  | Mis12IP\_Nocodazole\_MudPIT\_040709\_05.05447.05447.2 | 3.8846 | 0.4619 | 100.0% | 1555.2122 | 1555.6997 | 1 | 8.094 | 79.2% | 1 | K.EQLDNQLDAYMSK.T | 2 |

---

|  |  |  |  |  |  |  |  |  |
| --- | --- | --- | --- | --- | --- | --- | --- | --- |
| U | *gi|31645|emb|CAA25833* | 2 | 2 | 10.4% | 335 | 36054 | 8.2 | glyceraldehyde-3-phosphate dehydrogenase [Homo sapiens] |
| U | *gi|7669492|ref|NP\_002* | 2 | 2 | 10.4% | 335 | 36053 | 8.5 | glyceraldehyde-3-phosphate dehydrogenase [Homo sapiens] |
| U | *gi|54303910|gb|AAV333* | 2 | 2 | 10.4% | 335 | 36049 | 8.5 | aging-associated gene 9 protein [Homo sapiens] |
| U | *gi|35053|emb|CAA37794* | 2 | 2 | 10.6% | 331 | 35493 | 8.1 | uracil DNA glycosylase [Homo sapiens] |

| Filename XCorr DeltCN Conf% ObsM+H+ CalcM+H+ SpR ZScore Ion% # Sequence  | | | | | | | | | | | | |
| --- | --- | --- | --- | --- | --- | --- | --- | --- | --- | --- | --- | --- |
|  | Mis12IP\_Nocodazole\_MudPIT\_040709\_06.07567.07567.2 | 3.8001 | 0.4961 | 100.0% | 2277.7722 | 2278.495 | 1 | 8.579 | 45.0% | 1 | K.WGDAGAEYVVESTGVFTTMEK.A | 2 |
|  | Mis12IP\_Nocodazole\_MudPIT\_040709\_06.06978.06978.2 | 4.2926 | 0.5169 | 100.0% | 1764.0122 | 1764.8914 | 1 | 9.099 | 57.7% | 1 | K.LISWYDNEFGYSNR.V | 2 |

---

|  |  |  |  |  |  |  |  |  |
| --- | --- | --- | --- | --- | --- | --- | --- | --- |
| U | *gi|4757834|ref|NP\_004* | 2 | 2 | 10.4% | 211 | 23772 | 6.7 | BCL2-associated athanogene 2 [Homo sapiens] |
| U | *gi|49065418|emb|CAG38* | 2 | 2 | 10.4% | 211 | 23773 | 6.4 | BAG2 [Homo sapiens] |

| Filename XCorr DeltCN Conf% ObsM+H+ CalcM+H+ SpR ZScore Ion% # Sequence  | | | | | | | | | | | | |
| --- | --- | --- | --- | --- | --- | --- | --- | --- | --- | --- | --- | --- |
|  | Mis12IP\_Nocodazole\_MudPIT\_040709\_06.07034.07034.2 | 3.2982 | 0.4445 | 100.0% | 1329.1322 | 1329.5364 | 1 | 7.687 | 75.0% | 1 | R.LLESLDQLELR.V | 2 |
|  | Mis12IP\_Nocodazole\_MudPIT\_040709\_04.05327.05327.2 | 2.8485 | 0.4472 | 100.0% | 1307.8922 | 1308.3934 | 1 | 7.748 | 60.0% | 1 | K.TLQQNAESRFN.- | 2 |

---

|  |  |  |  |  |  |  |  |  |
| --- | --- | --- | --- | --- | --- | --- | --- | --- |
| U | *gi|27436946|ref|NP\_73* | 6 | 8 | 10.2% | 664 | 74140 | 7.0 | lamin A/C isoform 1 precursor [Homo sapiens] |
| U | *gi|57014043|gb|AAW325* | 6 | 8 | 10.2% | 664 | 74082 | 7.2 | lamin A/C transcript variant 1 [Homo sapiens] |

| Filename XCorr DeltCN Conf% ObsM+H+ CalcM+H+ SpR ZScore Ion% # Sequence  | | | | | | | | | | | | |
| --- | --- | --- | --- | --- | --- | --- | --- | --- | --- | --- | --- | --- |
|  | Mis12IP\_Nocodazole\_MudPIT\_040709\_06.05957.05957.1 | 1.942 | 0.2575 | 99.1% | 1028.46 | 1029.1814 | 4 | 5.165 | 62.5% | 1 | R.LADALQELR.A | 1 |
|  | Mis12IP\_Nocodazole\_MudPIT\_040709\_05.05338.05338.2 | 3.6106 | 0.2873 | 100.0% | 1029.0122 | 1029.1814 | 1 | 5.408 | 93.8% | 2 | R.LADALQELR.A | 2 |
|  | Mis12IP\_Nocodazole\_MudPIT\_040709\_06.06277.06277.2 | 3.8952 | 0.282 | 100.0% | 1430.9722 | 1431.6293 | 1 | 5.987 | 58.3% | 1 | R.IDSLSAQLSQLQK.Q | 2 |
|  | Mis12IP\_Nocodazole\_MudPIT\_040709\_06.07097.07097.2 | 4.5135 | 0.4548 | 100.0% | 1894.2922 | 1895.1346 | 1 | 8.693 | 75.0% | 1 | R.MQQQLDEYQELLDIK.L | 2 |
|  | Mis12IP\_Nocodazole\_MudPIT\_040709\_06.05767.05767.2 | 4.0237 | 0.5093 | 100.0% | 1492.1721 | 1492.6874 | 1 | 8.926 | 73.1% | 2 | R.TALINSTGEEVAMR.K | 2 |
|  | Mis12IP\_Nocodazole\_MudPIT\_040709\_06.05773.05773.2 | 4.3236 | 0.5478 | 100.0% | 1566.7522 | 1567.6555 | 1 | 9.822 | 56.2% | 1 | R.SVGGSGGGSFGDNLVTR.S | 2 |

---

|  |  |  |  |  |  |  |  |  |
| --- | --- | --- | --- | --- | --- | --- | --- | --- |
| U | *gi|4507513|ref|NP\_000* | 2 | 2 | 10.0% | 211 | 24145 | 8.7 | tissue inhibitor of metalloproteinase 3 precursor [Homo sapiens] |
| U | *gi|56202497|emb|CAI17* | 2 | 2 | 10.3% | 204 | 23226 | 8.9 | tissue inhibitor of metalloproteinase 3 (Sorsby fundus dystrophy, pseudoinflammatory) [Homo sapiens] |
| U | *gi|56202496|emb|CAI17* | 2 | 2 | 10.3% | 204 | 23114 | 8.9 | tissue inhibitor of metalloproteinase 3 (Sorsby fundus dystrophy, pseudoinflammatory) [Homo sapiens] |
| U | *gi|520932|emb|CAA8291* | 2 | 2 | 10.0% | 210 | 24069 | 9.1 | mig-5 [Homo sapiens] |

| Filename XCorr DeltCN Conf% ObsM+H+ CalcM+H+ SpR ZScore Ion% # Sequence  | | | | | | | | | | | | |
| --- | --- | --- | --- | --- | --- | --- | --- | --- | --- | --- | --- | --- |
|  | Mis12IP\_Nocodazole\_MudPIT\_040709\_06.07079.07079.2 | 2.1379 | 0.4233 | 99.9% | 1325.0922 | 1325.5474 | 2 | 6.708 | 59.1% | 1 | K.EGPFGTLVYTIK.Q | 2 |
|  | Mis12IP\_Nocodazole\_MudPIT\_040709\_06.05917.05917.2 | 2.6613 | 0.3328 | 99.9% | 1146.5122 | 1147.2761 | 1 | 5.281 | 75.0% | 1 | R.WDQLTLSQR.K | 2 |

---

|  |  |  |  |  |  |  |  |  |
| --- | --- | --- | --- | --- | --- | --- | --- | --- |
| U | *gi|1200072|emb|CAA316* | 3 | 3 | 9.8% | 469 | 51335 | 5.5 | keratin [Homo sapiens] |
| U | *gi|4688900|emb|CAB414* | 3 | 3 | 9.8% | 469 | 51414 | 5.7 | sarcolectin [Homo sapiens] |

| Filename XCorr DeltCN Conf% ObsM+H+ CalcM+H+ SpR ZScore Ion% # Sequence  | | | | | | | | | | | | |
| --- | --- | --- | --- | --- | --- | --- | --- | --- | --- | --- | --- | --- |
|  | Mis12IP\_Nocodazole\_MudPIT\_040709\_05.05262.05262.1 | 2.1158 | 0.1126 | 95.8% | 827.46 | 827.95544 | 11 | 4.725 | 66.7% | 1 | K.FASFIDK.V | 1111111 |
|  | Mis12IP\_Nocodazole\_MudPIT\_040709\_06.07776.07776.2 | 4.6715 | 0.4785 | 100.0% | 3012.392 | 3012.2698 | 1 | 8.417 | 40.4% | 1 | R.TLNETELTELQSQISDTSVVLSMDNSR.S | 2 |
|  | Mis12IP\_Nocodazole\_MudPIT\_040709\_06.07453.07453.2 | 3.6883 | 0.4321 | 100.0% | 1273.3322 | 1273.4692 | 1 | 7.465 | 72.7% | 1 | R.SLDLDGIIAEVK.A | 2 |

Similarities:
gi|4504919|ref|NP\_002(1:2)  
gi|17505189|ref|NP\_49(1:2)  
gi|15559584|gb|AAH141(1:2)  
gi|5031841|ref|NP\_005(1:2)  
gi|32567786|ref|NP\_78(1:2)  
gi|45597458|ref|NP\_77(1:2)  

---

|  |  |  |  |  |  |  |  |  |
| --- | --- | --- | --- | --- | --- | --- | --- | --- |
| U | *gi|14141152|ref|NP\_00* | 7 | 12 | 9.7% | 730 | 77516 | 8.7 | heterogeneous nuclear ribonucleoprotein M isoform a [Homo sapiens] |
| U | *gi|37747452|gb|AAH588* | 7 | 12 | 10.3% | 687 | 73096 | 8.7 | Unknown (protein for MGC:64929) [Homo sapiens] |
| U | *gi|16905456|gb|AAL313* | 7 | 12 | 10.3% | 691 | 73621 | 8.8 | ribonucleoprotein [Homo sapiens] |
| U | *gi|14141154|ref|NP\_11* | 7 | 12 | 10.3% | 691 | 73561 | 8.7 | heterogeneous nuclear ribonucleoprotein M isoform b [Homo sapiens] |

| Filename XCorr DeltCN Conf% ObsM+H+ CalcM+H+ SpR ZScore Ion% # Sequence  | | | | | | | | | | | | |
| --- | --- | --- | --- | --- | --- | --- | --- | --- | --- | --- | --- | --- |
|  | Mis12IP\_Nocodazole\_MudPIT\_040709\_06.07855.07855.2 | 3.875 | 0.5242 | 100.0% | 1753.1522 | 1754.0051 | 1 | 9.574 | 56.7% | 1 | K.VGEVTYVELLMDAEGK.S | 2 |
|  | Mis12IP\_Nocodazole\_MudPIT\_040709\_06.06181.06181.2 | 3.0093 | 0.1551 | 99.3% | 1115.0721 | 1115.3152 | 2 | 4.639 | 83.3% | 1 | R.INEILSNALK.R | 2 |
|  | Mis12IP\_Nocodazole\_MudPIT\_040709\_04.05284.05284.2 | 3.0048 | 0.5039 | 100.0% | 1284.5122 | 1285.3591 | 1 | 7.64 | 60.7% | 1 | K.QGGGGGGGSVPGIER.M | 2 |
|  | Mis12IP\_Nocodazole\_MudPIT\_040709\_04.05235.05235.1 | 1.7034 | 0.2322 | 98.7% | 773.43 | 773.9256 | 129 | 3.813 | 66.7% | 1 | R.MGPAIER.M | 1 |
|  | Mis12IP\_Nocodazole\_MudPIT\_040709\_04.05286.05286.1 | 1.7219 | 0.2466 | 99.3% | 805.39 | 805.9856 | 9 | 5.07 | 66.7% | 1 | R.MGPVMDR.M | 1 |
|  | Mis12IP\_Nocodazole\_MudPIT\_040709\_03.03829.03829.2 | 2.2688 | 0.2792 | 99.6% | 904.89215 | 905.0166 | 31 | 5.268 | 64.3% | 3 | R.MGANNLER.M | 2 |
|  | Mis12IP\_Nocodazole\_MudPIT\_040709\_03.04201.04201.2 | 2.5367 | 0.2634 | 99.8% | 877.83215 | 877.99097 | 1 | 6.299 | 78.6% | 4 | R.MGANSLER.M | 2 |

---

|  |  |  |  |  |  |  |  |  |
| --- | --- | --- | --- | --- | --- | --- | --- | --- |
| U | *gi|1167838|emb|CAA646* | 2 | 3 | 9.7% | 372 | 40090 | 9.8 | DNA-binding protein [Homo sapiens] |
| U | *gi|20070160|ref|NP\_00* | 2 | 3 | 9.7% | 372 | 40060 | 9.8 | cold shock domain protein A [Homo sapiens] |
| U | *gi|16198465|gb|AAH159* | 2 | 3 | 11.9% | 303 | 31947 | 9.7 | CSDA protein [Homo sapiens] |

| Filename XCorr DeltCN Conf% ObsM+H+ CalcM+H+ SpR ZScore Ion% # Sequence  | | | | | | | | | | | | |
| --- | --- | --- | --- | --- | --- | --- | --- | --- | --- | --- | --- | --- |
|  | Mis12IP\_Nocodazole\_MudPIT\_040709\_03.05379.05379.2 | 4.5521 | 0.5824 | 100.0% | 1796.1921 | 1796.8822 | 1 | 9.673 | 65.6% | 2 | R.SVGDGETVEFDVVEGEK.G | 22 |
|  | Mis12IP\_Nocodazole\_MudPIT\_040709\_02.03876.03876.2 | 2.0505 | 0.4275 | 99.8% | 1882.3722 | 1882.9353 | 1 | 6.376 | 36.1% | 1 | K.AGEAPTENPAPPTQQSSAE.- | 2 |

Similarities:
gi|116283293|gb|AAH18(1:1)  

---

|  |  |  |  |  |  |  |  |  |
| --- | --- | --- | --- | --- | --- | --- | --- | --- |
| U | *gi|16306492|ref|NP\_20* | 2 | 3 | 9.6% | 240 | 27503 | 7.1 | cell division cycle 2 protein isoform 2 [Homo sapiens] |
| U | *gi|59016744|emb|CAI46* | 2 | 3 | 7.6% | 303 | 34778 | 8.9 | hypothetical protein [Homo sapiens] |
| U | *gi|4502709|ref|NP\_001* | 2 | 3 | 7.7% | 297 | 34095 | 8.4 | cell division cycle 2 protein isoform 1 [Homo sapiens] |

| Filename XCorr DeltCN Conf% ObsM+H+ CalcM+H+ SpR ZScore Ion% # Sequence  | | | | | | | | | | | | |
| --- | --- | --- | --- | --- | --- | --- | --- | --- | --- | --- | --- | --- |
|  | Mis12IP\_Nocodazole\_MudPIT\_040709\_06.05723.05723.2 | 3.0928 | 0.3861 | 100.0% | 1185.4722 | 1186.3501 | 1 | 8.139 | 75.0% | 1 | K.IGEGTYGVVYK.G | 2 |
|  | Mis12IP\_Nocodazole\_MudPIT\_040709\_05.05929.05929.2 | 2.9881 | 0.4383 | 100.0% | 1331.2722 | 1331.4656 | 1 | 7.216 | 63.6% | 2 | K.NLDENGLDLLSK.M | 2 |

---

|  |  |  |  |  |  |  |  |  |
| --- | --- | --- | --- | --- | --- | --- | --- | --- |
| U | *gi|4502899|ref|NP\_001* | 2 | 4 | 9.6% | 218 | 23662 | 4.5 | clathrin, light polypeptide A isoform a [Homo sapiens] |

| Filename XCorr DeltCN Conf% ObsM+H+ CalcM+H+ SpR ZScore Ion% # Sequence  | | | | | | | | | | | | |
| --- | --- | --- | --- | --- | --- | --- | --- | --- | --- | --- | --- | --- |
| \* | Mis12IP\_Nocodazole\_MudPIT\_040709\_05.06052.06052.2 | 4.3279 | 0.4795 | 100.0% | 2352.392 | 2353.4175 | 1 | 8.609 | 55.0% | 3 | R.AAEEAFVNDIDESSPGTEWER.V | 2 |
| \* | Mis12IP\_Nocodazole\_MudPIT\_040709\_05.06061.06061.3 | 4.2407 | 0.3025 | 100.0% | 2353.0444 | 2353.4175 | 1 | 6.717 | 46.2% | 1 | R.AAEEAFVNDIDESSPGTEWER.V | 3 |

---

|  |  |  |  |  |  |  |  |  |
| --- | --- | --- | --- | --- | --- | --- | --- | --- |
| U | *gi|21619877|gb|AAH331* | 4 | 5 | 9.5% | 559 | 59120 | 6.8 | Similar to cleavage stimulation factor, 3' pre-RNA, subunit 2, 64kD [Homo sapiens] |
| U | *gi|4557493|ref|NP\_001* | 4 | 5 | 9.2% | 577 | 60959 | 6.8 | cleavage stimulation factor subunit 2 [Homo sapiens] |

| Filename XCorr DeltCN Conf% ObsM+H+ CalcM+H+ SpR ZScore Ion% # Sequence  | | | | | | | | | | | | |
| --- | --- | --- | --- | --- | --- | --- | --- | --- | --- | --- | --- | --- |
|  | Mis12IP\_Nocodazole\_MudPIT\_040709\_05.06088.06088.3 | 3.7266 | 0.372 | 100.0% | 2832.8044 | 2833.0754 | 1 | 6.775 | 30.6% | 1 | K.SLGTGAPVIESPYGETISPEDAPESISK.A | 3 |
|  | Mis12IP\_Nocodazole\_MudPIT\_040709\_05.06089.06089.2 | 3.07 | 0.3053 | 99.9% | 2833.892 | 2833.0754 | 1 | 5.778 | 33.3% | 2 | K.SLGTGAPVIESPYGETISPEDAPESISK.A | 2 |
|  | Mis12IP\_Nocodazole\_MudPIT\_040709\_03.04759.04759.2 | 2.9698 | 0.3719 | 100.0% | 1125.1522 | 1125.2266 | 1 | 6.61 | 80.0% | 1 | R.GLLGDAPNDPR.G | 2 |
|  | Mis12IP\_Nocodazole\_MudPIT\_040709\_06.06204.06204.2 | 3.6875 | 0.4356 | 100.0% | 1415.2322 | 1415.5864 | 1 | 6.868 | 73.1% | 1 | R.GGTLLSVTGEVEPR.G | 2 |

---

|  |  |  |  |  |  |  |  |  |
| --- | --- | --- | --- | --- | --- | --- | --- | --- |
| U | *gi|18088719|gb|AAH209* | 4 | 5 | 9.5% | 444 | 49672 | 4.9 | Tubulin, beta [Homo sapiens] |
| U | *gi|57209813|emb|CAI41* | 4 | 5 | 9.9% | 426 | 47767 | 4.8 | tubulin, beta polypeptide [Homo sapiens] |
| U | *gi|29788785|ref|NP\_82* | 4 | 5 | 9.5% | 444 | 49671 | 4.9 | tubulin, beta polypeptide [Homo sapiens] |

| Filename XCorr DeltCN Conf% ObsM+H+ CalcM+H+ SpR ZScore Ion% # Sequence  | | | | | | | | | | | | |
| --- | --- | --- | --- | --- | --- | --- | --- | --- | --- | --- | --- | --- |
|  | Mis12IP\_Nocodazole\_MudPIT\_040709\_05.04875.04875.1 | 1.9368 | 0.3402 | 98.3% | 1301.57 | 1302.4265 | 42 | 5.471 | 40.9% | 1 | R.ISVYYNEATGGK.Y | 1 |
|  | Mis12IP\_Nocodazole\_MudPIT\_040709\_05.04888.04888.2 | 2.1854 | 0.4013 | 99.9% | 1301.6921 | 1302.4265 | 1 | 6.347 | 68.2% | 1 | R.ISVYYNEATGGK.Y | 2 |
|  | Mis12IP\_Nocodazole\_MudPIT\_040709\_05.06424.06424.2 | 3.4394 | 0.4058 | 100.0% | 1617.7322 | 1616.8701 | 1 | 6.036 | 64.3% | 1 | R.AILVDLEPGTMDSVR.S | 2 |
|  | Mis12IP\_Nocodazole\_MudPIT\_040709\_06.07354.07354.2 | 3.7341 | 0.5113 | 100.0% | 1660.2722 | 1660.9078 | 1 | 8.403 | 67.9% | 2 | R.ALTVPELTQQVFDAK.D | 2 |

---

|  |  |  |  |  |  |  |  |  |
| --- | --- | --- | --- | --- | --- | --- | --- | --- |
| U | *gi|10433717|dbj|BAB14* | 3 | 4 | 9.4% | 446 | 49843 | 4.9 | unnamed protein product [Homo sapiens] |
| U | *gi|14210536|ref|NP\_11* | 3 | 4 | 9.4% | 446 | 49857 | 4.9 | tubulin, beta 6 [Homo sapiens] |

| Filename XCorr DeltCN Conf% ObsM+H+ CalcM+H+ SpR ZScore Ion% # Sequence  | | | | | | | | | | | | |
| --- | --- | --- | --- | --- | --- | --- | --- | --- | --- | --- | --- | --- |
|  | Mis12IP\_Nocodazole\_MudPIT\_040709\_04.05381.05381.2 | 3.4435 | 0.4116 | 100.0% | 1432.2722 | 1432.5297 | 1 | 7.551 | 68.2% | 1 | R.INVYYNESSSQK.Y | 2 |
|  | Mis12IP\_Nocodazole\_MudPIT\_040709\_06.06420.06420.2 | 3.4572 | 0.3463 | 100.0% | 1574.3722 | 1574.7894 | 1 | 6.357 | 60.7% | 2 | R.AALVDLEPGTMDSVR.S | 2 |
|  | Mis12IP\_Nocodazole\_MudPIT\_040709\_06.07536.07536.2 | 3.1135 | 0.4549 | 100.0% | 1720.2122 | 1720.9812 | 2 | 7.622 | 46.4% | 1 | R.ALTVPELTQQMFDAR.N | 2 |

---

|  |  |  |  |  |  |  |  |  |
| --- | --- | --- | --- | --- | --- | --- | --- | --- |
| U | *gi|12653279|gb|AAH004* | 2 | 2 | 9.3% | 397 | 41379 | 6.9 | ACAT2 protein [Homo sapiens] |
| U | *gi|62087566|dbj|BAD92* | 2 | 2 | 9.2% | 404 | 42136 | 7.4 | Acetyl-CoA acetyltransferase, cytosolic variant [Homo sapiens] |
| U | *gi|56204996|emb|CAI21* | 2 | 2 | 9.3% | 397 | 41351 | 6.9 | acetyl-Coenzyme A acetyltransferase 2 (acetoacetyl Coenzyme A thiolase) [Homo sapiens] |
| U | *gi|5174389|ref|NP\_005* | 2 | 2 | 9.3% | 397 | 41296 | 6.7 | acetyl-Coenzyme A acetyltransferase 2 [Homo sapiens] |
| U | *gi|19880019|gb|AAM002* | 2 | 2 | 9.3% | 397 | 41252 | 6.7 | acetyl CoA transferase-like protein [Homo sapiens] |

| Filename XCorr DeltCN Conf% ObsM+H+ CalcM+H+ SpR ZScore Ion% # Sequence  | | | | | | | | | | | | |
| --- | --- | --- | --- | --- | --- | --- | --- | --- | --- | --- | --- | --- |
|  | Mis12IP\_Nocodazole\_MudPIT\_040709\_06.07949.07949.2 | 2.7522 | 0.3547 | 99.9% | 2371.7922 | 2372.7673 | 4 | 6.138 | 32.6% | 1 | R.TIIGSFNGALAAVPVQDLGSTVIK.E | 2 |
|  | Mis12IP\_Nocodazole\_MudPIT\_040709\_06.05456.05456.2 | 3.6867 | 0.3746 | 100.0% | 1502.0721 | 1502.6243 | 1 | 6.881 | 75.0% | 1 | R.EDQDKVAVLSQNR.T | 2 |

---

|  |  |  |  |  |  |  |  |  |
| --- | --- | --- | --- | --- | --- | --- | --- | --- |
| U | *gi|13236565|ref|NP\_07* | 3 | 3 | 9.3% | 300 | 33786 | 7.7 | centromere protein O [Homo sapiens] |
| U | *gi|62822237|gb|AAY147* | 3 | 3 | 9.9% | 284 | 32118 | 7.9 | unknown [Homo sapiens] |

| Filename XCorr DeltCN Conf% ObsM+H+ CalcM+H+ SpR ZScore Ion% # Sequence  | | | | | | | | | | | | |
| --- | --- | --- | --- | --- | --- | --- | --- | --- | --- | --- | --- | --- |
|  | Mis12IP\_Nocodazole\_MudPIT\_040709\_05.04818.04818.3 | 2.6803 | 0.3426 | 100.0% | 3166.4944 | 3169.3896 | 3 | 5.068 | 22.2% | 1 | R.LETQVS\*RSRKQSEELQSVQAQEGALGTK.I | 3 |
|  | Mis12IP\_Nocodazole\_MudPIT\_040709\_05.04818.04818.2 | 3.4379 | 0.4925 | 100.0% | 2111.3323 | 2112.215 | 1 | 8.414 | 61.1% | 1 | R.KQS\*EELQSVQAQEGALGTK.I | 3 |
|  | Mis12IP\_Nocodazole\_MudPIT\_040709\_04.05499.05499.2 | 3.8369 | 0.3918 | 100.0% | 1903.3121 | 1904.041 | 1 | 8.439 | 52.9% | 1 | K.QSEELQSVQAQEGALGTK.I | 2 |

---

|  |  |  |  |  |  |  |  |  |
| --- | --- | --- | --- | --- | --- | --- | --- | --- |
| U | *gi|113425263|ref|XP\_0* | 2 | 2 | 9.3% | 204 | 22304 | 10.6 | PREDICTED: similar to 60S ribosomal protein L29 (Cell surface heparin-binding protein HIP) [Homo sapiens] |
| U | *gi|88982429|ref|XP\_49* | 2 | 2 | 12.3% | 155 | 17138 | 10.8 | PREDICTED: similar to 60S ribosomal protein L29 (Cell surface heparin-binding protein HIP) [Homo sapiens] |
| U | *gi|793843|emb|CAA8900* | 2 | 2 | 11.9% | 159 | 17667 | 11.6 | ribosomal protein L29 [Homo sapiens] |
| U | *gi|55959755|emb|CAI16* | 2 | 2 | 11.0% | 172 | 18712 | 11.0 | OTTHUMP00000017090 [Homo sapiens] |
| U | *gi|48734757|gb|AAH719* | 2 | 2 | 11.8% | 161 | 17951 | 11.7 | Ribosomal protein L29 [Homo sapiens] |
| U | *gi|4506629|ref|NP\_000* | 2 | 2 | 11.9% | 159 | 17752 | 11.7 | ribosomal protein L29 [Homo sapiens] |
| U | *gi|27482992|ref|XP\_21* | 2 | 2 | 12.3% | 155 | 17219 | 11.1 | PREDICTED: similar to 60S ribosomal protein L29 (Cell surface heparin-binding protein HIP) [Homo sapiens] |

| Filename XCorr DeltCN Conf% ObsM+H+ CalcM+H+ SpR ZScore Ion% # Sequence  | | | | | | | | | | | | |
| --- | --- | --- | --- | --- | --- | --- | --- | --- | --- | --- | --- | --- |
|  | Mis12IP\_Nocodazole\_MudPIT\_040709\_06.05370.05370.2 | 2.9731 | 0.3387 | 99.9% | 1850.3121 | 1851.0696 | 1 | 8.432 | 47.2% | 1 | K.DQTKAQAAAPASVPAQAPK.G | 2 |
|  | Mis12IP\_Nocodazole\_MudPIT\_040709\_05.04576.04576.2 | 3.9679 | 0.5349 | 100.0% | 1378.0721 | 1378.5712 | 1 | 8.212 | 75.0% | 1 | K.AQAAAPASVPAQAPK.G | 2 |

---

|  |  |  |  |  |  |  |  |  |
| --- | --- | --- | --- | --- | --- | --- | --- | --- |
| U | *gi|17388799|ref|NP\_49* | 2 | 2 | 9.2% | 326 | 36087 | 9.1 | DnaJ (Hsp40) homolog, subfamily B, member 6 isoform a [Homo sapiens] |
| U | *gi|62898934|dbj|BAD97* | 2 | 2 | 9.2% | 326 | 36117 | 9.1 | DnaJ (Hsp40) homolog, subfamily B, member 6 isoform a variant [Homo sapiens] |
| U | *gi|62089304|dbj|BAD93* | 2 | 2 | 9.0% | 335 | 36772 | 7.2 | DnaJ (Hsp40) homolog, subfamily B, member 6 isoform a variant [Homo sapiens] |

| Filename XCorr DeltCN Conf% ObsM+H+ CalcM+H+ SpR ZScore Ion% # Sequence  | | | | | | | | | | | | |
| --- | --- | --- | --- | --- | --- | --- | --- | --- | --- | --- | --- | --- |
|  | Mis12IP\_Nocodazole\_MudPIT\_040709\_05.05353.05353.2 | 2.6284 | 0.5194 | 100.0% | 1423.2722 | 1423.5627 | 9 | 7.74 | 45.8% | 1 | K.QVAEAYEVLSDAK.K | 2 |
|  | Mis12IP\_Nocodazole\_MudPIT\_040709\_04.06113.06113.2 | 2.9819 | 0.38 | 100.0% | 1789.4922 | 1789.8938 | 1 | 7.425 | 56.2% | 1 | K.SLTINGVADDDALAEER.M | 2 |

---

|  |  |  |  |  |  |  |  |  |
| --- | --- | --- | --- | --- | --- | --- | --- | --- |
| U | *gi|12697933|dbj|BAB21* | 5 | 10 | 8.9% | 757 | 84785 | 6.8 | KIAA1694 protein [Homo sapiens] |
| U | *gi|38229313|ref|NP\_93* | 5 | 10 | 9.1% | 739 | 82975 | 7.0 | c-Maf-inducing protein C-mip isoform [Homo sapiens] |
| U | *gi|29789255|ref|NP\_08* | 5 | 10 | 9.9% | 679 | 76033 | 6.2 | c-Maf-inducing protein Tc-mip isoform [Homo sapiens] |
| U | *gi|23271324|gb|AAH381* | 5 | 10 | 10.8% | 620 | 69288 | 6.4 | CMIP protein [Homo sapiens] |

| Filename XCorr DeltCN Conf% ObsM+H+ CalcM+H+ SpR ZScore Ion% # Sequence  | | | | | | | | | | | | |
| --- | --- | --- | --- | --- | --- | --- | --- | --- | --- | --- | --- | --- |
|  | Mis12IP\_Nocodazole\_MudPIT\_040709\_06.07913.07913.2 | 4.1174 | 0.4845 | 100.0% | 2799.8323 | 2800.1938 | 1 | 8.104 | 36.0% | 2 | R.TLVDMALTSPLQDDSINQAPLEIVSK.L | 2 |
|  | Mis12IP\_Nocodazole\_MudPIT\_040709\_06.07912.07912.3 | 5.9819 | 0.4713 | 100.0% | 2800.1643 | 2800.1938 | 1 | 9.66 | 38.0% | 4 | R.TLVDMALTSPLQDDSINQAPLEIVSK.L | 3 |
|  | Mis12IP\_Nocodazole\_MudPIT\_040709\_06.07632.07632.2 | 3.6806 | 0.4028 | 100.0% | 1604.0521 | 1604.9482 | 2 | 8.258 | 57.7% | 2 | R.SMVVIEVFTPVVQR.I | 2 |
|  | Mis12IP\_Nocodazole\_MudPIT\_040709\_04.06166.06166.2 | 3.585 | 0.3936 | 100.0% | 2224.912 | 2224.303 | 1 | 6.316 | 50.0% | 1 | R.LLHPS\*PDLVSQEATLS\*EAR.L | 2 |
|  | Mis12IP\_Nocodazole\_MudPIT\_040709\_02.03009.03009.1 | 1.7566 | 0.3005 | 98.9% | 848.38 | 848.88824 | 11 | 5.237 | 57.1% | 1 | K.STDADLAR.L | 1 |

---

|  |  |  |  |  |  |  |  |  |
| --- | --- | --- | --- | --- | --- | --- | --- | --- |
| U | *gi|15131402|emb|CAC48* | 5 | 6 | 8.9% | 708 | 82045 | 8.3 | dJ85M6.1 (cleavage stimulation factor, 3' pre-RNA, subunit 3, 77kD) [Homo sapiens] |
| U | *gi|4557495|ref|NP\_001* | 5 | 6 | 8.8% | 717 | 82922 | 8.1 | cleavage stimulation factor subunit 3 isoform 1 [Homo sapiens] |

| Filename XCorr DeltCN Conf% ObsM+H+ CalcM+H+ SpR ZScore Ion% # Sequence  | | | | | | | | | | | | |
| --- | --- | --- | --- | --- | --- | --- | --- | --- | --- | --- | --- | --- |
|  | Mis12IP\_Nocodazole\_MudPIT\_040709\_06.02970.02970.2 | 2.5944 | 0.0718 | 96.5% | 1012.9122 | 1013.11615 | 23 | 3.237 | 71.4% | 1 | R.SRDYMNAR.R | 2 |
|  | Mis12IP\_Nocodazole\_MudPIT\_040709\_05.05467.05467.2 | 3.6951 | 0.5285 | 100.0% | 1427.7322 | 1428.5413 | 1 | 8.776 | 77.3% | 1 | K.LFSDEAANIYER.A | 2 |
|  | Mis12IP\_Nocodazole\_MudPIT\_040709\_06.08769.08769.2 | 3.8651 | 0.5195 | 100.0% | 2138.8523 | 2139.555 | 1 | 8.585 | 58.8% | 2 | R.LLAIEDIDPTLVYIQYMK.F | 2 |
|  | Mis12IP\_Nocodazole\_MudPIT\_040709\_05.05841.05841.3 | 2.8689 | 0.3175 | 100.0% | 2436.2043 | 2437.7527 | 43 | 4.885 | 22.9% | 1 | R.IITGGAPELAVEGNGPVESNAVLTK.A | 3 |
|  | Mis12IP\_Nocodazole\_MudPIT\_040709\_06.06335.06335.2 | 4.1023 | 0.5708 | 100.0% | 2436.5122 | 2437.7527 | 1 | 9.399 | 43.8% | 1 | R.IITGGAPELAVEGNGPVESNAVLTK.A | 2 |

---

|  |  |  |  |  |  |  |  |  |
| --- | --- | --- | --- | --- | --- | --- | --- | --- |
| U | *gi|54781221|gb|AAV407* | 2 | 2 | 8.9% | 123 | 13179 | 8.7 | immunoglobulin kappa light chain variable region VK1 [Homo sapiens] |
| U | *gi|98956304|emb|CAI99* | 2 | 2 | 10.4% | 106 | 11565 | 9.6 | immunoglobulin kappa light chain variable region [Homo sapiens] |

| Filename XCorr DeltCN Conf% ObsM+H+ CalcM+H+ SpR ZScore Ion% # Sequence  | | | | | | | | | | | | |
| --- | --- | --- | --- | --- | --- | --- | --- | --- | --- | --- | --- | --- |
|  | Mis12IP\_Nocodazole\_MudPIT\_040709\_04.05258.05258.1 | 1.6592 | 0.3705 | 98.6% | 1045.55 | 1046.1686 | 2 | 5.213 | 60.0% | 1 | K.ASTLASGVPSR.F | 1 |
|  | Mis12IP\_Nocodazole\_MudPIT\_040709\_04.05253.05253.2 | 3.1266 | 0.3304 | 100.0% | 1045.8722 | 1046.1686 | 3 | 6.388 | 75.0% | 1 | K.ASTLASGVPSR.F | 2 |

---

|  |  |  |  |  |  |  |  |  |
| --- | --- | --- | --- | --- | --- | --- | --- | --- |
| U | *gi|11493459|gb|AAG355* | 5 | 5 | 8.8% | 499 | 56782 | 6.3 | PRO2619 [Homo sapiens] |
| U | *gi|62113341|gb|AAX634* | 5 | 5 | 7.2% | 609 | 69084 | 6.2 | serum albumin [Homo sapiens] |
| U | *gi|6013427|gb|AAF0133* | 5 | 5 | 7.2% | 609 | 69226 | 6.3 | serum albumin precursor [Homo sapiens] |
| U | *gi|51476390|emb|CAH18* | 5 | 5 | 7.2% | 609 | 69403 | 6.2 | hypothetical protein [Homo sapiens] |
| U | *gi|4502027|ref|NP\_000* | 5 | 5 | 7.2% | 609 | 69367 | 6.3 | albumin precursor [Homo sapiens] |
| U | *gi|28592|emb|CAA23754* | 5 | 5 | 7.2% | 609 | 69366 | 6.4 | serum albumin [Homo sapiens] |

| Filename XCorr DeltCN Conf% ObsM+H+ CalcM+H+ SpR ZScore Ion% # Sequence  | | | | | | | | | | | | |
| --- | --- | --- | --- | --- | --- | --- | --- | --- | --- | --- | --- | --- |
|  | Mis12IP\_Nocodazole\_MudPIT\_040709\_06.05902.05902.1 | 2.1002 | 0.3487 | 100.0% | 927.44 | 928.0758 | 10 | 5.788 | 66.7% | 1 | K.YLYEIAR.R | 1 |
|  | Mis12IP\_Nocodazole\_MudPIT\_040709\_06.05910.05910.2 | 2.6472 | 0.2781 | 99.9% | 927.9522 | 928.0758 | 1 | 5.559 | 83.3% | 1 | K.YLYEIAR.R | 2 |
|  | Mis12IP\_Nocodazole\_MudPIT\_040709\_06.06050.06050.2 | 2.834 | 0.1148 | 96.6% | 1512.4122 | 1512.7465 | 1 | 6.294 | 65.4% | 1 | K.VPQVSTPTLVEVSR.N | 2 |
|  | Mis12IP\_Nocodazole\_MudPIT\_040709\_06.07436.07436.2 | 2.7478 | 0.4837 | 100.0% | 1343.4521 | 1343.5372 | 1 | 7.635 | 63.6% | 1 | K.AVMDDFAAFVEK.C | 2 |
|  | Mis12IP\_Nocodazole\_MudPIT\_040709\_02.05132.05132.2 | 2.9452 | 0.5075 | 100.0% | 1013.4922 | 1014.21014 | 1 | 9.349 | 80.0% | 1 | K.LVAASQAALGL.- | 2 |

---

|  |  |  |  |  |  |  |  |  |
| --- | --- | --- | --- | --- | --- | --- | --- | --- |
| U | *gi|21626466|ref|NP\_06* | 6 | 7 | 8.7% | 847 | 94623 | 6.3 | matrin 3 [Homo sapiens] |
| U | *gi|40788339|dbj|BAA34* | 6 | 7 | 8.7% | 853 | 95227 | 6.3 | KIAA0723 protein [Homo sapiens] |

| Filename XCorr DeltCN Conf% ObsM+H+ CalcM+H+ SpR ZScore Ion% # Sequence  | | | | | | | | | | | | |
| --- | --- | --- | --- | --- | --- | --- | --- | --- | --- | --- | --- | --- |
|  | Mis12IP\_Nocodazole\_MudPIT\_040709\_04.05246.05246.2 | 2.5803 | 0.2606 | 99.7% | 1039.7122 | 1040.121 | 1 | 5.429 | 81.2% | 1 | K.SFQQSSLSR.D | 2 |
|  | Mis12IP\_Nocodazole\_MudPIT\_040709\_06.00010.00010.2 | 3.1148 | 0.4928 | 100.0% | 2371.8323 | 2372.7424 | 10 | 8.232 | 29.2% | 2 | R.DLSAAGIGLLAAATQSLSMPASLGR.M | 2 |
|  | Mis12IP\_Nocodazole\_MudPIT\_040709\_06.09596.09596.3 | 5.5267 | 0.5625 | 100.0% | 2372.4543 | 2372.7424 | 1 | 10.587 | 39.6% | 1 | R.DLSAAGIGLLAAATQSLSMPASLGR.M | 3 |
|  | Mis12IP\_Nocodazole\_MudPIT\_040709\_06.07777.07777.2 | 4.6953 | 0.5582 | 100.0% | 1793.4922 | 1793.931 | 1 | 9.665 | 64.7% | 1 | R.GDADQASNILASFGLSAR.D | 2 |
|  | Mis12IP\_Nocodazole\_MudPIT\_040709\_05.04602.04602.2 | 2.1497 | 0.2407 | 99.2% | 853.77216 | 853.95355 | 11 | 6.124 | 64.3% | 1 | R.GPGPLQER.S | 2 |
|  | Mis12IP\_Nocodazole\_MudPIT\_040709\_06.05410.05410.2 | 3.2953 | 0.4073 | 100.0% | 1325.2322 | 1325.4269 | 1 | 9.07 | 69.2% | 1 | R.GNLGAGNGNLQGPR.H | 2 |

---

|  |  |  |  |  |  |  |  |  |
| --- | --- | --- | --- | --- | --- | --- | --- | --- |
| U | *gi|10433819|dbj|BAB14* | 2 | 2 | 8.5% | 375 | 41862 | 5.8 | unnamed protein product [Homo sapiens] |
| U | *gi|116292170|ref|NP\_1* | 2 | 2 | 8.5% | 375 | 41891 | 6.0 | RUN domain containing 2A [Homo sapiens] |

| Filename XCorr DeltCN Conf% ObsM+H+ CalcM+H+ SpR ZScore Ion% # Sequence  | | | | | | | | | | | | |
| --- | --- | --- | --- | --- | --- | --- | --- | --- | --- | --- | --- | --- |
|  | Mis12IP\_Nocodazole\_MudPIT\_040709\_06.06114.06114.2 | 2.4909 | 0.2787 | 99.6% | 1211.1721 | 1211.317 | 1 | 5.747 | 65.0% | 1 | K.ESTQGVSSLFR.E | 2 |
|  | Mis12IP\_Nocodazole\_MudPIT\_040709\_05.06323.06323.2 | 4.5445 | 0.6342 | 100.0% | 2401.3523 | 2402.487 | 1 | 10.468 | 57.5% | 1 | K.VTNIISFDDEEDEQNSGDVFK.K | 2 |

---

|  |  |  |  |  |  |  |  |  |
| --- | --- | --- | --- | --- | --- | --- | --- | --- |
| U | *gi|4506661|ref|NP\_000* | 2 | 2 | 8.3% | 266 | 29996 | 10.6 | ribosomal protein L7a [Homo sapiens] |
| U | *gi|89047128|ref|XP\_37* | 2 | 2 | 8.3% | 266 | 30042 | 10.6 | PREDICTED: similar to 60S ribosomal protein L7a isoform 1 [Homo sapiens] |
| U | *gi|55958183|emb|CAI12* | 2 | 2 | 11.5% | 191 | 21545 | 11.0 | ribosomal protein L7a [Homo sapiens] |

| Filename XCorr DeltCN Conf% ObsM+H+ CalcM+H+ SpR ZScore Ion% # Sequence  | | | | | | | | | | | | |
| --- | --- | --- | --- | --- | --- | --- | --- | --- | --- | --- | --- | --- |
|  | Mis12IP\_Nocodazole\_MudPIT\_040709\_05.04603.04603.1 | 1.8485 | 0.3602 | 98.0% | 851.55 | 852.06476 | 2 | 6.234 | 68.8% | 1 | K.VAPAPAVVK.K | 1 |
|  | Mis12IP\_Nocodazole\_MudPIT\_040709\_06.05863.05863.2 | 2.576 | 0.3175 | 99.8% | 1346.0122 | 1346.5236 | 1 | 6.453 | 66.7% | 1 | R.AGVNTVTTLVENK.K | 2 |

---

|  |  |  |  |  |  |  |  |  |
| --- | --- | --- | --- | --- | --- | --- | --- | --- |
| U | *gi|47604944|ref|NP\_06* | 5 | 12 | 8.2% | 929 | 103709 | 8.2 | SCY1-like 2 protein [Homo sapiens] |

| Filename XCorr DeltCN Conf% ObsM+H+ CalcM+H+ SpR ZScore Ion% # Sequence  | | | | | | | | | | | | |
| --- | --- | --- | --- | --- | --- | --- | --- | --- | --- | --- | --- | --- |
| \* | Mis12IP\_Nocodazole\_MudPIT\_040709\_06.06004.06004.2 | 4.4626 | 0.4687 | 100.0% | 1618.2522 | 1618.8456 | 1 | 9.233 | 73.3% | 2 | K.VTADVTSAVMGNPVTR.E | 2 |
|  | Mis12IP\_Nocodazole\_MudPIT\_040709\_05.05439.05439.2 | 3.5608 | 0.5898 | 100.0% | 1694.6721 | 1695.9001 | 1 | 9.917 | 67.9% | 1 | K.SLDIGNQMNVSEEMK.V | 2 |
|  | Mis12IP\_Nocodazole\_MudPIT\_040709\_03.08698.08698.2 | 4.5714 | 0.5523 | 100.0% | 2356.7722 | 2357.6504 | 1 | 9.912 | 50.0% | 7 | K.DLTDTLMDNMSSLTSLSVSTPK.S | 2 |
|  | Mis12IP\_Nocodazole\_MudPIT\_040709\_02.08079.08079.3 | 2.495 | 0.2472 | 95.8% | 2359.5544 | 2357.6504 | 10 | 4.515 | 29.8% | 1 | K.DLTDTLMDNMSSLTSLSVSTPK.S | 3 |
|  | Mis12IP\_Nocodazole\_MudPIT\_040709\_04.08122.08122.2 | 2.1906 | 0.2421 | 97.6% | 2374.7922 | 2375.7021 | 2 | 5.133 | 38.6% | 1 | K.MTLGT#PPTLPNFNALSVPPAGAK.Q | 2 |

---

|  |  |  |  |  |  |  |  |  |
| --- | --- | --- | --- | --- | --- | --- | --- | --- |
| U | *gi|26996556|gb|AAH411* | 5 | 7 | 8.0% | 943 | 106868 | 9.4 | Centromere protein C 1 [Homo sapiens] |

| Filename XCorr DeltCN Conf% ObsM+H+ CalcM+H+ SpR ZScore Ion% # Sequence  | | | | | | | | | | | | |
| --- | --- | --- | --- | --- | --- | --- | --- | --- | --- | --- | --- | --- |
|  | Mis12IP\_Nocodazole\_MudPIT\_040709\_04.05451.05451.2 | 3.3531 | 0.4636 | 100.0% | 1284.8722 | 1285.3531 | 1 | 7.759 | 77.3% | 2 | K.SLANDFSTNSTK.S | 2 |
|  | Mis12IP\_Nocodazole\_MudPIT\_040709\_06.06293.06293.2 | 4.1716 | 0.3777 | 100.0% | 1777.2322 | 1777.9286 | 1 | 7.973 | 73.3% | 2 | K.EASLQFVVEPSEATNR.S | 2 |
|  | Mis12IP\_Nocodazole\_MudPIT\_040709\_03.04325.04325.2 | 2.3306 | 0.2198 | 97.6% | 1656.2322 | 1656.7441 | 3 | 4.532 | 53.6% | 1 | K.ILATDVSSKNT#PDSK.K | 2 |
| \* | Mis12IP\_Nocodazole\_MudPIT\_040709\_02.04865.04865.2 | 3.3418 | 0.5239 | 100.0% | 1558.2122 | 1558.6451 | 1 | 7.577 | 73.1% | 1 | R.TIS\*PAESTALFQGR.K | 2 |
|  | Mis12IP\_Nocodazole\_MudPIT\_040709\_06.07820.07820.2 | 4.1047 | 0.4819 | 100.0% | 2030.7322 | 2030.242 | 1 | 8.278 | 64.7% | 1 | K.TVLDTSYALIGETVNNYR.S | 2 |

---

|  |  |  |  |  |  |  |  |  |
| --- | --- | --- | --- | --- | --- | --- | --- | --- |
| U | *gi|14043271|gb|AAH076* | 7 | 10 | 8.0% | 400 | 44092 | 5.1 | Keratin 19 [Homo sapiens] |
| U | *gi|34039|emb|CAA68556* | 7 | 10 | 8.0% | 400 | 44106 | 5.1 | unnamed protein product [Homo sapiens] |

| Filename XCorr DeltCN Conf% ObsM+H+ CalcM+H+ SpR ZScore Ion% # Sequence  | | | | | | | | | | | | |
| --- | --- | --- | --- | --- | --- | --- | --- | --- | --- | --- | --- | --- |
|  | Mis12IP\_Nocodazole\_MudPIT\_040709\_03.04508.04508.1 | 1.9048 | 0.2501 | 99.0% | 809.5 | 809.93774 | 160 | 5.178 | 66.7% | 1 | R.LASYLDK.V | 1111111 |
|  | Mis12IP\_Nocodazole\_MudPIT\_040709\_05.05122.05122.2 | 2.6251 | 0.2664 | 99.8% | 1041.4722 | 1042.2235 | 1 | 5.746 | 87.5% | 1 | R.IVLQIDNAR.L | 222 |
|  | Mis12IP\_Nocodazole\_MudPIT\_040709\_06.05825.05825.1 | 2.4765 | 0.3556 | 100.0% | 1041.51 | 1042.2235 | 2 | 6.107 | 68.8% | 1 | R.IVLQIDNAR.L | 111 |
|  | Mis12IP\_Nocodazole\_MudPIT\_040709\_02.04220.04220.1 | 1.5217 | 0.3222 | 99.0% | 807.44 | 807.8815 | 2 | 4.741 | 58.3% | 1 | R.LAADDFR.T | 11111111 |
|  | Mis12IP\_Nocodazole\_MudPIT\_040709\_03.04543.04543.2 | 2.2948 | 0.233 | 99.3% | 808.0522 | 807.8815 | 1 | 5.671 | 91.7% | 3 | R.LAADDFR.T | 22222222 |
|  | Mis12IP\_Nocodazole\_MudPIT\_040709\_06.06210.06210.2 | 3.7407 | 0.4163 | 100.0% | 1030.0122 | 1030.2096 | 1 | 7.093 | 93.8% | 2 | R.VLDELTLAR.T | 22222 |
|  | Mis12IP\_Nocodazole\_MudPIT\_040709\_06.06209.06209.1 | 2.5279 | 0.3193 | 100.0% | 1030.56 | 1030.2096 | 6 | 5.521 | 62.5% | 1 | R.VLDELTLAR.T | 11111 |

Similarities:
gi|4557888|ref|NP\_000(4:3)  
gi|62897747|dbj|BAD96(2:5)  
gi|28317|emb|CAA32649(1:6)  
gi|24430192|ref|NP\_00(5:2)  
gi|12803709|gb|AAH026(5:2)  
gi|4557701|ref|NP\_000(5:2)  
gi|435476|emb|CAA8231(1:6)  
gi|24430190|ref|NP\_00(5:2)  
gi|34526448|dbj|BAC85(2:5)  
gi|85566621|gb|AAI119(2:5)  

---

|  |  |  |  |  |  |  |  |  |
| --- | --- | --- | --- | --- | --- | --- | --- | --- |
| U | *gi|2306915|gb|AAB6578* | 2 | 3 | 7.8% | 347 | 39717 | 9.5 | protein kinase [Homo sapiens] |
| U | *gi|83776600|ref|NP\_00* | 2 | 3 | 7.8% | 344 | 39311 | 9.3 | aurora kinase B [Homo sapiens] |
| U | *gi|5688866|dbj|BAA827* | 2 | 3 | 7.9% | 343 | 39147 | 9.2 | Aik2 [Homo sapiens] |
| U | *gi|38197155|gb|AAH004* | 2 | 3 | 7.4% | 364 | 41307 | 9.3 | AURKB protein [Homo sapiens] |
| U | *gi|33870194|gb|AAH133* | 2 | 3 | 7.3% | 371 | 42194 | 9.4 | AURKB protein [Homo sapiens] |
| U | *gi|2979630|gb|AAC1270* | 2 | 3 | 7.8% | 344 | 39280 | 9.3 | aurora-related kinase 2 [Homo sapiens] |

| Filename XCorr DeltCN Conf% ObsM+H+ CalcM+H+ SpR ZScore Ion% # Sequence  | | | | | | | | | | | | |
| --- | --- | --- | --- | --- | --- | --- | --- | --- | --- | --- | --- | --- |
|  | Mis12IP\_Nocodazole\_MudPIT\_040709\_06.05575.05575.2 | 2.0813 | 0.2569 | 98.1% | 1356.2122 | 1356.5216 | 1 | 4.787 | 58.3% | 1 | R.QTAPSGLSTLPQR.V | 2 |
|  | Mis12IP\_Nocodazole\_MudPIT\_040709\_06.05780.05780.2 | 3.6351 | 0.4416 | 100.0% | 1520.1921 | 1520.6978 | 1 | 7.7 | 65.4% | 2 | K.VMENSSGTPDILTR.H | 2 |

---

|  |  |  |  |  |  |  |  |  |
| --- | --- | --- | --- | --- | --- | --- | --- | --- |
| U | *gi|50417352|gb|AAH770* | 4 | 6 | 7.7% | 684 | 73910 | 6.6 | DPYSL3 protein [Homo sapiens] |

| Filename XCorr DeltCN Conf% ObsM+H+ CalcM+H+ SpR ZScore Ion% # Sequence  | | | | | | | | | | | | |
| --- | --- | --- | --- | --- | --- | --- | --- | --- | --- | --- | --- | --- |
| \* | Mis12IP\_Nocodazole\_MudPIT\_040709\_06.07469.07469.3 | 2.4435 | 0.2907 | 98.1% | 3525.2944 | 3526.9 | 10 | 4.961 | 17.2% | 1 | K.S\*DRLLIKGGRIVNDDQSFYADIYMEDGLIK.Q | 3 |
|  | Mis12IP\_Nocodazole\_MudPIT\_040709\_05.07367.07367.2 | 4.0573 | 0.3989 | 100.0% | 2349.4321 | 2350.602 | 1 | 7.299 | 47.4% | 2 | R.IVNDDQSFYADIYMEDGLIK.Q | 22 |
|  | Mis12IP\_Nocodazole\_MudPIT\_040709\_06.06136.06136.1 | 2.1294 | 0.1816 | 99.2% | 878.39 | 879.0619 | 9 | 5.554 | 66.7% | 1 | R.MSVIWDK.A | 11 |
|  | Mis12IP\_Nocodazole\_MudPIT\_040709\_05.05145.05145.2 | 5.4121 | 0.0873 | 99.9% | 1726.0521 | 1726.8988 | 2 | 9.357 | 60.0% | 2 | K.MDENQFVAVTSTNAAK.I | 222 |

Similarities:
gi|4503377|ref|NP\_001(3:1)  
gi|4503051|ref|NP\_001(1:3)  

---

|  |  |  |  |  |  |  |  |  |
| --- | --- | --- | --- | --- | --- | --- | --- | --- |
| U | *gi|20149594|ref|NP\_03* | 5 | 7 | 7.6% | 724 | 83264 | 5.0 | heat shock 90kDa protein 1, beta [Homo sapiens] |
| U | *gi|6807647|emb|CAB664* | 5 | 7 | 7.5% | 737 | 84843 | 5.4 | hypothetical protein [Homo sapiens] |

| Filename XCorr DeltCN Conf% ObsM+H+ CalcM+H+ SpR ZScore Ion% # Sequence  | | | | | | | | | | | | |
| --- | --- | --- | --- | --- | --- | --- | --- | --- | --- | --- | --- | --- |
|  | Mis12IP\_Nocodazole\_MudPIT\_040709\_06.05998.05998.2 | 2.3328 | 0.2108 | 98.9% | 1195.1522 | 1195.361 | 279 | 4.011 | 55.6% | 1 | K.IDIIPNPQER.T | 2 |
|  | Mis12IP\_Nocodazole\_MudPIT\_040709\_06.06371.06371.2 | 3.8076 | 0.4932 | 100.0% | 1242.7922 | 1243.4459 | 1 | 7.351 | 77.3% | 1 | K.ADLINNLGTIAK.S | 22 |
|  | Mis12IP\_Nocodazole\_MudPIT\_040709\_05.05446.05446.2 | 5.028 | 0.5461 | 100.0% | 1848.3121 | 1848.9171 | 1 | 10.28 | 82.1% | 3 | R.NPDDITQEEYGEFYK.S | 2 |
|  | Mis12IP\_Nocodazole\_MudPIT\_040709\_06.05790.05790.1 | 2.0043 | 0.2832 | 98.5% | 891.32 | 891.99884 | 2 | 5.117 | 75.0% | 1 | K.FYEAFSK.N | 1 |
|  | Mis12IP\_Nocodazole\_MudPIT\_040709\_03.04491.04491.2 | 2.0722 | 0.4553 | 99.9% | 1250.0721 | 1250.3538 | 8 | 6.85 | 50.0% | 1 | K.EQVANSAFVER.V | 2 |

Similarities:
gi|40254816|ref|NP\_00(1:4)  

---

|  |  |  |  |  |  |  |  |  |
| --- | --- | --- | --- | --- | --- | --- | --- | --- |
| U | *gi|48145673|emb|CAG33* | 2 | 3 | 7.6% | 449 | 49130 | 6.2 | HNRPH1 [Homo sapiens] |
| U | *gi|5031753|ref|NP\_005* | 2 | 3 | 7.6% | 449 | 49229 | 6.3 | heterogeneous nuclear ribonucleoprotein H1 [Homo sapiens] |

| Filename XCorr DeltCN Conf% ObsM+H+ CalcM+H+ SpR ZScore Ion% # Sequence  | | | | | | | | | | | | |
| --- | --- | --- | --- | --- | --- | --- | --- | --- | --- | --- | --- | --- |
|  | Mis12IP\_Nocodazole\_MudPIT\_040709\_06.07013.07013.2 | 4.6907 | 0.5584 | 100.0% | 1842.1721 | 1843.0001 | 1 | 10.304 | 68.8% | 2 | R.STGEAFVQFASQEIAEK.A | 2 |
|  | Mis12IP\_Nocodazole\_MudPIT\_040709\_06.08107.08107.2 | 4.5763 | 0.489 | 100.0% | 1997.3722 | 1998.2023 | 1 | 7.96 | 62.5% | 1 | R.ATENDIYNFFSPLNPVR.V | 22 |

Similarities:
gi|16876910|gb|AAH167(1:1)  

---

|  |  |  |  |  |  |  |  |  |
| --- | --- | --- | --- | --- | --- | --- | --- | --- |
| U | *gi|12654583|gb|AAH011* | 2 | 2 | 7.6% | 317 | 34274 | 5.6 | Ribosomal protein, large, P0 [Homo sapiens] |
| U | *gi|78070390|gb|AAI077* | 2 | 2 | 9.4% | 255 | 27436 | 8.2 | RPLP0 protein [Homo sapiens] |
| U | *gi|62896701|dbj|BAD96* | 2 | 2 | 7.6% | 317 | 34213 | 6.0 | ribosomal protein P0 variant [Homo sapiens] |
| U | *gi|47123412|gb|AAH701* | 2 | 2 | 9.4% | 254 | 27299 | 8.2 | RPLP0 protein [Homo sapiens] |
| U | *gi|4506667|ref|NP\_000* | 2 | 2 | 7.6% | 317 | 34274 | 6.0 | ribosomal protein P0 [Homo sapiens] |

| Filename XCorr DeltCN Conf% ObsM+H+ CalcM+H+ SpR ZScore Ion% # Sequence  | | | | | | | | | | | | |
| --- | --- | --- | --- | --- | --- | --- | --- | --- | --- | --- | --- | --- |
|  | Mis12IP\_Nocodazole\_MudPIT\_040709\_05.04861.04861.1 | 1.7786 | 0.2477 | 99.2% | 717.49 | 717.9451 | 1 | 6.484 | 75.0% | 1 | K.AVVLMGK.N | 1 |
|  | Mis12IP\_Nocodazole\_MudPIT\_040709\_06.07486.07486.2 | 2.8118 | 0.3903 | 100.0% | 1896.7522 | 1897.1754 | 1 | 7.341 | 50.0% | 1 | R.VLALSVETDYTFPLAEK.V | 2 |

---

|  |  |  |  |  |  |  |  |  |
| --- | --- | --- | --- | --- | --- | --- | --- | --- |
| U | *gi|109658796|gb|AAI17* | 7 | 9 | 7.4% | 1306 | 150352 | 5.7 | KTN1 protein [Homo sapiens] |
| U | *gi|40789066|dbj|BAA02* | 7 | 9 | 7.4% | 1307 | 150417 | 5.7 | KIAA0004 [Homo sapiens] |
| U | *gi|33620775|ref|NP\_89* | 7 | 9 | 7.1% | 1357 | 156275 | 5.6 | kinectin 1 [Homo sapiens] |

| Filename XCorr DeltCN Conf% ObsM+H+ CalcM+H+ SpR ZScore Ion% # Sequence  | | | | | | | | | | | | |
| --- | --- | --- | --- | --- | --- | --- | --- | --- | --- | --- | --- | --- |
|  | Mis12IP\_Nocodazole\_MudPIT\_040709\_06.07068.07068.3 | 3.8981 | 0.4694 | 100.0% | 2812.8843 | 2812.1033 | 1 | 6.832 | 30.8% | 2 | K.LSDALAVEDDQVAPVPLNVVETSSSVR.E | 3 |
|  | Mis12IP\_Nocodazole\_MudPIT\_040709\_03.04328.04328.1 | 1.691 | 0.2576 | 99.2% | 788.38 | 788.92236 | 26 | 5.276 | 66.7% | 1 | R.SNVVITR.M | 1 |
|  | Mis12IP\_Nocodazole\_MudPIT\_040709\_06.07939.07939.2 | 2.7857 | 0.3891 | 100.0% | 1372.7522 | 1373.5865 | 7 | 6.182 | 68.2% | 1 | K.SVEELLEAELLK.V | 2 |
|  | Mis12IP\_Nocodazole\_MudPIT\_040709\_06.06248.06248.2 | 3.4487 | 0.3334 | 100.0% | 1631.5922 | 1632.7673 | 1 | 7.638 | 66.7% | 1 | K.WLQDLQEENESLK.A | 2 |
|  | Mis12IP\_Nocodazole\_MudPIT\_040709\_06.06361.06361.2 | 4.0611 | 0.3869 | 100.0% | 1288.6122 | 1288.4839 | 1 | 6.556 | 72.7% | 1 | K.SVLAETEGILQK.L | 2 |
|  | Mis12IP\_Nocodazole\_MudPIT\_040709\_04.05559.05559.2 | 3.4548 | 0.4352 | 100.0% | 1788.0521 | 1787.8958 | 1 | 6.696 | 57.1% | 1 | K.QMQSSFTSSEQELER.L | 2 |
|  | Mis12IP\_Nocodazole\_MudPIT\_040709\_06.05758.05758.2 | 4.2594 | 0.3455 | 100.0% | 1245.0922 | 1245.4185 | 1 | 6.632 | 80.0% | 2 | K.AQQSLELIQSK.I | 2 |

---

|  |  |  |  |  |  |  |  |  |
| --- | --- | --- | --- | --- | --- | --- | --- | --- |
| U | *gi|24234696|ref|NP\_70* | 4 | 5 | 7.4% | 458 | 49586 | 5.0 | keratin 13 isoform a [Homo sapiens] |
| U | *gi|62897663|dbj|BAD96* | 4 | 5 | 7.4% | 458 | 49685 | 5.0 | keratin 13 isoform a variant [Homo sapiens] |
| U | *gi|50603586|gb|AAH777* | 4 | 5 | 7.2% | 471 | 50877 | 5.0 | KRT13 protein [Homo sapiens] |
| U | *gi|38114739|gb|AAH026* | 4 | 5 | 7.2% | 473 | 51061 | 5.0 | KRT13 protein [Homo sapiens] |
| U | *gi|34033|emb|CAA32786* | 4 | 5 | 7.4% | 458 | 49644 | 4.9 | keratin 13 [Homo sapiens] |

| Filename XCorr DeltCN Conf% ObsM+H+ CalcM+H+ SpR ZScore Ion% # Sequence  | | | | | | | | | | | | |
| --- | --- | --- | --- | --- | --- | --- | --- | --- | --- | --- | --- | --- |
|  | Mis12IP\_Nocodazole\_MudPIT\_040709\_06.05832.05832.1 | 2.8375 | 0.1798 | 98.8% | 1043.55 | 1043.2083 | 1 | 4.735 | 75.0% | 1 | R.VILEIDNAR.L | 11 |
|  | Mis12IP\_Nocodazole\_MudPIT\_040709\_06.05827.05827.2 | 3.4496 | 0.1026 | 99.5% | 1043.8522 | 1043.2083 | 1 | 5.399 | 87.5% | 1 | R.VILEIDNAR.L | 22 |
|  | Mis12IP\_Nocodazole\_MudPIT\_040709\_05.05008.05008.2 | 2.7343 | 0.3118 | 99.9% | 1201.9521 | 1202.3097 | 1 | 5.738 | 80.0% | 2 | R.QSVEADINGLR.R | 222 |
|  | Mis12IP\_Nocodazole\_MudPIT\_040709\_03.05257.05257.2 | 2.9548 | 0.4323 | 100.0% | 1473.0322 | 1473.6006 | 1 | 7.443 | 61.5% | 1 | K.MIGFPSSAGSVS\*PR.S | 2 |

Similarities:
gi|28317|emb|CAA32649(1:3)  
gi|24430190|ref|NP\_00(2:2)  
gi|85566621|gb|AAI119(1:3)  

---

|  |  |  |  |  |  |  |  |  |
| --- | --- | --- | --- | --- | --- | --- | --- | --- |
| U | *gi|40788895|dbj|BAA11* | 3 | 3 | 7.0% | 655 | 71447 | 7.0 | KIAA0171 [Homo sapiens] |
| U | *gi|7661968|ref|NP\_055* | 3 | 3 | 7.4% | 625 | 68259 | 6.4 | epsin 4 [Homo sapiens] |

| Filename XCorr DeltCN Conf% ObsM+H+ CalcM+H+ SpR ZScore Ion% # Sequence  | | | | | | | | | | | | |
| --- | --- | --- | --- | --- | --- | --- | --- | --- | --- | --- | --- | --- |
|  | Mis12IP\_Nocodazole\_MudPIT\_040709\_05.05207.05207.2 | 3.721 | 0.5093 | 100.0% | 1584.8722 | 1585.7693 | 1 | 9.698 | 65.4% | 1 | K.ATNVVMNYSEIESK.V | 2 |
|  | Mis12IP\_Nocodazole\_MudPIT\_040709\_06.06905.06905.2 | 2.4377 | 0.3505 | 99.7% | 2116.8323 | 2117.2947 | 68 | 5.32 | 34.2% | 1 | R.EATNDDPWGPSGQLMGEIAK.A | 2 |
|  | Mis12IP\_Nocodazole\_MudPIT\_040709\_06.06620.06620.2 | 1.9397 | 0.2191 | 95.7% | 1359.3322 | 1360.4686 | 70 | 5.1 | 50.0% | 1 | K.QDAFANFANFSK.- | 2 |

---

|  |  |  |  |  |  |  |  |  |
| --- | --- | --- | --- | --- | --- | --- | --- | --- |
| U | *gi|24430190|ref|NP\_00* | 7 | 10 | 7.0% | 456 | 49198 | 4.8 | keratin 15 [Homo sapiens] |
| U | *gi|34071|emb|CAA30535* | 7 | 10 | 7.0% | 456 | 49168 | 4.8 | unnamed protein product [Homo sapiens] |

| Filename XCorr DeltCN Conf% ObsM+H+ CalcM+H+ SpR ZScore Ion% # Sequence  | | | | | | | | | | | | |
| --- | --- | --- | --- | --- | --- | --- | --- | --- | --- | --- | --- | --- |
|  | Mis12IP\_Nocodazole\_MudPIT\_040709\_03.04508.04508.1 | 1.9048 | 0.2501 | 99.0% | 809.5 | 809.93774 | 160 | 5.178 | 66.7% | 1 | R.LASYLDK.V | 1111111 |
|  | Mis12IP\_Nocodazole\_MudPIT\_040709\_06.05832.05832.1 | 2.8375 | 0.1798 | 98.8% | 1043.55 | 1043.2083 | 1 | 4.735 | 75.0% | 1 | R.VILEIDNAR.L | 11 |
|  | Mis12IP\_Nocodazole\_MudPIT\_040709\_06.05827.05827.2 | 3.4496 | 0.1026 | 99.5% | 1043.8522 | 1043.2083 | 1 | 5.399 | 87.5% | 1 | R.VILEIDNAR.L | 22 |
|  | Mis12IP\_Nocodazole\_MudPIT\_040709\_02.04220.04220.1 | 1.5217 | 0.3222 | 99.0% | 807.44 | 807.8815 | 2 | 4.741 | 58.3% | 1 | R.LAADDFR.L | 11111111 |
|  | Mis12IP\_Nocodazole\_MudPIT\_040709\_03.04543.04543.2 | 2.2948 | 0.233 | 99.3% | 808.0522 | 807.8815 | 1 | 5.671 | 91.7% | 3 | R.LAADDFR.L | 22222222 |
|  | Mis12IP\_Nocodazole\_MudPIT\_040709\_06.06210.06210.2 | 3.7407 | 0.4163 | 100.0% | 1030.0122 | 1030.2096 | 1 | 7.093 | 93.8% | 2 | R.VLDELTLAR.T | 22222 |
|  | Mis12IP\_Nocodazole\_MudPIT\_040709\_06.06209.06209.1 | 2.5279 | 0.3193 | 100.0% | 1030.56 | 1030.2096 | 6 | 5.521 | 62.5% | 1 | R.VLDELTLAR.T | 11111 |

Similarities:
gi|4557888|ref|NP\_000(2:5)  
gi|28317|emb|CAA32649(1:6)  
gi|24430192|ref|NP\_00(5:2)  
gi|12803709|gb|AAH026(5:2)  
gi|4557701|ref|NP\_000(5:2)  
gi|435476|emb|CAA8231(1:6)  
gi|14043271|gb|AAH076(5:2)  
gi|24234696|ref|NP\_70(2:5)  
gi|34526448|dbj|BAC85(2:5)  
gi|85566621|gb|AAI119(2:5)  

---

|  |  |  |  |  |  |  |  |  |
| --- | --- | --- | --- | --- | --- | --- | --- | --- |
| U | *gi|17921989|ref|NP\_00* | 2 | 2 | 6.9% | 448 | 49924 | 5.1 | tubulin, alpha 1 [Homo sapiens] |
| U | *gi|32015|emb|CAA30026* | 2 | 2 | 6.9% | 447 | 49793 | 5.1 | alpha-tubulin [Homo sapiens] |

| Filename XCorr DeltCN Conf% ObsM+H+ CalcM+H+ SpR ZScore Ion% # Sequence  | | | | | | | | | | | | |
| --- | --- | --- | --- | --- | --- | --- | --- | --- | --- | --- | --- | --- |
|  | Mis12IP\_Nocodazole\_MudPIT\_040709\_06.08024.08024.2 | 4.0805 | 0.5597 | 100.0% | 2410.8123 | 2410.6885 | 1 | 9.93 | 47.5% | 1 | R.FDGALNVDLTEFQTNLVPYPR.I | 22 |
|  | Mis12IP\_Nocodazole\_MudPIT\_040709\_05.05243.05243.1 | 1.6696 | 0.3693 | 98.6% | 985.52 | 986.1564 | 12 | 5.022 | 55.6% | 1 | K.DVNAAIAAIK.T | 1 |

Similarities:
gi|14389309|ref|NP\_11(1:1)  

---

|  |  |  |  |  |  |  |  |  |
| --- | --- | --- | --- | --- | --- | --- | --- | --- |
| U | *gi|32567786|ref|NP\_78* | 5 | 9 | 6.7% | 535 | 57836 | 7.2 | keratin 6L [Homo sapiens] |

| Filename XCorr DeltCN Conf% ObsM+H+ CalcM+H+ SpR ZScore Ion% # Sequence  | | | | | | | | | | | | |
| --- | --- | --- | --- | --- | --- | --- | --- | --- | --- | --- | --- | --- |
|  | Mis12IP\_Nocodazole\_MudPIT\_040709\_05.05262.05262.1 | 2.1158 | 0.1126 | 95.8% | 827.46 | 827.95544 | 11 | 4.725 | 66.7% | 1 | K.FASFIDK.V | 1111111 |
|  | Mis12IP\_Nocodazole\_MudPIT\_040709\_05.04381.04381.2 | 2.3872 | 0.1809 | 99.0% | 945.6722 | 946.0513 | 10 | 5.236 | 78.6% | 2 | R.GRLDSELR.N | 22222222 |
|  | Mis12IP\_Nocodazole\_MudPIT\_040709\_06.07734.07734.1 | 2.6237 | 0.3674 | 100.0% | 1329.6 | 1330.5211 | 1 | 7.145 | 68.2% | 3 | R.NLDLDSIIAEVK.A | 111111111 |
|  | Mis12IP\_Nocodazole\_MudPIT\_040709\_06.07727.07727.2 | 4.4229 | 0.3694 | 100.0% | 1331.0122 | 1330.5211 | 1 | 7.672 | 81.8% | 2 | R.NLDLDSIIAEVK.A | 222222222 |
|  | Mis12IP\_Nocodazole\_MudPIT\_040709\_04.05859.05859.2 | 2.3297 | 0.2954 | 99.7% | 1139.6322 | 1140.2965 | 2 | 6.109 | 75.0% | 1 | R.DYQELMNVK.L | 22 |

Similarities:
gi|4504919|ref|NP\_002(1:4)  
gi|181402|gb|AAC83410(3:2)  
gi|27465517|ref|NP\_77(3:2)  
gi|46812692|gb|AAH692(3:2)  
gi|21961227|gb|AAH345(3:2)  
gi|17505189|ref|NP\_49(4:1)  
gi|15559584|gb|AAH141(4:1)  
gi|5031841|ref|NP\_005(4:1)  
gi|18999435|gb|AAH242(3:2)  
gi|1200072|emb|CAA316(1:4)  
gi|45597458|ref|NP\_77(1:4)  

---

|  |  |  |  |  |  |  |  |  |
| --- | --- | --- | --- | --- | --- | --- | --- | --- |
| U | *gi|117938834|gb|AAH17* | 5 | 9 | 6.5% | 827 | 92486 | 6.4 | Unknown (protein for IMAGE:3914054) [Homo sapiens] |
| U | *gi|4757878|ref|NP\_004* | 5 | 9 | 5.0% | 1085 | 122375 | 6.5 | BUB1 budding uninhibited by benzimidazoles 1 homolog [Homo sapiens] |
| U | *gi|2981233|gb|AAC0625* | 5 | 9 | 5.0% | 1085 | 122389 | 6.5 | mitotic checkpoint kinase Bub1 [Homo sapiens] |

| Filename XCorr DeltCN Conf% ObsM+H+ CalcM+H+ SpR ZScore Ion% # Sequence  | | | | | | | | | | | | |
| --- | --- | --- | --- | --- | --- | --- | --- | --- | --- | --- | --- | --- |
|  | Mis12IP\_Nocodazole\_MudPIT\_040709\_04.05511.05511.2 | 2.3605 | 0.1664 | 98.9% | 1111.7922 | 1112.2303 | 2 | 4.992 | 71.4% | 1 | R.EFLQQQYR.L | 2 |
|  | Mis12IP\_Nocodazole\_MudPIT\_040709\_06.06310.06310.2 | 3.2094 | 0.3426 | 100.0% | 1292.0521 | 1292.4314 | 24 | 6.748 | 70.0% | 2 | K.EANAFEEQLLK.Q | 2 |
|  | Mis12IP\_Nocodazole\_MudPIT\_040709\_06.05466.05466.2 | 3.0288 | 0.2602 | 99.8% | 1288.5922 | 1289.4491 | 2 | 4.855 | 72.7% | 3 | R.MGPSVGSQQELR.A | 2 |
|  | Mis12IP\_Nocodazole\_MudPIT\_040709\_06.06186.06186.2 | 4.2312 | 0.5269 | 100.0% | 1455.8322 | 1456.6538 | 1 | 10.189 | 80.8% | 2 | K.AQTVTDSMFAVASK.D | 2 |
|  | Mis12IP\_Nocodazole\_MudPIT\_040709\_04.05320.05320.2 | 2.898 | 0.2096 | 99.7% | 905.9522 | 905.98596 | 9 | 5.488 | 75.0% | 1 | R.SSGAWGVNK.I | 2 |

---

|  |  |  |  |  |  |  |  |  |
| --- | --- | --- | --- | --- | --- | --- | --- | --- |
| U | *gi|14327896|ref|NP\_11* | 2 | 2 | 6.5% | 433 | 48337 | 7.5 | cyclin B1 [Homo sapiens] |

| Filename XCorr DeltCN Conf% ObsM+H+ CalcM+H+ SpR ZScore Ion% # Sequence  | | | | | | | | | | | | |
| --- | --- | --- | --- | --- | --- | --- | --- | --- | --- | --- | --- | --- |
| \* | Mis12IP\_Nocodazole\_MudPIT\_040709\_06.05651.05651.1 | 2.3923 | 0.4821 | 100.0% | 1202.6 | 1203.4424 | 3 | 7.542 | 60.0% | 1 | K.NVVMVNQGLTK.H | 1 |
|  | Mis12IP\_Nocodazole\_MudPIT\_040709\_06.07374.07374.2 | 3.5333 | 0.5277 | 100.0% | 1811.0122 | 1812.1167 | 1 | 7.926 | 62.5% | 1 | K.ISTLPQLNSALVQDLAK.A | 2 |

---

|  |  |  |  |  |  |  |  |  |
| --- | --- | --- | --- | --- | --- | --- | --- | --- |
| U | *gi|14043024|ref|NP\_00* | 3 | 4 | 6.4% | 575 | 61595 | 7.0 | BCL2-associated athanogene 3 [Homo sapiens] |
| U | *gi|6808299|emb|CAB708* | 3 | 4 | 6.0% | 616 | 65842 | 8.0 | hypothetical protein [Homo sapiens] |
| U | *gi|6724086|gb|AAF2683* | 3 | 4 | 6.4% | 575 | 61648 | 7.1 | Bcl-2-binding protein BIS [Homo sapiens] |
| U | *gi|62897159|dbj|BAD96* | 3 | 4 | 6.4% | 575 | 61604 | 7.4 | BCL2-associated athanogene 3 variant [Homo sapiens] |
| U | *gi|5868898|gb|AAD1612* | 3 | 4 | 6.4% | 575 | 61623 | 7.2 | BAG-family molecular chaperone regulator-3; BAG-3 [Homo sapiens] |

| Filename XCorr DeltCN Conf% ObsM+H+ CalcM+H+ SpR ZScore Ion% # Sequence  | | | | | | | | | | | | |
| --- | --- | --- | --- | --- | --- | --- | --- | --- | --- | --- | --- | --- |
|  | Mis12IP\_Nocodazole\_MudPIT\_040709\_06.06415.06415.2 | 3.71 | 0.5115 | 100.0% | 1534.3722 | 1534.6659 | 1 | 9.377 | 65.4% | 1 | K.VQGLEQAVDNFEGK.K | 2 |
|  | Mis12IP\_Nocodazole\_MudPIT\_040709\_06.07387.07387.2 | 2.5599 | 0.2806 | 99.7% | 1302.7722 | 1303.5562 | 5 | 5.25 | 61.1% | 1 | K.YLMIEEYLTK.E | 2 |
|  | Mis12IP\_Nocodazole\_MudPIT\_040709\_05.06012.06012.2 | 4.0377 | 0.5069 | 100.0% | 1414.2922 | 1414.5553 | 1 | 8.724 | 75.0% | 2 | K.ELLALDSVDPEGR.A | 2 |

---

|  |  |  |  |  |  |  |  |  |
| --- | --- | --- | --- | --- | --- | --- | --- | --- |
| U | *gi|117320527|ref|NP\_0* | 4 | 5 | 6.3% | 899 | 96678 | 6.2 | nuclear factor of kappa light polypeptide gene enhancer in B-cells 2 isoform b [Homo sapiens] |
| U | *gi|117320531|ref|NP\_0* | 4 | 5 | 6.3% | 900 | 96749 | 6.2 | nuclear factor of kappa light polypeptide gene enhancer in B-cells 2 isoform a [Homo sapiens] |

| Filename XCorr DeltCN Conf% ObsM+H+ CalcM+H+ SpR ZScore Ion% # Sequence  | | | | | | | | | | | | |
| --- | --- | --- | --- | --- | --- | --- | --- | --- | --- | --- | --- | --- |
|  | Mis12IP\_Nocodazole\_MudPIT\_040709\_05.06148.06148.2 | 2.4954 | 0.4185 | 99.9% | 2053.8123 | 2054.3044 | 1 | 6.482 | 33.3% | 1 | K.EPAPETADGPYLVIVEQPK.Q | 2 |
|  | Mis12IP\_Nocodazole\_MudPIT\_040709\_06.06134.06134.2 | 3.787 | 0.4279 | 100.0% | 1264.8922 | 1265.4087 | 1 | 8.888 | 68.2% | 2 | R.ALLDYGVTADAR.A | 2 |
|  | Mis12IP\_Nocodazole\_MudPIT\_040709\_06.10249.10249.2 | 5.64 | 0.5717 | 100.0% | 2666.5723 | 2666.96 | 1 | 11.274 | 50.0% | 1 | R.SYELAGGDLAGLLEALSDMGLEEGVR.L | 2 |
|  | Mis12IP\_Nocodazole\_MudPIT\_040709\_06.10246.10246.3 | 5.3187 | 0.4773 | 100.0% | 2667.5942 | 2666.96 | 1 | 9.111 | 38.0% | 1 | R.SYELAGGDLAGLLEALSDMGLEEGVR.L | 3 |

---

|  |  |  |  |  |  |  |  |  |
| --- | --- | --- | --- | --- | --- | --- | --- | --- |
| U | *gi|1899055|gb|AAB4997* | 2 | 2 | 6.2% | 583 | 62852 | 6.3 | p66shc [Homo sapiens] |
| U | *gi|62087278|dbj|BAD92* | 2 | 2 | 9.5% | 377 | 40958 | 6.1 | SHC (Src homology 2 domain containing) transforming protein 1 isoform p66Shc variant [Homo sapiens] |
| U | *gi|55960116|emb|CAI13* | 2 | 2 | 9.4% | 384 | 41979 | 5.9 | SHC (Src homology 2 domain containing) transforming protein 1 [Homo sapiens] |
| U | *gi|55960113|emb|CAI13* | 2 | 2 | 6.2% | 584 | 62893 | 6.4 | SHC (Src homology 2 domain containing) transforming protein 1 [Homo sapiens] |
| U | *gi|55960112|emb|CAI13* | 2 | 2 | 6.2% | 583 | 62822 | 6.4 | SHC (Src homology 2 domain containing) transforming protein 1 [Homo sapiens] |
| U | *gi|55960107|emb|CAI13* | 2 | 2 | 12.2% | 296 | 32507 | 7.0 | SHC (Src homology 2 domain containing) transforming protein 1 [Homo sapiens] |
| U | *gi|52693921|ref|NP\_89* | 2 | 2 | 6.2% | 583 | 62849 | 6.5 | SHC (Src homology 2 domain containing) transforming protein 1 isoform p66Shc [Homo sapiens] |
| U | *gi|36454|emb|CAA48251* | 2 | 2 | 7.6% | 473 | 51611 | 7.2 | SHC transforming protein [Homo sapiens] |
| U | *gi|32261324|ref|NP\_00* | 2 | 2 | 7.6% | 474 | 51682 | 7.2 | SHC (Src homology 2 domain containing) transforming protein 1 isoform p52Shc [Homo sapiens] |
| U | *gi|21706900|gb|AAH339* | 2 | 2 | 9.8% | 369 | 40415 | 6.0 | SHC1 protein [Homo sapiens] |

| Filename XCorr DeltCN Conf% ObsM+H+ CalcM+H+ SpR ZScore Ion% # Sequence  | | | | | | | | | | | | |
| --- | --- | --- | --- | --- | --- | --- | --- | --- | --- | --- | --- | --- |
|  | Mis12IP\_Nocodazole\_MudPIT\_040709\_05.06209.06209.2 | 2.905 | 0.4079 | 100.0% | 1896.2522 | 1897.0483 | 1 | 6.916 | 60.0% | 1 | R.ELFDDPSYVNVQNLDK.A | 2 |
|  | Mis12IP\_Nocodazole\_MudPIT\_040709\_06.06428.06428.2 | 2.6114 | 0.3526 | 99.9% | 2093.2322 | 2093.298 | 1 | 5.957 | 42.1% | 1 | R.ESTTTPGQYVLTGLQSGQPK.H | 2 |

---

|  |  |  |  |  |  |  |  |  |
| --- | --- | --- | --- | --- | --- | --- | --- | --- |
| U | *gi|23398532|gb|AAH382* | 2 | 2 | 6.2% | 504 | 54462 | 5.2 | YAP1 protein [Homo sapiens] |
| U | *gi|5174751|ref|NP\_006* | 2 | 2 | 6.8% | 454 | 48755 | 5.1 | Yes-associated protein 1, 65 kD [Homo sapiens] |
| U | *gi|33086922|gb|AAP927* | 2 | 2 | 6.4% | 488 | 52748 | 5.1 | yes-associated protein 2 [Homo sapiens] |

| Filename XCorr DeltCN Conf% ObsM+H+ CalcM+H+ SpR ZScore Ion% # Sequence  | | | | | | | | | | | | |
| --- | --- | --- | --- | --- | --- | --- | --- | --- | --- | --- | --- | --- |
|  | Mis12IP\_Nocodazole\_MudPIT\_040709\_06.05503.05503.2 | 2.3813 | 0.221 | 99.0% | 1213.9321 | 1214.4253 | 23 | 4.465 | 60.0% | 1 | K.TANVPQTVPMR.L | 2 |
|  | Mis12IP\_Nocodazole\_MudPIT\_040709\_06.07643.07643.2 | 2.6362 | 0.422 | 100.0% | 2218.5522 | 2218.4858 | 8 | 5.627 | 34.2% | 1 | R.QSSFEIPDDVPLPAGWEMAK.T | 2 |

---

|  |  |  |  |  |  |  |  |  |
| --- | --- | --- | --- | --- | --- | --- | --- | --- |
| U | *gi|12667788|ref|NP\_00* | 8 | 10 | 6.1% | 1960 | 226530 | 5.6 | myosin, heavy polypeptide 9, non-muscle [Homo sapiens] |

| Filename XCorr DeltCN Conf% ObsM+H+ CalcM+H+ SpR ZScore Ion% # Sequence  | | | | | | | | | | | | |
| --- | --- | --- | --- | --- | --- | --- | --- | --- | --- | --- | --- | --- |
|  | Mis12IP\_Nocodazole\_MudPIT\_040709\_06.06797.06797.2 | 3.4365 | 0.4536 | 100.0% | 1752.8922 | 1753.0358 | 1 | 8.064 | 67.9% | 1 | R.LTEMETLQSQLMAEK.L | 2 |
|  | Mis12IP\_Nocodazole\_MudPIT\_040709\_06.06796.06796.3 | 3.926 | 0.3622 | 100.0% | 2333.9644 | 2334.4736 | 1 | 6.937 | 43.1% | 1 | K.MQQNIQELEEQLEEEESAR.Q | 3 |
|  | Mis12IP\_Nocodazole\_MudPIT\_040709\_04.05947.05947.2 | 2.769 | 0.4222 | 100.0% | 1653.4722 | 1654.7681 | 1 | 7.224 | 65.4% | 1 | R.IAEFTTNLTEEEEK.S | 2 |
|  | Mis12IP\_Nocodazole\_MudPIT\_040709\_06.07169.07169.2 | 3.6319 | 0.4247 | 100.0% | 1948.0922 | 1947.1498 | 1 | 8.151 | 47.1% | 2 | K.LQVELDNVTGLLSQSDSK.S | 2 |
|  | Mis12IP\_Nocodazole\_MudPIT\_040709\_05.06807.06807.2 | 2.3922 | 0.4343 | 99.9% | 1962.2722 | 1963.0594 | 1 | 7.027 | 43.8% | 1 | K.TQLEELEDELQATEDAK.L | 2 |
|  | Mis12IP\_Nocodazole\_MudPIT\_040709\_05.06124.06124.2 | 4.5384 | 0.5683 | 100.0% | 2472.612 | 2473.6099 | 1 | 9.749 | 50.0% | 1 | R.IAQLEEELEEEQGNTELINDR.L | 2 |
|  | Mis12IP\_Nocodazole\_MudPIT\_040709\_05.06125.06125.3 | 4.7018 | 0.3369 | 100.0% | 2472.9543 | 2473.6099 | 1 | 8.41 | 42.5% | 2 | R.IAQLEEELEEEQGNTELINDR.L | 3 |
|  | Mis12IP\_Nocodazole\_MudPIT\_040709\_06.06618.06618.2 | 4.5301 | 0.2735 | 100.0% | 1872.4321 | 1871.0574 | 1 | 7.29 | 70.0% | 1 | K.ANLQIDQINTDLNLER.S | 2 |

---

|  |  |  |  |  |  |  |  |  |
| --- | --- | --- | --- | --- | --- | --- | --- | --- |
| U | *gi|20809886|gb|AAH295* | 2 | 3 | 6.1% | 445 | 49808 | 4.9 | Tubulin, beta 2C [Homo sapiens] |
| U | *gi|5174735|ref|NP\_006* | 2 | 3 | 6.1% | 445 | 49831 | 4.9 | tubulin, beta, 2 [Homo sapiens] |
| U | *gi|27368062|gb|AAN873* | 2 | 3 | 6.1% | 445 | 49753 | 4.9 | class IVb beta tubulin [Homo sapiens] |
| U | *gi|23958133|gb|AAH240* | 2 | 3 | 6.1% | 445 | 49840 | 4.9 | Tubulin, beta 2C [Homo sapiens] |

| Filename XCorr DeltCN Conf% ObsM+H+ CalcM+H+ SpR ZScore Ion% # Sequence  | | | | | | | | | | | | |
| --- | --- | --- | --- | --- | --- | --- | --- | --- | --- | --- | --- | --- |
|  | Mis12IP\_Nocodazole\_MudPIT\_040709\_04.05496.05496.2 | 3.0833 | 0.3391 | 100.0% | 1328.6122 | 1329.4521 | 1 | 7.238 | 72.7% | 1 | R.INVYYNEATGGK.Y | 2 |
|  | Mis12IP\_Nocodazole\_MudPIT\_040709\_05.06130.06130.2 | 3.9734 | 0.3979 | 100.0% | 1602.1122 | 1602.8431 | 1 | 7.448 | 71.4% | 2 | R.AVLVDLEPGTMDSVR.S | 2 |

---

|  |  |  |  |  |  |  |  |  |
| --- | --- | --- | --- | --- | --- | --- | --- | --- |
| U | *gi|12652739|gb|AAH001* | 2 | 2 | 6.0% | 517 | 58241 | 7.5 | General transcription factor IIF, polypeptide 1, 74kDa [Homo sapiens] |
| U | *gi|48145713|emb|CAG33* | 2 | 2 | 6.0% | 517 | 58226 | 7.5 | GTF2F1 [Homo sapiens] |
| U | *gi|4504197|ref|NP\_002* | 2 | 2 | 6.0% | 517 | 58275 | 7.5 | general transcription factor IIF, polypeptide 1, 74kDa [Homo sapiens] |
| U | *gi|35871|emb|CAA45404* | 2 | 2 | 6.0% | 517 | 58255 | 7.5 | RAP74 [Homo sapiens] |

| Filename XCorr DeltCN Conf% ObsM+H+ CalcM+H+ SpR ZScore Ion% # Sequence  | | | | | | | | | | | | |
| --- | --- | --- | --- | --- | --- | --- | --- | --- | --- | --- | --- | --- |
|  | Mis12IP\_Nocodazole\_MudPIT\_040709\_03.04731.04731.2 | 4.7797 | 0.5412 | 100.0% | 2073.372 | 2074.1829 | 1 | 9.001 | 70.6% | 1 | K.IYQEEEMPESGAGSEFNR.K | 2 |
|  | Mis12IP\_Nocodazole\_MudPIT\_040709\_03.05104.05104.2 | 3.1899 | 0.4699 | 100.0% | 1593.1122 | 1593.6439 | 1 | 7.971 | 58.3% | 1 | R.TLTAEEAEEEWER.R | 2 |

---

|  |  |  |  |  |  |  |  |  |
| --- | --- | --- | --- | --- | --- | --- | --- | --- |
| U | *gi|10437975|dbj|BAB15* | 3 | 3 | 5.6% | 641 | 71086 | 6.5 | unnamed protein product [Homo sapiens] |
| U | *gi|106879206|ref|NP\_0* | 3 | 3 | 5.6% | 641 | 71086 | 6.5 | chromosome 14 open reading frame 169 [Homo sapiens] |

| Filename XCorr DeltCN Conf% ObsM+H+ CalcM+H+ SpR ZScore Ion% # Sequence  | | | | | | | | | | | | |
| --- | --- | --- | --- | --- | --- | --- | --- | --- | --- | --- | --- | --- |
|  | Mis12IP\_Nocodazole\_MudPIT\_040709\_05.05953.05953.2 | 3.0526 | 0.5086 | 100.0% | 2408.9321 | 2410.5999 | 1 | 7.756 | 34.0% | 1 | R.VESTADDLGDALPGGAAVAAVPDAAR.R | 2 |
|  | Mis12IP\_Nocodazole\_MudPIT\_040709\_05.05947.05947.3 | 2.9518 | 0.3354 | 100.0% | 2409.7444 | 2410.5999 | 2 | 5.374 | 29.0% | 1 | R.VESTADDLGDALPGGAAVAAVPDAAR.R | 3 |
|  | Mis12IP\_Nocodazole\_MudPIT\_040709\_06.05374.05374.1 | 2.2162 | 0.3383 | 97.6% | 978.61 | 979.16736 | 1 | 5.828 | 72.2% | 1 | R.LVPASAPPAR.L | 1 |

---

|  |  |  |  |  |  |  |  |  |
| --- | --- | --- | --- | --- | --- | --- | --- | --- |
| U | *gi|10433849|dbj|BAB14* | 2 | 2 | 5.5% | 622 | 68963 | 7.2 | unnamed protein product [Homo sapiens] |
| U | *gi|12383062|ref|NP\_07* | 2 | 2 | 5.5% | 622 | 68935 | 7.2 | kinesin light chain 2 [Homo sapiens] |
| U | *gi|113431408|ref|XP\_0* | 2 | 2 | 5.5% | 615 | 68209 | 7.0 | PREDICTED: similar to kinesin light chain 2 [Homo sapiens] |
| U | *gi|10434570|dbj|BAB14* | 2 | 2 | 5.5% | 622 | 68951 | 7.2 | unnamed protein product [Homo sapiens] |

| Filename XCorr DeltCN Conf% ObsM+H+ CalcM+H+ SpR ZScore Ion% # Sequence  | | | | | | | | | | | | |
| --- | --- | --- | --- | --- | --- | --- | --- | --- | --- | --- | --- | --- |
|  | Mis12IP\_Nocodazole\_MudPIT\_040709\_06.06667.06667.2 | 2.7303 | 0.3666 | 99.9% | 2105.9521 | 2106.3403 | 1 | 5.693 | 42.5% | 1 | R.ALLAPLVAPEAGEAEPGSQER.C | 2 |
|  | Mis12IP\_Nocodazole\_MudPIT\_040709\_05.04579.04579.2 | 2.1482 | 0.3227 | 99.3% | 1391.2322 | 1391.5265 | 1 | 5.794 | 54.2% | 1 | K.LQGGTPQEPPNPR.M | 2 |

---

|  |  |  |  |  |  |  |  |  |
| --- | --- | --- | --- | --- | --- | --- | --- | --- |
| U | *gi|112799849|ref|NP\_0* | 4 | 4 | 5.3% | 1134 | 126323 | 6.2 | tumor protein p53 binding protein, 2 isoform 1 [Homo sapiens] |
| U | *gi|4885643|ref|NP\_005* | 4 | 4 | 6.0% | 1005 | 111431 | 6.0 | tumor protein p53 binding protein, 2 isoform 2 [Homo sapiens] |
| U | *gi|37590181|gb|AAH589* | 4 | 4 | 5.3% | 1127 | 125485 | 6.1 | Tumor protein p53 binding protein, 2 [Homo sapiens] |
| U | *gi|16197705|emb|CAC83* | 4 | 4 | 5.3% | 1128 | 125616 | 6.1 | ASPP2 protein [Homo sapiens] |
| U | *gi|116283898|gb|AAH40* | 4 | 4 | 5.7% | 1048 | 116047 | 6.4 | TP53BP2 protein [Homo sapiens] |

| Filename XCorr DeltCN Conf% ObsM+H+ CalcM+H+ SpR ZScore Ion% # Sequence  | | | | | | | | | | | | |
| --- | --- | --- | --- | --- | --- | --- | --- | --- | --- | --- | --- | --- |
|  | Mis12IP\_Nocodazole\_MudPIT\_040709\_06.07223.07223.2 | 4.9487 | 0.4756 | 100.0% | 1608.1921 | 1608.8654 | 1 | 8.379 | 80.8% | 1 | R.MDLTLAELQEMASR.Q | 2 |
|  | Mis12IP\_Nocodazole\_MudPIT\_040709\_06.05648.05648.2 | 2.9158 | 0.3531 | 99.9% | 1755.3121 | 1755.9689 | 1 | 6.43 | 60.7% | 1 | R.QQQQIEAQQQLLATK.E | 2 |
|  | Mis12IP\_Nocodazole\_MudPIT\_040709\_06.07331.07331.2 | 3.7911 | 0.4653 | 100.0% | 1863.0322 | 1864.1235 | 1 | 8.197 | 60.7% | 1 | K.LVEEIEQMNNLFQQK.Q | 2 |
|  | Mis12IP\_Nocodazole\_MudPIT\_040709\_04.05345.05345.2 | 2.2312 | 0.2342 | 97.6% | 1667.2322 | 1668.8009 | 1 | 4.619 | 53.3% | 1 | R.SSITEPEGPNGPNIQK.L | 2 |

---

|  |  |  |  |  |  |  |  |  |
| --- | --- | --- | --- | --- | --- | --- | --- | --- |
| U | *gi|114325466|gb|AAH28* | 4 | 9 | 5.3% | 1042 | 117805 | 6.5 | Superkiller viralicidic activity 2-like 2 (S. cerevisiae) [Homo sapiens] |
| U | *gi|85397868|gb|AAI049* | 4 | 9 | 5.3% | 1042 | 117805 | 6.5 | Superkiller viralicidic activity 2-like 2 (S. cerevisiae) [Homo sapiens] |
| U | *gi|6633995|dbj|BAA061* | 4 | 9 | 5.3% | 1046 | 118256 | 6.6 | KIAA0052 protein [Homo sapiens] |
| U | *gi|40850929|gb|AAH652* | 4 | 9 | 5.3% | 1043 | 117933 | 6.6 | SKIV2L2 protein [Homo sapiens] |
| U | *gi|39930353|ref|NP\_05* | 4 | 9 | 5.3% | 1042 | 117761 | 6.7 | superkiller viralicidic activity 2-like 2 [Homo sapiens] |

| Filename XCorr DeltCN Conf% ObsM+H+ CalcM+H+ SpR ZScore Ion% # Sequence  | | | | | | | | | | | | |
| --- | --- | --- | --- | --- | --- | --- | --- | --- | --- | --- | --- | --- |
|  | Mis12IP\_Nocodazole\_MudPIT\_040709\_02.05403.05403.2 | 4.1032 | 0.5241 | 100.0% | 1569.1721 | 1569.6648 | 1 | 9.155 | 69.2% | 2 | R.DVDFEGTDEPIFGK.K | 2 |
|  | Mis12IP\_Nocodazole\_MudPIT\_040709\_06.08032.08032.2 | 3.9567 | 0.499 | 100.0% | 1932.5922 | 1933.181 | 1 | 7.976 | 56.2% | 5 | R.IEESITEDLSLADLMPR.V | 2 |
|  | Mis12IP\_Nocodazole\_MudPIT\_040709\_06.07927.07927.2 | 3.4477 | 0.3848 | 100.0% | 1399.0721 | 1399.589 | 1 | 7.275 | 75.0% | 1 | K.EYPFILDAFQR.E | 2 |
|  | Mis12IP\_Nocodazole\_MudPIT\_040709\_06.06467.06467.2 | 3.4959 | 0.5188 | 100.0% | 1397.4722 | 1398.6141 | 1 | 8.393 | 79.2% | 1 | R.LGFATSSDVIEMK.G | 22 |

Similarities:
gi|34783197|gb|AAH146(1:3)  

---

|  |  |  |  |  |  |  |  |  |
| --- | --- | --- | --- | --- | --- | --- | --- | --- |
| U | *gi|116063573|ref|NP\_0* | 10 | 10 | 5.2% | 2639 | 280016 | 6.0 | filamin 1 (actin-binding protein-280) [Homo sapiens] |
| U | *gi|57284166|emb|CAI43* | 10 | 10 | 5.2% | 2607 | 276548 | 6.0 | filamin A, alpha (actin binding protein 280) [Homo sapiens] |
| U | *gi|53791221|dbj|BAD52* | 10 | 10 | 5.2% | 2620 | 278224 | 6.1 | filamin A [Homo sapiens] |
| U | *gi|53791219|dbj|BAD52* | 10 | 10 | 5.2% | 2612 | 277503 | 6.0 | filamin A [Homo sapiens] |
| U | *gi|28243|emb|CAA37495* | 10 | 10 | 5.1% | 2647 | 280759 | 6.1 | unnamed protein product [Homo sapiens] |
| U | *gi|21748542|dbj|BAC03* | 10 | 10 | 5.1% | 2651 | 281428 | 6.2 | FLJ00343 protein [Homo sapiens] |
| U | *gi|1203969|gb|AAA9264* | 10 | 10 | 5.1% | 2647 | 280737 | 6.1 | filamin [Homo sapiens] |

| Filename XCorr DeltCN Conf% ObsM+H+ CalcM+H+ SpR ZScore Ion% # Sequence  | | | | | | | | | | | | |
| --- | --- | --- | --- | --- | --- | --- | --- | --- | --- | --- | --- | --- |
|  | Mis12IP\_Nocodazole\_MudPIT\_040709\_06.05726.05726.2 | 2.9534 | 0.342 | 99.9% | 1434.1921 | 1434.6501 | 1 | 6.275 | 61.5% | 1 | R.AYGPGIEPTGNMVK.K | 2 |
|  | Mis12IP\_Nocodazole\_MudPIT\_040709\_06.05879.05879.2 | 4.7021 | 0.5656 | 100.0% | 1571.3322 | 1571.7275 | 1 | 9.299 | 70.6% | 1 | R.GAGTGGLGLAVEGPSEAK.M | 2 |
|  | Mis12IP\_Nocodazole\_MudPIT\_040709\_06.05800.05800.2 | 3.5553 | 0.4825 | 100.0% | 1435.5122 | 1435.5767 | 1 | 8.123 | 79.2% | 1 | R.ANLPQSFQVDTSK.A | 2 |
|  | Mis12IP\_Nocodazole\_MudPIT\_040709\_05.05987.05987.3 | 3.138 | 0.4263 | 100.0% | 2545.4343 | 2545.7654 | 1 | 6.861 | 28.3% | 1 | K.GLVEPVDVVDNADGTQTVNYVPSR.E | 3 |
|  | Mis12IP\_Nocodazole\_MudPIT\_040709\_05.05989.05989.2 | 4.0687 | 0.4951 | 100.0% | 2546.2922 | 2545.7654 | 1 | 8.926 | 52.2% | 1 | K.GLVEPVDVVDNADGTQTVNYVPSR.E | 2 |
|  | Mis12IP\_Nocodazole\_MudPIT\_040709\_05.06329.06329.2 | 2.3553 | 0.3864 | 99.9% | 1911.3322 | 1911.0753 | 23 | 6.602 | 34.4% | 1 | R.EGPYSISVLYGDEEVPR.S | 2 |
|  | Mis12IP\_Nocodazole\_MudPIT\_040709\_06.07258.07258.3 | 3.4794 | 0.2758 | 100.0% | 2342.9644 | 2343.6392 | 1 | 5.825 | 33.7% | 1 | K.ASGPGLNTTGVPASLPVEFTIDAK.D | 3 |
|  | Mis12IP\_Nocodazole\_MudPIT\_040709\_06.07272.07272.2 | 2.5759 | 0.4487 | 100.0% | 2343.172 | 2343.6392 | 2 | 7.378 | 32.6% | 1 | K.ASGPGLNTTGVPASLPVEFTIDAK.D | 2 |
|  | Mis12IP\_Nocodazole\_MudPIT\_040709\_06.06426.06426.2 | 2.7554 | 0.1448 | 98.2% | 1400.9722 | 1401.5181 | 2 | 5.544 | 72.7% | 1 | K.YGGDEIPFSPYR.V | 2 |
|  | Mis12IP\_Nocodazole\_MudPIT\_040709\_06.07109.07109.2 | 2.1026 | 0.1939 | 95.5% | 1534.4521 | 1534.7117 | 12 | 4.196 | 53.8% | 1 | R.AEAGVPAEFSIWTR.E | 2 |

---

|  |  |  |  |  |  |  |  |  |
| --- | --- | --- | --- | --- | --- | --- | --- | --- |
| U | *gi|23273294|gb|AAH349* | 3 | 3 | 5.2% | 729 | 83643 | 6.7 | TANK-binding kinase 1 [Homo sapiens] |
| U | *gi|7019547|ref|NP\_037* | 3 | 3 | 5.2% | 729 | 83642 | 6.8 | TANK-binding kinase 1 [Homo sapiens] |

| Filename XCorr DeltCN Conf% ObsM+H+ CalcM+H+ SpR ZScore Ion% # Sequence  | | | | | | | | | | | | |
| --- | --- | --- | --- | --- | --- | --- | --- | --- | --- | --- | --- | --- |
|  | Mis12IP\_Nocodazole\_MudPIT\_040709\_05.05455.05455.2 | 2.9123 | 0.3458 | 100.0% | 1310.2522 | 1310.4467 | 1 | 6.502 | 70.0% | 1 | K.LFAIEEETTTR.H | 2 |
|  | Mis12IP\_Nocodazole\_MudPIT\_040709\_05.05535.05535.2 | 5.08 | 0.5392 | 100.0% | 1937.2722 | 1938.0557 | 1 | 9.306 | 64.7% | 1 | R.LSSSQGTIETSLQDIDSR.L | 2 |
|  | Mis12IP\_Nocodazole\_MudPIT\_040709\_06.05606.05606.1 | 1.7229 | 0.2906 | 99.1% | 941.53 | 942.11804 | 9 | 5.595 | 50.0% | 1 | K.MFTASSGIK.H | 1 |

---

|  |  |  |  |  |  |  |  |  |
| --- | --- | --- | --- | --- | --- | --- | --- | --- |
| U | *gi|10835010|ref|NP\_00* | 2 | 2 | 5.1% | 941 | 104958 | 6.3 | catenin (cadherin-associated protein), delta 1 [Homo sapiens] |
| U | *gi|51476280|emb|CAH18* | 2 | 2 | 5.1% | 938 | 104818 | 6.9 | hypothetical protein [Homo sapiens] |
| U | *gi|49899225|gb|AAH757* | 2 | 2 | 5.1% | 933 | 104098 | 7.0 | CTNND1 protein [Homo sapiens] |
| U | *gi|48257071|gb|AAH105* | 2 | 2 | 5.8% | 830 | 92388 | 7.2 | CTNND1 protein [Homo sapiens] |
| U | *gi|40788235|dbj|BAA20* | 2 | 2 | 5.0% | 967 | 107761 | 6.7 | KIAA0384 [Homo sapiens] |
| U | *gi|3152861|gb|AAC3982* | 2 | 2 | 5.0% | 968 | 108170 | 6.2 | p120 catenin isoform 1ABC [Homo sapiens] |
| U | *gi|3152855|gb|AAC3982* | 2 | 2 | 5.6% | 861 | 95868 | 6.3 | p120 catenin isoform 3AB [Homo sapiens] |
| U | *gi|3152853|gb|AAC3982* | 2 | 2 | 5.7% | 838 | 93496 | 7.0 | p120 catenin isoform 3AC [Homo sapiens] |
| U | *gi|3152843|gb|AAC3981* | 2 | 2 | 5.7% | 840 | 93478 | 6.3 | p120 catenin isoform 3B [Homo sapiens] |
| U | *gi|3152839|gb|AAC3981* | 2 | 2 | 5.8% | 832 | 92675 | 7.2 | p120 catenin isoform 3A [Homo sapiens] |
| U | *gi|3152835|gb|AAC3981* | 2 | 2 | 5.0% | 962 | 107349 | 6.3 | p120 catenin isoform 1AB [Homo sapiens] |
| U | *gi|3152833|gb|AAC3981* | 2 | 2 | 5.5% | 879 | 98049 | 7.5 | p120 catenin isoform 2A [Homo sapiens] |
| U | *gi|3152829|gb|AAC3981* | 2 | 2 | 5.4% | 887 | 98852 | 6.6 | p120 catenin isoform 2B [Homo sapiens] |
| U | *gi|3152827|gb|AAC3980* | 2 | 2 | 5.1% | 933 | 104156 | 7.0 | p120 catenin isoform 1A [Homo sapiens] |
| U | *gi|3152825|gb|AAC3980* | 2 | 2 | 5.5% | 867 | 96689 | 6.2 | p120 catenin isoform 3ABC [Homo sapiens] |
| U | *gi|3152823|gb|AAC3980* | 2 | 2 | 5.3% | 908 | 101242 | 6.6 | p120 catenin isoform 2AB [Homo sapiens] |
| U | *gi|3152821|gb|AAC3980* | 2 | 2 | 5.1% | 939 | 104977 | 6.9 | p120 catenin isoform 1AC [Homo sapiens] |
| U | *gi|3152819|gb|AAC3980* | 2 | 2 | 5.4% | 885 | 98870 | 7.3 | p120 catenin isoform 2AC [Homo sapiens] |
| U | *gi|3152817|gb|AAC3980* | 2 | 2 | 5.3% | 914 | 102063 | 6.5 | p120 catenin isoform 2ABC [Homo sapiens] |

| Filename XCorr DeltCN Conf% ObsM+H+ CalcM+H+ SpR ZScore Ion% # Sequence  | | | | | | | | | | | | |
| --- | --- | --- | --- | --- | --- | --- | --- | --- | --- | --- | --- | --- |
|  | Mis12IP\_Nocodazole\_MudPIT\_040709\_06.07033.07033.3 | 3.5631 | 0.3993 | 100.0% | 2987.6643 | 2988.3423 | 1 | 6.218 | 25.9% | 1 | R.TVQPVAMGPDGLPVDASSVSNNYIQTLGR.D | 3 |
|  | Mis12IP\_Nocodazole\_MudPIT\_040709\_05.06271.06271.2 | 4.5498 | 0.5981 | 100.0% | 2148.112 | 2149.3262 | 1 | 10.996 | 58.3% | 1 | R.SMGYDDLDYGMMSDYGTAR.R | 2 |

---

|  |  |  |  |  |  |  |  |  |
| --- | --- | --- | --- | --- | --- | --- | --- | --- |
| U | *gi|112180697|gb|AAH43* | 2 | 2 | 5.1% | 915 | 102094 | 6.8 | FBXO11 protein [Homo sapiens] |
| U | *gi|6573266|gb|AAF1761* | 2 | 2 | 24.5% | 192 | 21183 | 6.0 | F-box protein FBX11 [Homo sapiens] |
| U | *gi|6164741|gb|AAF0452* | 2 | 2 | 23.9% | 197 | 22336 | 8.2 | F-box protein Fbx11 [Homo sapiens] |
| U | *gi|56405842|gb|AAV873* | 2 | 2 | 5.1% | 927 | 103615 | 7.0 | F-box protein 11 [Homo sapiens] |

| Filename XCorr DeltCN Conf% ObsM+H+ CalcM+H+ SpR ZScore Ion% # Sequence  | | | | | | | | | | | | |
| --- | --- | --- | --- | --- | --- | --- | --- | --- | --- | --- | --- | --- |
|  | Mis12IP\_Nocodazole\_MudPIT\_040709\_06.05963.05963.3 | 8.194 | 0.5627 | 100.0% | 3412.2544 | 3412.4963 | 1 | 10.936 | 37.5% | 1 | R.NNVGERDDDVPADMVAEESGPGAQNS\*PYQLR.R | 3 |
|  | Mis12IP\_Nocodazole\_MudPIT\_040709\_05.05245.05245.2 | 2.6261 | 0.2785 | 99.4% | 1778.1921 | 1778.9133 | 1 | 6.54 | 53.3% | 1 | K.SQDLSAAPAEQYLQEK.L | 2 |

---

|  |  |  |  |  |  |  |  |  |
| --- | --- | --- | --- | --- | --- | --- | --- | --- |
| U | *gi|29792236|gb|AAH505* | 3 | 3 | 5.1% | 764 | 86549 | 8.2 | Rho guanine nucleotide exchange factor (GEF) 7 [Homo sapiens] |
| U | *gi|55957400|emb|CAI14* | 3 | 3 | 5.5% | 705 | 79832 | 6.7 | Rho guanine nucleotide exchange factor (GEF) 7 [Homo sapiens] |
| U | *gi|54633182|dbj|BAD66* | 3 | 3 | 5.7% | 680 | 77070 | 6.8 | KIAA0142 splice variant 2 [Homo sapiens] |
| U | *gi|4505573|ref|NP\_003* | 3 | 3 | 6.0% | 646 | 73140 | 7.2 | Rho guanine nucleotide exchange factor 7 isoform a [Homo sapiens] |
| U | *gi|40788881|dbj|BAA09* | 3 | 3 | 4.9% | 802 | 90263 | 8.2 | KIAA0142 [Homo sapiens] |

| Filename XCorr DeltCN Conf% ObsM+H+ CalcM+H+ SpR ZScore Ion% # Sequence  | | | | | | | | | | | | |
| --- | --- | --- | --- | --- | --- | --- | --- | --- | --- | --- | --- | --- |
|  | Mis12IP\_Nocodazole\_MudPIT\_040709\_06.06641.06641.2 | 2.9982 | 0.3855 | 100.0% | 1590.4321 | 1590.6921 | 1 | 7.736 | 57.7% | 1 | R.VEEGGWWEGTLNGR.T | 2 |
|  | Mis12IP\_Nocodazole\_MudPIT\_040709\_04.05989.05989.2 | 3.1609 | 0.2374 | 99.7% | 1389.3322 | 1389.5455 | 2 | 5.638 | 70.8% | 1 | K.STAALEEDAQILK.V | 2 |
|  | Mis12IP\_Nocodazole\_MudPIT\_040709\_02.05088.05088.2 | 2.4594 | 0.3651 | 99.9% | 1419.0521 | 1420.4932 | 1 | 6.02 | 72.7% | 1 | K.NMNDPAWDETNL.- | 2 |

---

|  |  |  |  |  |  |  |  |  |
| --- | --- | --- | --- | --- | --- | --- | --- | --- |
| U | *gi|34526448|dbj|BAC85* | 5 | 14 | 5.1% | 486 | 53025 | 5.5 | unnamed protein product [Homo sapiens] |

| Filename XCorr DeltCN Conf% ObsM+H+ CalcM+H+ SpR ZScore Ion% # Sequence  | | | | | | | | | | | | |
| --- | --- | --- | --- | --- | --- | --- | --- | --- | --- | --- | --- | --- |
|  | Mis12IP\_Nocodazole\_MudPIT\_040709\_04.05302.05302.2 | 2.5626 | 0.2281 | 99.5% | 1090.2922 | 1091.2273 | 1 | 5.497 | 81.2% | 2 | K.VTMQNLNDR.L | 2222 |
|  | Mis12IP\_Nocodazole\_MudPIT\_040709\_02.04220.04220.1 | 1.5217 | 0.3222 | 99.0% | 807.44 | 807.8815 | 2 | 4.741 | 58.3% | 1 | R.LAADDFR.L | 11111111 |
|  | Mis12IP\_Nocodazole\_MudPIT\_040709\_03.04543.04543.2 | 2.2948 | 0.233 | 99.3% | 808.0522 | 807.8815 | 1 | 5.671 | 91.7% | 3 | R.LAADDFR.L | 22222222 |
|  | Mis12IP\_Nocodazole\_MudPIT\_040709\_02.04797.04797.1 | 2.2793 | 0.4367 | 100.0% | 1109.51 | 1110.1681 | 2 | 7.211 | 62.5% | 4 | K.DAEAWFNEK.S | 11 |
|  | Mis12IP\_Nocodazole\_MudPIT\_040709\_03.05189.05189.2 | 3.1018 | 0.3908 | 100.0% | 1109.7522 | 1110.1681 | 1 | 6.675 | 75.0% | 4 | K.DAEAWFNEK.S | 22 |

Similarities:
gi|4557888|ref|NP\_000(2:3)  
gi|28317|emb|CAA32649(3:2)  
gi|24430192|ref|NP\_00(3:2)  
gi|12803709|gb|AAH026(3:2)  
gi|4557701|ref|NP\_000(2:3)  
gi|14043271|gb|AAH076(2:3)  
gi|24430190|ref|NP\_00(2:3)  
gi|85566621|gb|AAI119(2:3)  

---

|  |  |  |  |  |  |  |  |  |
| --- | --- | --- | --- | --- | --- | --- | --- | --- |
| U | *gi|34783197|gb|AAH146* | 2 | 2 | 5.0% | 706 | 80124 | 8.0 | SKIV2L2 protein [Homo sapiens] |

| Filename XCorr DeltCN Conf% ObsM+H+ CalcM+H+ SpR ZScore Ion% # Sequence  | | | | | | | | | | | | |
| --- | --- | --- | --- | --- | --- | --- | --- | --- | --- | --- | --- | --- |
| \* | Mis12IP\_Nocodazole\_MudPIT\_040709\_06.08712.08712.2 | 1.6622 | 0.2967 | 95.9% | 2398.172 | 2398.7334 | 64 | 3.887 | 21.4% | 1 | R.FPDGIPLLDPIDDMGIQDQGLK.K | 2 |
|  | Mis12IP\_Nocodazole\_MudPIT\_040709\_06.06467.06467.2 | 3.4959 | 0.5188 | 100.0% | 1397.4722 | 1398.6141 | 1 | 8.393 | 79.2% | 1 | R.LGFATSSDVIEMK.G | 22 |

Similarities:
gi|114325466|gb|AAH28(1:1)  

---

|  |  |  |  |  |  |  |  |  |
| --- | --- | --- | --- | --- | --- | --- | --- | --- |
| U | *gi|17511976|gb|AAH189* | 2 | 2 | 5.0% | 704 | 80110 | 8.2 | TNF receptor-associated protein 1 [Homo sapiens] |
| U | *gi|7706485|ref|NP\_057* | 2 | 2 | 5.0% | 704 | 80011 | 8.0 | TNF receptor-associated protein 1 [Homo sapiens] |
| U | *gi|62897971|dbj|BAD96* | 2 | 2 | 5.0% | 704 | 80091 | 8.0 | TNF receptor-associated protein 1 variant [Homo sapiens] |
| U | *gi|62897659|dbj|BAD96* | 2 | 2 | 5.0% | 704 | 80039 | 8.0 | TNF receptor-associated protein 1 variant [Homo sapiens] |
| U | *gi|62089196|dbj|BAD93* | 2 | 2 | 5.0% | 703 | 79993 | 8.2 | TNF receptor-associated protein 1 variant [Homo sapiens] |
| U | *gi|37589015|gb|AAH014* | 2 | 2 | 5.0% | 699 | 79357 | 7.8 | TRAP1 protein [Homo sapiens] |
| U | *gi|3273383|gb|AAC2472* | 2 | 2 | 5.7% | 616 | 70377 | 7.0 | TRAP1 [Homo sapiens] |
| U | *gi|21752190|dbj|BAC04* | 2 | 2 | 6.0% | 579 | 66047 | 7.3 | unnamed protein product [Homo sapiens] |

| Filename XCorr DeltCN Conf% ObsM+H+ CalcM+H+ SpR ZScore Ion% # Sequence  | | | | | | | | | | | | |
| --- | --- | --- | --- | --- | --- | --- | --- | --- | --- | --- | --- | --- |
|  | Mis12IP\_Nocodazole\_MudPIT\_040709\_06.06584.06584.2 | 2.5286 | 0.3524 | 99.8% | 1593.4922 | 1593.7336 | 1 | 6.055 | 42.9% | 1 | K.AFLDALQNQAEASSK.I | 2 |
|  | Mis12IP\_Nocodazole\_MudPIT\_040709\_05.06054.06054.2 | 2.8471 | 0.5357 | 100.0% | 2146.2722 | 2147.303 | 1 | 7.274 | 42.1% | 1 | R.YESSALPSGQLTSLSEYASR.M | 2 |

---

|  |  |  |  |  |  |  |  |  |
| --- | --- | --- | --- | --- | --- | --- | --- | --- |
| U | *gi|45597458|ref|NP\_77* | 3 | 3 | 5.0% | 578 | 61801 | 6.0 | keratin 1B [Homo sapiens] |

| Filename XCorr DeltCN Conf% ObsM+H+ CalcM+H+ SpR ZScore Ion% # Sequence  | | | | | | | | | | | | |
| --- | --- | --- | --- | --- | --- | --- | --- | --- | --- | --- | --- | --- |
|  | Mis12IP\_Nocodazole\_MudPIT\_040709\_05.05262.05262.1 | 2.1158 | 0.1126 | 95.8% | 827.46 | 827.95544 | 11 | 4.725 | 66.7% | 1 | K.FASFIDK.V | 1111111 |
|  | Mis12IP\_Nocodazole\_MudPIT\_040709\_06.05752.05752.2 | 4.2312 | 0.0778 | 99.6% | 1476.3522 | 1476.6726 | 3 | 7.319 | 86.4% | 1 | R.FLEQQNQVLQTK.W | 222 |
|  | Mis12IP\_Nocodazole\_MudPIT\_040709\_06.05678.05678.2 | 2.0976 | 0.2079 | 97.1% | 1177.9521 | 1179.3182 | 1 | 4.269 | 83.3% | 1 | K.YQELQITAGR.H | 2 |

Similarities:
gi|4504919|ref|NP\_002(1:2)  
gi|181402|gb|AAC83410(1:2)  
gi|17318569|ref|NP\_00(1:2)  
gi|17505189|ref|NP\_49(1:2)  
gi|15559584|gb|AAH141(1:2)  
gi|5031841|ref|NP\_005(1:2)  
gi|1200072|emb|CAA316(1:2)  
gi|32567786|ref|NP\_78(1:2)  

---

|  |  |  |  |  |  |  |  |  |
| --- | --- | --- | --- | --- | --- | --- | --- | --- |
| U | *gi|33286418|ref|NP\_00* | 2 | 2 | 4.9% | 531 | 57937 | 7.8 | pyruvate kinase 3 isoform 1 [Homo sapiens] |
| U | *gi|62897413|dbj|BAD96* | 2 | 2 | 4.9% | 531 | 57984 | 8.1 | pyruvate kinase 3 isoform 1 variant [Homo sapiens] |
| U | *gi|35505|emb|CAA39849* | 2 | 2 | 4.9% | 531 | 57878 | 7.7 | pyruvate kinase [Homo sapiens] |
| U | *gi|34782802|gb|AAH192* | 2 | 2 | 7.6% | 343 | 37276 | 8.2 | PKM2 protein [Homo sapiens] |
| U | *gi|33875497|gb|AAH004* | 2 | 2 | 4.6% | 565 | 61437 | 8.6 | PKM2 protein [Homo sapiens] |
| U | *gi|33873708|gb|AAH079* | 2 | 2 | 4.6% | 564 | 61380 | 8.6 | PKM2 protein [Homo sapiens] |
| U | *gi|33870117|gb|AAH128* | 2 | 2 | 4.6% | 564 | 61401 | 8.8 | PKM2 protein [Homo sapiens] |
| U | *gi|33286420|ref|NP\_87* | 2 | 2 | 4.9% | 531 | 58062 | 7.7 | pyruvate kinase 3 isoform 2 [Homo sapiens] |

| Filename XCorr DeltCN Conf% ObsM+H+ CalcM+H+ SpR ZScore Ion% # Sequence  | | | | | | | | | | | | |
| --- | --- | --- | --- | --- | --- | --- | --- | --- | --- | --- | --- | --- |
|  | Mis12IP\_Nocodazole\_MudPIT\_040709\_06.06812.06812.2 | 3.2384 | 0.3902 | 100.0% | 1779.8722 | 1780.9292 | 1 | 7.618 | 50.0% | 1 | K.GADFLVTEVENGGSLGSK.K | 2 |
|  | Mis12IP\_Nocodazole\_MudPIT\_040709\_06.05669.05669.1 | 1.254 | 0.3247 | 98.2% | 840.8 | 841.0415 | 82 | 5.058 | 50.0% | 1 | R.APIIAVTR.N | 1 |

---

|  |  |  |  |  |  |  |  |  |
| --- | --- | --- | --- | --- | --- | --- | --- | --- |
| U | *gi|2808511|emb|CAA721* | 2 | 3 | 4.7% | 471 | 54252 | 8.6 | p54nrb [Homo sapiens] |
| U | *gi|34932414|ref|NP\_03* | 2 | 3 | 4.7% | 471 | 54232 | 8.9 | non-POU domain containing, octamer-binding [Homo sapiens] |

| Filename XCorr DeltCN Conf% ObsM+H+ CalcM+H+ SpR ZScore Ion% # Sequence  | | | | | | | | | | | | |
| --- | --- | --- | --- | --- | --- | --- | --- | --- | --- | --- | --- | --- |
|  | Mis12IP\_Nocodazole\_MudPIT\_040709\_04.06887.06887.3 | 3.9114 | 0.3024 | 100.0% | 2244.5344 | 2244.4436 | 1 | 5.475 | 39.3% | 2 | R.FGQAATMEGIGAIGGT#PPAFNR.A | 3 |
|  | Mis12IP\_Nocodazole\_MudPIT\_040709\_03.06181.06181.2 | 2.4696 | 0.2018 | 97.8% | 2245.4321 | 2244.4436 | 2 | 4.11 | 33.3% | 1 | R.FGQAATMEGIGAIGGT#PPAFNR.A | 2 |

---

|  |  |  |  |  |  |  |  |  |
| --- | --- | --- | --- | --- | --- | --- | --- | --- |
| U | *gi|14595132|dbj|BAB61* | 4 | 11 | 4.6% | 758 | 85016 | 6.9 | Raichu404X [Homo sapiens] |

| Filename XCorr DeltCN Conf% ObsM+H+ CalcM+H+ SpR ZScore Ion% # Sequence  | | | | | | | | | | | | |
| --- | --- | --- | --- | --- | --- | --- | --- | --- | --- | --- | --- | --- |
| \* | Mis12IP\_Nocodazole\_MudPIT\_040709\_03.04526.04526.2 | 3.6811 | 0.5488 | 100.0% | 1506.0721 | 1504.5499 | 1 | 8.667 | 71.4% | 1 | K.FSVSGEGEGDATYGK.L | 2 |
| \* | Mis12IP\_Nocodazole\_MudPIT\_040709\_03.04494.04494.1 | 2.1715 | 0.2916 | 98.6% | 1266.58 | 1267.399 | 1 | 5.163 | 60.0% | 2 | K.SAMPEGYVQER.T | 1 |
| \* | Mis12IP\_Nocodazole\_MudPIT\_040709\_03.04488.04488.2 | 2.975 | 0.5252 | 100.0% | 1267.2122 | 1267.399 | 1 | 7.804 | 80.0% | 5 | K.SAMPEGYVQER.T | 2 |
| \* | Mis12IP\_Nocodazole\_MudPIT\_040709\_03.04578.04578.2 | 2.9231 | 0.3386 | 100.0% | 1050.4722 | 1051.1442 | 1 | 6.192 | 87.5% | 3 | K.FEGDTLVNR.I | 2 |

---

|  |  |  |  |  |  |  |  |  |
| --- | --- | --- | --- | --- | --- | --- | --- | --- |
| U | *gi|35570|emb|CAA68428* | 2 | 2 | 4.4% | 633 | 70324 | 9.5 | unnamed protein product [Homo sapiens] |
| U | *gi|693937|emb|CAA8840* | 2 | 2 | 5.4% | 522 | 58518 | 9.3 | polyadenylate binding protein II [Homo sapiens] |
| U | *gi|55959642|emb|CAI16* | 2 | 2 | 21.7% | 129 | 14395 | 8.8 | poly(A) binding protein, cytoplasmic 4 (inducible form) [Homo sapiens] |
| U | *gi|55959640|emb|CAI16* | 2 | 2 | 4.6% | 615 | 67971 | 9.5 | poly(A) binding protein, cytoplasmic 4 (inducible form) [Homo sapiens] |
| U | *gi|55959639|emb|CAI16* | 2 | 2 | 4.2% | 660 | 72391 | 9.3 | poly(A) binding protein, cytoplasmic 4 (inducible form) [Homo sapiens] |
| U | *gi|48734702|gb|AAH715* | 2 | 2 | 4.2% | 660 | 72361 | 9.3 | PABPC4 protein [Homo sapiens] |
| U | *gi|46367787|ref|NP\_00* | 2 | 2 | 4.4% | 636 | 70671 | 9.5 | poly(A) binding protein, cytoplasmic 1 [Homo sapiens] |
| U | *gi|4504715|ref|NP\_003* | 2 | 2 | 4.3% | 644 | 70783 | 9.3 | poly A binding protein, cytoplasmic 4 [Homo sapiens] |
| U | *gi|41388837|gb|AAH655* | 2 | 2 | 4.4% | 631 | 69607 | 9.5 | PABPC4 protein [Homo sapiens] |

| Filename XCorr DeltCN Conf% ObsM+H+ CalcM+H+ SpR ZScore Ion% # Sequence  | | | | | | | | | | | | |
| --- | --- | --- | --- | --- | --- | --- | --- | --- | --- | --- | --- | --- |
|  | Mis12IP\_Nocodazole\_MudPIT\_040709\_06.06365.06365.2 | 4.9457 | 0.5058 | 100.0% | 1930.5122 | 1930.0837 | 1 | 9.173 | 62.5% | 1 | R.SLGYAYVNFQQPADAER.A | 2 |
|  | Mis12IP\_Nocodazole\_MudPIT\_040709\_06.06714.06714.2 | 2.8159 | 0.2377 | 99.7% | 1266.4922 | 1267.4828 | 1 | 6.183 | 70.0% | 1 | R.ALDTMNFDVIK.G | 2 |

---

|  |  |  |  |  |  |  |  |  |
| --- | --- | --- | --- | --- | --- | --- | --- | --- |
| U | *gi|2772564|gb|AAB9634* | 2 | 2 | 4.4% | 298 | 32852 | 9.7 | ADP/ATP carrier protein (adenine nucleotide translocator 2) [Homo sapiens] |
| U | *gi|45829841|gb|AAH681* | 2 | 2 | 4.0% | 323 | 35294 | 9.8 | SLC25A5 protein [Homo sapiens] |
| U | *gi|4502099|ref|NP\_001* | 2 | 2 | 4.4% | 298 | 32895 | 9.7 | solute carrier family 25, member 5 [Homo sapiens] |

| Filename XCorr DeltCN Conf% ObsM+H+ CalcM+H+ SpR ZScore Ion% # Sequence  | | | | | | | | | | | | |
| --- | --- | --- | --- | --- | --- | --- | --- | --- | --- | --- | --- | --- |
|  | Mis12IP\_Nocodazole\_MudPIT\_040709\_06.06592.06592.2 | 2.3924 | 0.4659 | 100.0% | 1219.1322 | 1220.4111 | 1 | 7.398 | 79.2% | 1 | K.DFLAGGVAAAISK.T | 2 |
|  | Mis12IP\_Nocodazole\_MudPIT\_040709\_06.06596.06596.1 | 2.3365 | 0.4304 | 100.0% | 1219.61 | 1220.4111 | 1 | 7.381 | 58.3% | 1 | K.DFLAGGVAAAISK.T | 1 |

---

|  |  |  |  |  |  |  |  |  |
| --- | --- | --- | --- | --- | --- | --- | --- | --- |
| U | *gi|1296662|emb|CAA911* | 15 | 17 | 4.3% | 4684 | 531742 | 5.9 | plectin [Homo sapiens] |
| U | *gi|47607492|ref|NP\_00* | 15 | 17 | 4.4% | 4574 | 518478 | 5.7 | plectin 1 isoform 1 [Homo sapiens] |
| U | *gi|41322923|ref|NP\_95* | 15 | 17 | 4.4% | 4547 | 516204 | 5.8 | plectin 1 isoform 11 [Homo sapiens] |
| U | *gi|41322919|ref|NP\_95* | 15 | 17 | 4.4% | 4547 | 516282 | 5.8 | plectin 1 isoform 8 [Homo sapiens] |
| U | *gi|41322916|ref|NP\_95* | 15 | 17 | 4.3% | 4684 | 531796 | 6.0 | plectin 1 isoform 6 [Homo sapiens] |
| U | *gi|41322914|ref|NP\_95* | 15 | 17 | 4.4% | 4551 | 516484 | 5.8 | plectin 1 isoform 10 [Homo sapiens] |
| U | *gi|41322910|ref|NP\_95* | 15 | 17 | 4.5% | 4515 | 512609 | 5.8 | plectin 1 isoform 7 [Homo sapiens] |
| U | *gi|41322908|ref|NP\_95* | 15 | 17 | 4.4% | 4525 | 513712 | 5.8 | plectin 1 isoform 3 [Homo sapiens] |
| U | *gi|1477646|gb|AAB0542* | 15 | 17 | 4.4% | 4574 | 518494 | 5.7 | plectin [Homo sapiens] |

| Filename XCorr DeltCN Conf% ObsM+H+ CalcM+H+ SpR ZScore Ion% # Sequence  | | | | | | | | | | | | |
| --- | --- | --- | --- | --- | --- | --- | --- | --- | --- | --- | --- | --- |
|  | Mis12IP\_Nocodazole\_MudPIT\_040709\_06.06740.06740.2 | 3.4196 | 0.4657 | 100.0% | 1836.1122 | 1835.9683 | 1 | 7.962 | 56.7% | 1 | R.QTNLENLDQAFSVAER.D | 2 |
|  | Mis12IP\_Nocodazole\_MudPIT\_040709\_05.05355.05355.2 | 3.3885 | 0.3436 | 100.0% | 1708.5721 | 1709.8474 | 1 | 7.481 | 60.7% | 1 | R.LLDPEDVDVPQPDEK.S | 2 |
|  | Mis12IP\_Nocodazole\_MudPIT\_040709\_06.07292.07292.2 | 3.5186 | 0.4463 | 100.0% | 1529.4521 | 1529.6494 | 1 | 7.162 | 57.7% | 1 | R.ESADPLGAWLQDAR.R | 2 |
|  | Mis12IP\_Nocodazole\_MudPIT\_040709\_05.04795.04795.2 | 3.2635 | 0.3615 | 100.0% | 1243.2722 | 1243.4056 | 1 | 6.788 | 80.0% | 1 | R.QVQVALETAQR.S | 2 |
|  | Mis12IP\_Nocodazole\_MudPIT\_040709\_05.05104.05104.2 | 2.0672 | 0.3188 | 99.3% | 1287.5322 | 1288.4435 | 6 | 5.004 | 60.0% | 1 | K.AQVEQELTTLR.L | 2 |
|  | Mis12IP\_Nocodazole\_MudPIT\_040709\_06.06408.06408.2 | 3.6721 | 0.4985 | 100.0% | 1322.4321 | 1323.4454 | 1 | 8.921 | 80.0% | 1 | R.SQVEEELFSVR.V | 2 |
|  | Mis12IP\_Nocodazole\_MudPIT\_040709\_03.04238.04238.2 | 2.0482 | 0.3534 | 99.7% | 1015.9322 | 1016.1423 | 1 | 5.659 | 72.2% | 1 | R.LSVAAQEAAR.L | 2 |
|  | Mis12IP\_Nocodazole\_MudPIT\_040709\_06.06176.06176.2 | 4.9505 | 0.4576 | 100.0% | 1556.7522 | 1557.744 | 1 | 8.465 | 80.8% | 1 | R.LQEAGILSAEELQR.L | 2 |
|  | Mis12IP\_Nocodazole\_MudPIT\_040709\_04.05734.05734.2 | 2.9633 | 0.4115 | 100.0% | 1161.0322 | 1161.2311 | 1 | 6.902 | 87.5% | 2 | R.GYFDEEMNR.V | 22 |
|  | Mis12IP\_Nocodazole\_MudPIT\_040709\_06.06845.06845.2 | 2.4491 | 0.28 | 99.4% | 1284.9722 | 1285.4833 | 3 | 5.837 | 59.1% | 1 | R.SLVPAAELLESR.V | 2 |
|  | Mis12IP\_Nocodazole\_MudPIT\_040709\_05.05690.05690.2 | 2.3944 | 0.4018 | 99.9% | 1613.6921 | 1614.8363 | 1 | 6.898 | 53.3% | 1 | R.LLDAQLSTGGIVDPSK.S | 2 |
|  | Mis12IP\_Nocodazole\_MudPIT\_040709\_03.04628.04628.2 | 4.9743 | 0.6066 | 100.0% | 2070.4922 | 2071.1643 | 1 | 9.967 | 75.0% | 2 | K.AYSDPSTGEPATYGELQQR.C | 2 |
|  | Mis12IP\_Nocodazole\_MudPIT\_040709\_06.06475.06475.2 | 4.5171 | 0.4981 | 100.0% | 1540.1322 | 1539.7721 | 2 | 8.014 | 57.1% | 1 | R.LLDAQLATGGIVDPR.L | 2 |
|  | Mis12IP\_Nocodazole\_MudPIT\_040709\_02.04146.04146.2 | 3.8351 | 0.5051 | 100.0% | 1834.7522 | 1835.8792 | 1 | 7.677 | 70.6% | 1 | R.SSSVGSSSSYPIS\*PAVSR.T | 2 |
|  | Mis12IP\_Nocodazole\_MudPIT\_040709\_03.04532.04532.2 | 2.4368 | 0.3923 | 99.9% | 1079.3722 | 1079.2134 | 1 | 5.65 | 77.8% | 1 | R.SMVEEGTGLR.L | 2 |

Similarities:
gi|13876386|ref|NP\_11(1:14)  

---

|  |  |  |  |  |  |  |  |  |
| --- | --- | --- | --- | --- | --- | --- | --- | --- |
| U | *gi|15082586|gb|AAH121* | 2 | 3 | 4.3% | 257 | 28011 | 11.0 | Ribosomal protein L8 [Homo sapiens] |
| U | *gi|4506663|ref|NP\_000* | 2 | 3 | 4.3% | 257 | 28025 | 11.0 | ribosomal protein L8 [Homo sapiens] |
| U | *gi|38014625|gb|AAH000* | 2 | 3 | 5.2% | 211 | 22901 | 10.6 | RPL8 protein [Homo sapiens] |

| Filename XCorr DeltCN Conf% ObsM+H+ CalcM+H+ SpR ZScore Ion% # Sequence  | | | | | | | | | | | | |
| --- | --- | --- | --- | --- | --- | --- | --- | --- | --- | --- | --- | --- |
|  | Mis12IP\_Nocodazole\_MudPIT\_040709\_05.04704.04704.1 | 2.7459 | 0.4107 | 100.0% | 941.55 | 942.1062 | 1 | 7.323 | 65.0% | 2 | R.AVVGVVAGGGR.I | 1 |
|  | Mis12IP\_Nocodazole\_MudPIT\_040709\_05.04713.04713.2 | 3.6193 | 0.4612 | 100.0% | 941.9322 | 942.1062 | 1 | 7.709 | 90.0% | 1 | R.AVVGVVAGGGR.I | 2 |

---

|  |  |  |  |  |  |  |  |  |
| --- | --- | --- | --- | --- | --- | --- | --- | --- |
| U | *gi|30353925|gb|AAH518* | 4 | 4 | 4.1% | 1639 | 187889 | 5.7 | CLTC protein [Homo sapiens] |
| U | *gi|4758012|ref|NP\_004* | 4 | 4 | 4.0% | 1675 | 191613 | 5.7 | clathrin heavy chain 1 [Homo sapiens] |
| U | *gi|40788952|dbj|BAA04* | 4 | 4 | 4.0% | 1685 | 192685 | 5.6 | KIAA0034 [Homo sapiens] |
| U | *gi|34364629|emb|CAE45* | 4 | 4 | 4.0% | 1675 | 191523 | 5.7 | hypothetical protein [Homo sapiens] |

| Filename XCorr DeltCN Conf% ObsM+H+ CalcM+H+ SpR ZScore Ion% # Sequence  | | | | | | | | | | | | |
| --- | --- | --- | --- | --- | --- | --- | --- | --- | --- | --- | --- | --- |
|  | Mis12IP\_Nocodazole\_MudPIT\_040709\_03.04440.04440.2 | 2.7482 | 0.3326 | 99.9% | 1335.0122 | 1335.416 | 1 | 6.301 | 75.0% | 1 | K.IYIDSNNNPER.F | 2 |
|  | Mis12IP\_Nocodazole\_MudPIT\_040709\_05.07444.07444.2 | 2.7257 | 0.3288 | 99.9% | 2741.652 | 2741.9429 | 1 | 5.975 | 31.2% | 1 | K.ADDPSSYMEVVQAANTSGNWEELVK.Y | 2 |
|  | Mis12IP\_Nocodazole\_MudPIT\_040709\_06.08030.08030.2 | 2.4671 | 0.523 | 100.0% | 1613.6522 | 1613.848 | 3 | 7.022 | 46.2% | 1 | R.ESYVETELIFALAK.T | 2 |
|  | Mis12IP\_Nocodazole\_MudPIT\_040709\_05.06144.06144.2 | 3.8681 | 0.4972 | 100.0% | 1943.6122 | 1944.0648 | 1 | 8.284 | 68.8% | 1 | R.TSIDAYDNFDNISLAQR.L | 2 |

---

|  |  |  |  |  |  |  |  |  |
| --- | --- | --- | --- | --- | --- | --- | --- | --- |
| U | *gi|21359873|ref|NP\_00* | 2 | 3 | 4.1% | 603 | 68255 | 8.9 | polo-like kinase [Homo sapiens] |
| U | *gi|460769|emb|CAA5353* | 2 | 3 | 4.1% | 603 | 68311 | 8.8 | Serine/Threonine protein kinase [Homo sapiens] |

| Filename XCorr DeltCN Conf% ObsM+H+ CalcM+H+ SpR ZScore Ion% # Sequence  | | | | | | | | | | | | |
| --- | --- | --- | --- | --- | --- | --- | --- | --- | --- | --- | --- | --- |
|  | Mis12IP\_Nocodazole\_MudPIT\_040709\_05.05640.05640.2 | 2.045 | 0.2192 | 97.1% | 1167.1122 | 1167.3477 | 4 | 5.023 | 61.1% | 1 | K.EIPEVLVDPR.S | 2 |
|  | Mis12IP\_Nocodazole\_MudPIT\_040709\_05.06350.06350.2 | 2.7196 | 0.4124 | 100.0% | 1813.2522 | 1813.0172 | 1 | 7.07 | 46.4% | 2 | R.LILYNDGDSLQYIER.D | 2 |

---

|  |  |  |  |  |  |  |  |  |
| --- | --- | --- | --- | --- | --- | --- | --- | --- |
| U | *gi|2832596|emb|CAB097* | 2 | 2 | 4.0% | 650 | 72372 | 8.6 | OTTHUMP00000028920 [Homo sapiens] |
| U | *gi|62088770|dbj|BAD92* | 2 | 2 | 3.5% | 737 | 81068 | 7.9 | DEAD box polypeptide 17 isoform p82 variant [Homo sapiens] |
| U | *gi|52545677|emb|CAH10* | 2 | 2 | 4.0% | 652 | 72542 | 8.6 | hypothetical protein [Homo sapiens] |
| U | *gi|47678395|emb|CAG30* | 2 | 2 | 4.0% | 652 | 72557 | 8.7 | DDX17 [Homo sapiens] |
| U | *gi|38201710|ref|NP\_00* | 2 | 2 | 3.6% | 729 | 80273 | 8.3 | DEAD box polypeptide 17 isoform p82 [Homo sapiens] |

| Filename XCorr DeltCN Conf% ObsM+H+ CalcM+H+ SpR ZScore Ion% # Sequence  | | | | | | | | | | | | |
| --- | --- | --- | --- | --- | --- | --- | --- | --- | --- | --- | --- | --- |
|  | Mis12IP\_Nocodazole\_MudPIT\_040709\_06.06102.06102.2 | 3.7477 | 0.2993 | 100.0% | 1228.1721 | 1227.4465 | 2 | 6.856 | 72.7% | 1 | K.APILIATDVASR.G | 22 |
|  | Mis12IP\_Nocodazole\_MudPIT\_040709\_05.04711.04711.2 | 3.6954 | 0.4245 | 100.0% | 1456.3121 | 1456.5118 | 1 | 8.727 | 65.4% | 1 | R.SSQSSSQQFSGIGR.S | 2 |

Similarities:
gi|4758138|ref|NP\_004(1:1)  

---

|  |  |  |  |  |  |  |  |  |
| --- | --- | --- | --- | --- | --- | --- | --- | --- |
| U | *gi|19387846|ref|NP\_05* | 2 | 2 | 4.0% | 606 | 64954 | 9.3 | melanoma antigen family D, 2 [Homo sapiens] |
| U | *gi|57209937|emb|CAI42* | 2 | 2 | 4.4% | 550 | 59525 | 9.7 | melanoma antigen family D, 2 [Homo sapiens] |
| U | *gi|4928044|gb|AAD3339* | 2 | 2 | 4.0% | 606 | 64982 | 9.4 | breast cancer associated gene 1 protein [Homo sapiens] |
| U | *gi|4099969|gb|AAD0072* | 2 | 2 | 4.0% | 606 | 64944 | 9.3 | hepatocellular carcinoma associated protein [Homo sapiens] |
| U | *gi|21751066|dbj|BAC03* | 2 | 2 | 4.1% | 588 | 63183 | 9.3 | unnamed protein product [Homo sapiens] |

| Filename XCorr DeltCN Conf% ObsM+H+ CalcM+H+ SpR ZScore Ion% # Sequence  | | | | | | | | | | | | |
| --- | --- | --- | --- | --- | --- | --- | --- | --- | --- | --- | --- | --- |
|  | Mis12IP\_Nocodazole\_MudPIT\_040709\_05.05053.05053.3 | 5.0359 | 0.474 | 100.0% | 2475.9243 | 2476.6567 | 1 | 7.133 | 48.9% | 1 | R.EAPATQASSTTQLTDTQVLAAENK.S | 3 |
|  | Mis12IP\_Nocodazole\_MudPIT\_040709\_05.05056.05056.2 | 5.9955 | 0.5303 | 100.0% | 2476.7122 | 2476.6567 | 1 | 11.062 | 52.2% | 1 | R.EAPATQASSTTQLTDTQVLAAENK.S | 2 |

---

|  |  |  |  |  |  |  |  |  |
| --- | --- | --- | --- | --- | --- | --- | --- | --- |
| U | *gi|117938251|ref|NP\_0* | 4 | 4 | 3.9% | 869 | 100232 | 10.0 | BCL2-associated transcription factor 1 isoform 2 [Homo sapiens] |
| U | *gi|7661958|ref|NP\_055* | 4 | 4 | 3.7% | 920 | 106122 | 10.0 | BCL2-associated transcription factor 1 isoform 1 [Homo sapiens] |
| U | *gi|7582386|gb|AAF6430* | 4 | 4 | 3.9% | 869 | 100216 | 10.0 | Bcl-2-associated transcription factor short form [Homo sapiens] |
| U | *gi|40788889|dbj|BAA11* | 4 | 4 | 3.7% | 929 | 107220 | 10.0 | KIAA0164 [Homo sapiens] |
| U | *gi|39645648|gb|AAH638* | 4 | 4 | 4.8% | 705 | 80984 | 10.2 | BCLAF1 protein [Homo sapiens] |
| U | *gi|34783967|gb|AAH568* | 4 | 4 | 5.0% | 680 | 77948 | 10.2 | BCLAF1 protein [Homo sapiens] |
| U | *gi|28839788|gb|AAH478* | 4 | 4 | 4.9% | 689 | 79005 | 10.2 | BCLAF1 protein [Homo sapiens] |

| Filename XCorr DeltCN Conf% ObsM+H+ CalcM+H+ SpR ZScore Ion% # Sequence  | | | | | | | | | | | | |
| --- | --- | --- | --- | --- | --- | --- | --- | --- | --- | --- | --- | --- |
|  | Mis12IP\_Nocodazole\_MudPIT\_040709\_02.04115.04115.2 | 2.4042 | 0.2197 | 98.6% | 1502.1122 | 1502.534 | 1 | 4.861 | 57.7% | 1 | R.SSFYPDGGDQETAK.T | 2 |
|  | Mis12IP\_Nocodazole\_MudPIT\_040709\_06.05486.05486.2 | 4.1414 | 0.4742 | 100.0% | 1788.4122 | 1788.8651 | 1 | 7.79 | 59.4% | 1 | R.SSFYPDGGDQETAKTGK.F | 2 |
|  | Mis12IP\_Nocodazole\_MudPIT\_040709\_06.05255.05255.2 | 1.9614 | 0.354 | 99.7% | 876.6722 | 876.94745 | 9 | 6.687 | 64.3% | 1 | K.SFATASHR.N | 2 |
|  | Mis12IP\_Nocodazole\_MudPIT\_040709\_03.04395.04395.2 | 2.7363 | 0.3079 | 99.9% | 992.9922 | 993.1229 | 39 | 6.324 | 75.0% | 1 | K.SAAMTLNER.F | 2 |

---

|  |  |  |  |  |  |  |  |  |
| --- | --- | --- | --- | --- | --- | --- | --- | --- |
| U | *gi|1699027|gb|AAB3734* | 2 | 3 | 3.8% | 835 | 88536 | 5.8 | nuclear corepressor KAP-1 [Homo sapiens] |
| U | *gi|5032179|ref|NP\_005* | 2 | 3 | 3.8% | 835 | 88550 | 5.8 | tripartite motif-containing 28 protein [Homo sapiens] |
| U | *gi|33873597|gb|AAH073* | 2 | 3 | 8.6% | 370 | 38691 | 4.8 | TRIM28 protein [Homo sapiens] |
| U | *gi|31544959|gb|AAH529* | 2 | 3 | 4.2% | 753 | 79474 | 6.0 | TRIM28 protein [Homo sapiens] |

| Filename XCorr DeltCN Conf% ObsM+H+ CalcM+H+ SpR ZScore Ion% # Sequence  | | | | | | | | | | | | |
| --- | --- | --- | --- | --- | --- | --- | --- | --- | --- | --- | --- | --- |
|  | Mis12IP\_Nocodazole\_MudPIT\_040709\_05.06478.06478.2 | 3.8752 | 0.5005 | 100.0% | 1659.2322 | 1659.8767 | 1 | 8.475 | 67.9% | 2 | R.LDLDLTADSQPPVFK.V | 2 |
|  | Mis12IP\_Nocodazole\_MudPIT\_040709\_06.07030.07030.2 | 2.047 | 0.2887 | 98.9% | 1953.6322 | 1954.187 | 2 | 5.947 | 34.4% | 1 | K.VFPGSTTEDYNLIVIER.G | 2 |

---

|  |  |  |  |  |  |  |  |  |
| --- | --- | --- | --- | --- | --- | --- | --- | --- |
| U | *gi|40254816|ref|NP\_00* | 2 | 3 | 3.7% | 732 | 84674 | 5.0 | heat shock protein 90kDa alpha (cytosolic), class A member 1 isoform 2 [Homo sapiens] |
| U | *gi|83699649|gb|ABC407* | 2 | 3 | 3.2% | 854 | 98143 | 5.2 | heat shock 90kDa protein 1, alpha [Homo sapiens] |
| U | *gi|83318444|gb|AAI086* | 2 | 3 | 4.6% | 585 | 68372 | 5.2 | HSP90AA1 protein [Homo sapiens] |
| U | *gi|63029937|ref|NP\_00* | 2 | 3 | 3.2% | 854 | 98113 | 5.2 | heat shock protein 90kDa alpha (cytosolic), class A member 1 isoform 1 [Homo sapiens] |
| U | *gi|62914009|gb|AAH230* | 2 | 3 | 4.2% | 638 | 73827 | 5.2 | HSP90AA1 protein [Homo sapiens] |
| U | *gi|61656605|emb|CAI64* | 2 | 3 | 3.7% | 732 | 84660 | 5.0 | Heat shock protein HSP 90-alpha 4 [Homo sapiens] |

| Filename XCorr DeltCN Conf% ObsM+H+ CalcM+H+ SpR ZScore Ion% # Sequence  | | | | | | | | | | | | |
| --- | --- | --- | --- | --- | --- | --- | --- | --- | --- | --- | --- | --- |
|  | Mis12IP\_Nocodazole\_MudPIT\_040709\_06.06371.06371.2 | 3.8076 | 0.4932 | 100.0% | 1242.7922 | 1243.4459 | 1 | 7.351 | 77.3% | 1 | K.ADLINNLGTIAK.S | 22 |
|  | Mis12IP\_Nocodazole\_MudPIT\_040709\_05.05393.05393.2 | 4.8378 | 0.5152 | 100.0% | 1834.3322 | 1834.8903 | 1 | 9.046 | 75.0% | 2 | R.NPDDITNEEYGEFYK.S | 2 |

Similarities:
gi|20149594|ref|NP\_03(1:1)  

---

|  |  |  |  |  |  |  |  |  |
| --- | --- | --- | --- | --- | --- | --- | --- | --- |
| U | *gi|2394274|gb|AAB7025* | 2 | 2 | 3.6% | 781 | 91276 | 8.2 | vasopressin-activated calcium mobilizing putative receptor protein [Homo sapiens] |
| U | *gi|40254446|ref|NP\_00* | 2 | 2 | 3.6% | 780 | 90955 | 8.0 | Vasopressin-activated calcium-mobilizing receptor-1 [Homo sapiens] |

| Filename XCorr DeltCN Conf% ObsM+H+ CalcM+H+ SpR ZScore Ion% # Sequence  | | | | | | | | | | | | |
| --- | --- | --- | --- | --- | --- | --- | --- | --- | --- | --- | --- | --- |
|  | Mis12IP\_Nocodazole\_MudPIT\_040709\_06.05969.05969.2 | 4.4858 | 0.4942 | 100.0% | 1970.7522 | 1971.1951 | 1 | 8.155 | 59.4% | 1 | R.TQAPSYLQQNGVQNYMK.Y | 2 |
|  | Mis12IP\_Nocodazole\_MudPIT\_040709\_06.05993.05993.2 | 2.5824 | 0.3904 | 100.0% | 1141.1322 | 1141.3531 | 1 | 5.999 | 80.0% | 1 | K.LALPADSVNIK.I | 2 |

---

|  |  |  |  |  |  |  |  |  |
| --- | --- | --- | --- | --- | --- | --- | --- | --- |
| U | *gi|1469870|dbj|BAA097* | 2 | 2 | 3.5% | 983 | 103930 | 7.0 | KIAA0144 [Homo sapiens] |
| U | *gi|55665799|emb|CAH71* | 2 | 2 | 3.1% | 1087 | 114534 | 7.1 | ubiquitin associated protein 2-like [Homo sapiens] |
| U | *gi|40254861|ref|NP\_05* | 2 | 2 | 3.1% | 1087 | 114543 | 7.1 | ubiquitin associated protein 2-like [Homo sapiens] |

| Filename XCorr DeltCN Conf% ObsM+H+ CalcM+H+ SpR ZScore Ion% # Sequence  | | | | | | | | | | | | |
| --- | --- | --- | --- | --- | --- | --- | --- | --- | --- | --- | --- | --- |
|  | Mis12IP\_Nocodazole\_MudPIT\_040709\_04.06478.06478.2 | 2.4436 | 0.3845 | 99.9% | 2241.2322 | 2242.2708 | 1 | 6.949 | 38.9% | 1 | R.TATEEWGTEDWNEDLSETK.I | 2 |
|  | Mis12IP\_Nocodazole\_MudPIT\_040709\_03.04742.04742.2 | 3.4843 | 0.4583 | 100.0% | 1524.4922 | 1525.6122 | 1 | 7.494 | 60.7% | 1 | R.DGSLASNPYSGDLTK.F | 2 |

---

|  |  |  |  |  |  |  |  |  |
| --- | --- | --- | --- | --- | --- | --- | --- | --- |
| U | *gi|85566621|gb|AAI119* | 3 | 6 | 3.4% | 525 | 55087 | 5.0 | Keratin 24 [Homo sapiens] |
| U | *gi|9506669|ref|NP\_061* | 3 | 6 | 3.4% | 525 | 55145 | 4.9 | keratin 24 [Homo sapiens] |

| Filename XCorr DeltCN Conf% ObsM+H+ CalcM+H+ SpR ZScore Ion% # Sequence  | | | | | | | | | | | | |
| --- | --- | --- | --- | --- | --- | --- | --- | --- | --- | --- | --- | --- |
|  | Mis12IP\_Nocodazole\_MudPIT\_040709\_02.04220.04220.1 | 1.5217 | 0.3222 | 99.0% | 807.44 | 807.8815 | 2 | 4.741 | 58.3% | 1 | R.LAADDFR.L | 11111111 |
|  | Mis12IP\_Nocodazole\_MudPIT\_040709\_03.04543.04543.2 | 2.2948 | 0.233 | 99.3% | 808.0522 | 807.8815 | 1 | 5.671 | 91.7% | 3 | R.LAADDFR.L | 22222222 |
|  | Mis12IP\_Nocodazole\_MudPIT\_040709\_05.05008.05008.2 | 2.7343 | 0.3118 | 99.9% | 1201.9521 | 1202.3097 | 1 | 5.738 | 80.0% | 2 | R.QSVEADINGLR.K | 222 |

Similarities:
gi|4557888|ref|NP\_000(2:1)  
gi|28317|emb|CAA32649(1:2)  
gi|24430192|ref|NP\_00(2:1)  
gi|12803709|gb|AAH026(2:1)  
gi|4557701|ref|NP\_000(2:1)  
gi|14043271|gb|AAH076(2:1)  
gi|24234696|ref|NP\_70(1:2)  
gi|24430190|ref|NP\_00(2:1)  
gi|34526448|dbj|BAC85(2:1)  

---

|  |  |  |  |  |  |  |  |  |
| --- | --- | --- | --- | --- | --- | --- | --- | --- |
| U | *gi|58530840|ref|NP\_00* | 6 | 6 | 3.3% | 2871 | 331774 | 6.8 | desmoplakin isoform I [Homo sapiens] |
| U | *gi|68533039|dbj|BAE06* | 6 | 6 | 4.1% | 2319 | 265192 | 7.3 | DSP variant protein [Homo sapiens] |
| U | *gi|58530842|ref|NP\_00* | 6 | 6 | 4.2% | 2272 | 260116 | 7.0 | desmoplakin isoform II [Homo sapiens] |

| Filename XCorr DeltCN Conf% ObsM+H+ CalcM+H+ SpR ZScore Ion% # Sequence  | | | | | | | | | | | | |
| --- | --- | --- | --- | --- | --- | --- | --- | --- | --- | --- | --- | --- |
|  | Mis12IP\_Nocodazole\_MudPIT\_040709\_04.06202.06202.2 | 2.4012 | 0.0865 | 95.1% | 1129.9321 | 1130.2834 | 7 | 4.452 | 68.8% | 1 | K.IEVLEEELR.L | 2 |
|  | Mis12IP\_Nocodazole\_MudPIT\_040709\_06.06492.06492.2 | 2.7269 | 0.3931 | 100.0% | 1254.9521 | 1255.3696 | 1 | 6.349 | 68.2% | 1 | K.AITGFDDPFSGK.T | 2 |
|  | Mis12IP\_Nocodazole\_MudPIT\_040709\_06.07555.07555.2 | 2.6866 | 0.3097 | 99.7% | 2255.632 | 2254.6316 | 1 | 5.775 | 42.9% | 1 | R.LLEAQIASGGVVDPVNSVFLPK.D | 2 |
|  | Mis12IP\_Nocodazole\_MudPIT\_040709\_06.06554.06554.2 | 4.3119 | 0.5311 | 100.0% | 1539.3322 | 1539.8125 | 1 | 8.894 | 67.9% | 1 | R.LLEAQIATGGIIDPK.E | 2 |
|  | Mis12IP\_Nocodazole\_MudPIT\_040709\_05.07578.07578.2 | 2.6311 | 0.3357 | 99.8% | 2159.7122 | 2160.2524 | 1 | 5.556 | 38.9% | 1 | R.GYFNEELSEILSDPSDDTK.G | 2 |
|  | Mis12IP\_Nocodazole\_MudPIT\_040709\_06.06811.06811.2 | 2.4951 | 0.4234 | 99.9% | 2047.6721 | 2048.2756 | 1 | 6.056 | 44.4% | 1 | K.LSLQDAVSQGVIDQDMATR.L | 2 |

---

|  |  |  |  |  |  |  |  |  |
| --- | --- | --- | --- | --- | --- | --- | --- | --- |
| U | *gi|1045057|emb|CAA632* | 5 | 5 | 3.3% | 1972 | 218422 | 8.2 | unnamed protein product [Homo sapiens] |
| U | *gi|6633953|dbj|BAA078* | 5 | 5 | 3.2% | 2038 | 226034 | 7.9 | KIAA0097 protein [Homo sapiens] |
| U | *gi|57222563|ref|NP\_05* | 5 | 5 | 3.3% | 1972 | 218524 | 8.1 | colonic and hepatic tumor over-expressed protein isoform b [Homo sapiens] |
| U | *gi|57164942|ref|NP\_00* | 5 | 5 | 3.2% | 2032 | 225493 | 7.8 | colonic and hepatic tumor over-expressed protein isoform a [Homo sapiens] |
| U | *gi|111309288|gb|AAI20* | 5 | 5 | 3.4% | 1925 | 213571 | 7.7 | CKAP5 protein [Homo sapiens] |

| Filename XCorr DeltCN Conf% ObsM+H+ CalcM+H+ SpR ZScore Ion% # Sequence  | | | | | | | | | | | | |
| --- | --- | --- | --- | --- | --- | --- | --- | --- | --- | --- | --- | --- |
|  | Mis12IP\_Nocodazole\_MudPIT\_040709\_06.05264.05264.2 | 2.6086 | 0.2187 | 99.3% | 1169.2922 | 1170.3287 | 1 | 4.7 | 70.0% | 1 | K.MQGQSPPAPTR.G | 2 |
|  | Mis12IP\_Nocodazole\_MudPIT\_040709\_05.06491.06491.2 | 3.0479 | 0.4589 | 100.0% | 1444.2722 | 1444.5817 | 1 | 7.402 | 61.5% | 1 | K.EGLDEVAGIINDAK.F | 2 |
|  | Mis12IP\_Nocodazole\_MudPIT\_040709\_06.05518.05518.2 | 3.9293 | 0.3837 | 100.0% | 1319.0721 | 1319.4784 | 1 | 7.897 | 77.3% | 1 | R.AQNISSNANMLR.K | 2 |
|  | Mis12IP\_Nocodazole\_MudPIT\_040709\_05.06573.06573.2 | 3.5513 | 0.4892 | 100.0% | 2007.4722 | 2008.1058 | 2 | 8.194 | 50.0% | 1 | R.EFQLDLDEIENDNGTVR.C | 2 |
|  | Mis12IP\_Nocodazole\_MudPIT\_040709\_05.05993.05993.2 | 3.4187 | 0.5272 | 100.0% | 1297.8322 | 1298.435 | 1 | 8.164 | 85.0% | 1 | K.YSDADIEPFLK.N | 2 |

---

|  |  |  |  |  |  |  |  |  |
| --- | --- | --- | --- | --- | --- | --- | --- | --- |
| U | *gi|1679684|gb|AAB1920* | 3 | 3 | 3.3% | 1312 | 140140 | 9.6 | ataxin-2 [Homo sapiens] |
| U | *gi|90112010|gb|AAI145* | 3 | 3 | 4.3% | 1006 | 109037 | 8.7 | ATXN2 protein [Homo sapiens] |
| U | *gi|85057074|gb|AAI117* | 3 | 3 | 3.8% | 1127 | 122181 | 8.7 | ATXN2 protein [Homo sapiens] |
| U | *gi|51479160|ref|NP\_00* | 3 | 3 | 3.3% | 1313 | 140269 | 9.6 | ataxin 2 [Homo sapiens] |
| U | *gi|1770390|emb|CAA695* | 3 | 3 | 4.7% | 914 | 97862 | 9.4 | SCA2 [Homo sapiens] |

| Filename XCorr DeltCN Conf% ObsM+H+ CalcM+H+ SpR ZScore Ion% # Sequence  | | | | | | | | | | | | |
| --- | --- | --- | --- | --- | --- | --- | --- | --- | --- | --- | --- | --- |
|  | Mis12IP\_Nocodazole\_MudPIT\_040709\_05.05638.05638.2 | 2.4545 | 0.4359 | 99.9% | 1681.9321 | 1681.7966 | 21 | 6.722 | 50.0% | 1 | R.ANQLAEEIESSAQYK.A | 2 |
|  | Mis12IP\_Nocodazole\_MudPIT\_040709\_05.04564.04564.2 | 2.4533 | 0.3966 | 99.9% | 1191.8922 | 1192.3502 | 9 | 6.528 | 54.5% | 1 | R.MGQPGSGSMPSR.S | 2 |
|  | Mis12IP\_Nocodazole\_MudPIT\_040709\_06.06025.06025.2 | 2.2474 | 0.2063 | 96.6% | 1778.3322 | 1778.972 | 1 | 5.107 | 46.7% | 1 | R.LQPSSTSESMDQLLNK.N | 2 |

---

|  |  |  |  |  |  |  |  |  |
| --- | --- | --- | --- | --- | --- | --- | --- | --- |
| U | *gi|4758138|ref|NP\_004* | 2 | 2 | 3.3% | 614 | 69148 | 8.9 | DEAD (Asp-Glu-Ala-Asp) box polypeptide 5 [Homo sapiens] |

| Filename XCorr DeltCN Conf% ObsM+H+ CalcM+H+ SpR ZScore Ion% # Sequence  | | | | | | | | | | | | |
| --- | --- | --- | --- | --- | --- | --- | --- | --- | --- | --- | --- | --- |
|  | Mis12IP\_Nocodazole\_MudPIT\_040709\_06.06102.06102.2 | 3.7477 | 0.2993 | 100.0% | 1228.1721 | 1227.4465 | 2 | 6.856 | 72.7% | 1 | K.APILIATDVASR.G | 22 |
| \* | Mis12IP\_Nocodazole\_MudPIT\_040709\_06.06038.06038.1 | 2.0384 | 0.2918 | 98.5% | 985.62 | 986.1564 | 5 | 4.89 | 71.4% | 1 | K.LLQLVEDR.G | 1 |

Similarities:
gi|2832596|emb|CAB097(1:1)  

---

|  |  |  |  |  |  |  |  |  |
| --- | --- | --- | --- | --- | --- | --- | --- | --- |
| U | *gi|33350932|ref|NP\_00* | 10 | 11 | 3.2% | 4646 | 532412 | 6.4 | dynein, cytoplasmic, heavy polypeptide 1 [Homo sapiens] |
| U | *gi|71891782|dbj|BAA20* | 10 | 11 | 3.2% | 4658 | 533745 | 6.4 | KIAA0325 protein [Homo sapiens] |

| Filename XCorr DeltCN Conf% ObsM+H+ CalcM+H+ SpR ZScore Ion% # Sequence  | | | | | | | | | | | | |
| --- | --- | --- | --- | --- | --- | --- | --- | --- | --- | --- | --- | --- |
|  | Mis12IP\_Nocodazole\_MudPIT\_040709\_06.08501.08501.3 | 3.5272 | 0.469 | 100.0% | 2349.1743 | 2349.6836 | 1 | 7.508 | 31.8% | 2 | K.LVPLLLEDGGEAPAALEAALEEK.S | 3 |
|  | Mis12IP\_Nocodazole\_MudPIT\_040709\_06.08504.08504.2 | 4.1969 | 0.4799 | 100.0% | 2349.5923 | 2349.6836 | 1 | 7.597 | 47.7% | 1 | K.LVPLLLEDGGEAPAALEAALEEK.S | 2 |
|  | Mis12IP\_Nocodazole\_MudPIT\_040709\_05.05464.05464.2 | 3.9119 | 0.5372 | 100.0% | 1574.0922 | 1574.7925 | 1 | 8.48 | 61.5% | 1 | K.DSAIQQQVANLQMK.I | 2 |
|  | Mis12IP\_Nocodazole\_MudPIT\_040709\_05.06184.06184.2 | 2.8806 | 0.4173 | 100.0% | 1819.6921 | 1820.9481 | 2 | 6.861 | 46.9% | 1 | K.EALELTDTGLLSGSEER.V | 2 |
|  | Mis12IP\_Nocodazole\_MudPIT\_040709\_05.06626.06626.2 | 3.3131 | 0.4691 | 100.0% | 1957.7122 | 1958.2366 | 1 | 7.633 | 58.8% | 1 | R.DAATIMQPYFTSNGLVTK.A | 2 |
|  | Mis12IP\_Nocodazole\_MudPIT\_040709\_06.08497.08497.2 | 3.4592 | 0.4988 | 100.0% | 2445.912 | 2446.8616 | 1 | 8.262 | 38.6% | 1 | R.ALPDMEVVGLNFSSATTPELLLK.T | 2 |
|  | Mis12IP\_Nocodazole\_MudPIT\_040709\_06.08178.08178.2 | 3.5036 | 0.4276 | 100.0% | 2057.2322 | 2057.326 | 2 | 7.39 | 44.1% | 1 | K.IAFIMDESNVLDSGFLER.M | 2 |
|  | Mis12IP\_Nocodazole\_MudPIT\_040709\_06.08261.08261.2 | 3.4102 | 0.5244 | 100.0% | 2598.7722 | 2599.9463 | 2 | 8.702 | 36.4% | 1 | R.FGNPLLVQDVESYDPVLNPVLNR.E | 2 |
|  | Mis12IP\_Nocodazole\_MudPIT\_040709\_06.08270.08270.3 | 3.4628 | 0.3795 | 100.0% | 2599.0144 | 2599.9463 | 1 | 6.759 | 33.0% | 1 | R.FGNPLLVQDVESYDPVLNPVLNR.E | 3 |
|  | Mis12IP\_Nocodazole\_MudPIT\_040709\_06.06340.06340.2 | 3.9684 | 0.5971 | 100.0% | 1405.1721 | 1405.6929 | 1 | 9.104 | 75.0% | 1 | R.VLLTTQGVDMISK.M | 2 |

---

|  |  |  |  |  |  |  |  |  |
| --- | --- | --- | --- | --- | --- | --- | --- | --- |
| U | *gi|12803479|gb|AAH025* | 2 | 2 | 3.0% | 856 | 95785 | 6.9 | Heterogeneous nuclear ribonucleoprotein U-like 1 [Homo sapiens] |
| U | *gi|3319956|emb|CAA075* | 2 | 2 | 3.0% | 856 | 95810 | 6.9 | E1B-55kDa-associated protein [Homo sapiens] |
| U | *gi|21536326|ref|NP\_00* | 2 | 2 | 3.0% | 856 | 95739 | 6.9 | E1B-55kDa-associated protein 5 isoform a [Homo sapiens] |
| U | *gi|21536320|ref|NP\_65* | 2 | 2 | 3.4% | 756 | 84794 | 8.8 | E1B-55kDa-associated protein 5 isoform d [Homo sapiens] |
| U | *gi|20379474|gb|AAH277* | 2 | 2 | 3.2% | 804 | 90292 | 6.9 | HNRPUL1 protein [Homo sapiens] |

| Filename XCorr DeltCN Conf% ObsM+H+ CalcM+H+ SpR ZScore Ion% # Sequence  | | | | | | | | | | | | |
| --- | --- | --- | --- | --- | --- | --- | --- | --- | --- | --- | --- | --- |
|  | Mis12IP\_Nocodazole\_MudPIT\_040709\_06.05803.05803.2 | 2.1252 | 0.2188 | 97.4% | 1383.7722 | 1384.5315 | 11 | 4.005 | 65.0% | 1 | R.QNQFYDTQVIK.Q | 2 |
|  | Mis12IP\_Nocodazole\_MudPIT\_040709\_06.06085.06085.2 | 4.4856 | 0.4735 | 100.0% | 1743.6522 | 1742.8857 | 1 | 7.6 | 78.6% | 1 | R.NYILDQTNVYGSAQR.R | 2 |

---

|  |  |  |  |  |  |  |  |  |
| --- | --- | --- | --- | --- | --- | --- | --- | --- |
| U | *gi|13876386|ref|NP\_11* | 9 | 13 | 2.9% | 5065 | 553102 | 5.6 | epiplakin 1 [Homo sapiens] |
| U | *gi|37196760|dbj|BAC92* | 9 | 13 | 2.9% | 5061 | 552777 | 5.6 | epiplakin [Homo sapiens] |

| Filename XCorr DeltCN Conf% ObsM+H+ CalcM+H+ SpR ZScore Ion% # Sequence  | | | | | | | | | | | | |
| --- | --- | --- | --- | --- | --- | --- | --- | --- | --- | --- | --- | --- |
|  | Mis12IP\_Nocodazole\_MudPIT\_040709\_06.07157.07157.3 | 4.8254 | 0.3949 | 100.0% | 2447.1843 | 2448.753 | 1 | 7.668 | 45.2% | 2 | R.AVTGYTDPYTGQQISLFQAMQK.G | 3 |
|  | Mis12IP\_Nocodazole\_MudPIT\_040709\_06.07159.07159.2 | 4.9433 | 0.5507 | 100.0% | 2447.5923 | 2448.753 | 1 | 11.001 | 61.9% | 2 | R.AVTGYTDPYTGQQISLFQAMQK.G | 2 |
|  | Mis12IP\_Nocodazole\_MudPIT\_040709\_05.06603.06603.3 | 3.4996 | 0.3513 | 100.0% | 2791.1042 | 2790.9287 | 1 | 7.284 | 31.0% | 1 | R.GYVDQEMETALSSSSETFPTPDGQGR.T | 3 |
|  | Mis12IP\_Nocodazole\_MudPIT\_040709\_04.05734.05734.2 | 2.9633 | 0.4115 | 100.0% | 1161.0322 | 1161.2311 | 1 | 6.902 | 87.5% | 2 | C.GYFDEEMNR.I | 22 |
|  | Mis12IP\_Nocodazole\_MudPIT\_040709\_06.09992.09992.2 | 2.4539 | 0.2565 | 99.2% | 2523.372 | 2523.7607 | 2 | 3.92 | 27.5% | 1 | R.FADQVVSFWDLLSSPYFTEDR.K | 2 |
|  | Mis12IP\_Nocodazole\_MudPIT\_040709\_06.07842.07842.2 | 2.6516 | 0.4558 | 100.0% | 1618.3522 | 1619.8607 | 1 | 7.248 | 53.6% | 1 | R.AVPVWDVLASGYVSR.A | 2 |
|  | Mis12IP\_Nocodazole\_MudPIT\_040709\_06.08196.08196.2 | 2.8976 | 0.5001 | 100.0% | 2031.6721 | 2033.286 | 1 | 8.201 | 52.8% | 1 | R.EELLAEFGSGTLDLPALTR.R | 2 |
|  | Mis12IP\_Nocodazole\_MudPIT\_040709\_06.06935.06935.2 | 4.7504 | 0.4766 | 100.0% | 1885.8722 | 1886.1094 | 1 | 8.837 | 61.1% | 2 | R.LSVEEAVAAGVVGGEIQEK.L | 2 |
|  | Mis12IP\_Nocodazole\_MudPIT\_040709\_03.00095.00095.2 | 3.1704 | 0.4761 | 100.0% | 1818.5721 | 1819.1052 | 1 | 8.014 | 59.4% | 1 | R.ATLDPETGLLFLSLSLQ.- | 2 |

Similarities:
gi|1296662|emb|CAA911(1:8)  

---

|  |  |  |  |  |  |  |  |  |
| --- | --- | --- | --- | --- | --- | --- | --- | --- |
| U | *gi|24430146|ref|NP\_00* | 3 | 3 | 2.6% | 1475 | 153938 | 8.7 | nucleoporin 153kDa [Homo sapiens] |
| U | *gi|68533103|dbj|BAE06* | 3 | 3 | 2.7% | 1455 | 151461 | 8.9 | NUP153 variant protein [Homo sapiens] |
| U | *gi|406225|emb|CAA8098* | 3 | 3 | 2.6% | 1475 | 153889 | 8.7 | nuclear pore complex protein hnup153 [Homo sapiens] |
| U | *gi|31418202|gb|AAH529* | 3 | 3 | 2.6% | 1475 | 153985 | 8.8 | Nucleoporin 153kDa [Homo sapiens] |

| Filename XCorr DeltCN Conf% ObsM+H+ CalcM+H+ SpR ZScore Ion% # Sequence  | | | | | | | | | | | | |
| --- | --- | --- | --- | --- | --- | --- | --- | --- | --- | --- | --- | --- |
|  | Mis12IP\_Nocodazole\_MudPIT\_040709\_06.05982.05982.2 | 3.032 | 0.4029 | 100.0% | 1397.1122 | 1397.5272 | 1 | 6.541 | 62.5% | 1 | K.TSQLGDSPFYPGK.T | 2 |
|  | Mis12IP\_Nocodazole\_MudPIT\_040709\_03.04387.04387.2 | 2.2396 | 0.4078 | 99.9% | 1038.1721 | 1038.1484 | 1 | 6.327 | 70.0% | 1 | K.TTYGGAAAAVR.Q | 2 |
|  | Mis12IP\_Nocodazole\_MudPIT\_040709\_05.04732.04732.2 | 2.4608 | 0.4577 | 100.0% | 1556.3522 | 1556.6726 | 1 | 7.029 | 50.0% | 1 | K.QLSAQSYGVTSSTAR.R | 2 |

---

|  |  |  |  |  |  |  |  |  |
| --- | --- | --- | --- | --- | --- | --- | --- | --- |
| U | *gi|23986276|gb|AAM219* | 2 | 3 | 2.6% | 1265 | 144718 | 8.1 | tripin [Homo sapiens] |
| U | *gi|62990154|ref|NP\_68* | 2 | 3 | 2.6% | 1265 | 144797 | 7.8 | shugoshin-like 2 [Homo sapiens] |
| U | *gi|62988877|gb|AAY242* | 2 | 3 | 3.0% | 1103 | 125992 | 6.3 | unknown [Homo sapiens] |

| Filename XCorr DeltCN Conf% ObsM+H+ CalcM+H+ SpR ZScore Ion% # Sequence  | | | | | | | | | | | | |
| --- | --- | --- | --- | --- | --- | --- | --- | --- | --- | --- | --- | --- |
|  | Mis12IP\_Nocodazole\_MudPIT\_040709\_04.05946.05946.2 | 5.1577 | 0.575 | 100.0% | 1746.0521 | 1745.8959 | 1 | 10.755 | 70.0% | 1 | K.TVYDADMDLTASEVSK.I | 2 |
|  | Mis12IP\_Nocodazole\_MudPIT\_040709\_04.07754.07754.2 | 3.8192 | 0.3861 | 100.0% | 1850.9122 | 1852.008 | 1 | 8.777 | 56.2% | 2 | K.DSGNLYDSEIQNVLGVK.H | 2 |

---

|  |  |  |  |  |  |  |  |  |
| --- | --- | --- | --- | --- | --- | --- | --- | --- |
| U | *gi|20143967|ref|NP\_61* | 2 | 2 | 2.6% | 960 | 110059 | 8.5 | kinesin family member 23 isoform 1 [Homo sapiens] |
| U | *gi|6754472|ref|NP\_004* | 2 | 2 | 2.9% | 856 | 98105 | 8.5 | kinesin family member 23 isoform 2 [Homo sapiens] |
| U | *gi|34783297|gb|AAH177* | 2 | 2 | 2.6% | 956 | 109641 | 8.5 | KIF23 protein [Homo sapiens] |

| Filename XCorr DeltCN Conf% ObsM+H+ CalcM+H+ SpR ZScore Ion% # Sequence  | | | | | | | | | | | | |
| --- | --- | --- | --- | --- | --- | --- | --- | --- | --- | --- | --- | --- |
|  | Mis12IP\_Nocodazole\_MudPIT\_040709\_05.05419.05419.3 | 3.0183 | 0.3696 | 100.0% | 2737.5244 | 2739.0574 | 1 | 6.717 | 25.0% | 1 | R.SHSVFNIKLVQAPLDADGDNVLQEK.E | 3 |
|  | Mis12IP\_Nocodazole\_MudPIT\_040709\_05.05419.05419.2 | 3.3306 | 0.5269 | 100.0% | 1825.3522 | 1826.0135 | 1 | 8.568 | 62.5% | 1 | K.LVQAPLDADGDNVLQEK.E | 3 |

---

|  |  |  |  |  |  |  |  |  |
| --- | --- | --- | --- | --- | --- | --- | --- | --- |
| U | *gi|12025678|ref|NP\_00* | 2 | 2 | 2.2% | 911 | 104854 | 5.4 | actinin, alpha 4 [Homo sapiens] |
| U | *gi|94982457|gb|ABF500* | 2 | 2 | 2.2% | 914 | 105568 | 5.4 | actinin alpha 1 isoform b [Homo sapiens] |
| U | *gi|4501891|ref|NP\_001* | 2 | 2 | 2.2% | 892 | 103058 | 5.4 | actinin, alpha 1 [Homo sapiens] |
| U | *gi|3157976|gb|AAC1747* | 2 | 2 | 2.2% | 912 | 105224 | 5.7 | alpha actinin [Homo sapiens] |
| U | *gi|28334|emb|CAA33803* | 2 | 2 | 2.2% | 892 | 102974 | 5.4 | unnamed protein product [Homo sapiens] |
| U | *gi|2804273|dbj|BAA244* | 2 | 2 | 2.3% | 884 | 102268 | 5.4 | alpha actinin 4 [Homo sapiens] |

| Filename XCorr DeltCN Conf% ObsM+H+ CalcM+H+ SpR ZScore Ion% # Sequence  | | | | | | | | | | | | |
| --- | --- | --- | --- | --- | --- | --- | --- | --- | --- | --- | --- | --- |
|  | Mis12IP\_Nocodazole\_MudPIT\_040709\_06.06032.06032.1 | 1.3462 | 0.2722 | 95.8% | 864.43 | 865.01715 | 71 | 4.806 | 42.9% | 1 | K.ALDFIASK.G | 1 |
|  | Mis12IP\_Nocodazole\_MudPIT\_040709\_06.06097.06097.2 | 2.7107 | 0.2567 | 99.6% | 1429.4122 | 1430.6011 | 8 | 5.094 | 59.1% | 1 | R.TINEVENQILTR.D | 2 |

---

|  |  |  |  |  |  |  |  |  |
| --- | --- | --- | --- | --- | --- | --- | --- | --- |
| U | *gi|112382250|ref|NP\_0* | 3 | 3 | 2.0% | 2364 | 274608 | 5.6 | spectrin, beta, non-erythrocytic 1 isoform 1 [Homo sapiens] |
| U | *gi|62988842|gb|AAY242* | 3 | 3 | 2.0% | 2314 | 268714 | 5.6 | unknown [Homo sapiens] |
| U | *gi|62089082|dbj|BAD92* | 3 | 3 | 2.0% | 2377 | 276168 | 5.5 | spectrin, beta, non-erythrocytic 1 isoform 1 variant [Homo sapiens] |
| U | *gi|425553|gb|AAB28324* | 3 | 3 | 3.8% | 1252 | 146243 | 5.3 | beta-fodrin [Homo sapiens] |
| U | *gi|27462180|gb|AAO153* | 3 | 3 | 2.2% | 2155 | 251417 | 5.6 | beta-spectrin 2 isoform 2 [Homo sapiens] |
| U | *gi|112382252|ref|NP\_8* | 3 | 3 | 2.2% | 2155 | 251395 | 5.5 | spectrin, beta, non-erythrocytic 1 isoform 2 [Homo sapiens] |

| Filename XCorr DeltCN Conf% ObsM+H+ CalcM+H+ SpR ZScore Ion% # Sequence  | | | | | | | | | | | | |
| --- | --- | --- | --- | --- | --- | --- | --- | --- | --- | --- | --- | --- |
|  | Mis12IP\_Nocodazole\_MudPIT\_040709\_06.07351.07351.2 | 2.6769 | 0.1814 | 98.2% | 2022.3722 | 2023.25 | 1 | 5.851 | 41.7% | 1 | R.LVSQDNFGFDLPAVEAATK.K | 2 |
|  | Mis12IP\_Nocodazole\_MudPIT\_040709\_06.05524.05524.1 | 1.7318 | 0.2437 | 98.7% | 969.6 | 970.16 | 1 | 3.99 | 68.8% | 1 | R.VAVVNQIAR.Q | 1 |
|  | Mis12IP\_Nocodazole\_MudPIT\_040709\_05.05179.05179.2 | 2.71 | 0.4271 | 100.0% | 2050.5122 | 2051.23 | 1 | 6.85 | 36.1% | 1 | R.TQTAIASEDMPNTLTEAEK.L | 2 |

---

|  |  |  |  |  |  |  |  |  |
| --- | --- | --- | --- | --- | --- | --- | --- | --- |
| U | *gi|20521053|dbj|BAA23* | 3 | 3 | 1.9% | 1817 | 201641 | 8.2 | KIAA0440 [Homo sapiens] |
| U | *gi|7662126|ref|NP\_056* | 3 | 3 | 1.9% | 1804 | 200028 | 8.2 | signal-induced proliferation-associated 1 like 1 [Homo sapiens] |
| U | *gi|60219557|emb|CAI56* | 3 | 3 | 9.9% | 355 | 39597 | 7.3 | hypothetical protein [Homo sapiens] |
| U | *gi|4151328|gb|AAD1254* | 3 | 3 | 2.0% | 1783 | 197494 | 8.3 | high-risk human papilloma viruses E6 oncoproteins targeted protein E6TP1 alpha; putative GAP protein alpha [Homo sapiens] |

| Filename XCorr DeltCN Conf% ObsM+H+ CalcM+H+ SpR ZScore Ion% # Sequence  | | | | | | | | | | | | |
| --- | --- | --- | --- | --- | --- | --- | --- | --- | --- | --- | --- | --- |
|  | Mis12IP\_Nocodazole\_MudPIT\_040709\_06.08082.08082.2 | 3.3428 | 0.4114 | 100.0% | 2476.9722 | 2477.817 | 20 | 7.011 | 29.5% | 1 | R.ASLLDQALPNDVLFSSTYPSLPK.S | 2 |
|  | Mis12IP\_Nocodazole\_MudPIT\_040709\_06.08092.08092.3 | 2.9728 | 0.263 | 99.0% | 2477.3342 | 2477.817 | 1 | 5.917 | 38.6% | 1 | R.ASLLDQALPNDVLFSSTYPSLPK.S | 3 |
|  | Mis12IP\_Nocodazole\_MudPIT\_040709\_02.07469.07469.2 | 4.034 | 0.4004 | 100.0% | 1491.2522 | 1490.6704 | 1 | 8.622 | 72.7% | 1 | K.FTEWVFNTIDMS.- | 2 |

---

|  |  |  |  |  |  |  |  |  |
| --- | --- | --- | --- | --- | --- | --- | --- | --- |
| U | *gi|105990514|ref|NP\_0* | 2 | 2 | 1.7% | 2602 | 278162 | 5.7 | filamin B, beta (actin binding protein 278) [Homo sapiens] |
| U | *gi|62089364|dbj|BAD93* | 2 | 2 | 2.7% | 1613 | 170161 | 5.5 | filamin B, beta (actin binding protein 278) variant [Homo sapiens] |
| U | *gi|53791217|dbj|BAD52* | 2 | 2 | 1.7% | 2591 | 276936 | 5.7 | filamin B [Homo sapiens] |
| U | *gi|51491176|emb|CAH18* | 2 | 2 | 1.8% | 2409 | 256334 | 5.7 | hypothetical protein [Homo sapiens] |
| U | *gi|34365016|emb|CAE46* | 2 | 2 | 1.8% | 2422 | 257388 | 5.7 | hypothetical protein [Homo sapiens] |
| U | *gi|3298597|gb|AAC3984* | 2 | 2 | 1.7% | 2602 | 278193 | 5.7 | beta-filamin [Homo sapiens] |
| U | *gi|3282771|gb|AAC3384* | 2 | 2 | 1.7% | 2602 | 278190 | 5.7 | actin-binding protein homolog ABP-278 [Homo sapiens] |

| Filename XCorr DeltCN Conf% ObsM+H+ CalcM+H+ SpR ZScore Ion% # Sequence  | | | | | | | | | | | | |
| --- | --- | --- | --- | --- | --- | --- | --- | --- | --- | --- | --- | --- |
|  | Mis12IP\_Nocodazole\_MudPIT\_040709\_06.07706.07706.3 | 2.7173 | 0.2951 | 99.0% | 2548.2244 | 2548.8552 | 1 | 4.591 | 25.0% | 1 | K.VTASGPGLSSYGVPASLPVDFAIDAR.D | 3 |
|  | Mis12IP\_Nocodazole\_MudPIT\_040709\_06.07134.07134.2 | 3.1626 | 0.3104 | 99.9% | 2017.3322 | 2018.2916 | 1 | 6.713 | 47.1% | 1 | R.YMIGVTYGGDDIPLSPYR.I | 2 |

---

|  |  |  |  |  |  |  |  |  |
| --- | --- | --- | --- | --- | --- | --- | --- | --- |
| U | *gi|61743954|ref|NP\_00* | 6 | 6 | 1.6% | 5890 | 629114 | 6.1 | AHNAK nucleoprotein isoform 1 [Homo sapiens] |

| Filename XCorr DeltCN Conf% ObsM+H+ CalcM+H+ SpR ZScore Ion% # Sequence  | | | | | | | | | | | | |
| --- | --- | --- | --- | --- | --- | --- | --- | --- | --- | --- | --- | --- |
| \* | Mis12IP\_Nocodazole\_MudPIT\_040709\_06.06443.06443.2 | 2.7628 | 0.384 | 99.9% | 1598.6721 | 1599.7843 | 1 | 6.976 | 62.5% | 1 | R.GGVQVPAVDISSSLGGR.A | 2 |
| \* | Mis12IP\_Nocodazole\_MudPIT\_040709\_06.06450.06450.2 | 3.462 | 0.3237 | 100.0% | 1712.3322 | 1712.8975 | 1 | 7.771 | 66.7% | 1 | R.VDIETPNLEGTLTGPR.L | 2 |
| \* | Mis12IP\_Nocodazole\_MudPIT\_040709\_06.06284.06284.2 | 3.5811 | 0.3913 | 100.0% | 1474.1921 | 1474.7124 | 1 | 7.38 | 69.2% | 1 | R.ISMSEVDLNVAAPK.V | 2 |
| \* | Mis12IP\_Nocodazole\_MudPIT\_040709\_06.06276.06276.2 | 2.5665 | 0.3928 | 99.9% | 1610.7522 | 1611.7924 | 73 | 6.169 | 40.0% | 1 | K.VNVEAPDVNLEGLGGK.L | 2 |
| \* | Mis12IP\_Nocodazole\_MudPIT\_040709\_05.05650.05650.2 | 3.1181 | 0.4139 | 100.0% | 1639.2522 | 1638.815 | 1 | 7.83 | 56.7% | 1 | K.VGVEVPDVNIEGPEGK.L | 2 |
|  | Mis12IP\_Nocodazole\_MudPIT\_040709\_05.05766.05766.2 | 2.1269 | 0.352 | 99.3% | 1669.3522 | 1669.826 | 24 | 6.064 | 40.0% | 1 | K.VDVEVPDVSLEGPEGK.L | 2 |

---

|  |  |  |  |  |  |  |  |  |
| --- | --- | --- | --- | --- | --- | --- | --- | --- |
| U | *gi|20521848|dbj|BAA13* | 3 | 3 | 1.6% | 2675 | 293139 | 7.5 | KIAA0219 [Homo sapiens] |
| U | *gi|54607053|ref|NP\_00* | 3 | 3 | 1.6% | 2671 | 292708 | 7.4 | GCN1 general control of amino-acid synthesis 1-like 1 [Homo sapiens] |
| U | *gi|3970973|gb|AAC8318* | 3 | 3 | 1.8% | 2392 | 262292 | 6.9 | similar to human HsGCN1 U77700 (PID:g2282576); similar to yeast translation activator GCN1 (PID:g462168) [Homo sapiens] |

| Filename XCorr DeltCN Conf% ObsM+H+ CalcM+H+ SpR ZScore Ion% # Sequence  | | | | | | | | | | | | |
| --- | --- | --- | --- | --- | --- | --- | --- | --- | --- | --- | --- | --- |
|  | Mis12IP\_Nocodazole\_MudPIT\_040709\_06.05572.05572.2 | 3.2882 | 0.3116 | 99.9% | 1272.4722 | 1272.4037 | 1 | 6.52 | 70.8% | 1 | R.QAGAEALSQAVAR.Y | 2 |
|  | Mis12IP\_Nocodazole\_MudPIT\_040709\_06.06944.06944.2 | 3.4461 | 0.4828 | 100.0% | 1335.4722 | 1335.5431 | 1 | 8.656 | 68.2% | 1 | R.AYSDQAIVNLLK.M | 2 |
|  | Mis12IP\_Nocodazole\_MudPIT\_040709\_02.04648.04648.2 | 1.9725 | 0.2957 | 98.6% | 1807.4321 | 1807.906 | 1 | 5.597 | 43.8% | 1 | K.LASQADSTEQVDDTILT.- | 2 |

---

|  |  |  |  |  |  |  |  |  |
| --- | --- | --- | --- | --- | --- | --- | --- | --- |
| U | *gi|10946129|gb|AAG247* | 2 | 2 | 1.5% | 1679 | 188147 | 8.2 | SMARCA4 isoform 2 [Homo sapiens] |
| U | *gi|505088|dbj|BAA0514* | 2 | 2 | 1.6% | 1647 | 184585 | 7.8 | transcriptional activator hSNF2b [Homo sapiens] |
| U | *gi|21071056|ref|NP\_00* | 2 | 2 | 1.6% | 1647 | 184644 | 7.9 | SWI/SNF-related matrix-associated actin-dependent regulator of chromatin a4 [Homo sapiens] |

| Filename XCorr DeltCN Conf% ObsM+H+ CalcM+H+ SpR ZScore Ion% # Sequence  | | | | | | | | | | | | |
| --- | --- | --- | --- | --- | --- | --- | --- | --- | --- | --- | --- | --- |
|  | Mis12IP\_Nocodazole\_MudPIT\_040709\_04.05823.05823.2 | 2.9256 | 0.3178 | 99.9% | 1146.7322 | 1147.2706 | 1 | 6.705 | 75.0% | 1 | R.DTALETALNAK.A | 2 |
|  | Mis12IP\_Nocodazole\_MudPIT\_040709\_05.06605.06605.2 | 4.0768 | 0.4373 | 100.0% | 1746.4722 | 1746.8657 | 1 | 8.06 | 67.9% | 1 | K.AIEEGTLEEIEEEVR.Q | 2 |

---

|  |  |  |  |  |  |  |  |  |
| --- | --- | --- | --- | --- | --- | --- | --- | --- |
| U | *gi|113412358|ref|XP\_0* | 2 | 2 | 1.5% | 1885 | 188045 | 9.8 | PREDICTED: similar to Hornerin [Homo sapiens] |
| U | *gi|57864582|ref|NP\_00* | 2 | 2 | 1.0% | 2850 | 282389 | 10.0 | hornerin [Homo sapiens] |
| U | *gi|57546919|tpd|FAA00* | 2 | 2 | 1.0% | 2850 | 282365 | 10.0 | TPA: Hornerin [Homo sapiens] |
| U | *gi|40795897|gb|AAR916* | 2 | 2 | 1.0% | 2850 | 282360 | 10.0 | hornerin precursor [Homo sapiens] |

| Filename XCorr DeltCN Conf% ObsM+H+ CalcM+H+ SpR ZScore Ion% # Sequence  | | | | | | | | | | | | |
| --- | --- | --- | --- | --- | --- | --- | --- | --- | --- | --- | --- | --- |
|  | Mis12IP\_Nocodazole\_MudPIT\_040709\_06.05395.05395.2 | 2.42 | 0.3523 | 99.7% | 1748.6721 | 1748.766 | 1 | 7.283 | 38.9% | 1 | R.GSGSGQSPSSGQHGTGFGR.S | 2 |
|  | Mis12IP\_Nocodazole\_MudPIT\_040709\_04.04405.04405.2 | 2.0007 | 0.206 | 96.4% | 1125.6522 | 1126.171 | 2 | 3.993 | 61.1% | 1 | R.SSSRGPYESR.S | 2 |

---

|  |  |  |  |  |  |  |  |  |
| --- | --- | --- | --- | --- | --- | --- | --- | --- |
| U | *gi|21693128|dbj|BAC02* | 2 | 2 | 1.1% | 712 | 77595 | 9.1 | KIAA1991 protein [Homo sapiens] |
| U | *gi|89034999|ref|XP\_94* | 2 | 2 | 1.1% | 742 | 81084 | 9.2 | PREDICTED: hypothetical protein [Homo sapiens] |
| U | *gi|51468840|ref|XP\_49* | 2 | 2 | 1.1% | 742 | 80911 | 9.1 | PREDICTED: hypothetical protein [Homo sapiens] |

| Filename XCorr DeltCN Conf% ObsM+H+ CalcM+H+ SpR ZScore Ion% # Sequence  | | | | | | | | | | | | |
| --- | --- | --- | --- | --- | --- | --- | --- | --- | --- | --- | --- | --- |
|  | Mis12IP\_Nocodazole\_MudPIT\_040709\_04.04430.04430.1 | 2.1919 | 0.2879 | 98.1% | 865.51 | 865.88165 | 1 | 5.41 | 71.4% | 1 | R.CDETAAAK.T | 1 |
|  | Mis12IP\_Nocodazole\_MudPIT\_040709\_04.04360.04360.2 | 2.4136 | 0.1008 | 96.4% | 865.9322 | 865.88165 | 6 | 3.638 | 92.9% | 1 | R.CDETAAAK.T | 2 |

---

|  |  |  |  |  |  |  |  |  |
| --- | --- | --- | --- | --- | --- | --- | --- | --- |
| U | *gi|56417899|gb|AAV908* | 2 | 2 | 1.0% | 4374 | 481912 | 5.2 | ARF-binding protein 1 [Homo sapiens] |
| U | *gi|61676188|ref|NP\_11* | 2 | 2 | 1.0% | 4374 | 481896 | 5.2 | HECT, UBA and WWE domain containing 1 [Homo sapiens] |

| Filename XCorr DeltCN Conf% ObsM+H+ CalcM+H+ SpR ZScore Ion% # Sequence  | | | | | | | | | | | | |
| --- | --- | --- | --- | --- | --- | --- | --- | --- | --- | --- | --- | --- |
|  | Mis12IP\_Nocodazole\_MudPIT\_040709\_06.08090.08090.2 | 1.8264 | 0.2525 | 95.7% | 2135.612 | 2136.2952 | 31 | 5.296 | 30.0% | 1 | R.SSDPLGDTASNLGSAVDELMR.H | 2 |
|  | Mis12IP\_Nocodazole\_MudPIT\_040709\_06.09836.09836.2 | 1.7868 | 0.2686 | 95.8% | 2283.612 | 2283.4636 | 2 | 4.89 | 27.3% | 1 | K.AKQTGRLGS\*SGLGSASSIQAAVR.Q | 2 |

---

|  |  |  |  |  |  |  |  |  |
| --- | --- | --- | --- | --- | --- | --- | --- | --- |
| U | *gi|14571500|gb|AAK076* | 2 | 2 | 1.0% | 2386 | 259619 | 5.7 | NREBP [Homo sapiens] |
| U | *gi|36546|emb|CAA45282* | 2 | 2 | 1.6% | 1523 | 168281 | 9.0 | son-a [Homo sapiens] |
| U | *gi|33876681|gb|AAH024* | 2 | 2 | 1.8% | 1365 | 146836 | 4.8 | SON protein [Homo sapiens] |
| U | *gi|29421186|dbj|BAA82* | 2 | 2 | 1.1% | 2309 | 250917 | 5.6 | KIAA1019 protein [Homo sapiens] |
| U | *gi|21040326|ref|NP\_62* | 2 | 2 | 1.0% | 2426 | 263827 | 5.6 | SON DNA-binding protein isoform F [Homo sapiens] |
| U | *gi|21040314|ref|NP\_11* | 2 | 2 | 1.1% | 2303 | 250386 | 5.6 | SON DNA-binding protein isoform B [Homo sapiens] |
| U | *gi|17046383|gb|AAL345* | 2 | 2 | 1.0% | 2426 | 263843 | 5.6 | SON DNA binding protein isoform F [Homo sapiens] |
| U | *gi|17046381|gb|AAL345* | 2 | 2 | 1.2% | 2108 | 228195 | 5.4 | SON DNA binding protein isoform E [Homo sapiens] |
| U | *gi|17046377|gb|AAL344* | 2 | 2 | 1.1% | 2325 | 252267 | 5.4 | SON DNA binding protein isoform C [Homo sapiens] |
| U | *gi|17046375|gb|AAL344* | 2 | 2 | 1.1% | 2303 | 250402 | 5.6 | SON DNA binding protein isoform B [Homo sapiens] |
| U | *gi|17046373|gb|AAL344* | 2 | 2 | 1.2% | 2140 | 232322 | 6.0 | SON DNA binding protein isoform A [Homo sapiens] |

| Filename XCorr DeltCN Conf% ObsM+H+ CalcM+H+ SpR ZScore Ion% # Sequence  | | | | | | | | | | | | |
| --- | --- | --- | --- | --- | --- | --- | --- | --- | --- | --- | --- | --- |
|  | Mis12IP\_Nocodazole\_MudPIT\_040709\_04.05376.05376.2 | 3.2718 | 0.4199 | 100.0% | 1205.8121 | 1206.3308 | 1 | 9.152 | 85.0% | 1 | R.SMMSSYSAADR.S | 2 |
|  | Mis12IP\_Nocodazole\_MudPIT\_040709\_06.06265.06265.2 | 3.9347 | 0.5215 | 100.0% | 1355.2522 | 1355.5339 | 1 | 8.476 | 69.2% | 1 | R.AGIEGPLLASDVGR.D | 2 |

---

|  |  |  |  |  |  |  |  |  |
| --- | --- | --- | --- | --- | --- | --- | --- | --- |
| U | *gi|103472005|ref|NP\_0* | 2 | 2 | 0.7% | 3256 | 358695 | 9.4 | antigen identified by monoclonal antibody Ki-67 [Homo sapiens] |
| U | *gi|55664564|emb|CAH73* | 2 | 2 | 0.8% | 2896 | 319444 | 9.5 | antigen identified by monoclonal antibody Ki-67 [Homo sapiens] |
| U | *gi|415821|emb|CAA4652* | 2 | 2 | 0.8% | 2896 | 319496 | 9.5 | antigen of the monoclonal antibody Ki-67 [Homo sapiens] |
| U | *gi|415819|emb|CAA4651* | 2 | 2 | 0.7% | 3256 | 358747 | 9.4 | antigen of the monoclonal antibody Ki-67 [Homo sapiens] |

| Filename XCorr DeltCN Conf% ObsM+H+ CalcM+H+ SpR ZScore Ion% # Sequence  | | | | | | | | | | | | |
| --- | --- | --- | --- | --- | --- | --- | --- | --- | --- | --- | --- | --- |
|  | Mis12IP\_Nocodazole\_MudPIT\_040709\_04.05460.05460.2 | 3.4071 | 0.418 | 100.0% | 1159.5122 | 1160.3127 | 1 | 7.208 | 77.3% | 1 | R.SGASEANLIVAK.S | 2 |
|  | Mis12IP\_Nocodazole\_MudPIT\_040709\_05.05315.05315.2 | 2.6159 | 0.3557 | 99.9% | 1190.7122 | 1191.3239 | 1 | 6.507 | 80.0% | 1 | K.LDLTENLTGSK.R | 2 |

---

|  |  |  |  |  |  |  |  |  |
| --- | --- | --- | --- | --- | --- | --- | --- | --- |
| U | *gi|62822436|gb|AAY149* | 2 | 2 | 0.7% | 3224 | 358201 | 6.2 | unknown [Homo sapiens] |
| U | *gi|6382079|ref|NP\_006* | 2 | 2 | 0.7% | 3224 | 358173 | 6.2 | RAN binding protein 2 [Homo sapiens] |

| Filename XCorr DeltCN Conf% ObsM+H+ CalcM+H+ SpR ZScore Ion% # Sequence  | | | | | | | | | | | | |
| --- | --- | --- | --- | --- | --- | --- | --- | --- | --- | --- | --- | --- |
|  | Mis12IP\_Nocodazole\_MudPIT\_040709\_02.03942.03942.1 | 2.0715 | 0.1968 | 99.2% | 866.52 | 866.9897 | 17 | 4.939 | 75.0% | 1 | K.EYDLAKK.Y | 1 |
|  | Mis12IP\_Nocodazole\_MudPIT\_040709\_06.05984.05984.2 | 3.8437 | 0.5642 | 100.0% | 1814.2122 | 1814.9647 | 1 | 10.106 | 62.5% | 1 | K.NVSGISFTENMGSSQQK.N | 2 |

|  |  |  |  |
| --- | --- | --- | --- |
|  | Proteins | Peptide IDs | Spectra |
| Unfiltered | 60865 | 28747 | 119485 |
| Filtered | 191 | 912 | 1558 |
| Forward matches | 191 | 912 | 1558 |
| Decoy matches | 0 | 0 | 0 |
| Forward FP rate | 0.0% | 0.0% | 0.0% |

  
/nfs/cheeseman\_massspec/Jenny/Nocodazole1
