# Supplementary material for: Astrin-SKAP complex reconstitution reveals its kinetochore interaction with microtubule-bound Ndc80
Source: eLife. 2017 Aug 25;6:e26866. doi: 10.7554/eLife.26866 (PMC5602300; doi:10.7554/eLife.26866)
Supplement: Source data 1. — Complete mass spectrometry searches using methods described in (Washburn et al., 2001) for affinity purification/mass spectrometry data sets described in this paper (data from this study; [Kern et al., 2016] [Gascoigne et al., 2011]). Individual Astrin cross-linking immunoprecipitations are listed based on the order in Figure 4—figure supplement 1. These samples have not been pruned for common or antibody-specific contaminants. [file elife-26866-data1.zip › MYCBP-LAP.html]

D pDK268
DTASelect v2.0.21  
/nfs/cheeseman\_massspec/David/pDK268  
/nfs/cheeseman\_massspec/Databases/NCBI-RefSeq\_human\_na\_04-13-2009\_con\_reversed.fasta  
SEQUEST 3.0 in SQT format.  
-p 1  
 Jump  to the summary table.  
  
sequest.params modifications:

|  |  |  |
| --- | --- | --- |
| \* | S | 80.0 |
| # | T | 80.0 |
| @ | Y | 80.0 |
| Static | C | 57.0 |

|  |  |
| --- | --- |
| true | Use criteria |
| 0.0 | Minimum peptide confidence |
| 0.05 | Peptide false positive rate |
| 0.0 | Minimum protein confidence |
| 1.0 | Protein false positive rate |
| 1 | Minimum charge state |
| 16 | Maximum charge state |
| 0.0 | Minimum ion proportion |
| 1000 | Maximum Sp rank |
| -1.0 | Minimum Sp score |
| Include | Modified peptide inclusion |
| Any | Tryptic status requirement |
| false | Multiple, ambiguous IDs allowed |
| Ignore | Peptide validation handling |
| XCorr | Purge duplicate peptides by protein |
| false | Include only loci with unique peptide |
| true | Remove subset proteins |
| Ignore | Locus validation handling |
| 0 | Minimum modified peptides per locus |
| 1000 | Minimum redundancy for low coverage loci |
| 1 | Minimum peptides per locus |

#### Locus Key:

|  |  |  |  |  |  |  |  |  |
| --- | --- | --- | --- | --- | --- | --- | --- | --- |
| Validation Status | Locus | Sequence Count | Spectrum Count | Sequence Coverage | Length | MolWt | pI | Descriptive Name |

#### Similarity Key:

|  |  |  |
| --- | --- | --- |
| Locus | # of identical peptides | # of differing peptides |

---

|  |  |  |  |  |  |  |  |  |
| --- | --- | --- | --- | --- | --- | --- | --- | --- |
| U | *gi|4506671|ref|NP\_000* | 15 | 37 | 97.4% | 115 | 11665 | 4.5 | ribosomal protein P2 [Homo sapiens] |

| Filename XCorr DeltCN Conf% ObsM+H+ CalcM+H+ SpR ZScore Ion% # Sequence  | | | | | | | | | | | | |
| --- | --- | --- | --- | --- | --- | --- | --- | --- | --- | --- | --- | --- |
| \* | pDK268\_112012\_01.12729.12729.2 | 6.1025 | 0.607 | 100.0% | 2156.8123 | 2157.4924 | 1 | 9.883 | 65.0% | 1 | -.MRYVASYLLAALGGNSSPSAK.D | 2 |
| \* | pDK268\_112012\_01.12938.12938.2 | 4.9937 | 0.4868 | 100.0% | 1869.5922 | 1870.1124 | 1 | 9.11 | 63.9% | 3 | R.YVASYLLAALGGNSSPSAK.D | 2 |
| \* | pDK268\_112012\_01.12897.12897.3 | 3.952 | 0.3372 | 100.0% | 1870.4343 | 1870.1124 | 1 | 7.145 | 41.7% | 2 | R.YVASYLLAALGGNSSPSAK.D | 3 |
| \* | pDK268\_112012\_02.05457.05457.3 | 4.5668 | 0.4087 | 100.0% | 1902.0543 | 1902.1118 | 1 | 7.412 | 45.3% | 3 | K.KILDSVGIEADDDRLNK.V | 3 |
| \* | pDK268\_112012\_02.05673.05673.2 | 3.282 | 0.0063 | 97.6% | 1417.2922 | 1418.5004 | 2 | 7.079 | 70.8% | 2 | K.ILDSVGIEADDDR.L | 2 |
| \* | pDK268\_112012\_02.05089.05089.2 | 4.6566 | 0.4387 | 100.0% | 1773.2322 | 1773.9377 | 1 | 7.872 | 66.7% | 4 | K.ILDSVGIEADDDRLNK.V | 2 |
|  | pDK268\_112012\_01.04239.04239.1 | 1.9186 | 0.2651 | 100.0% | 859.51 | 859.99817 | 2 | 5.532 | 71.4% | 1 | K.VISELNGK.N | 1 |
| \* | pDK268\_112012\_01.10942.10942.2 | 4.6551 | 0.3808 | 100.0% | 1258.3722 | 1257.4294 | 1 | 7.3 | 81.8% | 4 | K.NIEDVIAQGIGK.L | 2 |
| \* | pDK268\_112012\_02.05053.05053.3 | 5.2659 | 0.5226 | 100.0% | 2775.6843 | 2776.0757 | 1 | 9.259 | 27.3% | 2 | K.LASVPAGGAVAVSAAPGSAAPAAGSAPAAAEEK.K | 3 |
| \* | pDK268\_112012\_01.07408.07408.2 | 4.3994 | 0.4112 | 100.0% | 2777.892 | 2776.0757 | 1 | 7.836 | 29.7% | 2 | K.LASVPAGGAVAVSAAPGSAAPAAGSAPAAAEEK.K | 2 |
| \* | pDK268\_112012\_01.06706.06706.3 | 2.882 | 0.2344 | 96.7% | 2903.6343 | 2904.2498 | 1 | 4.937 | 22.7% | 1 | K.LASVPAGGAVAVSAAPGSAAPAAGSAPAAAEEKK.D | 3 |
| \* | pDK268\_112012\_01.06224.06224.3 | 3.7285 | 0.4315 | 100.0% | 3276.5645 | 3276.628 | 1 | 6.371 | 21.5% | 1 | K.LASVPAGGAVAVSAAPGSAAPAAGSAPAAAEEKKDEK.K | 3 |
| \* | pDK268\_112012\_02.07791.07791.3 | 3.2282 | 0.2793 | 99.7% | 2452.2544 | 2451.5312 | 1 | 4.626 | 33.8% | 1 | K.KDEKKEESEESDDDMGFGLFD.- | 3 |
|  | pDK268\_112012\_01.12392.12392.2 | 5.2689 | 0.5852 | 100.0% | 1950.3121 | 1950.979 | 1 | 12.044 | 75.0% | 9 | K.KEESEESDDDMGFGLFD.- | 22 |
|  | pDK268\_112012\_02.09670.09670.2 | 3.4431 | 0.4262 | 100.0% | 2031.2722 | 2030.979 | 1 | 7.103 | 62.5% | 1 | K.KEES\*EESDDDMGFGLFD.- | 22 |

Similarities:
gi|4506669|ref|NP\_000(2:13)  

---

|  |  |  |  |  |  |  |  |  |
| --- | --- | --- | --- | --- | --- | --- | --- | --- |
| U | *gi|30795231|ref|NP\_00* | 21 | 44 | 89.0% | 227 | 22693 | 4.6 | brain abundant, membrane attached signal protein 1 [Homo sapiens] |

| Filename XCorr DeltCN Conf% ObsM+H+ CalcM+H+ SpR ZScore Ion% # Sequence  | | | | | | | | | | | | |
| --- | --- | --- | --- | --- | --- | --- | --- | --- | --- | --- | --- | --- |
| \* | pDK268\_112012\_01.03201.03201.2 | 4.172 | 0.4968 | 100.0% | 1661.2922 | 1661.7631 | 1 | 8.498 | 76.7% | 1 | K.DKKAEGAATEEEGTPK.E | 2 |
| \* | pDK268\_112012\_01.03203.03203.3 | 4.0215 | 0.3618 | 100.0% | 1661.6044 | 1661.7631 | 1 | 6.447 | 40.0% | 1 | K.DKKAEGAATEEEGTPK.E | 3 |
| \* | pDK268\_112012\_01.03189.03189.2 | 4.3902 | 0.4539 | 100.0% | 1418.2322 | 1418.5004 | 1 | 8.229 | 73.1% | 1 | K.KAEGAATEEEGTPK.E | 2 |
| \* | pDK268\_112012\_01.03350.03350.2 | 3.9714 | 0.4488 | 100.0% | 1290.0521 | 1290.3263 | 1 | 7.969 | 79.2% | 1 | K.AEGAATEEEGTPK.E | 2 |
| \* | pDK268\_112012\_02.03781.03781.3 | 5.8784 | 0.4856 | 100.0% | 2698.9443 | 2699.7986 | 1 | 8.211 | 32.7% | 3 | K.AEGAATEEEGTPKESEPQAAAEPAEAK.E | 3 |
| \* | pDK268\_112012\_01.03795.03795.2 | 3.4844 | 0.2383 | 99.9% | 1428.1921 | 1428.4955 | 1 | 6.175 | 73.1% | 4 | K.ESEPQAAAEPAEAK.E | 2 |
| \* | pDK268\_112012\_01.03123.03123.2 | 2.7162 | 0.341 | 99.9% | 1574.3121 | 1574.6415 | 1 | 6.111 | 76.9% | 1 | K.EKPDQDAEGKAEEK.E | 2 |
| \* | pDK268\_112012\_01.03552.03552.3 | 5.2679 | 0.4007 | 100.0% | 2001.7444 | 2002.1425 | 1 | 7.148 | 47.2% | 1 | K.AEEKEGEKDAAAAKEEAPK.A | 3 |
| \* | pDK268\_112012\_01.03558.03558.3 | 4.8593 | 0.4496 | 100.0% | 2413.6143 | 2413.6006 | 1 | 7.353 | 40.2% | 1 | K.DAAAAKEEAPKAEPEKTEGAAEAK.A | 3 |
| \* | pDK268\_112012\_01.04182.04182.3 | 4.4098 | 0.352 | 100.0% | 2298.8044 | 2299.5022 | 1 | 6.129 | 43.5% | 6 | K.AEPPKAPEQEQAAPGPAAGGEAPK.A | 3 |
| \* | pDK268\_112012\_01.04269.04269.2 | 3.7751 | 0.4017 | 100.0% | 2299.6921 | 2299.5022 | 1 | 6.609 | 58.7% | 2 | K.AEPPKAPEQEQAAPGPAAGGEAPK.A | 2 |
| \* | pDK268\_112012\_01.04511.04511.2 | 4.335 | 0.4308 | 100.0% | 2637.7122 | 2637.7734 | 1 | 7.577 | 40.7% | 1 | K.AAEAAAAPAESAAPAAGEEPSKEEGEPK.K | 2 |
| \* | pDK268\_112012\_01.03404.03404.2 | 4.8521 | 0.4773 | 100.0% | 1413.2722 | 1413.5706 | 1 | 8.513 | 84.6% | 3 | K.KTEAPAAPAAQETK.S | 2 |
| \* | pDK268\_112012\_01.03398.03398.3 | 2.6686 | 0.2867 | 99.3% | 1413.8944 | 1413.5706 | 18 | 4.819 | 38.5% | 1 | K.KTEAPAAPAAQETK.S | 3 |
| \* | pDK268\_112012\_01.03280.03280.2 | 4.6515 | 0.5559 | 100.0% | 1933.3121 | 1933.9806 | 1 | 8.412 | 60.0% | 1 | K.SDGAPASDSKPGSSEAAPSSK.E | 2 |
| \* | pDK268\_112012\_01.03284.03284.3 | 2.7147 | 0.4601 | 100.0% | 1934.0343 | 1933.9806 | 3 | 6.041 | 33.8% | 1 | K.SDGAPASDSKPGSSEAAPSSK.E | 3 |
| \* | pDK268\_112012\_01.03765.03765.2 | 3.4751 | 0.3945 | 100.0% | 1388.3722 | 1387.4863 | 10 | 6.741 | 53.8% | 3 | K.ETPAATEAPSSTPK.A | 2 |
| \* | pDK268\_112012\_02.03137.03137.2 | 2.6653 | 0.4176 | 100.0% | 1467.1522 | 1467.4863 | 1 | 6.58 | 57.7% | 1 | K.ETPAATEAPSST#PK.A | 2 |
| \* | pDK268\_112012\_01.03735.03735.2 | 2.4793 | 0.1946 | 98.2% | 1467.3722 | 1467.4863 | 1 | 4.189 | 65.4% | 1 | K.ETPAATEAPSS\*TPK.A | 2 |
| \* | pDK268\_112012\_01.05471.05471.3 | 5.7959 | 0.3938 | 100.0% | 2764.9744 | 2765.006 | 1 | 6.352 | 34.3% | 3 | K.AQGPAASAEEPKPVEAPAANSDQTVTVK.E | 3 |
| \* | pDK268\_112012\_02.04701.04701.3 | 6.3015 | 0.5427 | 100.0% | 2893.9443 | 2894.1216 | 1 | 9.113 | 33.0% | 7 | K.AQGPAASAEEPKPVEAPAANSDQTVTVKE.- | 3 |

---

|  |  |  |  |  |  |  |  |  |
| --- | --- | --- | --- | --- | --- | --- | --- | --- |
| U | *gi|4504517|ref|NP\_001* | 15 | 28 | 83.4% | 205 | 22783 | 6.4 | heat shock protein beta-1 [Homo sapiens] |

| Filename XCorr DeltCN Conf% ObsM+H+ CalcM+H+ SpR ZScore Ion% # Sequence  | | | | | | | | | | | | |
| --- | --- | --- | --- | --- | --- | --- | --- | --- | --- | --- | --- | --- |
| \* | pDK268\_112012\_01.10360.10360.2 | 2.0463 | 0.4217 | 99.9% | 962.1322 | 962.05255 | 2 | 7.488 | 78.6% | 2 | R.GPSWDPFR.D | 2 |
| \* | pDK268\_112012\_01.11358.11358.2 | 2.7827 | 0.2203 | 99.5% | 1903.2722 | 1904.0537 | 2 | 4.8 | 50.0% | 1 | R.GPSWDPFRDWYPHSR.L | 2 |
| \* | pDK268\_112012\_01.11372.11372.3 | 3.8037 | 0.3421 | 100.0% | 1904.0944 | 1904.0537 | 1 | 6.33 | 42.9% | 1 | R.GPSWDPFRDWYPHSR.L | 3 |
| \* | pDK268\_112012\_01.10952.10952.2 | 4.1449 | 0.4529 | 100.0% | 1164.1921 | 1164.3494 | 2 | 7.761 | 83.3% | 4 | R.LFDQAFGLPR.L | 2 |
| \* | pDK268\_112012\_01.14256.14256.3 | 4.8353 | 0.5157 | 100.0% | 4096.1943 | 4095.606 | 1 | 9.443 | 27.7% | 1 | R.LPEEWSQWLGGSSWPGYVRPLPPAAIESPAVAAPAYSR.A | 3 |
| \* | pDK268\_112012\_01.05078.05078.2 | 2.5596 | 0.127 | 98.4% | 1075.7322 | 1076.1948 | 1 | 4.959 | 77.8% | 2 | R.QLSSGVSEIR.H | 2 |
| \* | pDK268\_112012\_01.09939.09939.2 | 5.1858 | 0.5133 | 100.0% | 1784.2522 | 1785.0068 | 1 | 8.501 | 66.7% | 2 | R.VSLDVNHFAPDELTVK.T | 2 |
| \* | pDK268\_112012\_01.09932.09932.3 | 2.9651 | 0.4244 | 100.0% | 1785.5343 | 1785.0068 | 32 | 6.326 | 33.3% | 2 | R.VSLDVNHFAPDELTVK.T | 3 |
| \* | pDK268\_112012\_01.04224.04224.2 | 3.2694 | 0.4327 | 100.0% | 1146.4321 | 1147.314 | 1 | 7.302 | 85.0% | 1 | K.TKDGVVEITGK.H | 2 |
| \* | pDK268\_112012\_01.04070.04070.2 | 3.3539 | 0.3684 | 100.0% | 1469.5122 | 1469.5944 | 1 | 5.931 | 66.7% | 1 | K.DGVVEITGKHEER.Q | 2 |
| \* | pDK268\_112012\_01.03322.03322.3 | 3.4223 | 0.2773 | 100.0% | 1656.8644 | 1656.7117 | 1 | 5.455 | 47.9% | 1 | K.HEERQDEHGYISR.C | 3 |
| \* | pDK268\_112012\_01.11096.11096.3 | 6.3955 | 0.3237 | 100.0% | 3229.5244 | 3228.6821 | 1 | 6.276 | 32.5% | 5 | R.KYTLPPGVDPTQVSSSLSPEGTLTVEAPMPK.L | 32 |
| \* | pDK268\_112012\_01.10550.10550.2 | 3.8684 | 0.4798 | 100.0% | 1908.4922 | 1907.1307 | 1 | 8.051 | 59.4% | 3 | K.LATQSNEITIPVTFESR.A | 2 |
| \* | pDK268\_112012\_01.03544.03544.1 | 1.7802 | 0.3226 | 100.0% | 941.39 | 942.0599 | 1 | 5.73 | 55.6% | 1 | R.AQLGGPEAAK.S | 1 |
| \* | pDK268\_112012\_01.03566.03566.2 | 2.1718 | 0.1767 | 97.0% | 941.9922 | 942.0599 | 7 | 3.991 | 72.2% | 1 | R.AQLGGPEAAK.S | 2 |

---

|  |  |  |  |  |  |  |  |  |
| --- | --- | --- | --- | --- | --- | --- | --- | --- |
| U | *gi|57242777|ref|NP\_03* | 21 | 78 | 80.6% | 103 | 11967 | 5.9 | c-myc binding protein [Homo sapiens] |

| Filename XCorr DeltCN Conf% ObsM+H+ CalcM+H+ SpR ZScore Ion% # Sequence  | | | | | | | | | | | | |
| --- | --- | --- | --- | --- | --- | --- | --- | --- | --- | --- | --- | --- |
| \* | pDK268\_112012\_01.17012.17012.3 | 7.2624 | 0.5112 | 100.0% | 3725.1843 | 3725.3142 | 1 | 7.854 | 35.9% | 1 | R.YLEKSGVLDTLTKVLVALYEEPEKPNSALDFLK.H | 3 |
| \* | pDK268\_112012\_01.07760.07760.2 | 3.0717 | 0.3479 | 100.0% | 934.1122 | 934.07764 | 2 | 6.3 | 87.5% | 6 | K.SGVLDTLTK.V | 2 |
| \* | pDK268\_112012\_01.18152.18152.3 | 7.7445 | 0.4806 | 100.0% | 3191.7244 | 3191.6892 | 1 | 9.6 | 37.5% | 2 | K.SGVLDTLTKVLVALYEEPEKPNSALDFLK.H | 3 |
| \* | pDK268\_112012\_01.12038.12038.2 | 4.8595 | 0.4856 | 100.0% | 2275.8123 | 2276.6348 | 1 | 8.783 | 60.5% | 9 | K.VLVALYEEPEKPNSALDFLK.H | 2 |
| \* | pDK268\_112012\_02.08931.08931.3 | 5.0784 | 0.516 | 100.0% | 2277.5044 | 2276.6348 | 1 | 8.474 | 42.1% | 15 | K.VLVALYEEPEKPNSALDFLK.H | 3 |
| \* | pDK268\_112012\_01.07562.07562.2 | 4.343 | 0.3548 | 100.0% | 1897.5322 | 1898.1289 | 1 | 7.304 | 75.0% | 6 | K.HHLGAATPENPEIELLR.L | 2 |
| \* | pDK268\_112012\_01.07568.07568.3 | 4.9 | 0.3212 | 100.0% | 1898.4844 | 1898.1289 | 1 | 6.333 | 51.6% | 7 | K.HHLGAATPENPEIELLR.L | 3 |
| \* | pDK268\_112012\_01.11864.11864.3 | 4.5103 | 0.39 | 100.0% | 2713.2544 | 2713.124 | 1 | 6.917 | 40.2% | 2 | K.HHLGAATPENPEIELLRLELAEMK.E | 3 |
| \* | pDK268\_112012\_01.10936.10936.3 | 4.8352 | 0.4121 | 100.0% | 2970.2344 | 2970.4138 | 1 | 7.013 | 30.0% | 2 | K.HHLGAATPENPEIELLRLELAEMKEK.Y | 3 |
| \* | pDK268\_112012\_01.05168.05168.2 | 2.4176 | 0.1145 | 97.6% | 1090.7322 | 1091.3081 | 2 | 4.8 | 81.2% | 2 | R.LELAEMKEK.Y | 2 |
| \* | pDK268\_112012\_01.09328.09328.3 | 6.0586 | 0.5062 | 100.0% | 2295.2644 | 2295.6533 | 1 | 8.685 | 45.8% | 3 | R.LELAEMKEKYEAIVEENKK.L | 3 |
| \* | pDK268\_112012\_01.04794.04794.2 | 3.0288 | 0.341 | 100.0% | 1351.9922 | 1352.4839 | 1 | 6.831 | 75.0% | 1 | K.EKYEAIVEENK.K | 2 |
| \* | pDK268\_112012\_01.04154.04154.2 | 4.2298 | 0.3973 | 100.0% | 1480.1522 | 1480.658 | 2 | 7.544 | 68.2% | 3 | K.EKYEAIVEENKK.L | 2 |
| \* | pDK268\_112012\_01.04698.04698.1 | 2.144 | 0.3303 | 100.0% | 1094.45 | 1095.1943 | 2 | 6.231 | 68.8% | 1 | K.YEAIVEENK.K | 1 |
| \* | pDK268\_112012\_01.04702.04702.2 | 2.7175 | 0.3435 | 100.0% | 1096.0922 | 1095.1943 | 1 | 5.913 | 75.0% | 1 | K.YEAIVEENK.K | 2 |
| \* | pDK268\_112012\_01.03933.03933.1 | 2.7829 | 0.3567 | 100.0% | 1222.53 | 1223.3684 | 1 | 6.136 | 72.2% | 1 | K.YEAIVEENKK.L | 1 |
| \* | pDK268\_112012\_02.03268.03268.2 | 3.1338 | 0.385 | 100.0% | 1223.1122 | 1223.3684 | 1 | 6.3 | 83.3% | 3 | K.YEAIVEENKK.L | 2 |
| \* | pDK268\_112012\_01.03918.03918.3 | 2.3952 | 0.2545 | 98.3% | 1223.9944 | 1223.3684 | 3 | 5.033 | 50.0% | 2 | K.YEAIVEENKK.L | 3 |
| \* | pDK268\_112012\_01.04485.04485.2 | 4.5864 | 0.4883 | 100.0% | 1531.3522 | 1531.7056 | 1 | 8.998 | 70.8% | 1 | K.AKLAQYEPPQEEK.R | 2 |
| \* | pDK268\_112012\_01.04682.04682.2 | 3.2903 | 0.2591 | 100.0% | 1332.0922 | 1332.4528 | 1 | 5.718 | 75.0% | 8 | K.LAQYEPPQEEK.R | 2 |
| \* | pDK268\_112012\_01.04115.04115.2 | 3.3245 | 0.285 | 100.0% | 1488.1921 | 1488.6403 | 1 | 5.435 | 72.7% | 2 | K.LAQYEPPQEEKR.A | 2 |

---

|  |  |  |  |  |  |  |  |  |
| --- | --- | --- | --- | --- | --- | --- | --- | --- |
| U | *gi|4501885|ref|NP\_001* | 42 | 245 | 74.1% | 375 | 41737 | 5.5 | beta actin [Homo sapiens] |
| U | *gi|4501887|ref|NP\_001* | 42 | 245 | 74.1% | 375 | 41793 | 5.5 | actin, gamma 1 propeptide [Homo sapiens] |

| Filename XCorr DeltCN Conf% ObsM+H+ CalcM+H+ SpR ZScore Ion% # Sequence  | | | | | | | | | | | | |
| --- | --- | --- | --- | --- | --- | --- | --- | --- | --- | --- | --- | --- |
|  | pDK268\_112012\_01.04298.04298.1 | 1.8879 | 0.329 | 100.0% | 976.36 | 977.02136 | 191 | 5.657 | 44.4% | 2 | K.AGFAGDDAPR.A | 111 |
|  | pDK268\_112012\_02.03387.03387.2 | 3.4779 | 0.4692 | 100.0% | 976.89215 | 977.02136 | 1 | 7.775 | 88.9% | 11 | K.AGFAGDDAPR.A | 222 |
|  | pDK268\_112012\_01.09078.09078.3 | 3.194 | 0.2448 | 98.9% | 2157.2644 | 2157.4397 | 1 | 4.607 | 32.5% | 1 | K.AGFAGDDAPRAVFPSIVGRPR.H | 333 |
|  | pDK268\_112012\_01.07424.07424.2 | 2.9932 | 0.3571 | 100.0% | 1199.3522 | 1199.4415 | 1 | 6.426 | 75.0% | 9 | R.AVFPSIVGRPR.H | 222 |
|  | pDK268\_112012\_01.04100.04100.1 | 2.9097 | 0.2235 | 100.0% | 1173.65 | 1172.4058 | 1 | 4.493 | 65.0% | 5 | R.HQGVMVGMGQK.D | 11 |
|  | pDK268\_112012\_01.04095.04095.2 | 3.0844 | 0.138 | 99.7% | 1174.0721 | 1172.4058 | 1 | 4.962 | 75.0% | 8 | R.HQGVMVGMGQK.D | 22 |
|  | pDK268\_112012\_02.04623.04623.3 | 4.7125 | 0.5223 | 100.0% | 2352.2344 | 2352.5989 | 1 | 9.03 | 38.1% | 1 | R.HQGVMVGMGQKDSYVGDEAQSK.R | 33 |
|  | pDK268\_112012\_01.04865.04865.3 | 5.2731 | 0.4775 | 100.0% | 2508.3843 | 2508.7864 | 1 | 7.861 | 38.6% | 2 | R.HQGVMVGMGQKDSYVGDEAQSKR.G | 33 |
|  | pDK268\_112012\_01.03884.03884.2 | 3.4646 | 0.0536 | 99.6% | 1199.0122 | 1199.2163 | 1 | 7.147 | 85.0% | 7 | K.DSYVGDEAQSK.R | 22 |
|  | pDK268\_112012\_01.03692.03692.2 | 3.3148 | 0.1013 | 99.6% | 1355.1522 | 1355.4038 | 1 | 8.165 | 77.3% | 3 | K.DSYVGDEAQSKR.G | 22 |
|  | pDK268\_112012\_01.09533.09533.3 | 4.6255 | 0.2718 | 100.0% | 1947.5643 | 1948.1572 | 1 | 5.668 | 51.7% | 2 | K.YPIEHGIVTNWDDMEK.I | 3 |
|  | pDK268\_112012\_02.04842.04842.2 | 3.3544 | 0.4252 | 100.0% | 1516.7722 | 1516.7019 | 1 | 6.332 | 75.0% | 5 | K.IWHHTFYNELR.V | 222 |
|  | pDK268\_112012\_02.04830.04830.3 | 3.3578 | 0.2395 | 100.0% | 1517.0643 | 1516.7019 | 3 | 5.839 | 47.5% | 7 | K.IWHHTFYNELR.V | 333 |
|  | pDK268\_112012\_01.08396.08396.3 | 4.2833 | 0.0749 | 95.6% | 1955.3043 | 1955.2615 | 1 | 6.617 | 42.6% | 1 | R.VAPEEHPVLLTEAPLNPK.A | 3 |
|  | pDK268\_112012\_01.08284.08284.2 | 4.7064 | 0.2725 | 100.0% | 1955.6522 | 1955.2615 | 1 | 7.943 | 61.8% | 7 | R.VAPEEHPVLLTEAPLNPK.A | 2 |
|  | pDK268\_112012\_02.14613.14613.3 | 3.5378 | 0.2878 | 100.0% | 3511.7644 | 3513.122 | 11 | 4.799 | 19.2% | 3 | R.EKMTQIMFETFNTPAMYVAIQAVLSLYASGR.T | 3 |
|  | pDK268\_112012\_02.14697.14697.3 | 5.1785 | 0.4002 | 100.0% | 3256.1343 | 3255.8325 | 1 | 8.148 | 33.9% | 5 | K.MTQIMFETFNTPAMYVAIQAVLSLYASGR.T | 3 |
|  | pDK268\_112012\_01.11414.11414.3 | 7.2423 | 0.5293 | 100.0% | 3185.4844 | 3185.622 | 1 | 9.481 | 37.1% | 9 | R.TTGIVMDSGDGVTHTVPIYEGYALPHAILR.L | 32 |
|  | pDK268\_112012\_01.11594.11594.2 | 3.027 | 0.2986 | 99.9% | 1624.6921 | 1624.8927 | 1 | 5.628 | 61.5% | 2 | R.LDLAGRDLTDYLMK.I | 22 |
|  | pDK268\_112012\_01.11582.11582.3 | 3.101 | 0.3275 | 100.0% | 1625.0343 | 1624.8927 | 1 | 6.241 | 46.2% | 1 | R.LDLAGRDLTDYLMK.I | 33 |
|  | pDK268\_112012\_01.15548.15548.3 | 5.2814 | 0.3719 | 100.0% | 2238.5344 | 2237.6196 | 1 | 6.898 | 45.8% | 1 | R.LDLAGRDLTDYLMKILTER.G | 33 |
|  | pDK268\_112012\_02.06534.06534.1 | 2.1427 | 0.2707 | 100.0% | 998.28 | 999.167 | 2 | 5.469 | 71.4% | 3 | R.DLTDYLMK.I | 11 |
|  | pDK268\_112012\_01.10826.10826.2 | 2.7898 | 0.3169 | 100.0% | 999.21216 | 999.167 | 7 | 6.507 | 71.4% | 3 | R.DLTDYLMK.I | 22 |
|  | pDK268\_112012\_01.05852.05852.1 | 2.2021 | 0.4281 | 100.0% | 1132.42 | 1133.2029 | 18 | 5.437 | 50.0% | 2 | R.GYSFTTTAER.E | 1 |
|  | pDK268\_112012\_02.04913.04913.2 | 3.2932 | 0.4114 | 100.0% | 1134.1522 | 1133.2029 | 1 | 8.439 | 94.4% | 11 | R.GYSFTTTAER.E | 2 |
|  | pDK268\_112012\_01.10832.10832.2 | 4.9745 | 0.3219 | 100.0% | 1791.4122 | 1791.9554 | 1 | 9.534 | 86.7% | 14 | K.SYELPDGQVITIGNER.F | 222 |
|  | pDK268\_112012\_02.06969.06969.3 | 4.115 | 0.2183 | 100.0% | 1791.9844 | 1791.9554 | 1 | 7.677 | 50.0% | 3 | K.SYELPDGQVITIGNER.F | 333 |
|  | pDK268\_112012\_01.09904.09904.2 | 6.2228 | 0.6144 | 100.0% | 2344.5322 | 2344.6448 | 1 | 10.848 | 69.0% | 4 | R.KDLYANTVLSGGTTMYPGIADR.M | 2 |
|  | pDK268\_112012\_02.07305.07305.3 | 6.5086 | 0.5103 | 100.0% | 2345.7844 | 2344.6448 | 1 | 8.025 | 39.3% | 6 | R.KDLYANTVLSGGTTMYPGIADR.M | 3 |
|  | pDK268\_112012\_02.08314.08314.3 | 5.321 | 0.55 | 100.0% | 2215.5544 | 2216.4705 | 1 | 9.669 | 50.0% | 4 | K.DLYANTVLSGGTTMYPGIADR.M | 3 |
|  | pDK268\_112012\_01.11744.11744.2 | 5.9005 | 0.6578 | 100.0% | 2216.5122 | 2216.4705 | 1 | 11.158 | 60.0% | 77 | K.DLYANTVLSGGTTMYPGIADR.M | 2 |
|  | pDK268\_112012\_01.06398.06398.3 | 3.4178 | 0.2589 | 100.0% | 1549.4343 | 1549.8843 | 7 | 5.787 | 44.2% | 1 | R.MQKEITALAPSTMK.I | 33 |
|  | pDK268\_112012\_01.06963.06963.1 | 2.535 | 0.5055 | 100.0% | 1161.58 | 1162.3868 | 1 | 7.631 | 60.0% | 4 | K.EITALAPSTMK.I | 11 |
|  | pDK268\_112012\_01.06818.06818.2 | 2.9214 | 0.437 | 100.0% | 1162.1522 | 1162.3868 | 1 | 6.953 | 90.0% | 8 | K.EITALAPSTMK.I | 22 |
|  | pDK268\_112012\_01.05204.05204.2 | 2.4741 | 0.0524 | 96.4% | 1036.6522 | 1037.2908 | 11 | 4.453 | 62.5% | 1 | K.IKIIAPPER.K | 22 |
|  | pDK268\_112012\_01.04420.04420.2 | 2.5544 | 0.1031 | 97.7% | 1165.4122 | 1165.4648 | 51 | 4.464 | 61.1% | 1 | K.IKIIAPPERK.Y | 22 |
|  | pDK268\_112012\_02.12904.12904.3 | 5.9983 | 0.4479 | 100.0% | 3509.6943 | 3509.1467 | 1 | 7.822 | 33.3% | 1 | K.IIAPPERKYSVWIGGSILASLSTFQQMWISK.Q | 33 |
|  | pDK268\_112012\_02.14579.14579.2 | 3.9156 | 0.5639 | 100.0% | 2602.672 | 2604.0388 | 1 | 8.448 | 47.7% | 3 | K.YSVWIGGSILASLSTFQQMWISK.Q | 22 |
|  | pDK268\_112012\_02.13027.13027.3 | 2.4813 | 0.2894 | 97.4% | 4101.3545 | 4102.6104 | 182 | 7.313 | 20.7% | 1 | K.YSVWIGGSILASLSTFQQMWISKQEYDESGPSIVHR.K | 3 |
|  | pDK268\_112012\_02.03757.03757.2 | 2.5542 | 0.4757 | 100.0% | 1516.9922 | 1517.595 | 1 | 7.035 | 62.5% | 3 | K.QEYDESGPSIVHR.K | 22 |
|  | pDK268\_112012\_01.04904.04904.3 | 2.5139 | 0.3703 | 100.0% | 1517.6643 | 1517.595 | 1 | 6.026 | 50.0% | 2 | K.QEYDESGPSIVHR.K | 33 |
|  | pDK268\_112012\_01.04172.04172.3 | 2.2427 | 0.3292 | 98.9% | 1645.8243 | 1645.769 | 3 | 4.852 | 38.5% | 1 | K.QEYDESGPSIVHRK.C | 33 |

Similarities:
gi|4885049|ref|NP\_005(26:16)  
gi|134133226|ref|NP\_0(11:31)  

---

|  |  |  |  |  |  |  |  |  |
| --- | --- | --- | --- | --- | --- | --- | --- | --- |
| U | *gi|10864047|ref|NP\_06* | 60 | 99 | 73.1% | 864 | 94255 | 5.1 | epidermal growth factor receptor pathway substrate 15-like 1 [Homo sapiens] |

| Filename XCorr DeltCN Conf% ObsM+H+ CalcM+H+ SpR ZScore Ion% # Sequence  | | | | | | | | | | | | |
| --- | --- | --- | --- | --- | --- | --- | --- | --- | --- | --- | --- | --- |
| \* | pDK268\_112012\_01.03922.03922.2 | 1.786 | 0.3909 | 99.3% | 1006.5522 | 1007.091 | 7 | 6.478 | 62.5% | 1 | K.QVDPAYTGR.V | 2 |
| \* | pDK268\_112012\_01.09507.09507.2 | 3.2382 | 0.4502 | 100.0% | 1106.1322 | 1106.3073 | 1 | 8.158 | 85.0% | 3 | R.VGASEAALFLK.K | 2 |
| \* | pDK268\_112012\_02.05958.05958.2 | 3.3143 | 0.4483 | 100.0% | 1234.3922 | 1234.4813 | 1 | 6.788 | 72.7% | 3 | R.VGASEAALFLKK.S | 2 |
| \* | pDK268\_112012\_01.07524.07524.3 | 2.7595 | 0.3705 | 100.0% | 1234.4944 | 1234.4813 | 26 | 5.791 | 36.4% | 1 | R.VGASEAALFLKK.S | 3 |
| \* | pDK268\_112012\_01.08229.08229.2 | 2.3026 | 0.118 | 95.8% | 1131.3722 | 1131.358 | 9 | 4.084 | 75.0% | 1 | K.KSGLSDIILGK.I | 2 |
| \* | pDK268\_112012\_01.10784.10784.2 | 3.3717 | 0.3835 | 100.0% | 1003.15216 | 1003.1839 | 1 | 6.559 | 94.4% | 1 | K.SGLSDIILGK.I | 2 |
| \* | pDK268\_112012\_01.09729.09729.2 | 3.357 | 0.3062 | 100.0% | 1143.9122 | 1144.2694 | 4 | 7.665 | 72.2% | 2 | K.IWDLADPEGK.G | 2 |
| \* | pDK268\_112012\_01.09382.09382.3 | 3.6713 | 0.2196 | 99.1% | 2517.4744 | 2517.735 | 1 | 5.595 | 33.3% | 1 | K.FHDTSS\*PLMVTPPSAEAHWAVR.V | 3 |
| \* | pDK268\_112012\_01.15353.15353.2 | 5.6075 | 0.5993 | 100.0% | 2234.7122 | 2235.5852 | 1 | 10.049 | 70.0% | 2 | K.AKFDGIFESLLPINGLLSGDK.V | 2 |
| \* | pDK268\_112012\_01.15363.15363.3 | 3.7555 | 0.2522 | 99.9% | 2236.9143 | 2235.5852 | 1 | 4.626 | 38.8% | 1 | K.AKFDGIFESLLPINGLLSGDK.V | 3 |
| \* | pDK268\_112012\_01.16490.16490.2 | 4.5028 | 0.5731 | 100.0% | 2035.4321 | 2036.3324 | 1 | 9.71 | 50.0% | 2 | K.FDGIFESLLPINGLLSGDK.V | 2 |
| \* | pDK268\_112012\_01.09629.09629.1 | 1.758 | 0.2823 | 100.0% | 882.62 | 883.0788 | 1 | 5.418 | 71.4% | 1 | K.LPLDVLGR.V | 1 |
| \* | pDK268\_112012\_01.09598.09598.2 | 2.4162 | 0.187 | 99.5% | 882.7922 | 883.0788 | 1 | 5.006 | 85.7% | 2 | K.LPLDVLGR.V | 2 |
| \* | pDK268\_112012\_02.09577.09577.3 | 5.3886 | 0.496 | 100.0% | 3216.5645 | 3217.583 | 1 | 7.858 | 30.8% | 1 | R.VWDLSDIDKDGHLDRDEFAVAMHLVYR.A | 3 |
| \* | pDK268\_112012\_01.08204.08204.3 | 3.6096 | 0.3782 | 100.0% | 2227.5842 | 2227.6523 | 11 | 6.343 | 30.0% | 1 | R.ALEKEPVPSALPPSLIPPSKR.K | 32 |
| \* | pDK268\_112012\_01.10337.10337.2 | 4.2372 | 0.4785 | 100.0% | 2226.172 | 2226.5376 | 1 | 7.384 | 47.5% | 3 | K.TVFPGAVPVLPAS\*PPPKDSLR.S | 2 |
| \* | pDK268\_112012\_01.05130.05130.2 | 4.1623 | 0.4249 | 100.0% | 2010.3922 | 2011.0667 | 1 | 8.099 | 63.2% | 1 | R.STPSHGSVSSLNSTGSLS\*PK.H | 2 |
| \* | pDK268\_112012\_02.03873.03873.3 | 3.2109 | 0.3803 | 100.0% | 2010.4744 | 2011.0667 | 3 | 5.724 | 30.3% | 2 | R.STPSHGSVSSLNSTGSLS\*PK.H | 3 |
| \* | pDK268\_112012\_01.09693.09693.2 | 3.8493 | 0.3895 | 100.0% | 1683.3322 | 1682.917 | 1 | 7.699 | 60.7% | 3 | K.QTQPTVNWVVPVADK.M | 2 |
| \* | pDK268\_112012\_01.10866.10866.2 | 2.3967 | 0.2276 | 99.6% | 1198.6721 | 1199.4534 | 3 | 5.811 | 68.8% | 1 | K.MRFDEIFLK.T | 2 |
| \* | pDK268\_112012\_02.06726.06726.2 | 5.179 | 0.5226 | 100.0% | 1640.3922 | 1639.7563 | 1 | 9.573 | 82.1% | 2 | K.TDLDLDGYVSGQEVK.E | 2 |
| \* | pDK268\_112012\_01.15455.15455.3 | 4.4159 | 0.3772 | 100.0% | 2639.4243 | 2639.047 | 1 | 6.865 | 30.7% | 3 | K.EIFMHSGLTQNLLAHIWALADTR.Q | 3 |
| \* | pDK268\_112012\_02.10349.10349.2 | 4.3885 | 0.403 | 100.0% | 1603.3322 | 1603.8762 | 1 | 7.85 | 66.7% | 1 | K.DQFALAMYFIQQK.V | 2 |
| \* | pDK268\_112012\_01.10007.10007.2 | 2.7251 | 0.4559 | 100.0% | 1934.2522 | 1935.2024 | 1 | 6.384 | 47.1% | 2 | K.GIDPPQVLSPDMVPPSER.G | 2 |
| \* | pDK268\_112012\_02.05658.05658.2 | 2.6443 | 0.5095 | 100.0% | 1836.9922 | 1837.9377 | 1 | 8.031 | 57.9% | 1 | R.GTPGPDSSGSLGSGEFTGVK.E | 2 |
| \* | pDK268\_112012\_01.14421.14421.3 | 4.0641 | 0.4407 | 100.0% | 3477.7144 | 3477.7205 | 1 | 8.025 | 28.0% | 2 | R.GTPGPDSSGSLGSGEFTGVKELDDISQEIAQLQR.E | 3 |
| \* | pDK268\_112012\_01.11822.11822.2 | 4.6458 | 0.3252 | 100.0% | 1659.2722 | 1658.8058 | 1 | 6.283 | 76.9% | 2 | K.ELDDISQEIAQLQR.E | 2 |
| \* | pDK268\_112012\_01.05597.05597.2 | 2.5448 | 0.2738 | 99.8% | 1281.3922 | 1281.4081 | 1 | 6.596 | 72.2% | 2 | R.EKYSLEQDIR.E | 2 |
| \* | pDK268\_112012\_01.06707.06707.1 | 1.9364 | 0.3174 | 100.0% | 1023.41 | 1024.1185 | 1 | 5.121 | 71.4% | 1 | K.YSLEQDIR.E | 1 |
| \* | pDK268\_112012\_01.06711.06711.2 | 3.0873 | 0.1768 | 99.9% | 1024.1122 | 1024.1185 | 1 | 5.957 | 78.6% | 2 | K.YSLEQDIR.E | 2 |
| \* | pDK268\_112012\_01.09340.09340.2 | 3.0171 | 0.2669 | 99.9% | 1546.7522 | 1547.6189 | 1 | 5.829 | 79.2% | 1 | K.TSEVQELQNDLDR.E | 2 |
| \* | pDK268\_112012\_01.13125.13125.3 | 4.8057 | 0.4798 | 100.0% | 2891.2144 | 2892.06 | 1 | 8.271 | 33.3% | 1 | K.TSEVQELQNDLDRETSSLQELEAQK.Q | 3 |
| \* | pDK268\_112012\_02.04519.04519.2 | 2.8526 | 0.2502 | 99.8% | 1364.3121 | 1363.4644 | 1 | 6.21 | 59.1% | 2 | R.ETSSLQELEAQK.Q | 2 |
| \* | pDK268\_112012\_02.03856.03856.3 | 3.1386 | 0.2804 | 100.0% | 1720.1044 | 1720.8083 | 14 | 5.281 | 40.4% | 1 | K.QDAQDRLDEMDQQK.A | 3 |
| \* | pDK268\_112012\_02.03360.03360.2 | 3.1871 | 0.3811 | 100.0% | 1277.3722 | 1277.3739 | 1 | 6.779 | 85.0% | 2 | K.TQIQSQESDLK.S | 2 |
| \* | pDK268\_112012\_01.06017.06017.2 | 4.9383 | 0.4998 | 100.0% | 2234.5322 | 2235.3262 | 1 | 9.183 | 69.4% | 1 | K.TQIQSQESDLKSQEDDLNR.A | 2 |
| \* | pDK268\_112012\_01.06068.06068.3 | 4.2427 | 0.3514 | 100.0% | 2235.8943 | 2235.3262 | 1 | 7.026 | 41.7% | 2 | K.TQIQSQESDLKSQEDDLNR.A | 3 |
| \* | pDK268\_112012\_02.05901.05901.2 | 5.8334 | 0.4212 | 100.0% | 1858.4521 | 1859.003 | 1 | 8.656 | 83.3% | 4 | R.LQQEETQLEQSIQAGR.V | 2 |
| \* | pDK268\_112012\_02.05896.05896.3 | 3.7856 | 0.3225 | 100.0% | 1859.3043 | 1859.003 | 1 | 5.473 | 43.3% | 2 | R.LQQEETQLEQSIQAGR.V | 3 |
| \* | pDK268\_112012\_01.03720.03720.1 | 1.7209 | 0.2686 | 98.4% | 1161.45 | 1162.2015 | 21 | 5.078 | 44.4% | 1 | K.STQDEINQAR.S | 1 |
| \* | pDK268\_112012\_02.03160.03160.2 | 3.7569 | 0.3389 | 100.0% | 1162.8121 | 1162.2015 | 1 | 6.02 | 83.3% | 2 | K.STQDEINQAR.S | 2 |
| \* | pDK268\_112012\_01.03419.03419.2 | 3.3774 | 0.4343 | 100.0% | 1184.7322 | 1185.3256 | 1 | 7.35 | 77.8% | 1 | R.SKLSQLHESR.Q | 2 |
| \* | pDK268\_112012\_01.03405.03405.3 | 2.2682 | 0.2462 | 96.9% | 1185.3544 | 1185.3256 | 3 | 4.659 | 47.2% | 1 | R.SKLSQLHESR.Q | 3 |
| \* | pDK268\_112012\_02.09705.09705.3 | 6.1915 | 0.5332 | 100.0% | 3360.5645 | 3360.616 | 1 | 9.773 | 32.3% | 5 | R.SLEQYDQVLDGAHGASLTDLANLSEGVSLAER.G | 3 |
| \* | pDK268\_112012\_01.09756.09756.1 | 2.1525 | 0.3718 | 100.0% | 1171.39 | 1172.2977 | 2 | 7.045 | 60.0% | 1 | R.GSFGAMDDPFK.N | 1 |
| \* | pDK268\_112012\_01.09803.09803.2 | 3.0459 | 0.4492 | 100.0% | 1173.2722 | 1172.2977 | 1 | 6.931 | 80.0% | 2 | R.GSFGAMDDPFK.N | 2 |
| \* | pDK268\_112012\_01.08081.08081.2 | 3.1851 | 0.181 | 99.8% | 1413.9722 | 1414.5756 | 2 | 5.247 | 62.5% | 1 | R.GSFGAMDDPFKNK.A | 2 |
| \* | pDK268\_112012\_01.12291.12291.3 | 3.7098 | 0.4278 | 100.0% | 2688.9243 | 2689.941 | 1 | 6.455 | 30.7% | 1 | K.ALLFSNNTQELHPDPFQTEDPFK.S | 3 |
| \* | pDK268\_112012\_01.12434.12434.3 | 4.8099 | 0.3943 | 100.0% | 3264.6843 | 3264.575 | 2 | 6.106 | 22.2% | 2 | K.ALLFSNNTQELHPDPFQTEDPFKSDPFK.G | 3 |
| \* | pDK268\_112012\_01.13108.13108.3 | 5.2521 | 0.5009 | 100.0% | 3459.8044 | 3460.651 | 1 | 7.465 | 26.6% | 2 | K.GADPFKGDPFQNDPFAEQQTTSTDPFGGDPFK.E | 3 |
| \* | pDK268\_112012\_01.07664.07664.2 | 2.2967 | 0.2253 | 99.3% | 987.8522 | 988.0416 | 1 | 6.142 | 87.5% | 1 | R.GSATDDFFK.K | 2 |
| \* | pDK268\_112012\_01.05661.05661.2 | 2.7554 | 0.286 | 99.9% | 1116.5721 | 1116.2157 | 8 | 5.798 | 55.6% | 2 | R.GSATDDFFKK.Q | 2 |
| \* | pDK268\_112012\_01.09447.09447.2 | 2.9174 | 0.3813 | 100.0% | 1270.1921 | 1269.3531 | 1 | 5.643 | 60.0% | 2 | K.NDPFTSDPFTK.N | 23 |
| \* | pDK268\_112012\_01.09162.09162.2 | 3.0111 | 0.3907 | 100.0% | 1992.7322 | 1993.1803 | 1 | 6.638 | 52.9% | 1 | K.NDPFTSDPFTKNPSLPSK.L | 2 |
| \* | pDK268\_112012\_01.12393.12393.3 | 3.8951 | 0.3912 | 100.0% | 3877.0144 | 3878.1511 | 1 | 5.951 | 23.6% | 1 | K.NDPFTSDPFTKNPSLPSKLDPFESSDPFSSSSVSSK.G | 3 |
| \* | pDK268\_112012\_01.10937.10937.2 | 3.2382 | 0.3608 | 100.0% | 2627.612 | 2627.8213 | 1 | 6.761 | 37.5% | 1 | K.NPSLPSKLDPFESSDPFSSSSVSSK.G | 2 |
| \* | pDK268\_112012\_01.10517.10517.2 | 4.4987 | 0.5546 | 100.0% | 1904.3121 | 1903.994 | 1 | 10.173 | 64.7% | 2 | K.LDPFESSDPFSSSSVSSK.G | 2 |
| \* | pDK268\_112012\_01.03178.03178.2 | 2.3979 | 0.2446 | 98.9% | 1357.4321 | 1357.642 | 5 | 4.816 | 66.7% | 1 | K.KPAPPRPKPPSGK.S | 2 |
| \* | pDK268\_112012\_01.13414.13414.3 | 2.7447 | 0.298 | 98.8% | 3390.6843 | 3391.6287 | 49 | 4.341 | 16.4% | 1 | K.STPVSQLGSADFPEAPDPFQPLGADSGDPFQSK.K | 3 |
| \* | pDK268\_112012\_01.08511.08511.3 | 4.2945 | 0.213 | 99.9% | 2352.0544 | 2352.6519 | 1 | 4.861 | 31.8% | 1 | K.KGFGDPFSGKDPFVPSSAAKPSK.A | 3 |

---

|  |  |  |  |  |  |  |  |  |
| --- | --- | --- | --- | --- | --- | --- | --- | --- |
| U | *gi|10800130|ref|NP\_06* | 10 | 22 | 65.4% | 130 | 14107 | 10.9 | histone cluster 1, H2ad [Homo sapiens] |

| Filename XCorr DeltCN Conf% ObsM+H+ CalcM+H+ SpR ZScore Ion% # Sequence  | | | | | | | | | | | | |
| --- | --- | --- | --- | --- | --- | --- | --- | --- | --- | --- | --- | --- |
|  | pDK268\_112012\_01.08294.08294.2 | 3.1381 | 0.3918 | 100.0% | 944.65216 | 945.1093 | 1 | 6.245 | 87.5% | 3 | R.AGLQFPVGR.V | 22222 |
|  | pDK268\_112012\_01.20228.20228.3 | 2.2331 | 0.4878 | 100.0% | 4373.514 | 4374.7686 | 109 | 6.219 | 16.4% | 1 | R.LLRKGNY@S\*ERVGAGAPVY@LAAVLEYLTAEILELAGNAAR.D | 3 |
|  | pDK268\_112012\_02.15141.15141.3 | 5.54 | 0.4735 | 100.0% | 2916.9243 | 2917.3752 | 1 | 7.623 | 28.6% | 1 | R.VGAGAPVYLAAVLEYLTAEILELAGNAAR.D | 333 |
|  | pDK268\_112012\_01.20231.20231.2 | 4.4906 | 0.3206 | 100.0% | 2920.0923 | 2917.3752 | 1 | 5.892 | 41.1% | 2 | R.VGAGAPVYLAAVLEYLTAEILELAGNAAR.D | 222 |
|  | pDK268\_112012\_01.05180.05180.2 | 2.5905 | 0.2897 | 100.0% | 851.1922 | 851.0396 | 1 | 4.986 | 100.0% | 3 | R.HLQLAIR.N | 2222 |
|  | pDK268\_112012\_02.04950.04950.2 | 3.7402 | 0.4162 | 100.0% | 1693.0322 | 1693.9004 | 1 | 7.199 | 73.1% | 1 | R.HLQLAIRNDEELNK.L | 222 |
|  | pDK268\_112012\_02.04390.04390.3 | 2.6407 | 0.3436 | 100.0% | 1694.2444 | 1693.9004 | 3 | 5.163 | 40.4% | 1 | R.HLQLAIRNDEELNK.L | 333 |
|  | pDK268\_112012\_01.08084.08084.2 | 3.5907 | 0.3841 | 100.0% | 1273.1122 | 1273.4288 | 1 | 6.089 | 70.0% | 2 | R.NDEELNKLLGK.V | 22 |
|  | pDK268\_112012\_01.13778.13778.2 | 5.0117 | 0.5581 | 100.0% | 1931.8121 | 1932.3573 | 1 | 8.7 | 69.4% | 6 | K.VTIAQGGVLPNIQAVLLPK.K | 22 |
|  | pDK268\_112012\_01.13689.13689.3 | 4.6069 | 0.4764 | 100.0% | 1932.1144 | 1932.3573 | 1 | 8.262 | 51.4% | 2 | K.VTIAQGGVLPNIQAVLLPK.K | 33 |

Similarities:
gi|106775678|ref|NP\_0(7:3)  
gi|4504253|ref|NP\_002(6:4)  
gi|28195394|ref|NP\_77(3:7)  
gi|4504255|ref|NP\_002(2:8)  

---

|  |  |  |  |  |  |  |  |  |
| --- | --- | --- | --- | --- | --- | --- | --- | --- |
| U | *gi|12025678|ref|NP\_00* | 55 | 100 | 62.0% | 911 | 104854 | 5.4 | actinin, alpha 4 [Homo sapiens] |

| Filename XCorr DeltCN Conf% ObsM+H+ CalcM+H+ SpR ZScore Ion% # Sequence  | | | | | | | | | | | | |
| --- | --- | --- | --- | --- | --- | --- | --- | --- | --- | --- | --- | --- |
|  | pDK268\_112012\_01.11588.11588.2 | 2.7505 | 0.0539 | 97.6% | 1200.3322 | 1200.377 | 5 | 5.033 | 66.7% | 1 | R.DLLLDPAWEK.Q | 22 |
| \* | pDK268\_112012\_01.08660.08660.3 | 3.8164 | 0.3412 | 100.0% | 2050.5244 | 2050.2327 | 1 | 6.538 | 38.2% | 1 | R.KAGTQIENIDEDFRDGLK.L | 3 |
|  | pDK268\_112012\_01.15545.15545.2 | 4.4969 | 0.3334 | 100.0% | 1374.3322 | 1373.6941 | 1 | 7.133 | 81.8% | 3 | K.LMLLLEVISGER.L | 22 |
| \* | pDK268\_112012\_01.13527.13527.3 | 2.9152 | 0.261 | 98.9% | 2094.0842 | 2094.564 | 321 | 4.327 | 27.9% | 1 | K.LMLLLEVISGERLPKPER.G | 3 |
|  | pDK268\_112012\_01.07731.07731.2 | 2.8483 | 0.4277 | 100.0% | 865.0122 | 865.01715 | 1 | 7.269 | 92.9% | 1 | K.ALDFIASK.G | 22 |
| \* | pDK268\_112012\_01.08946.08946.2 | 4.2425 | 0.4518 | 100.0% | 1516.0322 | 1515.7037 | 1 | 8.568 | 82.1% | 3 | K.LVSIGAEEIVDGNAK.M | 2 |
|  | pDK268\_112012\_01.17678.17678.2 | 2.2663 | 0.2326 | 98.4% | 1449.0922 | 1448.8683 | 10 | 5.145 | 63.6% | 2 | K.MTLGMIWTIILR.F | 22 |
|  | pDK268\_112012\_01.09399.09399.2 | 4.5282 | 0.3891 | 100.0% | 1538.2522 | 1538.6947 | 1 | 9.071 | 80.8% | 2 | R.FAIQDISVEETSAK.E | 22 |
| \* | pDK268\_112012\_02.07125.07125.2 | 3.764 | 0.456 | 100.0% | 1486.6122 | 1486.6731 | 1 | 7.027 | 77.3% | 2 | K.NVNVQNFHISWK.D | 2 |
|  | pDK268\_112012\_02.07368.07368.2 | 2.8038 | 0.2919 | 99.9% | 1227.3722 | 1227.4086 | 1 | 6.021 | 85.0% | 3 | K.DGLAFNALIHR.H | 2 |
| \* | pDK268\_112012\_01.04586.04586.2 | 3.0537 | 0.2724 | 100.0% | 1300.2922 | 1300.4569 | 1 | 5.963 | 83.3% | 1 | R.HRPELIEYDK.L | 2 |
| \* | pDK268\_112012\_02.03649.03649.3 | 3.2913 | 0.1373 | 98.3% | 1300.8243 | 1300.4569 | 1 | 4.238 | 63.9% | 2 | R.HRPELIEYDK.L | 3 |
| \* | pDK268\_112012\_01.05410.05410.3 | 4.4942 | 0.3049 | 100.0% | 1570.4043 | 1569.8038 | 8 | 7.278 | 50.0% | 2 | R.HRPELIEYDKLR.K | 3 |
| \* | pDK268\_112012\_01.09404.09404.2 | 5.4165 | 0.5595 | 100.0% | 1904.3121 | 1905.0715 | 1 | 10.105 | 71.9% | 2 | R.KDDPVTNLNNAFEVAEK.Y | 2 |
| \* | pDK268\_112012\_01.09447.09447.3 | 3.3944 | 0.3169 | 100.0% | 1904.7843 | 1905.0715 | 1 | 5.088 | 48.4% | 1 | R.KDDPVTNLNNAFEVAEK.Y | 23 |
| \* | pDK268\_112012\_01.11147.11147.2 | 3.2993 | 0.223 | 99.9% | 1776.0521 | 1776.8975 | 1 | 6.655 | 53.3% | 2 | K.DDPVTNLNNAFEVAEK.Y | 2 |
| \* | pDK268\_112012\_01.08957.08957.2 | 4.3198 | 0.413 | 100.0% | 1817.3121 | 1818.0088 | 1 | 8.267 | 66.7% | 1 | K.MLDAEDIVNTARPDEK.A | 2 |
| \* | pDK268\_112012\_02.05897.05897.3 | 2.7282 | 0.3249 | 99.9% | 1817.8444 | 1818.0088 | 1 | 5.744 | 43.3% | 2 | K.MLDAEDIVNTARPDEK.A | 32 |
|  | pDK268\_112012\_01.13420.13420.2 | 5.057 | 0.4956 | 100.0% | 2008.3121 | 2009.2867 | 1 | 9.528 | 70.6% | 1 | K.AIMTYVSSFYHAFSGAQK.A | 22 |
|  | pDK268\_112012\_01.13448.13448.3 | 3.1893 | 0.366 | 100.0% | 2009.7244 | 2009.2867 | 1 | 6.723 | 38.2% | 2 | K.AIMTYVSSFYHAFSGAQK.A | 33 |
| \* | pDK268\_112012\_01.07842.07842.2 | 5.459 | 0.5359 | 100.0% | 2061.4321 | 2062.2585 | 1 | 9.714 | 68.8% | 1 | K.VLAVNQENEHLMEDYEK.L | 2 |
| \* | pDK268\_112012\_02.05895.05895.3 | 2.5075 | 0.2755 | 97.9% | 2061.5044 | 2062.2585 | 1 | 4.886 | 31.2% | 1 | K.VLAVNQENEHLMEDYEK.L | 3 |
|  | pDK268\_112012\_01.13638.13638.1 | 2.5564 | 0.293 | 100.0% | 1215.52 | 1216.4227 | 1 | 5.821 | 61.1% | 1 | K.LASDLLEWIR.R | 11 |
|  | pDK268\_112012\_01.13664.13664.2 | 3.4437 | 0.4167 | 100.0% | 1215.7722 | 1216.4227 | 1 | 7.564 | 88.9% | 2 | K.LASDLLEWIR.R | 22 |
| \* | pDK268\_112012\_01.09172.09172.2 | 2.5502 | 0.0945 | 96.7% | 1482.2322 | 1482.7227 | 28 | 4.259 | 54.5% | 2 | R.TIPWLEDRVPQK.T | 2 |
| \* | pDK268\_112012\_01.08658.08658.2 | 4.9811 | 0.4858 | 100.0% | 1999.3922 | 2000.1932 | 1 | 8.445 | 68.8% | 1 | K.MVSDINNGWQHLEQAEK.G | 2 |
|  | pDK268\_112012\_01.13160.13160.2 | 3.8297 | 0.4385 | 100.0% | 1422.1721 | 1422.5803 | 1 | 7.238 | 80.0% | 2 | K.GYEEWLLNEIR.R | 22 |
|  | pDK268\_112012\_01.04862.04862.2 | 3.4341 | 0.4239 | 100.0% | 1215.0922 | 1215.3079 | 1 | 6.831 | 65.0% | 1 | K.ASIHEAWTDGK.E | 22 |
| \* | pDK268\_112012\_01.07385.07385.3 | 2.7378 | 0.2407 | 97.8% | 1787.5443 | 1788.0283 | 1 | 4.373 | 45.0% | 1 | K.ASIHEAWTDGKEAMLK.H | 3 |
| \* | pDK268\_112012\_01.05218.05218.2 | 3.3037 | 0.3428 | 100.0% | 1549.2922 | 1549.6805 | 1 | 6.866 | 79.2% | 1 | K.HRDYETATLSDIK.A | 2 |
| \* | pDK268\_112012\_01.05219.05219.3 | 4.0805 | 0.3202 | 100.0% | 1549.8844 | 1549.6805 | 1 | 6.832 | 56.2% | 2 | K.HRDYETATLSDIK.A | 3 |
| \* | pDK268\_112012\_01.08176.08176.2 | 3.1511 | 0.4737 | 100.0% | 1256.1921 | 1256.3519 | 1 | 8.839 | 75.0% | 1 | R.DYETATLSDIK.A | 2 |
|  | pDK268\_112012\_01.04672.04672.3 | 4.4993 | 0.4701 | 100.0% | 1626.4143 | 1626.6824 | 1 | 8.234 | 55.8% | 2 | K.HEAFESDLAAHQDR.V | 33 |
| \* | pDK268\_112012\_02.09706.09706.3 | 4.3403 | 0.3931 | 100.0% | 2907.2644 | 2907.1228 | 1 | 6.674 | 31.2% | 2 | R.VEQIAAIAQELNELDYYDSHNVNTR.C | 3 |
| \* | pDK268\_112012\_01.09502.09502.2 | 3.4414 | 0.4402 | 100.0% | 1671.2722 | 1671.8907 | 1 | 6.899 | 57.7% | 2 | K.QLEAIDQLHLEYAK.R | 2 |
| \* | pDK268\_112012\_01.08732.08732.2 | 5.4474 | 0.4993 | 100.0% | 1920.5922 | 1921.1577 | 1 | 9.569 | 64.7% | 3 | K.LSGSNPYTTVTPQIINSK.W | 2 |
| \* | pDK268\_112012\_01.04310.04310.2 | 2.8857 | 0.0614 | 99.7% | 812.09216 | 812.0 | 14 | 4.306 | 91.7% | 3 | K.VQQLVPK.R | 2 |
| \* | pDK268\_112012\_01.03578.03578.2 | 4.1419 | 0.4253 | 100.0% | 1326.0521 | 1326.452 | 1 | 7.427 | 85.0% | 1 | K.RDHALLEEQSK.Q | 2 |
| \* | pDK268\_112012\_01.03573.03573.3 | 3.5584 | 0.2291 | 100.0% | 1326.9844 | 1326.452 | 1 | 4.726 | 52.5% | 1 | K.RDHALLEEQSK.Q | 3 |
| \* | pDK268\_112012\_01.04044.04044.2 | 3.0394 | 0.4367 | 100.0% | 1170.0721 | 1170.2645 | 1 | 6.625 | 88.9% | 1 | R.DHALLEEQSK.Q | 2 |
| \* | pDK268\_112012\_01.10238.10238.2 | 5.1664 | 0.5151 | 100.0% | 1774.5922 | 1775.0171 | 1 | 8.837 | 63.3% | 2 | R.QFASQANVVGPWIQTK.M | 2 |
| \* | pDK268\_112012\_01.11286.11286.2 | 4.4297 | 0.4474 | 100.0% | 1928.4122 | 1929.1957 | 1 | 8.859 | 50.0% | 2 | R.ISIEMNGTLEDQLSHLK.Q | 2 |
| \* | pDK268\_112012\_01.13742.13742.3 | 5.9748 | 0.501 | 100.0% | 3325.7644 | 3326.7742 | 1 | 8.256 | 34.3% | 3 | R.SIVDYKPNLDLLEQQHQLIQEALIFDNK.H | 3 |
|  | pDK268\_112012\_01.04023.04023.3 | 4.0352 | 0.3532 | 100.0% | 1303.7943 | 1302.4503 | 1 | 6.561 | 52.8% | 1 | K.HTNYTMEHIR.V | 33 |
|  | pDK268\_112012\_01.14414.14414.2 | 4.1948 | 0.3964 | 100.0% | 1386.9521 | 1387.6218 | 1 | 8.068 | 77.3% | 4 | R.VGWEQLLTTIAR.T | 22 |
|  | pDK268\_112012\_01.08912.08912.2 | 3.9714 | 0.3062 | 100.0% | 1431.4321 | 1430.6011 | 1 | 6.48 | 77.3% | 2 | R.TINEVENQILTR.D | 22 |
| \* | pDK268\_112012\_01.06816.06816.2 | 3.4591 | 0.5116 | 100.0% | 1353.0922 | 1353.4926 | 1 | 8.162 | 80.0% | 3 | K.GISQEQMQEFR.A | 2 |
| \* | pDK268\_112012\_01.06740.06740.3 | 3.9097 | 0.3136 | 100.0% | 2204.4844 | 2204.3628 | 1 | 6.295 | 46.1% | 2 | R.ASFNHFDKDHGGALGPEEFK.A | 3 |
| \* | pDK268\_112012\_01.17175.17175.3 | 3.5638 | 0.3162 | 100.0% | 2726.4243 | 2727.1682 | 1 | 4.949 | 27.2% | 2 | R.IMSLVDPNHSGLVTFQAFIDFMSR.E | 3 |
| \* | pDK268\_112012\_01.17092.17092.2 | 2.06 | 0.5619 | 100.0% | 2726.4922 | 2727.1682 | 1 | 7.895 | 50.0% | 1 | R.IMSLVDPNHSGLVTFQAFIDFMSR.E | 2 |
| \* | pDK268\_112012\_02.06678.06678.2 | 4.3949 | 0.4977 | 100.0% | 1742.3522 | 1742.834 | 1 | 7.946 | 63.3% | 4 | R.ETTDTDTADQVIASFK.V | 2 |
| \* | pDK268\_112012\_01.08586.08586.2 | 4.8113 | 0.4731 | 100.0% | 1676.3522 | 1676.9103 | 1 | 8.959 | 67.9% | 1 | K.VLAGDKNFITAEELR.R | 2 |
| \* | pDK268\_112012\_02.06435.06435.3 | 4.1734 | 0.2471 | 100.0% | 1676.9644 | 1676.9103 | 4 | 6.269 | 42.9% | 3 | K.VLAGDKNFITAEELR.R | 3 |
| \* | pDK268\_112012\_01.07360.07360.3 | 3.137 | 0.4385 | 100.0% | 1832.6643 | 1833.0977 | 20 | 6.127 | 35.0% | 1 | K.VLAGDKNFITAEELRR.E | 3 |
| \* | pDK268\_112012\_01.09098.09098.2 | 4.8401 | 0.5265 | 100.0% | 1793.3922 | 1794.032 | 1 | 9.229 | 75.0% | 3 | R.MAPYQGPDAVPGALDYK.S | 2 |

Similarities:
gi|194097350|ref|NP\_0(15:40)  

---

|  |  |  |  |  |  |  |  |  |
| --- | --- | --- | --- | --- | --- | --- | --- | --- |
| U | *gi|11415030|ref|NP\_06* | 13 | 24 | 60.2% | 103 | 11367 | 11.4 | histone cluster 1, H4j [Homo sapiens] |
| U | *gi|77539758|ref|NP\_00* | 13 | 24 | 60.2% | 103 | 11367 | 11.4 | histone cluster 2, H4b [Homo sapiens] |
| U | *gi|4504323|ref|NP\_003* | 13 | 24 | 60.2% | 103 | 11367 | 11.4 | histone cluster 2, H4a [Homo sapiens] |
| U | *gi|4504321|ref|NP\_003* | 13 | 24 | 60.2% | 103 | 11367 | 11.4 | histone cluster 1, H4i [Homo sapiens] |
| U | *gi|4504317|ref|NP\_003* | 13 | 24 | 60.2% | 103 | 11367 | 11.4 | histone cluster 1, H4l [Homo sapiens] |
| U | *gi|4504315|ref|NP\_003* | 13 | 24 | 60.2% | 103 | 11367 | 11.4 | histone cluster 1, H4e [Homo sapiens] |
| U | *gi|4504313|ref|NP\_003* | 13 | 24 | 60.2% | 103 | 11367 | 11.4 | histone cluster 1, H4b [Homo sapiens] |
| U | *gi|4504311|ref|NP\_003* | 13 | 24 | 60.2% | 103 | 11367 | 11.4 | histone cluster 1, H4h [Homo sapiens] |
| U | *gi|4504309|ref|NP\_003* | 13 | 24 | 60.2% | 103 | 11367 | 11.4 | histone cluster 1, H4c [Homo sapiens] |
| U | *gi|4504307|ref|NP\_003* | 13 | 24 | 60.2% | 103 | 11367 | 11.4 | histone cluster 1, H4k [Homo sapiens] |
| U | *gi|4504305|ref|NP\_003* | 13 | 24 | 60.2% | 103 | 11367 | 11.4 | histone cluster 1, H4f [Homo sapiens] |
| U | *gi|4504303|ref|NP\_003* | 13 | 24 | 60.2% | 103 | 11367 | 11.4 | histone cluster 1, H4d [Homo sapiens] |
| U | *gi|4504301|ref|NP\_003* | 13 | 24 | 60.2% | 103 | 11367 | 11.4 | histone cluster 1, H4a [Homo sapiens] |
| U | *gi|28173560|ref|NP\_77* | 13 | 24 | 60.2% | 103 | 11367 | 11.4 | histone cluster 4, H4 [Homo sapiens] |

| Filename XCorr DeltCN Conf% ObsM+H+ CalcM+H+ SpR ZScore Ion% # Sequence  | | | | | | | | | | | | |
| --- | --- | --- | --- | --- | --- | --- | --- | --- | --- | --- | --- | --- |
|  | pDK268\_112012\_01.05493.05493.2 | 3.4226 | 0.1757 | 99.9% | 1326.1721 | 1326.5387 | 1 | 6.53 | 77.3% | 4 | R.DNIQGITKPAIR.R | 2 |
|  | pDK268\_112012\_01.06430.06430.2 | 2.8759 | 0.2626 | 99.9% | 1337.1921 | 1337.5187 | 1 | 4.732 | 65.0% | 2 | K.RISGLIYEETR.G | 2 |
|  | pDK268\_112012\_01.07611.07611.1 | 2.4918 | 0.2246 | 98.4% | 1180.56 | 1181.3312 | 3 | 4.724 | 66.7% | 1 | R.ISGLIYEETR.G | 1 |
|  | pDK268\_112012\_02.05949.05949.2 | 3.4677 | 0.3502 | 100.0% | 1182.2722 | 1181.3312 | 1 | 6.303 | 94.4% | 3 | R.ISGLIYEETR.G | 2 |
|  | pDK268\_112012\_01.09776.09776.1 | 1.9629 | 0.2399 | 98.5% | 989.48 | 990.19055 | 60 | 5.231 | 57.1% | 1 | K.VFLENVIR.D | 1 |
|  | pDK268\_112012\_01.09758.09758.2 | 3.1385 | 0.3136 | 100.0% | 990.1922 | 990.19055 | 2 | 5.438 | 85.7% | 3 | K.VFLENVIR.D | 2 |
|  | pDK268\_112012\_01.03708.03708.1 | 2.0841 | 0.4455 | 100.0% | 1134.46 | 1135.2188 | 1 | 6.884 | 61.1% | 1 | R.DAVTYTEHAK.R | 1 |
|  | pDK268\_112012\_01.03687.03687.2 | 2.9959 | 0.3449 | 100.0% | 1134.9321 | 1135.2188 | 1 | 6.738 | 72.2% | 2 | R.DAVTYTEHAK.R | 2 |
|  | pDK268\_112012\_01.10403.10403.2 | 2.8972 | 0.2718 | 99.9% | 1440.5521 | 1439.7534 | 1 | 4.829 | 83.3% | 1 | R.KTVTAMDVVYALK.R | 2 |
|  | pDK268\_112012\_01.09398.09398.3 | 3.9004 | 0.4328 | 100.0% | 1595.9944 | 1595.9409 | 5 | 6.353 | 38.5% | 1 | R.KTVTAMDVVYALKR.Q | 3 |
|  | pDK268\_112012\_01.11978.11978.2 | 3.0997 | 0.4903 | 100.0% | 1311.1122 | 1311.5793 | 1 | 8.082 | 81.8% | 2 | K.TVTAMDVVYALK.R | 2 |
|  | pDK268\_112012\_01.10677.10677.2 | 3.4149 | 0.4899 | 100.0% | 1467.4321 | 1467.7667 | 1 | 7.395 | 79.2% | 1 | K.TVTAMDVVYALKR.Q | 2 |
|  | pDK268\_112012\_01.09657.09657.1 | 1.8138 | 0.4803 | 100.0% | 714.34 | 714.796 | 1 | 7.883 | 66.7% | 2 | R.TLYGFGG.- | 1 |

---

|  |  |  |  |  |  |  |  |  |
| --- | --- | --- | --- | --- | --- | --- | --- | --- |
| U | *gi|55956899|ref|NP\_00* | 40 | 77 | 59.6% | 623 | 62064 | 5.2 | keratin 9 [Homo sapiens] |

| Filename XCorr DeltCN Conf% ObsM+H+ CalcM+H+ SpR ZScore Ion% # Sequence  | | | | | | | | | | | | |
| --- | --- | --- | --- | --- | --- | --- | --- | --- | --- | --- | --- | --- |
| \* | pDK268\_112012\_01.04025.04025.1 | 2.6696 | 0.3928 | 100.0% | 1232.46 | 1233.2833 | 70 | 6.839 | 33.3% | 1 | R.SGGGGGGGLGSGGSIR.S | 1 |
| \* | pDK268\_112012\_02.03305.03305.2 | 4.5758 | 0.513 | 100.0% | 1233.4122 | 1233.2833 | 1 | 7.752 | 76.7% | 2 | R.SGGGGGGGLGSGGSIR.S | 2 |
|  | pDK268\_112012\_01.03706.03706.2 | 3.5672 | 0.4171 | 100.0% | 1235.3722 | 1236.2401 | 1 | 9.314 | 79.2% | 2 | R.FSSSSGYGGGSSR.V | 2 |
|  | pDK268\_112012\_02.06891.06891.3 | 4.2399 | 0.3759 | 100.0% | 2707.5544 | 2706.7605 | 1 | 6.243 | 24.2% | 1 | R.GGGGSFGYSYGGGSGGGFSASSLGGGFGGGSR.G | 3 |
|  | pDK268\_112012\_01.04191.04191.1 | 2.0334 | 0.2829 | 100.0% | 1065.44 | 1066.1742 | 38 | 5.127 | 50.0% | 1 | K.STMQELNSR.L | 1 |
|  | pDK268\_112012\_01.04232.04232.2 | 2.9254 | 0.2808 | 100.0% | 1066.1721 | 1066.1742 | 1 | 6.412 | 81.2% | 1 | K.STMQELNSR.L | 2 |
|  | pDK268\_112012\_01.10438.10438.3 | 2.7402 | 0.2287 | 95.6% | 2377.3743 | 2378.5981 | 7 | 3.998 | 28.8% | 1 | R.LASYLDKVQALEEANNDLENK.I | 3 |
|  | pDK268\_112012\_01.10456.10456.2 | 5.8735 | 0.551 | 100.0% | 2378.412 | 2378.5981 | 1 | 10.066 | 62.5% | 1 | R.LASYLDKVQALEEANNDLENK.I | 2 |
|  | pDK268\_112012\_01.13422.13422.3 | 5.243 | 0.4403 | 100.0% | 3326.9043 | 3327.6287 | 1 | 7.2 | 27.8% | 1 | R.LASYLDKVQALEEANNDLENKIQDWYDK.K | 3 |
|  | pDK268\_112012\_01.13127.13127.3 | 4.5873 | 0.3908 | 100.0% | 3455.1843 | 3455.8027 | 1 | 6.361 | 27.7% | 1 | R.LASYLDKVQALEEANNDLENKIQDWYDKK.G | 3 |
|  | pDK268\_112012\_01.06500.06500.2 | 5.2963 | 0.5146 | 100.0% | 1587.1921 | 1587.6836 | 1 | 9.941 | 84.6% | 4 | K.VQALEEANNDLENK.I | 2 |
|  | pDK268\_112012\_01.11578.11578.3 | 3.5657 | 0.3044 | 100.0% | 2536.1643 | 2536.714 | 1 | 5.146 | 32.5% | 1 | K.VQALEEANNDLENKIQDWYDK.K | 3 |
|  | pDK268\_112012\_01.11079.11079.3 | 4.0309 | 0.2733 | 100.0% | 2664.2344 | 2664.8882 | 1 | 5.551 | 35.7% | 1 | K.VQALEEANNDLENKIQDWYDKK.G | 3 |
|  | pDK268\_112012\_01.14420.14420.3 | 5.4913 | 0.4436 | 100.0% | 2903.2744 | 2904.1597 | 1 | 7.307 | 37.5% | 3 | K.NYSPYYNTIDDLKDQIVDLTVGNNK.T | 3 |
|  | pDK268\_112012\_01.07888.07888.1 | 2.3778 | 0.4265 | 100.0% | 1060.54 | 1061.1802 | 3 | 6.26 | 62.5% | 2 | K.TLLDIDNTR.M | 1 |
|  | pDK268\_112012\_01.07928.07928.2 | 2.7379 | 0.3141 | 100.0% | 1060.7722 | 1061.1802 | 1 | 6.144 | 87.5% | 3 | K.TLLDIDNTR.M | 2 |
|  | pDK268\_112012\_01.08580.08580.1 | 1.7157 | 0.2358 | 96.1% | 897.47 | 898.02155 | 42 | 5.433 | 66.7% | 1 | R.MTLDDFR.I | 1 |
|  | pDK268\_112012\_01.08600.08600.2 | 2.3265 | 0.4086 | 100.0% | 897.9922 | 898.02155 | 1 | 6.302 | 75.0% | 2 | R.MTLDDFR.I | 2 |
|  | pDK268\_112012\_02.05769.05769.3 | 3.4134 | 0.2107 | 100.0% | 1307.8444 | 1308.5383 | 2 | 4.979 | 52.8% | 2 | R.IKFEMEQNLR.Q | 3 |
|  | pDK268\_112012\_01.07293.07293.2 | 3.1484 | 0.3573 | 100.0% | 1308.1721 | 1308.5383 | 2 | 6.3 | 66.7% | 2 | R.IKFEMEQNLR.Q | 2 |
|  | pDK268\_112012\_01.06728.06728.2 | 3.2789 | 0.3668 | 100.0% | 1158.1522 | 1158.2566 | 1 | 6.551 | 80.0% | 3 | R.QGVDADINGLR.Q | 2 |
|  | pDK268\_112012\_01.07623.07623.2 | 2.5335 | 0.2012 | 99.5% | 1190.9922 | 1191.385 | 2 | 6.095 | 66.7% | 1 | R.QVLDNLTMEK.S | 2 |
|  | pDK268\_112012\_01.14117.14117.3 | 4.2797 | 0.3901 | 100.0% | 3472.5544 | 3473.006 | 1 | 5.757 | 22.3% | 1 | R.QVLDNLTMEKSDLEMQYETLQEELMALKK.N | 3 |
|  | pDK268\_112012\_02.10758.10758.2 | 5.4813 | 0.3553 | 100.0% | 2172.872 | 2172.4702 | 1 | 7.565 | 70.6% | 2 | K.SDLEMQYETLQEELMALK.K | 2 |
|  | pDK268\_112012\_01.13162.13162.2 | 5.1985 | 0.567 | 100.0% | 2299.5322 | 2300.6443 | 1 | 8.797 | 63.9% | 1 | K.SDLEMQYETLQEELMALKK.N | 2 |
|  | pDK268\_112012\_01.13145.13145.3 | 3.2653 | 0.2757 | 99.8% | 2301.8044 | 2300.6443 | 1 | 4.883 | 33.3% | 2 | K.SDLEMQYETLQEELMALKK.N | 3 |
|  | pDK268\_112012\_02.05610.05610.3 | 5.5946 | 0.4646 | 100.0% | 2895.7444 | 2897.1462 | 1 | 7.744 | 32.7% | 1 | K.NHKEEMSQLTGQNSGDVNVEINVAPGK.D | 32 |
|  | pDK268\_112012\_02.05788.05788.3 | 7.1549 | 0.4678 | 100.0% | 3353.9644 | 3354.6733 | 1 | 8.168 | 39.2% | 1 | K.NHKEEMSQLTGQNSGDVNVEINVAPGKDLTK.T | 3 |
|  | pDK268\_112012\_01.10618.10618.3 | 3.3766 | 0.1759 | 98.3% | 1852.5844 | 1853.1003 | 1 | 4.592 | 44.6% | 2 | K.TLNDMRQEYEQLIAK.N | 3 |
|  | pDK268\_112012\_01.10604.10604.2 | 3.123 | 0.1501 | 99.4% | 1853.6921 | 1853.1003 | 1 | 4.39 | 60.7% | 2 | K.TLNDMRQEYEQLIAK.N | 23 |
|  | pDK268\_112012\_01.06446.06446.2 | 2.0487 | 0.3705 | 99.8% | 1121.4321 | 1122.2633 | 2 | 5.956 | 62.5% | 3 | R.QEYEQLIAK.N | 2 |
|  | pDK268\_112012\_01.12699.12699.3 | 6.4308 | 0.4967 | 100.0% | 3266.5444 | 3266.413 | 1 | 8.58 | 33.0% | 3 | K.DIENQYETQITQIEHEVSSSGQEVQSSAK.E | 3 |
|  | pDK268\_112012\_01.11074.11074.2 | 6.1403 | 0.5865 | 100.0% | 1838.2522 | 1839.0557 | 1 | 10.619 | 76.7% | 3 | R.HGVQELEIELQSQLSK.K | 2 |
|  | pDK268\_112012\_02.08013.08013.3 | 3.9108 | 0.3166 | 100.0% | 1838.6344 | 1839.0557 | 1 | 6.127 | 41.7% | 2 | R.HGVQELEIELQSQLSK.K | 3 |
|  | pDK268\_112012\_02.07509.07509.3 | 6.3047 | 0.2938 | 100.0% | 1967.0643 | 1967.2297 | 1 | 7.138 | 53.1% | 4 | R.HGVQELEIELQSQLSKK.A | 3 |
|  | pDK268\_112012\_01.09677.09677.3 | 6.9565 | 0.283 | 100.0% | 2882.3044 | 2882.0667 | 1 | 6.827 | 34.0% | 1 | R.LEKEIETYHNLLEGGQEDFESSGAGK.I | 3 |
|  | pDK268\_112012\_01.09531.09531.2 | 4.8407 | 0.4631 | 100.0% | 2510.672 | 2511.6177 | 1 | 8.36 | 50.0% | 2 | K.EIETYHNLLEGGQEDFESSGAGK.I | 2 |
|  | pDK268\_112012\_02.06963.06963.3 | 5.9933 | 0.3578 | 100.0% | 2511.4143 | 2511.6177 | 1 | 7.996 | 40.9% | 5 | K.EIETYHNLLEGGQEDFESSGAGK.I | 3 |
|  | pDK268\_112012\_01.03570.03570.2 | 5.9173 | 0.4598 | 100.0% | 1793.2522 | 1792.7324 | 1 | 9.895 | 47.7% | 2 | R.GGSGGSYGGGGSGGGYGGGSGSR.G | 2 |
|  | pDK268\_112012\_02.04039.04039.3 | 8.6276 | 0.5866 | 100.0% | 3223.9443 | 3225.1118 | 1 | 9.838 | 31.4% | 3 | R.GGSGGSHGGGSGFGGESGGSYGGGEEASGSGGGYGGGSGK.S | 3 |

---

|  |  |  |  |  |  |  |  |  |
| --- | --- | --- | --- | --- | --- | --- | --- | --- |
| U | *GFP* | 27 | 208 | 59.2% | 238 | 26813 | 5.8 | no description |

| Filename XCorr DeltCN Conf% ObsM+H+ CalcM+H+ SpR ZScore Ion% # Sequence  | | | | | | | | | | | | |
| --- | --- | --- | --- | --- | --- | --- | --- | --- | --- | --- | --- | --- |
| \* | pDK268\_112012\_01.15710.15710.2 | 5.2313 | 0.6196 | 100.0% | 2437.5322 | 2438.7397 | 1 | 10.407 | 54.5% | 11 | K.GEELFTGVVPILVELDGDVNGHK.F | 2 |
| \* | pDK268\_112012\_01.15590.15590.3 | 4.0066 | 0.3615 | 100.0% | 2439.0842 | 2438.7397 | 1 | 7.031 | 30.7% | 11 | K.GEELFTGVVPILVELDGDVNGHK.F | 3 |
| \* | pDK268\_112012\_02.11302.11302.3 | 5.3057 | 0.5155 | 100.0% | 3923.2444 | 3924.2666 | 1 | 7.822 | 25.0% | 2 | K.GEELFTGVVPILVELDGDVNGHKFSVSGEGEGDATYGK.L | 3 |
| \* | pDK268\_112012\_02.04807.04807.3 | 4.2124 | 0.46 | 100.0% | 1504.7943 | 1504.5499 | 1 | 7.727 | 42.9% | 1 | K.FSVSGEGEGDATYGK.L | 3 |
| \* | pDK268\_112012\_02.05211.05211.2 | 4.7117 | 0.6163 | 100.0% | 1505.1122 | 1504.5499 | 1 | 9.806 | 71.4% | 87 | K.FSVSGEGEGDATYGK.L | 2 |
| \* | pDK268\_112012\_01.05558.05558.2 | 3.0094 | 0.4858 | 100.0% | 1266.9521 | 1267.399 | 1 | 8.147 | 75.0% | 9 | K.SAMPEGYVQER.T | 2 |
| \* | pDK268\_112012\_01.07005.07005.2 | 3.5481 | 0.4173 | 100.0% | 1349.2922 | 1348.4979 | 1 | 6.537 | 85.0% | 9 | R.TIFFKDDGNYK.T | 2 |
| \* | pDK268\_112012\_02.05044.05044.2 | 3.6764 | 0.4678 | 100.0% | 1604.8121 | 1605.7905 | 2 | 7.982 | 62.5% | 1 | R.TIFFKDDGNYKTR.A | 2 |
| \* | pDK268\_112012\_02.05015.05015.3 | 2.6978 | 0.3122 | 99.9% | 1605.1444 | 1605.7905 | 17 | 5.674 | 37.5% | 1 | R.TIFFKDDGNYKTR.A | 3 |
| \* | pDK268\_112012\_02.04935.04935.3 | 4.0197 | 0.3158 | 100.0% | 1735.9443 | 1735.9376 | 1 | 6.888 | 44.6% | 2 | K.TRAEVKFEGDTLVNR.I | 3 |
| \* | pDK268\_112012\_01.06789.06789.2 | 4.3546 | 0.4848 | 100.0% | 1478.2122 | 1478.6451 | 1 | 8.626 | 79.2% | 16 | R.AEVKFEGDTLVNR.I | 2 |
| \* | pDK268\_112012\_01.06746.06746.3 | 3.4736 | 0.3342 | 100.0% | 1478.9043 | 1478.6451 | 2 | 5.629 | 50.0% | 9 | R.AEVKFEGDTLVNR.I | 3 |
| \* | pDK268\_112012\_01.09293.09293.2 | 3.7554 | 0.3717 | 100.0% | 1961.3322 | 1962.2535 | 1 | 6.772 | 56.2% | 1 | R.AEVKFEGDTLVNRIELK.G | 2 |
| \* | pDK268\_112012\_02.06867.06867.3 | 4.1068 | 0.443 | 100.0% | 1963.0743 | 1962.2535 | 3 | 7.746 | 37.5% | 2 | R.AEVKFEGDTLVNRIELK.G | 3 |
| \* | pDK268\_112012\_01.05948.05948.1 | 2.1291 | 0.3666 | 100.0% | 1050.55 | 1051.1442 | 1 | 6.384 | 62.5% | 2 | K.FEGDTLVNR.I | 1 |
| \* | pDK268\_112012\_01.05906.05906.2 | 3.4278 | 0.4267 | 100.0% | 1051.1322 | 1051.1442 | 1 | 6.998 | 87.5% | 5 | K.FEGDTLVNR.I | 2 |
| \* | pDK268\_112012\_01.09335.09335.2 | 2.6971 | 0.1764 | 98.9% | 1534.3322 | 1534.7526 | 4 | 4.574 | 58.3% | 1 | K.FEGDTLVNRIELK.G | 2 |
| \* | pDK268\_112012\_01.07382.07382.2 | 4.4818 | 0.4926 | 100.0% | 1543.2322 | 1543.7196 | 1 | 9.741 | 73.1% | 8 | K.GIDFKEDGNILGHK.L | 2 |
| \* | pDK268\_112012\_02.05775.05775.3 | 3.8809 | 0.3988 | 100.0% | 1543.9443 | 1543.7196 | 1 | 6.313 | 46.2% | 5 | K.GIDFKEDGNILGHK.L | 3 |
| \* | pDK268\_112012\_02.07427.07427.3 | 5.6893 | 0.4449 | 100.0% | 3499.6743 | 3499.8792 | 1 | 7.053 | 32.8% | 2 | K.GIDFKEDGNILGHKLEYNYNSHNVYIMADK.Q | 3 |
| \* | pDK268\_112012\_01.03880.03880.1 | 2.0059 | 0.3499 | 100.0% | 982.55 | 983.06903 | 88 | 5.867 | 50.0% | 1 | K.EDGNILGHK.L | 1 |
| \* | pDK268\_112012\_02.06677.06677.3 | 4.5591 | 0.3963 | 100.0% | 2938.9744 | 2939.2288 | 1 | 6.642 | 30.2% | 2 | K.EDGNILGHKLEYNYNSHNVYIMADK.Q | 3 |
| \* | pDK268\_112012\_01.08066.08066.2 | 4.9406 | 0.5442 | 100.0% | 1974.4321 | 1975.1829 | 1 | 8.95 | 66.7% | 10 | K.LEYNYNSHNVYIMADK.Q | 2 |
| \* | pDK268\_112012\_02.06153.06153.3 | 3.5113 | 0.3791 | 100.0% | 1975.8844 | 1975.1829 | 27 | 6.334 | 38.3% | 6 | K.LEYNYNSHNVYIMADK.Q | 3 |
| \* | pDK268\_112012\_02.05445.05445.3 | 4.6412 | 0.4649 | 100.0% | 2231.0044 | 2231.4875 | 1 | 7.039 | 42.6% | 2 | K.LEYNYNSHNVYIMADKQK.N | 3 |
| \* | pDK268\_112012\_01.04851.04851.2 | 2.0766 | 0.2346 | 98.7% | 919.89215 | 920.09955 | 293 | 4.375 | 64.3% | 1 | K.NGIKVNFK.I | 2 |
| \* | pDK268\_112012\_01.18648.18648.2 | 2.3846 | 0.1414 | 95.6% | 2598.3323 | 2599.844 | 5 | 3.021 | 35.7% | 1 | R.DHMVLLEFVTAAGIT#LGMDELY@.- | 2 |

---

|  |  |  |  |  |  |  |  |  |
| --- | --- | --- | --- | --- | --- | --- | --- | --- |
| U | *gi|106775678|ref|NP\_0* | 8 | 20 | 57.7% | 130 | 14095 | 10.9 | histone cluster 2, H2aa4 [Homo sapiens] |
| U | *gi|4504251|ref|NP\_003* | 8 | 20 | 57.7% | 130 | 14095 | 10.9 | histone cluster 2, H2aa3 [Homo sapiens] |
| U | *gi|24638446|ref|NP\_00* | 8 | 20 | 58.1% | 129 | 13988 | 10.9 | histone cluster 2, H2ac [Homo sapiens] |

| Filename XCorr DeltCN Conf% ObsM+H+ CalcM+H+ SpR ZScore Ion% # Sequence  | | | | | | | | | | | | |
| --- | --- | --- | --- | --- | --- | --- | --- | --- | --- | --- | --- | --- |
|  | pDK268\_112012\_01.08294.08294.2 | 3.1381 | 0.3918 | 100.0% | 944.65216 | 945.1093 | 1 | 6.245 | 87.5% | 3 | R.AGLQFPVGR.V | 22222 |
|  | pDK268\_112012\_02.15027.15027.3 | 4.814 | 0.4628 | 100.0% | 2935.1042 | 2935.4082 | 1 | 8.183 | 31.2% | 2 | R.VGAGAPVYMAAVLEYLTAEILELAGNAAR.D | 3 |
|  | pDK268\_112012\_01.05180.05180.2 | 2.5905 | 0.2897 | 100.0% | 851.1922 | 851.0396 | 1 | 4.986 | 100.0% | 3 | R.HLQLAIR.N | 2222 |
|  | pDK268\_112012\_02.04950.04950.2 | 3.7402 | 0.4162 | 100.0% | 1693.0322 | 1693.9004 | 1 | 7.199 | 73.1% | 1 | R.HLQLAIRNDEELNK.L | 222 |
|  | pDK268\_112012\_02.04390.04390.3 | 2.6407 | 0.3436 | 100.0% | 1694.2444 | 1693.9004 | 3 | 5.163 | 40.4% | 1 | R.HLQLAIRNDEELNK.L | 333 |
|  | pDK268\_112012\_01.08084.08084.2 | 3.5907 | 0.3841 | 100.0% | 1273.1122 | 1273.4288 | 1 | 6.089 | 70.0% | 2 | R.NDEELNKLLGK.V | 22 |
|  | pDK268\_112012\_01.13778.13778.2 | 5.0117 | 0.5581 | 100.0% | 1931.8121 | 1932.3573 | 1 | 8.7 | 69.4% | 6 | K.VTIAQGGVLPNIQAVLLPK.K | 22 |
|  | pDK268\_112012\_01.13689.13689.3 | 4.6069 | 0.4764 | 100.0% | 1932.1144 | 1932.3573 | 1 | 8.262 | 51.4% | 2 | K.VTIAQGGVLPNIQAVLLPK.K | 33 |

Similarities:
gi|10800130|ref|NP\_06(7:1)  
gi|4504253|ref|NP\_002(4:4)  
gi|28195394|ref|NP\_77(1:7)  
gi|4504255|ref|NP\_002(2:6)  

---

|  |  |  |  |  |  |  |  |  |
| --- | --- | --- | --- | --- | --- | --- | --- | --- |
| U | *gi|40354195|ref|NP\_95* | 23 | 44 | 56.7% | 430 | 48058 | 5.5 | keratin 18 [Homo sapiens] |
| U | *gi|4557888|ref|NP\_000* | 23 | 44 | 56.7% | 430 | 48058 | 5.5 | keratin 18 [Homo sapiens] |

| Filename XCorr DeltCN Conf% ObsM+H+ CalcM+H+ SpR ZScore Ion% # Sequence  | | | | | | | | | | | | |
| --- | --- | --- | --- | --- | --- | --- | --- | --- | --- | --- | --- | --- |
|  | pDK268\_112012\_02.05795.05795.3 | 5.2466 | 0.5785 | 100.0% | 2856.0842 | 2856.0813 | 1 | 10.409 | 35.8% | 3 | R.SLGSVQAPSYGARPVSSAASVYAGAGGSGSR.I | 3 |
|  | pDK268\_112012\_02.08230.08230.2 | 5.127 | 0.5884 | 100.0% | 2261.5322 | 2262.561 | 1 | 10.089 | 54.0% | 2 | R.GGMGSGGLATGIAGGLAGMGGIQNEK.E | 2 |
|  | pDK268\_112012\_02.08271.08271.3 | 4.5694 | 0.287 | 100.0% | 2262.3843 | 2262.561 | 1 | 6.427 | 37.0% | 1 | R.GGMGSGGLATGIAGGLAGMGGIQNEK.E | 3 |
|  | pDK268\_112012\_01.11603.11603.3 | 5.5151 | 0.5326 | 100.0% | 3335.9944 | 3337.7224 | 1 | 8.833 | 25.7% | 2 | R.GGMGSGGLATGIAGGLAGMGGIQNEKETMQSLNDR.L | 3 |
|  | pDK268\_112012\_01.04216.04216.2 | 2.0433 | 0.3051 | 99.3% | 1093.8121 | 1094.1846 | 81 | 5.422 | 62.5% | 1 | K.ETMQSLNDR.L | 2 |
|  | pDK268\_112012\_01.06518.06518.2 | 3.6186 | 0.4366 | 100.0% | 1320.4122 | 1320.4478 | 1 | 8.721 | 81.8% | 2 | R.AQIFANTVDNAR.I | 2 |
|  | pDK268\_112012\_01.07130.07130.2 | 2.915 | 0.1721 | 99.8% | 1042.0721 | 1042.2235 | 2 | 5.963 | 87.5% | 2 | R.IVLQIDNAR.L | 2 |
|  | pDK268\_112012\_01.05343.05343.2 | 2.1162 | 0.2707 | 99.6% | 809.1722 | 807.8815 | 1 | 5.726 | 83.3% | 1 | R.LAADDFR.V | 2222 |
|  | pDK268\_112012\_01.05541.05541.2 | 2.8029 | 0.3036 | 100.0% | 1239.9722 | 1240.4601 | 2 | 5.899 | 77.8% | 2 | R.VKYETELAMR.Q | 2 |
|  | pDK268\_112012\_02.04233.04233.3 | 2.3569 | 0.2552 | 97.9% | 1241.6943 | 1240.4601 | 48 | 4.636 | 44.4% | 1 | R.VKYETELAMR.Q | 3 |
|  | pDK268\_112012\_01.04254.04254.2 | 2.4437 | 0.0712 | 96.0% | 1175.2522 | 1175.3274 | 27 | 4.349 | 61.1% | 1 | R.KVIDDTNITR.L | 2 |
|  | pDK268\_112012\_02.11603.11603.3 | 3.1166 | 0.362 | 100.0% | 2178.6543 | 2178.589 | 6 | 5.059 | 36.8% | 2 | R.LQLETEIEALKEELLFMK.K | 3 |
|  | pDK268\_112012\_01.15609.15609.2 | 6.0619 | 0.5023 | 100.0% | 2178.7122 | 2178.589 | 1 | 9.537 | 55.9% | 3 | R.LQLETEIEALKEELLFMK.K | 2 |
|  | pDK268\_112012\_02.07230.07230.2 | 4.0951 | 0.5463 | 100.0% | 1884.1322 | 1885.1246 | 1 | 9.081 | 61.1% | 1 | K.GLQAQIASSGLTVEVDAPK.S | 2 |
|  | pDK268\_112012\_02.08142.08142.2 | 3.4326 | 0.4901 | 100.0% | 1507.3722 | 1507.699 | 1 | 8.901 | 70.8% | 3 | R.TVQSLEIDLDSMR.N | 2 |
|  | pDK268\_112012\_01.04958.04958.2 | 2.6583 | 0.2414 | 99.9% | 889.4522 | 889.9841 | 4 | 5.03 | 85.7% | 1 | K.ASLENSLR.E | 2 |
|  | pDK268\_112012\_01.16481.16481.2 | 2.2993 | 0.3794 | 99.8% | 2670.8523 | 2672.0715 | 1 | 8.517 | 47.7% | 1 | R.YALQMEQLNGILLHLESELAQTR.A | 2 |
|  | pDK268\_112012\_01.16526.16526.3 | 6.278 | 0.5176 | 100.0% | 2672.4543 | 2672.0715 | 1 | 8.966 | 47.7% | 8 | R.YALQMEQLNGILLHLESELAQTR.A | 3 |
|  | pDK268\_112012\_01.10275.10275.2 | 3.1556 | 0.3966 | 100.0% | 1420.1522 | 1420.6055 | 2 | 7.052 | 68.2% | 2 | R.QAQEYEALLNIK.V | 2 |
|  | pDK268\_112012\_02.05579.05579.2 | 2.7646 | 0.3114 | 99.9% | 1294.6721 | 1293.5059 | 1 | 5.369 | 75.0% | 2 | K.VKLEAEIATYR.R | 2 |
|  | pDK268\_112012\_02.08068.08068.3 | 4.3364 | 0.4004 | 100.0% | 2897.5144 | 2898.128 | 1 | 6.892 | 29.0% | 1 | R.RLLEDGEDFNLGDALDSSNSMQTIQK.T | 3 |
|  | pDK268\_112012\_02.08650.08650.3 | 3.7483 | 0.4113 | 100.0% | 2740.2544 | 2741.9404 | 1 | 6.407 | 28.1% | 1 | R.LLEDGEDFNLGDALDSSNSMQTIQK.T | 3 |
|  | pDK268\_112012\_01.12060.12060.2 | 2.7203 | 0.5867 | 100.0% | 2741.612 | 2741.9404 | 1 | 10.567 | 47.9% | 1 | R.LLEDGEDFNLGDALDSSNSMQTIQK.T | 2 |

Similarities:
contaminant\_KERATIN03(1:22)  
gi|4557701|ref|NP\_000(1:22)  
gi|15431310|ref|NP\_00(1:22)  

---

|  |  |  |  |  |  |  |  |  |
| --- | --- | --- | --- | --- | --- | --- | --- | --- |
| U | *gi|4504919|ref|NP\_002* | 37 | 59 | 56.1% | 483 | 53704 | 5.6 | keratin 8 [Homo sapiens] |

| Filename XCorr DeltCN Conf% ObsM+H+ CalcM+H+ SpR ZScore Ion% # Sequence  | | | | | | | | | | | | |
| --- | --- | --- | --- | --- | --- | --- | --- | --- | --- | --- | --- | --- |
|  | pDK268\_112012\_01.07394.07394.2 | 2.6451 | 0.2517 | 99.9% | 827.9922 | 827.95544 | 6 | 5.29 | 91.7% | 2 | K.FASFIDK.V | 222 |
|  | pDK268\_112012\_01.07551.07551.2 | 2.566 | 0.1807 | 99.6% | 1083.1921 | 1083.2755 | 6 | 5.872 | 75.0% | 1 | K.FASFIDKVR.F | 222 |
|  | pDK268\_112012\_01.08331.08331.2 | 2.8844 | 0.1325 | 99.8% | 1031.2522 | 1031.1997 | 3 | 3.904 | 92.9% | 1 | K.WSLLQQQK.T | 2 |
|  | pDK268\_112012\_01.13658.13658.2 | 4.658 | 0.4978 | 100.0% | 1848.5122 | 1849.0431 | 1 | 8.084 | 71.4% | 1 | R.SNMDNMFESYINNLR.R | 2 |
|  | pDK268\_112012\_01.12656.12656.3 | 2.4282 | 0.2909 | 98.3% | 2005.3143 | 2005.2306 | 5 | 5.066 | 30.0% | 1 | R.SNMDNMFESYINNLRR.Q | 3 |
|  | pDK268\_112012\_02.09579.09579.2 | 6.0606 | 0.3096 | 100.0% | 2034.0122 | 2035.363 | 1 | 10.859 | 73.5% | 1 | K.LKLEAELGNMQGLVEDFK.N | 2 |
|  | pDK268\_112012\_02.09573.09573.3 | 4.9855 | 0.3244 | 100.0% | 2037.0543 | 2035.363 | 1 | 6.76 | 42.6% | 2 | K.LKLEAELGNMQGLVEDFK.N | 3 |
|  | pDK268\_112012\_01.12905.12905.2 | 3.2753 | 0.2421 | 99.9% | 1792.9122 | 1794.0295 | 1 | 7.359 | 53.3% | 1 | K.LEAELGNMQGLVEDFK.N | 2 |
|  | pDK268\_112012\_01.03894.03894.2 | 3.0681 | 0.3022 | 100.0% | 1309.1322 | 1309.4215 | 35 | 5.692 | 61.1% | 1 | K.NKYEDEINKR.T | 222 |
|  | pDK268\_112012\_01.03471.03471.2 | 2.0898 | 0.2063 | 98.2% | 1067.0922 | 1067.1436 | 79 | 4.956 | 57.1% | 1 | K.YEDEINKR.T | 2222 |
|  | pDK268\_112012\_02.07765.07765.2 | 2.8987 | 0.2732 | 99.9% | 1354.1721 | 1353.5732 | 2 | 5.497 | 65.0% | 2 | R.TEMENEFVLIK.K | 2 |
|  | pDK268\_112012\_01.08574.08574.2 | 3.1915 | 0.2639 | 99.9% | 1481.1122 | 1481.7473 | 1 | 5.883 | 68.2% | 1 | R.TEMENEFVLIKK.D | 2 |
|  | pDK268\_112012\_02.06468.06468.3 | 2.3008 | 0.3811 | 100.0% | 1481.3043 | 1481.7473 | 1 | 6.08 | 47.7% | 1 | R.TEMENEFVLIKK.D | 3 |
|  | pDK268\_112012\_02.05200.05200.3 | 2.9122 | 0.3475 | 100.0% | 1926.1444 | 1927.1365 | 5 | 5.674 | 28.3% | 1 | K.KDVDEAYMNKVELESR.L | 3 |
|  | pDK268\_112012\_01.07896.07896.2 | 4.3191 | 0.4873 | 100.0% | 1798.9321 | 1798.9623 | 1 | 8.364 | 75.0% | 2 | K.DVDEAYMNKVELESR.L | 2 |
|  | pDK268\_112012\_01.12417.12417.2 | 3.88 | 0.4829 | 100.0% | 1420.1721 | 1420.6055 | 1 | 9.057 | 90.9% | 2 | R.LEGLTDEINFLR.Q | 2 |
|  | pDK268\_112012\_02.04205.04205.2 | 2.0618 | 0.1819 | 97.2% | 1079.9722 | 1080.1827 | 8 | 4.035 | 71.4% | 1 | R.QLYEEEIR.E | 2 |
|  | pDK268\_112012\_02.07157.07157.2 | 3.773 | 0.4946 | 100.0% | 2109.0923 | 2110.3008 | 1 | 8.547 | 50.0% | 1 | R.ELQSQISDTSVVLSMDNSR.S | 2 |
|  | pDK268\_112012\_01.12176.12176.2 | 4.5824 | 0.451 | 100.0% | 1321.2122 | 1321.5286 | 1 | 8.188 | 81.8% | 3 | R.SLDMDSIIAEVK.A | 2 |
|  | pDK268\_112012\_01.04608.04608.1 | 2.1364 | 0.3359 | 100.0% | 1079.56 | 1080.1423 | 7 | 5.351 | 62.5% | 1 | K.AQYEDIANR.S | 1 |
|  | pDK268\_112012\_01.04583.04583.2 | 2.832 | 0.3202 | 100.0% | 1080.0521 | 1080.1423 | 1 | 6.214 | 81.2% | 2 | K.AQYEDIANR.S | 2 |
|  | pDK268\_112012\_02.06982.06982.3 | 3.7994 | 0.3357 | 100.0% | 2531.9043 | 2532.828 | 1 | 5.544 | 29.8% | 1 | R.SRAEAESMYQIKYEELQSLAGK.H | 3 |
|  | pDK268\_112012\_01.05974.05974.2 | 3.0037 | 0.4677 | 100.0% | 1169.5922 | 1170.3228 | 3 | 7.279 | 77.8% | 3 | R.AEAESMYQIK.Y | 2 |
|  | pDK268\_112012\_02.04602.04602.2 | 3.619 | 0.0861 | 99.9% | 1137.9321 | 1138.2627 | 1 | 7.484 | 83.3% | 4 | K.YEELQSLAGK.H | 2 |
|  | pDK268\_112012\_01.03800.03800.2 | 3.229 | 0.4761 | 100.0% | 1209.1921 | 1209.36 | 1 | 7.546 | 88.9% | 1 | R.TKTEISEMNR.N | 2 |
|  | pDK268\_112012\_01.06784.06784.2 | 2.4225 | 0.2086 | 99.5% | 1000.9922 | 1001.168 | 57 | 5.002 | 75.0% | 1 | R.LQAEIEGLK.G | 2 |
|  | pDK268\_112012\_01.05284.05284.2 | 3.2892 | 0.2668 | 100.0% | 1342.3121 | 1342.5381 | 1 | 7.296 | 81.8% | 1 | R.LQAEIEGLKGQR.A | 2 |
|  | pDK268\_112012\_02.07026.07026.2 | 3.7668 | 0.3964 | 100.0% | 1345.3322 | 1345.452 | 1 | 6.992 | 66.7% | 3 | R.ASLEAAIADAEQR.G | 2 |
|  | pDK268\_112012\_01.11928.11928.2 | 4.8193 | 0.4645 | 100.0% | 1956.4922 | 1957.1912 | 1 | 7.938 | 58.3% | 2 | R.ASLEAAIADAEQRGELAIK.D | 2 |
|  | pDK268\_112012\_01.11922.11922.3 | 4.0381 | 0.4104 | 100.0% | 1958.0643 | 1957.1912 | 1 | 6.813 | 45.8% | 2 | R.ASLEAAIADAEQRGELAIK.D | 3 |
|  | pDK268\_112012\_01.12413.12413.3 | 4.296 | 0.4428 | 100.0% | 2456.2744 | 2456.7153 | 1 | 7.236 | 30.4% | 1 | R.ASLEAAIADAEQRGELAIKDANAK.L | 3 |
|  | pDK268\_112012\_01.08576.08576.2 | 4.2114 | 0.2266 | 100.0% | 1130.2122 | 1130.2865 | 1 | 6.07 | 88.9% | 3 | K.LSELEAALQR.A | 2 |
|  | pDK268\_112012\_01.07060.07060.2 | 1.8669 | 0.2273 | 96.3% | 1153.9922 | 1154.3234 | 81 | 5.081 | 50.0% | 1 | R.EYQELMNVK.L | 22 |
|  | pDK268\_112012\_02.07212.07212.2 | 2.5836 | 0.3357 | 99.9% | 1407.2122 | 1406.6653 | 3 | 5.468 | 59.1% | 1 | K.LALDIEIATYRK.L | 2 |
|  | pDK268\_112012\_01.05133.05133.2 | 4.2467 | 0.537 | 100.0% | 1476.3121 | 1476.7058 | 1 | 8.747 | 87.5% | 1 | R.LESGMQNMSIHTK.T | 2 |
|  | pDK268\_112012\_02.03994.03994.3 | 2.8388 | 0.2353 | 98.7% | 1477.7344 | 1476.7058 | 13 | 4.349 | 39.6% | 1 | R.LESGMQNMSIHTK.T | 3 |
|  | pDK268\_112012\_01.06152.06152.2 | 3.3327 | 0.4244 | 100.0% | 1174.1522 | 1174.3367 | 1 | 7.066 | 80.0% | 4 | K.LVSESSDVLPK.- | 2 |

Similarities:
gi|119395750|ref|NP\_0(2:35)  
gi|119703753|ref|NP\_0(5:32)  
gi|47132620|ref|NP\_00(3:34)  

---

|  |  |  |  |  |  |  |  |  |
| --- | --- | --- | --- | --- | --- | --- | --- | --- |
| U | *gi|4506687|ref|NP\_001* | 4 | 12 | 55.2% | 145 | 17040 | 10.4 | ribosomal protein S15 [Homo sapiens] |

| Filename XCorr DeltCN Conf% ObsM+H+ CalcM+H+ SpR ZScore Ion% # Sequence  | | | | | | | | | | | | |
| --- | --- | --- | --- | --- | --- | --- | --- | --- | --- | --- | --- | --- |
|  | pDK268\_112012\_01.18117.18117.2 | 5.3742 | 0.543 | 100.0% | 2588.6921 | 2589.938 | 1 | 11.254 | 54.8% | 6 | R.GVDLDQLLDMSYEQLMQLYSAR.Q | 2 |
|  | pDK268\_112012\_01.04523.04523.2 | 2.1697 | 0.3799 | 99.8% | 1354.1721 | 1354.6044 | 6 | 5.335 | 63.6% | 1 | K.EAPPMEKPEVVK.T | 2 |
| \* | pDK268\_112012\_01.15404.15404.2 | 4.8521 | 0.5698 | 100.0% | 2054.5522 | 2054.4856 | 1 | 10.269 | 61.1% | 3 | R.DMIILPEMVGSMVGVYNGK.T | 2 |
| \* | pDK268\_112012\_02.09237.09237.3 | 6.1403 | 0.468 | 100.0% | 3168.8943 | 3170.6936 | 1 | 6.724 | 34.6% | 2 | K.TFNQVEIKPEMIGHYLGEFSITYKPVK.H | 32 |

---

|  |  |  |  |  |  |  |  |  |
| --- | --- | --- | --- | --- | --- | --- | --- | --- |
| U | *gi|119395750|ref|NP\_0* | 50 | 99 | 54.5% | 644 | 66039 | 8.1 | keratin 1 [Homo sapiens] |

| Filename XCorr DeltCN Conf% ObsM+H+ CalcM+H+ SpR ZScore Ion% # Sequence  | | | | | | | | | | | | |
| --- | --- | --- | --- | --- | --- | --- | --- | --- | --- | --- | --- | --- |
| \* | pDK268\_112012\_02.05868.05868.2 | 4.1461 | 0.4768 | 100.0% | 1658.3322 | 1658.7678 | 1 | 8.244 | 62.5% | 2 | R.SGGGFSSGSAGIINYQR.R | 2 |
| \* | pDK268\_112012\_01.05883.05883.1 | 1.5182 | 0.3202 | 100.0% | 874.44 | 875.0128 | 155 | 5.249 | 43.8% | 1 | R.SLVNLGGSK.S | 1 |
| \* | pDK268\_112012\_01.05865.05865.2 | 2.1074 | 0.3003 | 99.5% | 874.65216 | 875.0128 | 13 | 5.855 | 75.0% | 1 | R.SLVNLGGSK.S | 2 |
| \* | pDK268\_112012\_01.05805.05805.2 | 1.9328 | 0.1759 | 95.8% | 832.7522 | 832.9755 | 1 | 4.822 | 78.6% | 1 | K.SISISVAR.G | 2 |
|  | pDK268\_112012\_01.10652.10652.2 | 3.909 | 0.4534 | 100.0% | 1384.3121 | 1384.5315 | 1 | 7.583 | 81.8% | 2 | K.SLNNQFASFIDK.V | 2 |
|  | pDK268\_112012\_01.11304.11304.2 | 3.6978 | 0.4303 | 100.0% | 1639.3121 | 1639.8516 | 1 | 7.091 | 73.1% | 1 | K.SLNNQFASFIDKVR.F | 2 |
|  | pDK268\_112012\_01.11354.11354.3 | 2.3778 | 0.355 | 99.8% | 1640.0944 | 1639.8516 | 1 | 5.699 | 48.1% | 1 | K.SLNNQFASFIDKVR.F | 3 |
|  | pDK268\_112012\_01.06680.06680.2 | 4.3567 | 0.085 | 100.0% | 1476.3121 | 1476.6726 | 1 | 7.301 | 90.9% | 4 | R.FLEQQNQVLQTK.W | 22 |
|  | pDK268\_112012\_01.10443.10443.2 | 4.5499 | 0.4315 | 100.0% | 1477.6322 | 1476.6293 | 1 | 8.011 | 77.3% | 6 | K.WELLQQVDTSTR.T | 2 |
|  | pDK268\_112012\_01.13718.13718.2 | 4.709 | 0.523 | 100.0% | 1993.8522 | 1995.2017 | 1 | 9.032 | 80.0% | 3 | R.THNLEPYFESFINNLR.R | 2 |
|  | pDK268\_112012\_01.13694.13694.3 | 4.7419 | 0.3787 | 100.0% | 1995.5044 | 1995.2017 | 1 | 6.48 | 48.3% | 3 | R.THNLEPYFESFINNLR.R | 3 |
|  | pDK268\_112012\_01.12894.12894.2 | 3.1692 | 0.3901 | 100.0% | 2150.2922 | 2151.3892 | 4 | 6.29 | 40.6% | 1 | R.THNLEPYFESFINNLRR.R | 2 |
|  | pDK268\_112012\_01.12987.12987.3 | 3.0254 | 0.2811 | 99.7% | 2151.2043 | 2151.3892 | 33 | 4.767 | 26.6% | 2 | R.THNLEPYFESFINNLRR.R | 3 |
|  | pDK268\_112012\_01.08258.08258.2 | 3.5549 | 0.4499 | 100.0% | 1300.9122 | 1301.4316 | 2 | 7.652 | 72.2% | 3 | K.NMQDMVEDYR.N | 2 |
|  | pDK268\_112012\_01.03894.03894.2 | 3.0681 | 0.3022 | 100.0% | 1309.1322 | 1309.4215 | 35 | 5.692 | 61.1% | 1 | R.NKYEDEINKR.T | 222 |
|  | pDK268\_112012\_01.03471.03471.2 | 2.0898 | 0.2063 | 98.2% | 1067.0922 | 1067.1436 | 79 | 4.956 | 57.1% | 1 | K.YEDEINKR.T | 2222 |
|  | pDK268\_112012\_01.07265.07265.2 | 2.9679 | 0.3216 | 100.0% | 1266.3922 | 1266.3934 | 12 | 6.436 | 60.0% | 2 | R.TNAENEFVTIK.K | 2 |
|  | pDK268\_112012\_01.05423.05423.2 | 3.5366 | 0.3645 | 100.0% | 1394.1921 | 1394.5675 | 1 | 7.22 | 68.2% | 2 | R.TNAENEFVTIKK.D | 2 |
|  | pDK268\_112012\_01.05408.05408.3 | 2.6999 | 0.247 | 98.8% | 1394.7544 | 1394.5675 | 2 | 4.841 | 40.9% | 2 | R.TNAENEFVTIKK.D | 3 |
|  | pDK268\_112012\_01.04746.04746.1 | 1.992 | 0.318 | 100.0% | 999.28 | 1000.1114 | 1 | 6.774 | 62.5% | 1 | K.DVDGAYMTK.V | 1 |
| \* | pDK268\_112012\_01.13354.13354.2 | 4.7952 | 0.4775 | 100.0% | 1303.4722 | 1303.4955 | 1 | 8.842 | 86.4% | 3 | R.SLDLDSIIAEVK.A | 2 |
| \* | pDK268\_112012\_02.03414.03414.2 | 2.9029 | 0.3317 | 100.0% | 1065.6122 | 1066.1558 | 8 | 6.464 | 62.5% | 2 | K.AQYEDIAQK.S | 2 |
|  | pDK268\_112012\_01.03982.03982.2 | 4.448 | 0.4479 | 100.0% | 1341.1322 | 1341.4607 | 1 | 7.847 | 77.3% | 1 | K.SKAEAESLYQSK.Y | 2 |
|  | pDK268\_112012\_02.03315.03315.3 | 3.0189 | 0.21 | 98.8% | 1342.1344 | 1341.4607 | 5 | 4.267 | 43.2% | 1 | K.SKAEAESLYQSK.Y | 3 |
|  | pDK268\_112012\_01.04450.04450.2 | 3.3589 | 0.4267 | 100.0% | 1126.0322 | 1126.2084 | 1 | 8.098 | 83.3% | 1 | K.AEAESLYQSK.Y | 2 |
|  | pDK268\_112012\_01.06993.06993.1 | 2.5343 | 0.2346 | 98.3% | 1179.47 | 1180.303 | 3 | 5.963 | 61.1% | 1 | K.YEELQITAGR.H | 11 |
|  | pDK268\_112012\_01.07010.07010.2 | 3.945 | 0.3324 | 100.0% | 1180.1721 | 1180.303 | 1 | 7.576 | 88.9% | 9 | K.YEELQITAGR.H | 22 |
|  | pDK268\_112012\_01.06672.06672.1 | 2.0483 | 0.3304 | 100.0% | 973.49 | 974.102 | 1 | 5.096 | 78.6% | 1 | K.IEISELNR.V | 11 |
|  | pDK268\_112012\_01.06710.06710.2 | 3.0524 | 0.1862 | 99.9% | 974.09216 | 974.102 | 1 | 5.272 | 92.9% | 3 | K.IEISELNR.V | 22 |
|  | pDK268\_112012\_01.03501.03501.2 | 2.8939 | 0.2472 | 99.9% | 1202.2322 | 1202.3964 | 2 | 4.626 | 72.2% | 1 | R.LRSEIDNVKK.Q | 2 |
|  | pDK268\_112012\_01.08114.08114.2 | 4.5199 | 0.3873 | 100.0% | 1717.4922 | 1717.8333 | 1 | 7.959 | 57.1% | 2 | K.QISNLQQSISDAEQR.G | 2 |
|  | pDK268\_112012\_01.08136.08136.3 | 3.0461 | 0.2873 | 99.9% | 1718.1843 | 1717.8333 | 138 | 4.922 | 33.9% | 1 | K.QISNLQQSISDAEQR.G | 3 |
|  | pDK268\_112012\_01.10797.10797.3 | 3.1427 | 0.4082 | 100.0% | 2329.6443 | 2330.5168 | 14 | 5.57 | 28.8% | 1 | K.QISNLQQSISDAEQRGENALK.D | 3 |
|  | pDK268\_112012\_01.11043.11043.3 | 3.3163 | 0.3484 | 100.0% | 2644.7344 | 2644.8582 | 1 | 5.907 | 31.5% | 1 | K.QISNLQQSISDAEQRGENALKDAK.N | 3 |
|  | pDK268\_112012\_02.06258.06258.3 | 3.7362 | 0.3026 | 100.0% | 1600.9143 | 1600.769 | 1 | 5.373 | 51.9% | 3 | K.NKLNDLEDALQQAK.E | 3 |
|  | pDK268\_112012\_01.09506.09506.2 | 4.8705 | 0.409 | 100.0% | 1601.3322 | 1600.769 | 1 | 7.869 | 80.8% | 2 | K.NKLNDLEDALQQAK.E | 2 |
| \* | pDK268\_112012\_02.08385.08385.3 | 5.3729 | 0.4966 | 100.0% | 2184.7744 | 2185.399 | 1 | 7.624 | 47.2% | 3 | K.NKLNDLEDALQQAKEDLAR.L | 3 |
|  | pDK268\_112012\_01.09110.09110.2 | 3.2058 | 0.2957 | 100.0% | 1357.5521 | 1358.4912 | 2 | 5.036 | 77.3% | 2 | K.LNDLEDALQQAK.E | 2 |
| \* | pDK268\_112012\_01.12546.12546.2 | 4.6452 | 0.5103 | 100.0% | 1942.2922 | 1943.121 | 1 | 8.095 | 65.6% | 1 | K.LNDLEDALQQAKEDLAR.L | 2 |
| \* | pDK268\_112012\_01.12555.12555.3 | 3.5215 | 0.2408 | 99.8% | 1944.2644 | 1943.121 | 1 | 5.594 | 43.8% | 1 | K.LNDLEDALQQAKEDLAR.L | 32 |
|  | pDK268\_112012\_01.08132.08132.3 | 3.5218 | 0.3331 | 100.0% | 1524.6843 | 1524.7754 | 1 | 5.871 | 52.3% | 2 | R.LLRDYQELMNTK.L | 3 |
|  | pDK268\_112012\_01.08120.08120.2 | 3.7224 | 0.2403 | 100.0% | 1525.4922 | 1524.7754 | 1 | 5.261 | 72.7% | 2 | R.LLRDYQELMNTK.L | 2 |
|  | pDK268\_112012\_01.07032.07032.1 | 2.5204 | 0.2179 | 98.5% | 1141.42 | 1142.2689 | 25 | 6.808 | 62.5% | 1 | R.DYQELMNTK.L | 1 |
|  | pDK268\_112012\_02.04553.04553.2 | 2.7087 | 0.3546 | 100.0% | 1142.0721 | 1142.2689 | 14 | 7.403 | 62.5% | 3 | R.DYQELMNTK.L | 2 |
|  | pDK268\_112012\_01.04847.04847.1 | 2.1324 | 0.3446 | 100.0% | 1033.41 | 1034.1112 | 1 | 5.756 | 62.5% | 1 | R.TLLEGEESR.M | 1 |
|  | pDK268\_112012\_01.04842.04842.2 | 2.2414 | 0.2303 | 99.2% | 1034.1921 | 1034.1112 | 2 | 4.496 | 75.0% | 3 | R.TLLEGEESR.M | 2 |
| \* | pDK268\_112012\_02.04747.04747.3 | 3.2225 | 0.3723 | 100.0% | 2648.0344 | 2646.7253 | 1 | 5.211 | 28.0% | 1 | R.MS\*GECAPNVSVSVSTSHTTISGGGSR.G | 3 |
|  | pDK268\_112012\_02.03753.03753.3 | 7.1818 | 0.497 | 100.0% | 2384.4543 | 2385.298 | 1 | 11.385 | 40.0% | 1 | R.GGGGGGYGSGGSSYGSGGGSYGSGGGGGGGR.G | 3 |
|  | pDK268\_112012\_01.04259.04259.2 | 5.6189 | 0.5819 | 100.0% | 2384.5122 | 2385.298 | 1 | 10.666 | 43.3% | 3 | R.GGGGGGYGSGGSSYGSGGGSYGSGGGGGGGR.G | 2 |
| \* | pDK268\_112012\_02.04068.04068.3 | 6.242 | 0.2021 | 100.0% | 3314.8442 | 3314.2085 | 1 | 9.311 | 25.0% | 2 | R.GSYGSGGSSYGSGGGSYGSGGGGGGHGSYGSGSSSGGYR.G | 3 |

Similarities:
gi|4504919|ref|NP\_002(2:48)  
gi|119703753|ref|NP\_0(4:46)  
gi|47132620|ref|NP\_00(4:46)  

---

|  |  |  |  |  |  |  |  |  |
| --- | --- | --- | --- | --- | --- | --- | --- | --- |
| U | *gi|56243533|ref|NP\_07* | 7 | 13 | 53.8% | 221 | 23598 | 7.0 | stromal cell-derived factor 2-like 1 precursor [Homo sapiens] |

| Filename XCorr DeltCN Conf% ObsM+H+ CalcM+H+ SpR ZScore Ion% # Sequence  | | | | | | | | | | | | |
| --- | --- | --- | --- | --- | --- | --- | --- | --- | --- | --- | --- | --- |
| \* | pDK268\_112012\_02.05525.05525.3 | 4.5009 | 0.4431 | 100.0% | 2522.3044 | 2522.5603 | 1 | 7.793 | 31.5% | 3 | K.YGSGSGQQSVTGVEASDDANSYWR.I | 3 |
| \* | pDK268\_112012\_02.06209.06209.2 | 6.3618 | 0.586 | 100.0% | 2522.412 | 2522.5603 | 1 | 10.295 | 58.7% | 2 | K.YGSGSGQQSVTGVEASDDANSYWR.I | 2 |
| \* | pDK268\_112012\_01.11279.11279.3 | 5.4633 | 0.5042 | 100.0% | 3934.5544 | 3936.159 | 1 | 8.246 | 27.9% | 1 | K.NLHTHHFPSPLSNNQEVSAFGEDGEGDDLDLWTVR.C | 3 |
| \* | pDK268\_112012\_02.07887.07887.3 | 5.402 | 0.4368 | 100.0% | 2411.1543 | 2410.6917 | 1 | 8.294 | 38.1% | 1 | R.FQHVGTSVFLSVTGEQYGSPIR.G | 3 |
| \* | pDK268\_112012\_01.04169.04169.3 | 5.3903 | 0.4704 | 100.0% | 2032.3744 | 2032.2004 | 1 | 7.788 | 41.2% | 1 | R.GQHEVHGMPSANTHNTWK.A | 3 |
| \* | pDK268\_112012\_01.10010.10010.3 | 4.3985 | 0.4104 | 100.0% | 2128.3442 | 2128.4045 | 1 | 6.995 | 39.5% | 2 | K.AMEGIFIKPSVEPSAGHDEL.- | 3 |
| \* | pDK268\_112012\_01.09988.09988.2 | 5.1786 | 0.514 | 100.0% | 2128.4521 | 2128.4045 | 1 | 8.695 | 55.3% | 3 | K.AMEGIFIKPSVEPSAGHDEL.- | 23 |

---

|  |  |  |  |  |  |  |  |  |
| --- | --- | --- | --- | --- | --- | --- | --- | --- |
| U | *gi|29788785|ref|NP\_82* | 30 | 82 | 53.2% | 444 | 49671 | 4.9 | tubulin, beta [Homo sapiens] |

| Filename XCorr DeltCN Conf% ObsM+H+ CalcM+H+ SpR ZScore Ion% # Sequence  | | | | | | | | | | | | |
| --- | --- | --- | --- | --- | --- | --- | --- | --- | --- | --- | --- | --- |
| \* | pDK268\_112012\_02.07800.07800.3 | 6.3365 | 0.381 | 100.0% | 3102.6543 | 3104.2725 | 1 | 8.865 | 35.6% | 4 | K.FWEVISDEHGIDPTGTYHGDSDLQLDR.I | 3 |
| \* | pDK268\_112012\_01.06255.06255.2 | 3.4983 | 0.4713 | 100.0% | 1301.9922 | 1302.4265 | 1 | 8.97 | 86.4% | 4 | R.ISVYYNEATGGK.Y | 2 |
| \* | pDK268\_112012\_02.05573.05573.3 | 2.3817 | 0.3028 | 98.5% | 1817.3944 | 1818.0392 | 102 | 4.712 | 26.7% | 1 | R.ISVYYNEATGGKYVPR.A | 3 |
|  | pDK268\_112012\_01.10874.10874.2 | 4.8374 | 0.5405 | 100.0% | 1616.4321 | 1616.8701 | 1 | 8.91 | 78.6% | 6 | R.AILVDLEPGTMDSVR.S | 222 |
|  | pDK268\_112012\_01.13136.13136.3 | 7.9102 | 0.5123 | 100.0% | 2800.1643 | 2800.0647 | 1 | 9.259 | 46.0% | 5 | R.SGPFGQIFRPDNFVFGQSGAGNNWAK.G | 333 |
|  | pDK268\_112012\_01.13134.13134.2 | 4.3881 | 0.3308 | 100.0% | 2801.9321 | 2800.0647 | 3 | 5.679 | 36.0% | 1 | R.SGPFGQIFRPDNFVFGQSGAGNNWAK.G | 222 |
|  | pDK268\_112012\_01.13478.13478.2 | 7.2309 | 0.5963 | 100.0% | 1958.9321 | 1960.151 | 1 | 10.867 | 79.4% | 4 | K.GHYTEGAELVDSVLDVVR.K | 2222 |
|  | pDK268\_112012\_01.13460.13460.3 | 4.2635 | 0.3564 | 100.0% | 1960.4644 | 1960.151 | 1 | 6.094 | 41.2% | 2 | K.GHYTEGAELVDSVLDVVR.K | 3333 |
|  | pDK268\_112012\_01.12464.12464.3 | 4.2939 | 0.4255 | 100.0% | 2089.0144 | 2088.325 | 1 | 7.427 | 50.0% | 2 | K.GHYTEGAELVDSVLDVVRK.E | 3333 |
|  | pDK268\_112012\_01.12462.12462.2 | 4.7554 | 0.3831 | 100.0% | 2089.5923 | 2088.325 | 1 | 6.134 | 63.9% | 1 | K.GHYTEGAELVDSVLDVVRK.E | 2222 |
|  | pDK268\_112012\_01.03614.03614.2 | 2.9306 | 0.2373 | 100.0% | 1077.6122 | 1078.1698 | 2 | 4.833 | 85.7% | 2 | K.IREEYPDR.I | 222 |
|  | pDK268\_112012\_01.08960.08960.2 | 4.2436 | 0.39 | 100.0% | 1320.3322 | 1320.5896 | 1 | 7.358 | 81.8% | 6 | R.IMNTFSVVPSPK.V | 222 |
|  | pDK268\_112012\_01.07874.07874.2 | 2.8399 | 0.2733 | 99.9% | 1131.4722 | 1131.2767 | 1 | 4.886 | 88.9% | 5 | R.FPGQLNADLR.K | 22222 |
|  | pDK268\_112012\_01.06263.06263.3 | 2.7439 | 0.2398 | 98.9% | 1259.0643 | 1259.4508 | 21 | 5.025 | 42.5% | 1 | R.FPGQLNADLRK.L | 33333 |
|  | pDK268\_112012\_01.06137.06137.2 | 2.6599 | 0.2453 | 99.8% | 1259.1721 | 1259.4508 | 6 | 4.519 | 65.0% | 1 | R.FPGQLNADLRK.L | 22222 |
|  | pDK268\_112012\_01.08991.08991.2 | 3.3289 | 0.3674 | 100.0% | 1271.8522 | 1272.5945 | 1 | 8.084 | 80.0% | 2 | R.KLAVNMVPFPR.L | 22222 |
|  | pDK268\_112012\_01.10427.10427.1 | 2.0779 | 0.358 | 100.0% | 1143.58 | 1144.4204 | 51 | 6.116 | 55.6% | 1 | K.LAVNMVPFPR.L | 11111 |
|  | pDK268\_112012\_01.10442.10442.2 | 3.7495 | 0.4789 | 100.0% | 1144.3322 | 1144.4204 | 1 | 9.006 | 88.9% | 1 | K.LAVNMVPFPR.L | 22222 |
|  | pDK268\_112012\_01.12644.12644.3 | 3.8334 | 0.3162 | 100.0% | 1622.1843 | 1621.9403 | 1 | 5.671 | 55.8% | 2 | R.LHFFMPGFAPLTSR.G | 3333 |
|  | pDK268\_112012\_02.08489.08489.2 | 3.5612 | 0.4285 | 100.0% | 1622.4122 | 1621.9403 | 1 | 7.702 | 61.5% | 5 | R.LHFFMPGFAPLTSR.G | 2222 |
| \* | pDK268\_112012\_01.12440.12440.2 | 3.9191 | 0.5243 | 100.0% | 1660.4922 | 1660.9078 | 1 | 7.63 | 71.4% | 5 | R.ALTVPELTQQVFDAK.N | 2 |
|  | pDK268\_112012\_01.10763.10763.1 | 1.5476 | 0.4197 | 100.0% | 1039.7 | 1040.2505 | 1 | 6.004 | 68.8% | 1 | R.YLTVAAVFR.G | 11 |
|  | pDK268\_112012\_01.10821.10821.2 | 3.2186 | 0.4348 | 100.0% | 1040.2922 | 1040.2505 | 1 | 7.541 | 93.8% | 3 | R.YLTVAAVFR.G | 22 |
|  | pDK268\_112012\_01.06597.06597.2 | 4.388 | 0.253 | 100.0% | 1448.2722 | 1447.6031 | 1 | 6.034 | 81.8% | 4 | K.EVDEQMLNVQNK.N | 222 |
|  | pDK268\_112012\_01.12033.12033.2 | 3.9628 | 0.4064 | 100.0% | 1697.3322 | 1697.8877 | 1 | 8.146 | 76.9% | 2 | K.NSSYFVEWIPNNVK.T | 22222 |
| \* | pDK268\_112012\_01.14301.14301.2 | 5.5591 | 0.5527 | 100.0% | 1871.4122 | 1871.2018 | 1 | 9.973 | 75.0% | 2 | K.MAVTFIGNSTAIQELFK.R | 2 |
| \* | pDK268\_112012\_01.13287.13287.2 | 4.403 | 0.5215 | 100.0% | 2026.5521 | 2027.3893 | 1 | 8.186 | 44.1% | 1 | K.MAVTFIGNSTAIQELFKR.I | 2 |
| \* | pDK268\_112012\_01.13257.13257.3 | 3.5768 | 0.4391 | 100.0% | 2026.8844 | 2027.3893 | 1 | 7.21 | 33.8% | 2 | K.MAVTFIGNSTAIQELFKR.I | 3 |
| \* | pDK268\_112012\_01.19154.19154.3 | 3.9861 | 0.2393 | 99.8% | 3239.1843 | 3238.79 | 1 | 4.928 | 25.0% | 2 | K.MAVTFIGNSTAIQELFKRISEQFTAMFR.R | 3 |
|  | pDK268\_112012\_02.07869.07869.2 | 3.9817 | 0.4841 | 100.0% | 1230.2122 | 1230.4241 | 1 | 7.653 | 94.4% | 4 | R.ISEQFTAMFR.R | 2222 |

Similarities:
gi|5174735|ref|NP\_006(21:9)  
gi|29788768|ref|NP\_82(19:11)  
gi|14210536|ref|NP\_11(9:21)  
gi|50592996|ref|NP\_00(14:16)  

---

|  |  |  |  |  |  |  |  |  |
| --- | --- | --- | --- | --- | --- | --- | --- | --- |
| U | *gi|4504253|ref|NP\_002* | 7 | 12 | 52.4% | 143 | 15145 | 10.7 | H2A histone family, member X [Homo sapiens] |

| Filename XCorr DeltCN Conf% ObsM+H+ CalcM+H+ SpR ZScore Ion% # Sequence  | | | | | | | | | | | | |
| --- | --- | --- | --- | --- | --- | --- | --- | --- | --- | --- | --- | --- |
|  | pDK268\_112012\_01.08294.08294.2 | 3.1381 | 0.3918 | 100.0% | 944.65216 | 945.1093 | 1 | 6.245 | 87.5% | 3 | R.AGLQFPVGR.V | 22222 |
|  | pDK268\_112012\_02.15141.15141.3 | 5.54 | 0.4735 | 100.0% | 2916.9243 | 2917.3752 | 1 | 7.623 | 28.6% | 1 | R.VGAGAPVYLAAVLEYLTAEILELAGNAAR.D | 333 |
|  | pDK268\_112012\_01.20231.20231.2 | 4.4906 | 0.3206 | 100.0% | 2920.0923 | 2917.3752 | 1 | 5.892 | 41.1% | 2 | R.VGAGAPVYLAAVLEYLTAEILELAGNAAR.D | 222 |
|  | pDK268\_112012\_01.05180.05180.2 | 2.5905 | 0.2897 | 100.0% | 851.1922 | 851.0396 | 1 | 4.986 | 100.0% | 3 | R.HLQLAIR.N | 2222 |
|  | pDK268\_112012\_02.04950.04950.2 | 3.7402 | 0.4162 | 100.0% | 1693.0322 | 1693.9004 | 1 | 7.199 | 73.1% | 1 | R.HLQLAIRNDEELNK.L | 222 |
|  | pDK268\_112012\_02.04390.04390.3 | 2.6407 | 0.3436 | 100.0% | 1694.2444 | 1693.9004 | 3 | 5.163 | 40.4% | 1 | R.HLQLAIRNDEELNK.L | 333 |
|  | pDK268\_112012\_01.14816.14816.2 | 2.2465 | 0.2465 | 97.8% | 2270.7522 | 2272.78 | 1 | 5.855 | 40.9% | 1 | K.LLGGVTIAQGGVLPNIQAVLLPK.K | 22 |

Similarities:
gi|10800130|ref|NP\_06(6:1)  
gi|106775678|ref|NP\_0(4:3)  
gi|28195394|ref|NP\_77(4:3)  
gi|4504255|ref|NP\_002(2:5)  

---

|  |  |  |  |  |  |  |  |  |
| --- | --- | --- | --- | --- | --- | --- | --- | --- |
| U | *gi|28195394|ref|NP\_77* | 5 | 8 | 52.3% | 130 | 13995 | 10.9 | histone cluster 2, H2ab [Homo sapiens] |

| Filename XCorr DeltCN Conf% ObsM+H+ CalcM+H+ SpR ZScore Ion% # Sequence  | | | | | | | | | | | | |
| --- | --- | --- | --- | --- | --- | --- | --- | --- | --- | --- | --- | --- |
|  | pDK268\_112012\_01.08294.08294.2 | 3.1381 | 0.3918 | 100.0% | 944.65216 | 945.1093 | 1 | 6.245 | 87.5% | 3 | R.AGLQFPVGR.V | 22222 |
|  | pDK268\_112012\_02.15141.15141.3 | 5.54 | 0.4735 | 100.0% | 2916.9243 | 2917.3752 | 1 | 7.623 | 28.6% | 1 | R.VGAGAPVYLAAVLEYLTAEILELAGNAAR.D | 333 |
|  | pDK268\_112012\_01.20231.20231.2 | 4.4906 | 0.3206 | 100.0% | 2920.0923 | 2917.3752 | 1 | 5.892 | 41.1% | 2 | R.VGAGAPVYLAAVLEYLTAEILELAGNAAR.D | 222 |
| \* | pDK268\_112012\_01.04619.04619.2 | 2.1972 | 0.111 | 97.2% | 836.4122 | 837.01276 | 1 | 4.105 | 83.3% | 1 | R.HLQLAVR.N | 2 |
|  | pDK268\_112012\_01.14816.14816.2 | 2.2465 | 0.2465 | 97.8% | 2270.7522 | 2272.78 | 1 | 5.855 | 40.9% | 1 | K.LLGGVTIAQGGVLPNIQAVLLPK.K | 22 |

Similarities:
gi|10800130|ref|NP\_06(3:2)  
gi|106775678|ref|NP\_0(1:4)  
gi|4504253|ref|NP\_002(4:1)  
gi|4504255|ref|NP\_002(1:4)  

---

|  |  |  |  |  |  |  |  |  |
| --- | --- | --- | --- | --- | --- | --- | --- | --- |
| U | *gi|5174735|ref|NP\_006* | 26 | 64 | 52.1% | 445 | 49831 | 4.9 | tubulin, beta, 2 [Homo sapiens] |

| Filename XCorr DeltCN Conf% ObsM+H+ CalcM+H+ SpR ZScore Ion% # Sequence  | | | | | | | | | | | | |
| --- | --- | --- | --- | --- | --- | --- | --- | --- | --- | --- | --- | --- |
|  | pDK268\_112012\_01.10845.10845.3 | 4.2646 | 0.4273 | 100.0% | 3117.0244 | 3118.2996 | 1 | 6.364 | 32.7% | 1 | K.FWEVISDEHGIDPTGTYHGDSDLQLER.I | 3 |
| \* | pDK268\_112012\_01.06226.06226.2 | 3.0273 | 0.3727 | 100.0% | 1328.4122 | 1329.4521 | 1 | 6.816 | 72.7% | 3 | R.INVYYNEATGGK.Y | 2 |
|  | pDK268\_112012\_01.10353.10353.2 | 3.8574 | 0.4043 | 100.0% | 1602.1721 | 1602.8431 | 1 | 8.677 | 67.9% | 2 | R.AVLVDLEPGTMDSVR.S | 2 |
|  | pDK268\_112012\_01.13136.13136.3 | 7.9102 | 0.5123 | 100.0% | 2800.1643 | 2800.0647 | 1 | 9.259 | 46.0% | 5 | R.SGPFGQIFRPDNFVFGQSGAGNNWAK.G | 333 |
|  | pDK268\_112012\_01.13134.13134.2 | 4.3881 | 0.3308 | 100.0% | 2801.9321 | 2800.0647 | 3 | 5.679 | 36.0% | 1 | R.SGPFGQIFRPDNFVFGQSGAGNNWAK.G | 222 |
|  | pDK268\_112012\_01.13478.13478.2 | 7.2309 | 0.5963 | 100.0% | 1958.9321 | 1960.151 | 1 | 10.867 | 79.4% | 4 | K.GHYTEGAELVDSVLDVVR.K | 2222 |
|  | pDK268\_112012\_01.13460.13460.3 | 4.2635 | 0.3564 | 100.0% | 1960.4644 | 1960.151 | 1 | 6.094 | 41.2% | 2 | K.GHYTEGAELVDSVLDVVR.K | 3333 |
|  | pDK268\_112012\_01.12464.12464.3 | 4.2939 | 0.4255 | 100.0% | 2089.0144 | 2088.325 | 1 | 7.427 | 50.0% | 2 | K.GHYTEGAELVDSVLDVVRK.E | 3333 |
|  | pDK268\_112012\_01.12462.12462.2 | 4.7554 | 0.3831 | 100.0% | 2089.5923 | 2088.325 | 1 | 6.134 | 63.9% | 1 | K.GHYTEGAELVDSVLDVVRK.E | 2222 |
|  | pDK268\_112012\_01.03614.03614.2 | 2.9306 | 0.2373 | 100.0% | 1077.6122 | 1078.1698 | 2 | 4.833 | 85.7% | 2 | K.IREEYPDR.I | 222 |
|  | pDK268\_112012\_01.08960.08960.2 | 4.2436 | 0.39 | 100.0% | 1320.3322 | 1320.5896 | 1 | 7.358 | 81.8% | 6 | R.IMNTFSVVPSPK.V | 222 |
|  | pDK268\_112012\_01.07874.07874.2 | 2.8399 | 0.2733 | 99.9% | 1131.4722 | 1131.2767 | 1 | 4.886 | 88.9% | 5 | R.FPGQLNADLR.K | 22222 |
|  | pDK268\_112012\_01.06263.06263.3 | 2.7439 | 0.2398 | 98.9% | 1259.0643 | 1259.4508 | 21 | 5.025 | 42.5% | 1 | R.FPGQLNADLRK.L | 33333 |
|  | pDK268\_112012\_01.06137.06137.2 | 2.6599 | 0.2453 | 99.8% | 1259.1721 | 1259.4508 | 6 | 4.519 | 65.0% | 1 | R.FPGQLNADLRK.L | 22222 |
|  | pDK268\_112012\_01.08991.08991.2 | 3.3289 | 0.3674 | 100.0% | 1271.8522 | 1272.5945 | 1 | 8.084 | 80.0% | 2 | R.KLAVNMVPFPR.L | 22222 |
|  | pDK268\_112012\_01.10427.10427.1 | 2.0779 | 0.358 | 100.0% | 1143.58 | 1144.4204 | 51 | 6.116 | 55.6% | 1 | K.LAVNMVPFPR.L | 11111 |
|  | pDK268\_112012\_01.10442.10442.2 | 3.7495 | 0.4789 | 100.0% | 1144.3322 | 1144.4204 | 1 | 9.006 | 88.9% | 1 | K.LAVNMVPFPR.L | 22222 |
|  | pDK268\_112012\_01.12644.12644.3 | 3.8334 | 0.3162 | 100.0% | 1622.1843 | 1621.9403 | 1 | 5.671 | 55.8% | 2 | R.LHFFMPGFAPLTSR.G | 3333 |
|  | pDK268\_112012\_02.08489.08489.2 | 3.5612 | 0.4285 | 100.0% | 1622.4122 | 1621.9403 | 1 | 7.702 | 61.5% | 5 | R.LHFFMPGFAPLTSR.G | 2222 |
|  | pDK268\_112012\_01.12752.12752.2 | 3.621 | 0.4735 | 100.0% | 1692.0322 | 1692.9678 | 1 | 9.102 | 71.4% | 2 | R.ALTVPELTQQMFDAK.N | 22 |
|  | pDK268\_112012\_01.10763.10763.1 | 1.5476 | 0.4197 | 100.0% | 1039.7 | 1040.2505 | 1 | 6.004 | 68.8% | 1 | R.YLTVAAVFR.G | 11 |
|  | pDK268\_112012\_01.10821.10821.2 | 3.2186 | 0.4348 | 100.0% | 1040.2922 | 1040.2505 | 1 | 7.541 | 93.8% | 3 | R.YLTVAAVFR.G | 22 |
|  | pDK268\_112012\_01.06597.06597.2 | 4.388 | 0.253 | 100.0% | 1448.2722 | 1447.6031 | 1 | 6.034 | 81.8% | 4 | K.EVDEQMLNVQNK.N | 222 |
|  | pDK268\_112012\_01.12033.12033.2 | 3.9628 | 0.4064 | 100.0% | 1697.3322 | 1697.8877 | 1 | 8.146 | 76.9% | 2 | K.NSSYFVEWIPNNVK.T | 22222 |
|  | pDK268\_112012\_02.09277.09277.3 | 3.4682 | 0.1568 | 97.1% | 2015.4243 | 2015.335 | 1 | 6.218 | 44.1% | 1 | K.MSATFIGNSTAIQELFKR.I | 33 |
|  | pDK268\_112012\_02.07869.07869.2 | 3.9817 | 0.4841 | 100.0% | 1230.2122 | 1230.4241 | 1 | 7.653 | 94.4% | 4 | R.ISEQFTAMFR.R | 2222 |

Similarities:
gi|29788785|ref|NP\_82(21:5)  
gi|29788768|ref|NP\_82(19:7)  
gi|14210536|ref|NP\_11(9:17)  
gi|50592996|ref|NP\_00(14:12)  

---

|  |  |  |  |  |  |  |  |  |
| --- | --- | --- | --- | --- | --- | --- | --- | --- |
| U | *gi|4557469|ref|NP\_001* | 53 | 123 | 51.1% | 937 | 104553 | 5.4 | adaptor-related protein complex 2, beta 1 subunit isoform b [Homo sapiens] |
| U | *gi|71773106|ref|NP\_00* | 53 | 123 | 50.4% | 951 | 105692 | 5.3 | adaptor-related protein complex 2, beta 1 subunit isoform a [Homo sapiens] |

| Filename XCorr DeltCN Conf% ObsM+H+ CalcM+H+ SpR ZScore Ion% # Sequence  | | | | | | | | | | | | |
| --- | --- | --- | --- | --- | --- | --- | --- | --- | --- | --- | --- | --- |
|  | pDK268\_112012\_01.06543.06543.1 | 2.1833 | 0.3019 | 100.0% | 963.54 | 964.1497 | 65 | 4.574 | 57.1% | 1 | K.KGEIFELK.A | 11 |
|  | pDK268\_112012\_01.06572.06572.2 | 3.0848 | 0.1732 | 99.9% | 964.15216 | 964.1497 | 55 | 4.814 | 78.6% | 3 | K.KGEIFELK.A | 22 |
|  | pDK268\_112012\_01.02238.02238.2 | 2.2595 | 0.0851 | 96.3% | 945.71216 | 946.0483 | 21 | 3.685 | 78.6% | 1 | K.AELNNEKK.E | 2 |
|  | pDK268\_112012\_01.04486.04486.2 | 2.7867 | 0.231 | 99.8% | 1018.1722 | 1018.3031 | 1 | 5.347 | 83.3% | 1 | K.KVIAAMTVGK.D | 2 |
|  | pDK268\_112012\_01.11388.11388.2 | 3.3351 | 0.4915 | 100.0% | 1518.5521 | 1519.8854 | 1 | 8.074 | 72.7% | 1 | K.KLVYLYLMNYAK.S | 22 |
|  | pDK268\_112012\_02.09526.09526.2 | 4.3295 | 0.4217 | 100.0% | 1391.9722 | 1391.7113 | 1 | 8.836 | 85.0% | 4 | K.LVYLYLMNYAK.S | 22 |
|  | pDK268\_112012\_01.12278.12278.2 | 4.8322 | 0.4999 | 100.0% | 1638.4922 | 1638.9374 | 1 | 10.388 | 71.4% | 5 | K.SQPDMAIMAVNSFVK.D | 2 |
|  | pDK268\_112012\_01.12292.12292.3 | 3.2746 | 0.1777 | 98.1% | 1639.0144 | 1638.9374 | 40 | 4.78 | 37.5% | 1 | K.SQPDMAIMAVNSFVK.D | 3 |
|  | pDK268\_112012\_02.08493.08493.3 | 4.5245 | 0.404 | 100.0% | 2202.5044 | 2202.4468 | 1 | 7.43 | 40.3% | 3 | K.LHDINAQMVEDQGFLDSLR.D | 3 |
|  | pDK268\_112012\_01.11661.11661.2 | 6.6692 | 0.4464 | 100.0% | 2202.612 | 2202.4468 | 1 | 10.039 | 72.2% | 1 | K.LHDINAQMVEDQGFLDSLR.D | 2 |
|  | pDK268\_112012\_01.16244.16244.3 | 5.8779 | 0.4678 | 100.0% | 4231.5244 | 4230.6934 | 1 | 12.397 | 32.7% | 2 | R.DLIADSNPMVVANAVAALSEISESHPNSNLLDLNPQNINK.L | 3 |
|  | pDK268\_112012\_01.05554.05554.2 | 4.4871 | 0.4832 | 100.0% | 1396.1721 | 1396.6298 | 1 | 8.391 | 80.8% | 4 | R.LSHANSAVVLSAVK.V | 22 |
|  | pDK268\_112012\_01.05552.05552.3 | 4.0837 | 0.1741 | 99.8% | 1396.9143 | 1396.6298 | 17 | 4.663 | 46.2% | 1 | R.LSHANSAVVLSAVK.V | 33 |
|  | pDK268\_112012\_01.10364.10364.2 | 2.9709 | 0.0369 | 99.7% | 860.09216 | 860.08435 | 16 | 3.283 | 91.7% | 1 | K.FLELLPK.D | 2 |
|  | pDK268\_112012\_01.10637.10637.2 | 3.3014 | 0.2803 | 100.0% | 1262.3322 | 1262.4199 | 1 | 6.325 | 83.3% | 1 | K.DSDYYNMLLK.K | 2 |
|  | pDK268\_112012\_01.08948.08948.2 | 2.9912 | 0.2192 | 99.9% | 1390.3121 | 1390.594 | 3 | 5.261 | 65.0% | 1 | K.DSDYYNMLLKK.L | 2 |
|  | pDK268\_112012\_01.14063.14063.2 | 4.0511 | 0.4795 | 100.0% | 2393.7322 | 2394.86 | 1 | 8.011 | 52.4% | 1 | K.KLAPPLVTLLSGEPEVQYVALR.N | 2 |
|  | pDK268\_112012\_01.14003.14003.3 | 5.6316 | 0.4861 | 100.0% | 2395.7344 | 2394.86 | 1 | 9.128 | 47.6% | 2 | K.KLAPPLVTLLSGEPEVQYVALR.N | 3 |
|  | pDK268\_112012\_01.15536.15536.2 | 6.0681 | 0.6014 | 100.0% | 2265.7522 | 2266.686 | 1 | 9.934 | 70.0% | 6 | K.LAPPLVTLLSGEPEVQYVALR.N | 2 |
|  | pDK268\_112012\_01.15495.15495.3 | 4.911 | 0.4771 | 100.0% | 2267.1543 | 2266.686 | 1 | 8.779 | 42.5% | 3 | K.LAPPLVTLLSGEPEVQYVALR.N | 3 |
|  | pDK268\_112012\_01.06806.06806.1 | 2.2139 | 0.2222 | 98.6% | 941.42 | 942.1466 | 3 | 5.052 | 71.4% | 1 | R.NINLIVQK.R | 11 |
|  | pDK268\_112012\_01.06860.06860.2 | 2.8079 | 0.0413 | 98.7% | 942.1122 | 942.1466 | 8 | 4.164 | 78.6% | 3 | R.NINLIVQK.R | 22 |
|  | pDK268\_112012\_01.04442.04442.3 | 2.7284 | 0.177 | 96.7% | 1253.9644 | 1254.5155 | 1 | 4.384 | 58.3% | 1 | K.RPEILKQEIK.V | 3 |
|  | pDK268\_112012\_01.04454.04454.2 | 3.5601 | 0.3595 | 100.0% | 1254.1721 | 1254.5155 | 1 | 5.987 | 77.8% | 1 | K.RPEILKQEIK.V | 2 |
|  | pDK268\_112012\_01.06236.06236.2 | 2.6435 | 0.2908 | 99.9% | 1011.5722 | 1012.1503 | 8 | 6.08 | 85.7% | 3 | K.YNDPIYVK.L | 22 |
|  | pDK268\_112012\_01.07548.07548.2 | 2.8744 | 0.375 | 100.0% | 1382.3722 | 1382.5994 | 1 | 6.144 | 70.0% | 2 | K.YNDPIYVKLEK.L | 22 |
|  | pDK268\_112012\_01.13203.13203.2 | 4.9583 | 0.351 | 100.0% | 1570.5122 | 1569.8418 | 1 | 7.173 | 82.1% | 2 | R.LASQANIAQVLAELK.E | 22 |
|  | pDK268\_112012\_01.17144.17144.3 | 6.001 | 0.4907 | 100.0% | 2995.0444 | 2994.3716 | 1 | 9.974 | 35.6% | 3 | R.LASQANIAQVLAELKEYATEVDVDFVR.K | 33 |
|  | pDK268\_112012\_01.16204.16204.3 | 5.3306 | 0.5088 | 100.0% | 3122.0942 | 3122.5457 | 1 | 8.467 | 31.5% | 2 | R.LASQANIAQVLAELKEYATEVDVDFVRK.A | 33 |
|  | pDK268\_112012\_02.07043.07043.2 | 2.5636 | 0.4717 | 100.0% | 1442.2322 | 1443.553 | 1 | 7.0 | 63.6% | 3 | K.EYATEVDVDFVR.K | 22 |
|  | pDK268\_112012\_02.09514.09514.2 | 3.3519 | 0.5045 | 100.0% | 1509.3121 | 1509.7632 | 1 | 8.149 | 66.7% | 2 | R.AAMIWIVGEYAER.I | 22 |
|  | pDK268\_112012\_01.19592.19592.3 | 6.7162 | 0.536 | 100.0% | 3591.4443 | 3591.0063 | 1 | 9.186 | 35.5% | 3 | R.IDNADELLESFLEGFHDESTQVQLTLLTAIVK.L | 3 |
|  | pDK268\_112012\_01.14124.14124.3 | 5.1247 | 0.4762 | 100.0% | 3413.5745 | 3414.8376 | 1 | 7.857 | 31.0% | 1 | K.LFLKKPSETQELVQQVLSLATQDSDNPDLR.D | 3 |
|  | pDK268\_112012\_01.14577.14577.3 | 5.2073 | 0.4881 | 100.0% | 2912.2144 | 2913.168 | 1 | 8.397 | 33.0% | 1 | K.KPSETQELVQQVLSLATQDSDNPDLR.D | 3 |
|  | pDK268\_112012\_01.13870.13870.3 | 4.4846 | 0.3951 | 100.0% | 3182.9343 | 3184.444 | 1 | 7.508 | 32.4% | 1 | K.KPSETQELVQQVLSLATQDSDNPDLRDR.G | 3 |
|  | pDK268\_112012\_01.05330.05330.2 | 2.5284 | 0.3633 | 99.9% | 1045.0922 | 1045.2212 | 1 | 5.554 | 66.7% | 2 | R.LLSTDPVTAK.E | 2 |
|  | pDK268\_112012\_02.04941.04941.3 | 4.3222 | 0.5313 | 100.0% | 1417.7644 | 1417.6078 | 1 | 7.363 | 54.2% | 1 | K.AKGLEISGTFTHR.Q | 3 |
|  | pDK268\_112012\_02.05539.05539.2 | 2.8148 | 0.3635 | 100.0% | 1218.3121 | 1218.3549 | 2 | 6.435 | 65.0% | 5 | K.GLEISGTFTHR.Q | 2 |
|  | pDK268\_112012\_02.06161.06161.2 | 3.1038 | 0.4913 | 100.0% | 1613.3922 | 1613.8463 | 1 | 7.74 | 70.8% | 1 | R.QGHIYMEMNFTNK.A | 2 |
|  | pDK268\_112012\_02.06214.06214.3 | 4.2603 | 0.3259 | 100.0% | 1614.1444 | 1613.8463 | 1 | 5.554 | 43.8% | 4 | R.QGHIYMEMNFTNK.A | 3 |
|  | pDK268\_112012\_02.07209.07209.3 | 3.4999 | 0.3712 | 100.0% | 1664.9043 | 1664.9196 | 1 | 6.67 | 48.1% | 2 | K.ALQHMTDFAIQFNK.N | 3 |
|  | pDK268\_112012\_02.07152.07152.2 | 5.1227 | 0.4806 | 100.0% | 1665.0922 | 1664.9196 | 1 | 8.25 | 76.9% | 4 | K.ALQHMTDFAIQFNK.N | 2 |
|  | pDK268\_112012\_01.14756.14756.3 | 6.2234 | 0.4936 | 100.0% | 3901.6443 | 3901.6147 | 1 | 6.608 | 27.8% | 4 | K.NSFGVIPSTPLAIHTPLMPNQSIDVSLPLNTLGPVMK.M | 3 |
|  | pDK268\_112012\_01.08915.08915.2 | 3.29 | 0.3977 | 100.0% | 1356.2722 | 1356.6232 | 1 | 6.535 | 72.7% | 4 | K.MEPLNNLQVAVK.N | 22 |
|  | pDK268\_112012\_01.10126.10126.2 | 3.4037 | 0.4049 | 100.0% | 1587.7522 | 1588.7576 | 1 | 6.375 | 66.7% | 6 | K.DIPNENELQFQIK.E | 2 |
|  | pDK268\_112012\_01.05400.05400.2 | 3.586 | 0.3527 | 100.0% | 1279.1122 | 1278.4508 | 1 | 6.707 | 80.0% | 3 | K.LQNNNVYTIAK.R | 2 |
|  | pDK268\_112012\_01.07510.07510.2 | 4.4955 | 0.5085 | 100.0% | 1681.2722 | 1681.9042 | 1 | 7.876 | 69.2% | 1 | K.RNVEGQDMLYQSLK.L | 22 |
|  | pDK268\_112012\_01.09032.09032.2 | 4.1365 | 0.4613 | 100.0% | 1525.2122 | 1525.7168 | 1 | 8.913 | 75.0% | 4 | R.NVEGQDMLYQSLK.L | 22 |
|  | pDK268\_112012\_01.14522.14522.2 | 4.0416 | 0.4628 | 100.0% | 1398.6921 | 1399.6763 | 1 | 8.073 | 68.2% | 4 | K.LTNGIWILAELR.I | 2 |
|  | pDK268\_112012\_01.07982.07982.2 | 1.7924 | 0.3861 | 98.6% | 1445.1122 | 1445.6586 | 2 | 5.323 | 66.7% | 1 | R.IQPGNPNYTLSLK.C | 2 |
|  | pDK268\_112012\_01.15382.15382.2 | 4.4295 | 0.6579 | 100.0% | 2015.6322 | 2017.286 | 1 | 12.331 | 71.9% | 2 | R.APEVSQYIYQVYDSILK.N | 2 |
|  | pDK268\_112012\_01.15332.15332.3 | 2.7586 | 0.3449 | 100.0% | 2131.2244 | 2131.39 | 1 | 4.964 | 36.8% | 1 | R.APEVSQYIYQVYDSILKN.- | 32 |
|  | pDK268\_112012\_01.15338.15338.2 | 6.4847 | 0.5434 | 100.0% | 2131.4521 | 2131.39 | 1 | 9.31 | 70.6% | 2 | R.APEVSQYIYQVYDSILKN.- | 2 |

Similarities:
gi|22027651|ref|NP\_00(18:35)  

---

|  |  |  |  |  |  |  |  |  |
| --- | --- | --- | --- | --- | --- | --- | --- | --- |
| U | *Reverse\_gi|169172620|* | 1 | 1 | 48.9% | 47 | 5155 | 4.8 | PREDICTED: hypothetical protein [Homo sapiens] |
| U | *Reverse\_gi|169173017|* | 1 | 1 | 48.9% | 47 | 5155 | 4.8 | PREDICTED: hypothetical protein [Homo sapiens] |

| Filename XCorr DeltCN Conf% ObsM+H+ CalcM+H+ SpR ZScore Ion% # Sequence  | | | | | | | | | | | | |
| --- | --- | --- | --- | --- | --- | --- | --- | --- | --- | --- | --- | --- |
|  | pDK268\_112012\_02.11577.11577.3 | 2.8189 | 0.226 | 95.6% | 2540.0344 | 2542.503 | 215 | 4.217 | 21.6% | 1 | R.AS\*FLETGESEEDTSRNSSSSGRK.V | 3 |

---

|  |  |  |  |  |  |  |  |  |
| --- | --- | --- | --- | --- | --- | --- | --- | --- |
| U | *gi|14211889|ref|NP\_11* | 5 | 11 | 47.5% | 99 | 11250 | 4.9 | dpy-30-like protein [Homo sapiens] |

| Filename XCorr DeltCN Conf% ObsM+H+ CalcM+H+ SpR ZScore Ion% # Sequence  | | | | | | | | | | | | |
| --- | --- | --- | --- | --- | --- | --- | --- | --- | --- | --- | --- | --- |
| \* | pDK268\_112012\_01.05570.05570.2 | 2.2094 | 0.4203 | 99.9% | 1285.3922 | 1285.4862 | 153 | 6.002 | 55.0% | 1 | K.QKVDLQSLPTR.A | 2 |
| \* | pDK268\_112012\_01.19184.19184.2 | 5.3057 | 0.5972 | 100.0% | 2126.5322 | 2126.5876 | 1 | 9.846 | 71.1% | 2 | R.AYLDQTVVPILLQGLAVLAK.E | 2 |
| \* | pDK268\_112012\_01.19142.19142.3 | 6.6937 | 0.4393 | 100.0% | 2126.9343 | 2126.5876 | 1 | 9.375 | 50.0% | 3 | R.AYLDQTVVPILLQGLAVLAK.E | 3 |
| \* | pDK268\_112012\_01.16796.16796.2 | 4.6324 | 0.482 | 100.0% | 1887.9122 | 1888.2169 | 1 | 7.733 | 60.0% | 3 | K.ERPPNPIEFLASYLLK.N | 2 |
| \* | pDK268\_112012\_01.16838.16838.3 | 4.3294 | 0.2844 | 100.0% | 1888.8844 | 1888.2169 | 3 | 6.809 | 40.0% | 2 | K.ERPPNPIEFLASYLLK.N | 3 |

---

|  |  |  |  |  |  |  |  |  |
| --- | --- | --- | --- | --- | --- | --- | --- | --- |
| U | *gi|5729877|ref|NP\_006* | 35 | 65 | 47.2% | 646 | 70898 | 5.5 | heat shock 70kDa protein 8 isoform 1 [Homo sapiens] |

| Filename XCorr DeltCN Conf% ObsM+H+ CalcM+H+ SpR ZScore Ion% # Sequence  | | | | | | | | | | | | |
| --- | --- | --- | --- | --- | --- | --- | --- | --- | --- | --- | --- | --- |
|  | pDK268\_112012\_01.07984.07984.2 | 3.3856 | 0.4857 | 100.0% | 1488.2722 | 1488.5939 | 1 | 9.133 | 79.2% | 3 | R.TTPSYVAFTDTER.L | 2222 |
|  | pDK268\_112012\_01.08102.08102.2 | 4.9336 | 0.4944 | 100.0% | 1651.1322 | 1650.8468 | 1 | 9.93 | 85.7% | 3 | K.NQVAMNPTNTVFDAK.R | 2 |
|  | pDK268\_112012\_01.05854.05854.2 | 3.6081 | 0.4718 | 100.0% | 1411.1522 | 1411.5725 | 1 | 8.163 | 81.8% | 3 | R.RFDDAVVQSDMK.H | 2 |
|  | pDK268\_112012\_01.06698.06698.2 | 3.5728 | 0.4948 | 100.0% | 1255.8722 | 1255.385 | 1 | 8.98 | 90.0% | 2 | R.FDDAVVQSDMK.H | 2 |
|  | pDK268\_112012\_01.08579.08579.2 | 2.2001 | 0.3965 | 99.8% | 1654.1122 | 1654.9298 | 7 | 5.977 | 50.0% | 1 | K.HWPFMVVNDAGRPK.V | 2 |
|  | pDK268\_112012\_01.08654.08654.3 | 4.6828 | 0.4789 | 100.0% | 1654.6144 | 1654.9298 | 1 | 8.102 | 44.2% | 5 | K.HWPFMVVNDAGRPK.V | 3 |
|  | pDK268\_112012\_01.03650.03650.2 | 2.899 | 0.3125 | 100.0% | 1181.3121 | 1181.3312 | 1 | 5.967 | 77.8% | 1 | K.VQVEYKGETK.S | 2 |
|  | pDK268\_112012\_01.11471.11471.2 | 4.5151 | 0.514 | 100.0% | 1618.8922 | 1617.8542 | 1 | 7.759 | 84.6% | 3 | K.SFYPEEVSSMVLTK.M | 2 |
|  | pDK268\_112012\_01.06633.06633.2 | 3.9083 | 0.3096 | 100.0% | 1253.1322 | 1253.4993 | 1 | 6.194 | 90.0% | 1 | K.MKEIAEAYLGK.T | 2 |
|  | pDK268\_112012\_02.05409.05409.3 | 2.8332 | 0.3971 | 100.0% | 1253.6044 | 1253.4993 | 1 | 6.662 | 52.5% | 2 | K.MKEIAEAYLGK.T | 3 |
|  | pDK268\_112012\_01.10083.10083.2 | 4.2589 | 0.5209 | 100.0% | 1982.5922 | 1983.1882 | 1 | 8.665 | 70.6% | 2 | K.TVTNAVVTVPAYFNDSQR.Q | 2 |
|  | pDK268\_112012\_01.10095.10095.3 | 3.5418 | 0.288 | 100.0% | 1982.9043 | 1983.1882 | 1 | 6.544 | 47.1% | 1 | K.TVTNAVVTVPAYFNDSQR.Q | 3 |
|  | pDK268\_112012\_01.10463.10463.2 | 5.3485 | 0.5181 | 100.0% | 1661.5721 | 1660.9078 | 1 | 9.837 | 86.7% | 2 | R.IINEPTAAAIAYGLDK.K | 222 |
|  | pDK268\_112012\_01.09330.09330.2 | 4.7513 | 0.5226 | 100.0% | 1789.5122 | 1789.0819 | 1 | 9.249 | 71.9% | 1 | R.IINEPTAAAIAYGLDKK.V | 2 |
|  | pDK268\_112012\_01.05000.05000.2 | 4.5468 | 0.4817 | 100.0% | 1692.2722 | 1692.6958 | 1 | 8.252 | 66.7% | 1 | K.STAGDTHLGGEDFDNR.M | 2 |
|  | pDK268\_112012\_02.03825.03825.3 | 2.9327 | 0.5351 | 100.0% | 1692.3844 | 1692.6958 | 1 | 7.493 | 38.3% | 2 | K.STAGDTHLGGEDFDNR.M | 3 |
|  | pDK268\_112012\_02.06376.06376.2 | 3.4769 | 0.5524 | 100.0% | 1236.3121 | 1236.4741 | 1 | 9.186 | 88.9% | 3 | R.MVNHFIAEFK.R | 2 |
|  | pDK268\_112012\_01.08248.08248.3 | 2.9057 | 0.3301 | 100.0% | 1236.5944 | 1236.4741 | 1 | 6.357 | 44.4% | 3 | R.MVNHFIAEFK.R | 3 |
|  | pDK268\_112012\_01.09890.09890.2 | 3.9958 | 0.3908 | 100.0% | 1481.4321 | 1481.6511 | 1 | 7.847 | 77.3% | 1 | R.ARFEELNADLFR.G | 2 |
|  | pDK268\_112012\_01.09891.09891.3 | 3.9367 | 0.25 | 100.0% | 1481.9644 | 1481.6511 | 1 | 5.24 | 59.1% | 1 | R.ARFEELNADLFR.G | 3 |
|  | pDK268\_112012\_01.10964.10964.2 | 3.2199 | 0.4712 | 100.0% | 1253.8322 | 1254.3849 | 3 | 7.438 | 72.2% | 2 | R.FEELNADLFR.G | 2 |
|  | pDK268\_112012\_01.07544.07544.3 | 5.4036 | 0.4655 | 100.0% | 1839.5343 | 1839.1019 | 1 | 8.74 | 46.9% | 2 | K.LDKSQIHDIVLVGGSTR.I | 3 |
|  | pDK268\_112012\_01.07018.07018.2 | 4.8392 | 0.6012 | 100.0% | 1482.3522 | 1482.6798 | 1 | 9.791 | 80.8% | 4 | K.SQIHDIVLVGGSTR.I | 2 |
|  | pDK268\_112012\_02.05595.05595.3 | 3.4484 | 0.293 | 100.0% | 1483.8243 | 1482.6798 | 2 | 5.572 | 42.3% | 1 | K.SQIHDIVLVGGSTR.I | 3 |
|  | pDK268\_112012\_01.10481.10481.1 | 2.0842 | 0.3065 | 100.0% | 1081.49 | 1082.2444 | 1 | 5.17 | 75.0% | 1 | K.LLQDFFNGK.E | 11 |
|  | pDK268\_112012\_01.10515.10515.2 | 2.832 | 0.3774 | 100.0% | 1081.5721 | 1082.2444 | 1 | 6.603 | 81.2% | 2 | K.LLQDFFNGK.E | 22 |
|  | pDK268\_112012\_01.10048.10048.2 | 3.673 | 0.3305 | 100.0% | 1566.2322 | 1566.7972 | 2 | 5.917 | 66.7% | 1 | K.LLQDFFNGKELNK.S | 22 |
|  | pDK268\_112012\_02.10254.10254.2 | 2.8756 | 0.34 | 99.9% | 2259.9722 | 2261.4937 | 79 | 6.196 | 27.3% | 1 | K.SINPDEAVAYGAAVQAAILSGDK.S | 2 |
|  | pDK268\_112012\_02.14721.14721.3 | 4.4793 | 0.2344 | 100.0% | 3397.0444 | 3397.008 | 2 | 6.656 | 21.8% | 1 | K.SENVQDLLLLDVTPLSLGIETAGGVMTVLIKR.N | 3 |
|  | pDK268\_112012\_02.08373.08373.3 | 4.6555 | 0.3393 | 100.0% | 2775.0244 | 2775.9885 | 2 | 7.828 | 27.2% | 1 | K.QTQTFTTYSDNQPGVLIQVYEGER.A | 3 |
|  | pDK268\_112012\_01.11655.11655.2 | 3.2923 | 0.5768 | 100.0% | 2775.2922 | 2775.9885 | 16 | 9.724 | 37.0% | 3 | K.QTQTFTTYSDNQPGVLIQVYEGER.A | 2 |
|  | pDK268\_112012\_01.03453.03453.2 | 2.6857 | 0.3759 | 100.0% | 1018.2322 | 1018.1582 | 241 | 6.243 | 50.0% | 1 | K.ITITNDKGR.L | 2222 |
| \* | pDK268\_112012\_01.09482.09482.2 | 3.6589 | 0.4892 | 100.0% | 1304.9722 | 1304.4602 | 1 | 7.551 | 80.0% | 2 | K.NSLESYAFNMK.A | 2 |
| \* | pDK268\_112012\_01.03417.03417.2 | 4.3352 | 0.4121 | 100.0% | 1746.2922 | 1746.8308 | 1 | 7.864 | 73.1% | 1 | K.NQTAEKEEFEHQQK.E | 2 |
| \* | pDK268\_112012\_01.03424.03424.3 | 3.3877 | 0.235 | 99.8% | 1747.8243 | 1746.8308 | 1 | 5.793 | 51.9% | 1 | K.NQTAEKEEFEHQQK.E | 3 |

Similarities:
gi|16507237|ref|NP\_00(1:34)  
gi|167466173|ref|NP\_0(2:33)  
gi|124256496|ref|NP\_0(3:32)  
gi|34419635|ref|NP\_00(5:30)  

---

|  |  |  |  |  |  |  |  |  |
| --- | --- | --- | --- | --- | --- | --- | --- | --- |
| U | *gi|221316642|ref|NP\_0* | 12 | 17 | 47.2% | 335 | 37721 | 5.2 | nuclear distribution gene E homolog 1 [Homo sapiens] |
| U | *gi|8923110|ref|NP\_060* | 12 | 17 | 47.2% | 335 | 37721 | 5.2 | nuclear distribution gene E homolog 1 [Homo sapiens] |

| Filename XCorr DeltCN Conf% ObsM+H+ CalcM+H+ SpR ZScore Ion% # Sequence  | | | | | | | | | | | | |
| --- | --- | --- | --- | --- | --- | --- | --- | --- | --- | --- | --- | --- |
|  | pDK268\_112012\_01.08422.08422.2 | 3.1468 | 0.3772 | 100.0% | 1620.5122 | 1620.6691 | 2 | 6.437 | 58.3% | 2 | K.TFSSEEEEANYWK.D | 2 |
|  | pDK268\_112012\_02.08056.08056.2 | 4.073 | 0.3826 | 100.0% | 1980.0922 | 1981.1235 | 1 | 7.388 | 50.0% | 1 | R.EYEAELETQLQQIETR.N | 2 |
|  | pDK268\_112012\_01.04282.04282.2 | 2.41 | 0.4333 | 100.0% | 1252.1322 | 1252.3286 | 1 | 7.147 | 83.3% | 1 | K.FEVQHSEGYR.Q | 2 |
|  | pDK268\_112012\_01.08296.08296.2 | 3.7588 | 0.5067 | 100.0% | 1432.2322 | 1432.5707 | 1 | 7.633 | 70.8% | 1 | R.QISALEDDLAQTK.A | 2 |
|  | pDK268\_112012\_01.04824.04824.2 | 2.8988 | 0.2208 | 99.8% | 1332.0922 | 1332.3666 | 7 | 4.404 | 70.0% | 1 | R.ELEQANDDLER.A | 2 |
|  | pDK268\_112012\_01.10841.10841.2 | 3.171 | 0.3523 | 100.0% | 1440.2322 | 1440.6111 | 1 | 7.024 | 77.3% | 1 | R.ATIMSLEDFEQR.L | 2 |
|  | pDK268\_112012\_01.04786.04786.2 | 2.691 | 0.2991 | 99.9% | 1204.1522 | 1204.3405 | 1 | 5.591 | 75.0% | 1 | R.TPMPSSVEAER.T | 2 |
|  | pDK268\_112012\_02.04611.04611.3 | 3.3943 | 0.5148 | 100.0% | 1910.2444 | 1910.0941 | 1 | 7.931 | 37.5% | 3 | R.TDTAVQATGSVPSTPIAHR.G | 3 |
|  | pDK268\_112012\_01.05808.05808.2 | 3.6582 | 0.3639 | 100.0% | 1307.3322 | 1307.4056 | 1 | 9.404 | 70.8% | 1 | R.GPSSSLNTPGSFR.R | 2 |
|  | pDK268\_112012\_01.06376.06376.2 | 3.6562 | 0.4801 | 100.0% | 1529.1522 | 1529.647 | 1 | 8.089 | 63.3% | 2 | R.GLDDSTGGTPLTPAAR.I | 2 |
|  | pDK268\_112012\_01.15141.15141.2 | 4.8591 | 0.3887 | 100.0% | 1285.4321 | 1284.5419 | 1 | 7.047 | 90.9% | 2 | R.ISALNIVGDLLR.K | 2 |
|  | pDK268\_112012\_01.03892.03892.2 | 2.8198 | 0.3114 | 99.9% | 1244.1921 | 1244.3904 | 1 | 6.611 | 72.7% | 1 | R.RPSSTSVPLGDK.G | 2 |

---

|  |  |  |  |  |  |  |  |  |
| --- | --- | --- | --- | --- | --- | --- | --- | --- |
| U | *gi|4502549|ref|NP\_001* | 3 | 3 | 47.0% | 149 | 16838 | 4.2 | calmodulin 2 [Homo sapiens] |
| U | *gi|5901912|ref|NP\_008* | 3 | 3 | 47.0% | 149 | 16838 | 4.2 | calmodulin 1 [Homo sapiens] |
| U | *gi|58218968|ref|NP\_00* | 3 | 3 | 47.0% | 149 | 16838 | 4.2 | calmodulin 3 [Homo sapiens] |

| Filename XCorr DeltCN Conf% ObsM+H+ CalcM+H+ SpR ZScore Ion% # Sequence  | | | | | | | | | | | | |
| --- | --- | --- | --- | --- | --- | --- | --- | --- | --- | --- | --- | --- |
|  | pDK268\_112012\_01.09393.09393.2 | 4.3518 | 0.51 | 100.0% | 1845.3722 | 1846.0007 | 1 | 7.972 | 68.8% | 1 | K.EAFSLFDKDGDGTITTK.E | 2 |
|  | pDK268\_112012\_01.17674.17674.3 | 4.7924 | 0.2364 | 100.0% | 4075.2244 | 4072.4795 | 1 | 4.471 | 25.7% | 1 | R.SLGQNPTEAELQDMINEVDADGNGTIDFPEFLTMMAR.K | 3 |
|  | pDK268\_112012\_01.08054.08054.3 | 3.2418 | 0.1688 | 97.2% | 1755.9844 | 1755.9249 | 1 | 5.268 | 43.3% | 1 | R.VFDKDGNGYISAAELR.H | 3 |

---

|  |  |  |  |  |  |  |  |  |
| --- | --- | --- | --- | --- | --- | --- | --- | --- |
| U | *gi|4885049|ref|NP\_005* | 32 | 126 | 46.9% | 377 | 42019 | 5.4 | cardiac muscle alpha actin 1 proprotein [Homo sapiens] |

| Filename XCorr DeltCN Conf% ObsM+H+ CalcM+H+ SpR ZScore Ion% # Sequence  | | | | | | | | | | | | |
| --- | --- | --- | --- | --- | --- | --- | --- | --- | --- | --- | --- | --- |
|  | pDK268\_112012\_01.04298.04298.1 | 1.8879 | 0.329 | 100.0% | 976.36 | 977.02136 | 191 | 5.657 | 44.4% | 2 | K.AGFAGDDAPR.A | 111 |
|  | pDK268\_112012\_02.03387.03387.2 | 3.4779 | 0.4692 | 100.0% | 976.89215 | 977.02136 | 1 | 7.775 | 88.9% | 11 | K.AGFAGDDAPR.A | 222 |
|  | pDK268\_112012\_01.09078.09078.3 | 3.194 | 0.2448 | 98.9% | 2157.2644 | 2157.4397 | 1 | 4.607 | 32.5% | 1 | K.AGFAGDDAPRAVFPSIVGRPR.H | 333 |
|  | pDK268\_112012\_01.07424.07424.2 | 2.9932 | 0.3571 | 100.0% | 1199.3522 | 1199.4415 | 1 | 6.426 | 75.0% | 9 | R.AVFPSIVGRPR.H | 222 |
|  | pDK268\_112012\_01.04100.04100.1 | 2.9097 | 0.2235 | 100.0% | 1173.65 | 1172.4058 | 1 | 4.493 | 65.0% | 5 | R.HQGVMVGMGQK.D | 11 |
|  | pDK268\_112012\_01.04095.04095.2 | 3.0844 | 0.138 | 99.7% | 1174.0721 | 1172.4058 | 1 | 4.962 | 75.0% | 8 | R.HQGVMVGMGQK.D | 22 |
|  | pDK268\_112012\_02.04623.04623.3 | 4.7125 | 0.5223 | 100.0% | 2352.2344 | 2352.5989 | 1 | 9.03 | 38.1% | 1 | R.HQGVMVGMGQKDSYVGDEAQSK.R | 33 |
|  | pDK268\_112012\_01.04865.04865.3 | 5.2731 | 0.4775 | 100.0% | 2508.3843 | 2508.7864 | 1 | 7.861 | 38.6% | 2 | R.HQGVMVGMGQKDSYVGDEAQSKR.G | 33 |
|  | pDK268\_112012\_01.03884.03884.2 | 3.4646 | 0.0536 | 99.6% | 1199.0122 | 1199.2163 | 1 | 7.147 | 85.0% | 7 | K.DSYVGDEAQSK.R | 22 |
|  | pDK268\_112012\_01.03692.03692.2 | 3.3148 | 0.1013 | 99.6% | 1355.1522 | 1355.4038 | 1 | 8.165 | 77.3% | 3 | K.DSYVGDEAQSKR.G | 22 |
|  | pDK268\_112012\_01.11757.11757.3 | 3.957 | 0.3023 | 100.0% | 2588.0044 | 2587.9934 | 1 | 5.639 | 39.3% | 1 | R.GILTLKYPIEHGIITNWDDMEK.I | 3 |
|  | pDK268\_112012\_02.06381.06381.2 | 4.273 | 0.3805 | 100.0% | 1961.4122 | 1962.1841 | 1 | 6.268 | 60.0% | 6 | K.YPIEHGIITNWDDMEK.I | 2 |
|  | pDK268\_112012\_01.09722.09722.3 | 4.7348 | 0.1824 | 100.0% | 1962.4744 | 1962.1841 | 1 | 5.236 | 46.7% | 5 | K.YPIEHGIITNWDDMEK.I | 3 |
|  | pDK268\_112012\_01.13426.13426.3 | 5.6211 | 0.351 | 100.0% | 3459.2344 | 3459.8628 | 1 | 5.031 | 29.8% | 3 | K.YPIEHGIITNWDDMEKIWHHTFYNELR.V | 3 |
|  | pDK268\_112012\_02.04842.04842.2 | 3.3544 | 0.4252 | 100.0% | 1516.7722 | 1516.7019 | 1 | 6.332 | 75.0% | 5 | K.IWHHTFYNELR.V | 222 |
|  | pDK268\_112012\_02.04830.04830.3 | 3.3578 | 0.2395 | 100.0% | 1517.0643 | 1516.7019 | 3 | 5.839 | 47.5% | 7 | K.IWHHTFYNELR.V | 333 |
|  | pDK268\_112012\_01.08487.08487.2 | 4.457 | 0.4005 | 100.0% | 1958.4722 | 1957.234 | 7 | 6.883 | 47.1% | 3 | R.VAPEEHPTLLTEAPLNPK.A | 2 |
|  | pDK268\_112012\_02.05685.05685.3 | 3.7782 | 0.1505 | 98.1% | 1958.8744 | 1957.234 | 28 | 4.217 | 35.3% | 1 | R.VAPEEHPTLLTEAPLNPK.A | 3 |
|  | pDK268\_112012\_01.11594.11594.2 | 3.027 | 0.2986 | 99.9% | 1624.6921 | 1624.8927 | 1 | 5.628 | 61.5% | 2 | R.LDLAGRDLTDYLMK.I | 22 |
|  | pDK268\_112012\_01.11582.11582.3 | 3.101 | 0.3275 | 100.0% | 1625.0343 | 1624.8927 | 1 | 6.241 | 46.2% | 1 | R.LDLAGRDLTDYLMK.I | 33 |
|  | pDK268\_112012\_01.15548.15548.3 | 5.2814 | 0.3719 | 100.0% | 2238.5344 | 2237.6196 | 1 | 6.898 | 45.8% | 1 | R.LDLAGRDLTDYLMKILTER.G | 33 |
|  | pDK268\_112012\_02.06534.06534.1 | 2.1427 | 0.2707 | 100.0% | 998.28 | 999.167 | 2 | 5.469 | 71.4% | 3 | R.DLTDYLMK.I | 11 |
|  | pDK268\_112012\_01.10826.10826.2 | 2.7898 | 0.3169 | 100.0% | 999.21216 | 999.167 | 7 | 6.507 | 71.4% | 3 | R.DLTDYLMK.I | 22 |
|  | pDK268\_112012\_01.10832.10832.2 | 4.9745 | 0.3219 | 100.0% | 1791.4122 | 1791.9554 | 1 | 9.534 | 86.7% | 14 | K.SYELPDGQVITIGNER.F | 222 |
|  | pDK268\_112012\_02.06969.06969.3 | 4.115 | 0.2183 | 100.0% | 1791.9844 | 1791.9554 | 1 | 7.677 | 50.0% | 3 | K.SYELPDGQVITIGNER.F | 333 |
|  | pDK268\_112012\_01.06398.06398.3 | 3.4178 | 0.2589 | 100.0% | 1549.4343 | 1549.8843 | 7 | 5.787 | 44.2% | 1 | R.MQKEITALAPSTMK.I | 33 |
|  | pDK268\_112012\_01.06963.06963.1 | 2.535 | 0.5055 | 100.0% | 1161.58 | 1162.3868 | 1 | 7.631 | 60.0% | 4 | K.EITALAPSTMK.I | 11 |
|  | pDK268\_112012\_01.06818.06818.2 | 2.9214 | 0.437 | 100.0% | 1162.1522 | 1162.3868 | 1 | 6.953 | 90.0% | 8 | K.EITALAPSTMK.I | 22 |
|  | pDK268\_112012\_01.05204.05204.2 | 2.4741 | 0.0524 | 96.4% | 1036.6522 | 1037.2908 | 11 | 4.453 | 62.5% | 1 | K.IKIIAPPER.K | 22 |
|  | pDK268\_112012\_01.04420.04420.2 | 2.5544 | 0.1031 | 97.7% | 1165.4122 | 1165.4648 | 51 | 4.464 | 61.1% | 1 | K.IKIIAPPERK.Y | 22 |
|  | pDK268\_112012\_02.12904.12904.3 | 5.9983 | 0.4479 | 100.0% | 3509.6943 | 3509.1467 | 1 | 7.822 | 33.3% | 1 | K.IIAPPERKYSVWIGGSILASLSTFQQMWISK.Q | 33 |
|  | pDK268\_112012\_02.14579.14579.2 | 3.9156 | 0.5639 | 100.0% | 2602.672 | 2604.0388 | 1 | 8.448 | 47.7% | 3 | K.YSVWIGGSILASLSTFQQMWISK.Q | 22 |

Similarities:
gi|4501885|ref|NP\_001(26:6)  
gi|134133226|ref|NP\_0(8:24)  

---

|  |  |  |  |  |  |  |  |  |
| --- | --- | --- | --- | --- | --- | --- | --- | --- |
| U | *gi|34098946|ref|NP\_00* | 14 | 24 | 46.6% | 324 | 35924 | 9.9 | nuclease sensitive element binding protein 1 [Homo sapiens] |

| Filename XCorr DeltCN Conf% ObsM+H+ CalcM+H+ SpR ZScore Ion% # Sequence  | | | | | | | | | | | | |
| --- | --- | --- | --- | --- | --- | --- | --- | --- | --- | --- | --- | --- |
|  | pDK268\_112012\_01.08026.08026.2 | 2.0564 | 0.1284 | 95.1% | 942.1722 | 941.0342 | 3 | 4.601 | 71.4% | 1 | R.NGYGFINR.N | 22 |
|  | pDK268\_112012\_01.05208.05208.2 | 4.6974 | 0.4444 | 100.0% | 1745.2522 | 1745.9298 | 1 | 7.536 | 75.0% | 1 | R.NDTKEDVFVHQTAIK.K | 22 |
|  | pDK268\_112012\_02.04098.04098.3 | 3.8099 | 0.253 | 100.0% | 1746.2043 | 1745.9298 | 1 | 6.003 | 42.9% | 1 | R.NDTKEDVFVHQTAIK.K | 33 |
|  | pDK268\_112012\_01.04480.04480.2 | 4.8888 | 0.3907 | 100.0% | 1874.5122 | 1874.1039 | 1 | 6.397 | 76.7% | 1 | R.NDTKEDVFVHQTAIKK.N | 22 |
|  | pDK268\_112012\_01.04467.04467.3 | 4.1836 | 0.3947 | 100.0% | 1874.8143 | 1874.1039 | 1 | 7.133 | 48.3% | 1 | R.NDTKEDVFVHQTAIKK.N | 33 |
|  | pDK268\_112012\_02.06759.06759.2 | 4.3447 | 0.5209 | 100.0% | 1796.5922 | 1796.8822 | 1 | 9.551 | 68.8% | 6 | R.SVGDGETVEFDVVEGEK.G | 22 |
| \* | pDK268\_112012\_02.07057.07057.3 | 5.0419 | 0.3571 | 100.0% | 3473.9644 | 3474.7168 | 1 | 6.73 | 25.0% | 1 | R.SVGDGETVEFDVVEGEKGAEAANVTGPGGVPVQGSK.Y | 3 |
| \* | pDK268\_112012\_01.05002.05002.2 | 4.692 | 0.5164 | 100.0% | 1696.5322 | 1696.8577 | 1 | 8.554 | 63.9% | 3 | K.GAEAANVTGPGGVPVQGSK.Y | 2 |
| \* | pDK268\_112012\_02.03300.03300.3 | 5.9425 | 0.5396 | 100.0% | 3258.6543 | 3259.2566 | 1 | 8.459 | 36.6% | 1 | R.NYQQNYQNSESGEKNEGSESAPEGQAQQR.R | 3 |
| \* | pDK268\_112012\_01.07677.07677.2 | 2.6191 | 0.1434 | 99.6% | 1129.9122 | 1130.3525 | 9 | 3.74 | 71.4% | 2 | R.RFPPYYMR.R | 2 |
| \* | pDK268\_112012\_01.05699.05699.3 | 5.2712 | 0.4178 | 100.0% | 3224.4243 | 3225.4795 | 1 | 7.978 | 25.0% | 3 | R.RPQYSNPPVQGEVMEGADNQGAGEQGRPVR.Q | 3 |
| \* | pDK268\_112012\_01.03383.03383.2 | 3.8287 | 0.5099 | 100.0% | 2629.3323 | 2629.5835 | 1 | 8.158 | 47.7% | 1 | R.EDGNEEDKENQGDETQGQQPPQR.R | 2 |
| \* | pDK268\_112012\_01.03380.03380.3 | 4.3732 | 0.3852 | 100.0% | 2630.6643 | 2629.5835 | 1 | 6.626 | 34.1% | 1 | R.EDGNEEDKENQGDETQGQQPPQR.R | 3 |
| \* | pDK268\_112012\_01.03294.03294.3 | 2.9266 | 0.3951 | 100.0% | 2784.5044 | 2785.771 | 1 | 5.64 | 29.3% | 1 | R.EDGNEEDKENQGDETQGQQPPQRR.Y | 3 |

Similarities:
gi|224586884|ref|NP\_0(6:8)  

---

|  |  |  |  |  |  |  |  |  |
| --- | --- | --- | --- | --- | --- | --- | --- | --- |
| U | *gi|4504255|ref|NP\_002* | 5 | 9 | 46.1% | 128 | 13553 | 10.6 | H2A histone family, member Z [Homo sapiens] |
| U | *gi|6912616|ref|NP\_036* | 5 | 9 | 46.1% | 128 | 13509 | 10.6 | H2A histone family, member V isoform 1 [Homo sapiens] |

| Filename XCorr DeltCN Conf% ObsM+H+ CalcM+H+ SpR ZScore Ion% # Sequence  | | | | | | | | | | | | |
| --- | --- | --- | --- | --- | --- | --- | --- | --- | --- | --- | --- | --- |
|  | pDK268\_112012\_01.08294.08294.2 | 3.1381 | 0.3918 | 100.0% | 944.65216 | 945.1093 | 1 | 6.245 | 87.5% | 3 | R.AGLQFPVGR.I | 22222 |
|  | pDK268\_112012\_01.19778.19778.2 | 3.726 | 0.62 | 100.0% | 2896.912 | 2897.2952 | 5 | 10.167 | 39.3% | 1 | R.VGATAAVYSAAILEYLTAEVLELAGNASK.D | 2 |
|  | pDK268\_112012\_01.19772.19772.3 | 6.3823 | 0.5114 | 100.0% | 2897.6042 | 2897.2952 | 1 | 8.902 | 33.0% | 1 | R.VGATAAVYSAAILEYLTAEVLELAGNASK.D | 3 |
|  | pDK268\_112012\_01.05180.05180.2 | 2.5905 | 0.2897 | 100.0% | 851.1922 | 851.0396 | 1 | 4.986 | 100.0% | 3 | R.HLQLAIR.G | 2222 |
|  | pDK268\_112012\_01.05526.05526.2 | 3.5715 | 0.3295 | 100.0% | 1371.0721 | 1371.6255 | 1 | 6.408 | 57.7% | 1 | K.ATIAGGGVIPHIHK.S | 2 |

Similarities:
gi|10800130|ref|NP\_06(2:3)  
gi|106775678|ref|NP\_0(2:3)  
gi|4504253|ref|NP\_002(2:3)  
gi|28195394|ref|NP\_77(1:4)  

---

|  |  |  |  |  |  |  |  |  |
| --- | --- | --- | --- | --- | --- | --- | --- | --- |
| U | *gi|226530908|ref|NP\_0* | 20 | 40 | 46.0% | 285 | 30315 | 7.5 | protein-L-isoaspartate (D-aspartate) O-methyltransferase [Homo sapiens] |

| Filename XCorr DeltCN Conf% ObsM+H+ CalcM+H+ SpR ZScore Ion% # Sequence  | | | | | | | | | | | | |
| --- | --- | --- | --- | --- | --- | --- | --- | --- | --- | --- | --- | --- |
| \* | pDK268\_112012\_01.04461.04461.2 | 4.4242 | 0.3804 | 100.0% | 1478.2722 | 1478.6078 | 1 | 8.521 | 61.5% | 1 | K.SGGASHSELIHNLR.K | 2 |
| \* | pDK268\_112012\_01.04460.04460.3 | 4.3087 | 0.3259 | 100.0% | 1478.9644 | 1478.6078 | 2 | 6.499 | 44.2% | 2 | K.SGGASHSELIHNLR.K | 32 |
| \* | pDK268\_112012\_01.03983.03983.2 | 4.2541 | 0.5114 | 100.0% | 1606.2322 | 1606.7819 | 1 | 8.006 | 60.7% | 1 | K.SGGASHSELIHNLRK.N | 2 |
| \* | pDK268\_112012\_02.03299.03299.3 | 3.1694 | 0.16 | 96.4% | 1606.9744 | 1606.7819 | 2 | 5.144 | 37.5% | 1 | K.SGGASHSELIHNLRK.N | 3 |
| \* | pDK268\_112012\_02.07903.07903.3 | 3.2136 | 0.3967 | 100.0% | 2049.5344 | 2051.409 | 1 | 6.534 | 32.4% | 1 | K.NGIIKTDKVFEVMLATDR.S | 3 |
| \* | pDK268\_112012\_01.09591.09591.2 | 4.1869 | 0.515 | 100.0% | 1525.2522 | 1525.7601 | 1 | 10.014 | 79.2% | 5 | K.TDKVFEVMLATDR.S | 2 |
| \* | pDK268\_112012\_02.07249.07249.3 | 3.7541 | 0.3591 | 100.0% | 1526.2144 | 1525.7601 | 1 | 6.563 | 52.1% | 1 | K.TDKVFEVMLATDR.S | 3 |
| \* | pDK268\_112012\_01.09740.09740.2 | 3.4549 | 0.4862 | 100.0% | 1180.5721 | 1181.3923 | 1 | 8.571 | 88.9% | 5 | K.VFEVMLATDR.S | 2 |
| \* | pDK268\_112012\_01.05181.05181.2 | 2.5901 | 0.2653 | 99.9% | 894.9922 | 895.0898 | 1 | 5.623 | 92.9% | 3 | K.VIGIDHIK.E | 2 |
| \* | pDK268\_112012\_01.04488.04488.2 | 2.9707 | 0.423 | 100.0% | 1188.6322 | 1189.3109 | 1 | 7.241 | 80.0% | 2 | R.KDDPTLLSSGR.V | 2 |
| \* | pDK268\_112012\_02.04020.04020.2 | 2.1962 | 0.2224 | 98.4% | 1061.0521 | 1061.1368 | 1 | 5.083 | 77.8% | 1 | K.DDPTLLSSGR.V | 2 |
| \* | pDK268\_112012\_01.05487.05487.1 | 2.1915 | 0.291 | 100.0% | 942.53 | 943.091 | 1 | 6.519 | 68.8% | 2 | R.VQLVVGDGR.M | 1 |
| \* | pDK268\_112012\_02.03999.03999.2 | 2.9073 | 0.3587 | 100.0% | 943.1322 | 943.091 | 1 | 7.444 | 87.5% | 3 | R.VQLVVGDGR.M | 2 |
| \* | pDK268\_112012\_01.12705.12705.3 | 7.241 | 0.5777 | 100.0% | 3507.5044 | 3507.0015 | 1 | 9.63 | 34.1% | 3 | R.MGYAEEAPYDAIHVGAAAPVVPQALIDQLKPGGR.L | 3 |
| \* | pDK268\_112012\_01.11612.11612.2 | 4.5646 | 0.4297 | 100.0% | 2043.5521 | 2044.3734 | 1 | 7.755 | 63.9% | 3 | R.LILPVGPAGGNQMLEQYDK.L | 2 |
| \* | pDK268\_112012\_01.11576.11576.3 | 3.5337 | 0.2096 | 99.1% | 2044.5243 | 2044.3734 | 1 | 5.297 | 38.9% | 1 | R.LILPVGPAGGNQMLEQYDK.L | 3 |
| \* | pDK268\_112012\_01.11565.11565.3 | 2.6207 | 0.2278 | 96.9% | 1706.1843 | 1706.1549 | 3 | 5.18 | 37.5% | 1 | K.MKPLMGVIYVPLTDK.E | 3 |
| \* | pDK268\_112012\_01.11519.11519.2 | 4.0365 | 0.3929 | 100.0% | 1706.2922 | 1706.1549 | 1 | 7.602 | 64.3% | 1 | K.MKPLMGVIYVPLTDK.E | 2 |
| \* | pDK268\_112012\_01.10162.10162.2 | 4.4877 | 0.5826 | 100.0% | 1962.6921 | 1963.4445 | 1 | 9.637 | 68.8% | 2 | K.MKPLMGVIYVPLTDKEK.Q | 2 |
| \* | pDK268\_112012\_01.10184.10184.3 | 3.4821 | 0.3214 | 100.0% | 1963.9744 | 1963.4445 | 56 | 6.123 | 37.5% | 1 | K.MKPLMGVIYVPLTDKEK.Q | 3 |

---

|  |  |  |  |  |  |  |  |  |
| --- | --- | --- | --- | --- | --- | --- | --- | --- |
| U | *gi|14389309|ref|NP\_11* | 27 | 76 | 45.9% | 449 | 49895 | 5.1 | tubulin alpha 6 [Homo sapiens] |
| U | *gi|57013276|ref|NP\_00* | 27 | 76 | 45.7% | 451 | 50152 | 5.1 | tubulin, alpha, ubiquitous [Homo sapiens] |

| Filename XCorr DeltCN Conf% ObsM+H+ CalcM+H+ SpR ZScore Ion% # Sequence  | | | | | | | | | | | | |
| --- | --- | --- | --- | --- | --- | --- | --- | --- | --- | --- | --- | --- |
|  | pDK268\_112012\_02.08422.08422.2 | 5.8525 | 0.6505 | 100.0% | 2009.0322 | 2009.093 | 1 | 11.461 | 65.8% | 6 | K.TIGGGDDSFNTFFSETGAGK.H | 2 |
|  | pDK268\_112012\_02.08368.08368.3 | 3.5715 | 0.2318 | 99.3% | 2010.5343 | 2009.093 | 5 | 5.46 | 34.2% | 1 | K.TIGGGDDSFNTFFSETGAGK.H | 3 |
|  | pDK268\_112012\_01.12410.12410.2 | 4.8952 | 0.5278 | 100.0% | 1702.4521 | 1702.9451 | 1 | 8.586 | 78.6% | 6 | R.AVFVDLEPTVIDEVR.T | 2 |
|  | pDK268\_112012\_01.12377.12377.3 | 4.5109 | 0.4243 | 100.0% | 1703.2743 | 1702.9451 | 1 | 8.288 | 51.8% | 2 | R.AVFVDLEPTVIDEVR.T | 3 |
|  | pDK268\_112012\_01.09212.09212.2 | 1.6134 | 0.4066 | 97.8% | 1411.0322 | 1411.6439 | 78 | 6.183 | 59.1% | 1 | R.QLFHPEQLITGK.E | 22 |
|  | pDK268\_112012\_01.08756.08756.2 | 2.0447 | 0.4607 | 99.8% | 2415.4722 | 2416.6555 | 12 | 6.422 | 32.5% | 1 | R.QLFHPEQLITGKEDAANNYAR.G | 22 |
|  | pDK268\_112012\_01.08750.08750.3 | 4.1238 | 0.4883 | 100.0% | 2415.8943 | 2416.6555 | 1 | 7.217 | 36.2% | 3 | R.QLFHPEQLITGKEDAANNYAR.G | 33 |
|  | pDK268\_112012\_01.13277.13277.3 | 4.309 | 0.4737 | 100.0% | 1843.2544 | 1843.1332 | 1 | 8.099 | 46.7% | 1 | R.GHYTIGKEIIDLVLDR.I | 3 |
|  | pDK268\_112012\_01.11865.11865.2 | 2.6209 | 0.1838 | 99.7% | 1086.1322 | 1086.2737 | 8 | 5.019 | 75.0% | 1 | K.EIIDLVLDR.I | 2 |
|  | pDK268\_112012\_01.03926.03926.2 | 2.0667 | 0.1794 | 97.2% | 909.97217 | 910.05804 | 24 | 4.972 | 64.3% | 1 | R.LSVDYGKK.S | 22 |
|  | pDK268\_112012\_01.06032.06032.3 | 2.952 | 0.2267 | 98.5% | 1876.3744 | 1876.0824 | 1 | 4.679 | 41.1% | 1 | R.RNLDIERPTYTNLNR.L | 33 |
|  | pDK268\_112012\_01.07281.07281.2 | 3.2087 | 0.2207 | 99.9% | 1719.3322 | 1719.8949 | 1 | 4.93 | 61.5% | 2 | R.NLDIERPTYTNLNR.L | 22 |
|  | pDK268\_112012\_01.07274.07274.3 | 3.3116 | 0.3584 | 100.0% | 1720.0743 | 1719.8949 | 9 | 5.779 | 42.3% | 3 | R.NLDIERPTYTNLNR.L | 33 |
|  | pDK268\_112012\_02.11007.11007.2 | 4.1605 | 0.5215 | 100.0% | 1488.5122 | 1488.7678 | 1 | 9.06 | 73.1% | 13 | R.LISQIVSSITASLR.F | 22 |
|  | pDK268\_112012\_02.10969.10969.3 | 4.198 | 0.3431 | 100.0% | 1489.0743 | 1488.7678 | 2 | 6.289 | 48.1% | 2 | R.LISQIVSSITASLR.F | 33 |
|  | pDK268\_112012\_02.10194.10194.2 | 5.1564 | 0.5937 | 100.0% | 2409.7922 | 2410.6885 | 1 | 10.839 | 50.0% | 3 | R.FDGALNVDLTEFQTNLVPYPR.I | 22 |
|  | pDK268\_112012\_01.11078.11078.2 | 4.495 | 0.4985 | 100.0% | 1757.5721 | 1758.0703 | 1 | 9.063 | 76.7% | 6 | R.IHFPLATYAPVISAEK.A | 22 |
|  | pDK268\_112012\_01.10886.10886.3 | 3.4998 | 0.2408 | 99.9% | 1757.9644 | 1758.0703 | 1 | 5.498 | 43.3% | 3 | R.IHFPLATYAPVISAEK.A | 33 |
|  | pDK268\_112012\_01.07466.07466.1 | 1.7228 | 0.3655 | 100.0% | 1015.51 | 1016.1827 | 4 | 5.623 | 61.1% | 2 | K.DVNAAIATIK.T | 1 |
|  | pDK268\_112012\_01.07415.07415.2 | 3.2134 | 0.341 | 100.0% | 1016.9522 | 1016.1827 | 1 | 6.619 | 88.9% | 2 | K.DVNAAIATIK.T | 2 |
|  | pDK268\_112012\_01.09416.09416.2 | 3.8969 | 0.498 | 100.0% | 1825.3722 | 1826.1027 | 1 | 7.783 | 70.6% | 5 | K.VGINYQPPTVVPGGDLAK.V | 22 |
|  | pDK268\_112012\_01.07070.07070.2 | 3.2479 | 0.4165 | 100.0% | 1381.2522 | 1381.6324 | 1 | 6.343 | 80.0% | 1 | R.LDHKFDLMYAK.R | 22 |
|  | pDK268\_112012\_02.04967.04967.3 | 3.6743 | 0.339 | 100.0% | 1381.8243 | 1381.6324 | 1 | 6.207 | 52.5% | 2 | R.LDHKFDLMYAK.R | 33 |
|  | pDK268\_112012\_01.08716.08716.1 | 1.3964 | 0.263 | 96.1% | 887.29 | 888.0692 | 8 | 4.132 | 58.3% | 1 | K.FDLMYAK.R | 11 |
|  | pDK268\_112012\_01.08693.08693.2 | 2.3171 | 0.2151 | 99.7% | 887.83215 | 888.0692 | 2 | 4.947 | 91.7% | 1 | K.FDLMYAK.R | 22 |
|  | pDK268\_112012\_02.07229.07229.3 | 5.1213 | 0.4294 | 100.0% | 2488.1343 | 2487.7083 | 1 | 7.286 | 45.0% | 2 | K.RAFVHWYVGEGMEEGEFSEAR.E | 33 |
|  | pDK268\_112012\_02.08055.08055.3 | 4.5531 | 0.2662 | 100.0% | 2333.0044 | 2331.5208 | 1 | 5.518 | 40.8% | 4 | R.AFVHWYVGEGMEEGEFSEAR.E | 33 |

Similarities:
gi|17921989|ref|NP\_00(19:8)  

---

|  |  |  |  |  |  |  |  |  |
| --- | --- | --- | --- | --- | --- | --- | --- | --- |
| U | *gi|55770864|ref|NP\_00* | 9 | 28 | 45.5% | 257 | 26888 | 11.2 | THO complex 4 [Homo sapiens] |

| Filename XCorr DeltCN Conf% ObsM+H+ CalcM+H+ SpR ZScore Ion% # Sequence  | | | | | | | | | | | | |
| --- | --- | --- | --- | --- | --- | --- | --- | --- | --- | --- | --- | --- |
| \* | pDK268\_112012\_01.11592.11592.2 | 2.9539 | 0.3913 | 100.0% | 1181.0721 | 1181.4048 | 1 | 8.159 | 88.9% | 1 | K.MDMSLDDIIK.L | 2 |
| \* | pDK268\_112012\_02.07553.07553.3 | 4.4389 | 0.462 | 100.0% | 2704.2544 | 2704.915 | 1 | 7.093 | 30.0% | 3 | K.QLPDKWQHDLFDSGFGGGAGVETGGK.L | 3 |
| \* | pDK268\_112012\_02.07325.07325.3 | 3.8861 | 0.449 | 100.0% | 2122.4043 | 2123.2456 | 1 | 6.815 | 41.2% | 1 | K.WQHDLFDSGFGGGAGVETGGK.L | 3 |
| \* | pDK268\_112012\_02.13527.13527.3 | 4.9271 | 0.4876 | 100.0% | 2843.8442 | 2843.203 | 1 | 7.855 | 32.0% | 2 | K.LLVSNLDFGVSDADIQELFAEFGTLK.K | 3 |
| \* | pDK268\_112012\_01.16622.16622.3 | 4.6543 | 0.5114 | 100.0% | 2971.1643 | 2971.377 | 1 | 7.799 | 31.7% | 9 | K.LLVSNLDFGVSDADIQELFAEFGTLKK.A | 3 |
| \* | pDK268\_112012\_01.06026.06026.2 | 3.3867 | 0.4749 | 100.0% | 1232.0922 | 1232.3384 | 1 | 7.786 | 70.0% | 3 | R.SLGTADVHFER.K | 2 |
| \* | pDK268\_112012\_01.11174.11174.3 | 3.9018 | 0.3313 | 100.0% | 2814.5344 | 2815.1765 | 2 | 5.917 | 28.1% | 3 | K.QYNGVPLDGRPMNIQLVTSQIDAQR.R | 3 |
| \* | pDK268\_112012\_02.07252.07252.2 | 5.61 | 0.5175 | 100.0% | 2035.4722 | 2036.1626 | 1 | 9.587 | 67.6% | 4 | K.QQLSAEELDAQLDAYNAR.M | 2 |
| \* | pDK268\_112012\_02.06496.06496.3 | 4.3201 | 0.3436 | 100.0% | 2036.1543 | 2036.1626 | 1 | 6.967 | 45.6% | 2 | K.QQLSAEELDAQLDAYNAR.M | 3 |

---

|  |  |  |  |  |  |  |  |  |
| --- | --- | --- | --- | --- | --- | --- | --- | --- |
| U | *gi|5902102|ref|NP\_008* | 3 | 5 | 45.4% | 119 | 13282 | 11.6 | small nuclear ribonucleoprotein D1 polypeptide 16kDa [Homo sapiens] |

| Filename XCorr DeltCN Conf% ObsM+H+ CalcM+H+ SpR ZScore Ion% # Sequence  | | | | | | | | | | | | |
| --- | --- | --- | --- | --- | --- | --- | --- | --- | --- | --- | --- | --- |
| \* | pDK268\_112012\_02.05446.05446.3 | 5.6211 | 0.4534 | 100.0% | 2210.4543 | 2210.47 | 1 | 7.859 | 41.2% | 1 | K.NGTQVHGTITGVDVSMNTHLK.A | 3 |
|  | pDK268\_112012\_01.08085.08085.2 | 4.0823 | 0.4295 | 100.0% | 1555.3922 | 1555.7745 | 1 | 6.899 | 75.0% | 1 | K.NREPVQLETLSIR.G | 2 |
| \* | pDK268\_112012\_01.17246.17246.2 | 5.2633 | 0.5367 | 100.0% | 2288.4521 | 2288.6863 | 1 | 10.254 | 68.4% | 3 | R.YFILPDSLPLDTLLVDVEPK.V | 2 |

---

|  |  |  |  |  |  |  |  |  |
| --- | --- | --- | --- | --- | --- | --- | --- | --- |
| U | *gi|4506691|ref|NP\_001* | 9 | 19 | 45.2% | 146 | 16445 | 10.2 | ribosomal protein S16 [Homo sapiens] |

| Filename XCorr DeltCN Conf% ObsM+H+ CalcM+H+ SpR ZScore Ion% # Sequence  | | | | | | | | | | | | |
| --- | --- | --- | --- | --- | --- | --- | --- | --- | --- | --- | --- | --- |
|  | pDK268\_112012\_01.09314.09314.2 | 3.5609 | 0.5647 | 100.0% | 1187.9321 | 1188.372 | 1 | 8.746 | 75.0% | 4 | K.GPLQSVQVFGR.K | 2 |
| \* | pDK268\_112012\_01.07058.07058.3 | 3.5572 | 0.2167 | 100.0% | 1411.4343 | 1411.6622 | 1 | 5.191 | 50.0% | 3 | K.VNGRPLEMIEPR.T | 3 |
| \* | pDK268\_112012\_01.07055.07055.2 | 3.0133 | 0.272 | 99.9% | 1412.2722 | 1411.6622 | 2 | 4.863 | 59.1% | 2 | K.VNGRPLEMIEPR.T | 2 |
|  | pDK268\_112012\_01.12543.12543.2 | 2.3972 | 0.2559 | 99.6% | 1094.8322 | 1095.4111 | 1 | 6.433 | 72.2% | 2 | K.LLEPVLLLGK.E | 2 |
| \* | pDK268\_112012\_01.04868.04868.3 | 3.918 | 0.3274 | 100.0% | 1469.8444 | 1469.7299 | 1 | 6.33 | 48.1% | 2 | R.VKGGGHVAQIYAIR.Q | 3 |
| \* | pDK268\_112012\_01.05394.05394.2 | 2.7199 | 0.3251 | 99.9% | 1242.2522 | 1242.4232 | 1 | 5.959 | 77.3% | 1 | K.GGGHVAQIYAIR.Q | 2 |
| \* | pDK268\_112012\_01.09302.09302.2 | 2.4298 | 0.4367 | 100.0% | 1406.1921 | 1406.622 | 1 | 6.386 | 85.0% | 1 | K.EIKDILIQYDR.T | 2 |
| \* | pDK268\_112012\_01.08255.08255.2 | 2.2758 | 0.3578 | 99.9% | 1036.2122 | 1036.1729 | 3 | 5.915 | 85.7% | 1 | K.DILIQYDR.T | 2 |
| \* | pDK268\_112012\_01.06242.06242.2 | 2.2217 | 0.2388 | 99.5% | 885.03217 | 885.0513 | 6 | 5.706 | 71.4% | 3 | R.TLLVADPR.R | 2 |

---

|  |  |  |  |  |  |  |  |  |
| --- | --- | --- | --- | --- | --- | --- | --- | --- |
| U | *gi|5453597|ref|NP\_006* | 10 | 12 | 45.1% | 286 | 32923 | 5.7 | F-actin capping protein alpha-1 subunit [Homo sapiens] |

| Filename XCorr DeltCN Conf% ObsM+H+ CalcM+H+ SpR ZScore Ion% # Sequence  | | | | | | | | | | | | |
| --- | --- | --- | --- | --- | --- | --- | --- | --- | --- | --- | --- | --- |
| \* | pDK268\_112012\_01.10367.10367.2 | 3.051 | 0.3747 | 100.0% | 2089.7322 | 2090.3025 | 1 | 6.948 | 55.9% | 1 | K.FITHAPPGEFNEVFNDVR.L | 2 |
| \* | pDK268\_112012\_01.10400.10400.3 | 4.8552 | 0.3426 | 100.0% | 2090.0942 | 2090.3025 | 1 | 6.903 | 51.5% | 2 | K.FITHAPPGEFNEVFNDVR.L | 3 |
|  | pDK268\_112012\_01.09802.09802.2 | 3.1751 | 0.2575 | 100.0% | 1199.3121 | 1198.408 | 1 | 5.384 | 88.9% | 2 | R.LLLNNDNLLR.E | 22 |
| \* | pDK268\_112012\_02.06304.06304.3 | 3.8116 | 0.4805 | 100.0% | 2245.6443 | 2246.3953 | 6 | 7.342 | 28.9% | 1 | K.IEGYEDQVLITEHGDLGNSR.F | 3 |
| \* | pDK268\_112012\_01.04463.04463.2 | 3.2075 | 0.4448 | 100.0% | 1543.4521 | 1543.5841 | 3 | 6.469 | 57.1% | 1 | K.EASDPQPEEADGGLK.S | 2 |
|  | pDK268\_112012\_02.05341.05341.3 | 5.0621 | 0.4004 | 100.0% | 2029.9443 | 2030.2474 | 1 | 7.397 | 50.0% | 1 | K.IQVHYYEDGNVQLVSHK.D | 33 |
| \* | pDK268\_112012\_02.04846.04846.3 | 3.3823 | 0.3772 | 100.0% | 1706.2144 | 1706.804 | 1 | 6.308 | 41.7% | 1 | K.DVQDSLTVSNEAQTAK.E | 3 |
| \* | pDK268\_112012\_02.04816.04816.2 | 3.9651 | 0.5274 | 100.0% | 1706.3922 | 1706.804 | 1 | 9.208 | 60.0% | 1 | K.DVQDSLTVSNEAQTAK.E | 2 |
| \* | pDK268\_112012\_02.08333.08333.3 | 4.3514 | 0.3544 | 100.0% | 3041.1843 | 3042.2544 | 2 | 6.404 | 24.0% | 1 | K.IIENAENEYQTAISENYQTMSDTTFK.A | 3 |
|  | pDK268\_112012\_01.03442.03442.2 | 2.1766 | 0.0725 | 95.7% | 870.1122 | 870.04266 | 57 | 3.43 | 75.0% | 1 | R.RQLPVTR.T | 22 |

Similarities:
gi|5453599|ref|NP\_006(3:7)  

---

|  |  |  |  |  |  |  |  |  |
| --- | --- | --- | --- | --- | --- | --- | --- | --- |
| U | *gi|15809016|ref|NP\_29* | 7 | 10 | 44.8% | 172 | 19779 | 4.8 | myosin regulatory light chain MRCL2 isoform A [Homo sapiens] |
| U | *gi|5453740|ref|NP\_006* | 7 | 10 | 45.0% | 171 | 19794 | 4.8 | myosin, light chain 12A, regulatory, non-sarcomeric [Homo sapiens] |
| U | *gi|222144326|ref|NP\_0* | 7 | 10 | 44.8% | 172 | 19779 | 4.8 | myosin regulatory light chain MRCL2 isoform A [Homo sapiens] |
| U | *gi|222144324|ref|NP\_0* | 7 | 10 | 44.8% | 172 | 19779 | 4.8 | myosin regulatory light chain MRCL2 isoform A [Homo sapiens] |

| Filename XCorr DeltCN Conf% ObsM+H+ CalcM+H+ SpR ZScore Ion% # Sequence  | | | | | | | | | | | | |
| --- | --- | --- | --- | --- | --- | --- | --- | --- | --- | --- | --- | --- |
|  | pDK268\_112012\_01.13350.13350.2 | 3.9299 | 0.2175 | 100.0% | 2093.6921 | 2092.3308 | 1 | 5.015 | 58.8% | 2 | R.ATSNVFAMFDQSQIQEFK.E | 2 |
|  | pDK268\_112012\_01.12508.12508.3 | 2.6653 | 0.2708 | 98.3% | 2004.4744 | 2005.25 | 3 | 4.781 | 32.4% | 1 | R.DGFIDKEDLHDMLASLGK.N | 3 |
|  | pDK268\_112012\_01.06732.06732.2 | 3.0232 | 0.3847 | 100.0% | 1229.2122 | 1229.3324 | 1 | 6.538 | 90.0% | 1 | K.LNGTDPEDVIR.N | 2 |
|  | pDK268\_112012\_02.04488.04488.2 | 2.4551 | 0.3072 | 99.9% | 1036.1322 | 1036.1884 | 1 | 6.003 | 75.0% | 2 | R.ELLTTMGDR.F | 2 |
|  | pDK268\_112012\_01.11832.11832.3 | 4.3277 | 0.2832 | 100.0% | 2434.5544 | 2433.649 | 1 | 7.273 | 35.5% | 2 | R.ELLTTMGDRFTDEEVDELYR.E | 3 |
|  | pDK268\_112012\_01.08858.08858.2 | 4.3284 | 0.4392 | 100.0% | 1416.1522 | 1416.4839 | 1 | 8.444 | 80.0% | 1 | R.FTDEEVDELYR.E | 2 |
|  | pDK268\_112012\_01.09872.09872.2 | 3.0138 | 0.2271 | 99.9% | 1260.4521 | 1261.3794 | 1 | 6.72 | 77.8% | 1 | K.GNFNYIEFTR.I | 2 |

---

|  |  |  |  |  |  |  |  |  |
| --- | --- | --- | --- | --- | --- | --- | --- | --- |
| U | *gi|4506695|ref|NP\_001* | 11 | 15 | 44.8% | 145 | 16060 | 10.3 | ribosomal protein S19 [Homo sapiens] |

| Filename XCorr DeltCN Conf% ObsM+H+ CalcM+H+ SpR ZScore Ion% # Sequence  | | | | | | | | | | | | |
| --- | --- | --- | --- | --- | --- | --- | --- | --- | --- | --- | --- | --- |
| \* | pDK268\_112012\_01.05439.05439.2 | 2.993 | 0.2811 | 100.0% | 1135.0721 | 1135.2217 | 1 | 7.096 | 93.8% | 2 | K.DVNQQEFVR.A | 2 |
|  | pDK268\_112012\_01.09224.09224.2 | 2.3188 | 0.159 | 97.2% | 1313.8722 | 1314.5675 | 2 | 4.573 | 70.0% | 1 | K.LKVPEWVDTVK.L | 2 |
|  | pDK268\_112012\_01.09200.09200.3 | 3.6533 | 0.1716 | 99.7% | 1314.8944 | 1314.5675 | 9 | 6.0 | 47.5% | 1 | K.LKVPEWVDTVK.L | 3 |
|  | pDK268\_112012\_01.08223.08223.2 | 1.9981 | 0.2283 | 97.4% | 1073.0721 | 1073.234 | 1 | 5.079 | 68.8% | 2 | K.VPEWVDTVK.L | 2 |
| \* | pDK268\_112012\_01.09134.09134.2 | 4.1901 | 0.5228 | 100.0% | 1969.4321 | 1970.151 | 1 | 8.893 | 85.7% | 1 | K.HKELAPYDENWFYTR.A | 2 |
| \* | pDK268\_112012\_01.09158.09158.3 | 4.3336 | 0.3164 | 100.0% | 1970.4543 | 1970.151 | 1 | 6.86 | 50.0% | 3 | K.HKELAPYDENWFYTR.A | 3 |
| \* | pDK268\_112012\_01.11816.11816.2 | 2.7374 | 0.3949 | 100.0% | 1705.2122 | 1704.8358 | 1 | 6.246 | 54.2% | 1 | K.ELAPYDENWFYTR.A | 2 |
| \* | pDK268\_112012\_01.04704.04704.2 | 2.5044 | 0.232 | 99.7% | 1133.4521 | 1132.2823 | 4 | 4.687 | 72.2% | 1 | R.NGVMPSHFSR.G | 2 |
| \* | pDK268\_112012\_01.07262.07262.2 | 2.1461 | 0.1383 | 95.2% | 1127.3322 | 1127.3726 | 1 | 4.725 | 72.2% | 1 | R.RVLQALEGLK.M | 23 |
|  | pDK268\_112012\_01.08523.08523.2 | 2.4975 | 0.3296 | 99.9% | 971.0122 | 971.1851 | 1 | 5.98 | 87.5% | 1 | R.VLQALEGLK.M | 2 |
| \* | pDK268\_112012\_01.03335.03335.2 | 2.2373 | 0.19 | 97.8% | 944.27216 | 943.091 | 3 | 5.008 | 77.8% | 1 | R.IAGQVAAANK.K | 2 |

---

|  |  |  |  |  |  |  |  |  |
| --- | --- | --- | --- | --- | --- | --- | --- | --- |
| U | *gi|15718687|ref|NP\_00* | 8 | 17 | 44.4% | 243 | 26688 | 9.7 | ribosomal protein S3 [Homo sapiens] |

| Filename XCorr DeltCN Conf% ObsM+H+ CalcM+H+ SpR ZScore Ion% # Sequence  | | | | | | | | | | | | |
| --- | --- | --- | --- | --- | --- | --- | --- | --- | --- | --- | --- | --- |
| \* | pDK268\_112012\_01.09117.09117.2 | 3.1074 | 0.2589 | 100.0% | 1093.2322 | 1093.2249 | 1 | 6.763 | 87.5% | 2 | K.AELNEFLTR.E | 2 |
| \* | pDK268\_112012\_02.04559.04559.2 | 4.2426 | 0.4538 | 100.0% | 1425.1721 | 1424.5071 | 1 | 7.779 | 70.8% | 4 | R.ELAEDGYSGVEVR.V | 2 |
| \* | pDK268\_112012\_01.11030.11030.2 | 3.7897 | 0.5565 | 100.0% | 1573.4521 | 1573.7423 | 1 | 8.879 | 80.8% | 3 | R.FGFPEGSVELYAEK.V | 2 |
| \* | pDK268\_112012\_01.07576.07576.2 | 2.1465 | 0.1968 | 98.4% | 799.4322 | 799.0042 | 14 | 4.613 | 78.6% | 1 | K.LLGGLAVR.R | 2 |
| \* | pDK268\_112012\_02.08430.08430.3 | 4.5493 | 0.4044 | 100.0% | 2470.0144 | 2469.7742 | 1 | 7.076 | 38.1% | 2 | K.FVDGLMIHSGDPVNYYVDTAVR.H | 3 |
| \* | pDK268\_112012\_01.05278.05278.3 | 3.8869 | 0.2965 | 100.0% | 1460.9944 | 1459.7288 | 23 | 6.008 | 41.7% | 1 | K.KPLPDHVSIVEPK.D | 3 |
| \* | pDK268\_112012\_01.08181.08181.2 | 2.5131 | 0.1433 | 97.2% | 1471.5122 | 1471.6476 | 9 | 4.038 | 58.3% | 1 | K.DEILPTTPISEQK.G | 2 |
| \* | pDK268\_112012\_01.06922.06922.2 | 3.7393 | 0.4738 | 100.0% | 1574.4122 | 1574.8352 | 1 | 7.559 | 60.0% | 3 | K.GGKPEPPAMPQPVPTA.- | 2 |

---

|  |  |  |  |  |  |  |  |  |
| --- | --- | --- | --- | --- | --- | --- | --- | --- |
| U | *gi|10835063|ref|NP\_00* | 7 | 15 | 44.2% | 294 | 32575 | 4.8 | nucleophosmin 1 isoform 1 [Homo sapiens] |
| U | *gi|40353734|ref|NP\_95* | 7 | 15 | 49.1% | 265 | 29465 | 4.6 | nucleophosmin 1 isoform 2 [Homo sapiens] |

| Filename XCorr DeltCN Conf% ObsM+H+ CalcM+H+ SpR ZScore Ion% # Sequence  | | | | | | | | | | | | |
| --- | --- | --- | --- | --- | --- | --- | --- | --- | --- | --- | --- | --- |
|  | pDK268\_112012\_01.05940.05940.3 | 5.0096 | 0.2266 | 100.0% | 2573.8743 | 2574.7258 | 1 | 7.319 | 42.5% | 1 | K.ADKDYHFKVDNDENEHQLSLR.T | 3 |
|  | pDK268\_112012\_02.08771.08771.3 | 5.8562 | 0.4997 | 100.0% | 2929.1042 | 2931.2874 | 1 | 7.388 | 31.5% | 4 | R.TVSLGAGAKDELHIVEAEAMNYEGSPIK.V | 3 |
|  | pDK268\_112012\_01.13941.13941.2 | 4.7375 | 0.5914 | 100.0% | 2227.392 | 2228.655 | 1 | 9.47 | 57.5% | 3 | K.MSVQPTVSLGGFEITPPVVLR.L | 2 |
|  | pDK268\_112012\_02.05274.05274.3 | 7.0384 | 0.6258 | 100.0% | 4119.534 | 4120.7905 | 1 | 11.061 | 37.1% | 1 | K.LAADEDDDDDDEEDDDEDDDDDDFDDEEAEEKAPVK.K | 3 |
|  | pDK268\_112012\_02.04823.04823.3 | 3.8488 | 0.5224 | 100.0% | 4247.304 | 4248.9644 | 1 | 7.971 | 25.7% | 1 | K.LAADEDDDDDDEEDDDEDDDDDDFDDEEAEEKAPVKK.S | 3 |
|  | pDK268\_112012\_01.04227.04227.2 | 2.7213 | 0.3716 | 100.0% | 931.9322 | 932.0184 | 209 | 6.261 | 62.5% | 2 | K.GPSSVEDIK.A | 2 |
|  | pDK268\_112012\_01.13853.13853.2 | 3.6024 | 0.0825 | 99.6% | 1823.2922 | 1821.0172 | 1 | 3.673 | 69.2% | 3 | R.MTDQEAIQDLWQWR.K | 2 |

---

|  |  |  |  |  |  |  |  |  |
| --- | --- | --- | --- | --- | --- | --- | --- | --- |
| U | *gi|59859885|ref|NP\_00* | 8 | 15 | 44.1% | 295 | 32854 | 4.9 | ribosomal protein SA [Homo sapiens] |
| U | *gi|9845502|ref|NP\_002* | 8 | 15 | 44.1% | 295 | 32854 | 4.9 | ribosomal protein SA [Homo sapiens] |

| Filename XCorr DeltCN Conf% ObsM+H+ CalcM+H+ SpR ZScore Ion% # Sequence  | | | | | | | | | | | | |
| --- | --- | --- | --- | --- | --- | --- | --- | --- | --- | --- | --- | --- |
|  | pDK268\_112012\_02.08728.08728.3 | 6.046 | 0.4429 | 100.0% | 2619.2043 | 2618.9666 | 1 | 9.167 | 40.9% | 3 | K.FLAAGTHLGGTNLDFQMEQYIYK.R | 3 |
|  | pDK268\_112012\_01.08840.08840.2 | 3.2807 | 0.3235 | 100.0% | 1264.2722 | 1264.5077 | 1 | 6.265 | 80.0% | 1 | R.KSDGIYIINLK.R | 2 |
|  | pDK268\_112012\_01.10119.10119.2 | 3.7161 | 0.5565 | 100.0% | 1741.5521 | 1741.9823 | 1 | 9.815 | 56.2% | 3 | R.AIVAIENPADVSVISSR.N | 2 |
|  | pDK268\_112012\_02.04001.04001.2 | 3.3424 | 0.379 | 100.0% | 1205.4722 | 1204.3713 | 1 | 6.428 | 70.8% | 2 | K.FAAATGATPIAGR.F | 2 |
|  | pDK268\_112012\_02.08958.08958.2 | 3.8548 | 0.545 | 100.0% | 1700.5521 | 1699.9065 | 1 | 8.187 | 64.3% | 3 | R.FTPGTFTNQIQAAFR.E | 2 |
|  | pDK268\_112012\_01.06495.06495.2 | 2.5186 | 0.3141 | 99.9% | 912.71216 | 913.10504 | 1 | 6.909 | 85.7% | 1 | R.LLVVTDPR.A | 2 |
|  | pDK268\_112012\_02.10075.10075.2 | 4.3627 | 0.524 | 100.0% | 1615.7322 | 1615.9543 | 1 | 9.912 | 76.9% | 1 | K.GAHSVGLMWWMLAR.E | 2 |
|  | pDK268\_112012\_01.11952.11952.3 | 3.6942 | 0.4401 | 100.0% | 3579.1143 | 3580.9038 | 1 | 7.702 | 24.1% | 1 | R.EHPWEVMPDLYFYRDPEEIEKEEQAAAEK.A | 3 |

---

|  |  |  |  |  |  |  |  |  |
| --- | --- | --- | --- | --- | --- | --- | --- | --- |
| U | *gi|209862831|ref|NP\_0* | 15 | 22 | 43.4% | 339 | 38604 | 7.8 | annexin A2 isoform 2 [Homo sapiens] |
| U | *gi|50845388|ref|NP\_00* | 15 | 22 | 41.2% | 357 | 40411 | 8.4 | annexin A2 isoform 1 [Homo sapiens] |
| U | *gi|50845386|ref|NP\_00* | 15 | 22 | 43.4% | 339 | 38604 | 7.8 | annexin A2 isoform 2 [Homo sapiens] |
| U | *gi|4757756|ref|NP\_004* | 15 | 22 | 43.4% | 339 | 38604 | 7.8 | annexin A2 isoform 2 [Homo sapiens] |

| Filename XCorr DeltCN Conf% ObsM+H+ CalcM+H+ SpR ZScore Ion% # Sequence  | | | | | | | | | | | | |
| --- | --- | --- | --- | --- | --- | --- | --- | --- | --- | --- | --- | --- |
|  | pDK268\_112012\_01.06330.06330.2 | 4.6578 | 0.4841 | 100.0% | 1845.5322 | 1846.0038 | 1 | 8.969 | 61.8% | 1 | K.LSLEGDHSTPPSAYGSVK.A | 2 |
|  | pDK268\_112012\_01.05199.05199.2 | 2.329 | 0.4293 | 100.0% | 1086.7122 | 1087.1338 | 1 | 6.071 | 81.2% | 1 | K.AYTNFDAER.D | 2 |
|  | pDK268\_112012\_01.08852.08852.2 | 2.044 | 0.1802 | 96.0% | 1087.9521 | 1088.2462 | 1 | 5.281 | 72.2% | 1 | R.DALNIETAIK.T | 2 |
|  | pDK268\_112012\_01.11753.11753.2 | 2.8112 | 0.2985 | 99.8% | 1773.1522 | 1773.0397 | 1 | 5.114 | 53.3% | 1 | K.TKGVDEVTIVNILTNR.S | 23 |
|  | pDK268\_112012\_01.13329.13329.2 | 3.4452 | 0.3713 | 100.0% | 1543.4521 | 1543.7605 | 1 | 7.11 | 53.8% | 2 | K.GVDEVTIVNILTNR.S | 2 |
|  | pDK268\_112012\_01.07137.07137.2 | 2.5185 | 0.2964 | 99.9% | 1112.0122 | 1112.2303 | 1 | 5.565 | 81.2% | 1 | R.QDIAFAYQR.R | 2 |
|  | pDK268\_112012\_01.14715.14715.2 | 5.0141 | 0.4674 | 100.0% | 1651.6522 | 1651.9872 | 1 | 9.957 | 70.0% | 2 | K.SALSGHLETVILGLLK.T | 2 |
|  | pDK268\_112012\_02.11044.11044.3 | 4.4287 | 0.4172 | 100.0% | 1652.0944 | 1651.9872 | 1 | 6.943 | 46.7% | 2 | K.SALSGHLETVILGLLK.T | 3 |
|  | pDK268\_112012\_01.05122.05122.2 | 3.657 | 0.5295 | 100.0% | 1222.9521 | 1223.3251 | 1 | 8.806 | 85.0% | 2 | K.TPAQYDASELK.A | 2 |
|  | pDK268\_112012\_01.04793.04793.2 | 3.47 | 0.2807 | 100.0% | 1245.1721 | 1245.3347 | 2 | 6.041 | 77.8% | 2 | R.TNQELQEINR.V | 2 |
|  | pDK268\_112012\_01.09062.09062.3 | 4.7557 | 0.4595 | 100.0% | 1941.4143 | 1941.102 | 1 | 8.342 | 46.9% | 2 | K.TDLEKDIISDTSGDFRK.L | 3 |
|  | pDK268\_112012\_01.09282.09282.2 | 3.9903 | 0.3941 | 100.0% | 2065.5923 | 2066.1887 | 1 | 6.706 | 61.8% | 1 | R.RAEDGSVIDYELIDQDAR.D | 2 |
|  | pDK268\_112012\_01.09276.09276.3 | 3.7002 | 0.2314 | 99.8% | 2066.2144 | 2066.1887 | 1 | 5.066 | 52.9% | 2 | R.RAEDGSVIDYELIDQDAR.D | 3 |
|  | pDK268\_112012\_01.10308.10308.2 | 3.9243 | 0.4369 | 100.0% | 1909.1522 | 1910.0013 | 1 | 7.845 | 59.4% | 1 | R.AEDGSVIDYELIDQDAR.D | 2 |
|  | pDK268\_112012\_01.10758.10758.2 | 2.0712 | 0.2195 | 96.4% | 1588.6921 | 1589.8035 | 11 | 5.101 | 50.0% | 1 | K.SYSPYDMLESIRK.E | 2 |

---

|  |  |  |  |  |  |  |  |  |
| --- | --- | --- | --- | --- | --- | --- | --- | --- |
| U | *gi|194097350|ref|NP\_0* | 36 | 53 | 43.0% | 914 | 105568 | 5.4 | actinin, alpha 1 isoform a [Homo sapiens] |
| U | *gi|4501891|ref|NP\_001* | 36 | 53 | 44.1% | 892 | 103058 | 5.4 | actinin, alpha 1 isoform b [Homo sapiens] |
| U | *gi|194097352|ref|NP\_0* | 36 | 53 | 44.3% | 887 | 102709 | 5.5 | actinin, alpha 1 isoform c [Homo sapiens] |

| Filename XCorr DeltCN Conf% ObsM+H+ CalcM+H+ SpR ZScore Ion% # Sequence  | | | | | | | | | | | | |
| --- | --- | --- | --- | --- | --- | --- | --- | --- | --- | --- | --- | --- |
|  | pDK268\_112012\_01.11588.11588.2 | 2.7505 | 0.0539 | 97.6% | 1200.3322 | 1200.377 | 5 | 5.033 | 66.7% | 1 | R.DLLLDPAWEK.Q | 22 |
|  | pDK268\_112012\_01.15545.15545.2 | 4.4969 | 0.3334 | 100.0% | 1374.3322 | 1373.6941 | 1 | 7.133 | 81.8% | 3 | K.LMLLLEVISGER.L | 22 |
|  | pDK268\_112012\_01.07731.07731.2 | 2.8483 | 0.4277 | 100.0% | 865.0122 | 865.01715 | 1 | 7.269 | 92.9% | 1 | K.ALDFIASK.G | 22 |
|  | pDK268\_112012\_01.17678.17678.2 | 2.2663 | 0.2326 | 98.4% | 1449.0922 | 1448.8683 | 10 | 5.145 | 63.6% | 2 | K.MTLGMIWTIILR.F | 22 |
|  | pDK268\_112012\_01.09399.09399.2 | 4.5282 | 0.3891 | 100.0% | 1538.2522 | 1538.6947 | 1 | 9.071 | 80.8% | 2 | R.FAIQDISVEETSAK.E | 22 |
|  | pDK268\_112012\_01.10316.10316.2 | 3.3355 | 0.3687 | 100.0% | 1500.2722 | 1500.7001 | 1 | 6.611 | 63.6% | 1 | K.NVNIQNFHISWK.D | 2 |
|  | pDK268\_112012\_01.04701.04701.2 | 2.539 | 0.27 | 99.8% | 1228.4922 | 1228.3934 | 4 | 5.241 | 66.7% | 1 | R.HRPELIDYGK.L | 2 |
|  | pDK268\_112012\_01.08703.08703.2 | 4.0056 | 0.4541 | 100.0% | 1760.5922 | 1760.9568 | 1 | 8.102 | 60.0% | 1 | K.MLDAEDIVGTARPDEK.A | 2 |
|  | pDK268\_112012\_01.08667.08667.3 | 2.9671 | 0.2902 | 99.8% | 1761.3844 | 1760.9568 | 398 | 4.786 | 30.0% | 1 | K.MLDAEDIVGTARPDEK.A | 3 |
|  | pDK268\_112012\_01.13420.13420.2 | 5.057 | 0.4956 | 100.0% | 2008.3121 | 2009.2867 | 1 | 9.528 | 70.6% | 1 | K.AIMTYVSSFYHAFSGAQK.A | 22 |
|  | pDK268\_112012\_01.13448.13448.3 | 3.1893 | 0.366 | 100.0% | 2009.7244 | 2009.2867 | 1 | 6.723 | 38.2% | 2 | K.AIMTYVSSFYHAFSGAQK.A | 33 |
|  | pDK268\_112012\_02.06705.06705.2 | 5.6583 | 0.5339 | 100.0% | 2052.2522 | 2053.2483 | 1 | 9.633 | 68.8% | 2 | K.VLAVNQENEQLMEDYEK.L | 2 |
|  | pDK268\_112012\_01.19112.19112.3 | 4.8689 | 0.4614 | 100.0% | 3249.4443 | 3250.6477 | 1 | 7.359 | 29.8% | 1 | K.VLAVNQENEQLMEDYEKLASDLLEWIR.R | 3 |
|  | pDK268\_112012\_01.13638.13638.1 | 2.5564 | 0.293 | 100.0% | 1215.52 | 1216.4227 | 1 | 5.821 | 61.1% | 1 | K.LASDLLEWIR.R | 11 |
|  | pDK268\_112012\_01.13664.13664.2 | 3.4437 | 0.4167 | 100.0% | 1215.7722 | 1216.4227 | 1 | 7.564 | 88.9% | 2 | K.LASDLLEWIR.R | 22 |
|  | pDK268\_112012\_01.13160.13160.2 | 3.8297 | 0.4385 | 100.0% | 1422.1721 | 1422.5803 | 1 | 7.238 | 80.0% | 2 | K.GYEEWLLNEIR.R | 22 |
|  | pDK268\_112012\_01.04862.04862.2 | 3.4341 | 0.4239 | 100.0% | 1215.0922 | 1215.3079 | 1 | 6.831 | 65.0% | 1 | K.ASIHEAWTDGK.E | 22 |
|  | pDK268\_112012\_02.04651.04651.3 | 2.3418 | 0.2697 | 97.6% | 1526.2144 | 1526.6837 | 1 | 4.623 | 43.8% | 1 | R.QKDYETATLSEIK.A | 3 |
|  | pDK268\_112012\_01.04672.04672.3 | 4.4993 | 0.4701 | 100.0% | 1626.4143 | 1626.6824 | 1 | 8.234 | 55.8% | 2 | K.HEAFESDLAAHQDR.V | 33 |
|  | pDK268\_112012\_01.13844.13844.2 | 3.7158 | 0.4857 | 100.0% | 1712.6921 | 1712.9806 | 1 | 8.075 | 61.5% | 1 | K.LLETIDQLYLEYAK.R | 2 |
|  | pDK268\_112012\_01.19238.19238.3 | 5.4318 | 0.4857 | 100.0% | 4365.1143 | 4364.8477 | 1 | 7.507 | 25.7% | 1 | R.AAPFNNWMEGAMEDLQDTFIVHTIEEIQGLTTAHEQFK.A | 3 |
|  | pDK268\_112012\_01.03689.03689.2 | 2.1957 | 0.1526 | 96.6% | 1116.2522 | 1116.2163 | 1 | 4.157 | 77.8% | 1 | K.ATLPDADKER.L | 2 |
|  | pDK268\_112012\_01.07684.07684.2 | 3.9872 | 0.5476 | 100.0% | 1294.3722 | 1294.537 | 1 | 9.063 | 77.3% | 3 | R.LAILGIHNEVSK.I | 2 |
|  | pDK268\_112012\_01.03416.03416.2 | 2.0293 | 0.2199 | 97.0% | 1169.2922 | 1170.2241 | 1 | 4.564 | 83.3% | 1 | R.DQALTEEHAR.Q | 2 |
|  | pDK268\_112012\_02.07517.07517.2 | 3.4305 | 0.416 | 100.0% | 1979.4122 | 1980.2463 | 1 | 6.651 | 56.2% | 1 | R.ISIEMHGTLEDQLSHLR.Q | 2 |
|  | pDK268\_112012\_02.07527.07527.3 | 4.6107 | 0.4174 | 100.0% | 1979.7544 | 1980.2463 | 1 | 7.477 | 50.0% | 1 | R.ISIEMHGTLEDQLSHLR.Q | 3 |
|  | pDK268\_112012\_01.04082.04082.2 | 1.7727 | 0.3275 | 98.5% | 948.39215 | 949.13806 | 11 | 5.748 | 64.3% | 1 | K.SIVNYKPK.I | 2 |
|  | pDK268\_112012\_01.12186.12186.2 | 5.7611 | 0.4982 | 100.0% | 2339.7522 | 2340.595 | 1 | 10.342 | 68.4% | 1 | K.IDQLEGDHQLIQEALIFDNK.H | 2 |
|  | pDK268\_112012\_01.12226.12226.3 | 3.1604 | 0.2174 | 98.3% | 2340.0842 | 2340.595 | 2 | 4.006 | 32.9% | 1 | K.IDQLEGDHQLIQEALIFDNK.H | 3 |
|  | pDK268\_112012\_01.04023.04023.3 | 4.0352 | 0.3532 | 100.0% | 1303.7943 | 1302.4503 | 1 | 6.561 | 52.8% | 1 | K.HTNYTMEHIR.V | 33 |
|  | pDK268\_112012\_01.14414.14414.2 | 4.1948 | 0.3964 | 100.0% | 1386.9521 | 1387.6218 | 1 | 8.068 | 77.3% | 4 | R.VGWEQLLTTIAR.T | 22 |
|  | pDK268\_112012\_01.08912.08912.2 | 3.9714 | 0.3062 | 100.0% | 1431.4321 | 1430.6011 | 1 | 6.48 | 77.3% | 2 | R.TINEVENQILTR.D | 22 |
|  | pDK268\_112012\_01.06792.06792.2 | 2.0813 | 0.3778 | 99.8% | 1338.1721 | 1339.4656 | 36 | 6.026 | 55.0% | 1 | K.GISQEQMNEFR.A | 2 |
|  | pDK268\_112012\_02.07241.07241.2 | 4.3366 | 0.5339 | 100.0% | 1730.1322 | 1730.8408 | 1 | 10.186 | 70.0% | 2 | R.ETADTDTADQVMASFK.I | 2 |
|  | pDK268\_112012\_01.08951.08951.2 | 4.0854 | 0.3203 | 100.0% | 1752.3322 | 1753.0234 | 1 | 6.814 | 67.9% | 1 | K.ILAGDKNYITMDELR.R | 2 |
|  | pDK268\_112012\_02.06664.06664.3 | 3.948 | 0.3011 | 100.0% | 1752.3844 | 1753.0234 | 1 | 5.774 | 44.6% | 2 | K.ILAGDKNYITMDELR.R | 3 |

Similarities:
gi|12025678|ref|NP\_00(15:21)  

---

|  |  |  |  |  |  |  |  |  |
| --- | --- | --- | --- | --- | --- | --- | --- | --- |
| U | *gi|4506679|ref|NP\_001* | 7 | 13 | 43.0% | 165 | 18898 | 10.2 | ribosomal protein S10 [Homo sapiens] |

| Filename XCorr DeltCN Conf% ObsM+H+ CalcM+H+ SpR ZScore Ion% # Sequence  | | | | | | | | | | | | |
| --- | --- | --- | --- | --- | --- | --- | --- | --- | --- | --- | --- | --- |
|  | pDK268\_112012\_01.14306.14306.2 | 2.998 | 0.4895 | 100.0% | 1110.2722 | 1110.3818 | 1 | 7.806 | 87.5% | 2 | R.IAIYELLFK.E | 2 |
| \* | pDK268\_112012\_01.05715.05715.3 | 2.957 | 0.2939 | 99.8% | 1842.9543 | 1843.154 | 130 | 5.026 | 33.3% | 1 | K.HPELADKNVPNLHVMK.A | 3 |
| \* | pDK268\_112012\_02.09065.09065.2 | 5.2401 | 0.5617 | 100.0% | 2003.2922 | 2004.2548 | 1 | 11.027 | 78.6% | 2 | R.HFYWYLTNEGIQYLR.D | 2 |
| \* | pDK268\_112012\_01.10707.10707.3 | 3.223 | 0.3079 | 100.0% | 1890.3544 | 1891.2235 | 1 | 6.216 | 40.0% | 1 | R.DYLHLPPEIVPATLRR.S | 3 |
|  | pDK268\_112012\_02.05280.05280.2 | 4.8194 | 0.4495 | 100.0% | 1570.3922 | 1570.7019 | 1 | 8.703 | 85.7% | 2 | K.KAEAGAGSATEFQFR.G | 2 |
|  | pDK268\_112012\_02.04531.04531.3 | 3.9873 | 0.3085 | 100.0% | 1570.7644 | 1570.7019 | 1 | 5.443 | 46.4% | 1 | K.KAEAGAGSATEFQFR.G | 3 |
|  | pDK268\_112012\_02.05938.05938.2 | 3.9393 | 0.5024 | 100.0% | 1442.0322 | 1442.5278 | 1 | 8.433 | 73.1% | 4 | K.AEAGAGSATEFQFR.G | 2 |

---

|  |  |  |  |  |  |  |  |  |
| --- | --- | --- | --- | --- | --- | --- | --- | --- |
| U | *gi|62414289|ref|NP\_00* | 23 | 31 | 42.9% | 466 | 53652 | 5.1 | vimentin [Homo sapiens] |

| Filename XCorr DeltCN Conf% ObsM+H+ CalcM+H+ SpR ZScore Ion% # Sequence  | | | | | | | | | | | | |
| --- | --- | --- | --- | --- | --- | --- | --- | --- | --- | --- | --- | --- |
| \* | pDK268\_112012\_01.06392.06392.2 | 2.3843 | 0.2581 | 98.9% | 1496.3522 | 1496.6633 | 3 | 5.03 | 53.8% | 1 | R.TYSLGSALRPSTSR.S | 2 |
| \* | pDK268\_112012\_01.06554.06554.2 | 3.7963 | 0.4345 | 100.0% | 1430.1522 | 1429.5724 | 1 | 7.504 | 76.9% | 2 | R.SLYASSPGGVYATR.S | 2 |
| \* | pDK268\_112012\_01.07552.07552.2 | 2.8205 | 0.2929 | 99.9% | 1509.0721 | 1509.5724 | 1 | 5.167 | 65.4% | 1 | R.SLYASS\*PGGVYATR.S | 23 |
|  | pDK268\_112012\_01.05638.05638.2 | 3.8501 | 0.4107 | 100.0% | 1588.2122 | 1588.7147 | 1 | 7.041 | 75.0% | 1 | R.TNEKVELQELNDR.F | 2 |
|  | pDK268\_112012\_02.04249.04249.3 | 3.2111 | 0.2357 | 99.6% | 1588.6444 | 1588.7147 | 4 | 4.784 | 43.8% | 1 | R.TNEKVELQELNDR.F | 3 |
|  | pDK268\_112012\_01.06216.06216.2 | 2.7022 | 0.2201 | 99.8% | 1115.9122 | 1116.2163 | 1 | 5.447 | 93.8% | 1 | K.VELQELNDR.F | 2 |
| \* | pDK268\_112012\_01.11854.11854.2 | 3.3616 | 0.2993 | 100.0% | 1170.4122 | 1170.4349 | 4 | 6.653 | 72.2% | 1 | K.ILLAELEQLK.G | 2 |
| \* | pDK268\_112012\_01.09809.09809.2 | 3.2968 | 0.1522 | 99.7% | 1540.4122 | 1540.8436 | 1 | 5.722 | 69.2% | 1 | K.ILLAELEQLKGQGK.S | 2 |
| \* | pDK268\_112012\_01.07887.07887.2 | 3.5387 | 0.3417 | 100.0% | 1256.1921 | 1255.385 | 1 | 6.442 | 77.8% | 1 | R.LGDLYEEEMR.E | 2 |
| \* | pDK268\_112012\_01.09807.09807.2 | 2.3447 | 0.2312 | 99.5% | 1077.2322 | 1077.1975 | 2 | 5.991 | 68.8% | 2 | R.DNLAEDIMR.L | 2 |
| \* | pDK268\_112012\_01.05294.05294.2 | 2.6101 | 0.1245 | 99.3% | 1048.1522 | 1047.2146 | 40 | 3.89 | 78.6% | 1 | K.LQEEMLQR.E | 2 |
| \* | pDK268\_112012\_02.06925.06925.3 | 3.1534 | 0.2065 | 97.8% | 2351.6343 | 2352.581 | 52 | 5.06 | 30.6% | 1 | K.LQEEMLQREEAENTLQSFR.Q | 3 |
| \* | pDK268\_112012\_01.06988.06988.2 | 2.4593 | 0.2998 | 99.8% | 1323.8121 | 1324.3898 | 1 | 5.061 | 65.0% | 2 | R.EEAENTLQSFR.Q | 2 |
| \* | pDK268\_112012\_01.03993.03993.2 | 2.6138 | 0.3508 | 100.0% | 1088.9521 | 1089.1503 | 1 | 5.382 | 83.3% | 1 | R.QDVDNASLAR.L | 2 |
| \* | pDK268\_112012\_01.10559.10559.2 | 4.574 | 0.5059 | 100.0% | 1534.1322 | 1534.793 | 1 | 9.597 | 87.5% | 1 | R.KVESLQEEIAFLK.K | 2 |
| \* | pDK268\_112012\_01.09236.09236.3 | 4.6052 | 0.3707 | 100.0% | 1663.1344 | 1662.967 | 14 | 6.589 | 38.5% | 1 | R.KVESLQEEIAFLKK.L | 3 |
|  | pDK268\_112012\_02.05458.05458.2 | 2.0998 | 0.1883 | 96.8% | 1310.7322 | 1310.4056 | 9 | 4.763 | 55.6% | 2 | K.NLQEAEEWYK.S | 23 |
| \* | pDK268\_112012\_01.05858.05858.2 | 3.4943 | 0.4103 | 100.0% | 1093.6522 | 1094.1692 | 1 | 7.822 | 94.4% | 4 | K.FADLSEAANR.N | 2 |
| \* | pDK268\_112012\_02.07579.07579.2 | 5.827 | 0.6176 | 100.0% | 2187.2122 | 2188.33 | 1 | 10.578 | 66.7% | 1 | R.EMEENFAVEAANYQDTIGR.L | 2 |
| \* | pDK268\_112012\_02.07565.07565.3 | 3.8011 | 0.3626 | 100.0% | 2189.0344 | 2188.33 | 1 | 6.478 | 36.1% | 1 | R.EMEENFAVEAANYQDTIGR.L | 3 |
| \* | pDK268\_112012\_01.07762.07762.2 | 4.133 | 0.4094 | 100.0% | 1734.7722 | 1735.9679 | 1 | 7.698 | 69.2% | 1 | R.LQDEIQNMKEEMAR.H | 2 |
| \* | pDK268\_112012\_01.13859.13859.2 | 3.1874 | 0.4137 | 100.0% | 1572.0922 | 1571.8601 | 1 | 6.776 | 73.1% | 2 | R.ISLPLPNFSSLNLR.E | 2 |
| \* | pDK268\_112012\_01.04902.04902.2 | 3.6812 | 0.3774 | 100.0% | 1837.1921 | 1837.854 | 1 | 6.916 | 56.7% | 1 | R.DGQVINETSQHHDDLE.- | 2 |

---

|  |  |  |  |  |  |  |  |  |
| --- | --- | --- | --- | --- | --- | --- | --- | --- |
| U | *gi|225690529|ref|NP\_6* | 19 | 41 | 42.9% | 394 | 44993 | 7.1 | golgin, RAB6-interacting isoform a [Homo sapiens] |

| Filename XCorr DeltCN Conf% ObsM+H+ CalcM+H+ SpR ZScore Ion% # Sequence  | | | | | | | | | | | | |
| --- | --- | --- | --- | --- | --- | --- | --- | --- | --- | --- | --- | --- |
|  | pDK268\_112012\_01.12962.12962.2 | 5.9574 | 0.5491 | 100.0% | 2067.4321 | 2068.3752 | 1 | 9.91 | 68.4% | 5 | K.LGLQDGSTSLLPEQLLSAPK.Q | 2 |
|  | pDK268\_112012\_01.10328.10328.3 | 3.3358 | 0.4784 | 100.0% | 3802.7944 | 3804.2512 | 1 | 7.218 | 27.1% | 1 | R.VNVQKPPFSSPTLPSHFTLTSPVGDGQPQGIESQPK.E | 3 |
|  | pDK268\_112012\_01.10599.10599.3 | 4.7624 | 0.2968 | 100.0% | 3883.2244 | 3884.2512 | 1 | 6.005 | 27.1% | 1 | R.VNVQKPPFSSPTLPSHFTLTS\*PVGDGQPQGIESQPK.E | 3 |
|  | pDK268\_112012\_01.06215.06215.2 | 3.8352 | 0.2644 | 100.0% | 1459.2922 | 1459.6047 | 1 | 5.022 | 75.0% | 2 | K.SRWEVLQQEQR.L | 2 |
|  | pDK268\_112012\_01.06176.06176.3 | 4.1392 | 0.0877 | 98.9% | 1459.9744 | 1459.6047 | 2 | 4.059 | 55.0% | 1 | K.SRWEVLQQEQR.L | 3 |
|  | pDK268\_112012\_01.07181.07181.2 | 2.9934 | 0.2728 | 100.0% | 1216.1522 | 1216.339 | 1 | 5.481 | 81.2% | 1 | R.WEVLQQEQR.L | 2 |
|  | pDK268\_112012\_01.14682.14682.2 | 2.8212 | 0.2962 | 99.8% | 2230.132 | 2229.597 | 1 | 4.747 | 44.7% | 1 | R.IQKELQALDDMVSADIGILR.N | 2 |
|  | pDK268\_112012\_02.10641.10641.2 | 4.8442 | 0.4798 | 100.0% | 1859.7722 | 1860.1327 | 1 | 9.124 | 75.0% | 5 | K.ELQALDDMVSADIGILR.N | 2 |
|  | pDK268\_112012\_01.14397.14397.3 | 3.3594 | 0.2451 | 99.6% | 1860.5343 | 1860.1327 | 1 | 5.407 | 42.2% | 1 | K.ELQALDDMVSADIGILR.N | 3 |
|  | pDK268\_112012\_01.06526.06526.2 | 3.8377 | 0.4699 | 100.0% | 1672.1921 | 1672.7948 | 1 | 7.553 | 57.7% | 3 | R.NRIDQASLDYSYAR.K | 2 |
|  | pDK268\_112012\_02.05193.05193.3 | 4.0625 | 0.3784 | 100.0% | 1673.0343 | 1672.7948 | 1 | 7.019 | 46.2% | 3 | R.NRIDQASLDYSYAR.K | 3 |
|  | pDK268\_112012\_01.07120.07120.2 | 4.3937 | 0.5166 | 100.0% | 1402.2322 | 1402.5034 | 1 | 8.715 | 86.4% | 4 | R.IDQASLDYSYAR.K | 2 |
| \* | pDK268\_112012\_02.04738.04738.3 | 2.5981 | 0.2561 | 97.6% | 2102.4844 | 2105.3176 | 52 | 5.134 | 28.1% | 2 | R.IDQASLDYSYARKRFDR.A | 3 |
| \* | pDK268\_112012\_01.05686.05686.2 | 2.3962 | 0.1066 | 95.7% | 1384.0322 | 1384.5315 | 3 | 4.708 | 59.1% | 1 | R.FDRAEAEYIAAK.L | 2 |
| \* | pDK268\_112012\_01.04208.04208.2 | 2.2825 | 0.4228 | 100.0% | 965.4122 | 966.07886 | 1 | 6.801 | 87.5% | 1 | R.AEAEYIAAK.L | 2 |
| \* | pDK268\_112012\_02.11350.11350.3 | 3.9063 | 0.2224 | 99.3% | 3119.0942 | 3118.434 | 1 | 4.068 | 29.0% | 1 | K.KLEELMQQLDVEADEETLELEVEVER.L | 3 |
| \* | pDK268\_112012\_01.03903.03903.2 | 3.1551 | 0.3921 | 100.0% | 1240.1721 | 1240.3585 | 1 | 6.414 | 77.8% | 1 | R.LLHEQEVESR.R | 2 |
| \* | pDK268\_112012\_01.10116.10116.2 | 5.2195 | 0.5539 | 100.0% | 2091.5322 | 2092.3562 | 1 | 10.717 | 70.6% | 2 | R.LERPFQPAEESVTLEFAK.E | 2 |
| \* | pDK268\_112012\_01.10208.10208.3 | 5.0108 | 0.5 | 100.0% | 2092.4644 | 2092.3562 | 1 | 7.908 | 48.5% | 5 | R.LERPFQPAEESVTLEFAK.E | 3 |

---

|  |  |  |  |  |  |  |  |  |
| --- | --- | --- | --- | --- | --- | --- | --- | --- |
| U | *gi|63025212|ref|NP\_98* | 8 | 12 | 42.9% | 266 | 27202 | 4.9 | hypothetical protein LOC255374 [Homo sapiens] |

| Filename XCorr DeltCN Conf% ObsM+H+ CalcM+H+ SpR ZScore Ion% # Sequence  | | | | | | | | | | | | |
| --- | --- | --- | --- | --- | --- | --- | --- | --- | --- | --- | --- | --- |
| \* | pDK268\_112012\_01.08370.08370.2 | 2.1339 | 0.1963 | 96.3% | 1357.5122 | 1357.5498 | 75 | 4.129 | 45.8% | 1 | R.ADGSVTLVLPQTR.G | 2 |
| \* | pDK268\_112012\_01.06682.06682.2 | 4.486 | 0.3999 | 100.0% | 1290.0721 | 1289.3445 | 1 | 8.266 | 73.1% | 3 | R.GSGGAEAALEEAAR.G | 2 |
| \* | pDK268\_112012\_01.09903.09903.2 | 4.2859 | 0.5586 | 100.0% | 1338.7922 | 1339.537 | 1 | 9.33 | 75.0% | 3 | R.GPILVDTGGPWAR.E | 2 |
| \* | pDK268\_112012\_01.06479.06479.3 | 2.9534 | 0.2811 | 99.9% | 1437.3544 | 1437.6414 | 65 | 4.847 | 35.4% | 1 | R.YLPHGLGEGQPLR.L | 3 |
| \* | pDK268\_112012\_01.08765.08765.2 | 4.0124 | 0.5609 | 100.0% | 1732.6522 | 1732.9395 | 1 | 9.203 | 59.4% | 1 | R.LGPGLEVWATPGHGGQR.D | 2 |
| \* | pDK268\_112012\_01.08780.08780.3 | 2.82 | 0.3571 | 100.0% | 1733.0944 | 1732.9395 | 2 | 6.203 | 42.2% | 1 | R.LGPGLEVWATPGHGGQR.D | 3 |
| \* | pDK268\_112012\_01.10535.10535.2 | 4.1646 | 0.5077 | 100.0% | 1758.5122 | 1759.1044 | 1 | 8.741 | 56.2% | 1 | R.VLVVADVVVPGHGPPFR.V | 2 |
| \* | pDK268\_112012\_02.04572.04572.3 | 3.945 | 0.414 | 100.0% | 2792.1843 | 2792.8433 | 1 | 6.486 | 27.9% | 1 | R.EASQPETEGGGNSQQEPVVGDEEPALH.- | 3 |

---

|  |  |  |  |  |  |  |  |  |
| --- | --- | --- | --- | --- | --- | --- | --- | --- |
| U | *gi|4506741|ref|NP\_001* | 5 | 6 | 42.8% | 194 | 22127 | 10.1 | ribosomal protein S7 [Homo sapiens] |

| Filename XCorr DeltCN Conf% ObsM+H+ CalcM+H+ SpR ZScore Ion% # Sequence  | | | | | | | | | | | | |
| --- | --- | --- | --- | --- | --- | --- | --- | --- | --- | --- | --- | --- |
| \* | pDK268\_112012\_01.13917.13917.3 | 5.4466 | 0.4668 | 100.0% | 3332.0645 | 3332.7473 | 1 | 6.903 | 28.4% | 1 | K.IVKPNGEKPDEFESGISQALLELEMNSDLK.A | 3 |
| \* | pDK268\_112012\_01.13606.13606.2 | 2.1092 | 0.207 | 96.8% | 1338.2522 | 1338.7196 | 16 | 4.961 | 50.0% | 1 | K.AIIIFVPVPQLK.S | 2 |
| \* | pDK268\_112012\_01.14331.14331.2 | 4.2656 | 0.4364 | 100.0% | 2367.672 | 2368.7324 | 1 | 9.528 | 54.8% | 2 | R.TLTAVHDAILEDLVFPSEIVGK.R | 2 |
| \* | pDK268\_112012\_01.13640.13640.3 | 5.2849 | 0.3512 | 100.0% | 2525.7544 | 2524.92 | 1 | 7.202 | 43.2% | 1 | R.TLTAVHDAILEDLVFPSEIVGKR.I | 3 |
|  | pDK268\_112012\_01.06627.06627.3 | 3.2867 | 0.3123 | 100.0% | 2079.5044 | 2079.2769 | 1 | 4.924 | 39.7% | 1 | K.AQQNNVEHKVETFSGVYK.K | 3 |

---

|  |  |  |  |  |  |  |  |  |
| --- | --- | --- | --- | --- | --- | --- | --- | --- |
| U | *gi|4506439|ref|NP\_002* | 13 | 24 | 42.6% | 425 | 47820 | 5.0 | retinoblastoma binding protein 7 [Homo sapiens] |

| Filename XCorr DeltCN Conf% ObsM+H+ CalcM+H+ SpR ZScore Ion% # Sequence  | | | | | | | | | | | | |
| --- | --- | --- | --- | --- | --- | --- | --- | --- | --- | --- | --- | --- |
| \* | pDK268\_112012\_02.04653.04653.2 | 2.3754 | 0.3677 | 99.9% | 1286.0322 | 1285.3679 | 1 | 5.554 | 66.7% | 2 | K.EMFEDTVEER.V | 2 |
|  | pDK268\_112012\_01.03830.03830.2 | 2.2671 | 0.2807 | 99.8% | 894.3522 | 895.00006 | 4 | 5.031 | 83.3% | 2 | R.VINEEYK.I | 22 |
| \* | pDK268\_112012\_01.16582.16582.3 | 5.8649 | 0.5104 | 100.0% | 3841.8245 | 3841.4597 | 1 | 8.723 | 28.1% | 1 | K.NTPFLYDLVMTHALQWPSLTVQWLPEVTKPEGK.D | 3 |
| \* | pDK268\_112012\_02.08143.08143.3 | 5.8328 | 0.5139 | 100.0% | 2775.1143 | 2776.0837 | 1 | 8.706 | 43.5% | 2 | K.DYALHWLVLGTHTSDEQNHLVVAR.V | 3 |
| \* | pDK268\_112012\_01.05295.05295.2 | 2.7856 | 0.3771 | 100.0% | 1413.2122 | 1413.6776 | 1 | 5.822 | 72.7% | 1 | R.YMPQNPHIIATK.T | 2 |
|  | pDK268\_112012\_01.10179.10179.2 | 4.0312 | 0.5394 | 100.0% | 1473.1921 | 1472.6348 | 1 | 9.306 | 70.8% | 3 | K.TPSSDVLVFDYTK.H | 22 |
| \* | pDK268\_112012\_01.15005.15005.3 | 7.211 | 0.5366 | 100.0% | 3381.0244 | 3380.741 | 1 | 9.356 | 34.2% | 3 | K.AIFTGHSAVVEDVAWHLLHESLFGSVADDQK.L | 3 |
|  | pDK268\_112012\_01.09741.09741.2 | 2.284 | 0.1848 | 99.3% | 936.2522 | 935.129 | 10 | 4.06 | 83.3% | 1 | K.LMIWDTR.S | 22 |
|  | pDK268\_112012\_01.10785.10785.1 | 1.9496 | 0.3307 | 100.0% | 973.47 | 974.1478 | 2 | 5.225 | 71.4% | 1 | K.TVALWDLR.N | 11 |
|  | pDK268\_112012\_01.10822.10822.2 | 2.4879 | 0.1806 | 99.6% | 974.09216 | 974.1478 | 5 | 5.8 | 85.7% | 2 | K.TVALWDLR.N | 22 |
|  | pDK268\_112012\_01.08295.08295.2 | 2.7427 | 0.1728 | 99.8% | 1130.7722 | 1131.3201 | 4 | 4.485 | 68.8% | 2 | R.RLNVWDLSK.I | 22 |
|  | pDK268\_112012\_01.10073.10073.2 | 2.1647 | 0.3406 | 99.8% | 975.0722 | 975.13257 | 6 | 6.199 | 85.7% | 2 | R.LNVWDLSK.I | 22 |
| \* | pDK268\_112012\_01.09503.09503.3 | 5.2465 | 0.4884 | 100.0% | 2847.6243 | 2849.0398 | 1 | 8.267 | 35.6% | 2 | K.IGEEQSAEDAEDGPPELLFIHGGHTAK.I | 3 |

Similarities:
gi|207029415|ref|NP\_0(7:6)  

---

|  |  |  |  |  |  |  |  |  |
| --- | --- | --- | --- | --- | --- | --- | --- | --- |
| U | *gi|29788768|ref|NP\_82* | 21 | 53 | 42.5% | 445 | 49953 | 4.9 | tubulin, beta 2B [Homo sapiens] |
| U | *gi|4507729|ref|NP\_001* | 21 | 53 | 42.5% | 445 | 49907 | 4.9 | tubulin, beta 2 [Homo sapiens] |

| Filename XCorr DeltCN Conf% ObsM+H+ CalcM+H+ SpR ZScore Ion% # Sequence  | | | | | | | | | | | | |
| --- | --- | --- | --- | --- | --- | --- | --- | --- | --- | --- | --- | --- |
|  | pDK268\_112012\_01.10874.10874.2 | 4.8374 | 0.5405 | 100.0% | 1616.4321 | 1616.8701 | 1 | 8.91 | 78.6% | 6 | R.AILVDLEPGTMDSVR.S | 222 |
|  | pDK268\_112012\_01.13136.13136.3 | 7.9102 | 0.5123 | 100.0% | 2800.1643 | 2800.0647 | 1 | 9.259 | 46.0% | 5 | R.SGPFGQIFRPDNFVFGQSGAGNNWAK.G | 333 |
|  | pDK268\_112012\_01.13134.13134.2 | 4.3881 | 0.3308 | 100.0% | 2801.9321 | 2800.0647 | 3 | 5.679 | 36.0% | 1 | R.SGPFGQIFRPDNFVFGQSGAGNNWAK.G | 222 |
|  | pDK268\_112012\_01.13478.13478.2 | 7.2309 | 0.5963 | 100.0% | 1958.9321 | 1960.151 | 1 | 10.867 | 79.4% | 4 | K.GHYTEGAELVDSVLDVVR.K | 2222 |
|  | pDK268\_112012\_01.13460.13460.3 | 4.2635 | 0.3564 | 100.0% | 1960.4644 | 1960.151 | 1 | 6.094 | 41.2% | 2 | K.GHYTEGAELVDSVLDVVR.K | 3333 |
|  | pDK268\_112012\_01.12464.12464.3 | 4.2939 | 0.4255 | 100.0% | 2089.0144 | 2088.325 | 1 | 7.427 | 50.0% | 2 | K.GHYTEGAELVDSVLDVVRK.E | 3333 |
|  | pDK268\_112012\_01.12462.12462.2 | 4.7554 | 0.3831 | 100.0% | 2089.5923 | 2088.325 | 1 | 6.134 | 63.9% | 1 | K.GHYTEGAELVDSVLDVVRK.E | 2222 |
|  | pDK268\_112012\_02.08605.08605.3 | 5.4828 | 0.4494 | 100.0% | 3329.0044 | 3329.5925 | 1 | 6.735 | 29.8% | 1 | K.ESESCDCLQGFQLTHSLGGGTGSGMGTLLISK.I | 32 |
|  | pDK268\_112012\_01.03614.03614.2 | 2.9306 | 0.2373 | 100.0% | 1077.6122 | 1078.1698 | 2 | 4.833 | 85.7% | 2 | K.IREEYPDR.I | 222 |
|  | pDK268\_112012\_01.07874.07874.2 | 2.8399 | 0.2733 | 99.9% | 1131.4722 | 1131.2767 | 1 | 4.886 | 88.9% | 5 | R.FPGQLNADLR.K | 22222 |
|  | pDK268\_112012\_01.06263.06263.3 | 2.7439 | 0.2398 | 98.9% | 1259.0643 | 1259.4508 | 21 | 5.025 | 42.5% | 1 | R.FPGQLNADLRK.L | 33333 |
|  | pDK268\_112012\_01.06137.06137.2 | 2.6599 | 0.2453 | 99.8% | 1259.1721 | 1259.4508 | 6 | 4.519 | 65.0% | 1 | R.FPGQLNADLRK.L | 22222 |
|  | pDK268\_112012\_01.08991.08991.2 | 3.3289 | 0.3674 | 100.0% | 1271.8522 | 1272.5945 | 1 | 8.084 | 80.0% | 2 | R.KLAVNMVPFPR.L | 22222 |
|  | pDK268\_112012\_01.10427.10427.1 | 2.0779 | 0.358 | 100.0% | 1143.58 | 1144.4204 | 51 | 6.116 | 55.6% | 1 | K.LAVNMVPFPR.L | 11111 |
|  | pDK268\_112012\_01.10442.10442.2 | 3.7495 | 0.4789 | 100.0% | 1144.3322 | 1144.4204 | 1 | 9.006 | 88.9% | 1 | K.LAVNMVPFPR.L | 22222 |
|  | pDK268\_112012\_01.12644.12644.3 | 3.8334 | 0.3162 | 100.0% | 1622.1843 | 1621.9403 | 1 | 5.671 | 55.8% | 2 | R.LHFFMPGFAPLTSR.G | 3333 |
|  | pDK268\_112012\_02.08489.08489.2 | 3.5612 | 0.4285 | 100.0% | 1622.4122 | 1621.9403 | 1 | 7.702 | 61.5% | 5 | R.LHFFMPGFAPLTSR.G | 2222 |
|  | pDK268\_112012\_01.06597.06597.2 | 4.388 | 0.253 | 100.0% | 1448.2722 | 1447.6031 | 1 | 6.034 | 81.8% | 4 | K.EVDEQMLNVQNK.N | 222 |
|  | pDK268\_112012\_01.12033.12033.2 | 3.9628 | 0.4064 | 100.0% | 1697.3322 | 1697.8877 | 1 | 8.146 | 76.9% | 2 | K.NSSYFVEWIPNNVK.T | 22222 |
|  | pDK268\_112012\_02.09277.09277.3 | 3.4682 | 0.1568 | 97.1% | 2015.4243 | 2015.335 | 1 | 6.218 | 44.1% | 1 | K.MSATFIGNSTAIQELFKR.I | 33 |
|  | pDK268\_112012\_02.07869.07869.2 | 3.9817 | 0.4841 | 100.0% | 1230.2122 | 1230.4241 | 1 | 7.653 | 94.4% | 4 | R.ISEQFTAMFR.R | 2222 |

Similarities:
gi|29788785|ref|NP\_82(19:2)  
gi|5174735|ref|NP\_006(19:2)  
gi|14210536|ref|NP\_11(9:12)  
gi|50592996|ref|NP\_00(13:8)  

---

|  |  |  |  |  |  |  |  |  |
| --- | --- | --- | --- | --- | --- | --- | --- | --- |
| U | *gi|24234688|ref|NP\_00* | 26 | 52 | 42.1% | 679 | 73681 | 6.2 | heat shock 70kDa protein 9 precursor [Homo sapiens] |

| Filename XCorr DeltCN Conf% ObsM+H+ CalcM+H+ SpR ZScore Ion% # Sequence  | | | | | | | | | | | | |
| --- | --- | --- | --- | --- | --- | --- | --- | --- | --- | --- | --- | --- |
| \* | pDK268\_112012\_01.03542.03542.1 | 1.8017 | 0.2592 | 98.4% | 958.58 | 959.047 | 1 | 4.756 | 62.5% | 1 | K.VLENAEGAR.T | 1 |
| \* | pDK268\_112012\_01.03560.03560.2 | 3.1425 | 0.2453 | 100.0% | 958.9322 | 959.047 | 7 | 5.564 | 81.2% | 2 | K.VLENAEGAR.T | 2 |
| \* | pDK268\_112012\_02.06085.06085.2 | 3.4838 | 0.4508 | 100.0% | 1452.1322 | 1451.576 | 1 | 7.207 | 73.1% | 4 | R.TTPSVVAFTADGER.L | 2 |
| \* | pDK268\_112012\_01.05679.05679.2 | 3.4511 | 0.4517 | 100.0% | 1569.0322 | 1569.7141 | 1 | 7.743 | 69.2% | 3 | R.QAVTNPNNTFYATK.R | 2 |
| \* | pDK268\_112012\_01.03508.03508.2 | 2.9889 | 0.113 | 99.7% | 1151.1322 | 1150.2334 | 3 | 4.402 | 68.8% | 1 | R.RYDDPEVQK.D | 2 |
| \* | pDK268\_112012\_01.04365.04365.2 | 3.9133 | 0.4718 | 100.0% | 1341.9922 | 1342.4105 | 1 | 8.764 | 70.8% | 1 | R.ASNGDAWVEAHGK.L | 2 |
| \* | pDK268\_112012\_01.04356.04356.3 | 2.2404 | 0.2831 | 97.6% | 1342.4343 | 1342.4105 | 187 | 4.993 | 33.3% | 1 | R.ASNGDAWVEAHGK.L | 3 |
| \* | pDK268\_112012\_01.13238.13238.2 | 3.9299 | 0.5473 | 100.0% | 1554.1721 | 1554.8878 | 1 | 9.388 | 84.6% | 5 | K.LYSPSQIGAFVLMK.M | 2 |
| \* | pDK268\_112012\_01.04412.04412.3 | 4.1711 | 0.356 | 100.0% | 1593.2943 | 1593.7949 | 1 | 5.852 | 44.2% | 1 | K.MKETAENYLGHTAK.N | 3 |
| \* | pDK268\_112012\_01.04418.04418.2 | 4.6787 | 0.4849 | 100.0% | 1594.2922 | 1593.7949 | 1 | 8.485 | 76.9% | 1 | K.MKETAENYLGHTAK.N | 2 |
| \* | pDK268\_112012\_01.10125.10125.2 | 3.3755 | 0.4004 | 100.0% | 1695.3722 | 1695.8723 | 1 | 6.409 | 71.4% | 2 | K.NAVITVPAYFNDSQR.Q | 2 |
| \* | pDK268\_112012\_01.09350.09350.2 | 4.0387 | 0.3763 | 100.0% | 1243.6122 | 1243.4056 | 1 | 6.483 | 81.8% | 2 | K.DAGQISGLNVLR.V | 2 |
| \* | pDK268\_112012\_01.10276.10276.2 | 4.8738 | 1.0E-4 | 99.8% | 1646.2922 | 1646.881 | 1 | 9.365 | 80.0% | 1 | R.VINEPTAAALAYGLDK.S | 2 |
| \* | pDK268\_112012\_01.12449.12449.2 | 5.4921 | 0.5362 | 100.0% | 2056.412 | 2057.181 | 1 | 9.378 | 72.2% | 2 | K.STNGDTFLGGEDFDQALLR.H | 2 |
| \* | pDK268\_112012\_01.06982.06982.2 | 2.6691 | 0.3978 | 99.9% | 1691.4122 | 1691.8969 | 1 | 6.787 | 57.1% | 1 | R.ETGVDLTKDNMALQR.V | 2 |
| \* | pDK268\_112012\_01.12578.12578.2 | 4.2196 | 0.4606 | 100.0% | 1362.2722 | 1362.5687 | 1 | 8.894 | 77.3% | 4 | R.AQFEGIVTDLIR.R | 2 |
| \* | pDK268\_112012\_01.11285.11285.2 | 3.587 | 0.4016 | 100.0% | 1447.3322 | 1447.6898 | 1 | 7.274 | 76.9% | 3 | K.SDIGEVILVGGMTR.M | 2 |
| \* | pDK268\_112012\_01.09116.09116.2 | 3.6084 | 0.33 | 100.0% | 1291.1522 | 1291.4496 | 1 | 8.649 | 85.0% | 2 | K.VQQTVQDLFGR.A | 2 |
| \* | pDK268\_112012\_02.05663.05663.2 | 4.6857 | 0.511 | 100.0% | 1809.1122 | 1809.9707 | 1 | 9.349 | 62.5% | 1 | K.SQVFSTAADGQTQVEIK.V | 2 |
| \* | pDK268\_112012\_01.13137.13137.2 | 3.1258 | 0.3153 | 99.9% | 1594.4722 | 1593.9529 | 1 | 6.044 | 57.1% | 3 | K.LLGQFTLIGIPPAPR.G | 2 |
| \* | pDK268\_112012\_02.04989.04989.2 | 3.9265 | 0.3331 | 100.0% | 1475.7122 | 1474.6543 | 1 | 7.609 | 69.2% | 1 | R.EQQIVIQSSGGLSK.D | 2 |
| \* | pDK268\_112012\_02.06921.06921.3 | 3.1192 | 0.4024 | 100.0% | 2419.5842 | 2419.7095 | 1 | 6.015 | 31.0% | 3 | R.EQQIVIQSSGGLSKDDIENMVK.N | 3 |
| \* | pDK268\_112012\_01.10160.10160.3 | 5.8012 | 0.3302 | 100.0% | 2143.7644 | 2143.3765 | 1 | 7.24 | 54.2% | 2 | K.ERVEAVNMAEGIIHDTETK.M | 3 |
| \* | pDK268\_112012\_01.10640.10640.2 | 4.6428 | 0.5425 | 100.0% | 1857.4321 | 1858.0735 | 1 | 9.558 | 65.6% | 1 | R.VEAVNMAEGIIHDTETK.M | 2 |
| \* | pDK268\_112012\_01.10612.10612.3 | 2.9133 | 0.3946 | 100.0% | 1858.4944 | 1858.0735 | 1 | 7.016 | 48.4% | 2 | R.VEAVNMAEGIIHDTETK.M | 3 |
| \* | pDK268\_112012\_01.04577.04577.2 | 3.0923 | 0.4231 | 100.0% | 1232.5521 | 1232.3794 | 4 | 6.401 | 63.6% | 2 | R.QAASSLQQASLK.L | 2 |

---

|  |  |  |  |  |  |  |  |  |
| --- | --- | --- | --- | --- | --- | --- | --- | --- |
| U | *gi|4502491|ref|NP\_001* | 6 | 11 | 41.8% | 282 | 31362 | 4.8 | complement component 1, q subcomponent binding protein precursor [Homo sapiens] |

| Filename XCorr DeltCN Conf% ObsM+H+ CalcM+H+ SpR ZScore Ion% # Sequence  | | | | | | | | | | | | |
| --- | --- | --- | --- | --- | --- | --- | --- | --- | --- | --- | --- | --- |
| \* | pDK268\_112012\_01.11970.11970.2 | 3.8315 | 0.325 | 100.0% | 1699.2722 | 1698.87 | 1 | 5.874 | 73.1% | 1 | K.AFVDFLSDEIKEER.K | 2 |
| \* | pDK268\_112012\_02.07283.07283.2 | 5.1097 | 0.4836 | 100.0% | 1622.9922 | 1622.79 | 1 | 8.379 | 75.0% | 2 | K.MSGGWELELNGTEAK.L | 23 |
| \* | pDK268\_112012\_01.12454.12454.2 | 5.177 | 0.5133 | 100.0% | 2287.652 | 2288.5566 | 1 | 8.527 | 71.1% | 1 | K.VEEQEPELTSTPNFVVEVIK.N | 2 |
| \* | pDK268\_112012\_02.05909.05909.2 | 3.6103 | 0.4797 | 100.0% | 1514.4521 | 1514.5884 | 1 | 7.668 | 83.3% | 1 | R.EVSFQSTGESEWK.D | 2 |
| \* | pDK268\_112012\_01.17642.17642.3 | 3.932 | 0.4138 | 100.0% | 3120.4744 | 3120.3735 | 1 | 7.304 | 28.0% | 2 | K.DTNYTLNTDSLDWALYDHLMDFLADR.G | 3 |
| \* | pDK268\_112012\_01.18614.18614.3 | 6.3055 | 0.5836 | 100.0% | 3441.7144 | 3441.77 | 1 | 10.757 | 29.3% | 4 | R.GVDNTFADELVELSTALEHQEYITFLEDLK.S | 3 |

---

|  |  |  |  |  |  |  |  |  |
| --- | --- | --- | --- | --- | --- | --- | --- | --- |
| U | *gi|164519146|ref|NP\_0* | 20 | 28 | 41.4% | 633 | 68063 | 9.9 | GATA zinc finger domain containing 2A [Homo sapiens] |

| Filename XCorr DeltCN Conf% ObsM+H+ CalcM+H+ SpR ZScore Ion% # Sequence  | | | | | | | | | | | | |
| --- | --- | --- | --- | --- | --- | --- | --- | --- | --- | --- | --- | --- |
| \* | pDK268\_112012\_01.03827.03827.3 | 3.4901 | 0.3002 | 100.0% | 1732.9443 | 1732.8419 | 1 | 5.257 | 48.2% | 1 | R.ALERDPTEDDVESKK.I | 3 |
| \* | pDK268\_112012\_01.08636.08636.2 | 3.2084 | 0.4886 | 100.0% | 1478.2922 | 1478.6172 | 1 | 9.057 | 73.1% | 2 | R.GLLASDLNTDGDMR.V | 2 |
| \* | pDK268\_112012\_01.07346.07346.2 | 1.8725 | 0.3766 | 98.6% | 1493.2522 | 1493.7031 | 1 | 5.45 | 64.3% | 1 | R.VTPEPGAGPTQGLLR.A | 2 |
| \* | pDK268\_112012\_01.04874.04874.2 | 3.4244 | 0.5659 | 100.0% | 1109.2722 | 1110.2886 | 1 | 9.373 | 80.0% | 2 | R.ATEATAMAMGR.G | 2 |
| \* | pDK268\_112012\_01.07578.07578.2 | 3.5648 | 0.4266 | 100.0% | 1302.0922 | 1302.4448 | 1 | 8.123 | 75.0% | 3 | R.GEGLVGDGPVDMR.T | 2 |
| \* | pDK268\_112012\_01.07091.07091.3 | 3.503 | 0.3709 | 100.0% | 2375.4243 | 2375.5981 | 1 | 5.528 | 33.0% | 1 | K.EATAQKPTGSVGSTVTT#PPPLVR.G | 3 |
| \* | pDK268\_112012\_01.09434.09434.2 | 2.3909 | 0.2661 | 99.5% | 1263.6122 | 1263.5841 | 3 | 4.677 | 63.6% | 1 | R.MPGSVIPPPLVR.G | 2 |
| \* | pDK268\_112012\_01.09812.09812.2 | 3.7523 | 0.4604 | 100.0% | 1679.2522 | 1680.0184 | 1 | 9.0 | 56.7% | 2 | K.LGPQASSQVVMPPLVR.G | 2 |
| \* | pDK268\_112012\_01.03502.03502.2 | 3.1605 | 0.1939 | 99.9% | 1010.33215 | 1010.141 | 2 | 5.782 | 75.0% | 1 | R.GAQQIHSIR.Q | 2 |
| \* | pDK268\_112012\_01.06094.06094.2 | 1.9998 | 0.1756 | 96.6% | 940.5122 | 941.1618 | 2 | 5.222 | 85.7% | 1 | R.IIQQGLIR.V | 2 |
| \* | pDK268\_112012\_01.11400.11400.2 | 4.3239 | 0.5482 | 100.0% | 2273.7722 | 2274.6658 | 1 | 9.603 | 47.6% | 1 | R.VANVPNTSLLVNIPQPTPASLK.G | 2 |
| \* | pDK268\_112012\_01.06070.06070.2 | 2.7789 | 0.3765 | 100.0% | 936.27216 | 936.1143 | 1 | 7.263 | 87.5% | 1 | R.MSAATVLSR.E | 2 |
|  | pDK268\_112012\_01.04527.04527.2 | 2.8965 | 0.2159 | 99.8% | 1372.2922 | 1372.4777 | 2 | 5.13 | 75.0% | 1 | K.ALQQEQEIEQR.L | 2 |
| \* | pDK268\_112012\_01.03914.03914.2 | 3.3339 | 0.4363 | 100.0% | 1226.2922 | 1226.4185 | 1 | 7.097 | 72.7% | 2 | R.LLQQGTAPAQAK.A | 2 |
| \* | pDK268\_112012\_01.04311.04311.2 | 3.1389 | 0.42 | 100.0% | 1230.8522 | 1231.4374 | 2 | 6.059 | 63.6% | 1 | K.AEPTAAPHPVLK.Q | 2 |
| \* | pDK268\_112012\_02.07169.07169.2 | 4.2827 | 0.4258 | 100.0% | 1719.8322 | 1719.8516 | 1 | 6.623 | 80.0% | 1 | R.DWSNGAVLQASSQLSR.G | 2 |
| \* | pDK268\_112012\_01.05152.05152.2 | 2.5219 | 0.3685 | 99.9% | 1170.2322 | 1170.3538 | 3 | 6.702 | 70.0% | 1 | R.GVLHTFSPSPK.L | 2 |
| \* | pDK268\_112012\_02.04668.04668.2 | 4.0558 | 0.5152 | 100.0% | 1317.8922 | 1318.4728 | 1 | 8.687 | 75.0% | 2 | K.LQNSASATALVSR.T | 2 |
| \* | pDK268\_112012\_01.10322.10322.3 | 5.6932 | 0.4558 | 100.0% | 1984.3143 | 1984.303 | 1 | 7.304 | 46.1% | 2 | K.TPLSTGGTLAFVSPSLAVHK.S | 3 |
| \* | pDK268\_112012\_01.10359.10359.2 | 3.2335 | 0.3444 | 100.0% | 1986.0322 | 1984.303 | 31 | 5.243 | 28.9% | 1 | K.TPLSTGGTLAFVSPSLAVHK.S | 2 |

---

|  |  |  |  |  |  |  |  |  |
| --- | --- | --- | --- | --- | --- | --- | --- | --- |
| U | *gi|117190174|ref|NP\_0* | 11 | 26 | 41.0% | 293 | 32338 | 5.1 | heterogeneous nuclear ribonucleoprotein C isoform b [Homo sapiens] |
| U | *gi|117190254|ref|NP\_0* | 11 | 26 | 41.0% | 293 | 32338 | 5.1 | heterogeneous nuclear ribonucleoprotein C isoform b [Homo sapiens] |

| Filename XCorr DeltCN Conf% ObsM+H+ CalcM+H+ SpR ZScore Ion% # Sequence  | | | | | | | | | | | | |
| --- | --- | --- | --- | --- | --- | --- | --- | --- | --- | --- | --- | --- |
|  | pDK268\_112012\_01.11222.11222.2 | 3.7485 | 0.268 | 100.0% | 1317.3522 | 1317.6145 | 1 | 7.408 | 81.8% | 4 | R.VFIGNLNTLVVK.K | 2 |
|  | pDK268\_112012\_02.07096.07096.2 | 2.6459 | 0.3378 | 99.9% | 1446.2122 | 1445.7886 | 6 | 6.029 | 54.2% | 1 | R.VFIGNLNTLVVKK.S | 2 |
|  | pDK268\_112012\_01.06586.06586.2 | 2.8623 | 0.2779 | 99.9% | 1124.3322 | 1124.2792 | 3 | 6.299 | 72.2% | 2 | K.KSDVEAIFSK.Y | 2 |
|  | pDK268\_112012\_01.10611.10611.2 | 3.9345 | 0.5264 | 100.0% | 1330.2322 | 1330.4857 | 1 | 9.399 | 80.0% | 2 | K.GFAFVQYVNER.N | 2 |
|  | pDK268\_112012\_02.08265.08265.2 | 4.9653 | 0.3158 | 100.0% | 1684.3121 | 1684.0038 | 1 | 7.314 | 80.0% | 9 | R.MIAGQVLDINLAAEPK.V | 2 |
|  | pDK268\_112012\_02.08473.08473.2 | 5.714 | 0.6221 | 100.0% | 2103.112 | 2103.2239 | 1 | 10.917 | 70.6% | 1 | R.SAAEMYGSSFDLDYDFQR.D | 2 |
|  | pDK268\_112012\_02.03729.03729.2 | 1.7057 | 0.2956 | 96.6% | 944.15216 | 944.1649 | 29 | 5.3 | 50.0% | 2 | R.VPPPPPIAR.A | 2 |
|  | pDK268\_112012\_01.04028.04028.2 | 3.2085 | 0.354 | 100.0% | 1228.6921 | 1229.4624 | 2 | 6.662 | 75.0% | 1 | K.LKGDDLQAIKK.E | 2 |
|  | pDK268\_112012\_01.09027.09027.2 | 2.2316 | 0.3364 | 99.7% | 1415.7122 | 1416.6146 | 2 | 5.36 | 68.2% | 2 | K.QKVDSLLENLEK.I | 2 |
|  | pDK268\_112012\_01.03363.03363.3 | 4.5452 | 0.4196 | 100.0% | 2369.1843 | 2369.4583 | 1 | 7.101 | 45.0% | 1 | K.NDKSEEEQSSSSVKKDETNVK.M | 3 |
|  | pDK268\_112012\_01.03395.03395.3 | 3.175 | 0.2228 | 98.3% | 2448.9844 | 2449.4583 | 1 | 4.521 | 37.5% | 1 | K.NDKSEEEQSSS\*SVKKDETNVK.M | 3 |

---

|  |  |  |  |  |  |  |  |  |
| --- | --- | --- | --- | --- | --- | --- | --- | --- |
| U | *gi|17986258|ref|NP\_06* | 7 | 11 | 40.4% | 151 | 16930 | 4.7 | myosin, light chain 6, alkali, smooth muscle and non-muscle isoform 1 [Homo sapiens] |
| U | *gi|88999583|ref|NP\_52* | 7 | 11 | 40.4% | 151 | 16961 | 4.6 | myosin, light chain 6, alkali, smooth muscle and non-muscle isoform 2 [Homo sapiens] |

| Filename XCorr DeltCN Conf% ObsM+H+ CalcM+H+ SpR ZScore Ion% # Sequence  | | | | | | | | | | | | |
| --- | --- | --- | --- | --- | --- | --- | --- | --- | --- | --- | --- | --- |
|  | pDK268\_112012\_01.10074.10074.2 | 2.0978 | 0.1784 | 97.4% | 1026.5521 | 1026.1368 | 4 | 6.026 | 78.6% | 1 | K.EAFQLFDR.T | 2 |
|  | pDK268\_112012\_01.05888.05888.2 | 4.0281 | 0.3992 | 100.0% | 1356.2322 | 1355.5339 | 1 | 6.37 | 66.7% | 3 | R.ALGQNPTNAEVLK.V | 2 |
|  | pDK268\_112012\_01.14740.14740.2 | 4.5265 | 0.4976 | 100.0% | 1887.8922 | 1889.2628 | 1 | 8.471 | 66.7% | 2 | K.VLDFEHFLPMLQTVAK.N | 2 |
|  | pDK268\_112012\_01.14726.14726.3 | 4.5288 | 0.3244 | 100.0% | 1889.7843 | 1889.2628 | 1 | 6.369 | 53.3% | 2 | K.VLDFEHFLPMLQTVAK.N | 3 |
|  | pDK268\_112012\_01.08188.08188.2 | 4.4429 | 0.5019 | 100.0% | 1786.6721 | 1787.8804 | 1 | 9.142 | 64.3% | 1 | K.NKDQGTYEDYVEGLR.V | 2 |
|  | pDK268\_112012\_01.08178.08178.3 | 3.8326 | 0.3503 | 100.0% | 1787.3644 | 1787.8804 | 1 | 6.358 | 44.6% | 1 | K.NKDQGTYEDYVEGLR.V | 3 |
|  | pDK268\_112012\_01.05031.05031.2 | 2.9026 | 0.3407 | 100.0% | 996.2322 | 996.1949 | 1 | 6.97 | 75.0% | 1 | R.HVLVTLGEK.M | 2 |

---

|  |  |  |  |  |  |  |  |  |
| --- | --- | --- | --- | --- | --- | --- | --- | --- |
| U | *gi|7657649|ref|NP\_055* | 10 | 13 | 40.1% | 352 | 39595 | 5.2 | tropomodulin 3 (ubiquitous) [Homo sapiens] |

| Filename XCorr DeltCN Conf% ObsM+H+ CalcM+H+ SpR ZScore Ion% # Sequence  | | | | | | | | | | | | |
| --- | --- | --- | --- | --- | --- | --- | --- | --- | --- | --- | --- | --- |
| \* | pDK268\_112012\_01.11082.11082.2 | 6.1674 | 0.5926 | 100.0% | 2224.672 | 2225.4114 | 1 | 11.463 | 77.8% | 1 | K.YKDLDEDELLGNLSETELK.Q | 2 |
| \* | pDK268\_112012\_01.11061.11061.3 | 3.3447 | 0.1971 | 98.3% | 2225.0344 | 2225.4114 | 2 | 4.579 | 31.9% | 1 | K.YKDLDEDELLGNLSETELK.Q | 3 |
| \* | pDK268\_112012\_01.14963.14963.2 | 3.1566 | 0.3237 | 100.0% | 2326.7722 | 2327.5962 | 22 | 6.62 | 27.5% | 1 | K.QLETVLDDLDPENALLPAGFR.Q | 2 |
| \* | pDK268\_112012\_01.10416.10416.3 | 2.6843 | 0.3343 | 99.9% | 1993.7344 | 1994.2114 | 1 | 5.077 | 39.1% | 1 | K.STTGPFDREHLLSYLEK.E | 3 |
| \* | pDK268\_112012\_01.05429.05429.2 | 2.5014 | 0.124 | 97.0% | 1474.3522 | 1472.5511 | 3 | 3.55 | 63.6% | 1 | K.DREDYVPYTGEK.K | 2 |
| \* | pDK268\_112012\_01.07500.07500.3 | 5.2458 | 0.4012 | 100.0% | 1952.0944 | 1952.1747 | 1 | 8.015 | 48.4% | 1 | R.TKENDAHLVEVNLNNIK.N | 3 |
| \* | pDK268\_112012\_01.07518.07518.2 | 5.8526 | 0.4056 | 100.0% | 1952.6122 | 1952.1747 | 1 | 7.339 | 65.6% | 1 | R.TKENDAHLVEVNLNNIK.N | 2 |
| \* | pDK268\_112012\_01.13899.13899.2 | 4.0766 | 0.5811 | 100.0% | 1494.2122 | 1494.7028 | 1 | 9.25 | 69.2% | 2 | R.SNDPVATAFAEMLK.V | 2 |
| \* | pDK268\_112012\_01.18327.18327.3 | 3.6458 | 0.2153 | 98.8% | 3332.0344 | 3330.8035 | 72 | 4.404 | 18.3% | 2 | K.SLNVESNFITGVGILALIDALRDNETLAELK.I | 3 |
| \* | pDK268\_112012\_01.07260.07260.2 | 2.9949 | 0.2328 | 99.9% | 1205.1522 | 1205.4119 | 11 | 5.088 | 72.2% | 2 | K.MLEENTNILK.F | 2 |

---

|  |  |  |  |  |  |  |  |  |
| --- | --- | --- | --- | --- | --- | --- | --- | --- |
| U | *gi|15431295|ref|NP\_15* | 9 | 15 | 39.3% | 211 | 24261 | 11.7 | ribosomal protein L13 [Homo sapiens] |
| U | *gi|15431297|ref|NP\_00* | 9 | 15 | 39.3% | 211 | 24261 | 11.7 | ribosomal protein L13 [Homo sapiens] |

| Filename XCorr DeltCN Conf% ObsM+H+ CalcM+H+ SpR ZScore Ion% # Sequence  | | | | | | | | | | | | |
| --- | --- | --- | --- | --- | --- | --- | --- | --- | --- | --- | --- | --- |
|  | pDK268\_112012\_01.04320.04320.2 | 3.2091 | 0.2744 | 100.0% | 1308.2122 | 1308.5872 | 1 | 5.465 | 70.0% | 1 | R.NGMVLKPHFHK.D | 2 |
|  | pDK268\_112012\_01.06556.06556.2 | 3.4965 | 0.3223 | 100.0% | 1346.0922 | 1346.5344 | 1 | 5.825 | 90.0% | 1 | R.RVATWFNQPAR.K | 2 |
|  | pDK268\_112012\_02.05290.05290.2 | 2.3629 | 0.1574 | 97.9% | 1191.0122 | 1190.3469 | 1 | 4.333 | 72.2% | 1 | R.VATWFNQPAR.K | 2 |
|  | pDK268\_112012\_01.09728.09728.2 | 2.4004 | 0.4293 | 100.0% | 951.09216 | 951.0672 | 1 | 7.172 | 85.7% | 1 | R.GFSLEELR.V | 2 |
|  | pDK268\_112012\_01.06632.06632.2 | 2.2689 | 0.2785 | 99.7% | 958.0522 | 958.1026 | 1 | 4.926 | 75.0% | 2 | R.TIGISVDPR.R | 2 |
|  | pDK268\_112012\_01.04245.04245.2 | 3.7036 | 0.4138 | 100.0% | 1232.6721 | 1233.3237 | 1 | 6.486 | 85.0% | 2 | K.STESLQANVQR.L | 2 |
|  | pDK268\_112012\_02.07470.07470.3 | 4.7133 | 0.4768 | 100.0% | 2428.4644 | 2428.8064 | 1 | 7.344 | 36.4% | 3 | K.KGDSSAEELKLATQLTGPVMPVR.N | 3 |
|  | pDK268\_112012\_01.08720.08720.2 | 3.6967 | 0.4684 | 100.0% | 1383.2922 | 1383.6923 | 1 | 8.066 | 75.0% | 3 | K.LATQLTGPVMPVR.N | 2 |
|  | pDK268\_112012\_01.04344.04344.2 | 3.2524 | 0.3516 | 100.0% | 1236.9122 | 1237.3953 | 2 | 5.959 | 66.7% | 1 | R.VITEEEKNFK.A | 2 |

---

|  |  |  |  |  |  |  |  |  |
| --- | --- | --- | --- | --- | --- | --- | --- | --- |
| U | *gi|5032051|ref|NP\_005* | 6 | 12 | 39.1% | 151 | 16273 | 10.1 | ribosomal protein S14 [Homo sapiens] |
| U | *gi|68160922|ref|NP\_00* | 6 | 12 | 39.1% | 151 | 16273 | 10.1 | ribosomal protein S14 [Homo sapiens] |
| U | *gi|68160915|ref|NP\_00* | 6 | 12 | 39.1% | 151 | 16273 | 10.1 | ribosomal protein S14 [Homo sapiens] |

| Filename XCorr DeltCN Conf% ObsM+H+ CalcM+H+ SpR ZScore Ion% # Sequence  | | | | | | | | | | | | |
| --- | --- | --- | --- | --- | --- | --- | --- | --- | --- | --- | --- | --- |
|  | pDK268\_112012\_01.08741.08741.3 | 4.9489 | 0.4147 | 100.0% | 2493.5645 | 2493.754 | 1 | 6.803 | 38.6% | 1 | K.VKADRDESSPYAAMLAAQDVAQR.C | 32 |
|  | pDK268\_112012\_01.09520.09520.3 | 3.4951 | 0.2765 | 100.0% | 2265.5344 | 2266.4473 | 3 | 5.132 | 31.2% | 1 | K.ADRDESSPYAAMLAAQDVAQR.C | 3 |
|  | pDK268\_112012\_01.08492.08492.2 | 2.4662 | 0.3738 | 99.9% | 1095.2122 | 1095.3274 | 6 | 6.202 | 61.1% | 2 | K.ELGITALHIK.L | 2 |
|  | pDK268\_112012\_01.03756.03756.3 | 2.884 | 0.243 | 99.1% | 1284.8644 | 1284.4581 | 1 | 5.306 | 45.8% | 1 | R.TKTPGPGAQSALR.A | 3 |
|  | pDK268\_112012\_01.04113.04113.2 | 2.9521 | 0.5016 | 100.0% | 1054.9722 | 1055.179 | 1 | 7.885 | 80.0% | 3 | K.TPGPGAQSALR.A | 2 |
|  | pDK268\_112012\_02.04299.04299.2 | 3.8268 | 0.4072 | 100.0% | 1430.0721 | 1430.5547 | 1 | 7.706 | 62.5% | 4 | R.IEDVTPIPSDSTR.R | 2 |

---

|  |  |  |  |  |  |  |  |  |
| --- | --- | --- | --- | --- | --- | --- | --- | --- |
| U | *gi|4757834|ref|NP\_004* | 5 | 7 | 38.9% | 211 | 23772 | 6.7 | BCL2-associated athanogene 2 [Homo sapiens] |

| Filename XCorr DeltCN Conf% ObsM+H+ CalcM+H+ SpR ZScore Ion% # Sequence  | | | | | | | | | | | | |
| --- | --- | --- | --- | --- | --- | --- | --- | --- | --- | --- | --- | --- |
| \* | pDK268\_112012\_01.11303.11303.2 | 2.8588 | 0.301 | 99.9% | 1328.7522 | 1329.5364 | 1 | 6.671 | 75.0% | 1 | R.LLESLDQLELR.V | 2 |
| \* | pDK268\_112012\_01.14556.14556.3 | 6.0914 | 0.5009 | 100.0% | 3114.4143 | 3115.5007 | 1 | 8.856 | 32.7% | 2 | R.EAATAVEQEKEILLEMIHSIQNSQDMR.Q | 3 |
| \* | pDK268\_112012\_02.05115.05115.3 | 2.5932 | 0.3263 | 99.6% | 1846.1643 | 1845.964 | 14 | 5.03 | 35.0% | 1 | R.QISDGEREELNLTANR.L | 3 |
| \* | pDK268\_112012\_02.06491.06491.3 | 3.6563 | 0.4158 | 100.0% | 2399.8442 | 2400.6917 | 2 | 6.988 | 28.8% | 2 | R.TLTVEVSVETIRNPQQQESLK.H | 3 |
| \* | pDK268\_112012\_01.05327.05327.2 | 2.1821 | 0.0984 | 96.8% | 900.5522 | 901.09705 | 13 | 3.683 | 83.3% | 1 | R.RLETLLR.N | 2 |

---

|  |  |  |  |  |  |  |  |  |
| --- | --- | --- | --- | --- | --- | --- | --- | --- |
| U | *gi|22027651|ref|NP\_00* | 36 | 73 | 38.5% | 949 | 104637 | 5.1 | adaptor-related protein complex 1 beta 1 subunit isoform a [Homo sapiens] |
| U | *gi|22027653|ref|NP\_66* | 36 | 73 | 38.7% | 942 | 103921 | 5.1 | adaptor-related protein complex 1 beta 1 subunit isoform b [Homo sapiens] |

| Filename XCorr DeltCN Conf% ObsM+H+ CalcM+H+ SpR ZScore Ion% # Sequence  | | | | | | | | | | | | |
| --- | --- | --- | --- | --- | --- | --- | --- | --- | --- | --- | --- | --- |
|  | pDK268\_112012\_01.06543.06543.1 | 2.1833 | 0.3019 | 100.0% | 963.54 | 964.1497 | 65 | 4.574 | 57.1% | 1 | K.KGEIFELK.A | 11 |
|  | pDK268\_112012\_01.06572.06572.2 | 3.0848 | 0.1732 | 99.9% | 964.15216 | 964.1497 | 55 | 4.814 | 78.6% | 3 | K.KGEIFELK.A | 22 |
|  | pDK268\_112012\_01.11388.11388.2 | 3.3351 | 0.4915 | 100.0% | 1518.5521 | 1519.8854 | 1 | 8.074 | 72.7% | 1 | K.KLVYLYLMNYAK.S | 22 |
|  | pDK268\_112012\_02.09526.09526.2 | 4.3295 | 0.4217 | 100.0% | 1391.9722 | 1391.7113 | 1 | 8.836 | 85.0% | 4 | K.LVYLYLMNYAK.S | 22 |
|  | pDK268\_112012\_01.12459.12459.2 | 3.6487 | 0.4696 | 100.0% | 1652.4122 | 1652.9642 | 1 | 9.091 | 64.3% | 2 | K.SQPDMAIMAVNTFVK.D | 2 |
|  | pDK268\_112012\_01.05554.05554.2 | 4.4871 | 0.4832 | 100.0% | 1396.1721 | 1396.6298 | 1 | 8.391 | 80.8% | 4 | R.LSHANSAVVLSAVK.V | 22 |
|  | pDK268\_112012\_01.05552.05552.3 | 4.0837 | 0.1741 | 99.8% | 1396.9143 | 1396.6298 | 17 | 4.663 | 46.2% | 1 | R.LSHANSAVVLSAVK.V | 33 |
|  | pDK268\_112012\_01.16604.16604.2 | 5.3877 | 0.6009 | 100.0% | 2293.9722 | 2294.7397 | 1 | 9.857 | 55.0% | 3 | K.LAPPLVTLLSAEPELQYVALR.N | 2 |
|  | pDK268\_112012\_01.16617.16617.3 | 4.8706 | 0.5273 | 100.0% | 2295.4143 | 2294.7397 | 1 | 8.477 | 43.8% | 2 | K.LAPPLVTLLSAEPELQYVALR.N | 3 |
|  | pDK268\_112012\_01.06806.06806.1 | 2.2139 | 0.2222 | 98.6% | 941.42 | 942.1466 | 3 | 5.052 | 71.4% | 1 | R.NINLIVQK.R | 11 |
|  | pDK268\_112012\_01.06860.06860.2 | 2.8079 | 0.0413 | 98.7% | 942.1122 | 942.1466 | 8 | 4.164 | 78.6% | 3 | R.NINLIVQK.R | 22 |
|  | pDK268\_112012\_01.03828.03828.2 | 2.788 | 0.3461 | 100.0% | 1281.2122 | 1281.559 | 1 | 6.604 | 83.3% | 1 | K.RPEILKHEMK.V | 2 |
|  | pDK268\_112012\_01.06236.06236.2 | 2.6435 | 0.2908 | 99.9% | 1011.5722 | 1012.1503 | 8 | 6.08 | 85.7% | 3 | K.YNDPIYVK.L | 22 |
|  | pDK268\_112012\_01.07548.07548.2 | 2.8744 | 0.375 | 100.0% | 1382.3722 | 1382.5994 | 1 | 6.144 | 70.0% | 2 | K.YNDPIYVKLEK.L | 22 |
|  | pDK268\_112012\_01.13203.13203.2 | 4.9583 | 0.351 | 100.0% | 1570.5122 | 1569.8418 | 1 | 7.173 | 82.1% | 2 | R.LASQANIAQVLAELK.E | 22 |
|  | pDK268\_112012\_01.17144.17144.3 | 6.001 | 0.4907 | 100.0% | 2995.0444 | 2994.3716 | 1 | 9.974 | 35.6% | 3 | R.LASQANIAQVLAELKEYATEVDVDFVR.K | 33 |
|  | pDK268\_112012\_01.16204.16204.3 | 5.3306 | 0.5088 | 100.0% | 3122.0942 | 3122.5457 | 1 | 8.467 | 31.5% | 2 | R.LASQANIAQVLAELKEYATEVDVDFVRK.A | 33 |
|  | pDK268\_112012\_02.07043.07043.2 | 2.5636 | 0.4717 | 100.0% | 1442.2322 | 1443.553 | 1 | 7.0 | 63.6% | 2 | K.EYATEVDVDFVR.K | 22 |
|  | pDK268\_112012\_01.18762.18762.3 | 2.1275 | 0.3171 | 97.5% | 2006.5144 | 2006.3959 | 184 | 4.909 | 25.0% | 1 | K.VNYVVQEAIVVIKDIFR.K | 3 |
|  | pDK268\_112012\_02.09514.09514.2 | 3.3519 | 0.5045 | 100.0% | 1509.3121 | 1509.7632 | 1 | 8.149 | 66.7% | 2 | R.AAMIWIVGEYAER.I | 22 |
|  | pDK268\_112012\_01.19444.19444.3 | 7.0558 | 0.507 | 100.0% | 3618.2644 | 3618.032 | 1 | 9.989 | 37.1% | 2 | R.IDNADELLESFLEGFHDESTQVQLQLLTAIVK.L | 3 |
|  | pDK268\_112012\_01.13898.13898.3 | 4.4907 | 0.345 | 100.0% | 3198.5942 | 3198.471 | 1 | 5.867 | 29.6% | 1 | K.KPTETQELVQQVLSLATQDSDNPDLRDR.G | 3 |
|  | pDK268\_112012\_01.05283.05283.2 | 2.0793 | 0.2565 | 98.4% | 1014.5122 | 1015.1949 | 8 | 5.184 | 72.2% | 1 | R.LLSTDPVAAK.E | 2 |
|  | pDK268\_112012\_01.09696.09696.1 | 1.7548 | 0.2569 | 98.5% | 915.22 | 916.1693 | 1 | 4.995 | 71.4% | 1 | K.AVWLPAMK.A | 1 |
|  | pDK268\_112012\_01.08189.08189.2 | 2.8326 | 0.3281 | 100.0% | 1081.1721 | 1081.2139 | 5 | 6.822 | 61.1% | 2 | K.GLEISGTFTR.Q | 2 |
|  | pDK268\_112012\_02.07065.07065.2 | 3.7785 | 0.3359 | 100.0% | 1535.4321 | 1534.7681 | 2 | 6.181 | 53.8% | 2 | R.QVGSISMDLQLTNK.A | 2 |
|  | pDK268\_112012\_01.08915.08915.2 | 3.29 | 0.3977 | 100.0% | 1356.2722 | 1356.6232 | 1 | 6.535 | 72.7% | 4 | K.MEPLNNLQVAVK.N | 22 |
|  | pDK268\_112012\_01.16700.16700.3 | 2.7339 | 0.335 | 99.6% | 2746.3442 | 2746.1345 | 1 | 4.853 | 25.0% | 1 | K.NNIDVFYFSTLYPLHILFVEDGK.M | 3 |
|  | pDK268\_112012\_01.16157.16157.3 | 4.146 | 0.2416 | 100.0% | 3148.2544 | 3148.6033 | 1 | 5.449 | 30.0% | 2 | K.NNIDVFYFSTLYPLHILFVEDGKMDR.Q | 3 |
|  | pDK268\_112012\_01.08313.08313.2 | 3.3898 | 0.3849 | 100.0% | 1574.2922 | 1574.6903 | 1 | 7.208 | 70.8% | 3 | K.DIPNENEAQFQIR.D | 2 |
|  | pDK268\_112012\_02.06279.06279.2 | 2.6137 | 0.2775 | 99.8% | 1209.4922 | 1208.4001 | 4 | 4.908 | 70.0% | 2 | K.LQSSNIFTVAK.R | 2 |
|  | pDK268\_112012\_01.07510.07510.2 | 4.4955 | 0.5085 | 100.0% | 1681.2722 | 1681.9042 | 1 | 7.876 | 69.2% | 1 | K.RNVEGQDMLYQSLK.L | 22 |
|  | pDK268\_112012\_01.09032.09032.2 | 4.1365 | 0.4613 | 100.0% | 1525.2122 | 1525.7168 | 1 | 8.913 | 75.0% | 4 | R.NVEGQDMLYQSLK.L | 22 |
|  | pDK268\_112012\_02.10329.10329.2 | 3.8537 | 0.3727 | 100.0% | 1385.4521 | 1385.6493 | 1 | 7.928 | 72.7% | 2 | K.LTNGIWVLAELR.I | 2 |
|  | pDK268\_112012\_01.10425.10425.3 | 5.028 | 0.4755 | 100.0% | 1977.1444 | 1977.2242 | 1 | 9.032 | 53.1% | 1 | R.APEVSQHVYQAYETILK.N | 3 |
|  | pDK268\_112012\_01.10490.10490.2 | 4.4766 | 0.4801 | 100.0% | 2090.2922 | 2091.3281 | 1 | 8.533 | 58.8% | 1 | R.APEVSQHVYQAYETILKN.- | 2 |

Similarities:
gi|4557469|ref|NP\_001(18:18)  

---

|  |  |  |  |  |  |  |  |  |
| --- | --- | --- | --- | --- | --- | --- | --- | --- |
| U | *gi|5032161|ref|NP\_005* | 3 | 4 | 37.5% | 112 | 12473 | 4.8 | elongin C [Homo sapiens] |

| Filename XCorr DeltCN Conf% ObsM+H+ CalcM+H+ SpR ZScore Ion% # Sequence  | | | | | | | | | | | | |
| --- | --- | --- | --- | --- | --- | --- | --- | --- | --- | --- | --- | --- |
|  | pDK268\_112012\_01.07034.07034.2 | 3.6932 | 0.4304 | 100.0% | 1345.2722 | 1345.5382 | 1 | 7.573 | 77.3% | 1 | K.LISSDGHEFIVK.R | 2 |
|  | pDK268\_112012\_01.03760.03760.1 | 2.0854 | 0.4123 | 100.0% | 1056.53 | 1057.1918 | 1 | 7.501 | 72.2% | 1 | R.EHALTSGTIK.A | 1 |
| \* | pDK268\_112012\_01.10283.10283.2 | 6.2436 | 0.5376 | 100.0% | 2212.5522 | 2212.3984 | 1 | 10.234 | 57.9% | 2 | K.AMLSGPGQFAENETNEVNFR.E | 2 |

---

|  |  |  |  |  |  |  |  |  |
| --- | --- | --- | --- | --- | --- | --- | --- | --- |
| U | *gi|16507237|ref|NP\_00* | 20 | 27 | 37.2% | 654 | 72333 | 5.2 | heat shock 70kDa protein 5 [Homo sapiens] |

| Filename XCorr DeltCN Conf% ObsM+H+ CalcM+H+ SpR ZScore Ion% # Sequence  | | | | | | | | | | | | |
| --- | --- | --- | --- | --- | --- | --- | --- | --- | --- | --- | --- | --- |
|  | pDK268\_112012\_01.08894.08894.2 | 3.656 | 0.4944 | 100.0% | 1568.4122 | 1567.7386 | 1 | 9.578 | 65.4% | 1 | R.ITPSYVAFTPEGER.L | 2 |
|  | pDK268\_112012\_01.07827.07827.2 | 4.4818 | 0.4903 | 100.0% | 1678.3522 | 1678.796 | 1 | 9.02 | 78.6% | 1 | K.NQLTSNPENTVFDAK.R | 2 |
|  | pDK268\_112012\_01.06861.06861.2 | 3.5962 | 0.3274 | 100.0% | 1431.6322 | 1431.5449 | 1 | 6.452 | 77.3% | 2 | R.TWNDPSVQQDIK.F | 2 |
|  | pDK268\_112012\_01.06635.06635.2 | 3.6444 | 0.4628 | 100.0% | 1605.2322 | 1605.8314 | 1 | 6.863 | 85.7% | 1 | K.TKPYIQVDIGGGQTK.T | 2 |
|  | pDK268\_112012\_02.05371.05371.3 | 3.9568 | 0.2248 | 100.0% | 1607.2743 | 1605.8314 | 1 | 5.961 | 51.8% | 2 | K.TKPYIQVDIGGGQTK.T | 3 |
|  | pDK268\_112012\_01.12395.12395.2 | 4.3073 | 0.5191 | 100.0% | 1537.5521 | 1537.8114 | 1 | 8.579 | 80.8% | 3 | K.TFAPEEISAMVLTK.M | 2 |
|  | pDK268\_112012\_01.04353.04353.3 | 2.848 | 0.3693 | 100.0% | 1369.2544 | 1369.619 | 1 | 6.897 | 45.5% | 1 | K.MKETAEAYLGKK.V | 3 |
|  | pDK268\_112012\_01.08519.08519.3 | 3.9904 | 0.2204 | 100.0% | 1889.5144 | 1889.121 | 3 | 4.807 | 37.5% | 2 | K.VTHAVVTVPAYFNDAQR.Q | 3 |
|  | pDK268\_112012\_01.09303.09303.2 | 2.5853 | 0.2811 | 99.8% | 1218.1921 | 1218.4137 | 3 | 6.453 | 59.1% | 2 | K.DAGTIAGLNVMR.I | 2 |
|  | pDK268\_112012\_01.10463.10463.2 | 5.3485 | 0.5181 | 100.0% | 1661.5721 | 1660.9078 | 1 | 9.837 | 86.7% | 2 | R.IINEPTAAAIAYGLDK.R | 222 |
|  | pDK268\_112012\_01.11122.11122.2 | 3.3808 | 0.5006 | 100.0% | 1513.3522 | 1513.7516 | 1 | 8.663 | 72.7% | 1 | R.AKFEELNMDLFR.S | 2 |
|  | pDK268\_112012\_01.12429.12429.2 | 2.1408 | 0.3423 | 99.7% | 1314.8322 | 1314.4987 | 28 | 5.527 | 55.6% | 1 | K.FEELNMDLFR.S | 2 |
|  | pDK268\_112012\_02.06449.06449.2 | 4.5081 | 0.3058 | 100.0% | 1589.1122 | 1589.7863 | 1 | 8.169 | 78.6% | 1 | K.KSDIDEIVLVGGSTR.I | 2 |
|  | pDK268\_112012\_01.08151.08151.2 | 4.4406 | 0.4636 | 100.0% | 1837.3922 | 1838.0245 | 1 | 8.122 | 56.2% | 1 | K.SQIFSTASDNQPTVTIK.V | 2 |
|  | pDK268\_112012\_01.03917.03917.2 | 2.7282 | 0.426 | 100.0% | 1192.2722 | 1192.3574 | 1 | 7.078 | 83.3% | 1 | K.VYEGERPLTK.D | 2 |
|  | pDK268\_112012\_01.09245.09245.2 | 2.4543 | 0.1699 | 98.4% | 1317.0322 | 1317.4381 | 7 | 3.519 | 65.0% | 1 | R.NELESYAYSLK.N | 2 |
|  | pDK268\_112012\_02.07438.07438.3 | 4.3417 | 0.363 | 100.0% | 1976.7544 | 1976.1064 | 1 | 6.73 | 46.7% | 1 | K.IEWLESHQDADIEDFK.A | 3 |
|  | pDK268\_112012\_02.06721.06721.3 | 5.3993 | 0.4151 | 100.0% | 2174.7244 | 2175.3594 | 2 | 7.591 | 44.1% | 1 | K.IEWLESHQDADIEDFKAK.K | 3 |
|  | pDK268\_112012\_01.10025.10025.2 | 2.6132 | 0.2237 | 99.6% | 1398.0721 | 1398.6396 | 2 | 5.543 | 54.5% | 1 | K.ELEEIVQPIISK.L | 2 |
|  | pDK268\_112012\_01.07617.07617.2 | 5.0923 | 0.6002 | 100.0% | 2176.372 | 2177.283 | 1 | 11.1 | 52.5% | 1 | K.LYGSAGPPPTGEEDTAEKDEL.- | 2 |

Similarities:
gi|5729877|ref|NP\_006(1:19)  
gi|124256496|ref|NP\_0(1:19)  

---

|  |  |  |  |  |  |  |  |  |
| --- | --- | --- | --- | --- | --- | --- | --- | --- |
| U | *gi|31542947|ref|NP\_00* | 15 | 21 | 36.1% | 573 | 61055 | 5.9 | chaperonin [Homo sapiens] |
| U | *gi|41399285|ref|NP\_95* | 15 | 21 | 36.1% | 573 | 61055 | 5.9 | chaperonin [Homo sapiens] |

| Filename XCorr DeltCN Conf% ObsM+H+ CalcM+H+ SpR ZScore Ion% # Sequence  | | | | | | | | | | | | |
| --- | --- | --- | --- | --- | --- | --- | --- | --- | --- | --- | --- | --- |
|  | pDK268\_112012\_01.17948.17948.3 | 4.8308 | 0.4366 | 100.0% | 2113.8843 | 2114.5667 | 1 | 8.681 | 47.5% | 3 | R.ALMLQGVDLLADAVAVTMGPK.G | 3 |
|  | pDK268\_112012\_02.13584.13584.2 | 4.0053 | 0.4968 | 100.0% | 2115.2122 | 2114.5667 | 1 | 7.983 | 47.5% | 2 | R.ALMLQGVDLLADAVAVTMGPK.G | 2 |
|  | pDK268\_112012\_01.08234.08234.2 | 3.088 | 0.4062 | 100.0% | 1345.7722 | 1345.5382 | 1 | 6.596 | 72.7% | 1 | R.TVIIEQSWGSPK.V | 2 |
|  | pDK268\_112012\_02.10798.10798.2 | 3.5866 | 0.3007 | 100.0% | 1559.5721 | 1557.9324 | 1 | 6.14 | 64.3% | 1 | R.GVMLAVDAVIAELKK.Q | 2 |
|  | pDK268\_112012\_01.12510.12510.2 | 4.591 | 0.2021 | 100.0% | 1506.1522 | 1505.7235 | 2 | 8.122 | 70.8% | 1 | K.TLNDELEIIEGMK.F | 2 |
|  | pDK268\_112012\_01.10413.10413.3 | 4.0281 | 0.3272 | 100.0% | 2047.1344 | 2048.3933 | 1 | 6.071 | 38.9% | 1 | K.KISSIQSIVPALEIANAHR.K | 3 |
|  | pDK268\_112012\_02.06556.06556.3 | 3.0919 | 0.3272 | 100.0% | 1632.1743 | 1631.9684 | 2 | 6.643 | 39.3% | 3 | K.VGEVIVTKDDAMLLK.G | 3 |
|  | pDK268\_112012\_02.07979.07979.3 | 3.0139 | 0.2334 | 98.3% | 2295.4744 | 2296.5334 | 4 | 4.07 | 31.9% | 1 | R.IQEIIEQLDVTTSEYEKEK.L | 3 |
|  | pDK268\_112012\_01.03873.03873.2 | 2.9948 | 0.3536 | 100.0% | 1233.9722 | 1234.3055 | 1 | 7.015 | 72.7% | 2 | K.VGGTSDVEVNEK.K | 2 |
|  | pDK268\_112012\_01.03477.03477.2 | 3.2816 | 0.2456 | 99.9% | 1362.1322 | 1362.4796 | 1 | 5.276 | 75.0% | 1 | K.VGGTSDVEVNEKK.D | 2 |
|  | pDK268\_112012\_01.04053.04053.2 | 2.389 | 0.3347 | 99.9% | 960.2922 | 961.0629 | 1 | 6.577 | 81.2% | 1 | R.VTDALNATR.A | 2 |
|  | pDK268\_112012\_01.06149.06149.2 | 1.9816 | 0.2053 | 97.1% | 844.9922 | 845.0881 | 3 | 4.217 | 78.6% | 1 | K.IPAMTIAK.N | 2 |
|  | pDK268\_112012\_02.05358.05358.2 | 2.5056 | 0.3167 | 99.8% | 1215.4321 | 1216.377 | 3 | 6.748 | 54.5% | 1 | K.NAGVEGSLIVEK.I | 2 |
|  | pDK268\_112012\_02.09717.09717.3 | 5.0173 | 0.4491 | 100.0% | 2509.5544 | 2509.8235 | 1 | 8.681 | 46.6% | 1 | K.IMQSSSEVGYDAMAGDFVNMVEK.G | 3 |
|  | pDK268\_112012\_02.13561.13561.3 | 3.3187 | 0.4002 | 100.0% | 2870.3044 | 2870.3105 | 1 | 6.334 | 27.8% | 1 | R.TALLDAAGVASLLTTAEVVVTEIPKEEK.D | 3 |

---

|  |  |  |  |  |  |  |  |  |
| --- | --- | --- | --- | --- | --- | --- | --- | --- |
| U | *gi|16933546|ref|NP\_44* | 9 | 11 | 36.0% | 317 | 34274 | 6.0 | ribosomal protein P0 [Homo sapiens] |
| U | *gi|4506667|ref|NP\_000* | 9 | 11 | 36.0% | 317 | 34274 | 6.0 | ribosomal protein P0 [Homo sapiens] |

| Filename XCorr DeltCN Conf% ObsM+H+ CalcM+H+ SpR ZScore Ion% # Sequence  | | | | | | | | | | | | |
| --- | --- | --- | --- | --- | --- | --- | --- | --- | --- | --- | --- | --- |
|  | pDK268\_112012\_01.10334.10334.2 | 3.233 | 0.316 | 100.0% | 1217.7722 | 1218.4357 | 1 | 7.779 | 88.9% | 1 | K.IIQLLDDYPK.C | 2 |
|  | pDK268\_112012\_01.03826.03826.2 | 3.5433 | 0.3739 | 100.0% | 1223.1721 | 1222.3433 | 1 | 7.201 | 75.0% | 1 | R.GHLENNPALEK.L | 2 |
|  | pDK268\_112012\_02.07798.07798.3 | 3.4577 | 0.4274 | 100.0% | 1824.6843 | 1826.0593 | 1 | 6.905 | 45.0% | 1 | R.GNVGFVFTKEDLTEIR.D | 3 |
|  | pDK268\_112012\_01.11573.11573.3 | 3.8699 | 0.3365 | 100.0% | 2226.9243 | 2227.584 | 1 | 5.303 | 35.5% | 1 | K.EDLTEIRDMLLANKVPAAAR.A | 3 |
|  | pDK268\_112012\_01.07100.07100.2 | 3.5557 | 0.4345 | 100.0% | 1370.3322 | 1370.6531 | 1 | 6.758 | 75.0% | 1 | R.DMLLANKVPAAAR.A | 2 |
|  | pDK268\_112012\_02.09535.09535.2 | 3.5276 | 0.4264 | 100.0% | 1314.4321 | 1314.5242 | 1 | 9.132 | 77.3% | 2 | K.TSFFQALGITTK.I | 2 |
|  | pDK268\_112012\_02.09251.09251.2 | 3.0091 | 0.4057 | 100.0% | 1895.6122 | 1897.1754 | 1 | 7.421 | 40.6% | 1 | R.VLALSVETDYTFPLAEK.V | 2 |
|  | pDK268\_112012\_01.12456.12456.2 | 5.1454 | 0.5094 | 100.0% | 2754.9922 | 2754.1582 | 1 | 7.754 | 48.3% | 2 | K.AFLADPSAFVAAAPVAAATTAAPAAAAAPAK.V | 2 |
|  | pDK268\_112012\_01.11799.11799.3 | 3.7786 | 0.2866 | 100.0% | 3180.5044 | 3181.6592 | 4 | 4.734 | 19.1% | 1 | K.AFLADPSAFVAAAPVAAATTAAPAAAAAPAKVEAK.E | 3 |

---

|  |  |  |  |  |  |  |  |  |
| --- | --- | --- | --- | --- | --- | --- | --- | --- |
| U | *gi|15431288|ref|NP\_00* | 9 | 14 | 35.9% | 217 | 24831 | 9.9 | ribosomal protein L10a [Homo sapiens] |

| Filename XCorr DeltCN Conf% ObsM+H+ CalcM+H+ SpR ZScore Ion% # Sequence  | | | | | | | | | | | | |
| --- | --- | --- | --- | --- | --- | --- | --- | --- | --- | --- | --- | --- |
| \* | pDK268\_112012\_02.09233.09233.2 | 2.8163 | 0.2744 | 99.9% | 1421.0322 | 1420.6891 | 3 | 5.298 | 68.2% | 2 | K.FLETVELQISLK.N | 2 |
|  | pDK268\_112012\_01.10097.10097.2 | 2.4476 | 0.3433 | 99.8% | 1452.0122 | 1452.7087 | 1 | 5.875 | 62.5% | 1 | K.AVDIPHMDIEALK.K | 2 |
|  | pDK268\_112012\_01.08790.08790.2 | 2.5218 | 0.3886 | 99.9% | 1579.4521 | 1580.8828 | 1 | 6.428 | 61.5% | 1 | K.AVDIPHMDIEALKK.L | 2 |
|  | pDK268\_112012\_01.09680.09680.2 | 4.0861 | 0.4561 | 100.0% | 1485.5122 | 1485.7203 | 1 | 8.816 | 75.0% | 4 | K.KYDAFLASESLIK.Q | 2 |
|  | pDK268\_112012\_02.07259.07259.3 | 2.6686 | 0.2323 | 97.8% | 1485.6843 | 1485.7203 | 1 | 4.689 | 45.8% | 1 | K.KYDAFLASESLIK.Q | 3 |
|  | pDK268\_112012\_01.07169.07169.3 | 3.5942 | 0.3652 | 100.0% | 1857.4143 | 1858.1656 | 2 | 6.075 | 37.5% | 2 | K.AGKFPSLLTHNENMVAK.V | 3 |
|  | pDK268\_112012\_01.08400.08400.2 | 2.9755 | 0.2886 | 99.9% | 1601.8522 | 1601.8608 | 4 | 5.281 | 53.8% | 1 | K.FPSLLTHNENMVAK.V | 2 |
|  | pDK268\_112012\_02.15013.15013.2 | 2.2726 | 0.3563 | 99.7% | 2547.912 | 2549.0002 | 9 | 6.157 | 33.3% | 1 | K.MTDDELVYNIHLAVNFLVSLLK.K | 2 |
|  | pDK268\_112012\_02.14982.14982.3 | 3.7067 | 0.3447 | 100.0% | 2548.4343 | 2549.0002 | 1 | 7.33 | 33.3% | 1 | K.MTDDELVYNIHLAVNFLVSLLK.K | 3 |

---

|  |  |  |  |  |  |  |  |  |
| --- | --- | --- | --- | --- | --- | --- | --- | --- |
| U | *gi|17921989|ref|NP\_00* | 20 | 56 | 35.7% | 448 | 49924 | 5.1 | tubulin, alpha 4a [Homo sapiens] |

| Filename XCorr DeltCN Conf% ObsM+H+ CalcM+H+ SpR ZScore Ion% # Sequence  | | | | | | | | | | | | |
| --- | --- | --- | --- | --- | --- | --- | --- | --- | --- | --- | --- | --- |
| \* | pDK268\_112012\_01.13156.13156.2 | 2.4881 | 0.2197 | 98.5% | 1716.6122 | 1716.9719 | 1 | 5.055 | 53.6% | 1 | R.AVFVDLEPTVIDEIR.N | 2 |
|  | pDK268\_112012\_01.09212.09212.2 | 1.6134 | 0.4066 | 97.8% | 1411.0322 | 1411.6439 | 78 | 6.183 | 59.1% | 1 | R.QLFHPEQLITGK.E | 22 |
|  | pDK268\_112012\_01.08756.08756.2 | 2.0447 | 0.4607 | 99.8% | 2415.4722 | 2416.6555 | 12 | 6.422 | 32.5% | 1 | R.QLFHPEQLITGKEDAANNYAR.G | 22 |
|  | pDK268\_112012\_01.08750.08750.3 | 4.1238 | 0.4883 | 100.0% | 2415.8943 | 2416.6555 | 1 | 7.217 | 36.2% | 3 | R.QLFHPEQLITGKEDAANNYAR.G | 33 |
|  | pDK268\_112012\_01.03926.03926.2 | 2.0667 | 0.1794 | 97.2% | 909.97217 | 910.05804 | 24 | 4.972 | 64.3% | 1 | R.LSVDYGKK.S | 22 |
|  | pDK268\_112012\_01.06032.06032.3 | 2.952 | 0.2267 | 98.5% | 1876.3744 | 1876.0824 | 1 | 4.679 | 41.1% | 1 | R.RNLDIERPTYTNLNR.L | 33 |
|  | pDK268\_112012\_01.07281.07281.2 | 3.2087 | 0.2207 | 99.9% | 1719.3322 | 1719.8949 | 1 | 4.93 | 61.5% | 2 | R.NLDIERPTYTNLNR.L | 22 |
|  | pDK268\_112012\_01.07274.07274.3 | 3.3116 | 0.3584 | 100.0% | 1720.0743 | 1719.8949 | 9 | 5.779 | 42.3% | 3 | R.NLDIERPTYTNLNR.L | 33 |
|  | pDK268\_112012\_02.11007.11007.2 | 4.1605 | 0.5215 | 100.0% | 1488.5122 | 1488.7678 | 1 | 9.06 | 73.1% | 13 | R.LISQIVSSITASLR.F | 22 |
|  | pDK268\_112012\_02.10969.10969.3 | 4.198 | 0.3431 | 100.0% | 1489.0743 | 1488.7678 | 2 | 6.289 | 48.1% | 2 | R.LISQIVSSITASLR.F | 33 |
|  | pDK268\_112012\_02.10194.10194.2 | 5.1564 | 0.5937 | 100.0% | 2409.7922 | 2410.6885 | 1 | 10.839 | 50.0% | 3 | R.FDGALNVDLTEFQTNLVPYPR.I | 22 |
|  | pDK268\_112012\_01.11078.11078.2 | 4.495 | 0.4985 | 100.0% | 1757.5721 | 1758.0703 | 1 | 9.063 | 76.7% | 6 | R.IHFPLATYAPVISAEK.A | 22 |
|  | pDK268\_112012\_01.10886.10886.3 | 3.4998 | 0.2408 | 99.9% | 1757.9644 | 1758.0703 | 1 | 5.498 | 43.3% | 3 | R.IHFPLATYAPVISAEK.A | 33 |
|  | pDK268\_112012\_01.09416.09416.2 | 3.8969 | 0.498 | 100.0% | 1825.3722 | 1826.1027 | 1 | 7.783 | 70.6% | 5 | K.VGINYQPPTVVPGGDLAK.V | 22 |
|  | pDK268\_112012\_01.07070.07070.2 | 3.2479 | 0.4165 | 100.0% | 1381.2522 | 1381.6324 | 1 | 6.343 | 80.0% | 1 | R.LDHKFDLMYAK.R | 22 |
|  | pDK268\_112012\_02.04967.04967.3 | 3.6743 | 0.339 | 100.0% | 1381.8243 | 1381.6324 | 1 | 6.207 | 52.5% | 2 | R.LDHKFDLMYAK.R | 33 |
|  | pDK268\_112012\_01.08716.08716.1 | 1.3964 | 0.263 | 96.1% | 887.29 | 888.0692 | 8 | 4.132 | 58.3% | 1 | K.FDLMYAK.R | 11 |
|  | pDK268\_112012\_01.08693.08693.2 | 2.3171 | 0.2151 | 99.7% | 887.83215 | 888.0692 | 2 | 4.947 | 91.7% | 1 | K.FDLMYAK.R | 22 |
|  | pDK268\_112012\_02.07229.07229.3 | 5.1213 | 0.4294 | 100.0% | 2488.1343 | 2487.7083 | 1 | 7.286 | 45.0% | 2 | K.RAFVHWYVGEGMEEGEFSEAR.E | 33 |
|  | pDK268\_112012\_02.08055.08055.3 | 4.5531 | 0.2662 | 100.0% | 2333.0044 | 2331.5208 | 1 | 5.518 | 40.8% | 4 | R.AFVHWYVGEGMEEGEFSEAR.E | 33 |

Similarities:
gi|14389309|ref|NP\_11(19:1)  

---

|  |  |  |  |  |  |  |  |  |
| --- | --- | --- | --- | --- | --- | --- | --- | --- |
| U | *gi|4506645|ref|NP\_000* | 6 | 13 | 35.7% | 70 | 8218 | 10.1 | ribosomal protein L38 [Homo sapiens] |
| U | *gi|78214522|ref|NP\_00* | 6 | 13 | 35.7% | 70 | 8218 | 10.1 | ribosomal protein L38 [Homo sapiens] |

| Filename XCorr DeltCN Conf% ObsM+H+ CalcM+H+ SpR ZScore Ion% # Sequence  | | | | | | | | | | | | |
| --- | --- | --- | --- | --- | --- | --- | --- | --- | --- | --- | --- | --- |
|  | pDK268\_112012\_01.09926.09926.2 | 4.7925 | 0.4065 | 100.0% | 1575.7122 | 1576.8766 | 1 | 7.693 | 79.2% | 2 | R.KIEEIKDFLLTAR.R | 2 |
|  | pDK268\_112012\_01.09874.09874.3 | 4.3196 | 0.4459 | 100.0% | 1576.9143 | 1576.8766 | 1 | 7.005 | 56.2% | 3 | R.KIEEIKDFLLTAR.R | 3 |
|  | pDK268\_112012\_01.10852.10852.2 | 3.6716 | 0.3732 | 100.0% | 1447.6921 | 1448.7025 | 1 | 6.969 | 72.7% | 1 | K.IEEIKDFLLTAR.R | 2 |
|  | pDK268\_112012\_02.07803.07803.2 | 2.3148 | 0.2174 | 98.9% | 1228.8322 | 1229.4589 | 2 | 4.441 | 72.2% | 1 | R.YLYTLVITDK.E | 2 |
|  | pDK268\_112012\_02.06777.06777.3 | 3.0126 | 0.3423 | 100.0% | 1487.0643 | 1486.7484 | 1 | 5.801 | 52.3% | 2 | R.YLYTLVITDKEK.A | 3 |
|  | pDK268\_112012\_01.08962.08962.2 | 4.0419 | 0.3563 | 100.0% | 1487.2522 | 1486.7484 | 1 | 7.084 | 86.4% | 4 | R.YLYTLVITDKEK.A | 2 |

---

|  |  |  |  |  |  |  |  |  |
| --- | --- | --- | --- | --- | --- | --- | --- | --- |
| U | *gi|14141170|ref|NP\_00* | 17 | 21 | 35.5% | 668 | 75023 | 9.7 | metastasis-associated protein 2 [Homo sapiens] |

| Filename XCorr DeltCN Conf% ObsM+H+ CalcM+H+ SpR ZScore Ion% # Sequence  | | | | | | | | | | | | |
| --- | --- | --- | --- | --- | --- | --- | --- | --- | --- | --- | --- | --- |
| \* | pDK268\_112012\_01.10703.10703.2 | 4.2227 | 0.5316 | 100.0% | 2109.132 | 2110.287 | 1 | 9.696 | 70.6% | 1 | R.VGDYVYFENSSSNPYLVR.R | 2 |
| \* | pDK268\_112012\_01.09450.09450.2 | 4.2748 | 0.5504 | 100.0% | 1550.0521 | 1550.6226 | 1 | 9.027 | 78.6% | 2 | R.DISSSLNSLADSNAR.E | 2 |
| \* | pDK268\_112012\_01.04125.04125.2 | 3.1625 | 0.3719 | 100.0% | 1907.6322 | 1907.9884 | 1 | 6.488 | 63.3% | 1 | R.EFEEESKQPGVSEQQR.H | 2 |
| \* | pDK268\_112012\_01.06322.06322.2 | 2.6648 | 0.337 | 99.9% | 1298.4122 | 1299.4722 | 6 | 5.922 | 65.0% | 1 | R.QFESLPATHIR.G | 2 |
| \* | pDK268\_112012\_01.08936.08936.2 | 2.9716 | 0.2673 | 99.9% | 1328.1522 | 1328.424 | 2 | 6.535 | 75.0% | 1 | K.VWDPDNPLTDR.Q | 2 |
| \* | pDK268\_112012\_01.11517.11517.2 | 4.0686 | 0.506 | 100.0% | 1561.1122 | 1561.7961 | 1 | 8.686 | 75.0% | 2 | R.DITLFHAMDTLQR.N | 2 |
|  | pDK268\_112012\_01.11189.11189.2 | 3.0929 | 0.1956 | 99.7% | 2043.2522 | 2043.289 | 1 | 4.815 | 50.0% | 1 | K.YGKDFNDIRQDFLPWK.S | 2 |
| \* | pDK268\_112012\_01.15629.15629.2 | 2.5693 | 0.3553 | 99.9% | 1637.2322 | 1636.9489 | 1 | 5.739 | 50.0% | 2 | K.SLASIVQFYYMWK.T | 2 |
| \* | pDK268\_112012\_01.05043.05043.3 | 4.4325 | 0.3711 | 100.0% | 2172.4143 | 2173.35 | 1 | 6.437 | 42.1% | 1 | R.GHLSRPEAQSLSPYTTSANR.A | 3 |
| \* | pDK268\_112012\_01.05532.05532.3 | 4.2603 | 0.2406 | 100.0% | 2252.9944 | 2253.35 | 1 | 7.358 | 46.1% | 1 | R.GHLSRPEAQSLS\*PYTTSANR.A | 3 |
| \* | pDK268\_112012\_01.05253.05253.3 | 3.5591 | 0.2616 | 100.0% | 1328.5743 | 1328.557 | 1 | 5.525 | 47.7% | 1 | R.RPYAPINANAIK.A | 3 |
| \* | pDK268\_112012\_01.08205.08205.2 | 2.1348 | 0.1692 | 97.5% | 854.7522 | 855.10876 | 79 | 5.514 | 64.3% | 1 | R.LPLATIVK.D | 2 |
| \* | pDK268\_112012\_01.05357.05357.2 | 3.1589 | 0.1884 | 99.9% | 1181.2322 | 1180.4331 | 2 | 5.008 | 75.0% | 1 | K.DLVAQAPLKPK.T | 2 |
| \* | pDK268\_112012\_01.09358.09358.3 | 3.6787 | 0.3904 | 100.0% | 2393.9644 | 2394.7104 | 53 | 6.138 | 22.8% | 1 | R.AYETMAGAGVPFSANGRPLASGIR.S | 3 |
| \* | pDK268\_112012\_01.08862.08862.3 | 2.9834 | 0.2925 | 99.7% | 1910.0343 | 1910.2236 | 198 | 4.867 | 26.5% | 1 | R.QKLNPADAPNPVVFVATK.D | 3 |
| \* | pDK268\_112012\_01.10323.10323.2 | 3.7728 | 0.6154 | 100.0% | 1653.4122 | 1653.9188 | 1 | 9.122 | 70.0% | 2 | K.LNPADAPNPVVFVATK.D | 2 |
| \* | pDK268\_112012\_01.10602.10602.3 | 4.9858 | 0.4367 | 100.0% | 3256.0444 | 3256.813 | 1 | 7.958 | 26.7% | 1 | K.VKPTLIAVRPPVPLPAPSHPASTNEPIVLED.- | 3 |

---

|  |  |  |  |  |  |  |  |  |
| --- | --- | --- | --- | --- | --- | --- | --- | --- |
| U | *gi|15431303|ref|NP\_00* | 6 | 9 | 34.9% | 192 | 21863 | 10.0 | ribosomal protein L9 [Homo sapiens] |
| U | *gi|67944630|ref|NP\_00* | 6 | 9 | 34.9% | 192 | 21863 | 10.0 | ribosomal protein L9 [Homo sapiens] |

| Filename XCorr DeltCN Conf% ObsM+H+ CalcM+H+ SpR ZScore Ion% # Sequence  | | | | | | | | | | | | |
| --- | --- | --- | --- | --- | --- | --- | --- | --- | --- | --- | --- | --- |
|  | pDK268\_112012\_02.08227.08227.2 | 4.9984 | 0.5471 | 100.0% | 2113.9922 | 2114.401 | 1 | 9.283 | 66.7% | 2 | K.TILSNQTVDIPENVDITLK.G | 2 |
|  | pDK268\_112012\_02.07345.07345.2 | 2.4161 | 0.183 | 97.2% | 1755.7122 | 1756.0146 | 15 | 4.283 | 42.9% | 1 | R.RDFNHINVELSLLGK.K | 2 |
|  | pDK268\_112012\_01.11835.11835.2 | 4.3937 | 0.5138 | 100.0% | 1598.9722 | 1599.8271 | 1 | 8.896 | 69.2% | 3 | R.DFNHINVELSLLGK.K | 2 |
|  | pDK268\_112012\_02.09316.09316.3 | 3.7252 | 0.2692 | 100.0% | 2485.5544 | 2485.8455 | 1 | 4.78 | 33.3% | 1 | R.SVYAHFPINVVIQENGSLVEIR.N | 3 |
|  | pDK268\_112012\_01.08001.08001.2 | 2.9109 | 0.1655 | 99.7% | 1299.3322 | 1299.5095 | 1 | 4.843 | 75.0% | 1 | R.KFLDGIYVSEK.G | 2 |
|  | pDK268\_112012\_01.09368.09368.2 | 3.1453 | 0.3833 | 100.0% | 1171.5922 | 1171.3354 | 1 | 6.839 | 77.8% | 1 | K.FLDGIYVSEK.G | 2 |

---

|  |  |  |  |  |  |  |  |  |
| --- | --- | --- | --- | --- | --- | --- | --- | --- |
| U | *gi|4506699|ref|NP\_001* | 5 | 9 | 34.9% | 83 | 9111 | 8.5 | ribosomal protein S21 [Homo sapiens] |

| Filename XCorr DeltCN Conf% ObsM+H+ CalcM+H+ SpR ZScore Ion% # Sequence  | | | | | | | | | | | | |
| --- | --- | --- | --- | --- | --- | --- | --- | --- | --- | --- | --- | --- |
| \* | pDK268\_112012\_02.05196.05196.2 | 2.5123 | 0.2784 | 99.6% | 1556.5721 | 1557.7185 | 15 | 5.62 | 46.2% | 1 | K.DHASIQMNVAEVDK.V | 2 |
| \* | pDK268\_112012\_02.05201.05201.3 | 3.1973 | 0.2765 | 100.0% | 1557.1743 | 1557.7185 | 17 | 5.394 | 40.4% | 1 | K.DHASIQMNVAEVDK.V | 3 |
| \* | pDK268\_112012\_02.06015.06015.3 | 5.5433 | 0.603 | 100.0% | 1971.0543 | 1971.1956 | 1 | 9.561 | 47.1% | 4 | K.DHASIQMNVAEVDKVTGR.F | 3 |
| \* | pDK268\_112012\_01.05009.05009.2 | 2.7797 | 0.2882 | 99.9% | 1279.1322 | 1279.4106 | 1 | 4.936 | 80.0% | 1 | R.RMGESDDSILR.L | 2 |
| \* | pDK268\_112012\_01.06249.06249.2 | 3.0869 | 0.4975 | 100.0% | 1122.8322 | 1123.2231 | 1 | 8.254 | 83.3% | 2 | R.MGESDDSILR.L | 2 |

---

|  |  |  |  |  |  |  |  |  |
| --- | --- | --- | --- | --- | --- | --- | --- | --- |
| U | *gi|14043070|ref|NP\_11* | 13 | 22 | 34.7% | 372 | 38747 | 9.1 | heterogeneous nuclear ribonucleoprotein A1 isoform b [Homo sapiens] |
| U | *gi|4504445|ref|NP\_002* | 13 | 22 | 40.3% | 320 | 34196 | 9.2 | heterogeneous nuclear ribonucleoprotein A1 isoform a [Homo sapiens] |

| Filename XCorr DeltCN Conf% ObsM+H+ CalcM+H+ SpR ZScore Ion% # Sequence  | | | | | | | | | | | | |
| --- | --- | --- | --- | --- | --- | --- | --- | --- | --- | --- | --- | --- |
|  | pDK268\_112012\_01.03813.03813.2 | 2.5773 | 0.2507 | 99.8% | 1300.1122 | 1300.4111 | 4 | 4.484 | 70.0% | 1 | K.SESPKEPEQLR.K | 2 |
|  | pDK268\_112012\_01.03452.03452.2 | 2.4764 | 0.1797 | 98.4% | 1428.2522 | 1428.5852 | 2 | 4.193 | 72.7% | 1 | K.SESPKEPEQLRK.L | 2 |
|  | pDK268\_112012\_01.03448.03448.3 | 2.7828 | 0.2292 | 98.7% | 1428.3544 | 1428.5852 | 14 | 4.509 | 40.9% | 1 | K.SESPKEPEQLRK.L | 3 |
|  | pDK268\_112012\_02.08353.08353.2 | 4.2256 | 0.4486 | 100.0% | 1913.4521 | 1914.1656 | 1 | 6.988 | 68.8% | 1 | R.KLFIGGLSFETTDESLR.S | 2 |
|  | pDK268\_112012\_01.12926.12926.2 | 4.8358 | 0.486 | 100.0% | 1785.6522 | 1785.9916 | 1 | 8.421 | 70.0% | 5 | K.LFIGGLSFETTDESLR.S | 2 |
|  | pDK268\_112012\_02.09501.09501.3 | 2.8978 | 0.4229 | 100.0% | 2510.3342 | 2511.8145 | 1 | 6.692 | 29.5% | 1 | R.GFGFVTYATVEEVDAAMNARPHK.V | 3 |
|  | pDK268\_112012\_01.03354.03354.2 | 2.9657 | 0.2509 | 99.8% | 1566.3322 | 1566.7574 | 6 | 4.504 | 53.8% | 1 | R.EDSQRPGAHLTVKK.I | 2 |
|  | pDK268\_112012\_01.10900.10900.2 | 3.7967 | 0.4105 | 100.0% | 1218.6921 | 1219.4387 | 1 | 7.144 | 83.3% | 2 | K.IEVIEIMTDR.G | 2 |
|  | pDK268\_112012\_01.10326.10326.2 | 4.3727 | 0.5352 | 100.0% | 1699.9722 | 1700.8016 | 1 | 8.942 | 64.3% | 3 | R.GFAFVTFDDHDSVDK.I | 2 |
|  | pDK268\_112012\_01.11102.11102.3 | 3.6494 | 0.2815 | 100.0% | 2281.4043 | 2282.5579 | 1 | 7.155 | 38.2% | 1 | R.GFAFVTFDDHDSVDKIVIQK.Y | 3 |
|  | pDK268\_112012\_01.11139.11139.2 | 3.9351 | 0.415 | 100.0% | 2281.672 | 2282.5579 | 1 | 7.215 | 52.6% | 1 | R.GFAFVTFDDHDSVDKIVIQK.Y | 2 |
|  | pDK268\_112012\_01.05399.05399.2 | 3.7301 | 0.5223 | 100.0% | 1628.6522 | 1629.7721 | 1 | 8.043 | 60.0% | 2 | R.SSGPYGGGGQYFAKPR.N | 2 |
|  | pDK268\_112012\_01.03647.03647.2 | 5.0912 | 0.571 | 100.0% | 1695.1522 | 1695.6561 | 1 | 10.598 | 67.6% | 2 | R.NQGGYGGSSSSSSYGSGR.R | 2 |

---

|  |  |  |  |  |  |  |  |  |
| --- | --- | --- | --- | --- | --- | --- | --- | --- |
| U | *gi|4506723|ref|NP\_000* | 9 | 10 | 34.5% | 264 | 29945 | 9.7 | ribosomal protein S3a [Homo sapiens] |

| Filename XCorr DeltCN Conf% ObsM+H+ CalcM+H+ SpR ZScore Ion% # Sequence  | | | | | | | | | | | | |
| --- | --- | --- | --- | --- | --- | --- | --- | --- | --- | --- | --- | --- |
| \* | pDK268\_112012\_01.08283.08283.2 | 2.6723 | 0.4645 | 100.0% | 920.1322 | 920.1174 | 2 | 7.745 | 85.7% | 2 | K.APAMFNIR.N | 2 |
|  | pDK268\_112012\_02.09653.09653.2 | 3.2935 | 0.4241 | 100.0% | 1953.3922 | 1953.1589 | 1 | 7.193 | 43.8% | 1 | R.VFEVSLADLQNDEVAFR.K | 2 |
|  | pDK268\_112012\_02.08699.08699.3 | 3.0928 | 0.3854 | 100.0% | 2081.6042 | 2081.333 | 1 | 6.64 | 42.6% | 1 | R.VFEVSLADLQNDEVAFRK.F | 3 |
|  | pDK268\_112012\_01.06372.06372.2 | 2.5915 | 0.2632 | 99.9% | 939.2322 | 939.0562 | 1 | 5.071 | 85.7% | 1 | K.TTDGYLLR.L | 2 |
|  | pDK268\_112012\_01.06755.06755.2 | 2.0177 | 0.1256 | 95.8% | 913.1322 | 912.1684 | 2 | 4.979 | 75.0% | 1 | K.MMEIMTR.E | 2 |
|  | pDK268\_112012\_01.05297.05297.2 | 3.6425 | 0.3503 | 100.0% | 1516.2122 | 1516.6915 | 2 | 6.181 | 66.7% | 1 | R.EVQTNDLKEVVNK.L | 2 |
| \* | pDK268\_112012\_01.07128.07128.2 | 2.2394 | 0.2338 | 98.3% | 1328.1721 | 1328.5486 | 15 | 4.993 | 54.5% | 1 | K.LIPDSIGKDIEK.A | 2 |
| \* | pDK268\_112012\_01.04241.04241.2 | 3.0817 | 0.4599 | 100.0% | 1332.1921 | 1332.4712 | 1 | 6.987 | 75.0% | 1 | K.LMELHGEGSSSGK.A | 2 |
| \* | pDK268\_112012\_01.07624.07624.1 | 1.5381 | 0.3214 | 100.0% | 1291.63 | 1291.3568 | 2 | 6.766 | 45.5% | 1 | R.ADGYEPPVQESV.- | 1 |

---

|  |  |  |  |  |  |  |  |  |
| --- | --- | --- | --- | --- | --- | --- | --- | --- |
| U | *gi|10800140|ref|NP\_06* | 4 | 10 | 34.1% | 126 | 13950 | 10.3 | histone cluster 1, H2bb [Homo sapiens] |
| U | *gi|4504277|ref|NP\_003* | 4 | 10 | 34.1% | 126 | 13920 | 10.3 | histone cluster 2, H2be [Homo sapiens] |
| U | *gi|28173554|ref|NP\_77* | 4 | 10 | 34.1% | 126 | 13908 | 10.3 | histone cluster 3, H2bb [Homo sapiens] |
| U | *gi|20336754|ref|NP\_06* | 4 | 10 | 34.1% | 126 | 13904 | 10.3 | histone cluster 1, H2bj [Homo sapiens] |
| U | *gi|16306566|ref|NP\_00* | 4 | 10 | 34.1% | 126 | 13906 | 10.3 | histone cluster 1, H2bo [Homo sapiens] |

| Filename XCorr DeltCN Conf% ObsM+H+ CalcM+H+ SpR ZScore Ion% # Sequence  | | | | | | | | | | | | |
| --- | --- | --- | --- | --- | --- | --- | --- | --- | --- | --- | --- | --- |
|  | pDK268\_112012\_01.06356.06356.2 | 3.0622 | 0.3652 | 100.0% | 1280.1322 | 1280.4631 | 1 | 6.411 | 77.8% | 1 | R.KESYSIYVYK.V | 2 |
|  | pDK268\_112012\_01.03429.03429.1 | 1.6018 | 0.2567 | 96.0% | 1168.61 | 1169.2798 | 1 | 5.493 | 70.0% | 1 | K.QVHPDTGISSK.A | 11 |
|  | pDK268\_112012\_01.14924.14924.2 | 5.1303 | 0.4906 | 100.0% | 1744.2722 | 1745.0211 | 1 | 9.306 | 78.6% | 7 | K.AMGIMNSFVNDIFER.I | 22 |
|  | pDK268\_112012\_01.02926.02926.2 | 2.1651 | 0.1345 | 97.5% | 901.7522 | 902.0439 | 13 | 4.455 | 83.3% | 1 | R.LAHYNKR.S | 22 |

Similarities:
gi|10800138|ref|NP\_06(3:1)  

---

|  |  |  |  |  |  |  |  |  |
| --- | --- | --- | --- | --- | --- | --- | --- | --- |
| U | *gi|15011936|ref|NP\_00* | 4 | 5 | 33.9% | 115 | 13015 | 11.0 | ribosomal protein S26 [Homo sapiens] |
| U | *gi|169167044|ref|XP\_0* | 4 | 5 | 33.9% | 115 | 13015 | 11.0 | PREDICTED: similar to ribosomal protein S26 [Homo sapiens] |
| U | *gi|169166765|ref|XP\_0* | 4 | 5 | 33.9% | 115 | 13015 | 11.0 | PREDICTED: similar to ribosomal protein S26 [Homo sapiens] |
| U | *gi|169166210|ref|XP\_0* | 4 | 5 | 33.9% | 115 | 13015 | 11.0 | PREDICTED: hypothetical protein LOC728937 [Homo sapiens] |

| Filename XCorr DeltCN Conf% ObsM+H+ CalcM+H+ SpR ZScore Ion% # Sequence  | | | | | | | | | | | | |
| --- | --- | --- | --- | --- | --- | --- | --- | --- | --- | --- | --- | --- |
|  | pDK268\_112012\_01.05286.05286.1 | 2.0172 | 0.2658 | 100.0% | 942.46 | 943.091 | 1 | 5.745 | 62.5% | 1 | R.NIVEAAAVR.D | 1 |
|  | pDK268\_112012\_01.05276.05276.2 | 2.7459 | 0.3128 | 100.0% | 943.1322 | 943.091 | 1 | 6.228 | 87.5% | 1 | R.NIVEAAAVR.D | 2 |
|  | pDK268\_112012\_01.12845.12845.2 | 2.6181 | 0.2669 | 99.6% | 1654.2922 | 1654.8572 | 1 | 5.211 | 60.7% | 2 | R.DISEASVFDAYVLPK.L | 2 |
|  | pDK268\_112012\_01.04590.04590.2 | 2.9419 | 0.3962 | 100.0% | 1590.2522 | 1590.9298 | 1 | 6.797 | 67.9% | 1 | R.FRPAGAAPRPPPKPM.- | 2 |

---

|  |  |  |  |  |  |  |  |  |
| --- | --- | --- | --- | --- | --- | --- | --- | --- |
| U | *gi|167466173|ref|NP\_0* | 20 | 37 | 33.7% | 641 | 70052 | 5.6 | heat shock 70kDa protein 1B [Homo sapiens] |
| U | *gi|194248072|ref|NP\_0* | 20 | 37 | 33.7% | 641 | 70052 | 5.6 | heat shock 70kDa protein 1A [Homo sapiens] |

| Filename XCorr DeltCN Conf% ObsM+H+ CalcM+H+ SpR ZScore Ion% # Sequence  | | | | | | | | | | | | |
| --- | --- | --- | --- | --- | --- | --- | --- | --- | --- | --- | --- | --- |
|  | pDK268\_112012\_01.07984.07984.2 | 3.3856 | 0.4857 | 100.0% | 1488.2722 | 1488.5939 | 1 | 9.133 | 79.2% | 3 | R.TTPSYVAFTDTER.L | 2222 |
|  | pDK268\_112012\_01.08312.08312.2 | 4.7653 | 0.3713 | 100.0% | 1659.2122 | 1659.8394 | 1 | 9.105 | 75.0% | 2 | K.NQVALNPQNTVFDAK.R | 2 |
|  | pDK268\_112012\_01.05398.05398.2 | 2.7813 | 0.3964 | 100.0% | 1351.1721 | 1351.5603 | 1 | 7.422 | 68.2% | 1 | R.KFGDPVVQSDMK.H | 2 |
|  | pDK268\_112012\_01.06560.06560.2 | 3.4927 | 0.5227 | 100.0% | 1222.8922 | 1223.3862 | 1 | 8.831 | 75.0% | 1 | K.FGDPVVQSDMK.H | 2 |
|  | pDK268\_112012\_01.08271.08271.2 | 3.0201 | 0.2862 | 99.9% | 1681.3722 | 1681.8912 | 1 | 7.122 | 61.5% | 2 | K.HWPFQVINDGDKPK.V | 2 |
|  | pDK268\_112012\_01.08288.08288.3 | 4.9768 | 0.4269 | 100.0% | 1681.9744 | 1681.8912 | 1 | 6.428 | 55.8% | 3 | K.HWPFQVINDGDKPK.V | 3 |
|  | pDK268\_112012\_01.12598.12598.2 | 3.2433 | 0.5149 | 100.0% | 1615.4321 | 1615.8817 | 1 | 7.547 | 69.2% | 3 | K.AFYPEEISSMVLTK.M | 22 |
|  | pDK268\_112012\_01.13282.13282.3 | 4.4464 | 0.3657 | 100.0% | 3263.8743 | 3262.7046 | 5 | 6.419 | 23.2% | 2 | K.MKEIAEAYLGYPVTNAVITVPAYFNDSQR.Q | 3 |
|  | pDK268\_112012\_02.08087.08087.2 | 3.5783 | 0.4019 | 100.0% | 1198.2522 | 1198.408 | 1 | 7.736 | 86.4% | 3 | K.DAGVIAGLNVLR.I | 22 |
|  | pDK268\_112012\_01.10697.10697.2 | 5.13 | 0.4959 | 100.0% | 1689.5122 | 1688.9213 | 1 | 10.445 | 83.3% | 3 | R.IINEPTAAAIAYGLDR.T | 22 |
|  | pDK268\_112012\_01.05019.05019.2 | 4.3957 | 0.5202 | 100.0% | 1676.1921 | 1676.6964 | 1 | 8.648 | 73.3% | 1 | K.ATAGDTHLGGEDFDNR.L | 222 |
|  | pDK268\_112012\_01.05050.05050.3 | 3.4865 | 0.5332 | 100.0% | 1676.5144 | 1676.6964 | 1 | 7.452 | 50.0% | 1 | K.ATAGDTHLGGEDFDNR.L | 333 |
|  | pDK268\_112012\_01.07822.07822.2 | 2.9404 | 0.3827 | 100.0% | 1262.2122 | 1262.4508 | 1 | 7.121 | 77.8% | 2 | R.LVNHFVEEFK.R | 2 |
|  | pDK268\_112012\_01.06611.06611.3 | 2.3816 | 0.2889 | 98.9% | 1418.8444 | 1418.6383 | 2 | 5.74 | 42.5% | 1 | R.LVNHFVEEFKR.K | 3 |
|  | pDK268\_112012\_01.07965.07965.3 | 4.3186 | 0.3898 | 100.0% | 1823.4543 | 1823.1025 | 1 | 6.899 | 43.8% | 2 | K.LDKAQIHDLVLVGGSTR.I | 3 |
|  | pDK268\_112012\_01.10684.10684.2 | 2.6514 | 0.2753 | 99.9% | 1110.2122 | 1110.2578 | 1 | 6.412 | 87.5% | 2 | K.LLQDFFNGR.D | 2 |
|  | pDK268\_112012\_01.03453.03453.2 | 2.6857 | 0.3759 | 100.0% | 1018.2322 | 1018.1582 | 241 | 6.243 | 50.0% | 1 | K.ITITNDKGR.L | 2222 |
|  | pDK268\_112012\_01.03443.03443.2 | 3.2272 | 0.4434 | 100.0% | 1137.9521 | 1138.2224 | 1 | 7.475 | 81.2% | 1 | K.YKAEDEVQR.E | 22 |
|  | pDK268\_112012\_01.09651.09651.2 | 3.2188 | 0.4826 | 100.0% | 1287.5922 | 1288.4608 | 1 | 8.154 | 80.0% | 2 | K.NALESYAFNMK.S | 22 |
|  | pDK268\_112012\_01.03984.03984.2 | 2.4119 | 0.3457 | 99.9% | 947.9322 | 948.01776 | 1 | 6.323 | 81.2% | 1 | K.SAVEDEGLK.G | 2 |

Similarities:
gi|5729877|ref|NP\_006(2:18)  
gi|124256496|ref|NP\_0(8:12)  
gi|34419635|ref|NP\_00(5:15)  

---

|  |  |  |  |  |  |  |  |  |
| --- | --- | --- | --- | --- | --- | --- | --- | --- |
| U | *gi|4506707|ref|NP\_001* | 6 | 10 | 33.6% | 125 | 13742 | 10.1 | ribosomal protein S25 [Homo sapiens] |

| Filename XCorr DeltCN Conf% ObsM+H+ CalcM+H+ SpR ZScore Ion% # Sequence  | | | | | | | | | | | | |
| --- | --- | --- | --- | --- | --- | --- | --- | --- | --- | --- | --- | --- |
| \* | pDK268\_112012\_01.09539.09539.2 | 3.4315 | 0.2815 | 100.0% | 1319.4122 | 1319.5437 | 3 | 5.753 | 70.0% | 2 | R.DKLNNLVLFDK.A | 2 |
| \* | pDK268\_112012\_01.09950.09950.2 | 3.4928 | 0.426 | 100.0% | 1076.3322 | 1076.281 | 1 | 7.032 | 81.2% | 2 | K.LNNLVLFDK.A | 2 |
| \* | pDK268\_112012\_01.07088.07088.2 | 2.3411 | 0.3966 | 99.9% | 1085.1522 | 1085.289 | 1 | 5.938 | 77.8% | 2 | K.LITPAVVSER.L | 2 |
| \* | pDK268\_112012\_01.09076.09076.3 | 2.6108 | 0.2205 | 96.6% | 1459.1344 | 1457.7135 | 25 | 4.918 | 30.8% | 1 | R.GSLARAALQELLSK.G | 3 |
| \* | pDK268\_112012\_01.09076.09076.2 | 3.1841 | 0.3066 | 100.0% | 973.09216 | 973.1576 | 1 | 7.041 | 87.5% | 2 | R.AALQELLSK.G | 3 |
| \* | pDK268\_112012\_01.04108.04108.2 | 1.7559 | 0.2156 | 95.3% | 851.2322 | 850.9933 | 7 | 4.829 | 75.0% | 1 | R.AQVIYTR.N | 2 |

---

|  |  |  |  |  |  |  |  |  |
| --- | --- | --- | --- | --- | --- | --- | --- | --- |
| U | *gi|224586844|ref|NP\_0* | 19 | 25 | 33.5% | 644 | 71686 | 9.5 | sorbin and SH3 domain containing 2 isoform 3 [Homo sapiens] |

| Filename XCorr DeltCN Conf% ObsM+H+ CalcM+H+ SpR ZScore Ion% # Sequence  | | | | | | | | | | | | |
| --- | --- | --- | --- | --- | --- | --- | --- | --- | --- | --- | --- | --- |
|  | pDK268\_112012\_01.06262.06262.2 | 2.2912 | 0.2027 | 97.8% | 1213.3722 | 1213.3793 | 66 | 4.984 | 50.0% | 1 | R.VQSSPNLLAAGR.D | 22 |
|  | pDK268\_112012\_02.05615.05615.2 | 5.3786 | 0.5818 | 100.0% | 2319.9922 | 2320.388 | 1 | 10.527 | 57.1% | 1 | R.SYNDGNQETLNGDATYSSLAAK.G | 22 |
|  | pDK268\_112012\_02.05596.05596.3 | 4.2585 | 0.4289 | 100.0% | 2320.2244 | 2320.388 | 1 | 7.094 | 34.5% | 1 | R.SYNDGNQETLNGDATYSSLAAK.G | 33 |
|  | pDK268\_112012\_01.03324.03324.3 | 3.3855 | 0.2456 | 100.0% | 1213.5543 | 1213.3823 | 1 | 5.296 | 58.3% | 1 | R.SVRPNLQDKR.S | 33 |
|  | pDK268\_112012\_01.09306.09306.2 | 3.0216 | 0.4398 | 100.0% | 2047.7922 | 2048.3062 | 1 | 6.751 | 47.4% | 1 | K.APHYPGIGPVDESGIPTAIR.T | 22 |
|  | pDK268\_112012\_01.09278.09278.3 | 4.3869 | 0.4169 | 100.0% | 2048.6343 | 2048.3062 | 1 | 6.313 | 38.2% | 2 | K.APHYPGIGPVDESGIPTAIR.T | 33 |
|  | pDK268\_112012\_01.03700.03700.2 | 1.9921 | 0.248 | 97.9% | 1093.5122 | 1094.256 | 5 | 4.442 | 62.5% | 1 | K.TQTYRPLSK.S | 22 |
|  | pDK268\_112012\_01.06843.06843.3 | 3.9449 | 0.3799 | 100.0% | 2188.0144 | 2188.405 | 1 | 6.465 | 43.1% | 2 | K.SSILQHERPASLYQSSIDR.S | 3 |
|  | pDK268\_112012\_01.07479.07479.2 | 2.6316 | 0.3018 | 99.8% | 1771.3522 | 1772.9886 | 1 | 5.856 | 56.7% | 1 | R.SLERPMSSASMASDFR.K | 22 |
|  | pDK268\_112012\_01.06196.06196.3 | 4.7367 | 0.4319 | 100.0% | 1900.6144 | 1901.1627 | 1 | 7.648 | 43.8% | 1 | R.SLERPMSSASMASDFRK.R | 33 |
|  | pDK268\_112012\_01.06932.06932.2 | 2.637 | 0.4401 | 100.0% | 1218.0922 | 1218.324 | 1 | 7.68 | 80.0% | 1 | R.SYSSTLTDMGR.S | 22 |
|  | pDK268\_112012\_01.05574.05574.2 | 2.8984 | 0.2794 | 100.0% | 1065.4122 | 1065.2578 | 2 | 5.596 | 75.0% | 1 | K.KGDTVYILR.K | 22 |
|  | pDK268\_112012\_01.11759.11759.2 | 2.5329 | 0.3917 | 100.0% | 1252.1522 | 1252.4961 | 1 | 6.85 | 70.0% | 3 | R.VGIFPISYVEK.L | 22 |
|  | pDK268\_112012\_01.06353.06353.2 | 5.1389 | 0.5174 | 100.0% | 2095.5322 | 2096.394 | 1 | 9.851 | 55.0% | 1 | K.AQPARPPPPAQPGEIGEAIAK.Y | 22 |
|  | pDK268\_112012\_02.15478.15478.2 | 2.1344 | 0.208 | 96.3% | 1656.6322 | 1656.7924 | 1 | 4.436 | 46.2% | 1 | K.YNFNADTNVELSLR.K | 22 |
|  | pDK268\_112012\_01.06545.06545.2 | 2.5607 | 0.2541 | 99.8% | 1138.9521 | 1139.2096 | 1 | 6.08 | 81.2% | 1 | R.VDQNWYEGK.I | 22 |
|  | pDK268\_112012\_01.11982.11982.2 | 2.4078 | 0.2582 | 99.3% | 1466.4321 | 1465.7325 | 30 | 4.712 | 45.8% | 1 | R.QGIFPVSYVEVVK.K | 22 |
|  | pDK268\_112012\_01.06058.06058.2 | 2.3615 | 0.2695 | 99.8% | 1018.0522 | 1018.0685 | 10 | 5.489 | 85.7% | 2 | R.NEDELELR.E | 22 |
|  | pDK268\_112012\_01.10343.10343.2 | 2.316 | 0.1871 | 97.9% | 1277.6921 | 1277.465 | 1 | 4.873 | 70.0% | 2 | K.FFGTFPGNYVK.R | 22 |

Similarities:
gi|224586846|ref|NP\_0(18:1)  

---

|  |  |  |  |  |  |  |  |  |
| --- | --- | --- | --- | --- | --- | --- | --- | --- |
| U | *gi|14141193|ref|NP\_00* | 8 | 8 | 33.5% | 194 | 22591 | 10.7 | ribosomal protein S9 [Homo sapiens] |

| Filename XCorr DeltCN Conf% ObsM+H+ CalcM+H+ SpR ZScore Ion% # Sequence  | | | | | | | | | | | | |
| --- | --- | --- | --- | --- | --- | --- | --- | --- | --- | --- | --- | --- |
| \* | pDK268\_112012\_01.07431.07431.2 | 1.867 | 0.2063 | 96.0% | 921.1122 | 921.0843 | 1 | 5.384 | 78.6% | 1 | K.LIGEYGLR.N | 2 |
| \* | pDK268\_112012\_01.07994.07994.2 | 2.5627 | 0.1912 | 99.5% | 1189.2722 | 1189.4031 | 1 | 4.728 | 72.2% | 1 | R.RLFEGNALLR.R | 2 |
| \* | pDK268\_112012\_01.09143.09143.2 | 2.1881 | 0.349 | 99.8% | 1033.2522 | 1033.2156 | 1 | 5.859 | 93.8% | 1 | R.LFEGNALLR.R | 2 |
| \* | pDK268\_112012\_01.05370.05370.2 | 2.171 | 0.3533 | 99.9% | 830.5122 | 830.95667 | 2 | 6.433 | 71.4% | 1 | R.IGVLDEGK.M | 2 |
| \* | pDK268\_112012\_01.10706.10706.2 | 2.7281 | 0.2095 | 99.8% | 1194.6122 | 1194.5182 | 1 | 4.814 | 77.8% | 1 | K.MKLDYILGLK.I | 2 |
| \* | pDK268\_112012\_01.08220.08220.2 | 1.6557 | 0.2579 | 95.4% | 921.9122 | 922.0257 | 37 | 4.641 | 66.7% | 1 | K.IEDFLER.R | 23 |
| \* | pDK268\_112012\_01.09688.09688.2 | 3.0131 | 0.2709 | 99.9% | 1400.8322 | 1400.7074 | 2 | 5.782 | 63.6% | 1 | R.KQVVNIPSFIVR.L | 2 |
| \* | pDK268\_112012\_01.03252.03252.2 | 2.8067 | 0.1581 | 99.6% | 1003.9922 | 1004.0934 | 2 | 5.461 | 72.2% | 1 | R.SPYGGGRPGR.V | 2 |

---

|  |  |  |  |  |  |  |  |  |
| --- | --- | --- | --- | --- | --- | --- | --- | --- |
| U | *gi|46367787|ref|NP\_00* | 26 | 44 | 33.3% | 636 | 70671 | 9.5 | poly(A) binding protein, cytoplasmic 1 [Homo sapiens] |

| Filename XCorr DeltCN Conf% ObsM+H+ CalcM+H+ SpR ZScore Ion% # Sequence  | | | | | | | | | | | | |
| --- | --- | --- | --- | --- | --- | --- | --- | --- | --- | --- | --- | --- |
|  | pDK268\_112012\_01.10106.10106.2 | 2.7156 | 0.4078 | 100.0% | 1157.8522 | 1158.3861 | 1 | 7.001 | 80.0% | 2 | K.FSPAGPILSIR.V | 2 |
|  | pDK268\_112012\_02.06241.06241.3 | 3.4809 | 0.318 | 100.0% | 2086.2244 | 2086.2712 | 2 | 5.482 | 36.8% | 1 | R.RSLGYAYVNFQQPADAER.A | 33 |
|  | pDK268\_112012\_01.09561.09561.2 | 4.7786 | 0.4793 | 100.0% | 1929.3121 | 1930.0837 | 1 | 8.371 | 56.2% | 3 | R.SLGYAYVNFQQPADAER.A | 22 |
|  | pDK268\_112012\_02.07006.07006.3 | 3.361 | 0.3456 | 100.0% | 1929.7743 | 1930.0837 | 1 | 5.605 | 45.3% | 1 | R.SLGYAYVNFQQPADAER.A | 33 |
|  | pDK268\_112012\_01.10653.10653.2 | 3.3927 | 0.5503 | 100.0% | 1266.5322 | 1267.4828 | 1 | 9.095 | 85.0% | 1 | R.ALDTMNFDVIK.G | 22 |
|  | pDK268\_112012\_01.06250.06250.2 | 2.4618 | 0.1976 | 99.2% | 1063.1522 | 1063.2853 | 19 | 5.002 | 61.1% | 1 | R.KSGVGNIFIK.N | 2 |
|  | pDK268\_112012\_01.08517.08517.2 | 2.8307 | 0.3067 | 100.0% | 935.27216 | 935.1112 | 1 | 6.178 | 81.2% | 2 | K.SGVGNIFIK.N | 2 |
| \* | pDK268\_112012\_02.06274.06274.2 | 4.5685 | 0.4992 | 100.0% | 1741.2922 | 1741.857 | 1 | 9.306 | 78.6% | 2 | K.GYGFVHFETQEAAER.A | 2 |
| \* | pDK268\_112012\_02.06285.06285.3 | 3.9316 | 0.3721 | 100.0% | 1741.9143 | 1741.857 | 1 | 7.618 | 53.6% | 1 | K.GYGFVHFETQEAAER.A | 3 |
|  | pDK268\_112012\_01.06940.06940.2 | 2.6508 | 0.2937 | 99.9% | 1063.6721 | 1064.263 | 2 | 5.323 | 75.0% | 2 | K.MNGMLLNDR.K | 22 |
|  | pDK268\_112012\_01.05027.05027.2 | 2.5104 | 0.2514 | 99.8% | 1192.1721 | 1192.437 | 28 | 5.165 | 61.1% | 1 | K.MNGMLLNDRK.V | 22 |
|  | pDK268\_112012\_01.05764.05764.2 | 2.7974 | 0.2854 | 99.9% | 1213.1522 | 1213.4191 | 2 | 5.834 | 72.2% | 3 | R.AKEFTNVYIK.N | 22 |
|  | pDK268\_112012\_01.05789.05789.3 | 3.6643 | 0.3443 | 100.0% | 1213.5243 | 1213.4191 | 3 | 7.04 | 55.6% | 1 | R.AKEFTNVYIK.N | 33 |
|  | pDK268\_112012\_02.03897.03897.2 | 2.8496 | 0.4037 | 100.0% | 1227.8121 | 1228.2323 | 1 | 6.266 | 77.8% | 2 | K.NFGEDMDDER.L | 2 |
|  | pDK268\_112012\_01.10494.10494.2 | 3.7804 | 0.4846 | 100.0% | 2029.2322 | 2030.2164 | 1 | 8.305 | 46.9% | 1 | K.NFGEDMDDERLKDLFGK.F | 2 |
|  | pDK268\_112012\_01.10978.10978.2 | 3.2234 | 0.4461 | 100.0% | 1046.2122 | 1046.1704 | 1 | 8.822 | 75.0% | 2 | K.GFGFVSFER.H | 2 |
|  | pDK268\_112012\_01.07322.07322.2 | 2.7928 | 0.1967 | 99.8% | 1083.6921 | 1084.2603 | 2 | 6.521 | 81.2% | 2 | R.YQGVNLYVK.N | 2 |
|  | pDK268\_112012\_01.05252.05252.2 | 3.1232 | 0.4266 | 100.0% | 1162.0322 | 1162.1553 | 1 | 7.419 | 94.4% | 1 | K.NLDDGIDDER.L | 2 |
| \* | pDK268\_112012\_01.08211.08211.2 | 3.2648 | 0.4249 | 100.0% | 1413.1921 | 1413.6134 | 1 | 6.649 | 66.7% | 2 | R.KEFSPFGTITSAK.V | 2 |
| \* | pDK268\_112012\_01.10112.10112.2 | 2.5369 | 0.4794 | 100.0% | 1285.2122 | 1285.4393 | 1 | 7.463 | 63.6% | 1 | K.EFSPFGTITSAK.V | 2 |
|  | pDK268\_112012\_01.08426.08426.2 | 4.3323 | 0.456 | 100.0% | 1543.4521 | 1543.8931 | 1 | 8.59 | 73.1% | 3 | R.IVATKPLYVALAQR.K | 2 |
|  | pDK268\_112012\_01.08463.08463.3 | 3.6706 | 0.2294 | 100.0% | 1545.0243 | 1543.8931 | 85 | 5.236 | 40.4% | 2 | R.IVATKPLYVALAQR.K | 3 |
|  | pDK268\_112012\_01.11787.11787.2 | 3.6218 | 0.4894 | 100.0% | 1637.0922 | 1638.0239 | 1 | 7.199 | 64.3% | 1 | R.LFPLIQAMHPTLAGK.I | 2 |
|  | pDK268\_112012\_01.15494.15494.3 | 5.6071 | 0.4119 | 100.0% | 2742.5344 | 2742.175 | 1 | 7.586 | 40.2% | 3 | K.ITGMLLEIDNSELLHMLESPESLR.S | 332 |
|  | pDK268\_112012\_01.06975.06975.2 | 4.8014 | 0.6003 | 100.0% | 1694.4321 | 1694.9285 | 1 | 10.71 | 83.3% | 1 | R.SKVDEAVAVLQAHQAK.E | 2 |
|  | pDK268\_112012\_01.06980.06980.3 | 5.0443 | 0.4814 | 100.0% | 1694.5743 | 1694.9285 | 1 | 9.447 | 56.7% | 2 | R.SKVDEAVAVLQAHQAK.E | 3 |

Similarities:
gi|208431833|ref|NP\_0(9:17)  

---

|  |  |  |  |  |  |  |  |  |
| --- | --- | --- | --- | --- | --- | --- | --- | --- |
| U | *gi|10800138|ref|NP\_06* | 5 | 11 | 33.3% | 126 | 13936 | 10.3 | histone cluster 1, H2bd [Homo sapiens] |
| U | *gi|66912162|ref|NP\_00* | 5 | 11 | 33.3% | 126 | 13920 | 10.3 | histone cluster 2, H2bf [Homo sapiens] |
| U | *gi|4504271|ref|NP\_003* | 5 | 11 | 33.3% | 126 | 13906 | 10.3 | histone cluster 1, H2bi [Homo sapiens] |
| U | *gi|4504269|ref|NP\_003* | 5 | 11 | 33.3% | 126 | 13892 | 10.3 | histone cluster 1, H2bh [Homo sapiens] |
| U | *gi|4504265|ref|NP\_003* | 4 | 10 | 33.3% | 126 | 13906 | 10.3 | histone cluster 1, H2bf [Homo sapiens] |
| U | *gi|4504263|ref|NP\_003* | 5 | 11 | 33.3% | 126 | 13989 | 10.3 | histone cluster 1, H2bm [Homo sapiens] |
| U | *gi|4504261|ref|NP\_003* | 5 | 11 | 33.3% | 126 | 13922 | 10.3 | histone cluster 1, H2bn [Homo sapiens] |
| U | *gi|4504259|ref|NP\_003* | 5 | 11 | 33.3% | 126 | 13952 | 10.3 | histone cluster 1, H2bl [Homo sapiens] |
| U | *gi|4504257|ref|NP\_003* | 5 | 11 | 33.3% | 126 | 13906 | 10.3 | histone cluster 1, H2bg [Homo sapiens] |
| U | *gi|21396484|ref|NP\_00* | 5 | 11 | 33.3% | 126 | 13906 | 10.3 | histone cluster 1, H2be [Homo sapiens] |
| U | *gi|21166389|ref|NP\_00* | 5 | 11 | 33.3% | 126 | 13906 | 10.3 | histone cluster 1, H2bc [Homo sapiens] |
| U | *gi|20336752|ref|NP\_61* | 5 | 11 | 33.3% | 126 | 13936 | 10.3 | histone cluster 1, H2bd [Homo sapiens] |
| U | *gi|18105048|ref|NP\_54* | 5 | 11 | 33.3% | 126 | 13890 | 10.3 | histone cluster 1, H2bk [Homo sapiens] |

| Filename XCorr DeltCN Conf% ObsM+H+ CalcM+H+ SpR ZScore Ion% # Sequence  | | | | | | | | | | | | |
| --- | --- | --- | --- | --- | --- | --- | --- | --- | --- | --- | --- | --- |
|  | pDK268\_112012\_01.07124.07124.1 | 1.835 | 0.2721 | 100.0% | 1137.6 | 1138.2622 | 1 | 5.987 | 68.8% | 1 | K.ESYSVYVYK.V | 1 |
|  | pDK268\_112012\_01.07112.07112.2 | 2.0605 | 0.2689 | 98.7% | 1138.3121 | 1138.2622 | 6 | 5.248 | 56.2% | 1 | K.ESYSVYVYK.V | 2 |
|  | pDK268\_112012\_01.03429.03429.1 | 1.6018 | 0.2567 | 96.0% | 1168.61 | 1169.2798 | 1 | 5.493 | 70.0% | 1 | K.QVHPDTGISSK.A | 11 |
|  | pDK268\_112012\_01.14924.14924.2 | 5.1303 | 0.4906 | 100.0% | 1744.2722 | 1745.0211 | 1 | 9.306 | 78.6% | 7 | K.AMGIMNSFVNDIFER.I | 22 |
|  | pDK268\_112012\_01.02926.02926.2 | 2.1651 | 0.1345 | 97.5% | 901.7522 | 902.0439 | 13 | 4.455 | 83.3% | 1 | R.LAHYNKR.S | 22 |

Similarities:
gi|10800140|ref|NP\_06(3:2)  

---

|  |  |  |  |  |  |  |  |  |
| --- | --- | --- | --- | --- | --- | --- | --- | --- |
| U | *contaminant\_gi|746301* | 10 | 35 | 33.1% | 269 | 27961 | 6.7 | lysyl endopeptidase (EC 3.4.21.50) - Lysobacter enzymogenes |

| Filename XCorr DeltCN Conf% ObsM+H+ CalcM+H+ SpR ZScore Ion% # Sequence  | | | | | | | | | | | | |
| --- | --- | --- | --- | --- | --- | --- | --- | --- | --- | --- | --- | --- |
| \* | pDK268\_112012\_02.04395.04395.3 | 4.7718 | 0.4523 | 100.0% | 2262.4744 | 2262.355 | 1 | 8.516 | 39.6% | 6 | R.APGSSSSGANGDGSLAQSQTGAVVR.A | 3 |
| \* | pDK268\_112012\_01.05115.05115.2 | 6.324 | 0.5981 | 100.0% | 2262.4922 | 2262.355 | 1 | 10.89 | 56.2% | 5 | R.APGSSSSGANGDGSLAQSQTGAVVR.A | 2 |
| \* | pDK268\_112012\_01.16800.16800.3 | 5.5514 | 0.4508 | 100.0% | 3315.8342 | 3315.6257 | 1 | 6.536 | 27.6% | 5 | R.ATNAASDFTLLELNTAANPAYNLFWAGWDR.R | 3 |
| \* | pDK268\_112012\_02.11817.11817.3 | 5.1718 | 0.4841 | 100.0% | 3472.3145 | 3471.813 | 1 | 7.477 | 25.8% | 4 | R.ATNAASDFTLLELNTAANPAYNLFWAGWDRR.D | 3 |
| \* | pDK268\_112012\_01.04558.04558.3 | 3.9773 | 0.4003 | 100.0% | 2076.6843 | 2077.2668 | 1 | 7.014 | 34.7% | 1 | R.RDQNFAGATAIHHPNVAEK.R | 3 |
| \* | pDK268\_112012\_01.04331.04331.3 | 4.4262 | 0.3863 | 100.0% | 2232.9243 | 2233.4543 | 1 | 6.027 | 38.2% | 1 | R.RDQNFAGATAIHHPNVAEKR.I | 3 |
| \* | pDK268\_112012\_01.05206.05206.2 | 4.3681 | 0.5366 | 100.0% | 1920.5721 | 1921.0793 | 1 | 9.481 | 58.8% | 2 | R.DQNFAGATAIHHPNVAEK.R | 2 |
| \* | pDK268\_112012\_01.04781.04781.3 | 3.9878 | 0.4305 | 100.0% | 2077.3442 | 2077.2668 | 1 | 6.912 | 33.3% | 3 | R.DQNFAGATAIHHPNVAEKR.I | 3 |
| \* | pDK268\_112012\_01.04748.04748.2 | 5.1918 | 0.3851 | 100.0% | 2077.4722 | 2077.2668 | 1 | 6.84 | 58.3% | 1 | R.DQNFAGATAIHHPNVAEKR.I | 2 |
| \* | pDK268\_112012\_01.07232.07232.2 | 4.5612 | 0.5365 | 100.0% | 1428.1721 | 1428.5443 | 1 | 8.753 | 76.9% | 7 | R.VFTSWTGGGTSATR.L | 2 |

---

|  |  |  |  |  |  |  |  |  |
| --- | --- | --- | --- | --- | --- | --- | --- | --- |
| U | *gi|4506685|ref|NP\_001* | 4 | 5 | 33.1% | 151 | 17222 | 10.5 | ribosomal protein S13 [Homo sapiens] |

| Filename XCorr DeltCN Conf% ObsM+H+ CalcM+H+ SpR ZScore Ion% # Sequence  | | | | | | | | | | | | |
| --- | --- | --- | --- | --- | --- | --- | --- | --- | --- | --- | --- | --- |
| \* | pDK268\_112012\_01.06370.06370.2 | 2.5136 | 0.3049 | 99.9% | 1091.9122 | 1092.2401 | 3 | 6.185 | 66.7% | 1 | K.GLSQSALPYR.R | 2 |
| \* | pDK268\_112012\_01.05903.05903.2 | 3.9791 | 0.3846 | 100.0% | 1439.2522 | 1439.6055 | 1 | 7.932 | 77.3% | 1 | K.LTSDDVKEQIYK.L | 2 |
| \* | pDK268\_112012\_01.08921.08921.2 | 3.6254 | 0.3587 | 100.0% | 1382.4722 | 1382.6896 | 1 | 6.484 | 70.8% | 1 | K.KGLTPSQIGVILR.D | 2 |
| \* | pDK268\_112012\_01.13234.13234.2 | 2.6853 | 0.3816 | 99.9% | 1694.2322 | 1694.9683 | 1 | 6.056 | 53.6% | 2 | K.GLAPDLPEDLYHLIK.K | 2 |

---

|  |  |  |  |  |  |  |  |  |
| --- | --- | --- | --- | --- | --- | --- | --- | --- |
| U | *gi|150417986|ref|NP\_0* | 55 | 86 | 32.9% | 1785 | 202037 | 6.3 | ADP-ribosylation factor guanine nucleotide-exchange factor 2 [Homo sapiens] |

| Filename XCorr DeltCN Conf% ObsM+H+ CalcM+H+ SpR ZScore Ion% # Sequence  | | | | | | | | | | | | |
| --- | --- | --- | --- | --- | --- | --- | --- | --- | --- | --- | --- | --- |
| \* | pDK268\_112012\_01.02934.02934.2 | 3.1293 | 0.3092 | 100.0% | 1022.2322 | 1022.155 | 1 | 5.341 | 92.9% | 1 | K.RPQHSQLR.R | 2 |
| \* | pDK268\_112012\_02.05019.05019.3 | 3.2028 | 0.1706 | 95.3% | 1839.6543 | 1840.0458 | 27 | 3.918 | 34.7% | 1 | K.LIAYGHITGNAPDSGAPGK.R | 3 |
| \* | pDK268\_112012\_01.05234.05234.3 | 4.3367 | 0.3066 | 100.0% | 1995.3243 | 1996.2333 | 1 | 6.608 | 40.8% | 3 | K.LIAYGHITGNAPDSGAPGKR.L | 3 |
| \* | pDK268\_112012\_01.16028.16028.2 | 3.465 | 0.2989 | 100.0% | 1508.3522 | 1508.8193 | 1 | 6.417 | 70.8% | 3 | K.ATLTQMLNVIFTR.M | 2 |
| \* | pDK268\_112012\_01.05198.05198.2 | 2.84 | 0.1552 | 99.7% | 1218.1122 | 1218.3702 | 1 | 4.523 | 94.4% | 1 | R.MENQVLQEAR.E | 2 |
| \* | pDK268\_112012\_02.04588.04588.3 | 3.3867 | 0.3819 | 100.0% | 2605.5544 | 2606.8499 | 1 | 5.197 | 30.7% | 1 | R.ELEKPIQSKPQS\*PVIQAAAVS\*PK.F | 3 |
| \* | pDK268\_112012\_01.03423.03423.3 | 4.0176 | 0.2214 | 99.6% | 2855.9944 | 2856.0383 | 2 | 5.748 | 34.4% | 1 | R.LKHSQAQSKPTT#PEKTDLTNGEHAR.S | 3 |
| \* | pDK268\_112012\_01.03450.03450.3 | 4.3697 | 0.3089 | 100.0% | 2613.5344 | 2614.7048 | 1 | 6.523 | 39.8% | 1 | K.HSQAQSKPTT#PEKTDLTNGEHAR.S | 3 |
| \* | pDK268\_112012\_01.16421.16421.3 | 3.4241 | 0.2833 | 99.8% | 3119.4243 | 3120.3948 | 7 | 4.576 | 19.8% | 1 | R.ERGSSLSGTDDGAQEVVKDILEDVVTSAIK.E | 3 |
| \* | pDK268\_112012\_01.17306.17306.3 | 5.5029 | 0.5155 | 100.0% | 2835.2344 | 2835.0918 | 1 | 7.548 | 36.1% | 2 | R.GSSLSGTDDGAQEVVKDILEDVVTSAIK.E | 3 |
| \* | pDK268\_112012\_01.18404.18404.3 | 4.7985 | 0.4796 | 100.0% | 2915.0645 | 2915.0918 | 1 | 6.945 | 30.6% | 2 | R.GSS\*LSGTDDGAQEVVKDILEDVVTSAIK.E | 3 |
| \* | pDK268\_112012\_01.17574.17574.3 | 4.5637 | 0.481 | 100.0% | 3361.6143 | 3363.6545 | 1 | 7.215 | 28.9% | 1 | R.GSSLSGTDDGAQEVVKDILEDVVTSAIKEAAEK.H | 3 |
| \* | pDK268\_112012\_01.18363.18363.3 | 3.6482 | 0.2691 | 99.9% | 3442.4944 | 3443.6545 | 2 | 4.587 | 21.9% | 1 | R.GS\*SLSGTDDGAQEVVKDILEDVVTSAIKEAAEK.H | 3 |
| \* | pDK268\_112012\_01.13649.13649.2 | 3.5801 | 0.5458 | 100.0% | 1303.2922 | 1303.4955 | 1 | 9.429 | 72.7% | 1 | K.DILEDVVTSAIK.E | 2 |
| \* | pDK268\_112012\_01.03384.03384.1 | 1.6263 | 0.3406 | 100.0% | 938.38 | 939.01587 | 2 | 5.683 | 64.3% | 1 | K.HGLTEPER.V | 1 |
| \* | pDK268\_112012\_01.05829.05829.2 | 2.4905 | 0.4793 | 100.0% | 2184.3323 | 2185.2717 | 12 | 7.472 | 37.5% | 1 | R.QSLSSADNLESDAQGHQVAAR.F | 2 |
| \* | pDK268\_112012\_01.05847.05847.3 | 3.7315 | 0.4833 | 100.0% | 2185.1042 | 2185.2717 | 1 | 7.843 | 30.0% | 4 | R.QSLSSADNLESDAQGHQVAAR.F | 3 |
| \* | pDK268\_112012\_01.03818.03818.2 | 2.2799 | 0.0922 | 97.4% | 859.09216 | 859.01587 | 2 | 3.757 | 91.7% | 1 | R.FSHVLQK.D | 2 |
|  | pDK268\_112012\_01.11236.11236.1 | 2.1735 | 0.3764 | 100.0% | 867.79 | 868.0232 | 2 | 5.872 | 66.7% | 1 | K.DAFLVFR.S | 11 |
| \* | pDK268\_112012\_01.06290.06290.3 | 3.1975 | 0.3298 | 100.0% | 1466.7544 | 1466.7357 | 1 | 5.398 | 46.2% | 1 | K.LSMKPLGEGPPDPK.S | 3 |
| \* | pDK268\_112012\_01.16380.16380.2 | 5.8101 | 0.4049 | 100.0% | 2269.9321 | 2269.736 | 1 | 8.837 | 65.0% | 1 | R.SKVVSLQLLLSVLQNAGPVFR.T | 2 |
|  | pDK268\_112012\_01.03945.03945.2 | 2.2229 | 0.1256 | 97.8% | 789.8522 | 788.9194 | 29 | 4.99 | 66.7% | 1 | R.LVNDLSK.I | 22 |
| \* | pDK268\_112012\_01.10389.10389.2 | 4.6927 | 0.4529 | 100.0% | 1769.3922 | 1769.0258 | 1 | 7.778 | 63.3% | 2 | R.SGHELGMTPLQELSLR.K | 2 |
| \* | pDK268\_112012\_01.10449.10449.3 | 3.8747 | 0.3466 | 100.0% | 1769.4543 | 1769.0258 | 3 | 6.644 | 36.7% | 3 | R.SGHELGMTPLQELSLR.K | 3 |
| \* | pDK268\_112012\_01.05985.05985.2 | 4.4801 | 0.5128 | 100.0% | 1871.3121 | 1872.0043 | 1 | 8.092 | 70.0% | 1 | K.DLYVNPNHQTSLGQER.L | 2 |
| \* | pDK268\_112012\_02.04305.04305.3 | 2.4828 | 0.2805 | 98.3% | 1872.3844 | 1872.0043 | 4 | 4.936 | 36.7% | 1 | K.DLYVNPNHQTSLGQER.L | 3 |
| \* | pDK268\_112012\_01.03999.03999.2 | 2.8509 | 0.4136 | 100.0% | 1075.5122 | 1076.1486 | 2 | 6.795 | 72.2% | 2 | R.LTDQEIGDGK.G | 2 |
| \* | pDK268\_112012\_01.09269.09269.2 | 4.5945 | 0.4471 | 100.0% | 1443.3121 | 1442.6549 | 1 | 6.914 | 90.9% | 1 | K.EIIEHGIELFNK.K | 2 |
| \* | pDK268\_112012\_01.15059.15059.3 | 7.396 | 0.4504 | 100.0% | 3261.2944 | 3261.6367 | 1 | 8.978 | 38.9% | 3 | K.RGIQFLQEQGMLGTSVEDIAQFLHQEER.L | 3 |
| \* | pDK268\_112012\_02.11920.11920.3 | 5.5649 | 0.5278 | 100.0% | 3104.4844 | 3105.4492 | 1 | 7.425 | 38.5% | 3 | R.GIQFLQEQGMLGTSVEDIAQFLHQEER.L | 3 |
| \* | pDK268\_112012\_01.09987.09987.2 | 5.028 | 0.5711 | 100.0% | 1582.2322 | 1581.6793 | 1 | 9.944 | 78.6% | 3 | R.LDSTQVGDFLGDSAR.F | 2 |
| \* | pDK268\_112012\_01.14084.14084.3 | 6.6966 | 0.447 | 100.0% | 2757.9844 | 2758.008 | 1 | 6.743 | 34.8% | 1 | R.GINDSKDLPEEYLSSIYEEIEGKK.I | 3 |
| \* | pDK268\_112012\_01.15620.15620.2 | 4.8825 | 0.4748 | 100.0% | 2015.4722 | 2015.178 | 1 | 7.165 | 65.6% | 2 | K.DLPEEYLSSIYEEIEGK.K | 2 |
| \* | pDK268\_112012\_01.14560.14560.2 | 3.6396 | 0.2465 | 100.0% | 2144.612 | 2143.352 | 1 | 5.025 | 50.0% | 1 | K.DLPEEYLSSIYEEIEGKK.I | 2 |
|  | pDK268\_112012\_02.06829.06829.2 | 2.667 | 0.1964 | 99.2% | 1639.1522 | 1639.9685 | 2 | 4.391 | 62.5% | 1 | R.RLLYNLEMEQMAK.T | 22 |
|  | pDK268\_112012\_01.10382.10382.2 | 4.1105 | 0.4186 | 100.0% | 1483.5122 | 1483.781 | 1 | 7.887 | 81.8% | 4 | R.LLYNLEMEQMAK.T | 22 |
| \* | pDK268\_112012\_01.04481.04481.2 | 2.7295 | 0.3527 | 100.0% | 1056.5521 | 1057.253 | 1 | 6.832 | 77.8% | 1 | K.ALMEAVSHAK.A | 2 |
| \* | pDK268\_112012\_01.07221.07221.3 | 3.459 | 0.296 | 100.0% | 1955.9343 | 1956.2726 | 1 | 6.231 | 42.2% | 1 | K.APFTSATHLDHVRPMFK.L | 3 |
|  | pDK268\_112012\_01.06569.06569.2 | 2.1792 | 0.2526 | 99.2% | 1006.4922 | 1007.1344 | 1 | 6.388 | 81.2% | 1 | R.DAYVQALAR.F | 22 |
| \* | pDK268\_112012\_02.08625.08625.2 | 3.7394 | 0.5672 | 100.0% | 1515.4722 | 1515.762 | 1 | 8.876 | 84.6% | 2 | R.FSLLTASSSITEMK.Q | 2 |
|  | pDK268\_112012\_01.11198.11198.3 | 5.3323 | 0.5255 | 100.0% | 2483.0344 | 2483.7861 | 1 | 8.351 | 40.5% | 4 | K.TLITVAHTDGNYLGNSWHEILK.C | 33 |
| \* | pDK268\_112012\_01.12026.12026.3 | 3.0672 | 0.4127 | 100.0% | 2517.3843 | 2515.8467 | 6 | 6.21 | 27.1% | 1 | K.GHTLAGEEFMGLGLGNLVSGGVDKR.Q | 3 |
|  | pDK268\_112012\_01.11036.11036.2 | 3.5281 | 0.2082 | 100.0% | 1219.3121 | 1219.383 | 1 | 5.872 | 90.0% | 2 | R.LDGNAIVDFVR.W | 22 |
| \* | pDK268\_112012\_01.08734.08734.2 | 3.4765 | 0.3491 | 100.0% | 1403.2722 | 1402.608 | 1 | 6.492 | 75.0% | 1 | K.IVEISYYNMNR.I | 2 |
| \* | pDK268\_112012\_01.07067.07067.2 | 3.1675 | 0.3565 | 100.0% | 1365.2722 | 1366.5651 | 1 | 6.004 | 80.0% | 1 | R.IWHVIGDHFNK.V | 2 |
|  | pDK268\_112012\_01.07836.07836.2 | 2.9261 | 0.3885 | 100.0% | 1324.2922 | 1324.5223 | 1 | 5.765 | 80.0% | 1 | K.FLEKGELANFR.F | 22 |
|  | pDK268\_112012\_01.07816.07816.3 | 2.8678 | 0.4239 | 100.0% | 1324.9143 | 1324.5223 | 2 | 6.096 | 47.5% | 1 | K.FLEKGELANFR.F | 33 |
|  | pDK268\_112012\_01.10670.10670.2 | 2.6565 | 0.2477 | 99.8% | 1433.4922 | 1433.7112 | 1 | 5.025 | 80.0% | 1 | K.DFLRPFEHIMK.K | 22 |
|  | pDK268\_112012\_01.10719.10719.3 | 2.521 | 0.27 | 98.9% | 1433.5743 | 1433.7112 | 2 | 4.527 | 57.5% | 1 | K.DFLRPFEHIMK.K | 33 |
| \* | pDK268\_112012\_02.05493.05493.2 | 5.15 | 0.5481 | 100.0% | 1910.3922 | 1911.0503 | 1 | 8.777 | 56.2% | 2 | R.VLQEYTSDDMNVAPGDR.V | 2 |
| \* | pDK268\_112012\_01.11024.11024.3 | 2.7398 | 0.3404 | 99.8% | 2526.3245 | 2526.867 | 22 | 5.89 | 23.8% | 1 | K.TTIPHVLLTWRPVGMEEDSSEK.H | 3 |
| \* | pDK268\_112012\_02.03928.03928.2 | 2.6022 | 0.2254 | 99.8% | 983.03217 | 983.06903 | 4 | 6.689 | 71.4% | 2 | K.HLDVDLDR.Q | 2 |
| \* | pDK268\_112012\_01.04202.04202.2 | 1.9356 | 0.4082 | 99.6% | 1461.2522 | 1461.5718 | 170 | 6.728 | 50.0% | 1 | R.QSLSSIDKNPSER.G | 2 |
| \* | pDK268\_112012\_01.06266.06266.2 | 4.7627 | 0.5143 | 100.0% | 1563.2922 | 1563.6207 | 1 | 8.409 | 73.1% | 2 | R.GQSQLSNPTDDSWK.G | 2 |
| \* | pDK268\_112012\_01.04215.04215.2 | 3.101 | 0.4351 | 100.0% | 1128.8722 | 1129.1741 | 1 | 7.561 | 81.2% | 1 | K.AFNSNYEQR.T | 2 |

Similarities:
gi|51479145|ref|NP\_00(11:44)  

---

|  |  |  |  |  |  |  |  |  |
| --- | --- | --- | --- | --- | --- | --- | --- | --- |
| U | *gi|11968182|ref|NP\_07* | 6 | 10 | 32.9% | 152 | 17719 | 11.0 | ribosomal protein S18 [Homo sapiens] |
| U | *gi|169168597|ref|XP\_0* | 6 | 10 | 32.9% | 152 | 17719 | 11.0 | PREDICTED: hypothetical protein [Homo sapiens] |

| Filename XCorr DeltCN Conf% ObsM+H+ CalcM+H+ SpR ZScore Ion% # Sequence  | | | | | | | | | | | | |
| --- | --- | --- | --- | --- | --- | --- | --- | --- | --- | --- | --- | --- |
|  | pDK268\_112012\_01.04785.04785.2 | 2.35 | 0.2184 | 99.4% | 1004.2322 | 1002.1154 | 12 | 4.549 | 75.0% | 1 | R.VLNTNIDGR.R | 2 |
|  | pDK268\_112012\_01.04240.04240.2 | 3.2664 | 0.3904 | 100.0% | 1403.9922 | 1404.4764 | 1 | 6.291 | 81.8% | 1 | K.RAGELTEDEVER.V | 2 |
|  | pDK268\_112012\_01.04858.04858.2 | 3.0655 | 0.3219 | 100.0% | 1248.2922 | 1248.2891 | 1 | 6.169 | 80.0% | 1 | R.AGELTEDEVER.V | 2 |
|  | pDK268\_112012\_01.06507.06507.2 | 2.8729 | 0.333 | 100.0% | 1072.4922 | 1072.311 | 1 | 6.815 | 75.0% | 3 | R.VITIMQNPR.Q | 2 |
|  | pDK268\_112012\_01.11492.11492.2 | 2.8213 | 0.2997 | 100.0% | 1061.0721 | 1061.2285 | 1 | 6.841 | 92.9% | 2 | K.IPDWFLNR.Q | 2 |
|  | pDK268\_112012\_01.06340.06340.2 | 4.3161 | 0.3938 | 100.0% | 1322.3121 | 1322.4606 | 1 | 8.067 | 72.7% | 2 | K.YSQVLANGLDNK.L | 2 |

---

|  |  |  |  |  |  |  |  |  |
| --- | --- | --- | --- | --- | --- | --- | --- | --- |
| U | *gi|18087855|ref|NP\_54* | 2 | 2 | 32.6% | 89 | 10350 | 7.4 | dynein, light chain, LC8-type 2 [Homo sapiens] |
| U | *gi|83267868|ref|NP\_00* | 2 | 2 | 32.6% | 89 | 10366 | 7.4 | dynein light chain 1 [Homo sapiens] |
| U | *gi|83267866|ref|NP\_00* | 2 | 2 | 32.6% | 89 | 10366 | 7.4 | dynein light chain 1 [Homo sapiens] |
| U | *gi|4505813|ref|NP\_003* | 2 | 2 | 32.6% | 89 | 10366 | 7.4 | dynein light chain 1 [Homo sapiens] |

| Filename XCorr DeltCN Conf% ObsM+H+ CalcM+H+ SpR ZScore Ion% # Sequence  | | | | | | | | | | | | |
| --- | --- | --- | --- | --- | --- | --- | --- | --- | --- | --- | --- | --- |
|  | pDK268\_112012\_01.05004.05004.2 | 3.1801 | 0.3794 | 100.0% | 1282.8722 | 1283.383 | 1 | 6.85 | 70.0% | 1 | R.NFGSYVTHETK.H | 2 |
|  | pDK268\_112012\_02.13416.13416.3 | 3.1818 | 0.3228 | 100.0% | 3380.5444 | 3381.9011 | 18 | 6.379 | 19.6% | 1 | R.NFGSYVTHETKHFIYFYLGQVAILLFKSG.- | 3 |

---

|  |  |  |  |  |  |  |  |  |
| --- | --- | --- | --- | --- | --- | --- | --- | --- |
| U | *gi|73760405|ref|NP\_00* | 17 | 39 | 32.4% | 454 | 50670 | 9.4 | thymopoietin isoform beta [Homo sapiens] |

| Filename XCorr DeltCN Conf% ObsM+H+ CalcM+H+ SpR ZScore Ion% # Sequence  | | | | | | | | | | | | |
| --- | --- | --- | --- | --- | --- | --- | --- | --- | --- | --- | --- | --- |
|  | pDK268\_112012\_01.07064.07064.2 | 5.1845 | 0.4616 | 100.0% | 1939.4922 | 1940.207 | 1 | 9.143 | 76.5% | 1 | K.LKSELVANNVTLPAGEQR.K | 2 |
|  | pDK268\_112012\_01.07048.07048.3 | 3.3856 | 0.2716 | 99.9% | 1941.1444 | 1940.207 | 1 | 4.744 | 41.2% | 2 | K.LKSELVANNVTLPAGEQR.K | 3 |
|  | pDK268\_112012\_01.06064.06064.3 | 3.6216 | 0.3459 | 100.0% | 2067.4744 | 2068.381 | 2 | 5.332 | 36.1% | 1 | K.LKSELVANNVTLPAGEQRK.D | 3 |
|  | pDK268\_112012\_01.07437.07437.2 | 4.3017 | 0.4322 | 100.0% | 1698.4122 | 1698.8735 | 1 | 8.528 | 66.7% | 1 | K.SELVANNVTLPAGEQR.K | 2 |
|  | pDK268\_112012\_01.06232.06232.2 | 3.4142 | 0.3752 | 100.0% | 1825.7922 | 1827.0476 | 1 | 6.933 | 53.1% | 1 | K.SELVANNVTLPAGEQRK.D | 2 |
|  | pDK268\_112012\_01.11948.11948.2 | 4.7552 | 0.5237 | 100.0% | 1719.3922 | 1719.9811 | 1 | 8.97 | 73.1% | 7 | K.DVYVQLYLQHLTAR.N | 2 |
|  | pDK268\_112012\_02.08681.08681.3 | 3.1166 | 0.3975 | 100.0% | 1720.1943 | 1719.9811 | 1 | 7.55 | 44.2% | 4 | K.DVYVQLYLQHLTAR.N | 3 |
|  | pDK268\_112012\_02.03135.03135.2 | 2.6282 | 0.2492 | 99.7% | 1252.0922 | 1252.4159 | 1 | 5.082 | 72.7% | 3 | R.NRPPLPAGTNSK.G | 2 |
|  | pDK268\_112012\_01.07798.07798.3 | 5.3939 | 0.5128 | 100.0% | 2571.4443 | 2571.7197 | 1 | 9.788 | 40.0% | 2 | K.GPPDFSSDEEREPTPVLGSGAAAAGR.S | 3 |
|  | pDK268\_112012\_01.08363.08363.3 | 4.9343 | 0.3936 | 100.0% | 2651.5144 | 2651.7197 | 1 | 6.531 | 34.0% | 1 | K.GPPDFSS\*DEEREPTPVLGSGAAAAGR.S | 3 |
|  | pDK268\_112012\_01.09090.09090.3 | 5.2382 | 0.4875 | 100.0% | 2730.7144 | 2731.7197 | 1 | 7.718 | 38.0% | 2 | K.GPPDFS\*S\*DEEREPTPVLGSGAAAAGR.S | 3 |
|  | pDK268\_112012\_01.13334.13334.3 | 4.1 | 0.1407 | 97.7% | 2689.9143 | 2689.8872 | 2 | 3.998 | 31.8% | 2 | R.QEDKDDLDVTELTNEDLLDQLVK.Y | 3 |
|  | pDK268\_112012\_01.13288.13288.2 | 4.6138 | 0.4013 | 100.0% | 2690.3523 | 2689.8872 | 1 | 7.608 | 45.5% | 2 | R.QEDKDDLDVTELTNEDLLDQLVK.Y | 2 |
|  | pDK268\_112012\_01.06962.06962.2 | 3.2671 | 0.5091 | 100.0% | 1331.2922 | 1331.5143 | 1 | 8.178 | 83.3% | 4 | K.YGVNPGPIVGTTR.K | 2 |
|  | pDK268\_112012\_01.06741.06741.2 | 3.6309 | 0.3594 | 100.0% | 1649.4122 | 1648.7673 | 1 | 7.329 | 66.7% | 4 | R.SSTPLPTISSSAENTR.Q | 2 |
| \* | pDK268\_112012\_02.04974.04974.3 | 4.7548 | 0.4511 | 100.0% | 2683.0144 | 2683.8076 | 1 | 8.134 | 32.6% | 1 | R.RVEHNQSYSQAGITETEWTSGSSK.G | 3 |
| \* | pDK268\_112012\_02.05332.05332.3 | 4.9008 | 0.5179 | 100.0% | 2527.1943 | 2527.62 | 1 | 8.572 | 35.2% | 1 | R.VEHNQSYSQAGITETEWTSGSSK.G | 3 |

---

|  |  |  |  |  |  |  |  |  |
| --- | --- | --- | --- | --- | --- | --- | --- | --- |
| U | *gi|149999611|ref|NP\_0* | 2 | 2 | 32.4% | 136 | 14570 | 10.0 | signal recognition particle 14kDa (homologous Alu RNA binding protein) [Homo sapiens] |

| Filename XCorr DeltCN Conf% ObsM+H+ CalcM+H+ SpR ZScore Ion% # Sequence  | | | | | | | | | | | | |
| --- | --- | --- | --- | --- | --- | --- | --- | --- | --- | --- | --- | --- |
| \* | pDK268\_112012\_01.03534.03534.3 | 2.7678 | 0.2248 | 97.6% | 1809.2043 | 1810.1156 | 38 | 4.259 | 28.6% | 1 | -.MVLLESEQFLTELTR.L | 32 |
| \* | pDK268\_112012\_01.07913.07913.3 | 3.6683 | 0.415 | 100.0% | 2369.6042 | 2369.5938 | 1 | 6.805 | 28.6% | 1 | K.AAAAAAAAAPAAAATAPTTAATTAATAAQ.- | 3 |

---

|  |  |  |  |  |  |  |  |  |
| --- | --- | --- | --- | --- | --- | --- | --- | --- |
| U | *gi|14043072|ref|NP\_11* | 9 | 10 | 32.3% | 353 | 37430 | 8.9 | heterogeneous nuclear ribonucleoprotein A2/B1 isoform B1 [Homo sapiens] |
| U | *gi|4504447|ref|NP\_002* | 9 | 10 | 33.4% | 341 | 36006 | 8.6 | heterogeneous nuclear ribonucleoprotein A2/B1 isoform A2 [Homo sapiens] |

| Filename XCorr DeltCN Conf% ObsM+H+ CalcM+H+ SpR ZScore Ion% # Sequence  | | | | | | | | | | | | |
| --- | --- | --- | --- | --- | --- | --- | --- | --- | --- | --- | --- | --- |
|  | pDK268\_112012\_01.13073.13073.2 | 3.6339 | 0.3378 | 100.0% | 1800.7722 | 1800.0184 | 1 | 5.728 | 63.3% | 2 | K.LFIGGLSFETTEESLR.N | 2 |
|  | pDK268\_112012\_01.06720.06720.2 | 1.9551 | 0.2451 | 98.1% | 1088.0122 | 1088.1644 | 2 | 4.586 | 78.6% | 1 | R.NYYEQWGK.L | 2 |
|  | pDK268\_112012\_01.09707.09707.2 | 1.8241 | 0.2845 | 97.0% | 1189.1122 | 1189.3513 | 1 | 5.181 | 66.7% | 1 | K.IDTIEIITDR.Q | 2 |
|  | pDK268\_112012\_01.10433.10433.2 | 3.4654 | 0.4764 | 100.0% | 1695.6522 | 1696.8132 | 1 | 7.193 | 75.0% | 1 | R.GFGFVTFDDHDPVDK.I | 2 |
|  | pDK268\_112012\_01.11738.11738.3 | 3.6081 | 0.4418 | 100.0% | 2278.5544 | 2278.5693 | 1 | 7.257 | 34.2% | 1 | R.GFGFVTFDDHDPVDKIVLQK.Y | 3 |
|  | pDK268\_112012\_01.03572.03572.2 | 2.9821 | 0.3218 | 100.0% | 1222.1921 | 1222.3153 | 16 | 7.098 | 72.2% | 1 | R.QEMQEVQSSR.S | 2 |
|  | pDK268\_112012\_01.06380.06380.2 | 2.2787 | 0.1953 | 98.3% | 1013.9522 | 1014.0421 | 1 | 5.279 | 77.8% | 1 | R.GGNFGFGDSR.G | 2 |
|  | pDK268\_112012\_01.06194.06194.2 | 2.794 | 0.4747 | 100.0% | 1377.9521 | 1378.4465 | 1 | 6.439 | 53.6% | 1 | R.GGGGNFGPGPGSNFR.G | 2 |
|  | pDK268\_112012\_01.06297.06297.2 | 5.5641 | 0.6469 | 100.0% | 2190.412 | 2191.2554 | 1 | 11.8 | 47.9% | 1 | R.NMGGPYGGGNYGPGGSGGSGGYGGR.S | 2 |

---

|  |  |  |  |  |  |  |  |  |
| --- | --- | --- | --- | --- | --- | --- | --- | --- |
| U | *gi|38016911|ref|NP\_00* | 5 | 10 | 32.3% | 288 | 31731 | 7.9 | stomatin isoform a [Homo sapiens] |

| Filename XCorr DeltCN Conf% ObsM+H+ CalcM+H+ SpR ZScore Ion% # Sequence  | | | | | | | | | | | | |
| --- | --- | --- | --- | --- | --- | --- | --- | --- | --- | --- | --- | --- |
| \* | pDK268\_112012\_02.06223.06223.2 | 5.562 | 0.4411 | 100.0% | 1930.5322 | 1931.113 | 1 | 10.269 | 66.7% | 1 | R.VQNATLAVANITNADSATR.L | 2 |
| \* | pDK268\_112012\_01.14123.14123.3 | 4.9281 | 0.4764 | 100.0% | 3371.2144 | 3373.6758 | 1 | 7.649 | 27.6% | 1 | K.NLSQILSDREEIAHNMQSTLDDATDAWGIK.V | 3 |
|  | pDK268\_112012\_02.04176.04176.2 | 3.3657 | 0.5037 | 100.0% | 1247.9722 | 1248.3966 | 1 | 8.853 | 77.3% | 3 | K.VIAAEGEMNASR.A | 2 |
|  | pDK268\_112012\_01.08758.08758.2 | 3.5676 | 0.3999 | 100.0% | 1352.3922 | 1352.5707 | 1 | 7.675 | 81.8% | 2 | R.YLQTLTTIAAEK.N | 2 |
|  | pDK268\_112012\_01.17744.17744.2 | 4.0662 | 0.3341 | 100.0% | 2128.5522 | 2128.5781 | 1 | 6.116 | 57.9% | 3 | K.NSTIVFPLPIDMLQGIIGAK.H | 2 |

---

|  |  |  |  |  |  |  |  |  |
| --- | --- | --- | --- | --- | --- | --- | --- | --- |
| U | *gi|4503529|ref|NP\_001* | 9 | 11 | 32.0% | 406 | 46154 | 5.5 | eukaryotic translation initiation factor 4A isoform 1 [Homo sapiens] |

| Filename XCorr DeltCN Conf% ObsM+H+ CalcM+H+ SpR ZScore Ion% # Sequence  | | | | | | | | | | | | |
| --- | --- | --- | --- | --- | --- | --- | --- | --- | --- | --- | --- | --- |
| \* | pDK268\_112012\_01.17212.17212.3 | 5.0953 | 0.6154 | 100.0% | 4168.284 | 4169.451 | 1 | 9.771 | 33.3% | 1 | R.SRDNGPDGMEPEGVIESNWNEIVDSFDDMNLSESLLR.G | 3 |
|  | pDK268\_112012\_01.07944.07944.3 | 3.1278 | 0.3905 | 100.0% | 1828.4343 | 1829.0654 | 1 | 6.931 | 38.3% | 1 | R.GIYAYGFEKPSAIQQR.A | 33 |
|  | pDK268\_112012\_01.07950.07950.2 | 4.4991 | 0.4585 | 100.0% | 1829.5521 | 1829.0654 | 1 | 7.592 | 66.7% | 1 | R.GIYAYGFEKPSAIQQR.A | 22 |
| \* | pDK268\_112012\_01.08518.08518.3 | 3.4826 | 0.4738 | 100.0% | 1619.4844 | 1619.9225 | 19 | 6.866 | 41.1% | 1 | K.LQMEAPHIIVGTPGR.V | 3 |
|  | pDK268\_112012\_01.13197.13197.2 | 3.2059 | 0.1425 | 99.7% | 1558.5521 | 1556.789 | 1 | 4.333 | 66.7% | 1 | K.MFVLDEADEMLSR.G | 2 |
| \* | pDK268\_112012\_01.11169.11169.2 | 3.5764 | 0.1861 | 99.9% | 1502.4321 | 1502.71 | 1 | 5.965 | 72.7% | 1 | R.GFKDQIYDIFQK.L | 2 |
|  | pDK268\_112012\_01.04854.04854.2 | 1.9774 | 0.2163 | 96.9% | 1174.3922 | 1175.3732 | 5 | 4.093 | 68.8% | 1 | R.RKVDWLTEK.M | 22 |
|  | pDK268\_112012\_01.07163.07163.2 | 3.3263 | 0.376 | 100.0% | 1583.5322 | 1582.7432 | 1 | 6.33 | 65.4% | 2 | R.DFTVSAMHGDMDQK.E | 23 |
| \* | pDK268\_112012\_02.04529.04529.3 | 3.5458 | 0.3172 | 100.0% | 1590.9543 | 1590.8352 | 3 | 5.52 | 40.4% | 2 | R.KGVAINMVTEEDKR.T | 3 |

Similarities:
gi|7661920|ref|NP\_055(3:6)  

---

|  |  |  |  |  |  |  |  |  |
| --- | --- | --- | --- | --- | --- | --- | --- | --- |
| U | *gi|207029415|ref|NP\_0* | 10 | 18 | 31.8% | 424 | 47585 | 4.9 | retinoblastoma binding protein 4 isoform b [Homo sapiens] |
| U | *gi|5032027|ref|NP\_005* | 10 | 18 | 31.8% | 425 | 47656 | 4.9 | retinoblastoma binding protein 4 isoform a [Homo sapiens] |

| Filename XCorr DeltCN Conf% ObsM+H+ CalcM+H+ SpR ZScore Ion% # Sequence  | | | | | | | | | | | | |
| --- | --- | --- | --- | --- | --- | --- | --- | --- | --- | --- | --- | --- |
|  | pDK268\_112012\_01.03830.03830.2 | 2.2671 | 0.2807 | 99.8% | 894.3522 | 895.00006 | 4 | 5.031 | 83.3% | 2 | R.VINEEYK.I | 22 |
|  | pDK268\_112012\_01.16760.16760.3 | 5.3933 | 0.4015 | 100.0% | 3827.9343 | 3828.3772 | 1 | 5.826 | 23.4% | 2 | K.NTPFLYDLVMTHALEWPSLTAQWLPDVTRPEGK.D | 3 |
|  | pDK268\_112012\_01.10179.10179.2 | 4.0312 | 0.5394 | 100.0% | 1473.1921 | 1472.6348 | 1 | 9.306 | 70.8% | 3 | K.TPSSDVLVFDYTK.H | 22 |
|  | pDK268\_112012\_01.13937.13937.3 | 5.9222 | 0.5981 | 100.0% | 3440.3643 | 3440.7937 | 1 | 9.523 | 29.2% | 1 | K.TIFTGHTAVVEDVSWHLLHESLFGSVADDQK.L | 3 |
|  | pDK268\_112012\_01.09741.09741.2 | 2.284 | 0.1848 | 99.3% | 936.2522 | 935.129 | 10 | 4.06 | 83.3% | 1 | K.LMIWDTR.S | 22 |
|  | pDK268\_112012\_01.10785.10785.1 | 1.9496 | 0.3307 | 100.0% | 973.47 | 974.1478 | 2 | 5.225 | 71.4% | 1 | K.TVALWDLR.N | 11 |
|  | pDK268\_112012\_01.10822.10822.2 | 2.4879 | 0.1806 | 99.6% | 974.09216 | 974.1478 | 5 | 5.8 | 85.7% | 2 | K.TVALWDLR.N | 22 |
|  | pDK268\_112012\_01.08295.08295.2 | 2.7427 | 0.1728 | 99.8% | 1130.7722 | 1131.3201 | 4 | 4.485 | 68.8% | 2 | R.RLNVWDLSK.I | 22 |
|  | pDK268\_112012\_01.10073.10073.2 | 2.1647 | 0.3406 | 99.8% | 975.0722 | 975.13257 | 6 | 6.199 | 85.7% | 2 | R.LNVWDLSK.I | 22 |
|  | pDK268\_112012\_01.09468.09468.3 | 4.9518 | 0.4075 | 100.0% | 2874.6543 | 2875.0776 | 1 | 6.544 | 28.8% | 2 | K.IGEEQSPEDAEDGPPELLFIHGGHTAK.I | 3 |

Similarities:
gi|4506439|ref|NP\_002(7:3)  

---

|  |  |  |  |  |  |  |  |  |
| --- | --- | --- | --- | --- | --- | --- | --- | --- |
| U | *gi|4506725|ref|NP\_000* | 8 | 11 | 31.6% | 263 | 29598 | 10.2 | ribosomal protein S4, X-linked X isoform [Homo sapiens] |

| Filename XCorr DeltCN Conf% ObsM+H+ CalcM+H+ SpR ZScore Ion% # Sequence  | | | | | | | | | | | | |
| --- | --- | --- | --- | --- | --- | --- | --- | --- | --- | --- | --- | --- |
| \* | pDK268\_112012\_01.13613.13613.2 | 3.3883 | 0.5099 | 100.0% | 1885.7122 | 1887.1548 | 1 | 8.047 | 56.2% | 1 | R.TDITYPAGFMDVISIDK.T | 2 |
|  | pDK268\_112012\_01.04448.04448.2 | 3.0243 | 0.4812 | 100.0% | 1216.2322 | 1216.3854 | 1 | 7.037 | 80.0% | 1 | K.GIPHLVTHDAR.T | 2 |
|  | pDK268\_112012\_01.07590.07590.3 | 3.4887 | 0.3038 | 100.0% | 1217.2144 | 1216.4661 | 2 | 5.718 | 58.3% | 1 | R.TIRYPDPLIK.V | 3 |
| \* | pDK268\_112012\_02.06463.06463.2 | 4.0594 | 0.3451 | 100.0% | 1448.3722 | 1446.5975 | 1 | 7.346 | 79.2% | 2 | K.VNDTIQIDLETGK.I | 2 |
|  | pDK268\_112012\_01.04611.04611.3 | 3.135 | 0.2188 | 99.1% | 1507.7344 | 1507.692 | 1 | 5.312 | 47.9% | 1 | R.ERHPGSFDVVHVK.D | 3 |
|  | pDK268\_112012\_01.04889.04889.3 | 2.8292 | 0.2598 | 99.6% | 1222.7644 | 1222.3892 | 5 | 5.125 | 37.5% | 1 | R.HPGSFDVVHVK.D | 3 |
| \* | pDK268\_112012\_01.10084.10084.2 | 3.1571 | 0.417 | 100.0% | 991.27216 | 991.2187 | 1 | 7.149 | 87.5% | 3 | R.LSNIFVIGK.G | 2 |
| \* | pDK268\_112012\_01.07989.07989.2 | 2.2856 | 0.1966 | 98.4% | 1168.3922 | 1168.3842 | 1 | 5.413 | 66.7% | 1 | K.GNKPWISLPR.G | 2 |

---

|  |  |  |  |  |  |  |  |  |
| --- | --- | --- | --- | --- | --- | --- | --- | --- |
| U | *gi|14141152|ref|NP\_00* | 20 | 37 | 31.5% | 730 | 77516 | 8.7 | heterogeneous nuclear ribonucleoprotein M isoform a [Homo sapiens] |
| U | *gi|157412270|ref|NP\_1* | 20 | 37 | 33.3% | 691 | 73621 | 8.8 | heterogeneous nuclear ribonucleoprotein M isoform b [Homo sapiens] |

| Filename XCorr DeltCN Conf% ObsM+H+ CalcM+H+ SpR ZScore Ion% # Sequence  | | | | | | | | | | | | |
| --- | --- | --- | --- | --- | --- | --- | --- | --- | --- | --- | --- | --- |
|  | pDK268\_112012\_01.11818.11818.2 | 3.083 | 0.5111 | 100.0% | 1265.4122 | 1265.4949 | 1 | 8.417 | 80.0% | 2 | R.AFITNIPFDVK.W | 2 |
|  | pDK268\_112012\_02.07410.07410.2 | 3.6496 | 0.5485 | 100.0% | 1426.9122 | 1427.6403 | 1 | 8.525 | 58.3% | 3 | R.LGSTVFVANLDYK.V | 2 |
|  | pDK268\_112012\_01.12909.12909.2 | 5.3019 | 0.5493 | 100.0% | 2178.5723 | 2179.5752 | 1 | 10.408 | 56.8% | 4 | K.GIGMGNIGPAGMGMEGIGFGINK.M | 2 |
|  | pDK268\_112012\_01.10114.10114.2 | 4.2323 | 0.5816 | 100.0% | 1715.2322 | 1715.9724 | 1 | 9.604 | 68.8% | 3 | K.MGGMEGPFGGGMENMGR.F | 2 |
|  | pDK268\_112012\_01.05205.05205.2 | 2.2528 | 0.4782 | 100.0% | 956.7922 | 957.11017 | 4 | 8.003 | 62.5% | 2 | R.FGSGMNMGR.I | 2 |
|  | pDK268\_112012\_01.08810.08810.2 | 3.0781 | 0.3493 | 100.0% | 1115.3322 | 1115.3152 | 1 | 5.603 | 88.9% | 2 | R.INEILSNALK.R | 2 |
|  | pDK268\_112012\_01.04518.04518.2 | 3.6128 | 0.4699 | 100.0% | 1285.3922 | 1285.3591 | 1 | 8.117 | 64.3% | 1 | K.QGGGGGGGSVPGIER.M | 2 |
|  | pDK268\_112012\_01.04238.04238.2 | 3.4769 | 0.4337 | 100.0% | 1101.9321 | 1102.2714 | 1 | 7.924 | 90.0% | 1 | R.MGAGLGHGMDR.V | 2 |
|  | pDK268\_112012\_01.06388.06388.2 | 2.2797 | 0.2623 | 99.8% | 821.9322 | 822.0283 | 2 | 6.374 | 83.3% | 1 | R.MGLVMDR.M | 2 |
|  | pDK268\_112012\_01.09297.09297.3 | 2.9102 | 0.4045 | 100.0% | 1614.3844 | 1614.875 | 1 | 6.632 | 41.1% | 2 | R.MGPLGLDHMASSIER.M | 3 |
|  | pDK268\_112012\_01.09304.09304.2 | 4.4943 | 0.4436 | 100.0% | 1615.5521 | 1614.875 | 1 | 8.212 | 71.4% | 1 | R.MGPLGLDHMASSIER.M | 2 |
|  | pDK268\_112012\_02.06631.06631.2 | 3.4852 | 0.5106 | 100.0% | 1126.0721 | 1126.3337 | 1 | 9.031 | 75.0% | 2 | R.MGAGMGFGLER.M | 2 |
|  | pDK268\_112012\_01.06965.06965.2 | 2.6669 | 0.4279 | 100.0% | 1189.3322 | 1189.4333 | 6 | 6.164 | 68.2% | 1 | R.MVPAGMGAGLER.M | 2 |
|  | pDK268\_112012\_01.04478.04478.2 | 1.9524 | 0.1699 | 96.7% | 805.8722 | 805.9856 | 5 | 4.917 | 83.3% | 1 | R.MGPVMDR.M | 2 |
|  | pDK268\_112012\_01.03897.03897.2 | 1.9708 | 0.3216 | 99.5% | 904.47217 | 905.0166 | 1 | 5.506 | 85.7% | 1 | R.MGANNLER.M | 2 |
|  | pDK268\_112012\_01.04054.04054.2 | 2.334 | 0.2098 | 99.5% | 878.1122 | 877.99097 | 61 | 5.168 | 78.6% | 2 | R.MGANSLER.M | 2 |
|  | pDK268\_112012\_01.08702.08702.2 | 3.4759 | 0.4443 | 100.0% | 1428.3722 | 1428.7076 | 1 | 7.474 | 78.6% | 2 | R.MGPAMGPALGAGIER.M | 2 |
|  | pDK268\_112012\_02.06159.06159.2 | 3.9611 | 0.4125 | 100.0% | 1383.5521 | 1384.5677 | 1 | 8.986 | 78.6% | 3 | R.MGLAMGGGGGASFDR.A | 2 |
|  | pDK268\_112012\_01.08424.08424.2 | 5.1335 | 0.5339 | 100.0% | 2035.0721 | 2036.1735 | 1 | 9.813 | 43.2% | 1 | R.GNFGGSFAGSFGGAGGHAPGVAR.K | 2 |
|  | pDK268\_112012\_02.06345.06345.3 | 3.7651 | 0.4523 | 100.0% | 2035.3444 | 2036.1735 | 1 | 7.164 | 33.0% | 2 | R.GNFGGSFAGSFGGAGGHAPGVAR.K | 3 |

---

|  |  |  |  |  |  |  |  |  |
| --- | --- | --- | --- | --- | --- | --- | --- | --- |
| U | *gi|7706495|ref|NP\_057* | 8 | 10 | 31.3% | 358 | 40514 | 6.2 | DnaJ (Hsp40) homolog, subfamily B, member 11 precursor [Homo sapiens] |

| Filename XCorr DeltCN Conf% ObsM+H+ CalcM+H+ SpR ZScore Ion% # Sequence  | | | | | | | | | | | | |
| --- | --- | --- | --- | --- | --- | --- | --- | --- | --- | --- | --- | --- |
| \* | pDK268\_112012\_01.05572.05572.3 | 5.0941 | 0.319 | 100.0% | 2185.8843 | 2186.3892 | 1 | 6.986 | 40.3% | 1 | K.LALQLHPDRNPDDPQAQEK.F | 3 |
| \* | pDK268\_112012\_01.11350.11350.2 | 5.797 | 0.4614 | 100.0% | 1772.4521 | 1772.906 | 1 | 8.72 | 83.3% | 2 | K.FQDLGAAYEVLSDSEK.R | 2 |
| \* | pDK268\_112012\_01.08238.08238.2 | 2.6122 | 0.3155 | 99.9% | 1241.9122 | 1242.4148 | 30 | 5.519 | 60.0% | 1 | R.TLEVEIEPGVR.D | 2 |
| \* | pDK268\_112012\_01.10959.10959.2 | 2.2925 | 0.2793 | 98.7% | 2516.4521 | 2517.6855 | 4 | 6.237 | 40.9% | 1 | R.DGMEYPFIGEGEPHVDGEPGDLR.F | 2 |
| \* | pDK268\_112012\_01.06620.06620.3 | 3.0974 | 0.3052 | 100.0% | 1560.6244 | 1560.7068 | 57 | 5.376 | 42.3% | 1 | K.KGEGLPNFDNNNIK.G | 3 |
| \* | pDK268\_112012\_01.06582.06582.2 | 3.6498 | 0.328 | 100.0% | 1561.1122 | 1560.7068 | 1 | 5.779 | 76.9% | 1 | K.KGEGLPNFDNNNIK.G | 2 |
| \* | pDK268\_112012\_02.09394.09394.3 | 3.7136 | 0.3586 | 100.0% | 2410.4343 | 2409.6982 | 1 | 6.967 | 37.5% | 2 | K.GSLIITFDVDFPKEQLTEEAR.E | 3 |
| \* | pDK268\_112012\_01.07931.07931.1 | 1.6058 | 0.2442 | 96.3% | 913.31 | 914.00555 | 24 | 4.626 | 57.1% | 1 | K.VYNGLQGY.- | 1 |

---

|  |  |  |  |  |  |  |  |  |
| --- | --- | --- | --- | --- | --- | --- | --- | --- |
| U | *gi|12667788|ref|NP\_00* | 44 | 54 | 31.2% | 1960 | 226530 | 5.6 | myosin, heavy polypeptide 9, non-muscle [Homo sapiens] |

| Filename XCorr DeltCN Conf% ObsM+H+ CalcM+H+ SpR ZScore Ion% # Sequence  | | | | | | | | | | | | |
| --- | --- | --- | --- | --- | --- | --- | --- | --- | --- | --- | --- | --- |
| \* | pDK268\_112012\_01.11529.11529.2 | 4.6166 | 0.4055 | 100.0% | 1673.4722 | 1673.8687 | 1 | 8.069 | 82.1% | 1 | K.NFINNPLAQADWAAK.K | 2 |
| \* | pDK268\_112012\_02.08837.08837.3 | 5.4019 | 0.4264 | 100.0% | 2789.8442 | 2790.0967 | 1 | 8.076 | 39.0% | 2 | K.SGFEPASLKEEVGEEAIVELVENGKK.V | 3 |
| \* | pDK268\_112012\_01.12316.12316.2 | 2.457 | 0.2581 | 99.3% | 1729.8922 | 1728.9978 | 1 | 4.632 | 50.0% | 1 | K.NLPIYSEEIVEMYK.G | 2 |
|  | pDK268\_112012\_01.13408.13408.2 | 2.1277 | 0.3957 | 99.7% | 1726.7722 | 1728.0012 | 1 | 5.757 | 43.3% | 1 | R.QLLQANPILEAFGNAK.T | 2 |
| \* | pDK268\_112012\_02.09813.09813.3 | 3.6194 | 0.2778 | 100.0% | 1996.0443 | 1997.3037 | 5 | 5.893 | 37.5% | 1 | R.TFHIFYYLLSGAGEHLK.T | 32 |
| \* | pDK268\_112012\_01.11408.11408.2 | 2.7405 | 0.2997 | 99.8% | 1615.3922 | 1616.9313 | 1 | 6.306 | 69.2% | 2 | R.IMGIPEEEQMGLLR.V | 2 |
| \* | pDK268\_112012\_01.13829.13829.2 | 4.4528 | 0.4294 | 100.0% | 1487.9922 | 1487.8259 | 1 | 8.177 | 76.9% | 1 | R.VISGVLQLGNIVFK.K | 2 |
|  | pDK268\_112012\_01.03748.03748.2 | 4.8446 | 0.4261 | 100.0% | 1592.1322 | 1592.6776 | 1 | 7.417 | 71.4% | 2 | R.NTDQASMPDNTAAQK.V | 2 |
| \* | pDK268\_112012\_01.10584.10584.2 | 4.4571 | 0.4123 | 100.0% | 1571.8121 | 1572.8044 | 1 | 9.598 | 76.9% | 1 | K.VSHLLGINVTDFTR.G | 2 |
| \* | pDK268\_112012\_01.10566.10566.3 | 2.8316 | 0.3621 | 100.0% | 1572.2043 | 1572.8044 | 1 | 6.143 | 46.2% | 1 | K.VSHLLGINVTDFTR.G | 3 |
|  | pDK268\_112012\_02.08981.08981.3 | 2.6451 | 0.2635 | 97.8% | 2468.6042 | 2468.7893 | 20 | 4.787 | 27.8% | 1 | K.LQQLFNHTMFILEQEEYQR.E | 33 |
| \* | pDK268\_112012\_01.13361.13361.2 | 4.8837 | 0.4303 | 100.0% | 2019.6921 | 2019.3636 | 1 | 9.682 | 57.9% | 1 | R.IIGLDQVAGMSETALPGAFK.T | 2 |
|  | pDK268\_112012\_01.10362.10362.2 | 2.2409 | 0.3705 | 99.8% | 1319.3522 | 1319.5468 | 7 | 6.046 | 65.0% | 1 | K.LDPHLVLDQLR.C | 22 |
| \* | pDK268\_112012\_01.08754.08754.2 | 2.5239 | 0.3445 | 99.9% | 1194.2922 | 1194.33 | 3 | 6.547 | 66.7% | 1 | K.ALELDSNLYR.I | 2 |
|  | pDK268\_112012\_01.06648.06648.2 | 3.3071 | 0.4243 | 100.0% | 1223.3322 | 1224.3591 | 1 | 8.155 | 80.0% | 1 | R.AGVLAHLEEER.D | 22 |
| \* | pDK268\_112012\_02.08035.08035.2 | 3.7783 | 0.5051 | 100.0% | 1751.5122 | 1753.0358 | 1 | 8.998 | 67.9% | 2 | R.LTEMETLQSQLMAEK.L | 2 |
| \* | pDK268\_112012\_01.11346.11346.2 | 5.3739 | 0.4716 | 100.0% | 2333.392 | 2334.4736 | 1 | 10.706 | 63.9% | 1 | K.MQQNIQELEEQLEEEESAR.Q | 2 |
| \* | pDK268\_112012\_02.06135.06135.2 | 3.318 | 0.2473 | 99.9% | 1653.3722 | 1654.7681 | 1 | 5.853 | 69.2% | 2 | R.IAEFTTNLTEEEEK.S | 2 |
| \* | pDK268\_112012\_01.05322.05322.2 | 3.6984 | 0.4717 | 100.0% | 1585.5521 | 1586.76 | 1 | 9.075 | 75.0% | 1 | K.NKHEAMITDLEER.L | 2 |
| \* | pDK268\_112012\_01.13517.13517.3 | 3.9187 | 0.281 | 100.0% | 2772.3843 | 2773.0696 | 1 | 5.513 | 29.2% | 1 | R.RKLEGDSTDLSDQIAELQAQIAELK.M | 3 |
| \* | pDK268\_112012\_01.17216.17216.3 | 3.1729 | 0.286 | 99.3% | 3018.7444 | 3019.2434 | 1 | 5.51 | 31.7% | 1 | R.DLGEELEALKTELEDTLDSTAAQQELR.S | 3 |
| \* | pDK268\_112012\_01.09574.09574.2 | 5.3968 | 0.4611 | 100.0% | 1840.0521 | 1840.9847 | 1 | 9.318 | 73.3% | 1 | K.HSQAVEELAEQLEQTK.R | 2 |
| \* | pDK268\_112012\_01.08738.08738.3 | 5.4346 | 0.387 | 100.0% | 1997.4844 | 1997.1722 | 1 | 8.238 | 48.4% | 1 | K.HSQAVEELAEQLEQTKR.V | 3 |
| \* | pDK268\_112012\_01.06264.06264.2 | 4.5558 | 0.3963 | 100.0% | 1413.0521 | 1413.6573 | 1 | 8.049 | 86.4% | 1 | K.KVEAQLQELQVK.F | 2 |
| \* | pDK268\_112012\_02.08664.08664.2 | 5.6978 | 0.4773 | 100.0% | 1947.7722 | 1947.1498 | 1 | 8.026 | 76.5% | 1 | K.LQVELDNVTGLLSQSDSK.S | 2 |
| \* | pDK268\_112012\_01.13966.13966.2 | 4.4699 | 0.5236 | 100.0% | 2493.5522 | 2494.631 | 1 | 9.788 | 62.5% | 1 | K.DFSALESQLQDTQELLQEENR.Q | 2 |
| \* | pDK268\_112012\_01.12749.12749.3 | 4.1379 | 0.3561 | 100.0% | 1950.9243 | 1951.1436 | 1 | 6.204 | 43.3% | 2 | R.LQQELDDLLVDLDHQR.Q | 3 |
| \* | pDK268\_112012\_01.12795.12795.2 | 4.085 | 0.2064 | 100.0% | 1951.7122 | 1951.1436 | 3 | 6.774 | 56.7% | 2 | R.LQQELDDLLVDLDHQR.Q | 2 |
|  | pDK268\_112012\_01.06365.06365.2 | 3.1252 | 0.2552 | 100.0% | 1221.2722 | 1221.3959 | 3 | 4.856 | 77.8% | 1 | K.KFDQLLAEEK.T | 22 |
| \* | pDK268\_112012\_01.04541.04541.2 | 2.8057 | 0.2507 | 99.9% | 1050.1721 | 1049.1841 | 2 | 5.438 | 75.0% | 1 | R.ALEEAMEQK.A | 2 |
| \* | pDK268\_112012\_02.04455.04455.2 | 2.7015 | 0.2937 | 99.9% | 1205.8522 | 1205.3685 | 233 | 6.81 | 61.1% | 2 | R.ALEQQVEEMK.T | 2 |
| \* | pDK268\_112012\_01.15296.15296.3 | 3.843 | 0.2102 | 99.1% | 3149.3643 | 3149.4048 | 1 | 4.991 | 25.0% | 1 | R.ALEQQVEEMKTQLEELEDELQATEDAK.L | 3 |
|  | pDK268\_112012\_01.12014.12014.2 | 5.5917 | 0.4806 | 100.0% | 1963.2922 | 1963.0594 | 1 | 8.879 | 75.0% | 1 | K.TQLEELEDELQATEDAK.L | 22 |
| \* | pDK268\_112012\_01.08195.08195.2 | 2.288 | 0.3324 | 99.8% | 1046.0922 | 1046.2701 | 1 | 5.763 | 81.2% | 1 | R.LEVNLQAMK.A | 2 |
| \* | pDK268\_112012\_01.04588.04588.2 | 2.6845 | 0.1065 | 98.1% | 1212.3121 | 1213.2896 | 2 | 5.672 | 85.0% | 1 | K.DLEAHIDSANK.N | 2 |
| \* | pDK268\_112012\_01.04302.04302.2 | 2.8962 | 0.2289 | 99.8% | 1215.8522 | 1216.3799 | 18 | 4.827 | 60.0% | 1 | R.ASREEILAQAK.E | 2 |
| \* | pDK268\_112012\_01.14025.14025.2 | 3.301 | 0.463 | 100.0% | 2049.152 | 2050.3064 | 1 | 7.201 | 44.1% | 1 | K.SMEAEMIQLQEELAAAER.A | 2 |
| \* | pDK268\_112012\_01.06801.06801.3 | 3.758 | 0.3948 | 100.0% | 2090.0645 | 2090.168 | 1 | 6.648 | 36.1% | 1 | R.QAQQERDELADEIANSSGK.G | 3 |
| \* | pDK268\_112012\_02.07701.07701.3 | 4.509 | 0.4527 | 100.0% | 2473.0144 | 2473.6099 | 1 | 7.567 | 36.2% | 1 | R.IAQLEEELEEEQGNTELINDR.L | 3 |
| \* | pDK268\_112012\_01.10642.10642.2 | 5.5411 | 0.5158 | 100.0% | 1870.4521 | 1871.0574 | 1 | 10.517 | 76.7% | 1 | K.ANLQIDQINTDLNLER.S | 2 |
| \* | pDK268\_112012\_02.05664.05664.2 | 4.6043 | 0.4327 | 100.0% | 1531.1921 | 1531.6598 | 1 | 8.448 | 83.3% | 2 | K.IAQLEEQLDNETK.E | 2 |
| \* | pDK268\_112012\_01.04174.04174.2 | 3.189 | 0.1789 | 99.9% | 1333.2722 | 1332.3666 | 6 | 4.677 | 70.0% | 1 | R.QLEEAEEEAQR.A | 2 |
| \* | pDK268\_112012\_01.06108.06108.2 | 4.3021 | 0.5664 | 100.0% | 1565.9922 | 1566.6367 | 1 | 9.594 | 69.2% | 2 | R.ELEDATETADAMNR.E | 2 |
| \* | pDK268\_112012\_01.08241.08241.2 | 3.3052 | 0.1864 | 99.9% | 1156.2322 | 1156.3732 | 1 | 5.926 | 88.9% | 1 | R.RGDLPFVVPR.R | 2 |

Similarities:
gi|41406064|ref|NP\_00(5:39)  

---

|  |  |  |  |  |  |  |  |  |
| --- | --- | --- | --- | --- | --- | --- | --- | --- |
| U | *gi|4506693|ref|NP\_001* | 3 | 4 | 31.1% | 135 | 15550 | 9.8 | ribosomal protein S17 [Homo sapiens] |

| Filename XCorr DeltCN Conf% ObsM+H+ CalcM+H+ SpR ZScore Ion% # Sequence  | | | | | | | | | | | | |
| --- | --- | --- | --- | --- | --- | --- | --- | --- | --- | --- | --- | --- |
| \* | pDK268\_112012\_01.03347.03347.2 | 3.0642 | 0.2926 | 100.0% | 1202.1522 | 1202.3152 | 1 | 5.47 | 83.3% | 1 | R.LGNDFHTNKR.V | 2 |
| \* | pDK268\_112012\_01.06485.06485.2 | 2.5526 | 0.2858 | 99.8% | 1132.4922 | 1133.3942 | 1 | 5.887 | 77.8% | 1 | K.IAGYVTHLMK.R | 2 |
| \* | pDK268\_112012\_01.13690.13690.2 | 4.7981 | 0.5254 | 100.0% | 2410.9722 | 2410.7908 | 1 | 9.054 | 57.1% | 2 | K.LLDFGSLSNLQVTQPTVGMNFK.T | 2 |

---

|  |  |  |  |  |  |  |  |  |
| --- | --- | --- | --- | --- | --- | --- | --- | --- |
| U | *gi|169160598|ref|XP\_0* | 3 | 5 | 31.0% | 84 | 9461 | 9.5 | PREDICTED: similar to hCG1783679 [Homo sapiens] |
| U | *gi|4506711|ref|NP\_001* | 3 | 5 | 31.0% | 84 | 9461 | 9.5 | ribosomal protein S27 [Homo sapiens] |
| U | *gi|169161552|ref|XP\_0* | 3 | 5 | 31.0% | 84 | 9461 | 9.5 | PREDICTED: similar to hCG1783679 [Homo sapiens] |
| U | *gi|169161255|ref|XP\_0* | 3 | 5 | 31.0% | 84 | 9461 | 9.5 | PREDICTED: hypothetical protein [Homo sapiens] |

| Filename XCorr DeltCN Conf% ObsM+H+ CalcM+H+ SpR ZScore Ion% # Sequence  | | | | | | | | | | | | |
| --- | --- | --- | --- | --- | --- | --- | --- | --- | --- | --- | --- | --- |
|  | pDK268\_112012\_01.05484.05484.2 | 2.5922 | 0.2828 | 99.8% | 1294.0122 | 1294.4038 | 4 | 5.356 | 70.0% | 1 | K.DLLHPSPEEEK.R | 2 |
|  | pDK268\_112012\_01.04090.04090.2 | 2.5185 | 0.2519 | 99.5% | 1578.4922 | 1578.7654 | 1 | 5.317 | 62.5% | 1 | K.DLLHPSPEEEKRK.H | 2 |
|  | pDK268\_112012\_01.09548.09548.2 | 3.457 | 0.4963 | 100.0% | 1528.3322 | 1528.7632 | 1 | 8.283 | 75.0% | 3 | R.LVQSPNSYFMDVK.C | 2 |

---

|  |  |  |  |  |  |  |  |  |
| --- | --- | --- | --- | --- | --- | --- | --- | --- |
| U | *gi|15431293|ref|NP\_00* | 7 | 9 | 30.9% | 204 | 24146 | 11.6 | ribosomal protein L15 [Homo sapiens] |
| U | *gi|88998868|ref|XP\_94* | 7 | 9 | 30.9% | 204 | 24174 | 11.6 | PREDICTED: hypothetical protein isoform 4 [Homo sapiens] |
| U | *gi|88992455|ref|XP\_93* | 7 | 9 | 30.9% | 204 | 24174 | 11.6 | PREDICTED: hypothetical protein isoform 1 [Homo sapiens] |
| U | *gi|169169711|ref|XP\_0* | 7 | 9 | 30.9% | 204 | 24174 | 11.6 | PREDICTED: hypothetical protein [Homo sapiens] |

| Filename XCorr DeltCN Conf% ObsM+H+ CalcM+H+ SpR ZScore Ion% # Sequence  | | | | | | | | | | | | |
| --- | --- | --- | --- | --- | --- | --- | --- | --- | --- | --- | --- | --- |
|  | pDK268\_112012\_01.09107.09107.2 | 2.2629 | 0.0852 | 97.0% | 1008.1722 | 1008.1649 | 4 | 5.12 | 91.7% | 1 | K.YIQELWR.K | 2 |
|  | pDK268\_112012\_01.03473.03473.2 | 4.196 | 0.5092 | 100.0% | 1705.8322 | 1706.945 | 1 | 8.757 | 63.3% | 1 | K.GATYGKPVHHGVNQLK.F | 2 |
|  | pDK268\_112012\_02.03064.03064.3 | 3.1876 | 0.3967 | 100.0% | 1707.1144 | 1706.945 | 1 | 6.356 | 43.3% | 1 | K.GATYGKPVHHGVNQLK.F | 3 |
|  | pDK268\_112012\_01.09196.09196.2 | 4.544 | 0.4869 | 100.0% | 1661.5322 | 1661.8083 | 1 | 7.902 | 61.5% | 2 | R.VLNSYWVGEDSTYK.F | 2 |
|  | pDK268\_112012\_01.14074.14074.3 | 2.2863 | 0.2602 | 97.0% | 1506.1444 | 1505.7997 | 1 | 5.744 | 47.7% | 1 | K.FFEVILIDPFHK.A | 3 |
|  | pDK268\_112012\_01.14072.14072.2 | 3.6168 | 0.4298 | 100.0% | 1506.2122 | 1505.7997 | 1 | 7.193 | 68.2% | 2 | K.FFEVILIDPFHK.A | 2 |
|  | pDK268\_112012\_01.04664.04664.3 | 2.9031 | 0.2272 | 98.6% | 1721.0944 | 1720.9718 | 1 | 5.091 | 44.2% | 1 | R.RNPDTQWITKPVHK.H | 3 |

---

|  |  |  |  |  |  |  |  |  |
| --- | --- | --- | --- | --- | --- | --- | --- | --- |
| U | *gi|13904870|ref|NP\_00* | 4 | 6 | 30.9% | 204 | 22876 | 9.7 | ribosomal protein S5 [Homo sapiens] |

| Filename XCorr DeltCN Conf% ObsM+H+ CalcM+H+ SpR ZScore Ion% # Sequence  | | | | | | | | | | | | |
| --- | --- | --- | --- | --- | --- | --- | --- | --- | --- | --- | --- | --- |
| \* | pDK268\_112012\_02.09426.09426.2 | 5.368 | 0.4815 | 100.0% | 2324.2922 | 2324.5493 | 1 | 9.606 | 60.5% | 1 | K.WSTDDVQINDISLQDYIAVK.E | 2 |
| \* | pDK268\_112012\_01.04402.04402.2 | 2.9468 | 0.2608 | 99.9% | 1179.4922 | 1178.428 | 1 | 5.136 | 83.3% | 1 | R.LTNSMMMHGR.N | 2 |
| \* | pDK268\_112012\_01.18885.18885.3 | 5.937 | 0.5507 | 100.0% | 3066.8342 | 3067.5613 | 1 | 10.494 | 39.8% | 3 | K.HAFEIIHLLTGENPLQVLVNAIINSGPR.E | 3 |
| \* | pDK268\_112012\_01.17837.17837.3 | 6.0219 | 0.5705 | 100.0% | 3654.9844 | 3656.1362 | 1 | 10.043 | 28.9% | 1 | K.HAFEIIHLLTGENPLQVLVNAIINSGPREDSTR.I | 3 |

---

|  |  |  |  |  |  |  |  |  |
| --- | --- | --- | --- | --- | --- | --- | --- | --- |
| U | *gi|222352151|ref|NP\_0* | 6 | 10 | 30.6% | 356 | 37498 | 7.1 | poly(rC) binding protein 1 [Homo sapiens] |

| Filename XCorr DeltCN Conf% ObsM+H+ CalcM+H+ SpR ZScore Ion% # Sequence  | | | | | | | | | | | | |
| --- | --- | --- | --- | --- | --- | --- | --- | --- | --- | --- | --- | --- |
| \* | pDK268\_112012\_01.11176.11176.2 | 2.0017 | 0.2503 | 96.8% | 1388.4321 | 1389.6781 | 7 | 4.767 | 50.0% | 1 | R.IITLTGPTNAIFK.A | 2 |
| \* | pDK268\_112012\_01.14514.14514.3 | 3.9191 | 0.3139 | 100.0% | 3381.5344 | 3380.8562 | 1 | 4.696 | 29.2% | 3 | K.AFAMIIDKLEEDINSSMTNSTAASRPPVTLR.L | 3 |
|  | pDK268\_112012\_01.07991.07991.2 | 5.7124 | 0.4336 | 100.0% | 2091.0723 | 2091.2573 | 1 | 8.628 | 55.3% | 3 | R.ESTGAQVQVAGDMLPNSTER.A | 22 |
| \* | pDK268\_112012\_02.06370.06370.3 | 3.3983 | 0.3212 | 100.0% | 2686.5244 | 2687.875 | 1 | 5.456 | 32.3% | 1 | R.QQSHFAMMHGGTGFAGIDSSS\*PEVK.G | 3 |
| \* | pDK268\_112012\_01.04557.04557.2 | 2.1554 | 0.1578 | 96.9% | 1015.2322 | 1015.1142 | 235 | 4.37 | 62.5% | 1 | R.QGANINEIR.Q | 2 |
| \* | pDK268\_112012\_01.04217.04217.2 | 2.8261 | 0.4235 | 100.0% | 1087.5322 | 1087.1777 | 6 | 6.861 | 65.0% | 1 | K.IANPVEGSSGR.Q | 2 |

Similarities:
gi|14141166|ref|NP\_11(1:5)  

---

|  |  |  |  |  |  |  |  |  |
| --- | --- | --- | --- | --- | --- | --- | --- | --- |
| U | *gi|4506743|ref|NP\_001* | 5 | 7 | 30.3% | 208 | 24205 | 10.3 | ribosomal protein S8 [Homo sapiens] |

| Filename XCorr DeltCN Conf% ObsM+H+ CalcM+H+ SpR ZScore Ion% # Sequence  | | | | | | | | | | | | |
| --- | --- | --- | --- | --- | --- | --- | --- | --- | --- | --- | --- | --- |
| \* | pDK268\_112012\_01.04035.04035.2 | 3.2027 | 0.3278 | 100.0% | 1219.4321 | 1220.3707 | 1 | 6.003 | 75.0% | 1 | K.YELGRPAANTK.I | 2 |
| \* | pDK268\_112012\_02.07023.07023.3 | 3.972 | 0.3155 | 100.0% | 1721.1244 | 1719.9353 | 1 | 5.955 | 44.6% | 1 | R.IIDVVYNASNNELVR.T | 3 |
| \* | pDK268\_112012\_01.09380.09380.3 | 2.8665 | 0.2036 | 97.8% | 1620.8944 | 1620.8076 | 246 | 4.346 | 39.6% | 1 | R.QWYESHYALPLGR.K | 3 |
|  | pDK268\_112012\_01.07450.07450.2 | 2.4942 | 0.39 | 99.9% | 1315.0721 | 1315.4631 | 1 | 5.338 | 80.0% | 1 | K.LTPEEEEILNK.K | 2 |
| \* | pDK268\_112012\_02.06689.06689.2 | 4.3461 | 0.4032 | 100.0% | 1508.2122 | 1507.6836 | 1 | 7.8 | 70.8% | 3 | K.ISSLLEEQFQQGK.L | 2 |

---

|  |  |  |  |  |  |  |  |  |
| --- | --- | --- | --- | --- | --- | --- | --- | --- |
| U | *gi|4506621|ref|NP\_000* | 6 | 6 | 30.3% | 145 | 17258 | 10.6 | ribosomal protein L26 [Homo sapiens] |

| Filename XCorr DeltCN Conf% ObsM+H+ CalcM+H+ SpR ZScore Ion% # Sequence  | | | | | | | | | | | | |
| --- | --- | --- | --- | --- | --- | --- | --- | --- | --- | --- | --- | --- |
|  | pDK268\_112012\_01.08350.08350.2 | 2.807 | 0.1814 | 99.7% | 1341.9321 | 1342.5554 | 1 | 6.05 | 70.0% | 1 | -.MKFNPFVTSDR.S | 2 |
|  | pDK268\_112012\_01.08468.08468.2 | 2.2121 | 0.1929 | 98.2% | 1083.1522 | 1083.1887 | 7 | 4.713 | 75.0% | 1 | K.FNPFVTSDR.S | 2 |
| \* | pDK268\_112012\_01.03808.03808.2 | 2.4951 | 0.2057 | 99.6% | 1079.2722 | 1079.2064 | 2 | 5.057 | 68.8% | 1 | R.HFNAPSHIR.R | 2 |
|  | pDK268\_112012\_01.03724.03724.2 | 2.4265 | 0.2406 | 99.7% | 1087.9922 | 1088.2058 | 9 | 5.316 | 75.0% | 1 | R.KDDEVQVVR.G | 2 |
|  | pDK268\_112012\_01.07979.07979.2 | 2.1852 | 0.1961 | 99.0% | 956.21216 | 956.1295 | 11 | 5.723 | 83.3% | 1 | K.YVIYIER.V | 2 |
| \* | pDK268\_112012\_01.03428.03428.2 | 2.4658 | 0.1029 | 98.3% | 1040.2522 | 1040.1583 | 8 | 4.195 | 78.6% | 1 | K.YKEETIEK.M | 2 |

---

|  |  |  |  |  |  |  |  |  |
| --- | --- | --- | --- | --- | --- | --- | --- | --- |
| U | *gi|15431301|ref|NP\_00* | 8 | 11 | 30.2% | 248 | 29226 | 10.7 | ribosomal protein L7 [Homo sapiens] |

| Filename XCorr DeltCN Conf% ObsM+H+ CalcM+H+ SpR ZScore Ion% # Sequence  | | | | | | | | | | | | |
| --- | --- | --- | --- | --- | --- | --- | --- | --- | --- | --- | --- | --- |
|  | pDK268\_112012\_01.05435.05435.2 | 3.0795 | 0.121 | 99.5% | 1321.3922 | 1321.5187 | 1 | 5.264 | 77.3% | 1 | R.KAGNFYVPAEPK.L | 2 |
|  | pDK268\_112012\_01.07050.07050.2 | 2.5639 | 0.2054 | 99.4% | 1192.9722 | 1193.3446 | 3 | 4.711 | 60.0% | 1 | K.AGNFYVPAEPK.L | 2 |
|  | pDK268\_112012\_01.05952.05952.2 | 1.9501 | 0.2309 | 98.2% | 805.0522 | 804.983 | 1 | 5.966 | 91.7% | 1 | K.ASINMLR.I | 2 |
|  | pDK268\_112012\_01.12284.12284.2 | 3.59 | 0.3679 | 100.0% | 1663.2522 | 1663.9567 | 1 | 7.353 | 57.7% | 1 | R.IVEPYIAWGYPNLK.S | 2 |
|  | pDK268\_112012\_01.08872.08872.2 | 2.934 | 0.4664 | 100.0% | 1171.2722 | 1171.3823 | 1 | 7.544 | 90.0% | 4 | R.IALTDNALIAR.S | 2 |
|  | pDK268\_112012\_01.12087.12087.2 | 3.1463 | 0.3068 | 100.0% | 1541.2122 | 1541.7924 | 2 | 6.526 | 72.7% | 1 | R.FKEANNFLWPFK.L | 2 |
|  | pDK268\_112012\_01.04329.04329.2 | 4.4484 | 0.3509 | 100.0% | 2116.372 | 2117.1992 | 1 | 6.874 | 52.8% | 1 | K.TTHFVEGGDAGNREDQINR.L | 2 |
|  | pDK268\_112012\_01.04324.04324.3 | 2.5693 | 0.3171 | 99.1% | 2116.7644 | 2117.1992 | 2 | 5.75 | 31.9% | 1 | K.TTHFVEGGDAGNREDQINR.L | 3 |

---

|  |  |  |  |  |  |  |  |  |
| --- | --- | --- | --- | --- | --- | --- | --- | --- |
| U | *gi|4506623|ref|NP\_000* | 3 | 4 | 30.1% | 136 | 15798 | 10.6 | ribosomal protein L27 [Homo sapiens] |

| Filename XCorr DeltCN Conf% ObsM+H+ CalcM+H+ SpR ZScore Ion% # Sequence  | | | | | | | | | | | | |
| --- | --- | --- | --- | --- | --- | --- | --- | --- | --- | --- | --- | --- |
| \* | pDK268\_112012\_01.07301.07301.3 | 3.9199 | 0.4107 | 100.0% | 2273.8442 | 2273.4238 | 1 | 6.242 | 36.2% | 1 | K.NIDDGTSDRPYSHALVAGIDR.Y | 3 |
|  | pDK268\_112012\_01.06634.06634.2 | 3.3582 | 0.415 | 100.0% | 1408.3722 | 1408.6177 | 1 | 7.724 | 85.0% | 1 | K.VYNYNHLMPTR.Y | 2 |
| \* | pDK268\_112012\_01.08530.08530.2 | 2.1263 | 0.3212 | 99.7% | 1050.0122 | 1050.1968 | 1 | 6.43 | 75.0% | 2 | R.YSVDIPLDK.T | 2 |

---

|  |  |  |  |  |  |  |  |  |
| --- | --- | --- | --- | --- | --- | --- | --- | --- |
| U | *gi|5174447|ref|NP\_006* | 8 | 12 | 30.0% | 317 | 35077 | 7.7 | guanine nucleotide binding protein (G protein), beta polypeptide 2-like 1 [Homo sapiens] |

| Filename XCorr DeltCN Conf% ObsM+H+ CalcM+H+ SpR ZScore Ion% # Sequence  | | | | | | | | | | | | |
| --- | --- | --- | --- | --- | --- | --- | --- | --- | --- | --- | --- | --- |
| \* | pDK268\_112012\_01.12813.12813.3 | 3.1209 | 0.2395 | 98.3% | 2627.4844 | 2628.9653 | 1 | 4.602 | 27.2% | 1 | K.GHNGWVTQIATTPQFPDMILSASR.D | 3 |
| \* | pDK268\_112012\_01.12767.12767.2 | 5.6993 | 0.6312 | 100.0% | 2628.7922 | 2628.9653 | 1 | 10.571 | 52.2% | 1 | K.GHNGWVTQIATTPQFPDMILSASR.D | 2 |
| \* | pDK268\_112012\_01.05662.05662.2 | 2.9858 | 0.3552 | 100.0% | 1193.0922 | 1193.2584 | 1 | 6.84 | 83.3% | 1 | R.DETNYGIPQR.A | 2 |
| \* | pDK268\_112012\_01.09225.09225.2 | 2.8393 | 0.4345 | 100.0% | 1265.2922 | 1265.4087 | 1 | 7.427 | 80.0% | 1 | R.LWDLTTGTTTR.R | 2 |
| \* | pDK268\_112012\_02.07253.07253.2 | 2.4119 | 0.3659 | 99.9% | 1309.9922 | 1310.4062 | 1 | 6.437 | 68.2% | 1 | K.DVLSVAFSSDNR.Q | 2 |
|  | pDK268\_112012\_01.10748.10748.2 | 3.4392 | 0.3618 | 100.0% | 1476.9722 | 1477.6318 | 1 | 6.571 | 58.3% | 2 | K.DGQAMLWDLNEGK.H | 2 |
| \* | pDK268\_112012\_01.09436.09436.2 | 5.0367 | 0.3403 | 100.0% | 1790.2722 | 1790.0642 | 1 | 8.179 | 70.0% | 3 | K.IIVDELKQEVISTSSK.A | 2 |
| \* | pDK268\_112012\_01.08362.08362.2 | 2.3508 | 0.2416 | 99.6% | 1060.1721 | 1060.2412 | 2 | 5.519 | 68.8% | 2 | R.VWQVTIGTR.- | 2 |

---

|  |  |  |  |  |  |  |  |  |
| --- | --- | --- | --- | --- | --- | --- | --- | --- |
| U | *gi|14916501|ref|NP\_14* | 3 | 7 | 30.0% | 130 | 15069 | 10.9 | ribosomal protein S24 isoform a [Homo sapiens] |
| U | *gi|4506703|ref|NP\_001* | 3 | 7 | 29.3% | 133 | 15423 | 10.8 | ribosomal protein S24 isoform c [Homo sapiens] |
| U | *gi|214829241|ref|NP\_0* | 3 | 7 | 29.8% | 131 | 15197 | 10.9 | ribosomal protein S24 isoform b [Homo sapiens] |
| U | *gi|214010226|ref|NP\_0* | 3 | 7 | 13.5% | 289 | 32431 | 10.2 | ribosomal protein S24 isoform d [Homo sapiens] |
| U | *gi|214010224|ref|NP\_0* | 3 | 7 | 29.8% | 131 | 15197 | 10.9 | ribosomal protein S24 isoform f [Homo sapiens] |
| U | *gi|214010222|ref|NP\_0* | 3 | 7 | 29.5% | 132 | 15325 | 10.9 | ribosomal protein S24 isoform e [Homo sapiens] |

| Filename XCorr DeltCN Conf% ObsM+H+ CalcM+H+ SpR ZScore Ion% # Sequence  | | | | | | | | | | | | |
| --- | --- | --- | --- | --- | --- | --- | --- | --- | --- | --- | --- | --- |
|  | pDK268\_112012\_01.06429.06429.3 | 3.7414 | 0.312 | 100.0% | 1367.9644 | 1365.6769 | 3 | 5.613 | 50.0% | 1 | R.KQMVIDVLHPGK.A | 3 |
|  | pDK268\_112012\_02.10689.10689.2 | 2.9161 | 0.6209 | 100.0% | 1399.9321 | 1399.6323 | 1 | 9.223 | 63.6% | 3 | K.TTPDVIFVFGFR.T | 2 |
|  | pDK268\_112012\_01.12542.12542.2 | 4.344 | 0.5632 | 100.0% | 1682.8522 | 1682.8854 | 1 | 9.629 | 75.0% | 3 | K.TTGFGMIYDSLDYAK.K | 2 |

---

|  |  |  |  |  |  |  |  |  |
| --- | --- | --- | --- | --- | --- | --- | --- | --- |
| U | *gi|224586846|ref|NP\_0* | 19 | 24 | 29.8% | 731 | 80966 | 9.4 | sorbin and SH3 domain containing 2 isoform 4 [Homo sapiens] |

| Filename XCorr DeltCN Conf% ObsM+H+ CalcM+H+ SpR ZScore Ion% # Sequence  | | | | | | | | | | | | |
| --- | --- | --- | --- | --- | --- | --- | --- | --- | --- | --- | --- | --- |
|  | pDK268\_112012\_01.06262.06262.2 | 2.2912 | 0.2027 | 97.8% | 1213.3722 | 1213.3793 | 66 | 4.984 | 50.0% | 1 | R.VQSSPNLLAAGR.D | 22 |
|  | pDK268\_112012\_02.05615.05615.2 | 5.3786 | 0.5818 | 100.0% | 2319.9922 | 2320.388 | 1 | 10.527 | 57.1% | 1 | R.SYNDGNQETLNGDATYSSLAAK.G | 22 |
|  | pDK268\_112012\_02.05596.05596.3 | 4.2585 | 0.4289 | 100.0% | 2320.2244 | 2320.388 | 1 | 7.094 | 34.5% | 1 | R.SYNDGNQETLNGDATYSSLAAK.G | 33 |
|  | pDK268\_112012\_01.03324.03324.3 | 3.3855 | 0.2456 | 100.0% | 1213.5543 | 1213.3823 | 1 | 5.296 | 58.3% | 1 | R.SVRPNLQDKR.S | 33 |
|  | pDK268\_112012\_01.09306.09306.2 | 3.0216 | 0.4398 | 100.0% | 2047.7922 | 2048.3062 | 1 | 6.751 | 47.4% | 1 | K.APHYPGIGPVDESGIPTAIR.T | 22 |
|  | pDK268\_112012\_01.09278.09278.3 | 4.3869 | 0.4169 | 100.0% | 2048.6343 | 2048.3062 | 1 | 6.313 | 38.2% | 2 | K.APHYPGIGPVDESGIPTAIR.T | 33 |
|  | pDK268\_112012\_01.03700.03700.2 | 1.9921 | 0.248 | 97.9% | 1093.5122 | 1094.256 | 5 | 4.442 | 62.5% | 1 | K.TQTYRPLSK.S | 22 |
|  | pDK268\_112012\_01.07592.07592.3 | 3.5761 | 0.3256 | 100.0% | 2321.9944 | 2321.6848 | 1 | 5.821 | 37.5% | 1 | K.SSILQHERPPPLPTTPTPVPR.E | 3 |
|  | pDK268\_112012\_01.07479.07479.2 | 2.6316 | 0.3018 | 99.8% | 1771.3522 | 1772.9886 | 1 | 5.856 | 56.7% | 1 | R.SLERPMSSASMASDFR.K | 22 |
|  | pDK268\_112012\_01.06196.06196.3 | 4.7367 | 0.4319 | 100.0% | 1900.6144 | 1901.1627 | 1 | 7.648 | 43.8% | 1 | R.SLERPMSSASMASDFRK.R | 33 |
|  | pDK268\_112012\_01.06932.06932.2 | 2.637 | 0.4401 | 100.0% | 1218.0922 | 1218.324 | 1 | 7.68 | 80.0% | 1 | R.SYSSTLTDMGR.S | 22 |
|  | pDK268\_112012\_01.05574.05574.2 | 2.8984 | 0.2794 | 100.0% | 1065.4122 | 1065.2578 | 2 | 5.596 | 75.0% | 1 | K.KGDTVYILR.K | 22 |
|  | pDK268\_112012\_01.11759.11759.2 | 2.5329 | 0.3917 | 100.0% | 1252.1522 | 1252.4961 | 1 | 6.85 | 70.0% | 3 | R.VGIFPISYVEK.L | 22 |
|  | pDK268\_112012\_01.06353.06353.2 | 5.1389 | 0.5174 | 100.0% | 2095.5322 | 2096.394 | 1 | 9.851 | 55.0% | 1 | K.AQPARPPPPAQPGEIGEAIAK.Y | 22 |
|  | pDK268\_112012\_02.15478.15478.2 | 2.1344 | 0.208 | 96.3% | 1656.6322 | 1656.7924 | 1 | 4.436 | 46.2% | 1 | K.YNFNADTNVELSLR.K | 22 |
|  | pDK268\_112012\_01.06545.06545.2 | 2.5607 | 0.2541 | 99.8% | 1138.9521 | 1139.2096 | 1 | 6.08 | 81.2% | 1 | R.VDQNWYEGK.I | 22 |
|  | pDK268\_112012\_01.11982.11982.2 | 2.4078 | 0.2582 | 99.3% | 1466.4321 | 1465.7325 | 30 | 4.712 | 45.8% | 1 | R.QGIFPVSYVEVVK.K | 22 |
|  | pDK268\_112012\_01.06058.06058.2 | 2.3615 | 0.2695 | 99.8% | 1018.0522 | 1018.0685 | 10 | 5.489 | 85.7% | 2 | R.NEDELELR.E | 22 |
|  | pDK268\_112012\_01.10343.10343.2 | 2.316 | 0.1871 | 97.9% | 1277.6921 | 1277.465 | 1 | 4.873 | 70.0% | 2 | K.FFGTFPGNYVK.R | 22 |

Similarities:
gi|224586844|ref|NP\_0(18:1)  

---

|  |  |  |  |  |  |  |  |  |
| --- | --- | --- | --- | --- | --- | --- | --- | --- |
| U | *gi|21493033|ref|NP\_00* | 13 | 17 | 29.8% | 662 | 73818 | 6.4 | A-kinase anchor protein 10 precursor [Homo sapiens] |

| Filename XCorr DeltCN Conf% ObsM+H+ CalcM+H+ SpR ZScore Ion% # Sequence  | | | | | | | | | | | | |
| --- | --- | --- | --- | --- | --- | --- | --- | --- | --- | --- | --- | --- |
| \* | pDK268\_112012\_01.04797.04797.3 | 3.2377 | 0.2598 | 99.7% | 1782.2043 | 1781.9866 | 4 | 6.296 | 38.3% | 1 | K.KQPSHMEAAHFGDLGR.S | 3 |
| \* | pDK268\_112012\_01.07587.07587.3 | 4.272 | 0.342 | 100.0% | 1722.0543 | 1721.8644 | 1 | 6.706 | 41.1% | 1 | K.HETTASFLTDSLDKR.L | 3 |
| \* | pDK268\_112012\_02.07044.07044.3 | 3.885 | 0.3939 | 100.0% | 2435.5144 | 2435.6274 | 1 | 7.561 | 34.5% | 1 | R.LEDSGSAQLFMTHSEGIDLNNR.T | 3 |
| \* | pDK268\_112012\_02.04705.04705.3 | 3.901 | 0.407 | 100.0% | 2307.0544 | 2308.4822 | 1 | 7.558 | 40.5% | 3 | R.AGTHQVSMETQESSSTLTVASR.N | 3 |
| \* | pDK268\_112012\_01.09209.09209.2 | 3.2652 | 0.4349 | 100.0% | 1353.1522 | 1353.4717 | 1 | 8.354 | 77.3% | 2 | K.SIEQDAVNTFTK.Y | 2 |
| \* | pDK268\_112012\_01.08540.08540.2 | 4.0543 | 0.584 | 100.0% | 1873.2522 | 1874.2054 | 1 | 9.385 | 62.5% | 1 | K.YISPDAAKPIPITEAMR.N | 2 |
| \* | pDK268\_112012\_01.11088.11088.2 | 4.7714 | 0.5483 | 100.0% | 1965.4922 | 1966.2035 | 1 | 9.784 | 65.6% | 1 | K.YFSLQATHPLGFDDVVR.L | 2 |
| \* | pDK268\_112012\_01.13796.13796.2 | 4.0038 | 0.328 | 100.0% | 1649.3522 | 1648.942 | 1 | 7.68 | 80.8% | 2 | K.VFLPGFLSSNLYYK.Y | 2 |
| \* | pDK268\_112012\_01.08300.08300.3 | 2.3572 | 0.2829 | 98.9% | 1230.6543 | 1230.4093 | 1 | 5.293 | 47.2% | 1 | K.YLNDLIHSVR.G | 3 |
| \* | pDK268\_112012\_01.09003.09003.2 | 3.0377 | 0.3235 | 100.0% | 1035.0922 | 1035.1881 | 2 | 7.027 | 87.5% | 1 | R.VSDLGQFIR.E | 2 |
| \* | pDK268\_112012\_01.06514.06514.2 | 2.1807 | 0.2603 | 99.3% | 987.9922 | 987.1768 | 2 | 5.183 | 68.8% | 1 | K.GSMFSQAMK.K | 2 |
| \* | pDK268\_112012\_02.06766.06766.2 | 5.4177 | 0.5594 | 100.0% | 1905.1322 | 1905.0306 | 1 | 10.571 | 80.0% | 1 | K.WVQGNTDEAQEELAWK.I | 2 |
| \* | pDK268\_112012\_01.11446.11446.2 | 5.2659 | 0.5622 | 100.0% | 2138.4321 | 2138.4585 | 1 | 9.273 | 70.6% | 1 | K.MIVSDIMQQAQYDQPLEK.S | 2 |

---

|  |  |  |  |  |  |  |  |  |
| --- | --- | --- | --- | --- | --- | --- | --- | --- |
| U | *gi|74136883|ref|NP\_11* | 24 | 32 | 29.6% | 825 | 90585 | 6.0 | heterogeneous nuclear ribonucleoprotein U isoform a [Homo sapiens] |

| Filename XCorr DeltCN Conf% ObsM+H+ CalcM+H+ SpR ZScore Ion% # Sequence  | | | | | | | | | | | | |
| --- | --- | --- | --- | --- | --- | --- | --- | --- | --- | --- | --- | --- |
|  | pDK268\_112012\_01.04400.04400.2 | 2.6203 | 0.1329 | 99.2% | 1075.0922 | 1075.2474 | 3 | 5.078 | 75.0% | 1 | K.VSELKEELK.K | 2 |
|  | pDK268\_112012\_01.03855.03855.2 | 3.1318 | 0.1178 | 99.8% | 1203.2922 | 1203.4215 | 3 | 4.653 | 77.8% | 1 | K.VSELKEELKK.R | 2 |
|  | pDK268\_112012\_02.06268.06268.3 | 5.8371 | 0.4988 | 100.0% | 3128.0942 | 3128.311 | 1 | 8.0 | 27.4% | 3 | R.LQAALDDEEAGGRPAMEPGNGSLDLGGDSAGR.S | 3 |
|  | pDK268\_112012\_01.10875.10875.2 | 2.7417 | 0.4591 | 100.0% | 1715.3922 | 1715.9469 | 1 | 6.35 | 47.1% | 1 | K.SSGPTSLFAVTVAPPGAR.Q | 2 |
| \* | pDK268\_112012\_01.03058.03058.3 | 3.3742 | 0.2792 | 99.7% | 2282.9644 | 2282.4783 | 1 | 5.067 | 34.4% | 1 | K.KKAEGGGGGGRPGAPAAGDGKTEQK.G | 3 |
| \* | pDK268\_112012\_01.03095.03095.3 | 3.7727 | 0.3485 | 100.0% | 2153.9944 | 2154.3042 | 1 | 5.282 | 33.7% | 1 | K.KAEGGGGGGRPGAPAAGDGKTEQK.G | 3 |
| \* | pDK268\_112012\_01.03173.03173.2 | 3.6802 | 0.558 | 100.0% | 1538.7922 | 1539.6047 | 1 | 9.169 | 61.1% | 1 | K.AEGGGGGGRPGAPAAGDGK.T | 2 |
| \* | pDK268\_112012\_01.03150.03150.3 | 5.4186 | 0.5333 | 100.0% | 2025.7144 | 2026.13 | 1 | 8.939 | 42.0% | 1 | K.AEGGGGGGRPGAPAAGDGKTEQK.G | 3 |
|  | pDK268\_112012\_01.08806.08806.2 | 4.2572 | 0.5077 | 100.0% | 1698.1522 | 1698.8291 | 1 | 7.81 | 79.2% | 1 | R.GYFEYIEENKYSR.A | 2 |
|  | pDK268\_112012\_02.06549.06549.3 | 2.2722 | 0.3138 | 98.8% | 1698.6843 | 1698.8291 | 1 | 6.139 | 45.8% | 1 | R.GYFEYIEENKYSR.A | 3 |
|  | pDK268\_112012\_01.05153.05153.2 | 2.49 | 0.2669 | 99.9% | 997.39215 | 997.0959 | 13 | 5.88 | 78.6% | 1 | K.DIDIHEVR.I | 2 |
|  | pDK268\_112012\_01.06753.06753.2 | 2.6007 | 0.3171 | 99.9% | 1049.1122 | 1049.1716 | 3 | 7.212 | 61.1% | 1 | K.NGQDLGVAFK.I | 2 |
|  | pDK268\_112012\_01.13076.13076.3 | 5.3691 | 0.3984 | 100.0% | 2724.9543 | 2726.0576 | 1 | 6.899 | 29.8% | 2 | K.EKPYFPIPEEYTFIQNVPLEDR.V | 3 |
|  | pDK268\_112012\_01.07787.07787.2 | 5.6972 | 0.5058 | 100.0% | 2187.7722 | 2188.4631 | 1 | 10.182 | 63.2% | 1 | K.HAAENPGKYNILGTNTIMDK.M | 2 |
|  | pDK268\_112012\_01.07780.07780.3 | 4.8946 | 0.4784 | 100.0% | 2188.5244 | 2188.4631 | 1 | 8.844 | 40.8% | 1 | K.HAAENPGKYNILGTNTIMDK.M | 3 |
|  | pDK268\_112012\_01.09784.09784.2 | 4.01 | 0.3845 | 100.0% | 1384.4922 | 1383.6025 | 1 | 6.815 | 77.3% | 1 | K.YNILGTNTIMDK.M | 2 |
|  | pDK268\_112012\_01.04820.04820.2 | 2.3252 | 0.3377 | 99.9% | 911.9922 | 912.19635 | 1 | 6.212 | 85.7% | 1 | K.MMVAGFKK.Q | 2 |
|  | pDK268\_112012\_02.04146.04146.2 | 2.1056 | 0.1204 | 96.8% | 857.47217 | 858.0287 | 152 | 3.603 | 66.7% | 1 | K.LNTLLQR.A | 2 |
|  | pDK268\_112012\_01.06593.06593.2 | 2.4589 | 0.0963 | 98.7% | 819.9522 | 819.97925 | 194 | 3.825 | 75.0% | 2 | K.FIEIAAR.K | 2 |
|  | pDK268\_112012\_02.06528.06528.2 | 4.9523 | 0.4698 | 100.0% | 1648.1322 | 1648.816 | 1 | 8.253 | 82.1% | 4 | R.NFILDQTNVSAAAQR.R | 2 |
|  | pDK268\_112012\_02.06535.06535.3 | 4.4455 | 0.3199 | 100.0% | 1648.8844 | 1648.816 | 1 | 6.027 | 48.2% | 1 | R.NFILDQTNVSAAAQR.R | 3 |
|  | pDK268\_112012\_01.05086.05086.2 | 2.1144 | 0.2754 | 99.3% | 1022.1922 | 1022.1894 | 2 | 4.89 | 81.2% | 1 | K.DLPEHAVLK.M | 2 |
|  | pDK268\_112012\_01.03900.03900.2 | 3.6456 | 0.4135 | 100.0% | 1395.4122 | 1395.5957 | 1 | 7.18 | 80.0% | 1 | K.LLEQYKEESKK.A | 2 |
|  | pDK268\_112012\_01.10124.10124.3 | 4.8408 | 0.5052 | 100.0% | 3659.8442 | 3660.8645 | 1 | 8.26 | 27.7% | 2 | K.NQSQGYNQWQQGQFWGQKPWSQHYHQGYY.- | 3 |

---

|  |  |  |  |  |  |  |  |  |
| --- | --- | --- | --- | --- | --- | --- | --- | --- |
| U | *gi|68799814|ref|NP\_00* | 12 | 14 | 29.3% | 433 | 49389 | 9.5 | adaptor-related protein complex 2, mu 1 subunit isoform b [Homo sapiens] |

| Filename XCorr DeltCN Conf% ObsM+H+ CalcM+H+ SpR ZScore Ion% # Sequence  | | | | | | | | | | | | |
| --- | --- | --- | --- | --- | --- | --- | --- | --- | --- | --- | --- | --- |
|  | pDK268\_112012\_01.09550.09550.2 | 3.8115 | 0.3998 | 100.0% | 1293.1522 | 1293.5695 | 1 | 7.635 | 85.0% | 1 | -.MIGGLFIYNHK.G | 2 |
|  | pDK268\_112012\_01.09549.09549.3 | 2.2945 | 0.3652 | 100.0% | 1293.9243 | 1293.5695 | 35 | 6.44 | 40.0% | 1 | -.MIGGLFIYNHK.G | 3 |
|  | pDK268\_112012\_01.03696.03696.2 | 2.2561 | 0.1616 | 98.3% | 994.5722 | 994.0953 | 39 | 4.322 | 71.4% | 1 | R.VYRDDIGR.N | 2 |
|  | pDK268\_112012\_01.04203.04203.2 | 2.1451 | 0.2492 | 99.3% | 857.53217 | 857.9853 | 17 | 5.197 | 78.6% | 1 | R.SPVTNIAR.T | 2 |
|  | pDK268\_112012\_01.06305.06305.2 | 2.5611 | 0.3297 | 99.9% | 1036.0721 | 1036.2163 | 3 | 6.336 | 62.5% | 1 | K.TFITQQGIK.S | 2 |
| \* | pDK268\_112012\_02.06341.06341.3 | 3.3388 | 0.3564 | 100.0% | 2391.4143 | 2392.5876 | 1 | 5.558 | 31.2% | 1 | K.SQTKEEQSQITSQVTGQIGWR.R | 3 |
|  | pDK268\_112012\_01.17016.17016.3 | 6.2921 | 0.4402 | 100.0% | 3309.0842 | 3310.7961 | 1 | 6.863 | 31.0% | 1 | R.RNELFLDVLESVNLLMSPQGQVLSAHVSGR.V | 3 |
|  | pDK268\_112012\_02.13722.13722.3 | 4.0288 | 0.468 | 100.0% | 3153.8342 | 3154.6086 | 1 | 7.95 | 24.1% | 1 | R.NELFLDVLESVNLLMSPQGQVLSAHVSGR.V | 3 |
|  | pDK268\_112012\_01.05704.05704.2 | 3.2359 | 0.2802 | 100.0% | 1233.2922 | 1233.4532 | 1 | 6.231 | 80.0% | 2 | K.SNFKPSLLAQK.I | 2 |
|  | pDK268\_112012\_01.06606.06606.2 | 3.1549 | 0.378 | 100.0% | 1017.27216 | 1018.1576 | 1 | 7.193 | 87.5% | 2 | K.ASENAIVWK.I | 2 |
|  | pDK268\_112012\_01.13011.13011.2 | 3.4553 | 0.3165 | 100.0% | 2245.632 | 2245.647 | 1 | 6.607 | 52.6% | 1 | K.WARPPISMNFEVPFAPSGLK.V | 2 |
|  | pDK268\_112012\_01.13034.13034.3 | 4.088 | 0.321 | 100.0% | 2246.4543 | 2245.647 | 1 | 5.562 | 43.4% | 1 | K.WARPPISMNFEVPFAPSGLK.V | 3 |

---

|  |  |  |  |  |  |  |  |  |
| --- | --- | --- | --- | --- | --- | --- | --- | --- |
| U | *gi|14141166|ref|NP\_11* | 6 | 12 | 29.3% | 362 | 38222 | 6.8 | poly(rC) binding protein 2 isoform b [Homo sapiens] |
| U | *gi|193083112|ref|NP\_0* | 6 | 12 | 31.6% | 335 | 35347 | 8.0 | poly(rC) binding protein 2 isoform f [Homo sapiens] |
| U | *gi|193083110|ref|NP\_0* | 6 | 12 | 29.4% | 361 | 38151 | 6.8 | poly(rC) binding protein 2 isoform e [Homo sapiens] |
| U | *gi|193083108|ref|NP\_0* | 6 | 12 | 29.0% | 365 | 38580 | 6.8 | poly(rC) binding protein 2 isoform d [Homo sapiens] |
| U | *gi|148833484|ref|NP\_0* | 6 | 12 | 32.0% | 331 | 34917 | 8.0 | poly(rC) binding protein 2 isoform c [Homo sapiens] |
| U | *gi|14141168|ref|NP\_00* | 6 | 12 | 29.0% | 366 | 38651 | 6.8 | poly(rC) binding protein 2 isoform a [Homo sapiens] |

| Filename XCorr DeltCN Conf% ObsM+H+ CalcM+H+ SpR ZScore Ion% # Sequence  | | | | | | | | | | | | |
| --- | --- | --- | --- | --- | --- | --- | --- | --- | --- | --- | --- | --- |
|  | pDK268\_112012\_01.11282.11282.2 | 3.5762 | 0.4388 | 100.0% | 1360.2922 | 1359.6519 | 1 | 7.497 | 66.7% | 3 | R.IITLAGPTNAIFK.A | 2 |
|  | pDK268\_112012\_02.11260.11260.3 | 4.8724 | 0.4034 | 100.0% | 3352.9143 | 3353.8306 | 1 | 7.041 | 28.3% | 2 | K.AFAMIIDKLEEDISSSMTNSTAASRPPVTLR.L | 3 |
|  | pDK268\_112012\_02.06009.06009.3 | 4.1821 | 0.4325 | 100.0% | 2462.9944 | 2463.7222 | 1 | 7.442 | 37.5% | 1 | K.LEEDISSSMTNSTAASRPPVTLR.L | 3 |
|  | pDK268\_112012\_01.07991.07991.2 | 5.7124 | 0.4336 | 100.0% | 2091.0723 | 2091.2573 | 1 | 8.628 | 55.3% | 3 | R.ESTGAQVQVAGDMLPNSTER.A | 22 |
|  | pDK268\_112012\_01.08106.08106.3 | 6.1848 | 0.5847 | 100.0% | 3383.7844 | 3384.7656 | 1 | 9.364 | 28.3% | 1 | K.LHQLAMQQSHFPMTHGNTGFSGIESSSPEVK.G | 3 |
|  | pDK268\_112012\_01.04514.04514.2 | 2.6798 | 0.3888 | 100.0% | 1159.5922 | 1159.2413 | 2 | 6.18 | 80.0% | 2 | K.IANPVEGSTDR.Q | 2 |

Similarities:
gi|222352151|ref|NP\_0(1:5)  

---

|  |  |  |  |  |  |  |  |  |
| --- | --- | --- | --- | --- | --- | --- | --- | --- |
| U | *gi|4506669|ref|NP\_000* | 3 | 14 | 28.9% | 114 | 11514 | 4.3 | ribosomal protein P1 isoform 1 [Homo sapiens] |

| Filename XCorr DeltCN Conf% ObsM+H+ CalcM+H+ SpR ZScore Ion% # Sequence  | | | | | | | | | | | | |
| --- | --- | --- | --- | --- | --- | --- | --- | --- | --- | --- | --- | --- |
|  | pDK268\_112012\_01.14024.14024.2 | 4.2273 | 0.46 | 100.0% | 1704.6522 | 1703.9811 | 1 | 8.312 | 53.3% | 4 | K.AAGVNVEPFWPGLFAK.A | 2 |
|  | pDK268\_112012\_01.12392.12392.2 | 5.2689 | 0.5852 | 100.0% | 1950.3121 | 1950.979 | 1 | 12.044 | 75.0% | 9 | K.KEESEESDDDMGFGLFD.- | 22 |
|  | pDK268\_112012\_02.09670.09670.2 | 3.4431 | 0.4262 | 100.0% | 2031.2722 | 2030.979 | 1 | 7.103 | 62.5% | 1 | K.KEES\*EESDDDMGFGLFD.- | 22 |

Similarities:
gi|4506671|ref|NP\_000(2:1)  

---

|  |  |  |  |  |  |  |  |  |
| --- | --- | --- | --- | --- | --- | --- | --- | --- |
| U | *gi|27436946|ref|NP\_73* | 14 | 16 | 28.8% | 664 | 74140 | 7.0 | lamin A/C isoform 1 precursor [Homo sapiens] |

| Filename XCorr DeltCN Conf% ObsM+H+ CalcM+H+ SpR ZScore Ion% # Sequence  | | | | | | | | | | | | |
| --- | --- | --- | --- | --- | --- | --- | --- | --- | --- | --- | --- | --- |
|  | pDK268\_112012\_01.05300.05300.3 | 3.3033 | 0.1808 | 98.7% | 1630.8544 | 1630.7521 | 16 | 4.455 | 45.8% | 1 | R.LQEKEDLQELNDR.L | 3 |
|  | pDK268\_112012\_01.04994.04994.2 | 2.5905 | 0.1821 | 99.4% | 1044.0322 | 1044.1527 | 1 | 5.063 | 77.8% | 1 | K.EGDLIAAQAR.L | 2 |
|  | pDK268\_112012\_01.07445.07445.2 | 3.1116 | 0.3275 | 100.0% | 1183.1122 | 1183.3066 | 3 | 6.33 | 77.8% | 1 | R.TLEGELHDLR.G | 2 |
|  | pDK268\_112012\_01.04572.04572.2 | 2.9668 | 0.2271 | 99.9% | 902.0722 | 902.03546 | 9 | 4.936 | 75.0% | 1 | K.LEAALGEAK.K | 2 |
|  | pDK268\_112012\_01.08199.08199.2 | 2.9271 | 0.302 | 100.0% | 1029.1721 | 1029.1814 | 1 | 6.296 | 87.5% | 2 | R.LADALQELR.A | 2 |
|  | pDK268\_112012\_01.03288.03288.2 | 2.6152 | 0.2252 | 99.6% | 1505.1322 | 1503.6115 | 1 | 5.097 | 72.7% | 1 | R.AQHEDQVEQYKK.E | 2 |
|  | pDK268\_112012\_01.06352.06352.2 | 4.3176 | 0.5188 | 100.0% | 1753.3922 | 1753.8693 | 1 | 9.192 | 66.7% | 1 | R.NSNLVGAAHEELQQSR.I | 2 |
|  | pDK268\_112012\_01.10146.10146.2 | 4.5515 | 0.4037 | 100.0% | 1700.6322 | 1700.9762 | 1 | 6.852 | 78.6% | 1 | R.IRIDSLSAQLSQLQK.Q | 2 |
|  | pDK268\_112012\_01.06201.06201.2 | 2.7546 | 0.1863 | 99.7% | 1188.3922 | 1188.3262 | 1 | 4.655 | 83.3% | 1 | K.LRDLEDSLAR.E | 2 |
|  | pDK268\_112012\_01.12009.12009.2 | 4.4774 | 0.542 | 100.0% | 1894.4521 | 1895.1346 | 1 | 9.727 | 75.0% | 1 | R.MQQQLDEYQELLDIK.L | 2 |
|  | pDK268\_112012\_02.05296.05296.3 | 2.1451 | 0.3886 | 99.8% | 1607.0944 | 1606.7728 | 1 | 5.598 | 34.6% | 1 | R.VAVEEVDEEGKFVR.L | 3 |
|  | pDK268\_112012\_02.05700.05700.2 | 3.9676 | 0.4931 | 100.0% | 1493.1921 | 1492.6874 | 1 | 8.384 | 69.2% | 2 | R.TALINSTGEEVAMR.K | 2 |
|  | pDK268\_112012\_02.04815.04815.3 | 3.4385 | 0.3043 | 100.0% | 2365.7644 | 2366.504 | 2 | 5.585 | 27.9% | 1 | K.ASASGSGAQVGGPISSGSSASSVTVTR.S | 3 |
|  | pDK268\_112012\_02.05628.05628.2 | 4.3865 | 0.5051 | 100.0% | 1567.0922 | 1567.6555 | 1 | 9.275 | 59.4% | 1 | R.SVGGSGGGSFGDNLVTR.S | 2 |

---

|  |  |  |  |  |  |  |  |  |
| --- | --- | --- | --- | --- | --- | --- | --- | --- |
| U | *gi|17105394|ref|NP\_00* | 4 | 4 | 28.8% | 156 | 17695 | 10.4 | ribosomal protein L23a [Homo sapiens] |

| Filename XCorr DeltCN Conf% ObsM+H+ CalcM+H+ SpR ZScore Ion% # Sequence  | | | | | | | | | | | | |
| --- | --- | --- | --- | --- | --- | --- | --- | --- | --- | --- | --- | --- |
|  | pDK268\_112012\_01.03266.03266.2 | 2.0787 | 0.2953 | 98.8% | 1109.2922 | 1109.2676 | 1 | 6.097 | 70.0% | 1 | K.EAPAPPKAEAK.A | 2 |
|  | pDK268\_112012\_01.05978.05978.2 | 2.3299 | 0.3023 | 99.8% | 1064.4122 | 1065.2548 | 3 | 5.101 | 68.8% | 1 | K.KLYDIDVAK.V | 2 |
|  | pDK268\_112012\_01.03875.03875.2 | 2.8249 | 0.2734 | 99.9% | 1370.0922 | 1370.5919 | 3 | 5.454 | 63.6% | 1 | K.VNTLIRPDGEKK.A | 2 |
| \* | pDK268\_112012\_01.08403.08403.2 | 3.5427 | 0.4823 | 100.0% | 1405.1921 | 1405.5474 | 1 | 7.74 | 75.0% | 1 | R.LAPDYDALDVANK.I | 2 |

---

|  |  |  |  |  |  |  |  |  |
| --- | --- | --- | --- | --- | --- | --- | --- | --- |
| U | *gi|4758302|ref|NP\_004* | 4 | 6 | 28.8% | 104 | 12259 | 5.9 | enhancer of rudimentary homolog [Homo sapiens] |

| Filename XCorr DeltCN Conf% ObsM+H+ CalcM+H+ SpR ZScore Ion% # Sequence  | | | | | | | | | | | | |
| --- | --- | --- | --- | --- | --- | --- | --- | --- | --- | --- | --- | --- |
| \* | pDK268\_112012\_01.03584.03584.2 | 2.4053 | 0.0703 | 97.0% | 1108.0322 | 1106.2848 | 4 | 3.294 | 78.6% | 1 | K.MYEEHLKR.M | 2 |
| \* | pDK268\_112012\_01.04107.04107.2 | 2.8002 | 0.3836 | 100.0% | 1329.2522 | 1329.4087 | 32 | 5.755 | 60.0% | 1 | R.ADTQTYQPYNK.D | 2 |
| \* | pDK268\_112012\_01.07485.07485.2 | 3.7317 | 0.2726 | 100.0% | 1871.4321 | 1872.0441 | 1 | 6.279 | 64.3% | 3 | R.ADTQTYQPYNKDWIK.E | 2 |
| \* | pDK268\_112012\_01.06402.06402.2 | 2.0618 | 0.1451 | 96.9% | 932.6322 | 933.185 | 1 | 4.668 | 91.7% | 1 | K.IYVLLRR.Q | 2 |

---

|  |  |  |  |  |  |  |  |  |
| --- | --- | --- | --- | --- | --- | --- | --- | --- |
| U | *gi|19913410|ref|NP\_05* | 13 | 15 | 28.6% | 893 | 99327 | 5.5 | major vault protein [Homo sapiens] |
| U | *gi|19913412|ref|NP\_00* | 13 | 15 | 28.6% | 893 | 99327 | 5.5 | major vault protein [Homo sapiens] |

| Filename XCorr DeltCN Conf% ObsM+H+ CalcM+H+ SpR ZScore Ion% # Sequence  | | | | | | | | | | | | |
| --- | --- | --- | --- | --- | --- | --- | --- | --- | --- | --- | --- | --- |
|  | pDK268\_112012\_01.07817.07817.3 | 3.9952 | 0.4233 | 100.0% | 2153.5144 | 2153.405 | 1 | 7.595 | 36.8% | 1 | R.IPPYHYIHVLDQNSNVSR.V | 3 |
|  | pDK268\_112012\_01.12333.12333.2 | 3.987 | 0.4556 | 100.0% | 1816.4722 | 1817.0918 | 1 | 6.995 | 73.3% | 1 | R.LAQDPFPLYPGEVLEK.D | 2 |
|  | pDK268\_112012\_01.12402.12402.2 | 3.5333 | 0.3849 | 100.0% | 1872.8722 | 1873.2462 | 4 | 6.553 | 43.8% | 1 | K.DITPLQVVLPNTALHLK.A | 2 |
|  | pDK268\_112012\_02.09899.09899.3 | 4.7771 | 0.4558 | 100.0% | 3353.2744 | 3354.6973 | 1 | 5.876 | 28.4% | 2 | K.ALLDFEDKDGDKVVAGDEWLFEGPGTYIPR.K | 3 |
|  | pDK268\_112012\_01.13595.13595.2 | 5.4658 | 0.4829 | 100.0% | 1741.2922 | 1741.0813 | 1 | 8.581 | 71.4% | 2 | R.KEVEVVEIIQATIIR.Q | 2 |
|  | pDK268\_112012\_01.08802.08802.3 | 3.5431 | 0.378 | 100.0% | 2887.8843 | 2888.1228 | 1 | 5.273 | 27.1% | 1 | R.ALQPLEEGEDEEKVSHQAGDHWLIR.G | 3 |
|  | pDK268\_112012\_01.09405.09405.2 | 4.2448 | 0.5002 | 100.0% | 1931.4922 | 1932.1376 | 1 | 8.299 | 59.4% | 1 | R.QAIPLDENEGIYVQDVK.T | 2 |
|  | pDK268\_112012\_02.09235.09235.2 | 3.6293 | 0.3344 | 100.0% | 2083.7122 | 2084.3918 | 1 | 7.176 | 47.1% | 1 | R.AVIGSTYMLTQDEVLWEK.E | 2 |
|  | pDK268\_112012\_01.09023.09023.2 | 2.4151 | 0.2325 | 99.1% | 1286.9321 | 1287.4119 | 1 | 6.813 | 72.7% | 1 | R.TAVFGFETSEAK.G | 2 |
|  | pDK268\_112012\_01.05085.05085.2 | 3.5429 | 0.3258 | 100.0% | 1131.1921 | 1131.2712 | 3 | 7.157 | 70.0% | 1 | R.IEGEGSVLQAK.L | 2 |
|  | pDK268\_112012\_01.07742.07742.2 | 2.864 | 0.2843 | 99.9% | 1259.3922 | 1258.4755 | 10 | 5.453 | 63.6% | 1 | R.DLAVAGPEMQVK.L | 2 |
|  | pDK268\_112012\_01.17990.17990.3 | 4.2117 | 0.5064 | 100.0% | 3275.7244 | 3276.732 | 1 | 8.203 | 24.2% | 1 | K.STLITDGSTPINLFNTAFGLLGMGPEGQPLGR.R | 3 |
|  | pDK268\_112012\_01.08129.08129.3 | 5.6564 | 0.5387 | 100.0% | 3120.5645 | 3121.4368 | 1 | 9.211 | 28.2% | 1 | R.VASGPSPGEGISPQSAQAPQAPGDNHVVPVLR.- | 3 |

---

|  |  |  |  |  |  |  |  |  |
| --- | --- | --- | --- | --- | --- | --- | --- | --- |
| U | *gi|169211725|ref|XP\_9* | 4 | 7 | 28.4% | 88 | 9949 | 11.3 | PREDICTED: similar to 40S ribosomal protein S28 [Homo sapiens] |
| U | *gi|4506715|ref|NP\_001* | 4 | 7 | 36.2% | 69 | 7841 | 10.7 | ribosomal protein S28 [Homo sapiens] |
| U | *gi|169212081|ref|XP\_0* | 4 | 7 | 27.5% | 91 | 10272 | 11.2 | PREDICTED: similar to 40S ribosomal protein S28 [Homo sapiens] |

| Filename XCorr DeltCN Conf% ObsM+H+ CalcM+H+ SpR ZScore Ion% # Sequence  | | | | | | | | | | | | |
| --- | --- | --- | --- | --- | --- | --- | --- | --- | --- | --- | --- | --- |
|  | pDK268\_112012\_01.06293.06293.1 | 1.8648 | 0.3359 | 100.0% | 1099.39 | 1100.1884 | 1 | 5.589 | 62.5% | 1 | R.VEFMDDTSR.S | 1 |
|  | pDK268\_112012\_01.06284.06284.2 | 2.4551 | 0.3798 | 100.0% | 1100.0521 | 1100.1884 | 1 | 6.677 | 87.5% | 2 | R.VEFMDDTSR.S | 2 |
|  | pDK268\_112012\_01.11183.11183.2 | 3.3427 | 0.2214 | 99.9% | 1770.5122 | 1770.9805 | 1 | 5.903 | 56.7% | 1 | K.GPVREGDVLTLLESER.E | 2 |
|  | pDK268\_112012\_01.12092.12092.2 | 4.2677 | 0.498 | 100.0% | 1361.3322 | 1361.4918 | 1 | 7.881 | 81.8% | 3 | R.EGDVLTLLESER.E | 2 |

---

|  |  |  |  |  |  |  |  |  |
| --- | --- | --- | --- | --- | --- | --- | --- | --- |
| U | *gi|20149594|ref|NP\_03* | 15 | 23 | 28.2% | 724 | 83264 | 5.0 | heat shock 90kDa protein 1, beta [Homo sapiens] |

| Filename XCorr DeltCN Conf% ObsM+H+ CalcM+H+ SpR ZScore Ion% # Sequence  | | | | | | | | | | | | |
| --- | --- | --- | --- | --- | --- | --- | --- | --- | --- | --- | --- | --- |
|  | pDK268\_112012\_01.07101.07101.2 | 2.9383 | 0.4427 | 100.0% | 1276.1322 | 1276.3861 | 1 | 7.505 | 68.2% | 1 | R.ELISNASDALDK.I | 2 |
|  | pDK268\_112012\_01.09048.09048.2 | 3.5362 | 0.3286 | 100.0% | 1544.5922 | 1545.733 | 1 | 7.215 | 61.5% | 1 | R.ELISNASDALDKIR.Y | 2 |
|  | pDK268\_112012\_01.05892.05892.2 | 3.2528 | 0.26 | 99.9% | 1540.2322 | 1540.6672 | 1 | 6.297 | 65.4% | 1 | R.YESLTDPSKLDSGK.E | 22 |
|  | pDK268\_112012\_01.09603.09603.2 | 3.2078 | 0.1872 | 99.8% | 1245.3922 | 1243.4459 | 1 | 5.645 | 77.3% | 1 | K.ADLINNLGTIAK.S | 22 |
|  | pDK268\_112012\_02.05733.05733.3 | 3.7817 | 0.397 | 100.0% | 2017.3444 | 2016.2584 | 1 | 6.464 | 43.3% | 1 | K.VILHLKEDQTEYLEER.R | 33 |
| \* | pDK268\_112012\_01.12081.12081.2 | 4.7962 | 0.4667 | 100.0% | 1809.1522 | 1810.1027 | 1 | 8.46 | 78.6% | 2 | K.HSQFIGYPITLYLEK.E | 2 |
|  | pDK268\_112012\_01.04817.04817.2 | 2.2323 | 0.1635 | 97.6% | 1152.1322 | 1152.2462 | 1 | 4.198 | 81.2% | 1 | K.YIDQEELNK.T | 22 |
| \* | pDK268\_112012\_01.09105.09105.2 | 5.1506 | 0.4975 | 100.0% | 1847.9321 | 1848.9171 | 1 | 9.488 | 78.6% | 2 | R.NPDDITQEEYGEFYK.S | 2 |
|  | pDK268\_112012\_02.06611.06611.2 | 3.9605 | 0.4265 | 100.0% | 1527.8322 | 1528.6616 | 1 | 8.401 | 70.8% | 2 | K.SLTNDWEDHLAVK.H | 22 |
|  | pDK268\_112012\_02.06427.06427.2 | 3.0508 | 0.4105 | 100.0% | 1349.1322 | 1349.4886 | 1 | 6.947 | 70.0% | 3 | K.HFSVEGQLEFR.A | 22 |
| \* | pDK268\_112012\_01.09635.09635.2 | 3.177 | 0.1552 | 99.9% | 1237.3922 | 1237.4008 | 1 | 4.752 | 88.9% | 2 | R.RAPFDLFENK.K | 2 |
| \* | pDK268\_112012\_02.06454.06454.3 | 4.0706 | 0.564 | 100.0% | 2177.8145 | 2178.2915 | 1 | 8.838 | 40.3% | 2 | R.YHTSQSGDEMTSLSEYVSR.M | 3 |
| \* | pDK268\_112012\_01.05296.05296.2 | 2.3669 | 0.4182 | 99.9% | 1250.4922 | 1250.3538 | 7 | 7.602 | 55.0% | 1 | K.EQVANSAFVER.V | 2 |
| \* | pDK268\_112012\_01.07808.07808.3 | 4.4973 | 0.5261 | 100.0% | 1784.2743 | 1784.025 | 1 | 8.467 | 48.2% | 2 | K.HLEINPDHPIVETLR.Q | 3 |
| \* | pDK268\_112012\_02.14145.14145.3 | 4.3876 | 0.2959 | 100.0% | 3287.5144 | 3288.725 | 1 | 6.785 | 24.1% | 1 | K.AVKDLVVLLFETALLSSGFSLEDPQTHSNR.I | 3 |

Similarities:
gi|153792590|ref|NP\_0(6:9)  

---

|  |  |  |  |  |  |  |  |  |
| --- | --- | --- | --- | --- | --- | --- | --- | --- |
| U | *gi|32189394|ref|NP\_00* | 10 | 11 | 28.0% | 529 | 56560 | 5.4 | mitochondrial ATP synthase beta subunit precursor [Homo sapiens] |

| Filename XCorr DeltCN Conf% ObsM+H+ CalcM+H+ SpR ZScore Ion% # Sequence  | | | | | | | | | | | | |
| --- | --- | --- | --- | --- | --- | --- | --- | --- | --- | --- | --- | --- |
| \* | pDK268\_112012\_02.06847.06847.2 | 3.1876 | 0.545 | 100.0% | 1650.0521 | 1651.9034 | 1 | 8.621 | 67.9% | 1 | R.LVLEVAQHLGESTVR.T | 2 |
| \* | pDK268\_112012\_02.06826.06826.3 | 2.2708 | 0.3208 | 98.7% | 1651.3444 | 1651.9034 | 2 | 5.48 | 35.7% | 1 | R.LVLEVAQHLGESTVR.T | 3 |
| \* | pDK268\_112012\_02.05124.05124.2 | 2.4095 | 0.3406 | 99.8% | 1263.2722 | 1263.4515 | 4 | 5.942 | 68.2% | 1 | R.TIAMDGTEGLVR.G | 2 |
| \* | pDK268\_112012\_01.09995.09995.3 | 3.4802 | 0.4803 | 100.0% | 1920.3844 | 1920.2596 | 1 | 7.33 | 36.1% | 1 | K.VLDSGAPIKIPVGPETLGR.I | 3 |
| \* | pDK268\_112012\_01.08882.08882.2 | 4.0646 | 0.3389 | 100.0% | 1386.5322 | 1386.6061 | 3 | 6.384 | 72.7% | 1 | R.IMNVIGEPIDER.G | 23 |
| \* | pDK268\_112012\_01.19032.19032.3 | 3.9055 | 0.3486 | 100.0% | 3344.4844 | 3344.7917 | 3 | 5.27 | 24.1% | 1 | R.VALTGLTVAEYFRDQEGQDVLLFIDNIFR.F | 3 |
| \* | pDK268\_112012\_01.18047.18047.2 | 4.3095 | 0.4406 | 100.0% | 1923.7522 | 1923.1326 | 3 | 7.496 | 46.7% | 2 | R.DQEGQDVLLFIDNIFR.F | 2 |
| \* | pDK268\_112012\_01.10068.10068.2 | 5.0518 | 0.6373 | 100.0% | 2266.4321 | 2267.577 | 1 | 10.637 | 67.5% | 1 | R.IPSAVGYQPTLATDMGTMQER.I | 2 |
| \* | pDK268\_112012\_01.07755.07755.3 | 2.6016 | 0.2602 | 98.1% | 1817.3344 | 1817.0264 | 24 | 4.659 | 33.3% | 1 | R.IMDPNIVGSEHYDVAR.G | 3 |
| \* | pDK268\_112012\_02.11955.11955.3 | 3.0455 | 0.279 | 99.1% | 2677.5244 | 2677.0393 | 4 | 4.54 | 22.8% | 1 | K.SLQDIIAILGMDELSEEDKLTVSR.A | 3 |

---

|  |  |  |  |  |  |  |  |  |
| --- | --- | --- | --- | --- | --- | --- | --- | --- |
| U | *gi|4506605|ref|NP\_000* | 6 | 14 | 27.9% | 140 | 14865 | 10.5 | ribosomal protein L23 [Homo sapiens] |

| Filename XCorr DeltCN Conf% ObsM+H+ CalcM+H+ SpR ZScore Ion% # Sequence  | | | | | | | | | | | | |
| --- | --- | --- | --- | --- | --- | --- | --- | --- | --- | --- | --- | --- |
| \* | pDK268\_112012\_01.10468.10468.3 | 3.0832 | 0.2866 | 99.8% | 1845.3544 | 1844.2408 | 6 | 4.858 | 36.8% | 1 | R.LNRLPAAGVGDMVMATVK.K | 3 |
| \* | pDK268\_112012\_02.07924.07924.2 | 4.639 | 0.5367 | 100.0% | 1461.2922 | 1460.7902 | 1 | 9.921 | 78.6% | 8 | R.LPAAGVGDMVMATVK.K | 2 |
| \* | pDK268\_112012\_02.07031.07031.3 | 2.7633 | 0.3384 | 100.0% | 1589.1843 | 1588.9642 | 1 | 5.213 | 40.0% | 2 | R.LPAAGVGDMVMATVKK.G | 3 |
| \* | pDK268\_112012\_01.03348.03348.2 | 2.5992 | 0.2204 | 99.7% | 1146.6122 | 1147.4525 | 2 | 5.822 | 72.2% | 1 | R.KKVHPAVVIR.Q | 2 |
| \* | pDK268\_112012\_01.03974.03974.1 | 1.234 | 0.2975 | 98.6% | 900.57 | 901.0507 | 92 | 4.921 | 33.3% | 1 | K.GSAITGPVAK.E | 1 |
| \* | pDK268\_112012\_01.03965.03965.2 | 2.6411 | 0.2879 | 99.9% | 901.03217 | 901.0507 | 5 | 5.268 | 72.2% | 1 | K.GSAITGPVAK.E | 2 |

---

|  |  |  |  |  |  |  |  |  |
| --- | --- | --- | --- | --- | --- | --- | --- | --- |
| U | *gi|224586884|ref|NP\_0* | 8 | 13 | 27.7% | 303 | 31947 | 9.7 | cold shock domain protein A isoform b [Homo sapiens] |

| Filename XCorr DeltCN Conf% ObsM+H+ CalcM+H+ SpR ZScore Ion% # Sequence  | | | | | | | | | | | | |
| --- | --- | --- | --- | --- | --- | --- | --- | --- | --- | --- | --- | --- |
|  | pDK268\_112012\_01.08026.08026.2 | 2.0564 | 0.1284 | 95.1% | 942.1722 | 941.0342 | 3 | 4.601 | 71.4% | 1 | R.NGYGFINR.N | 22 |
|  | pDK268\_112012\_01.05208.05208.2 | 4.6974 | 0.4444 | 100.0% | 1745.2522 | 1745.9298 | 1 | 7.536 | 75.0% | 1 | R.NDTKEDVFVHQTAIK.K | 22 |
|  | pDK268\_112012\_02.04098.04098.3 | 3.8099 | 0.253 | 100.0% | 1746.2043 | 1745.9298 | 1 | 6.003 | 42.9% | 1 | R.NDTKEDVFVHQTAIK.K | 33 |
|  | pDK268\_112012\_01.04480.04480.2 | 4.8888 | 0.3907 | 100.0% | 1874.5122 | 1874.1039 | 1 | 6.397 | 76.7% | 1 | R.NDTKEDVFVHQTAIKK.N | 22 |
|  | pDK268\_112012\_01.04467.04467.3 | 4.1836 | 0.3947 | 100.0% | 1874.8143 | 1874.1039 | 1 | 7.133 | 48.3% | 1 | R.NDTKEDVFVHQTAIKK.N | 33 |
|  | pDK268\_112012\_02.06759.06759.2 | 4.3447 | 0.5209 | 100.0% | 1796.5922 | 1796.8822 | 1 | 9.551 | 68.8% | 6 | R.SVGDGETVEFDVVEGEK.G | 22 |
|  | pDK268\_112012\_01.05916.05916.2 | 4.6781 | 0.5739 | 100.0% | 1783.3322 | 1783.8925 | 1 | 10.283 | 58.3% | 1 | K.GAEAANVTGPDGVPVEGSR.Y | 2 |
| \* | pDK268\_112012\_01.07074.07074.3 | 5.65 | 0.4597 | 100.0% | 2490.3843 | 2490.7534 | 1 | 7.846 | 33.7% | 1 | R.NAGEIGEMKDGVPEGAQLQGPVHR.N | 3 |

Similarities:
gi|34098946|ref|NP\_00(6:2)  

---

|  |  |  |  |  |  |  |  |  |
| --- | --- | --- | --- | --- | --- | --- | --- | --- |
| U | *gi|25777713|ref|NP\_73* | 3 | 3 | 27.6% | 163 | 18658 | 4.5 | S-phase kinase-associated protein 1 isoform b [Homo sapiens] |

| Filename XCorr DeltCN Conf% ObsM+H+ CalcM+H+ SpR ZScore Ion% # Sequence  | | | | | | | | | | | | |
| --- | --- | --- | --- | --- | --- | --- | --- | --- | --- | --- | --- | --- |
|  | pDK268\_112012\_01.11118.11118.2 | 2.2185 | 0.1885 | 96.4% | 1763.6522 | 1762.9597 | 3 | 3.83 | 50.0% | 1 | K.RTDDIPVWDQEFLK.V | 2 |
|  | pDK268\_112012\_02.12844.12844.2 | 2.8029 | 0.1602 | 98.6% | 2136.9722 | 2137.481 | 3 | 5.066 | 44.4% | 1 | K.VDQGTLFELILAANYLDIK.G | 2 |
| \* | pDK268\_112012\_01.06252.06252.2 | 2.4765 | 0.1476 | 97.5% | 1467.2322 | 1467.4888 | 1 | 4.718 | 72.7% | 1 | K.NDFTEEEEAQVR.K | 2 |

---

|  |  |  |  |  |  |  |  |  |
| --- | --- | --- | --- | --- | --- | --- | --- | --- |
| U | *gi|169201338|ref|XP\_0* | 4 | 8 | 27.5% | 160 | 18565 | 10.5 | PREDICTED: hypothetical protein [Homo sapiens] |
| U | *gi|18104948|ref|NP\_00* | 4 | 8 | 27.5% | 160 | 18565 | 10.5 | ribosomal protein L21 [Homo sapiens] |
| U | *gi|169210381|ref|XP\_0* | 4 | 8 | 27.5% | 160 | 18535 | 10.6 | PREDICTED: hypothetical protein isoform 2 [Homo sapiens] |
| U | *gi|169210379|ref|XP\_0* | 4 | 8 | 27.5% | 160 | 18535 | 10.6 | PREDICTED: hypothetical protein isoform 3 [Homo sapiens] |
| U | *gi|169210377|ref|XP\_0* | 4 | 8 | 27.5% | 160 | 18535 | 10.6 | PREDICTED: hypothetical protein isoform 1 [Homo sapiens] |
| U | *gi|169201750|ref|XP\_0* | 4 | 8 | 27.5% | 160 | 18550 | 10.5 | PREDICTED: hypothetical protein [Homo sapiens] |

| Filename XCorr DeltCN Conf% ObsM+H+ CalcM+H+ SpR ZScore Ion% # Sequence  | | | | | | | | | | | | |
| --- | --- | --- | --- | --- | --- | --- | --- | --- | --- | --- | --- | --- |
|  | pDK268\_112012\_01.07646.07646.2 | 2.6441 | 0.4176 | 100.0% | 1244.0322 | 1244.4973 | 1 | 7.678 | 70.0% | 2 | K.HGVVPLATYMR.I | 2 |
|  | pDK268\_112012\_01.07190.07190.2 | 4.8762 | 0.4166 | 100.0% | 1641.3922 | 1641.9108 | 1 | 8.948 | 78.6% | 2 | R.VYNVTQHAVGIVVNK.Q | 2 |
|  | pDK268\_112012\_02.05626.05626.3 | 4.1883 | 0.4689 | 100.0% | 1642.2843 | 1641.9108 | 1 | 8.904 | 48.2% | 2 | R.VYNVTQHAVGIVVNK.Q | 3 |
|  | pDK268\_112012\_01.13736.13736.2 | 4.0354 | 0.513 | 100.0% | 2079.2122 | 2079.3723 | 1 | 8.304 | 52.9% | 2 | R.TNGKEPELLEPIPYEFMA.- | 2 |

---

|  |  |  |  |  |  |  |  |  |
| --- | --- | --- | --- | --- | --- | --- | --- | --- |
| U | *gi|24234747|ref|NP\_00* | 10 | 11 | 27.2% | 390 | 43062 | 5.3 | interleukin enhancer binding factor 2 [Homo sapiens] |

| Filename XCorr DeltCN Conf% ObsM+H+ CalcM+H+ SpR ZScore Ion% # Sequence  | | | | | | | | | | | | |
| --- | --- | --- | --- | --- | --- | --- | --- | --- | --- | --- | --- | --- |
| \* | pDK268\_112012\_01.08838.08838.2 | 2.4554 | 0.264 | 99.2% | 1732.3922 | 1732.9714 | 1 | 5.656 | 50.0% | 1 | R.VKPAPDETSFSEALLK.R | 2 |
| \* | pDK268\_112012\_01.08847.08847.3 | 3.7632 | 0.4327 | 100.0% | 1732.6144 | 1732.9714 | 1 | 7.251 | 46.7% | 1 | R.VKPAPDETSFSEALLK.R | 3 |
| \* | pDK268\_112012\_01.12122.12122.2 | 4.947 | 0.566 | 100.0% | 2100.372 | 2100.3335 | 1 | 9.59 | 65.8% | 1 | R.NQDLAPNSAEQASILSLVTK.I | 2 |
| \* | pDK268\_112012\_02.07210.07210.2 | 3.15 | 0.2903 | 99.9% | 1898.3722 | 1898.2714 | 1 | 5.481 | 41.2% | 1 | K.KGTMTTGHNVADLVVILK.I | 2 |
| \* | pDK268\_112012\_02.08034.08034.2 | 3.7887 | 0.4076 | 100.0% | 1768.6721 | 1770.0973 | 1 | 6.78 | 65.6% | 2 | K.GTMTTGHNVADLVVILK.I | 2 |
| \* | pDK268\_112012\_02.08004.08004.3 | 4.3811 | 0.3761 | 100.0% | 1770.1144 | 1770.0973 | 1 | 6.856 | 53.1% | 1 | K.GTMTTGHNVADLVVILK.I | 3 |
| \* | pDK268\_112012\_01.12134.12134.2 | 2.3926 | 0.2885 | 99.5% | 1409.7122 | 1410.697 | 1 | 5.911 | 61.5% | 1 | K.ILPTLEAVAALGNK.V | 2 |
| \* | pDK268\_112012\_01.08187.08187.2 | 3.1179 | 0.3778 | 100.0% | 1042.5521 | 1042.2668 | 1 | 6.845 | 88.9% | 1 | K.VLQSALAAIR.H | 2 |
| \* | pDK268\_112012\_01.17296.17296.3 | 3.6048 | 0.332 | 100.0% | 3267.6543 | 3269.8345 | 1 | 5.305 | 24.1% | 1 | R.IRFPGFEPLTPWILDLLGHYAVMNNPTR.Q | 3 |
| \* | pDK268\_112012\_01.18251.18251.3 | 5.7253 | 0.544 | 100.0% | 2999.4844 | 3000.4875 | 1 | 9.411 | 32.0% | 1 | R.FPGFEPLTPWILDLLGHYAVMNNPTR.Q | 3 |

---

|  |  |  |  |  |  |  |  |  |
| --- | --- | --- | --- | --- | --- | --- | --- | --- |
| U | *gi|18426902|ref|NP\_06* | 14 | 17 | 26.9% | 665 | 72133 | 6.1 | Werner helicase interacting protein isoform 1 [Homo sapiens] |
| U | *gi|18426904|ref|NP\_56* | 14 | 17 | 28.0% | 640 | 69459 | 6.3 | Werner helicase interacting protein isoform 2 [Homo sapiens] |

| Filename XCorr DeltCN Conf% ObsM+H+ CalcM+H+ SpR ZScore Ion% # Sequence  | | | | | | | | | | | | |
| --- | --- | --- | --- | --- | --- | --- | --- | --- | --- | --- | --- | --- |
|  | pDK268\_112012\_01.03198.03198.2 | 2.5648 | 0.1471 | 97.1% | 1582.8922 | 1583.7476 | 295 | 4.363 | 38.2% | 1 | K.GSGKRPAAAAAAGSASPR.S | 2 |
|  | pDK268\_112012\_01.03263.03263.2 | 3.8599 | 0.4866 | 100.0% | 1253.6322 | 1254.3915 | 1 | 8.978 | 84.6% | 1 | K.RPAAAAAAGSASPR.S | 2 |
|  | pDK268\_112012\_01.10294.10294.3 | 3.6989 | 0.409 | 100.0% | 2869.1643 | 2870.2678 | 2 | 6.571 | 29.2% | 1 | R.QMLQGKPLADTMRPDTLQDYFGQSK.A | 3 |
|  | pDK268\_112012\_01.05636.05636.2 | 2.9114 | 0.27 | 100.0% | 973.2922 | 973.11725 | 1 | 5.471 | 81.2% | 1 | K.AVGQDTLLR.S | 2 |
|  | pDK268\_112012\_01.05759.05759.2 | 2.3923 | 0.3107 | 99.8% | 1051.6122 | 1052.2157 | 1 | 6.22 | 88.9% | 1 | R.FVTLSATNAK.T | 2 |
|  | pDK268\_112012\_01.10170.10170.2 | 2.8575 | 0.3793 | 100.0% | 1257.4321 | 1257.4752 | 2 | 6.976 | 72.2% | 1 | K.TILFIDEIHR.F | 2 |
|  | pDK268\_112012\_01.09221.09221.2 | 2.8459 | 0.2737 | 99.8% | 1466.5122 | 1467.5754 | 1 | 6.41 | 53.8% | 1 | K.AVDTLAYLSDGDAR.A | 2 |
|  | pDK268\_112012\_01.11481.11481.2 | 3.7345 | 0.4068 | 100.0% | 1296.5122 | 1296.5558 | 1 | 7.732 | 66.7% | 1 | R.AGLNGLQLAVLAR.L | 2 |
|  | pDK268\_112012\_01.05412.05412.2 | 2.0882 | 0.296 | 99.4% | 1030.5721 | 1031.1943 | 145 | 6.465 | 50.0% | 1 | R.VLITENDVK.E | 2 |
|  | pDK268\_112012\_02.07763.07763.2 | 3.5295 | 0.3973 | 100.0% | 1481.4122 | 1481.6078 | 1 | 7.692 | 66.7% | 3 | R.GSDQNASLYWLAR.M | 2 |
|  | pDK268\_112012\_01.08855.08855.2 | 3.9699 | 0.4819 | 100.0% | 1450.2522 | 1450.6495 | 1 | 8.038 | 87.5% | 1 | R.MLEGGEDPLYVAR.R | 2 |
|  | pDK268\_112012\_01.07300.07300.2 | 3.6787 | 0.4483 | 100.0% | 1387.3121 | 1387.5321 | 1 | 6.938 | 72.7% | 2 | K.SIEVYSAYNNVK.A | 2 |
|  | pDK268\_112012\_01.07504.07504.3 | 3.1848 | 0.4628 | 100.0% | 1576.9443 | 1575.8569 | 1 | 6.961 | 40.4% | 1 | R.NHQGPLPPVPLHLR.N | 3 |
|  | pDK268\_112012\_01.11554.11554.2 | 3.7881 | 0.5564 | 100.0% | 2372.412 | 2373.596 | 1 | 9.246 | 50.0% | 1 | K.YNPMYSEPVDQEYLPEELR.G | 2 |

---

|  |  |  |  |  |  |  |  |  |
| --- | --- | --- | --- | --- | --- | --- | --- | --- |
| U | *gi|4506607|ref|NP\_000* | 5 | 8 | 26.6% | 188 | 21634 | 11.7 | ribosomal protein L18 [Homo sapiens] |

| Filename XCorr DeltCN Conf% ObsM+H+ CalcM+H+ SpR ZScore Ion% # Sequence  | | | | | | | | | | | | |
| --- | --- | --- | --- | --- | --- | --- | --- | --- | --- | --- | --- | --- |
| \* | pDK268\_112012\_01.04755.04755.2 | 2.5018 | 0.1935 | 99.3% | 1140.5322 | 1141.3158 | 1 | 4.652 | 72.2% | 1 | R.TNRPPLSLSR.M | 2 |
| \* | pDK268\_112012\_01.08168.08168.2 | 3.3424 | 0.3796 | 100.0% | 1346.6322 | 1346.5236 | 1 | 7.445 | 79.2% | 3 | K.TAVVVGTITDDVR.V | 2 |
| \* | pDK268\_112012\_01.11782.11782.2 | 3.8682 | 0.3906 | 100.0% | 1463.7722 | 1461.6982 | 1 | 6.719 | 70.8% | 2 | K.ILTFDQLALDSPK.G | 2 |
| \* | pDK268\_112012\_01.03207.03207.2 | 3.1115 | 0.388 | 100.0% | 1548.3722 | 1548.7446 | 1 | 7.626 | 65.4% | 1 | K.APGTPHSHTKPYVR.S | 2 |
| \* | pDK268\_112012\_01.03202.03202.3 | 3.0924 | 0.3011 | 100.0% | 1549.8544 | 1548.7446 | 4 | 5.661 | 40.4% | 1 | K.APGTPHSHTKPYVR.S | 3 |

---

|  |  |  |  |  |  |  |  |  |
| --- | --- | --- | --- | --- | --- | --- | --- | --- |
| U | *gi|148746199|ref|NP\_0* | 3 | 3 | 26.6% | 128 | 14632 | 10.8 | ribosomal protein L31 isoform 2 [Homo sapiens] |
| U | *gi|4506633|ref|NP\_000* | 3 | 3 | 27.2% | 125 | 14463 | 10.5 | ribosomal protein L31 isoform 1 [Homo sapiens] |
| U | *gi|153252132|ref|NP\_0* | 3 | 3 | 28.1% | 121 | 13995 | 10.8 | ribosomal protein L31 isoform 3 [Homo sapiens] |

| Filename XCorr DeltCN Conf% ObsM+H+ CalcM+H+ SpR ZScore Ion% # Sequence  | | | | | | | | | | | | |
| --- | --- | --- | --- | --- | --- | --- | --- | --- | --- | --- | --- | --- |
|  | pDK268\_112012\_01.05138.05138.2 | 2.972 | 0.3006 | 100.0% | 988.7322 | 989.11664 | 1 | 6.491 | 93.8% | 1 | R.SAINEVVTR.E | 2 |
|  | pDK268\_112012\_01.11363.11363.3 | 5.321 | 0.4925 | 100.0% | 2958.9844 | 2959.3257 | 1 | 8.74 | 38.5% | 1 | R.KRNEDEDSPNKLYTLVTYVPVTTFK.I | 3 |
|  | pDK268\_112012\_01.12550.12550.2 | 4.7434 | 0.4844 | 100.0% | 1646.5322 | 1645.9794 | 1 | 9.071 | 76.9% | 1 | K.LYTLVTYVPVTTFK.I | 2 |

---

|  |  |  |  |  |  |  |  |  |
| --- | --- | --- | --- | --- | --- | --- | --- | --- |
| U | *gi|28875797|ref|NP\_05* | 6 | 12 | 26.2% | 248 | 26397 | 12.2 | hypothetical protein LOC26097 [Homo sapiens] |

| Filename XCorr DeltCN Conf% ObsM+H+ CalcM+H+ SpR ZScore Ion% # Sequence  | | | | | | | | | | | | |
| --- | --- | --- | --- | --- | --- | --- | --- | --- | --- | --- | --- | --- |
| \* | pDK268\_112012\_01.04098.04098.2 | 4.4086 | 0.3944 | 100.0% | 1448.3322 | 1447.6091 | 5 | 7.178 | 62.5% | 1 | R.ASMQQQQQLASAR.N | 2 |
| \* | pDK268\_112012\_01.05124.05124.3 | 3.3177 | 0.1651 | 97.1% | 1941.0543 | 1941.259 | 4 | 3.937 | 35.9% | 1 | R.RLAQQMENRPSVQAALK.L | 3 |
| \* | pDK268\_112012\_01.05537.05537.2 | 4.6637 | 0.2907 | 100.0% | 1784.4321 | 1785.0715 | 1 | 6.869 | 66.7% | 2 | R.LAQQMENRPSVQAALK.L | 2 |
| \* | pDK268\_112012\_02.04257.04257.3 | 3.6728 | 0.3983 | 100.0% | 1785.1144 | 1785.0715 | 1 | 6.772 | 43.3% | 4 | R.LAQQMENRPSVQAALK.L | 3 |
| \* | pDK268\_112012\_01.09194.09194.2 | 3.9874 | 0.3693 | 100.0% | 1556.2522 | 1555.6997 | 1 | 7.397 | 70.8% | 3 | K.EQLDNQLDAYMSK.T | 2 |
| \* | pDK268\_112012\_02.06796.06796.3 | 3.2526 | 0.2212 | 98.3% | 2436.3542 | 2436.566 | 1 | 5.583 | 31.0% | 1 | K.TKGHLDAELDAYMAQTDPETND.- | 3 |

---

|  |  |  |  |  |  |  |  |  |
| --- | --- | --- | --- | --- | --- | --- | --- | --- |
| U | *Reverse\_gi|169216482|* | 1 | 1 | 26.2% | 103 | 11699 | 8.0 | PREDICTED: similar to hCG1808463 [Homo sapiens] |

| Filename XCorr DeltCN Conf% ObsM+H+ CalcM+H+ SpR ZScore Ion% # Sequence  | | | | | | | | | | | | |
| --- | --- | --- | --- | --- | --- | --- | --- | --- | --- | --- | --- | --- |
| \* | pDK268\_112012\_01.15555.15555.3 | 3.2771 | 0.238 | 98.6% | 3402.6543 | 3404.4583 | 86 | 4.492 | 18.3% | 1 | R.HNGS\*FPSLVVSNLSVLPILSY@Y@NNY@ER.W | 3 |

---

|  |  |  |  |  |  |  |  |  |
| --- | --- | --- | --- | --- | --- | --- | --- | --- |
| U | *gi|4506609|ref|NP\_000* | 5 | 7 | 26.0% | 196 | 23466 | 11.5 | ribosomal protein L19 [Homo sapiens] |

| Filename XCorr DeltCN Conf% ObsM+H+ CalcM+H+ SpR ZScore Ion% # Sequence  | | | | | | | | | | | | |
| --- | --- | --- | --- | --- | --- | --- | --- | --- | --- | --- | --- | --- |
| \* | pDK268\_112012\_01.09228.09228.2 | 4.9733 | 0.5369 | 100.0% | 1943.4122 | 1944.0679 | 1 | 10.232 | 68.8% | 2 | K.VWLDPNETNEIANANSR.Q | 2 |
| \* | pDK268\_112012\_01.02748.02748.2 | 1.9495 | 0.2558 | 98.2% | 923.3122 | 924.09094 | 1 | 5.452 | 100.0% | 1 | R.KPVTVHSR.A | 2 |
| \* | pDK268\_112012\_01.04871.04871.2 | 1.8934 | 0.2484 | 97.0% | 1193.3121 | 1192.421 | 1 | 4.887 | 62.5% | 1 | R.HMYHSLYLK.V | 2 |
| \* | pDK268\_112012\_01.04262.04262.2 | 2.4786 | 0.2363 | 99.8% | 1021.4122 | 1021.2658 | 2 | 5.32 | 78.6% | 1 | R.ILMEHIHK.L | 2 |
| \* | pDK268\_112012\_02.03346.03346.2 | 2.6706 | 0.3285 | 100.0% | 987.3122 | 987.10077 | 72 | 6.215 | 68.8% | 2 | K.LLADQAEAR.R | 2 |

---

|  |  |  |  |  |  |  |  |  |
| --- | --- | --- | --- | --- | --- | --- | --- | --- |
| U | *gi|221307584|ref|NP\_0* | 5 | 7 | 25.8% | 299 | 33296 | 9.8 | prohibitin 2 isoform 1 [Homo sapiens] |
| U | *gi|6005854|ref|NP\_009* | 5 | 7 | 25.8% | 299 | 33296 | 9.8 | prohibitin 2 isoform 2 [Homo sapiens] |

| Filename XCorr DeltCN Conf% ObsM+H+ CalcM+H+ SpR ZScore Ion% # Sequence  | | | | | | | | | | | | |
| --- | --- | --- | --- | --- | --- | --- | --- | --- | --- | --- | --- | --- |
|  | pDK268\_112012\_02.08001.08001.3 | 3.8787 | 0.366 | 100.0% | 1854.6843 | 1855.1038 | 1 | 7.514 | 48.4% | 2 | R.IGGVQQDTILAEGLHFR.I | 3 |
|  | pDK268\_112012\_01.15053.15053.2 | 3.9195 | 0.3893 | 100.0% | 1724.7122 | 1725.0428 | 1 | 8.297 | 75.0% | 2 | R.IPWFQYPIIYDIR.A | 2 |
|  | pDK268\_112012\_01.07139.07139.3 | 3.3927 | 0.2339 | 99.6% | 1890.2644 | 1890.1675 | 394 | 4.395 | 30.0% | 1 | R.VLSRPNAQELPSMYQR.L | 3 |
|  | pDK268\_112012\_01.03840.03840.2 | 4.2144 | 0.521 | 100.0% | 1216.4722 | 1216.3336 | 1 | 8.561 | 86.4% | 1 | K.IVQAEGEAEAAK.M | 2 |
|  | pDK268\_112012\_01.13722.13722.2 | 3.4998 | 0.4621 | 100.0% | 2226.4521 | 2226.4912 | 1 | 7.497 | 50.0% | 1 | R.IYLTADNLVLNLQDESFTR.G | 2 |

---

|  |  |  |  |  |  |  |  |  |
| --- | --- | --- | --- | --- | --- | --- | --- | --- |
| U | *gi|167234419|ref|NP\_0* | 25 | 30 | 25.7% | 955 | 108666 | 10.2 | thyroid hormone receptor associated protein 3 [Homo sapiens] |

| Filename XCorr DeltCN Conf% ObsM+H+ CalcM+H+ SpR ZScore Ion% # Sequence  | | | | | | | | | | | | |
| --- | --- | --- | --- | --- | --- | --- | --- | --- | --- | --- | --- | --- |
| \* | pDK268\_112012\_01.03135.03135.2 | 3.647 | 0.3455 | 100.0% | 1746.3722 | 1746.7446 | 1 | 7.189 | 56.2% | 1 | K.DSRPSQAAGDNQGDEAK.E | 2 |
| \* | pDK268\_112012\_01.03129.03129.3 | 4.7662 | 0.4349 | 100.0% | 1746.5343 | 1746.7446 | 2 | 7.781 | 43.8% | 1 | K.DSRPSQAAGDNQGDEAK.E | 3 |
| \* | pDK268\_112012\_01.05741.05741.3 | 3.8735 | 0.3263 | 100.0% | 2056.3145 | 2056.1528 | 1 | 5.762 | 38.2% | 1 | K.ASESSKPWPDATYGTGSASR.A | 3 |
| \* | pDK268\_112012\_01.05068.05068.2 | 2.6213 | 0.306 | 99.9% | 1017.4522 | 1017.1271 | 5 | 5.811 | 66.7% | 1 | R.ASAVSELSPR.E | 2 |
| \* | pDK268\_112012\_01.06444.06444.2 | 2.5542 | 0.2062 | 99.7% | 986.2322 | 985.17163 | 8 | 5.295 | 68.8% | 1 | K.SPLQSVVVR.R | 2 |
| \* | pDK268\_112012\_01.03230.03230.3 | 3.2213 | 0.3398 | 100.0% | 1966.7344 | 1968.1277 | 3 | 5.686 | 36.7% | 1 | R.YLEEQKTENGKDKEQK.Q | 3 |
| \* | pDK268\_112012\_01.03171.03171.2 | 3.6249 | 0.3755 | 100.0% | 1967.4321 | 1968.1277 | 1 | 6.033 | 73.3% | 1 | R.YLEEQKTENGKDKEQK.Q | 2 |
| \* | pDK268\_112012\_02.03875.03875.2 | 2.8001 | 0.5221 | 100.0% | 1141.3922 | 1141.1797 | 1 | 8.241 | 68.2% | 1 | K.GSFSDTGLGDGK.M | 2 |
| \* | pDK268\_112012\_01.04004.04004.3 | 4.8181 | 0.3199 | 100.0% | 1982.9944 | 1983.1317 | 1 | 6.489 | 53.3% | 2 | K.MADFHKEEMDDQDKDK.A | 3 |
| \* | pDK268\_112012\_01.07877.07877.2 | 4.1345 | 0.4939 | 100.0% | 1490.1721 | 1489.7141 | 1 | 8.726 | 76.9% | 1 | K.SGKWEGLVYAPPGK.E | 2 |
| \* | pDK268\_112012\_01.05795.05795.2 | 4.8466 | 0.4683 | 100.0% | 1623.1721 | 1623.7136 | 1 | 8.783 | 70.8% | 1 | R.KTEELEEESFPER.S | 2 |
| \* | pDK268\_112012\_01.05862.05862.3 | 2.7102 | 0.2932 | 99.6% | 1623.5044 | 1623.7136 | 1 | 5.059 | 45.8% | 1 | R.KTEELEEESFPER.S | 3 |
| \* | pDK268\_112012\_01.07092.07092.2 | 3.2822 | 0.4083 | 100.0% | 1495.0721 | 1495.5396 | 1 | 7.681 | 77.3% | 1 | K.TEELEEESFPER.S | 2 |
| \* | pDK268\_112012\_01.10236.10236.3 | 3.6875 | 0.3627 | 100.0% | 2152.9143 | 2151.3557 | 1 | 5.837 | 38.9% | 2 | R.MDSFDEDLARPSGLLAQER.K | 3 |
| \* | pDK268\_112012\_01.06171.06171.2 | 4.053 | 0.4859 | 100.0% | 1530.1921 | 1530.6835 | 1 | 7.838 | 70.8% | 2 | R.SIFQHIQSAQSQR.S | 2 |
| \* | pDK268\_112012\_01.06106.06106.3 | 3.4562 | 0.2739 | 100.0% | 1531.1643 | 1530.6835 | 2 | 5.328 | 41.7% | 2 | R.SIFQHIQSAQSQR.S | 3 |
| \* | pDK268\_112012\_01.09917.09917.3 | 5.0974 | 0.4989 | 100.0% | 2042.9043 | 2043.3762 | 1 | 8.888 | 44.1% | 2 | R.SPSELFAQHIVTIVHHVK.E | 3 |
| \* | pDK268\_112012\_01.04086.04086.2 | 3.3758 | 0.4528 | 100.0% | 1625.0721 | 1625.7588 | 1 | 8.246 | 73.1% | 1 | K.EHHFGSSGMTLHER.F | 2 |
| \* | pDK268\_112012\_01.04078.04078.3 | 3.2426 | 0.3865 | 100.0% | 1625.8143 | 1625.7588 | 90 | 6.186 | 34.6% | 1 | K.EHHFGSSGMTLHER.F | 3 |
| \* | pDK268\_112012\_01.03365.03365.2 | 2.3986 | 0.2953 | 99.7% | 1458.1721 | 1458.5486 | 1 | 6.744 | 77.3% | 1 | K.HGLAHDEMKS\*PR.E | 2 |
| \* | pDK268\_112012\_01.04948.04948.2 | 2.3412 | 0.1323 | 97.5% | 1121.3522 | 1121.2352 | 1 | 4.133 | 75.0% | 1 | K.YKDDPVDLR.L | 2 |
| \* | pDK268\_112012\_02.04829.04829.3 | 4.329 | 0.4836 | 100.0% | 2380.8245 | 2381.379 | 1 | 7.991 | 39.5% | 1 | K.AEEYTEETEEREESTTGFDK.S | 3 |
| \* | pDK268\_112012\_01.06260.06260.2 | 3.359 | 0.3864 | 100.0% | 1693.3522 | 1693.7667 | 2 | 6.707 | 70.8% | 1 | R.NREEEWDPEYTPK.S | 2 |
| \* | pDK268\_112012\_02.04494.04494.3 | 3.2925 | 0.1986 | 99.1% | 1694.2444 | 1693.7667 | 409 | 4.594 | 31.2% | 1 | R.NREEEWDPEYTPK.S | 3 |
|  | pDK268\_112012\_01.07530.07530.2 | 2.4273 | 0.3787 | 99.9% | 1423.1921 | 1423.4755 | 3 | 6.045 | 55.0% | 1 | R.EEEWDPEYTPK.S | 22 |

Similarities:
gi|7661958|ref|NP\_055(1:24)  

---

|  |  |  |  |  |  |  |  |  |
| --- | --- | --- | --- | --- | --- | --- | --- | --- |
| U | *gi|142976675|ref|NP\_0* | 12 | 25 | 25.7% | 713 | 77745 | 5.3 | nuclear autoantigen isoform 2 [Homo sapiens] |

| Filename XCorr DeltCN Conf% ObsM+H+ CalcM+H+ SpR ZScore Ion% # Sequence  | | | | | | | | | | | | |
| --- | --- | --- | --- | --- | --- | --- | --- | --- | --- | --- | --- | --- |
|  | pDK268\_112012\_01.05606.05606.2 | 3.6379 | 0.4602 | 100.0% | 1238.0721 | 1238.3397 | 1 | 7.747 | 90.0% | 2 | K.YGTELNQGDLK.M | 22 |
|  | pDK268\_112012\_01.08967.08967.3 | 5.764 | 0.4153 | 100.0% | 2966.9043 | 2968.2183 | 1 | 8.056 | 37.0% | 2 | K.MPTFESEETKDTEAPTAPQNSQLTWK.Q | 33 |
|  | pDK268\_112012\_02.08416.08416.2 | 3.386 | 0.2692 | 99.9% | 1912.8522 | 1914.1223 | 1 | 6.012 | 56.7% | 2 | R.QYLQEVGYTDTILDVR.S | 22 |
|  | pDK268\_112012\_01.08112.08112.2 | 4.7283 | 0.5606 | 100.0% | 1732.3922 | 1732.8853 | 1 | 9.819 | 65.6% | 1 | R.SLLGLSNSEPNGSVETK.N | 22 |
|  | pDK268\_112012\_01.09456.09456.2 | 3.6265 | 0.3459 | 100.0% | 1400.2522 | 1399.5437 | 1 | 6.151 | 75.0% | 1 | K.NLEQILNGGESPK.Q | 22 |
| \* | pDK268\_112012\_01.10310.10310.2 | 4.1213 | 0.6113 | 100.0% | 1980.3722 | 1981.0825 | 1 | 10.129 | 36.8% | 1 | R.SSGDGTEWAEPITFPSGGGK.S | 2 |
|  | pDK268\_112012\_02.07255.07255.3 | 5.8819 | 0.4704 | 100.0% | 2275.2844 | 2275.6096 | 1 | 8.501 | 45.0% | 5 | R.ALAFHPVEPVLVTASEDHTLK.L | 33 |
|  | pDK268\_112012\_01.10842.10842.2 | 4.3577 | 0.3601 | 100.0% | 1499.2522 | 1498.6757 | 1 | 7.263 | 75.0% | 2 | K.SASLDVEPIYTFR.A | 22 |
|  | pDK268\_112012\_01.06464.06464.3 | 4.6023 | 0.4214 | 100.0% | 1873.1344 | 1873.1191 | 1 | 7.262 | 42.2% | 3 | R.VVSHPTLPVTITAHEDR.H | 33 |
|  | pDK268\_112012\_01.07750.07750.3 | 3.0774 | 0.4079 | 100.0% | 1867.2843 | 1868.0966 | 1 | 6.131 | 40.0% | 1 | R.KKLDESIYDVAFHSSK.A | 33 |
|  | pDK268\_112012\_01.08663.08663.3 | 3.9623 | 0.5427 | 100.0% | 1738.0144 | 1739.9225 | 1 | 8.433 | 48.2% | 1 | K.KLDESIYDVAFHSSK.A | 33 |
|  | pDK268\_112012\_01.06497.06497.2 | 3.9442 | 0.4314 | 100.0% | 1222.1522 | 1222.3837 | 1 | 8.664 | 83.3% | 4 | K.AYIASAGADALAK.V | 22 |

Similarities:
gi|142976686|ref|NP\_0(11:1)  

---

|  |  |  |  |  |  |  |  |  |
| --- | --- | --- | --- | --- | --- | --- | --- | --- |
| U | *gi|4826659|ref|NP\_004* | 6 | 8 | 25.7% | 272 | 30629 | 6.0 | F-actin capping protein beta subunit [Homo sapiens] |

| Filename XCorr DeltCN Conf% ObsM+H+ CalcM+H+ SpR ZScore Ion% # Sequence  | | | | | | | | | | | | |
| --- | --- | --- | --- | --- | --- | --- | --- | --- | --- | --- | --- | --- |
| \* | pDK268\_112012\_01.03695.03695.2 | 2.5312 | 0.2174 | 99.7% | 1109.5122 | 1109.314 | 4 | 4.183 | 75.0% | 1 | R.RLPPQQIEK.N | 2 |
| \* | pDK268\_112012\_01.07371.07371.2 | 3.8112 | 0.2673 | 100.0% | 1698.0922 | 1697.8448 | 1 | 5.708 | 76.9% | 1 | R.KLEVEANNAFDQYR.D | 2 |
| \* | pDK268\_112012\_01.06688.06688.2 | 4.1848 | 0.4632 | 100.0% | 1338.1322 | 1338.4784 | 1 | 7.636 | 73.1% | 3 | K.SGSGTMNLGGSLTR.Q | 2 |
| \* | pDK268\_112012\_01.09104.09104.2 | 3.0559 | 0.3347 | 100.0% | 1172.3522 | 1172.3232 | 12 | 6.688 | 66.7% | 1 | R.STLNEIYFGK.T | 2 |
| \* | pDK268\_112012\_01.04079.04079.2 | 1.7284 | 0.3547 | 98.6% | 896.0122 | 895.9878 | 56 | 5.486 | 71.4% | 1 | R.SVQTFADK.S | 2 |
| \* | pDK268\_112012\_01.07894.07894.3 | 3.0826 | 0.3538 | 100.0% | 1686.1743 | 1686.9463 | 1 | 5.54 | 41.1% | 1 | K.SKQEALKNDLVEALK.R | 3 |

---

|  |  |  |  |  |  |  |  |  |
| --- | --- | --- | --- | --- | --- | --- | --- | --- |
| U | *gi|13654278|ref|NP\_11* | 2 | 2 | 25.7% | 109 | 12349 | 10.2 | SRA stem-loop-interacting RNA-binding protein [Homo sapiens] |

| Filename XCorr DeltCN Conf% ObsM+H+ CalcM+H+ SpR ZScore Ion% # Sequence  | | | | | | | | | | | | |
| --- | --- | --- | --- | --- | --- | --- | --- | --- | --- | --- | --- | --- |
| \* | pDK268\_112012\_02.08508.08508.2 | 3.6732 | 0.3891 | 100.0% | 1565.3722 | 1565.7257 | 1 | 6.744 | 65.4% | 1 | R.GLGWVQFSSEEGLR.N | 2 |
| \* | pDK268\_112012\_01.06622.06622.2 | 4.1539 | 0.189 | 100.0% | 1579.3922 | 1579.7532 | 1 | 7.099 | 69.2% | 1 | R.NALQQENHIIDGVK.V | 2 |

---

|  |  |  |  |  |  |  |  |  |
| --- | --- | --- | --- | --- | --- | --- | --- | --- |
| U | *gi|142976686|ref|NP\_0* | 13 | 26 | 25.5% | 797 | 87209 | 5.4 | nuclear autoantigen isoform 1 [Homo sapiens] |

| Filename XCorr DeltCN Conf% ObsM+H+ CalcM+H+ SpR ZScore Ion% # Sequence  | | | | | | | | | | | | |
| --- | --- | --- | --- | --- | --- | --- | --- | --- | --- | --- | --- | --- |
|  | pDK268\_112012\_01.05606.05606.2 | 3.6379 | 0.4602 | 100.0% | 1238.0721 | 1238.3397 | 1 | 7.747 | 90.0% | 2 | K.YGTELNQGDLK.M | 22 |
|  | pDK268\_112012\_01.08967.08967.3 | 5.764 | 0.4153 | 100.0% | 2966.9043 | 2968.2183 | 1 | 8.056 | 37.0% | 2 | K.MPTFESEETKDTEAPTAPQNSQLTWK.Q | 33 |
|  | pDK268\_112012\_02.08416.08416.2 | 3.386 | 0.2692 | 99.9% | 1912.8522 | 1914.1223 | 1 | 6.012 | 56.7% | 2 | R.QYLQEVGYTDTILDVR.S | 22 |
|  | pDK268\_112012\_01.08112.08112.2 | 4.7283 | 0.5606 | 100.0% | 1732.3922 | 1732.8853 | 1 | 9.819 | 65.6% | 1 | R.SLLGLSNSEPNGSVETK.N | 22 |
|  | pDK268\_112012\_01.09456.09456.2 | 3.6265 | 0.3459 | 100.0% | 1400.2522 | 1399.5437 | 1 | 6.151 | 75.0% | 1 | K.NLEQILNGGESPK.Q | 22 |
| \* | pDK268\_112012\_01.13486.13486.3 | 4.186 | 0.4368 | 100.0% | 2783.3943 | 2784.1133 | 1 | 6.452 | 31.2% | 1 | K.LYDMIADLGDDELPHIPSGIINQSR.S | 3 |
| \* | pDK268\_112012\_01.07347.07347.2 | 2.9876 | 0.412 | 100.0% | 1490.1721 | 1490.6097 | 1 | 6.936 | 50.0% | 1 | R.AEEAEPITFPSGGGK.S | 2 |
|  | pDK268\_112012\_02.07255.07255.3 | 5.8819 | 0.4704 | 100.0% | 2275.2844 | 2275.6096 | 1 | 8.501 | 45.0% | 5 | R.ALAFHPVEPVLVTASEDHTLK.L | 33 |
|  | pDK268\_112012\_01.10842.10842.2 | 4.3577 | 0.3601 | 100.0% | 1499.2522 | 1498.6757 | 1 | 7.263 | 75.0% | 2 | K.SASLDVEPIYTFR.A | 22 |
|  | pDK268\_112012\_01.06464.06464.3 | 4.6023 | 0.4214 | 100.0% | 1873.1344 | 1873.1191 | 1 | 7.262 | 42.2% | 3 | R.VVSHPTLPVTITAHEDR.H | 33 |
|  | pDK268\_112012\_01.07750.07750.3 | 3.0774 | 0.4079 | 100.0% | 1867.2843 | 1868.0966 | 1 | 6.131 | 40.0% | 1 | R.KKLDESIYDVAFHSSK.A | 33 |
|  | pDK268\_112012\_01.08663.08663.3 | 3.9623 | 0.5427 | 100.0% | 1738.0144 | 1739.9225 | 1 | 8.433 | 48.2% | 1 | K.KLDESIYDVAFHSSK.A | 33 |
|  | pDK268\_112012\_01.06497.06497.2 | 3.9442 | 0.4314 | 100.0% | 1222.1522 | 1222.3837 | 1 | 8.664 | 83.3% | 4 | K.AYIASAGADALAK.V | 22 |

Similarities:
gi|142976675|ref|NP\_0(11:2)  

---

|  |  |  |  |  |  |  |  |  |
| --- | --- | --- | --- | --- | --- | --- | --- | --- |
| U | *gi|209862851|ref|NP\_0* | 11 | 14 | 25.4% | 630 | 70811 | 5.6 | plastin 3 [Homo sapiens] |
| U | *gi|7549809|ref|NP\_005* | 11 | 14 | 25.4% | 630 | 70811 | 5.6 | plastin 3 [Homo sapiens] |

| Filename XCorr DeltCN Conf% ObsM+H+ CalcM+H+ SpR ZScore Ion% # Sequence  | | | | | | | | | | | | |
| --- | --- | --- | --- | --- | --- | --- | --- | --- | --- | --- | --- | --- |
|  | pDK268\_112012\_01.08925.08925.2 | 3.0636 | 0.0585 | 97.6% | 1641.4722 | 1641.8822 | 1 | 5.736 | 61.5% | 1 | R.HVIPMNPNTDDLFK.A | 2 |
|  | pDK268\_112012\_01.10186.10186.2 | 3.8371 | 0.2972 | 100.0% | 1504.1522 | 1503.7106 | 7 | 7.195 | 58.3% | 2 | K.MINLSVPDTIDER.A | 2 |
|  | pDK268\_112012\_01.09579.09579.3 | 3.5703 | 0.3673 | 100.0% | 1415.7544 | 1415.6781 | 1 | 6.543 | 50.0% | 1 | K.AYFHLLNQIAPK.G | 33 |
|  | pDK268\_112012\_01.09581.09581.2 | 3.4401 | 0.3525 | 100.0% | 1416.3121 | 1415.6781 | 1 | 7.549 | 63.6% | 2 | K.AYFHLLNQIAPK.G | 22 |
|  | pDK268\_112012\_02.06786.06786.3 | 3.317 | 0.2175 | 99.1% | 1868.6344 | 1869.0564 | 10 | 5.066 | 35.0% | 1 | R.IDINMSGFNETDDLKR.A | 3 |
|  | pDK268\_112012\_01.08302.08302.2 | 3.1792 | 0.4101 | 100.0% | 1460.0122 | 1459.6421 | 1 | 6.781 | 73.1% | 2 | R.QFVTPADVVSGNPK.L | 22 |
|  | pDK268\_112012\_01.14687.14687.2 | 2.3155 | 0.1927 | 97.7% | 1364.1721 | 1364.6304 | 12 | 5.687 | 54.5% | 1 | K.LNLAFVANLFNK.Y | 2 |
|  | pDK268\_112012\_01.11297.11297.3 | 4.9191 | 0.5293 | 100.0% | 2591.0942 | 2590.849 | 1 | 9.436 | 38.1% | 1 | K.YPALTKPENQDIDWTLLEGETR.E | 3 |
|  | pDK268\_112012\_01.16724.16724.3 | 5.4956 | 0.4855 | 100.0% | 3636.5344 | 3638.1382 | 1 | 8.431 | 30.8% | 1 | R.NWMNSLGVNPHVNHLYADLQDALVILQLYER.I | 3 |
|  | pDK268\_112012\_01.11868.11868.2 | 4.0624 | 0.4764 | 100.0% | 1565.4722 | 1565.7203 | 1 | 9.469 | 76.9% | 1 | R.YTLNVLEDLGDGQK.A | 2 |
|  | pDK268\_112012\_01.10974.10974.2 | 3.0123 | 0.3983 | 100.0% | 1429.5322 | 1429.5754 | 1 | 6.097 | 54.5% | 1 | K.ANDDIIVNWVNR.T | 2 |

Similarities:
gi|223718246|ref|NP\_0(3:8)  

---

|  |  |  |  |  |  |  |  |  |
| --- | --- | --- | --- | --- | --- | --- | --- | --- |
| U | *gi|4758012|ref|NP\_004* | 31 | 38 | 25.3% | 1675 | 191613 | 5.7 | clathrin heavy chain 1 [Homo sapiens] |

| Filename XCorr DeltCN Conf% ObsM+H+ CalcM+H+ SpR ZScore Ion% # Sequence  | | | | | | | | | | | | |
| --- | --- | --- | --- | --- | --- | --- | --- | --- | --- | --- | --- | --- |
|  | pDK268\_112012\_01.11998.11998.2 | 2.7921 | 0.4719 | 100.0% | 1237.1122 | 1237.4999 | 2 | 7.484 | 66.7% | 1 | K.TLQIFNIEMK.S | 2 |
| \* | pDK268\_112012\_01.08994.08994.2 | 3.0221 | 0.5707 | 100.0% | 1452.1721 | 1452.6245 | 1 | 9.157 | 72.7% | 1 | K.AHTMTDDVTFWK.W | 2 |
| \* | pDK268\_112012\_02.08941.08941.2 | 2.0651 | 0.3286 | 98.9% | 1500.7722 | 1500.741 | 1 | 5.353 | 45.8% | 1 | K.WLLLTGISAQQNR.V | 2 |
|  | pDK268\_112012\_01.08748.08748.2 | 2.4698 | 0.3325 | 99.8% | 1337.4521 | 1338.5646 | 2 | 5.573 | 59.1% | 1 | R.VVGAMQLYSVDR.K | 2 |
| \* | pDK268\_112012\_02.05256.05256.3 | 3.4195 | 0.3478 | 100.0% | 1845.2943 | 1846.096 | 1 | 6.693 | 35.9% | 1 | R.KVSQPIEGHAASFAQFK.M | 3 |
| \* | pDK268\_112012\_02.07771.07771.3 | 3.8581 | 0.4404 | 100.0% | 2355.2043 | 2354.6682 | 1 | 6.901 | 28.4% | 2 | R.ISGETIFVTAPHEATAGIIGVNR.K | 3 |
| \* | pDK268\_112012\_01.09893.09893.2 | 4.1905 | 0.4335 | 100.0% | 1305.5922 | 1305.4331 | 1 | 7.715 | 81.8% | 2 | R.NNLAGAEELFAR.K | 2 |
| \* | pDK268\_112012\_02.06063.06063.2 | 5.2136 | 0.4978 | 100.0% | 1758.9521 | 1759.9591 | 1 | 8.924 | 70.0% | 2 | R.KFNALFAQGNYSEAAK.V | 2 |
| \* | pDK268\_112012\_02.06052.06052.3 | 4.7585 | 0.3322 | 100.0% | 1759.6144 | 1759.9591 | 1 | 7.008 | 51.7% | 1 | R.KFNALFAQGNYSEAAK.V | 3 |
| \* | pDK268\_112012\_02.06744.06744.2 | 4.794 | 0.567 | 100.0% | 1631.1522 | 1631.785 | 1 | 9.727 | 82.1% | 1 | K.FNALFAQGNYSEAAK.V | 2 |
|  | pDK268\_112012\_01.15125.15125.2 | 3.2978 | 0.4411 | 100.0% | 1480.4122 | 1480.7496 | 1 | 7.433 | 72.7% | 1 | K.VGYTPDWIFLLR.N | 2 |
| \* | pDK268\_112012\_01.14032.14032.3 | 6.6913 | 0.5163 | 100.0% | 3129.4443 | 3130.6248 | 1 | 9.596 | 35.6% | 1 | R.LLEMNLMHAPQVADAILGNQMFTHYDR.A | 3 |
| \* | pDK268\_112012\_01.08584.08584.3 | 2.9661 | 0.36 | 100.0% | 1622.3944 | 1621.8333 | 1 | 5.926 | 45.8% | 1 | R.ALEHFTDLYDIKR.A | 3 |
| \* | pDK268\_112012\_01.13880.13880.2 | 1.8191 | 0.232 | 95.8% | 1111.6322 | 1111.3727 | 1 | 4.757 | 87.5% | 1 | K.LLLPWLEAR.I | 2 |
| \* | pDK268\_112012\_01.04860.04860.2 | 3.1391 | 0.2992 | 100.0% | 1335.9122 | 1335.416 | 4 | 6.352 | 70.0% | 1 | K.IYIDSNNNPER.F | 2 |
| \* | pDK268\_112012\_02.08122.08122.3 | 4.9477 | 0.4699 | 100.0% | 2160.5044 | 2160.4375 | 1 | 7.486 | 45.6% | 1 | R.RKDPELWGSVLLESNPYR.R | 3 |
|  | pDK268\_112012\_01.16821.16821.2 | 4.0237 | 0.4349 | 100.0% | 1947.2522 | 1948.2819 | 1 | 7.568 | 59.4% | 2 | K.AFMTADLPNELIELLEK.I | 2 |
|  | pDK268\_112012\_01.13737.13737.2 | 4.0127 | 0.4402 | 100.0% | 1354.3522 | 1354.6763 | 1 | 8.423 | 72.7% | 1 | R.NLQNLLILTAIK.A | 2 |
| \* | pDK268\_112012\_01.14033.14033.3 | 6.9752 | 0.5016 | 100.0% | 2369.7844 | 2369.6829 | 1 | 9.221 | 45.0% | 2 | R.KFDVNTSAVQVLIEHIGNLDR.A | 3 |
| \* | pDK268\_112012\_01.15204.15204.2 | 2.6758 | 0.3986 | 99.9% | 2241.2922 | 2241.5088 | 2 | 5.659 | 34.2% | 1 | K.FDVNTSAVQVLIEHIGNLDR.A | 2 |
| \* | pDK268\_112012\_01.10408.10408.3 | 3.3713 | 0.2134 | 98.3% | 2465.3044 | 2465.6848 | 1 | 5.269 | 34.5% | 1 | R.LAELEEFINGPNNAHIQQVGDR.C | 3 |
| \* | pDK268\_112012\_01.08624.08624.2 | 2.9975 | 0.4404 | 100.0% | 1296.4722 | 1297.4563 | 1 | 8.234 | 75.0% | 2 | K.LLYNNVSNFGR.L | 2 |
| \* | pDK268\_112012\_01.09305.09305.2 | 4.8177 | 0.555 | 100.0% | 1971.5322 | 1972.2083 | 1 | 9.372 | 63.9% | 1 | R.LASTLVHLGEYQAAVDGAR.K | 2 |
| \* | pDK268\_112012\_02.06873.06873.3 | 3.9162 | 0.4785 | 100.0% | 1972.8243 | 1972.2083 | 1 | 7.937 | 41.7% | 2 | R.LASTLVHLGEYQAAVDGAR.K | 3 |
|  | pDK268\_112012\_02.09675.09675.2 | 3.4726 | 0.4405 | 100.0% | 1712.4122 | 1713.0626 | 1 | 7.742 | 60.7% | 1 | R.AHMGMFTELAILYSK.F | 2 |
| \* | pDK268\_112012\_01.12866.12866.2 | 4.7677 | 0.5331 | 100.0% | 2355.672 | 2356.551 | 1 | 9.86 | 52.6% | 1 | K.SVNESLNNLFITEEDYQALR.T | 2 |
| \* | pDK268\_112012\_01.10593.10593.2 | 2.9709 | 0.0669 | 97.1% | 1944.3722 | 1944.0648 | 1 | 3.831 | 62.5% | 1 | R.TSIDAYDNFDNISLAQR.L | 2 |
| \* | pDK268\_112012\_01.06052.06052.3 | 3.7594 | 0.2602 | 100.0% | 1314.3544 | 1314.5272 | 33 | 5.468 | 44.4% | 1 | R.LEKHELIEFR.R | 3 |
| \* | pDK268\_112012\_01.16102.16102.3 | 5.4283 | 0.5011 | 100.0% | 3389.0942 | 3389.715 | 1 | 7.386 | 35.2% | 1 | K.DAMQYASESKDTELAEELLQWFLQEEKR.E | 3 |
| \* | pDK268\_112012\_01.15400.15400.2 | 3.6649 | 0.5184 | 100.0% | 1985.8522 | 1986.4152 | 1 | 9.94 | 53.3% | 1 | R.HNIMDFAMPYFIQVMK.E | 2 |
| \* | pDK268\_112012\_01.04706.04706.2 | 2.5944 | 0.1783 | 99.2% | 1233.7722 | 1233.3641 | 22 | 4.671 | 65.0% | 1 | K.VDKLDASESLR.K | 2 |

---

|  |  |  |  |  |  |  |  |  |
| --- | --- | --- | --- | --- | --- | --- | --- | --- |
| U | *gi|16753227|ref|NP\_00* | 6 | 6 | 25.0% | 288 | 32728 | 10.6 | ribosomal protein L6 [Homo sapiens] |
| U | *gi|67189747|ref|NP\_00* | 6 | 6 | 25.0% | 288 | 32728 | 10.6 | ribosomal protein L6 [Homo sapiens] |

| Filename XCorr DeltCN Conf% ObsM+H+ CalcM+H+ SpR ZScore Ion% # Sequence  | | | | | | | | | | | | |
| --- | --- | --- | --- | --- | --- | --- | --- | --- | --- | --- | --- | --- |
|  | pDK268\_112012\_01.04151.04151.2 | 2.8634 | 0.4491 | 100.0% | 1285.0122 | 1285.5266 | 1 | 6.793 | 62.5% | 1 | K.VLATVTKPVGGDK.N | 2 |
|  | pDK268\_112012\_01.05873.05873.2 | 1.7898 | 0.2969 | 97.4% | 1141.1522 | 1140.2377 | 1 | 4.859 | 87.5% | 1 | R.YYPTEDVPR.K | 2 |
|  | pDK268\_112012\_01.15968.15968.2 | 4.5286 | 0.5594 | 100.0% | 1526.4521 | 1526.8601 | 1 | 9.446 | 78.6% | 1 | R.ASITPGTILIILTGR.H | 2 |
|  | pDK268\_112012\_01.14040.14040.2 | 3.8134 | 0.388 | 100.0% | 1764.9722 | 1765.1497 | 1 | 7.435 | 56.2% | 1 | K.QLASGLLLVTGPLVLNR.V | 2 |
|  | pDK268\_112012\_01.05472.05472.2 | 2.2113 | 0.2431 | 99.5% | 994.4922 | 995.1228 | 1 | 5.263 | 78.6% | 1 | K.HLTDAYFK.K | 2 |
|  | pDK268\_112012\_01.09620.09620.2 | 2.5593 | 0.2933 | 99.9% | 1159.2522 | 1159.3738 | 2 | 5.644 | 83.3% | 1 | K.AIPQLQGYLR.S | 2 |

---

|  |  |  |  |  |  |  |  |  |
| --- | --- | --- | --- | --- | --- | --- | --- | --- |
| U | *gi|154759259|ref|NP\_0* | 36 | 40 | 24.6% | 2472 | 284538 | 5.3 | spectrin, alpha, non-erythrocytic 1 (alpha-fodrin) isoform 2 [Homo sapiens] |
| U | *gi|194595509|ref|NP\_0* | 36 | 40 | 24.6% | 2477 | 285093 | 5.4 | spectrin, alpha, non-erythrocytic 1 (alpha-fodrin) isoform 1 [Homo sapiens] |

| Filename XCorr DeltCN Conf% ObsM+H+ CalcM+H+ SpR ZScore Ion% # Sequence  | | | | | | | | | | | | |
| --- | --- | --- | --- | --- | --- | --- | --- | --- | --- | --- | --- | --- |
|  | pDK268\_112012\_01.06129.06129.3 | 3.5188 | 0.3796 | 100.0% | 1867.4343 | 1869.0155 | 1 | 6.181 | 44.6% | 1 | K.KFEEFQTDMAAHEER.V | 3 |
|  | pDK268\_112012\_02.04219.04219.3 | 3.2322 | 0.2417 | 99.6% | 1679.0343 | 1678.9291 | 5 | 5.271 | 37.5% | 2 | R.LQQSHPLSATQIQVK.R | 3 |
|  | pDK268\_112012\_01.15724.15724.2 | 4.6399 | 0.4098 | 100.0% | 2127.3323 | 2128.344 | 1 | 9.928 | 65.0% | 1 | K.ALINADELASDVAGAEALLDR.H | 2 |
|  | pDK268\_112012\_01.08831.08831.3 | 3.5201 | 0.1991 | 98.5% | 2148.3245 | 2148.2505 | 1 | 4.969 | 43.8% | 1 | K.SADESGQALLAAGHYASDEVR.E | 3 |
|  | pDK268\_112012\_01.16368.16368.2 | 3.6623 | 0.4394 | 100.0% | 2419.6921 | 2420.6758 | 1 | 8.075 | 47.6% | 1 | K.QEAFLLNEDLGDSLDSVEALLK.K | 2 |
|  | pDK268\_112012\_01.08778.08778.2 | 2.5757 | 0.2551 | 99.8% | 1109.1122 | 1109.2645 | 187 | 5.93 | 50.0% | 1 | K.ITALDEFATK.L | 2 |
|  | pDK268\_112012\_02.04980.04980.3 | 3.0237 | 0.3096 | 100.0% | 1775.0643 | 1775.9769 | 4 | 4.708 | 35.7% | 1 | K.LIQNNHYAMEDVATR.R | 3 |
|  | pDK268\_112012\_01.12072.12072.2 | 4.4621 | 0.462 | 100.0% | 1709.4521 | 1708.9169 | 1 | 7.986 | 80.8% | 1 | R.AQLADSFHLQQFFR.D | 2 |
|  | pDK268\_112012\_02.05026.05026.3 | 3.3095 | 0.2376 | 99.6% | 1715.8143 | 1714.9187 | 5 | 5.883 | 37.5% | 1 | K.LIDVNHYAKDEVAAR.M | 3 |
|  | pDK268\_112012\_01.15678.15678.3 | 4.6401 | 0.5649 | 100.0% | 3849.5942 | 3850.187 | 1 | 8.554 | 26.6% | 1 | R.NVEDIELWLYEVEGHLASDDYGKDLTNVQNLQK.K | 3 |
|  | pDK268\_112012\_02.04295.04295.3 | 4.6159 | 0.485 | 100.0% | 1546.8844 | 1546.6829 | 1 | 8.159 | 51.9% | 1 | K.HALLEADVAAHQDR.I | 3 |
|  | pDK268\_112012\_01.03396.03396.2 | 1.9602 | 0.2368 | 96.8% | 1216.3922 | 1216.3365 | 1 | 4.167 | 70.0% | 1 | R.EKEPIAASTNR.G | 2 |
|  | pDK268\_112012\_02.05239.05239.2 | 3.3252 | 0.3573 | 100.0% | 1704.5521 | 1704.8517 | 1 | 6.321 | 56.7% | 1 | K.GNAMVEEGHFAAEDVK.A | 2 |
|  | pDK268\_112012\_01.08628.08628.3 | 4.0994 | 0.2987 | 100.0% | 2495.0044 | 2495.6997 | 1 | 6.636 | 36.4% | 1 | R.EKEPIVGSTDYGKDEDSAEALLK.K | 3 |
|  | pDK268\_112012\_01.12005.12005.3 | 3.5337 | 0.2187 | 98.8% | 2755.7644 | 2755.0076 | 23 | 3.956 | 22.8% | 1 | R.QQVAPTDDETGKELVLALYDYQEK.S | 3 |
|  | pDK268\_112012\_01.12378.12378.3 | 4.5496 | 0.467 | 100.0% | 2032.7344 | 2033.3348 | 1 | 7.766 | 45.3% | 1 | K.KGDILTLLNSTNKDWWK.V | 3 |
|  | pDK268\_112012\_01.10390.10390.2 | 3.3344 | 0.2585 | 100.0% | 1502.2922 | 1502.6238 | 4 | 5.348 | 59.1% | 1 | R.EANELQQWINEK.E | 2 |
|  | pDK268\_112012\_01.08747.08747.3 | 3.625 | 0.3495 | 100.0% | 2328.6243 | 2329.4912 | 1 | 6.834 | 35.0% | 1 | K.NQALNTDNYGHDLASVQALQR.K | 3 |
|  | pDK268\_112012\_01.13572.13572.2 | 4.5274 | 0.3655 | 100.0% | 2175.632 | 2174.413 | 1 | 6.566 | 55.0% | 1 | R.GLVSSDELAKDVTGAEALLER.H | 2 |
|  | pDK268\_112012\_01.14169.14169.3 | 4.1621 | 0.491 | 100.0% | 1972.7644 | 1973.2822 | 1 | 8.265 | 38.9% | 1 | K.IAALQAFADQLIAAGHYAK.G | 3 |
|  | pDK268\_112012\_02.04301.04301.3 | 3.5846 | 0.3072 | 100.0% | 1608.9543 | 1609.6981 | 10 | 5.874 | 38.5% | 1 | K.HQAFEAELHANADR.I | 3 |
|  | pDK268\_112012\_01.04804.04804.2 | 3.5361 | 0.1681 | 99.9% | 1609.7122 | 1609.6981 | 2 | 6.652 | 69.2% | 1 | K.HQAFEAELHANADR.I | 2 |
|  | pDK268\_112012\_02.06238.06238.2 | 2.381 | 0.2296 | 98.8% | 1304.2722 | 1304.5042 | 12 | 4.801 | 54.5% | 1 | R.GVIDMGNSLIER.G | 2 |
|  | pDK268\_112012\_02.09381.09381.2 | 3.9515 | 0.4119 | 100.0% | 1631.0721 | 1631.915 | 1 | 7.509 | 73.1% | 3 | R.LAALADQWQFLVQK.S | 2 |
|  | pDK268\_112012\_02.04719.04719.3 | 4.0622 | 0.3466 | 100.0% | 1634.9043 | 1634.7458 | 2 | 7.006 | 42.3% | 1 | K.HQLLEADISAHEDR.L | 3 |
|  | pDK268\_112012\_01.15460.15460.2 | 4.3835 | 0.526 | 100.0% | 2209.372 | 2210.5476 | 1 | 10.13 | 47.5% | 1 | K.MTLVASEDYGDTLAAIQGLLK.K | 2 |
|  | pDK268\_112012\_01.16143.16143.3 | 5.769 | 0.3759 | 100.0% | 3235.9443 | 3234.6335 | 1 | 8.201 | 30.2% | 1 | K.QETFDAGLQAFQQEGIANITALKDQLLAAK.H | 3 |
|  | pDK268\_112012\_01.07311.07311.2 | 2.8968 | 0.3241 | 100.0% | 1216.9521 | 1217.3701 | 1 | 5.894 | 65.0% | 1 | R.WSQLLANSAAR.K | 2 |
|  | pDK268\_112012\_01.11403.11403.2 | 3.3546 | 0.4557 | 100.0% | 1310.9521 | 1311.5638 | 1 | 8.131 | 75.0% | 1 | R.KVEDLFLTFAK.K | 2 |
|  | pDK268\_112012\_01.11703.11703.2 | 5.6991 | 0.5217 | 100.0% | 1952.4521 | 1953.0728 | 1 | 9.352 | 76.5% | 1 | R.SSLSSAQADFNQLAELDR.Q | 2 |
|  | pDK268\_112012\_01.15927.15927.2 | 4.288 | 0.5809 | 100.0% | 2331.2322 | 2332.5923 | 1 | 11.151 | 50.0% | 1 | R.VASNPYTWFTMEALEETWR.N | 2 |
|  | pDK268\_112012\_01.08645.08645.3 | 3.4506 | 0.3069 | 100.0% | 2241.9844 | 2242.4177 | 5 | 5.795 | 32.4% | 1 | R.QEFAQHANAFHQWIQETR.T | 3 |
|  | pDK268\_112012\_02.08443.08443.3 | 4.9498 | 0.4309 | 100.0% | 2577.2344 | 2577.831 | 1 | 6.844 | 36.9% | 1 | K.YTEHSTVGLAQQWDQLDQLGMR.M | 3 |
|  | pDK268\_112012\_01.04886.04886.2 | 4.0626 | 0.4839 | 100.0% | 1496.2322 | 1496.6842 | 1 | 7.694 | 77.3% | 1 | R.MQHNLEQQIQAR.N | 2 |
|  | pDK268\_112012\_01.08051.08051.3 | 4.6156 | 0.4598 | 100.0% | 2427.3542 | 2427.7166 | 1 | 7.541 | 42.5% | 1 | R.ALSSEGKPYVTKEELYQNLTR.E | 3 |
|  | pDK268\_112012\_01.12443.12443.2 | 2.6845 | 0.158 | 98.6% | 1590.2122 | 1588.757 | 1 | 4.791 | 66.7% | 2 | R.ELPTAFDYVEFTR.S | 2 |

---

|  |  |  |  |  |  |  |  |  |
| --- | --- | --- | --- | --- | --- | --- | --- | --- |
| U | *gi|14165435|ref|NP\_11* | 9 | 10 | 24.6% | 463 | 50976 | 5.5 | heterogeneous nuclear ribonucleoprotein K isoform b [Homo sapiens] |
| U | *gi|14165439|ref|NP\_00* | 9 | 10 | 24.6% | 464 | 51028 | 5.3 | heterogeneous nuclear ribonucleoprotein K isoform a [Homo sapiens] |
| U | *gi|14165437|ref|NP\_11* | 9 | 10 | 24.6% | 464 | 51028 | 5.3 | heterogeneous nuclear ribonucleoprotein K isoform a [Homo sapiens] |

| Filename XCorr DeltCN Conf% ObsM+H+ CalcM+H+ SpR ZScore Ion% # Sequence  | | | | | | | | | | | | |
| --- | --- | --- | --- | --- | --- | --- | --- | --- | --- | --- | --- | --- |
|  | pDK268\_112012\_01.06374.06374.2 | 2.7496 | 0.2312 | 99.9% | 1107.0122 | 1107.2238 | 3 | 4.745 | 81.2% | 1 | R.NTDEMVELR.I | 2 |
|  | pDK268\_112012\_01.06082.06082.2 | 3.1573 | 0.3929 | 100.0% | 1781.4122 | 1781.8302 | 1 | 7.36 | 56.2% | 1 | R.TDYNASVSVPDSSGPER.I | 2 |
|  | pDK268\_112012\_02.07075.07075.2 | 4.0632 | 0.5547 | 100.0% | 1519.3322 | 1519.8711 | 1 | 9.608 | 75.0% | 1 | R.LLIHQSLAGGIIGVK.G | 2 |
|  | pDK268\_112012\_02.07050.07050.3 | 3.8718 | 0.4495 | 100.0% | 1519.4944 | 1519.8711 | 2 | 7.219 | 46.4% | 1 | R.LLIHQSLAGGIIGVK.G | 3 |
|  | pDK268\_112012\_01.12686.12686.2 | 3.7952 | 0.4596 | 100.0% | 1341.2122 | 1341.6311 | 1 | 8.635 | 90.9% | 1 | K.IILDLISESPIK.G | 2 |
|  | pDK268\_112012\_01.10876.10876.2 | 4.4968 | 0.5509 | 100.0% | 1917.5122 | 1918.1974 | 1 | 10.377 | 58.3% | 2 | R.GSYGDLGGPIITTQVTIPK.D | 2 |
|  | pDK268\_112012\_02.05086.05086.3 | 2.9294 | 0.2247 | 97.6% | 2068.6143 | 2070.1772 | 13 | 5.083 | 27.8% | 1 | R.HESGASIKIDEPLEGSEDR.I | 3 |
|  | pDK268\_112012\_01.12833.12833.2 | 5.6991 | 0.513 | 100.0% | 2590.632 | 2590.9365 | 1 | 10.219 | 47.7% | 1 | R.IITITGTQDQIQNAQYLLQNSVK.Q | 2 |
|  | pDK268\_112012\_02.09243.09243.3 | 4.8037 | 0.537 | 100.0% | 2591.0344 | 2590.9365 | 1 | 9.331 | 33.0% | 1 | R.IITITGTQDQIQNAQYLLQNSVK.Q | 3 |

---

|  |  |  |  |  |  |  |  |  |
| --- | --- | --- | --- | --- | --- | --- | --- | --- |
| U | *gi|5902076|ref|NP\_008* | 7 | 8 | 24.6% | 248 | 27745 | 10.4 | splicing factor, arginine/serine-rich 1 isoform 1 [Homo sapiens] |

| Filename XCorr DeltCN Conf% ObsM+H+ CalcM+H+ SpR ZScore Ion% # Sequence  | | | | | | | | | | | | |
| --- | --- | --- | --- | --- | --- | --- | --- | --- | --- | --- | --- | --- |
|  | pDK268\_112012\_01.08834.08834.2 | 2.3849 | 0.277 | 99.6% | 1257.0122 | 1257.4752 | 51 | 5.331 | 55.0% | 1 | R.IYVGNLPPDIR.T | 2 |
|  | pDK268\_112012\_01.07652.07652.3 | 3.448 | 0.1031 | 97.5% | 1257.8043 | 1258.4137 | 1 | 3.626 | 63.9% | 1 | R.TKDIEDVFYK.Y | 3 |
|  | pDK268\_112012\_01.07610.07610.2 | 2.979 | 0.3611 | 100.0% | 1258.5122 | 1258.4137 | 1 | 6.257 | 77.8% | 2 | R.TKDIEDVFYK.Y | 2 |
|  | pDK268\_112012\_01.12605.12605.2 | 3.3798 | 0.5016 | 100.0% | 1565.5122 | 1565.7252 | 1 | 8.718 | 53.8% | 1 | R.GGPPFAFVEFEDPR.D | 2 |
|  | pDK268\_112012\_01.06393.06393.2 | 2.0018 | 0.1275 | 95.6% | 917.27216 | 917.0989 | 1 | 3.838 | 91.7% | 1 | R.LRVEFPR.S | 2 |
|  | pDK268\_112012\_01.04194.04194.2 | 2.5408 | 0.2704 | 99.9% | 1113.2322 | 1113.2738 | 1 | 5.417 | 87.5% | 1 | R.KEDMTYAVR.K | 2 |
| \* | pDK268\_112012\_01.03896.03896.2 | 2.6305 | 0.2529 | 99.8% | 1162.5721 | 1163.2322 | 1 | 6.165 | 88.9% | 1 | R.SHEGETAYIR.V | 2 |

---

|  |  |  |  |  |  |  |  |  |
| --- | --- | --- | --- | --- | --- | --- | --- | --- |
| U | *gi|51479145|ref|NP\_00* | 35 | 48 | 24.5% | 1849 | 208766 | 5.9 | brefeldin A-inhibited guanine nucleotide-exchange protein 1 [Homo sapiens] |

| Filename XCorr DeltCN Conf% ObsM+H+ CalcM+H+ SpR ZScore Ion% # Sequence  | | | | | | | | | | | | |
| --- | --- | --- | --- | --- | --- | --- | --- | --- | --- | --- | --- | --- |
| \* | pDK268\_112012\_02.04751.04751.3 | 3.3711 | 0.3435 | 100.0% | 2043.5343 | 2042.2993 | 5 | 5.402 | 32.9% | 1 | K.LIAYGHLTGNAPDSTTPGKK.L | 3 |
| \* | pDK268\_112012\_01.16174.16174.2 | 3.7271 | 0.4518 | 100.0% | 1479.4722 | 1478.793 | 1 | 8.245 | 66.7% | 2 | K.ATLTQMLNVIFAR.M | 2 |
| \* | pDK268\_112012\_01.04036.04036.2 | 3.6794 | 0.3249 | 100.0% | 1161.7122 | 1162.3031 | 2 | 6.134 | 83.3% | 1 | R.MENQALQEAK.Q | 2 |
|  | pDK268\_112012\_01.11236.11236.1 | 2.1735 | 0.3764 | 100.0% | 867.79 | 868.0232 | 2 | 5.872 | 66.7% | 1 | K.DAFLVFR.S | 11 |
| \* | pDK268\_112012\_01.19581.19581.3 | 3.3586 | 0.4618 | 100.0% | 2110.2244 | 2110.5913 | 1 | 6.335 | 38.9% | 1 | K.ILSLQLLLSILQNAGPIFR.T | 3 |
|  | pDK268\_112012\_01.03945.03945.2 | 2.2229 | 0.1256 | 97.8% | 789.8522 | 788.9194 | 29 | 4.99 | 66.7% | 1 | R.LVNDLSK.I | 22 |
| \* | pDK268\_112012\_02.07013.07013.2 | 4.8313 | 0.5049 | 100.0% | 1748.1721 | 1748.9489 | 1 | 8.773 | 73.3% | 1 | R.GSQELGMSNVQELSLR.K | 2 |
| \* | pDK268\_112012\_02.08393.08393.3 | 3.8391 | 0.4326 | 100.0% | 3701.5144 | 3702.9363 | 1 | 6.016 | 22.8% | 1 | R.YGSLNSLESTSSSGIGSYSTQMSGTDNPEQFEVLK.Q | 3 |
| \* | pDK268\_112012\_01.11352.11352.2 | 4.1022 | 0.4186 | 100.0% | 1418.7522 | 1419.6177 | 1 | 7.716 | 72.7% | 1 | K.EIIEQGIDLFNK.K | 2 |
| \* | pDK268\_112012\_01.13024.13024.3 | 6.8409 | 0.4959 | 100.0% | 3288.4443 | 3289.647 | 1 | 9.575 | 37.0% | 1 | K.RGIQYLQEQGMLGTTPEDIAQFLHQEER.L | 3 |
| \* | pDK268\_112012\_01.13842.13842.3 | 4.5785 | 0.3656 | 100.0% | 3133.0444 | 3133.4595 | 1 | 7.062 | 26.0% | 1 | R.GIQYLQEQGMLGTTPEDIAQFLHQEER.L | 3 |
| \* | pDK268\_112012\_01.15832.15832.3 | 3.8975 | 0.2723 | 100.0% | 3045.9844 | 3048.313 | 1 | 5.291 | 26.0% | 1 | K.FNKEVMYAYVDQHDFS\*GKDFVSALR.M | 3 |
| \* | pDK268\_112012\_01.11037.11037.3 | 4.218 | 0.4799 | 100.0% | 2578.9143 | 2578.8584 | 1 | 7.376 | 34.5% | 2 | K.EVMYAYVDQHDFSGKDFVSALR.M | 3 |
| \* | pDK268\_112012\_01.11448.11448.2 | 2.9054 | 0.3512 | 100.0% | 1622.8322 | 1623.9104 | 1 | 5.914 | 65.4% | 1 | R.MFLEGFRLPGEAQK.I | 2 |
| \* | pDK268\_112012\_01.14698.14698.3 | 6.1095 | 0.4218 | 100.0% | 2668.1042 | 2668.9604 | 1 | 6.303 | 40.2% | 1 | R.GINDSKDLPEEYLSAIYNEIAGKK.I | 3 |
| \* | pDK268\_112012\_01.16364.16364.2 | 4.6537 | 0.5092 | 100.0% | 1925.3922 | 1926.1302 | 1 | 8.652 | 62.5% | 2 | K.DLPEEYLSAIYNEIAGK.K | 2 |
| \* | pDK268\_112012\_01.13787.13787.3 | 2.7972 | 0.226 | 96.5% | 2539.4644 | 2539.4973 | 23 | 4.141 | 27.6% | 1 | K.ET#KELT#IPTKS\*S\*KQNVASEK.Q | 3 |
|  | pDK268\_112012\_02.06829.06829.2 | 2.667 | 0.1964 | 99.2% | 1639.1522 | 1639.9685 | 2 | 4.391 | 62.5% | 1 | R.RLLYNLEMEQMAK.T | 22 |
|  | pDK268\_112012\_01.10382.10382.2 | 4.1105 | 0.4186 | 100.0% | 1483.5122 | 1483.781 | 1 | 7.887 | 81.8% | 4 | R.LLYNLEMEQMAK.T | 22 |
|  | pDK268\_112012\_01.06569.06569.2 | 2.1792 | 0.2526 | 99.2% | 1006.4922 | 1007.1344 | 1 | 6.388 | 81.2% | 1 | R.DAYVQALAR.F | 22 |
| \* | pDK268\_112012\_01.12174.12174.2 | 3.4873 | 0.3429 | 100.0% | 1528.5122 | 1527.8163 | 1 | 7.276 | 65.4% | 1 | R.FTLLTVSSGITEMK.Q | 2 |
|  | pDK268\_112012\_01.11198.11198.3 | 5.3323 | 0.5255 | 100.0% | 2483.0344 | 2483.7861 | 1 | 8.351 | 40.5% | 4 | K.TLITVAHTDGNYLGNSWHEILK.C | 33 |
|  | pDK268\_112012\_01.11036.11036.2 | 3.5281 | 0.2082 | 100.0% | 1219.3121 | 1219.383 | 1 | 5.872 | 90.0% | 2 | R.LDGNAIVDFVR.W | 22 |
| \* | pDK268\_112012\_01.09071.09071.2 | 2.2204 | 0.1354 | 95.5% | 1345.1322 | 1345.556 | 46 | 4.389 | 55.0% | 1 | K.IVEISYYNMGR.I | 2 |
| \* | pDK268\_112012\_01.09346.09346.2 | 3.3831 | 0.4287 | 100.0% | 1357.6522 | 1358.5394 | 1 | 6.763 | 75.0% | 1 | R.IWEVIGDHFNK.V | 2 |
|  | pDK268\_112012\_01.07836.07836.2 | 2.9261 | 0.3885 | 100.0% | 1324.2922 | 1324.5223 | 1 | 5.765 | 80.0% | 1 | K.FLEKGELANFR.F | 22 |
|  | pDK268\_112012\_01.07816.07816.3 | 2.8678 | 0.4239 | 100.0% | 1324.9143 | 1324.5223 | 2 | 6.096 | 47.5% | 1 | K.FLEKGELANFR.F | 33 |
|  | pDK268\_112012\_01.10670.10670.2 | 2.6565 | 0.2477 | 99.8% | 1433.4922 | 1433.7112 | 1 | 5.025 | 80.0% | 1 | K.DFLRPFEHIMK.R | 22 |
|  | pDK268\_112012\_01.10719.10719.3 | 2.521 | 0.27 | 98.9% | 1433.5743 | 1433.7112 | 2 | 4.527 | 57.5% | 1 | K.DFLRPFEHIMK.R | 33 |
| \* | pDK268\_112012\_01.19574.19574.3 | 7.2686 | 0.6297 | 100.0% | 3907.4944 | 3908.3997 | 1 | 10.432 | 32.4% | 2 | K.NIFSVFHLAASDQDESIVELAFQTTGHIVTLVFEK.H | 3 |
| \* | pDK268\_112012\_01.04284.04284.2 | 2.5767 | 0.3661 | 100.0% | 1210.9521 | 1211.3629 | 1 | 6.779 | 72.2% | 1 | K.YVSDRPQAFK.E | 2 |
| \* | pDK268\_112012\_01.04936.04936.2 | 2.9148 | 0.3804 | 100.0% | 1267.2122 | 1267.3843 | 1 | 7.142 | 70.0% | 1 | K.SVDIHDSIQPR.S | 2 |
| \* | pDK268\_112012\_01.07157.07157.2 | 5.6877 | 0.5884 | 100.0% | 2298.672 | 2298.515 | 1 | 10.347 | 54.8% | 1 | R.SVDNRPQAPLVSASAVNEEVSK.I | 2 |
| \* | pDK268\_112012\_02.04998.04998.3 | 4.7299 | 0.4471 | 100.0% | 2299.1943 | 2298.515 | 1 | 8.581 | 44.0% | 3 | R.SVDNRPQAPLVSASAVNEEVSK.I | 3 |
| \* | pDK268\_112012\_01.03819.03819.2 | 3.7611 | 0.2865 | 100.0% | 1316.2722 | 1316.4136 | 1 | 6.835 | 81.8% | 1 | K.KEDAENLAAAQR.D | 2 |

Similarities:
gi|150417986|ref|NP\_0(11:24)  

---

|  |  |  |  |  |  |  |  |  |
| --- | --- | --- | --- | --- | --- | --- | --- | --- |
| U | *gi|66346679|ref|NP\_00* | 8 | 9 | 24.5% | 408 | 44965 | 8.6 | SERPINE1 mRNA binding protein 1 isoform 1 [Homo sapiens] |
| U | *gi|66346683|ref|NP\_00* | 8 | 9 | 25.4% | 393 | 43135 | 8.4 | SERPINE1 mRNA binding protein 1 isoform 3 [Homo sapiens] |

| Filename XCorr DeltCN Conf% ObsM+H+ CalcM+H+ SpR ZScore Ion% # Sequence  | | | | | | | | | | | | |
| --- | --- | --- | --- | --- | --- | --- | --- | --- | --- | --- | --- | --- |
|  | pDK268\_112012\_01.13827.13827.2 | 5.1669 | 0.4839 | 100.0% | 1944.1921 | 1945.0894 | 1 | 9.435 | 76.7% | 2 | R.FDQLFDDESDPFEVLK.A | 2 |
|  | pDK268\_112012\_01.04630.04630.3 | 3.9988 | 0.33 | 100.0% | 1478.5743 | 1478.7751 | 2 | 6.599 | 44.2% | 1 | R.KNPLPPSVGVVDKK.E | 3 |
|  | pDK268\_112012\_01.04055.04055.2 | 2.2281 | 0.2611 | 98.9% | 1239.7122 | 1240.4423 | 92 | 4.545 | 70.0% | 1 | K.EETQPPVALKK.E | 2 |
|  | pDK268\_112012\_01.03326.03326.2 | 3.9768 | 0.4654 | 100.0% | 1256.2322 | 1256.361 | 1 | 7.664 | 75.0% | 1 | R.RPDQQLQGEGK.I | 2 |
|  | pDK268\_112012\_01.03699.03699.2 | 2.2752 | 0.1297 | 97.0% | 1176.1921 | 1176.358 | 324 | 3.798 | 56.2% | 1 | R.RFEKPLEEK.G | 2 |
|  | pDK268\_112012\_01.04799.04799.2 | 2.6801 | 0.3989 | 100.0% | 1113.6122 | 1113.2151 | 1 | 7.177 | 77.8% | 1 | R.SSFSHYSGLK.H | 2 |
|  | pDK268\_112012\_01.03514.03514.2 | 2.9738 | 0.3332 | 100.0% | 1230.0521 | 1230.3225 | 1 | 5.946 | 75.0% | 1 | R.KPNEGADGQWK.K | 2 |
|  | pDK268\_112012\_01.04666.04666.3 | 5.0995 | 0.4095 | 100.0% | 2113.0444 | 2113.2256 | 1 | 7.604 | 52.9% | 1 | K.SKSEEAHAEDSVMDHHFR.K | 3 |

---

|  |  |  |  |  |  |  |  |  |
| --- | --- | --- | --- | --- | --- | --- | --- | --- |
| U | *gi|7657326|ref|NP\_055* | 3 | 11 | 24.3% | 185 | 19905 | 4.2 | male-enhanced antigen [Homo sapiens] |

| Filename XCorr DeltCN Conf% ObsM+H+ CalcM+H+ SpR ZScore Ion% # Sequence  | | | | | | | | | | | | |
| --- | --- | --- | --- | --- | --- | --- | --- | --- | --- | --- | --- | --- |
| \* | pDK268\_112012\_01.08268.08268.2 | 4.0648 | 0.4366 | 100.0% | 1534.1921 | 1534.7859 | 1 | 8.0 | 78.6% | 2 | R.MATVVLGGDTMGPER.I | 2 |
| \* | pDK268\_112012\_01.11438.11438.2 | 4.1567 | 0.4955 | 100.0% | 1681.4122 | 1681.9934 | 1 | 8.228 | 71.9% | 4 | R.TMAGVSLPAPGVPAWAR.E | 2 |
| \* | pDK268\_112012\_02.05763.05763.2 | 4.3524 | 0.4851 | 100.0% | 1547.0922 | 1547.6616 | 1 | 8.359 | 70.8% | 5 | R.EISDAQWEDVVQK.A | 2 |

---

|  |  |  |  |  |  |  |  |  |
| --- | --- | --- | --- | --- | --- | --- | --- | --- |
| U | *gi|4506597|ref|NP\_000* | 5 | 15 | 24.2% | 165 | 17819 | 9.4 | ribosomal protein L12 [Homo sapiens] |

| Filename XCorr DeltCN Conf% ObsM+H+ CalcM+H+ SpR ZScore Ion% # Sequence  | | | | | | | | | | | | |
| --- | --- | --- | --- | --- | --- | --- | --- | --- | --- | --- | --- | --- |
| \* | pDK268\_112012\_01.07421.07421.1 | 1.7249 | 0.2408 | 96.2% | 881.58 | 882.091 | 108 | 5.785 | 50.0% | 2 | K.IGPLGLSPK.K | 1 |
| \* | pDK268\_112012\_02.04953.04953.2 | 2.4179 | 0.3844 | 100.0% | 881.9922 | 882.091 | 21 | 7.517 | 68.8% | 1 | K.IGPLGLSPK.K | 2 |
|  | pDK268\_112012\_01.10977.10977.2 | 4.1638 | 0.2236 | 100.0% | 1668.4722 | 1667.9866 | 1 | 6.987 | 66.7% | 5 | R.QAQIEVVPSASALIIK.A | 2 |
| \* | pDK268\_112012\_02.08199.08199.3 | 3.1297 | 0.201 | 98.3% | 1686.0844 | 1686.865 | 1 | 4.314 | 46.4% | 1 | K.HSGNITFDEIVNIAR.Q | 3 |
| \* | pDK268\_112012\_01.11205.11205.2 | 5.5118 | 0.6351 | 100.0% | 1687.0922 | 1686.865 | 1 | 10.947 | 82.1% | 6 | K.HSGNITFDEIVNIAR.Q | 2 |

---

|  |  |  |  |  |  |  |  |  |
| --- | --- | --- | --- | --- | --- | --- | --- | --- |
| U | *gi|14210536|ref|NP\_11* | 12 | 23 | 24.0% | 446 | 49857 | 4.9 | tubulin, beta 6 [Homo sapiens] |

| Filename XCorr DeltCN Conf% ObsM+H+ CalcM+H+ SpR ZScore Ion% # Sequence  | | | | | | | | | | | | |
| --- | --- | --- | --- | --- | --- | --- | --- | --- | --- | --- | --- | --- |
| \* | pDK268\_112012\_01.13823.13823.3 | 3.6279 | 0.281 | 100.0% | 2827.9443 | 2828.1184 | 1 | 5.443 | 29.0% | 1 | R.SGPFGQLFRPDNFIFGQTGAGNNWAK.G | 3 |
|  | pDK268\_112012\_01.07874.07874.2 | 2.8399 | 0.2733 | 99.9% | 1131.4722 | 1131.2767 | 1 | 4.886 | 88.9% | 5 | R.FPGQLNADLR.K | 22222 |
|  | pDK268\_112012\_01.06263.06263.3 | 2.7439 | 0.2398 | 98.9% | 1259.0643 | 1259.4508 | 21 | 5.025 | 42.5% | 1 | R.FPGQLNADLRK.L | 33333 |
|  | pDK268\_112012\_01.06137.06137.2 | 2.6599 | 0.2453 | 99.8% | 1259.1721 | 1259.4508 | 6 | 4.519 | 65.0% | 1 | R.FPGQLNADLRK.L | 22222 |
|  | pDK268\_112012\_01.08991.08991.2 | 3.3289 | 0.3674 | 100.0% | 1271.8522 | 1272.5945 | 1 | 8.084 | 80.0% | 2 | R.KLAVNMVPFPR.L | 22222 |
|  | pDK268\_112012\_01.10427.10427.1 | 2.0779 | 0.358 | 100.0% | 1143.58 | 1144.4204 | 51 | 6.116 | 55.6% | 1 | K.LAVNMVPFPR.L | 11111 |
|  | pDK268\_112012\_01.10442.10442.2 | 3.7495 | 0.4789 | 100.0% | 1144.3322 | 1144.4204 | 1 | 9.006 | 88.9% | 1 | K.LAVNMVPFPR.L | 22222 |
|  | pDK268\_112012\_01.12644.12644.3 | 3.8334 | 0.3162 | 100.0% | 1622.1843 | 1621.9403 | 1 | 5.671 | 55.8% | 2 | R.LHFFMPGFAPLTSR.G | 3333 |
|  | pDK268\_112012\_02.08489.08489.2 | 3.5612 | 0.4285 | 100.0% | 1622.4122 | 1621.9403 | 1 | 7.702 | 61.5% | 5 | R.LHFFMPGFAPLTSR.G | 2222 |
| \* | pDK268\_112012\_01.13025.13025.2 | 2.7636 | 0.1038 | 97.0% | 1720.1921 | 1720.9812 | 2 | 6.083 | 50.0% | 1 | R.ALTVPELTQQMFDAR.N | 2 |
|  | pDK268\_112012\_01.12033.12033.2 | 3.9628 | 0.4064 | 100.0% | 1697.3322 | 1697.8877 | 1 | 8.146 | 76.9% | 2 | K.NSSYFVEWIPNNVK.V | 22222 |
| \* | pDK268\_112012\_01.13766.13766.2 | 3.992 | 0.0024 | 99.2% | 1858.4321 | 1859.1475 | 1 | 6.884 | 62.5% | 1 | K.MASTFIGNSTAIQELFK.R | 2 |

Similarities:
gi|29788785|ref|NP\_82(9:3)  
gi|5174735|ref|NP\_006(9:3)  
gi|29788768|ref|NP\_82(9:3)  
gi|50592996|ref|NP\_00(7:5)  

---

|  |  |  |  |  |  |  |  |  |
| --- | --- | --- | --- | --- | --- | --- | --- | --- |
| U | *gi|41349449|ref|NP\_05* | 3 | 6 | 24.0% | 225 | 26032 | 5.8 | Mps One Binder kinase activator-like 3 isoform 1 [Homo sapiens] |

| Filename XCorr DeltCN Conf% ObsM+H+ CalcM+H+ SpR ZScore Ion% # Sequence  | | | | | | | | | | | | |
| --- | --- | --- | --- | --- | --- | --- | --- | --- | --- | --- | --- | --- |
| \* | pDK268\_112012\_02.11160.11160.3 | 4.8308 | 0.4848 | 100.0% | 3654.1443 | 3653.9148 | 1 | 8.121 | 29.3% | 3 | K.AQDFYNWPDESFDEMDSTLAVQQYIQQNIR.A | 3 |
|  | pDK268\_112012\_01.08616.08616.2 | 2.875 | 0.3238 | 99.9% | 1597.1721 | 1597.7649 | 9 | 5.643 | 50.0% | 2 | K.ILEPPEGQDEGVWK.Y | 2 |
|  | pDK268\_112012\_01.04408.04408.2 | 3.2984 | 0.432 | 100.0% | 1314.6122 | 1315.4795 | 1 | 7.332 | 77.8% | 1 | R.IFSHAYFHHR.Q | 2 |

---

|  |  |  |  |  |  |  |  |  |
| --- | --- | --- | --- | --- | --- | --- | --- | --- |
| U | *gi|17471847|ref|XP\_06* | 2 | 3 | 23.9% | 117 | 13370 | 9.5 | PREDICTED: similar to hCG2040270 [Homo sapiens] |
| U | *gi|4759158|ref|NP\_004* | 2 | 3 | 23.7% | 118 | 13527 | 9.9 | small nuclear ribonucleoprotein polypeptide D2 [Homo sapiens] |
| U | *gi|29294624|ref|NP\_80* | 2 | 3 | 23.7% | 118 | 13527 | 9.9 | small nuclear ribonucleoprotein polypeptide D2 [Homo sapiens] |

| Filename XCorr DeltCN Conf% ObsM+H+ CalcM+H+ SpR ZScore Ion% # Sequence  | | | | | | | | | | | | |
| --- | --- | --- | --- | --- | --- | --- | --- | --- | --- | --- | --- | --- |
|  | pDK268\_112012\_02.03811.03811.2 | 2.5442 | 0.3515 | 99.9% | 1192.0922 | 1192.3264 | 68 | 5.196 | 55.6% | 2 | K.SEMTPEELQK.R | 2 |
|  | pDK268\_112012\_01.12918.12918.2 | 2.6086 | 0.2954 | 99.8% | 2007.5922 | 2008.1895 | 5 | 5.092 | 41.2% | 1 | R.EEEEFNTGPLSVLTQSVK.N | 2 |

---

|  |  |  |  |  |  |  |  |  |
| --- | --- | --- | --- | --- | --- | --- | --- | --- |
| U | *Reverse\_gi|169163469|* | 1 | 2 | 23.8% | 101 | 10439 | 7.7 | PREDICTED: hypothetical protein [Homo sapiens] |
| U | *Reverse\_gi|169164858|* | 1 | 2 | 23.8% | 101 | 10439 | 7.7 | PREDICTED: hypothetical protein [Homo sapiens] |
| U | *Reverse\_gi|169164526|* | 1 | 2 | 23.8% | 101 | 10439 | 7.7 | PREDICTED: hypothetical protein [Homo sapiens] |

| Filename XCorr DeltCN Conf% ObsM+H+ CalcM+H+ SpR ZScore Ion% # Sequence  | | | | | | | | | | | | |
| --- | --- | --- | --- | --- | --- | --- | --- | --- | --- | --- | --- | --- |
|  | pDK268\_112012\_01.18051.18051.2 | 2.3489 | 0.2247 | 97.9% | 2381.7322 | 2383.5696 | 22 | 4.117 | 28.3% | 2 | R.HMQGEPAGLFPS\*SGAQGGSVLGSM.- | 2 |

---

|  |  |  |  |  |  |  |  |  |
| --- | --- | --- | --- | --- | --- | --- | --- | --- |
| U | *gi|50592996|ref|NP\_00* | 15 | 40 | 23.6% | 450 | 50433 | 4.9 | tubulin, beta, 4 [Homo sapiens] |

| Filename XCorr DeltCN Conf% ObsM+H+ CalcM+H+ SpR ZScore Ion% # Sequence  | | | | | | | | | | | | |
| --- | --- | --- | --- | --- | --- | --- | --- | --- | --- | --- | --- | --- |
|  | pDK268\_112012\_01.10874.10874.2 | 4.8374 | 0.5405 | 100.0% | 1616.4321 | 1616.8701 | 1 | 8.91 | 78.6% | 6 | R.AILVDLEPGTMDSVR.S | 222 |
|  | pDK268\_112012\_01.13478.13478.2 | 7.2309 | 0.5963 | 100.0% | 1958.9321 | 1960.151 | 1 | 10.867 | 79.4% | 4 | K.GHYTEGAELVDSVLDVVR.K | 2222 |
|  | pDK268\_112012\_01.13460.13460.3 | 4.2635 | 0.3564 | 100.0% | 1960.4644 | 1960.151 | 1 | 6.094 | 41.2% | 2 | K.GHYTEGAELVDSVLDVVR.K | 3333 |
|  | pDK268\_112012\_01.12464.12464.3 | 4.2939 | 0.4255 | 100.0% | 2089.0144 | 2088.325 | 1 | 7.427 | 50.0% | 2 | K.GHYTEGAELVDSVLDVVRK.E | 3333 |
|  | pDK268\_112012\_01.12462.12462.2 | 4.7554 | 0.3831 | 100.0% | 2089.5923 | 2088.325 | 1 | 6.134 | 63.9% | 1 | K.GHYTEGAELVDSVLDVVRK.E | 2222 |
|  | pDK268\_112012\_01.08960.08960.2 | 4.2436 | 0.39 | 100.0% | 1320.3322 | 1320.5896 | 1 | 7.358 | 81.8% | 6 | R.IMNTFSVVPSPK.V | 222 |
|  | pDK268\_112012\_01.07874.07874.2 | 2.8399 | 0.2733 | 99.9% | 1131.4722 | 1131.2767 | 1 | 4.886 | 88.9% | 5 | R.FPGQLNADLR.K | 22222 |
|  | pDK268\_112012\_01.06263.06263.3 | 2.7439 | 0.2398 | 98.9% | 1259.0643 | 1259.4508 | 21 | 5.025 | 42.5% | 1 | R.FPGQLNADLRK.L | 33333 |
|  | pDK268\_112012\_01.06137.06137.2 | 2.6599 | 0.2453 | 99.8% | 1259.1721 | 1259.4508 | 6 | 4.519 | 65.0% | 1 | R.FPGQLNADLRK.L | 22222 |
|  | pDK268\_112012\_01.08991.08991.2 | 3.3289 | 0.3674 | 100.0% | 1271.8522 | 1272.5945 | 1 | 8.084 | 80.0% | 2 | R.KLAVNMVPFPR.L | 22222 |
|  | pDK268\_112012\_01.10427.10427.1 | 2.0779 | 0.358 | 100.0% | 1143.58 | 1144.4204 | 51 | 6.116 | 55.6% | 1 | K.LAVNMVPFPR.L | 11111 |
|  | pDK268\_112012\_01.10442.10442.2 | 3.7495 | 0.4789 | 100.0% | 1144.3322 | 1144.4204 | 1 | 9.006 | 88.9% | 1 | K.LAVNMVPFPR.L | 22222 |
|  | pDK268\_112012\_01.12752.12752.2 | 3.621 | 0.4735 | 100.0% | 1692.0322 | 1692.9678 | 1 | 9.102 | 71.4% | 2 | R.ALTVPELTQQMFDAK.N | 22 |
|  | pDK268\_112012\_01.12033.12033.2 | 3.9628 | 0.4064 | 100.0% | 1697.3322 | 1697.8877 | 1 | 8.146 | 76.9% | 2 | K.NSSYFVEWIPNNVK.V | 22222 |
|  | pDK268\_112012\_02.07869.07869.2 | 3.9817 | 0.4841 | 100.0% | 1230.2122 | 1230.4241 | 1 | 7.653 | 94.4% | 4 | R.ISEQFTAMFR.R | 2222 |

Similarities:
gi|29788785|ref|NP\_82(14:1)  
gi|5174735|ref|NP\_006(14:1)  
gi|29788768|ref|NP\_82(13:2)  
gi|14210536|ref|NP\_11(7:8)  

---

|  |  |  |  |  |  |  |  |  |
| --- | --- | --- | --- | --- | --- | --- | --- | --- |
| U | *gi|5454102|ref|NP\_006* | 20 | 28 | 23.5% | 838 | 90360 | 5.0 | transforming, acidic coiled-coil containing protein 3 [Homo sapiens] |

| Filename XCorr DeltCN Conf% ObsM+H+ CalcM+H+ SpR ZScore Ion% # Sequence  | | | | | | | | | | | | |
| --- | --- | --- | --- | --- | --- | --- | --- | --- | --- | --- | --- | --- |
| \* | pDK268\_112012\_01.03099.03099.2 | 2.875 | 0.3285 | 100.0% | 1126.0922 | 1126.2981 | 1 | 6.294 | 61.1% | 1 | R.VSQKENVPPK.N | 2 |
| \* | pDK268\_112012\_01.05510.05510.3 | 2.9288 | 0.2064 | 97.8% | 1697.1843 | 1696.9065 | 5 | 4.831 | 36.5% | 1 | K.VTFQTPLRDPQTHR.I | 3 |
| \* | pDK268\_112012\_01.10982.10982.3 | 3.6486 | 0.3045 | 100.0% | 2626.9744 | 2627.8699 | 1 | 5.963 | 35.2% | 1 | K.LEAPFTQDDTLGLENSHPVWTQK.E | 3 |
| \* | pDK268\_112012\_01.11002.11002.2 | 3.4398 | 0.4452 | 100.0% | 2627.7722 | 2627.8699 | 1 | 8.07 | 45.5% | 1 | K.LEAPFTQDDTLGLENSHPVWTQK.E | 2 |
| \* | pDK268\_112012\_02.06322.06322.3 | 3.8042 | 0.4338 | 100.0% | 2131.4944 | 2132.282 | 1 | 7.721 | 38.2% | 1 | R.AMTLS\*PQEEVAAGQMASSSR.S | 3 |
| \* | pDK268\_112012\_01.08973.08973.2 | 5.1319 | 0.3345 | 100.0% | 2132.3123 | 2132.282 | 1 | 8.58 | 57.9% | 2 | R.AMTLS\*PQEEVAAGQMASSSR.S | 2 |
| \* | pDK268\_112012\_01.07996.07996.2 | 4.5197 | 0.4822 | 100.0% | 1738.2322 | 1737.9042 | 1 | 8.557 | 65.6% | 2 | R.SGPVKLEFDVSDGATSK.R | 2 |
| \* | pDK268\_112012\_02.04817.04817.2 | 2.3737 | 0.2488 | 99.2% | 1268.5122 | 1269.3507 | 1 | 5.455 | 77.3% | 1 | K.LEFDVSDGATSK.R | 2 |
| \* | pDK268\_112012\_01.03488.03488.2 | 3.0226 | 0.35 | 100.0% | 1245.0122 | 1245.245 | 1 | 7.215 | 75.0% | 1 | K.APQEVEEDDGR.S | 2 |
| \* | pDK268\_112012\_01.04930.04930.3 | 4.9718 | 0.4425 | 100.0% | 2498.1843 | 2498.597 | 1 | 7.494 | 38.0% | 2 | K.APQEVEEDDGRSGAGEDPPMPASR.G | 3 |
| \* | pDK268\_112012\_01.04210.04210.2 | 3.1267 | 0.3507 | 100.0% | 1272.1322 | 1272.3752 | 6 | 6.325 | 70.8% | 1 | R.SGAGEDPPMPASR.G | 2 |
| \* | pDK268\_112012\_02.10140.10140.3 | 4.3348 | 0.4504 | 100.0% | 3209.3342 | 3210.4346 | 1 | 7.509 | 27.7% | 2 | K.EESFRDPAEVLGTGAEVDYLEQFGTSSFK.E | 3 |
| \* | pDK268\_112012\_01.14433.14433.2 | 5.1072 | 0.4905 | 100.0% | 2244.8323 | 2245.5703 | 1 | 8.227 | 70.6% | 1 | K.IMDRFEEVVYQAMEEVQK.Q | 2 |
| \* | pDK268\_112012\_01.14481.14481.3 | 4.7341 | 0.3712 | 100.0% | 2246.1543 | 2245.5703 | 1 | 7.447 | 44.1% | 2 | K.IMDRFEEVVYQAMEEVQK.Q | 3 |
| \* | pDK268\_112012\_01.11362.11362.2 | 4.3888 | 0.4526 | 100.0% | 1730.3322 | 1729.9423 | 1 | 9.072 | 76.9% | 1 | R.FEEVVYQAMEEVQK.Q | 2 |
| \* | pDK268\_112012\_01.07204.07204.2 | 4.1893 | 0.3439 | 100.0% | 1652.2322 | 1652.8138 | 1 | 6.722 | 65.4% | 1 | K.EKDQLTTDLNSMEK.S | 2 |
| \* | pDK268\_112012\_01.09033.09033.2 | 2.805 | 0.3776 | 100.0% | 1395.1921 | 1395.5243 | 1 | 6.35 | 81.8% | 1 | K.DQLTTDLNSMEK.S | 2 |
| \* | pDK268\_112012\_02.06094.06094.2 | 3.7506 | 0.4512 | 100.0% | 1384.2522 | 1384.5754 | 1 | 8.04 | 81.8% | 4 | K.LQLANEEIAQVR.S | 2 |
| \* | pDK268\_112012\_02.05454.05454.3 | 4.1378 | 0.3597 | 100.0% | 1685.8744 | 1685.9645 | 1 | 5.619 | 55.0% | 1 | R.SKAQAEALALQASLRK.E | 3 |
| \* | pDK268\_112012\_02.06448.06448.2 | 2.4721 | 0.1467 | 97.1% | 1342.2122 | 1342.5381 | 1 | 4.61 | 70.8% | 1 | K.AQAEALALQASLR.K | 2 |

---

|  |  |  |  |  |  |  |  |  |
| --- | --- | --- | --- | --- | --- | --- | --- | --- |
| U | *gi|56699409|ref|NP\_00* | 12 | 22 | 23.5% | 391 | 42332 | 10.1 | RNA binding motif protein, X-linked [Homo sapiens] |

| Filename XCorr DeltCN Conf% ObsM+H+ CalcM+H+ SpR ZScore Ion% # Sequence  | | | | | | | | | | | | |
| --- | --- | --- | --- | --- | --- | --- | --- | --- | --- | --- | --- | --- |
|  | pDK268\_112012\_01.08914.08914.2 | 4.0754 | 0.2509 | 100.0% | 1437.2322 | 1436.6049 | 1 | 6.159 | 83.3% | 5 | K.LFIGGLNTETNEK.A | 2 |
| \* | pDK268\_112012\_01.07583.07583.1 | 1.6426 | 0.2642 | 98.5% | 834.35 | 834.99084 | 3 | 5.367 | 64.3% | 1 | K.ALEAVFGK.Y | 1 |
| \* | pDK268\_112012\_01.07533.07533.2 | 2.6131 | 0.1773 | 99.8% | 834.9522 | 834.99084 | 2 | 5.53 | 85.7% | 1 | K.ALEAVFGK.Y | 2 |
|  | pDK268\_112012\_01.09846.09846.1 | 1.8783 | 0.2275 | 96.2% | 944.45 | 945.2488 | 9 | 5.654 | 71.4% | 1 | R.IVEVLLMK.D | 1 |
|  | pDK268\_112012\_01.09909.09909.2 | 2.3275 | 0.1672 | 98.7% | 944.83215 | 945.2488 | 2 | 5.682 | 85.7% | 2 | R.IVEVLLMK.D | 2 |
|  | pDK268\_112012\_01.11620.11620.2 | 4.9094 | 0.4103 | 100.0% | 1488.3522 | 1487.6519 | 1 | 8.156 | 76.9% | 2 | R.GFAFVTFESPADAK.D | 2 |
| \* | pDK268\_112012\_01.04720.04720.2 | 4.6359 | 0.459 | 100.0% | 1748.5122 | 1748.9768 | 2 | 8.362 | 60.0% | 1 | K.AIKVEQATKPSFESGR.R | 2 |
| \* | pDK268\_112012\_01.04708.04708.3 | 3.9101 | 0.4019 | 100.0% | 1750.1643 | 1748.9768 | 1 | 6.543 | 45.0% | 2 | K.AIKVEQATKPSFESGR.R | 3 |
| \* | pDK268\_112012\_01.03962.03962.2 | 3.9197 | 0.4871 | 100.0% | 1436.1921 | 1436.5645 | 1 | 8.461 | 70.8% | 1 | K.VEQATKPSFESGR.R | 2 |
| \* | pDK268\_112012\_01.03950.03950.3 | 2.8059 | 0.2267 | 98.3% | 1437.0543 | 1436.5645 | 1 | 4.87 | 39.6% | 1 | K.VEQATKPSFESGR.R | 3 |
|  | pDK268\_112012\_02.05292.05292.3 | 3.978 | 0.3736 | 100.0% | 2051.8743 | 2051.1873 | 1 | 6.545 | 36.1% | 4 | R.GGHMDDGGYSMNFNMSSSR.G | 3 |
|  | pDK268\_112012\_01.03496.03496.3 | 3.2359 | 0.2488 | 99.8% | 1612.4043 | 1612.612 | 1 | 5.053 | 46.2% | 1 | R.DRDYSDHPSGGSYR.D | 3 |

---

|  |  |  |  |  |  |  |  |  |
| --- | --- | --- | --- | --- | --- | --- | --- | --- |
| U | *gi|5174449|ref|NP\_006* | 5 | 8 | 23.5% | 213 | 22487 | 10.8 | H1 histone family, member X [Homo sapiens] |

| Filename XCorr DeltCN Conf% ObsM+H+ CalcM+H+ SpR ZScore Ion% # Sequence  | | | | | | | | | | | | |
| --- | --- | --- | --- | --- | --- | --- | --- | --- | --- | --- | --- | --- |
| \* | pDK268\_112012\_01.06670.06670.3 | 2.4357 | 0.3955 | 100.0% | 1364.7544 | 1364.5876 | 4 | 5.59 | 40.0% | 1 | K.YSQLVVETIRR.L | 3 |
| \* | pDK268\_112012\_01.06929.06929.2 | 2.0868 | 0.2613 | 98.2% | 1376.0322 | 1375.5295 | 2 | 4.821 | 60.0% | 1 | K.KVPWFDQQNGR.T | 2 |
| \* | pDK268\_112012\_02.06513.06513.2 | 3.3667 | 0.3917 | 100.0% | 1342.2922 | 1342.5785 | 1 | 6.889 | 77.3% | 4 | K.ALVQNDTLLQVK.G | 2 |
| \* | pDK268\_112012\_01.03296.03296.3 | 3.1621 | 0.3357 | 100.0% | 1488.6843 | 1488.6896 | 1 | 6.249 | 50.0% | 1 | R.RGAPAAATAPAPTAHK.A | 3 |
| \* | pDK268\_112012\_01.03440.03440.2 | 4.4503 | 0.4574 | 100.0% | 1332.2922 | 1332.5021 | 1 | 8.52 | 71.4% | 1 | R.GAPAAATAPAPTAHK.A | 2 |

---

|  |  |  |  |  |  |  |  |  |
| --- | --- | --- | --- | --- | --- | --- | --- | --- |
| U | *gi|4506649|ref|NP\_000* | 8 | 9 | 23.1% | 403 | 46109 | 10.2 | ribosomal protein L3 isoform a [Homo sapiens] |
| U | *gi|76496472|ref|NP\_00* | 8 | 9 | 26.3% | 354 | 40152 | 10.2 | ribosomal protein L3 isoform b [Homo sapiens] |

| Filename XCorr DeltCN Conf% ObsM+H+ CalcM+H+ SpR ZScore Ion% # Sequence  | | | | | | | | | | | | |
| --- | --- | --- | --- | --- | --- | --- | --- | --- | --- | --- | --- | --- |
|  | pDK268\_112012\_01.03802.03802.2 | 2.1294 | 0.2585 | 99.3% | 884.83215 | 885.07214 | 7 | 5.528 | 78.6% | 1 | K.AGMTHIVR.E | 2 |
|  | pDK268\_112012\_02.05020.05020.3 | 3.5182 | 0.2438 | 99.8% | 1825.8544 | 1826.1211 | 1 | 5.452 | 39.1% | 1 | K.KAHLMEIQVNGGTVAEK.L | 3 |
|  | pDK268\_112012\_02.04966.04966.2 | 3.6004 | 0.3865 | 100.0% | 1698.1522 | 1697.947 | 1 | 6.565 | 46.7% | 1 | K.AHLMEIQVNGGTVAEK.L | 2 |
|  | pDK268\_112012\_02.08868.08868.3 | 5.155 | 0.3652 | 100.0% | 2971.8843 | 2973.371 | 1 | 8.43 | 31.0% | 2 | R.ERLEQQVPVNQVFGQDEMIDVIGVTK.G | 3 |
|  | pDK268\_112012\_01.13062.13062.2 | 3.6463 | 0.6483 | 100.0% | 2687.4922 | 2688.068 | 1 | 11.914 | 50.0% | 1 | R.LEQQVPVNQVFGQDEMIDVIGVTK.G | 2 |
|  | pDK268\_112012\_01.06494.06494.2 | 2.066 | 0.254 | 98.9% | 891.6122 | 892.0861 | 1 | 4.943 | 78.6% | 1 | K.IGQGYLIK.D | 2 |
|  | pDK268\_112012\_01.04733.04733.2 | 3.017 | 0.3224 | 100.0% | 1343.0521 | 1343.3464 | 1 | 6.627 | 68.2% | 1 | K.NNASTDYDLSDK.S | 2 |
|  | pDK268\_112012\_02.09976.09976.3 | 4.4427 | 0.523 | 100.0% | 2438.2744 | 2438.8035 | 1 | 8.078 | 42.9% | 1 | K.SINPLGGFVHYGEVTNDFVMLK.G | 3 |

---

|  |  |  |  |  |  |  |  |  |
| --- | --- | --- | --- | --- | --- | --- | --- | --- |
| U | *gi|16418357|ref|NP\_44* | 7 | 9 | 23.1% | 368 | 41089 | 5.0 | ankyrin repeat domain 40 [Homo sapiens] |

| Filename XCorr DeltCN Conf% ObsM+H+ CalcM+H+ SpR ZScore Ion% # Sequence  | | | | | | | | | | | | |
| --- | --- | --- | --- | --- | --- | --- | --- | --- | --- | --- | --- | --- |
| \* | pDK268\_112012\_01.07552.07552.3 | 4.1391 | 0.3492 | 100.0% | 2263.1042 | 2262.5835 | 1 | 5.542 | 38.8% | 1 | K.SGADKEILTTKGEMPVQLTSR.R | 23 |
| \* | pDK268\_112012\_01.06585.06585.2 | 2.9121 | 0.4124 | 100.0% | 1118.1122 | 1118.2933 | 1 | 6.389 | 83.3% | 2 | K.GEMPVQLTSR.R | 2 |
| \* | pDK268\_112012\_01.08177.08177.3 | 4.1676 | 0.4503 | 100.0% | 2549.2744 | 2549.6333 | 1 | 7.535 | 38.1% | 2 | K.IMGVEEEDDDDDDDDNLPQLKK.E | 3 |
| \* | pDK268\_112012\_02.08158.08158.2 | 2.693 | 0.3074 | 99.9% | 1393.2922 | 1393.493 | 64 | 5.754 | 50.0% | 1 | R.ENDFIEIELDR.Q | 2 |
| \* | pDK268\_112012\_02.10302.10302.3 | 3.553 | 0.3022 | 100.0% | 2667.1743 | 2667.9324 | 273 | 5.955 | 25.0% | 1 | R.ENDFIEIELDRQELTYQELLR.V | 3 |
| \* | pDK268\_112012\_01.18711.18711.2 | 3.6336 | 0.2356 | 99.9% | 2599.7522 | 2600.0044 | 70 | 4.932 | 32.5% | 1 | R.LQDFQELELVLMISENNFLFR.N | 2 |
| \* | pDK268\_112012\_01.18686.18686.3 | 3.1914 | 0.2206 | 98.3% | 2601.4143 | 2600.0044 | 3 | 4.368 | 31.2% | 1 | R.LQDFQELELVLMISENNFLFR.N | 3 |

---

|  |  |  |  |  |  |  |  |  |
| --- | --- | --- | --- | --- | --- | --- | --- | --- |
| U | *gi|4506625|ref|NP\_000* | 3 | 3 | 23.0% | 148 | 16561 | 11.0 | ribosomal protein L27a [Homo sapiens] |

| Filename XCorr DeltCN Conf% ObsM+H+ CalcM+H+ SpR ZScore Ion% # Sequence  | | | | | | | | | | | | |
| --- | --- | --- | --- | --- | --- | --- | --- | --- | --- | --- | --- | --- |
| \* | pDK268\_112012\_01.07487.07487.3 | 2.5264 | 0.2204 | 95.9% | 1586.7544 | 1586.7899 | 3 | 4.925 | 41.7% | 1 | R.INFDKYHPGYFGK.V | 3 |
| \* | pDK268\_112012\_01.09527.09527.2 | 2.3554 | 0.2979 | 99.8% | 1233.4722 | 1233.4099 | 1 | 6.119 | 77.8% | 1 | K.LWTLVSEQTR.V | 2 |
| \* | pDK268\_112012\_01.08391.08391.2 | 2.9663 | 0.3325 | 100.0% | 1113.3522 | 1112.3146 | 1 | 7.608 | 90.0% | 1 | K.TGAAPIIDVVR.S | 2 |

---

|  |  |  |  |  |  |  |  |  |
| --- | --- | --- | --- | --- | --- | --- | --- | --- |
| U | *gi|169161177|ref|XP\_0* | 3 | 3 | 22.8% | 114 | 12783 | 9.9 | PREDICTED: similar to hCG1994130 [Homo sapiens] |
| U | *gi|169162482|ref|XP\_0* | 3 | 3 | 22.8% | 114 | 12783 | 9.9 | PREDICTED: similar to hCG1994130 [Homo sapiens] |
| U | *gi|169162007|ref|XP\_0* | 3 | 3 | 22.8% | 114 | 12783 | 9.9 | PREDICTED: hypothetical protein [Homo sapiens] |

| Filename XCorr DeltCN Conf% ObsM+H+ CalcM+H+ SpR ZScore Ion% # Sequence  | | | | | | | | | | | | |
| --- | --- | --- | --- | --- | --- | --- | --- | --- | --- | --- | --- | --- |
|  | pDK268\_112012\_01.09321.09321.2 | 2.7596 | 0.2497 | 99.9% | 975.09216 | 975.19135 | 2 | 6.482 | 87.5% | 1 | -.MNVLADALK.S | 2 |
|  | pDK268\_112012\_01.06694.06694.2 | 2.0682 | 0.1834 | 97.2% | 872.1922 | 872.05554 | 1 | 4.481 | 85.7% | 1 | K.IVVNLTGR.L | 2 |
|  | pDK268\_112012\_01.07953.07953.2 | 2.4819 | 0.2515 | 99.8% | 1128.2522 | 1128.276 | 17 | 4.49 | 75.0% | 1 | K.WQNNLLPSR.Q | 2 |

---

|  |  |  |  |  |  |  |  |  |
| --- | --- | --- | --- | --- | --- | --- | --- | --- |
| U | *gi|5031753|ref|NP\_005* | 9 | 14 | 22.5% | 449 | 49229 | 6.3 | heterogeneous nuclear ribonucleoprotein H1 [Homo sapiens] |

| Filename XCorr DeltCN Conf% ObsM+H+ CalcM+H+ SpR ZScore Ion% # Sequence  | | | | | | | | | | | | |
| --- | --- | --- | --- | --- | --- | --- | --- | --- | --- | --- | --- | --- |
| \* | pDK268\_112012\_01.03497.03497.2 | 2.7751 | 0.2787 | 99.9% | 957.0722 | 957.0775 | 7 | 5.169 | 81.2% | 1 | K.IQNGAQGIR.F | 2 |
| \* | pDK268\_112012\_01.10628.10628.2 | 3.5526 | 0.3078 | 100.0% | 1335.2322 | 1335.5176 | 2 | 7.081 | 65.0% | 2 | K.SNNVEMDWVLK.H | 2 |
|  | pDK268\_112012\_01.04508.04508.2 | 3.2281 | 0.3642 | 100.0% | 1685.1322 | 1685.7501 | 2 | 5.931 | 63.3% | 1 | K.HTGPNSPDTANDGFVR.L | 2 |
|  | pDK268\_112012\_01.04515.04515.3 | 2.9617 | 0.1897 | 96.5% | 1685.6643 | 1685.7501 | 2 | 4.788 | 40.0% | 1 | K.HTGPNSPDTANDGFVR.L | 3 |
|  | pDK268\_112012\_02.08691.08691.2 | 4.6779 | 0.5218 | 100.0% | 1842.1522 | 1843.0001 | 1 | 9.996 | 65.6% | 4 | R.STGEAFVQFASQEIAEK.A | 2 |
|  | pDK268\_112012\_02.08634.08634.3 | 3.634 | 0.4749 | 100.0% | 1842.9243 | 1843.0001 | 1 | 7.612 | 37.5% | 1 | R.STGEAFVQFASQEIAEK.A | 3 |
|  | pDK268\_112012\_01.13982.13982.2 | 4.0333 | 0.4606 | 100.0% | 1997.4722 | 1998.2023 | 1 | 8.234 | 53.1% | 2 | R.ATENDIYNFFSPLNPVR.V | 22 |
|  | pDK268\_112012\_01.05273.05273.2 | 2.9644 | 0.4902 | 100.0% | 1093.2722 | 1093.2278 | 2 | 8.087 | 66.7% | 1 | R.VHIEIGPDGR.V | 22 |
| \* | pDK268\_112012\_02.06388.06388.3 | 2.7699 | 0.2319 | 96.6% | 2179.3743 | 2179.363 | 1 | 4.735 | 26.2% | 1 | R.VTGEADVEFATHEDAVAAMSK.D | 3 |

Similarities:
gi|148470397|ref|NP\_0(2:7)  

---

|  |  |  |  |  |  |  |  |  |
| --- | --- | --- | --- | --- | --- | --- | --- | --- |
| U | *gi|116235460|ref|NP\_6* | 9 | 13 | 22.4% | 585 | 63861 | 9.0 | YTH domain family, member 3 [Homo sapiens] |

| Filename XCorr DeltCN Conf% ObsM+H+ CalcM+H+ SpR ZScore Ion% # Sequence  | | | | | | | | | | | | |
| --- | --- | --- | --- | --- | --- | --- | --- | --- | --- | --- | --- | --- |
| \* | pDK268\_112012\_01.03461.03461.3 | 2.2759 | 0.272 | 97.8% | 1196.1543 | 1197.3365 | 39 | 4.504 | 35.0% | 1 | K.VSVQNGSIHQK.D | 3 |
| \* | pDK268\_112012\_01.03459.03459.2 | 2.5882 | 0.2536 | 99.8% | 1196.4521 | 1197.3365 | 4 | 4.76 | 60.0% | 1 | K.VSVQNGSIHQK.D | 2 |
| \* | pDK268\_112012\_01.07024.07024.2 | 4.7587 | 0.5198 | 100.0% | 1594.7922 | 1595.7062 | 1 | 9.536 | 80.0% | 1 | R.AITDGQAGFGNDTLSK.V | 2 |
| \* | pDK268\_112012\_01.10834.10834.2 | 4.0703 | 0.5399 | 100.0% | 1517.3722 | 1517.7809 | 1 | 9.196 | 78.6% | 3 | K.VPGISSIEQGMTGLK.I | 2 |
| \* | pDK268\_112012\_02.04404.04404.2 | 2.9116 | 0.4569 | 100.0% | 1045.8922 | 1046.209 | 1 | 8.173 | 70.0% | 3 | K.IGGDLTAAVTK.T | 2 |
| \* | pDK268\_112012\_02.07975.07975.3 | 5.0514 | 0.5418 | 100.0% | 3573.5044 | 3574.0435 | 1 | 9.368 | 25.0% | 1 | K.TVGTALSSSGMTSIATNSVPPVSSAAPKPTSWAAIAR.K | 3 |
| \* | pDK268\_112012\_01.07515.07515.2 | 4.0759 | 0.4472 | 100.0% | 1460.3121 | 1460.7166 | 1 | 7.831 | 63.3% | 1 | K.GNVGIGGSAVPPPPIK.H | 2 |
| \* | pDK268\_112012\_01.06197.06197.2 | 2.6013 | 0.3904 | 100.0% | 1344.1721 | 1345.4723 | 1 | 7.081 | 70.0% | 1 | K.HNMNIGTWDEK.G | 2 |
|  | pDK268\_112012\_01.09665.09665.3 | 3.672 | 0.4063 | 100.0% | 1712.0343 | 1711.8279 | 1 | 6.839 | 44.2% | 1 | K.HTTSIFDDFAHYEK.R | 3 |

---

|  |  |  |  |  |  |  |  |  |
| --- | --- | --- | --- | --- | --- | --- | --- | --- |
| U | *gi|148470397|ref|NP\_0* | 6 | 10 | 22.2% | 415 | 45672 | 5.6 | heterogeneous nuclear ribonucleoprotein F [Homo sapiens] |
| U | *gi|4826760|ref|NP\_004* | 6 | 10 | 22.2% | 415 | 45672 | 5.6 | heterogeneous nuclear ribonucleoprotein F [Homo sapiens] |
| U | *gi|148470406|ref|NP\_0* | 6 | 10 | 22.2% | 415 | 45672 | 5.6 | heterogeneous nuclear ribonucleoprotein F [Homo sapiens] |
| U | *gi|148470404|ref|NP\_0* | 6 | 10 | 22.2% | 415 | 45672 | 5.6 | heterogeneous nuclear ribonucleoprotein F [Homo sapiens] |
| U | *gi|148470402|ref|NP\_0* | 6 | 10 | 22.2% | 415 | 45672 | 5.6 | heterogeneous nuclear ribonucleoprotein F [Homo sapiens] |
| U | *gi|148470400|ref|NP\_0* | 6 | 10 | 22.2% | 415 | 45672 | 5.6 | heterogeneous nuclear ribonucleoprotein F [Homo sapiens] |

| Filename XCorr DeltCN Conf% ObsM+H+ CalcM+H+ SpR ZScore Ion% # Sequence  | | | | | | | | | | | | |
| --- | --- | --- | --- | --- | --- | --- | --- | --- | --- | --- | --- | --- |
|  | pDK268\_112012\_02.06365.06365.2 | 2.0678 | 0.3065 | 98.3% | 1709.3722 | 1710.7919 | 2 | 4.956 | 40.0% | 1 | R.QSGEAFVELGSEDDVK.M | 2 |
|  | pDK268\_112012\_01.13516.13516.2 | 5.1525 | 0.3504 | 100.0% | 1868.7122 | 1869.0813 | 1 | 8.604 | 68.8% | 4 | K.ITGEAFVQFASQELAEK.A | 2 |
|  | pDK268\_112012\_02.09789.09789.3 | 4.3541 | 0.4271 | 100.0% | 1869.1743 | 1869.0813 | 1 | 7.478 | 42.2% | 1 | K.ITGEAFVQFASQELAEK.A | 3 |
|  | pDK268\_112012\_02.09456.09456.3 | 6.5403 | 0.638 | 100.0% | 3474.9543 | 3476.7114 | 1 | 12.703 | 37.1% | 1 | R.MRPGAYSTGYGGYEEYSGLSDGYGFTTDLFGR.D | 32 |
|  | pDK268\_112012\_01.13982.13982.2 | 4.0333 | 0.4606 | 100.0% | 1997.4722 | 1998.2023 | 1 | 8.234 | 53.1% | 2 | K.ATENDIYNFFSPLNPVR.V | 22 |
|  | pDK268\_112012\_01.05273.05273.2 | 2.9644 | 0.4902 | 100.0% | 1093.2722 | 1093.2278 | 2 | 8.087 | 66.7% | 1 | R.VHIEIGPDGR.V | 22 |

Similarities:
gi|5031753|ref|NP\_005(2:4)  

---

|  |  |  |  |  |  |  |  |  |
| --- | --- | --- | --- | --- | --- | --- | --- | --- |
| U | *gi|115270970|ref|NP\_0* | 9 | 11 | 22.1% | 551 | 62023 | 5.6 | Mid-1-related chloride channel 1 isoform 1 [Homo sapiens] |

| Filename XCorr DeltCN Conf% ObsM+H+ CalcM+H+ SpR ZScore Ion% # Sequence  | | | | | | | | | | | | |
| --- | --- | --- | --- | --- | --- | --- | --- | --- | --- | --- | --- | --- |
|  | pDK268\_112012\_01.04768.04768.3 | 3.3847 | 0.2605 | 100.0% | 1755.8344 | 1755.8845 | 4 | 5.804 | 40.4% | 1 | K.KREDYESQSNPVFR.R | 3 |
|  | pDK268\_112012\_01.06891.06891.2 | 3.3521 | 0.4601 | 100.0% | 1470.7122 | 1471.523 | 1 | 7.381 | 68.2% | 2 | R.EDYESQSNPVFR.R | 2 |
| \* | pDK268\_112012\_01.16496.16496.3 | 4.954 | 0.4862 | 100.0% | 2692.8843 | 2693.0283 | 1 | 8.828 | 39.1% | 1 | K.FLNGEDWKPGALDDALSDILINFK.F | 3 |
|  | pDK268\_112012\_01.14769.14769.2 | 3.1898 | 0.3554 | 100.0% | 1741.2322 | 1742.9922 | 1 | 8.259 | 70.8% | 1 | K.KMDWTGSIWEWFR.S | 2 |
|  | pDK268\_112012\_01.16191.16191.2 | 3.1338 | 0.3925 | 100.0% | 2058.7922 | 2059.4993 | 1 | 6.34 | 46.9% | 1 | K.YYELLLVNPIWLVPPTK.A | 2 |
|  | pDK268\_112012\_01.04464.04464.2 | 3.5596 | 0.393 | 100.0% | 1364.1921 | 1364.5157 | 1 | 6.789 | 62.5% | 2 | R.GQMGPTEQGPYAK.T | 2 |
|  | pDK268\_112012\_01.04960.04960.2 | 3.2724 | 0.2712 | 100.0% | 1455.7522 | 1456.5553 | 6 | 6.032 | 63.6% | 1 | R.FQTGNKS\*PEVLR.A | 2 |
|  | pDK268\_112012\_01.06640.06640.2 | 1.9424 | 0.2621 | 97.4% | 1090.9122 | 1091.1655 | 163 | 5.155 | 55.6% | 1 | R.AFDVPDAEAR.E | 2 |
|  | pDK268\_112012\_01.04062.04062.3 | 2.7162 | 0.3508 | 100.0% | 2177.7244 | 2177.422 | 1 | 5.933 | 30.6% | 1 | R.EHPTVVPSHKS\*PVLDTKPK.E | 3 |

---

|  |  |  |  |  |  |  |  |  |
| --- | --- | --- | --- | --- | --- | --- | --- | --- |
| U | *gi|7669492|ref|NP\_002* | 4 | 4 | 22.1% | 335 | 36053 | 8.5 | glyceraldehyde-3-phosphate dehydrogenase [Homo sapiens] |

| Filename XCorr DeltCN Conf% ObsM+H+ CalcM+H+ SpR ZScore Ion% # Sequence  | | | | | | | | | | | | |
| --- | --- | --- | --- | --- | --- | --- | --- | --- | --- | --- | --- | --- |
| \* | pDK268\_112012\_02.13497.13497.3 | 3.1742 | 0.25 | 98.7% | 3310.4043 | 3310.7634 | 22 | 4.686 | 20.4% | 1 | K.VDIVAINDPFIDLNYMVYMFQYDSTHGK.F | 3 |
| \* | pDK268\_112012\_01.14817.14817.3 | 6.2081 | 0.5146 | 100.0% | 2596.7344 | 2597.0044 | 1 | 9.961 | 41.3% | 1 | K.VIHDNFGIVEGLMTTVHAITATQK.T | 32 |
| \* | pDK268\_112012\_01.08440.08440.2 | 2.5264 | 0.2535 | 99.3% | 1412.1122 | 1412.6292 | 2 | 4.534 | 50.0% | 1 | R.GALQNIIPASTGAAK.A | 2 |
|  | pDK268\_112012\_01.06407.06407.2 | 1.8009 | 0.2733 | 97.8% | 795.9522 | 795.97504 | 4 | 4.867 | 75.0% | 1 | K.LTGMAFR.V | 2 |

---

|  |  |  |  |  |  |  |  |  |
| --- | --- | --- | --- | --- | --- | --- | --- | --- |
| U | *gi|21626466|ref|NP\_06* | 13 | 18 | 22.0% | 847 | 94623 | 6.3 | matrin 3 [Homo sapiens] |
| U | *gi|62750354|ref|NP\_95* | 13 | 18 | 22.0% | 847 | 94623 | 6.3 | matrin 3 [Homo sapiens] |

| Filename XCorr DeltCN Conf% ObsM+H+ CalcM+H+ SpR ZScore Ion% # Sequence  | | | | | | | | | | | | |
| --- | --- | --- | --- | --- | --- | --- | --- | --- | --- | --- | --- | --- |
|  | pDK268\_112012\_01.04325.04325.2 | 2.0662 | 0.286 | 99.2% | 1040.0122 | 1040.121 | 1 | 6.019 | 68.8% | 1 | K.SFQQSSLSR.D | 2 |
|  | pDK268\_112012\_02.12377.12377.2 | 3.7414 | 0.3806 | 100.0% | 2372.5923 | 2372.7424 | 1 | 8.463 | 39.6% | 1 | R.DLSAAGIGLLAAATQSLSMPASLGR.M | 2 |
|  | pDK268\_112012\_02.09778.09778.2 | 4.293 | 0.5423 | 100.0% | 1793.0521 | 1793.931 | 1 | 9.411 | 52.9% | 2 | R.GDADQASNILASFGLSAR.D | 2 |
|  | pDK268\_112012\_01.07098.07098.2 | 3.0623 | 0.1622 | 99.7% | 1480.3522 | 1480.5713 | 6 | 4.649 | 68.2% | 1 | R.DLDELSRYPEDK.I | 2 |
|  | pDK268\_112012\_01.14469.14469.2 | 3.6296 | 0.3063 | 100.0% | 1620.6522 | 1620.9731 | 1 | 6.008 | 69.2% | 2 | K.ITPENLPQILLQLK.R | 2 |
|  | pDK268\_112012\_01.05464.05464.2 | 2.5794 | 0.3358 | 99.9% | 1210.1921 | 1210.2859 | 1 | 5.296 | 65.0% | 1 | R.TEEGPTLSYGR.D | 2 |
|  | pDK268\_112012\_01.08975.08975.3 | 3.9081 | 0.2116 | 99.5% | 2363.5144 | 2363.462 | 1 | 4.956 | 37.5% | 1 | R.DSFDDRGPSLNPVLDYDHGSR.S | 3 |
|  | pDK268\_112012\_01.03978.03978.2 | 1.9147 | 0.2714 | 98.3% | 854.2522 | 853.95355 | 1 | 6.146 | 78.6% | 1 | R.GPGPLQER.S | 2 |
|  | pDK268\_112012\_01.15358.15358.2 | 5.1996 | 0.5498 | 100.0% | 2439.0322 | 2439.9036 | 1 | 8.509 | 55.0% | 1 | R.YQLLQLVEPFGVISNHLILNK.I | 2 |
|  | pDK268\_112012\_01.15392.15392.3 | 4.1006 | 0.4497 | 100.0% | 2439.6243 | 2439.9036 | 1 | 7.03 | 32.5% | 2 | R.YQLLQLVEPFGVISNHLILNK.I | 3 |
|  | pDK268\_112012\_01.07737.07737.3 | 3.0671 | 0.3755 | 100.0% | 2037.3544 | 2038.3109 | 1 | 5.538 | 36.1% | 2 | R.VIHLSNLPHSGYSDSAVLK.L | 3 |
|  | pDK268\_112012\_02.05897.05897.2 | 1.8645 | 0.4084 | 99.6% | 1212.2322 | 1212.363 | 1 | 5.777 | 66.7% | 1 | K.SQAFIEMETR.E | 32 |
|  | pDK268\_112012\_01.11966.11966.2 | 4.254 | 0.4896 | 100.0% | 1970.4321 | 1970.319 | 1 | 8.104 | 64.7% | 2 | R.IGPYQPNVPVGIDYVIPK.T | 2 |

---

|  |  |  |  |  |  |  |  |  |
| --- | --- | --- | --- | --- | --- | --- | --- | --- |
| U | *gi|169212979|ref|XP\_0* | 3 | 4 | 21.7% | 184 | 21397 | 10.2 | PREDICTED: hypothetical protein [Homo sapiens] |
| U | *gi|78000186|ref|NP\_00* | 3 | 4 | 21.7% | 184 | 21397 | 10.2 | ribosomal protein L17 [Homo sapiens] |
| U | *gi|4506617|ref|NP\_000* | 3 | 4 | 21.7% | 184 | 21397 | 10.2 | ribosomal protein L17 [Homo sapiens] |

| Filename XCorr DeltCN Conf% ObsM+H+ CalcM+H+ SpR ZScore Ion% # Sequence  | | | | | | | | | | | | |
| --- | --- | --- | --- | --- | --- | --- | --- | --- | --- | --- | --- | --- |
|  | pDK268\_112012\_01.06341.06341.2 | 2.8527 | 0.201 | 99.8% | 1165.0322 | 1164.2572 | 1 | 5.859 | 66.7% | 2 | R.YSLDPENPTK.S | 2 |
|  | pDK268\_112012\_02.08148.08148.2 | 4.4541 | 0.4504 | 100.0% | 1779.5922 | 1780.0311 | 1 | 8.8 | 63.3% | 1 | K.GLDVDSLVIEHIQVNK.A | 2 |
|  | pDK268\_112012\_01.04974.04974.2 | 3.2675 | 0.271 | 99.9% | 1624.2922 | 1624.8314 | 1 | 6.143 | 65.4% | 1 | K.EQIVPKPEEEVAQK.K | 2 |

---

|  |  |  |  |  |  |  |  |  |
| --- | --- | --- | --- | --- | --- | --- | --- | --- |
| U | *gi|72534660|ref|NP\_00* | 5 | 7 | 21.4% | 238 | 27367 | 11.8 | splicing factor, arginine/serine-rich 7 [Homo sapiens] |

| Filename XCorr DeltCN Conf% ObsM+H+ CalcM+H+ SpR ZScore Ion% # Sequence  | | | | | | | | | | | | |
| --- | --- | --- | --- | --- | --- | --- | --- | --- | --- | --- | --- | --- |
| \* | pDK268\_112012\_02.05129.05129.2 | 3.2253 | 0.4354 | 100.0% | 1720.1122 | 1720.923 | 1 | 6.421 | 50.0% | 1 | K.VYVGNLGTGAGKGELER.A | 2 |
| \* | pDK268\_112012\_02.05104.05104.3 | 2.1843 | 0.3628 | 99.1% | 1721.6943 | 1720.923 | 1 | 5.692 | 37.5% | 1 | K.VYVGNLGTGAGKGELER.A | 3 |
| \* | pDK268\_112012\_01.08826.08826.2 | 2.2057 | 0.269 | 99.5% | 1074.3322 | 1074.2242 | 3 | 6.86 | 75.0% | 1 | R.AFSYYGPLR.T | 2 |
|  | pDK268\_112012\_01.12224.12224.2 | 3.5877 | 0.4551 | 100.0% | 1621.9122 | 1622.7771 | 1 | 7.658 | 65.4% | 2 | R.NPPGFAFVEFEDPR.D | 22 |
| \* | pDK268\_112012\_01.06066.06066.2 | 2.6379 | 0.3369 | 99.9% | 1244.5322 | 1245.4827 | 1 | 6.207 | 75.0% | 2 | R.VRVELSTGMPR.R | 2 |

Similarities:
gi|4506901|ref|NP\_003(1:4)  

---

|  |  |  |  |  |  |  |  |  |
| --- | --- | --- | --- | --- | --- | --- | --- | --- |
| U | *gi|7661958|ref|NP\_055* | 16 | 17 | 21.1% | 920 | 106122 | 10.0 | BCL2-associated transcription factor 1 isoform 1 [Homo sapiens] |

| Filename XCorr DeltCN Conf% ObsM+H+ CalcM+H+ SpR ZScore Ion% # Sequence  | | | | | | | | | | | | |
| --- | --- | --- | --- | --- | --- | --- | --- | --- | --- | --- | --- | --- |
|  | pDK268\_112012\_01.03869.03869.2 | 2.7935 | 0.3336 | 99.9% | 1522.0721 | 1522.5658 | 8 | 6.106 | 58.3% | 1 | K.KAEGEPQEES\*PLK.S | 2 |
|  | pDK268\_112012\_01.07395.07395.3 | 4.168 | 0.2391 | 100.0% | 2610.2344 | 2609.6758 | 27 | 4.922 | 28.6% | 1 | K.SQEEPKDTFEHDPSESIDEFNK.S | 3 |
|  | pDK268\_112012\_01.03124.03124.3 | 2.6428 | 0.3865 | 100.0% | 1922.6344 | 1922.9254 | 2 | 6.054 | 38.3% | 1 | K.NTPSQHSHSIQHS\*PER.S | 3 |
|  | pDK268\_112012\_01.05361.05361.2 | 3.0656 | 0.3232 | 100.0% | 1503.1721 | 1502.534 | 1 | 7.071 | 69.2% | 1 | R.SSFYPDGGDQETAK.T | 2 |
|  | pDK268\_112012\_02.06807.06807.3 | 4.4668 | 0.4502 | 100.0% | 2727.0842 | 2727.7278 | 1 | 6.859 | 39.8% | 1 | K.GRAEGEWEDQEALDYFS\*DKESGK.Q | 3 |
|  | pDK268\_112012\_02.07379.07379.2 | 3.118 | 0.4424 | 100.0% | 2513.5322 | 2514.4883 | 1 | 6.835 | 45.0% | 1 | R.AEGEWEDQEALDYFS\*DKESGK.Q | 2 |
|  | pDK268\_112012\_02.07359.07359.3 | 3.5195 | 0.3251 | 100.0% | 2514.0544 | 2514.4883 | 1 | 5.267 | 30.0% | 1 | R.AEGEWEDQEALDYFS\*DKESGK.Q | 3 |
|  | pDK268\_112012\_01.04379.04379.2 | 4.5014 | 0.4255 | 100.0% | 1708.4321 | 1708.9524 | 1 | 8.841 | 57.1% | 1 | K.LKETGYVVERPSTTK.D | 2 |
|  | pDK268\_112012\_01.04188.04188.2 | 3.4112 | 0.4535 | 100.0% | 1467.2722 | 1467.6189 | 1 | 6.628 | 54.2% | 1 | K.ETGYVVERPSTTK.D | 2 |
|  | pDK268\_112012\_01.09814.09814.2 | 3.37 | 0.3411 | 100.0% | 1653.3922 | 1653.832 | 1 | 6.537 | 62.5% | 1 | K.LKDLFDYS\*PPLHK.N | 2 |
|  | pDK268\_112012\_01.03533.03533.2 | 3.9152 | 0.3466 | 100.0% | 1370.2522 | 1370.5664 | 1 | 6.364 | 72.7% | 1 | K.MIASDSHRPEVK.L | 2 |
|  | pDK268\_112012\_01.06562.06562.2 | 3.7326 | 0.4095 | 100.0% | 1812.3922 | 1813.079 | 1 | 8.187 | 62.5% | 1 | K.MAPVPLDDSNRPASLTK.D | 2 |
|  | pDK268\_112012\_01.06981.06981.2 | 2.9909 | 0.3485 | 100.0% | 1168.4922 | 1168.4221 | 1 | 7.426 | 75.0% | 1 | R.LLASTLVHSVK.K | 2 |
|  | pDK268\_112012\_01.12422.12422.3 | 3.1523 | 0.3876 | 100.0% | 2049.8943 | 2049.3372 | 1 | 6.238 | 38.2% | 1 | K.STSESFIQHIVSLVHHVK.E | 3 |
|  | pDK268\_112012\_01.04457.04457.2 | 2.9723 | 0.3592 | 100.0% | 994.39215 | 993.1229 | 7 | 7.168 | 75.0% | 2 | K.SAAMTLNER.F | 2 |
|  | pDK268\_112012\_01.07530.07530.2 | 2.4273 | 0.3787 | 99.9% | 1423.1921 | 1423.4755 | 3 | 6.045 | 55.0% | 1 | K.EEEWDPEYTPK.S | 22 |

Similarities:
gi|167234419|ref|NP\_0(1:15)  

---

|  |  |  |  |  |  |  |  |  |
| --- | --- | --- | --- | --- | --- | --- | --- | --- |
| U | *gi|221136753|ref|NP\_0* | 6 | 7 | 20.9% | 412 | 45135 | 8.7 | chromodomain protein, Y chromosome-like isoform d [Homo sapiens] |
| U | *gi|221307494|ref|NP\_0* | 6 | 7 | 15.8% | 544 | 60609 | 9.2 | chromodomain protein, Y chromosome-like isoform a [Homo sapiens] |

| Filename XCorr DeltCN Conf% ObsM+H+ CalcM+H+ SpR ZScore Ion% # Sequence  | | | | | | | | | | | | |
| --- | --- | --- | --- | --- | --- | --- | --- | --- | --- | --- | --- | --- |
|  | pDK268\_112012\_01.11990.11990.3 | 4.8255 | 0.404 | 100.0% | 2441.0344 | 2439.9219 | 1 | 6.158 | 35.4% | 1 | R.IHPLVPQVPGPVTAAMATGLAVNGK.G | 3 |
|  | pDK268\_112012\_01.05994.05994.2 | 2.5245 | 0.3406 | 99.9% | 1476.9122 | 1477.589 | 1 | 5.344 | 66.7% | 1 | K.SSENNSLNPEVMR.E | 2 |
|  | pDK268\_112012\_01.08850.08850.2 | 3.5232 | 0.4983 | 100.0% | 1506.6721 | 1507.7632 | 1 | 7.872 | 67.9% | 2 | K.IMGGASANEMLLSGR.K | 2 |
|  | pDK268\_112012\_01.14488.14488.2 | 4.3844 | 0.3049 | 100.0% | 2182.7522 | 2182.5452 | 1 | 6.832 | 61.1% | 1 | K.GLVSQVFWPGTFTQEVMVR.I | 2 |
|  | pDK268\_112012\_01.08679.08679.2 | 2.3161 | 0.3587 | 99.8% | 1552.0521 | 1552.8469 | 236 | 5.825 | 42.3% | 1 | K.KIWGSAQGMDSMLK.Y | 2 |
|  | pDK268\_112012\_01.10197.10197.2 | 2.5166 | 0.2713 | 99.6% | 1423.5322 | 1424.6729 | 1 | 6.052 | 70.8% | 1 | K.IWGSAQGMDSMLK.Y | 2 |

---

|  |  |  |  |  |  |  |  |  |
| --- | --- | --- | --- | --- | --- | --- | --- | --- |
| U | *gi|20149675|ref|NP\_07* | 5 | 5 | 20.8% | 240 | 26697 | 5.2 | EF-hand domain family, member D2 [Homo sapiens] |

| Filename XCorr DeltCN Conf% ObsM+H+ CalcM+H+ SpR ZScore Ion% # Sequence  | | | | | | | | | | | | |
| --- | --- | --- | --- | --- | --- | --- | --- | --- | --- | --- | --- | --- |
| \* | pDK268\_112012\_01.05244.05244.2 | 4.27 | 0.5013 | 100.0% | 1569.1721 | 1569.6714 | 1 | 8.493 | 67.9% | 1 | R.ADLNQGIGEPQSPSR.R | 2 |
|  | pDK268\_112012\_01.05302.05302.2 | 3.0007 | 0.3503 | 100.0% | 1135.3322 | 1135.3518 | 6 | 6.653 | 65.0% | 1 | K.LGAPQTHLGLK.N | 2 |
| \* | pDK268\_112012\_01.06539.06539.2 | 3.3476 | 0.3172 | 100.0% | 1263.2922 | 1263.3873 | 1 | 6.595 | 72.7% | 1 | R.LSEIDVSSEGVK.G | 2 |
| \* | pDK268\_112012\_02.04185.04185.3 | 2.5597 | 0.2667 | 98.8% | 1537.7043 | 1537.6232 | 11 | 5.283 | 40.9% | 1 | R.FEEEIKAEQEER.K | 3 |
| \* | pDK268\_112012\_01.04721.04721.2 | 4.1491 | 0.2851 | 100.0% | 1538.0122 | 1537.6232 | 1 | 6.554 | 81.8% | 1 | R.FEEEIKAEQEER.K | 2 |

---

|  |  |  |  |  |  |  |  |  |
| --- | --- | --- | --- | --- | --- | --- | --- | --- |
| U | *gi|4885375|ref|NP\_005* | 8 | 18 | 20.7% | 213 | 21365 | 10.9 | histone cluster 1, H1c [Homo sapiens] |

| Filename XCorr DeltCN Conf% ObsM+H+ CalcM+H+ SpR ZScore Ion% # Sequence  | | | | | | | | | | | | |
| --- | --- | --- | --- | --- | --- | --- | --- | --- | --- | --- | --- | --- |
|  | pDK268\_112012\_01.06146.06146.2 | 4.3905 | 0.47 | 100.0% | 1327.5322 | 1327.5638 | 1 | 8.361 | 79.2% | 3 | R.KASGPPVSELITK.A | 2 |
|  | pDK268\_112012\_01.06110.06110.3 | 4.173 | 0.3428 | 100.0% | 1327.7943 | 1327.5638 | 1 | 6.447 | 54.2% | 1 | R.KASGPPVSELITK.A | 3 |
|  | pDK268\_112012\_01.07695.07695.2 | 3.8866 | 0.511 | 100.0% | 1198.7522 | 1199.3898 | 1 | 8.089 | 68.2% | 3 | K.ASGPPVSELITK.A | 2 |
|  | pDK268\_112012\_01.07133.07133.2 | 2.6731 | 0.248 | 99.9% | 845.5722 | 846.01465 | 1 | 5.339 | 87.5% | 1 | R.SGVSLAALK.K | 2 |
|  | pDK268\_112012\_01.05210.05210.2 | 2.8134 | 0.1461 | 99.6% | 973.89215 | 974.1887 | 1 | 5.56 | 88.9% | 2 | R.SGVSLAALKK.A | 2 |
|  | pDK268\_112012\_01.05411.05411.1 | 2.8876 | 0.478 | 100.0% | 1107.45 | 1108.2365 | 3 | 8.428 | 60.0% | 1 | K.ALAAAGYDVEK.N | 1 |
|  | pDK268\_112012\_01.05444.05444.2 | 3.7734 | 0.4962 | 100.0% | 1108.0322 | 1108.2365 | 1 | 8.108 | 75.0% | 4 | K.ALAAAGYDVEK.N | 2 |
| \* | pDK268\_112012\_01.02408.02408.2 | 2.9116 | 0.3615 | 100.0% | 1015.3522 | 1015.2413 | 1 | 6.301 | 77.8% | 3 | K.KPAAATVTKK.V | 2 |

---

|  |  |  |  |  |  |  |  |  |
| --- | --- | --- | --- | --- | --- | --- | --- | --- |
| U | *gi|4505119|ref|NP\_003* | 7 | 9 | 20.6% | 291 | 32844 | 5.3 | methyl-CpG binding domain protein 3 [Homo sapiens] |

| Filename XCorr DeltCN Conf% ObsM+H+ CalcM+H+ SpR ZScore Ion% # Sequence  | | | | | | | | | | | | |
| --- | --- | --- | --- | --- | --- | --- | --- | --- | --- | --- | --- | --- |
| \* | pDK268\_112012\_02.09163.09163.2 | 4.1742 | 0.5443 | 100.0% | 1610.5122 | 1609.7937 | 1 | 9.785 | 65.4% | 2 | R.YLGGSMDLSTFDFR.T | 2 |
| \* | pDK268\_112012\_01.13589.13589.2 | 4.4398 | 0.5053 | 100.0% | 1747.4321 | 1748.0294 | 1 | 8.931 | 70.0% | 1 | K.KLSGLNAFDIAEELVK.T | 2 |
| \* | pDK268\_112012\_01.14829.14829.2 | 5.1096 | 0.5273 | 100.0% | 1619.8121 | 1619.8553 | 1 | 10.084 | 82.1% | 1 | K.LSGLNAFDIAEELVK.T | 2 |
| \* | pDK268\_112012\_01.08280.08280.2 | 3.2978 | 0.2691 | 100.0% | 1198.0922 | 1197.3484 | 1 | 7.843 | 83.3% | 1 | K.AFMVTDEDIR.K | 2 |
| \* | pDK268\_112012\_01.13959.13959.3 | 6.7757 | 0.5187 | 100.0% | 2327.8743 | 2326.7346 | 1 | 8.204 | 56.6% | 2 | R.KRLEEALMADMLAHVEELAR.D | 3 |
| \* | pDK268\_112012\_01.14793.14793.3 | 5.6425 | 0.4956 | 100.0% | 2198.5444 | 2198.5603 | 1 | 9.691 | 59.7% | 1 | K.RLEEALMADMLAHVEELAR.D | 3 |
| \* | pDK268\_112012\_01.15506.15506.3 | 3.9467 | 0.4277 | 100.0% | 2042.4844 | 2042.3729 | 1 | 8.021 | 47.1% | 1 | R.LEEALMADMLAHVEELAR.D | 3 |

---

|  |  |  |  |  |  |  |  |  |
| --- | --- | --- | --- | --- | --- | --- | --- | --- |
| U | *gi|24308111|ref|NP\_05* | 3 | 3 | 20.6% | 253 | 29426 | 6.0 | FGFR1 oncogene partner 2 [Homo sapiens] |

| Filename XCorr DeltCN Conf% ObsM+H+ CalcM+H+ SpR ZScore Ion% # Sequence  | | | | | | | | | | | | |
| --- | --- | --- | --- | --- | --- | --- | --- | --- | --- | --- | --- | --- |
| \* | pDK268\_112012\_02.05551.05551.2 | 2.9555 | 0.2853 | 99.9% | 1376.4122 | 1376.5707 | 2 | 6.286 | 68.2% | 1 | R.STLVMGIQQENR.Q | 2 |
| \* | pDK268\_112012\_02.07692.07692.2 | 2.9035 | 0.379 | 100.0% | 1816.9922 | 1817.0643 | 1 | 6.444 | 56.7% | 1 | R.TSLEEHQSALELIMSK.Y | 2 |
| \* | pDK268\_112012\_02.08693.08693.3 | 3.2334 | 0.2032 | 97.1% | 2749.1643 | 2750.0767 | 1 | 5.047 | 30.4% | 1 | R.HLEANQNELQAHVDQITEMAAVMR.K | 3 |

---

|  |  |  |  |  |  |  |  |  |
| --- | --- | --- | --- | --- | --- | --- | --- | --- |
| U | *gi|4758138|ref|NP\_004* | 11 | 19 | 20.4% | 614 | 69148 | 8.9 | DEAD (Asp-Glu-Ala-Asp) box polypeptide 5 [Homo sapiens] |

| Filename XCorr DeltCN Conf% ObsM+H+ CalcM+H+ SpR ZScore Ion% # Sequence  | | | | | | | | | | | | |
| --- | --- | --- | --- | --- | --- | --- | --- | --- | --- | --- | --- | --- |
| \* | pDK268\_112012\_01.10680.10680.2 | 3.2236 | 0.2362 | 99.9% | 1418.9521 | 1419.6201 | 109 | 4.688 | 50.0% | 1 | K.WNLDELPKFEK.N | 2 |
| \* | pDK268\_112012\_01.05740.05740.2 | 3.728 | 0.4124 | 100.0% | 1390.3522 | 1390.4978 | 2 | 6.8 | 65.0% | 1 | K.NFYQEHPDLAR.R | 2 |
| \* | pDK268\_112012\_01.08798.08798.2 | 3.2721 | 0.4925 | 100.0% | 1295.8322 | 1296.4198 | 1 | 9.494 | 80.0% | 2 | R.TTYLVLDEADR.M | 2 |
|  | pDK268\_112012\_01.10587.10587.2 | 4.1287 | 0.4085 | 100.0% | 1338.4521 | 1337.5946 | 1 | 7.078 | 85.0% | 1 | R.MLDMGFEPQIR.K | 222 |
|  | pDK268\_112012\_01.11267.11267.2 | 3.1834 | 0.4163 | 100.0% | 1350.2522 | 1349.5902 | 1 | 6.253 | 70.0% | 2 | R.QTLMWSATWPK.E | 22 |
| \* | pDK268\_112012\_01.07547.07547.2 | 2.7653 | 0.3134 | 100.0% | 1110.1721 | 1110.3259 | 4 | 6.591 | 75.0% | 1 | R.LMEEIMSEK.E | 2 |
|  | pDK268\_112012\_01.08402.08402.2 | 3.8561 | 0.239 | 100.0% | 1227.3121 | 1227.4465 | 2 | 7.574 | 86.4% | 4 | K.APILIATDVASR.G | 22 |
| \* | pDK268\_112012\_01.09406.09406.3 | 3.0915 | 0.2631 | 99.3% | 2132.9644 | 2133.281 | 17 | 4.606 | 35.9% | 1 | K.FVINYDYPNSSEDYIHR.I | 3 |
| \* | pDK268\_112012\_01.10307.10307.2 | 3.0785 | 0.1669 | 99.6% | 1576.6522 | 1575.7612 | 1 | 5.211 | 61.5% | 2 | K.TGTAYTFFTPNNIK.Q | 2 |
| \* | pDK268\_112012\_01.11123.11123.2 | 1.8292 | 0.2868 | 97.1% | 1130.2722 | 1130.3298 | 1 | 4.304 | 77.8% | 1 | K.QVSDLISVLR.E | 2 |
| \* | pDK268\_112012\_01.08012.08012.2 | 2.9071 | 0.273 | 100.0% | 985.9522 | 986.1564 | 2 | 6.151 | 85.7% | 3 | K.LLQLVEDR.G | 2 |

Similarities:
gi|148613856|ref|NP\_0(3:8)  
gi|87196351|ref|NP\_00(1:10)  

---

|  |  |  |  |  |  |  |  |  |
| --- | --- | --- | --- | --- | --- | --- | --- | --- |
| U | *gi|8923942|ref|NP\_061* | 1 | 1 | 20.3% | 64 | 7706 | 10.0 | nucleolar protein family A, member 3 [Homo sapiens] |

| Filename XCorr DeltCN Conf% ObsM+H+ CalcM+H+ SpR ZScore Ion% # Sequence  | | | | | | | | | | | | |
| --- | --- | --- | --- | --- | --- | --- | --- | --- | --- | --- | --- | --- |
| \* | pDK268\_112012\_01.11606.11606.2 | 2.4867 | 0.3115 | 99.8% | 1677.6721 | 1677.872 | 1 | 4.702 | 54.2% | 1 | -.MFLQYYLNEQGDR.V | 2 |

---

|  |  |  |  |  |  |  |  |  |
| --- | --- | --- | --- | --- | --- | --- | --- | --- |
| U | *gi|4503519|ref|NP\_003* | 4 | 4 | 20.2% | 357 | 37564 | 5.4 | eukaryotic translation initiation factor 3, subunit 5 epsilon, 47kDa [Homo sapiens] |

| Filename XCorr DeltCN Conf% ObsM+H+ CalcM+H+ SpR ZScore Ion% # Sequence  | | | | | | | | | | | | |
| --- | --- | --- | --- | --- | --- | --- | --- | --- | --- | --- | --- | --- |
| \* | pDK268\_112012\_02.06477.06477.2 | 4.86 | 0.5099 | 100.0% | 1658.5521 | 1658.8522 | 1 | 10.141 | 71.9% | 1 | R.VIGLSSDLQQVGGASAR.I | 2 |
| \* | pDK268\_112012\_01.19425.19425.2 | 4.8481 | 0.6488 | 100.0% | 2050.392 | 2051.3013 | 1 | 10.622 | 61.1% | 1 | R.IQDALSTVLQYAEDVLSGK.V | 23 |
| \* | pDK268\_112012\_01.19446.19446.3 | 2.5624 | 0.2491 | 96.6% | 2051.4243 | 2051.3013 | 6 | 5.393 | 31.9% | 1 | R.IQDALSTVLQYAEDVLSGK.V | 3 |
| \* | pDK268\_112012\_01.19310.19310.3 | 3.0521 | 0.2979 | 99.3% | 4082.0044 | 4083.6584 | 2 | 4.892 | 20.0% | 1 | K.IVPDDFETMLNSNINDLLMVTYLANLTQSQIALNEK.L | 3 |

---
[truncated: 685,038 more chars]
