# Supplementary material for: Astrin-SKAP complex reconstitution reveals its kinetochore interaction with microtubule-bound Ndc80
Source: eLife. 2017 Aug 25;6:e26866. doi: 10.7554/eLife.26866 (PMC5602300; doi:10.7554/eLife.26866)
Supplement: Source data 1. — Complete mass spectrometry searches using methods described in (Washburn et al., 2001) for affinity purification/mass spectrometry data sets described in this paper (data from this study; [Kern et al., 2016] [Gascoigne et al., 2011]). Individual Astrin cross-linking immunoprecipitations are listed based on the order in Figure 4—figure supplement 1. These samples have not been pruned for common or antibody-specific contaminants. [file elife-26866-data1.zip › PooledAstrinSKAPIPs_Kern2016.html]

D PooledAstrinSKAP\_preps
DTASelect v2.0.21  
/nfs/cheeseman\_massspec/David/PooledAstrinSKAP\_preps  
/nfs/cheeseman\_massspec/Databases/NCBI-RefSeq\_human\_na\_04-13-2009\_con\_reversed.fasta  
SEQUEST 3.0 in SQT format.  
  
 Jump  to the summary table.  
  
sequest.params modifications:

|  |  |  |
| --- | --- | --- |
| \* | S | 80.0 |
| # | T | 80.0 |
| @ | Y | 80.0 |
| Static | C | 57.0 |

|  |  |
| --- | --- |
| true | Use criteria |
| 0.0 | Minimum peptide confidence |
| 0.05 | Peptide false positive rate |
| 0.0 | Minimum protein confidence |
| 1.0 | Protein false positive rate |
| 1 | Minimum charge state |
| 16 | Maximum charge state |
| 0.0 | Minimum ion proportion |
| 1000 | Maximum Sp rank |
| -1.0 | Minimum Sp score |
| Include | Modified peptide inclusion |
| Any | Tryptic status requirement |
| false | Multiple, ambiguous IDs allowed |
| Ignore | Peptide validation handling |
| XCorr | Purge duplicate peptides by protein |
| false | Include only loci with unique peptide |
| true | Remove subset proteins |
| Ignore | Locus validation handling |
| 0 | Minimum modified peptides per locus |
| 1000 | Minimum redundancy for low coverage loci |
| 2 | Minimum peptides per locus |

#### Locus Key:

|  |  |  |  |  |  |  |  |  |
| --- | --- | --- | --- | --- | --- | --- | --- | --- |
| Validation Status | Locus | Sequence Count | Spectrum Count | Sequence Coverage | Length | MolWt | pI | Descriptive Name |

#### Similarity Key:

|  |  |  |
| --- | --- | --- |
| Locus | # of identical peptides | # of differing peptides |

---

|  |  |  |  |  |  |  |  |  |
| --- | --- | --- | --- | --- | --- | --- | --- | --- |
| U | *gi|4506671|ref|NP\_000* | 29 | 346 | 97.4% | 115 | 11665 | 4.5 | ribosomal protein P2 [Homo sapiens] |

| Filename XCorr DeltCN Conf% ObsM+H+ CalcM+H+ SpR ZScore Ion% # Sequence  | | | | | | | | | | | | |
| --- | --- | --- | --- | --- | --- | --- | --- | --- | --- | --- | --- | --- |
| \* | pJS43\_100mM\_120812\_01.12230.12230.2 | 6.379 | 0.6025 | 100.0% | 2156.1921 | 2157.4924 | 1 | 10.37 | 70.0% | 1 | -.MRYVASYLLAALGGNSSPSAK.D | 2 |
| \* | pJS43\_100mM\_120812\_02.08169.08169.3 | 3.5171 | 0.3747 | 100.0% | 2157.3542 | 2157.4924 | 1 | 7.196 | 35.0% | 5 | -.MRYVASYLLAALGGNSSPSAK.D | 3 |
| \* | pDK365N\_100mM\_082713\_01.11088.11088.2 | 4.9605 | 0.5047 | 100.0% | 1869.8322 | 1870.1124 | 1 | 9.847 | 69.4% | 26 | R.YVASYLLAALGGNSSPSAK.D | 2 |
| \* | pJS43\_100mM\_120812\_01.12312.12312.3 | 4.676 | 0.4264 | 100.0% | 1870.1943 | 1870.1124 | 1 | 8.077 | 44.4% | 7 | R.YVASYLLAALGGNSSPSAK.D | 3 |
| \* | pJS43\_100mM\_120812\_02.08514.08514.2 | 4.0682 | 0.3002 | 100.0% | 1949.5322 | 1950.1124 | 1 | 7.045 | 58.3% | 3 | R.YVASYLLAALGGNSS\*PSAK.D | 2 |
| \* | pDK365N\_100mM\_082813\_04.08529.08529.3 | 3.9853 | 0.2923 | 99.9% | 1950.2943 | 1950.1124 | 1 | 4.968 | 40.3% | 1 | R.YVASYLLAALGGNSS\*PSAK.D | 3 |
| \* | pDK365N\_100mM\_082713\_02.08554.08554.3 | 3.4609 | 0.2947 | 99.8% | 1950.7444 | 1950.1124 | 1 | 6.065 | 45.8% | 1 | R.YVASYLLAALGGNSSPS\*AK.D | 3 |
| \* | pJS43\_100mM\_120812\_02.05065.05065.3 | 3.6967 | 0.3159 | 100.0% | 1546.8544 | 1546.6744 | 1 | 5.525 | 50.0% | 1 | K.KILDSVGIEADDDR.L | 3 |
| \* | pJS43\_100mM\_120812\_01.06875.06875.2 | 4.1193 | 0.4635 | 100.0% | 1901.4722 | 1902.1118 | 1 | 7.275 | 50.0% | 2 | K.KILDSVGIEADDDRLNK.V | 2 |
| \* | pDK365N\_100mM\_082813\_03.05831.05831.3 | 5.458 | 0.3832 | 100.0% | 1903.1044 | 1902.1118 | 1 | 8.256 | 50.0% | 11 | K.KILDSVGIEADDDRLNK.V | 3 |
| \* | pDK365N\_100mM\_082713\_01.06234.06234.2 | 4.4745 | 0.5432 | 100.0% | 1419.3322 | 1418.5004 | 1 | 9.998 | 75.0% | 21 | K.ILDSVGIEADDDR.L | 2 |
| \* | pJS43\_100mM\_120812\_01.07302.07302.2 | 4.7487 | 0.4394 | 100.0% | 1773.2722 | 1773.9377 | 1 | 8.409 | 70.0% | 12 | K.ILDSVGIEADDDRLNK.V | 2 |
| \* | pJS43\_100mM\_120812\_01.07237.07237.3 | 2.4457 | 0.2515 | 95.4% | 1775.6943 | 1773.9377 | 1 | 4.751 | 43.3% | 1 | K.ILDSVGIEADDDRLNK.V | 3 |
| \* | pDK365N\_100mM\_082813\_03.11051.11051.3 | 5.5819 | 0.444 | 100.0% | 2614.1042 | 2614.9126 | 1 | 8.017 | 33.7% | 3 | K.ILDSVGIEADDDRLNKVISELNGK.N | 3 |
|  | pJS43\_100mM\_120812\_01.03968.03968.1 | 1.9666 | 0.2233 | 97.9% | 859.4 | 859.99817 | 1 | 5.565 | 71.4% | 2 | K.VISELNGK.N | 1 |
|  | pJS43\_100mM\_120812\_01.03953.03953.2 | 2.078 | 0.3046 | 99.1% | 860.09216 | 859.99817 | 215 | 5.265 | 64.3% | 1 | K.VISELNGK.N | 2 |
| \* | pDK365N\_100mM\_082713\_01.10176.10176.3 | 4.99 | 0.4571 | 100.0% | 2098.4944 | 2098.4045 | 1 | 7.489 | 43.4% | 2 | K.VISELNGKNIEDVIAQGIGK.L | 3 |
| \* | pDK365N\_100mM\_082813\_03.09731.09731.1 | 2.8845 | 0.4507 | 98.6% | 1256.76 | 1257.4294 | 1 | 7.475 | 63.6% | 2 | K.NIEDVIAQGIGK.L | 1 |
| \* | pJS43\_100mM\_120812\_01.10781.10781.2 | 4.763 | 0.4957 | 100.0% | 1257.1921 | 1257.4294 | 1 | 8.551 | 81.8% | 39 | K.NIEDVIAQGIGK.L | 2 |
| \* | pDK365N\_100mM\_082713\_01.06060.06060.2 | 4.7035 | 0.6434 | 100.0% | 2776.0923 | 2776.0757 | 1 | 11.022 | 39.1% | 7 | K.LASVPAGGAVAVSAAPGSAAPAAGSAPAAAEEK.K | 2 |
| \* | pDK365N\_100mM\_082713\_02.04904.04904.3 | 6.8005 | 0.5284 | 100.0% | 2776.3145 | 2776.0757 | 1 | 10.399 | 32.0% | 26 | K.LASVPAGGAVAVSAAPGSAAPAAGSAPAAAEEK.K | 3 |
| \* | pJS43\_100mM\_120812\_02.05190.05190.3 | 3.986 | 0.3152 | 99.9% | 2856.3245 | 2856.0757 | 1 | 5.28 | 22.7% | 1 | K.LASVPAGGAVAVSAAPGS\*AAPAAGSAPAAAEEK.K | 3 |
| \* | pJS43\_100mM\_120812\_01.06417.06417.3 | 3.3596 | 0.367 | 99.9% | 2903.7844 | 2904.2498 | 2 | 6.339 | 22.0% | 5 | K.LASVPAGGAVAVSAAPGSAAPAAGSAPAAAEEKK.D | 3 |
| \* | pDK365N\_100mM\_082713\_01.05028.05028.3 | 4.3963 | 0.5216 | 100.0% | 3275.8743 | 3276.628 | 1 | 9.24 | 29.2% | 4 | K.LASVPAGGAVAVSAAPGSAAPAAGSAPAAAEEKKDEK.K | 3 |
| \* | pDK365N\_100mM\_082813\_03.09329.09329.3 | 4.0584 | 0.4548 | 100.0% | 2452.6143 | 2451.5312 | 1 | 6.969 | 36.2% | 6 | K.KDEKKEESEESDDDMGFGLFD.- | 3 |
|  | pJS43\_100mM\_120812\_01.11939.11939.2 | 5.1632 | 0.5738 | 100.0% | 1950.1522 | 1950.979 | 1 | 11.209 | 81.2% | 145 | K.KEESEESDDDMGFGLFD.- | 22 |
|  | pJS43\_100mM\_120812\_02.08410.08410.2 | 3.7525 | 0.4679 | 100.0% | 2030.3322 | 2030.979 | 1 | 8.972 | 56.2% | 5 | K.KEES\*EESDDDMGFGLFD.- | 22 |
|  | pJS43\_100mM\_120812\_01.12672.12672.2 | 3.6849 | 0.4023 | 100.0% | 2030.9922 | 2030.979 | 1 | 7.695 | 59.4% | 4 | K.KEESEES\*DDDMGFGLFD.- | 22 |
|  | pJS43\_100mM\_120812\_02.16231.16231.2 | 2.2632 | 0.4024 | 99.6% | 2110.152 | 2110.979 | 1 | 6.076 | 43.8% | 2 | K.KEES\*EES\*DDDMGFGLFD.- | 22 |

Similarities:
gi|4506669|ref|NP\_000(4:25)  

---

|  |  |  |  |  |  |  |  |  |
| --- | --- | --- | --- | --- | --- | --- | --- | --- |
| U | *gi|73623035|ref|NP\_00* | 262 | 4813 | 91.9% | 1193 | 134422 | 5.0 | sperm associated antigen 5 [Homo sapiens] |

| Filename XCorr DeltCN Conf% ObsM+H+ CalcM+H+ SpR ZScore Ion% # Sequence  | | | | | | | | | | | | |
| --- | --- | --- | --- | --- | --- | --- | --- | --- | --- | --- | --- | --- |
| \* | 100326\_pJS43\_01.04040.04040.2 | 5.1306 | 0.5633 | 100.0% | 1914.4521 | 1915.2585 | 1 | 9.463 | 61.8% | 10 | K.KLSLSLSPSPQTGKPSMR.T | 2 |
| \* | 100326\_pJS43\_01.04358.04358.3 | 4.9272 | 0.5104 | 100.0% | 1915.2244 | 1915.2585 | 1 | 8.501 | 47.1% | 17 | K.KLSLSLSPSPQTGKPSMR.T | 3 |
| \* | SKAPIP\_tube2\_041314\_01.06244.06244.3 | 2.8295 | 0.2134 | 95.3% | 1994.8744 | 1995.2585 | 1 | 4.567 | 39.7% | 1 | K.KLSLSLSPS\*PQTGKPSMR.T | 3 |
| \* | AstrinIP\_MS2\_022614\_01.07025.07025.3 | 3.1631 | 0.3421 | 99.9% | 1995.4744 | 1995.2585 | 27 | 5.443 | 32.4% | 1 | K.KLS\*LSLSPSPQTGKPSMR.T | 3 |
| \* | AstrinIP\_MS1\_022614\_01.07020.07020.3 | 2.7341 | 0.3242 | 99.4% | 2073.4744 | 2075.2585 | 58 | 4.879 | 32.4% | 1 | K.KLSLS\*LSPS\*PQTGKPSMR.T | 3 |
| \* | AstrinIP\_MS2\_022614\_01.07470.07470.3 | 2.8101 | 0.2209 | 95.7% | 2074.1643 | 2075.2585 | 21 | 3.96 | 32.4% | 1 | K.KLS\*LSLS\*PSPQTGKPSMR.T | 3 |
| \* | 100326\_pJS43\_01.04691.04691.2 | 3.9989 | 0.4723 | 100.0% | 1786.2322 | 1787.0845 | 1 | 8.803 | 68.8% | 23 | K.LSLSLSPSPQTGKPSMR.T | 2 |
| \* | AstrinIP\_MS1\_022614\_01.06824.06824.3 | 2.7904 | 0.2139 | 95.4% | 1786.8844 | 1787.0845 | 5 | 5.207 | 34.4% | 5 | K.LSLSLSPSPQTGKPSMR.T | 3 |
| \* | AstrinIP\_MS1\_022614\_01.08363.08363.3 | 2.8097 | 0.2127 | 95.4% | 1946.7843 | 1947.0845 | 1 | 4.416 | 40.6% | 1 | K.LSLSLS\*PS\*PQTGKPSMR.T | 3 |
| \* | 100326\_pJS43\_01.06248.06248.3 | 3.5218 | 0.3398 | 100.0% | 1895.8744 | 1897.182 | 1 | 5.992 | 36.8% | 3 | R.TPLRELTLQPGALTNSGK.R | 3 |
| \* | 100326\_pJS43\_01.06206.06206.2 | 5.8016 | 0.548 | 100.0% | 1896.5521 | 1897.182 | 1 | 10.371 | 70.6% | 4 | R.TPLRELTLQPGALTNSGK.R | 2 |
| \* | 100326\_pJS43\_01.05538.05538.3 | 4.7602 | 0.4523 | 100.0% | 2051.7544 | 2053.3694 | 1 | 7.877 | 45.8% | 7 | R.TPLRELTLQPGALTNSGKR.S | 3 |
| \* | 100326\_pJS43\_01.05512.05512.2 | 3.5451 | 0.3345 | 100.0% | 2053.5122 | 2053.3694 | 1 | 6.119 | 63.9% | 1 | R.TPLRELTLQPGALTNSGKR.S | 2 |
| \* | AstrinIP\_MS1\_022614\_01.07388.07388.2 | 3.8288 | 0.3591 | 100.0% | 1429.2322 | 1429.6133 | 1 | 7.364 | 80.8% | 27 | R.ELTLQPGALTNSGK.R | 2 |
| \* | AstrinIP\_MS2\_022614\_01.07793.07793.1 | 3.349 | 0.4471 | 97.8% | 1429.68 | 1429.6133 | 1 | 8.373 | 61.5% | 8 | R.ELTLQPGALTNSGK.R | 1 |
| \* | AstrinIP\_MS2\_022614\_01.08207.08207.2 | 2.9224 | 0.2636 | 99.6% | 1509.2322 | 1509.6133 | 1 | 5.252 | 69.2% | 1 | R.ELTLQPGALTNS\*GK.R | 2 |
| \* | 100326\_pJS43\_01.04484.04484.2 | 3.4848 | 0.2915 | 99.9% | 1586.3522 | 1585.8008 | 2 | 5.515 | 60.7% | 21 | R.ELTLQPGALTNSGKR.S | 2 |
| \* | AstrinIP\_MS1\_022614\_01.05100.05100.2 | 2.2895 | 0.3336 | 99.1% | 1643.7722 | 1644.7113 | 1 | 5.335 | 65.4% | 1 | K.RS\*PACSSLTPSLCK.L | 2 |
| \* | AstrinIP\_MS1\_022614\_01.05463.05463.2 | 3.5969 | 0.4869 | 100.0% | 1407.6322 | 1408.5238 | 1 | 8.354 | 66.7% | 3 | R.SPACSSLTPSLCK.L | 2 |
| \* | 100326\_pJS43\_01.06500.06500.2 | 5.7398 | 0.5895 | 100.0% | 2018.8322 | 2020.1631 | 1 | 10.671 | 61.1% | 15 | K.LGLQEGSNNSSPVDFVNNK.R | 2 |
| \* | AstrinIP\_MS2\_022614\_01.09896.09896.2 | 4.5442 | 0.5172 | 100.0% | 2099.5923 | 2100.163 | 1 | 7.477 | 58.3% | 2 | K.LGLQEGS\*NNSSPVDFVNNK.R | 2 |
| \* | 100326\_pJS43\_01.05667.05667.2 | 4.0608 | 0.5487 | 100.0% | 2175.2922 | 2176.3506 | 1 | 9.256 | 50.0% | 18 | K.LGLQEGSNNSSPVDFVNNKR.T | 2 |
| \* | SKAPIP\_041314\_01.11129.11129.3 | 4.341 | 0.4324 | 100.0% | 2175.5942 | 2176.3506 | 1 | 7.096 | 39.5% | 42 | K.LGLQEGSNNSSPVDFVNNKR.T | 3 |
| \* | AstrinIP\_MS1\_022614\_01.09509.09509.2 | 4.1979 | 0.5317 | 100.0% | 2179.0122 | 2180.163 | 1 | 7.249 | 58.3% | 1 | K.LGLQEGS\*NNS\*SPVDFVNNK.R | 2 |
| \* | AstrinIP\_MS2\_022614\_01.09977.09977.2 | 4.5047 | 0.5245 | 100.0% | 2179.2722 | 2180.163 | 1 | 7.247 | 61.1% | 2 | K.LGLQEGS\*NNSS\*PVDFVNNK.R | 2 |
| \* | AstrinIP\_MS1\_022614\_01.07667.07667.2 | 3.4257 | 0.2772 | 99.9% | 2255.152 | 2256.3506 | 2 | 4.919 | 39.5% | 1 | K.LGLQEGSNNSS\*PVDFVNNKR.T | 2 |
| \* | AstrinIP\_MS2\_022614\_01.08055.08055.2 | 3.1136 | 0.3941 | 100.0% | 2255.4321 | 2256.3506 | 1 | 6.82 | 42.1% | 1 | K.LGLQEGSNNS\*SPVDFVNNKR.T | 2 |
| \* | pDK339othertube\_033013\_01.07157.07157.3 | 3.1296 | 0.3406 | 99.9% | 2255.7544 | 2256.3506 | 2 | 5.16 | 34.2% | 1 | K.LGLQEGSNNSS\*PVDFVNNKR.T | 3 |
| \* | SKAPIP\_tube2\_041314\_01.08097.08097.3 | 4.0067 | 0.4128 | 100.0% | 2257.1042 | 2256.3506 | 13 | 6.062 | 32.9% | 14 | K.LGLQEGSNNS\*SPVDFVNNKR.T | 3 |
| \* | AstrinIP\_MS2\_022614\_01.09014.09014.3 | 2.7406 | 0.2613 | 97.5% | 2335.5244 | 2336.3506 | 12 | 4.48 | 28.9% | 1 | K.LGLQEGS\*NNSS\*PVDFVNNKR.T | 3 |
| \* | AstrinIP\_MS2\_022614\_01.08948.08948.3 | 3.0926 | 0.3932 | 100.0% | 2335.8843 | 2336.3506 | 2 | 5.343 | 31.6% | 4 | K.LGLQEGSNNS\*S\*PVDFVNNKR.T | 3 |
| \* | pJS43\_100mM\_120812\_01.03312.03312.2 | 3.6134 | 0.4923 | 100.0% | 1618.2922 | 1618.7031 | 1 | 7.968 | 69.2% | 4 | K.RTDLSSEHFSHSSK.W | 2 |
| \* | pJS43\_100mM\_120812\_01.03306.03306.3 | 3.6058 | 0.2995 | 100.0% | 1619.0944 | 1618.7031 | 3 | 5.484 | 42.3% | 4 | K.RTDLSSEHFSHSSK.W | 3 |
| \* | AstrinIP\_MS2\_022614\_01.03586.03586.1 | 2.4768 | 0.5135 | 97.7% | 1461.57 | 1462.5156 | 1 | 8.196 | 50.0% | 1 | R.TDLSSEHFSHSSK.W | 1 |
| \* | 100326\_pJS43\_01.00440.00440.2 | 4.6217 | 0.4906 | 100.0% | 1462.3322 | 1462.5156 | 1 | 7.843 | 79.2% | 15 | R.TDLSSEHFSHSSK.W | 2 |
| \* | AstrinIP\_MS2\_022614\_01.03579.03579.3 | 2.1094 | 0.3215 | 97.8% | 1462.9143 | 1462.5156 | 72 | 5.323 | 35.4% | 1 | R.TDLSSEHFSHSSK.W | 3 |
| \* | AstrinIP\_MS1\_022614\_01.09384.09384.2 | 4.0326 | 0.4516 | 100.0% | 2936.7922 | 2937.1555 | 1 | 7.482 | 43.8% | 1 | K.WLETCQHESDEQPLDPIPQISSTPK.T | 2 |
| \* | 100326\_pJS43\_01.07461.07461.3 | 5.0896 | 0.3369 | 100.0% | 2938.3145 | 2937.1555 | 1 | 6.084 | 37.5% | 8 | K.WLETCQHESDEQPLDPIPQISSTPK.T | 3 |
| \* | AstrinIP\_MS1\_022614\_02.06375.06375.3 | 4.2486 | 0.275 | 99.9% | 3016.1943 | 3017.1555 | 1 | 5.415 | 38.5% | 1 | K.WLETCQHESDEQPLDPIPQISST#PK.T | 3 |
| \* | AstrinIP\_MS1\_022614\_01.09970.09970.2 | 3.2596 | 0.2857 | 99.9% | 3017.412 | 3017.1555 | 2 | 5.321 | 33.3% | 1 | K.WLETCQHESDEQPLDPIPQIS\*STPK.T | 2 |
| \* | 100326\_pJS43\_01.07220.07220.2 | 4.6542 | 0.4436 | 100.0% | 1652.7122 | 1653.8445 | 1 | 9.068 | 67.9% | 43 | K.TSEEAVDPLGNYMVK.T | 2 |
| \* | AstrinIP\_MS1\_022614\_01.09051.09051.1 | 3.6463 | 0.4038 | 98.6% | 1653.72 | 1653.8445 | 1 | 7.996 | 60.7% | 15 | K.TSEEAVDPLGNYMVK.T | 1 |
| \* | AstrinIP\_MS2\_022614\_01.13024.13024.2 | 4.4861 | 0.5096 | 100.0% | 2243.372 | 2243.6262 | 1 | 8.728 | 63.2% | 17 | K.TIVLVPSPLGQQQDMIFEAR.L | 2 |
| \* | 100326\_pJS43\_01.10286.10286.3 | 3.6673 | 0.4942 | 100.0% | 2244.1143 | 2243.6262 | 1 | 8.427 | 44.7% | 6 | K.TIVLVPSPLGQQQDMIFEAR.L | 3 |
| \* | AstrinIP\_MS1\_022614\_01.12884.12884.3 | 3.2797 | 0.4519 | 100.0% | 2323.1042 | 2323.6262 | 1 | 7.818 | 40.8% | 8 | K.TIVLVPS\*PLGQQQDMIFEAR.L | 3 |
| \* | AstrinIP\_MS1\_022614\_01.12878.12878.2 | 4.1129 | 0.5463 | 100.0% | 2323.672 | 2323.6262 | 1 | 8.499 | 60.5% | 37 | K.TIVLVPS\*PLGQQQDMIFEAR.L | 2 |
| \* | 100326\_pJS43\_01.07101.07101.2 | 5.6326 | 0.5071 | 100.0% | 1832.3322 | 1833.0668 | 1 | 8.943 | 71.9% | 63 | R.LDTMAETNSISLNGPLR.T | 2 |
| \* | AstrinIP\_MS2\_022614\_02.06374.06374.3 | 3.4187 | 0.3039 | 99.8% | 1832.9043 | 1833.0668 | 4 | 5.184 | 39.1% | 3 | R.LDTMAETNSISLNGPLR.T | 3 |
| \* | AstrinIP\_MS2\_022614\_01.10202.10202.2 | 5.016 | 0.4416 | 100.0% | 1913.2522 | 1913.0668 | 1 | 7.129 | 59.4% | 15 | R.LDTMAETNSIS\*LNGPLR.T | 2 |
| \* | AstrinIP\_MS2\_022614\_01.10037.10037.2 | 3.6717 | 0.2334 | 99.9% | 1913.5122 | 1913.0668 | 2 | 4.984 | 53.1% | 3 | R.LDTMAETNS\*ISLNGPLR.T | 2 |
| \* | AstrinIP\_MS1\_022614\_01.09806.09806.3 | 4.7475 | 0.4664 | 100.0% | 2531.5444 | 2532.8286 | 1 | 8.649 | 35.2% | 36 | R.LDTMAETNSISLNGPLRTDDLVR.E | 3 |
| \* | AstrinIP\_MS1\_022614\_01.10205.10205.3 | 3.1319 | 0.2866 | 99.2% | 2612.2744 | 2612.8286 | 8 | 4.489 | 26.1% | 1 | R.LDTMAETNS\*ISLNGPLRTDDLVR.E | 3 |
| \* | SKAPIP\_tube2\_041314\_02.07793.07793.3 | 4.127 | 0.3152 | 100.0% | 2612.4243 | 2612.8286 | 1 | 5.307 | 31.8% | 9 | R.LDTMAETNSIS\*LNGPLRTDDLVR.E | 3 |
| \* | SKAPIP\_tube2\_041314\_01.06264.06264.2 | 3.4035 | 0.3284 | 100.0% | 1863.1721 | 1864.0033 | 1 | 6.687 | 53.3% | 2 | R.TDDLVREEVAPCMGDR.F | 2 |
| \* | SKAPIP\_tube2\_041314\_01.06246.06246.3 | 3.0576 | 0.2695 | 99.3% | 1864.1643 | 1864.0033 | 19 | 5.608 | 33.3% | 1 | R.TDDLVREEVAPCMGDR.F | 3 |
| \* | AstrinIP\_MS1\_022614\_01.04096.04096.2 | 2.6123 | 0.3161 | 99.8% | 1164.0322 | 1164.2416 | 3 | 5.344 | 72.2% | 3 | R.EEVAPCMGDR.F | 2 |
| \* | SKAPIP\_tube2\_041314\_01.14330.14330.3 | 7.2538 | 0.5129 | 100.0% | 3779.1243 | 3778.2102 | 1 | 9.456 | 25.0% | 23 | R.TEAVREDLVPSESNAFLPSSVLWLSPSTALAADFR.V | 3 |
| \* | SKAPIP\_tube2\_041314\_01.14338.14338.3 | 6.2488 | 0.2155 | 100.0% | 3857.4844 | 3858.2102 | 2 | 7.21 | 32.4% | 1 | R.TEAVREDLVPSESNAFLPSSVLWLSPS\*TALAADFR.V | 3 |
| \* | pJS43\_100mM\_120812\_02.09630.09630.3 | 6.5786 | 0.1612 | 99.9% | 3857.6643 | 3858.2102 | 1 | 7.569 | 34.6% | 41 | R.TEAVREDLVPSESNAFLPSSVLWLS\*PSTALAADFR.V | 3 |
| \* | AstrinIP\_MS1\_022614\_01.15592.15592.3 | 4.9313 | 0.1078 | 97.6% | 3937.1643 | 3938.2102 | 2 | 5.439 | 21.3% | 1 | R.TEAVREDLVPS\*ESNAFLPSSVLWLS\*PSTALAADFR.V | 3 |
| \* | AstrinIP\_MS2\_022614\_01.16102.16102.3 | 5.5998 | 0.0524 | 96.3% | 3937.1943 | 3938.2102 | 3 | 4.978 | 22.1% | 1 | R.TEAVREDLVPSES\*NAFLPSSVLWLSPST#ALAADFR.V | 3 |
| \* | SKAPIP\_tube2\_041314\_01.14453.14453.3 | 5.4226 | 0.0485 | 95.3% | 3937.7344 | 3938.2102 | 1 | 5.481 | 26.5% | 1 | R.TEAVREDLVPSESNAFLPSS\*VLWLS\*PSTALAADFR.V | 3 |
| \* | SKAPIP\_tube2\_041314\_01.15078.15078.3 | 5.0457 | 0.0719 | 95.5% | 3937.9744 | 3938.2102 | 1 | 4.641 | 26.5% | 1 | R.TEAVREDLVPSES\*NAFLPSSVLWLS\*PSTALAADFR.V | 3 |
| \* | AstrinIP\_MS2\_022614\_01.16437.16437.2 | 4.1564 | 0.5354 | 100.0% | 3221.0322 | 3221.5908 | 1 | 8.807 | 36.2% | 2 | R.EDLVPSESNAFLPSSVLWLSPSTALAADFR.V | 2 |
| \* | AstrinIP\_MS1\_022614\_01.16130.16130.2 | 3.9306 | 0.5132 | 100.0% | 3301.412 | 3301.5908 | 1 | 6.883 | 34.5% | 7 | R.EDLVPSESNAFLPSSVLWLS\*PSTALAADFR.V | 2 |
| \* | AstrinIP\_MS1\_022614\_01.06591.06591.2 | 5.988 | 0.5626 | 100.0% | 2219.5322 | 2220.3752 | 1 | 10.714 | 72.2% | 36 | R.VNHVDPEEEIVEHGAMEER.E | 2 |
| \* | SKAPIP\_041314\_02.05518.05518.3 | 5.4795 | 0.4329 | 100.0% | 2220.9243 | 2220.3752 | 1 | 7.376 | 47.2% | 143 | R.VNHVDPEEEIVEHGAMEER.E | 3 |
| \* | SKAPIP\_041314\_01.05289.05289.2 | 2.1566 | 0.2947 | 99.0% | 1142.8922 | 1143.3489 | 54 | 4.956 | 50.0% | 2 | R.EMRFPTHPK.E | 2 |
| \* | AstrinIP\_MS1\_022614\_01.16982.16982.3 | 5.1975 | 0.5728 | 100.0% | 4634.574 | 4633.9536 | 1 | 8.461 | 20.8% | 1 | K.ESETEDQALVSSVEDILSTCLTPNLVEMESQEAPGPAVEDVGR.I | 3 |
| \* | SKAPIP\_041314\_01.15008.15008.2 | 6.2027 | 0.5679 | 100.0% | 2063.5923 | 2064.3606 | 1 | 9.878 | 64.7% | 42 | R.ILGSDTESWMSPLAWLEK.G | 2 |
| \* | AstrinIP\_MS2\_022614\_01.16112.16112.3 | 4.8082 | 0.4108 | 100.0% | 2065.2544 | 2064.3606 | 1 | 7.147 | 41.2% | 8 | R.ILGSDTESWMSPLAWLEK.G | 3 |
| \* | SKAPIP\_tube2\_041314\_01.15584.15584.2 | 5.3447 | 0.5338 | 100.0% | 2143.392 | 2144.3606 | 1 | 7.866 | 67.6% | 30 | R.ILGSDTESWMS\*PLAWLEK.G | 2 |
| \* | SKAPIP\_041314\_01.16124.16124.2 | 4.0896 | 0.4197 | 100.0% | 2143.4321 | 2144.3606 | 1 | 6.828 | 58.8% | 4 | R.ILGS\*DTESWMSPLAWLEK.G | 2 |
| \* | SKAPIP\_tube2\_041314\_01.16192.16192.2 | 4.263 | 0.5236 | 100.0% | 2143.612 | 2144.3606 | 2 | 7.069 | 50.0% | 5 | R.ILGSDT#ESWMSPLAWLEK.G | 2 |
| \* | AstrinIP\_MS2\_022614\_01.16673.16673.3 | 4.6072 | 0.489 | 100.0% | 2143.9443 | 2144.3606 | 1 | 7.817 | 42.6% | 3 | R.ILGSDTESWMS\*PLAWLEK.G | 3 |
| \* | AstrinIP\_MS2\_022614\_01.17200.17200.2 | 3.8022 | 0.436 | 100.0% | 2144.3323 | 2144.3606 | 2 | 6.004 | 44.1% | 2 | R.ILGSDTES\*WMSPLAWLEK.G | 2 |
| \* | AstrinIP\_MS1\_022614\_01.17181.17181.2 | 3.9689 | 0.2116 | 99.9% | 2223.872 | 2224.3606 | 1 | 6.127 | 55.9% | 3 | R.ILGS\*DTESWMS\*PLAWLEK.G | 2 |
| \* | AstrinIP\_MS2\_022614\_01.17648.17648.2 | 3.8999 | 0.2026 | 99.9% | 2224.4321 | 2224.3606 | 2 | 5.808 | 50.0% | 7 | R.ILGSDT#ESWMS\*PLAWLEK.G | 2 |
| \* | AstrinIP\_MS2\_022614\_01.09345.09345.1 | 2.5466 | 0.3536 | 98.7% | 1332.6 | 1333.5457 | 1 | 5.431 | 54.5% | 9 | K.GVNTSVMLENLR.Q | 1 |
| \* | 100326\_pJS43\_01.06872.06872.2 | 4.3522 | 0.4645 | 100.0% | 1333.3522 | 1333.5457 | 1 | 8.239 | 81.8% | 34 | K.GVNTSVMLENLR.Q | 2 |
| \* | AstrinIP\_MS1\_022614\_01.09999.09999.1 | 1.4677 | 0.4635 | 98.7% | 1412.5 | 1413.5457 | 248 | 6.537 | 36.4% | 2 | K.GVNT#SVMLENLR.Q | 1 |
| \* | AstrinIP\_MS1\_022614\_01.09934.09934.1 | 1.3523 | 0.3386 | 98.6% | 1412.52 | 1413.5457 | 240 | 6.654 | 40.9% | 1 | K.GVNTS\*VMLENLR.Q | 1 |
| \* | AstrinIP\_MS2\_022614\_01.10556.10556.2 | 3.9941 | 0.4087 | 100.0% | 1413.1522 | 1413.5457 | 1 | 6.552 | 77.3% | 18 | K.GVNTS\*VMLENLR.Q | 2 |
| \* | AstrinIP\_MS2\_022614\_01.13907.13907.3 | 3.2737 | 0.1883 | 95.6% | 2607.9243 | 2606.886 | 13 | 4.404 | 27.4% | 3 | K.GVNTS\*VMLENLRQS\*LSLPSMLR.D | 3 |
| \* | AstrinIP\_MS2\_022614\_01.10346.10346.1 | 1.2377 | 0.2982 | 96.4% | 1131.5 | 1132.3635 | 137 | 5.224 | 50.0% | 1 | R.QSLSLPSMLR.D | 1 |
| \* | AstrinIP\_MS1\_022614\_02.06207.06207.2 | 2.3303 | 0.2343 | 98.6% | 1132.1322 | 1132.3635 | 99 | 5.396 | 61.1% | 16 | R.QSLSLPSMLR.D | 2 |
| \* | SKAPIP\_tube2\_041314\_02.09490.09490.3 | 3.2926 | 0.3212 | 99.7% | 2755.8542 | 2757.9795 | 1 | 5.615 | 29.0% | 1 | R.DAAIGTTPFSTCSVGTWFTPSAPQEK.S | 3 |
| \* | SKAPIP\_tube2\_041314\_01.11901.11901.2 | 4.7149 | 0.5369 | 100.0% | 2757.7122 | 2757.9795 | 1 | 9.302 | 46.0% | 9 | R.DAAIGTTPFSTCSVGTWFTPSAPQEK.S | 2 |
| \* | 100326\_pJS43\_01.00449.00449.1 | 1.9948 | 0.3491 | 98.5% | 1293.34 | 1294.4044 | 17 | 5.696 | 45.8% | 3 | K.STNTSQTGLVGTK.H | 1 |
| \* | AstrinIP\_MS2\_022614\_01.03752.03752.2 | 3.6058 | 0.464 | 100.0% | 1294.2722 | 1294.4044 | 1 | 8.287 | 75.0% | 12 | K.STNTSQTGLVGTK.H | 2 |
| \* | AstrinIP\_MS2\_022614\_01.04119.04119.1 | 1.5937 | 0.2575 | 96.6% | 1373.51 | 1374.4044 | 19 | 4.365 | 45.8% | 1 | K.STNT#SQTGLVGTK.H | 1 |
| \* | SKAPIP\_041314\_01.04923.04923.2 | 3.3542 | 0.1433 | 99.3% | 1374.2122 | 1374.4044 | 2 | 6.004 | 70.8% | 10 | K.STNT#SQTGLVGTK.H | 2 |
| \* | SKAPIP\_041314\_01.04592.04592.2 | 2.9253 | 0.1496 | 98.5% | 1374.2322 | 1374.4044 | 1 | 5.077 | 75.0% | 7 | K.STNTS\*QTGLVGTK.H | 2 |
| \* | AstrinIP\_MS1\_022614\_01.08702.08702.3 | 3.4855 | 0.3019 | 99.8% | 2469.4443 | 2469.68 | 1 | 6.185 | 39.3% | 11 | K.HSTSETEQLLCGRPPDLTALSR.H | 3 |
| \* | SKAPIP\_tube2\_041314\_01.08333.08333.2 | 3.5073 | 0.4305 | 100.0% | 2469.652 | 2469.68 | 1 | 7.286 | 35.7% | 5 | K.HSTSETEQLLCGRPPDLTALSR.H | 2 |
| \* | 100326\_pJS43\_01.13934.13934.2 | 7.7524 | 0.6617 | 100.0% | 2165.0322 | 2166.4795 | 1 | 11.31 | 83.3% | 229 | R.HDLEDNLLSSLVILEVLSR.Q | 2 |
| \* | pDK339othertube\_033013\_01.15926.15926.3 | 3.8514 | 0.4313 | 100.0% | 2166.9243 | 2166.4795 | 1 | 6.975 | 41.7% | 50 | R.HDLEDNLLSSLVILEVLSR.Q | 3 |
| \* | pDK339\_033013\_01.04448.04448.3 | 5.6598 | 0.5185 | 100.0% | 2867.0645 | 2868.0 | 1 | 7.792 | 31.7% | 61 | K.SQLAVPHPETQDSSTQTDTSHSGITNK.L | 3 |
| \* | AstrinIP\_MS1\_022614\_01.04246.04246.2 | 4.4118 | 0.4895 | 100.0% | 2867.5923 | 2868.0 | 1 | 7.831 | 42.3% | 13 | K.SQLAVPHPETQDSSTQTDTSHSGITNK.L | 2 |
| \* | AstrinIP\_MS1\_022614\_01.04749.04749.3 | 4.084 | 0.1638 | 97.6% | 2947.7644 | 2948.0 | 5 | 5.366 | 30.8% | 2 | K.SQLAVPHPETQDSSTQTDTS\*HSGITNK.L | 3 |
| \* | AstrinIP\_MS1\_022614\_01.03996.03996.2 | 6.1689 | 0.5315 | 100.0% | 2104.2722 | 2105.3794 | 1 | 10.664 | 70.6% | 26 | K.LQHLKESHEMGQALQQAR.N | 2 |
| \* | pSKT11\_1\_020812\_01.06344.06344.3 | 6.1265 | 0.4543 | 100.0% | 2105.6042 | 2105.3794 | 2 | 7.847 | 48.5% | 32 | K.LQHLKESHEMGQALQQAR.N | 3 |
| \* | SKAPIP\_041314\_01.05669.05669.2 | 4.0189 | 0.3812 | 100.0% | 1485.1322 | 1485.6146 | 1 | 6.897 | 75.0% | 14 | K.ESHEMGQALQQAR.N | 2 |
| \* | SKAPIP\_041314\_01.05780.05780.3 | 4.2985 | 0.0842 | 97.9% | 1485.7144 | 1485.6146 | 5 | 4.93 | 52.1% | 6 | K.ESHEMGQALQQAR.N | 3 |
| \* | AstrinIP\_MS1\_022614\_01.03860.03860.1 | 3.5338 | 0.1684 | 98.7% | 1486.58 | 1485.6146 | 1 | 5.349 | 66.7% | 9 | K.ESHEMGQALQQAR.N | 1 |
| \* | SKAPIP\_tube2\_041314\_01.10688.10688.2 | 4.3078 | 0.3539 | 100.0% | 1306.5122 | 1305.578 | 1 | 6.772 | 75.0% | 52 | R.NVMQSWVLISK.E | 2 |
| \* | AstrinIP\_MS2\_022614\_01.19937.19937.3 | 7.2736 | 0.5772 | 100.0% | 3288.5645 | 3289.9011 | 1 | 9.618 | 32.4% | 1 | R.NVMQSWVLISKELISLLHLSLLHLEEDK.T | 3 |
| \* | AstrinIP\_MS2\_022614\_01.19653.19653.3 | 7.3529 | 0.5958 | 100.0% | 4177.344 | 4178.834 | 1 | 9.486 | 25.7% | 5 | R.NVMQSWVLISKELISLLHLSLLHLEEDKTTVSQESR.R | 3 |
| \* | AstrinIP\_MS2\_022614\_01.12981.12981.3 | 4.2115 | 0.2478 | 99.7% | 4337.514 | 4338.834 | 1 | 4.94 | 20.0% | 4 | R.NVMQS\*WVLIS\*KELISLLHLSLLHLEEDKTTVSQESR.R | 3 |
| \* | AstrinIP\_MS2\_022614\_01.13869.13869.2 | 4.9283 | 0.44 | 100.0% | 2003.4922 | 2003.3464 | 1 | 8.669 | 56.2% | 3 | K.ELISLLHLSLLHLEEDK.T | 2 |
| \* | AstrinIP\_MS2\_022614\_01.13862.13862.3 | 4.2828 | 0.4175 | 100.0% | 2003.5443 | 2003.3464 | 1 | 7.235 | 42.2% | 9 | K.ELISLLHLSLLHLEEDK.T | 3 |
| \* | AstrinIP\_MS2\_022614\_01.12981.12981.2 | 5.6176 | 0.4711 | 100.0% | 2892.0122 | 2892.2793 | 1 | 9.277 | 41.7% | 10 | K.ELISLLHLSLLHLEEDKTTVSQESR.R | 3 |
| \* | AstrinIP\_MS1\_022614\_01.12320.12320.3 | 7.682 | 0.432 | 100.0% | 2892.0842 | 2892.2793 | 1 | 7.99 | 37.5% | 207 | K.ELISLLHLSLLHLEEDKTTVSQESR.R | 3 |
| \* | pSKT11\_1\_020812\_02.06579.06579.3 | 5.1683 | 0.4674 | 100.0% | 3046.7644 | 3048.4668 | 1 | 6.617 | 27.0% | 3 | K.ELISLLHLSLLHLEEDKTTVSQESRR.A | 3 |
| \* | AstrinIP\_MS1\_022614\_01.09844.09844.2 | 4.0451 | 0.378 | 100.0% | 1785.3522 | 1785.9766 | 1 | 7.205 | 76.9% | 5 | R.RAETLVCCCFDLLK.K | 2 |
| \* | AstrinIP\_MS1\_022614\_01.08481.08481.2 | 3.4297 | 0.2285 | 99.7% | 1913.5322 | 1914.1506 | 1 | 4.974 | 67.9% | 1 | R.RAETLVCCCFDLLKK.L | 2 |
| \* | SKAPIP\_tube2\_041314\_02.06693.06693.3 | 4.5313 | 0.3968 | 100.0% | 1914.5643 | 1914.1506 | 2 | 6.711 | 48.2% | 6 | R.RAETLVCCCFDLLKK.L | 3 |
| \* | AstrinIP\_MS1\_022614\_02.07071.07071.2 | 3.9651 | 0.4831 | 100.0% | 1629.1921 | 1629.7891 | 1 | 8.232 | 62.5% | 8 | R.AETLVCCCFDLLK.K | 2 |
| \* | AstrinIP\_MS1\_022614\_01.09621.09621.2 | 3.9803 | 0.3261 | 100.0% | 1756.9521 | 1757.9631 | 1 | 6.653 | 61.5% | 2 | R.AETLVCCCFDLLKK.L | 2 |
| \* | 100326\_pJS43\_01.00366.00366.2 | 3.0351 | 0.3359 | 100.0% | 1145.4321 | 1144.3597 | 1 | 5.978 | 77.8% | 2 | R.AKLQSLKAER.E | 2 |
| \* | 100326\_pJS43\_01.00360.00360.2 | 3.3378 | 0.2806 | 99.9% | 1629.4722 | 1629.8569 | 1 | 6.026 | 65.4% | 13 | R.AKLQSLKAEREEAR.H | 2 |
| \* | 100326\_pJS43\_01.00364.00364.3 | 3.5093 | 0.1953 | 98.9% | 1629.8644 | 1629.8569 | 4 | 5.469 | 36.5% | 3 | R.AKLQSLKAEREEAR.H | 3 |
| \* | 100326\_pJS43\_01.00353.00353.2 | 3.0246 | 0.1235 | 98.6% | 1430.8722 | 1430.6041 | 2 | 5.774 | 63.6% | 3 | K.LQSLKAEREEAR.H | 2 |
| \* | 100326\_pJS43\_01.00428.00428.2 | 2.5751 | 0.174 | 99.1% | 1042.4122 | 1042.201 | 3 | 5.458 | 78.6% | 10 | R.HREEMALR.G | 2 |
| \* | AstrinIP\_MS1\_022614\_01.11522.11522.2 | 6.7759 | 0.5354 | 100.0% | 2073.412 | 2074.267 | 1 | 10.369 | 75.0% | 4 | R.GKDAAEIVLEAFCAHASQR.I | 2 |
| \* | SKAPIP\_tube2\_041314\_01.11120.11120.3 | 5.9643 | 0.5463 | 100.0% | 2074.6443 | 2074.267 | 1 | 8.268 | 45.8% | 18 | R.GKDAAEIVLEAFCAHASQR.I | 3 |
| \* | AstrinIP\_MS1\_022614\_01.12567.12567.3 | 4.0364 | 0.4531 | 100.0% | 1888.5844 | 1889.041 | 1 | 8.202 | 46.9% | 13 | K.DAAEIVLEAFCAHASQR.I | 3 |
| \* | AstrinIP\_MS1\_022614\_01.12371.12371.2 | 5.6245 | 0.4487 | 100.0% | 1889.1122 | 1889.041 | 1 | 8.65 | 71.9% | 27 | K.DAAEIVLEAFCAHASQR.I | 2 |
| \* | 100326\_pJS43\_01.07568.07568.2 | 4.1227 | 0.4021 | 100.0% | 1391.3322 | 1391.5823 | 1 | 8.359 | 68.2% | 50 | R.ISQLEQDLASMR.E | 2 |
| \* | SKAPIP\_tube2\_041314\_01.09621.09621.1 | 3.0675 | 0.4197 | 99.0% | 1391.62 | 1391.5823 | 14 | 6.667 | 50.0% | 21 | R.ISQLEQDLASMR.E | 1 |
| \* | AstrinIP\_MS1\_022614\_01.10863.10863.2 | 3.097 | 0.1785 | 99.0% | 1823.4722 | 1824.0618 | 1 | 4.69 | 46.4% | 10 | R.ISQLEQDLASMREFR.G | 2 |
| \* | AstrinIP\_MS2\_022614\_01.11494.11494.3 | 3.3642 | 0.3995 | 100.0% | 1824.2644 | 1824.0618 | 2 | 6.028 | 42.9% | 9 | R.ISQLEQDLASMREFR.G | 3 |
| \* | 100326\_pJS43\_01.06308.06308.2 | 4.9299 | 0.5237 | 100.0% | 1692.4722 | 1692.9994 | 1 | 8.909 | 63.3% | 13 | R.GLLKDAQTQLVGLHAK.Q | 2 |
| \* | AstrinIP\_MS2\_022614\_01.08228.08228.3 | 4.7497 | 0.3302 | 100.0% | 1693.2544 | 1692.9994 | 1 | 6.756 | 45.0% | 20 | R.GLLKDAQTQLVGLHAK.Q | 3 |
| \* | pSKT11\_1\_020812\_02.08116.08116.3 | 7.6375 | 0.5843 | 100.0% | 4064.9343 | 4064.5884 | 1 | 9.39 | 31.4% | 4 | R.GLLKDAQTQLVGLHAKQEELVQQTVSLTSTLQQDWR.S | 3 |
| \* | pSKT11\_1\_020812\_01.06368.06368.2 | 4.1564 | 0.3951 | 100.0% | 1280.7722 | 1281.4545 | 1 | 6.804 | 77.3% | 49 | K.DAQTQLVGLHAK.Q | 2 |
| \* | SKAPIP\_041314\_02.04000.04000.3 | 2.9856 | 0.2998 | 99.8% | 1281.5944 | 1281.4545 | 3 | 5.189 | 40.9% | 4 | K.DAQTQLVGLHAK.Q | 3 |
| \* | AstrinIP\_MS2\_022614\_01.04817.04817.1 | 3.1433 | 0.2695 | 98.4% | 1282.54 | 1281.4545 | 1 | 5.076 | 63.6% | 14 | K.DAQTQLVGLHAK.Q | 1 |
| \* | pSKT11\_1\_020812\_01.10241.10241.3 | 6.2487 | 0.6069 | 100.0% | 3651.5044 | 3653.0435 | 1 | 10.722 | 29.0% | 2 | K.DAQTQLVGLHAKQEELVQQTVSLTSTLQQDWR.S | 3 |
| \* | AstrinIP\_MS2\_022614\_01.13670.13670.2 | 5.9251 | 0.6546 | 100.0% | 2390.0322 | 2390.612 | 1 | 12.065 | 65.8% | 118 | K.QEELVQQTVSLTSTLQQDWR.S | 2 |
| \* | SKAPIP\_041314\_02.09850.09850.3 | 5.7193 | 0.5352 | 100.0% | 2390.9944 | 2390.612 | 1 | 9.57 | 39.5% | 30 | K.QEELVQQTVSLTSTLQQDWR.S | 3 |
| \* | AstrinIP\_MS2\_022614\_02.13348.13348.3 | 5.3831 | 0.5287 | 100.0% | 4157.784 | 4158.6294 | 1 | 8.929 | 25.0% | 4 | K.QEELVQQTVSLTSTLQQDWRSMQLDYTTWTALLSR.S | 3 |
| \* | AstrinIP\_MS2\_022614\_01.14061.14061.1 | 3.3013 | 0.4595 | 97.4% | 1786.81 | 1787.0405 | 1 | 6.97 | 50.0% | 9 | R.SMQLDYTTWTALLSR.S | 1 |
| \* | pSKT11\_1\_020812\_01.10281.10281.2 | 6.1507 | 0.4113 | 100.0% | 1787.7922 | 1787.0405 | 1 | 8.278 | 78.6% | 112 | R.SMQLDYTTWTALLSR.S | 2 |
| \* | 100326\_pJS43\_02.08099.08099.3 | 4.1866 | 0.3644 | 100.0% | 1788.1144 | 1787.0405 | 1 | 5.794 | 55.4% | 3 | R.SMQLDYTTWTALLSR.S | 3 |
| \* | AstrinIP\_MS2\_022614\_01.03840.03840.3 | 3.5759 | 0.1729 | 99.2% | 1303.5243 | 1303.5448 | 37 | 3.818 | 45.0% | 2 | R.SRQLTEKLTVK.S | 3 |
| \* | AstrinIP\_MS2\_022614\_01.03834.03834.2 | 3.65 | 0.1581 | 99.8% | 1303.6522 | 1303.5448 | 1 | 5.621 | 80.0% | 1 | R.SRQLTEKLTVK.S | 2 |
| \* | 100326\_pJS43\_01.03336.03336.1 | 2.1474 | 0.1747 | 96.5% | 958.68 | 960.0348 | 2 | 3.848 | 71.4% | 1 | K.SQQALQER.D | 1 |
| \* | AstrinIP\_MS2\_022614\_01.03304.03304.2 | 2.5927 | 0.1366 | 98.7% | 959.9522 | 960.0348 | 1 | 4.693 | 85.7% | 2 | K.SQQALQER.D | 2 |
| \* | pSKT11\_1\_020812\_01.06038.06038.3 | 2.1899 | 0.285 | 95.5% | 1745.0343 | 1744.8992 | 21 | 5.041 | 32.1% | 1 | K.SQQALQERDVAIEEK.Q | 3 |
| \* | pSKT11\_1\_020812\_01.05998.05998.2 | 4.2637 | 0.3549 | 100.0% | 1745.4521 | 1744.8992 | 2 | 6.287 | 60.7% | 5 | K.SQQALQERDVAIEEK.Q | 2 |
| \* | pSKT11\_1\_020812\_01.07948.07948.2 | 4.5029 | 0.5265 | 100.0% | 2343.4321 | 2344.5437 | 1 | 8.132 | 50.0% | 1 | K.SQQALQERDVAIEEKQEVSR.V | 2 |
| \* | pSKT11\_1\_020812\_01.07983.07983.3 | 6.0479 | 0.4734 | 100.0% | 2344.2844 | 2344.5437 | 1 | 7.412 | 47.4% | 23 | K.SQQALQERDVAIEEKQEVSR.V | 3 |
| \* | 100326\_pJS43\_01.00480.00480.1 | 2.0032 | 0.1724 | 95.8% | 803.46 | 803.88763 | 1 | 4.738 | 75.0% | 1 | R.DVAIEEK.Q | 1 |
| \* | AstrinIP\_MS1\_022614\_01.03827.03827.1 | 3.0295 | 0.4037 | 99.2% | 1402.71 | 1403.5321 | 1 | 7.4 | 63.6% | 2 | R.DVAIEEKQEVSR.V | 1 |
| \* | pDK339\_033013\_01.03805.03805.3 | 3.3342 | 0.3578 | 100.0% | 1403.2743 | 1403.5321 | 1 | 5.764 | 47.7% | 15 | R.DVAIEEKQEVSR.V | 3 |
| \* | 100326\_pJS43\_01.00747.00747.2 | 4.1811 | 0.4089 | 100.0% | 1404.3121 | 1403.5321 | 1 | 7.252 | 86.4% | 71 | R.DVAIEEKQEVSR.V | 2 |
| \* | AstrinIP\_MS1\_022614\_01.07025.07025.1 | 3.6356 | 0.4606 | 100.0% | 1532.55 | 1533.6849 | 1 | 9.183 | 54.2% | 1 | R.VLEQVSAQLEECK.G | 1 |
| \* | AstrinIP\_MS1\_022614\_01.07040.07040.2 | 4.9305 | 0.4726 | 100.0% | 1533.1322 | 1533.6849 | 1 | 9.052 | 70.8% | 29 | R.VLEQVSAQLEECK.G | 2 |
| \* | SKAPIP\_041314\_02.05633.05633.3 | 3.6232 | 0.2275 | 99.7% | 1533.7743 | 1533.6849 | 2 | 5.724 | 54.2% | 1 | R.VLEQVSAQLEECK.G | 3 |
| \* | AstrinIP\_MS1\_022614\_01.08493.08493.2 | 5.0393 | 0.5318 | 100.0% | 2918.2122 | 2919.1382 | 1 | 9.126 | 41.7% | 4 | R.VLEQVSAQLEECKGQTEQLELENSR.L | 2 |
| \* | 100326\_pJS43\_02.05228.05228.3 | 7.1159 | 0.5509 | 100.0% | 2918.9343 | 2919.1382 | 1 | 9.819 | 41.7% | 19 | R.VLEQVSAQLEECKGQTEQLELENSR.L | 3 |
| \* | AstrinIP\_MS1\_022614\_01.04659.04659.2 | 4.4176 | 0.4966 | 100.0% | 1404.0521 | 1404.4764 | 1 | 7.631 | 81.8% | 25 | K.GQTEQLELENSR.L | 2 |
| \* | AstrinIP\_MS2\_022614\_01.04784.04784.1 | 3.0534 | 0.4097 | 99.1% | 1404.68 | 1404.4764 | 1 | 6.26 | 54.5% | 8 | K.GQTEQLELENSR.L | 1 |
| \* | AstrinIP\_MS1\_022614\_01.10761.10761.1 | 3.8457 | 0.5052 | 100.0% | 1572.68 | 1573.848 | 1 | 8.588 | 65.4% | 11 | R.AQLQILANMDSQLK.E | 1 |
| \* | SKAPIP\_041314\_01.12153.12153.2 | 5.7391 | 0.4398 | 100.0% | 1573.5521 | 1573.848 | 1 | 8.421 | 80.8% | 238 | R.AQLQILANMDSQLK.E | 2 |
| \* | SKAPIP\_041314\_02.08309.08309.3 | 5.1994 | 0.395 | 100.0% | 1573.7344 | 1573.848 | 1 | 7.298 | 53.8% | 6 | R.AQLQILANMDSQLK.E | 3 |
| \* | AstrinIP\_MS1\_022614\_01.03758.03758.2 | 3.9237 | 0.2675 | 100.0% | 1897.2122 | 1898.0668 | 1 | 7.604 | 60.0% | 1 | K.ELQSQHTHCAQDLAMK.D | 2 |
| \* | SKAPIP\_041314\_01.05297.05297.3 | 3.0077 | 0.3499 | 99.9% | 1897.7644 | 1898.0668 | 120 | 5.484 | 31.7% | 3 | K.ELQSQHTHCAQDLAMK.D | 3 |
| \* | AstrinIP\_MS1\_022614\_01.09574.09574.3 | 6.3411 | 0.5332 | 100.0% | 4298.3945 | 4299.6045 | 1 | 10.187 | 25.0% | 2 | K.ELQSQHTHCAQDLAMKDELLCQLTQSNEEQAAQWQK.E | 3 |
| \* | AstrinIP\_MS2\_022614\_02.07248.07248.3 | 4.2459 | 0.3902 | 100.0% | 2418.0244 | 2420.561 | 1 | 6.494 | 36.8% | 2 | K.DELLCQLTQSNEEQAAQWQK.E | 3 |
| \* | AstrinIP\_MS1\_022614\_01.10371.10371.2 | 5.9619 | 0.5426 | 100.0% | 2419.612 | 2420.561 | 1 | 10.069 | 60.5% | 1 | K.DELLCQLTQSNEEQAAQWQK.E | 2 |
| \* | 100326\_pJS43\_02.03345.03345.2 | 5.9163 | 0.5782 | 100.0% | 1723.3121 | 1723.9879 | 1 | 10.24 | 89.3% | 209 | K.HMQAELQQQQAVLAK.E | 2 |
| \* | 100326\_pJS43\_01.01488.01488.1 | 4.3033 | 0.4935 | 100.0% | 1723.7 | 1723.9879 | 1 | 8.114 | 64.3% | 9 | K.HMQAELQQQQAVLAK.E | 1 |
| \* | SKAPIP\_tube2\_041314\_02.04520.04520.3 | 5.2962 | 0.3769 | 100.0% | 1724.5144 | 1723.9879 | 1 | 6.48 | 58.9% | 95 | K.HMQAELQQQQAVLAK.E | 3 |
| \* | AstrinIP\_MS1\_022614\_02.04988.04988.3 | 3.0664 | 0.2033 | 96.4% | 2108.3342 | 2108.4233 | 1 | 4.56 | 38.2% | 1 | K.HMQAELQQQQAVLAKEVR.D | 3 |
| \* | AstrinIP\_MS1\_022614\_02.08822.08822.3 | 7.0438 | 0.5196 | 100.0% | 3286.7344 | 3286.5862 | 1 | 8.212 | 34.3% | 29 | R.DLKETLEFADQENQVAHLELGQVECQLK.T | 3 |
| \* | AstrinIP\_MS1\_022614\_01.11154.11154.2 | 4.9416 | 0.438 | 100.0% | 2929.9321 | 2930.1638 | 1 | 7.623 | 39.6% | 3 | K.ETLEFADQENQVAHLELGQVECQLK.T | 2 |
| \* | AstrinIP\_MS1\_022614\_02.07651.07651.3 | 6.0255 | 0.5251 | 100.0% | 2930.3342 | 2930.1638 | 1 | 9.119 | 37.5% | 10 | K.ETLEFADQENQVAHLELGQVECQLK.T | 3 |
| \* | 100326\_pJS43\_01.02897.02897.1 | 1.6727 | 0.2082 | 95.5% | 831.46 | 831.9878 | 3 | 3.973 | 66.7% | 4 | K.TTLEVLR.E | 1 |
| \* | SKAPIP\_tube2\_041314\_01.05660.05660.2 | 2.4536 | 0.2173 | 99.5% | 831.89215 | 831.9878 | 16 | 5.181 | 83.3% | 16 | K.TTLEVLR.E | 2 |
| \* | pSKT11\_1\_020812\_01.06905.06905.2 | 2.1109 | 0.1863 | 95.3% | 1117.2322 | 1117.2908 | 15 | 4.475 | 68.8% | 1 | K.TTLEVLRER.S | 2 |
| \* | AstrinIP\_MS1\_022614\_01.07887.07887.3 | 3.3816 | 0.3084 | 99.8% | 2249.3943 | 2249.4492 | 1 | 5.094 | 37.5% | 1 | R.ERSLQCENLKDTVENLTAK.L | 3 |
| \* | AstrinIP\_MS1\_022614\_01.08564.08564.2 | 5.6114 | 0.5769 | 100.0% | 1963.4722 | 1964.1462 | 1 | 10.064 | 68.8% | 9 | R.SLQCENLKDTVENLTAK.L | 2 |
| \* | SKAPIP\_041314\_01.11438.11438.3 | 4.7194 | 0.3297 | 100.0% | 1965.4443 | 1964.1462 | 1 | 6.279 | 50.0% | 8 | R.SLQCENLKDTVENLTAK.L | 3 |
| \* | AstrinIP\_MS1\_022614\_01.04083.04083.1 | 2.023 | 0.4048 | 99.5% | 990.49 | 991.0862 | 1 | 6.146 | 62.5% | 6 | K.DTVENLTAK.L | 1 |
| \* | SKAPIP\_041314\_01.06260.06260.2 | 2.5487 | 0.2956 | 99.7% | 991.03217 | 991.0862 | 1 | 5.087 | 93.8% | 7 | K.DTVENLTAK.L | 2 |
| \* | 100326\_pJS43\_02.03104.03104.2 | 4.9416 | 0.4653 | 100.0% | 1675.4122 | 1675.7899 | 1 | 7.293 | 71.4% | 128 | K.LASTIADNQEQDLEK.T | 2 |
| \* | AstrinIP\_MS1\_022614\_01.05140.05140.1 | 3.467 | 0.4086 | 98.7% | 1675.83 | 1675.7899 | 1 | 6.475 | 53.6% | 13 | K.LASTIADNQEQDLEK.T | 1 |
| \* | AstrinIP\_MS1\_022614\_01.05301.05301.3 | 4.4723 | 0.2692 | 100.0% | 1676.1543 | 1675.7899 | 1 | 6.169 | 48.2% | 8 | K.LASTIADNQEQDLEK.T | 3 |
| \* | 100326\_pJS43\_01.02013.02013.2 | 4.6099 | 0.5491 | 100.0% | 1932.3922 | 1933.0825 | 1 | 8.304 | 53.1% | 30 | K.LASTIADNQEQDLEKTR.Q | 2 |
| \* | SKAPIP\_041314\_02.04116.04116.3 | 2.853 | 0.3349 | 99.7% | 1934.6344 | 1933.0825 | 1 | 6.291 | 37.5% | 3 | K.LASTIADNQEQDLEKTR.Q | 3 |
| \* | SKAPIP\_tube2\_041314\_02.10463.10463.3 | 4.7177 | 0.3413 | 100.0% | 2939.3943 | 2939.4253 | 1 | 6.148 | 32.3% | 1 | K.TRQYSQKLGLLTEQLQSLTLFLQTK.L | 3 |
| \* | SKAPIP\_041314\_01.14565.14565.3 | 3.5233 | 0.2355 | 98.8% | 2681.5745 | 2682.1326 | 1 | 5.078 | 31.8% | 3 | R.QYSQKLGLLTEQLQSLTLFLQTK.L | 3 |
| \* | pSKT11\_1\_020812\_01.11534.11534.2 | 6.1449 | 0.4959 | 100.0% | 2046.3121 | 2047.443 | 1 | 10.764 | 64.7% | 481 | K.LGLLTEQLQSLTLFLQTK.L | 2 |
| \* | SKAPIP\_tube2\_041314\_02.12014.12014.3 | 5.7297 | 0.3995 | 100.0% | 2048.5444 | 2047.443 | 1 | 8.821 | 48.5% | 126 | K.LGLLTEQLQSLTLFLQTK.L | 3 |
| \* | AstrinIP\_MS1\_022614\_01.07575.07575.3 | 4.5102 | 0.3809 | 100.0% | 3005.4844 | 3006.2622 | 1 | 6.655 | 28.0% | 3 | K.EKTEQETLLLSTACPPTQEHPLPNDR.T | 3 |
| \* | AstrinIP\_MS1\_022614\_01.08174.08174.2 | 4.1464 | 0.5364 | 100.0% | 2747.5522 | 2748.9727 | 1 | 9.043 | 39.1% | 6 | K.TEQETLLLSTACPPTQEHPLPNDR.T | 2 |
| \* | AstrinIP\_MS1\_022614\_01.08246.08246.3 | 3.5343 | 0.3261 | 99.9% | 2748.9543 | 2748.9727 | 1 | 5.699 | 31.5% | 5 | K.TEQETLLLSTACPPTQEHPLPNDR.T | 3 |
| \* | SKAPIP\_tube2\_041314\_01.15428.15428.2 | 5.3773 | 0.5813 | 100.0% | 2787.5122 | 2788.121 | 1 | 10.378 | 38.5% | 60 | R.TFLGSILTAVADEEPESTPVPLLGSDK.S | 2 |
| \* | AstrinIP\_MS2\_022614\_01.16496.16496.3 | 6.6632 | 0.6039 | 100.0% | 2788.4644 | 2788.121 | 1 | 10.343 | 34.6% | 17 | R.TFLGSILTAVADEEPESTPVPLLGSDK.S | 3 |
| \* | AstrinIP\_MS1\_022614\_01.16653.16653.2 | 4.9802 | 0.5714 | 100.0% | 2867.652 | 2868.121 | 1 | 9.226 | 44.2% | 7 | R.TFLGSILTAVADEEPESTPVPLLGS\*DK.S | 2 |
| \* | pDK339othertube\_033013\_01.15333.15333.3 | 3.754 | 0.3301 | 99.9% | 2868.8044 | 2868.121 | 29 | 5.935 | 24.0% | 1 | R.TFLGSILTAVADEEPESTPVPLLGS\*DK.S | 3 |
| \* | pSKT11\_1\_020812\_01.10990.10990.2 | 3.4959 | 0.5242 | 100.0% | 3350.2922 | 3350.7473 | 1 | 7.912 | 24.2% | 1 | R.TFLGSILTAVADEEPESTPVPLLGSDKSAFTR.V | 2 |
| \* | SKAPIP\_tube2\_041314\_01.14763.14763.3 | 5.5875 | 0.5623 | 100.0% | 3350.3044 | 3350.7473 | 1 | 10.136 | 31.5% | 6 | R.TFLGSILTAVADEEPESTPVPLLGSDKSAFTR.V | 3 |
| \* | AstrinIP\_MS2\_022614\_01.16389.16389.3 | 5.7185 | 0.3671 | 100.0% | 3429.5645 | 3430.7473 | 2 | 8.674 | 25.8% | 11 | R.TFLGSILTAVADEEPESTPVPLLGSDKS\*AFTR.V | 3 |
| \* | pDK339othertube\_033013\_01.14888.14888.3 | 4.7588 | 0.2933 | 100.0% | 3430.3442 | 3430.7473 | 1 | 6.892 | 28.2% | 7 | R.TFLGSILTAVADEEPESTPVPLLGS\*DKSAFTR.V | 3 |
| \* | AstrinIP\_MS1\_022614\_02.12577.12577.3 | 5.7757 | 0.4825 | 100.0% | 4358.8745 | 4358.957 | 1 | 8.346 | 24.4% | 9 | R.VASMVSLQPAETPGMEESLAEMSIMTTELQSLCSLLQESK.E | 3 |
| \* | SKAPIP\_tube2\_041314\_01.16665.16665.3 | 5.1927 | 0.4728 | 100.0% | 4958.2744 | 4957.614 | 1 | 8.304 | 21.6% | 7 | R.VASMVSLQPAETPGMEESLAEMSIMTTELQSLCSLLQESKEEAIR.T | 3 |
| \* | AstrinIP\_MS1\_022614\_01.03462.03462.1 | 2.32 | 0.294 | 98.8% | 1594.71 | 1595.7092 | 18 | 4.755 | 45.8% | 1 | R.LQAQEEQHQEVQK.A | 1 |
| \* | 100326\_pJS43\_01.00333.00333.2 | 4.8689 | 0.3998 | 100.0% | 1594.9922 | 1595.7092 | 1 | 7.172 | 87.5% | 23 | R.LQAQEEQHQEVQK.A | 2 |
| \* | AstrinIP\_MS1\_022614\_02.03145.03145.3 | 2.149 | 0.2773 | 95.2% | 1595.8744 | 1595.7092 | 8 | 4.266 | 41.7% | 1 | R.LQAQEEQHQEVQK.A | 3 |
| \* | SKAPIP\_tube2\_041314\_01.07611.07611.3 | 4.8497 | 0.3534 | 100.0% | 1873.3143 | 1873.126 | 1 | 7.168 | 46.7% | 6 | K.AKEADIEKLNQALCLR.Y | 3 |
| \* | SKAPIP\_041314\_01.11248.11248.2 | 3.2482 | 0.0387 | 95.4% | 1874.6322 | 1873.126 | 1 | 4.417 | 53.3% | 1 | K.AKEADIEKLNQALCLR.Y | 2 |
| \* | AstrinIP\_MS2\_022614\_01.06875.06875.3 | 3.2499 | 0.2766 | 99.5% | 2162.9644 | 2164.476 | 1 | 5.012 | 30.9% | 1 | K.AKEADIEKLNQALCLRYK.N | 3 |
| \* | SKAPIP\_tube2\_041314\_01.08753.08753.2 | 4.4469 | 0.4315 | 100.0% | 1673.4122 | 1673.873 | 1 | 7.81 | 76.9% | 4 | K.EADIEKLNQALCLR.Y | 2 |
| \* | SKAPIP\_041314\_01.11577.11577.3 | 3.0956 | 0.2981 | 99.8% | 1673.9644 | 1673.873 | 103 | 5.43 | 34.6% | 2 | K.EADIEKLNQALCLR.Y | 3 |
| \* | SKAPIP\_tube2\_041314\_01.05224.05224.1 | 2.0325 | 0.2383 | 98.7% | 987.59 | 988.1412 | 1 | 5.201 | 78.6% | 4 | K.LNQALCLR.Y | 1 |
| \* | AstrinIP\_MS1\_022614\_01.05582.05582.2 | 3.1789 | 0.3339 | 100.0% | 987.97217 | 988.1412 | 1 | 5.524 | 85.7% | 7 | K.LNQALCLR.Y | 2 |
| \* | AstrinIP\_MS2\_022614\_01.05144.05144.2 | 6.3385 | 0.4233 | 100.0% | 2148.5322 | 2149.3652 | 1 | 8.339 | 68.8% | 25 | R.YKNEKELQEVIQQQNEK.I | 2 |
| \* | pSKT11\_1\_020812\_01.08158.08158.3 | 6.7943 | 0.3223 | 100.0% | 2149.5244 | 2149.3652 | 1 | 6.276 | 54.7% | 28 | R.YKNEKELQEVIQQQNEK.I | 3 |
| \* | pSKT11\_1\_020812\_01.05927.05927.1 | 3.6259 | 0.2069 | 98.8% | 1485.57 | 1486.622 | 1 | 6.147 | 72.7% | 15 | K.ELQEVIQQQNEK.I | 1 |
| \* | AstrinIP\_MS1\_022614\_01.04826.04826.2 | 4.7936 | 0.2338 | 100.0% | 1486.1721 | 1486.622 | 1 | 6.568 | 86.4% | 53 | K.ELQEVIQQQNEK.I | 2 |
| \* | SKAPIP\_041314\_02.03954.03954.3 | 3.4065 | 0.1763 | 98.7% | 1487.6643 | 1486.622 | 6 | 4.921 | 47.7% | 1 | K.ELQEVIQQQNEK.I | 3 |
| \* | AstrinIP\_MS1\_022614\_01.13077.13077.3 | 3.5247 | 0.2058 | 97.6% | 3181.3442 | 3182.5986 | 277 | 4.059 | 20.2% | 1 | K.ELQEVIQQQNEKILEQIDKSGELISLR.E | 3 |
| \* | SKAPIP\_041314\_01.09050.09050.2 | 3.0069 | 0.0752 | 99.4% | 859.1722 | 859.01044 | 5 | 3.938 | 83.3% | 15 | K.ILEQIDK.S | 2 |
| \* | SKAPIP\_tube2\_041314\_01.08792.08792.2 | 5.248 | 0.4678 | 100.0% | 1714.4321 | 1715.0 | 1 | 9.12 | 78.6% | 14 | K.ILEQIDKSGELISLR.E | 2 |
| \* | 100326\_pJS43\_01.07228.07228.3 | 4.9598 | 0.3463 | 100.0% | 1716.2344 | 1715.0 | 1 | 7.56 | 57.1% | 12 | K.ILEQIDKSGELISLR.E | 3 |
| \* | AstrinIP\_MS2\_022614\_01.11559.11559.3 | 5.7572 | 0.3338 | 100.0% | 2680.8843 | 2681.0618 | 1 | 6.959 | 40.9% | 26 | K.ILEQIDKSGELISLREEVTHLTR.S | 3 |
| \* | AstrinIP\_MS1\_022614\_01.06956.06956.1 | 2.0036 | 0.1888 | 96.4% | 874.39 | 875.0128 | 88 | 4.993 | 57.1% | 5 | K.SGELISLR.E | 1 |
| \* | AstrinIP\_MS2\_022614\_01.07298.07298.2 | 3.1831 | 0.1752 | 99.9% | 874.9922 | 875.0128 | 2 | 5.294 | 85.7% | 14 | K.SGELISLR.E | 2 |
| \* | 100326\_pJS43\_01.07424.07424.2 | 4.5341 | 0.3989 | 100.0% | 1840.3922 | 1841.0745 | 1 | 6.599 | 60.0% | 29 | K.SGELISLREEVTHLTR.S | 2 |
| \* | AstrinIP\_MS2\_022614\_01.09950.09950.3 | 5.243 | 0.4348 | 100.0% | 1841.6044 | 1841.0745 | 1 | 7.777 | 53.3% | 69 | K.SGELISLREEVTHLTR.S | 3 |
| \* | AstrinIP\_MS1\_022614\_01.10754.10754.3 | 6.1017 | 0.5778 | 100.0% | 2845.3145 | 2846.1506 | 1 | 11.176 | 40.6% | 9 | K.VLQEALAGQLDSNCQPMATNWIQEK.V | 3 |
| \* | SKAPIP\_tube2\_041314\_01.10361.10361.2 | 6.1504 | 0.6029 | 100.0% | 2846.5522 | 2846.1506 | 1 | 10.958 | 54.2% | 6 | K.VLQEALAGQLDSNCQPMATNWIQEK.V | 2 |
| \* | AstrinIP\_MS2\_022614\_01.07324.07324.1 | 1.8376 | 0.3668 | 98.4% | 1103.52 | 1104.248 | 110 | 5.328 | 50.0% | 2 | K.VWLSQEVDK.L | 1 |
| \* | SKAPIP\_tube2\_041314\_01.06705.06705.2 | 3.0724 | 0.3083 | 100.0% | 1104.0721 | 1104.248 | 1 | 5.68 | 81.2% | 17 | K.VWLSQEVDK.L | 2 |
| \* | AstrinIP\_MS1\_022614\_01.08694.08694.1 | 2.7102 | 0.3291 | 98.4% | 1372.61 | 1373.595 | 1 | 6.502 | 70.0% | 4 | K.VWLSQEVDKLR.V | 1 |
| \* | AstrinIP\_MS2\_022614\_01.09164.09164.2 | 3.6908 | 0.3736 | 100.0% | 1373.3121 | 1373.595 | 1 | 7.077 | 90.0% | 24 | K.VWLSQEVDKLR.V | 2 |
| \* | pDK339\_033013\_01.10324.10324.1 | 2.117 | 0.1597 | 95.8% | 897.75 | 898.16644 | 3 | 4.931 | 75.0% | 1 | R.VMFLEMK.N | 1 |
| \* | AstrinIP\_MS1\_022614\_01.09423.09423.2 | 2.8747 | 0.2493 | 99.9% | 898.1922 | 898.16644 | 1 | 7.002 | 83.3% | 16 | R.VMFLEMK.N | 2 |
| \* | AstrinIP\_MS1\_022614\_01.07124.07124.2 | 3.0715 | 0.4444 | 100.0% | 1268.9922 | 1269.5598 | 1 | 7.659 | 77.8% | 7 | R.VMFLEMKNEK.E | 2 |
| \* | AstrinIP\_MS2\_022614\_01.06146.06146.3 | 2.596 | 0.3913 | 99.9% | 1526.6943 | 1526.8494 | 11 | 5.675 | 36.4% | 4 | R.VMFLEMKNEKEK.L | 3 |
| \* | pSKT11\_1\_020812\_01.08378.08378.2 | 3.7552 | 0.2825 | 100.0% | 1527.3121 | 1526.8494 | 1 | 5.291 | 81.8% | 7 | R.VMFLEMKNEKEK.L | 2 |
| \* | AstrinIP\_MS1\_022614\_01.07755.07755.3 | 3.5078 | 0.3246 | 100.0% | 2012.5144 | 2012.5349 | 1 | 5.324 | 43.3% | 4 | R.VMFLEMKNEKEKLMIK.F | 3 |
| \* | pSKT11\_1\_020812\_01.08643.08643.1 | 2.4325 | 0.2317 | 98.8% | 1000.49 | 1001.1277 | 2 | 4.413 | 78.6% | 15 | R.NILEENLR.R | 1 |
| \* | AstrinIP\_MS2\_022614\_01.07214.07214.2 | 2.7519 | 0.0113 | 95.6% | 1000.9522 | 1001.1277 | 1 | 4.118 | 85.7% | 5 | R.NILEENLR.R | 2 |
| \* | AstrinIP\_MS1\_022614\_01.09371.09371.2 | 5.6666 | 0.4707 | 100.0% | 2230.4521 | 2230.526 | 1 | 8.37 | 67.6% | 11 | R.RSDKELEKLDDIVQHIYK.T | 2 |
| \* | SKAPIP\_tube2\_041314\_01.09100.09100.3 | 6.1797 | 0.4445 | 100.0% | 2232.5645 | 2230.526 | 1 | 8.455 | 51.5% | 28 | R.RSDKELEKLDDIVQHIYK.T | 3 |
| \* | SKAPIP\_tube2\_041314\_01.09987.09987.2 | 5.7013 | 0.5529 | 100.0% | 2073.3123 | 2074.3384 | 1 | 10.52 | 68.8% | 7 | R.SDKELEKLDDIVQHIYK.T | 2 |
| \* | SKAPIP\_tube2\_041314\_01.10028.10028.3 | 5.7671 | 0.3786 | 100.0% | 2075.1243 | 2074.3384 | 2 | 7.237 | 43.8% | 15 | R.SDKELEKLDDIVQHIYK.T | 3 |
| \* | AstrinIP\_MS1\_022614\_01.17732.17732.3 | 4.2141 | 0.2909 | 99.9% | 3181.0444 | 3182.6848 | 14 | 5.709 | 22.1% | 1 | R.SDKELEKLDDIVQHIYKTLLSIPEVVR.G | 3 |
| \* | AstrinIP\_MS1\_022614\_01.09848.09848.2 | 3.8856 | 0.4612 | 100.0% | 1743.4922 | 1743.9977 | 1 | 7.385 | 73.1% | 7 | K.ELEKLDDIVQHIYK.T | 2 |
| \* | AstrinIP\_MS2\_022614\_01.10436.10436.3 | 3.8692 | 0.4427 | 100.0% | 1743.8944 | 1743.9977 | 1 | 7.103 | 50.0% | 11 | K.ELEKLDDIVQHIYK.T | 3 |
| \* | AstrinIP\_MS1\_022614\_01.07084.07084.1 | 1.8541 | 0.2864 | 98.6% | 1243.54 | 1244.4331 | 1 | 5.086 | 55.6% | 3 | K.LDDIVQHIYK.T | 1 |
| \* | AstrinIP\_MS1\_022614\_01.07100.07100.2 | 3.6514 | 0.3931 | 100.0% | 1243.7122 | 1244.4331 | 1 | 7.542 | 83.3% | 12 | K.LDDIVQHIYK.T | 2 |
| \* | AstrinIP\_MS2\_022614\_01.07413.07413.3 | 3.1639 | 0.2336 | 99.6% | 1244.6044 | 1244.4331 | 7 | 5.11 | 55.6% | 2 | K.LDDIVQHIYK.T | 3 |
| \* | AstrinIP\_MS1\_022614\_01.10322.10322.2 | 2.8109 | 0.3081 | 99.9% | 1127.1522 | 1127.3696 | 1 | 5.729 | 83.3% | 23 | K.TLLSIPEVVR.G | 2 |
| \* | AstrinIP\_MS1\_022614\_01.10158.10158.1 | 1.9365 | 0.1954 | 95.2% | 1128.62 | 1127.3696 | 36 | 3.825 | 50.0% | 8 | K.TLLSIPEVVR.G | 1 |
| \* | SKAPIP\_tube2\_041314\_01.13796.13796.2 | 3.6797 | 0.4836 | 100.0% | 1494.4521 | 1494.6941 | 1 | 8.042 | 75.0% | 4 | R.GCKELQGLLEFLS.- | 2 |
| \* | AstrinIP\_MS2\_022614\_01.16244.16244.2 | 2.6394 | 0.409 | 100.0% | 1149.1322 | 1149.3293 | 1 | 6.738 | 66.7% | 3 | K.ELQGLLEFLS.- | 2 |
| \* | SKAPIP\_041314\_02.11923.11923.1 | 2.1068 | 0.319 | 98.7% | 1149.42 | 1149.3293 | 1 | 5.001 | 61.1% | 17 | K.ELQGLLEFLS.- | 1 |

---

|  |  |  |  |  |  |  |  |  |
| --- | --- | --- | --- | --- | --- | --- | --- | --- |
| U | *gi|4504517|ref|NP\_001* | 13 | 114 | 90.7% | 205 | 22783 | 6.4 | heat shock protein beta-1 [Homo sapiens] |

| Filename XCorr DeltCN Conf% ObsM+H+ CalcM+H+ SpR ZScore Ion% # Sequence  | | | | | | | | | | | | |
| --- | --- | --- | --- | --- | --- | --- | --- | --- | --- | --- | --- | --- |
| \* | pDK339\_033013\_01.09122.09122.2 | 2.6799 | 0.1245 | 98.8% | 988.21216 | 988.22107 | 2 | 4.852 | 85.7% | 2 | R.RVPFSLLR.G | 2 |
| \* | pJS43\_100mM\_120812\_01.10225.10225.2 | 2.1391 | 0.3664 | 99.7% | 961.9522 | 962.05255 | 1 | 6.514 | 78.6% | 1 | R.GPSWDPFR.D | 2 |
| \* | AstrinIP\_MS2\_022614\_01.11550.11550.3 | 3.11 | 0.2727 | 99.5% | 1903.7943 | 1904.0537 | 1 | 5.364 | 42.9% | 6 | R.GPSWDPFRDWYPHSR.L | 3 |
| \* | pDK365N\_300mM\_082713\_01.10054.10054.2 | 4.0269 | 0.3764 | 100.0% | 1165.3522 | 1164.3494 | 1 | 7.928 | 88.9% | 18 | R.LFDQAFGLPR.L | 2 |
| \* | pDK365N\_300mM\_082713\_01.12750.12750.3 | 7.0669 | 0.599 | 100.0% | 4096.2544 | 4095.606 | 1 | 11.159 | 29.7% | 9 | R.LPEEWSQWLGGSSWPGYVRPLPPAAIESPAVAAPAYSR.A | 3 |
| \* | pDK339\_033013\_01.04547.04547.2 | 2.5197 | 0.1937 | 98.6% | 1076.2122 | 1076.1948 | 116 | 4.358 | 61.1% | 5 | R.QLSSGVSEIR.H | 2 |
| \* | AstrinIP\_MS2\_022614\_01.09747.09747.2 | 5.0939 | 0.5101 | 100.0% | 1784.1921 | 1785.0068 | 1 | 8.618 | 63.3% | 12 | R.VSLDVNHFAPDELTVK.T | 2 |
| \* | pDK365N\_100mM\_082713\_01.08366.08366.3 | 3.414 | 0.4242 | 100.0% | 1784.7544 | 1785.0068 | 52 | 7.104 | 33.3% | 12 | R.VSLDVNHFAPDELTVK.T | 3 |
| \* | AstrinIP\_MS2\_022614\_01.03936.03936.2 | 2.5763 | 0.2376 | 99.1% | 1147.4321 | 1147.314 | 1 | 4.931 | 75.0% | 2 | K.TKDGVVEITGK.H | 2 |
| \* | pDk339\_033013\_02.03578.03578.3 | 3.937 | 0.3019 | 99.9% | 2785.2844 | 2785.0024 | 1 | 5.774 | 29.3% | 3 | K.TKDGVVEITGKHEERQDEHGYISR.C | 3 |
| \* | pDK365N\_100mM\_082813\_04.06952.06952.3 | 6.1367 | 0.4316 | 100.0% | 3228.1443 | 3228.6821 | 1 | 6.642 | 30.8% | 21 | R.KYTLPPGVDPTQVSSSLSPEGTLTVEAPMPK.L | 3 |
| \* | pDK339othertube\_033013\_01.09284.09284.2 | 3.9071 | 0.577 | 100.0% | 1906.3722 | 1907.1307 | 1 | 8.907 | 59.4% | 22 | K.LATQSNEITIPVTFESR.A | 2 |
| \* | AstrinNocIP\_020510\_02.03401.03401.3 | 3.6913 | 0.3477 | 100.0% | 3532.4343 | 3532.8865 | 1 | 6.223 | 22.7% | 1 | K.LATQSNEITIPVTFESRAQLGGPEAAKSDETAAK.- | 3 |

---

|  |  |  |  |  |  |  |  |  |
| --- | --- | --- | --- | --- | --- | --- | --- | --- |
| U | *gi|30795231|ref|NP\_00* | 24 | 116 | 90.3% | 227 | 22693 | 4.6 | brain abundant, membrane attached signal protein 1 [Homo sapiens] |

| Filename XCorr DeltCN Conf% ObsM+H+ CalcM+H+ SpR ZScore Ion% # Sequence  | | | | | | | | | | | | |
| --- | --- | --- | --- | --- | --- | --- | --- | --- | --- | --- | --- | --- |
| \* | SKAPIP\_041314\_01.04908.04908.3 | 7.0295 | 0.526 | 100.0% | 3071.3943 | 3071.2354 | 1 | 9.731 | 32.8% | 1 | K.DKKAEGAATEEEGTPKESEPQAAAEPAEAK.E | 3 |
| \* | SKAPIP\_041314\_01.05082.05082.3 | 5.1614 | 0.455 | 100.0% | 2827.7944 | 2827.9727 | 1 | 8.018 | 34.3% | 1 | K.KAEGAATEEEGTPKESEPQAAAEPAEAK.E | 3 |
| \* | SKAPIP\_041314\_01.07772.07772.3 | 6.3005 | 0.5571 | 100.0% | 2699.2444 | 2699.7986 | 1 | 9.172 | 35.6% | 10 | K.AEGAATEEEGTPKESEPQAAAEPAEAK.E | 3 |
| \* | SKAPIP\_tube2\_041314\_01.03893.03893.3 | 4.0414 | 0.3777 | 100.0% | 2778.5344 | 2779.7986 | 1 | 6.874 | 30.8% | 1 | K.AEGAATEEEGT#PKESEPQAAAEPAEAK.E | 3 |
| \* | SKAPIP\_041314\_01.04052.04052.2 | 3.8262 | 0.3143 | 100.0% | 1428.1522 | 1428.4955 | 1 | 6.532 | 73.1% | 5 | K.ESEPQAAAEPAEAK.E | 2 |
| \* | SKAPIP\_041314\_01.03978.03978.2 | 3.7098 | 0.4936 | 100.0% | 1742.2122 | 1742.837 | 1 | 8.261 | 65.6% | 1 | K.ESEPQAAAEPAEAKEGK.E | 2 |
| \* | pJS43\_100mM\_120812\_01.02877.02877.2 | 2.9548 | 0.3101 | 99.8% | 1574.3322 | 1574.6415 | 1 | 6.69 | 73.1% | 1 | K.EKPDQDAEGKAEEK.E | 2 |
| \* | SKAPIP\_041314\_01.03947.03947.3 | 3.1691 | 0.2989 | 99.4% | 2545.4043 | 2545.6763 | 40 | 5.76 | 26.1% | 1 | K.EKPDQDAEGKAEEKEGEKDAAAAK.E | 32 |
| \* | SKAPIP\_041314\_01.10407.10407.3 | 4.3995 | 0.43 | 100.0% | 3099.8342 | 3100.2769 | 1 | 7.575 | 30.4% | 2 | K.EKPDQDAEGKAEEKEGEKDAAAAKEEAPK.A | 3 |
| \* | SKAPIP\_041314\_01.03791.03791.3 | 5.5205 | 0.4418 | 100.0% | 2002.1643 | 2002.1425 | 1 | 7.254 | 52.8% | 1 | K.AEEKEGEKDAAAAKEEAPK.A | 3 |
| \* | SKAPIP\_041314\_01.03882.03882.3 | 5.2711 | 0.4671 | 100.0% | 2857.0444 | 2857.0576 | 1 | 7.84 | 26.9% | 1 | K.EGEKDAAAAKEEAPKAEPEKTEGAAEAK.A | 3 |
| \* | SKAPIP\_041314\_01.03830.03830.3 | 5.0601 | 0.4345 | 100.0% | 2413.0444 | 2413.6006 | 1 | 7.88 | 38.0% | 1 | K.DAAAAKEEAPKAEPEKTEGAAEAK.A | 3 |
| \* | SKAPIP\_041314\_01.06264.06264.2 | 3.734 | 0.4758 | 100.0% | 2298.5723 | 2299.5022 | 1 | 7.353 | 56.5% | 5 | K.AEPPKAPEQEQAAPGPAAGGEAPK.A | 2 |
| \* | SKAPIP\_041314\_01.06081.06081.3 | 4.8592 | 0.4102 | 100.0% | 2300.0942 | 2299.5022 | 1 | 6.953 | 46.7% | 35 | K.AEPPKAPEQEQAAPGPAAGGEAPK.A | 3 |
| \* | SKAPIP\_041314\_01.09801.09801.2 | 4.7306 | 0.447 | 100.0% | 2636.7922 | 2637.7734 | 1 | 9.046 | 46.3% | 5 | K.AAEAAAAPAESAAPAAGEEPSKEEGEPK.K | 2 |
| \* | SKAPIP\_041314\_02.03683.03683.3 | 3.0844 | 0.4115 | 100.0% | 2638.2244 | 2637.7734 | 1 | 6.344 | 31.5% | 6 | K.AAEAAAAPAESAAPAAGEEPSKEEGEPK.K | 3 |
| \* | SKAPIP\_041314\_01.10336.10336.3 | 5.9649 | 0.5389 | 100.0% | 4031.9043 | 4032.3208 | 1 | 9.246 | 23.2% | 2 | K.AAEAAAAPAESAAPAAGEEPSKEEGEPKKTEAPAAPAAQETK.S | 3 |
| \* | pJS43\_100mM\_120812\_01.03105.03105.2 | 3.9324 | 0.5156 | 100.0% | 1412.3522 | 1413.5706 | 1 | 8.275 | 76.9% | 1 | K.KTEAPAAPAAQETK.S | 2 |
| \* | AstrinIP\_MS2\_022614\_01.03381.03381.3 | 2.5467 | 0.3511 | 99.4% | 1934.0044 | 1933.9806 | 12 | 5.377 | 28.8% | 1 | K.SDGAPASDSKPGSSEAAPSSK.E | 3 |
| \* | pDK365N\_300mM\_082713\_01.03534.03534.3 | 4.3337 | 0.2441 | 99.7% | 3302.6343 | 3302.4436 | 1 | 4.441 | 29.4% | 1 | K.SDGAPASDSKPGSSEAAPSSKETPAATEAPSSTPK.A | 3 |
| \* | pJS43\_100mM\_120812\_01.03404.03404.2 | 2.8837 | 0.3601 | 99.9% | 1387.3121 | 1387.4863 | 1 | 6.817 | 65.4% | 2 | K.ETPAATEAPSSTPK.A | 2 |
| \* | SKAPIP\_tube2\_041314\_02.04383.04383.3 | 5.3705 | 0.4893 | 100.0% | 2764.8542 | 2765.006 | 1 | 6.961 | 35.2% | 5 | K.AQGPAASAEEPKPVEAPAANSDQTVTVK.E | 3 |
| \* | SKAPIP\_tube2\_041314\_01.04648.04648.2 | 5.1072 | 0.5303 | 100.0% | 2893.672 | 2894.1216 | 1 | 9.94 | 37.5% | 2 | K.AQGPAASAEEPKPVEAPAANSDQTVTVKE.- | 2 |
| \* | SKAPIP\_041314\_02.04426.04426.3 | 6.2618 | 0.5067 | 100.0% | 2894.1243 | 2894.1216 | 1 | 8.431 | 33.9% | 25 | K.AQGPAASAEEPKPVEAPAANSDQTVTVKE.- | 3 |

---

|  |  |  |  |  |  |  |  |  |
| --- | --- | --- | --- | --- | --- | --- | --- | --- |
| U | *gi|4505813|ref|NP\_003* | 17 | 215 | 89.9% | 89 | 10366 | 7.4 | dynein light chain 1 [Homo sapiens] |
| U | *gi|83267868|ref|NP\_00* | 17 | 215 | 89.9% | 89 | 10366 | 7.4 | dynein light chain 1 [Homo sapiens] |
| U | *gi|83267866|ref|NP\_00* | 17 | 215 | 89.9% | 89 | 10366 | 7.4 | dynein light chain 1 [Homo sapiens] |

| Filename XCorr DeltCN Conf% ObsM+H+ CalcM+H+ SpR ZScore Ion% # Sequence  | | | | | | | | | | | | |
| --- | --- | --- | --- | --- | --- | --- | --- | --- | --- | --- | --- | --- |
|  | SKAPIP\_tube2\_041314\_01.09196.09196.2 | 6.4814 | 0.5538 | 100.0% | 2514.7722 | 2515.6462 | 1 | 11.849 | 61.9% | 8 | K.NADMSEEMQQDSVECATQALEK.Y | 2 |
|  | AstrinIP\_MS1\_022614\_02.06595.06595.3 | 6.0162 | 0.4855 | 100.0% | 2515.0444 | 2515.6462 | 1 | 9.507 | 45.2% | 7 | K.NADMSEEMQQDSVECATQALEK.Y | 3 |
|  | SKAPIP\_tube2\_041314\_01.10834.10834.3 | 6.027 | 0.5182 | 100.0% | 3163.1042 | 3163.375 | 1 | 7.976 | 29.8% | 1 | K.NADMSEEMQQDSVECATQALEKYNIEK.D | 3 |
|  | SKAPIP\_041314\_01.11147.11147.2 | 4.3323 | 0.3562 | 100.0% | 1416.1322 | 1415.6322 | 1 | 6.733 | 81.8% | 13 | K.YNIEKDIAAHIK.K | 2 |
|  | SKAPIP\_tube2\_041314\_01.07233.07233.3 | 3.5307 | 0.3288 | 100.0% | 1416.2344 | 1415.6322 | 1 | 5.692 | 63.6% | 10 | K.YNIEKDIAAHIK.K | 3 |
|  | SKAPIP\_tube2\_041314\_01.06212.06212.2 | 4.4101 | 0.4566 | 100.0% | 1542.7722 | 1543.8064 | 1 | 8.04 | 79.2% | 12 | K.YNIEKDIAAHIKK.E | 2 |
|  | SKAPIP\_tube2\_041314\_01.06200.06200.3 | 4.3956 | 0.2709 | 100.0% | 1544.3043 | 1543.8064 | 1 | 5.732 | 54.2% | 22 | K.YNIEKDIAAHIKK.E | 3 |
|  | SKAPIP\_tube2\_041314\_01.08397.08397.3 | 4.4055 | 0.3428 | 100.0% | 2191.7644 | 2191.5352 | 1 | 6.661 | 44.1% | 2 | K.YNIEKDIAAHIKKEFDKK.Y | 3 |
|  | pJS43\_100mM\_120812\_01.03614.03614.1 | 1.3965 | 0.2925 | 97.9% | 767.42 | 767.90344 | 29 | 4.753 | 50.0% | 1 | K.DIAAHIK.K | 1 |
|  | pJS43\_100mM\_120812\_01.03254.03254.2 | 2.4044 | 0.1265 | 97.3% | 896.03217 | 896.0775 | 6 | 4.379 | 78.6% | 1 | K.DIAAHIKK.E | 2 |
|  | SKAPIP\_041314\_01.10835.10835.3 | 2.9433 | 0.4029 | 100.0% | 1532.3944 | 1531.7234 | 1 | 6.77 | 56.8% | 1 | K.KYNPTWHCIVGR.N | 33 |
|  | SKAPIP\_041314\_01.11072.11072.2 | 3.5438 | 0.4953 | 100.0% | 1403.2122 | 1403.5493 | 1 | 8.101 | 75.0% | 6 | K.YNPTWHCIVGR.N | 22 |
|  | SKAPIP\_tube2\_041314\_01.04293.04293.1 | 2.1619 | 0.4004 | 99.5% | 1282.72 | 1283.383 | 1 | 6.709 | 60.0% | 4 | R.NFGSYVTHETK.H | 11 |
|  | SKAPIP\_041314\_01.10570.10570.2 | 3.6058 | 0.3859 | 100.0% | 1284.1322 | 1283.383 | 3 | 6.788 | 65.0% | 13 | R.NFGSYVTHETK.H | 22 |
|  | pSKT11\_1\_020812\_02.09868.09868.3 | 5.9448 | 0.5117 | 100.0% | 3237.0842 | 3237.771 | 1 | 8.667 | 40.4% | 62 | R.NFGSYVTHETKHFIYFYLGQVAILLFK.S | 33 |
|  | SKAPIP\_041314\_02.13295.13295.3 | 5.2249 | 0.4365 | 100.0% | 3381.5344 | 3381.9011 | 1 | 6.941 | 30.4% | 39 | R.NFGSYVTHETKHFIYFYLGQVAILLFKSG.- | 33 |
|  | 100326\_pJS43\_02.10540.10540.2 | 5.0541 | 0.5173 | 100.0% | 1974.2922 | 1973.4111 | 1 | 10.255 | 73.3% | 13 | K.HFIYFYLGQVAILLFK.S | 22 |

Similarities:
gi|18087855|ref|NP\_54(7:10)  

---

|  |  |  |  |  |  |  |  |  |
| --- | --- | --- | --- | --- | --- | --- | --- | --- |
| U | *gi|7657381|ref|NP\_055* | 49 | 717 | 85.3% | 504 | 55181 | 6.6 | PRP19/PSO4 pre-mRNA processing factor 19 homolog [Homo sapiens] |

| Filename XCorr DeltCN Conf% ObsM+H+ CalcM+H+ SpR ZScore Ion% # Sequence  | | | | | | | | | | | | |
| --- | --- | --- | --- | --- | --- | --- | --- | --- | --- | --- | --- | --- |
| \* | SKAPIP\_tube2\_041314\_01.10176.10176.3 | 3.8816 | 0.2554 | 99.6% | 3199.1943 | 3199.5823 | 1 | 5.069 | 30.6% | 1 | R.LIEKYIAENGTDPINNQPLSEEQLIDIK.V | 3 |
| \* | pDK365N\_100mM\_082713\_01.09447.09447.2 | 4.9597 | 0.5533 | 100.0% | 2715.9922 | 2715.9739 | 1 | 9.895 | 50.0% | 20 | K.YIAENGTDPINNQPLSEEQLIDIK.V | 2 |
| \* | pDK365N\_300mM\_082713\_01.10130.10130.3 | 5.1023 | 0.4112 | 100.0% | 2716.1943 | 2715.9739 | 1 | 7.812 | 48.9% | 6 | K.YIAENGTDPINNQPLSEEQLIDIK.V | 3 |
| \* | SKAPIP\_tube2\_041314\_01.05973.05973.2 | 3.672 | 0.5499 | 100.0% | 2093.7122 | 2094.5515 | 1 | 8.882 | 50.0% | 6 | K.VAHPIRPKPPSATSIPAILK.A | 2 |
| \* | pDK365N\_300mM\_082713\_03.05834.05834.3 | 3.742 | 0.3545 | 100.0% | 2094.3245 | 2094.5515 | 1 | 6.014 | 34.2% | 26 | K.VAHPIRPKPPSATSIPAILK.A | 3 |
| \* | SKAPIP\_tube2\_041314\_01.13046.13046.2 | 5.7271 | 0.5772 | 100.0% | 2031.9321 | 2033.3092 | 1 | 9.7 | 71.9% | 59 | K.ALQDEWDAVMLHSFTLR.Q | 2 |
| \* | SKAPIP\_tube2\_041314\_01.13040.13040.3 | 3.9841 | 0.5061 | 100.0% | 2033.3644 | 2033.3092 | 1 | 7.928 | 43.8% | 36 | K.ALQDEWDAVMLHSFTLR.Q | 3 |
| \* | SKAPIP\_041314\_01.13348.13348.3 | 3.4274 | 0.3054 | 99.7% | 3048.2644 | 3049.2585 | 1 | 5.736 | 26.1% | 3 | K.ALQDEWDAVMLHSFTLRQQLQT#T#R.Q | 3 |
| \* | pDK365N\_300mM\_082713\_01.00976.00976.2 | 2.1173 | 0.2278 | 98.5% | 875.0522 | 874.9725 | 189 | 4.759 | 75.0% | 1 | R.QQLQTTR.Q | 2 |
| \* | SKAPIP\_041314\_01.09138.09138.2 | 3.0408 | 0.4322 | 100.0% | 1799.1122 | 1799.9066 | 1 | 6.769 | 64.3% | 5 | R.QELSHALYQHDAACR.V | 2 |
| \* | SKAPIP\_041314\_01.09314.09314.3 | 2.9812 | 0.2956 | 99.6% | 1800.2943 | 1799.9066 | 1 | 6.651 | 44.6% | 9 | R.QELSHALYQHDAACR.V | 3 |
| \* | SKAPIP\_tube2\_041314\_01.13076.13076.3 | 5.4322 | 0.5122 | 100.0% | 4770.264 | 4769.567 | 1 | 8.573 | 23.4% | 6 | R.EALATLKPQAGLIVPQAVPSSQPSVVGAGEPMDLGELVGMTPEIIQK.L | 3 |
| \* | pDK365N\_300mM\_082713\_03.03789.03789.2 | 4.0431 | 0.4507 | 100.0% | 1375.3922 | 1375.5651 | 1 | 7.007 | 81.8% | 9 | K.LQDKATVLTTER.K | 2 |
| \* | SKAPIP\_041314\_01.07364.07364.3 | 3.8578 | 0.3412 | 100.0% | 1375.7043 | 1375.5651 | 1 | 6.561 | 52.3% | 13 | K.LQDKATVLTTER.K | 3 |
| \* | SKAPIP\_041314\_01.04227.04227.3 | 3.1299 | 0.2603 | 99.6% | 1503.7743 | 1503.7391 | 73 | 5.176 | 33.3% | 2 | K.LQDKATVLTTERK.K | 3 |
| \* | SKAPIP\_041314\_02.04439.04439.3 | 3.9695 | 0.4527 | 100.0% | 1783.7344 | 1784.0599 | 1 | 6.97 | 43.3% | 14 | R.GKTVPEELVKPEELSK.Y | 3 |
| \* | SKAPIP\_tube2\_041314\_01.05435.05435.2 | 4.5591 | 0.4455 | 100.0% | 1784.5521 | 1784.0599 | 1 | 7.615 | 66.7% | 5 | R.GKTVPEELVKPEELSK.Y | 2 |
| \* | pDK365N\_300mM\_082713\_03.06154.06154.2 | 3.4202 | 0.4447 | 100.0% | 1598.4922 | 1598.834 | 1 | 7.745 | 61.5% | 24 | K.TVPEELVKPEELSK.Y | 2 |
| \* | SKAPIP\_tube2\_041314\_01.06453.06453.1 | 3.4283 | 0.3481 | 99.3% | 1598.94 | 1598.834 | 1 | 7.442 | 57.7% | 2 | K.TVPEELVKPEELSK.Y | 1 |
| \* | SKAPIP\_041314\_01.12051.12051.3 | 6.7187 | 0.5038 | 100.0% | 2900.9944 | 2902.2432 | 1 | 10.61 | 31.5% | 5 | R.QVASHVGLHSASIPGILALDLCPSDTNK.I | 3 |
| \* | SKAPIP\_tube2\_041314\_01.10373.10373.2 | 3.2303 | 0.4769 | 100.0% | 2901.9521 | 2902.2432 | 1 | 7.64 | 35.2% | 2 | R.QVASHVGLHSASIPGILALDLCPSDTNK.I | 2 |
| \* | SKAPIP\_tube2\_041314\_01.06378.06378.1 | 3.4475 | 0.4496 | 97.6% | 1575.81 | 1576.8333 | 1 | 7.475 | 57.1% | 1 | K.ILTGGADKNVVVFDK.S | 1 |
| \* | pDK365N\_300mM\_082713\_01.06441.06441.2 | 4.8268 | 0.5588 | 100.0% | 1576.3722 | 1576.8333 | 1 | 9.777 | 78.6% | 24 | K.ILTGGADKNVVVFDK.S | 2 |
| \* | pDK365N\_300mM\_082713\_03.06188.06188.3 | 3.8266 | 0.4371 | 100.0% | 1577.2144 | 1576.8333 | 1 | 6.246 | 42.9% | 23 | K.ILTGGADKNVVVFDK.S | 3 |
| \* | SKAPIP\_tube2\_041314\_01.10756.10756.2 | 5.0454 | 0.5307 | 100.0% | 2647.2322 | 2648.072 | 1 | 10.41 | 41.7% | 1 | K.ILTGGADKNVVVFDKSSEQILATLK.G | 2 |
| \* | SKAPIP\_tube2\_041314\_01.10766.10766.3 | 6.6518 | 0.4775 | 100.0% | 2647.2844 | 2648.072 | 1 | 8.405 | 45.8% | 22 | K.ILTGGADKNVVVFDKSSEQILATLK.G | 3 |
| \* | SKAPIP\_tube2\_041314\_01.04670.04670.1 | 1.9617 | 0.2498 | 98.6% | 820.5 | 820.964 | 10 | 6.166 | 66.7% | 2 | K.NVVVFDK.S | 1 |
| \* | SKAPIP\_041314\_02.08255.08255.2 | 5.635 | 0.4839 | 100.0% | 1891.6721 | 1892.2029 | 1 | 10.304 | 78.1% | 3 | K.NVVVFDKSSEQILATLK.G | 2 |
| \* | SKAPIP\_tube2\_041314\_02.08061.08061.3 | 4.1487 | 0.4587 | 100.0% | 1892.4844 | 1892.2029 | 1 | 6.784 | 46.9% | 4 | K.NVVVFDKSSEQILATLK.G | 3 |
| \* | SKAPIP\_tube2\_041314\_02.07295.07295.3 | 3.9297 | 0.3124 | 100.0% | 2316.5942 | 2315.675 | 1 | 5.868 | 35.0% | 2 | K.NVVVFDKSSEQILATLKGHTK.K | 3 |
| \* | SKAPIP\_tube2\_041314\_02.06210.06210.2 | 3.2496 | 0.2845 | 100.0% | 1091.3322 | 1090.2621 | 2 | 5.29 | 72.2% | 27 | K.SSEQILATLK.G | 2 |
| \* | SKAPIP\_tube2\_041314\_01.05648.05648.3 | 2.4214 | 0.3908 | 99.8% | 1514.2444 | 1513.7343 | 41 | 6.772 | 34.6% | 1 | K.SSEQILATLKGHTK.K | 3 |
| \* | SKAPIP\_tube2\_041314\_01.08672.08672.2 | 5.9173 | 0.5482 | 100.0% | 2602.8123 | 2602.95 | 1 | 10.434 | 58.7% | 6 | K.KVTSVVFHPSQDLVFSASPDATIR.I | 2 |
| \* | SKAPIP\_tube2\_041314\_02.07046.07046.3 | 6.4315 | 0.5051 | 100.0% | 2603.1243 | 2602.95 | 1 | 8.616 | 48.9% | 28 | K.KVTSVVFHPSQDLVFSASPDATIR.I | 3 |
| \* | SKAPIP\_041314\_01.11817.11817.2 | 5.9317 | 0.6271 | 100.0% | 2473.7922 | 2474.776 | 1 | 11.563 | 54.5% | 8 | K.VTSVVFHPSQDLVFSASPDATIR.I | 2 |
| \* | SKAPIP\_tube2\_041314\_01.09747.09747.3 | 4.7469 | 0.4111 | 100.0% | 2474.1543 | 2474.776 | 2 | 7.223 | 35.2% | 15 | K.VTSVVFHPSQDLVFSASPDATIR.I | 3 |
| \* | SKAPIP\_tube2\_041314\_01.08706.08706.2 | 4.0484 | 0.3722 | 100.0% | 1616.2322 | 1615.8389 | 1 | 8.512 | 76.9% | 5 | R.IWSVPNASCVQVVR.A | 2 |
| \* | SKAPIP\_tube2\_041314\_01.11584.11584.3 | 7.7057 | 0.6147 | 100.0% | 3900.0842 | 3901.1511 | 1 | 11.147 | 34.3% | 27 | R.AHESAVTGLSLHATGDYLLSSSDDQYWAFSDIQTGR.V | 3 |
| \* | SKAPIP\_041314\_02.08417.08417.3 | 5.9227 | 0.4215 | 100.0% | 3475.4043 | 3475.7378 | 1 | 7.47 | 29.8% | 3 | K.VTDETSGCSLTCAQFHPDGLIFGTGTMDSQIK.I | 3 |
| \* | SKAPIP\_tube2\_041314\_01.05309.05309.2 | 2.5956 | 0.1566 | 99.3% | 959.5522 | 960.1209 | 35 | 4.717 | 75.0% | 2 | K.IWDLKER.T | 2 |
| \* | pDK365N\_300mM\_082713\_01.10592.10592.3 | 7.3659 | 0.5232 | 100.0% | 3603.6843 | 3603.8804 | 1 | 9.533 | 31.6% | 166 | R.TNVANFPGHSGPITSIAFSENGYYLATAADDSSVK.L | 3 |
| \* | SKAPIP\_tube2\_041314\_02.09713.09713.3 | 5.8706 | 0.4205 | 100.0% | 4286.7544 | 4287.6885 | 1 | 6.436 | 23.1% | 2 | R.TNVANFPGHSGPITSIAFSENGYYLATAADDSSVKLWDLR.K | 3 |
| \* | SKAPIP\_tube2\_041314\_02.06258.06258.3 | 3.8085 | 0.3203 | 100.0% | 1952.0343 | 1952.2609 | 1 | 4.865 | 40.0% | 1 | K.LKNFKTLQLDNNFEVK.S | 3 |
| \* | SKAPIP\_tube2\_041314\_01.07882.07882.1 | 2.8569 | 0.2457 | 98.8% | 1320.53 | 1321.4729 | 1 | 5.22 | 70.0% | 3 | K.TLQLDNNFEVK.S | 1 |
| \* | pDK365N\_300mM\_082713\_02.06062.06062.2 | 3.8749 | 0.3939 | 100.0% | 1321.4722 | 1321.4729 | 1 | 7.609 | 85.0% | 58 | K.TLQLDNNFEVK.S | 2 |
| \* | SKAPIP\_041314\_02.07583.07583.3 | 5.0968 | 0.4568 | 100.0% | 2805.1743 | 2806.1123 | 1 | 8.448 | 32.3% | 3 | K.QWTEILHFTEHSGLTTGVAFGHHAK.F | 3 |
| \* | SKAPIP\_041314\_01.10047.10047.1 | 2.4497 | 0.4455 | 99.1% | 997.46 | 998.14185 | 1 | 6.36 | 75.0% | 7 | K.FIASTGMDR.S | 1 |
| \* | pDK365N\_300mM\_082713\_01.04226.04226.2 | 3.2459 | 0.2602 | 100.0% | 997.9322 | 998.14185 | 1 | 6.672 | 81.2% | 16 | K.FIASTGMDR.S | 2 |
| \* | pDK365N\_300mM\_082713\_01.09428.09428.2 | 2.4325 | 0.4613 | 100.0% | 858.0122 | 858.0251 | 2 | 7.374 | 83.3% | 3 | R.SLKFYSL.- | 2 |

---

|  |  |  |  |  |  |  |  |  |
| --- | --- | --- | --- | --- | --- | --- | --- | --- |
| U | *gi|4501885|ref|NP\_001* | 47 | 355 | 83.7% | 375 | 41737 | 5.5 | beta actin [Homo sapiens] |
| U | *gi|4501887|ref|NP\_001* | 47 | 355 | 83.7% | 375 | 41793 | 5.5 | actin, gamma 1 propeptide [Homo sapiens] |

| Filename XCorr DeltCN Conf% ObsM+H+ CalcM+H+ SpR ZScore Ion% # Sequence  | | | | | | | | | | | | |
| --- | --- | --- | --- | --- | --- | --- | --- | --- | --- | --- | --- | --- |
|  | SKAPIP\_041314\_01.04413.04413.2 | 3.3044 | 0.4393 | 100.0% | 976.4122 | 977.02136 | 1 | 7.48 | 77.8% | 7 | K.AGFAGDDAPR.A | 222 |
|  | AstrinIP\_MS2\_022614\_01.04036.04036.1 | 1.9667 | 0.3834 | 98.5% | 976.48 | 977.02136 | 6 | 5.951 | 55.6% | 1 | K.AGFAGDDAPR.A | 111 |
|  | SKAPIP\_tube2\_041314\_01.08159.08159.3 | 4.1697 | 0.4663 | 100.0% | 2156.9644 | 2157.4397 | 1 | 6.941 | 35.0% | 2 | K.AGFAGDDAPRAVFPSIVGRPR.H | 333 |
|  | SKAPIP\_041314\_01.11156.11156.2 | 3.215 | 0.3094 | 100.0% | 1199.2922 | 1199.4415 | 1 | 6.647 | 75.0% | 27 | R.AVFPSIVGRPR.H | 222 |
|  | AstrinIP\_MS2\_022614\_01.03903.03903.1 | 2.5908 | 0.3969 | 99.4% | 1171.48 | 1172.4058 | 1 | 7.283 | 65.0% | 1 | R.HQGVMVGMGQK.D | 111 |
|  | pDK365N\_300mM\_082713\_01.03760.03760.2 | 2.6602 | 0.3292 | 99.8% | 1172.0122 | 1172.4058 | 1 | 6.253 | 75.0% | 2 | R.HQGVMVGMGQK.D | 222 |
|  | SKAPIP\_tube2\_041314\_02.04470.04470.3 | 4.4137 | 0.58 | 100.0% | 2350.8245 | 2352.5989 | 1 | 9.895 | 38.1% | 1 | R.HQGVMVGMGQKDSYVGDEAQSK.R | 33 |
|  | pSKT11\_1\_020812\_01.07479.07479.3 | 5.1607 | 0.4976 | 100.0% | 2508.0842 | 2508.7864 | 1 | 7.555 | 36.4% | 3 | R.HQGVMVGMGQKDSYVGDEAQSKR.G | 33 |
|  | pJS43\_100mM\_120812\_01.03359.03359.2 | 3.2799 | 0.1437 | 99.4% | 1355.0922 | 1355.4038 | 1 | 7.295 | 77.3% | 2 | K.DSYVGDEAQSKR.G | 22 |
|  | SKAPIP\_tube2\_041314\_01.05751.05751.3 | 3.5086 | 0.2783 | 99.9% | 1514.7244 | 1516.7019 | 1 | 6.019 | 60.0% | 16 | K.IWHHTFYNELR.V | 333 |
|  | SKAPIP\_041314\_01.10924.10924.2 | 3.3602 | 0.3431 | 100.0% | 1517.4122 | 1516.7019 | 1 | 5.935 | 70.0% | 18 | K.IWHHTFYNELR.V | 222 |
|  | SKAPIP\_tube2\_041314\_01.05764.05764.1 | 3.335 | 0.2365 | 98.6% | 1517.72 | 1516.7019 | 1 | 4.979 | 70.0% | 4 | K.IWHHTFYNELR.V | 111 |
|  | SKAPIP\_tube2\_041314\_01.07464.07464.1 | 4.1073 | 0.2076 | 98.5% | 1955.02 | 1955.2615 | 1 | 9.605 | 58.8% | 2 | R.VAPEEHPVLLTEAPLNPK.A | 1 |
|  | SKAPIP\_tube2\_041314\_02.05867.05867.3 | 3.5382 | 0.1486 | 95.7% | 1955.1244 | 1955.2615 | 1 | 6.026 | 41.2% | 2 | R.VAPEEHPVLLTEAPLNPK.A | 3 |
|  | pDK339othertube\_033013\_01.07389.07389.2 | 4.6168 | 0.2996 | 100.0% | 1955.3722 | 1955.2615 | 1 | 7.982 | 58.8% | 20 | R.VAPEEHPVLLTEAPLNPK.A | 2 |
|  | SKAPIP\_041314\_01.18028.18028.3 | 4.3072 | 0.345 | 100.0% | 3512.3342 | 3513.122 | 1 | 6.417 | 23.3% | 2 | R.EKMTQIMFETFNTPAMYVAIQAVLSLYASGR.T | 3 |
|  | SKAPIP\_041314\_02.14963.14963.3 | 4.4839 | 0.3888 | 100.0% | 3256.9744 | 3255.8325 | 1 | 6.703 | 28.6% | 4 | K.MTQIMFETFNTPAMYVAIQAVLSLYASGR.T | 3 |
|  | SKAPIP\_tube2\_041314\_01.18501.18501.2 | 4.962 | 0.5487 | 100.0% | 3257.0122 | 3255.8325 | 1 | 9.851 | 44.6% | 3 | K.MTQIMFETFNTPAMYVAIQAVLSLYASGR.T | 2 |
|  | SKAPIP\_tube2\_041314\_01.10418.10418.3 | 7.1714 | 0.642 | 100.0% | 3183.1743 | 3185.622 | 1 | 10.627 | 38.8% | 20 | R.TTGIVMDSGDGVTHTVPIYEGYALPHAILR.L | 3 |
|  | SKAPIP\_tube2\_041314\_01.10475.10475.2 | 4.6444 | 0.5666 | 100.0% | 3184.2122 | 3185.622 | 1 | 9.152 | 34.5% | 2 | R.TTGIVMDSGDGVTHTVPIYEGYALPHAILR.L | 2 |
|  | SKAPIP\_tube2\_041314\_01.10616.10616.2 | 3.4989 | 0.3548 | 100.0% | 1624.5122 | 1624.8927 | 1 | 6.273 | 61.5% | 5 | R.LDLAGRDLTDYLMK.I | 222 |
|  | SKAPIP\_tube2\_041314\_01.10557.10557.3 | 3.2649 | 0.3522 | 100.0% | 1625.0944 | 1624.8927 | 1 | 6.655 | 46.2% | 4 | R.LDLAGRDLTDYLMK.I | 333 |
|  | SKAPIP\_tube2\_041314\_01.09831.09831.1 | 2.1541 | 0.2631 | 98.8% | 998.54 | 999.167 | 1 | 5.467 | 78.6% | 7 | R.DLTDYLMK.I | 111 |
|  | SKAPIP\_tube2\_041314\_01.09902.09902.2 | 2.4407 | 0.316 | 99.8% | 999.15216 | 999.167 | 5 | 5.876 | 78.6% | 4 | R.DLTDYLMK.I | 222 |
|  | SKAPIP\_041314\_01.10814.10814.1 | 2.0148 | 0.3027 | 98.9% | 1132.49 | 1133.2029 | 104 | 5.148 | 44.4% | 4 | R.GYSFTTTAER.E | 1 |
|  | pDK339othertube\_033013\_01.05081.05081.2 | 3.1499 | 0.4903 | 100.0% | 1132.7522 | 1133.2029 | 1 | 10.236 | 88.9% | 27 | R.GYSFTTTAER.E | 2 |
|  | SKAPIP\_tube2\_041314\_01.07283.07283.2 | 3.1099 | 0.2023 | 99.4% | 1630.3722 | 1630.7979 | 1 | 4.244 | 57.7% | 1 | R.GYSFTTTAEREIVR.D | 2 |
|  | SKAPIP\_tube2\_041314\_02.09814.09814.2 | 5.1658 | 0.6069 | 100.0% | 2550.5322 | 2551.791 | 1 | 11.476 | 56.8% | 1 | K.LCYVALDFEQEMATAASSSSLEK.S | 22 |
|  | SKAPIP\_tube2\_041314\_01.09797.09797.1 | 2.4357 | 0.1615 | 95.9% | 1791.9 | 1791.9554 | 1 | 5.61 | 46.7% | 1 | K.SYELPDGQVITIGNER.F | 1111 |
|  | pDK339othertube\_033013\_01.09500.09500.2 | 4.9277 | 0.3112 | 100.0% | 1792.3322 | 1791.9554 | 1 | 8.517 | 83.3% | 34 | K.SYELPDGQVITIGNER.F | 2222 |
|  | SKAPIP\_tube2\_041314\_02.09196.09196.3 | 7.443 | 0.487 | 100.0% | 3536.1843 | 3536.9744 | 1 | 9.775 | 38.8% | 2 | R.FRCPEALFQPSFLGMESCGIHETTFNSIMK.C | 3 |
|  | SKAPIP\_041314\_02.09922.09922.3 | 5.1237 | 0.3499 | 100.0% | 3233.6943 | 3233.6104 | 1 | 5.944 | 29.6% | 4 | R.CPEALFQPSFLGMESCGIHETTFNSIMK.C | 3 |
|  | SKAPIP\_tube2\_041314\_02.07066.07066.2 | 6.0534 | 0.6539 | 100.0% | 2343.7522 | 2344.6448 | 1 | 11.648 | 66.7% | 4 | R.KDLYANTVLSGGTTMYPGIADR.M | 2 |
|  | SKAPIP\_tube2\_041314\_02.07106.07106.3 | 6.8872 | 0.5212 | 100.0% | 2344.8245 | 2344.6448 | 1 | 10.223 | 51.2% | 17 | R.KDLYANTVLSGGTTMYPGIADR.M | 3 |
|  | SKAPIP\_041314\_02.08273.08273.2 | 5.2296 | 0.624 | 100.0% | 2216.5723 | 2216.4705 | 1 | 10.563 | 55.0% | 32 | K.DLYANTVLSGGTTMYPGIADR.M | 2 |
|  | SKAPIP\_041314\_02.08266.08266.3 | 5.4024 | 0.5871 | 100.0% | 2216.7244 | 2216.4705 | 1 | 10.672 | 48.8% | 3 | K.DLYANTVLSGGTTMYPGIADR.M | 3 |
|  | SKAPIP\_tube2\_041314\_01.05690.05690.3 | 3.9322 | 0.2558 | 99.9% | 1550.0044 | 1549.8843 | 3 | 5.468 | 48.1% | 6 | R.MQKEITALAPSTMK.I | 33 |
|  | SKAPIP\_tube2\_041314\_01.05672.05672.2 | 3.9543 | 0.2632 | 100.0% | 1550.4321 | 1549.8843 | 1 | 5.333 | 80.8% | 5 | R.MQKEITALAPSTMK.I | 22 |
|  | pDK339\_033013\_01.06337.06337.2 | 2.9107 | 0.3944 | 100.0% | 1162.0922 | 1162.3868 | 3 | 6.612 | 60.0% | 24 | K.EITALAPSTMK.I | 22 |
|  | SKAPIP\_041314\_01.11040.11040.1 | 2.8116 | 0.1393 | 96.5% | 1163.59 | 1162.3868 | 1 | 4.735 | 65.0% | 13 | K.EITALAPSTMK.I | 11 |
|  | SKAPIP\_tube2\_041314\_01.04526.04526.2 | 2.3655 | 0.1818 | 98.0% | 1037.3121 | 1037.2908 | 4 | 4.889 | 75.0% | 1 | K.IKIIAPPER.K | 222 |
|  | SKAPIP\_041314\_01.06362.06362.2 | 2.5702 | 0.1639 | 98.3% | 1165.0322 | 1165.4648 | 13 | 4.518 | 72.2% | 1 | K.IKIIAPPERK.Y | 222 |
|  | SKAPIP\_tube2\_041314\_02.14154.14154.2 | 4.7851 | 0.558 | 100.0% | 2603.8123 | 2604.0388 | 1 | 8.3 | 40.9% | 3 | K.YSVWIGGSILASLSTFQQMWISK.Q | 222 |
|  | SKAPIP\_tube2\_041314\_02.12364.12364.3 | 4.9227 | 0.3638 | 100.0% | 4101.264 | 4102.6104 | 1 | 6.958 | 23.6% | 3 | K.YSVWIGGSILASLSTFQQMWISKQEYDESGPSIVHR.K | 3 |
|  | SKAPIP\_041314\_01.10370.10370.2 | 2.2537 | 0.4043 | 99.7% | 1517.1721 | 1517.595 | 1 | 6.963 | 75.0% | 5 | K.QEYDESGPSIVHR.K | 22 |
|  | SKAPIP\_041314\_01.10376.10376.3 | 2.2316 | 0.2948 | 97.3% | 1517.5743 | 1517.595 | 6 | 5.036 | 37.5% | 2 | K.QEYDESGPSIVHR.K | 33 |
|  | SKAPIP\_041314\_01.05542.05542.3 | 2.4506 | 0.2525 | 96.2% | 1644.5643 | 1645.769 | 103 | 4.682 | 30.8% | 3 | K.QEYDESGPSIVHRK.C | 33 |

Similarities:
gi|4885049|ref|NP\_005(25:22)  
gi|63055057|ref|NP\_00(11:36)  
gi|134133226|ref|NP\_0(13:34)  

---

|  |  |  |  |  |  |  |  |  |
| --- | --- | --- | --- | --- | --- | --- | --- | --- |
| U | *gi|62414289|ref|NP\_00* | 57 | 345 | 79.0% | 466 | 53652 | 5.1 | vimentin [Homo sapiens] |

| Filename XCorr DeltCN Conf% ObsM+H+ CalcM+H+ SpR ZScore Ion% # Sequence  | | | | | | | | | | | | |
| --- | --- | --- | --- | --- | --- | --- | --- | --- | --- | --- | --- | --- |
| \* | pDK339othertube\_033013\_01.03737.03737.2 | 2.6983 | 0.2061 | 98.4% | 1495.3522 | 1495.6531 | 66 | 4.623 | 46.4% | 4 | R.MFGGPGTASRPSSSR.S | 2 |
| \* | pDK339\_033013\_01.06122.06122.2 | 2.4809 | 0.3034 | 99.2% | 1496.2122 | 1496.6633 | 27 | 5.527 | 46.2% | 7 | R.TYSLGSALRPSTSR.S | 2 |
| \* | SKAPIP\_tube2\_041314\_02.04643.04643.3 | 2.4072 | 0.2751 | 97.4% | 1496.5144 | 1496.6633 | 1 | 5.008 | 40.4% | 2 | R.TYSLGSALRPSTSR.S | 3 |
| \* | pDK339othertube\_033013\_01.05679.05679.2 | 4.673 | 0.5094 | 100.0% | 1429.8922 | 1429.5724 | 1 | 8.518 | 80.8% | 15 | R.SLYASSPGGVYATR.S | 2 |
| \* | pDK339\_033013\_01.06782.06782.2 | 4.034 | 0.4545 | 100.0% | 1509.2522 | 1509.5724 | 1 | 6.86 | 73.1% | 8 | R.SLYASS\*PGGVYATR.S | 2 |
| \* | pDk339\_033013\_02.09385.09385.2 | 5.2682 | 0.5575 | 100.0% | 2127.0122 | 2127.3557 | 1 | 8.841 | 63.9% | 7 | R.LLQDSVDFSLADAINTEFK.N | 2 |
|  | SKAPIP\_tube2\_041314\_01.04917.04917.2 | 4.2486 | 0.4481 | 100.0% | 1589.2322 | 1588.7147 | 1 | 7.394 | 79.2% | 7 | R.TNEKVELQELNDR.F | 2 |
|  | pDK339othertube\_033013\_01.04977.04977.3 | 4.2038 | 0.2824 | 100.0% | 1589.6344 | 1588.7147 | 1 | 5.837 | 54.2% | 8 | R.TNEKVELQELNDR.F | 3 |
|  | pDK339\_033013\_01.05474.05474.2 | 3.0742 | 0.2826 | 100.0% | 1117.3522 | 1116.2163 | 1 | 5.626 | 87.5% | 11 | K.VELQELNDR.F | 2 |
| \* | pDK339\_033013\_01.04756.04756.1 | 2.0358 | 0.1591 | 95.2% | 870.54 | 870.9805 | 12 | 3.936 | 66.7% | 2 | R.FANYIDK.V | 1 |
| \* | pDK339othertube\_033013\_01.04701.04701.2 | 2.5137 | 0.3294 | 99.9% | 871.0122 | 870.9805 | 1 | 5.296 | 91.7% | 3 | R.FANYIDK.V | 2 |
| \* | pDK339\_033013\_01.05501.05501.2 | 2.868 | 0.3555 | 100.0% | 1125.2522 | 1126.3005 | 1 | 6.081 | 81.2% | 4 | R.FANYIDKVR.F | 2 |
| \* | SKAPIP\_tube2\_041314\_01.10824.10824.3 | 5.3744 | 0.3356 | 100.0% | 2429.4243 | 2428.8345 | 1 | 6.981 | 41.2% | 1 | R.FLEQQNKILLAELEQLKGQGK.S | 3 |
| \* | AstrinIP\_MS2\_022614\_01.12065.12065.2 | 3.6176 | 0.2915 | 100.0% | 1170.0122 | 1170.4349 | 1 | 7.296 | 83.3% | 11 | K.ILLAELEQLK.G | 2 |
| \* | pDK339othertube\_033013\_01.08510.08510.3 | 2.0844 | 0.3164 | 96.8% | 1540.6444 | 1540.8436 | 5 | 5.064 | 38.5% | 3 | K.ILLAELEQLKGQGK.S | 3 |
| \* | pDK339\_033013\_01.10177.10177.2 | 4.2319 | 0.3762 | 100.0% | 1540.6721 | 1540.8436 | 1 | 7.437 | 76.9% | 14 | K.ILLAELEQLKGQGK.S | 2 |
| \* | SKAPIP\_tube2\_041314\_01.07366.07366.2 | 3.5113 | 0.4606 | 100.0% | 1498.2322 | 1498.6508 | 1 | 7.792 | 81.8% | 4 | K.SRLGDLYEEEMR.E | 2 |
| \* | SKAPIP\_041314\_01.11247.11247.2 | 3.7763 | 0.4928 | 100.0% | 1255.2122 | 1255.385 | 1 | 8.561 | 77.8% | 17 | R.LGDLYEEEMR.E | 2 |
| \* | SKAPIP\_tube2\_041314\_01.07794.07794.2 | 3.2995 | 0.3271 | 100.0% | 1689.1721 | 1689.881 | 1 | 6.356 | 61.5% | 6 | R.VEVERDNLAEDIMR.L | 2 |
| \* | SKAPIP\_tube2\_041314\_01.07840.07840.3 | 3.7058 | 0.3248 | 100.0% | 1690.8844 | 1689.881 | 2 | 5.816 | 42.3% | 7 | R.VEVERDNLAEDIMR.L | 3 |
| \* | pDK365N\_100mM\_082813\_03.08442.08442.2 | 2.8877 | 0.1998 | 99.7% | 1077.1921 | 1077.1975 | 1 | 6.231 | 75.0% | 7 | R.DNLAEDIMR.L | 2 |
| \* | pJS43\_100mM\_120812\_01.05072.05072.2 | 3.0463 | 0.2406 | 99.8% | 1304.3922 | 1304.5042 | 9 | 5.262 | 66.7% | 1 | R.EKLQEEMLQR.E | 2 |
| \* | pDK339\_033013\_01.09520.09520.3 | 3.7852 | 0.4096 | 100.0% | 2609.0645 | 2609.8706 | 1 | 7.181 | 32.5% | 1 | R.EKLQEEMLQREEAENTLQSFR.Q | 3 |
| \* | SKAPIP\_tube2\_041314\_01.04402.04402.2 | 2.8444 | 0.1523 | 99.5% | 1048.2722 | 1047.2146 | 6 | 3.807 | 85.7% | 3 | K.LQEEMLQR.E | 2 |
| \* | SKAPIP\_tube2\_041314\_02.06764.06764.3 | 4.5824 | 0.3812 | 100.0% | 2351.8442 | 2352.581 | 1 | 7.368 | 45.8% | 9 | K.LQEEMLQREEAENTLQSFR.Q | 3 |
| \* | pDK339othertube\_033013\_01.06069.06069.2 | 3.4022 | 0.4204 | 100.0% | 1325.0122 | 1324.3898 | 1 | 7.872 | 75.0% | 13 | R.EEAENTLQSFR.Q | 2 |
| \* | SKAPIP\_tube2\_041314\_01.09377.09377.3 | 4.0545 | 0.4561 | 100.0% | 2394.2944 | 2394.5168 | 1 | 7.36 | 31.2% | 7 | R.EEAENTLQSFRQDVDNASLAR.L | 3 |
| \* | SKAPIP\_tube2\_041314\_01.09389.09389.2 | 3.6418 | 0.2871 | 100.0% | 2394.6921 | 2394.5168 | 11 | 5.743 | 37.5% | 2 | R.EEAENTLQSFRQDVDNASLAR.L | 2 |
| \* | pJS43\_100mM\_120812\_01.03656.03656.2 | 2.4276 | 0.3533 | 99.8% | 1088.4521 | 1089.1503 | 1 | 6.051 | 83.3% | 2 | R.QDVDNASLAR.L | 2 |
| \* | SKAPIP\_041314\_01.11885.11885.2 | 4.8638 | 0.4424 | 100.0% | 1534.5521 | 1534.793 | 1 | 8.778 | 87.5% | 5 | R.KVESLQEEIAFLK.K | 2 |
| \* | pDK339othertube\_033013\_01.09503.09503.3 | 4.1797 | 0.2665 | 100.0% | 1534.6144 | 1534.793 | 1 | 5.357 | 54.2% | 1 | R.KVESLQEEIAFLK.K | 3 |
| \* | AstrinIP\_MS2\_022614\_01.09126.09126.2 | 5.1348 | 0.4893 | 100.0% | 1662.4521 | 1662.967 | 1 | 8.701 | 84.6% | 4 | R.KVESLQEEIAFLKK.L | 2 |
| \* | pDK339othertube\_033013\_01.08098.08098.3 | 4.4721 | 0.3193 | 100.0% | 1663.0743 | 1662.967 | 3 | 6.357 | 44.2% | 18 | R.KVESLQEEIAFLKK.L | 3 |
| \* | AstrinIP\_MS2\_022614\_01.12185.12185.2 | 2.6305 | 0.3162 | 99.7% | 1405.8522 | 1406.6189 | 1 | 5.63 | 72.7% | 1 | K.VESLQEEIAFLK.K | 2 |
| \* | SKAPIP\_tube2\_041314\_01.10770.10770.3 | 8.3714 | 0.5832 | 100.0% | 3924.4744 | 3925.3445 | 1 | 11.086 | 34.8% | 2 | K.LHEEEIQELQAQIQEQHVQIDVDVSKPDLTAALR.D | 3 |
|  | SKAPIP\_tube2\_041314\_01.07614.07614.1 | 2.1401 | 0.2535 | 98.6% | 1309.6 | 1310.4056 | 1 | 4.894 | 66.7% | 2 | K.NLQEAEEWYK.S | 1 |
|  | pDK339\_033013\_01.08665.08665.2 | 3.5223 | 0.3377 | 100.0% | 1310.0122 | 1310.4056 | 1 | 5.954 | 77.8% | 16 | K.NLQEAEEWYK.S | 2 |
| \* | pDK339\_033013\_01.05069.05069.1 | 2.1803 | 0.311 | 98.7% | 1093.41 | 1094.1692 | 4 | 5.279 | 50.0% | 1 | K.FADLSEAANR.N | 1 |
| \* | pJS43\_100mM\_120812\_01.05525.05525.2 | 4.1969 | 0.434 | 100.0% | 1094.1122 | 1094.1692 | 1 | 8.021 | 94.4% | 21 | K.FADLSEAANR.N | 2 |
| \* | pDK339othertube\_033013\_01.05666.05666.3 | 3.6156 | 0.4934 | 100.0% | 1777.7943 | 1777.8912 | 1 | 7.814 | 45.0% | 4 | K.FADLSEAANRNNDALR.Q | 3 |
| \* | SKAPIP\_041314\_01.11430.11430.3 | 2.4476 | 0.2642 | 95.1% | 2377.4944 | 2378.5647 | 378 | 4.308 | 20.0% | 1 | R.QVQSLTCEVDALKGTNESLER.Q | 3 |
| \* | pDk339\_033013\_02.06749.06749.2 | 6.4474 | 0.5841 | 100.0% | 2187.392 | 2188.33 | 1 | 10.455 | 69.4% | 10 | R.EMEENFAVEAANYQDTIGR.L | 2 |
| \* | AstrinIP\_MS2\_022614\_02.06828.06828.3 | 4.2674 | 0.3896 | 100.0% | 2187.8044 | 2188.33 | 1 | 6.919 | 45.8% | 4 | R.EMEENFAVEAANYQDTIGR.L | 3 |
| \* | SKAPIP\_041314\_01.11099.11099.2 | 4.7613 | 0.4629 | 100.0% | 1735.3922 | 1735.9679 | 1 | 8.348 | 76.9% | 9 | R.LQDEIQNMKEEMAR.H | 2 |
|  | pDK339\_033013\_01.07526.07526.2 | 3.1575 | 0.2377 | 99.8% | 1528.1522 | 1528.7513 | 1 | 5.01 | 72.7% | 2 | R.HLREYQDLLNVK.M | 22 |
|  | SKAPIP\_tube2\_041314\_01.06459.06459.3 | 4.4288 | 0.4198 | 100.0% | 1529.7244 | 1528.7513 | 1 | 7.233 | 54.5% | 9 | R.HLREYQDLLNVK.M | 33 |
|  | pDK339\_033013\_01.08609.08609.1 | 2.4485 | 0.3393 | 98.4% | 1121.57 | 1122.2633 | 2 | 5.424 | 75.0% | 1 | R.EYQDLLNVK.M | 111 |
|  | SKAPIP\_tube2\_041314\_01.07604.07604.2 | 2.4982 | 0.1578 | 98.2% | 1122.1322 | 1122.2633 | 2 | 5.688 | 75.0% | 2 | R.EYQDLLNVK.M | 222 |
|  | SKAPIP\_041314\_02.08045.08045.2 | 3.7762 | 0.3569 | 100.0% | 1296.6322 | 1296.5243 | 1 | 8.295 | 85.0% | 6 | K.MALDIEIATYR.K | 2 |
|  | SKAPIP\_tube2\_041314\_02.06844.06844.2 | 3.6248 | 0.4012 | 100.0% | 1424.2722 | 1424.6984 | 1 | 6.783 | 68.2% | 2 | K.MALDIEIATYRK.L | 2 |
|  | SKAPIP\_041314\_02.06839.06839.3 | 2.9344 | 0.341 | 99.9% | 1425.0243 | 1424.6984 | 1 | 5.238 | 47.7% | 2 | K.MALDIEIATYRK.L | 3 |
|  | pJS43\_100mM\_120812\_01.03584.03584.1 | 2.3838 | 0.1736 | 97.7% | 932.52 | 933.00616 | 42 | 5.42 | 64.3% | 1 | K.LLEGEESR.I | 111 |
|  | pJS43\_100mM\_120812\_01.03569.03569.2 | 2.2842 | 0.1217 | 95.4% | 932.6122 | 933.00616 | 36 | 4.304 | 71.4% | 1 | K.LLEGEESR.I | 222 |
| \* | SKAPIP\_041314\_01.13185.13185.2 | 3.3034 | 0.4796 | 100.0% | 1571.6122 | 1571.8601 | 1 | 7.586 | 80.8% | 12 | R.ISLPLPNFSSLNLR.E | 2 |
| \* | pJS43\_100mM\_120812\_01.08474.08474.2 | 3.1471 | 0.3566 | 99.9% | 1669.3722 | 1669.829 | 1 | 5.668 | 57.1% | 6 | R.ETNLDSLPLVDTHSK.R | 2 |
| \* | SKAPIP\_tube2\_041314\_01.06567.06567.2 | 3.6823 | 0.3636 | 100.0% | 1825.4722 | 1826.0165 | 4 | 7.239 | 46.7% | 4 | R.ETNLDSLPLVDTHSKR.T | 2 |
| \* | pJS43\_100mM\_120812\_01.04514.04514.2 | 3.934 | 0.4 | 100.0% | 1837.2922 | 1837.854 | 1 | 6.738 | 60.0% | 12 | R.DGQVINETSQHHDDLE.- | 2 |

Similarities:
gi|4504919|ref|NP\_002(2:55)  
gi|67782365|ref|NP\_00(2:55)  
gi|157738641|ref|NP\_0(4:53)  
gi|32483416|ref|NP\_06(2:55)  

---

|  |  |  |  |  |  |  |  |  |
| --- | --- | --- | --- | --- | --- | --- | --- | --- |
| U | *gi|57242777|ref|NP\_03* | 24 | 231 | 78.6% | 103 | 11967 | 5.9 | c-myc binding protein [Homo sapiens] |

| Filename XCorr DeltCN Conf% ObsM+H+ CalcM+H+ SpR ZScore Ion% # Sequence  | | | | | | | | | | | | |
| --- | --- | --- | --- | --- | --- | --- | --- | --- | --- | --- | --- | --- |
| \* | pDK339\_033013\_01.07561.07561.2 | 3.181 | 0.301 | 100.0% | 933.9922 | 934.07764 | 3 | 6.297 | 81.2% | 21 | K.SGVLDTLTK.V | 2 |
| \* | pSKT11\_1\_020812\_01.08681.08681.1 | 2.0375 | 0.2185 | 98.0% | 934.61 | 934.07764 | 1 | 4.337 | 62.5% | 9 | K.SGVLDTLTK.V | 1 |
| \* | 100326\_pJS43\_01.09447.09447.2 | 4.963 | 0.482 | 100.0% | 2276.3323 | 2276.6348 | 1 | 7.993 | 57.9% | 16 | K.VLVALYEEPEKPNSALDFLK.H | 2 |
| \* | SKAPIP\_041314\_01.12422.12422.3 | 4.9092 | 0.5468 | 100.0% | 2276.4243 | 2276.6348 | 1 | 8.98 | 43.4% | 39 | K.VLVALYEEPEKPNSALDFLK.H | 3 |
| \* | SKAPIP\_tube2\_041314\_01.11838.11838.3 | 6.7129 | 0.5576 | 100.0% | 4154.6343 | 4155.74 | 1 | 9.345 | 26.4% | 2 | K.VLVALYEEPEKPNSALDFLKHHLGAATPENPEIELLR.L | 3 |
| \* | pDK339\_033013\_01.08263.08263.2 | 4.8557 | 0.4153 | 100.0% | 1897.4922 | 1898.1289 | 1 | 7.827 | 75.0% | 17 | K.HHLGAATPENPEIELLR.L | 2 |
| \* | pDK365N\_300mM\_082713\_03.06672.06672.3 | 4.8084 | 0.2294 | 100.0% | 1899.6543 | 1898.1289 | 1 | 5.28 | 51.6% | 20 | K.HHLGAATPENPEIELLR.L | 3 |
| \* | 100326\_pJS43\_01.09392.09392.3 | 4.5099 | 0.3659 | 100.0% | 2712.6243 | 2713.124 | 1 | 7.052 | 37.0% | 5 | K.HHLGAATPENPEIELLRLELAEMK.E | 3 |
| \* | pSKT11\_1\_020812\_01.09279.09279.3 | 4.0774 | 0.2768 | 99.8% | 2969.8442 | 2970.4138 | 1 | 5.579 | 31.0% | 2 | K.HHLGAATPENPEIELLRLELAEMKEK.Y | 3 |
| \* | pSKT11\_1\_020812\_01.04240.04240.2 | 2.7085 | 0.121 | 98.5% | 1091.1721 | 1091.3081 | 10 | 4.683 | 68.8% | 4 | R.LELAEMKEK.Y | 2 |
| \* | pSKT11\_1\_020812\_02.05345.05345.3 | 4.2074 | 0.2527 | 99.9% | 2168.2744 | 2167.4792 | 4 | 4.88 | 32.4% | 1 | R.LELAEMKEKYEAIVEENK.K | 3 |
| \* | pSKT11\_1\_020812\_02.04954.04954.2 | 5.5002 | 0.4532 | 100.0% | 2295.392 | 2295.6533 | 1 | 8.592 | 61.1% | 1 | R.LELAEMKEKYEAIVEENKK.L | 2 |
| \* | pSKT11\_1\_020812\_02.04952.04952.3 | 6.4469 | 0.4037 | 100.0% | 2295.4744 | 2295.6533 | 1 | 7.823 | 43.1% | 12 | R.LELAEMKEKYEAIVEENKK.L | 3 |
| \* | SKAPIP\_041314\_01.10240.10240.2 | 3.4913 | 0.419 | 100.0% | 1352.2522 | 1352.4839 | 1 | 7.577 | 80.0% | 4 | K.EKYEAIVEENK.K | 2 |
| \* | pDK339\_033013\_01.03808.03808.2 | 4.214 | 0.3704 | 100.0% | 1481.0721 | 1480.658 | 1 | 7.561 | 68.2% | 10 | K.EKYEAIVEENKK.L | 2 |
| \* | SKAPIP\_041314\_01.06480.06480.2 | 2.7966 | 0.4706 | 100.0% | 1094.7922 | 1095.1943 | 1 | 7.324 | 81.2% | 3 | K.YEAIVEENK.K | 2 |
| \* | AstrinIP\_MS2\_022614\_01.03710.03710.2 | 3.0158 | 0.2761 | 99.9% | 1224.1322 | 1223.3684 | 1 | 5.907 | 88.9% | 5 | K.YEAIVEENKK.L | 2 |
| \* | SKAPIP\_041314\_01.08163.08163.2 | 4.2175 | 0.4578 | 100.0% | 1531.0322 | 1531.7056 | 1 | 7.803 | 70.8% | 13 | K.AKLAQYEPPQEEK.R | 2 |
| \* | SKAPIP\_041314\_01.07977.07977.3 | 2.679 | 0.2232 | 96.6% | 1531.8844 | 1531.7056 | 1 | 4.144 | 39.6% | 1 | K.AKLAQYEPPQEEK.R | 3 |
| \* | pSKT11\_1\_020812\_01.03746.03746.2 | 4.916 | 0.4715 | 100.0% | 1887.3322 | 1888.0874 | 1 | 8.503 | 56.7% | 2 | K.AKLAQYEPPQEEKRAE.- | 2 |
| \* | pSKT11\_1\_020812\_01.03752.03752.3 | 4.6016 | 0.5019 | 100.0% | 1888.2244 | 1888.0874 | 1 | 7.778 | 41.7% | 2 | K.AKLAQYEPPQEEKRAE.- | 3 |
| \* | SKAPIP\_041314\_01.07539.07539.2 | 3.5265 | 0.3249 | 100.0% | 1332.2722 | 1332.4528 | 3 | 5.363 | 70.0% | 36 | K.LAQYEPPQEEK.R | 2 |
| \* | SKAPIP\_041314\_01.05382.05382.2 | 3.4749 | 0.2923 | 100.0% | 1488.3522 | 1488.6403 | 1 | 5.788 | 68.2% | 2 | K.LAQYEPPQEEKR.A | 2 |
| \* | AstrinNocIP\_020510\_01.01845.01845.2 | 3.1895 | 0.3632 | 100.0% | 1688.1322 | 1688.8345 | 1 | 6.399 | 53.8% | 4 | K.LAQYEPPQEEKRAE.- | 2 |

---

|  |  |  |  |  |  |  |  |  |
| --- | --- | --- | --- | --- | --- | --- | --- | --- |
| U | *GFP* | 44 | 1008 | 76.5% | 238 | 26813 | 5.8 | no description |

| Filename XCorr DeltCN Conf% ObsM+H+ CalcM+H+ SpR ZScore Ion% # Sequence  | | | | | | | | | | | | |
| --- | --- | --- | --- | --- | --- | --- | --- | --- | --- | --- | --- | --- |
| \* | pDK365N\_300mM\_082713\_01.14006.14006.2 | 5.0789 | 0.5816 | 100.0% | 2437.9722 | 2438.7397 | 1 | 10.637 | 52.3% | 16 | K.GEELFTGVVPILVELDGDVNGHK.F | 2 |
| \* | pJS43\_100mM\_120812\_02.10032.10032.3 | 3.9984 | 0.3778 | 100.0% | 2439.0244 | 2438.7397 | 1 | 6.129 | 34.1% | 40 | K.GEELFTGVVPILVELDGDVNGHK.F | 3 |
| \* | pSKT11\_1\_020812\_01.10628.10628.3 | 4.2607 | 0.3934 | 100.0% | 3924.4744 | 3924.2666 | 1 | 5.884 | 22.3% | 3 | K.GEELFTGVVPILVELDGDVNGHKFSVSGEGEGDATYGK.L | 3 |
| \* | pSKT11\_1\_020812\_02.08150.08150.3 | 4.3633 | 0.4338 | 100.0% | 4378.494 | 4379.8647 | 1 | 8.189 | 17.1% | 1 | K.GEELFTGVVPILVELDGDVNGHKFSVSGEGEGDATYGKLTLK.F | 3 |
| \* | pSKT11\_1\_020812\_02.03252.03252.1 | 2.0418 | 0.4164 | 99.5% | 1503.51 | 1504.5499 | 1 | 7.579 | 50.0% | 1 | K.FSVSGEGEGDATYGK.L | 1 |
| \* | 100326\_pJS43\_02.03434.03434.2 | 4.721 | 0.6408 | 100.0% | 1504.2922 | 1504.5499 | 1 | 10.677 | 71.4% | 266 | K.FSVSGEGEGDATYGK.L | 2 |
| \* | pSKT11\_1\_020812\_02.04370.04370.2 | 5.038 | 0.5362 | 100.0% | 1959.3722 | 1960.148 | 1 | 9.817 | 63.9% | 8 | K.FSVSGEGEGDATYGKLTLK.F | 2 |
| \* | pSKT11\_1\_020812\_02.04376.04376.3 | 2.8265 | 0.2283 | 96.1% | 1960.6743 | 1960.148 | 2 | 5.735 | 34.7% | 4 | K.FSVSGEGEGDATYGKLTLK.F | 3 |
| \* | pSKT11\_1\_020812\_01.07322.07322.2 | 4.1617 | 0.4512 | 100.0% | 1592.9922 | 1593.7998 | 1 | 6.947 | 86.4% | 14 | R.YPDHMKQHDFFK.S | 2 |
| \* | pSKT11\_1\_020812\_01.07286.07286.3 | 3.4595 | 0.2398 | 99.7% | 1594.2244 | 1593.7998 | 1 | 5.635 | 50.0% | 11 | R.YPDHMKQHDFFK.S | 3 |
| \* | pSKT11\_1\_020812\_01.06632.06632.2 | 3.1209 | 0.4111 | 100.0% | 1266.9321 | 1267.399 | 1 | 7.543 | 75.0% | 60 | K.SAMPEGYVQER.T | 2 |
| \* | pSKT11\_1\_020812\_01.06687.06687.1 | 2.2325 | 0.1936 | 96.9% | 1268.44 | 1267.399 | 4 | 3.823 | 45.0% | 20 | K.SAMPEGYVQER.T | 1 |
| \* | pSKT11\_1\_020812\_01.08504.08504.1 | 2.6362 | 0.4609 | 98.8% | 1347.53 | 1348.4979 | 1 | 7.724 | 70.0% | 4 | R.TIFFKDDGNYK.T | 1 |
| \* | pDK365N\_300mM\_082713\_01.05756.05756.2 | 3.6082 | 0.4422 | 100.0% | 1349.2722 | 1348.4979 | 1 | 7.503 | 85.0% | 64 | R.TIFFKDDGNYK.T | 2 |
| \* | pSKT11\_1\_020812\_01.08184.08184.1 | 2.3172 | 0.5474 | 100.0% | 1604.73 | 1605.7905 | 8 | 8.212 | 45.8% | 1 | R.TIFFKDDGNYKTR.A | 1 |
| \* | pDK365N\_300mM\_082713\_01.04607.04607.2 | 3.8966 | 0.4506 | 100.0% | 1605.4321 | 1605.7905 | 1 | 7.857 | 70.8% | 13 | R.TIFFKDDGNYKTR.A | 2 |
| \* | pSKT11\_1\_020812\_01.08210.08210.3 | 3.0966 | 0.4329 | 100.0% | 1606.1643 | 1605.7905 | 1 | 6.136 | 45.8% | 20 | R.TIFFKDDGNYKTR.A | 3 |
| \* | pSKT11\_1\_020812\_02.03281.03281.2 | 4.1583 | 0.4056 | 100.0% | 1735.3722 | 1735.9376 | 1 | 7.048 | 57.1% | 4 | K.TRAEVKFEGDTLVNR.I | 2 |
| \* | 100326\_pJS43\_02.03560.03560.3 | 4.5973 | 0.385 | 100.0% | 1735.8544 | 1735.9376 | 1 | 8.167 | 50.0% | 18 | K.TRAEVKFEGDTLVNR.I | 3 |
| \* | pSKT11\_1\_020812\_02.04539.04539.2 | 3.6271 | 0.4264 | 100.0% | 2218.5723 | 2219.5461 | 2 | 7.441 | 41.7% | 1 | K.TRAEVKFEGDTLVNRIELK.G | 2 |
| \* | pSKT11\_1\_020812\_02.04532.04532.3 | 5.0432 | 0.3966 | 100.0% | 2219.7244 | 2219.5461 | 1 | 6.506 | 43.1% | 4 | K.TRAEVKFEGDTLVNRIELK.G | 3 |
| \* | 100326\_pJS43\_01.04468.04468.1 | 2.4599 | 0.4742 | 98.8% | 1477.42 | 1478.6451 | 3 | 7.793 | 50.0% | 3 | R.AEVKFEGDTLVNR.I | 1 |
| \* | 100326\_pJS43\_01.04526.04526.2 | 4.5049 | 0.5143 | 100.0% | 1477.6921 | 1478.6451 | 1 | 9.18 | 79.2% | 93 | R.AEVKFEGDTLVNR.I | 2 |
| \* | pDK339othertube\_033013\_01.05870.05870.3 | 3.3934 | 0.292 | 99.9% | 1480.2244 | 1478.6451 | 2 | 5.42 | 43.8% | 47 | R.AEVKFEGDTLVNR.I | 3 |
| \* | 100326\_pJS43\_02.05306.05306.3 | 4.4306 | 0.3921 | 100.0% | 1960.5844 | 1962.2535 | 1 | 7.104 | 45.3% | 32 | R.AEVKFEGDTLVNRIELK.G | 3 |
| \* | 100326\_pJS43\_01.06728.06728.2 | 5.0311 | 0.3239 | 100.0% | 1962.3922 | 1962.2535 | 1 | 6.85 | 56.2% | 16 | R.AEVKFEGDTLVNRIELK.G | 2 |
| \* | pSKT11\_1\_020812\_01.07205.07205.1 | 2.295 | 0.4357 | 99.3% | 1050.42 | 1051.1442 | 1 | 6.684 | 68.8% | 12 | K.FEGDTLVNR.I | 1 |
| \* | pDK365N\_100mM\_082713\_01.04842.04842.2 | 3.3002 | 0.4551 | 100.0% | 1051.0922 | 1051.1442 | 3 | 7.133 | 87.5% | 58 | K.FEGDTLVNR.I | 2 |
| \* | 100326\_pJS43\_02.05320.05320.2 | 3.1421 | 0.2615 | 99.8% | 1534.5322 | 1534.7526 | 3 | 4.904 | 62.5% | 7 | K.FEGDTLVNRIELK.G | 2 |
| \* | pDK339othertube\_033013\_01.06440.06440.3 | 4.4032 | 0.3784 | 100.0% | 1543.6444 | 1543.7196 | 1 | 5.836 | 46.2% | 40 | K.GIDFKEDGNILGHK.L | 3 |
| \* | pDK365N\_300mM\_082713\_01.06598.06598.2 | 4.5642 | 0.5071 | 100.0% | 1544.3121 | 1543.7196 | 1 | 8.667 | 73.1% | 22 | K.GIDFKEDGNILGHK.L | 2 |
| \* | 100326\_pJS43\_01.07658.07658.3 | 6.7878 | 0.5726 | 100.0% | 3499.4644 | 3499.8792 | 1 | 9.766 | 31.9% | 9 | K.GIDFKEDGNILGHKLEYNYNSHNVYIMADK.Q | 3 |
| \* | 100326\_pJS43\_01.07048.07048.3 | 5.2839 | 0.5397 | 100.0% | 3755.5745 | 3756.184 | 1 | 8.056 | 28.2% | 6 | K.GIDFKEDGNILGHKLEYNYNSHNVYIMADKQK.N | 3 |
| \* | pJS43\_100mM\_120812\_01.03615.03615.1 | 1.8676 | 0.2486 | 98.3% | 982.46 | 983.06903 | 128 | 5.513 | 43.8% | 1 | K.EDGNILGHK.L | 1 |
| \* | 100326\_pJS43\_01.06543.06543.3 | 4.7147 | 0.3443 | 100.0% | 2939.4844 | 2939.2288 | 1 | 5.227 | 33.3% | 4 | K.EDGNILGHKLEYNYNSHNVYIMADK.Q | 3 |
| \* | 100326\_pJS43\_01.05934.05934.3 | 4.1526 | 0.5111 | 100.0% | 3195.1743 | 3195.5334 | 1 | 7.692 | 26.9% | 2 | K.EDGNILGHKLEYNYNSHNVYIMADKQK.N | 3 |
| \* | 100326\_pJS43\_01.05648.05648.2 | 5.1342 | 0.502 | 100.0% | 1975.2322 | 1975.1829 | 1 | 8.178 | 63.3% | 27 | K.LEYNYNSHNVYIMADK.Q | 2 |
| \* | pDK365N\_300mM\_082713\_03.06761.06761.3 | 3.6929 | 0.4501 | 100.0% | 1976.0343 | 1975.1829 | 121 | 7.531 | 33.3% | 30 | K.LEYNYNSHNVYIMADK.Q | 3 |
| \* | pSKT11\_1\_020812\_02.03722.03722.2 | 4.9391 | 0.4352 | 100.0% | 2230.5522 | 2231.4875 | 1 | 6.567 | 61.8% | 6 | K.LEYNYNSHNVYIMADKQK.N | 2 |
| \* | pSKT11\_1\_020812\_01.08422.08422.3 | 4.6245 | 0.3662 | 100.0% | 2232.5044 | 2231.4875 | 1 | 6.325 | 42.6% | 16 | K.LEYNYNSHNVYIMADKQK.N | 3 |
| \* | 100326\_pJS43\_01.01050.01050.1 | 2.2733 | 0.2365 | 98.7% | 919.39 | 920.09955 | 34 | 5.644 | 64.3% | 1 | K.NGIKVNFK.I | 1 |
| \* | 100326\_pJS43\_01.01043.01043.2 | 2.4875 | 0.2931 | 99.8% | 920.5522 | 920.09955 | 23 | 5.767 | 71.4% | 1 | K.NGIKVNFK.I | 2 |
| \* | pSKT11\_1\_020812\_02.05429.05429.3 | 2.9418 | 0.3083 | 99.2% | 4472.784 | 4474.844 | 252 | 5.525 | 14.4% | 1 | R.HNIEDGSVQLADHYQQNTPIGDGPVLLPDNHYLSTQSALSK.D | 3 |
| \* | 100326\_pJS43\_01.06888.06888.3 | 4.1715 | 0.3115 | 100.0% | 5213.0044 | 5214.6304 | 1 | 4.973 | 17.9% | 1 | R.HNIEDGSVQLADHYQQNTPIGDGPVLLPDNHYLSTQSALSKDPNEKR.D | 3 |

---

|  |  |  |  |  |  |  |  |  |
| --- | --- | --- | --- | --- | --- | --- | --- | --- |
| U | *gi|21361144|ref|NP\_00* | 35 | 88 | 72.7% | 439 | 49204 | 5.2 | proteasome 26S ATPase subunit 3 [Homo sapiens] |

| Filename XCorr DeltCN Conf% ObsM+H+ CalcM+H+ SpR ZScore Ion% # Sequence  | | | | | | | | | | | | |
| --- | --- | --- | --- | --- | --- | --- | --- | --- | --- | --- | --- | --- |
| \* | SKAPIP\_tube2\_041314\_01.11098.11098.2 | 6.4692 | 0.5442 | 100.0% | 2121.372 | 2121.3235 | 1 | 9.899 | 69.4% | 3 | K.MATVWDEAEQDGIGEEVLK.M | 2 |
| \* | SKAPIP\_tube2\_041314\_01.05258.05258.2 | 3.2921 | 0.2785 | 100.0% | 1107.0122 | 1107.2671 | 1 | 6.096 | 87.5% | 6 | K.MSTEEIIQR.T | 2 |
| \* | SKAPIP\_tube2\_041314\_01.04194.04194.3 | 4.0686 | 0.3602 | 100.0% | 2129.2444 | 2129.4363 | 3 | 5.909 | 33.8% | 1 | R.VTHELQAMKDKIKENSEK.I | 3 |
| \* | SKAPIP\_tube2\_041314\_01.15303.15303.3 | 4.9355 | 0.5106 | 100.0% | 3829.8245 | 3830.1511 | 1 | 8.126 | 25.0% | 1 | K.TLPYLVSNVIELLDVDPNDQEEDGANIDLDSQRK.G | 3 |
| \* | SKAPIP\_tube2\_041314\_01.13508.13508.2 | 3.4791 | 0.4986 | 100.0% | 1693.4521 | 1693.9806 | 1 | 8.196 | 71.4% | 4 | R.QTYFLPVIGLVDAEK.L | 2 |
| \* | SKAPIP\_tube2\_041314\_01.05092.05092.2 | 1.8698 | 0.2945 | 96.0% | 1140.2122 | 1140.3683 | 15 | 4.603 | 60.0% | 1 | K.LKPGDLVGVNK.D | 2 |
| \* | SKAPIP\_tube2\_041314\_01.11696.11696.3 | 3.6708 | 0.1817 | 96.7% | 3038.7244 | 3037.437 | 1 | 4.502 | 26.9% | 2 | K.LKPGDLVGVNKDSYLILETLPTEYDSR.V | 3 |
| \* | SKAPIP\_tube2\_041314\_01.06796.06796.3 | 5.4625 | 0.4506 | 100.0% | 2254.0444 | 2254.4304 | 1 | 8.005 | 44.7% | 2 | K.AMEVDERPTEQYSDIGGLDK.Q | 3 |
| \* | SKAPIP\_tube2\_041314\_01.06808.06808.2 | 4.6839 | 0.4313 | 100.0% | 2254.4722 | 2254.4304 | 1 | 9.002 | 57.9% | 1 | K.AMEVDERPTEQYSDIGGLDK.Q | 2 |
| \* | SKAPIP\_tube2\_041314\_01.15684.15684.3 | 4.7894 | 0.293 | 100.0% | 4098.8643 | 4098.633 | 1 | 6.345 | 22.1% | 1 | K.AMEVDERPTEQYSDIGGLDKQIQELVEAIVLPMNHK.E | 3 |
| \* | SKAPIP\_tube2\_041314\_01.12111.12111.2 | 4.0865 | 0.4127 | 100.0% | 1862.6322 | 1863.2256 | 1 | 7.243 | 60.0% | 3 | K.QIQELVEAIVLPMNHK.E | 2 |
| \* | SKAPIP\_041314\_01.12914.12914.3 | 3.4888 | 0.2021 | 98.8% | 1863.9543 | 1863.2256 | 2 | 5.029 | 41.7% | 2 | K.QIQELVEAIVLPMNHK.E | 3 |
| \* | SKAPIP\_041314\_01.11008.11008.2 | 3.7016 | 0.2946 | 100.0% | 1401.8922 | 1400.6177 | 7 | 5.571 | 63.6% | 3 | K.EKFENLGIQPPK.G | 2 |
| \* | SKAPIP\_tube2\_041314\_01.08948.08948.3 | 3.7739 | 0.3545 | 100.0% | 2560.1343 | 2558.9983 | 1 | 5.205 | 30.4% | 1 | K.EKFENLGIQPPKGVLMYGPPGTGK.T | 3 |
| \* | SKAPIP\_tube2\_041314\_01.06954.06954.2 | 2.5596 | 0.2746 | 99.6% | 1143.2322 | 1143.328 | 1 | 6.565 | 77.8% | 1 | K.FENLGIQPPK.G | 2 |
| \* | SKAPIP\_tube2\_041314\_01.06718.06718.2 | 3.2007 | 0.3862 | 100.0% | 1176.6921 | 1177.4039 | 1 | 9.015 | 77.3% | 6 | K.GVLMYGPPGTGK.T | 2 |
| \* | SKAPIP\_tube2\_041314\_01.12920.12920.2 | 5.1218 | 0.5088 | 100.0% | 1645.5322 | 1645.9574 | 1 | 9.952 | 70.0% | 6 | K.LAGPQLVQMFIGDGAK.L | 2 |
| \* | SKAPIP\_041314\_01.13008.13008.2 | 4.445 | 0.5834 | 100.0% | 1959.8121 | 1961.2628 | 3 | 9.359 | 47.1% | 5 | K.EKAPSIIFIDELDAIGTK.R | 2 |
| \* | SKAPIP\_tube2\_041314\_01.12524.12524.3 | 5.18 | 0.4889 | 100.0% | 1961.6643 | 1961.2628 | 1 | 8.589 | 47.1% | 6 | K.EKAPSIIFIDELDAIGTK.R | 3 |
| \* | SKAPIP\_tube2\_041314\_01.11510.11510.3 | 2.8277 | 0.3051 | 99.3% | 2118.1743 | 2117.4502 | 1 | 4.879 | 33.3% | 1 | K.EKAPSIIFIDELDAIGTKR.F | 3 |
| \* | SKAPIP\_041314\_01.13407.13407.2 | 4.4608 | 0.487 | 100.0% | 1704.3322 | 1703.9733 | 1 | 9.522 | 60.0% | 2 | K.APSIIFIDELDAIGTK.R | 2 |
| \* | SKAPIP\_041314\_02.09678.09678.3 | 3.1893 | 0.382 | 100.0% | 1860.5643 | 1860.1606 | 67 | 6.127 | 31.2% | 1 | K.APSIIFIDELDAIGTKR.F | 3 |
| \* | SKAPIP\_tube2\_041314\_01.13442.13442.2 | 4.8214 | 0.5117 | 100.0% | 2189.7722 | 2190.5195 | 1 | 8.027 | 72.2% | 4 | R.TMLELLNQLDGFQPNTQVK.V | 2 |
| \* | SKAPIP\_tube2\_041314\_01.13420.13420.3 | 6.2068 | 0.4914 | 100.0% | 2190.5942 | 2190.5195 | 1 | 8.865 | 56.9% | 2 | R.TMLELLNQLDGFQPNTQVK.V | 3 |
| \* | SKAPIP\_tube2\_041314\_01.09999.09999.2 | 3.6831 | 0.3808 | 100.0% | 1850.7522 | 1851.1997 | 1 | 6.015 | 59.4% | 1 | K.VIAATNRVDILDPALLR.S | 2 |
| \* | SKAPIP\_041314\_01.11993.11993.3 | 5.4196 | 0.3818 | 100.0% | 1851.0844 | 1851.1997 | 1 | 7.015 | 57.8% | 5 | K.VIAATNRVDILDPALLR.S | 3 |
| \* | SKAPIP\_041314\_01.12401.12401.2 | 3.521 | 0.3039 | 100.0% | 1125.3522 | 1125.3538 | 3 | 5.649 | 83.3% | 2 | R.VDILDPALLR.S | 2 |
| \* | SKAPIP\_tube2\_041314\_01.06994.06994.2 | 3.6907 | 0.3308 | 100.0% | 1460.5322 | 1461.6758 | 1 | 6.031 | 68.2% | 3 | R.KIEFPMPNEEAR.A | 2 |
| \* | SKAPIP\_tube2\_041314\_01.06911.06911.3 | 3.0667 | 0.3148 | 99.9% | 1462.1943 | 1461.6758 | 1 | 5.582 | 59.1% | 2 | R.KIEFPMPNEEAR.A | 3 |
| \* | SKAPIP\_041314\_01.11080.11080.3 | 3.8374 | 0.3113 | 100.0% | 1765.9443 | 1765.9788 | 1 | 5.617 | 48.2% | 5 | R.KMNVSPDVNYEELAR.C | 3 |
| \* | SKAPIP\_tube2\_041314\_01.06791.06791.2 | 4.5305 | 0.3925 | 100.0% | 1766.2322 | 1765.9788 | 1 | 7.519 | 78.6% | 1 | R.KMNVSPDVNYEELAR.C | 2 |
| \* | SKAPIP\_tube2\_041314\_01.08070.08070.2 | 3.8083 | 0.4922 | 100.0% | 1637.4521 | 1637.8047 | 1 | 7.968 | 84.6% | 1 | K.MNVSPDVNYEELAR.C | 2 |
| \* | SKAPIP\_tube2\_041314\_02.07140.07140.2 | 2.9913 | 0.3594 | 100.0% | 1289.9521 | 1290.5299 | 2 | 6.729 | 68.2% | 1 | K.AVCVEAGMIALR.R | 2 |
| \* | SKAPIP\_tube2\_041314\_02.07466.07466.3 | 3.737 | 0.2154 | 99.0% | 2236.6743 | 2235.4707 | 1 | 4.537 | 36.8% | 1 | R.GATELTHEDYMEGILEVQAK.K | 3 |
| \* | SKAPIP\_tube2\_041314\_01.06428.06428.1 | 1.4339 | 0.4224 | 98.6% | 842.36 | 842.92676 | 1 | 6.128 | 66.7% | 1 | K.ANLQYYA.- | 1 |

---

|  |  |  |  |  |  |  |  |  |
| --- | --- | --- | --- | --- | --- | --- | --- | --- |
| U | *gi|10864047|ref|NP\_06* | 58 | 154 | 72.0% | 864 | 94255 | 5.1 | epidermal growth factor receptor pathway substrate 15-like 1 [Homo sapiens] |

| Filename XCorr DeltCN Conf% ObsM+H+ CalcM+H+ SpR ZScore Ion% # Sequence  | | | | | | | | | | | | |
| --- | --- | --- | --- | --- | --- | --- | --- | --- | --- | --- | --- | --- |
| \* | pSKT11\_1\_020812\_01.09126.09126.2 | 3.2731 | 0.45 | 100.0% | 1106.1921 | 1106.3073 | 1 | 8.636 | 85.0% | 5 | R.VGASEAALFLK.K | 2 |
| \* | pSKT11\_1\_020812\_01.08666.08666.2 | 3.5951 | 0.4046 | 100.0% | 1234.1122 | 1234.4813 | 1 | 7.663 | 72.7% | 3 | R.VGASEAALFLKK.S | 2 |
| \* | pSKT11\_1\_020812\_02.08994.08994.3 | 6.025 | 0.4349 | 100.0% | 3624.2344 | 3624.174 | 1 | 7.542 | 28.9% | 2 | K.SGLSDIILGKIWDLADPEGKGFLDKQGFYVALR.L | 3 |
| \* | pDK339\_033013\_01.09969.09969.2 | 2.8622 | 0.2907 | 99.9% | 1144.0521 | 1144.2694 | 4 | 6.402 | 77.8% | 2 | K.IWDLADPEGK.G | 2 |
| \* | pSKT11\_1\_020812\_02.06694.06694.3 | 6.3493 | 0.4658 | 100.0% | 2639.6343 | 2640.0134 | 1 | 8.158 | 39.8% | 2 | K.IWDLADPEGKGFLDKQGFYVALR.L | 3 |
| \* | pSKT11\_1\_020812\_02.05680.05680.2 | 3.3275 | 0.2928 | 99.9% | 1515.5322 | 1514.7672 | 1 | 5.982 | 70.8% | 1 | K.GFLDKQGFYVALR.L | 2 |
| \* | pDK339othertube\_033013\_01.14016.14016.2 | 5.1074 | 0.5575 | 100.0% | 2234.5522 | 2235.5852 | 1 | 8.918 | 55.0% | 4 | K.AKFDGIFESLLPINGLLSGDK.V | 2 |
| \* | pDK339\_033013\_01.14985.14985.3 | 4.2845 | 0.4615 | 100.0% | 2235.3843 | 2235.5852 | 1 | 6.916 | 37.5% | 2 | K.AKFDGIFESLLPINGLLSGDK.V | 3 |
| \* | pDK339othertube\_033013\_01.14886.14886.2 | 4.3905 | 0.5417 | 100.0% | 2035.4321 | 2036.3324 | 1 | 8.643 | 55.6% | 3 | K.FDGIFESLLPINGLLSGDK.V | 2 |
| \* | pDK339othertube\_033013\_01.08397.08397.2 | 2.1635 | 0.3001 | 99.3% | 882.6322 | 883.0788 | 1 | 5.653 | 78.6% | 1 | K.LPLDVLGR.V | 2 |
| \* | pSKT11\_1\_020812\_01.09822.09822.3 | 5.1928 | 0.4321 | 100.0% | 3217.2244 | 3217.583 | 3 | 7.05 | 25.0% | 3 | R.VWDLSDIDKDGHLDRDEFAVAMHLVYR.A | 3 |
| \* | pSKT11\_1\_020812\_01.08742.08742.3 | 5.2049 | 0.4641 | 100.0% | 2228.2144 | 2227.6523 | 1 | 7.407 | 41.2% | 4 | R.ALEKEPVPSALPPSLIPPSKR.K | 3 |
| \* | pSKT11\_1\_020812\_01.09232.09232.2 | 3.367 | 0.4559 | 100.0% | 2225.5723 | 2226.5376 | 1 | 7.094 | 50.0% | 1 | K.TVFPGAVPVLPAS\*PPPKDSLR.S | 2 |
| \* | pSKT11\_1\_020812\_01.06083.06083.2 | 4.608 | 0.5617 | 100.0% | 1930.4122 | 1931.0667 | 1 | 9.694 | 65.8% | 3 | R.STPSHGSVSSLNSTGSLSPK.H | 2 |
| \* | pSKT11\_1\_020812\_01.07070.07070.2 | 4.67 | 0.4942 | 100.0% | 2010.1721 | 2011.0667 | 1 | 7.469 | 68.4% | 6 | R.STPSHGSVSSLNSTGSLS\*PK.H | 2 |
| \* | pSKT11\_1\_020812\_01.07202.07202.3 | 3.0887 | 0.3399 | 99.8% | 2010.2043 | 2011.0667 | 7 | 5.753 | 30.3% | 4 | R.STPSHGSVSSLNSTGSLS\*PK.H | 3 |
| \* | pDK365N\_300mM\_082713\_03.08448.08448.2 | 3.6691 | 0.5063 | 100.0% | 1683.3722 | 1682.917 | 1 | 7.037 | 57.1% | 2 | K.QTQPTVNWVVPVADK.M | 2 |
| \* | pDk339\_033013\_02.06062.06062.2 | 4.5554 | 0.526 | 100.0% | 1638.8121 | 1639.7563 | 1 | 9.132 | 78.6% | 4 | K.TDLDLDGYVSGQEVK.E | 2 |
| \* | pDK339\_033013\_01.15543.15543.3 | 5.3797 | 0.4562 | 100.0% | 2638.3743 | 2639.047 | 1 | 8.529 | 37.5% | 2 | K.EIFMHSGLTQNLLAHIWALADTR.Q | 3 |
| \* | pSKT11\_1\_020812\_02.07593.07593.2 | 4.2971 | 0.4324 | 100.0% | 1603.3522 | 1603.8762 | 2 | 7.64 | 70.8% | 2 | K.DQFALAMYFIQQK.V | 2 |
| \* | pSKT11\_1\_020812\_01.09162.09162.2 | 3.0058 | 0.4348 | 100.0% | 1934.5322 | 1935.2024 | 1 | 7.009 | 52.9% | 1 | K.GIDPPQVLSPDMVPPSER.G | 2 |
| \* | pDK339othertube\_033013\_01.06334.06334.2 | 3.0774 | 0.5152 | 100.0% | 1837.0322 | 1837.9377 | 1 | 9.455 | 60.5% | 2 | R.GTPGPDSSGSLGSGEFTGVK.E | 2 |
| \* | pDK339\_033013\_01.13899.13899.3 | 4.4622 | 0.4955 | 100.0% | 3476.6643 | 3477.7205 | 1 | 8.312 | 25.8% | 3 | R.GTPGPDSSGSLGSGEFTGVKELDDISQEIAQLQR.E | 3 |
| \* | pJS43\_100mM\_120812\_01.11462.11462.2 | 4.3292 | 0.302 | 100.0% | 1658.3922 | 1658.8058 | 1 | 6.727 | 73.1% | 6 | K.ELDDISQEIAQLQR.E | 2 |
| \* | pSKT11\_1\_020812\_01.07953.07953.2 | 3.2707 | 0.2426 | 99.9% | 1282.3322 | 1281.4081 | 1 | 6.1 | 77.8% | 1 | R.EKYSLEQDIR.E | 2 |
| \* | pSKT11\_1\_020812\_01.08546.08546.2 | 4.5452 | 0.311 | 100.0% | 2136.5322 | 2137.3542 | 1 | 5.727 | 50.0% | 1 | R.EKYSLEQDIREKEEAIR.Q | 2 |
| \* | pSKT11\_1\_020812\_02.04008.04008.3 | 3.2061 | 0.3192 | 99.9% | 2137.2244 | 2137.3542 | 1 | 5.717 | 40.6% | 1 | R.EKYSLEQDIREKEEAIR.Q | 3 |
| \* | pSKT11\_1\_020812\_01.08458.08458.3 | 4.2216 | 0.5061 | 100.0% | 2392.9744 | 2393.6592 | 1 | 7.682 | 40.3% | 1 | R.EKYSLEQDIREKEEAIRQK.T | 3 |
| \* | pDK339othertube\_033013\_01.05880.05880.2 | 2.4765 | 0.2098 | 99.2% | 1024.0922 | 1024.1185 | 1 | 5.844 | 78.6% | 3 | K.YSLEQDIR.E | 2 |
| \* | pSKT11\_1\_020812\_01.08723.08723.2 | 3.4407 | 0.2428 | 99.8% | 1879.4722 | 1880.0648 | 1 | 5.162 | 60.7% | 1 | K.YSLEQDIREKEEAIR.Q | 2 |
| \* | pSKT11\_1\_020812\_01.08697.08697.3 | 3.2084 | 0.2522 | 99.3% | 1880.5144 | 1880.0648 | 1 | 5.552 | 39.3% | 1 | K.YSLEQDIREKEEAIR.Q | 3 |
| \* | pSKT11\_1\_020812\_01.08610.08610.3 | 3.349 | 0.2848 | 99.7% | 2136.4443 | 2136.3696 | 1 | 5.694 | 35.9% | 1 | K.YSLEQDIREKEEAIRQK.T | 3 |
| \* | pDK339othertube\_033013\_01.08081.08081.2 | 3.9027 | 0.315 | 100.0% | 1547.2122 | 1547.6189 | 1 | 7.036 | 79.2% | 1 | K.TSEVQELQNDLDR.E | 2 |
| \* | pDK365N\_300mM\_082713\_03.11799.11799.3 | 3.9641 | 0.3367 | 100.0% | 2892.3245 | 2892.06 | 1 | 6.243 | 32.3% | 1 | K.TSEVQELQNDLDRETSSLQELEAQK.Q | 3 |
| \* | pSKT11\_1\_020812\_01.03882.03882.3 | 2.991 | 0.2881 | 99.5% | 1920.0543 | 1920.0613 | 225 | 5.085 | 30.0% | 1 | K.QDAQDRLDEMDQQKAK.L | 3 |
| \* | pSKT11\_1\_020812\_01.08297.08297.2 | 2.5156 | 0.1822 | 98.7% | 1105.0922 | 1105.2976 | 6 | 4.819 | 68.8% | 1 | K.LRDMLSDVR.Q | 2 |
| \* | pDK339othertube\_033013\_01.03644.03644.2 | 3.1009 | 0.4169 | 100.0% | 1276.6322 | 1277.3739 | 1 | 7.588 | 70.0% | 1 | K.TQIQSQESDLK.S | 2 |
| \* | pSKT11\_1\_020812\_01.08180.08180.2 | 4.9509 | 0.4704 | 100.0% | 2235.2522 | 2235.3262 | 1 | 7.927 | 66.7% | 2 | K.TQIQSQESDLKSQEDDLNR.A | 2 |
| \* | pDK339othertube\_033013\_01.05079.05079.3 | 4.8623 | 0.48 | 100.0% | 2235.2944 | 2235.3262 | 1 | 7.754 | 43.1% | 6 | K.TQIQSQESDLKSQEDDLNR.A | 3 |
| \* | pSKT11\_1\_020812\_01.08144.08144.2 | 4.3864 | 0.4535 | 100.0% | 2433.392 | 2434.579 | 1 | 7.428 | 60.0% | 1 | K.TQIQSQESDLKSQEDDLNRAK.S | 2 |
| \* | pSKT11\_1\_020812\_01.08140.08140.3 | 4.8266 | 0.354 | 100.0% | 2434.7644 | 2434.579 | 1 | 6.429 | 37.5% | 1 | K.TQIQSQESDLKSQEDDLNRAK.S | 3 |
| \* | pSKT11\_1\_020812\_01.09344.09344.2 | 4.7757 | 0.3946 | 100.0% | 2457.8123 | 2458.6475 | 1 | 6.714 | 50.0% | 1 | K.SELNRLQQEETQLEQSIQAGR.V | 2 |
| \* | pDK339othertube\_033013\_01.06813.06813.2 | 5.8344 | 0.4112 | 100.0% | 1858.3722 | 1859.003 | 1 | 8.157 | 83.3% | 17 | R.LQQEETQLEQSIQAGR.V | 2 |
| \* | pDK339othertube\_033013\_02.05472.05472.3 | 4.241 | 0.2708 | 100.0% | 1859.3644 | 1859.003 | 1 | 6.259 | 51.7% | 6 | R.LQQEETQLEQSIQAGR.V | 3 |
| \* | pJS43\_100mM\_120812\_01.03410.03410.2 | 3.3687 | 0.3202 | 100.0% | 1162.1122 | 1162.2015 | 1 | 6.235 | 83.3% | 1 | K.STQDEINQAR.S | 2 |
| \* | pSKT11\_1\_020812\_01.03545.03545.2 | 2.7282 | 0.1895 | 98.6% | 1591.2722 | 1591.7268 | 1 | 4.596 | 50.0% | 2 | K.LSQLHESRQEAHR.S | 2 |
| \* | pSKT11\_1\_020812\_01.09939.09939.2 | 5.2998 | 0.5274 | 100.0% | 3360.0322 | 3360.616 | 1 | 7.94 | 37.1% | 2 | R.SLEQYDQVLDGAHGASLTDLANLSEGVSLAER.G | 2 |
| \* | pSKT11\_1\_020812\_02.07190.07190.3 | 6.8268 | 0.5135 | 100.0% | 3360.4744 | 3360.616 | 1 | 9.45 | 35.5% | 6 | R.SLEQYDQVLDGAHGASLTDLANLSEGVSLAER.G | 3 |
| \* | pSKT11\_1\_020812\_01.08732.08732.2 | 3.5656 | 0.2641 | 99.9% | 1414.1522 | 1414.5756 | 1 | 6.442 | 62.5% | 2 | R.GSFGAMDDPFKNK.A | 2 |
| \* | pJS43\_100mM\_120812\_01.11869.11869.3 | 2.8687 | 0.3308 | 99.5% | 2688.7144 | 2689.941 | 1 | 5.517 | 26.1% | 2 | K.ALLFSNNTQELHPDPFQTEDPFK.S | 3 |
| \* | pDK339\_033013\_01.12226.12226.3 | 5.7036 | 0.4438 | 100.0% | 3263.9043 | 3264.575 | 1 | 7.465 | 27.8% | 3 | K.ALLFSNNTQELHPDPFQTEDPFKSDPFK.G | 3 |
| \* | pDK339othertube\_033013\_01.11781.11781.3 | 5.8719 | 0.5019 | 100.0% | 3459.6243 | 3460.651 | 1 | 10.088 | 29.8% | 3 | K.GADPFKGDPFQNDPFAEQQTTSTDPFGGDPFK.E | 3 |
| \* | pSKT11\_1\_020812\_01.05306.05306.2 | 3.0113 | 0.2018 | 99.7% | 1116.1522 | 1116.2157 | 2 | 6.029 | 66.7% | 9 | R.GSATDDFFKK.Q | 2 |
| \* | pDK339othertube\_033013\_01.08248.08248.2 | 2.9322 | 0.4208 | 100.0% | 1269.2322 | 1269.3531 | 1 | 7.428 | 75.0% | 3 | K.NDPFTSDPFTK.N | 2 |
| \* | pDK339othertube\_033013\_01.09663.09663.3 | 4.1713 | 0.3765 | 100.0% | 2627.0344 | 2627.8213 | 1 | 5.782 | 30.2% | 1 | K.NPSLPSKLDPFESSDPFSSSSVSSK.G | 3 |
| \* | pJS43\_100mM\_120812\_01.10335.10335.2 | 3.5216 | 0.546 | 100.0% | 1902.3922 | 1903.994 | 1 | 8.958 | 58.8% | 1 | K.LDPFESSDPFSSSSVSSK.G | 2 |
| \* | pSKT11\_1\_020812\_01.09974.09974.2 | 3.5682 | 0.4159 | 100.0% | 3390.8123 | 3391.6287 | 1 | 7.256 | 28.1% | 1 | K.STPVSQLGSADFPEAPDPFQPLGADSGDPFQSK.K | 2 |
| \* | pSKT11\_1\_020812\_01.08763.08763.3 | 4.0264 | 0.3209 | 100.0% | 2351.5444 | 2352.6519 | 1 | 5.481 | 34.1% | 1 | K.KGFGDPFSGKDPFVPSSAAKPSK.A | 3 |

---

|  |  |  |  |  |  |  |  |  |
| --- | --- | --- | --- | --- | --- | --- | --- | --- |
| U | *gi|40354195|ref|NP\_95* | 49 | 331 | 71.4% | 430 | 48058 | 5.5 | keratin 18 [Homo sapiens] |
| U | *gi|4557888|ref|NP\_000* | 49 | 331 | 71.4% | 430 | 48058 | 5.5 | keratin 18 [Homo sapiens] |

| Filename XCorr DeltCN Conf% ObsM+H+ CalcM+H+ SpR ZScore Ion% # Sequence  | | | | | | | | | | | | |
| --- | --- | --- | --- | --- | --- | --- | --- | --- | --- | --- | --- | --- |
|  | SKAPIP\_041314\_01.07178.07178.2 | 1.7452 | 0.3419 | 97.9% | 975.97217 | 976.0336 | 1 | 6.682 | 78.6% | 1 | R.STFSTNYR.S | 2 |
|  | SKAPIP\_tube2\_041314\_01.06756.06756.2 | 5.1434 | 0.5396 | 100.0% | 2854.8323 | 2856.0813 | 1 | 9.236 | 33.3% | 2 | R.SLGSVQAPSYGARPVSSAASVYAGAGGSGSR.I | 2 |
|  | pDK365N\_300mM\_082713\_04.05108.05108.3 | 5.464 | 0.5424 | 100.0% | 2856.3245 | 2856.0813 | 1 | 9.685 | 31.7% | 30 | R.SLGSVQAPSYGARPVSSAASVYAGAGGSGSR.I | 3 |
|  | SKAPIP\_tube2\_041314\_02.05862.05862.3 | 4.0203 | 0.3816 | 100.0% | 2935.6743 | 2936.0813 | 1 | 6.578 | 28.3% | 2 | R.SLGSVQAPSYGARPVSSAAS\*VYAGAGGSGSR.I | 3 |
|  | pDK365N\_100mM\_082713\_02.07204.07204.3 | 5.4692 | 0.457 | 100.0% | 2262.3843 | 2262.561 | 1 | 7.879 | 37.0% | 7 | R.GGMGSGGLATGIAGGLAGMGGIQNEK.E | 3 |
|  | SKAPIP\_041314\_02.08210.08210.2 | 5.8151 | 0.473 | 100.0% | 2262.8523 | 2262.561 | 1 | 8.428 | 54.0% | 6 | R.GGMGSGGLATGIAGGLAGMGGIQNEK.E | 2 |
|  | SKAPIP\_tube2\_041314\_02.08111.08111.3 | 6.3714 | 0.5246 | 100.0% | 3337.7344 | 3337.7224 | 1 | 8.672 | 25.0% | 15 | R.GGMGSGGLATGIAGGLAGMGGIQNEKETMQSLNDR.L | 3 |
|  | pJS43\_100mM\_120812\_01.03861.03861.2 | 2.7492 | 0.307 | 99.9% | 1094.0521 | 1094.1846 | 2 | 5.424 | 75.0% | 1 | K.ETMQSLNDR.L | 2 |
|  | SKAPIP\_041314\_01.10334.10334.2 | 2.0056 | 0.276 | 98.7% | 838.0522 | 837.9511 | 8 | 5.553 | 83.3% | 3 | R.LASYLDR.V | 2 |
|  | SKAPIP\_tube2\_041314\_01.08039.08039.1 | 1.7493 | 0.2598 | 98.7% | 982.52 | 983.0709 | 2 | 5.263 | 66.7% | 2 | R.DWSHYFK.I | 1 |
|  | SKAPIP\_tube2\_041314\_01.08072.08072.2 | 2.2851 | 0.2062 | 98.9% | 982.5722 | 983.0709 | 4 | 4.74 | 83.3% | 4 | R.DWSHYFK.I | 2 |
|  | pDK365N\_300mM\_082713\_04.06779.06779.3 | 4.1494 | 0.4424 | 100.0% | 2060.3943 | 2060.3176 | 1 | 7.23 | 36.8% | 2 | K.IIEDLRAQIFANTVDNAR.I | 3 |
|  | SKAPIP\_tube2\_041314\_01.05739.05739.1 | 2.029 | 0.2862 | 98.8% | 1319.65 | 1320.4478 | 24 | 5.528 | 45.5% | 1 | R.AQIFANTVDNAR.I | 1 |
|  | SKAPIP\_041314\_01.10968.10968.2 | 4.0312 | 0.5253 | 100.0% | 1320.4122 | 1320.4478 | 1 | 9.269 | 81.8% | 22 | R.AQIFANTVDNAR.I | 2 |
|  | AstrinIP\_MS2\_022614\_01.06845.06845.1 | 2.5181 | 0.3095 | 98.6% | 1041.62 | 1042.2235 | 1 | 6.391 | 68.8% | 3 | R.IVLQIDNAR.L | 11 |
|  | SKAPIP\_tube2\_041314\_01.06338.06338.2 | 3.2711 | 0.0745 | 99.3% | 1042.2722 | 1042.2235 | 1 | 5.647 | 87.5% | 16 | R.IVLQIDNAR.L | 22 |
|  | SKAPIP\_041314\_01.10178.10178.1 | 1.6254 | 0.229 | 96.5% | 807.56 | 807.8815 | 13 | 4.849 | 58.3% | 1 | R.LAADDFR.V | 1111111 |
|  | SKAPIP\_041314\_01.10160.10160.2 | 2.4333 | 0.3828 | 100.0% | 807.6122 | 807.8815 | 1 | 6.683 | 83.3% | 13 | R.LAADDFR.V | 2222222 |
|  | SKAPIP\_tube2\_041314\_01.04851.04851.2 | 3.1927 | 0.5164 | 100.0% | 1240.0721 | 1240.4601 | 5 | 8.198 | 72.2% | 10 | R.VKYETELAMR.Q | 2 |
|  | SKAPIP\_041314\_02.04150.04150.3 | 3.218 | 0.2046 | 99.3% | 1240.8844 | 1240.4601 | 15 | 4.588 | 47.2% | 6 | R.VKYETELAMR.Q | 3 |
|  | pDK365N\_300mM\_082713\_03.04942.04942.2 | 2.3348 | 0.3225 | 99.7% | 1013.1722 | 1013.1535 | 1 | 5.735 | 78.6% | 1 | K.YETELAMR.Q | 2 |
|  | SKAPIP\_041314\_01.10710.10710.2 | 1.7227 | 0.4111 | 98.5% | 1267.3322 | 1268.372 | 136 | 6.422 | 60.0% | 1 | R.QSVENDIHGLR.K | 2 |
|  | SKAPIP\_041314\_01.07960.07960.2 | 1.7844 | 0.3559 | 97.3% | 1396.2522 | 1396.546 | 311 | 5.966 | 50.0% | 2 | R.QSVENDIHGLRK.V | 2 |
|  | SKAPIP\_041314\_01.05199.05199.2 | 3.4019 | 0.2708 | 100.0% | 1175.2322 | 1175.3274 | 2 | 6.57 | 88.9% | 9 | R.KVIDDTNITR.L | 2 |
|  | SKAPIP\_tube2\_041314\_01.16377.16377.3 | 3.6483 | 0.2779 | 99.6% | 3333.0244 | 3334.8933 | 1 | 4.431 | 23.1% | 1 | R.KVIDDTNITRLQLETEIEALKEELLFMK.K | 3 |
|  | SKAPIP\_041314\_01.06314.06314.2 | 2.8037 | 0.4084 | 100.0% | 1046.5922 | 1047.1533 | 1 | 7.455 | 87.5% | 3 | K.VIDDTNITR.L | 2 |
|  | SKAPIP\_041314\_01.06294.06294.1 | 2.4169 | 0.3134 | 98.4% | 1046.6 | 1047.1533 | 1 | 6.131 | 62.5% | 2 | K.VIDDTNITR.L | 1 |
|  | SKAPIP\_041314\_01.14590.14590.2 | 6.1841 | 0.4785 | 100.0% | 2177.892 | 2178.589 | 1 | 9.531 | 58.8% | 10 | R.LQLETEIEALKEELLFMK.K | 2 |
|  | pJS43\_100mM\_120812\_01.14169.14169.3 | 2.9737 | 0.3521 | 99.8% | 2177.9944 | 2178.589 | 79 | 5.219 | 32.4% | 11 | R.LQLETEIEALKEELLFMK.K | 3 |
|  | SKAPIP\_tube2\_041314\_02.06753.06753.3 | 5.8166 | 0.4559 | 100.0% | 2750.4844 | 2751.0227 | 1 | 8.095 | 37.0% | 8 | K.NHEEEVKGLQAQIASSGLTVEVDAPK.S | 3 |
|  | SKAPIP\_tube2\_041314\_01.09033.09033.2 | 5.4045 | 0.5828 | 100.0% | 1884.4321 | 1885.1246 | 1 | 10.09 | 66.7% | 7 | K.GLQAQIASSGLTVEVDAPK.S | 2 |
|  | SKAPIP\_041314\_01.07932.07932.1 | 1.8979 | 0.3224 | 98.7% | 965.49 | 966.0385 | 44 | 5.258 | 50.0% | 3 | R.AQYDELAR.K | 1 |
|  | SKAPIP\_041314\_01.08090.08090.2 | 2.3829 | 0.092 | 95.1% | 965.5722 | 966.0385 | 4 | 5.284 | 71.4% | 1 | R.AQYDELAR.K | 2 |
|  | SKAPIP\_041314\_02.07064.07064.2 | 2.4914 | 0.3797 | 99.7% | 1662.3922 | 1663.8865 | 1 | 6.442 | 46.2% | 1 | R.RTVQSLEIDLDSMR.N | 2 |
|  | SKAPIP\_tube2\_041314\_01.10248.10248.1 | 2.1056 | 0.534 | 98.0% | 1506.67 | 1507.699 | 1 | 7.817 | 58.3% | 1 | R.TVQSLEIDLDSMR.N | 1 |
|  | pJS43\_100mM\_120812\_02.07214.07214.2 | 4.0695 | 0.4897 | 100.0% | 1508.2922 | 1507.699 | 1 | 8.254 | 75.0% | 26 | R.TVQSLEIDLDSMR.N | 2 |
|  | SKAPIP\_tube2\_041314\_01.08592.08592.3 | 2.718 | 0.3509 | 99.8% | 1830.3243 | 1830.0513 | 1 | 5.931 | 36.7% | 1 | R.NLKASLENSLREVEAR.Y | 3 |
|  | SKAPIP\_041314\_01.08330.08330.2 | 2.8554 | 0.1669 | 99.6% | 891.15216 | 889.9841 | 3 | 5.465 | 85.7% | 6 | K.ASLENSLR.E | 2 |
|  | SKAPIP\_041314\_01.15428.15428.3 | 6.6438 | 0.5246 | 100.0% | 2671.9744 | 2672.0715 | 1 | 8.679 | 45.5% | 47 | R.YALQMEQLNGILLHLESELAQTR.A | 3 |
|  | SKAPIP\_041314\_01.15382.15382.2 | 6.1416 | 0.4732 | 100.0% | 2671.9922 | 2672.0715 | 1 | 9.092 | 56.8% | 6 | R.YALQMEQLNGILLHLESELAQTR.A | 2 |
|  | SKAPIP\_tube2\_041314\_01.09443.09443.1 | 2.1157 | 0.2572 | 98.6% | 1419.62 | 1420.6055 | 64 | 4.882 | 54.5% | 1 | R.QAQEYEALLNIK.V | 1 |
|  | SKAPIP\_tube2\_041314\_02.06574.06574.2 | 3.6857 | 0.3563 | 100.0% | 1420.7122 | 1420.6055 | 8 | 6.28 | 68.2% | 15 | R.QAQEYEALLNIK.V | 2 |
|  | SKAPIP\_041314\_02.05471.05471.2 | 3.844 | 0.443 | 100.0% | 1292.6921 | 1293.5059 | 1 | 8.359 | 85.0% | 10 | K.VKLEAEIATYR.R | 2 |
|  | pJS43\_100mM\_120812\_02.05253.05253.3 | 2.8238 | 0.1813 | 96.0% | 1294.1943 | 1293.5059 | 35 | 4.539 | 47.5% | 1 | K.VKLEAEIATYR.R | 3 |
|  | SKAPIP\_tube2\_041314\_02.04901.04901.2 | 2.5023 | 0.1938 | 97.9% | 1449.6721 | 1449.6934 | 28 | 4.326 | 50.0% | 1 | K.VKLEAEIATYRR.L | 2 |
|  | pJS43\_100mM\_120812\_01.06146.06146.2 | 3.2361 | 0.2543 | 100.0% | 1067.4722 | 1066.1992 | 1 | 5.23 | 87.5% | 3 | K.LEAEIATYR.R | 2 |
|  | SKAPIP\_041314\_02.08005.08005.3 | 5.0823 | 0.5075 | 100.0% | 2897.2744 | 2898.128 | 1 | 8.537 | 29.0% | 3 | R.RLLEDGEDFNLGDALDSSNSMQTIQK.T | 3 |
|  | pJS43\_100mM\_120812\_01.11647.11647.3 | 3.8665 | 0.3176 | 99.9% | 2741.1843 | 2741.9404 | 7 | 6.695 | 27.1% | 1 | R.LLEDGEDFNLGDALDSSNSMQTIQK.T | 3 |
|  | SKAPIP\_tube2\_041314\_01.10995.10995.2 | 6.4374 | 0.6228 | 100.0% | 2741.5122 | 2741.9404 | 1 | 11.408 | 56.2% | 2 | R.LLEDGEDFNLGDALDSSNSMQTIQK.T | 2 |

Similarities:
gi|4557701|ref|NP\_000(2:47)  
contaminant\_KERATIN03(2:47)  
gi|15431310|ref|NP\_00(2:47)  
gi|24430192|ref|NP\_00(2:47)  
gi|24234699|ref|NP\_00(4:45)  
gi|131412225|ref|NP\_7(2:47)  

---

|  |  |  |  |  |  |  |  |  |
| --- | --- | --- | --- | --- | --- | --- | --- | --- |
| U | *TEV-Speptide* | 9 | 160 | 70.6% | 51 | 5423 | 9.4 | no description |

| Filename XCorr DeltCN Conf% ObsM+H+ CalcM+H+ SpR ZScore Ion% # Sequence  | | | | | | | | | | | | |
| --- | --- | --- | --- | --- | --- | --- | --- | --- | --- | --- | --- | --- |
| \* | pJS43\_100mM\_120812\_01.03241.03241.2 | 3.2095 | 0.326 | 100.0% | 1279.9922 | 1280.2993 | 1 | 6.054 | 66.7% | 1 | -.SGGDRWSSTGGGR.S | 2 |
| \* | pSKT11\_1\_020812\_01.04646.04646.2 | 4.303 | 0.3395 | 100.0% | 1384.4722 | 1384.5345 | 1 | 7.631 | 77.3% | 83 | R.SRENLYFQGAAK.F | 2 |
| \* | pJS43\_100mM\_120812\_01.04956.04956.3 | 4.0982 | 0.438 | 100.0% | 1384.9744 | 1384.5345 | 47 | 7.039 | 45.5% | 8 | R.SRENLYFQGAAK.F | 3 |
| \* | pSKT11\_1\_020812\_01.04652.04652.1 | 3.4336 | 0.235 | 98.6% | 1385.63 | 1384.5345 | 139 | 5.15 | 54.5% | 17 | R.SRENLYFQGAAK.F | 1 |
| \* | 100326\_pJS43\_01.03224.03224.1 | 2.8838 | 0.2859 | 98.4% | 1140.65 | 1141.2688 | 2 | 5.83 | 61.1% | 12 | R.ENLYFQGAAK.F | 1 |
| \* | pJS43\_100mM\_120812\_01.06455.06455.2 | 2.6967 | 0.2378 | 99.5% | 1141.1721 | 1141.2688 | 1 | 5.705 | 83.3% | 5 | R.ENLYFQGAAK.F | 2 |
| \* | 100326\_pJS43\_01.00650.00650.2 | 3.4934 | 0.3644 | 100.0% | 1297.8922 | 1298.4844 | 1 | 6.805 | 70.0% | 20 | K.FKETAAAKFER.Q | 2 |
| \* | 100326\_pJS43\_01.00644.00644.3 | 3.8781 | 0.3487 | 100.0% | 1298.5144 | 1298.4844 | 1 | 6.206 | 55.0% | 13 | K.FKETAAAKFER.Q | 3 |
| \* | pSKT11\_1\_020812\_02.03563.03563.2 | 2.2264 | 0.3747 | 99.7% | 1022.5722 | 1023.1338 | 70 | 5.731 | 56.2% | 1 | K.ETAAAKFER.Q | 2 |

---

|  |  |  |  |  |  |  |  |  |
| --- | --- | --- | --- | --- | --- | --- | --- | --- |
| U | *gi|150456457|ref|NP\_9* | 34 | 159 | 70.3% | 347 | 39929 | 5.6 | HMT1 hnRNP methyltransferase-like 2 isoform 2 [Homo sapiens] |
| U | *gi|154759421|ref|NP\_0* | 34 | 158 | 65.8% | 371 | 42462 | 5.3 | HMT1 hnRNP methyltransferase-like 2 isoform 1 [Homo sapiens] |
| U | *gi|151301219|ref|NP\_9* | 34 | 159 | 69.1% | 353 | 40548 | 5.5 | HMT1 hnRNP methyltransferase-like 2 isoform 3 [Homo sapiens] |

| Filename XCorr DeltCN Conf% ObsM+H+ CalcM+H+ SpR ZScore Ion% # Sequence  | | | | | | | | | | | | |
| --- | --- | --- | --- | --- | --- | --- | --- | --- | --- | --- | --- | --- |
|  | AstrinIP\_MS2\_022614\_02.07336.07336.3 | 3.9822 | 0.4443 | 100.0% | 2266.4343 | 2266.489 | 1 | 6.553 | 38.2% | 2 | K.DYYFDSYAHFGIHEEMLK.D | 3 |
|  | AstrinIP\_MS2\_022614\_01.10786.10786.3 | 5.7399 | 0.4798 | 100.0% | 2765.0942 | 2766.0132 | 1 | 9.358 | 42.9% | 4 | K.DYYFDSYAHFGIHEEMLKDEVR.T | 3 |
|  | AstrinIP\_MS1\_022614\_02.09387.09387.2 | 5.599 | 0.3636 | 100.0% | 1838.3522 | 1839.1665 | 1 | 9.781 | 76.5% | 4 | K.VVLDVGSGTGILCMFAAK.A | 2 |
|  | AstrinIP\_MS1\_022614\_02.05624.05624.2 | 4.0734 | 0.4925 | 100.0% | 1769.1522 | 1769.999 | 1 | 7.745 | 53.3% | 2 | R.KVIGIECSSISDYAVK.I | 2 |
|  | AstrinIP\_MS1\_022614\_01.08768.08768.2 | 4.7529 | 0.583 | 100.0% | 1641.0721 | 1641.825 | 1 | 10.547 | 78.6% | 5 | K.VIGIECSSISDYAVK.I | 2 |
|  | AstrinIP\_MS1\_022614\_01.05465.05465.1 | 2.4014 | 0.3473 | 98.5% | 1350.75 | 1351.6322 | 10 | 7.099 | 54.5% | 2 | K.ANKLDHVVTIIK.G | 1 |
|  | AstrinIP\_MS2\_022614\_01.05487.05487.2 | 3.5365 | 0.3062 | 100.0% | 1350.9122 | 1351.6322 | 1 | 6.432 | 68.2% | 8 | K.ANKLDHVVTIIK.G | 2 |
|  | AstrinIP\_MS2\_022614\_01.05522.05522.3 | 4.7075 | 0.4344 | 100.0% | 1351.6144 | 1351.6322 | 1 | 7.951 | 61.4% | 9 | K.ANKLDHVVTIIK.G | 3 |
|  | AstrinIP\_MS1\_022614\_01.05514.05514.1 | 2.1191 | 0.2518 | 98.6% | 1037.6 | 1038.2755 | 4 | 6.453 | 62.5% | 3 | K.LDHVVTIIK.G | 1 |
|  | AstrinIP\_MS2\_022614\_01.05690.05690.2 | 2.7381 | 0.2985 | 99.9% | 1038.1721 | 1038.2755 | 1 | 5.333 | 87.5% | 4 | K.LDHVVTIIK.G | 2 |
|  | AstrinIP\_MS2\_022614\_01.05769.05769.2 | 4.0198 | 0.4017 | 100.0% | 1356.0521 | 1356.559 | 1 | 7.664 | 72.7% | 6 | K.GKVEEVELPVEK.V | 2 |
|  | AstrinIP\_MS2\_022614\_01.11870.11870.2 | 3.9373 | 0.4127 | 100.0% | 1643.1721 | 1643.8827 | 1 | 6.68 | 76.9% | 5 | R.DKWLAPDGLIFPDR.A | 2 |
|  | AstrinIP\_MS2\_022614\_01.11859.11859.3 | 3.335 | 0.2613 | 99.7% | 1644.5044 | 1643.8827 | 3 | 4.589 | 44.2% | 1 | R.DKWLAPDGLIFPDR.A | 3 |
|  | AstrinIP\_MS2\_022614\_01.13599.13599.3 | 4.0914 | 0.4927 | 100.0% | 2876.7544 | 2877.2695 | 1 | 7.246 | 30.2% | 3 | R.DKWLAPDGLIFPDRATLYVTAIEDR.Q | 3 |
|  | AstrinIP\_MS1\_022614\_01.12065.12065.2 | 3.3967 | 0.4255 | 100.0% | 1401.0322 | 1400.6201 | 1 | 6.398 | 77.3% | 3 | K.WLAPDGLIFPDR.A | 2 |
|  | AstrinIP\_MS1\_022614\_01.13560.13560.3 | 3.1355 | 0.2383 | 97.9% | 2633.6042 | 2634.0068 | 1 | 4.662 | 29.5% | 1 | K.WLAPDGLIFPDRATLYVTAIEDR.Q | 32 |
|  | AstrinIP\_MS2\_022614\_01.08541.08541.1 | 2.4844 | 0.4572 | 99.0% | 1251.61 | 1252.4099 | 1 | 7.745 | 60.0% | 4 | R.ATLYVTAIEDR.Q | 1 |
|  | AstrinIP\_MS2\_022614\_01.08606.08606.2 | 3.698 | 0.499 | 100.0% | 1252.3522 | 1252.4099 | 1 | 8.595 | 70.0% | 15 | R.ATLYVTAIEDR.Q | 2 |
|  | AstrinIP\_MS1\_022614\_01.12456.12456.2 | 4.8219 | 0.56 | 100.0% | 2085.4722 | 2086.3381 | 1 | 9.468 | 76.7% | 1 | K.IHWWENVYGFDMSCIK.D | 2 |
|  | AstrinIP\_MS2\_022614\_01.09356.09356.2 | 4.0252 | 0.4007 | 100.0% | 1637.5122 | 1637.914 | 1 | 7.433 | 75.0% | 4 | K.DVAIKEPLVDVVDPK.Q | 2 |
|  | AstrinIP\_MS1\_022614\_01.08808.08808.3 | 4.0459 | 0.379 | 100.0% | 1637.5443 | 1637.914 | 1 | 6.235 | 42.9% | 2 | K.DVAIKEPLVDVVDPK.Q | 3 |
|  | AstrinIP\_MS1\_022614\_01.06593.06593.2 | 2.2905 | 0.2908 | 99.2% | 1159.6122 | 1160.3655 | 44 | 6.22 | 61.1% | 1 | K.QLVTNACLIK.E | 2 |
|  | AstrinIP\_MS1\_022614\_01.06090.06090.1 | 1.9368 | 0.2407 | 98.6% | 966.52 | 967.107 | 35 | 4.763 | 57.1% | 2 | K.EVDIYTVK.V | 1 |
|  | AstrinIP\_MS1\_022614\_01.12130.12130.2 | 3.9424 | 0.4397 | 100.0% | 1784.3322 | 1785.0131 | 1 | 7.458 | 57.1% | 2 | K.VEDLTFTSPFCLQVK.R | 2 |
|  | AstrinIP\_MS1\_022614\_01.13992.13992.2 | 5.506 | 0.5518 | 100.0% | 2228.9521 | 2229.5027 | 1 | 9.625 | 67.6% | 5 | K.RNDYVHALVAYFNIEFTR.C | 2 |
|  | AstrinIP\_MS2\_022614\_02.09932.09932.3 | 5.6392 | 0.3744 | 100.0% | 2230.0745 | 2229.5027 | 1 | 9.184 | 51.5% | 10 | K.RNDYVHALVAYFNIEFTR.C | 3 |
|  | AstrinIP\_MS2\_022614\_02.10772.10772.3 | 3.8466 | 0.4324 | 100.0% | 2072.4543 | 2073.3152 | 1 | 7.045 | 43.8% | 2 | R.NDYVHALVAYFNIEFTR.C | 3 |
|  | AstrinIP\_MS2\_022614\_01.15808.15808.2 | 5.5933 | 0.6045 | 100.0% | 2072.5122 | 2073.3152 | 1 | 10.304 | 75.0% | 4 | R.NDYVHALVAYFNIEFTR.C | 2 |
|  | AstrinIP\_MS2\_022614\_01.07292.07292.2 | 3.6137 | 0.4566 | 100.0% | 1725.1721 | 1725.8547 | 1 | 7.163 | 67.9% | 5 | R.TGFSTSPESPYTHWK.Q | 2 |
|  | AstrinIP\_MS2\_022614\_01.07263.07263.3 | 2.7425 | 0.4543 | 100.0% | 1725.7144 | 1725.8547 | 13 | 6.343 | 33.9% | 4 | R.TGFSTSPESPYTHWK.Q | 3 |
|  | AstrinIP\_MS1\_022614\_02.08221.08221.2 | 4.0451 | 0.4192 | 100.0% | 1637.1921 | 1637.8878 | 1 | 8.282 | 70.8% | 20 | K.QTVFYMEDYLTVK.T | 2 |
|  | AstrinIP\_MS2\_022614\_02.06036.06036.2 | 4.1698 | 0.3586 | 100.0% | 1721.3522 | 1721.969 | 1 | 7.226 | 56.7% | 6 | K.TGEEIFGTIGMRPNAK.N | 2 |
|  | AstrinIP\_MS2\_022614\_02.06056.06056.3 | 3.3341 | 0.3725 | 100.0% | 1721.8744 | 1721.969 | 15 | 6.365 | 35.0% | 6 | K.TGEEIFGTIGMRPNAK.N | 3 |
|  | AstrinIP\_MS1\_022614\_01.06135.06135.2 | 4.1438 | 0.5344 | 100.0% | 1589.1122 | 1589.6313 | 1 | 9.203 | 75.0% | 4 | K.GQLCELSCSTDYR.M | 2 |

---

|  |  |  |  |  |  |  |  |  |
| --- | --- | --- | --- | --- | --- | --- | --- | --- |
| U | *gi|4504919|ref|NP\_002* | 58 | 393 | 69.2% | 483 | 53704 | 5.6 | keratin 8 [Homo sapiens] |

| Filename XCorr DeltCN Conf% ObsM+H+ CalcM+H+ SpR ZScore Ion% # Sequence  | | | | | | | | | | | | |
| --- | --- | --- | --- | --- | --- | --- | --- | --- | --- | --- | --- | --- |
| \* | SKAPIP\_tube2\_041314\_02.10868.10868.3 | 5.5441 | 0.3834 | 100.0% | 3928.8245 | 3927.465 | 1 | 5.667 | 25.6% | 3 | R.GGLGGGYGGASGMGGITAVTVNQSLLSPLVLEVDPNIQAVR.T | 3 |
|  | pJS43\_100mM\_120812\_01.07265.07265.2 | 2.4075 | 0.2649 | 99.7% | 828.09216 | 827.95544 | 2 | 5.168 | 91.7% | 6 | K.FASFIDK.V | 222222222 |
|  | SKAPIP\_tube2\_041314\_01.06813.06813.2 | 2.9746 | 0.2137 | 99.8% | 1082.7522 | 1083.2755 | 2 | 7.239 | 75.0% | 6 | K.FASFIDKVR.F | 22222222 |
|  | pDK365N\_300mM\_082713\_03.07352.07352.2 | 3.2173 | 0.1275 | 99.7% | 1031.1921 | 1031.1997 | 2 | 3.83 | 92.9% | 17 | K.WSLLQQQK.T | 2 |
|  | pJS43\_100mM\_120812\_01.12732.12732.2 | 4.4663 | 0.5373 | 100.0% | 1848.2722 | 1849.0431 | 1 | 9.863 | 67.9% | 10 | R.SNMDNMFESYINNLR.R | 2 |
|  | SKAPIP\_041314\_01.04898.04898.2 | 2.1423 | 0.2569 | 98.5% | 1045.9122 | 1046.1655 | 32 | 4.912 | 68.8% | 6 | R.QLETLGQEK.L | 2 |
|  | SKAPIP\_tube2\_041314\_02.09232.09232.2 | 6.6993 | 0.3178 | 100.0% | 2034.8922 | 2035.363 | 1 | 10.732 | 76.5% | 4 | K.LKLEAELGNMQGLVEDFK.N | 2 |
|  | SKAPIP\_041314\_02.09563.09563.3 | 5.4875 | 0.2538 | 100.0% | 2035.6444 | 2035.363 | 1 | 8.311 | 50.0% | 22 | K.LKLEAELGNMQGLVEDFK.N | 3 |
|  | SKAPIP\_tube2\_041314\_01.12413.12413.3 | 5.5223 | 0.4371 | 100.0% | 3325.3442 | 3325.7612 | 1 | 7.866 | 29.6% | 2 | K.LKLEAELGNMQGLVEDFKNKYEDEINKR.T | 3 |
|  | pJS43\_100mM\_120812\_02.08112.08112.2 | 3.8108 | 0.2579 | 99.9% | 1792.4922 | 1794.0295 | 1 | 9.677 | 63.3% | 5 | K.LEAELGNMQGLVEDFK.N | 2 |
|  | SKAPIP\_041314\_01.04274.04274.2 | 2.9252 | 0.238 | 99.8% | 1310.0322 | 1309.4215 | 154 | 4.901 | 55.6% | 7 | K.NKYEDEINKR.T | 22222 |
|  | SKAPIP\_tube2\_041314\_02.05802.05802.3 | 3.6103 | 0.2733 | 99.9% | 1636.8243 | 1637.9348 | 1 | 5.553 | 54.2% | 2 | K.RTEMENEFVLIKK.D | 3 |
|  | SKAPIP\_tube2\_041314\_01.09689.09689.2 | 3.7582 | 0.4832 | 100.0% | 1353.3522 | 1353.5732 | 1 | 7.79 | 80.0% | 10 | R.TEMENEFVLIK.K | 2 |
|  | pJS43\_100mM\_120812\_02.05982.05982.3 | 2.1568 | 0.3826 | 99.6% | 1482.0243 | 1481.7473 | 196 | 6.174 | 29.5% | 2 | R.TEMENEFVLIKK.D | 3 |
|  | SKAPIP\_tube2\_041314\_01.07649.07649.2 | 3.7735 | 0.2809 | 100.0% | 1482.3522 | 1481.7473 | 1 | 5.967 | 68.2% | 8 | R.TEMENEFVLIKK.D | 2 |
|  | SKAPIP\_041314\_02.05006.05006.3 | 4.0466 | 0.4548 | 100.0% | 1926.7144 | 1927.1365 | 1 | 7.524 | 41.7% | 1 | K.KDVDEAYMNKVELESR.L | 3 |
|  | SKAPIP\_041314\_01.06849.06849.2 | 2.5652 | 0.3887 | 100.0% | 1084.9521 | 1085.1737 | 12 | 6.062 | 68.8% | 4 | K.DVDEAYMNK.V | 2 |
|  | SKAPIP\_tube2\_041314\_02.05798.05798.2 | 4.3114 | 0.5322 | 100.0% | 1798.3522 | 1798.9623 | 1 | 8.844 | 75.0% | 9 | K.DVDEAYMNKVELESR.L | 2 |
|  | SKAPIP\_041314\_02.05752.05752.3 | 4.4777 | 0.4199 | 100.0% | 1799.4543 | 1798.9623 | 1 | 6.335 | 53.6% | 17 | K.DVDEAYMNKVELESR.L | 3 |
|  | SKAPIP\_tube2\_041314\_01.11489.11489.1 | 2.7891 | 0.2989 | 98.6% | 1419.63 | 1420.6055 | 1 | 5.616 | 68.2% | 1 | R.LEGLTDEINFLR.Q | 1 |
|  | SKAPIP\_tube2\_041314\_01.11522.11522.2 | 4.0044 | 0.4895 | 100.0% | 1420.4922 | 1420.6055 | 1 | 8.183 | 86.4% | 20 | R.LEGLTDEINFLR.Q | 2 |
|  | AstrinIP\_MS1\_022614\_01.05772.05772.2 | 2.3103 | 0.1976 | 98.5% | 1081.8522 | 1080.1827 | 1 | 4.224 | 78.6% | 3 | R.QLYEEEIR.E | 2 |
|  | SKAPIP\_tube2\_041314\_02.07038.07038.2 | 6.3814 | 0.5876 | 100.0% | 2109.5122 | 2110.3008 | 1 | 10.421 | 72.2% | 5 | R.ELQSQISDTSVVLSMDNSR.S | 2 |
|  | SKAPIP\_041314\_01.12484.12484.1 | 2.5878 | 0.3394 | 98.4% | 1320.57 | 1321.5286 | 1 | 6.728 | 63.6% | 3 | R.SLDMDSIIAEVK.A | 1 |
|  | SKAPIP\_tube2\_041314\_01.11270.11270.2 | 4.3996 | 0.4611 | 100.0% | 1321.2322 | 1321.5286 | 1 | 8.379 | 81.8% | 15 | R.SLDMDSIIAEVK.A | 2 |
|  | SKAPIP\_tube2\_041314\_01.14285.14285.2 | 2.0743 | 0.3425 | 98.5% | 2381.892 | 2382.6477 | 29 | 4.792 | 25.0% | 1 | R.SLDMDSIIAEVKAQYEDIANR.S | 2 |
|  | SKAPIP\_tube2\_041314\_01.14255.14255.3 | 3.9744 | 0.3965 | 100.0% | 2382.4143 | 2382.6477 | 1 | 7.017 | 37.5% | 2 | R.SLDMDSIIAEVKAQYEDIANR.S | 3 |
|  | SKAPIP\_041314\_01.06515.06515.1 | 2.0181 | 0.3244 | 98.7% | 1079.51 | 1080.1423 | 8 | 5.477 | 62.5% | 4 | K.AQYEDIANR.S | 11 |
|  | SKAPIP\_041314\_01.06350.06350.2 | 3.1953 | 0.259 | 100.0% | 1080.0922 | 1080.1423 | 1 | 6.865 | 87.5% | 11 | K.AQYEDIANR.S | 22 |
|  | SKAPIP\_041314\_01.10468.10468.2 | 3.5898 | 0.321 | 100.0% | 1413.3121 | 1413.5884 | 1 | 5.795 | 81.8% | 4 | R.SRAEAESMYQIK.Y | 2 |
|  | SKAPIP\_tube2\_041314\_02.06808.06808.3 | 6.2695 | 0.3822 | 100.0% | 2533.3743 | 2532.828 | 1 | 7.785 | 40.5% | 6 | R.SRAEAESMYQIKYEELQSLAGK.H | 3 |
|  | pJS43\_100mM\_120812\_01.05659.05659.2 | 3.4691 | 0.4884 | 100.0% | 1170.0122 | 1170.3228 | 1 | 7.373 | 83.3% | 8 | R.AEAESMYQIK.Y | 2 |
|  | SKAPIP\_tube2\_041314\_01.09340.09340.2 | 4.8316 | 0.4604 | 100.0% | 2288.892 | 2289.5623 | 1 | 8.504 | 47.4% | 1 | R.AEAESMYQIKYEELQSLAGK.H | 2 |
|  | SKAPIP\_tube2\_041314\_01.09329.09329.3 | 5.686 | 0.4881 | 100.0% | 2289.9243 | 2289.5623 | 1 | 9.028 | 39.5% | 10 | R.AEAESMYQIKYEELQSLAGK.H | 3 |
|  | SKAPIP\_041314\_01.11013.11013.2 | 3.8846 | 0.0676 | 99.7% | 1138.2922 | 1138.2627 | 1 | 7.213 | 83.3% | 14 | K.YEELQSLAGK.H | 2 |
|  | pJS43\_100mM\_120812\_01.03509.03509.2 | 3.2823 | 0.4224 | 100.0% | 1209.1322 | 1209.36 | 1 | 7.241 | 94.4% | 2 | R.TKTEISEMNR.N | 2 |
|  | SKAPIP\_tube2\_041314\_01.05939.05939.1 | 2.1557 | 0.2646 | 98.8% | 1000.49 | 1001.168 | 8 | 5.449 | 68.8% | 3 | R.LQAEIEGLK.G | 1 |
|  | SKAPIP\_tube2\_041314\_01.05992.05992.2 | 3.1603 | 0.2032 | 99.9% | 1000.9922 | 1001.168 | 49 | 5.327 | 75.0% | 7 | R.LQAEIEGLK.G | 2 |
|  | SKAPIP\_041314\_01.10703.10703.2 | 3.6117 | 0.2912 | 100.0% | 1343.4521 | 1342.5381 | 1 | 7.227 | 81.8% | 9 | R.LQAEIEGLKGQR.A | 2 |
|  | pJS43\_100mM\_120812\_01.09479.09479.2 | 4.2248 | 0.42 | 100.0% | 1346.1322 | 1345.452 | 1 | 7.207 | 66.7% | 12 | R.ASLEAAIADAEQR.G | 2 |
|  | SKAPIP\_041314\_01.12267.12267.2 | 5.5242 | 0.5159 | 100.0% | 1956.4722 | 1957.1912 | 1 | 9.442 | 66.7% | 10 | R.ASLEAAIADAEQRGELAIK.D | 2 |
|  | pJS43\_100mM\_120812\_02.07639.07639.3 | 4.2657 | 0.4018 | 100.0% | 1958.0944 | 1957.1912 | 1 | 7.081 | 45.8% | 7 | R.ASLEAAIADAEQRGELAIK.D | 3 |
|  | SKAPIP\_tube2\_041314\_01.11432.11432.3 | 5.3422 | 0.5051 | 100.0% | 2456.1843 | 2456.7153 | 1 | 7.965 | 32.6% | 11 | R.ASLEAAIADAEQRGELAIKDANAK.L | 3 |
|  | AstrinIP\_MS2\_022614\_01.03687.03687.2 | 3.2598 | 0.3466 | 100.0% | 1130.1522 | 1130.2865 | 3 | 6.237 | 70.0% | 1 | R.GELAIKDANAK.L | 2 |
|  | AstrinIP\_MS2\_022614\_01.08400.08400.1 | 2.2917 | 0.2629 | 98.8% | 1129.59 | 1130.2865 | 4 | 5.525 | 55.6% | 4 | K.LSELEAALQR.A | 1 |
|  | SKAPIP\_tube2\_041314\_01.07706.07706.2 | 4.1681 | 0.2245 | 100.0% | 1130.2522 | 1130.2865 | 1 | 6.375 | 83.3% | 21 | K.LSELEAALQR.A | 2 |
|  | SKAPIP\_tube2\_041314\_01.06975.06975.2 | 3.9247 | 0.3477 | 100.0% | 1551.1122 | 1551.801 | 1 | 6.526 | 72.7% | 1 | R.QLREYQELMNVK.L | 2 |
|  | AstrinIP\_MS2\_022614\_01.06780.06780.1 | 2.5379 | 0.2335 | 98.8% | 1153.47 | 1154.3234 | 178 | 5.811 | 56.2% | 2 | R.EYQELMNVK.L | 111 |
|  | SKAPIP\_tube2\_041314\_01.06290.06290.2 | 2.8709 | 0.2637 | 99.9% | 1154.2522 | 1154.3234 | 3 | 6.393 | 68.8% | 5 | R.EYQELMNVK.L | 222 |
|  | SKAPIP\_tube2\_041314\_01.08620.08620.2 | 2.9744 | 0.3975 | 100.0% | 1406.8922 | 1406.6653 | 2 | 6.859 | 63.6% | 2 | K.LALDIEIATYRK.L | 2222 |
|  | SKAPIP\_tube2\_041314\_01.07323.07323.3 | 5.0749 | 0.5179 | 100.0% | 2517.8943 | 2518.8628 | 1 | 8.199 | 41.7% | 1 | R.KLLEGEESRLESGMQNMSIHTK.T | 3 |
|  | pJS43\_100mM\_120812\_01.03584.03584.1 | 2.3838 | 0.1736 | 97.7% | 932.52 | 933.00616 | 42 | 5.42 | 64.3% | 1 | K.LLEGEESR.L | 111 |
|  | pJS43\_100mM\_120812\_01.03569.03569.2 | 2.2842 | 0.1217 | 95.4% | 932.6122 | 933.00616 | 36 | 4.304 | 71.4% | 1 | K.LLEGEESR.L | 222 |
|  | SKAPIP\_tube2\_041314\_01.08098.08098.3 | 4.7267 | 0.3471 | 100.0% | 2389.6443 | 2390.6887 | 1 | 7.209 | 36.2% | 2 | K.LLEGEESRLESGMQNMSIHTK.T | 3 |
|  | pJS43\_100mM\_120812\_01.04880.04880.2 | 4.4521 | 0.5026 | 100.0% | 1475.9521 | 1476.7058 | 1 | 8.032 | 83.3% | 7 | R.LESGMQNMSIHTK.T | 2 |
|  | SKAPIP\_tube2\_041314\_01.04455.04455.3 | 3.3049 | 0.4229 | 100.0% | 1476.4744 | 1476.7058 | 3 | 6.146 | 43.8% | 8 | R.LESGMQNMSIHTK.T | 3 |
|  | SKAPIP\_tube2\_041314\_01.05604.05604.2 | 4.0266 | 0.4066 | 100.0% | 1474.1921 | 1474.6512 | 1 | 8.021 | 73.1% | 2 | R.DGKLVSESSDVLPK.- | 2 |
|  | SKAPIP\_tube2\_041314\_01.05241.05241.2 | 3.6077 | 0.4216 | 100.0% | 1174.1322 | 1174.3367 | 1 | 7.492 | 80.0% | 25 | K.LVSESSDVLPK.- | 2 |

Similarities:
gi|62414289|ref|NP\_00(2:56)  
gi|119395750|ref|NP\_0(1:57)  
gi|47132620|ref|NP\_00(2:56)  
gi|67782365|ref|NP\_00(5:53)  
gi|119395754|ref|NP\_0(3:55)  
gi|155969697|ref|NP\_7(5:53)  
gi|119703753|ref|NP\_0(5:53)  
gi|32567786|ref|NP\_78(2:56)  
gi|153791158|ref|NP\_0(4:54)  
gi|125628632|ref|NP\_0(1:57)  
gi|109255249|ref|NP\_0(2:56)  

---

|  |  |  |  |  |  |  |  |  |
| --- | --- | --- | --- | --- | --- | --- | --- | --- |
| U | *gi|5031653|ref|NP\_005* | 21 | 92 | 68.0% | 225 | 26131 | 5.6 | breast carcinoma amplified sequence 2 [Homo sapiens] |

| Filename XCorr DeltCN Conf% ObsM+H+ CalcM+H+ SpR ZScore Ion% # Sequence  | | | | | | | | | | | | |
| --- | --- | --- | --- | --- | --- | --- | --- | --- | --- | --- | --- | --- |
| \* | pDK365N\_300mM\_082713\_01.05528.05528.2 | 4.089 | 0.4541 | 100.0% | 1289.1322 | 1289.3849 | 1 | 8.922 | 86.4% | 9 | R.EAAAALVEEETR.R | 2 |
| \* | SKAPIP\_tube2\_041314\_01.05630.05630.2 | 3.1063 | 0.2669 | 99.8% | 1445.5122 | 1445.5724 | 2 | 5.056 | 62.5% | 8 | R.EAAAALVEEETRR.Y | 2 |
| \* | SKAPIP\_041314\_01.13552.13552.2 | 4.6448 | 0.571 | 100.0% | 2371.652 | 2371.6233 | 1 | 9.936 | 68.4% | 6 | K.NYLSYLTAPDYSAFETDIMR.N | 2 |
| \* | SKAPIP\_041314\_01.13570.13570.3 | 3.7893 | 0.3124 | 99.9% | 2371.8245 | 2371.6233 | 1 | 5.915 | 38.2% | 1 | K.NYLSYLTAPDYSAFETDIMR.N | 3 |
| \* | pDK365N\_300mM\_082713\_03.08236.08236.3 | 2.7302 | 0.3269 | 99.7% | 1472.3344 | 1470.8137 | 1 | 5.485 | 45.8% | 1 | R.LAARQPIELLSMK.R | 3 |
| \* | SKAPIP\_tube2\_041314\_01.08777.08777.1 | 1.3477 | 0.2628 | 95.1% | 1058.57 | 1059.3092 | 15 | 4.246 | 68.8% | 1 | R.QPIELLSMK.R | 1 |
| \* | SKAPIP\_041314\_01.08792.08792.2 | 4.2833 | 0.3826 | 100.0% | 1333.1721 | 1333.4868 | 1 | 7.257 | 81.8% | 11 | K.RYELPAPSSGQK.N | 2 |
| \* | SKAPIP\_tube2\_041314\_01.04587.04587.2 | 2.4222 | 0.3938 | 99.9% | 1176.5721 | 1177.2994 | 6 | 6.954 | 60.0% | 3 | R.YELPAPSSGQK.N | 2 |
| \* | SKAPIP\_041314\_01.13031.13031.3 | 4.2393 | 0.4705 | 100.0% | 2715.8643 | 2715.9233 | 2 | 7.716 | 29.5% | 1 | K.NDITAWQECVNNSMAQLEHQAVR.I | 3 |
| \* | SKAPIP\_041314\_01.11254.11254.3 | 3.0128 | 0.3849 | 100.0% | 1826.0343 | 1826.0802 | 46 | 5.58 | 32.1% | 3 | K.VYNENLVHMIEHAQK.E | 3 |
| \* | SKAPIP\_tube2\_041314\_01.07590.07590.2 | 4.5009 | 0.3836 | 100.0% | 1826.5122 | 1826.0802 | 1 | 6.736 | 53.6% | 1 | K.VYNENLVHMIEHAQK.E | 2 |
| \* | SKAPIP\_tube2\_041314\_01.09705.09705.3 | 4.2406 | 0.4374 | 100.0% | 2324.4243 | 2324.66 | 1 | 7.653 | 45.8% | 1 | K.VYNENLVHMIEHAQKELQK.L | 3 |
| \* | SKAPIP\_041314\_01.09921.09921.2 | 2.8141 | 0.4067 | 100.0% | 1337.5322 | 1338.5118 | 1 | 6.48 | 83.3% | 3 | R.KHIQDLNWQR.K | 2 |
| \* | SKAPIP\_041314\_01.09974.09974.3 | 4.1608 | 0.2155 | 100.0% | 1340.1843 | 1338.5118 | 34 | 4.997 | 55.6% | 3 | R.KHIQDLNWQR.K | 3 |
| \* | SKAPIP\_tube2\_041314\_01.05174.05174.2 | 2.5532 | 0.1315 | 97.9% | 1210.2122 | 1210.3378 | 2 | 5.629 | 75.0% | 4 | K.HIQDLNWQR.K | 2 |
| \* | SKAPIP\_041314\_01.10146.10146.2 | 2.6583 | 0.185 | 98.9% | 1338.2922 | 1338.5118 | 1 | 5.571 | 72.2% | 1 | K.HIQDLNWQRK.N | 2 |
| \* | SKAPIP\_tube2\_041314\_01.09063.09063.2 | 4.2111 | 0.444 | 100.0% | 1678.5122 | 1678.944 | 1 | 7.136 | 69.2% | 3 | K.LREMESNWVSLVSK.N | 2 |
| \* | pDK365N\_300mM\_082713\_03.08812.08812.3 | 3.7228 | 0.3268 | 100.0% | 1679.3344 | 1678.944 | 1 | 6.21 | 44.2% | 5 | K.LREMESNWVSLVSK.N | 3 |
| \* | SKAPIP\_041314\_02.07954.07954.2 | 3.4941 | 0.3727 | 100.0% | 1409.6522 | 1409.597 | 1 | 7.58 | 77.3% | 13 | R.EMESNWVSLVSK.N | 2 |
| \* | SKAPIP\_tube2\_041314\_01.11150.11150.2 | 4.1653 | 0.3586 | 100.0% | 1592.0322 | 1591.8448 | 2 | 6.684 | 70.8% | 13 | R.TIVQLENEIYQIK.Q | 2 |
| \* | SKAPIP\_041314\_01.06101.06101.2 | 2.6993 | 0.4 | 99.9% | 1814.2322 | 1814.912 | 2 | 6.44 | 46.4% | 1 | K.QQHGEANKENIRQDF.- | 2 |

---

|  |  |  |  |  |  |  |  |  |
| --- | --- | --- | --- | --- | --- | --- | --- | --- |
| U | *contaminant\_gi|746301* | 19 | 675 | 67.7% | 269 | 27961 | 6.7 | lysyl endopeptidase (EC 3.4.21.50) - Lysobacter enzymogenes |

| Filename XCorr DeltCN Conf% ObsM+H+ CalcM+H+ SpR ZScore Ion% # Sequence  | | | | | | | | | | | | |
| --- | --- | --- | --- | --- | --- | --- | --- | --- | --- | --- | --- | --- |
| \* | pDK339\_033013\_01.04465.04465.2 | 6.6753 | 0.6177 | 100.0% | 2261.4321 | 2262.355 | 1 | 11.082 | 58.3% | 94 | R.APGSSSSGANGDGSLAQSQTGAVVR.A | 2 |
| \* | AstrinIP\_MS2\_022614\_01.04539.04539.3 | 4.9517 | 0.4334 | 100.0% | 2262.0544 | 2262.355 | 1 | 7.946 | 38.5% | 59 | R.APGSSSSGANGDGSLAQSQTGAVVR.A | 3 |
| \* | pJS43\_100mM\_120812\_02.10356.10356.3 | 6.9865 | 0.5177 | 100.0% | 3315.7144 | 3315.6257 | 1 | 8.528 | 27.6% | 57 | R.ATNAASDFTLLELNTAANPAYNLFWAGWDR.R | 3 |
| \* | AstrinIP\_MS2\_022614\_01.17097.17097.2 | 5.4131 | 0.5201 | 100.0% | 3315.9521 | 3315.6257 | 1 | 9.768 | 41.4% | 24 | R.ATNAASDFTLLELNTAANPAYNLFWAGWDR.R | 2 |
| \* | pDK365N\_300mM\_082713\_01.14120.14120.3 | 6.6201 | 0.5686 | 100.0% | 3471.7744 | 3471.813 | 1 | 10.178 | 30.0% | 106 | R.ATNAASDFTLLELNTAANPAYNLFWAGWDRR.D | 3 |
| \* | AstrinIP\_MS2\_022614\_01.15992.15992.3 | 4.3121 | 0.2183 | 99.5% | 3475.2544 | 3475.6257 | 40 | 4.07 | 19.8% | 5 | R.ATNAASDFTLLELNT#AANPAY@NLFWAGWDR.R | 3 |
| \* | AstrinIP\_MS1\_022614\_01.03960.03960.2 | 4.0013 | 0.44 | 100.0% | 2076.2522 | 2077.2668 | 1 | 7.221 | 47.2% | 3 | R.RDQNFAGATAIHHPNVAEK.R | 2 |
| \* | SKAPIP\_041314\_01.10232.10232.3 | 5.1678 | 0.4279 | 100.0% | 2078.1843 | 2077.2668 | 1 | 7.114 | 47.2% | 15 | R.RDQNFAGATAIHHPNVAEK.R | 3 |
| \* | pDK339\_033013\_01.03842.03842.3 | 5.0402 | 0.3698 | 100.0% | 2233.5842 | 2233.4543 | 1 | 6.732 | 42.1% | 23 | R.RDQNFAGATAIHHPNVAEKR.I | 3 |
| \* | SKAPIP\_tube2\_041314\_01.04328.04328.2 | 4.9133 | 0.5313 | 100.0% | 1920.5122 | 1921.0793 | 1 | 9.493 | 61.8% | 18 | R.DQNFAGATAIHHPNVAEK.R | 2 |
| \* | pJS43\_100mM\_120812\_01.04859.04859.3 | 3.0634 | 0.3228 | 99.8% | 1921.1643 | 1921.0793 | 2 | 5.606 | 36.8% | 7 | R.DQNFAGATAIHHPNVAEK.R | 3 |
| \* | pSKT11\_1\_020812\_01.06134.06134.2 | 5.912 | 0.4419 | 100.0% | 2076.4922 | 2077.2668 | 1 | 7.53 | 55.6% | 23 | R.DQNFAGATAIHHPNVAEKR.I | 23 |
| \* | SKAPIP\_041314\_01.10478.10478.3 | 4.8927 | 0.4911 | 100.0% | 2078.0942 | 2077.2668 | 1 | 8.924 | 41.7% | 34 | R.DQNFAGATAIHHPNVAEKR.I | 3 |
| \* | SKAPIP\_tube2\_041314\_02.06897.06897.3 | 5.539 | 0.5339 | 100.0% | 4946.3345 | 4947.345 | 1 | 10.089 | 23.4% | 2 | R.ISHSTVATEISGYNGATGTSHLHVFWQASGGVTEPGSSGSPIYSPEKR.V | 3 |
| \* | SKAPIP\_041314\_01.09422.09422.2 | 4.8161 | 0.4896 | 100.0% | 1870.2522 | 1870.983 | 1 | 8.239 | 61.1% | 10 | R.VLGQLHGGPSSCSATGADR.S | 2 |
| \* | SKAPIP\_041314\_01.09411.09411.3 | 4.639 | 0.5065 | 100.0% | 1870.4944 | 1870.983 | 1 | 8.319 | 43.1% | 13 | R.VLGQLHGGPSSCSATGADR.S | 3 |
| \* | SKAPIP\_tube2\_041314\_01.06478.06478.1 | 2.4966 | 0.3091 | 98.4% | 1429.67 | 1428.5443 | 1 | 4.796 | 46.2% | 14 | R.VFTSWTGGGTSATR.L | 1 |
| \* | pDK365N\_100mM\_082713\_01.06091.06091.2 | 5.2646 | 0.4795 | 100.0% | 1429.8922 | 1428.5443 | 1 | 8.647 | 80.8% | 161 | R.VFTSWTGGGTSATR.L | 2 |
| \* | SKAPIP\_041314\_01.14399.14399.2 | 4.7554 | 0.5089 | 100.0% | 2605.652 | 2605.8174 | 1 | 8.436 | 42.0% | 7 | R.LSDWLDAAGTGAQFIDGLDSTGTPPV.- | 2 |

---

|  |  |  |  |  |  |  |  |  |
| --- | --- | --- | --- | --- | --- | --- | --- | --- |
| U | *gi|218505827|ref|NP\_1* | 76 | 1303 | 67.1% | 316 | 35438 | 6.3 | TRAF4 associated factor 1 isoform a [Homo sapiens] |

| Filename XCorr DeltCN Conf% ObsM+H+ CalcM+H+ SpR ZScore Ion% # Sequence  | | | | | | | | | | | | |
| --- | --- | --- | --- | --- | --- | --- | --- | --- | --- | --- | --- | --- |
|  | pDK339othertube\_033013\_01.07484.07484.3 | 5.7577 | 0.4978 | 100.0% | 2275.1343 | 2275.4802 | 1 | 8.389 | 48.8% | 69 | K.TVYSLQPPSALSGGQPADTQTR.A | 3 |
|  | pDK365N\_300mM\_082713\_03.07560.07560.2 | 6.0545 | 0.5656 | 100.0% | 2275.2122 | 2275.4802 | 1 | 9.558 | 61.9% | 132 | K.TVYSLQPPSALSGGQPADTQTR.A | 2 |
|  | 100326\_pJS43\_01.08402.08402.3 | 3.2557 | 0.1923 | 95.7% | 2757.6543 | 2755.4802 | 400 | 3.841 | 19.0% | 1 | K.T#VY@S\*LQPPS\*ALS\*GGQPADT#QTR.A | 32 |
|  | AstrinIP\_MS2\_022614\_01.08486.08486.3 | 3.003 | 0.2502 | 97.5% | 3411.2644 | 3408.7502 | 265 | 4.86 | 18.5% | 1 | K.TVYSLQPPSALSGGQPADTQT#RATSKSLLPVR.S | 3 |
|  | pDK365N\_300mM\_082713\_01.07601.07601.3 | 3.3838 | 0.2667 | 99.1% | 3411.5044 | 3408.7502 | 86 | 5.282 | 17.7% | 4 | K.TVYSLQPPSALSGGQPADTQTRATSKS\*LLPVR.S | 3 |
|  | SKAPIP\_041314\_01.10025.10025.2 | 2.0239 | 0.2502 | 96.5% | 1072.1522 | 1072.2932 | 14 | 4.401 | 61.1% | 1 | R.ATSKSLLPVR.S | 2 |
|  | pJS43\_100mM\_120812\_01.02750.02750.1 | 2.1661 | 0.2033 | 97.9% | 892.18 | 891.997 | 3 | 5.163 | 64.3% | 4 | R.SKEVDVSK.Q | 1 |
|  | SKAPIP\_tube2\_041314\_01.03940.03940.2 | 6.4552 | 0.4653 | 100.0% | 2254.5322 | 2255.4465 | 1 | 9.39 | 67.5% | 5 | R.SKEVDVSKQLHSGGPENDVTK.I | 2 |
|  | pDK365N\_300mM\_082713\_01.03761.03761.3 | 6.1051 | 0.4282 | 100.0% | 2255.2744 | 2255.4465 | 1 | 7.75 | 46.2% | 13 | R.SKEVDVSKQLHSGGPENDVTK.I | 3 |
|  | pSKT11\_1\_020812\_01.08056.08056.3 | 6.4767 | 0.485 | 100.0% | 2597.6943 | 2597.8853 | 1 | 8.642 | 38.0% | 3 | R.SKEVDVSKQLHSGGPENDVTKITK.L | 3 |
|  | pSKT11\_1\_020812\_01.07458.07458.2 | 4.1365 | 0.4674 | 100.0% | 2039.3121 | 2040.1943 | 1 | 9.654 | 66.7% | 1 | K.EVDVSKQLHSGGPENDVTK.I | 2 |
|  | SKAPIP\_tube2\_041314\_01.04263.04263.3 | 3.5457 | 0.5205 | 100.0% | 2040.2344 | 2040.1943 | 1 | 7.765 | 40.3% | 3 | K.EVDVSKQLHSGGPENDVTK.I | 3 |
|  | pDK365N\_300mM\_082713\_01.03459.03459.2 | 2.3638 | 0.2788 | 98.7% | 1382.2322 | 1382.4728 | 19 | 5.965 | 54.2% | 11 | K.QLHSGGPENDVTK.I | 2 |
|  | pJS43\_100mM\_120812\_01.03235.03235.3 | 2.7872 | 0.1926 | 95.4% | 1382.6643 | 1382.4728 | 71 | 4.899 | 43.8% | 4 | K.QLHSGGPENDVTK.I | 3 |
|  | SKAPIP\_041314\_01.07037.07037.2 | 2.6108 | 0.3555 | 99.7% | 1724.5122 | 1724.9114 | 7 | 6.539 | 50.0% | 3 | K.QLHSGGPENDVTKITK.L | 2 |
|  | SKAPIP\_041314\_01.06983.06983.3 | 2.9513 | 0.3261 | 99.8% | 1724.9944 | 1724.9114 | 3 | 5.192 | 36.7% | 2 | K.QLHSGGPENDVTKITK.L | 3 |
|  | SKAPIP\_tube2\_041314\_01.03578.03578.2 | 2.6625 | 0.1536 | 99.1% | 921.15216 | 921.1277 | 5 | 4.851 | 85.7% | 2 | R.KGYKPLSK.Q | 2 |
|  | SKAPIP\_tube2\_041314\_02.04689.04689.2 | 3.9058 | 0.4219 | 100.0% | 2243.8323 | 2244.507 | 1 | 6.884 | 47.2% | 28 | K.QKSEEELKDKNQLLEAVNK.Q | 2 |
|  | SKAPIP\_tube2\_041314\_01.04966.04966.3 | 3.7045 | 0.267 | 99.7% | 2243.9944 | 2244.507 | 10 | 6.288 | 30.6% | 72 | K.QKSEEELKDKNQLLEAVNK.Q | 3 |
|  | pDK365N\_300mM\_082713\_03.05393.05393.3 | 2.4884 | 0.2544 | 95.2% | 2323.7644 | 2324.507 | 1 | 5.179 | 37.5% | 3 | K.QKS\*EEELKDKNQLLEAVNK.Q | 3 |
|  | SKAPIP\_tube2\_041314\_01.06842.06842.3 | 4.2543 | 0.399 | 100.0% | 2878.7043 | 2879.2432 | 38 | 6.337 | 23.9% | 2 | K.QKSEEELKDKNQLLEAVNKQLHQK.L | 3 |
|  | AstrinIP\_MS2\_022614\_01.06431.06431.2 | 5.2517 | 0.447 | 100.0% | 1987.4922 | 1988.2023 | 1 | 9.133 | 65.6% | 27 | K.SEEELKDKNQLLEAVNK.Q | 2 |
|  | SKAPIP\_tube2\_041314\_02.05240.05240.3 | 5.7393 | 0.4607 | 100.0% | 1988.5144 | 1988.2023 | 1 | 8.169 | 43.8% | 85 | K.SEEELKDKNQLLEAVNK.Q | 3 |
|  | SKAPIP\_tube2\_041314\_01.07589.07589.3 | 5.5596 | 0.5028 | 100.0% | 2622.1443 | 2622.9382 | 1 | 7.779 | 41.7% | 2 | K.SEEELKDKNQLLEAVNKQLHQK.L | 3 |
|  | AstrinIP\_MS1\_022614\_01.05512.05512.1 | 2.1761 | 0.2415 | 98.7% | 1271.52 | 1272.4441 | 1 | 5.11 | 65.0% | 1 | K.DKNQLLEAVNK.Q | 1 |
|  | SKAPIP\_041314\_01.10871.10871.2 | 3.9048 | 0.2857 | 100.0% | 1272.1721 | 1272.4441 | 1 | 6.11 | 85.0% | 22 | K.DKNQLLEAVNK.Q | 2 |
|  | AstrinIP\_MS2\_022614\_01.05985.05985.1 | 2.1877 | 0.2289 | 98.7% | 1028.53 | 1029.1814 | 20 | 4.994 | 62.5% | 23 | K.NQLLEAVNK.Q | 1 |
|  | pDK365N\_100mM\_082713\_01.05202.05202.2 | 3.3051 | 0.2246 | 99.9% | 1029.2722 | 1029.1814 | 2 | 4.906 | 87.5% | 56 | K.NQLLEAVNK.Q | 2 |
|  | SKAPIP\_tube2\_041314\_01.07518.07518.2 | 3.0855 | 0.2206 | 99.5% | 1663.9722 | 1663.9175 | 3 | 5.831 | 57.7% | 2 | K.NQLLEAVNKQLHQK.L | 23 |
|  | AstrinIP\_MS2\_022614\_01.03794.03794.2 | 2.799 | 0.2947 | 99.9% | 1019.1122 | 1019.13995 | 1 | 6.817 | 87.5% | 3 | K.LTETQGELK.D | 2 |
|  | pJS43\_100mM\_120812\_01.06080.06080.3 | 2.1676 | 0.3699 | 99.3% | 1603.0144 | 1604.7979 | 8 | 6.189 | 40.4% | 2 | K.LTETQGELKDLTQK.V | 3 |
|  | AstrinIP\_MS1\_022614\_01.05594.05594.2 | 4.388 | 0.4022 | 100.0% | 1604.4321 | 1604.7979 | 1 | 7.253 | 65.4% | 44 | K.LTETQGELKDLTQK.V | 2 |
|  | SKAPIP\_tube2\_041314\_01.05423.05423.1 | 3.5862 | 0.2776 | 98.3% | 1605.78 | 1604.7979 | 1 | 4.835 | 53.8% | 6 | K.LTETQGELKDLTQK.V | 1 |
|  | SKAPIP\_tube2\_041314\_01.10119.10119.2 | 6.0793 | 0.4911 | 100.0% | 2315.8323 | 2316.6543 | 1 | 9.368 | 55.3% | 24 | K.LTETQGELKDLTQKVELLEK.F | 2 |
|  | SKAPIP\_tube2\_041314\_01.10124.10124.3 | 5.6648 | 0.3883 | 100.0% | 2316.8643 | 2316.6543 | 1 | 7.213 | 47.4% | 53 | K.LTETQGELKDLTQKVELLEK.F | 3 |
|  | SKAPIP\_tube2\_041314\_01.07404.07404.1 | 2.8416 | 0.2048 | 98.5% | 1316.68 | 1316.5376 | 1 | 4.651 | 65.0% | 2 | K.DLTQKVELLEK.F | 1 |
|  | SKAPIP\_tube2\_041314\_02.06192.06192.3 | 3.1097 | 0.2293 | 99.3% | 1317.0243 | 1316.5376 | 9 | 5.353 | 52.5% | 8 | K.DLTQKVELLEK.F | 3 |
|  | SKAPIP\_041314\_02.06144.06144.2 | 4.0736 | 0.255 | 100.0% | 1317.3322 | 1316.5376 | 1 | 5.311 | 75.0% | 42 | K.DLTQKVELLEK.F | 2 |
|  | SKAPIP\_041314\_02.08504.08504.3 | 3.5775 | 0.3498 | 100.0% | 2178.8643 | 2178.4998 | 1 | 5.883 | 42.6% | 1 | K.VELLEKFRDNCLAILESK.G | 3 |
|  | SKAPIP\_041314\_01.11355.11355.2 | 3.7073 | 0.3548 | 100.0% | 1466.2522 | 1466.6434 | 1 | 6.12 | 77.3% | 13 | K.FRDNCLAILESK.G | 2 |
|  | SKAPIP\_041314\_01.11290.11290.3 | 3.7453 | 0.1996 | 99.6% | 1467.9543 | 1466.6434 | 21 | 4.868 | 43.2% | 6 | K.FRDNCLAILESK.G | 3 |
|  | SKAPIP\_tube2\_041314\_01.08172.08172.1 | 2.1995 | 0.2947 | 98.8% | 1162.63 | 1163.2793 | 9 | 5.954 | 55.6% | 5 | R.DNCLAILESK.G | 1 |
|  | SKAPIP\_tube2\_041314\_01.08163.08163.2 | 3.7941 | 0.2505 | 100.0% | 1164.2122 | 1163.2793 | 1 | 5.877 | 77.8% | 6 | R.DNCLAILESK.G | 2 |
|  | AstrinIP\_MS1\_022614\_01.07517.07517.1 | 1.5464 | 0.3522 | 98.9% | 1386.65 | 1387.5327 | 4 | 6.414 | 50.0% | 4 | K.GLDPALGSETLASR.Q | 1 |
|  | 100326\_pJS43\_01.05642.05642.2 | 4.4576 | 0.5961 | 100.0% | 1387.3522 | 1387.5327 | 1 | 10.405 | 84.6% | 199 | K.GLDPALGSETLASR.Q | 2 |
|  | SKAPIP\_041314\_02.05057.05057.3 | 2.5489 | 0.2978 | 98.8% | 1387.6743 | 1387.5327 | 1 | 5.466 | 38.5% | 1 | K.GLDPALGSETLASR.Q | 3 |
|  | AstrinIP\_MS1\_022614\_01.07611.07611.2 | 2.4204 | 0.2862 | 98.9% | 1466.7322 | 1467.5327 | 3 | 5.526 | 57.7% | 1 | K.GLDPALGS\*ETLASR.Q | 2 |
|  | AstrinIP\_MS2\_022614\_01.07966.07966.2 | 2.9295 | 0.4656 | 100.0% | 1467.3522 | 1467.5327 | 1 | 6.645 | 69.2% | 1 | K.GLDPALGSET#LASR.Q | 2 |
|  | pSKT11\_1\_020812\_02.07733.07733.3 | 5.328 | 0.5109 | 100.0% | 3959.2744 | 3961.4023 | 1 | 7.685 | 25.0% | 1 | K.GLDPALGSETLASRQESTTDHMDSMLLLETLQEELK.L | 3 |
|  | pSKT11\_1\_020812\_01.10762.10762.3 | 3.303 | 0.32 | 99.7% | 4764.594 | 4765.3154 | 18 | 5.495 | 15.5% | 1 | K.GLDPALGSETLASRQESTTDHMDSMLLLETLQEELKLFNETAK.K | 3 |
|  | SKAPIP\_tube2\_041314\_01.13605.13605.2 | 5.3662 | 0.4345 | 100.0% | 2591.7122 | 2592.8928 | 1 | 6.965 | 50.0% | 50 | R.QESTTDHMDSMLLLETLQEELK.L | 2 |
|  | SKAPIP\_041314\_01.13502.13502.3 | 4.3937 | 0.2897 | 100.0% | 2592.1743 | 2592.8928 | 12 | 4.949 | 33.3% | 8 | R.QESTTDHMDSMLLLETLQEELK.L | 3 |
|  | SKAPIP\_041314\_01.15004.15004.3 | 7.0032 | 0.5515 | 100.0% | 3396.5645 | 3396.8062 | 1 | 9.043 | 33.0% | 63 | R.QESTTDHMDSMLLLETLQEELKLFNETAK.K | 3 |
|  | SKAPIP\_tube2\_041314\_01.14919.14919.3 | 3.7582 | 0.2942 | 99.8% | 3475.8245 | 3476.8062 | 24 | 4.805 | 20.5% | 1 | R.QESTTDHMDS\*MLLLETLQEELKLFNETAK.K | 3 |
|  | SKAPIP\_tube2\_041314\_01.14169.14169.3 | 4.767 | 0.3873 | 100.0% | 3522.4143 | 3524.9802 | 1 | 7.103 | 25.0% | 12 | R.QESTTDHMDSMLLLETLQEELKLFNETAKK.Q | 3 |
|  | AstrinIP\_MS2\_022614\_01.03956.03956.1 | 1.8115 | 0.2288 | 98.0% | 822.35 | 822.9365 | 23 | 4.387 | 66.7% | 3 | K.LFNETAK.K | 1 |
|  | pJS43\_100mM\_120812\_01.03354.03354.2 | 2.3187 | 0.1243 | 96.1% | 950.4922 | 951.1106 | 9 | 4.62 | 78.6% | 1 | K.LFNETAKK.Q | 2 |
| \* | pSKT11\_1\_020812\_01.05421.05421.1 | 2.8539 | 0.1335 | 96.4% | 1217.54 | 1218.454 | 195 | 4.098 | 55.6% | 7 | K.KQMEELQALK.V | 1 |
| \* | pSKT11\_1\_020812\_01.05408.05408.2 | 3.6264 | 0.2507 | 100.0% | 1218.0521 | 1218.454 | 27 | 5.155 | 72.2% | 51 | K.KQMEELQALK.V | 2 |
| \* | SKAPIP\_041314\_02.04060.04060.3 | 4.3481 | 0.1687 | 99.9% | 1219.8844 | 1218.454 | 14 | 4.485 | 58.3% | 15 | K.KQMEELQALK.V | 3 |
| \* | pSKT11\_1\_020812\_01.08111.08111.2 | 4.2098 | 0.3754 | 100.0% | 1445.1921 | 1445.7606 | 1 | 6.955 | 72.7% | 2 | K.KQMEELQALKVK.L | 2 |
| \* | pDK365N\_300mM\_082713\_01.04368.04368.3 | 3.3483 | 0.2622 | 99.8% | 1447.4043 | 1445.7606 | 11 | 4.609 | 43.2% | 3 | K.KQMEELQALKVK.L | 3 |
| \* | SKAPIP\_tube2\_041314\_01.06333.06333.2 | 2.1535 | 0.354 | 99.3% | 1317.2322 | 1317.5865 | 100 | 5.276 | 70.0% | 1 | K.QMEELQALKVK.L | 2 |
| \* | pSKT11\_1\_020812\_02.04641.04641.3 | 4.6605 | 0.4724 | 100.0% | 2233.1343 | 2233.6465 | 1 | 7.354 | 41.2% | 1 | K.QMEELQALKVKLEMKEER.V | 3 |
| \* | AstrinIP\_MS2\_022614\_01.03627.03627.1 | 2.1887 | 0.216 | 98.3% | 1161.52 | 1162.3899 | 6 | 5.022 | 68.8% | 1 | K.VKLEMKEER.V | 1 |
| \* | SKAPIP\_tube2\_041314\_01.03754.03754.2 | 3.362 | 0.3218 | 100.0% | 1161.8322 | 1162.3899 | 1 | 6.721 | 87.5% | 25 | K.VKLEMKEER.V | 2 |
| \* | SKAPIP\_tube2\_041314\_01.03671.03671.2 | 2.4151 | 0.1352 | 98.4% | 935.1122 | 935.0832 | 36 | 3.979 | 75.0% | 3 | K.LEMKEER.V | 2 |
| \* | SKAPIP\_041314\_01.11823.11823.3 | 3.9286 | 0.1419 | 95.3% | 3525.6543 | 3522.9648 | 2 | 4.951 | 20.5% | 4 | K.LEMKEERVRFLEQQTLCNNQVNDLTTALK.E | 3 |
| \* | SKAPIP\_tube2\_041314\_01.09438.09438.2 | 4.9117 | 0.4849 | 100.0% | 2606.1921 | 2606.9048 | 1 | 8.811 | 54.8% | 1 | R.VRFLEQQTLCNNQVNDLTTALK.E | 2 |
| \* | SKAPIP\_041314\_02.07474.07474.3 | 6.4992 | 0.4408 | 100.0% | 2606.2744 | 2606.9048 | 1 | 8.269 | 40.5% | 5 | R.VRFLEQQTLCNNQVNDLTTALK.E | 3 |
| \* | SKAPIP\_tube2\_041314\_01.16938.16938.2 | 3.485 | 0.4372 | 100.0% | 3610.392 | 3611.086 | 1 | 7.941 | 31.0% | 1 | R.VRFLEQQTLCNNQVNDLTTALKEMEQLLEM.- | 2 |
| \* | SKAPIP\_041314\_02.13637.13637.3 | 4.6206 | 0.2109 | 99.6% | 3610.6443 | 3611.086 | 1 | 5.699 | 22.4% | 8 | R.VRFLEQQTLCNNQVNDLTTALKEMEQLLEM.- | 3 |
| \* | AstrinIP\_MS1\_022614\_01.10166.10166.2 | 6.323 | 0.5723 | 100.0% | 2350.5522 | 2351.5847 | 1 | 10.59 | 63.2% | 16 | R.FLEQQTLCNNQVNDLTTALK.E | 2 |
| \* | AstrinIP\_MS1\_022614\_01.10130.10130.3 | 6.1915 | 0.4025 | 100.0% | 2351.8743 | 2351.5847 | 1 | 7.753 | 57.9% | 10 | R.FLEQQTLCNNQVNDLTTALK.E | 3 |
| \* | AstrinIP\_MS1\_022614\_01.19169.19169.3 | 3.6619 | 0.292 | 99.7% | 3356.2444 | 3355.7659 | 8 | 5.333 | 23.1% | 4 | R.FLEQQTLCNNQVNDLTTALKEMEQLLEM.- | 3 |
| \* | SKAPIP\_tube2\_041314\_01.11354.11354.2 | 2.033 | 0.2657 | 98.4% | 1023.1122 | 1023.2044 | 1 | 4.972 | 71.4% | 2 | K.EMEQLLEM.- | 2 |

---

|  |  |  |  |  |  |  |  |  |
| --- | --- | --- | --- | --- | --- | --- | --- | --- |
| U | *gi|34147513|ref|NP\_00* | 11 | 28 | 67.1% | 207 | 23490 | 6.7 | RAB7, member RAS oncogene family [Homo sapiens] |

| Filename XCorr DeltCN Conf% ObsM+H+ CalcM+H+ SpR ZScore Ion% # Sequence  | | | | | | | | | | | | |
| --- | --- | --- | --- | --- | --- | --- | --- | --- | --- | --- | --- | --- |
| \* | pDK365N\_300mM\_082713\_01.06544.06544.2 | 3.2064 | 0.3303 | 100.0% | 1058.2922 | 1058.2633 | 1 | 6.451 | 80.0% | 3 | K.VIILGDSGVGK.T | 2 |
| \* | pDK365N\_300mM\_082713\_01.04121.04121.2 | 2.5212 | 0.1295 | 96.0% | 1326.2322 | 1326.5536 | 6 | 3.727 | 60.0% | 1 | K.TSLMNQYVNKK.F | 2 |
| \* | pDK365N\_300mM\_082713\_03.07773.07773.2 | 2.3376 | 0.3451 | 99.7% | 1037.1721 | 1037.201 | 1 | 5.83 | 83.3% | 3 | K.ATIGADFLTK.E | 2 |
| \* | pDK365N\_100mM\_082713\_01.09236.09236.2 | 3.3993 | 0.162 | 99.5% | 1649.6322 | 1648.8743 | 1 | 5.583 | 61.5% | 3 | R.LVTMQIWDTAGQER.F | 2 |
| \* | pDK365N\_100mM\_082813\_03.08978.08978.2 | 2.3783 | 0.2646 | 99.1% | 1188.4922 | 1188.3715 | 1 | 5.762 | 77.8% | 1 | R.FQSLGVAFYR.G | 2 |
| \* | pDK365N\_300mM\_082713\_04.07143.07143.3 | 4.0265 | 0.3666 | 100.0% | 1935.4143 | 1935.1466 | 2 | 6.179 | 38.3% | 4 | K.TLDSWRDEFLIQASPR.D | 3 |
| \* | pDK365N\_300mM\_082713\_03.11273.11273.2 | 3.1493 | 0.3596 | 100.0% | 1476.2122 | 1476.672 | 1 | 7.199 | 54.2% | 2 | R.DPENFPFVVLGNK.I | 2 |
| \* | pDK365N\_100mM\_082713\_01.11284.11284.3 | 3.4633 | 0.2885 | 99.7% | 2218.2844 | 2217.4863 | 34 | 5.84 | 29.2% | 2 | R.DPENFPFVVLGNKIDLENR.Q | 3 |
| \* | pDK365N\_300mM\_082713\_01.07046.07046.2 | 2.5662 | 0.1753 | 98.2% | 1284.2722 | 1284.4111 | 4 | 5.04 | 65.0% | 2 | K.NNIPYFETSAK.E | 2 |
| \* | pDK365N\_100mM\_082713\_01.10358.10358.2 | 4.0565 | 0.4306 | 100.0% | 1590.9321 | 1590.7764 | 1 | 8.585 | 73.1% | 6 | K.EAINVEQAFQTIAR.N | 2 |
| \* | pDK365N\_300mM\_082713\_02.07206.07206.3 | 5.5649 | 0.3971 | 100.0% | 2749.3145 | 2749.0898 | 1 | 6.64 | 38.6% | 1 | R.NALKQETEVELYNEFPEPIKLDK.N | 3 |

---

|  |  |  |  |  |  |  |  |  |
| --- | --- | --- | --- | --- | --- | --- | --- | --- |
| U | *gi|119395750|ref|NP\_0* | 53 | 304 | 66.1% | 644 | 66039 | 8.1 | keratin 1 [Homo sapiens] |

| Filename XCorr DeltCN Conf% ObsM+H+ CalcM+H+ SpR ZScore Ion% # Sequence  | | | | | | | | | | | | |
| --- | --- | --- | --- | --- | --- | --- | --- | --- | --- | --- | --- | --- |
| \* | pJS43\_100mM\_120812\_02.05469.05469.2 | 4.2338 | 0.4933 | 100.0% | 1657.5122 | 1658.7678 | 1 | 7.992 | 59.4% | 6 | R.SGGGFSSGSAGIINYQR.R | 2 |
| \* | 100326\_pJS43\_02.04115.04115.2 | 3.7887 | 0.4238 | 100.0% | 1765.4722 | 1766.79 | 1 | 8.071 | 50.0% | 2 | R.FSSCGGGGGSFGAGGGFGSR.S | 2 |
| \* | pSKT11\_1\_020812\_01.04534.04534.1 | 1.5886 | 0.3304 | 98.8% | 874.35 | 875.0128 | 18 | 5.302 | 56.2% | 4 | R.SLVNLGGSK.S | 1 |
| \* | pJS43\_100mM\_120812\_01.05552.05552.2 | 2.3128 | 0.2378 | 98.8% | 875.2522 | 875.0128 | 1 | 4.558 | 75.0% | 1 | R.SLVNLGGSK.S | 2 |
|  | pJS43\_100mM\_120812\_01.10643.10643.2 | 3.8833 | 0.4203 | 100.0% | 1385.3922 | 1384.5315 | 1 | 7.05 | 81.8% | 17 | K.SLNNQFASFIDK.V | 2 |
|  | 100326\_pJS43\_01.08648.08648.2 | 4.4525 | 0.3681 | 100.0% | 1639.6122 | 1639.8516 | 1 | 7.137 | 76.9% | 4 | K.SLNNQFASFIDKVR.F | 2 |
|  | pDK365N\_300mM\_082713\_03.10149.10149.3 | 1.9356 | 0.3318 | 96.3% | 1639.8544 | 1639.8516 | 2 | 5.128 | 42.3% | 1 | K.SLNNQFASFIDKVR.F | 3 |
|  | pDK339\_033013\_01.05947.05947.2 | 4.5619 | 0.0649 | 99.9% | 1476.1921 | 1476.6726 | 1 | 7.056 | 90.9% | 25 | R.FLEQQNQVLQTK.W | 222 |
|  | pSKT11\_1\_020812\_02.06225.06225.2 | 4.2399 | 0.4409 | 100.0% | 2933.8323 | 2934.2786 | 1 | 8.521 | 39.1% | 1 | R.FLEQQNQVLQTKWELLQQVDTSTR.T | 2 |
|  | pDK365N\_300mM\_082713\_04.07424.07424.3 | 6.0077 | 0.4667 | 100.0% | 2933.8743 | 2934.2786 | 1 | 9.146 | 37.0% | 6 | R.FLEQQNQVLQTKWELLQQVDTSTR.T | 3 |
|  | 100326\_pJS43\_01.07731.07731.2 | 4.6938 | 0.5292 | 100.0% | 1476.0521 | 1476.6293 | 1 | 9.848 | 77.3% | 18 | K.WELLQQVDTSTR.T | 2 |
|  | 100326\_pJS43\_01.10766.10766.2 | 4.7387 | 0.459 | 100.0% | 1994.6522 | 1995.2017 | 1 | 8.598 | 76.7% | 6 | R.THNLEPYFESFINNLR.R | 2 |
|  | pDK365N\_300mM\_082713\_03.12519.12519.3 | 4.1515 | 0.3625 | 100.0% | 1994.7544 | 1995.2017 | 1 | 6.957 | 45.0% | 5 | R.THNLEPYFESFINNLR.R | 3 |
|  | 100326\_pJS43\_01.10020.10020.2 | 2.9479 | 0.2875 | 99.7% | 2151.412 | 2151.3892 | 8 | 4.967 | 37.5% | 1 | R.THNLEPYFESFINNLRR.R | 2 |
|  | pDK365N\_300mM\_082713\_03.11769.11769.3 | 3.0615 | 0.2493 | 98.8% | 2151.7444 | 2151.3892 | 38 | 4.733 | 29.7% | 5 | R.THNLEPYFESFINNLRR.R | 3 |
|  | 100326\_pJS43\_01.08076.08076.2 | 3.7412 | 0.5424 | 100.0% | 1986.1122 | 1987.2068 | 1 | 8.135 | 56.7% | 1 | R.LDSELKNMQDMVEDYR.N | 2 |
|  | 100326\_pJS43\_01.05505.05505.2 | 2.579 | 0.3243 | 99.8% | 1301.2722 | 1301.4316 | 1 | 5.426 | 72.2% | 2 | K.NMQDMVEDYR.N | 2 |
|  | 100326\_pJS43\_01.06968.06968.3 | 4.3387 | 0.4891 | 100.0% | 2591.3943 | 2591.8298 | 1 | 7.868 | 35.5% | 2 | K.NMQDMVEDYRNKYEDEINKR.T | 3 |
|  | SKAPIP\_041314\_01.04274.04274.2 | 2.9252 | 0.238 | 99.8% | 1310.0322 | 1309.4215 | 154 | 4.901 | 55.6% | 7 | R.NKYEDEINKR.T | 22222 |
|  | pDK339\_033013\_01.07129.07129.2 | 3.2492 | 0.3945 | 100.0% | 1267.2122 | 1266.3934 | 1 | 7.639 | 80.0% | 6 | R.TNAENEFVTIK.K | 2 |
|  | pDK339\_033013\_01.04946.04946.2 | 3.69 | 0.3186 | 100.0% | 1394.1721 | 1394.5675 | 1 | 6.874 | 72.7% | 14 | R.TNAENEFVTIKK.D | 2 |
|  | pSKT11\_1\_020812\_01.13013.13013.3 | 3.1367 | 0.2245 | 96.6% | 5184.534 | 5184.7993 | 1 | 4.679 | 14.2% | 1 | K.VDLQAKLDNLQQEIDFLTALYQAELSQMQTQISETNVILSMDNNR.S | 3 |
|  | 100326\_pJS43\_01.15584.15584.3 | 2.8796 | 0.2903 | 98.7% | 4528.4346 | 4530.035 | 2 | 5.27 | 16.4% | 1 | K.LDNLQQEIDFLTALYQAELSQMQTQISETNVILSMDNNR.S | 3 |
| \* | 100326\_pJS43\_01.10446.10446.1 | 2.4426 | 0.3292 | 98.6% | 1302.54 | 1303.4955 | 23 | 6.047 | 50.0% | 3 | R.SLDLDSIIAEVK.A | 1 |
| \* | 100326\_pJS43\_01.10496.10496.2 | 4.569 | 0.4105 | 100.0% | 1303.1721 | 1303.4955 | 1 | 8.983 | 81.8% | 14 | R.SLDLDSIIAEVK.A | 2 |
| \* | pSKT11\_1\_020812\_01.11008.11008.2 | 2.8396 | 0.373 | 99.9% | 2349.7322 | 2350.6282 | 9 | 6.389 | 32.5% | 1 | R.SLDLDSIIAEVKAQYEDIAQK.S | 2 |
| \* | 100326\_pJS43\_01.00707.00707.1 | 1.9224 | 0.2691 | 98.6% | 1065.3 | 1066.1558 | 212 | 5.024 | 43.8% | 1 | K.AQYEDIAQK.S | 1 |
| \* | SKAPIP\_041314\_01.05000.05000.2 | 2.9425 | 0.3139 | 100.0% | 1066.0721 | 1066.1558 | 34 | 5.727 | 56.2% | 6 | K.AQYEDIAQK.S | 2 |
|  | pJS43\_100mM\_120812\_01.03719.03719.2 | 4.3133 | 0.469 | 100.0% | 1341.0122 | 1341.4607 | 1 | 8.126 | 77.3% | 4 | K.SKAEAESLYQSK.Y | 2 |
|  | 100326\_pJS43\_01.05735.05735.3 | 6.1717 | 0.5261 | 100.0% | 2501.8442 | 2502.7405 | 1 | 9.954 | 41.7% | 12 | K.SKAEAESLYQSKYEELQITAGR.H | 3 |
|  | pSKT11\_1\_020812\_01.08670.08670.2 | 5.3639 | 0.3923 | 100.0% | 2502.8523 | 2502.7405 | 1 | 7.826 | 54.8% | 2 | K.SKAEAESLYQSKYEELQITAGR.H | 2 |
|  | SKAPIP\_041314\_01.05423.05423.2 | 2.7118 | 0.3081 | 99.8% | 1126.3922 | 1126.2084 | 1 | 6.035 | 77.8% | 2 | K.AEAESLYQSK.Y | 2 |
|  | pDK365N\_300mM\_082713\_04.05764.05764.3 | 3.7461 | 0.3405 | 100.0% | 2286.8044 | 2287.4883 | 1 | 5.8 | 36.8% | 3 | K.AEAESLYQSKYEELQITAGR.H | 3 |
|  | pSKT11\_1\_020812\_02.04601.04601.2 | 4.0326 | 0.3849 | 100.0% | 2288.4321 | 2287.4883 | 1 | 7.641 | 44.7% | 1 | K.AEAESLYQSKYEELQITAGR.H | 2 |
|  | 100326\_pJS43\_02.03363.03363.2 | 3.962 | 0.271 | 100.0% | 1180.5521 | 1180.303 | 1 | 7.834 | 94.4% | 37 | K.YEELQITAGR.H | 22 |
|  | pSKT11\_1\_020812\_01.07875.07875.2 | 2.7906 | 0.3087 | 99.8% | 1303.3922 | 1303.4581 | 2 | 5.084 | 80.0% | 3 | R.NSKIEISELNR.V | 2 |
|  | pDK339\_033013\_01.06043.06043.2 | 2.8783 | 0.1411 | 99.5% | 974.1322 | 974.102 | 1 | 4.646 | 92.9% | 5 | K.IEISELNR.V | 222 |
|  | pJS43\_100mM\_120812\_01.07956.07956.2 | 4.8829 | 0.442 | 100.0% | 1717.5122 | 1717.8333 | 1 | 7.817 | 71.4% | 4 | K.QISNLQQSISDAEQR.G | 2 |
|  | 100326\_pJS43\_01.08319.08319.3 | 3.3969 | 0.2499 | 98.8% | 2645.0344 | 2644.8582 | 1 | 4.275 | 31.5% | 1 | K.QISNLQQSISDAEQRGENALKDAK.N | 3 |
|  | pJS43\_100mM\_120812\_01.09500.09500.2 | 4.7373 | 0.4645 | 100.0% | 1600.5721 | 1600.769 | 1 | 7.975 | 80.8% | 7 | K.NKLNDLEDALQQAK.E | 2 |
| \* | 100326\_pJS43\_01.09546.09546.2 | 5.6063 | 0.4867 | 100.0% | 2184.4722 | 2185.399 | 1 | 9.404 | 77.8% | 1 | K.NKLNDLEDALQQAKEDLAR.L | 2 |
| \* | 100326\_pJS43\_01.09584.09584.3 | 5.9102 | 0.4148 | 100.0% | 2186.0942 | 2185.399 | 1 | 7.473 | 45.8% | 14 | K.NKLNDLEDALQQAKEDLAR.L | 3 |
|  | AstrinIP\_MS1\_022614\_01.08430.08430.2 | 3.6926 | 0.3227 | 100.0% | 1358.2922 | 1358.4912 | 1 | 6.566 | 81.8% | 4 | K.LNDLEDALQQAK.E | 2 |
| \* | 100326\_pJS43\_01.09605.09605.2 | 2.7854 | 0.3207 | 99.7% | 1942.0922 | 1943.121 | 17 | 5.371 | 34.4% | 1 | K.LNDLEDALQQAKEDLAR.L | 2 |
|  | 100326\_pJS43\_01.05704.05704.3 | 2.8642 | 0.339 | 99.9% | 1524.1743 | 1524.7754 | 31 | 5.624 | 38.6% | 3 | R.LLRDYQELMNTK.L | 3 |
|  | 100326\_pJS43\_01.05734.05734.2 | 3.6359 | 0.2657 | 100.0% | 1524.3121 | 1524.7754 | 1 | 6.42 | 68.2% | 5 | R.LLRDYQELMNTK.L | 2 |
|  | SKAPIP\_041314\_01.06816.06816.1 | 2.2119 | 0.4203 | 99.4% | 1033.54 | 1034.1112 | 5 | 6.56 | 56.2% | 2 | R.TLLEGEESR.M | 1 |
|  | SKAPIP\_041314\_01.06812.06812.2 | 2.4784 | 0.4003 | 100.0% | 1033.6322 | 1034.1112 | 4 | 5.786 | 75.0% | 6 | R.TLLEGEESR.M | 2 |
| \* | SKAPIP\_041314\_02.04841.04841.3 | 3.5114 | 0.184 | 96.1% | 2566.2544 | 2566.7253 | 1 | 5.621 | 29.0% | 1 | R.MSGECAPNVSVSVSTSHTTISGGGSR.G | 3 |
|  | pDK339\_033013\_01.03809.03809.2 | 6.1342 | 0.6029 | 100.0% | 2384.2922 | 2385.298 | 1 | 12.233 | 41.7% | 2 | R.GGGGGGYGSGGSSYGSGGGSYGSGGGGGGGR.G | 2 |
|  | pDK339\_033013\_01.03806.03806.3 | 7.6429 | 0.5434 | 100.0% | 2384.8743 | 2385.298 | 1 | 11.433 | 40.0% | 2 | R.GGGGGGYGSGGSSYGSGGGSYGSGGGGGGGR.G | 3 |
| \* | pJS43\_100mM\_120812\_02.03822.03822.3 | 4.145 | 0.2972 | 99.9% | 3313.5842 | 3314.2085 | 3 | 6.512 | 19.1% | 7 | R.GSYGSGGSSYGSGGGSYGSGGGGGGHGSYGSGSSSGGYR.G | 3 |
| \* | pDK365N\_100mM\_082813\_03.08795.08795.3 | 4.2329 | 0.2781 | 99.9% | 2240.6343 | 2241.0396 | 1 | 4.965 | 36.1% | 14 | R.GGSGGGGGGS\*S\*GGRGSGGGSSGGSIGGR.G | 3 |

Similarities:
gi|4504919|ref|NP\_002(1:52)  
gi|47132620|ref|NP\_00(2:51)  
contaminant\_KERATIN22(2:51)  
gi|119395754|ref|NP\_0(1:52)  
gi|155969697|ref|NP\_7(1:52)  
gi|119703753|ref|NP\_0(2:51)  

---

|  |  |  |  |  |  |  |  |  |
| --- | --- | --- | --- | --- | --- | --- | --- | --- |
| U | *gi|11415030|ref|NP\_06* | 11 | 60 | 66.0% | 103 | 11367 | 11.4 | histone cluster 1, H4j [Homo sapiens] |
| U | *gi|77539758|ref|NP\_00* | 11 | 60 | 66.0% | 103 | 11367 | 11.4 | histone cluster 2, H4b [Homo sapiens] |
| U | *gi|4504323|ref|NP\_003* | 11 | 60 | 66.0% | 103 | 11367 | 11.4 | histone cluster 2, H4a [Homo sapiens] |
| U | *gi|4504321|ref|NP\_003* | 11 | 60 | 66.0% | 103 | 11367 | 11.4 | histone cluster 1, H4i [Homo sapiens] |
| U | *gi|4504317|ref|NP\_003* | 11 | 60 | 66.0% | 103 | 11367 | 11.4 | histone cluster 1, H4l [Homo sapiens] |
| U | *gi|4504315|ref|NP\_003* | 11 | 60 | 66.0% | 103 | 11367 | 11.4 | histone cluster 1, H4e [Homo sapiens] |
| U | *gi|4504313|ref|NP\_003* | 11 | 60 | 66.0% | 103 | 11367 | 11.4 | histone cluster 1, H4b [Homo sapiens] |
| U | *gi|4504311|ref|NP\_003* | 11 | 60 | 66.0% | 103 | 11367 | 11.4 | histone cluster 1, H4h [Homo sapiens] |
| U | *gi|4504309|ref|NP\_003* | 11 | 60 | 66.0% | 103 | 11367 | 11.4 | histone cluster 1, H4c [Homo sapiens] |
| U | *gi|4504307|ref|NP\_003* | 11 | 60 | 66.0% | 103 | 11367 | 11.4 | histone cluster 1, H4k [Homo sapiens] |
| U | *gi|4504305|ref|NP\_003* | 11 | 60 | 66.0% | 103 | 11367 | 11.4 | histone cluster 1, H4f [Homo sapiens] |
| U | *gi|4504303|ref|NP\_003* | 11 | 60 | 66.0% | 103 | 11367 | 11.4 | histone cluster 1, H4d [Homo sapiens] |
| U | *gi|4504301|ref|NP\_003* | 11 | 60 | 66.0% | 103 | 11367 | 11.4 | histone cluster 1, H4a [Homo sapiens] |
| U | *gi|28173560|ref|NP\_77* | 11 | 60 | 66.0% | 103 | 11367 | 11.4 | histone cluster 4, H4 [Homo sapiens] |

| Filename XCorr DeltCN Conf% ObsM+H+ CalcM+H+ SpR ZScore Ion% # Sequence  | | | | | | | | | | | | |
| --- | --- | --- | --- | --- | --- | --- | --- | --- | --- | --- | --- | --- |
|  | 100326\_pJS43\_01.04206.04206.3 | 2.9273 | 0.2021 | 96.4% | 1694.6943 | 1695.0182 | 116 | 4.083 | 32.1% | 1 | K.VLRDNIQGITKPAIR.R | 3 |
|  | pDK365N\_300mM\_082713\_03.04571.04571.2 | 3.5475 | 0.2391 | 99.9% | 1326.0922 | 1326.5387 | 1 | 7.123 | 72.7% | 14 | R.DNIQGITKPAIR.R | 2 |
|  | pDK339othertube\_033013\_01.06668.06668.2 | 3.5714 | 0.4691 | 100.0% | 1180.6122 | 1181.3312 | 1 | 8.3 | 88.9% | 21 | R.ISGLIYEETR.G | 2 |
|  | AstrinNocIP\_020510\_01.05721.05721.2 | 2.0593 | 0.2385 | 95.1% | 1386.9321 | 1387.7086 | 3 | 5.133 | 50.0% | 1 | R.GVLKVFLENVIR.D | 2 |
|  | pDK339othertube\_033013\_01.08541.08541.2 | 3.2636 | 0.2682 | 100.0% | 990.1922 | 990.19055 | 3 | 5.824 | 85.7% | 11 | K.VFLENVIR.D | 2 |
|  | pDK365N\_100mM\_082713\_01.11830.11830.3 | 5.0563 | 0.3792 | 100.0% | 2107.7644 | 2106.386 | 1 | 6.695 | 38.2% | 1 | K.VFLENVIRDAVTYTEHAK.R | 3 |
|  | pJS43\_100mM\_120812\_01.03401.03401.2 | 3.0129 | 0.2983 | 99.9% | 1135.0721 | 1135.2188 | 1 | 6.816 | 77.8% | 1 | R.DAVTYTEHAK.R | 2 |
|  | pDK365N\_300mM\_082713\_01.08862.08862.3 | 2.6865 | 0.2377 | 97.3% | 1596.0543 | 1595.9409 | 28 | 4.715 | 36.5% | 1 | R.KTVTAMDVVYALKR.Q | 3 |
|  | pJS43\_100mM\_120812\_01.11709.11709.2 | 3.7436 | 0.4008 | 100.0% | 1312.1522 | 1311.5793 | 1 | 7.045 | 81.8% | 3 | K.TVTAMDVVYALK.R | 2 |
|  | pJS43\_100mM\_120812\_01.10729.10729.2 | 3.645 | 0.4309 | 100.0% | 1467.4321 | 1467.7667 | 1 | 7.979 | 83.3% | 3 | K.TVTAMDVVYALKR.Q | 2 |
|  | AstrinNocIP\_020510\_01.02966.02966.1 | 2.4225 | 0.5172 | 100.0% | 715.36 | 714.796 | 1 | 8.111 | 66.7% | 3 | R.TLYGFGG.- | 1 |

---

|  |  |  |  |  |  |  |  |  |
| --- | --- | --- | --- | --- | --- | --- | --- | --- |
| U | *gi|10800130|ref|NP\_06* | 10 | 72 | 65.4% | 130 | 14107 | 10.9 | histone cluster 1, H2ad [Homo sapiens] |

| Filename XCorr DeltCN Conf% ObsM+H+ CalcM+H+ SpR ZScore Ion% # Sequence  | | | | | | | | | | | | |
| --- | --- | --- | --- | --- | --- | --- | --- | --- | --- | --- | --- | --- |
|  | pDK365N\_100mM\_082813\_03.07151.07151.2 | 3.2969 | 0.3218 | 100.0% | 945.0122 | 945.1093 | 3 | 5.574 | 81.2% | 13 | R.AGLQFPVGR.V | 2222 |
|  | pDK339othertube\_033013\_01.18681.18681.3 | 2.6301 | 0.4084 | 99.9% | 4373.4243 | 4374.7686 | 1 | 6.177 | 17.1% | 2 | R.LLRKGNY@S\*ERVGAGAPVY@LAAVLEYLTAEILELAGNAAR.D | 3 |
|  | pJS43\_100mM\_120812\_01.18071.18071.3 | 5.2267 | 0.4872 | 100.0% | 2916.7144 | 2917.3752 | 1 | 8.614 | 33.9% | 8 | R.VGAGAPVYLAAVLEYLTAEILELAGNAAR.D | 3 |
|  | 100326\_pJS43\_01.16467.16467.2 | 5.2028 | 0.4892 | 100.0% | 2917.892 | 2917.3752 | 1 | 8.316 | 39.3% | 6 | R.VGAGAPVYLAAVLEYLTAEILELAGNAAR.D | 2 |
|  | pJS43\_100mM\_120812\_01.05135.05135.2 | 2.5063 | 0.2795 | 99.8% | 851.1722 | 851.0396 | 1 | 5.65 | 91.7% | 4 | R.HLQLAIR.N | 222 |
|  | pDK365N\_300mM\_082713\_02.04718.04718.3 | 3.2892 | 0.3261 | 99.9% | 1693.8243 | 1693.9004 | 5 | 6.383 | 38.5% | 9 | R.HLQLAIRNDEELNK.L | 33 |
|  | pDK365N\_100mM\_082713\_01.08028.08028.3 | 4.5718 | 0.4939 | 100.0% | 2105.6042 | 2105.4453 | 1 | 7.619 | 44.1% | 2 | R.HLQLAIRNDEELNKLLGK.V | 33 |
|  | pDK365N\_100mM\_082713\_01.06878.06878.2 | 3.7745 | 0.3591 | 100.0% | 1273.9722 | 1273.4288 | 1 | 6.538 | 80.0% | 7 | R.NDEELNKLLGK.V | 22 |
|  | pJS43\_100mM\_120812\_01.12803.12803.2 | 5.2767 | 0.5529 | 100.0% | 1931.6522 | 1932.3573 | 1 | 8.855 | 63.9% | 19 | K.VTIAQGGVLPNIQAVLLPK.K | 22 |
|  | pJS43\_100mM\_120812\_01.12773.12773.3 | 4.3713 | 0.3264 | 100.0% | 1932.8944 | 1932.3573 | 1 | 6.635 | 52.8% | 2 | K.VTIAQGGVLPNIQAVLLPK.K | 33 |

Similarities:
gi|106775678|ref|NP\_0(7:3)  
gi|4504255|ref|NP\_002(2:8)  
gi|113425815|ref|XP\_9(1:9)  

---

|  |  |  |  |  |  |  |  |  |
| --- | --- | --- | --- | --- | --- | --- | --- | --- |
| U | *gi|5729877|ref|NP\_006* | 68 | 840 | 63.2% | 646 | 70898 | 5.5 | heat shock 70kDa protein 8 isoform 1 [Homo sapiens] |

| Filename XCorr DeltCN Conf% ObsM+H+ CalcM+H+ SpR ZScore Ion% # Sequence  | | | | | | | | | | | | |
| --- | --- | --- | --- | --- | --- | --- | --- | --- | --- | --- | --- | --- |
|  | AstrinIP\_MS1\_022614\_02.06931.06931.3 | 3.9696 | 0.4267 | 100.0% | 2266.0144 | 2264.509 | 1 | 7.14 | 36.9% | 8 | K.GPAVGIDLGTTYSCVGVFQHGK.V | 3 |
|  | AstrinIP\_MS2\_022614\_01.07734.07734.1 | 2.2714 | 0.4224 | 99.4% | 1487.62 | 1488.5939 | 1 | 6.368 | 50.0% | 7 | R.TTPSYVAFTDTER.L | 11111 |
|  | AstrinIP\_MS1\_022614\_02.04868.04868.2 | 3.849 | 0.5598 | 100.0% | 1489.2922 | 1488.5939 | 1 | 9.39 | 79.2% | 43 | R.TTPSYVAFTDTER.L | 22222 |
|  | AstrinIP\_MS1\_022614\_01.07349.07349.2 | 5.1458 | 0.5614 | 100.0% | 1650.2122 | 1650.8468 | 1 | 10.415 | 82.1% | 40 | K.NQVAMNPTNTVFDAK.R | 2 |
|  | AstrinIP\_MS2\_022614\_01.06663.06663.2 | 4.6058 | 0.3665 | 100.0% | 1806.3522 | 1807.0343 | 1 | 7.549 | 60.0% | 11 | K.NQVAMNPTNTVFDAKR.L | 2 |
|  | AstrinIP\_MS1\_022614\_01.05186.05186.2 | 3.6406 | 0.4442 | 100.0% | 1411.1721 | 1411.5725 | 1 | 7.27 | 77.3% | 18 | R.RFDDAVVQSDMK.H | 2 |
|  | SKAPIP\_tube2\_041314\_01.09735.09735.3 | 6.0387 | 0.4818 | 100.0% | 3046.3442 | 3047.4792 | 1 | 7.361 | 36.0% | 5 | R.RFDDAVVQSDMKHWPFMVVNDAGRPK.V | 3 |
|  | SKAPIP\_tube2\_041314\_01.05789.05789.2 | 4.4067 | 0.3288 | 100.0% | 1256.0322 | 1255.385 | 1 | 7.888 | 90.0% | 13 | R.FDDAVVQSDMK.H | 2 |
|  | SKAPIP\_tube2\_041314\_01.10397.10397.3 | 4.6412 | 0.3964 | 100.0% | 2890.4343 | 2891.2917 | 2 | 6.922 | 28.1% | 2 | R.FDDAVVQSDMKHWPFMVVNDAGRPK.V | 3 |
|  | AstrinIP\_MS2\_022614\_01.08706.08706.2 | 3.2984 | 0.5125 | 100.0% | 1654.0922 | 1654.9298 | 1 | 7.831 | 65.4% | 11 | K.HWPFMVVNDAGRPK.V | 2 |
|  | SKAPIP\_tube2\_041314\_01.07922.07922.3 | 4.399 | 0.4498 | 100.0% | 1654.8544 | 1654.9298 | 1 | 7.264 | 46.2% | 34 | K.HWPFMVVNDAGRPK.V | 3 |
|  | AstrinIP\_MS2\_022614\_01.03561.03561.1 | 2.2284 | 0.2726 | 98.8% | 1180.55 | 1181.3312 | 1 | 5.221 | 66.7% | 1 | K.VQVEYKGETK.S | 11 |
|  | AstrinIP\_MS2\_022614\_01.03554.03554.2 | 3.2438 | 0.3839 | 100.0% | 1181.1721 | 1181.3312 | 1 | 6.258 | 77.8% | 6 | K.VQVEYKGETK.S | 22 |
|  | AstrinIP\_MS1\_022614\_01.10876.10876.1 | 2.7527 | 0.5396 | 100.0% | 1616.56 | 1617.8542 | 1 | 8.889 | 53.8% | 4 | K.SFYPEEVSSMVLTK.M | 1 |
|  | pDK365N\_300mM\_082713\_01.10388.10388.2 | 5.2889 | 0.5851 | 100.0% | 1618.3922 | 1617.8542 | 1 | 10.246 | 84.6% | 40 | K.SFYPEEVSSMVLTK.M | 2 |
|  | SKAPIP\_tube2\_041314\_01.05879.05879.1 | 2.5591 | 0.3483 | 98.7% | 1252.62 | 1253.4993 | 1 | 6.717 | 75.0% | 4 | K.MKEIAEAYLGK.T | 1 |
|  | SKAPIP\_tube2\_041314\_02.05344.05344.2 | 4.0131 | 0.318 | 100.0% | 1253.4922 | 1253.4993 | 1 | 6.581 | 90.0% | 37 | K.MKEIAEAYLGK.T | 2 |
|  | pDK339othertube\_033013\_01.05930.05930.3 | 3.3343 | 0.1714 | 98.6% | 1253.7544 | 1253.4993 | 52 | 4.876 | 42.5% | 15 | K.MKEIAEAYLGK.T | 3 |
|  | AstrinIP\_MS2\_022614\_01.06263.06263.1 | 2.1455 | 0.3922 | 99.5% | 993.36 | 994.1326 | 8 | 6.49 | 56.2% | 1 | K.EIAEAYLGK.T | 1 |
|  | AstrinIP\_MS2\_022614\_01.06258.06258.2 | 2.0109 | 0.2233 | 95.8% | 993.9522 | 994.1326 | 10 | 5.016 | 56.2% | 1 | K.EIAEAYLGK.T | 2 |
|  | pDK365N\_300mM\_082713\_01.09352.09352.2 | 4.1931 | 0.4275 | 100.0% | 1982.6122 | 1983.1882 | 1 | 7.17 | 70.6% | 35 | K.TVTNAVVTVPAYFNDSQR.Q | 2 |
|  | AstrinIP\_MS2\_022614\_01.09929.09929.3 | 4.5313 | 0.3892 | 100.0% | 1983.5643 | 1983.1882 | 1 | 7.124 | 45.6% | 15 | K.TVTNAVVTVPAYFNDSQR.Q | 3 |
|  | SKAPIP\_tube2\_041314\_01.09597.09597.2 | 5.3704 | 0.5527 | 100.0% | 1660.5322 | 1660.9078 | 1 | 10.193 | 83.3% | 26 | R.IINEPTAAAIAYGLDK.K | 22222 |
|  | pDK365N\_300mM\_082713\_03.09334.09334.3 | 3.8002 | 0.3215 | 100.0% | 1661.3043 | 1660.9078 | 1 | 6.407 | 51.7% | 1 | R.IINEPTAAAIAYGLDK.K | 33333 |
|  | pDK365N\_300mM\_082713\_01.08710.08710.2 | 4.8794 | 0.5104 | 100.0% | 1789.4321 | 1789.0819 | 1 | 8.201 | 78.1% | 16 | R.IINEPTAAAIAYGLDKK.V | 222 |
|  | pSKT11\_1\_020812\_01.05468.05468.2 | 4.7145 | 0.471 | 100.0% | 1692.0322 | 1692.6958 | 1 | 8.284 | 66.7% | 25 | K.STAGDTHLGGEDFDNR.M | 22 |
|  | SKAPIP\_tube2\_041314\_01.04275.04275.3 | 3.2573 | 0.4826 | 100.0% | 1693.9143 | 1692.6958 | 1 | 7.169 | 41.7% | 17 | K.STAGDTHLGGEDFDNR.M | 33 |
|  | SKAPIP\_tube2\_041314\_01.07432.07432.1 | 2.2146 | 0.3836 | 98.7% | 1235.53 | 1236.4741 | 1 | 6.846 | 61.1% | 1 | R.MVNHFIAEFK.R | 1 |
|  | pDK339othertube\_033013\_01.07352.07352.2 | 3.5709 | 0.5422 | 100.0% | 1236.1721 | 1236.4741 | 1 | 8.784 | 88.9% | 32 | R.MVNHFIAEFK.R | 2 |
|  | AstrinIP\_MS2\_022614\_01.08230.08230.3 | 3.4825 | 0.4315 | 100.0% | 1237.7344 | 1236.4741 | 1 | 6.984 | 58.3% | 26 | R.MVNHFIAEFK.R | 3 |
|  | AstrinIP\_MS1\_022614\_01.06455.06455.2 | 3.0328 | 0.4531 | 100.0% | 1391.9922 | 1392.6616 | 1 | 7.251 | 80.0% | 2 | R.MVNHFIAEFKR.K | 2 |
|  | SKAPIP\_tube2\_041314\_02.05560.05560.3 | 3.6765 | 0.4012 | 100.0% | 1393.4343 | 1392.6616 | 1 | 7.773 | 57.5% | 7 | R.MVNHFIAEFKR.K | 3 |
|  | 100326\_pJS43\_02.11478.11478.3 | 3.3718 | 0.2013 | 96.5% | 2998.7944 | 2999.255 | 1 | 4.227 | 26.0% | 1 | R.TLSSSTQASIEIDSLYEGIDFYTSITR.A | 3 |
|  | SKAPIP\_tube2\_041314\_02.11348.11348.2 | 4.0646 | 0.611 | 100.0% | 2999.0522 | 2999.255 | 1 | 10.672 | 26.9% | 3 | R.TLSSSTQASIEIDSLYEGIDFYTSITR.A | 2 |
|  | 100326\_pJS43\_01.07274.07274.2 | 4.1452 | 0.3207 | 100.0% | 1481.3922 | 1481.6511 | 1 | 7.03 | 77.3% | 22 | R.ARFEELNADLFR.G | 22 |
|  | SKAPIP\_tube2\_041314\_01.09082.09082.3 | 4.347 | 0.3293 | 100.0% | 1482.1743 | 1481.6511 | 1 | 5.773 | 56.8% | 14 | R.ARFEELNADLFR.G | 33 |
|  | AstrinIP\_MS2\_022614\_01.11079.11079.1 | 2.2045 | 0.4317 | 99.4% | 1253.55 | 1254.3849 | 5 | 6.105 | 50.0% | 1 | R.FEELNADLFR.G | 11 |
|  | pDK365N\_300mM\_082713\_01.10052.10052.2 | 3.5741 | 0.3498 | 100.0% | 1254.0122 | 1254.3849 | 2 | 7.449 | 77.8% | 19 | R.FEELNADLFR.G | 22 |
|  | 100326\_pJS43\_01.05345.05345.2 | 4.977 | 0.3839 | 100.0% | 1839.3522 | 1839.1019 | 2 | 9.686 | 68.8% | 13 | K.LDKSQIHDIVLVGGSTR.I | 2 |
|  | 100326\_pJS43\_02.04442.04442.3 | 5.6478 | 0.3349 | 100.0% | 1840.8243 | 1839.1019 | 2 | 8.328 | 45.3% | 38 | K.LDKSQIHDIVLVGGSTR.I | 3 |
|  | SKAPIP\_tube2\_041314\_01.06210.06210.1 | 3.6994 | 0.5249 | 100.0% | 1481.76 | 1482.6798 | 1 | 9.721 | 65.4% | 3 | K.SQIHDIVLVGGSTR.I | 1 |
|  | SKAPIP\_041314\_02.05449.05449.2 | 4.8246 | 0.6219 | 100.0% | 1483.1522 | 1482.6798 | 1 | 10.673 | 80.8% | 34 | K.SQIHDIVLVGGSTR.I | 2 |
|  | pDK365N\_300mM\_082713\_01.05726.05726.3 | 3.6395 | 0.3174 | 100.0% | 1483.7644 | 1482.6798 | 1 | 5.083 | 48.1% | 12 | K.SQIHDIVLVGGSTR.I | 3 |
|  | SKAPIP\_tube2\_041314\_02.05735.05735.3 | 3.8926 | 0.4466 | 100.0% | 1821.6543 | 1821.13 | 1 | 6.478 | 40.6% | 1 | K.SQIHDIVLVGGSTRIPK.I | 3 |
|  | AstrinIP\_MS1\_022614\_01.09870.09870.1 | 2.2447 | 0.2928 | 98.8% | 1081.36 | 1082.2444 | 1 | 4.936 | 75.0% | 7 | K.LLQDFFNGK.E | 111 |
|  | SKAPIP\_tube2\_041314\_01.09698.09698.2 | 3.13 | 0.3738 | 100.0% | 1082.1921 | 1082.2444 | 1 | 5.924 | 81.2% | 23 | K.LLQDFFNGK.E | 222 |
|  | SKAPIP\_tube2\_041314\_01.09189.09189.2 | 4.5852 | 0.3422 | 100.0% | 1566.0922 | 1566.7972 | 1 | 6.242 | 75.0% | 17 | K.LLQDFFNGKELNK.S | 222 |
|  | SKAPIP\_tube2\_041314\_01.12923.12923.2 | 4.9185 | 0.6389 | 100.0% | 2260.6921 | 2261.4937 | 1 | 10.361 | 47.7% | 16 | K.SINPDEAVAYGAAVQAAILSGDK.S | 2 |
|  | AstrinIP\_MS2\_022614\_01.13952.13952.3 | 5.3519 | 0.5915 | 100.0% | 2261.2144 | 2261.4937 | 1 | 9.58 | 37.5% | 8 | K.SINPDEAVAYGAAVQAAILSGDK.S | 3 |
|  | AstrinIP\_MS1\_022614\_01.19506.19506.2 | 4.2739 | 0.4207 | 100.0% | 3239.9722 | 3240.8206 | 3 | 7.115 | 25.0% | 1 | K.SENVQDLLLLDVTPLSLGIETAGGVMTVLIK.R | 2 |
|  | AstrinIP\_MS2\_022614\_01.20078.20078.3 | 3.5807 | 0.3187 | 99.9% | 3239.9944 | 3240.8206 | 1 | 5.513 | 28.3% | 1 | K.SENVQDLLLLDVTPLSLGIETAGGVMTVLIK.R | 3 |
|  | SKAPIP\_041314\_01.18338.18338.3 | 4.9097 | 0.1977 | 99.7% | 3397.7644 | 3397.008 | 2 | 7.055 | 22.6% | 4 | K.SENVQDLLLLDVTPLSLGIETAGGVMTVLIKR.N | 3 |
|  | pDK365N\_300mM\_082713\_03.09546.09546.3 | 5.6308 | 0.3746 | 100.0% | 3531.1743 | 3531.8577 | 1 | 8.272 | 32.5% | 3 | R.NTTIPTKQTQTFTTYSDNQPGVLIQVYEGER.A | 3 |
|  | AstrinIP\_MS2\_022614\_01.11600.11600.2 | 4.7896 | 0.4483 | 100.0% | 2774.8523 | 2775.9885 | 1 | 10.122 | 45.7% | 17 | K.QTQTFTTYSDNQPGVLIQVYEGER.A | 2 |
|  | 100326\_pJS43\_02.06500.06500.3 | 6.0481 | 0.4846 | 100.0% | 2776.8843 | 2775.9885 | 1 | 9.022 | 35.9% | 14 | K.QTQTFTTYSDNQPGVLIQVYEGER.A | 3 |
|  | 100326\_pJS43\_01.00359.00359.2 | 2.6233 | 0.3699 | 100.0% | 1018.47217 | 1018.1582 | 122 | 5.98 | 56.2% | 2 | K.ITITNDKGR.L | 22222 |
| \* | AstrinIP\_MS2\_022614\_01.03588.03588.2 | 2.5772 | 0.1194 | 98.3% | 989.6122 | 990.10144 | 5 | 4.764 | 85.7% | 1 | R.LSKEDIER.M | 2 |
|  | AstrinIP\_MS2\_022614\_01.03545.03545.2 | 1.9886 | 0.2475 | 96.6% | 1126.0122 | 1126.313 | 7 | 5.056 | 68.8% | 1 | R.MVQEAEKYK.A | 22 |
| \* | SKAPIP\_041314\_01.03947.03947.2 | 2.6631 | 0.223 | 98.7% | 1697.2722 | 1698.8854 | 1 | 5.043 | 65.4% | 1 | R.MVQEAEKYKAEDEK.Q | 32 |
| \* | SKAPIP\_041314\_01.03923.03923.3 | 3.0279 | 0.1665 | 95.0% | 1699.2544 | 1698.8854 | 1 | 4.25 | 46.2% | 1 | R.MVQEAEKYKAEDEK.Q | 3 |
| \* | AstrinIP\_MS2\_022614\_01.03512.03512.2 | 5.1515 | 0.4402 | 100.0% | 1982.3121 | 1983.2036 | 1 | 6.723 | 73.3% | 2 | R.MVQEAEKYKAEDEKQR.D | 2 |
| \* | SKAPIP\_041314\_01.03812.03812.3 | 4.9909 | 0.3906 | 100.0% | 1982.9043 | 1983.2036 | 1 | 7.539 | 53.3% | 7 | R.MVQEAEKYKAEDEKQR.D | 3 |
| \* | AstrinIP\_MS2\_022614\_01.03509.03509.2 | 2.0975 | 0.2922 | 98.8% | 1168.3322 | 1167.2639 | 62 | 4.777 | 62.5% | 1 | K.YKAEDEKQR.D | 2 |
| \* | SKAPIP\_tube2\_041314\_02.06093.06093.3 | 4.0263 | 0.3796 | 100.0% | 1949.0044 | 1949.1859 | 1 | 6.635 | 37.5% | 1 | R.DKVSSKNSLESYAFNMK.A | 3 |
| \* | AstrinIP\_MS2\_022614\_01.09354.09354.1 | 2.9995 | 0.4368 | 98.7% | 1303.43 | 1304.4602 | 1 | 6.957 | 65.0% | 3 | K.NSLESYAFNMK.A | 1 |
| \* | SKAPIP\_041314\_01.11603.11603.2 | 3.9484 | 0.3835 | 100.0% | 1305.5721 | 1304.4602 | 1 | 6.653 | 85.0% | 29 | K.NSLESYAFNMK.A | 2 |
| \* | SKAPIP\_041314\_01.09092.09092.3 | 3.8917 | 0.3797 | 100.0% | 2246.2444 | 2246.3953 | 1 | 6.748 | 41.2% | 6 | K.NQTAEKEEFEHQQKELEK.V | 3 |
| \* | SKAPIP\_041314\_01.08078.08078.2 | 2.5537 | 0.1971 | 99.3% | 945.0522 | 945.11316 | 20 | 3.956 | 78.6% | 9 | K.VCNPIITK.L | 2 |

Similarities:
gi|16507237|ref|NP\_00(2:66)  
gi|167466173|ref|NP\_0(4:64)  
gi|13676857|ref|NP\_06(17:51)  
gi|124256496|ref|NP\_0(5:63)  
gi|34419635|ref|NP\_00(6:62)  
contaminant\_GR78\_MAIZ(3:65)  

---

|  |  |  |  |  |  |  |  |  |
| --- | --- | --- | --- | --- | --- | --- | --- | --- |
| U | *gi|15147335|ref|NP\_06* | 30 | 86 | 62.9% | 579 | 65536 | 9.6 | nuclear receptor coactivator 5 [Homo sapiens] |

| Filename XCorr DeltCN Conf% ObsM+H+ CalcM+H+ SpR ZScore Ion% # Sequence  | | | | | | | | | | | | |
| --- | --- | --- | --- | --- | --- | --- | --- | --- | --- | --- | --- | --- |
| \* | SKAPIP\_041314\_01.07796.07796.2 | 2.7042 | 0.1761 | 98.9% | 1170.2722 | 1170.2266 | 15 | 4.62 | 66.7% | 3 | R.RDPYGFGDSR.D | 2 |
| \* | SKAPIP\_041314\_01.09730.09730.2 | 2.787 | 0.155 | 98.6% | 1521.1522 | 1521.6322 | 2 | 5.118 | 75.0% | 2 | R.RKDDSYFDRYR.D | 2 |
| \* | SKAPIP\_041314\_01.09782.09782.3 | 3.2656 | 0.2438 | 99.7% | 1521.5044 | 1521.6322 | 1 | 4.917 | 50.0% | 4 | R.RKDDSYFDRYR.D | 3 |
| \* | SKAPIP\_tube2\_041314\_01.03996.03996.2 | 1.7777 | 0.3755 | 97.2% | 2008.4321 | 2009.1019 | 1 | 4.976 | 41.2% | 1 | R.YRDSFDGRGPPGPESQSR.A | 2 |
| \* | SKAPIP\_tube2\_041314\_01.03974.03974.3 | 3.0663 | 0.3165 | 99.7% | 2009.5144 | 2009.1019 | 1 | 5.813 | 36.8% | 1 | R.YRDSFDGRGPPGPESQSR.A | 3 |
| \* | SKAPIP\_041314\_01.07466.07466.3 | 3.2644 | 0.2021 | 98.1% | 1689.6843 | 1689.7385 | 1 | 5.595 | 51.7% | 7 | R.DSFDGRGPPGPESQSR.A | 3 |
| \* | SKAPIP\_tube2\_041314\_01.05931.05931.1 | 1.6056 | 0.2395 | 96.5% | 1112.46 | 1113.2151 | 30 | 4.667 | 64.3% | 1 | R.QYFEEIQR.R | 1 |
| \* | SKAPIP\_tube2\_041314\_01.05870.05870.3 | 3.5572 | 0.251 | 99.6% | 2005.4043 | 2005.2476 | 1 | 5.032 | 40.6% | 2 | R.RFDAERPVDCSVIVVNK.Q | 3 |
| \* | SKAPIP\_tube2\_041314\_01.06713.06713.3 | 2.9376 | 0.2474 | 98.5% | 1849.3143 | 1849.06 | 2 | 4.804 | 36.7% | 1 | R.FDAERPVDCSVIVVNK.Q | 3 |
| \* | SKAPIP\_tube2\_041314\_01.18543.18543.3 | 5.086 | 0.4635 | 100.0% | 3119.4844 | 3120.5884 | 1 | 8.666 | 29.6% | 3 | K.VRDLGMVVDLIFLNTEVSLSQALEDVSR.G | 3 |
| \* | SKAPIP\_041314\_01.18900.18900.2 | 5.3865 | 0.5874 | 100.0% | 2865.1921 | 2865.2683 | 1 | 10.712 | 40.0% | 2 | R.DLGMVVDLIFLNTEVSLSQALEDVSR.G | 2 |
| \* | SKAPIP\_tube2\_041314\_01.07544.07544.2 | 3.3172 | 0.3125 | 99.9% | 1888.7322 | 1890.155 | 2 | 6.271 | 46.9% | 1 | R.GGSPFAIVITQQHQIHR.S | 2 |
| \* | SKAPIP\_tube2\_041314\_01.08588.08588.1 | 2.4727 | 0.2522 | 98.8% | 1415.65 | 1416.6967 | 2 | 6.517 | 45.8% | 1 | R.NMPQADAMVLVAR.N | 1 |
| \* | SKAPIP\_tube2\_041314\_02.06956.06956.2 | 3.4855 | 0.2661 | 99.9% | 1416.3922 | 1416.6967 | 1 | 6.492 | 75.0% | 3 | R.NMPQADAMVLVAR.N | 2 |
| \* | SKAPIP\_tube2\_041314\_01.05940.05940.1 | 1.8576 | 0.289 | 98.5% | 1175.36 | 1176.3301 | 59 | 4.708 | 55.6% | 1 | K.MADEAILQER.E | 1 |
| \* | SKAPIP\_tube2\_041314\_01.06044.06044.2 | 4.2793 | 0.3562 | 100.0% | 1175.9722 | 1176.3301 | 1 | 7.039 | 88.9% | 8 | K.MADEAILQER.E | 2 |
| \* | SKAPIP\_tube2\_041314\_01.12914.12914.2 | 5.4953 | 0.4502 | 100.0% | 1886.4722 | 1887.1489 | 1 | 8.615 | 70.6% | 4 | R.GGHPPAIQSLINLLADNR.Y | 2 |
| \* | SKAPIP\_tube2\_041314\_01.12932.12932.3 | 4.5597 | 0.2867 | 100.0% | 1886.9043 | 1887.1489 | 4 | 6.125 | 44.1% | 3 | R.GGHPPAIQSLINLLADNR.Y | 3 |
| \* | SKAPIP\_tube2\_041314\_01.14842.14842.3 | 3.9719 | 0.3781 | 100.0% | 3709.8843 | 3711.2126 | 1 | 6.426 | 25.8% | 2 | R.GGHPPAIQSLINLLADNRYLTAEETDKIINYLR.E | 3 |
| \* | SKAPIP\_tube2\_041314\_01.10760.10760.2 | 5.1705 | 0.4979 | 100.0% | 1842.4521 | 1843.0868 | 1 | 8.841 | 71.4% | 5 | R.YLTAEETDKIINYLR.E | 2 |
| \* | SKAPIP\_tube2\_041314\_01.10748.10748.3 | 4.0518 | 0.4418 | 100.0% | 1843.1943 | 1843.0868 | 1 | 8.09 | 58.9% | 6 | R.YLTAEETDKIINYLR.E | 3 |
| \* | SKAPIP\_tube2\_041314\_01.05228.05228.2 | 3.3917 | 0.2849 | 100.0% | 1217.2922 | 1217.3213 | 1 | 5.833 | 86.4% | 6 | R.SSTDSLPGPISR.Q | 2 |
| \* | SKAPIP\_041314\_01.08108.08108.2 | 1.9105 | 0.299 | 96.3% | 1129.5721 | 1130.2865 | 4 | 6.489 | 50.0% | 1 | R.QPLGATSGASLK.T | 2 |
| \* | SKAPIP\_041314\_01.07780.07780.1 | 1.3908 | 0.2659 | 95.4% | 1129.59 | 1130.2865 | 14 | 5.508 | 59.1% | 1 | R.QPLGATSGASLK.T | 1 |
| \* | SKAPIP\_tube2\_041314\_01.07068.07068.3 | 6.1782 | 0.4977 | 100.0% | 3489.2644 | 3488.8333 | 1 | 7.735 | 31.8% | 1 | K.TQPSSQPLQSGQVLPSATPTPSAPPTSQQELQAK.I | 3 |
| \* | SKAPIP\_tube2\_041314\_02.07535.07535.3 | 4.9707 | 0.531 | 100.0% | 4223.4546 | 4224.504 | 1 | 10.929 | 23.2% | 3 | K.ILSLFNSGTVTANSSSASPSVAAGNTPNQNFSTAANSQPQQR.S | 3 |
| \* | SKAPIP\_tube2\_041314\_01.08294.08294.3 | 4.9463 | 0.4592 | 100.0% | 3807.5942 | 3808.137 | 1 | 7.773 | 25.0% | 3 | R.SQASGNQPPSILGQGGSAQNMGPRPGAPSQGLFGQPSSR.L | 3 |
| \* | SKAPIP\_tube2\_041314\_01.06872.06872.2 | 4.0887 | 0.4915 | 100.0% | 2847.8523 | 2848.1602 | 1 | 7.841 | 38.5% | 1 | R.LAPASNMTSQRPVSSTGINFDNPSVQK.A | 2 |
| \* | SKAPIP\_041314\_01.11067.11067.3 | 5.6 | 0.5338 | 100.0% | 2847.9243 | 2848.1602 | 1 | 9.039 | 34.6% | 3 | R.LAPASNMTSQRPVSSTGINFDNPSVQK.A | 3 |
| \* | SKAPIP\_tube2\_041314\_01.12195.12195.3 | 5.9453 | 0.55 | 100.0% | 3684.0544 | 3685.1646 | 1 | 8.873 | 33.1% | 6 | K.ALDTLIQSGPALSHLVSQTTAQMGQPQAPMGSYQR.H | 3 |

---

|  |  |  |  |  |  |  |  |  |
| --- | --- | --- | --- | --- | --- | --- | --- | --- |
| U | *gi|221316642|ref|NP\_0* | 20 | 63 | 62.7% | 335 | 37721 | 5.2 | nuclear distribution gene E homolog 1 [Homo sapiens] |
| U | *gi|8923110|ref|NP\_060* | 20 | 63 | 62.7% | 335 | 37721 | 5.2 | nuclear distribution gene E homolog 1 [Homo sapiens] |

| Filename XCorr DeltCN Conf% ObsM+H+ CalcM+H+ SpR ZScore Ion% # Sequence  | | | | | | | | | | | | |
| --- | --- | --- | --- | --- | --- | --- | --- | --- | --- | --- | --- | --- |
|  | pDK339othertube\_033013\_01.07228.07228.2 | 3.6018 | 0.4309 | 100.0% | 1619.9922 | 1620.6691 | 2 | 7.787 | 62.5% | 2 | K.TFSSEEEEANYWK.D | 2 |
|  | pDK339othertube\_033013\_01.10792.10792.3 | 2.9726 | 0.3417 | 99.8% | 2443.0745 | 2443.6436 | 3 | 4.973 | 30.3% | 1 | K.TFSSEEEEANYWKDLAMTYK.Q | 3 |
|  | pDK339othertube\_033013\_01.05918.05918.3 | 3.4166 | 0.4675 | 100.0% | 1923.8644 | 1923.9908 | 1 | 7.191 | 45.0% | 3 | R.AENTQEELREFQEGSR.E | 3 |
|  | pSKT11\_1\_020812\_01.04038.04038.2 | 3.3517 | 0.1267 | 99.3% | 1500.3522 | 1500.6578 | 22 | 4.475 | 59.1% | 2 | R.NRDLLSENNRLR.M | 2 |
|  | pSKT11\_1\_020812\_01.04065.04065.3 | 3.5465 | 0.2033 | 99.5% | 1501.1943 | 1500.6578 | 13 | 4.722 | 43.2% | 2 | R.NRDLLSENNRLR.M | 3 |
|  | pSKT11\_1\_020812\_02.04258.04258.3 | 4.0565 | 0.1756 | 98.8% | 2354.8442 | 2354.64 | 4 | 4.587 | 40.3% | 1 | R.MELETIKEKFEVQHSEGYR.Q | 3 |
|  | pDK339\_033013\_01.03838.03838.2 | 2.92 | 0.3527 | 100.0% | 1251.6721 | 1252.3286 | 1 | 6.291 | 83.3% | 2 | K.FEVQHSEGYR.Q | 2 |
|  | pDK365N\_300mM\_082713\_01.07534.07534.2 | 4.0476 | 0.5256 | 100.0% | 1432.4321 | 1432.5707 | 1 | 8.347 | 75.0% | 6 | R.QISALEDDLAQTK.A | 2 |
|  | pDK339othertube\_033013\_01.04133.04133.2 | 3.0228 | 0.2354 | 99.7% | 1332.3121 | 1332.3666 | 3 | 4.994 | 65.0% | 2 | R.ELEQANDDLER.A | 2 |
|  | pDK339othertube\_033013\_01.09689.09689.2 | 3.7984 | 0.3296 | 100.0% | 1441.3322 | 1440.6111 | 1 | 6.02 | 77.3% | 5 | R.ATIMSLEDFEQR.L | 2 |
|  | pDK365N\_300mM\_082713\_04.07150.07150.3 | 3.9848 | 0.4552 | 100.0% | 2364.3843 | 2364.5713 | 1 | 7.484 | 35.5% | 4 | R.NAFLESELDEKENLLESVQR.L | 3 |
|  | pSKT11\_1\_020812\_02.05954.05954.2 | 5.4086 | 0.4926 | 100.0% | 2364.4521 | 2364.5713 | 1 | 8.558 | 55.3% | 1 | R.NAFLESELDEKENLLESVQR.L | 2 |
|  | pDK339othertube\_033013\_01.05520.05520.2 | 3.0418 | 0.1741 | 99.4% | 1328.1921 | 1328.5112 | 1 | 4.199 | 75.0% | 2 | R.DLRQELAVQQK.Q | 2 |
|  | pSKT11\_1\_020812\_01.07005.07005.3 | 4.1797 | 0.3761 | 100.0% | 1967.8744 | 1967.2357 | 2 | 6.767 | 45.0% | 2 | R.DLRQELAVQQKQEKPR.T | 3 |
|  | pDK339othertube\_033013\_01.04169.04169.2 | 2.5619 | 0.3286 | 99.7% | 1204.2922 | 1204.3405 | 1 | 5.71 | 75.0% | 4 | R.TPMPSSVEAER.T | 2 |
|  | pDk339\_033013\_02.04883.04883.3 | 5.6839 | 0.5174 | 100.0% | 3094.9744 | 3095.4114 | 1 | 8.476 | 29.3% | 2 | R.TPMPSSVEAERTDTAVQATGSVPSTPIAHR.G | 3 |
|  | pDK339othertube\_033013\_01.04634.04634.3 | 3.9898 | 0.5171 | 100.0% | 1909.6743 | 1910.0941 | 1 | 7.77 | 38.9% | 4 | R.TDTAVQATGSVPSTPIAHR.G | 3 |
|  | pDK365N\_300mM\_082713\_03.04857.04857.2 | 3.208 | 0.3937 | 100.0% | 1307.2522 | 1307.4056 | 1 | 8.318 | 70.8% | 5 | R.GPSSSLNTPGSFR.R | 2 |
|  | pDK365N\_300mM\_082713\_03.05297.05297.2 | 3.8389 | 0.5002 | 100.0% | 1529.2722 | 1529.647 | 1 | 8.065 | 73.3% | 6 | R.GLDDSTGGTPLTPAAR.I | 2 |
|  | pDK365N\_300mM\_082713\_03.13720.13720.2 | 4.6213 | 0.4285 | 100.0% | 1284.4521 | 1284.5419 | 1 | 8.013 | 90.9% | 7 | R.ISALNIVGDLLR.K | 2 |

---

|  |  |  |  |  |  |  |  |  |
| --- | --- | --- | --- | --- | --- | --- | --- | --- |
| U | *gi|4505773|ref|NP\_002* | 16 | 72 | 62.5% | 272 | 29804 | 5.8 | prohibitin [Homo sapiens] |

| Filename XCorr DeltCN Conf% ObsM+H+ CalcM+H+ SpR ZScore Ion% # Sequence  | | | | | | | | | | | | |
| --- | --- | --- | --- | --- | --- | --- | --- | --- | --- | --- | --- | --- |
| \* | pDK365N\_300mM\_082713\_02.09220.09220.3 | 3.643 | 0.3633 | 100.0% | 2371.8245 | 2372.689 | 1 | 5.727 | 30.4% | 1 | K.FGLALAVAGGVVNSALYNVDAGHR.A | 3 |
| \* | pDK365N\_300mM\_082713\_04.06201.06201.3 | 3.3575 | 0.3729 | 100.0% | 2082.5344 | 2082.408 | 2 | 6.491 | 31.9% | 3 | R.NVPVITGSKDLQNVNITLR.I | 3 |
| \* | pDK365N\_300mM\_082713\_01.07790.07790.2 | 3.3091 | 0.295 | 100.0% | 1186.3722 | 1186.3536 | 1 | 6.255 | 88.9% | 3 | K.DLQNVNITLR.I | 2 |
| \* | SKAPIP\_tube2\_041314\_01.07433.07433.2 | 2.5025 | 0.1967 | 98.0% | 1397.3121 | 1397.7067 | 3 | 4.87 | 63.6% | 1 | R.ILFRPVASQLPR.I | 2 |
| \* | pDK365N\_300mM\_082713\_01.07779.07779.3 | 3.2188 | 0.2418 | 99.5% | 1397.8444 | 1397.7067 | 1 | 5.728 | 45.5% | 6 | R.ILFRPVASQLPR.I | 3 |
| \* | pDK365N\_300mM\_082713\_03.07072.07072.2 | 3.7821 | 0.5257 | 100.0% | 1444.9922 | 1445.5255 | 1 | 8.848 | 81.8% | 13 | R.IFTSIGEDYDER.V | 2 |
| \* | pDK365N\_300mM\_082713\_03.07604.07604.2 | 3.3938 | 0.4507 | 100.0% | 1149.6921 | 1150.2767 | 1 | 7.975 | 77.8% | 12 | R.FDAGELITQR.E | 2 |
| \* | pDK365N\_100mM\_082813\_04.09245.09245.3 | 2.8841 | 0.3478 | 99.7% | 2120.9644 | 2120.4534 | 5 | 5.574 | 30.3% | 1 | R.AATFGLILDDVSLTHLTFGK.E | 3 |
| \* | pDK365N\_300mM\_082713\_02.09766.09766.3 | 3.8904 | 0.5204 | 100.0% | 3123.7444 | 3125.5457 | 3 | 8.493 | 24.1% | 4 | R.AATFGLILDDVSLTHLTFGKEFTEAVEAK.Q | 3 |
| \* | pDK365N\_300mM\_082713\_01.03356.03356.2 | 2.7473 | 0.2029 | 99.1% | 1334.9722 | 1334.5583 | 1 | 5.41 | 75.0% | 1 | R.FVVEKAEQQKK.A | 2 |
| \* | pDK365N\_100mM\_082713\_02.08516.08516.3 | 5.4262 | 0.4771 | 100.0% | 1999.1643 | 1999.2719 | 1 | 9.306 | 46.1% | 6 | K.AAELIANSLATAGDGLIELR.K | 3 |
| \* | pDK365N\_100mM\_082713\_02.08514.08514.2 | 4.5491 | 0.3144 | 100.0% | 1999.7922 | 1999.2719 | 1 | 7.503 | 60.5% | 5 | K.AAELIANSLATAGDGLIELR.K | 2 |
| \* | pDK365N\_300mM\_082713\_02.06542.06542.3 | 3.8791 | 0.2135 | 99.7% | 1607.5144 | 1607.804 | 19 | 5.567 | 36.5% | 5 | R.KLEAAEDIAYQLSR.S | 3 |
| \* | pDK365N\_300mM\_082713\_03.08309.08309.2 | 5.4575 | 0.4885 | 100.0% | 1608.2122 | 1607.804 | 1 | 8.794 | 84.6% | 8 | R.KLEAAEDIAYQLSR.S | 2 |
| \* | pDK365N\_300mM\_082713\_04.06790.06790.2 | 3.2533 | 0.2746 | 99.9% | 1479.4122 | 1479.6299 | 1 | 6.668 | 70.8% | 1 | K.LEAAEDIAYQLSR.S | 2 |
| \* | pDK365N\_300mM\_082713\_01.11028.11028.2 | 3.8236 | 0.2786 | 100.0% | 2099.612 | 2099.438 | 1 | 6.687 | 63.9% | 2 | R.SRNITYLPAGQSVLLQLPQ.- | 2 |

---

|  |  |  |  |  |  |  |  |  |
| --- | --- | --- | --- | --- | --- | --- | --- | --- |
| U | *gi|4506645|ref|NP\_000* | 11 | 75 | 61.4% | 70 | 8218 | 10.1 | ribosomal protein L38 [Homo sapiens] |
| U | *gi|78214522|ref|NP\_00* | 11 | 75 | 61.4% | 70 | 8218 | 10.1 | ribosomal protein L38 [Homo sapiens] |

| Filename XCorr DeltCN Conf% ObsM+H+ CalcM+H+ SpR ZScore Ion% # Sequence  | | | | | | | | | | | | |
| --- | --- | --- | --- | --- | --- | --- | --- | --- | --- | --- | --- | --- |
|  | pJS43\_100mM\_120812\_01.10020.10020.2 | 5.0857 | 0.4356 | 100.0% | 1576.4521 | 1576.8766 | 1 | 8.585 | 83.3% | 7 | R.KIEEIKDFLLTAR.R | 2 |
|  | pJS43\_100mM\_120812\_01.10161.10161.3 | 4.4222 | 0.3878 | 100.0% | 1577.2444 | 1576.8766 | 1 | 7.18 | 54.2% | 13 | R.KIEEIKDFLLTAR.R | 3 |
|  | pDK365N\_100mM\_082813\_03.09866.09866.2 | 4.0024 | 0.458 | 100.0% | 1448.5922 | 1448.7025 | 1 | 7.596 | 77.3% | 5 | K.IEEIKDFLLTAR.R | 2 |
|  | pDK365N\_100mM\_082813\_03.09893.09893.3 | 4.1346 | 0.2982 | 100.0% | 1449.1444 | 1448.7025 | 1 | 5.492 | 50.0% | 6 | K.IEEIKDFLLTAR.R | 3 |
|  | pJS43\_100mM\_120812\_01.10445.10445.2 | 3.4918 | 0.4577 | 100.0% | 1230.2322 | 1229.4589 | 1 | 7.539 | 83.3% | 4 | R.YLYTLVITDK.E | 2 |
|  | pJS43\_100mM\_120812\_01.09048.09048.2 | 3.9415 | 0.419 | 100.0% | 1485.7122 | 1486.7484 | 1 | 7.835 | 86.4% | 20 | R.YLYTLVITDKEK.A | 2 |
|  | pJS43\_100mM\_120812\_01.08976.08976.3 | 2.8337 | 0.3964 | 100.0% | 1486.7344 | 1486.7484 | 1 | 6.329 | 52.3% | 7 | R.YLYTLVITDKEK.A | 3 |
|  | 100326\_pJS43\_02.04644.04644.2 | 3.1045 | 0.3067 | 99.8% | 1814.7522 | 1815.1168 | 1 | 5.982 | 50.0% | 1 | R.YLYTLVITDKEKAEK.L | 2 |
|  | AstrinNocIP\_020510\_01.02424.02424.3 | 2.8178 | 0.2924 | 99.0% | 1950.9243 | 1950.3727 | 127 | 4.547 | 26.5% | 1 | K.AEKLKQSLPPGLAVKELK.- | 3 |
|  | pDK365N\_100mM\_082713\_01.05265.05265.2 | 3.2441 | 0.2386 | 99.8% | 1251.5721 | 1251.5553 | 9 | 6.022 | 63.6% | 4 | K.LKQSLPPGLAVK.E | 2 |
|  | pJS43\_100mM\_120812\_01.06629.06629.2 | 2.1015 | 0.289 | 98.5% | 1010.15216 | 1010.22174 | 35 | 5.826 | 66.7% | 7 | K.QSLPPGLAVK.E | 2 |

---

|  |  |  |  |  |  |  |  |  |
| --- | --- | --- | --- | --- | --- | --- | --- | --- |
| U | *gi|27436946|ref|NP\_73* | 56 | 290 | 61.3% | 664 | 74140 | 7.0 | lamin A/C isoform 1 precursor [Homo sapiens] |

| Filename XCorr DeltCN Conf% ObsM+H+ CalcM+H+ SpR ZScore Ion% # Sequence  | | | | | | | | | | | | |
| --- | --- | --- | --- | --- | --- | --- | --- | --- | --- | --- | --- | --- |
|  | pDK339othertube\_033013\_01.03662.03662.2 | 3.542 | 0.4267 | 100.0% | 1440.1522 | 1440.4667 | 1 | 7.646 | 69.2% | 2 | R.SGAQASSTPLS\*PTR.I | 2 |
|  | pDK365N\_300mM\_082713\_03.04383.04383.2 | 4.8748 | 0.3502 | 100.0% | 1630.3922 | 1630.7521 | 1 | 7.613 | 87.5% | 5 | R.LQEKEDLQELNDR.L | 2 |
|  | pDK339\_033013\_01.04687.04687.3 | 4.3309 | 0.2262 | 99.9% | 1631.7843 | 1630.7521 | 2 | 4.836 | 56.2% | 7 | R.LQEKEDLQELNDR.L | 3 |
|  | pDK365N\_300mM\_082713\_01.10019.10019.3 | 3.3863 | 0.2621 | 99.4% | 2462.5745 | 2461.7344 | 1 | 4.785 | 34.2% | 1 | R.LQEKEDLQELNDRLAVYIDR.V | 3 |
|  | pDK339\_033013\_01.05890.05890.1 | 1.7857 | 0.2237 | 97.5% | 849.41 | 850.0055 | 8 | 5.382 | 66.7% | 1 | R.LAVYIDR.V | 1 |
|  | pDK339othertube\_033013\_01.04107.04107.2 | 3.11 | 0.3603 | 100.0% | 1089.9122 | 1090.1783 | 1 | 6.764 | 83.3% | 6 | R.SLETENAGLR.L | 2 |
|  | pDK339othertube\_033013\_02.04426.04426.2 | 3.9439 | 0.4322 | 100.0% | 1417.7922 | 1418.5901 | 1 | 7.622 | 81.8% | 3 | R.LRITESEEVVSR.E | 2 |
|  | pDK365N\_300mM\_082713\_04.04192.04192.3 | 3.0922 | 0.2581 | 99.6% | 1418.2144 | 1418.5901 | 48 | 4.841 | 40.9% | 1 | R.LRITESEEVVSR.E | 3 |
|  | pDK339othertube\_033013\_01.03694.03694.2 | 3.5344 | 0.4896 | 100.0% | 1149.0322 | 1149.2432 | 1 | 8.606 | 88.9% | 5 | R.ITESEEVVSR.E | 2 |
|  | pDK365N\_300mM\_082713\_03.04486.04486.2 | 3.1971 | 0.1981 | 99.7% | 1167.3121 | 1166.2328 | 1 | 8.258 | 80.0% | 6 | K.AAYEAELGDAR.K | 2 |
|  | pDK339\_033013\_01.04113.04113.2 | 3.4426 | 0.3408 | 100.0% | 1293.7322 | 1294.4069 | 1 | 7.484 | 77.3% | 2 | K.AAYEAELGDARK.T | 2 |
|  | AstrinIP\_MS2\_022614\_01.13389.13389.3 | 2.935 | 0.2319 | 97.1% | 2346.2344 | 2346.56 | 4 | 3.875 | 29.2% | 1 | R.KT#LDSVAKERARLQLELS\*K.V | 3 |
|  | pJS43\_100mM\_120812\_01.04663.04663.2 | 2.9294 | 0.3127 | 99.9% | 1043.9722 | 1044.1527 | 2 | 6.724 | 83.3% | 1 | K.EGDLIAAQAR.L | 2 |
|  | pDK339\_033013\_01.09905.09905.3 | 3.4981 | 0.1022 | 95.7% | 1244.5743 | 1244.474 | 10 | 4.737 | 50.0% | 2 | R.LKDLEALLNSK.E | 3 |
|  | pDK339\_033013\_01.09890.09890.2 | 3.8299 | 0.3342 | 100.0% | 1245.3322 | 1244.474 | 1 | 6.449 | 85.0% | 7 | R.LKDLEALLNSK.E | 2 |
|  | pDK339\_033013\_01.05631.05631.2 | 2.6187 | 0.3908 | 99.9% | 1119.8522 | 1120.245 | 8 | 6.71 | 65.0% | 5 | K.EAALSTALSEK.R | 2 |
|  | pSKT11\_1\_020812\_01.05292.05292.2 | 3.1686 | 0.2745 | 99.9% | 1277.1522 | 1276.4325 | 1 | 6.251 | 68.2% | 7 | K.EAALSTALSEKR.T | 2 |
|  | pDK339othertube\_033013\_01.06540.06540.2 | 2.9717 | 0.3344 | 100.0% | 1182.7122 | 1183.3066 | 1 | 7.088 | 88.9% | 3 | R.TLEGELHDLR.G | 2 |
|  | pDK339\_033013\_01.08429.08429.2 | 3.8938 | 0.2609 | 100.0% | 1666.4521 | 1666.8748 | 1 | 6.308 | 67.9% | 1 | R.TLEGELHDLRGQVAK.L | 2 |
|  | pJS43\_100mM\_120812\_01.03585.03585.2 | 2.6608 | 0.2391 | 99.5% | 1030.0721 | 1030.2096 | 1 | 5.712 | 83.3% | 1 | K.LEAALGEAKK.Q | 2 |
|  | pDK339\_033013\_01.04357.04357.2 | 2.6429 | 0.2916 | 99.8% | 1160.9722 | 1161.3617 | 1 | 5.489 | 81.2% | 2 | K.KQLQDEMLR.R | 2 |
|  | pDK339\_033013\_01.06967.06967.2 | 3.7017 | 0.3436 | 100.0% | 1510.2122 | 1510.7455 | 1 | 6.962 | 81.8% | 5 | R.LQTMKEELDFQK.N | 2 |
|  | pDK339\_033013\_01.07015.07015.3 | 3.4132 | 0.198 | 99.2% | 1510.9443 | 1510.7455 | 3 | 4.77 | 45.5% | 3 | R.LQTMKEELDFQK.N | 3 |
|  | pDK339othertube\_033013\_01.05468.05468.2 | 2.1196 | 0.2358 | 98.2% | 1023.89215 | 1024.1185 | 3 | 4.938 | 71.4% | 2 | K.NIYSEELR.E | 2 |
|  | pDK339\_033013\_01.04859.04859.2 | 2.737 | 0.2643 | 99.6% | 1382.5122 | 1382.5132 | 1 | 5.794 | 65.0% | 1 | K.NIYSEELRETK.R | 2 |
|  | pDK339\_033013\_01.08408.08408.1 | 1.9923 | 0.2286 | 97.9% | 1028.43 | 1029.1814 | 6 | 5.643 | 56.2% | 1 | R.LADALQELR.A | 1 |
|  | pDK339othertube\_033013\_01.07196.07196.2 | 3.8907 | 0.287 | 100.0% | 1029.1721 | 1029.1814 | 1 | 6.244 | 87.5% | 12 | R.LADALQELR.A | 2 |
|  | pJS43\_100mM\_120812\_01.03039.03039.2 | 3.7612 | 0.4479 | 100.0% | 1502.4922 | 1503.6115 | 1 | 6.874 | 68.2% | 1 | R.AQHEDQVEQYKK.E | 2 |
|  | pJS43\_100mM\_120812\_01.03033.03033.3 | 3.3711 | 0.3612 | 100.0% | 1503.9844 | 1503.6115 | 155 | 5.807 | 40.9% | 1 | R.AQHEDQVEQYKK.E | 3 |
|  | pDK339\_033013\_01.05833.05833.2 | 5.0412 | 0.5008 | 100.0% | 1752.6921 | 1753.8693 | 1 | 8.441 | 76.7% | 10 | R.NSNLVGAAHEELQQSR.I | 2 |
|  | pDk339\_033013\_02.04662.04662.3 | 3.0105 | 0.2783 | 99.3% | 1753.9143 | 1753.8693 | 27 | 5.69 | 33.3% | 3 | R.NSNLVGAAHEELQQSR.I | 3 |
|  | pDK339\_033013\_01.10491.10491.2 | 4.6916 | 0.4074 | 100.0% | 1700.6122 | 1700.9762 | 1 | 7.594 | 67.9% | 6 | R.IRIDSLSAQLSQLQK.Q | 2 |
|  | pDK365N\_300mM\_082713\_03.08927.08927.3 | 3.4908 | 0.3061 | 99.9% | 1700.6943 | 1700.9762 | 1 | 5.712 | 46.4% | 3 | R.IRIDSLSAQLSQLQK.Q | 3 |
|  | pDK365N\_300mM\_082713\_01.08744.08744.2 | 3.7421 | 0.2612 | 100.0% | 1431.2722 | 1431.6293 | 1 | 5.591 | 79.2% | 6 | R.IDSLSAQLSQLQK.Q | 2 |
|  | pDK339\_033013\_01.05625.05625.2 | 3.0964 | 0.3236 | 100.0% | 1188.4122 | 1188.3262 | 1 | 5.95 | 88.9% | 13 | K.LRDLEDSLAR.E | 2 |
|  | pDK365N\_300mM\_082713\_01.04894.04894.3 | 3.0766 | 0.1367 | 95.7% | 1188.7444 | 1188.3262 | 1 | 4.446 | 58.3% | 1 | K.LRDLEDSLAR.E | 3 |
|  | pDK365N\_300mM\_082713\_03.05303.05303.2 | 2.1636 | 0.1748 | 96.4% | 918.8522 | 918.97925 | 5 | 4.067 | 78.6% | 2 | R.DLEDSLAR.E | 2 |
|  | pDK339\_033013\_01.11677.11677.2 | 5.1505 | 0.4712 | 100.0% | 1894.3322 | 1895.1346 | 1 | 8.451 | 78.6% | 12 | R.MQQQLDEYQELLDIK.L | 2 |
|  | pDK339\_033013\_01.09344.09344.2 | 2.4849 | 0.3643 | 99.8% | 1331.9922 | 1332.5603 | 1 | 6.13 | 60.0% | 1 | K.LALDMEIHAYR.K | 2 |
|  | AstrinIP\_MS2\_022614\_01.03624.03624.2 | 2.3554 | 0.1304 | 95.8% | 1103.4521 | 1103.2175 | 12 | 4.01 | 68.8% | 1 | R.KLLEGEEER.L | 222 |
|  | pJS43\_100mM\_120812\_01.03761.03761.2 | 2.4416 | 0.1907 | 98.9% | 974.9122 | 975.0434 | 42 | 3.925 | 71.4% | 1 | K.LLEGEEER.L | 222 |
|  | pDK365N\_300mM\_082713\_03.04283.04283.2 | 2.3497 | 0.1869 | 96.9% | 1401.8922 | 1402.4208 | 6 | 5.108 | 70.0% | 3 | R.LRLS\*PS\*PTSQR.S | 2 |
|  | pDK339\_033013\_01.03890.03890.2 | 3.5715 | 0.4505 | 100.0% | 1203.8922 | 1204.2762 | 1 | 8.895 | 85.0% | 1 | R.VAVEEVDEEGK.F | 2 |
|  | pDK339\_033013\_01.06037.06037.2 | 4.2421 | 0.477 | 100.0% | 1606.4321 | 1606.7728 | 1 | 9.194 | 76.9% | 9 | R.VAVEEVDEEGKFVR.L | 2 |
|  | pDk339\_033013\_02.04721.04721.3 | 2.7615 | 0.305 | 99.5% | 1606.8544 | 1606.7728 | 9 | 5.05 | 36.5% | 7 | R.VAVEEVDEEGKFVR.L | 3 |
|  | pDK365N\_300mM\_082713\_03.04290.04290.3 | 4.2401 | 0.3425 | 100.0% | 1935.5343 | 1936.1094 | 1 | 6.751 | 41.7% | 2 | R.NKSNEDQSMGNWQIKR.Q | 3 |
|  | pDK365N\_300mM\_082713\_01.06899.06899.2 | 4.1135 | 0.359 | 100.0% | 1537.4922 | 1537.644 | 2 | 6.183 | 62.5% | 8 | K.SNEDQSMGNWQIK.R | 2 |
|  | pDK339\_033013\_01.05641.05641.3 | 2.9224 | 0.3281 | 99.8% | 1693.9443 | 1693.8315 | 13 | 5.457 | 34.6% | 2 | K.SNEDQSMGNWQIKR.Q | 3 |
|  | pDK365N\_300mM\_082713\_03.06887.06887.2 | 2.6502 | 0.4591 | 100.0% | 1292.4922 | 1292.391 | 1 | 6.96 | 60.0% | 8 | R.QNGDDPLLTYR.F | 2 |
|  | pSKT11\_1\_020812\_02.06239.06239.2 | 4.1897 | 0.5451 | 100.0% | 2533.3323 | 2534.8772 | 1 | 8.874 | 39.6% | 1 | K.AGQVVTIWAAGAGATHSPPTDLVWK.A | 2 |
|  | pDk339\_033013\_02.07696.07696.3 | 4.879 | 0.4719 | 100.0% | 2534.4844 | 2534.8772 | 1 | 8.878 | 38.5% | 15 | K.AGQVVTIWAAGAGATHSPPTDLVWK.A | 3 |
|  | pJS43\_100mM\_120812\_02.05322.05322.2 | 4.4432 | 0.5404 | 100.0% | 1492.2722 | 1492.6874 | 1 | 9.951 | 76.9% | 30 | R.TALINSTGEEVAMR.K | 2 |
|  | pDK339othertube\_033013\_02.04416.04416.3 | 4.9677 | 0.6098 | 100.0% | 2365.2244 | 2366.504 | 1 | 10.127 | 42.3% | 14 | K.ASASGSGAQVGGPISSGSSASSVTVTR.S | 3 |
|  | pDK339othertube\_033013\_01.05067.05067.2 | 5.9432 | 0.5938 | 100.0% | 2365.4922 | 2366.504 | 1 | 11.155 | 61.5% | 5 | K.ASASGSGAQVGGPISSGSSASSVTVTR.S | 2 |
|  | pDK339othertube\_033013\_02.05169.05169.2 | 4.5149 | 0.5504 | 100.0% | 1567.4321 | 1567.6555 | 1 | 9.125 | 59.4% | 21 | R.SVGGSGGGSFGDNLVTR.S | 2 |
|  | pDk339\_033013\_02.05326.05326.2 | 4.3662 | 0.4409 | 100.0% | 1647.0721 | 1647.6555 | 1 | 7.194 | 59.4% | 10 | R.SVGGSGGGS\*FGDNLVTR.S | 2 |

Similarities:
gi|5031877|ref|NP\_005(2:54)  
gi|27436951|ref|NP\_11(2:54)  

---

|  |  |  |  |  |  |  |  |  |
| --- | --- | --- | --- | --- | --- | --- | --- | --- |
| U | *gi|32189394|ref|NP\_00* | 29 | 190 | 60.9% | 529 | 56560 | 5.4 | mitochondrial ATP synthase beta subunit precursor [Homo sapiens] |

| Filename XCorr DeltCN Conf% ObsM+H+ CalcM+H+ SpR ZScore Ion% # Sequence  | | | | | | | | | | | | |
| --- | --- | --- | --- | --- | --- | --- | --- | --- | --- | --- | --- | --- |
| \* | pDK365N\_100mM\_082713\_01.07880.07880.2 | 4.4153 | 0.5427 | 100.0% | 1651.4321 | 1651.9034 | 1 | 9.616 | 71.4% | 11 | R.LVLEVAQHLGESTVR.T | 2 |
| \* | pDK365N\_300mM\_082713\_01.08558.08558.3 | 3.9957 | 0.5103 | 100.0% | 1652.7244 | 1651.9034 | 1 | 8.117 | 53.6% | 13 | R.LVLEVAQHLGESTVR.T | 3 |
| \* | pDK365N\_300mM\_082713\_01.06914.06914.2 | 3.6405 | 0.494 | 100.0% | 1264.3121 | 1263.4515 | 1 | 9.03 | 86.4% | 9 | R.TIAMDGTEGLVR.G | 2 |
| \* | SKAPIP\_tube2\_041314\_01.08964.08964.2 | 3.534 | 0.464 | 100.0% | 1920.5922 | 1920.2596 | 1 | 7.289 | 58.3% | 2 | K.VLDSGAPIKIPVGPETLGR.I | 2 |
| \* | pDK365N\_100mM\_082813\_03.08807.08807.3 | 5.1204 | 0.4521 | 100.0% | 1921.4043 | 1920.2596 | 1 | 9.021 | 43.1% | 16 | K.VLDSGAPIKIPVGPETLGR.I | 3 |
| \* | pDK365N\_300mM\_082713\_01.05474.05474.2 | 2.037 | 0.2732 | 97.7% | 1039.0721 | 1039.2198 | 3 | 5.823 | 61.1% | 3 | K.IPVGPETLGR.I | 2 |
| \* | SKAPIP\_tube2\_041314\_01.08033.08033.2 | 4.5961 | 0.3826 | 100.0% | 1387.3522 | 1386.6061 | 1 | 6.88 | 81.8% | 10 | R.IMNVIGEPIDER.G | 2 |
| \* | SKAPIP\_tube2\_041314\_01.07752.07752.2 | 2.3728 | 0.2332 | 97.3% | 1780.9321 | 1782.1083 | 2 | 5.376 | 46.7% | 1 | R.IMNVIGEPIDERGPIK.T | 2 |
| \* | pDK365N\_300mM\_082713\_01.08067.08067.3 | 2.923 | 0.3435 | 99.9% | 1782.4744 | 1782.1083 | 1 | 5.157 | 41.7% | 5 | R.IMNVIGEPIDERGPIK.T | 3 |
| \* | pDK365N\_300mM\_082713\_01.09322.09322.2 | 2.691 | 0.3241 | 99.9% | 1089.3922 | 1089.3202 | 2 | 6.039 | 72.2% | 4 | K.VVDLLAPYAK.G | 2 |
| \* | pDK365N\_300mM\_082713\_01.08282.08282.2 | 3.2179 | 0.4 | 100.0% | 976.1722 | 976.1637 | 1 | 7.294 | 75.0% | 7 | K.IGLFGGAGVGK.T | 2 |
| \* | pDK365N\_100mM\_082713\_01.13036.13036.2 | 4.8017 | 0.3308 | 100.0% | 1459.5521 | 1458.7998 | 1 | 7.744 | 79.2% | 10 | K.TVLIMELINNVAK.A | 2 |
| \* | pDK365N\_300mM\_082713\_01.06101.06101.2 | 4.1432 | 0.4437 | 100.0% | 1407.6721 | 1407.5283 | 1 | 10.158 | 73.1% | 3 | K.AHGGYSVFAGVGER.T | 2 |
| \* | pDK365N\_300mM\_082713\_02.05303.05303.3 | 2.8644 | 0.254 | 98.8% | 1407.8644 | 1407.5283 | 14 | 5.334 | 40.4% | 4 | K.AHGGYSVFAGVGER.T | 3 |
| \* | pDK365N\_100mM\_082813\_03.10037.10037.3 | 4.9525 | 0.2465 | 100.0% | 2319.7744 | 2319.5945 | 4 | 5.543 | 38.2% | 2 | R.TREGNDLYHEMIESGVINLK.D | 3 |
| \* | pDK365N\_100mM\_082813\_03.11019.11019.2 | 2.2948 | 0.2567 | 97.5% | 2061.412 | 2062.302 | 180 | 5.112 | 29.4% | 1 | R.EGNDLYHEMIESGVINLK.D | 2 |
| \* | pDK365N\_300mM\_082713\_02.07872.07872.3 | 4.0693 | 0.279 | 99.9% | 2063.9343 | 2062.302 | 3 | 5.241 | 38.2% | 4 | R.EGNDLYHEMIESGVINLK.D | 3 |
| \* | pDK365N\_100mM\_082713\_01.06606.06606.2 | 4.4056 | 0.3977 | 100.0% | 1602.5322 | 1602.8486 | 1 | 8.762 | 82.1% | 8 | K.VALVYGQMNEPPGAR.A | 2 |
| \* | pDK365N\_300mM\_082713\_03.11638.11638.2 | 3.9379 | 0.5443 | 100.0% | 1440.7522 | 1440.6824 | 1 | 9.697 | 66.7% | 2 | R.VALTGLTVAEYFR.D | 2 |
| \* | pDK365N\_300mM\_082713\_02.12410.12410.3 | 4.6337 | 0.5031 | 100.0% | 3344.3044 | 3344.7917 | 1 | 7.665 | 28.6% | 4 | R.VALTGLTVAEYFRDQEGQDVLLFIDNIFR.F | 3 |
| \* | pDK365N\_100mM\_082713\_01.14996.14996.2 | 4.9267 | 0.6324 | 100.0% | 1922.8722 | 1923.1326 | 1 | 10.562 | 66.7% | 18 | R.DQEGQDVLLFIDNIFR.F | 2 |
| \* | pDK365N\_300mM\_082713\_01.09563.09563.2 | 4.5418 | 0.4467 | 100.0% | 1437.3922 | 1436.6078 | 1 | 8.618 | 73.1% | 12 | R.FTQAGSEVSALLGR.I | 2 |
| \* | pDK365N\_100mM\_082813\_03.08805.08805.2 | 5.7205 | 0.5645 | 100.0% | 2266.7722 | 2267.577 | 1 | 10.811 | 65.0% | 5 | R.IPSAVGYQPTLATDMGTMQER.I | 2 |
| \* | pDK365N\_300mM\_082713\_01.12225.12225.3 | 5.7729 | 0.5204 | 100.0% | 3717.0244 | 3717.124 | 1 | 8.902 | 23.6% | 8 | K.GSITSVQAIYVPADDLTDPAPATTFAHLDATTVLSR.A | 3 |
| \* | pDK365N\_100mM\_082813\_03.10974.10974.2 | 4.1965 | 0.4624 | 100.0% | 1988.5521 | 1989.2328 | 1 | 9.231 | 52.8% | 12 | R.AIAELGIYPAVDPLDSTSR.I | 2 |
| \* | pDK365N\_300mM\_082713\_03.06488.06488.3 | 2.9303 | 0.4167 | 100.0% | 1817.5144 | 1817.0264 | 2 | 5.816 | 40.0% | 4 | R.IMDPNIVGSEHYDVAR.G | 3 |
| \* | pDK365N\_100mM\_082813\_03.13902.13902.3 | 3.4962 | 0.3578 | 100.0% | 2675.9944 | 2677.0393 | 1 | 6.69 | 34.8% | 4 | K.SLQDIIAILGMDELSEEDKLTVSR.A | 3 |
| \* | pDK365N\_300mM\_082713\_03.14398.14398.2 | 3.5368 | 0.6072 | 100.0% | 2677.0322 | 2677.0393 | 1 | 10.805 | 50.0% | 2 | K.SLQDIIAILGMDELSEEDKLTVSR.A | 2 |
| \* | pDK365N\_300mM\_082713\_02.08542.08542.3 | 3.1622 | 0.2937 | 99.6% | 2024.3043 | 2024.3447 | 2 | 5.841 | 35.3% | 6 | R.FLSQPFQVAEVFTGHMGK.L | 3 |

---

|  |  |  |  |  |  |  |  |  |
| --- | --- | --- | --- | --- | --- | --- | --- | --- |
| U | *gi|55956899|ref|NP\_00* | 37 | 168 | 60.8% | 623 | 62064 | 5.2 | keratin 9 [Homo sapiens] |

| Filename XCorr DeltCN Conf% ObsM+H+ CalcM+H+ SpR ZScore Ion% # Sequence  | | | | | | | | | | | | |
| --- | --- | --- | --- | --- | --- | --- | --- | --- | --- | --- | --- | --- |
| \* | AstrinIP\_MS2\_022614\_01.03843.03843.2 | 4.5157 | 0.499 | 100.0% | 1233.0721 | 1233.2833 | 1 | 8.465 | 70.0% | 5 | R.SGGGGGGGLGSGGSIR.S | 2 |
| \* | pSKT11\_1\_020812\_01.03684.03684.2 | 2.3222 | 0.2916 | 98.6% | 2198.8123 | 2200.2021 | 24 | 5.476 | 25.0% | 1 | R.FSSSGGGGGGGRFSSSSGYGGGSSR.V | 2 |
| \* | pDk339\_033013\_02.03592.03592.3 | 3.8521 | 0.3602 | 100.0% | 2199.5645 | 2200.2021 | 1 | 6.965 | 33.3% | 2 | R.FSSSGGGGGGGRFSSSSGYGGGSSR.V | 3 |
|  | pSKT11\_1\_020812\_02.05475.05475.2 | 5.5024 | 0.5975 | 100.0% | 2705.3323 | 2706.7605 | 1 | 11.812 | 37.1% | 5 | R.GGGGSFGYSYGGGSGGGFSASSLGGGFGGGSR.G | 2 |
|  | 100326\_pJS43\_02.05930.05930.3 | 5.557 | 0.5593 | 100.0% | 2707.0745 | 2706.7605 | 1 | 8.685 | 36.3% | 8 | R.GGGGSFGYSYGGGSGGGFSASSLGGGFGGGSR.G | 3 |
|  | pJS43\_100mM\_120812\_01.03870.03870.2 | 2.8701 | 0.1985 | 99.6% | 1066.0122 | 1066.1742 | 1 | 6.434 | 87.5% | 1 | K.STMQELNSR.L | 2 |
|  | SKAPIP\_041314\_01.07463.07463.1 | 1.8759 | 0.3024 | 98.8% | 809.61 | 809.93774 | 30 | 5.035 | 66.7% | 7 | R.LASYLDK.V | 111111 |
|  | 100326\_pJS43\_01.07829.07829.2 | 6.0137 | 0.4991 | 100.0% | 2377.412 | 2378.5981 | 1 | 10.991 | 62.5% | 1 | R.LASYLDKVQALEEANNDLENK.I | 2 |
|  | pJS43\_100mM\_120812\_01.10416.10416.3 | 4.7275 | 0.3674 | 100.0% | 2378.9644 | 2378.5981 | 1 | 7.121 | 38.8% | 1 | R.LASYLDKVQALEEANNDLENK.I | 3 |
|  | 100326\_pJS43\_01.10473.10473.3 | 4.8993 | 0.4452 | 100.0% | 3326.8442 | 3327.6287 | 1 | 6.117 | 33.3% | 1 | R.LASYLDKVQALEEANNDLENKIQDWYDK.K | 3 |
|  | 100326\_pJS43\_01.10263.10263.3 | 4.1831 | 0.3319 | 100.0% | 3456.8342 | 3455.8027 | 1 | 5.595 | 31.2% | 1 | R.LASYLDKVQALEEANNDLENKIQDWYDKK.G | 3 |
|  | pJS43\_100mM\_120812\_01.06217.06217.2 | 5.2003 | 0.4746 | 100.0% | 1588.3922 | 1587.6836 | 1 | 9.06 | 84.6% | 3 | K.VQALEEANNDLENK.I | 2 |
|  | 100326\_pJS43\_01.11400.11400.2 | 4.4183 | 0.575 | 100.0% | 2903.2722 | 2904.1597 | 1 | 10.644 | 41.7% | 2 | K.NYSPYYNTIDDLKDQIVDLTVGNNK.T | 2 |
|  | 100326\_pJS43\_01.11354.11354.3 | 5.1433 | 0.4454 | 100.0% | 2903.8743 | 2904.1597 | 1 | 7.035 | 35.4% | 3 | K.NYSPYYNTIDDLKDQIVDLTVGNNK.T | 3 |
|  | pDK339\_033013\_01.07791.07791.1 | 2.4825 | 0.4036 | 99.3% | 1060.51 | 1061.1802 | 1 | 6.018 | 62.5% | 2 | K.TLLDIDNTR.M | 1 |
|  | pJS43\_100mM\_120812\_01.07667.07667.2 | 2.91 | 0.3651 | 100.0% | 1061.0521 | 1061.1802 | 2 | 6.097 | 81.2% | 8 | K.TLLDIDNTR.M | 2 |
|  | 100326\_pJS43\_01.05036.05036.2 | 2.8754 | 0.3855 | 100.0% | 1307.4922 | 1308.5383 | 2 | 6.41 | 66.7% | 4 | R.IKFEMEQNLR.Q | 2 |
|  | pJS43\_100mM\_120812\_01.06422.06422.2 | 3.3145 | 0.3514 | 100.0% | 1159.3522 | 1158.2566 | 14 | 6.003 | 65.0% | 12 | R.QGVDADINGLR.Q | 2 |
|  | 100326\_pJS43\_01.11184.11184.3 | 4.4552 | 0.4312 | 100.0% | 3472.1643 | 3473.006 | 1 | 7.382 | 25.0% | 1 | R.QVLDNLTMEKSDLEMQYETLQEELMALKK.N | 3 |
|  | 100326\_pJS43\_01.10256.10256.2 | 4.2785 | 0.539 | 100.0% | 2299.112 | 2300.6443 | 1 | 8.589 | 61.1% | 1 | K.SDLEMQYETLQEELMALKK.N | 2 |
|  | 100326\_pJS43\_02.04290.04290.3 | 5.0912 | 0.4567 | 100.0% | 2898.0244 | 2897.1462 | 1 | 7.171 | 31.7% | 2 | K.NHKEEMSQLTGQNSGDVNVEINVAPGK.D | 3 |
|  | 100326\_pJS43\_02.04448.04448.3 | 6.8862 | 0.4894 | 100.0% | 3355.1042 | 3354.6733 | 1 | 7.878 | 37.5% | 14 | K.NHKEEMSQLTGQNSGDVNVEINVAPGKDLTK.T | 3 |
|  | 100326\_pJS43\_02.05129.05129.3 | 3.4186 | 0.4214 | 100.0% | 2973.5044 | 2975.2544 | 8 | 6.139 | 24.1% | 1 | K.EEMSQLTGQNSGDVNVEINVAPGKDLTK.T | 3 |
|  | 100326\_pJS43\_01.07911.07911.2 | 3.6586 | 0.2903 | 100.0% | 1853.2122 | 1853.1003 | 1 | 5.403 | 71.4% | 3 | K.TLNDMRQEYEQLIAK.N | 2 |
|  | 100326\_pJS43\_01.07904.07904.3 | 3.8897 | 0.2696 | 99.9% | 1853.5443 | 1853.1003 | 1 | 4.991 | 44.6% | 1 | K.TLNDMRQEYEQLIAK.N | 3 |
|  | 100326\_pJS43\_01.08231.08231.3 | 6.9092 | 0.5674 | 100.0% | 3664.4043 | 3664.8784 | 1 | 9.224 | 29.0% | 1 | K.NRKDIENQYETQITQIEHEVSSSGQEVQSSAK.E | 3 |
|  | 100326\_pJS43\_01.09832.09832.3 | 7.0021 | 0.5632 | 100.0% | 3264.7444 | 3266.413 | 1 | 9.671 | 34.8% | 8 | K.DIENQYETQITQIEHEVSSSGQEVQSSAK.E | 3 |
|  | 100326\_pJS43\_01.09732.09732.2 | 4.6907 | 0.544 | 100.0% | 3265.372 | 3266.413 | 1 | 9.624 | 37.5% | 1 | K.DIENQYETQITQIEHEVSSSGQEVQSSAK.E | 2 |
|  | 100326\_pJS43\_01.08402.08402.2 | 6.4982 | 0.5325 | 100.0% | 1838.7722 | 1839.0557 | 1 | 9.594 | 83.3% | 9 | R.HGVQELEIELQSQLSK.K | 32 |
|  | pJS43\_100mM\_120812\_02.07190.07190.3 | 4.2574 | 0.3219 | 100.0% | 1839.9243 | 1839.0557 | 1 | 6.345 | 41.7% | 2 | R.HGVQELEIELQSQLSK.K | 3 |
|  | pSKT11\_1\_020812\_02.05392.05392.2 | 6.3085 | 0.3156 | 100.0% | 1966.3322 | 1967.2297 | 1 | 8.932 | 75.0% | 3 | R.HGVQELEIELQSQLSKK.A | 2 |
|  | 100326\_pJS43\_02.05870.05870.3 | 6.5412 | 0.339 | 100.0% | 1967.7544 | 1967.2297 | 1 | 7.722 | 53.1% | 15 | R.HGVQELEIELQSQLSKK.A | 3 |
|  | 100326\_pJS43\_01.07049.07049.3 | 7.0048 | 0.5322 | 100.0% | 2881.5842 | 2882.0667 | 1 | 9.631 | 41.0% | 1 | R.LEKEIETYHNLLEGGQEDFESSGAGK.I | 3 |
|  | pJS43\_100mM\_120812\_01.09494.09494.2 | 4.9502 | 0.4544 | 100.0% | 2511.412 | 2511.6177 | 1 | 8.591 | 50.0% | 4 | K.EIETYHNLLEGGQEDFESSGAGK.I | 2 |
|  | 100326\_pJS43\_02.05354.05354.3 | 5.634 | 0.3068 | 100.0% | 2511.9243 | 2511.6177 | 1 | 7.705 | 38.6% | 15 | K.EIETYHNLLEGGQEDFESSGAGK.I | 3 |
|  | pJS43\_100mM\_120812\_01.03288.03288.2 | 5.6067 | 0.4514 | 100.0% | 1792.2922 | 1792.7324 | 1 | 11.43 | 59.1% | 1 | R.GGSGGSYGGGGSGGGYGGGSGSR.G | 2 |
|  | pDk339\_033013\_02.03731.03731.3 | 7.6684 | 0.5539 | 100.0% | 3224.7844 | 3225.1118 | 1 | 9.51 | 28.8% | 18 | R.GGSGGSHGGGSGFGGESGGSYGGGEEASGSGGGYGGGSGK.S | 3 |

Similarities:
gi|4557701|ref|NP\_000(1:36)  
contaminant\_KERATIN03(1:36)  
gi|15431310|ref|NP\_00(1:36)  
gi|24430192|ref|NP\_00(1:36)  
gi|24234699|ref|NP\_00(1:36)  

---

|  |  |  |  |  |  |  |  |  |
| --- | --- | --- | --- | --- | --- | --- | --- | --- |
| U | *gi|18087855|ref|NP\_54* | 11 | 149 | 60.7% | 89 | 10350 | 7.4 | dynein, light chain, LC8-type 2 [Homo sapiens] |

| Filename XCorr DeltCN Conf% ObsM+H+ CalcM+H+ SpR ZScore Ion% # Sequence  | | | | | | | | | | | | |
| --- | --- | --- | --- | --- | --- | --- | --- | --- | --- | --- | --- | --- |
| \* | SKAPIP\_tube2\_041314\_01.09809.09809.2 | 4.2029 | 0.4745 | 100.0% | 1441.4722 | 1441.6671 | 1 | 8.22 | 81.8% | 1 | K.YNIEKDIAAYIK.K | 2 |
| \* | 100326\_pJS43\_01.06921.06921.2 | 3.6873 | 0.3719 | 100.0% | 1569.0721 | 1569.8412 | 1 | 6.613 | 79.2% | 1 | K.YNIEKDIAAYIKK.E | 2 |
| \* | SKAPIP\_tube2\_041314\_01.08512.08512.3 | 4.297 | 0.3648 | 100.0% | 1570.5844 | 1569.8412 | 1 | 6.855 | 62.5% | 5 | K.YNIEKDIAAYIKK.E | 3 |
| \* | AstrinIP\_MS2\_022614\_01.07606.07606.1 | 1.9958 | 0.2366 | 98.6% | 793.5 | 793.93835 | 3 | 6.283 | 75.0% | 4 | K.DIAAYIK.K | 1 |
|  | SKAPIP\_041314\_01.10835.10835.3 | 2.9433 | 0.4029 | 100.0% | 1532.3944 | 1531.7234 | 1 | 6.77 | 56.8% | 1 | K.KYNPTWHCIVGR.N | 33 |
|  | SKAPIP\_041314\_01.11072.11072.2 | 3.5438 | 0.4953 | 100.0% | 1403.2122 | 1403.5493 | 1 | 8.101 | 75.0% | 6 | K.YNPTWHCIVGR.N | 22 |
|  | SKAPIP\_tube2\_041314\_01.04293.04293.1 | 2.1619 | 0.4004 | 99.5% | 1282.72 | 1283.383 | 1 | 6.709 | 60.0% | 4 | R.NFGSYVTHETK.H | 11 |
|  | SKAPIP\_041314\_01.10570.10570.2 | 3.6058 | 0.3859 | 100.0% | 1284.1322 | 1283.383 | 3 | 6.788 | 65.0% | 13 | R.NFGSYVTHETK.H | 22 |
|  | pSKT11\_1\_020812\_02.09868.09868.3 | 5.9448 | 0.5117 | 100.0% | 3237.0842 | 3237.771 | 1 | 8.667 | 40.4% | 62 | R.NFGSYVTHETKHFIYFYLGQVAILLFK.S | 33 |
|  | SKAPIP\_041314\_02.13295.13295.3 | 5.2249 | 0.4365 | 100.0% | 3381.5344 | 3381.9011 | 1 | 6.941 | 30.4% | 39 | R.NFGSYVTHETKHFIYFYLGQVAILLFKSG.- | 33 |
|  | 100326\_pJS43\_02.10540.10540.2 | 5.0541 | 0.5173 | 100.0% | 1974.2922 | 1973.4111 | 1 | 10.255 | 73.3% | 13 | K.HFIYFYLGQVAILLFK.S | 22 |

Similarities:
gi|4505813|ref|NP\_003(7:4)  

---

|  |  |  |  |  |  |  |  |  |
| --- | --- | --- | --- | --- | --- | --- | --- | --- |
| U | *gi|57013276|ref|NP\_00* | 31 | 335 | 60.5% | 451 | 50152 | 5.1 | tubulin, alpha, ubiquitous [Homo sapiens] |

| Filename XCorr DeltCN Conf% ObsM+H+ CalcM+H+ SpR ZScore Ion% # Sequence  | | | | | | | | | | | | |
| --- | --- | --- | --- | --- | --- | --- | --- | --- | --- | --- | --- | --- |
|  | pDK365N\_300mM\_082713\_02.07532.07532.2 | 5.9094 | 0.6089 | 100.0% | 2008.4122 | 2009.093 | 1 | 11.774 | 57.9% | 38 | K.TIGGGDDSFNTFFSETGAGK.H | 22 |
|  | pDK339\_033013\_01.12157.12157.2 | 5.3622 | 0.4904 | 100.0% | 1702.5122 | 1702.9451 | 1 | 8.482 | 78.6% | 36 | R.AVFVDLEPTVIDEVR.T | 22 |
|  | AstrinIP\_MS2\_022614\_01.09048.09048.2 | 2.6573 | 0.4517 | 100.0% | 1410.3121 | 1411.6439 | 51 | 6.74 | 54.5% | 4 | R.QLFHPEQLITGK.E | 222 |
|  | 100326\_pJS43\_01.06218.06218.2 | 2.6061 | 0.2557 | 98.8% | 2416.2122 | 2416.6555 | 5 | 5.618 | 35.0% | 3 | R.QLFHPEQLITGKEDAANNYAR.G | 222 |
|  | pDK365N\_300mM\_082713\_01.08105.08105.3 | 4.2433 | 0.3985 | 100.0% | 2416.2844 | 2416.6555 | 1 | 7.004 | 30.0% | 21 | R.QLFHPEQLITGKEDAANNYAR.G | 333 |
|  | 100326\_pJS43\_01.10718.10718.2 | 4.127 | 0.5314 | 100.0% | 1843.7922 | 1843.1332 | 1 | 8.607 | 76.7% | 4 | R.GHYTIGKEIIDLVLDR.I | 22 |
|  | 100326\_pJS43\_01.10701.10701.3 | 5.0142 | 0.4478 | 100.0% | 1843.8844 | 1843.1332 | 1 | 7.44 | 50.0% | 2 | R.GHYTIGKEIIDLVLDR.I | 33 |
|  | 100326\_pJS43\_01.09051.09051.1 | 2.1194 | 0.4161 | 99.4% | 1085.48 | 1086.2737 | 78 | 6.268 | 56.2% | 2 | K.EIIDLVLDR.I | 11 |
|  | 100326\_pJS43\_01.09026.09026.2 | 2.6652 | 0.3008 | 99.9% | 1086.0721 | 1086.2737 | 32 | 5.508 | 62.5% | 5 | K.EIIDLVLDR.I | 22 |
|  | AstrinIP\_MS2\_022614\_01.03693.03693.2 | 2.3076 | 0.2225 | 98.9% | 909.9922 | 910.05804 | 2 | 5.244 | 78.6% | 1 | R.LSVDYGKK.S | 222 |
|  | AstrinIP\_MS2\_022614\_01.05537.05537.3 | 3.1267 | 0.2098 | 98.1% | 1876.0443 | 1876.0824 | 1 | 5.415 | 41.1% | 2 | R.RNLDIERPTYTNLNR.L | 333 |
|  | 100326\_pJS43\_01.04940.04940.2 | 3.4438 | 0.1692 | 99.5% | 1719.1122 | 1719.8949 | 1 | 5.078 | 65.4% | 19 | R.NLDIERPTYTNLNR.L | 222 |
|  | pDK365N\_300mM\_082713\_01.06254.06254.3 | 2.956 | 0.3024 | 99.7% | 1720.4644 | 1719.8949 | 8 | 5.659 | 38.5% | 7 | R.NLDIERPTYTNLNR.L | 333 |
|  | 100326\_pJS43\_02.08678.08678.2 | 4.9741 | 0.4878 | 100.0% | 1488.2122 | 1488.7678 | 1 | 9.092 | 73.1% | 27 | R.LISQIVSSITASLR.F | 222 |
|  | 100326\_pJS43\_01.11114.11114.2 | 5.2561 | 0.5725 | 100.0% | 2409.4521 | 2410.6885 | 1 | 10.409 | 52.5% | 20 | R.FDGALNVDLTEFQTNLVPYPR.I | 222 |
|  | pDK365N\_300mM\_082713\_02.08931.08931.3 | 3.2455 | 0.2341 | 98.4% | 2411.6643 | 2410.6885 | 1 | 4.972 | 35.0% | 2 | R.FDGALNVDLTEFQTNLVPYPR.I | 333 |
|  | 100326\_pJS43\_01.08403.08403.3 | 3.956 | 0.3773 | 100.0% | 1757.6044 | 1758.0703 | 1 | 6.72 | 43.3% | 11 | R.IHFPLATYAPVISAEK.A | 333 |
|  | 100326\_pJS43\_01.08450.08450.2 | 4.3366 | 0.4911 | 100.0% | 1758.3922 | 1758.0703 | 1 | 8.686 | 73.3% | 21 | R.IHFPLATYAPVISAEK.A | 222 |
|  | 100326\_pJS43\_02.06020.06020.3 | 3.1035 | 0.2679 | 98.7% | 2752.2844 | 2752.0369 | 33 | 5.392 | 22.8% | 1 | K.AYHEQLSVAEITNACFEPANQMVK.C | 33 |
|  | AstrinIP\_MS1\_022614\_01.08429.08429.2 | 2.7335 | 0.3316 | 99.9% | 1249.9922 | 1250.4304 | 1 | 6.82 | 81.2% | 1 | K.YMACCLLYR.G | 222 |
|  | pDK339\_033013\_01.06838.06838.1 | 1.8415 | 0.4374 | 99.5% | 1015.55 | 1016.1827 | 3 | 6.618 | 61.1% | 13 | K.DVNAAIATIK.T | 11 |
|  | pDK339\_033013\_01.06872.06872.2 | 3.3091 | 0.3518 | 100.0% | 1016.0522 | 1016.1827 | 1 | 7.202 | 88.9% | 16 | K.DVNAAIATIK.T | 22 |
|  | SKAPIP\_041314\_01.12542.12542.2 | 2.6367 | 0.2083 | 98.6% | 1587.6122 | 1585.7656 | 1 | 5.003 | 66.7% | 1 | R.SIQFVDWCPTGFK.V | 22 |
|  | AstrinIP\_MS2\_022614\_01.09147.09147.2 | 4.5299 | 0.4948 | 100.0% | 1825.4722 | 1826.1027 | 1 | 7.664 | 61.8% | 31 | K.VGINYQPPTVVPGGDLAK.V | 222 |
|  | pDK339\_033013\_01.06961.06961.2 | 3.5035 | 0.4054 | 100.0% | 1381.2522 | 1381.6324 | 1 | 6.607 | 80.0% | 8 | R.LDHKFDLMYAK.R | 222 |
|  | pDK365N\_100mM\_082813\_03.06041.06041.3 | 4.3182 | 0.3902 | 100.0% | 1381.9143 | 1381.6324 | 2 | 7.156 | 62.5% | 20 | R.LDHKFDLMYAK.R | 333 |
|  | AstrinIP\_MS2\_022614\_01.06849.06849.1 | 2.8794 | 0.1799 | 98.7% | 1382.19 | 1381.6324 | 1 | 4.024 | 65.0% | 1 | R.LDHKFDLMYAK.R | 111 |
|  | pSKT11\_1\_020812\_01.08280.08280.3 | 3.0442 | 0.2407 | 99.2% | 1538.1843 | 1537.82 | 35 | 4.538 | 38.6% | 1 | R.LDHKFDLMYAKR.A | 333 |
|  | 100326\_pJS43\_02.05668.05668.3 | 6.1453 | 0.4595 | 100.0% | 2487.8044 | 2487.7083 | 1 | 8.41 | 47.5% | 3 | K.RAFVHWYVGEGMEEGEFSEAR.E | 333 |
|  | pDK339othertube\_033013\_02.07266.07266.3 | 5.3032 | 0.4274 | 100.0% | 2331.2043 | 2331.5208 | 1 | 8.132 | 44.7% | 13 | R.AFVHWYVGEGMEEGEFSEAR.E | 333 |
|  | pDK339othertube\_033013\_02.05510.05510.2 | 3.552 | 0.5712 | 100.0% | 2349.2322 | 2350.2751 | 1 | 11.002 | 52.5% | 1 | K.DYEEVGVDSVEGEGEEEGEEY.- | 2 |

Similarities:
gi|14389309|ref|NP\_11(28:3)  
gi|17921989|ref|NP\_00(22:9)  

---

|  |  |  |  |  |  |  |  |  |
| --- | --- | --- | --- | --- | --- | --- | --- | --- |
| U | *gi|4504445|ref|NP\_002* | 18 | 42 | 60.0% | 320 | 34196 | 9.2 | heterogeneous nuclear ribonucleoprotein A1 isoform a [Homo sapiens] |

| Filename XCorr DeltCN Conf% ObsM+H+ CalcM+H+ SpR ZScore Ion% # Sequence  | | | | | | | | | | | | |
| --- | --- | --- | --- | --- | --- | --- | --- | --- | --- | --- | --- | --- |
|  | pJS43\_100mM\_120812\_01.03228.03228.2 | 2.9366 | 0.1854 | 99.2% | 1428.3522 | 1428.5852 | 1 | 4.828 | 77.3% | 1 | K.SESPKEPEQLRK.L | 2 |
|  | pJS43\_100mM\_120812\_01.03222.03222.3 | 3.032 | 0.215 | 98.7% | 1428.8043 | 1428.5852 | 25 | 4.876 | 40.9% | 1 | K.SESPKEPEQLRK.L | 3 |
|  | pDK365N\_100mM\_082713\_02.07324.07324.3 | 4.057 | 0.3882 | 100.0% | 1913.9944 | 1914.1656 | 1 | 6.491 | 43.8% | 1 | R.KLFIGGLSFETTDESLR.S | 3 |
|  | pSKT11\_1\_020812\_02.06826.06826.2 | 5.2395 | 0.4952 | 100.0% | 1786.4321 | 1785.9916 | 1 | 8.177 | 73.3% | 5 | K.LFIGGLSFETTDESLR.S | 2 |
|  | pSKT11\_1\_020812\_02.06400.06400.3 | 4.1487 | 0.3481 | 100.0% | 2755.4944 | 2755.08 | 1 | 5.255 | 28.1% | 1 | R.SRGFGFVTYATVEEVDAAMNARPHK.V | 3 |
|  | pSKT11\_1\_020812\_02.06124.06124.3 | 2.8926 | 0.2618 | 97.6% | 3181.5544 | 3182.5408 | 1 | 4.878 | 23.2% | 1 | R.SRGFGFVTYATVEEVDAAMNARPHKVDGR.V | 3 |
|  | pSKT11\_1\_020812\_02.06615.06615.3 | 3.4879 | 0.4094 | 100.0% | 2938.8542 | 2939.275 | 1 | 7.146 | 25.0% | 1 | R.GFGFVTYATVEEVDAAMNARPHKVDGR.V | 3 |
|  | pSKT11\_1\_020812\_02.06999.06999.3 | 4.5704 | 0.3264 | 100.0% | 4112.2144 | 4112.603 | 1 | 5.812 | 20.5% | 1 | K.IFVGGIKEDTEEHHLRDYFEQYGKIEVIEIMTDR.G | 3 |
|  | pJS43\_100mM\_120812\_01.10776.10776.2 | 3.8552 | 0.3805 | 100.0% | 1219.7522 | 1219.4387 | 1 | 7.314 | 88.9% | 6 | K.IEVIEIMTDR.G | 2 |
|  | pSKT11\_1\_020812\_02.04961.04961.3 | 5.1543 | 0.3145 | 100.0% | 2566.0745 | 2566.9194 | 1 | 7.239 | 35.7% | 1 | K.KRGFAFVTFDDHDSVDKIVIQK.Y | 3 |
|  | pJS43\_100mM\_120812\_02.06324.06324.3 | 4.252 | 0.3692 | 100.0% | 1857.1144 | 1856.989 | 1 | 6.672 | 41.7% | 1 | K.RGFAFVTFDDHDSVDK.I | 3 |
|  | pSKT11\_1\_020812\_02.05452.05452.3 | 4.674 | 0.4187 | 100.0% | 2437.3145 | 2438.7454 | 1 | 6.37 | 36.2% | 1 | K.RGFAFVTFDDHDSVDKIVIQK.Y | 3 |
|  | pJS43\_100mM\_120812\_01.10250.10250.2 | 4.1482 | 0.5095 | 100.0% | 1700.4321 | 1700.8016 | 1 | 8.669 | 71.4% | 2 | R.GFAFVTFDDHDSVDK.I | 2 |
|  | pSKT11\_1\_020812\_02.05900.05900.2 | 4.9582 | 0.4913 | 100.0% | 2281.612 | 2282.5579 | 1 | 9.387 | 57.9% | 1 | R.GFAFVTFDDHDSVDKIVIQK.Y | 2 |
|  | pDK365N\_100mM\_082713\_01.09452.09452.3 | 4.2118 | 0.2429 | 99.8% | 2282.9644 | 2282.5579 | 1 | 7.672 | 34.2% | 11 | R.GFAFVTFDDHDSVDKIVIQK.Y | 3 |
| \* | pDK365N\_100mM\_082713\_02.06759.06759.3 | 4.2477 | 0.4442 | 100.0% | 4408.074 | 4408.4175 | 37 | 7.502 | 19.3% | 1 | R.GGGGYGGSGDGYNGFGNDGSNFGGGGSYNDFGNYNNQSSNFGPMK.G | 3 |
|  | pJS43\_100mM\_120812\_01.05281.05281.2 | 3.8031 | 0.4403 | 100.0% | 1629.1122 | 1629.7721 | 1 | 8.221 | 63.3% | 5 | R.SSGPYGGGGQYFAKPR.N | 2 |
|  | pJS43\_100mM\_120812\_01.03347.03347.2 | 4.8906 | 0.4871 | 100.0% | 1696.0922 | 1695.6561 | 1 | 10.206 | 64.7% | 1 | R.NQGGYGGSSSSSSYGSGR.R | 2 |

---

|  |  |  |  |  |  |  |  |  |
| --- | --- | --- | --- | --- | --- | --- | --- | --- |
| U | *gi|47132620|ref|NP\_00* | 32 | 123 | 59.9% | 639 | 65433 | 8.0 | keratin 2 [Homo sapiens] |

| Filename XCorr DeltCN Conf% ObsM+H+ CalcM+H+ SpR ZScore Ion% # Sequence  | | | | | | | | | | | | |
| --- | --- | --- | --- | --- | --- | --- | --- | --- | --- | --- | --- | --- |
|  | pDK365N\_300mM\_082713\_04.04625.04625.3 | 2.5257 | 0.2655 | 95.6% | 1900.8844 | 1901.0054 | 3 | 4.839 | 28.6% | 1 | R.GGGGGGFRGFSSGSAVVSGGSR.R | 33 |
|  | pDK365N\_300mM\_082713\_04.04222.04222.3 | 3.3938 | 0.2608 | 99.2% | 2056.8542 | 2057.1929 | 1 | 5.327 | 28.4% | 1 | R.GGGGGGFRGFSSGSAVVSGGSRR.S | 33 |
|  | SKAPIP\_041314\_01.07953.07953.2 | 4.4386 | 0.4671 | 100.0% | 1255.3522 | 1255.3298 | 1 | 9.752 | 84.6% | 8 | R.GFSSGSAVVSGGSR.R | 22 |
|  | SKAPIP\_041314\_01.06838.06838.2 | 4.8279 | 0.6645 | 100.0% | 1320.6122 | 1321.3542 | 1 | 12.006 | 90.0% | 5 | R.HGGGGGGFGGGGFGSR.S | 22 |
|  | AstrinIP\_MS1\_022614\_01.04997.04997.1 | 1.5068 | 0.2943 | 98.3% | 831.42 | 831.9878 | 3 | 5.18 | 50.0% | 1 | R.SLVGLGGTK.S | 11 |
|  | pDK365N\_300mM\_082713\_03.04326.04326.2 | 2.3649 | 0.3059 | 99.6% | 833.27216 | 831.9878 | 18 | 6.16 | 68.8% | 1 | R.SLVGLGGTK.S | 22 |
|  | pDK365N\_300mM\_082713\_04.06518.06518.2 | 4.2935 | 0.436 | 100.0% | 1840.9722 | 1840.0055 | 1 | 7.36 | 50.0% | 4 | K.SISISVAGGGGGFGAAGGFGGR.G | 22 |
| \* | pDK365N\_300mM\_082713\_03.09396.09396.2 | 5.8693 | 0.5991 | 100.0% | 2399.7322 | 2400.4446 | 1 | 11.215 | 48.3% | 3 | R.GGGFGGGSSFGGGSGFSGGGFGGGGFGGGR.F | 2 |
| \* | pJS43\_100mM\_120812\_02.06914.06914.3 | 6.053 | 0.4688 | 100.0% | 2400.8342 | 2400.4446 | 1 | 9.502 | 45.7% | 4 | R.GGGFGGGSSFGGGSGFSGGGFGGGGFGGGR.F | 3 |
|  | AstrinIP\_MS1\_022614\_01.12798.12798.3 | 5.8301 | 0.4425 | 100.0% | 4094.9043 | 4094.5786 | 1 | 8.393 | 20.3% | 3 | R.FGGFGGPGGVGGLGGPGGFGPGGYPGGIHEVSVNQSLLQPLNVK.V | 33 |
|  | pJS43\_100mM\_120812\_01.07265.07265.2 | 2.4075 | 0.2649 | 99.7% | 828.09216 | 827.95544 | 2 | 5.168 | 91.7% | 6 | K.FASFIDK.V | 222222222 |
|  | SKAPIP\_tube2\_041314\_01.06813.06813.2 | 2.9746 | 0.2137 | 99.8% | 1082.7522 | 1083.2755 | 2 | 7.239 | 75.0% | 6 | K.FASFIDKVR.F | 22222222 |
|  | pDK339\_033013\_01.05947.05947.2 | 4.5619 | 0.0649 | 99.9% | 1476.1921 | 1476.6726 | 1 | 7.056 | 90.9% | 25 | R.FLEQQNQVLQTK.W | 222 |
|  | pDK365N\_300mM\_082713\_03.05800.05800.2 | 2.8563 | 0.2855 | 99.9% | 1038.1522 | 1038.1454 | 1 | 6.007 | 81.2% | 3 | R.YLDGLTAER.T | 22 |
|  | SKAPIP\_041314\_01.11547.11547.3 | 3.4712 | 0.3926 | 100.0% | 2257.8843 | 2257.4338 | 4 | 5.772 | 31.9% | 1 | R.TSQNSELNNMQDLVEDYKK.K | 33 |
|  | pSKT11\_1\_020812\_01.07779.07779.2 | 3.6316 | 0.3783 | 100.0% | 1337.1522 | 1337.5156 | 1 | 6.696 | 81.8% | 4 | R.TAAENDFVTLKK.D | 22 |
|  | pDK365N\_300mM\_082713\_03.11824.11824.2 | 3.9989 | 0.3052 | 100.0% | 1461.5721 | 1461.6982 | 1 | 6.586 | 77.3% | 7 | K.VDLLNQEIEFLK.V | 22 |
|  | SKAPIP\_041314\_01.13030.13030.2 | 4.2714 | 0.4084 | 100.0% | 1330.6522 | 1330.5211 | 1 | 7.867 | 86.4% | 8 | R.NLDLDSIIAEVK.A | 2222222 |
|  | SKAPIP\_041314\_01.06429.06429.2 | 3.0935 | 0.0675 | 98.8% | 1108.0721 | 1108.196 | 1 | 6.67 | 81.2% | 4 | K.AQYEEIAQR.S | 22222 |
|  | pSKT11\_1\_020812\_02.04094.04094.3 | 5.8891 | 0.4769 | 100.0% | 2567.6643 | 2567.815 | 1 | 7.53 | 36.9% | 2 | R.SKEEAEALYHSKYEELQVTVGR.H | 33 |
|  | pJS43\_100mM\_120812\_02.05199.05199.2 | 3.4388 | 0.3338 | 100.0% | 1194.2522 | 1194.33 | 1 | 7.95 | 83.3% | 6 | K.YEELQVTVGR.H | 22 |
|  | pDK339\_033013\_01.06043.06043.2 | 2.8783 | 0.1411 | 99.5% | 974.1322 | 974.102 | 1 | 4.646 | 92.9% | 5 | K.IEISELNR.V | 222 |
|  | SKAPIP\_041314\_01.11010.11010.2 | 2.6348 | 0.2363 | 99.1% | 1330.6921 | 1330.3971 | 2 | 4.731 | 63.6% | 1 | K.NVQDAIADAEQR.G | 22 |
|  | AstrinIP\_MS1\_022614\_01.09212.09212.2 | 3.1819 | 0.3522 | 100.0% | 1614.6522 | 1614.796 | 1 | 6.342 | 61.5% | 2 | R.NKLNDLEEALQQAK.E | 22 |
|  | SKAPIP\_041314\_01.12492.12492.2 | 3.2308 | 0.4644 | 100.0% | 2198.9722 | 2199.4258 | 1 | 6.852 | 44.4% | 1 | R.NKLNDLEEALQQAKEDLAR.L | 22 |
|  | SKAPIP\_041314\_01.12503.12503.3 | 5.3615 | 0.4435 | 100.0% | 2199.6243 | 2199.4258 | 1 | 7.665 | 44.4% | 4 | R.NKLNDLEEALQQAKEDLAR.L | 33 |
|  | pDK339\_033013\_01.09919.09919.2 | 2.8332 | 0.3549 | 99.9% | 1371.4722 | 1372.5181 | 1 | 6.592 | 68.2% | 1 | K.LNDLEEALQQAK.E | 22 |
|  | SKAPIP\_041314\_02.07718.07718.2 | 2.9806 | 0.3812 | 100.0% | 1264.5922 | 1264.4644 | 1 | 7.611 | 75.0% | 2 | K.LALDVEIATYR.K | 2222222 |
|  | pSKT11\_1\_020812\_02.04722.04722.2 | 3.2655 | 0.3735 | 100.0% | 1392.3922 | 1392.6384 | 9 | 6.788 | 59.1% | 1 | K.LALDVEIATYRK.L | 2222222 |
|  | pDK365N\_300mM\_082713\_03.03638.03638.3 | 6.1975 | 0.4766 | 100.0% | 2502.4443 | 2502.4893 | 1 | 11.128 | 39.3% | 1 | K.AAFGGSGGRGSSSGGGYSSGSSSYGSGGR.Q | 33 |
|  | pDK339\_033013\_01.03423.03423.2 | 3.4056 | 0.4658 | 100.0% | 1197.7322 | 1198.2346 | 1 | 7.391 | 60.7% | 1 | K.GGSISGGGYGSGGGK.H | 22 |
|  | pDK339\_033013\_01.03271.03271.3 | 3.7834 | 0.2859 | 99.8% | 2315.7544 | 2315.2913 | 1 | 5.569 | 33.3% | 1 | K.HSSGGGSRGGSSSGGGYGSGGGGSSSVK.G | 33 |

Similarities:
gi|4504919|ref|NP\_002(2:30)  
gi|119395750|ref|NP\_0(2:30)  
contaminant\_KERATIN22(28:4)  
gi|67782365|ref|NP\_00(2:30)  
gi|119395754|ref|NP\_0(5:27)  
gi|155969697|ref|NP\_7(6:26)  
gi|119703753|ref|NP\_0(6:26)  
gi|32567786|ref|NP\_78(5:27)  
gi|153791158|ref|NP\_0(5:27)  
gi|109255249|ref|NP\_0(2:30)  

---

|  |  |  |  |  |  |  |  |  |
| --- | --- | --- | --- | --- | --- | --- | --- | --- |
| U | *contaminant\_KERATIN22* | 29 | 105 | 58.9% | 645 | 65865 | 8.0 | no description |

| Filename XCorr DeltCN Conf% ObsM+H+ CalcM+H+ SpR ZScore Ion% # Sequence  | | | | | | | | | | | | |
| --- | --- | --- | --- | --- | --- | --- | --- | --- | --- | --- | --- | --- |
|  | pDK365N\_300mM\_082713\_04.04625.04625.3 | 2.5257 | 0.2655 | 95.6% | 1900.8844 | 1901.0054 | 3 | 4.839 | 28.6% | 1 | R.GGGGGGFRGFSSGSAVVSGGSR.R | 33 |
|  | pDK365N\_300mM\_082713\_04.04222.04222.3 | 3.3938 | 0.2608 | 99.2% | 2056.8542 | 2057.1929 | 1 | 5.327 | 28.4% | 1 | R.GGGGGGFRGFSSGSAVVSGGSRR.S | 33 |
|  | SKAPIP\_041314\_01.07953.07953.2 | 4.4386 | 0.4671 | 100.0% | 1255.3522 | 1255.3298 | 1 | 9.752 | 84.6% | 8 | R.GFSSGSAVVSGGSR.R | 22 |
|  | SKAPIP\_041314\_01.06838.06838.2 | 4.8279 | 0.6645 | 100.0% | 1320.6122 | 1321.3542 | 1 | 12.006 | 90.0% | 5 | R.HGGGGGGFGGGGFGSR.S | 22 |
|  | AstrinIP\_MS1\_022614\_01.04997.04997.1 | 1.5068 | 0.2943 | 98.3% | 831.42 | 831.9878 | 3 | 5.18 | 50.0% | 1 | R.SLVGLGGTK.S | 11 |
|  | pDK365N\_300mM\_082713\_03.04326.04326.2 | 2.3649 | 0.3059 | 99.6% | 833.27216 | 831.9878 | 18 | 6.16 | 68.8% | 1 | R.SLVGLGGTK.S | 22 |
|  | pDK365N\_300mM\_082713\_04.06518.06518.2 | 4.2935 | 0.436 | 100.0% | 1840.9722 | 1840.0055 | 1 | 7.36 | 50.0% | 4 | K.SISISVAGGGGGFGAAGGFGGR.G | 22 |
| \* | pSKT11\_1\_020812\_02.06040.06040.3 | 3.9734 | 0.2518 | 99.6% | 2832.6543 | 2832.8809 | 1 | 5.297 | 22.1% | 1 | R.GGGFGGGSGFGGGSGFGGGSGFSGGGFGGGGFGGGR.F | 3 |
|  | AstrinIP\_MS1\_022614\_01.12798.12798.3 | 5.8301 | 0.4425 | 100.0% | 4094.9043 | 4094.5786 | 1 | 8.393 | 20.3% | 3 | R.FGGFGGPGGVGGLGGPGGFGPGGYPGGIHEVSVNQSLLQPLNVK.V | 33 |
|  | pDK339\_033013\_01.05947.05947.2 | 4.5619 | 0.0649 | 99.9% | 1476.1921 | 1476.6726 | 1 | 7.056 | 90.9% | 25 | R.FLEQQNQVLQTK.W | 222 |
|  | pDK365N\_300mM\_082713\_03.05800.05800.2 | 2.8563 | 0.2855 | 99.9% | 1038.1522 | 1038.1454 | 1 | 6.007 | 81.2% | 3 | R.YLDGLTAER.T | 22 |
|  | SKAPIP\_041314\_01.11547.11547.3 | 3.4712 | 0.3926 | 100.0% | 2257.8843 | 2257.4338 | 4 | 5.772 | 31.9% | 1 | R.TSQNSELNNMQDLVEDYKK.K | 33 |
|  | pSKT11\_1\_020812\_01.07779.07779.2 | 3.6316 | 0.3783 | 100.0% | 1337.1522 | 1337.5156 | 1 | 6.696 | 81.8% | 4 | R.TAAENDFVTLKK.D | 22 |
|  | pDK365N\_300mM\_082713\_03.11824.11824.2 | 3.9989 | 0.3052 | 100.0% | 1461.5721 | 1461.6982 | 1 | 6.586 | 77.3% | 7 | K.VDLLNQEIEFLK.V | 22 |
|  | SKAPIP\_041314\_01.13030.13030.2 | 4.2714 | 0.4084 | 100.0% | 1330.6522 | 1330.5211 | 1 | 7.867 | 86.4% | 8 | R.NLDLDSIIAEVK.A | 2222222 |
|  | SKAPIP\_041314\_01.06429.06429.2 | 3.0935 | 0.0675 | 98.8% | 1108.0721 | 1108.196 | 1 | 6.67 | 81.2% | 4 | K.AQYEEIAQR.S | 22222 |
|  | pSKT11\_1\_020812\_02.04094.04094.3 | 5.8891 | 0.4769 | 100.0% | 2567.6643 | 2567.815 | 1 | 7.53 | 36.9% | 2 | R.SKEEAEALYHSKYEELQVTVGR.H | 33 |
|  | pJS43\_100mM\_120812\_02.05199.05199.2 | 3.4388 | 0.3338 | 100.0% | 1194.2522 | 1194.33 | 1 | 7.95 | 83.3% | 6 | K.YEELQVTVGR.H | 22 |
|  | pDK339\_033013\_01.06043.06043.2 | 2.8783 | 0.1411 | 99.5% | 974.1322 | 974.102 | 1 | 4.646 | 92.9% | 5 | K.IEISELNR.V | 222 |
|  | SKAPIP\_041314\_01.11010.11010.2 | 2.6348 | 0.2363 | 99.1% | 1330.6921 | 1330.3971 | 2 | 4.731 | 63.6% | 1 | K.NVQDAIADAEQR.G | 22 |
|  | AstrinIP\_MS1\_022614\_01.09212.09212.2 | 3.1819 | 0.3522 | 100.0% | 1614.6522 | 1614.796 | 1 | 6.342 | 61.5% | 2 | R.NKLNDLEEALQQAK.E | 22 |
|  | SKAPIP\_041314\_01.12492.12492.2 | 3.2308 | 0.4644 | 100.0% | 2198.9722 | 2199.4258 | 1 | 6.852 | 44.4% | 1 | R.NKLNDLEEALQQAKEDLAR.L | 22 |
|  | SKAPIP\_041314\_01.12503.12503.3 | 5.3615 | 0.4435 | 100.0% | 2199.6243 | 2199.4258 | 1 | 7.665 | 44.4% | 4 | R.NKLNDLEEALQQAKEDLAR.L | 33 |
|  | pDK339\_033013\_01.09919.09919.2 | 2.8332 | 0.3549 | 99.9% | 1371.4722 | 1372.5181 | 1 | 6.592 | 68.2% | 1 | K.LNDLEEALQQAK.E | 22 |
|  | SKAPIP\_041314\_02.07718.07718.2 | 2.9806 | 0.3812 | 100.0% | 1264.5922 | 1264.4644 | 1 | 7.611 | 75.0% | 2 | K.LALDVEIATYR.K | 2222222 |
|  | pSKT11\_1\_020812\_02.04722.04722.2 | 3.2655 | 0.3735 | 100.0% | 1392.3922 | 1392.6384 | 9 | 6.788 | 59.1% | 1 | K.LALDVEIATYRK.L | 2222222 |
|  | pDK365N\_300mM\_082713\_03.03638.03638.3 | 6.1975 | 0.4766 | 100.0% | 2502.4443 | 2502.4893 | 1 | 11.128 | 39.3% | 1 | K.AAFGGSGGRGSSSGGGYSSGSSSYGSGGR.Q | 33 |
|  | pDK339\_033013\_01.03423.03423.2 | 3.4056 | 0.4658 | 100.0% | 1197.7322 | 1198.2346 | 1 | 7.391 | 60.7% | 1 | K.GGSISGGGYGSGGGK.H | 22 |
|  | pDK339\_033013\_01.03271.03271.3 | 3.7834 | 0.2859 | 99.8% | 2315.7544 | 2315.2913 | 1 | 5.569 | 33.3% | 1 | K.HSSGGGSRGGSSSGGGYGSGGGGSSSVK.G | 33 |

Similarities:
gi|119395750|ref|NP\_0(2:27)  
gi|47132620|ref|NP\_00(28:1)  
gi|119395754|ref|NP\_0(3:26)  
gi|155969697|ref|NP\_7(4:25)  
gi|119703753|ref|NP\_0(4:25)  
gi|32567786|ref|NP\_78(3:26)  
gi|153791158|ref|NP\_0(3:26)  
gi|109255249|ref|NP\_0(1:28)  

---

|  |  |  |  |  |  |  |  |  |
| --- | --- | --- | --- | --- | --- | --- | --- | --- |
| U | *gi|5032161|ref|NP\_005* | 7 | 98 | 58.9% | 112 | 12473 | 4.8 | elongin C [Homo sapiens] |

| Filename XCorr DeltCN Conf% ObsM+H+ CalcM+H+ SpR ZScore Ion% # Sequence  | | | | | | | | | | | | |
| --- | --- | --- | --- | --- | --- | --- | --- | --- | --- | --- | --- | --- |
| \* | SKAPIP\_tube2\_041314\_01.05757.05757.2 | 4.2809 | 0.4494 | 100.0% | 1548.1921 | 1548.6736 | 1 | 8.403 | 80.8% | 3 | K.TYGGCEGPDAMYVK.L | 2 |
|  | SKAPIP\_tube2\_041314\_01.06176.06176.2 | 4.291 | 0.3362 | 100.0% | 1346.4722 | 1345.5382 | 1 | 6.938 | 72.7% | 12 | K.LISSDGHEFIVK.R | 2 |
|  | SKAPIP\_tube2\_041314\_01.04870.04870.2 | 3.9173 | 0.4527 | 100.0% | 1502.1921 | 1501.7257 | 1 | 7.188 | 83.3% | 5 | K.LISSDGHEFIVKR.E | 2 |
|  | pJS43\_100mM\_120812\_01.03468.03468.1 | 2.4341 | 0.4504 | 99.1% | 1056.5 | 1057.1918 | 1 | 7.752 | 72.2% | 1 | R.EHALTSGTIK.A | 1 |
| \* | pJS43\_100mM\_120812\_02.06680.06680.2 | 6.6938 | 0.5833 | 100.0% | 2211.3523 | 2212.3984 | 1 | 10.759 | 60.5% | 71 | K.AMLSGPGQFAENETNEVNFR.E | 2 |
| \* | pDK339othertube\_033013\_02.06696.06696.3 | 4.8904 | 0.3444 | 100.0% | 2212.2844 | 2212.3984 | 13 | 5.902 | 36.8% | 5 | K.AMLSGPGQFAENETNEVNFR.E | 3 |
| \* | pSKT11\_1\_020812\_01.09215.09215.3 | 3.8065 | 0.3487 | 100.0% | 3202.3743 | 3203.5537 | 1 | 4.956 | 23.2% | 1 | K.AMLSGPGQFAENETNEVNFREIPSHVLSK.V | 3 |

---

|  |  |  |  |  |  |  |  |  |
| --- | --- | --- | --- | --- | --- | --- | --- | --- |
| U | *gi|7705300|ref|NP\_057* | 2 | 2 | 58.8% | 85 | 9118 | 9.3 | ubiquitin-fold modifier 1 [Homo sapiens] |

| Filename XCorr DeltCN Conf% ObsM+H+ CalcM+H+ SpR ZScore Ion% # Sequence  | | | | | | | | | | | | |
| --- | --- | --- | --- | --- | --- | --- | --- | --- | --- | --- | --- | --- |
| \* | SKAPIP\_041314\_01.12345.12345.2 | 2.0561 | 0.3056 | 97.3% | 1588.8522 | 1588.8846 | 47 | 5.028 | 42.9% | 1 | K.VLSVPESTPFTAVLK.F | 2 |
| \* | SKAPIP\_tube2\_041314\_01.12401.12401.3 | 5.7604 | 0.4463 | 100.0% | 3579.3843 | 3578.0593 | 1 | 8.378 | 31.6% | 1 | K.FAAEEFKVPAATSAIITNDGIGINPAQTAGNVFLK.H | 3 |

---

|  |  |  |  |  |  |  |  |  |
| --- | --- | --- | --- | --- | --- | --- | --- | --- |
| U | *gi|14211889|ref|NP\_11* | 7 | 67 | 58.6% | 99 | 11250 | 4.9 | dpy-30-like protein [Homo sapiens] |

| Filename XCorr DeltCN Conf% ObsM+H+ CalcM+H+ SpR ZScore Ion% # Sequence  | | | | | | | | | | | | |
| --- | --- | --- | --- | --- | --- | --- | --- | --- | --- | --- | --- | --- |
| \* | pSKT11\_1\_020812\_01.03123.03123.2 | 2.4118 | 0.2907 | 99.3% | 1287.5122 | 1287.4557 | 28 | 5.57 | 60.0% | 2 | R.IVENEKINAEK.S | 2 |
| \* | pSKT11\_1\_020812\_01.08054.08054.2 | 2.3223 | 0.4094 | 99.8% | 1285.3922 | 1285.4862 | 1 | 5.658 | 75.0% | 4 | K.QKVDLQSLPTR.A | 2 |
| \* | pDK365N\_100mM\_082713\_01.05617.05617.2 | 2.2856 | 0.2335 | 98.7% | 1029.0721 | 1029.1814 | 8 | 5.125 | 75.0% | 1 | K.VDLQSLPTR.A | 2 |
| \* | pSKT11\_1\_020812\_01.12896.12896.2 | 5.9703 | 0.6312 | 100.0% | 2126.7322 | 2126.5876 | 1 | 10.901 | 81.6% | 18 | R.AYLDQTVVPILLQGLAVLAK.E | 2 |
| \* | pDK365N\_300mM\_082713\_03.16796.16796.3 | 6.4697 | 0.4238 | 100.0% | 2127.7444 | 2126.5876 | 1 | 9.437 | 48.7% | 18 | R.AYLDQTVVPILLQGLAVLAK.E | 3 |
| \* | pSKT11\_1\_020812\_01.11541.11541.2 | 4.4026 | 0.4657 | 100.0% | 1887.5721 | 1888.2169 | 1 | 7.418 | 60.0% | 11 | K.ERPPNPIEFLASYLLK.N | 2 |
| \* | pJS43\_100mM\_120812\_01.15090.15090.3 | 4.5396 | 0.4358 | 100.0% | 1889.3344 | 1888.2169 | 2 | 6.781 | 40.0% | 13 | K.ERPPNPIEFLASYLLK.N | 3 |

---

|  |  |  |  |  |  |  |  |  |
| --- | --- | --- | --- | --- | --- | --- | --- | --- |
| U | *gi|10800138|ref|NP\_06* | 12 | 83 | 57.9% | 126 | 13936 | 10.3 | histone cluster 1, H2bd [Homo sapiens] |
| U | *gi|66912162|ref|NP\_00* | 13 | 84 | 57.9% | 126 | 13920 | 10.3 | histone cluster 2, H2bf [Homo sapiens] |
| U | *gi|4504271|ref|NP\_003* | 13 | 84 | 57.9% | 126 | 13906 | 10.3 | histone cluster 1, H2bi [Homo sapiens] |
| U | *gi|4504269|ref|NP\_003* | 13 | 84 | 57.9% | 126 | 13892 | 10.3 | histone cluster 1, H2bh [Homo sapiens] |
| U | *gi|4504265|ref|NP\_003* | 13 | 84 | 57.9% | 126 | 13906 | 10.3 | histone cluster 1, H2bf [Homo sapiens] |
| U | *gi|4504263|ref|NP\_003* | 13 | 84 | 57.9% | 126 | 13989 | 10.3 | histone cluster 1, H2bm [Homo sapiens] |
| U | *gi|4504261|ref|NP\_003* | 13 | 84 | 57.9% | 126 | 13922 | 10.3 | histone cluster 1, H2bn [Homo sapiens] |
| U | *gi|4504259|ref|NP\_003* | 13 | 84 | 57.9% | 126 | 13952 | 10.3 | histone cluster 1, H2bl [Homo sapiens] |
| U | *gi|4504257|ref|NP\_003* | 13 | 84 | 57.9% | 126 | 13906 | 10.3 | histone cluster 1, H2bg [Homo sapiens] |
| U | *gi|21396484|ref|NP\_00* | 13 | 84 | 57.9% | 126 | 13906 | 10.3 | histone cluster 1, H2be [Homo sapiens] |
| U | *gi|21166389|ref|NP\_00* | 13 | 84 | 57.9% | 126 | 13906 | 10.3 | histone cluster 1, H2bc [Homo sapiens] |
| U | *gi|20336752|ref|NP\_61* | 13 | 84 | 57.9% | 126 | 13936 | 10.3 | histone cluster 1, H2bd [Homo sapiens] |

| Filename XCorr DeltCN Conf% ObsM+H+ CalcM+H+ SpR ZScore Ion% # Sequence  | | | | | | | | | | | | |
| --- | --- | --- | --- | --- | --- | --- | --- | --- | --- | --- | --- | --- |
|  | pDK365N\_100mM\_082713\_01.04012.04012.3 | 3.6816 | 0.2531 | 99.9% | 1510.1044 | 1509.7019 | 2 | 6.027 | 45.5% | 1 | R.SRKESYSVYVYK.V | 33 |
|  | pDK365N\_100mM\_082713\_01.04680.04680.2 | 2.988 | 0.3879 | 100.0% | 1266.1522 | 1266.4363 | 1 | 6.381 | 77.8% | 4 | R.KESYSVYVYK.V | 22 |
|  | pJS43\_100mM\_120812\_01.06943.06943.1 | 1.7773 | 0.2842 | 98.6% | 1137.5 | 1138.2622 | 3 | 5.838 | 62.5% | 1 | K.ESYSVYVYK.V | 11 |
|  | pJS43\_100mM\_120812\_01.06947.06947.2 | 1.9933 | 0.4824 | 99.9% | 1137.7322 | 1138.2622 | 2 | 7.097 | 56.2% | 1 | K.ESYSVYVYK.V | 22 |
|  | pDK365N\_100mM\_082713\_01.12750.12750.2 | 5.0897 | 0.4342 | 100.0% | 1745.7322 | 1745.0211 | 1 | 8.753 | 71.4% | 34 | K.AMGIMNSFVNDIFER.I | 2222 |
|  | pJS43\_100mM\_120812\_01.02709.02709.2 | 2.2213 | 0.1555 | 97.4% | 901.9922 | 902.0439 | 12 | 3.881 | 83.3% | 1 | R.LAHYNKR.S | 2222 |
|  | AstrinNocIP\_020510\_01.06332.06332.3 | 3.0197 | 0.3576 | 99.9% | 2396.7844 | 2397.821 | 1 | 5.978 | 31.0% | 1 | R.STITSREIQTAVRLLLPGELAK.H | 3333 |
|  | AstrinNocIP\_020510\_01.04893.04893.2 | 3.9454 | 0.2645 | 100.0% | 1752.4922 | 1752.1075 | 1 | 6.034 | 60.0% | 2 | R.EIQTAVRLLLPGELAK.H | 2222 |
|  | pJS43\_100mM\_120812\_01.09215.09215.2 | 2.2344 | 0.1613 | 95.8% | 954.3122 | 954.19794 | 19 | 4.455 | 68.8% | 1 | R.LLLPGELAK.H | 2222 |
|  | pJS43\_100mM\_120812\_01.01053.01053.2 | 2.3399 | 0.2029 | 98.7% | 829.4522 | 828.9004 | 5 | 5.39 | 71.4% | 1 | K.HAVSEGTK.A | 2222 |
|  | AstrinNocIP\_020510\_01.01827.01827.2 | 4.752 | 0.5783 | 100.0% | 1794.4122 | 1795.0024 | 1 | 11.118 | 68.8% | 9 | K.HAVSEGTKAVTKYTSSK.- | 22 |
|  | AstrinNocIP\_020510\_01.01730.01730.3 | 4.8535 | 0.5208 | 100.0% | 1795.3444 | 1795.0024 | 1 | 9.44 | 42.2% | 27 | K.HAVSEGTKAVTKYTSSK.- | 33 |

Similarities:
gi|18105048|ref|NP\_54(10:2)  
gi|10800140|ref|NP\_06(8:4)  
gi|20336754|ref|NP\_06(6:6)  

---

|  |  |  |  |  |  |  |  |  |
| --- | --- | --- | --- | --- | --- | --- | --- | --- |
| U | *gi|18105048|ref|NP\_54* | 12 | 53 | 57.9% | 126 | 13890 | 10.3 | histone cluster 1, H2bk [Homo sapiens] |

| Filename XCorr DeltCN Conf% ObsM+H+ CalcM+H+ SpR ZScore Ion% # Sequence  | | | | | | | | | | | | |
| --- | --- | --- | --- | --- | --- | --- | --- | --- | --- | --- | --- | --- |
|  | pDK365N\_100mM\_082713\_01.04012.04012.3 | 3.6816 | 0.2531 | 99.9% | 1510.1044 | 1509.7019 | 2 | 6.027 | 45.5% | 1 | R.SRKESYSVYVYK.V | 33 |
|  | pDK365N\_100mM\_082713\_01.04680.04680.2 | 2.988 | 0.3879 | 100.0% | 1266.1522 | 1266.4363 | 1 | 6.381 | 77.8% | 4 | R.KESYSVYVYK.V | 22 |
|  | pJS43\_100mM\_120812\_01.06943.06943.1 | 1.7773 | 0.2842 | 98.6% | 1137.5 | 1138.2622 | 3 | 5.838 | 62.5% | 1 | K.ESYSVYVYK.V | 11 |
|  | pJS43\_100mM\_120812\_01.06947.06947.2 | 1.9933 | 0.4824 | 99.9% | 1137.7322 | 1138.2622 | 2 | 7.097 | 56.2% | 1 | K.ESYSVYVYK.V | 22 |
|  | pDK365N\_100mM\_082713\_01.12750.12750.2 | 5.0897 | 0.4342 | 100.0% | 1745.7322 | 1745.0211 | 1 | 8.753 | 71.4% | 34 | K.AMGIMNSFVNDIFER.I | 2222 |
|  | pJS43\_100mM\_120812\_01.02709.02709.2 | 2.2213 | 0.1555 | 97.4% | 901.9922 | 902.0439 | 12 | 3.881 | 83.3% | 1 | R.LAHYNKR.S | 2222 |
|  | AstrinNocIP\_020510\_01.06332.06332.3 | 3.0197 | 0.3576 | 99.9% | 2396.7844 | 2397.821 | 1 | 5.978 | 31.0% | 1 | R.STITSREIQTAVRLLLPGELAK.H | 3333 |
|  | AstrinNocIP\_020510\_01.04893.04893.2 | 3.9454 | 0.2645 | 100.0% | 1752.4922 | 1752.1075 | 1 | 6.034 | 60.0% | 2 | R.EIQTAVRLLLPGELAK.H | 2222 |
|  | pJS43\_100mM\_120812\_01.09215.09215.2 | 2.2344 | 0.1613 | 95.8% | 954.3122 | 954.19794 | 19 | 4.455 | 68.8% | 1 | R.LLLPGELAK.H | 2222 |
|  | pJS43\_100mM\_120812\_01.01053.01053.2 | 2.3399 | 0.2029 | 98.7% | 829.4522 | 828.9004 | 5 | 5.39 | 71.4% | 1 | K.HAVSEGTK.A | 2222 |
|  | AstrinNocIP\_020510\_01.01761.01761.3 | 3.7363 | 0.507 | 100.0% | 1777.7344 | 1779.003 | 1 | 8.724 | 40.6% | 4 | K.HAVSEGTKAVTKYTSAK.- | 33 |
|  | AstrinNocIP\_020510\_01.01803.01803.2 | 4.6925 | 0.6285 | 100.0% | 1778.3522 | 1779.003 | 1 | 11.72 | 62.5% | 2 | K.HAVSEGTKAVTKYTSAK.- | 22 |

Similarities:
gi|10800138|ref|NP\_06(10:2)  
gi|10800140|ref|NP\_06(6:6)  
gi|20336754|ref|NP\_06(8:4)  

---

|  |  |  |  |  |  |  |  |  |
| --- | --- | --- | --- | --- | --- | --- | --- | --- |
| U | *gi|51479152|ref|NP\_00* | 6 | 16 | 57.7% | 137 | 15773 | 7.2 | ATP synthase, H+ transporting, mitochondrial F0 complex, subunit d isoform b [Homo sapiens] |
| U | *gi|5453559|ref|NP\_006* | 6 | 16 | 49.1% | 161 | 18491 | 5.3 | ATP synthase, H+ transporting, mitochondrial F0 complex, subunit d isoform a [Homo sapiens] |

| Filename XCorr DeltCN Conf% ObsM+H+ CalcM+H+ SpR ZScore Ion% # Sequence  | | | | | | | | | | | | |
| --- | --- | --- | --- | --- | --- | --- | --- | --- | --- | --- | --- | --- |
|  | pDK365N\_100mM\_082713\_01.05398.05398.2 | 2.4335 | 0.2135 | 98.9% | 1093.8322 | 1094.1692 | 2 | 5.163 | 81.2% | 3 | K.SWNETLTSR.L | 2 |
|  | pDK365N\_300mM\_082713\_01.10809.10809.2 | 3.9333 | 0.4302 | 100.0% | 1933.3522 | 1933.2139 | 1 | 7.94 | 59.4% | 5 | R.LAALPENPPAIDWAYYK.A | 2 |
|  | SKAPIP\_tube2\_041314\_01.05886.05886.2 | 2.3787 | 0.3613 | 99.8% | 1122.2722 | 1122.2633 | 2 | 5.877 | 72.2% | 1 | K.AGLVDDFEKK.V | 2 |
|  | pDK365N\_100mM\_082713\_01.03890.03890.3 | 2.0614 | 0.2741 | 95.2% | 1299.0543 | 1298.4938 | 201 | 4.862 | 30.6% | 1 | R.IVEYEKEMEK.M | 3 |
|  | pDK365N\_300mM\_082713\_01.13223.13223.2 | 4.6903 | 0.4553 | 100.0% | 2466.1921 | 2466.7651 | 1 | 8.534 | 57.5% | 3 | K.NLIPFDQMTIEDLNEAFPETK.L | 2 |
|  | pDK365N\_300mM\_082713\_03.09965.09965.2 | 3.4663 | 0.3761 | 100.0% | 1557.3121 | 1557.7484 | 1 | 6.416 | 68.2% | 3 | K.YPYWPHQPIENL.- | 2 |

---

|  |  |  |  |  |  |  |  |  |
| --- | --- | --- | --- | --- | --- | --- | --- | --- |
| U | *gi|106775678|ref|NP\_0* | 9 | 66 | 57.7% | 130 | 14095 | 10.9 | histone cluster 2, H2aa4 [Homo sapiens] |
| U | *gi|4504251|ref|NP\_003* | 9 | 66 | 57.7% | 130 | 14095 | 10.9 | histone cluster 2, H2aa3 [Homo sapiens] |
| U | *gi|24638446|ref|NP\_00* | 9 | 66 | 58.1% | 129 | 13988 | 10.9 | histone cluster 2, H2ac [Homo sapiens] |

| Filename XCorr DeltCN Conf% ObsM+H+ CalcM+H+ SpR ZScore Ion% # Sequence  | | | | | | | | | | | | |
| --- | --- | --- | --- | --- | --- | --- | --- | --- | --- | --- | --- | --- |
|  | pDK365N\_100mM\_082813\_03.07151.07151.2 | 3.2969 | 0.3218 | 100.0% | 945.0122 | 945.1093 | 3 | 5.574 | 81.2% | 13 | R.AGLQFPVGR.V | 2222 |
|  | pDK339othertube\_033013\_01.18528.18528.2 | 4.8728 | 0.542 | 100.0% | 2935.0522 | 2935.4082 | 1 | 10.175 | 41.1% | 4 | R.VGAGAPVYMAAVLEYLTAEILELAGNAAR.D | 2 |
|  | pDK339othertube\_033013\_01.18524.18524.3 | 5.1266 | 0.4266 | 100.0% | 2935.4644 | 2935.4082 | 1 | 7.428 | 26.8% | 6 | R.VGAGAPVYMAAVLEYLTAEILELAGNAAR.D | 3 |
|  | pJS43\_100mM\_120812\_01.05135.05135.2 | 2.5063 | 0.2795 | 99.8% | 851.1722 | 851.0396 | 1 | 5.65 | 91.7% | 4 | R.HLQLAIR.N | 222 |
|  | pDK365N\_300mM\_082713\_02.04718.04718.3 | 3.2892 | 0.3261 | 99.9% | 1693.8243 | 1693.9004 | 5 | 6.383 | 38.5% | 9 | R.HLQLAIRNDEELNK.L | 33 |
|  | pDK365N\_100mM\_082713\_01.08028.08028.3 | 4.5718 | 0.4939 | 100.0% | 2105.6042 | 2105.4453 | 1 | 7.619 | 44.1% | 2 | R.HLQLAIRNDEELNKLLGK.V | 33 |
|  | pDK365N\_100mM\_082713\_01.06878.06878.2 | 3.7745 | 0.3591 | 100.0% | 1273.9722 | 1273.4288 | 1 | 6.538 | 80.0% | 7 | R.NDEELNKLLGK.V | 22 |
|  | pJS43\_100mM\_120812\_01.12803.12803.2 | 5.2767 | 0.5529 | 100.0% | 1931.6522 | 1932.3573 | 1 | 8.855 | 63.9% | 19 | K.VTIAQGGVLPNIQAVLLPK.K | 22 |
|  | pJS43\_100mM\_120812\_01.12773.12773.3 | 4.3713 | 0.3264 | 100.0% | 1932.8944 | 1932.3573 | 1 | 6.635 | 52.8% | 2 | K.VTIAQGGVLPNIQAVLLPK.K | 33 |

Similarities:
gi|10800130|ref|NP\_06(7:2)  
gi|4504255|ref|NP\_002(2:7)  
gi|113425815|ref|XP\_9(1:8)  

---

|  |  |  |  |  |  |  |  |  |
| --- | --- | --- | --- | --- | --- | --- | --- | --- |
| U | *gi|20127519|ref|NP\_03* | 71 | 241 | 57.6% | 747 | 85653 | 9.2 | TPX2, microtubule-associated protein homolog [Homo sapiens] |

| Filename XCorr DeltCN Conf% ObsM+H+ CalcM+H+ SpR ZScore Ion% # Sequence  | | | | | | | | | | | | |
| --- | --- | --- | --- | --- | --- | --- | --- | --- | --- | --- | --- | --- |
| \* | AstrinIP\_MS2\_022614\_02.10014.10014.3 | 4.6398 | 0.391 | 100.0% | 3811.4944 | 3810.8867 | 1 | 6.615 | 22.7% | 2 | K.SSYSYDAPSDFINFSSLDDEGDTQNIDSWFEEK.A | 3 |
| \* | AstrinIP\_MS1\_022614\_01.07678.07678.2 | 4.7396 | 0.4451 | 100.0% | 2404.4521 | 2405.7996 | 1 | 8.689 | 52.5% | 1 | R.KANLQQAIVTPLKPVDNTYYK.E | 2 |
| \* | AstrinIP\_MS1\_022614\_01.07695.07695.3 | 5.3433 | 0.4179 | 100.0% | 2405.2444 | 2405.7996 | 1 | 8.823 | 47.5% | 5 | R.KANLQQAIVTPLKPVDNTYYK.E | 3 |
| \* | AstrinIP\_MS1\_022614\_01.08775.08775.2 | 4.8721 | 0.5495 | 100.0% | 2276.4722 | 2277.6255 | 1 | 9.633 | 52.6% | 3 | K.ANLQQAIVTPLKPVDNTYYK.E | 2 |
| \* | AstrinIP\_MS1\_022614\_01.08776.08776.3 | 4.1651 | 0.4447 | 100.0% | 2278.8843 | 2277.6255 | 1 | 7.064 | 32.9% | 5 | K.ANLQQAIVTPLKPVDNTYYK.E | 3 |
| \* | AstrinIP\_MS2\_022614\_01.03555.03555.2 | 2.7307 | 0.0644 | 95.8% | 1159.6322 | 1160.3127 | 18 | 3.854 | 66.7% | 1 | R.LSAQKDLEQK.E | 2 |
| \* | AstrinIP\_MS2\_022614\_01.03599.03599.1 | 1.9249 | 0.2718 | 98.6% | 1092.46 | 1093.1327 | 47 | 4.774 | 56.2% | 1 | K.STEEQELEK.S | 1 |
| \* | AstrinIP\_MS2\_022614\_01.04857.04857.1 | 2.2504 | 0.2856 | 98.8% | 1149.56 | 1150.3534 | 5 | 5.457 | 68.8% | 1 | K.MQQEVVEMR.K | 1 |
| \* | AstrinIP\_MS2\_022614\_01.04893.04893.2 | 3.4648 | 0.4406 | 100.0% | 1150.0322 | 1150.3534 | 1 | 7.802 | 87.5% | 6 | K.MQQEVVEMR.K | 2 |
| \* | AstrinIP\_MS2\_022614\_01.04607.04607.2 | 2.8108 | 0.2506 | 99.5% | 1324.1721 | 1323.6652 | 1 | 4.823 | 66.7% | 1 | K.KLALAGIGQPVKK.S | 2 |
| \* | AstrinIP\_MS2\_022614\_01.08036.08036.2 | 3.0987 | 0.4169 | 100.0% | 1066.5521 | 1067.317 | 1 | 7.858 | 85.0% | 2 | K.LALAGIGQPVK.K | 2 |
| \* | AstrinIP\_MS2\_022614\_01.06171.06171.1 | 2.2437 | 0.4259 | 99.4% | 1194.61 | 1195.4911 | 5 | 6.679 | 54.5% | 2 | K.LALAGIGQPVKK.S | 1 |
| \* | AstrinIP\_MS1\_022614\_01.05871.05871.2 | 3.3658 | 0.3305 | 100.0% | 1196.1322 | 1195.4911 | 2 | 6.345 | 63.6% | 7 | K.LALAGIGQPVKK.S | 2 |
| \* | AstrinIP\_MS2\_022614\_01.06598.06598.2 | 1.9998 | 0.1958 | 96.2% | 907.6322 | 908.0043 | 119 | 5.275 | 66.7% | 3 | K.SVDFHFR.T | 2 |
| \* | AstrinIP\_MS2\_022614\_01.07900.07900.2 | 5.078 | 0.4497 | 100.0% | 1887.3522 | 1887.013 | 1 | 7.971 | 75.0% | 4 | K.NQEEYKEVNFTSELR.K | 2 |
| \* | AstrinIP\_MS1\_022614\_01.07551.07551.3 | 4.4822 | 0.3629 | 100.0% | 1887.6543 | 1887.013 | 1 | 6.578 | 50.0% | 9 | K.NQEEYKEVNFTSELR.K | 3 |
| \* | AstrinIP\_MS2\_022614\_01.06656.06656.3 | 3.4628 | 0.298 | 99.9% | 2014.8544 | 2015.187 | 3 | 5.03 | 41.7% | 2 | K.NQEEYKEVNFTSELRK.H | 3 |
| \* | AstrinIP\_MS1\_022614\_01.07589.07589.2 | 2.1579 | 0.1991 | 96.6% | 1095.2122 | 1095.1974 | 5 | 5.079 | 68.8% | 2 | K.EVNFTSELR.K | 2 |
| \* | AstrinIP\_MS1\_022614\_01.06651.06651.2 | 3.5916 | 0.4332 | 100.0% | 1548.6322 | 1549.7765 | 1 | 7.155 | 69.2% | 3 | K.GCTIVKPFNLSQGK.K | 2 |
| \* | AstrinIP\_MS2\_022614\_01.12090.12090.3 | 3.7636 | 0.4099 | 100.0% | 2454.8342 | 2455.683 | 1 | 6.58 | 36.2% | 2 | R.TFDETVSTYVPLAQQVEDFHK.R | 3 |
| \* | AstrinIP\_MS1\_022614\_01.10625.10625.2 | 3.7433 | 0.519 | 100.0% | 2611.0923 | 2611.8706 | 1 | 8.422 | 40.5% | 4 | R.TFDETVSTYVPLAQQVEDFHKR.T | 2 |
| \* | AstrinIP\_MS1\_022614\_01.10629.10629.3 | 3.8858 | 0.4129 | 100.0% | 2611.9143 | 2611.8706 | 1 | 6.213 | 39.3% | 2 | R.TFDETVSTYVPLAQQVEDFHKR.T | 3 |
| \* | AstrinIP\_MS1\_022614\_01.04384.04384.2 | 3.9178 | 0.2449 | 100.0% | 1357.8722 | 1358.5779 | 1 | 6.529 | 77.3% | 4 | R.SKKDDINLLPSK.S | 2 |
| \* | AstrinIP\_MS1\_022614\_01.04332.04332.3 | 3.7321 | 0.156 | 98.9% | 1359.1444 | 1358.5779 | 3 | 4.356 | 52.3% | 2 | R.SKKDDINLLPSK.S | 3 |
| \* | AstrinIP\_MS2\_022614\_01.05835.05835.2 | 2.9136 | 0.2637 | 99.8% | 1143.4922 | 1143.3256 | 1 | 4.949 | 83.3% | 4 | K.KDDINLLPSK.S | 2 |
| \* | AstrinIP\_MS1\_022614\_01.04049.04049.2 | 3.7012 | 0.3636 | 100.0% | 1556.4922 | 1556.7686 | 1 | 6.449 | 70.8% | 2 | K.ICRDPQTPVLQTK.H | 2 |
| \* | AstrinIP\_MS2\_022614\_01.06484.06484.1 | 2.5128 | 0.2801 | 98.7% | 1348.45 | 1349.4344 | 12 | 5.488 | 54.5% | 2 | K.STAELEAEELEK.L | 1 |
| \* | AstrinIP\_MS2\_022614\_01.06518.06518.2 | 4.0747 | 0.2758 | 100.0% | 1349.0322 | 1349.4344 | 2 | 5.96 | 77.3% | 9 | K.STAELEAEELEK.L | 2 |
| \* | AstrinIP\_MS1\_022614\_01.09256.09256.2 | 5.6275 | 0.5352 | 100.0% | 2009.4122 | 2010.2053 | 1 | 9.407 | 65.6% | 4 | K.STAELEAEELEKLQQYK.F | 2 |
| \* | AstrinIP\_MS2\_022614\_02.06526.06526.3 | 3.5092 | 0.3244 | 99.9% | 2010.8344 | 2010.2053 | 1 | 7.29 | 35.9% | 1 | K.STAELEAEELEKLQQYK.F | 3 |
| \* | AstrinIP\_MS2\_022614\_01.07756.07756.2 | 3.489 | 0.318 | 100.0% | 1037.2522 | 1037.2877 | 1 | 6.113 | 83.3% | 8 | R.ILEGGPILPK.K | 2 |
| \* | AstrinIP\_MS2\_022614\_01.08992.08992.2 | 4.2063 | 0.5033 | 100.0% | 2134.872 | 2135.5083 | 1 | 9.203 | 61.1% | 2 | K.KPPVKPPTEPIGFDLEIEK.R | 2 |
| \* | AstrinIP\_MS2\_022614\_01.09056.09056.3 | 4.2665 | 0.4667 | 100.0% | 2135.4243 | 2135.5083 | 1 | 6.66 | 50.0% | 9 | K.KPPVKPPTEPIGFDLEIEK.R | 3 |
| \* | AstrinIP\_MS1\_022614\_01.07666.07666.2 | 3.7128 | 0.4866 | 100.0% | 2290.392 | 2291.6958 | 1 | 8.732 | 50.0% | 1 | K.KPPVKPPTEPIGFDLEIEKR.I | 2 |
| \* | AstrinIP\_MS1\_022614\_01.07665.07665.3 | 5.4295 | 0.5133 | 100.0% | 2291.2144 | 2291.6958 | 1 | 8.591 | 44.7% | 7 | K.KPPVKPPTEPIGFDLEIEKR.I | 3 |
| \* | AstrinIP\_MS1\_022614\_01.03658.03658.2 | 4.7578 | 0.4487 | 100.0% | 2273.5522 | 2274.4639 | 1 | 9.119 | 55.9% | 1 | K.KKTEDEHFEFHSRPCPTK.I | 2 |
| \* | AstrinIP\_MS2\_022614\_01.07923.07923.2 | 3.735 | 0.4049 | 100.0% | 1198.5521 | 1198.402 | 1 | 7.21 | 85.0% | 4 | K.ILEDVVGVPEK.K | 2 |
| \* | AstrinIP\_MS2\_022614\_01.06477.06477.2 | 3.7808 | 0.2957 | 100.0% | 1326.2322 | 1326.576 | 1 | 5.976 | 77.3% | 9 | K.ILEDVVGVPEKK.V | 2 |
| \* | AstrinIP\_MS2\_022614\_01.06548.06548.3 | 3.2353 | 0.3375 | 100.0% | 1327.1344 | 1326.576 | 2 | 5.792 | 47.7% | 4 | K.ILEDVVGVPEKK.V | 3 |
| \* | AstrinIP\_MS1\_022614\_01.10329.10329.2 | 3.9888 | 0.5225 | 100.0% | 1661.4122 | 1661.9823 | 1 | 8.015 | 67.9% | 3 | K.VLPITVPKS\*PAFALK.N | 2 |
| \* | AstrinIP\_MS2\_022614\_01.05678.05678.1 | 1.8089 | 0.2566 | 98.7% | 733.39 | 733.8858 | 4 | 5.162 | 75.0% | 2 | K.SPAFALK.N | 1 |
| \* | AstrinIP\_MS1\_022614\_01.05934.05934.2 | 4.212 | 0.4857 | 100.0% | 2157.412 | 2158.4285 | 1 | 8.128 | 55.9% | 1 | R.IRMPTKEDEEEDEPVVIK.A | 2 |
| \* | AstrinIP\_MS2\_022614\_01.06305.06305.3 | 5.1504 | 0.2843 | 100.0% | 2157.7144 | 2158.4285 | 1 | 7.081 | 42.6% | 7 | R.IRMPTKEDEEEDEPVVIK.A | 3 |
| \* | AstrinIP\_MS2\_022614\_01.05117.05117.2 | 5.3279 | 0.4055 | 100.0% | 1888.5322 | 1889.0815 | 1 | 8.47 | 80.0% | 6 | R.MPTKEDEEEDEPVVIK.A | 2 |
| \* | AstrinIP\_MS2\_022614\_01.05150.05150.3 | 5.6037 | 0.3985 | 100.0% | 1888.6144 | 1889.0815 | 1 | 7.054 | 53.3% | 9 | R.MPTKEDEEEDEPVVIK.A | 3 |
| \* | AstrinIP\_MS2\_022614\_01.05668.05668.2 | 2.9808 | 0.3253 | 99.9% | 1431.0922 | 1431.4932 | 1 | 5.642 | 68.2% | 1 | K.EDEEEDEPVVIK.A | 2 |
| \* | AstrinIP\_MS1\_022614\_01.07510.07510.2 | 4.2334 | 0.4404 | 100.0% | 2132.5923 | 2132.473 | 1 | 8.547 | 58.3% | 4 | K.AQPVPHYGVPFKPQIPEAR.T | 2 |
| \* | AstrinIP\_MS2\_022614\_01.07887.07887.3 | 3.2943 | 0.4056 | 100.0% | 2133.5942 | 2132.473 | 5 | 6.794 | 34.7% | 4 | K.AQPVPHYGVPFKPQIPEAR.T | 3 |
| \* | AstrinIP\_MS1\_022614\_01.10346.10346.2 | 3.2327 | 0.4748 | 100.0% | 1458.1921 | 1458.5769 | 1 | 7.282 | 77.3% | 3 | R.TVEICPFSFDSR.D | 2 |
| \* | AstrinIP\_MS2\_022614\_01.11105.11105.2 | 2.9747 | 0.4068 | 100.0% | 1705.3322 | 1705.9945 | 1 | 7.301 | 67.9% | 1 | K.ALPLPHFDTINLPEK.K | 2 |
| \* | AstrinIP\_MS1\_022614\_01.09232.09232.2 | 3.6338 | 0.3049 | 100.0% | 1832.6322 | 1834.1686 | 1 | 6.883 | 56.7% | 2 | K.ALPLPHFDTINLPEKK.V | 2 |
| \* | AstrinIP\_MS2\_022614\_01.03942.03942.2 | 3.1103 | 0.2243 | 99.9% | 1054.1122 | 1054.1478 | 3 | 5.625 | 78.6% | 2 | K.HQLEEELR.Q | 2 |
| \* | AstrinIP\_MS1\_022614\_01.03719.03719.2 | 3.7585 | 0.3689 | 100.0% | 1438.2722 | 1438.5834 | 1 | 6.763 | 85.0% | 2 | K.HQLEEELRQQK.E | 2 |
| \* | AstrinIP\_MS1\_022614\_01.03722.03722.3 | 3.0048 | 0.2653 | 99.6% | 1438.3444 | 1438.5834 | 5 | 5.19 | 42.5% | 1 | K.HQLEEELRQQK.E | 3 |
| \* | AstrinIP\_MS1\_022614\_01.06712.06712.2 | 3.7302 | 0.2801 | 100.0% | 1683.3722 | 1683.9481 | 3 | 5.743 | 57.1% | 6 | K.ARPNTVISQEPFVPK.K | 2 |
| \* | AstrinIP\_MS1\_022614\_01.05106.05106.3 | 4.6843 | 0.3798 | 100.0% | 1811.5443 | 1812.1222 | 1 | 7.108 | 48.3% | 8 | K.ARPNTVISQEPFVPKK.E | 3 |
| \* | AstrinIP\_MS1\_022614\_01.05154.05154.2 | 2.5724 | 0.3831 | 99.8% | 1811.8121 | 1812.1222 | 1 | 6.193 | 53.3% | 1 | K.ARPNTVISQEPFVPKK.E | 2 |
| \* | AstrinIP\_MS2\_022614\_01.04455.04455.3 | 3.9697 | 0.263 | 99.8% | 2069.6943 | 2069.4116 | 6 | 4.528 | 38.2% | 2 | K.ARPNTVISQEPFVPKKEK.K | 3 |
| \* | AstrinIP\_MS2\_022614\_01.11092.11092.2 | 6.1658 | 0.5663 | 100.0% | 2318.632 | 2319.617 | 1 | 10.498 | 64.3% | 2 | K.KSVAEGLSGSLVQEPFQLATEK.R | 2 |
| \* | AstrinIP\_MS2\_022614\_01.11076.11076.3 | 5.8678 | 0.3715 | 100.0% | 2320.1343 | 2319.617 | 1 | 6.762 | 44.0% | 3 | K.KSVAEGLSGSLVQEPFQLATEK.R | 3 |
| \* | AstrinIP\_MS1\_022614\_01.09674.09674.3 | 5.8494 | 0.4898 | 100.0% | 2475.6843 | 2475.8044 | 1 | 8.474 | 46.6% | 4 | K.KSVAEGLSGSLVQEPFQLATEKR.A | 3 |
| \* | AstrinIP\_MS1\_022614\_01.11435.11435.2 | 6.1693 | 0.5699 | 100.0% | 2190.5522 | 2191.4429 | 1 | 11.006 | 57.5% | 3 | K.SVAEGLSGSLVQEPFQLATEK.R | 2 |
| \* | AstrinIP\_MS1\_022614\_01.11436.11436.3 | 4.5058 | 0.337 | 100.0% | 2192.9043 | 2191.4429 | 1 | 6.443 | 37.5% | 4 | K.SVAEGLSGSLVQEPFQLATEK.R | 3 |
| \* | AstrinIP\_MS2\_022614\_01.11193.11193.2 | 4.838 | 0.5717 | 100.0% | 2347.632 | 2347.6304 | 1 | 9.513 | 47.6% | 2 | K.SVAEGLSGSLVQEPFQLATEKR.A | 2 |
| \* | AstrinIP\_MS1\_022614\_01.10526.10526.3 | 4.4897 | 0.3939 | 100.0% | 2348.0344 | 2347.6304 | 1 | 7.532 | 32.1% | 4 | K.SVAEGLSGSLVQEPFQLATEKR.A | 3 |
| \* | AstrinIP\_MS1\_022614\_01.04932.04932.3 | 4.1403 | 0.3928 | 100.0% | 1832.4543 | 1832.0386 | 1 | 6.608 | 41.7% | 3 | R.MAEVEAQKAQQLEEAR.L | 3 |
| \* | AstrinIP\_MS2\_022614\_01.03476.03476.2 | 5.3049 | 0.3426 | 100.0% | 1630.2922 | 1630.7954 | 1 | 7.907 | 83.3% | 1 | R.LQEEEQKKEELAR.L | 2 |
| \* | AstrinIP\_MS2\_022614\_01.03464.03464.3 | 3.5398 | 0.2529 | 99.8% | 1631.1843 | 1630.7954 | 1 | 5.467 | 52.1% | 1 | R.LQEEEQKKEELAR.L | 3 |
| \* | AstrinIP\_MS2\_022614\_01.06144.06144.2 | 3.3468 | 0.122 | 99.1% | 1356.5922 | 1355.5309 | 1 | 5.002 | 66.7% | 2 | K.SSDQPLTVPVSPK.F | 2 |
| \* | AstrinIP\_MS1\_022614\_01.06477.06477.1 | 1.5295 | 0.2481 | 95.5% | 1434.49 | 1435.5309 | 2 | 4.049 | 58.3% | 1 | K.SSDQPLTVPVS\*PK.F | 1 |
| \* | AstrinIP\_MS1\_022614\_01.06464.06464.2 | 3.3637 | 0.341 | 100.0% | 1434.8922 | 1435.5309 | 1 | 6.42 | 75.0% | 5 | K.SSDQPLTVPVS\*PK.F | 2 |

---

|  |  |  |  |  |  |  |  |  |
| --- | --- | --- | --- | --- | --- | --- | --- | --- |
| U | *gi|16507237|ref|NP\_00* | 60 | 634 | 57.6% | 654 | 72333 | 5.2 | heat shock 70kDa protein 5 [Homo sapiens] |

| Filename XCorr DeltCN Conf% ObsM+H+ CalcM+H+ SpR ZScore Ion% # Sequence  | | | | | | | | | | | | |
| --- | --- | --- | --- | --- | --- | --- | --- | --- | --- | --- | --- | --- |
|  | SKAPIP\_041314\_01.05758.05758.2 | 3.5283 | 0.3135 | 100.0% | 1556.4122 | 1556.6786 | 1 | 5.812 | 65.4% | 4 | K.NGRVEIIANDQGNR.I | 22 |
|  | SKAPIP\_041314\_01.05769.05769.3 | 3.9798 | 0.2813 | 100.0% | 1556.8143 | 1556.6786 | 1 | 5.794 | 50.0% | 2 | K.NGRVEIIANDQGNR.I | 33 |
|  | AstrinIP\_MS1\_022614\_01.08324.08324.2 | 3.7808 | 0.4627 | 100.0% | 1568.1522 | 1567.7386 | 1 | 9.238 | 69.2% | 25 | R.ITPSYVAFTPEGER.L | 2 |
|  | SKAPIP\_tube2\_041314\_01.06866.06866.2 | 4.9788 | 0.4857 | 100.0% | 1679.2922 | 1678.796 | 1 | 8.978 | 78.6% | 25 | K.NQLTSNPENTVFDAK.R | 2 |
|  | AstrinIP\_MS2\_022614\_01.06448.06448.2 | 4.4444 | 0.4873 | 100.0% | 1834.4922 | 1834.9835 | 1 | 8.799 | 70.0% | 3 | K.NQLTSNPENTVFDAKR.L | 2 |
|  | SKAPIP\_tube2\_041314\_01.06032.06032.2 | 3.9778 | 0.4032 | 100.0% | 1431.2122 | 1431.5449 | 1 | 7.357 | 81.8% | 23 | R.TWNDPSVQQDIK.F | 2 |
|  | SKAPIP\_tube2\_041314\_02.04673.04673.3 | 3.758 | 0.3286 | 100.0% | 1733.8143 | 1734.0055 | 1 | 6.57 | 36.7% | 3 | K.KTKPYIQVDIGGGQTK.T | 3 |
|  | SKAPIP\_tube2\_041314\_01.05865.05865.2 | 4.3593 | 0.5068 | 100.0% | 1604.8722 | 1605.8314 | 1 | 8.699 | 85.7% | 24 | K.TKPYIQVDIGGGQTK.T | 2 |
|  | SKAPIP\_tube2\_041314\_02.05330.05330.3 | 4.3714 | 0.2722 | 100.0% | 1607.0643 | 1605.8314 | 1 | 6.728 | 50.0% | 10 | K.TKPYIQVDIGGGQTK.T | 3 |
|  | AstrinIP\_MS2\_022614\_01.12558.12558.1 | 2.4231 | 0.4899 | 98.4% | 1536.62 | 1537.8114 | 11 | 7.966 | 42.3% | 1 | K.TFAPEEISAMVLTK.M | 1 |
|  | pDK365N\_300mM\_082713\_03.11228.11228.2 | 4.7961 | 0.5668 | 100.0% | 1537.6122 | 1537.8114 | 1 | 8.705 | 80.8% | 29 | K.TFAPEEISAMVLTK.M | 2 |
|  | SKAPIP\_041314\_01.10533.10533.2 | 3.6295 | 0.3907 | 100.0% | 1241.4722 | 1241.445 | 1 | 7.031 | 85.0% | 5 | K.MKETAEAYLGK.K | 2 |
|  | SKAPIP\_041314\_02.04369.04369.3 | 3.1064 | 0.2758 | 99.8% | 1242.2043 | 1241.445 | 1 | 5.634 | 45.0% | 3 | K.MKETAEAYLGK.K | 3 |
|  | SKAPIP\_041314\_01.05991.05991.2 | 3.7805 | 0.2503 | 100.0% | 1369.2122 | 1369.619 | 1 | 6.729 | 72.7% | 5 | K.MKETAEAYLGKK.V | 2 |
|  | SKAPIP\_tube2\_041314\_02.03989.03989.3 | 4.2907 | 0.3718 | 100.0% | 1369.4043 | 1369.619 | 1 | 7.063 | 56.8% | 10 | K.MKETAEAYLGKK.V | 3 |
|  | SKAPIP\_tube2\_041314\_01.06473.06473.2 | 4.7232 | 0.4836 | 100.0% | 2016.3922 | 2017.295 | 1 | 9.606 | 64.7% | 1 | K.KVTHAVVTVPAYFNDAQR.Q | 22 |
|  | 100326\_pJS43\_01.06020.06020.2 | 4.8547 | 0.4423 | 100.0% | 1889.5122 | 1889.121 | 1 | 7.768 | 71.9% | 10 | K.VTHAVVTVPAYFNDAQR.Q | 22 |
|  | pDK365N\_300mM\_082713\_03.07299.07299.3 | 5.1924 | 0.3256 | 100.0% | 1890.0844 | 1889.121 | 2 | 6.852 | 40.6% | 17 | K.VTHAVVTVPAYFNDAQR.Q | 33 |
|  | SKAPIP\_tube2\_041314\_01.08423.08423.1 | 2.4881 | 0.4155 | 99.3% | 1217.56 | 1218.4137 | 198 | 6.916 | 50.0% | 2 | K.DAGTIAGLNVMR.I | 1 |
|  | AstrinIP\_MS1\_022614\_01.08596.08596.2 | 3.8689 | 0.4284 | 100.0% | 1218.1522 | 1218.4137 | 1 | 7.466 | 86.4% | 23 | K.DAGTIAGLNVMR.I | 2 |
|  | SKAPIP\_tube2\_041314\_01.09597.09597.2 | 5.3704 | 0.5527 | 100.0% | 1660.5322 | 1660.9078 | 1 | 10.193 | 83.3% | 26 | R.IINEPTAAAIAYGLDK.R | 22222 |
|  | pDK365N\_300mM\_082713\_03.09334.09334.3 | 3.8002 | 0.3215 | 100.0% | 1661.3043 | 1660.9078 | 1 | 6.407 | 51.7% | 1 | R.IINEPTAAAIAYGLDK.R | 33333 |
|  | SKAPIP\_tube2\_041314\_01.08583.08583.2 | 5.2935 | 0.5702 | 100.0% | 1816.3722 | 1817.0953 | 1 | 9.108 | 78.1% | 9 | R.IINEPTAAAIAYGLDKR.E | 2 |
|  | AstrinIP\_MS1\_022614\_01.08889.08889.3 | 2.7471 | 0.2552 | 97.9% | 1817.0643 | 1817.0953 | 92 | 5.521 | 26.6% | 1 | R.IINEPTAAAIAYGLDKR.E | 3 |
|  | SKAPIP\_041314\_01.09603.09603.2 | 2.2804 | 0.1308 | 97.2% | 904.97217 | 904.115 | 12 | 4.605 | 75.0% | 4 | R.VMEHFIK.L | 2 |
|  | AstrinIP\_MS2\_022614\_02.08158.08158.2 | 5.9482 | 0.603 | 100.0% | 2165.5522 | 2166.3025 | 1 | 11.014 | 73.5% | 31 | R.IEIESFYEGEDFSETLTR.A | 2 |
|  | SKAPIP\_tube2\_041314\_01.10244.10244.2 | 4.0889 | 0.5093 | 100.0% | 1513.4521 | 1513.7516 | 1 | 8.219 | 72.7% | 16 | R.AKFEELNMDLFR.S | 2 |
|  | pDK365N\_300mM\_082713\_01.10146.10146.3 | 4.6528 | 0.3377 | 100.0% | 1514.1244 | 1513.7516 | 1 | 6.362 | 59.1% | 8 | R.AKFEELNMDLFR.S | 3 |
|  | pDK365N\_300mM\_082713\_01.11303.11303.2 | 3.7155 | 0.4514 | 100.0% | 1315.2922 | 1314.4987 | 1 | 7.478 | 77.8% | 6 | K.FEELNMDLFR.S | 2 |
|  | AstrinIP\_MS1\_022614\_01.03948.03948.1 | 1.9054 | 0.2407 | 98.4% | 918.34 | 919.0196 | 2 | 5.479 | 71.4% | 2 | K.VLEDSDLK.K | 1 |
|  | SKAPIP\_041314\_01.05224.05224.2 | 2.3127 | 0.2127 | 98.7% | 919.21216 | 919.0196 | 122 | 4.616 | 57.1% | 2 | K.VLEDSDLK.K | 2 |
|  | SKAPIP\_tube2\_041314\_01.08660.08660.3 | 5.6975 | 0.4759 | 100.0% | 2490.8943 | 2489.7827 | 1 | 7.63 | 44.3% | 13 | K.VLEDSDLKKSDIDEIVLVGGSTR.I | 3 |
|  | SKAPIP\_tube2\_041314\_02.06411.06411.2 | 4.9458 | 0.2969 | 100.0% | 1588.9521 | 1589.7863 | 1 | 9.691 | 82.1% | 19 | K.KSDIDEIVLVGGSTR.I | 2 |
|  | pDK365N\_300mM\_082713\_02.06676.06676.2 | 3.5832 | 0.0452 | 98.2% | 1462.3922 | 1461.6122 | 1 | 6.646 | 65.4% | 5 | K.SDIDEIVLVGGSTR.I | 2 |
|  | SKAPIP\_041314\_01.09327.09327.2 | 2.9128 | 0.2711 | 99.9% | 1211.0922 | 1211.3195 | 3 | 5.546 | 66.7% | 11 | K.EFFNGKEPSR.G | 22 |
|  | pDK339othertube\_033013\_01.07019.07019.2 | 5.448 | 0.501 | 100.0% | 1837.4922 | 1838.0245 | 1 | 9.641 | 75.0% | 34 | K.SQIFSTASDNQPTVTIK.V | 22 |
|  | pDK365N\_300mM\_082713\_03.06935.06935.3 | 3.0168 | 0.2427 | 98.5% | 1840.0743 | 1838.0245 | 4 | 4.373 | 34.4% | 1 | K.SQIFSTASDNQPTVTIK.V | 33 |
|  | pDK365N\_300mM\_082713\_01.03552.03552.2 | 2.7685 | 0.4548 | 100.0% | 1191.6522 | 1192.3574 | 1 | 7.136 | 83.3% | 4 | K.VYEGERPLTK.D | 22 |
|  | AstrinIP\_MS1\_022614\_01.11088.11088.2 | 4.1652 | 0.564 | 100.0% | 1934.5122 | 1935.19 | 1 | 9.377 | 61.8% | 16 | K.DNHLLGTFDLTGIPPAPR.G | 22 |
|  | pDK365N\_300mM\_082713\_03.10484.10484.3 | 2.5731 | 0.28 | 97.9% | 1935.9243 | 1935.19 | 201 | 5.304 | 29.4% | 1 | K.DNHLLGTFDLTGIPPAPR.G | 33 |
|  | SKAPIP\_tube2\_041314\_02.11004.11004.2 | 3.4152 | 0.4421 | 100.0% | 2000.4122 | 2000.3024 | 1 | 7.794 | 44.1% | 6 | R.GVPQIEVTFEIDVNGILR.V | 22 |
|  | AstrinIP\_MS2\_022614\_01.03741.03741.2 | 2.5616 | 0.2861 | 99.7% | 1075.0322 | 1075.1667 | 5 | 6.002 | 75.0% | 1 | K.ITITNDQNR.L | 22 |
|  | SKAPIP\_041314\_01.09665.09665.2 | 4.3023 | 0.3916 | 100.0% | 1654.2922 | 1654.8323 | 1 | 7.962 | 76.9% | 6 | R.MVNDAEKFAEEDKK.L | 22 |
|  | SKAPIP\_041314\_01.09428.09428.3 | 3.7114 | 0.4453 | 100.0% | 1656.3243 | 1654.8323 | 1 | 6.745 | 44.2% | 9 | R.MVNDAEKFAEEDKK.L | 33 |
|  | pDK365N\_300mM\_082713\_01.04076.04076.3 | 4.2362 | 0.3324 | 100.0% | 1896.5643 | 1896.1658 | 11 | 6.35 | 36.7% | 3 | R.MVNDAEKFAEEDKKLK.E | 33 |
|  | pSKT11\_1\_020812\_01.07800.07800.3 | 4.7512 | 0.451 | 100.0% | 2180.7244 | 2181.4688 | 1 | 8.039 | 44.1% | 1 | R.MVNDAEKFAEEDKKLKER.I | 3 |
|  | SKAPIP\_tube2\_041314\_01.08654.08654.2 | 3.1903 | 0.4626 | 100.0% | 1802.2122 | 1802.9788 | 1 | 6.405 | 71.4% | 2 | R.IDTRNELESYAYSLK.N | 2 |
|  | AstrinIP\_MS2\_022614\_01.09395.09395.3 | 3.8743 | 0.2334 | 99.8% | 1804.1344 | 1802.9788 | 1 | 4.226 | 46.4% | 5 | R.IDTRNELESYAYSLK.N | 3 |
|  | SKAPIP\_tube2\_041314\_01.08374.08374.1 | 2.4271 | 0.249 | 98.8% | 1316.55 | 1317.4381 | 1 | 4.795 | 70.0% | 2 | R.NELESYAYSLK.N | 1 |
|  | pDK365N\_300mM\_082713\_03.07983.07983.2 | 3.8381 | 0.3572 | 100.0% | 1318.4321 | 1317.4381 | 1 | 6.261 | 90.0% | 23 | R.NELESYAYSLK.N | 2 |
|  | SKAPIP\_tube2\_041314\_02.07172.07172.3 | 5.1219 | 0.3889 | 100.0% | 2532.5645 | 2532.723 | 1 | 6.901 | 43.8% | 11 | K.AVEEKIEWLESHQDADIEDFK.A | 3 |
|  | pSKT11\_1\_020812\_02.04929.04929.3 | 6.0236 | 0.5236 | 100.0% | 2731.1643 | 2731.9756 | 1 | 7.459 | 34.1% | 5 | K.AVEEKIEWLESHQDADIEDFKAK.K | 3 |
|  | AstrinIP\_MS2\_022614\_01.10173.10173.2 | 5.1961 | 0.3803 | 100.0% | 1975.4122 | 1976.1064 | 1 | 8.593 | 66.7% | 5 | K.IEWLESHQDADIEDFK.A | 2 |
|  | pDK365N\_100mM\_082713\_02.06537.06537.3 | 4.4542 | 0.3652 | 100.0% | 1975.7043 | 1976.1064 | 1 | 5.808 | 50.0% | 25 | K.IEWLESHQDADIEDFK.A | 3 |
|  | SKAPIP\_tube2\_041314\_02.06606.06606.2 | 5.4982 | 0.5189 | 100.0% | 2175.4922 | 2175.3594 | 1 | 8.87 | 64.7% | 1 | K.IEWLESHQDADIEDFKAK.K | 2 |
|  | pDK365N\_300mM\_082713\_02.06213.06213.3 | 5.4498 | 0.3704 | 100.0% | 2175.6543 | 2175.3594 | 1 | 7.082 | 47.1% | 23 | K.IEWLESHQDADIEDFKAK.K | 3 |
|  | SKAPIP\_041314\_01.11124.11124.2 | 5.3597 | 0.34 | 100.0% | 1654.6322 | 1654.9878 | 1 | 7.778 | 88.5% | 10 | K.KKELEEIVQPIISK.L | 2 |
|  | pDK365N\_300mM\_082713\_03.06692.06692.3 | 4.4179 | 0.2235 | 99.9% | 1656.2043 | 1654.9878 | 28 | 4.954 | 42.3% | 13 | K.KKELEEIVQPIISK.L | 3 |
|  | pDK365N\_300mM\_082713\_03.08781.08781.2 | 4.2143 | 0.3963 | 100.0% | 1399.5122 | 1398.6396 | 1 | 6.717 | 81.8% | 20 | K.ELEEIVQPIISK.L | 2 |
|  | SKAPIP\_tube2\_041314\_01.06584.06584.2 | 6.0714 | 0.6488 | 100.0% | 2176.412 | 2177.283 | 1 | 11.349 | 60.0% | 28 | K.LYGSAGPPPTGEEDTAEKDEL.- | 2 |

Similarities:
gi|5729877|ref|NP\_006(2:58)  
contaminant\_GR78\_PIG(11:49)  
gi|13676857|ref|NP\_06(2:58)  
gi|124256496|ref|NP\_0(2:58)  
contaminant\_GR78\_KLUL(3:57)  
contaminant\_GR78\_MAIZ(2:58)  
contaminant\_GR78\_SCHP(2:58)  

---

|  |  |  |  |  |  |  |  |  |
| --- | --- | --- | --- | --- | --- | --- | --- | --- |
| U | *gi|226530908|ref|NP\_0* | 34 | 540 | 57.5% | 285 | 30315 | 7.5 | protein-L-isoaspartate (D-aspartate) O-methyltransferase [Homo sapiens] |

| Filename XCorr DeltCN Conf% ObsM+H+ CalcM+H+ SpR ZScore Ion% # Sequence  | | | | | | | | | | | | |
| --- | --- | --- | --- | --- | --- | --- | --- | --- | --- | --- | --- | --- |
| \* | SKAPIP\_041314\_01.06434.06434.2 | 4.5562 | 0.4083 | 100.0% | 1478.2922 | 1478.6078 | 1 | 8.409 | 61.5% | 17 | K.SGGASHSELIHNLR.K | 2 |
| \* | SKAPIP\_041314\_01.06470.06470.3 | 4.389 | 0.3534 | 100.0% | 1479.0844 | 1478.6078 | 3 | 7.212 | 44.2% | 27 | K.SGGASHSELIHNLR.K | 3 |
| \* | pSKT11\_1\_020812\_01.03219.03219.2 | 4.2283 | 0.5182 | 100.0% | 1605.4521 | 1606.7819 | 1 | 8.07 | 60.7% | 31 | K.SGGASHSELIHNLRK.N | 2 |
| \* | AstrinIP\_MS2\_022614\_01.03662.03662.3 | 3.888 | 0.3738 | 100.0% | 1607.6643 | 1606.7819 | 1 | 6.65 | 42.9% | 28 | K.SGGASHSELIHNLRK.N | 3 |
| \* | pSKT11\_1\_020812\_02.05079.05079.3 | 4.7447 | 0.4728 | 100.0% | 2178.8342 | 2179.583 | 1 | 7.974 | 37.5% | 1 | R.KNGIIKTDKVFEVMLATDR.S | 3 |
| \* | pSKT11\_1\_020812\_01.09267.09267.2 | 5.9203 | 0.5231 | 100.0% | 2051.652 | 2051.409 | 1 | 9.388 | 67.6% | 6 | K.NGIIKTDKVFEVMLATDR.S | 2 |
| \* | pSKT11\_1\_020812\_02.05708.05708.3 | 4.4842 | 0.5088 | 100.0% | 2051.7244 | 2051.409 | 1 | 8.551 | 41.2% | 30 | K.NGIIKTDKVFEVMLATDR.S | 3 |
| \* | SKAPIP\_tube2\_041314\_01.08504.08504.3 | 3.1399 | 0.2923 | 99.3% | 2636.7544 | 2638.057 | 1 | 4.715 | 29.5% | 1 | K.NGIIKTDKVFEVMLATDRSHYAK.C | 3 |
| \* | SKAPIP\_tube2\_041314\_01.08728.08728.1 | 2.8639 | 0.5036 | 96.9% | 1524.66 | 1525.7601 | 1 | 7.64 | 58.3% | 3 | K.TDKVFEVMLATDR.S | 1 |
| \* | AstrinIP\_MS1\_022614\_01.08930.08930.2 | 4.7151 | 0.5257 | 100.0% | 1526.0521 | 1525.7601 | 1 | 9.142 | 83.3% | 45 | K.TDKVFEVMLATDR.S | 2 |
| \* | pDK365N\_300mM\_082713\_02.06666.06666.3 | 4.2288 | 0.3978 | 100.0% | 1526.5443 | 1525.7601 | 1 | 6.475 | 52.1% | 17 | K.TDKVFEVMLATDR.S | 3 |
| \* | SKAPIP\_tube2\_041314\_01.07332.07332.3 | 2.7143 | 0.2605 | 97.7% | 2112.0244 | 2112.4082 | 8 | 4.396 | 30.9% | 1 | K.TDKVFEVMLATDRSHYAK.C | 3 |
| \* | AstrinIP\_MS1\_022614\_01.09096.09096.1 | 2.4875 | 0.4445 | 99.2% | 1180.6 | 1181.3923 | 5 | 6.713 | 61.1% | 5 | K.VFEVMLATDR.S | 1 |
| \* | pDK365N\_300mM\_082713\_03.08540.08540.2 | 4.1975 | 0.5756 | 100.0% | 1181.2322 | 1181.3923 | 1 | 9.435 | 88.9% | 45 | K.VFEVMLATDR.S | 2 |
| \* | AstrinIP\_MS1\_022614\_02.07321.07321.2 | 5.8616 | 0.6678 | 100.0% | 1695.1921 | 1695.8792 | 1 | 12.258 | 81.2% | 14 | K.ALDVGSGSGILTACFAR.M | 2 |
| \* | SKAPIP\_041314\_02.07979.07979.3 | 3.1479 | 0.2793 | 99.5% | 1696.4944 | 1695.8792 | 3 | 5.024 | 39.1% | 1 | K.ALDVGSGSGILTACFAR.M | 3 |
| \* | pSKT11\_1\_020812\_01.04496.04496.1 | 1.5311 | 0.293 | 98.5% | 894.49 | 895.0898 | 18 | 4.781 | 64.3% | 5 | K.VIGIDHIK.E | 1 |
| \* | pSKT11\_1\_020812\_01.04532.04532.2 | 3.0543 | 0.3393 | 100.0% | 895.09216 | 895.0898 | 1 | 6.116 | 92.9% | 12 | K.VIGIDHIK.E | 2 |
| \* | AstrinIP\_MS2\_022614\_01.04185.04185.1 | 1.6336 | 0.4457 | 98.7% | 1188.55 | 1189.3109 | 2 | 6.803 | 50.0% | 4 | R.KDDPTLLSSGR.V | 1 |
| \* | AstrinIP\_MS2\_022614\_01.04160.04160.2 | 3.0462 | 0.4016 | 100.0% | 1188.6721 | 1189.3109 | 1 | 6.999 | 75.0% | 24 | R.KDDPTLLSSGR.V | 2 |
| \* | SKAPIP\_041314\_01.05600.05600.3 | 3.2935 | 0.3873 | 100.0% | 1189.3444 | 1189.3109 | 3 | 6.421 | 47.5% | 10 | R.KDDPTLLSSGR.V | 3 |
| \* | pDK365N\_300mM\_082713\_03.04538.04538.2 | 2.9819 | 0.2887 | 99.9% | 1061.0922 | 1061.1368 | 1 | 5.484 | 88.9% | 6 | K.DDPTLLSSGR.V | 2 |
| \* | pJS43\_100mM\_120812\_01.05193.05193.1 | 2.3093 | 0.3823 | 99.5% | 942.57 | 943.091 | 5 | 7.789 | 62.5% | 21 | R.VQLVVGDGR.M | 1 |
| \* | pDK339\_033013\_01.04807.04807.2 | 3.157 | 0.3348 | 100.0% | 942.9922 | 943.091 | 1 | 7.361 | 87.5% | 33 | R.VQLVVGDGR.M | 2 |
| \* | SKAPIP\_tube2\_041314\_01.11613.11613.2 | 4.2124 | 0.5227 | 100.0% | 3506.2322 | 3507.0015 | 1 | 9.759 | 27.3% | 3 | R.MGYAEEAPYDAIHVGAAAPVVPQALIDQLKPGGR.L | 2 |
| \* | pDK339\_033013\_01.12643.12643.3 | 7.5943 | 0.614 | 100.0% | 3507.3542 | 3507.0015 | 1 | 10.397 | 31.8% | 36 | R.MGYAEEAPYDAIHVGAAAPVVPQALIDQLKPGGR.L | 3 |
| \* | AstrinIP\_MS1\_022614\_01.10929.10929.3 | 3.4857 | 0.4517 | 100.0% | 2043.9543 | 2044.3734 | 1 | 6.309 | 38.9% | 3 | R.LILPVGPAGGNQMLEQYDK.L | 3 |
| \* | SKAPIP\_041314\_01.12230.12230.2 | 5.1605 | 0.4668 | 100.0% | 2044.6122 | 2044.3734 | 1 | 8.398 | 75.0% | 35 | R.LILPVGPAGGNQMLEQYDK.L | 2 |
| \* | AstrinIP\_MS1\_022614\_01.11471.11471.2 | 4.8964 | 0.507 | 100.0% | 2785.2122 | 2786.2158 | 1 | 8.933 | 40.0% | 11 | R.LILPVGPAGGNQMLEQYDKLQDGSIK.M | 2 |
| \* | pDK365N\_300mM\_082713\_03.10466.10466.3 | 4.1859 | 0.3087 | 100.0% | 1706.2444 | 1706.1549 | 1 | 6.988 | 51.8% | 9 | K.MKPLMGVIYVPLTDK.E | 3 |
| \* | AstrinIP\_MS2\_022614\_01.11631.11631.2 | 4.4542 | 0.4427 | 100.0% | 1706.3922 | 1706.1549 | 1 | 7.488 | 78.6% | 15 | K.MKPLMGVIYVPLTDK.E | 2 |
| \* | AstrinIP\_MS1\_022614\_01.09602.09602.2 | 4.9935 | 0.6105 | 100.0% | 1962.5322 | 1963.4445 | 1 | 10.072 | 71.9% | 15 | K.MKPLMGVIYVPLTDKEK.Q | 2 |
| \* | pJS43\_100mM\_120812\_01.10283.10283.3 | 4.1426 | 0.339 | 100.0% | 1963.3444 | 1963.4445 | 1 | 7.241 | 42.2% | 25 | K.MKPLMGVIYVPLTDKEK.Q | 3 |
| \* | pSKT11\_1\_020812\_01.09147.09147.3 | 3.3433 | 0.4334 | 100.0% | 2521.0745 | 2521.054 | 1 | 6.413 | 38.8% | 1 | K.MKPLMGVIYVPLTDKEKQWSR.W | 3 |

---

|  |  |  |  |  |  |  |  |  |
| --- | --- | --- | --- | --- | --- | --- | --- | --- |
| U | *gi|10800140|ref|NP\_06* | 10 | 79 | 56.3% | 126 | 13950 | 10.3 | histone cluster 1, H2bb [Homo sapiens] |
| U | *gi|4504277|ref|NP\_003* | 10 | 79 | 56.3% | 126 | 13920 | 10.3 | histone cluster 2, H2be [Homo sapiens] |
| U | *gi|16306566|ref|NP\_00* | 10 | 79 | 56.3% | 126 | 13906 | 10.3 | histone cluster 1, H2bo [Homo sapiens] |

| Filename XCorr DeltCN Conf% ObsM+H+ CalcM+H+ SpR ZScore Ion% # Sequence  | | | | | | | | | | | | |
| --- | --- | --- | --- | --- | --- | --- | --- | --- | --- | --- | --- | --- |
|  | pJS43\_100mM\_120812\_01.06355.06355.2 | 2.9417 | 0.3672 | 100.0% | 1280.2722 | 1280.4631 | 1 | 6.51 | 77.8% | 2 | R.KESYSIYVYK.V | 22 |
|  | pDK365N\_100mM\_082713\_01.12750.12750.2 | 5.0897 | 0.4342 | 100.0% | 1745.7322 | 1745.0211 | 1 | 8.753 | 71.4% | 34 | K.AMGIMNSFVNDIFER.I | 2222 |
|  | pJS43\_100mM\_120812\_01.02709.02709.2 | 2.2213 | 0.1555 | 97.4% | 901.9922 | 902.0439 | 12 | 3.881 | 83.3% | 1 | R.LAHYNKR.S | 2222 |
|  | AstrinNocIP\_020510\_01.06332.06332.3 | 3.0197 | 0.3576 | 99.9% | 2396.7844 | 2397.821 | 1 | 5.978 | 31.0% | 1 | R.STITSREIQTAVRLLLPGELAK.H | 3333 |
|  | AstrinNocIP\_020510\_01.04893.04893.2 | 3.9454 | 0.2645 | 100.0% | 1752.4922 | 1752.1075 | 1 | 6.034 | 60.0% | 2 | R.EIQTAVRLLLPGELAK.H | 2222 |
|  | pJS43\_100mM\_120812\_01.09215.09215.2 | 2.2344 | 0.1613 | 95.8% | 954.3122 | 954.19794 | 19 | 4.455 | 68.8% | 1 | R.LLLPGELAK.H | 2222 |
|  | AstrinNocIP\_020510\_01.04779.04779.3 | 3.4319 | 0.2162 | 97.8% | 2729.9644 | 2730.1772 | 293 | 4.247 | 23.0% | 1 | R.LLLPGELAKHAVSEGTKAVTKYTSSK.- | 3 |
|  | pJS43\_100mM\_120812\_01.01053.01053.2 | 2.3399 | 0.2029 | 98.7% | 829.4522 | 828.9004 | 5 | 5.39 | 71.4% | 1 | K.HAVSEGTK.A | 2222 |
|  | AstrinNocIP\_020510\_01.01827.01827.2 | 4.752 | 0.5783 | 100.0% | 1794.4122 | 1795.0024 | 1 | 11.118 | 68.8% | 9 | K.HAVSEGTKAVTKYTSSK.- | 22 |
|  | AstrinNocIP\_020510\_01.01730.01730.3 | 4.8535 | 0.5208 | 100.0% | 1795.3444 | 1795.0024 | 1 | 9.44 | 42.2% | 27 | K.HAVSEGTKAVTKYTSSK.- | 33 |

Similarities:
gi|10800138|ref|NP\_06(8:2)  
gi|18105048|ref|NP\_54(6:4)  
gi|20336754|ref|NP\_06(7:3)  

---

|  |  |  |  |  |  |  |  |  |
| --- | --- | --- | --- | --- | --- | --- | --- | --- |
| U | *gi|20336754|ref|NP\_06* | 9 | 48 | 56.3% | 126 | 13904 | 10.3 | histone cluster 1, H2bj [Homo sapiens] |

| Filename XCorr DeltCN Conf% ObsM+H+ CalcM+H+ SpR ZScore Ion% # Sequence  | | | | | | | | | | | | |
| --- | --- | --- | --- | --- | --- | --- | --- | --- | --- | --- | --- | --- |
|  | pJS43\_100mM\_120812\_01.06355.06355.2 | 2.9417 | 0.3672 | 100.0% | 1280.2722 | 1280.4631 | 1 | 6.51 | 77.8% | 2 | R.KESYSIYVYK.V | 22 |
|  | pDK365N\_100mM\_082713\_01.12750.12750.2 | 5.0897 | 0.4342 | 100.0% | 1745.7322 | 1745.0211 | 1 | 8.753 | 71.4% | 34 | K.AMGIMNSFVNDIFER.I | 2222 |
|  | pJS43\_100mM\_120812\_01.02709.02709.2 | 2.2213 | 0.1555 | 97.4% | 901.9922 | 902.0439 | 12 | 3.881 | 83.3% | 1 | R.LAHYNKR.S | 2222 |
|  | AstrinNocIP\_020510\_01.06332.06332.3 | 3.0197 | 0.3576 | 99.9% | 2396.7844 | 2397.821 | 1 | 5.978 | 31.0% | 1 | R.STITSREIQTAVRLLLPGELAK.H | 3333 |
|  | AstrinNocIP\_020510\_01.04893.04893.2 | 3.9454 | 0.2645 | 100.0% | 1752.4922 | 1752.1075 | 1 | 6.034 | 60.0% | 2 | R.EIQTAVRLLLPGELAK.H | 2222 |
|  | pJS43\_100mM\_120812\_01.09215.09215.2 | 2.2344 | 0.1613 | 95.8% | 954.3122 | 954.19794 | 19 | 4.455 | 68.8% | 1 | R.LLLPGELAK.H | 2222 |
|  | pJS43\_100mM\_120812\_01.01053.01053.2 | 2.3399 | 0.2029 | 98.7% | 829.4522 | 828.9004 | 5 | 5.39 | 71.4% | 1 | K.HAVSEGTK.A | 2222 |
|  | AstrinNocIP\_020510\_01.01761.01761.3 | 3.7363 | 0.507 | 100.0% | 1777.7344 | 1779.003 | 1 | 8.724 | 40.6% | 4 | K.HAVSEGTKAVTKYTSAK.- | 33 |
|  | AstrinNocIP\_020510\_01.01803.01803.2 | 4.6925 | 0.6285 | 100.0% | 1778.3522 | 1779.003 | 1 | 11.72 | 62.5% | 2 | K.HAVSEGTKAVTKYTSAK.- | 22 |

Similarities:
gi|10800138|ref|NP\_06(6:3)  
gi|18105048|ref|NP\_54(8:1)  
gi|10800140|ref|NP\_06(7:2)  

---

|  |  |  |  |  |  |  |  |  |
| --- | --- | --- | --- | --- | --- | --- | --- | --- |
| U | *gi|4506691|ref|NP\_001* | 13 | 52 | 56.2% | 146 | 16445 | 10.2 | ribosomal protein S16 [Homo sapiens] |

| Filename XCorr DeltCN Conf% ObsM+H+ CalcM+H+ SpR ZScore Ion% # Sequence  | | | | | | | | | | | | |
| --- | --- | --- | --- | --- | --- | --- | --- | --- | --- | --- | --- | --- |
|  | pJS43\_100mM\_120812\_01.09281.09281.2 | 4.038 | 0.5382 | 100.0% | 1188.3121 | 1188.372 | 1 | 8.329 | 75.0% | 15 | K.GPLQSVQVFGR.K | 2 |
| \* | pJS43\_100mM\_120812\_01.06948.06948.3 | 3.5405 | 0.1896 | 99.2% | 1411.1344 | 1411.6622 | 1 | 5.163 | 50.0% | 8 | K.VNGRPLEMIEPR.T | 3 |
| \* | pJS43\_100mM\_120812\_01.06956.06956.2 | 3.2092 | 0.291 | 99.9% | 1412.5721 | 1411.6622 | 1 | 5.308 | 68.2% | 2 | K.VNGRPLEMIEPR.T | 2 |
| \* | AstrinNocIP\_020510\_01.05472.05472.3 | 3.3936 | 0.3141 | 99.9% | 2014.7644 | 2014.4594 | 1 | 6.004 | 39.1% | 1 | R.TLQYKLLEPVLLLGKER.F | 3 |
|  | pDK365N\_100mM\_082813\_03.11454.11454.2 | 2.7467 | 0.3154 | 99.9% | 1095.2922 | 1095.4111 | 1 | 6.52 | 72.2% | 6 | K.LLEPVLLLGK.E | 2 |
| \* | pJS43\_100mM\_120812\_02.04278.04278.3 | 3.852 | 0.3231 | 100.0% | 1470.0543 | 1469.7299 | 1 | 6.202 | 46.2% | 3 | R.VKGGGHVAQIYAIR.Q | 3 |
| \* | pJS43\_100mM\_120812\_01.05275.05275.2 | 3.4565 | 0.4645 | 100.0% | 1242.1122 | 1242.4232 | 3 | 7.526 | 68.2% | 2 | K.GGGHVAQIYAIR.Q | 2 |
|  | pSKT11\_1\_020812\_01.05638.05638.1 | 1.8911 | 0.2098 | 96.5% | 955.48 | 956.1295 | 7 | 4.83 | 64.3% | 1 | K.ALVAYYQK.Y | 1 |
| \* | pJS43\_100mM\_120812\_01.09203.09203.2 | 2.5307 | 0.3494 | 99.8% | 1406.6322 | 1406.622 | 6 | 5.74 | 75.0% | 3 | K.EIKDILIQYDR.T | 2 |
| \* | pJS43\_100mM\_120812\_01.09264.09264.3 | 3.5095 | 0.1929 | 99.4% | 1407.3844 | 1406.622 | 9 | 4.829 | 45.0% | 5 | K.EIKDILIQYDR.T | 3 |
| \* | pDK365N\_100mM\_082713\_01.06909.06909.2 | 2.3158 | 0.2757 | 99.5% | 1037.0122 | 1036.1729 | 5 | 5.53 | 85.7% | 2 | K.DILIQYDR.T | 2 |
| \* | pJS43\_100mM\_120812\_01.05934.05934.2 | 2.3433 | 0.2082 | 98.8% | 885.0122 | 885.0513 | 9 | 5.332 | 71.4% | 3 | R.TLLVADPR.R | 2 |
| \* | pSKT11\_1\_020812\_01.04199.04199.2 | 2.1068 | 0.2188 | 96.9% | 1041.0922 | 1041.2388 | 1 | 4.559 | 75.0% | 1 | R.TLLVADPRR.C | 2 |

---

|  |  |  |  |  |  |  |  |  |
| --- | --- | --- | --- | --- | --- | --- | --- | --- |
| U | *gi|4757926|ref|NP\_004* | 39 | 133 | 56.1% | 524 | 58657 | 10.1 | RNA binding motif protein 39 isoform b [Homo sapiens] |

| Filename XCorr DeltCN Conf% ObsM+H+ CalcM+H+ SpR ZScore Ion% # Sequence  | | | | | | | | | | | | |
| --- | --- | --- | --- | --- | --- | --- | --- | --- | --- | --- | --- | --- |
|  | SKAPIP\_041314\_01.10671.10671.2 | 2.6228 | 0.2043 | 99.5% | 865.0122 | 865.0635 | 2 | 5.073 | 78.6% | 3 | K.IGLPHSIK.L | 2 |
|  | SKAPIP\_tube2\_041314\_01.04684.04684.3 | 3.1285 | 0.3032 | 99.7% | 2204.2444 | 2204.3586 | 1 | 5.382 | 39.7% | 2 | R.KDKS\*PVREPIDNLTPEER.D | 3 |
|  | SKAPIP\_041314\_02.04429.04429.3 | 4.4269 | 0.485 | 100.0% | 2075.6943 | 2076.1846 | 1 | 7.627 | 46.9% | 5 | K.DKS\*PVREPIDNLTPEER.D | 3 |
|  | SKAPIP\_041314\_02.06754.06754.2 | 3.3239 | 0.469 | 100.0% | 1197.3322 | 1197.404 | 1 | 7.288 | 88.9% | 4 | R.TVFCMQLAAR.I | 22 |
|  | SKAPIP\_tube2\_041314\_01.10685.10685.3 | 2.5475 | 0.2425 | 96.0% | 1796.3043 | 1795.0482 | 1 | 4.187 | 44.6% | 1 | R.IRPRDLEEFFSTVGK.V | 3 |
|  | SKAPIP\_tube2\_041314\_01.12255.12255.1 | 2.2084 | 0.4093 | 99.5% | 1271.45 | 1272.3972 | 1 | 6.995 | 65.0% | 3 | R.DLEEFFSTVGK.V | 1 |
|  | SKAPIP\_tube2\_041314\_01.12236.12236.2 | 3.756 | 0.4979 | 100.0% | 1272.1921 | 1272.3972 | 1 | 7.9 | 70.0% | 7 | R.DLEEFFSTVGK.V | 2 |
|  | SKAPIP\_041314\_01.12779.12779.2 | 2.7621 | 0.3551 | 99.9% | 1528.3322 | 1527.7172 | 61 | 6.377 | 41.7% | 1 | R.DLEEFFSTVGKVR.D | 2 |
|  | SKAPIP\_tube2\_041314\_01.12826.12826.2 | 5.2543 | 0.562 | 100.0% | 2607.2522 | 2608.0098 | 1 | 9.517 | 54.2% | 2 | R.SKGIAYVEFVDVSSVPLAIGLTGQR.V | 2 |
|  | SKAPIP\_tube2\_041314\_02.09801.09801.3 | 3.8495 | 0.1837 | 97.8% | 2608.1943 | 2608.0098 | 1 | 4.74 | 30.2% | 1 | R.SKGIAYVEFVDVSSVPLAIGLTGQR.V | 3 |
|  | SKAPIP\_tube2\_041314\_01.13934.13934.2 | 4.6236 | 0.6306 | 100.0% | 2391.912 | 2392.7576 | 1 | 10.846 | 54.5% | 7 | K.GIAYVEFVDVSSVPLAIGLTGQR.V | 2 |
|  | SKAPIP\_041314\_02.11092.11092.3 | 6.3591 | 0.5772 | 100.0% | 2393.3044 | 2392.7576 | 1 | 11.091 | 42.0% | 5 | K.GIAYVEFVDVSSVPLAIGLTGQR.V | 3 |
|  | SKAPIP\_tube2\_041314\_01.09798.09798.1 | 2.8717 | 0.4655 | 98.3% | 1551.96 | 1552.8546 | 1 | 6.832 | 53.6% | 1 | R.VLGVPIIVQASQAEK.N | 1 |
|  | SKAPIP\_tube2\_041314\_01.09818.09818.2 | 4.5948 | 0.5618 | 100.0% | 1552.4521 | 1552.8546 | 1 | 9.27 | 78.6% | 15 | R.VLGVPIIVQASQAEK.N | 2 |
|  | SKAPIP\_tube2\_041314\_01.11529.11529.2 | 4.1924 | 0.3693 | 100.0% | 1909.4722 | 1909.2103 | 1 | 8.159 | 73.3% | 6 | R.LYVGSLHFNITEDMLR.G | 22 |
|  | SKAPIP\_041314\_02.09220.09220.3 | 3.8767 | 0.436 | 100.0% | 1910.4844 | 1909.2103 | 2 | 6.721 | 46.7% | 5 | R.LYVGSLHFNITEDMLR.G | 33 |
|  | SKAPIP\_tube2\_041314\_01.09620.09620.1 | 1.8115 | 0.2341 | 97.8% | 921.9 | 923.05927 | 23 | 5.399 | 50.0% | 1 | R.GIFEPFGR.I | 1 |
|  | SKAPIP\_041314\_01.12010.12010.2 | 2.1261 | 0.3431 | 99.5% | 922.71216 | 923.05927 | 2 | 6.72 | 71.4% | 2 | R.GIFEPFGR.I | 2 |
|  | SKAPIP\_041314\_01.11878.11878.2 | 5.0534 | 0.5324 | 100.0% | 1610.4922 | 1610.8378 | 1 | 9.536 | 80.8% | 11 | R.IESIQLMMDSETGR.S | 2 |
|  | SKAPIP\_tube2\_041314\_02.06549.06549.3 | 3.8692 | 0.378 | 100.0% | 1798.1044 | 1797.925 | 1 | 6.675 | 48.3% | 3 | R.SKGYGFITFSDSECAK.K | 3 |
|  | SKAPIP\_041314\_02.05860.05860.3 | 3.9885 | 0.3762 | 100.0% | 1926.5343 | 1926.0991 | 2 | 6.642 | 40.6% | 3 | R.SKGYGFITFSDSECAKK.A | 3 |
|  | SKAPIP\_041314\_02.07661.07661.2 | 4.163 | 0.577 | 100.0% | 1582.3322 | 1582.6729 | 1 | 9.679 | 65.4% | 2 | K.GYGFITFSDSECAK.K | 2 |
|  | SKAPIP\_tube2\_041314\_02.06699.06699.2 | 3.7444 | 0.3985 | 100.0% | 1709.7122 | 1710.8469 | 1 | 7.061 | 50.0% | 1 | K.GYGFITFSDSECAKK.A | 2 |
|  | SKAPIP\_041314\_02.06736.06736.3 | 2.2222 | 0.3742 | 99.4% | 1711.9744 | 1710.8469 | 1 | 5.851 | 41.1% | 1 | K.GYGFITFSDSECAKK.A | 3 |
|  | SKAPIP\_tube2\_041314\_01.07338.07338.2 | 4.8242 | 0.4525 | 100.0% | 1902.3922 | 1903.25 | 1 | 8.043 | 62.5% | 1 | K.KALEQLNGFELAGRPMK.V | 2 |
|  | SKAPIP\_tube2\_041314\_01.07316.07316.3 | 4.7256 | 0.3518 | 100.0% | 1902.7743 | 1903.25 | 2 | 7.033 | 42.2% | 2 | K.KALEQLNGFELAGRPMK.V | 3 |
|  | SKAPIP\_tube2\_041314\_01.08542.08542.3 | 2.9142 | 0.2917 | 99.4% | 1775.4243 | 1775.0758 | 2 | 4.66 | 41.7% | 1 | K.ALEQLNGFELAGRPMK.V | 3 |
|  | SKAPIP\_041314\_01.11530.11530.2 | 4.1512 | 0.3929 | 100.0% | 1775.5122 | 1775.0758 | 1 | 6.47 | 53.3% | 2 | K.ALEQLNGFELAGRPMK.V | 2 |
|  | SKAPIP\_tube2\_041314\_02.06842.06842.2 | 5.6556 | 0.5363 | 100.0% | 1830.2322 | 1830.8566 | 1 | 10.569 | 71.9% | 10 | R.TDASSASSFLDSDELER.T | 2 |
|  | SKAPIP\_tube2\_041314\_02.08073.08073.3 | 5.261 | 0.5132 | 100.0% | 2802.4744 | 2802.9226 | 1 | 7.932 | 33.7% | 3 | R.TDASSASSFLDSDELERTGIDLGTTGR.L | 3 |
|  | SKAPIP\_041314\_01.10659.10659.1 | 2.1717 | 0.3619 | 98.5% | 990.52 | 991.0892 | 16 | 6.429 | 55.6% | 2 | R.TGIDLGTTGR.L | 1 |
|  | SKAPIP\_041314\_01.10655.10655.2 | 3.5659 | 0.291 | 100.0% | 992.1922 | 991.0892 | 1 | 7.234 | 83.3% | 7 | R.TGIDLGTTGR.L | 2 |
| \* | SKAPIP\_tube2\_041314\_02.12006.12006.3 | 3.8015 | 0.2605 | 99.6% | 3413.8442 | 3412.886 | 69 | 5.981 | 18.2% | 1 | R.LAEGTGLQIPPAAQQALQMSGSLAFGAVADLQTR.L | 3 |
|  | SKAPIP\_041314\_01.10558.10558.2 | 3.997 | 0.5228 | 100.0% | 1237.7122 | 1238.4319 | 1 | 9.959 | 90.0% | 1 | K.HGGVIHIYVDK.N | 2 |
|  | SKAPIP\_tube2\_041314\_02.04515.04515.3 | 3.7175 | 0.2778 | 100.0% | 1238.4243 | 1238.4319 | 2 | 5.655 | 50.0% | 3 | K.HGGVIHIYVDK.N | 3 |
|  | SKAPIP\_tube2\_041314\_02.05032.05032.3 | 3.34 | 0.3208 | 99.8% | 2298.9543 | 2299.5942 | 4 | 5.091 | 32.5% | 1 | K.HGGVIHIYVDKNSAQGNVYVK.C | 3 |
|  | SKAPIP\_tube2\_041314\_01.11171.11171.3 | 5.675 | 0.4722 | 100.0% | 1692.8944 | 1692.9249 | 1 | 8.721 | 56.2% | 3 | K.CPSIAAAIAAVNALHGR.W | 3 |
|  | SKAPIP\_041314\_01.12376.12376.2 | 5.4224 | 0.5381 | 100.0% | 1692.9321 | 1692.9249 | 1 | 10.3 | 71.9% | 2 | K.CPSIAAAIAAVNALHGR.W | 2 |
|  | SKAPIP\_041314\_01.12976.12976.3 | 3.3148 | 0.2079 | 96.6% | 3507.2043 | 3506.1182 | 1 | 4.906 | 27.5% | 2 | K.MITAAYVPLPTYHNLFPDSMTATQLLVPSRR.- | 3 |

Similarities:
gi|116734694|ref|NP\_0(3:36)  

---

|  |  |  |  |  |  |  |  |  |
| --- | --- | --- | --- | --- | --- | --- | --- | --- |
| U | *gi|8923557|ref|NP\_060* | 9 | 26 | 55.7% | 228 | 26749 | 5.0 | chromosome 20 open reading frame 11 [Homo sapiens] |

| Filename XCorr DeltCN Conf% ObsM+H+ CalcM+H+ SpR ZScore Ion% # Sequence  | | | | | | | | | | | | |
| --- | --- | --- | --- | --- | --- | --- | --- | --- | --- | --- | --- | --- |
| \* | pJS43\_100mM\_120812\_02.08034.08034.2 | 3.9786 | 0.5696 | 100.0% | 1428.1322 | 1428.7296 | 1 | 10.536 | 86.4% | 2 | R.LIMNYLVTEGFK.E | 2 |
| \* | pJS43\_100mM\_120812\_02.06763.06763.2 | 4.8249 | 0.4127 | 100.0% | 1921.0922 | 1921.0834 | 1 | 8.257 | 65.6% | 4 | R.MESGIEPSVDLETLDER.I | 2 |
| \* | pJS43\_100mM\_120812\_01.13680.13680.3 | 5.1336 | 0.4695 | 100.0% | 2447.4543 | 2446.766 | 2 | 8.616 | 33.3% | 3 | K.GQIQEAIALINSLHPELLDTNR.Y | 3 |
| \* | pJS43\_100mM\_120812\_01.09720.09720.3 | 4.8329 | 0.4396 | 100.0% | 2622.5942 | 2622.765 | 1 | 7.434 | 37.5% | 1 | R.QRETEAALEFAQTQLAEQGEESR.E | 3 |
| \* | pJS43\_100mM\_120812\_02.06571.06571.3 | 5.7256 | 0.5116 | 100.0% | 2338.6443 | 2338.4468 | 1 | 9.191 | 46.2% | 1 | R.ETEAALEFAQTQLAEQGEESR.E | 3 |
| \* | pJS43\_100mM\_120812\_01.14148.14148.3 | 3.5496 | 0.3507 | 99.9% | 2688.7444 | 2690.043 | 1 | 6.296 | 27.2% | 2 | R.TLALLAFDSPEESPFGDLLHTMQR.Q | 3 |
| \* | pDK339\_033013\_01.10730.10730.3 | 3.3728 | 0.1906 | 97.9% | 2079.4143 | 2079.2769 | 8 | 4.488 | 35.9% | 1 | R.QKVWSEVNQAVLDYENR.E | 3 |
| \* | pJS43\_100mM\_120812\_02.06925.06925.2 | 4.8437 | 0.4654 | 100.0% | 1823.1522 | 1822.9719 | 1 | 8.331 | 75.0% | 3 | K.VWSEVNQAVLDYENR.E | 2 |
| \* | pJS43\_100mM\_120812\_02.07369.07369.2 | 4.1415 | 0.3705 | 100.0% | 1472.3121 | 1471.6964 | 1 | 6.704 | 81.8% | 9 | K.LLLWAQNELDQK.K | 2 |

---

|  |  |  |  |  |  |  |  |  |
| --- | --- | --- | --- | --- | --- | --- | --- | --- |
| U | *contaminant\_GR78\_PIG* | 12 | 93 | 55.5% | 200 | 21709 | 5.0 | owl|P34935| 78 KD GLUCOSE REGULATED PROTEIN (GRP 78) (IMMUNOGLOBULIN HEAVY... |

| Filename XCorr DeltCN Conf% ObsM+H+ CalcM+H+ SpR ZScore Ion% # Sequence  | | | | | | | | | | | | |
| --- | --- | --- | --- | --- | --- | --- | --- | --- | --- | --- | --- | --- |
| \* | AstrinIP\_MS1\_022614\_01.17182.17182.2 | 3.1606 | 0.1676 | 99.2% | 1450.1122 | 1449.6475 | 12 | 4.0 | 58.3% | 1 | -.EIVLVGGST#RIPK.I | 2 |
|  | SKAPIP\_041314\_01.09327.09327.2 | 2.9128 | 0.2711 | 99.9% | 1211.0922 | 1211.3195 | 3 | 5.546 | 66.7% | 11 | K.EFFNGKEPSR.G | 22 |
|  | pDK339othertube\_033013\_01.07019.07019.2 | 5.448 | 0.501 | 100.0% | 1837.4922 | 1838.0245 | 1 | 9.641 | 75.0% | 34 | K.SQIFSTASDNQPTVTIK.V | 22 |
|  | pDK365N\_300mM\_082713\_03.06935.06935.3 | 3.0168 | 0.2427 | 98.5% | 1840.0743 | 1838.0245 | 4 | 4.373 | 34.4% | 1 | K.SQIFSTASDNQPTVTIK.V | 33 |
|  | pDK365N\_300mM\_082713\_01.03552.03552.2 | 2.7685 | 0.4548 | 100.0% | 1191.6522 | 1192.3574 | 1 | 7.136 | 83.3% | 4 | K.VYEGERPLTK.D | 22 |
|  | AstrinIP\_MS1\_022614\_01.11088.11088.2 | 4.1652 | 0.564 | 100.0% | 1934.5122 | 1935.19 | 1 | 9.377 | 61.8% | 16 | K.DNHLLGTFDLTGIPPAPR.G | 22 |
|  | pDK365N\_300mM\_082713\_03.10484.10484.3 | 2.5731 | 0.28 | 97.9% | 1935.9243 | 1935.19 | 201 | 5.304 | 29.4% | 1 | K.DNHLLGTFDLTGIPPAPR.G | 33 |
|  | SKAPIP\_tube2\_041314\_02.11004.11004.2 | 3.4152 | 0.4421 | 100.0% | 2000.4122 | 2000.3024 | 1 | 7.794 | 44.1% | 6 | R.GVPQIEVTFEIDVNGILR.V | 22 |
|  | AstrinIP\_MS2\_022614\_01.03741.03741.2 | 2.5616 | 0.2861 | 99.7% | 1075.0322 | 1075.1667 | 5 | 6.002 | 75.0% | 1 | K.ITITNDQNR.L | 22 |
|  | SKAPIP\_041314\_01.09665.09665.2 | 4.3023 | 0.3916 | 100.0% | 1654.2922 | 1654.8323 | 1 | 7.962 | 76.9% | 6 | R.MVNDAEKFAEEDKK.L | 22 |
|  | SKAPIP\_041314\_01.09428.09428.3 | 3.7114 | 0.4453 | 100.0% | 1656.3243 | 1654.8323 | 1 | 6.745 | 44.2% | 9 | R.MVNDAEKFAEEDKK.L | 33 |
|  | pDK365N\_300mM\_082713\_01.04076.04076.3 | 4.2362 | 0.3324 | 100.0% | 1896.5643 | 1896.1658 | 11 | 6.35 | 36.7% | 3 | R.MVNDAEKFAEEDKKLK.E | 33 |

Similarities:
gi|16507237|ref|NP\_00(11:1)  

---

|  |  |  |  |  |  |  |  |  |
| --- | --- | --- | --- | --- | --- | --- | --- | --- |
| U | *gi|4885049|ref|NP\_005* | 35 | 233 | 55.4% | 377 | 42019 | 5.4 | cardiac muscle alpha actin 1 proprotein [Homo sapiens] |

| Filename XCorr DeltCN Conf% ObsM+H+ CalcM+H+ SpR ZScore Ion% # Sequence  | | | | | | | | | | | | |
| --- | --- | --- | --- | --- | --- | --- | --- | --- | --- | --- | --- | --- |
|  | SKAPIP\_041314\_01.04413.04413.2 | 3.3044 | 0.4393 | 100.0% | 976.4122 | 977.02136 | 1 | 7.48 | 77.8% | 7 | K.AGFAGDDAPR.A | 222 |
|  | AstrinIP\_MS2\_022614\_01.04036.04036.1 | 1.9667 | 0.3834 | 98.5% | 976.48 | 977.02136 | 6 | 5.951 | 55.6% | 1 | K.AGFAGDDAPR.A | 111 |
|  | SKAPIP\_tube2\_041314\_01.08159.08159.3 | 4.1697 | 0.4663 | 100.0% | 2156.9644 | 2157.4397 | 1 | 6.941 | 35.0% | 2 | K.AGFAGDDAPRAVFPSIVGRPR.H | 333 |
|  | SKAPIP\_041314\_01.11156.11156.2 | 3.215 | 0.3094 | 100.0% | 1199.2922 | 1199.4415 | 1 | 6.647 | 75.0% | 27 | R.AVFPSIVGRPR.H | 222 |
|  | AstrinIP\_MS2\_022614\_01.03903.03903.1 | 2.5908 | 0.3969 | 99.4% | 1171.48 | 1172.4058 | 1 | 7.283 | 65.0% | 1 | R.HQGVMVGMGQK.D | 111 |
|  | pDK365N\_300mM\_082713\_01.03760.03760.2 | 2.6602 | 0.3292 | 99.8% | 1172.0122 | 1172.4058 | 1 | 6.253 | 75.0% | 2 | R.HQGVMVGMGQK.D | 222 |
|  | SKAPIP\_tube2\_041314\_02.04470.04470.3 | 4.4137 | 0.58 | 100.0% | 2350.8245 | 2352.5989 | 1 | 9.895 | 38.1% | 1 | R.HQGVMVGMGQKDSYVGDEAQSK.R | 33 |
|  | pSKT11\_1\_020812\_01.07479.07479.3 | 5.1607 | 0.4976 | 100.0% | 2508.0842 | 2508.7864 | 1 | 7.555 | 36.4% | 3 | R.HQGVMVGMGQKDSYVGDEAQSKR.G | 33 |
|  | pJS43\_100mM\_120812\_01.03359.03359.2 | 3.2799 | 0.1437 | 99.4% | 1355.0922 | 1355.4038 | 1 | 7.295 | 77.3% | 2 | K.DSYVGDEAQSKR.G | 22 |
|  | SKAPIP\_tube2\_041314\_01.10804.10804.3 | 4.1061 | 0.2894 | 99.9% | 2588.2744 | 2587.9934 | 1 | 5.371 | 34.5% | 1 | R.GILTLKYPIEHGIITNWDDMEK.I | 3 |
|  | SKAPIP\_tube2\_041314\_01.08656.08656.1 | 3.431 | 0.2819 | 98.4% | 1961.98 | 1962.1841 | 1 | 5.581 | 50.0% | 1 | K.YPIEHGIITNWDDMEK.I | 1 |
|  | SKAPIP\_tube2\_041314\_01.08584.08584.2 | 4.3785 | 0.4105 | 100.0% | 1962.3121 | 1962.1841 | 1 | 7.294 | 63.3% | 13 | K.YPIEHGIITNWDDMEK.I | 2 |
|  | SKAPIP\_tube2\_041314\_01.08630.08630.3 | 4.5022 | 0.1765 | 99.7% | 1963.3143 | 1962.1841 | 1 | 5.475 | 50.0% | 13 | K.YPIEHGIITNWDDMEK.I | 3 |
|  | SKAPIP\_tube2\_041314\_01.12422.12422.3 | 6.1423 | 0.4752 | 100.0% | 3458.7844 | 3459.8628 | 1 | 7.776 | 36.5% | 5 | K.YPIEHGIITNWDDMEKIWHHTFYNELR.V | 3 |
|  | SKAPIP\_tube2\_041314\_01.12425.12425.2 | 3.7137 | 0.4797 | 100.0% | 3459.5322 | 3459.8628 | 1 | 8.455 | 30.8% | 2 | K.YPIEHGIITNWDDMEKIWHHTFYNELR.V | 2 |
|  | SKAPIP\_tube2\_041314\_01.05751.05751.3 | 3.5086 | 0.2783 | 99.9% | 1514.7244 | 1516.7019 | 1 | 6.019 | 60.0% | 16 | K.IWHHTFYNELR.V | 333 |
|  | SKAPIP\_041314\_01.10924.10924.2 | 3.3602 | 0.3431 | 100.0% | 1517.4122 | 1516.7019 | 1 | 5.935 | 70.0% | 18 | K.IWHHTFYNELR.V | 222 |
|  | SKAPIP\_tube2\_041314\_01.05764.05764.1 | 3.335 | 0.2365 | 98.6% | 1517.72 | 1516.7019 | 1 | 4.979 | 70.0% | 4 | K.IWHHTFYNELR.V | 111 |
|  | SKAPIP\_tube2\_041314\_01.07451.07451.2 | 4.8162 | 0.4245 | 100.0% | 1958.2122 | 1957.234 | 16 | 7.063 | 41.2% | 3 | R.VAPEEHPTLLTEAPLNPK.A | 2 |
|  | SKAPIP\_tube2\_041314\_02.05692.05692.3 | 3.2395 | 0.1871 | 96.5% | 1959.9243 | 1957.234 | 12 | 4.097 | 33.8% | 1 | R.VAPEEHPTLLTEAPLNPK.A | 3 |
|  | SKAPIP\_tube2\_041314\_01.10616.10616.2 | 3.4989 | 0.3548 | 100.0% | 1624.5122 | 1624.8927 | 1 | 6.273 | 61.5% | 5 | R.LDLAGRDLTDYLMK.I | 222 |
|  | SKAPIP\_tube2\_041314\_01.10557.10557.3 | 3.2649 | 0.3522 | 100.0% | 1625.0944 | 1624.8927 | 1 | 6.655 | 46.2% | 4 | R.LDLAGRDLTDYLMK.I | 333 |
|  | SKAPIP\_tube2\_041314\_01.09831.09831.1 | 2.1541 | 0.2631 | 98.8% | 998.54 | 999.167 | 1 | 5.467 | 78.6% | 7 | R.DLTDYLMK.I | 111 |
|  | SKAPIP\_tube2\_041314\_01.09902.09902.2 | 2.4407 | 0.316 | 99.8% | 999.15216 | 999.167 | 5 | 5.876 | 78.6% | 4 | R.DLTDYLMK.I | 222 |
|  | SKAPIP\_tube2\_041314\_01.18627.18627.2 | 3.0742 | 0.3067 | 99.8% | 2537.9321 | 2537.764 | 1 | 5.897 | 31.8% | 1 | K.LCYVALDFENEMATAASSSSLEK.S | 2 |
|  | SKAPIP\_tube2\_041314\_01.09797.09797.1 | 2.4357 | 0.1615 | 95.9% | 1791.9 | 1791.9554 | 1 | 5.61 | 46.7% | 1 | K.SYELPDGQVITIGNER.F | 1111 |
|  | pDK339othertube\_033013\_01.09500.09500.2 | 4.9277 | 0.3112 | 100.0% | 1792.3322 | 1791.9554 | 1 | 8.517 | 83.3% | 34 | K.SYELPDGQVITIGNER.F | 2222 |
|  | SKAPIP\_tube2\_041314\_01.05690.05690.3 | 3.9322 | 0.2558 | 99.9% | 1550.0044 | 1549.8843 | 3 | 5.468 | 48.1% | 6 | R.MQKEITALAPSTMK.I | 33 |
|  | SKAPIP\_tube2\_041314\_01.05672.05672.2 | 3.9543 | 0.2632 | 100.0% | 1550.4321 | 1549.8843 | 1 | 5.333 | 80.8% | 5 | R.MQKEITALAPSTMK.I | 22 |
|  | pDK339\_033013\_01.06337.06337.2 | 2.9107 | 0.3944 | 100.0% | 1162.0922 | 1162.3868 | 3 | 6.612 | 60.0% | 24 | K.EITALAPSTMK.I | 22 |
|  | SKAPIP\_041314\_01.11040.11040.1 | 2.8116 | 0.1393 | 96.5% | 1163.59 | 1162.3868 | 1 | 4.735 | 65.0% | 13 | K.EITALAPSTMK.I | 11 |
|  | SKAPIP\_tube2\_041314\_01.04526.04526.2 | 2.3655 | 0.1818 | 98.0% | 1037.3121 | 1037.2908 | 4 | 4.889 | 75.0% | 1 | K.IKIIAPPER.K | 222 |
|  | SKAPIP\_041314\_01.06362.06362.2 | 2.5702 | 0.1639 | 98.3% | 1165.0322 | 1165.4648 | 13 | 4.518 | 72.2% | 1 | K.IKIIAPPERK.Y | 222 |
|  | SKAPIP\_tube2\_041314\_02.14154.14154.2 | 4.7851 | 0.558 | 100.0% | 2603.8123 | 2604.0388 | 1 | 8.3 | 40.9% | 3 | K.YSVWIGGSILASLSTFQQMWISK.Q | 222 |
|  | SKAPIP\_041314\_01.10594.10594.2 | 2.9052 | 0.1511 | 98.2% | 1628.5521 | 1629.7697 | 12 | 4.694 | 46.2% | 1 | K.QEYDEAGPSIVHRK.C | 2 |

Similarities:
gi|4501885|ref|NP\_001(25:10)  
gi|63055057|ref|NP\_00(11:24)  
gi|134133226|ref|NP\_0(9:26)  

---

|  |  |  |  |  |  |  |  |  |
| --- | --- | --- | --- | --- | --- | --- | --- | --- |
| U | *gi|4506699|ref|NP\_001* | 8 | 57 | 55.4% | 83 | 9111 | 8.5 | ribosomal protein S21 [Homo sapiens] |

| Filename XCorr DeltCN Conf% ObsM+H+ CalcM+H+ SpR ZScore Ion% # Sequence  | | | | | | | | | | | | |
| --- | --- | --- | --- | --- | --- | --- | --- | --- | --- | --- | --- | --- |
| \* | pSKT11\_1\_020812\_02.04065.04065.3 | 5.2223 | 0.4455 | 100.0% | 2454.4443 | 2453.8193 | 1 | 7.136 | 33.0% | 1 | R.IIGAKDHASIQMNVAEVDKVTGR.F | 3 |
| \* | pDK365N\_100mM\_082713\_02.04577.04577.2 | 4.4569 | 0.488 | 100.0% | 1557.4321 | 1557.7185 | 1 | 9.273 | 65.4% | 7 | K.DHASIQMNVAEVDK.V | 2 |
| \* | pJS43\_100mM\_120812\_02.04999.04999.3 | 3.1023 | 0.202 | 97.9% | 1557.8944 | 1557.7185 | 11 | 4.481 | 46.2% | 2 | K.DHASIQMNVAEVDK.V | 3 |
| \* | pSKT11\_1\_020812\_02.04180.04180.2 | 5.7021 | 0.4891 | 100.0% | 1970.7122 | 1971.1956 | 1 | 10.694 | 76.5% | 3 | K.DHASIQMNVAEVDKVTGR.F | 2 |
| \* | pJS43\_100mM\_120812\_02.05628.05628.3 | 5.6137 | 0.5978 | 100.0% | 1971.2344 | 1971.1956 | 1 | 10.18 | 48.5% | 32 | K.DHASIQMNVAEVDKVTGR.F | 3 |
| \* | pJS43\_100mM\_120812\_01.04729.04729.2 | 2.986 | 0.2598 | 99.8% | 1279.0922 | 1279.4106 | 1 | 5.372 | 75.0% | 1 | R.RMGESDDSILR.L | 2 |
| \* | pJS43\_100mM\_120812\_01.05952.05952.2 | 3.5212 | 0.489 | 100.0% | 1123.0521 | 1123.2231 | 1 | 9.155 | 88.9% | 10 | R.MGESDDSILR.L | 2 |
| \* | AstrinNocIP\_020510\_01.04997.04997.3 | 3.0399 | 0.39 | 99.9% | 2367.0544 | 2367.6794 | 1 | 5.379 | 29.8% | 1 | R.MGESDDSILRLAKADGIVSKNF.- | 3 |

---

|  |  |  |  |  |  |  |  |  |
| --- | --- | --- | --- | --- | --- | --- | --- | --- |
| U | *gi|31542947|ref|NP\_00* | 31 | 160 | 55.0% | 573 | 61055 | 5.9 | chaperonin [Homo sapiens] |
| U | *gi|41399285|ref|NP\_95* | 31 | 160 | 55.0% | 573 | 61055 | 5.9 | chaperonin [Homo sapiens] |

| Filename XCorr DeltCN Conf% ObsM+H+ CalcM+H+ SpR ZScore Ion% # Sequence  | | | | | | | | | | | | |
| --- | --- | --- | --- | --- | --- | --- | --- | --- | --- | --- | --- | --- |
|  | AstrinIP\_MS2\_022614\_01.18516.18516.2 | 4.6211 | 0.4826 | 100.0% | 2114.9722 | 2114.5667 | 1 | 9.137 | 55.0% | 22 | R.ALMLQGVDLLADAVAVTMGPK.G | 2 |
|  | pDK365N\_300mM\_082713\_02.11594.11594.3 | 5.633 | 0.4435 | 100.0% | 2115.2043 | 2114.5667 | 1 | 8.327 | 46.2% | 22 | R.ALMLQGVDLLADAVAVTMGPK.G | 3 |
|  | pDK365N\_300mM\_082713\_01.07426.07426.2 | 3.8299 | 0.4455 | 100.0% | 1346.1322 | 1345.5382 | 1 | 7.141 | 81.8% | 19 | R.TVIIEQSWGSPK.V | 2 |
|  | SKAPIP\_tube2\_041314\_02.05486.05486.2 | 6.1738 | 0.6551 | 100.0% | 2560.872 | 2561.7222 | 1 | 13.266 | 60.4% | 8 | K.LVQDVANNTNEEAGDGTTTATVLAR.S | 2 |
|  | pDk339\_033013\_02.05032.05032.3 | 4.984 | 0.5217 | 100.0% | 2562.1443 | 2561.7222 | 1 | 9.614 | 33.3% | 7 | K.LVQDVANNTNEEAGDGTTTATVLAR.S | 3 |
|  | pSKT11\_1\_020812\_02.07906.07906.2 | 5.2757 | 0.438 | 100.0% | 1558.1721 | 1557.9324 | 1 | 8.096 | 75.0% | 5 | R.GVMLAVDAVIAELKK.Q | 2 |
|  | pDK339othertube\_033013\_01.11139.11139.2 | 4.518 | 0.3169 | 100.0% | 1505.3722 | 1505.7235 | 1 | 8.35 | 70.8% | 4 | K.TLNDELEIIEGMK.F | 2 |
|  | pDK339\_033013\_01.09955.09955.2 | 3.4852 | 0.4594 | 100.0% | 1390.4321 | 1390.5786 | 1 | 7.674 | 81.8% | 5 | R.GYISPYFINTSK.G | 2 |
|  | pDK339\_033013\_01.11471.11471.2 | 2.6207 | 0.3182 | 99.5% | 1918.7722 | 1920.2192 | 31 | 6.02 | 32.4% | 2 | K.ISSIQSIVPALEIANAHR.K | 2 |
|  | pDK365N\_300mM\_082713\_03.10514.10514.3 | 3.1781 | 0.3934 | 100.0% | 1920.3243 | 1920.2192 | 1 | 6.435 | 41.2% | 4 | K.ISSIQSIVPALEIANAHR.K | 3 |
|  | pSKT11\_1\_020812\_02.08385.08385.3 | 5.3909 | 0.3712 | 100.0% | 4266.9243 | 4267.9565 | 1 | 7.365 | 23.1% | 1 | K.ISSIQSIVPALEIANAHRKPLVIIAEDVDGEALSTLVLNR.L | 3 |
|  | pSKT11\_1\_020812\_02.07427.07427.2 | 3.1278 | 0.4461 | 100.0% | 2366.8323 | 2366.7605 | 1 | 7.79 | 50.0% | 1 | R.KPLVIIAEDVDGEALSTLVLNR.L | 2 |
|  | pDK365N\_300mM\_082713\_04.08663.08663.3 | 3.0272 | 0.2379 | 97.5% | 2367.5942 | 2366.7605 | 10 | 4.645 | 29.8% | 1 | R.KPLVIIAEDVDGEALSTLVLNR.L | 3 |
|  | pSKT11\_1\_020812\_02.04157.04157.2 | 2.4203 | 0.449 | 100.0% | 1153.5322 | 1154.4819 | 1 | 6.698 | 70.0% | 1 | R.LKVGLQVVAVK.A | 2 |
|  | pDK365N\_300mM\_082713\_01.05974.05974.2 | 2.7093 | 0.2186 | 99.5% | 914.2522 | 913.14844 | 1 | 6.105 | 87.5% | 3 | K.VGLQVVAVK.A | 2 |
|  | pSKT11\_1\_020812\_01.09808.09808.3 | 3.973 | 0.2347 | 99.3% | 3582.5645 | 3583.0076 | 1 | 4.257 | 22.7% | 1 | K.NQLKDMAIATGGAVFGEEGLTLNLEDVQPHDLGK.V | 3 |
|  | pSKT11\_1\_020812\_02.04664.04664.2 | 4.4315 | 0.4808 | 100.0% | 1631.3922 | 1631.9684 | 1 | 8.553 | 67.9% | 4 | K.VGEVIVTKDDAMLLK.G | 2 |
|  | pDK365N\_300mM\_082713\_01.08174.08174.3 | 3.7157 | 0.4396 | 100.0% | 1631.9944 | 1631.9684 | 1 | 7.49 | 48.2% | 14 | K.VGEVIVTKDDAMLLK.G | 3 |
|  | pSKT11\_1\_020812\_02.06028.06028.3 | 3.2076 | 0.2931 | 99.6% | 2450.4243 | 2452.721 | 383 | 4.822 | 25.0% | 1 | K.RIQEIIEQLDVTTSEYEKEK.L | 3 |
|  | pSKT11\_1\_020812\_02.05760.05760.2 | 5.8796 | 0.4563 | 100.0% | 2296.2922 | 2296.5334 | 1 | 7.813 | 52.8% | 1 | R.IQEIIEQLDVTTSEYEKEK.L | 2 |
|  | SKAPIP\_041314\_02.07936.07936.3 | 3.4415 | 0.1944 | 97.9% | 2296.3145 | 2296.5334 | 7 | 4.167 | 34.7% | 2 | R.IQEIIEQLDVTTSEYEKEK.L | 3 |
|  | pSKT11\_1\_020812\_02.05783.05783.3 | 4.5601 | 0.3093 | 100.0% | 2806.7644 | 2809.0996 | 1 | 6.317 | 34.1% | 1 | R.IQEIIEQLDVTTSEYEKEKLNER.L | 3 |
|  | pSKT11\_1\_020812\_02.05751.05751.2 | 3.8058 | 0.481 | 100.0% | 2808.2722 | 2809.0996 | 1 | 8.458 | 34.1% | 1 | R.IQEIIEQLDVTTSEYEKEKLNER.L | 2 |
|  | SKAPIP\_tube2\_041314\_01.05250.05250.2 | 3.048 | 0.2779 | 100.0% | 902.03217 | 902.0788 | 2 | 7.191 | 93.8% | 2 | K.LSDGVAVLK.V | 2 |
|  | pJS43\_100mM\_120812\_01.03552.03552.2 | 3.4612 | 0.4647 | 100.0% | 1233.8922 | 1234.3055 | 1 | 7.977 | 72.7% | 1 | K.VGGTSDVEVNEK.K | 2 |
|  | pJS43\_100mM\_120812\_01.03781.03781.2 | 2.4851 | 0.2588 | 99.5% | 961.9322 | 961.0629 | 73 | 4.994 | 75.0% | 2 | R.VTDALNATR.A | 2 |
|  | pDK365N\_300mM\_082713\_02.05001.05001.2 | 3.7764 | 0.3469 | 100.0% | 1216.1122 | 1216.377 | 1 | 6.977 | 81.8% | 20 | K.NAGVEGSLIVEK.I | 2 |
|  | pSKT11\_1\_020812\_02.07068.07068.2 | 6.0544 | 0.6547 | 100.0% | 2508.4722 | 2509.8235 | 1 | 12.299 | 52.3% | 2 | K.IMQSSSEVGYDAMAGDFVNMVEK.G | 2 |
|  | pSKT11\_1\_020812\_02.07076.07076.3 | 5.0555 | 0.4405 | 100.0% | 2509.0745 | 2509.8235 | 1 | 7.968 | 39.8% | 1 | K.IMQSSSEVGYDAMAGDFVNMVEK.G | 3 |
|  | AstrinIP\_MS1\_022614\_02.13645.13645.3 | 4.5074 | 0.2664 | 99.9% | 2483.8743 | 2483.9055 | 1 | 6.588 | 36.5% | 1 | R.TALLDAAGVASLLTTAEVVVTEIPK.E | 3 |
|  | pJS43\_100mM\_120812\_02.11064.11064.3 | 2.4826 | 0.3091 | 97.7% | 2870.5444 | 2870.3105 | 3 | 5.472 | 23.1% | 1 | R.TALLDAAGVASLLTTAEVVVTEIPKEEK.D | 3 |

---

|  |  |  |  |  |  |  |  |  |
| --- | --- | --- | --- | --- | --- | --- | --- | --- |
| U | *gi|5902102|ref|NP\_008* | 6 | 39 | 54.6% | 119 | 13282 | 11.6 | small nuclear ribonucleoprotein D1 polypeptide 16kDa [Homo sapiens] |

| Filename XCorr DeltCN Conf% ObsM+H+ CalcM+H+ SpR ZScore Ion% # Sequence  | | | | | | | | | | | | |
| --- | --- | --- | --- | --- | --- | --- | --- | --- | --- | --- | --- | --- |
| \* | pDk339\_033013\_02.04801.04801.2 | 2.4134 | 0.2504 | 98.8% | 1269.8922 | 1270.4686 | 10 | 5.153 | 55.0% | 1 | K.LSHETVTIELK.N | 2 |
| \* | pJS43\_100mM\_120812\_02.04950.04950.3 | 3.3102 | 0.2823 | 99.9% | 1270.4043 | 1270.4686 | 111 | 5.41 | 40.0% | 1 | K.LSHETVTIELK.N | 3 |
| \* | pJS43\_100mM\_120812\_02.05124.05124.3 | 4.9398 | 0.4266 | 100.0% | 2210.0044 | 2210.47 | 1 | 7.733 | 45.0% | 5 | K.NGTQVHGTITGVDVSMNTHLK.A | 3 |
|  | pJS43\_100mM\_120812\_01.08138.08138.2 | 3.9926 | 0.5079 | 100.0% | 1555.0721 | 1555.7745 | 1 | 7.73 | 70.8% | 6 | K.NREPVQLETLSIR.G | 2 |
|  | pJS43\_100mM\_120812\_01.08125.08125.3 | 4.4895 | 0.3294 | 100.0% | 1555.7943 | 1555.7745 | 4 | 6.278 | 45.8% | 4 | K.NREPVQLETLSIR.G | 3 |
| \* | pDK365N\_100mM\_082713\_01.14514.14514.2 | 5.599 | 0.5531 | 100.0% | 2288.9521 | 2288.6863 | 1 | 10.479 | 71.1% | 22 | R.YFILPDSLPLDTLLVDVEPK.V | 2 |

---

|  |  |  |  |  |  |  |  |  |
| --- | --- | --- | --- | --- | --- | --- | --- | --- |
| U | *gi|29788785|ref|NP\_82* | 33 | 347 | 54.1% | 444 | 49671 | 4.9 | tubulin, beta [Homo sapiens] |

| Filename XCorr DeltCN Conf% ObsM+H+ CalcM+H+ SpR ZScore Ion% # Sequence  | | | | | | | | | | | | |
| --- | --- | --- | --- | --- | --- | --- | --- | --- | --- | --- | --- | --- |
| \* | 100326\_pJS43\_02.06152.06152.3 | 6.845 | 0.4483 | 100.0% | 3102.6543 | 3104.2725 | 1 | 11.148 | 38.5% | 13 | K.FWEVISDEHGIDPTGTYHGDSDLQLDR.I | 3 |
| \* | pDK339\_033013\_01.05470.05470.1 | 2.0451 | 0.4315 | 99.4% | 1301.52 | 1302.4265 | 13 | 6.685 | 50.0% | 1 | R.ISVYYNEATGGK.Y | 1 |
| \* | pDK339othertube\_033013\_01.05366.05366.2 | 3.7774 | 0.5462 | 100.0% | 1302.0922 | 1302.4265 | 1 | 9.475 | 86.4% | 26 | R.ISVYYNEATGGK.Y | 2 |
| \* | 100326\_pJS43\_02.04157.04157.2 | 3.1953 | 0.4064 | 100.0% | 1817.7322 | 1818.0392 | 1 | 7.818 | 50.0% | 3 | R.ISVYYNEATGGKYVPR.A | 2 |
| \* | pDk339\_033013\_02.05050.05050.3 | 2.54 | 0.3909 | 99.9% | 1818.3243 | 1818.0392 | 3 | 5.72 | 33.3% | 3 | R.ISVYYNEATGGKYVPR.A | 3 |
|  | 100326\_pJS43\_01.08288.08288.2 | 4.382 | 0.3854 | 100.0% | 1617.6721 | 1616.8701 | 1 | 6.875 | 78.6% | 31 | R.AILVDLEPGTMDSVR.S | 22 |
|  | 100326\_pJS43\_01.10197.10197.2 | 5.5004 | 0.5198 | 100.0% | 2798.5522 | 2800.0647 | 1 | 8.823 | 42.0% | 4 | R.SGPFGQIFRPDNFVFGQSGAGNNWAK.G | 22 |
|  | 100326\_pJS43\_01.10196.10196.3 | 7.8655 | 0.5039 | 100.0% | 2799.9243 | 2800.0647 | 1 | 8.789 | 44.0% | 26 | R.SGPFGQIFRPDNFVFGQSGAGNNWAK.G | 33 |
|  | pDK339othertube\_033013\_01.12321.12321.2 | 7.1963 | 0.5639 | 100.0% | 1959.5922 | 1960.151 | 1 | 11.054 | 79.4% | 11 | K.GHYTEGAELVDSVLDVVR.K | 222 |
|  | pDK339othertube\_033013\_01.12374.12374.3 | 3.9746 | 0.3545 | 100.0% | 1960.0144 | 1960.151 | 1 | 5.866 | 42.6% | 8 | K.GHYTEGAELVDSVLDVVR.K | 333 |
|  | 100326\_pJS43\_01.09680.09680.2 | 6.0516 | 0.5778 | 100.0% | 2087.372 | 2088.325 | 1 | 10.101 | 69.4% | 4 | K.GHYTEGAELVDSVLDVVRK.E | 222 |
|  | pDK339othertube\_033013\_01.11294.11294.3 | 4.8073 | 0.4721 | 100.0% | 2087.9644 | 2088.325 | 1 | 8.016 | 45.8% | 16 | K.GHYTEGAELVDSVLDVVRK.E | 333 |
|  | pJS43\_100mM\_120812\_01.03371.03371.2 | 2.8494 | 0.3232 | 100.0% | 1077.6921 | 1078.1698 | 1 | 5.745 | 85.7% | 2 | K.IREEYPDR.I | 22 |
|  | 100326\_pJS43\_01.06554.06554.2 | 4.5776 | 0.3956 | 100.0% | 1320.5322 | 1320.5896 | 1 | 7.817 | 77.3% | 29 | R.IMNTFSVVPSPK.V | 222 |
|  | pDK339\_033013\_01.08258.08258.2 | 2.5483 | 0.2033 | 98.8% | 1131.2122 | 1131.2767 | 3 | 5.089 | 72.2% | 4 | R.FPGQLNADLR.K | 2222 |
|  | 100326\_pJS43\_01.06578.06578.2 | 3.7419 | 0.3673 | 100.0% | 1272.5122 | 1272.5945 | 1 | 7.434 | 75.0% | 9 | R.KLAVNMVPFPR.L | 2222 |
|  | 100326\_pJS43\_01.07836.07836.1 | 1.9611 | 0.2769 | 98.5% | 1143.47 | 1144.4204 | 11 | 5.674 | 61.1% | 1 | K.LAVNMVPFPR.L | 1111 |
|  | pDK339othertube\_033013\_01.09272.09272.2 | 3.9416 | 0.4873 | 100.0% | 1144.1122 | 1144.4204 | 1 | 9.149 | 94.4% | 17 | K.LAVNMVPFPR.L | 2222 |
|  | pDK365N\_300mM\_082713\_03.11541.11541.3 | 4.2348 | 0.3148 | 100.0% | 1622.0643 | 1621.9403 | 2 | 6.293 | 50.0% | 4 | R.LHFFMPGFAPLTSR.G | 333 |
|  | pDK339\_033013\_01.12823.12823.2 | 3.7781 | 0.4034 | 100.0% | 1622.2722 | 1621.9403 | 1 | 8.323 | 80.8% | 19 | R.LHFFMPGFAPLTSR.G | 222 |
| \* | pDK365N\_300mM\_082713\_01.11228.11228.2 | 3.9716 | 0.4777 | 100.0% | 1661.4321 | 1660.9078 | 1 | 7.753 | 67.9% | 20 | R.ALTVPELTQQVFDAK.N | 2 |
|  | 100326\_pJS43\_01.08229.08229.1 | 1.556 | 0.3461 | 98.9% | 1039.46 | 1040.2505 | 1 | 5.469 | 68.8% | 3 | R.YLTVAAVFR.G | 11 |
|  | 100326\_pJS43\_01.08205.08205.2 | 2.7179 | 0.4304 | 100.0% | 1040.0922 | 1040.2505 | 1 | 7.712 | 87.5% | 4 | R.YLTVAAVFR.G | 22 |
|  | 100326\_pJS43\_02.04928.04928.2 | 4.9757 | 0.5158 | 100.0% | 1924.5521 | 1925.2405 | 1 | 8.985 | 56.7% | 2 | R.MSMKEVDEQMLNVQNK.N | 22 |
|  | pDK339othertube\_033013\_02.05941.05941.3 | 4.8367 | 0.3253 | 100.0% | 1925.0944 | 1925.2405 | 1 | 6.493 | 51.7% | 12 | R.MSMKEVDEQMLNVQNK.N | 33 |
|  | pDK365N\_300mM\_082713\_03.05374.05374.2 | 4.1533 | 0.2361 | 100.0% | 1448.2122 | 1447.6031 | 1 | 5.848 | 68.2% | 13 | K.EVDEQMLNVQNK.N | 22 |
|  | pDK339othertube\_033013\_01.10778.10778.2 | 3.9613 | 0.4453 | 100.0% | 1697.3121 | 1697.8877 | 1 | 7.642 | 65.4% | 13 | K.NSSYFVEWIPNNVK.T | 2222 |
| \* | 100326\_pJS43\_02.08268.08268.2 | 5.1694 | 0.4035 | 100.0% | 1871.5721 | 1871.2018 | 1 | 8.922 | 75.0% | 9 | K.MAVTFIGNSTAIQELFK.R | 2 |
| \* | pSKT11\_1\_020812\_02.07130.07130.2 | 4.6658 | 0.489 | 100.0% | 2026.9521 | 2027.3893 | 1 | 8.433 | 52.9% | 3 | K.MAVTFIGNSTAIQELFKR.I | 2 |
| \* | 100326\_pJS43\_02.07610.07610.3 | 4.1244 | 0.3335 | 100.0% | 2029.1643 | 2027.3893 | 1 | 6.432 | 42.6% | 13 | K.MAVTFIGNSTAIQELFKR.I | 3 |
|  | 100326\_pJS43\_01.06864.06864.2 | 3.1178 | 0.3686 | 100.0% | 1386.2922 | 1386.6116 | 2 | 7.406 | 65.0% | 4 | K.RISEQFTAMFR.R | 222 |
|  | 100326\_pJS43\_01.07953.07953.1 | 2.031 | 0.3168 | 98.8% | 1229.52 | 1230.4241 | 1 | 5.359 | 66.7% | 1 | R.ISEQFTAMFR.R | 111 |
|  | pDK339othertube\_033013\_02.07069.07069.2 | 3.8553 | 0.4627 | 100.0% | 1231.1721 | 1230.4241 | 1 | 7.816 | 88.9% | 19 | R.ISEQFTAMFR.R | 222 |

Similarities:
gi|5174735|ref|NP\_006(23:10)  
gi|50592996|ref|NP\_00(14:19)  
gi|14210536|ref|NP\_11(7:26)  

---

|  |  |  |  |  |  |  |  |  |
| --- | --- | --- | --- | --- | --- | --- | --- | --- |
| U | *gi|4504255|ref|NP\_002* | 7 | 30 | 53.9% | 128 | 13553 | 10.6 | H2A histone family, member Z [Homo sapiens] |
| U | *gi|6912616|ref|NP\_036* | 7 | 30 | 53.9% | 128 | 13509 | 10.6 | H2A histone family, member V isoform 1 [Homo sapiens] |

| Filename XCorr DeltCN Conf% ObsM+H+ CalcM+H+ SpR ZScore Ion% # Sequence  | | | | | | | | | | | | |
| --- | --- | --- | --- | --- | --- | --- | --- | --- | --- | --- | --- | --- |
|  | pDK365N\_100mM\_082813\_03.07151.07151.2 | 3.2969 | 0.3218 | 100.0% | 945.0122 | 945.1093 | 3 | 5.574 | 81.2% | 13 | R.AGLQFPVGR.I | 2222 |
|  | pSKT11\_1\_020812\_01.13881.13881.2 | 3.6468 | 0.5273 | 100.0% | 2896.632 | 2897.2952 | 19 | 8.339 | 25.0% | 1 | R.VGATAAVYSAAILEYLTAEVLELAGNASK.D | 2 |
|  | pJS43\_100mM\_120812\_01.17579.17579.3 | 6.2097 | 0.5252 | 100.0% | 2898.4443 | 2897.2952 | 1 | 9.207 | 35.7% | 7 | R.VGATAAVYSAAILEYLTAEVLELAGNASK.D | 3 |
|  | pJS43\_100mM\_120812\_01.05135.05135.2 | 2.5063 | 0.2795 | 99.8% | 851.1722 | 851.0396 | 1 | 5.65 | 91.7% | 4 | R.HLQLAIR.G | 222 |
|  | pDK365N\_100mM\_082713\_02.06755.06755.3 | 3.8291 | 0.4671 | 100.0% | 1951.2843 | 1951.2303 | 1 | 8.066 | 40.6% | 3 | R.HLQLAIRGDEELDSLIK.A | 3 |
|  | pJS43\_100mM\_120812\_01.08972.08972.2 | 2.794 | 0.2894 | 99.8% | 1119.0122 | 1119.2139 | 1 | 6.012 | 77.8% | 1 | R.GDEELDSLIK.A | 2 |
|  | pDK339\_033013\_01.05272.05272.2 | 2.8978 | 0.2688 | 99.6% | 1371.8121 | 1371.6255 | 3 | 5.816 | 50.0% | 1 | K.ATIAGGGVIPHIHK.S | 2 |

Similarities:
gi|10800130|ref|NP\_06(2:5)  
gi|106775678|ref|NP\_0(2:5)  
gi|113425815|ref|XP\_9(1:6)  

---

|  |  |  |  |  |  |  |  |  |
| --- | --- | --- | --- | --- | --- | --- | --- | --- |
| U | *gi|56243533|ref|NP\_07* | 10 | 35 | 53.8% | 221 | 23598 | 7.0 | stromal cell-derived factor 2-like 1 precursor [Homo sapiens] |

| Filename XCorr DeltCN Conf% ObsM+H+ CalcM+H+ SpR ZScore Ion% # Sequence  | | | | | | | | | | | | |
| --- | --- | --- | --- | --- | --- | --- | --- | --- | --- | --- | --- | --- |
| \* | pSKT11\_1\_020812\_02.04364.04364.2 | 6.1472 | 0.6117 | 100.0% | 2521.5122 | 2522.5603 | 1 | 10.421 | 52.2% | 6 | K.YGSGSGQQSVTGVEASDDANSYWR.I | 2 |
| \* | pDk339\_033013\_02.05603.05603.3 | 4.5844 | 0.4989 | 100.0% | 2521.9143 | 2522.5603 | 1 | 8.367 | 34.8% | 4 | K.YGSGSGQQSVTGVEASDDANSYWR.I | 3 |
| \* | pSKT11\_1\_020812\_02.06053.06053.3 | 4.6935 | 0.4099 | 100.0% | 3933.8643 | 3936.159 | 1 | 7.687 | 25.0% | 3 | K.NLHTHHFPSPLSNNQEVSAFGEDGEGDDLDLWTVR.C | 3 |
| \* | pSKT11\_1\_020812\_02.05721.05721.2 | 6.3477 | 0.6775 | 100.0% | 2410.4922 | 2410.6917 | 1 | 12.056 | 66.7% | 1 | R.FQHVGTSVFLSVTGEQYGSPIR.G | 2 |
| \* | pDK365N\_100mM\_082713\_02.06981.06981.3 | 4.5013 | 0.3287 | 100.0% | 2410.7944 | 2410.6917 | 1 | 6.367 | 35.7% | 6 | R.FQHVGTSVFLSVTGEQYGSPIR.G | 3 |
| \* | pSKT11\_1\_020812\_02.04640.04640.3 | 3.5784 | 0.2689 | 99.5% | 4423.554 | 4423.8687 | 1 | 4.717 | 20.5% | 1 | R.FQHVGTSVFLSVTGEQYGSPIRGQHEVHGMPSANTHNTWK.A | 3 |
| \* | pSKT11\_1\_020812\_01.03627.03627.2 | 4.1385 | 0.5383 | 100.0% | 2031.2522 | 2032.2004 | 1 | 9.099 | 55.9% | 1 | R.GQHEVHGMPSANTHNTWK.A | 2 |
| \* | pDK339\_033013\_01.03813.03813.3 | 4.9734 | 0.4055 | 100.0% | 2032.7344 | 2032.2004 | 1 | 7.766 | 50.0% | 2 | R.GQHEVHGMPSANTHNTWK.A | 3 |
| \* | pSKT11\_1\_020812\_01.09132.09132.2 | 4.2763 | 0.4929 | 100.0% | 2127.4722 | 2128.4045 | 1 | 8.907 | 55.3% | 1 | K.AMEGIFIKPSVEPSAGHDEL.- | 2 |
| \* | pJS43\_100mM\_120812\_01.10063.10063.3 | 4.9648 | 0.4384 | 100.0% | 2129.3943 | 2128.4045 | 1 | 7.637 | 40.8% | 10 | K.AMEGIFIKPSVEPSAGHDEL.- | 3 |

---

|  |  |  |  |  |  |  |  |  |
| --- | --- | --- | --- | --- | --- | --- | --- | --- |
| U | *gi|24234688|ref|NP\_00* | 51 | 485 | 53.6% | 679 | 73681 | 6.2 | heat shock 70kDa protein 9 precursor [Homo sapiens] |

| Filename XCorr DeltCN Conf% ObsM+H+ CalcM+H+ SpR ZScore Ion% # Sequence  | | | | | | | | | | | | |
| --- | --- | --- | --- | --- | --- | --- | --- | --- | --- | --- | --- | --- |
| \* | pJS43\_100mM\_120812\_01.03263.03263.2 | 2.9541 | 0.2243 | 99.8% | 958.7922 | 959.047 | 4 | 5.546 | 81.2% | 1 | K.VLENAEGAR.T | 2 |
| \* | SKAPIP\_tube2\_041314\_01.07091.07091.1 | 2.9493 | 0.5807 | 100.0% | 1450.59 | 1451.576 | 1 | 8.5 | 50.0% | 1 | R.TTPSVVAFTADGER.L | 1 |
| \* | SKAPIP\_tube2\_041314\_01.07088.07088.2 | 3.9154 | 0.548 | 100.0% | 1451.2122 | 1451.576 | 1 | 9.37 | 73.1% | 24 | R.TTPSVVAFTADGER.L | 2 |
| \* | SKAPIP\_041314\_01.10170.10170.2 | 4.5432 | 0.4557 | 100.0% | 1725.2322 | 1725.9016 | 1 | 8.304 | 60.7% | 1 | K.RQAVTNPNNTFYATK.R | 2 |
| \* | SKAPIP\_tube2\_041314\_01.04863.04863.2 | 4.036 | 0.5212 | 100.0% | 1569.3121 | 1569.7141 | 1 | 9.029 | 73.1% | 24 | R.QAVTNPNNTFYATK.R | 2 |
| \* | pSKT11\_1\_020812\_01.05054.05054.2 | 3.1225 | 0.4884 | 100.0% | 1724.5721 | 1725.9016 | 2 | 6.525 | 50.0% | 3 | R.QAVTNPNNTFYATKR.L | 2 |
| \* | pSKT11\_1\_020812\_01.05061.05061.3 | 3.032 | 0.2339 | 98.6% | 1725.2644 | 1725.9016 | 40 | 4.411 | 33.9% | 3 | R.QAVTNPNNTFYATKR.L | 3 |
| \* | pJS43\_100mM\_120812\_01.03255.03255.2 | 3.0613 | 0.2799 | 100.0% | 1151.0322 | 1150.2334 | 1 | 5.468 | 75.0% | 1 | R.RYDDPEVQK.D | 2 |
| \* | SKAPIP\_041314\_01.05451.05451.2 | 3.6143 | 0.376 | 100.0% | 1506.1921 | 1506.6555 | 1 | 6.566 | 77.3% | 5 | R.RYDDPEVQKDIK.N | 2 |
| \* | SKAPIP\_041314\_01.07926.07926.2 | 2.7868 | 0.2614 | 99.7% | 1350.2122 | 1350.468 | 2 | 5.455 | 70.0% | 2 | R.YDDPEVQKDIK.N | 2 |
| \* | SKAPIP\_041314\_01.05818.05818.2 | 4.6903 | 0.5322 | 100.0% | 1342.1122 | 1342.4105 | 1 | 9.172 | 66.7% | 8 | R.ASNGDAWVEAHGK.L | 2 |
| \* | SKAPIP\_041314\_01.05756.05756.3 | 2.1208 | 0.3378 | 98.5% | 1343.2444 | 1342.4105 | 54 | 5.536 | 37.5% | 1 | R.ASNGDAWVEAHGK.L | 3 |
| \* | AstrinIP\_MS2\_022614\_01.13407.13407.2 | 4.1916 | 0.4909 | 100.0% | 1554.4722 | 1554.8878 | 1 | 8.014 | 76.9% | 29 | K.LYSPSQIGAFVLMK.M | 2 |
| \* | pDK365N\_300mM\_082713\_03.12048.12048.3 | 3.6716 | 0.3518 | 100.0% | 1555.4944 | 1554.8878 | 1 | 6.524 | 50.0% | 1 | K.LYSPSQIGAFVLMK.M | 3 |
| \* | SKAPIP\_041314\_01.06926.06926.2 | 4.9855 | 0.4709 | 100.0% | 1593.2322 | 1593.7949 | 1 | 8.912 | 80.8% | 14 | K.MKETAENYLGHTAK.N | 2 |
| \* | SKAPIP\_041314\_01.06920.06920.3 | 4.4348 | 0.3195 | 100.0% | 1593.8043 | 1593.7949 | 1 | 6.413 | 48.1% | 15 | K.MKETAENYLGHTAK.N | 3 |
| \* | SKAPIP\_041314\_01.07505.07505.2 | 3.1187 | 0.2497 | 99.8% | 1335.3322 | 1334.4282 | 409 | 4.948 | 50.0% | 2 | K.ETAENYLGHTAK.N | 2 |
| \* | pDK365N\_300mM\_082713\_03.08864.08864.2 | 3.4163 | 0.4325 | 100.0% | 1696.3522 | 1695.8723 | 1 | 7.088 | 75.0% | 18 | K.NAVITVPAYFNDSQR.Q | 2 |
| \* | AstrinIP\_MS2\_022614\_01.09132.09132.1 | 2.625 | 0.3212 | 98.6% | 1242.56 | 1243.4056 | 1 | 5.397 | 63.6% | 2 | K.DAGQISGLNVLR.V | 1 |
| \* | SKAPIP\_tube2\_041314\_01.08498.08498.2 | 4.219 | 0.3407 | 100.0% | 1242.8522 | 1243.4056 | 1 | 7.085 | 81.8% | 27 | K.DAGQISGLNVLR.V | 2 |
| \* | pDK365N\_100mM\_082713\_01.08737.08737.2 | 4.9126 | 0.0198 | 99.6% | 1646.6522 | 1646.881 | 1 | 9.054 | 76.7% | 4 | R.VINEPTAAALAYGLDK.S | 2 |
| \* | SKAPIP\_tube2\_041314\_02.10629.10629.3 | 7.6658 | 0.6382 | 100.0% | 4338.8345 | 4339.927 | 1 | 11.052 | 30.0% | 4 | R.VINEPTAAALAYGLDKSEDKVIAVYDLGGGTFDISILEIQK.G | 3 |
| \* | SKAPIP\_tube2\_041314\_01.11494.11494.2 | 6.2355 | 0.5005 | 100.0% | 2057.5122 | 2057.181 | 1 | 7.94 | 63.9% | 22 | K.STNGDTFLGGEDFDQALLR.H | 2 |
| \* | pDK365N\_300mM\_082713\_03.04875.04875.3 | 3.0163 | 0.2386 | 98.5% | 1847.5144 | 1848.0844 | 25 | 5.003 | 38.3% | 1 | K.RETGVDLTKDNMALQR.V | 3 |
| \* | 100326\_pJS43\_01.04115.04115.2 | 3.4578 | 0.4062 | 100.0% | 1691.4922 | 1691.8969 | 1 | 7.063 | 64.3% | 11 | R.ETGVDLTKDNMALQR.V | 2 |
| \* | pDK365N\_100mM\_082813\_03.05723.05723.3 | 3.381 | 0.4076 | 100.0% | 1692.8644 | 1691.8969 | 1 | 6.766 | 39.3% | 4 | R.ETGVDLTKDNMALQR.V | 3 |
| \* | pDK365N\_100mM\_082813\_03.11453.11453.2 | 4.4431 | 0.419 | 100.0% | 1362.5521 | 1362.5687 | 1 | 8.653 | 72.7% | 30 | R.AQFEGIVTDLIR.R | 2 |
| \* | 100326\_pJS43\_01.08726.08726.2 | 2.4247 | 0.2488 | 98.5% | 1518.7322 | 1518.7562 | 1 | 5.053 | 54.2% | 1 | R.AQFEGIVTDLIRR.T | 2 |
| \* | pDK365N\_300mM\_082713\_01.10424.10424.3 | 2.1953 | 0.3376 | 98.8% | 1518.9243 | 1518.7562 | 25 | 5.983 | 35.4% | 1 | R.AQFEGIVTDLIRR.T | 3 |
| \* | pDK365N\_300mM\_082713\_01.01674.01674.2 | 1.9338 | 0.3098 | 98.2% | 979.1122 | 979.093 | 6 | 5.296 | 75.0% | 1 | K.AMQDAEVSK.S | 2 |
| \* | pSKT11\_1\_020812\_02.05792.05792.2 | 5.2725 | 0.5215 | 100.0% | 2407.4521 | 2407.7595 | 1 | 9.496 | 52.3% | 4 | K.AMQDAEVSKSDIGEVILVGGMTR.M | 2 |
| \* | pDK365N\_100mM\_082813\_03.09918.09918.3 | 4.1366 | 0.4067 | 100.0% | 2408.2744 | 2407.7595 | 1 | 6.542 | 30.7% | 8 | K.AMQDAEVSKSDIGEVILVGGMTR.M | 3 |
| \* | pDK365N\_300mM\_082713\_03.09998.09998.2 | 4.1689 | 0.4721 | 100.0% | 1447.3922 | 1447.6898 | 1 | 8.082 | 76.9% | 19 | K.SDIGEVILVGGMTR.M | 2 |
| \* | SKAPIP\_tube2\_041314\_01.08301.08301.1 | 2.6007 | 0.3578 | 99.5% | 1290.68 | 1291.4496 | 1 | 6.409 | 70.0% | 1 | K.VQQTVQDLFGR.A | 1 |
| \* | AstrinIP\_MS2\_022614\_01.08984.08984.2 | 3.8971 | 0.1755 | 99.9% | 1292.3322 | 1291.4496 | 1 | 6.618 | 80.0% | 32 | K.VQQTVQDLFGR.A | 2 |
| \* | pSKT11\_1\_020812\_02.03450.03450.2 | 3.5706 | 0.4525 | 100.0% | 1937.2122 | 1938.1448 | 1 | 8.428 | 61.8% | 1 | K.KSQVFSTAADGQTQVEIK.V | 2 |
| \* | SKAPIP\_041314\_02.04912.04912.3 | 4.7714 | 0.4312 | 100.0% | 1938.2043 | 1938.1448 | 1 | 7.235 | 41.2% | 5 | K.KSQVFSTAADGQTQVEIK.V | 3 |
| \* | pDK365N\_300mM\_082713\_02.05252.05252.2 | 5.6757 | 0.5914 | 100.0% | 1809.2922 | 1809.9707 | 1 | 10.502 | 78.1% | 39 | K.SQVFSTAADGQTQVEIK.V | 2 |
| \* | 100326\_pJS43\_02.04325.04325.3 | 4.8061 | 0.5036 | 100.0% | 1810.3444 | 1809.9707 | 1 | 9.159 | 43.8% | 5 | K.SQVFSTAADGQTQVEIK.V | 3 |
| \* | SKAPIP\_tube2\_041314\_01.12152.12152.2 | 3.7218 | 0.467 | 100.0% | 1593.8722 | 1593.9529 | 1 | 7.91 | 78.6% | 15 | K.LLGQFTLIGIPPAPR.G | 2 |
| \* | 100326\_pJS43\_02.12642.12642.2 | 2.6079 | 0.4229 | 99.9% | 2309.7122 | 2310.612 | 2 | 6.353 | 31.0% | 1 | R.GVPQIEVTFDIDANGIVHVSAK.D | 2 |
| \* | pSKT11\_1\_020812\_02.04571.04571.3 | 4.9847 | 0.3832 | 100.0% | 3033.3245 | 3034.3684 | 1 | 6.422 | 33.3% | 1 | K.DKGTGREQQIVIQSSGGLSKDDIENMVK.N | 3 |
| \* | pDK365N\_300mM\_082713\_04.04392.04392.2 | 4.2504 | 0.4498 | 100.0% | 1475.3522 | 1474.6543 | 1 | 8.052 | 73.1% | 14 | R.EQQIVIQSSGGLSK.D | 2 |
| \* | 100326\_pJS43\_01.06946.06946.2 | 5.1663 | 0.5532 | 100.0% | 2419.3123 | 2419.7095 | 1 | 10.068 | 57.1% | 4 | R.EQQIVIQSSGGLSKDDIENMVK.N | 2 |
| \* | AstrinIP\_MS2\_022614\_02.06212.06212.3 | 3.6321 | 0.4908 | 100.0% | 2420.3044 | 2419.7095 | 1 | 7.418 | 36.9% | 22 | R.EQQIVIQSSGGLSKDDIENMVK.N | 3 |
| \* | SKAPIP\_tube2\_041314\_02.07280.07280.3 | 5.5 | 0.4078 | 100.0% | 2142.9844 | 2143.3765 | 1 | 8.108 | 50.0% | 14 | K.ERVEAVNMAEGIIHDTETK.M | 3 |
| \* | AstrinIP\_MS1\_022614\_01.10040.10040.3 | 3.4367 | 0.3716 | 100.0% | 1857.8944 | 1858.0735 | 3 | 6.613 | 40.6% | 13 | R.VEAVNMAEGIIHDTETK.M | 3 |
| \* | AstrinIP\_MS2\_022614\_01.10781.10781.2 | 4.8579 | 0.4808 | 100.0% | 1858.2722 | 1858.0735 | 1 | 8.011 | 75.0% | 5 | R.VEAVNMAEGIIHDTETK.M | 2 |
| \* | SKAPIP\_041314\_01.06777.06777.2 | 3.434 | 0.4427 | 100.0% | 1233.1322 | 1232.3794 | 1 | 7.358 | 72.7% | 17 | R.QAASSLQQASLK.L | 2 |
| \* | pDK365N\_300mM\_082713\_03.07150.07150.2 | 2.3707 | 0.1053 | 97.1% | 901.8122 | 902.0961 | 148 | 4.17 | 83.3% | 2 | K.LFEMAYK.K | 2 |
| \* | SKAPIP\_tube2\_041314\_01.05601.05601.2 | 2.536 | 0.1926 | 99.2% | 1030.1122 | 1030.2701 | 85 | 4.851 | 78.6% | 2 | K.LFEMAYKK.M | 2 |

---

|  |  |  |  |  |  |  |  |  |
| --- | --- | --- | --- | --- | --- | --- | --- | --- |
| U | *gi|209862831|ref|NP\_0* | 15 | 47 | 53.4% | 339 | 38604 | 7.8 | annexin A2 isoform 2 [Homo sapiens] |
| U | *gi|50845388|ref|NP\_00* | 15 | 47 | 50.7% | 357 | 40411 | 8.4 | annexin A2 isoform 1 [Homo sapiens] |
| U | *gi|50845386|ref|NP\_00* | 15 | 47 | 53.4% | 339 | 38604 | 7.8 | annexin A2 isoform 2 [Homo sapiens] |
| U | *gi|4757756|ref|NP\_004* | 15 | 47 | 53.4% | 339 | 38604 | 7.8 | annexin A2 isoform 2 [Homo sapiens] |

| Filename XCorr DeltCN Conf% ObsM+H+ CalcM+H+ SpR ZScore Ion% # Sequence  | | | | | | | | | | | | |
| --- | --- | --- | --- | --- | --- | --- | --- | --- | --- | --- | --- | --- |
|  | SKAPIP\_tube2\_041314\_01.05519.05519.2 | 3.1739 | 0.3066 | 99.9% | 1845.4321 | 1846.0038 | 1 | 6.393 | 52.9% | 1 | K.LSLEGDHSTPPSAYGSVK.A | 2 |
|  | SKAPIP\_041314\_01.10427.10427.2 | 1.7026 | 0.341 | 96.7% | 1087.7322 | 1087.1338 | 132 | 5.594 | 68.8% | 1 | K.AYTNFDAER.D | 2 |
|  | pDK365N\_100mM\_082713\_01.09240.09240.3 | 3.0469 | 0.2595 | 98.8% | 2157.3245 | 2156.357 | 7 | 4.816 | 30.6% | 2 | K.AYTNFDAERDALNIETAIK.T | 3 |
|  | SKAPIP\_tube2\_041314\_01.12474.12474.2 | 4.0694 | 0.439 | 100.0% | 1543.4722 | 1543.7605 | 1 | 7.741 | 65.4% | 6 | K.GVDEVTIVNILTNR.S | 2 |
|  | pDK365N\_100mM\_082813\_03.05976.05976.2 | 2.5458 | 0.3408 | 99.9% | 1112.3922 | 1112.2303 | 5 | 5.497 | 68.8% | 2 | R.QDIAFAYQR.R | 2 |
|  | SKAPIP\_tube2\_041314\_01.13709.13709.2 | 4.9313 | 0.4777 | 100.0% | 1651.4922 | 1651.9872 | 1 | 9.565 | 63.3% | 3 | K.SALSGHLETVILGLLK.T | 2 |
|  | SKAPIP\_tube2\_041314\_02.10560.10560.3 | 3.9848 | 0.4219 | 100.0% | 1652.6943 | 1651.9872 | 2 | 6.466 | 45.0% | 4 | K.SALSGHLETVILGLLK.T | 3 |
|  | pDK339othertube\_033013\_01.04385.04385.2 | 3.5757 | 0.4937 | 100.0% | 1223.0922 | 1223.3251 | 1 | 8.32 | 80.0% | 6 | K.TPAQYDASELK.A | 2 |
|  | SKAPIP\_041314\_01.08853.08853.2 | 3.5872 | 0.2705 | 100.0% | 1245.3722 | 1245.3347 | 2 | 5.965 | 83.3% | 7 | R.TNQELQEINR.V | 2 |
|  | pDK365N\_100mM\_082713\_01.07543.07543.3 | 4.7457 | 0.4555 | 100.0% | 1940.7843 | 1941.102 | 1 | 8.365 | 43.8% | 7 | K.TDLEKDIISDTSGDFRK.L | 3 |
|  | pDK365N\_100mM\_082713\_01.07831.07831.3 | 3.0595 | 0.1917 | 95.5% | 2066.6643 | 2066.1887 | 1 | 4.292 | 38.2% | 2 | R.RAEDGSVIDYELIDQDAR.D | 3 |
|  | pDK365N\_100mM\_082713\_01.08666.08666.2 | 4.376 | 0.4507 | 100.0% | 1909.3722 | 1910.0013 | 1 | 8.639 | 56.2% | 3 | R.AEDGSVIDYELIDQDAR.D | 2 |
|  | SKAPIP\_041314\_01.06334.06334.2 | 2.0284 | 0.2196 | 95.8% | 1037.2922 | 1037.1606 | 9 | 4.724 | 75.0% | 1 | R.DLYDAGVKR.K | 2 |
|  | SKAPIP\_041314\_01.15252.15252.3 | 2.7514 | 0.2493 | 95.8% | 2839.7043 | 2840.1743 | 135 | 4.699 | 21.7% | 1 | K.GDLENAFLNLVQCIQNKPLYFADR.L | 3 |
|  | SKAPIP\_tube2\_041314\_02.06480.06480.2 | 4.2638 | 0.4633 | 100.0% | 2013.7522 | 2014.1986 | 1 | 8.523 | 63.3% | 1 | K.SLYYYIQQDTKGDYQK.A | 2 |

---

|  |  |  |  |  |  |  |  |  |
| --- | --- | --- | --- | --- | --- | --- | --- | --- |
| U | *gi|14389309|ref|NP\_11* | 29 | 331 | 53.2% | 449 | 49895 | 5.1 | tubulin alpha 6 [Homo sapiens] |

| Filename XCorr DeltCN Conf% ObsM+H+ CalcM+H+ SpR ZScore Ion% # Sequence  | | | | | | | | | | | | |
| --- | --- | --- | --- | --- | --- | --- | --- | --- | --- | --- | --- | --- |
|  | pDK365N\_300mM\_082713\_02.07532.07532.2 | 5.9094 | 0.6089 | 100.0% | 2008.4122 | 2009.093 | 1 | 11.774 | 57.9% | 38 | K.TIGGGDDSFNTFFSETGAGK.H | 22 |
|  | pDK339\_033013\_01.12157.12157.2 | 5.3622 | 0.4904 | 100.0% | 1702.5122 | 1702.9451 | 1 | 8.482 | 78.6% | 36 | R.AVFVDLEPTVIDEVR.T | 22 |
|  | AstrinIP\_MS2\_022614\_01.09048.09048.2 | 2.6573 | 0.4517 | 100.0% | 1410.3121 | 1411.6439 | 51 | 6.74 | 54.5% | 4 | R.QLFHPEQLITGK.E | 222 |
|  | 100326\_pJS43\_01.06218.06218.2 | 2.6061 | 0.2557 | 98.8% | 2416.2122 | 2416.6555 | 5 | 5.618 | 35.0% | 3 | R.QLFHPEQLITGKEDAANNYAR.G | 222 |
|  | pDK365N\_300mM\_082713\_01.08105.08105.3 | 4.2433 | 0.3985 | 100.0% | 2416.2844 | 2416.6555 | 1 | 7.004 | 30.0% | 21 | R.QLFHPEQLITGKEDAANNYAR.G | 333 |
|  | 100326\_pJS43\_01.10718.10718.2 | 4.127 | 0.5314 | 100.0% | 1843.7922 | 1843.1332 | 1 | 8.607 | 76.7% | 4 | R.GHYTIGKEIIDLVLDR.I | 22 |
|  | 100326\_pJS43\_01.10701.10701.3 | 5.0142 | 0.4478 | 100.0% | 1843.8844 | 1843.1332 | 1 | 7.44 | 50.0% | 2 | R.GHYTIGKEIIDLVLDR.I | 33 |
|  | 100326\_pJS43\_01.09051.09051.1 | 2.1194 | 0.4161 | 99.4% | 1085.48 | 1086.2737 | 78 | 6.268 | 56.2% | 2 | K.EIIDLVLDR.I | 11 |
|  | 100326\_pJS43\_01.09026.09026.2 | 2.6652 | 0.3008 | 99.9% | 1086.0721 | 1086.2737 | 32 | 5.508 | 62.5% | 5 | K.EIIDLVLDR.I | 22 |
|  | AstrinIP\_MS2\_022614\_01.03693.03693.2 | 2.3076 | 0.2225 | 98.9% | 909.9922 | 910.05804 | 2 | 5.244 | 78.6% | 1 | R.LSVDYGKK.S | 222 |
|  | AstrinIP\_MS2\_022614\_01.05537.05537.3 | 3.1267 | 0.2098 | 98.1% | 1876.0443 | 1876.0824 | 1 | 5.415 | 41.1% | 2 | R.RNLDIERPTYTNLNR.L | 333 |
|  | 100326\_pJS43\_01.04940.04940.2 | 3.4438 | 0.1692 | 99.5% | 1719.1122 | 1719.8949 | 1 | 5.078 | 65.4% | 19 | R.NLDIERPTYTNLNR.L | 222 |
|  | pDK365N\_300mM\_082713\_01.06254.06254.3 | 2.956 | 0.3024 | 99.7% | 1720.4644 | 1719.8949 | 8 | 5.659 | 38.5% | 6 | R.NLDIERPTYTNLNR.L | 333 |
|  | 100326\_pJS43\_02.08678.08678.2 | 4.9741 | 0.4878 | 100.0% | 1488.2122 | 1488.7678 | 1 | 9.092 | 73.1% | 27 | R.LISQIVSSITASLR.F | 222 |
|  | 100326\_pJS43\_01.11114.11114.2 | 5.2561 | 0.5725 | 100.0% | 2409.4521 | 2410.6885 | 1 | 10.409 | 52.5% | 20 | R.FDGALNVDLTEFQTNLVPYPR.I | 222 |
|  | pDK365N\_300mM\_082713\_02.08931.08931.3 | 3.2455 | 0.2341 | 98.4% | 2411.6643 | 2410.6885 | 1 | 4.972 | 35.0% | 2 | R.FDGALNVDLTEFQTNLVPYPR.I | 333 |
|  | 100326\_pJS43\_01.08403.08403.3 | 3.956 | 0.3773 | 100.0% | 1757.6044 | 1758.0703 | 1 | 6.72 | 43.3% | 11 | R.IHFPLATYAPVISAEK.A | 333 |
|  | 100326\_pJS43\_01.08450.08450.2 | 4.3366 | 0.4911 | 100.0% | 1758.3922 | 1758.0703 | 1 | 8.686 | 73.3% | 21 | R.IHFPLATYAPVISAEK.A | 222 |
| \* | 100326\_pJS43\_02.06082.06082.3 | 3.1115 | 0.2955 | 99.3% | 2765.6643 | 2766.064 | 9 | 4.443 | 26.1% | 1 | K.AYHEQLTVAEITNACFEPANQMVK.C | 3 |
|  | AstrinIP\_MS1\_022614\_01.08429.08429.2 | 2.7335 | 0.3316 | 99.9% | 1249.9922 | 1250.4304 | 1 | 6.82 | 81.2% | 1 | K.YMACCLLYR.G | 222 |
|  | pDK339\_033013\_01.06838.06838.1 | 1.8415 | 0.4374 | 99.5% | 1015.55 | 1016.1827 | 3 | 6.618 | 61.1% | 13 | K.DVNAAIATIK.T | 11 |
|  | pDK339\_033013\_01.06872.06872.2 | 3.3091 | 0.3518 | 100.0% | 1016.0522 | 1016.1827 | 1 | 7.202 | 88.9% | 16 | K.DVNAAIATIK.T | 22 |
|  | AstrinIP\_MS2\_022614\_01.09147.09147.2 | 4.5299 | 0.4948 | 100.0% | 1825.4722 | 1826.1027 | 1 | 7.664 | 61.8% | 31 | K.VGINYQPPTVVPGGDLAK.V | 222 |
|  | pDK339\_033013\_01.06961.06961.2 | 3.5035 | 0.4054 | 100.0% | 1381.2522 | 1381.6324 | 1 | 6.607 | 80.0% | 8 | R.LDHKFDLMYAK.R | 222 |
|  | pDK365N\_100mM\_082813\_03.06041.06041.3 | 4.3182 | 0.3902 | 100.0% | 1381.9143 | 1381.6324 | 2 | 7.156 | 62.5% | 19 | R.LDHKFDLMYAK.R | 333 |
|  | AstrinIP\_MS2\_022614\_01.06849.06849.1 | 2.8794 | 0.1799 | 98.7% | 1382.19 | 1381.6324 | 1 | 4.024 | 65.0% | 1 | R.LDHKFDLMYAK.R | 111 |
|  | pSKT11\_1\_020812\_01.08280.08280.3 | 3.0442 | 0.2407 | 99.2% | 1538.1843 | 1537.82 | 35 | 4.538 | 38.6% | 1 | R.LDHKFDLMYAKR.A | 333 |
|  | 100326\_pJS43\_02.05668.05668.3 | 6.1453 | 0.4595 | 100.0% | 2487.8044 | 2487.7083 | 1 | 8.41 | 47.5% | 3 | K.RAFVHWYVGEGMEEGEFSEAR.E | 333 |
|  | pDK339othertube\_033013\_02.07266.07266.3 | 5.3032 | 0.4274 | 100.0% | 2331.2043 | 2331.5208 | 1 | 8.132 | 44.7% | 13 | R.AFVHWYVGEGMEEGEFSEAR.E | 333 |

Similarities:
gi|57013276|ref|NP\_00(28:1)  
gi|17921989|ref|NP\_00(20:9)  

---

|  |  |  |  |  |  |  |  |  |
| --- | --- | --- | --- | --- | --- | --- | --- | --- |
| U | *gi|34098946|ref|NP\_00* | 17 | 111 | 53.1% | 324 | 35924 | 9.9 | nuclease sensitive element binding protein 1 [Homo sapiens] |

| Filename XCorr DeltCN Conf% ObsM+H+ CalcM+H+ SpR ZScore Ion% # Sequence  | | | | | | | | | | | | |
| --- | --- | --- | --- | --- | --- | --- | --- | --- | --- | --- | --- | --- |
|  | pDK365N\_100mM\_082813\_03.05529.05529.2 | 2.2663 | 0.3358 | 99.7% | 942.3522 | 941.0342 | 1 | 6.403 | 78.6% | 4 | R.NGYGFINR.N | 222 |
|  | pSKT11\_1\_020812\_02.04103.04103.3 | 4.6094 | 0.4282 | 100.0% | 2667.2344 | 2667.9407 | 1 | 7.198 | 29.5% | 2 | R.NGYGFINRNDTKEDVFVHQTAIK.K | 333 |
|  | pSKT11\_1\_020812\_02.03669.03669.3 | 4.5426 | 0.3818 | 100.0% | 2795.2444 | 2796.1147 | 1 | 5.985 | 31.5% | 1 | R.NGYGFINRNDTKEDVFVHQTAIKK.N | 333 |
|  | AstrinIP\_MS2\_022614\_01.04626.04626.3 | 3.3734 | 0.3198 | 99.9% | 1745.8744 | 1745.9298 | 1 | 5.888 | 42.9% | 8 | R.NDTKEDVFVHQTAIK.K | 333 |
|  | SKAPIP\_041314\_01.08574.08574.2 | 4.855 | 0.3996 | 100.0% | 1873.4722 | 1874.1039 | 1 | 6.888 | 70.0% | 5 | R.NDTKEDVFVHQTAIKK.N | 222 |
|  | pSKT11\_1\_020812\_01.04060.04060.3 | 4.701 | 0.3475 | 100.0% | 1875.2943 | 1874.1039 | 1 | 7.021 | 50.0% | 12 | R.NDTKEDVFVHQTAIKK.N | 333 |
|  | pJS43\_100mM\_120812\_02.06264.06264.2 | 4.9339 | 0.4881 | 100.0% | 1796.1921 | 1796.8822 | 1 | 9.316 | 68.8% | 27 | R.SVGDGETVEFDVVEGEK.G | 222 |
| \* | pDK365N\_100mM\_082813\_04.06288.06288.3 | 5.9661 | 0.4703 | 100.0% | 3474.2344 | 3474.7168 | 1 | 8.677 | 27.9% | 8 | R.SVGDGETVEFDVVEGEKGAEAANVTGPGGVPVQGSK.Y | 3 |
| \* | SKAPIP\_041314\_01.10530.10530.2 | 4.8467 | 0.535 | 100.0% | 1696.2922 | 1696.8577 | 1 | 8.884 | 63.9% | 9 | K.GAEAANVTGPGGVPVQGSK.Y | 2 |
| \* | pDK339othertube\_033013\_01.03510.03510.3 | 6.4456 | 0.5024 | 100.0% | 3258.1743 | 3259.2566 | 1 | 7.942 | 37.5% | 13 | R.NYQQNYQNSESGEKNEGSESAPEGQAQQR.R | 3 |
| \* | pSKT11\_1\_020812\_01.03562.03562.2 | 3.0863 | 0.406 | 100.0% | 3258.7922 | 3259.2566 | 1 | 6.223 | 23.2% | 2 | R.NYQQNYQNSESGEKNEGSESAPEGQAQQR.R | 2 |
| \* | pDK365N\_300mM\_082713\_01.05056.05056.3 | 3.1433 | 0.18 | 98.8% | 1288.1943 | 1286.54 | 3 | 4.324 | 59.4% | 3 | R.RRFPPYYMR.R | 3 |
| \* | pDK365N\_300mM\_082713\_01.04299.04299.3 | 5.3496 | 0.4557 | 100.0% | 3224.7844 | 3225.4795 | 1 | 9.057 | 29.3% | 9 | R.RPQYSNPPVQGEVMEGADNQGAGEQGRPVR.Q | 3 |
| \* | pJS43\_100mM\_120812\_01.03109.03109.2 | 1.9371 | 0.3314 | 97.1% | 2628.372 | 2629.5835 | 1 | 6.863 | 50.0% | 1 | R.EDGNEEDKENQGDETQGQQPPQR.R | 2 |
| \* | AstrinIP\_MS2\_022614\_01.03422.03422.3 | 4.9179 | 0.3841 | 100.0% | 2629.8542 | 2629.5835 | 1 | 7.317 | 42.0% | 3 | R.EDGNEEDKENQGDETQGQQPPQR.R | 3 |
| \* | AstrinIP\_MS2\_022614\_01.03368.03368.3 | 3.1245 | 0.3993 | 100.0% | 2784.8342 | 2785.771 | 1 | 6.639 | 28.3% | 3 | R.EDGNEEDKENQGDETQGQQPPQRR.Y | 3 |
| \* | pDK365N\_300mM\_082713\_03.03530.03530.2 | 2.8543 | 0.4633 | 100.0% | 1898.4122 | 1898.8914 | 5 | 8.281 | 36.8% | 1 | K.AADPPAENSSAPEAEQGGAE.- | 2 |

Similarities:
gi|224586884|ref|NP\_0(7:10)  
gi|224586882|ref|NP\_0(7:10)  

---

|  |  |  |  |  |  |  |  |  |
| --- | --- | --- | --- | --- | --- | --- | --- | --- |
| U | *gi|67782365|ref|NP\_00* | 29 | 87 | 52.2% | 469 | 51386 | 5.5 | keratin 7 [Homo sapiens] |

| Filename XCorr DeltCN Conf% ObsM+H+ CalcM+H+ SpR ZScore Ion% # Sequence  | | | | | | | | | | | | |
| --- | --- | --- | --- | --- | --- | --- | --- | --- | --- | --- | --- | --- |
|  | SKAPIP\_tube2\_041314\_01.06233.06233.3 | 3.9529 | 0.3606 | 100.0% | 2248.4343 | 2247.519 | 119 | 5.804 | 25.0% | 4 | R.LSSARPGGLGSSSLYGLGASRPR.V | 3 |
|  | SKAPIP\_tube2\_041314\_01.04744.04744.2 | 2.9433 | 0.3202 | 99.9% | 1106.1721 | 1105.2388 | 1 | 5.125 | 72.7% | 7 | R.SAYGGPVGAGIR.E | 2 |
|  | SKAPIP\_tube2\_041314\_01.11379.11379.3 | 3.96 | 0.394 | 100.0% | 2451.6543 | 2450.7979 | 1 | 7.521 | 38.1% | 1 | R.EVTINQSLLAPLRLDADPSLQR.V | 3 |
|  | SKAPIP\_041314\_01.07775.07775.2 | 2.7245 | 0.1644 | 99.1% | 1014.5522 | 1015.1112 | 1 | 4.68 | 81.2% | 4 | R.LDADPSLQR.V | 2 |
|  | pJS43\_100mM\_120812\_01.07265.07265.2 | 2.4075 | 0.2649 | 99.7% | 828.09216 | 827.95544 | 2 | 5.168 | 91.7% | 6 | K.FASFIDK.V | 222222222 |
|  | SKAPIP\_tube2\_041314\_01.06813.06813.2 | 2.9746 | 0.2137 | 99.8% | 1082.7522 | 1083.2755 | 2 | 7.239 | 75.0% | 6 | K.FASFIDKVR.F | 22222222 |
|  | SKAPIP\_tube2\_041314\_01.07800.07800.2 | 2.9171 | 0.201 | 99.8% | 1045.7722 | 1046.2114 | 3 | 5.081 | 78.6% | 5 | K.WTLLQEQK.S | 2 |
|  | SKAPIP\_tube2\_041314\_01.11106.11106.2 | 3.5725 | 0.2563 | 99.9% | 1773.3522 | 1774.0299 | 1 | 5.279 | 56.7% | 1 | K.SSRLPDIFEAQIAGLR.G | 2 |
|  | SKAPIP\_041314\_01.12861.12861.2 | 4.0975 | 0.4384 | 100.0% | 1443.6522 | 1443.686 | 1 | 9.229 | 83.3% | 7 | R.LPDIFEAQIAGLR.G | 2 |
|  | AstrinIP\_MS2\_022614\_01.06519.06519.1 | 2.2516 | 0.2656 | 98.8% | 1242.63 | 1243.3622 | 2 | 5.261 | 54.5% | 1 | R.GQLEALQVDGGR.L | 1 |
|  | pDK339othertube\_033013\_01.05850.05850.2 | 3.342 | 0.4777 | 100.0% | 1243.3722 | 1243.3622 | 1 | 8.499 | 81.8% | 3 | R.GQLEALQVDGGR.L | 2 |
| \* | SKAPIP\_041314\_02.07810.07810.2 | 3.3942 | 0.2376 | 99.8% | 1953.6921 | 1955.1783 | 2 | 5.715 | 44.1% | 1 | R.GQLEALQVDGGRLEAELR.S | 2 |
| \* | SKAPIP\_041314\_02.07805.07805.3 | 4.2409 | 0.4575 | 100.0% | 1955.7544 | 1955.1783 | 1 | 7.783 | 42.6% | 3 | R.GQLEALQVDGGRLEAELR.S | 3 |
|  | SKAPIP\_tube2\_041314\_01.10692.10692.2 | 4.2872 | 0.0544 | 99.7% | 1420.4321 | 1419.5773 | 1 | 6.893 | 81.8% | 6 | K.VDALNDEINFLR.T | 2 |
|  | SKAPIP\_041314\_01.13193.13193.3 | 5.8748 | 0.0377 | 96.2% | 3172.0444 | 3172.2698 | 4 | 5.662 | 31.7% | 3 | R.TLNET#ELT#ELQSQISDTSVVLSMDNSR.S | 3 |
|  | SKAPIP\_tube2\_041314\_01.11819.11819.2 | 4.0571 | 0.4831 | 100.0% | 1273.3121 | 1273.4692 | 1 | 8.461 | 86.4% | 1 | R.SLDLDGIIAEVK.A | 2 |
|  | SKAPIP\_tube2\_041314\_01.05447.05447.2 | 2.2784 | 0.3513 | 99.6% | 1196.6721 | 1197.2897 | 2 | 6.242 | 72.2% | 1 | R.AEAEAWYQTK.F | 22 |
|  | SKAPIP\_041314\_01.08450.08450.2 | 3.5761 | 0.3989 | 100.0% | 1092.9122 | 1093.2249 | 1 | 7.422 | 77.8% | 8 | K.FETLQAQAGK.H | 2 |
|  | pSKT11\_1\_020812\_01.05910.05910.2 | 2.4419 | 0.294 | 99.3% | 1442.0521 | 1442.6151 | 1 | 5.013 | 63.6% | 3 | R.LQAEIDNIKNQR.A | 2 |
|  | SKAPIP\_tube2\_041314\_01.04253.04253.3 | 2.2257 | 0.3451 | 99.2% | 1442.7244 | 1442.6151 | 1 | 5.122 | 43.2% | 1 | R.LQAEIDNIKNQR.A | 3 |
| \* | SKAPIP\_tube2\_041314\_02.05405.05405.3 | 3.4649 | 0.2584 | 99.8% | 1401.9243 | 1401.5596 | 4 | 5.081 | 47.9% | 1 | R.AKLEAAIAEAEER.G | 3 |
| \* | SKAPIP\_041314\_02.07760.07760.2 | 4.4866 | 0.4581 | 100.0% | 2012.9521 | 2013.2987 | 1 | 7.314 | 52.8% | 1 | R.AKLEAAIAEAEERGELALK.D | 2 |
| \* | pDk339\_033013\_02.07070.07070.3 | 4.3132 | 0.4112 | 100.0% | 2014.1943 | 2013.2987 | 1 | 6.929 | 45.8% | 3 | R.AKLEAAIAEAEERGELALK.D | 3 |
| \* | SKAPIP\_tube2\_041314\_01.10203.10203.3 | 4.0085 | 0.4458 | 100.0% | 2355.3245 | 2355.6536 | 1 | 6.673 | 36.9% | 1 | R.AKLEAAIAEAEERGELALKDAR.A | 3 |
| \* | pDK339othertube\_033013\_01.05184.05184.2 | 2.6616 | 0.1801 | 98.6% | 1202.1522 | 1202.3066 | 1 | 5.42 | 80.0% | 1 | K.LEAAIAEAEER.G | 2 |
|  | SKAPIP\_tube2\_041314\_01.04972.04972.2 | 3.9816 | 0.3921 | 100.0% | 1386.3522 | 1386.548 | 2 | 6.787 | 72.7% | 4 | R.AKQEELEAALQR.G | 2 |
|  | SKAPIP\_tube2\_041314\_01.08620.08620.2 | 2.9744 | 0.3975 | 100.0% | 1406.8922 | 1406.6653 | 2 | 6.859 | 63.6% | 2 | K.LALDIEIATYRK.L | 2222 |
|  | pJS43\_100mM\_120812\_01.03584.03584.1 | 2.3838 | 0.1736 | 97.7% | 932.52 | 933.00616 | 42 | 5.42 | 64.3% | 1 | K.LLEGEESR.L | 111 |
|  | pJS43\_100mM\_120812\_01.03569.03569.2 | 2.2842 | 0.1217 | 95.4% | 932.6122 | 933.00616 | 36 | 4.304 | 71.4% | 1 | K.LLEGEESR.L | 222 |

Similarities:
gi|62414289|ref|NP\_00(2:27)  
gi|4504919|ref|NP\_002(5:24)  
gi|47132620|ref|NP\_00(2:27)  
gi|119395754|ref|NP\_0(2:27)  
gi|155969697|ref|NP\_7(2:27)  
gi|119703753|ref|NP\_0(2:27)  
gi|32567786|ref|NP\_78(3:26)  
gi|153791158|ref|NP\_0(2:27)  
gi|125628632|ref|NP\_0(1:28)  
gi|109255249|ref|NP\_0(2:27)  

---

|  |  |  |  |  |  |  |  |  |
| --- | --- | --- | --- | --- | --- | --- | --- | --- |
| U | *gi|19923935|ref|NP\_61* | 13 | 36 | 52.2% | 293 | 33238 | 8.1 | coiled-coil domain containing 101 [Homo sapiens] |

| Filename XCorr DeltCN Conf% ObsM+H+ CalcM+H+ SpR ZScore Ion% # Sequence  | | | | | | | | | | | | |
| --- | --- | --- | --- | --- | --- | --- | --- | --- | --- | --- | --- | --- |
| \* | SKAPIP\_tube2\_041314\_01.12651.12651.2 | 4.5355 | 0.3923 | 100.0% | 1521.5922 | 1521.8406 | 1 | 7.852 | 79.2% | 4 | R.IAELLTELHQLIK.Q | 2 |
| \* | SKAPIP\_041314\_01.13064.13064.3 | 3.1364 | 0.1874 | 97.8% | 1522.4944 | 1521.8406 | 1 | 5.303 | 54.2% | 3 | R.IAELLTELHQLIK.Q | 3 |
| \* | pDK365N\_300mM\_082713\_01.03614.03614.3 | 3.2887 | 0.2387 | 99.6% | 1425.4744 | 1425.5875 | 13 | 5.946 | 47.7% | 1 | R.SRSEHNLVNIQK.T | 3 |
| \* | SKAPIP\_041314\_01.06008.06008.2 | 3.2332 | 0.3628 | 100.0% | 1182.0521 | 1182.3219 | 1 | 6.337 | 72.2% | 7 | R.SEHNLVNIQK.T | 2 |
| \* | SKAPIP\_tube2\_041314\_01.05062.05062.2 | 2.8526 | 0.4383 | 100.0% | 1610.2522 | 1610.7386 | 1 | 6.553 | 72.7% | 1 | R.MQTENKIS\*PYYR.T | 2 |
| \* | SKAPIP\_041314\_01.09036.09036.2 | 2.9723 | 0.2355 | 99.8% | 1129.3322 | 1129.3855 | 1 | 5.261 | 72.2% | 1 | R.KALDKIAEIK.S | 2 |
| \* | SKAPIP\_tube2\_041314\_01.05454.05454.2 | 2.9135 | 0.5502 | 100.0% | 1332.1322 | 1332.4557 | 1 | 8.468 | 59.1% | 1 | K.IAGLYNDSEPPR.K | 2 |
| \* | SKAPIP\_041314\_01.10053.10053.2 | 3.4896 | 0.4135 | 100.0% | 1460.0322 | 1460.6298 | 1 | 6.967 | 66.7% | 6 | K.IAGLYNDSEPPRK.T | 2 |
| \* | SKAPIP\_041314\_01.10077.10077.3 | 2.6863 | 0.3012 | 99.4% | 1462.4043 | 1460.6298 | 1 | 5.093 | 47.9% | 1 | K.IAGLYNDSEPPRK.T | 3 |
| \* | SKAPIP\_tube2\_041314\_02.09099.09099.3 | 4.1538 | 0.1934 | 98.8% | 4012.5544 | 4012.2458 | 1 | 5.568 | 25.0% | 1 | K.AVDGDEQWILAEVVSYSHATNKYEVDDIDEEGKER.H | 3 |
| \* | pDK365N\_300mM\_082713\_03.07332.07332.2 | 2.3451 | 0.2613 | 99.2% | 1137.4521 | 1137.4135 | 1 | 4.923 | 68.8% | 1 | R.RVIPLPQWK.A | 2 |
| \* | SKAPIP\_041314\_01.11489.11489.2 | 3.6502 | 0.4901 | 100.0% | 1460.1322 | 1460.5835 | 1 | 7.323 | 66.7% | 6 | K.ANPETDPEALFQK.E | 2 |
| \* | SKAPIP\_tube2\_041314\_02.08049.08049.3 | 5.956 | 0.4032 | 100.0% | 4033.4043 | 4033.4026 | 1 | 7.081 | 27.9% | 3 | R.ALIHAPPQRPQDDYSVLFEDTSYADGYSPPLNVAQR.Y | 3 |

---

|  |  |  |  |  |  |  |  |  |
| --- | --- | --- | --- | --- | --- | --- | --- | --- |
| U | *gi|4506695|ref|NP\_001* | 13 | 60 | 51.7% | 145 | 16060 | 10.3 | ribosomal protein S19 [Homo sapiens] |

| Filename XCorr DeltCN Conf% ObsM+H+ CalcM+H+ SpR ZScore Ion% # Sequence  | | | | | | | | | | | | |
| --- | --- | --- | --- | --- | --- | --- | --- | --- | --- | --- | --- | --- |
| \* | pJS43\_100mM\_120812\_01.05017.05017.1 | 2.0611 | 0.2085 | 97.5% | 1134.46 | 1135.2217 | 1 | 4.567 | 68.8% | 1 | K.DVNQQEFVR.A | 1 |
| \* | pJS43\_100mM\_120812\_01.05057.05057.2 | 3.4561 | 0.2234 | 100.0% | 1135.2322 | 1135.2217 | 1 | 7.28 | 87.5% | 8 | K.DVNQQEFVR.A | 2 |
| \* | pDK365N\_100mM\_082713\_01.05250.05250.2 | 2.5295 | 0.3632 | 100.0% | 861.5722 | 862.1032 | 1 | 7.261 | 85.7% | 3 | R.ALAAFLKK.S | 2 |
|  | pDK365N\_100mM\_082813\_03.08069.08069.3 | 3.8382 | 0.1986 | 99.8% | 1314.9243 | 1314.5675 | 5 | 6.264 | 50.0% | 6 | K.LKVPEWVDTVK.L | 3 |
|  | pDK365N\_100mM\_082713\_01.06996.06996.2 | 2.585 | 0.3013 | 99.8% | 1073.1522 | 1073.234 | 1 | 7.59 | 81.2% | 4 | K.VPEWVDTVK.L | 2 |
| \* | pJS43\_100mM\_120812\_01.09223.09223.2 | 4.0686 | 0.5333 | 100.0% | 1969.4521 | 1970.151 | 1 | 8.199 | 75.0% | 2 | K.HKELAPYDENWFYTR.A | 2 |
| \* | pDK365N\_100mM\_082713\_01.07764.07764.3 | 4.9191 | 0.3821 | 100.0% | 1969.9143 | 1970.151 | 1 | 7.432 | 57.1% | 13 | K.HKELAPYDENWFYTR.A | 3 |
| \* | pJS43\_100mM\_120812\_01.11473.11473.2 | 3.4244 | 0.3753 | 100.0% | 1704.3522 | 1704.8358 | 1 | 7.581 | 75.0% | 7 | K.ELAPYDENWFYTR.A | 2 |
| \* | pJS43\_100mM\_120812\_01.07349.07349.2 | 2.7141 | 0.2141 | 99.4% | 1127.1721 | 1127.3726 | 1 | 5.824 | 77.8% | 6 | R.RVLQALEGLK.M | 2 |
|  | pJS43\_100mM\_120812\_01.08454.08454.1 | 2.0529 | 0.2896 | 98.9% | 970.54 | 971.1851 | 1 | 5.578 | 75.0% | 1 | R.VLQALEGLK.M | 1 |
|  | pJS43\_100mM\_120812\_01.08525.08525.2 | 3.1147 | 0.3749 | 100.0% | 970.97217 | 971.1851 | 2 | 6.183 | 81.2% | 7 | R.VLQALEGLK.M | 2 |
|  | pJS43\_100mM\_120812\_01.02773.02773.2 | 1.8893 | 0.2653 | 95.3% | 1134.1522 | 1135.2372 | 2 | 5.359 | 72.2% | 1 | K.MVEKDQDGGR.K | 2 |
| \* | pJS43\_100mM\_120812\_01.02843.02843.2 | 3.0213 | 0.3523 | 100.0% | 1208.3322 | 1208.4061 | 2 | 6.045 | 63.6% | 1 | R.IAGQVAAANKKH.- | 2 |

---

|  |  |  |  |  |  |  |  |  |
| --- | --- | --- | --- | --- | --- | --- | --- | --- |
| U | *gi|4758792|ref|NP\_004* | 4 | 5 | 50.8% | 124 | 13712 | 8.3 | NADH dehydrogenase (ubiquinone) Fe-S protein 6, 13kDa (NADH-coenzyme Q reductase) [Homo sapiens] |

| Filename XCorr DeltCN Conf% ObsM+H+ CalcM+H+ SpR ZScore Ion% # Sequence  | | | | | | | | | | | | |
| --- | --- | --- | --- | --- | --- | --- | --- | --- | --- | --- | --- | --- |
| \* | AstrinIP\_MS1\_022614\_01.03639.03639.3 | 3.1152 | 0.3403 | 99.9% | 1698.1444 | 1697.8015 | 1 | 5.367 | 38.5% | 1 | K.VTHTGQVYDDKDYR.R | 3 |
| \* | AstrinIP\_MS2\_022614\_01.11836.11836.3 | 3.7494 | 0.2252 | 98.9% | 2759.8743 | 2760.03 | 11 | 4.406 | 25.0% | 1 | R.QKEVNENFAIDLIAEQPVSEVETR.V | 3 |
| \* | AstrinIP\_MS1\_022614\_01.03749.03749.2 | 3.4197 | 0.4126 | 100.0% | 1409.4922 | 1409.5511 | 1 | 7.125 | 71.4% | 1 | R.VIACDGGGGALGHPK.V | 2 |
| \* | AstrinIP\_MS1\_022614\_01.04727.04727.2 | 3.0558 | 0.216 | 99.8% | 1223.4722 | 1223.4117 | 4 | 4.995 | 66.7% | 2 | K.VYINLDKETK.T | 2 |

---

|  |  |  |  |  |  |  |  |  |
| --- | --- | --- | --- | --- | --- | --- | --- | --- |
| U | *gi|4757810|ref|NP\_004* | 24 | 123 | 50.5% | 553 | 59751 | 9.1 | ATP synthase, H+ transporting, mitochondrial F1 complex, alpha subunit precursor [Homo sapiens] |
| U | *gi|50345984|ref|NP\_00* | 24 | 124 | 50.5% | 553 | 59751 | 9.1 | ATP synthase, H+ transporting, mitochondrial F1 complex, alpha subunit precursor [Homo sapiens] |

| Filename XCorr DeltCN Conf% ObsM+H+ CalcM+H+ SpR ZScore Ion% # Sequence  | | | | | | | | | | | | |
| --- | --- | --- | --- | --- | --- | --- | --- | --- | --- | --- | --- | --- |
|  | pDK365N\_300mM\_082713\_03.07664.07664.2 | 4.0293 | 0.4707 | 100.0% | 1424.0721 | 1424.5659 | 1 | 7.834 | 70.8% | 8 | K.TGTAEMSSILEER.I | 2 |
|  | pDK365N\_300mM\_082713\_01.07443.07443.2 | 5.1572 | 0.5766 | 100.0% | 1576.5322 | 1576.7007 | 1 | 10.815 | 85.7% | 23 | R.ILGADTSVDLEETGR.V | 2 |
|  | pDK365N\_300mM\_082713\_03.06584.06584.2 | 2.7348 | 0.342 | 99.9% | 1000.71216 | 1001.171 | 1 | 7.131 | 88.9% | 5 | R.VLSIGDGIAR.V | 2 |
|  | pDK365N\_100mM\_082713\_02.06618.06618.2 | 5.0624 | 0.534 | 100.0% | 1668.3722 | 1668.8591 | 1 | 10.923 | 67.9% | 7 | R.NVQAEEMVEFSSGLK.G | 2 |
|  | pDK365N\_300mM\_082713\_01.11580.11580.2 | 5.4273 | 0.5033 | 100.0% | 2105.7922 | 2105.3704 | 1 | 8.114 | 57.9% | 2 | K.GMSLNLEPDNVGVVVFGNDK.L | 2 |
|  | pDK365N\_300mM\_082713\_01.10539.10539.2 | 4.3686 | 0.3607 | 100.0% | 1626.5721 | 1625.8625 | 1 | 7.45 | 80.0% | 9 | R.TGAIVDVPVGEELLGR.V | 2 |
|  | pDK365N\_300mM\_082713\_03.06143.06143.2 | 3.7851 | 0.455 | 100.0% | 1173.4521 | 1172.3237 | 1 | 8.476 | 81.8% | 8 | R.VVDALGNAIDGK.G | 2 |
|  | pDK365N\_300mM\_082713\_03.06699.06699.3 | 2.9149 | 0.3294 | 99.7% | 1713.2043 | 1711.956 | 3 | 5.754 | 35.3% | 3 | R.VVDALGNAIDGKGPIGSK.T | 3 |
|  | SKAPIP\_tube2\_041314\_01.06191.06191.2 | 2.3711 | 0.2742 | 99.0% | 1121.0922 | 1121.4116 | 5 | 4.557 | 60.0% | 1 | R.VGLKAPGIIPR.I | 2 |
|  | pDK365N\_300mM\_082713\_03.04659.04659.3 | 2.7208 | 0.3498 | 99.9% | 1358.9043 | 1359.6268 | 9 | 5.333 | 38.6% | 2 | R.ISVREPMQTGIK.A | 3 |
|  | pDK365N\_300mM\_082713\_03.04635.04635.2 | 2.6382 | 0.1098 | 95.4% | 1359.3922 | 1359.6268 | 65 | 4.208 | 54.5% | 1 | R.ISVREPMQTGIK.A | 2 |
|  | pDK365N\_300mM\_082713\_01.07352.07352.2 | 2.7896 | 0.4761 | 100.0% | 1026.6122 | 1027.2089 | 1 | 8.592 | 77.8% | 2 | K.AVDSLVPIGR.G | 2 |
|  | pDK365N\_100mM\_082813\_04.06280.06280.2 | 3.6794 | 0.3555 | 100.0% | 1317.0521 | 1317.5254 | 1 | 6.438 | 72.7% | 7 | K.TSIAIDTIINQK.R | 2 |
|  | pDK365N\_300mM\_082713\_03.07335.07335.2 | 3.0867 | 0.2725 | 99.8% | 1474.1122 | 1473.7129 | 1 | 5.664 | 66.7% | 1 | K.TSIAIDTIINQKR.F | 2 |
|  | pDK365N\_300mM\_082713\_04.05645.05645.2 | 3.6502 | 0.4236 | 100.0% | 1288.4521 | 1288.4863 | 1 | 8.465 | 80.0% | 12 | K.HALIIYDDLSK.Q | 2 |
|  | SKAPIP\_tube2\_041314\_01.08212.08212.2 | 2.7953 | 0.3054 | 99.7% | 1554.6522 | 1554.7019 | 1 | 6.587 | 62.5% | 1 | R.EAYPGDVFYLHSR.L | 2 |
|  | 100326\_pJS43\_02.04583.04583.2 | 2.5292 | 0.2617 | 98.8% | 1439.8722 | 1439.7012 | 1 | 5.266 | 53.8% | 1 | K.GIRPAINVGLSVSR.V | 2 |
|  | pDK365N\_100mM\_082713\_02.05354.05354.3 | 3.9343 | 0.3954 | 100.0% | 1440.0243 | 1439.7012 | 1 | 6.95 | 50.0% | 6 | K.GIRPAINVGLSVSR.V | 3 |
|  | pDK365N\_300mM\_082713\_02.10104.10104.2 | 7.0711 | 0.5471 | 100.0% | 2339.2122 | 2339.567 | 1 | 10.984 | 64.3% | 8 | R.EVAAFAQFGSDLDAATQQLLSR.G | 2 |
|  | pDK365N\_300mM\_082713\_04.09884.09884.3 | 3.8589 | 0.3097 | 99.9% | 2339.4543 | 2339.567 | 1 | 6.557 | 36.9% | 2 | R.EVAAFAQFGSDLDAATQQLLSR.G | 3 |
|  | pDK365N\_300mM\_082713\_04.09004.09004.2 | 4.8278 | 0.5237 | 100.0% | 2309.912 | 2310.63 | 1 | 9.424 | 60.0% | 3 | K.QGQYSPMAIEEQVAVIYAGVR.G | 2 |
|  | pDK365N\_300mM\_082713\_01.05256.05256.2 | 1.9203 | 0.3051 | 97.6% | 1150.2322 | 1150.3171 | 1 | 5.383 | 72.2% | 1 | R.GYLDKLEPSK.I | 2 |
|  | pDK365N\_100mM\_082713\_01.09945.09945.3 | 4.9509 | 0.5444 | 100.0% | 2370.0544 | 2368.7007 | 1 | 8.936 | 40.0% | 6 | K.FENAFLSHVVSQHQALLGTIR.A | 3 |
|  | pDK365N\_300mM\_082713\_04.08895.08895.2 | 3.8978 | 0.5705 | 100.0% | 1552.7122 | 1552.8107 | 1 | 10.356 | 73.1% | 4 | K.LKEIVTNFLAGFEA.- | 2 |

---

|  |  |  |  |  |  |  |  |  |
| --- | --- | --- | --- | --- | --- | --- | --- | --- |
| U | *gi|4557469|ref|NP\_001* | 64 | 245 | 50.3% | 937 | 104553 | 5.4 | adaptor-related protein complex 2, beta 1 subunit isoform b [Homo sapiens] |
| U | *gi|71773106|ref|NP\_00* | 64 | 245 | 49.5% | 951 | 105692 | 5.3 | adaptor-related protein complex 2, beta 1 subunit isoform a [Homo sapiens] |

| Filename XCorr DeltCN Conf% ObsM+H+ CalcM+H+ SpR ZScore Ion% # Sequence  | | | | | | | | | | | | |
| --- | --- | --- | --- | --- | --- | --- | --- | --- | --- | --- | --- | --- |
|  | pSKT11\_1\_020812\_02.03966.03966.3 | 3.8248 | 0.3302 | 100.0% | 2645.8442 | 2646.0154 | 1 | 5.677 | 33.3% | 1 | K.YFTTNKKGEIFELKAELNNEKK.E | 3 |
|  | pDK339othertube\_033013\_01.05972.05972.2 | 2.9796 | 0.1618 | 99.7% | 963.9922 | 964.1497 | 23 | 4.18 | 78.6% | 6 | K.KGEIFELK.A | 22 |
|  | pSKT11\_1\_020812\_02.03780.03780.2 | 4.2849 | 0.4524 | 100.0% | 1890.5721 | 1891.1747 | 1 | 7.892 | 60.0% | 1 | K.KGEIFELKAELNNEKK.E | 2 |
|  | pSKT11\_1\_020812\_02.03777.03777.3 | 3.5155 | 0.2584 | 99.7% | 1891.1943 | 1891.1747 | 2 | 5.245 | 40.0% | 1 | K.KGEIFELKAELNNEKK.E | 3 |
|  | pSKT11\_1\_020812\_02.03467.03467.2 | 4.4315 | 0.4066 | 100.0% | 2148.4722 | 2148.4644 | 1 | 7.56 | 50.0% | 1 | K.KGEIFELKAELNNEKKEK.R | 2 |
|  | pSKT11\_1\_020812\_02.03476.03476.3 | 4.458 | 0.3276 | 100.0% | 2148.5645 | 2148.4644 | 1 | 5.697 | 38.2% | 2 | K.KGEIFELKAELNNEKKEK.R | 3 |
|  | pJS43\_100mM\_120812\_01.05099.05099.2 | 2.0281 | 0.299 | 98.6% | 889.8122 | 890.129 | 32 | 6.113 | 56.2% | 1 | K.VIAAMTVGK.D | 2 |
|  | pSKT11\_1\_020812\_01.09512.09512.2 | 3.7374 | 0.3104 | 100.0% | 1519.8322 | 1519.8854 | 1 | 6.005 | 81.8% | 3 | K.KLVYLYLMNYAK.S | 22 |
|  | pSKT11\_1\_020812\_01.09946.09946.1 | 2.4137 | 0.3071 | 98.6% | 1390.5 | 1391.7113 | 1 | 5.637 | 60.0% | 1 | K.LVYLYLMNYAK.S | 11 |
|  | pSKT11\_1\_020812\_02.06910.06910.2 | 3.8663 | 0.3014 | 100.0% | 1392.1122 | 1391.7113 | 1 | 7.742 | 80.0% | 11 | K.LVYLYLMNYAK.S | 22 |
|  | pJS43\_100mM\_120812\_01.11911.11911.2 | 4.2808 | 0.3824 | 100.0% | 1638.2922 | 1638.9374 | 1 | 9.633 | 67.9% | 2 | K.SQPDMAIMAVNSFVK.D | 2 |
|  | pJS43\_100mM\_120812\_02.07489.07489.3 | 4.7723 | 0.408 | 100.0% | 2202.0842 | 2202.4468 | 1 | 7.878 | 47.2% | 4 | K.LHDINAQMVEDQGFLDSLR.D | 3 |
|  | pSKT11\_1\_020812\_01.08211.08211.1 | 3.0983 | 0.3205 | 98.7% | 1395.62 | 1396.6298 | 1 | 8.183 | 65.4% | 1 | R.LSHANSAVVLSAVK.V | 11 |
|  | pDK339\_033013\_01.05288.05288.2 | 4.3856 | 0.3935 | 100.0% | 1396.3121 | 1396.6298 | 1 | 7.519 | 76.9% | 6 | R.LSHANSAVVLSAVK.V | 22 |
|  | pDK365N\_300mM\_082713\_03.04923.04923.3 | 3.9129 | 0.1845 | 99.4% | 1397.1244 | 1396.6298 | 6 | 4.969 | 46.2% | 2 | R.LSHANSAVVLSAVK.V | 33 |
|  | pSKT11\_1\_020812\_02.05866.05866.2 | 2.9659 | 0.5911 | 100.0% | 1867.5521 | 1868.2885 | 3 | 8.788 | 41.2% | 1 | R.LSHANSAVVLSAVKVLMK.F | 22 |
|  | pSKT11\_1\_020812\_02.05877.05877.3 | 2.9457 | 0.2154 | 96.4% | 1868.2144 | 1868.2885 | 1 | 4.592 | 38.2% | 1 | R.LSHANSAVVLSAVKVLMK.F | 33 |
|  | pSKT11\_1\_020812\_01.09546.09546.3 | 3.6732 | 0.228 | 99.3% | 2232.4143 | 2231.655 | 1 | 4.989 | 39.7% | 1 | K.FLELLPKDSDYYNMLLKK.L | 3 |
|  | pJS43\_100mM\_120812\_01.10422.10422.2 | 2.9003 | 0.2238 | 99.7% | 1262.2122 | 1262.4199 | 2 | 5.385 | 72.2% | 1 | K.DSDYYNMLLK.K | 2 |
|  | pSKT11\_1\_020812\_01.08903.08903.2 | 2.4804 | 0.2071 | 98.5% | 1390.3722 | 1390.594 | 35 | 4.494 | 60.0% | 2 | K.DSDYYNMLLKK.L | 2 |
|  | pSKT11\_1\_020812\_01.10182.10182.2 | 3.489 | 0.2131 | 99.7% | 2396.5122 | 2394.86 | 1 | 5.269 | 52.4% | 1 | K.KLAPPLVTLLSGEPEVQYVALR.N | 2 |
|  | pJS43\_100mM\_120812\_01.14102.14102.2 | 5.5257 | 0.6062 | 100.0% | 2265.8323 | 2266.686 | 1 | 11.263 | 62.5% | 17 | K.LAPPLVTLLSGEPEVQYVALR.N | 2 |
|  | pDK339othertube\_033013\_01.14102.14102.3 | 4.1825 | 0.4436 | 100.0% | 2266.2544 | 2266.686 | 1 | 7.829 | 38.8% | 3 | K.LAPPLVTLLSGEPEVQYVALR.N | 3 |
|  | pSKT11\_1\_020812\_01.08512.08512.1 | 2.2073 | 0.2068 | 98.3% | 941.58 | 942.1466 | 1 | 5.032 | 71.4% | 1 | R.NINLIVQK.R | 11 |
|  | pDK339\_033013\_01.06446.06446.2 | 2.4579 | 0.0916 | 96.2% | 941.9522 | 942.1466 | 4 | 4.465 | 85.7% | 1 | R.NINLIVQK.R | 22 |
|  | pSKT11\_1\_020812\_01.08577.08577.3 | 3.8231 | 0.3238 | 100.0% | 2177.3044 | 2177.639 | 1 | 5.754 | 36.8% | 1 | R.NINLIVQKRPEILKQEIK.V | 3 |
|  | pDK339othertube\_033013\_01.03821.03821.3 | 3.1009 | 0.1638 | 97.8% | 1254.1144 | 1254.5155 | 1 | 4.392 | 63.9% | 1 | K.RPEILKQEIK.V | 3 |
|  | pSKT11\_1\_020812\_02.07024.07024.3 | 3.2065 | 0.3794 | 100.0% | 2743.1643 | 2745.3386 | 1 | 5.606 | 31.0% | 1 | K.VFFVKYNDPIYVKLEKLDIMIR.L | 33 |
|  | pDK339othertube\_033013\_01.05433.05433.2 | 2.4979 | 0.1786 | 98.9% | 1012.1322 | 1012.1503 | 35 | 4.88 | 64.3% | 2 | K.YNDPIYVK.L | 22 |
|  | pSKT11\_1\_020812\_01.08652.08652.2 | 2.8671 | 0.4184 | 100.0% | 1382.1522 | 1382.5994 | 1 | 7.183 | 80.0% | 2 | K.YNDPIYVKLEK.L | 22 |
|  | pJS43\_100mM\_120812\_01.12444.12444.2 | 4.5631 | 0.3647 | 100.0% | 1569.6921 | 1569.8418 | 1 | 8.005 | 82.1% | 2 | R.LASQANIAQVLAELK.E | 22 |
|  | pDK339\_033013\_01.16705.16705.3 | 5.4736 | 0.5231 | 100.0% | 2993.4243 | 2994.3716 | 1 | 10.412 | 31.7% | 15 | R.LASQANIAQVLAELKEYATEVDVDFVR.K | 33 |
|  | pSKT11\_1\_020812\_01.11656.11656.2 | 4.7462 | 0.3269 | 100.0% | 2995.872 | 2994.3716 | 1 | 7.312 | 38.5% | 2 | R.LASQANIAQVLAELKEYATEVDVDFVR.K | 22 |
|  | pSKT11\_1\_020812\_01.11168.11168.3 | 4.9137 | 0.5565 | 100.0% | 3122.0645 | 3122.5457 | 1 | 9.482 | 32.4% | 2 | R.LASQANIAQVLAELKEYATEVDVDFVRK.A | 33 |
|  | pSKT11\_1\_020812\_01.11127.11127.2 | 4.3503 | 0.4493 | 100.0% | 3122.112 | 3122.5457 | 1 | 7.171 | 33.3% | 1 | R.LASQANIAQVLAELKEYATEVDVDFVRK.A | 22 |
|  | pJS43\_100mM\_120812\_02.08130.08130.2 | 4.2456 | 0.5562 | 100.0% | 1509.2722 | 1509.7632 | 1 | 9.669 | 75.0% | 2 | R.AAMIWIVGEYAER.I | 22 |
|  | pSKT11\_1\_020812\_02.11690.11690.3 | 3.817 | 0.3525 | 100.0% | 5080.1943 | 5081.746 | 1 | 6.083 | 18.2% | 1 | R.AAMIWIVGEYAERIDNADELLESFLEGFHDESTQVQLTLLTAIVK.L | 3 |
|  | pJS43\_100mM\_120812\_02.12408.12408.3 | 8.1373 | 0.613 | 100.0% | 3590.8442 | 3591.0063 | 1 | 11.627 | 37.1% | 9 | R.IDNADELLESFLEGFHDESTQVQLTLLTAIVK.L | 3 |
|  | pJS43\_100mM\_120812\_01.13418.13418.3 | 3.2945 | 0.2715 | 99.0% | 2913.4143 | 2913.168 | 19 | 4.369 | 23.0% | 1 | K.KPSETQELVQQVLSLATQDSDNPDLR.D | 3 |
|  | pSKT11\_1\_020812\_01.05096.05096.1 | 1.8499 | 0.2413 | 97.6% | 1044.54 | 1045.2212 | 1 | 5.689 | 61.1% | 1 | R.LLSTDPVTAK.E | 1 |
|  | pSKT11\_1\_020812\_01.04820.04820.2 | 2.7974 | 0.3537 | 100.0% | 1045.2122 | 1045.2212 | 2 | 5.471 | 66.7% | 8 | R.LLSTDPVTAK.E | 2 |
|  | pSKT11\_1\_020812\_01.09096.09096.1 | 2.1469 | 0.1619 | 95.4% | 883.63 | 884.1093 | 51 | 4.599 | 57.1% | 2 | K.AVWLPAVK.A | 1 |
|  | pSKT11\_1\_020812\_01.08104.08104.2 | 3.6808 | 0.5786 | 100.0% | 1417.2122 | 1417.6078 | 1 | 8.93 | 75.0% | 2 | K.AKGLEISGTFTHR.Q | 2 |
|  | pSKT11\_1\_020812\_01.08094.08094.3 | 3.879 | 0.4016 | 100.0% | 1417.6444 | 1417.6078 | 1 | 6.851 | 45.8% | 1 | K.AKGLEISGTFTHR.Q | 3 |
|  | pSKT11\_1\_020812\_02.03842.03842.2 | 2.9565 | 0.3301 | 99.9% | 1219.2922 | 1218.3549 | 1 | 5.886 | 75.0% | 7 | K.GLEISGTFTHR.Q | 2 |
|  | pSKT11\_1\_020812\_02.04328.04328.2 | 2.2049 | 0.2276 | 96.1% | 1614.2322 | 1613.8463 | 1 | 4.535 | 75.0% | 1 | R.QGHIYMEMNFTNK.A | 2 |
|  | pJS43\_100mM\_120812\_02.05736.05736.3 | 3.8952 | 0.3141 | 100.0% | 1614.2344 | 1613.8463 | 1 | 5.454 | 47.9% | 2 | R.QGHIYMEMNFTNK.A | 3 |
|  | pSKT11\_1\_020812\_01.09033.09033.2 | 4.9433 | 0.4947 | 100.0% | 1664.4922 | 1664.9196 | 1 | 8.325 | 76.9% | 7 | K.ALQHMTDFAIQFNK.N | 2 |
|  | pDK365N\_100mM\_082813\_04.06407.06407.3 | 4.0538 | 0.3131 | 100.0% | 1664.6643 | 1664.9196 | 1 | 5.561 | 50.0% | 6 | K.ALQHMTDFAIQFNK.N | 3 |
|  | pDK339\_033013\_01.14308.14308.3 | 6.3506 | 0.5478 | 100.0% | 3900.2043 | 3901.6147 | 1 | 8.115 | 27.8% | 9 | K.NSFGVIPSTPLAIHTPLMPNQSIDVSLPLNTLGPVMK.M | 3 |
|  | pJS43\_100mM\_120812\_01.08975.08975.2 | 3.4377 | 0.393 | 100.0% | 1356.3322 | 1356.6232 | 1 | 6.776 | 86.4% | 11 | K.MEPLNNLQVAVK.N | 22 |
|  | pSKT11\_1\_020812\_01.09219.09219.1 | 1.9387 | 0.2491 | 98.7% | 992.52 | 993.19366 | 1 | 5.704 | 78.6% | 1 | R.QVFLATWK.D | 1 |
|  | pDK339othertube\_033013\_01.08693.08693.2 | 3.8036 | 0.3391 | 100.0% | 1589.0922 | 1588.7576 | 2 | 6.305 | 66.7% | 13 | K.DIPNENELQFQIK.E | 2 |
|  | pSKT11\_1\_020812\_01.06526.06526.2 | 3.6476 | 0.4286 | 100.0% | 1277.9722 | 1278.4508 | 1 | 7.376 | 70.0% | 24 | K.LQNNNVYTIAK.R | 2 |
|  | pSKT11\_1\_020812\_01.04158.04158.1 | 2.9193 | 0.2755 | 98.5% | 1433.7 | 1434.6383 | 99 | 4.967 | 40.9% | 2 | K.LQNNNVYTIAKR.N | 1 |
|  | pSKT11\_1\_020812\_01.04256.04256.2 | 3.7231 | 0.3433 | 100.0% | 1434.1921 | 1434.6383 | 1 | 6.587 | 81.8% | 5 | K.LQNNNVYTIAKR.N | 2 |
|  | pSKT11\_1\_020812\_01.08642.08642.2 | 4.3337 | 0.3098 | 100.0% | 1681.2122 | 1681.9042 | 1 | 6.481 | 69.2% | 1 | K.RNVEGQDMLYQSLK.L | 22 |
|  | pJS43\_100mM\_120812\_01.08951.08951.2 | 4.2654 | 0.4355 | 100.0% | 1525.3121 | 1525.7168 | 1 | 8.35 | 83.3% | 11 | R.NVEGQDMLYQSLK.L | 22 |
|  | pSKT11\_1\_020812\_02.09300.09300.3 | 4.2041 | 0.3759 | 100.0% | 2905.1643 | 2906.3696 | 1 | 6.659 | 25.0% | 1 | R.NVEGQDMLYQSLKLTNGIWILAELR.I | 3 |
|  | pSKT11\_1\_020812\_01.11316.11316.3 | 3.5233 | 0.2332 | 98.7% | 4332.9844 | 4333.005 | 1 | 4.38 | 16.2% | 2 | R.NVEGQDMLYQSLKLTNGIWILAELRIQPGNPNYTLSLK.C | 3 |
|  | pJS43\_100mM\_120812\_02.09110.09110.2 | 4.1557 | 0.4166 | 100.0% | 1399.2722 | 1399.6763 | 1 | 7.51 | 72.7% | 6 | K.LTNGIWILAELR.I | 2 |
|  | pDK339othertube\_033013\_01.06920.06920.2 | 2.3063 | 0.2684 | 98.4% | 1445.5721 | 1445.6586 | 167 | 4.497 | 45.8% | 2 | R.IQPGNPNYTLSLK.C | 2 |
|  | pDK365N\_300mM\_082713\_01.13738.13738.2 | 2.9573 | 0.3674 | 99.9% | 2018.4122 | 2017.286 | 1 | 6.019 | 40.6% | 2 | R.APEVSQYIYQVYDSILK.N | 2 |
|  | pDK339\_033013\_01.14759.14759.2 | 5.9217 | 0.4887 | 100.0% | 2131.5122 | 2131.39 | 1 | 9.715 | 64.7% | 4 | R.APEVSQYIYQVYDSILKN.- | 2 |

Similarities:
gi|22027651|ref|NP\_00(23:41)  

---

|  |  |  |  |  |  |  |  |  |
| --- | --- | --- | --- | --- | --- | --- | --- | --- |
| U | *gi|15718687|ref|NP\_00* | 10 | 40 | 50.2% | 243 | 26688 | 9.7 | ribosomal protein S3 [Homo sapiens] |

| Filename XCorr DeltCN Conf% ObsM+H+ CalcM+H+ SpR ZScore Ion% # Sequence  | | | | | | | | | | | | |
| --- | --- | --- | --- | --- | --- | --- | --- | --- | --- | --- | --- | --- |
| \* | pDK365N\_100mM\_082713\_02.09080.09080.3 | 4.9076 | 0.4541 | 100.0% | 1972.2843 | 1971.2633 | 1 | 6.719 | 51.6% | 1 | K.FVADGIFKAELNEFLTR.E | 3 |
| \* | pJS43\_100mM\_120812\_01.08977.08977.2 | 2.8236 | 0.2247 | 99.7% | 1093.2922 | 1093.2249 | 1 | 5.995 | 87.5% | 6 | K.AELNEFLTR.E | 2 |
| \* | pJS43\_100mM\_120812\_02.04914.04914.2 | 4.2963 | 0.4052 | 100.0% | 1425.4122 | 1424.5071 | 1 | 8.54 | 75.0% | 12 | R.ELAEDGYSGVEVR.V | 2 |
| \* | pDK365N\_100mM\_082813\_03.07859.07859.3 | 2.8688 | 0.3568 | 99.9% | 1584.3544 | 1584.8998 | 35 | 5.335 | 36.5% | 1 | R.VTPTRTEIIILATR.T | 3 |
| \* | pJS43\_100mM\_120812\_01.09147.09147.2 | 2.568 | 0.3298 | 99.9% | 1030.0922 | 1030.2529 | 1 | 6.604 | 87.5% | 1 | R.TEIIILATR.T | 2 |
| \* | pJS43\_100mM\_120812\_02.07137.07137.2 | 3.681 | 0.5753 | 100.0% | 1573.2322 | 1573.7423 | 1 | 8.751 | 76.9% | 8 | R.FGFPEGSVELYAEK.V | 2 |
| \* | pJS43\_100mM\_120812\_02.07441.07441.3 | 4.6584 | 0.4572 | 100.0% | 2469.5645 | 2469.7742 | 1 | 8.08 | 41.7% | 2 | K.FVDGLMIHSGDPVNYYVDTAVR.H | 3 |
| \* | pJS43\_100mM\_120812\_01.05167.05167.3 | 3.5902 | 0.254 | 99.8% | 1459.9443 | 1459.7288 | 6 | 6.296 | 43.8% | 2 | K.KPLPDHVSIVEPK.D | 3 |
| \* | pJS43\_100mM\_120812\_01.07910.07910.2 | 3.335 | 0.3133 | 100.0% | 1472.4122 | 1471.6476 | 1 | 5.652 | 75.0% | 3 | K.DEILPTTPISEQK.G | 2 |
| \* | pJS43\_100mM\_120812\_01.06737.06737.2 | 3.8899 | 0.4759 | 100.0% | 1574.3722 | 1574.8352 | 2 | 7.709 | 53.3% | 4 | K.GGKPEPPAMPQPVPTA.- | 2 |

---

|  |  |  |  |  |  |  |  |  |
| --- | --- | --- | --- | --- | --- | --- | --- | --- |
| U | *gi|63025212|ref|NP\_98* | 10 | 63 | 50.0% | 266 | 27202 | 4.9 | hypothetical protein LOC255374 [Homo sapiens] |

| Filename XCorr DeltCN Conf% ObsM+H+ CalcM+H+ SpR ZScore Ion% # Sequence  | | | | | | | | | | | | |
| --- | --- | --- | --- | --- | --- | --- | --- | --- | --- | --- | --- | --- |
| \* | pDK365N\_100mM\_082713\_01.07236.07236.2 | 3.3779 | 0.3462 | 100.0% | 1357.6122 | 1357.5498 | 32 | 6.41 | 54.2% | 7 | R.ADGSVTLVLPQTR.G | 2 |
| \* | pDK365N\_100mM\_082713\_01.05616.05616.2 | 4.1235 | 0.3654 | 100.0% | 1289.1122 | 1289.3445 | 1 | 8.612 | 73.1% | 10 | R.GSGGAEAALEEAAR.G | 2 |
| \* | pJS43\_100mM\_120812\_01.09786.09786.2 | 4.3028 | 0.4949 | 100.0% | 1340.2122 | 1339.537 | 1 | 8.831 | 66.7% | 14 | R.GPILVDTGGPWAR.E | 2 |
| \* | pDK365N\_100mM\_082713\_01.05544.05544.3 | 3.4866 | 0.2684 | 99.9% | 1438.2244 | 1437.6414 | 1 | 5.48 | 54.2% | 3 | R.YLPHGLGEGQPLR.L | 3 |
| \* | pJS43\_100mM\_120812\_01.08749.08749.2 | 4.0681 | 0.5102 | 100.0% | 1732.4722 | 1732.9395 | 1 | 8.095 | 62.5% | 4 | R.LGPGLEVWATPGHGGQR.D | 2 |
| \* | pDK365N\_100mM\_082813\_03.07661.07661.3 | 3.3416 | 0.4464 | 100.0% | 1733.0343 | 1732.9395 | 1 | 6.988 | 40.6% | 12 | R.LGPGLEVWATPGHGGQR.D | 3 |
| \* | pJS43\_100mM\_120812\_01.09090.09090.2 | 6.1729 | 0.5692 | 100.0% | 2119.5122 | 2120.107 | 1 | 10.375 | 61.1% | 2 | R.DGDEDSWQALSEDPAAQER.S | 2 |
| \* | pSKT11\_1\_020812\_02.05667.05667.2 | 3.9744 | 0.4547 | 100.0% | 1758.9722 | 1759.1044 | 1 | 6.999 | 62.5% | 2 | R.VLVVADVVVPGHGPPFR.V | 2 |
| \* | pJS43\_100mM\_120812\_01.10541.10541.3 | 3.8834 | 0.3918 | 100.0% | 1760.6344 | 1759.1044 | 1 | 6.849 | 40.6% | 8 | R.VLVVADVVVPGHGPPFR.V | 3 |
| \* | pDK365N\_100mM\_082813\_03.05379.05379.3 | 3.3639 | 0.3319 | 99.8% | 2792.4844 | 2792.8433 | 1 | 5.881 | 26.0% | 1 | R.EASQPETEGGGNSQQEPVVGDEEPALH.- | 3 |

---

|  |  |  |  |  |  |  |  |  |
| --- | --- | --- | --- | --- | --- | --- | --- | --- |
| U | *gi|5174735|ref|NP\_006* | 27 | 245 | 49.2% | 445 | 49831 | 4.9 | tubulin, beta, 2 [Homo sapiens] |

| Filename XCorr DeltCN Conf% ObsM+H+ CalcM+H+ SpR ZScore Ion% # Sequence  | | | | | | | | | | | | |
| --- | --- | --- | --- | --- | --- | --- | --- | --- | --- | --- | --- | --- |
|  | 100326\_pJS43\_02.06146.06146.3 | 6.2947 | 0.4483 | 100.0% | 3117.5044 | 3118.2996 | 1 | 8.951 | 34.6% | 5 | K.FWEVISDEHGIDPTGTYHGDSDLQLER.I | 3 |
| \* | pDK339\_033013\_01.05557.05557.2 | 3.7491 | 0.3198 | 100.0% | 1329.0721 | 1329.4521 | 1 | 7.207 | 86.4% | 5 | R.INVYYNEATGGK.Y | 2 |
|  | pDK365N\_100mM\_082713\_01.08811.08811.2 | 4.3947 | 0.4488 | 100.0% | 1602.6122 | 1602.8431 | 1 | 8.503 | 71.4% | 7 | R.AVLVDLEPGTMDSVR.S | 2 |
|  | 100326\_pJS43\_01.10197.10197.2 | 5.5004 | 0.5198 | 100.0% | 2798.5522 | 2800.0647 | 1 | 8.823 | 42.0% | 4 | R.SGPFGQIFRPDNFVFGQSGAGNNWAK.G | 22 |
|  | 100326\_pJS43\_01.10196.10196.3 | 7.8655 | 0.5039 | 100.0% | 2799.9243 | 2800.0647 | 1 | 8.789 | 44.0% | 26 | R.SGPFGQIFRPDNFVFGQSGAGNNWAK.G | 33 |
|  | pDK339othertube\_033013\_01.12321.12321.2 | 7.1963 | 0.5639 | 100.0% | 1959.5922 | 1960.151 | 1 | 11.054 | 79.4% | 11 | K.GHYTEGAELVDSVLDVVR.K | 222 |
|  | pDK339othertube\_033013\_01.12374.12374.3 | 3.9746 | 0.3545 | 100.0% | 1960.0144 | 1960.151 | 1 | 5.866 | 42.6% | 8 | K.GHYTEGAELVDSVLDVVR.K | 333 |
|  | 100326\_pJS43\_01.09680.09680.2 | 6.0516 | 0.5778 | 100.0% | 2087.372 | 2088.325 | 1 | 10.101 | 69.4% | 4 | K.GHYTEGAELVDSVLDVVRK.E | 222 |
|  | pDK339othertube\_033013\_01.11294.11294.3 | 4.8073 | 0.4721 | 100.0% | 2087.9644 | 2088.325 | 1 | 8.016 | 45.8% | 16 | K.GHYTEGAELVDSVLDVVRK.E | 333 |
|  | pJS43\_100mM\_120812\_01.03371.03371.2 | 2.8494 | 0.3232 | 100.0% | 1077.6921 | 1078.1698 | 1 | 5.745 | 85.7% | 2 | K.IREEYPDR.I | 22 |
|  | 100326\_pJS43\_01.06554.06554.2 | 4.5776 | 0.3956 | 100.0% | 1320.5322 | 1320.5896 | 1 | 7.817 | 77.3% | 29 | R.IMNTFSVVPSPK.V | 222 |
|  | pDK339\_033013\_01.08258.08258.2 | 2.5483 | 0.2033 | 98.8% | 1131.2122 | 1131.2767 | 3 | 5.089 | 72.2% | 4 | R.FPGQLNADLR.K | 2222 |
|  | 100326\_pJS43\_01.06578.06578.2 | 3.7419 | 0.3673 | 100.0% | 1272.5122 | 1272.5945 | 1 | 7.434 | 75.0% | 9 | R.KLAVNMVPFPR.L | 2222 |
|  | 100326\_pJS43\_01.07836.07836.1 | 1.9611 | 0.2769 | 98.5% | 1143.47 | 1144.4204 | 11 | 5.674 | 61.1% | 1 | K.LAVNMVPFPR.L | 1111 |
|  | pDK339othertube\_033013\_01.09272.09272.2 | 3.9416 | 0.4873 | 100.0% | 1144.1122 | 1144.4204 | 1 | 9.149 | 94.4% | 17 | K.LAVNMVPFPR.L | 2222 |
|  | pDK365N\_300mM\_082713\_03.11541.11541.3 | 4.2348 | 0.3148 | 100.0% | 1622.0643 | 1621.9403 | 2 | 6.293 | 50.0% | 4 | R.LHFFMPGFAPLTSR.G | 333 |
|  | pDK339\_033013\_01.12823.12823.2 | 3.7781 | 0.4034 | 100.0% | 1622.2722 | 1621.9403 | 1 | 8.323 | 80.8% | 19 | R.LHFFMPGFAPLTSR.G | 222 |
|  | pDK339othertube\_033013\_01.11469.11469.2 | 2.9558 | 0.3475 | 99.9% | 1692.4722 | 1692.9678 | 2 | 7.158 | 53.6% | 3 | R.ALTVPELTQQMFDAK.N | 22 |
|  | 100326\_pJS43\_01.08229.08229.1 | 1.556 | 0.3461 | 98.9% | 1039.46 | 1040.2505 | 1 | 5.469 | 68.8% | 3 | R.YLTVAAVFR.G | 11 |
|  | 100326\_pJS43\_01.08205.08205.2 | 2.7179 | 0.4304 | 100.0% | 1040.0922 | 1040.2505 | 1 | 7.712 | 87.5% | 4 | R.YLTVAAVFR.G | 22 |
|  | 100326\_pJS43\_02.04928.04928.2 | 4.9757 | 0.5158 | 100.0% | 1924.5521 | 1925.2405 | 1 | 8.985 | 56.7% | 2 | R.MSMKEVDEQMLNVQNK.N | 22 |
|  | pDK339othertube\_033013\_02.05941.05941.3 | 4.8367 | 0.3253 | 100.0% | 1925.0944 | 1925.2405 | 1 | 6.493 | 51.7% | 12 | R.MSMKEVDEQMLNVQNK.N | 33 |
|  | pDK365N\_300mM\_082713\_03.05374.05374.2 | 4.1533 | 0.2361 | 100.0% | 1448.2122 | 1447.6031 | 1 | 5.848 | 68.2% | 13 | K.EVDEQMLNVQNK.N | 22 |
|  | pDK339othertube\_033013\_01.10778.10778.2 | 3.9613 | 0.4453 | 100.0% | 1697.3121 | 1697.8877 | 1 | 7.642 | 65.4% | 13 | K.NSSYFVEWIPNNVK.T | 2222 |
|  | 100326\_pJS43\_01.06864.06864.2 | 3.1178 | 0.3686 | 100.0% | 1386.2922 | 1386.6116 | 2 | 7.406 | 65.0% | 4 | K.RISEQFTAMFR.R | 222 |
|  | 100326\_pJS43\_01.07953.07953.1 | 2.031 | 0.3168 | 98.8% | 1229.52 | 1230.4241 | 1 | 5.359 | 66.7% | 1 | R.ISEQFTAMFR.R | 111 |
|  | pDK339othertube\_033013\_02.07069.07069.2 | 3.8553 | 0.4627 | 100.0% | 1231.1721 | 1230.4241 | 1 | 7.816 | 88.9% | 19 | R.ISEQFTAMFR.R | 222 |

Similarities:
gi|29788785|ref|NP\_82(23:4)  
gi|50592996|ref|NP\_00(14:13)  
gi|14210536|ref|NP\_11(7:20)  

---

|  |  |  |  |  |  |  |  |  |
| --- | --- | --- | --- | --- | --- | --- | --- | --- |
| U | *gi|221307584|ref|NP\_0* | 13 | 73 | 49.2% | 299 | 33296 | 9.8 | prohibitin 2 isoform 1 [Homo sapiens] |
| U | *gi|6005854|ref|NP\_009* | 13 | 73 | 49.2% | 299 | 33296 | 9.8 | prohibitin 2 isoform 2 [Homo sapiens] |

| Filename XCorr DeltCN Conf% ObsM+H+ CalcM+H+ SpR ZScore Ion% # Sequence  | | | | | | | | | | | | |
| --- | --- | --- | --- | --- | --- | --- | --- | --- | --- | --- | --- | --- |
|  | pDK365N\_100mM\_082713\_02.06581.06581.2 | 4.3407 | 0.4803 | 100.0% | 1260.1522 | 1260.5222 | 1 | 9.273 | 83.3% | 8 | K.LLLGAGAVAYGVR.E | 2 |
|  | 100326\_pJS43\_01.08172.08172.2 | 3.3061 | 0.2059 | 99.5% | 1855.6721 | 1855.1038 | 1 | 6.414 | 53.1% | 1 | R.IGGVQQDTILAEGLHFR.I | 2 |
|  | pDK365N\_300mM\_082713\_02.07275.07275.3 | 5.437 | 0.4875 | 100.0% | 1856.1543 | 1855.1038 | 1 | 9.231 | 53.1% | 16 | R.IGGVQQDTILAEGLHFR.I | 3 |
|  | pDK365N\_100mM\_082713\_01.12991.12991.2 | 4.2199 | 0.4208 | 100.0% | 1724.8121 | 1725.0428 | 1 | 7.95 | 75.0% | 11 | R.IPWFQYPIIYDIR.A | 2 |
|  | pDK365N\_100mM\_082713\_01.07028.07028.3 | 3.6299 | 0.3311 | 100.0% | 2075.6042 | 2075.4314 | 5 | 5.52 | 33.3% | 3 | R.KISSPTGSKDLQMVNISLR.V | 3 |
|  | pDK365N\_300mM\_082713\_01.09698.09698.2 | 3.3865 | 0.3445 | 100.0% | 1189.4122 | 1189.4155 | 1 | 6.831 | 94.4% | 3 | K.DLQMVNISLR.V | 2 |
|  | pDK365N\_300mM\_082713\_03.05942.05942.3 | 4.1589 | 0.2774 | 100.0% | 1889.8744 | 1890.1675 | 29 | 5.142 | 41.7% | 4 | R.VLSRPNAQELPSMYQR.L | 3 |
|  | pDK365N\_300mM\_082713\_01.05180.05180.2 | 2.6277 | 0.2989 | 99.9% | 995.2922 | 995.077 | 1 | 6.799 | 85.7% | 3 | R.LGLDYEER.V | 2 |
|  | pDK365N\_100mM\_082813\_03.07219.07219.2 | 3.5712 | 0.3012 | 100.0% | 1178.5322 | 1178.3335 | 1 | 7.161 | 88.9% | 4 | K.FNASQLITQR.A | 2 |
|  | pDK365N\_300mM\_082713\_04.09593.09593.3 | 3.4337 | 0.3533 | 100.0% | 2241.2043 | 2241.5461 | 1 | 6.023 | 36.8% | 2 | R.AKDFSLILDDVAITELSFSR.E | 3 |
|  | pDK365N\_300mM\_082713\_01.03516.03516.2 | 2.7269 | 0.2892 | 99.7% | 1215.8322 | 1216.3336 | 1 | 6.575 | 72.7% | 2 | K.IVQAEGEAEAAK.M | 2 |
|  | pDK365N\_100mM\_082713\_01.11756.11756.2 | 5.4194 | 0.586 | 100.0% | 2226.672 | 2226.4912 | 1 | 9.719 | 63.9% | 12 | R.IYLTADNLVLNLQDESFTR.G | 2 |
|  | pDK365N\_300mM\_082713\_02.08788.08788.3 | 6.192 | 0.4414 | 100.0% | 2227.9143 | 2226.4912 | 1 | 7.651 | 52.8% | 4 | R.IYLTADNLVLNLQDESFTR.G | 3 |

---

|  |  |  |  |  |  |  |  |  |
| --- | --- | --- | --- | --- | --- | --- | --- | --- |
| U | *gi|25777713|ref|NP\_73* | 7 | 14 | 49.1% | 163 | 18658 | 4.5 | S-phase kinase-associated protein 1 isoform b [Homo sapiens] |

| Filename XCorr DeltCN Conf% ObsM+H+ CalcM+H+ SpR ZScore Ion% # Sequence  | | | | | | | | | | | | |
| --- | --- | --- | --- | --- | --- | --- | --- | --- | --- | --- | --- | --- |
|  | AstrinIP\_MS2\_022614\_01.12743.12743.3 | 3.838 | 0.3449 | 100.0% | 3126.3245 | 3127.5056 | 1 | 7.514 | 23.2% | 2 | K.TMLEDLGMDDEGDDDPVPLPNVNAAILKK.V | 3 |
|  | AstrinIP\_MS2\_022614\_01.11214.11214.2 | 3.5726 | 0.487 | 100.0% | 1762.3121 | 1762.9597 | 1 | 7.531 | 61.5% | 1 | K.RTDDIPVWDQEFLK.V | 2 |
|  | pSKT11\_1\_020812\_01.12504.12504.3 | 3.6782 | 0.2144 | 98.5% | 3880.5244 | 3881.4175 | 1 | 5.235 | 20.3% | 2 | K.RTDDIPVWDQEFLKVDQGTLFELILAANYLDIK.G | 3 |
|  | pJS43\_100mM\_120812\_02.10503.10503.2 | 4.2542 | 0.3454 | 100.0% | 2137.632 | 2137.481 | 2 | 7.016 | 44.4% | 1 | K.VDQGTLFELILAANYLDIK.G | 2 |
| \* | pDK365N\_300mM\_082713\_04.05762.05762.3 | 4.0515 | 0.3241 | 100.0% | 2071.7043 | 2071.2078 | 1 | 5.411 | 42.2% | 4 | K.TFNIKNDFTEEEEAQVR.K | 3 |
| \* | AstrinIP\_MS2\_022614\_02.05417.05417.3 | 3.4187 | 0.2581 | 99.5% | 2199.8643 | 2199.3818 | 22 | 5.722 | 32.4% | 1 | K.TFNIKNDFTEEEEAQVRK.E | 3 |
| \* | pDk339\_033013\_02.04453.04453.2 | 2.4919 | 0.2352 | 98.7% | 1466.3121 | 1467.4888 | 4 | 5.01 | 59.1% | 3 | K.NDFTEEEEAQVR.K | 2 |

---

|  |  |  |  |  |  |  |  |  |
| --- | --- | --- | --- | --- | --- | --- | --- | --- |
| U | *gi|4506707|ref|NP\_001* | 9 | 38 | 48.8% | 125 | 13742 | 10.1 | ribosomal protein S25 [Homo sapiens] |

| Filename XCorr DeltCN Conf% ObsM+H+ CalcM+H+ SpR ZScore Ion% # Sequence  | | | | | | | | | | | | |
| --- | --- | --- | --- | --- | --- | --- | --- | --- | --- | --- | --- | --- |
| \* | pJS43\_100mM\_120812\_01.01110.01110.2 | 2.4649 | 0.1829 | 98.9% | 943.71216 | 944.07574 | 5 | 4.906 | 85.7% | 1 | K.KDKDPVNK.S | 2 |
| \* | pDK365N\_100mM\_082813\_03.07140.07140.3 | 3.4537 | 0.2513 | 99.7% | 1575.1743 | 1574.8638 | 26 | 5.487 | 35.4% | 2 | K.VRDKLNNLVLFDK.A | 3 |
| \* | pJS43\_100mM\_120812\_01.09563.09563.2 | 3.686 | 0.3006 | 100.0% | 1319.4321 | 1319.5437 | 2 | 5.791 | 80.0% | 9 | R.DKLNNLVLFDK.A | 2 |
| \* | pDK365N\_100mM\_082713\_01.07891.07891.3 | 3.9026 | 0.454 | 100.0% | 1897.5543 | 1898.1663 | 1 | 7.47 | 41.7% | 1 | R.DKLNNLVLFDKATYDK.L | 3 |
| \* | pJS43\_100mM\_120812\_01.09906.09906.2 | 3.5164 | 0.4104 | 100.0% | 1075.8922 | 1076.281 | 1 | 6.921 | 81.2% | 5 | K.LNNLVLFDK.A | 2 |
| \* | pJS43\_100mM\_120812\_01.06917.06917.2 | 2.698 | 0.371 | 100.0% | 1086.1322 | 1085.289 | 2 | 6.739 | 77.8% | 7 | K.LITPAVVSER.L | 2 |
| \* | pJS43\_100mM\_120812\_01.09101.09101.3 | 2.5458 | 0.2956 | 98.8% | 1458.6244 | 1457.7135 | 6 | 4.864 | 30.8% | 1 | R.GSLARAALQELLSK.G | 3 |
| \* | pJS43\_100mM\_120812\_01.09035.09035.2 | 3.3524 | 0.2495 | 100.0% | 973.1122 | 973.1576 | 1 | 6.272 | 87.5% | 11 | R.AALQELLSK.G | 2 |
| \* | pJS43\_100mM\_120812\_01.03060.03060.1 | 2.3383 | 0.4843 | 98.8% | 931.07 | 930.90356 | 1 | 6.707 | 55.0% | 1 | K.GGDAPAAGEDA.- | 1 |

---

|  |  |  |  |  |  |  |  |  |
| --- | --- | --- | --- | --- | --- | --- | --- | --- |
| U | *gi|4506679|ref|NP\_001* | 10 | 37 | 48.5% | 165 | 18898 | 10.2 | ribosomal protein S10 [Homo sapiens] |

| Filename XCorr DeltCN Conf% ObsM+H+ CalcM+H+ SpR ZScore Ion% # Sequence  | | | | | | | | | | | | |
| --- | --- | --- | --- | --- | --- | --- | --- | --- | --- | --- | --- | --- |
|  | pJS43\_100mM\_120812\_01.13218.13218.2 | 3.5858 | 0.4933 | 100.0% | 1110.1522 | 1110.3818 | 1 | 7.46 | 87.5% | 4 | R.IAIYELLFK.E | 2 |
| \* | pJS43\_100mM\_120812\_01.05647.05647.3 | 3.4365 | 0.3314 | 100.0% | 1843.2544 | 1843.154 | 3 | 6.448 | 36.7% | 1 | K.HPELADKNVPNLHVMK.A | 3 |
| \* | pJS43\_100mM\_120812\_01.11935.11935.2 | 5.4512 | 0.5152 | 100.0% | 2003.7122 | 2004.2548 | 1 | 10.018 | 82.1% | 3 | R.HFYWYLTNEGIQYLR.D | 2 |
| \* | pDK365N\_100mM\_082713\_02.07868.07868.3 | 4.3883 | 0.384 | 100.0% | 2004.6843 | 2004.2548 | 1 | 6.137 | 48.2% | 2 | R.HFYWYLTNEGIQYLR.D | 3 |
| \* | pJS43\_100mM\_120812\_01.11721.11721.2 | 2.9303 | 0.4323 | 100.0% | 1734.4521 | 1735.036 | 1 | 6.372 | 67.9% | 1 | R.DYLHLPPEIVPATLR.R | 2 |
| \* | pDK365N\_100mM\_082713\_01.09235.09235.3 | 3.0441 | 0.2189 | 97.9% | 1891.5543 | 1891.2235 | 1 | 5.355 | 38.3% | 1 | R.DYLHLPPEIVPATLRR.S | 3 |
|  | pJS43\_100mM\_120812\_01.03200.03200.2 | 2.127 | 0.1931 | 95.9% | 984.97217 | 985.0879 | 161 | 4.571 | 50.0% | 1 | K.GLEGERPAR.L | 2 |
|  | pJS43\_100mM\_120812\_02.04980.04980.3 | 4.0283 | 0.1723 | 99.3% | 1570.8243 | 1570.7019 | 2 | 5.475 | 44.6% | 8 | K.KAEAGAGSATEFQFR.G | 3 |
|  | pJS43\_100mM\_120812\_02.04969.04969.2 | 5.0674 | 0.5039 | 100.0% | 1571.1721 | 1570.7019 | 1 | 8.818 | 82.1% | 3 | K.KAEAGAGSATEFQFR.G | 2 |
|  | pJS43\_100mM\_120812\_02.05532.05532.2 | 4.1121 | 0.5403 | 100.0% | 1441.6522 | 1442.5278 | 1 | 9.403 | 76.9% | 13 | K.AEAGAGSATEFQFR.G | 2 |

---

|  |  |  |  |  |  |  |  |  |
| --- | --- | --- | --- | --- | --- | --- | --- | --- |
| U | *gi|7669492|ref|NP\_002* | 11 | 46 | 48.4% | 335 | 36053 | 8.5 | glyceraldehyde-3-phosphate dehydrogenase [Homo sapiens] |

| Filename XCorr DeltCN Conf% ObsM+H+ CalcM+H+ SpR ZScore Ion% # Sequence  | | | | | | | | | | | | |
| --- | --- | --- | --- | --- | --- | --- | --- | --- | --- | --- | --- | --- |
| \* | SKAPIP\_041314\_01.09368.09368.2 | 2.14 | 0.265 | 98.8% | 805.9922 | 805.912 | 2 | 5.752 | 78.6% | 1 | K.VGVNGFGR.I | 2 |
| \* | pDK339othertube\_033013\_01.16167.16167.3 | 3.9958 | 0.258 | 99.7% | 3310.7344 | 3310.7634 | 1 | 3.827 | 26.9% | 6 | K.VDIVAINDPFIDLNYMVYMFQYDSTHGK.F | 3 |
| \* | AstrinIP\_MS1\_022614\_01.11776.11776.2 | 3.3021 | 0.3396 | 100.0% | 1614.3522 | 1614.8851 | 1 | 7.692 | 65.4% | 1 | K.LVINGNPITIFQER.D | 2 |
| \* | pSKT11\_1\_020812\_02.06958.06958.2 | 3.0622 | 0.4033 | 100.0% | 2277.3323 | 2278.495 | 1 | 6.719 | 37.5% | 1 | K.WGDAGAEYVVESTGVFTTMEK.A | 2 |
| \* | pSKT11\_1\_020812\_01.10535.10535.2 | 5.7085 | 0.6182 | 100.0% | 2595.5723 | 2597.0044 | 1 | 10.574 | 45.7% | 1 | K.VIHDNFGIVEGLMTTVHAITATQK.T | 2 |
| \* | pJS43\_100mM\_120812\_01.13621.13621.3 | 5.6829 | 0.5702 | 100.0% | 2597.8145 | 2597.0044 | 1 | 9.616 | 37.0% | 13 | K.VIHDNFGIVEGLMTTVHAITATQK.T | 3 |
| \* | pJS43\_100mM\_120812\_01.08393.08393.2 | 3.9787 | 0.3307 | 100.0% | 1412.4122 | 1412.6292 | 1 | 5.896 | 71.4% | 8 | R.GALQNIIPASTGAAK.A | 2 |
| \* | AstrinIP\_MS1\_022614\_01.08200.08200.2 | 2.8188 | 0.375 | 99.9% | 1532.3322 | 1531.7155 | 1 | 6.09 | 57.7% | 1 | R.VPTANVSVVDLTCR.L | 2 |
| \* | AstrinIP\_MS2\_022614\_01.03526.03526.3 | 2.669 | 0.2 | 95.4% | 1448.3043 | 1448.7025 | 1 | 4.333 | 47.7% | 1 | R.LEKPAKYDDIKK.V | 3 |
|  | SKAPIP\_tube2\_041314\_01.10528.10528.2 | 4.4204 | 0.4999 | 100.0% | 1764.4722 | 1764.8914 | 1 | 8.566 | 65.4% | 10 | K.LISWYDNEFGYSNR.V | 2 |
| \* | AstrinIP\_MS2\_022614\_01.07092.07092.2 | 2.9041 | 0.4028 | 100.0% | 1331.1122 | 1331.5879 | 8 | 6.555 | 54.5% | 3 | R.VVDLMAHMASKE.- | 2 |

---

|  |  |  |  |  |  |  |  |  |
| --- | --- | --- | --- | --- | --- | --- | --- | --- |
| U | *gi|225690529|ref|NP\_6* | 28 | 135 | 48.2% | 394 | 44993 | 7.1 | golgin, RAB6-interacting isoform a [Homo sapiens] |

| Filename XCorr DeltCN Conf% ObsM+H+ CalcM+H+ SpR ZScore Ion% # Sequence  | | | | | | | | | | | | |
| --- | --- | --- | --- | --- | --- | --- | --- | --- | --- | --- | --- | --- |
|  | pSKT11\_1\_020812\_02.05961.05961.3 | 5.9363 | 0.5134 | 100.0% | 3208.2244 | 3209.6648 | 1 | 7.214 | 28.4% | 1 | R.EKALVEQSQKLGLQDGSTSLLPEQLLSAPK.Q | 3 |
|  | pSKT11\_1\_020812\_01.09881.09881.2 | 5.6659 | 0.5958 | 100.0% | 2067.892 | 2068.3752 | 1 | 10.192 | 55.3% | 15 | K.LGLQDGSTSLLPEQLLSAPK.Q | 2 |
|  | pSKT11\_1\_020812\_01.09108.09108.3 | 4.9878 | 0.4432 | 100.0% | 3803.6643 | 3804.2512 | 1 | 7.56 | 28.6% | 1 | R.VNVQKPPFSSPTLPSHFTLTSPVGDGQPQGIESQPK.E | 3 |
|  | pDK365N\_300mM\_082713\_03.05282.05282.3 | 3.678 | 0.0784 | 95.3% | 1459.1344 | 1459.6047 | 6 | 3.751 | 50.0% | 1 | K.SRWEVLQQEQR.L | 3 |
|  | pJS43\_100mM\_120812\_01.06085.06085.2 | 3.5024 | 0.1766 | 99.8% | 1459.3121 | 1459.6047 | 1 | 4.702 | 75.0% | 6 | K.SRWEVLQQEQR.L | 2 |
|  | pDK365N\_300mM\_082713\_01.06042.06042.2 | 2.9196 | 0.3153 | 100.0% | 1216.5922 | 1216.339 | 1 | 6.035 | 87.5% | 7 | R.WEVLQQEQR.L | 2 |
|  | pSKT11\_1\_020812\_01.10420.10420.2 | 5.9256 | 0.6073 | 100.0% | 2228.7122 | 2229.597 | 1 | 11.868 | 71.1% | 3 | R.IQKELQALDDMVSADIGILR.N | 2 |
|  | pSKT11\_1\_020812\_01.10419.10419.3 | 5.3616 | 0.3811 | 100.0% | 2229.4744 | 2229.597 | 1 | 7.496 | 43.4% | 2 | R.IQKELQALDDMVSADIGILR.N | 3 |
|  | pJS43\_100mM\_120812\_02.09007.09007.2 | 4.388 | 0.5039 | 100.0% | 1859.1522 | 1860.1327 | 1 | 8.488 | 53.1% | 16 | K.ELQALDDMVSADIGILR.N | 2 |
|  | pJS43\_100mM\_120812\_02.04898.04898.3 | 4.3 | 0.4302 | 100.0% | 1672.8243 | 1672.7948 | 1 | 7.595 | 50.0% | 6 | R.NRIDQASLDYSYAR.K | 3 |
|  | pSKT11\_1\_020812\_01.08343.08343.2 | 4.1768 | 0.4108 | 100.0% | 1673.2922 | 1672.7948 | 1 | 6.803 | 69.2% | 5 | R.NRIDQASLDYSYAR.K | 2 |
| \* | pSKT11\_1\_020812\_01.08002.08002.3 | 4.7395 | 0.399 | 100.0% | 1801.1643 | 1800.9689 | 1 | 7.15 | 57.1% | 1 | R.NRIDQASLDYSYARK.R | 3 |
|  | pDK365N\_300mM\_082713\_01.05764.05764.2 | 4.3413 | 0.537 | 100.0% | 1402.4321 | 1402.5034 | 1 | 9.147 | 90.9% | 11 | R.IDQASLDYSYAR.K | 2 |
| \* | pSKT11\_1\_020812\_01.04448.04448.2 | 3.2256 | 0.2561 | 99.8% | 1668.0922 | 1668.8931 | 1 | 5.736 | 73.1% | 16 | R.KRFDRAEAEYIAAK.L | 2 |
| \* | pSKT11\_1\_020812\_01.04628.04628.3 | 3.4379 | 0.3776 | 100.0% | 1669.6743 | 1668.8931 | 1 | 5.334 | 46.2% | 8 | R.KRFDRAEAEYIAAK.L | 3 |
| \* | pSKT11\_1\_020812\_02.04444.04444.3 | 5.1098 | 0.5033 | 100.0% | 2293.3442 | 2294.6187 | 1 | 8.364 | 40.3% | 1 | R.KRFDRAEAEYIAAKLDIQR.K | 3 |
| \* | pSKT11\_1\_020812\_01.07248.07248.3 | 3.7635 | 0.2443 | 99.8% | 1541.2144 | 1540.719 | 1 | 5.934 | 52.1% | 1 | K.RFDRAEAEYIAAK.L | 3 |
| \* | pSKT11\_1\_020812\_01.08060.08060.2 | 3.0108 | 0.1648 | 99.1% | 1384.4321 | 1384.5315 | 1 | 4.972 | 68.2% | 1 | R.FDRAEAEYIAAK.L | 2 |
| \* | pJS43\_100mM\_120812\_01.03896.03896.2 | 2.1537 | 0.2605 | 98.5% | 965.9522 | 966.07886 | 1 | 5.865 | 87.5% | 1 | R.AEAEYIAAK.L | 2 |
| \* | pSKT11\_1\_020812\_02.04512.04512.2 | 3.2235 | 0.302 | 99.9% | 1590.7122 | 1591.8046 | 1 | 5.651 | 53.8% | 2 | R.AEAEYIAAKLDIQR.K | 2 |
| \* | pSKT11\_1\_020812\_02.08228.08228.3 | 5.9464 | 0.4662 | 100.0% | 3317.6643 | 3317.687 | 1 | 8.333 | 36.1% | 2 | K.AKKLEELMQQLDVEADEETLELEVEVER.L | 3 |
| \* | pSKT11\_1\_020812\_01.10703.10703.2 | 5.8119 | 0.4754 | 100.0% | 3117.3523 | 3118.434 | 1 | 9.74 | 46.0% | 2 | K.KLEELMQQLDVEADEETLELEVEVER.L | 2 |
| \* | pJS43\_100mM\_120812\_02.09554.09554.3 | 3.7114 | 0.3492 | 100.0% | 3118.0144 | 3118.434 | 1 | 5.908 | 25.0% | 1 | K.KLEELMQQLDVEADEETLELEVEVER.L | 3 |
| \* | pJS43\_100mM\_120812\_01.03577.03577.2 | 2.9629 | 0.3756 | 100.0% | 1240.1721 | 1240.3585 | 3 | 6.507 | 72.2% | 2 | R.LLHEQEVESR.R | 2 |
| \* | pSKT11\_1\_020812\_01.09134.09134.2 | 5.2127 | 0.5392 | 100.0% | 2091.3123 | 2092.3562 | 1 | 10.078 | 67.6% | 5 | R.LERPFQPAEESVTLEFAK.E | 2 |
| \* | pDK339othertube\_033013\_01.09032.09032.3 | 5.6883 | 0.5253 | 100.0% | 2093.2144 | 2092.3562 | 1 | 9.755 | 50.0% | 16 | R.LERPFQPAEESVTLEFAK.E | 3 |
| \* | pSKT11\_1\_020812\_01.08930.08930.3 | 4.7435 | 0.3463 | 100.0% | 2493.3843 | 2491.763 | 3 | 5.631 | 32.5% | 1 | R.LERPFQPAEESVTLEFAKENR.K | 3 |
| \* | pSKT11\_1\_020812\_02.04533.04533.3 | 5.719 | 0.4479 | 100.0% | 2619.0842 | 2619.937 | 1 | 7.124 | 33.3% | 1 | R.LERPFQPAEESVTLEFAKENRK.C | 3 |

---

|  |  |  |  |  |  |  |  |  |
| --- | --- | --- | --- | --- | --- | --- | --- | --- |
| U | *gi|117190174|ref|NP\_0* | 22 | 167 | 48.1% | 293 | 32338 | 5.1 | heterogeneous nuclear ribonucleoprotein C isoform b [Homo sapiens] |
| U | *gi|117190254|ref|NP\_0* | 22 | 167 | 48.1% | 293 | 32338 | 5.1 | heterogeneous nuclear ribonucleoprotein C isoform b [Homo sapiens] |

| Filename XCorr DeltCN Conf% ObsM+H+ CalcM+H+ SpR ZScore Ion% # Sequence  | | | | | | | | | | | | |
| --- | --- | --- | --- | --- | --- | --- | --- | --- | --- | --- | --- | --- |
|  | pDK365N\_300mM\_082713\_04.07226.07226.3 | 2.9003 | 0.2817 | 99.1% | 1975.8243 | 1973.2548 | 6 | 4.909 | 32.8% | 4 | R.SMNS\*RVFIGNLNTLVVK.K | 3 |
|  | pJS43\_100mM\_120812\_01.11081.11081.2 | 4.0292 | 0.3065 | 100.0% | 1317.4122 | 1317.6145 | 1 | 7.456 | 86.4% | 23 | R.VFIGNLNTLVVK.K | 22 |
|  | SKAPIP\_041314\_02.06996.06996.2 | 2.8939 | 0.2982 | 99.8% | 1446.2522 | 1445.7886 | 9 | 6.78 | 58.3% | 1 | R.VFIGNLNTLVVKK.S | 22 |
|  | SKAPIP\_tube2\_041314\_01.05946.05946.2 | 3.5832 | 0.4276 | 100.0% | 1125.2722 | 1124.2792 | 1 | 6.377 | 77.8% | 15 | K.KSDVEAIFSK.Y | 22 |
|  | pDK365N\_100mM\_082713\_01.06985.06985.2 | 2.6759 | 0.3856 | 100.0% | 996.1122 | 996.10516 | 1 | 8.077 | 75.0% | 4 | K.SDVEAIFSK.Y | 22 |
|  | pDK365N\_100mM\_082713\_01.09068.09068.2 | 4.3566 | 0.4786 | 100.0% | 1331.3722 | 1330.4857 | 1 | 8.645 | 80.0% | 19 | K.GFAFVQYVNER.N | 2 |
|  | SKAPIP\_tube2\_041314\_01.10342.10342.1 | 2.4197 | 0.2268 | 98.6% | 1682.74 | 1684.0038 | 1 | 4.666 | 43.3% | 1 | R.MIAGQVLDINLAAEPK.V | 1 |
|  | pJS43\_100mM\_120812\_01.11088.11088.2 | 5.5508 | 0.4476 | 100.0% | 1683.4521 | 1684.0038 | 1 | 8.981 | 83.3% | 50 | R.MIAGQVLDINLAAEPK.V | 2 |
|  | SKAPIP\_041314\_02.08173.08173.3 | 4.147 | 0.3952 | 100.0% | 1683.6543 | 1684.0038 | 1 | 6.772 | 51.7% | 3 | R.MIAGQVLDINLAAEPK.V | 3 |
|  | pJS43\_100mM\_120812\_02.11586.11586.3 | 3.019 | 0.2192 | 95.6% | 2969.3643 | 2972.128 | 13 | 5.138 | 22.8% | 1 | K.RSAAEMYGSSFDLDYDFQRDYYDR.M | 3 |
|  | pJS43\_100mM\_120812\_02.07440.07440.2 | 5.9995 | 0.6187 | 100.0% | 2103.5322 | 2103.2239 | 1 | 10.679 | 67.6% | 10 | R.SAAEMYGSSFDLDYDFQR.D | 2 |
|  | pSKT11\_1\_020812\_02.06167.06167.3 | 3.9639 | 0.4052 | 100.0% | 2814.7144 | 2815.9404 | 1 | 6.79 | 33.0% | 8 | R.SAAEMYGSSFDLDYDFQRDYYDR.M | 3 |
|  | pSKT11\_1\_020812\_02.06183.06183.2 | 3.0458 | 0.3319 | 99.9% | 2815.5322 | 2815.9404 | 3 | 5.269 | 27.3% | 1 | R.SAAEMYGSSFDLDYDFQRDYYDR.M | 2 |
|  | SKAPIP\_041314\_01.06945.06945.2 | 2.0329 | 0.197 | 96.7% | 888.21216 | 888.0289 | 1 | 4.387 | 83.3% | 1 | R.MYSYPAR.V | 2 |
|  | pDK339\_033013\_01.04317.04317.2 | 2.0199 | 0.325 | 98.9% | 943.53217 | 944.1649 | 2 | 5.675 | 68.8% | 2 | R.VPPPPPIAR.A | 2 |
|  | SKAPIP\_041314\_01.04337.04337.2 | 3.1852 | 0.3552 | 100.0% | 1229.5322 | 1229.4624 | 1 | 5.77 | 75.0% | 5 | K.LKGDDLQAIKK.E | 2 |
|  | pDK365N\_300mM\_082713\_01.03632.03632.3 | 3.1479 | 0.2163 | 99.1% | 1229.9944 | 1229.4624 | 190 | 5.17 | 37.5% | 4 | K.LKGDDLQAIKK.E | 3 |
|  | pJS43\_100mM\_120812\_01.09008.09008.2 | 2.4165 | 0.3883 | 99.8% | 1416.2122 | 1416.6146 | 6 | 5.745 | 63.6% | 8 | K.QKVDSLLENLEK.I | 22 |
|  | pDK365N\_300mM\_082713\_01.08421.08421.3 | 3.2125 | 0.1825 | 98.2% | 1417.5844 | 1416.6146 | 190 | 4.759 | 45.5% | 1 | K.QKVDSLLENLEK.I | 33 |
|  | SKAPIP\_tube2\_041314\_01.09537.09537.3 | 2.535 | 0.2649 | 97.5% | 1788.2344 | 1787.0636 | 203 | 5.214 | 32.1% | 2 | K.QKVDSLLENLEKIEK.E | 33 |
|  | pDK365N\_300mM\_082713\_03.08722.08722.2 | 2.5019 | 0.2776 | 99.5% | 1159.7522 | 1160.3098 | 1 | 5.701 | 77.8% | 2 | K.VDSLLENLEK.I | 22 |
|  | pJS43\_100mM\_120812\_01.03125.03125.3 | 4.6429 | 0.347 | 100.0% | 2368.9143 | 2369.4583 | 1 | 6.386 | 46.2% | 2 | K.NDKSEEEQSSSSVKKDETNVK.M | 3 |

Similarities:
gi|169217907|ref|XP\_0(8:14)  

---

|  |  |  |  |  |  |  |  |  |
| --- | --- | --- | --- | --- | --- | --- | --- | --- |
| U | *gi|5901926|ref|NP\_008* | 9 | 26 | 48.0% | 227 | 26227 | 8.8 | cleavage and polyadenylation specific factor 5 [Homo sapiens] |

| Filename XCorr DeltCN Conf% ObsM+H+ CalcM+H+ SpR ZScore Ion% # Sequence  | | | | | | | | | | | | |
| --- | --- | --- | --- | --- | --- | --- | --- | --- | --- | --- | --- | --- |
| \* | pJS43\_100mM\_120812\_01.05283.05283.2 | 3.3272 | 0.3555 | 100.0% | 1490.2122 | 1490.7428 | 1 | 6.096 | 68.2% | 1 | K.YIQQTKPLTLER.T | 2 |
| \* | pDK365N\_100mM\_082713\_01.04698.04698.3 | 3.0461 | 0.2511 | 99.4% | 1491.0243 | 1490.7428 | 1 | 5.37 | 59.1% | 5 | K.YIQQTKPLTLER.T | 3 |
| \* | pDK365N\_100mM\_082713\_01.10670.10670.2 | 2.708 | 0.254 | 99.1% | 1747.7122 | 1747.0007 | 1 | 4.755 | 57.1% | 1 | R.TINLYPLTNYTFGTK.E | 2 |
| \* | pJS43\_100mM\_120812\_01.13411.13411.2 | 1.9168 | 0.3013 | 95.3% | 1728.7322 | 1728.1307 | 50 | 5.526 | 35.7% | 1 | R.LPHVLLLQLGTTFFK.L | 2 |
| \* | pJS43\_100mM\_120812\_01.07541.07541.3 | 4.1391 | 0.3251 | 100.0% | 1911.0243 | 1910.0911 | 1 | 6.779 | 47.1% | 6 | K.LPGGELNPGEDEVEGLKR.L | 3 |
| \* | pJS43\_100mM\_120812\_01.08268.08268.2 | 2.8936 | 0.2517 | 99.9% | 933.83215 | 933.15405 | 3 | 5.281 | 78.6% | 2 | R.LMTEILGR.Q | 2 |
| \* | pDK365N\_100mM\_082713\_01.09014.09014.2 | 2.842 | 0.2922 | 99.9% | 1117.9922 | 1118.3617 | 4 | 5.805 | 81.2% | 3 | K.LFLVQLQEK.A | 2 |
| \* | pDK365N\_100mM\_082713\_01.13954.13954.3 | 5.7438 | 0.5524 | 100.0% | 3522.2644 | 3522.0813 | 1 | 9.706 | 33.1% | 1 | K.NYKLVAAPLFELYDNAPGYGPIISSLPQLLSR.F | 3 |
| \* | pDK339othertube\_033013\_01.15680.15680.3 | 4.0843 | 0.3158 | 99.9% | 3116.0942 | 3116.6274 | 1 | 5.039 | 25.0% | 6 | K.LVAAPLFELYDNAPGYGPIISSLPQLLSR.F | 3 |

---

|  |  |  |  |  |  |  |  |  |
| --- | --- | --- | --- | --- | --- | --- | --- | --- |
| U | *gi|190885499|ref|NP\_0* | 6 | 32 | 48.0% | 150 | 16762 | 6.8 | cytochrome c oxidase subunit Va precursor [Homo sapiens] |

| Filename XCorr DeltCN Conf% ObsM+H+ CalcM+H+ SpR ZScore Ion% # Sequence  | | | | | | | | | | | | |
| --- | --- | --- | --- | --- | --- | --- | --- | --- | --- | --- | --- | --- |
| \* | pDK365N\_100mM\_082813\_03.11190.11190.3 | 3.0475 | 0.4507 | 100.0% | 2054.6943 | 2054.3123 | 1 | 6.886 | 38.3% | 3 | R.WVTYFNKPDIDAWELR.K | 3 |
| \* | pDK365N\_300mM\_082713\_03.09737.09737.3 | 4.3913 | 0.3832 | 100.0% | 2183.0645 | 2182.4863 | 1 | 6.555 | 42.2% | 6 | R.WVTYFNKPDIDAWELRK.G | 3 |
| \* | pDK365N\_100mM\_082813\_03.09623.09623.2 | 4.5026 | 0.5123 | 100.0% | 1678.3922 | 1677.9531 | 1 | 8.698 | 71.4% | 5 | K.GINTLVTYDMVPEPK.I | 2 |
| \* | pDK365N\_100mM\_082813\_03.04527.04527.2 | 3.3198 | 0.3091 | 100.0% | 1179.3922 | 1179.3213 | 16 | 5.481 | 72.2% | 3 | R.RLNDFASTVR.I | 2 |
| \* | pDK365N\_100mM\_082713\_01.04956.04956.2 | 3.1983 | 0.3972 | 100.0% | 1023.07214 | 1023.1338 | 1 | 7.107 | 87.5% | 6 | R.LNDFASTVR.I | 2 |
| \* | pDK365N\_100mM\_082713\_01.13374.13374.3 | 6.6478 | 0.5076 | 100.0% | 3431.1843 | 3430.9202 | 1 | 10.434 | 29.3% | 9 | K.EIYPYVIQELRPTLNELGISTPEELGLDKV.- | 3 |

---

|  |  |  |  |  |  |  |  |  |
| --- | --- | --- | --- | --- | --- | --- | --- | --- |
| U | *gi|167466173|ref|NP\_0* | 42 | 316 | 47.9% | 641 | 70052 | 5.6 | heat shock 70kDa protein 1B [Homo sapiens] |
| U | *gi|194248072|ref|NP\_0* | 42 | 316 | 47.9% | 641 | 70052 | 5.6 | heat shock 70kDa protein 1A [Homo sapiens] |

| Filename XCorr DeltCN Conf% ObsM+H+ CalcM+H+ SpR ZScore Ion% # Sequence  | | | | | | | | | | | | |
| --- | --- | --- | --- | --- | --- | --- | --- | --- | --- | --- | --- | --- |
|  | AstrinIP\_MS2\_022614\_01.07734.07734.1 | 2.2714 | 0.4224 | 99.4% | 1487.62 | 1488.5939 | 1 | 6.368 | 50.0% | 7 | R.TTPSYVAFTDTER.L | 11111 |
|  | AstrinIP\_MS1\_022614\_02.04868.04868.2 | 3.849 | 0.5598 | 100.0% | 1489.2922 | 1488.5939 | 1 | 9.39 | 79.2% | 43 | R.TTPSYVAFTDTER.L | 22222 |
|  | pDK339othertube\_033013\_01.07256.07256.2 | 5.1399 | 0.4418 | 100.0% | 1659.3522 | 1659.8394 | 1 | 8.816 | 78.6% | 21 | K.NQVALNPQNTVFDAK.R | 2 |
|  | SKAPIP\_tube2\_041314\_01.06353.06353.2 | 3.9523 | 0.4514 | 100.0% | 1815.6322 | 1816.0269 | 1 | 6.905 | 70.0% | 1 | K.NQVALNPQNTVFDAKR.L | 2 |
|  | pDK365N\_300mM\_082713\_01.06332.06332.3 | 2.695 | 0.2505 | 97.5% | 1817.5743 | 1816.0269 | 4 | 4.899 | 33.3% | 1 | K.NQVALNPQNTVFDAKR.L | 3 |
|  | SKAPIP\_tube2\_041314\_01.04701.04701.2 | 3.2449 | 0.3873 | 100.0% | 1351.0521 | 1351.5603 | 1 | 7.212 | 72.7% | 3 | R.KFGDPVVQSDMK.H | 2 |
|  | pDK365N\_300mM\_082713\_01.05192.05192.2 | 3.1855 | 0.3538 | 100.0% | 1222.9122 | 1223.3862 | 1 | 7.785 | 75.0% | 11 | K.FGDPVVQSDMK.H | 2 |
|  | AstrinIP\_MS2\_022614\_01.08219.08219.2 | 4.0316 | 0.4162 | 100.0% | 1681.5322 | 1681.8912 | 1 | 7.259 | 73.1% | 5 | K.HWPFQVINDGDKPK.V | 2 |
|  | pDK365N\_300mM\_082713\_01.07676.07676.3 | 4.844 | 0.4408 | 100.0% | 1682.0044 | 1681.8912 | 1 | 6.45 | 51.9% | 25 | K.HWPFQVINDGDKPK.V | 3 |
|  | AstrinIP\_MS1\_022614\_01.11918.11918.2 | 4.8916 | 0.5504 | 100.0% | 1616.3722 | 1615.8817 | 1 | 8.96 | 84.6% | 18 | K.AFYPEEISSMVLTK.M | 22 |
|  | SKAPIP\_tube2\_041314\_01.12322.12322.2 | 4.6765 | 0.6123 | 100.0% | 3261.7322 | 3262.7046 | 1 | 10.116 | 41.1% | 1 | K.MKEIAEAYLGYPVTNAVITVPAYFNDSQR.Q | 2 |
|  | SKAPIP\_tube2\_041314\_01.12285.12285.3 | 5.0477 | 0.4669 | 100.0% | 3262.2844 | 3262.7046 | 1 | 7.721 | 26.8% | 15 | K.MKEIAEAYLGYPVTNAVITVPAYFNDSQR.Q | 3 |
|  | SKAPIP\_tube2\_041314\_01.10259.10259.1 | 2.426 | 0.389 | 99.5% | 1197.59 | 1198.408 | 34 | 6.193 | 45.5% | 4 | K.DAGVIAGLNVLR.I | 11 |
|  | pDK365N\_300mM\_082713\_03.09927.09927.2 | 3.9879 | 0.3027 | 100.0% | 1198.4521 | 1198.408 | 2 | 6.715 | 81.8% | 26 | K.DAGVIAGLNVLR.I | 22 |
|  | SKAPIP\_tube2\_041314\_01.09867.09867.2 | 5.4807 | 0.4785 | 100.0% | 1688.2722 | 1688.9213 | 1 | 10.707 | 83.3% | 21 | R.IINEPTAAAIAYGLDR.T | 22 |
|  | pDK339othertube\_033013\_01.09546.09546.3 | 3.6626 | 0.1363 | 96.4% | 1688.6044 | 1688.9213 | 1 | 5.242 | 45.0% | 3 | R.IINEPTAAAIAYGLDR.T | 33 |
|  | SKAPIP\_tube2\_041314\_01.04282.04282.2 | 4.5795 | 0.5335 | 100.0% | 1676.2122 | 1676.6964 | 1 | 8.053 | 73.3% | 8 | K.ATAGDTHLGGEDFDNR.L | 222 |
|  | pDK365N\_300mM\_082713\_01.04028.04028.3 | 3.4806 | 0.4364 | 100.0% | 1677.2043 | 1676.6964 | 1 | 7.175 | 43.3% | 8 | K.ATAGDTHLGGEDFDNR.L | 333 |
|  | 100326\_pJS43\_01.09070.09070.3 | 3.4672 | 0.203 | 97.1% | 2919.8643 | 2920.124 | 2 | 4.623 | 25.0% | 1 | K.ATAGDTHLGGEDFDNRLVNHFVEEFK.R | 3 |
|  | pDK365N\_300mM\_082713\_03.06743.06743.2 | 3.4375 | 0.4935 | 100.0% | 1263.2922 | 1262.4508 | 1 | 7.828 | 72.2% | 9 | R.LVNHFVEEFK.R | 2 |
|  | pDK365N\_300mM\_082713\_01.07090.07090.3 | 2.6784 | 0.4084 | 100.0% | 1263.8644 | 1262.4508 | 45 | 6.376 | 38.9% | 5 | R.LVNHFVEEFK.R | 3 |
|  | AstrinIP\_MS2\_022614\_01.06551.06551.2 | 2.9311 | 0.3484 | 100.0% | 1418.2322 | 1418.6383 | 4 | 6.379 | 60.0% | 2 | R.LVNHFVEEFKR.K | 2 |
|  | pDK365N\_300mM\_082713\_01.05408.05408.3 | 2.862 | 0.262 | 99.4% | 1418.9043 | 1418.6383 | 1 | 5.362 | 45.0% | 4 | R.LVNHFVEEFKR.K | 3 |
|  | SKAPIP\_tube2\_041314\_01.09444.09444.2 | 3.3839 | 0.3891 | 100.0% | 1543.2722 | 1543.6855 | 1 | 6.85 | 81.8% | 2 | R.ARFEELCSDLFR.S | 22 |
|  | AstrinIP\_MS1\_022614\_01.10366.10366.2 | 3.1778 | 0.4564 | 100.0% | 1316.2522 | 1316.4193 | 1 | 6.925 | 83.3% | 2 | R.FEELCSDLFR.S | 22 |
|  | SKAPIP\_tube2\_041314\_02.06040.06040.2 | 4.3812 | 0.609 | 100.0% | 1822.6322 | 1823.1025 | 1 | 10.24 | 68.8% | 3 | K.LDKAQIHDLVLVGGSTR.I | 2 |
|  | SKAPIP\_041314\_02.05971.05971.3 | 4.8704 | 0.3722 | 100.0% | 1822.7344 | 1823.1025 | 1 | 7.496 | 48.4% | 11 | K.LDKAQIHDLVLVGGSTR.I | 3 |
|  | AstrinIP\_MS2\_022614\_01.10762.10762.1 | 2.0368 | 0.176 | 95.1% | 1109.5 | 1110.2578 | 3 | 4.061 | 56.2% | 1 | K.LLQDFFNGR.D | 1 |
|  | SKAPIP\_041314\_01.12038.12038.2 | 3.1782 | 0.3475 | 100.0% | 1110.2322 | 1110.2578 | 1 | 6.413 | 87.5% | 14 | K.LLQDFFNGR.D | 2 |
|  | AstrinIP\_MS2\_022614\_02.09924.09924.3 | 3.7164 | 0.4297 | 100.0% | 2305.0444 | 2305.608 | 1 | 6.568 | 33.0% | 1 | K.SINPDEAVAYGAAVQAAILMGDK.S | 33 |
|  | pDK365N\_300mM\_082713\_04.09172.09172.2 | 2.9331 | 0.3912 | 99.9% | 2305.3323 | 2305.608 | 2 | 7.23 | 29.5% | 1 | K.SINPDEAVAYGAAVQAAILMGDK.S | 22 |
|  | AstrinIP\_MS1\_022614\_01.19508.19508.2 | 5.0635 | 0.4581 | 100.0% | 3182.0322 | 3182.7407 | 1 | 7.491 | 35.0% | 1 | K.SENVQDLLLLDVAPLSLGLETAGGVMTALIK.R | 2 |
|  | pJS43\_100mM\_120812\_01.17592.17592.3 | 3.6203 | 0.2307 | 98.8% | 3182.9043 | 3182.7407 | 44 | 4.185 | 20.0% | 2 | K.SENVQDLLLLDVAPLSLGLETAGGVMTALIK.R | 3 |
|  | AstrinIP\_MS1\_022614\_01.11703.11703.2 | 4.922 | 0.5638 | 100.0% | 2787.7122 | 2788.043 | 1 | 10.506 | 41.3% | 3 | K.QTQIFTTYSDNQPGVLIQVYEGER.A | 22 |
|  | 100326\_pJS43\_01.00359.00359.2 | 2.6233 | 0.3699 | 100.0% | 1018.47217 | 1018.1582 | 122 | 5.98 | 56.2% | 2 | K.ITITNDKGR.L | 22222 |
|  | AstrinIP\_MS2\_022614\_01.03545.03545.2 | 1.9886 | 0.2475 | 96.6% | 1126.0122 | 1126.313 | 7 | 5.056 | 68.8% | 1 | R.MVQEAEKYK.A | 22 |
|  | pSKT11\_1\_020812\_01.04032.04032.2 | 4.5128 | 0.4006 | 100.0% | 1952.7922 | 1954.1621 | 1 | 8.224 | 66.7% | 1 | R.MVQEAEKYKAEDEVQR.E | 2 |
|  | pDK365N\_300mM\_082713\_01.03680.03680.3 | 3.835 | 0.2723 | 99.9% | 1954.7043 | 1954.1621 | 1 | 6.752 | 41.7% | 8 | R.MVQEAEKYKAEDEVQR.E | 3 |
|  | pSKT11\_1\_020812\_01.04295.04295.2 | 3.2412 | 0.3763 | 100.0% | 2238.4722 | 2239.465 | 1 | 6.481 | 47.1% | 2 | R.MVQEAEKYKAEDEVQRER.V | 2 |
|  | pSKT11\_1\_020812\_01.04233.04233.3 | 3.9863 | 0.3398 | 100.0% | 2239.0144 | 2239.465 | 1 | 5.818 | 45.6% | 2 | R.MVQEAEKYKAEDEVQRER.V | 3 |
|  | pJS43\_100mM\_120812\_01.03205.03205.2 | 3.2062 | 0.4301 | 100.0% | 1137.6522 | 1138.2224 | 1 | 7.617 | 81.2% | 1 | K.YKAEDEVQR.E | 22 |
|  | pDK365N\_300mM\_082713\_01.08883.08883.2 | 3.905 | 0.3786 | 100.0% | 1289.4922 | 1288.4608 | 1 | 6.866 | 80.0% | 16 | K.NALESYAFNMK.S | 22 |

Similarities:
gi|5729877|ref|NP\_006(4:38)  
gi|13676857|ref|NP\_06(3:39)  
gi|124256496|ref|NP\_0(13:29)  
gi|34419635|ref|NP\_00(9:33)  

---

|  |  |  |  |  |  |  |  |  |
| --- | --- | --- | --- | --- | --- | --- | --- | --- |
| U | *gi|11067747|ref|NP\_00* | 33 | 107 | 47.6% | 802 | 92251 | 8.2 | CDC5-like [Homo sapiens] |

| Filename XCorr DeltCN Conf% ObsM+H+ CalcM+H+ SpR ZScore Ion% # Sequence  | | | | | | | | | | | | |
| --- | --- | --- | --- | --- | --- | --- | --- | --- | --- | --- | --- | --- |
| \* | pDK365N\_300mM\_082713\_01.11214.11214.2 | 2.5977 | 0.2587 | 99.5% | 1337.0721 | 1337.5175 | 1 | 5.444 | 72.2% | 1 | R.WYEWLDPSIK.K | 2 |
| \* | pDK365N\_300mM\_082713\_01.08265.08265.2 | 3.2993 | 0.2521 | 99.9% | 1383.3322 | 1382.5126 | 2 | 4.868 | 72.7% | 4 | R.GVDYNAEIPFEK.K | 2 |
| \* | pDK365N\_300mM\_082713\_04.07028.07028.3 | 4.2256 | 0.1618 | 98.5% | 2552.6643 | 2551.7283 | 1 | 5.454 | 36.9% | 4 | K.KPALGFYDTSEENYQALDADFR.K | 3 |
| \* | SKAPIP\_041314\_02.07292.07292.3 | 5.2495 | 0.4085 | 100.0% | 2679.9844 | 2679.9023 | 1 | 7.048 | 39.8% | 4 | K.KPALGFYDTSEENYQALDADFRK.L | 3 |
| \* | pDK365N\_300mM\_082713\_01.09408.09408.3 | 5.0931 | 0.4359 | 100.0% | 2266.2844 | 2266.5535 | 1 | 7.941 | 46.2% | 4 | R.KKESDLPSAILQTSGVSEFTK.K | 3 |
| \* | pDK365N\_300mM\_082713\_03.10875.10875.2 | 4.6917 | 0.5373 | 100.0% | 2009.9722 | 2010.2053 | 1 | 9.625 | 47.2% | 4 | K.ESDLPSAILQTSGVSEFTK.K | 2 |
| \* | pDK365N\_300mM\_082713\_03.09812.09812.3 | 4.7336 | 0.508 | 100.0% | 2165.9343 | 2165.5352 | 1 | 8.241 | 40.8% | 1 | R.SKLVLPAPQISDAELQEVVK.V | 3 |
| \* | pDK365N\_300mM\_082713\_03.10959.10959.2 | 3.6903 | 0.5573 | 100.0% | 1949.3722 | 1950.283 | 1 | 10.193 | 73.5% | 4 | K.LVLPAPQISDAELQEVVK.V | 2 |
| \* | pDK365N\_300mM\_082713\_02.07301.07301.3 | 5.3555 | 0.4405 | 100.0% | 3157.5842 | 3158.3599 | 2 | 7.86 | 24.1% | 1 | R.QTAEESGITNSASSTLLSEYNVTNNSVALR.T | 3 |
| \* | pDK365N\_300mM\_082713\_03.11628.11628.2 | 5.9232 | 0.5032 | 100.0% | 2113.7722 | 2113.4773 | 1 | 9.82 | 69.4% | 1 | R.ILQEAQNLMALTNVDTPLK.G | 2 |
| \* | pDK365N\_300mM\_082713\_01.07164.07164.3 | 3.8619 | 0.3028 | 99.9% | 2013.3844 | 2013.1741 | 1 | 5.323 | 36.1% | 3 | K.GGLNTPLHESDFSGVTPQR.Q | 3 |
| \* | pDK365N\_300mM\_082713\_03.08382.08382.2 | 3.9263 | 0.4478 | 100.0% | 1687.3722 | 1687.9364 | 2 | 8.477 | 60.7% | 6 | R.QVVQTPNTVLSTPFR.T | 2 |
| \* | SKAPIP\_041314\_01.05312.05312.2 | 3.423 | 0.4803 | 100.0% | 1200.2322 | 1200.2938 | 1 | 8.341 | 81.8% | 4 | R.TPSNGAEGLTPR.S | 2 |
| \* | SKAPIP\_041314\_01.04916.04916.2 | 2.2266 | 0.2841 | 97.9% | 1513.2722 | 1512.7062 | 1 | 5.194 | 53.6% | 1 | R.SGTTPKPVINSTPGR.T | 2 |
| \* | SKAPIP\_041314\_01.04904.04904.3 | 2.6998 | 0.3453 | 99.7% | 1514.0044 | 1512.7062 | 21 | 5.555 | 37.5% | 2 | R.SGTTPKPVINSTPGR.T | 3 |
| \* | pDK365N\_300mM\_082713\_03.07589.07589.3 | 5.1707 | 0.391 | 100.0% | 2372.9944 | 2372.5654 | 1 | 7.474 | 40.0% | 1 | R.DKLNINPEDGMADYSDPSYVK.Q | 3 |
| \* | pDK365N\_300mM\_082713\_03.08243.08243.2 | 5.2538 | 0.4965 | 100.0% | 2128.652 | 2129.3027 | 1 | 9.018 | 69.4% | 2 | K.LNINPEDGMADYSDPSYVK.Q | 2 |
| \* | SKAPIP\_tube2\_041314\_01.09808.09808.1 | 2.1763 | 0.3476 | 98.6% | 978.66 | 979.25104 | 1 | 6.932 | 77.8% | 1 | R.LGLLGLPAPK.N | 1 |
| \* | pDK365N\_300mM\_082713\_03.09561.09561.2 | 3.4521 | 0.3618 | 100.0% | 979.3122 | 979.25104 | 1 | 7.784 | 94.4% | 4 | R.LGLLGLPAPK.N | 2 |
| \* | pDK365N\_300mM\_082713\_03.08961.08961.2 | 4.0079 | 0.3599 | 100.0% | 1518.3722 | 1518.6635 | 1 | 7.167 | 70.8% | 4 | K.NDFEIVLPENAEK.E | 2 |
| \* | pDK365N\_300mM\_082713\_02.06275.06275.2 | 4.93 | 0.4868 | 100.0% | 1811.1721 | 1811.8535 | 1 | 10.152 | 66.7% | 5 | R.EIDDTYIEDAADVDAR.K | 2 |
| \* | pDK365N\_300mM\_082713\_03.10650.10650.3 | 3.8426 | 0.3529 | 100.0% | 3085.6143 | 3085.5278 | 1 | 6.121 | 30.8% | 3 | K.DLPRPSEVNETILRPLNVEPPLTDLQK.S | 3 |
| \* | SKAPIP\_041314\_02.05933.05933.3 | 3.887 | 0.2927 | 99.9% | 2550.7744 | 2551.7312 | 1 | 6.695 | 28.6% | 6 | K.TVGFGTNNSEHITYLEHNPYEK.F | 3 |
| \* | pDK365N\_300mM\_082713\_04.06682.06682.2 | 4.5814 | 0.5646 | 100.0% | 1488.5922 | 1488.7394 | 1 | 9.461 | 75.0% | 10 | K.AQDVLVQEMEVVK.Q | 2 |
| \* | pDK365N\_300mM\_082713\_03.05768.05768.2 | 2.5276 | 0.3067 | 99.8% | 1006.97217 | 1007.17773 | 1 | 5.288 | 87.5% | 4 | K.ILLGGYQSR.A | 2 |
| \* | SKAPIP\_tube2\_041314\_01.12341.12341.2 | 3.6346 | 0.4338 | 100.0% | 2121.9922 | 2122.3452 | 1 | 7.351 | 56.2% | 1 | K.QLNDLWDQIEQAHLELR.T | 2 |
| \* | SKAPIP\_tube2\_041314\_01.12338.12338.3 | 3.8541 | 0.3343 | 100.0% | 2123.3943 | 2122.3452 | 1 | 5.776 | 42.2% | 8 | K.QLNDLWDQIEQAHLELR.T | 3 |
| \* | SKAPIP\_tube2\_041314\_01.04446.04446.2 | 4.1296 | 0.4388 | 100.0% | 1800.7722 | 1801.0092 | 1 | 7.404 | 67.9% | 1 | R.TFEELKKHEDSAIPR.R | 2 |
| \* | pDK365N\_300mM\_082713\_01.04122.04122.3 | 3.8544 | 0.4864 | 100.0% | 1801.6743 | 1801.0092 | 1 | 8.342 | 50.0% | 3 | R.TFEELKKHEDSAIPR.R | 3 |
| \* | SKAPIP\_041314\_01.05963.05963.3 | 3.3788 | 0.2488 | 99.8% | 1446.9844 | 1446.6128 | 1 | 5.337 | 55.0% | 2 | R.RLECLKEDVQR.Q | 3 |
| \* | pDK365N\_300mM\_082713\_03.07575.07575.2 | 2.934 | 0.2513 | 99.9% | 965.2322 | 965.13446 | 1 | 6.805 | 85.7% | 5 | R.YADLLLEK.E | 2 |
| \* | SKAPIP\_tube2\_041314\_01.07756.07756.2 | 4.3587 | 0.4783 | 100.0% | 1436.5922 | 1436.6886 | 1 | 8.971 | 90.9% | 2 | R.YADLLLEKETLK.S | 2 |
| \* | pDK365N\_300mM\_082713\_03.07508.07508.3 | 2.3565 | 0.3195 | 99.0% | 1436.8143 | 1436.6886 | 1 | 6.095 | 45.5% | 1 | R.YADLLLEKETLK.S | 3 |

---

|  |  |  |  |  |  |  |  |  |
| --- | --- | --- | --- | --- | --- | --- | --- | --- |
| U | *gi|7706322|ref|NP\_057* | 11 | 24 | 47.1% | 244 | 28068 | 6.7 | homeobox prox 1 [Homo sapiens] |

| Filename XCorr DeltCN Conf% ObsM+H+ CalcM+H+ SpR ZScore Ion% # Sequence  | | | | | | | | | | | | |
| --- | --- | --- | --- | --- | --- | --- | --- | --- | --- | --- | --- | --- |
| \* | SKAPIP\_041314\_01.13017.13017.2 | 3.1933 | 0.3824 | 100.0% | 1292.2122 | 1292.4772 | 5 | 6.645 | 72.2% | 3 | R.NFIVWLEDQK.I | 2 |
| \* | SKAPIP\_tube2\_041314\_01.14553.14553.2 | 2.39 | 0.3698 | 99.7% | 1485.3121 | 1484.7385 | 3 | 5.897 | 58.3% | 1 | R.QEAIDWLLGLAVR.L | 2 |
| \* | SKAPIP\_tube2\_041314\_01.09959.09959.2 | 3.7288 | 0.2073 | 99.8% | 1813.4722 | 1814.0049 | 1 | 6.392 | 63.3% | 3 | K.NAEPLINLDVNNPDFK.A | 2 |
| \* | SKAPIP\_041314\_01.13182.13182.2 | 3.3698 | 0.279 | 99.9% | 1498.5922 | 1498.8271 | 1 | 5.406 | 65.4% | 2 | K.AGVMALANLLQIQR.H | 2 |
| \* | SKAPIP\_tube2\_041314\_01.07859.07859.2 | 3.5179 | 0.4423 | 100.0% | 1135.2522 | 1134.3356 | 1 | 7.924 | 87.5% | 5 | R.HDDYLVMLK.A | 2 |
| \* | SKAPIP\_tube2\_041314\_01.13877.13877.3 | 7.159 | 0.4778 | 100.0% | 3078.4744 | 3077.5076 | 1 | 8.95 | 35.7% | 2 | K.EGLPVALDKHILGFDTGDAVLNEAAQILR.L | 3 |
| \* | SKAPIP\_tube2\_041314\_01.13575.13575.2 | 6.0293 | 0.6021 | 100.0% | 2153.412 | 2154.4307 | 1 | 11.07 | 76.3% | 1 | K.HILGFDTGDAVLNEAAQILR.L | 2 |
| \* | SKAPIP\_tube2\_041314\_01.13582.13582.3 | 3.804 | 0.303 | 99.9% | 2154.7144 | 2154.4307 | 1 | 4.873 | 40.8% | 1 | K.HILGFDTGDAVLNEAAQILR.L | 3 |
| \* | SKAPIP\_tube2\_041314\_01.06599.06599.2 | 2.3783 | 0.2295 | 99.2% | 1023.1122 | 1023.2205 | 1 | 5.702 | 85.7% | 1 | R.LLHIEELR.E | 2 |
| \* | SKAPIP\_041314\_02.09773.09773.3 | 2.8727 | 0.2983 | 99.4% | 1665.5044 | 1665.9707 | 1 | 5.601 | 48.3% | 1 | K.INEAIVAVQAIIADPK.T | 3 |
| \* | SKAPIP\_041314\_02.09750.09750.2 | 4.8788 | 0.4377 | 100.0% | 1666.1921 | 1665.9707 | 1 | 8.821 | 80.0% | 4 | K.INEAIVAVQAIIADPK.T | 2 |

---

|  |  |  |  |  |  |  |  |  |
| --- | --- | --- | --- | --- | --- | --- | --- | --- |
| U | *gi|4758302|ref|NP\_004* | 10 | 31 | 47.1% | 104 | 12259 | 5.9 | enhancer of rudimentary homolog [Homo sapiens] |

| Filename XCorr DeltCN Conf% ObsM+H+ CalcM+H+ SpR ZScore Ion% # Sequence  | | | | | | | | | | | | |
| --- | --- | --- | --- | --- | --- | --- | --- | --- | --- | --- | --- | --- |
| \* | SKAPIP\_041314\_02.05952.05952.3 | 4.2847 | 0.469 | 100.0% | 2055.8643 | 2056.1375 | 1 | 7.497 | 51.6% | 1 | R.TYADYESVNECMEGVCK.M | 3 |
| \* | SKAPIP\_041314\_02.05920.05920.2 | 5.6845 | 0.5893 | 100.0% | 2056.3323 | 2056.1375 | 1 | 10.934 | 81.2% | 2 | R.TYADYESVNECMEGVCK.M | 2 |
| \* | pJS43\_100mM\_120812\_01.03611.03611.2 | 2.064 | 0.2622 | 98.8% | 950.0122 | 950.0973 | 27 | 4.808 | 66.7% | 1 | K.MYEEHLK.R | 2 |
| \* | pJS43\_100mM\_120812\_01.03341.03341.2 | 2.756 | 0.2606 | 99.9% | 1106.1322 | 1106.2848 | 2 | 5.29 | 78.6% | 2 | K.MYEEHLKR.M | 2 |
| \* | SKAPIP\_041314\_01.04421.04421.2 | 3.1853 | 0.361 | 100.0% | 1330.0322 | 1329.4087 | 2 | 6.319 | 65.0% | 5 | R.ADTQTYQPYNK.D | 2 |
| \* | SKAPIP\_tube2\_041314\_01.06572.06572.2 | 3.8742 | 0.3625 | 100.0% | 1871.5521 | 1872.0441 | 1 | 6.633 | 64.3% | 12 | R.ADTQTYQPYNKDWIK.E | 2 |
| \* | SKAPIP\_041314\_01.11051.11051.3 | 2.4499 | 0.2559 | 96.1% | 1871.9043 | 1872.0441 | 16 | 4.639 | 33.9% | 1 | R.ADTQTYQPYNKDWIK.E | 3 |
| \* | SKAPIP\_tube2\_041314\_01.05560.05560.2 | 3.2649 | 0.165 | 99.1% | 2128.4321 | 2129.3337 | 1 | 4.528 | 53.1% | 1 | R.ADTQTYQPYNKDWIKEK.I | 2 |
| \* | pDK365N\_100mM\_082813\_03.05339.05339.3 | 3.1635 | 0.2565 | 99.1% | 2129.5444 | 2129.3337 | 2 | 5.461 | 34.4% | 3 | R.ADTQTYQPYNKDWIKEK.I | 3 |
| \* | SKAPIP\_tube2\_041314\_01.05804.05804.2 | 2.2752 | 0.1216 | 96.6% | 934.2922 | 933.185 | 1 | 4.014 | 91.7% | 3 | K.IYVLLRR.Q | 2 |

---

|  |  |  |  |  |  |  |  |  |
| --- | --- | --- | --- | --- | --- | --- | --- | --- |
| U | *gi|4502549|ref|NP\_001* | 4 | 11 | 47.0% | 149 | 16838 | 4.2 | calmodulin 2 [Homo sapiens] |
| U | *gi|5901912|ref|NP\_008* | 4 | 11 | 47.0% | 149 | 16838 | 4.2 | calmodulin 1 [Homo sapiens] |
| U | *gi|58218968|ref|NP\_00* | 4 | 11 | 47.0% | 149 | 16838 | 4.2 | calmodulin 3 [Homo sapiens] |

| Filename XCorr DeltCN Conf% ObsM+H+ CalcM+H+ SpR ZScore Ion% # Sequence  | | | | | | | | | | | | |
| --- | --- | --- | --- | --- | --- | --- | --- | --- | --- | --- | --- | --- |
|  | SKAPIP\_tube2\_041314\_01.08528.08528.2 | 4.4232 | 0.4438 | 100.0% | 1846.5521 | 1846.0007 | 3 | 7.967 | 50.0% | 5 | K.EAFSLFDKDGDGTITTK.E | 2 |
|  | SKAPIP\_041314\_01.11543.11543.3 | 2.8678 | 0.2619 | 98.6% | 1846.7344 | 1846.0007 | 121 | 6.28 | 29.7% | 1 | K.EAFSLFDKDGDGTITTK.E | 3 |
|  | SKAPIP\_041314\_01.16368.16368.3 | 4.2303 | 0.3111 | 100.0% | 4071.9844 | 4072.4795 | 1 | 5.039 | 18.8% | 3 | R.SLGQNPTEAELQDMINEVDADGNGTIDFPEFLTMMAR.K | 3 |
|  | pDK365N\_100mM\_082713\_01.06681.06681.3 | 3.9655 | 0.3128 | 100.0% | 1756.2843 | 1755.9249 | 1 | 6.304 | 48.3% | 2 | R.VFDKDGNGYISAAELR.H | 3 |

---

|  |  |  |  |  |  |  |  |  |
| --- | --- | --- | --- | --- | --- | --- | --- | --- |
| U | *gi|5454152|ref|NP\_006* | 4 | 12 | 46.8% | 111 | 13530 | 8.8 | ubiquinol-cytochrome c reductase binding protein [Homo sapiens] |

| Filename XCorr DeltCN Conf% ObsM+H+ CalcM+H+ SpR ZScore Ion% # Sequence  | | | | | | | | | | | | |
| --- | --- | --- | --- | --- | --- | --- | --- | --- | --- | --- | --- | --- |
| \* | pDK365N\_300mM\_082713\_01.07894.07894.2 | 2.7928 | 0.3068 | 99.9% | 1234.2322 | 1234.3562 | 1 | 7.597 | 72.2% | 5 | K.WYYNAAGFNK.L | 2 |
| \* | 100326\_pJS43\_02.05127.05127.3 | 4.0278 | 0.3503 | 100.0% | 2383.7043 | 2382.6477 | 9 | 5.656 | 27.6% | 2 | K.LGLMRDDTIYEDEDVKEAIR.R | 3 |
|  | pDK365N\_100mM\_082813\_03.05256.05256.2 | 2.2718 | 0.168 | 96.5% | 1134.3121 | 1134.234 | 121 | 4.131 | 62.5% | 2 | R.LPENLYNDR.M | 2 |
|  | pDK365N\_300mM\_082713\_03.10474.10474.2 | 4.176 | 0.4088 | 100.0% | 1738.7922 | 1737.9031 | 1 | 7.035 | 83.3% | 3 | K.YEEENFYLEPYLK.E | 2 |

---

|  |  |  |  |  |  |  |  |  |
| --- | --- | --- | --- | --- | --- | --- | --- | --- |
| U | *gi|55770864|ref|NP\_00* | 12 | 90 | 46.7% | 257 | 26888 | 11.2 | THO complex 4 [Homo sapiens] |

| Filename XCorr DeltCN Conf% ObsM+H+ CalcM+H+ SpR ZScore Ion% # Sequence  | | | | | | | | | | | | |
| --- | --- | --- | --- | --- | --- | --- | --- | --- | --- | --- | --- | --- |
| \* | pJS43\_100mM\_120812\_01.11327.11327.2 | 3.2142 | 0.3822 | 100.0% | 1181.0521 | 1181.4048 | 1 | 8.37 | 88.9% | 4 | K.MDMSLDDIIK.L | 2 |
| \* | pDK365N\_100mM\_082713\_01.08788.08788.3 | 4.6651 | 0.4753 | 100.0% | 2704.2844 | 2704.915 | 16 | 7.084 | 27.0% | 6 | K.QLPDKWQHDLFDSGFGGGAGVETGGK.L | 3 |
| \* | pJS43\_100mM\_120812\_02.06612.06612.3 | 4.882 | 0.4901 | 100.0% | 2122.6743 | 2123.2456 | 1 | 8.503 | 42.5% | 5 | K.WQHDLFDSGFGGGAGVETGGK.L | 3 |
| \* | pJS43\_100mM\_120812\_01.15948.15948.3 | 4.3036 | 0.353 | 100.0% | 2843.2744 | 2843.203 | 1 | 7.367 | 29.0% | 3 | K.LLVSNLDFGVSDADIQELFAEFGTLK.K | 3 |
| \* | pDK365N\_100mM\_082713\_02.11247.11247.2 | 3.4755 | 0.66 | 100.0% | 2843.4321 | 2843.203 | 1 | 11.118 | 46.0% | 1 | K.LLVSNLDFGVSDADIQELFAEFGTLK.K | 2 |
| \* | pJS43\_100mM\_120812\_01.14957.14957.3 | 4.762 | 0.501 | 100.0% | 2971.1643 | 2971.377 | 1 | 8.019 | 29.8% | 18 | K.LLVSNLDFGVSDADIQELFAEFGTLKK.A | 3 |
| \* | pDK365N\_100mM\_082813\_03.14300.14300.2 | 2.2985 | 0.4004 | 99.6% | 2972.2722 | 2971.377 | 1 | 6.292 | 32.7% | 1 | K.LLVSNLDFGVSDADIQELFAEFGTLKK.A | 2 |
| \* | pJS43\_100mM\_120812\_01.05765.05765.2 | 3.3413 | 0.4886 | 100.0% | 1232.1721 | 1232.3384 | 1 | 8.133 | 75.0% | 13 | R.SLGTADVHFER.K | 2 |
| \* | pDK339othertube\_033013\_01.09848.09848.3 | 4.3651 | 0.3526 | 100.0% | 2814.7744 | 2815.1765 | 1 | 6.268 | 26.0% | 13 | K.QYNGVPLDGRPMNIQLVTSQIDAQR.R | 3 |
| \* | pJS43\_100mM\_120812\_01.08867.08867.3 | 4.0524 | 0.3988 | 100.0% | 2365.0144 | 2365.5186 | 1 | 6.95 | 33.8% | 1 | R.NSKQQLSAEELDAQLDAYNAR.M | 3 |
| \* | pJS43\_100mM\_120812\_02.06522.06522.2 | 6.2135 | 0.5589 | 100.0% | 2035.3722 | 2036.1626 | 1 | 10.305 | 70.6% | 16 | K.QQLSAEELDAQLDAYNAR.M | 2 |
| \* | pJS43\_100mM\_120812\_02.06585.06585.3 | 5.3902 | 0.4465 | 100.0% | 2036.9043 | 2036.1626 | 1 | 8.206 | 51.5% | 9 | K.QQLSAEELDAQLDAYNAR.M | 3 |

---

|  |  |  |  |  |  |  |  |  |
| --- | --- | --- | --- | --- | --- | --- | --- | --- |
| U | *gi|4557701|ref|NP\_000* | 25 | 97 | 46.5% | 432 | 48106 | 5.0 | keratin 17 [Homo sapiens] |

| Filename XCorr DeltCN Conf% ObsM+H+ CalcM+H+ SpR ZScore Ion% # Sequence  | | | | | | | | | | | | |
| --- | --- | --- | --- | --- | --- | --- | --- | --- | --- | --- | --- | --- |
|  | SKAPIP\_041314\_01.07463.07463.1 | 1.8759 | 0.3024 | 98.8% | 809.61 | 809.93774 | 30 | 5.035 | 66.7% | 7 | R.LASYLDK.V | 111111 |
|  | pSKT11\_1\_020812\_01.07860.07860.2 | 3.1681 | 0.1606 | 99.7% | 1066.2322 | 1065.2578 | 3 | 5.49 | 75.0% | 8 | R.LASYLDKVR.A | 22222 |
|  | pDK339othertube\_033013\_02.04740.04740.2 | 4.0363 | 0.3955 | 100.0% | 1346.2922 | 1346.4772 | 1 | 7.502 | 72.7% | 15 | R.ALEEANTELEVK.I | 2 |
|  | SKAPIP\_041314\_01.10325.10325.2 | 2.4245 | 0.1328 | 98.4% | 1037.2522 | 1037.1661 | 24 | 4.143 | 75.0% | 1 | K.IRDWYQR.Q | 222 |
|  | SKAPIP\_tube2\_041314\_01.11410.11410.2 | 5.1791 | 0.4208 | 100.0% | 2068.5923 | 2069.366 | 1 | 7.896 | 63.9% | 1 | K.ILTATVDNANILLQIDNAR.L | 2 |
|  | SKAPIP\_041314\_02.08957.08957.3 | 4.9241 | 0.3424 | 100.0% | 2069.1543 | 2069.366 | 1 | 7.147 | 48.6% | 1 | K.ILTATVDNANILLQIDNAR.L | 3 |
|  | SKAPIP\_041314\_01.10178.10178.1 | 1.6254 | 0.229 | 96.5% | 807.56 | 807.8815 | 13 | 4.849 | 58.3% | 1 | R.LAADDFR.T | 1111111 |
|  | SKAPIP\_041314\_01.10160.10160.2 | 2.4333 | 0.3828 | 100.0% | 807.6122 | 807.8815 | 1 | 6.683 | 83.3% | 13 | R.LAADDFR.T | 2222222 |
|  | SKAPIP\_041314\_01.06444.06444.2 | 2.9995 | 0.3535 | 100.0% | 1222.4922 | 1223.3715 | 35 | 5.673 | 66.7% | 5 | R.TKFETEQALR.L | 22 |
|  | SKAPIP\_041314\_01.06544.06544.3 | 3.3228 | 0.1834 | 99.0% | 1223.5743 | 1223.3715 | 4 | 4.987 | 50.0% | 1 | R.TKFETEQALR.L | 33 |
|  | SKAPIP\_tube2\_041314\_01.07814.07814.2 | 3.6074 | 0.4399 | 100.0% | 1030.1122 | 1030.2096 | 1 | 7.439 | 93.8% | 8 | R.VLDELTLAR.A | 2222 |
|  | SKAPIP\_tube2\_041314\_01.11847.11847.3 | 4.2922 | 0.4094 | 100.0% | 2279.4543 | 2279.6538 | 1 | 6.992 | 36.1% | 4 | R.ADLEMQIENLKEELAYLKK.N | 3 |
[truncated: 1,320,315 more chars]
