# Supplementary material for: Astrin-SKAP complex reconstitution reveals its kinetochore interaction with microtubule-bound Ndc80
Source: eLife. 2017 Aug 25;6:e26866. doi: 10.7554/eLife.26866 (PMC5602300; doi:10.7554/eLife.26866)
Supplement: Transparent reporting form [file elife-26866-transrepform.docx]

***eLife’s* transparent reporting form**

We encourage authors to provide detailed information *within their submission* to facilitate the interpretation and replication of experiments. If you have any questions, please contact us: [editorial@elifesciences.org](mailto:editorial@elifesciences.org).

**Sample-size estimation**

- You should state whether an appropriate sample size was computed when the study was being designed
- You should state the statistical method of sample size computation and any required assumptions
- If no explicit power analysis was used, you should describe how you decided what sample (replicate) size (number) to use

Please outline where this information can be found within the submission (e.g., page numbers or figure legends), or explain why this information doesn’t apply to your submission:

A subset of experiments in this paper (for example, protein localization studies) are qualitative in nature, and behaved consistently across multiple sample replicates, preventing the need for a statistical analysis. For the core microtubule binding assays, experiments were repeated in duplicate or triplicate for quantification purposes. Based on our extensive prior experience using this co-sedimentation assay, it is very reproducible between replicates (as also attested by the data). The experiment also occurs over a range of different concentrations, providing reproducibility within the experiment itself. Based on prior experience, we find that this number of replicates allows for an appropriate sense of variability from pipetting or other scientist errors. For our main cross-linking mass spectrometry on isolating Astrin from human cells, we performed the technique 6 times to assess its reproducibility for the Ndc80 interaction and to test variations on cross-linking time.

**Replicates**

- You should report how often each experiment was performed
- You should include a definition of biological versus technical replication
- The data obtained should be provided and sufficient information should be provided to indicate the number of independent biological and/or technical replicates
- If you encountered any outliers, you should describe how these were handled
- Criteria for exclusion/inclusion of data should be clearly stated
- High-throughput sequence data should be uploaded before submission, with a private link for reviewers provided (these are available from both GEO and ArrayExpress)

Please outline where this information can be found within the submission (e.g., page numbers or figure legends), or explain why this information doesn’t apply to your submission:

Experiments were performed multiple times to ensure reproducibility and consistency as indicated in the methods and figure legends. We show data for the six cross-linking mass spectrometry replicates in Figure 4 - Supplement 1. We indicate the number of microtubule-binding assay replicates in the legends of Figures 2 and 5. We also provide more details about these replicates in the methods sections for the microtubule binding assays.

**Statistical reporting**

- Statistical analysis methods should be described and justified
- Raw data should be presented in figures whenever informative to do so (typically when N per group is less than 10)
- For each experiment, you should identify the statistical tests used, exact values of N, definitions of center, methods of multiple test correction, and dispersion and precision measures (e.g., mean, median, SD, SEM, confidence intervals; and, for the major substantive results, a measure of effect size (e.g., Pearson's r, Cohen's d)
- Report exact p-values wherever possible alongside the summary statistics and 95% confidence intervals. These should be reported for all key questions and not only when the p-value is less than 0.05.

We present microtubule binding quantification data with both mean and standard deviation values as indicated in the figure legends for Figures 2 and 5. P values are not provided for this or other data in this paper given the nature of this data and experiments. However, the apparent affinity and curve fits for the binding data from the microtubule binding experiments is used for a comparison of this binding behavior. These values were generated from the binding data using the Prism graphing software, as indicated in the figure legends.

Please outline where this information can be found within the submission (e.g., page numbers or figure legends), or explain why this information doesn’t apply to your submission:

(For large datasets, or papers with a very large number of statistical tests, you may upload a single table file with tests, Ns, etc., with reference to page numbers in the manuscript.)

**Additional data files (“source data”)**

- We encourage you to upload relevant additional data files, such as numerical data that are represented as a graph in a figure, or as a summary table
- Where provided, these should be in the most useful format, and they can be uploaded as “Source data” files linked to a main figure or table
- Include model definition files including the full list of parameters used
- Include code used for data analysis (e.g., R, MatLab)
- Avoid stating that data files are “available upon request”

Please indicate the figures or tables for which source data files have been provided:

The only tables that rely on a larger set of source data are the mass spectrometry searches and graphs for microtubule binding assays. We have provided expanded data for the mass spec searches in Figure 4 - Supplement 1 to fully represent this data. We believe that the microtubule binding graphs fully represent the data from these experiments without the need for the precise values from each individual binding point (and provide better clarity). However, if the journal policy encourages this raw data or if the reviewers would find this useful, we could be happy to provide this information.
